# Supplementary material for: Assessing the Causal Relationship Between Plasma Proteins and Pulmonary Fibrosis: A Systematic Analysis Based on Mendelian Randomization
Source: Biology (Basel). 2025 Feb 14;14(2):200. doi: 10.3390/biology14020200 (PMC11852313; doi:10.3390/biology14020200)
Supplement: Supplementary file 1 [file biology-14-00200-s001.zip › Supplementary Material S1/Figure S12.pdf]

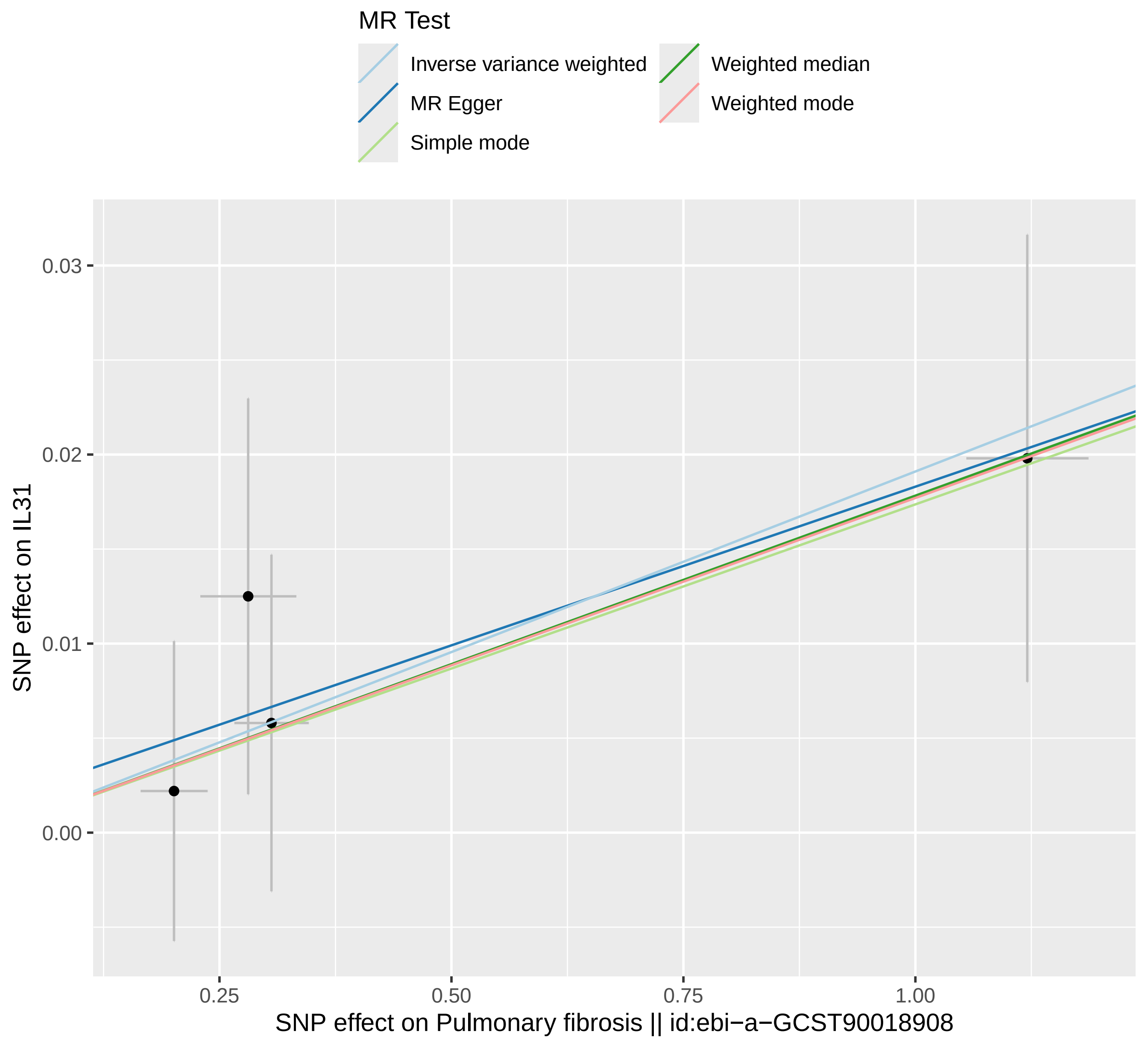
MR Test
Inverse variance weighted
MR Egger
Simple mode
Weighted median
Weighted mode
0.03
0.02
0.01
0.00
SNP effect on IL31
0.25
0.50
0.75
1.00
SNP effect on Pulmonary fibrosis || id:ebi−a−GCST90018908
