# Supplementary figures and images for: Assessing the Causal Relationship Between Plasma Proteins and Pulmonary Fibrosis: A Systematic Analysis Based on Mendelian Randomization
Source: Biology (Basel). 2025 Feb 14;14(2):200. doi: 10.3390/biology14020200 (PMC11852313; doi:10.3390/biology14020200)

# MR Method

- Inverse variance weighted
- MR Egger

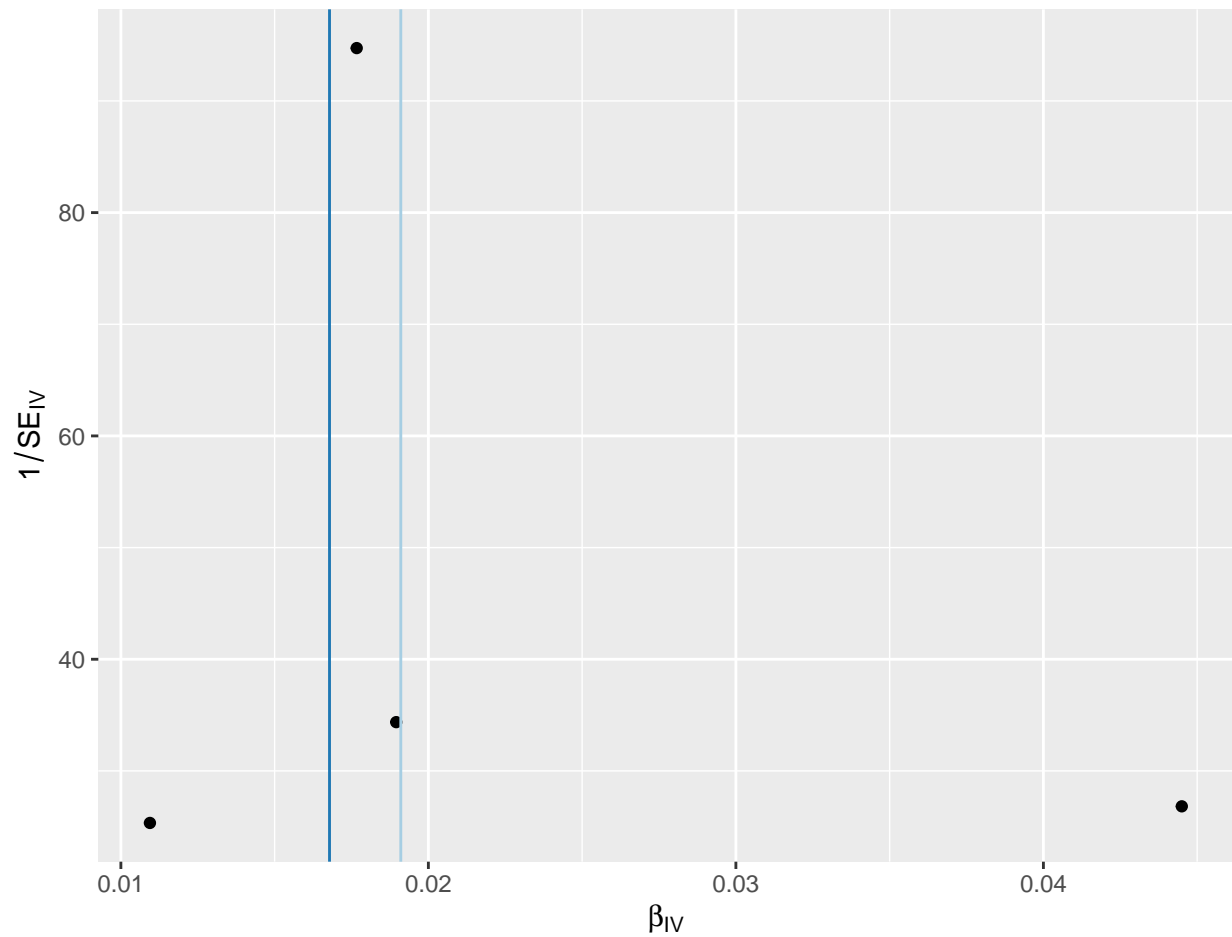

Supplement: Supplementary file 1 [file biology-14-00200-s001.zip › Supplementary Material S1/Figure S10.pdf]

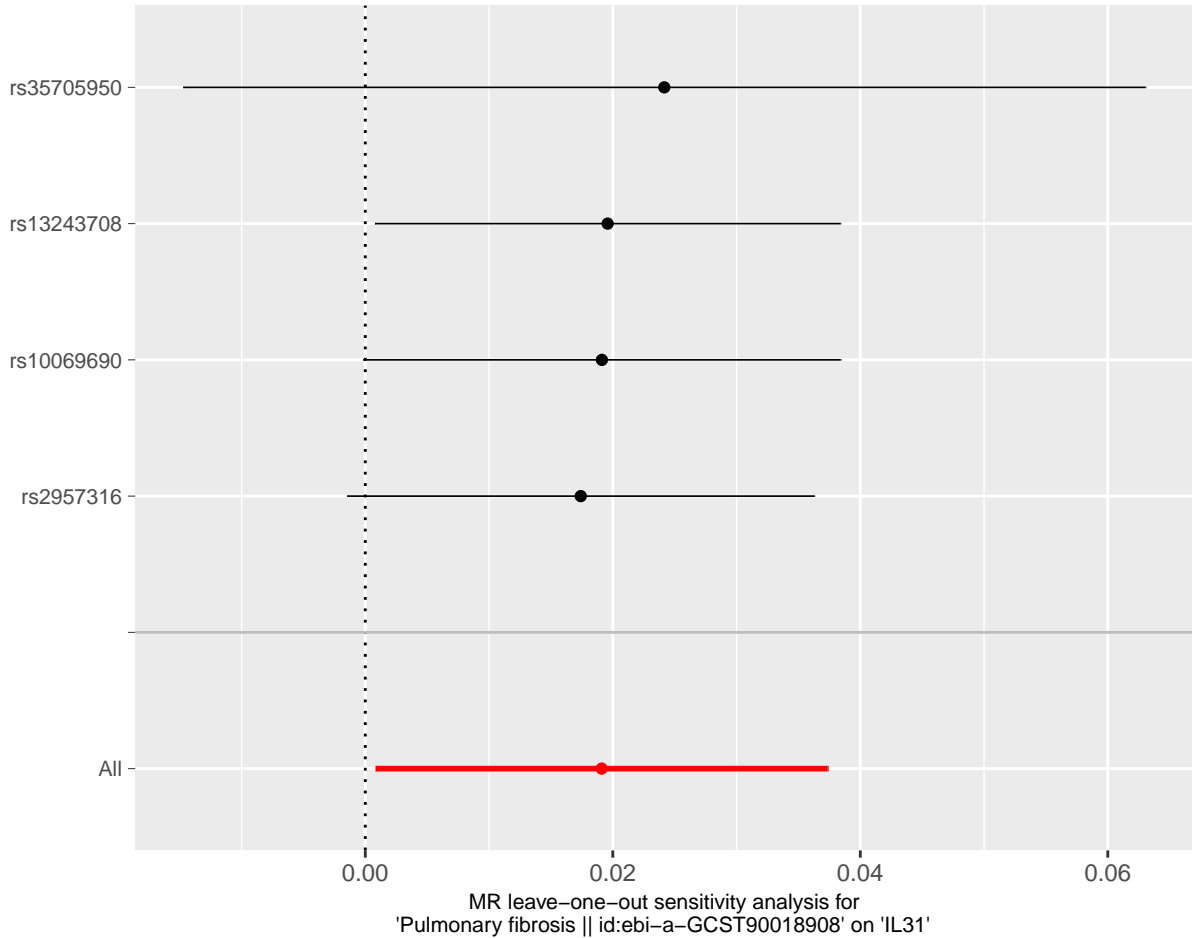

Supplement: Supplementary file 1 [file biology-14-00200-s001.zip › Supplementary Material S1/Figure S11.pdf]

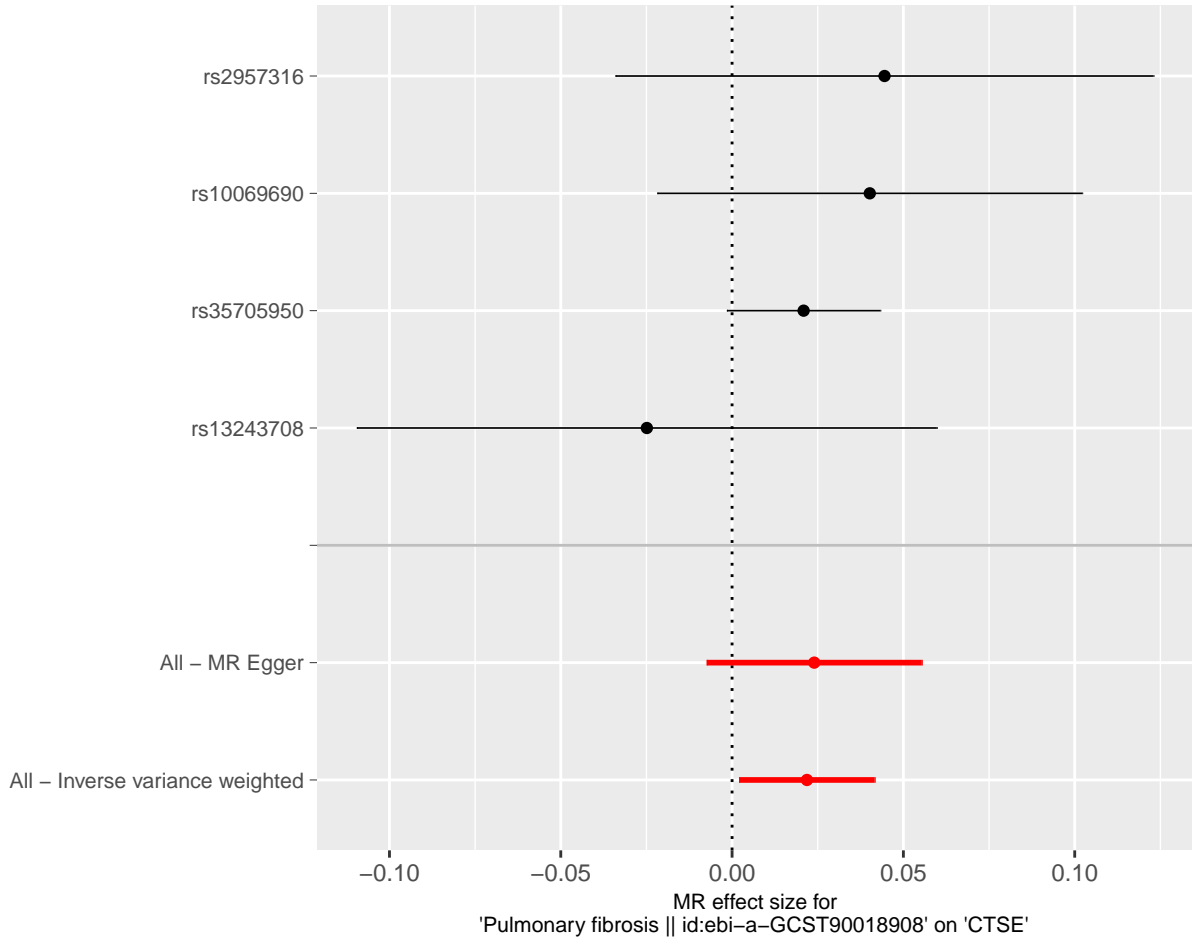

Supplement: Supplementary file 1 [file biology-14-00200-s001.zip › Supplementary Material S1/Figure S13.pdf]

# MR Method

- Inverse variance weighted
- MR Egger

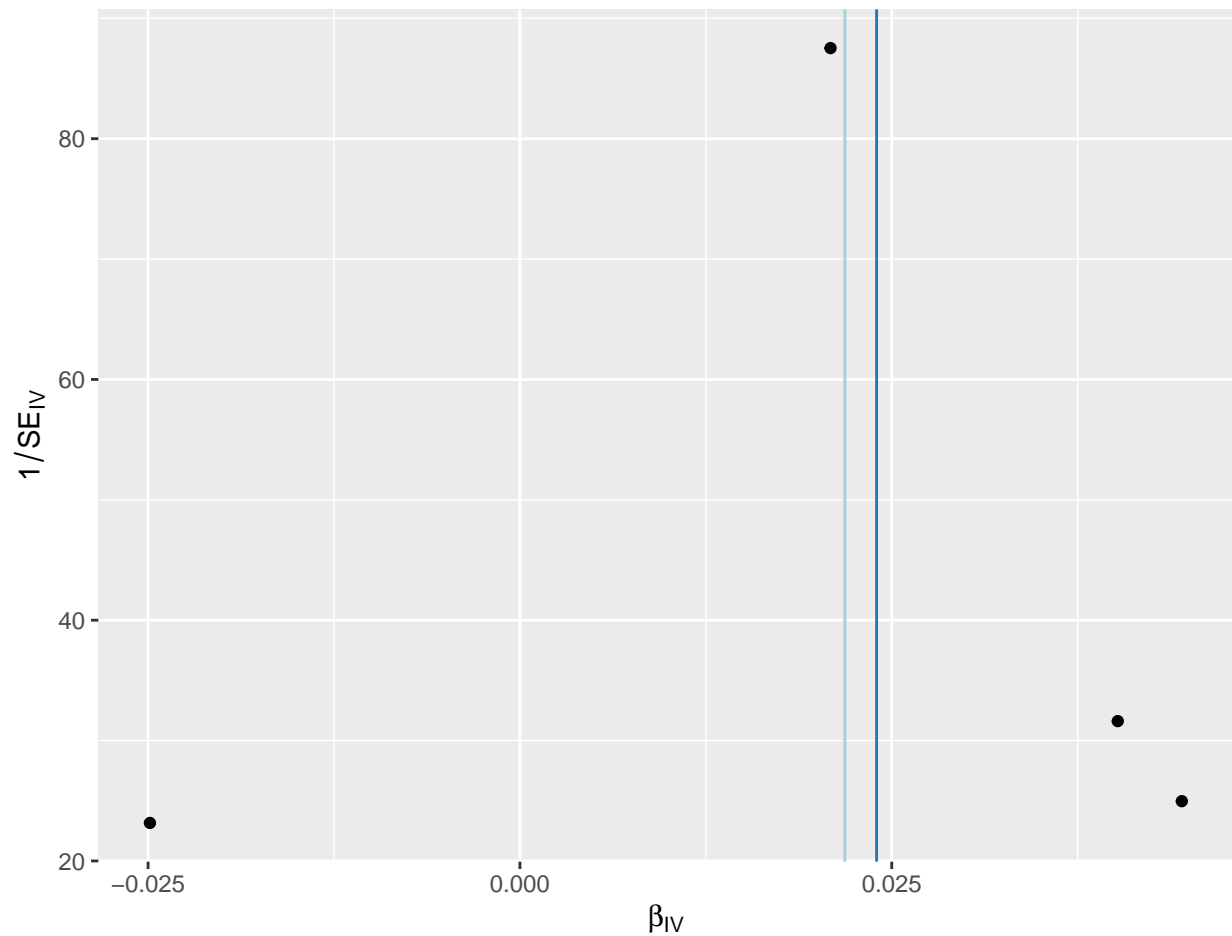

Supplement: Supplementary file 1 [file biology-14-00200-s001.zip › Supplementary Material S1/Figure S14.pdf]

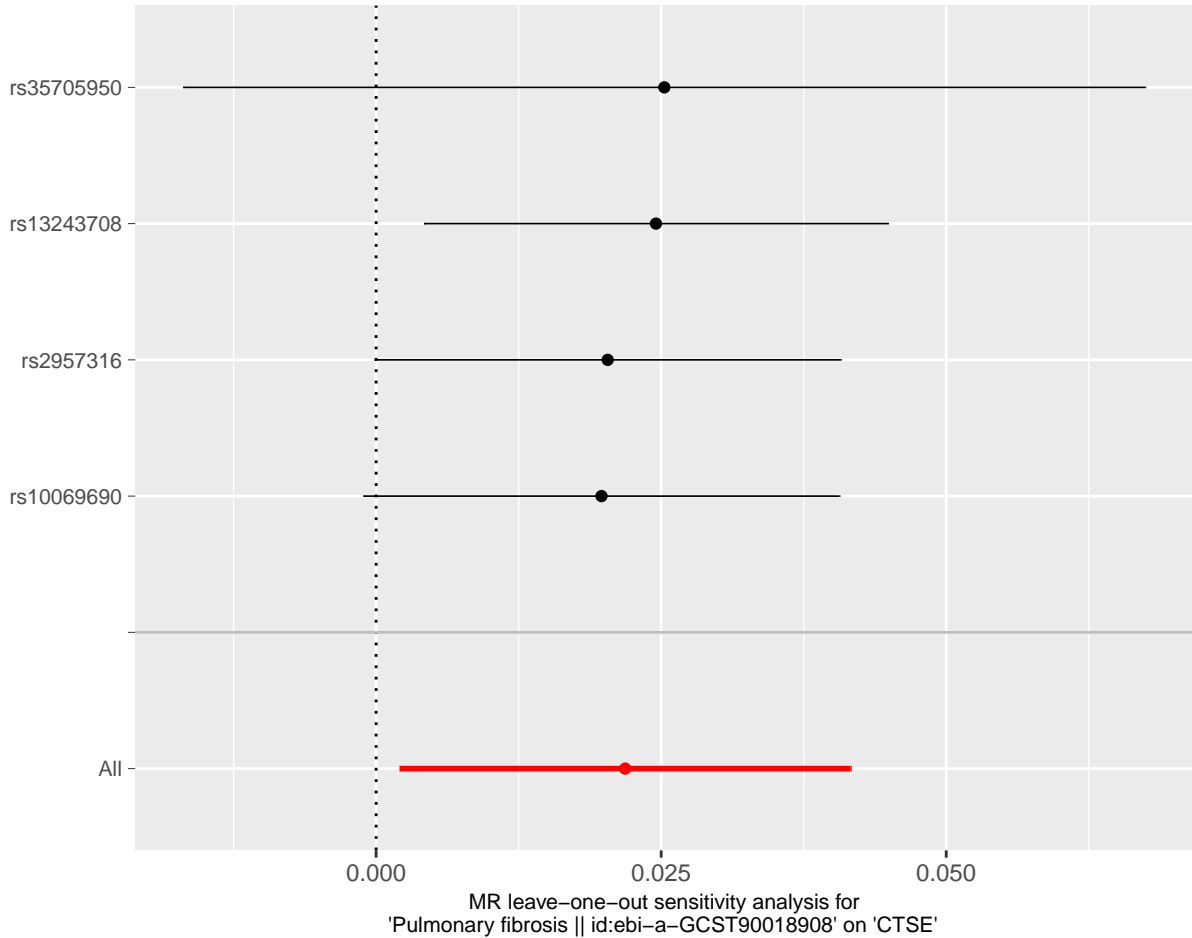

Supplement: Supplementary file 1 [file biology-14-00200-s001.zip › Supplementary Material S1/Figure S15.pdf]

# MR Test

- Inverse variance weighted
- MR Egger
- Simple mode
- Weighted median
- Weighted mode

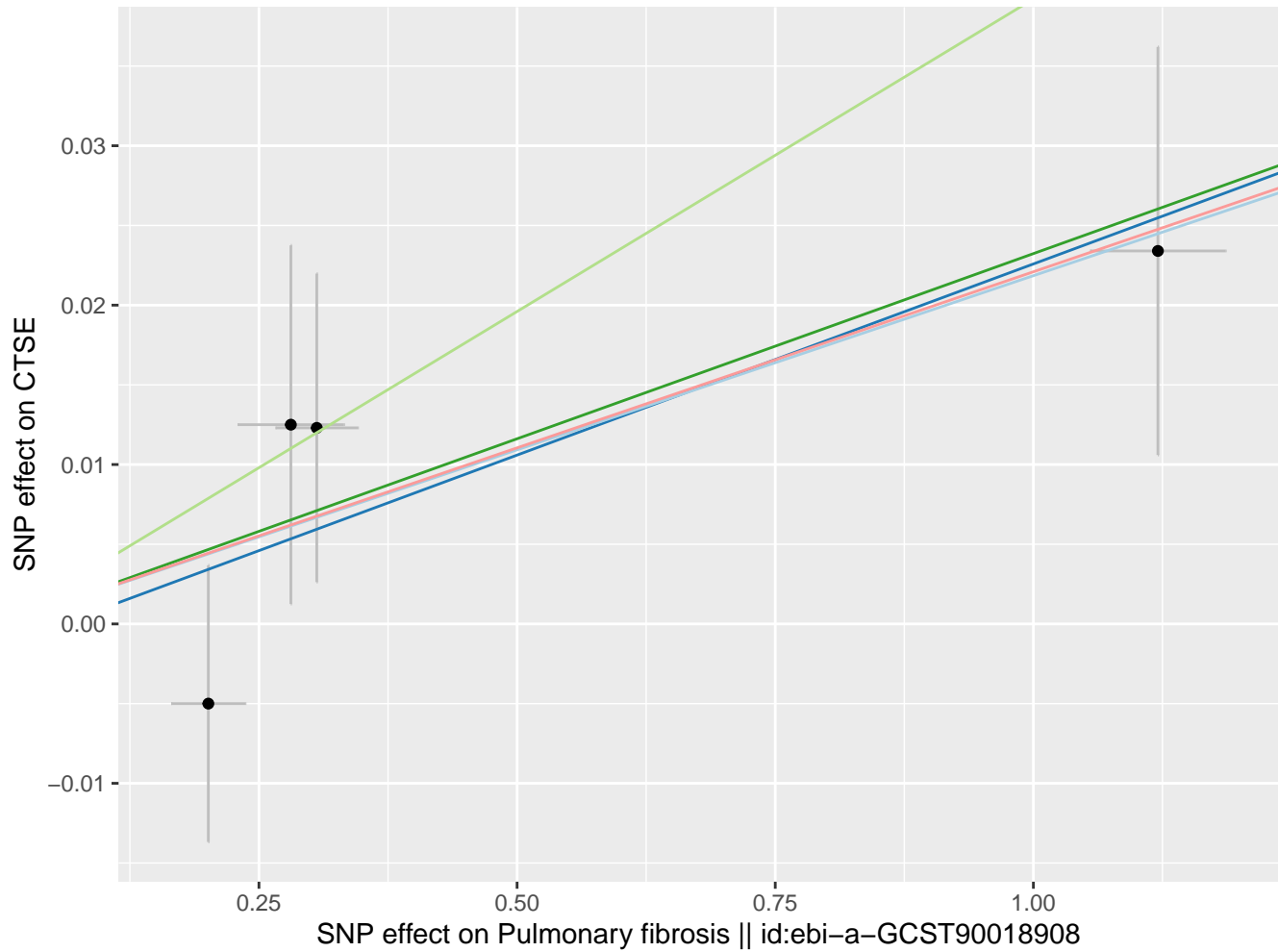

Supplement: Supplementary file 1 [file biology-14-00200-s001.zip › Supplementary Material S1/Figure S16.pdf]

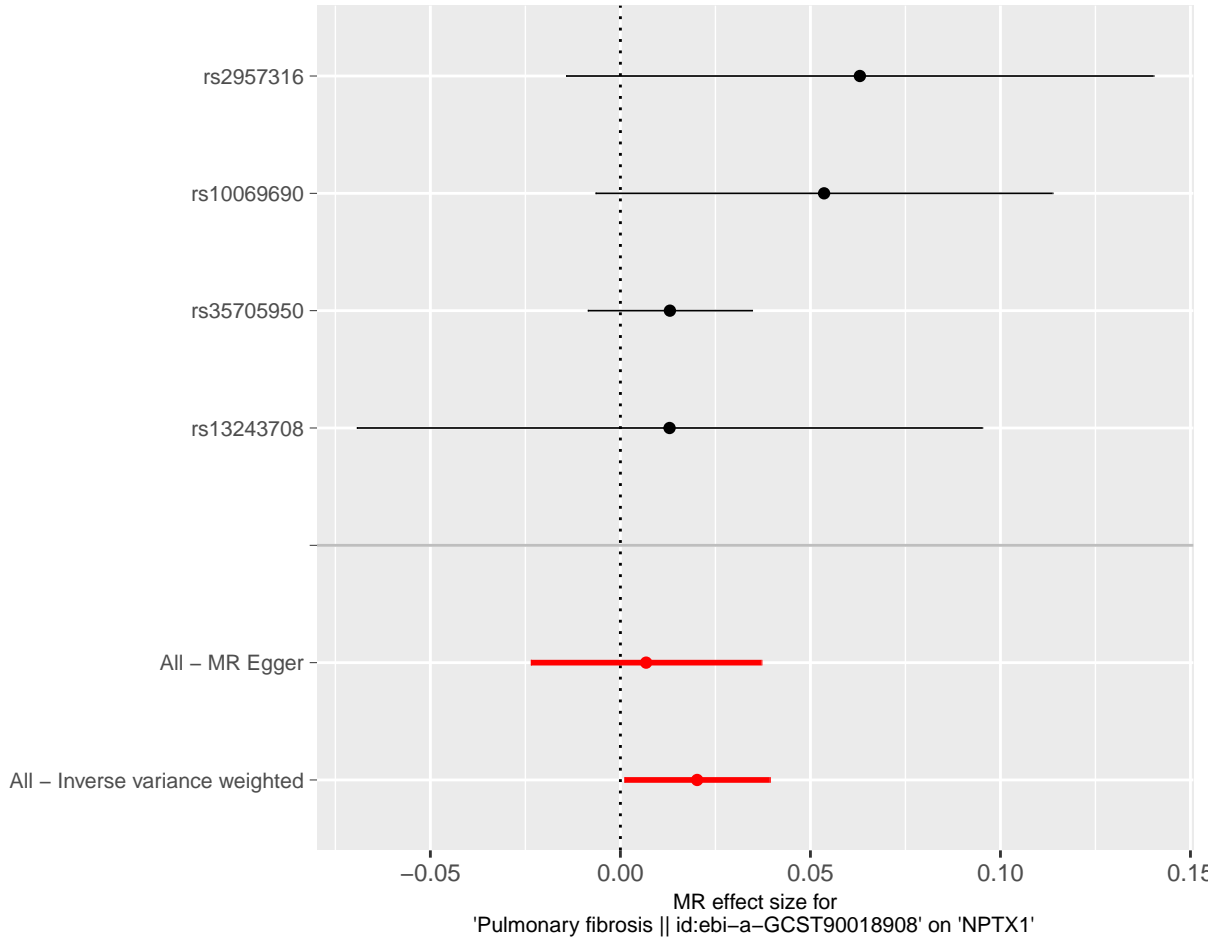

Supplement: Supplementary file 1 [file biology-14-00200-s001.zip › Supplementary Material S1/Figure S5.pdf]

# MR Method

- Inverse variance weighted
- MR Egger

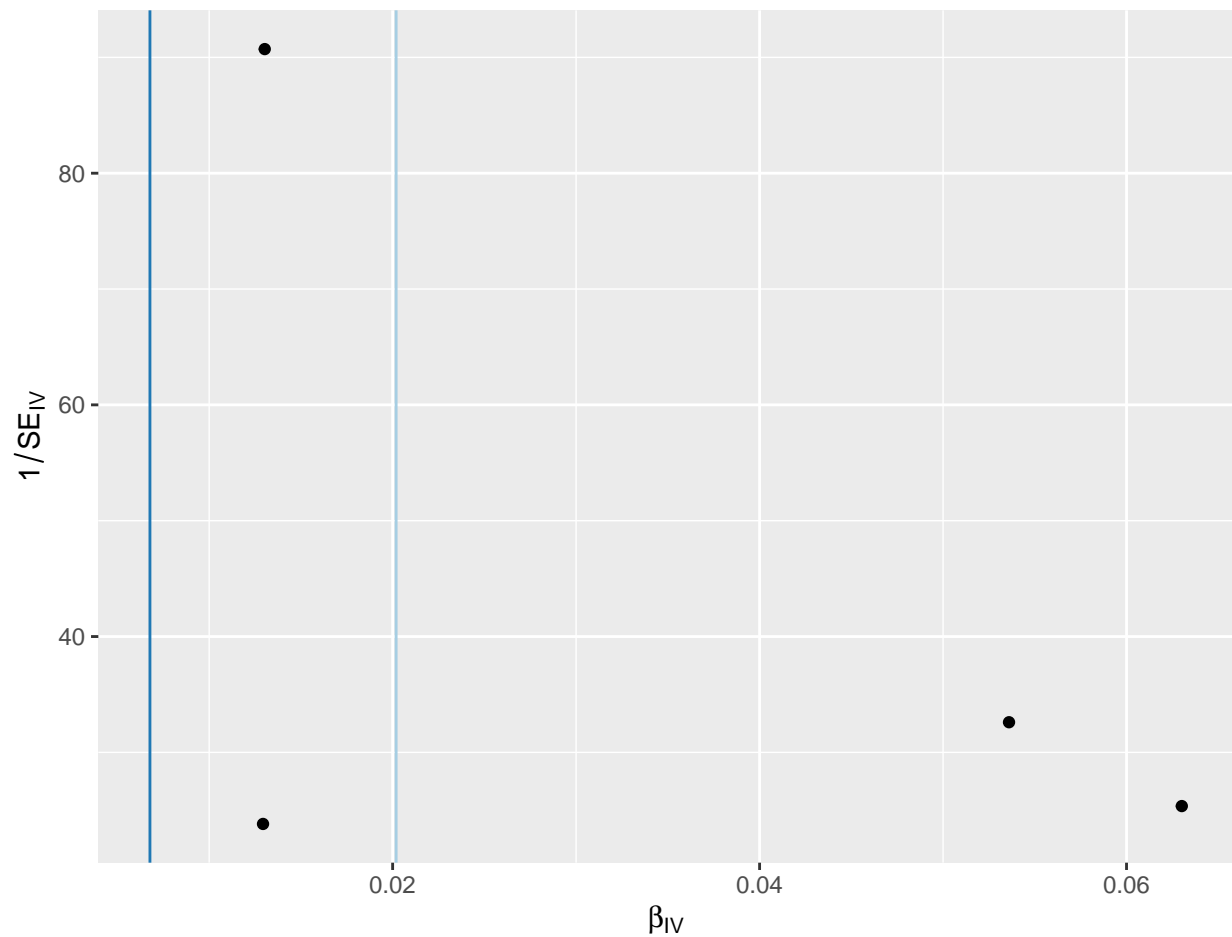

Supplement: Supplementary file 1 [file biology-14-00200-s001.zip › Supplementary Material S1/Figure S6.pdf]

rs35705950

rs13243708

rs2957316

rs10069690

All

0.000

0.025

0.050

0.075

MR leave-one-out sensitivity analysis for  
'Pulmonary fibrosis || id:ebi-a-GCST90018908' on 'NPTX1'

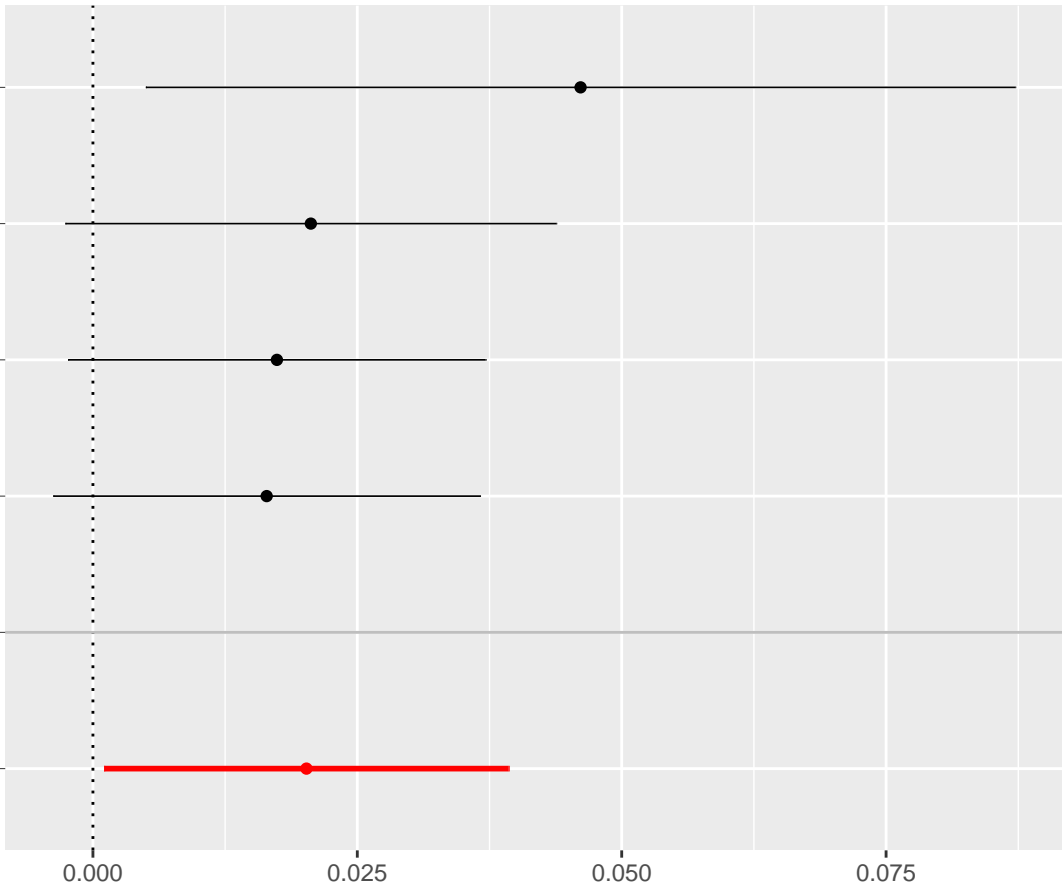

Supplement: Supplementary file 1 [file biology-14-00200-s001.zip › Supplementary Material S1/Figure S7.pdf]

# MR Test

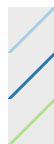

Inverse variance weighted

MR Egger

Simple mode

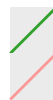

Weighted median

Weighted mode

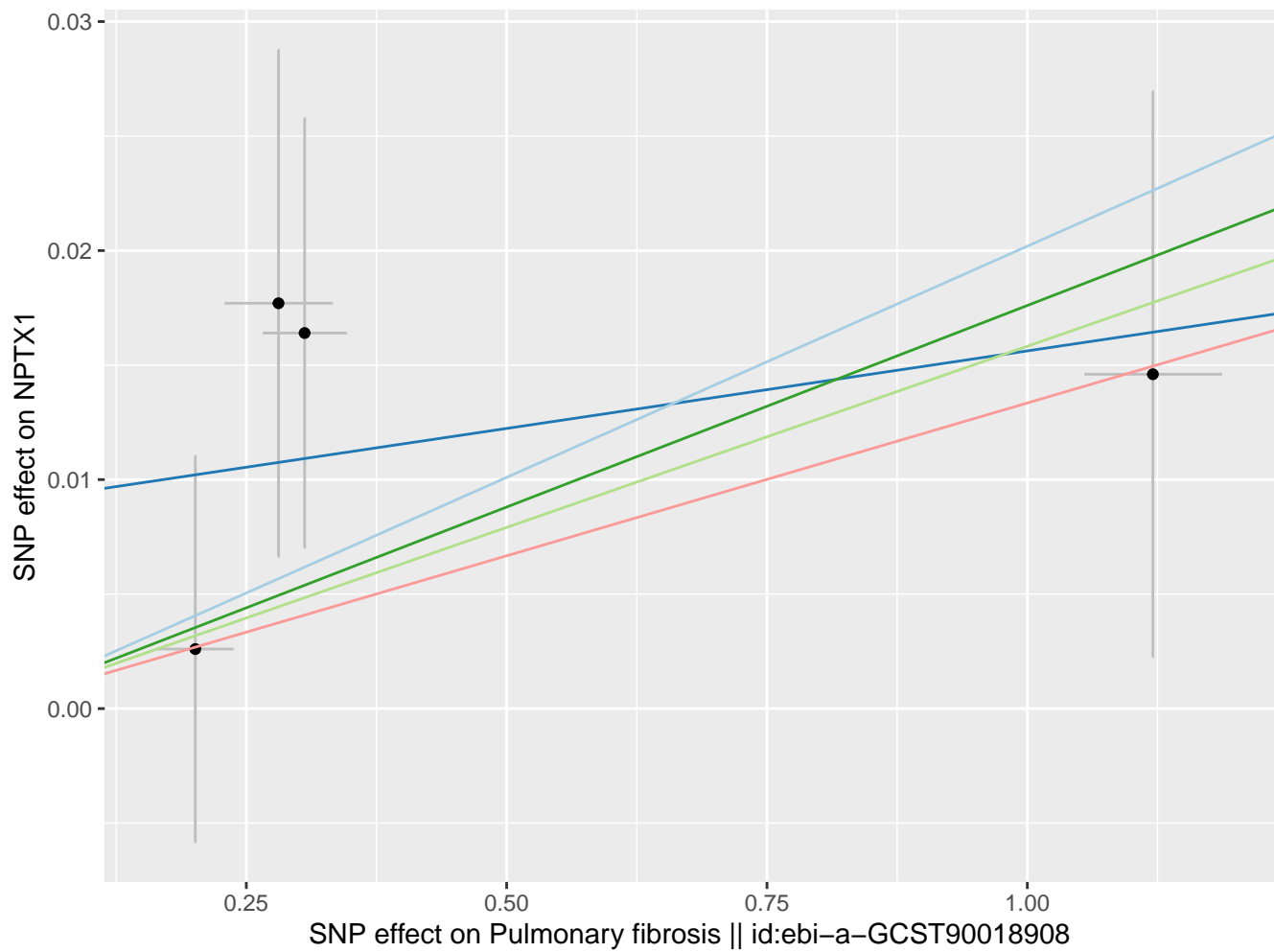

Supplement: Supplementary file 1 [file biology-14-00200-s001.zip › Supplementary Material S1/Figure S8.pdf]

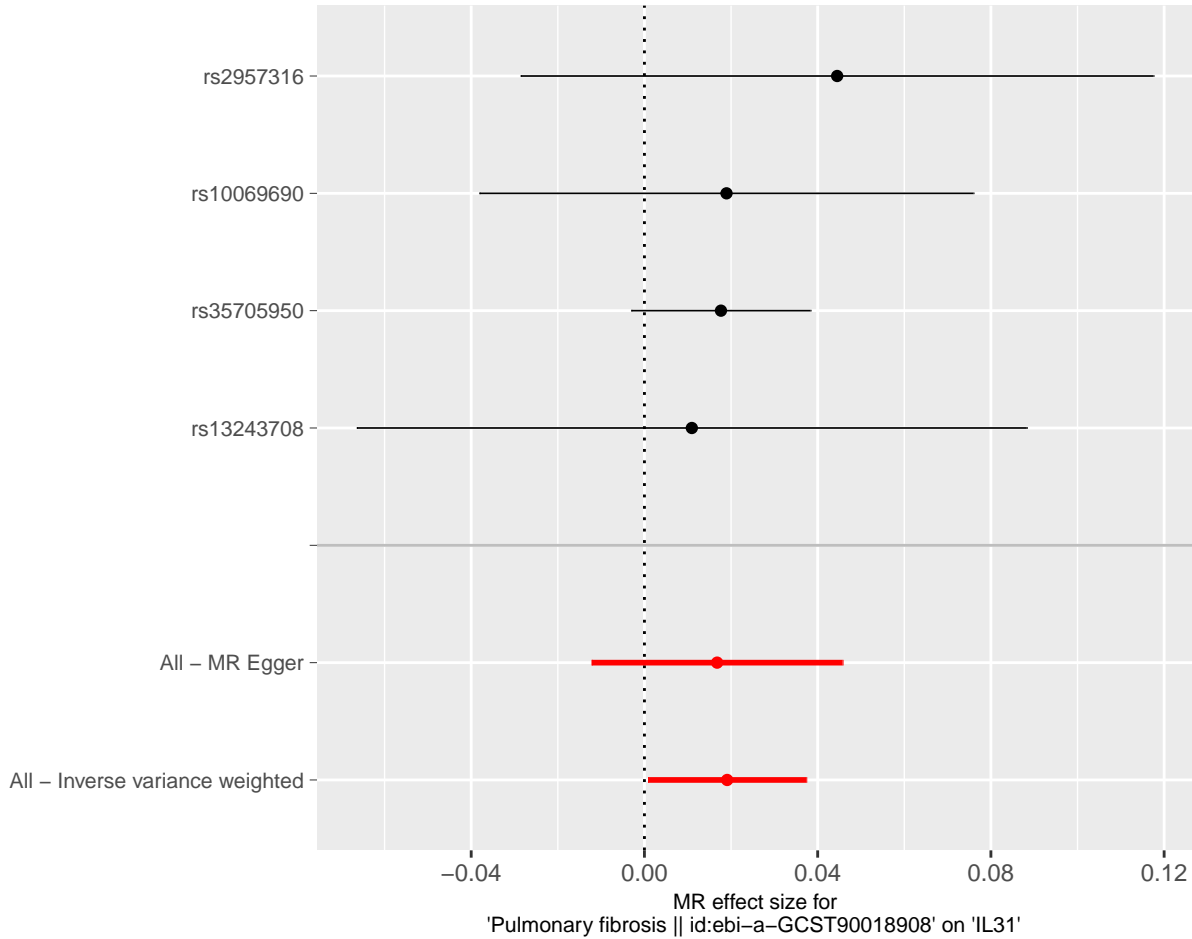

Supplement: Supplementary file 1 [file biology-14-00200-s001.zip › Supplementary Material S1/Figure S9.pdf]
