# Supplementary material for: Assessing the Causal Relationship Between Plasma Proteins and Pulmonary Fibrosis: A Systematic Analysis Based on Mendelian Randomization
Source: Biology (Basel). 2025 Feb 14;14(2):200. doi: 10.3390/biology14020200 (PMC11852313; doi:10.3390/biology14020200)
Supplement: Supplementary file 1 [file Figure S1.pdf › Supplementary Material S1/Figure S1.pdf]

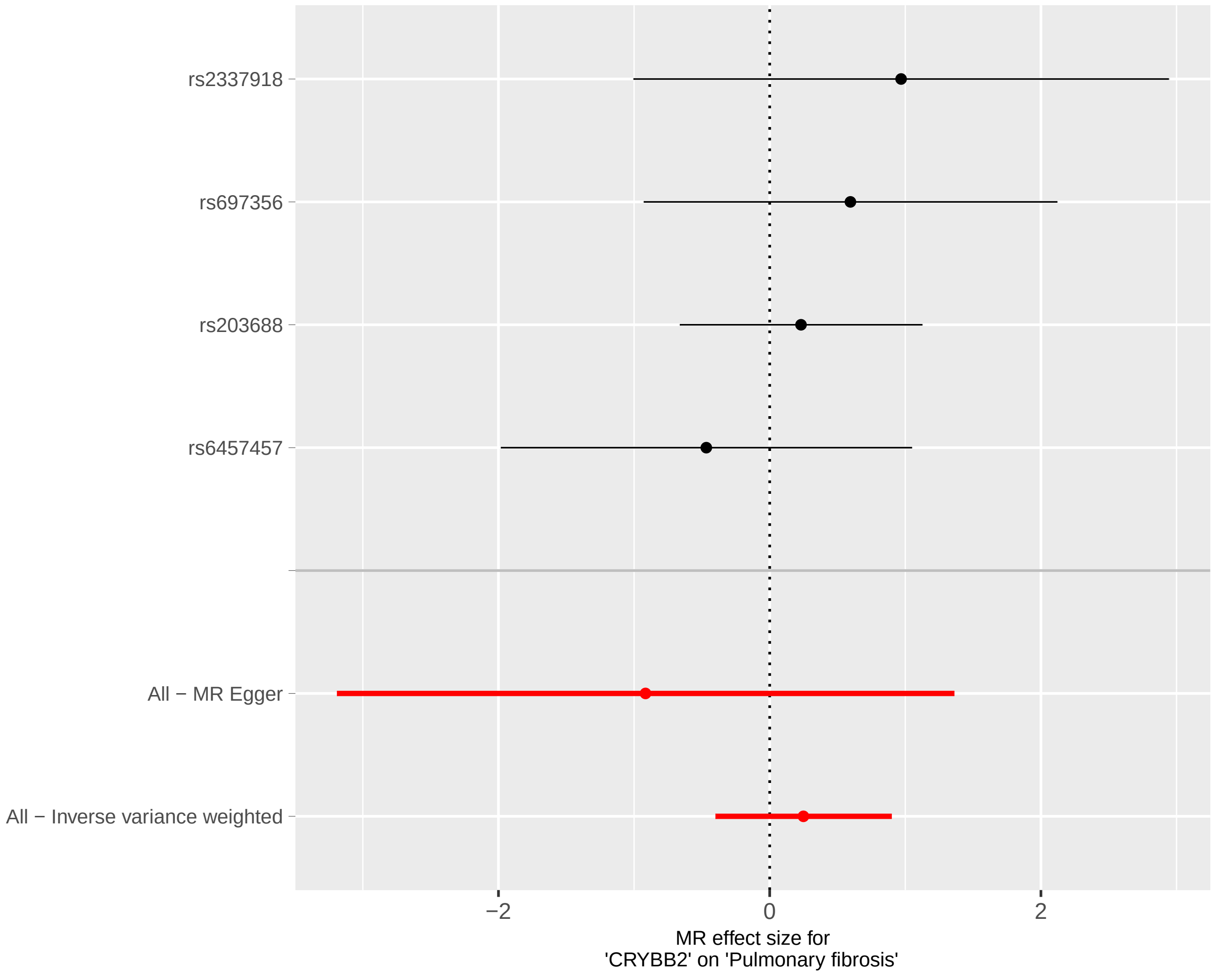
rs2337918
rs697356
rs203688
rs6457457
All − MR Egger
All − Inverse variance weighted
−2
0
2
MR effect size for
'CRYBB2' on 'Pulmonary fibrosis'

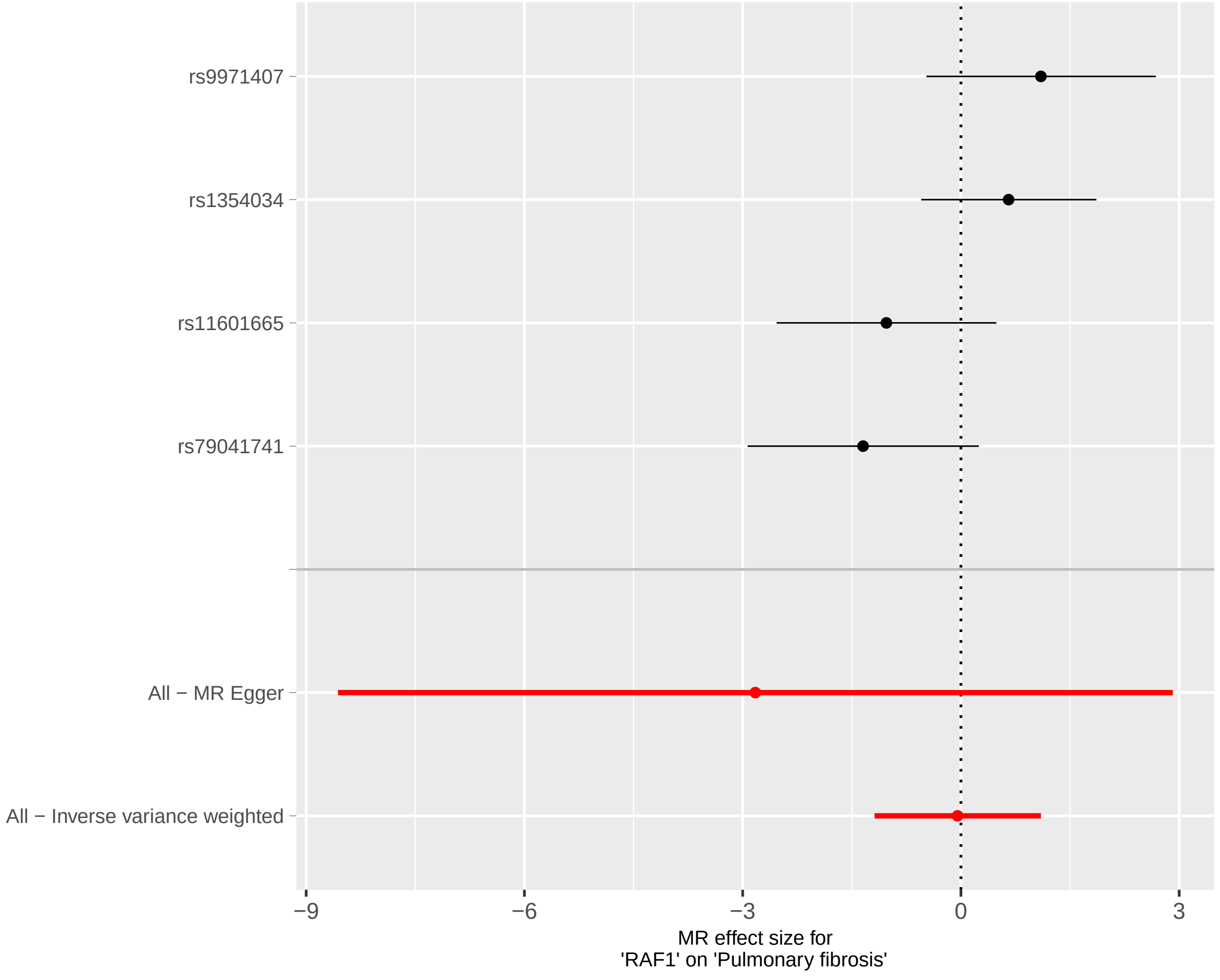
rs9971407
rs1354034
rs11601665
rs79041741
All − MR Egger
All − Inverse variance weighted
−9
−6
−3
0
3
MR effect size for
'RAF1' on 'Pulmonary fibrosis'

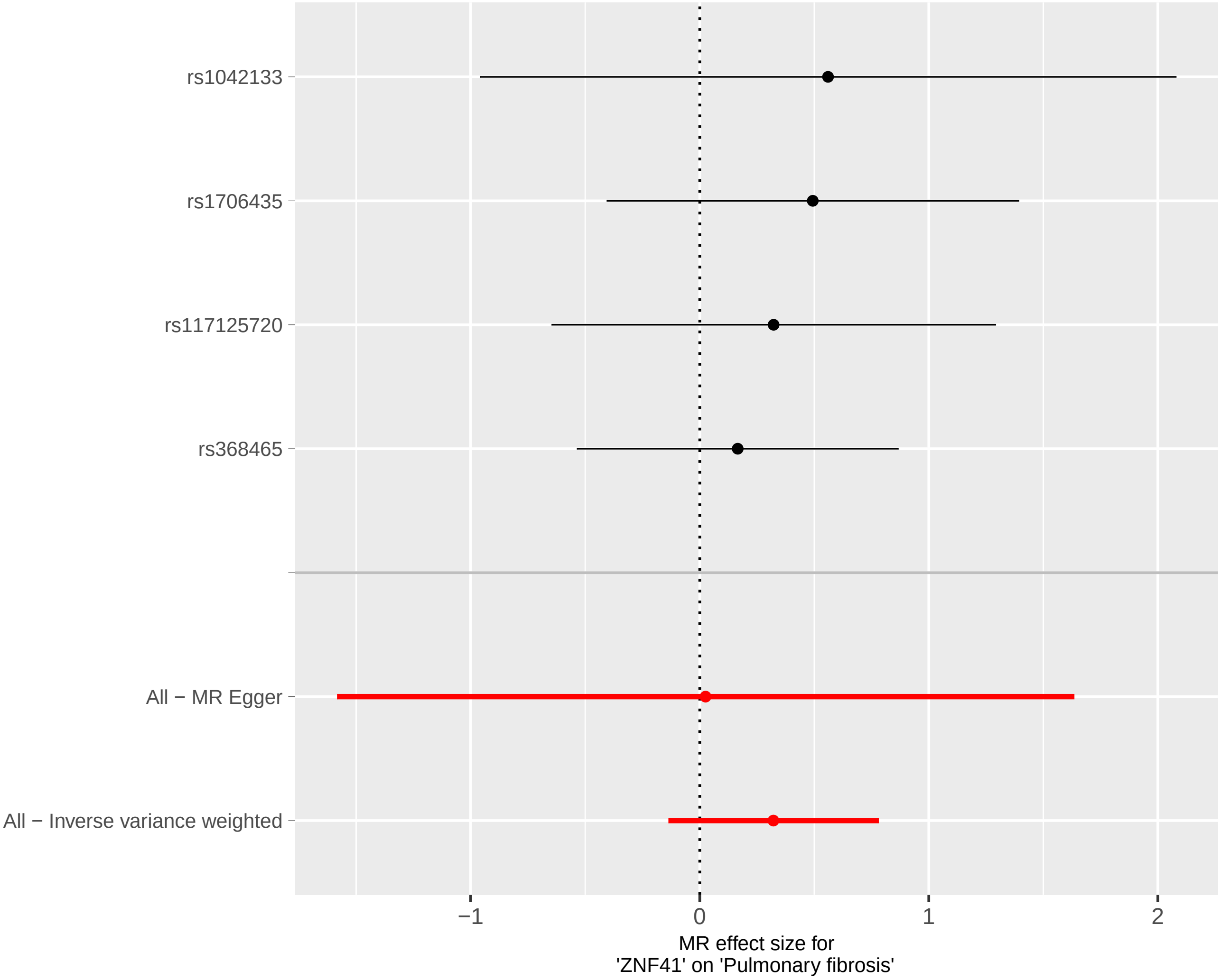

rs1042133
rs1706435
rs117125720
rs368465
All − MR Egger
All − Inverse variance weighted
−1
0
1
2
MR effect size for
'ZNF41' on 'Pulmonary fibrosis'

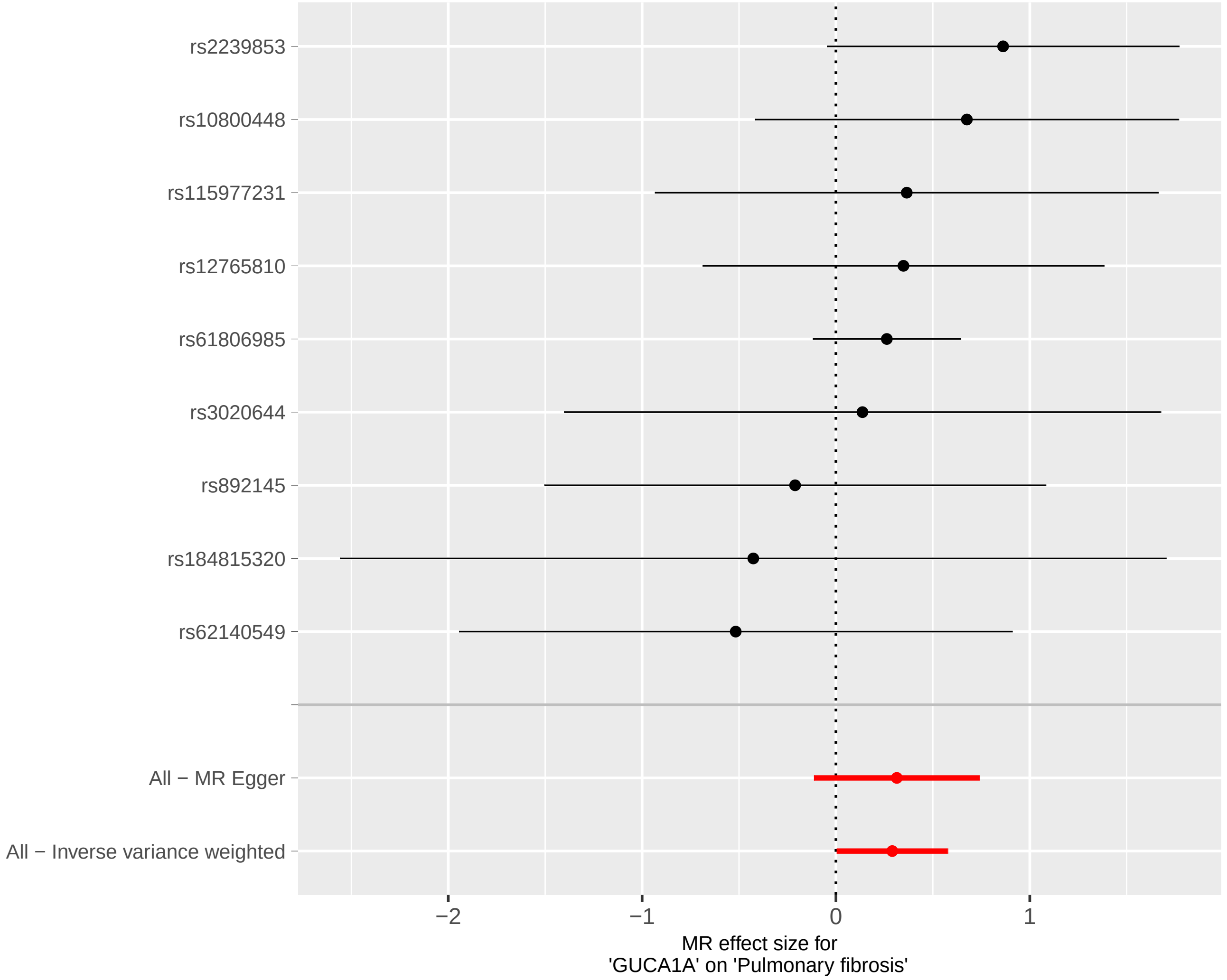
rs2239853
rs10800448
rs115977231
rs12765810
rs61806985
rs3020644
rs892145
rs184815320
rs62140549
All − MR Egger
All − Inverse variance weighted
−2
−1
0
1
MR effect size for
'GUCA1A' on 'Pulmonary fibrosis'

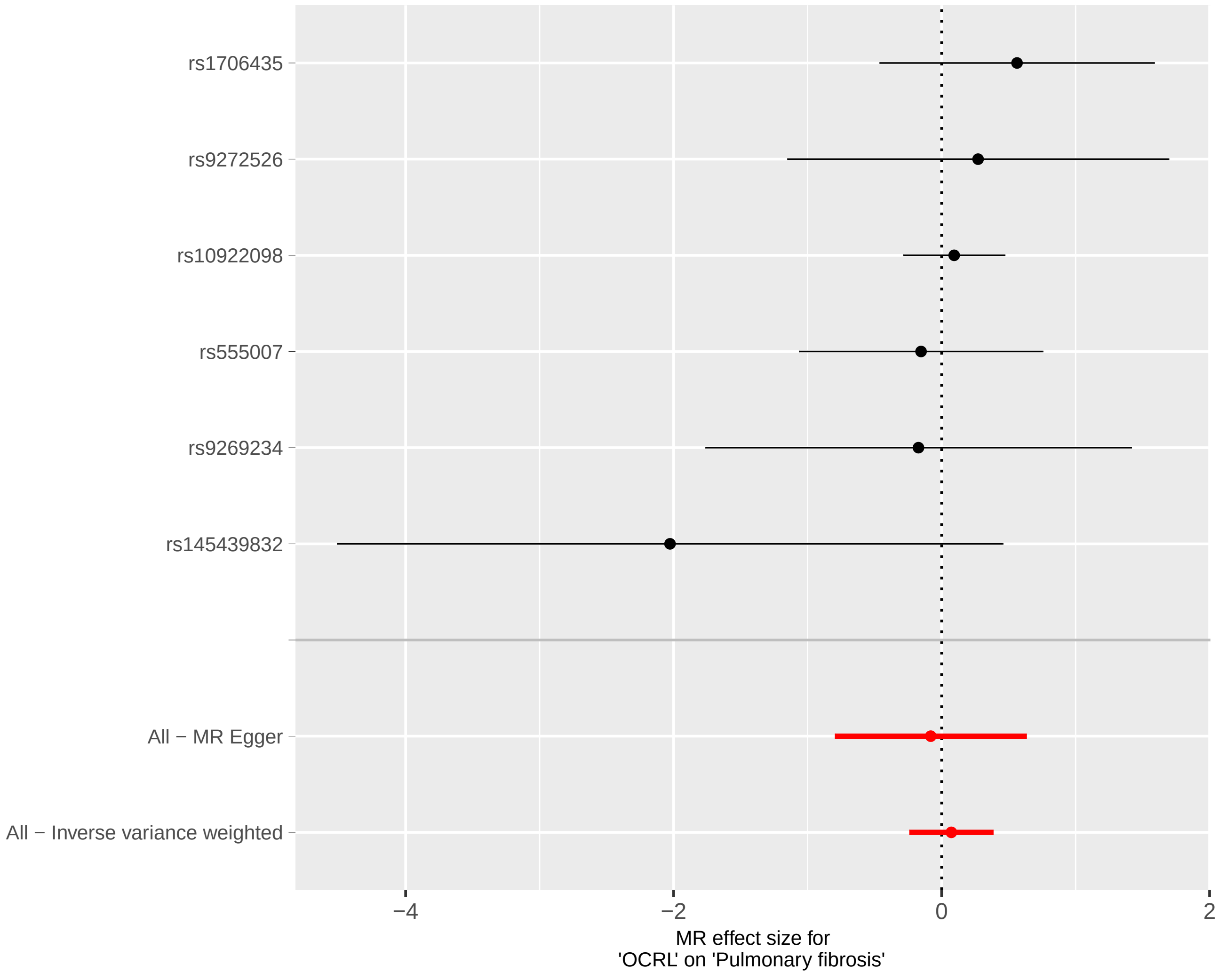
rs1706435
rs9272526
rs10922098
rs555007
rs9269234
rs145439832
All − MR Egger
All − Inverse variance weighted
−4
−2
0
2
MR effect size for
'OCRL' on 'Pulmonary fibrosis'

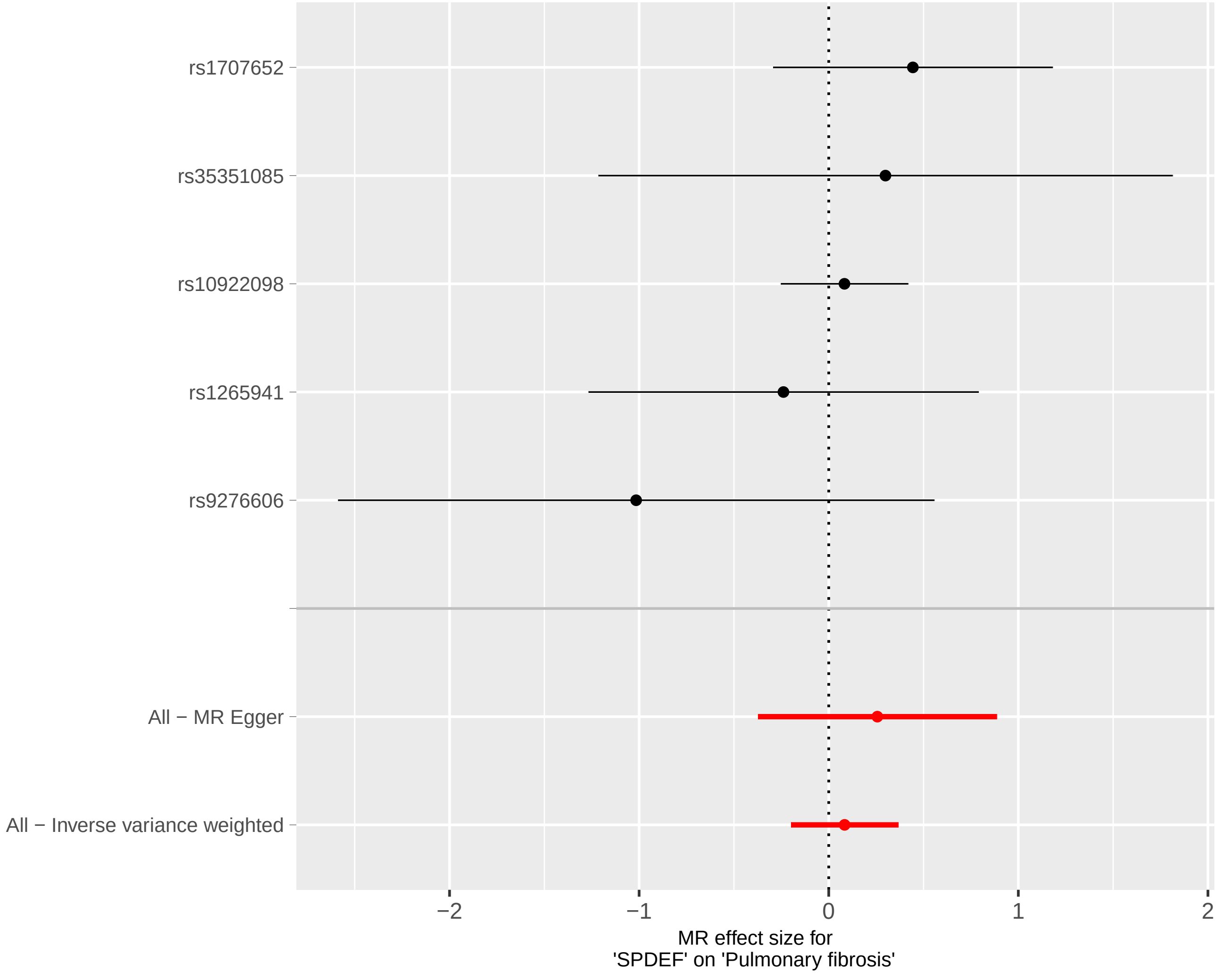
rs1707652
rs35351085
rs10922098
rs1265941
rs9276606
All − MR Egger
All − Inverse variance weighted
−2
−1
0
1
2
MR effect size for
'SPDEF' on 'Pulmonary fibrosis'

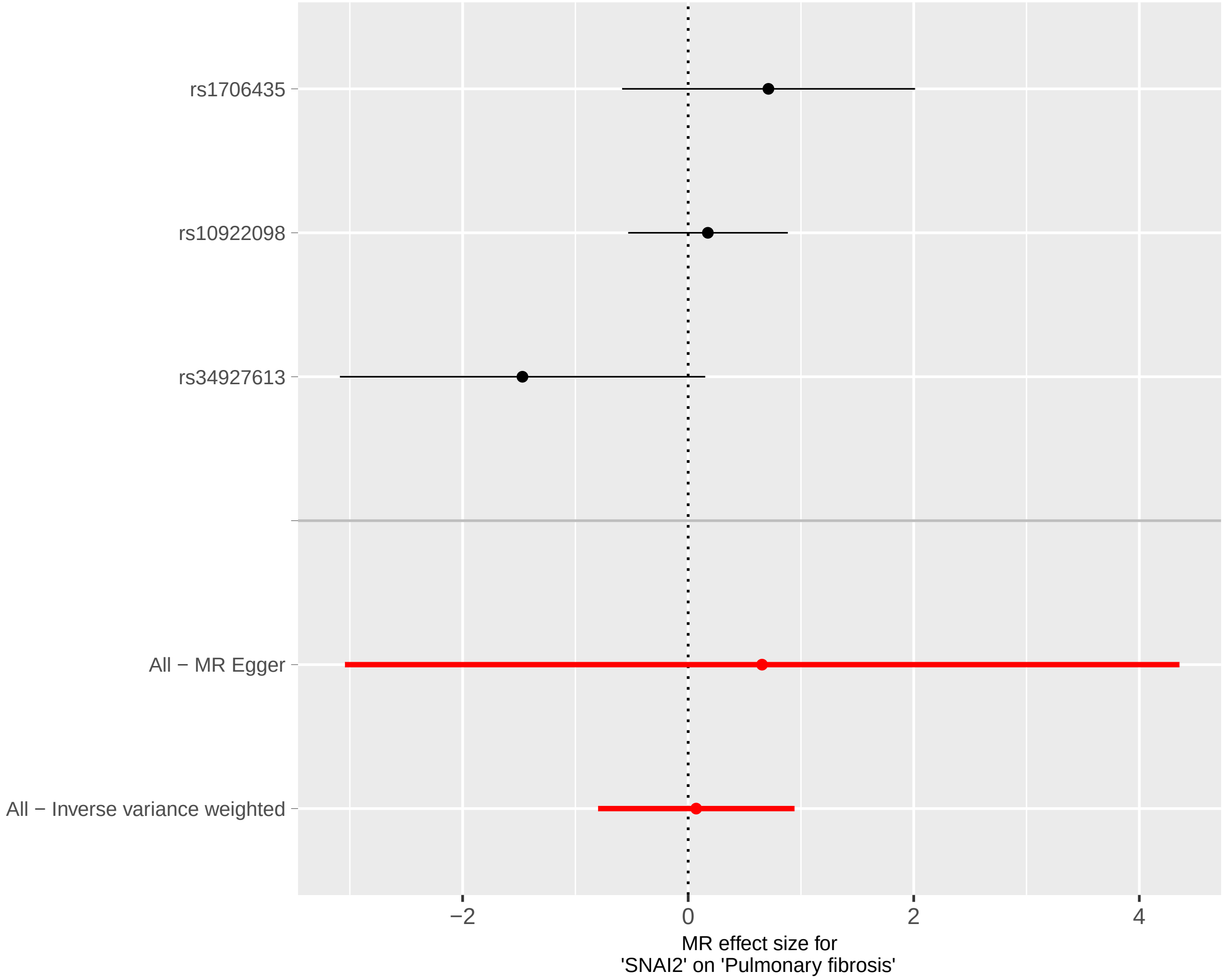
rs1706435
rs10922098
rs34927613
All − MR Egger
All − Inverse variance weighted
−2
0
2
4
MR effect size for
'SNAI2' on 'Pulmonary fibrosis'

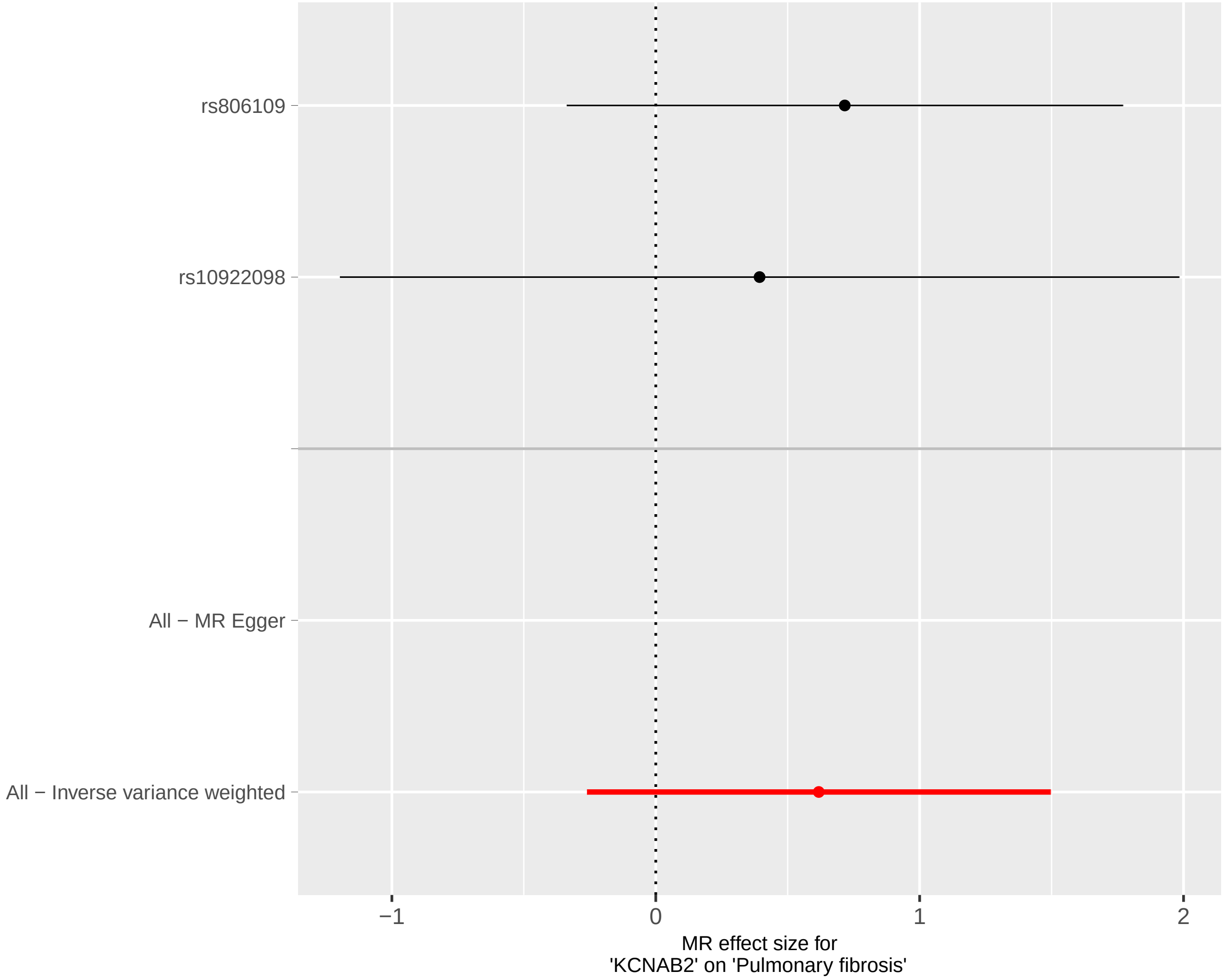

rs806109
rs10922098
All − MR Egger
All − Inverse variance weighted
−1
0
1
2
MR effect size for
'KCNAB2' on 'Pulmonary fibrosis'

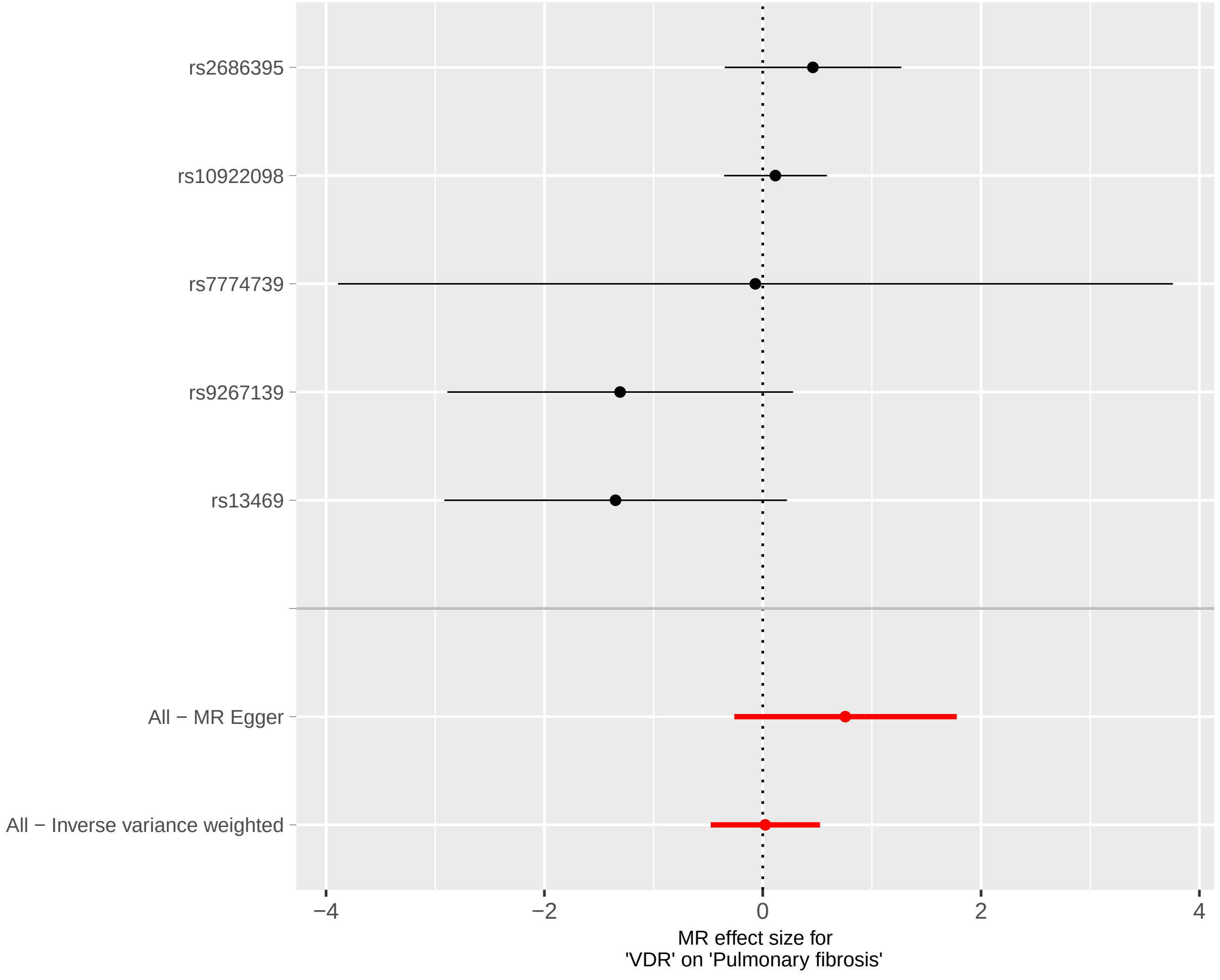
rs2686395
rs10922098
rs7774739
rs9267139
rs13469
All − MR Egger
All − Inverse variance weighted
−4
−2
0
2
4
MR effect size for
'VDR' on 'Pulmonary fibrosis'

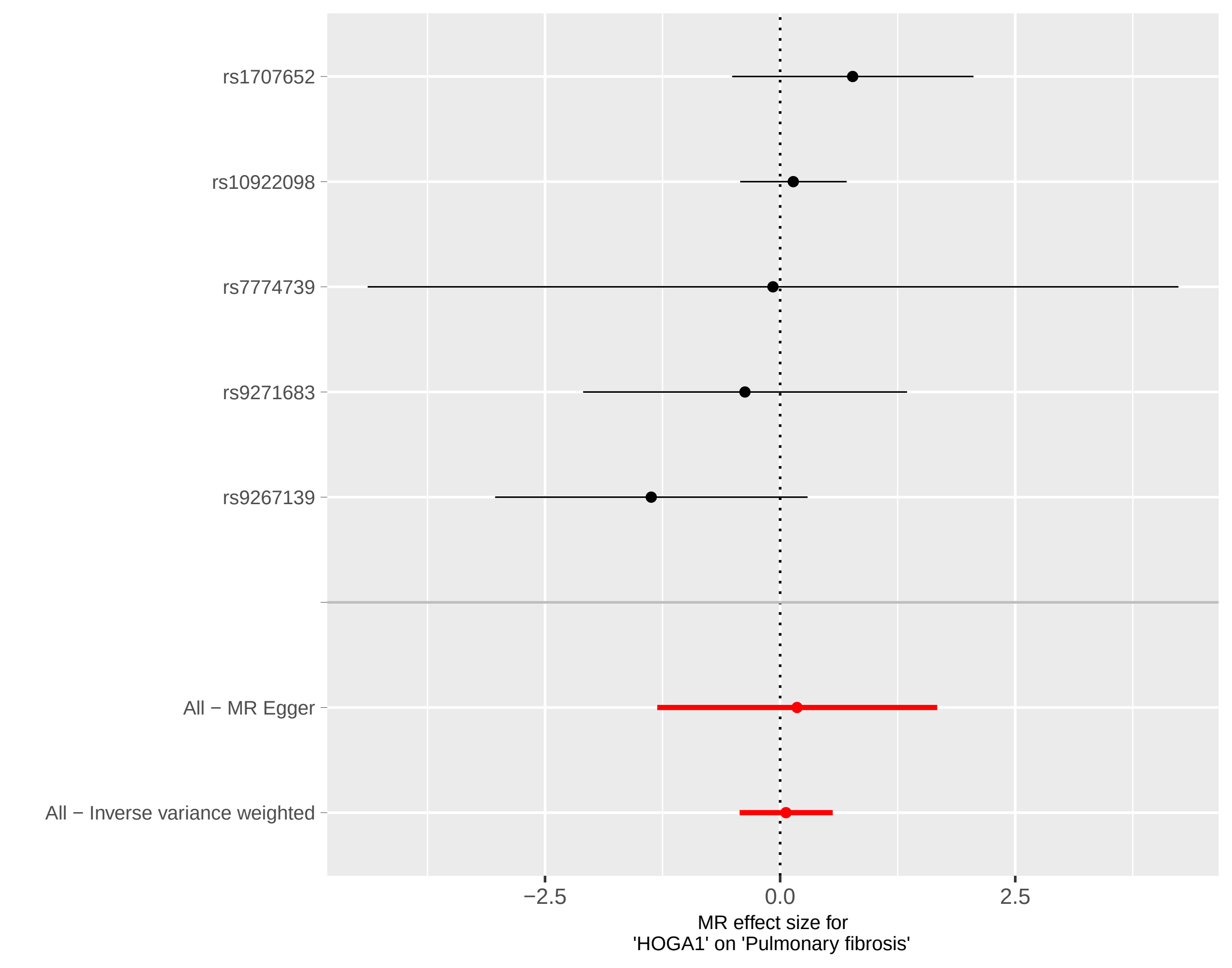
rs1707652
rs10922098
rs7774739
rs9271683
rs9267139
All − MR Egger
All − Inverse variance weighted
−2.5
0.0
2.5
MR effect size for
'HOGA1' on 'Pulmonary fibrosis'

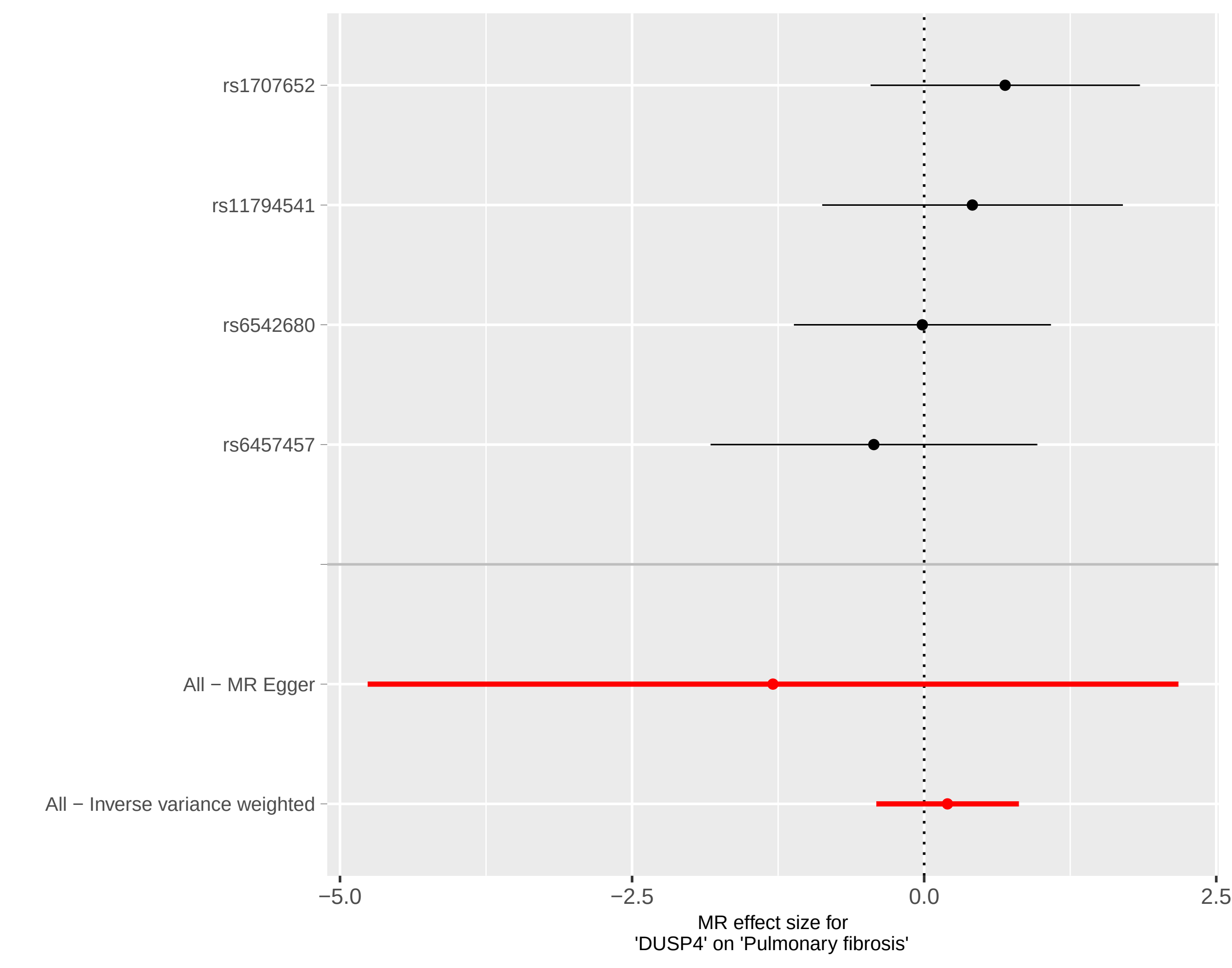
rs1707652
rs11794541
rs6542680
rs6457457
All − MR Egger
All − Inverse variance weighted
−5.0
−2.5
0.0
2.5
MR effect size for
'DUSP4' on 'Pulmonary fibrosis'

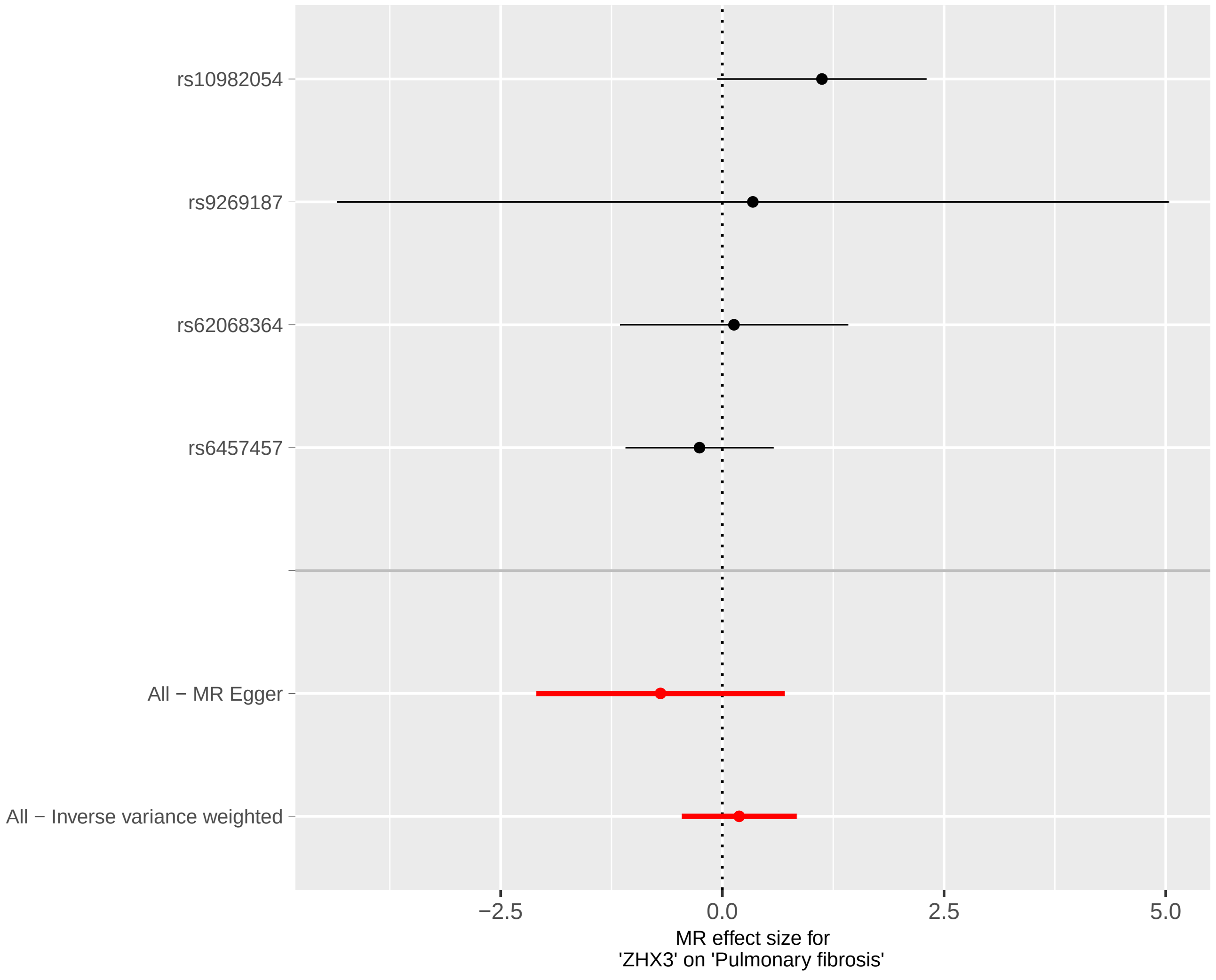

rs10982054
rs9269187
rs62068364
rs6457457
All − MR Egger
All − Inverse variance weighted
−2.5
0.0
2.5
5.0
MR effect size for
'ZHX3' on 'Pulmonary fibrosis'

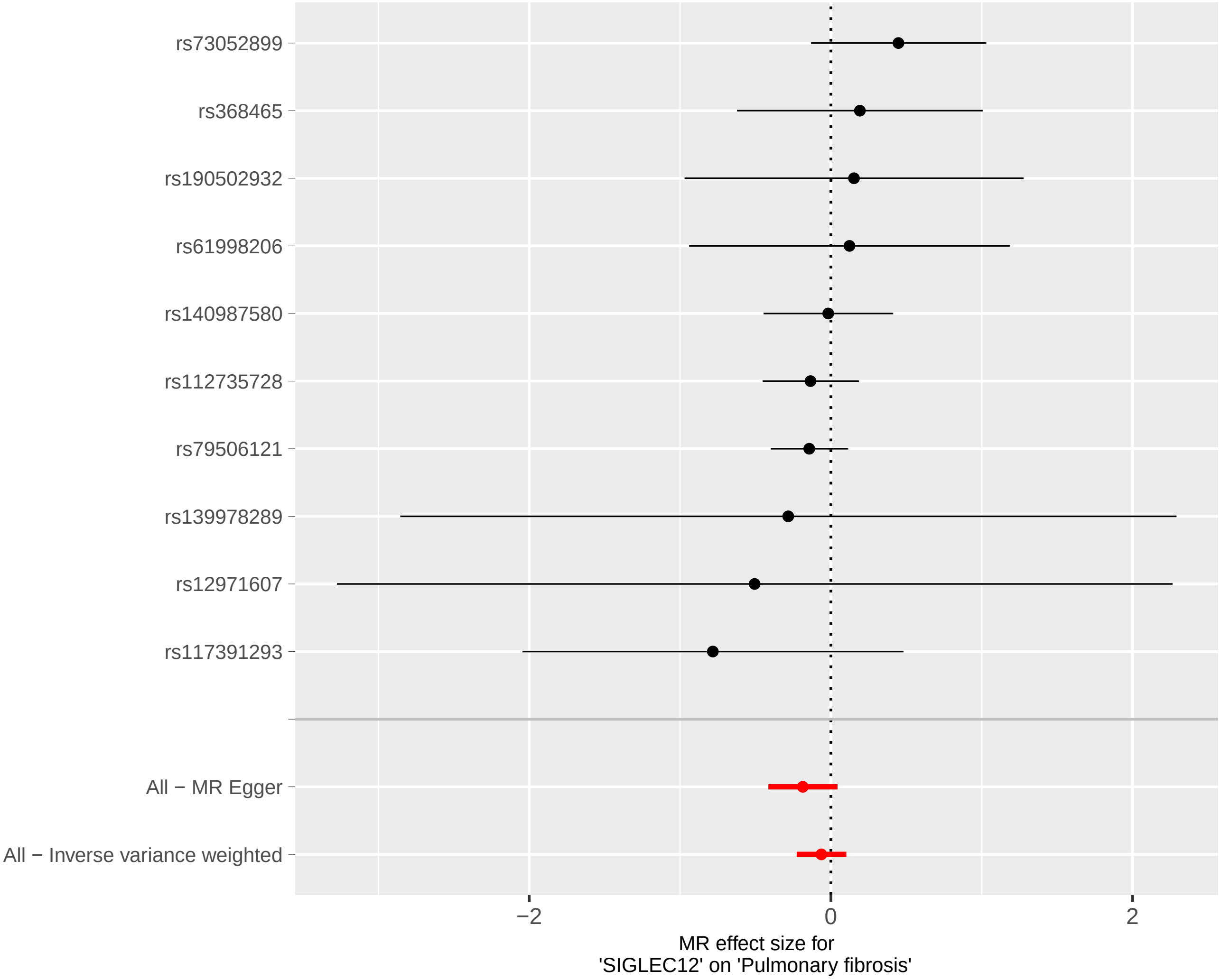
rs73052899
rs368465
rs190502932
rs61998206
rs140987580
rs112735728
rs79506121
rs139978289
rs12971607
rs117391293
All − MR Egger
All − Inverse variance weighted
−2
0
2
MR effect size for
'SIGLEC12' on 'Pulmonary fibrosis'

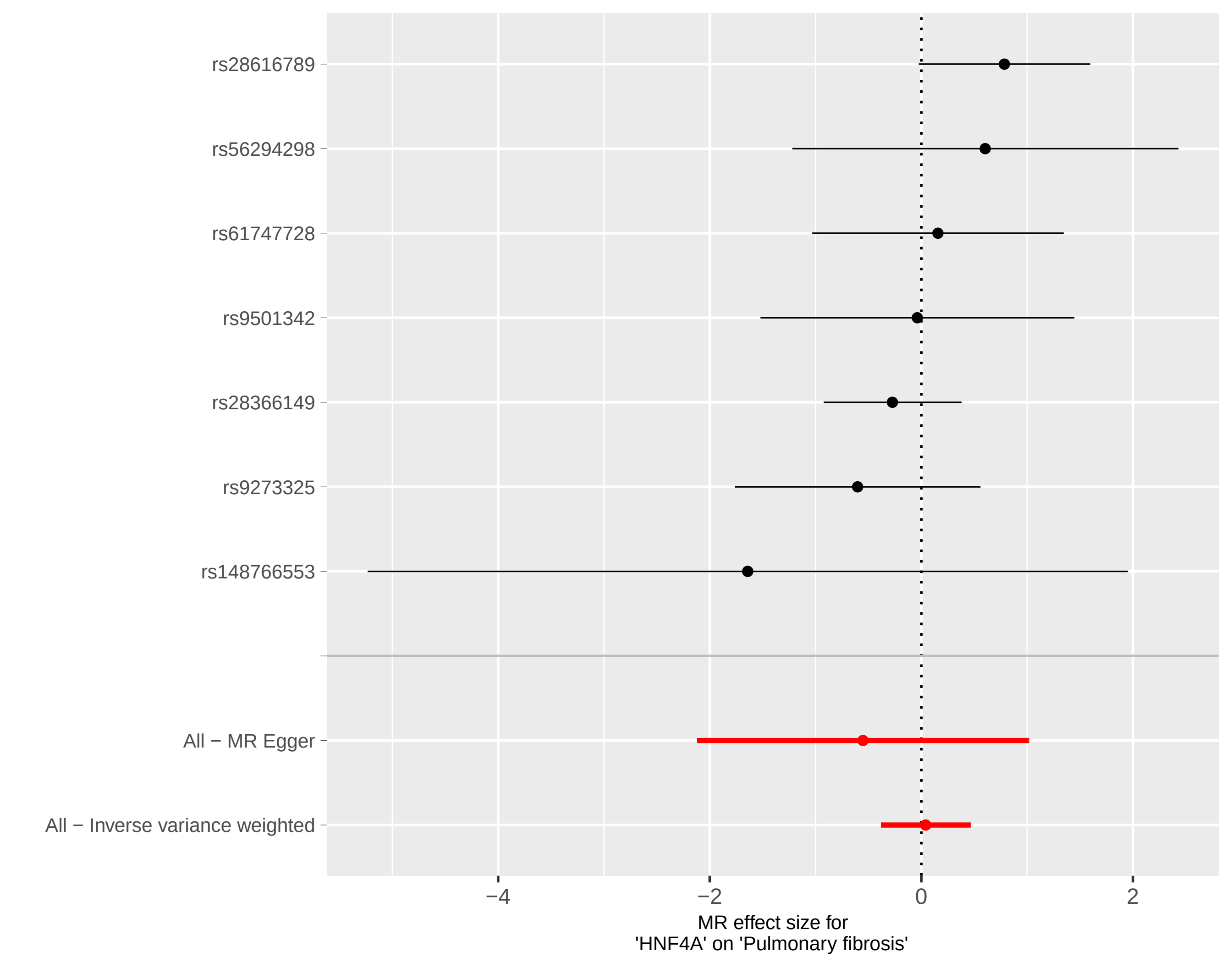
rs28616789
rs56294298
rs61747728
rs9501342
rs28366149
rs9273325
rs148766553
All − MR Egger
All − Inverse variance weighted
−4
−2
0
2
MR effect size for
'HNF4A' on 'Pulmonary fibrosis'

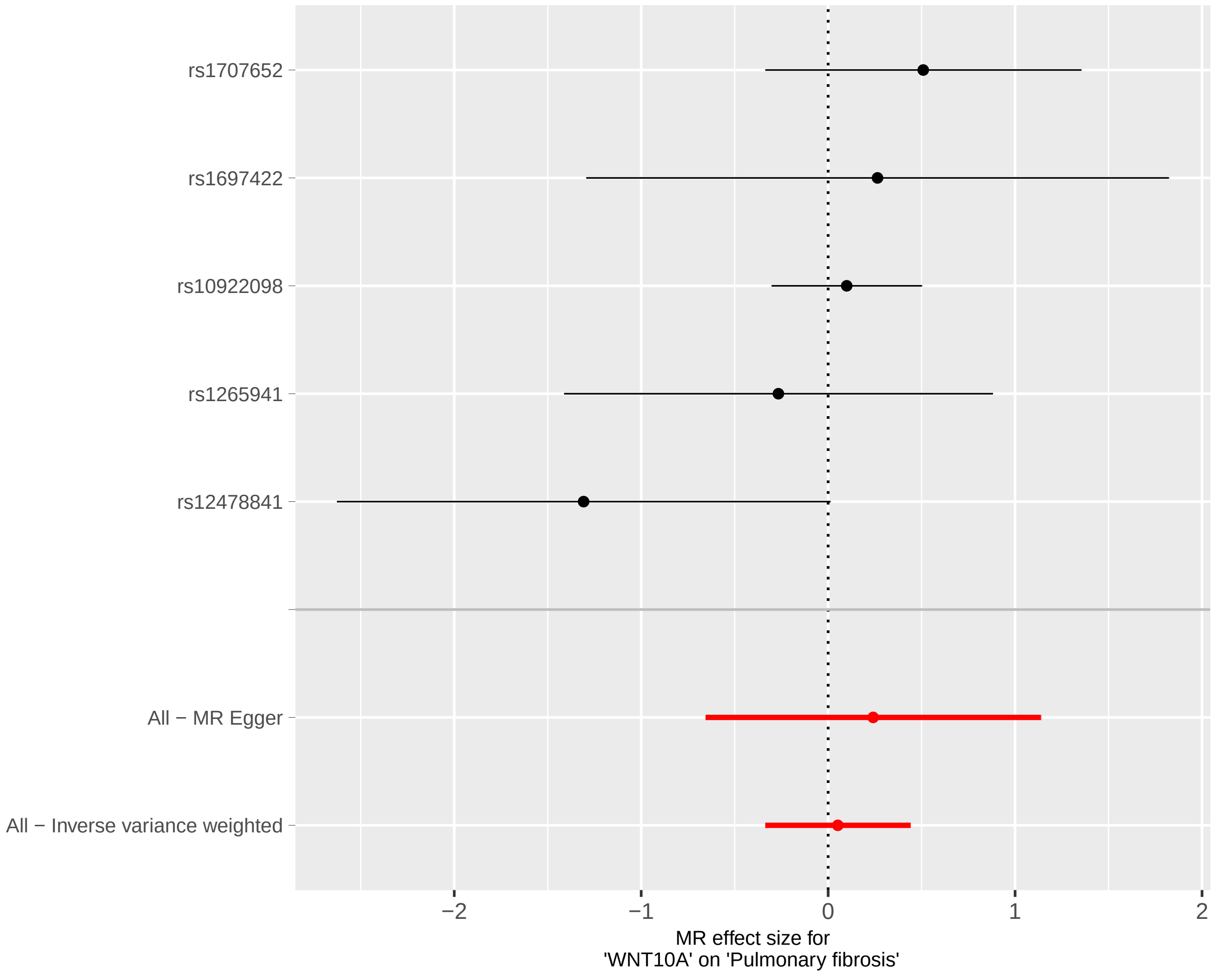
rs1707652
rs1697422
rs10922098
rs1265941
rs12478841
All − MR Egger
All − Inverse variance weighted
−2
−1
0
1
2
MR effect size for
'WNT10A' on 'Pulmonary fibrosis'

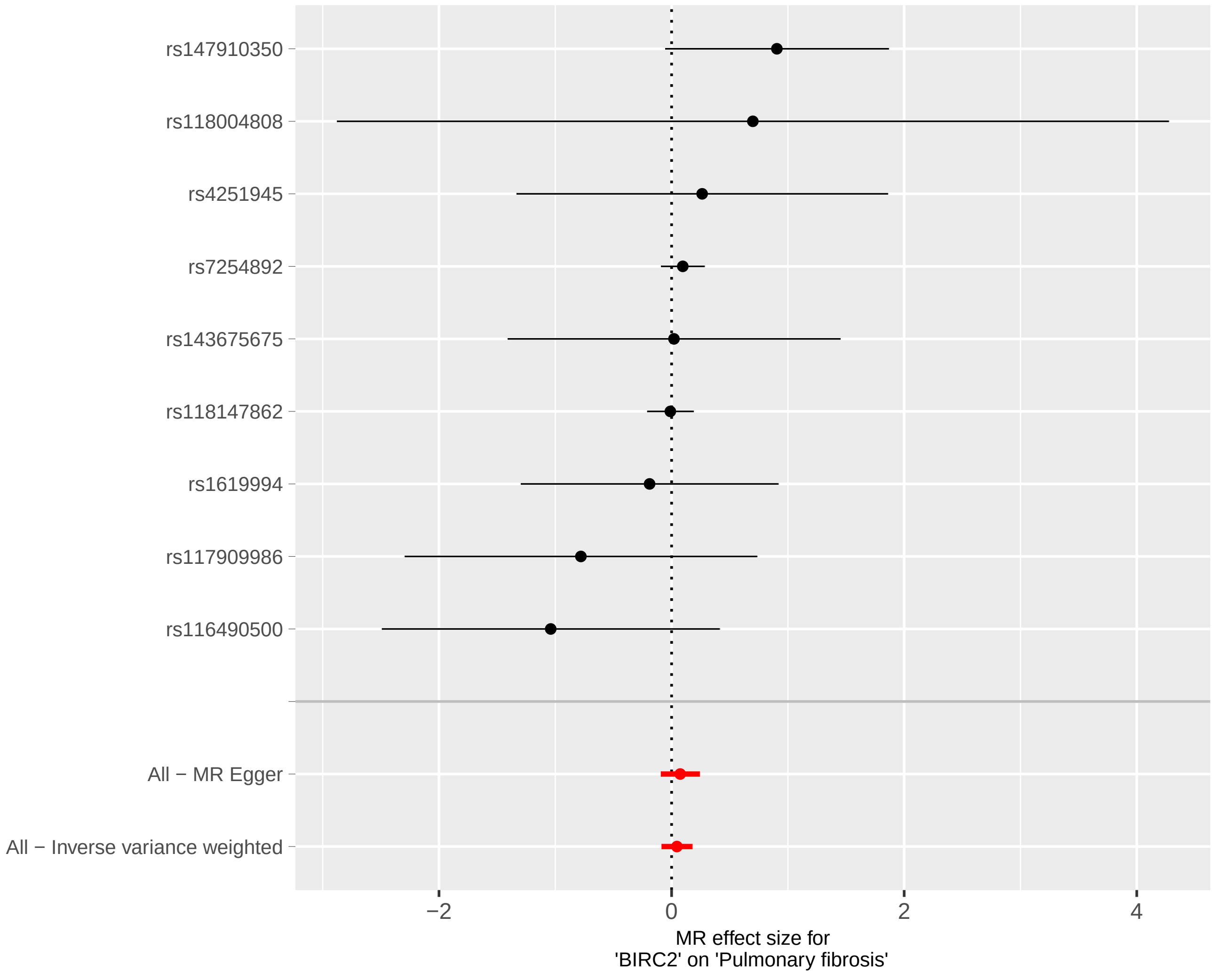
rs147910350
rs118004808
rs4251945
rs7254892
rs143675675
rs118147862
rs1619994
rs117909986
rs116490500
All − MR Egger
All − Inverse variance weighted
−2
0
2
4
MR effect size for
'BIRC2' on 'Pulmonary fibrosis'

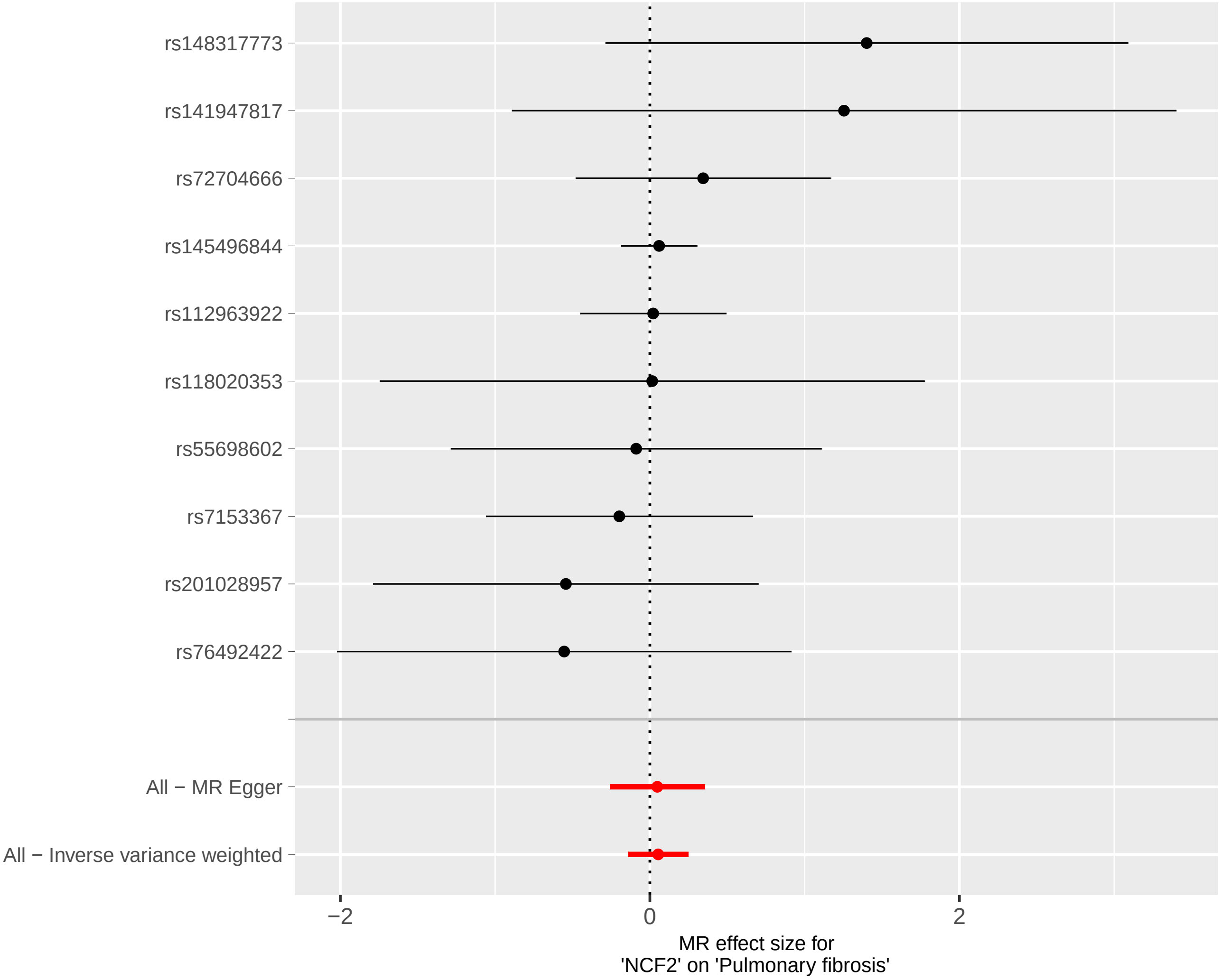
rs148317773
rs141947817
rs72704666
rs145496844
rs112963922
rs118020353
rs55698602
rs7153367
rs201028957
rs76492422
All − MR Egger
All − Inverse variance weighted
−2
0
2
MR effect size for
'NCF2' on 'Pulmonary fibrosis'

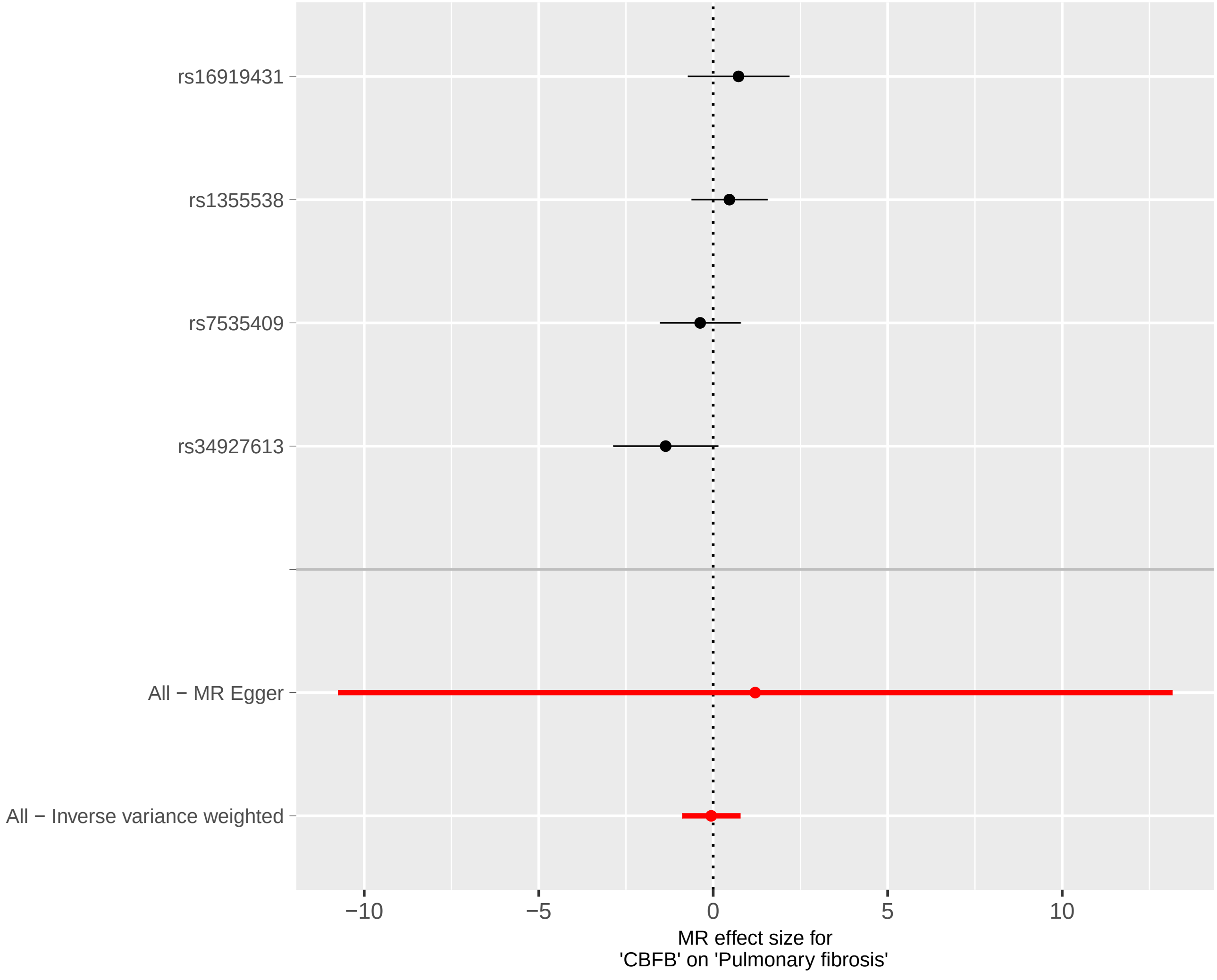

rs16919431
rs1355538
rs7535409
rs34927613
All − MR Egger
All − Inverse variance weighted
−10
−5
0
5
10
MR effect size for
'CBFB' on 'Pulmonary fibrosis'

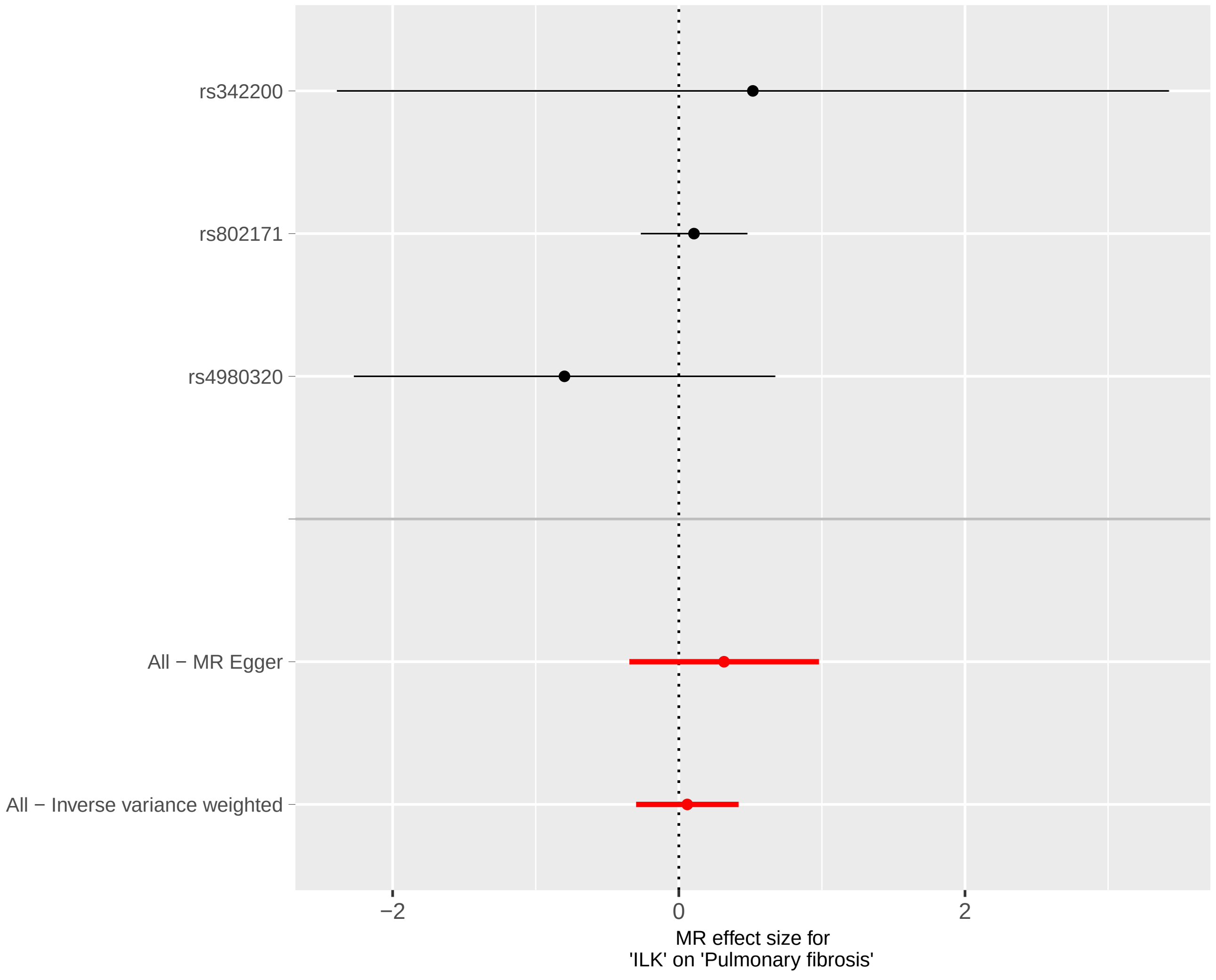
rs342200
rs802171
rs4980320
All − MR Egger
All − Inverse variance weighted
−2
0
2
MR effect size for
'ILK' on 'Pulmonary fibrosis'

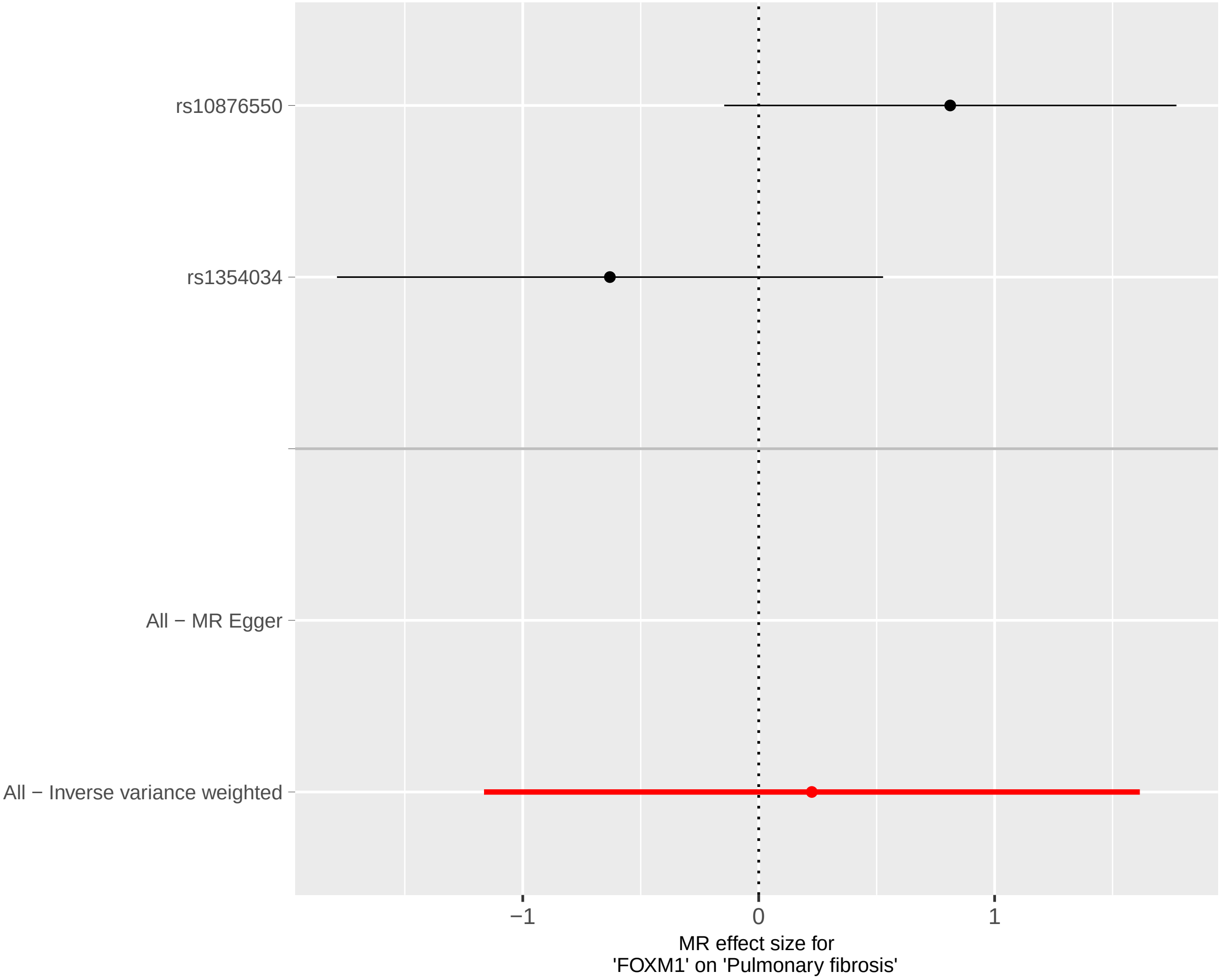

rs10876550
rs1354034
All − MR Egger
All − Inverse variance weighted
−1
0
1
MR effect size for
'FOXM1' on 'Pulmonary fibrosis'

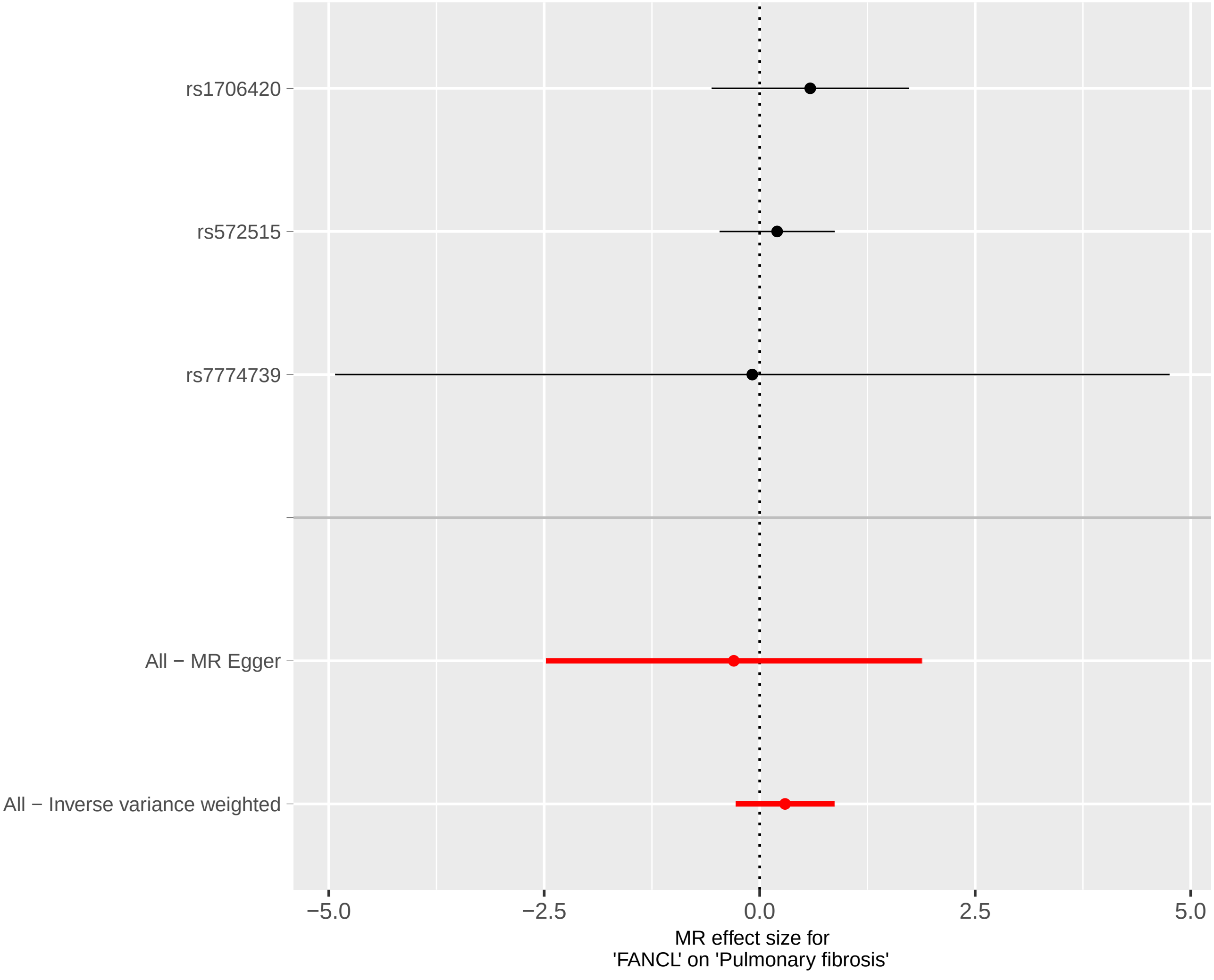

rs1706420
rs572515
rs7774739
All − MR Egger
All − Inverse variance weighted
−5.0
−2.5
0.0
2.5
5.0
MR effect size for
'FANCL' on 'Pulmonary fibrosis'

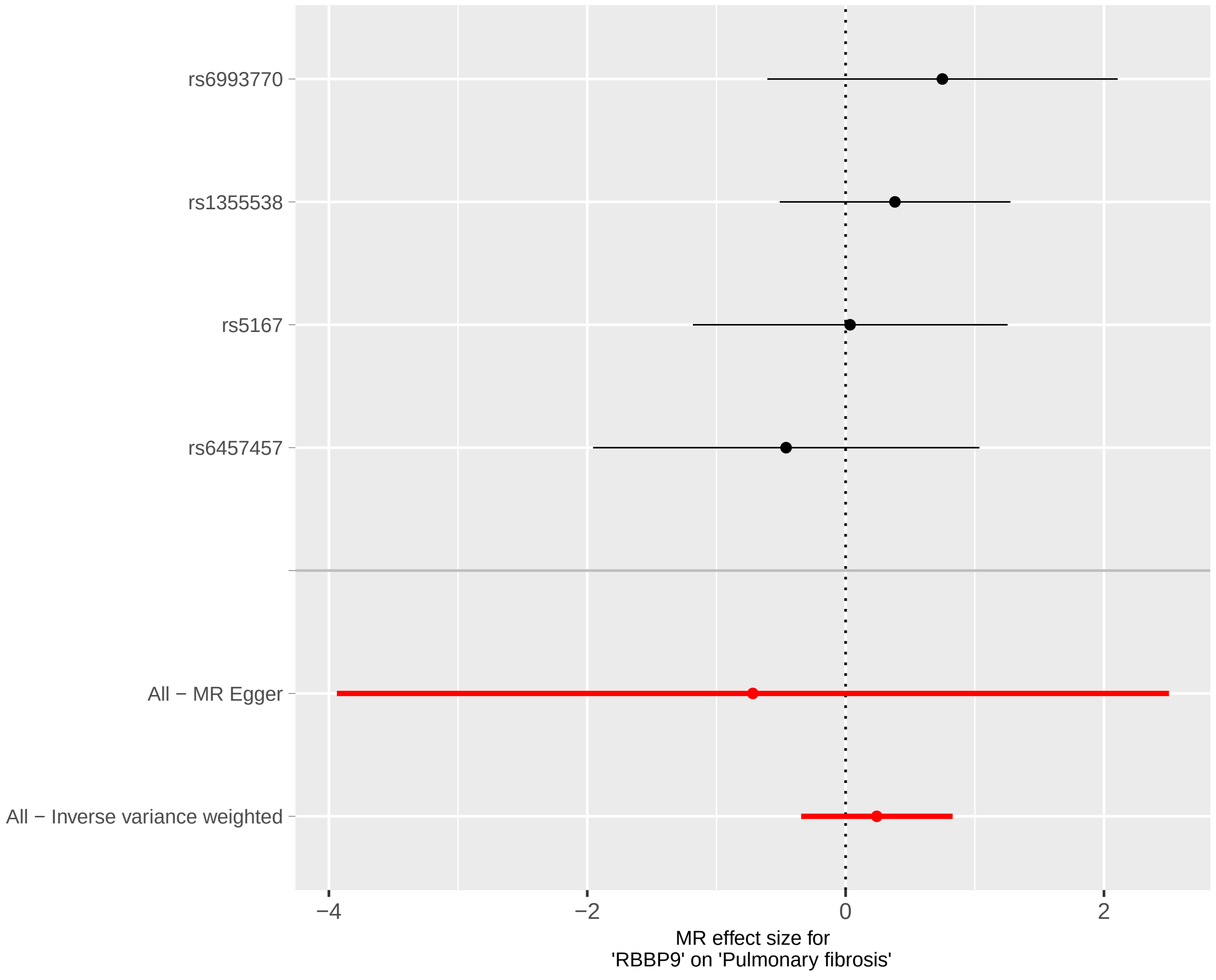

rs6993770
rs1355538
rs5167
rs6457457
All − MR Egger
All − Inverse variance weighted
−4
−2
0
2
MR effect size for
'RBBP9' on 'Pulmonary fibrosis'

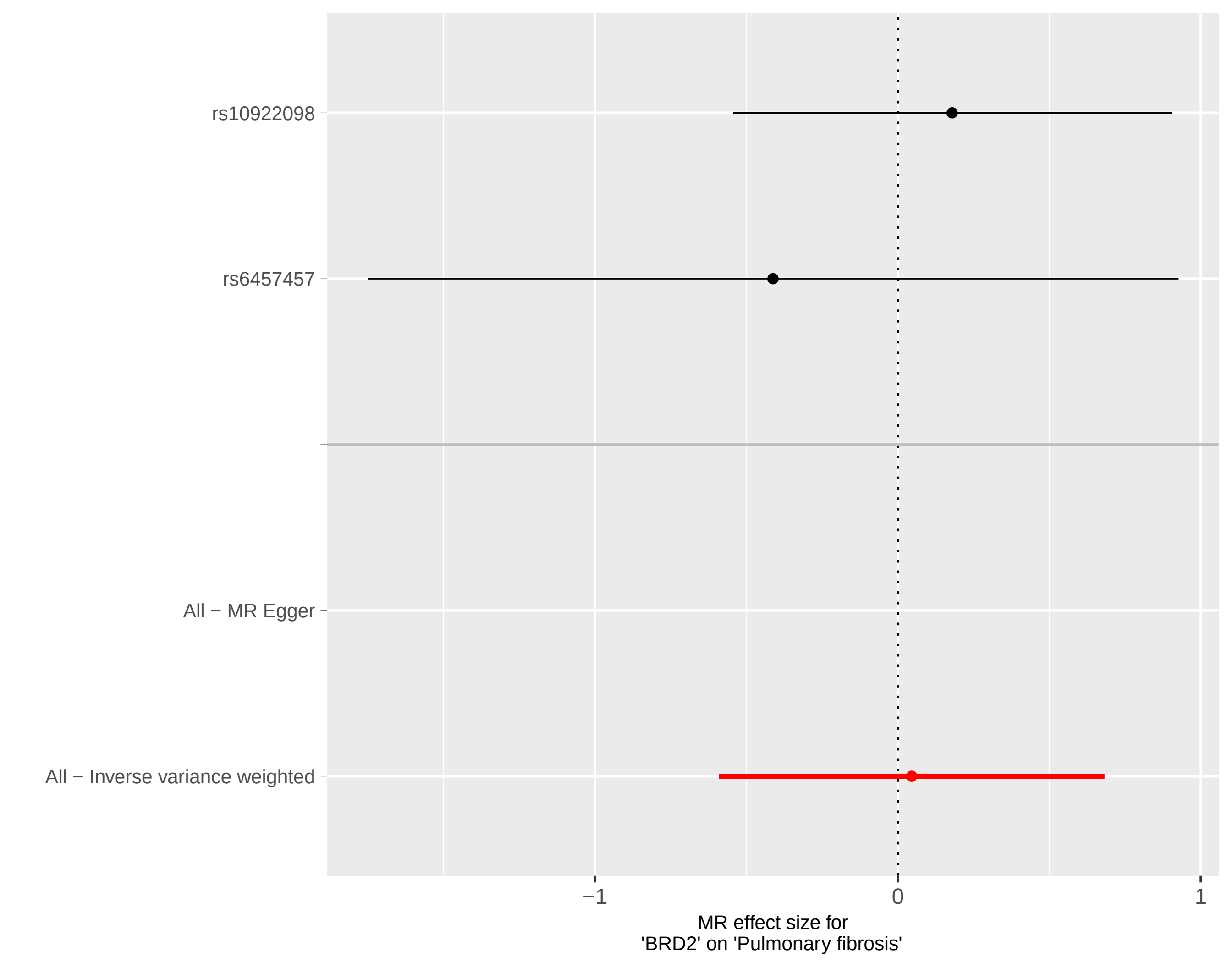
rs10922098
rs6457457
All − MR Egger
All − Inverse variance weighted
−1
0
1
MR effect size for
'BRD2' on 'Pulmonary fibrosis'

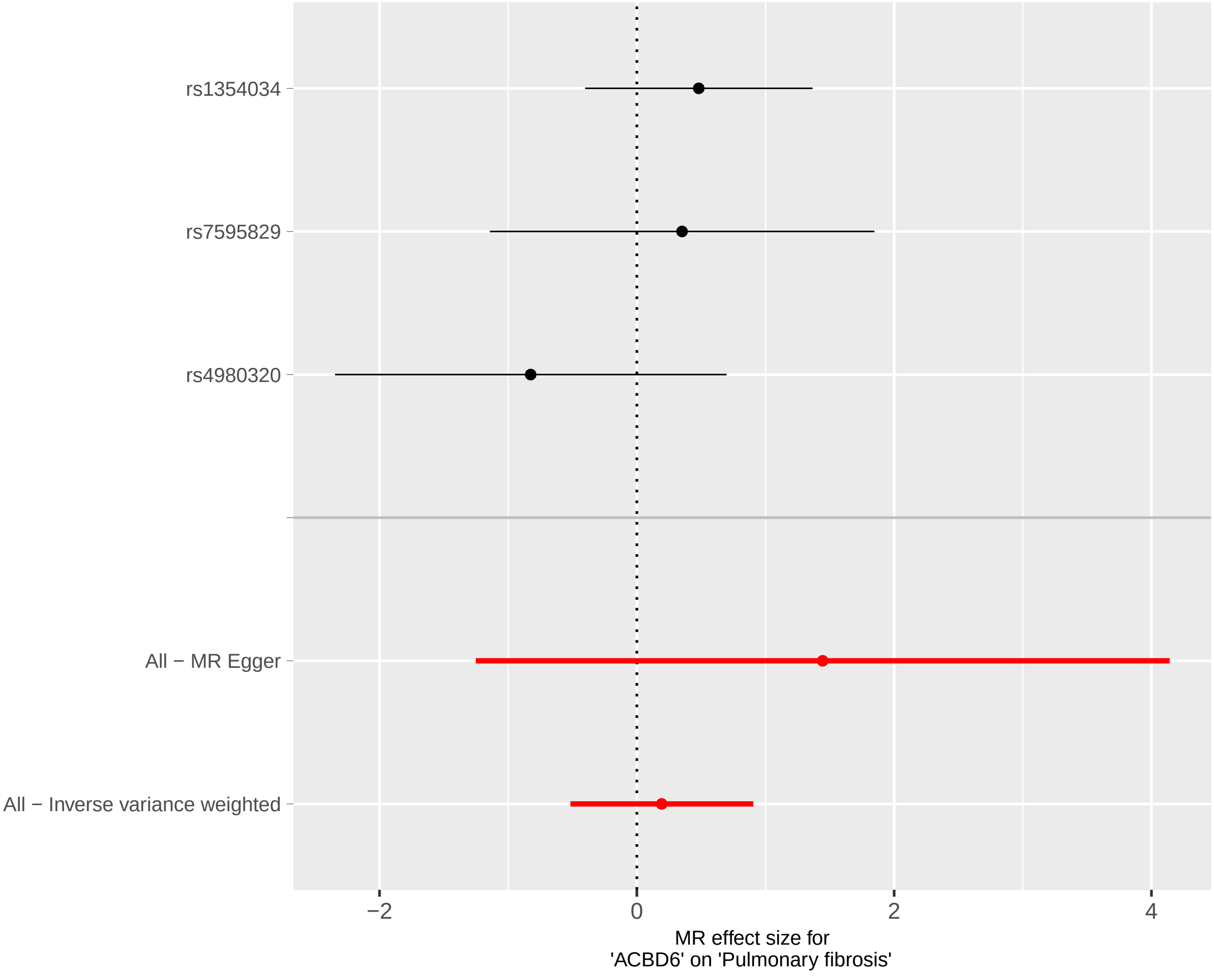
rs1354034
rs7595829
rs4980320
All − MR Egger
All − Inverse variance weighted
−2
0
2
4
MR effect size for
'ACBD6' on 'Pulmonary fibrosis'

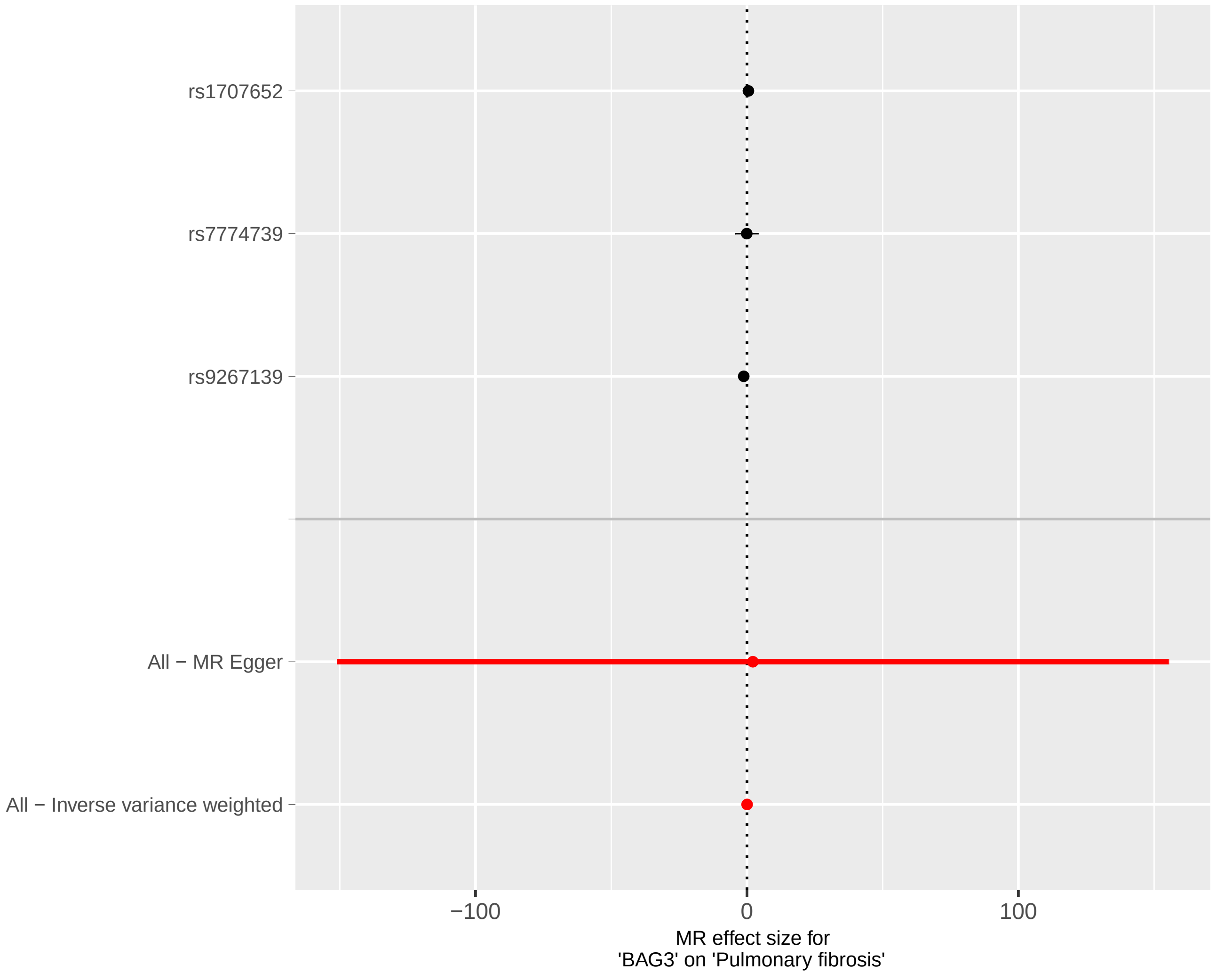
rs1707652
rs7774739
rs9267139
All − MR Egger
All − Inverse variance weighted
−100
0
100
MR effect size for
'BAG3' on 'Pulmonary fibrosis'

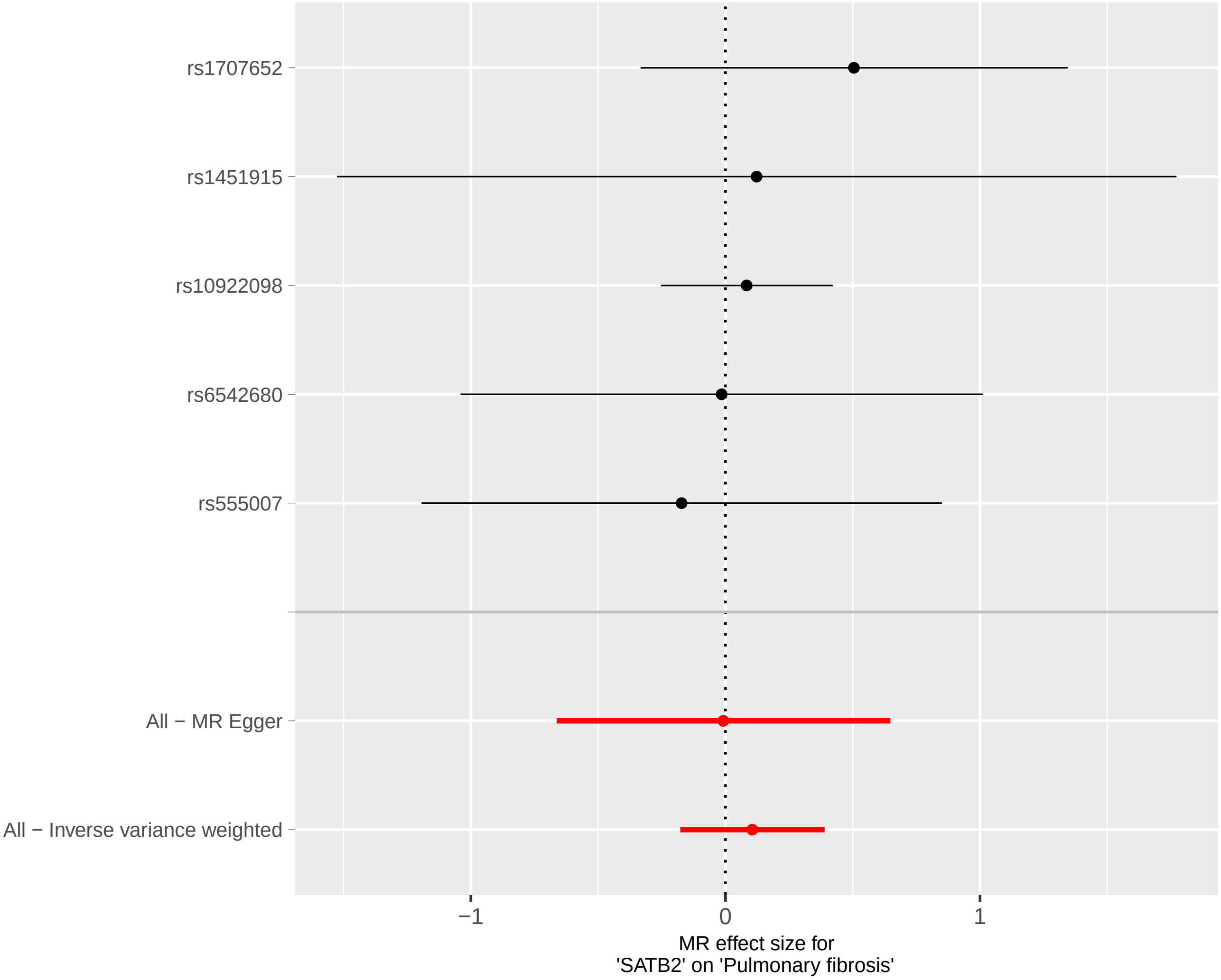

rs1707652
rs1451915
rs10922098
rs6542680
rs555007
All − MR Egger
All − Inverse variance weighted
−1
0
1
MR effect size for
'SATB2' on 'Pulmonary fibrosis'

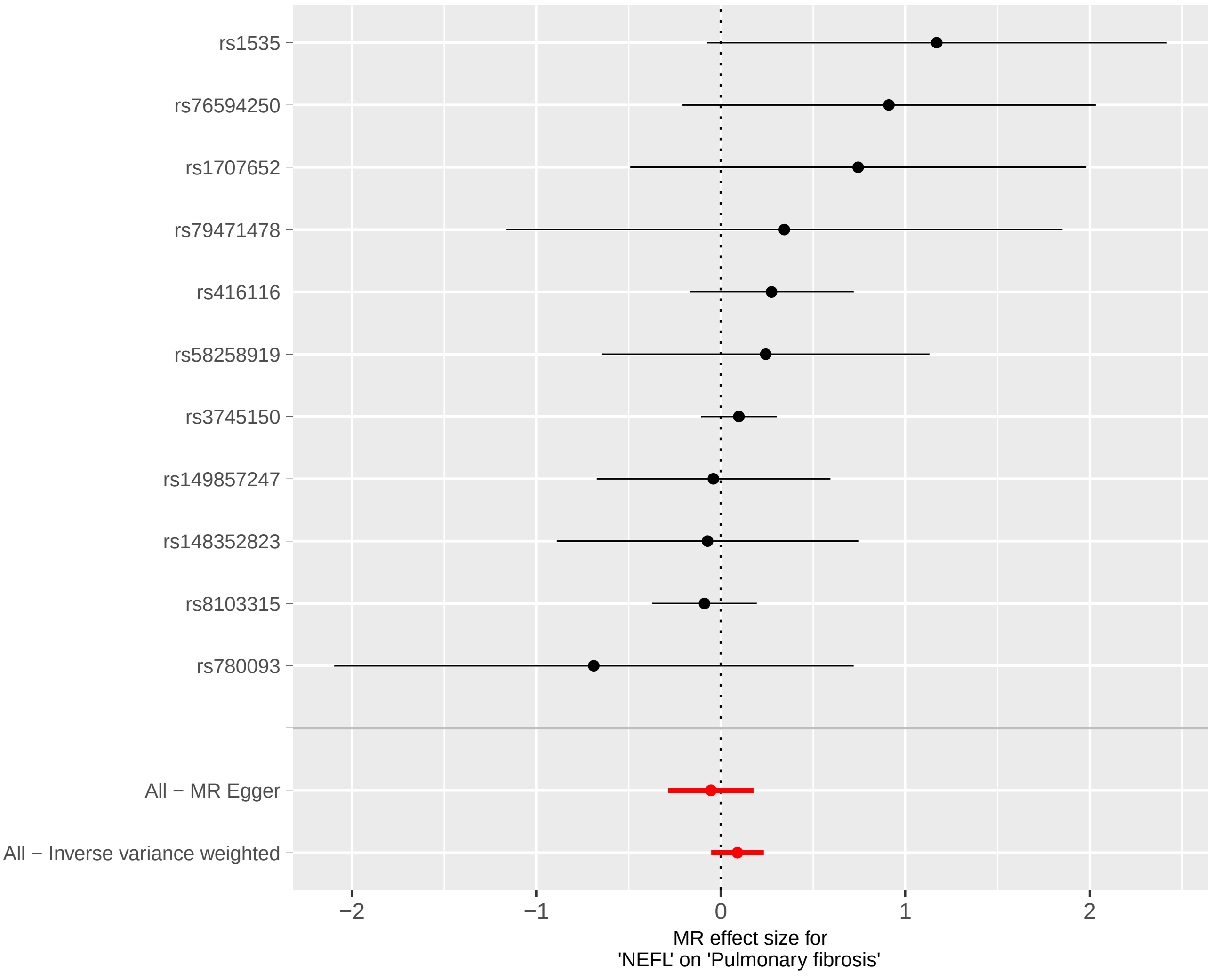
rs1535
rs76594250
rs1707652
rs79471478
rs416116
rs58258919
rs3745150
rs149857247
rs148352823
rs8103315
rs780093
All − MR Egger
All − Inverse variance weighted
−2
−1
0
1
2
MR effect size for
'NEFL' on 'Pulmonary fibrosis'

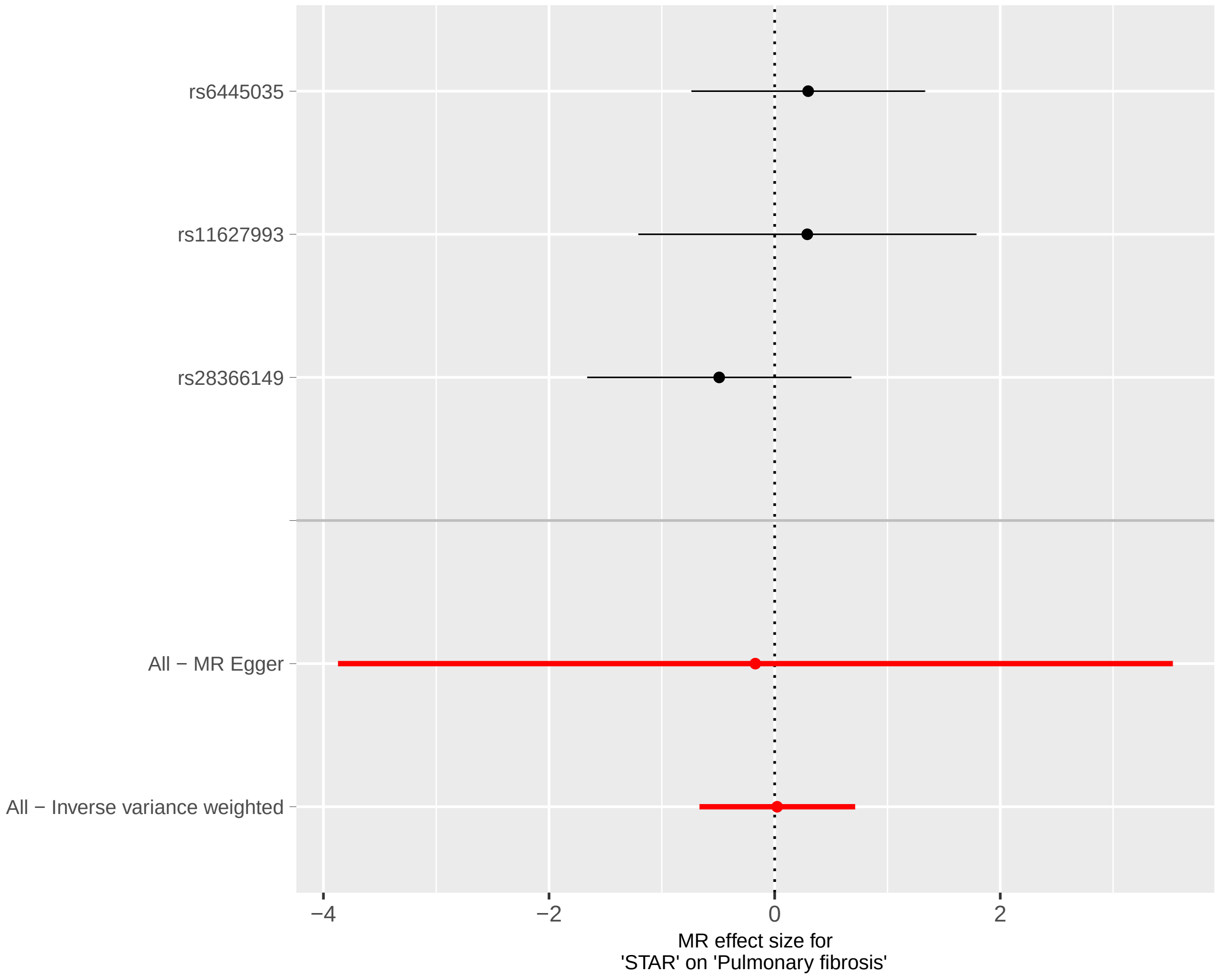
rs6445035
rs11627993
rs28366149
All − MR Egger
All − Inverse variance weighted
−4
−2
0
2
MR effect size for
'STAR' on 'Pulmonary fibrosis'

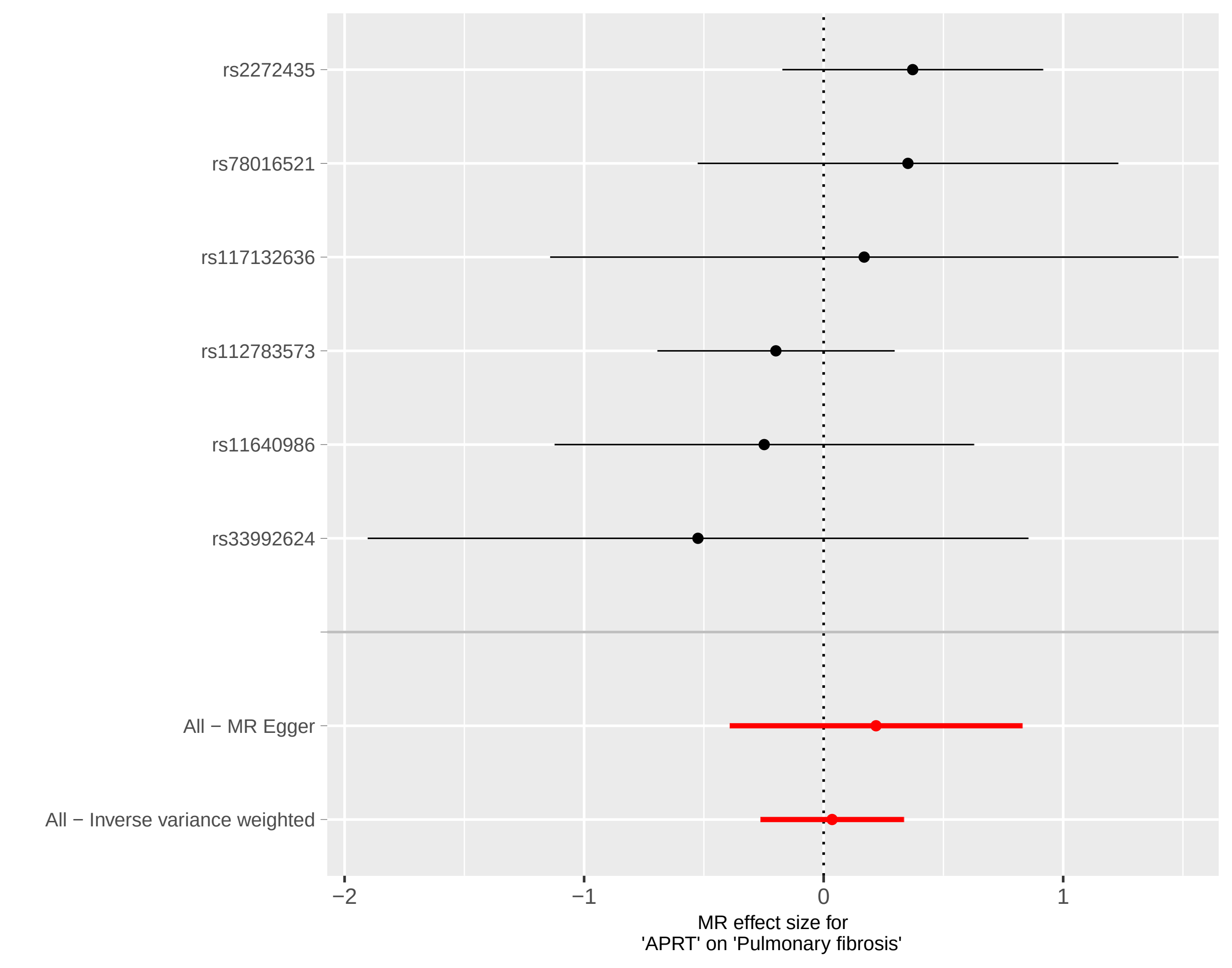
rs2272435
rs78016521
rs117132636
rs112783573
rs11640986
rs33992624
All − MR Egger
All − Inverse variance weighted
−2
−1
0
1
MR effect size for
'APRT' on 'Pulmonary fibrosis'

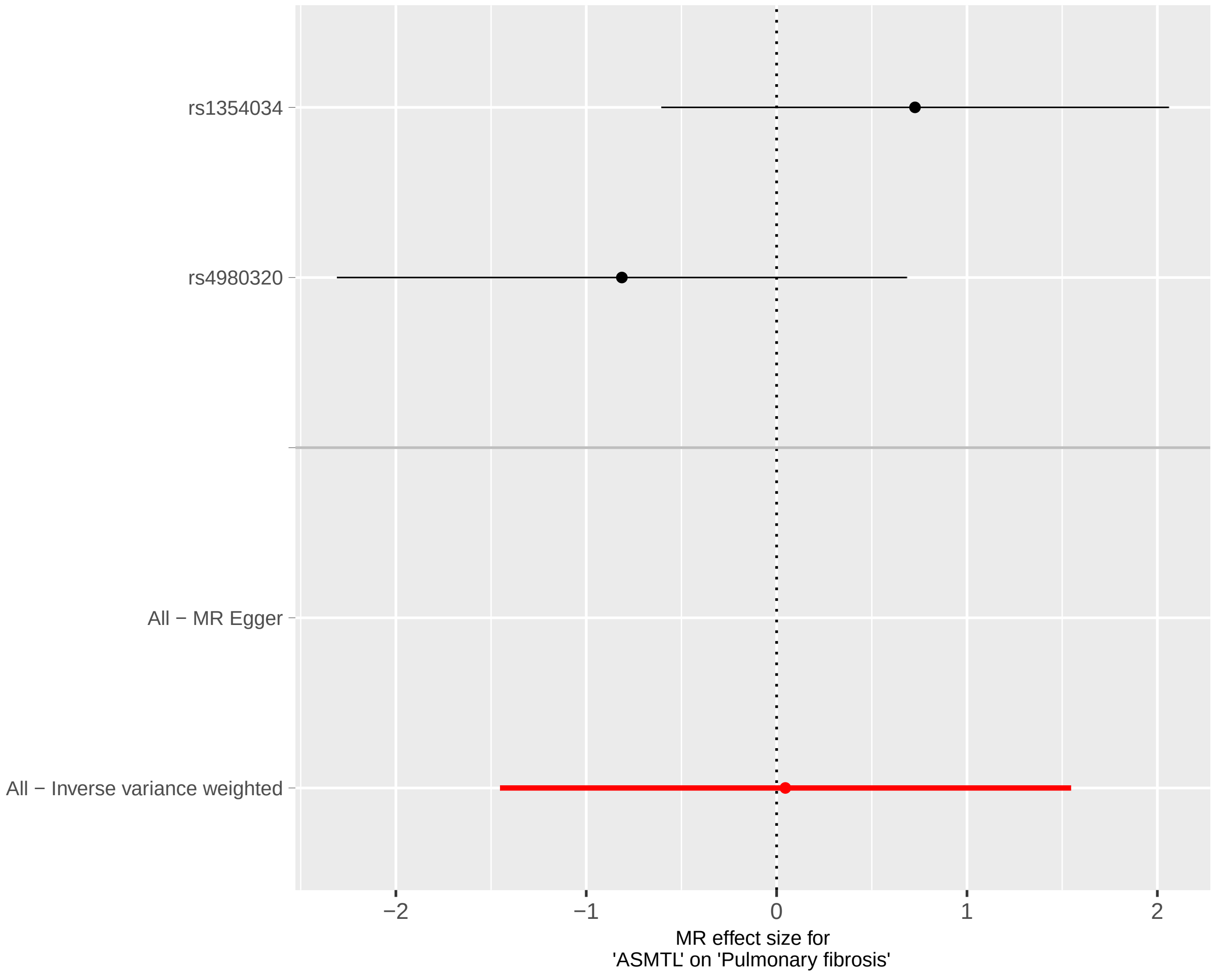
rs1354034
rs4980320
All − MR Egger
All − Inverse variance weighted
−2
−1
0
1
2
MR effect size for
'ASMTL' on 'Pulmonary fibrosis'

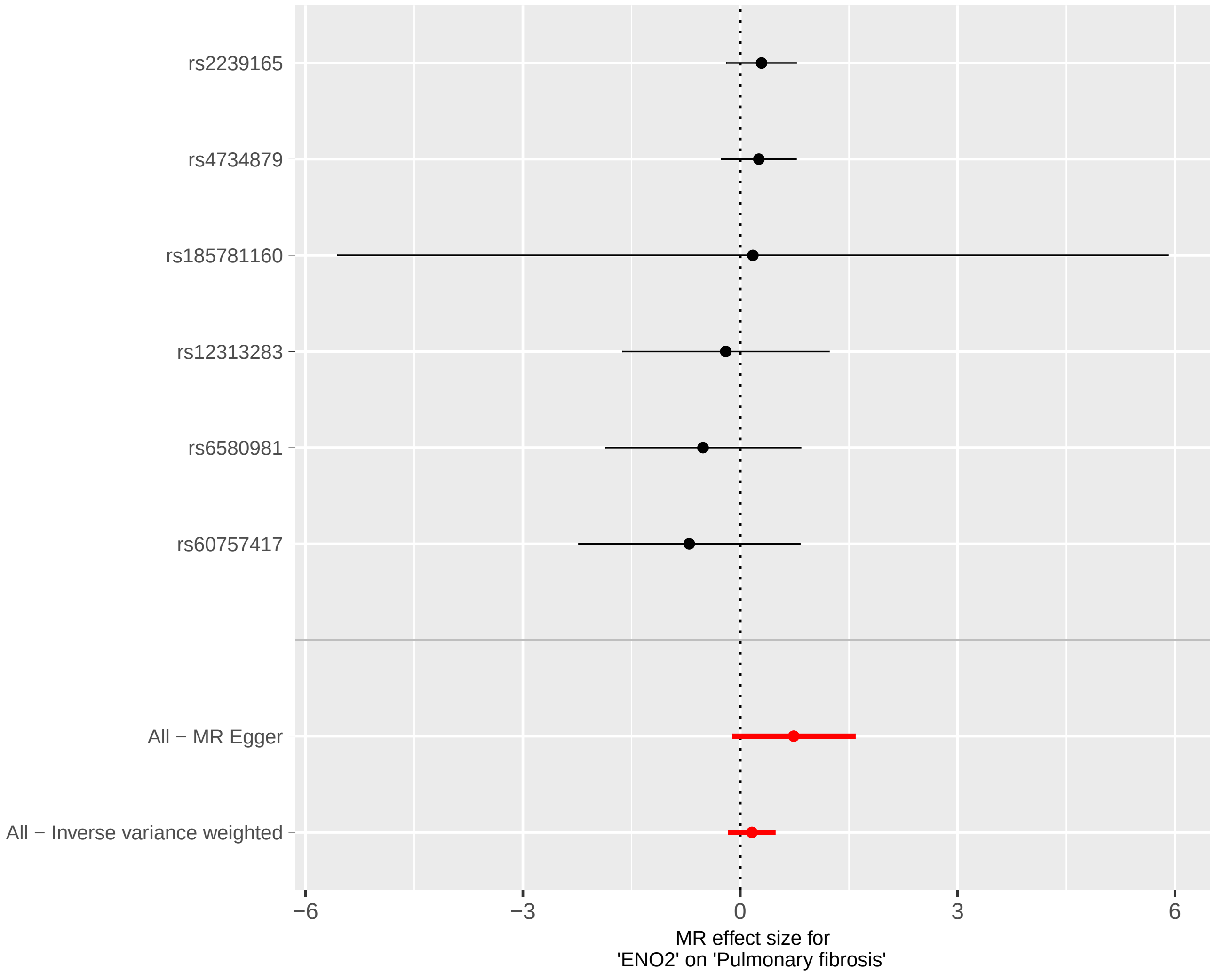

rs2239165
rs4734879
rs185781160
rs12313283
rs6580981
rs60757417
All − MR Egger
All − Inverse variance weighted
−6
−3
0
3
6
MR effect size for
'ENO2' on 'Pulmonary fibrosis'

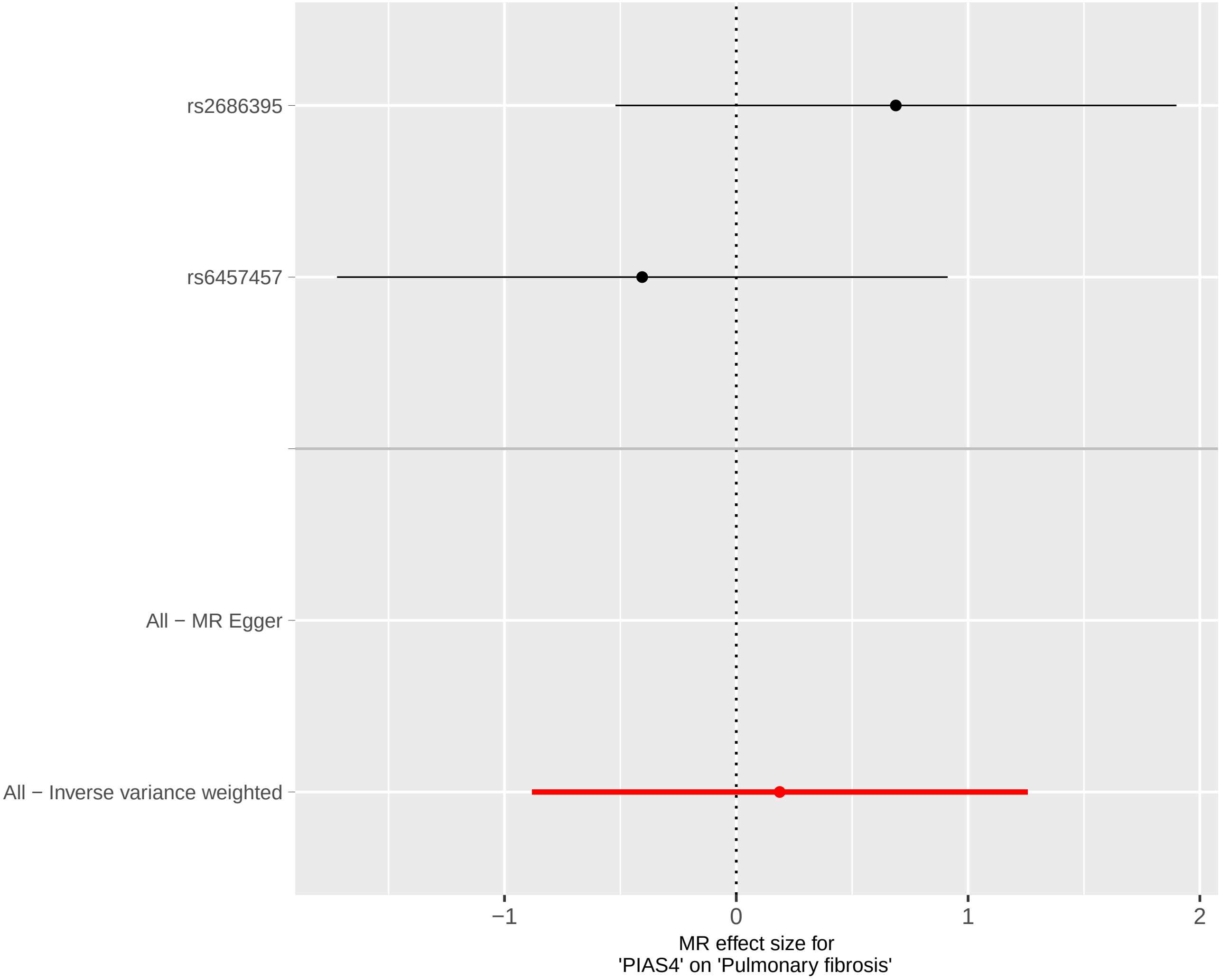
rs2686395
rs6457457
All − MR Egger
All − Inverse variance weighted
−1
0
1
2
MR effect size for
'PIAS4' on 'Pulmonary fibrosis'

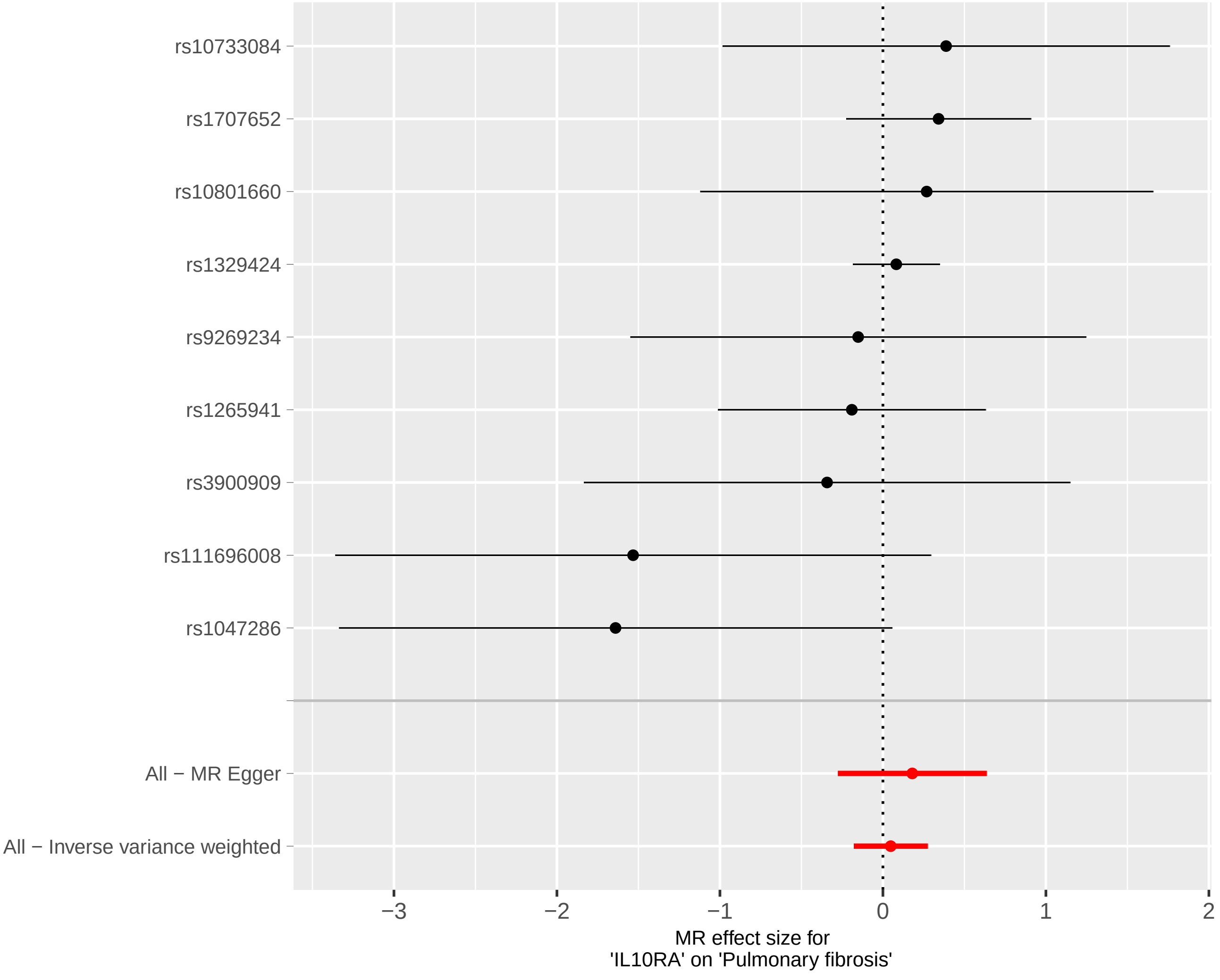

rs10733084
rs1707652
rs10801660
rs1329424
rs9269234
rs1265941
rs3900909
rs111696008
rs1047286
All − MR Egger
All − Inverse variance weighted
−3
−2
−1
0
1
2
MR effect size for
'IL10RA' on 'Pulmonary fibrosis'

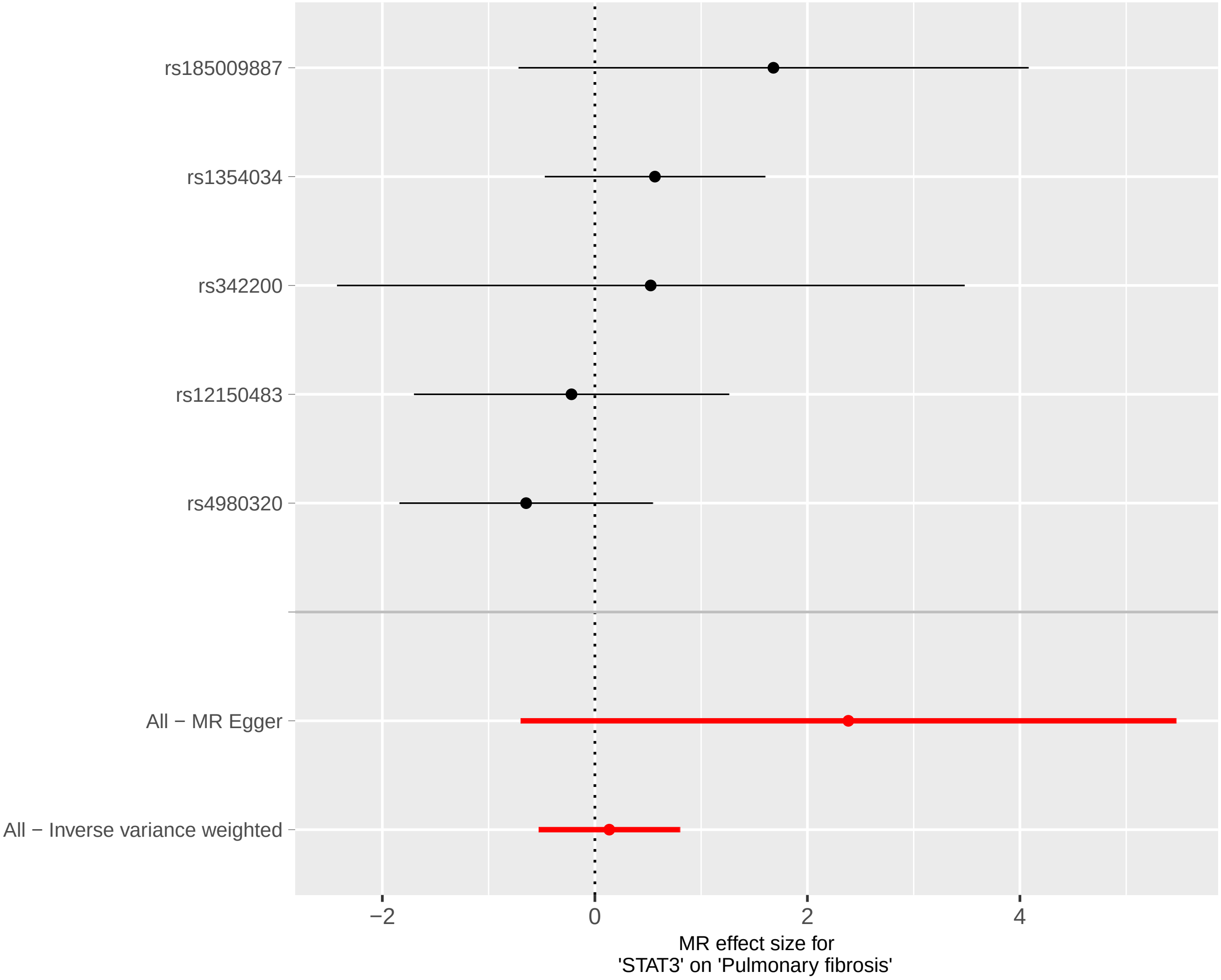
rs185009887
rs1354034
rs342200
rs12150483
rs4980320
All − MR Egger
All − Inverse variance weighted
−2
0
2
4
MR effect size for
'STAT3' on 'Pulmonary fibrosis'

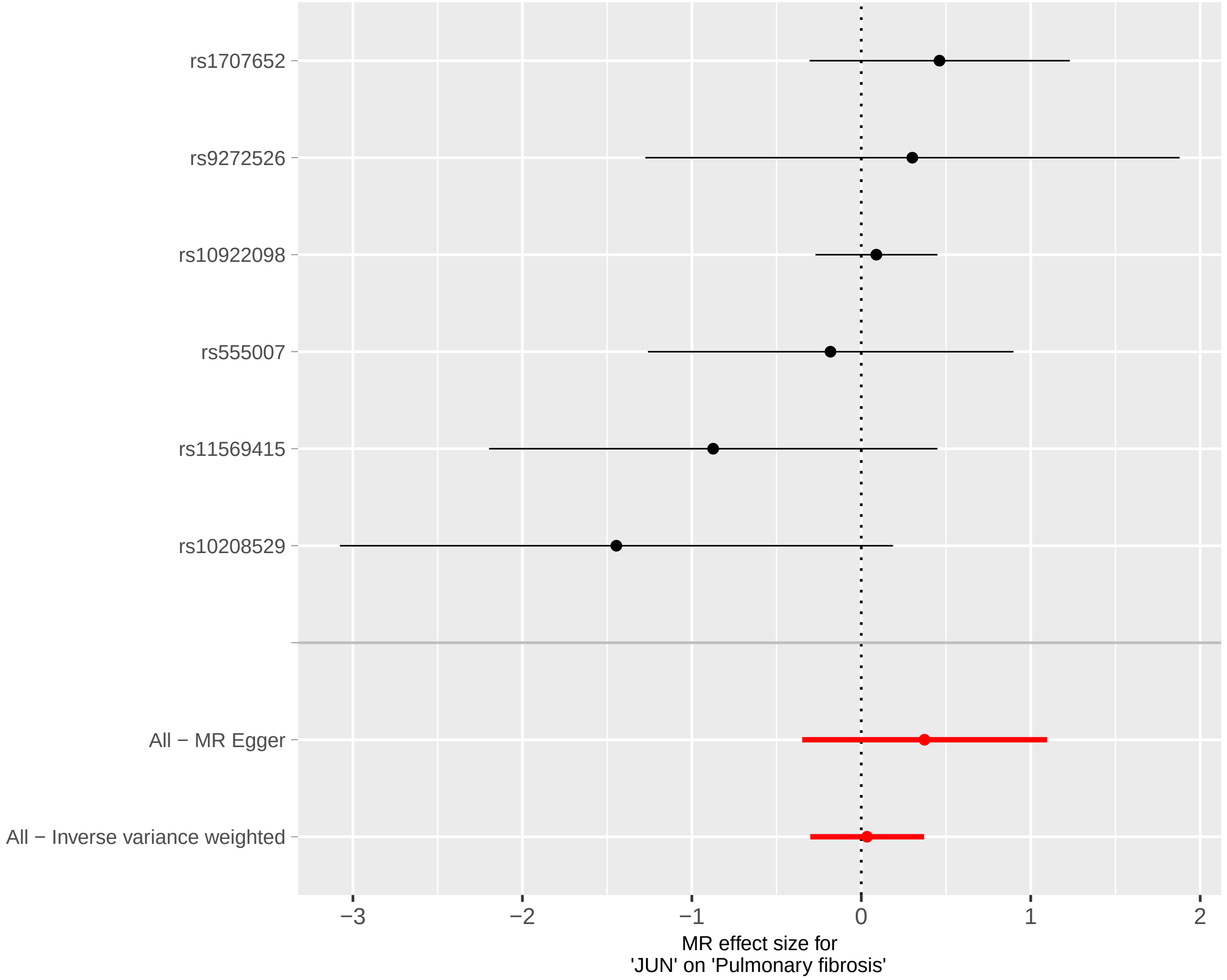
rs1707652
rs9272526
rs10922098
rs555007
rs11569415
rs10208529
All − MR Egger
All − Inverse variance weighted
−3
−2
−1
0
1
2
MR effect size for
'JUN' on 'Pulmonary fibrosis'

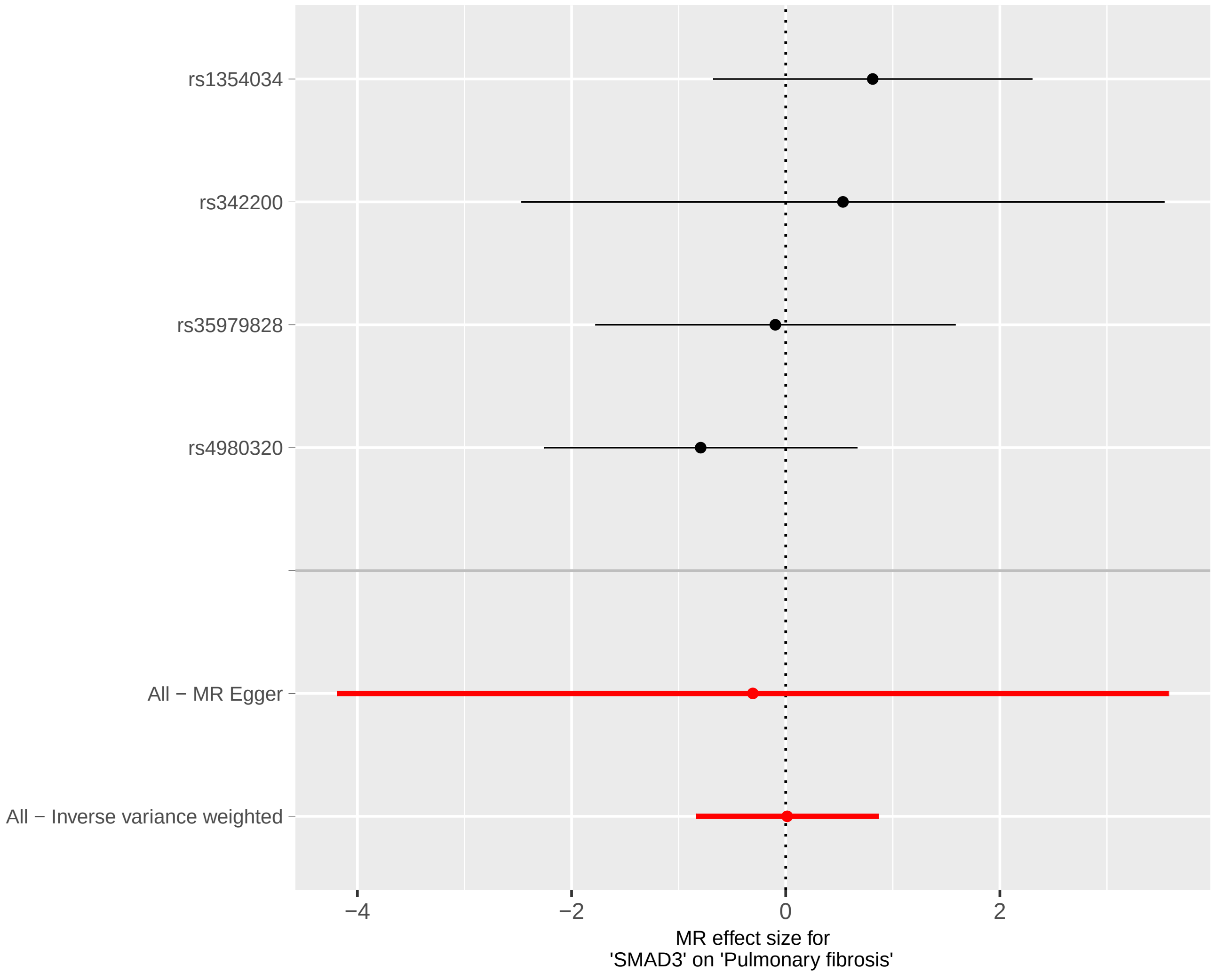
rs1354034
rs342200
rs35979828
rs4980320
All − MR Egger
All − Inverse variance weighted
−4
−2
0
2
MR effect size for
'SMAD3' on 'Pulmonary fibrosis'

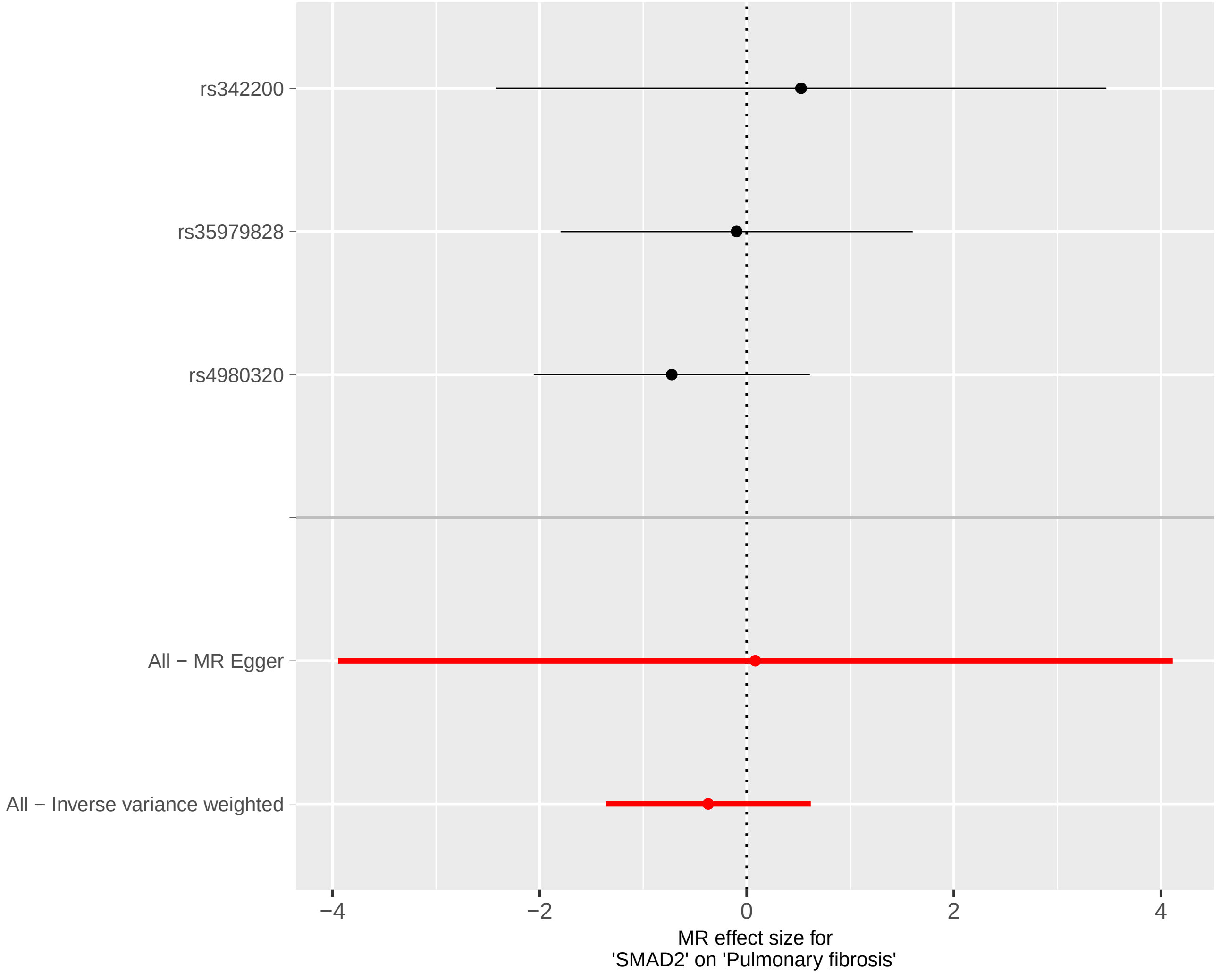

rs342200
rs35979828
rs4980320
All − MR Egger
All − Inverse variance weighted
−4
−2
0
2
4
MR effect size for
'SMAD2' on 'Pulmonary fibrosis'

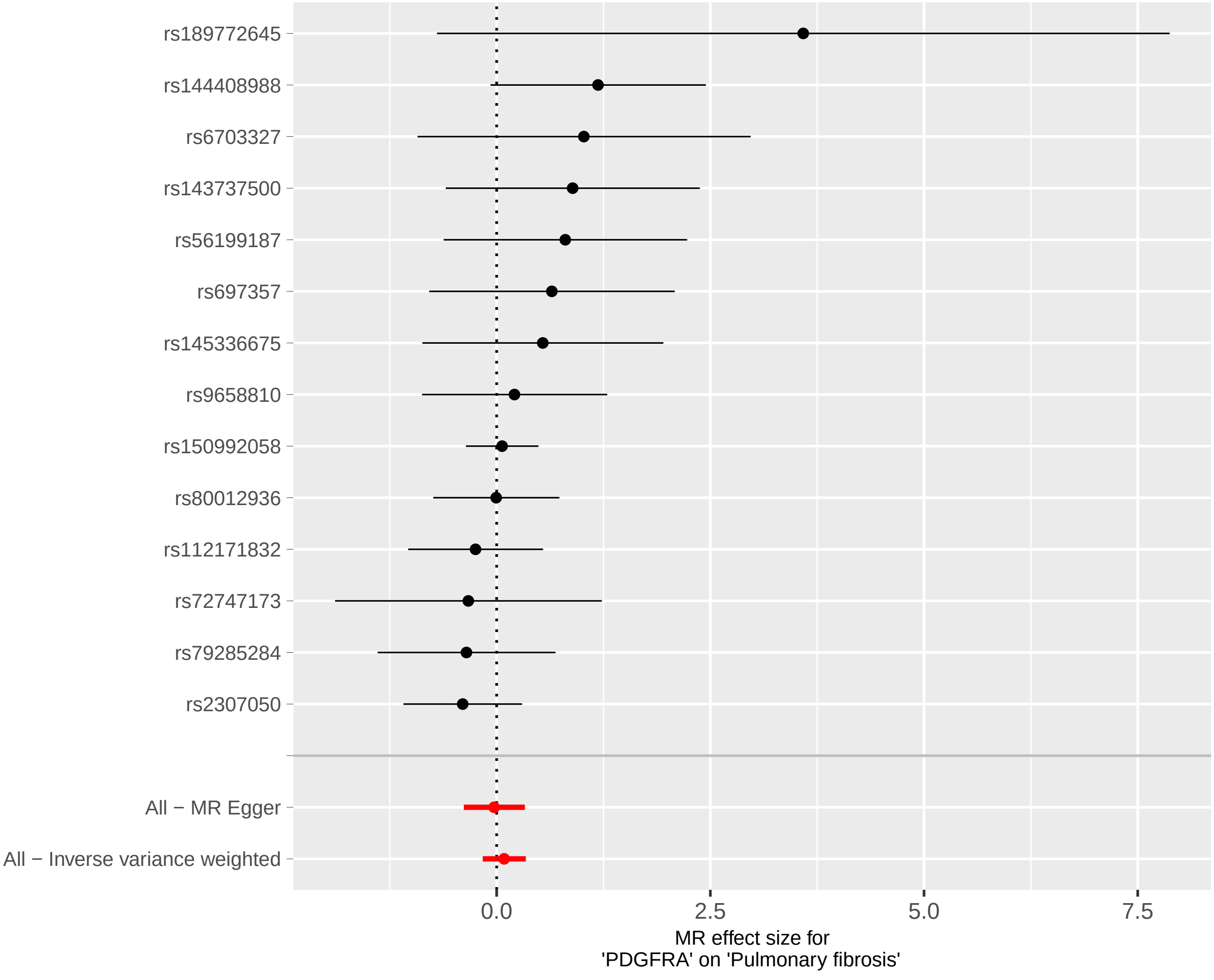

rs189772645
rs144408988
rs6703327
rs143737500
rs56199187
rs697357
rs145336675
rs9658810
rs150992058
rs80012936
rs112171832
rs72747173
rs79285284
rs2307050
All − MR Egger
All − Inverse variance weighted
0.0
2.5
5.0
7.5
MR effect size for
'PDGFRA' on 'Pulmonary fibrosis'

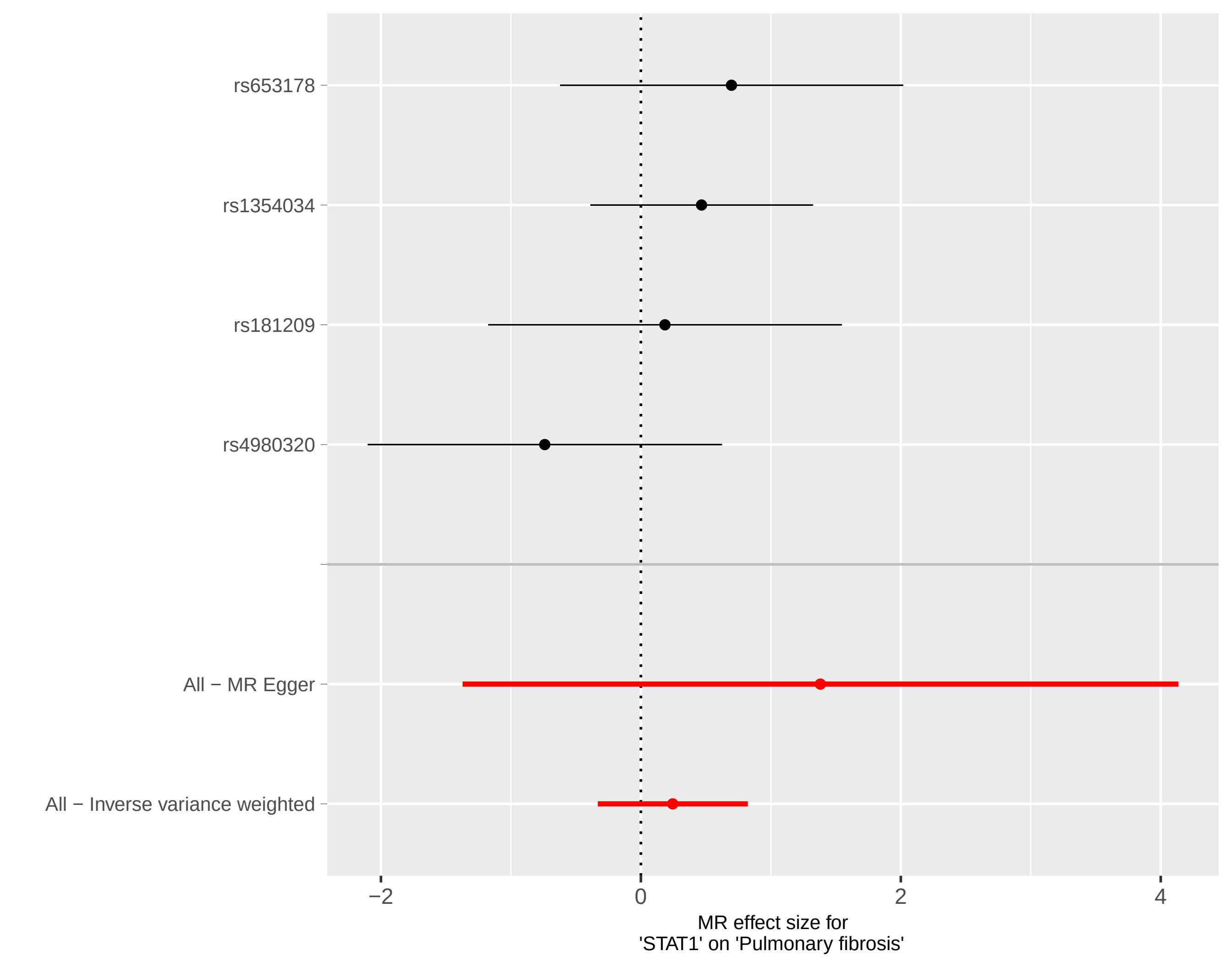

rs653178
rs1354034
rs181209
rs4980320
All − MR Egger
All − Inverse variance weighted
−2
0
2
4
MR effect size for
'STAT1' on 'Pulmonary fibrosis'

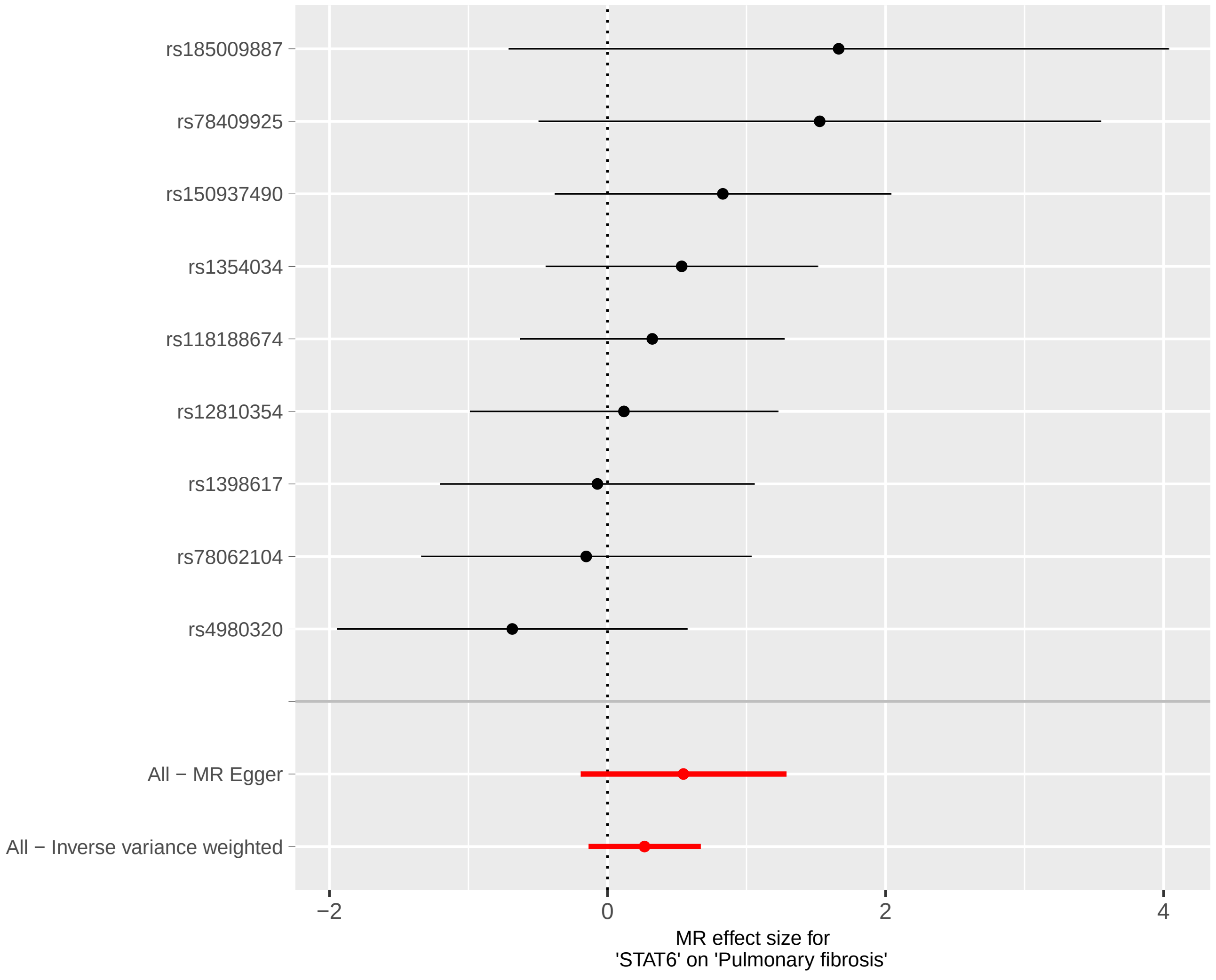

rs185009887
rs78409925
rs150937490
rs1354034
rs118188674
rs12810354
rs1398617
rs78062104
rs4980320
All − MR Egger
All − Inverse variance weighted
−2
0
2
4
MR effect size for
'STAT6' on 'Pulmonary fibrosis'

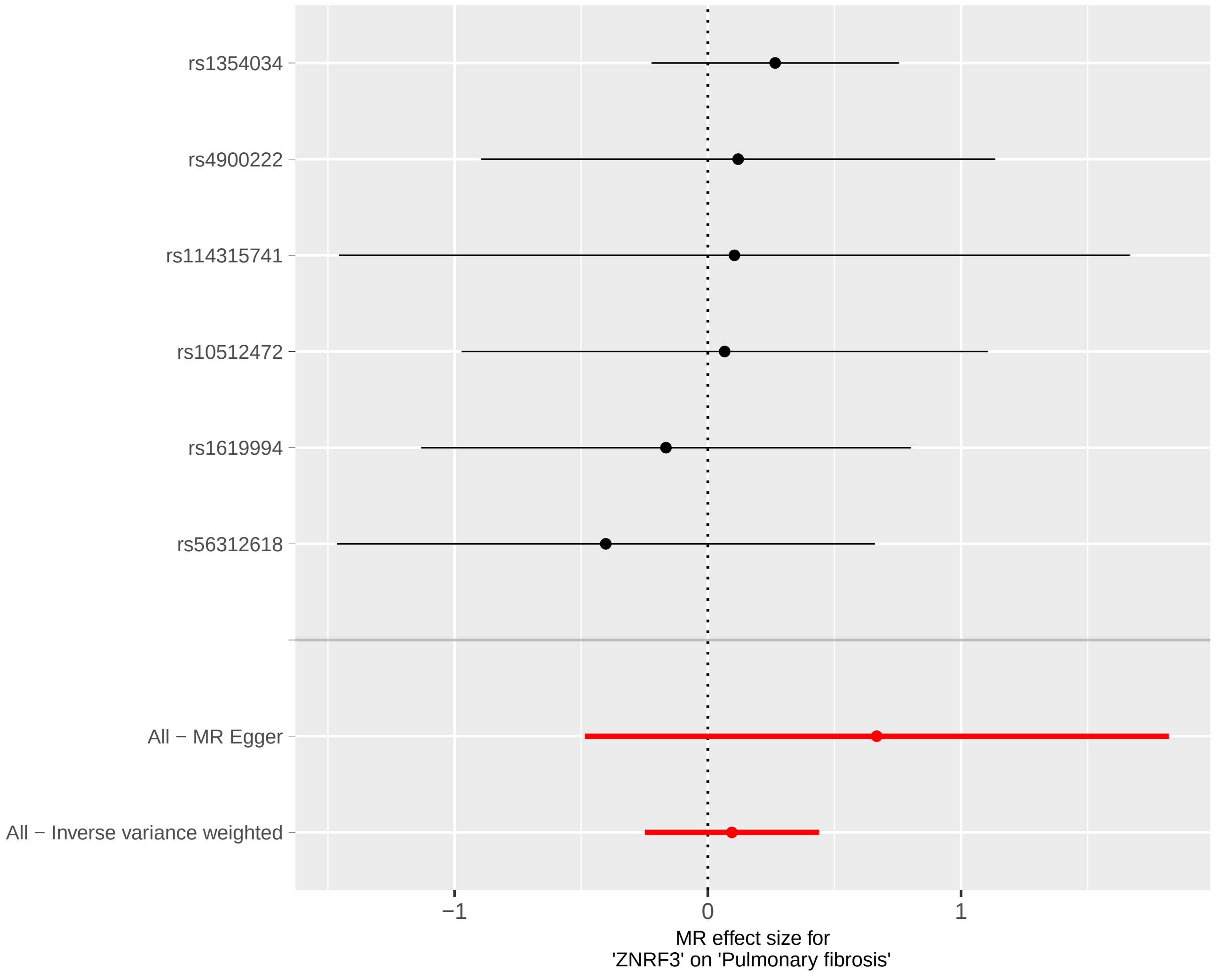
rs1354034
rs4900222
rs114315741
rs10512472
rs1619994
rs56312618
All − MR Egger
All − Inverse variance weighted
−1
0
1
MR effect size for
'ZNRF3' on 'Pulmonary fibrosis'

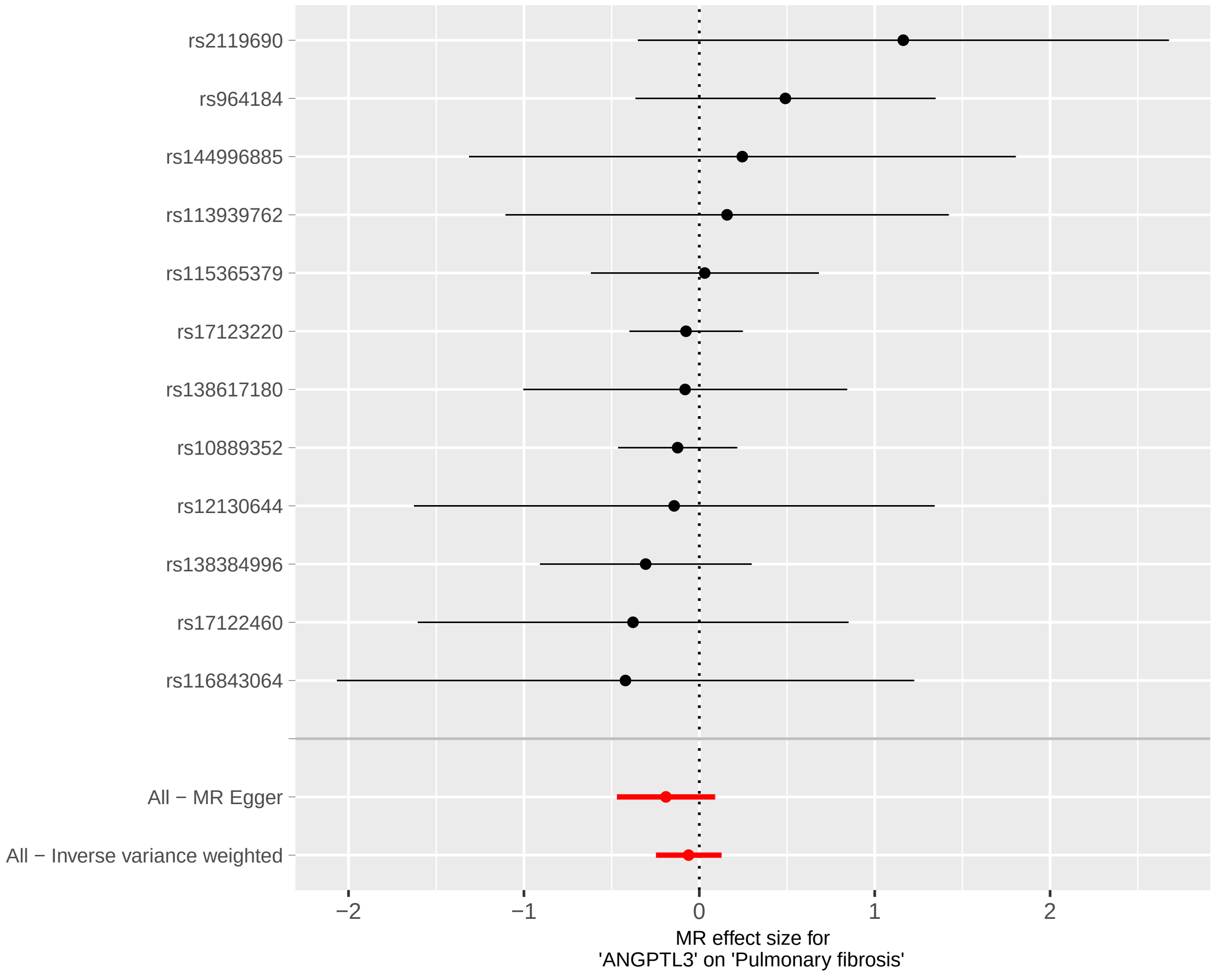
rs2119690
rs964184
rs144996885
rs113939762
rs115365379
rs17123220
rs138617180
rs10889352
rs12130644
rs138384996
rs17122460
rs116843064
All − MR Egger
All − Inverse variance weighted
−2
−1
0
1
2
MR effect size for
'ANGPTL3' on 'Pulmonary fibrosis'

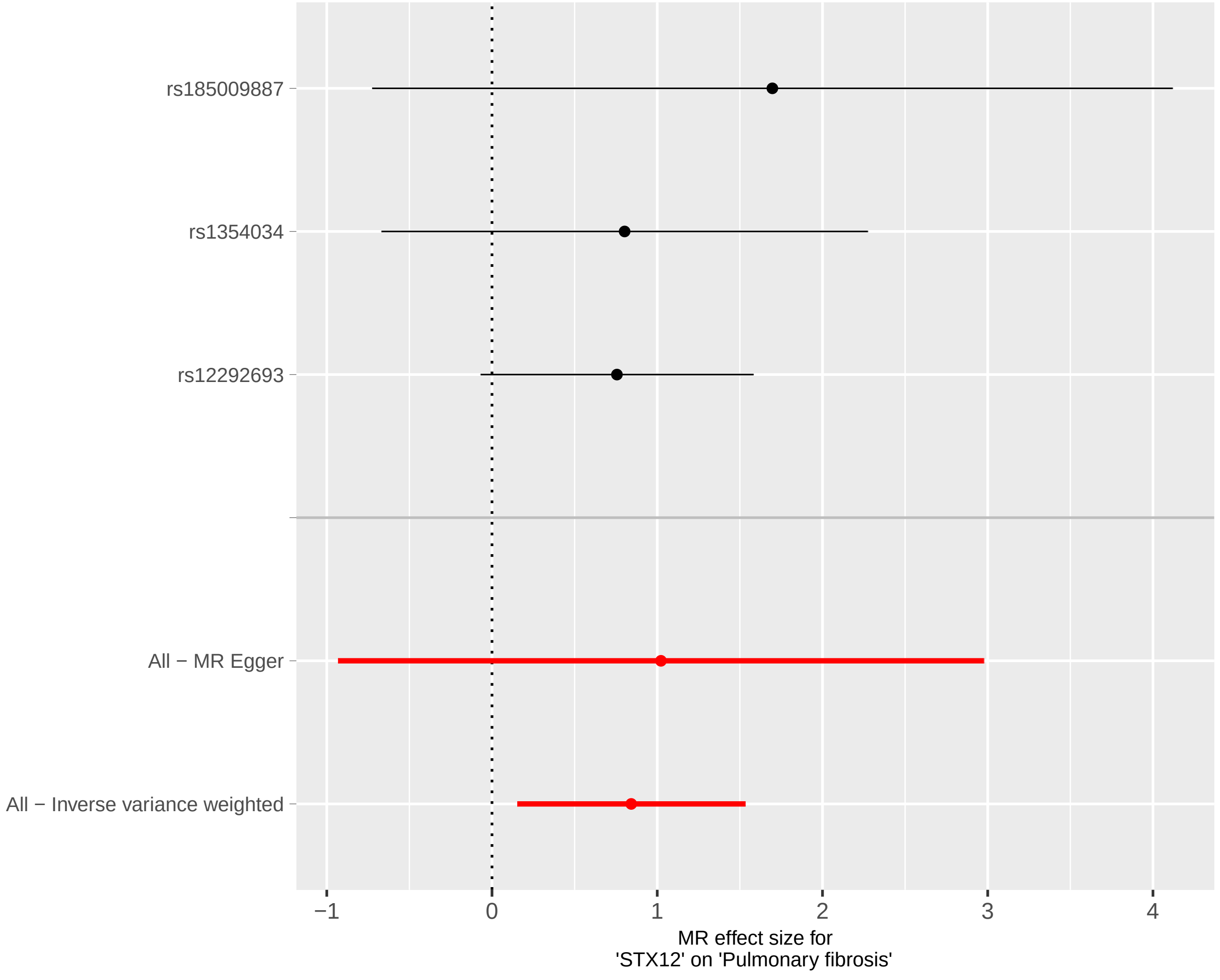
rs185009887
rs1354034
rs12292693
All − MR Egger
All − Inverse variance weighted
−1
0
1
2
3
4
MR effect size for
'STX12' on 'Pulmonary fibrosis'

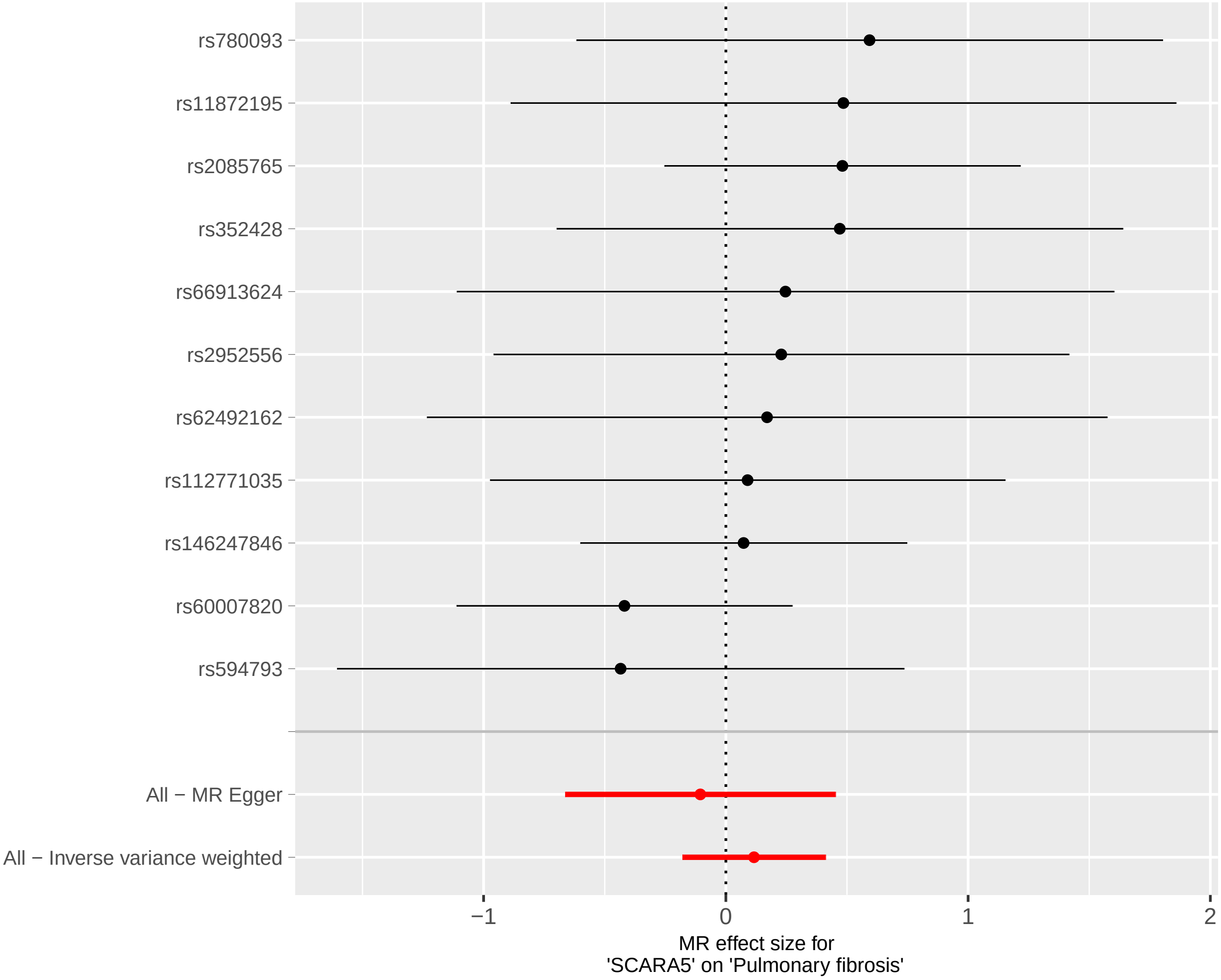
rs780093
rs11872195
rs2085765
rs352428
rs66913624
rs2952556
rs62492162
rs112771035
rs146247846
rs60007820
rs594793
All − MR Egger
All − Inverse variance weighted
−1
0
1
2
MR effect size for
'SCARA5' on 'Pulmonary fibrosis'

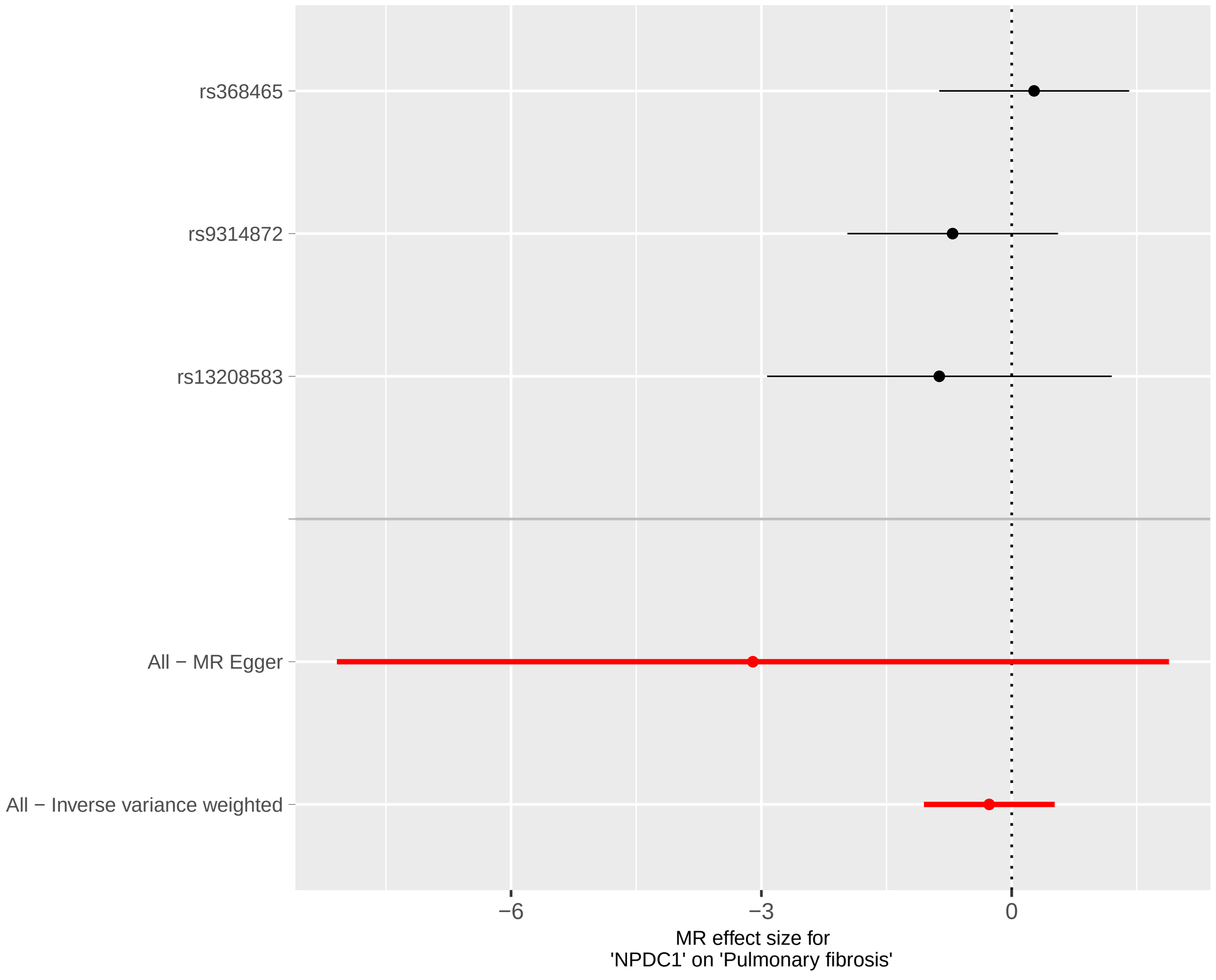
rs368465
rs9314872
rs13208583
All − MR Egger
All − Inverse variance weighted
−6
−3
0
MR effect size for
'NPDC1' on 'Pulmonary fibrosis'

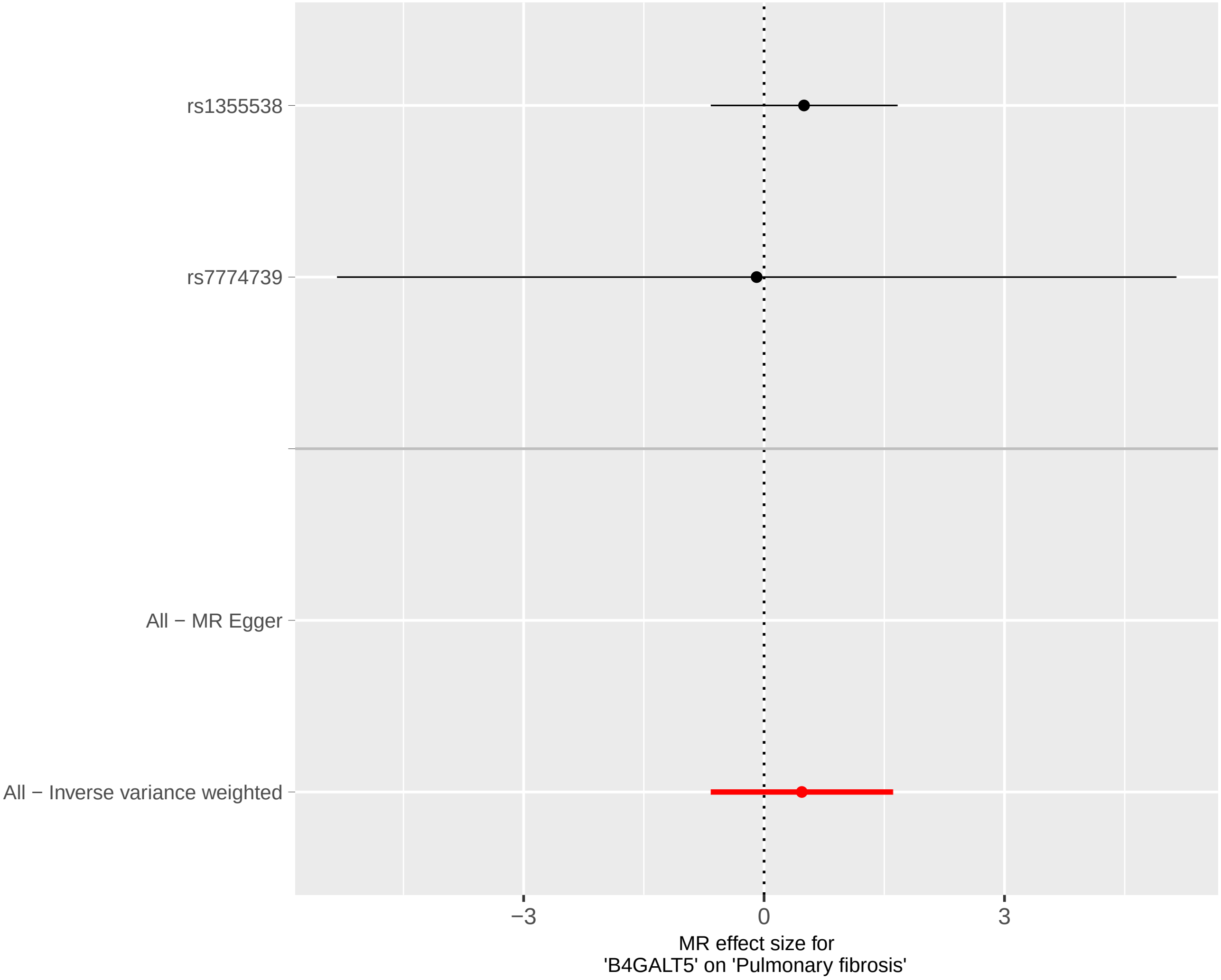
rs1355538
rs7774739
All − MR Egger
All − Inverse variance weighted
−3
0
3
MR effect size for
'B4GALT5' on 'Pulmonary fibrosis'

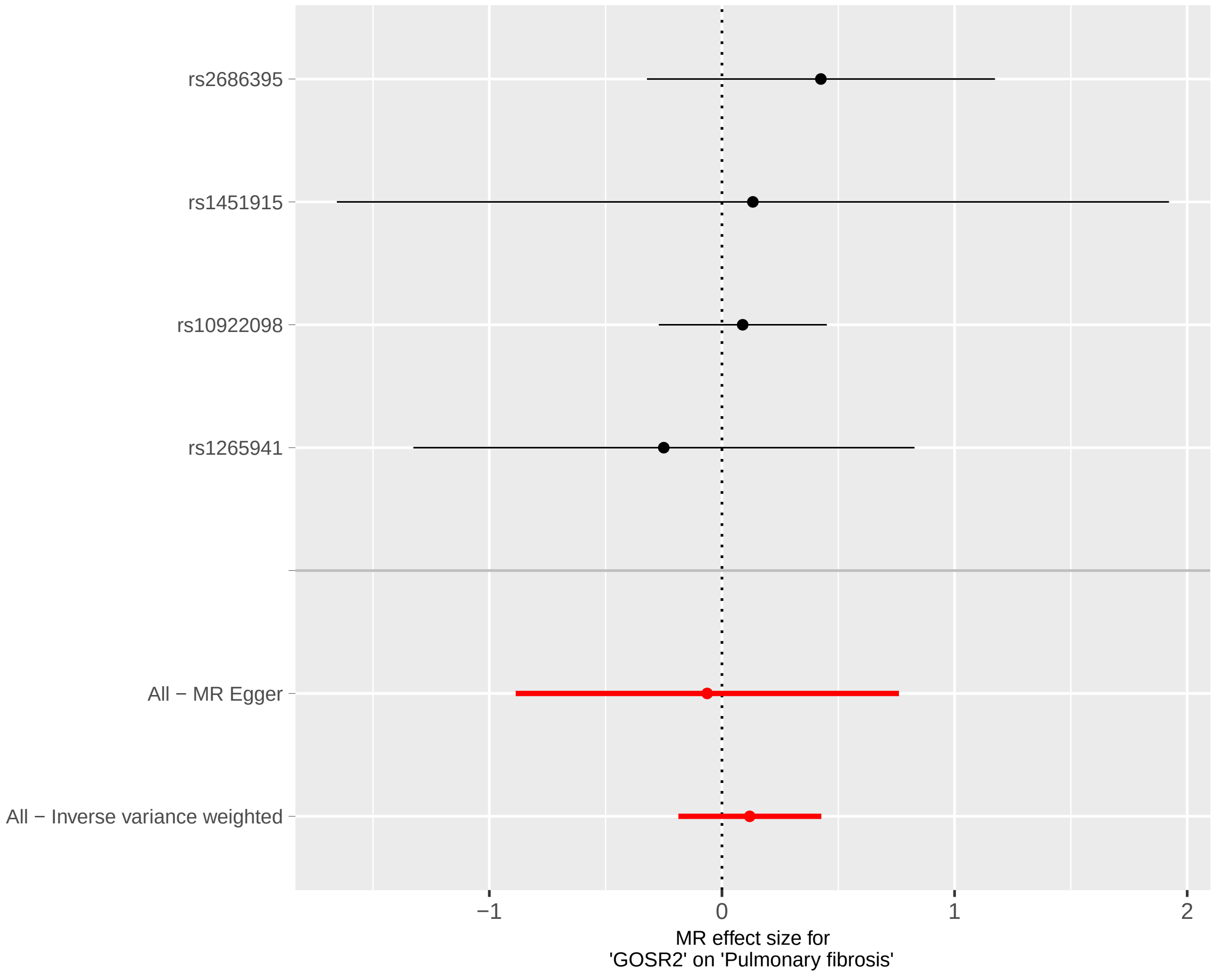
rs2686395
rs1451915
rs10922098
rs1265941
All − MR Egger
All − Inverse variance weighted
−1
0
1
2
MR effect size for
'GOSR2' on 'Pulmonary fibrosis'

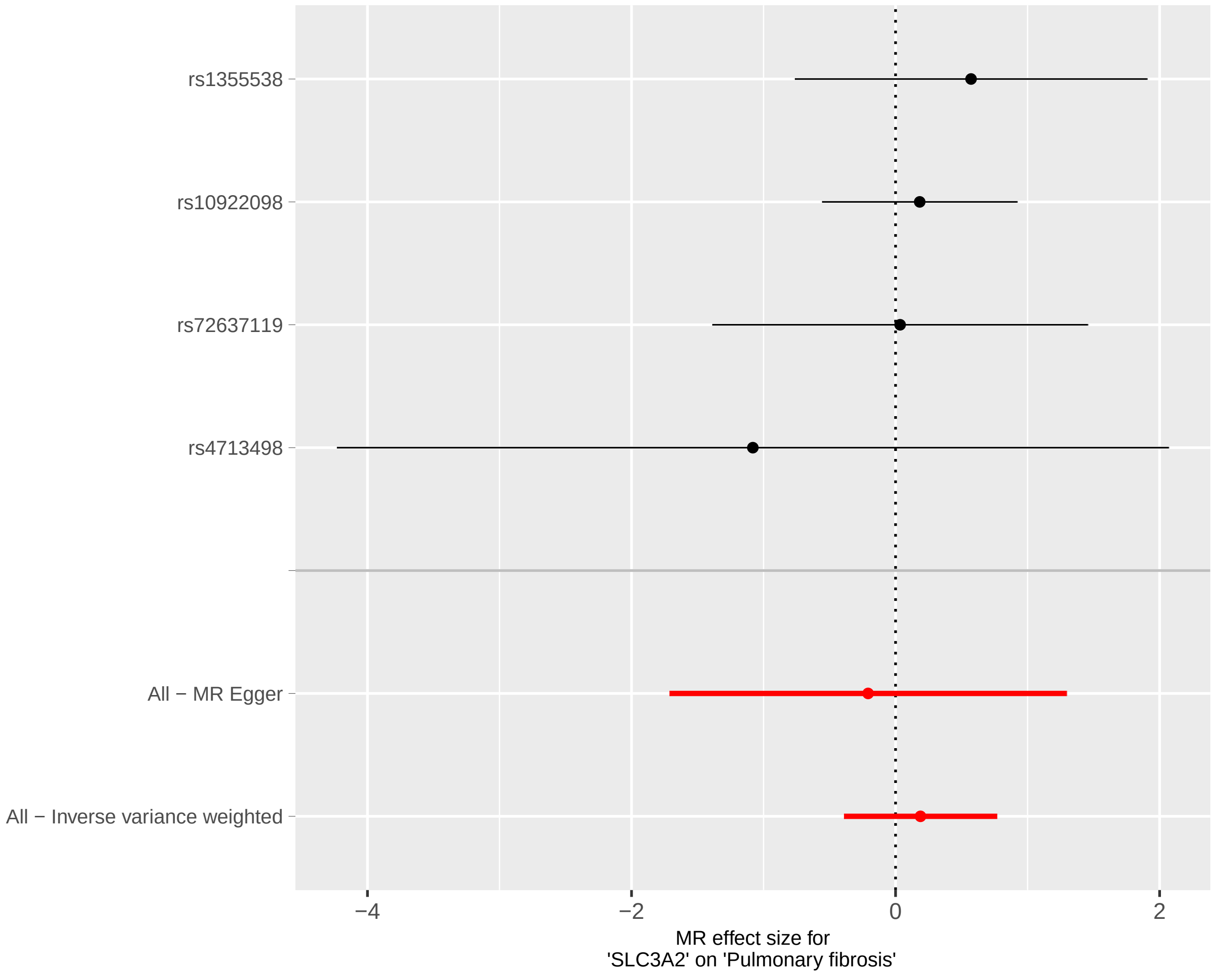
rs1355538
rs10922098
rs72637119
rs4713498
All − MR Egger
All − Inverse variance weighted
−4
−2
0
2
MR effect size for
'SLC3A2' on 'Pulmonary fibrosis'

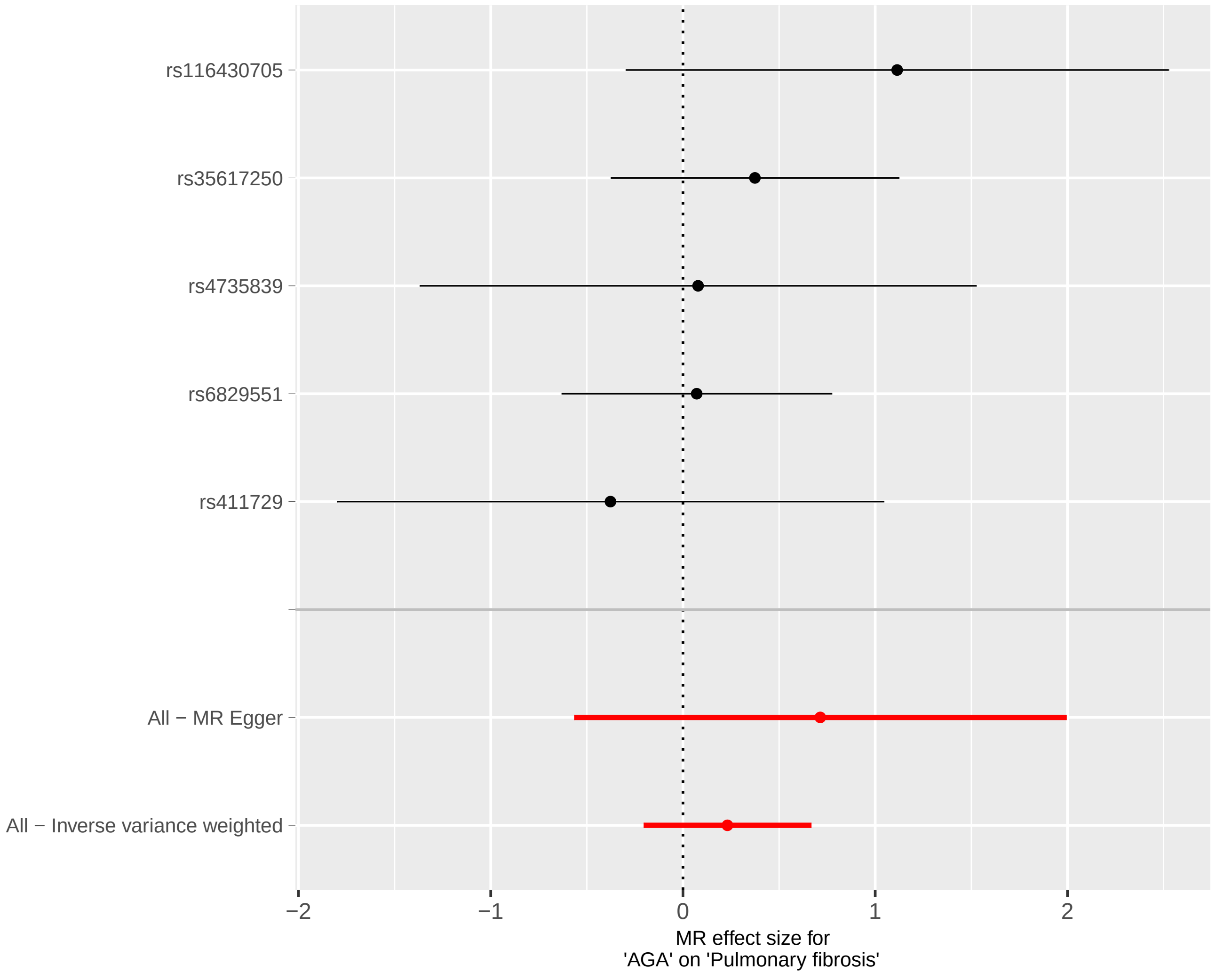

rs116430705
rs35617250
rs4735839
rs6829551
rs411729
All − MR Egger
All − Inverse variance weighted
−2
−1
0
1
2
MR effect size for
'AGA' on 'Pulmonary fibrosis'

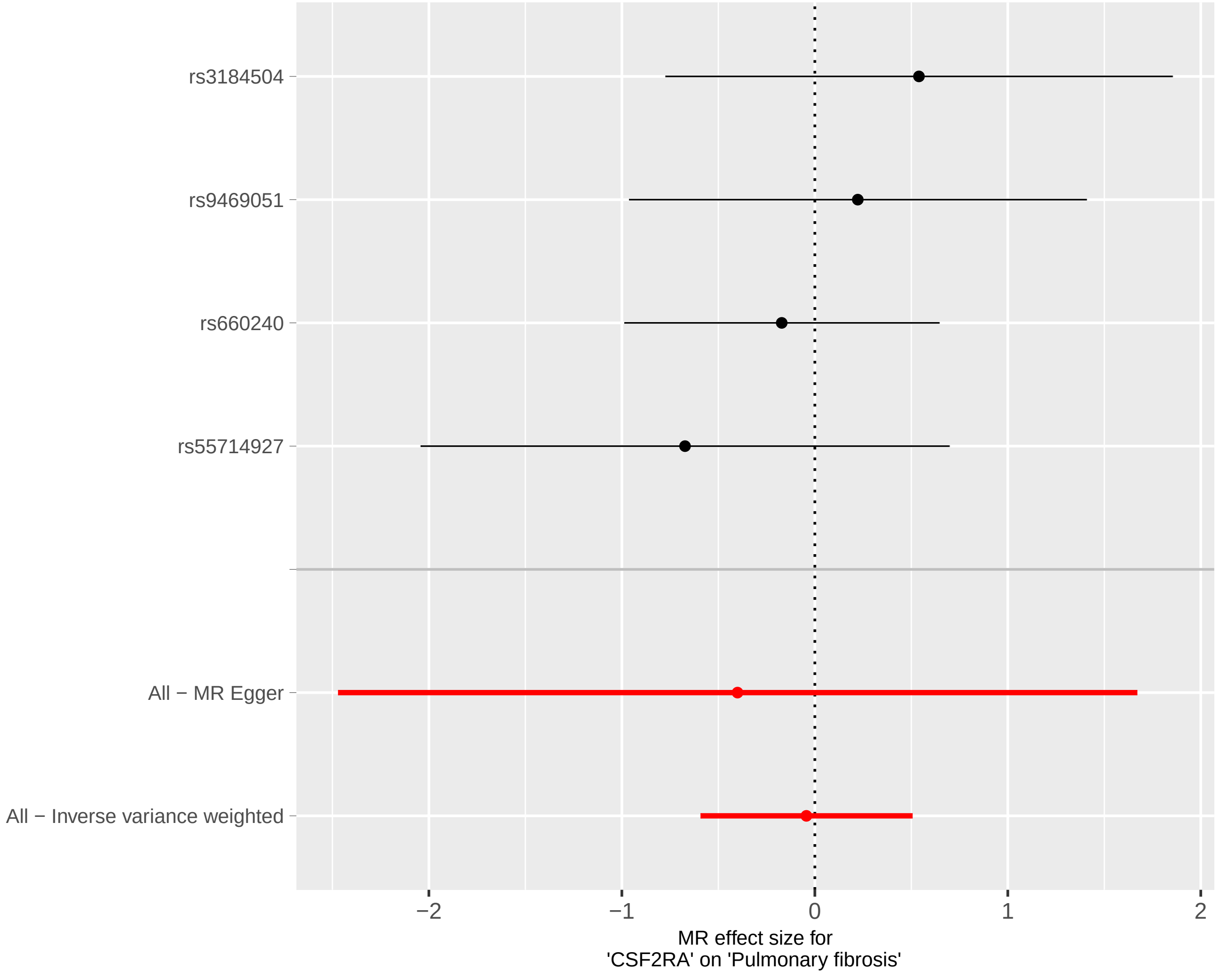

rs3184504
rs9469051
rs660240
rs55714927
All − MR Egger
All − Inverse variance weighted
−2
−1
0
1
2
MR effect size for
'CSF2RA' on 'Pulmonary fibrosis'

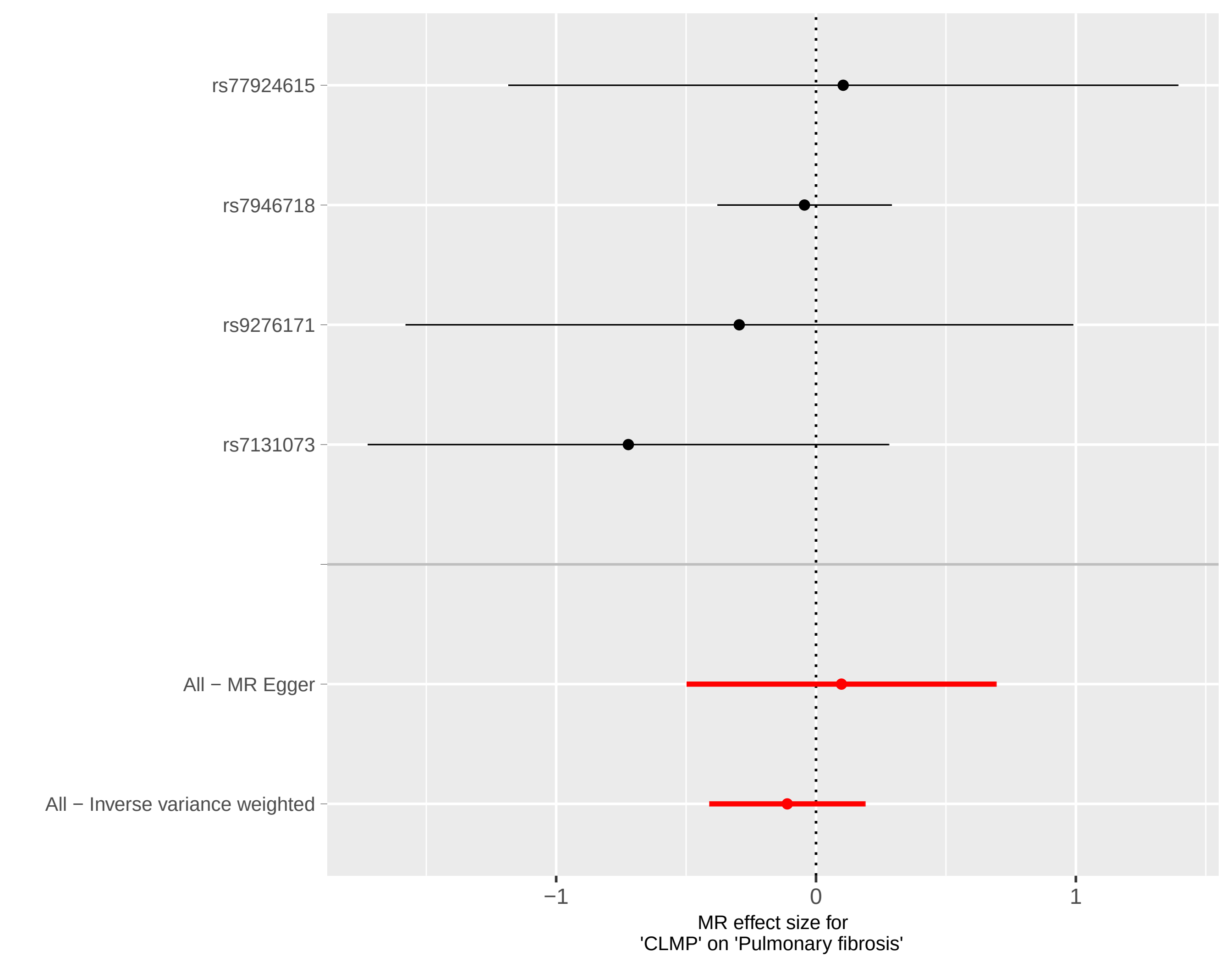

rs77924615
rs7946718
rs9276171
rs7131073
All − MR Egger
All − Inverse variance weighted
−1
0
1
MR effect size for
'CLMP' on 'Pulmonary fibrosis'

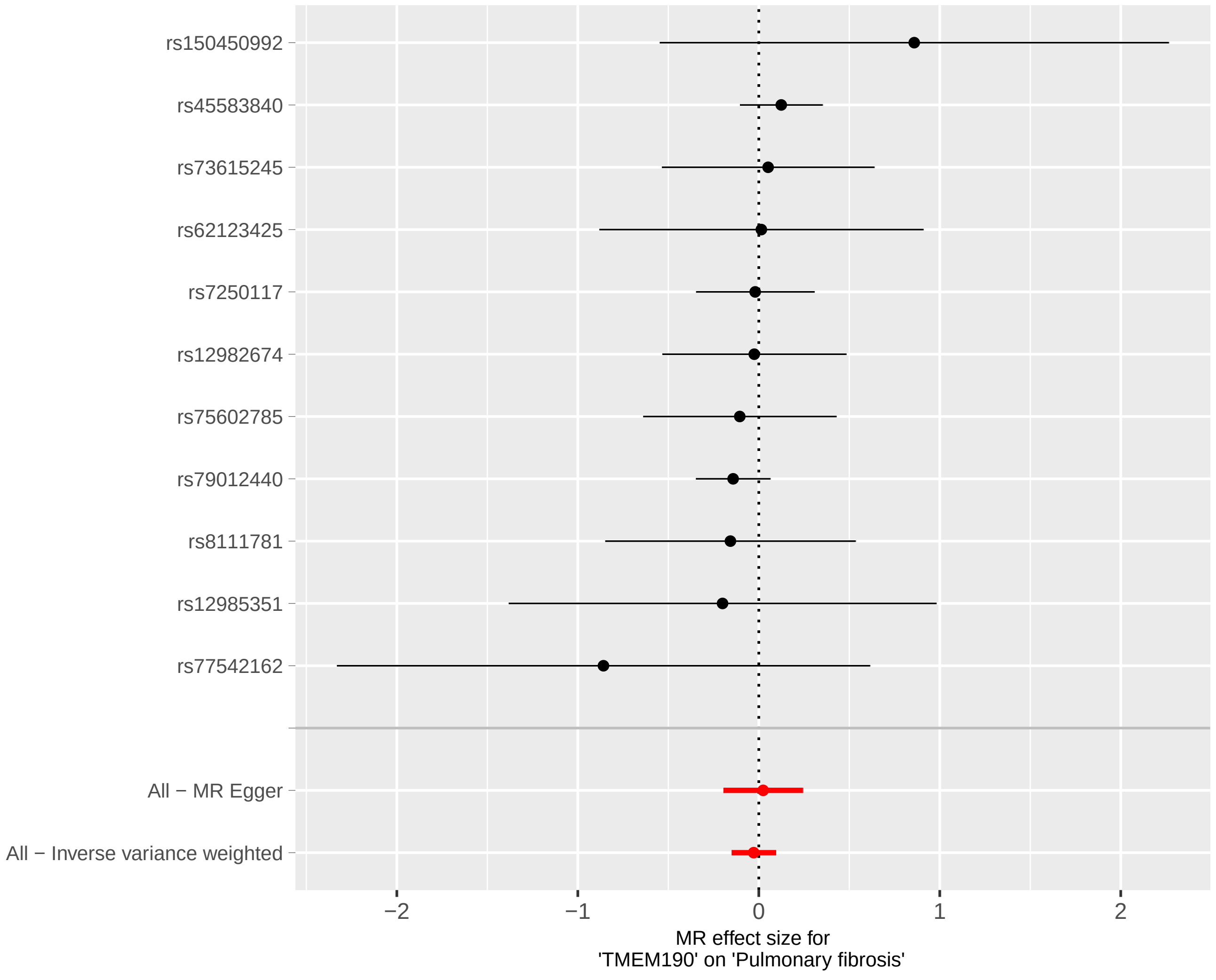
rs150450992
rs45583840
rs73615245
rs62123425
rs7250117
rs12982674
rs75602785
rs79012440
rs8111781
rs12985351
rs77542162
All − MR Egger
All − Inverse variance weighted
−2
−1
0
1
2
MR effect size for
'TMEM190' on 'Pulmonary fibrosis'

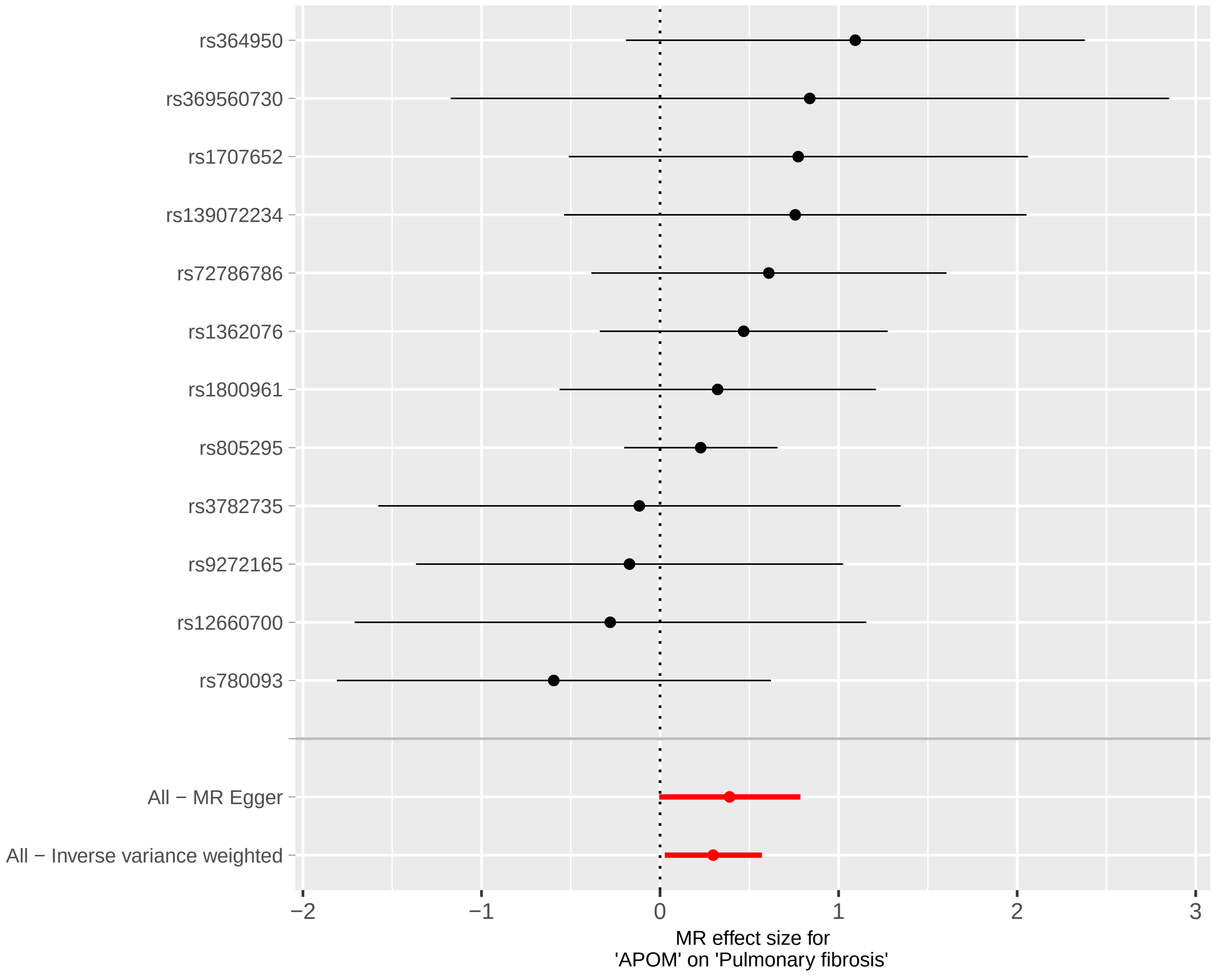
rs364950
rs369560730
rs1707652
rs139072234
rs72786786
rs1362076
rs1800961
rs805295
rs3782735
rs9272165
rs12660700
rs780093
All − MR Egger
All − Inverse variance weighted
−2
−1
0
1
2
3
MR effect size for
'APOM' on 'Pulmonary fibrosis'

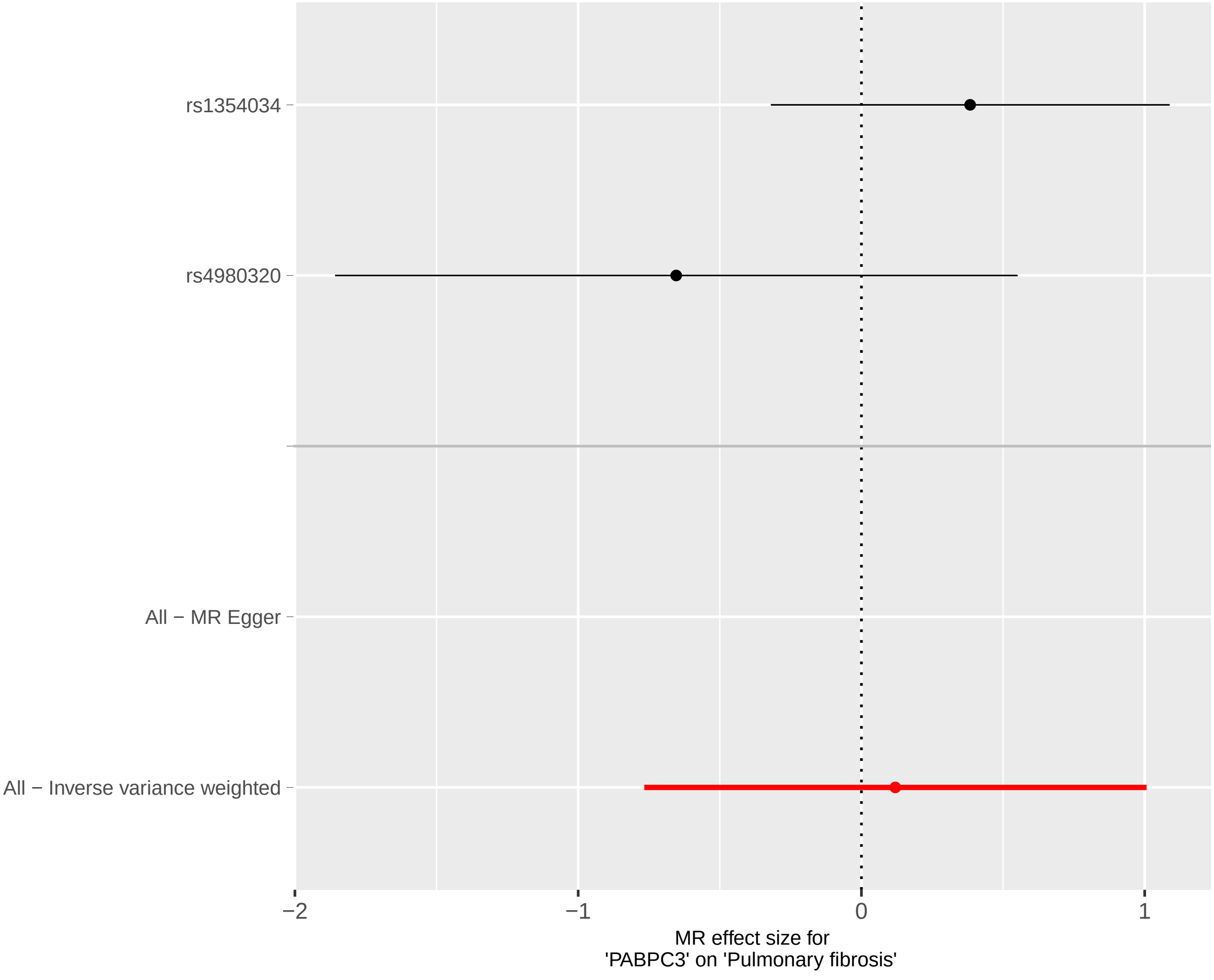
rs1354034
rs4980320
All − MR Egger
All − Inverse variance weighted
−2
−1
0
1
MR effect size for
'PABPC3' on 'Pulmonary fibrosis'

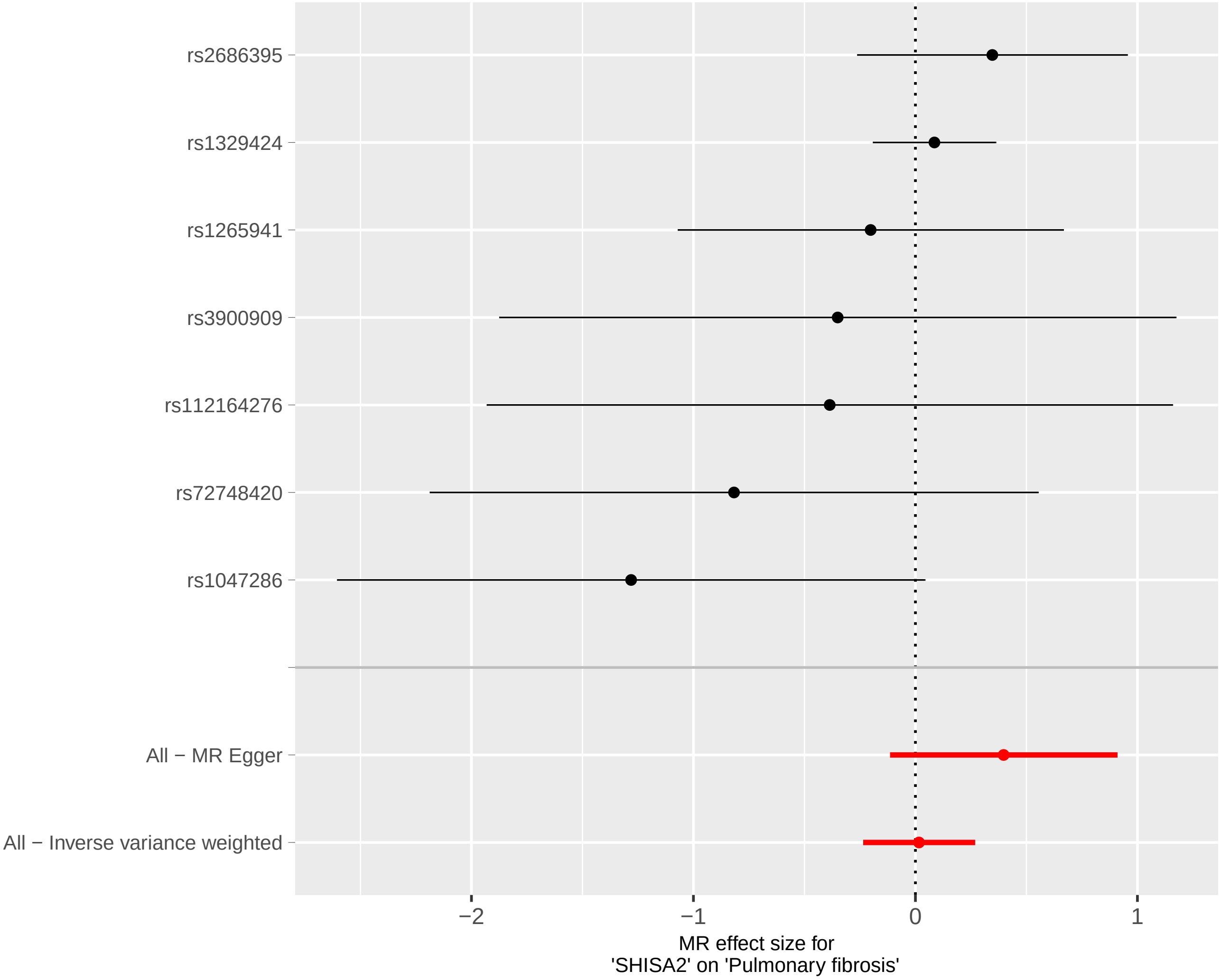

rs2686395
rs1329424
rs1265941
rs3900909
rs112164276
rs72748420
rs1047286
All − MR Egger
All − Inverse variance weighted
−2
−1
0
1
MR effect size for
'SHISA2' on 'Pulmonary fibrosis'

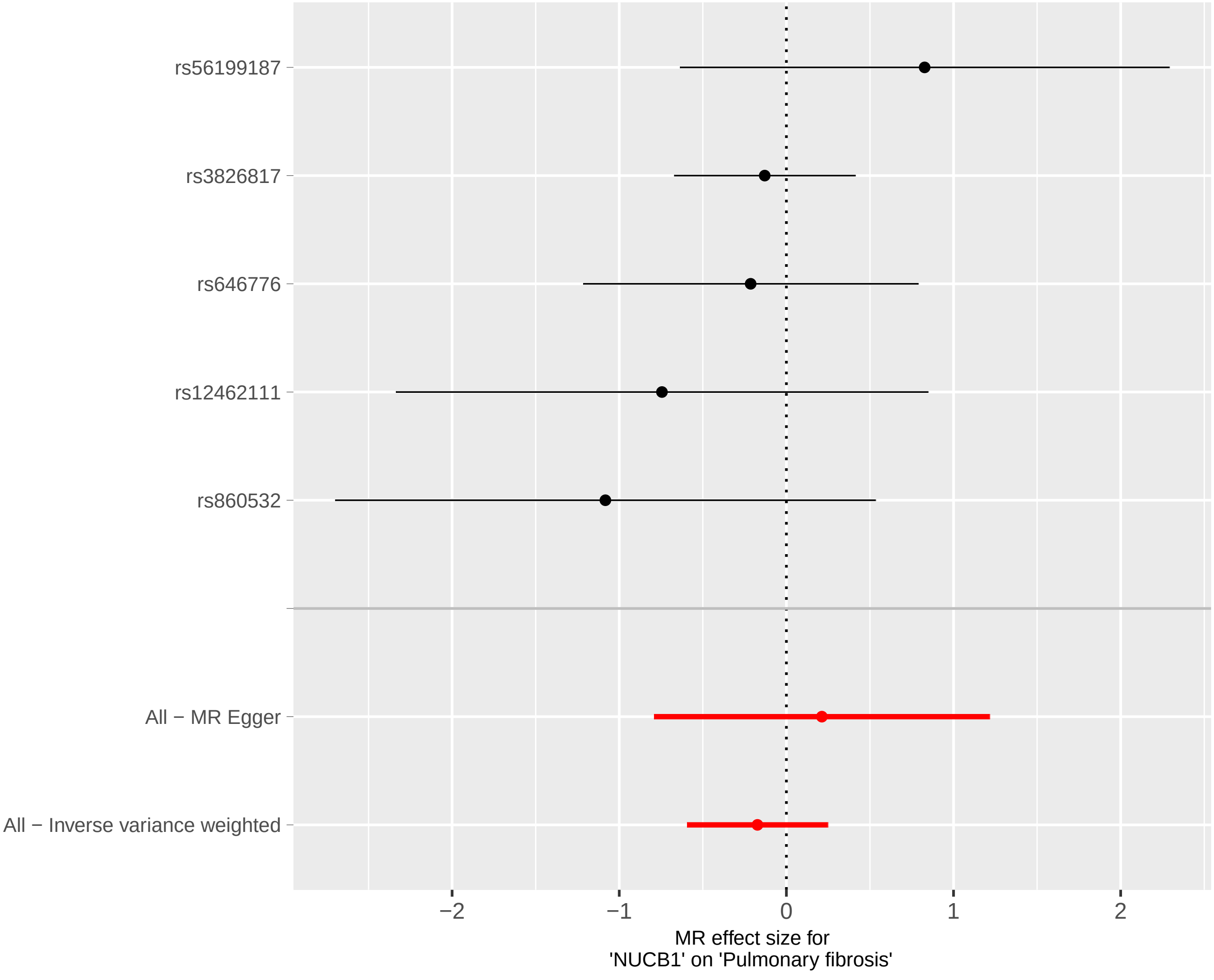
rs56199187
rs3826817
rs646776
rs12462111
rs860532
All − MR Egger
All − Inverse variance weighted
−2
−1
0
1
2
MR effect size for
'NUCB1' on 'Pulmonary fibrosis'

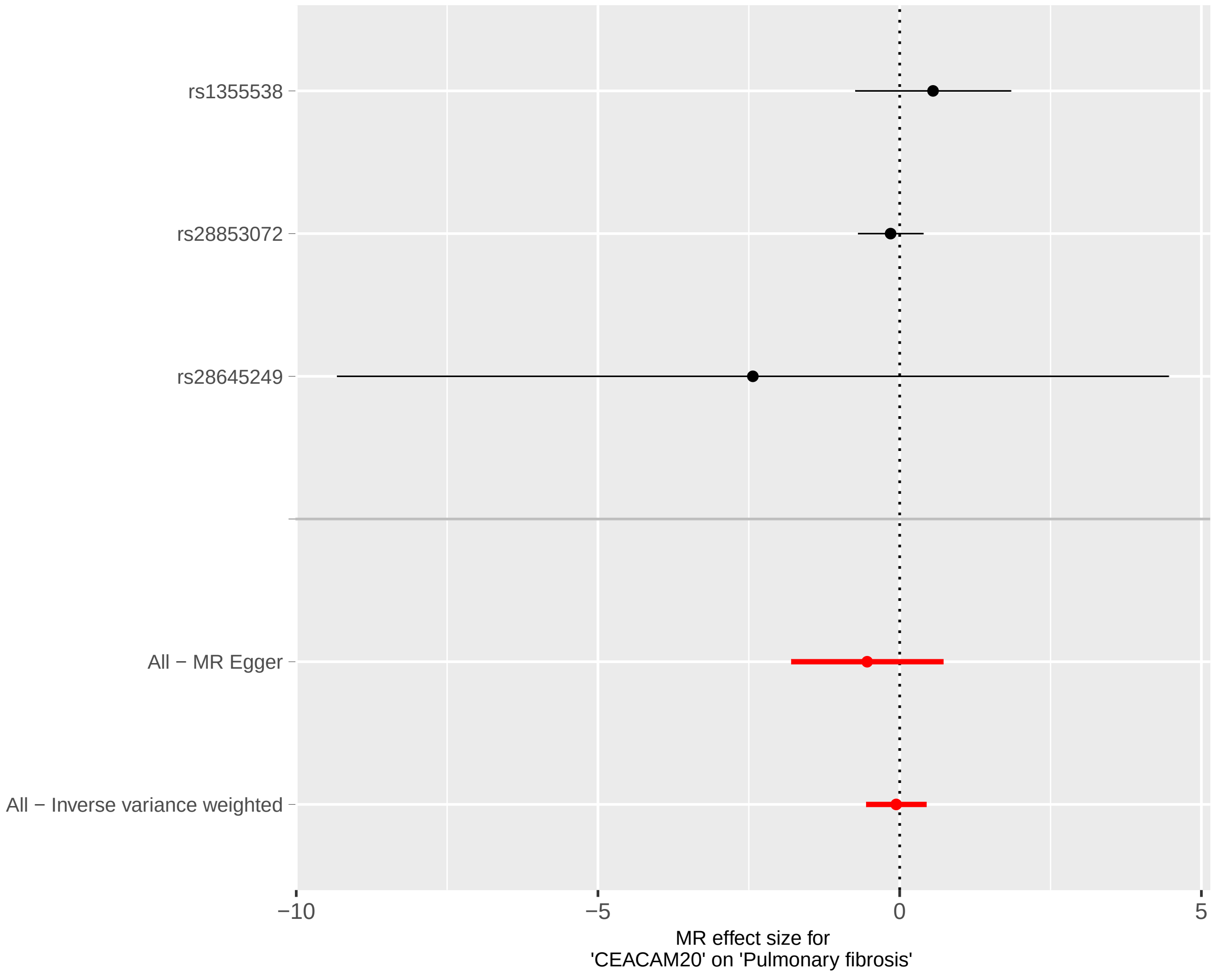
rs1355538
rs28853072
rs28645249
All − MR Egger
All − Inverse variance weighted
−10
−5
0
5
MR effect size for
'CEACAM20' on 'Pulmonary fibrosis'

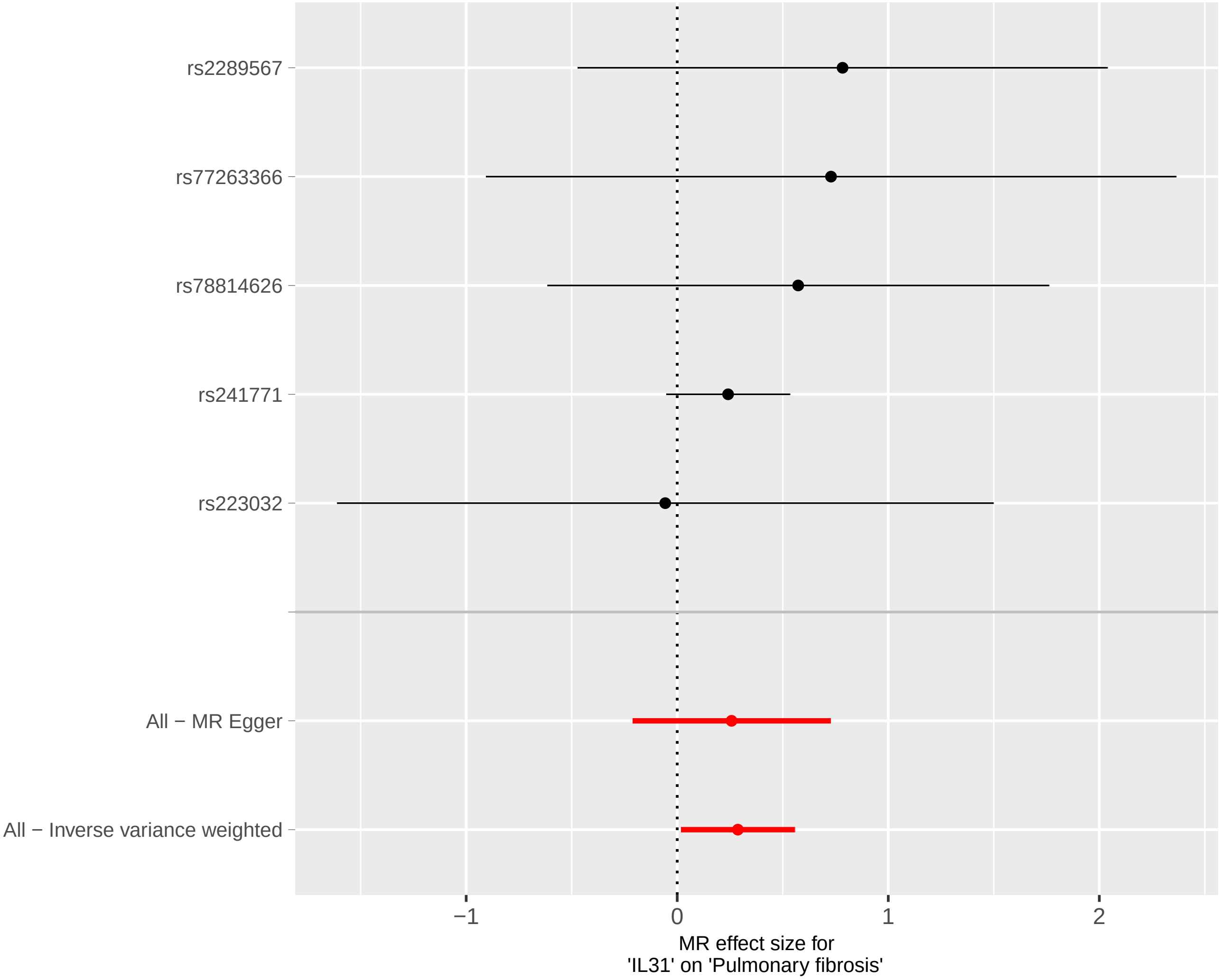
rs2289567
rs77263366
rs78814626
rs241771
rs223032
All − MR Egger
All − Inverse variance weighted
−1
0
1
2
MR effect size for
'IL31' on 'Pulmonary fibrosis'

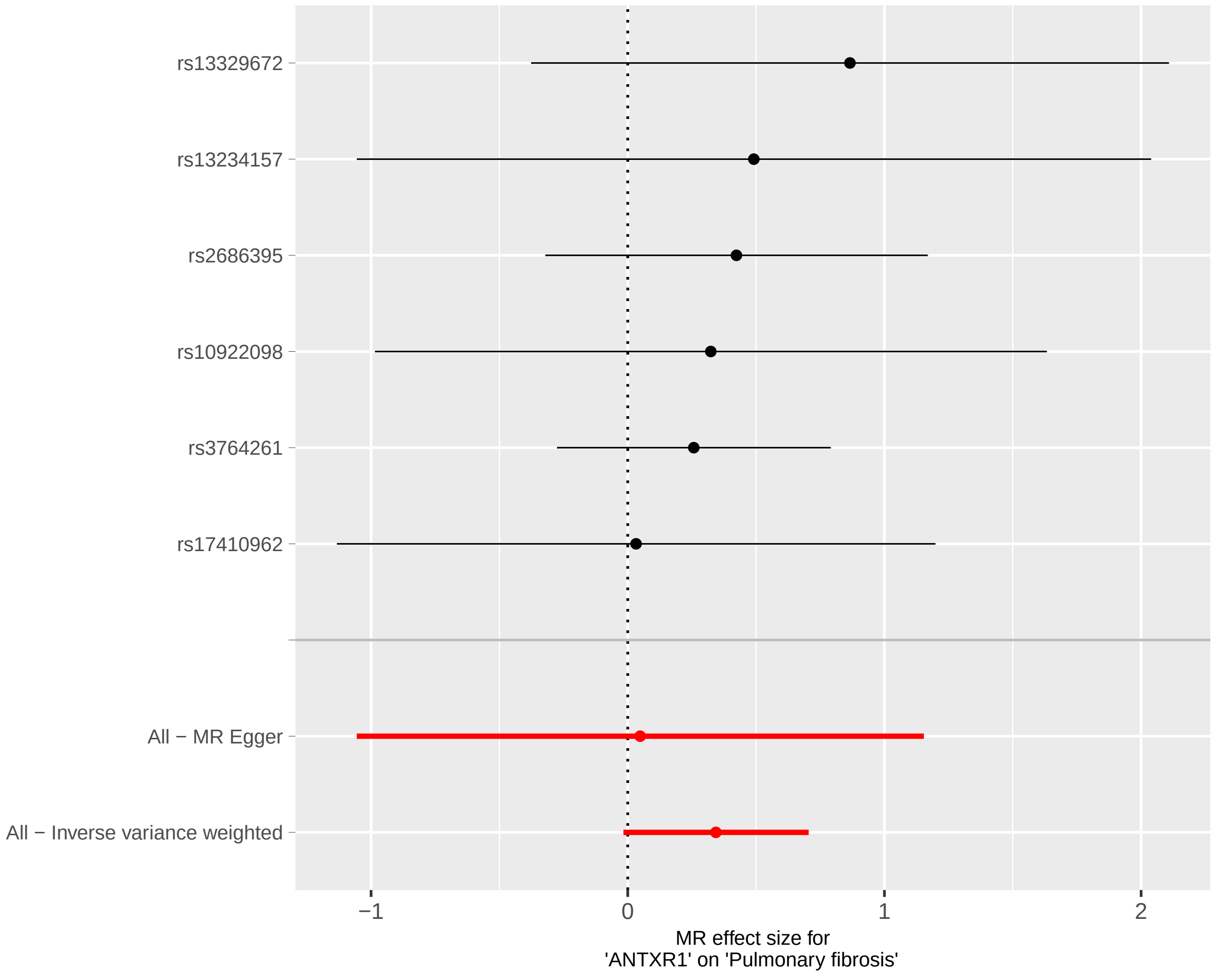
rs13329672
rs13234157
rs2686395
rs10922098
rs3764261
rs17410962
All − MR Egger
All − Inverse variance weighted
−1
0
1
2
MR effect size for
'ANTXR1' on 'Pulmonary fibrosis'

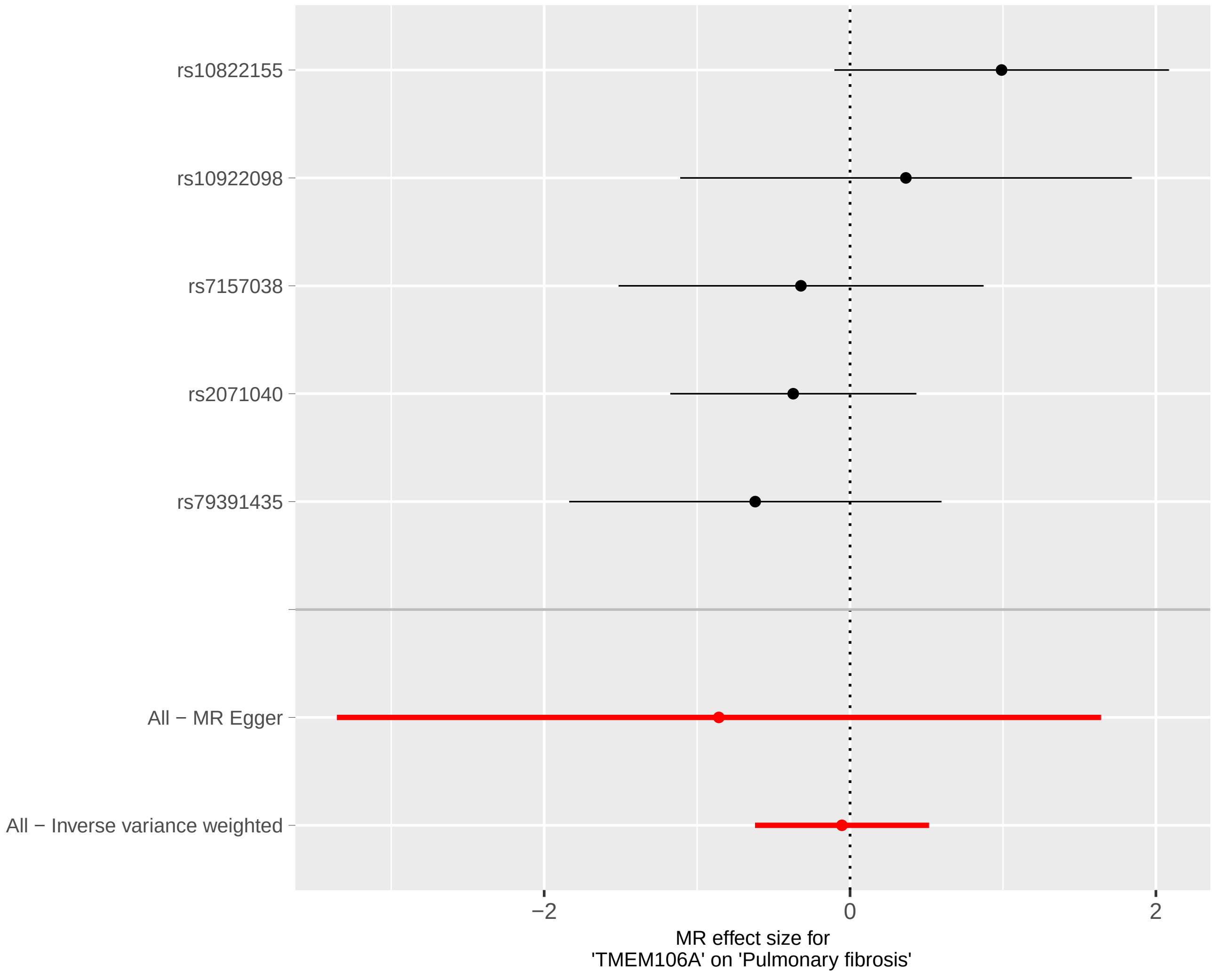
rs10822155
rs10922098
rs7157038
rs2071040
rs79391435
All − MR Egger
All − Inverse variance weighted
−2
0
2
MR effect size for
'TMEM106A' on 'Pulmonary fibrosis'

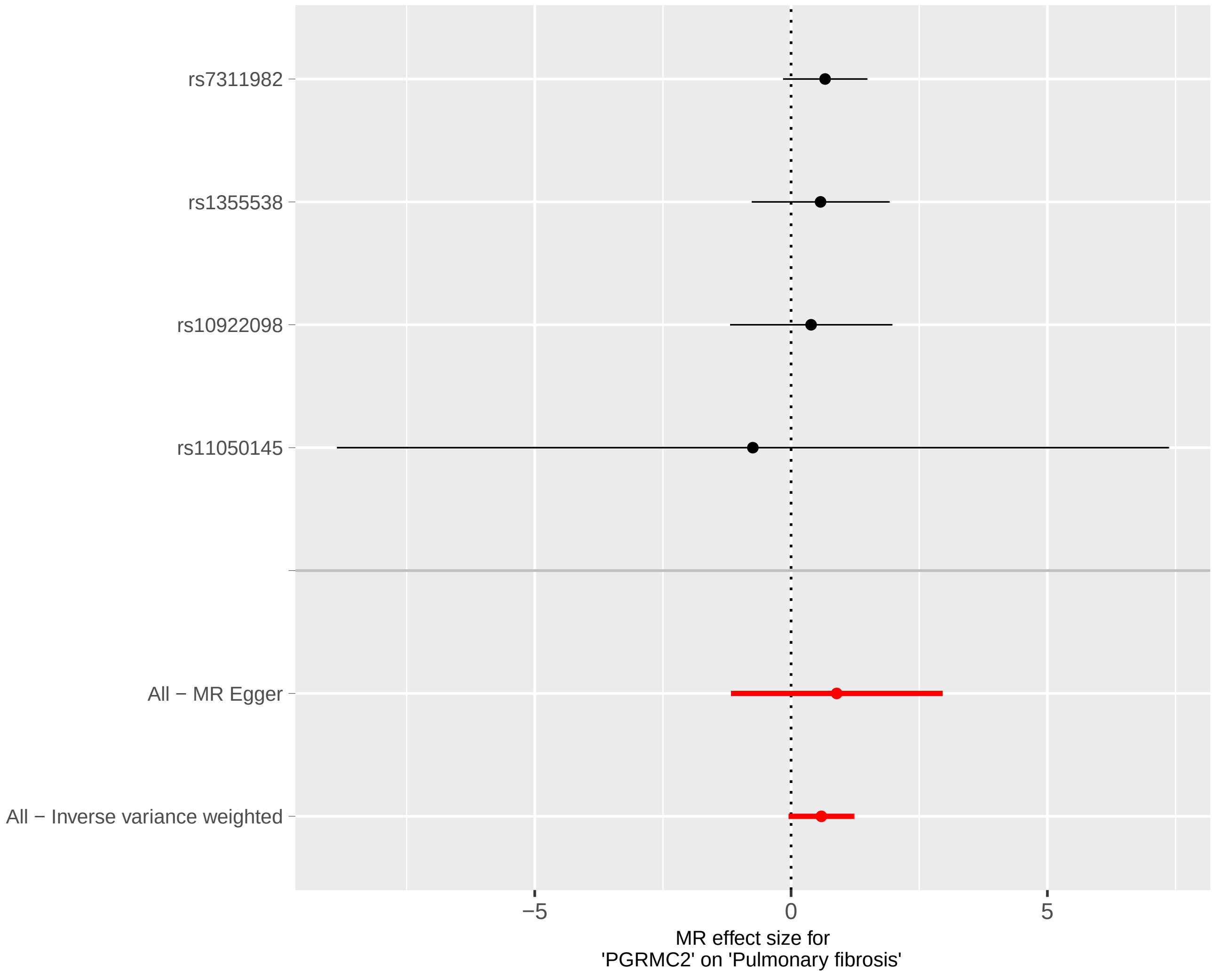

rs7311982
rs1355538
rs10922098
rs11050145
All − MR Egger
All − Inverse variance weighted
−5
0
5
MR effect size for
'PGRMC2' on 'Pulmonary fibrosis'

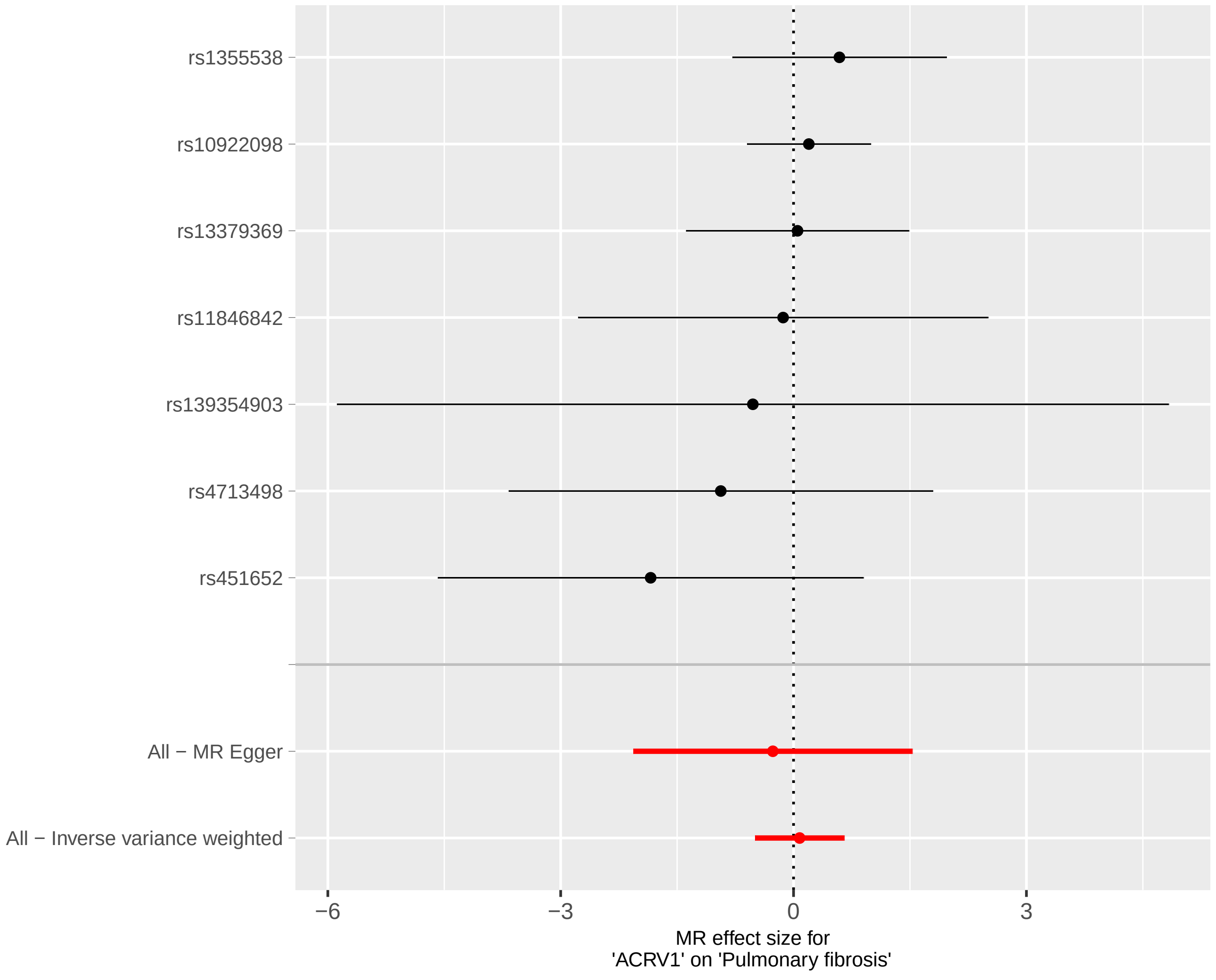

rs1355538
rs10922098
rs13379369
rs11846842
rs139354903
rs4713498
rs451652
All − MR Egger
All − Inverse variance weighted
−6
−3
0
3
MR effect size for
'ACRV1' on 'Pulmonary fibrosis'

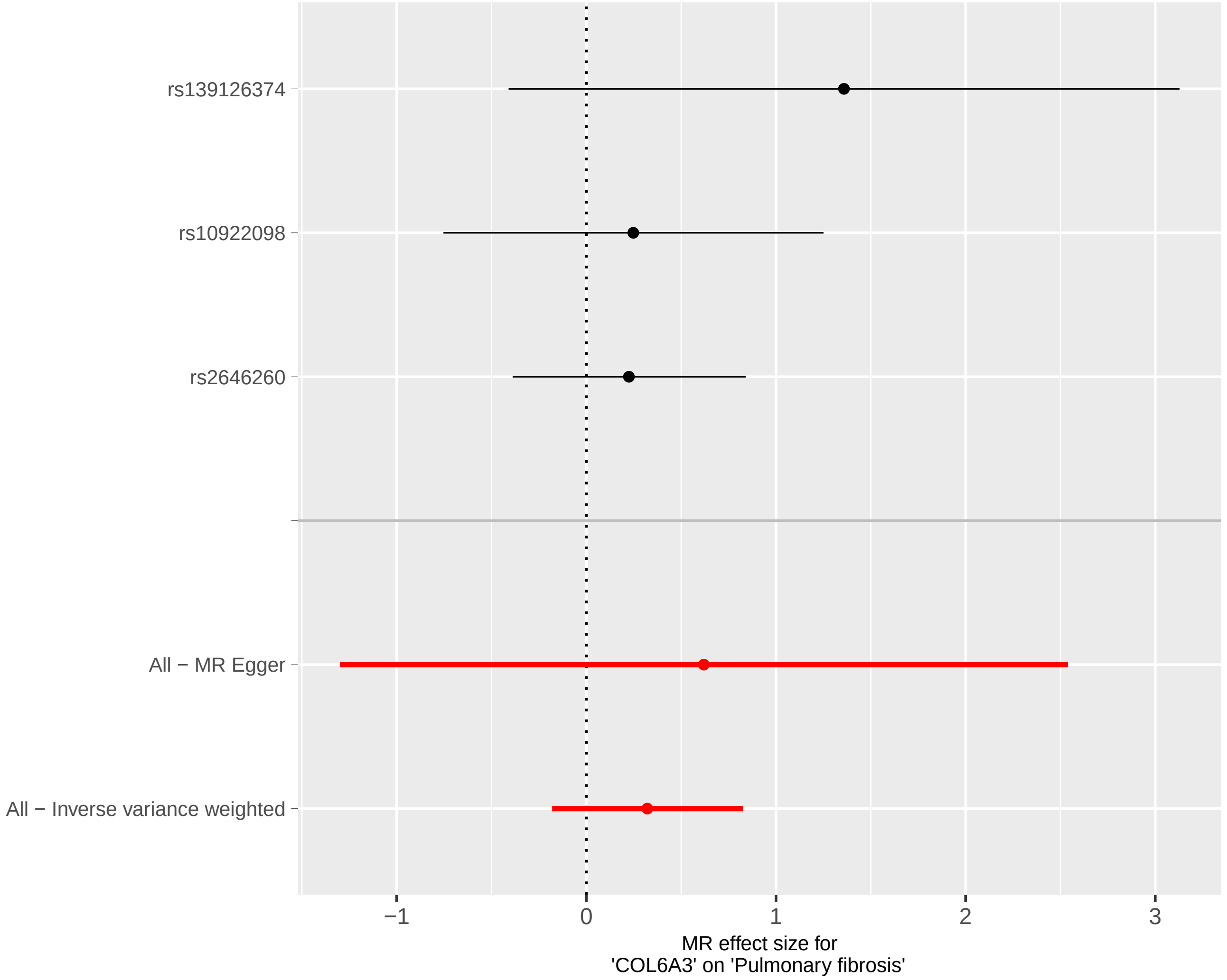
rs139126374
rs10922098
rs2646260
All − MR Egger
All − Inverse variance weighted
−1
0
1
2
3
MR effect size for
'COL6A3' on 'Pulmonary fibrosis'

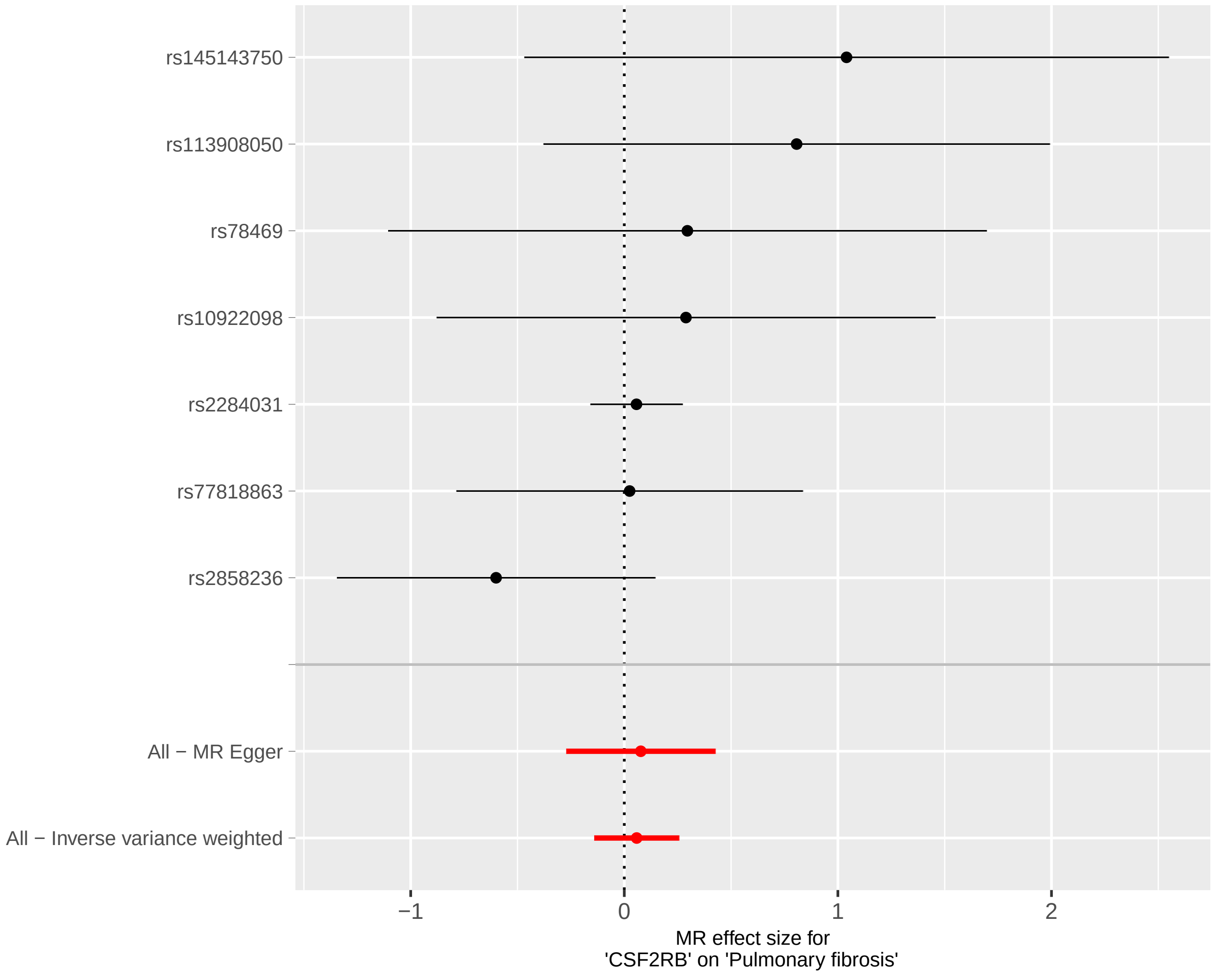
rs145143750
rs113908050
rs78469
rs10922098
rs2284031
rs77818863
rs2858236
All − MR Egger
All − Inverse variance weighted
−1
0
1
2
MR effect size for
'CSF2RB' on 'Pulmonary fibrosis'

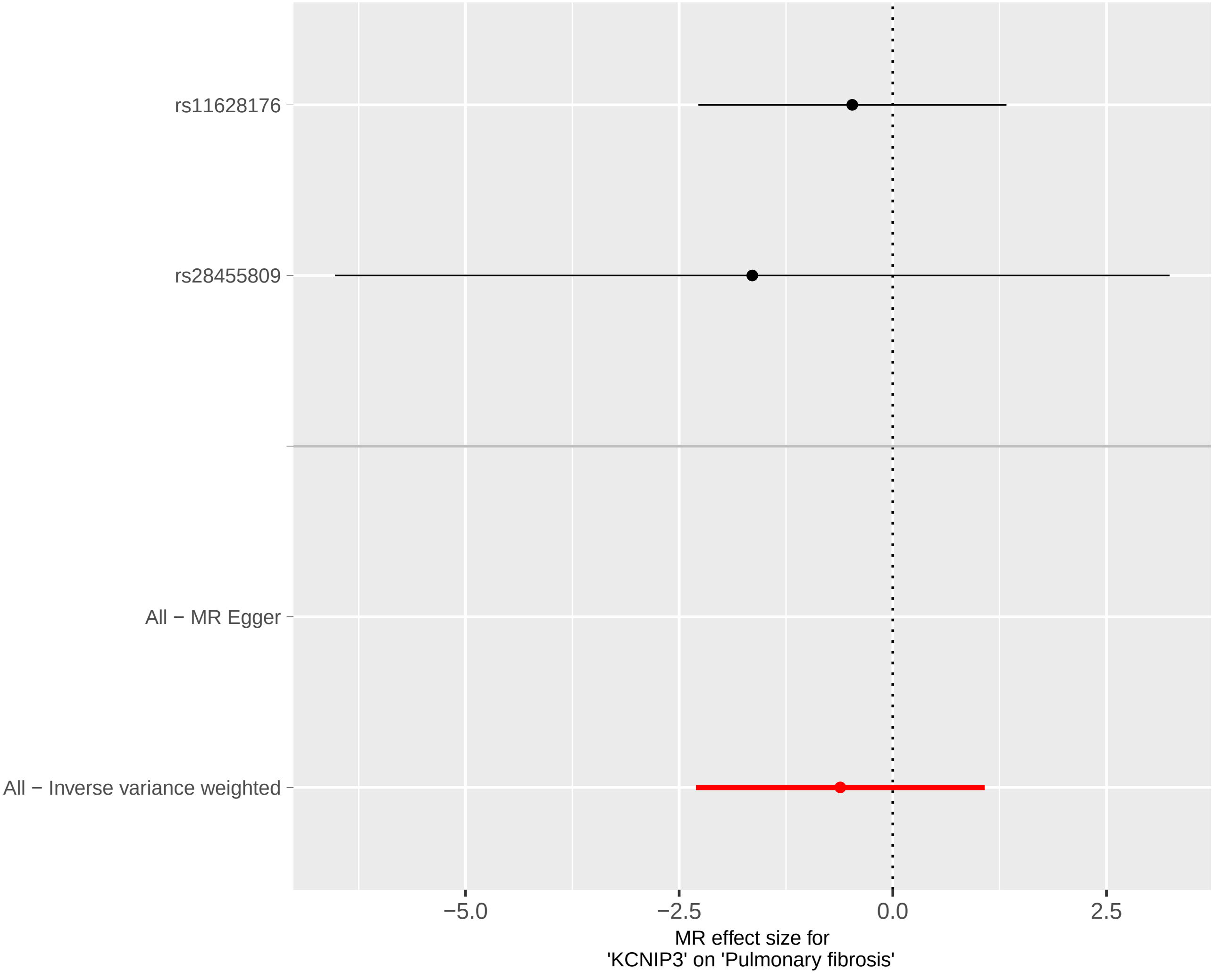
rs11628176
rs28455809
All − MR Egger
All − Inverse variance weighted
−5.0
−2.5
0.0
2.5
MR effect size for
'KCNIP3' on 'Pulmonary fibrosis'

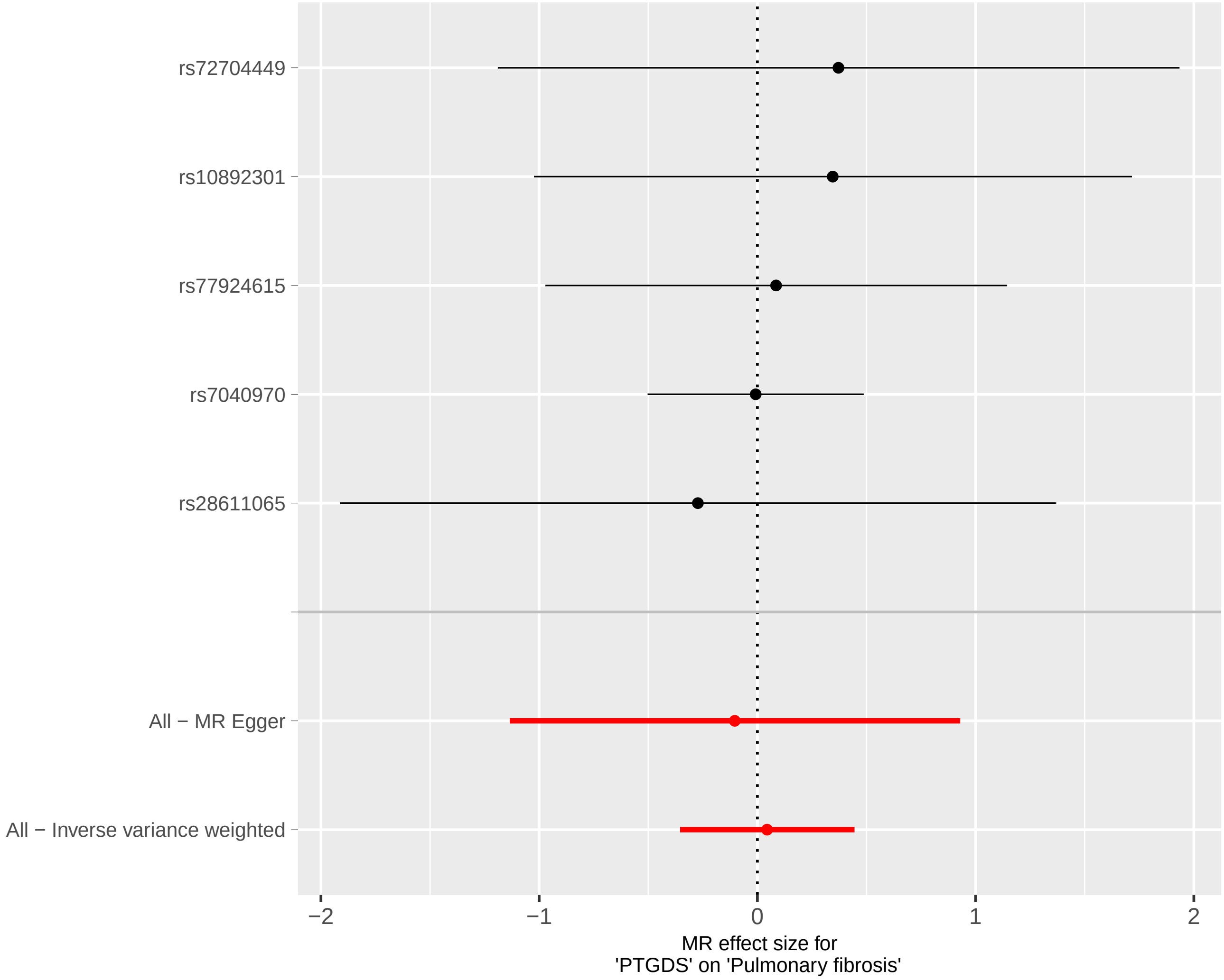
rs72704449
rs10892301
rs77924615
rs7040970
rs28611065
All − MR Egger
All − Inverse variance weighted
−2
−1
0
1
2
MR effect size for
'PTGDS' on 'Pulmonary fibrosis'

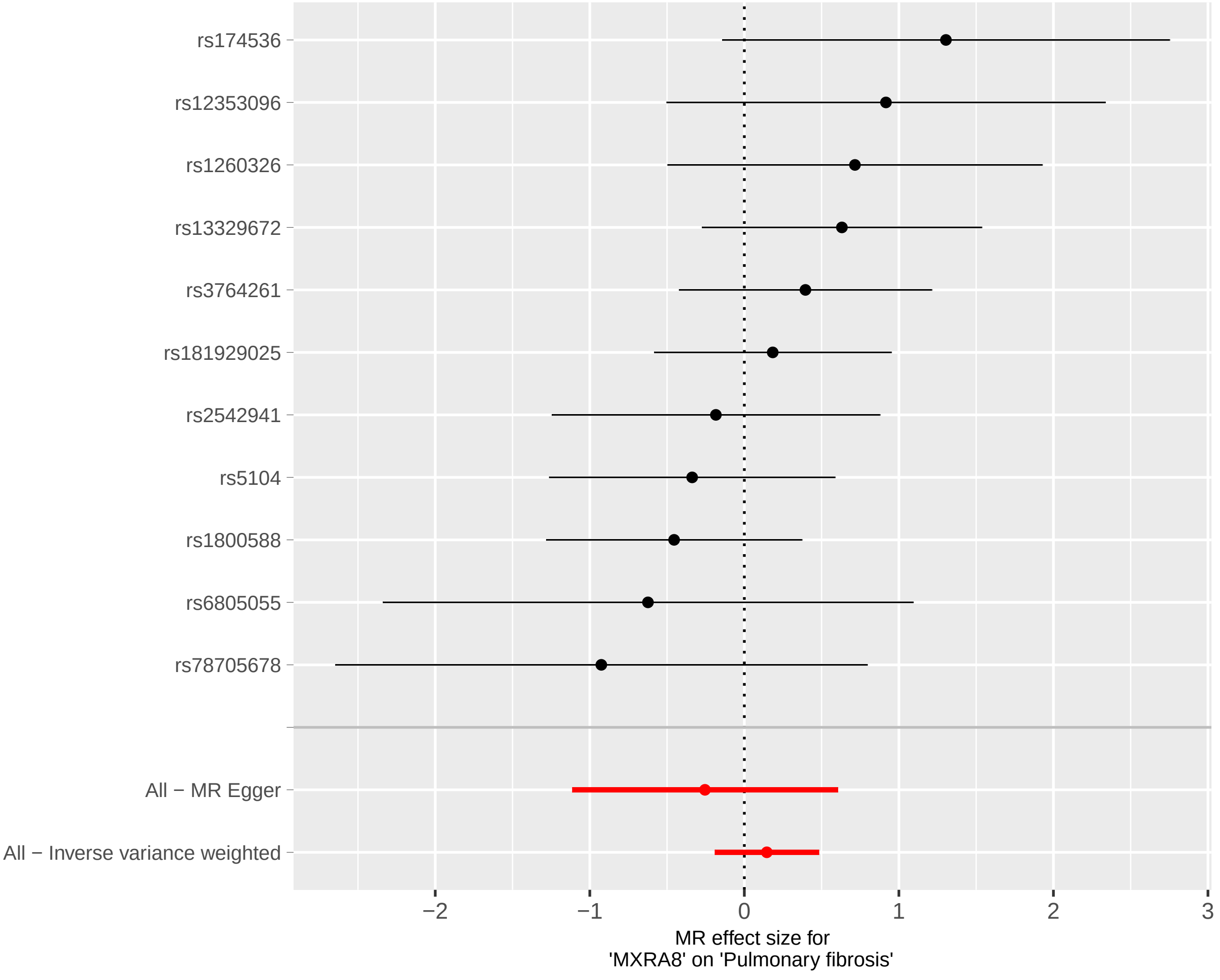
rs174536
rs12353096
rs1260326
rs13329672
rs3764261
rs181929025
rs2542941
rs5104
rs1800588
rs6805055
rs78705678
All − MR Egger
All − Inverse variance weighted
−2
−1
0
1
2
3
MR effect size for
'MXRA8' on 'Pulmonary fibrosis'

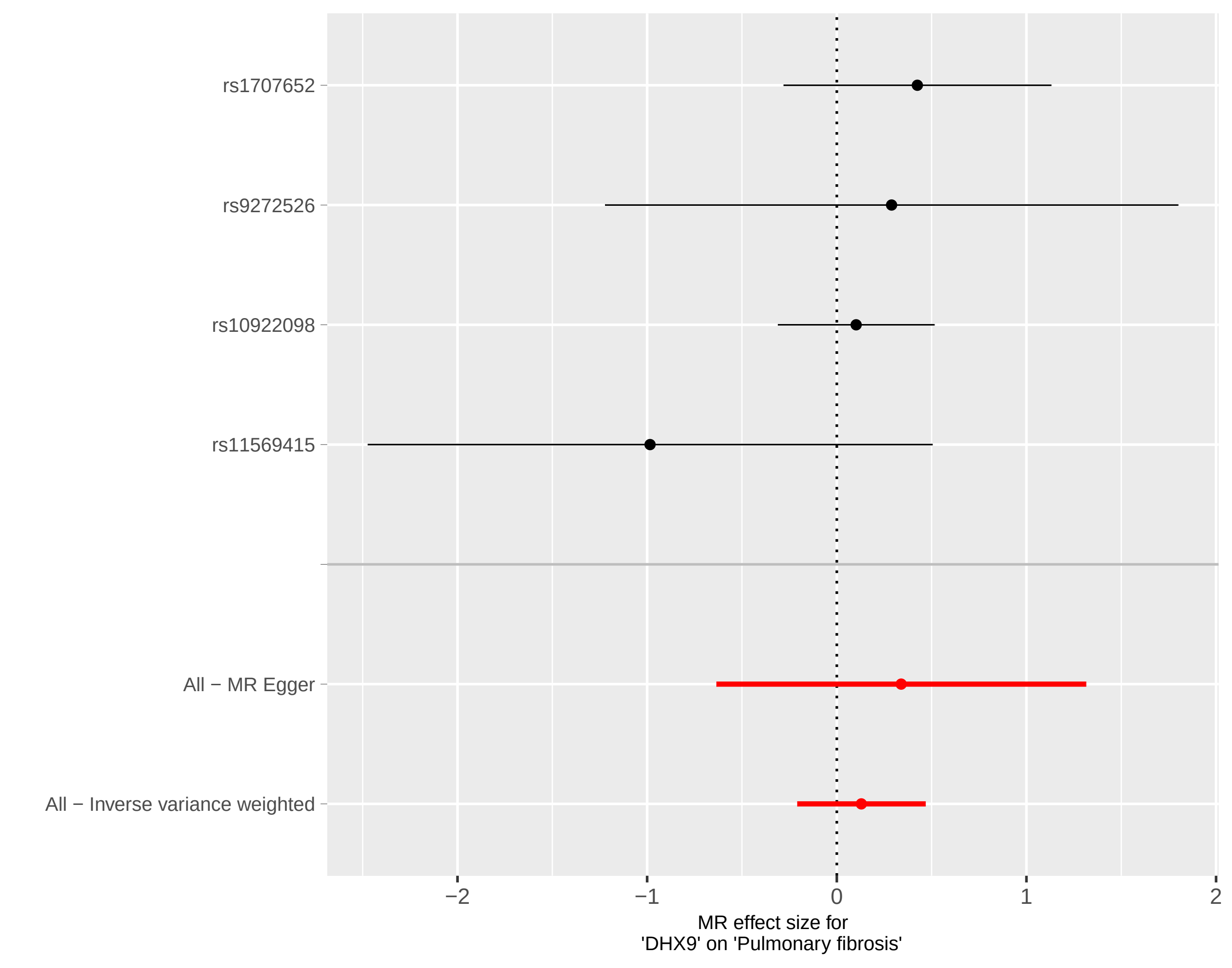

rs1707652
rs9272526
rs10922098
rs11569415
All − MR Egger
All − Inverse variance weighted
−2
−1
0
1
2
MR effect size for
'DHX9' on 'Pulmonary fibrosis'

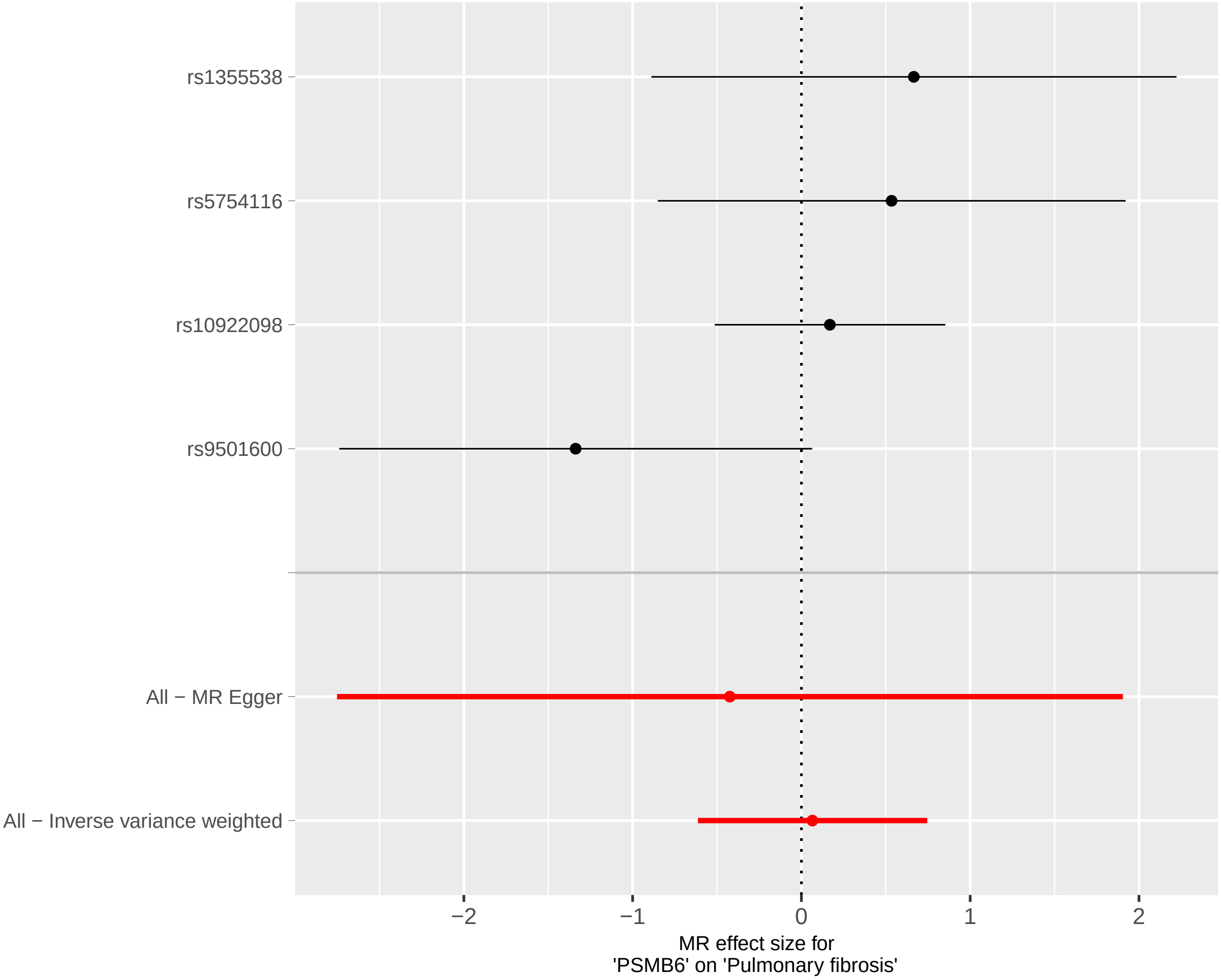
rs1355538
rs5754116
rs10922098
rs9501600
All − MR Egger
All − Inverse variance weighted
−2
−1
0
1
2
MR effect size for
'PSMB6' on 'Pulmonary fibrosis'

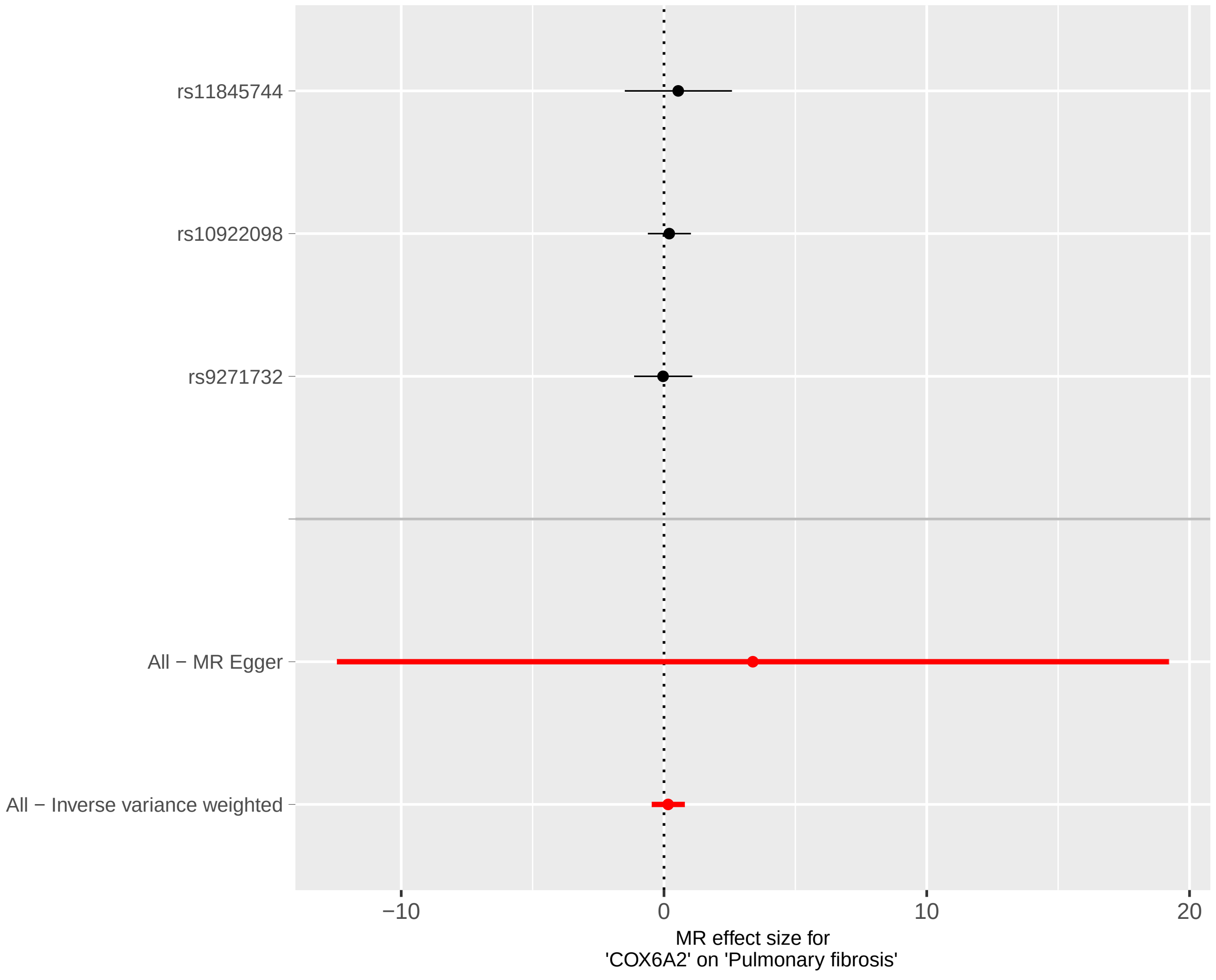

rs11845744
rs10922098
rs9271732
All − MR Egger
All − Inverse variance weighted
−10
0
10
20
MR effect size for
'COX6A2' on 'Pulmonary fibrosis'

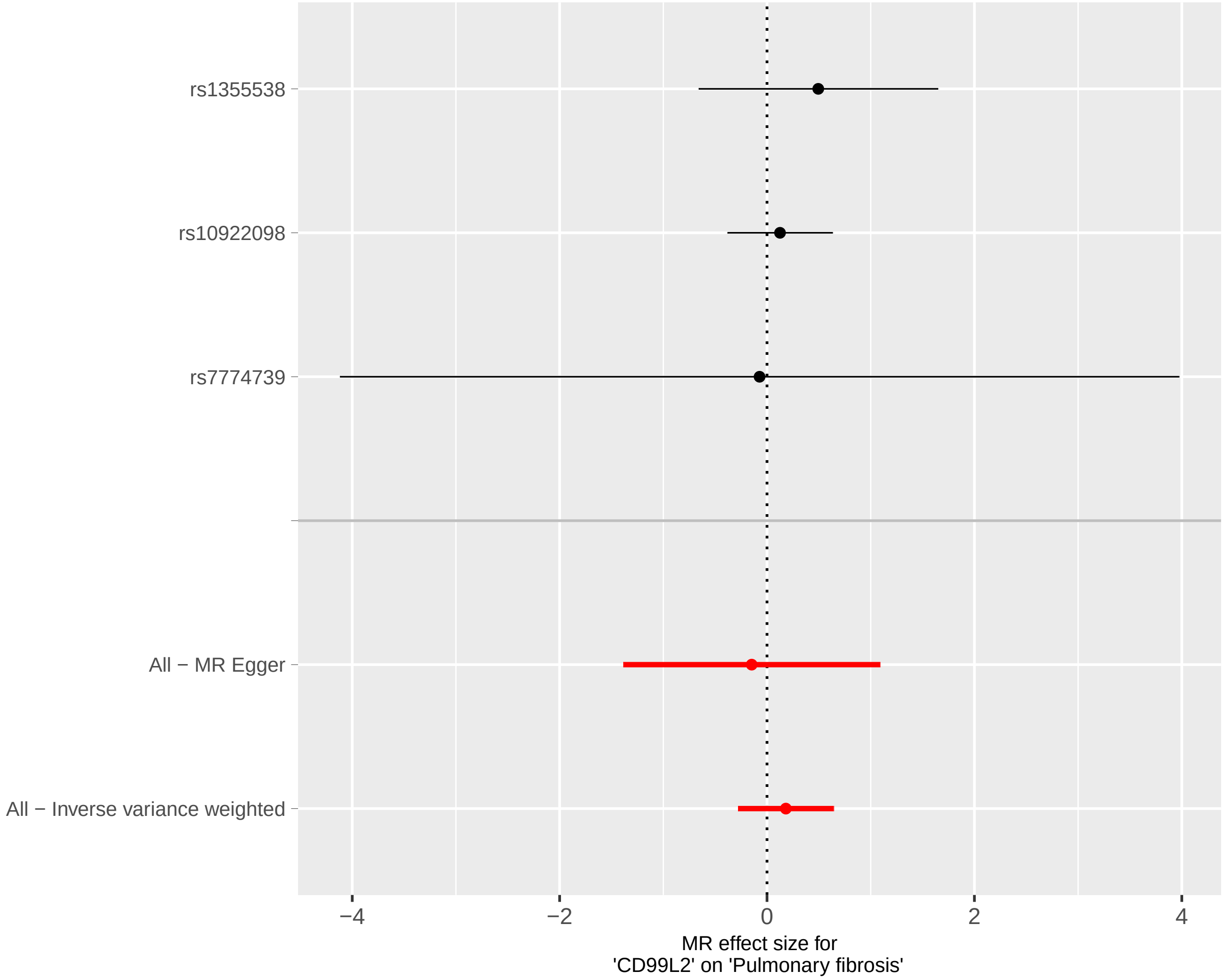
rs1355538
rs10922098
rs7774739
All − MR Egger
All − Inverse variance weighted
−4
−2
0
2
4
MR effect size for
'CD99L2' on 'Pulmonary fibrosis'

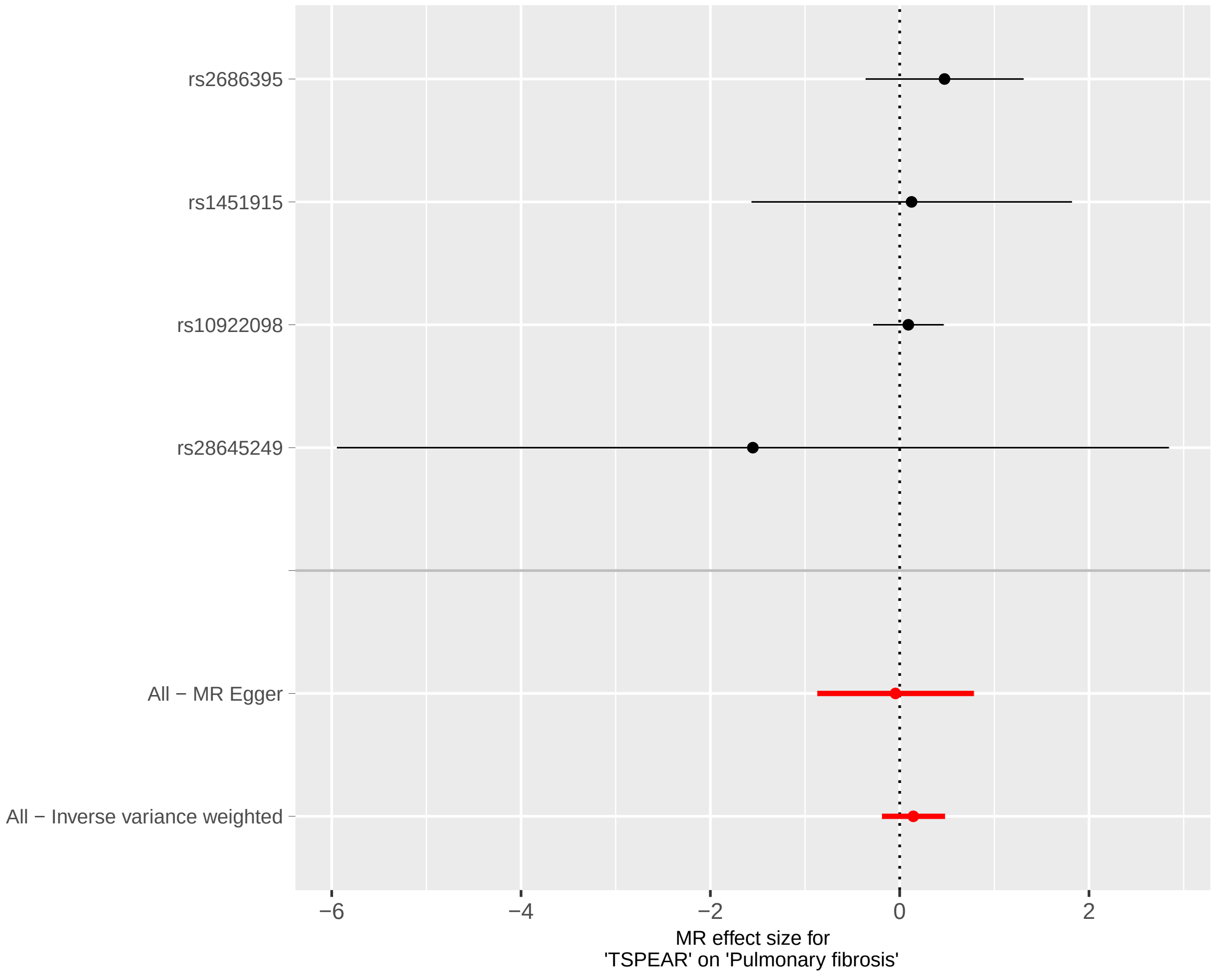

rs2686395
rs1451915
rs10922098
rs28645249
All − MR Egger
All − Inverse variance weighted
−6
−4
−2
0
2
MR effect size for
'TSPEAR' on 'Pulmonary fibrosis'

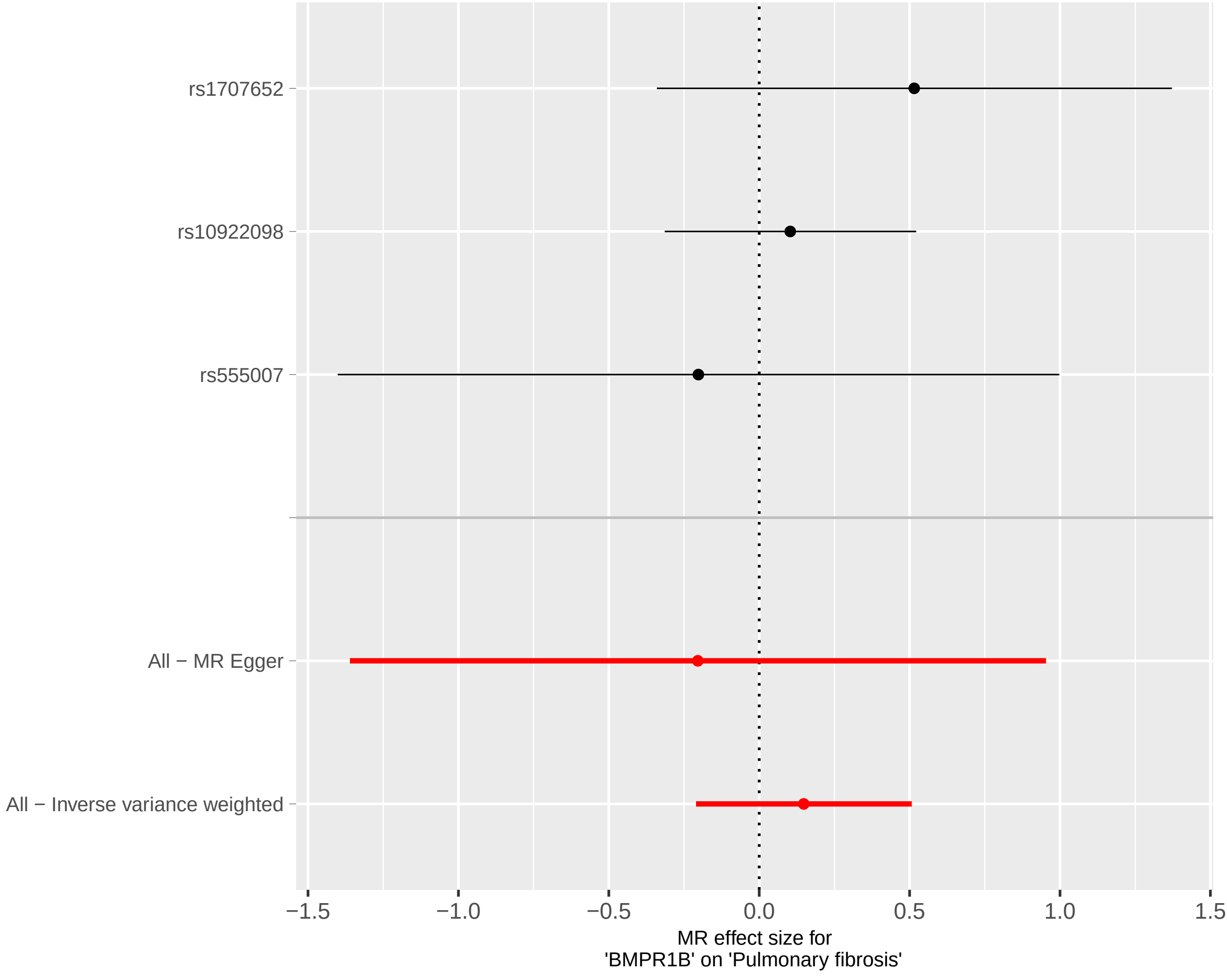
rs1707652
rs10922098
rs555007
All − MR Egger
All − Inverse variance weighted
−1.5
−1.0
−0.5
0.0
0.5
1.0
1.5
MR effect size for
'BMPR1B' on 'Pulmonary fibrosis'

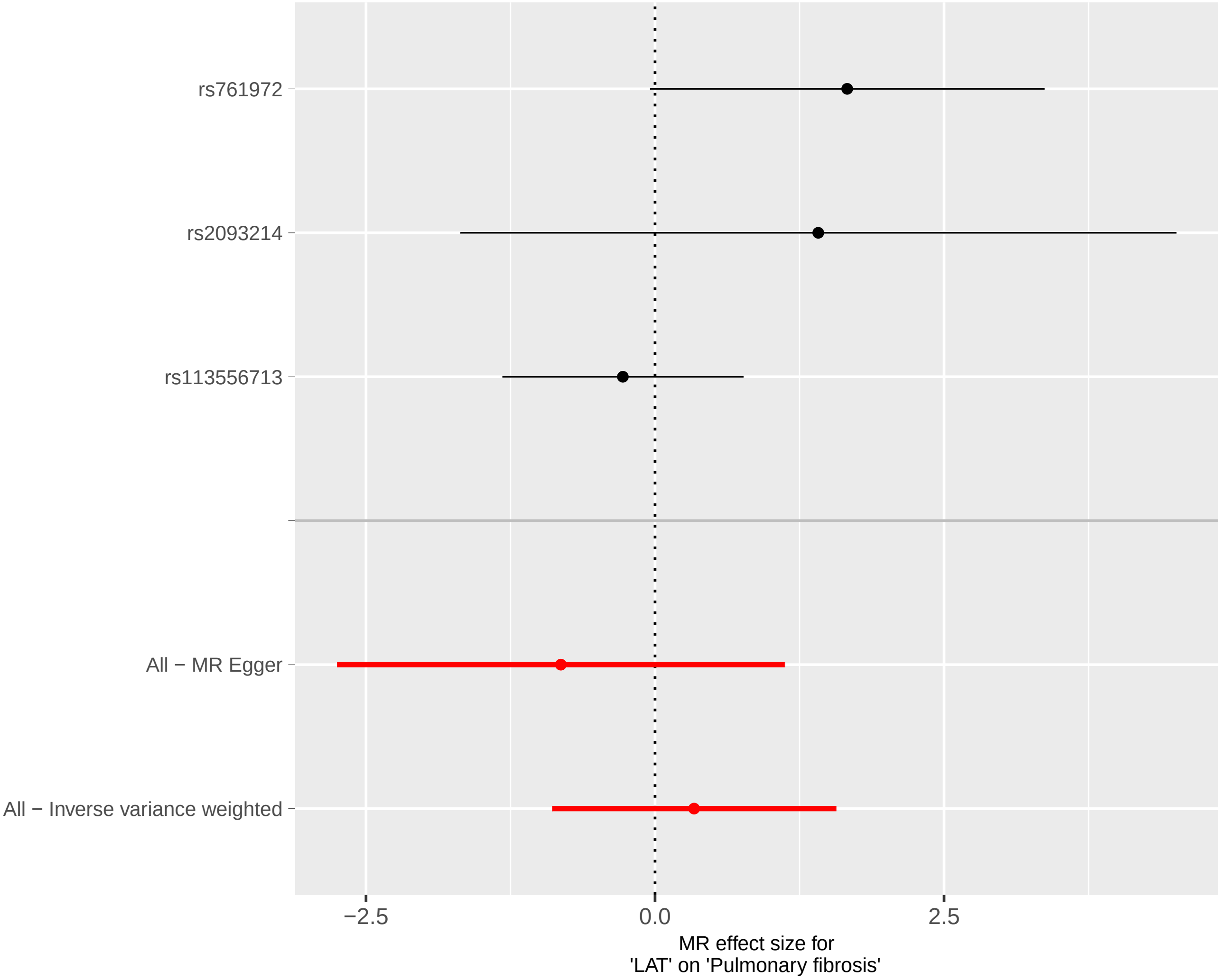
rs761972
rs2093214
rs113556713
All − MR Egger
All − Inverse variance weighted
−2.5
0.0
2.5
MR effect size for
'LAT' on 'Pulmonary fibrosis'

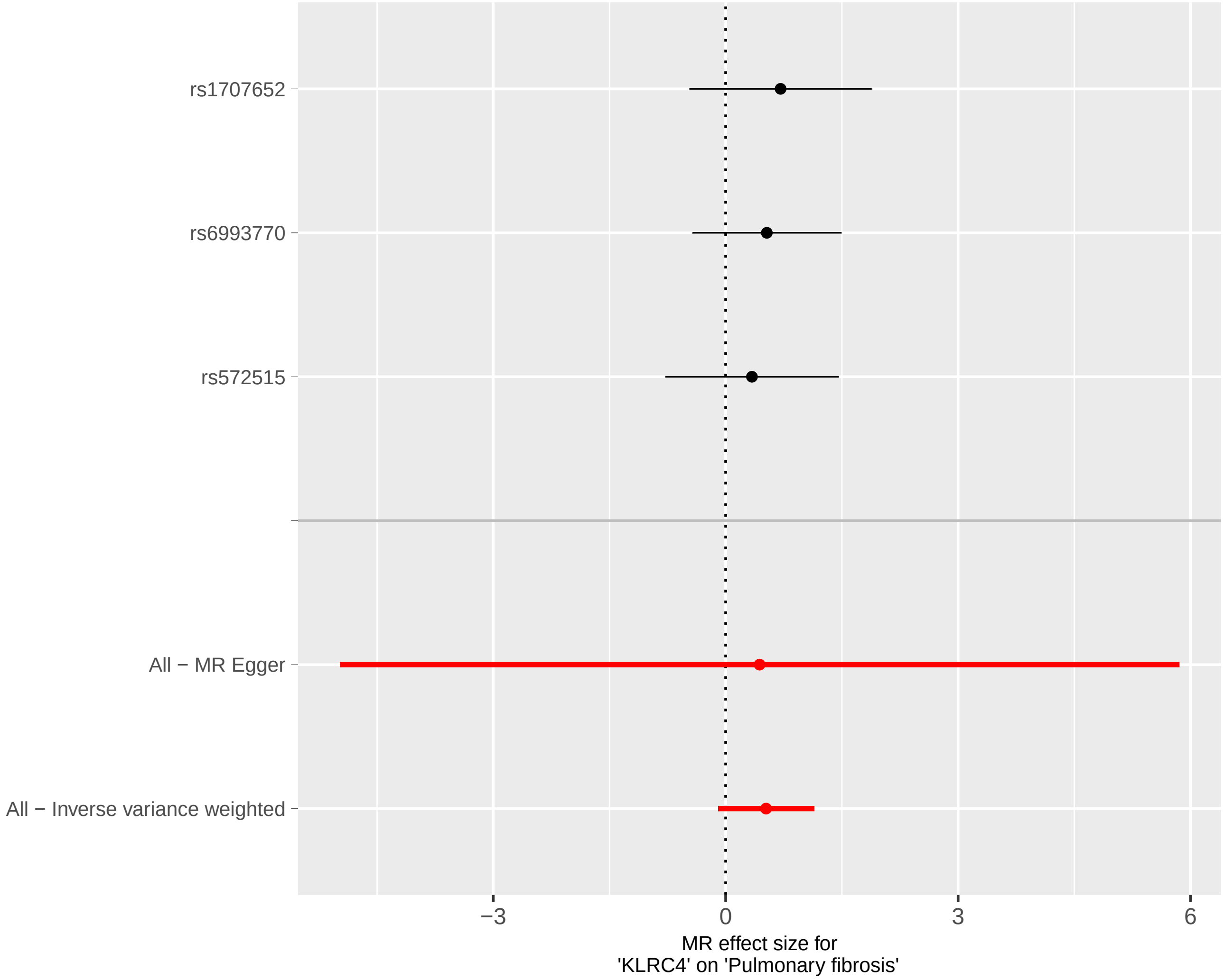
rs1707652
rs6993770
rs572515
All − MR Egger
All − Inverse variance weighted
−3
0
3
6
MR effect size for
'KLRC4' on 'Pulmonary fibrosis'

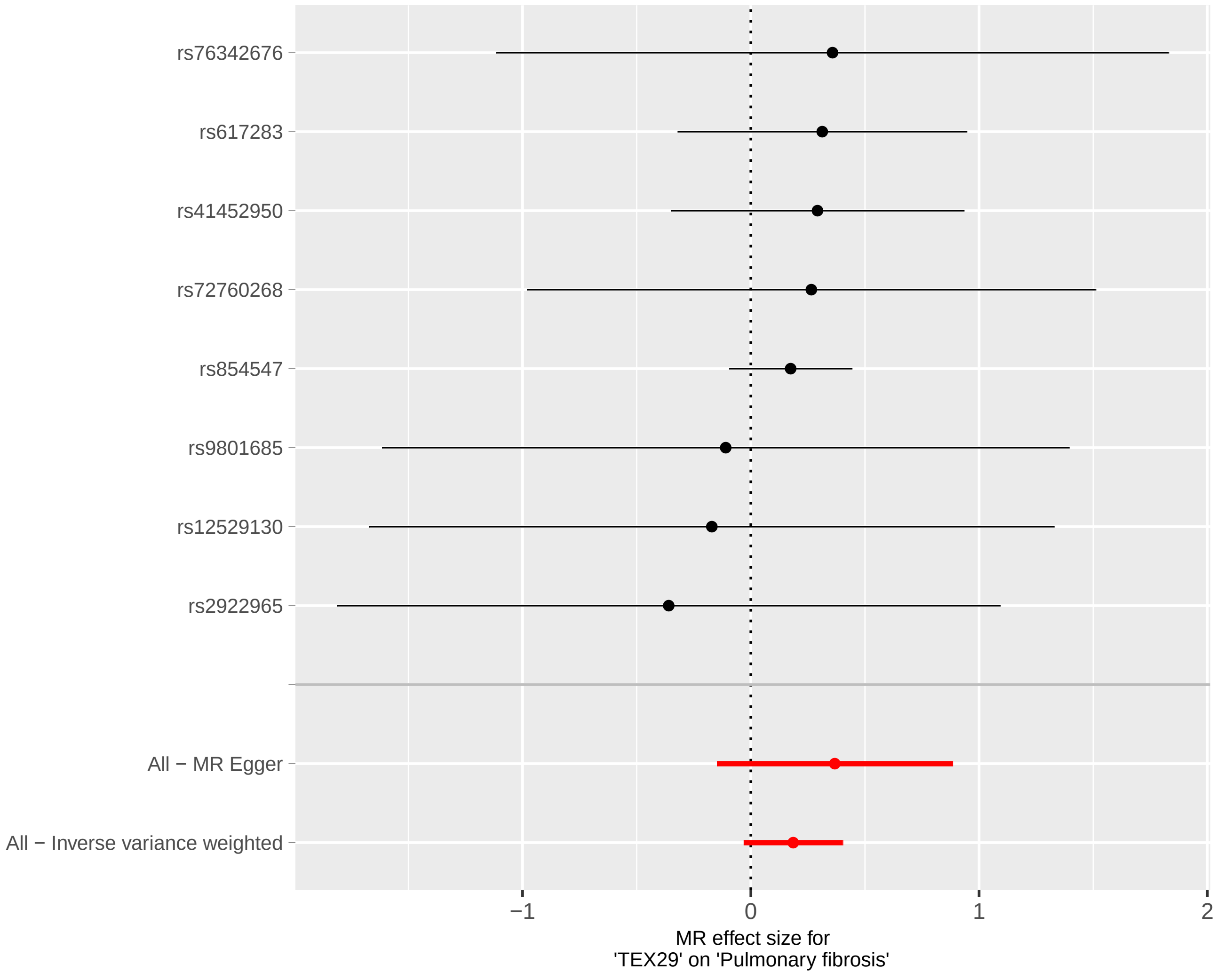
rs76342676
rs617283
rs41452950
rs72760268
rs854547
rs9801685
rs12529130
rs2922965
All − MR Egger
All − Inverse variance weighted
−1
0
1
2
MR effect size for
'TEX29' on 'Pulmonary fibrosis'

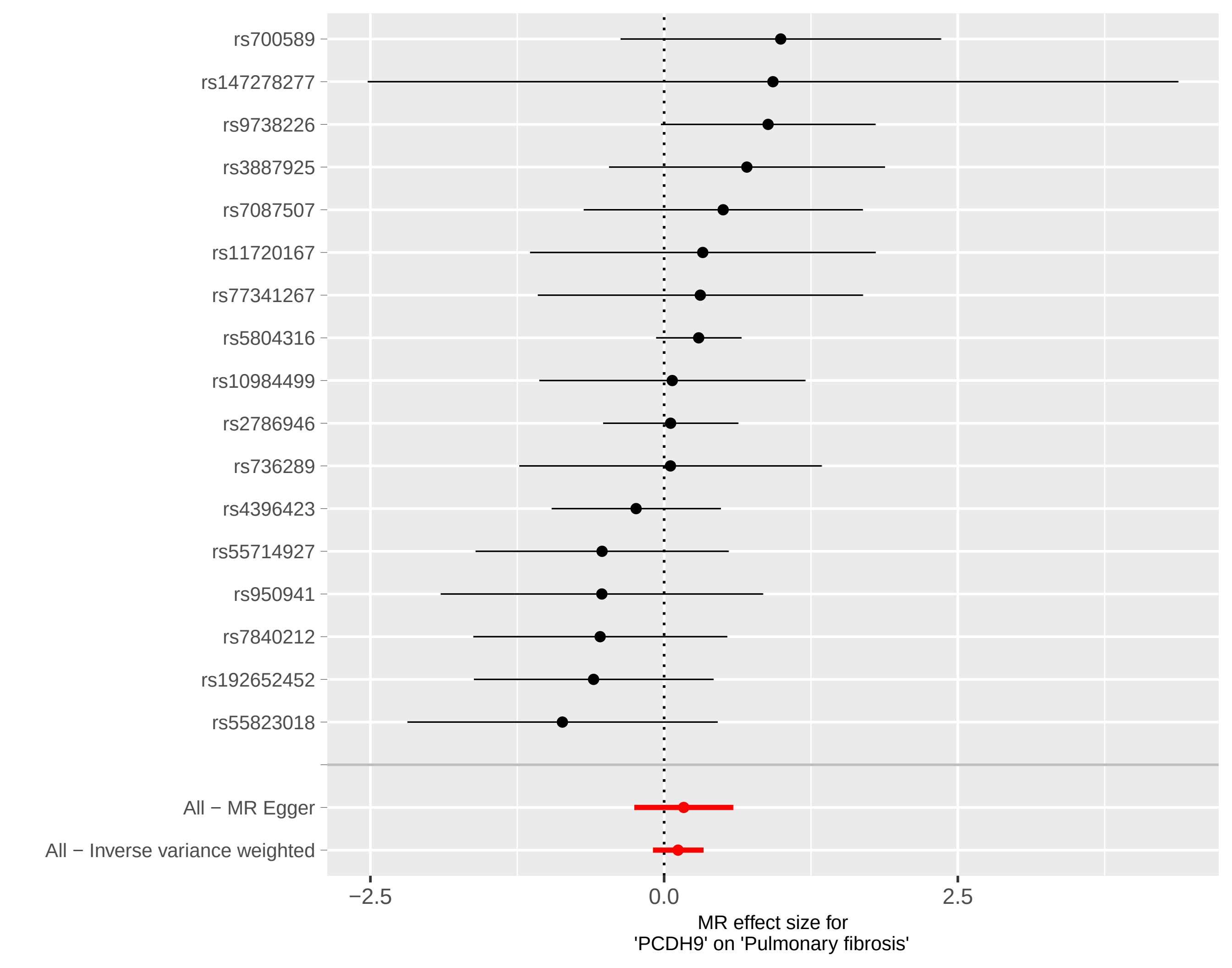
rs700589
rs147278277
rs9738226
rs3887925
rs7087507
rs11720167
rs77341267
rs5804316
rs10984499
rs2786946
rs736289
rs4396423
rs55714927
rs950941
rs7840212
rs192652452
rs55823018
All − MR Egger
All − Inverse variance weighted
−2.5
0.0
2.5
MR effect size for
'PCDH9' on 'Pulmonary fibrosis'

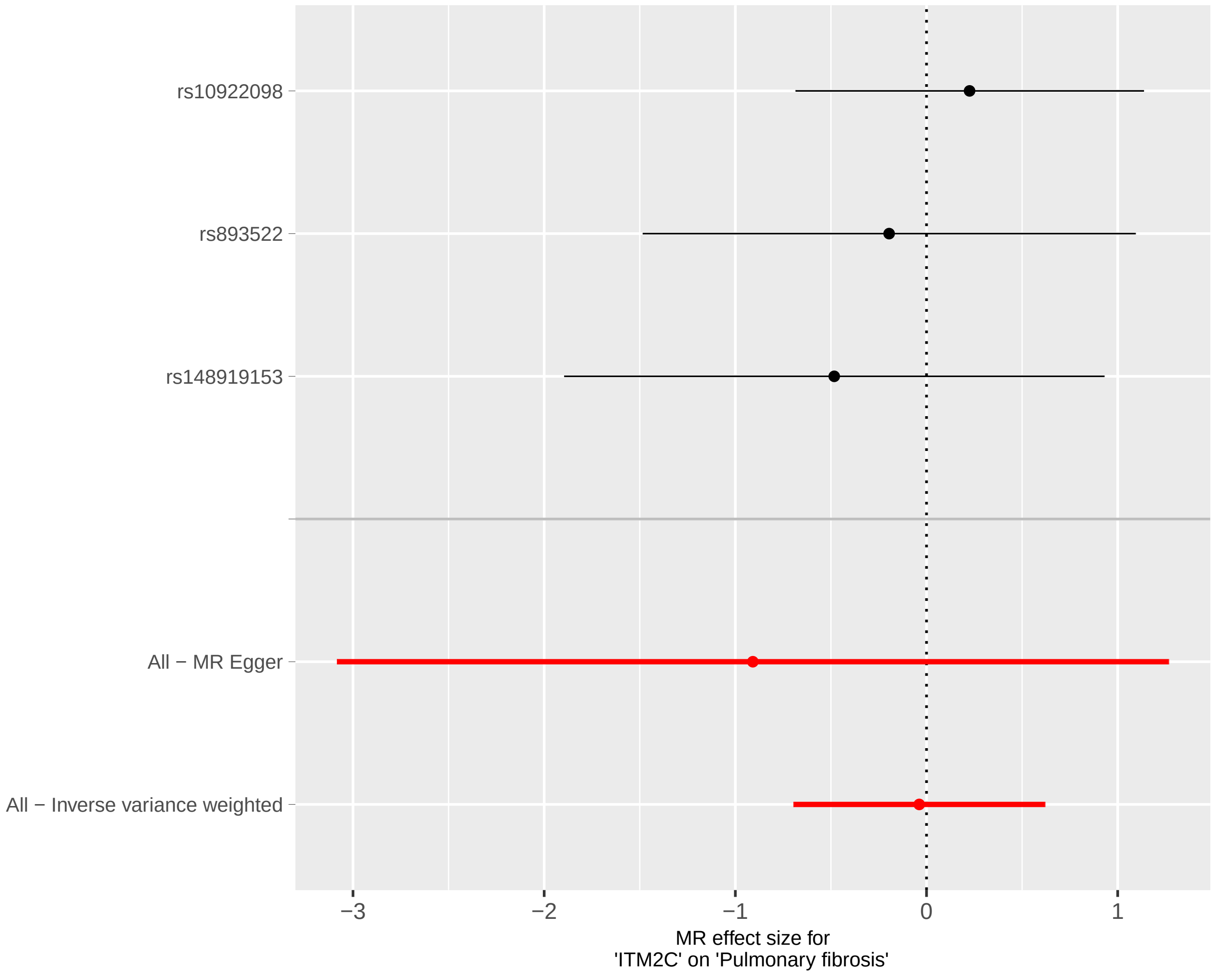
rs10922098
rs893522
rs148919153
All − MR Egger
All − Inverse variance weighted
−3
−2
−1
0
1
MR effect size for
'ITM2C' on 'Pulmonary fibrosis'

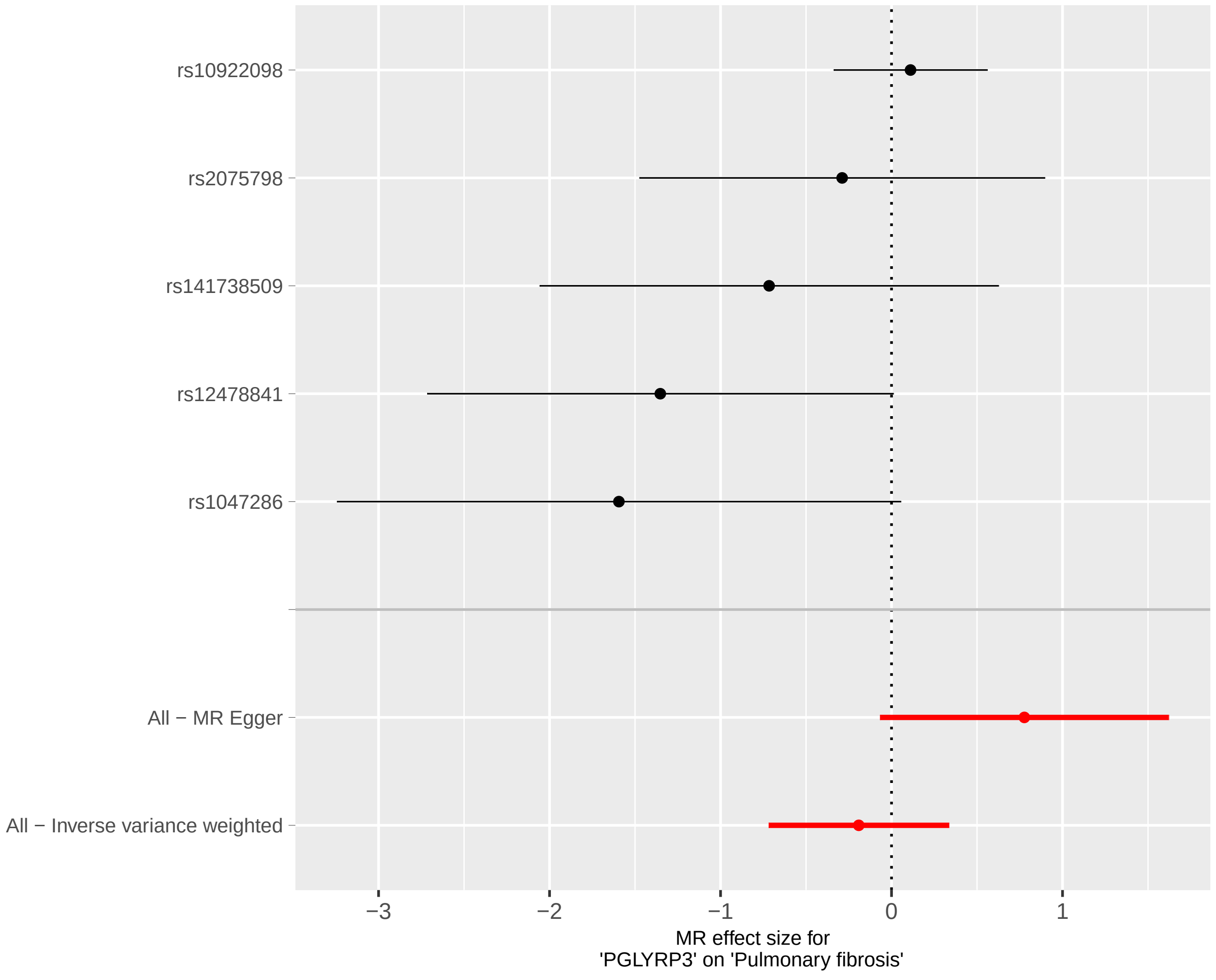
rs10922098
rs2075798
rs141738509
rs12478841
rs1047286
All − MR Egger
All − Inverse variance weighted
−3
−2
−1
0
1
MR effect size for
'PGLYRP3' on 'Pulmonary fibrosis'

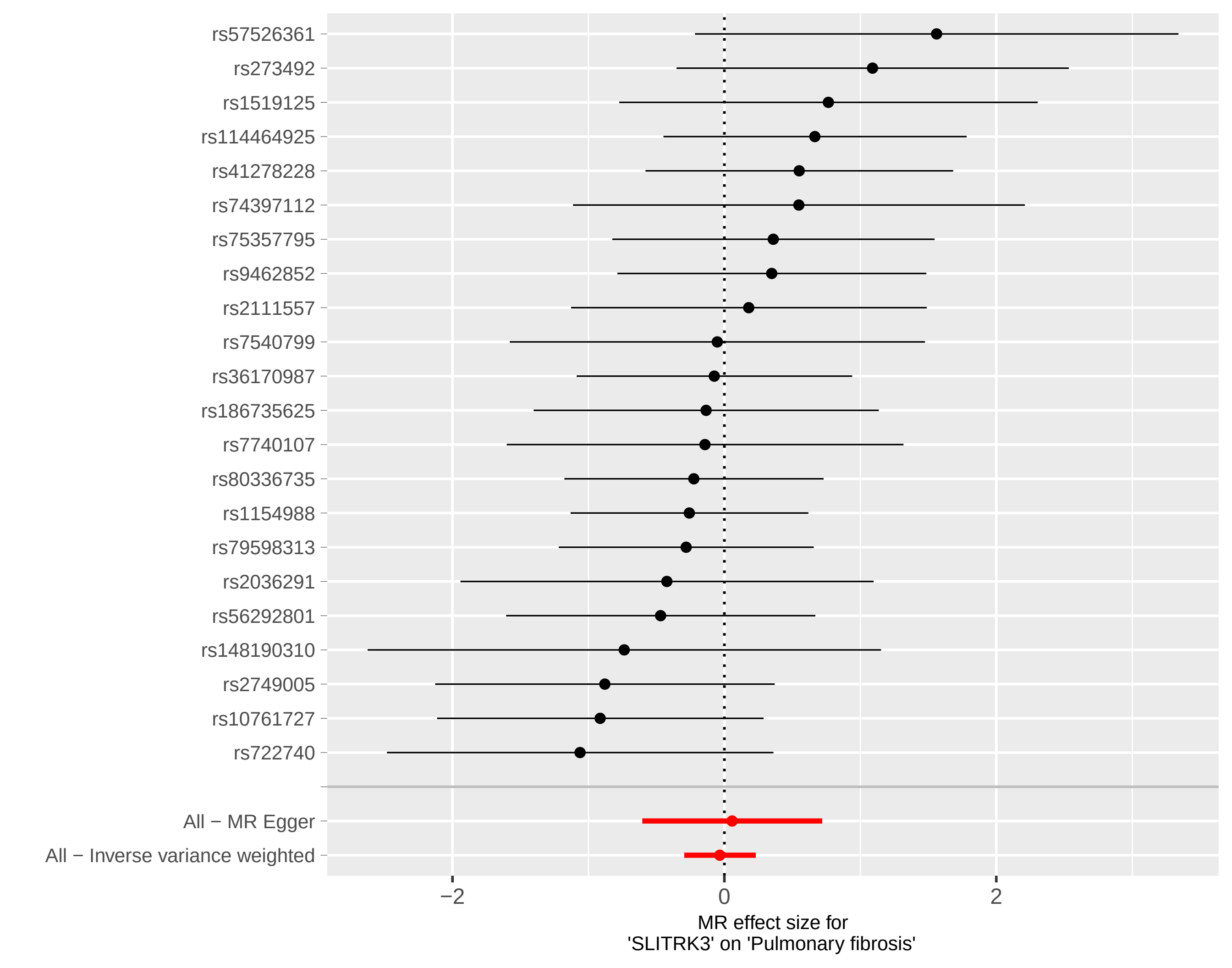
rs57526361
rs273492
rs1519125
rs114464925
rs41278228
rs74397112
rs75357795
rs9462852
rs2111557
rs7540799
rs36170987
rs186735625
rs7740107
rs80336735
rs1154988
rs79598313
rs2036291
rs56292801
rs148190310
rs2749005
rs10761727
rs722740
All − MR Egger
All − Inverse variance weighted
−2
0
2
MR effect size for
'SLITRK3' on 'Pulmonary fibrosis'

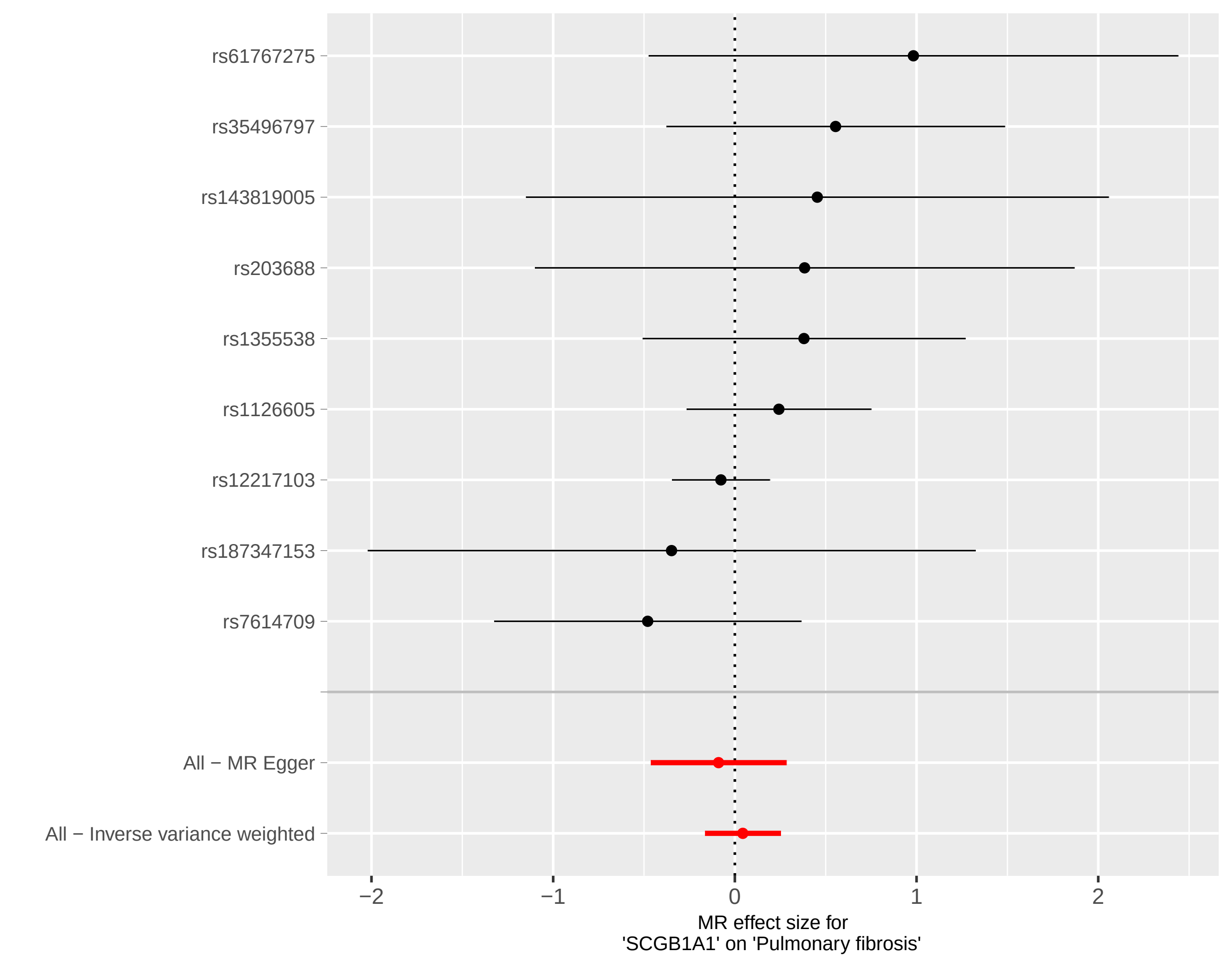

rs61767275
rs35496797
rs143819005
rs203688
rs1355538
rs1126605
rs12217103
rs187347153
rs7614709
All − MR Egger
All − Inverse variance weighted
−2
−1
0
1
2
MR effect size for
'SCGB1A1' on 'Pulmonary fibrosis'

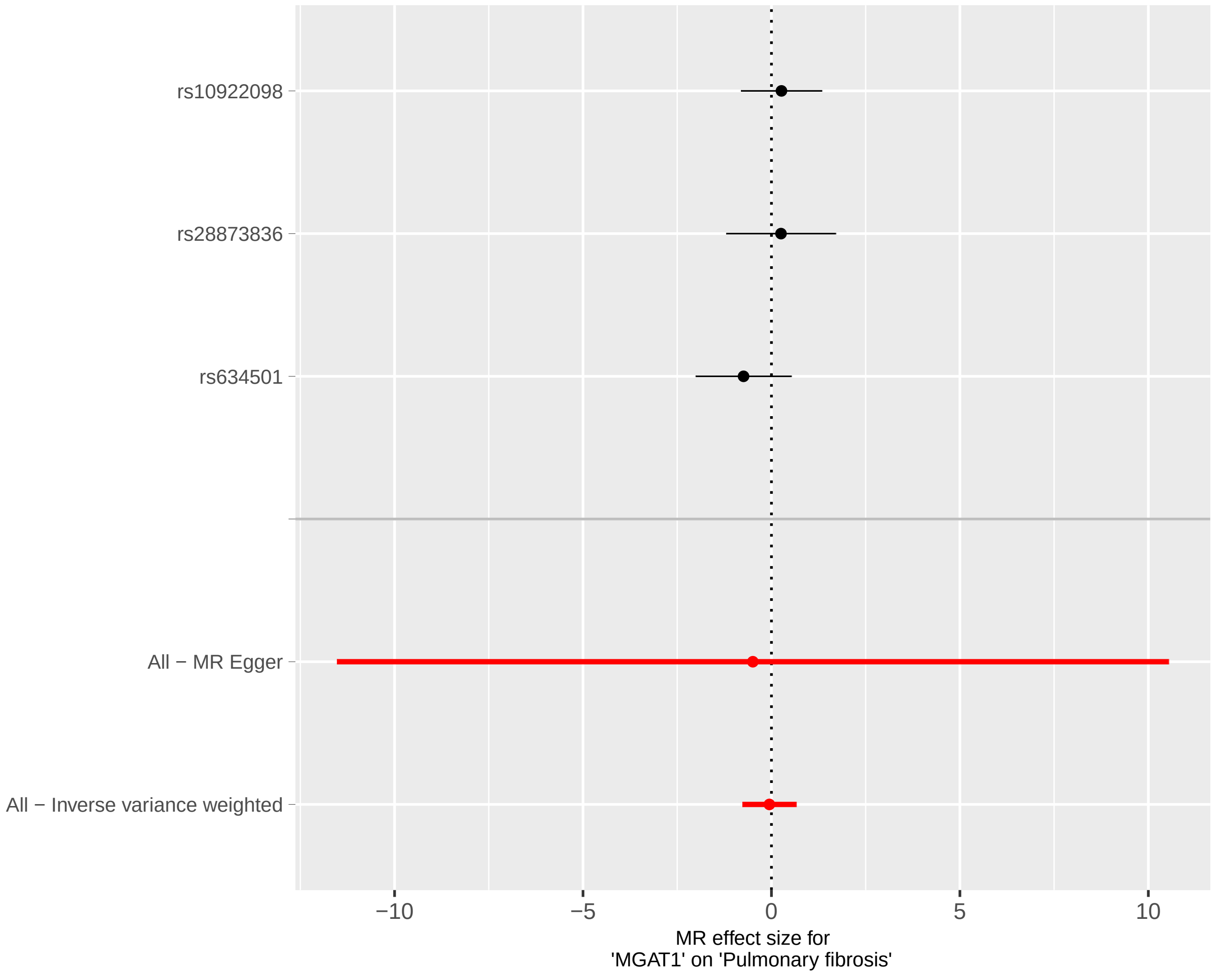
rs10922098
rs28873836
rs634501
All − MR Egger
All − Inverse variance weighted
−10
−5
0
5
10
MR effect size for
'MGAT1' on 'Pulmonary fibrosis'

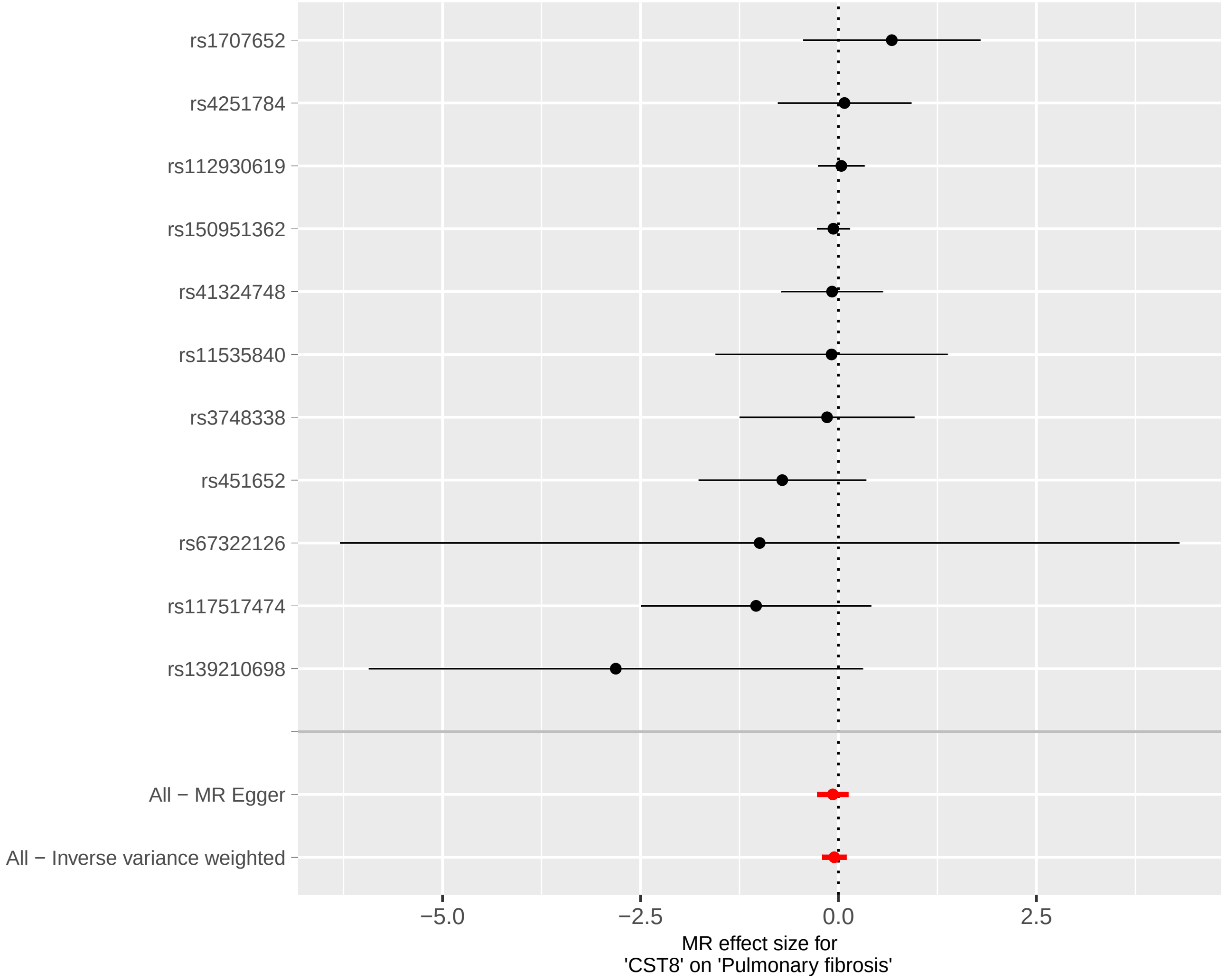
rs1707652
rs4251784
rs112930619
rs150951362
rs41324748
rs11535840
rs3748338
rs451652
rs67322126
rs117517474
rs139210698
All − MR Egger
All − Inverse variance weighted
−5.0
−2.5
0.0
2.5
MR effect size for
'CST8' on 'Pulmonary fibrosis'

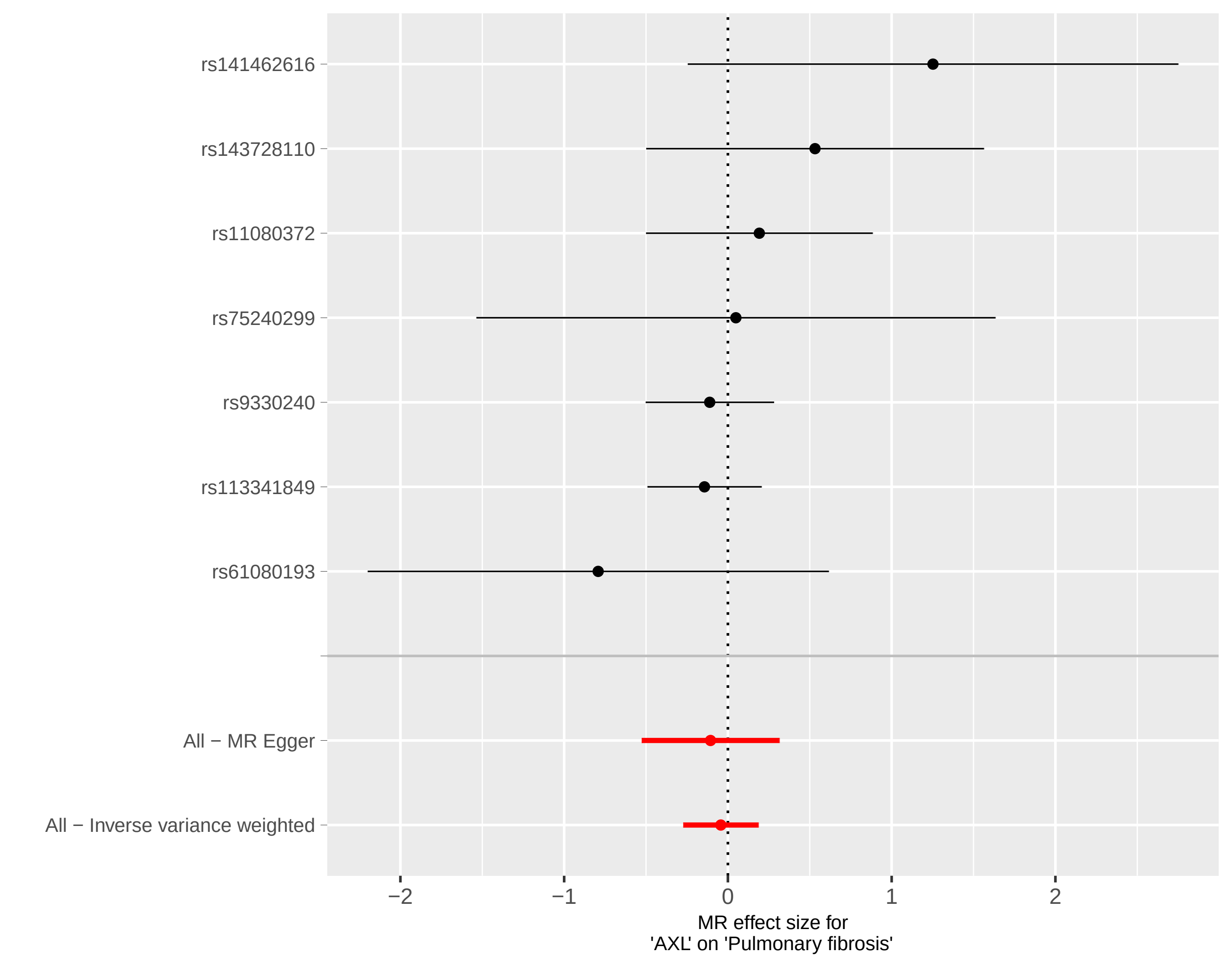

rs141462616
rs143728110
rs11080372
rs75240299
rs9330240
rs113341849
rs61080193
All − MR Egger
All − Inverse variance weighted
−2
−1
0
1
2
MR effect size for
'AXL' on 'Pulmonary fibrosis'

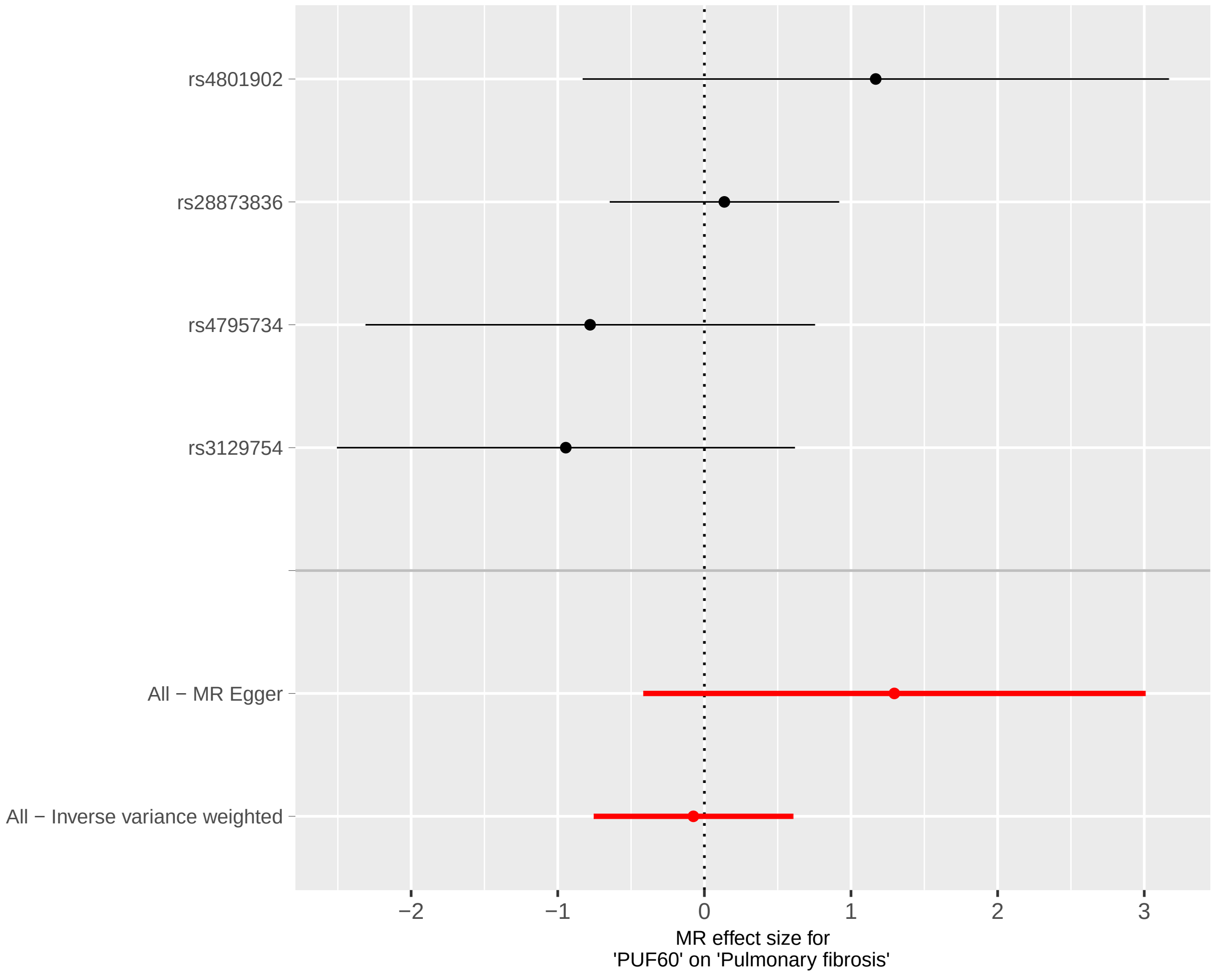
rs4801902
rs28873836
rs4795734
rs3129754
All − MR Egger
All − Inverse variance weighted
−2
−1
0
1
2
3
MR effect size for
'PUF60' on 'Pulmonary fibrosis'

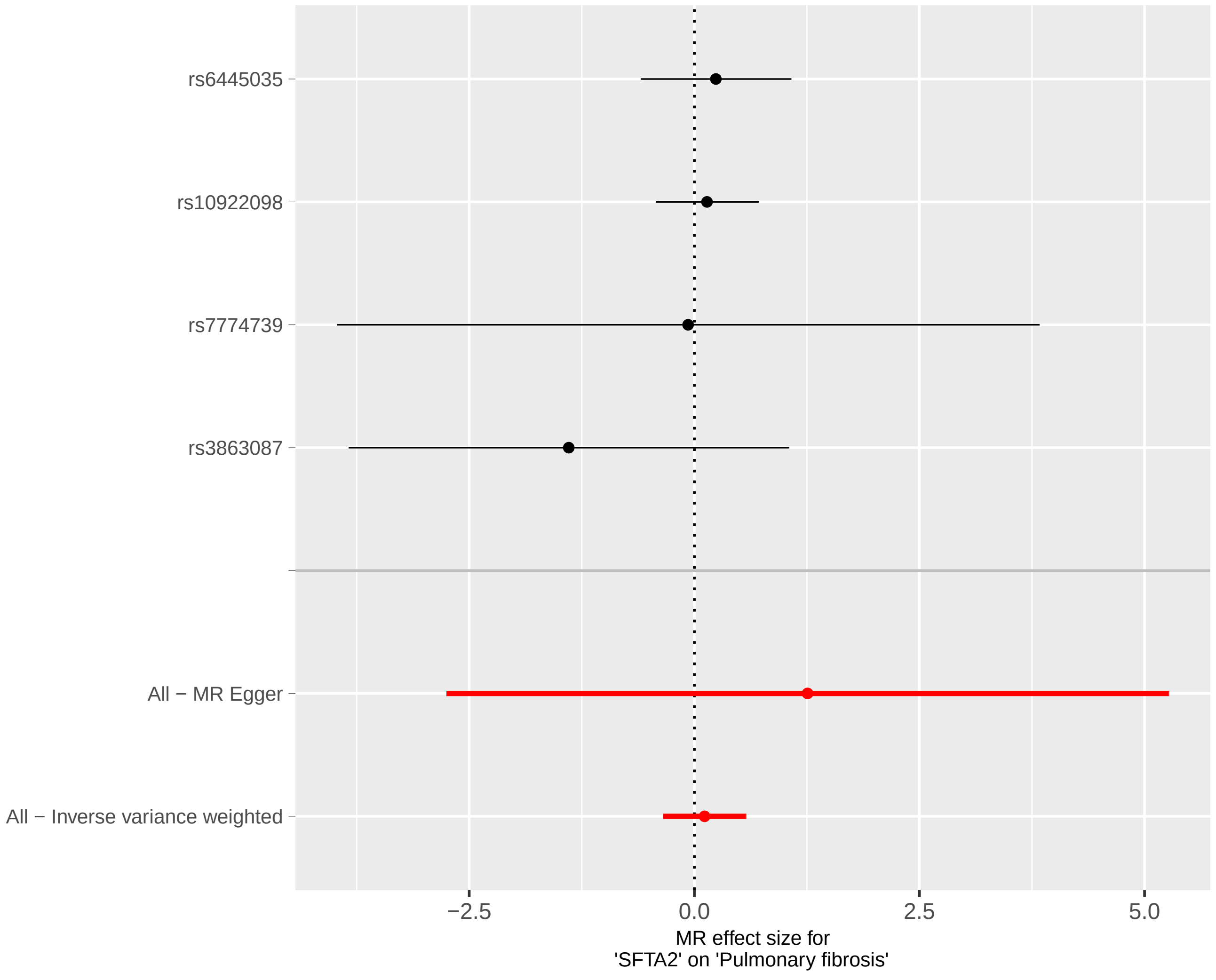
rs6445035
rs10922098
rs7774739
rs3863087
All − MR Egger
All − Inverse variance weighted
−2.5
0.0
2.5
5.0
MR effect size for
'SFTA2' on 'Pulmonary fibrosis'

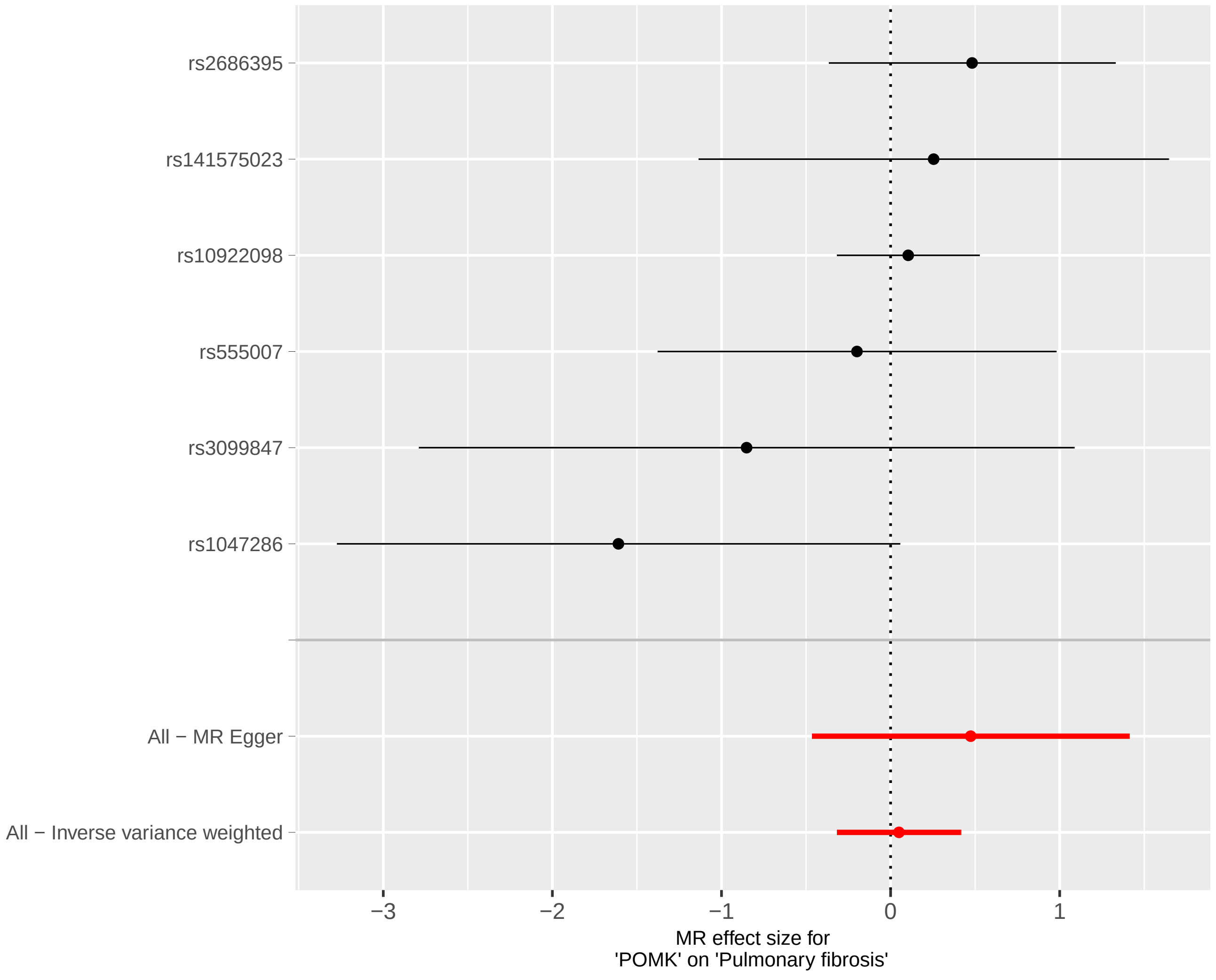

rs2686395
rs141575023
rs10922098
rs555007
rs3099847
rs1047286
All − MR Egger
All − Inverse variance weighted
−3
−2
−1
0
1
MR effect size for
'POMK' on 'Pulmonary fibrosis'

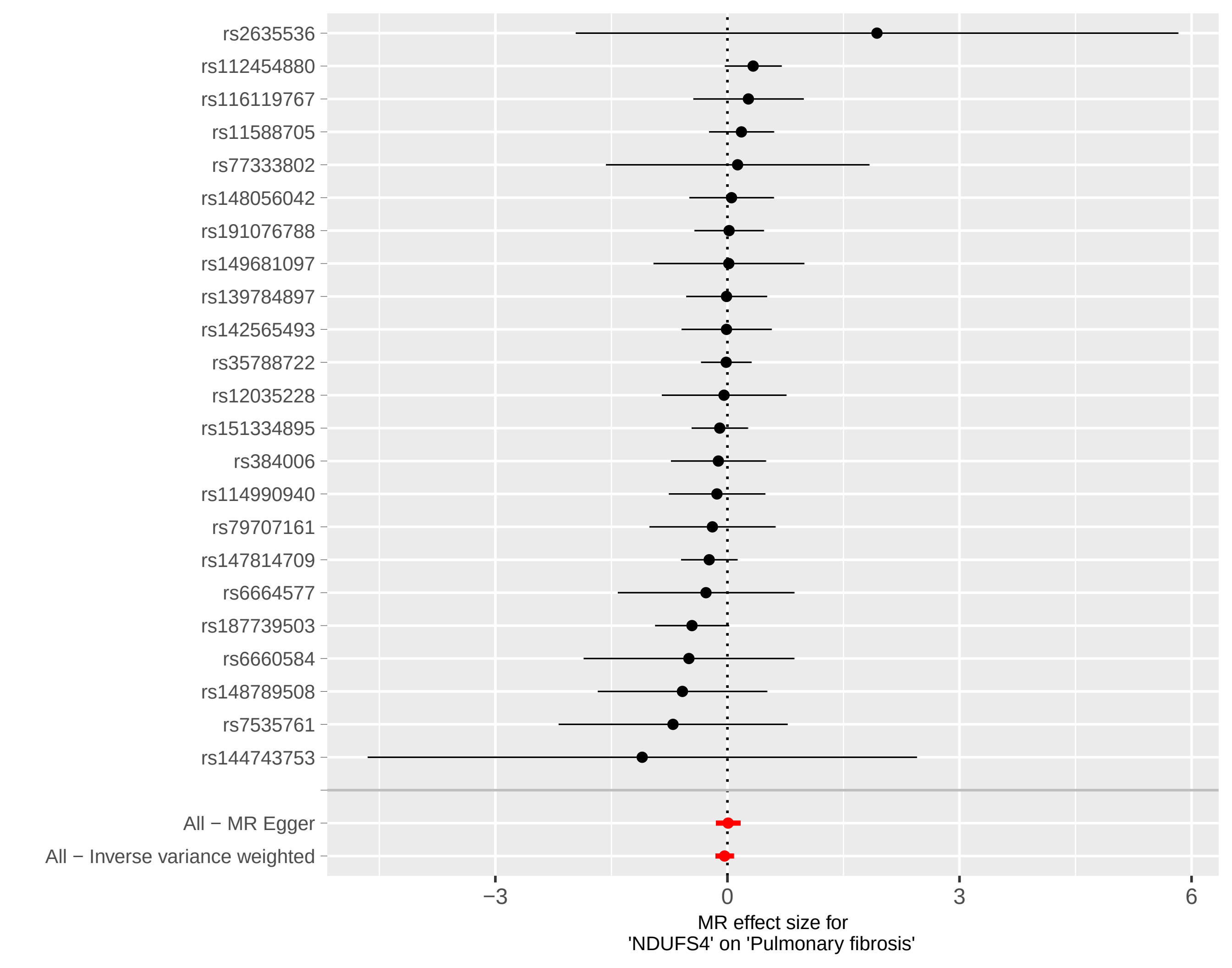

rs2635536
rs112454880
rs116119767
rs11588705
rs77333802
rs148056042
rs191076788
rs149681097
rs139784897
rs142565493
rs35788722
rs12035228
rs151334895
rs384006
rs114990940
rs79707161
rs147814709
rs6664577
rs187739503
rs6660584
rs148789508
rs7535761
rs144743753
All − MR Egger
All − Inverse variance weighted
−3
0
3
6
MR effect size for
'NDUFS4' on 'Pulmonary fibrosis'

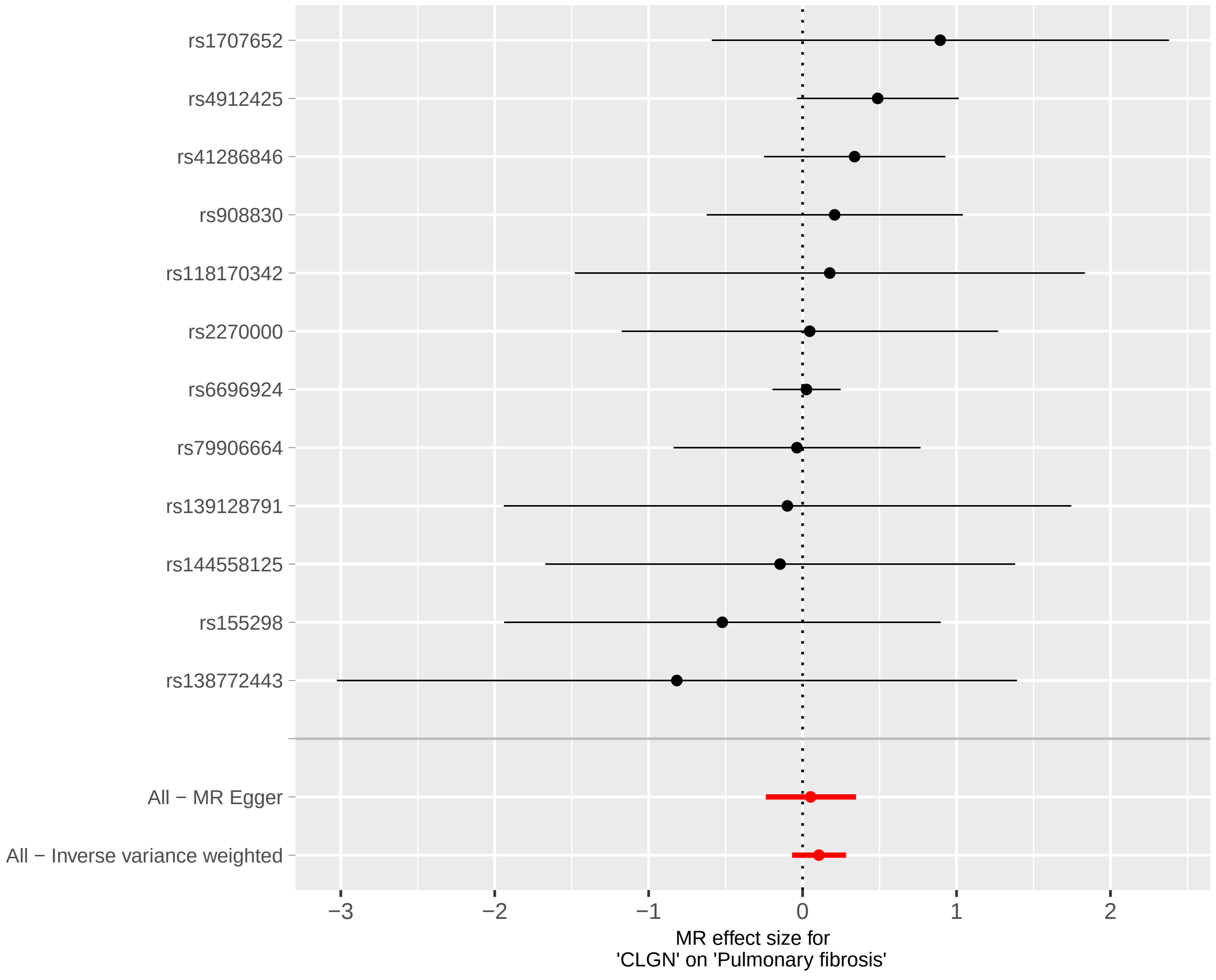
rs1707652
rs4912425
rs41286846
rs908830
rs118170342
rs2270000
rs6696924
rs79906664
rs139128791
rs144558125
rs155298
rs138772443
All − MR Egger
All − Inverse variance weighted
−3
−2
−1
0
1
2
MR effect size for
'CLGN' on 'Pulmonary fibrosis'

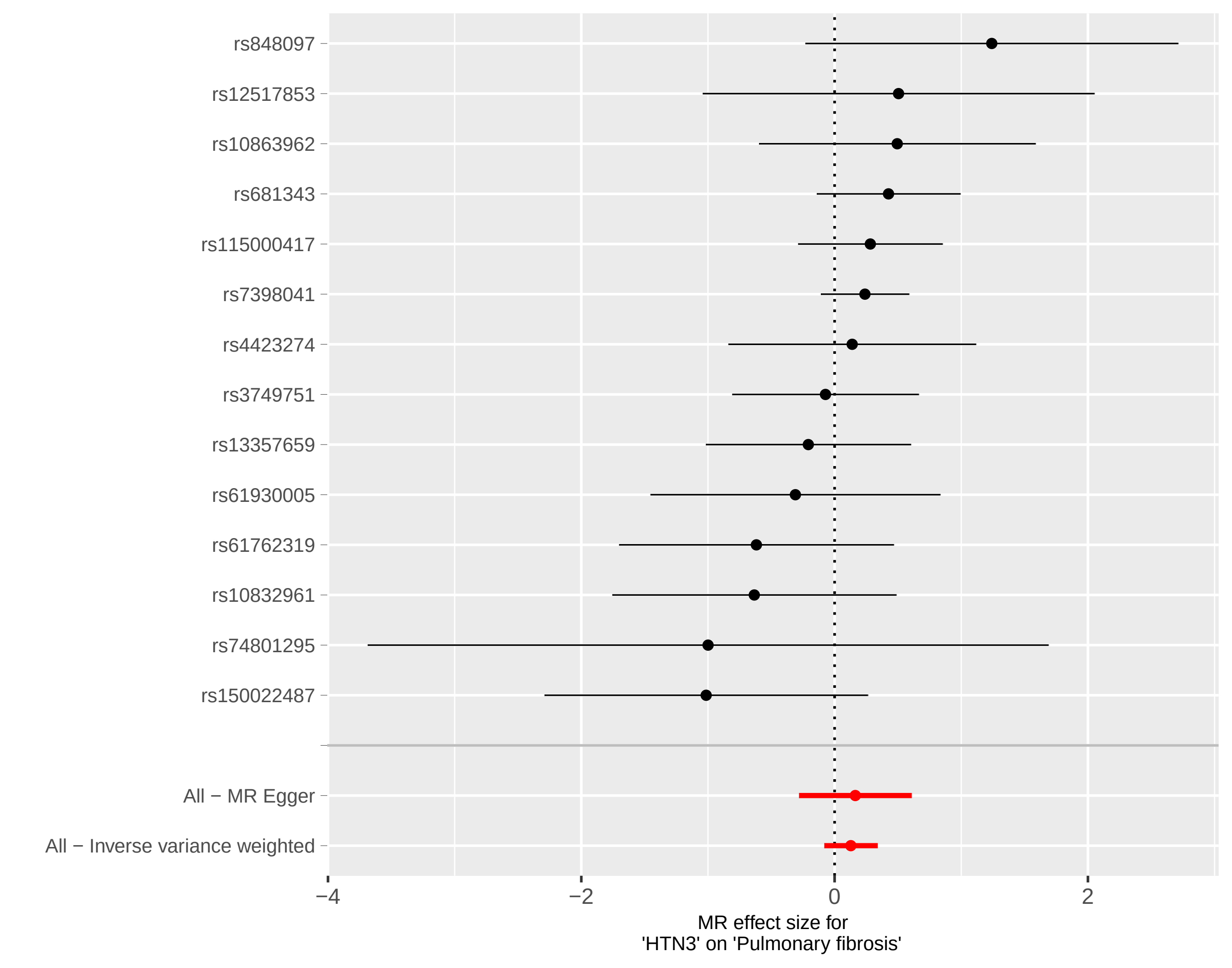

rs848097
rs12517853
rs10863962
rs681343
rs115000417
rs7398041
rs4423274
rs3749751
rs13357659
rs61930005
rs61762319
rs10832961
rs74801295
rs150022487
All − MR Egger
All − Inverse variance weighted
−4
−2
0
2
MR effect size for
'HTN3' on 'Pulmonary fibrosis'

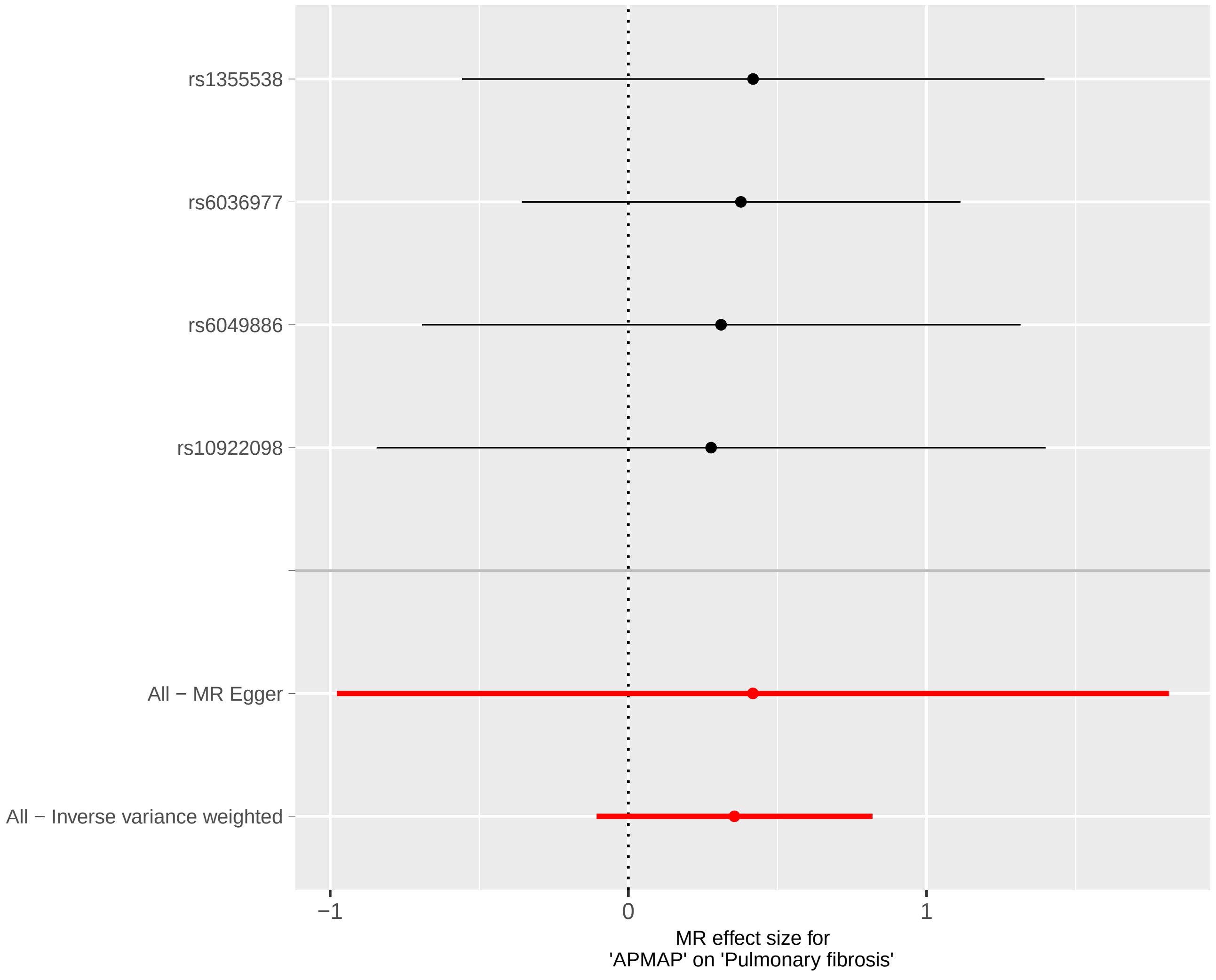
rs1355538
rs6036977
rs6049886
rs10922098
All − MR Egger
All − Inverse variance weighted
−1
0
1
MR effect size for
'APMAP' on 'Pulmonary fibrosis'

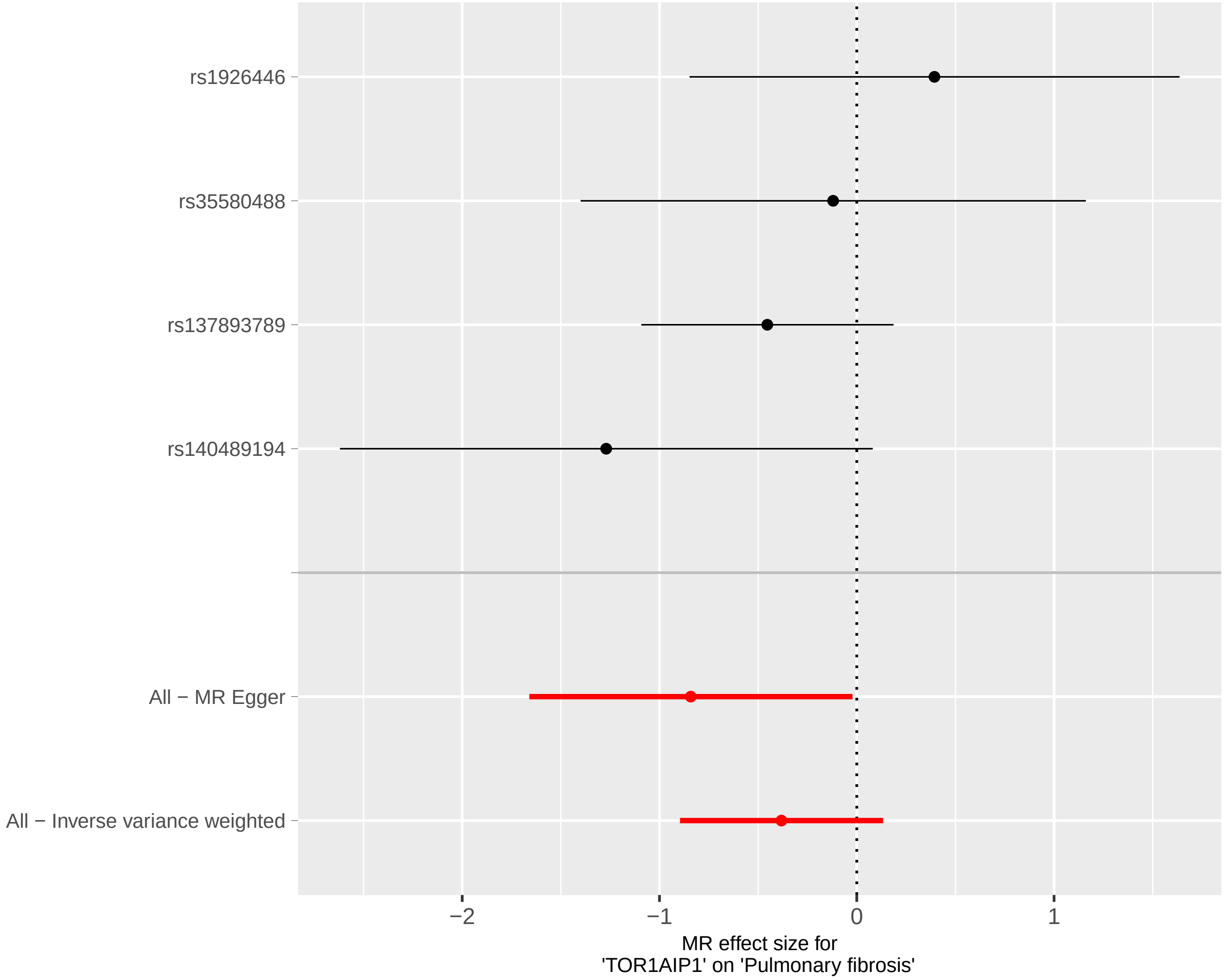

rs1926446
rs35580488
rs137893789
rs140489194
All − MR Egger
All − Inverse variance weighted
−2
−1
0
1
MR effect size for
'TOR1AIP1' on 'Pulmonary fibrosis'

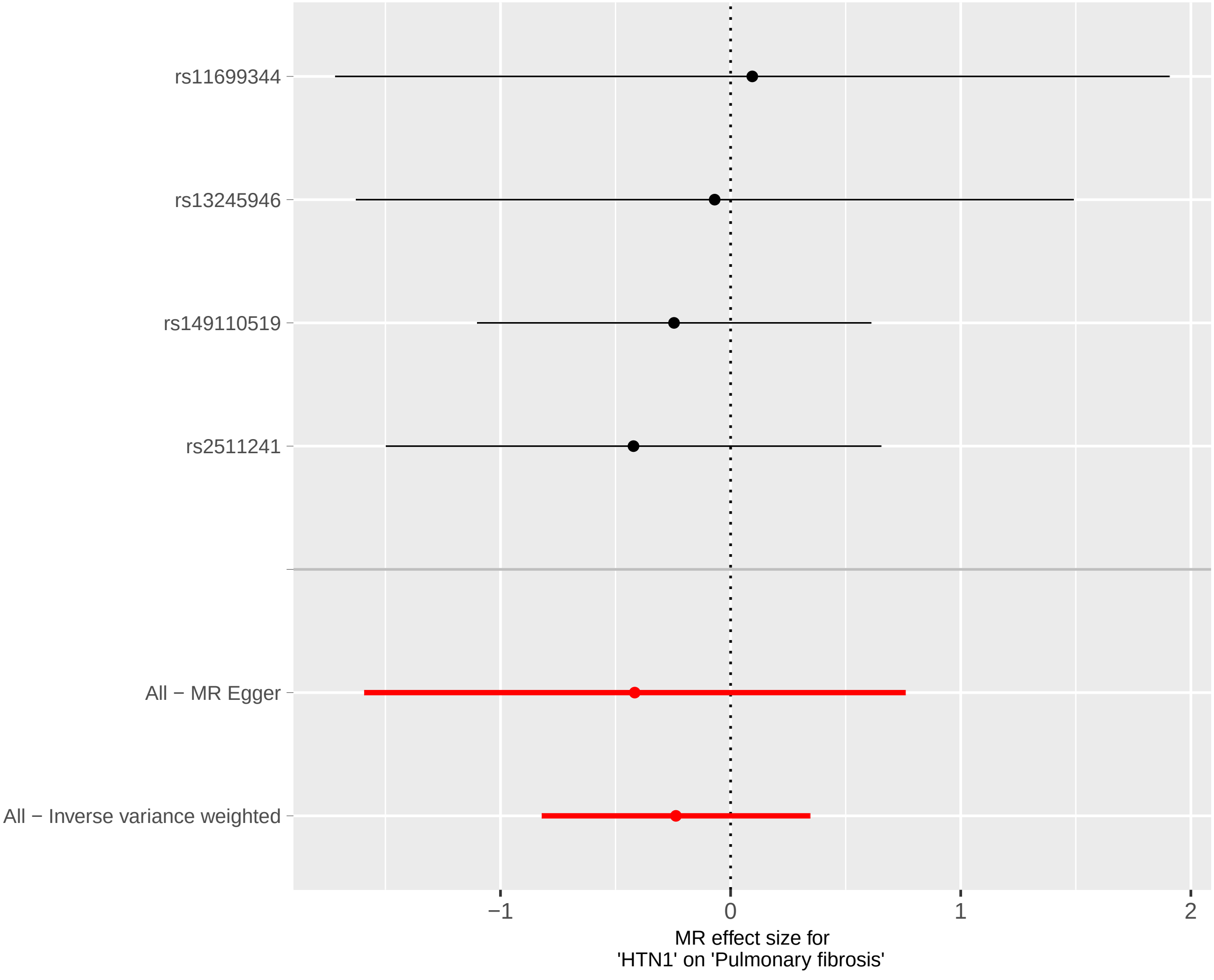
rs11699344
rs13245946
rs149110519
rs2511241
All − MR Egger
All − Inverse variance weighted
−1
0
1
2
MR effect size for
'HTN1' on 'Pulmonary fibrosis'

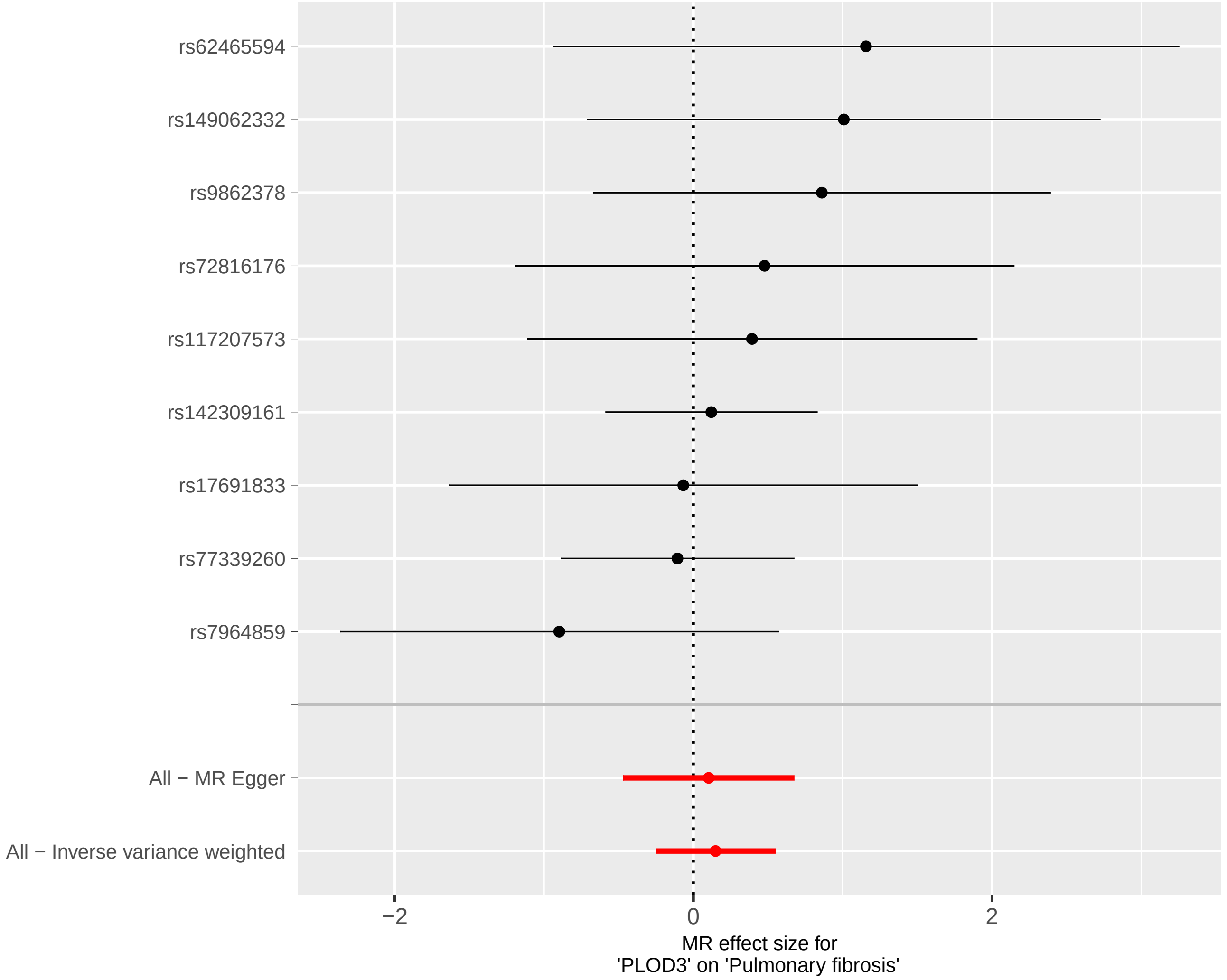
rs62465594
rs149062332
rs9862378
rs72816176
rs117207573
rs142309161
rs17691833
rs77339260
rs7964859
All − MR Egger
All − Inverse variance weighted
−2
0
2
MR effect size for
'PLOD3' on 'Pulmonary fibrosis'

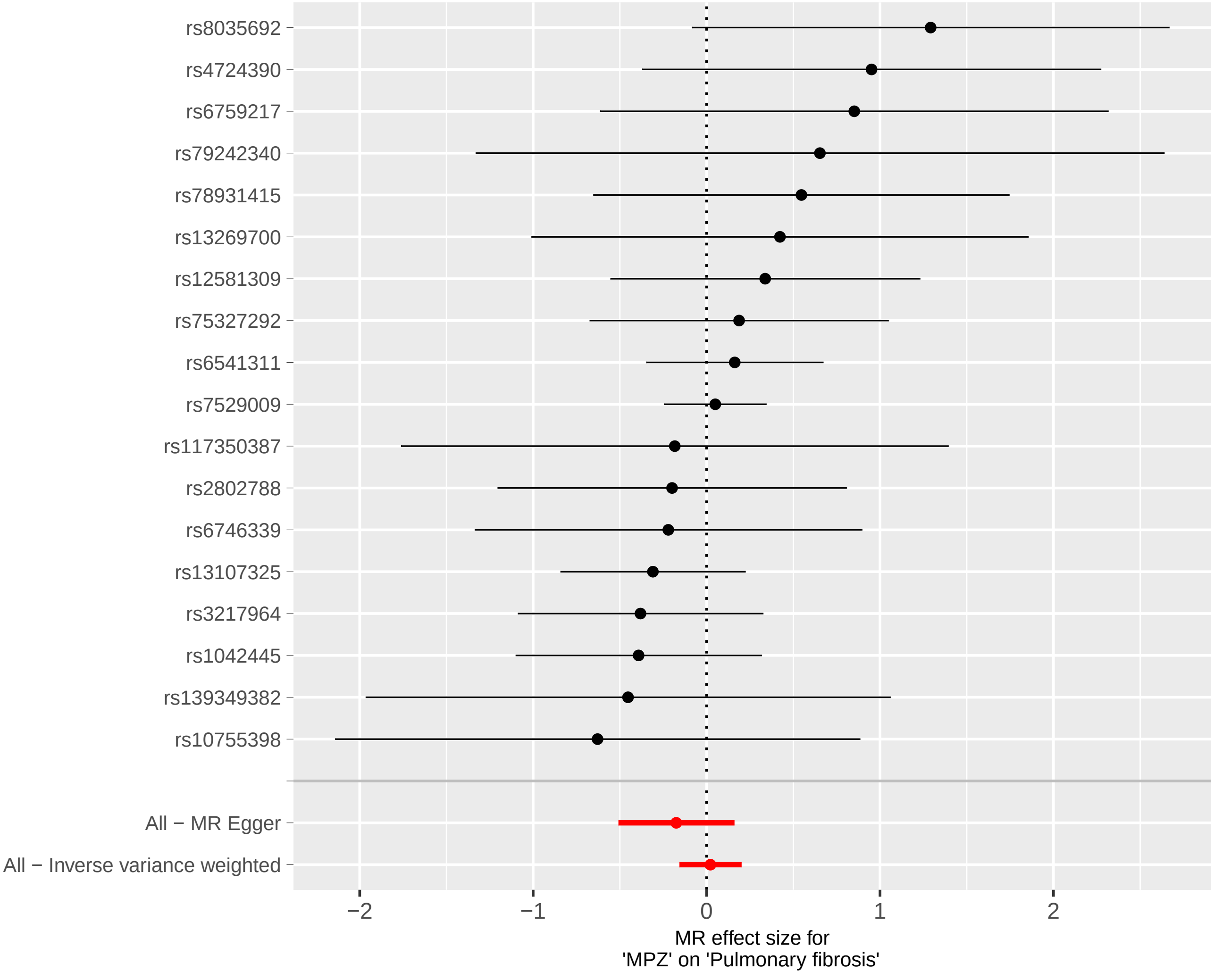
rs8035692
rs4724390
rs6759217
rs79242340
rs78931415
rs13269700
rs12581309
rs75327292
rs6541311
rs7529009
rs117350387
rs2802788
rs6746339
rs13107325
rs3217964
rs1042445
rs139349382
rs10755398
All − MR Egger
All − Inverse variance weighted
−2
−1
0
1
2
MR effect size for
'MPZ' on 'Pulmonary fibrosis'

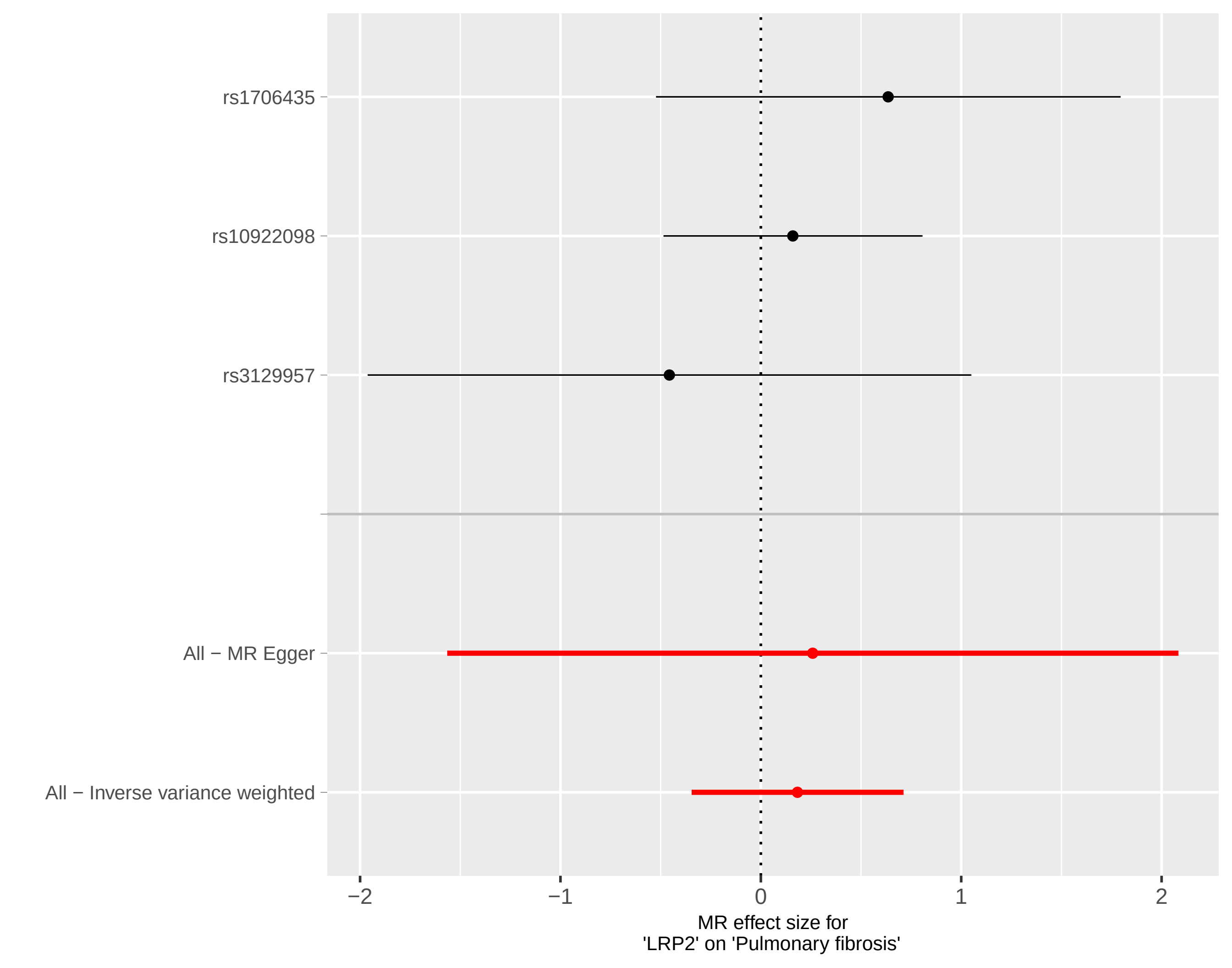
rs1706435
rs10922098
rs3129957
All − MR Egger
All − Inverse variance weighted
−2
−1
0
1
2
MR effect size for
'LRP2' on 'Pulmonary fibrosis'

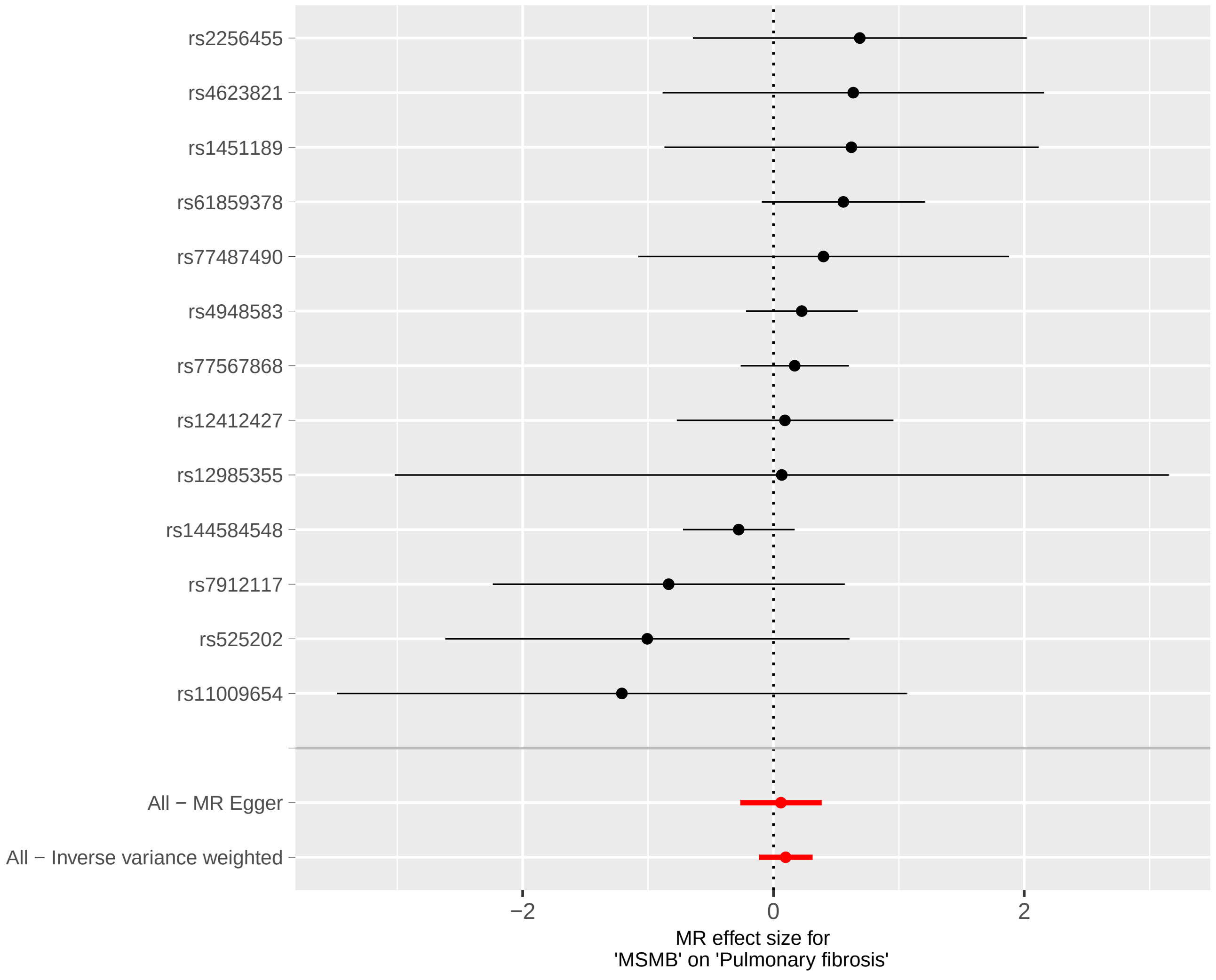
rs2256455
rs4623821
rs1451189
rs61859378
rs77487490
rs4948583
rs77567868
rs12412427
rs12985355
rs144584548
rs7912117
rs525202
rs11009654
All − MR Egger
All − Inverse variance weighted
−2
0
2
MR effect size for
'MSMB' on 'Pulmonary fibrosis'

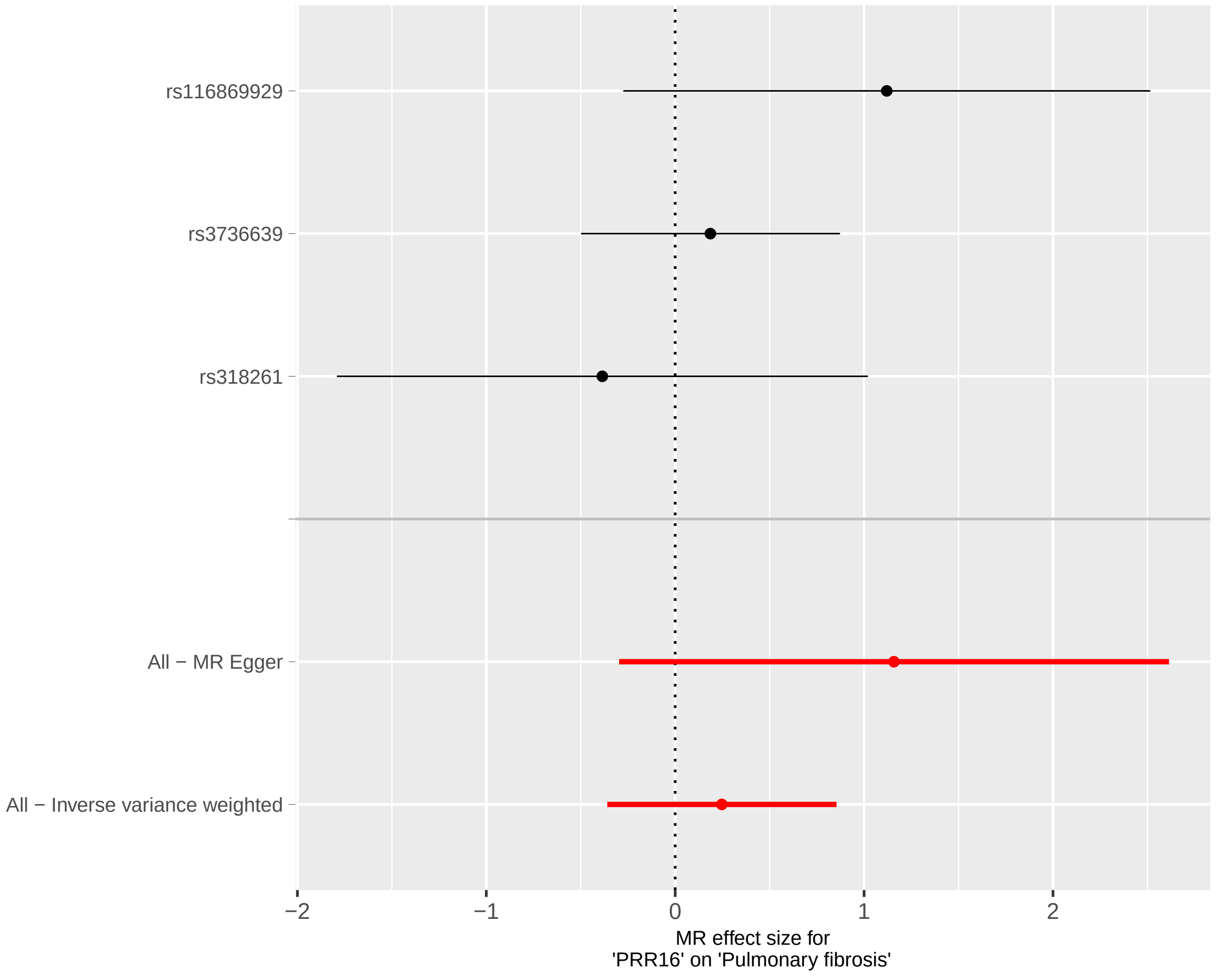

rs116869929
rs3736639
rs318261
All − MR Egger
All − Inverse variance weighted
−2
−1
0
1
2
MR effect size for
'PRR16' on 'Pulmonary fibrosis'

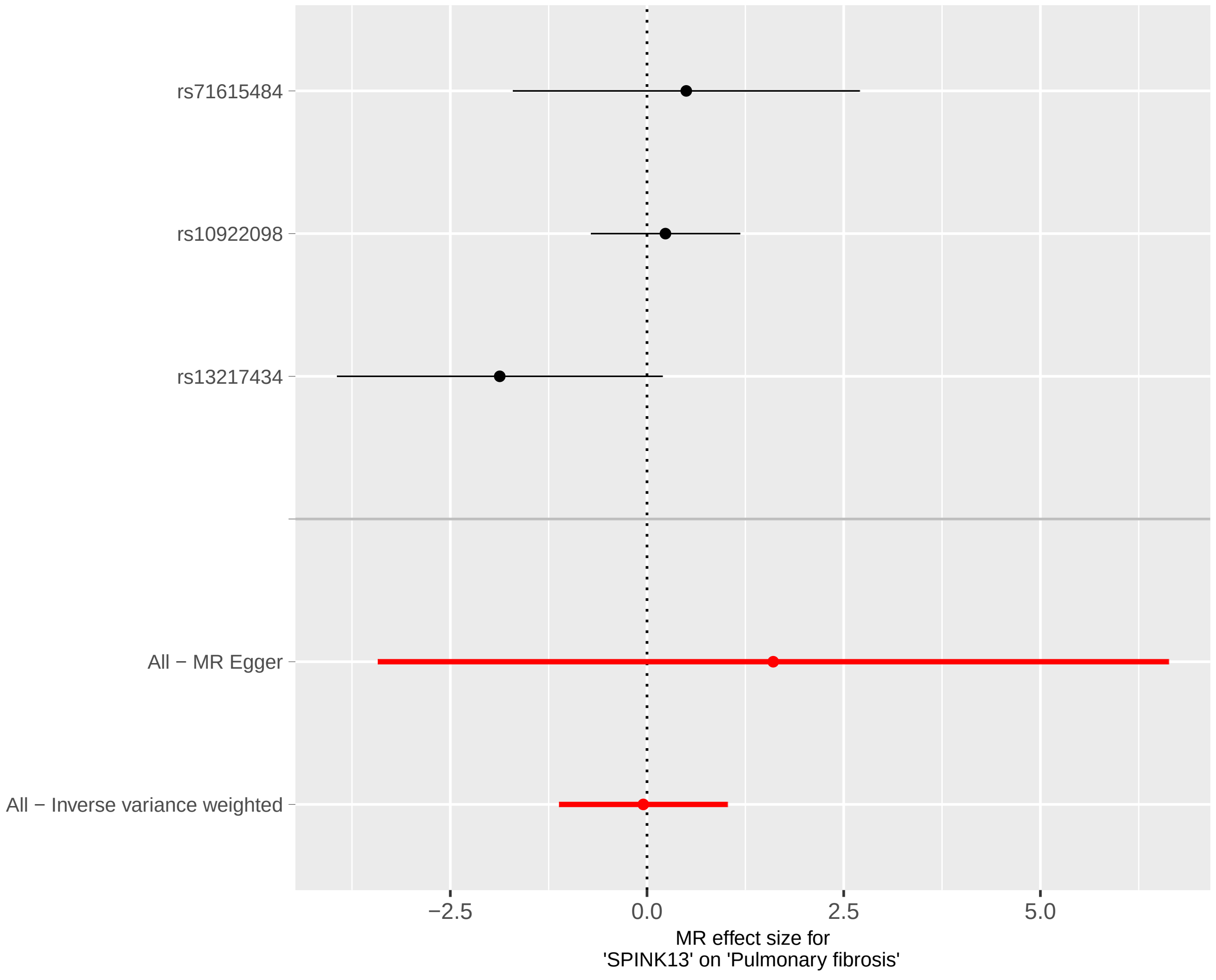

rs71615484
rs10922098
rs13217434
All − MR Egger
All − Inverse variance weighted
−2.5
0.0
2.5
5.0
MR effect size for
'SPINK13' on 'Pulmonary fibrosis'

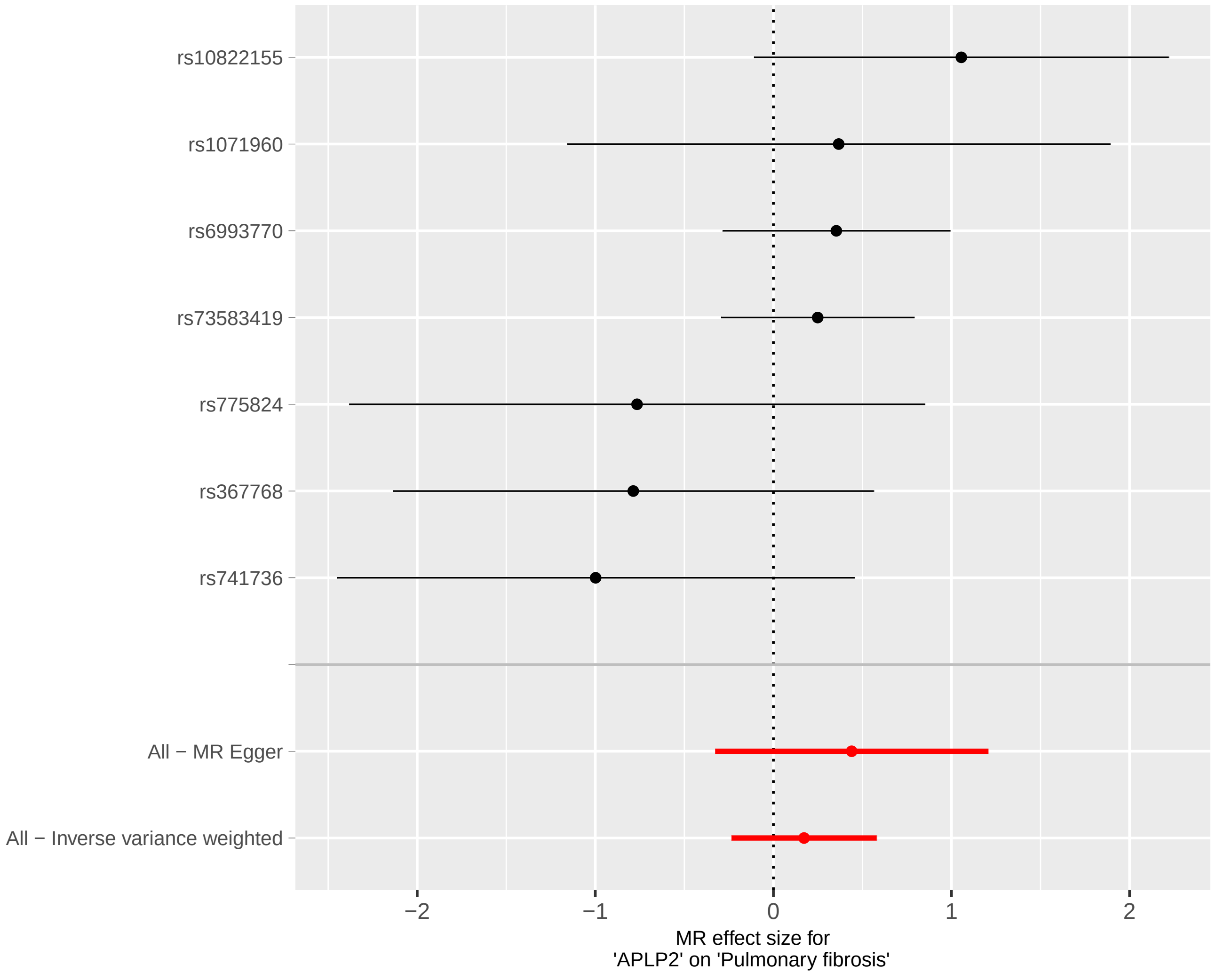
rs10822155
rs1071960
rs6993770
rs73583419
rs775824
rs367768
rs741736
All − MR Egger
All − Inverse variance weighted
−2
−1
0
1
2
MR effect size for
'APLP2' on 'Pulmonary fibrosis'

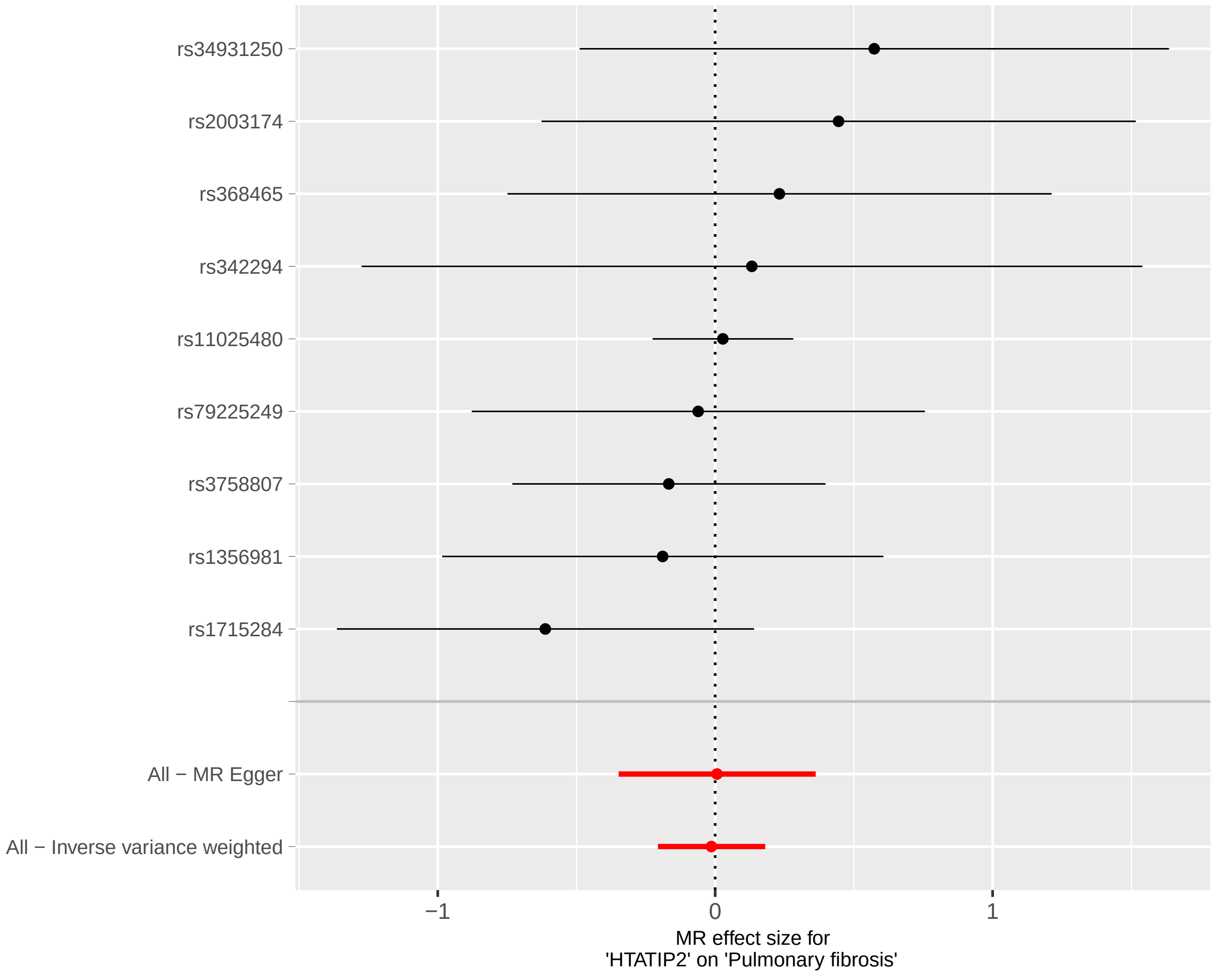

rs34931250
rs2003174
rs368465
rs342294
rs11025480
rs79225249
rs3758807
rs1356981
rs1715284
All − MR Egger
All − Inverse variance weighted
−1
0
1
MR effect size for
'HTATIP2' on 'Pulmonary fibrosis'

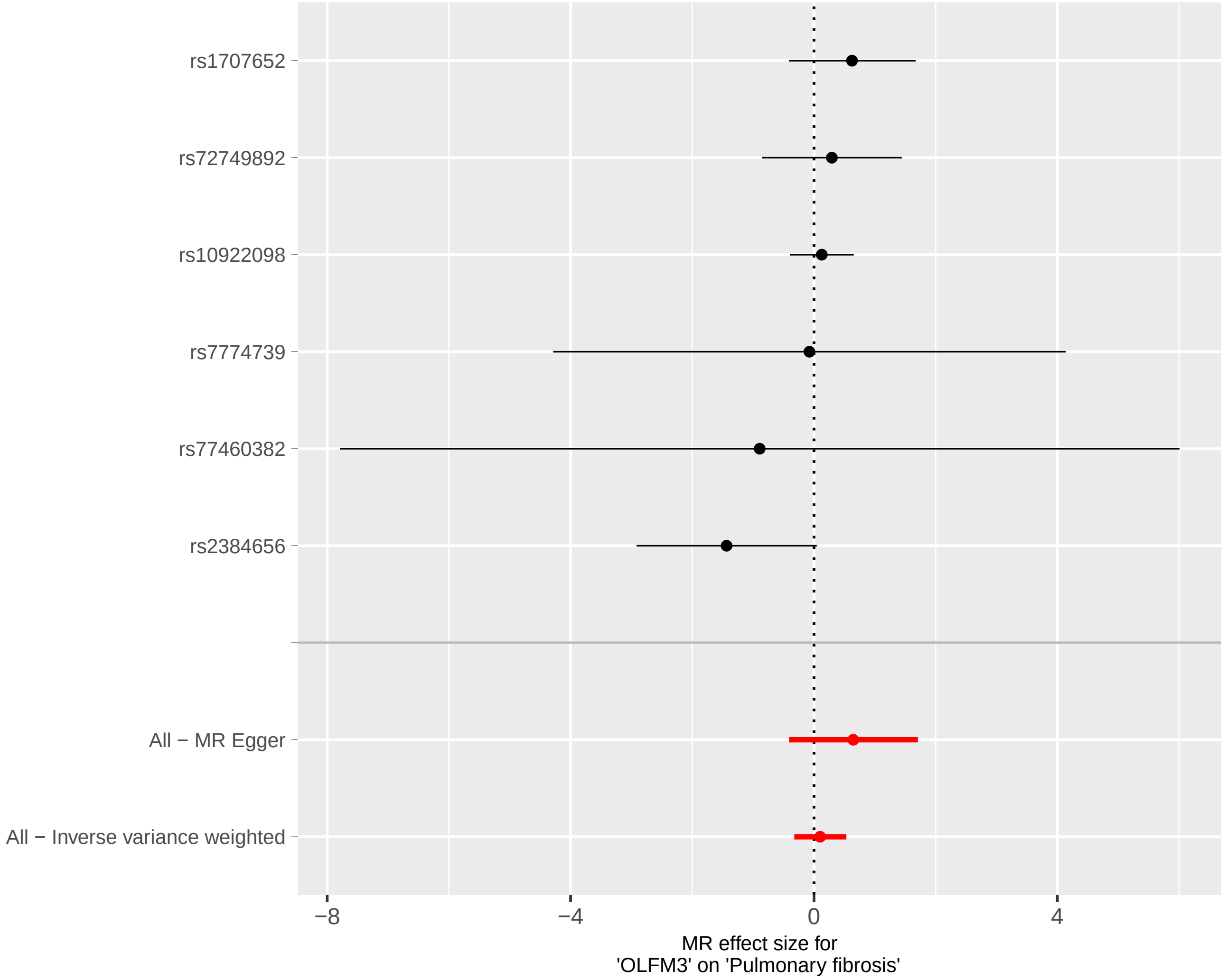
rs1707652
rs72749892
rs10922098
rs7774739
rs77460382
rs2384656
All − MR Egger
All − Inverse variance weighted
−8
−4
0
4
MR effect size for
'OLFM3' on 'Pulmonary fibrosis'

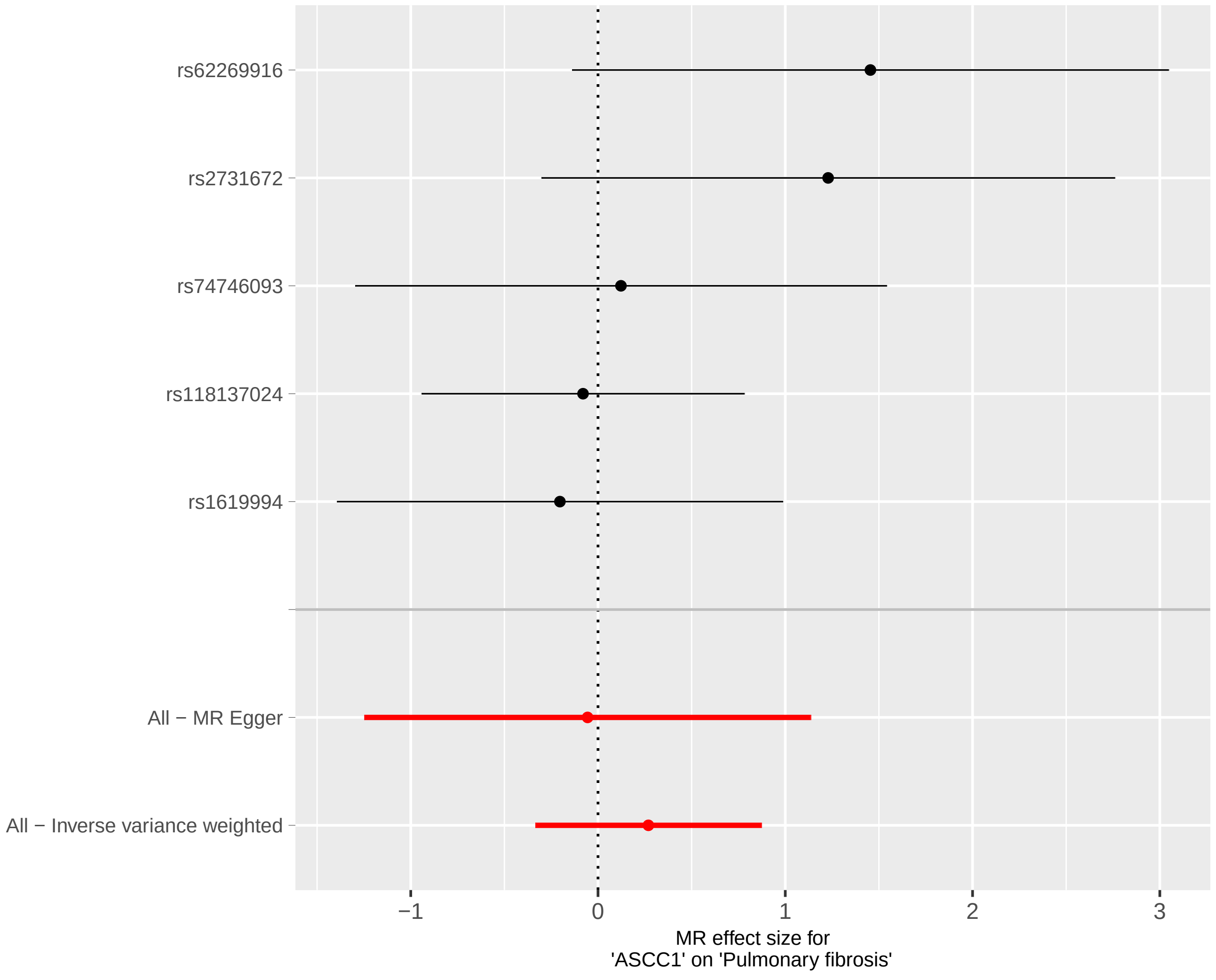

rs62269916
rs2731672
rs74746093
rs118137024
rs1619994
All − MR Egger
All − Inverse variance weighted
−1
0
1
2
3
MR effect size for
'ASCC1' on 'Pulmonary fibrosis'

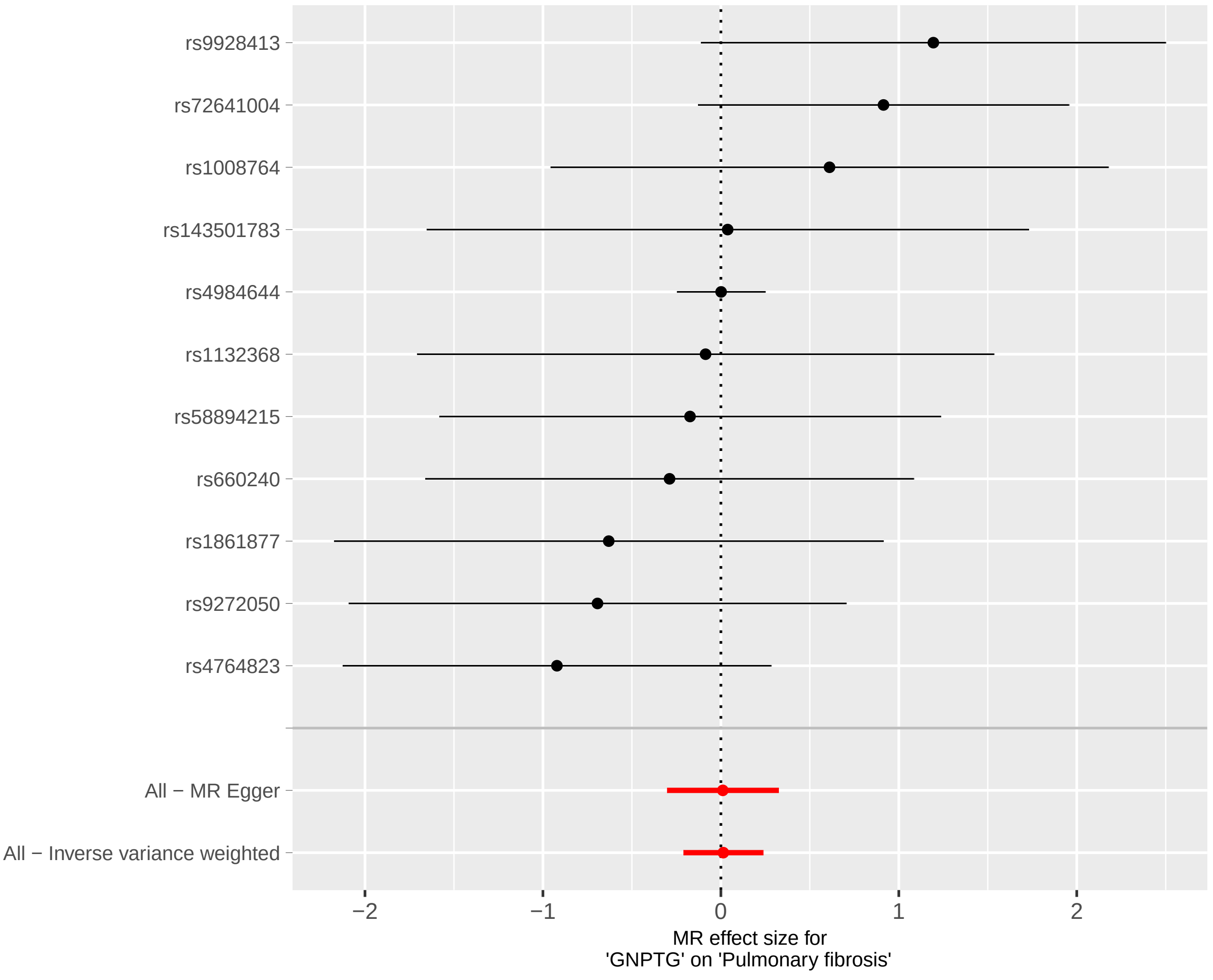
rs9928413
rs72641004
rs1008764
rs143501783
rs4984644
rs1132368
rs58894215
rs660240
rs1861877
rs9272050
rs4764823
All − MR Egger
All − Inverse variance weighted
−2
−1
0
1
2
MR effect size for
'GNPTG' on 'Pulmonary fibrosis'

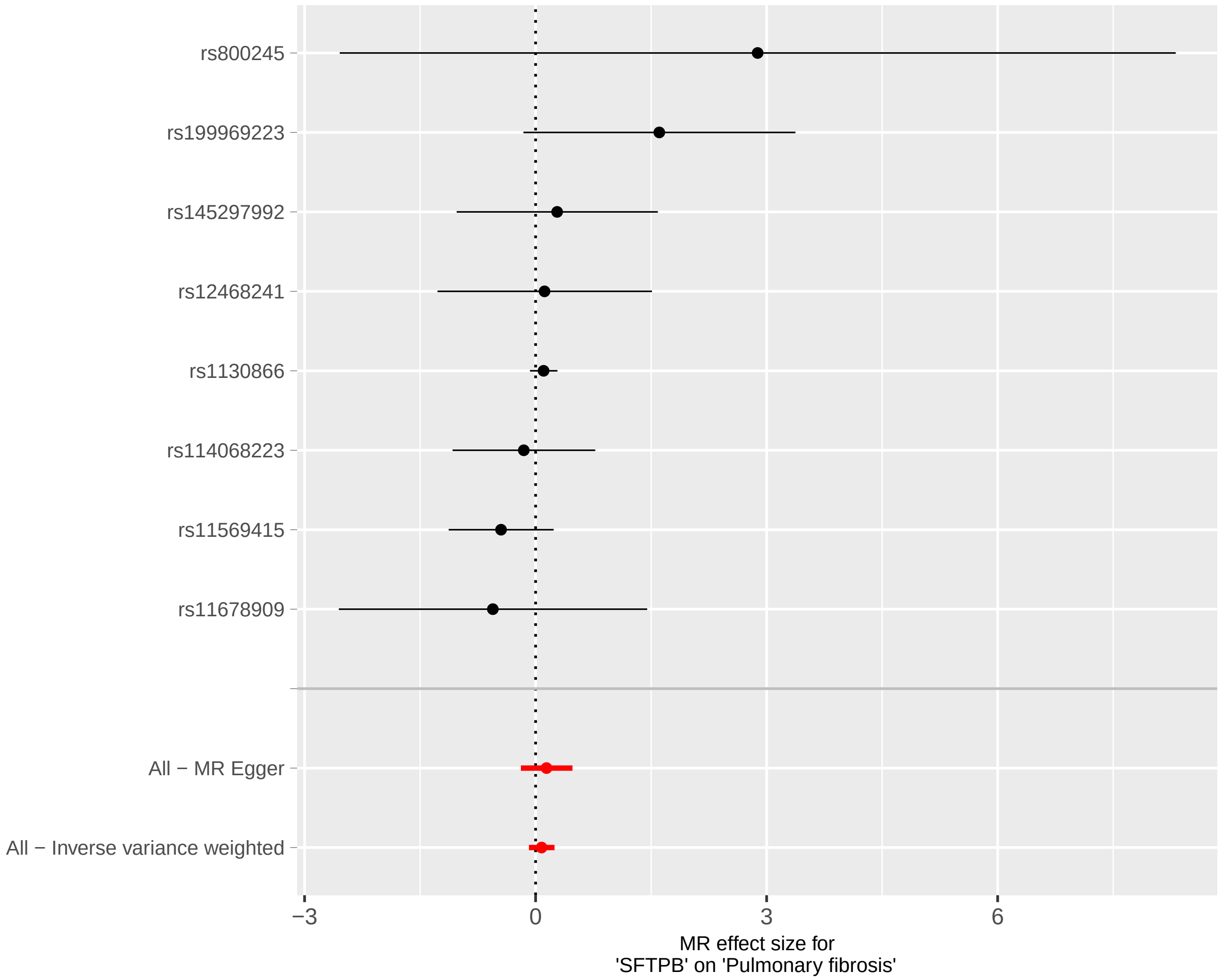

rs800245
rs199969223
rs145297992
rs12468241
rs1130866
rs114068223
rs11569415
rs11678909
All − MR Egger
All − Inverse variance weighted
−3
0
3
6
MR effect size for
'SFTPB' on 'Pulmonary fibrosis'

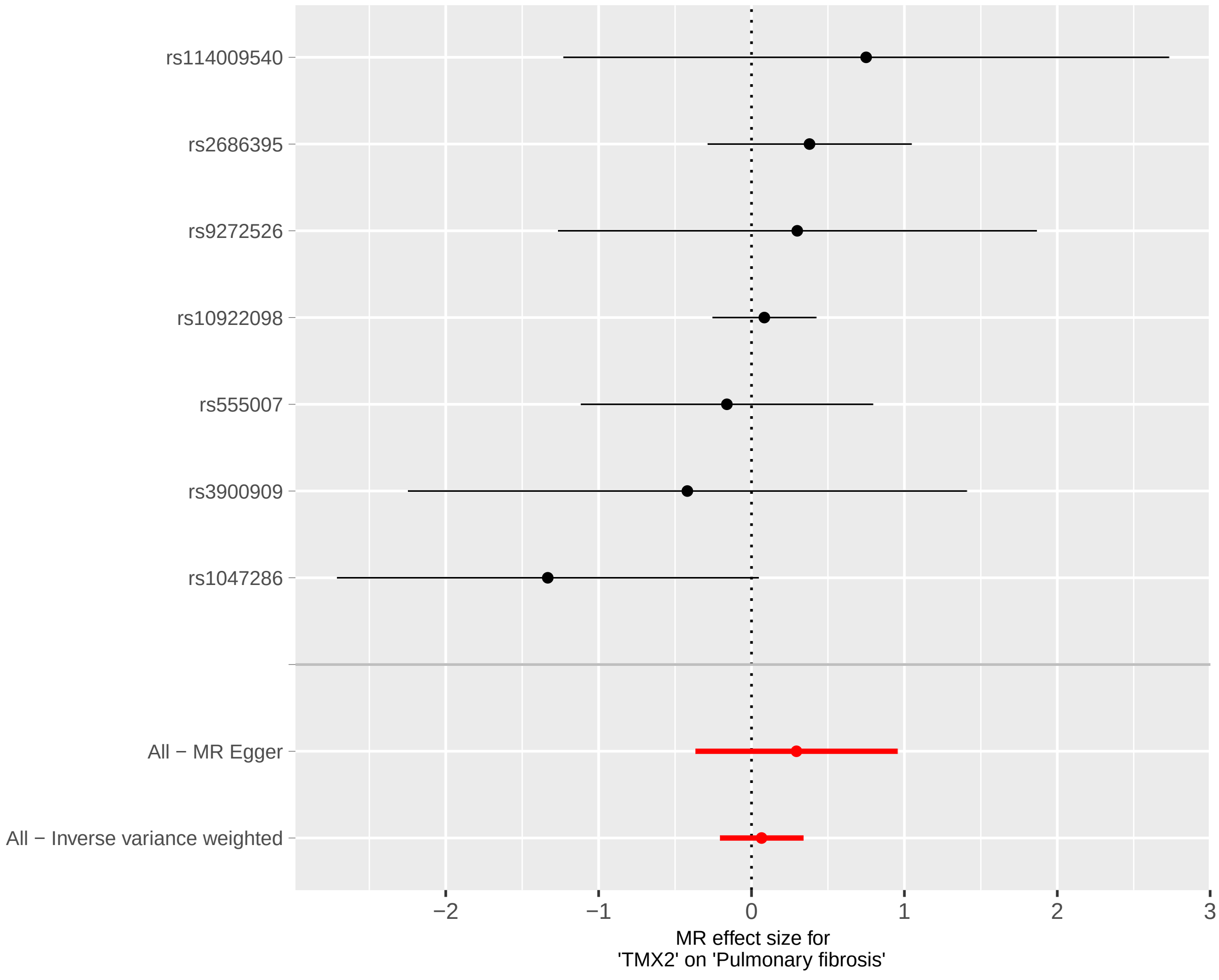

rs114009540
rs2686395
rs9272526
rs10922098
rs555007
rs3900909
rs1047286
All − MR Egger
All − Inverse variance weighted
−2
−1
0
1
2
3
MR effect size for
'TMX2' on 'Pulmonary fibrosis'

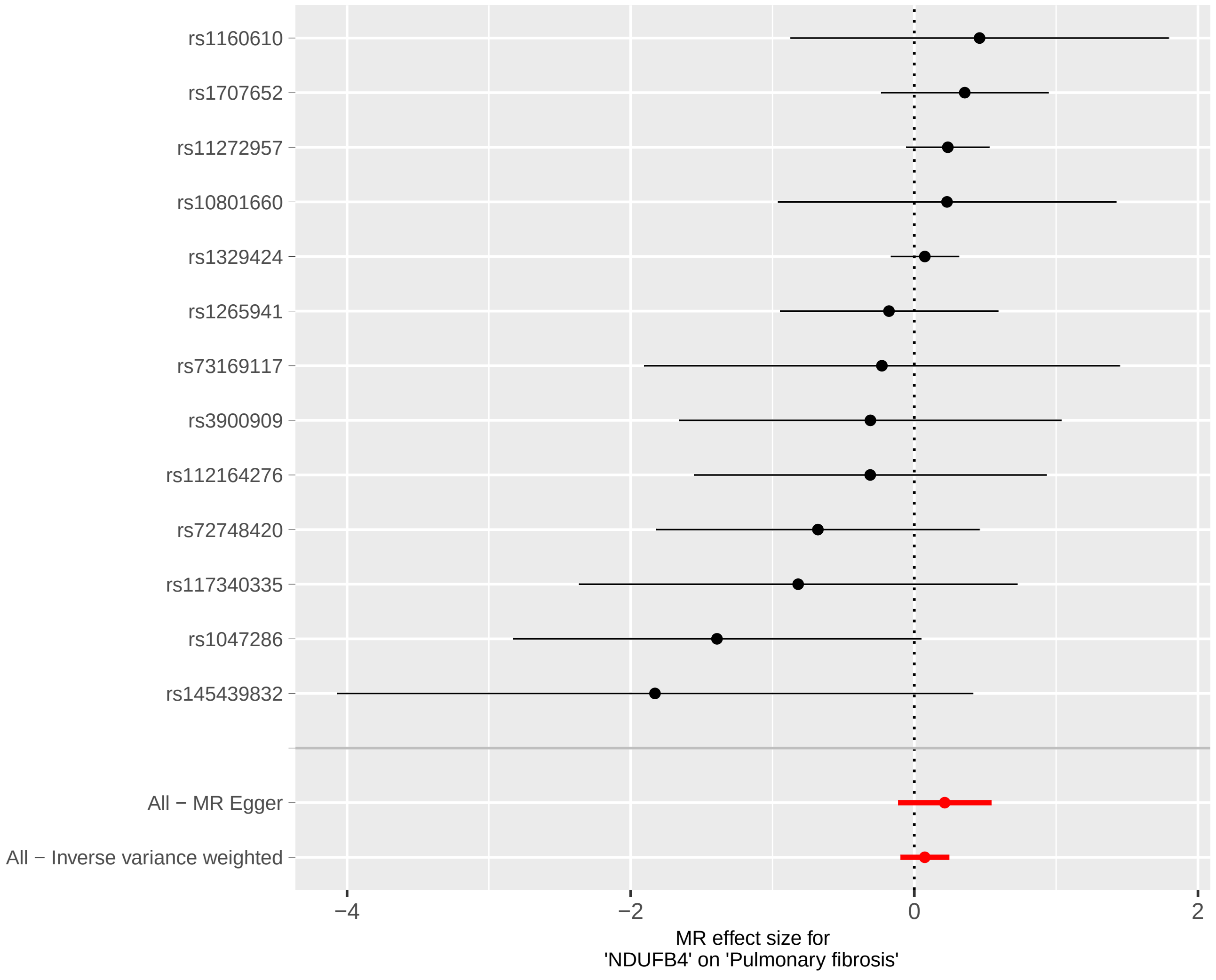

rs1160610
rs1707652
rs11272957
rs10801660
rs1329424
rs1265941
rs73169117
rs3900909
rs112164276
rs72748420
rs117340335
rs1047286
rs145439832
All − MR Egger
All − Inverse variance weighted
−4
−2
0
2
MR effect size for
'NDUFB4' on 'Pulmonary fibrosis'

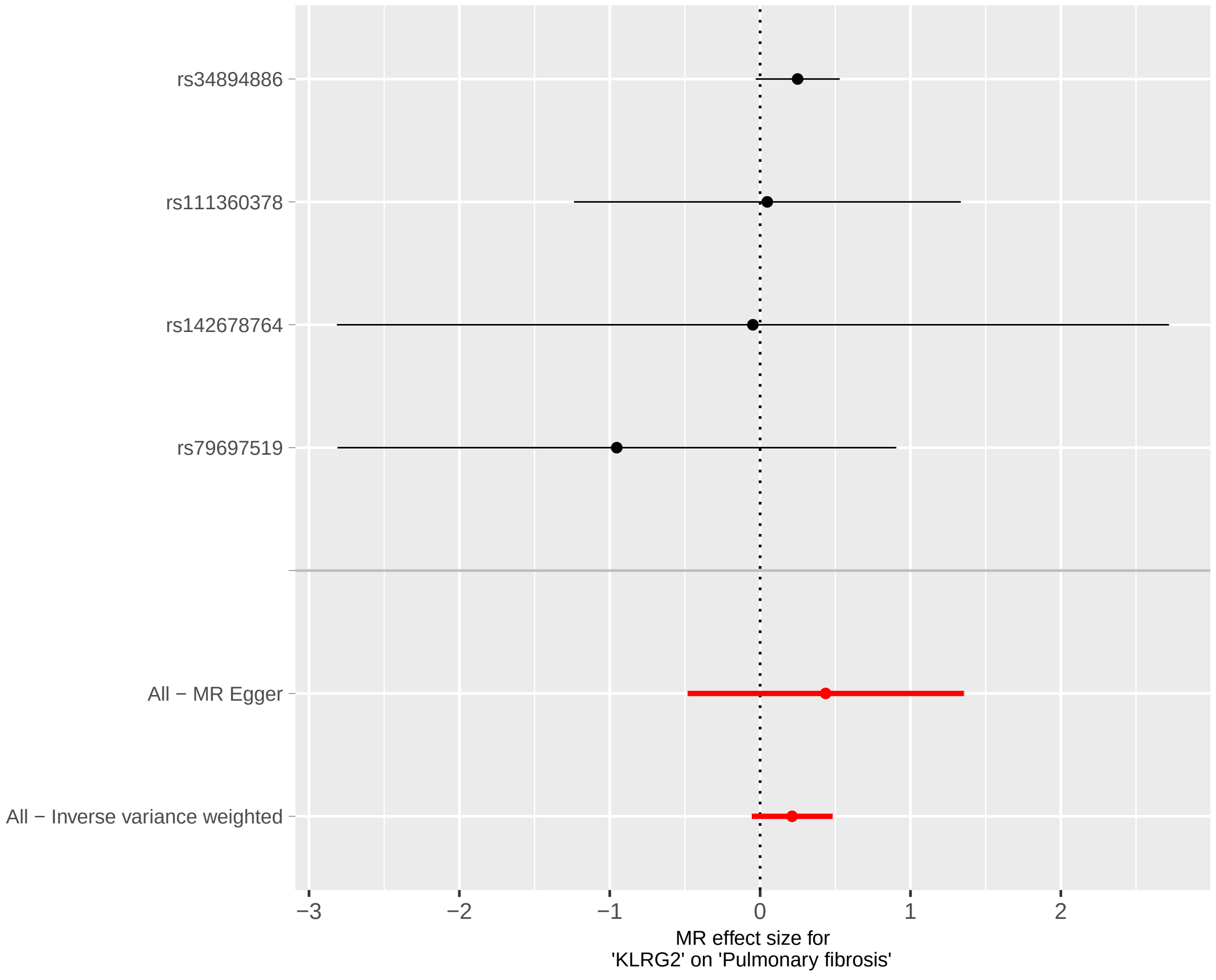
rs34894886
rs111360378
rs142678764
rs79697519
All − MR Egger
All − Inverse variance weighted
−3
−2
−1
0
1
2
MR effect size for
'KLRG2' on 'Pulmonary fibrosis'

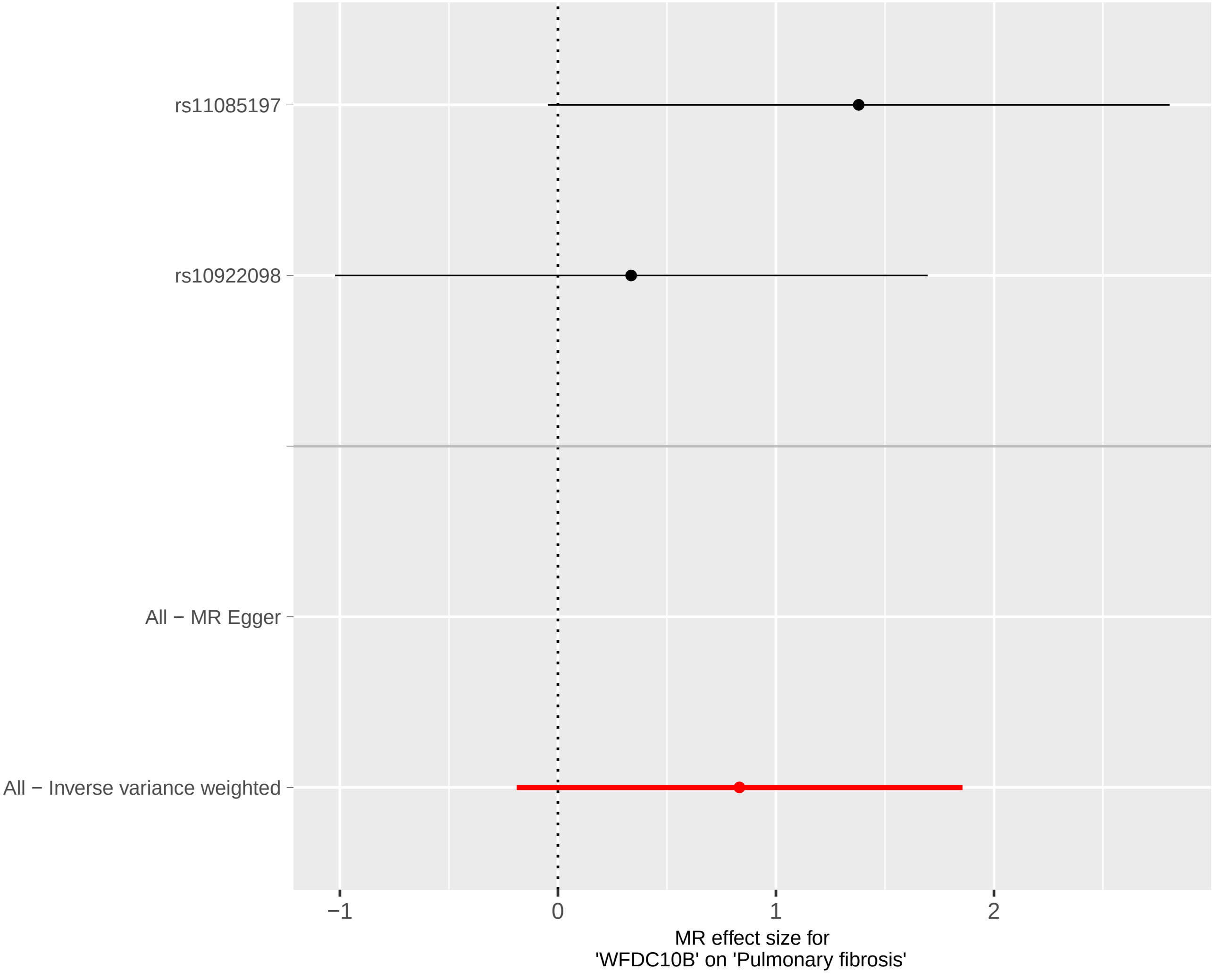
rs11085197
rs10922098
All − MR Egger
All − Inverse variance weighted
−1
0
1
2
MR effect size for
'WFDC10B' on 'Pulmonary fibrosis'

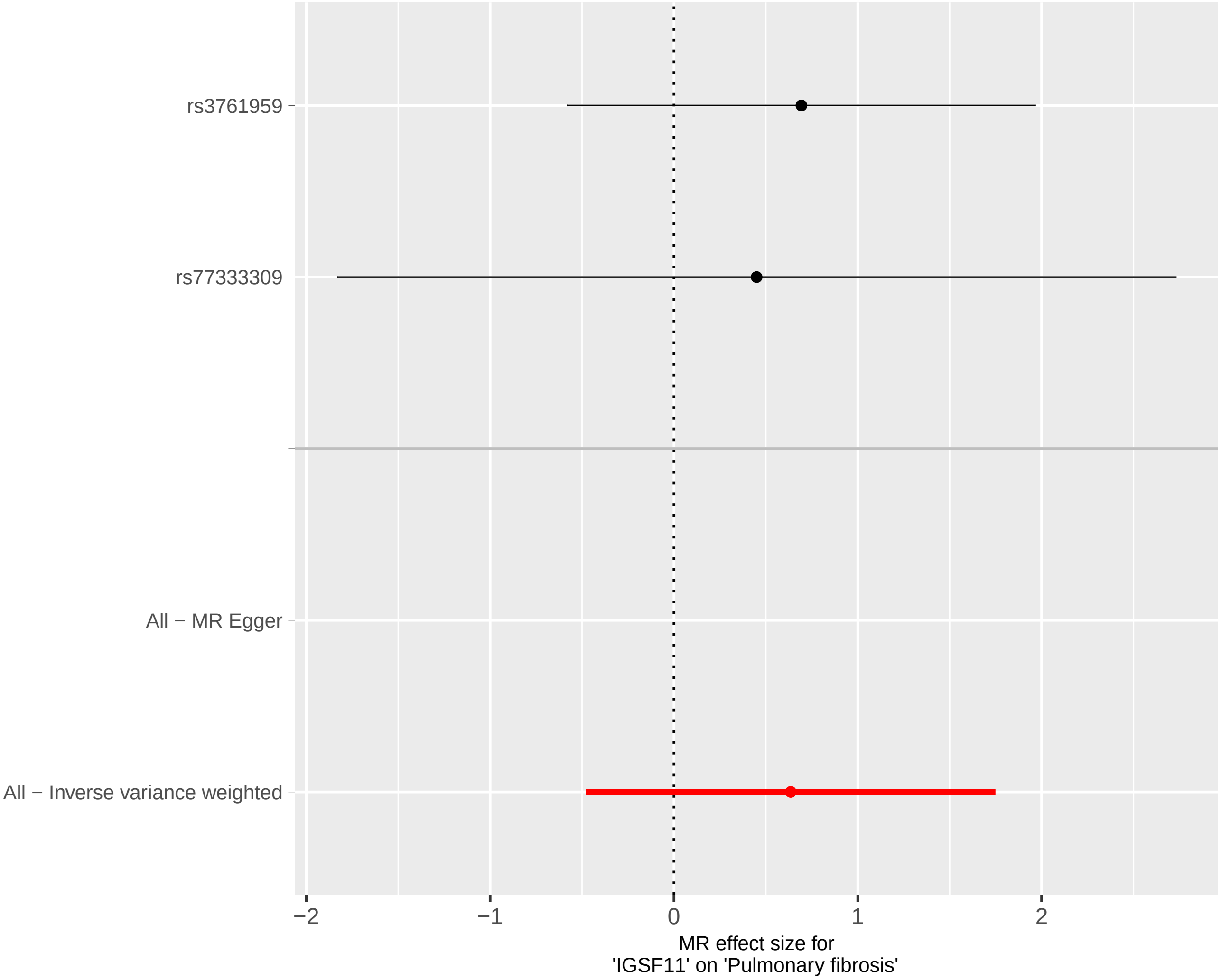
rs3761959
rs77333309
All − MR Egger
All − Inverse variance weighted
−2
−1
0
1
2
MR effect size for
'IGSF11' on 'Pulmonary fibrosis'

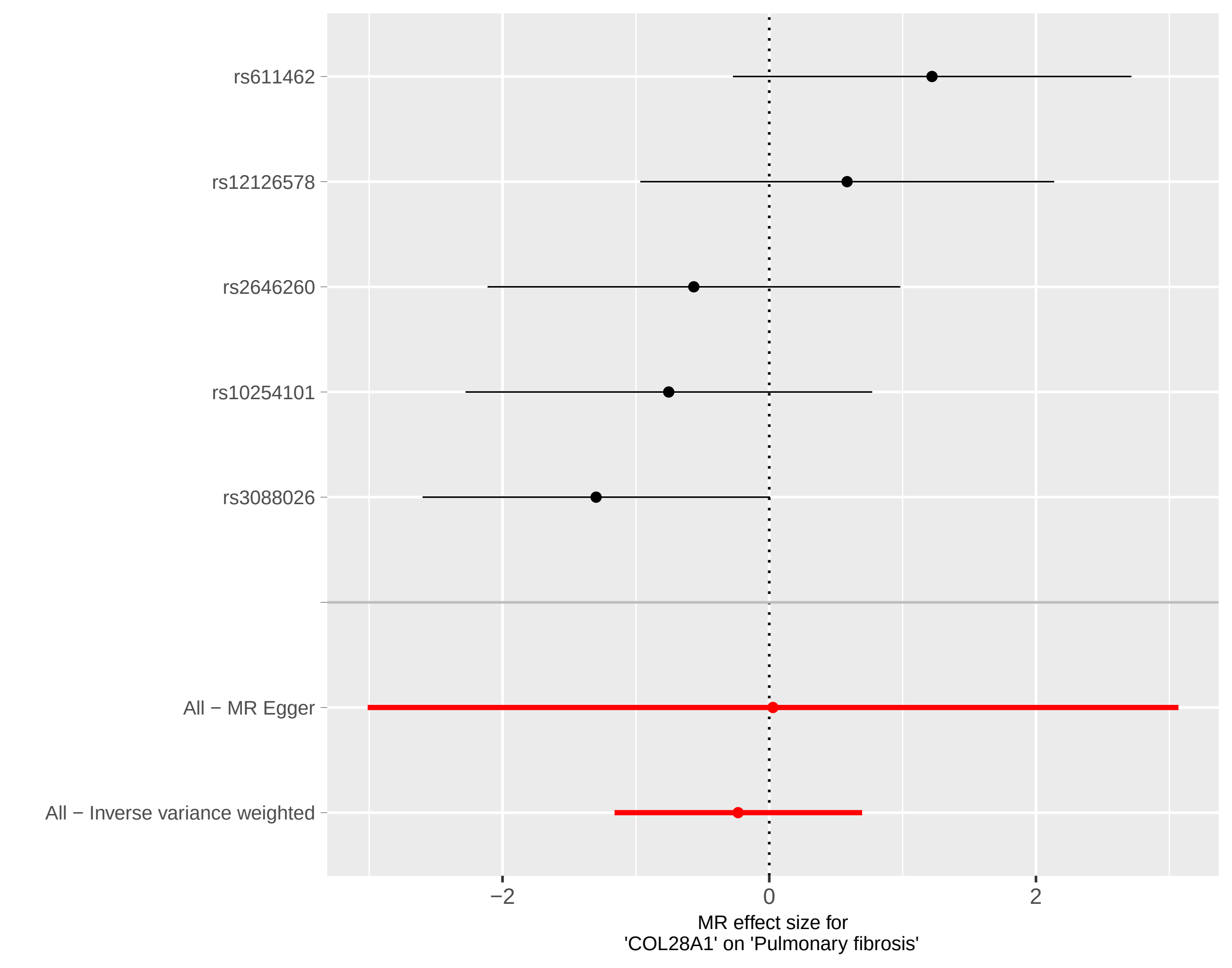
rs611462
rs12126578
rs2646260
rs10254101
rs3088026
All − MR Egger
All − Inverse variance weighted
−2
0
2
MR effect size for
'COL28A1' on 'Pulmonary fibrosis'

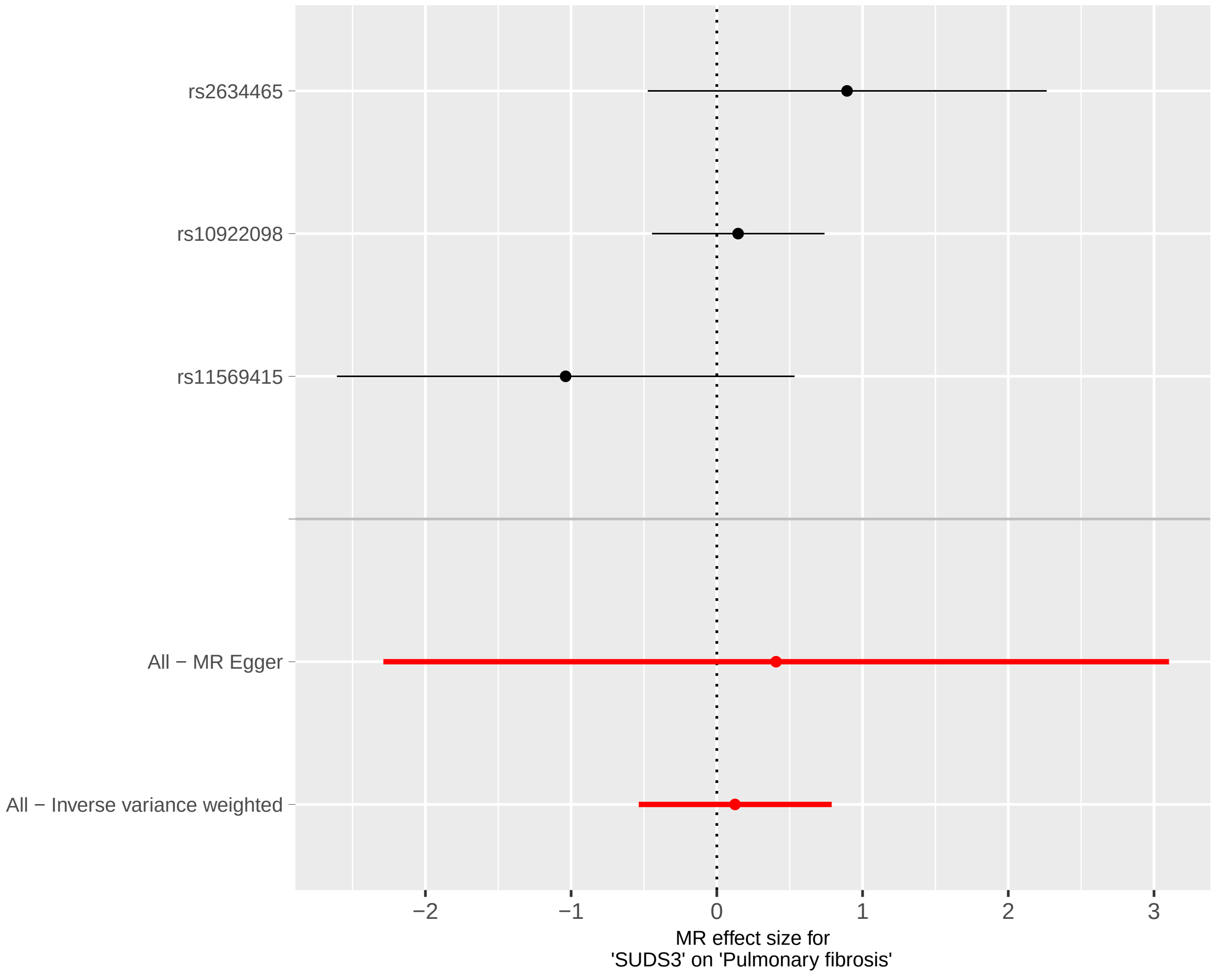
rs2634465
rs10922098
rs11569415
All − MR Egger
All − Inverse variance weighted
−2
−1
0
1
2
3
MR effect size for
'SUDS3' on 'Pulmonary fibrosis'

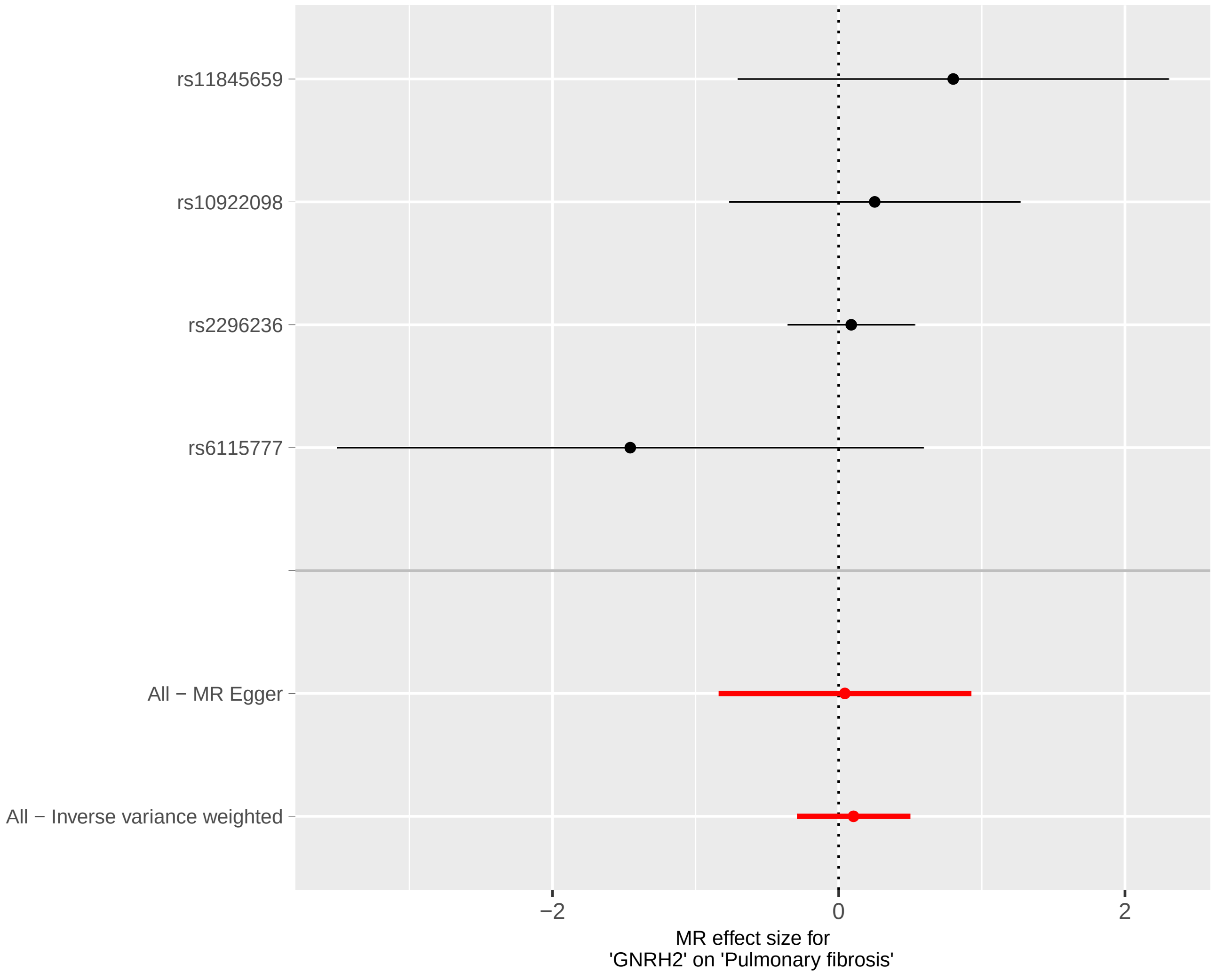
rs11845659
rs10922098
rs2296236
rs6115777
All − MR Egger
All − Inverse variance weighted
−2
0
2
MR effect size for
'GNRH2' on 'Pulmonary fibrosis'

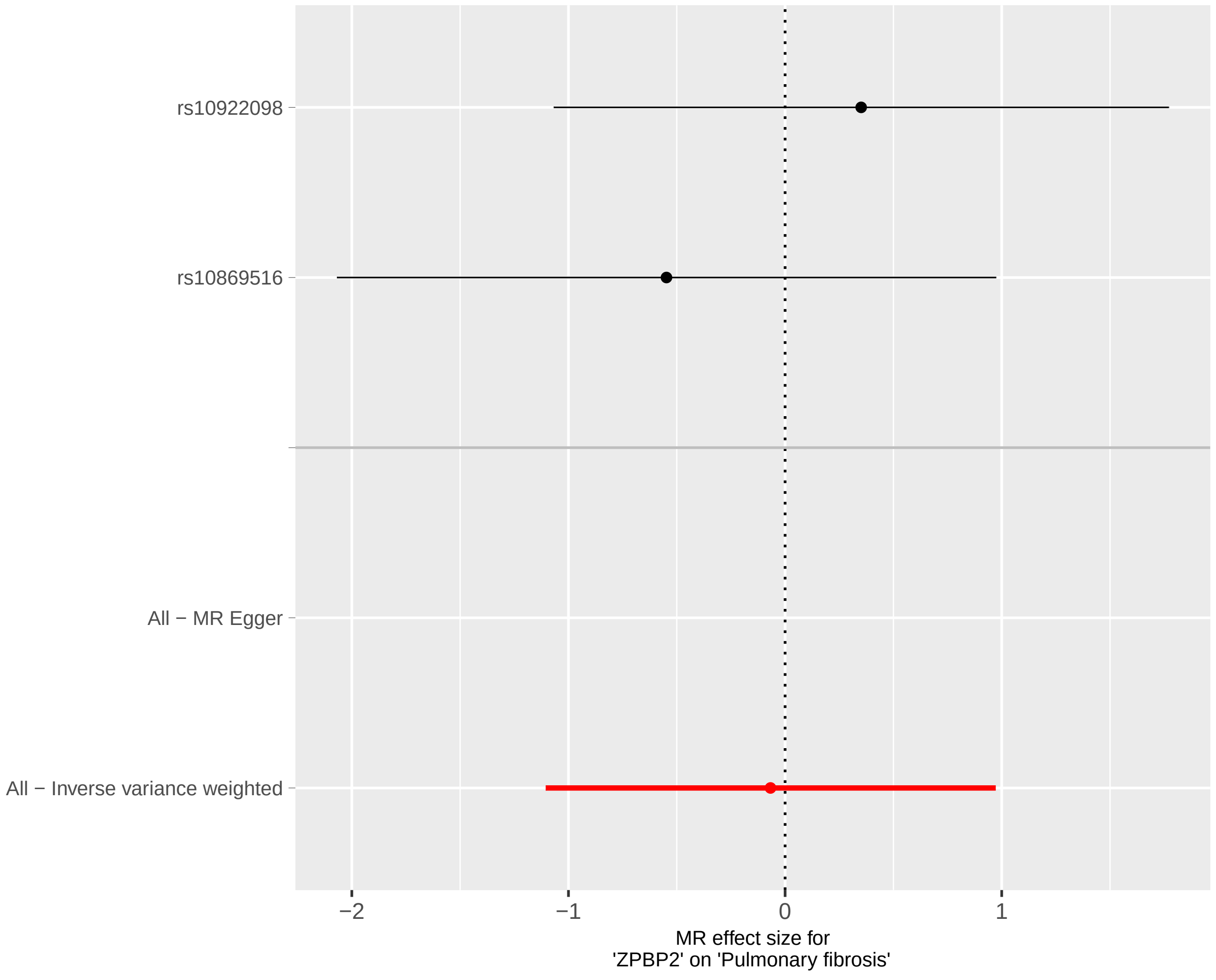
rs10922098
rs10869516
All − MR Egger
All − Inverse variance weighted
−2
−1
0
1
MR effect size for
'ZPBP2' on 'Pulmonary fibrosis'

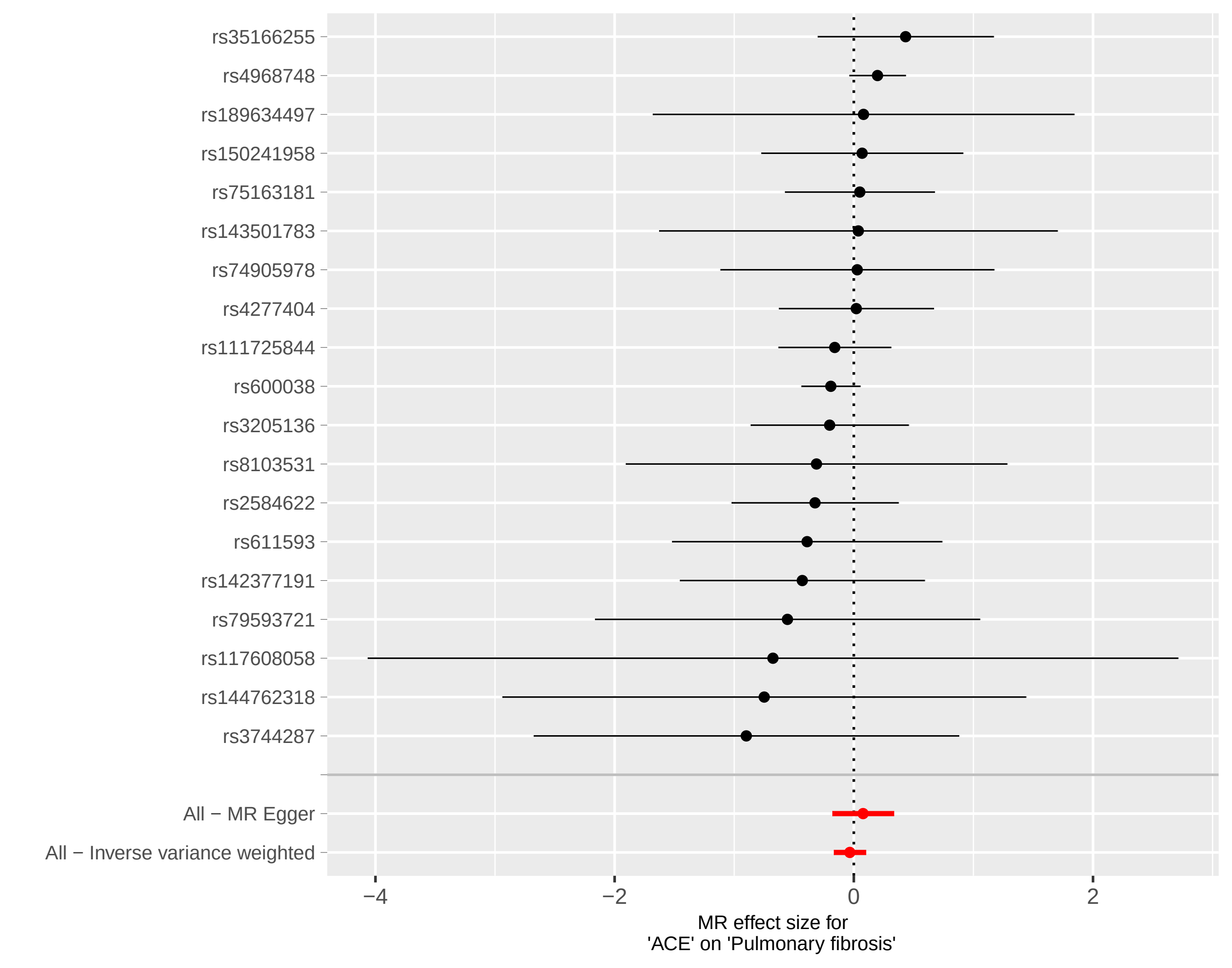
rs35166255
rs4968748
rs189634497
rs150241958
rs75163181
rs143501783
rs74905978
rs4277404
rs111725844
rs600038
rs3205136
rs8103531
rs2584622
rs611593
rs142377191
rs79593721
rs117608058
rs144762318
rs3744287
All − MR Egger
All − Inverse variance weighted
−4
−2
0
2
MR effect size for
'ACE' on 'Pulmonary fibrosis'

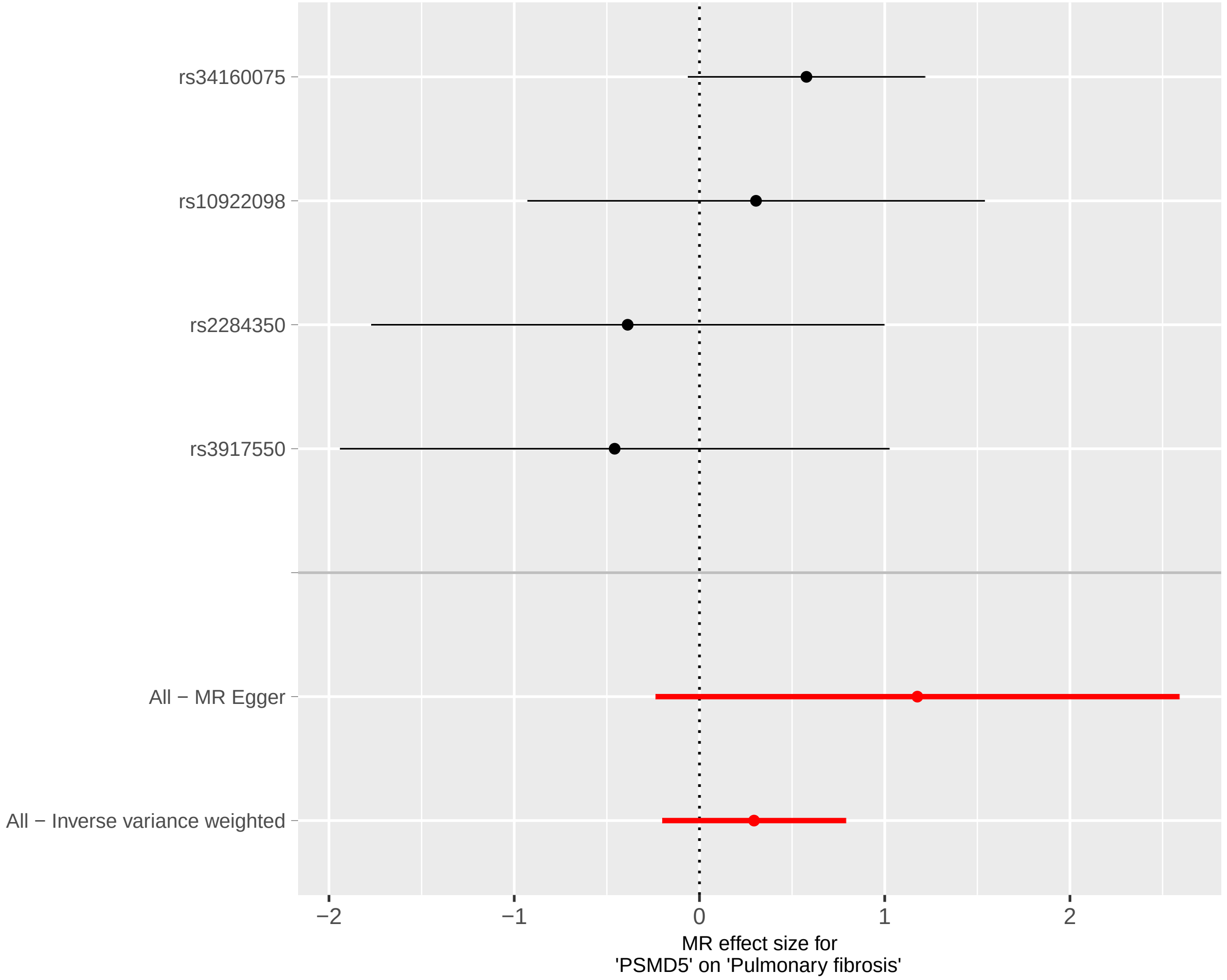

rs34160075
rs10922098
rs2284350
rs3917550
All − MR Egger
All − Inverse variance weighted
−2
−1
0
1
2
MR effect size for
'PSMD5' on 'Pulmonary fibrosis'

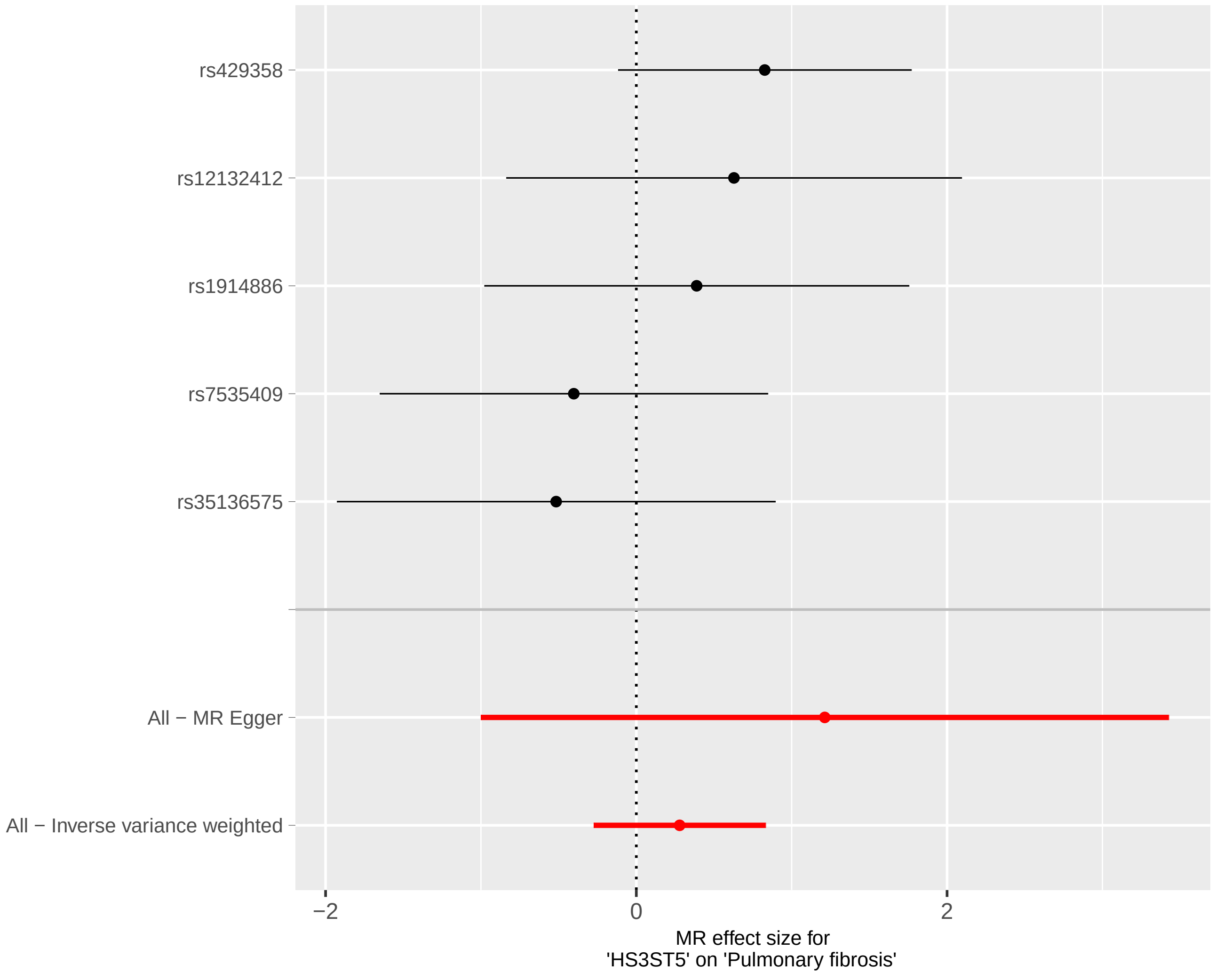

rs429358
rs12132412
rs1914886
rs7535409
rs35136575
All − MR Egger
All − Inverse variance weighted
−2
0
2
MR effect size for
'HS3ST5' on 'Pulmonary fibrosis'

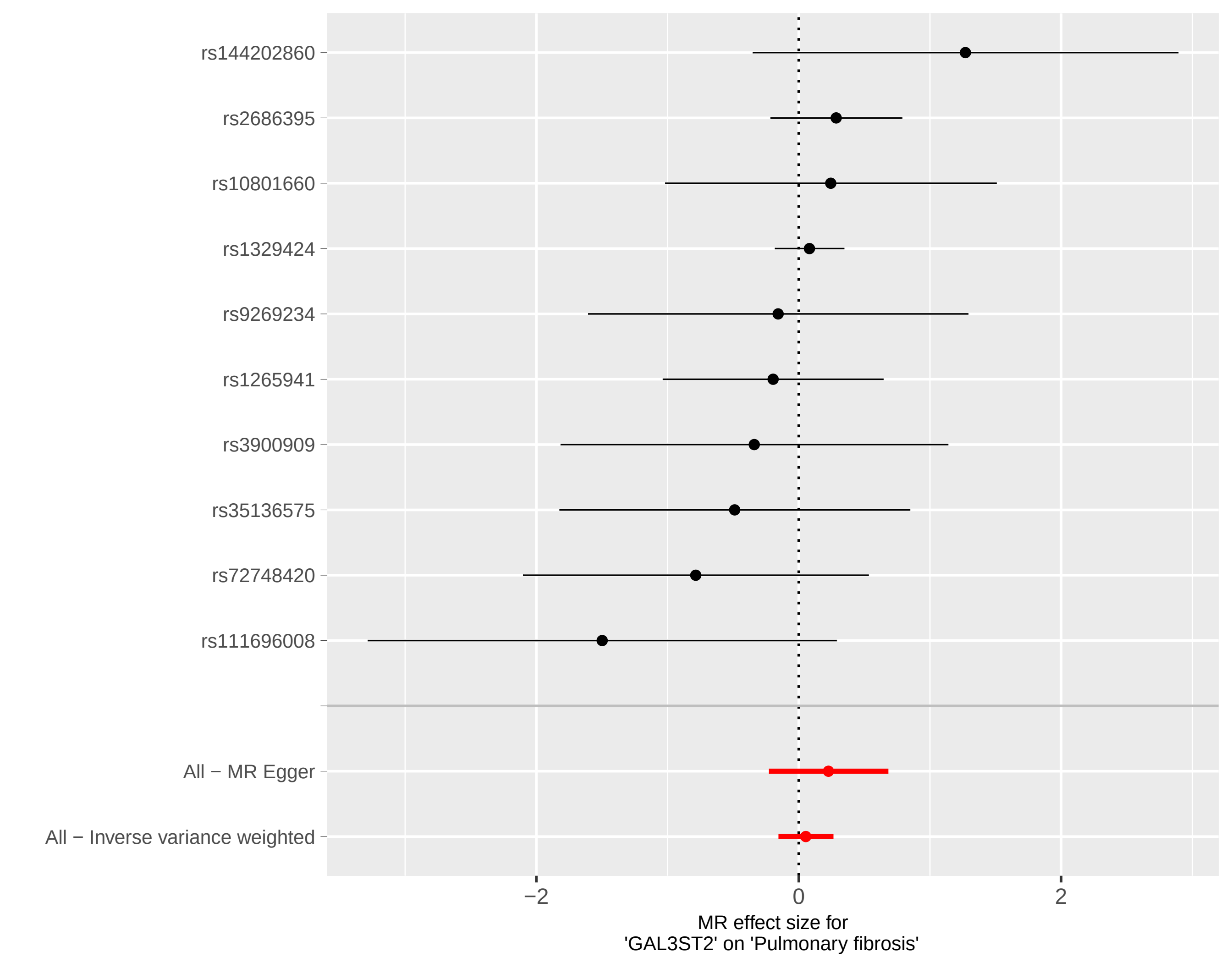

rs144202860
rs2686395
rs10801660
rs1329424
rs9269234
rs1265941
rs3900909
rs35136575
rs72748420
rs111696008
All − MR Egger
All − Inverse variance weighted
−2
0
2
MR effect size for
'GAL3ST2' on 'Pulmonary fibrosis'

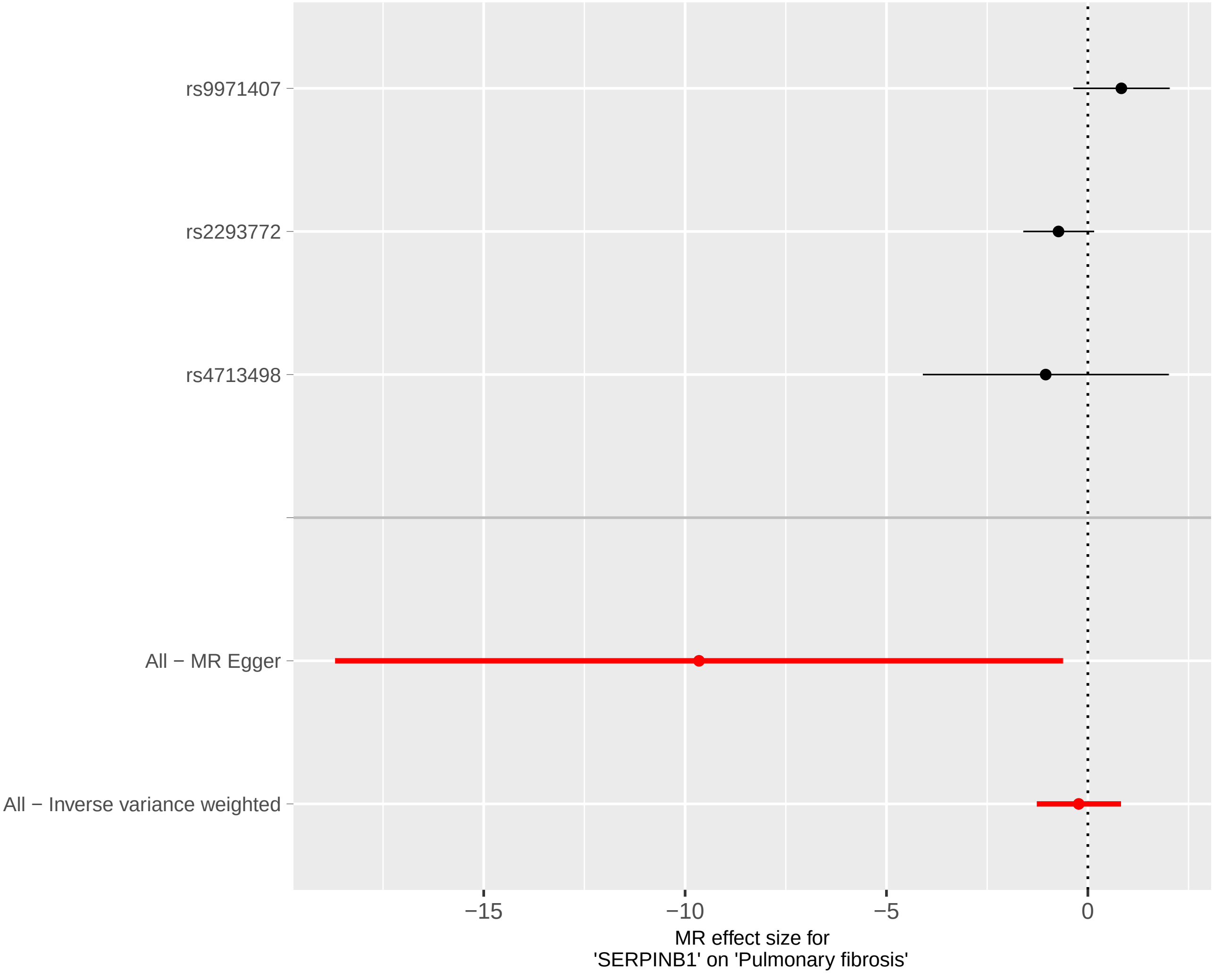
rs9971407
rs2293772
rs4713498
All − MR Egger
All − Inverse variance weighted
−15
−10
−5
0
MR effect size for
'SERPINB1' on 'Pulmonary fibrosis'

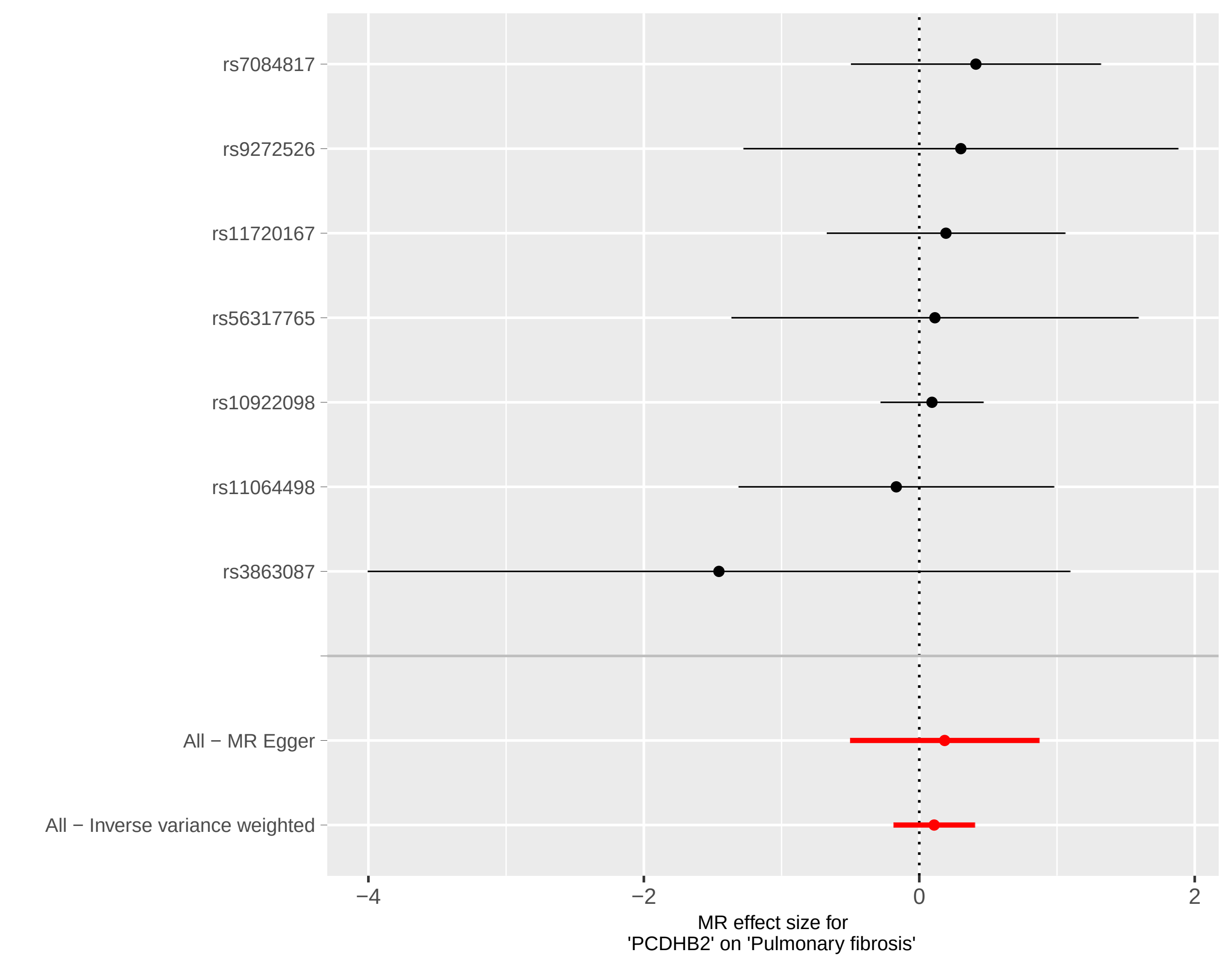

rs7084817
rs9272526
rs11720167
rs56317765
rs10922098
rs11064498
rs3863087
All − MR Egger
All − Inverse variance weighted
−4
−2
0
2
MR effect size for
'PCDHB2' on 'Pulmonary fibrosis'

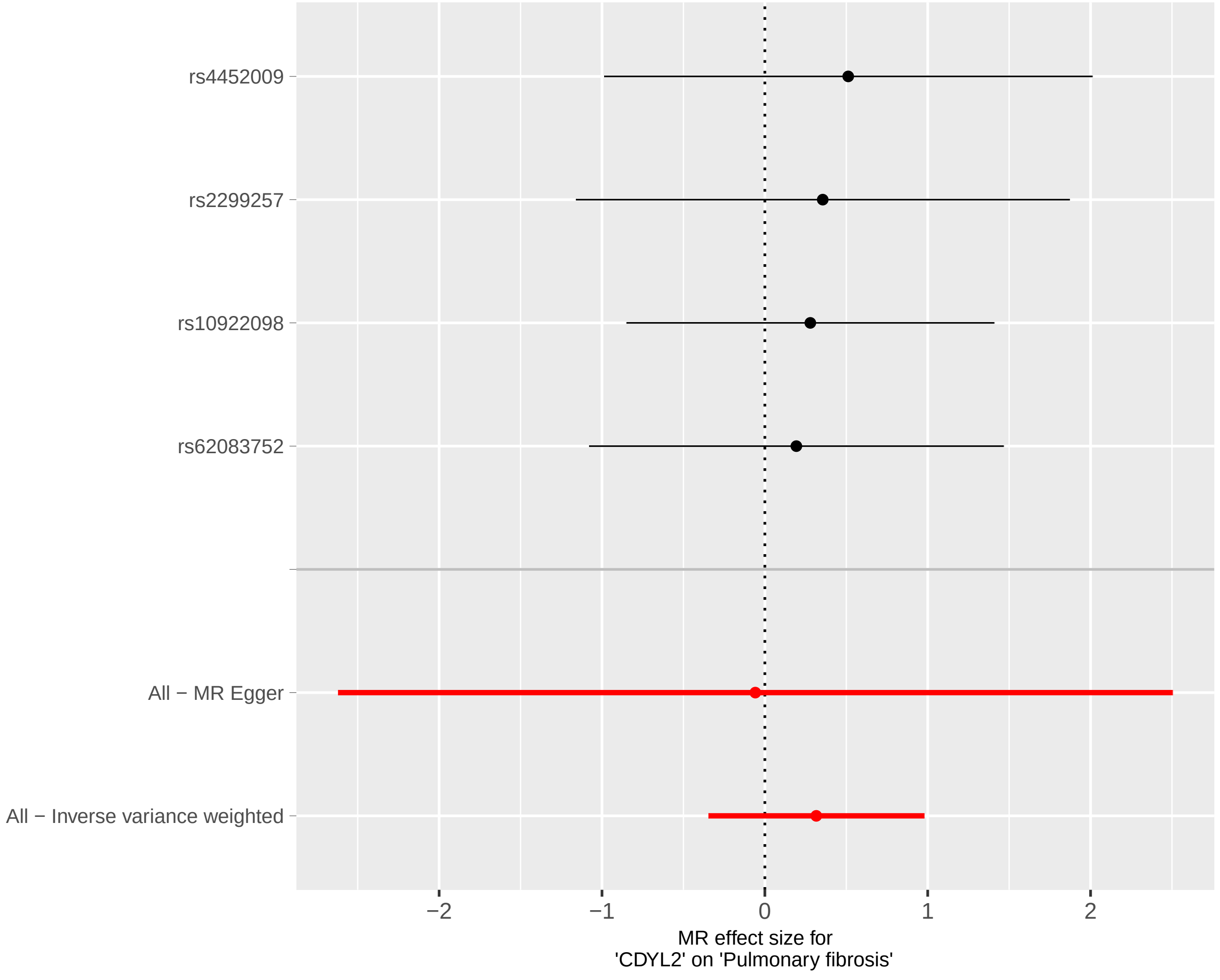

rs4452009
rs2299257
rs10922098
rs62083752
All − MR Egger
All − Inverse variance weighted
−2
−1
0
1
2
MR effect size for
'CDYL2' on 'Pulmonary fibrosis'

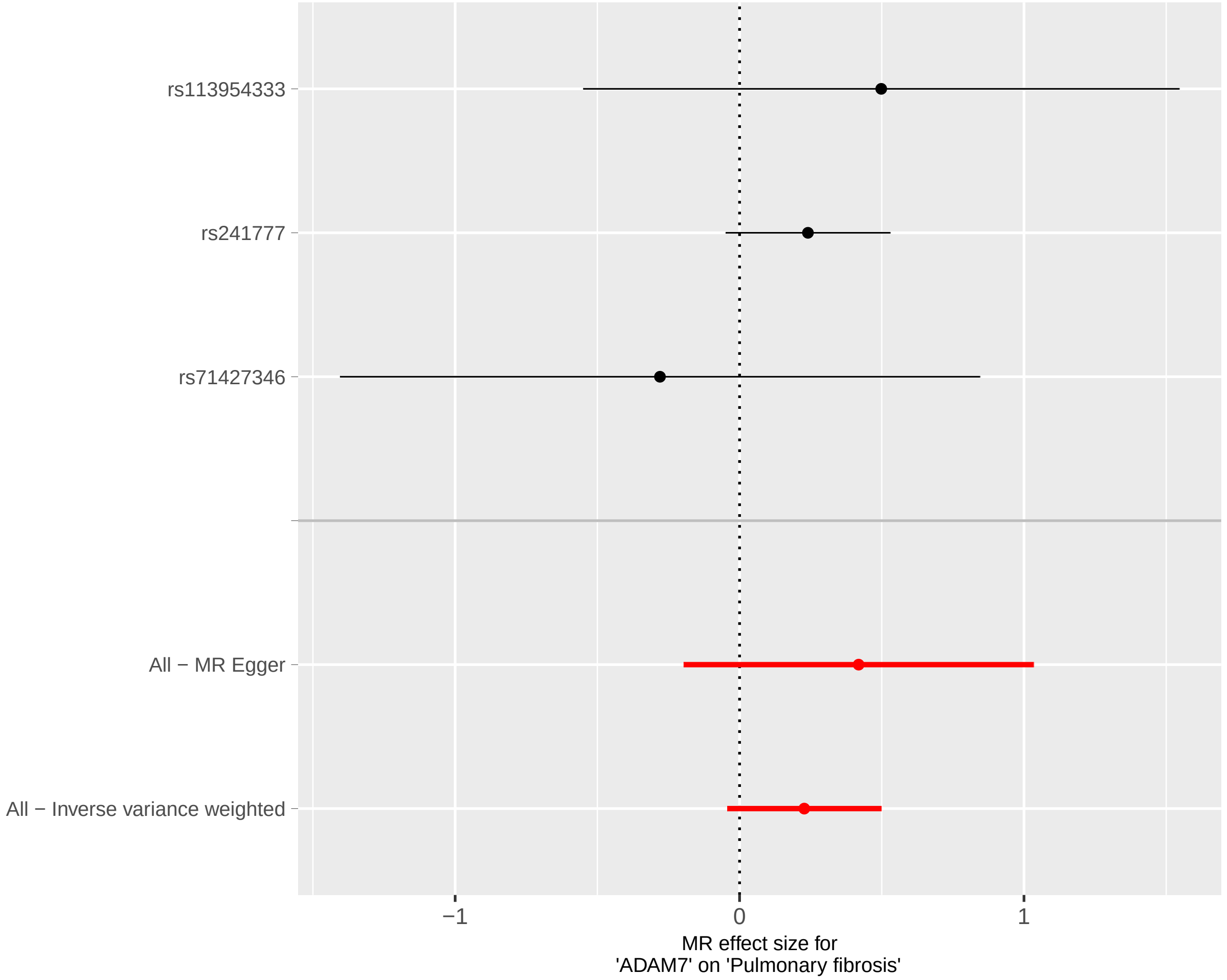
rs113954333
rs241777
rs71427346
All − MR Egger
All − Inverse variance weighted
−1
0
1
MR effect size for
'ADAM7' on 'Pulmonary fibrosis'

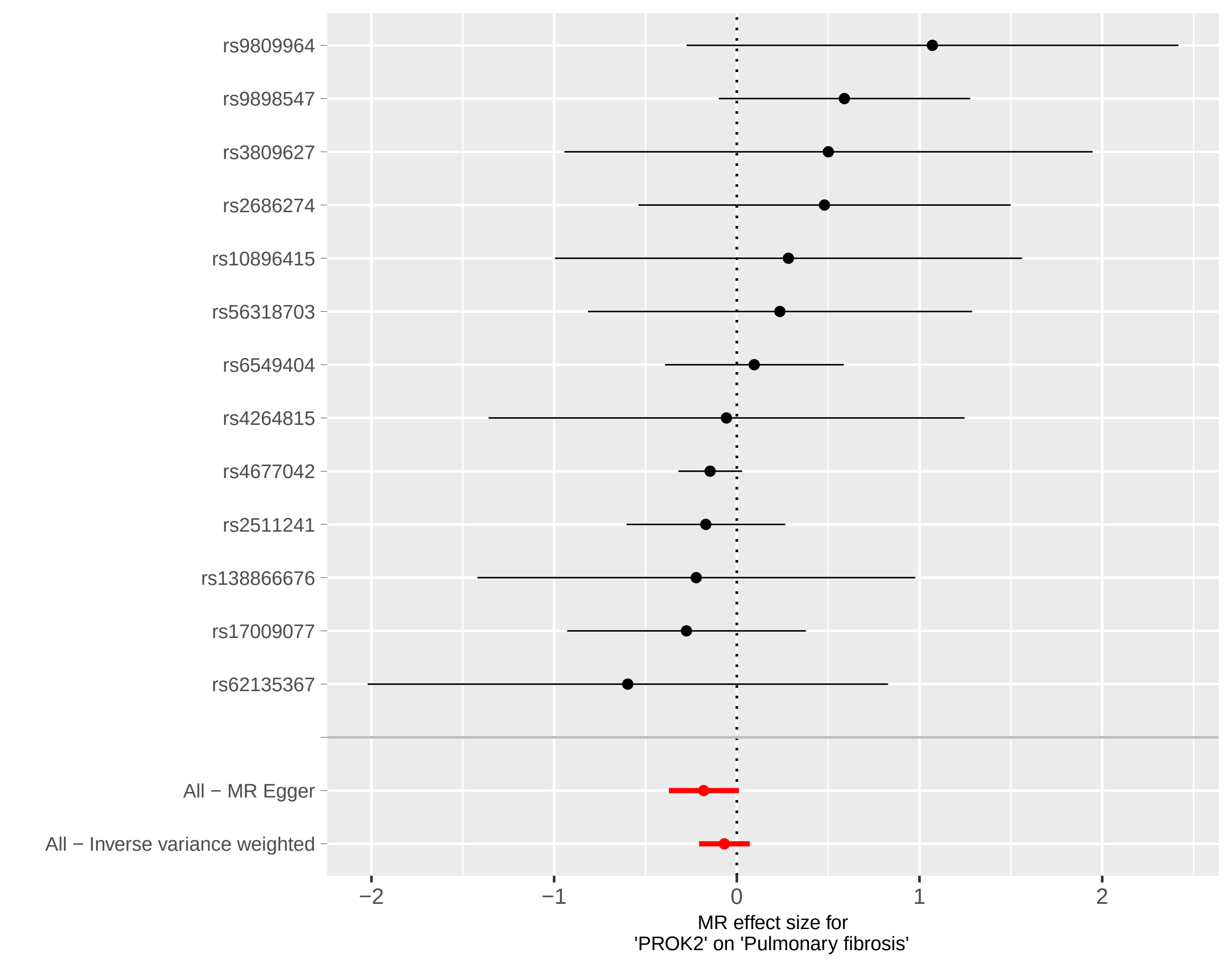
rs9809964
rs9898547
rs3809627
rs2686274
rs10896415
rs56318703
rs6549404
rs4264815
rs4677042
rs2511241
rs138866676
rs17009077
rs62135367
All − MR Egger
All − Inverse variance weighted
−2
−1
0
1
2
MR effect size for
'PROK2' on 'Pulmonary fibrosis'

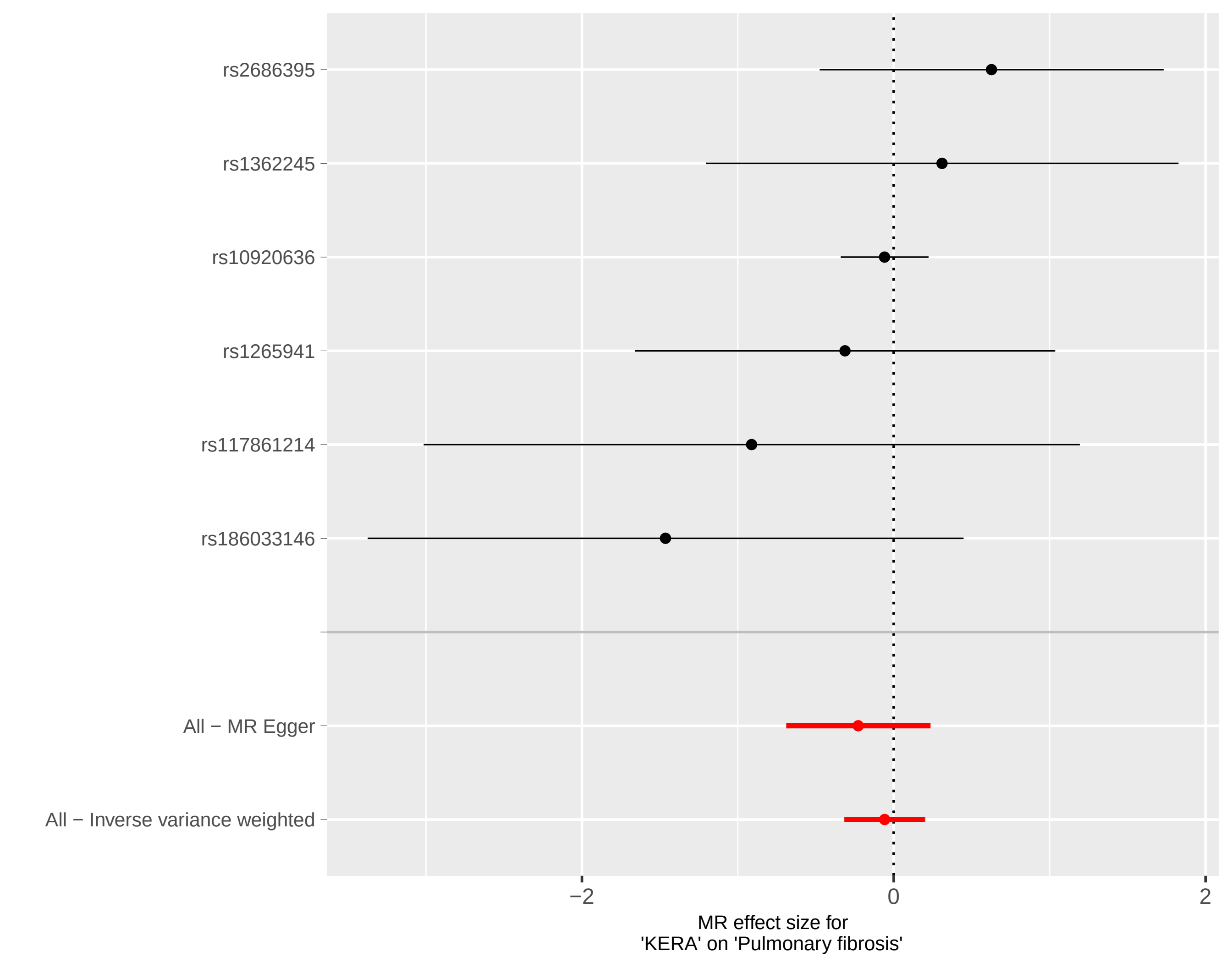
rs2686395
rs1362245
rs10920636
rs1265941
rs117861214
rs186033146
All − MR Egger
All − Inverse variance weighted
−2
0
2
MR effect size for
'KERA' on 'Pulmonary fibrosis'

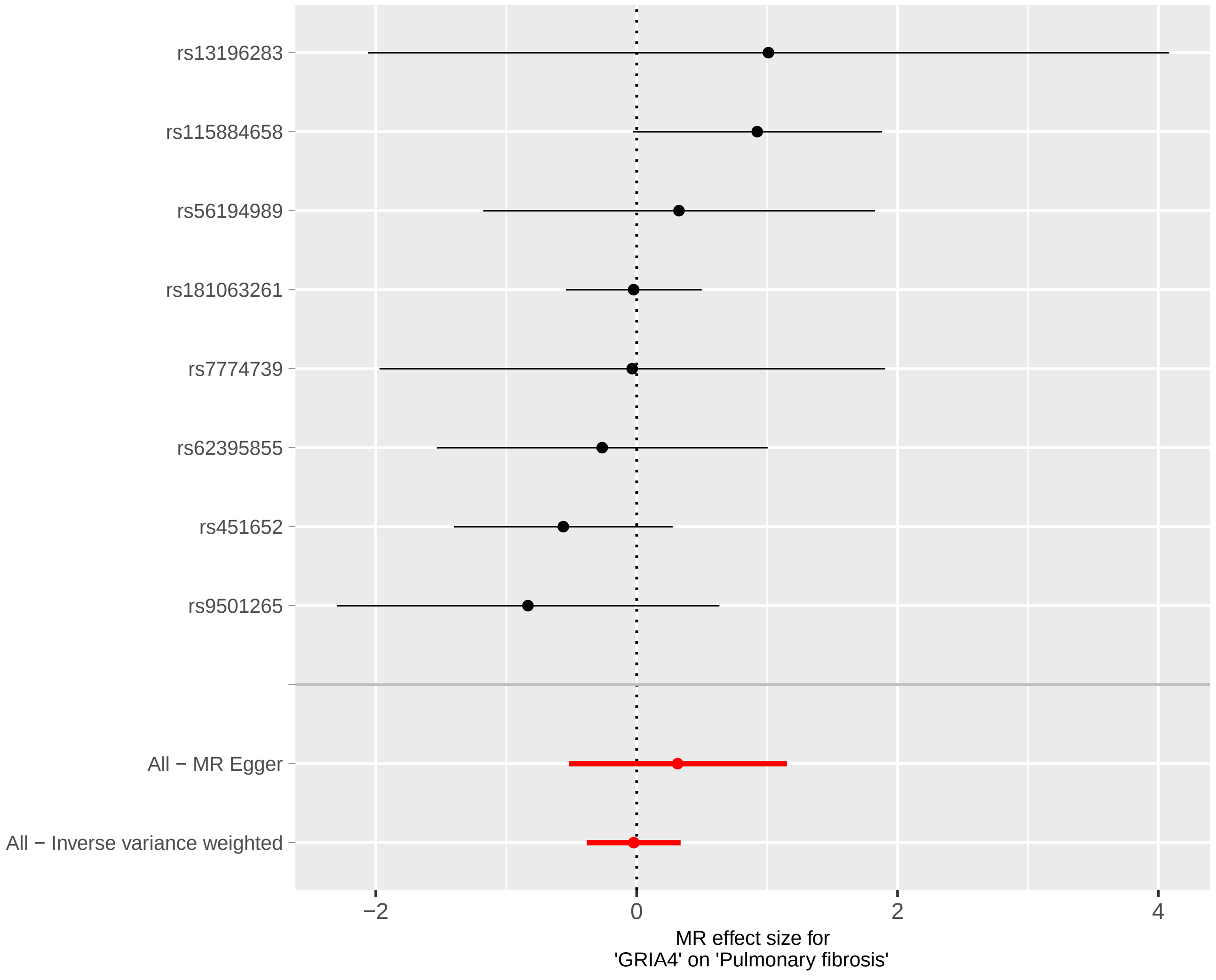
rs13196283
rs115884658
rs56194989
rs181063261
rs7774739
rs62395855
rs451652
rs9501265
All − MR Egger
All − Inverse variance weighted
−2
0
2
4
MR effect size for
'GRIA4' on 'Pulmonary fibrosis'

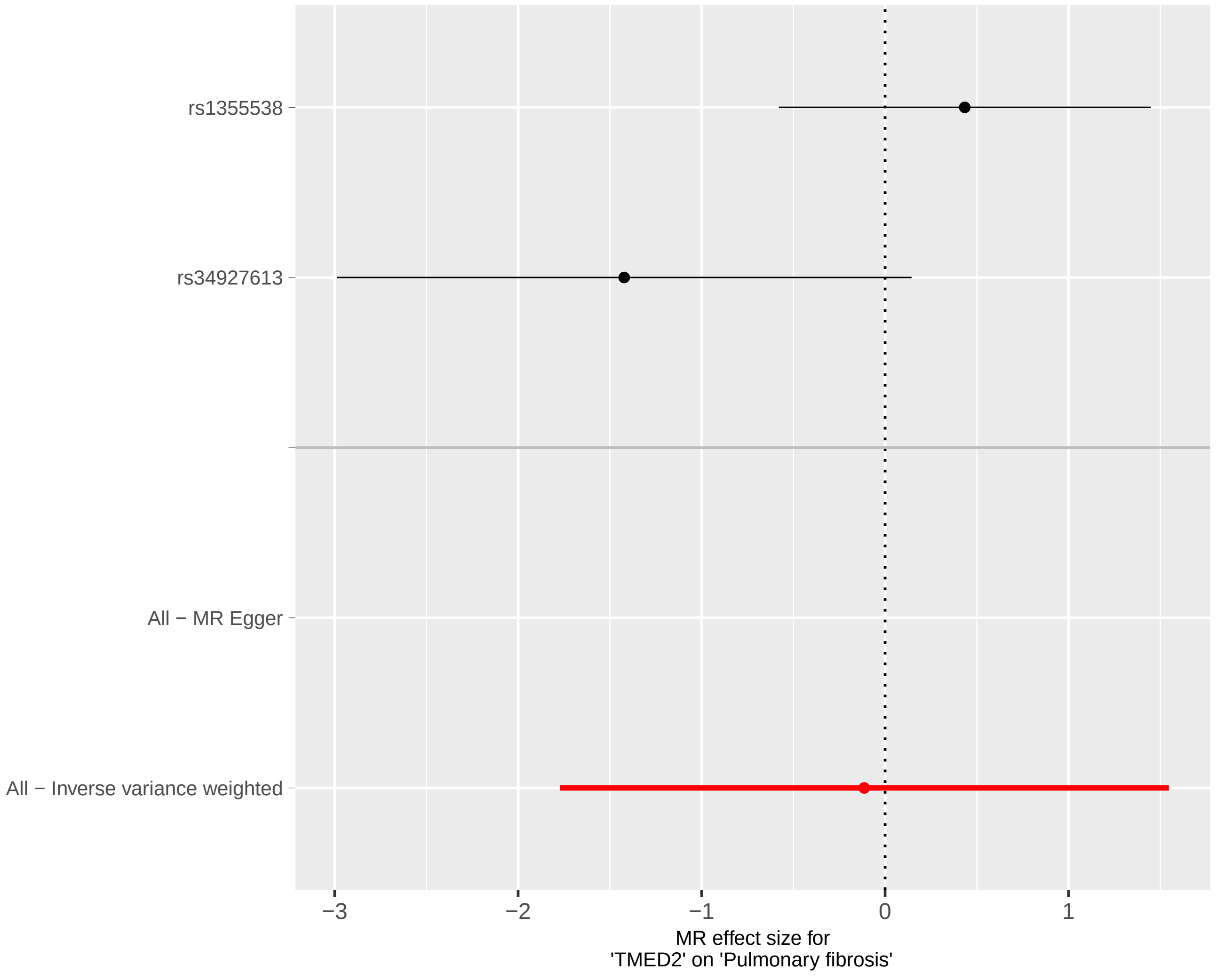
rs1355538
rs34927613
All − MR Egger
All − Inverse variance weighted
−3
−2
−1
0
1
MR effect size for
'TMED2' on 'Pulmonary fibrosis'

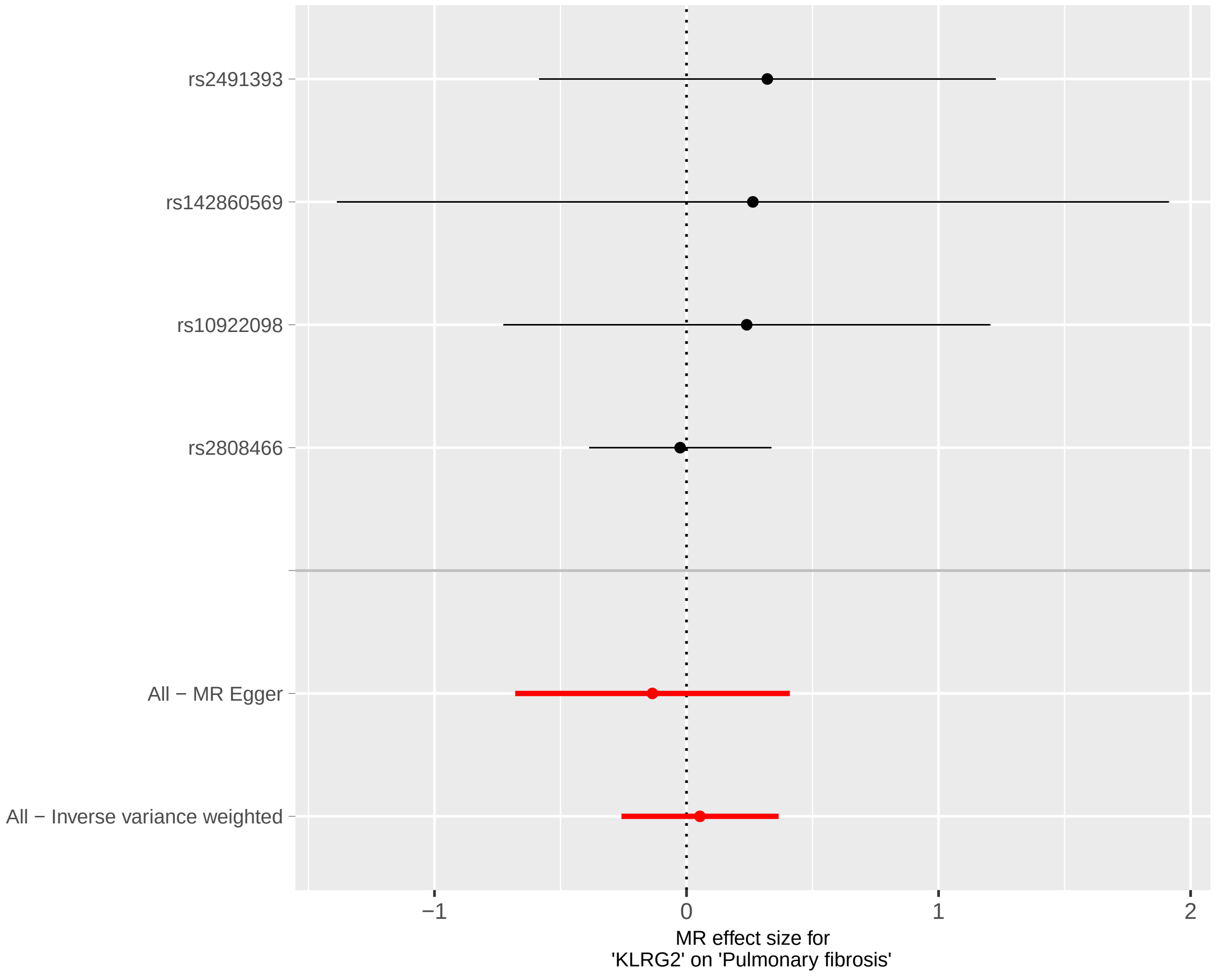
rs2491393
rs142860569
rs10922098
rs2808466
All − MR Egger
All − Inverse variance weighted
−1
0
1
2
MR effect size for
'KLRG2' on 'Pulmonary fibrosis'

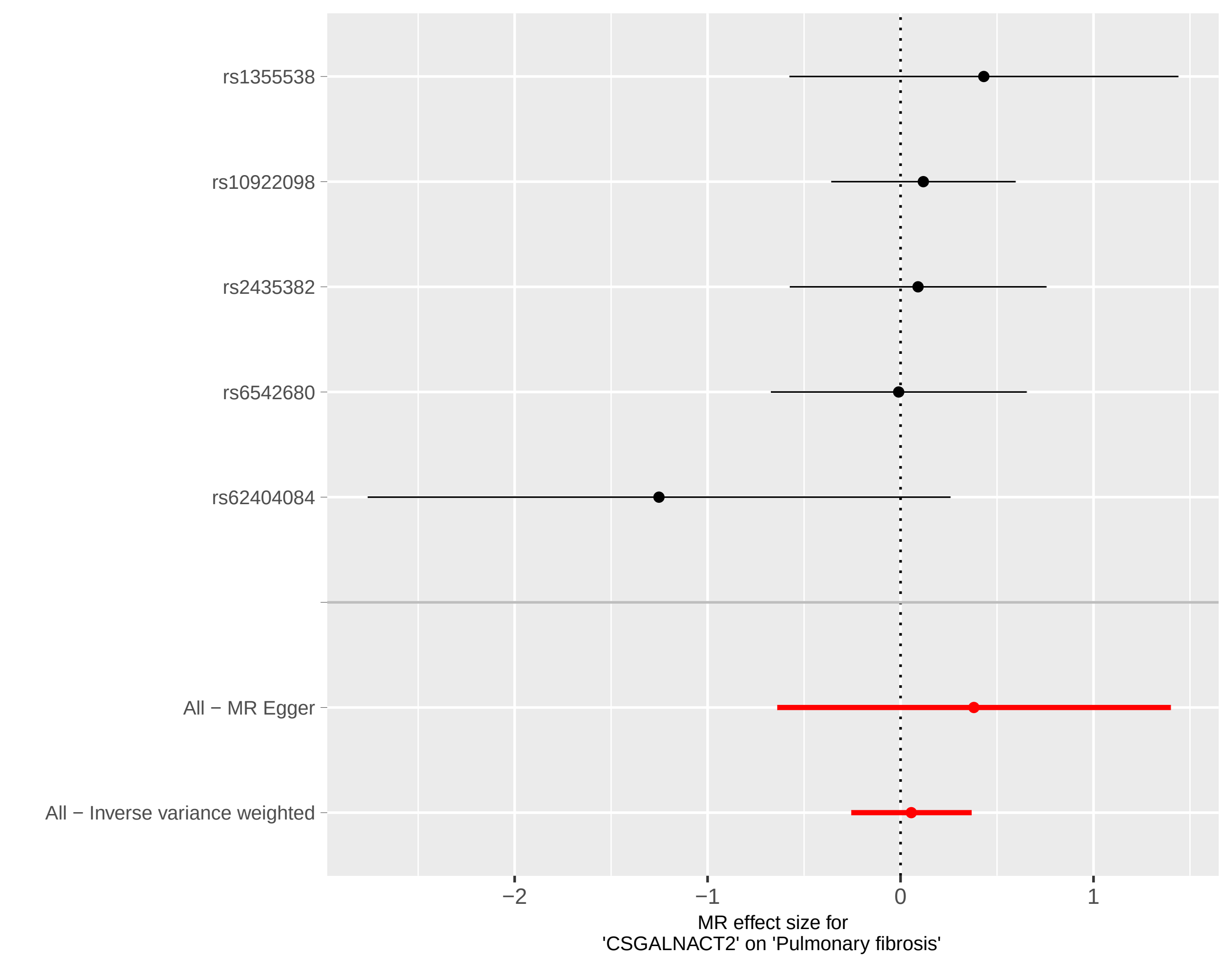

rs1355538
rs10922098
rs2435382
rs6542680
rs62404084
All − MR Egger
All − Inverse variance weighted
−2
−1
0
1
MR effect size for
'CSGALNACT2' on 'Pulmonary fibrosis'

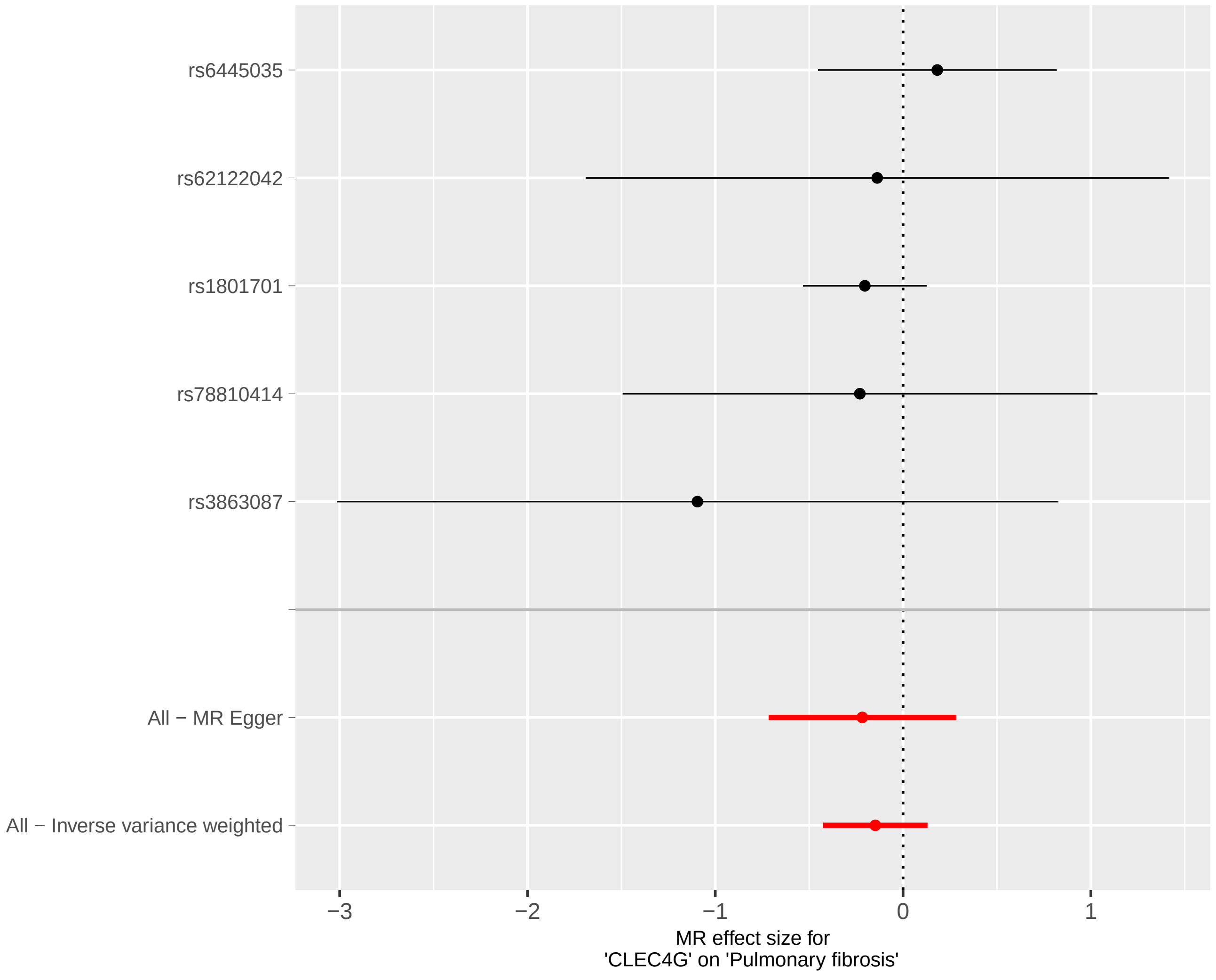
rs6445035
rs62122042
rs1801701
rs78810414
rs3863087
All − MR Egger
All − Inverse variance weighted
−3
−2
−1
0
1
MR effect size for
'CLEC4G' on 'Pulmonary fibrosis'

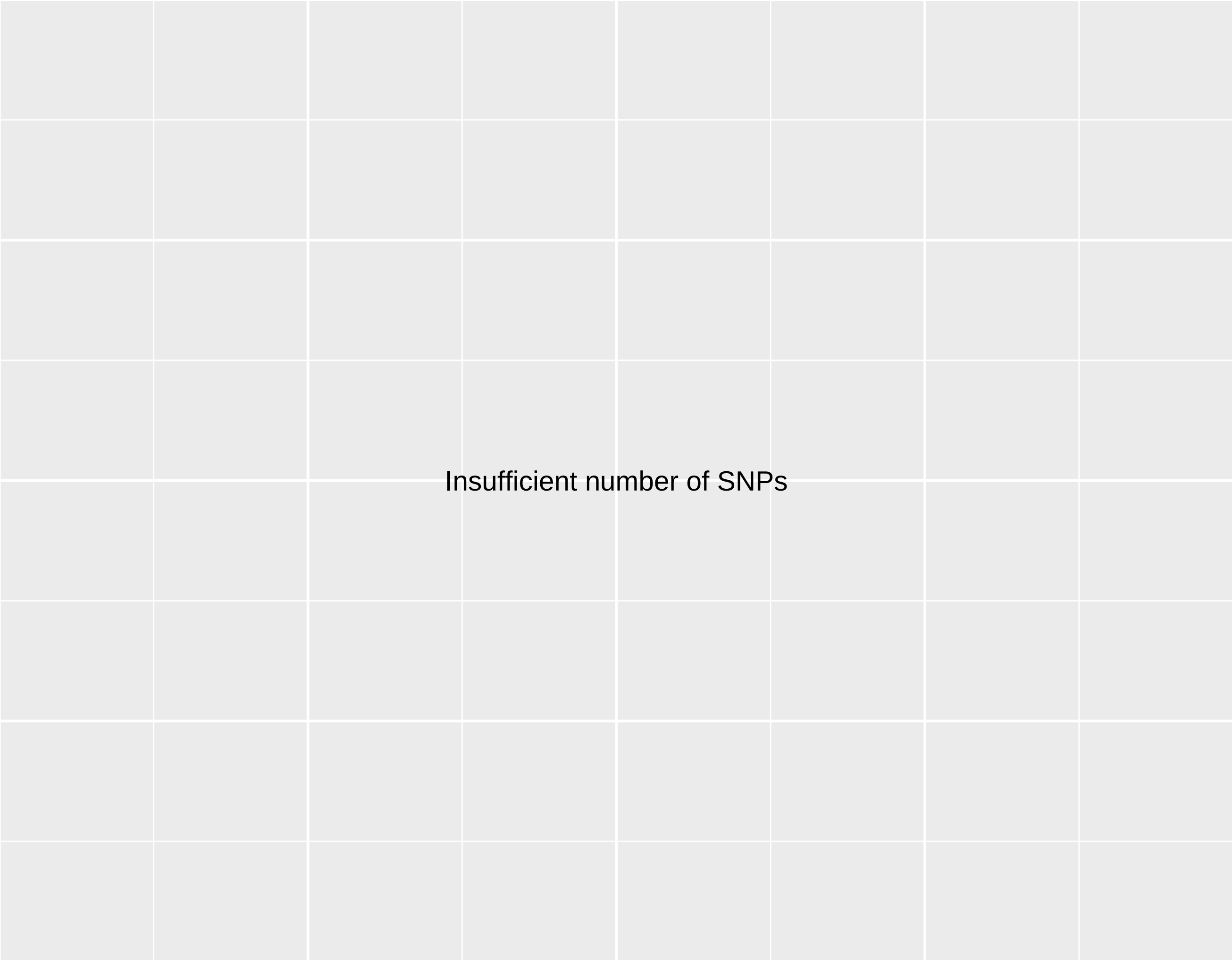
Insufficient number of SNPs

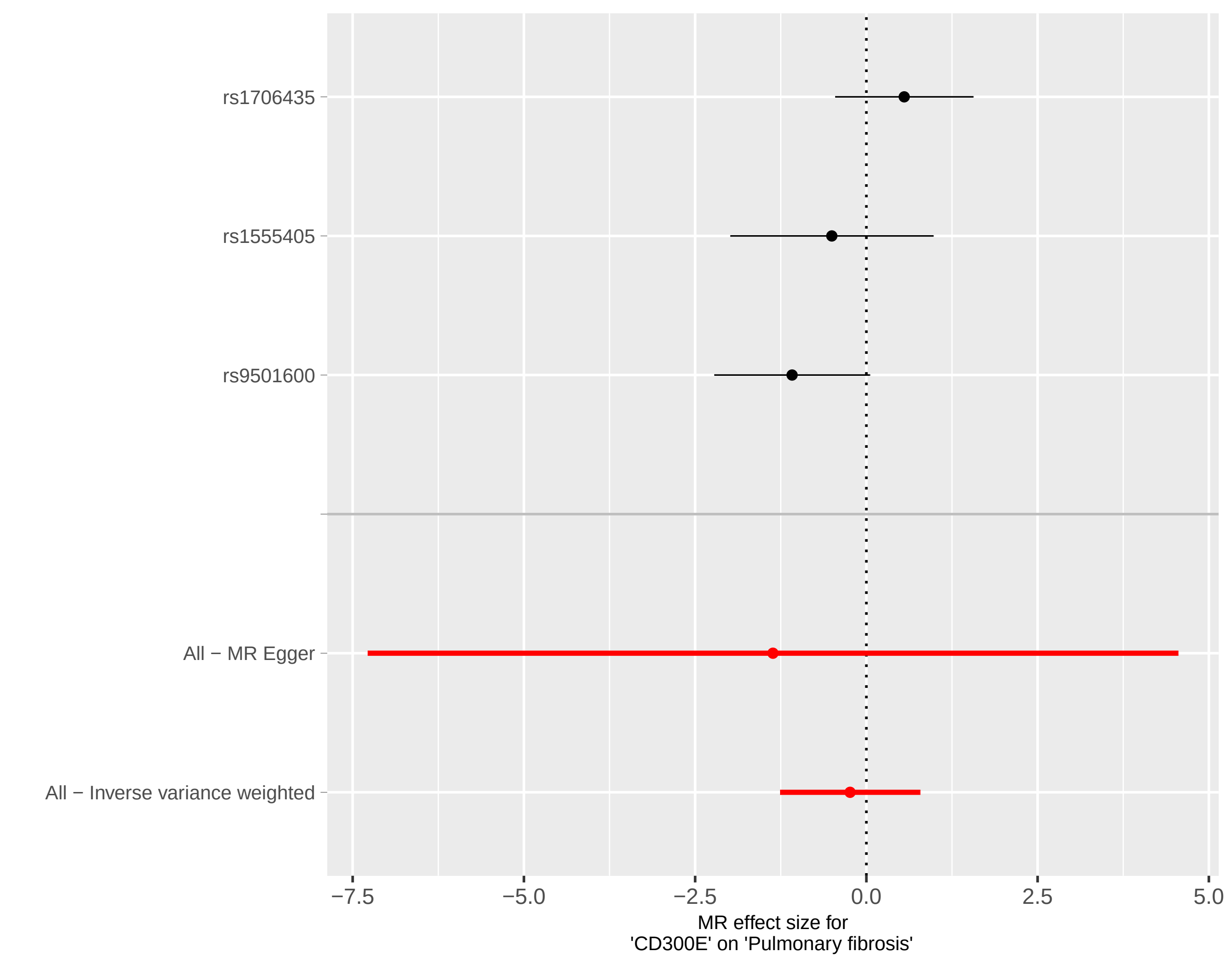

rs1706435
rs1555405
rs9501600
All − MR Egger
All − Inverse variance weighted
−7.5
−5.0
−2.5
0.0
2.5
5.0
MR effect size for
'CD300E' on 'Pulmonary fibrosis'

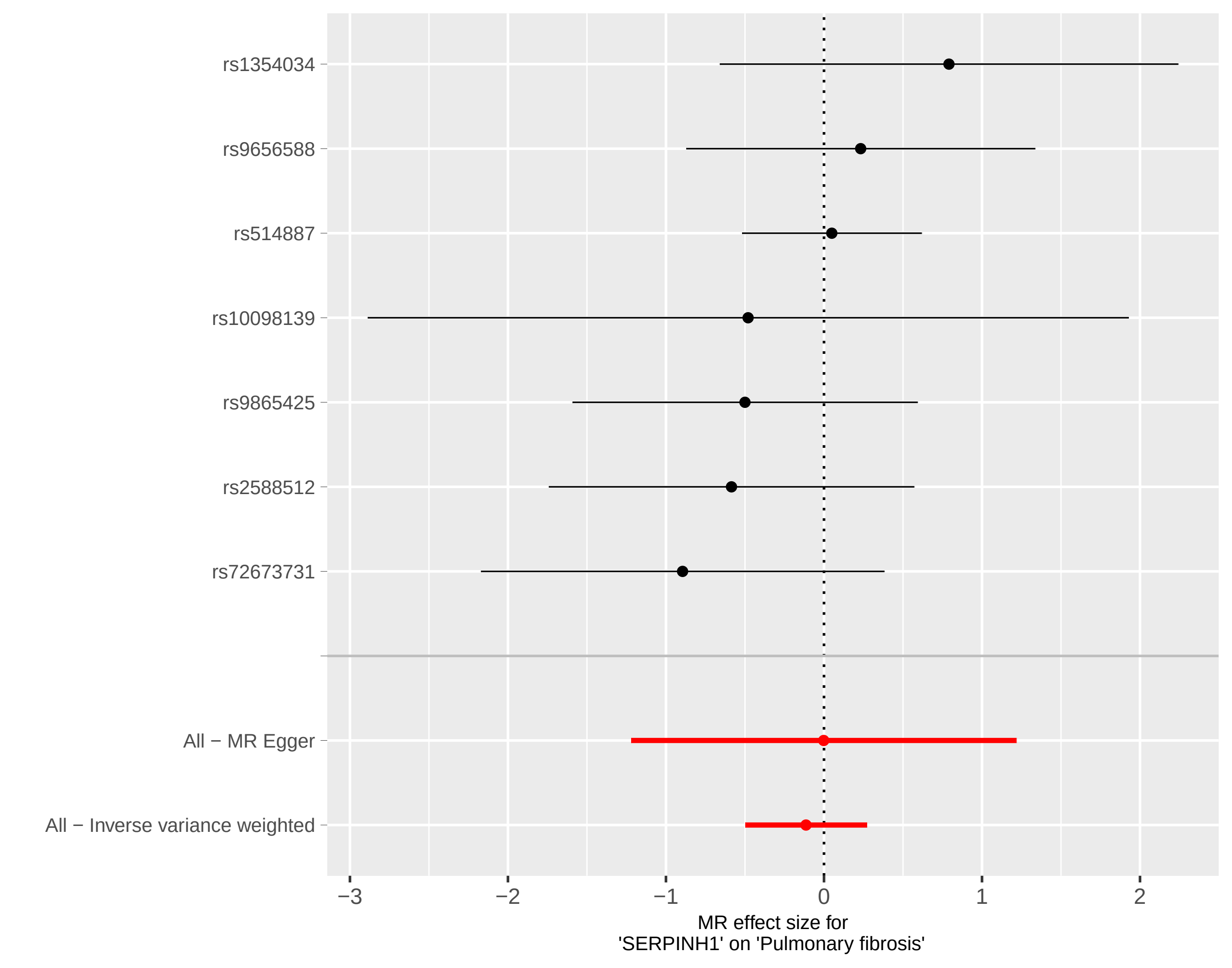
rs1354034
rs9656588
rs514887
rs10098139
rs9865425
rs2588512
rs72673731
All − MR Egger
All − Inverse variance weighted
−3
−2
−1
0
1
2
MR effect size for
'SERPINH1' on 'Pulmonary fibrosis'

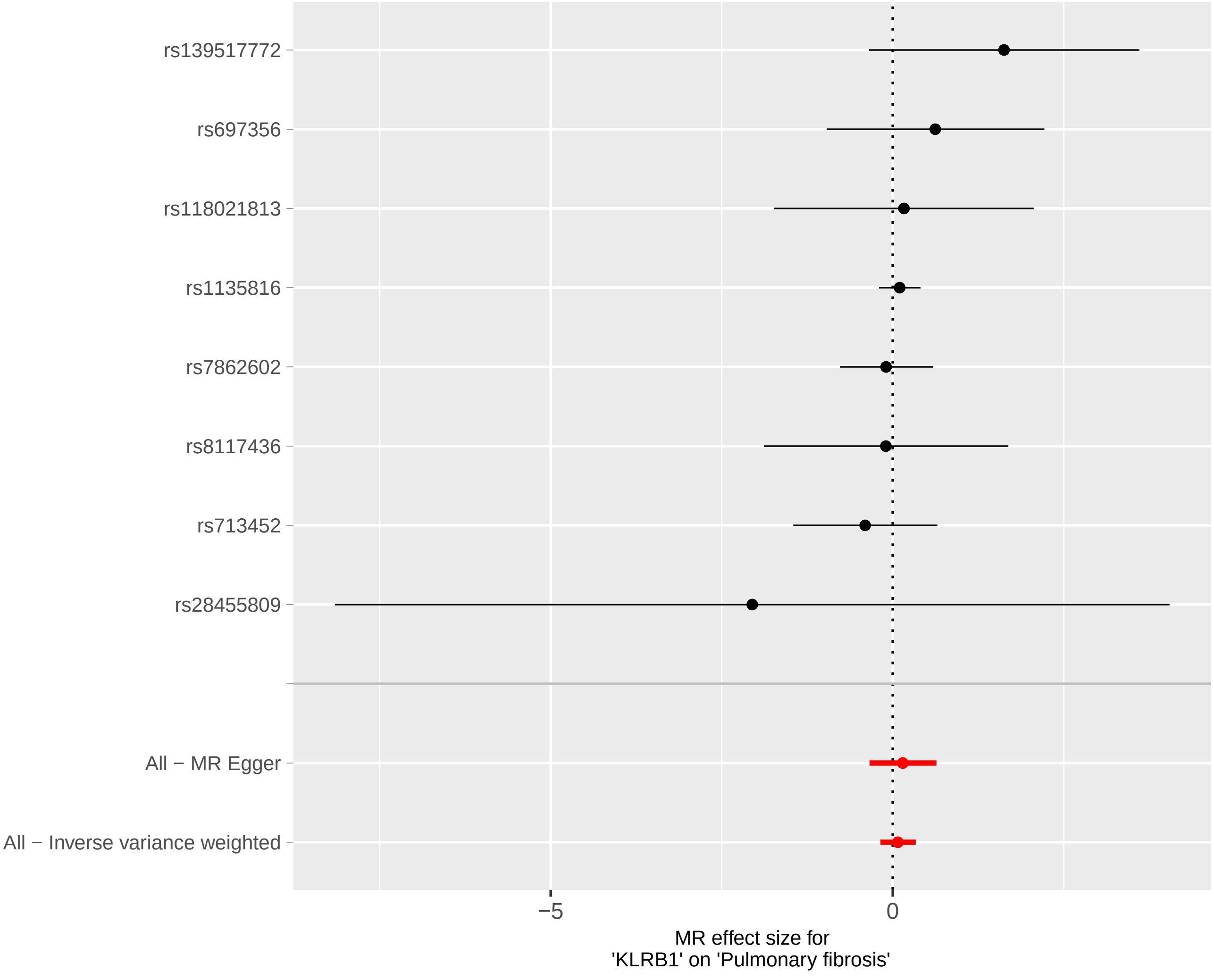
rs139517772
rs697356
rs118021813
rs1135816
rs7862602
rs8117436
rs713452
rs28455809
All − MR Egger
All − Inverse variance weighted
−5
0
MR effect size for
'KLRB1' on 'Pulmonary fibrosis'

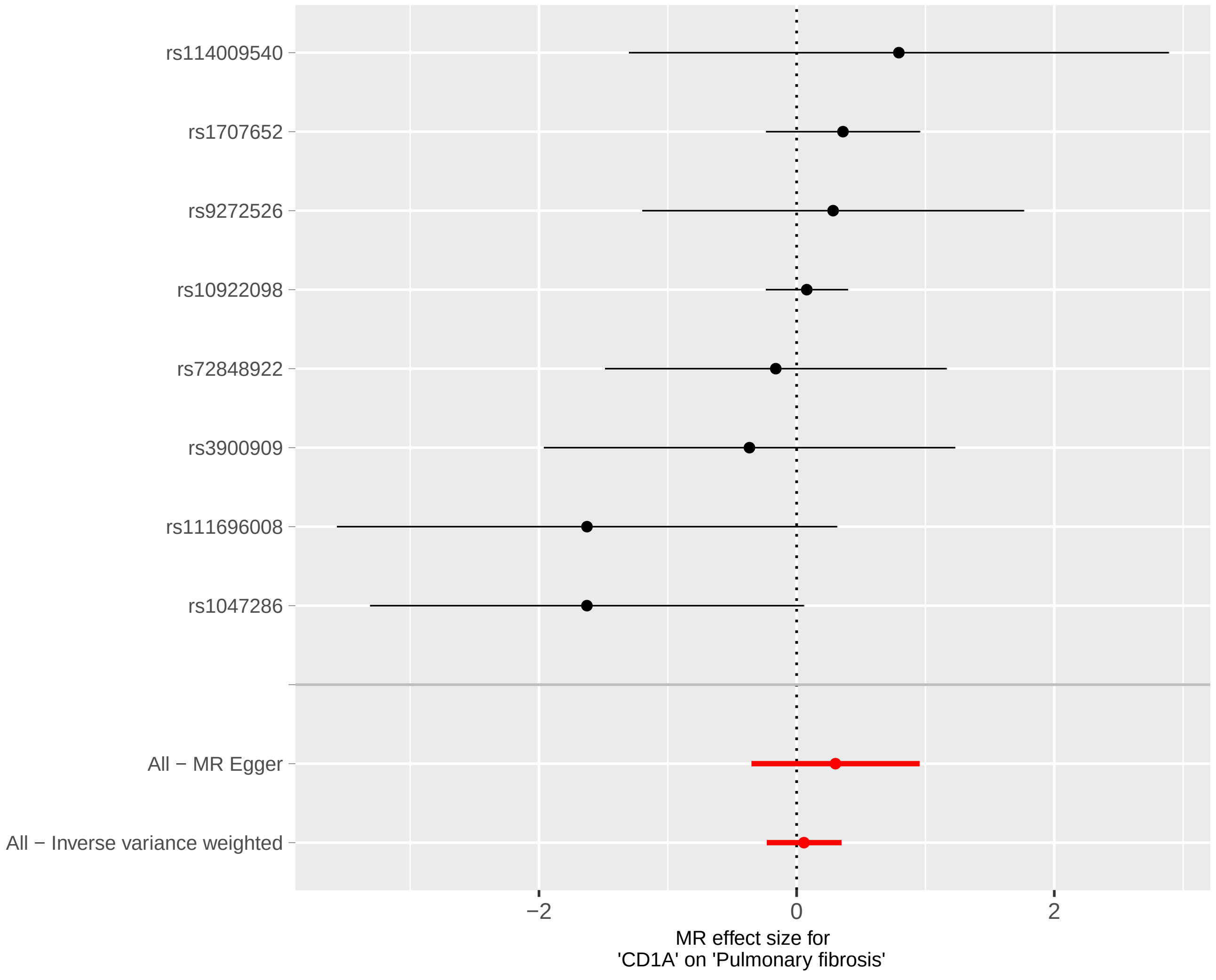
rs114009540
rs1707652
rs9272526
rs10922098
rs72848922
rs3900909
rs111696008
rs1047286
All − MR Egger
All − Inverse variance weighted
−2
0
2
MR effect size for
'CD1A' on 'Pulmonary fibrosis'

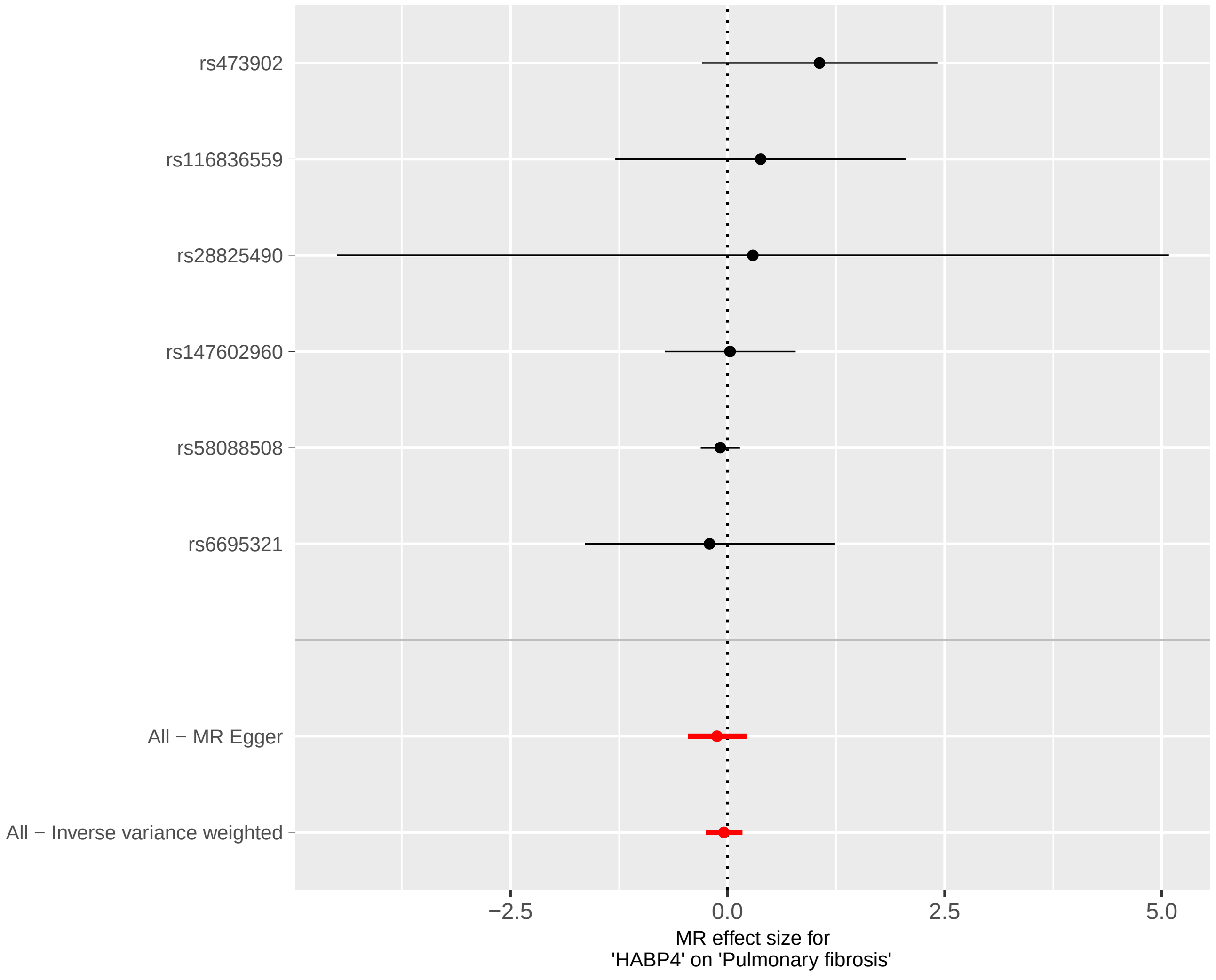
rs473902
rs116836559
rs28825490
rs147602960
rs58088508
rs6695321
All − MR Egger
All − Inverse variance weighted
−2.5
0.0
2.5
5.0
MR effect size for
'HABP4' on 'Pulmonary fibrosis'

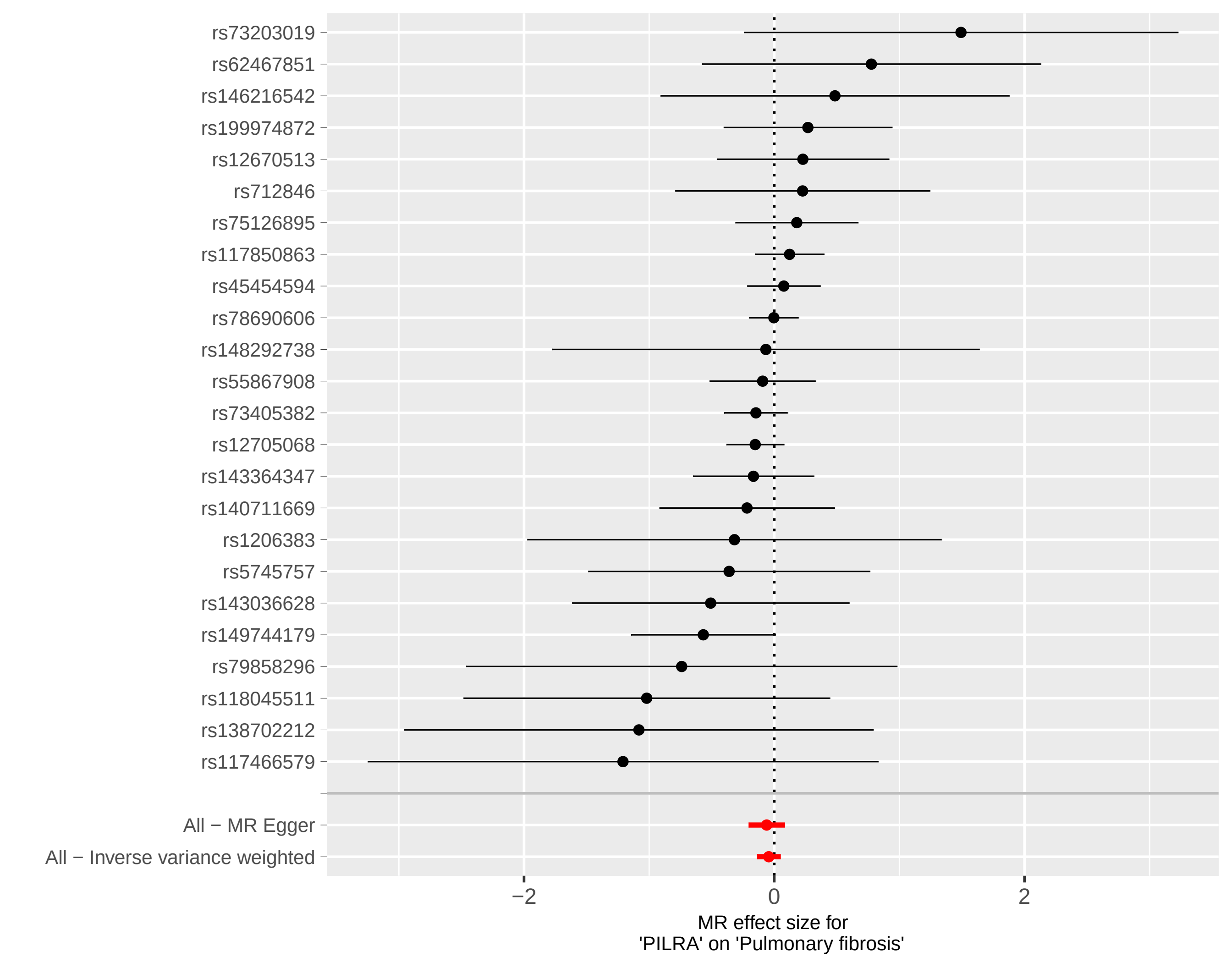

rs73203019
rs62467851
rs146216542
rs199974872
rs12670513
rs712846
rs75126895
rs117850863
rs45454594
rs78690606
rs148292738
rs55867908
rs73405382
rs12705068
rs143364347
rs140711669
rs1206383
rs5745757
rs143036628
rs149744179
rs79858296
rs118045511
rs138702212
rs117466579
All − MR Egger
All − Inverse variance weighted
−2
0
2
MR effect size for
'PILRA' on 'Pulmonary fibrosis'

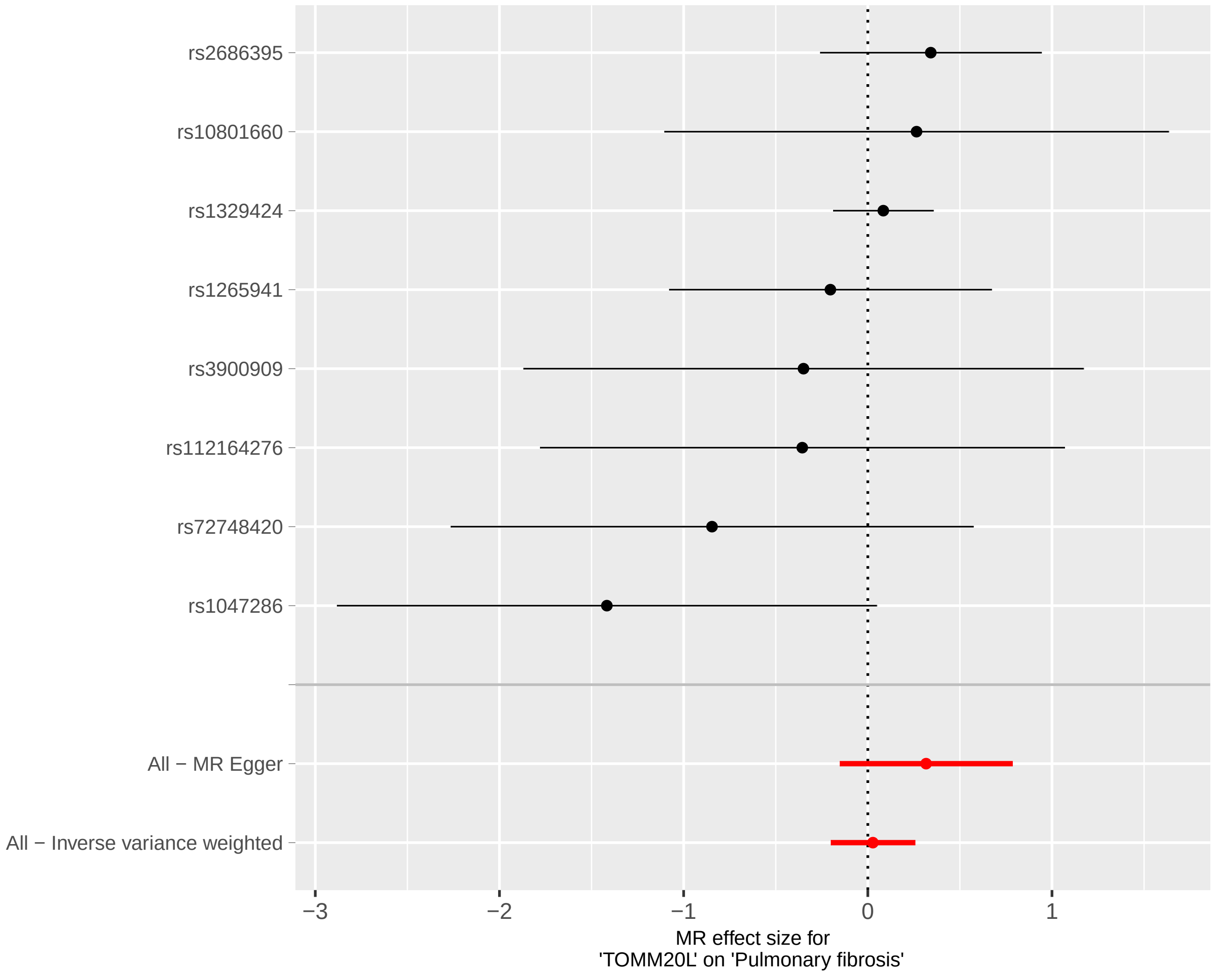
rs2686395
rs10801660
rs1329424
rs1265941
rs3900909
rs112164276
rs72748420
rs1047286
All − MR Egger
All − Inverse variance weighted
−3
−2
−1
0
1
MR effect size for
'TOMM20L' on 'Pulmonary fibrosis'

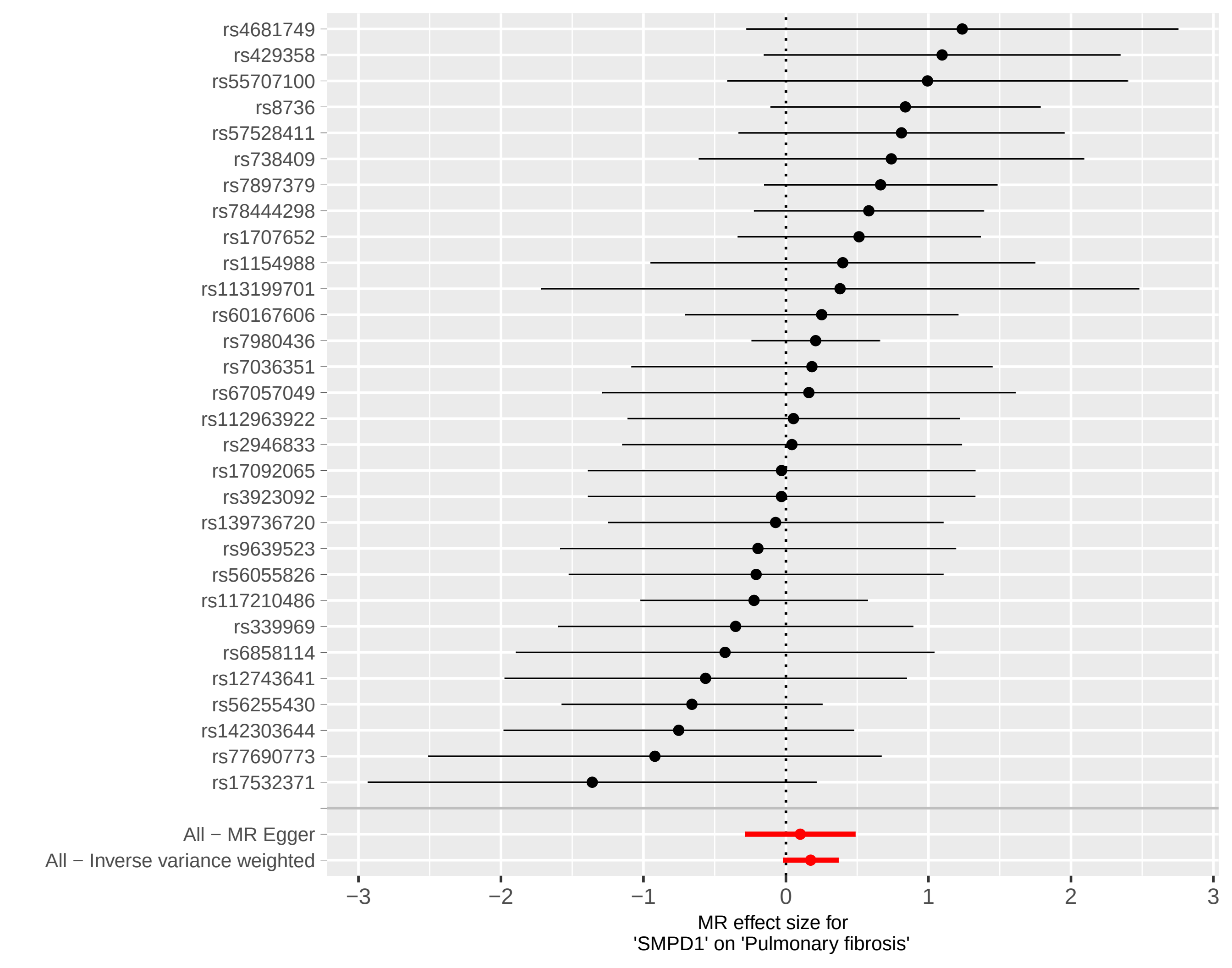
rs4681749
rs429358
rs55707100
rs8736
rs57528411
rs738409
rs7897379
rs78444298
rs1707652
rs1154988
rs113199701
rs60167606
rs7980436
rs7036351
rs67057049
rs112963922
rs2946833
rs17092065
rs3923092
rs139736720
rs9639523
rs56055826
rs117210486
rs339969
rs6858114
rs12743641
rs56255430
rs142303644
rs77690773
rs17532371
All − MR Egger
All − Inverse variance weighted
−3
−2
−1
0
1
2
3
MR effect size for
'SMPD1' on 'Pulmonary fibrosis'

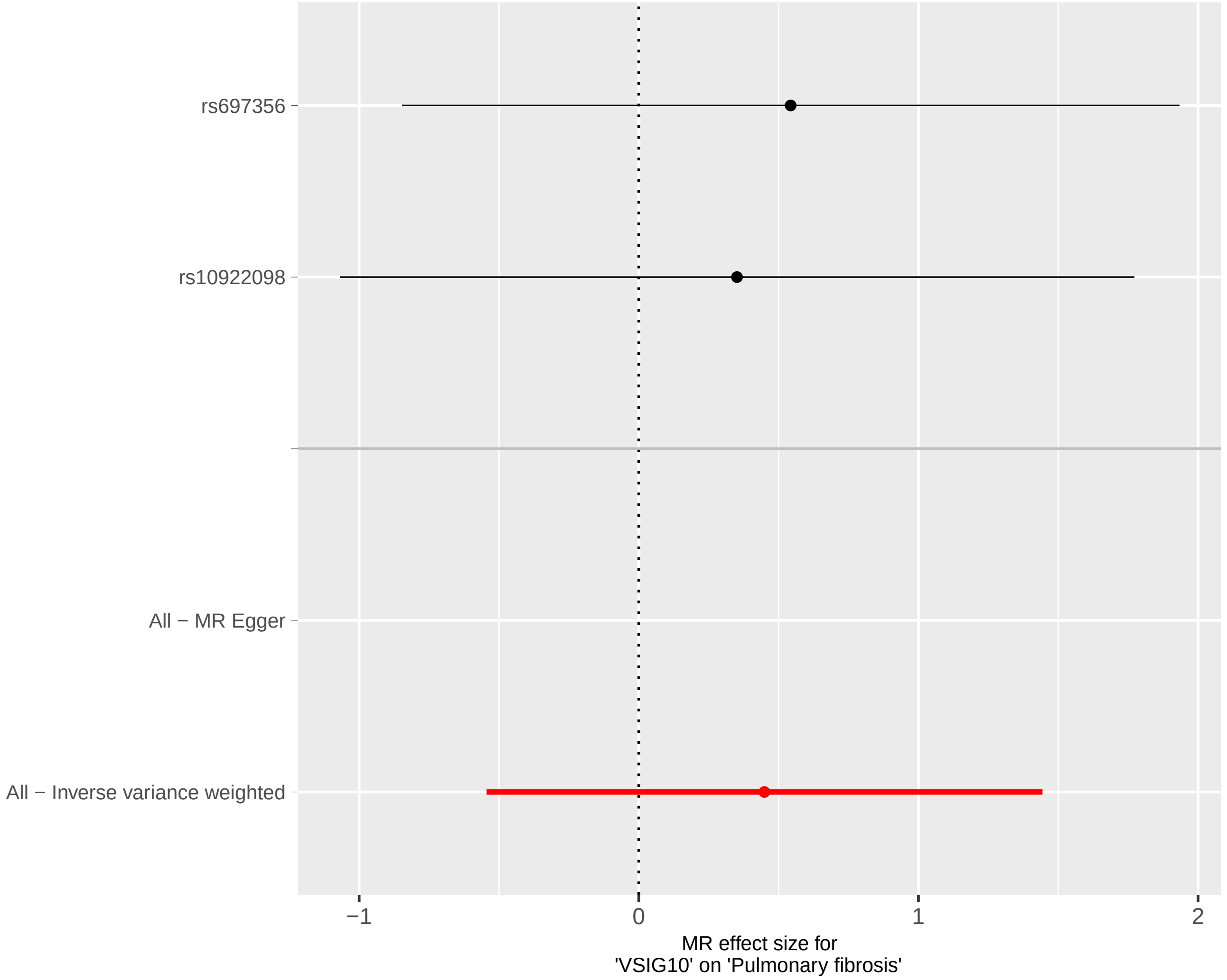
rs697356
rs10922098
All − MR Egger
All − Inverse variance weighted
−1
0
1
2
MR effect size for
'VSIG10' on 'Pulmonary fibrosis'

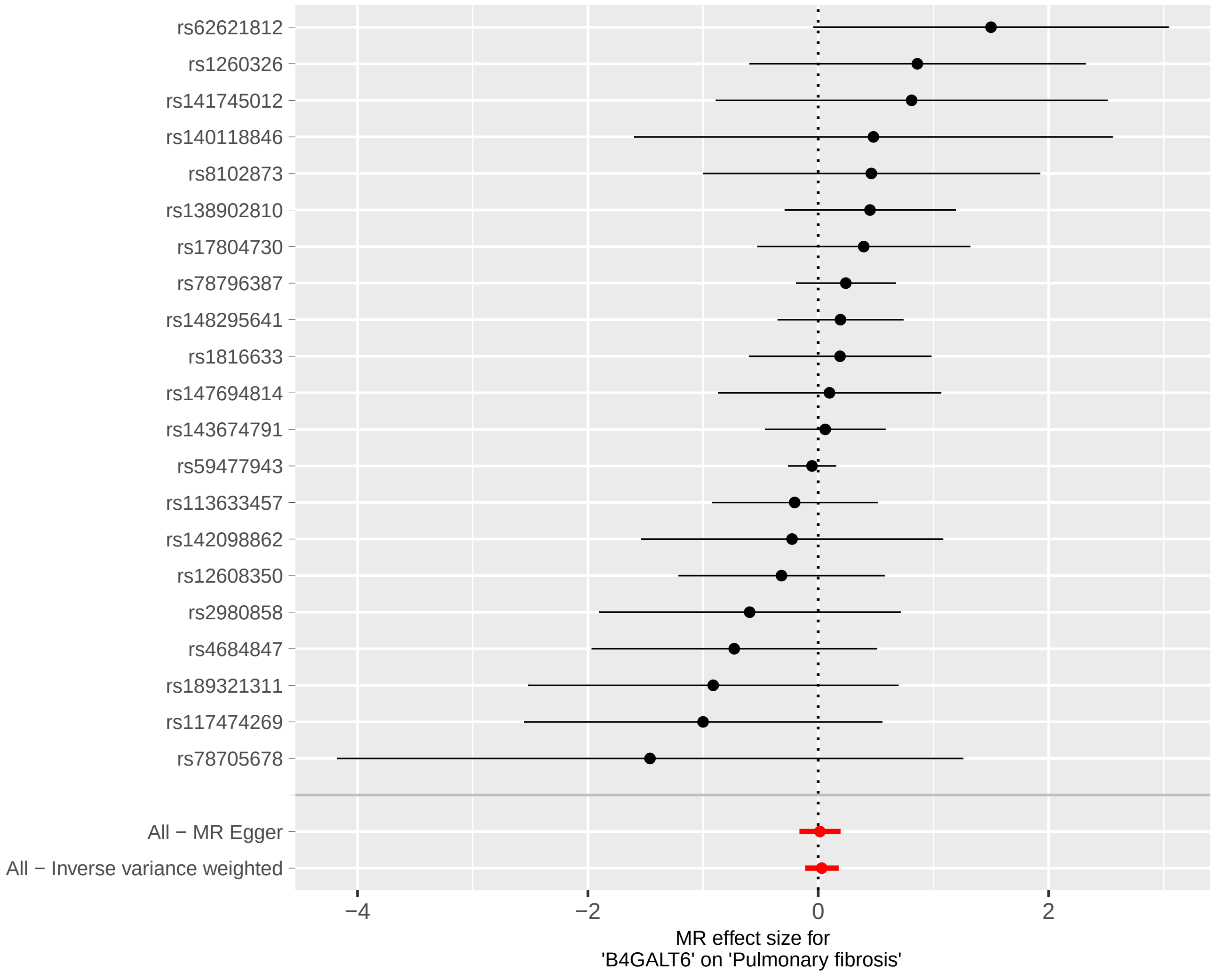
rs62621812
rs1260326
rs141745012
rs140118846
rs8102873
rs138902810
rs17804730
rs78796387
rs148295641
rs1816633
rs147694814
rs143674791
rs59477943
rs113633457
rs142098862
rs12608350
rs2980858
rs4684847
rs189321311
rs117474269
rs78705678
All − MR Egger
All − Inverse variance weighted
−4
−2
0
2
MR effect size for
'B4GALT6' on 'Pulmonary fibrosis'

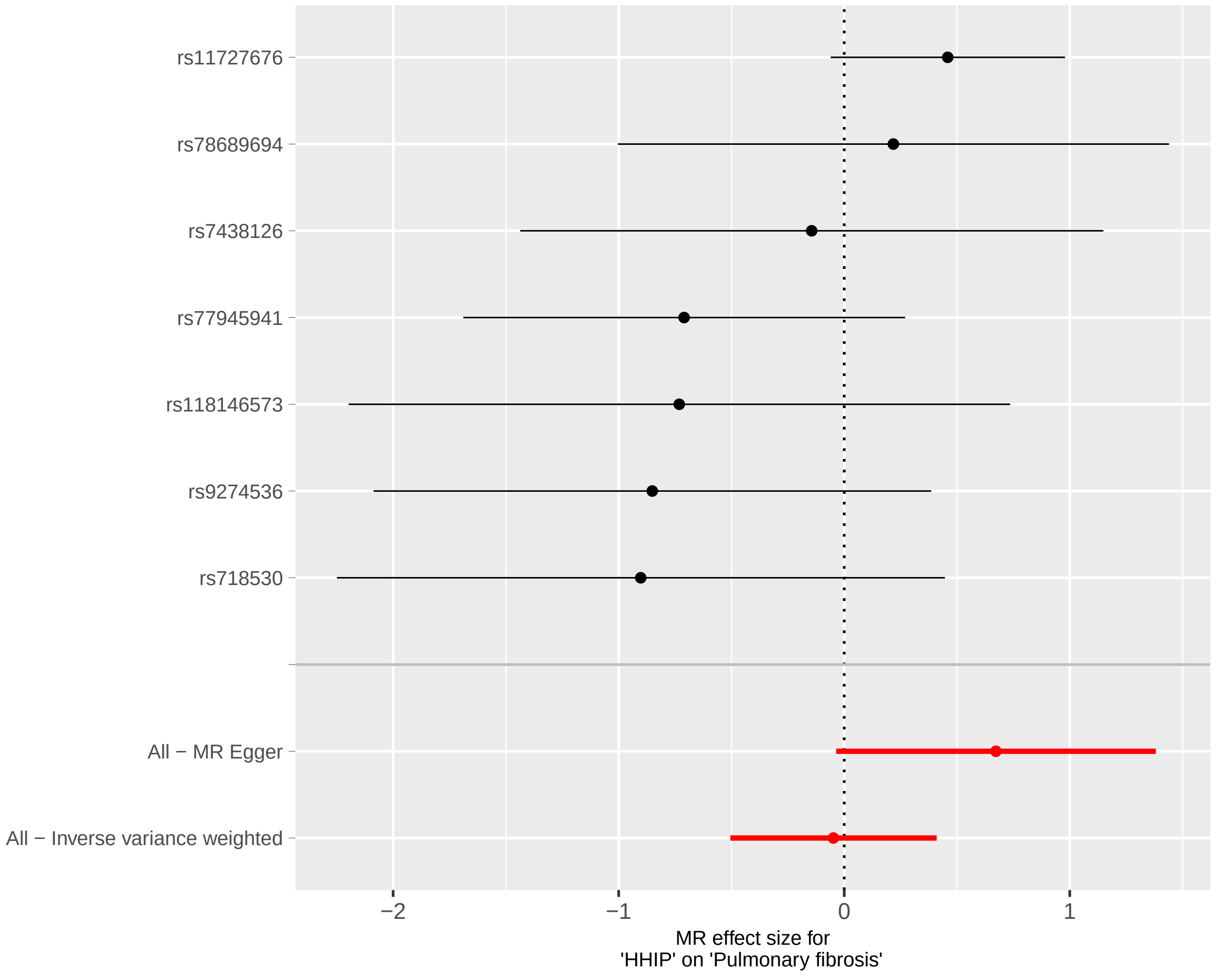

rs11727676
rs78689694
rs7438126
rs77945941
rs118146573
rs9274536
rs718530
All − MR Egger
All − Inverse variance weighted
−2
−1
0
1
MR effect size for
'HHIP' on 'Pulmonary fibrosis'

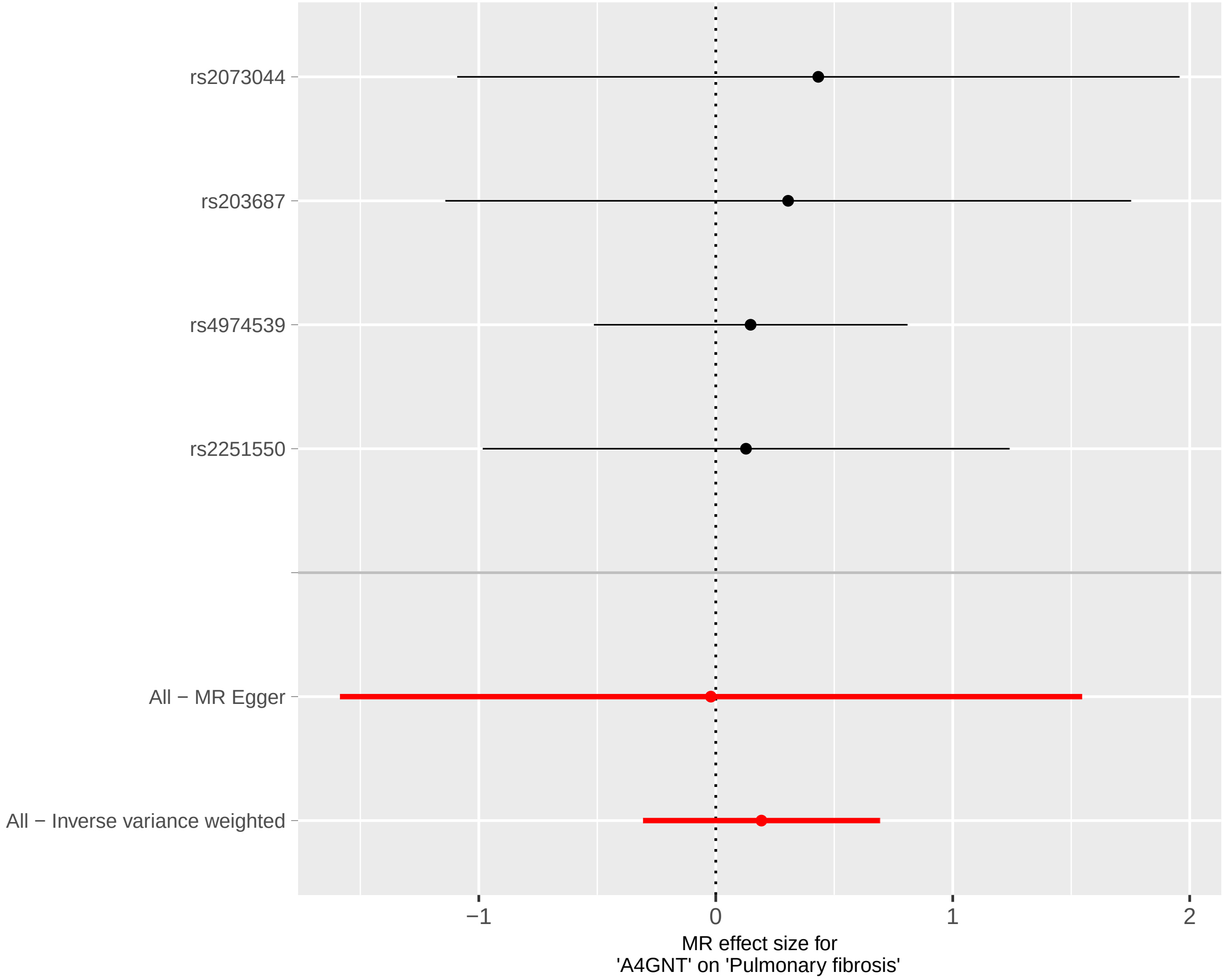

rs2073044
rs203687
rs4974539
rs2251550
All − MR Egger
All − Inverse variance weighted
−1
0
1
2
MR effect size for
'A4GNT' on 'Pulmonary fibrosis'

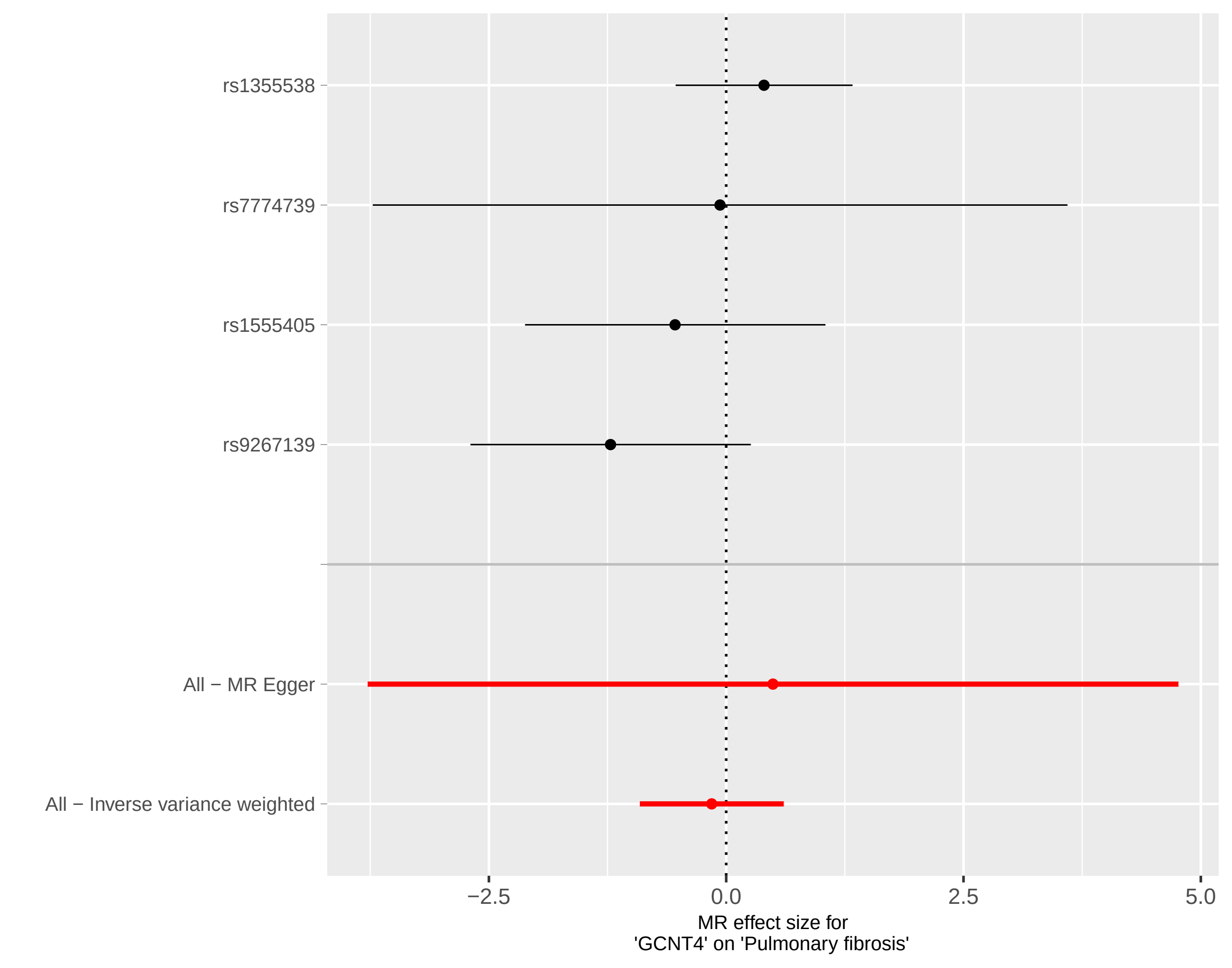
rs1355538
rs7774739
rs1555405
rs9267139
All − MR Egger
All − Inverse variance weighted
−2.5
0.0
2.5
5.0
MR effect size for
'GCNT4' on 'Pulmonary fibrosis'

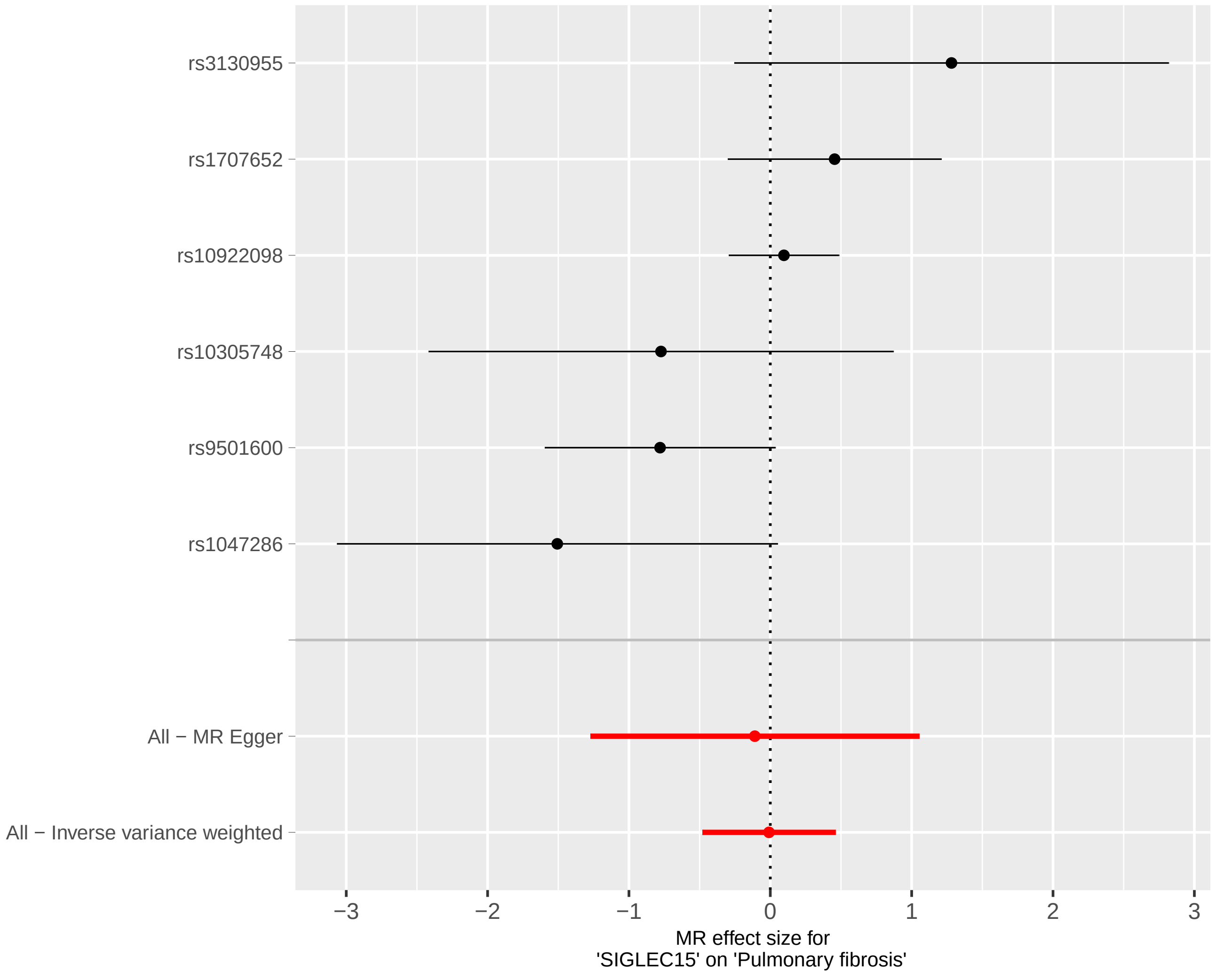
rs3130955
rs1707652
rs10922098
rs10305748
rs9501600
rs1047286
All − MR Egger
All − Inverse variance weighted
−3
−2
−1
0
1
2
3
MR effect size for
'SIGLEC15' on 'Pulmonary fibrosis'

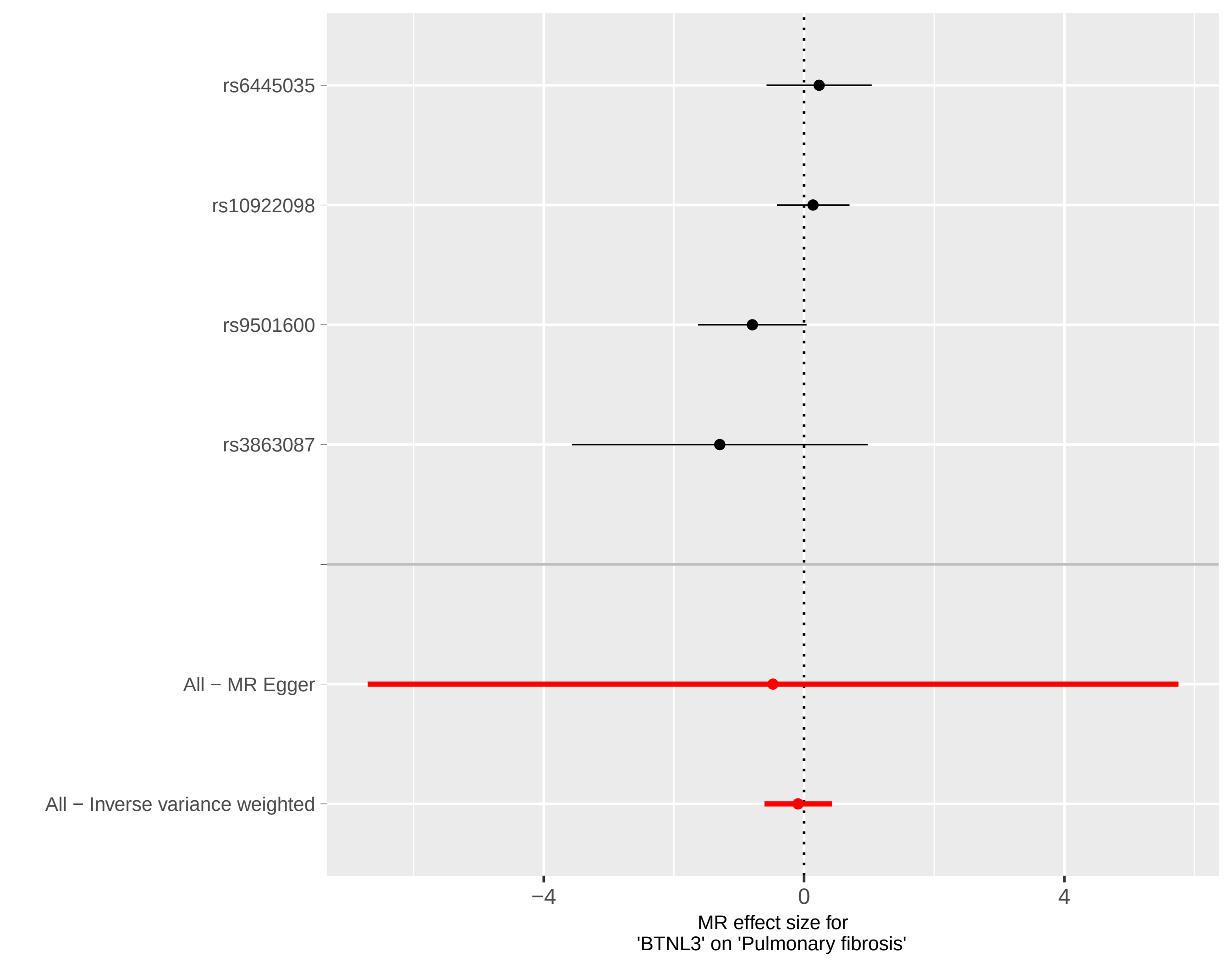
rs6445035
rs10922098
rs9501600
rs3863087
All − MR Egger
All − Inverse variance weighted
−4
0
4
MR effect size for
'BTNL3' on 'Pulmonary fibrosis'

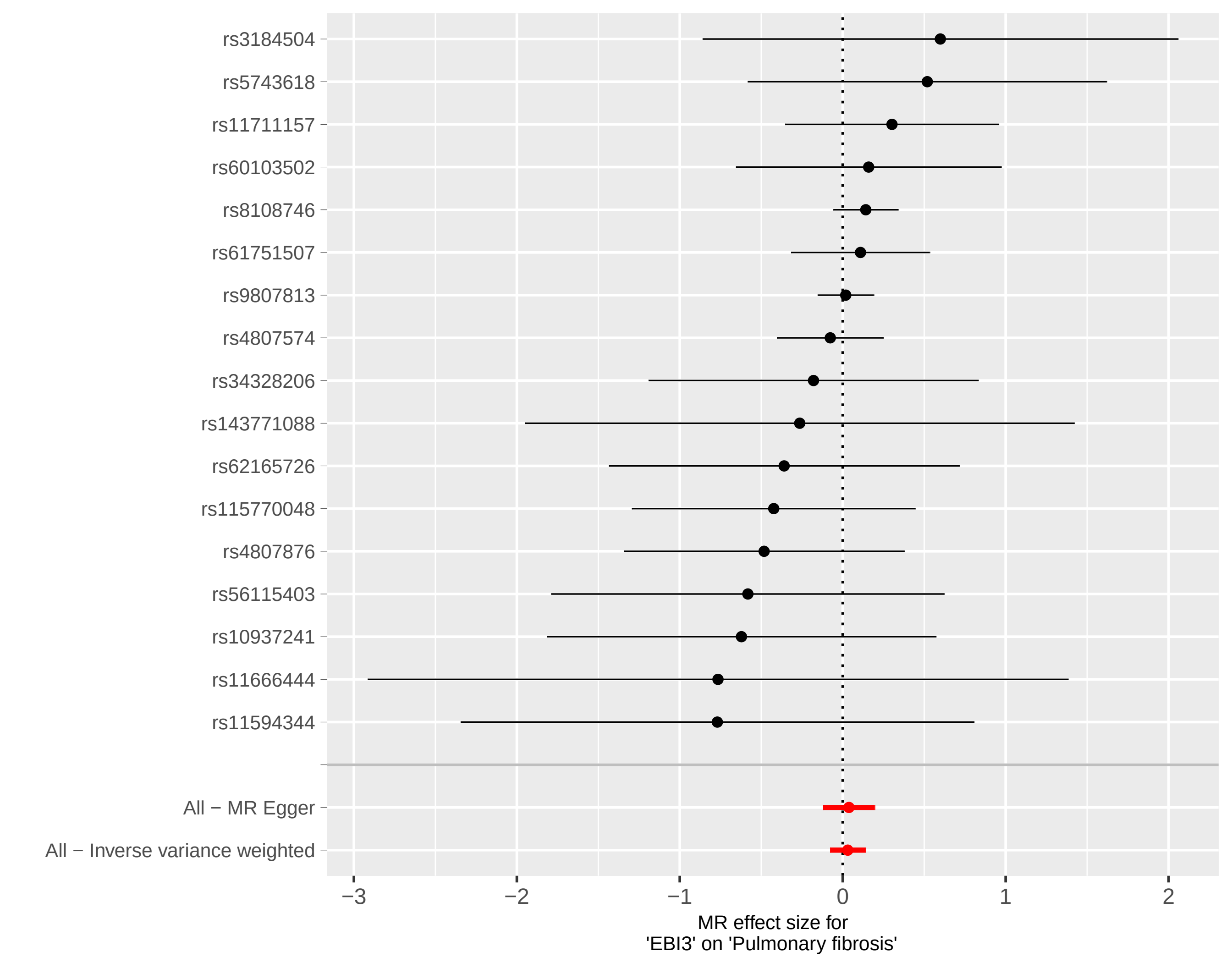
rs3184504
rs5743618
rs11711157
rs60103502
rs8108746
rs61751507
rs9807813
rs4807574
rs34328206
rs143771088
rs62165726
rs115770048
rs4807876
rs56115403
rs10937241
rs11666444
rs11594344
All − MR Egger
All − Inverse variance weighted
−3
−2
−1
0
1
2
MR effect size for
'EBI3' on 'Pulmonary fibrosis'

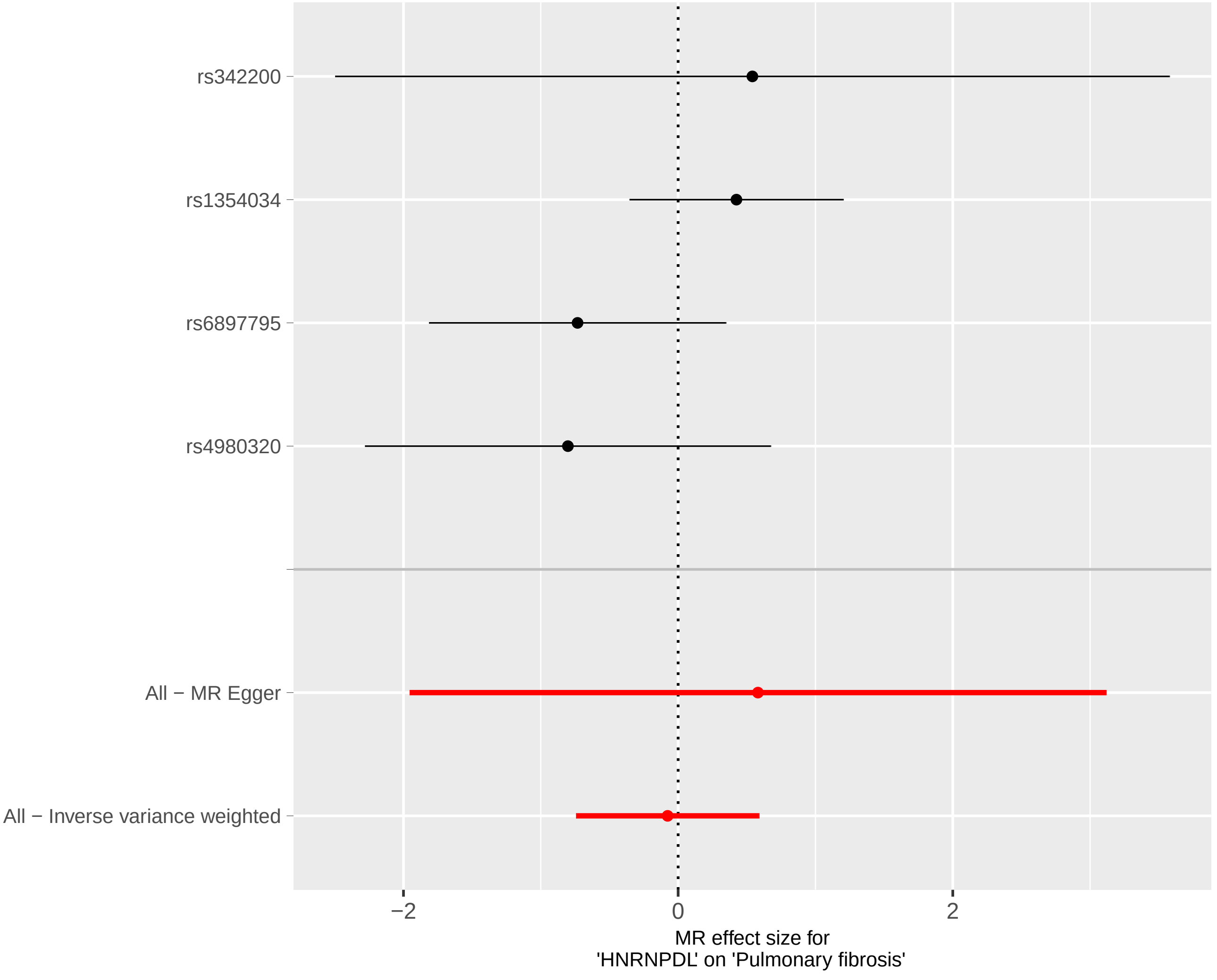

rs342200
rs1354034
rs6897795
rs4980320
All − MR Egger
All − Inverse variance weighted
−2
0
2
MR effect size for
'HNRNPDL' on 'Pulmonary fibrosis'

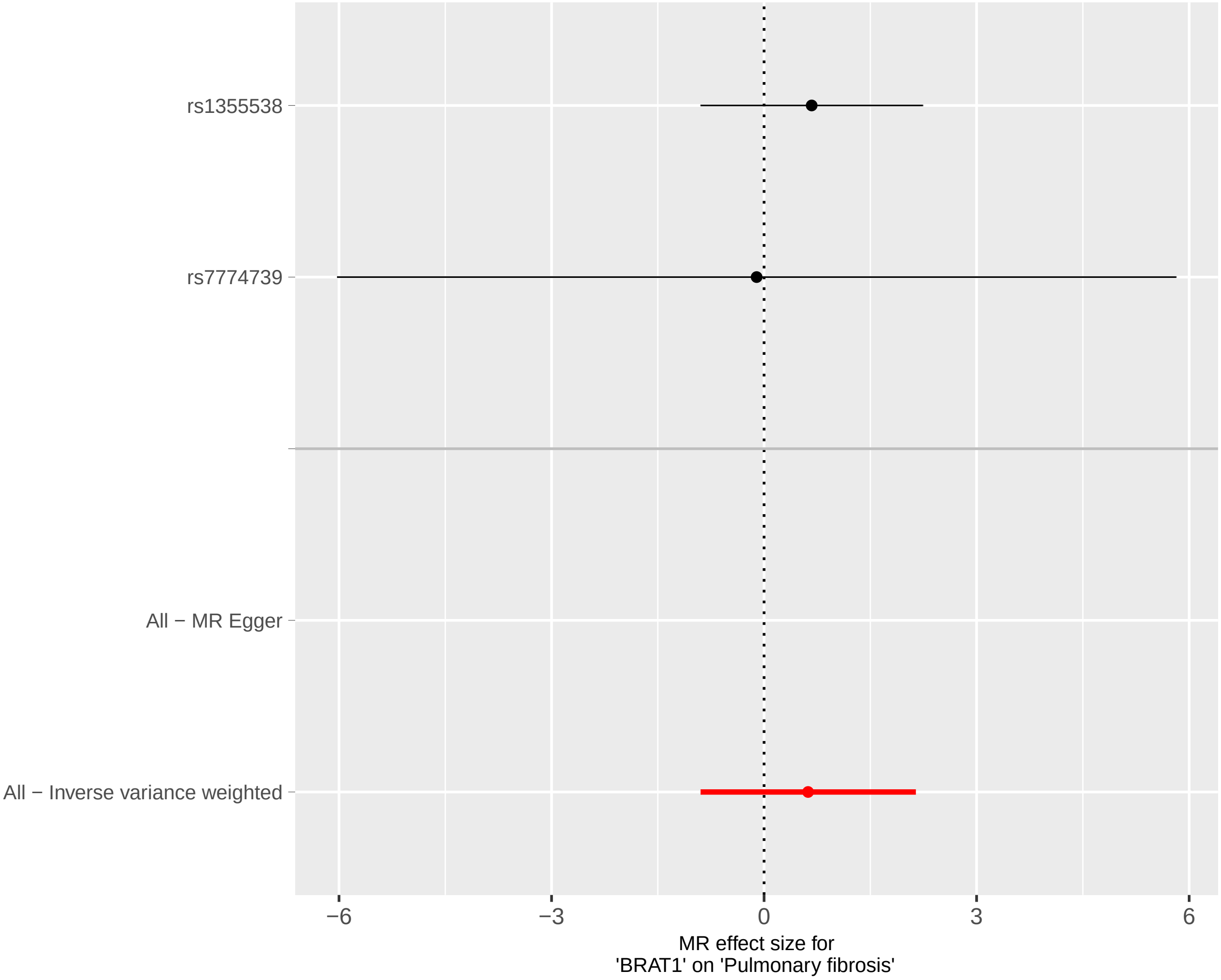
rs1355538
rs7774739
All − MR Egger
All − Inverse variance weighted
−6
−3
0
3
6
MR effect size for
'BRAT1' on 'Pulmonary fibrosis'

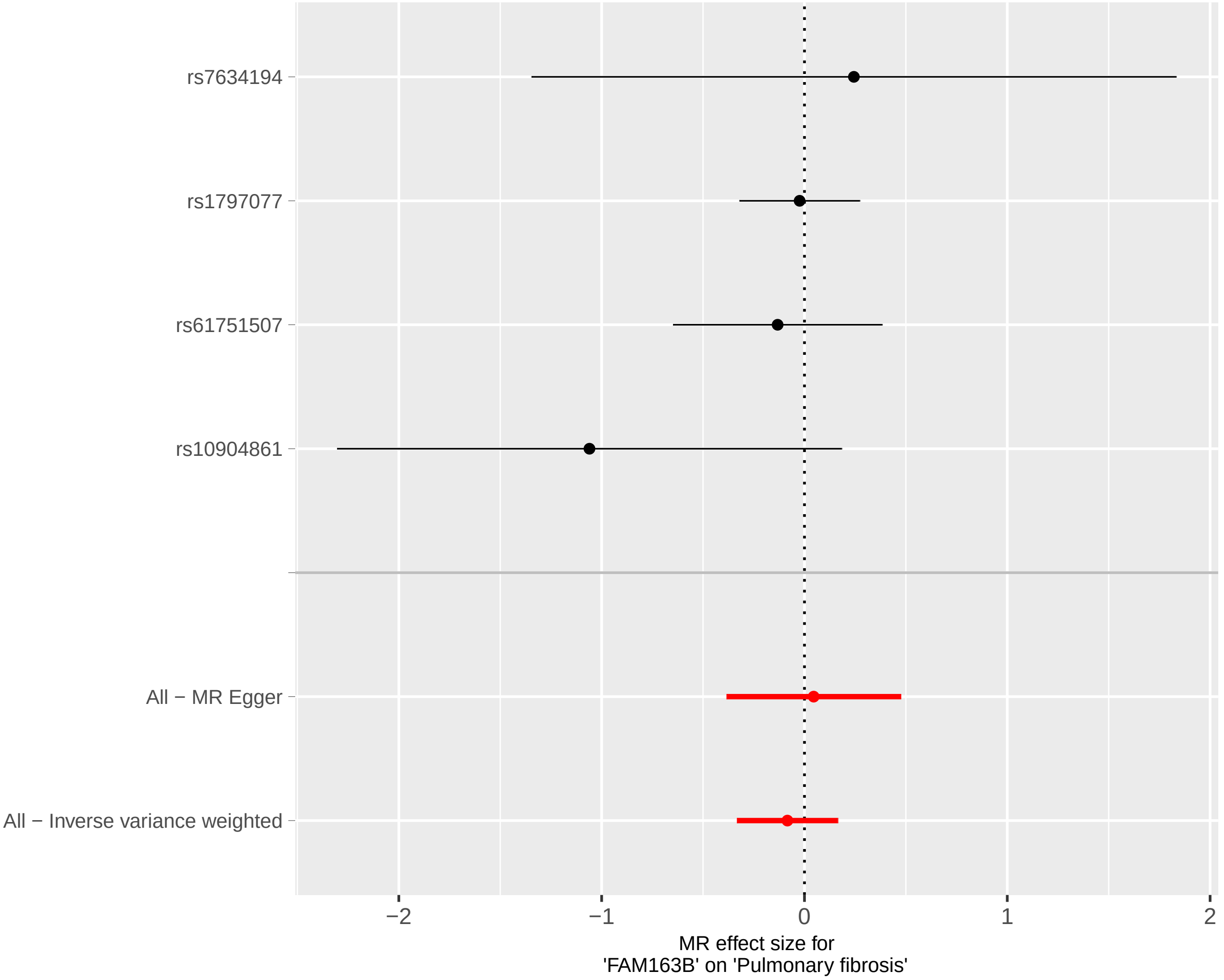
rs7634194
rs1797077
rs61751507
rs10904861
All − MR Egger
All − Inverse variance weighted
−2
−1
0
1
2
MR effect size for
'FAM163B' on 'Pulmonary fibrosis'

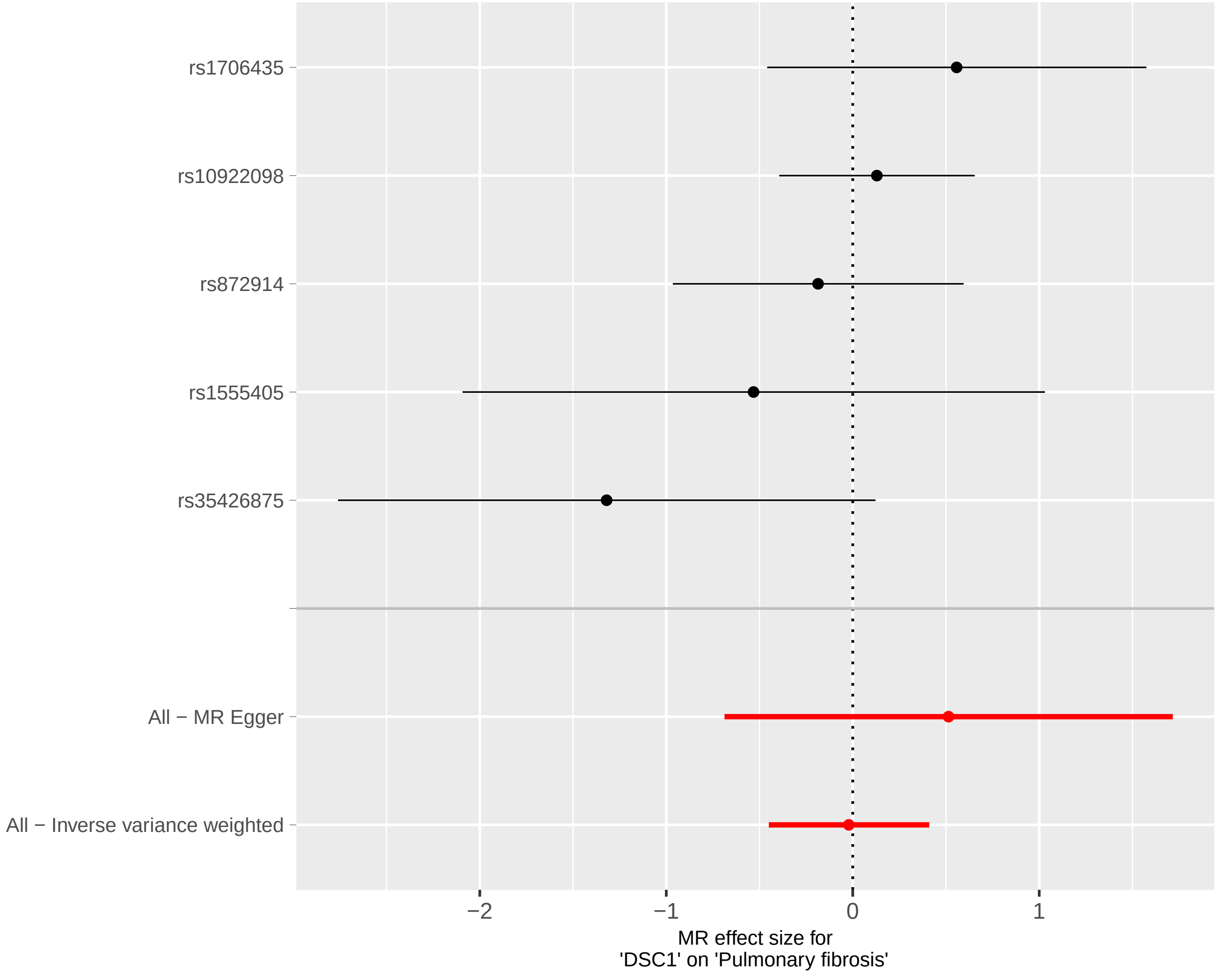
rs1706435
rs10922098
rs872914
rs1555405
rs35426875
All − MR Egger
All − Inverse variance weighted
−2
−1
0
1
MR effect size for
'DSC1' on 'Pulmonary fibrosis'

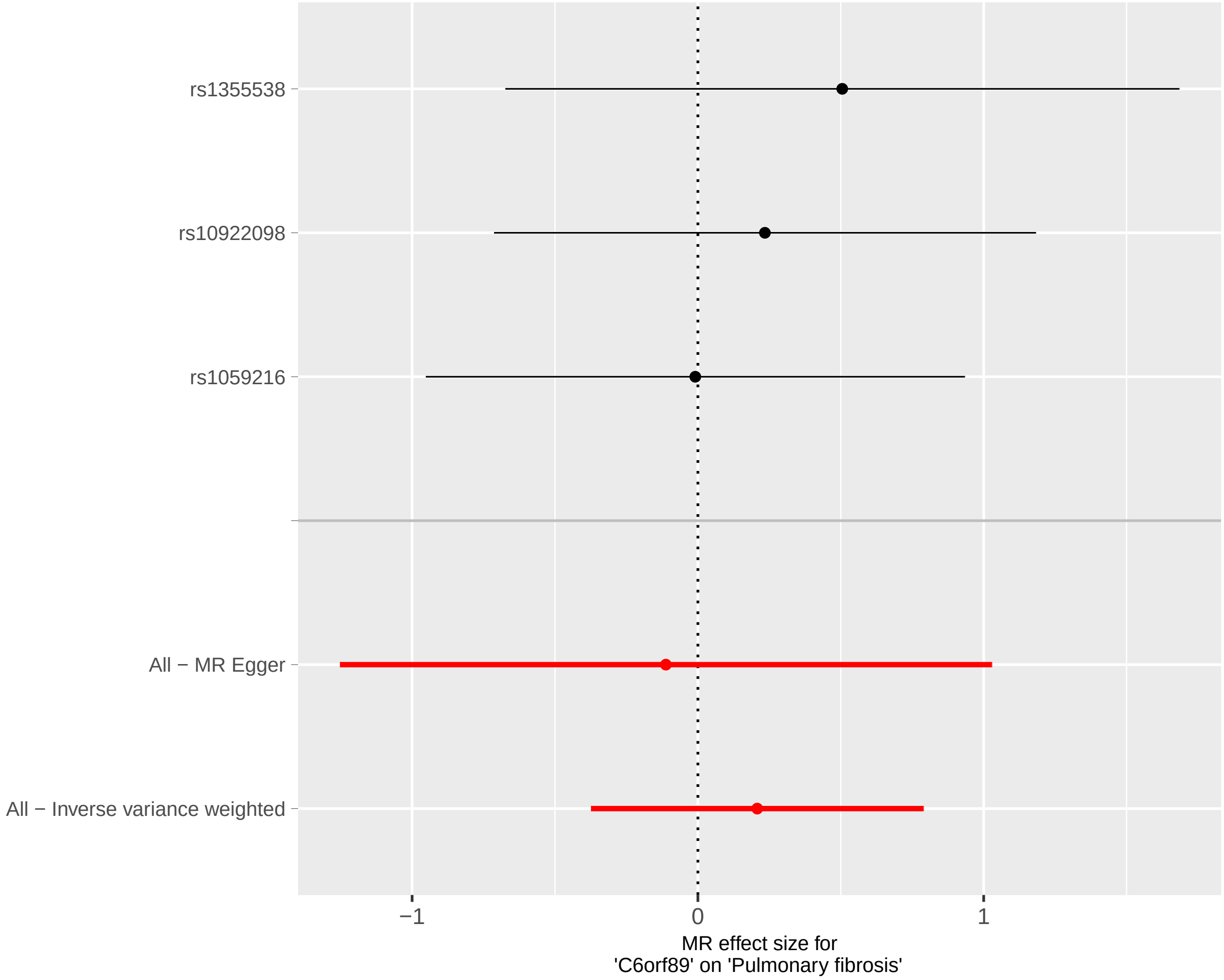
rs1355538
rs10922098
rs1059216
All − MR Egger
All − Inverse variance weighted
−1
0
1
MR effect size for
'C6orf89' on 'Pulmonary fibrosis'

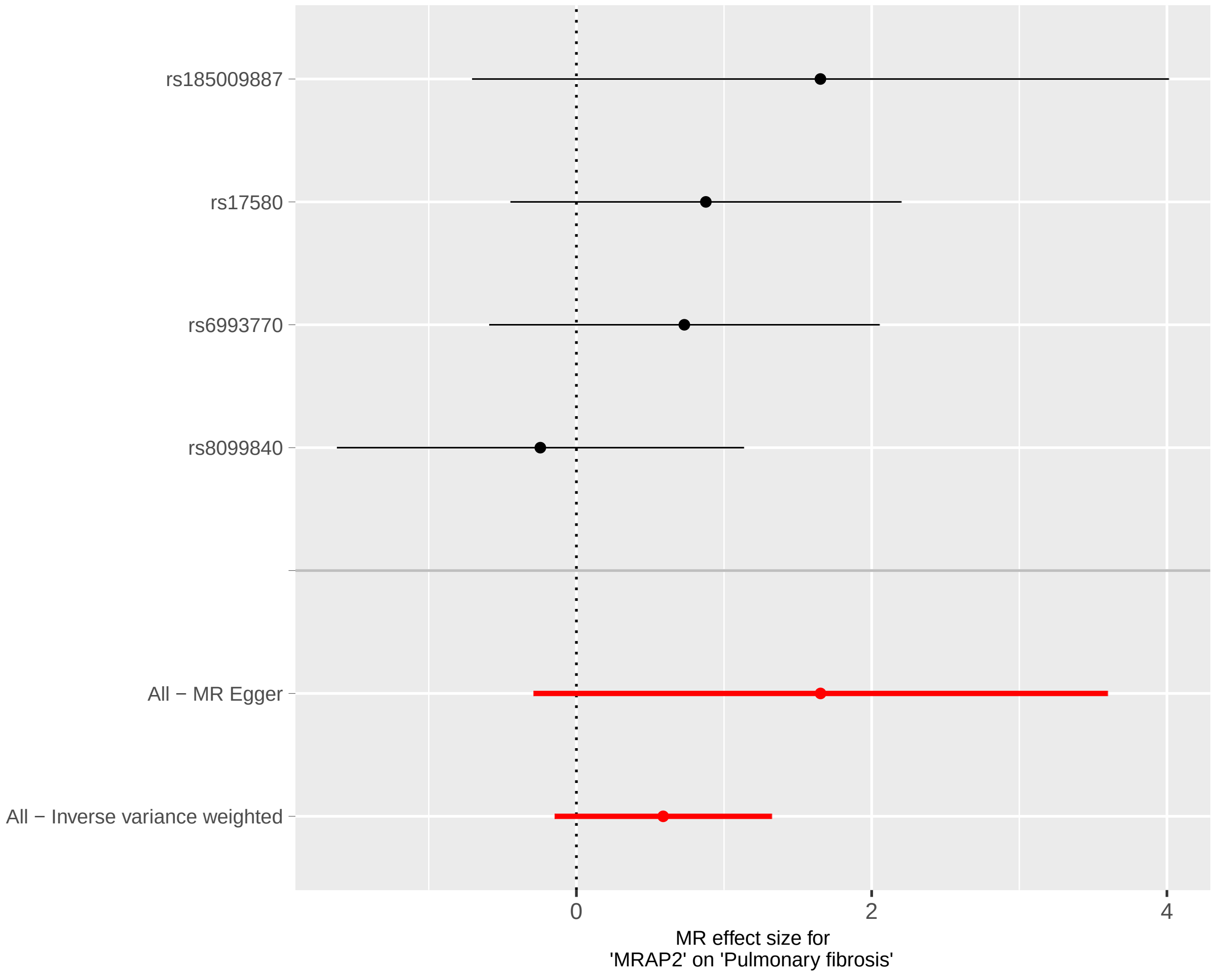
rs185009887
rs17580
rs6993770
rs8099840
All − MR Egger
All − Inverse variance weighted
0
2
4
MR effect size for
'MRAP2' on 'Pulmonary fibrosis'

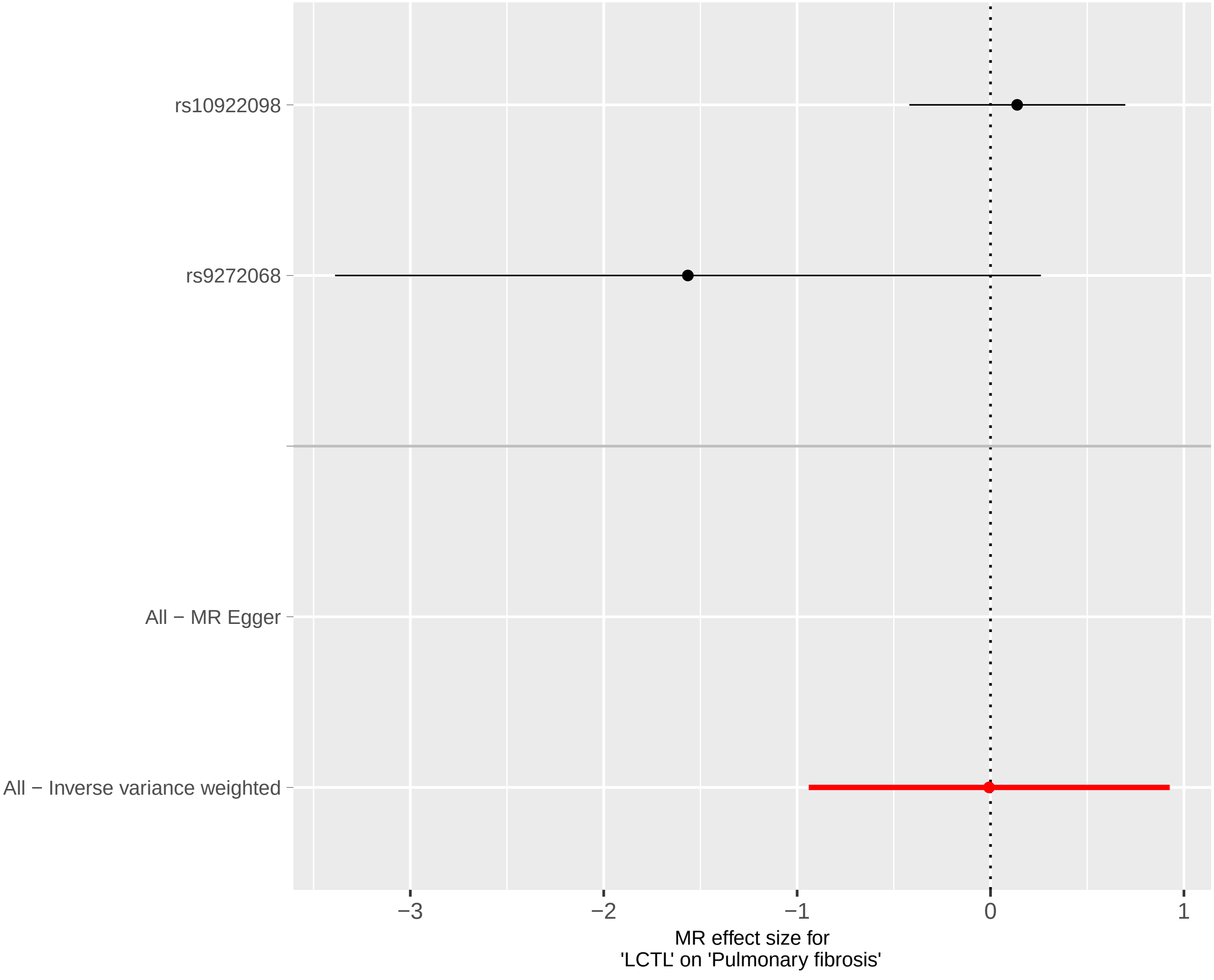
rs10922098
rs9272068
All − MR Egger
All − Inverse variance weighted
−3
−2
−1
0
1
MR effect size for
'LCTL' on 'Pulmonary fibrosis'

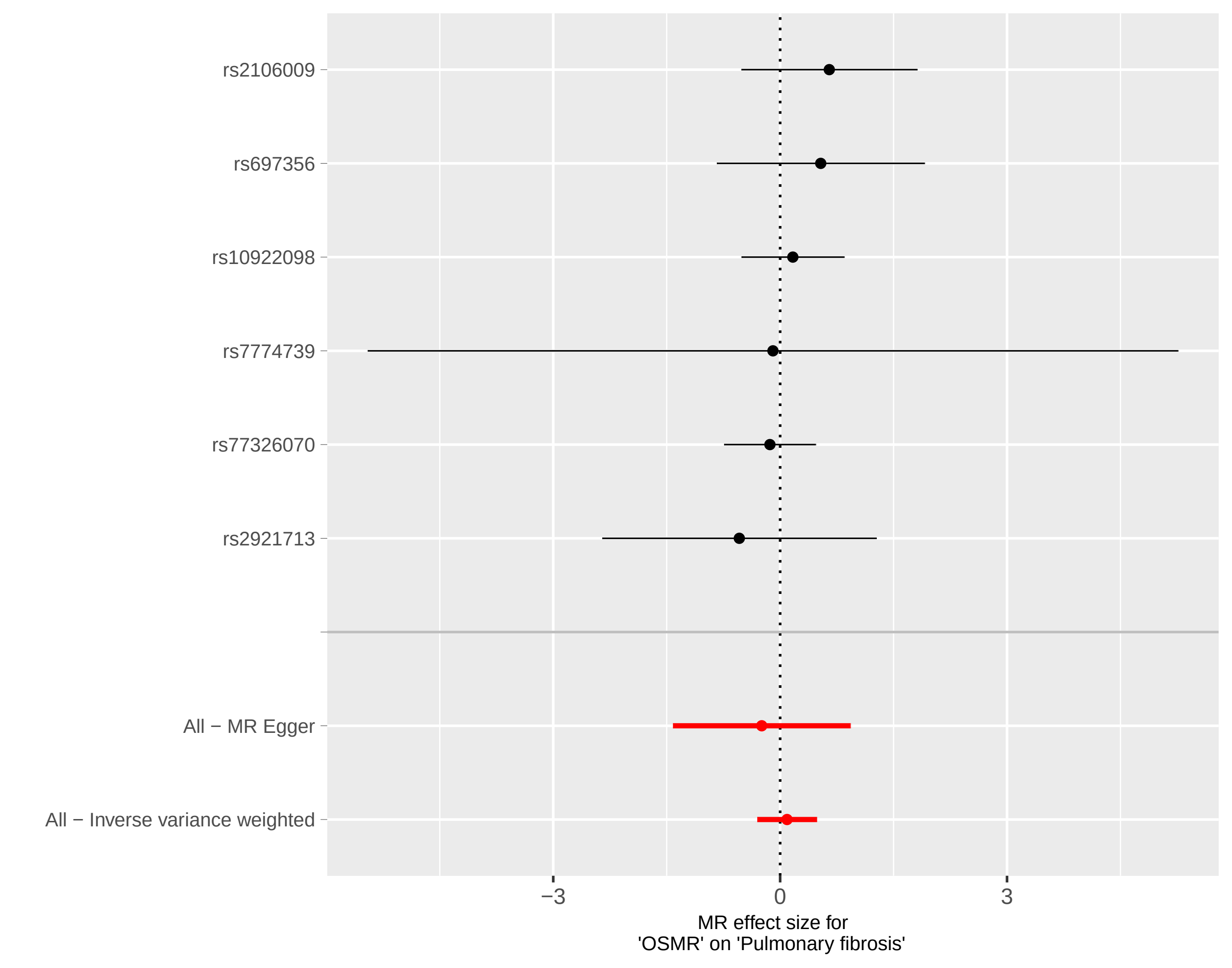

rs2106009
rs697356
rs10922098
rs7774739
rs77326070
rs2921713
All − MR Egger
All − Inverse variance weighted
−3
0
3
MR effect size for
'OSMR' on 'Pulmonary fibrosis'

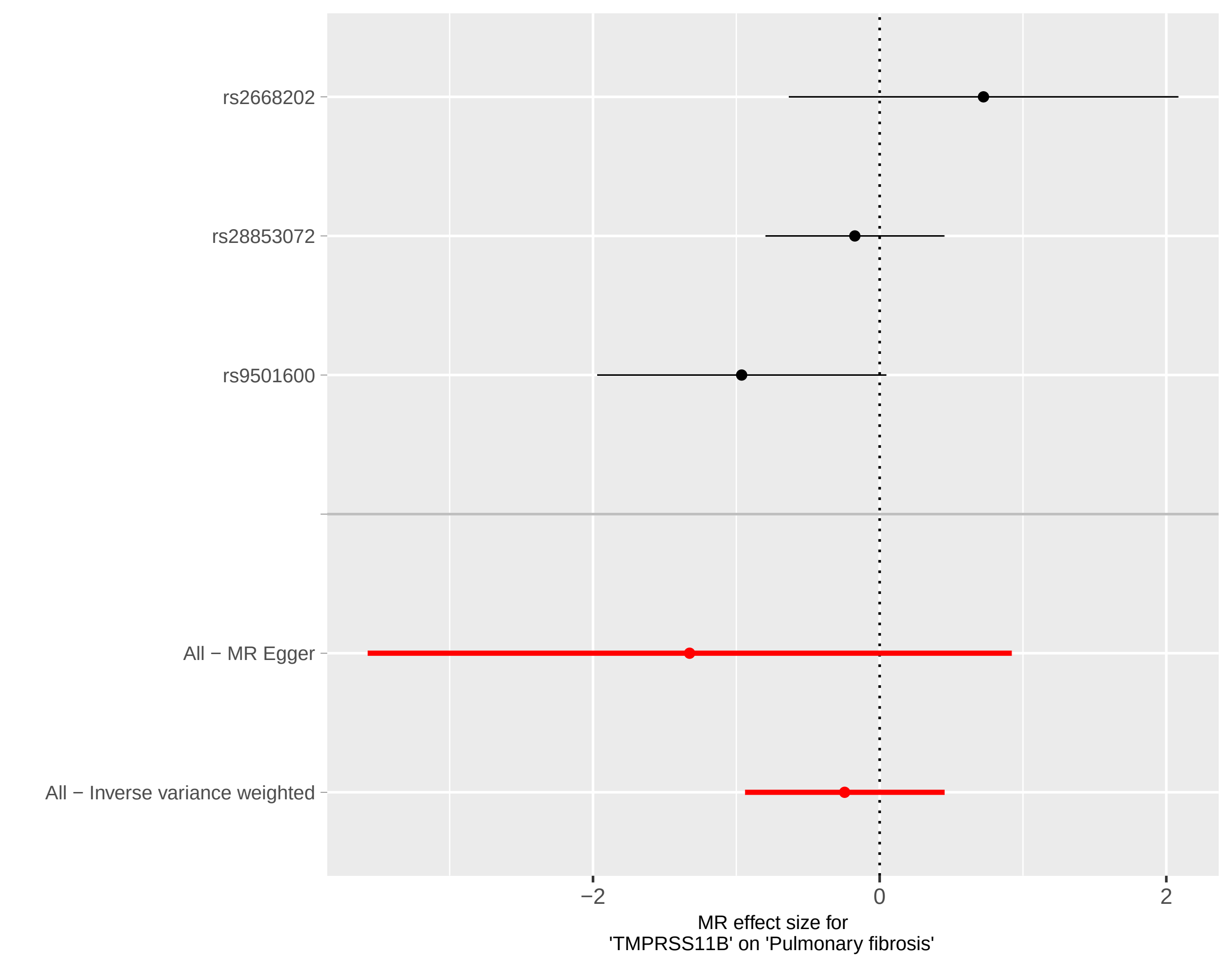
rs2668202
rs28853072
rs9501600
All − MR Egger
All − Inverse variance weighted
−2
0
2
MR effect size for
'TMPRSS11B' on 'Pulmonary fibrosis'

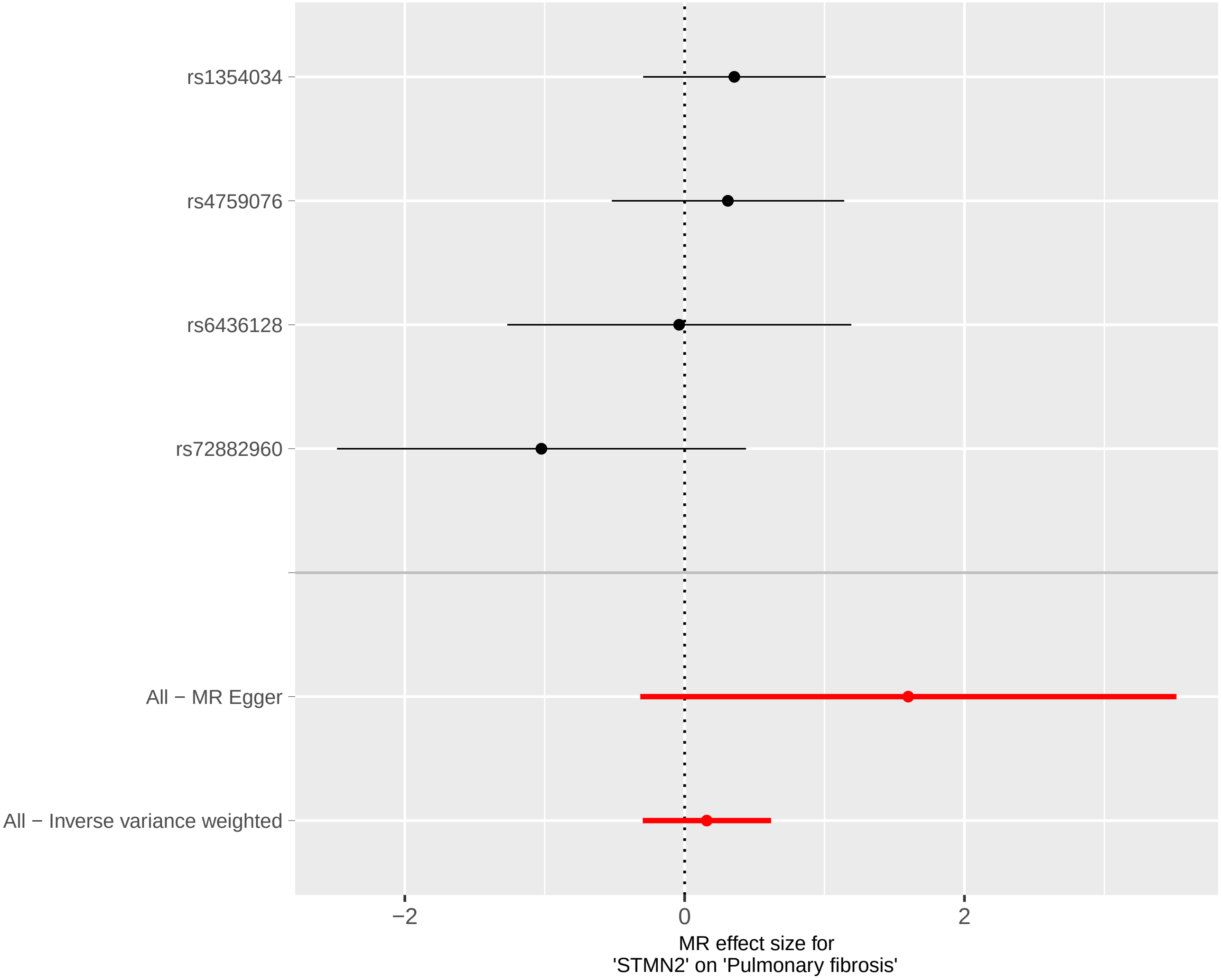
rs1354034
rs4759076
rs6436128
rs72882960
All − MR Egger
All − Inverse variance weighted
−2
0
2
MR effect size for
'STMN2' on 'Pulmonary fibrosis'

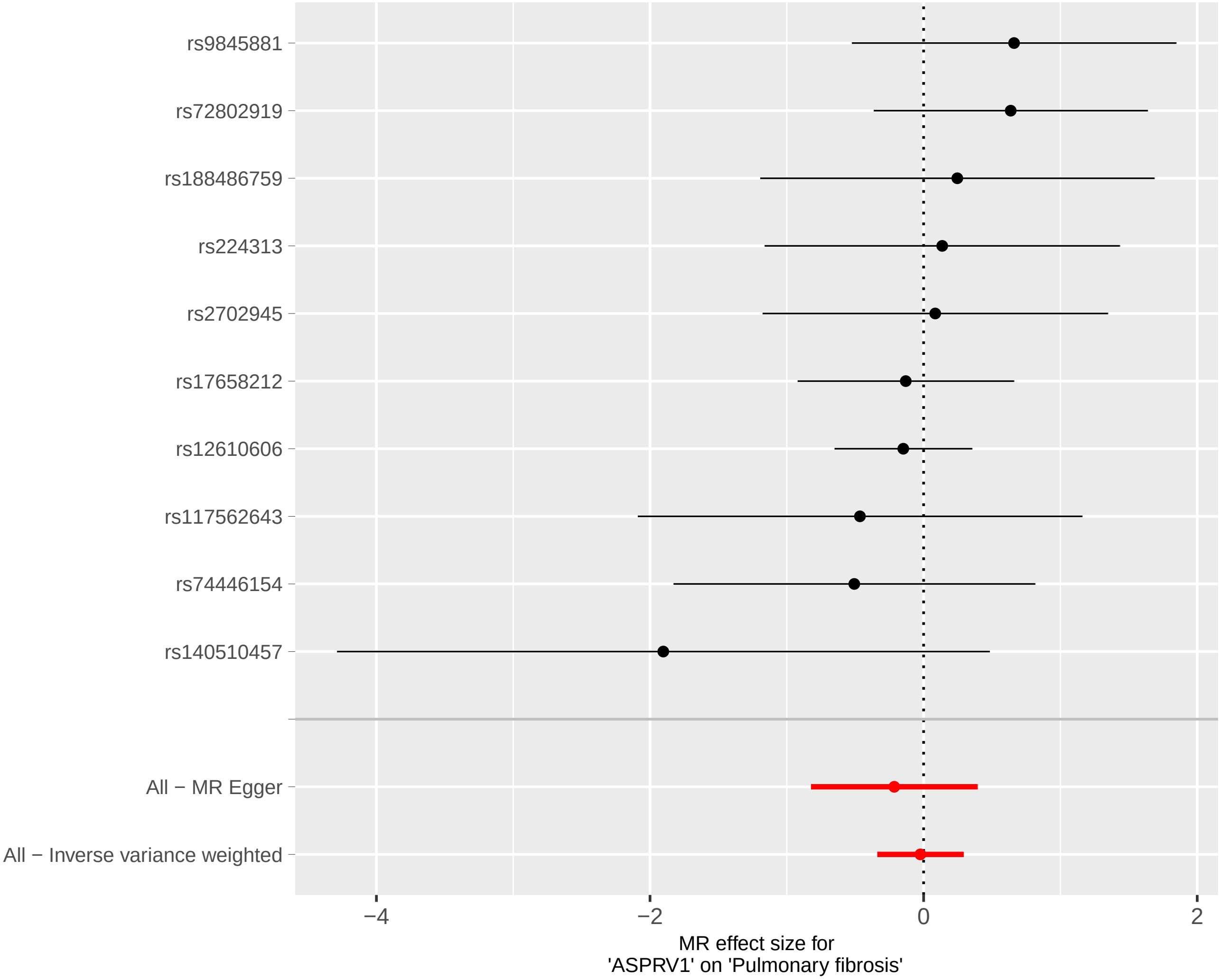
rs9845881
rs72802919
rs188486759
rs224313
rs2702945
rs17658212
rs12610606
rs117562643
rs74446154
rs140510457
All − MR Egger
All − Inverse variance weighted
−4
−2
0
2
MR effect size for
'ASPRV1' on 'Pulmonary fibrosis'

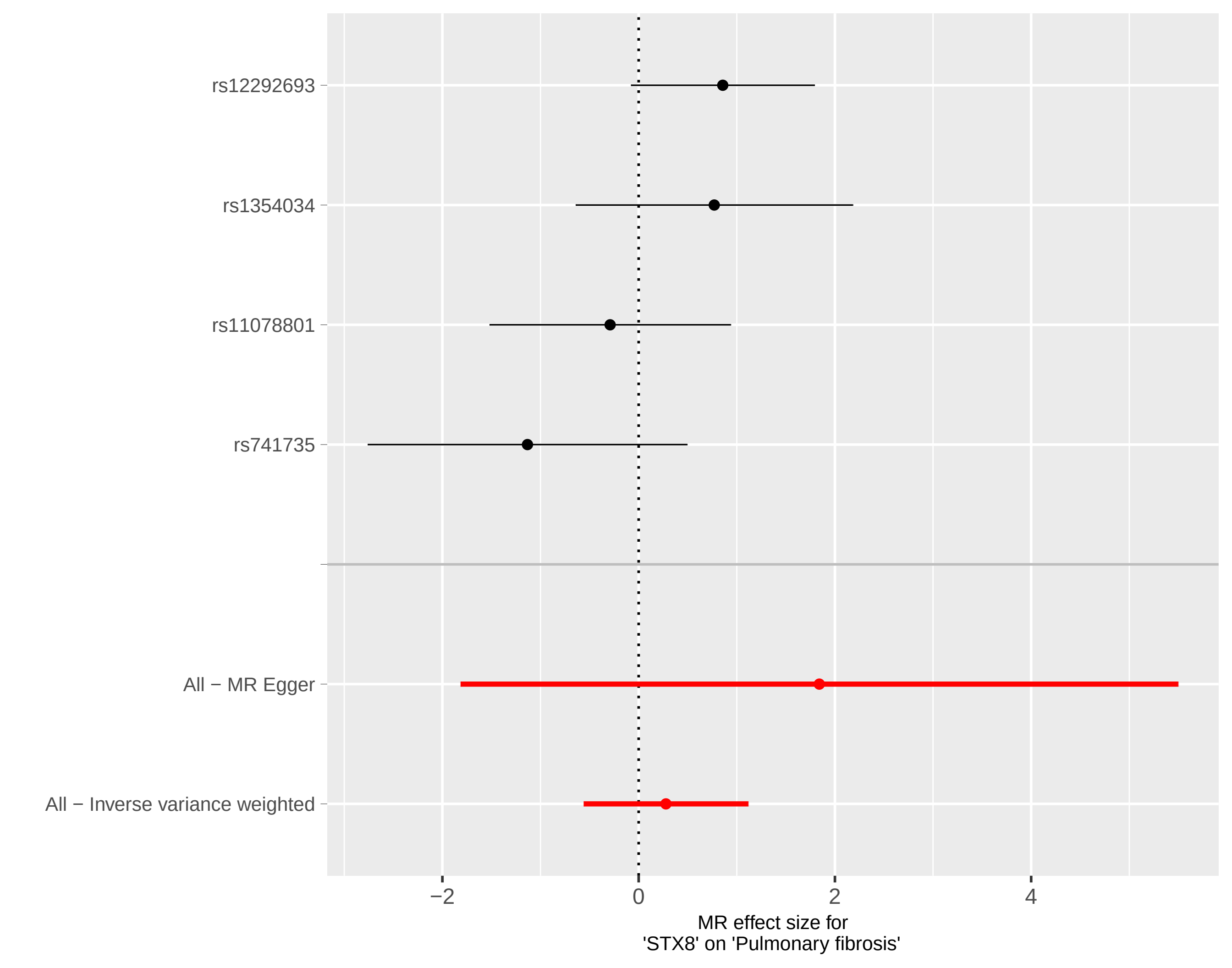
rs12292693
rs1354034
rs11078801
rs741735
All − MR Egger
All − Inverse variance weighted
−2
0
2
4
MR effect size for
'STX8' on 'Pulmonary fibrosis'

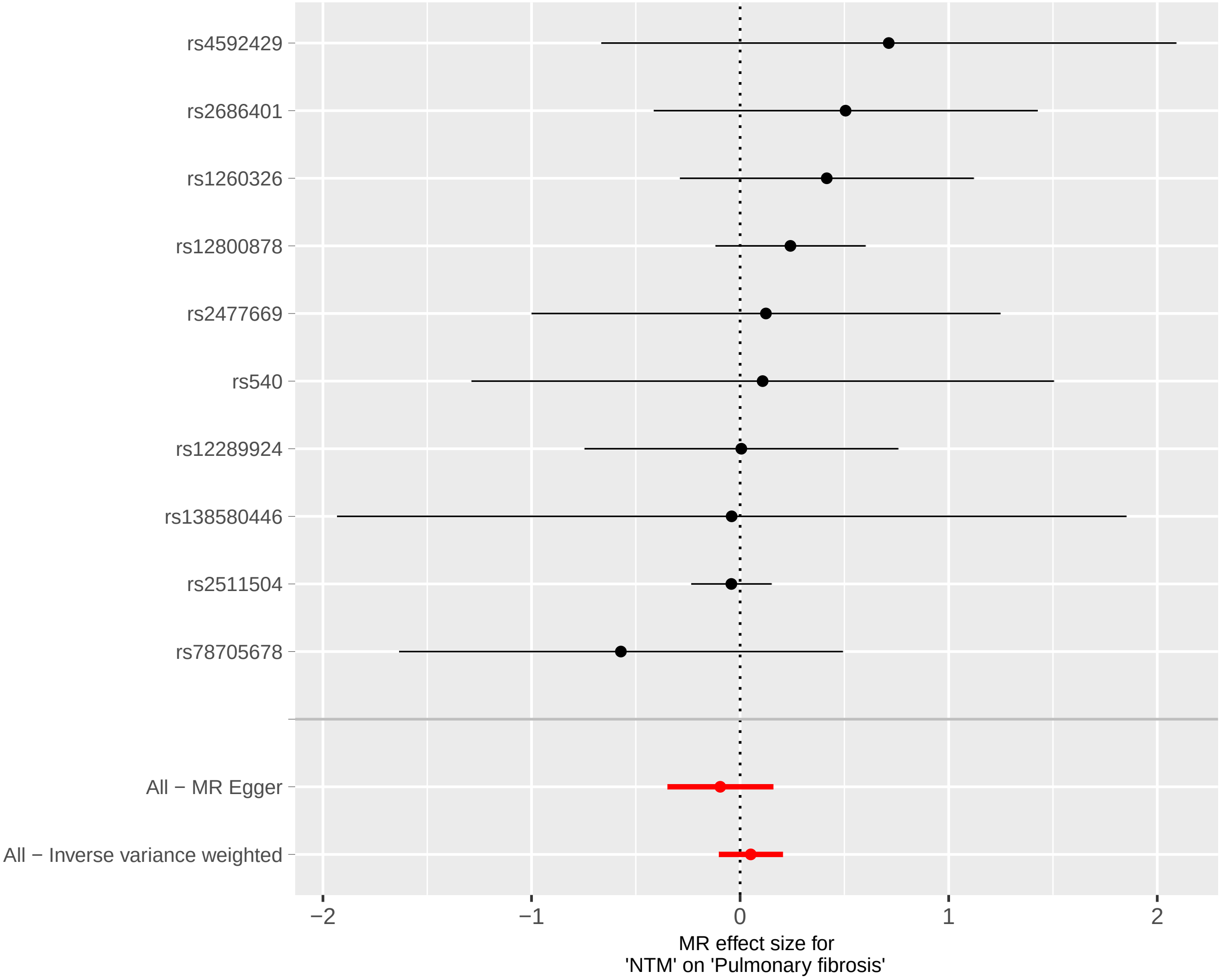
rs4592429
rs2686401
rs1260326
rs12800878
rs2477669
rs540
rs12289924
rs138580446
rs2511504
rs78705678
All − MR Egger
All − Inverse variance weighted
−2
−1
0
1
2
MR effect size for
'NTM' on 'Pulmonary fibrosis'

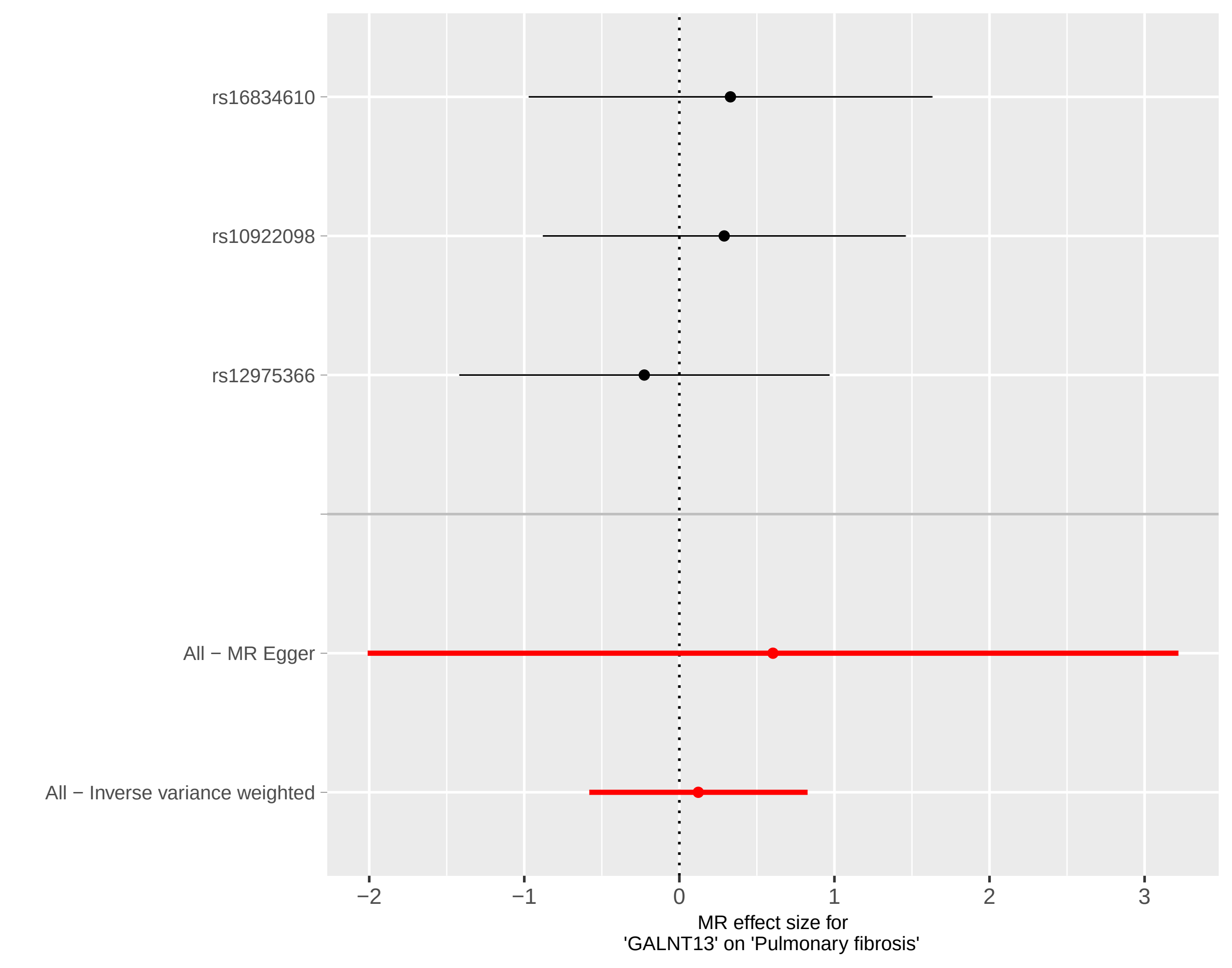

rs16834610
rs10922098
rs12975366
All − MR Egger
All − Inverse variance weighted
−2
−1
0
1
2
3
MR effect size for
'GALNT13' on 'Pulmonary fibrosis'

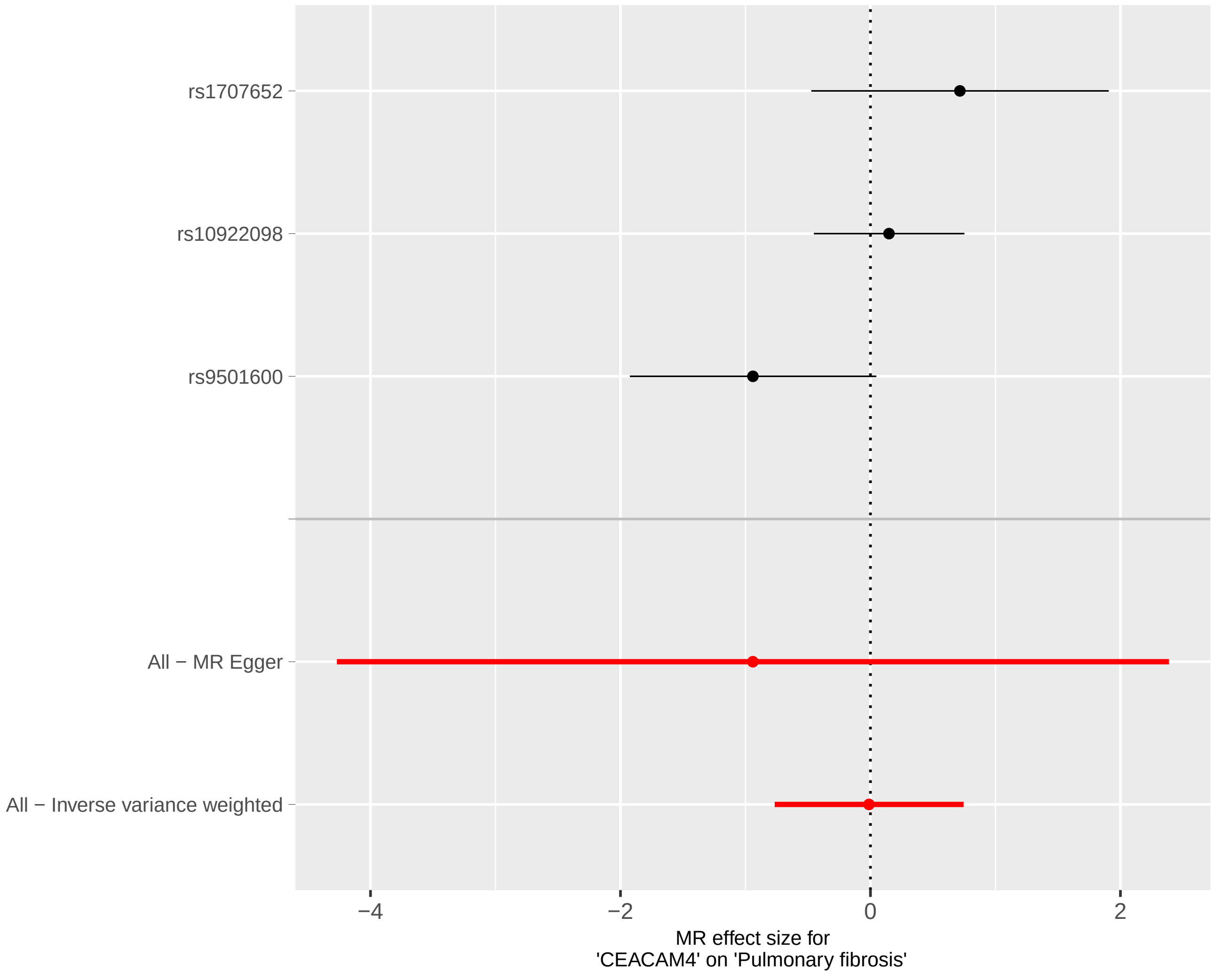
rs1707652
rs10922098
rs9501600
All − MR Egger
All − Inverse variance weighted
−4
−2
0
2
MR effect size for
'CEACAM4' on 'Pulmonary fibrosis'

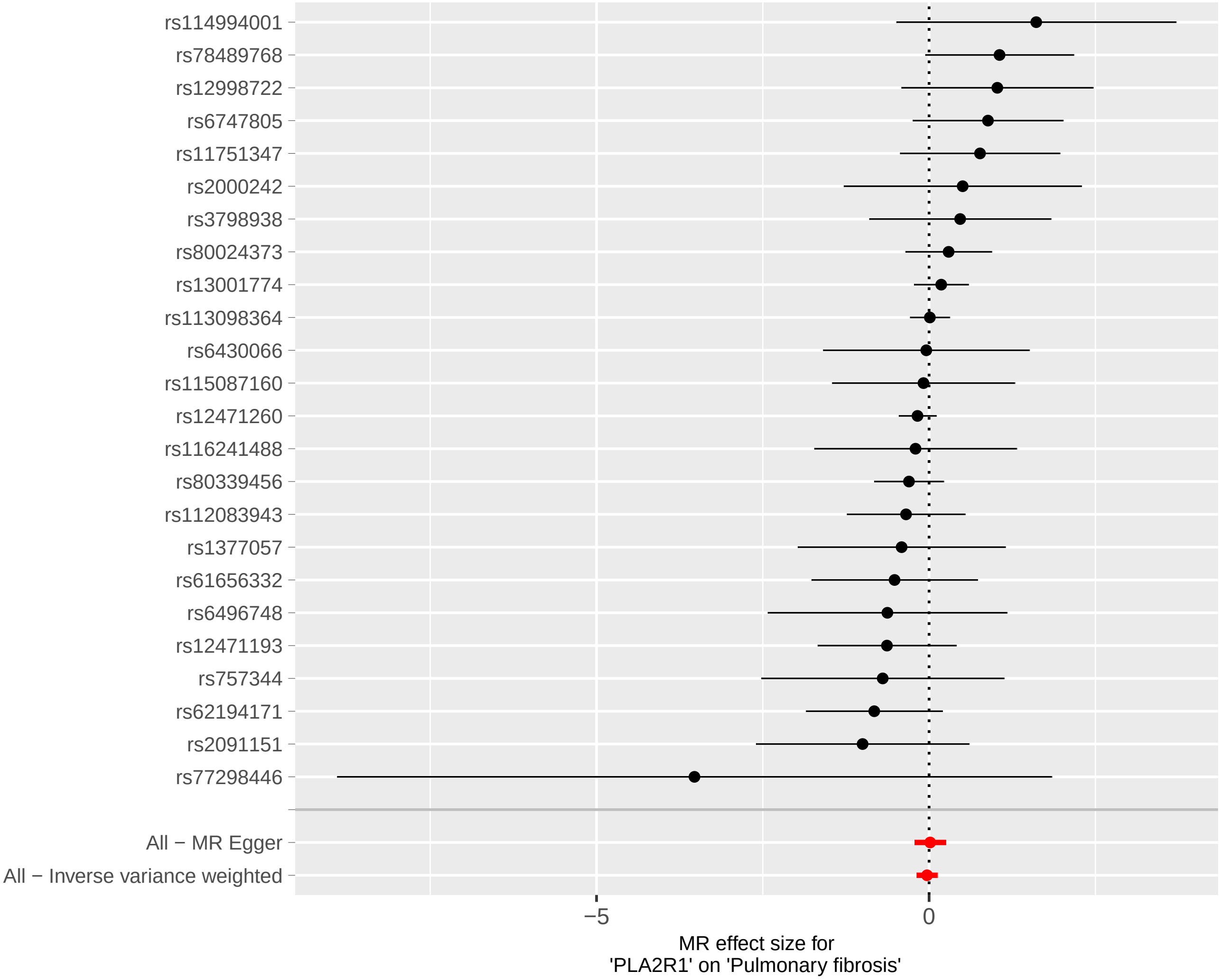

rs114994001
rs78489768
rs12998722
rs6747805
rs11751347
rs2000242
rs3798938
rs80024373
rs13001774
rs113098364
rs6430066
rs115087160
rs12471260
rs116241488
rs80339456
rs112083943
rs1377057
rs61656332
rs6496748
rs12471193
rs757344
rs62194171
rs2091151
rs77298446
All − MR Egger
All − Inverse variance weighted
−5
0
MR effect size for
'PLA2R1' on 'Pulmonary fibrosis'

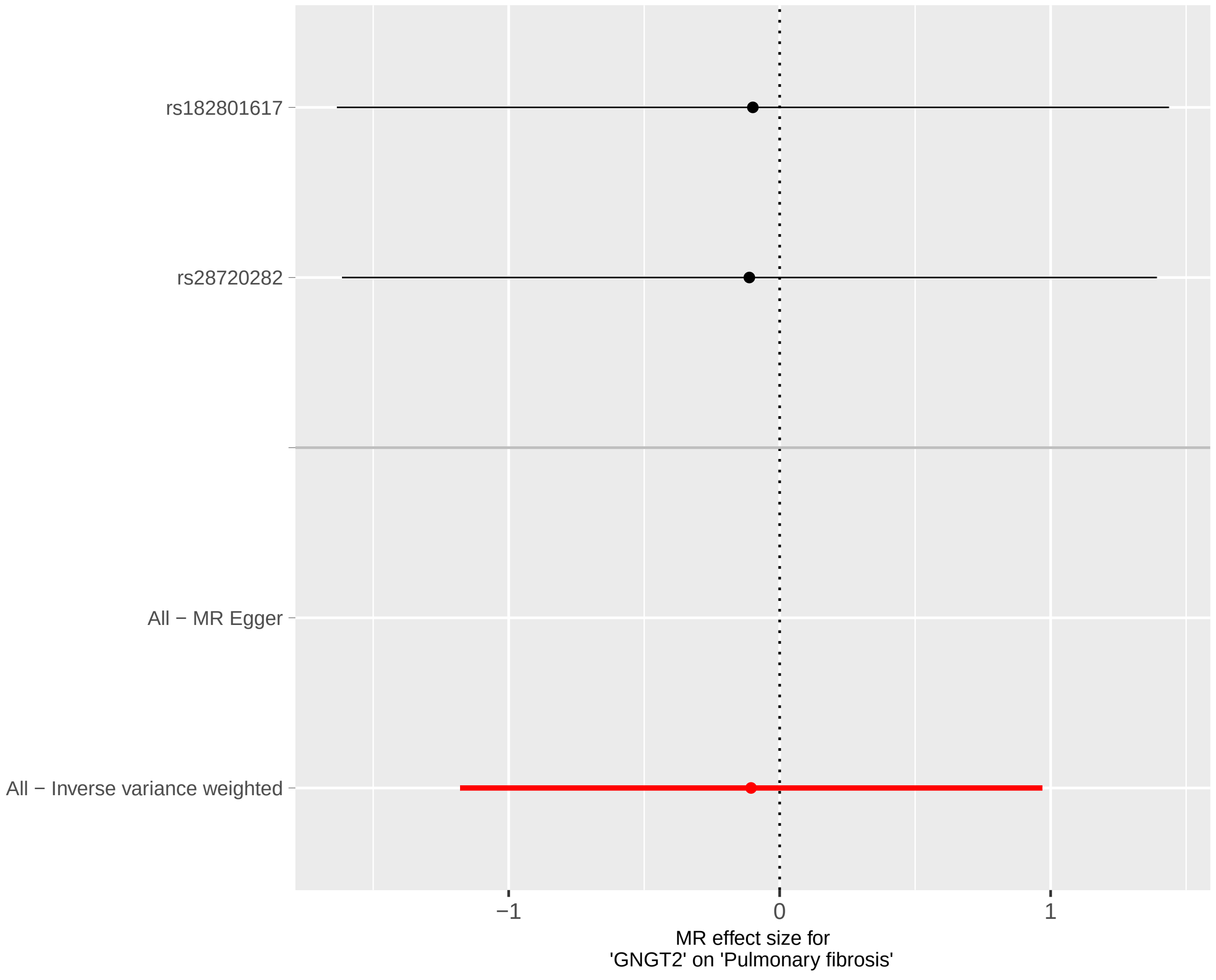
rs182801617
rs28720282
All − MR Egger
All − Inverse variance weighted
−1
0
1
MR effect size for
'GNGT2' on 'Pulmonary fibrosis'

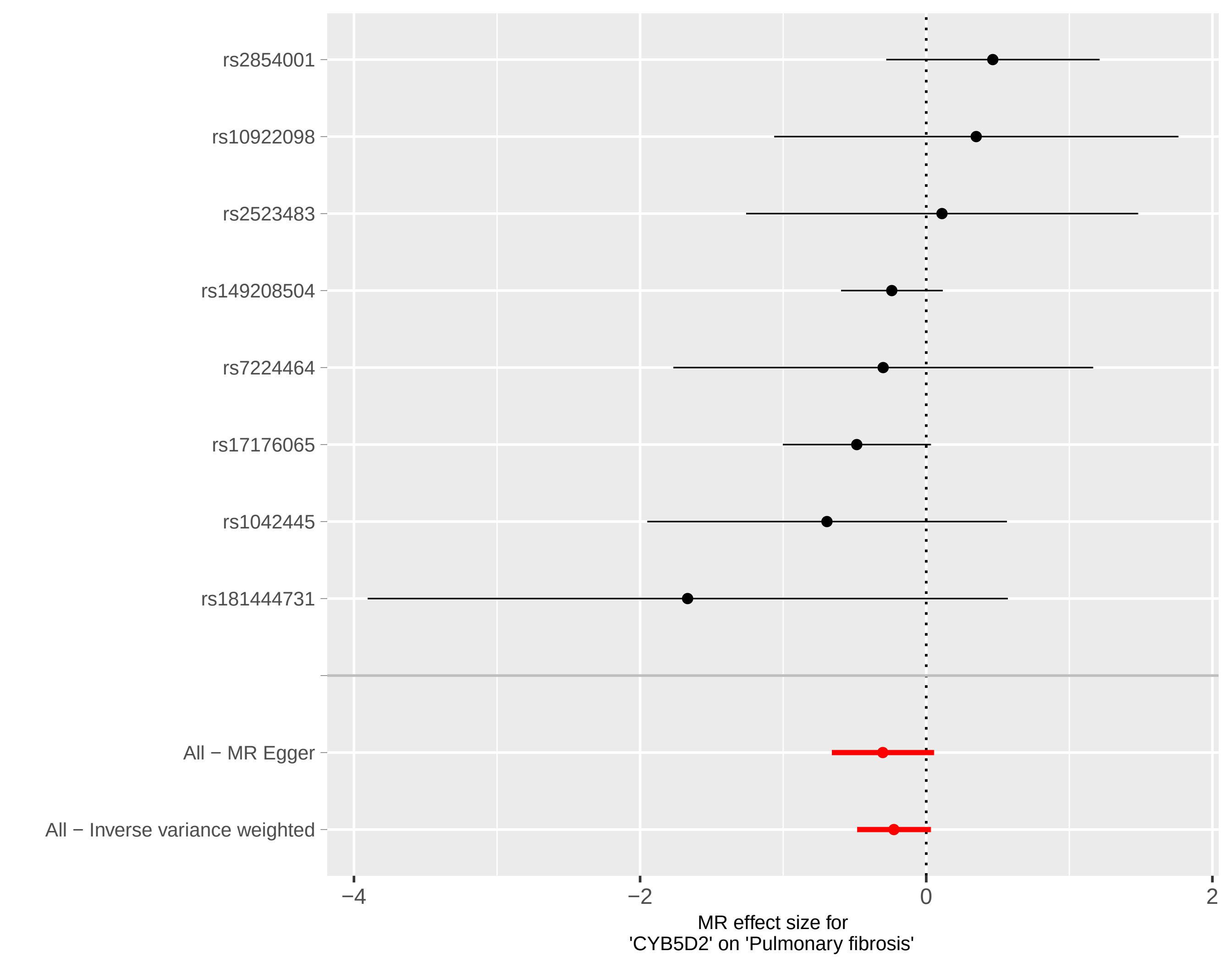

rs2854001
rs10922098
rs2523483
rs149208504
rs7224464
rs17176065
rs1042445
rs181444731
All − MR Egger
All − Inverse variance weighted
−4
−2
0
2
MR effect size for
'CYB5D2' on 'Pulmonary fibrosis'

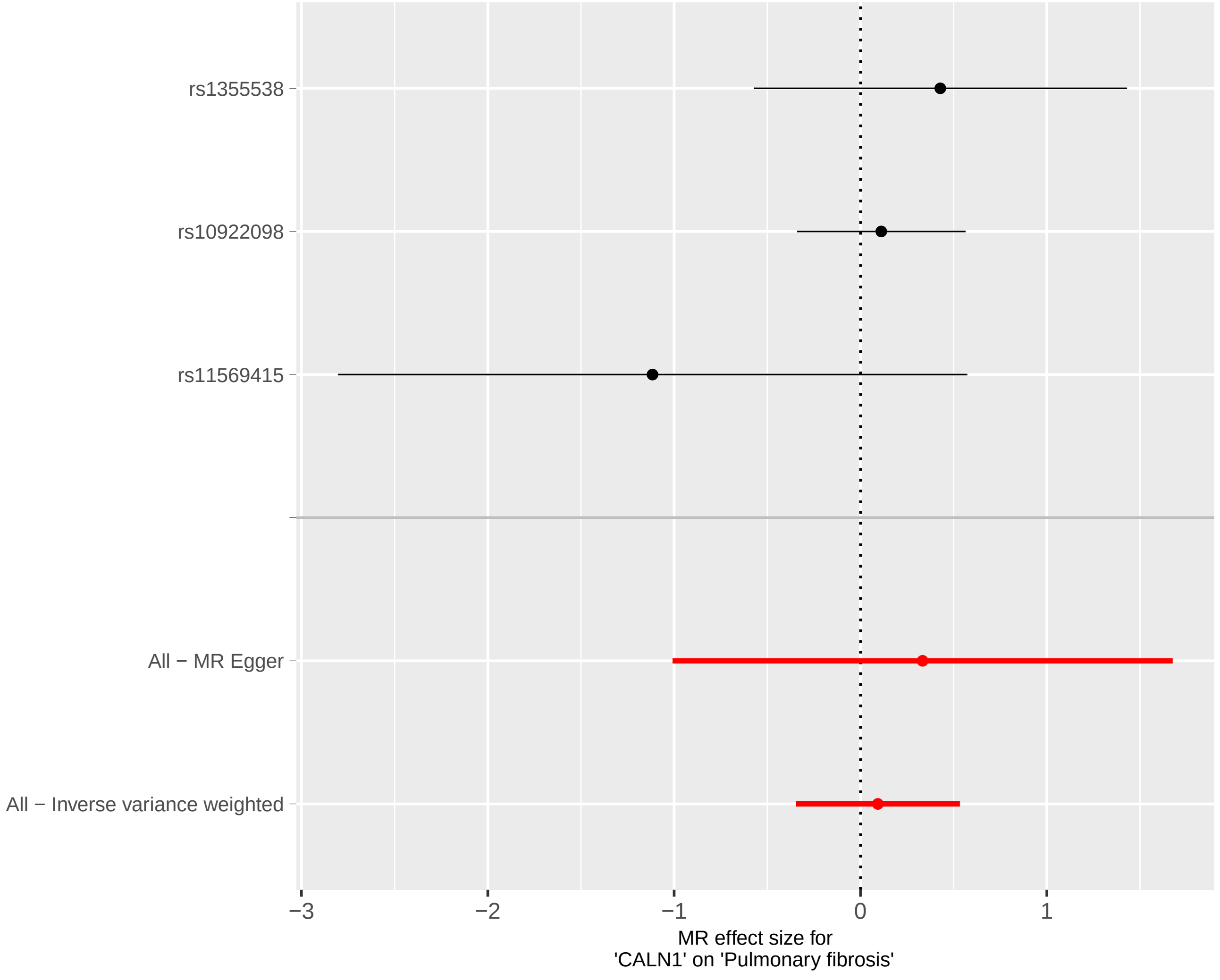
rs1355538
rs10922098
rs11569415
All − MR Egger
All − Inverse variance weighted
−3
−2
−1
0
1
MR effect size for
'CALN1' on 'Pulmonary fibrosis'

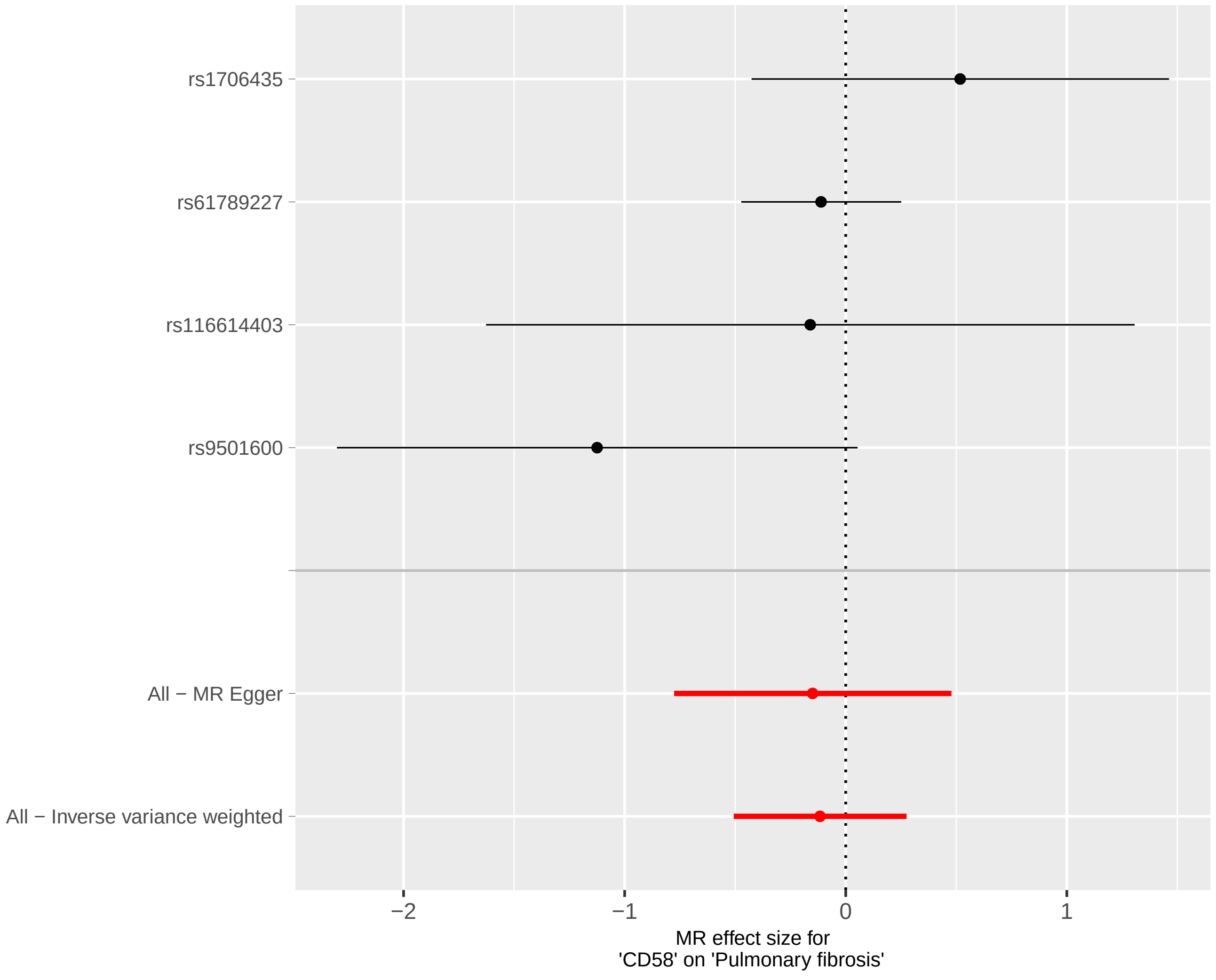
rs1706435
rs61789227
rs116614403
rs9501600
All − MR Egger
All − Inverse variance weighted
−2
−1
0
1
MR effect size for
'CD58' on 'Pulmonary fibrosis'

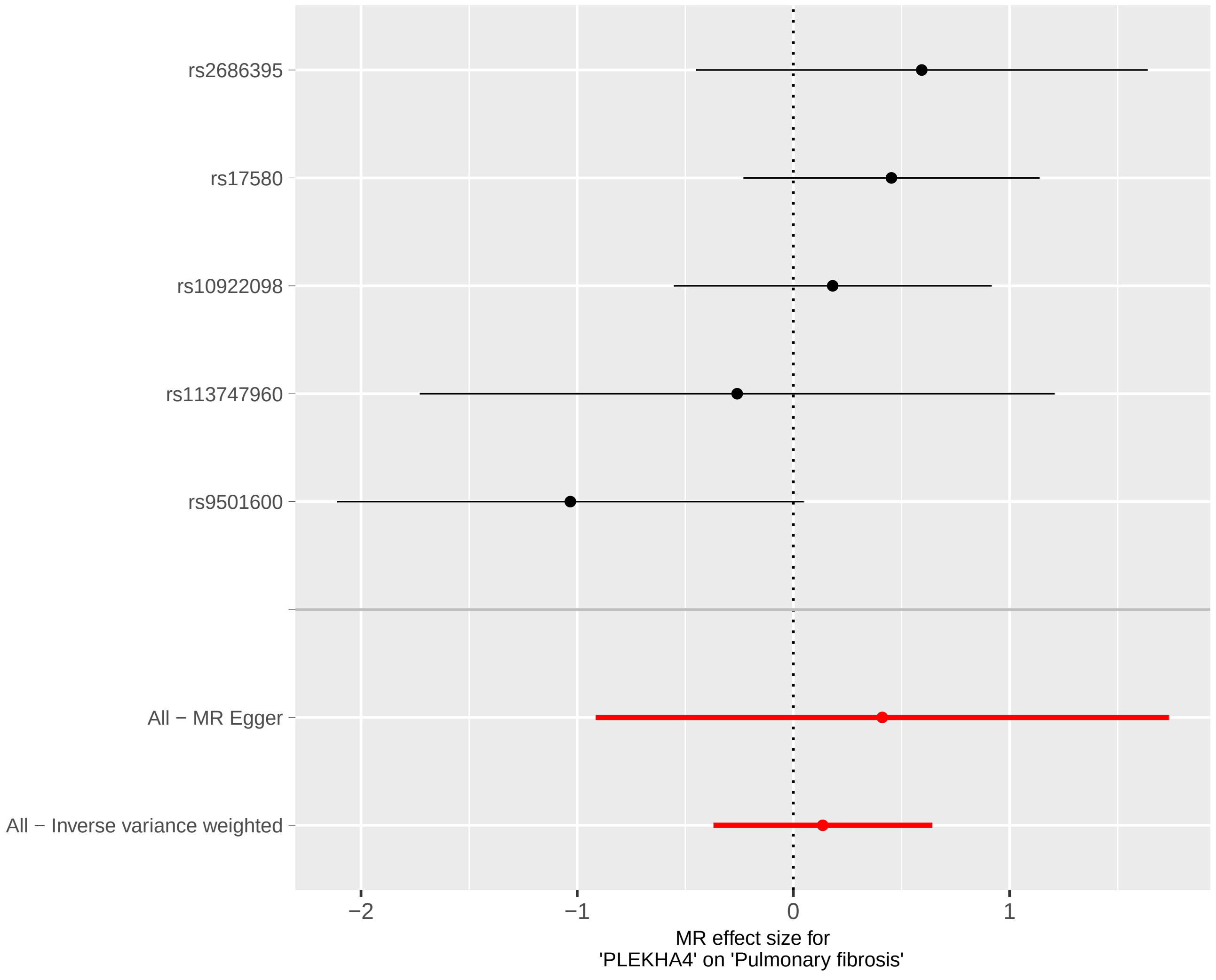
rs2686395
rs17580
rs10922098
rs113747960
rs9501600
All − MR Egger
All − Inverse variance weighted
−2
−1
0
1
MR effect size for
'PLEKHA4' on 'Pulmonary fibrosis'

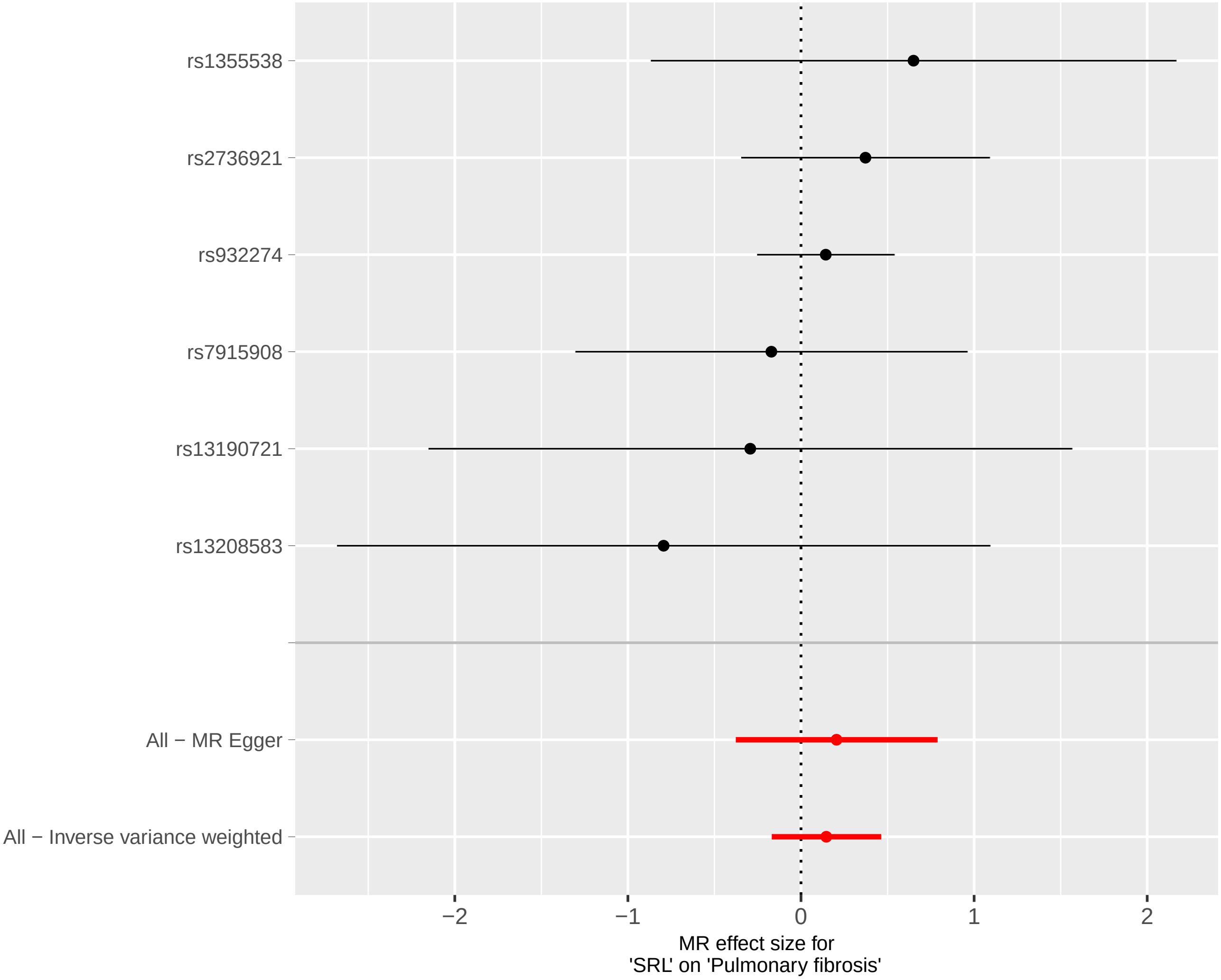

rs1355538
rs2736921
rs932274
rs7915908
rs13190721
rs13208583
All − MR Egger
All − Inverse variance weighted
−2
−1
0
1
2
MR effect size for
'SRL' on 'Pulmonary fibrosis'

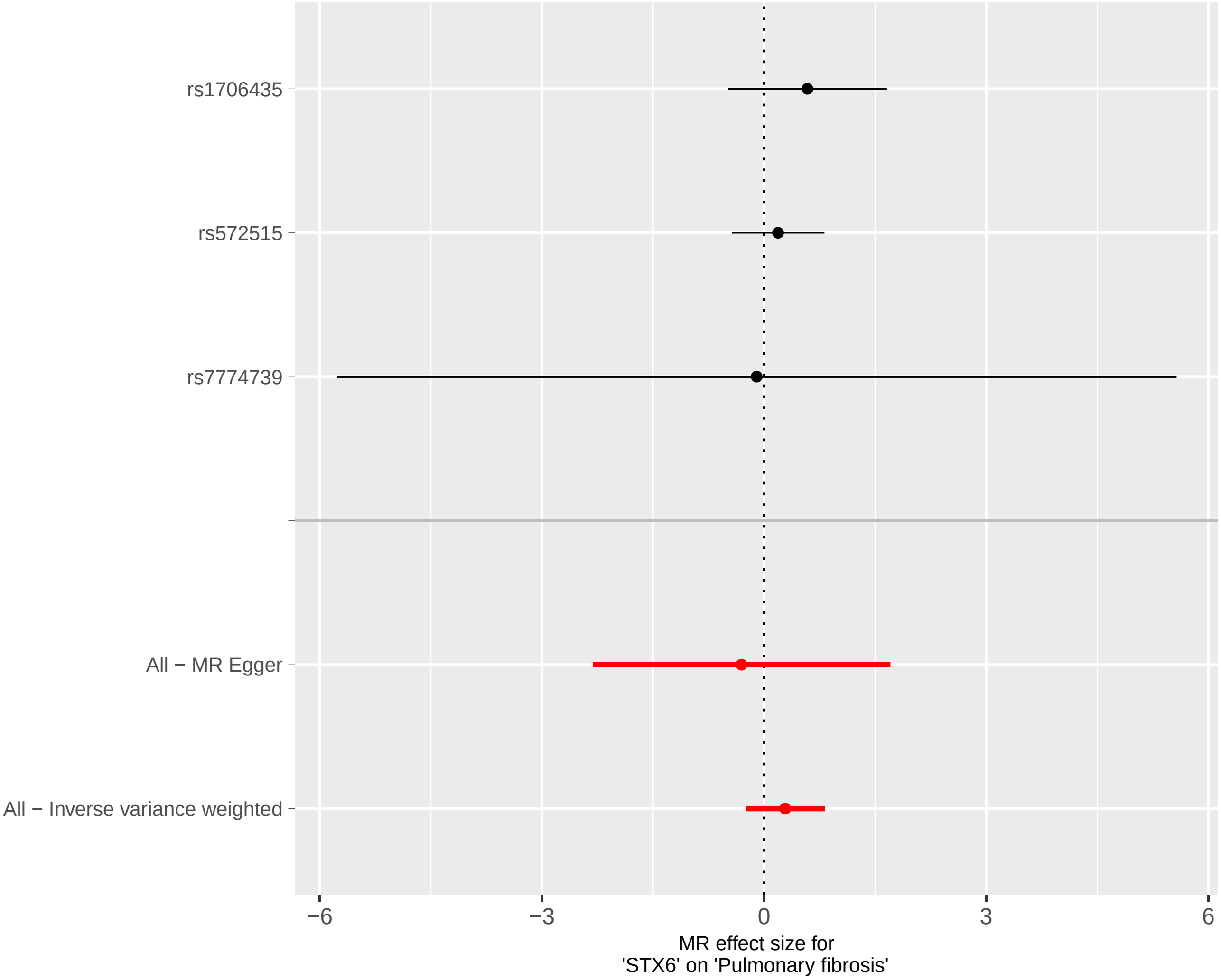

rs1706435
rs572515
rs7774739
All − MR Egger
All − Inverse variance weighted
−6
−3
0
3
6
MR effect size for
'STX6' on 'Pulmonary fibrosis'

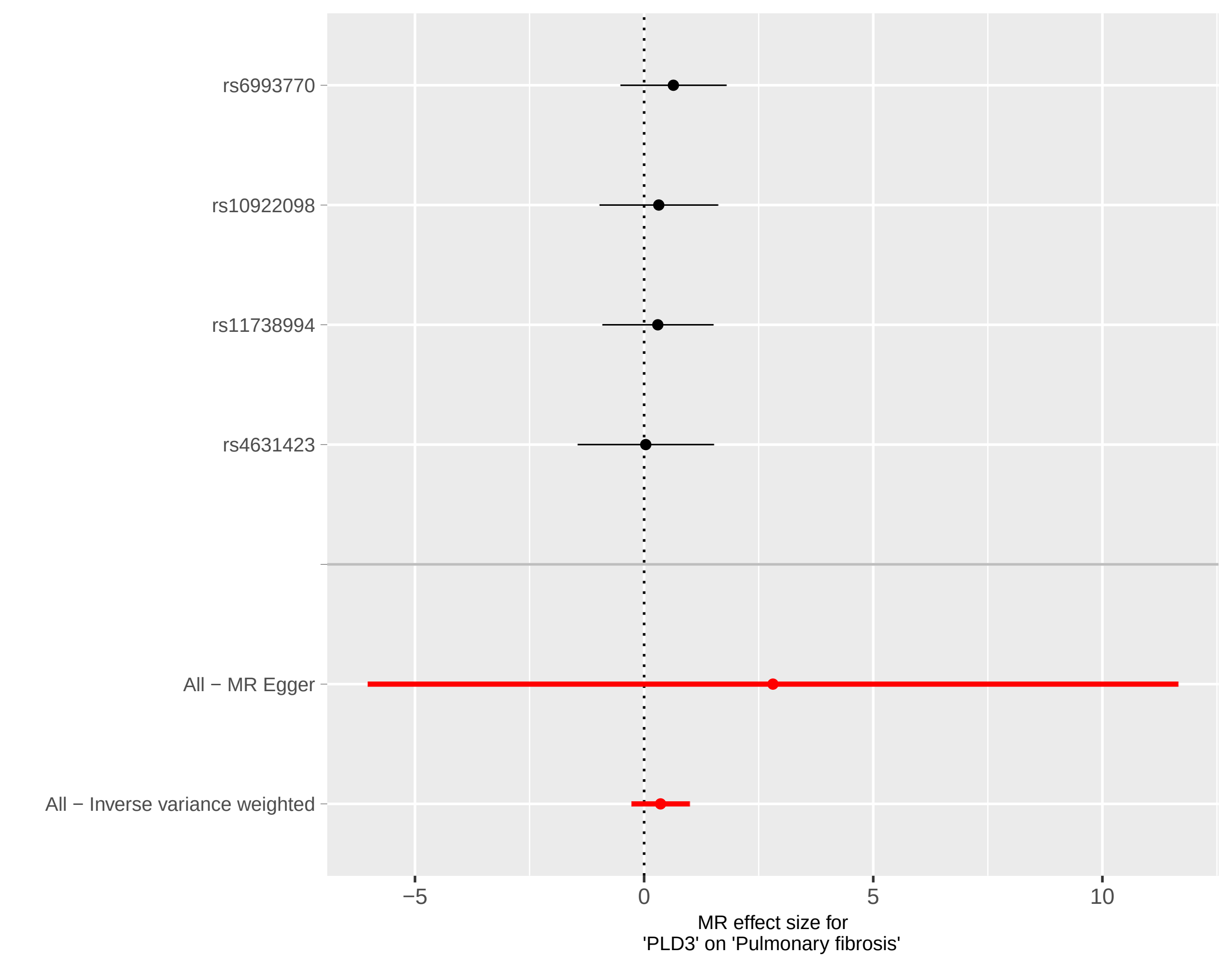
rs6993770
rs10922098
rs11738994
rs4631423
All − MR Egger
All − Inverse variance weighted
−5
0
5
10
MR effect size for
'PLD3' on 'Pulmonary fibrosis'

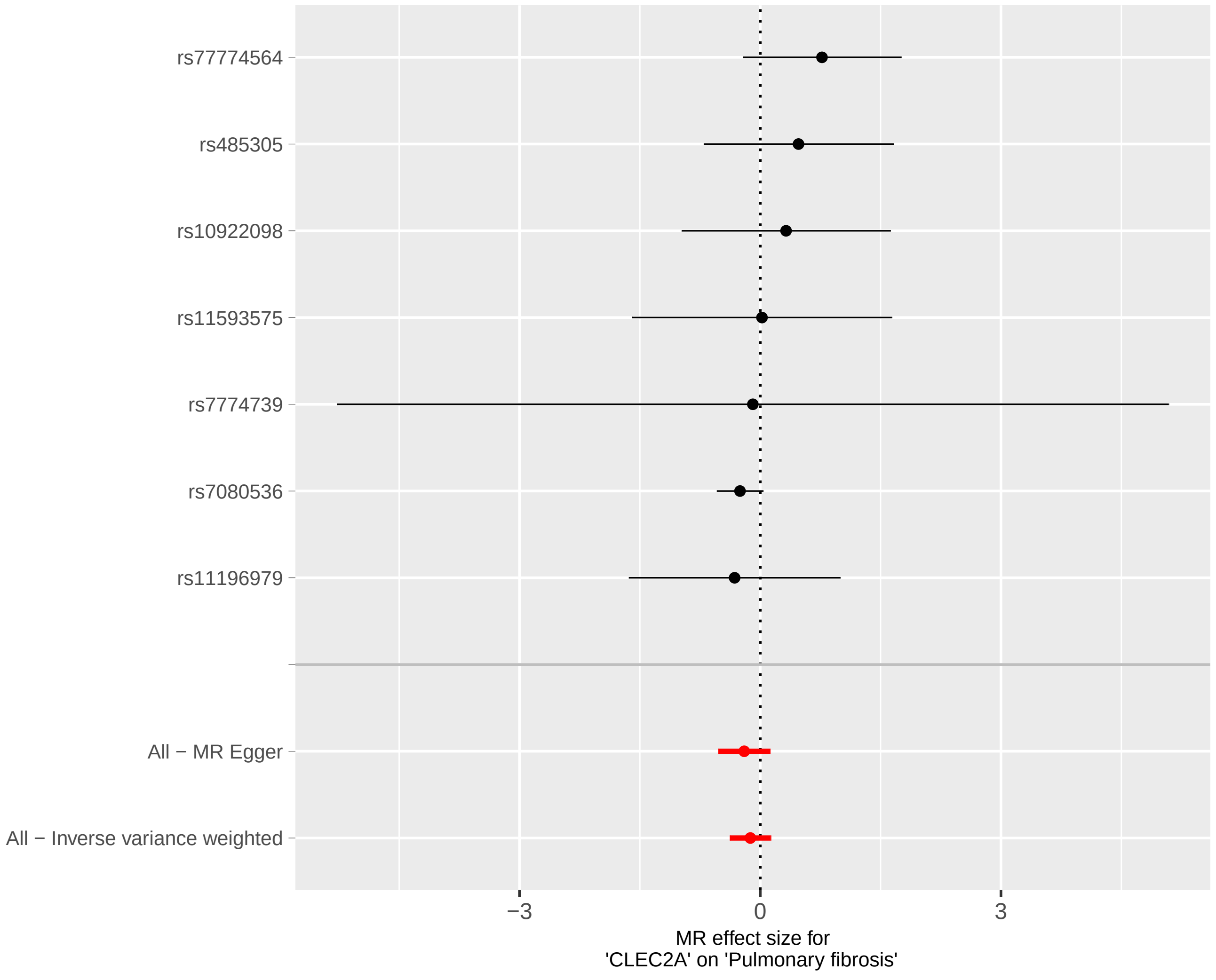
rs77774564
rs485305
rs10922098
rs11593575
rs7774739
rs7080536
rs11196979
All − MR Egger
All − Inverse variance weighted
−3
0
3
MR effect size for
'CLEC2A' on 'Pulmonary fibrosis'

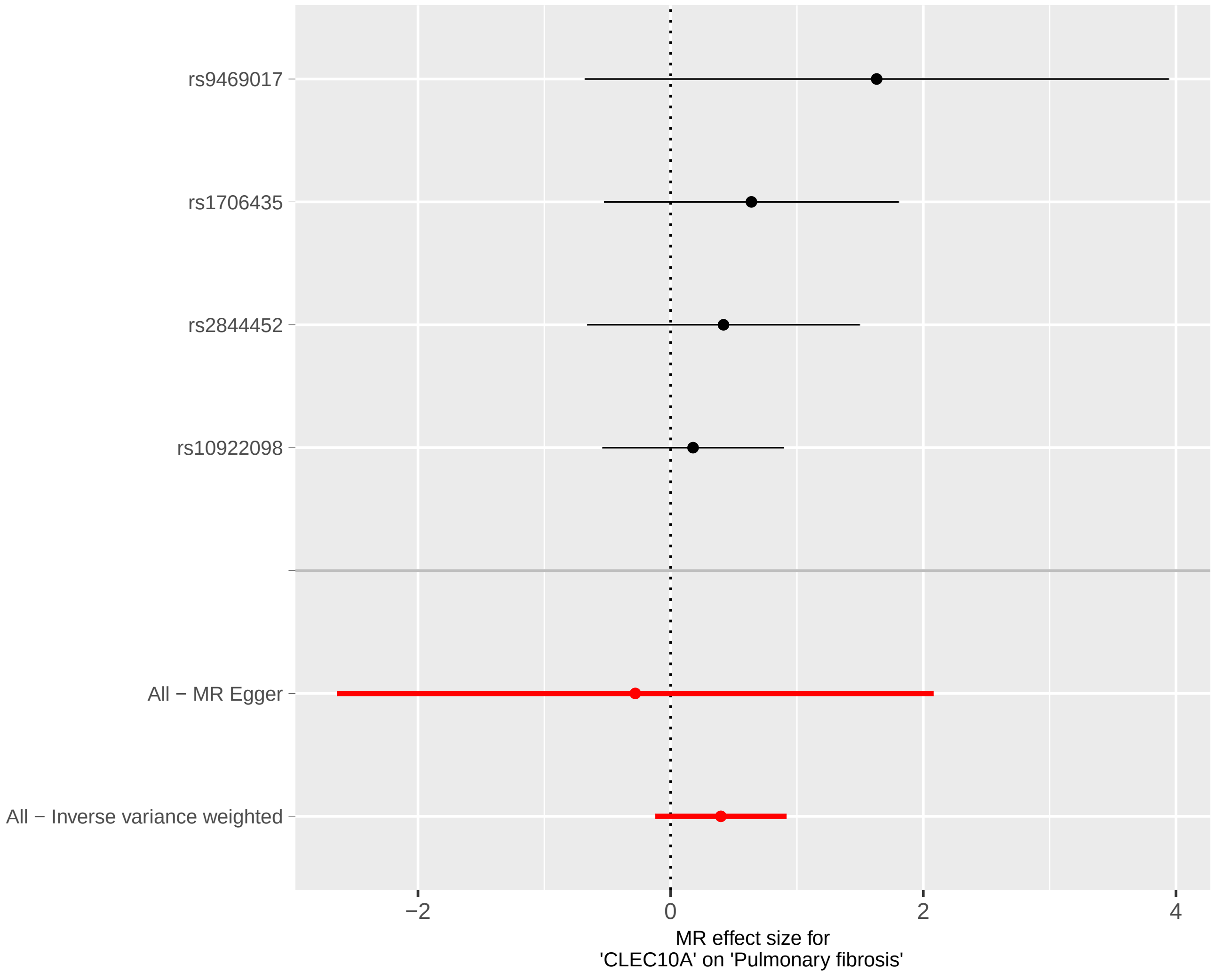

rs9469017
rs1706435
rs2844452
rs10922098
All − MR Egger
All − Inverse variance weighted
−2
0
2
4
MR effect size for
'CLEC10A' on 'Pulmonary fibrosis'

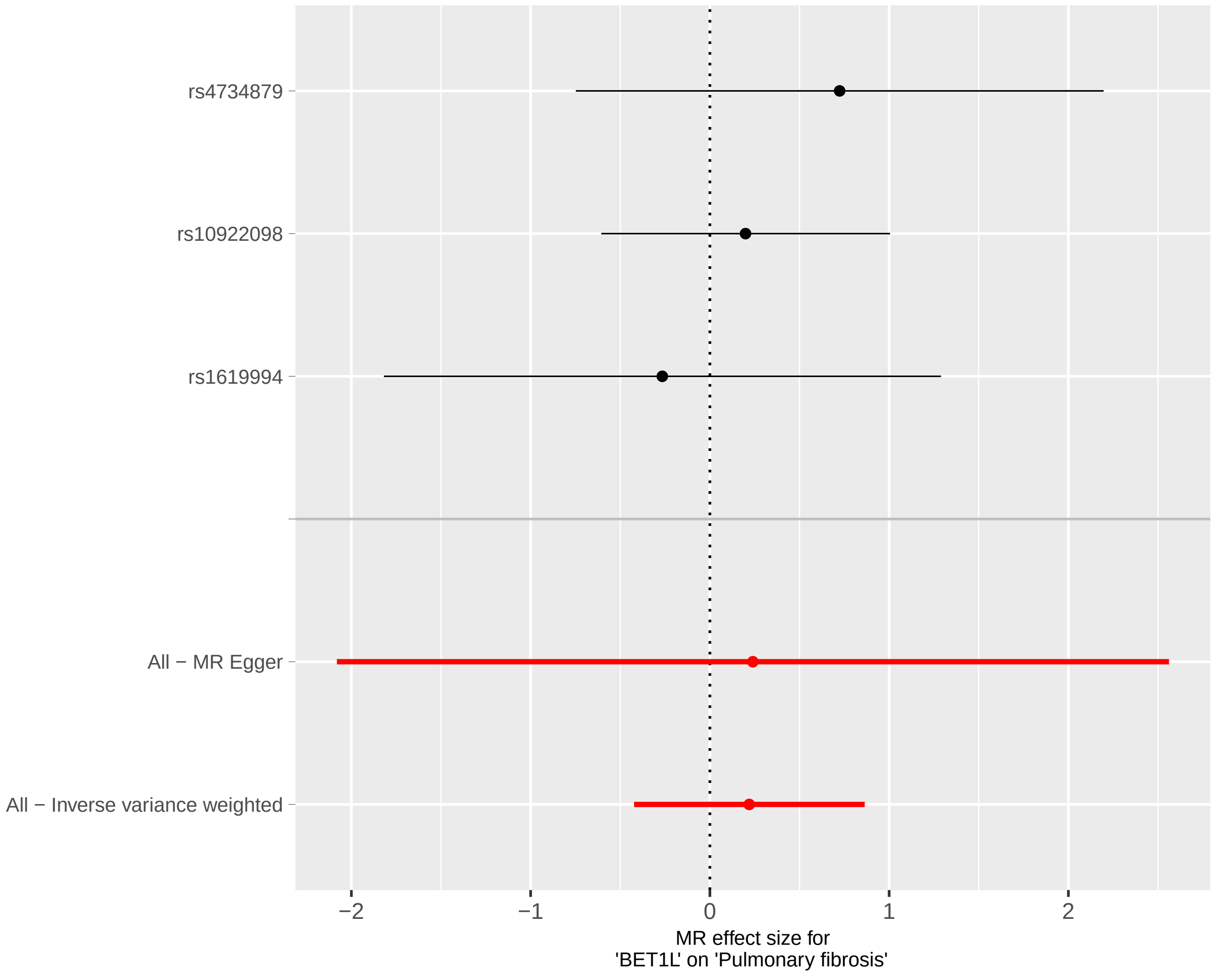
rs4734879
rs10922098
rs1619994
All − MR Egger
All − Inverse variance weighted
−2
−1
0
1
2
MR effect size for
'BET1L' on 'Pulmonary fibrosis'

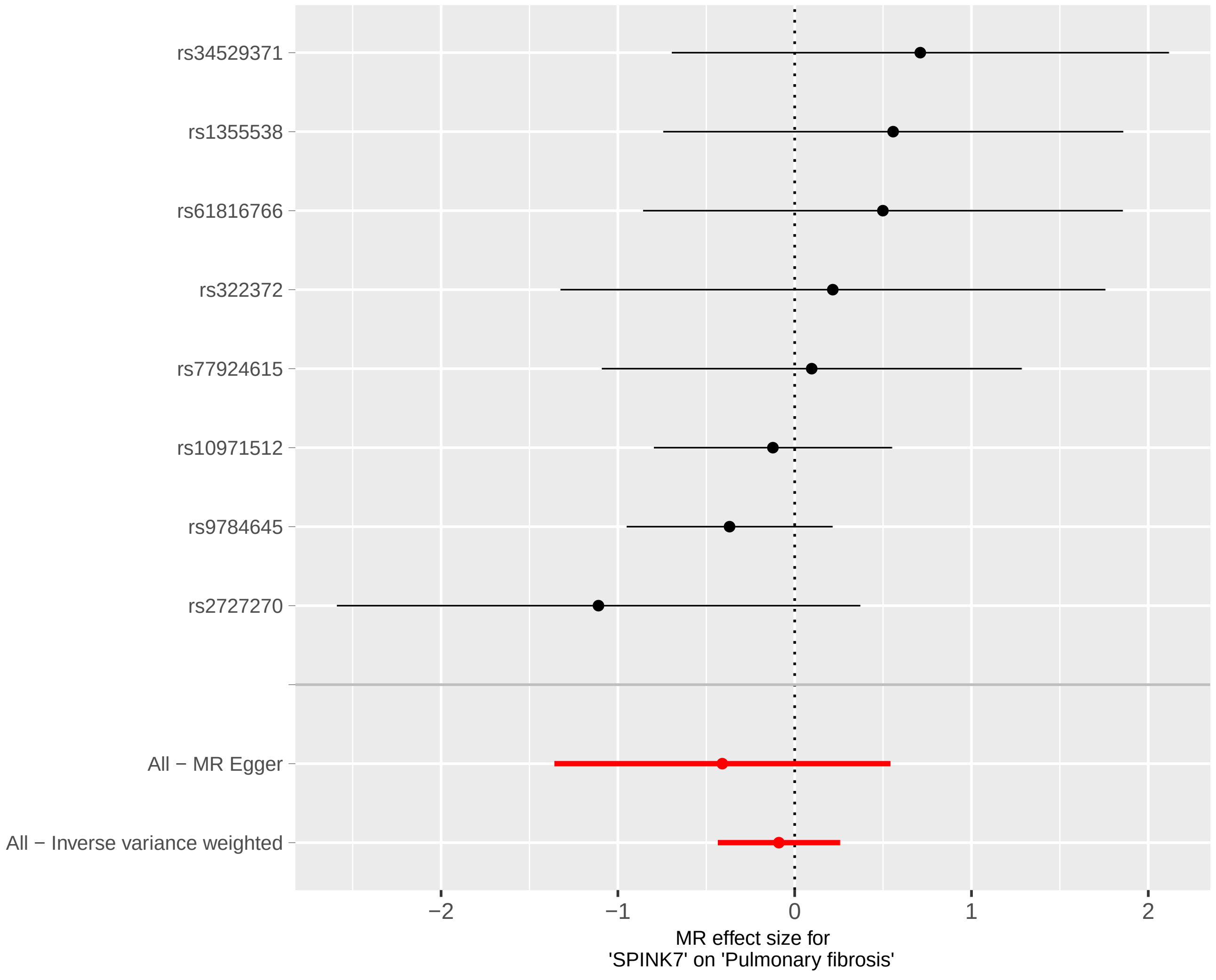
rs34529371
rs1355538
rs61816766
rs322372
rs77924615
rs10971512
rs9784645
rs2727270
All − MR Egger
All − Inverse variance weighted
−2
−1
0
1
2
MR effect size for
'SPINK7' on 'Pulmonary fibrosis'

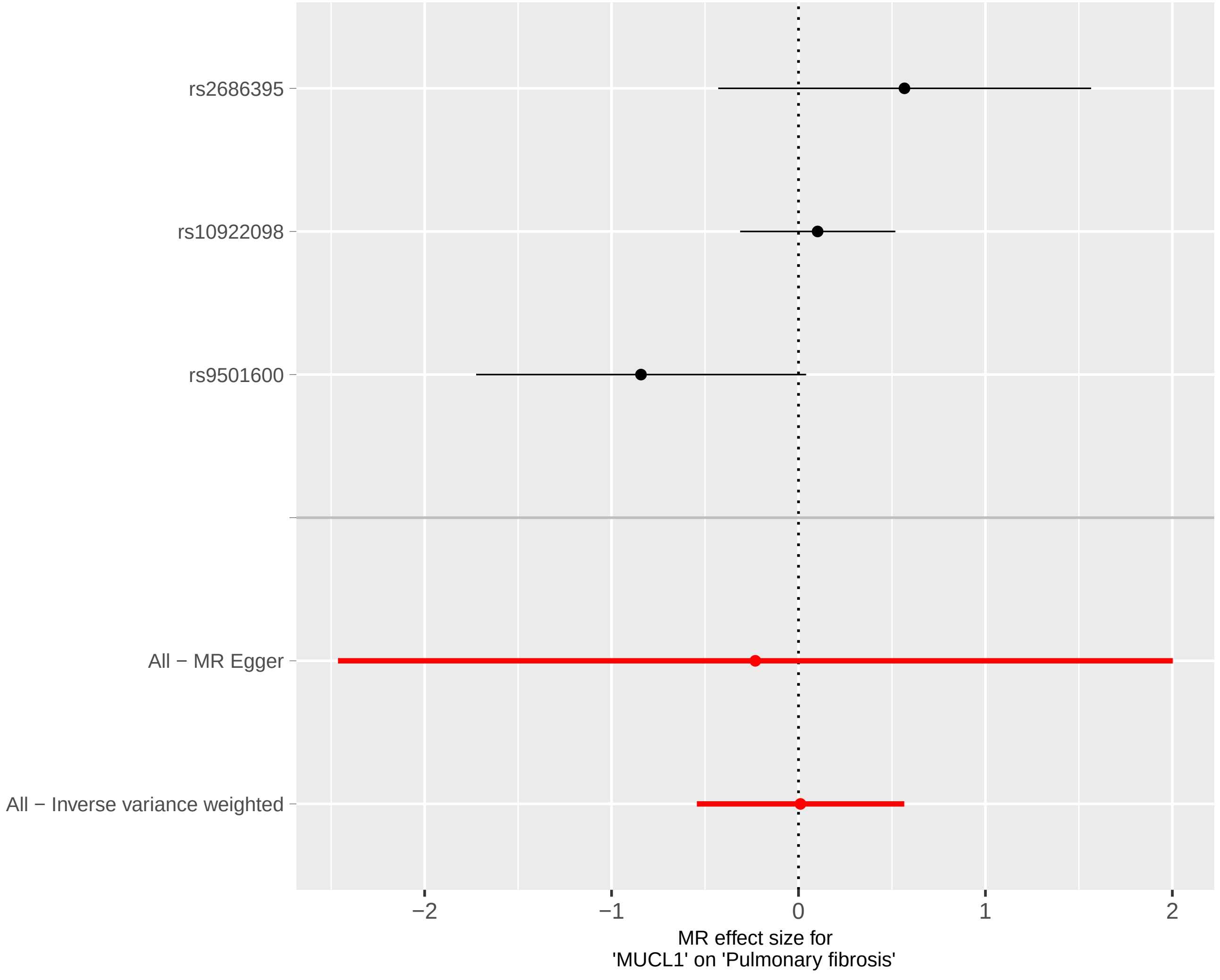

rs2686395
rs10922098
rs9501600
All − MR Egger
All − Inverse variance weighted
−2
−1
0
1
2
MR effect size for
'MUCL1' on 'Pulmonary fibrosis'

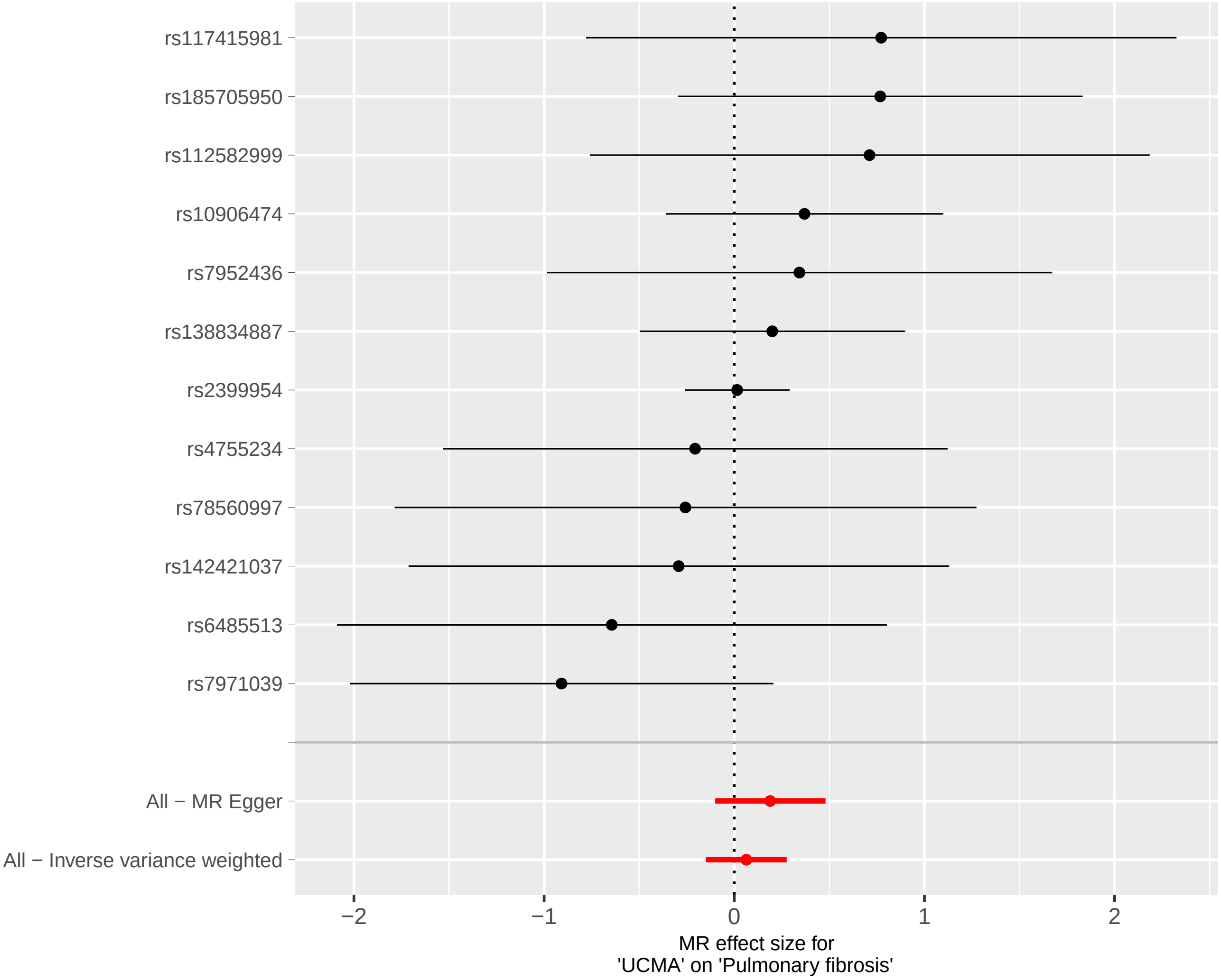
rs117415981
rs185705950
rs112582999
rs10906474
rs7952436
rs138834887
rs2399954
rs4755234
rs78560997
rs142421037
rs6485513
rs7971039
All − MR Egger
All − Inverse variance weighted
−2
−1
0
1
2
MR effect size for
'UCMA' on 'Pulmonary fibrosis'

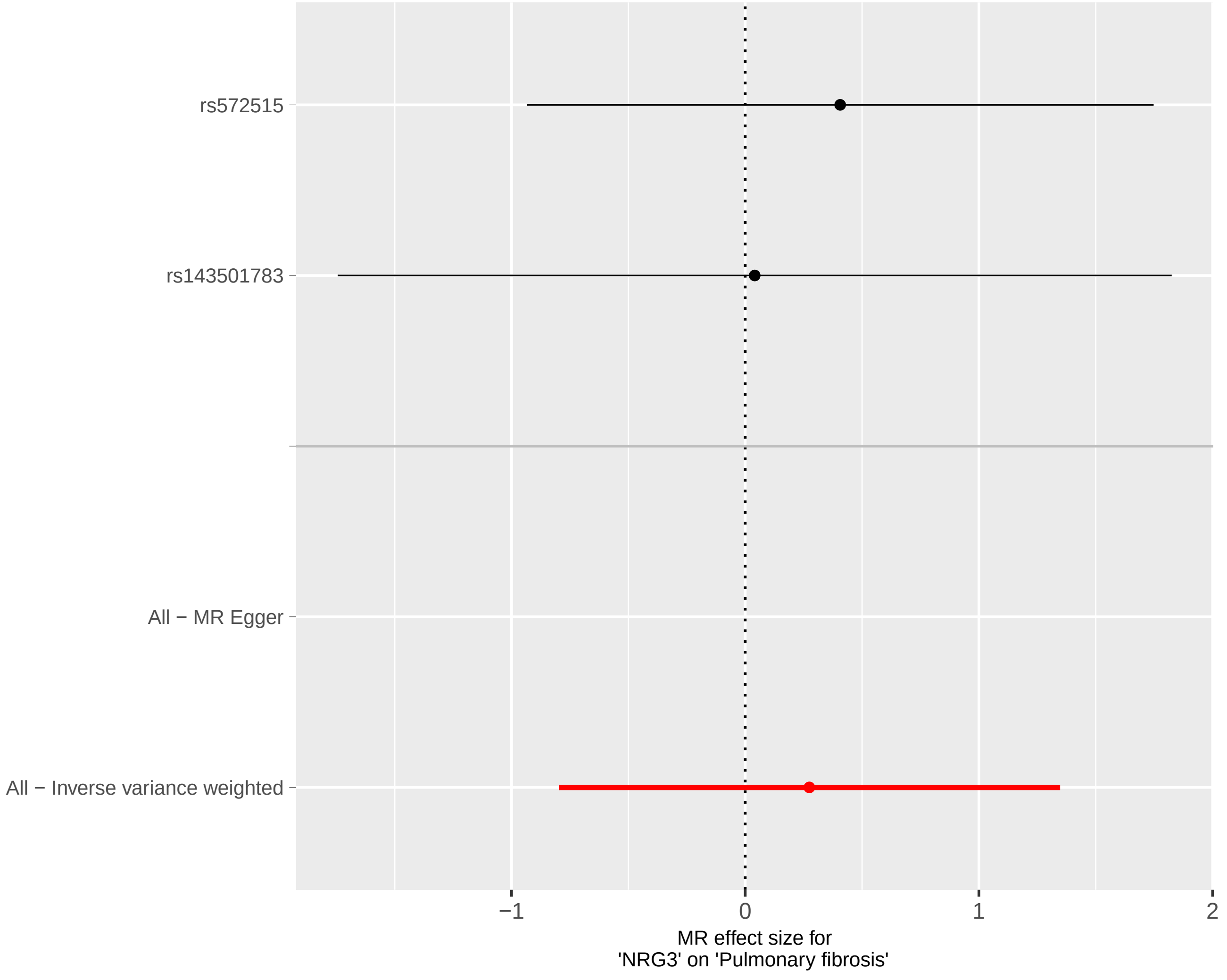

rs572515
rs143501783
All − MR Egger
All − Inverse variance weighted
−1
0
1
2
MR effect size for
'NRG3' on 'Pulmonary fibrosis'

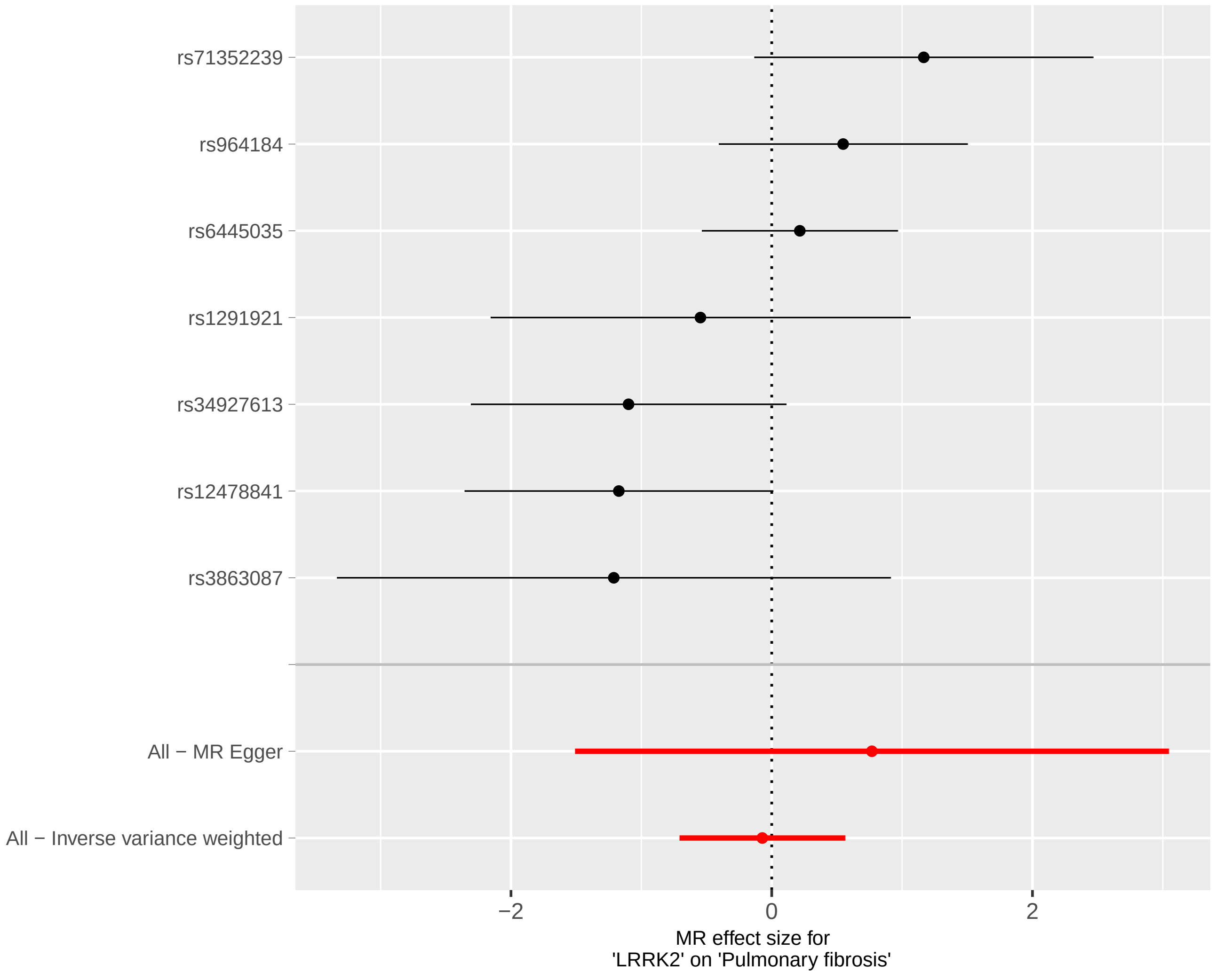
rs71352239
rs964184
rs6445035
rs1291921
rs34927613
rs12478841
rs3863087
All − MR Egger
All − Inverse variance weighted
−2
0
2
MR effect size for
'LRRK2' on 'Pulmonary fibrosis'

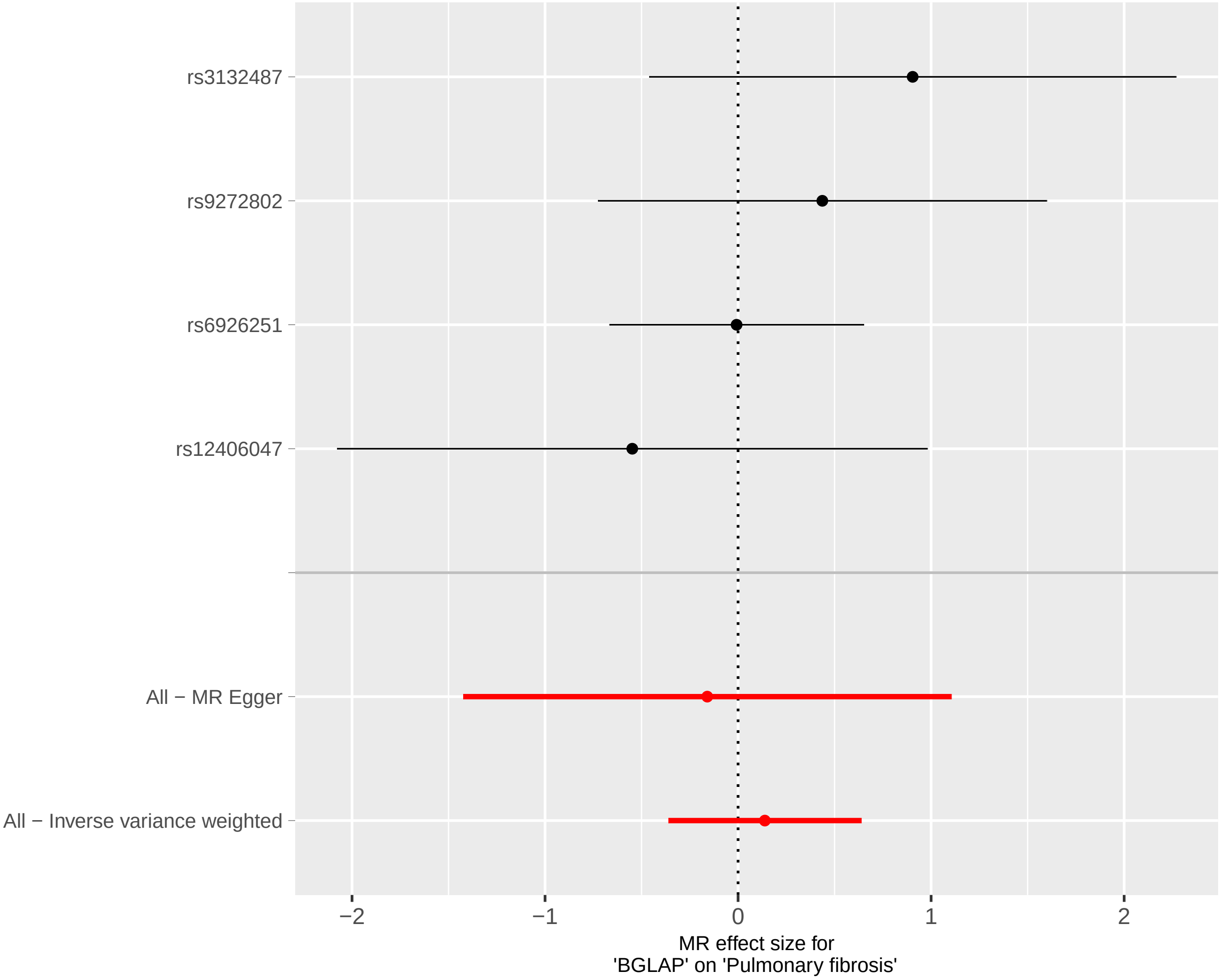

rs3132487
rs9272802
rs6926251
rs12406047
All − MR Egger
All − Inverse variance weighted
−2
−1
0
1
2
MR effect size for
'BGLAP' on 'Pulmonary fibrosis'

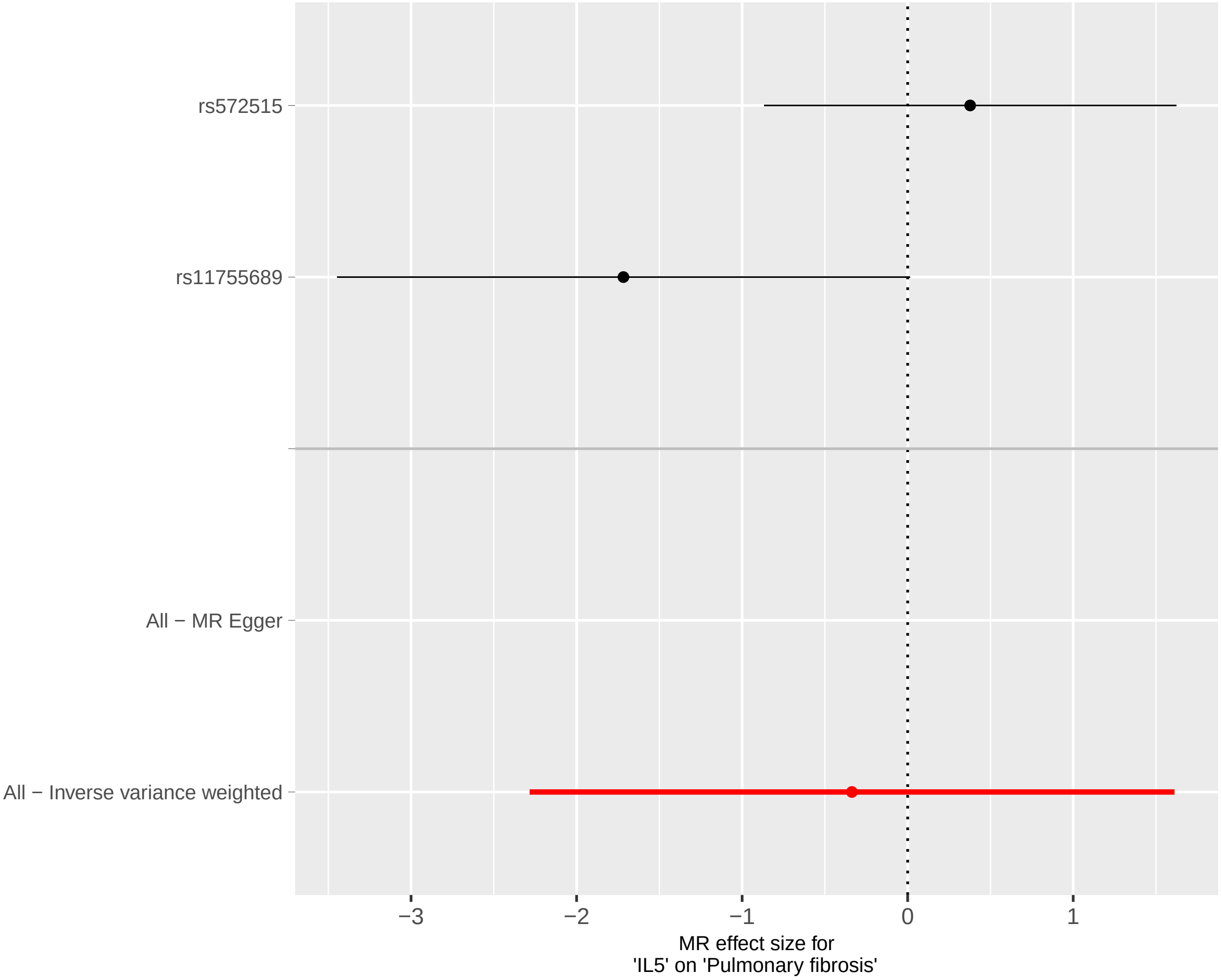
rs572515
rs11755689
All − MR Egger
All − Inverse variance weighted
−3
−2
−1
0
1
MR effect size for
'IL5' on 'Pulmonary fibrosis'

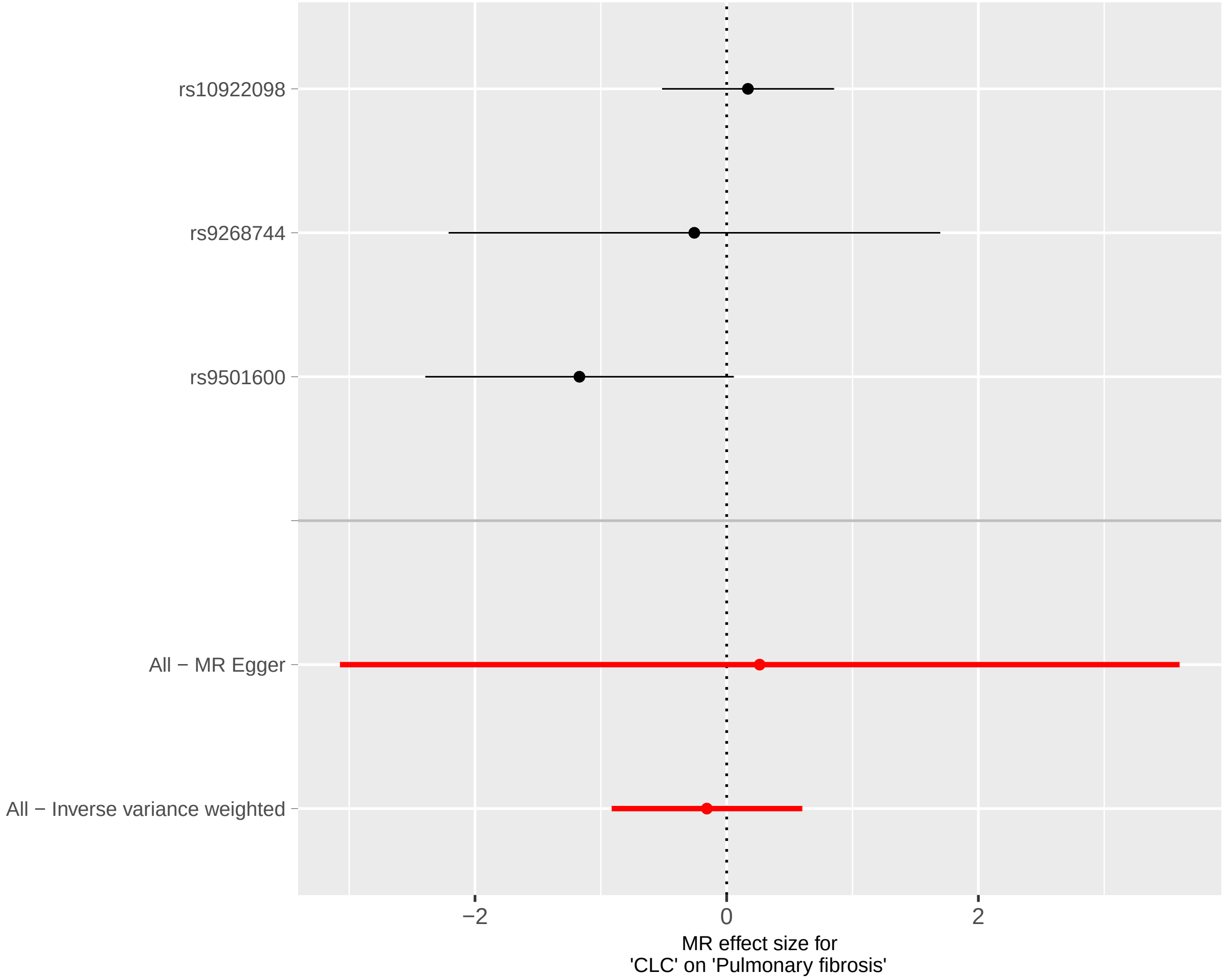

rs10922098
rs9268744
rs9501600
All − MR Egger
All − Inverse variance weighted
−2
0
2
MR effect size for
'CLC' on 'Pulmonary fibrosis'

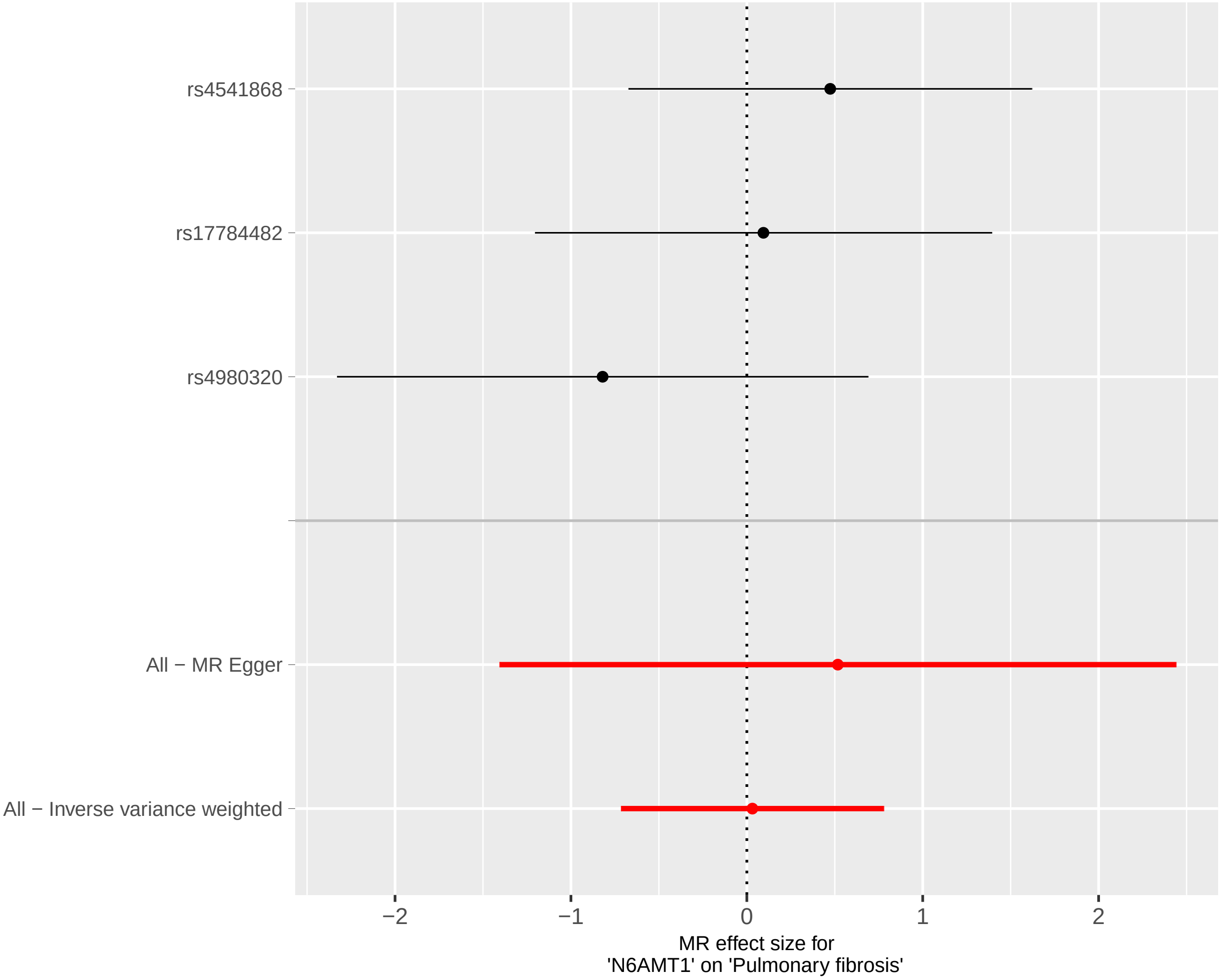

rs4541868
rs17784482
rs4980320
All − MR Egger
All − Inverse variance weighted
−2
−1
0
1
2
MR effect size for
'N6AMT1' on 'Pulmonary fibrosis'

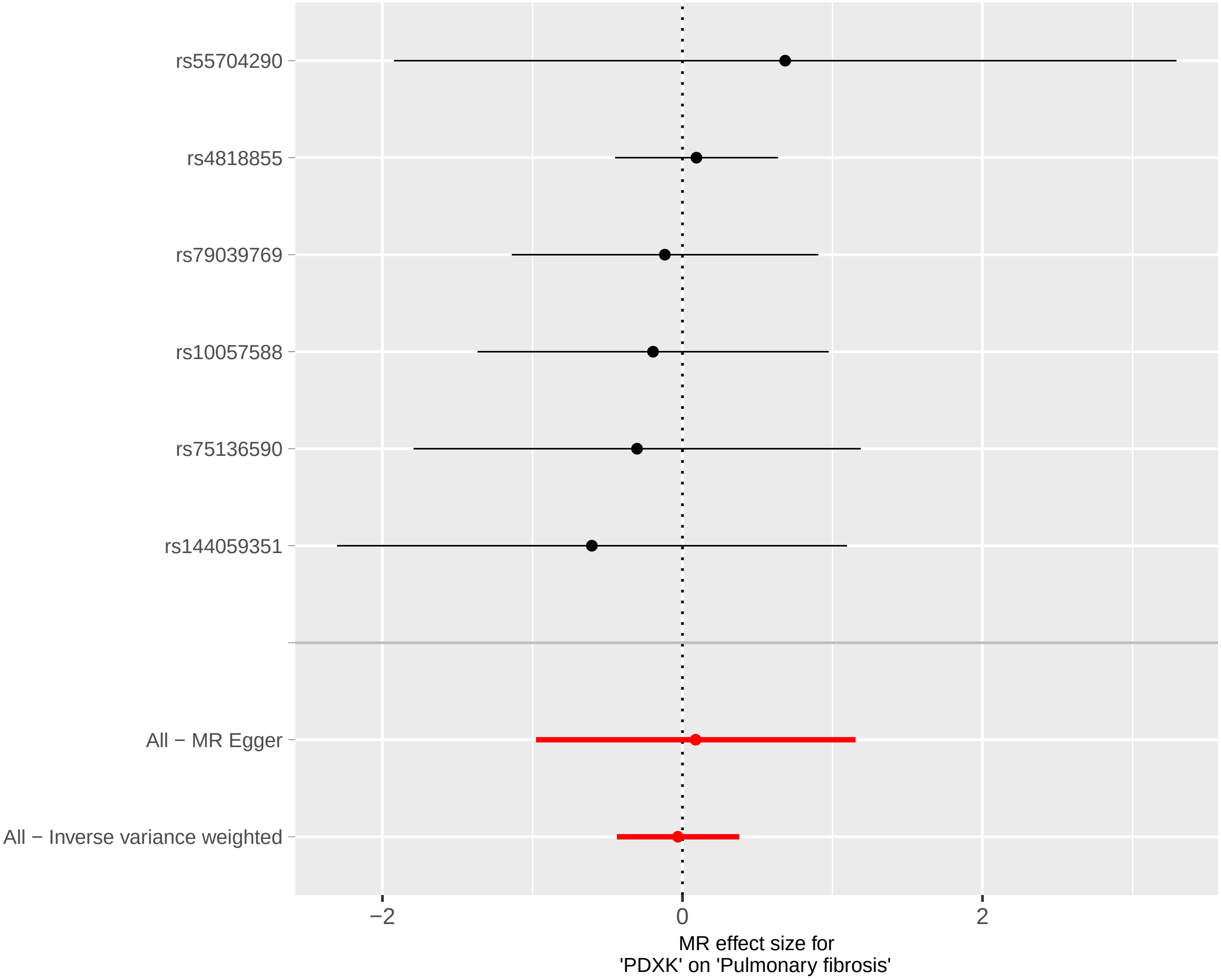

rs55704290
rs4818855
rs79039769
rs10057588
rs75136590
rs144059351
All − MR Egger
All − Inverse variance weighted
−2
0
2
MR effect size for
'PDXK' on 'Pulmonary fibrosis'

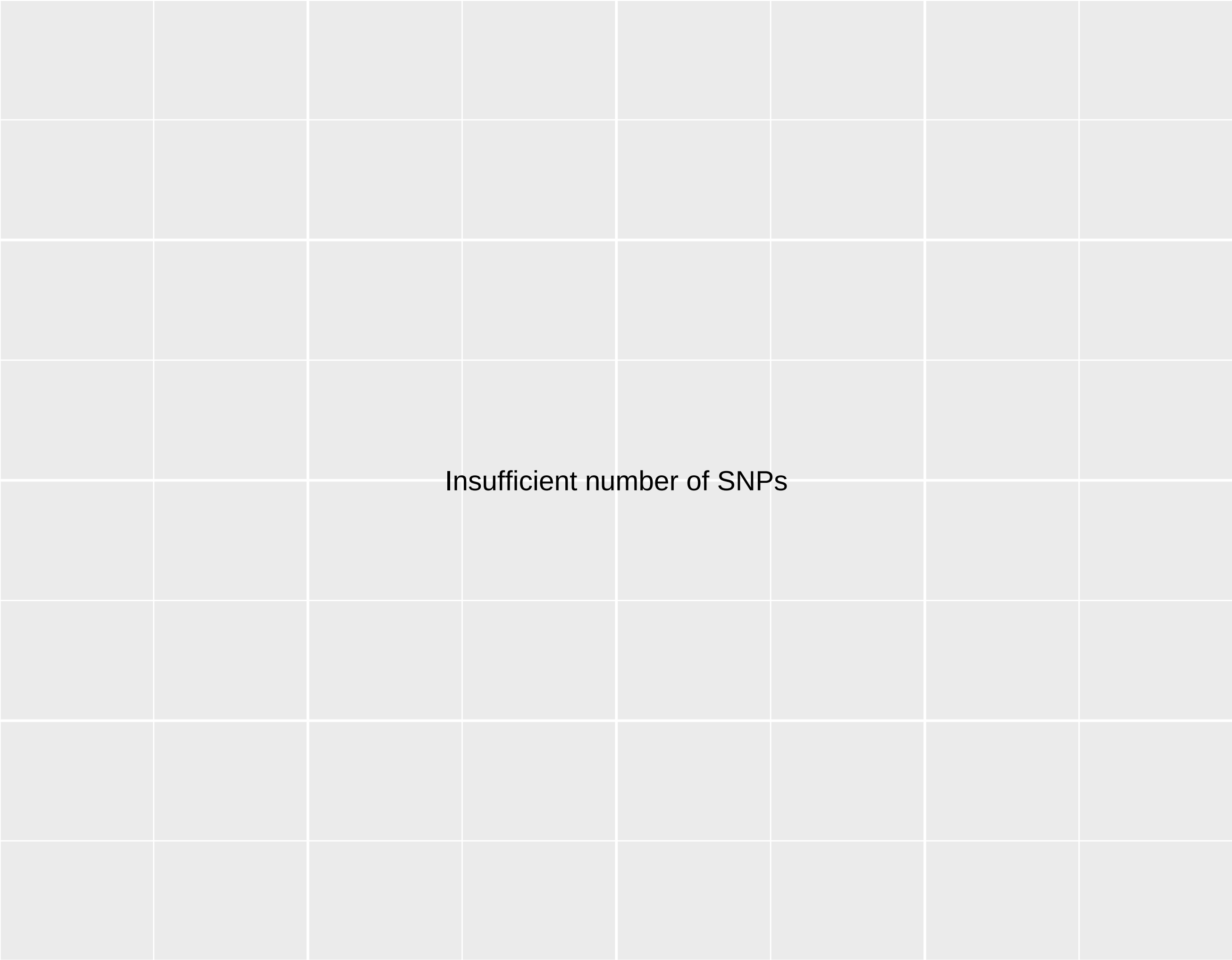
Insufficient number of SNPs

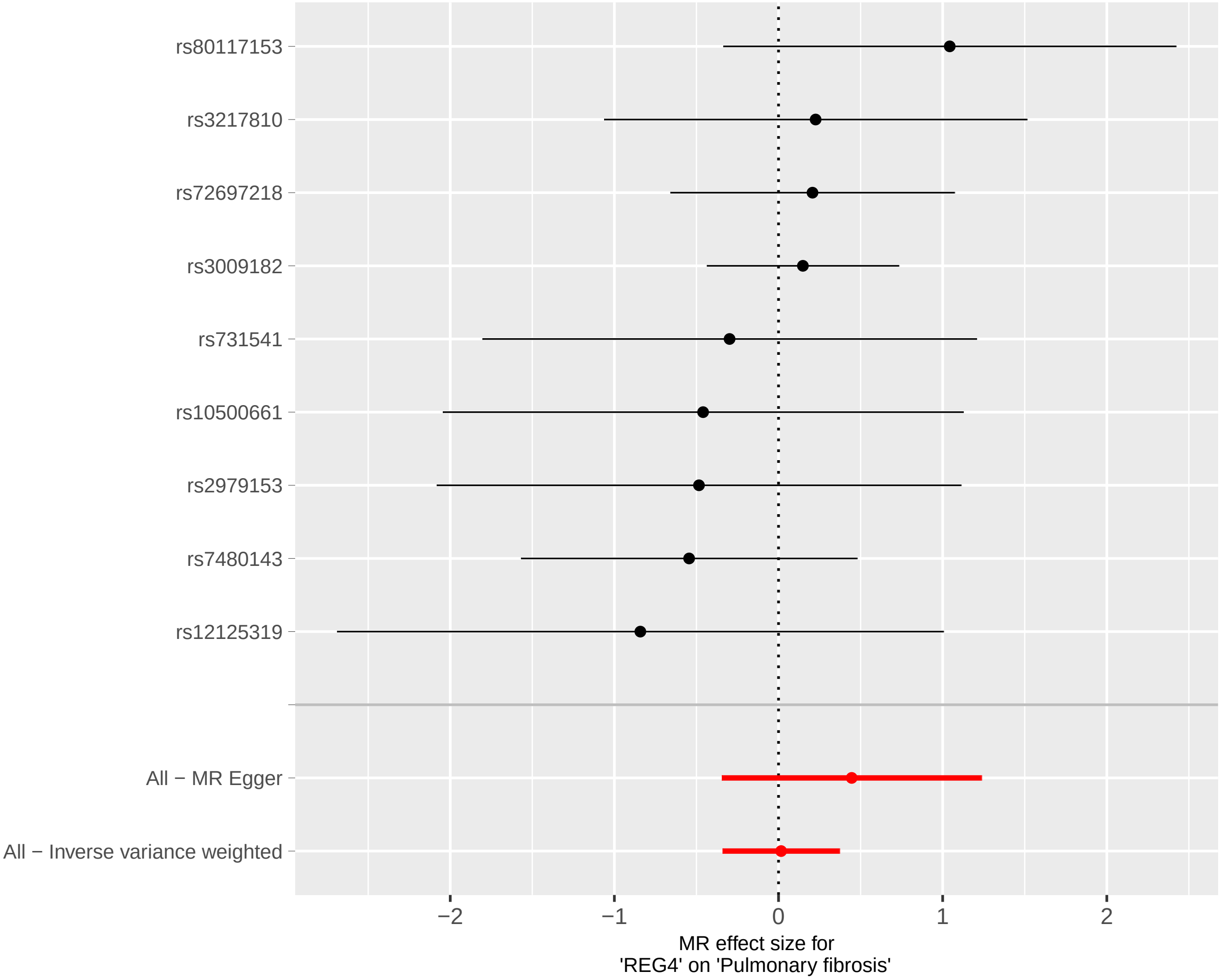
rs80117153
rs3217810
rs72697218
rs3009182
rs731541
rs10500661
rs2979153
rs7480143
rs12125319
All − MR Egger
All − Inverse variance weighted
−2
−1
0
1
2
MR effect size for
'REG4' on 'Pulmonary fibrosis'

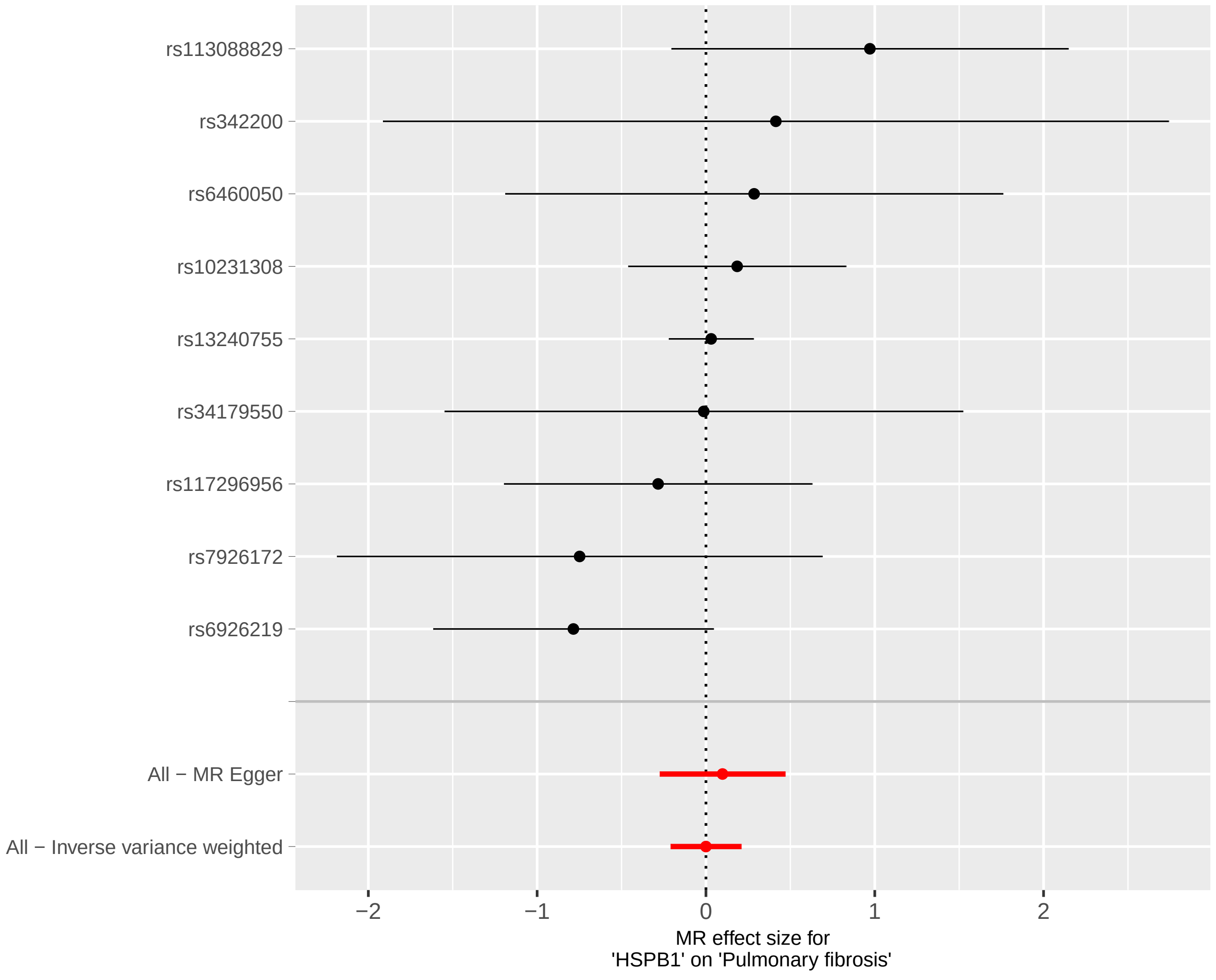

rs113088829
rs342200
rs6460050
rs10231308
rs13240755
rs34179550
rs117296956
rs7926172
rs6926219
All − MR Egger
All − Inverse variance weighted
−2
−1
0
1
2
MR effect size for
'HSPB1' on 'Pulmonary fibrosis'

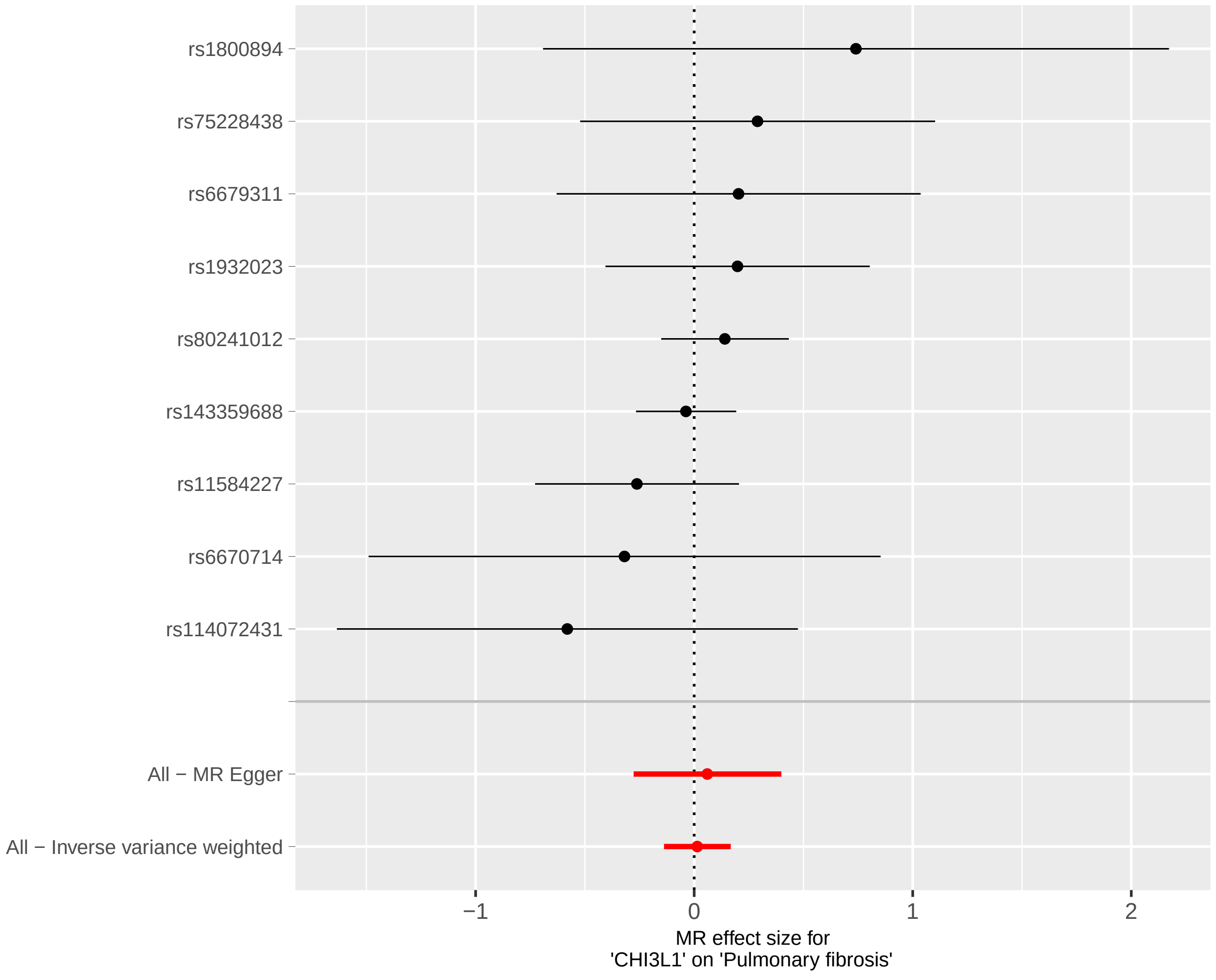
rs1800894
rs75228438
rs6679311
rs1932023
rs80241012
rs143359688
rs11584227
rs6670714
rs114072431
All − MR Egger
All − Inverse variance weighted
−1
0
1
2
MR effect size for
'CHI3L1' on 'Pulmonary fibrosis'

Insufficient number of SNPs

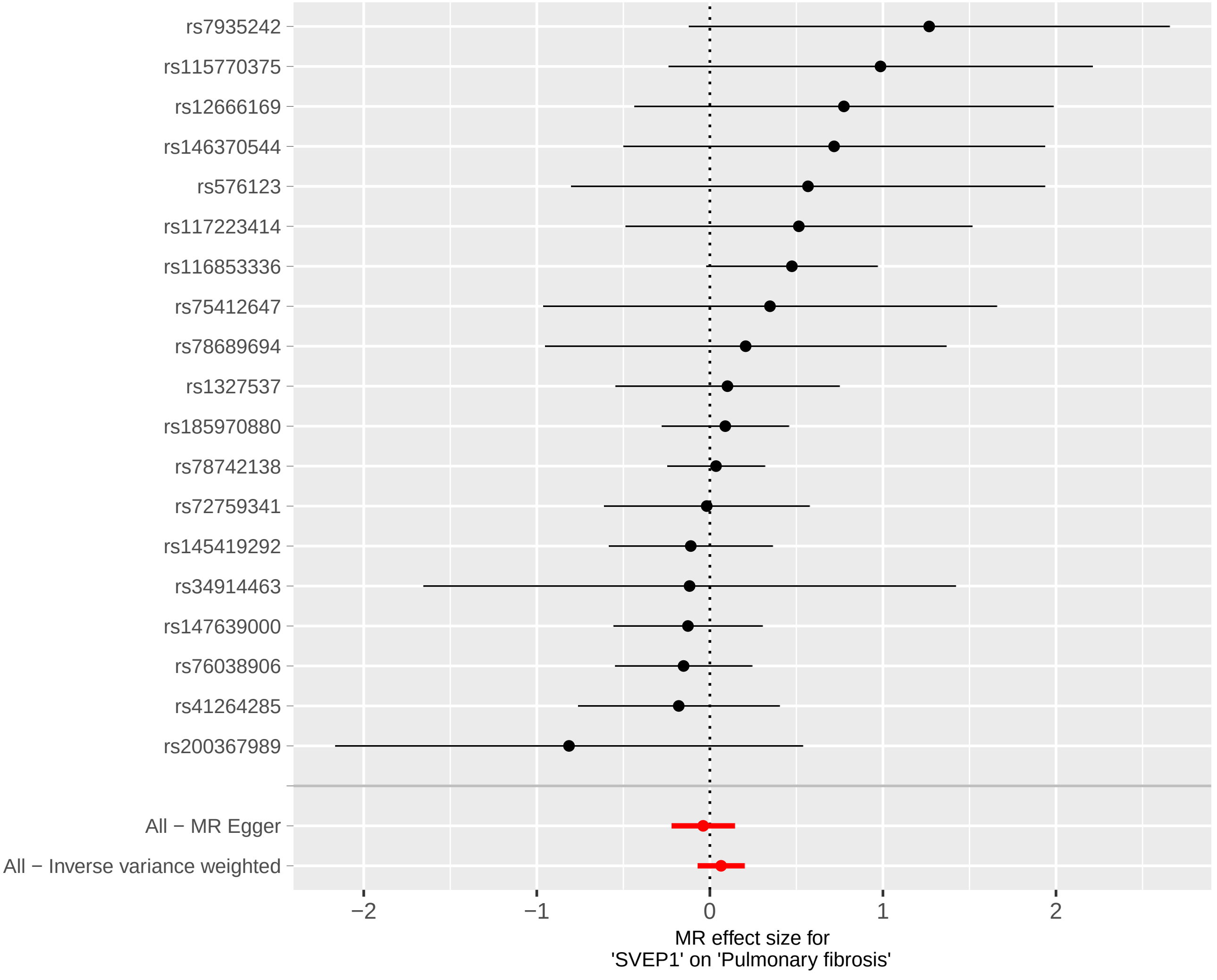
rs7935242
rs115770375
rs12666169
rs146370544
rs576123
rs117223414
rs116853336
rs75412647
rs78689694
rs1327537
rs185970880
rs78742138
rs72759341
rs145419292
rs34914463
rs147639000
rs76038906
rs41264285
rs200367989
All − MR Egger
All − Inverse variance weighted
−2
−1
0
1
2
MR effect size for
'SVEP1' on 'Pulmonary fibrosis'

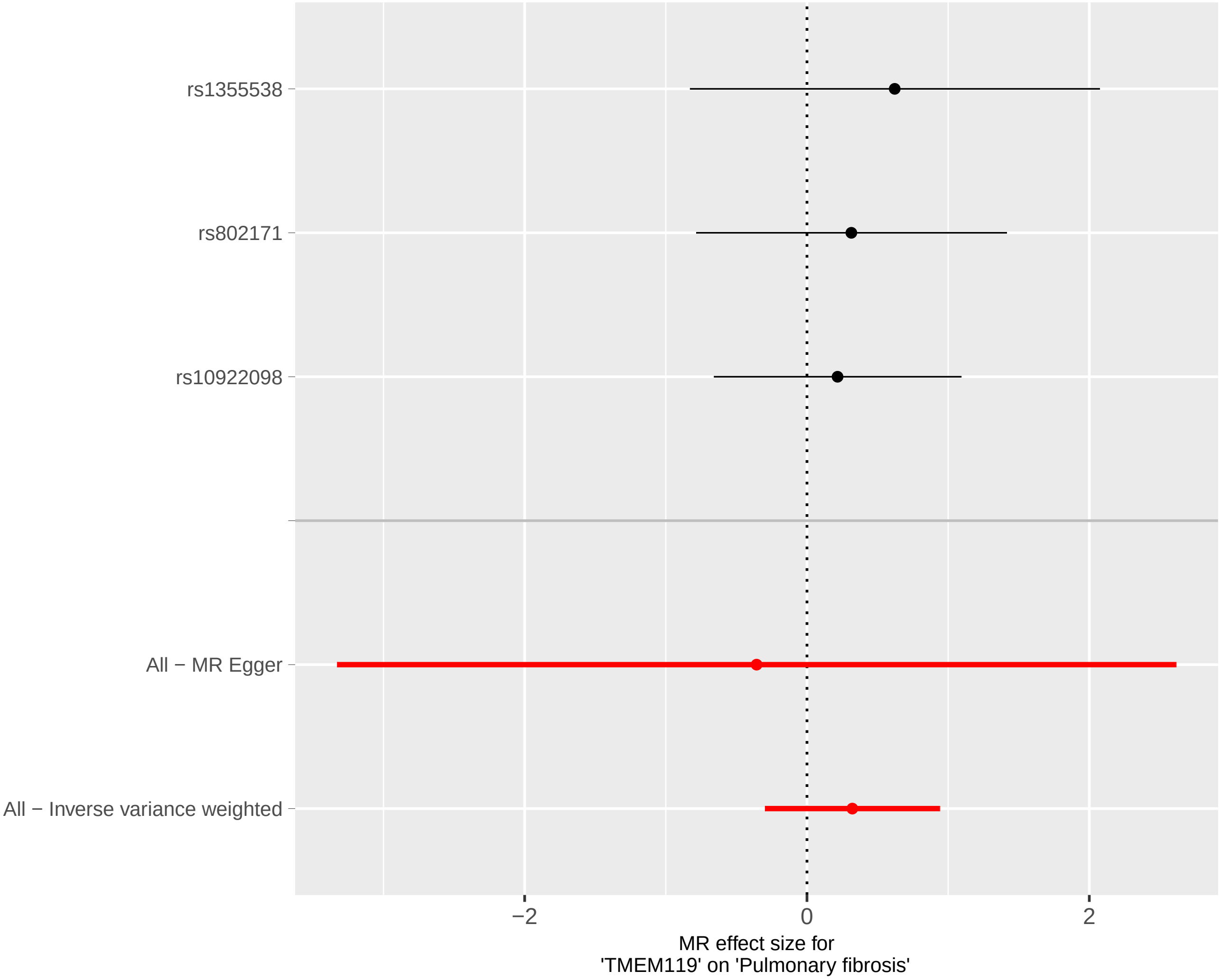
rs1355538
rs802171
rs10922098
All − MR Egger
All − Inverse variance weighted
−2
0
2
MR effect size for
'TMEM119' on 'Pulmonary fibrosis'

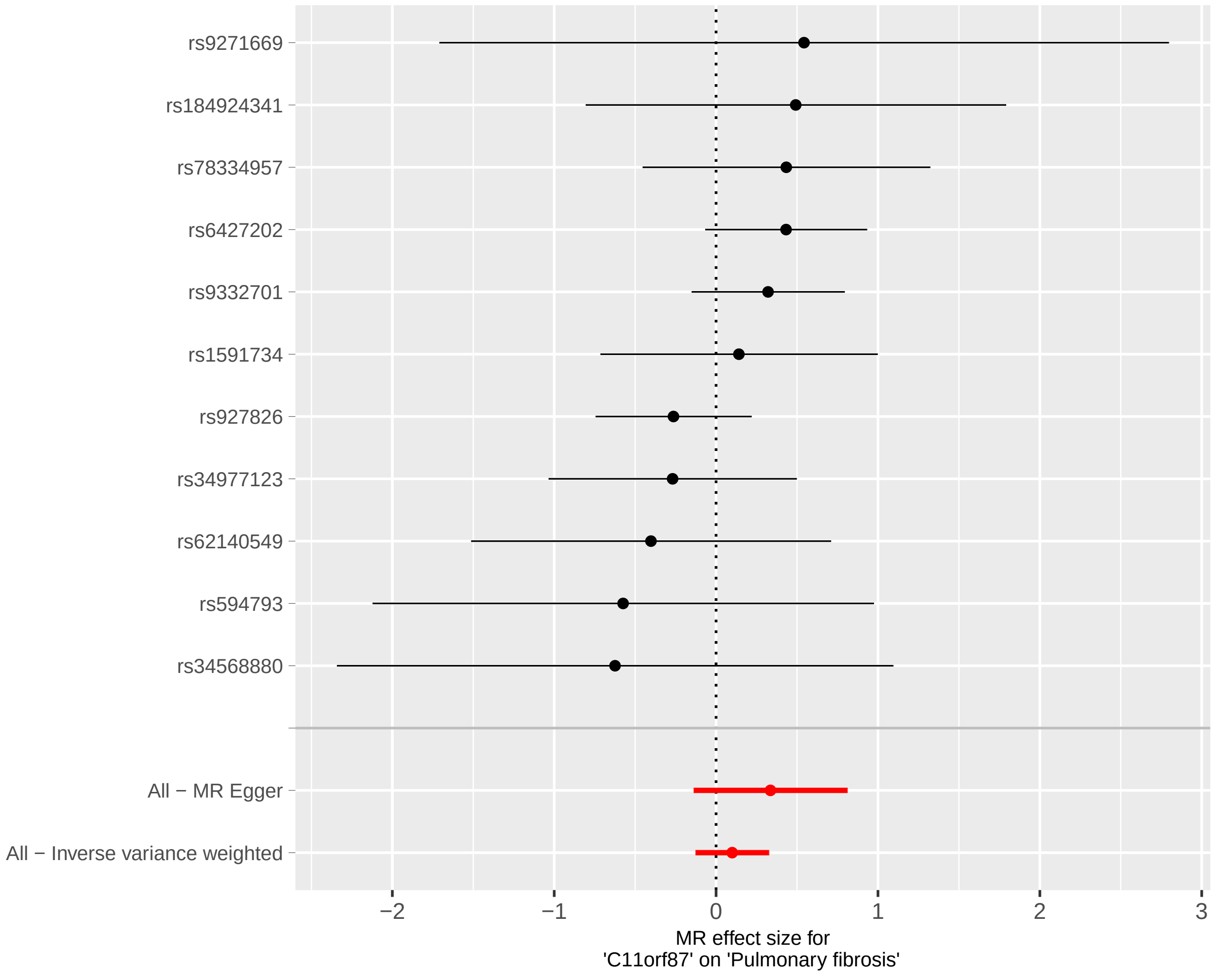
rs9271669
rs184924341
rs78334957
rs6427202
rs9332701
rs1591734
rs927826
rs34977123
rs62140549
rs594793
rs34568880
All − MR Egger
All − Inverse variance weighted
−2
−1
0
1
2
3
MR effect size for
'C11orf87' on 'Pulmonary fibrosis'

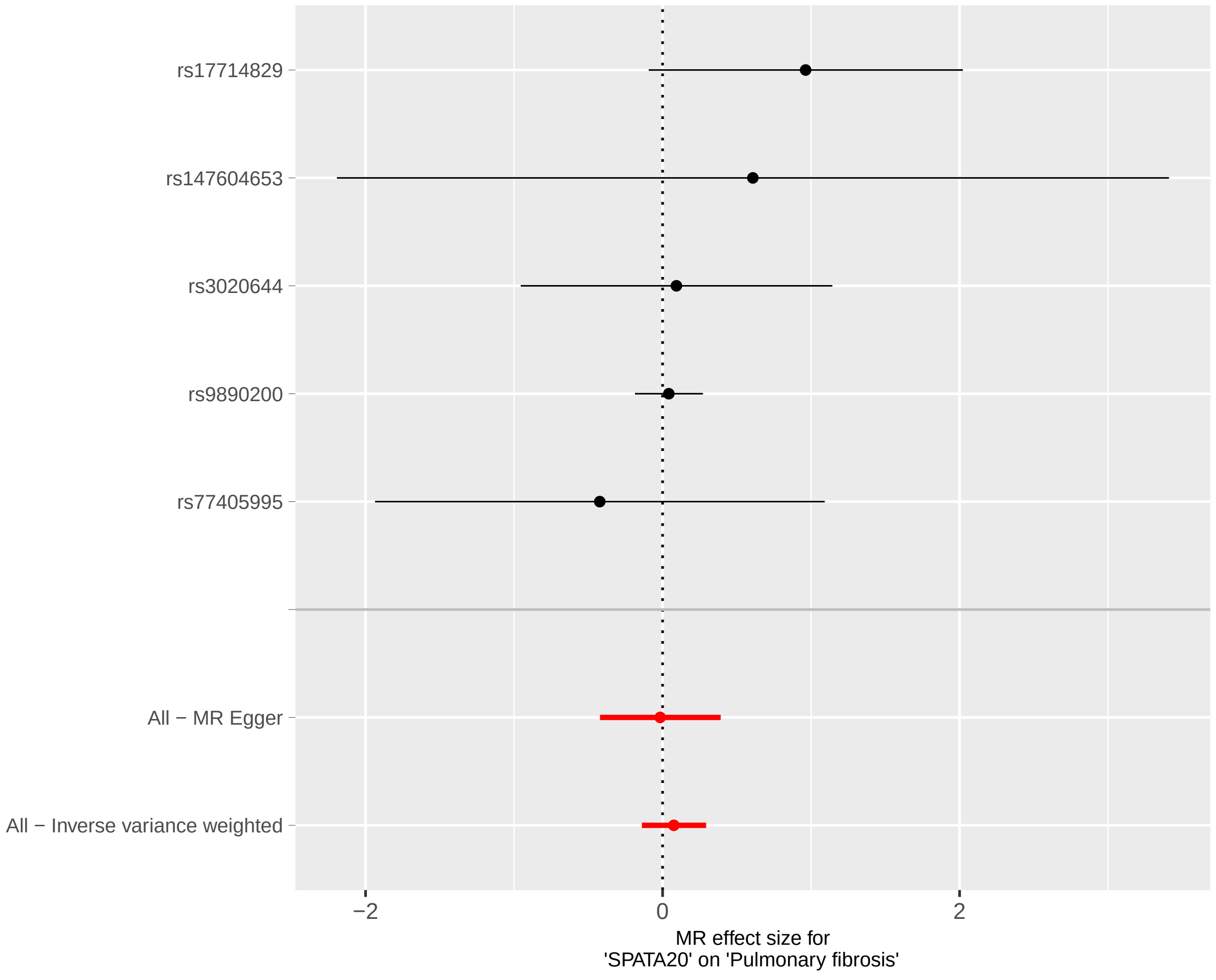
rs17714829
rs147604653
rs3020644
rs9890200
rs77405995
All − MR Egger
All − Inverse variance weighted
−2
0
2
MR effect size for
'SPATA20' on 'Pulmonary fibrosis'

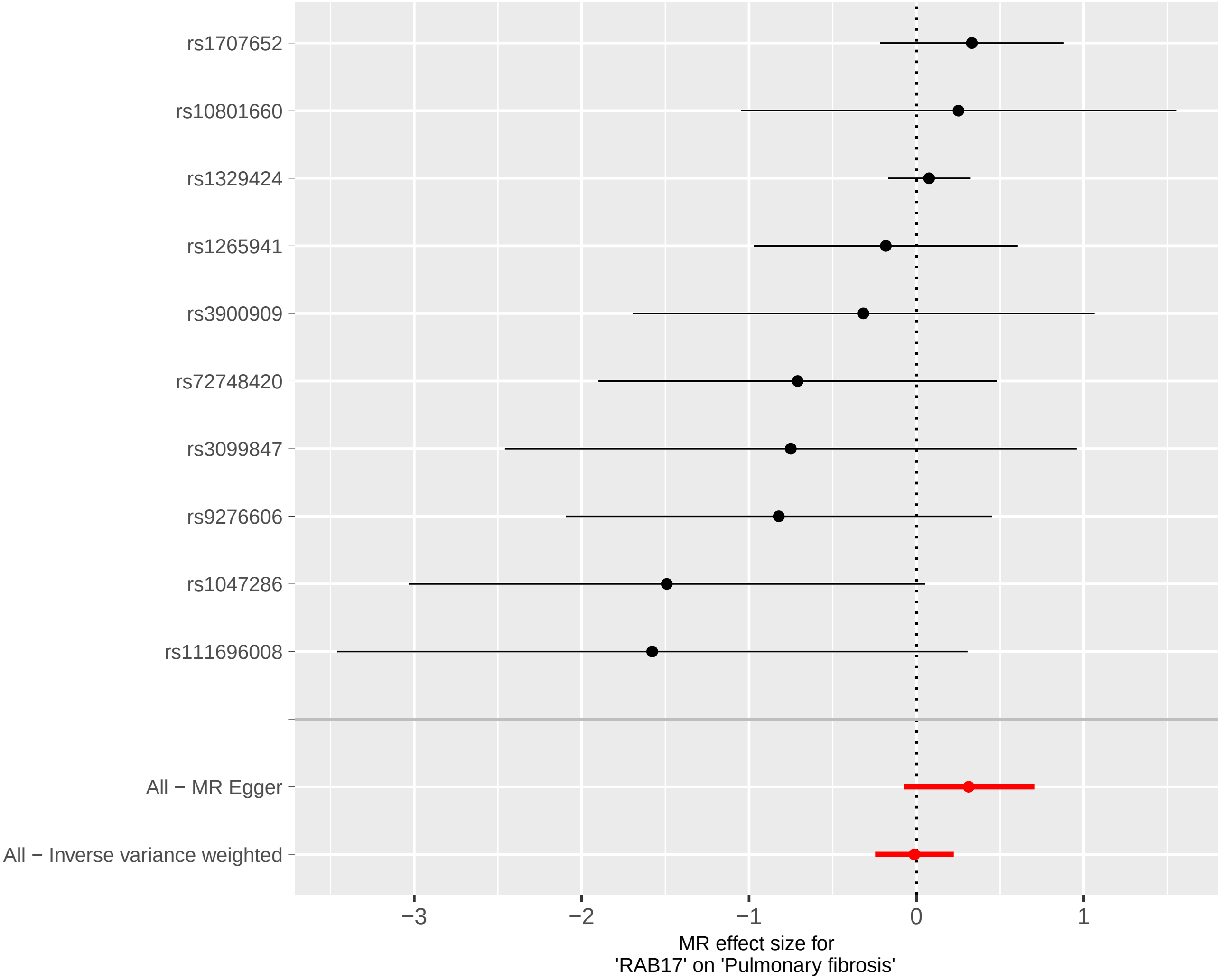

rs1707652
rs10801660
rs1329424
rs1265941
rs3900909
rs72748420
rs3099847
rs9276606
rs1047286
rs111696008
All − MR Egger
All − Inverse variance weighted
−3
−2
−1
0
1
MR effect size for
'RAB17' on 'Pulmonary fibrosis'

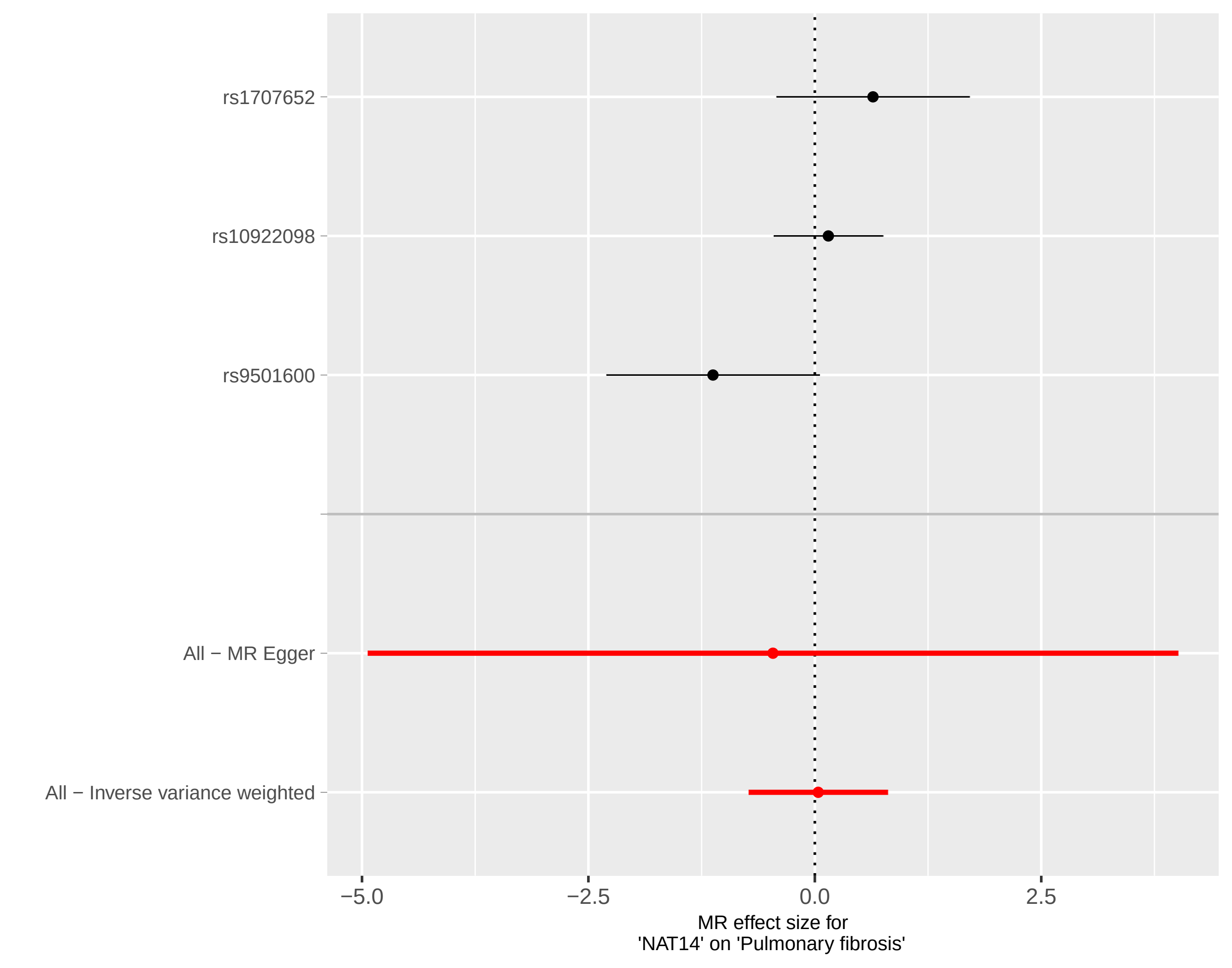

rs1707652
rs10922098
rs9501600
All − MR Egger
All − Inverse variance weighted
−5.0
−2.5
0.0
2.5
MR effect size for
'NAT14' on 'Pulmonary fibrosis'

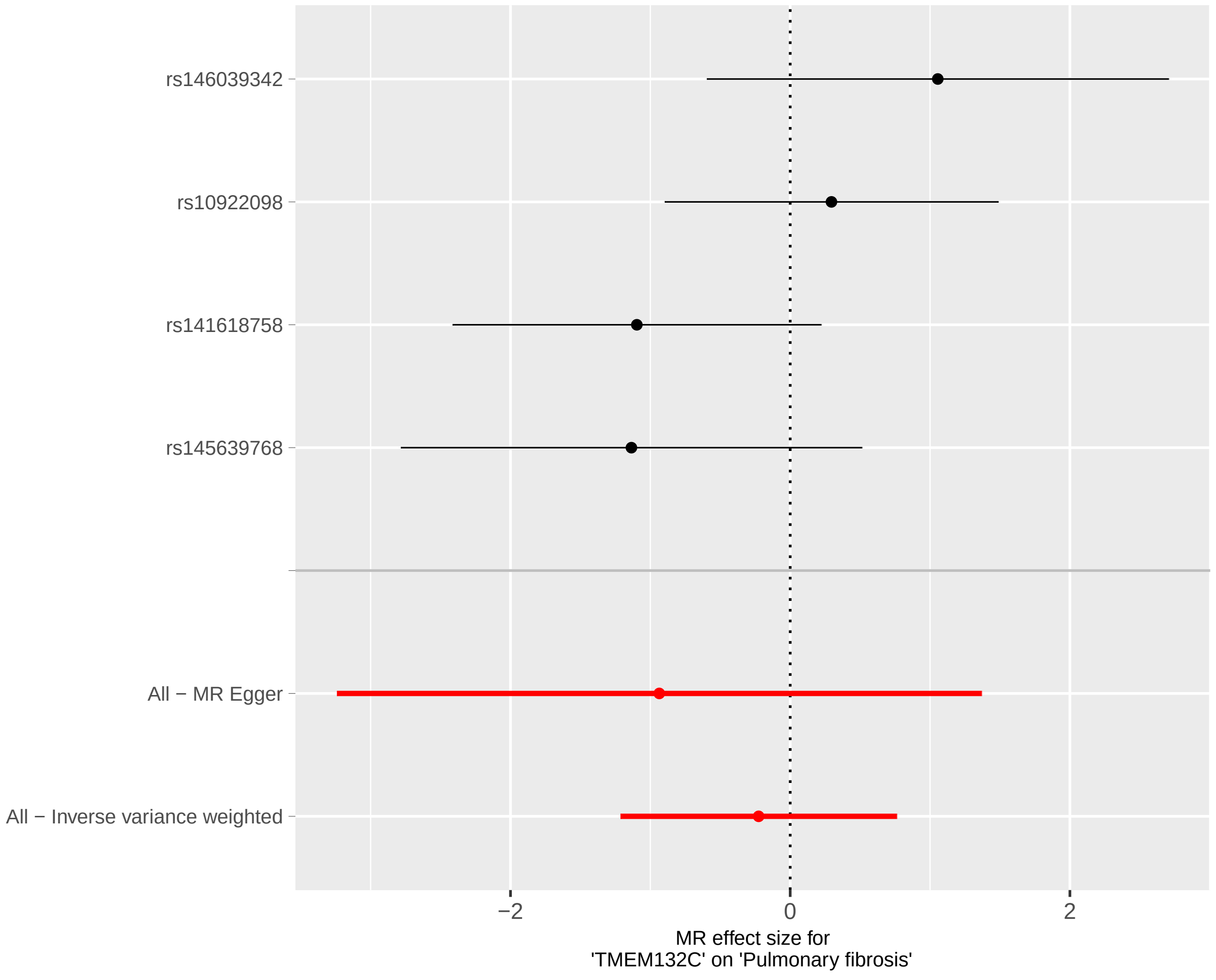
rs146039342
rs10922098
rs141618758
rs145639768
All − MR Egger
All − Inverse variance weighted
−2
0
2
MR effect size for
'TMEM132C' on 'Pulmonary fibrosis'

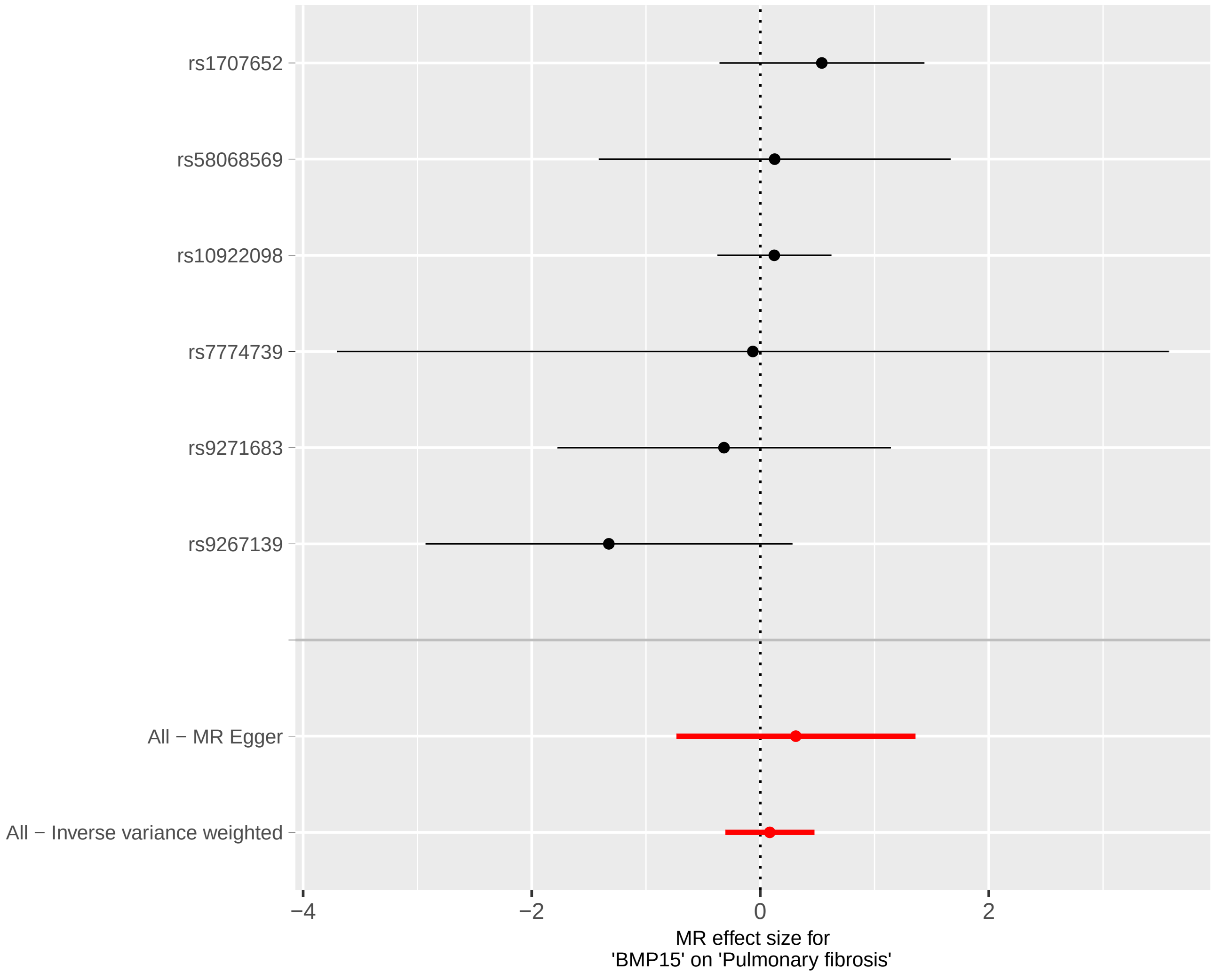
rs1707652
rs58068569
rs10922098
rs7774739
rs9271683
rs9267139
All − MR Egger
All − Inverse variance weighted
−4
−2
0
2
MR effect size for
'BMP15' on 'Pulmonary fibrosis'

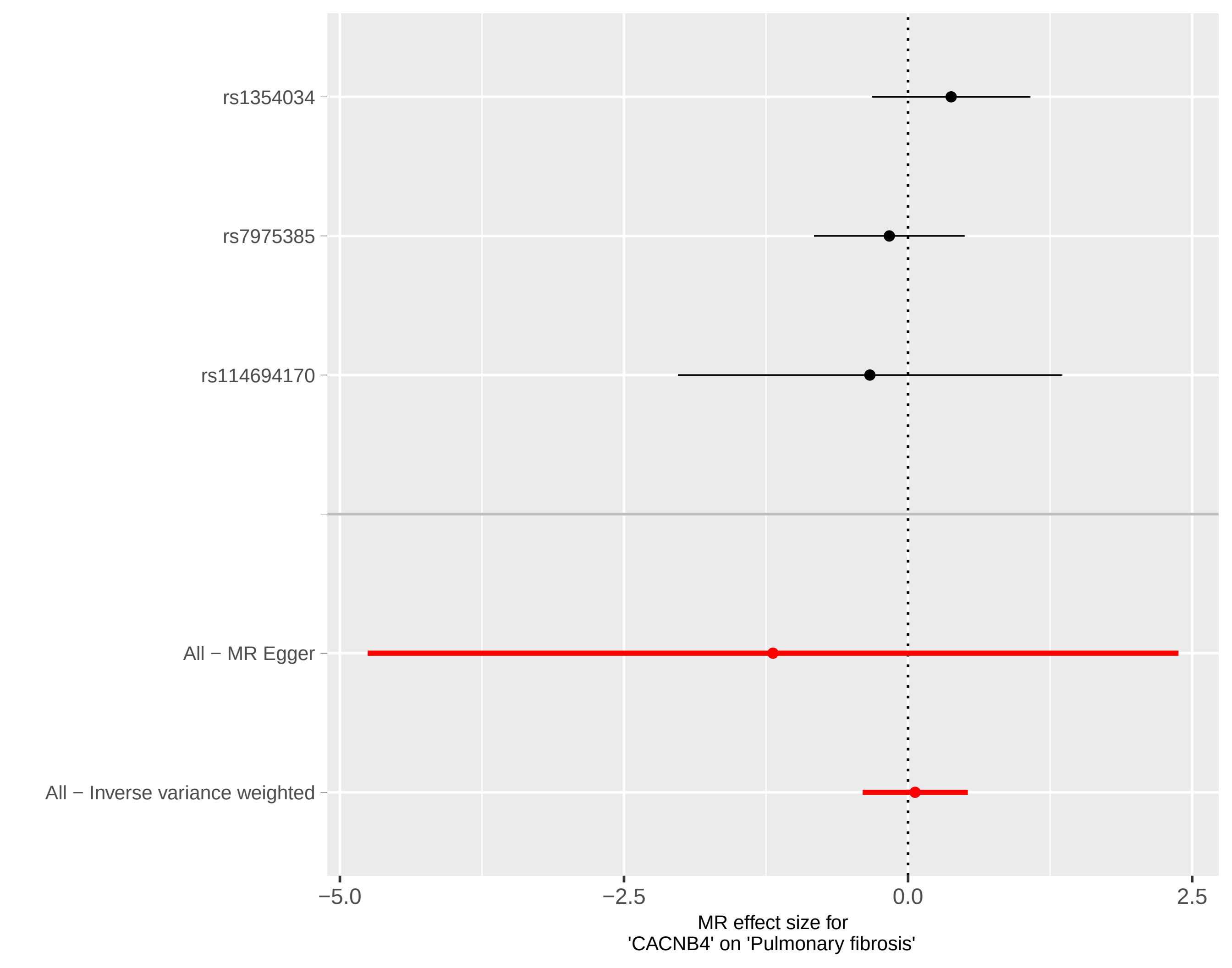

rs1354034
rs7975385
rs114694170
All − MR Egger
All − Inverse variance weighted
−5.0
−2.5
0.0
2.5
MR effect size for
'CACNB4' on 'Pulmonary fibrosis'

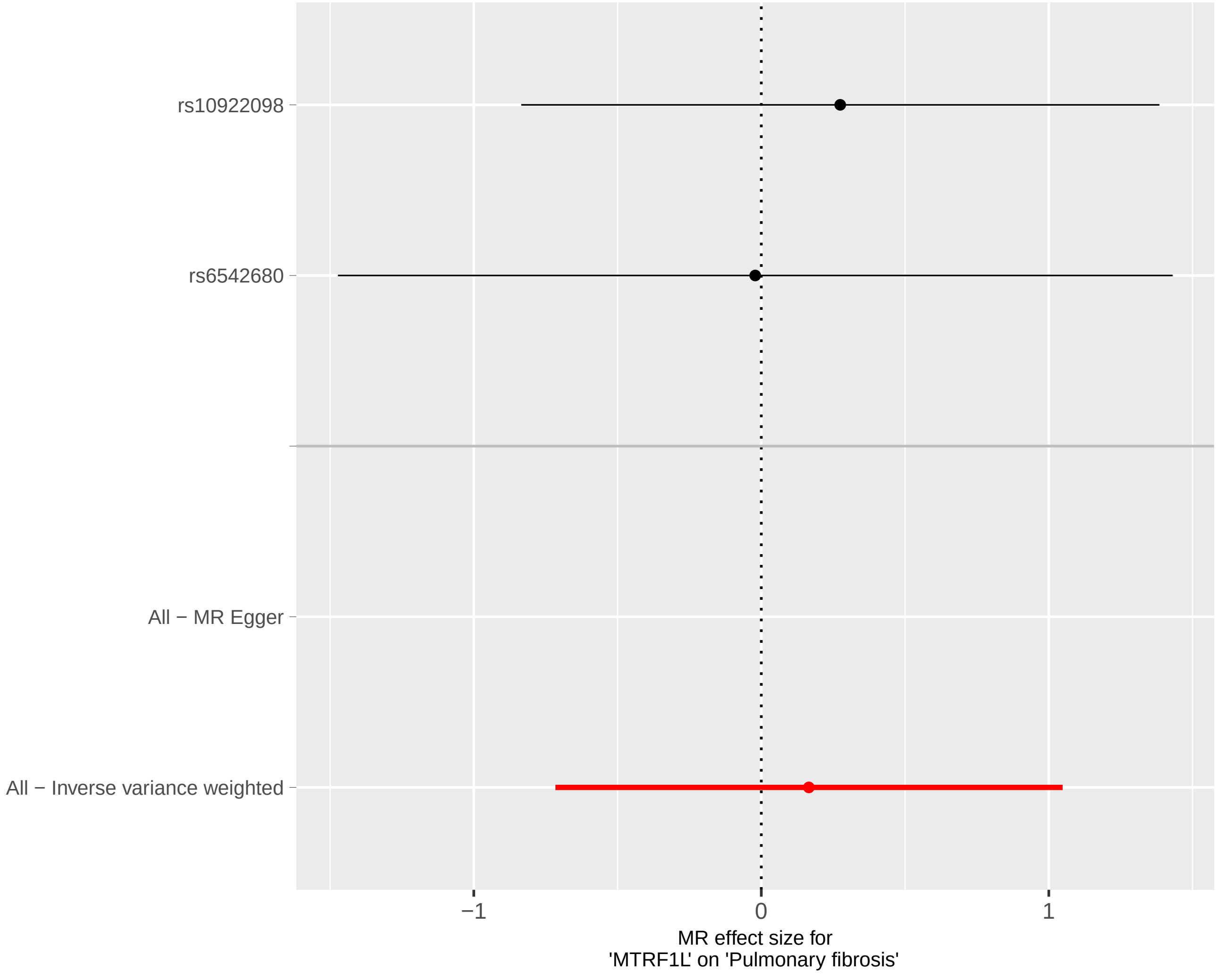

rs10922098
rs6542680
All − MR Egger
All − Inverse variance weighted
−1
0
1
MR effect size for
'MTRF1L' on 'Pulmonary fibrosis'

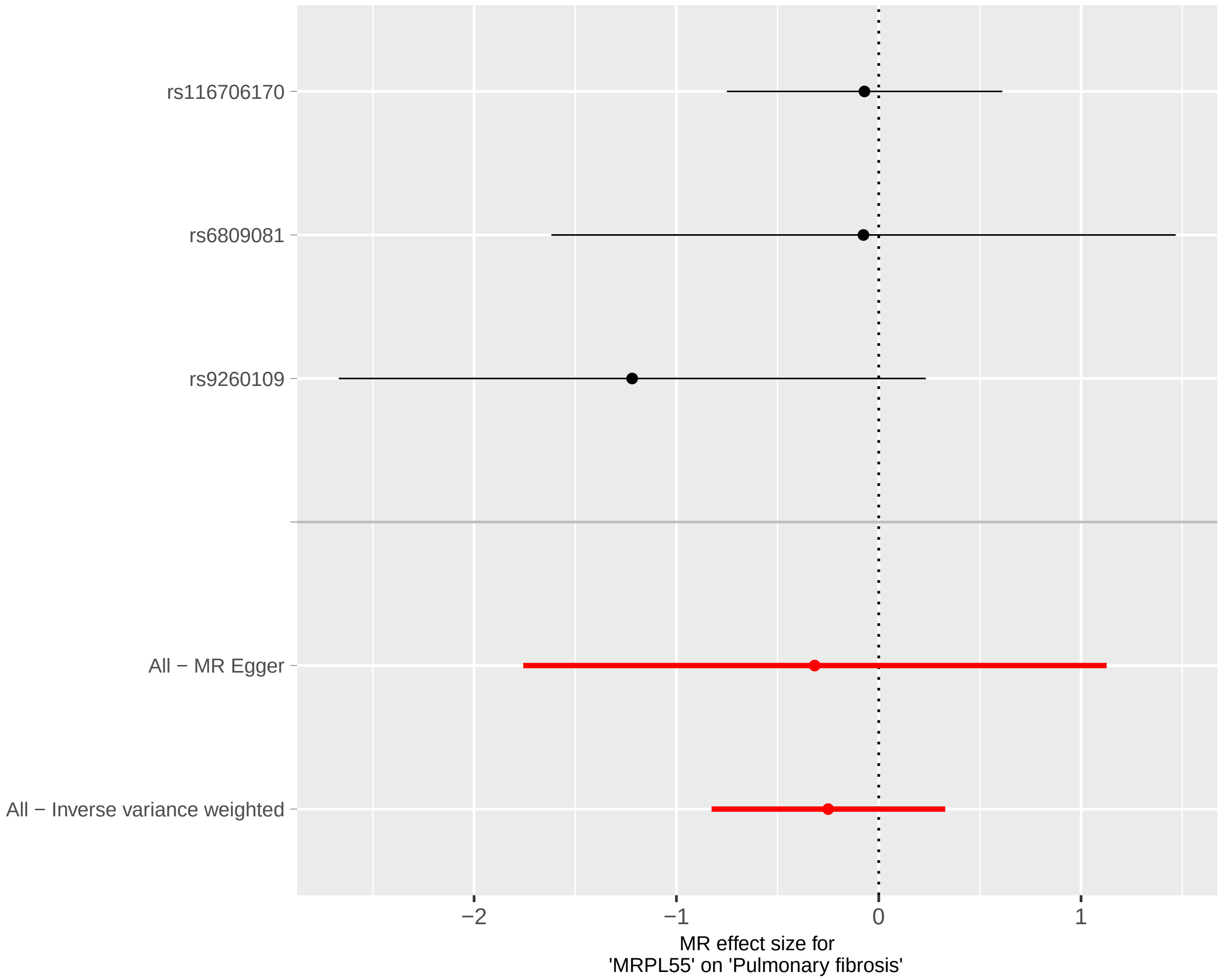

rs116706170
rs6809081
rs9260109
All − MR Egger
All − Inverse variance weighted
−2
−1
0
1
MR effect size for
'MRPL55' on 'Pulmonary fibrosis'

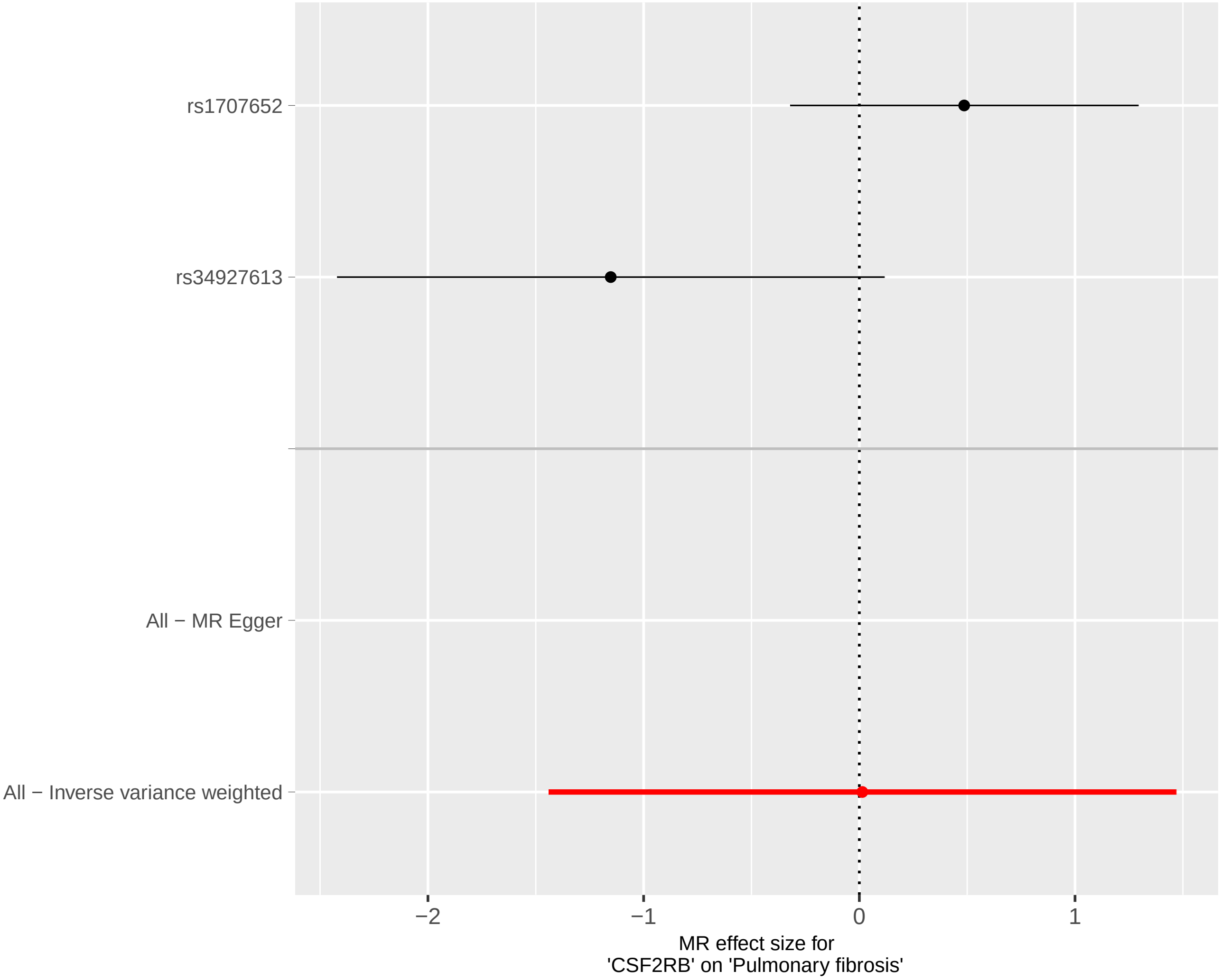
rs1707652
rs34927613
All − MR Egger
All − Inverse variance weighted
−2
−1
0
1
MR effect size for
'CSF2RB' on 'Pulmonary fibrosis'

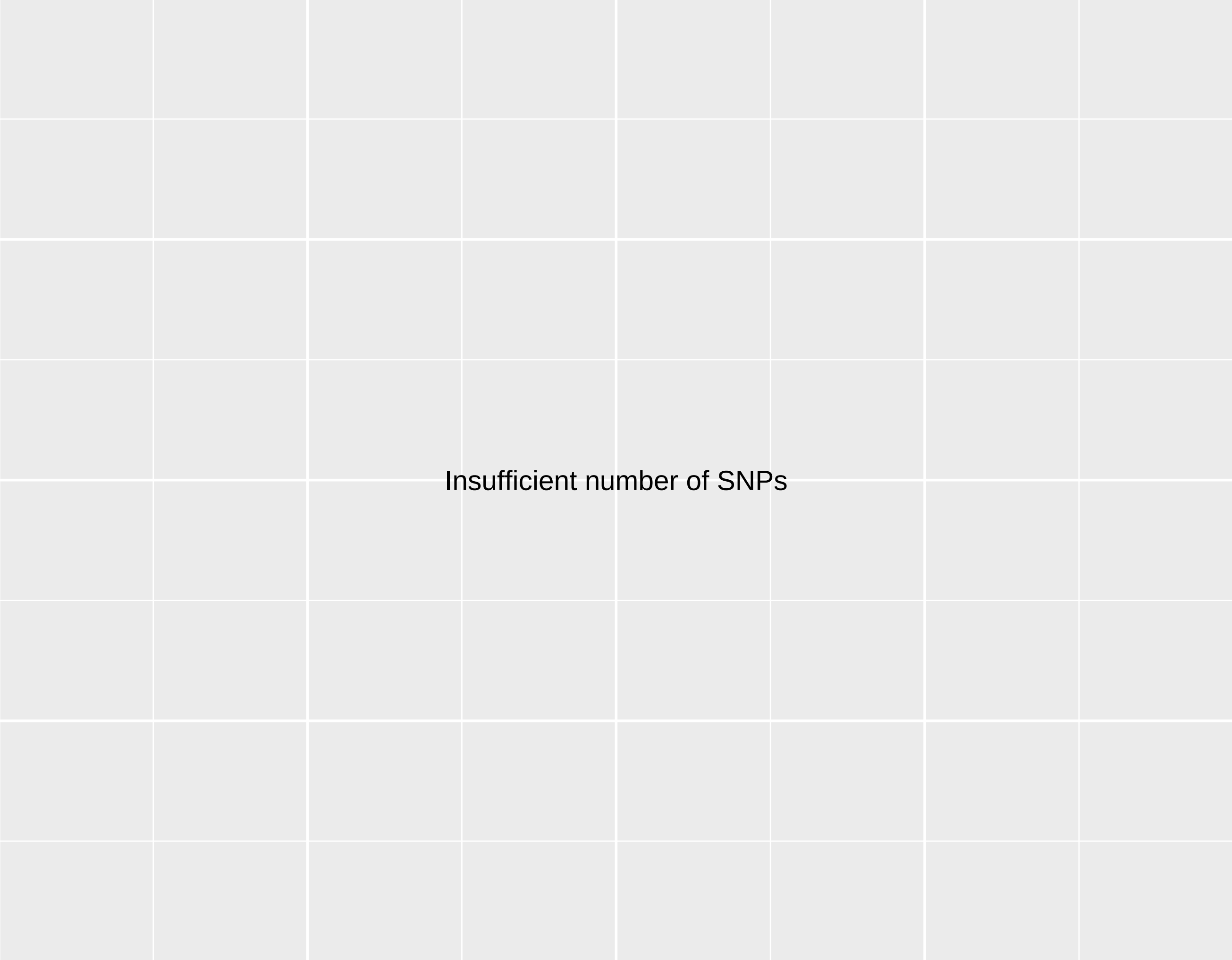
Insufficient number of SNPs

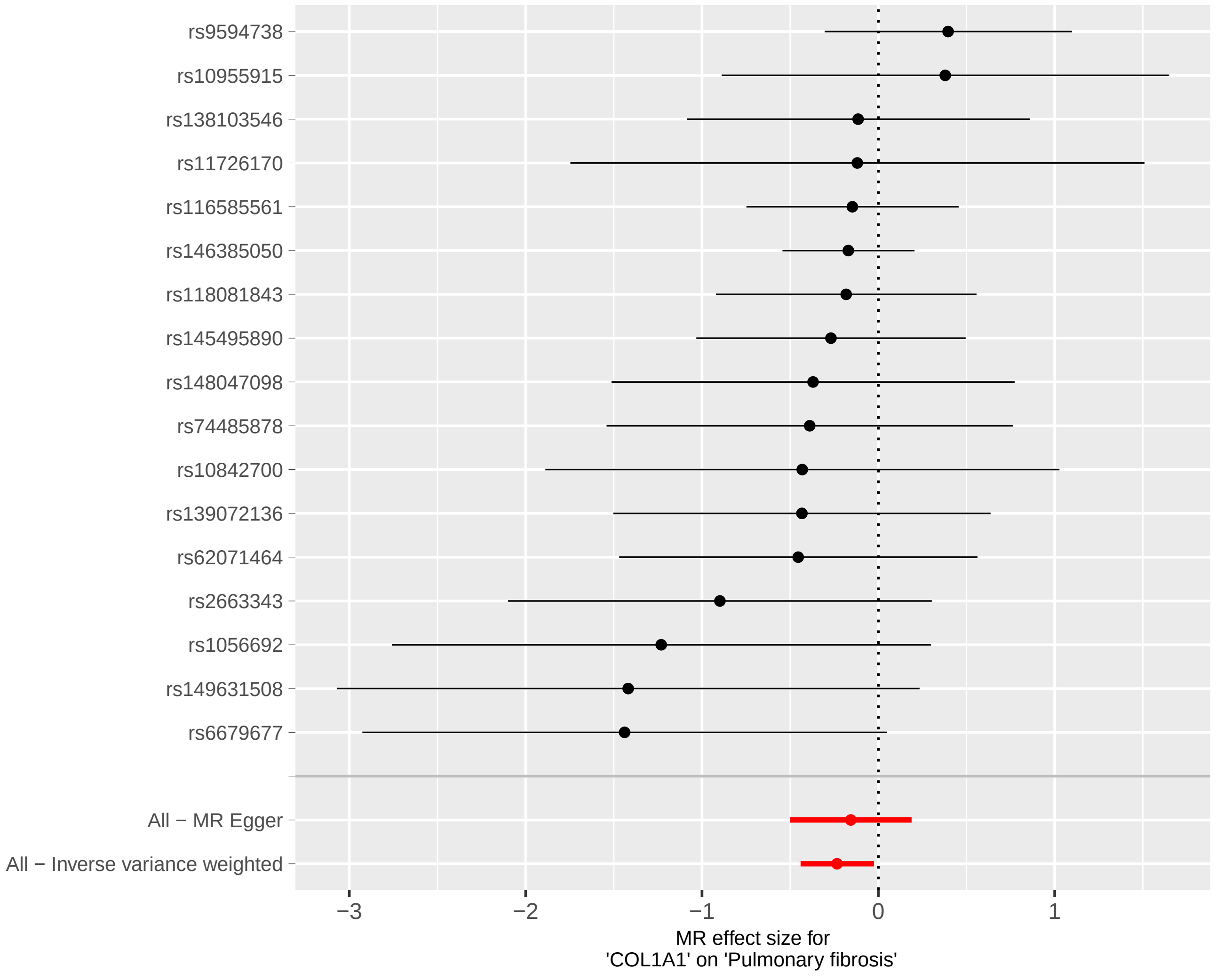
rs9594738
rs10955915
rs138103546
rs11726170
rs116585561
rs146385050
rs118081843
rs145495890
rs148047098
rs74485878
rs10842700
rs139072136
rs62071464
rs2663343
rs1056692
rs149631508
rs6679677
All − MR Egger
All − Inverse variance weighted
−3
−2
−1
0
1
MR effect size for
'COL1A1' on 'Pulmonary fibrosis'

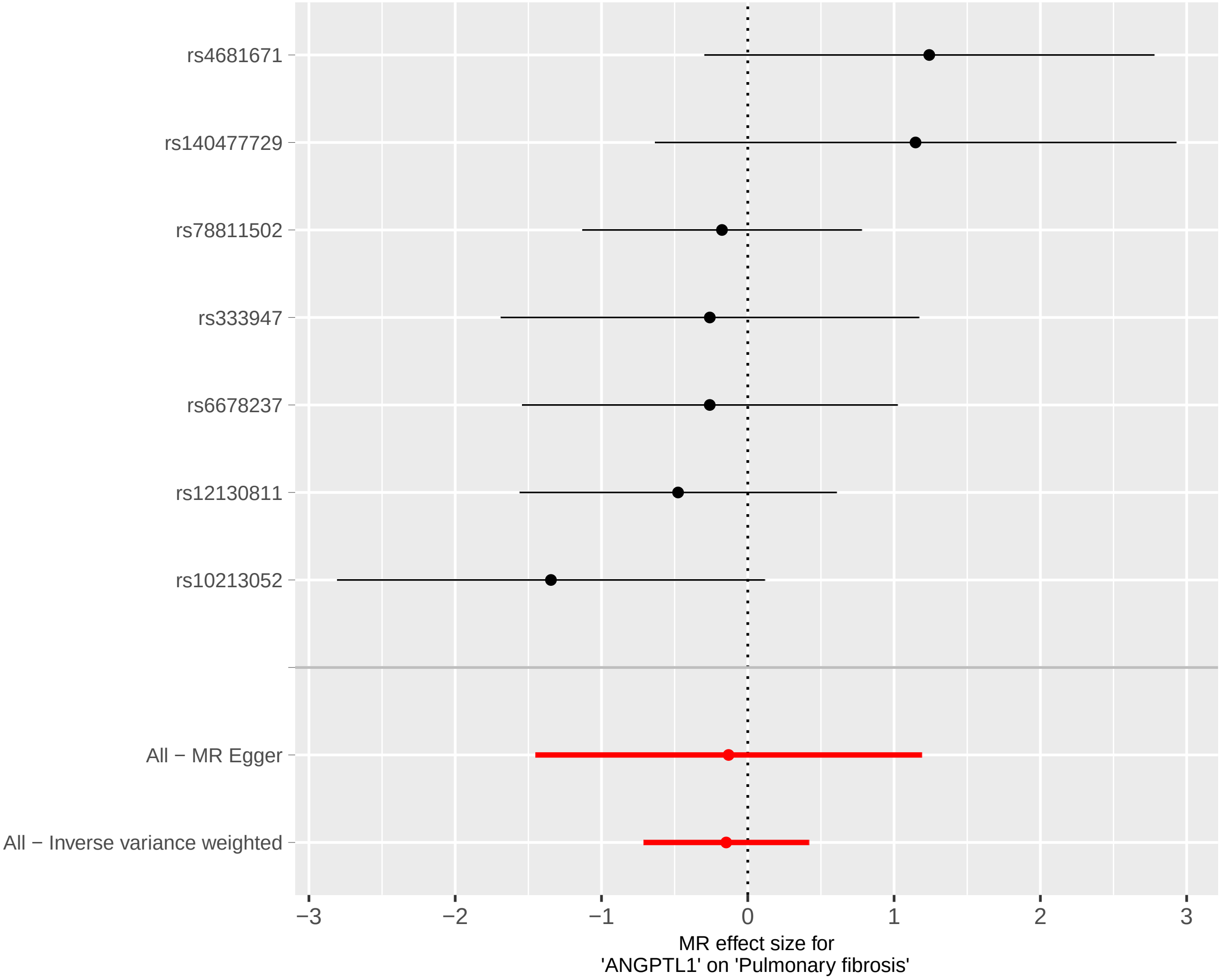
rs4681671
rs140477729
rs78811502
rs333947
rs6678237
rs12130811
rs10213052
All − MR Egger
All − Inverse variance weighted
−3
−2
−1
0
1
2
3
MR effect size for
'ANGPTL1' on 'Pulmonary fibrosis'

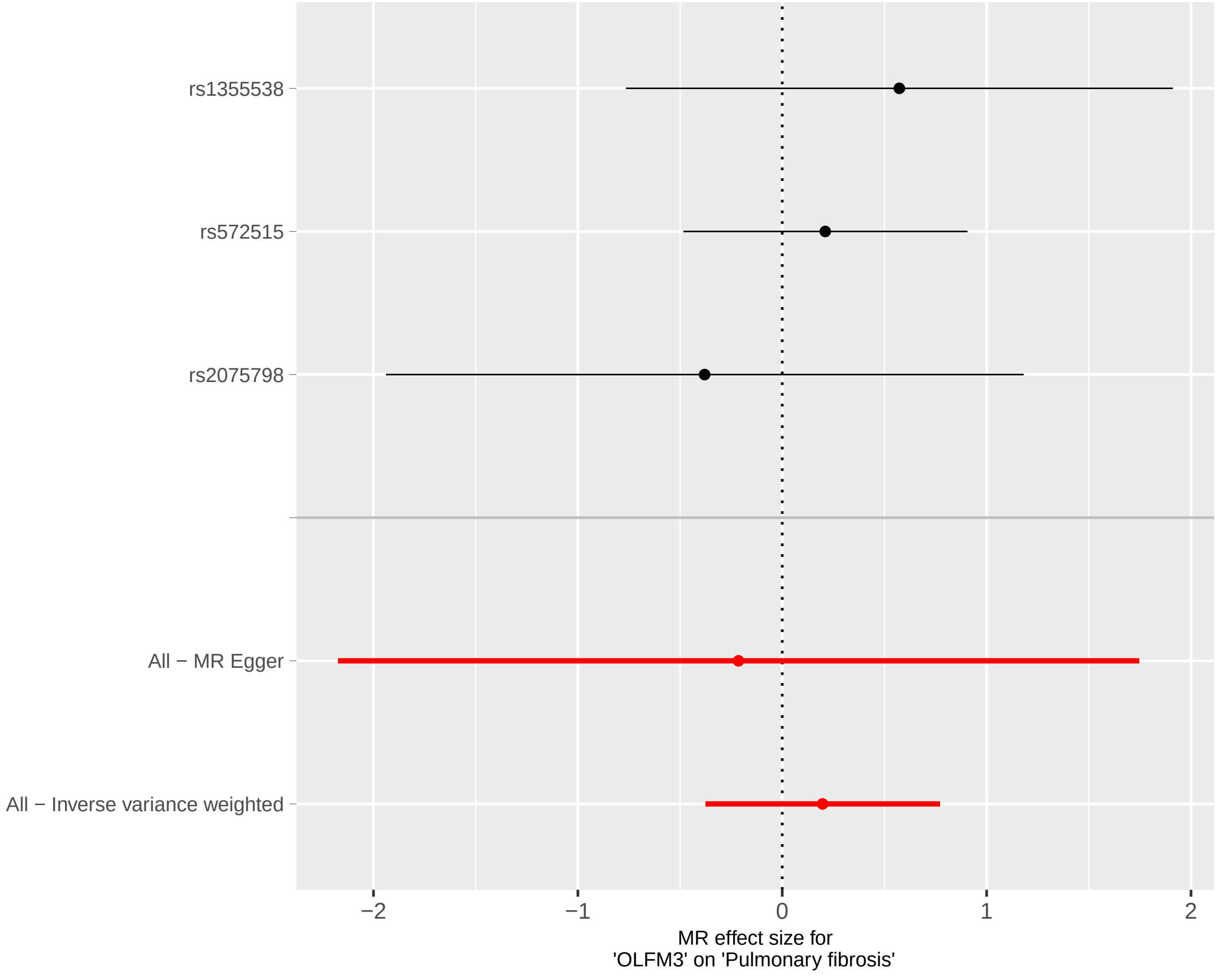
rs1355538
rs572515
rs2075798
All − MR Egger
All − Inverse variance weighted
−2
−1
0
1
2
MR effect size for
'OLFM3' on 'Pulmonary fibrosis'

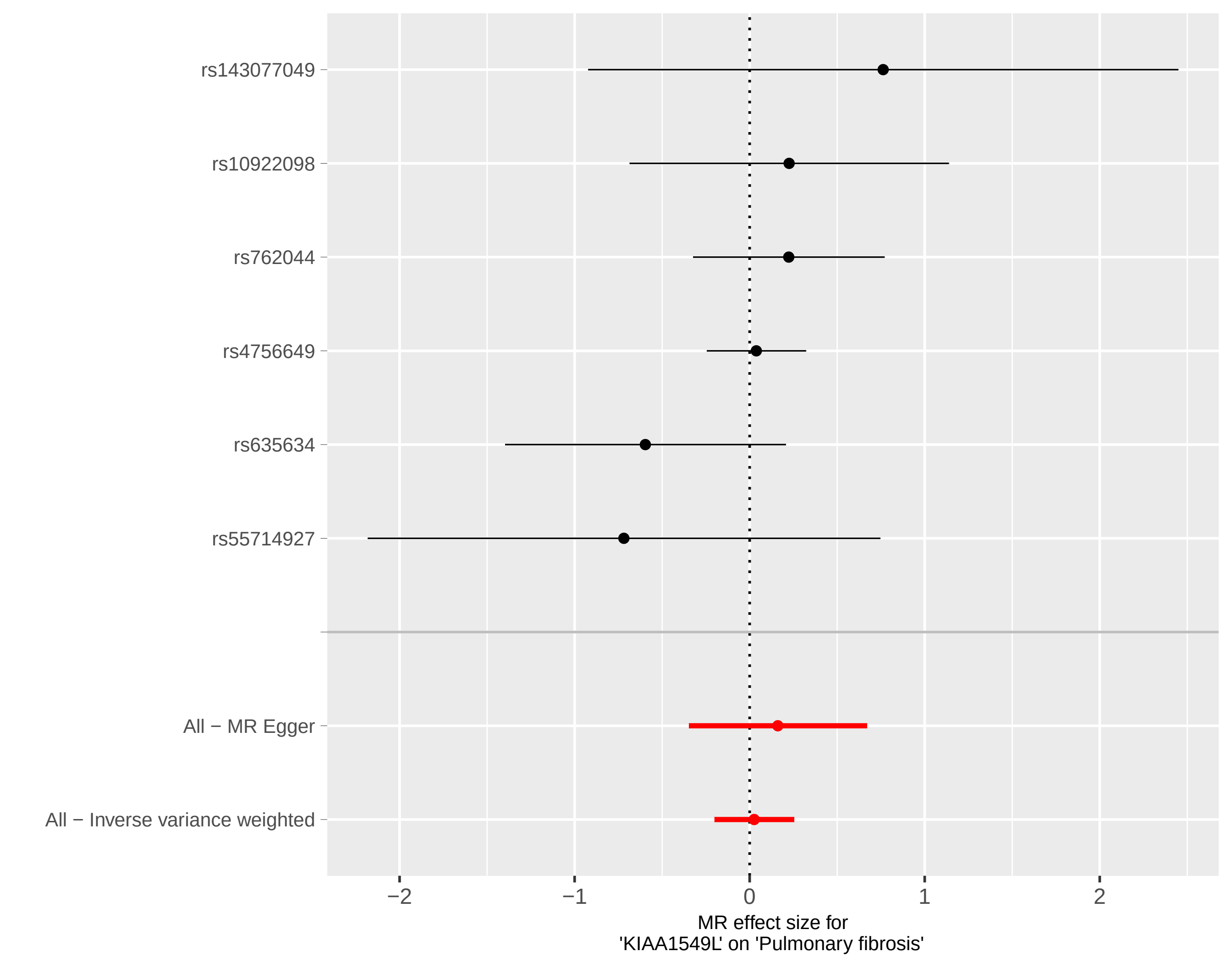
rs143077049
rs10922098
rs762044
rs4756649
rs635634
rs55714927
All − MR Egger
All − Inverse variance weighted
−2
−1
0
1
2
MR effect size for
'KIAA1549L' on 'Pulmonary fibrosis'

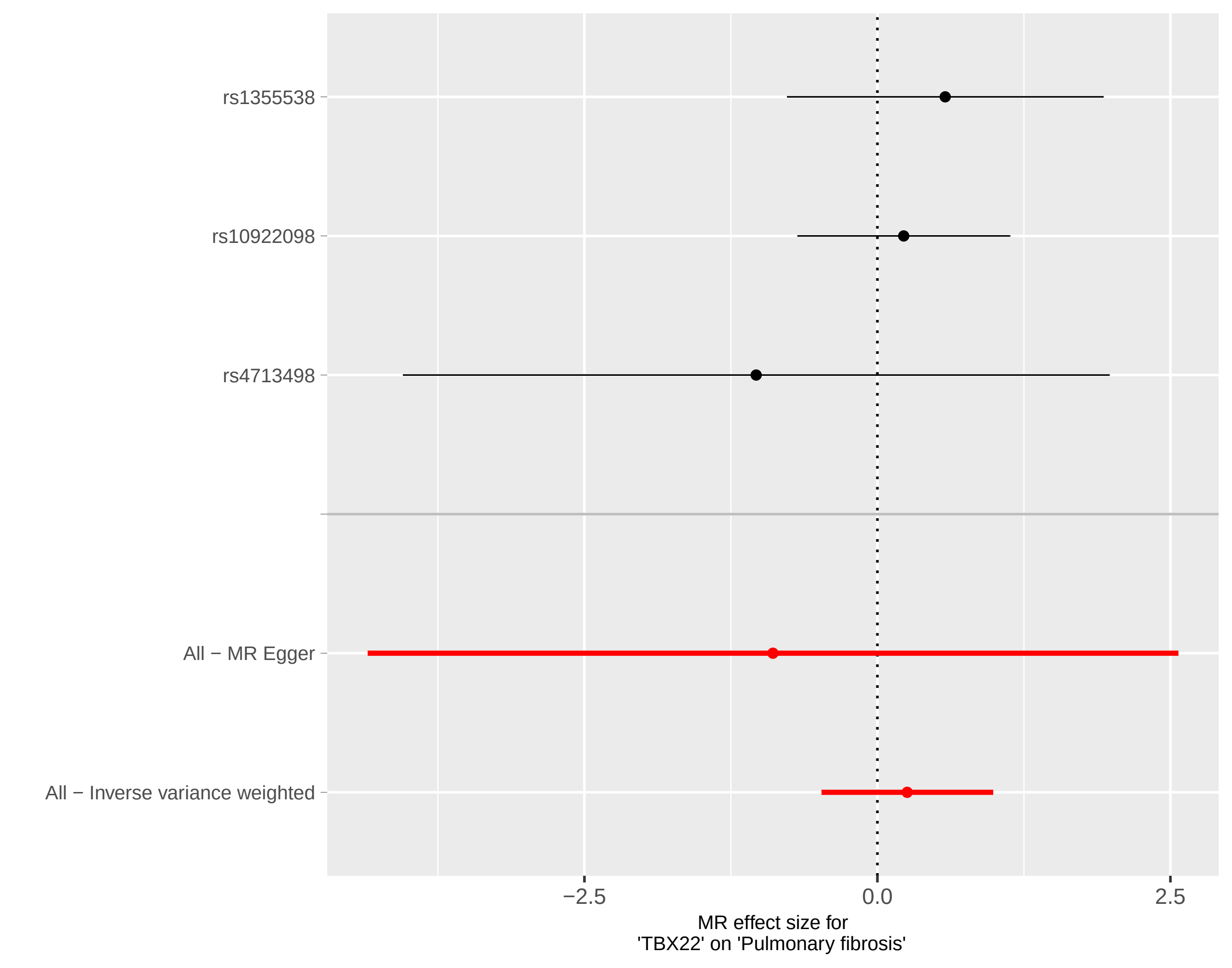
rs1355538
rs10922098
rs4713498
All − MR Egger
All − Inverse variance weighted
−2.5
0.0
2.5
MR effect size for
'TBX22' on 'Pulmonary fibrosis'

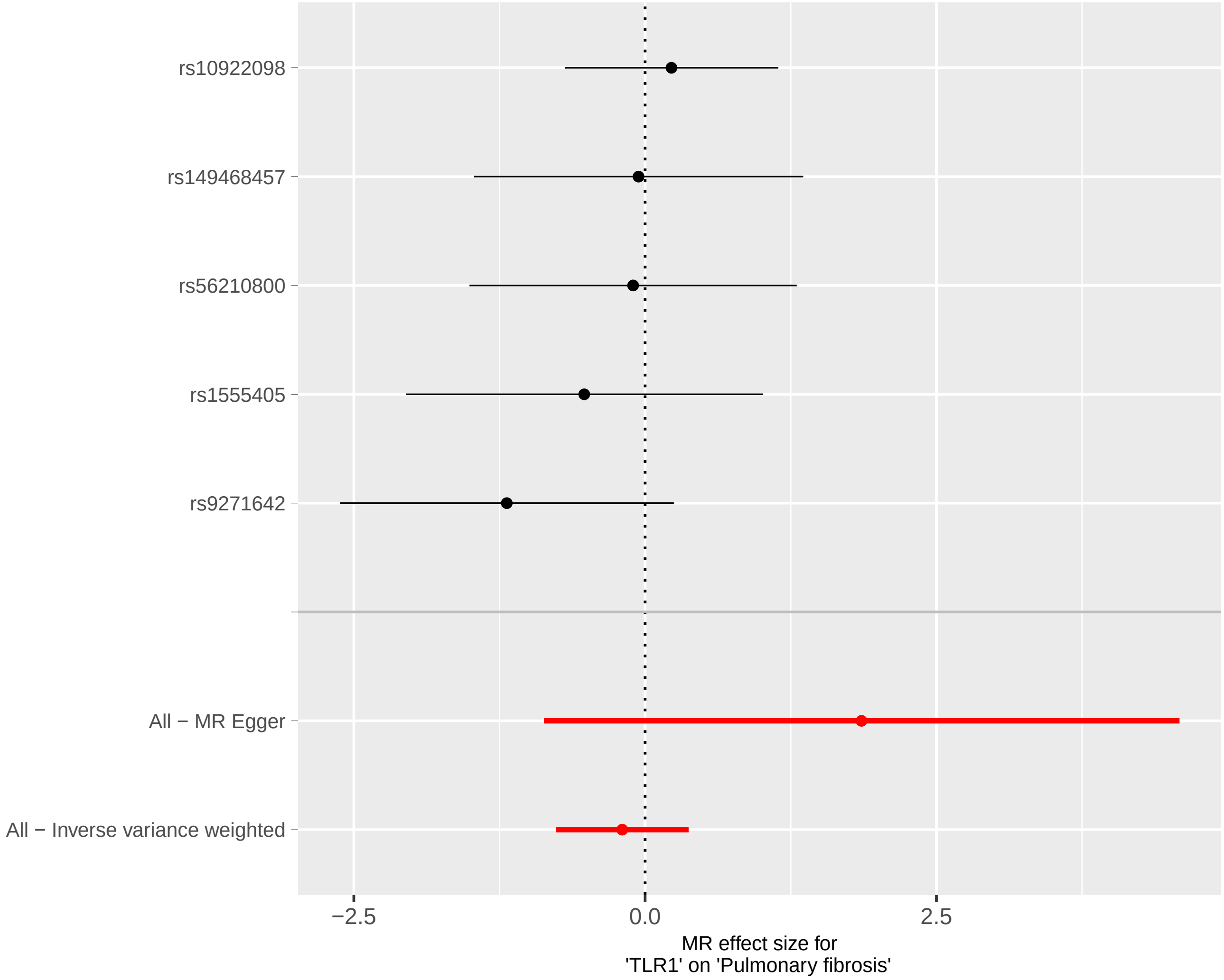

rs10922098
rs149468457
rs56210800
rs1555405
rs9271642
All − MR Egger
All − Inverse variance weighted
−2.5
0.0
2.5
MR effect size for
'TLR1' on 'Pulmonary fibrosis'

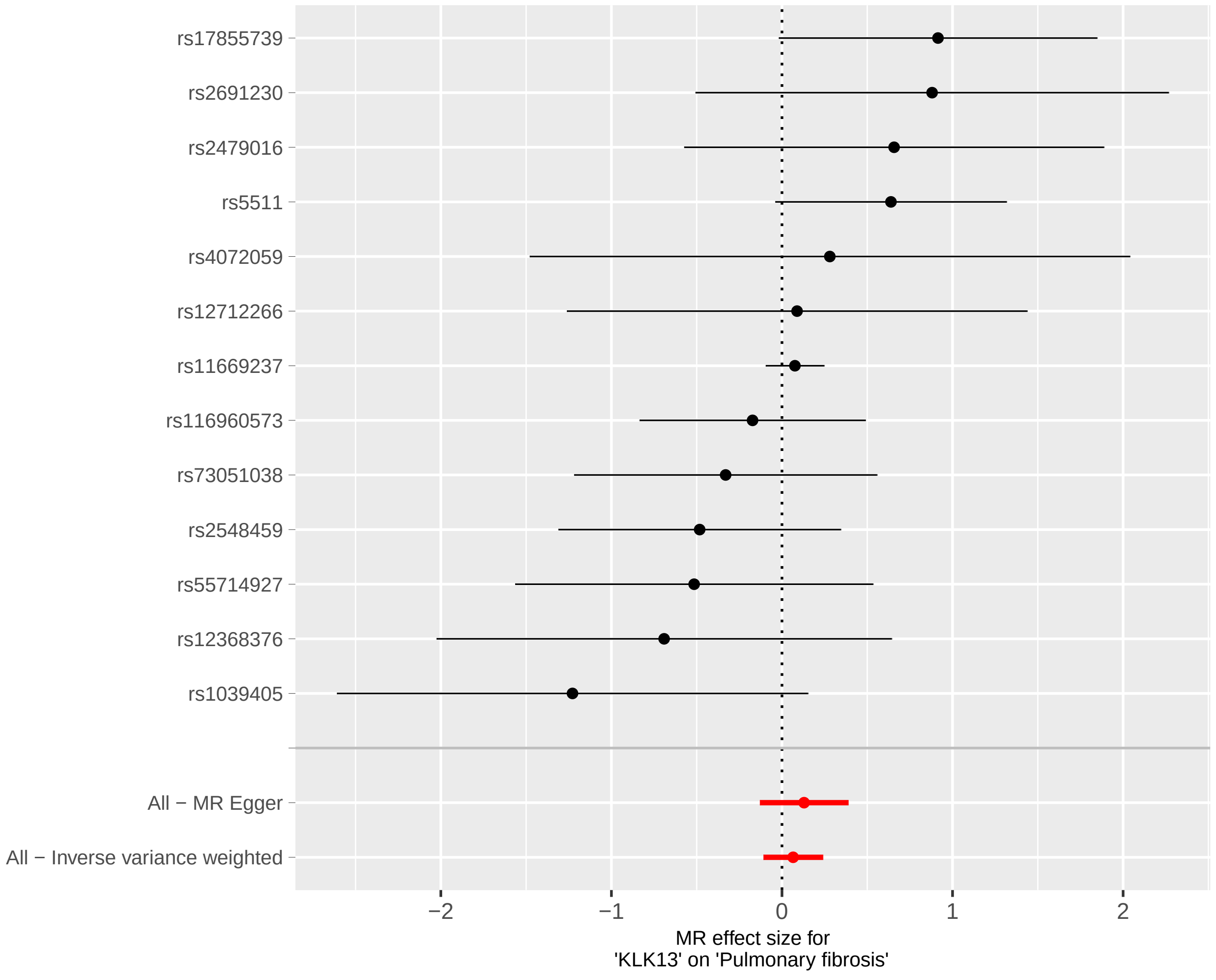

rs17855739
rs2691230
rs2479016
rs5511
rs4072059
rs12712266
rs11669237
rs116960573
rs73051038
rs2548459
rs55714927
rs12368376
rs1039405
All − MR Egger
All − Inverse variance weighted
−2
−1
0
1
2
MR effect size for
'KLK13' on 'Pulmonary fibrosis'

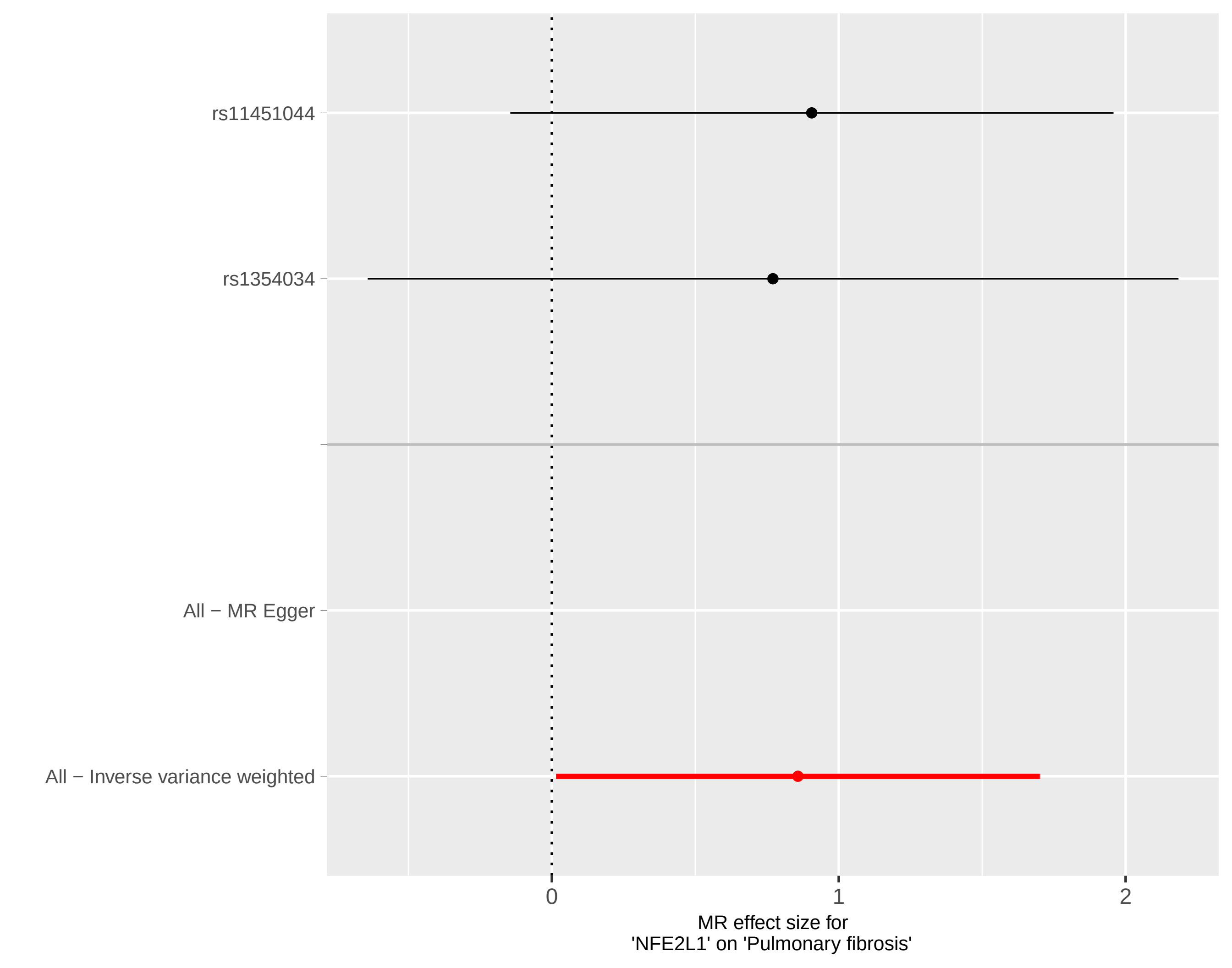
rs11451044
rs1354034
All − MR Egger
All − Inverse variance weighted
0
1
2
MR effect size for
'NFE2L1' on 'Pulmonary fibrosis'

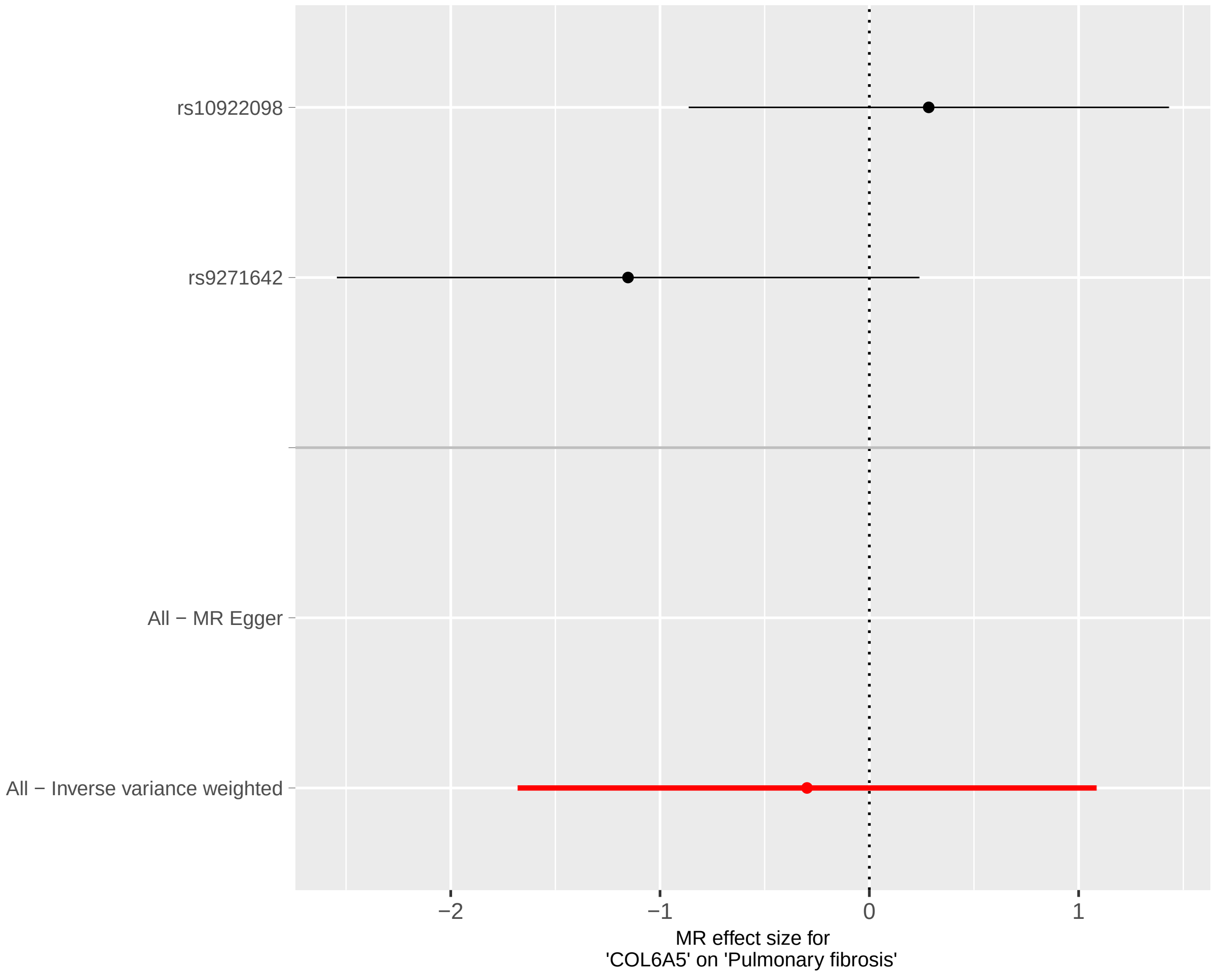
rs10922098
rs9271642
All − MR Egger
All − Inverse variance weighted
−2
−1
0
1
MR effect size for
'COL6A5' on 'Pulmonary fibrosis'

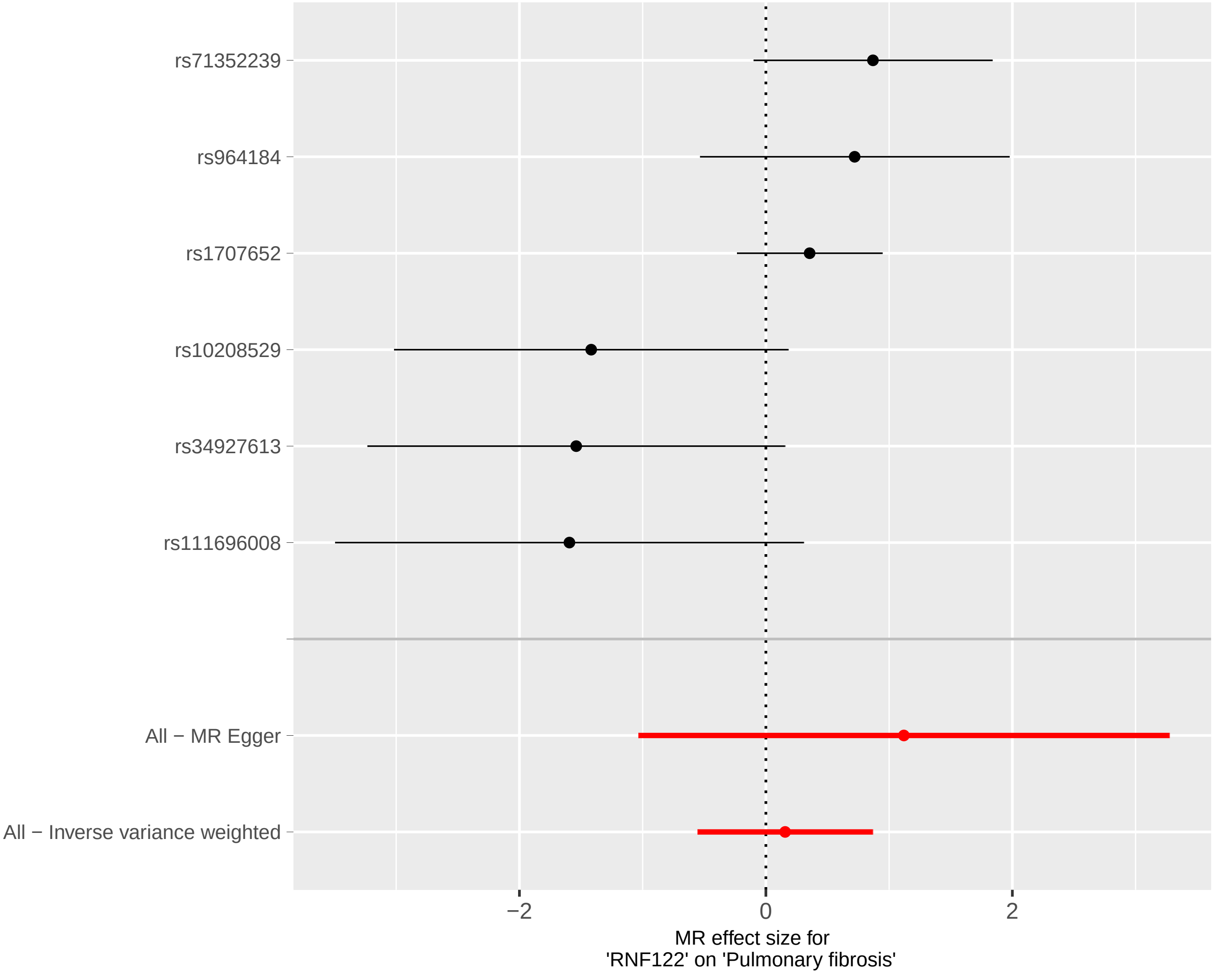
rs71352239
rs964184
rs1707652
rs10208529
rs34927613
rs111696008
All − MR Egger
All − Inverse variance weighted
−2
0
2
MR effect size for
'RNF122' on 'Pulmonary fibrosis'

Insufficient number of SNPs

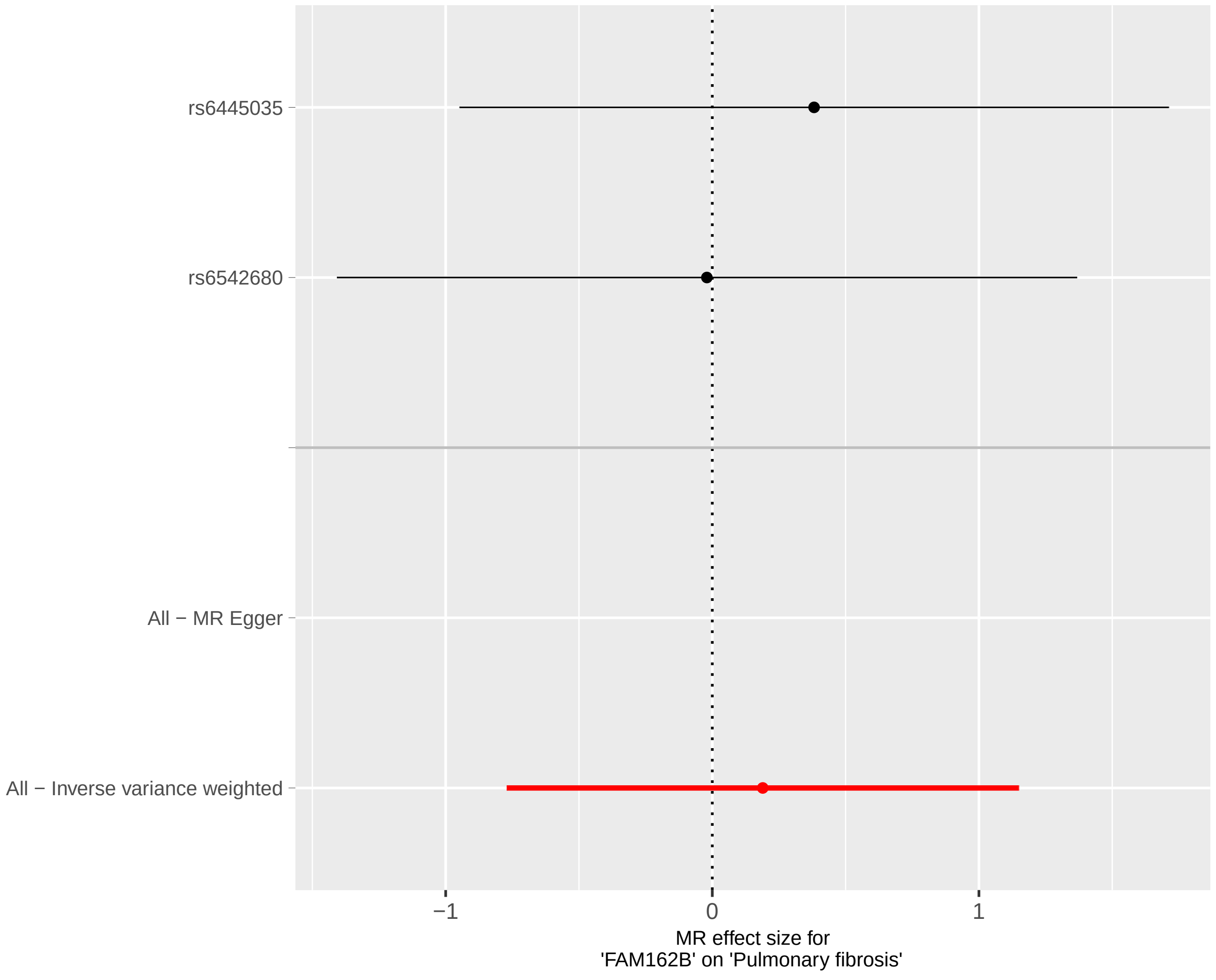
rs6445035
rs6542680
All − MR Egger
All − Inverse variance weighted
−1
0
1
MR effect size for
'FAM162B' on 'Pulmonary fibrosis'

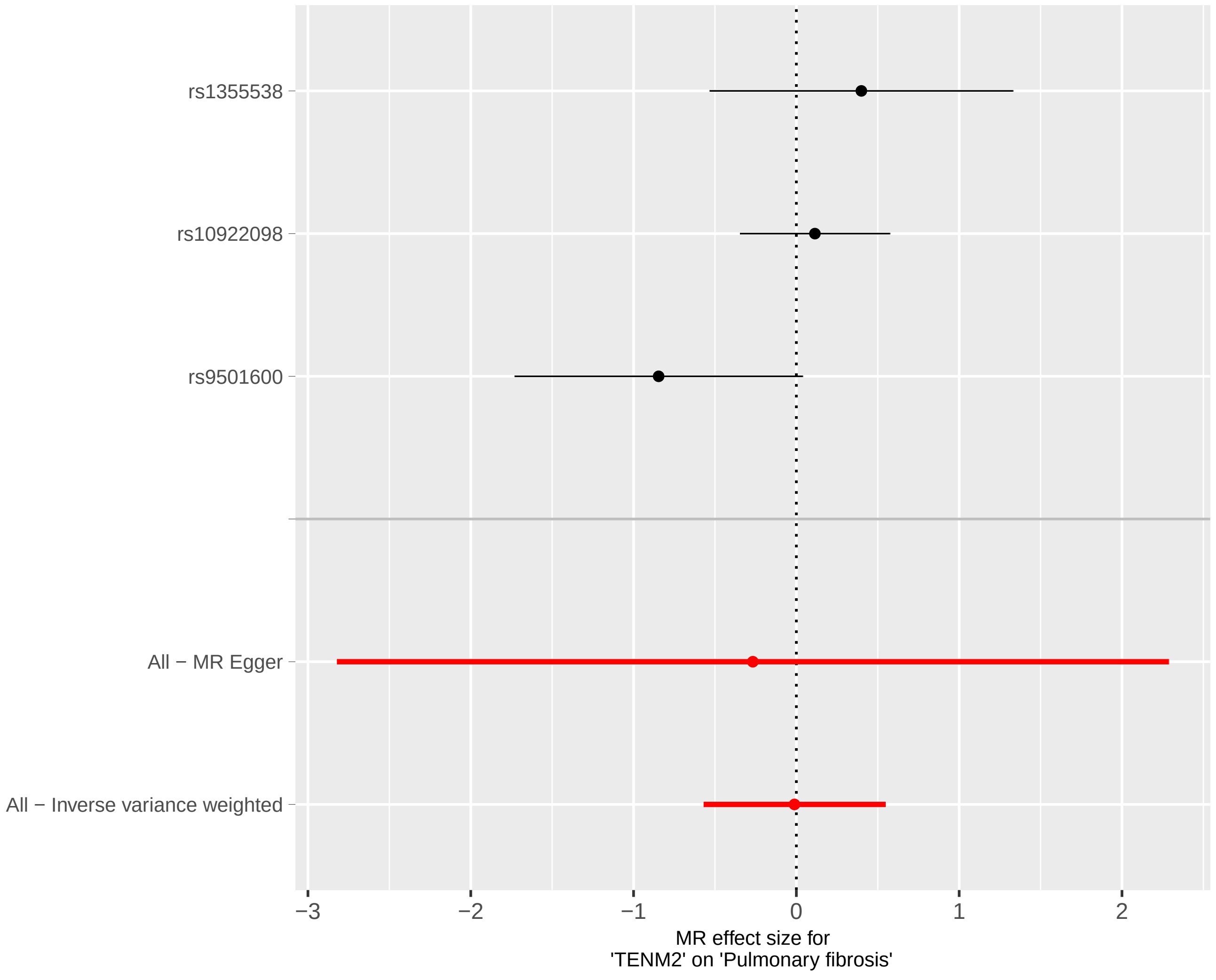
rs1355538
rs10922098
rs9501600
All − MR Egger
All − Inverse variance weighted
−3
−2
−1
0
1
2
MR effect size for
'TENM2' on 'Pulmonary fibrosis'

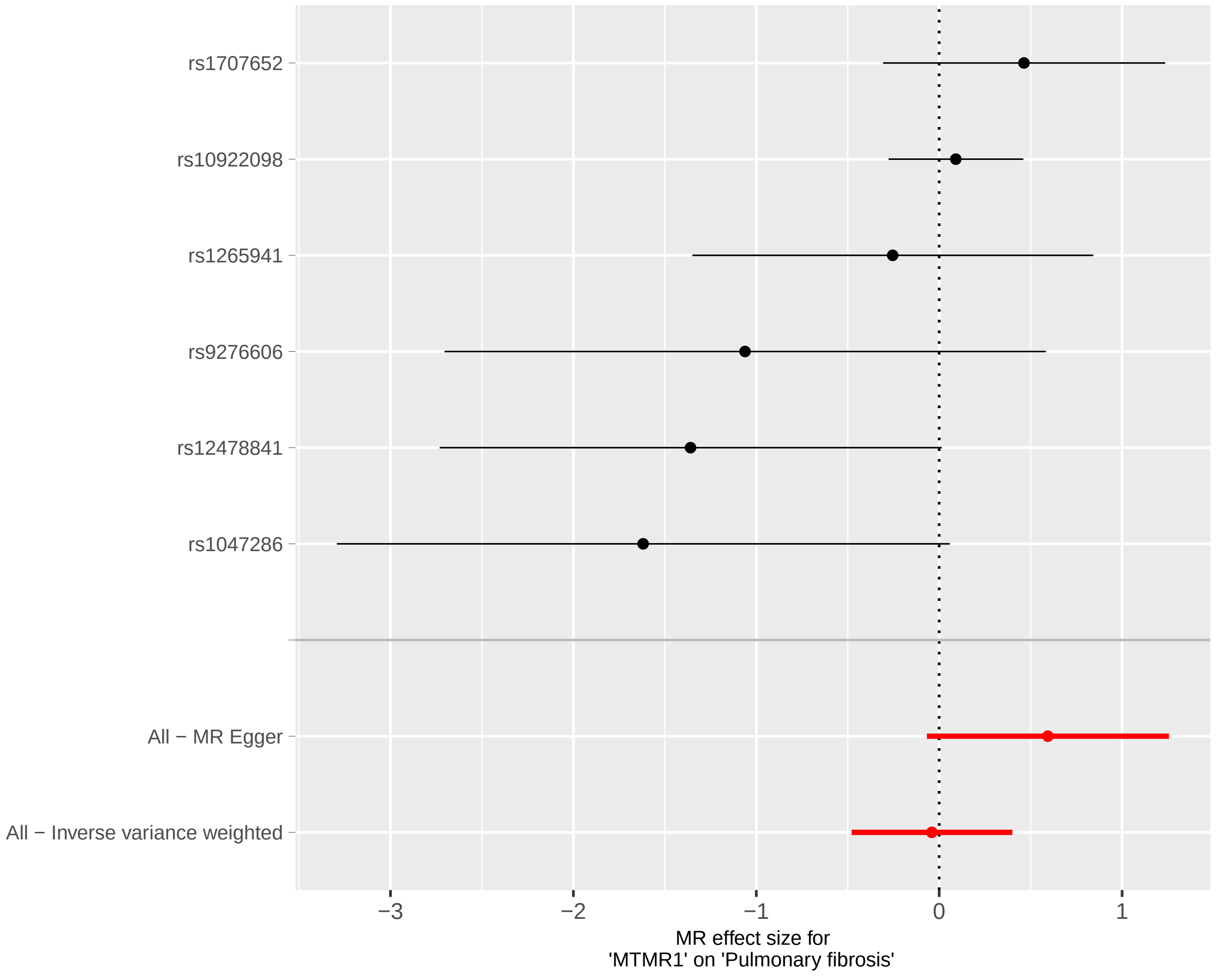
rs1707652
rs10922098
rs1265941
rs9276606
rs12478841
rs1047286
All − MR Egger
All − Inverse variance weighted
−3
−2
−1
0
1
MR effect size for
'MTMR1' on 'Pulmonary fibrosis'

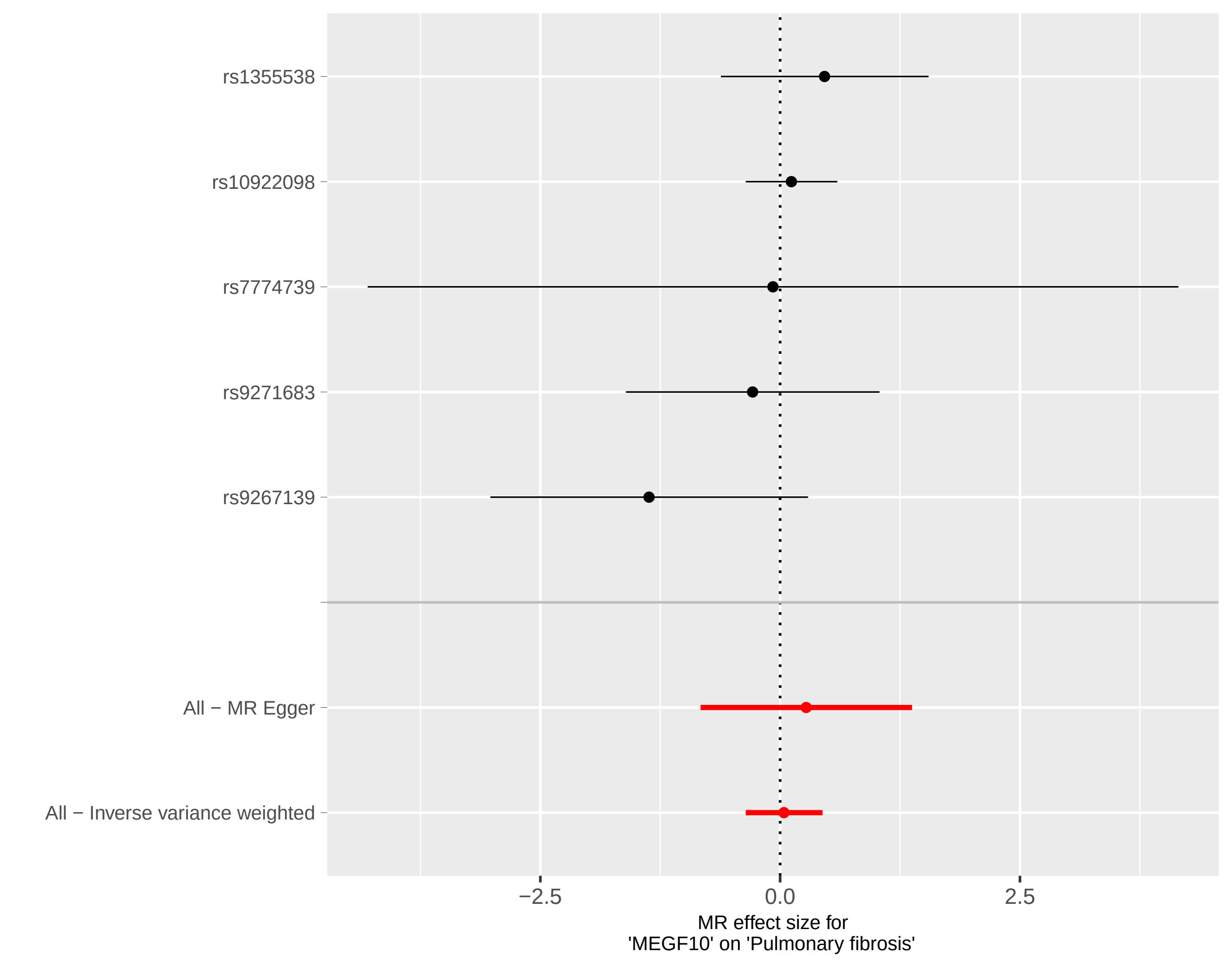
rs1355538
rs10922098
rs7774739
rs9271683
rs9267139
All − MR Egger
All − Inverse variance weighted
−2.5
0.0
2.5
MR effect size for
'MEGF10' on 'Pulmonary fibrosis'

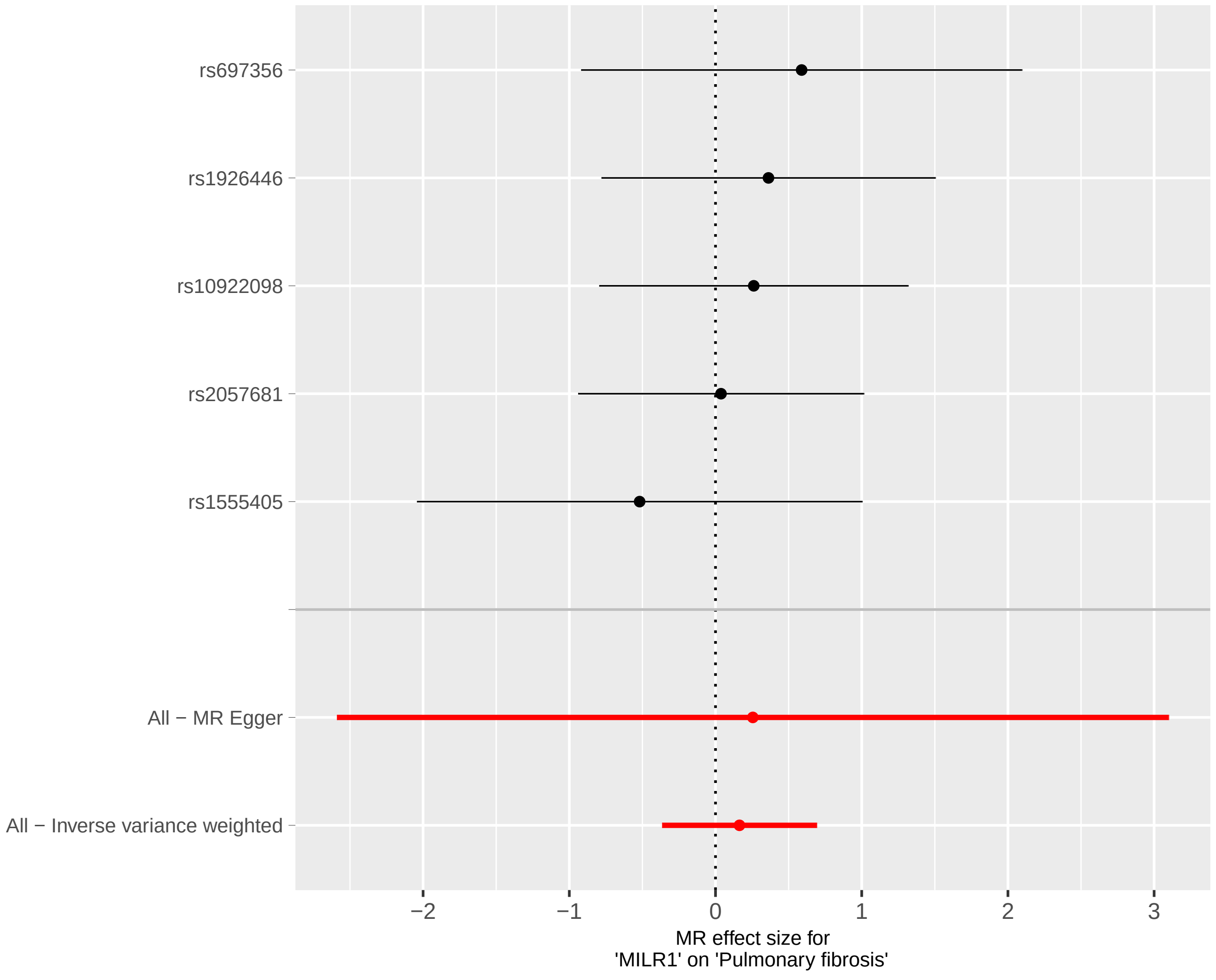
rs697356
rs1926446
rs10922098
rs2057681
rs1555405
All − MR Egger
All − Inverse variance weighted
−2
−1
0
1
2
3
MR effect size for
'MILR1' on 'Pulmonary fibrosis'

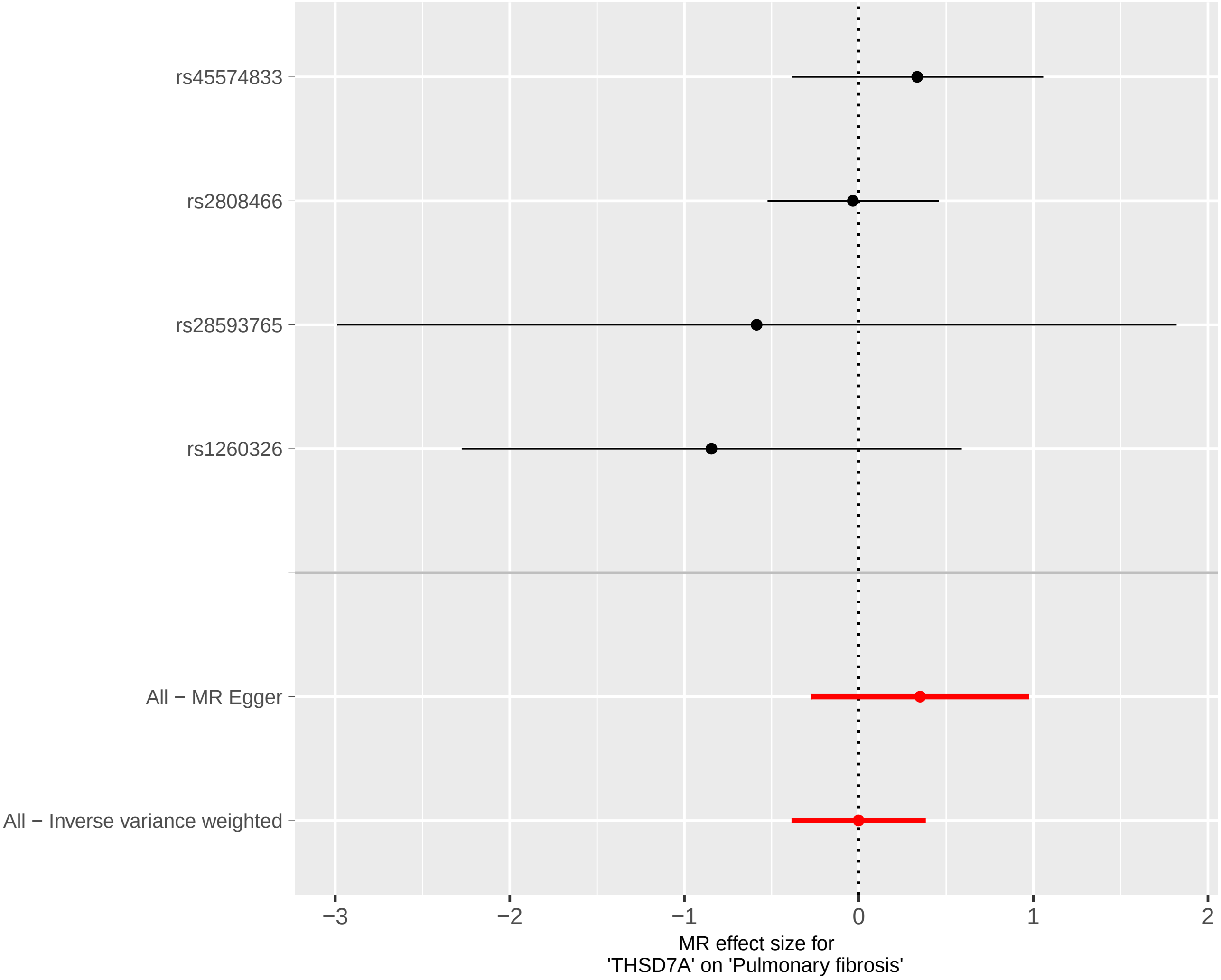
rs45574833
rs2808466
rs28593765
rs1260326
All − MR Egger
All − Inverse variance weighted
−3
−2
−1
0
1
2
MR effect size for
'THSD7A' on 'Pulmonary fibrosis'

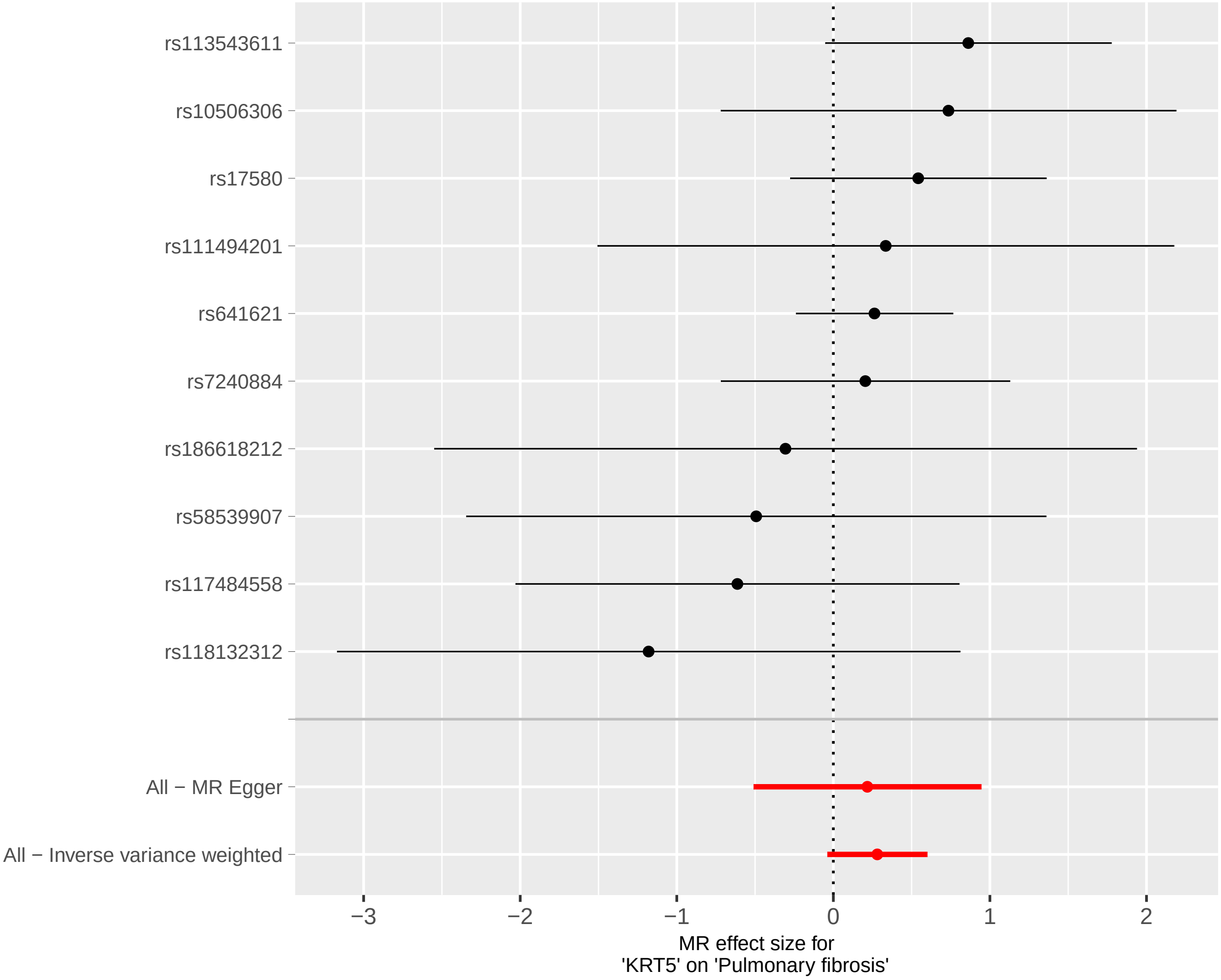

rs113543611
rs10506306
rs17580
rs111494201
rs641621
rs7240884
rs186618212
rs58539907
rs117484558
rs118132312
All − MR Egger
All − Inverse variance weighted
−3
−2
−1
0
1
2
MR effect size for
'KRT5' on 'Pulmonary fibrosis'

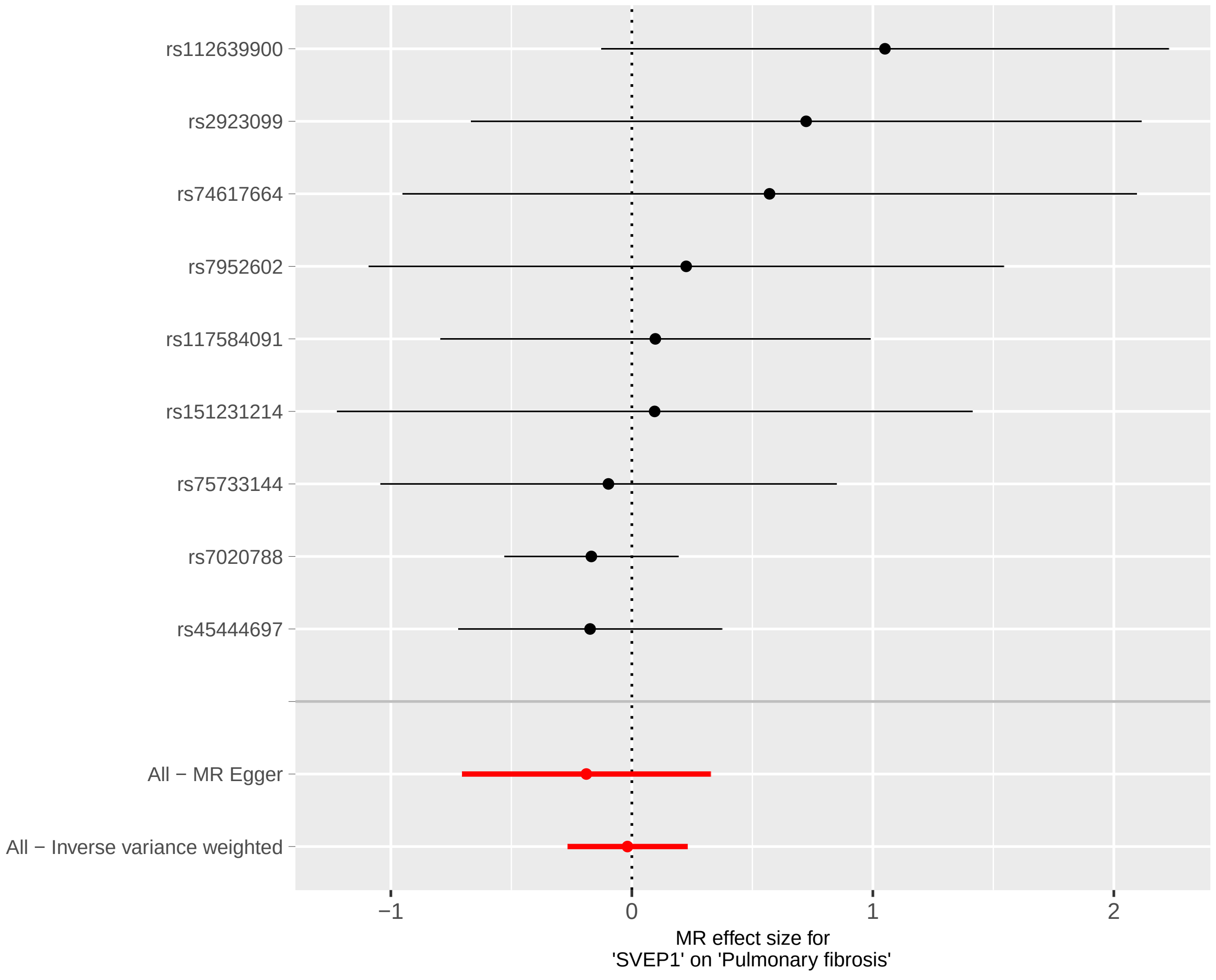
rs112639900
rs2923099
rs74617664
rs7952602
rs117584091
rs151231214
rs75733144
rs7020788
rs45444697
All − MR Egger
All − Inverse variance weighted
−1
0
1
2
MR effect size for
'SVEP1' on 'Pulmonary fibrosis'

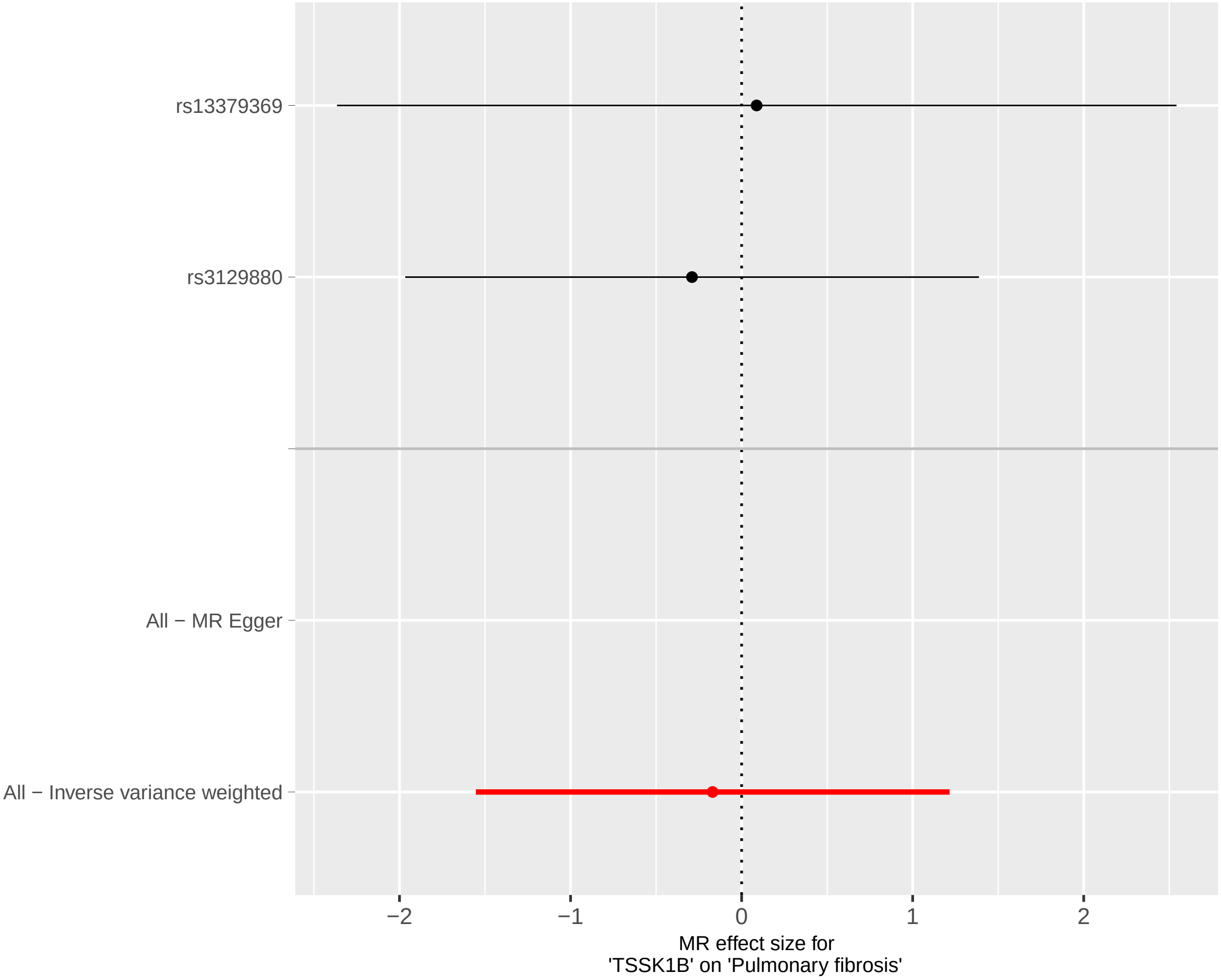

rs13379369
rs3129880
All − MR Egger
All − Inverse variance weighted
−2
−1
0
1
2
MR effect size for
'TSSK1B' on 'Pulmonary fibrosis'

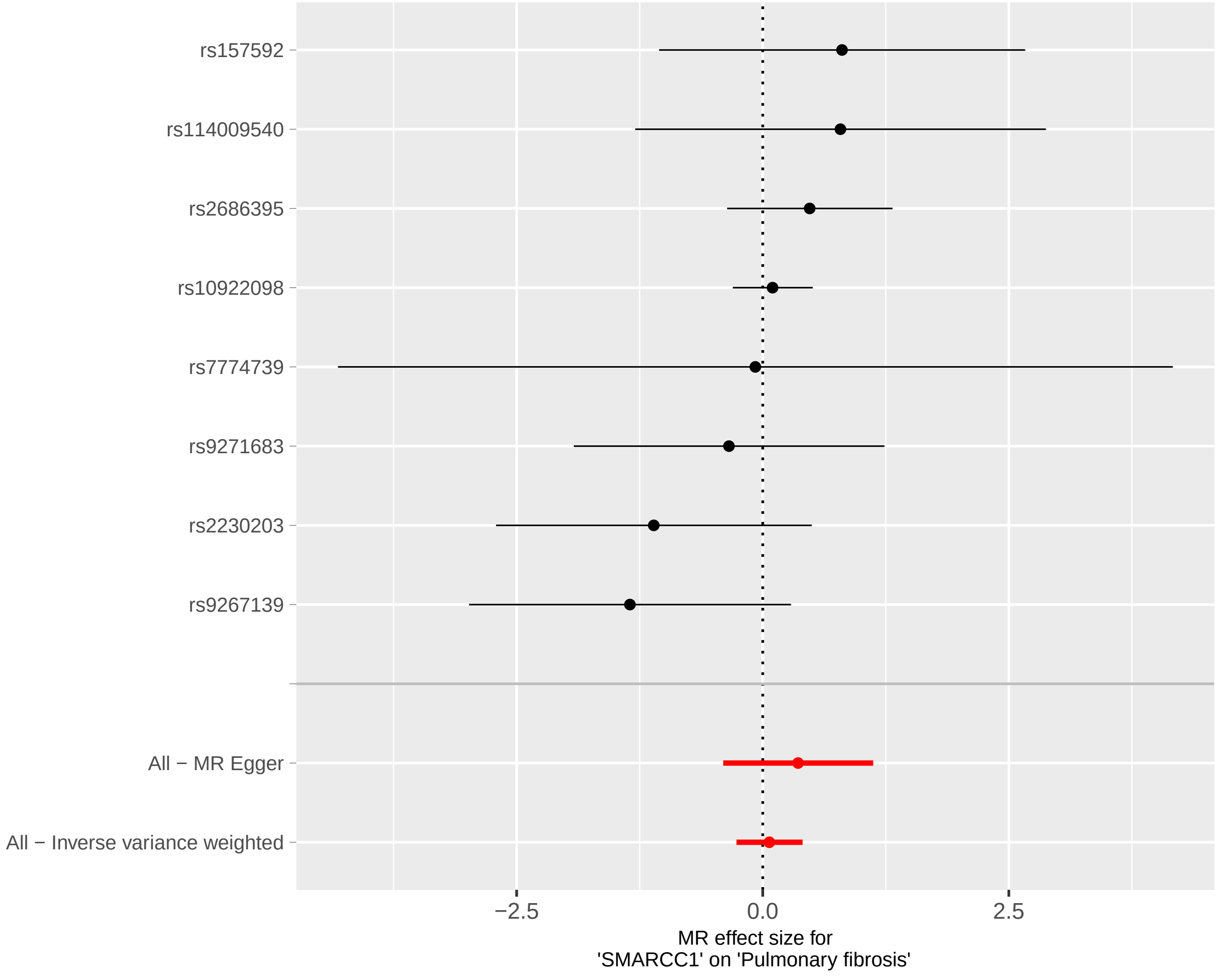
rs157592
rs114009540
rs2686395
rs10922098
rs7774739
rs9271683
rs2230203
rs9267139
All − MR Egger
All − Inverse variance weighted
−2.5
0.0
2.5
MR effect size for
'SMARCC1' on 'Pulmonary fibrosis'

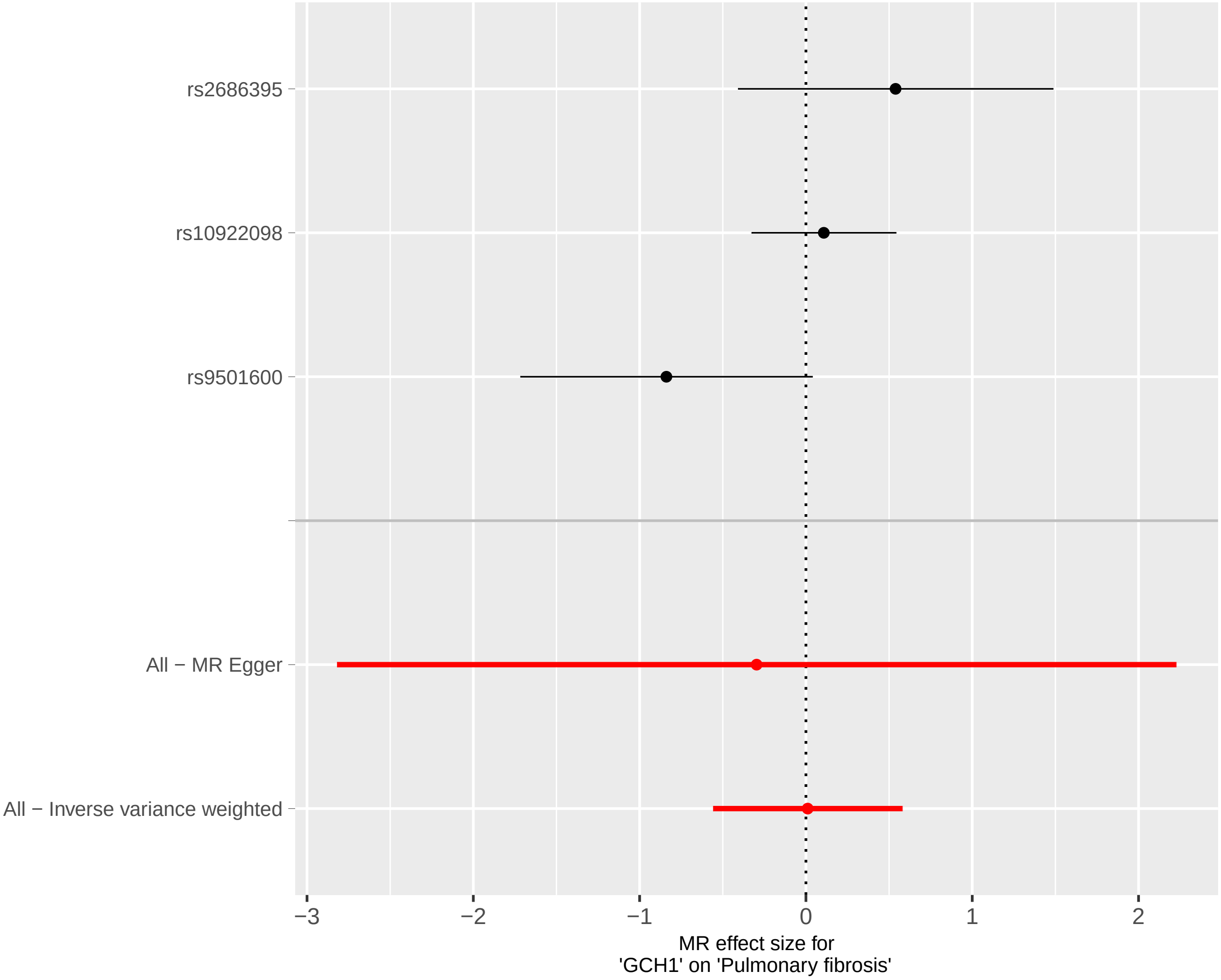

rs2686395
rs10922098
rs9501600
All − MR Egger
All − Inverse variance weighted
−3
−2
−1
0
1
2
MR effect size for
'GCH1' on 'Pulmonary fibrosis'

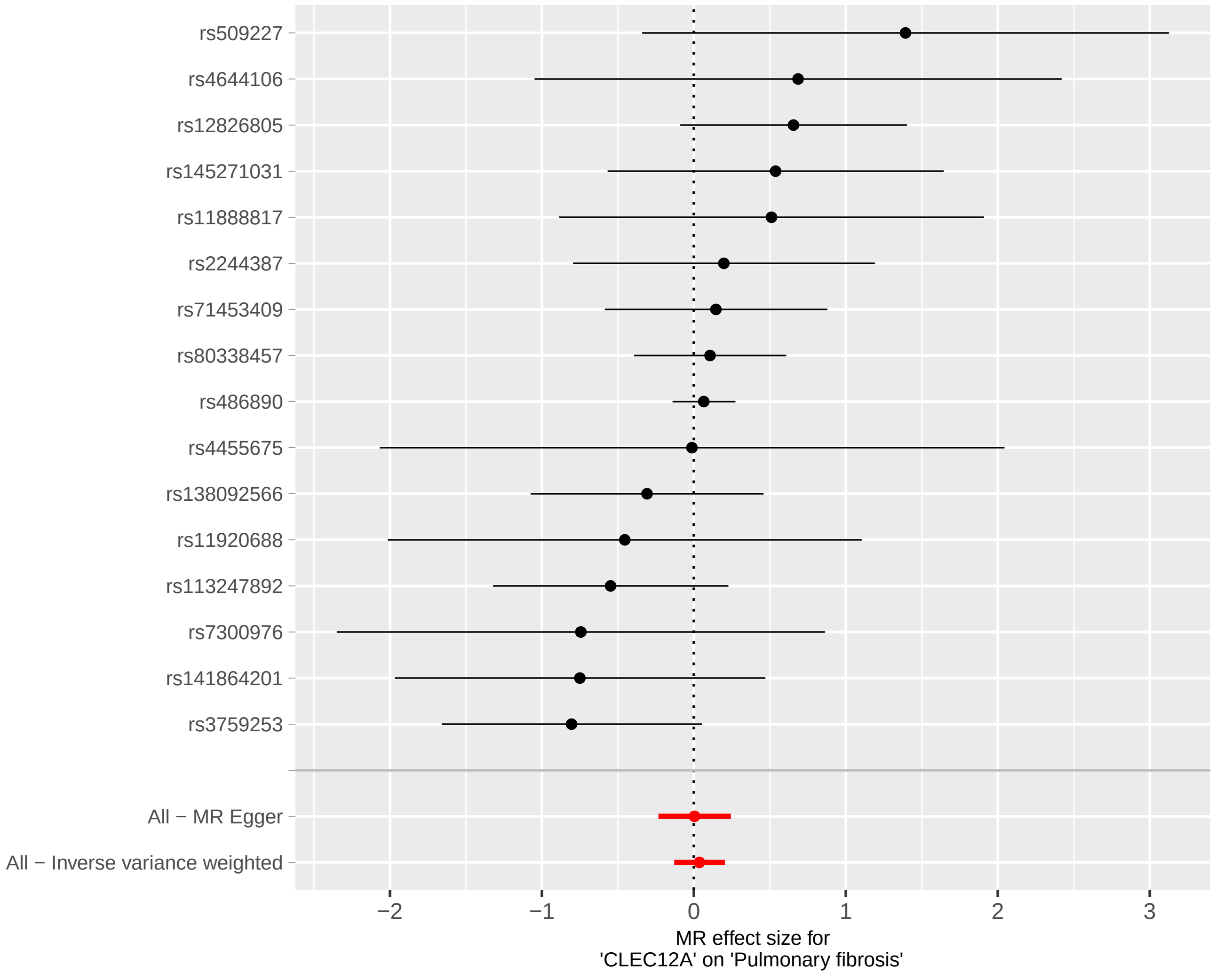
rs509227
rs4644106
rs12826805
rs145271031
rs11888817
rs2244387
rs71453409
rs80338457
rs486890
rs4455675
rs138092566
rs11920688
rs113247892
rs7300976
rs141864201
rs3759253
All − MR Egger
All − Inverse variance weighted
−2
−1
0
1
2
3
MR effect size for
'CLEC12A' on 'Pulmonary fibrosis'

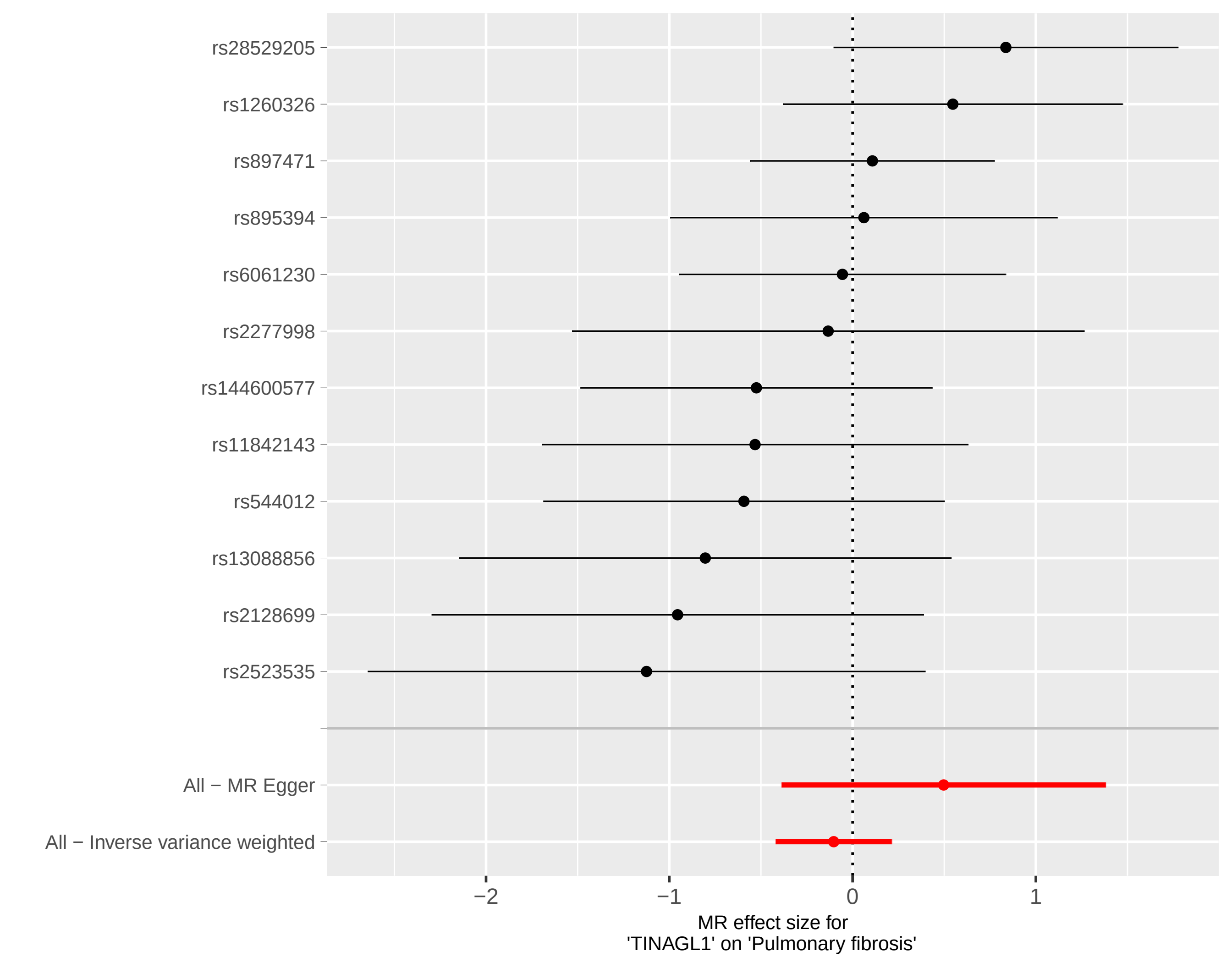
rs28529205
rs1260326
rs897471
rs895394
rs6061230
rs2277998
rs144600577
rs11842143
rs544012
rs13088856
rs2128699
rs2523535
All − MR Egger
All − Inverse variance weighted
−2
−1
0
1
MR effect size for
'TINAGL1' on 'Pulmonary fibrosis'

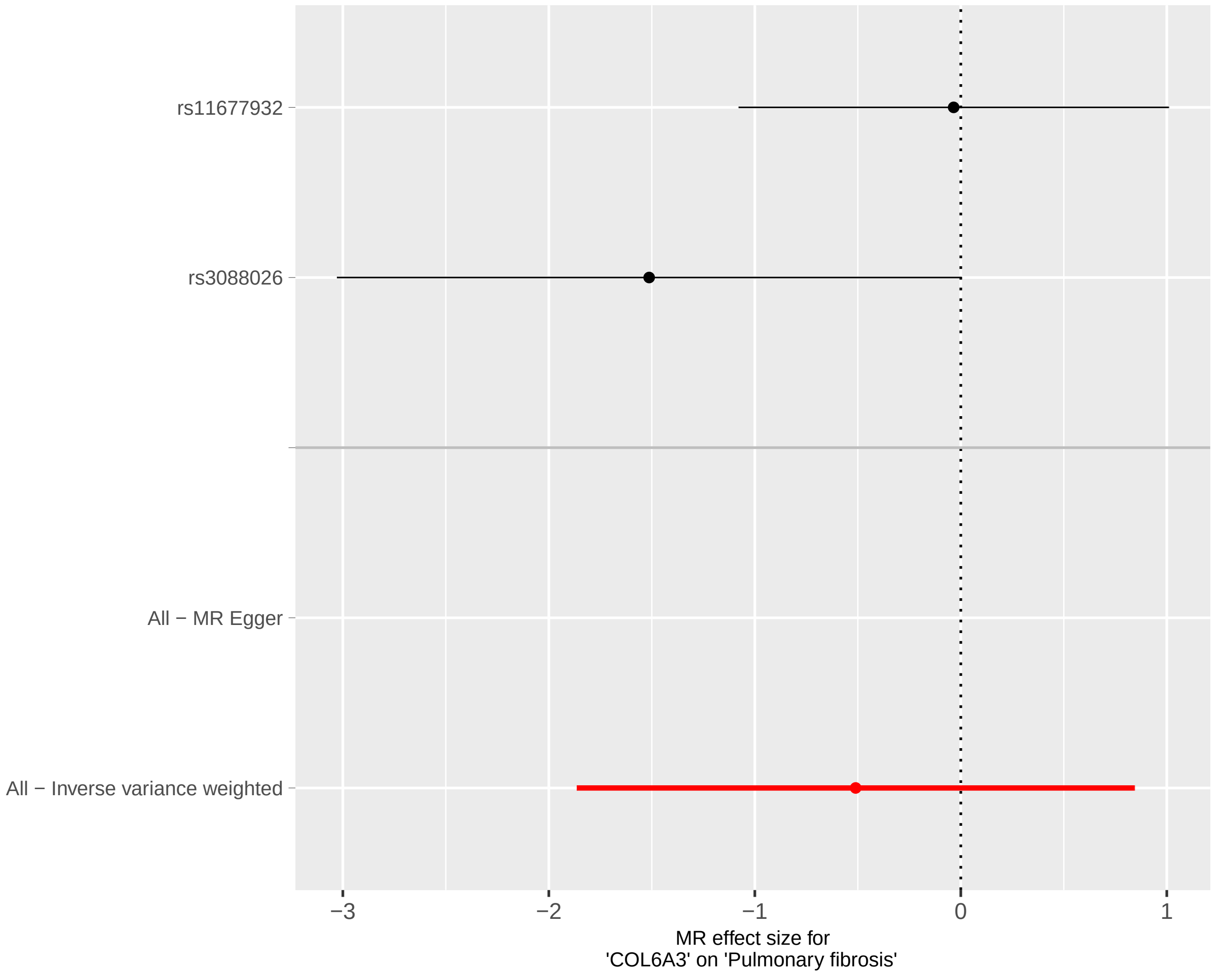
rs11677932
rs3088026
All − MR Egger
All − Inverse variance weighted
−3
−2
−1
0
1
MR effect size for
'COL6A3' on 'Pulmonary fibrosis'

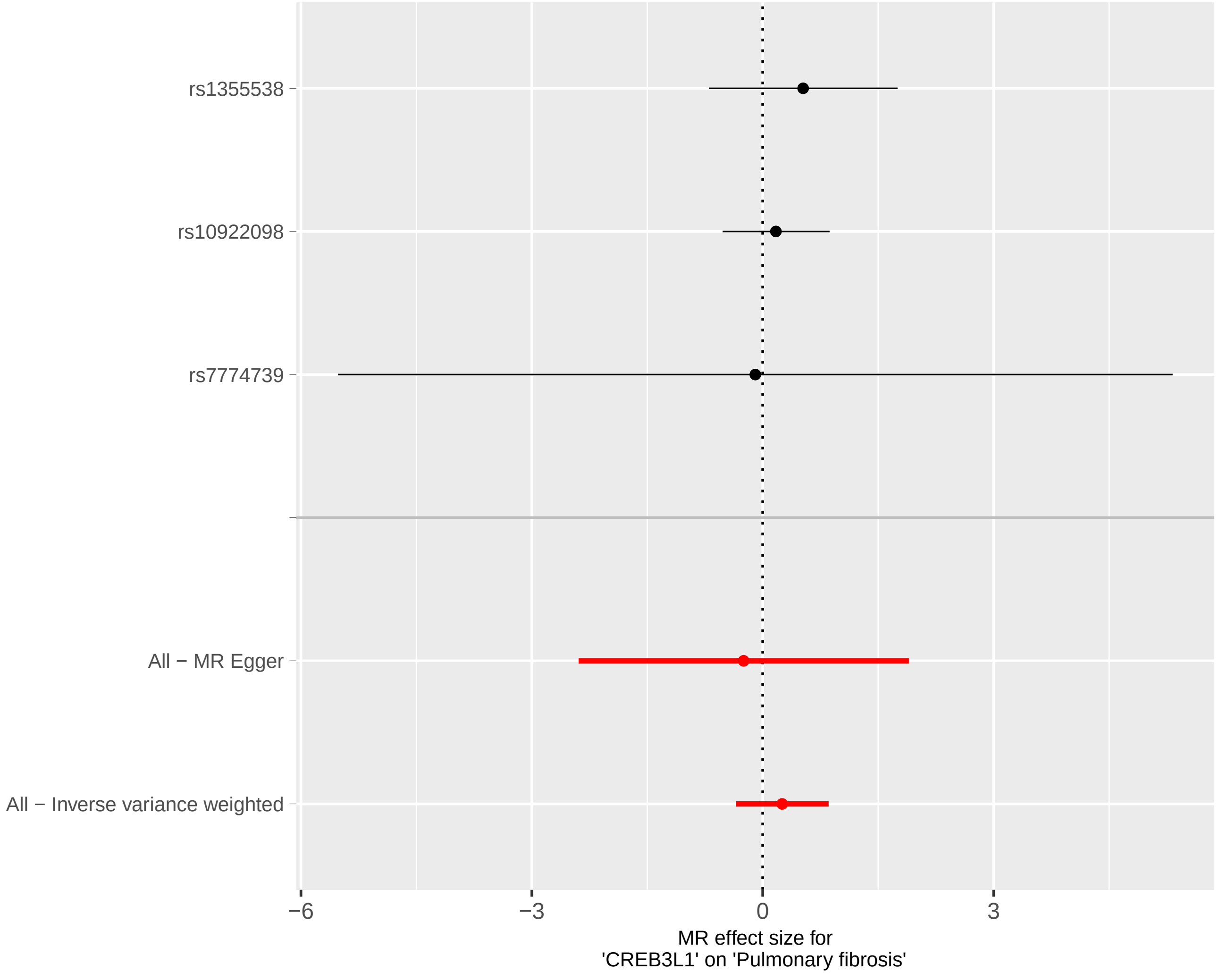

rs1355538
rs10922098
rs7774739
All − MR Egger
All − Inverse variance weighted
−6
−3
0
3
MR effect size for
'CREB3L1' on 'Pulmonary fibrosis'

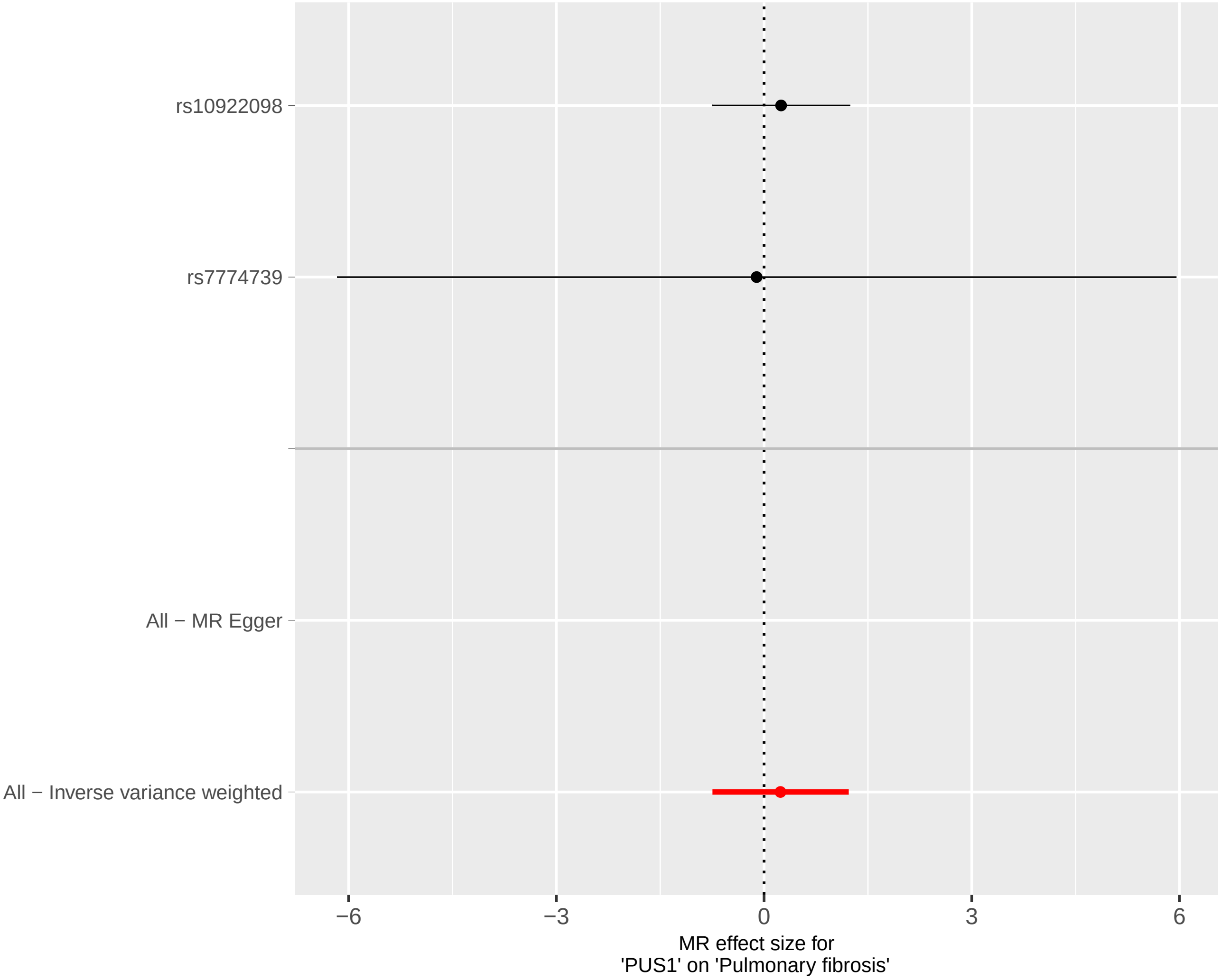

rs10922098
rs7774739
All − MR Egger
All − Inverse variance weighted
−6
−3
0
3
6
MR effect size for
'PUS1' on 'Pulmonary fibrosis'

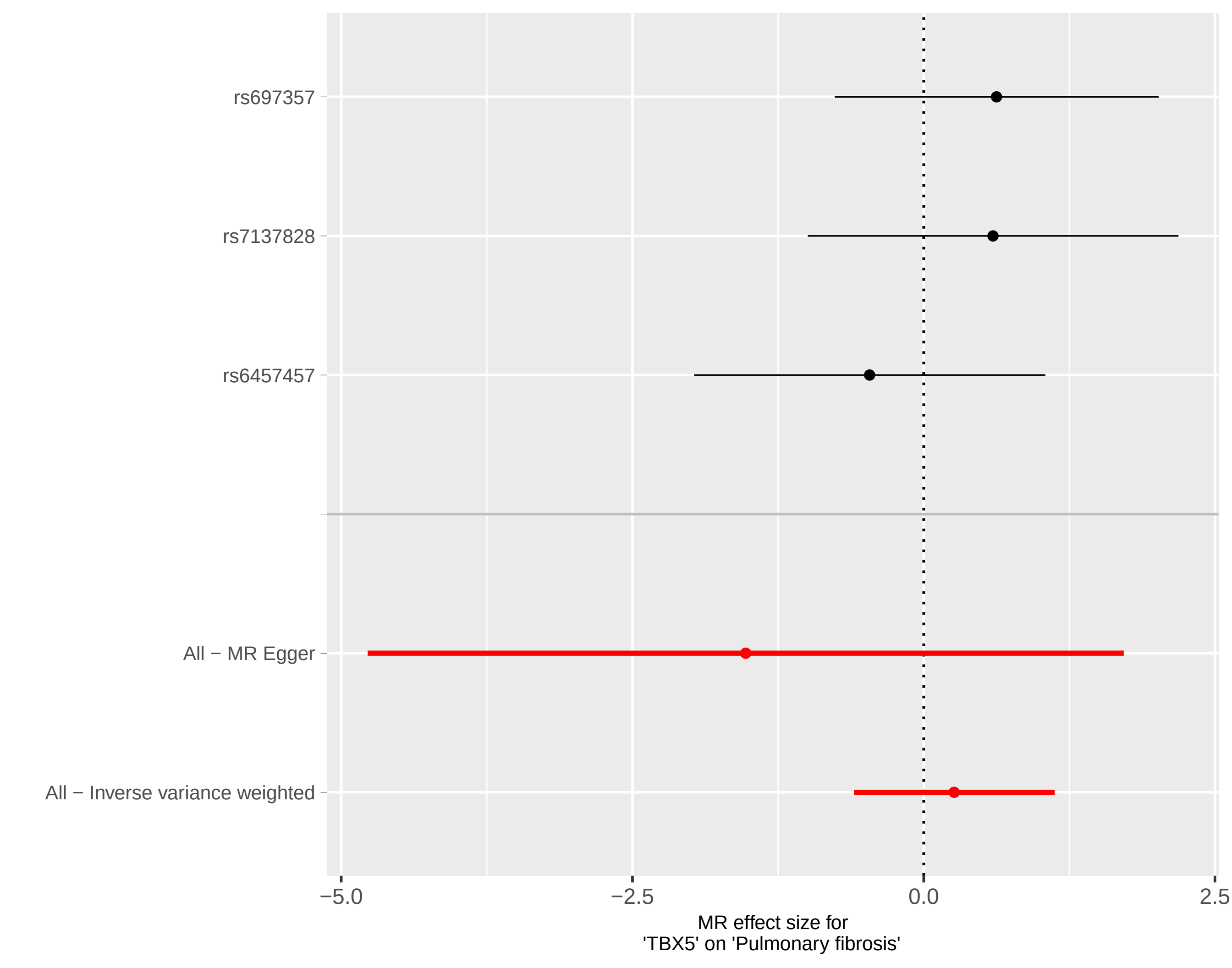
rs697357
rs7137828
rs6457457
All − MR Egger
All − Inverse variance weighted
−5.0
−2.5
0.0
2.5
MR effect size for
'TBX5' on 'Pulmonary fibrosis'

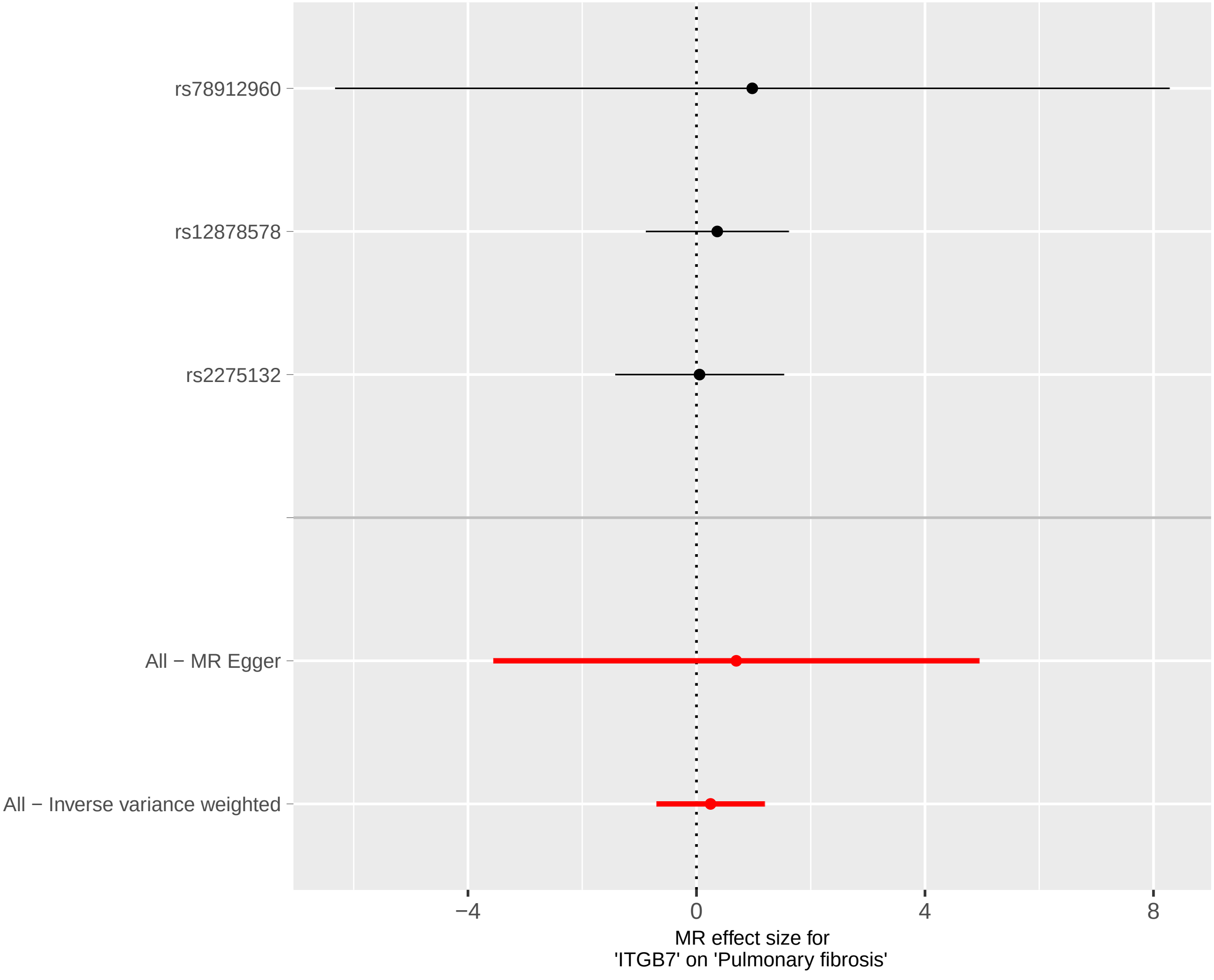

rs78912960
rs12878578
rs2275132
All − MR Egger
All − Inverse variance weighted
−4
0
4
8
MR effect size for
'ITGB7' on 'Pulmonary fibrosis'

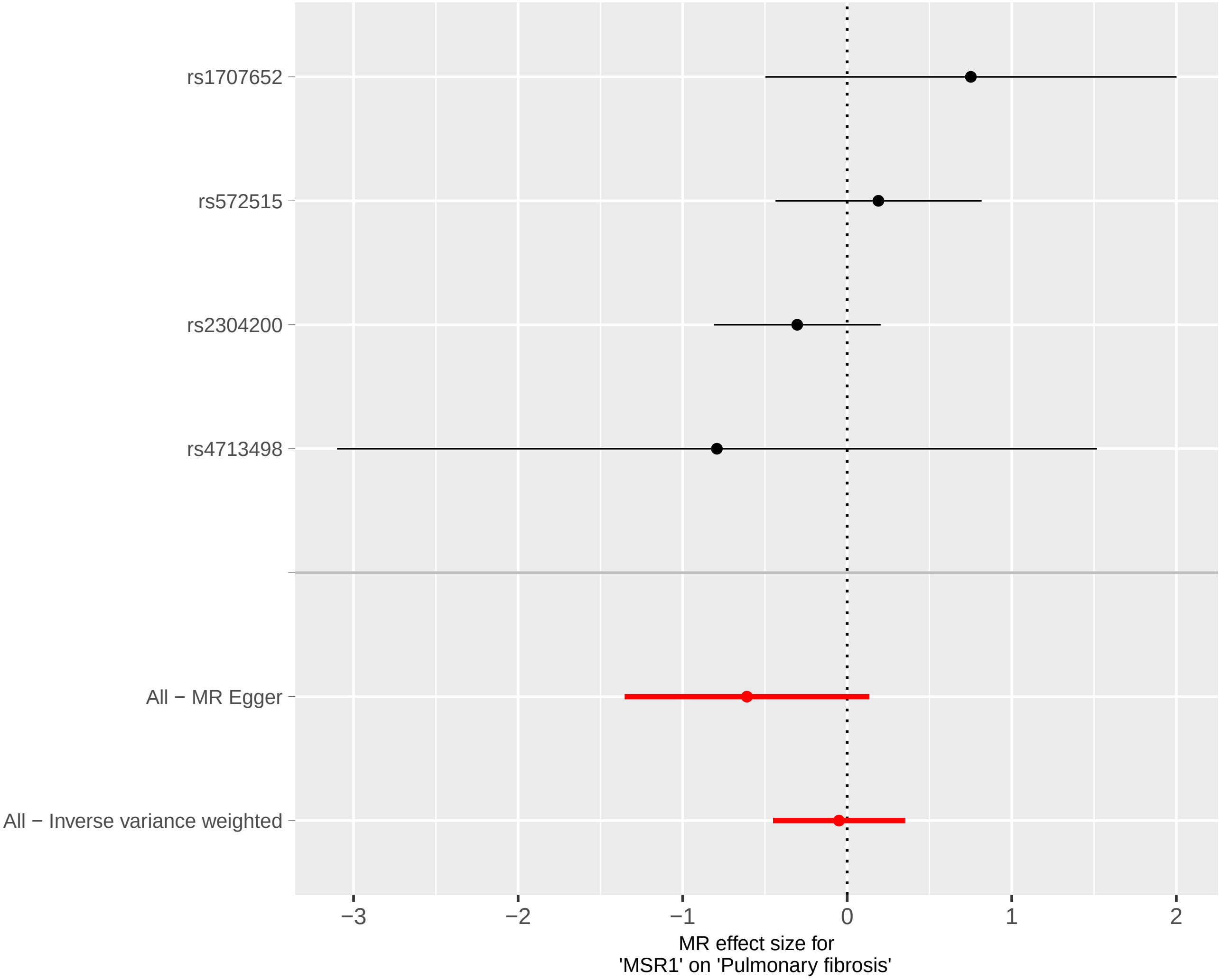
rs1707652
rs572515
rs2304200
rs4713498
All − MR Egger
All − Inverse variance weighted
−3
−2
−1
0
1
2
MR effect size for
'MSR1' on 'Pulmonary fibrosis'

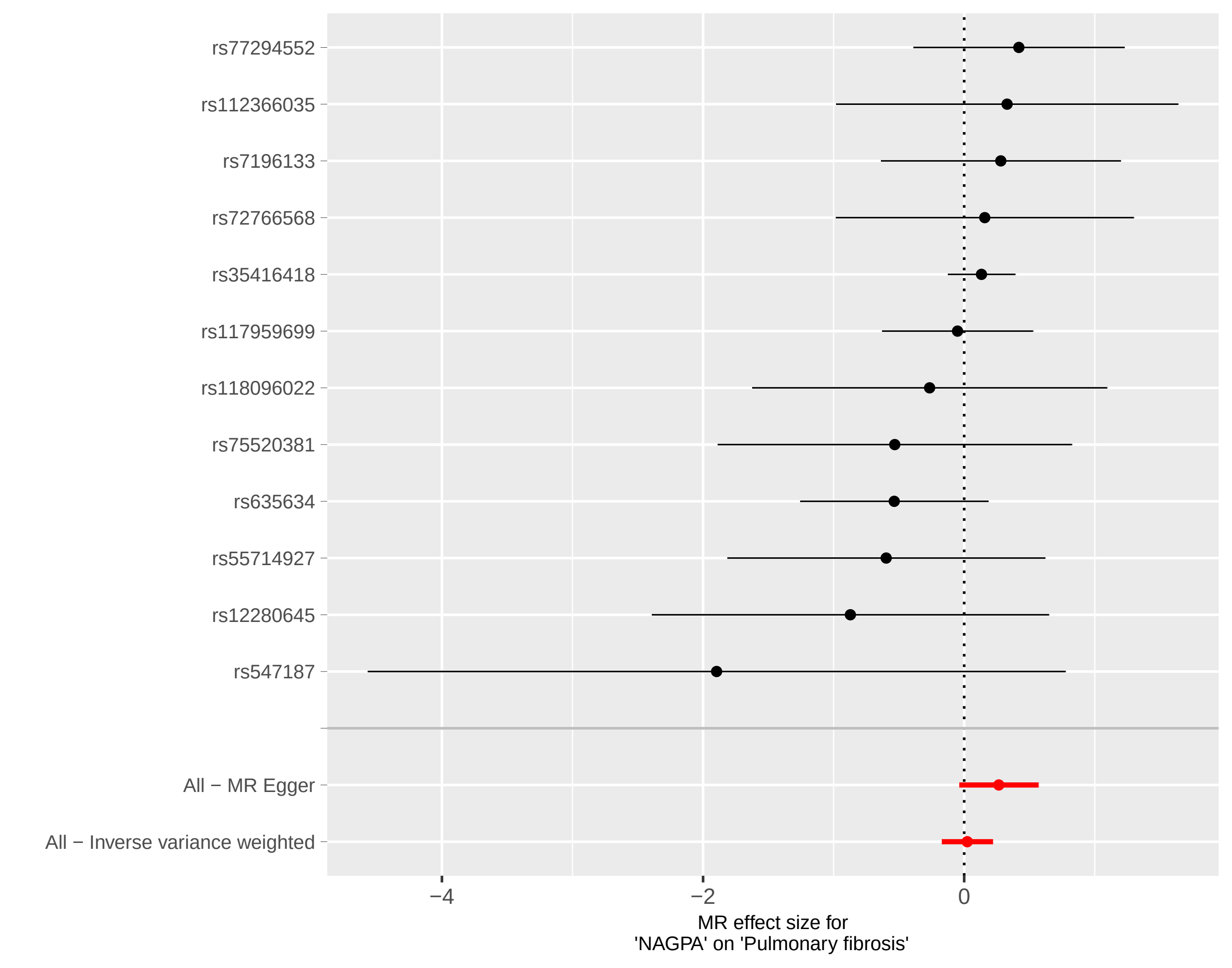

rs77294552
rs112366035
rs7196133
rs72766568
rs35416418
rs117959699
rs118096022
rs75520381
rs635634
rs55714927
rs12280645
rs547187
All − MR Egger
All − Inverse variance weighted
−4
−2
0
MR effect size for
'NAGPA' on 'Pulmonary fibrosis'

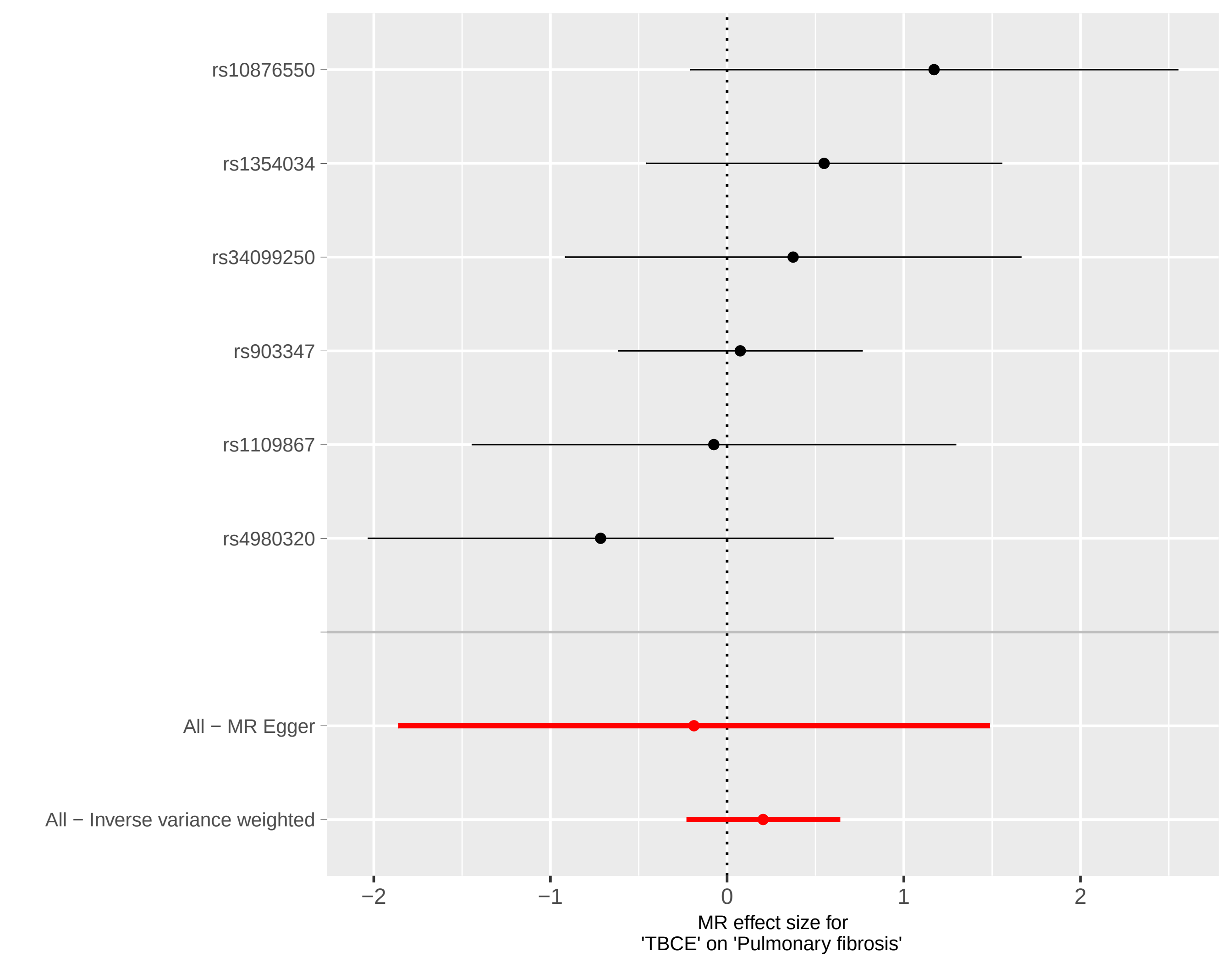
rs10876550
rs1354034
rs34099250
rs903347
rs1109867
rs4980320
All − MR Egger
All − Inverse variance weighted
−2
−1
0
1
2
MR effect size for
'TBCE' on 'Pulmonary fibrosis'

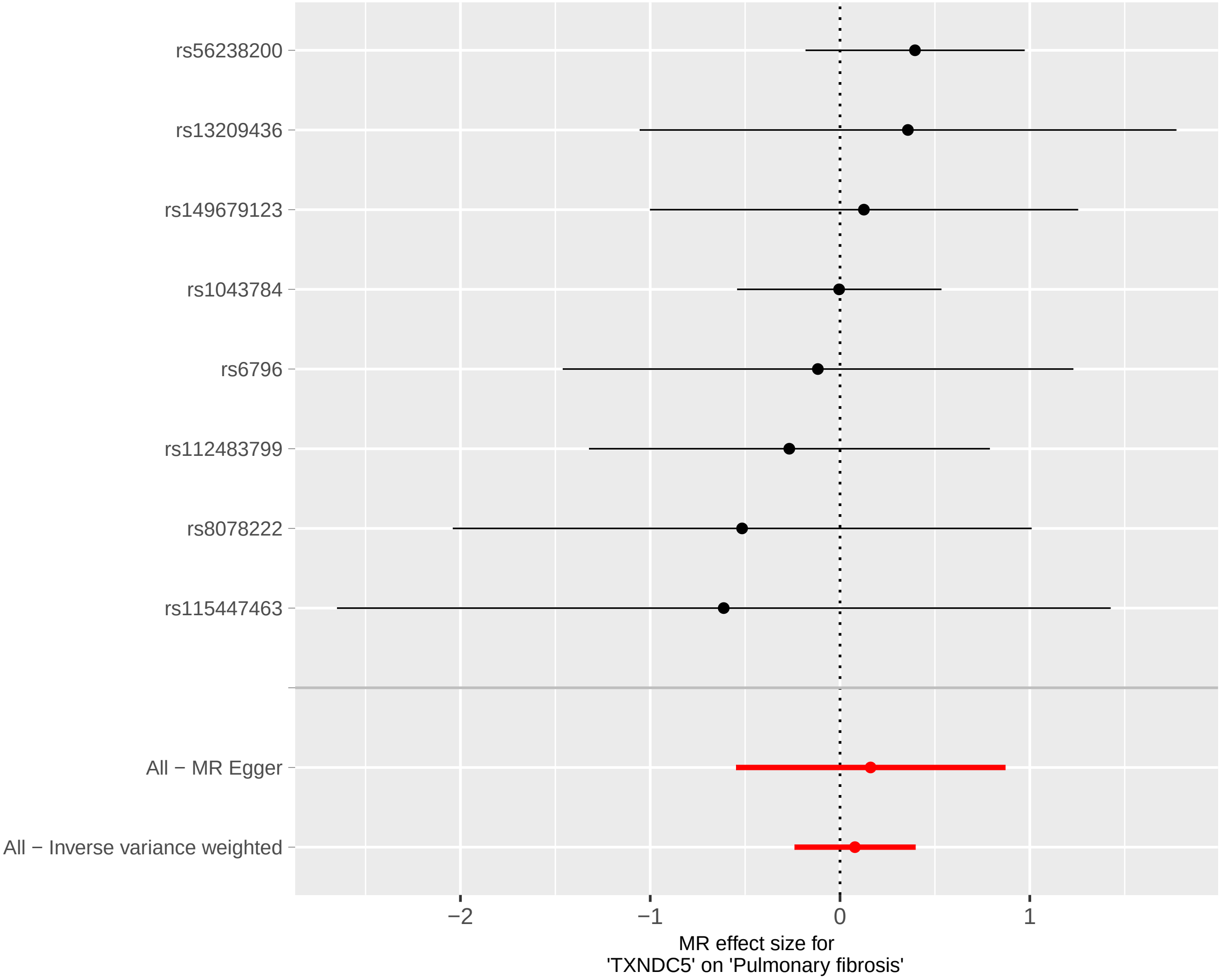
rs56238200
rs13209436
rs149679123
rs1043784
rs6796
rs112483799
rs8078222
rs115447463
All − MR Egger
All − Inverse variance weighted
−2
−1
0
1
MR effect size for
'TXNDC5' on 'Pulmonary fibrosis'

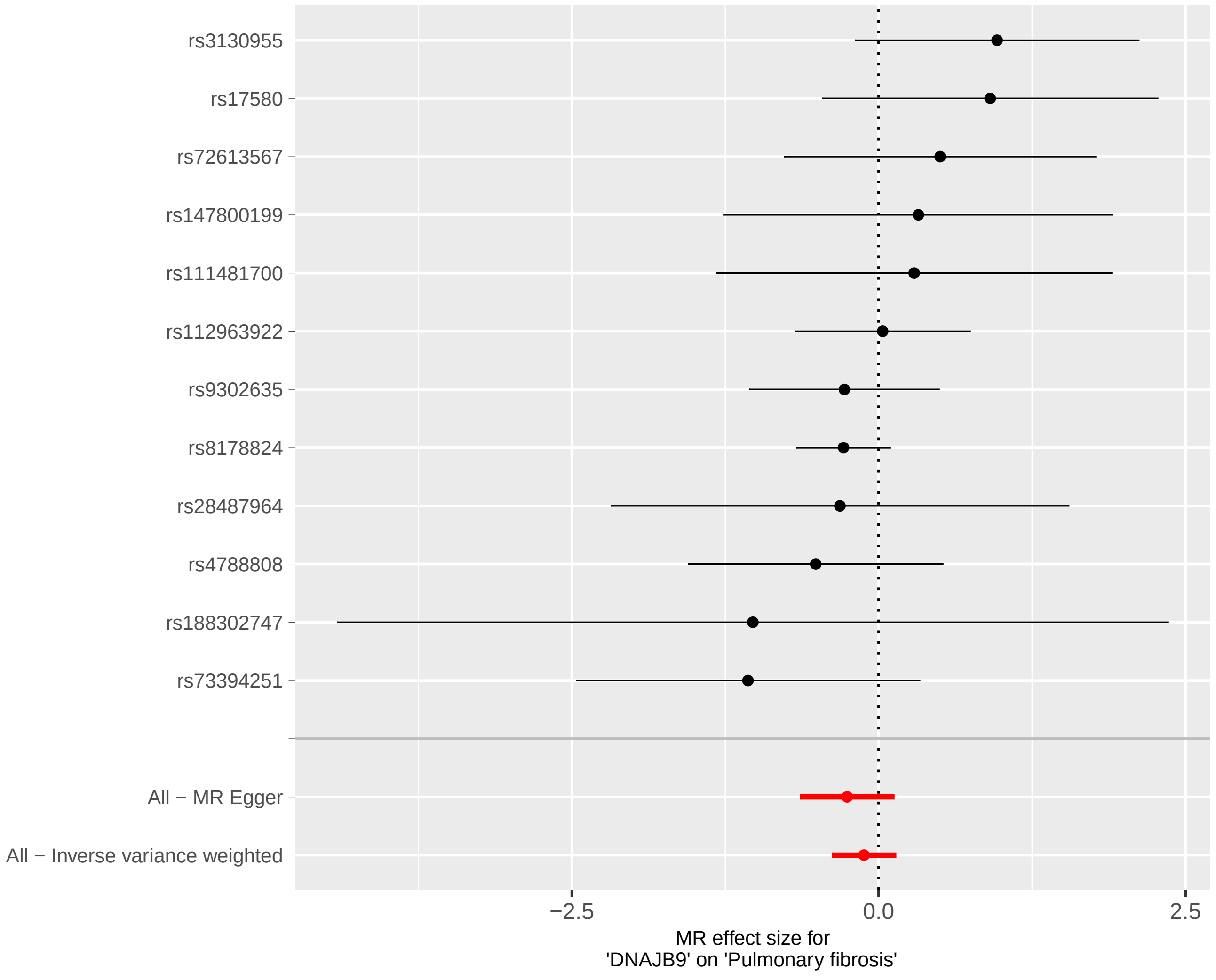

rs3130955
rs17580
rs72613567
rs147800199
rs111481700
rs112963922
rs9302635
rs8178824
rs28487964
rs4788808
rs188302747
rs73394251
All − MR Egger
All − Inverse variance weighted
−2.5
0.0
2.5
MR effect size for
'DNAJB9' on 'Pulmonary fibrosis'

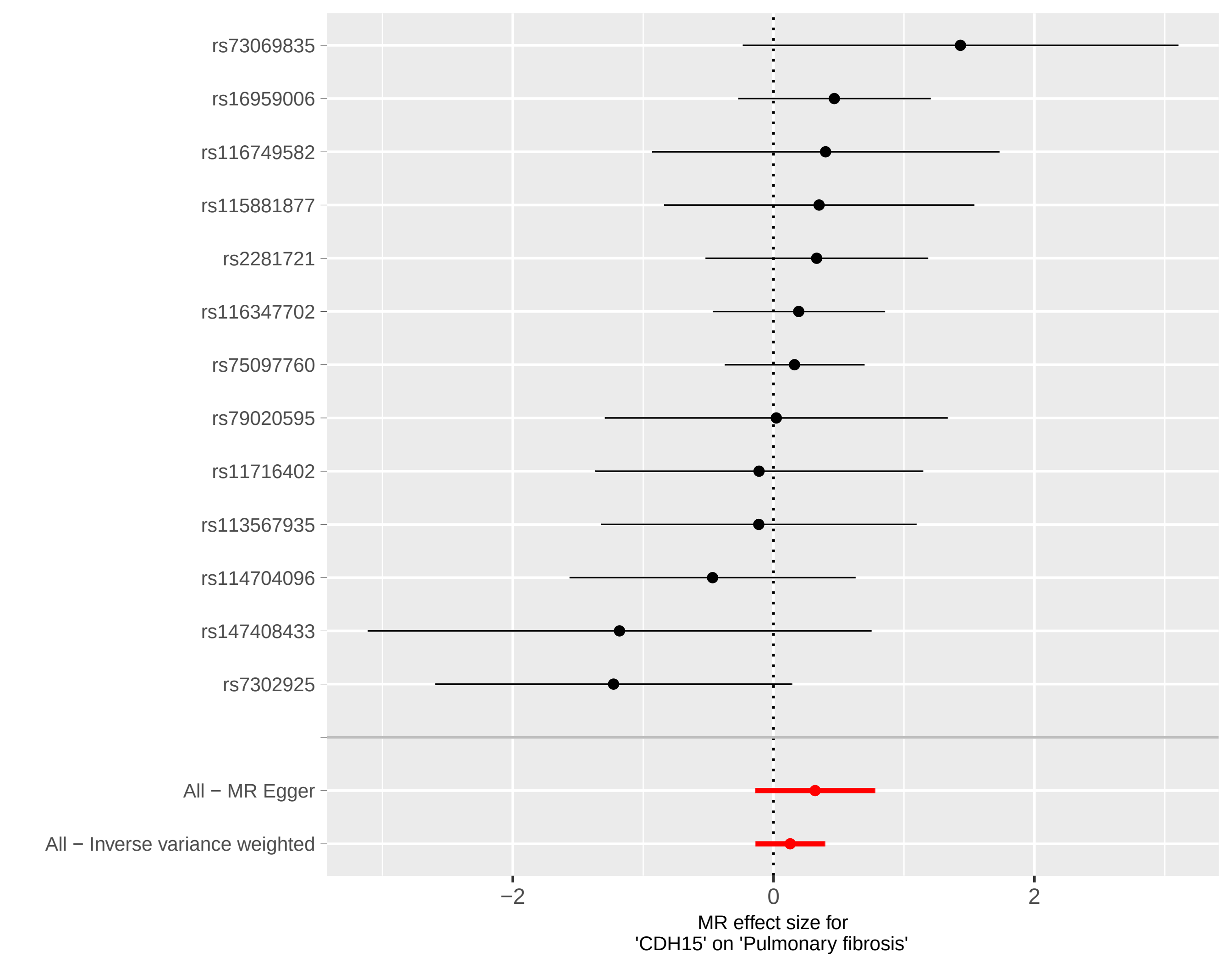
rs73069835
rs16959006
rs116749582
rs115881877
rs2281721
rs116347702
rs75097760
rs79020595
rs11716402
rs113567935
rs114704096
rs147408433
rs7302925
All − MR Egger
All − Inverse variance weighted
−2
0
2
MR effect size for
'CDH15' on 'Pulmonary fibrosis'

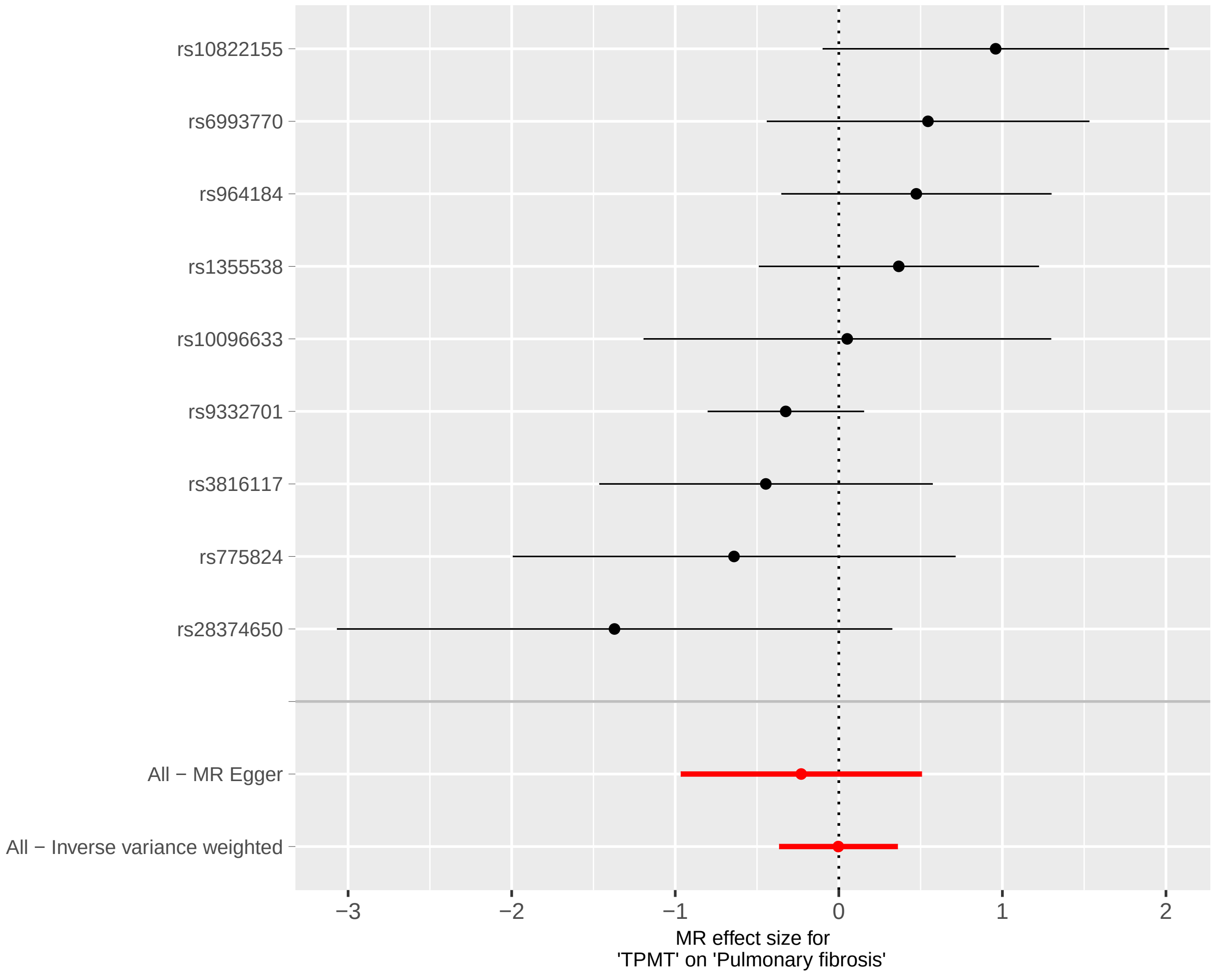
rs10822155
rs6993770
rs964184
rs1355538
rs10096633
rs9332701
rs3816117
rs775824
rs28374650
All − MR Egger
All − Inverse variance weighted
−3
−2
−1
0
1
2
MR effect size for
'TPMT' on 'Pulmonary fibrosis'

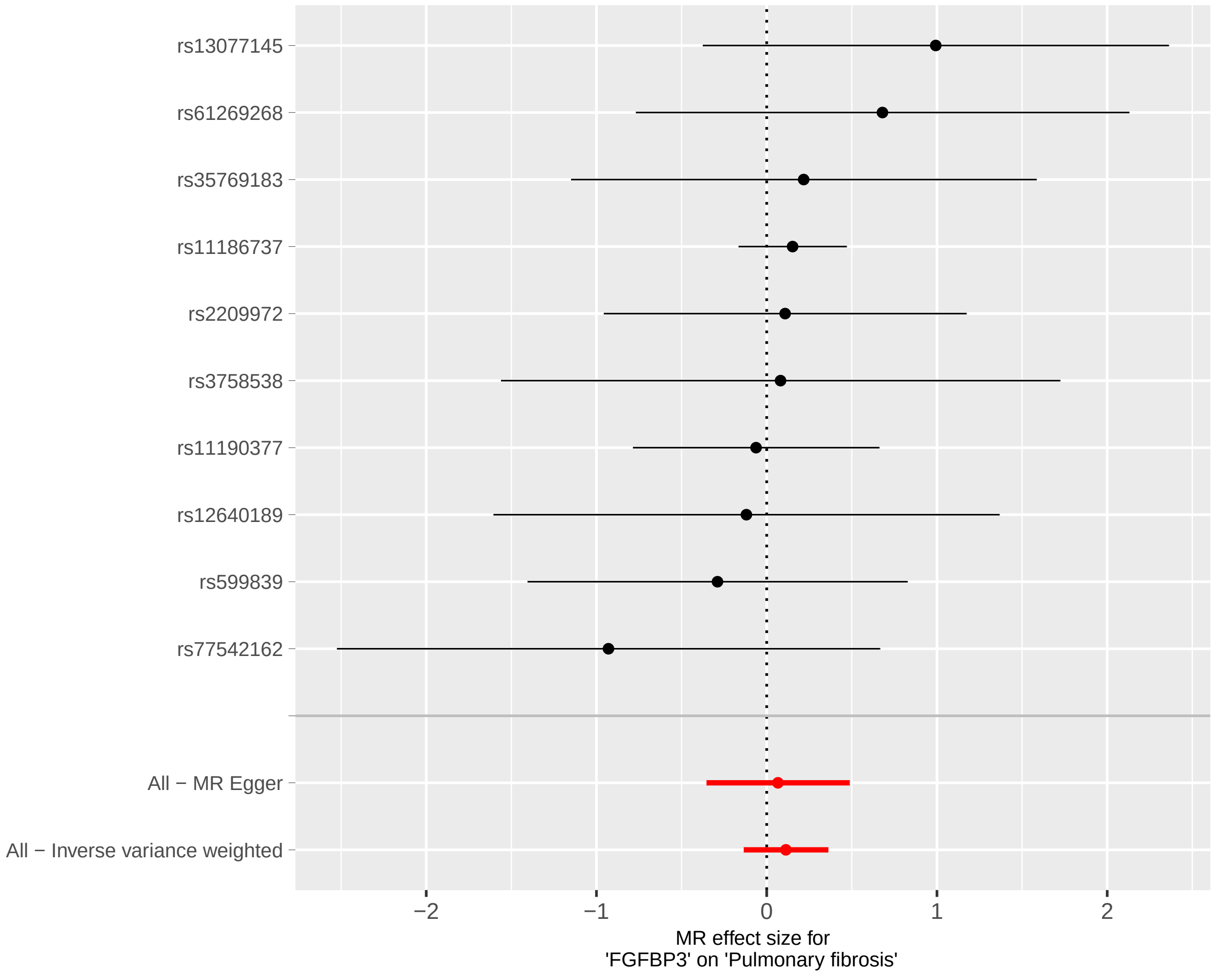

rs13077145
rs61269268
rs35769183
rs11186737
rs2209972
rs3758538
rs11190377
rs12640189
rs599839
rs77542162
All − MR Egger
All − Inverse variance weighted
−2
−1
0
1
2
MR effect size for
'FGFBP3' on 'Pulmonary fibrosis'

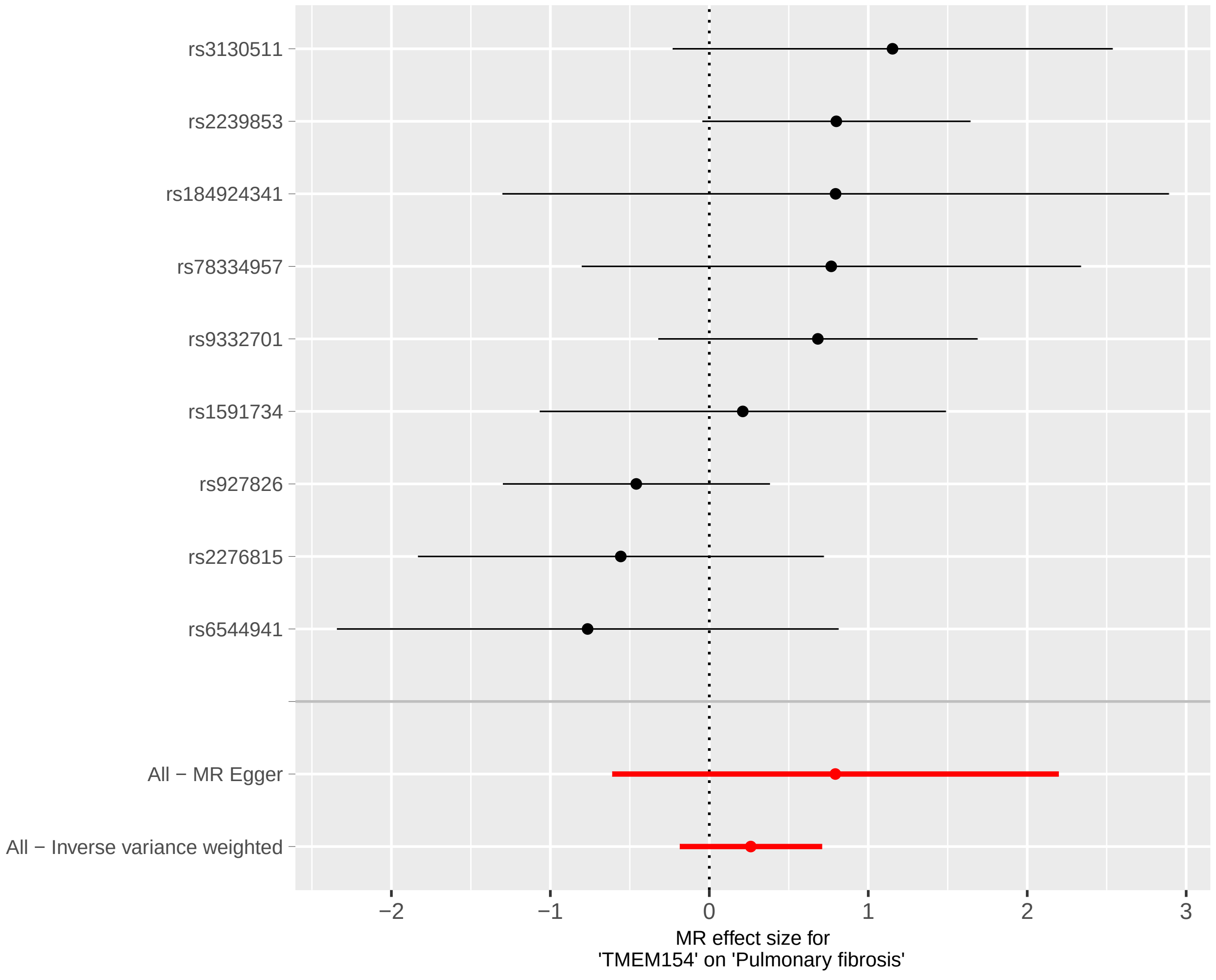

rs3130511
rs2239853
rs184924341
rs78334957
rs9332701
rs1591734
rs927826
rs2276815
rs6544941
All − MR Egger
All − Inverse variance weighted
−2
−1
0
1
2
3
MR effect size for
'TMEM154' on 'Pulmonary fibrosis'

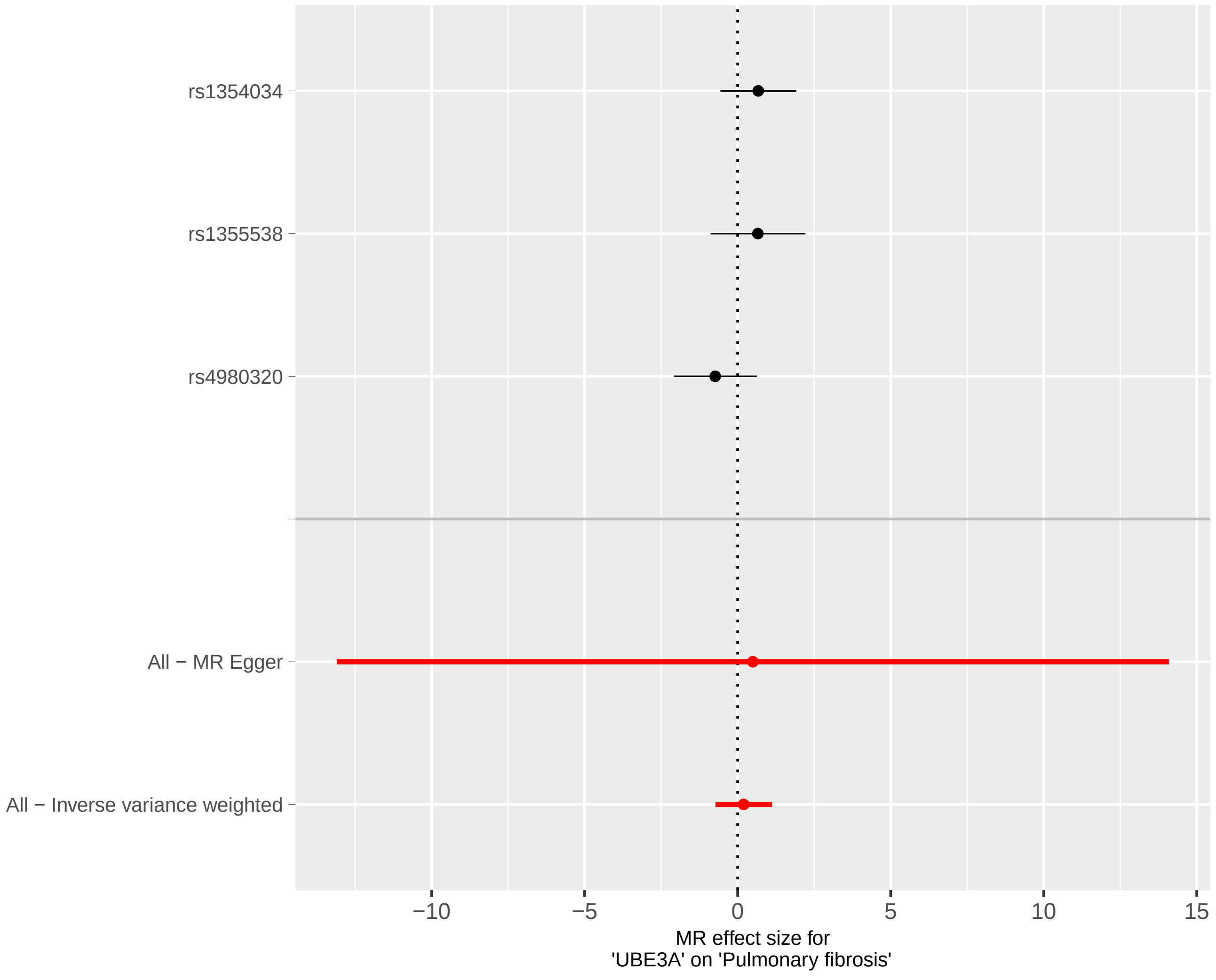

rs1354034
rs1355538
rs4980320
All − MR Egger
All − Inverse variance weighted
−10
−5
0
5
10
15
MR effect size for
'UBE3A' on 'Pulmonary fibrosis'

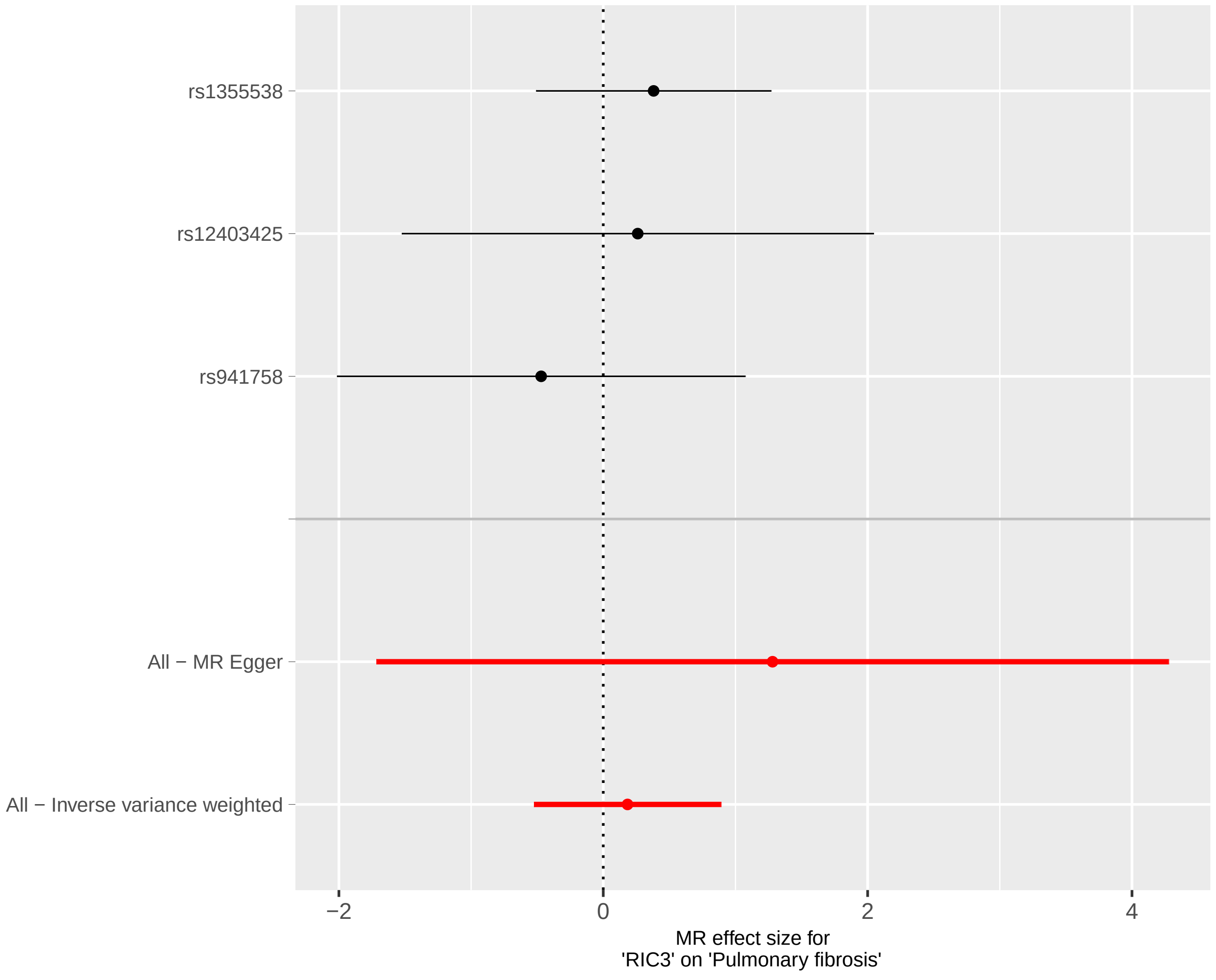
rs1355538
rs12403425
rs941758
All − MR Egger
All − Inverse variance weighted
−2
0
2
4
MR effect size for
'RIC3' on 'Pulmonary fibrosis'

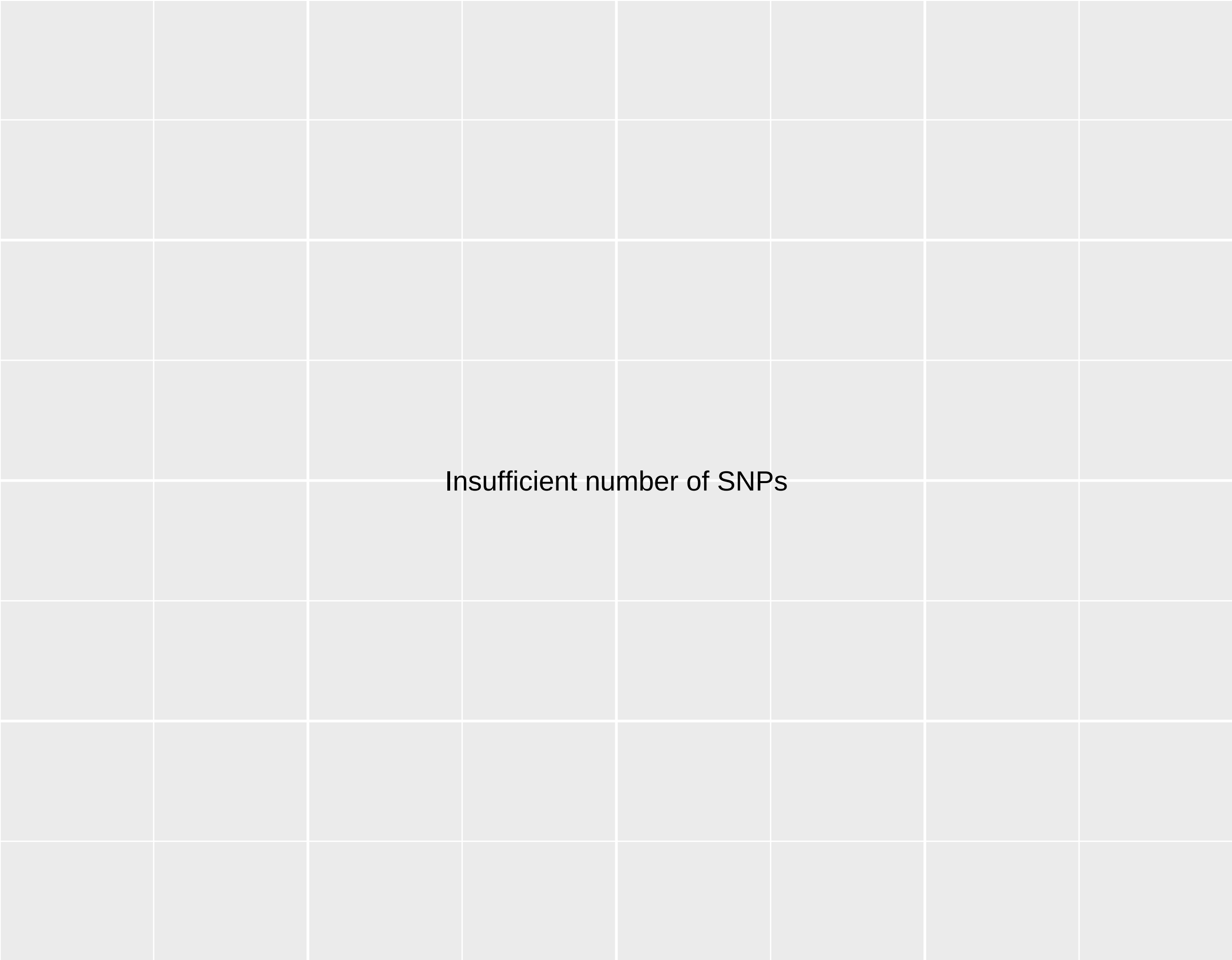
Insufficient number of SNPs

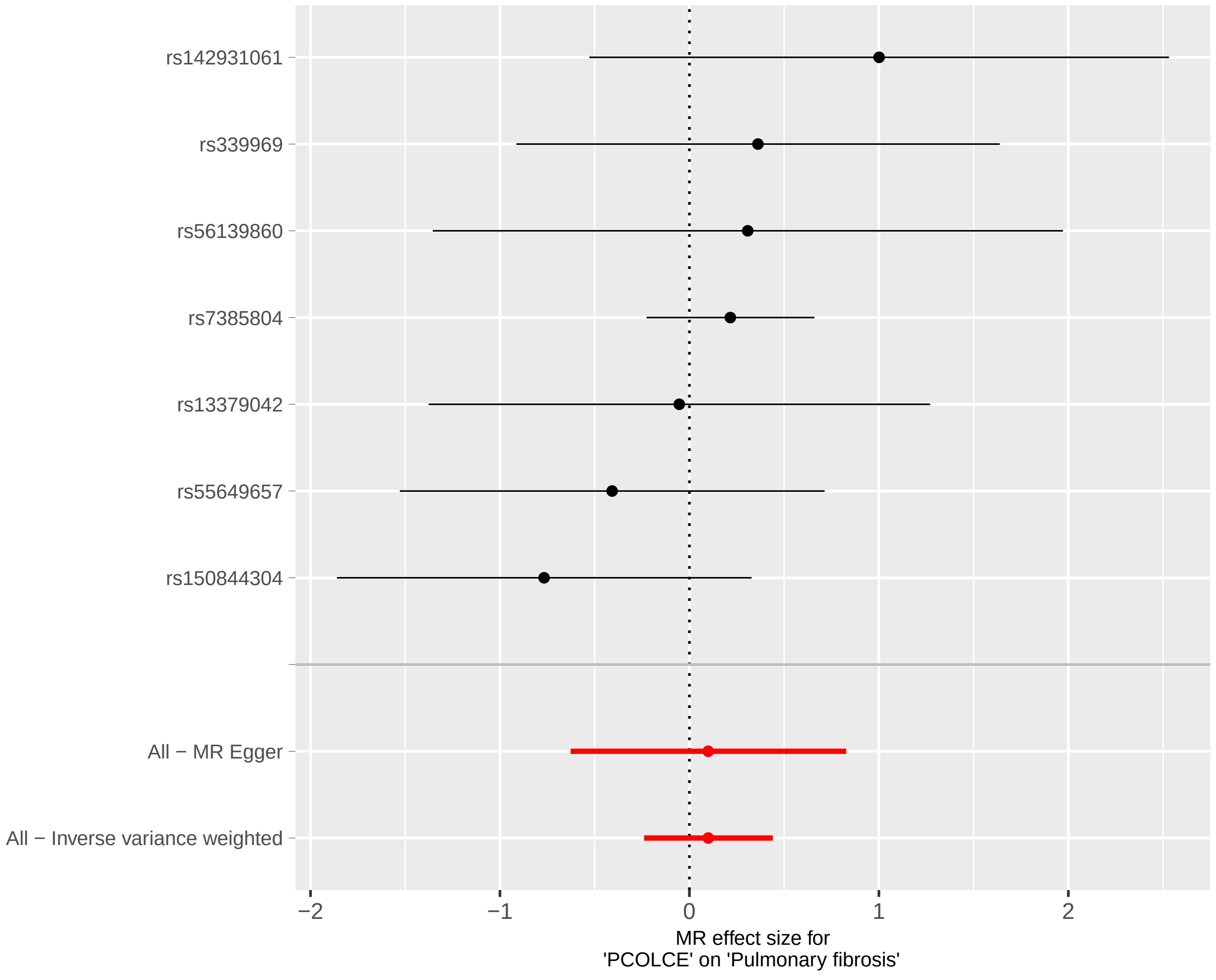
rs142931061
rs339969
rs56139860
rs7385804
rs13379042
rs55649657
rs150844304
All − MR Egger
All − Inverse variance weighted
−2
−1
0
1
2
MR effect size for
'PCOLCE' on 'Pulmonary fibrosis'

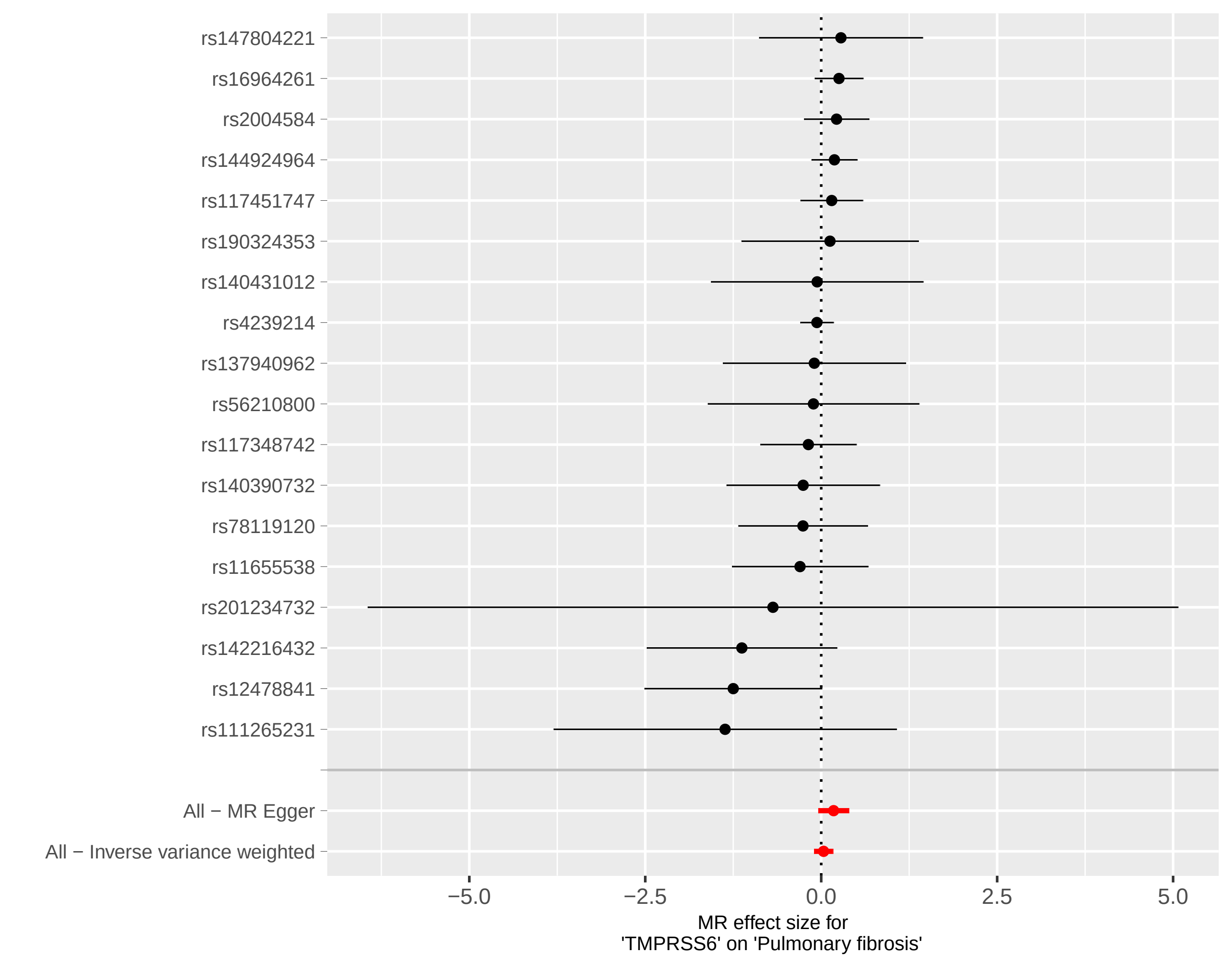

rs147804221
rs16964261
rs2004584
rs144924964
rs117451747
rs190324353
rs140431012
rs4239214
rs137940962
rs56210800
rs117348742
rs140390732
rs78119120
rs11655538
rs201234732
rs142216432
rs12478841
rs111265231
All − MR Egger
All − Inverse variance weighted
−5.0
−2.5
0.0
2.5
5.0
MR effect size for
'TMPRSS6' on 'Pulmonary fibrosis'

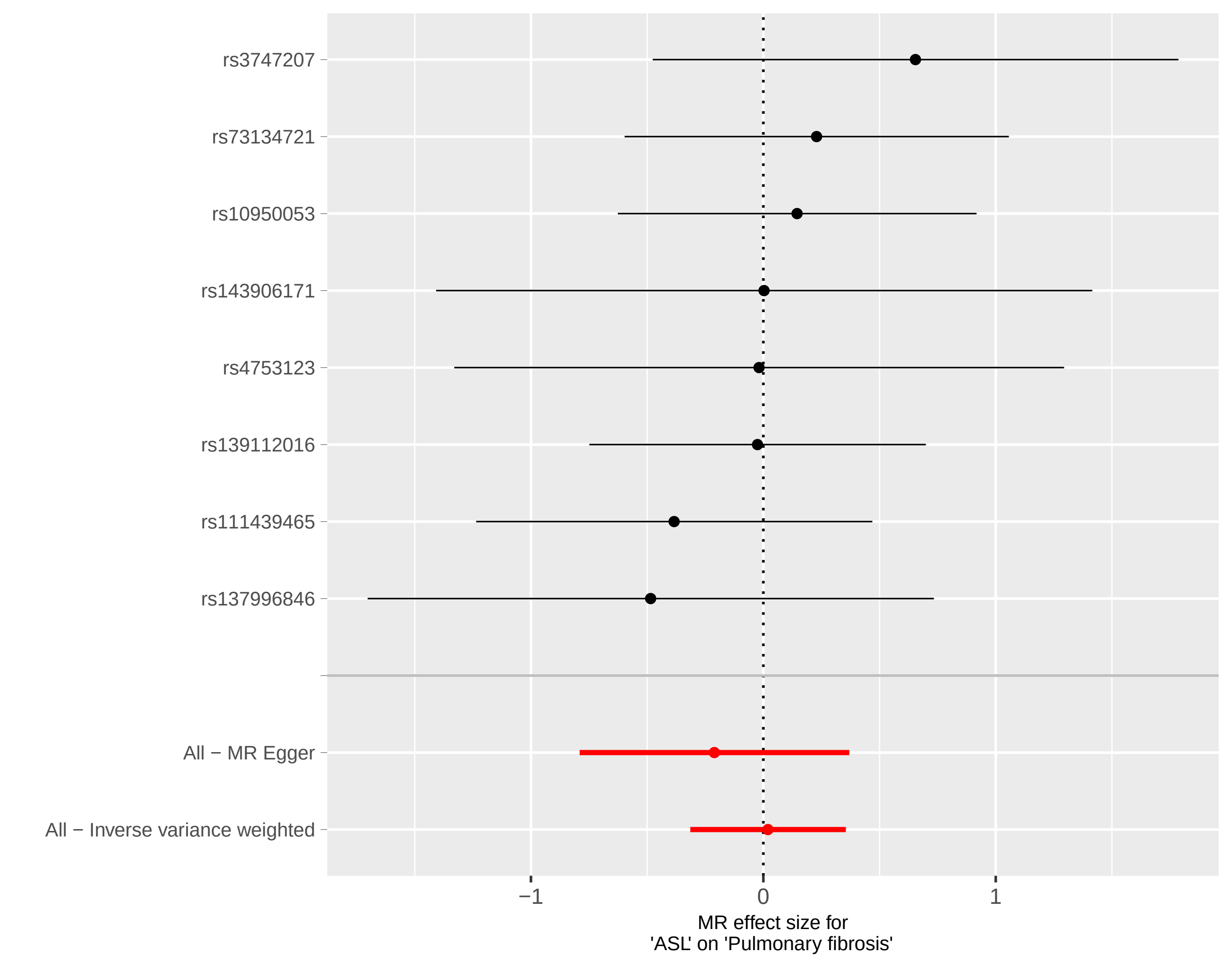

rs3747207
rs73134721
rs10950053
rs143906171
rs4753123
rs139112016
rs111439465
rs137996846
All − MR Egger
All − Inverse variance weighted
−1
0
1
MR effect size for
'ASL' on 'Pulmonary fibrosis'

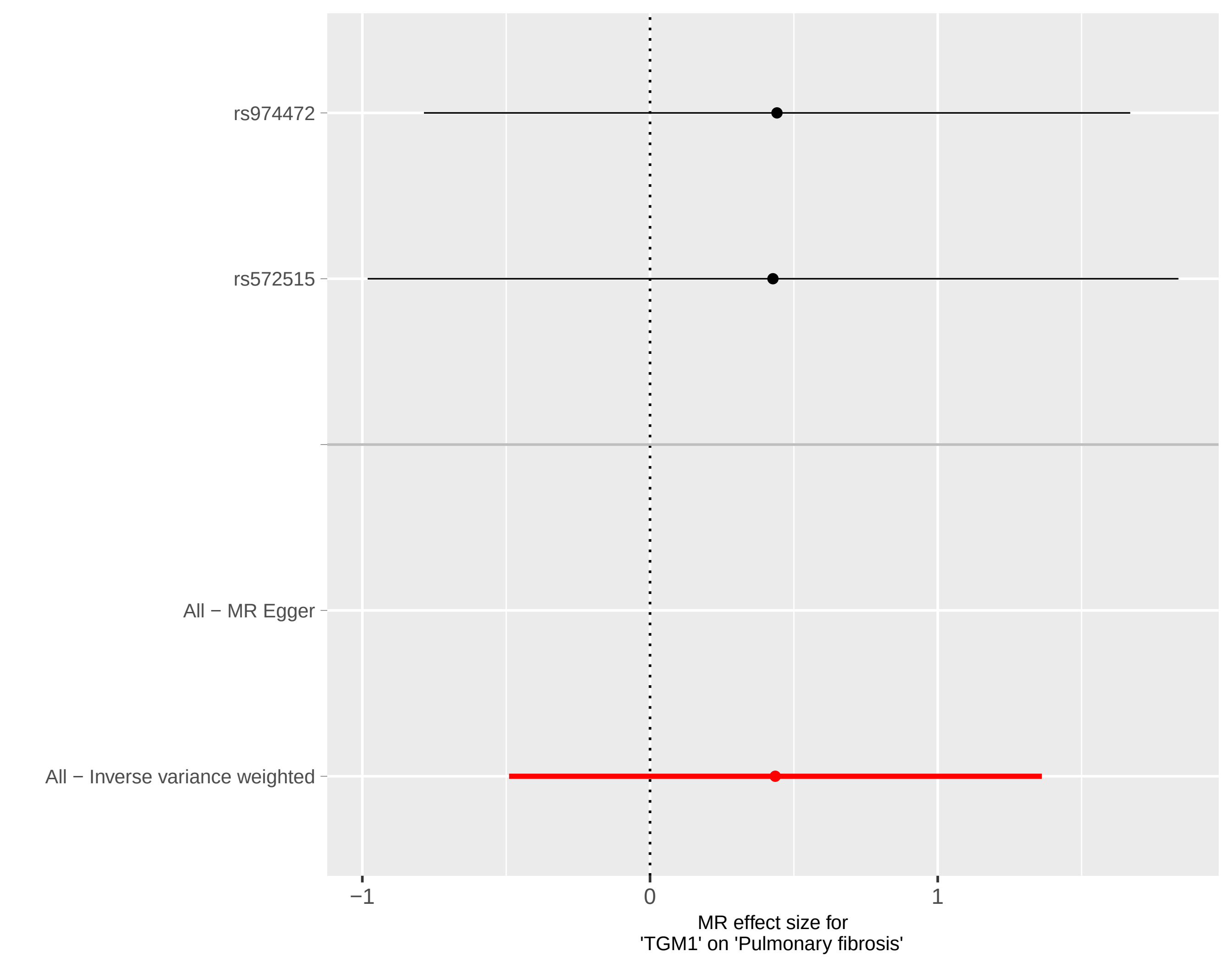

rs974472
rs572515
All − MR Egger
All − Inverse variance weighted
−1
0
1
MR effect size for
'TGM1' on 'Pulmonary fibrosis'

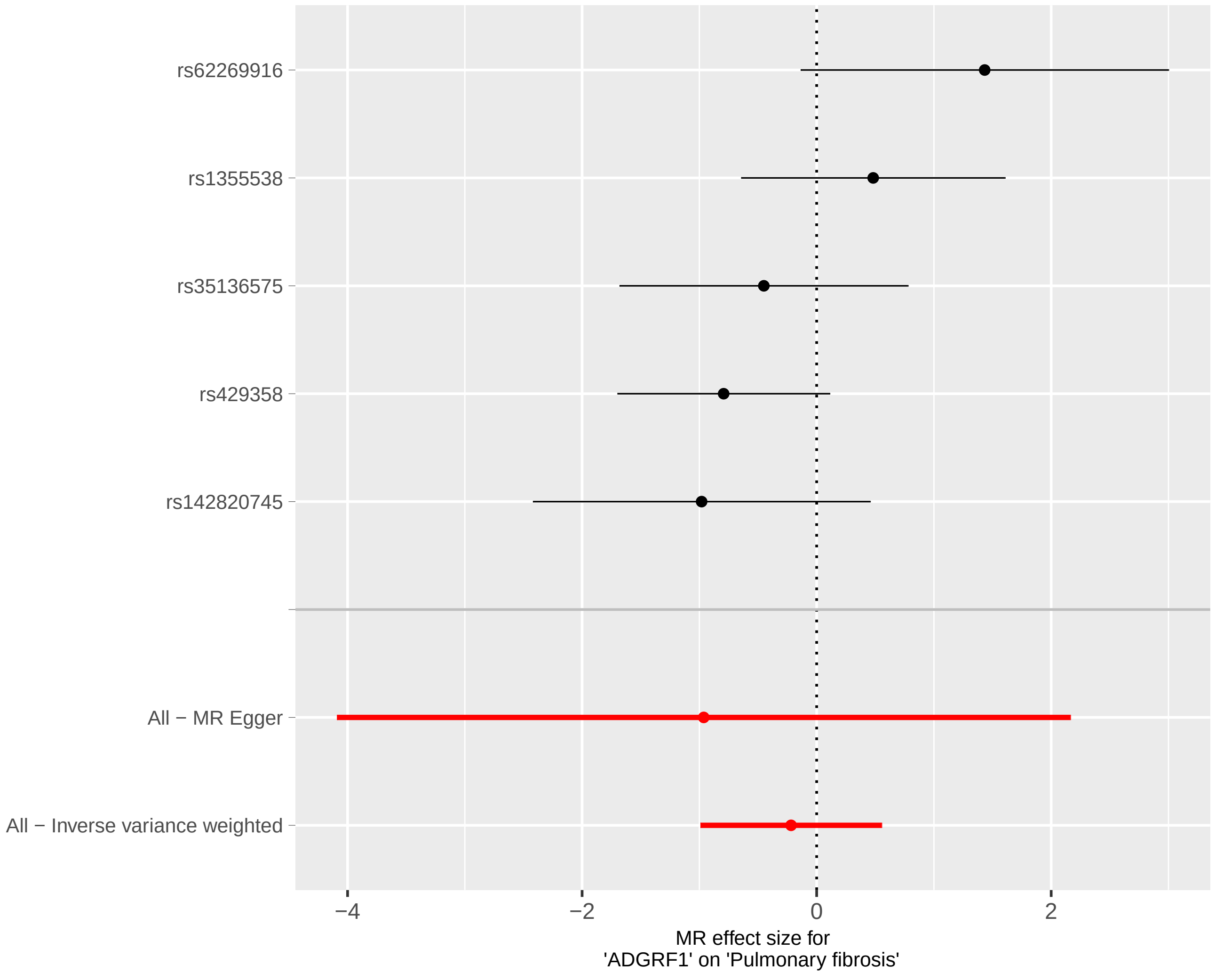
rs62269916
rs1355538
rs35136575
rs429358
rs142820745
All − MR Egger
All − Inverse variance weighted
−4
−2
0
2
MR effect size for
'ADGRF1' on 'Pulmonary fibrosis'

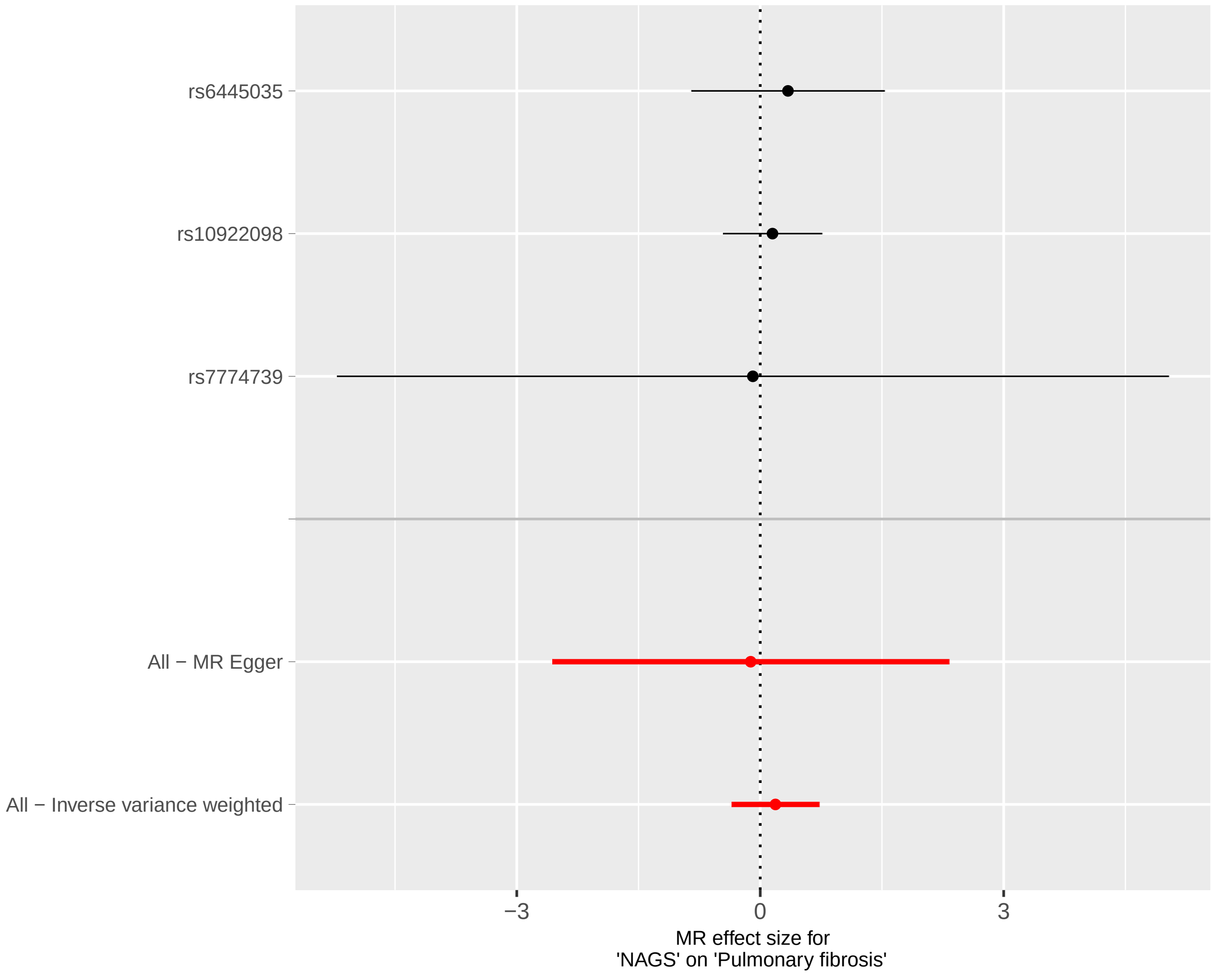
rs6445035
rs10922098
rs7774739
All − MR Egger
All − Inverse variance weighted
−3
0
3
MR effect size for
'NAGS' on 'Pulmonary fibrosis'

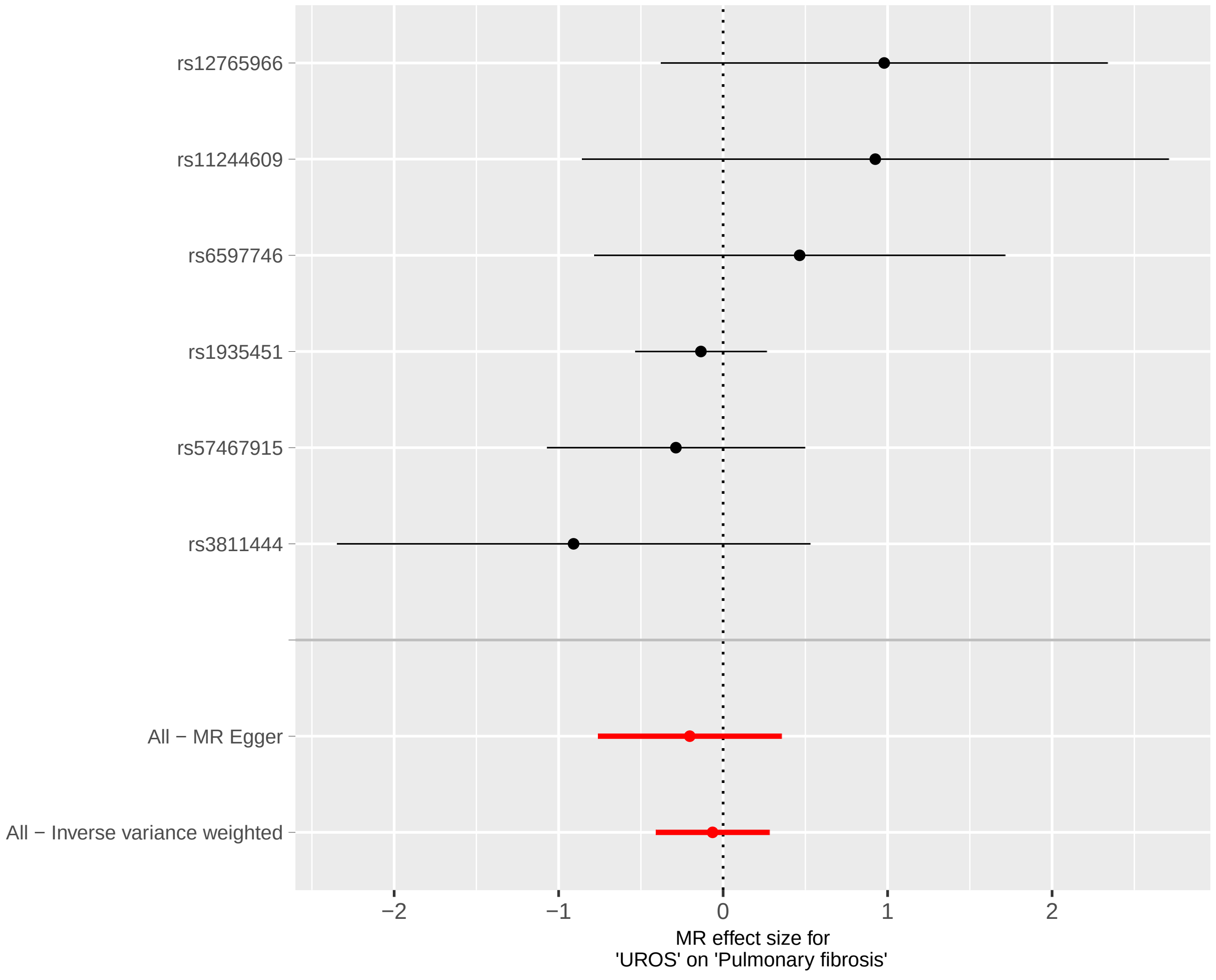
rs12765966
rs11244609
rs6597746
rs1935451
rs57467915
rs3811444
All − MR Egger
All − Inverse variance weighted
−2
−1
0
1
2
MR effect size for
'UROS' on 'Pulmonary fibrosis'

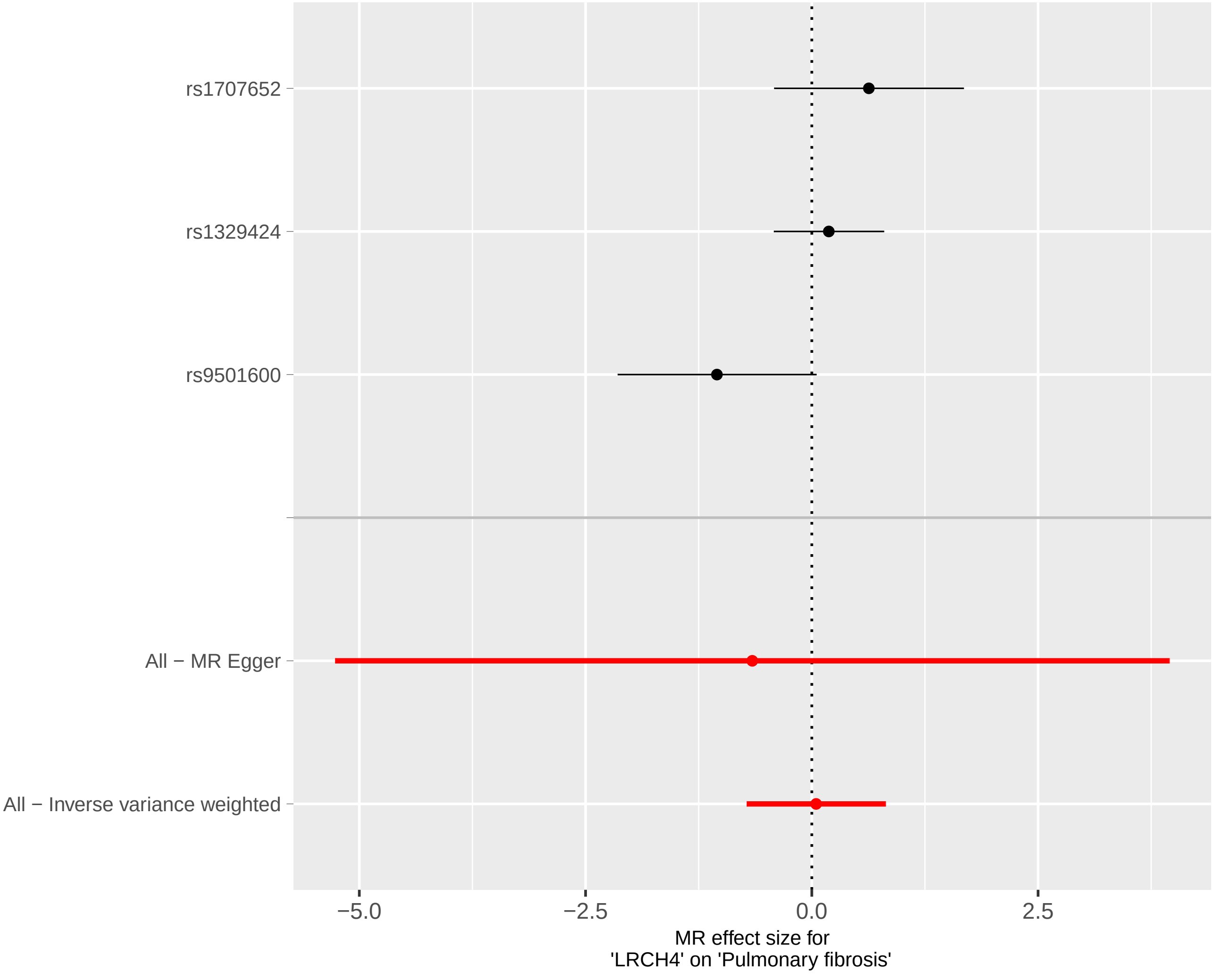
rs1707652
rs1329424
rs9501600
All − MR Egger
All − Inverse variance weighted
−5.0
−2.5
0.0
2.5
MR effect size for
'LRCH4' on 'Pulmonary fibrosis'

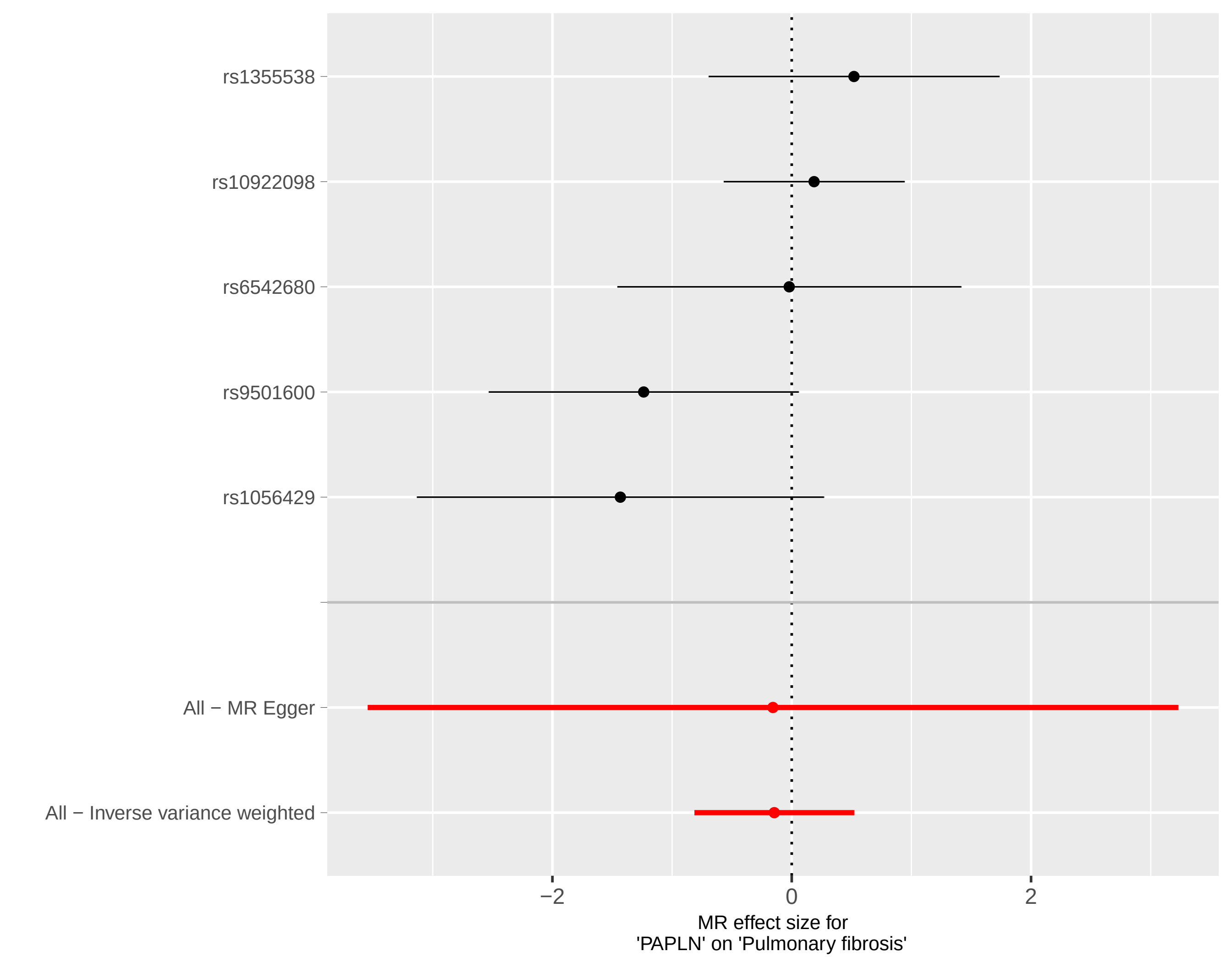
rs1355538
rs10922098
rs6542680
rs9501600
rs1056429
All − MR Egger
All − Inverse variance weighted
−2
0
2
MR effect size for
'PAPLN' on 'Pulmonary fibrosis'

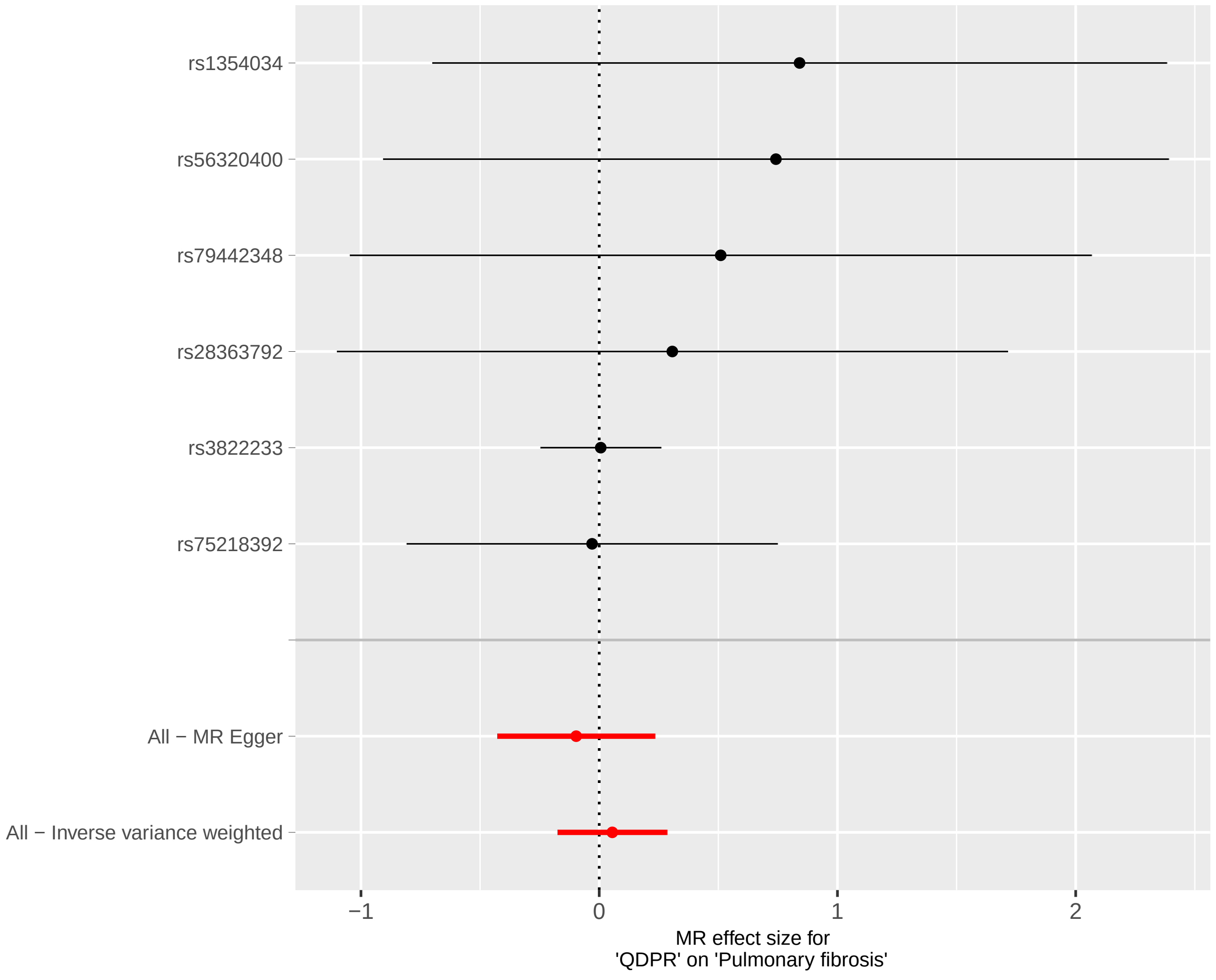

rs1354034
rs56320400
rs79442348
rs28363792
rs3822233
rs75218392
All − MR Egger
All − Inverse variance weighted
−1
0
1
2
MR effect size for
'QDPR' on 'Pulmonary fibrosis'

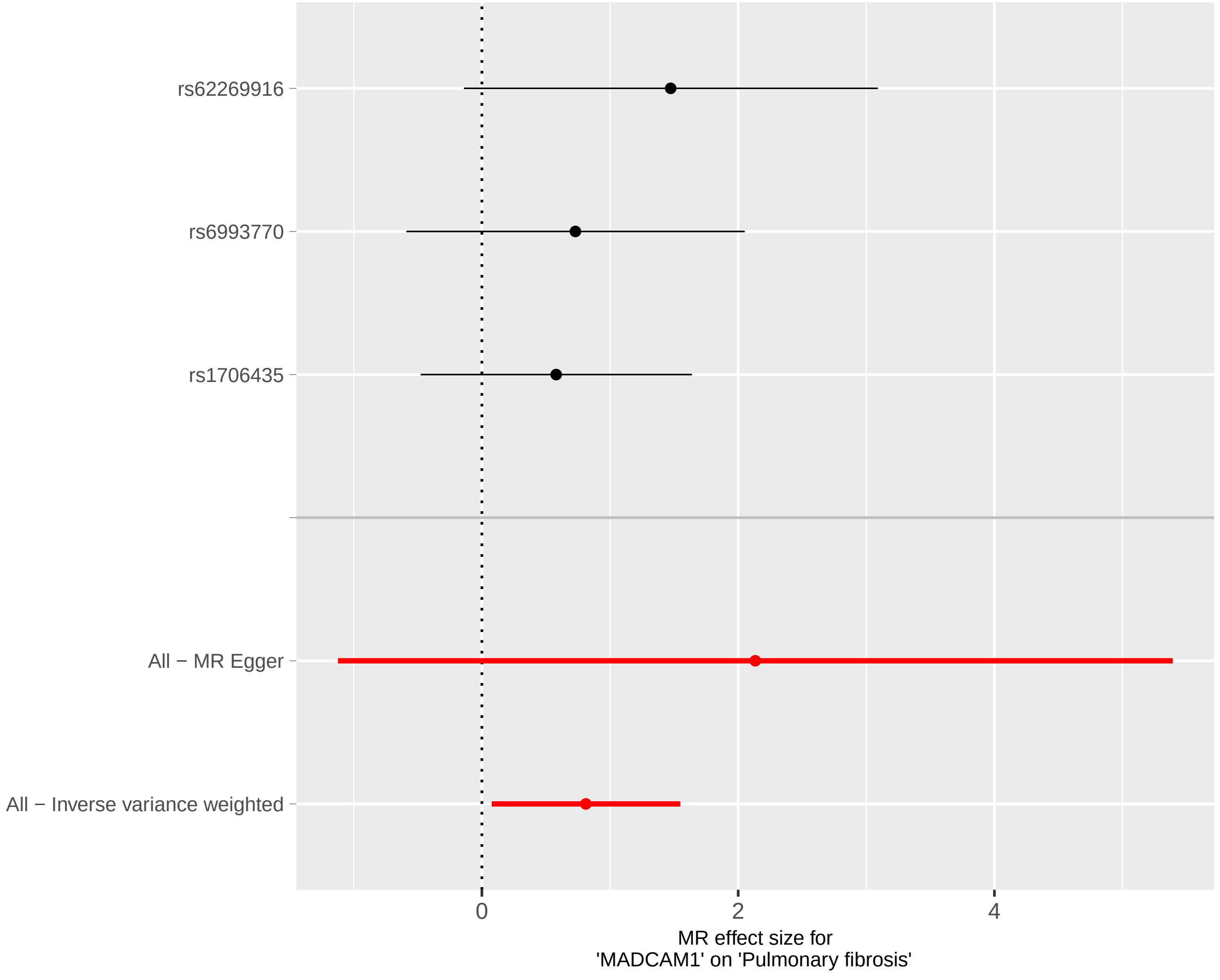

rs62269916
rs6993770
rs1706435
All − MR Egger
All − Inverse variance weighted
0
2
4
MR effect size for
'MADCAM1' on 'Pulmonary fibrosis'

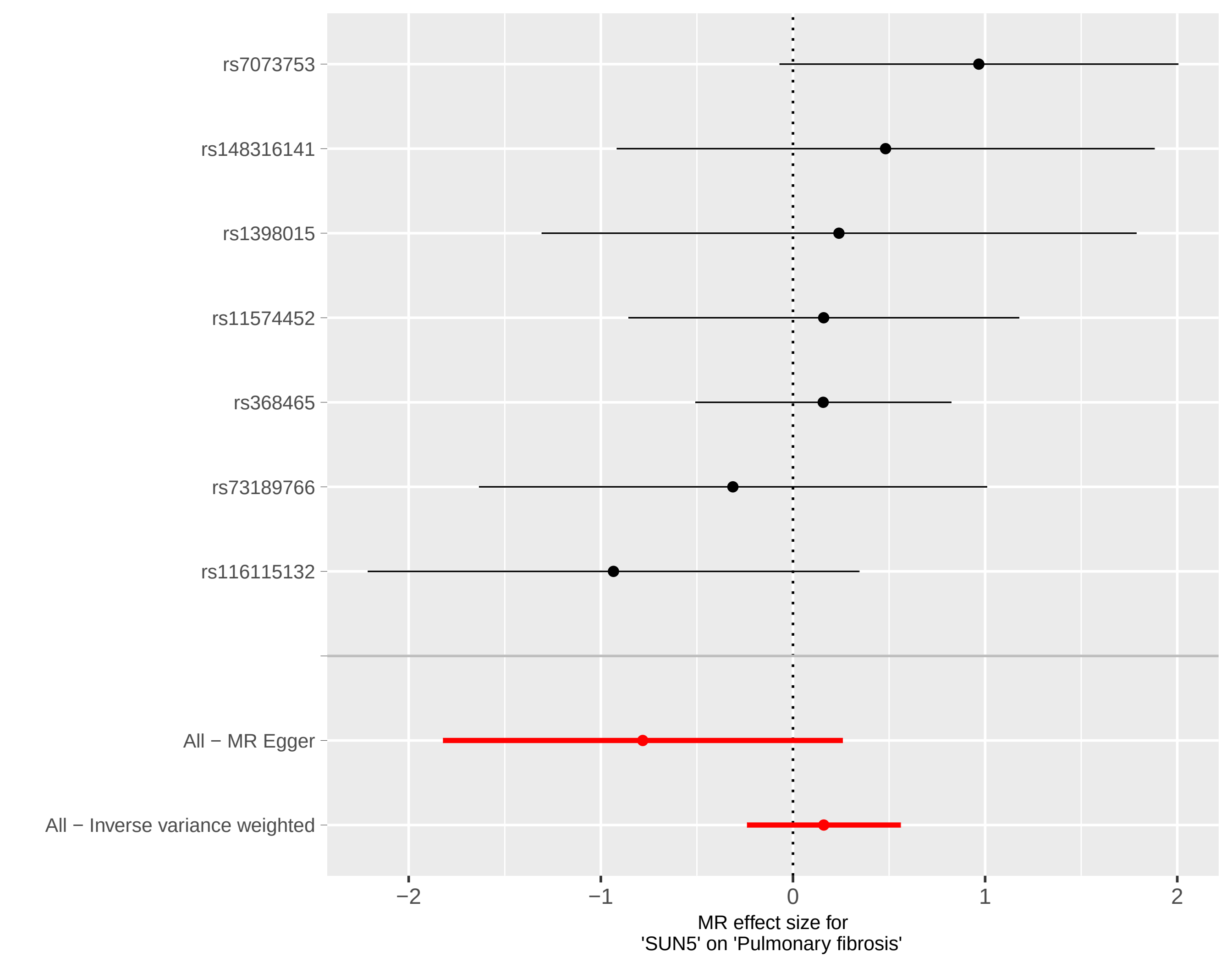
rs7073753
rs148316141
rs1398015
rs11574452
rs368465
rs73189766
rs116115132
All − MR Egger
All − Inverse variance weighted
−2
−1
0
1
2
MR effect size for
'SUN5' on 'Pulmonary fibrosis'

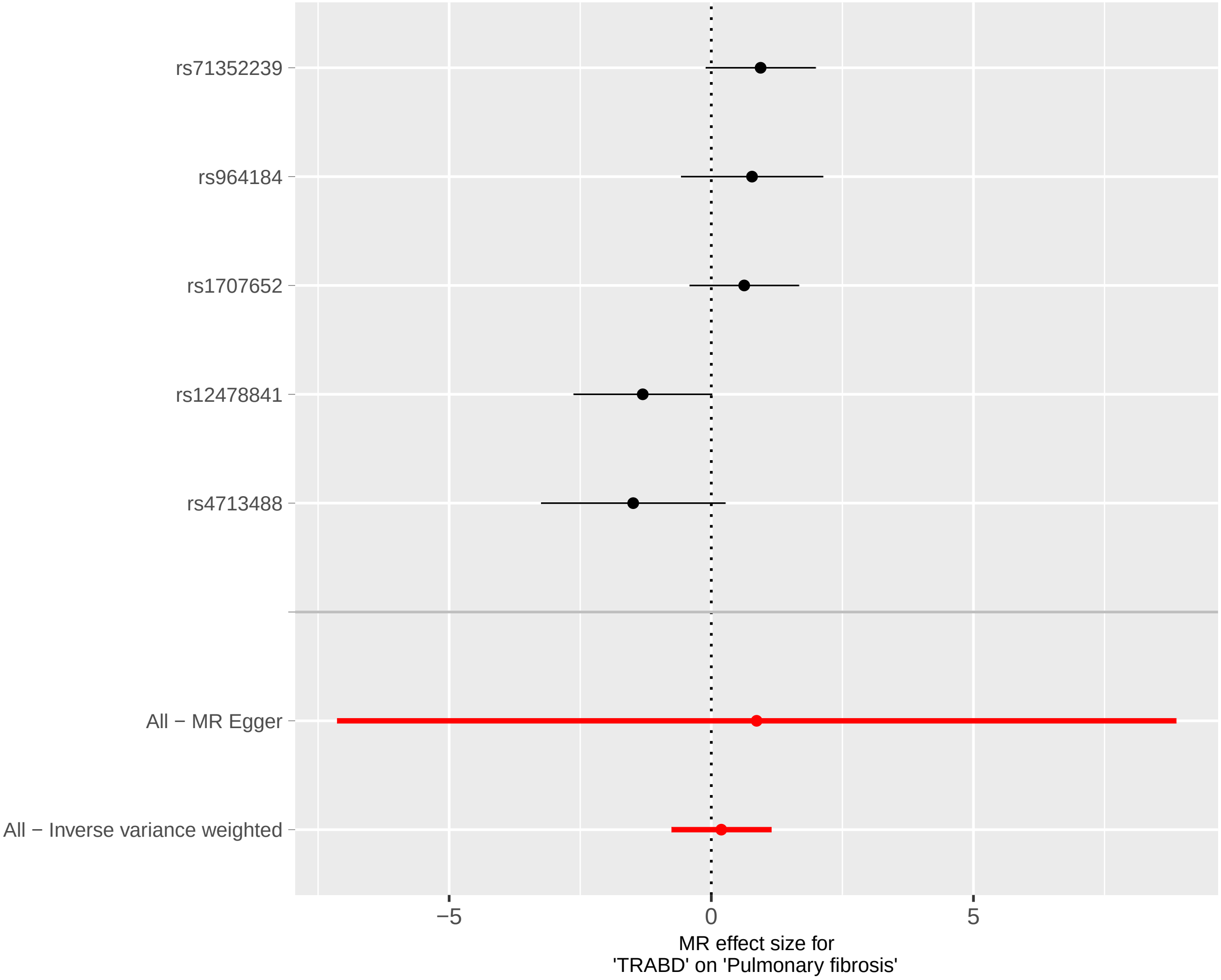

rs71352239
rs964184
rs1707652
rs12478841
rs4713488
All − MR Egger
All − Inverse variance weighted
−5
0
5
MR effect size for
'TRABD' on 'Pulmonary fibrosis'

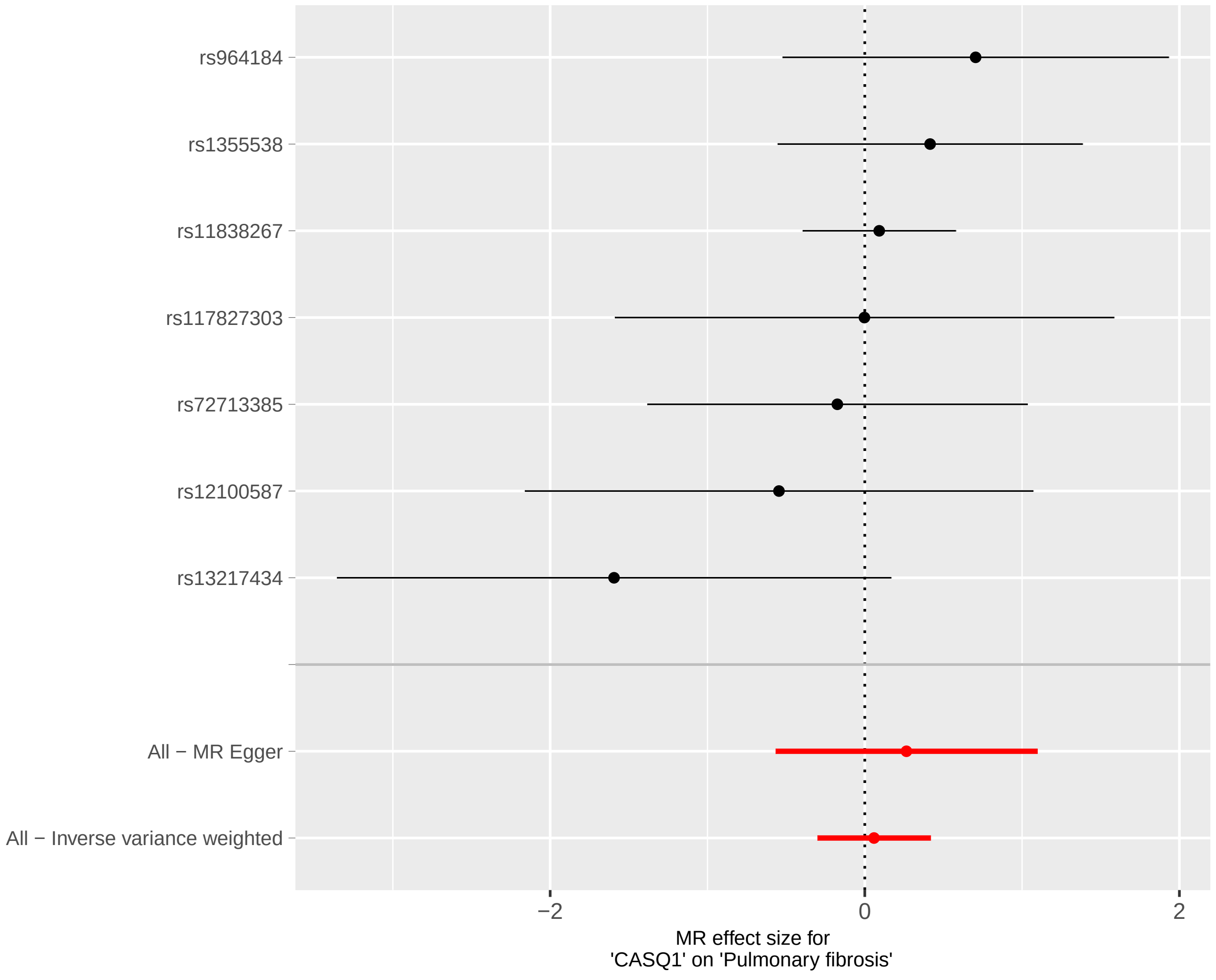
rs964184
rs1355538
rs11838267
rs117827303
rs72713385
rs12100587
rs13217434
All − MR Egger
All − Inverse variance weighted
−2
0
2
MR effect size for
'CASQ1' on 'Pulmonary fibrosis'

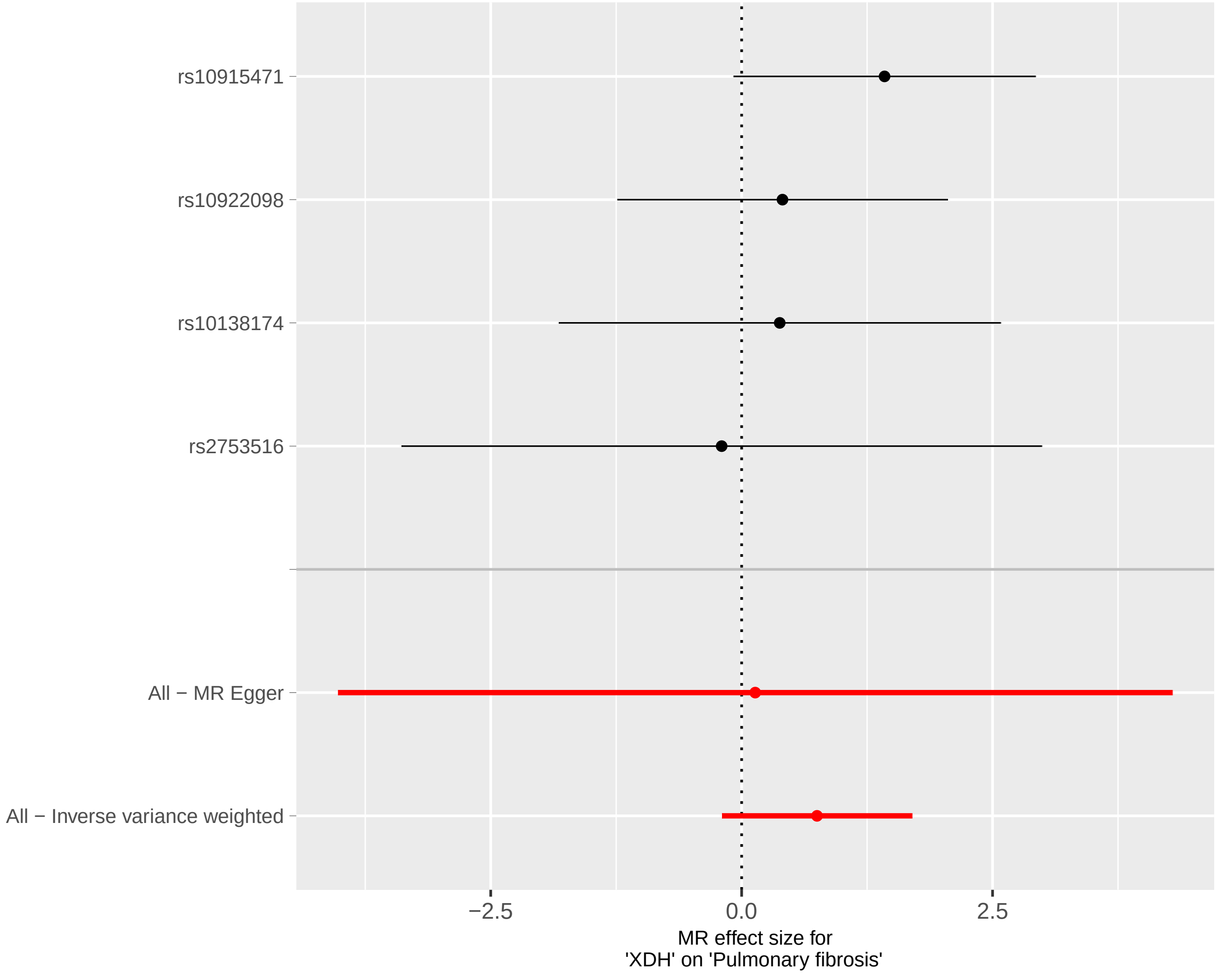
rs10915471
rs10922098
rs10138174
rs2753516
All − MR Egger
All − Inverse variance weighted
−2.5
0.0
2.5
MR effect size for
'XDH' on 'Pulmonary fibrosis'

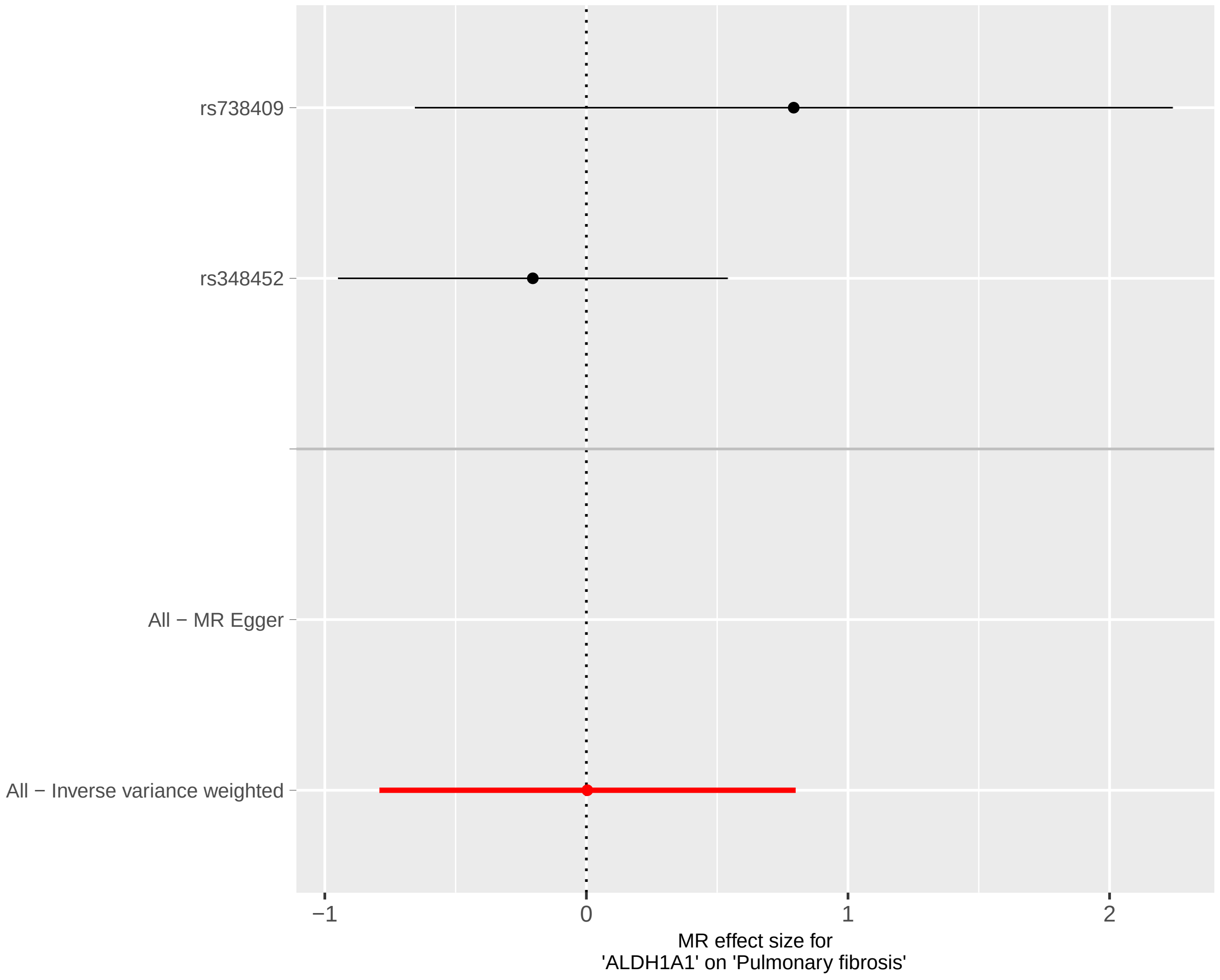
rs738409
rs348452
All − MR Egger
All − Inverse variance weighted
−1
0
1
2
MR effect size for
'ALDH1A1' on 'Pulmonary fibrosis'

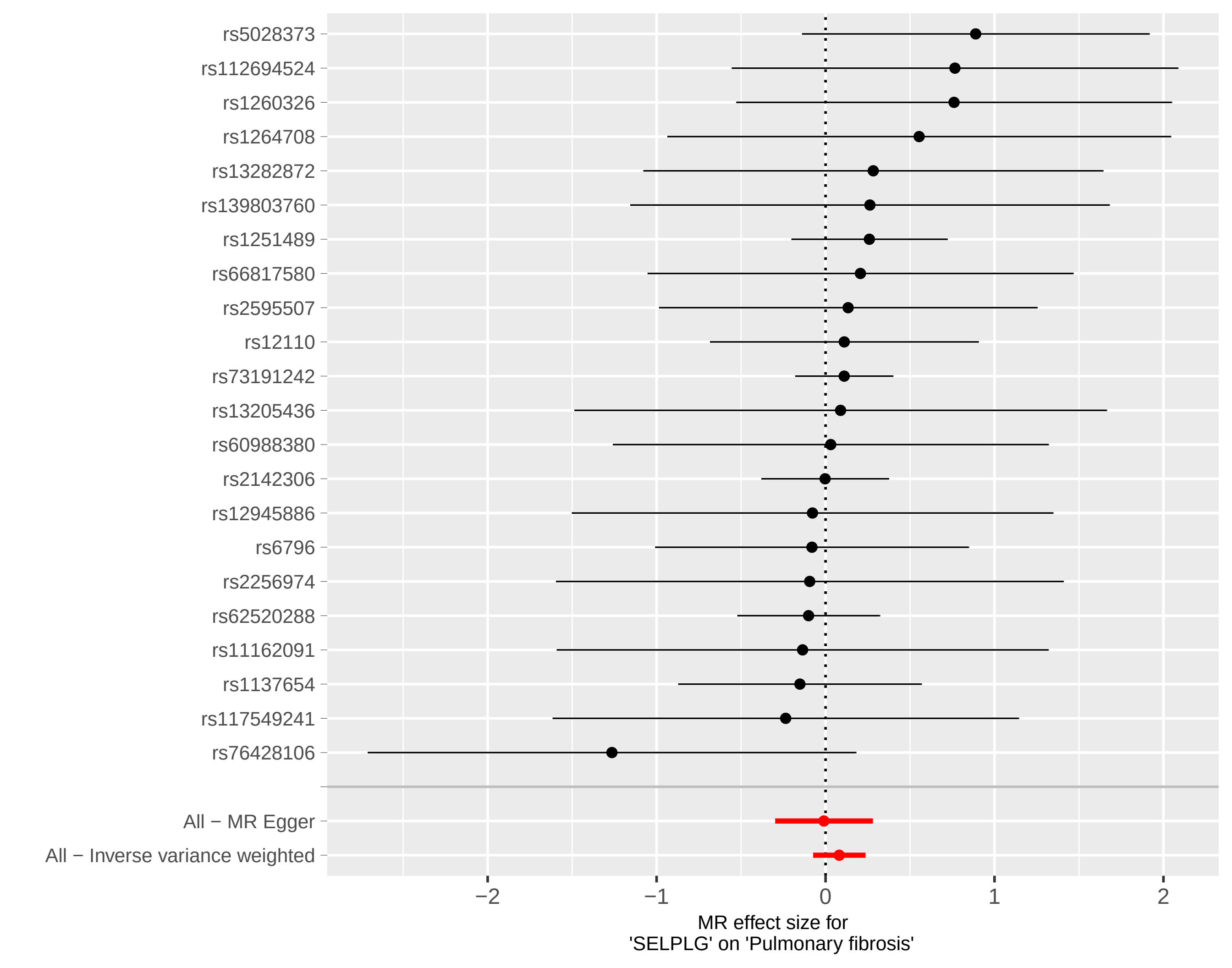
rs5028373
rs112694524
rs1260326
rs1264708
rs13282872
rs139803760
rs1251489
rs66817580
rs2595507
rs12110
rs73191242
rs13205436
rs60988380
rs2142306
rs12945886
rs6796
rs2256974
rs62520288
rs11162091
rs1137654
rs117549241
rs76428106
All − MR Egger
All − Inverse variance weighted
−2
−1
0
1
2
MR effect size for
'SELPLG' on 'Pulmonary fibrosis'

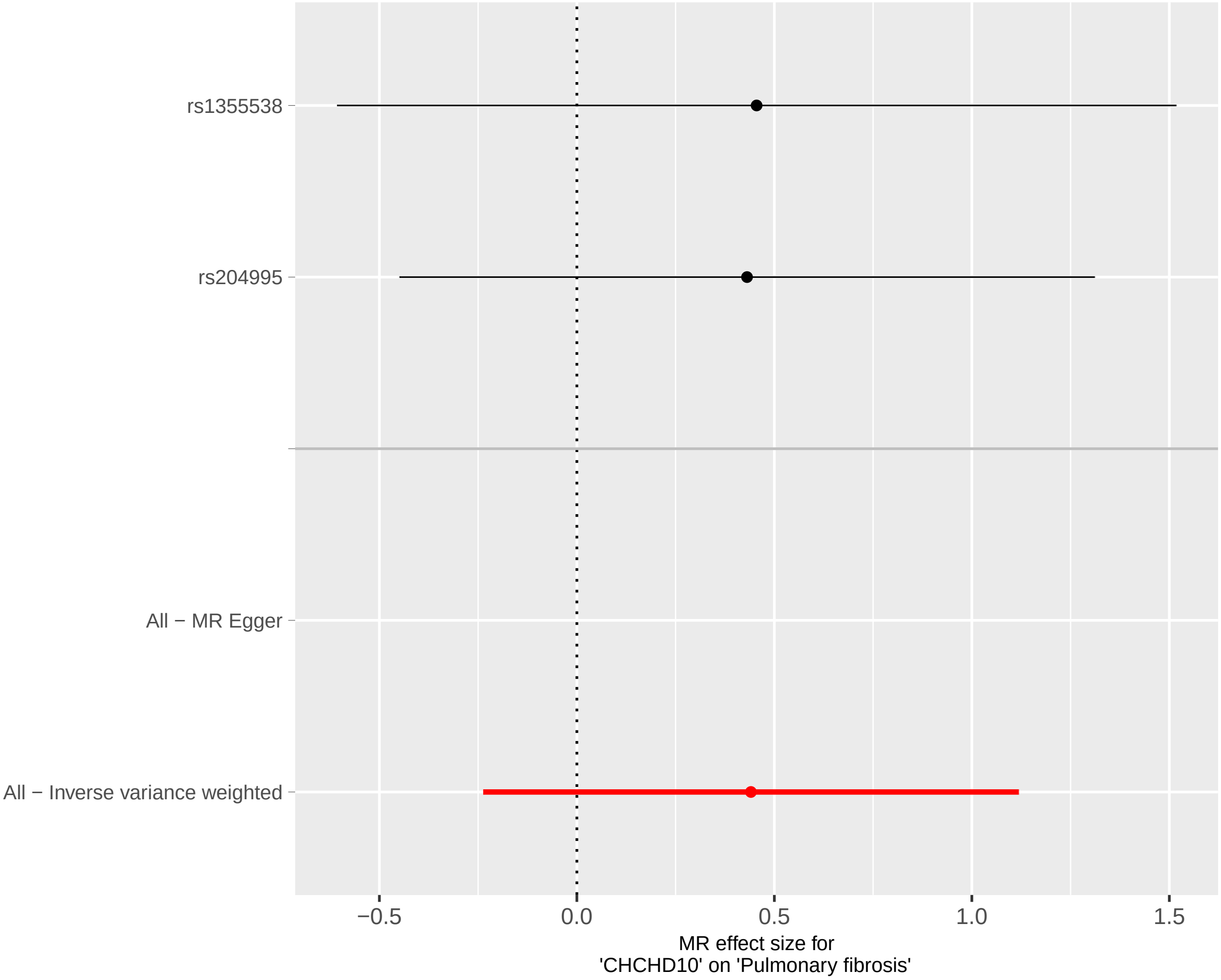
rs1355538
rs204995
All − MR Egger
All − Inverse variance weighted
−0.5
0.0
0.5
1.0
1.5
MR effect size for
'CHCHD10' on 'Pulmonary fibrosis'

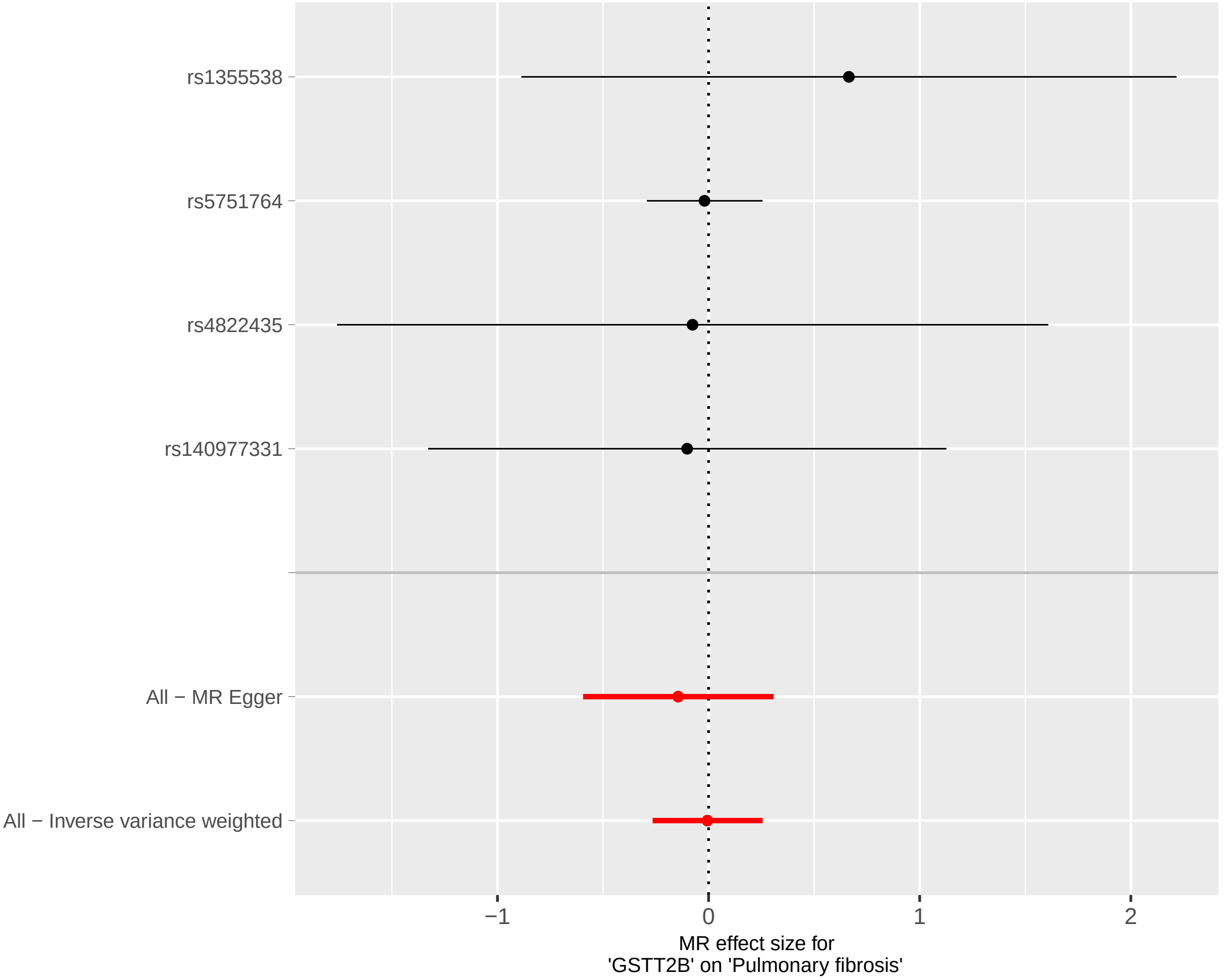
rs1355538
rs5751764
rs4822435
rs140977331
All − MR Egger
All − Inverse variance weighted
−1
0
1
2
MR effect size for
'GSTT2B' on 'Pulmonary fibrosis'

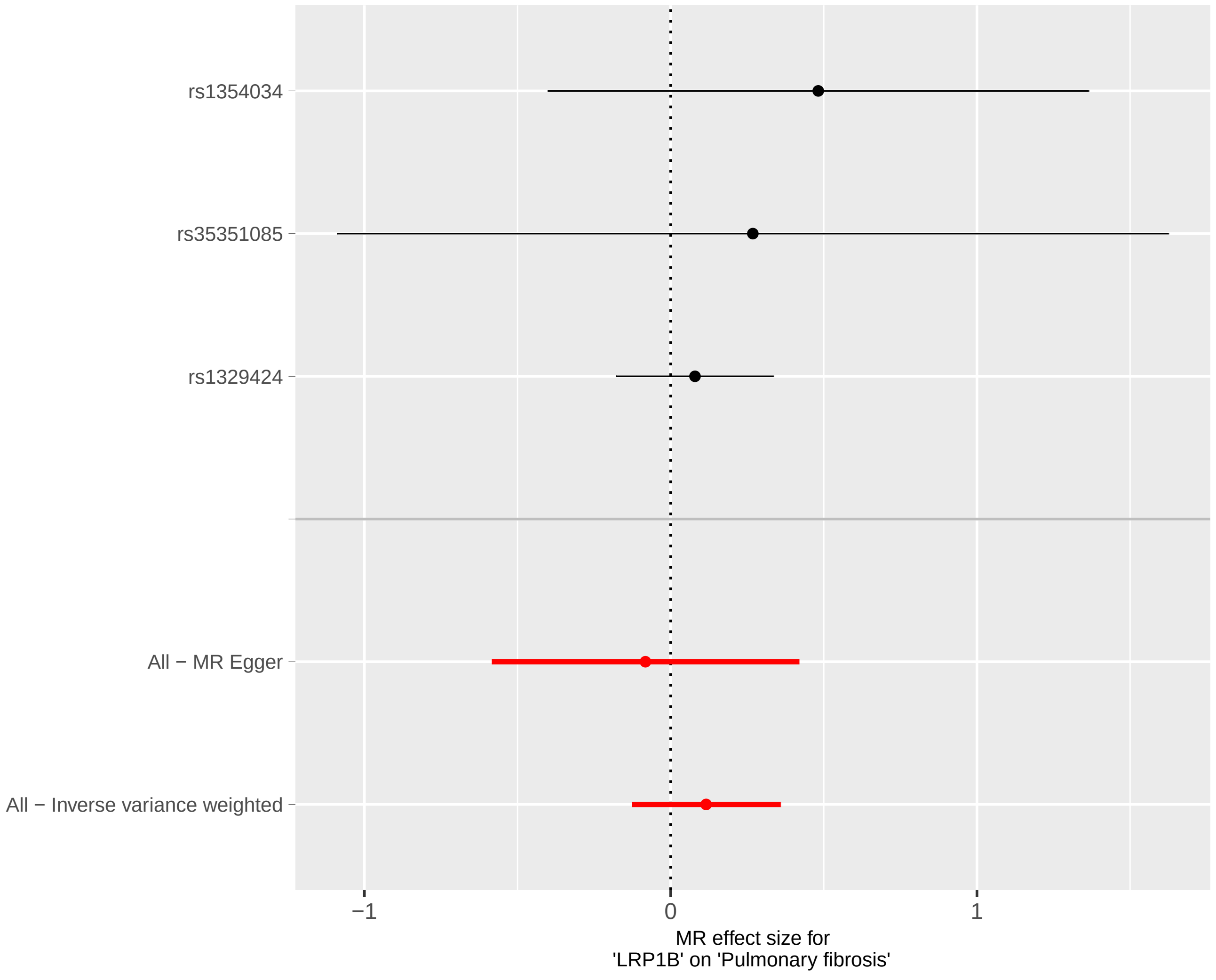

rs1354034
rs35351085
rs1329424
All − MR Egger
All − Inverse variance weighted
−1
0
1
MR effect size for
'LRP1B' on 'Pulmonary fibrosis'

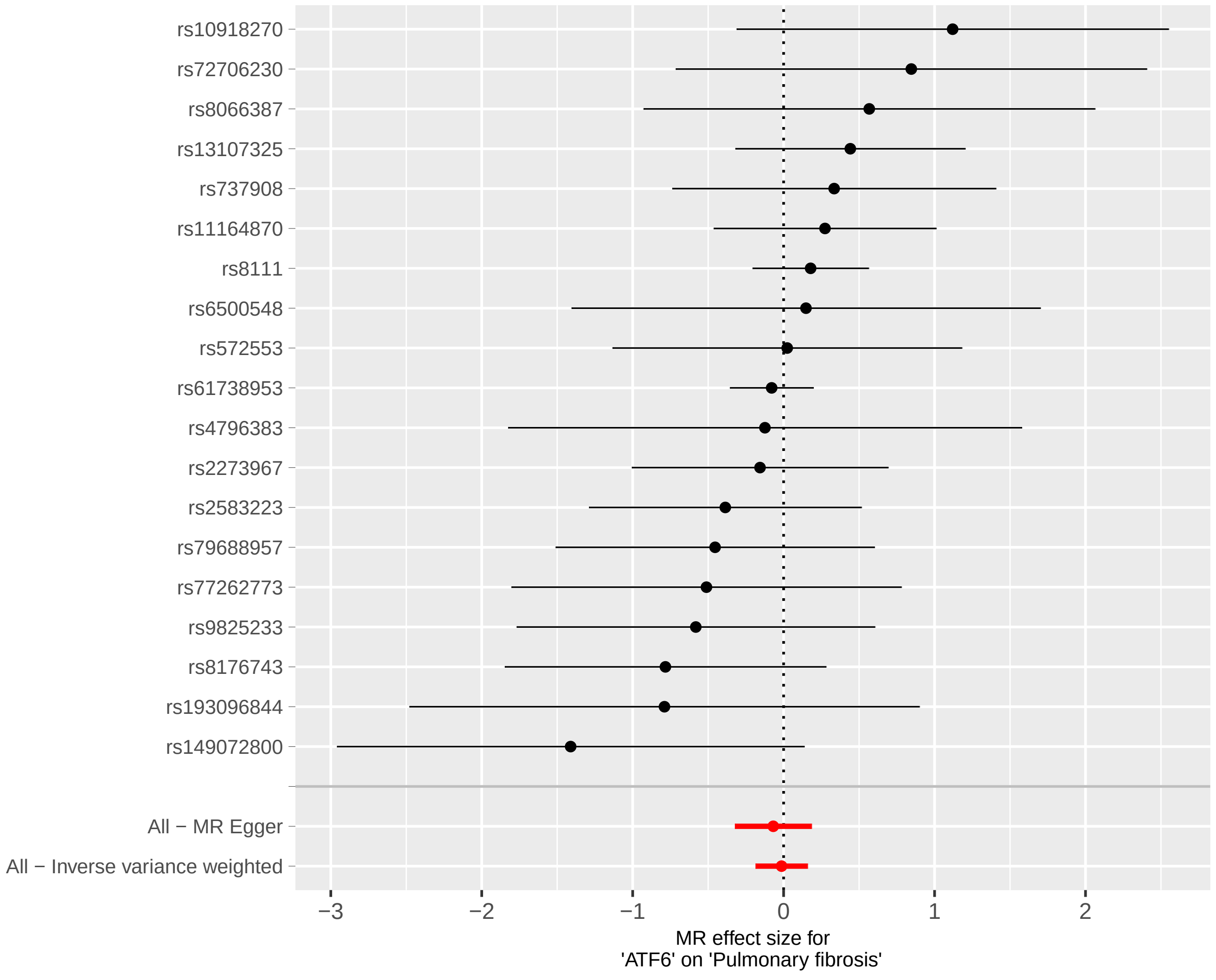

rs10918270
rs72706230
rs8066387
rs13107325
rs737908
rs11164870
rs8111
rs6500548
rs572553
rs61738953
rs4796383
rs2273967
rs2583223
rs79688957
rs77262773
rs9825233
rs8176743
rs193096844
rs149072800
All − MR Egger
All − Inverse variance weighted
−3
−2
−1
0
1
2
MR effect size for
'ATF6' on 'Pulmonary fibrosis'

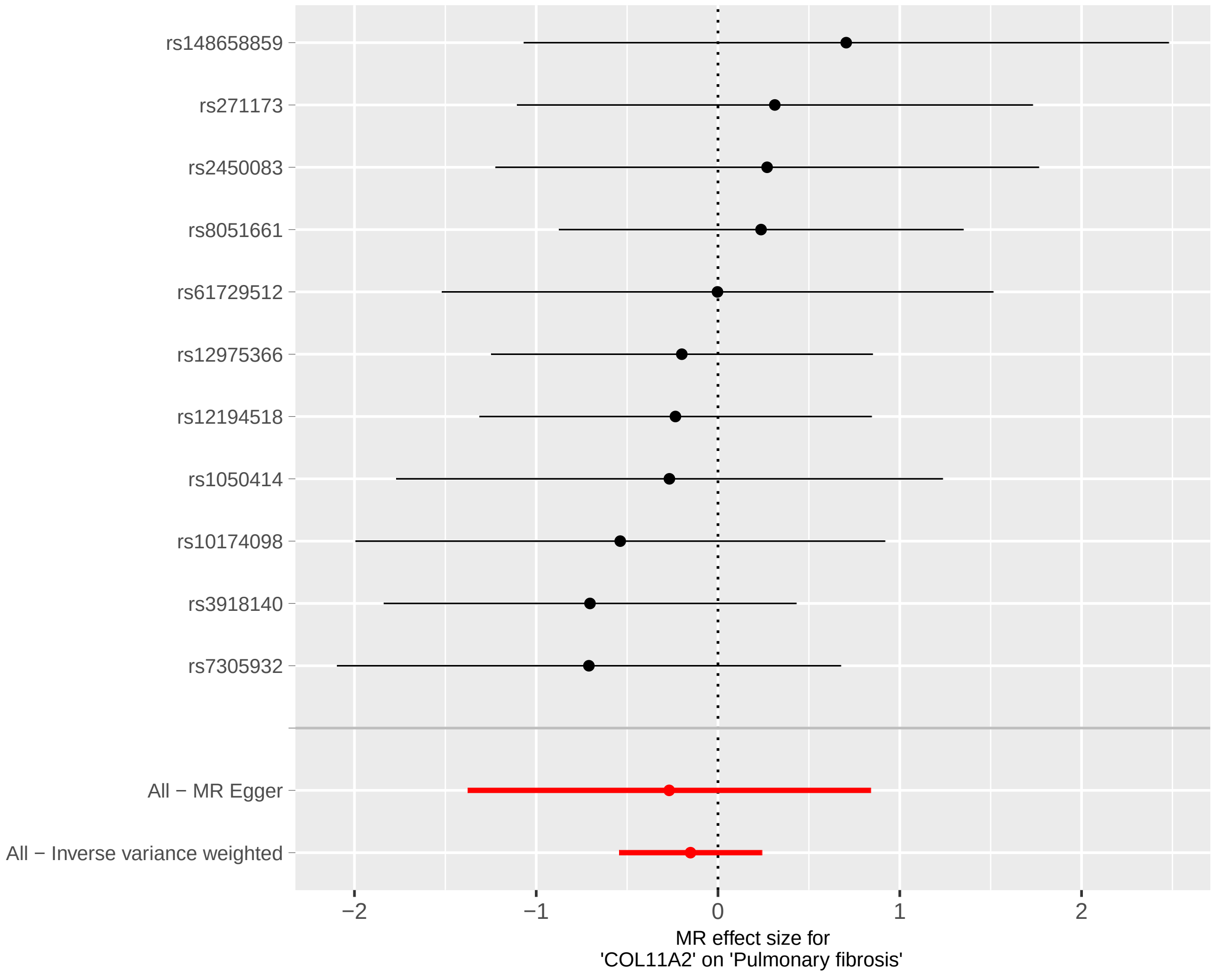
rs148658859
rs271173
rs2450083
rs8051661
rs61729512
rs12975366
rs12194518
rs1050414
rs10174098
rs3918140
rs7305932
All − MR Egger
All − Inverse variance weighted
−2
−1
0
1
2
MR effect size for
'COL11A2' on 'Pulmonary fibrosis'

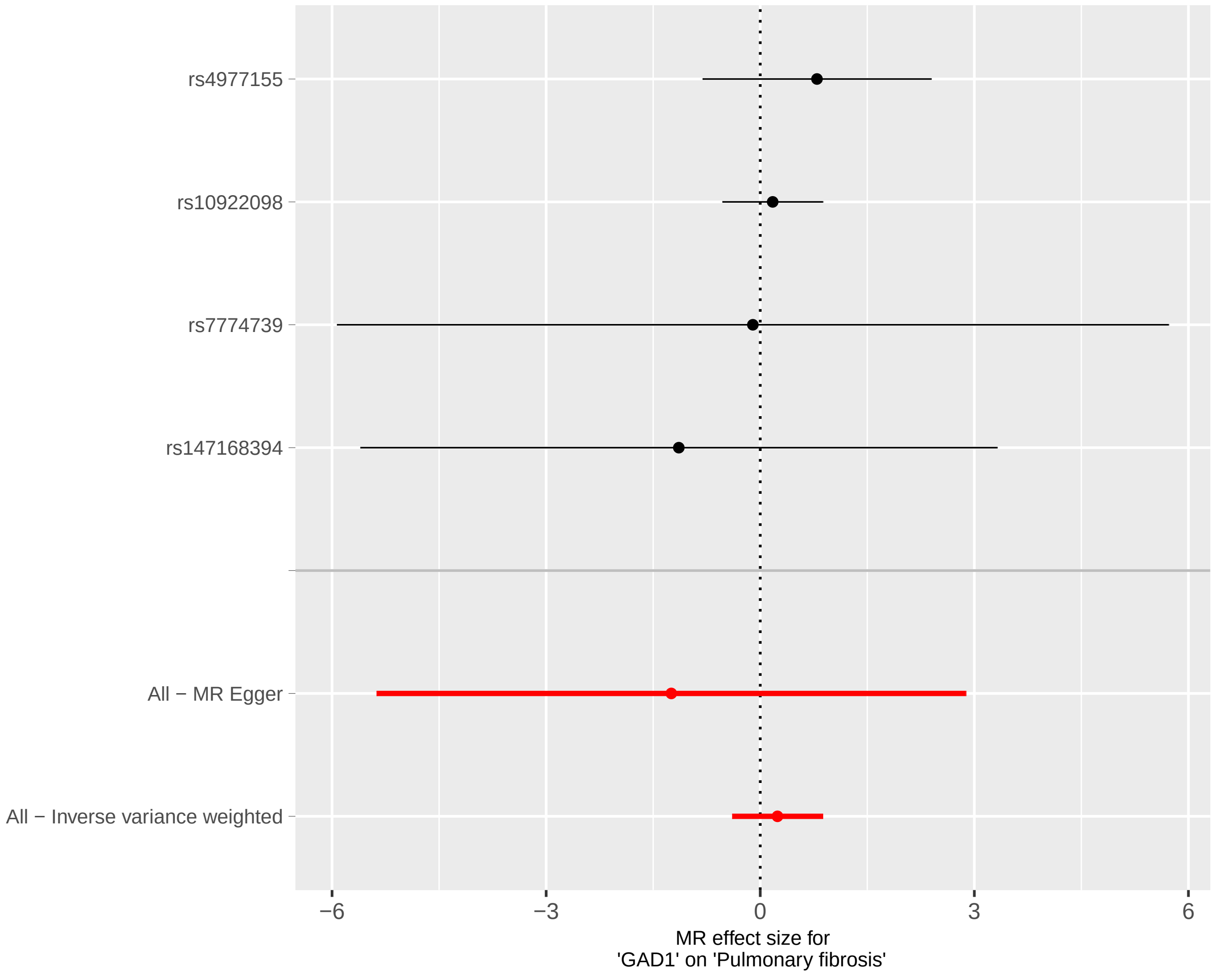
rs4977155
rs10922098
rs7774739
rs147168394
All − MR Egger
All − Inverse variance weighted
−6
−3
0
3
6
MR effect size for
'GAD1' on 'Pulmonary fibrosis'

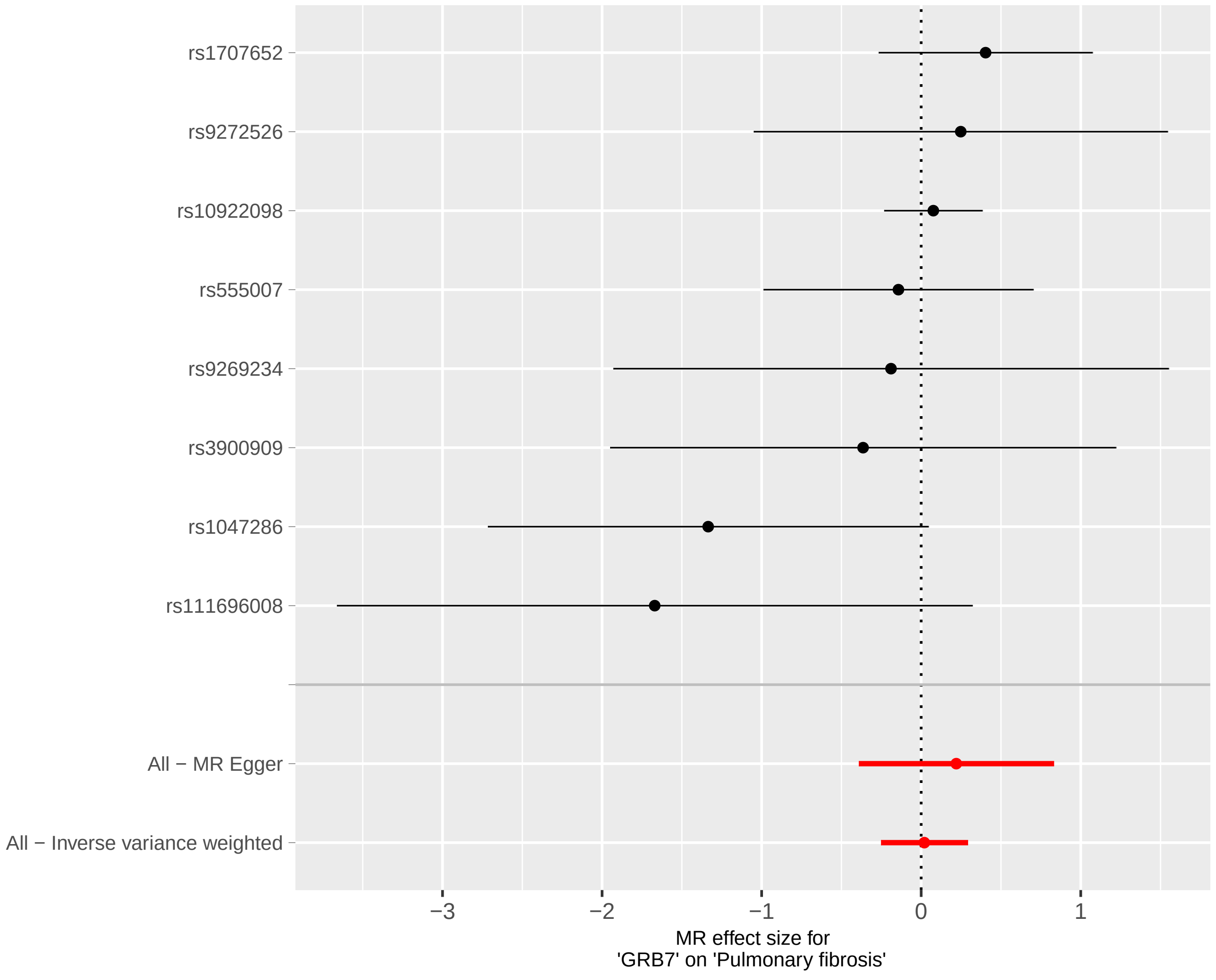
rs1707652
rs9272526
rs10922098
rs555007
rs9269234
rs3900909
rs1047286
rs111696008
All − MR Egger
All − Inverse variance weighted
−3
−2
−1
0
1
MR effect size for
'GRB7' on 'Pulmonary fibrosis'

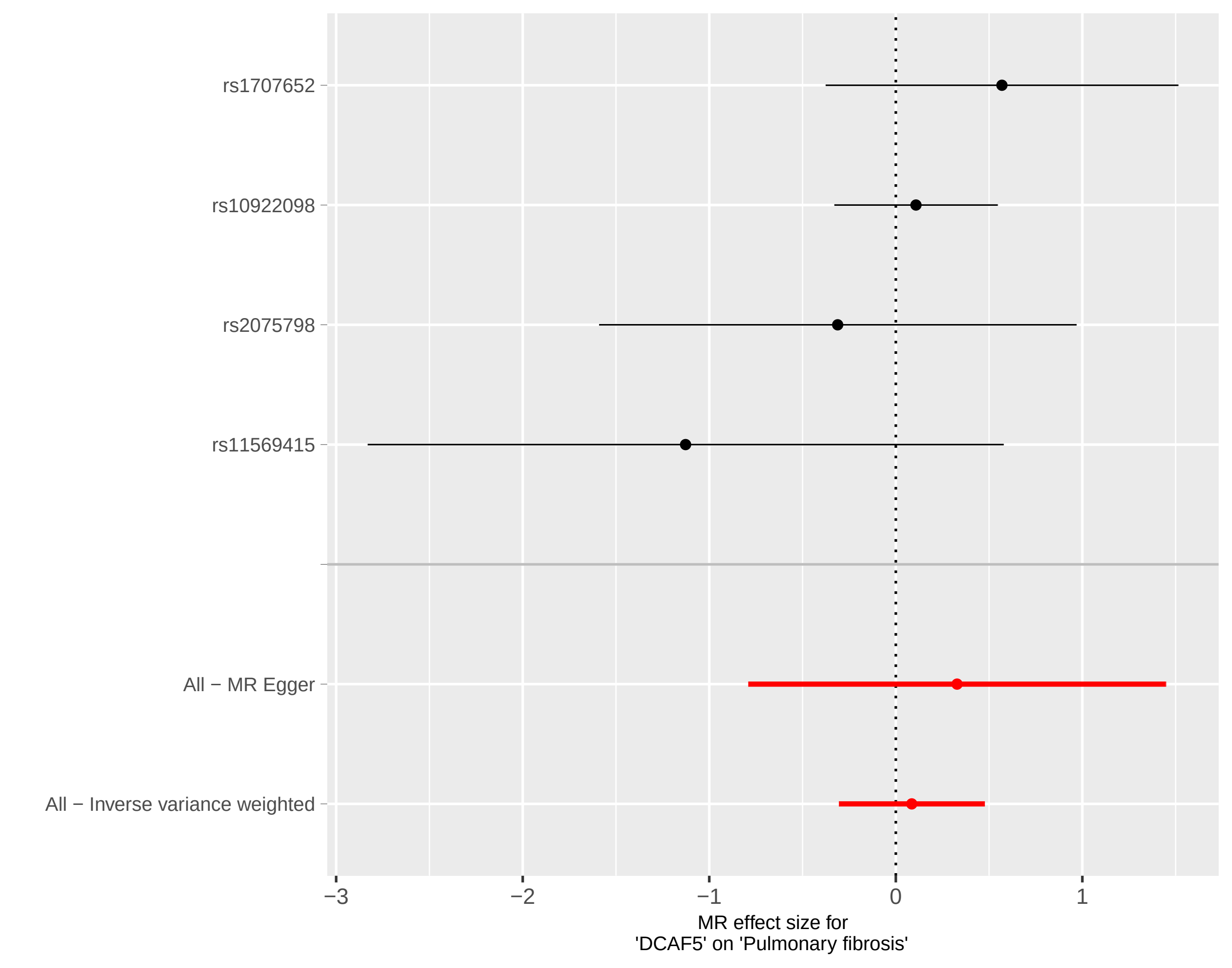
rs1707652
rs10922098
rs2075798
rs11569415
All − MR Egger
All − Inverse variance weighted
−3
−2
−1
0
1
MR effect size for
'DCAF5' on 'Pulmonary fibrosis'

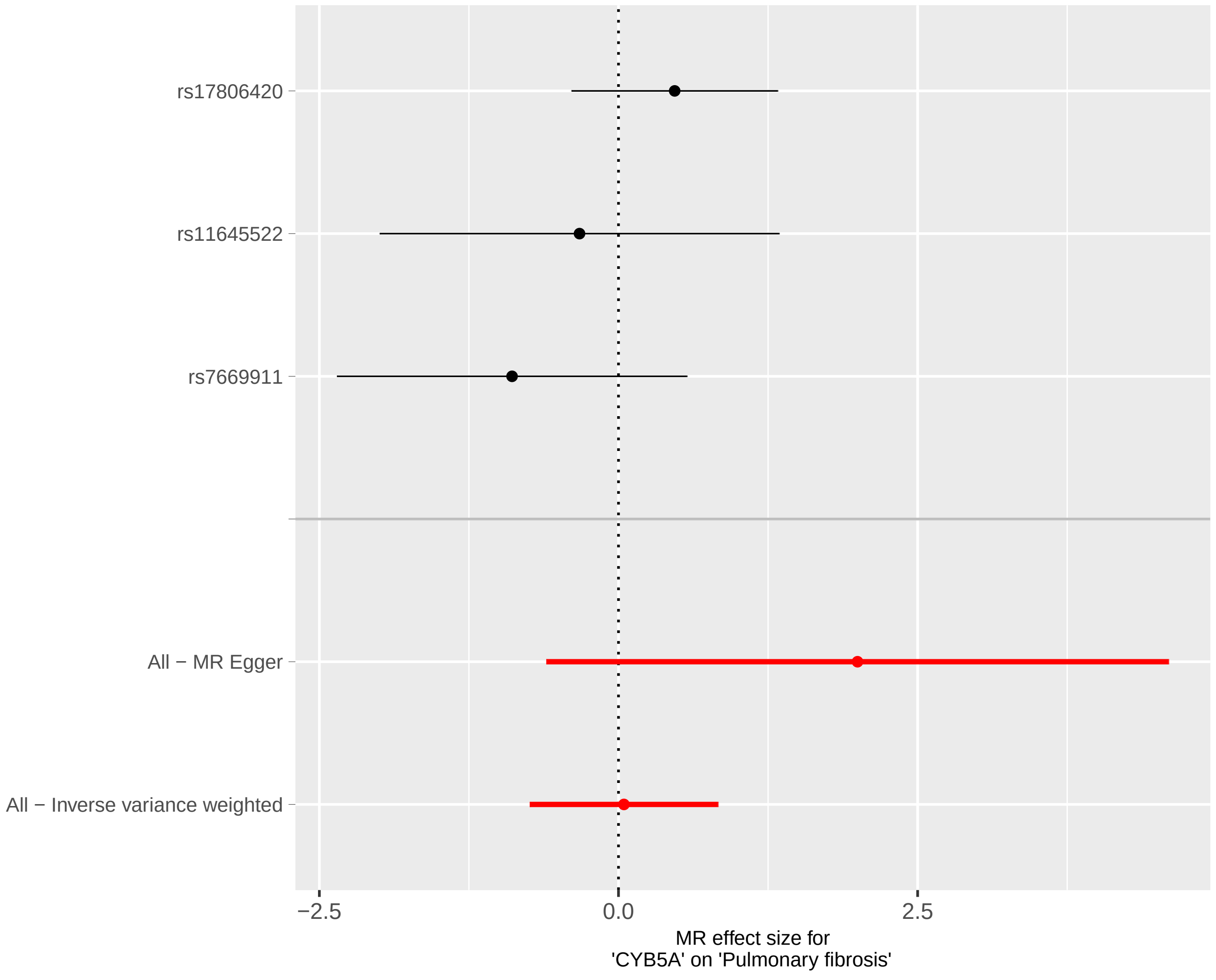
rs17806420
rs11645522
rs7669911
All − MR Egger
All − Inverse variance weighted
−2.5
0.0
2.5
MR effect size for
'CYB5A' on 'Pulmonary fibrosis'

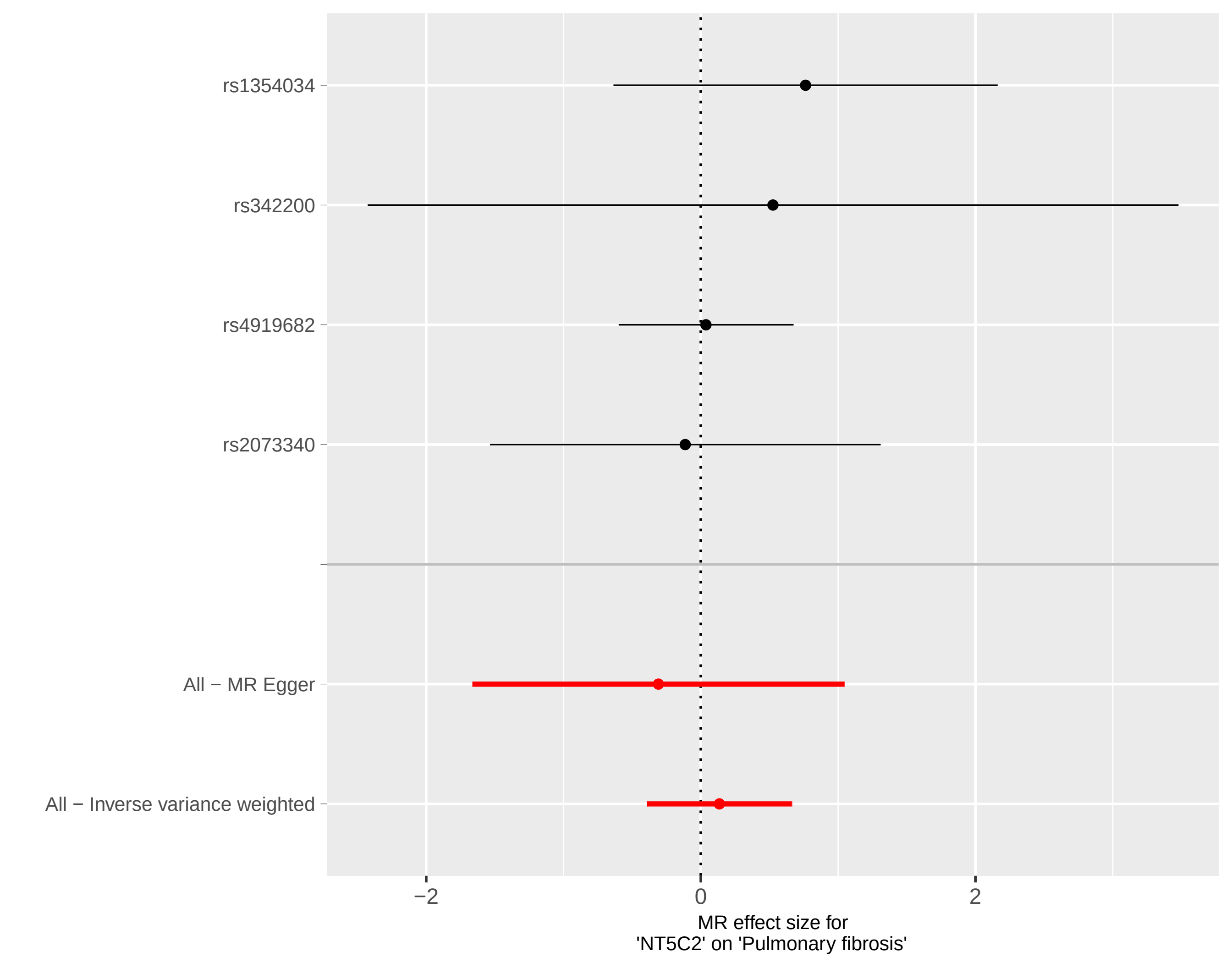
rs1354034
rs342200
rs4919682
rs2073340
All − MR Egger
All − Inverse variance weighted
−2
0
2
MR effect size for
'NT5C2' on 'Pulmonary fibrosis'

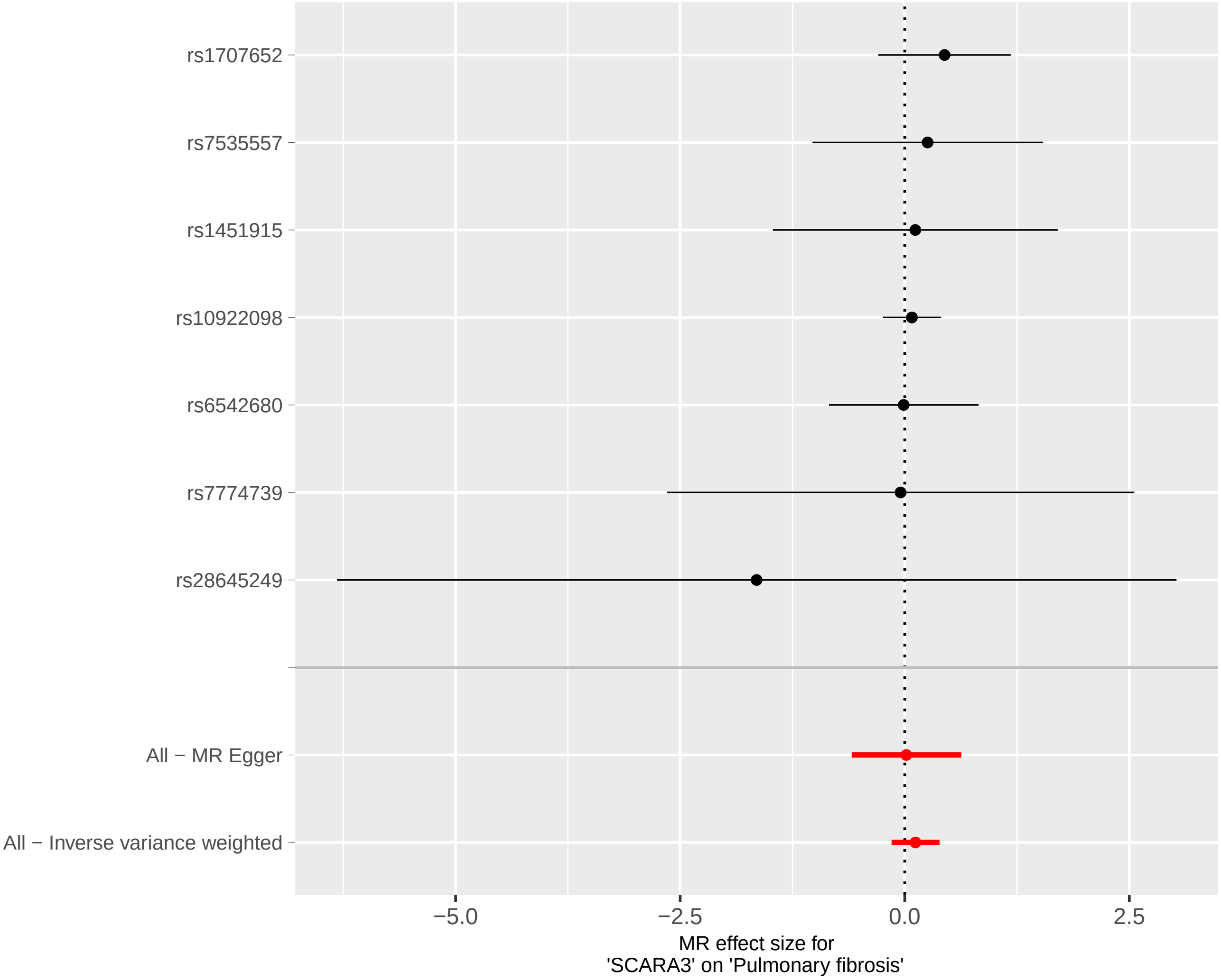
rs1707652
rs7535557
rs1451915
rs10922098
rs6542680
rs7774739
rs28645249
All − MR Egger
All − Inverse variance weighted
−5.0
−2.5
0.0
2.5
MR effect size for
'SCARA3' on 'Pulmonary fibrosis'

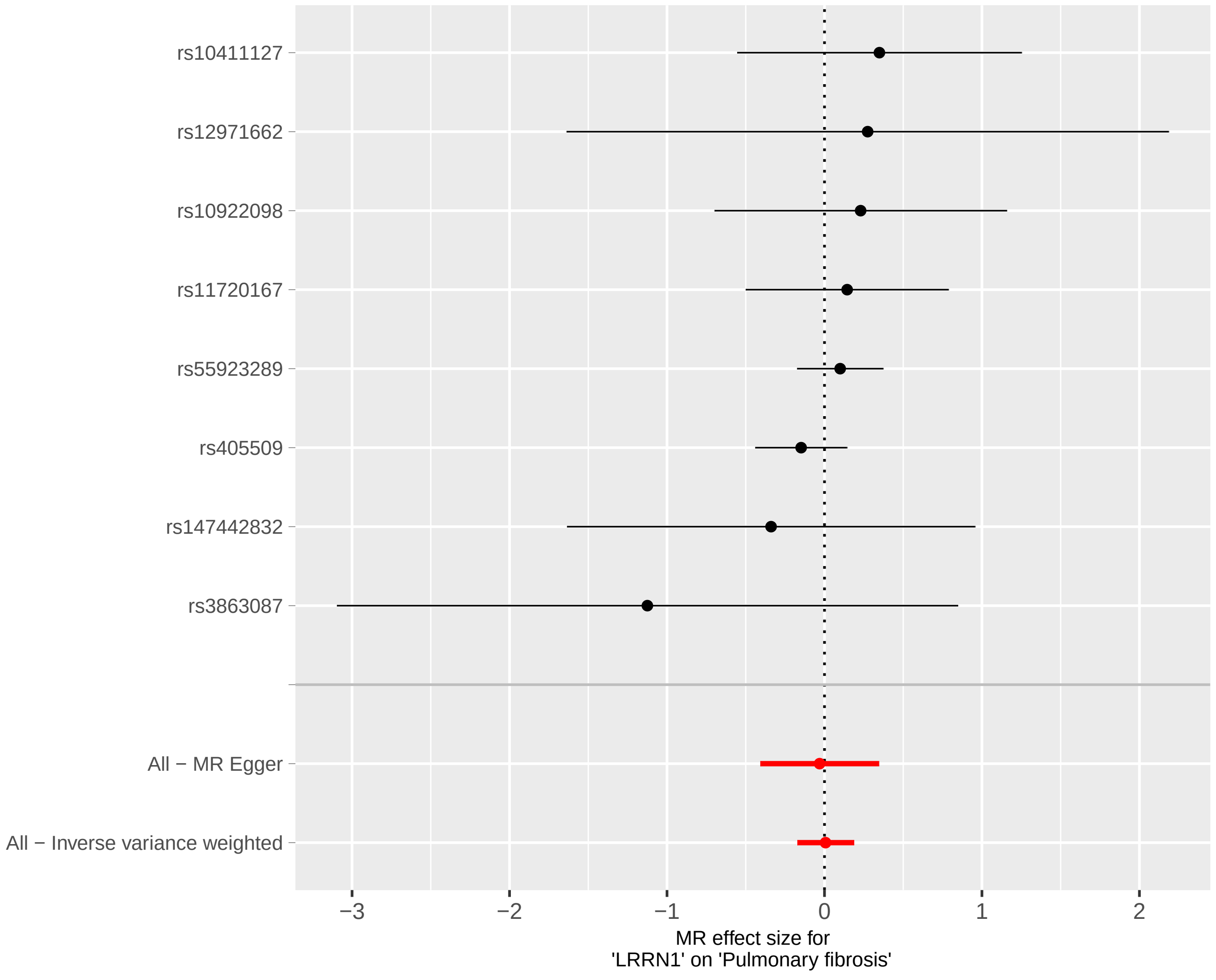
rs10411127
rs12971662
rs10922098
rs11720167
rs55923289
rs405509
rs147442832
rs3863087
All − MR Egger
All − Inverse variance weighted
−3
−2
−1
0
1
2
MR effect size for
'LRRN1' on 'Pulmonary fibrosis'

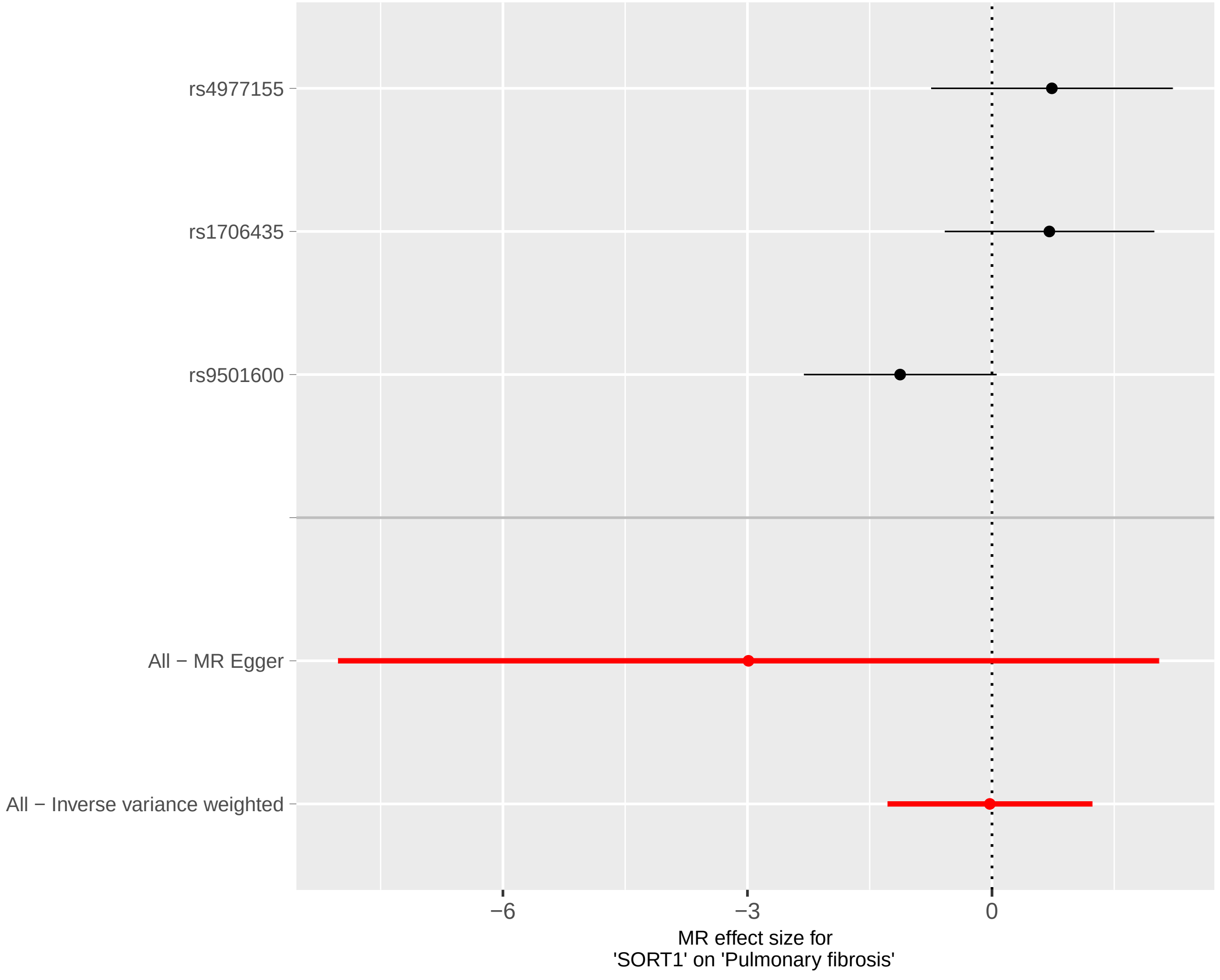
rs4977155
rs1706435
rs9501600
All − MR Egger
All − Inverse variance weighted
−6
−3
0
MR effect size for
'SORT1' on 'Pulmonary fibrosis'

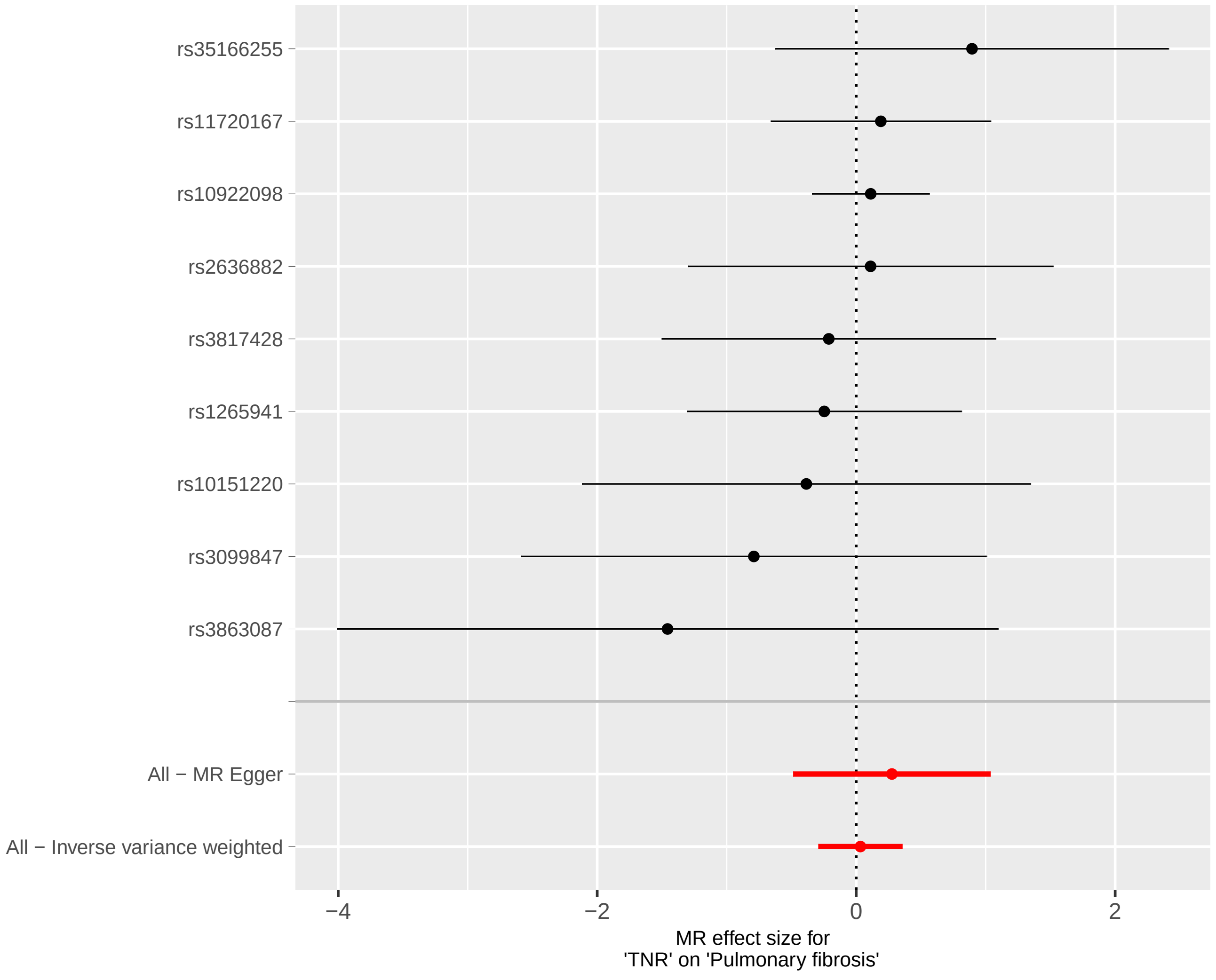
rs35166255
rs11720167
rs10922098
rs2636882
rs3817428
rs1265941
rs10151220
rs3099847
rs3863087
All − MR Egger
All − Inverse variance weighted
−4
−2
0
2
MR effect size for
'TNR' on 'Pulmonary fibrosis'

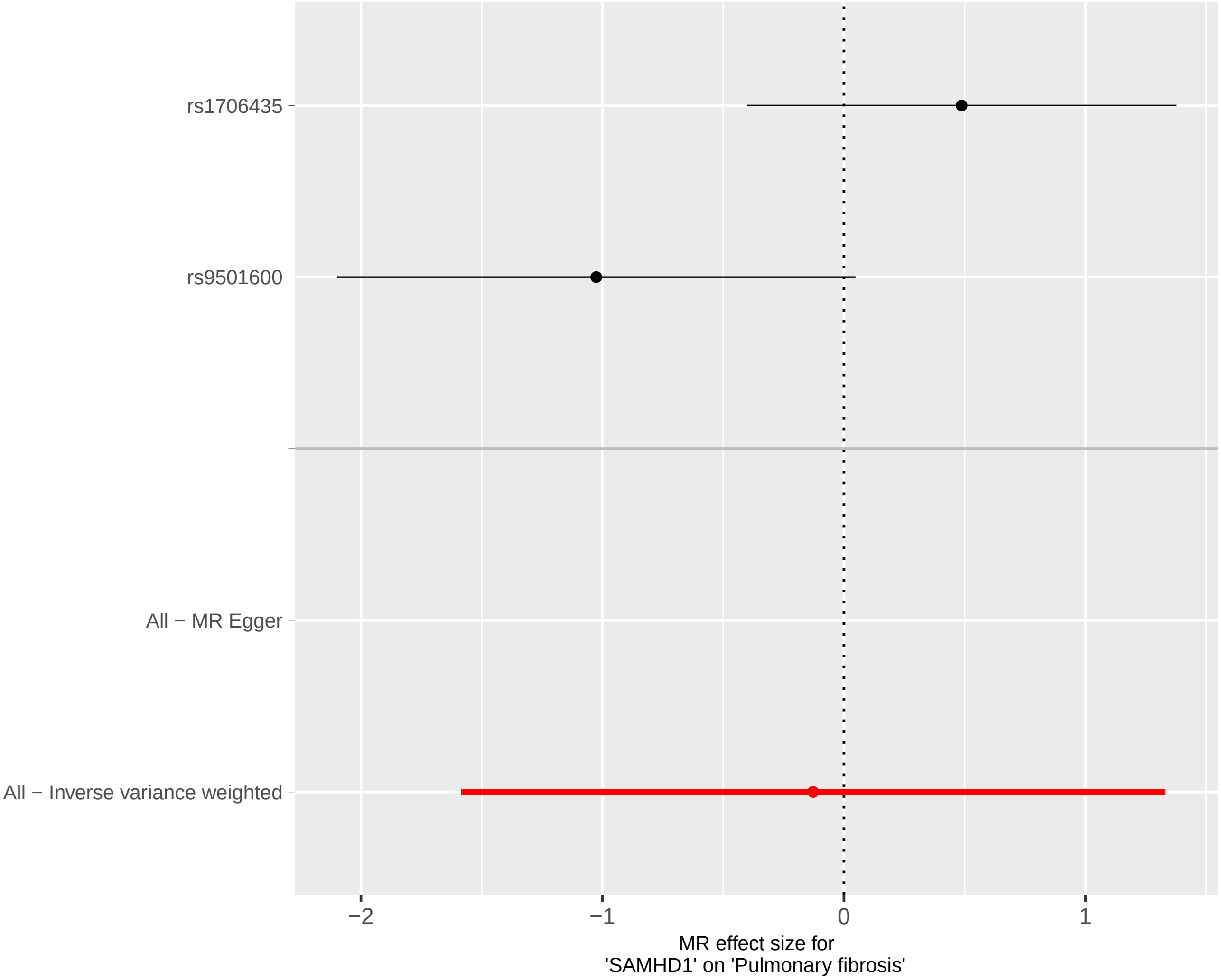
rs1706435
rs9501600
All − MR Egger
All − Inverse variance weighted
−2
−1
0
1
MR effect size for
'SAMHD1' on 'Pulmonary fibrosis'

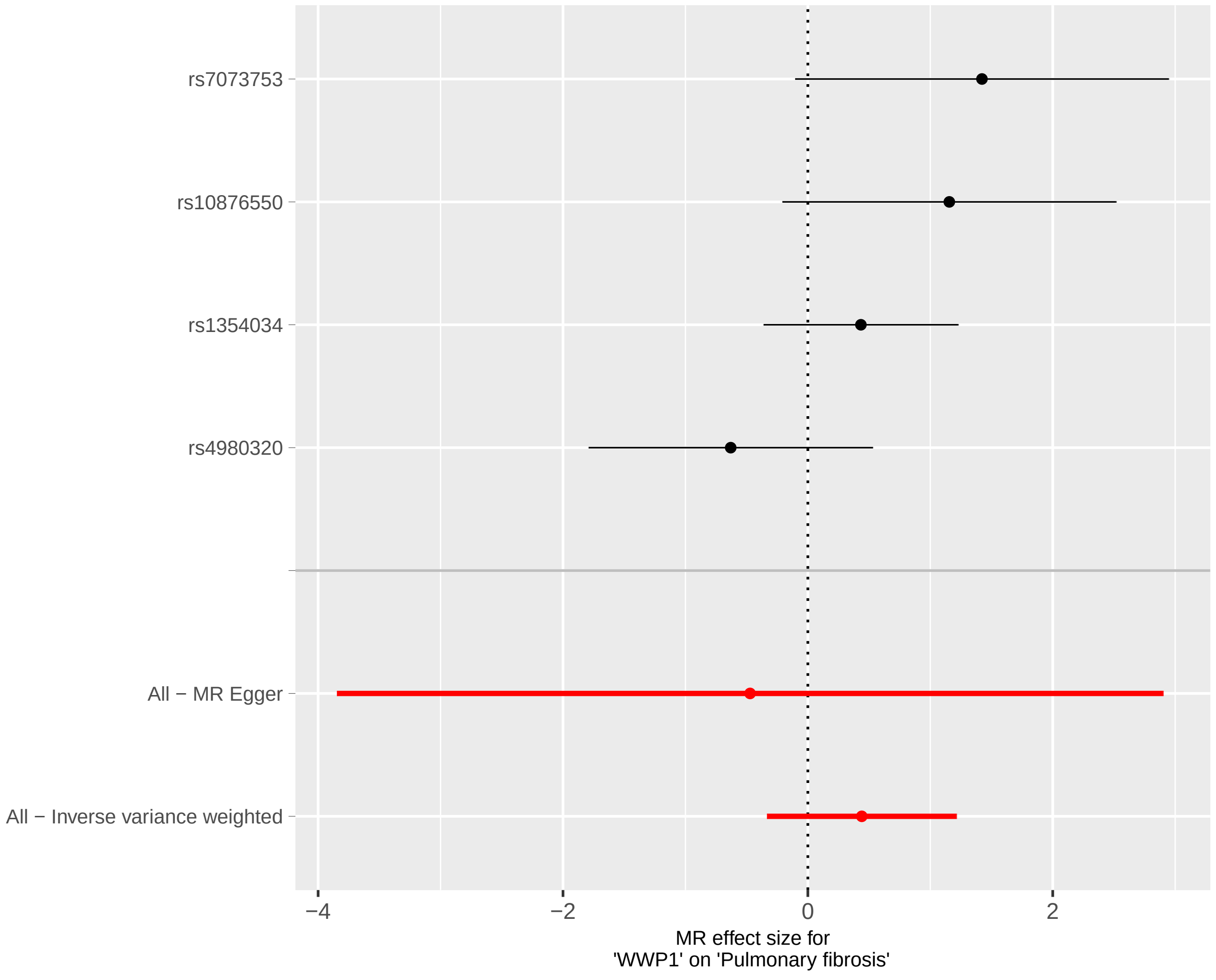
rs7073753
rs10876550
rs1354034
rs4980320
All − MR Egger
All − Inverse variance weighted
−4
−2
0
2
MR effect size for
'WWP1' on 'Pulmonary fibrosis'

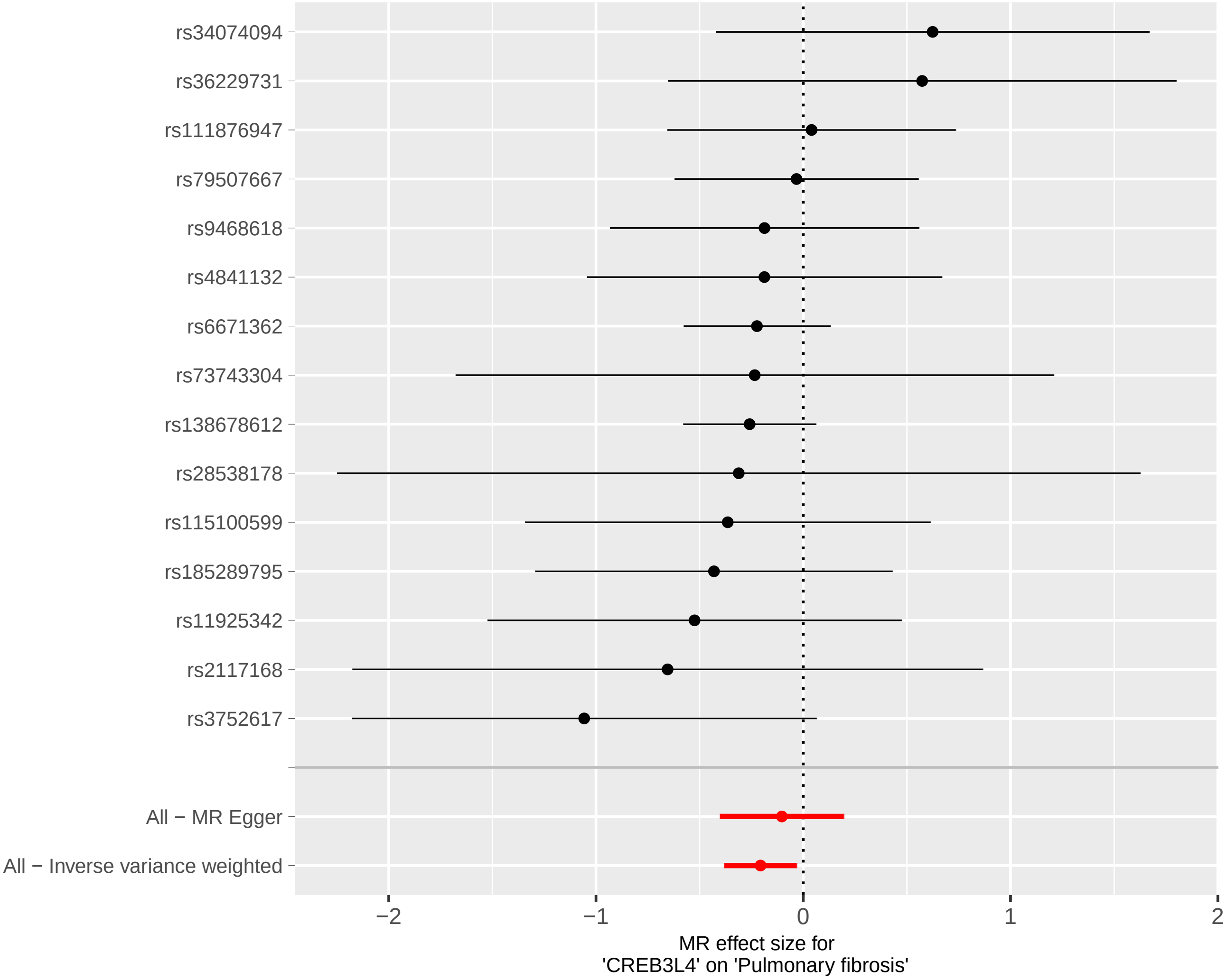
rs34074094
rs36229731
rs111876947
rs79507667
rs9468618
rs4841132
rs6671362
rs73743304
rs138678612
rs28538178
rs115100599
rs185289795
rs11925342
rs2117168
rs3752617
All − MR Egger
All − Inverse variance weighted
−2
−1
0
1
2
MR effect size for
'CREB3L4' on 'Pulmonary fibrosis'

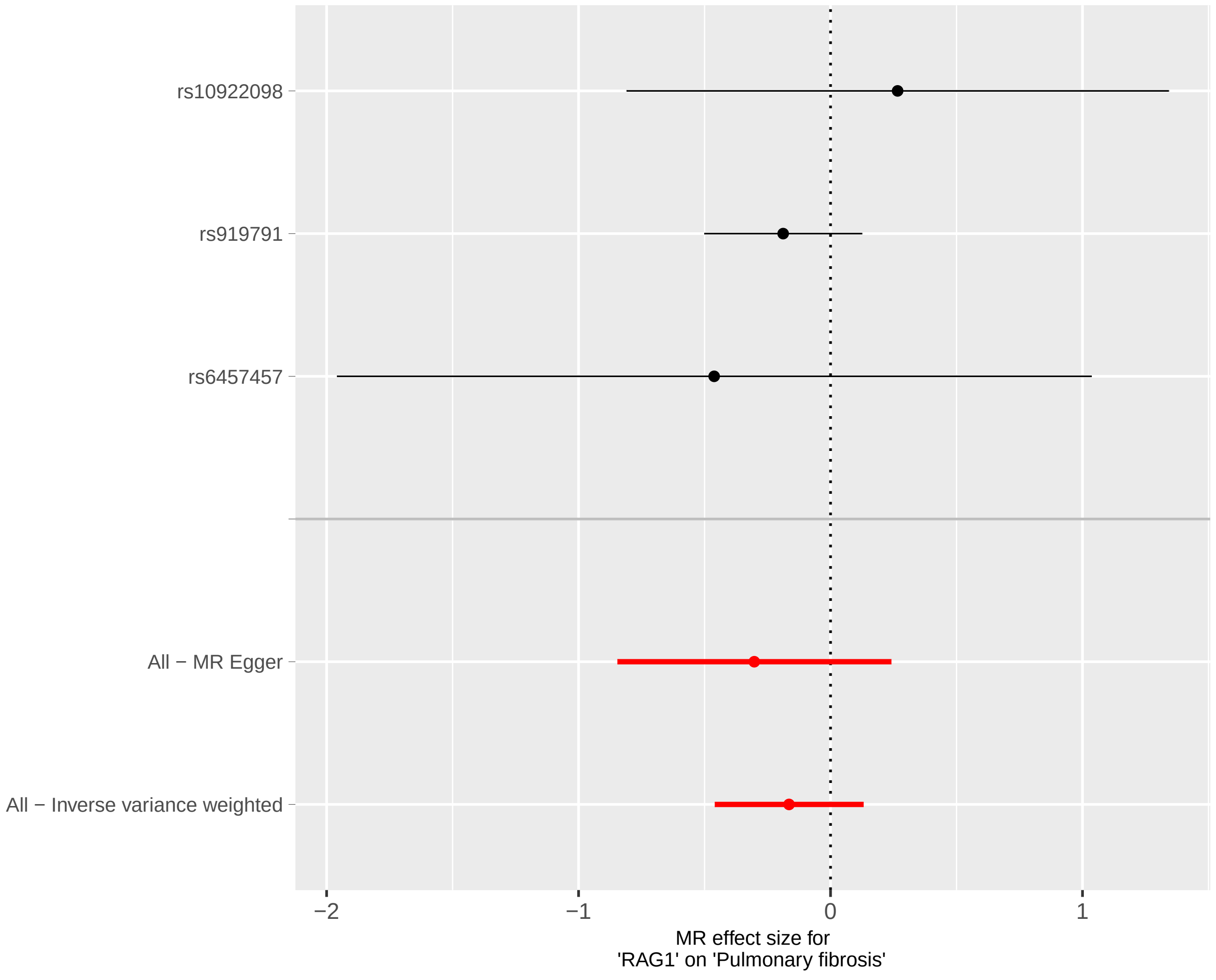

rs10922098
rs919791
rs6457457
All − MR Egger
All − Inverse variance weighted
−2
−1
0
1
MR effect size for
'RAG1' on 'Pulmonary fibrosis'

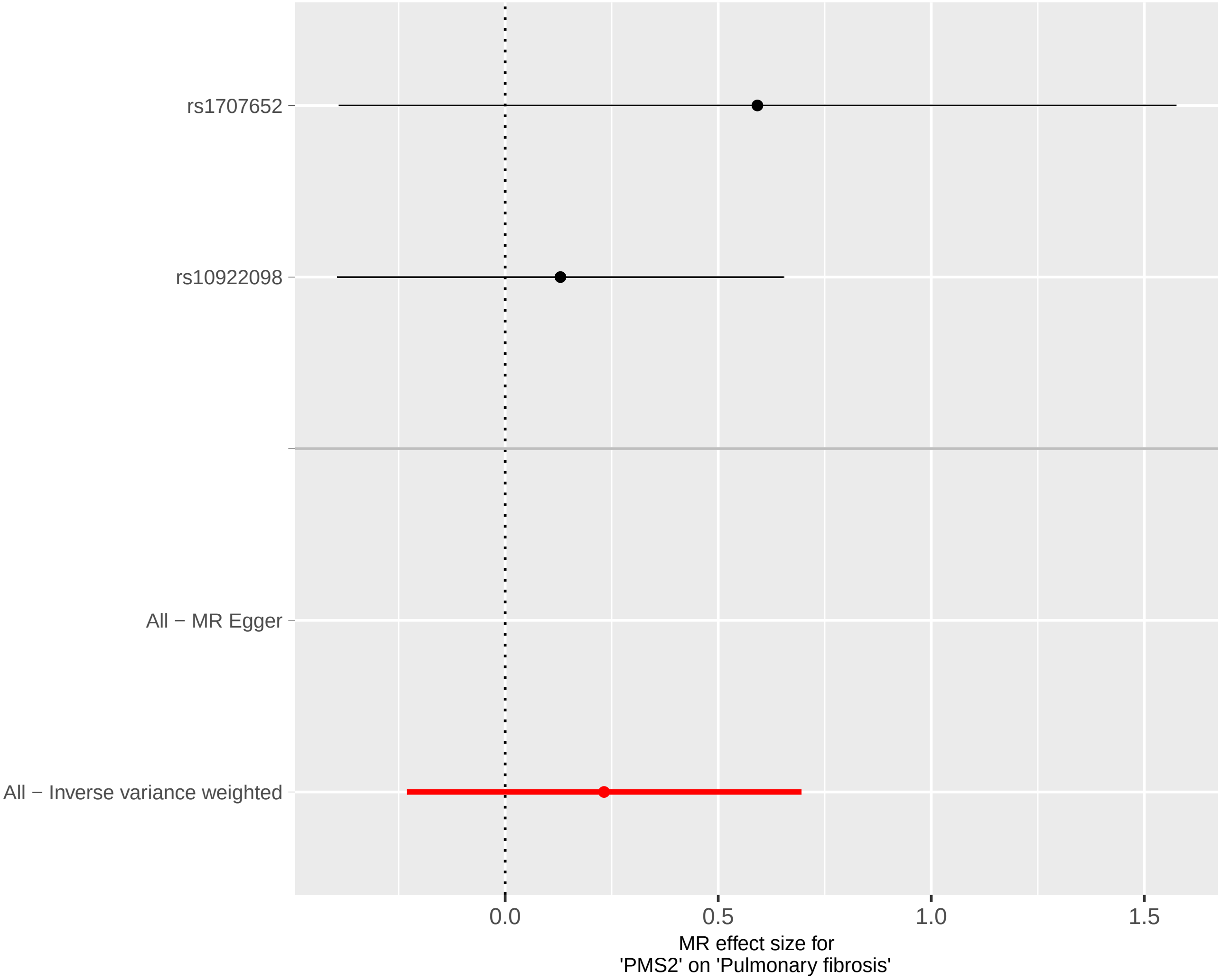
rs1707652
rs10922098
All − MR Egger
All − Inverse variance weighted
0.0
0.5
1.0
1.5
MR effect size for
'PMS2' on 'Pulmonary fibrosis'

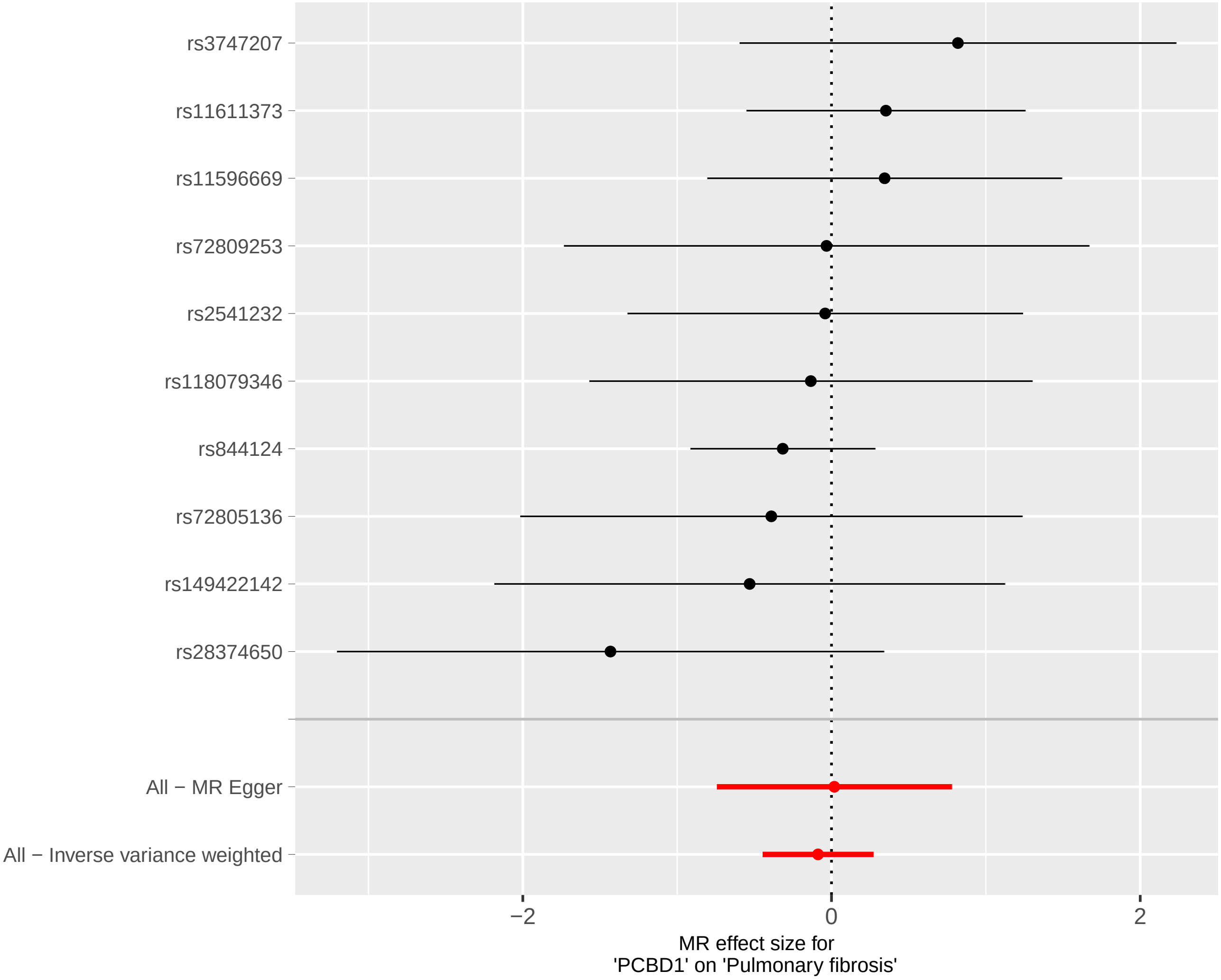

rs3747207
rs11611373
rs11596669
rs72809253
rs2541232
rs118079346
rs844124
rs72805136
rs149422142
rs28374650
All − MR Egger
All − Inverse variance weighted
−2
0
2
MR effect size for
'PCBD1' on 'Pulmonary fibrosis'

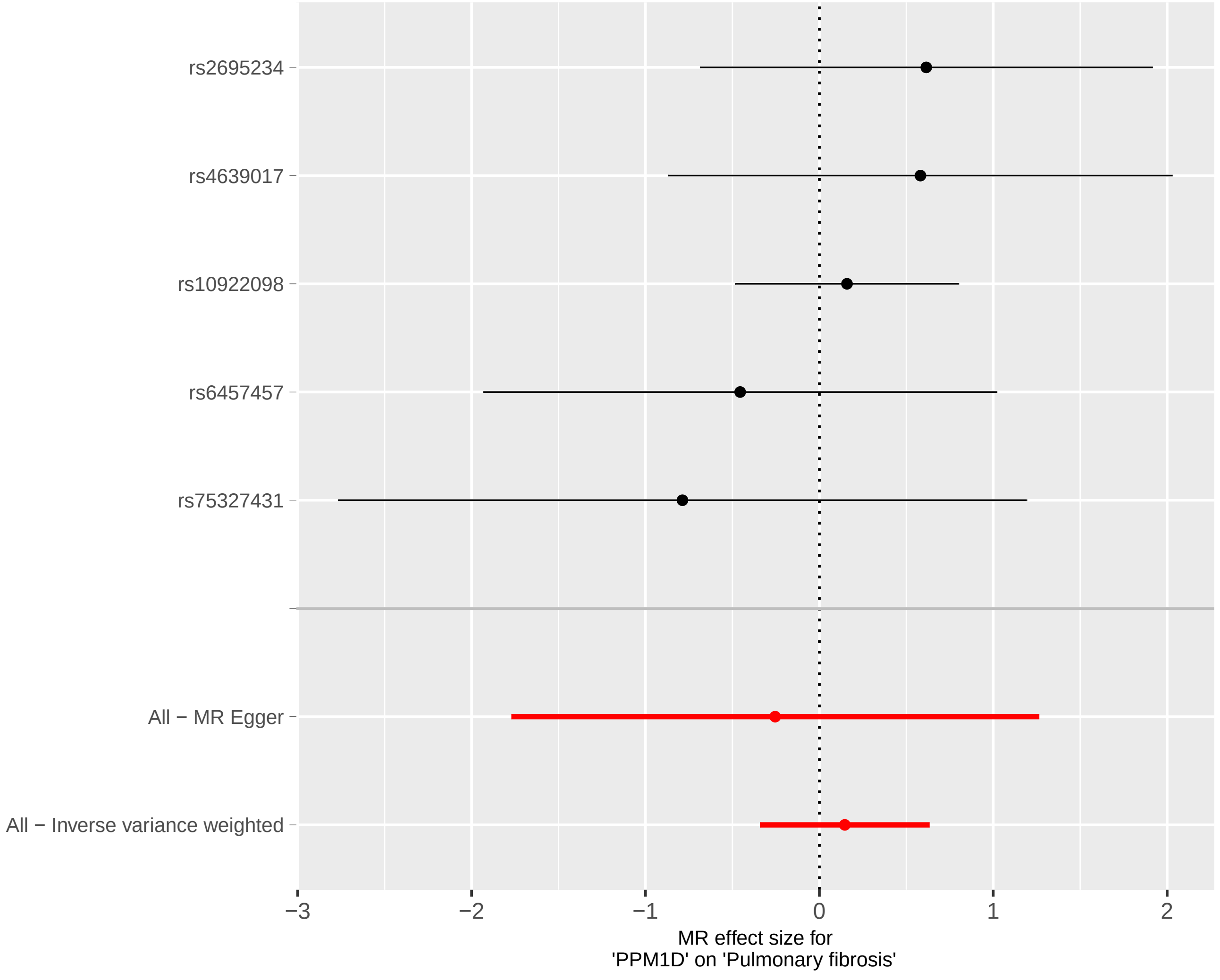
rs2695234
rs4639017
rs10922098
rs6457457
rs75327431
All − MR Egger
All − Inverse variance weighted
−3
−2
−1
0
1
2
MR effect size for
'PPM1D' on 'Pulmonary fibrosis'

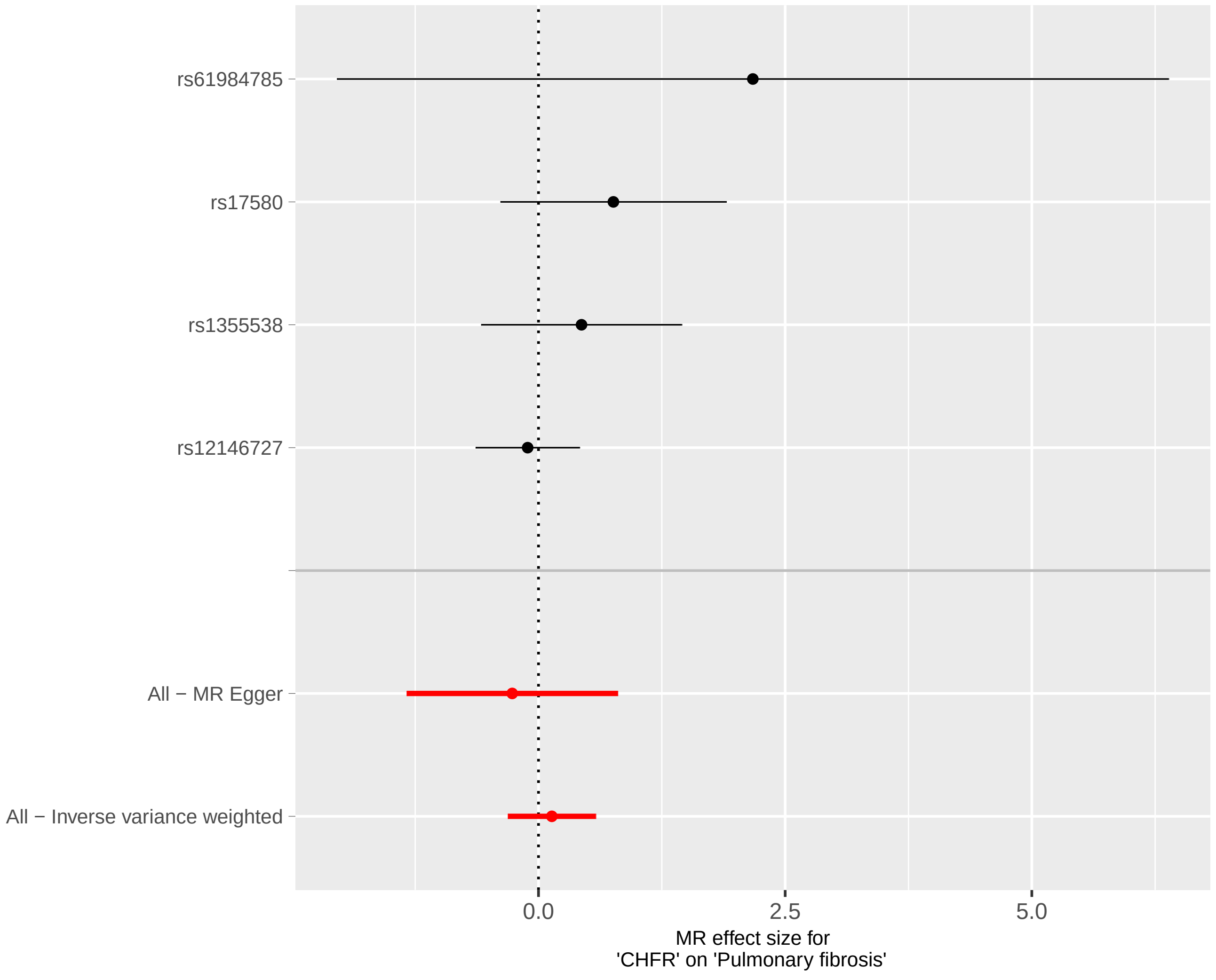
rs61984785
rs17580
rs1355538
rs12146727
All − MR Egger
All − Inverse variance weighted
0.0
2.5
5.0
MR effect size for
'CHFR' on 'Pulmonary fibrosis'

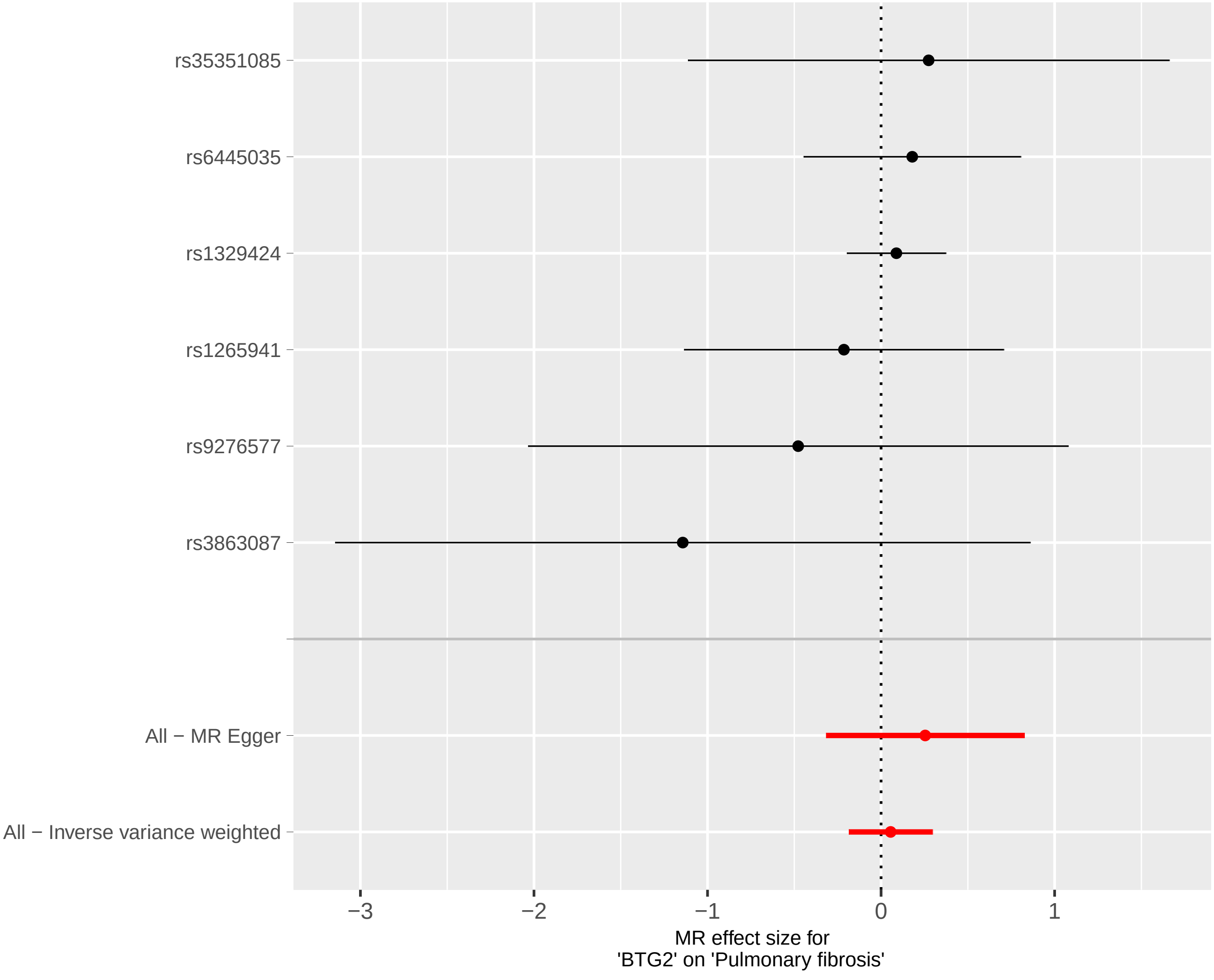
rs35351085
rs6445035
rs1329424
rs1265941
rs9276577
rs3863087
All − MR Egger
All − Inverse variance weighted
−3
−2
−1
0
1
MR effect size for
'BTG2' on 'Pulmonary fibrosis'

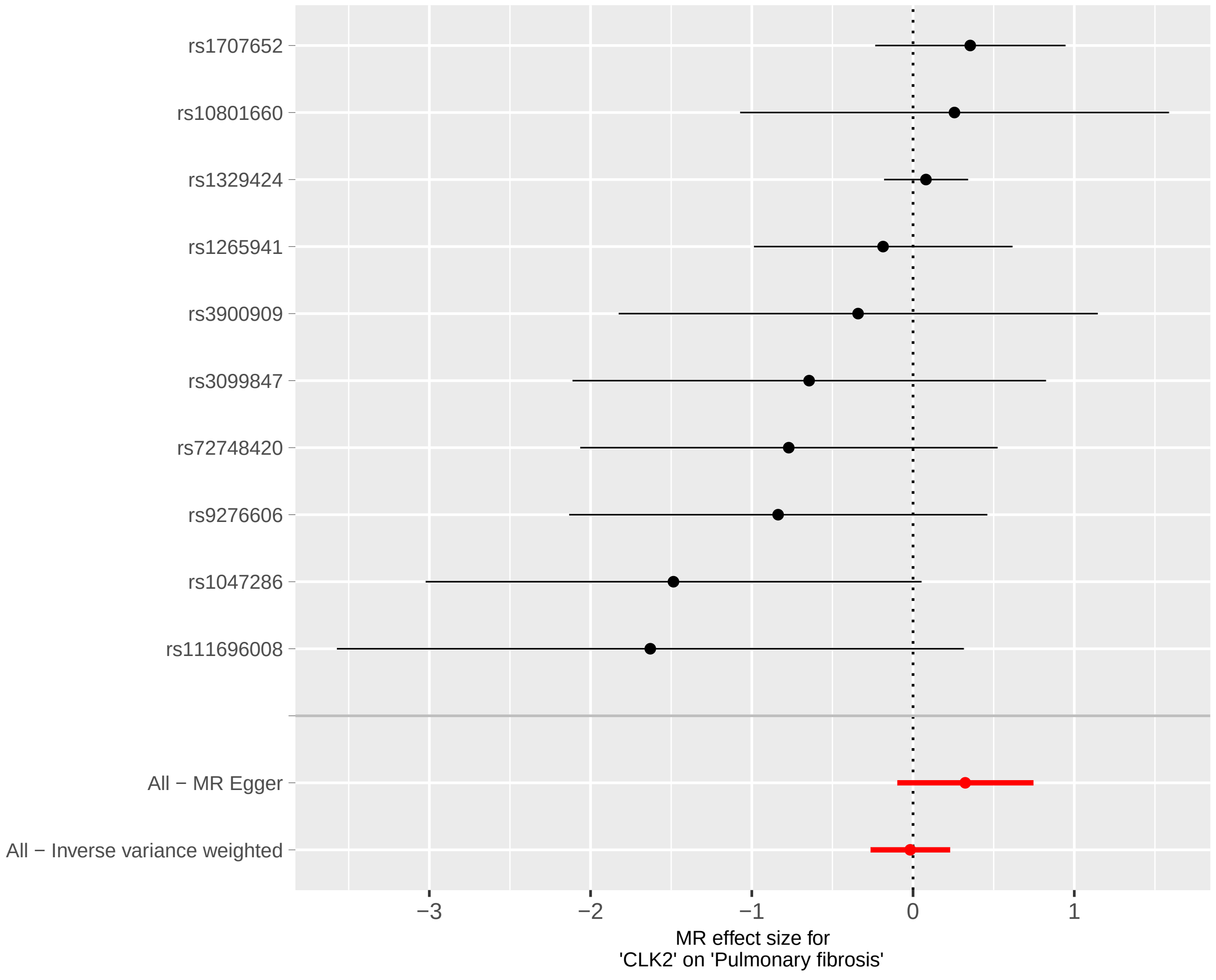
rs1707652
rs10801660
rs1329424
rs1265941
rs3900909
rs3099847
rs72748420
rs9276606
rs1047286
rs111696008
All − MR Egger
All − Inverse variance weighted
−3
−2
−1
0
1
MR effect size for
'CLK2' on 'Pulmonary fibrosis'

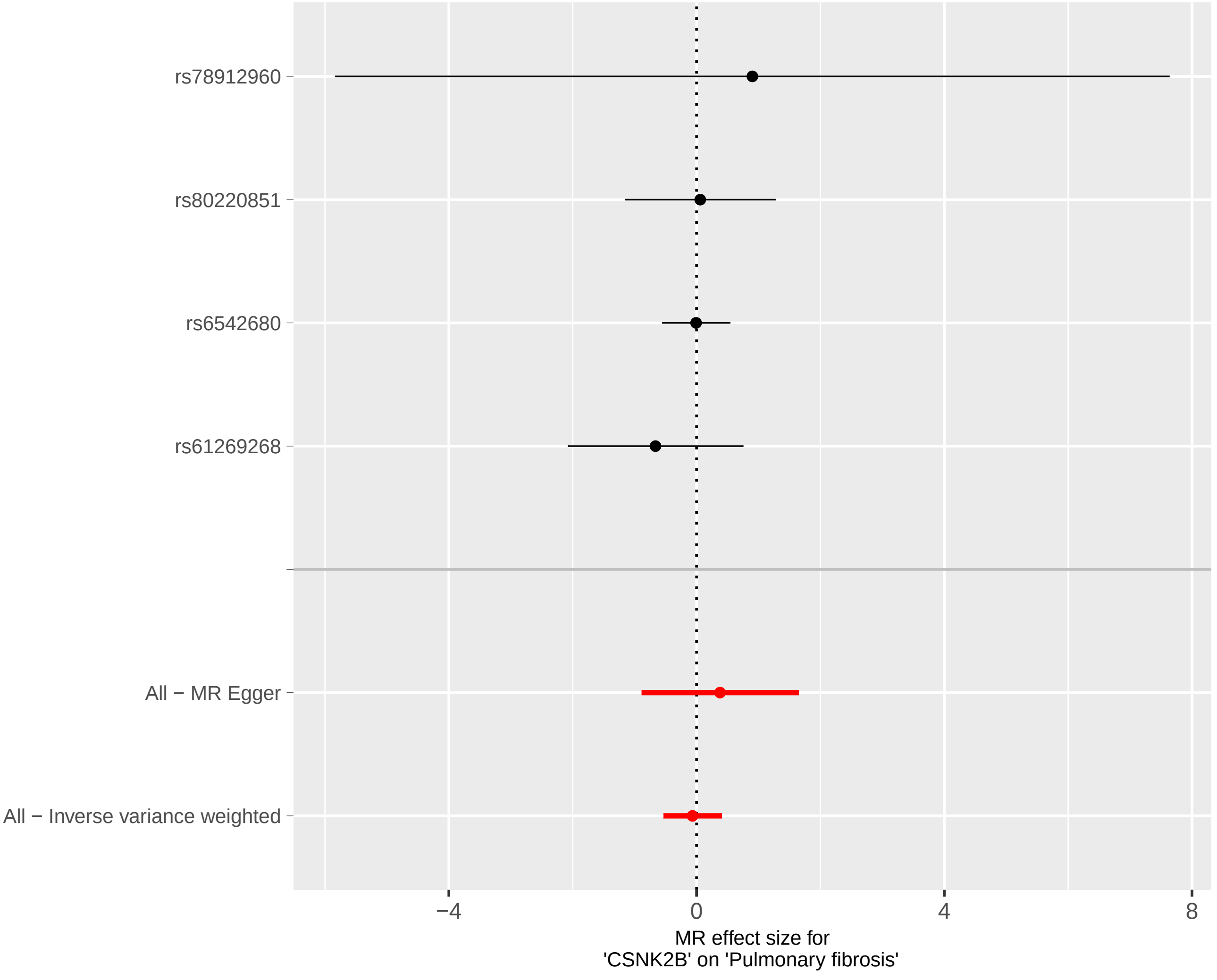
rs78912960
rs80220851
rs6542680
rs61269268
All − MR Egger
All − Inverse variance weighted
−4
0
4
8
MR effect size for
'CSNK2B' on 'Pulmonary fibrosis'

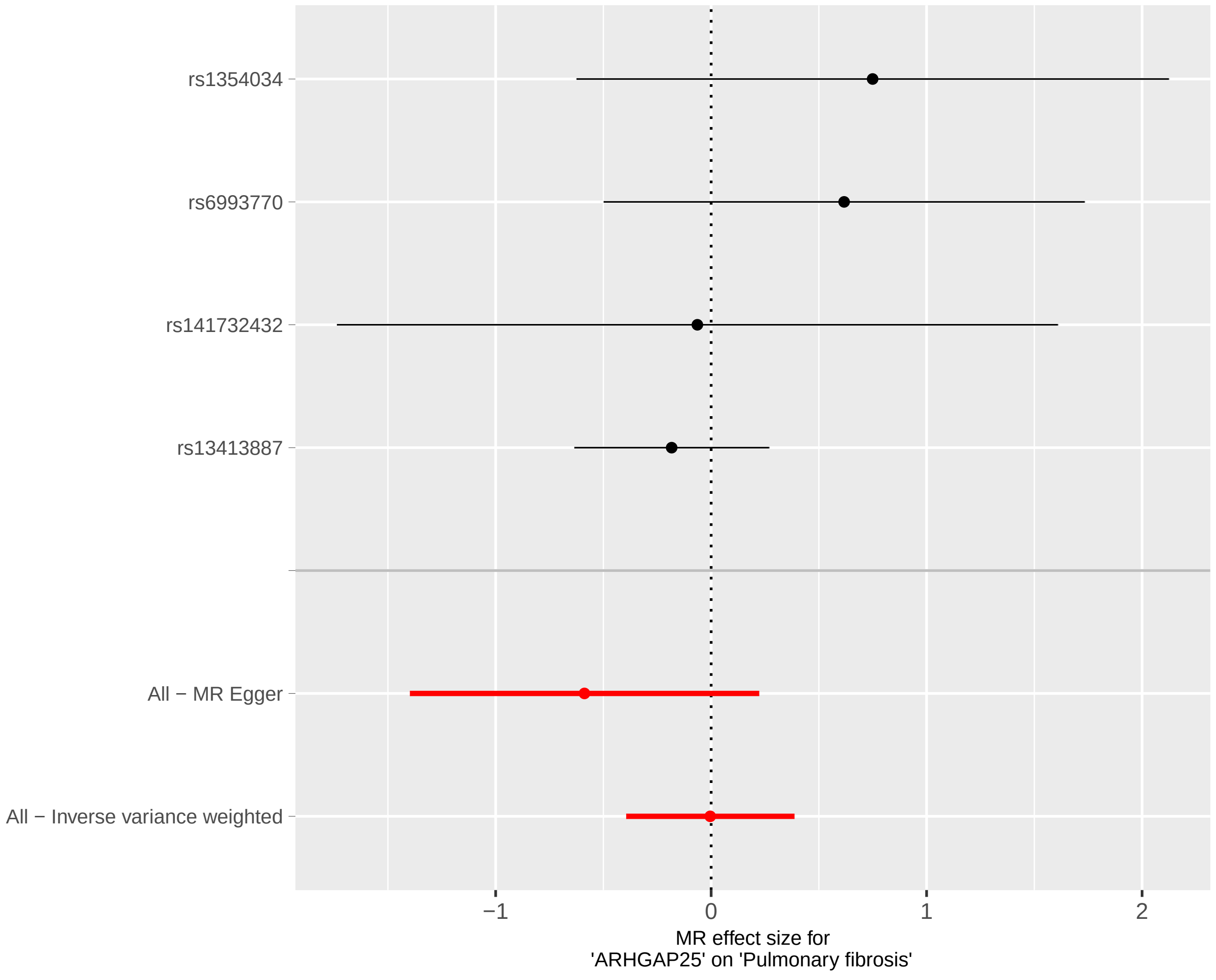
rs1354034
rs6993770
rs141732432
rs13413887
All − MR Egger
All − Inverse variance weighted
−1
0
1
2
MR effect size for
'ARHGAP25' on 'Pulmonary fibrosis'

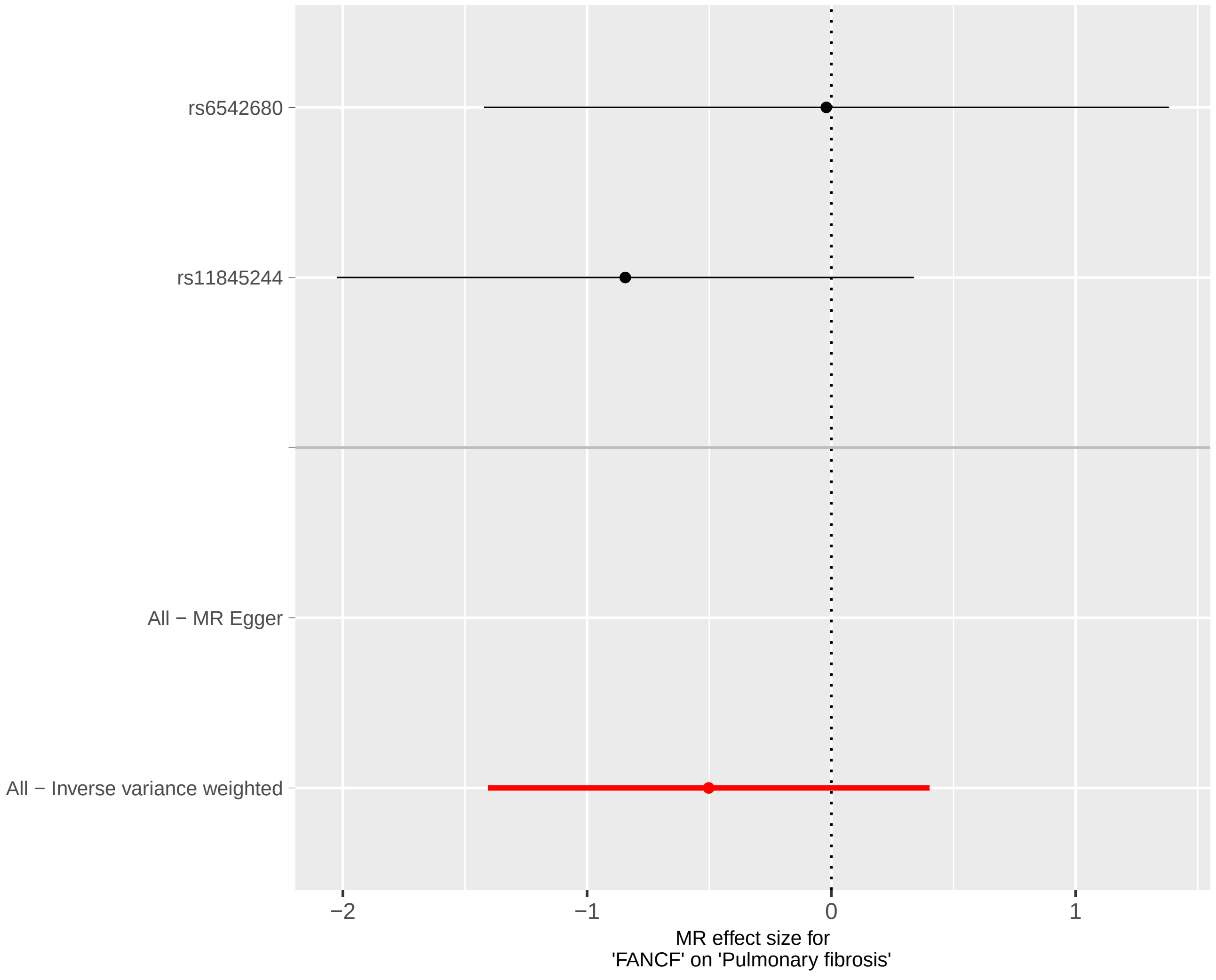

rs6542680
rs11845244
All − MR Egger
All − Inverse variance weighted
−2
−1
0
1
MR effect size for
'FANCF' on 'Pulmonary fibrosis'

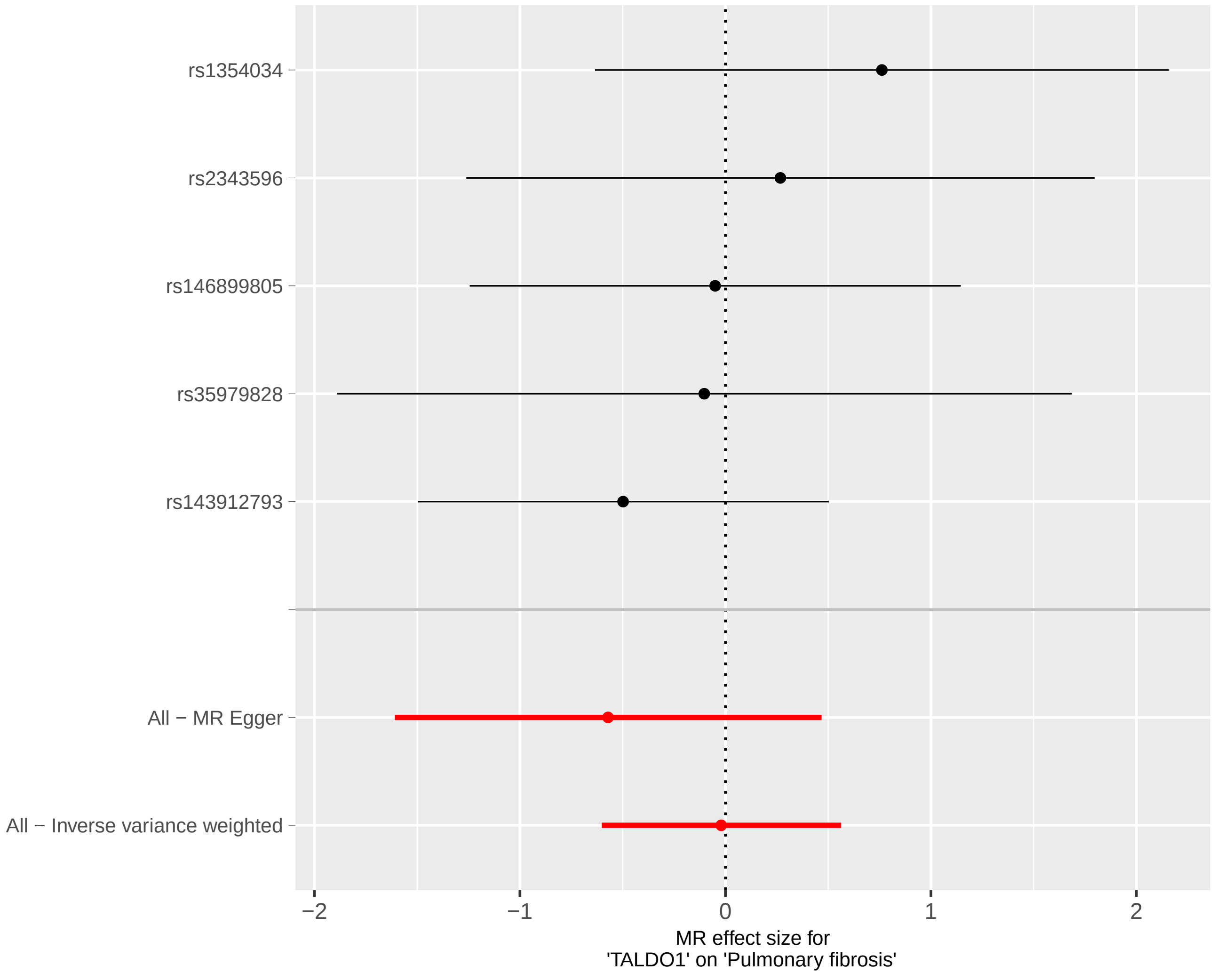
rs1354034
rs2343596
rs146899805
rs35979828
rs143912793
All − MR Egger
All − Inverse variance weighted
−2
−1
0
1
2
MR effect size for
'TALDO1' on 'Pulmonary fibrosis'

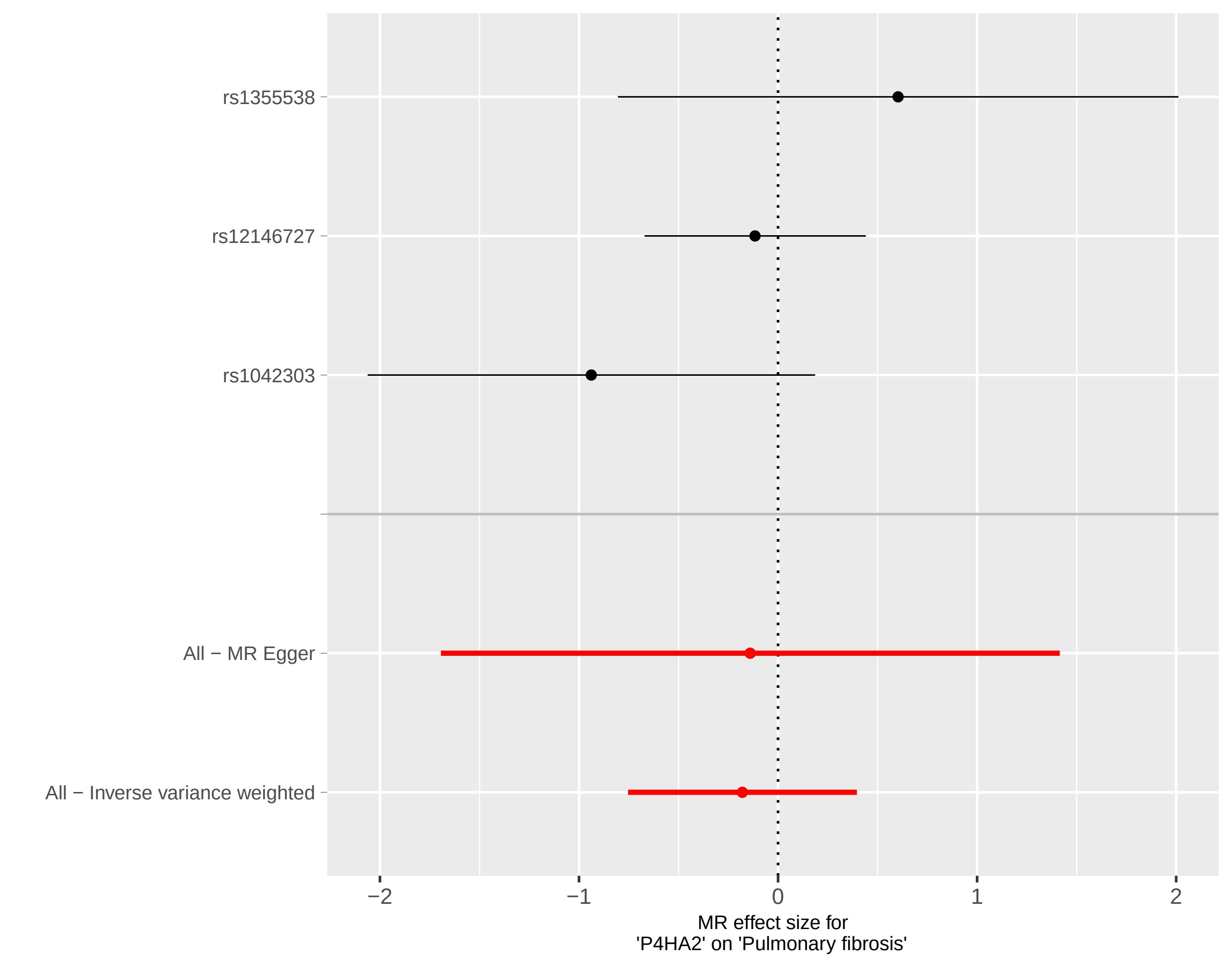

rs1355538
rs12146727
rs1042303
All − MR Egger
All − Inverse variance weighted
−2
−1
0
1
2
MR effect size for
'P4HA2' on 'Pulmonary fibrosis'

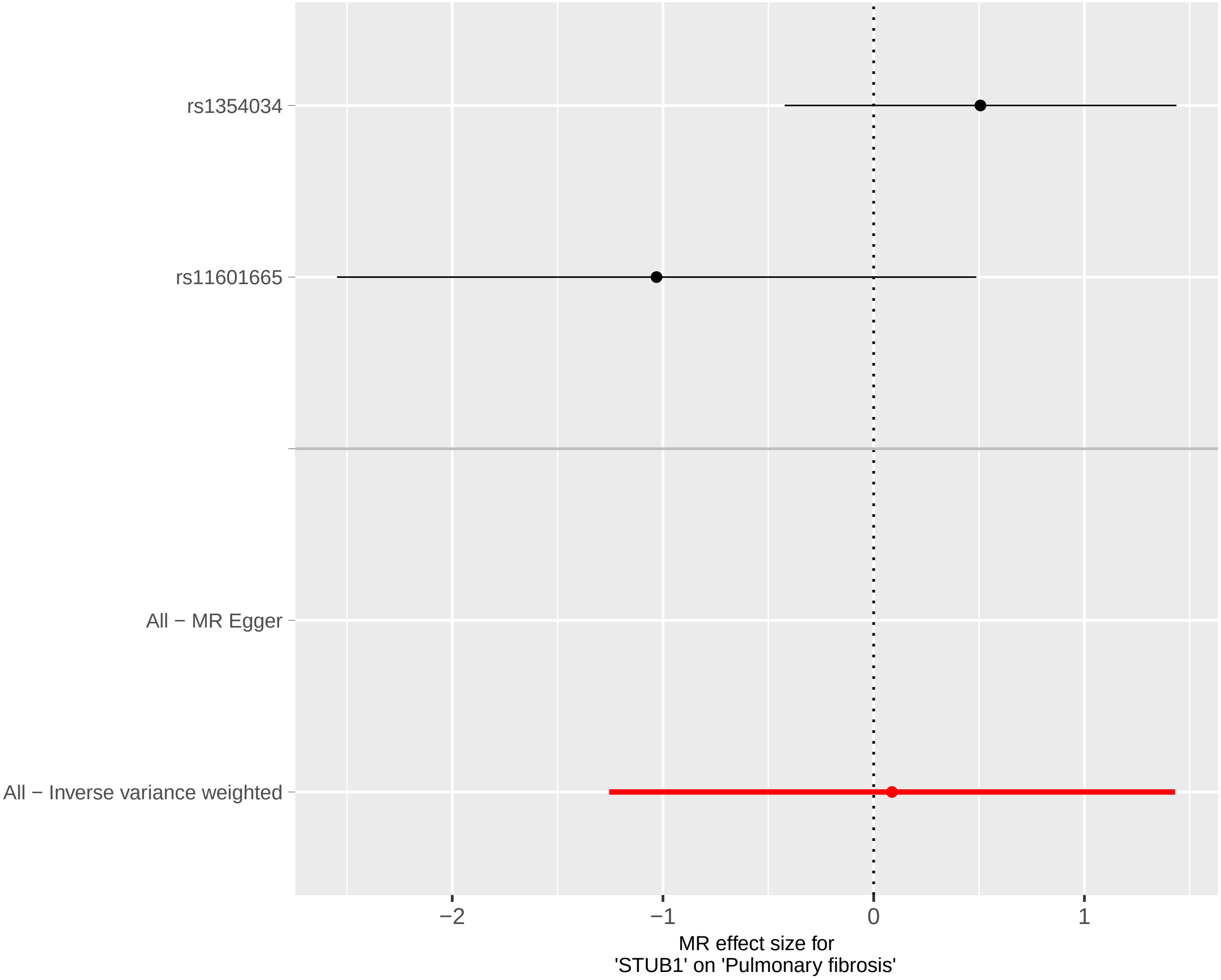
rs1354034
rs11601665
All − MR Egger
All − Inverse variance weighted
−2
−1
0
1
MR effect size for
'STUB1' on 'Pulmonary fibrosis'

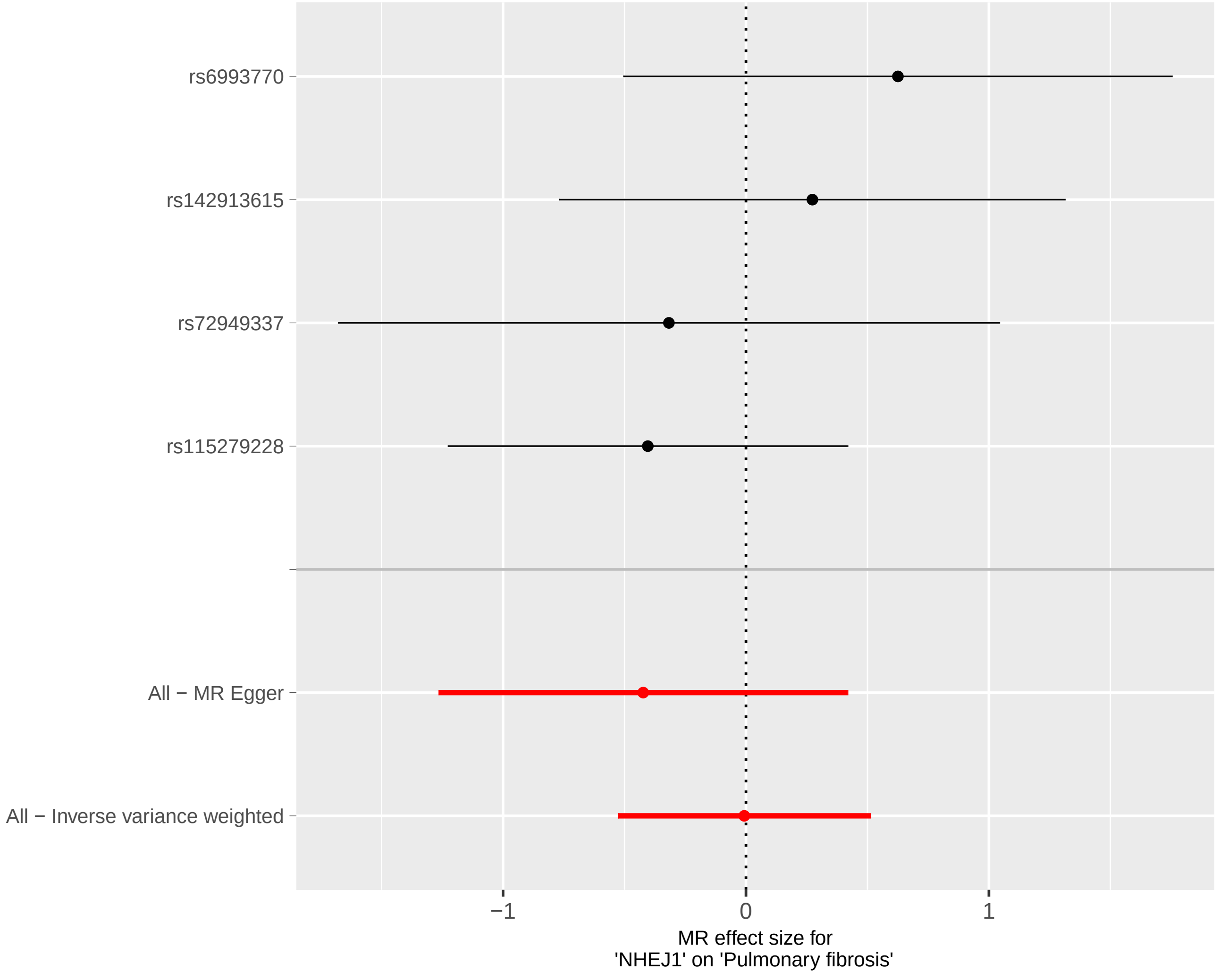
rs6993770
rs142913615
rs72949337
rs115279228
All − MR Egger
All − Inverse variance weighted
−1
0
1
MR effect size for
'NHEJ1' on 'Pulmonary fibrosis'

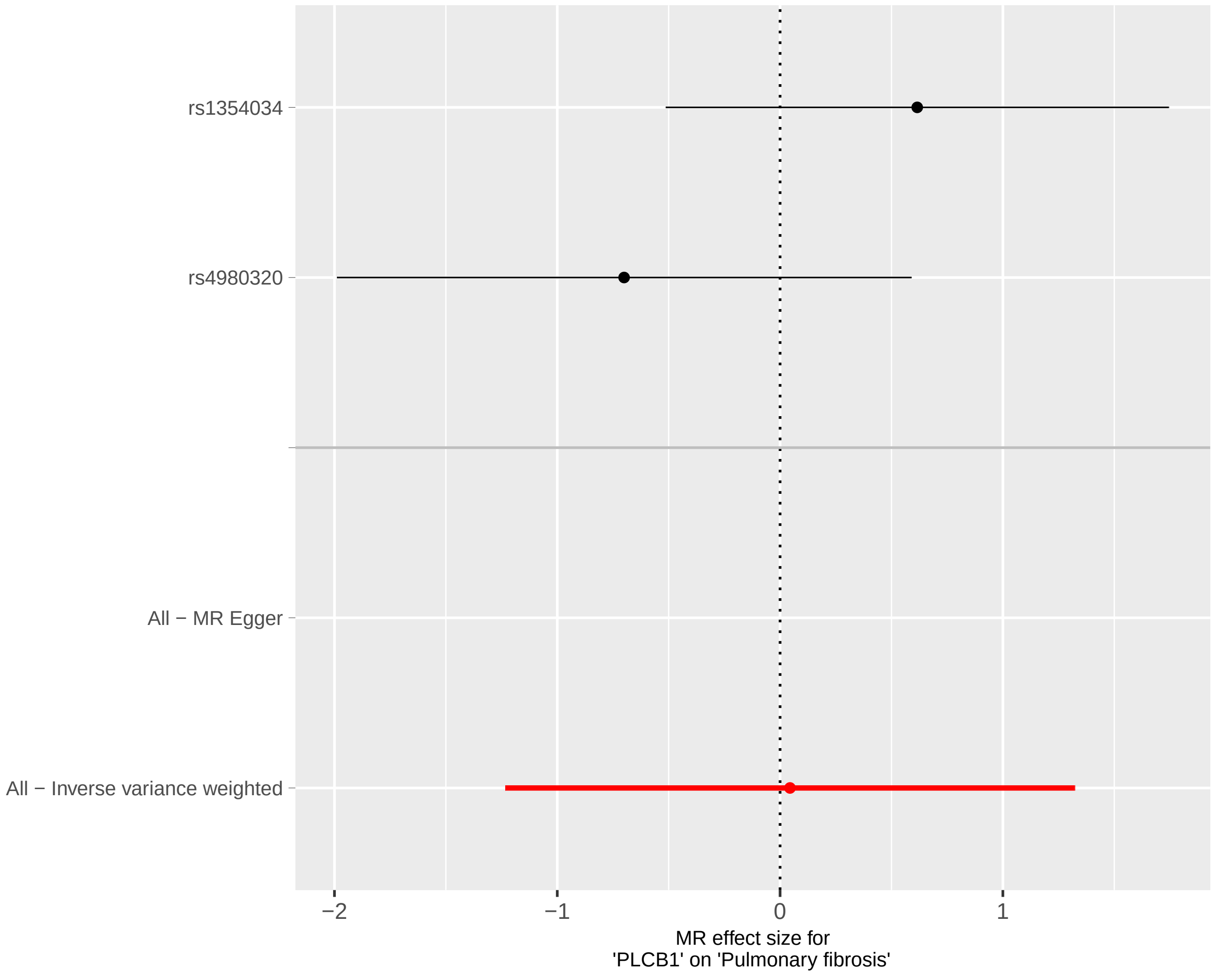

rs1354034
rs4980320
All − MR Egger
All − Inverse variance weighted
−2
−1
0
1
MR effect size for
'PLCB1' on 'Pulmonary fibrosis'

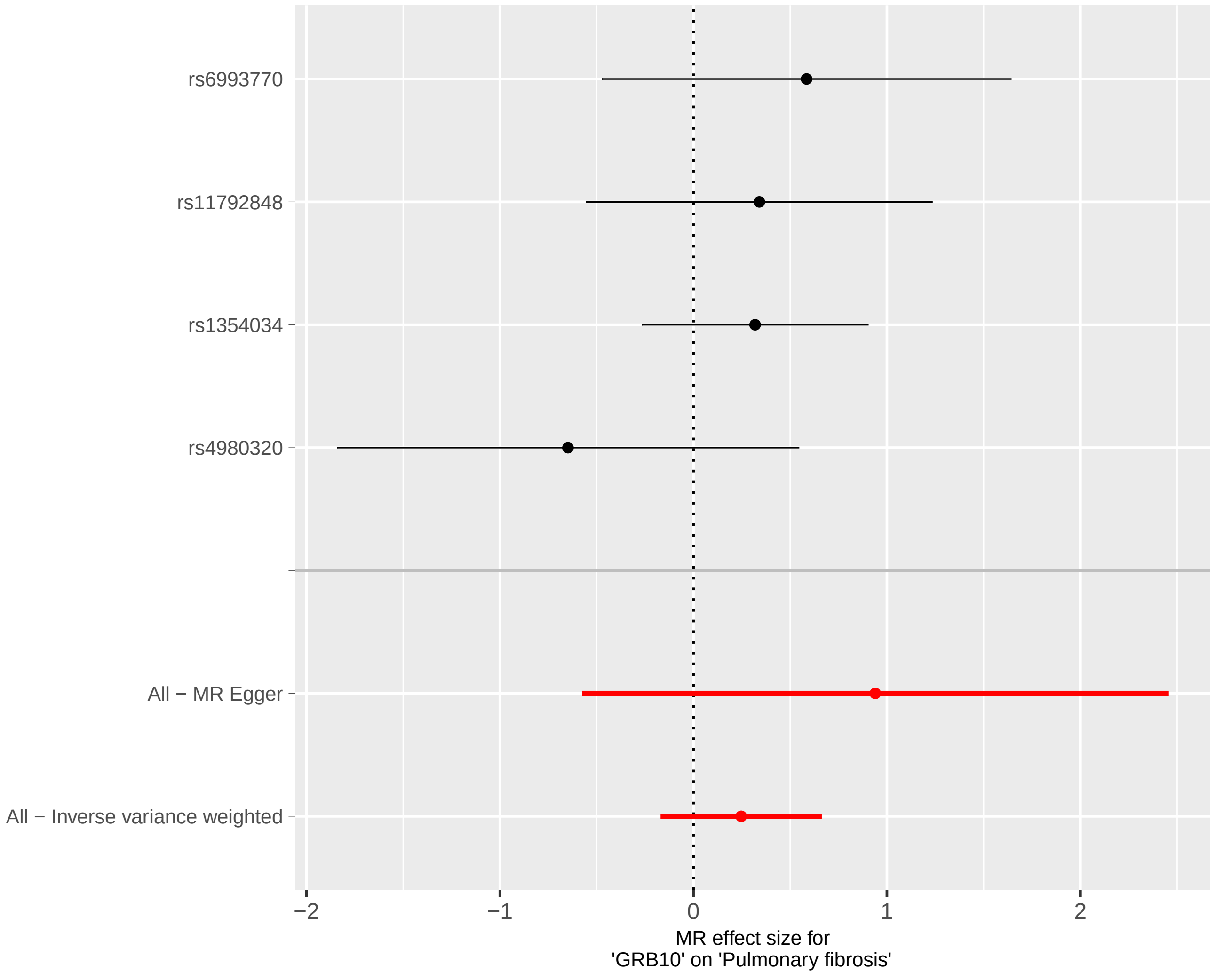

rs6993770
rs11792848
rs1354034
rs4980320
All − MR Egger
All − Inverse variance weighted
−2
−1
0
1
2
MR effect size for
'GRB10' on 'Pulmonary fibrosis'

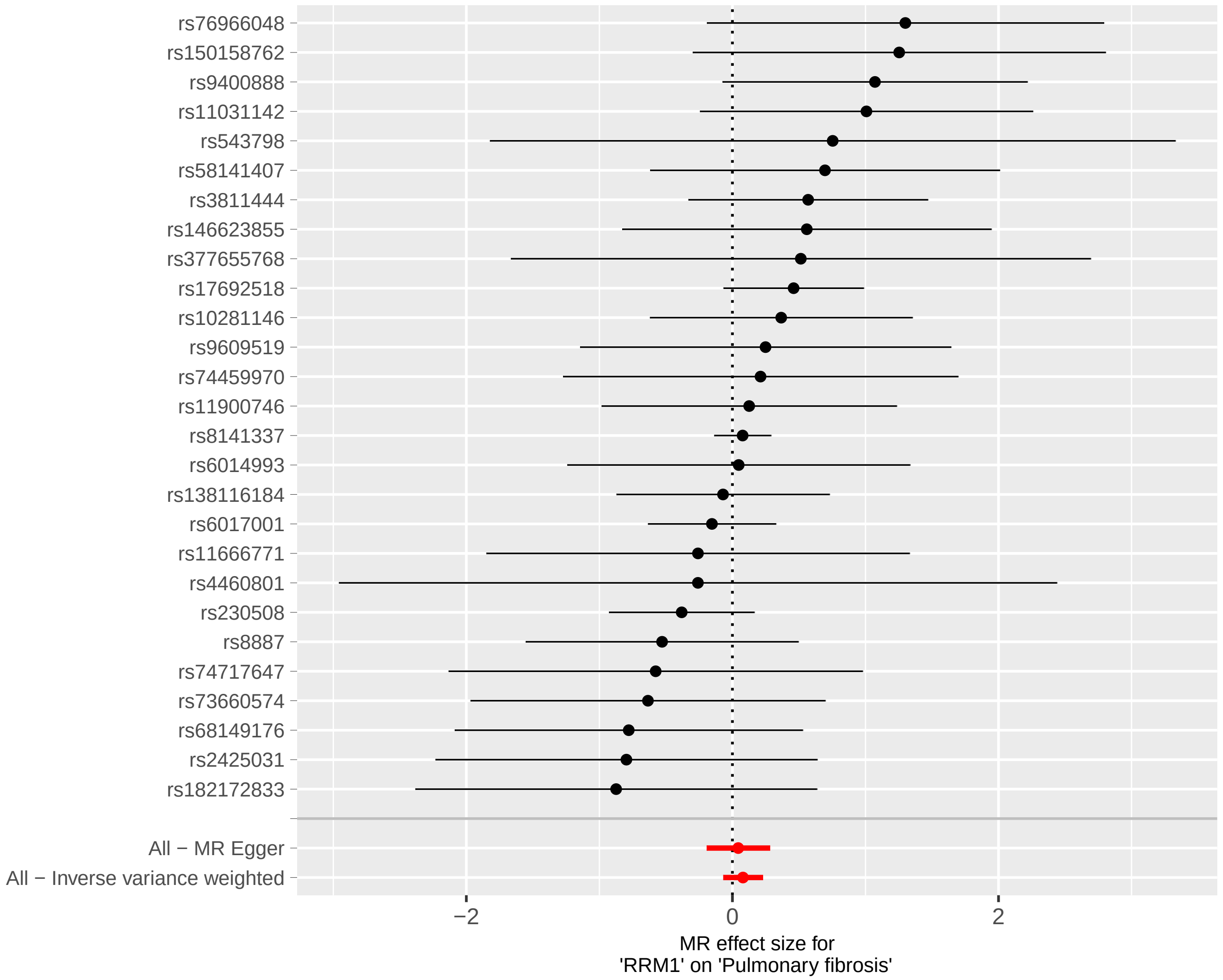
rs76966048
rs150158762
rs9400888
rs11031142
rs543798
rs58141407
rs3811444
rs146623855
rs377655768
rs17692518
rs10281146
rs9609519
rs74459970
rs11900746
rs8141337
rs6014993
rs138116184
rs6017001
rs11666771
rs4460801
rs230508
rs8887
rs74717647
rs73660574
rs68149176
rs2425031
rs182172833
All − MR Egger
All − Inverse variance weighted
−2
0
2
MR effect size for
'RRM1' on 'Pulmonary fibrosis'

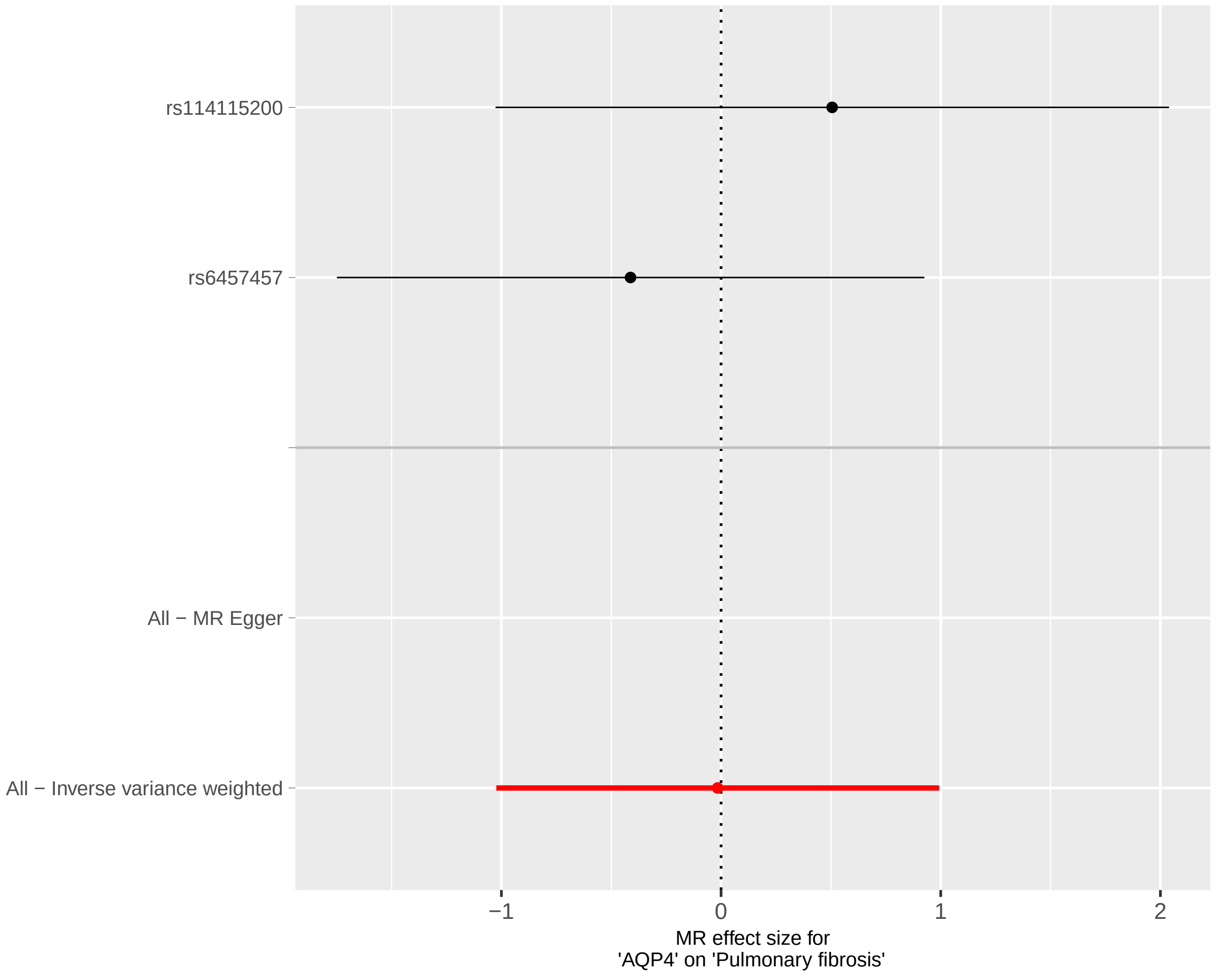
rs114115200
rs6457457
All − MR Egger
All − Inverse variance weighted
−1
0
1
2
MR effect size for
'AQP4' on 'Pulmonary fibrosis'

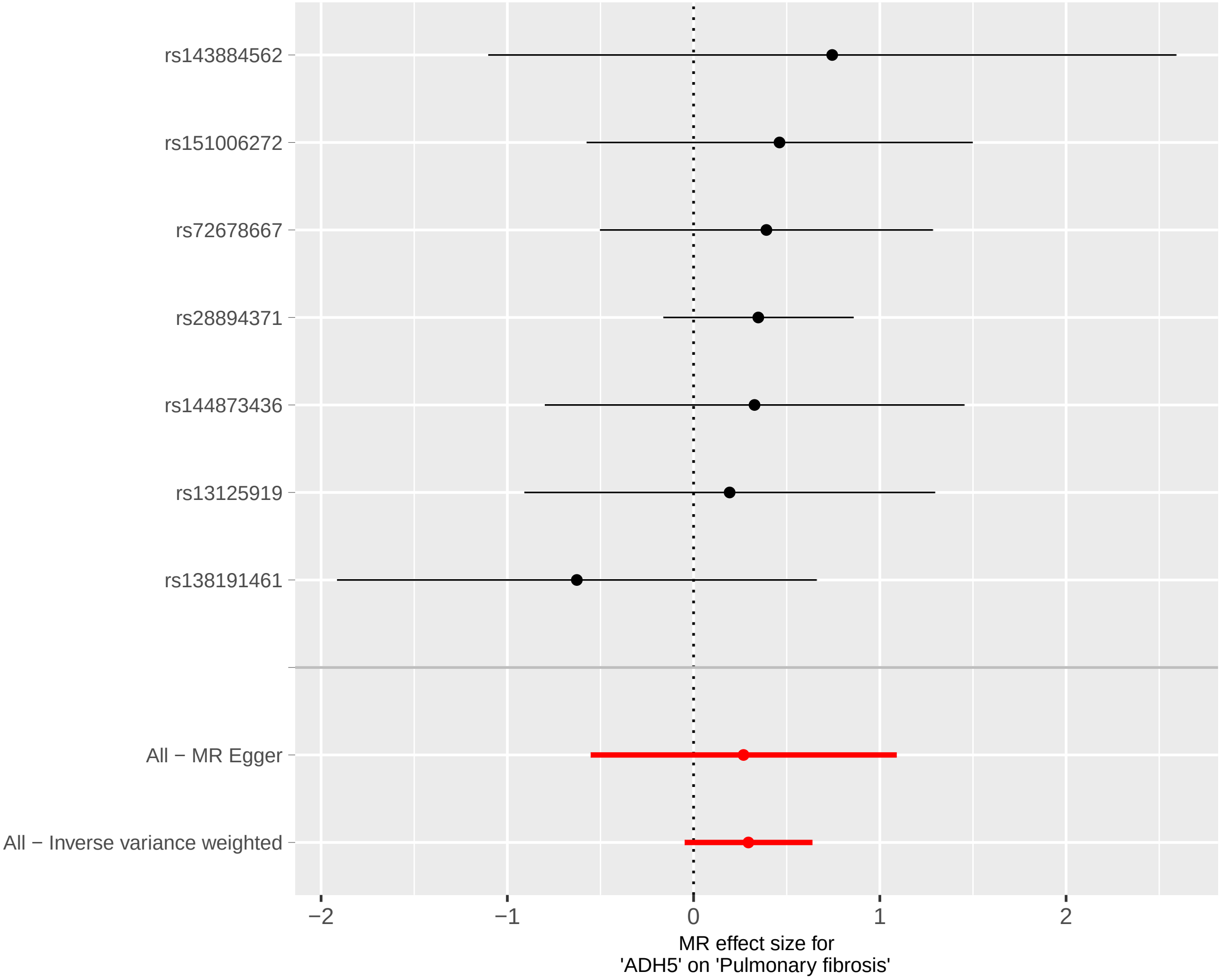

rs143884562
rs151006272
rs72678667
rs28894371
rs144873436
rs13125919
rs138191461
All − MR Egger
All − Inverse variance weighted
−2
−1
0
1
2
MR effect size for
'ADH5' on 'Pulmonary fibrosis'

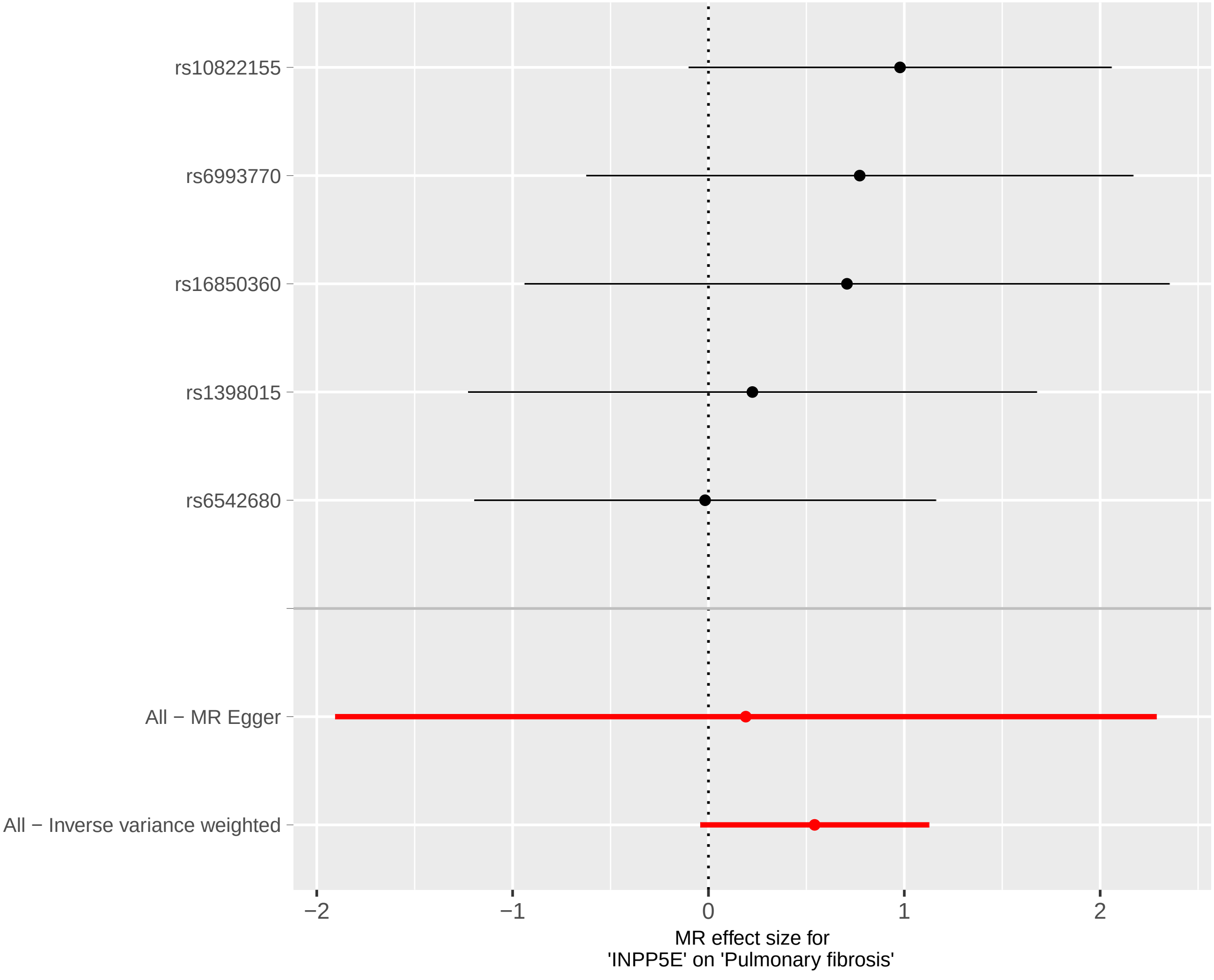
rs10822155
rs6993770
rs16850360
rs1398015
rs6542680
All − MR Egger
All − Inverse variance weighted
−2
−1
0
1
2
MR effect size for
'INPP5E' on 'Pulmonary fibrosis'

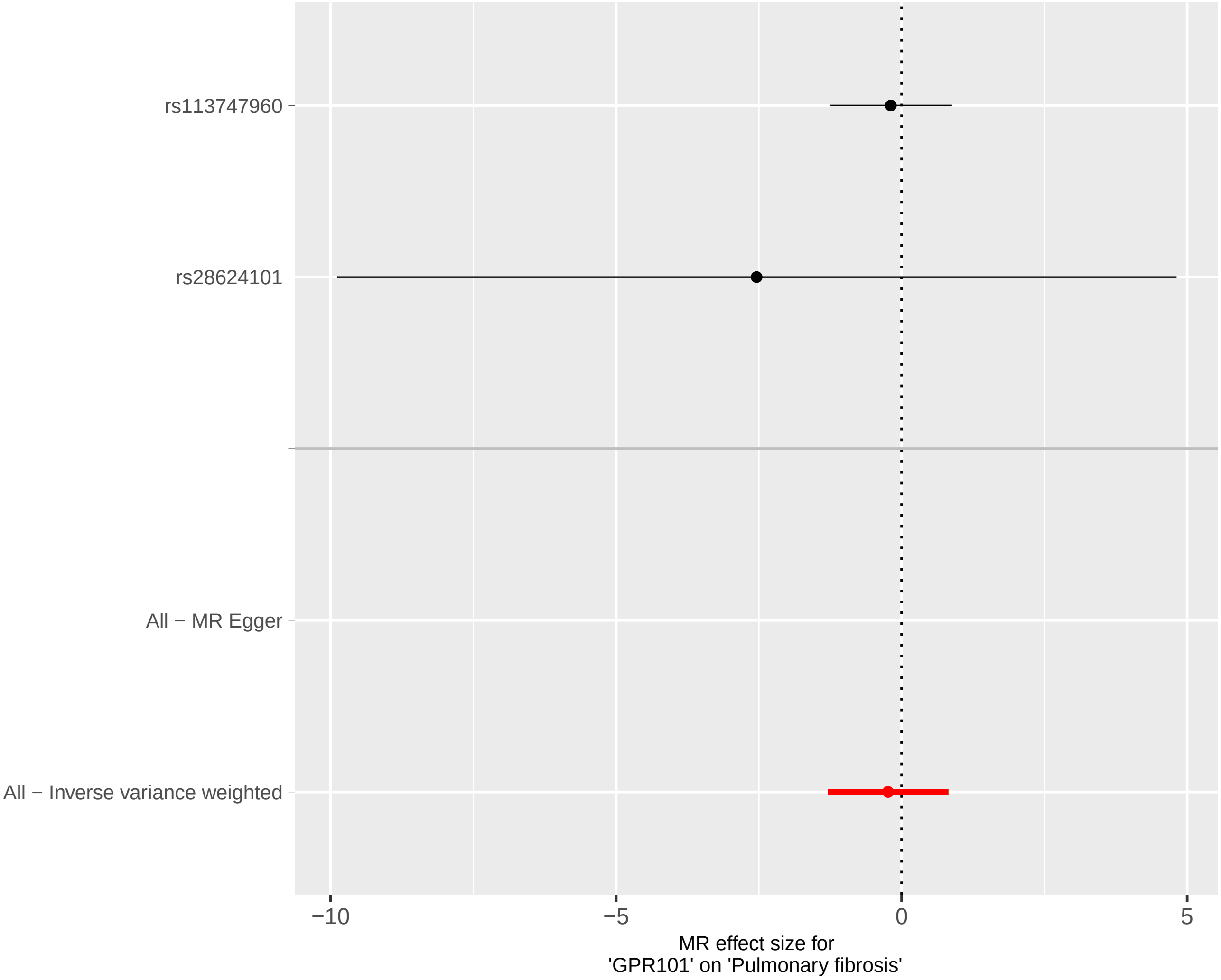

rs113747960
rs28624101
All − MR Egger
All − Inverse variance weighted
−10
−5
0
5
MR effect size for
'GPR101' on 'Pulmonary fibrosis'

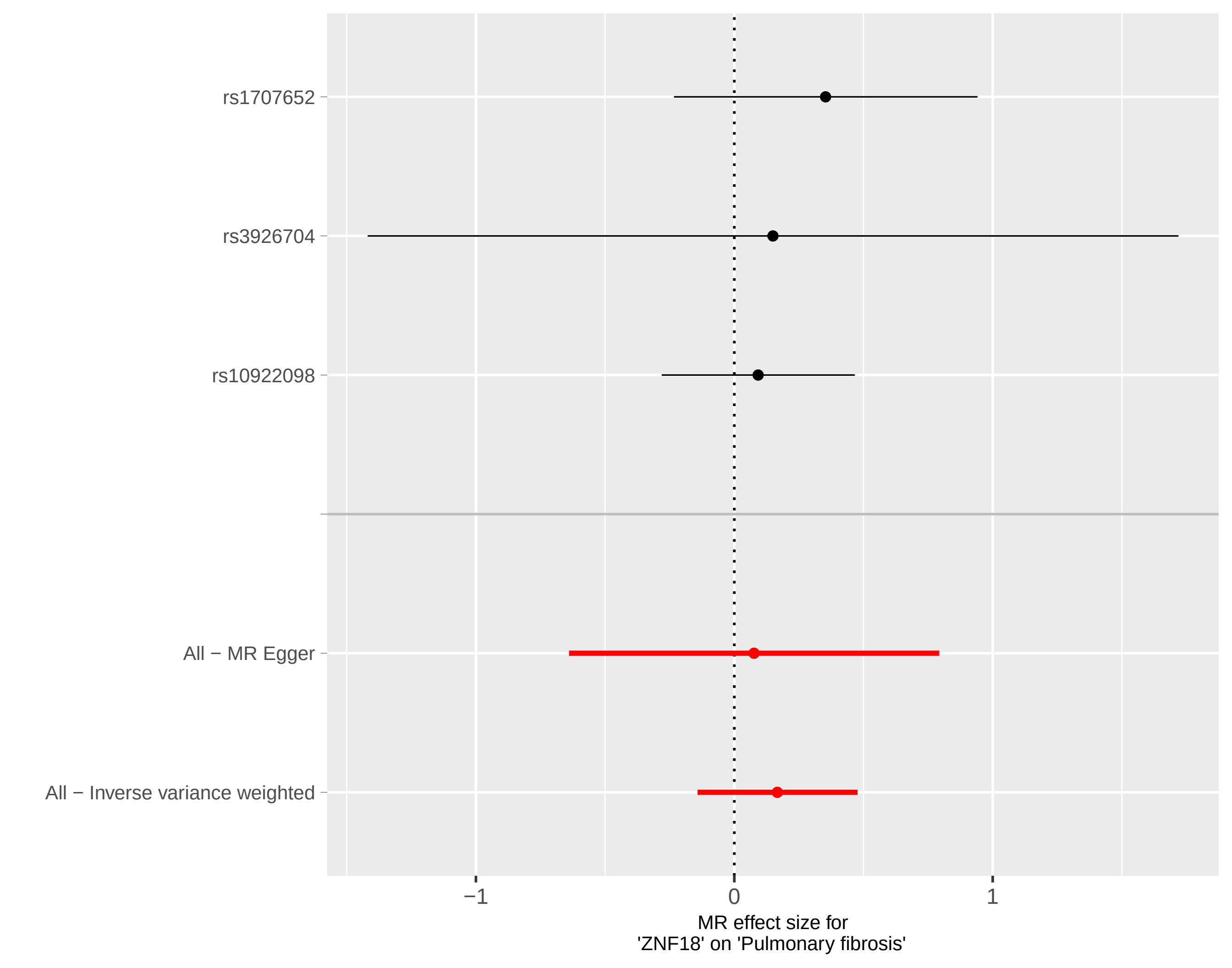
rs1707652
rs3926704
rs10922098
All − MR Egger
All − Inverse variance weighted
−1
0
1
MR effect size for
'ZNF18' on 'Pulmonary fibrosis'

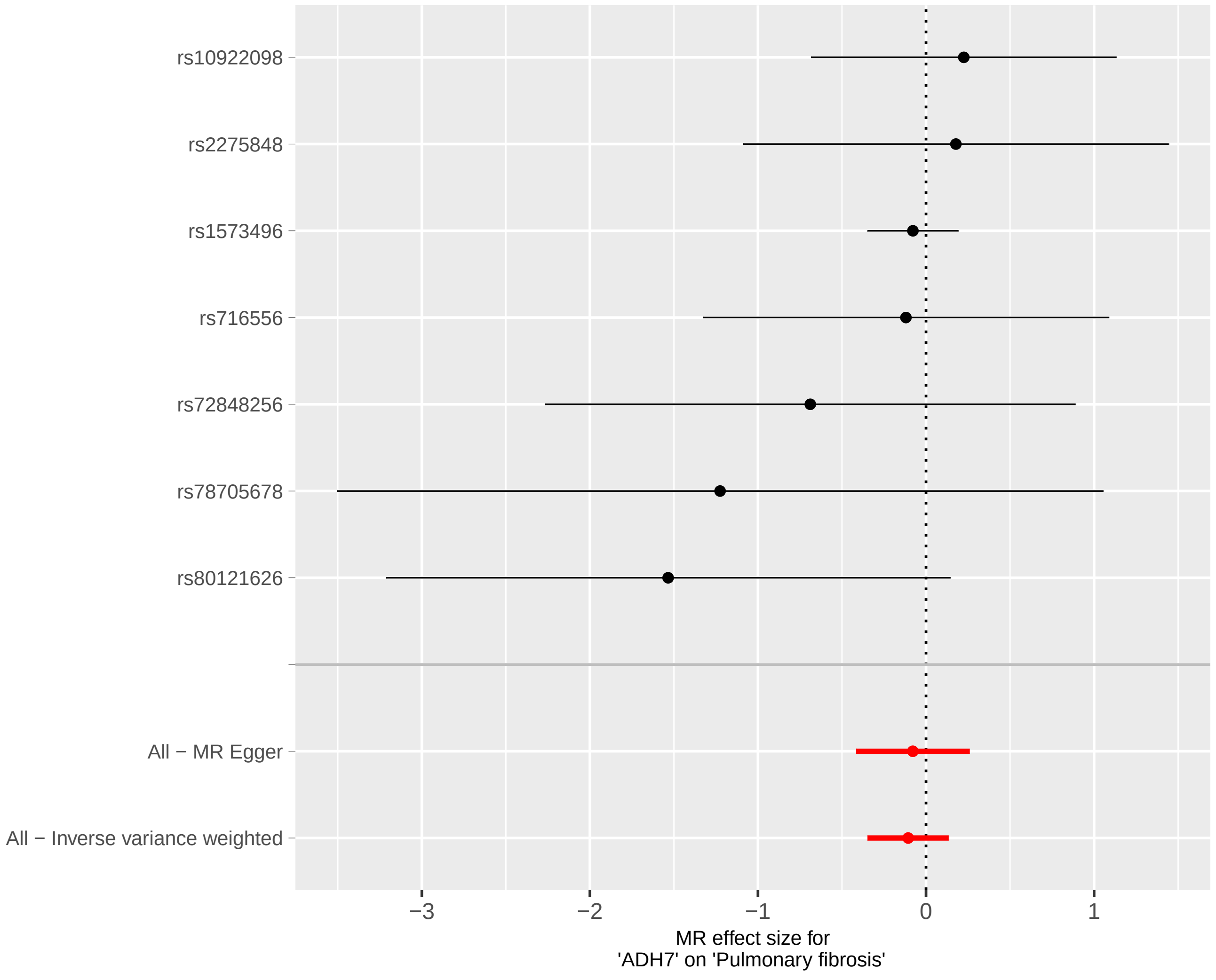
rs10922098
rs2275848
rs1573496
rs716556
rs72848256
rs78705678
rs80121626
All − MR Egger
All − Inverse variance weighted
−3
−2
−1
0
1
MR effect size for
'ADH7' on 'Pulmonary fibrosis'

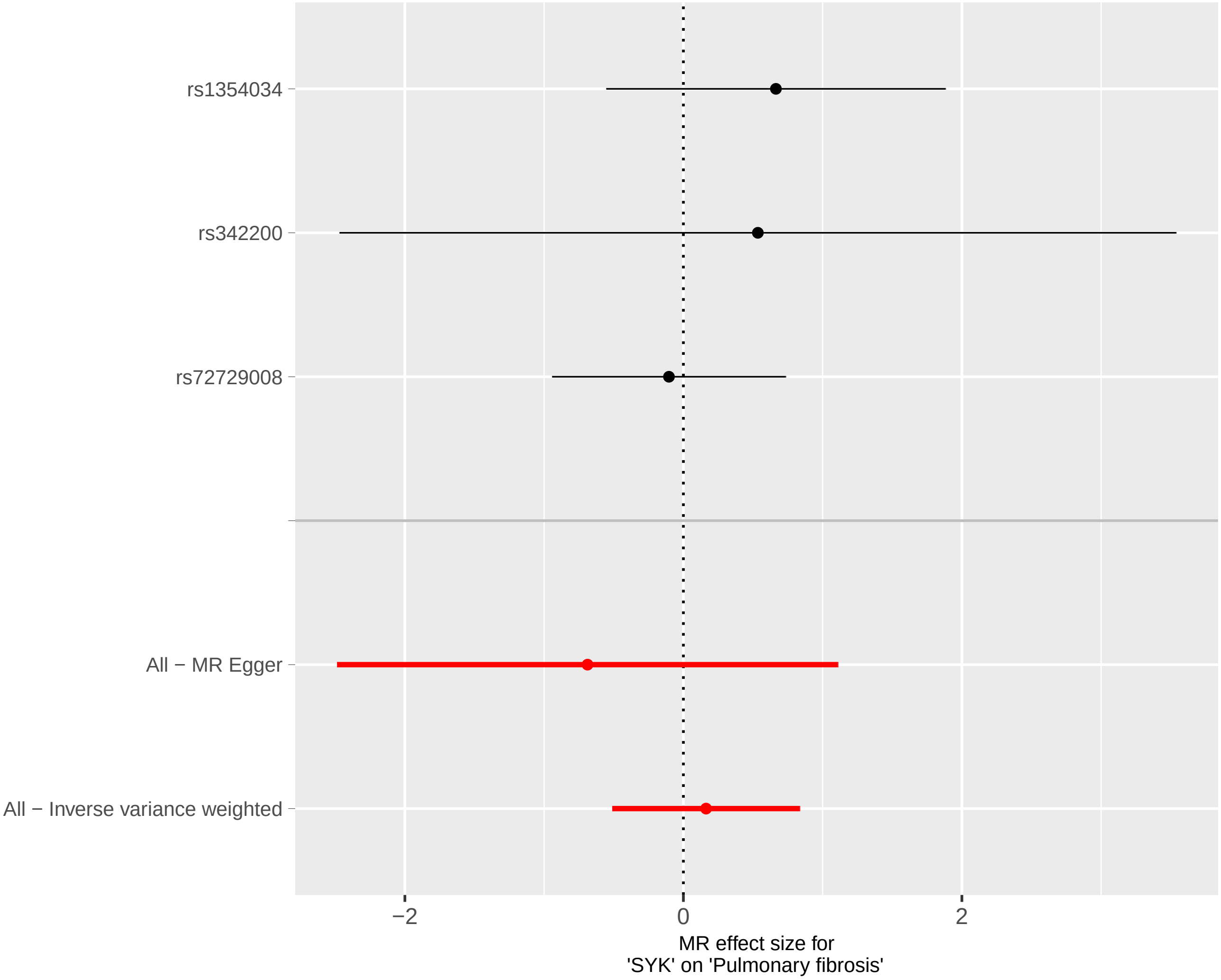

rs1354034
rs342200
rs72729008
All − MR Egger
All − Inverse variance weighted
−2
0
2
MR effect size for
'SYK' on 'Pulmonary fibrosis'

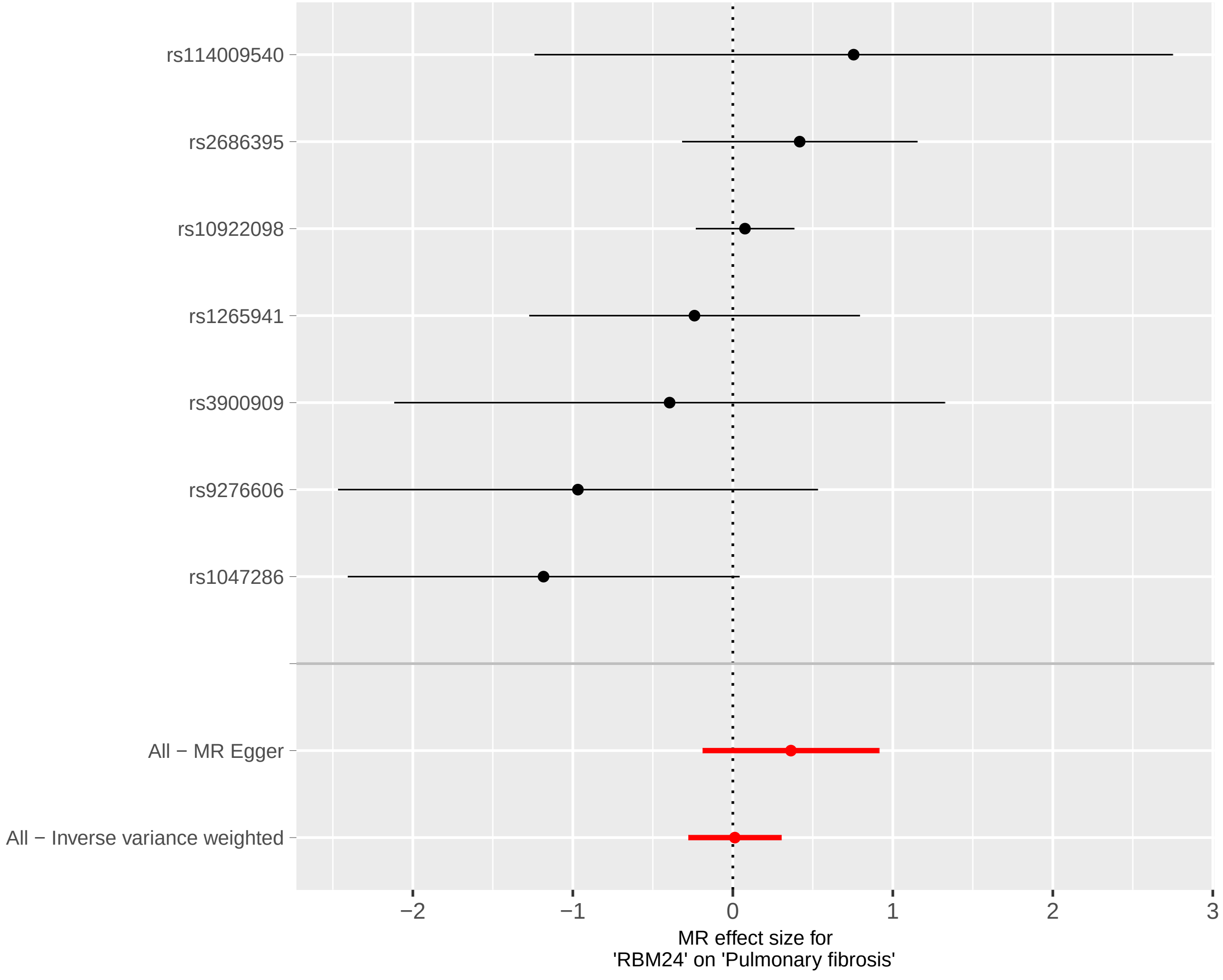
rs114009540
rs2686395
rs10922098
rs1265941
rs3900909
rs9276606
rs1047286
All − MR Egger
All − Inverse variance weighted
−2
−1
0
1
2
3
MR effect size for
'RBM24' on 'Pulmonary fibrosis'

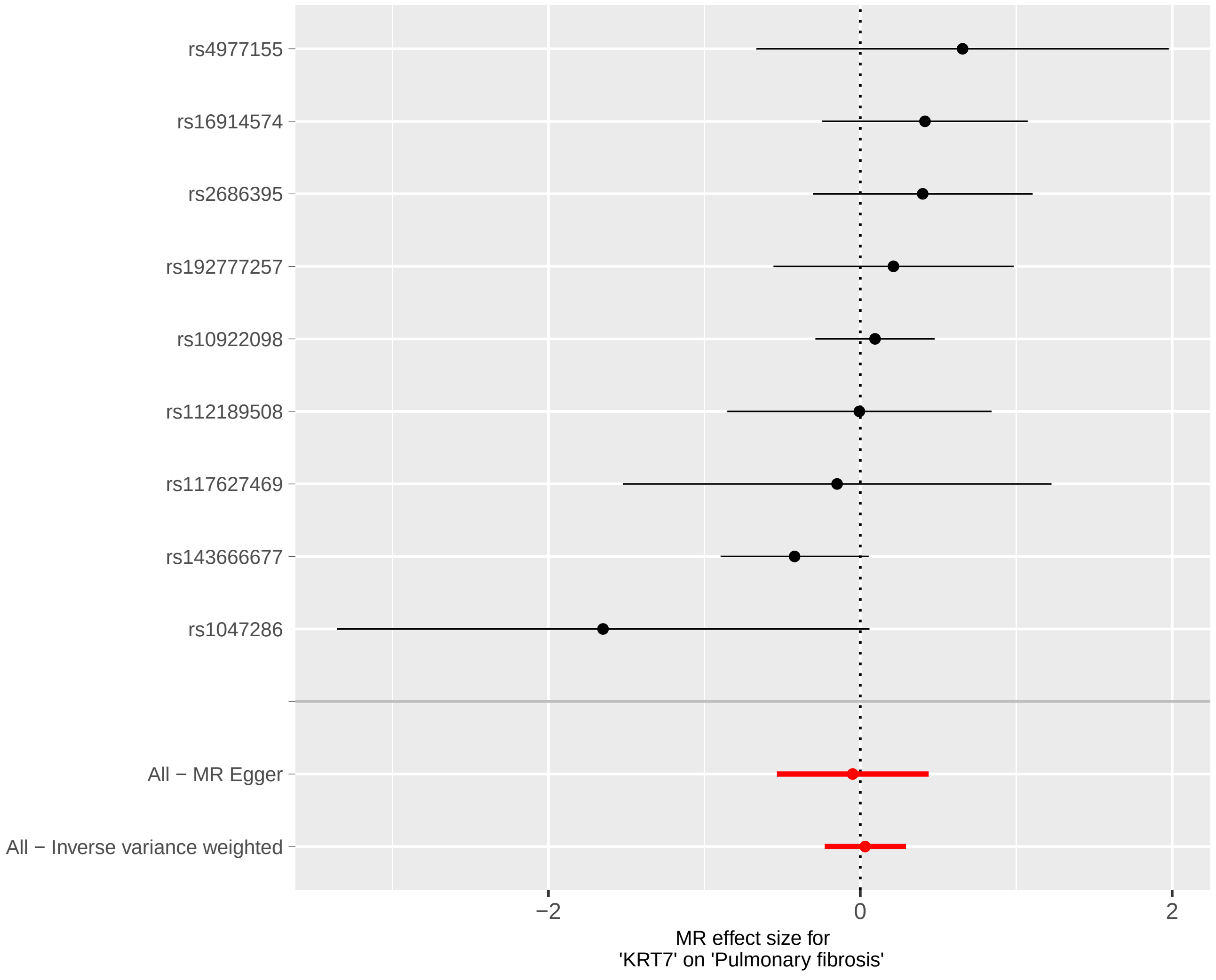
rs4977155
rs16914574
rs2686395
rs192777257
rs10922098
rs112189508
rs117627469
rs143666677
rs1047286
All − MR Egger
All − Inverse variance weighted
−2
0
2
MR effect size for
'KRT7' on 'Pulmonary fibrosis'

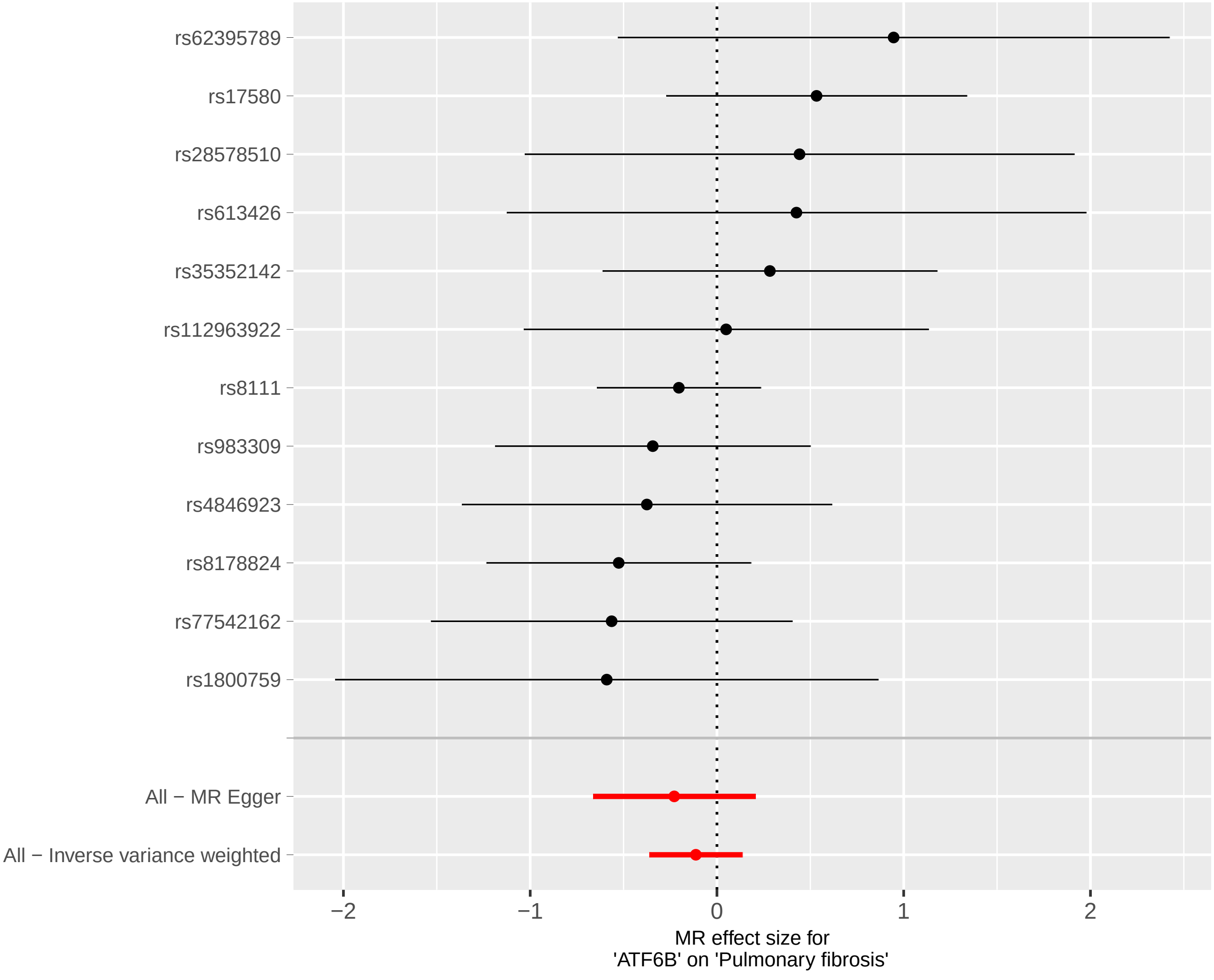

rs62395789
rs17580
rs28578510
rs613426
rs35352142
rs112963922
rs8111
rs983309
rs4846923
rs8178824
rs77542162
rs1800759
All − MR Egger
All − Inverse variance weighted
−2
−1
0
1
2
MR effect size for
'ATF6B' on 'Pulmonary fibrosis'

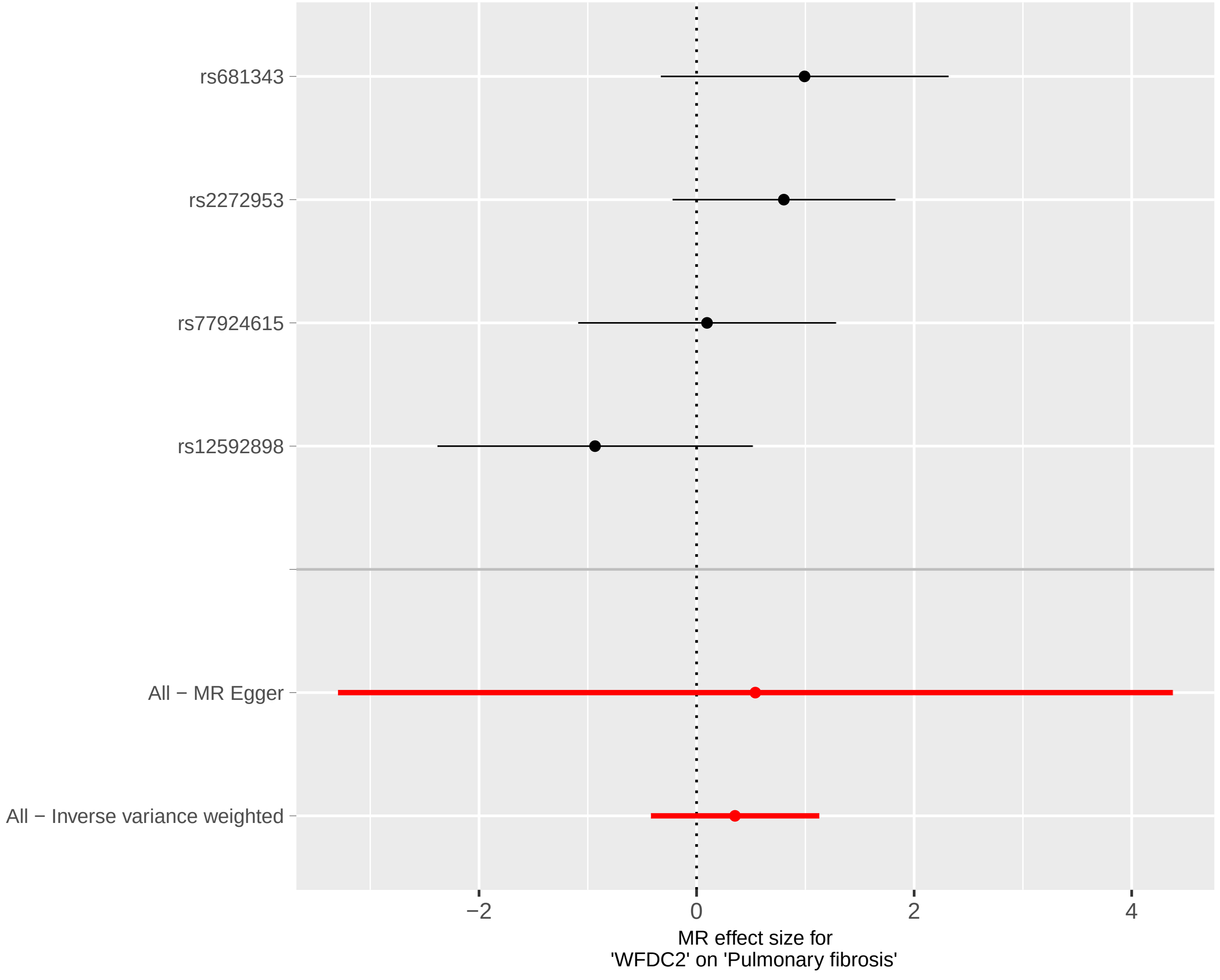

rs681343
rs2272953
rs77924615
rs12592898
All − MR Egger
All − Inverse variance weighted
−2
0
2
4
MR effect size for
'WFDC2' on 'Pulmonary fibrosis'

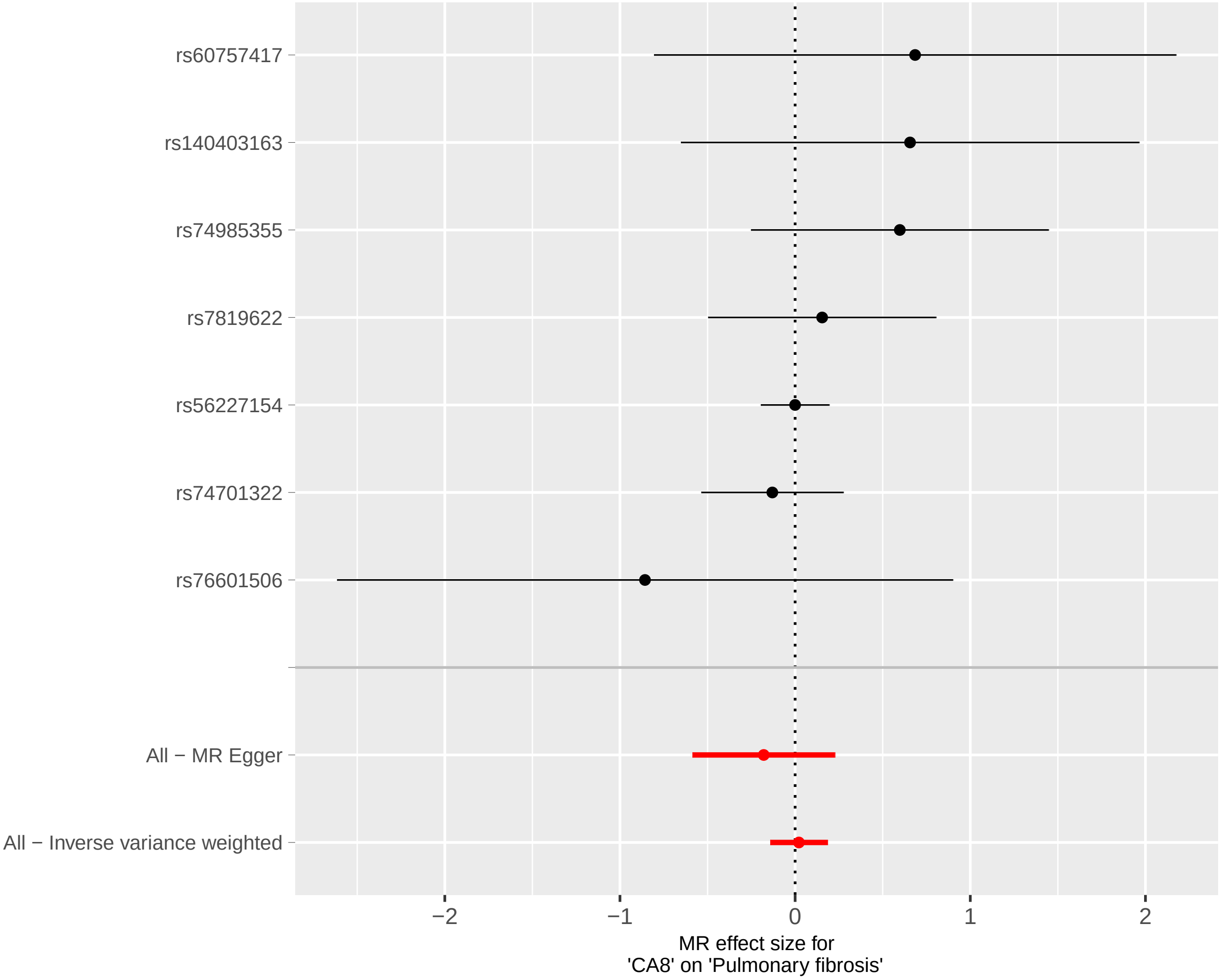

rs60757417
rs140403163
rs74985355
rs7819622
rs56227154
rs74701322
rs76601506
All − MR Egger
All − Inverse variance weighted
−2
−1
0
1
2
MR effect size for
'CA8' on 'Pulmonary fibrosis'

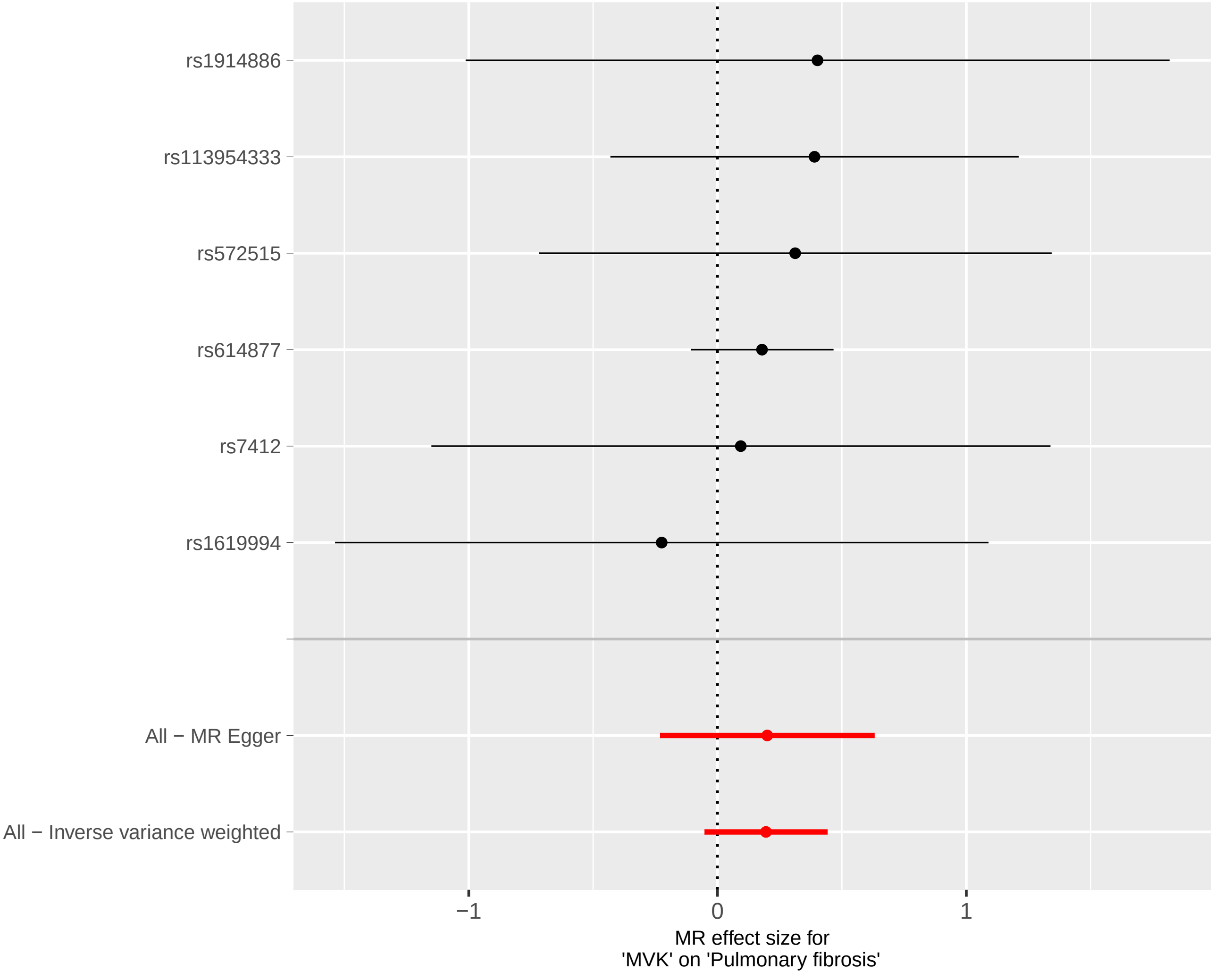

rs1914886
rs113954333
rs572515
rs614877
rs7412
rs1619994
All − MR Egger
All − Inverse variance weighted
−1
0
1
MR effect size for
'MVK' on 'Pulmonary fibrosis'

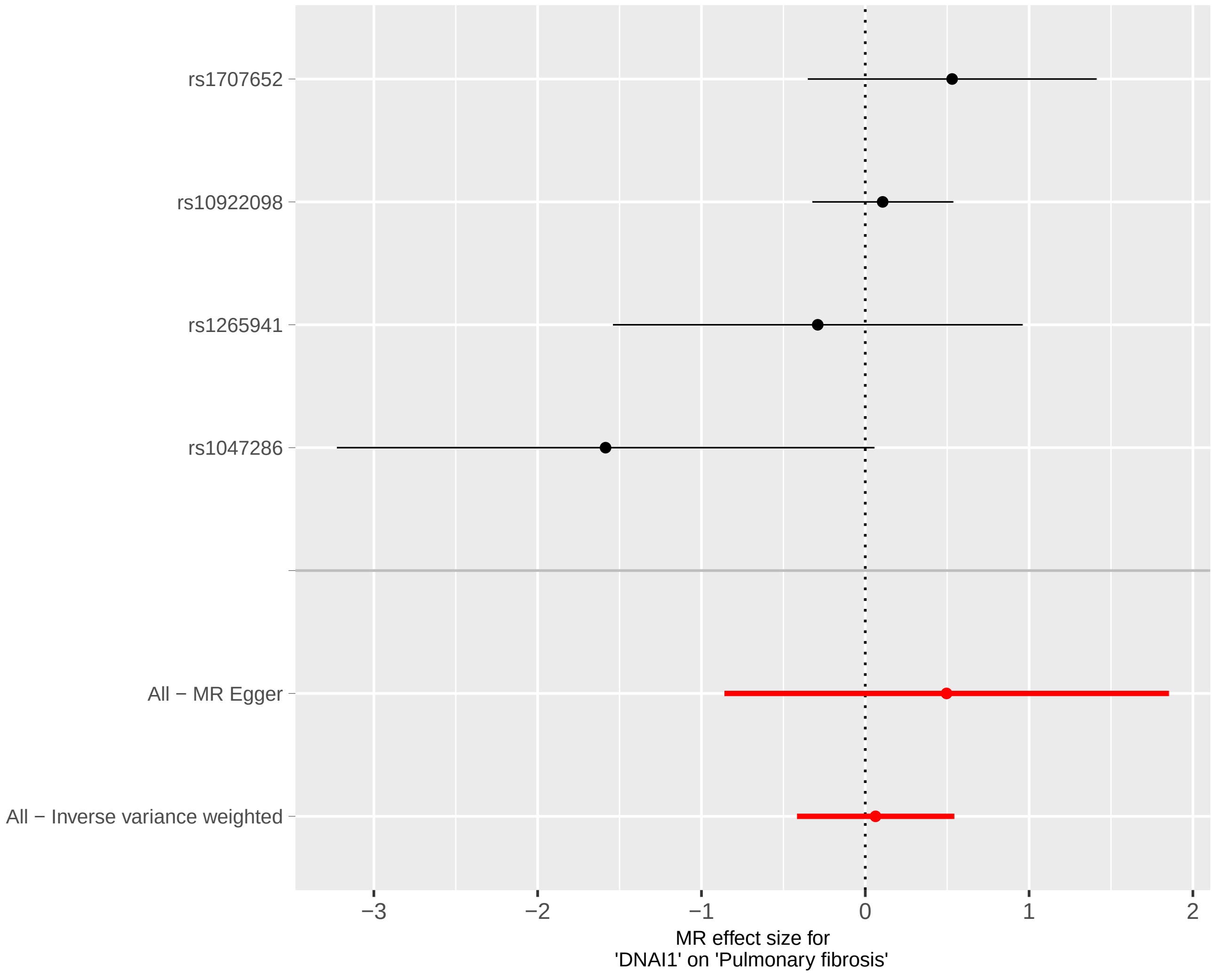
rs1707652
rs10922098
rs1265941
rs1047286
All − MR Egger
All − Inverse variance weighted
−3
−2
−1
0
1
2
MR effect size for
'DNAI1' on 'Pulmonary fibrosis'

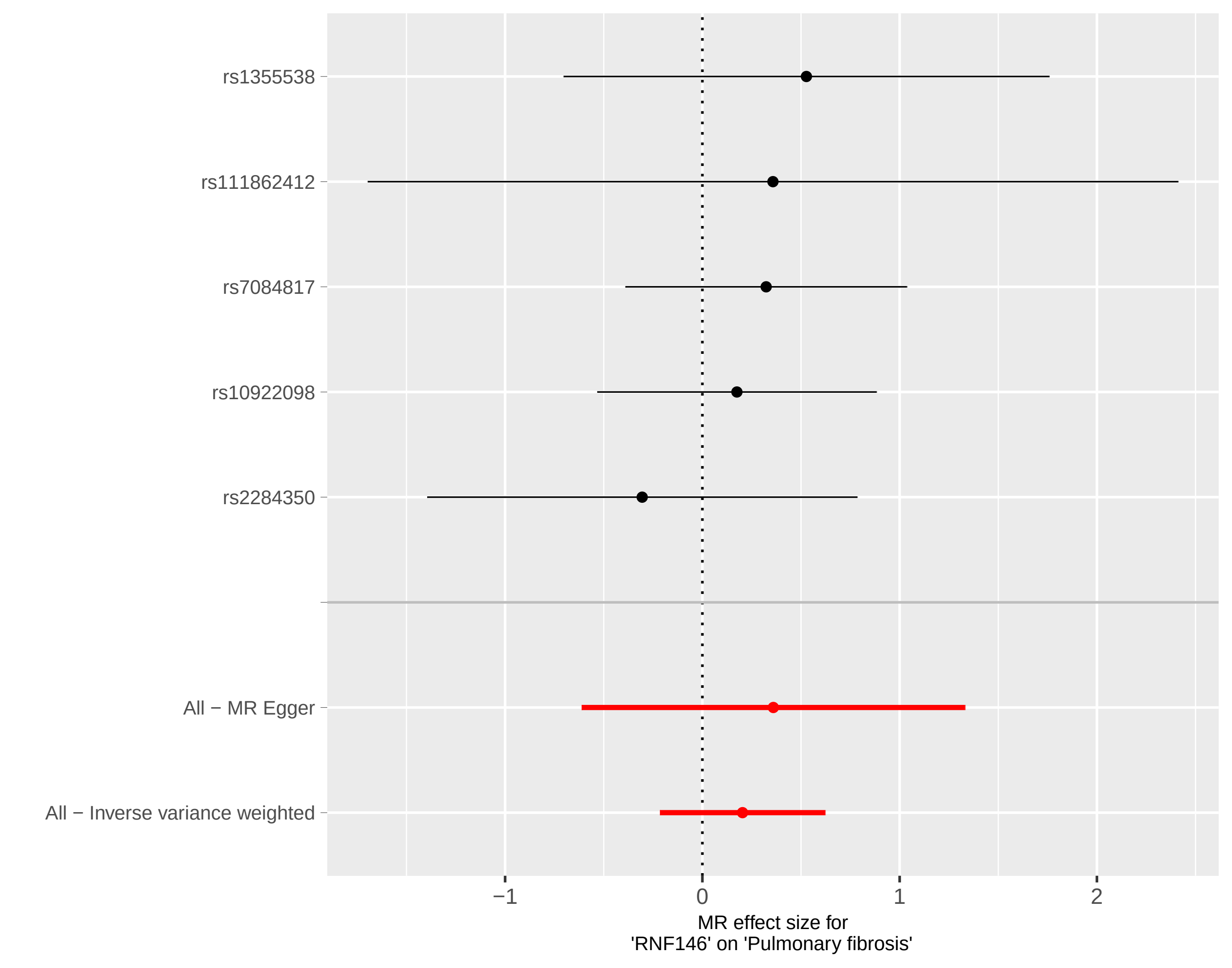
rs1355538
rs111862412
rs7084817
rs10922098
rs2284350
All − MR Egger
All − Inverse variance weighted
−1
0
1
2
MR effect size for
'RNF146' on 'Pulmonary fibrosis'

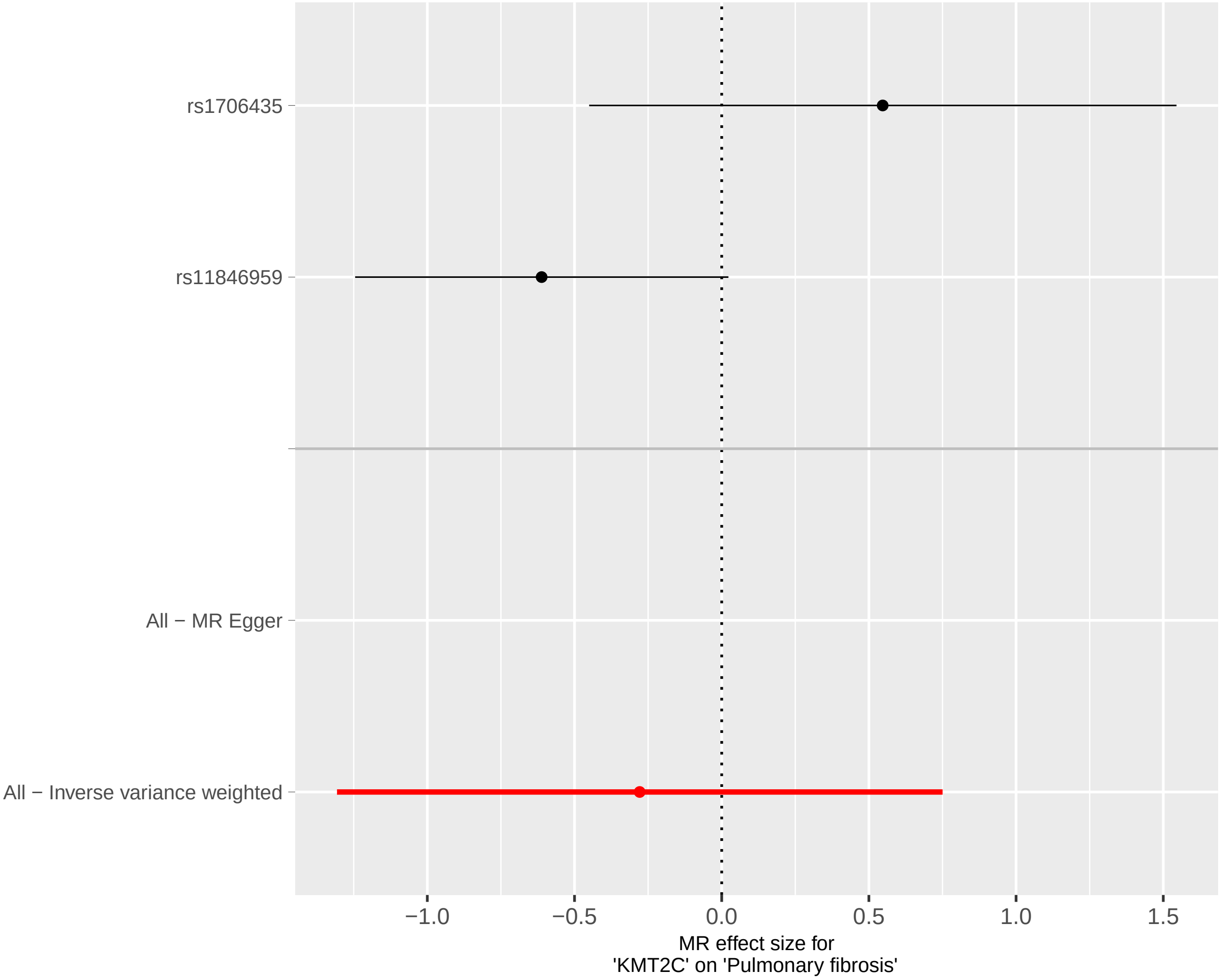
rs1706435
rs11846959
All − MR Egger
All − Inverse variance weighted
−1.0
−0.5
0.0
0.5
1.0
1.5
MR effect size for
'KMT2C' on 'Pulmonary fibrosis'

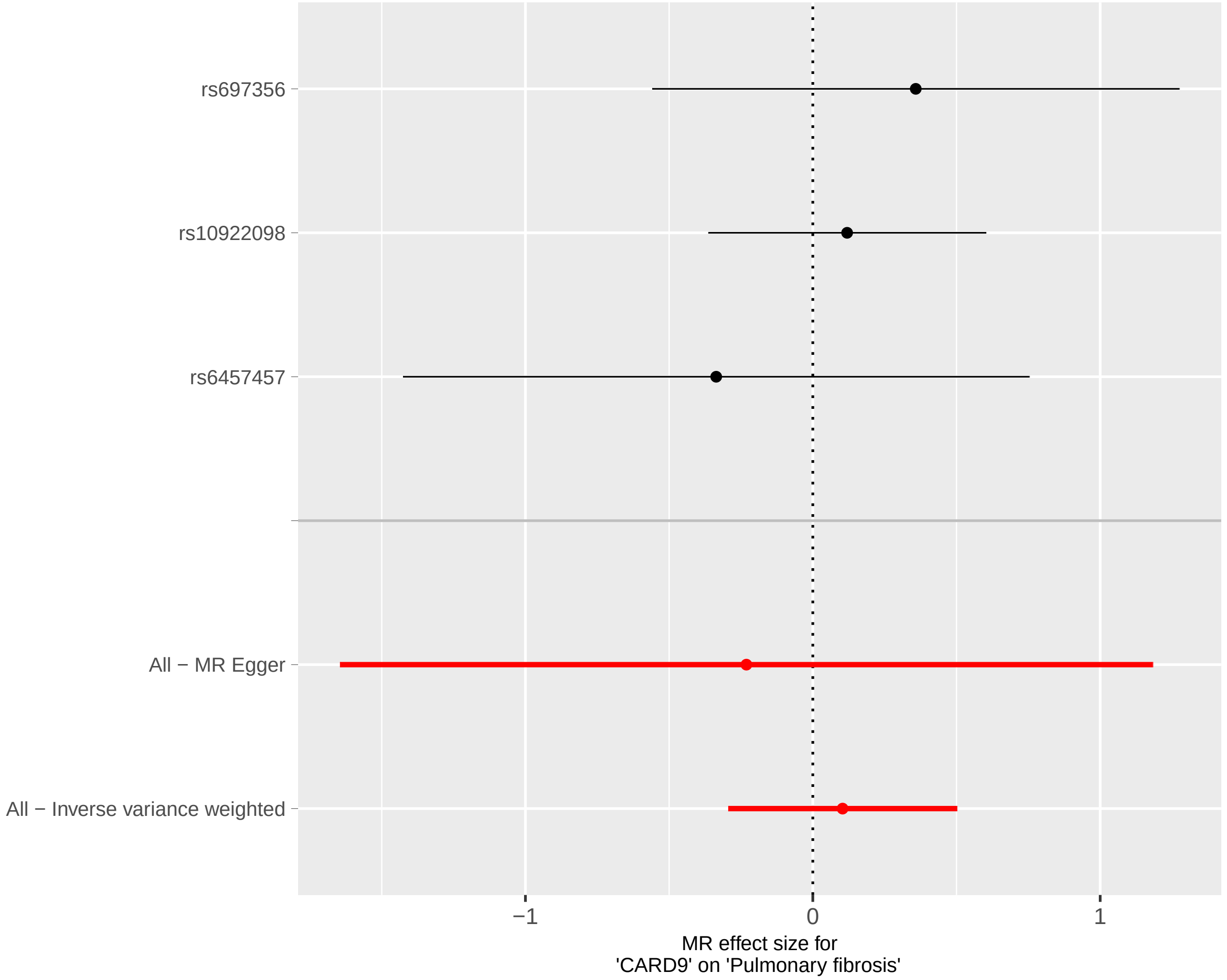

rs697356
rs10922098
rs6457457
All − MR Egger
All − Inverse variance weighted
−1
0
1
MR effect size for
'CARD9' on 'Pulmonary fibrosis'

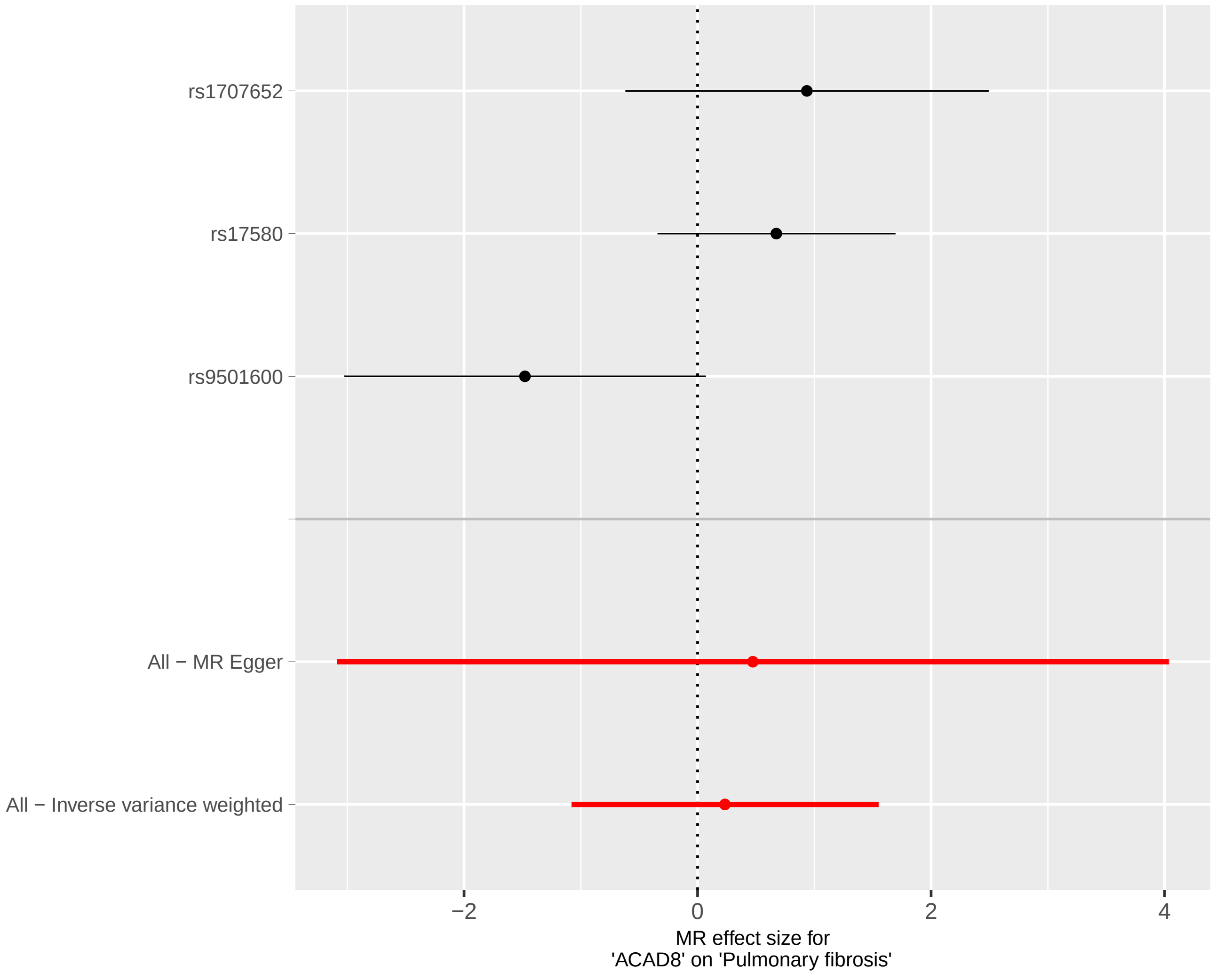
rs1707652
rs17580
rs9501600
All − MR Egger
All − Inverse variance weighted
−2
0
2
4
MR effect size for
'ACAD8' on 'Pulmonary fibrosis'

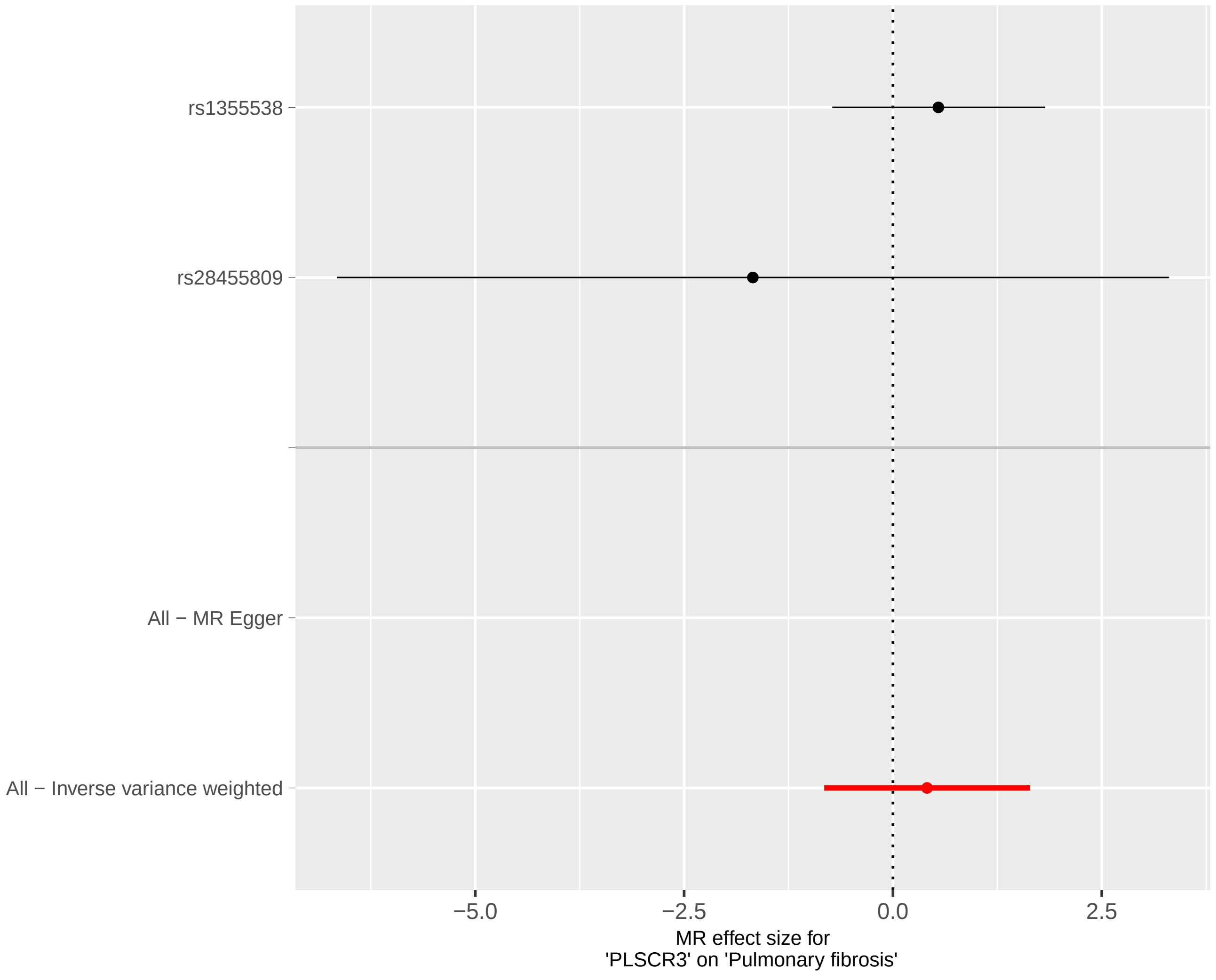

rs1355538
rs28455809
All − MR Egger
All − Inverse variance weighted
−5.0
−2.5
0.0
2.5
MR effect size for
'PLSCR3' on 'Pulmonary fibrosis'

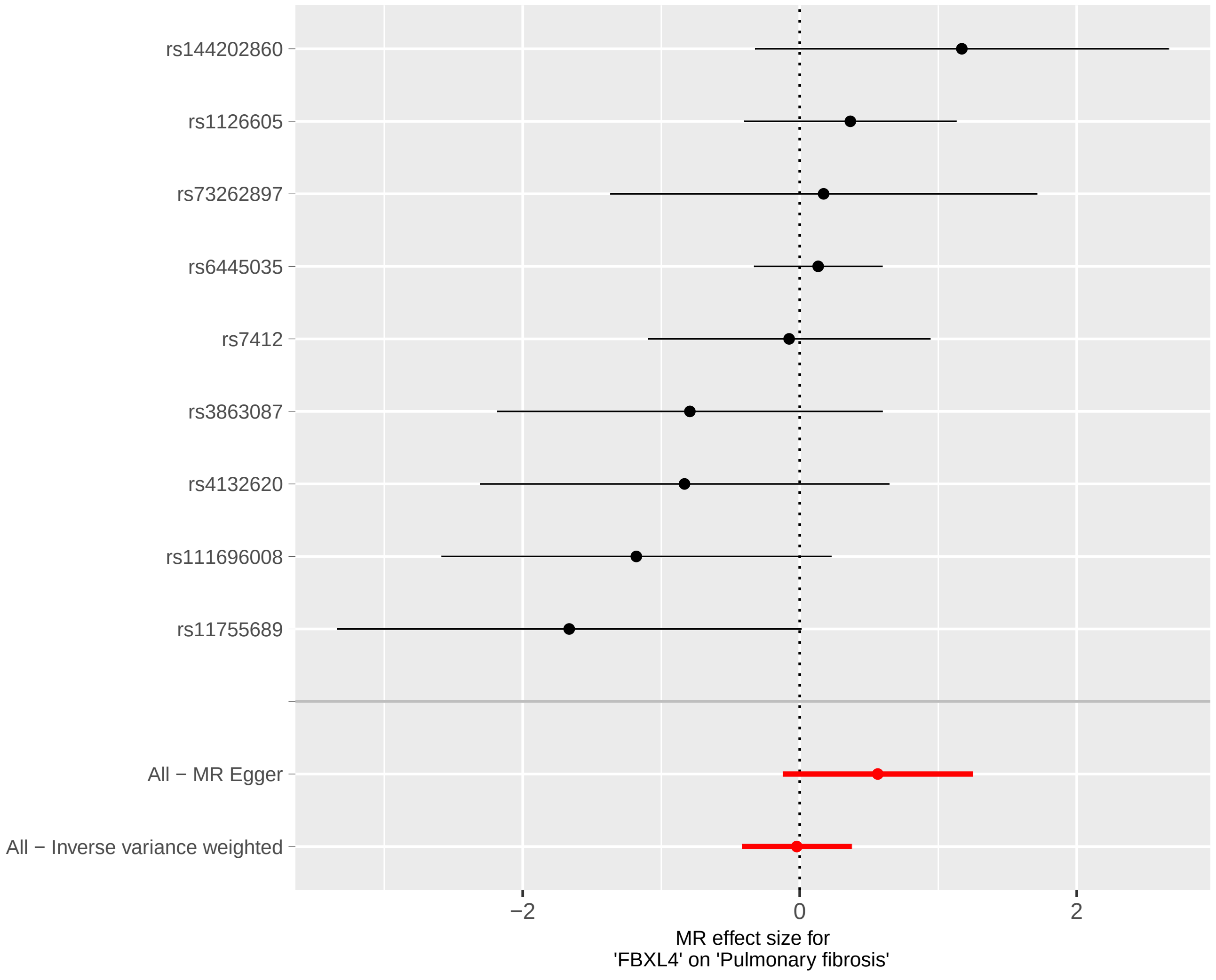
rs144202860
rs1126605
rs73262897
rs6445035
rs7412
rs3863087
rs4132620
rs111696008
rs11755689
All − MR Egger
All − Inverse variance weighted
−2
0
2
MR effect size for
'FBXL4' on 'Pulmonary fibrosis'

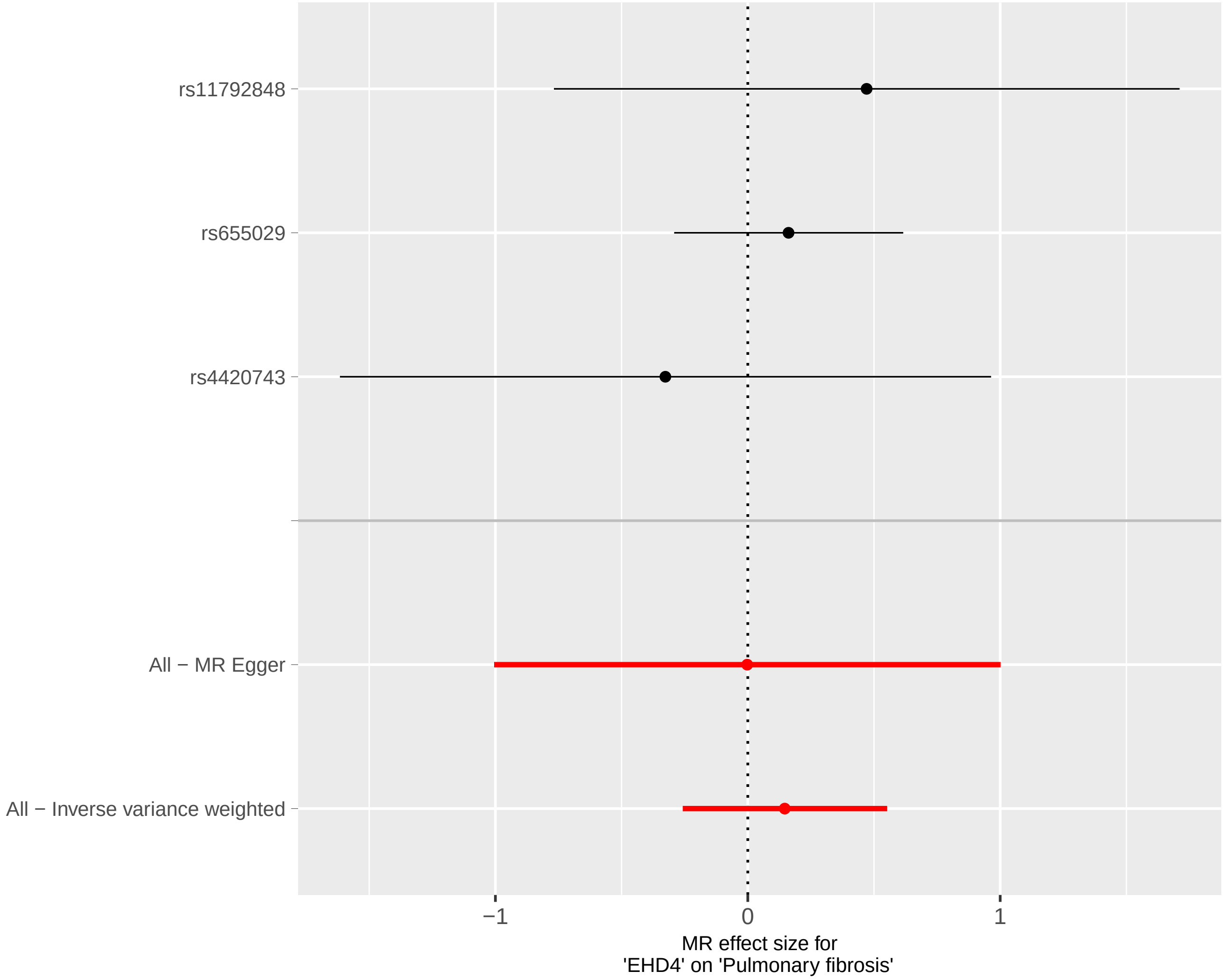

rs11792848
rs655029
rs4420743
All − MR Egger
All − Inverse variance weighted
−1
0
1
MR effect size for
'EHD4' on 'Pulmonary fibrosis'

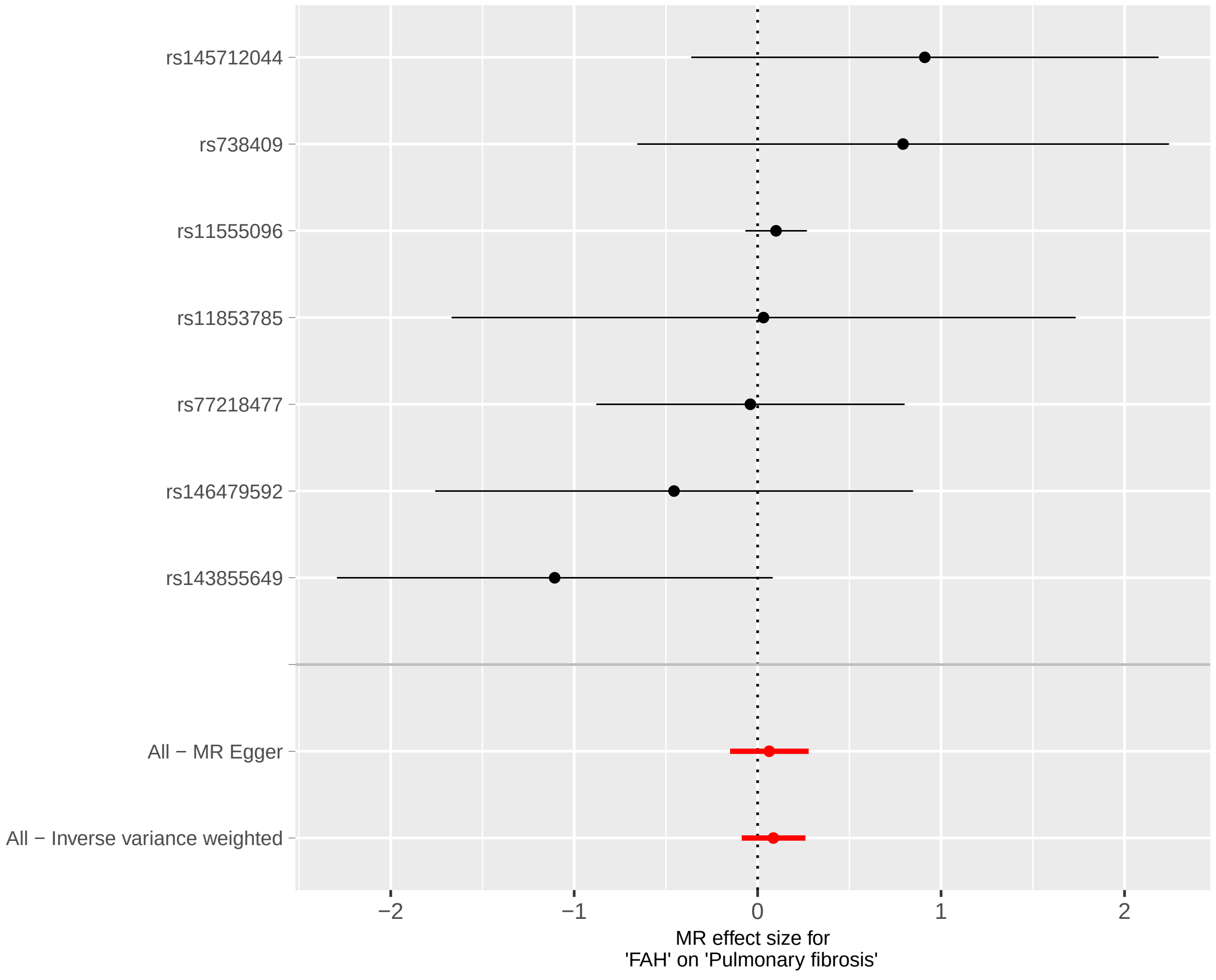

rs145712044
rs738409
rs11555096
rs11853785
rs77218477
rs146479592
rs143855649
All − MR Egger
All − Inverse variance weighted
−2
−1
0
1
2
MR effect size for
'FAH' on 'Pulmonary fibrosis'

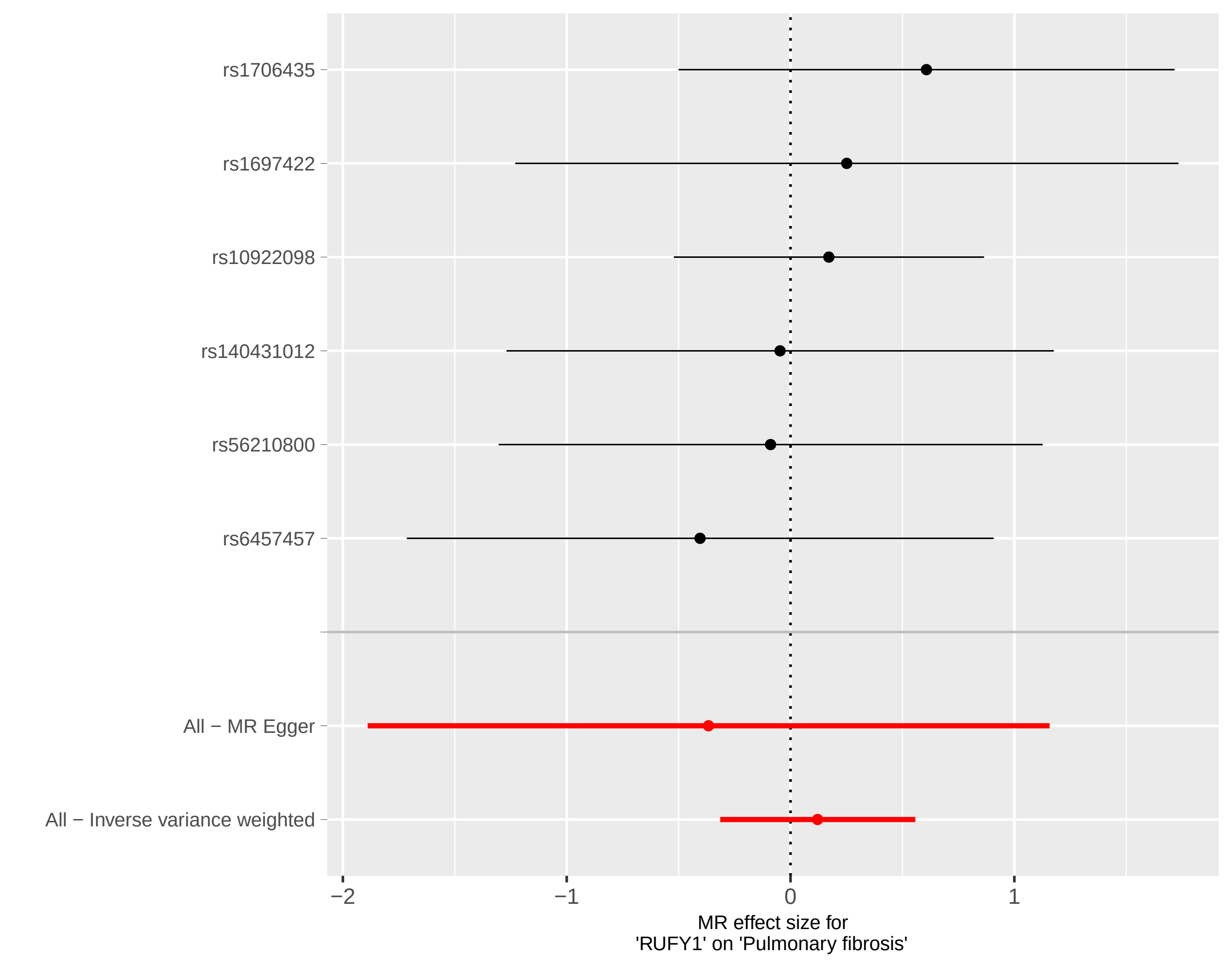
rs1706435
rs1697422
rs10922098
rs140431012
rs56210800
rs6457457
All − MR Egger
All − Inverse variance weighted
−2
−1
0
1
MR effect size for
'RUFY1' on 'Pulmonary fibrosis'

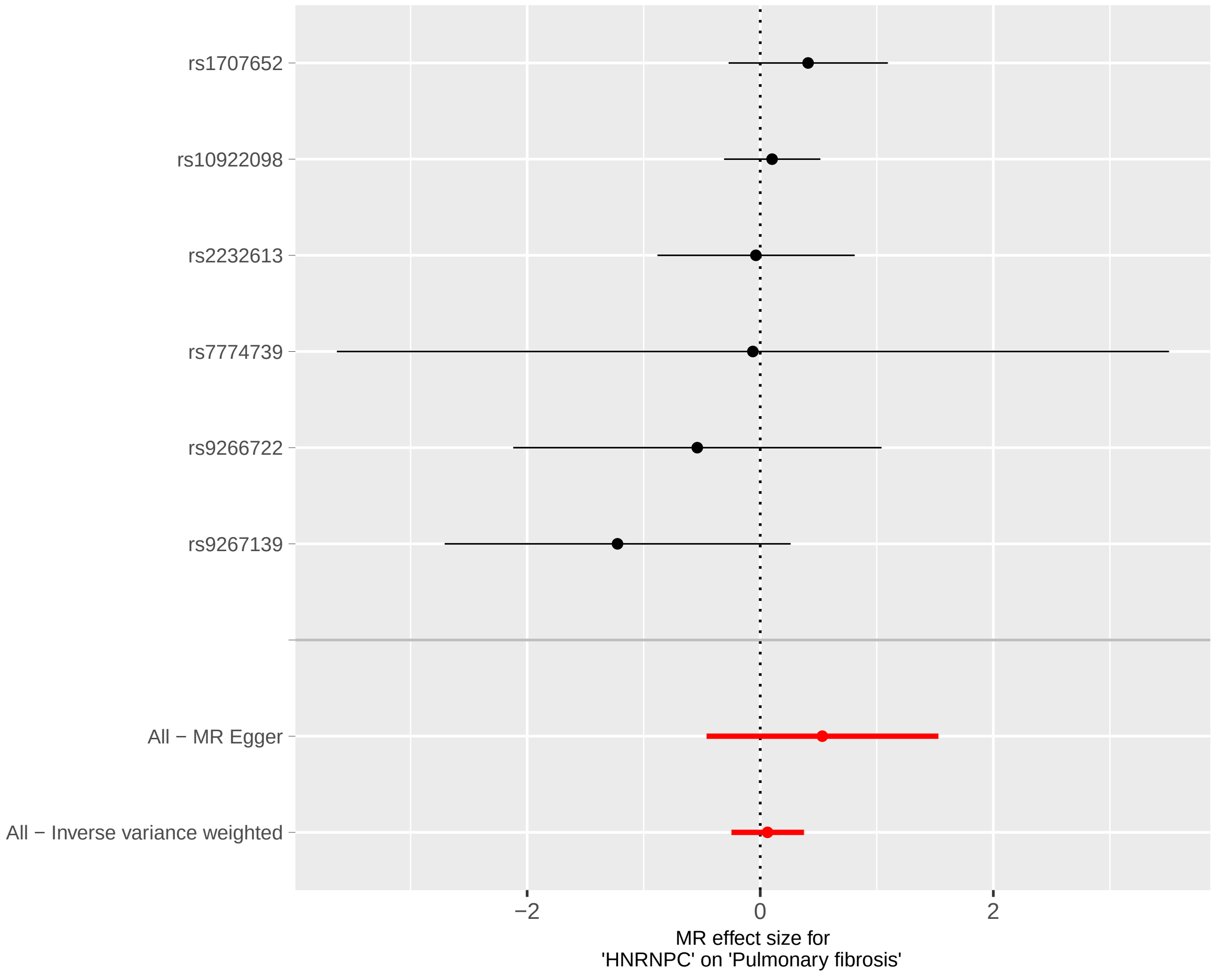

rs1707652
rs10922098
rs2232613
rs7774739
rs9266722
rs9267139
All − MR Egger
All − Inverse variance weighted
−2
0
2
MR effect size for
'HNRNPC' on 'Pulmonary fibrosis'

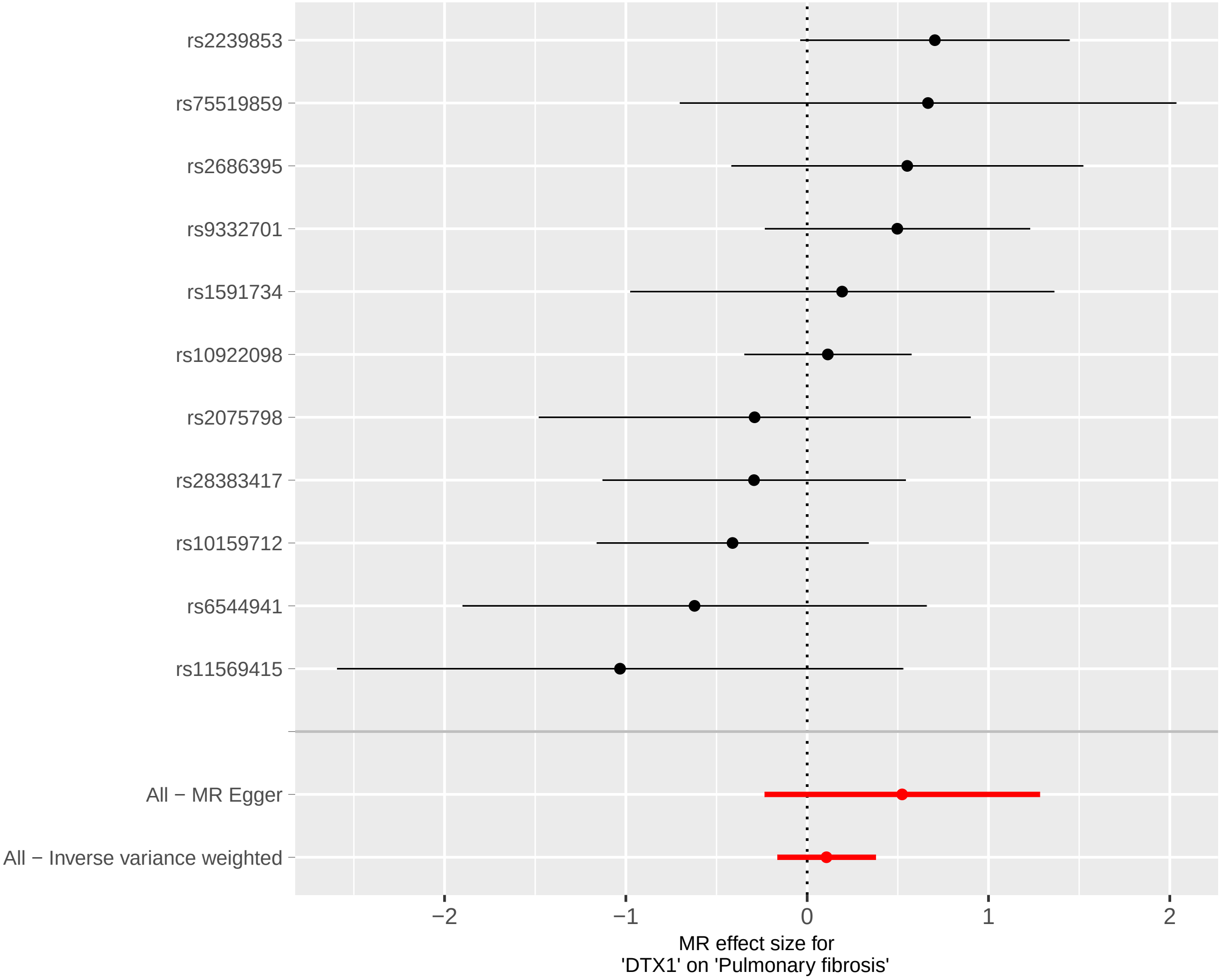
rs2239853
rs75519859
rs2686395
rs9332701
rs1591734
rs10922098
rs2075798
rs28383417
rs10159712
rs6544941
rs11569415
All − MR Egger
All − Inverse variance weighted
−2
−1
0
1
2
MR effect size for
'DTX1' on 'Pulmonary fibrosis'

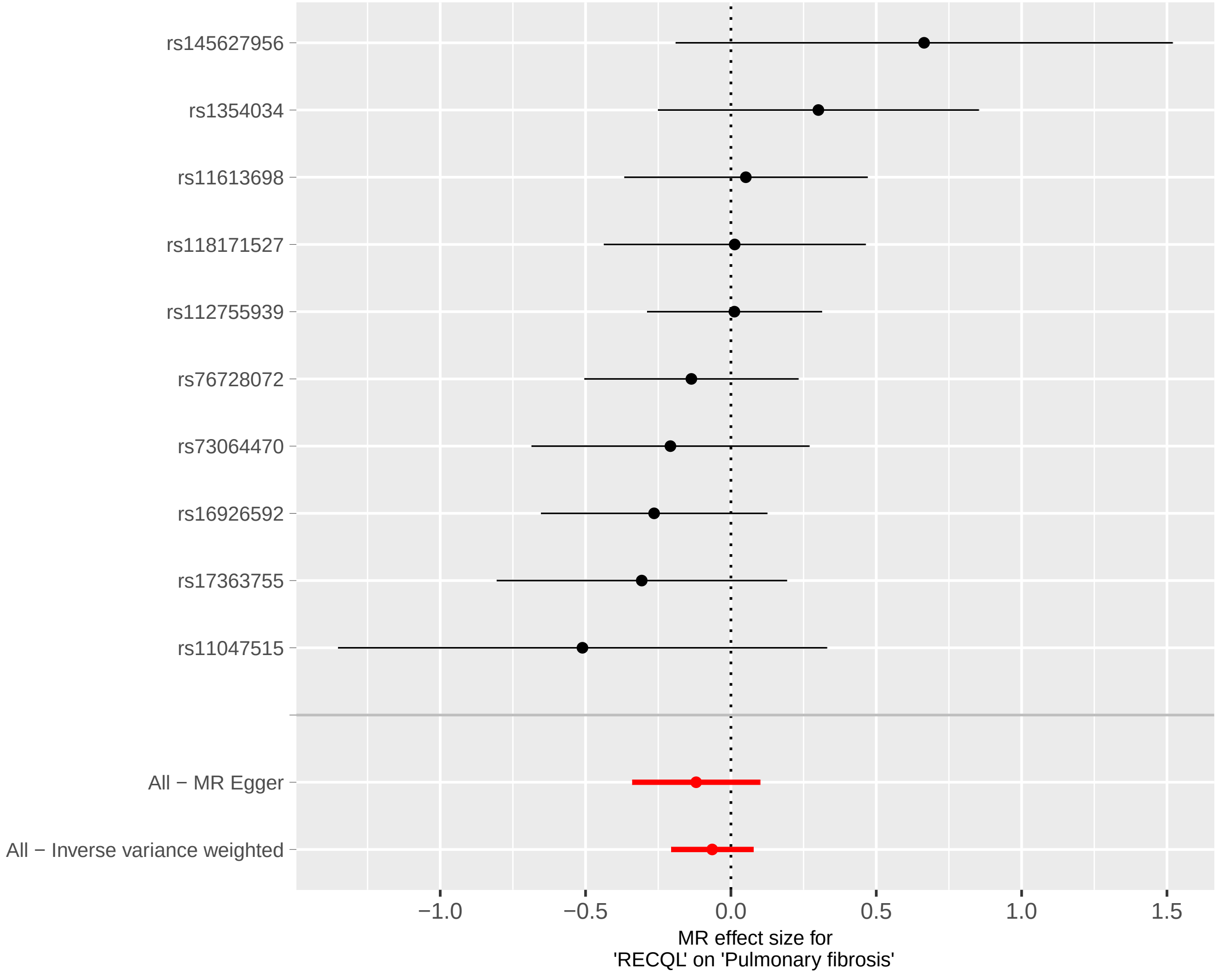

rs145627956
rs1354034
rs11613698
rs118171527
rs112755939
rs76728072
rs73064470
rs16926592
rs17363755
rs11047515
All − MR Egger
All − Inverse variance weighted
−1.0
−0.5
0.0
0.5
1.0
1.5
MR effect size for
'RECQL' on 'Pulmonary fibrosis'

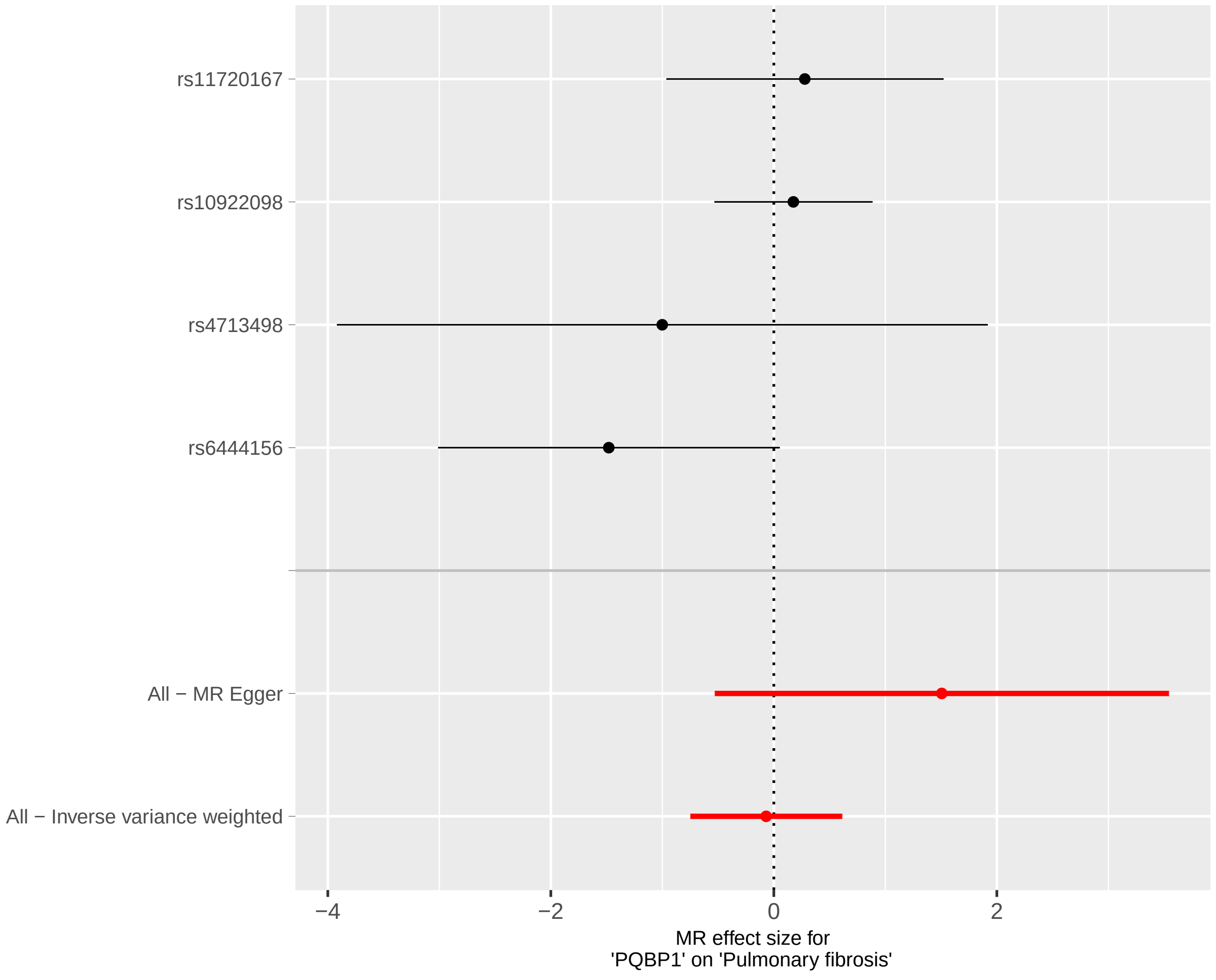

rs11720167
rs10922098
rs4713498
rs6444156
All − MR Egger
All − Inverse variance weighted
−4
−2
0
2
MR effect size for
'PQBP1' on 'Pulmonary fibrosis'

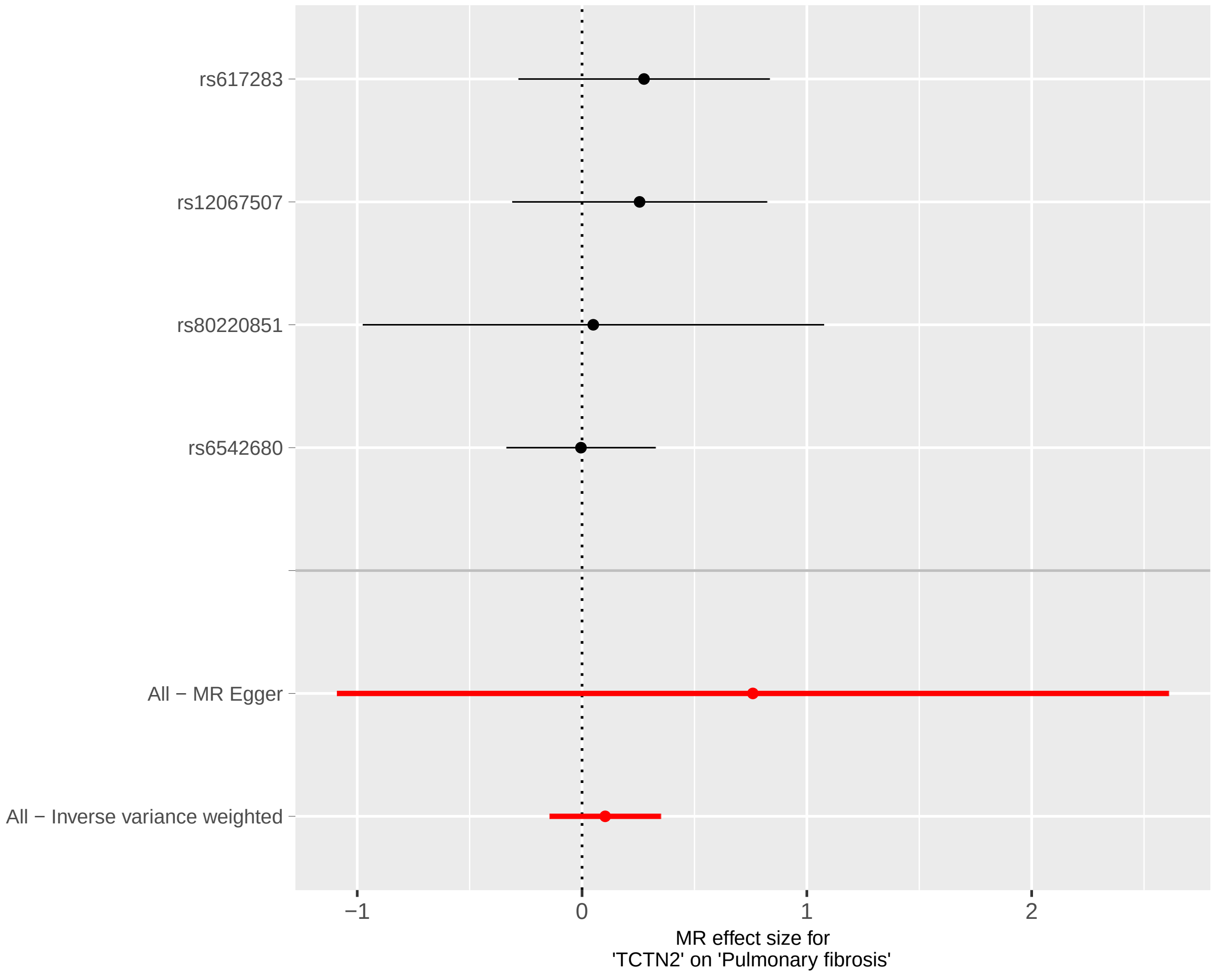
rs617283
rs12067507
rs80220851
rs6542680
All − MR Egger
All − Inverse variance weighted
−1
0
1
2
MR effect size for
'TCTN2' on 'Pulmonary fibrosis'

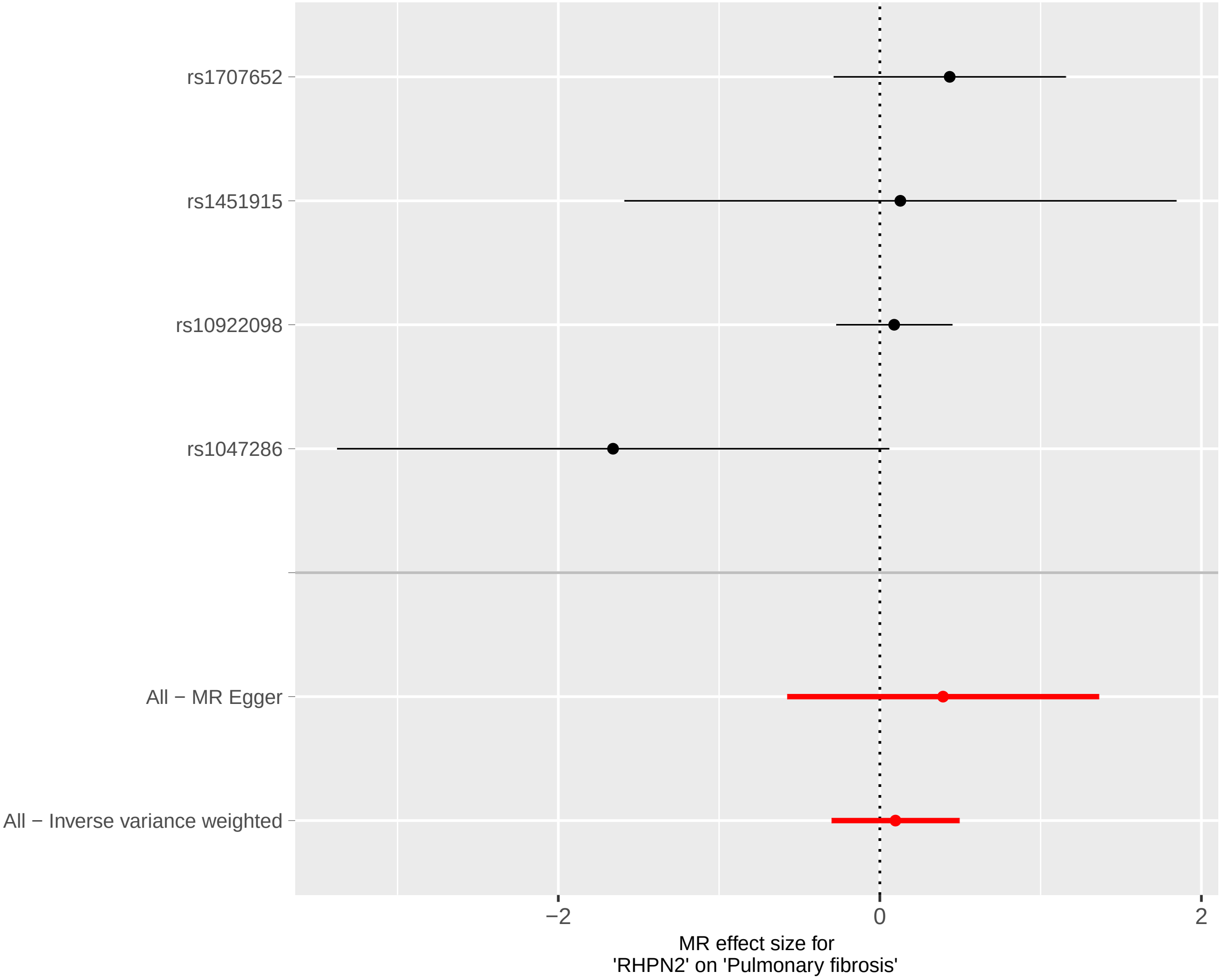
rs1707652
rs1451915
rs10922098
rs1047286
All − MR Egger
All − Inverse variance weighted
−2
0
2
MR effect size for
'RHPN2' on 'Pulmonary fibrosis'

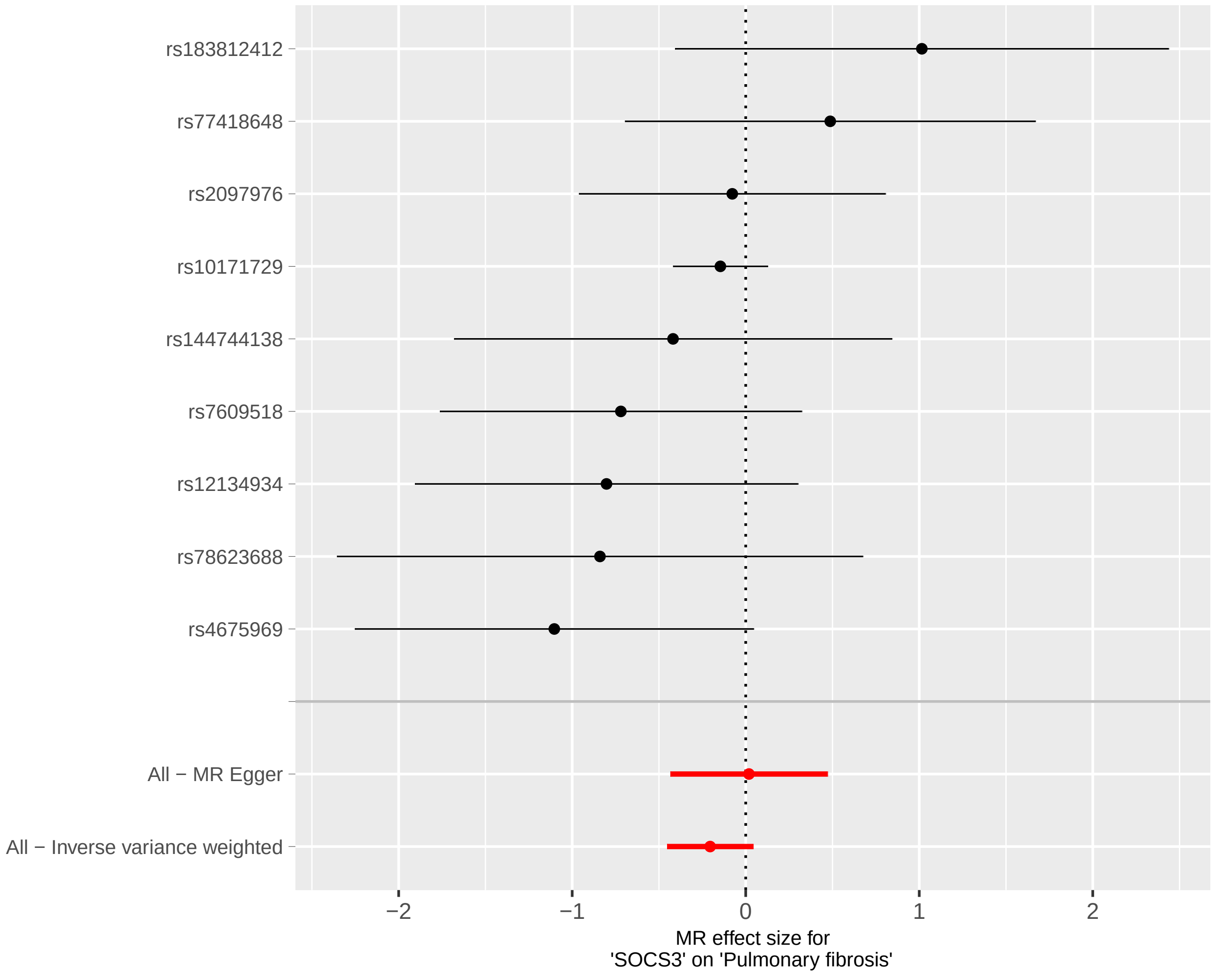
rs183812412
rs77418648
rs2097976
rs10171729
rs144744138
rs7609518
rs12134934
rs78623688
rs4675969
All − MR Egger
All − Inverse variance weighted
−2
−1
0
1
2
MR effect size for
'SOCS3' on 'Pulmonary fibrosis'

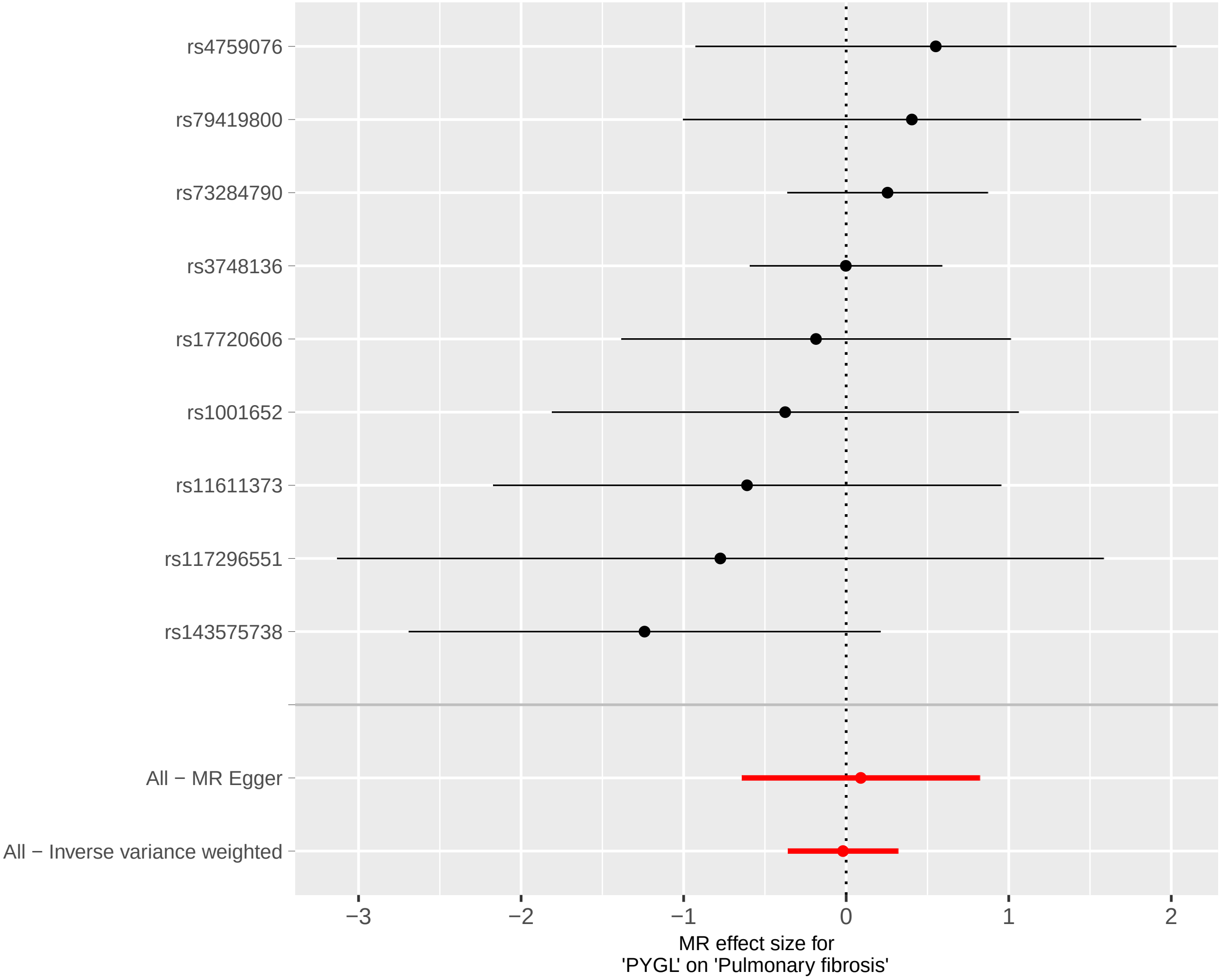
rs4759076
rs79419800
rs73284790
rs3748136
rs17720606
rs1001652
rs11611373
rs117296551
rs143575738
All − MR Egger
All − Inverse variance weighted
−3
−2
−1
0
1
2
MR effect size for
'PYGL' on 'Pulmonary fibrosis'

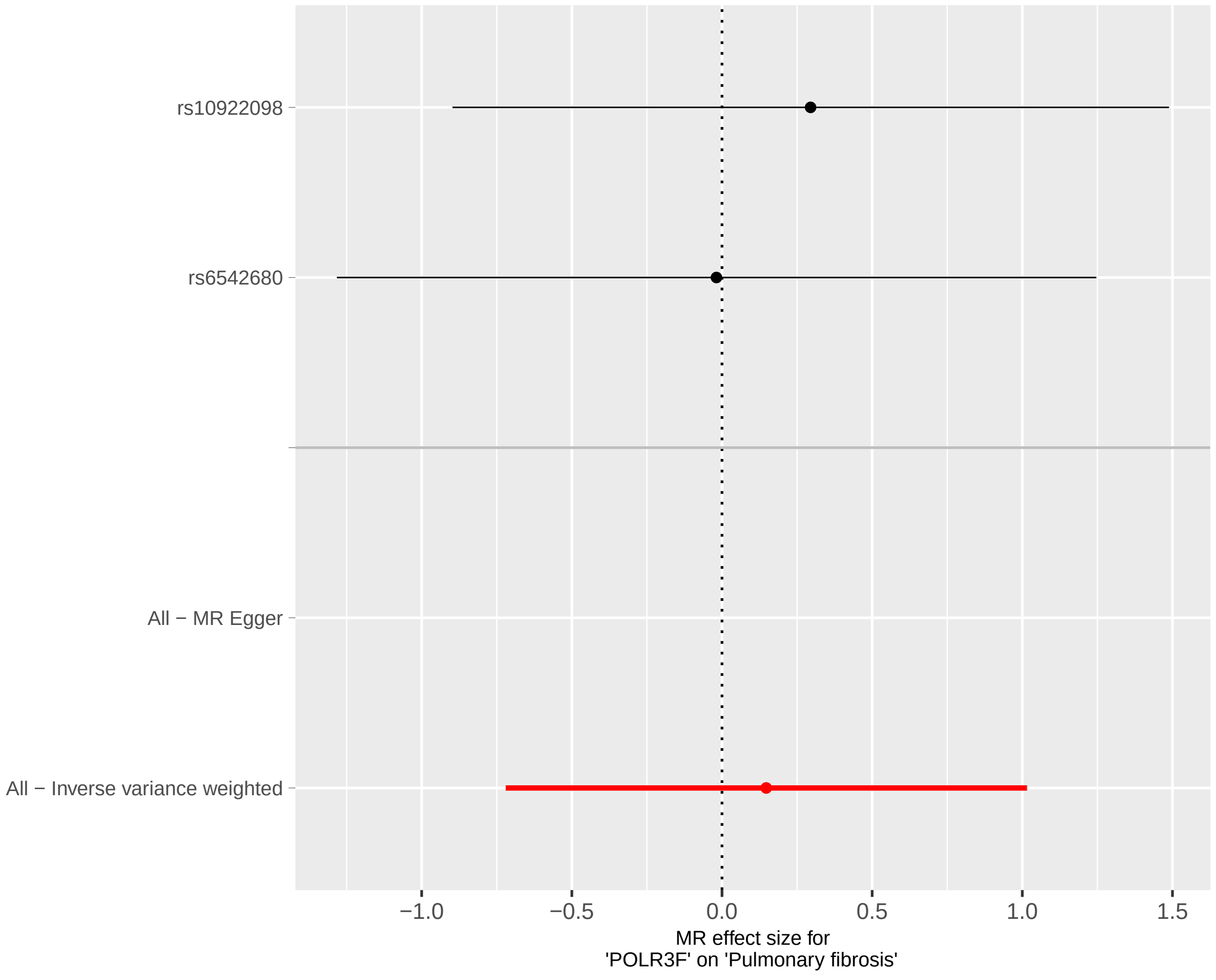
rs10922098
rs6542680
All − MR Egger
All − Inverse variance weighted
−1.0
−0.5
0.0
0.5
1.0
1.5
MR effect size for
'POLR3F' on 'Pulmonary fibrosis'

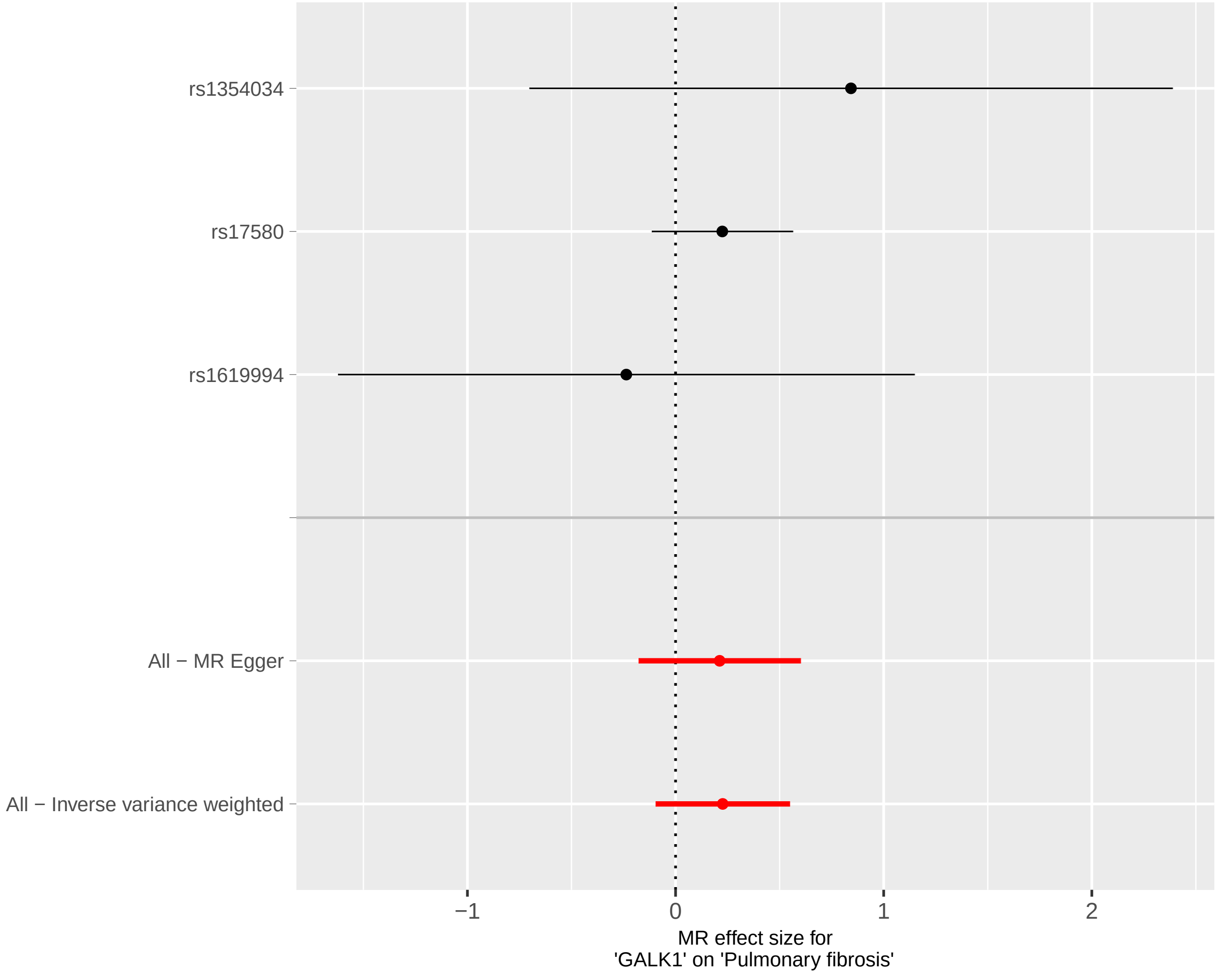
rs1354034
rs17580
rs1619994
All − MR Egger
All − Inverse variance weighted
−1
0
1
2
MR effect size for
'GALK1' on 'Pulmonary fibrosis'

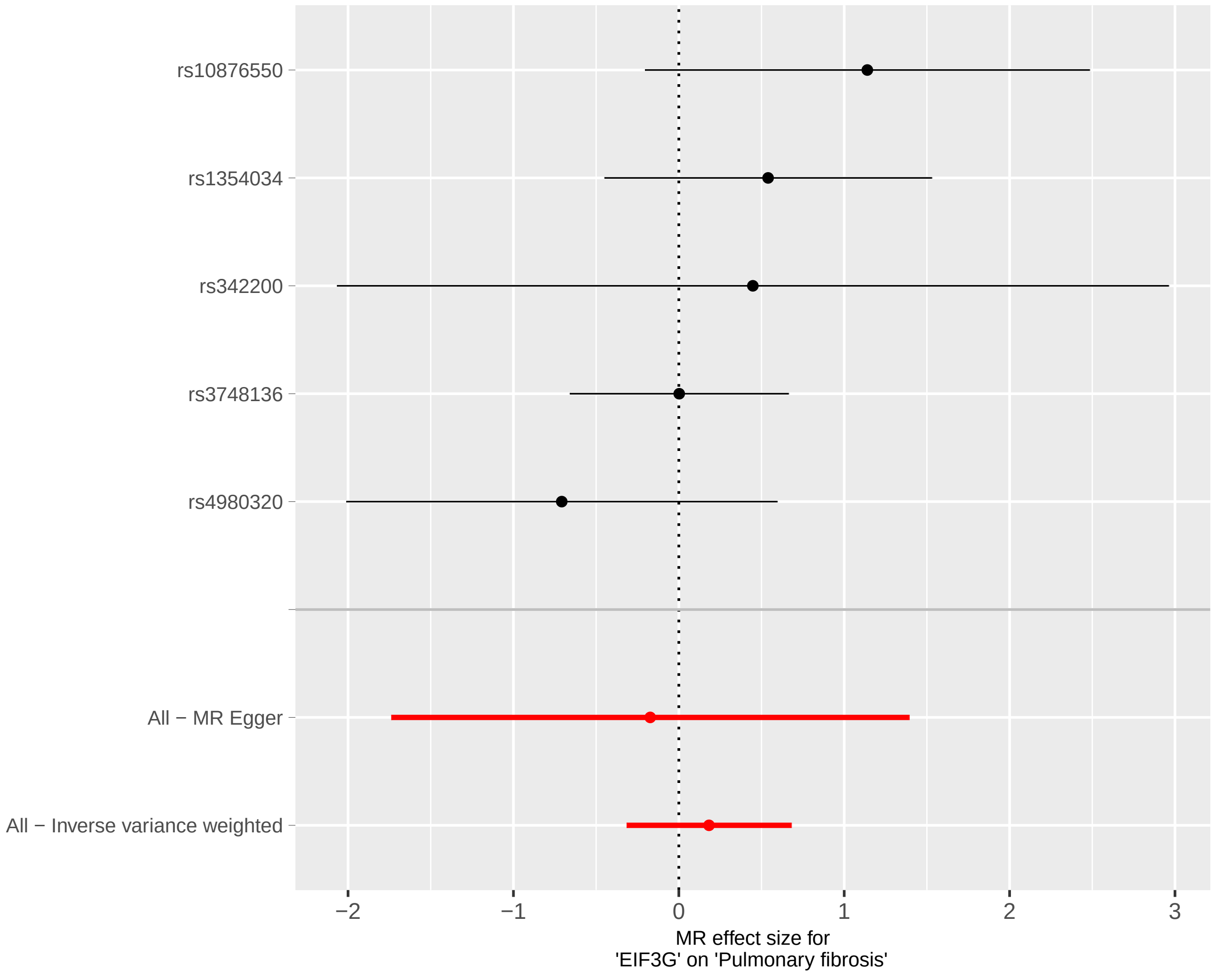
rs10876550
rs1354034
rs342200
rs3748136
rs4980320
All − MR Egger
All − Inverse variance weighted
−2
−1
0
1
2
3
MR effect size for
'EIF3G' on 'Pulmonary fibrosis'

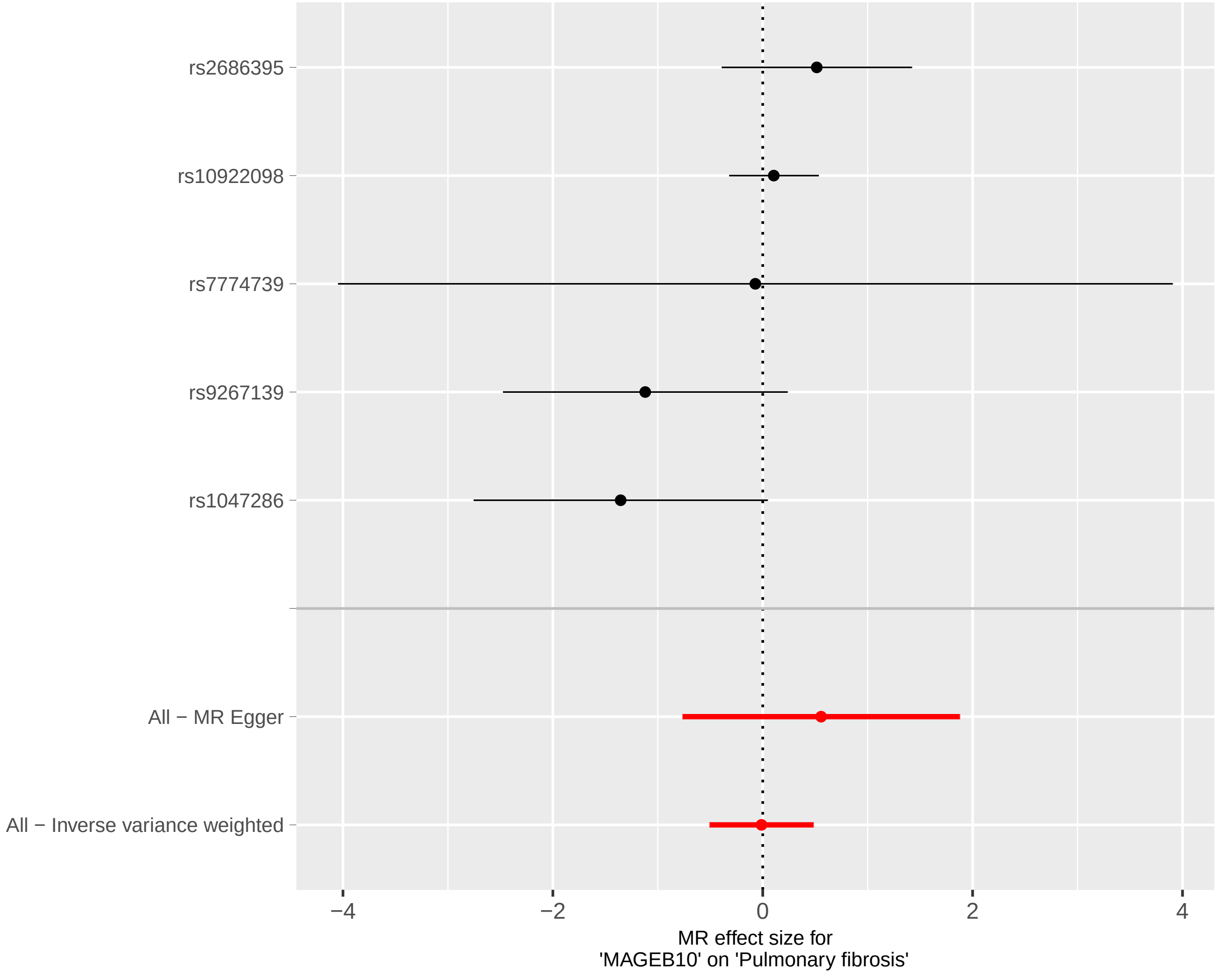
rs2686395
rs10922098
rs7774739
rs9267139
rs1047286
All − MR Egger
All − Inverse variance weighted
−4
−2
0
2
4
MR effect size for
'MAGEB10' on 'Pulmonary fibrosis'

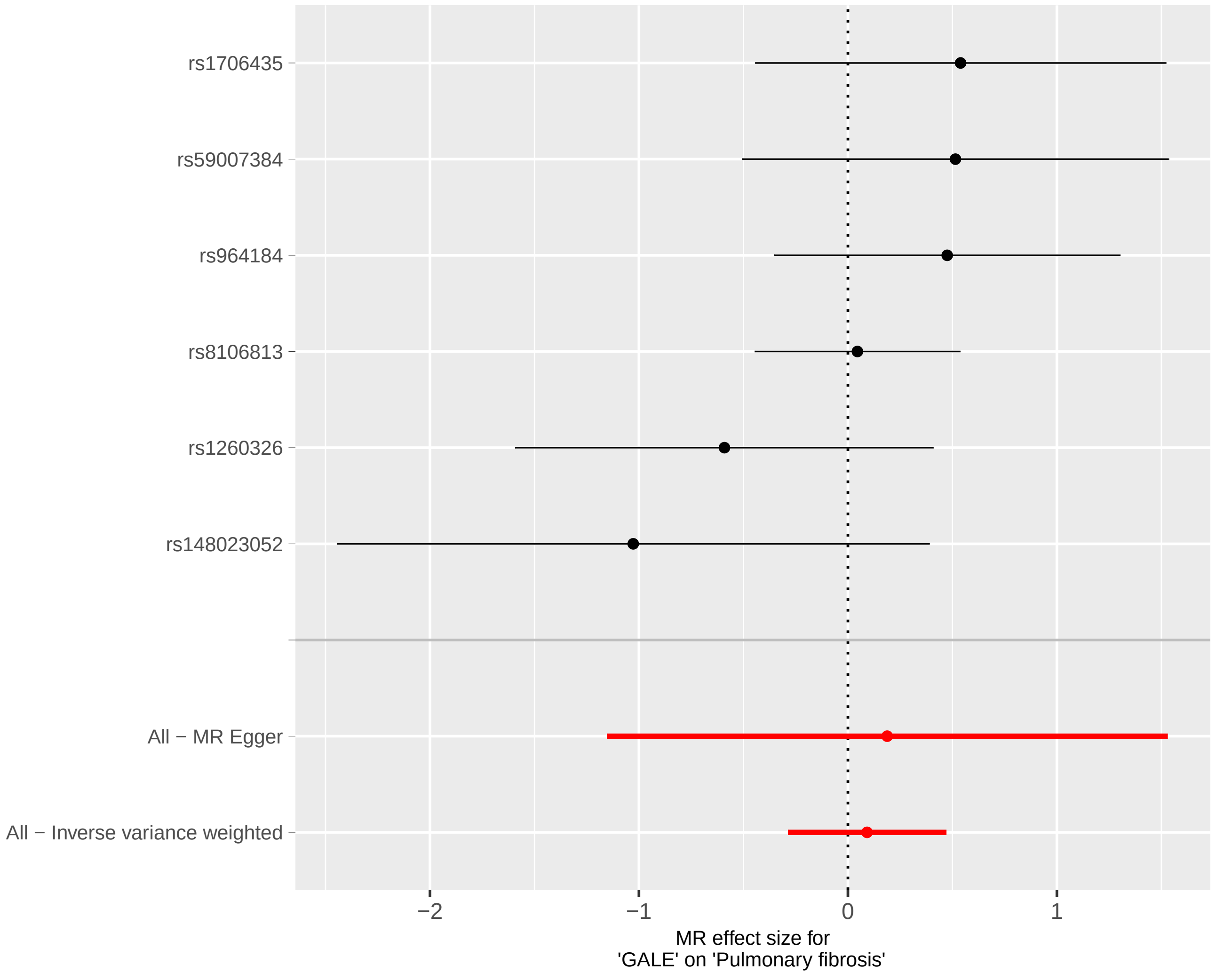
rs1706435
rs59007384
rs964184
rs8106813
rs1260326
rs148023052
All − MR Egger
All − Inverse variance weighted
−2
−1
0
1
MR effect size for
'GALE' on 'Pulmonary fibrosis'

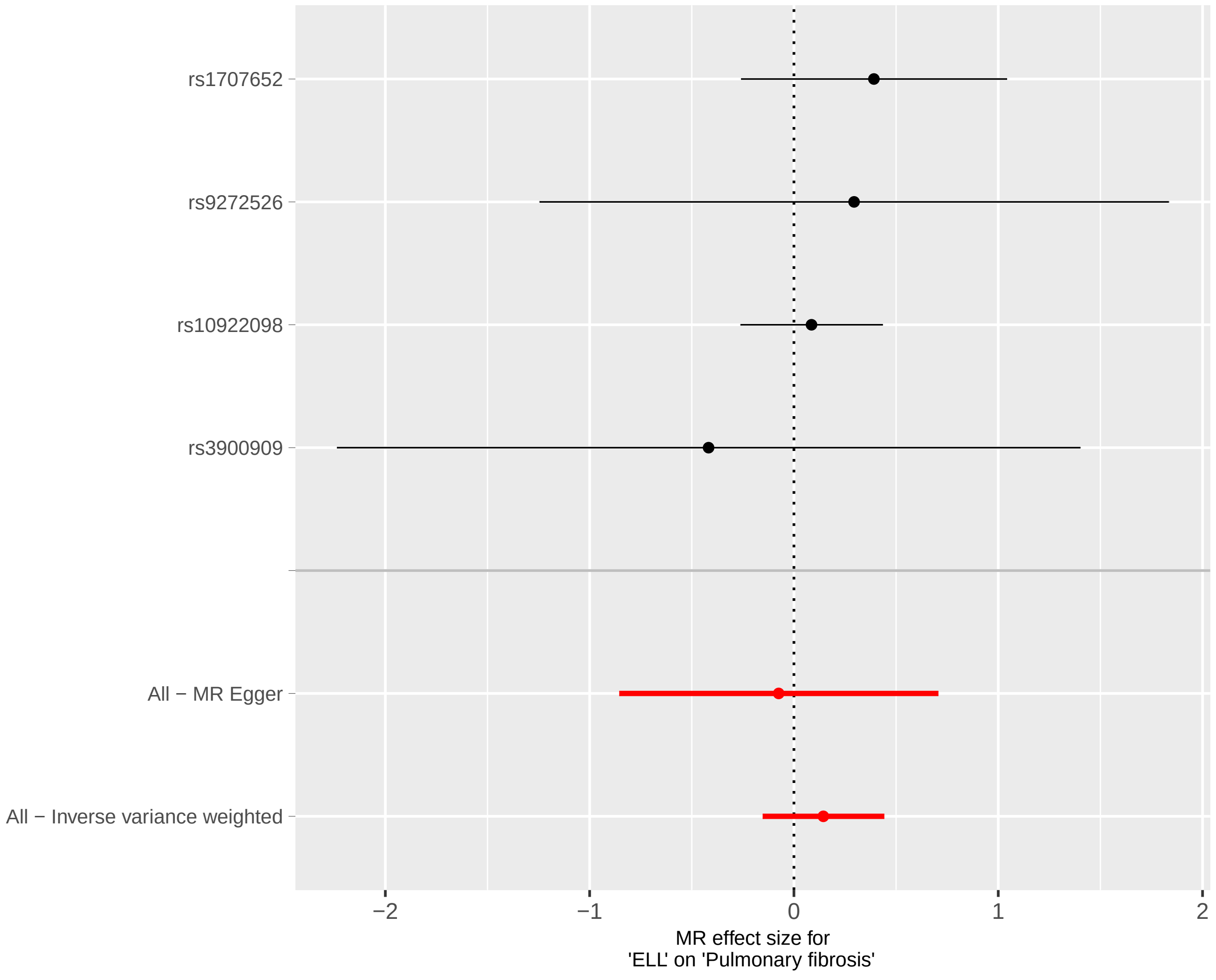
rs1707652
rs9272526
rs10922098
rs3900909
All − MR Egger
All − Inverse variance weighted
−2
−1
0
1
2
MR effect size for
'ELL' on 'Pulmonary fibrosis'

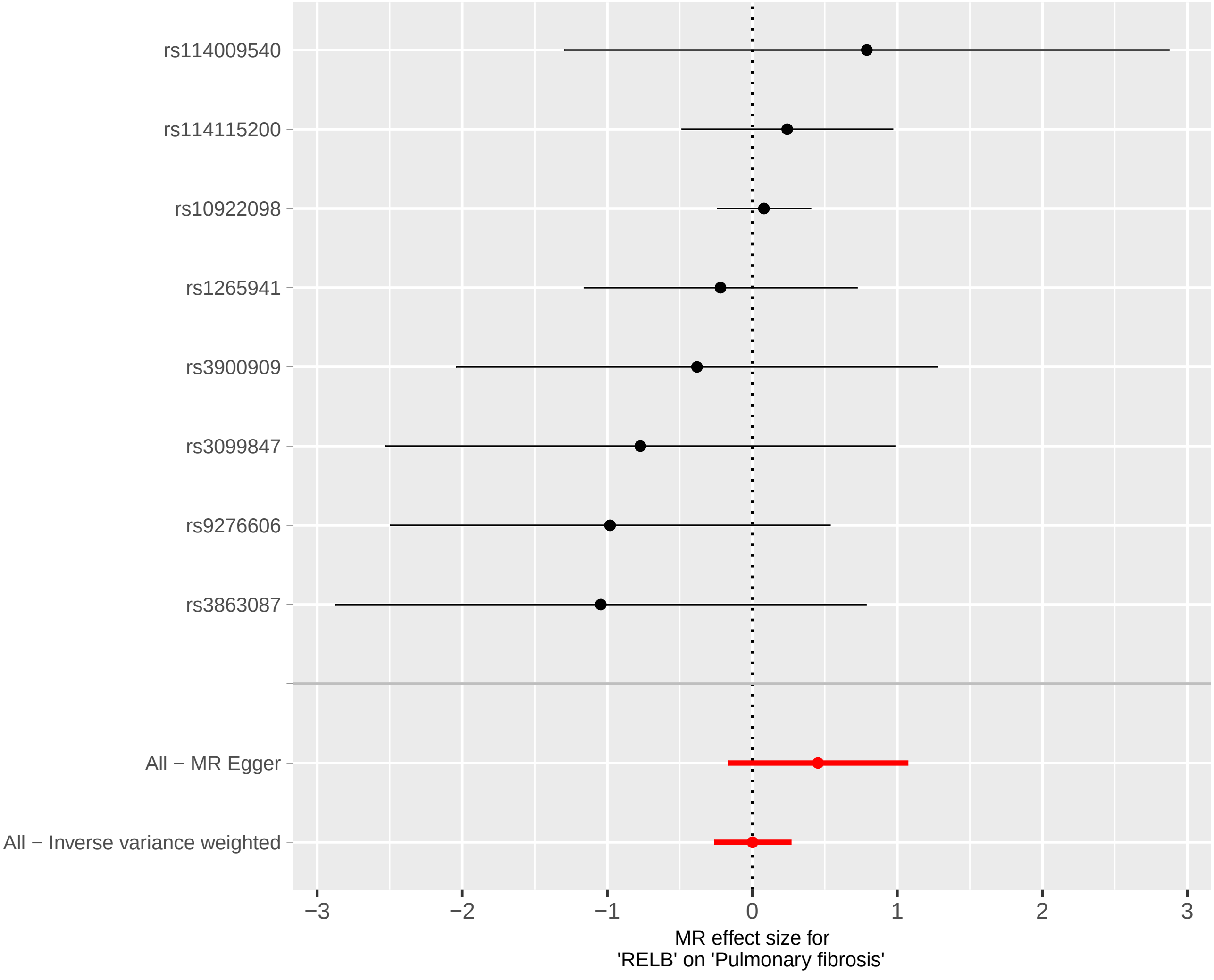
rs114009540
rs114115200
rs10922098
rs1265941
rs3900909
rs3099847
rs9276606
rs3863087
All − MR Egger
All − Inverse variance weighted
−3
−2
−1
0
1
2
3
MR effect size for
'RELB' on 'Pulmonary fibrosis'

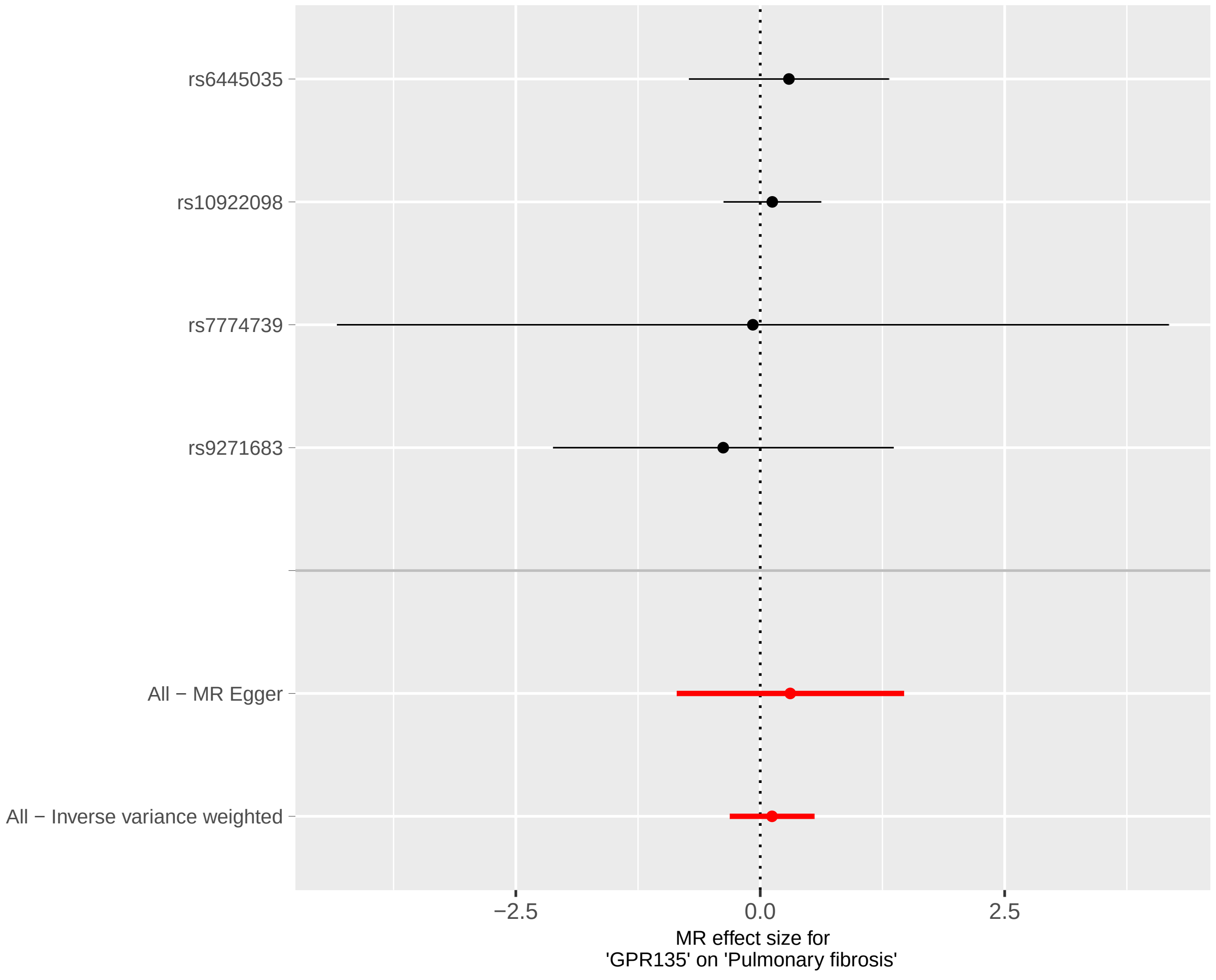

rs6445035
rs10922098
rs7774739
rs9271683
All − MR Egger
All − Inverse variance weighted
−2.5
0.0
2.5
MR effect size for
'GPR135' on 'Pulmonary fibrosis'

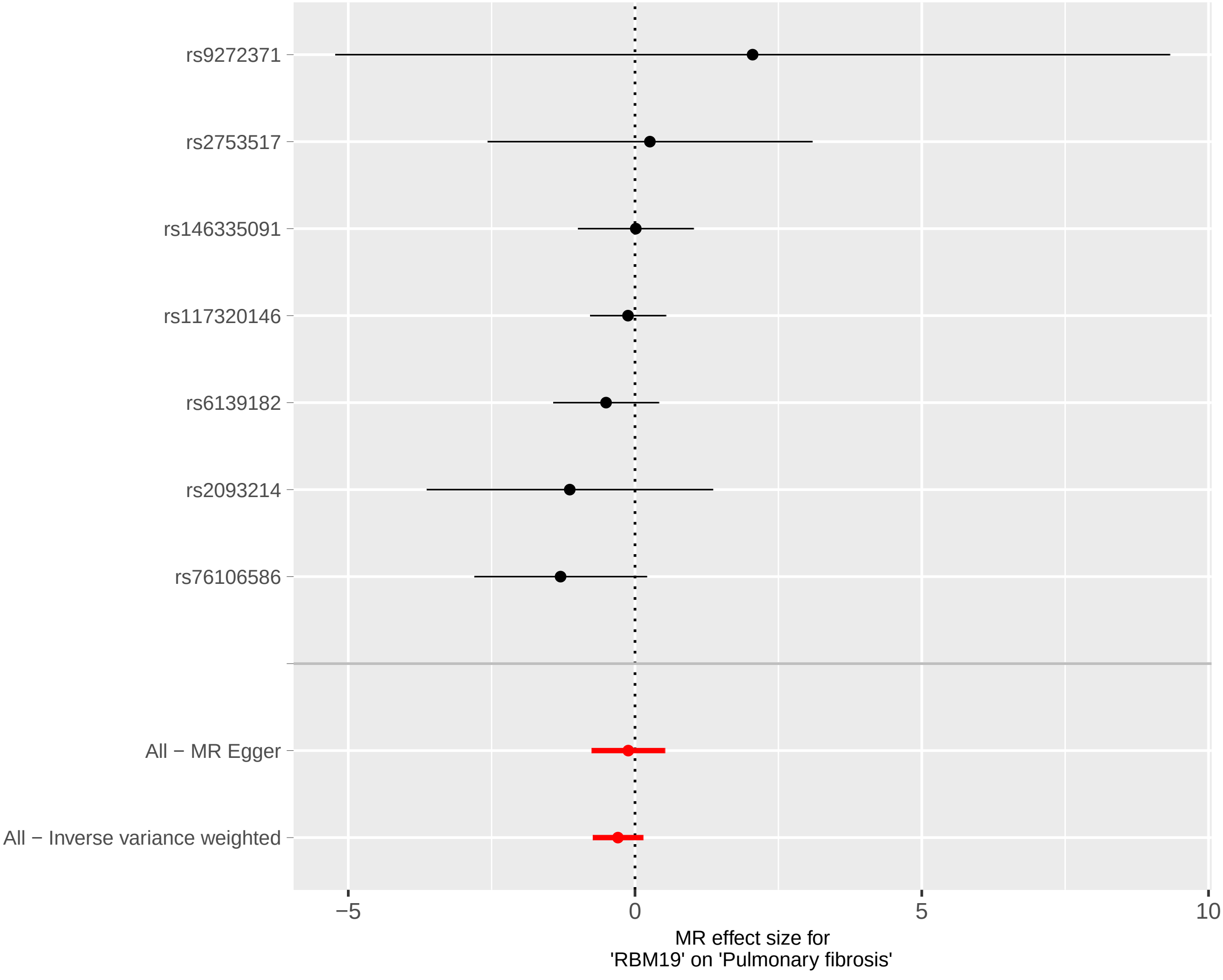
rs9272371
rs2753517
rs146335091
rs117320146
rs6139182
rs2093214
rs76106586
All − MR Egger
All − Inverse variance weighted
−5
0
5
10
MR effect size for
'RBM19' on 'Pulmonary fibrosis'

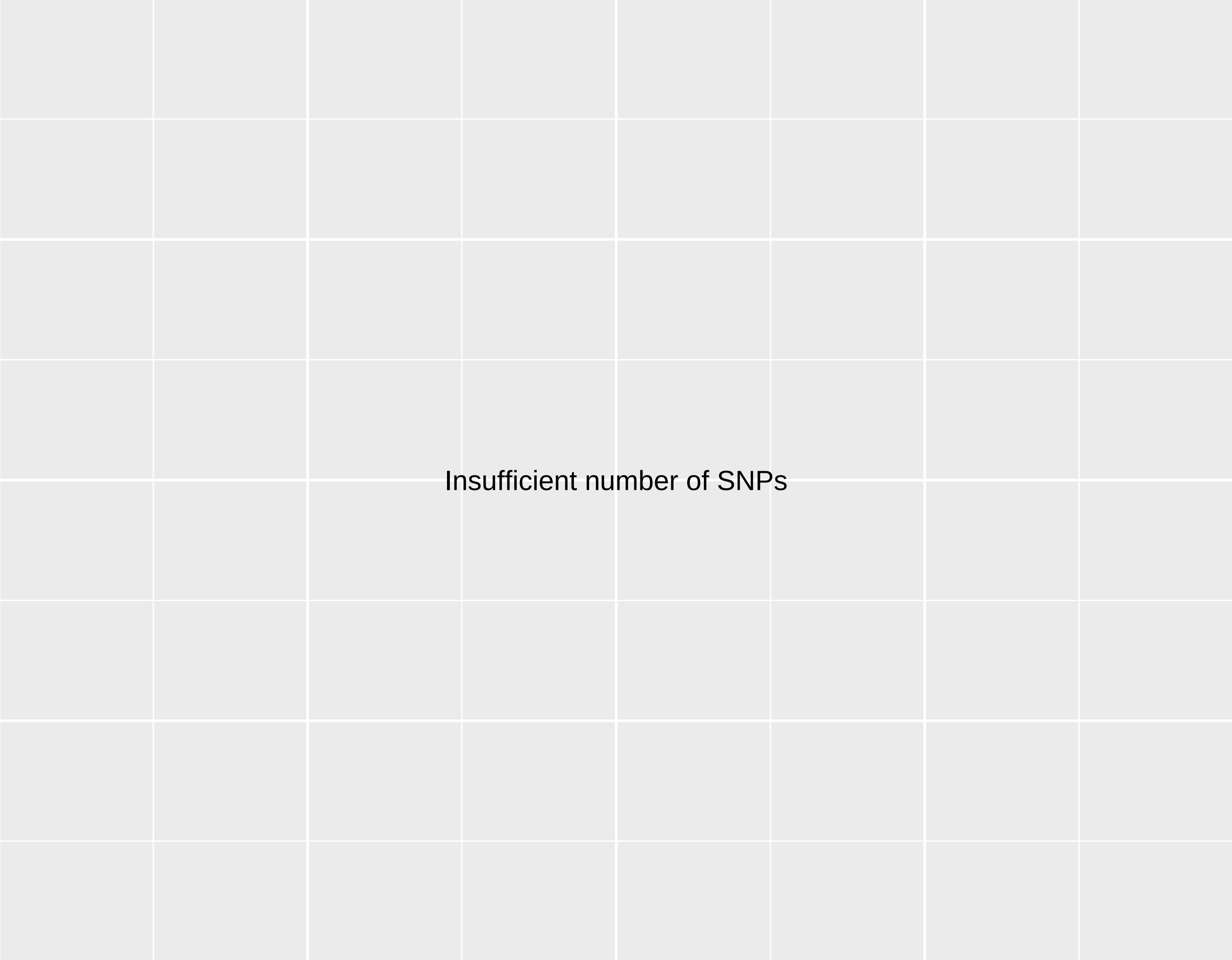
Insufficient number of SNPs

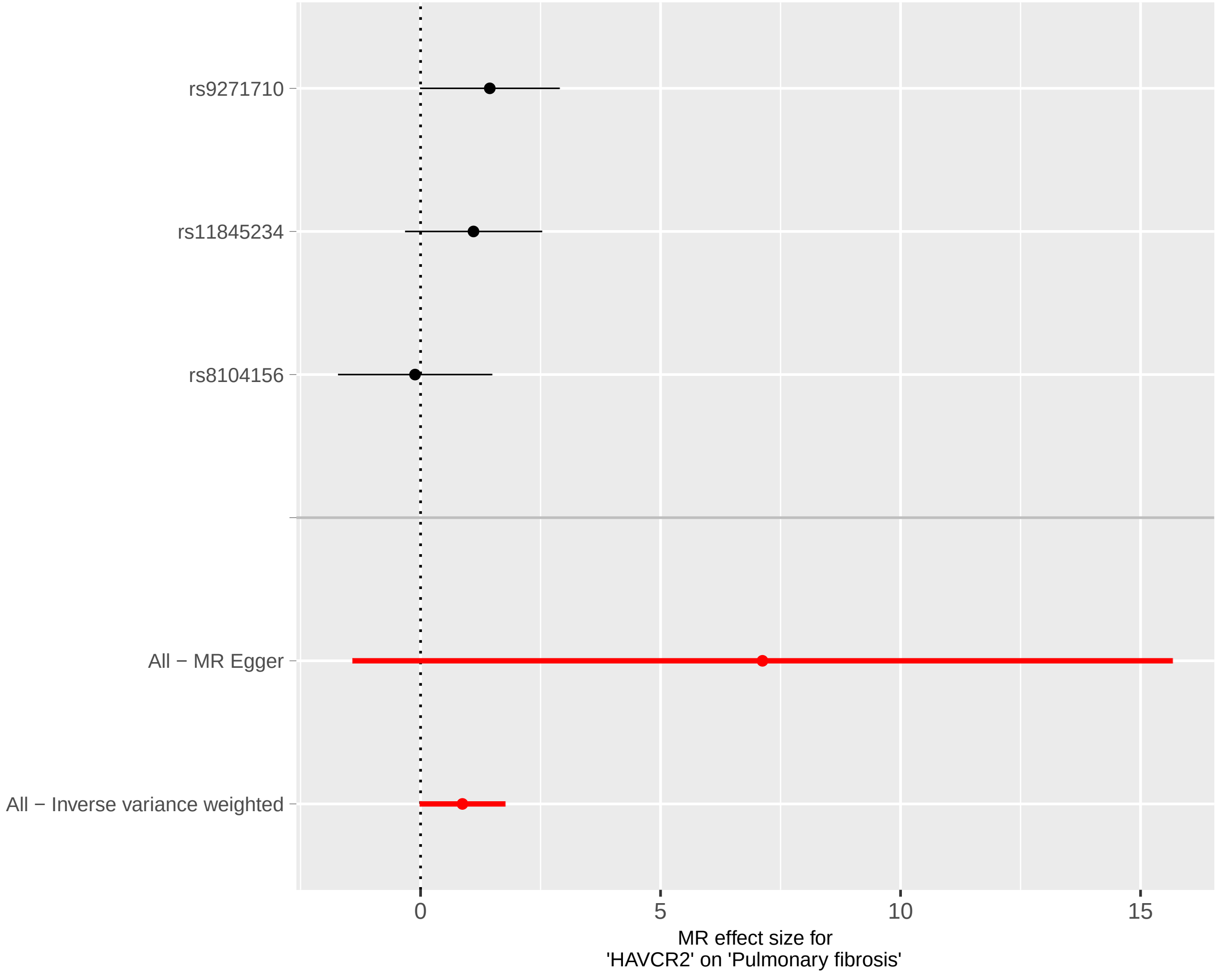
rs9271710
rs11845234
rs8104156
All − MR Egger
All − Inverse variance weighted
0
5
10
15
MR effect size for
'HAVCR2' on 'Pulmonary fibrosis'

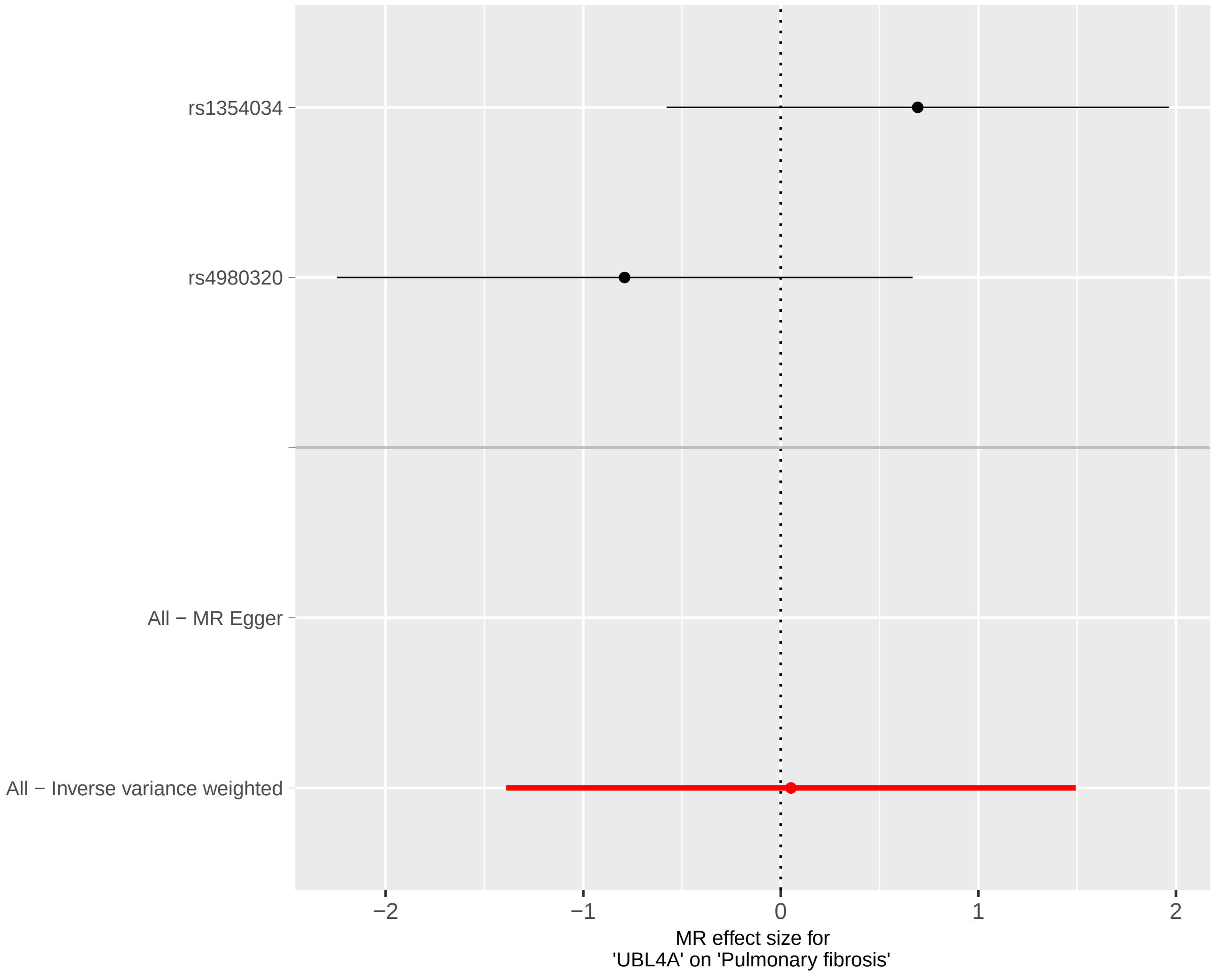
rs1354034
rs4980320
All − MR Egger
All − Inverse variance weighted
−2
−1
0
1
2
MR effect size for
'UBL4A' on 'Pulmonary fibrosis'

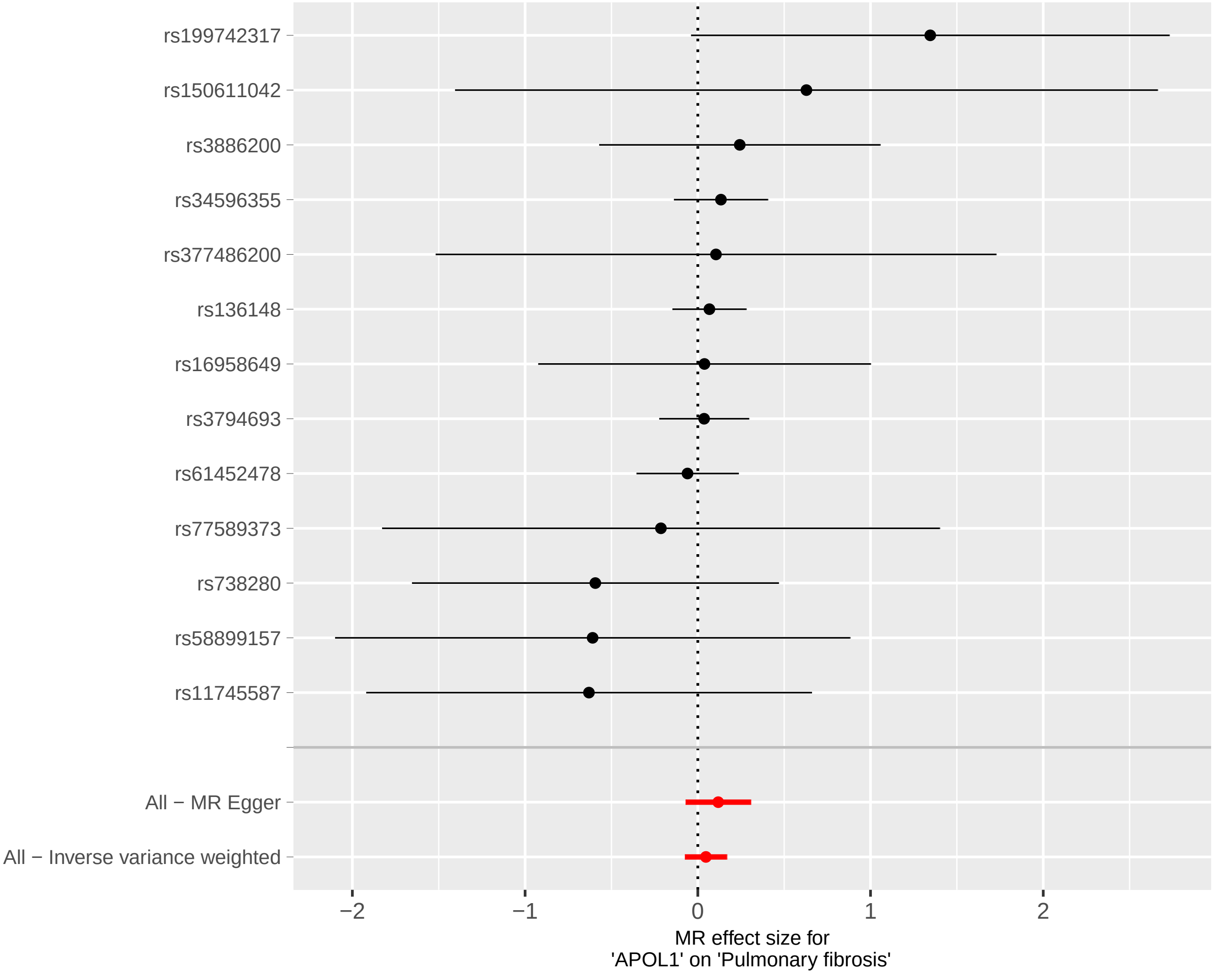
rs199742317
rs150611042
rs3886200
rs34596355
rs377486200
rs136148
rs16958649
rs3794693
rs61452478
rs77589373
rs738280
rs58899157
rs11745587
All − MR Egger
All − Inverse variance weighted
−2
−1
0
1
2
MR effect size for
'APOL1' on 'Pulmonary fibrosis'

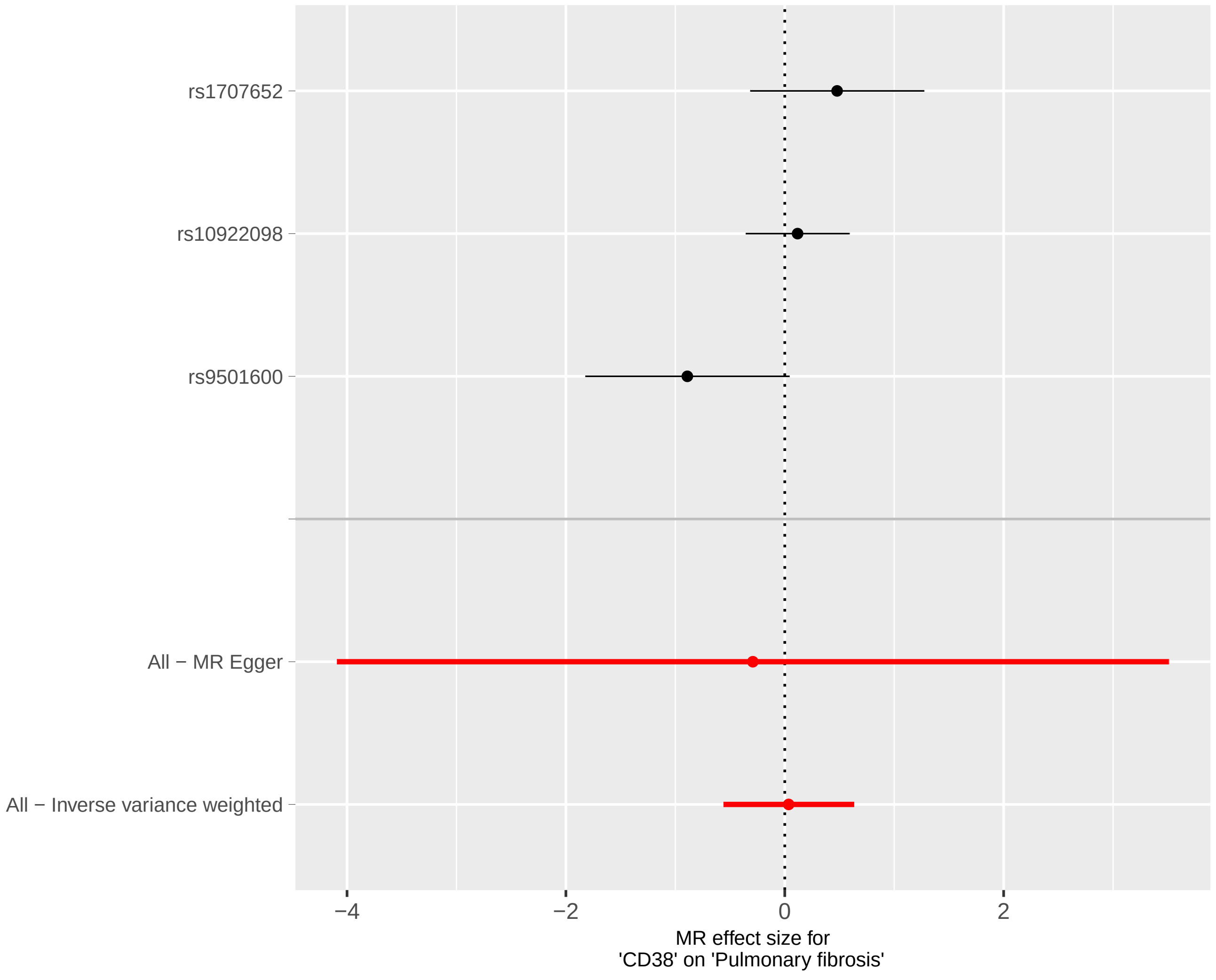
rs1707652
rs10922098
rs9501600
All − MR Egger
All − Inverse variance weighted
−4
−2
0
2
MR effect size for
'CD38' on 'Pulmonary fibrosis'

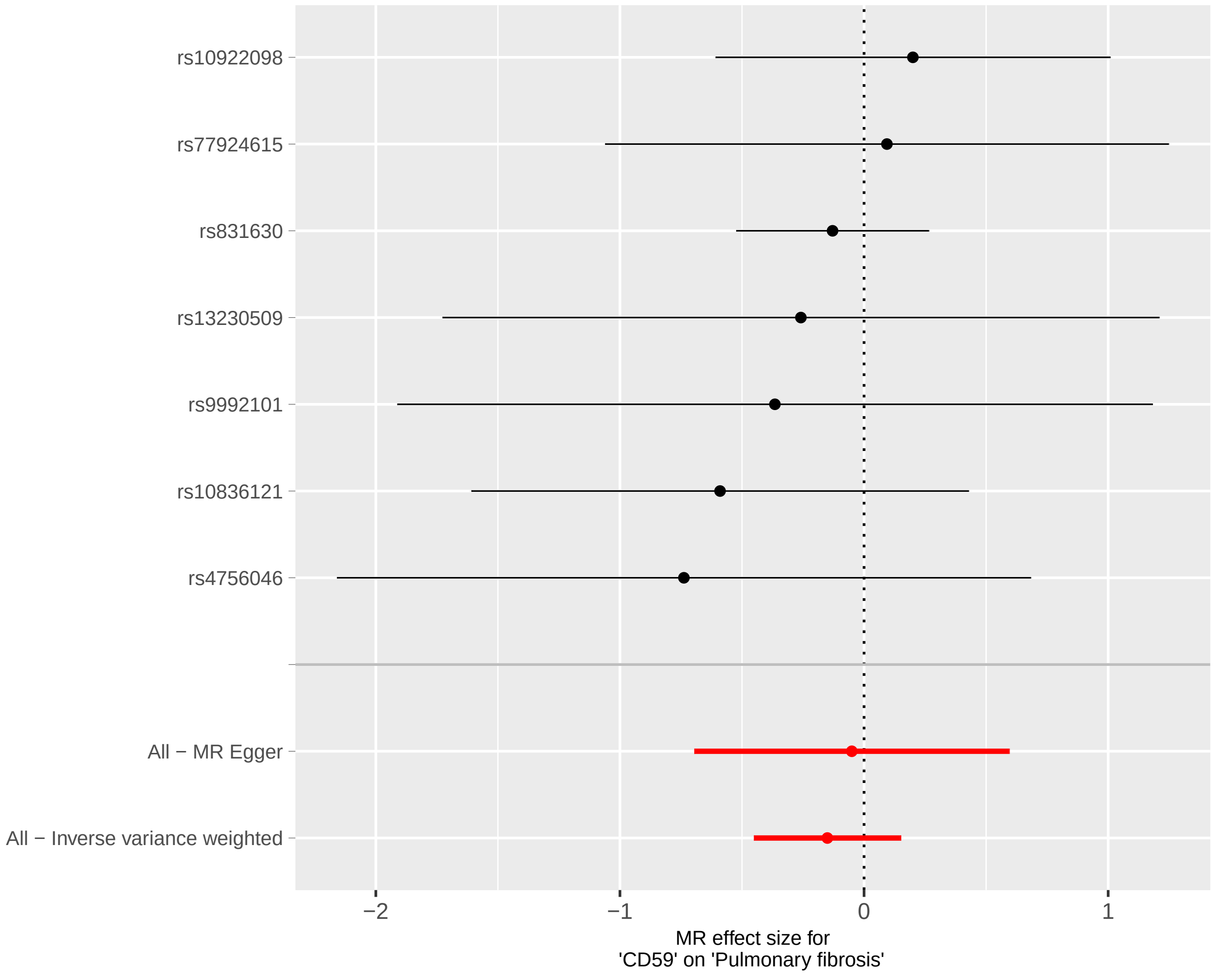

rs10922098
rs77924615
rs831630
rs13230509
rs9992101
rs10836121
rs4756046
All − MR Egger
All − Inverse variance weighted
−2
−1
0
1
MR effect size for
'CD59' on 'Pulmonary fibrosis'

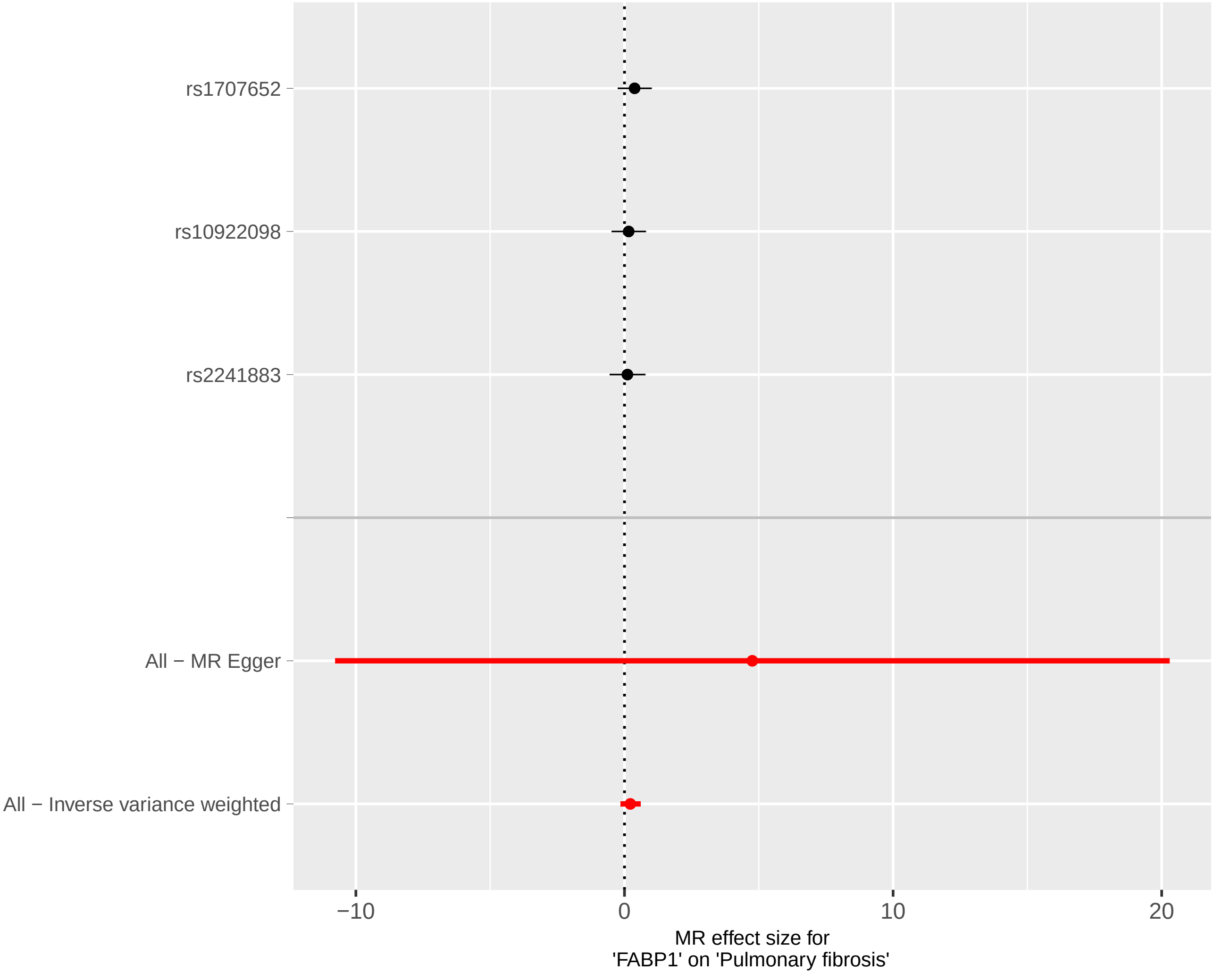
rs1707652
rs10922098
rs2241883
All − MR Egger
All − Inverse variance weighted
−10
0
10
20
MR effect size for
'FABP1' on 'Pulmonary fibrosis'

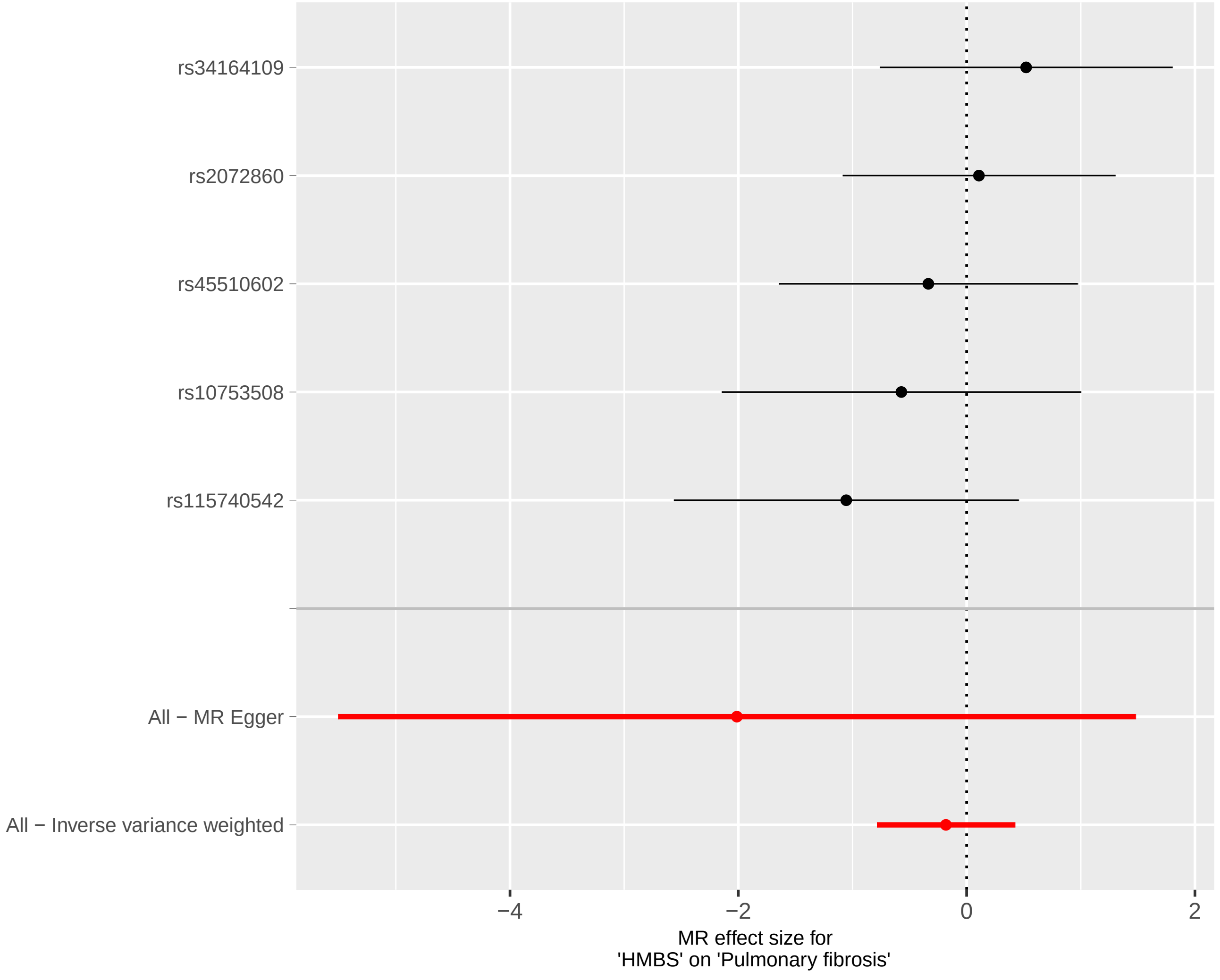

rs34164109
rs2072860
rs45510602
rs10753508
rs115740542
All − MR Egger
All − Inverse variance weighted
−4
−2
0
2
MR effect size for
'HMBS' on 'Pulmonary fibrosis'

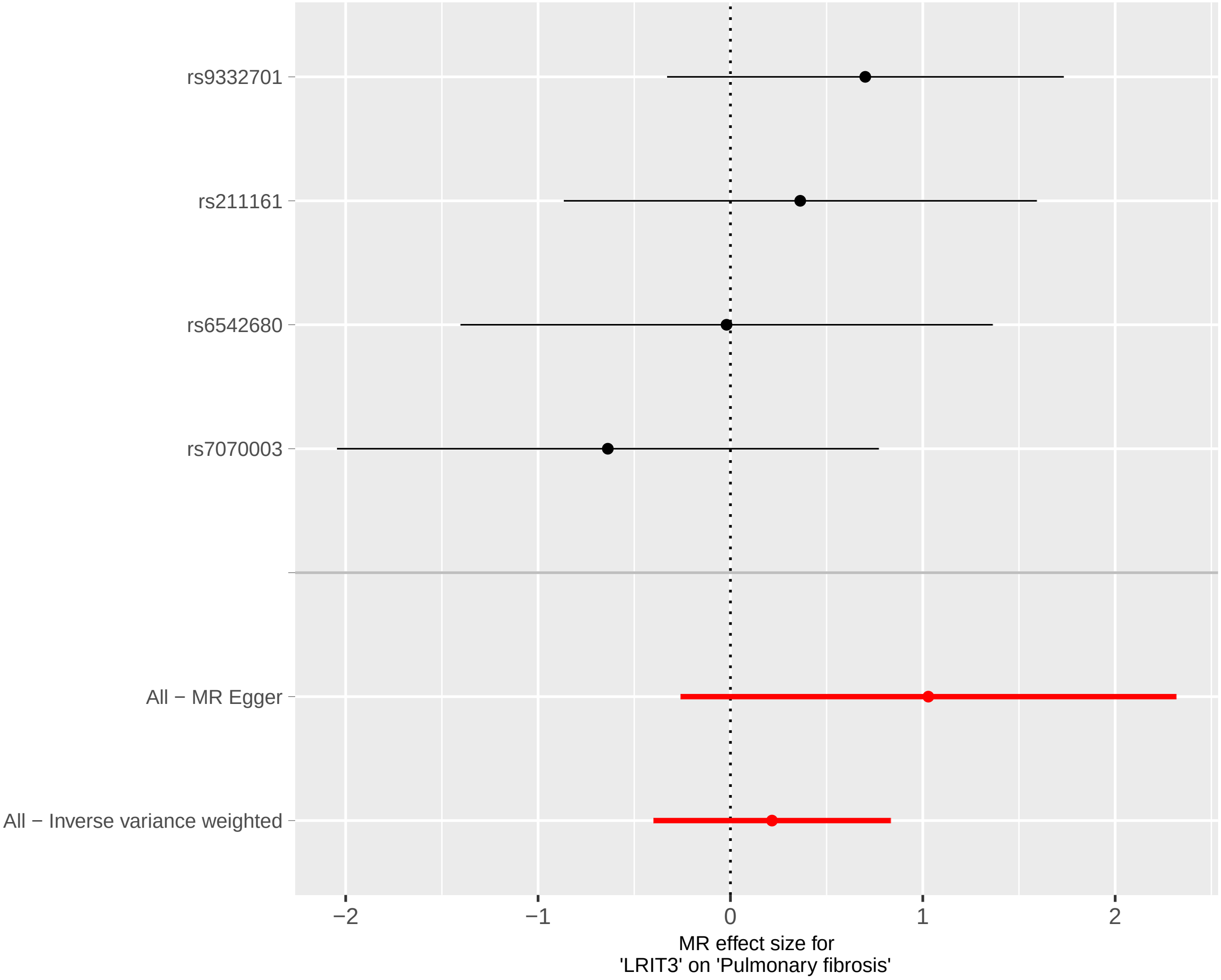

rs9332701
rs211161
rs6542680
rs7070003
All − MR Egger
All − Inverse variance weighted
−2
−1
0
1
2
MR effect size for
'LRIT3' on 'Pulmonary fibrosis'

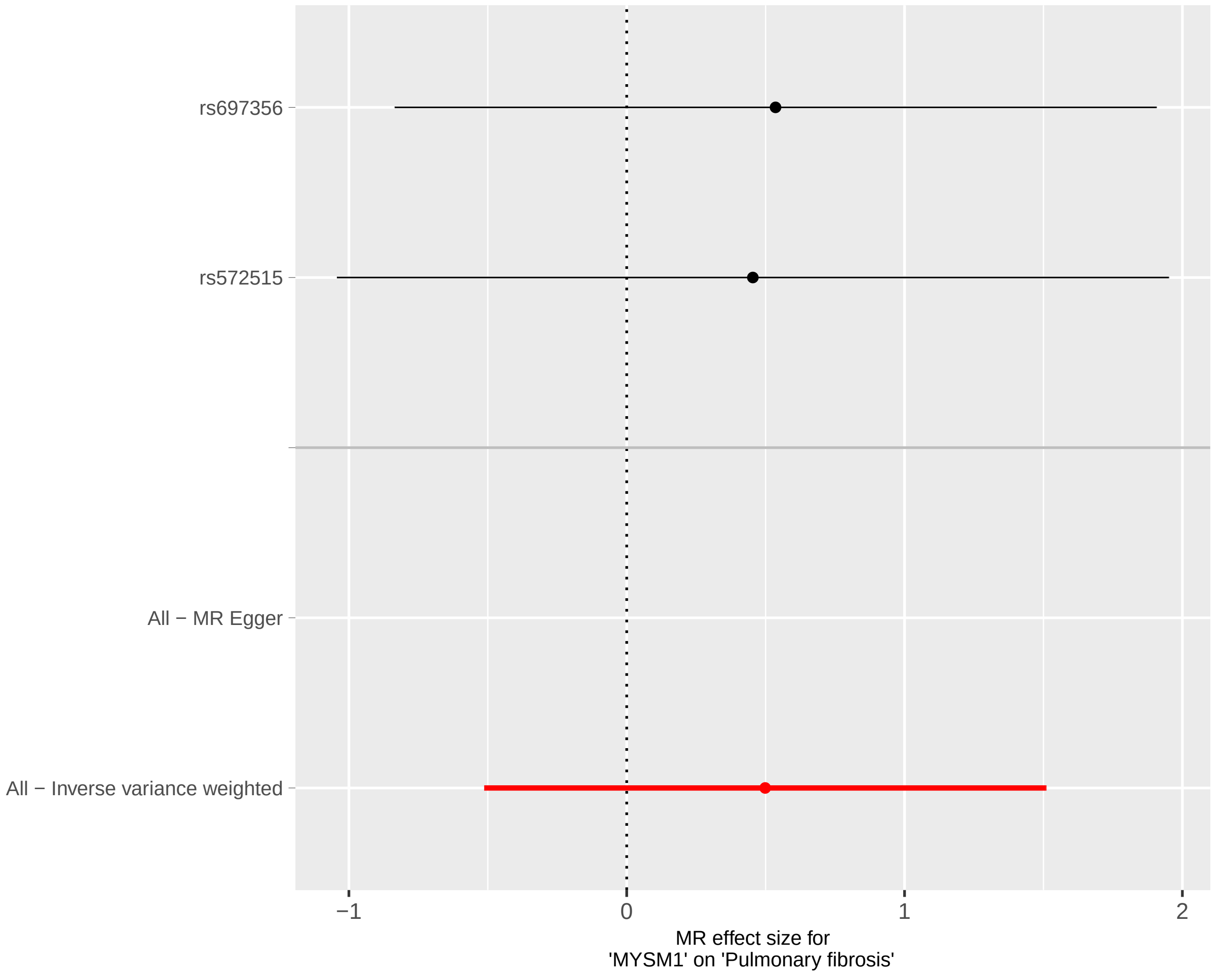
rs697356
rs572515
All − MR Egger
All − Inverse variance weighted
−1
0
1
2
MR effect size for
'MYSM1' on 'Pulmonary fibrosis'

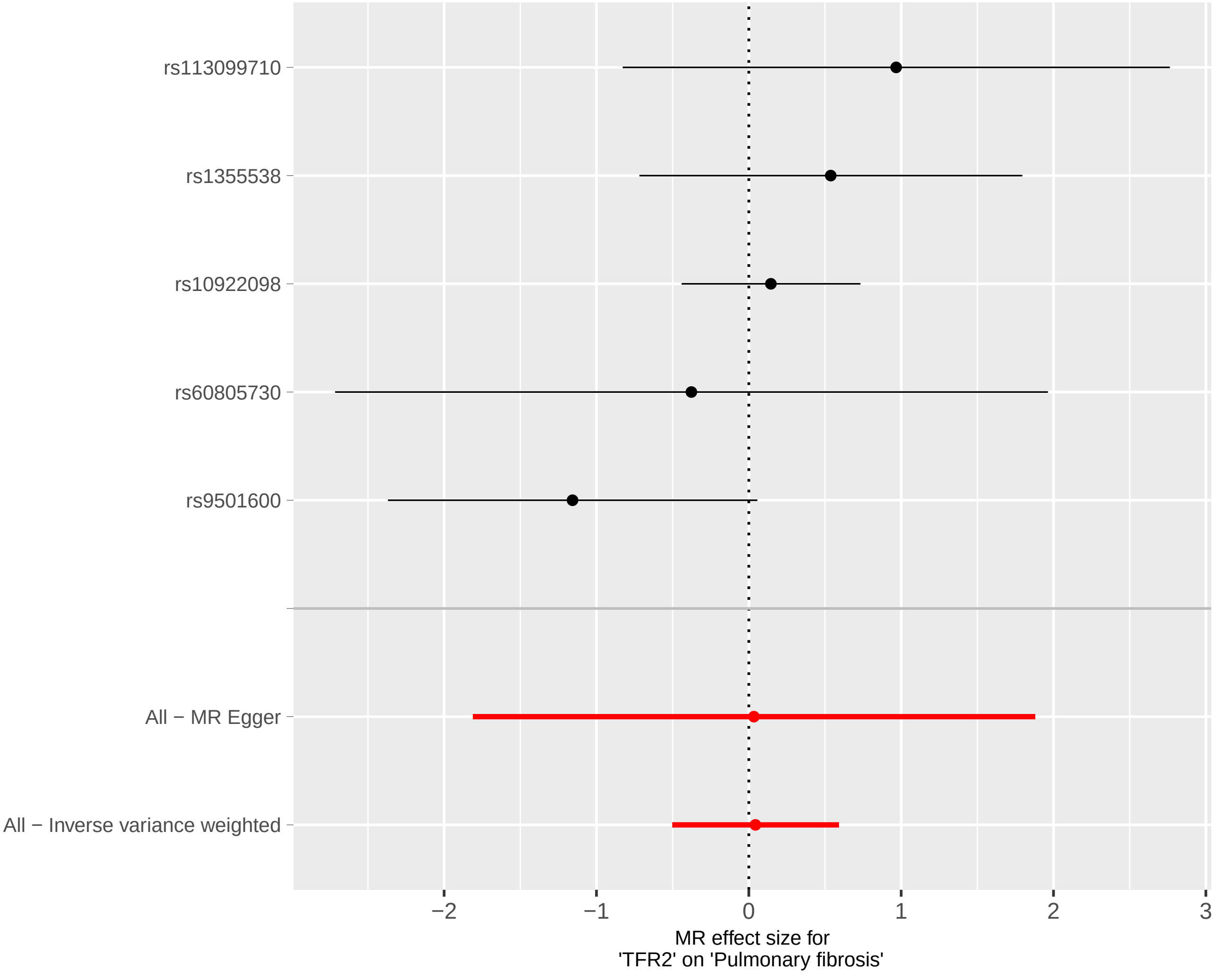
rs113099710
rs1355538
rs10922098
rs60805730
rs9501600
All − MR Egger
All − Inverse variance weighted
−2
−1
0
1
2
3
MR effect size for
'TFR2' on 'Pulmonary fibrosis'

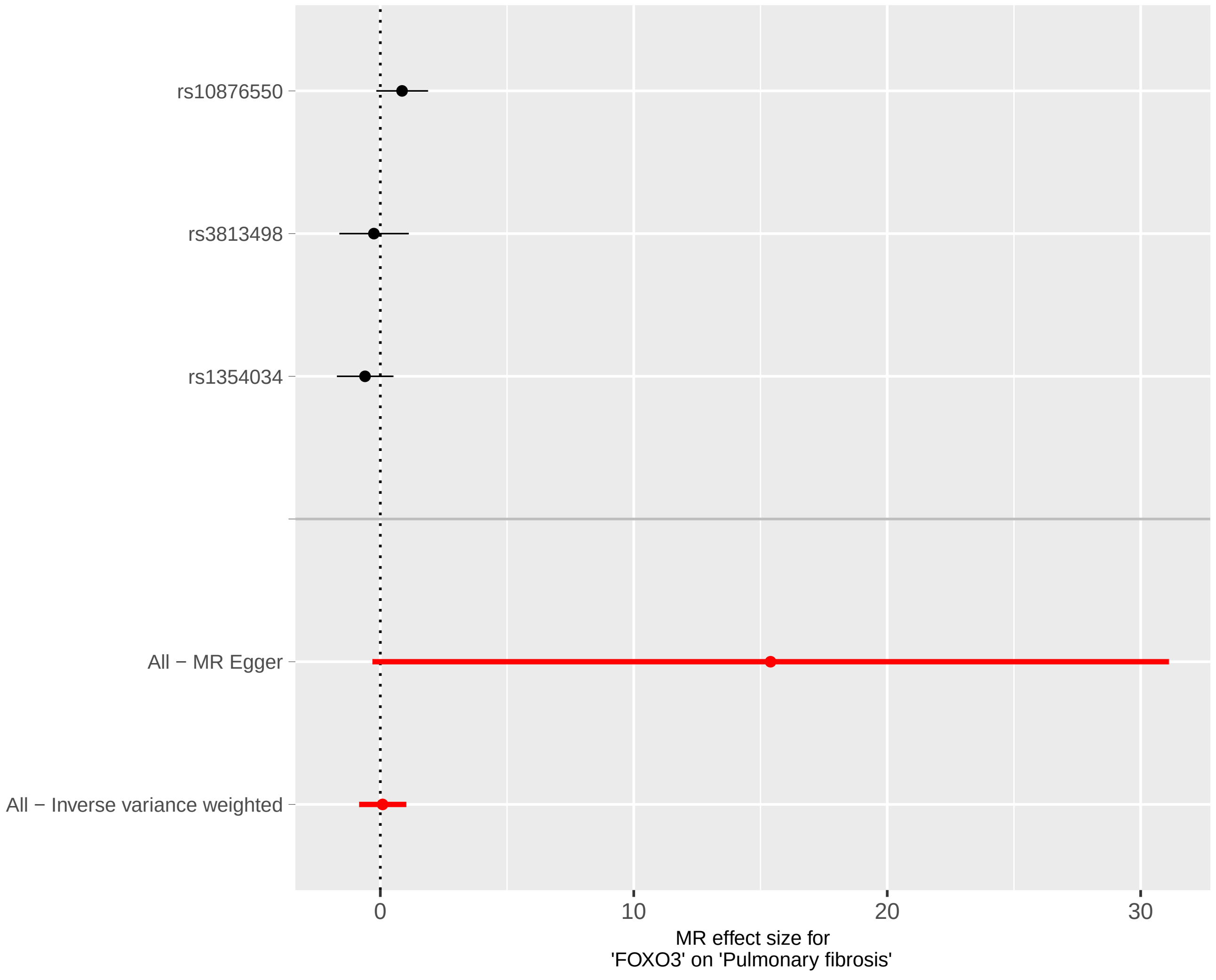
rs10876550
rs3813498
rs1354034
All − MR Egger
All − Inverse variance weighted
0
10
20
30
MR effect size for
'FOXO3' on 'Pulmonary fibrosis'

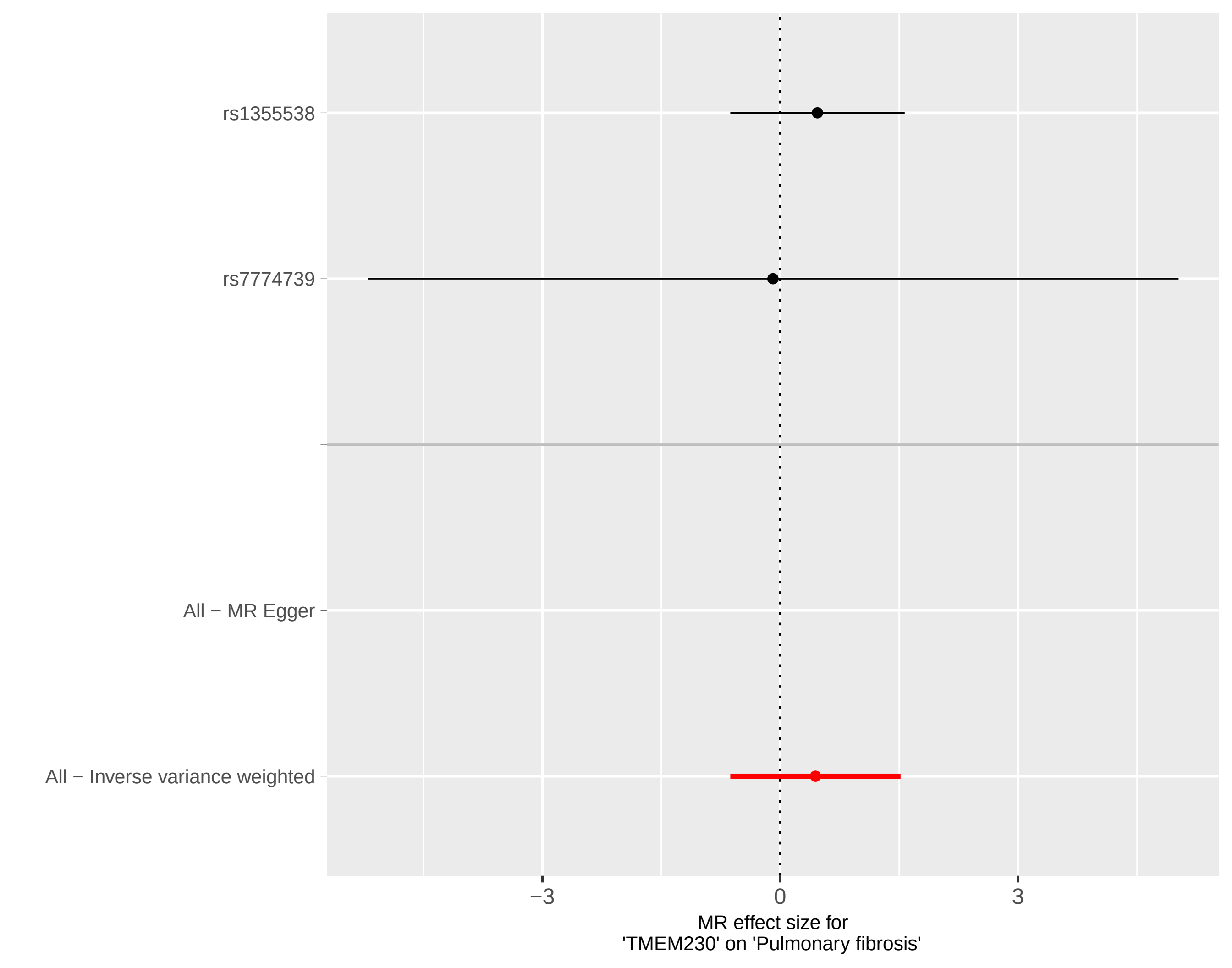
rs1355538
rs7774739
All − MR Egger
All − Inverse variance weighted
−3
0
3
MR effect size for
'TMEM230' on 'Pulmonary fibrosis'

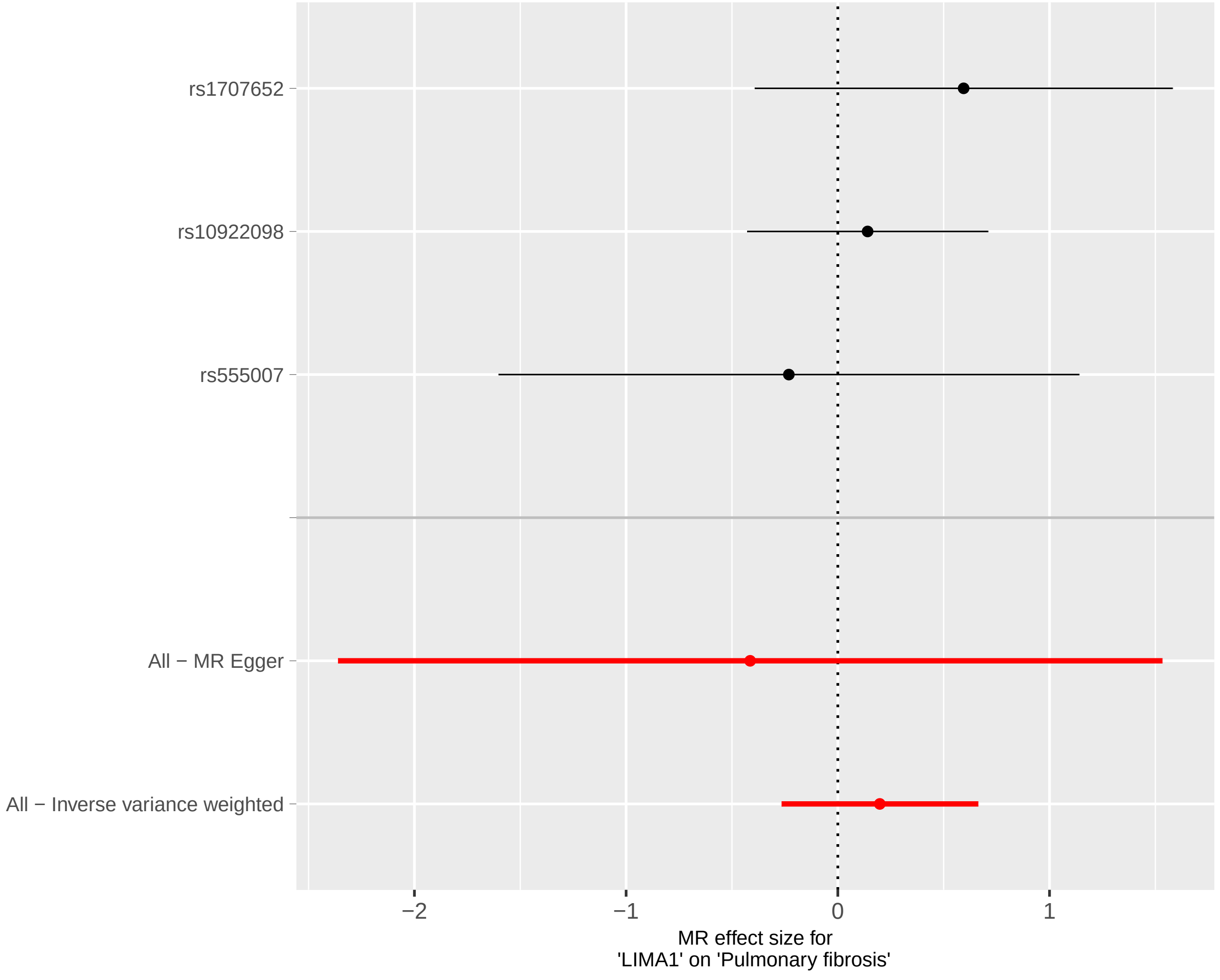
rs1707652
rs10922098
rs555007
All − MR Egger
All − Inverse variance weighted
−2
−1
0
1
MR effect size for
'LIMA1' on 'Pulmonary fibrosis'

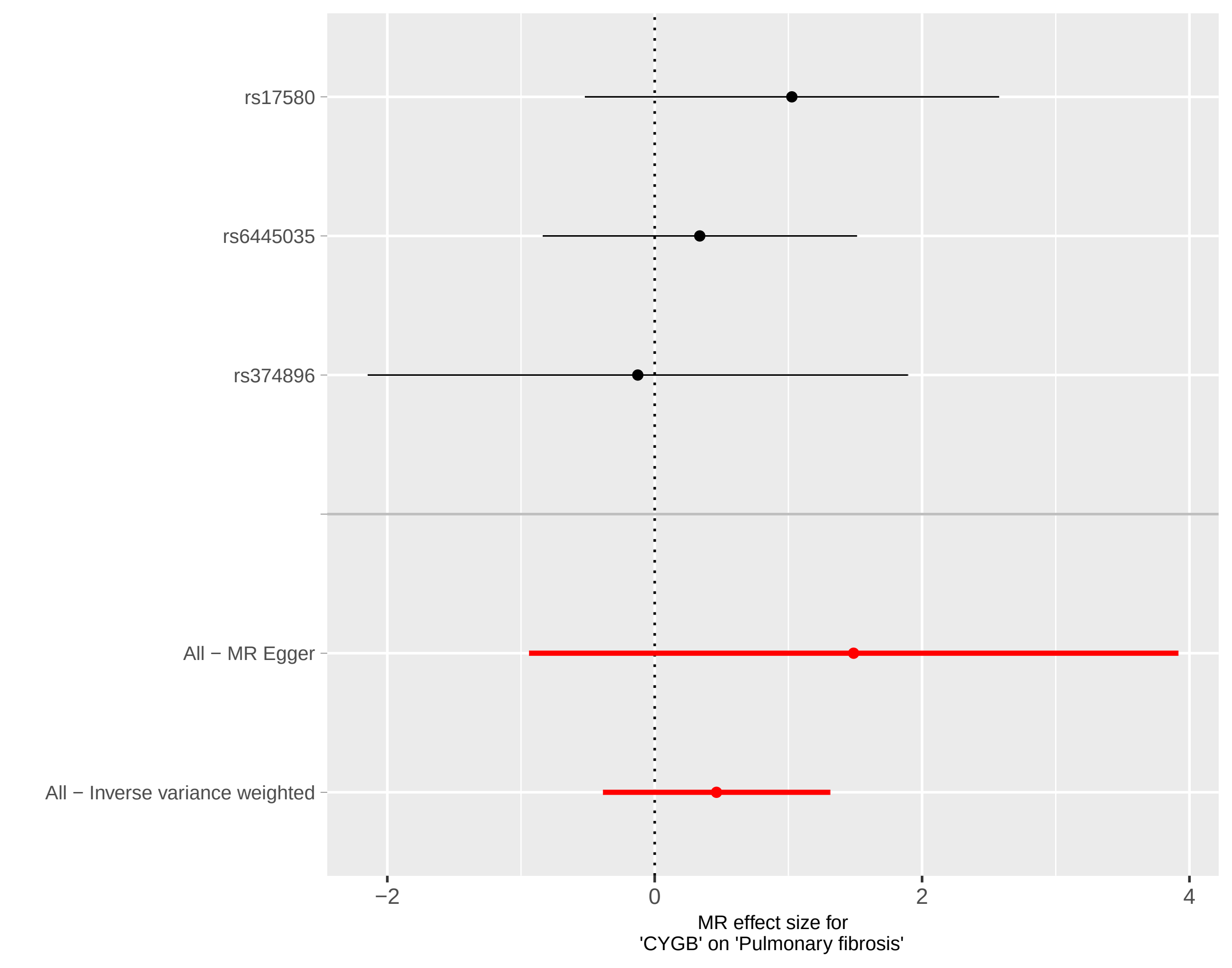
rs17580
rs6445035
rs374896
All − MR Egger
All − Inverse variance weighted
−2
0
2
4
MR effect size for
'CYGB' on 'Pulmonary fibrosis'

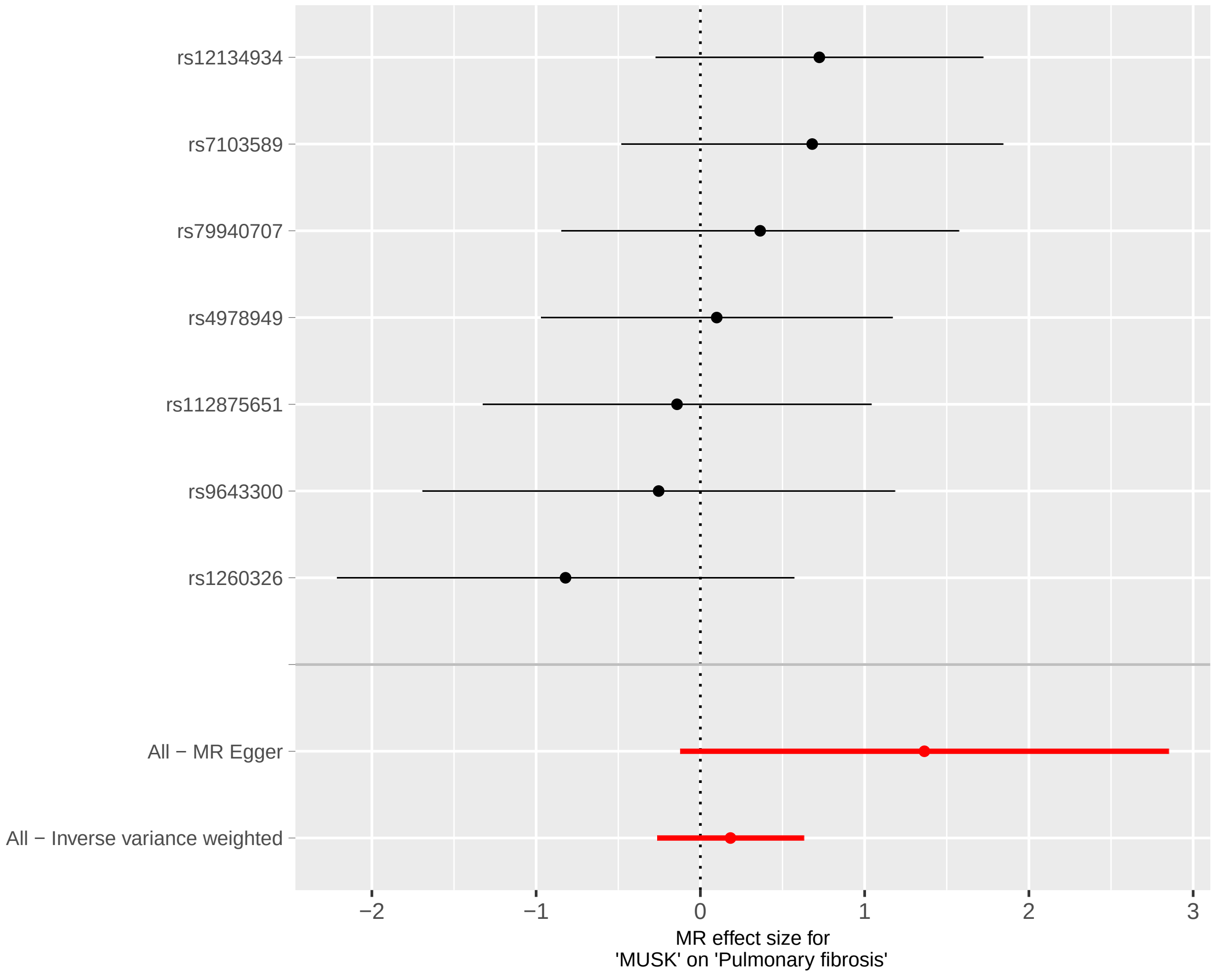
rs12134934
rs7103589
rs79940707
rs4978949
rs112875651
rs9643300
rs1260326
All − MR Egger
All − Inverse variance weighted
−2
−1
0
1
2
3
MR effect size for
'MUSK' on 'Pulmonary fibrosis'

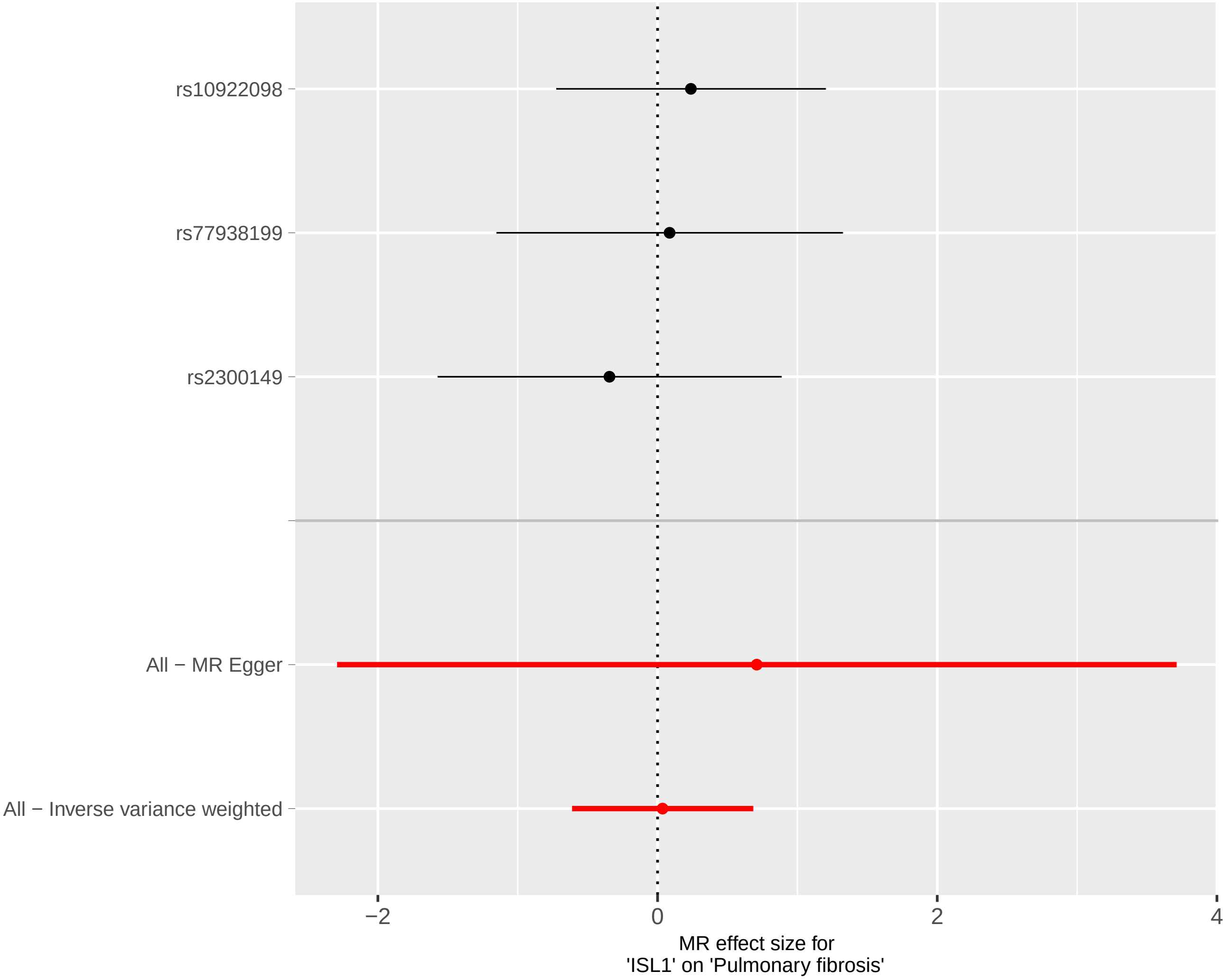
rs10922098
rs77938199
rs2300149
All − MR Egger
All − Inverse variance weighted
−2
0
2
4
MR effect size for
'ISL1' on 'Pulmonary fibrosis'

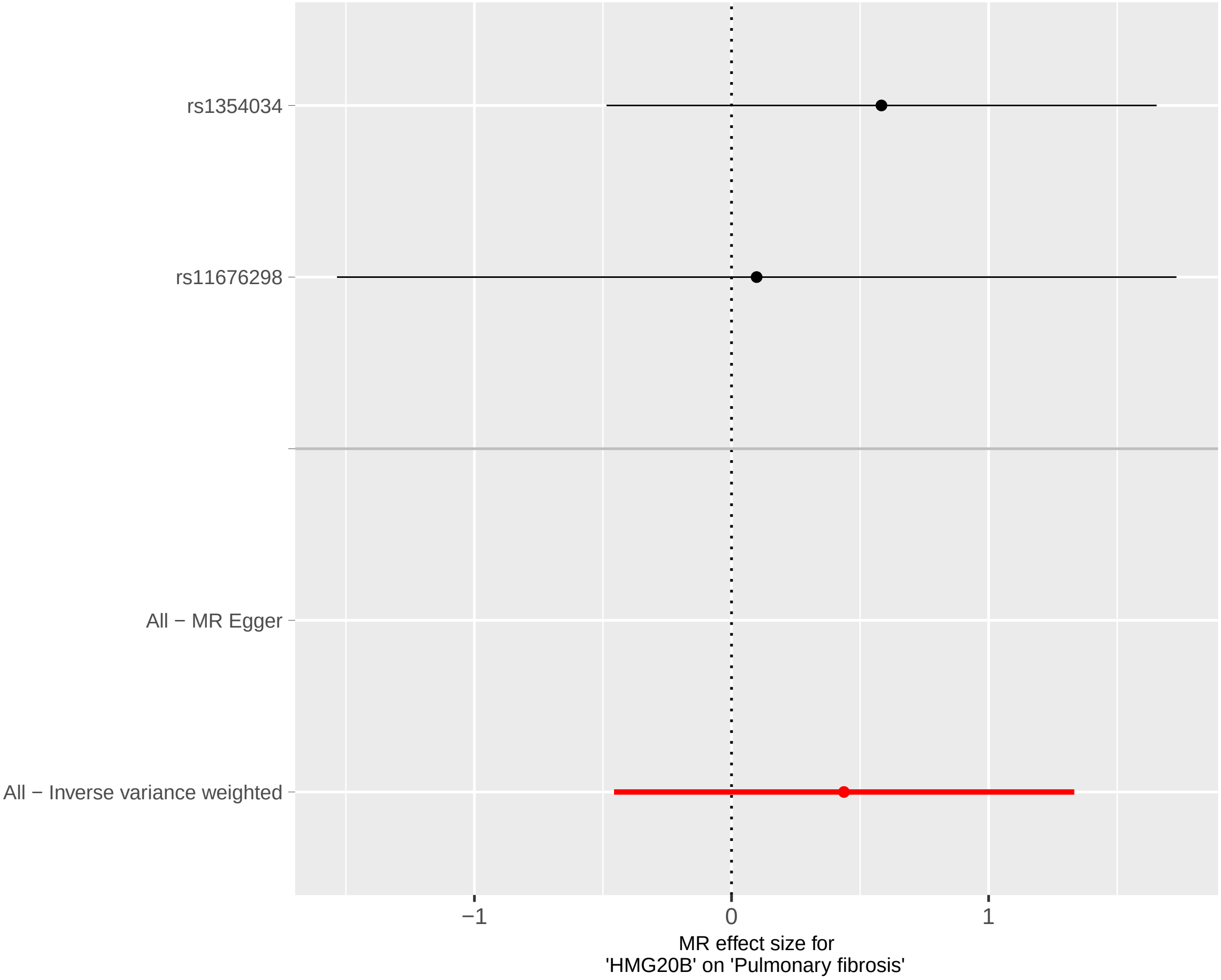
rs1354034
rs11676298
All − MR Egger
All − Inverse variance weighted
−1
0
1
MR effect size for
'HMG20B' on 'Pulmonary fibrosis'

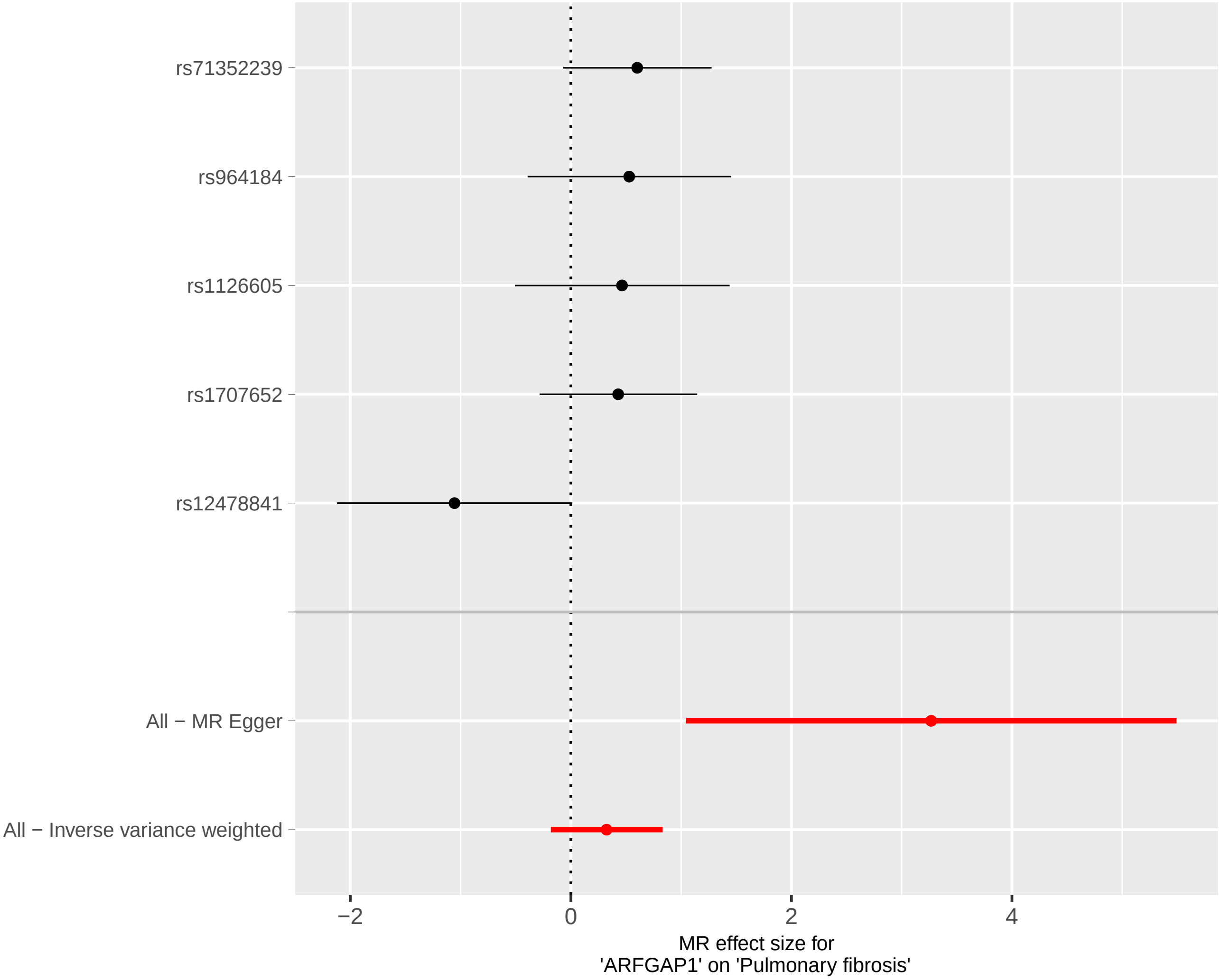
rs71352239
rs964184
rs1126605
rs1707652
rs12478841
All − MR Egger
All − Inverse variance weighted
−2
0
2
4
MR effect size for
'ARFGAP1' on 'Pulmonary fibrosis'

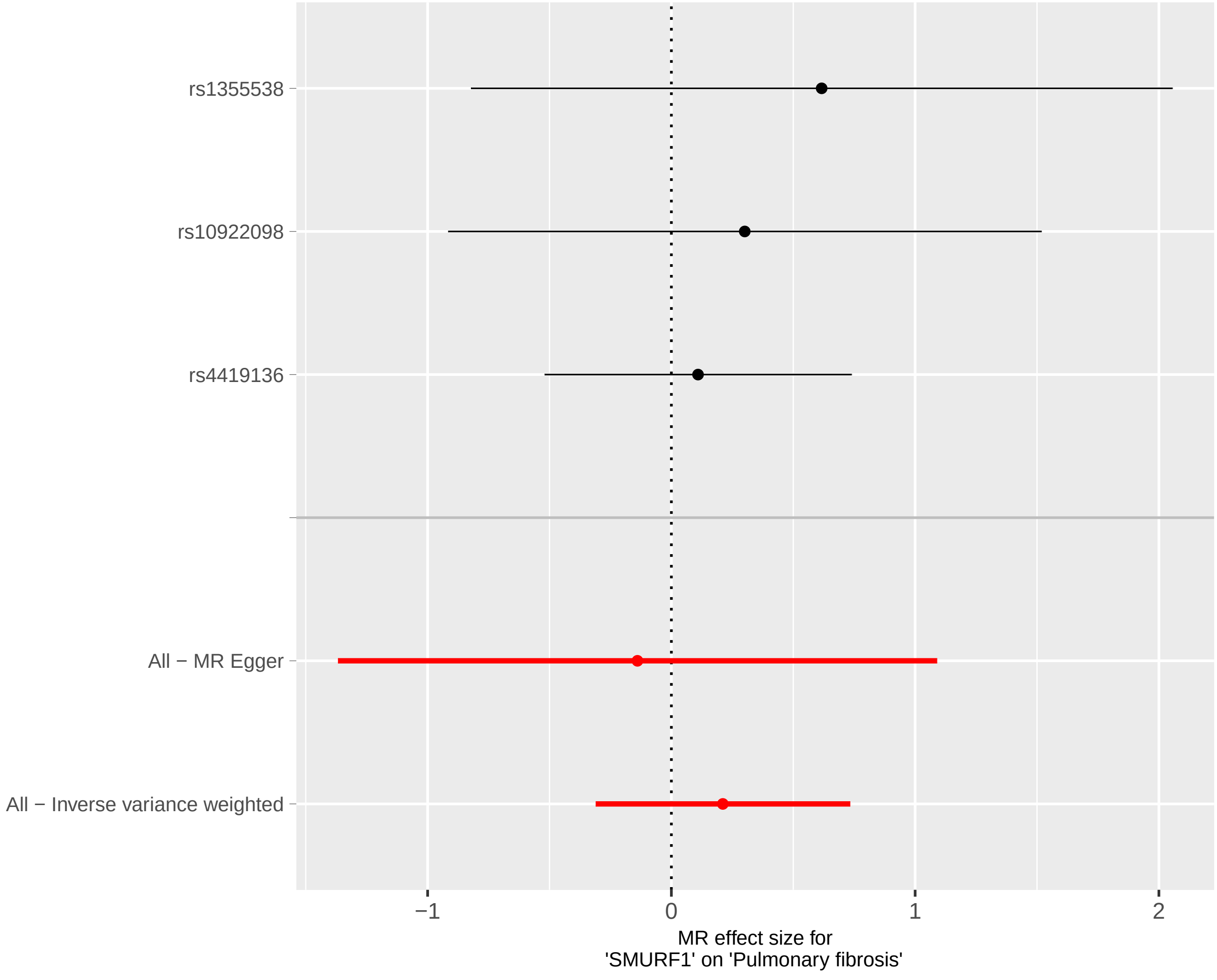
rs1355538
rs10922098
rs4419136
All − MR Egger
All − Inverse variance weighted
−1
0
1
2
MR effect size for
'SMURF1' on 'Pulmonary fibrosis'

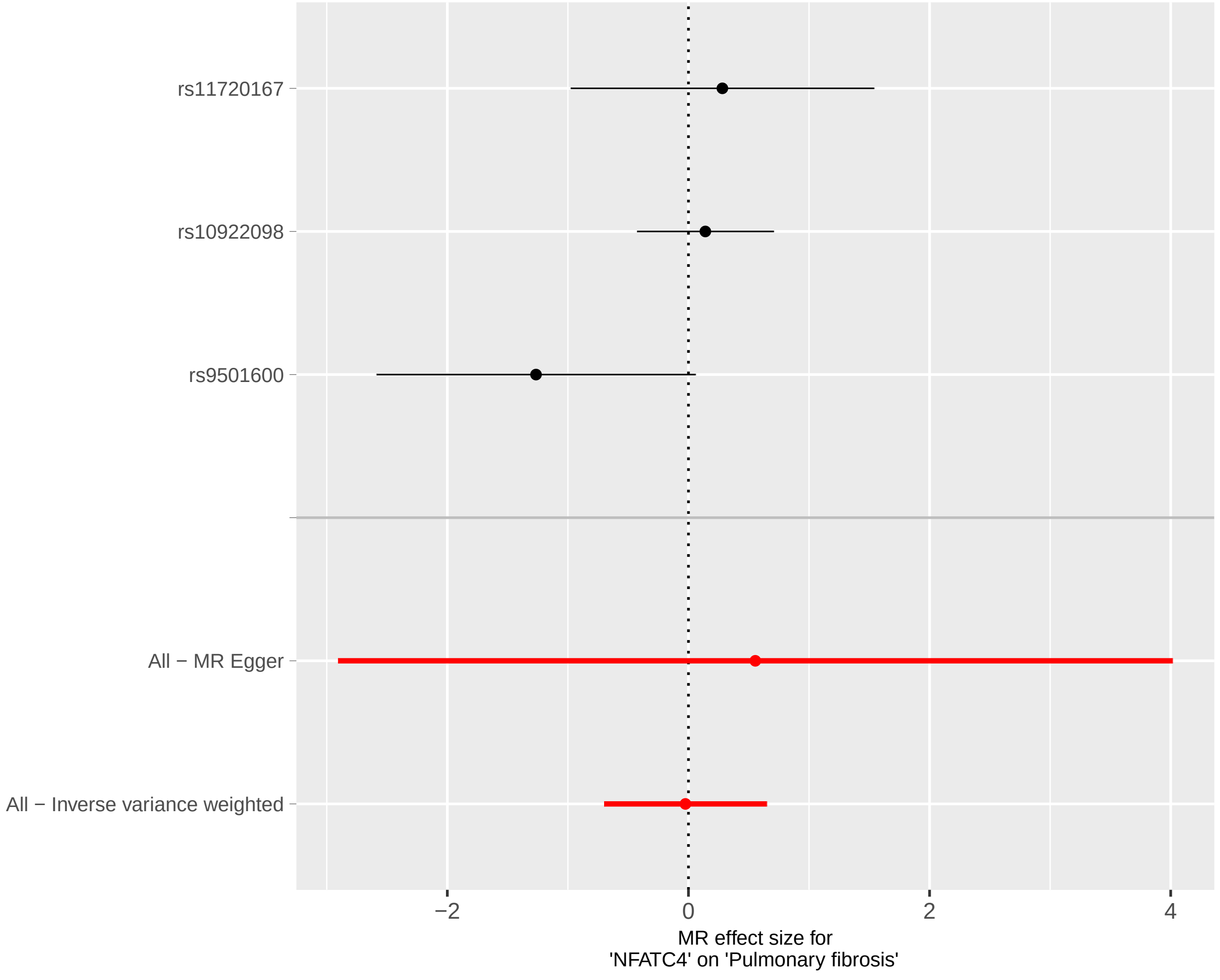

rs11720167
rs10922098
rs9501600
All − MR Egger
All − Inverse variance weighted
−2
0
2
4
MR effect size for
'NFATC4' on 'Pulmonary fibrosis'

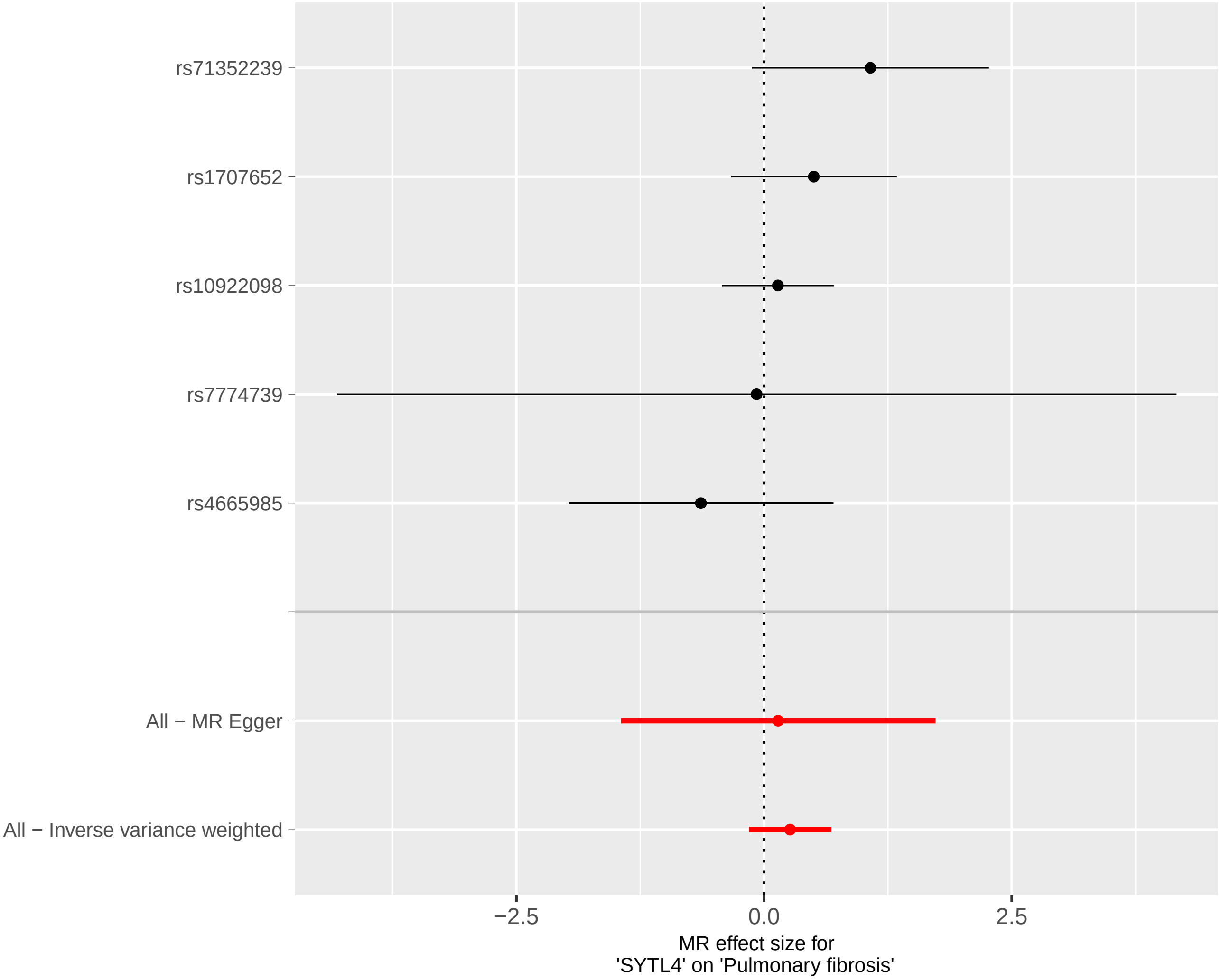

rs71352239
rs1707652
rs10922098
rs7774739
rs4665985
All − MR Egger
All − Inverse variance weighted
−2.5
0.0
2.5
MR effect size for
'SYTL4' on 'Pulmonary fibrosis'

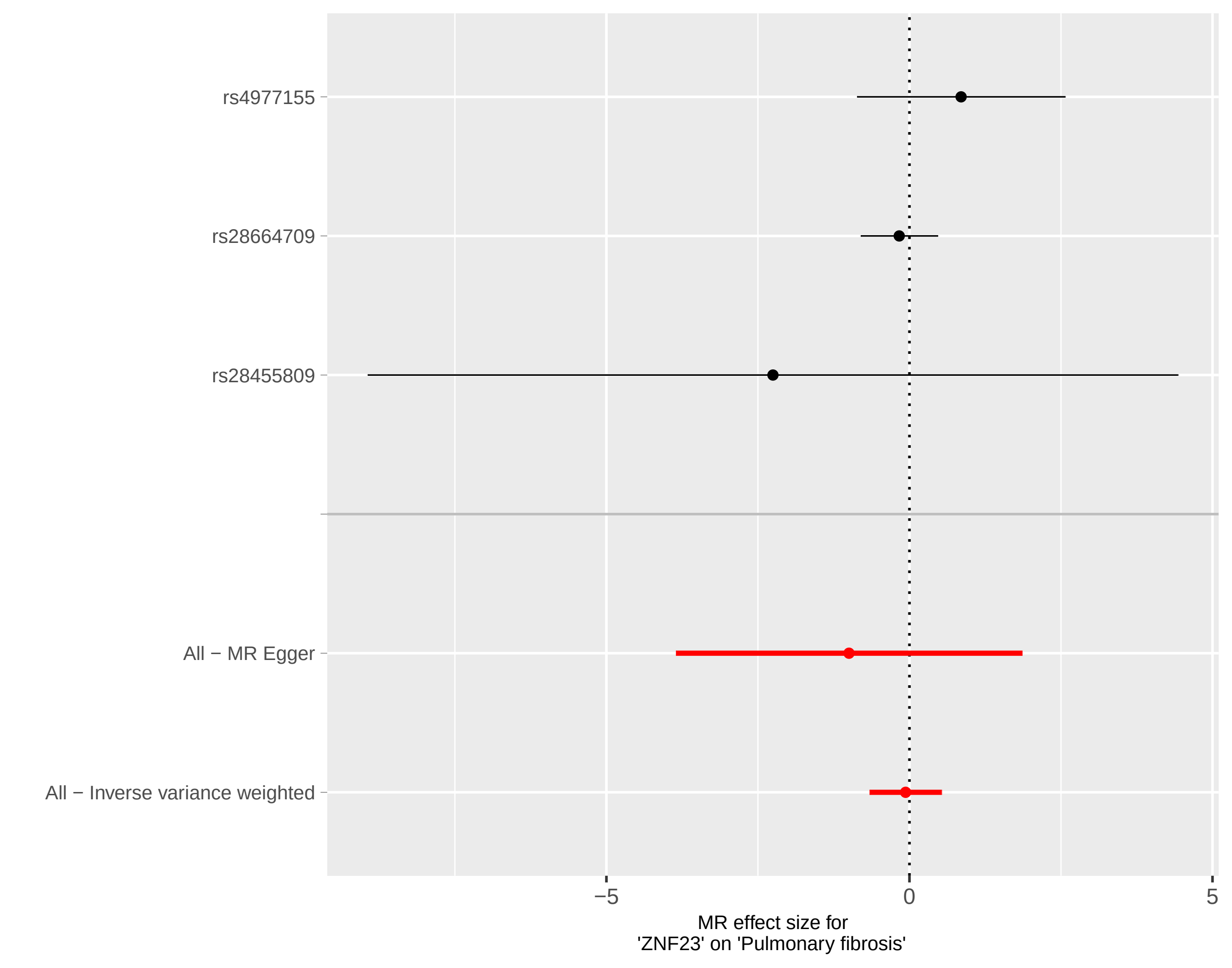
rs4977155
rs28664709
rs28455809
All − MR Egger
All − Inverse variance weighted
−5
0
5
MR effect size for
'ZNF23' on 'Pulmonary fibrosis'

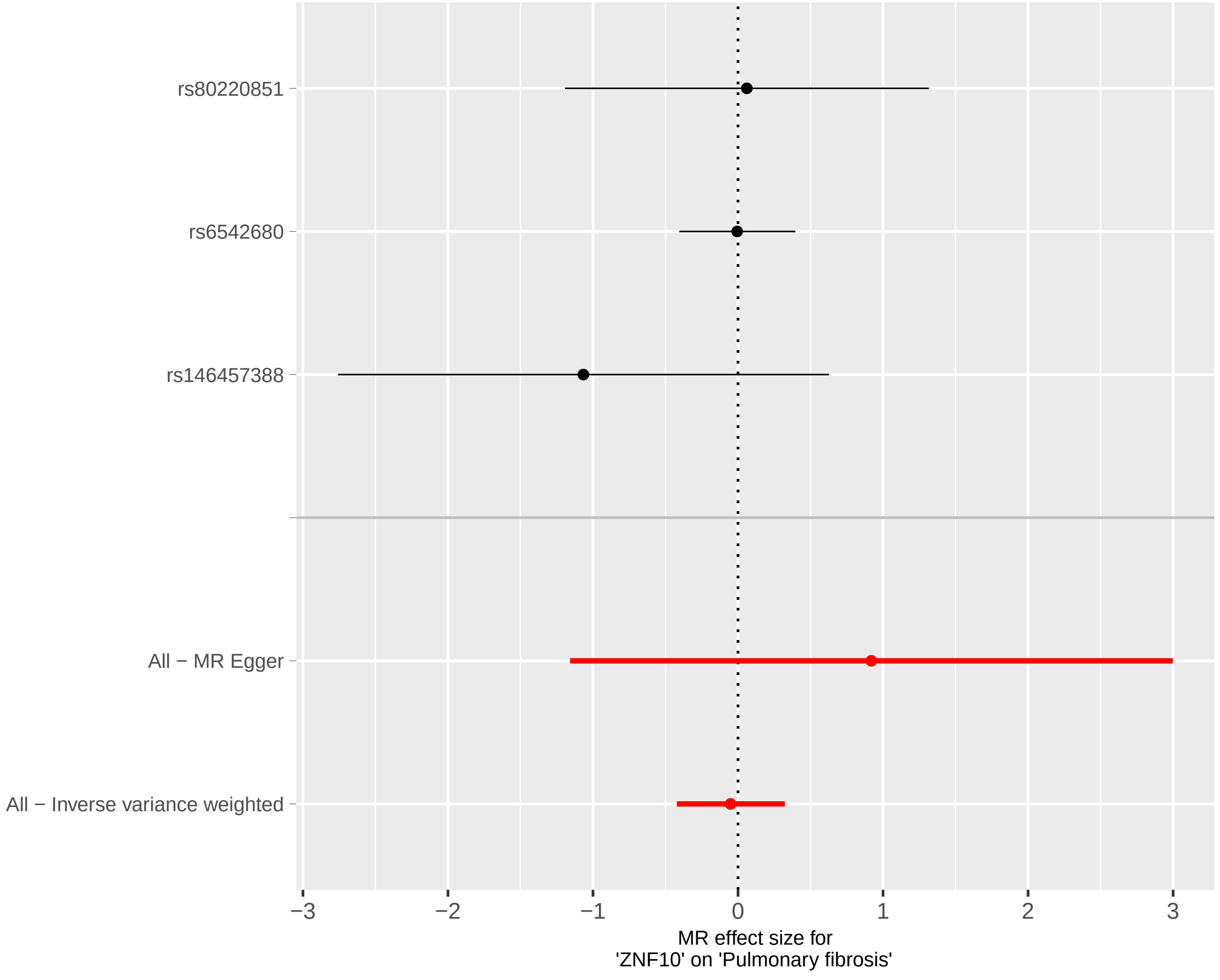
rs80220851
rs6542680
rs146457388
All − MR Egger
All − Inverse variance weighted
−3
−2
−1
0
1
2
3
MR effect size for
'ZNF10' on 'Pulmonary fibrosis'

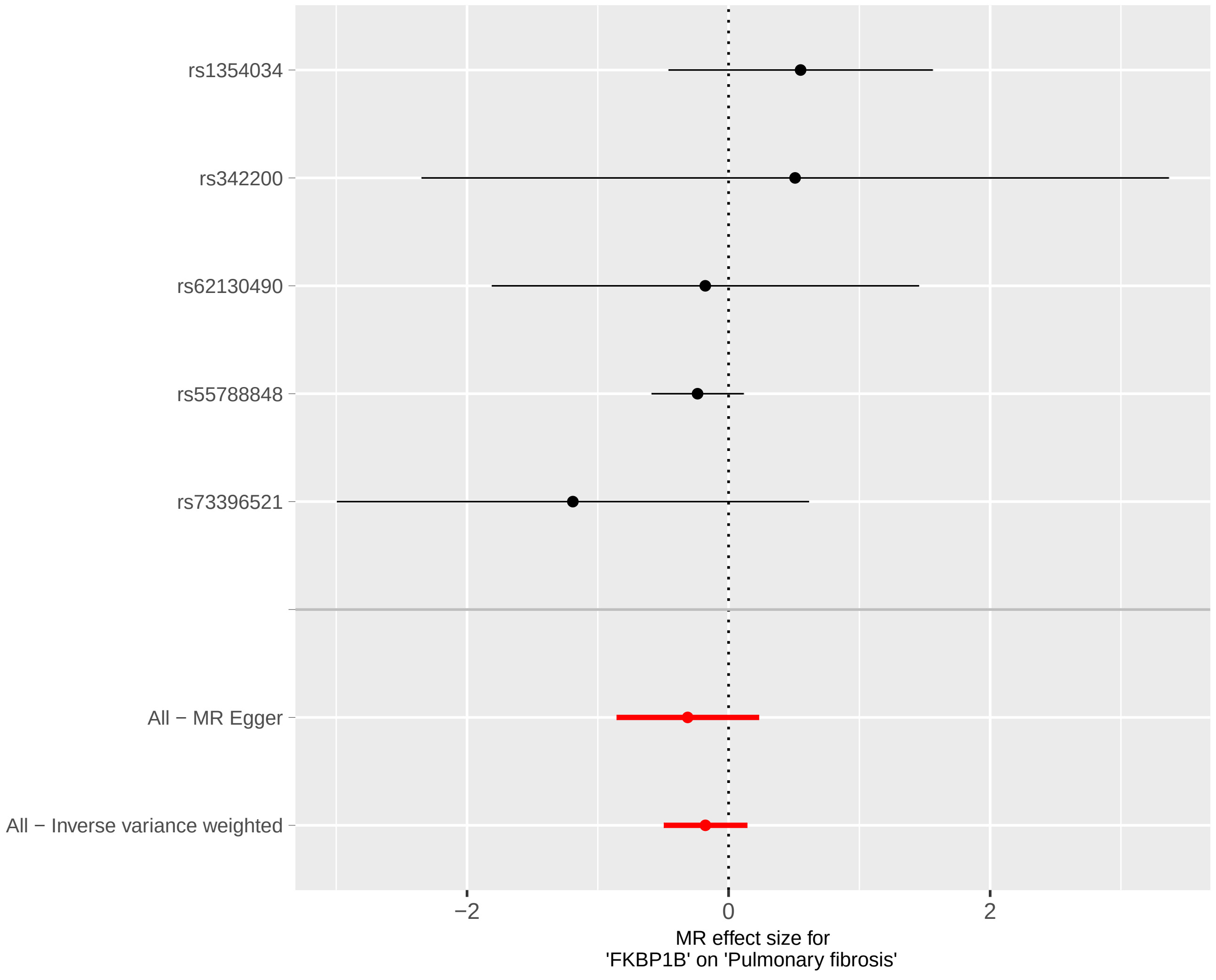
rs1354034
rs342200
rs62130490
rs55788848
rs73396521
All − MR Egger
All − Inverse variance weighted
−2
0
2
MR effect size for
'FKBP1B' on 'Pulmonary fibrosis'

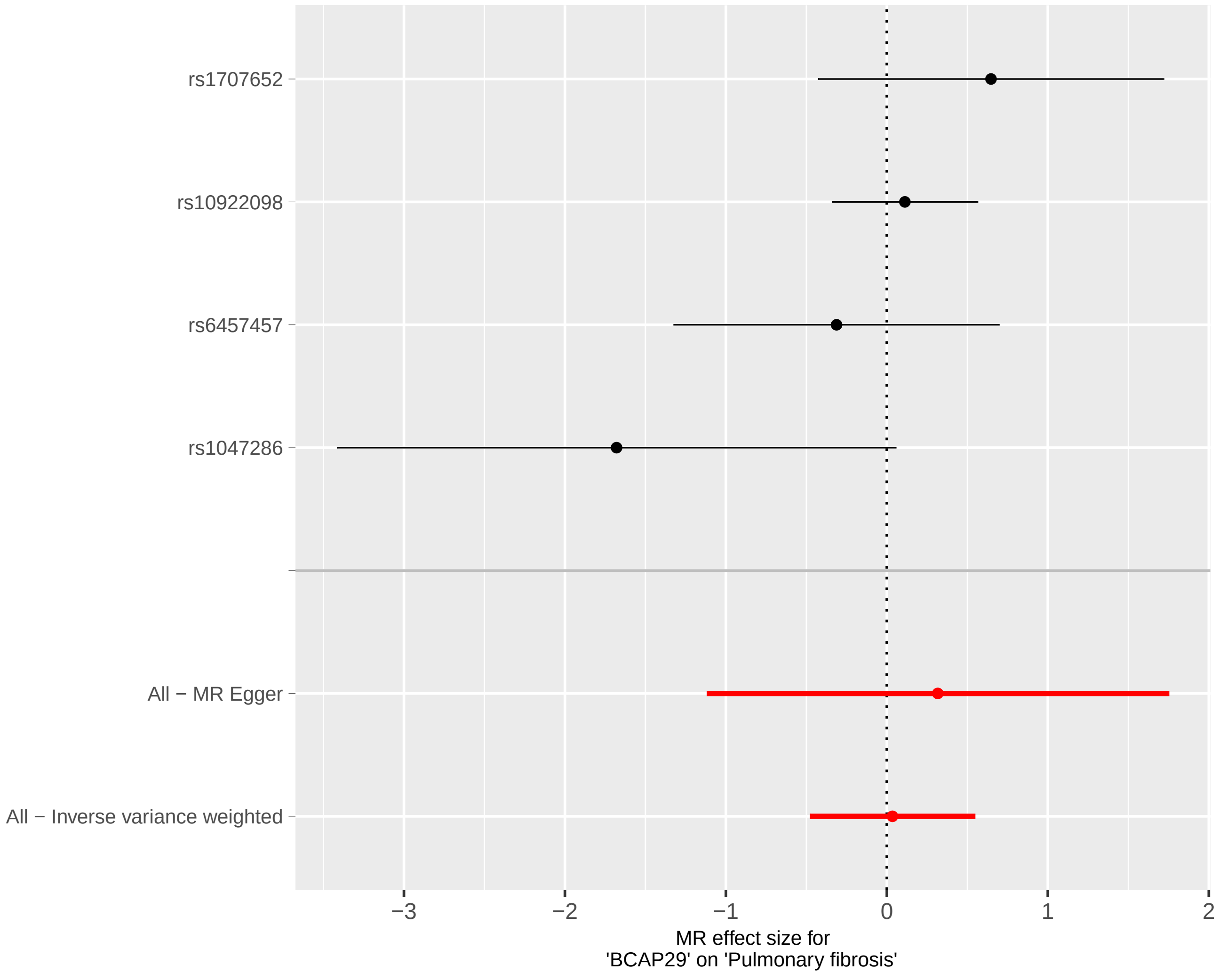
rs1707652
rs10922098
rs6457457
rs1047286
All − MR Egger
All − Inverse variance weighted
−3
−2
−1
0
1
2
MR effect size for
'BCAP29' on 'Pulmonary fibrosis'

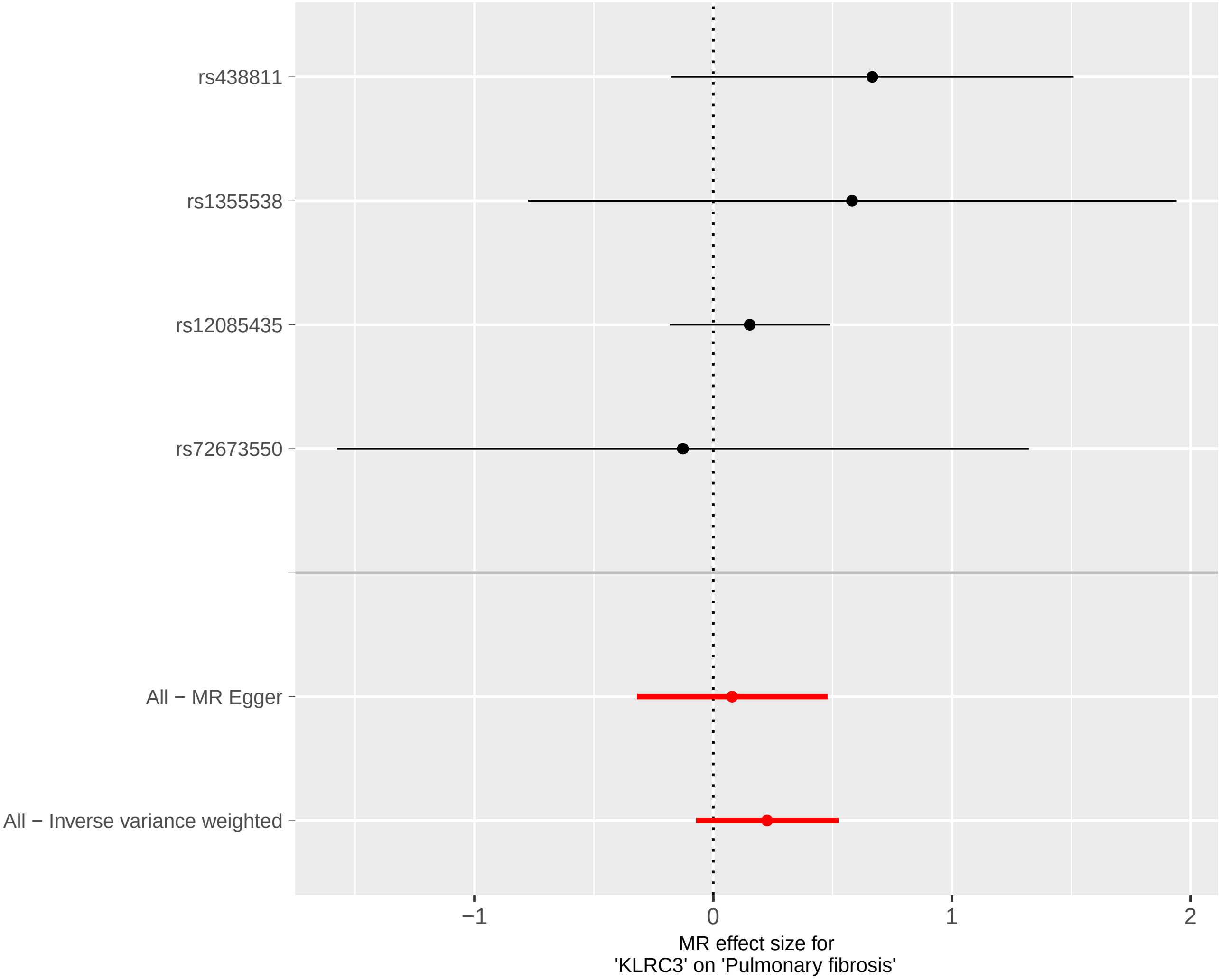

rs438811
rs1355538
rs12085435
rs72673550
All − MR Egger
All − Inverse variance weighted
−1
0
1
2
MR effect size for
'KLRC3' on 'Pulmonary fibrosis'

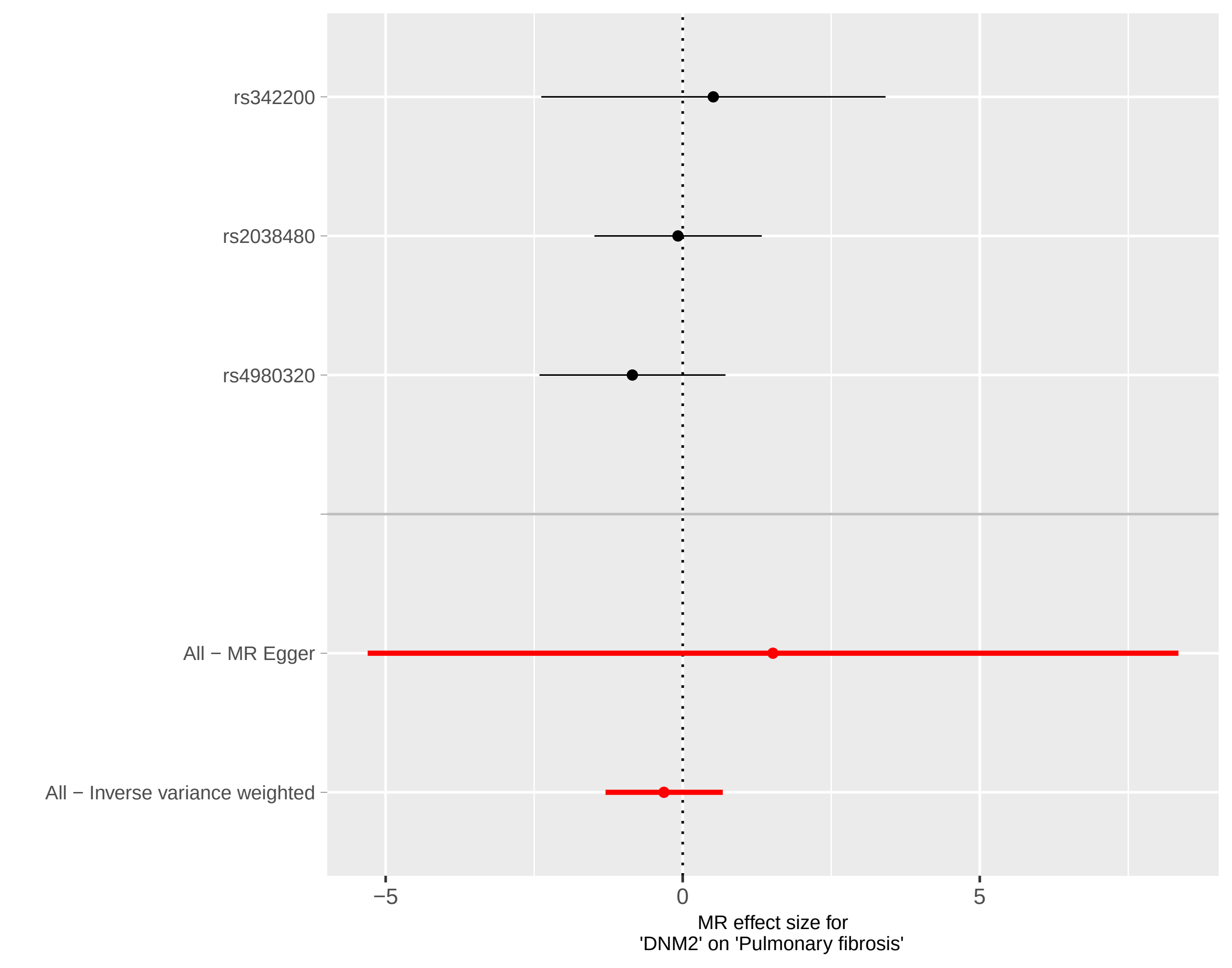
rs342200
rs2038480
rs4980320
All − MR Egger
All − Inverse variance weighted
−5
0
5
MR effect size for
'DNM2' on 'Pulmonary fibrosis'

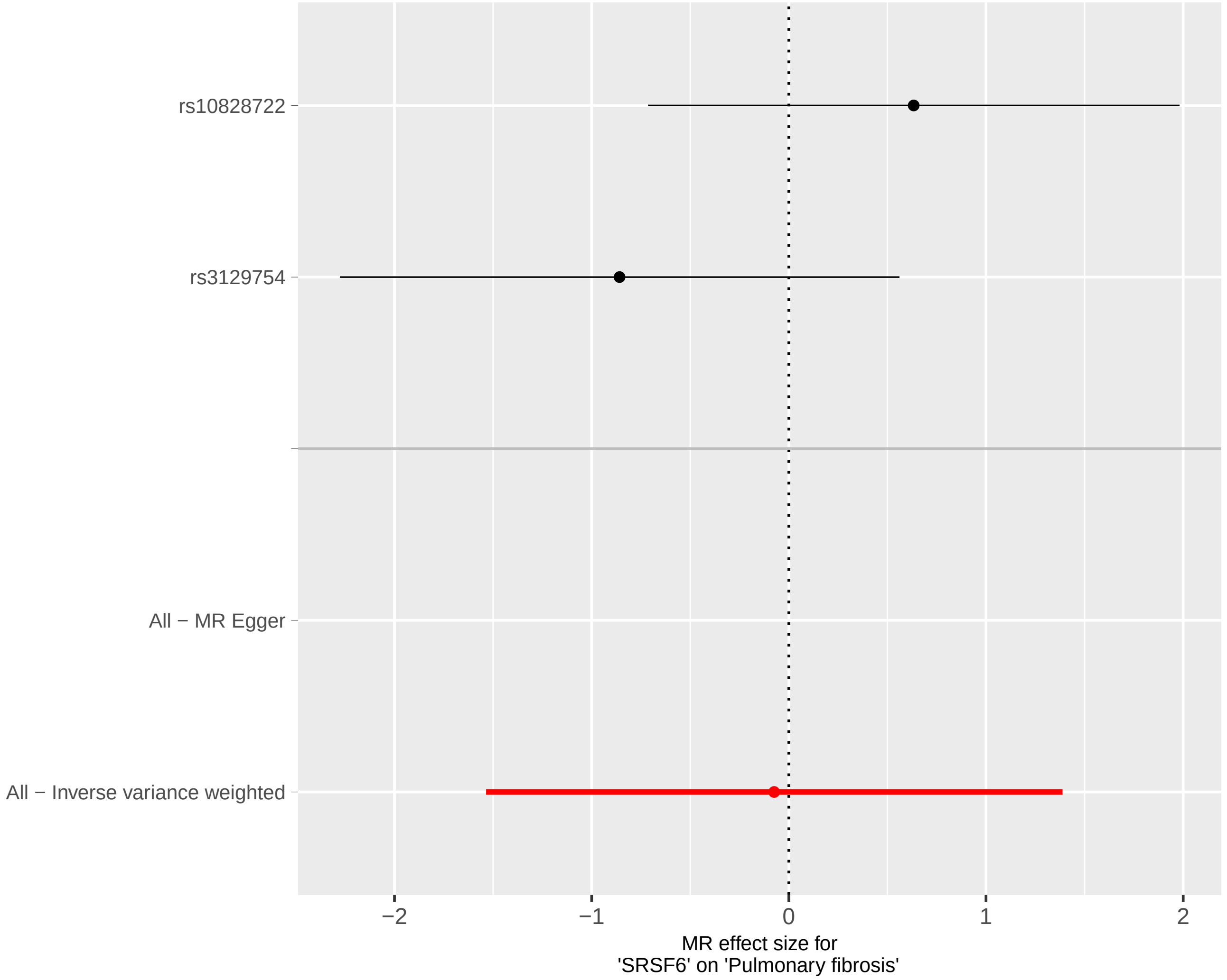

rs10828722
rs3129754
All − MR Egger
All − Inverse variance weighted
−2
−1
0
1
2
MR effect size for
'SRSF6' on 'Pulmonary fibrosis'

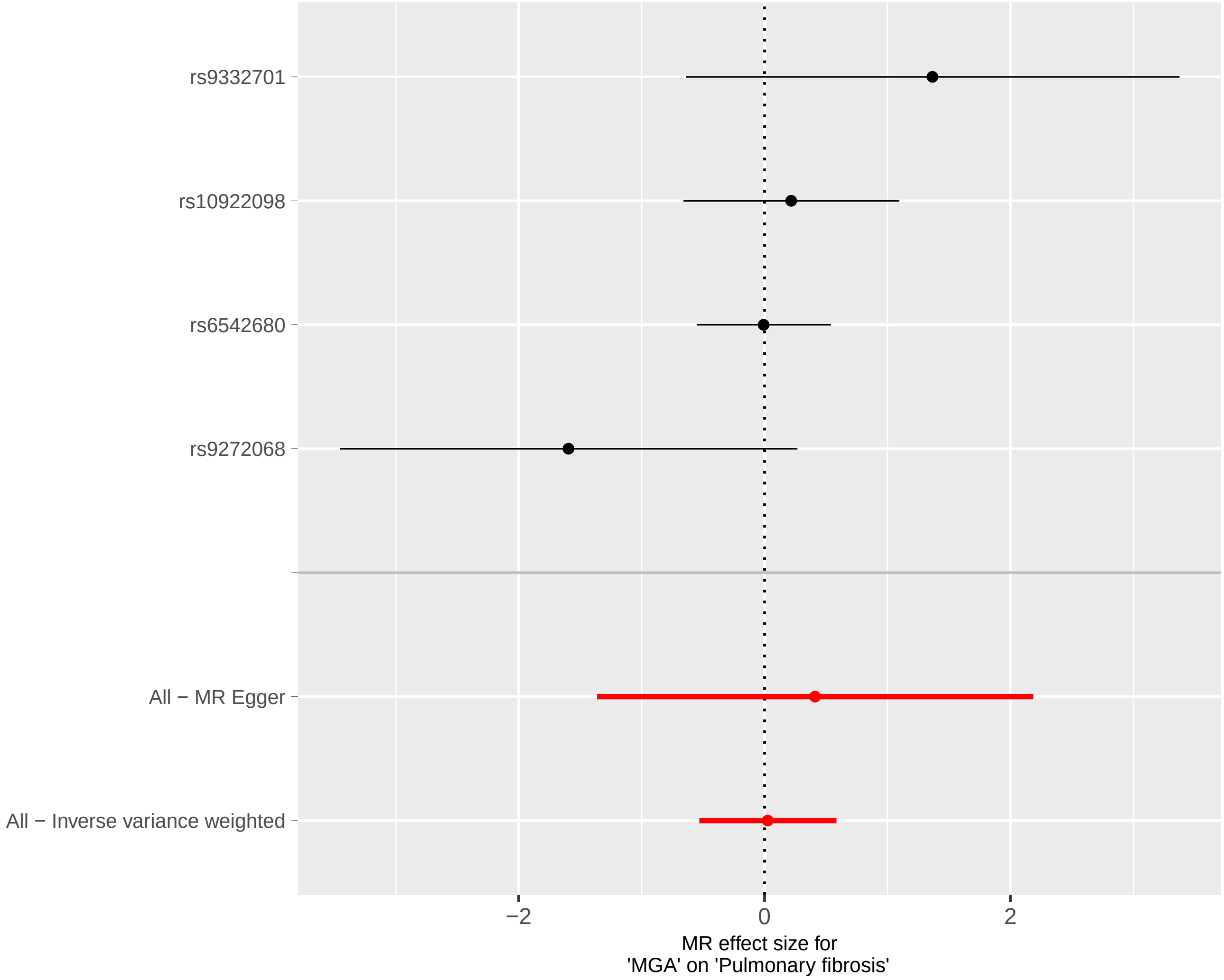
rs9332701
rs10922098
rs6542680
rs9272068
All − MR Egger
All − Inverse variance weighted
−2
0
2
MR effect size for
'MGA' on 'Pulmonary fibrosis'

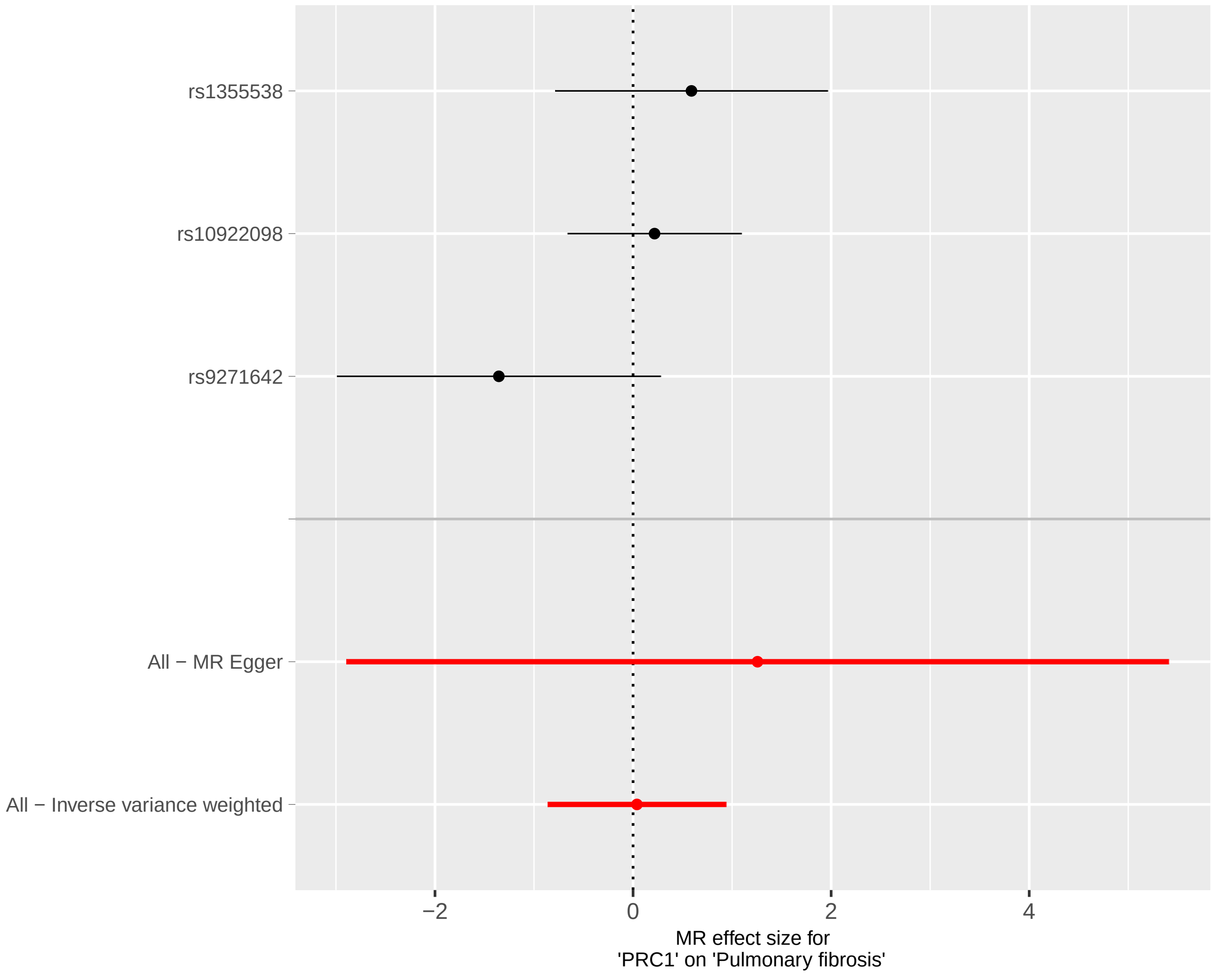
rs1355538
rs10922098
rs9271642
All − MR Egger
All − Inverse variance weighted
−2
0
2
4
MR effect size for
'PRC1' on 'Pulmonary fibrosis'

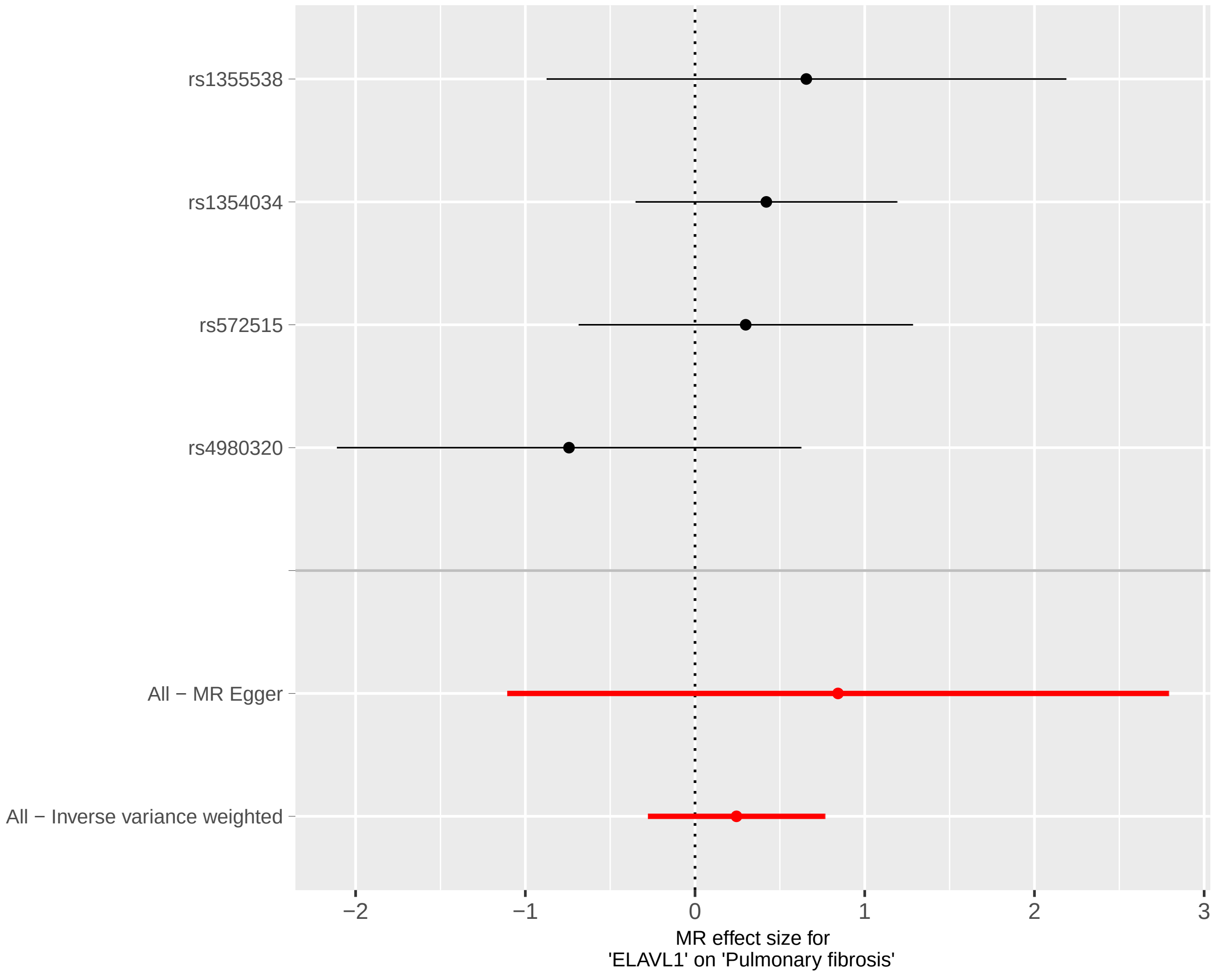

rs1355538
rs1354034
rs572515
rs4980320
All − MR Egger
All − Inverse variance weighted
−2
−1
0
1
2
3
MR effect size for
'ELAVL1' on 'Pulmonary fibrosis'

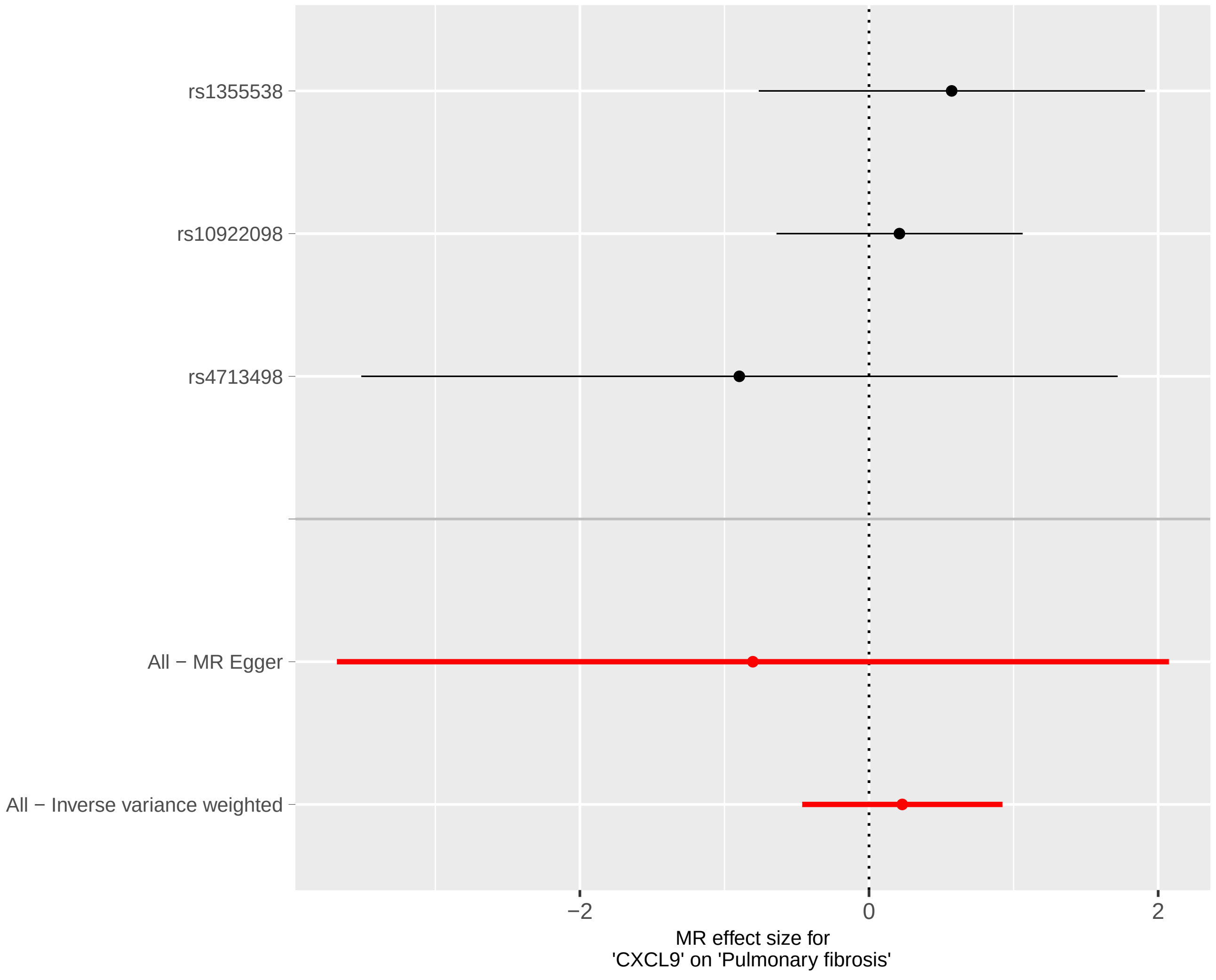

rs1355538
rs10922098
rs4713498
All − MR Egger
All − Inverse variance weighted
−2
0
2
MR effect size for
'CXCL9' on 'Pulmonary fibrosis'

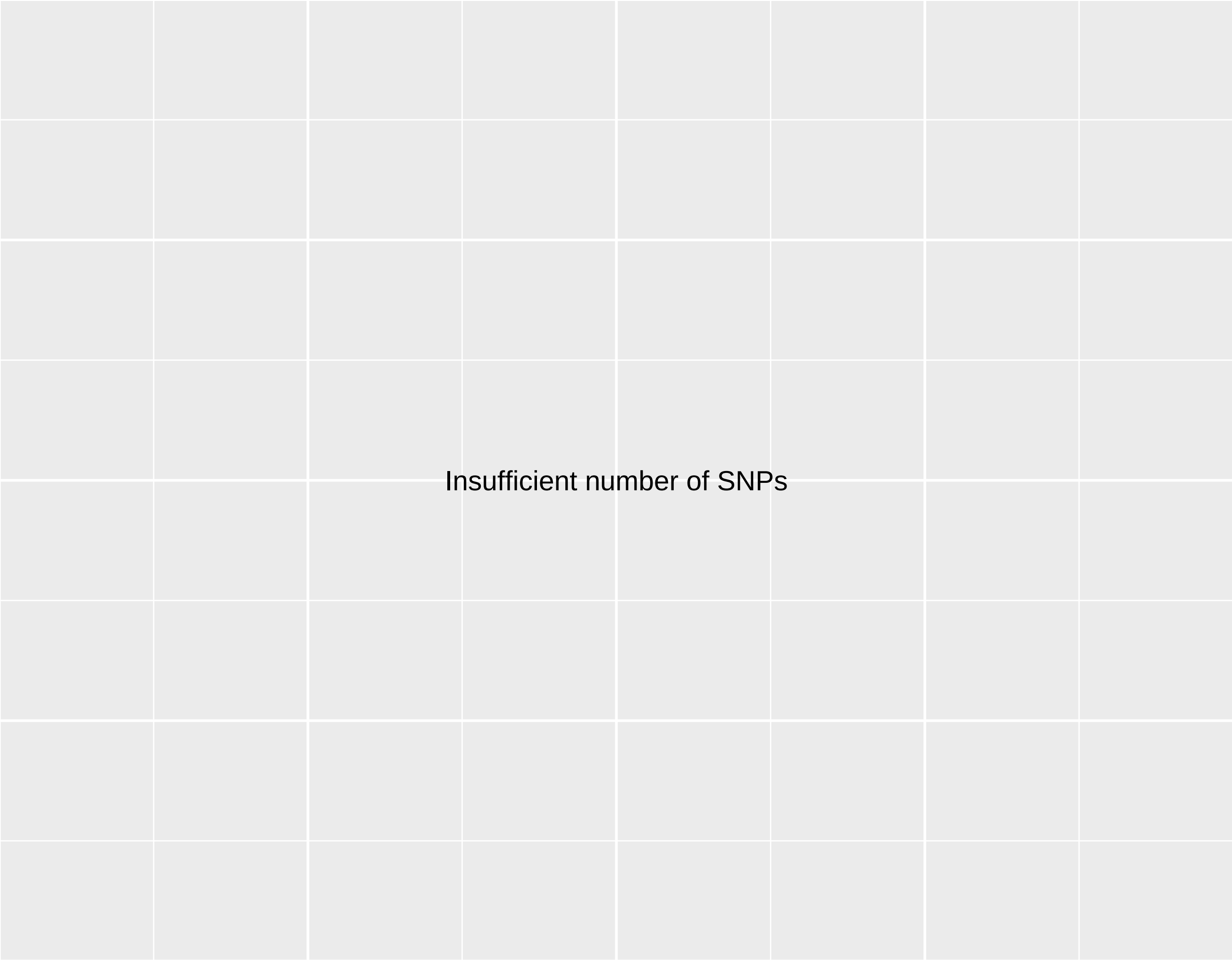
Insufficient number of SNPs

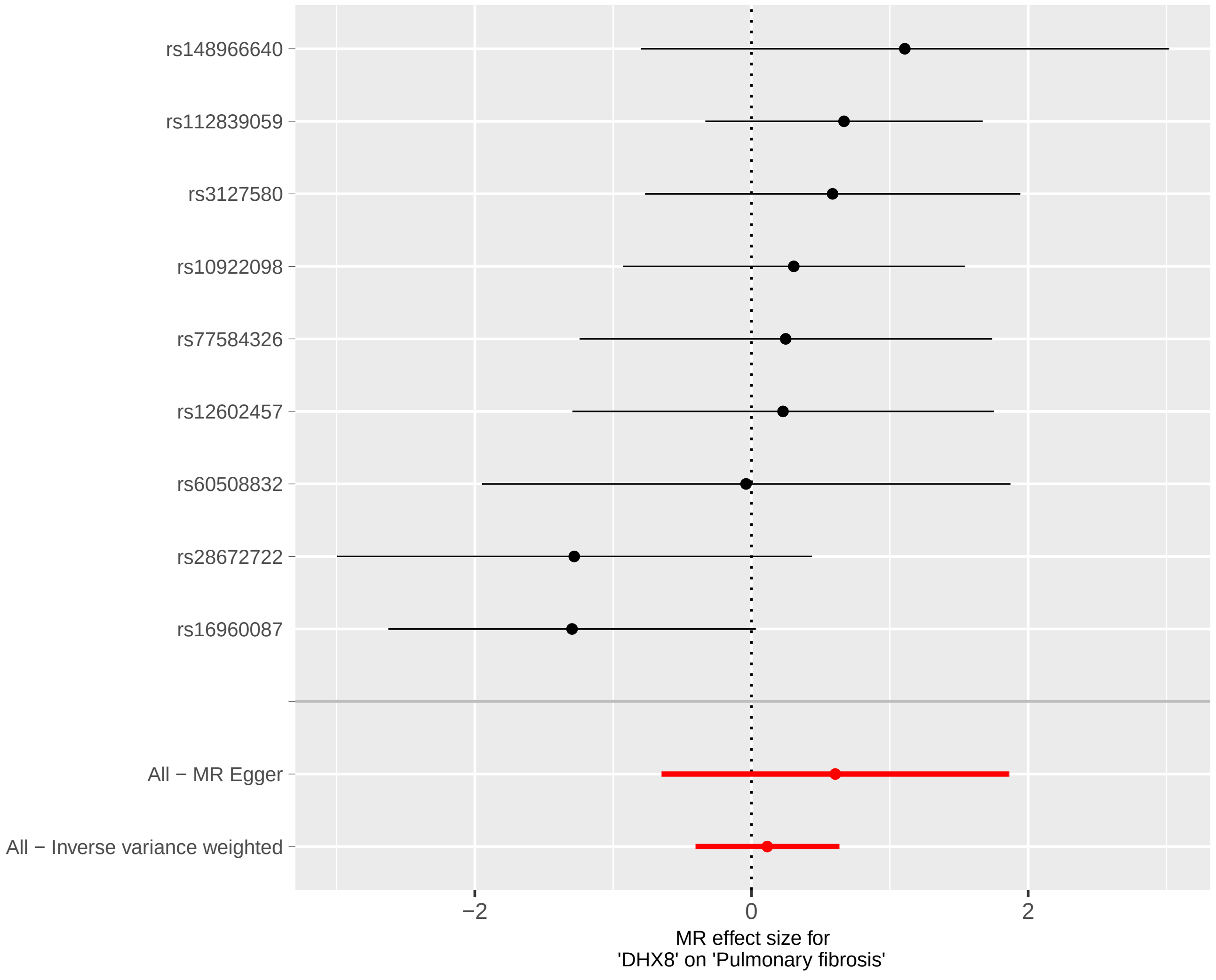
rs148966640
rs112839059
rs3127580
rs10922098
rs77584326
rs12602457
rs60508832
rs28672722
rs16960087
All − MR Egger
All − Inverse variance weighted
−2
0
2
MR effect size for
'DHX8' on 'Pulmonary fibrosis'

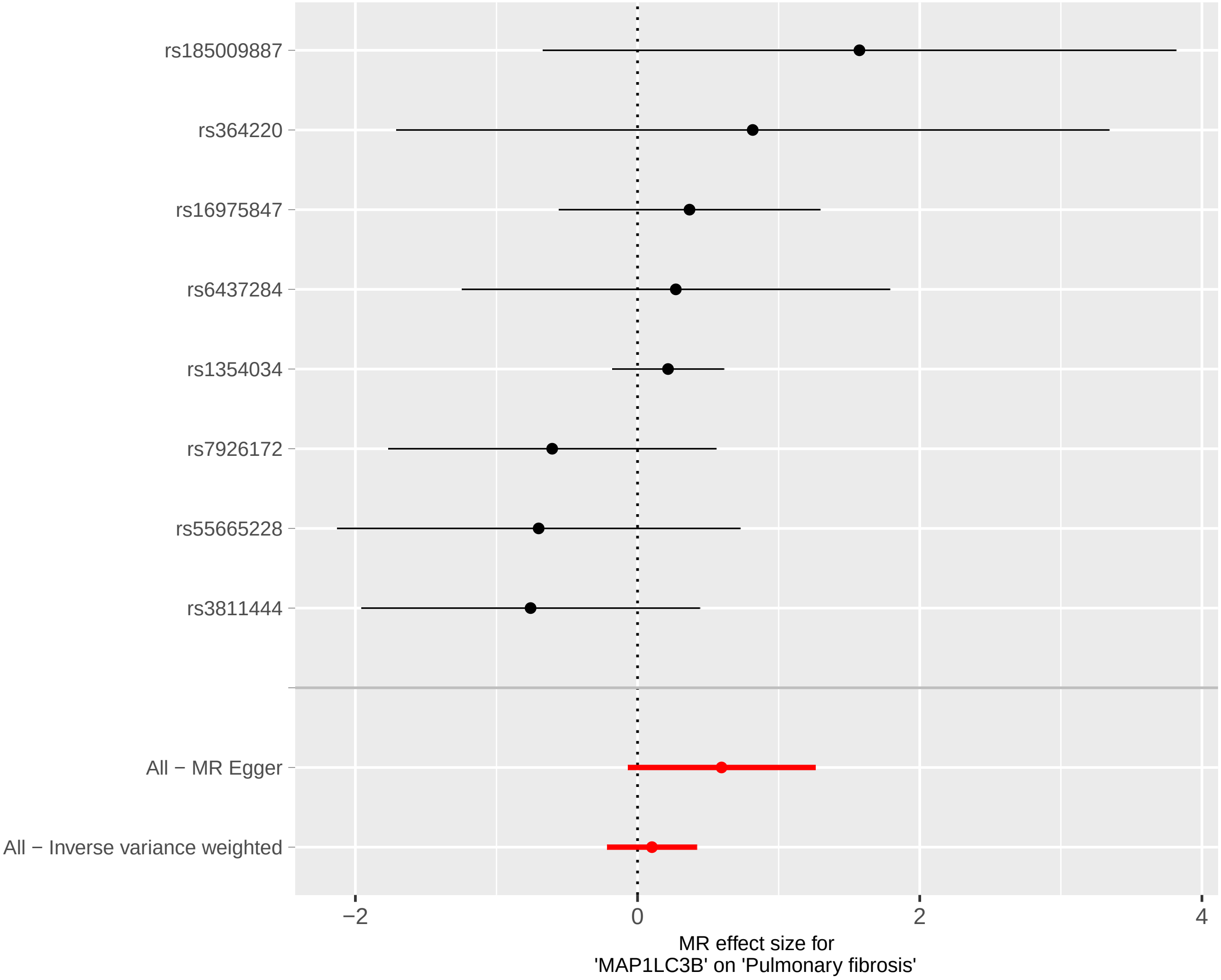

rs185009887
rs364220
rs16975847
rs6437284
rs1354034
rs7926172
rs55665228
rs3811444
All − MR Egger
All − Inverse variance weighted
−2
0
2
4
MR effect size for
'MAP1LC3B' on 'Pulmonary fibrosis'

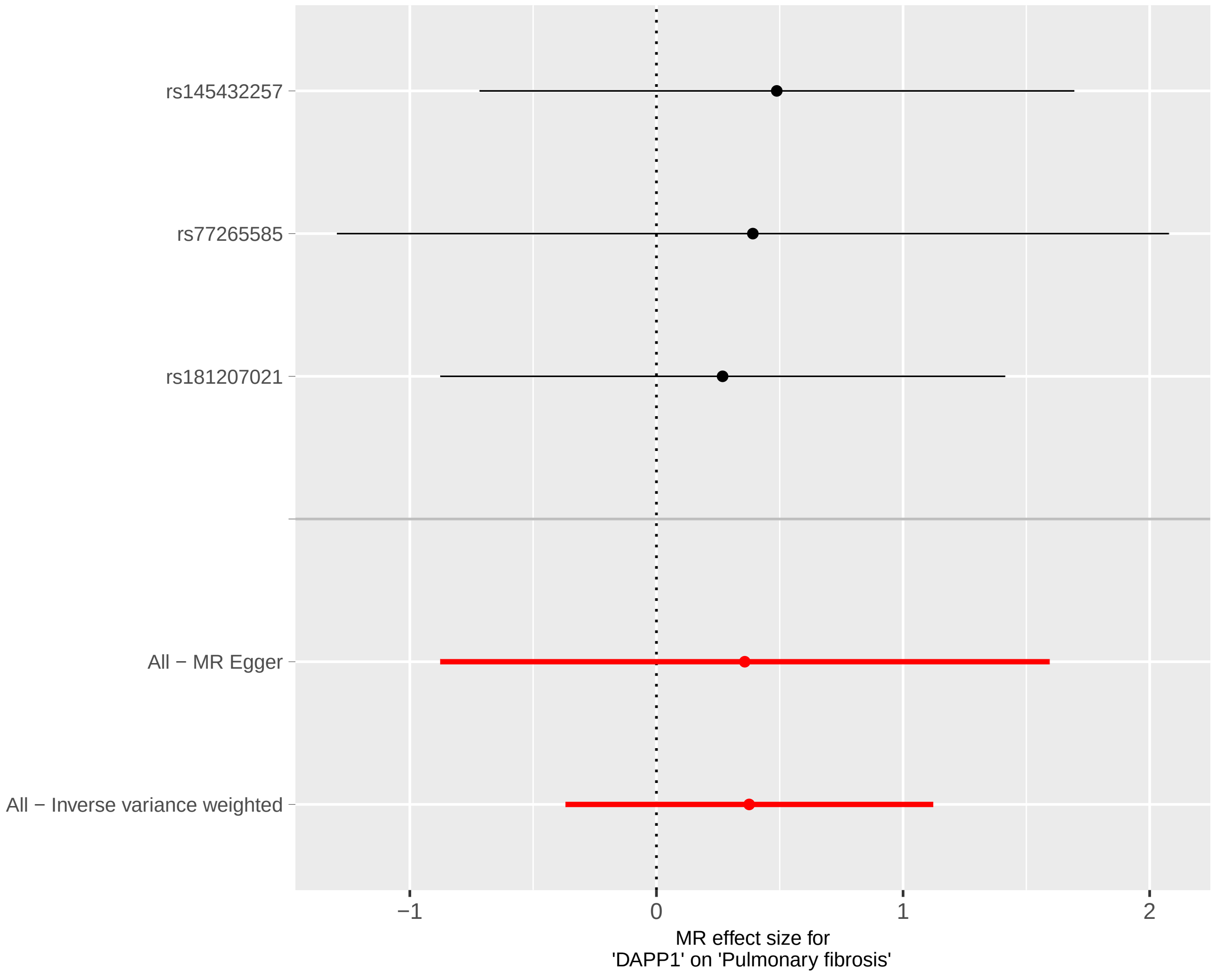
rs145432257
rs77265585
rs181207021
All − MR Egger
All − Inverse variance weighted
−1
0
1
2
MR effect size for
'DAPP1' on 'Pulmonary fibrosis'

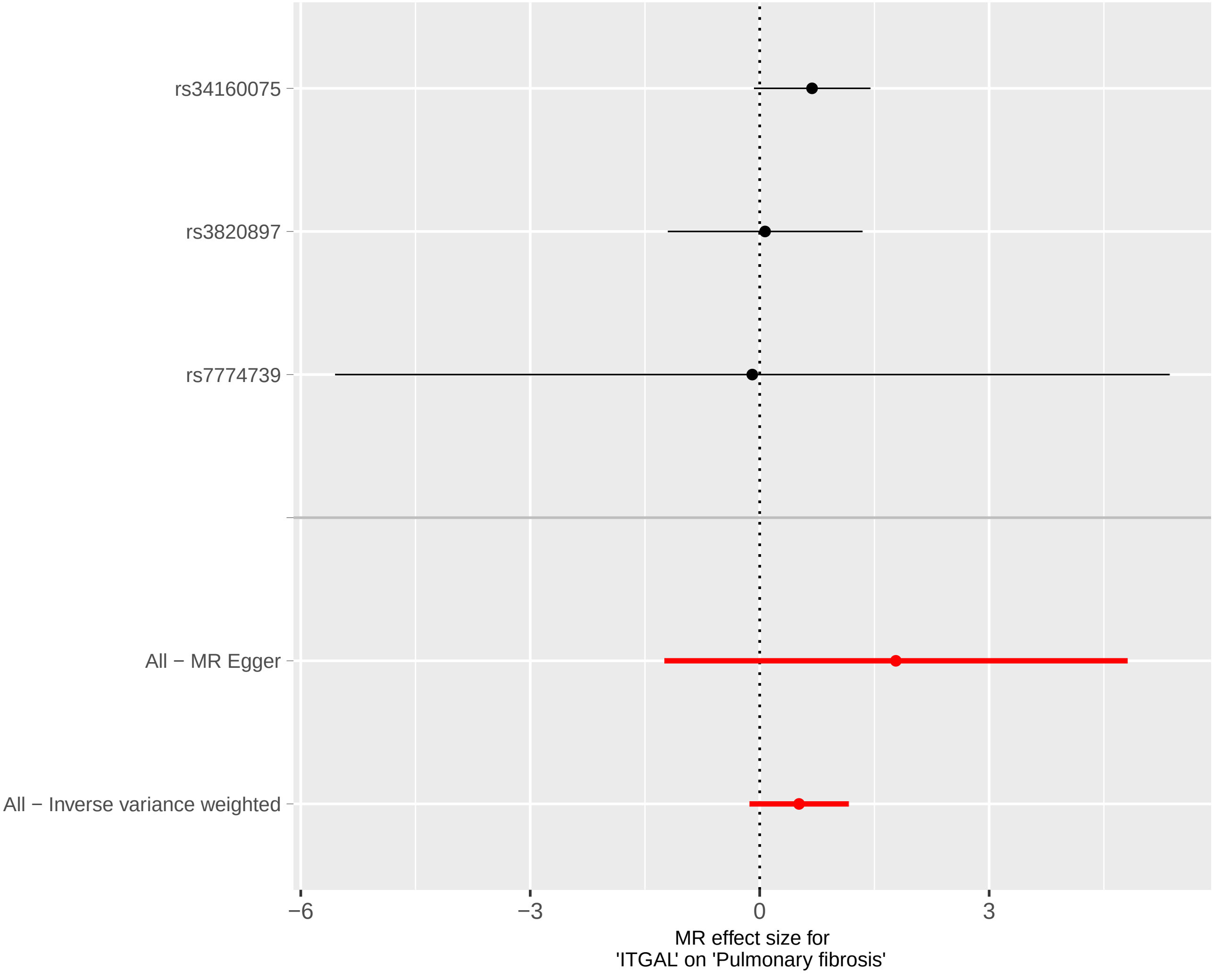
rs34160075
rs3820897
rs7774739
All − MR Egger
All − Inverse variance weighted
−6
−3
0
3
MR effect size for
'ITGAL' on 'Pulmonary fibrosis'

Insufficient number of SNPs

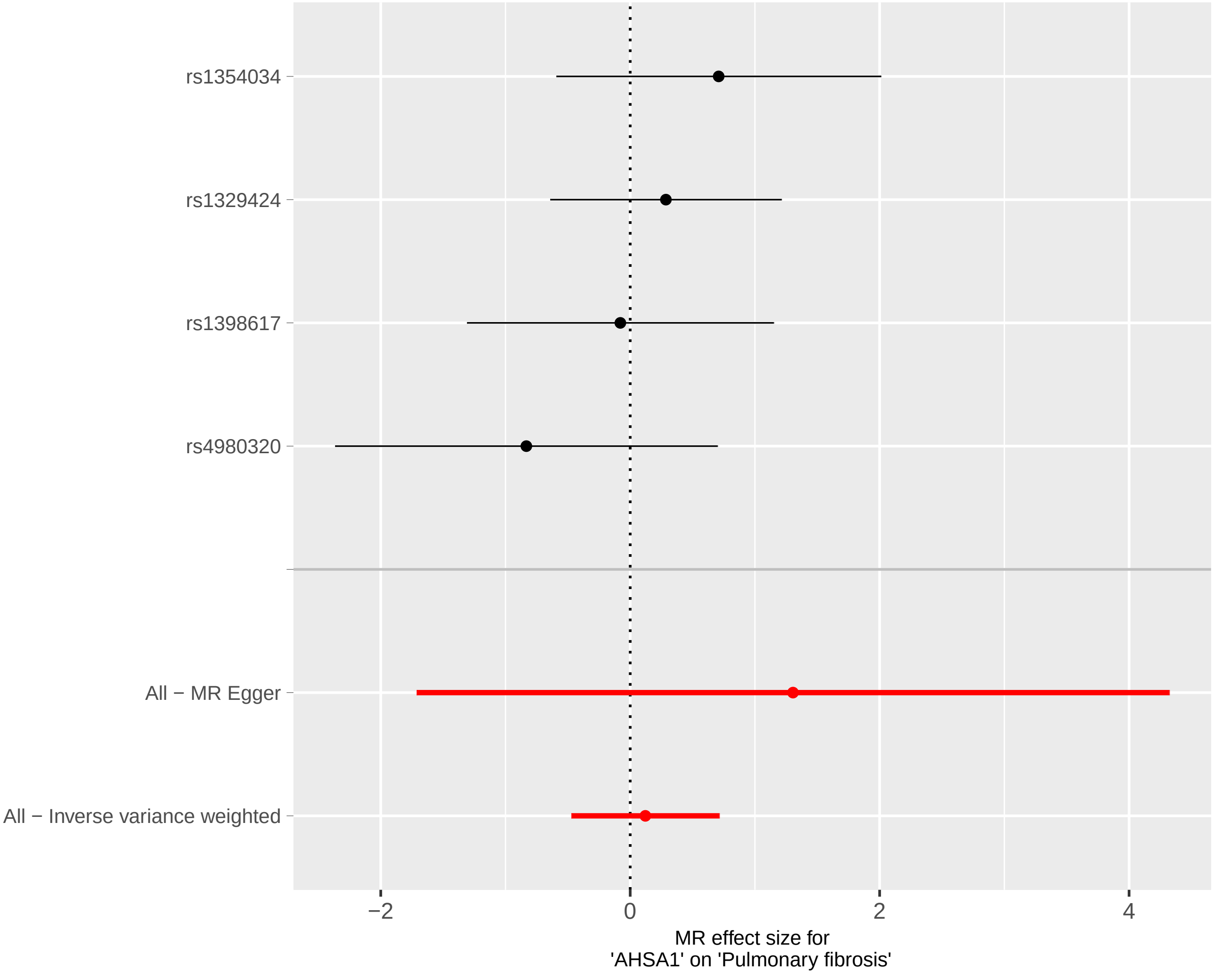
rs1354034
rs1329424
rs1398617
rs4980320
All − MR Egger
All − Inverse variance weighted
−2
0
2
4
MR effect size for
'AHSA1' on 'Pulmonary fibrosis'

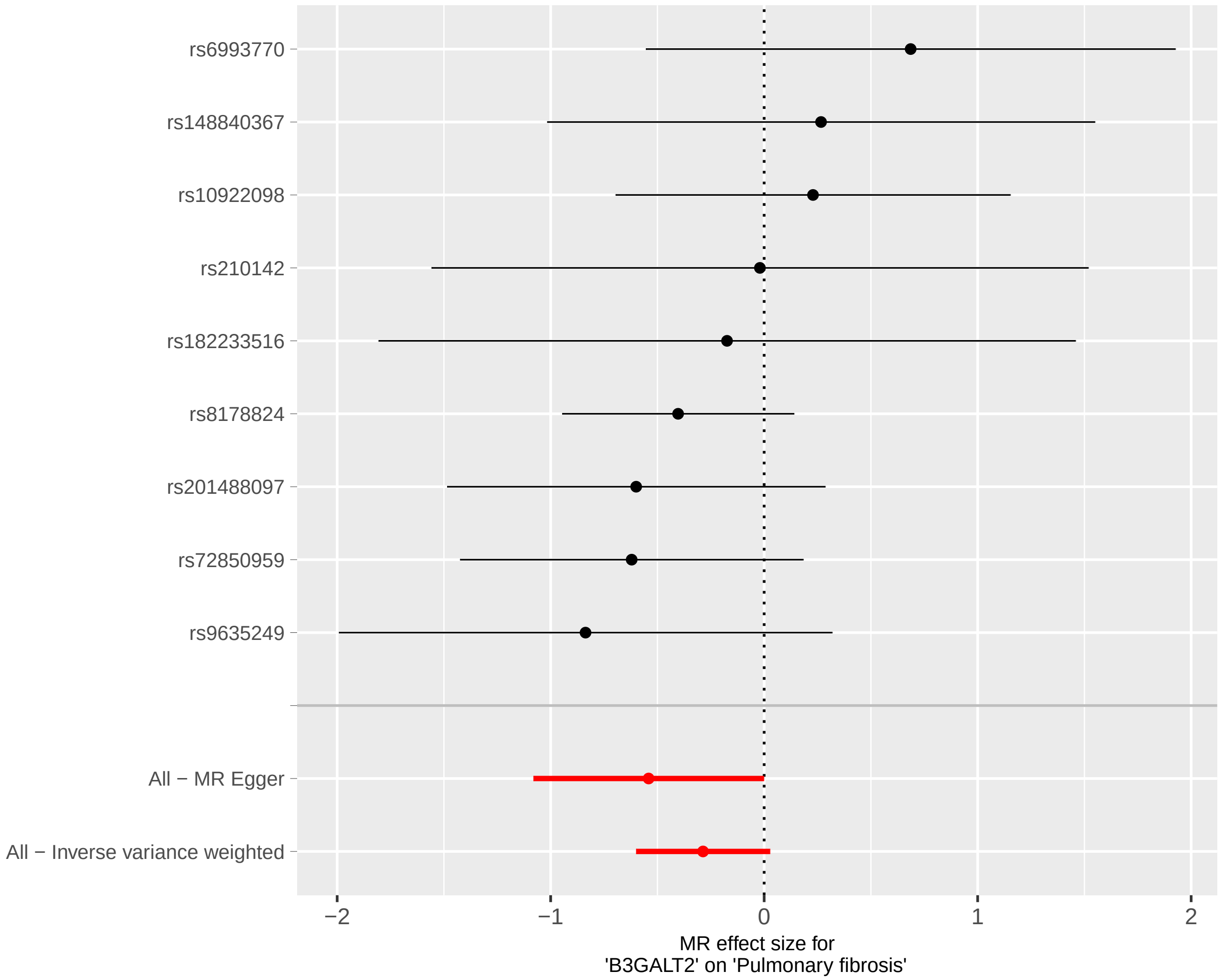

rs6993770
rs148840367
rs10922098
rs210142
rs182233516
rs8178824
rs201488097
rs72850959
rs9635249
All − MR Egger
All − Inverse variance weighted
−2
−1
0
1
2
MR effect size for
'B3GALT2' on 'Pulmonary fibrosis'

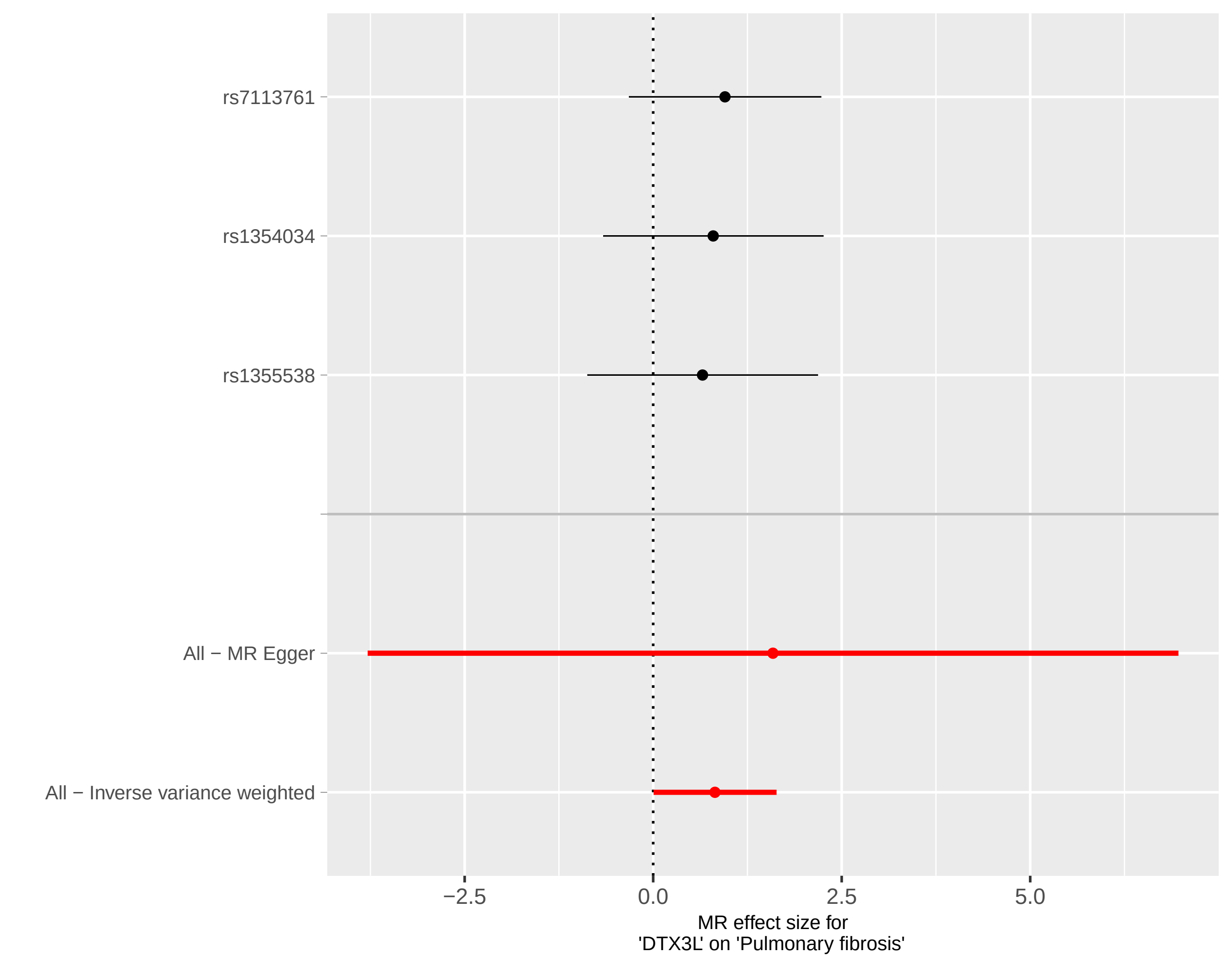
rs7113761
rs1354034
rs1355538
All − MR Egger
All − Inverse variance weighted
−2.5
0.0
2.5
5.0
MR effect size for
'DTX3L' on 'Pulmonary fibrosis'

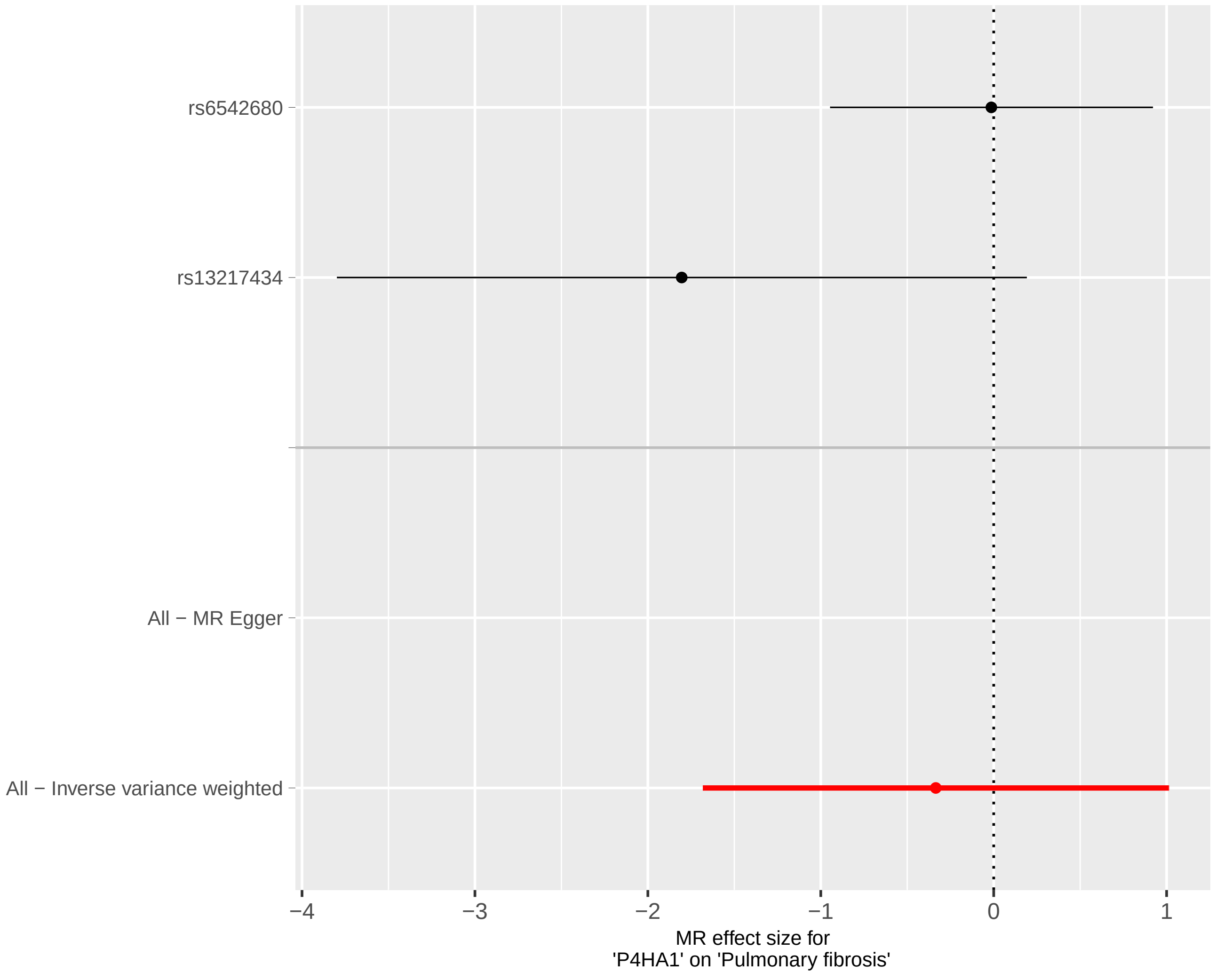
rs6542680
rs13217434
All − MR Egger
All − Inverse variance weighted
−4
−3
−2
−1
0
1
MR effect size for
'P4HA1' on 'Pulmonary fibrosis'

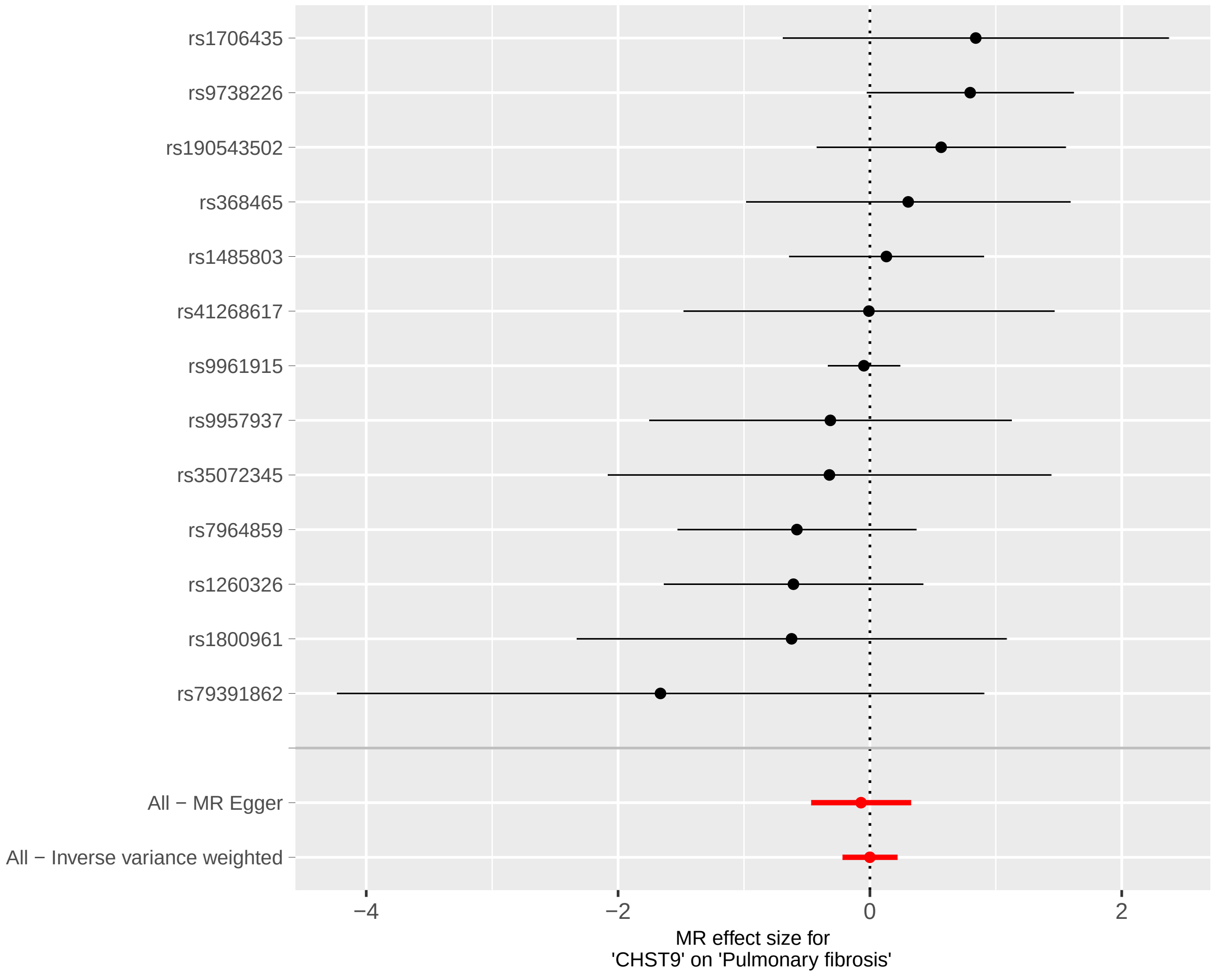

rs1706435
rs9738226
rs190543502
rs368465
rs1485803
rs41268617
rs9961915
rs9957937
rs35072345
rs7964859
rs1260326
rs1800961
rs79391862
All − MR Egger
All − Inverse variance weighted
−4
−2
0
2
MR effect size for
'CHST9' on 'Pulmonary fibrosis'

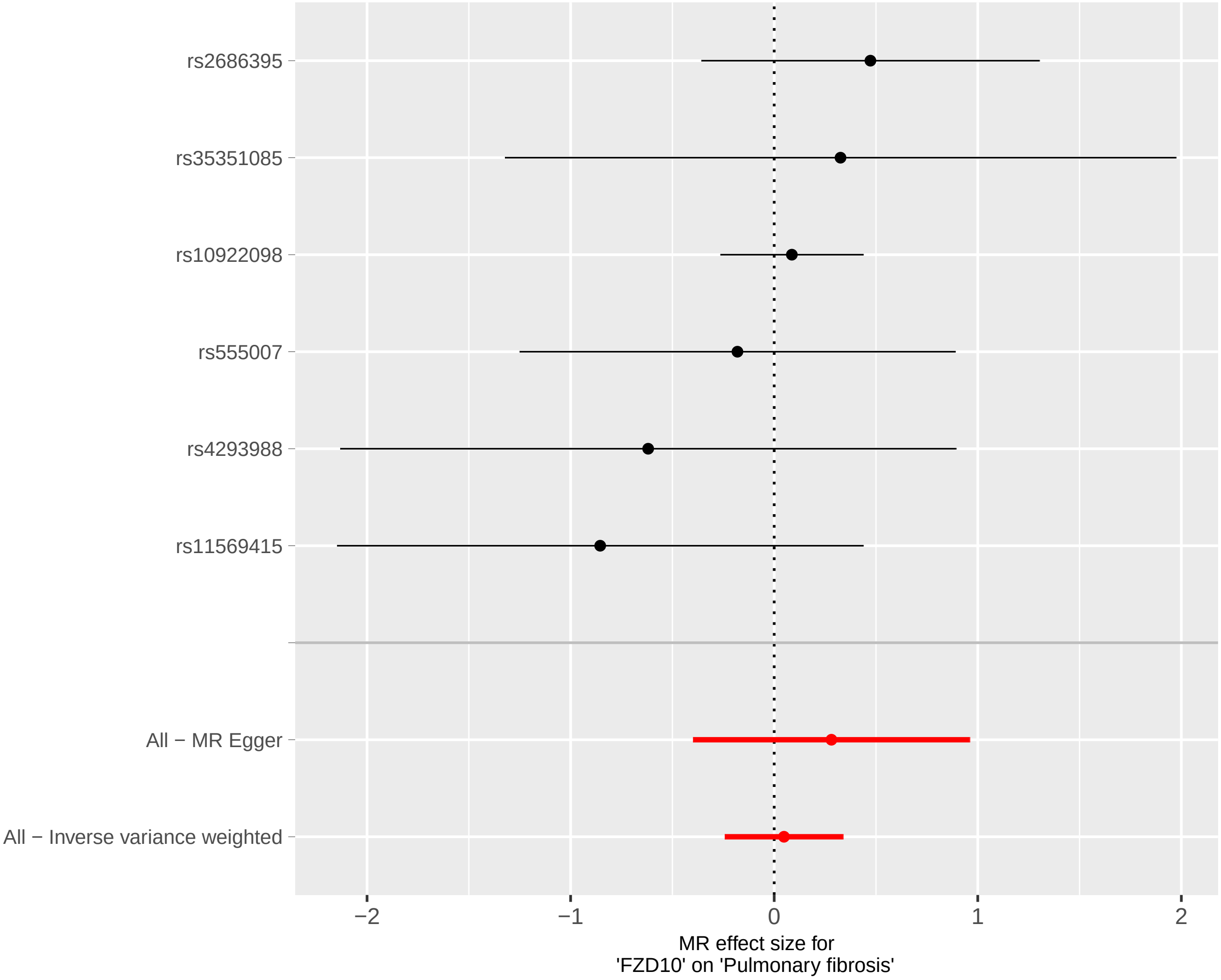

rs2686395
rs35351085
rs10922098
rs555007
rs4293988
rs11569415
All − MR Egger
All − Inverse variance weighted
−2
−1
0
1
2
MR effect size for
'FZD10' on 'Pulmonary fibrosis'

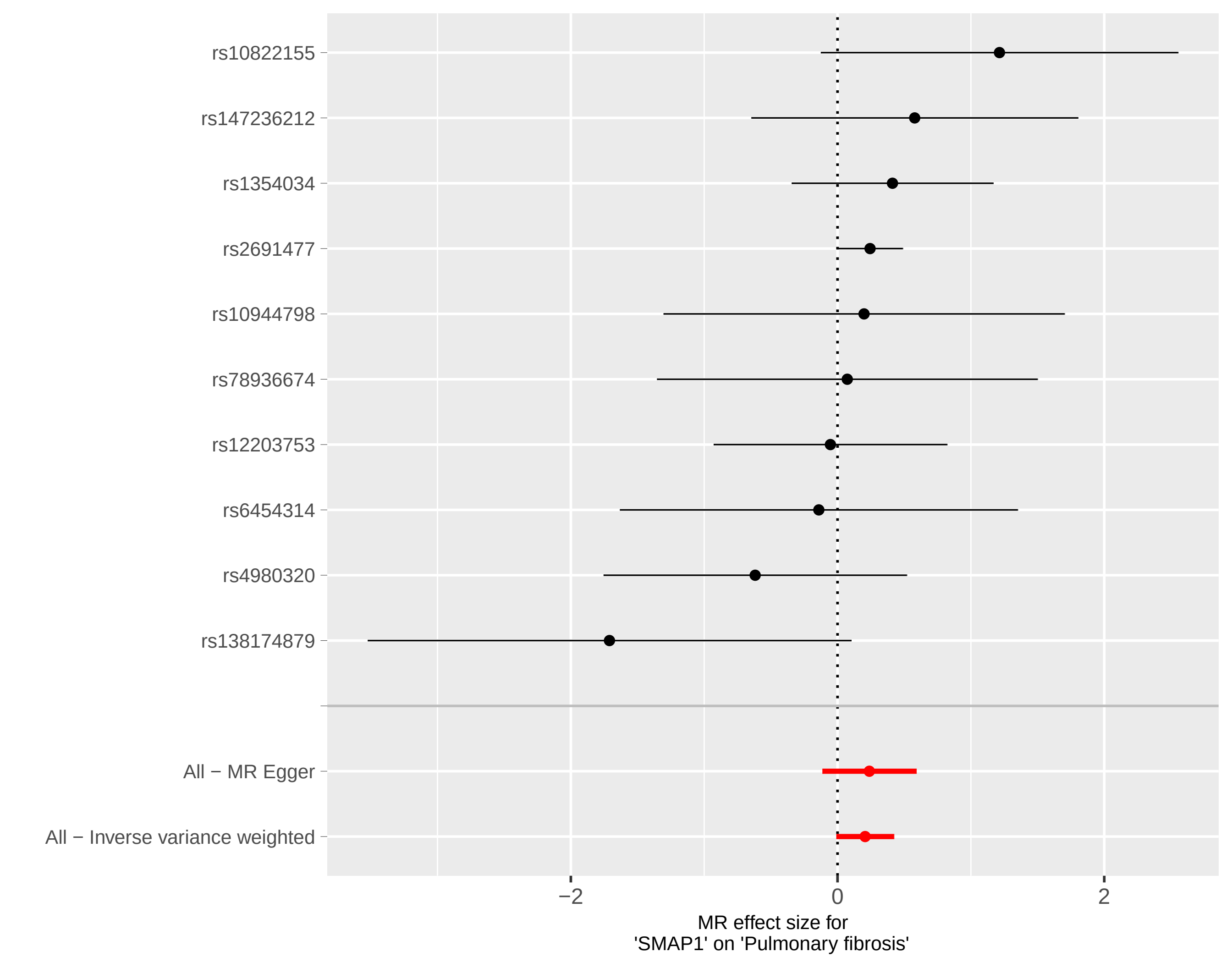
rs10822155
rs147236212
rs1354034
rs2691477
rs10944798
rs78936674
rs12203753
rs6454314
rs4980320
rs138174879
All − MR Egger
All − Inverse variance weighted
−2
0
2
MR effect size for
'SMAP1' on 'Pulmonary fibrosis'

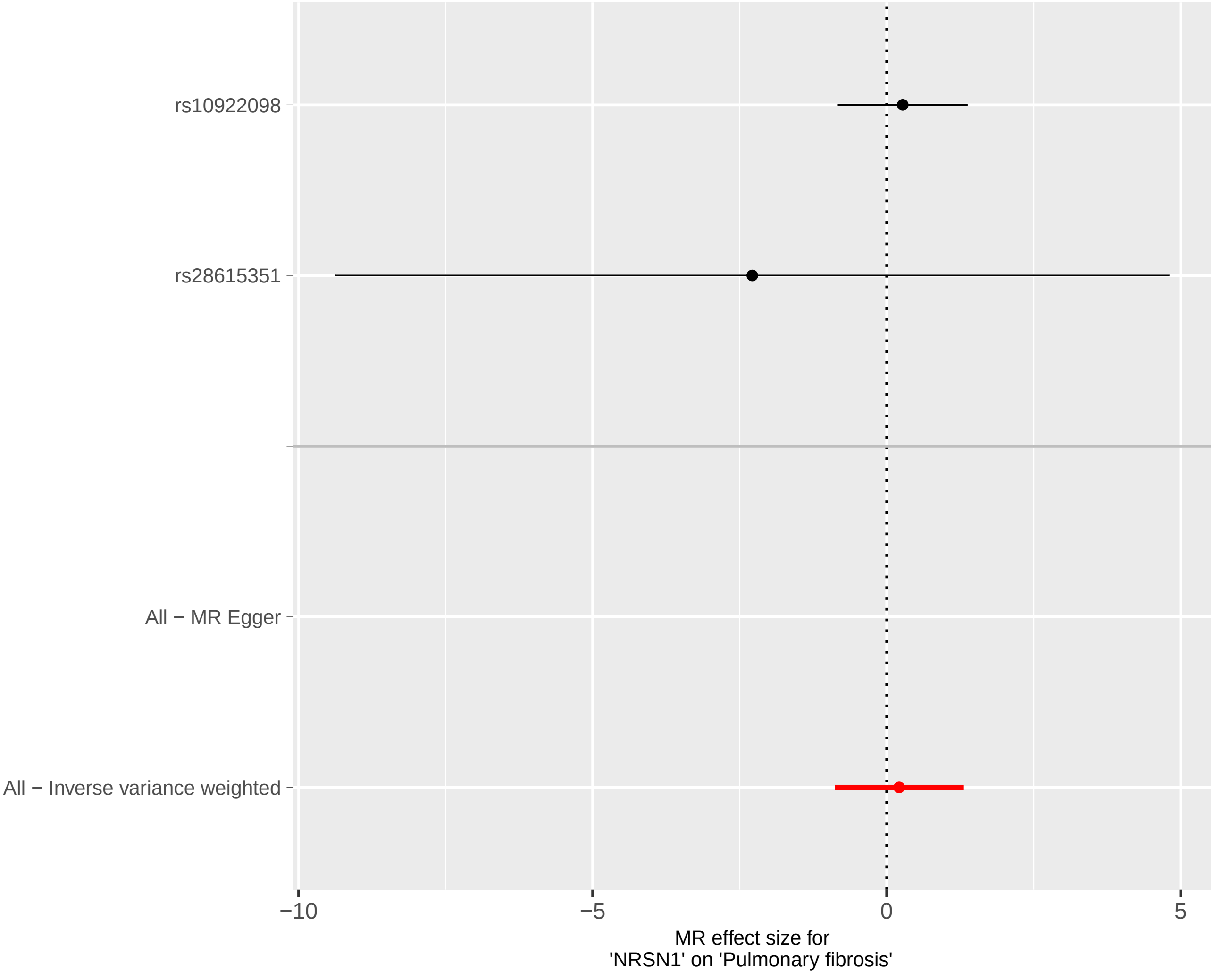

rs10922098
rs28615351
All − MR Egger
All − Inverse variance weighted
−10
−5
0
5
MR effect size for
'NRSN1' on 'Pulmonary fibrosis'

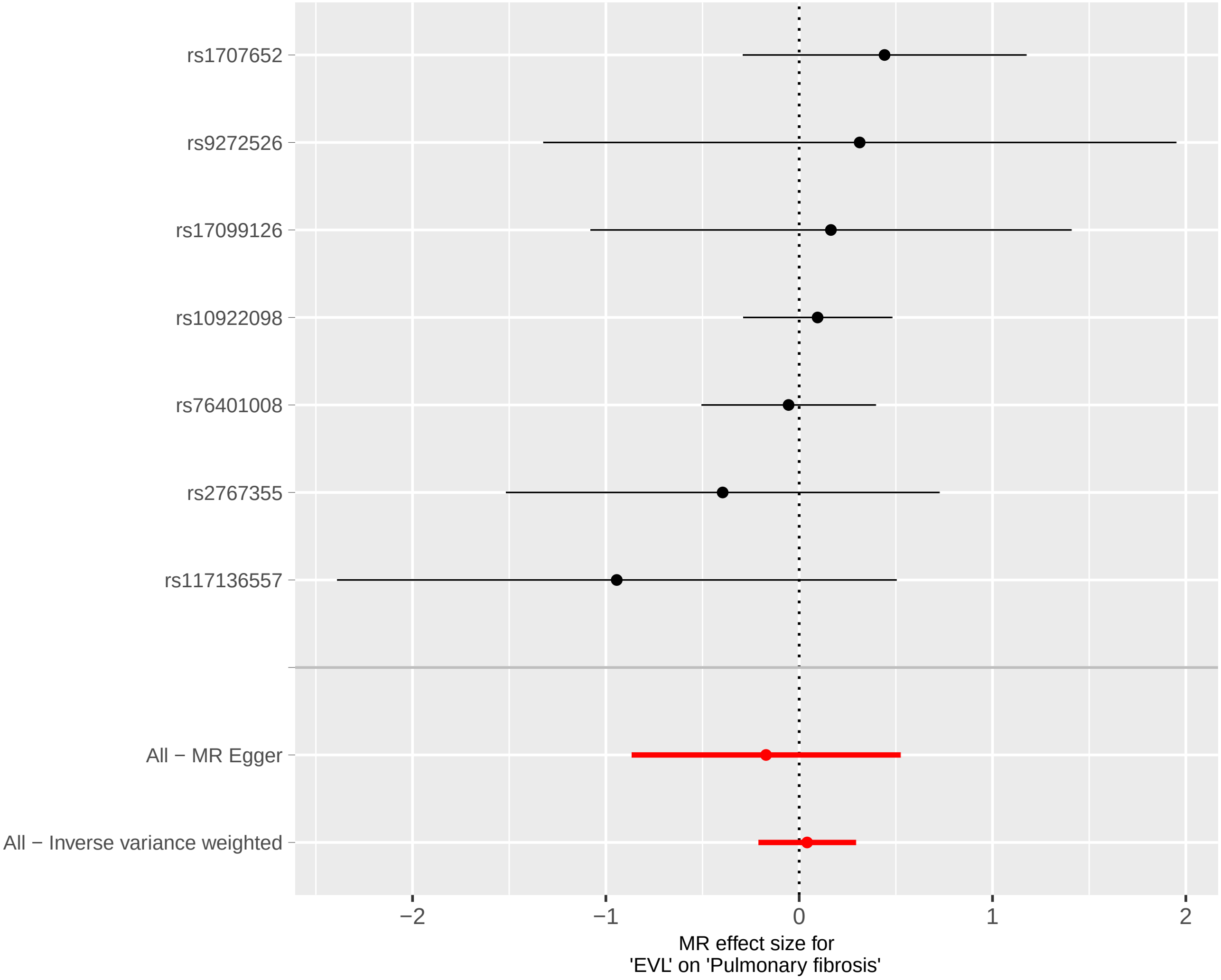
rs1707652
rs9272526
rs17099126
rs10922098
rs76401008
rs2767355
rs117136557
All − MR Egger
All − Inverse variance weighted
−2
−1
0
1
2
MR effect size for
'EVL' on 'Pulmonary fibrosis'

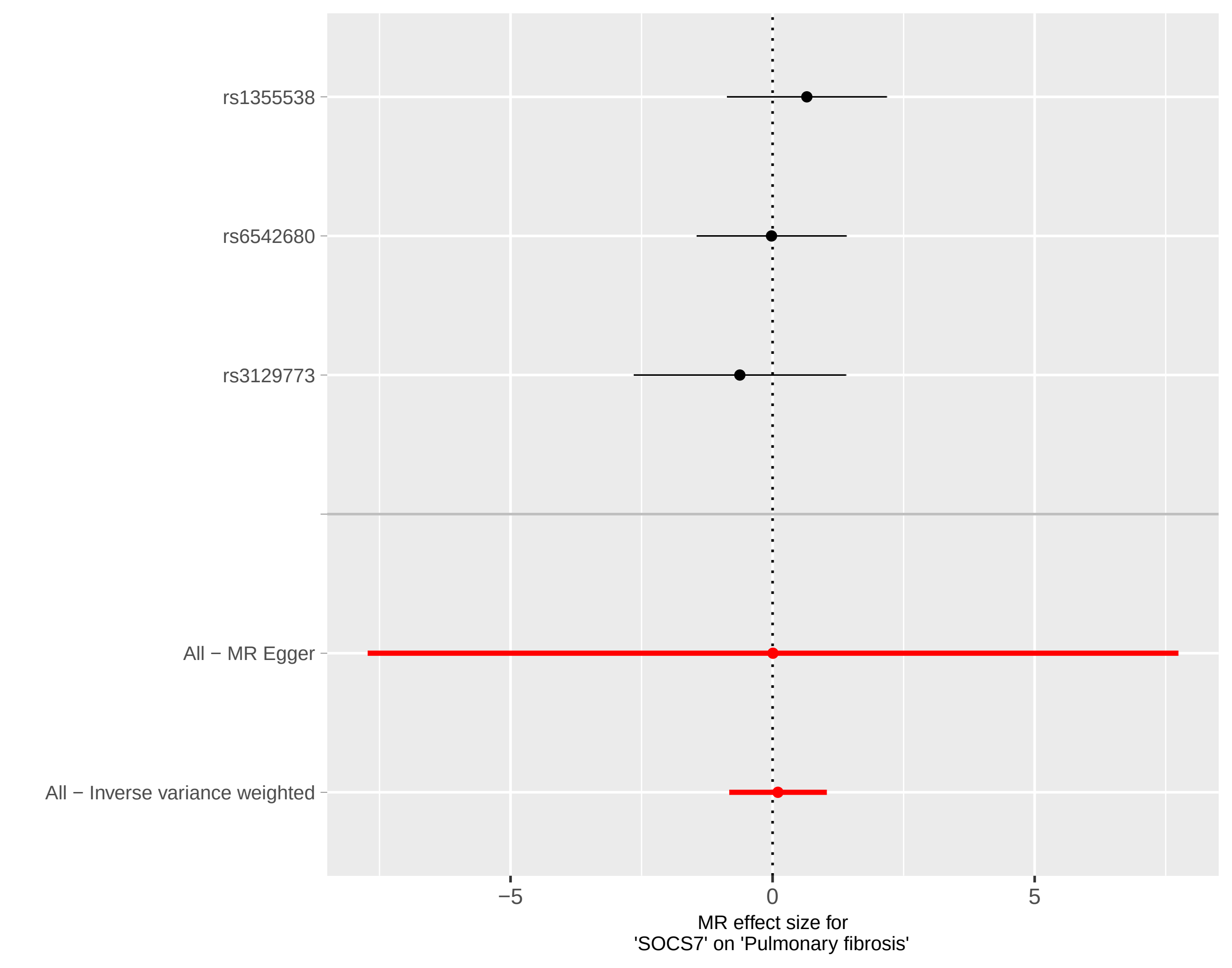
rs1355538
rs6542680
rs3129773
All − MR Egger
All − Inverse variance weighted
−5
0
5
MR effect size for
'SOCS7' on 'Pulmonary fibrosis'

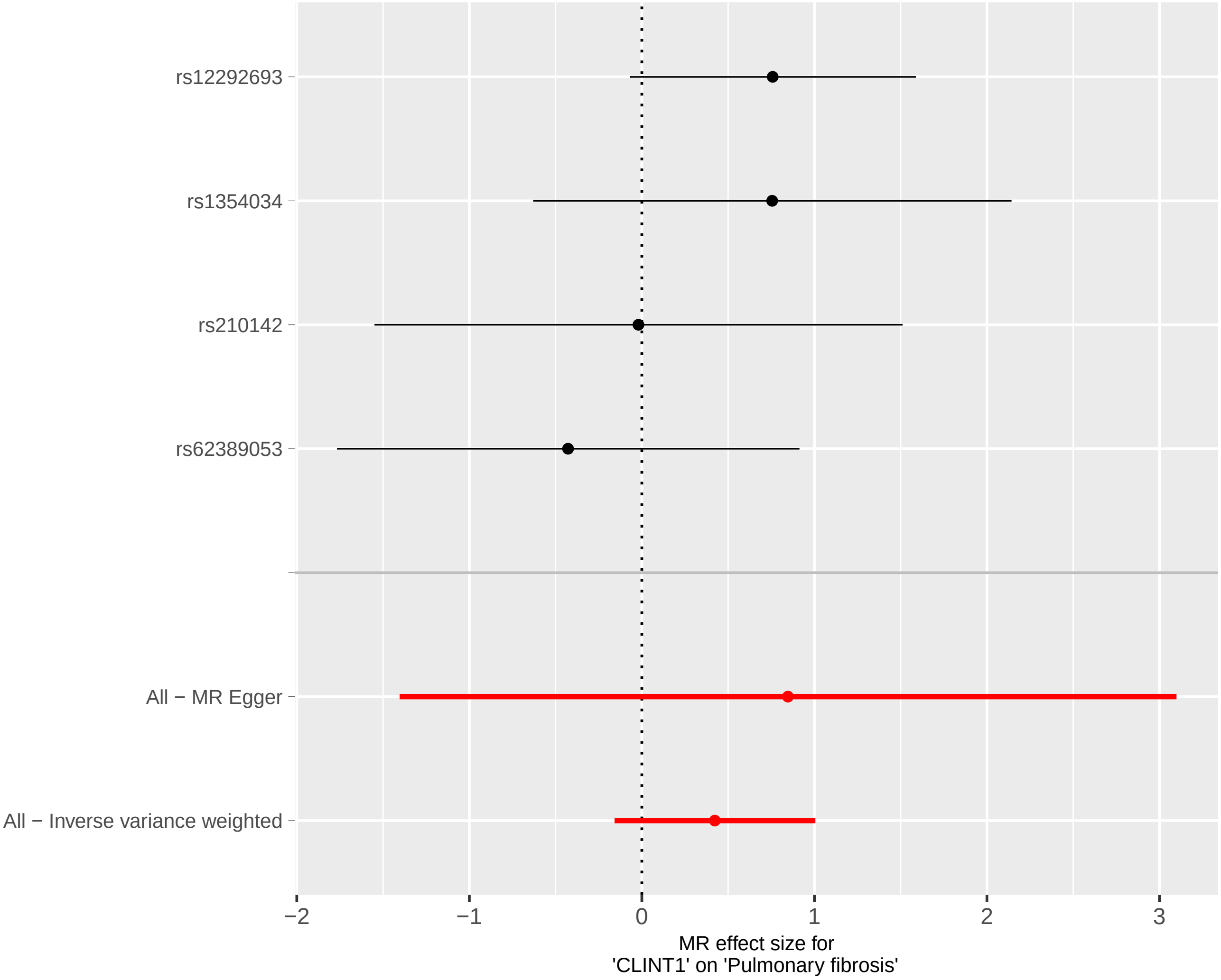

rs12292693
rs1354034
rs210142
rs62389053
All − MR Egger
All − Inverse variance weighted
−2
−1
0
1
2
3
MR effect size for
'CLINT1' on 'Pulmonary fibrosis'

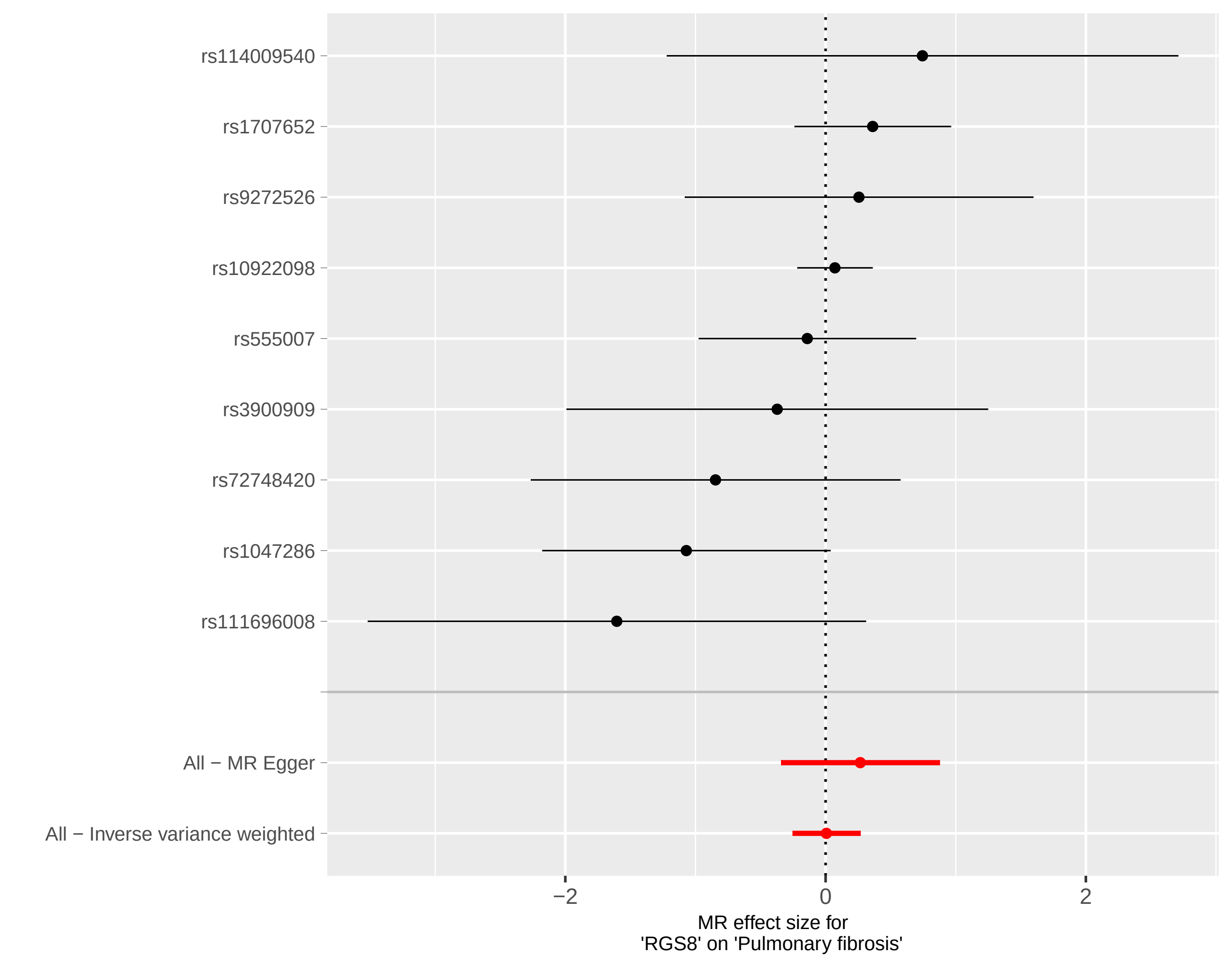
rs114009540
rs1707652
rs9272526
rs10922098
rs555007
rs3900909
rs72748420
rs1047286
rs111696008
All − MR Egger
All − Inverse variance weighted
−2
0
2
MR effect size for
'RGS8' on 'Pulmonary fibrosis'

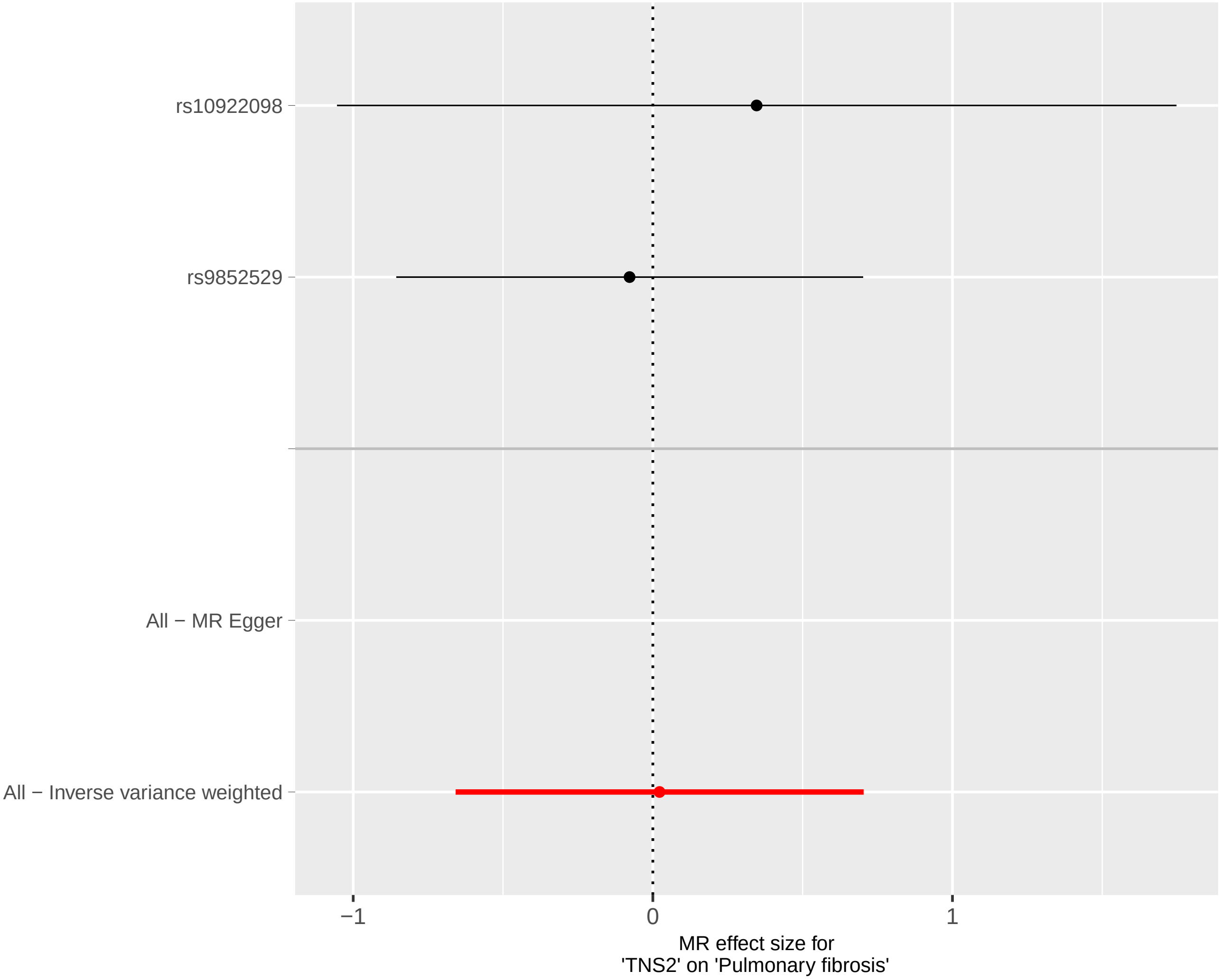

rs10922098
rs9852529
All − MR Egger
All − Inverse variance weighted
−1
0
1
MR effect size for
'TNS2' on 'Pulmonary fibrosis'

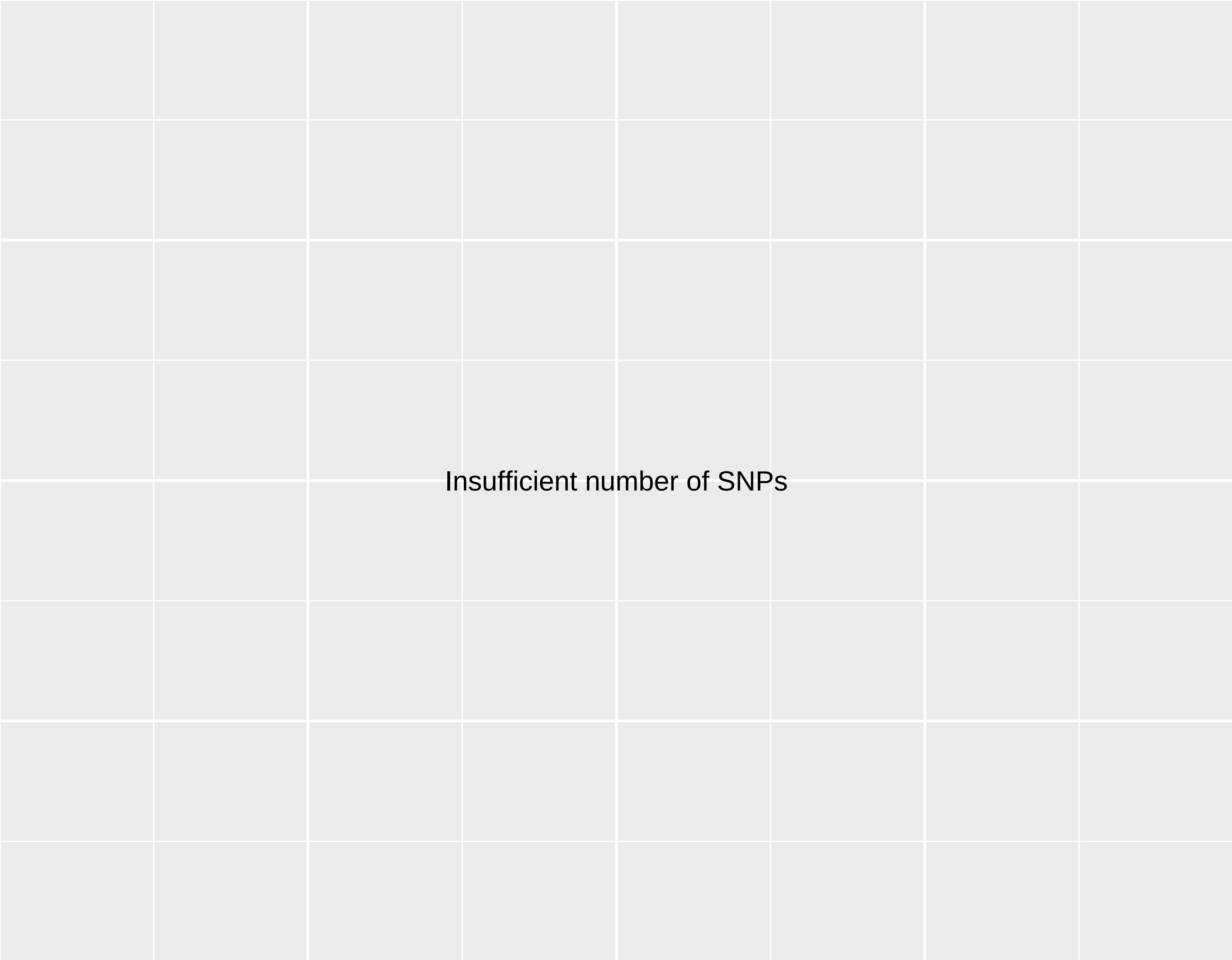
Insufficient number of SNPs

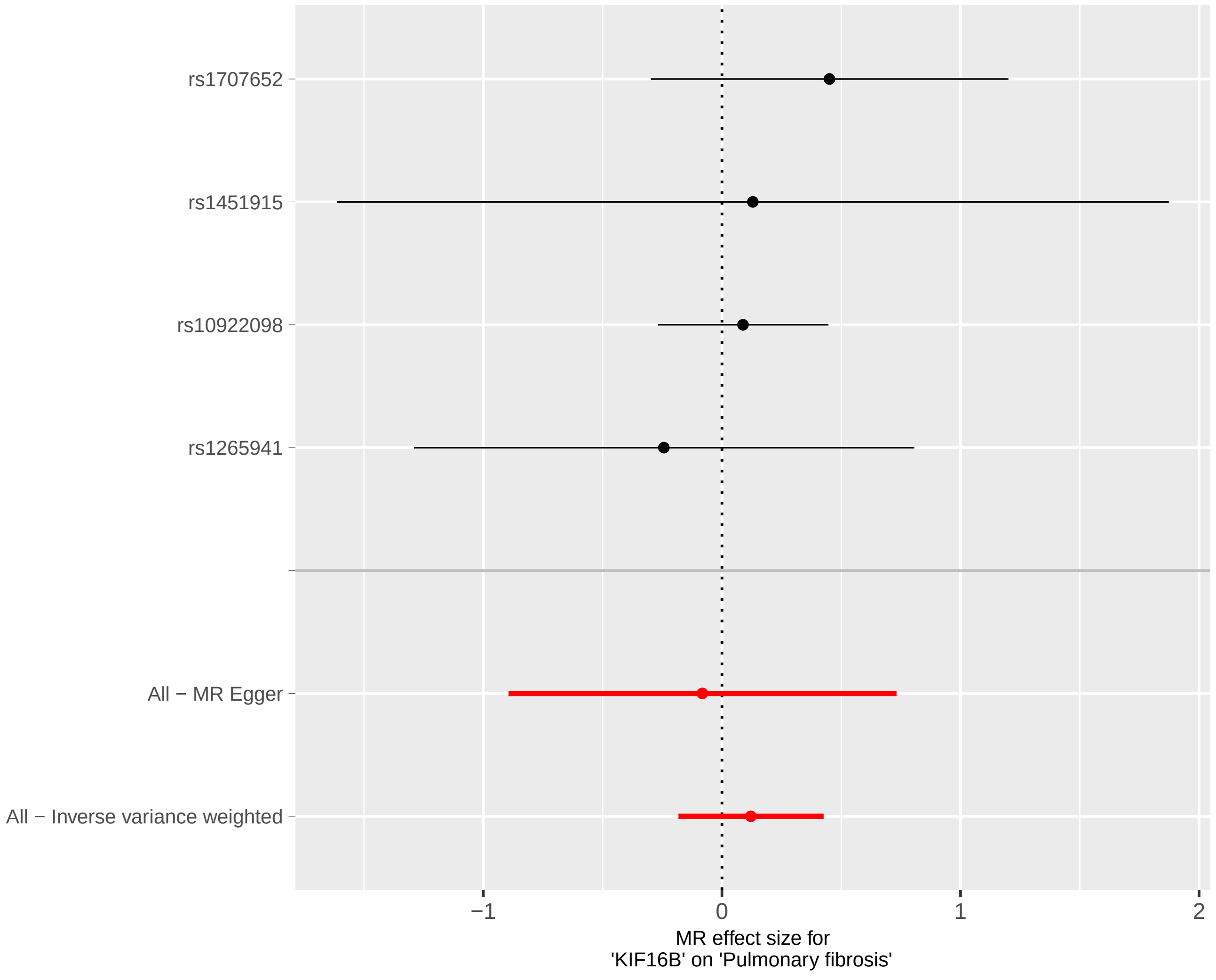
rs1707652
rs1451915
rs10922098
rs1265941
All − MR Egger
All − Inverse variance weighted
−1
0
1
2
MR effect size for
'KIF16B' on 'Pulmonary fibrosis'

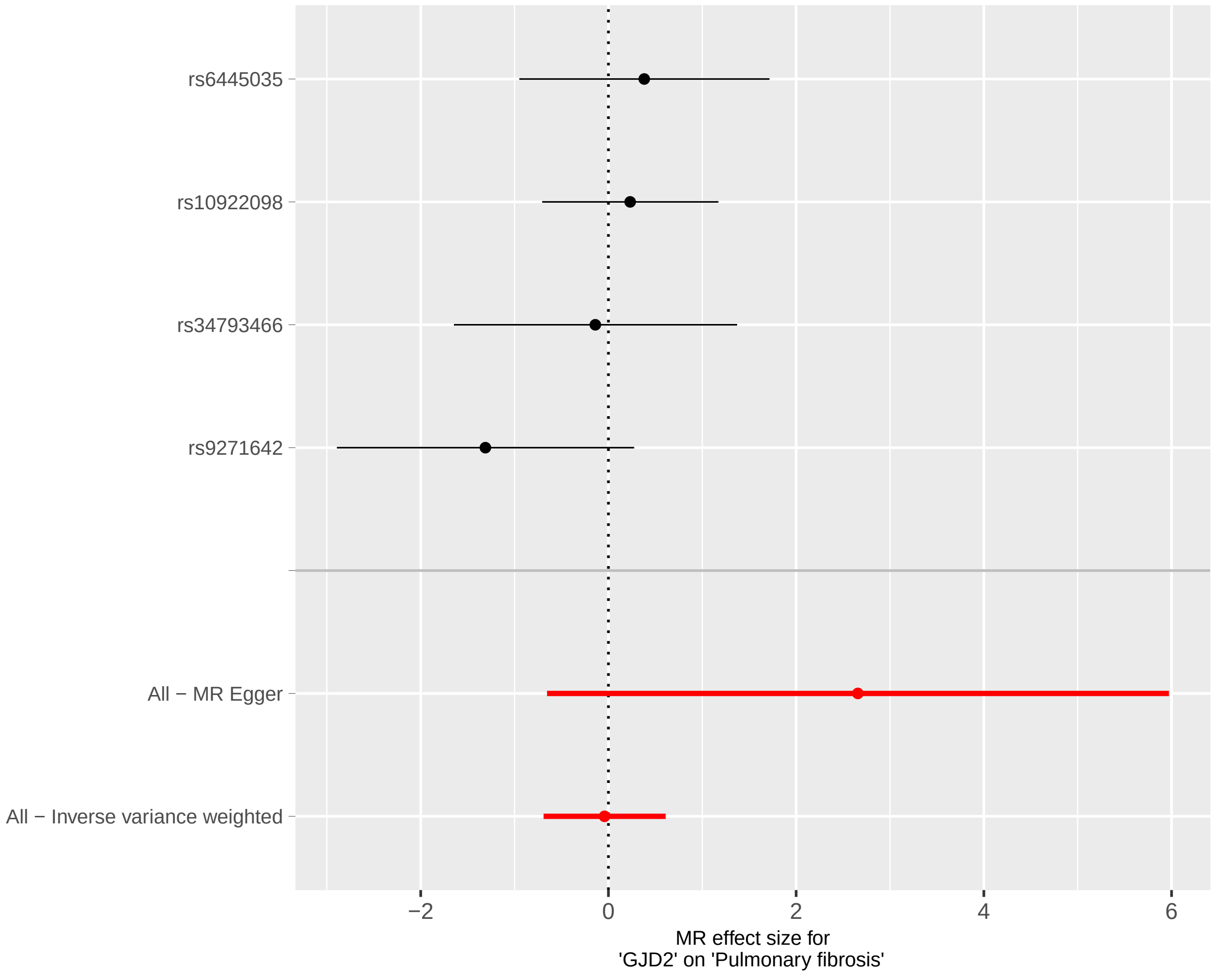

rs6445035
rs10922098
rs34793466
rs9271642
All − MR Egger
All − Inverse variance weighted
−2
0
2
4
6
MR effect size for
'GJD2' on 'Pulmonary fibrosis'

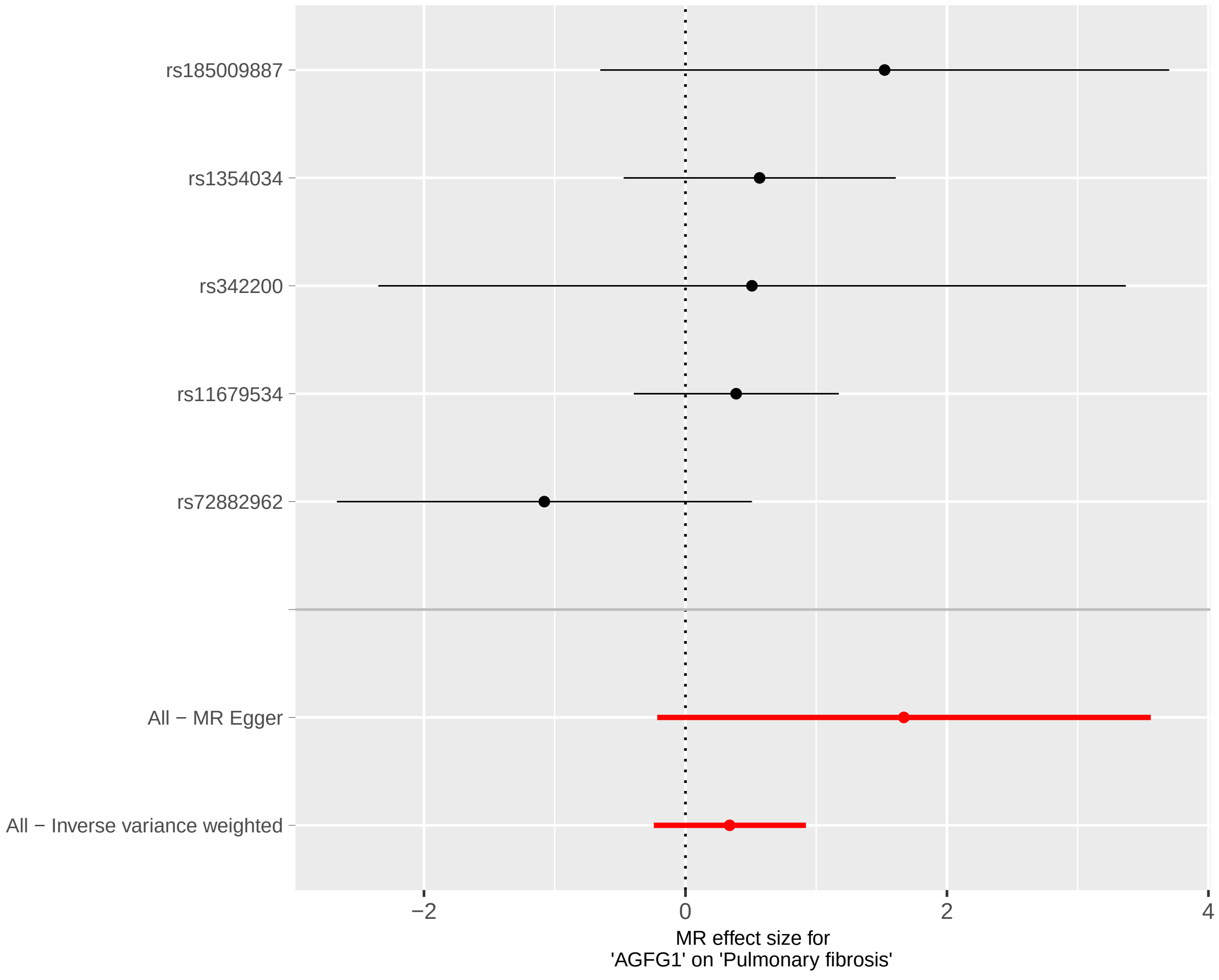

rs185009887
rs1354034
rs342200
rs11679534
rs72882962
All − MR Egger
All − Inverse variance weighted
−2
0
2
4
MR effect size for
'AGFG1' on 'Pulmonary fibrosis'

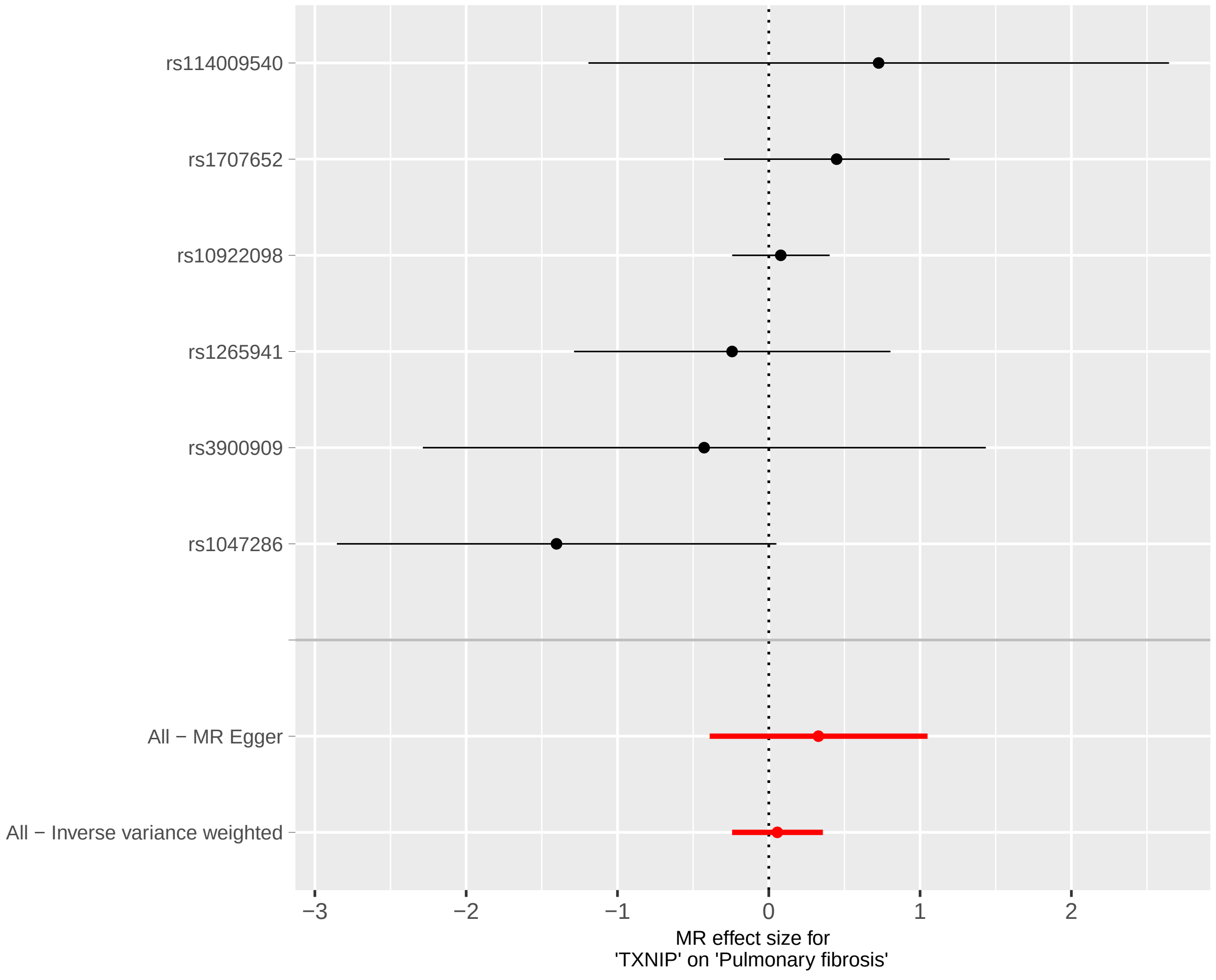
rs114009540
rs1707652
rs10922098
rs1265941
rs3900909
rs1047286
All − MR Egger
All − Inverse variance weighted
−3
−2
−1
0
1
2
MR effect size for
'TXNIP' on 'Pulmonary fibrosis'

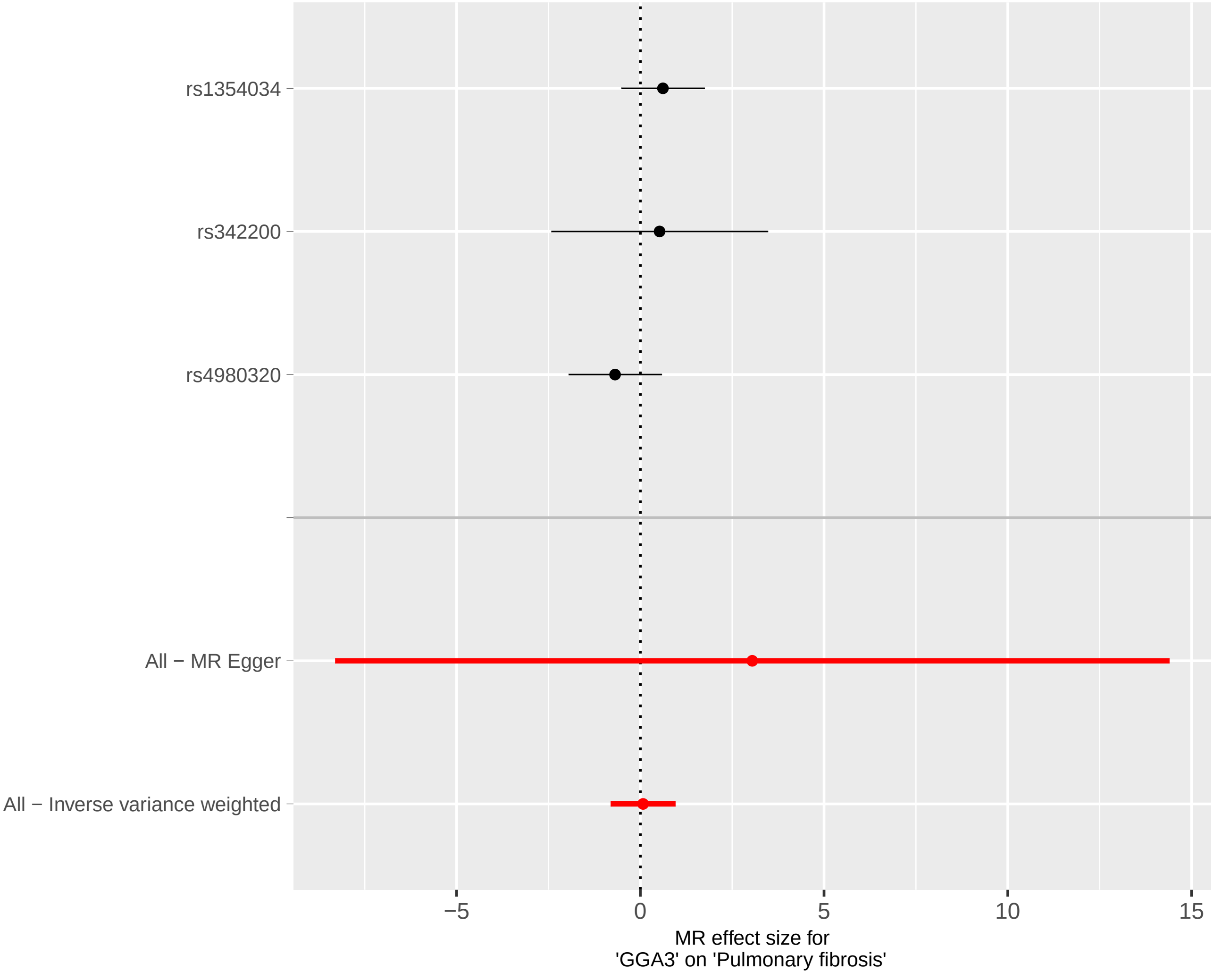

rs1354034
rs342200
rs4980320
All − MR Egger
All − Inverse variance weighted
−5
0
5
10
15
MR effect size for
'GGA3' on 'Pulmonary fibrosis'

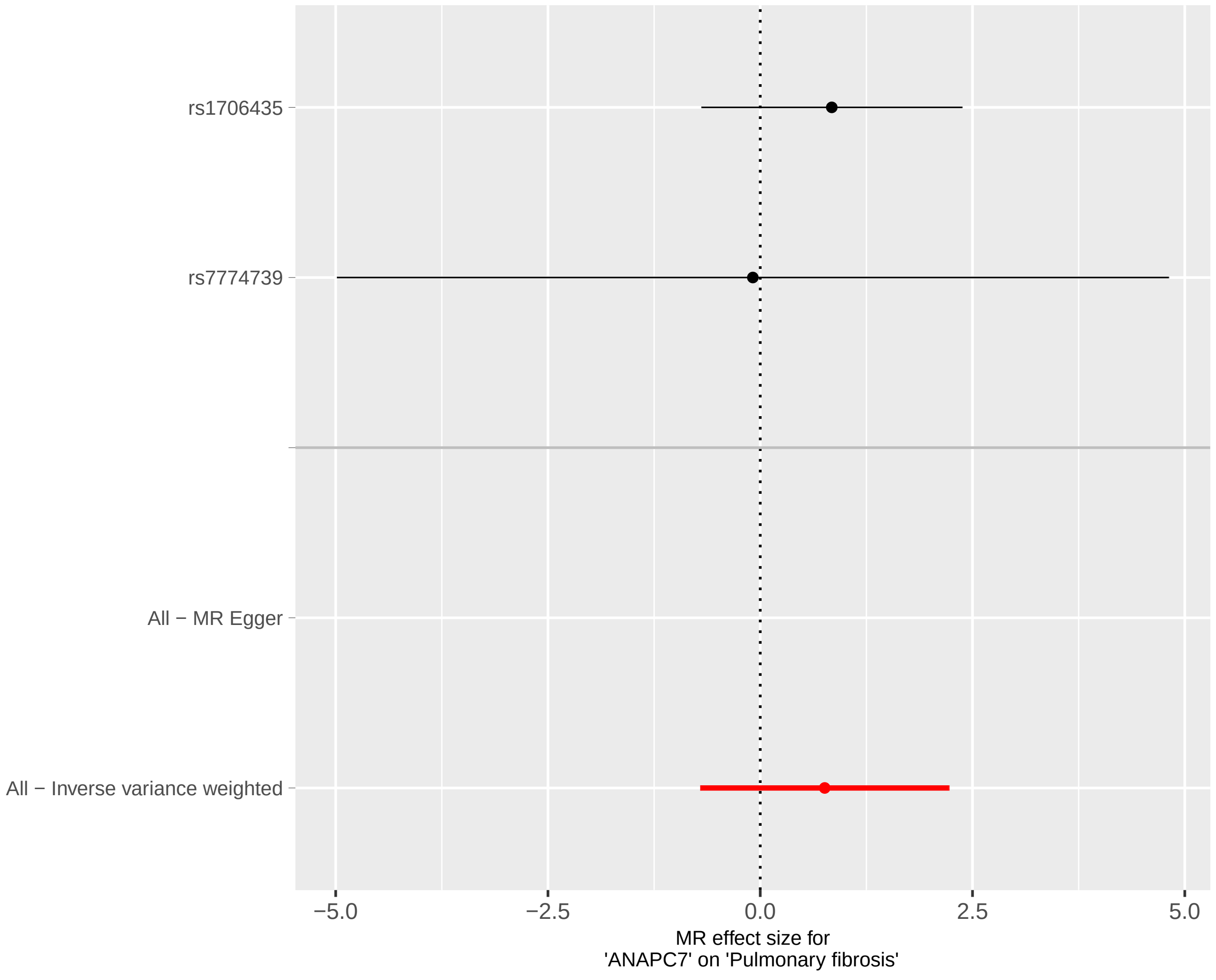
rs1706435
rs7774739
All − MR Egger
All − Inverse variance weighted
−5.0
−2.5
0.0
2.5
5.0
MR effect size for
'ANAPC7' on 'Pulmonary fibrosis'

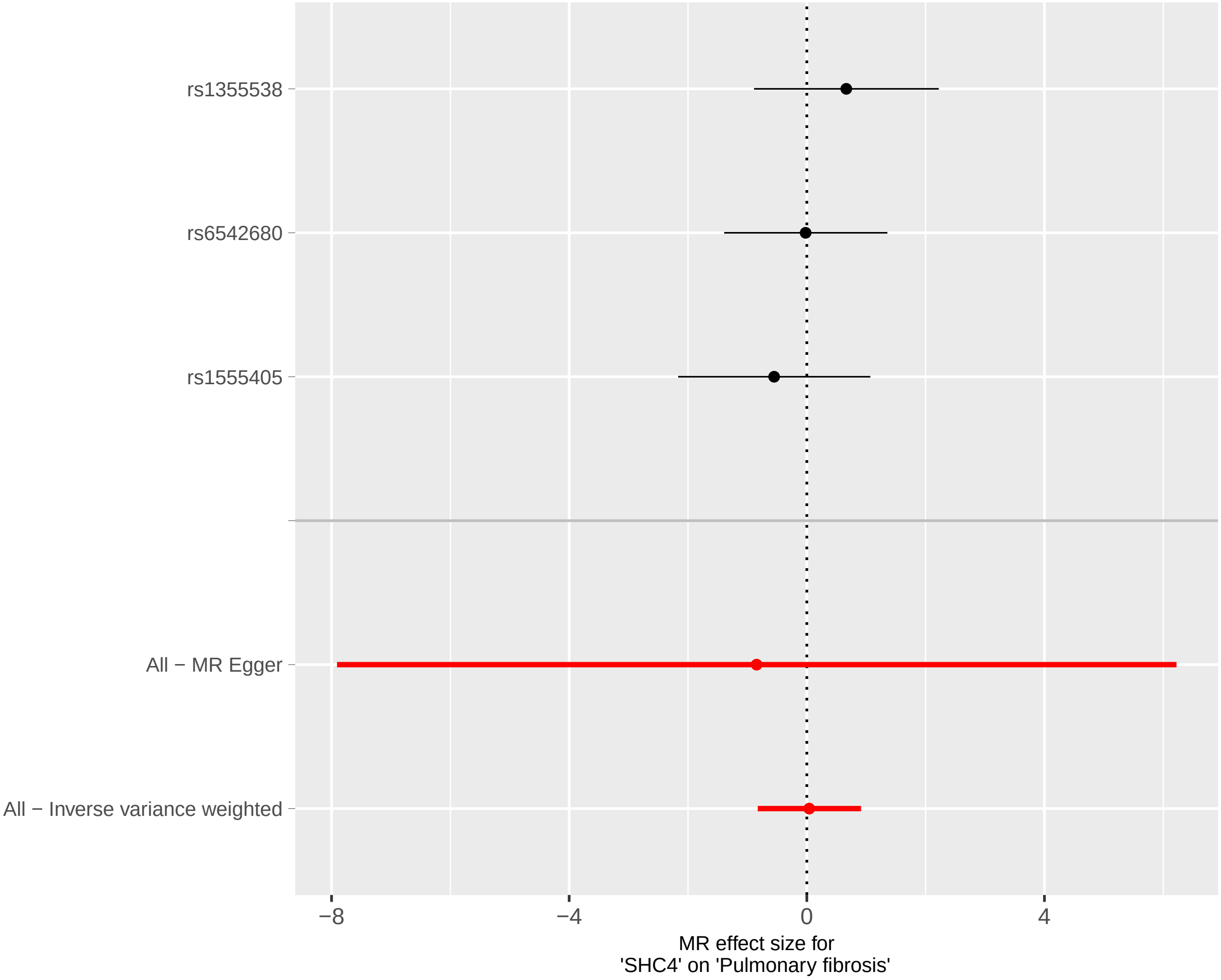

rs1355538
rs6542680
rs1555405
All − MR Egger
All − Inverse variance weighted
−8
−4
0
4
MR effect size for
'SHC4' on 'Pulmonary fibrosis'

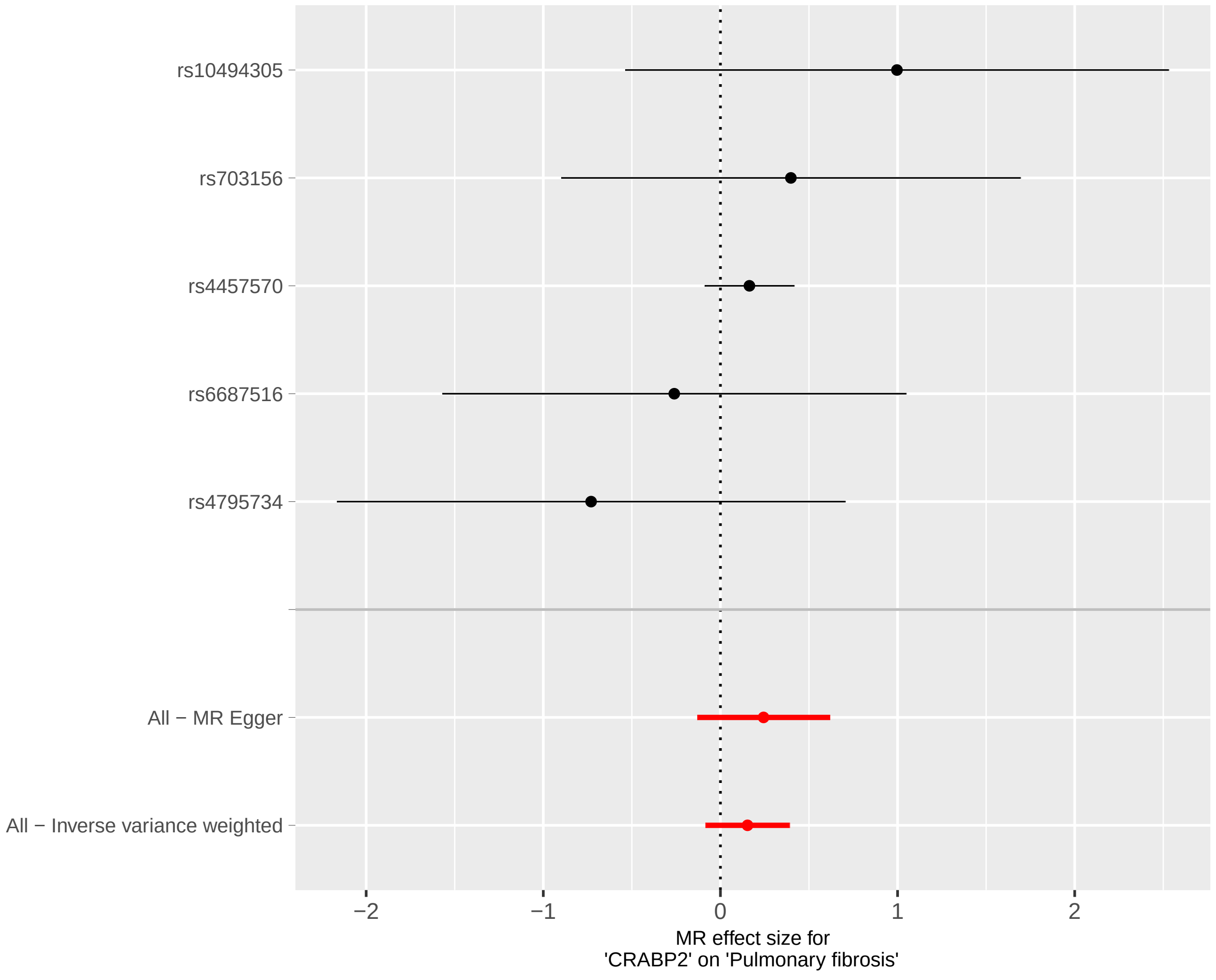
rs10494305
rs703156
rs4457570
rs6687516
rs4795734
All − MR Egger
All − Inverse variance weighted
−2
−1
0
1
2
MR effect size for
'CRABP2' on 'Pulmonary fibrosis'

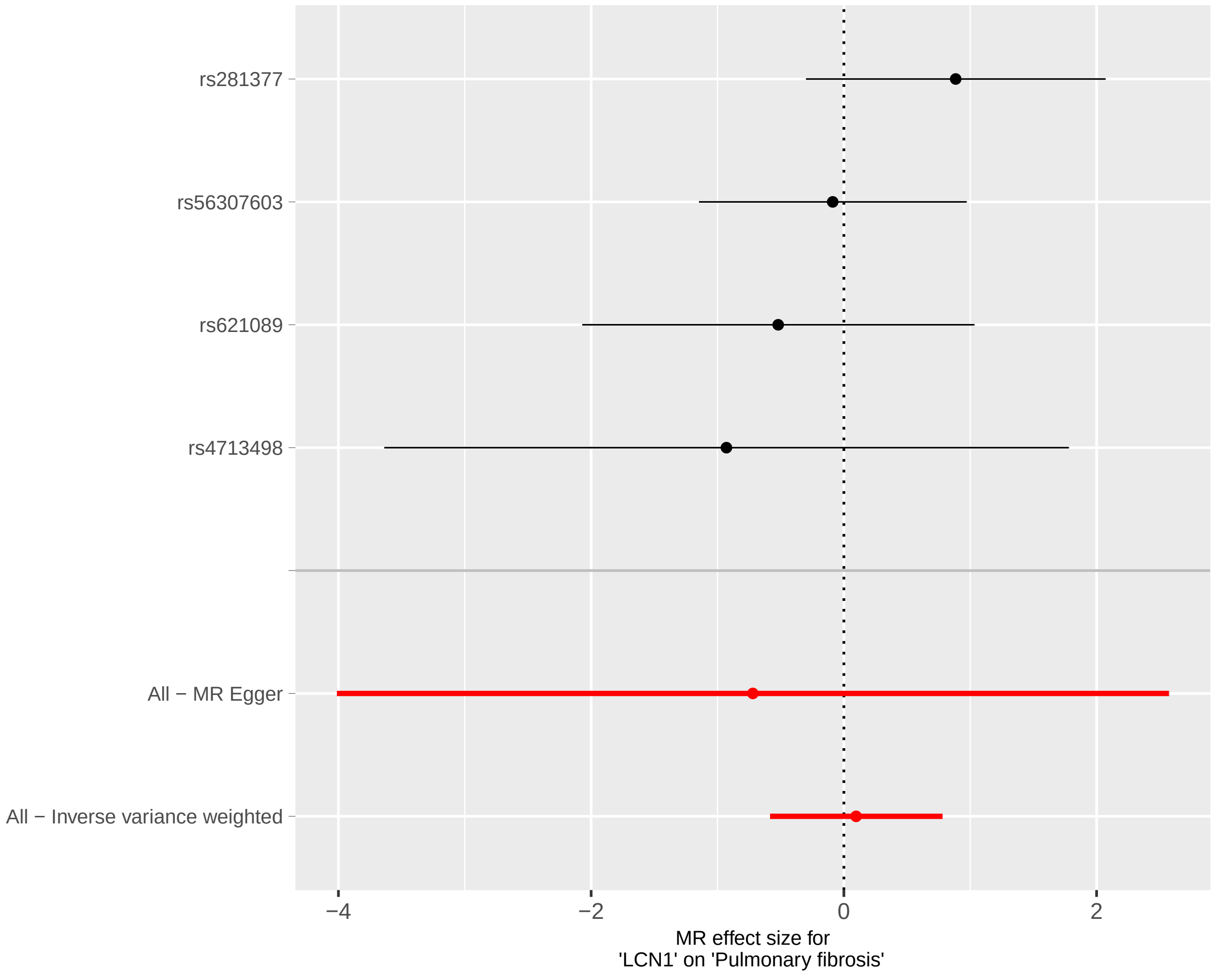
rs281377
rs56307603
rs621089
rs4713498
All − MR Egger
All − Inverse variance weighted
−4
−2
0
2
MR effect size for
'LCN1' on 'Pulmonary fibrosis'

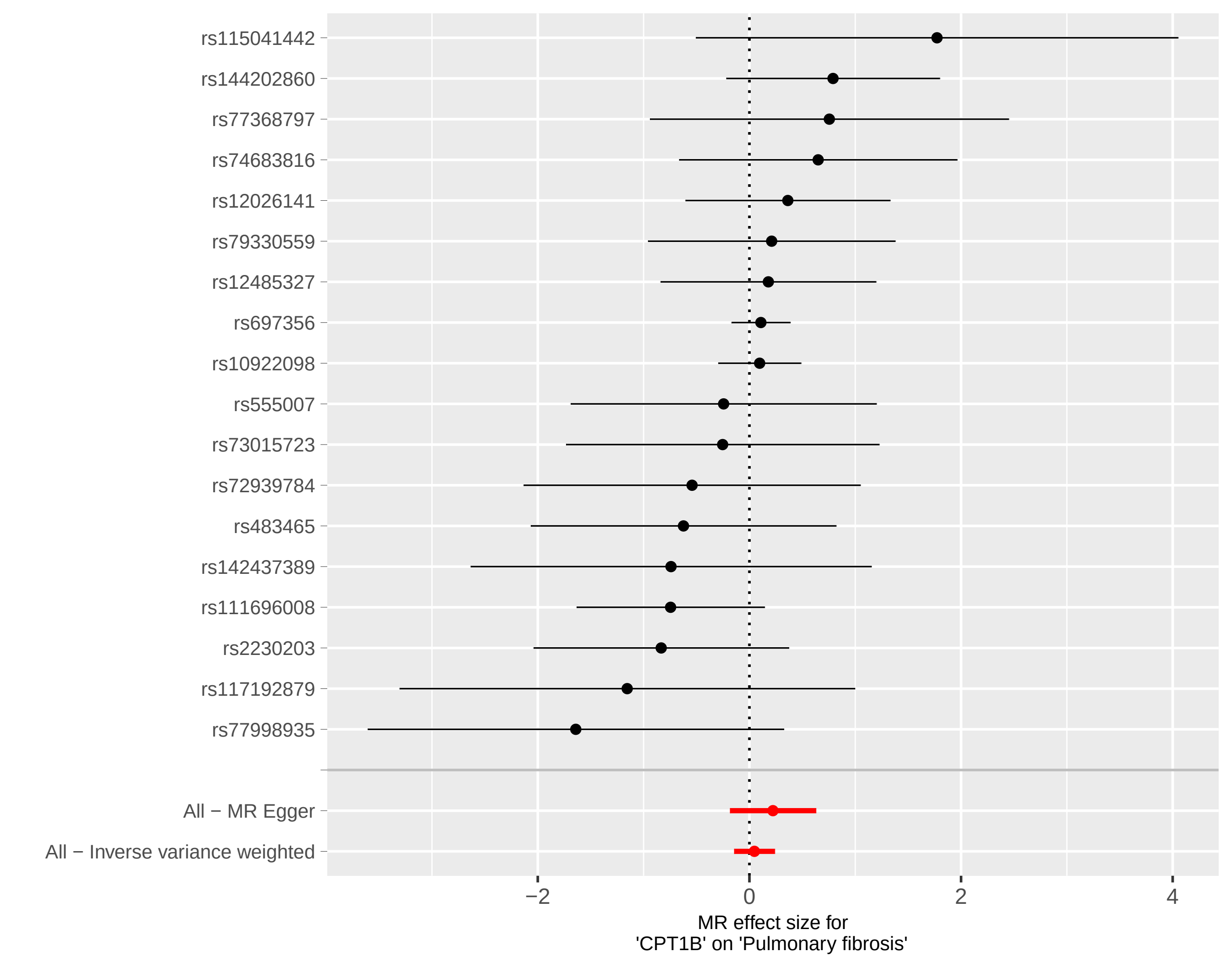

rs115041442
rs144202860
rs77368797
rs74683816
rs12026141
rs79330559
rs12485327
rs697356
rs10922098
rs555007
rs73015723
rs72939784
rs483465
rs142437389
rs111696008
rs2230203
rs117192879
rs77998935
All − MR Egger
All − Inverse variance weighted
−2
0
2
4
MR effect size for
'CPT1B' on 'Pulmonary fibrosis'

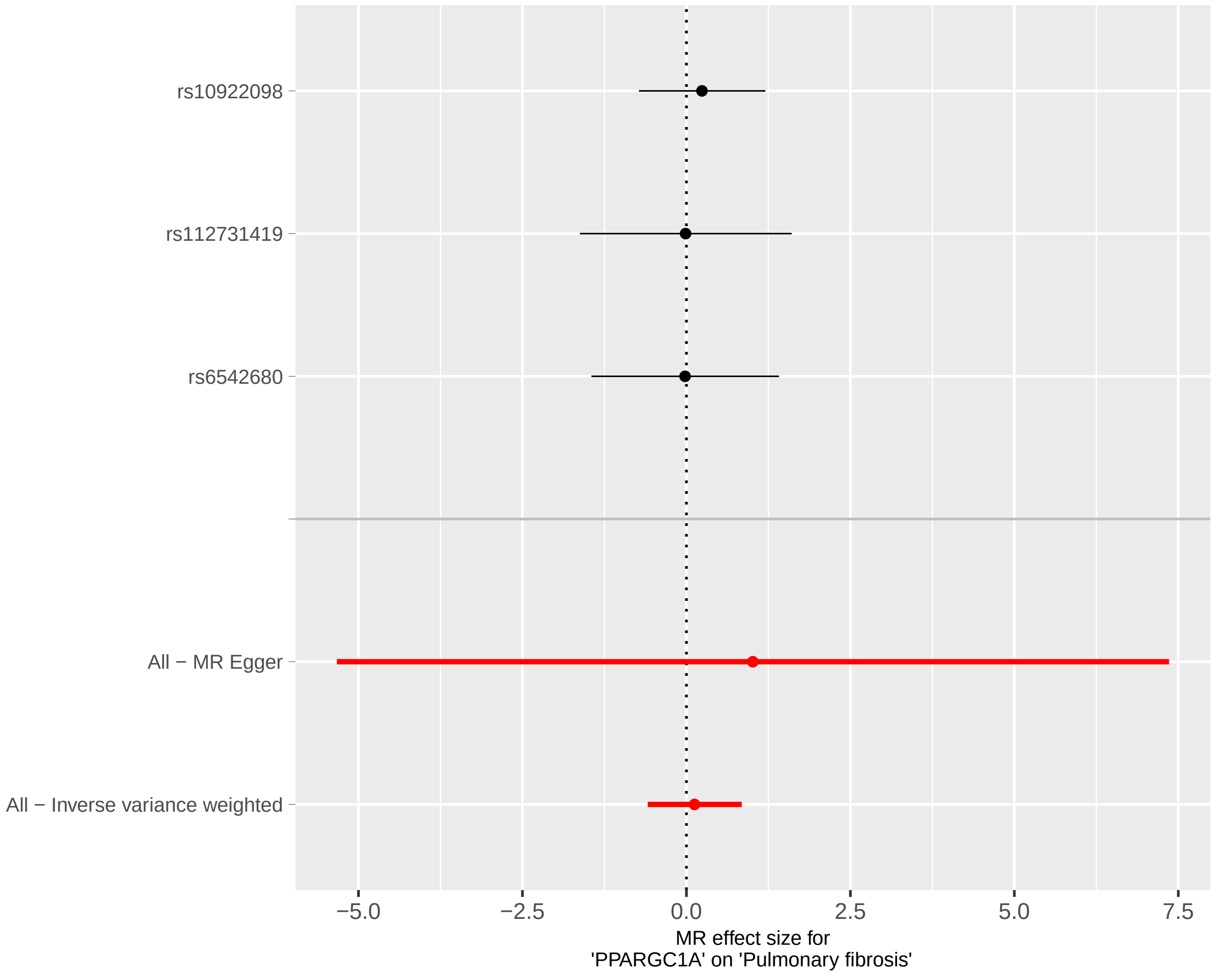
rs10922098
rs112731419
rs6542680
All − MR Egger
All − Inverse variance weighted
−5.0
−2.5
0.0
2.5
5.0
7.5
MR effect size for
'PPARGC1A' on 'Pulmonary fibrosis'

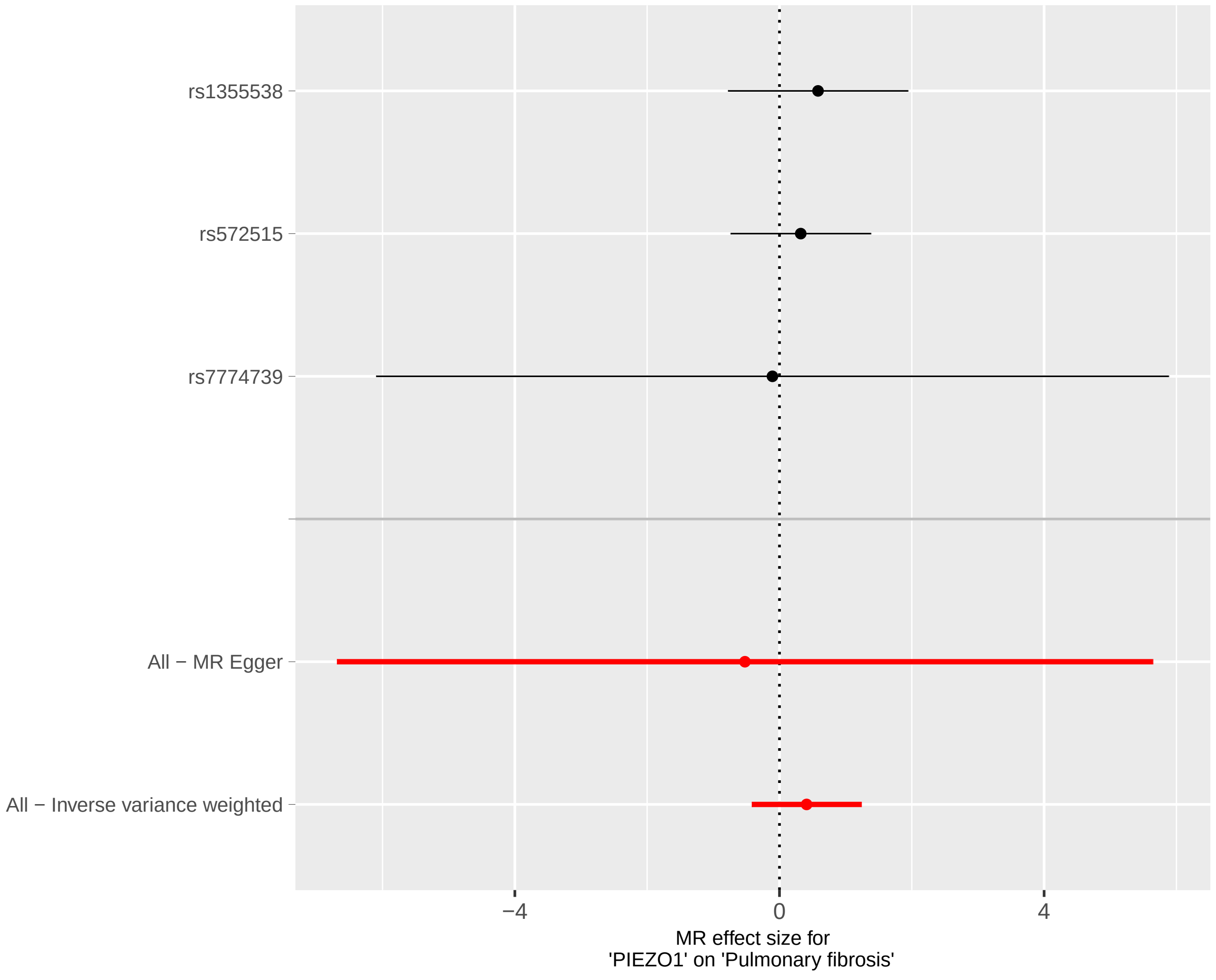

rs1355538
rs572515
rs7774739
All − MR Egger
All − Inverse variance weighted
−4
0
4
MR effect size for
'PIEZO1' on 'Pulmonary fibrosis'

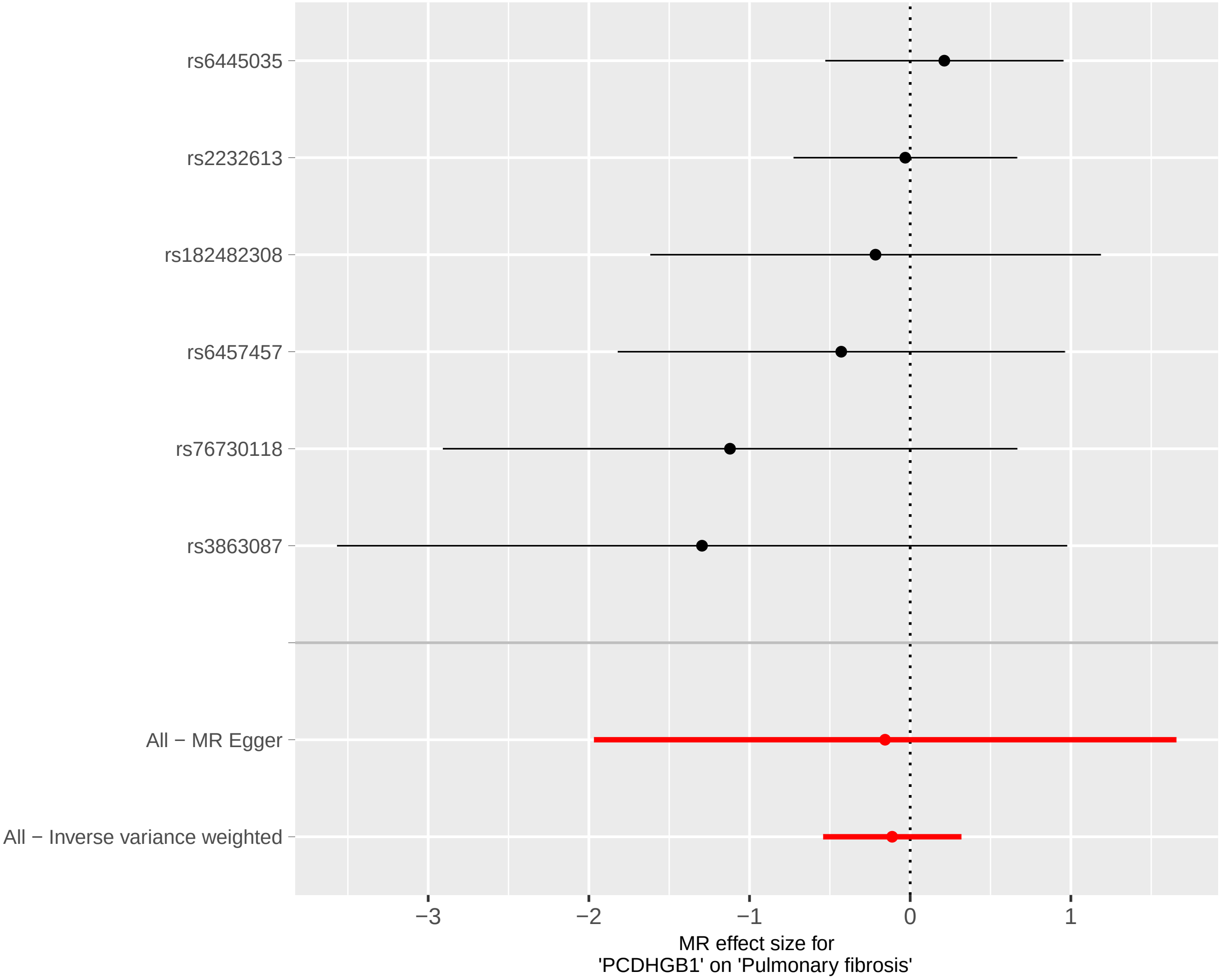
rs6445035
rs2232613
rs182482308
rs6457457
rs76730118
rs3863087
All − MR Egger
All − Inverse variance weighted
−3
−2
−1
0
1
MR effect size for
'PCDHGB1' on 'Pulmonary fibrosis'

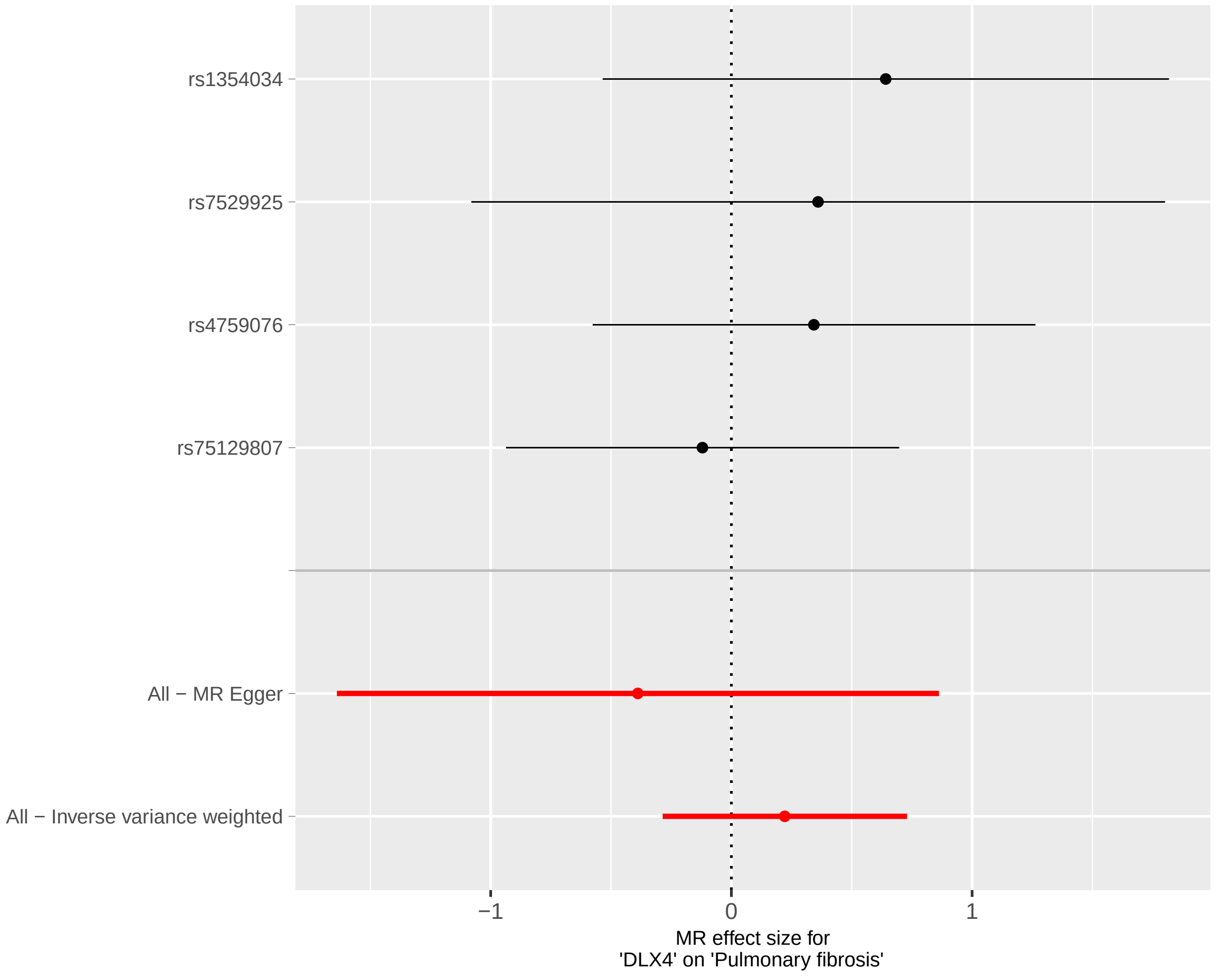
rs1354034
rs7529925
rs4759076
rs75129807
All − MR Egger
All − Inverse variance weighted
−1
0
1
MR effect size for
'DLX4' on 'Pulmonary fibrosis'

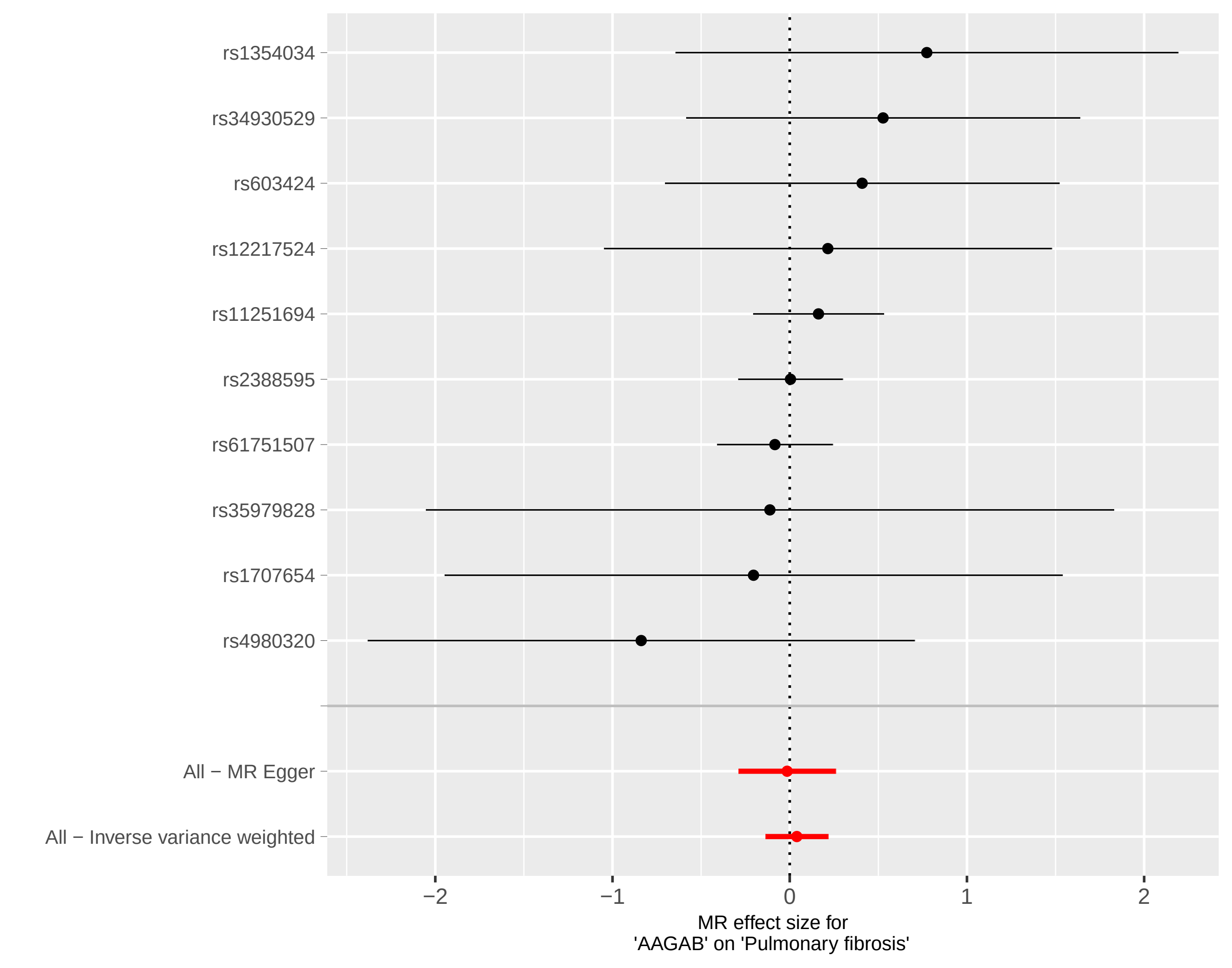
rs1354034
rs34930529
rs603424
rs12217524
rs11251694
rs2388595
rs61751507
rs35979828
rs1707654
rs4980320
All − MR Egger
All − Inverse variance weighted
−2
−1
0
1
2
MR effect size for
'AAGAB' on 'Pulmonary fibrosis'

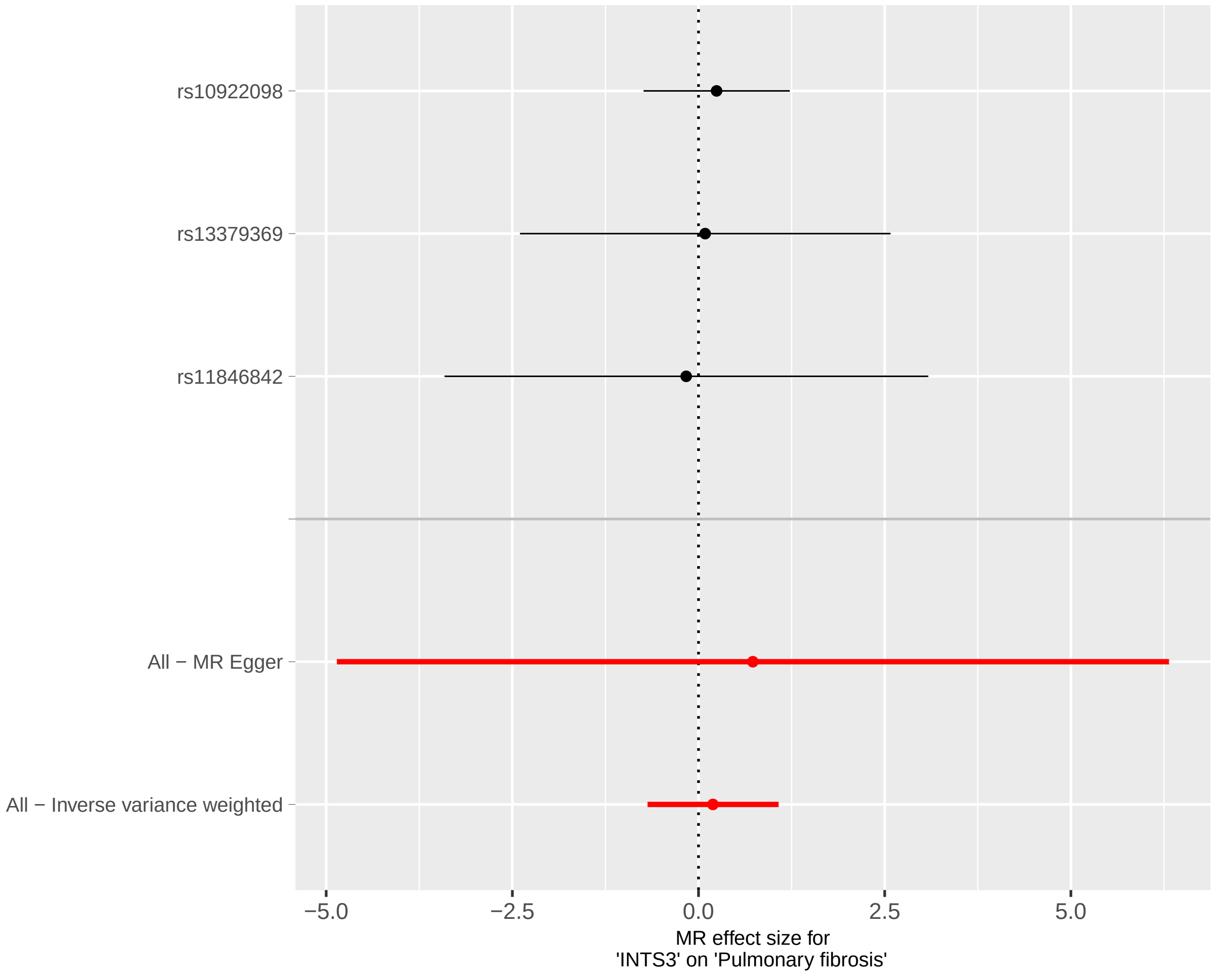
rs10922098
rs13379369
rs11846842
All − MR Egger
All − Inverse variance weighted
−5.0
−2.5
0.0
2.5
5.0
MR effect size for
'INTS3' on 'Pulmonary fibrosis'

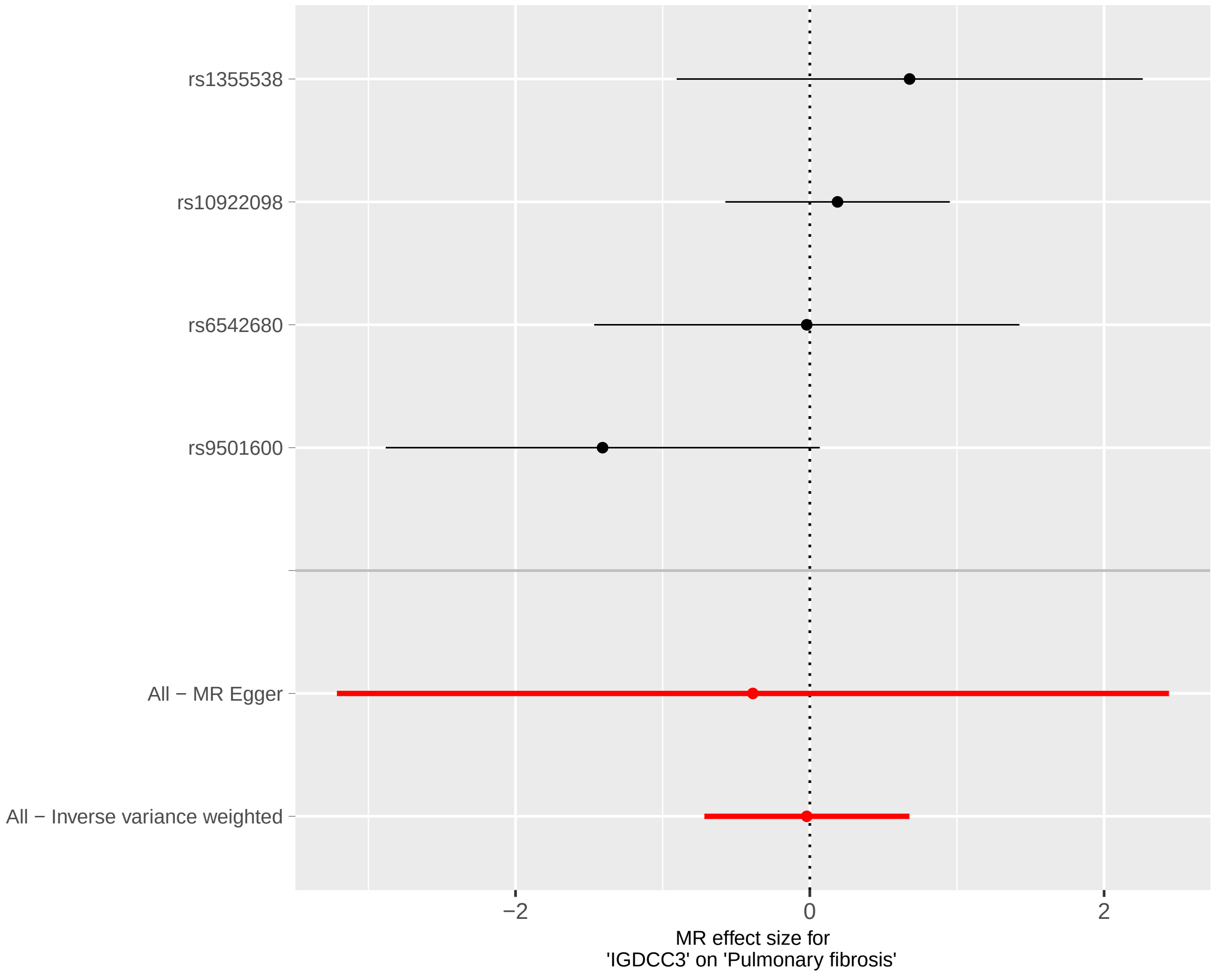
rs1355538
rs10922098
rs6542680
rs9501600
All − MR Egger
All − Inverse variance weighted
−2
0
2
MR effect size for
'IGDCC3' on 'Pulmonary fibrosis'

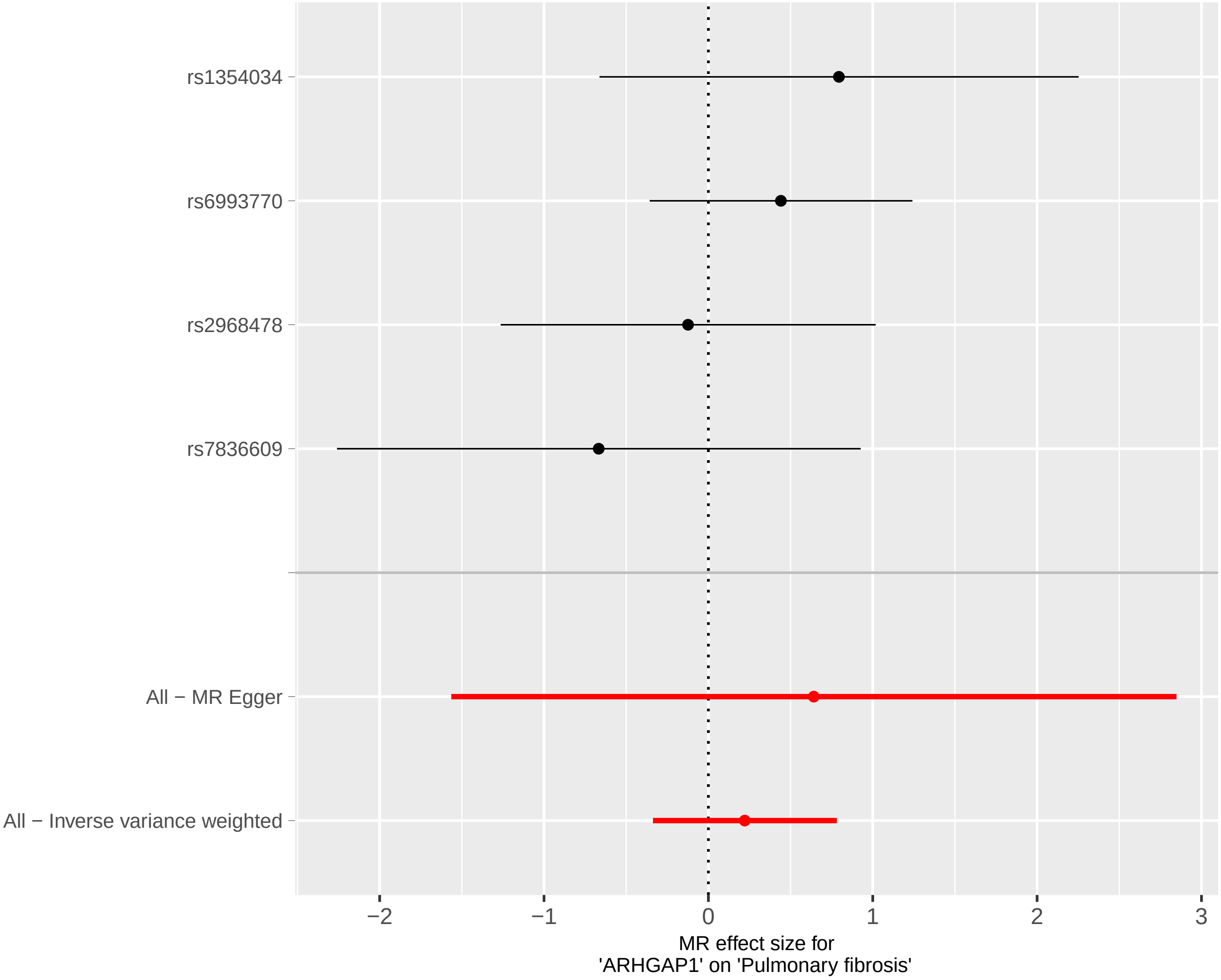
rs1354034
rs6993770
rs2968478
rs7836609
All − MR Egger
All − Inverse variance weighted
−2
−1
0
1
2
3
MR effect size for
'ARHGAP1' on 'Pulmonary fibrosis'

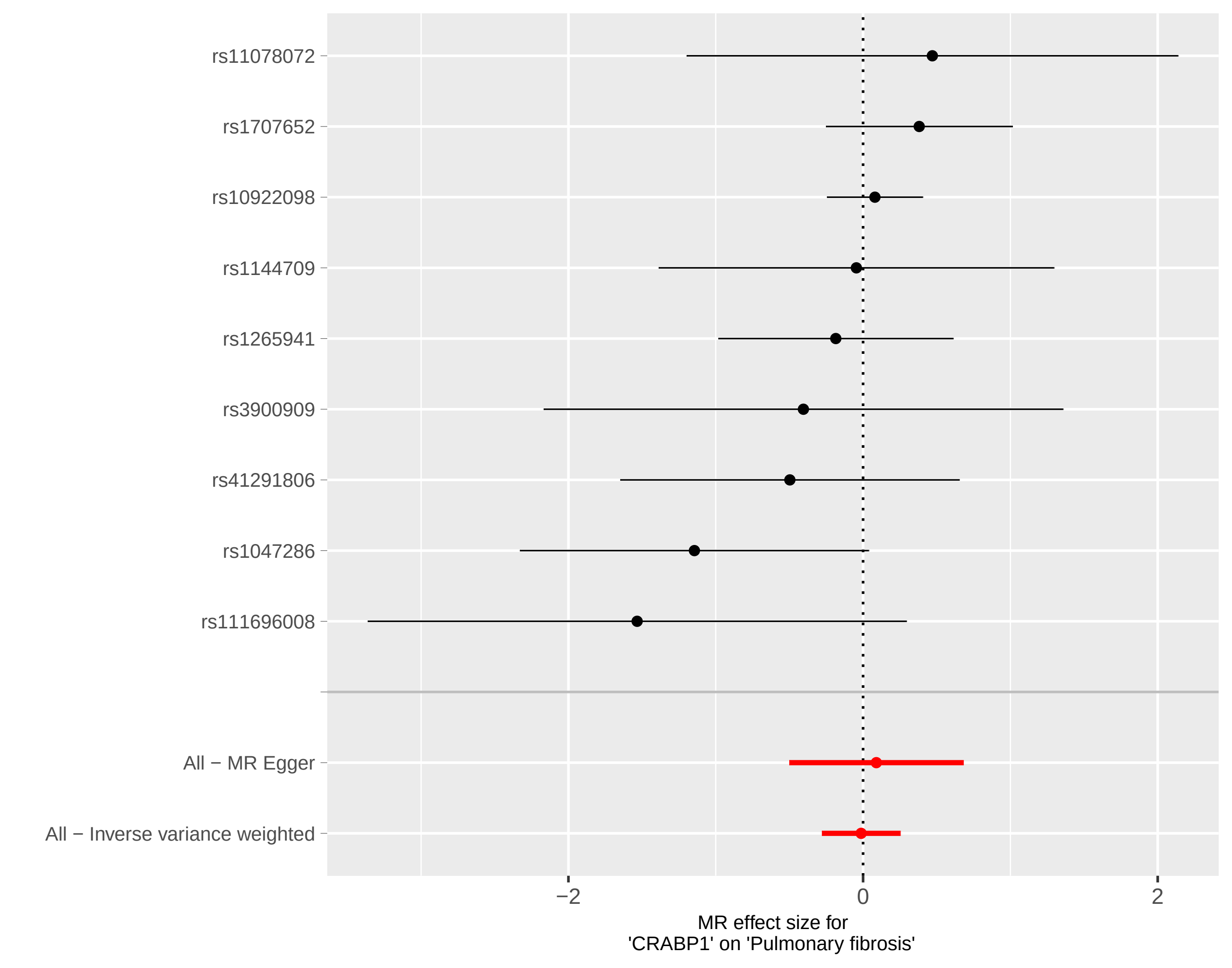
rs11078072
rs1707652
rs10922098
rs1144709
rs1265941
rs3900909
rs41291806
rs1047286
rs111696008
All − MR Egger
All − Inverse variance weighted
−2
0
2
MR effect size for
'CRABP1' on 'Pulmonary fibrosis'

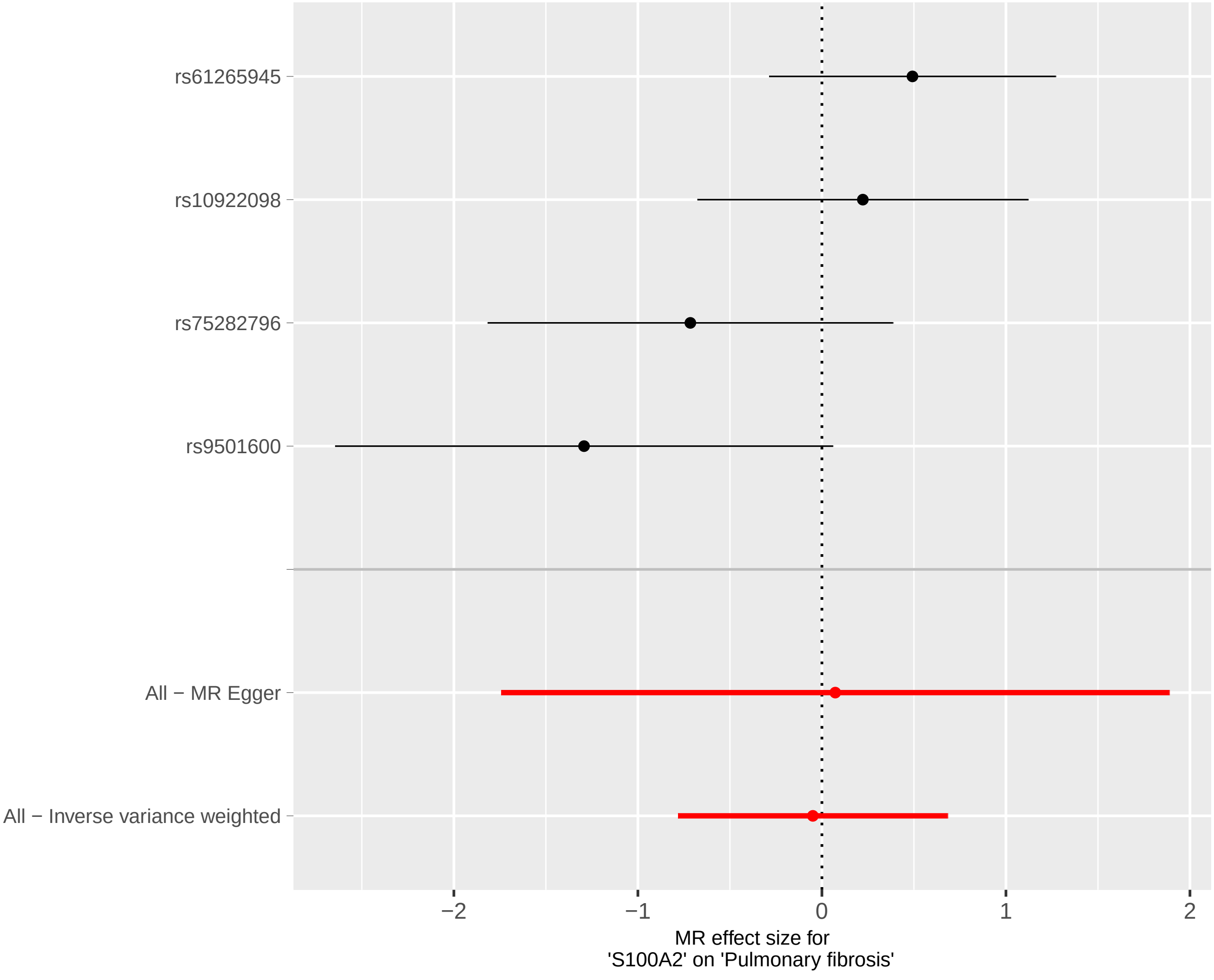
rs61265945
rs10922098
rs75282796
rs9501600
All − MR Egger
All − Inverse variance weighted
−2
−1
0
1
2
MR effect size for
'S100A2' on 'Pulmonary fibrosis'

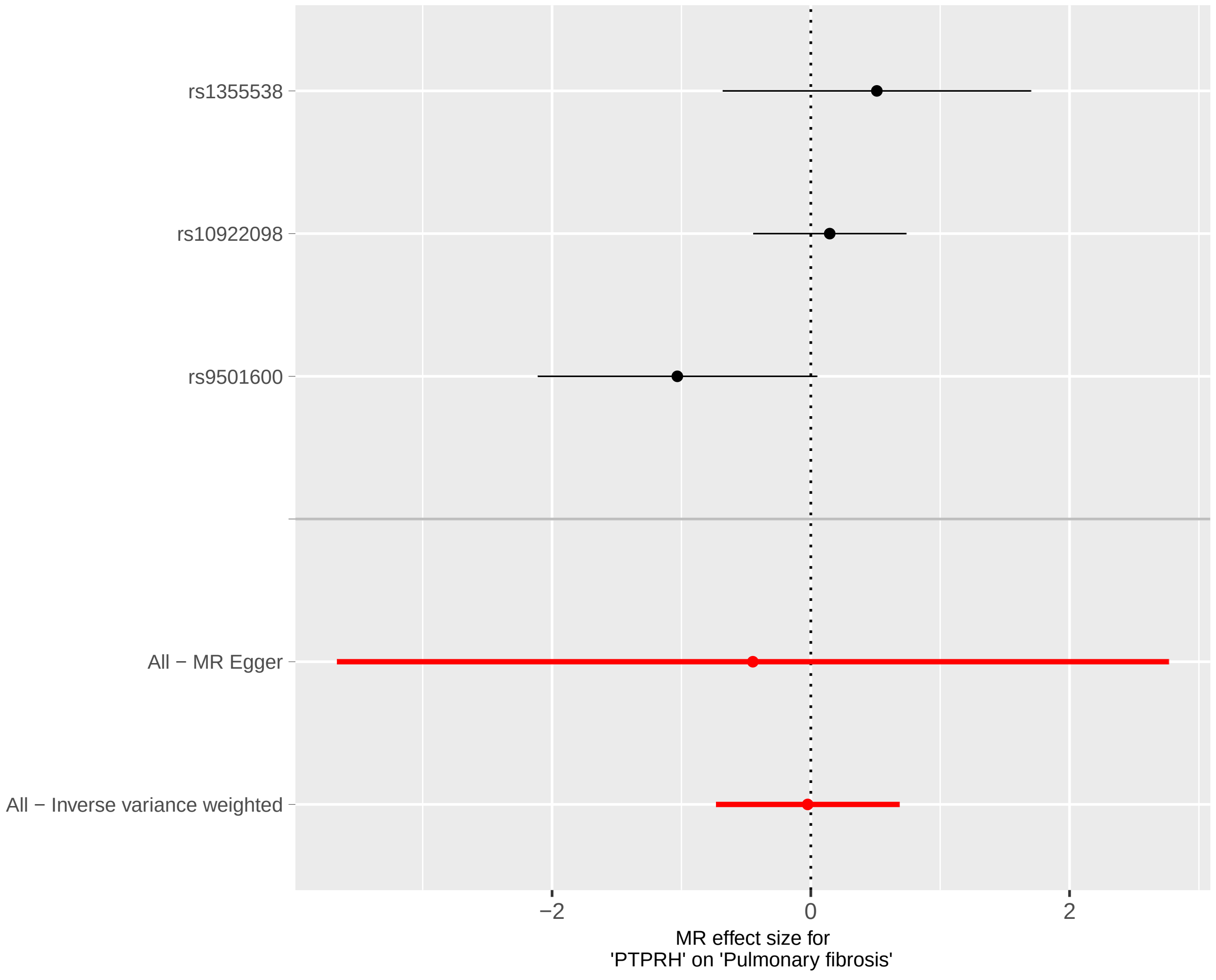

rs1355538
rs10922098
rs9501600
All − MR Egger
All − Inverse variance weighted
−2
0
2
MR effect size for
'PTPRH' on 'Pulmonary fibrosis'

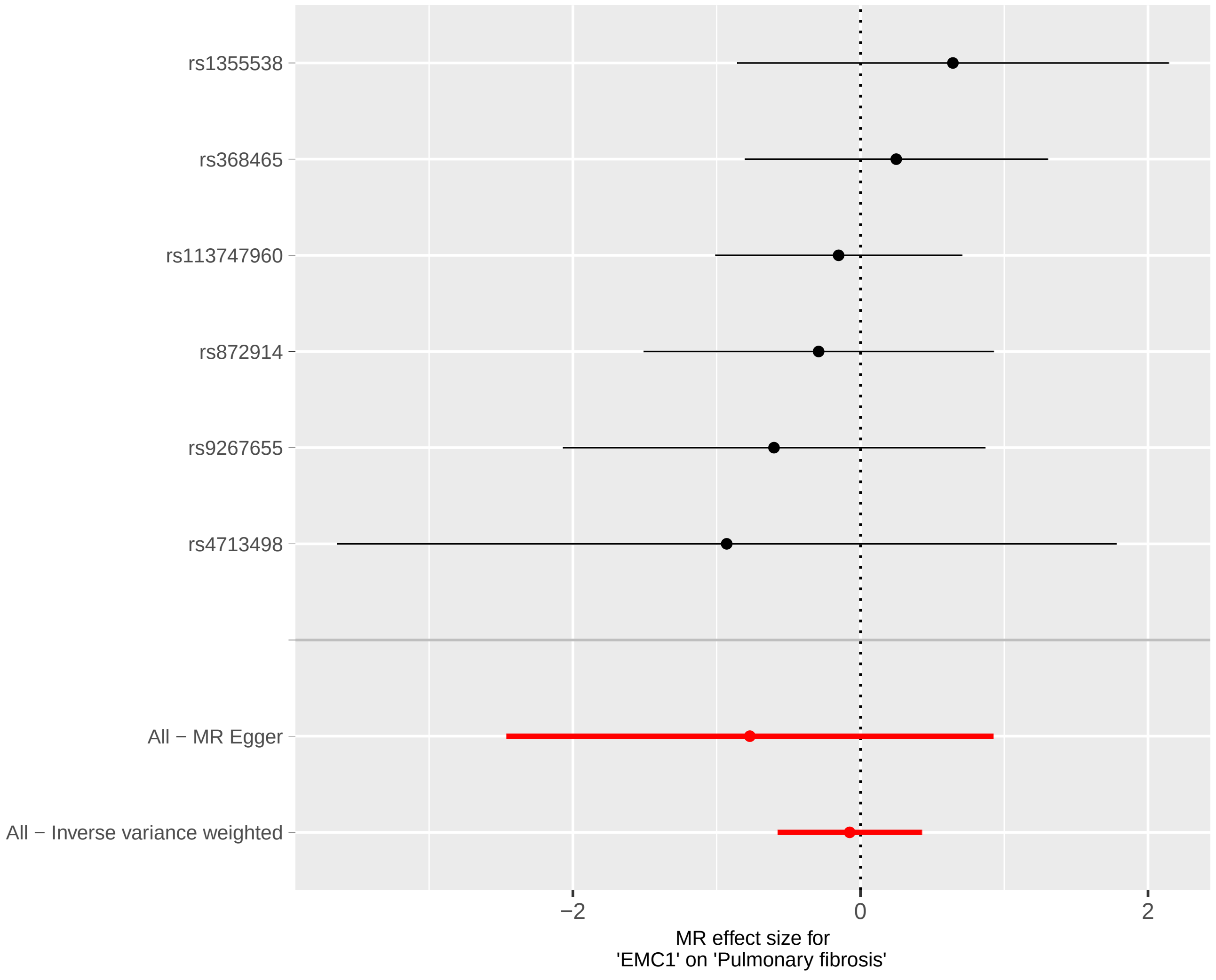

rs1355538
rs368465
rs113747960
rs872914
rs9267655
rs4713498
All − MR Egger
All − Inverse variance weighted
−2
0
2
MR effect size for
'EMC1' on 'Pulmonary fibrosis'

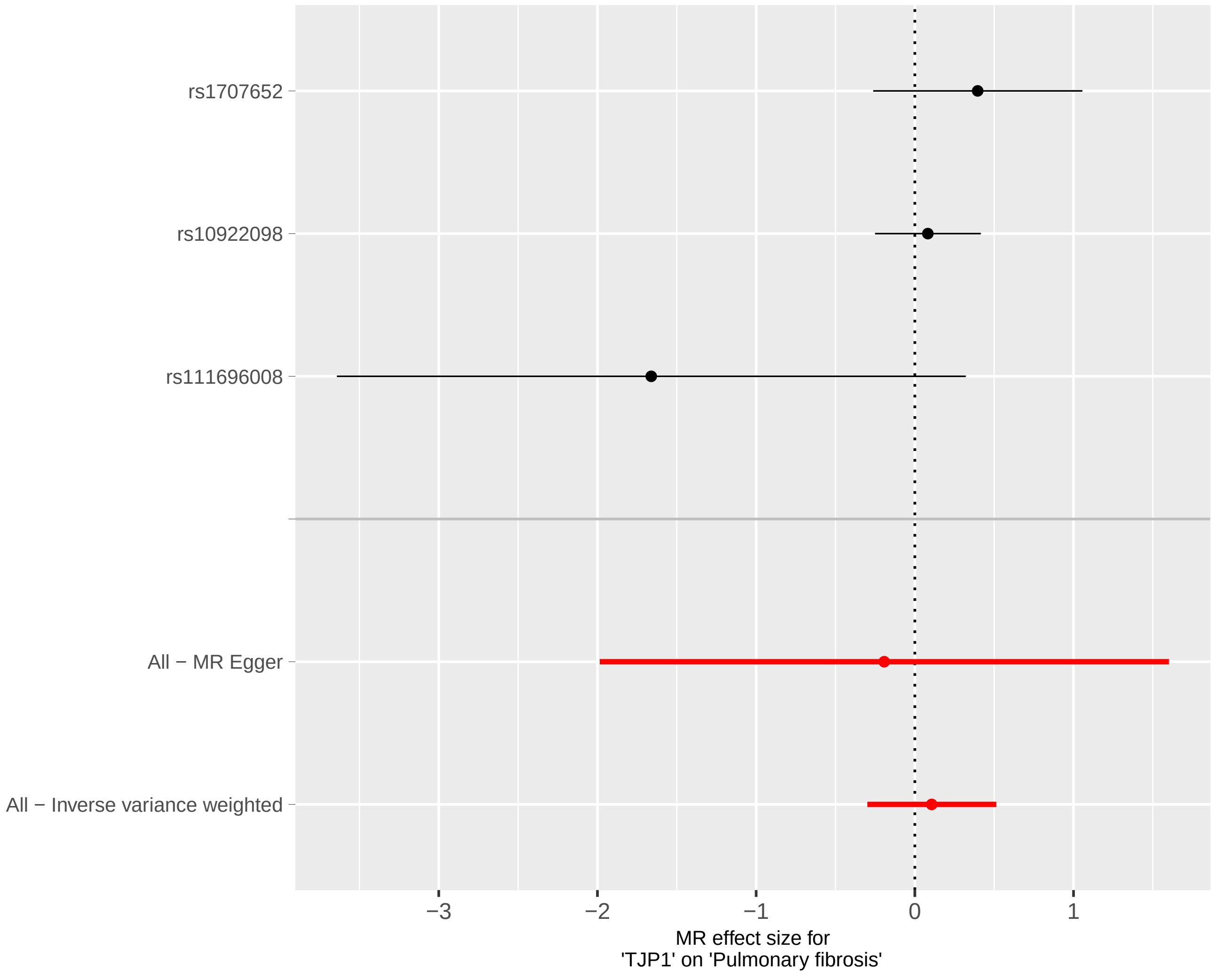
rs1707652
rs10922098
rs111696008
All − MR Egger
All − Inverse variance weighted
−3
−2
−1
0
1
MR effect size for
'TJP1' on 'Pulmonary fibrosis'

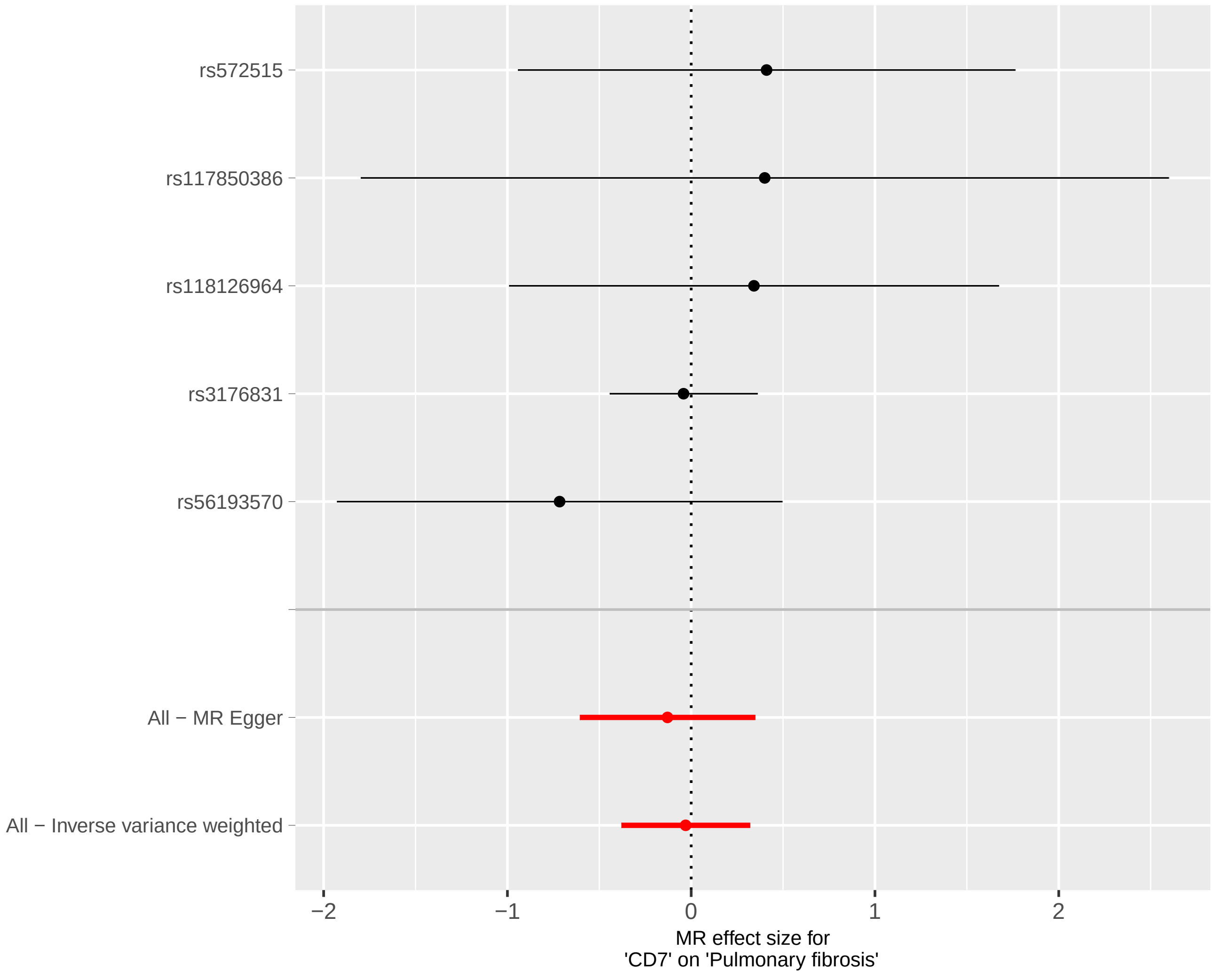

rs572515
rs117850386
rs118126964
rs3176831
rs56193570
All − MR Egger
All − Inverse variance weighted
−2
−1
0
1
2
MR effect size for
'CD7' on 'Pulmonary fibrosis'

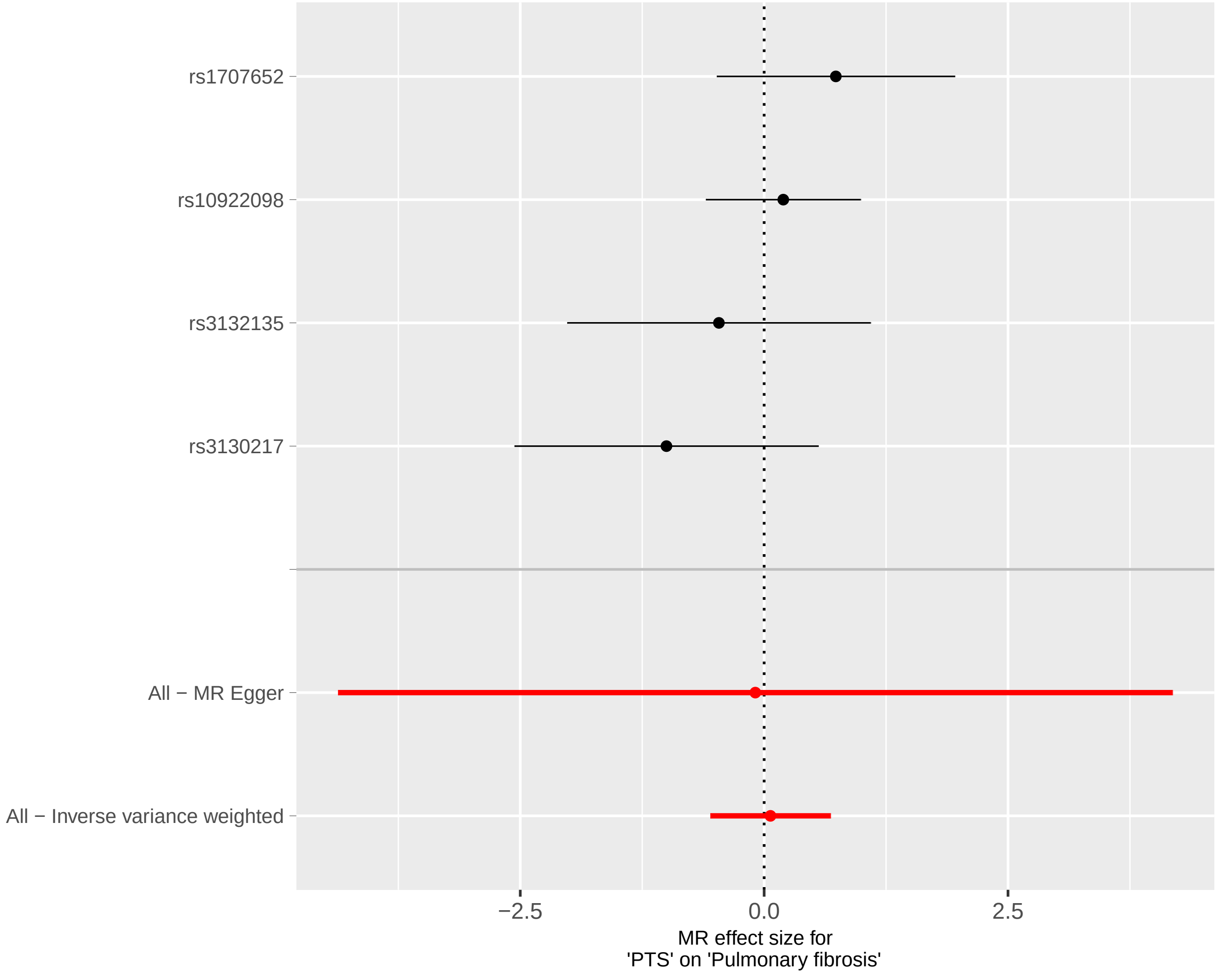
rs1707652
rs10922098
rs3132135
rs3130217
All − MR Egger
All − Inverse variance weighted
−2.5
0.0
2.5
MR effect size for
'PTS' on 'Pulmonary fibrosis'

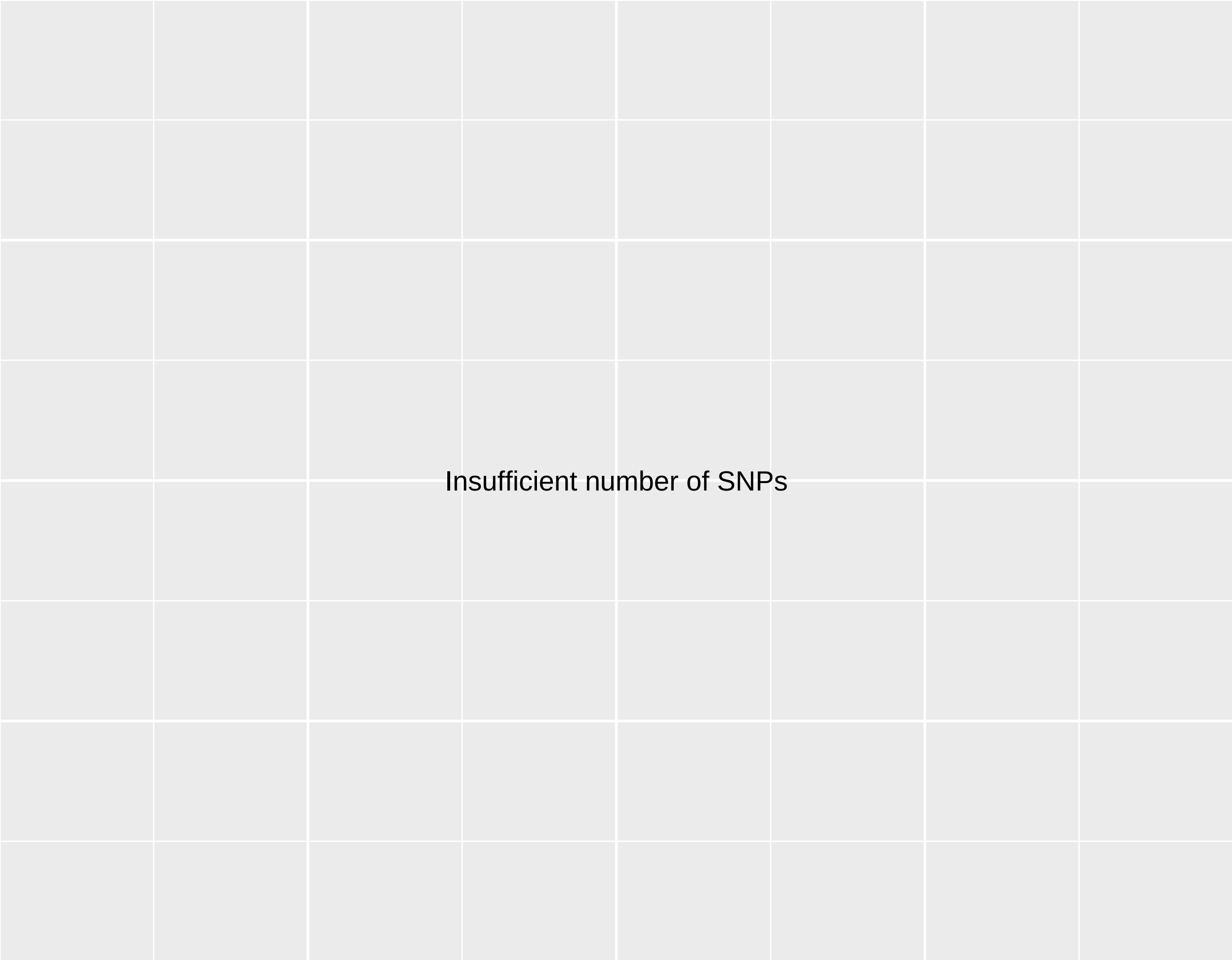
Insufficient number of SNPs

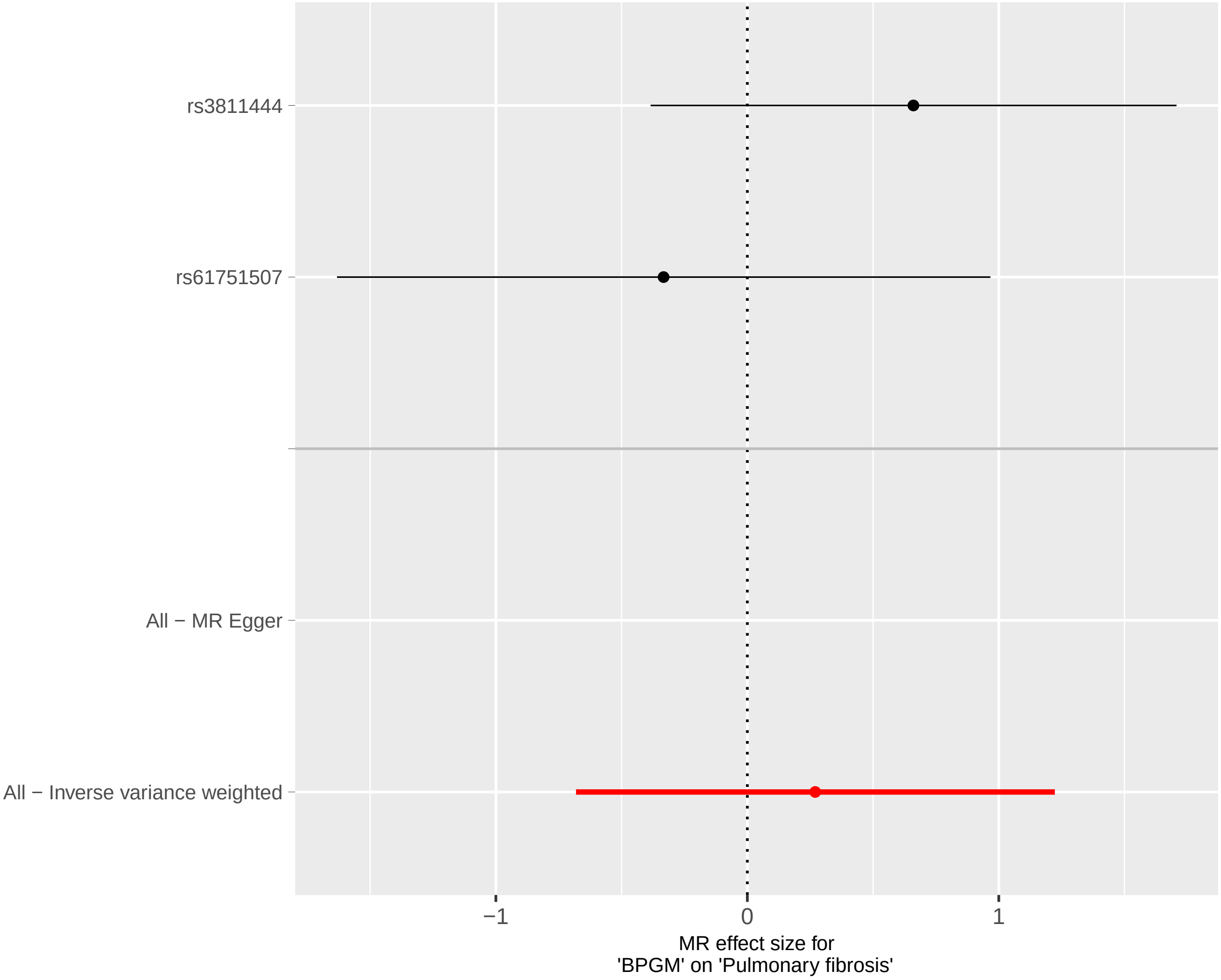
rs3811444
rs61751507
All − MR Egger
All − Inverse variance weighted
−1
0
1
MR effect size for
'BPGM' on 'Pulmonary fibrosis'

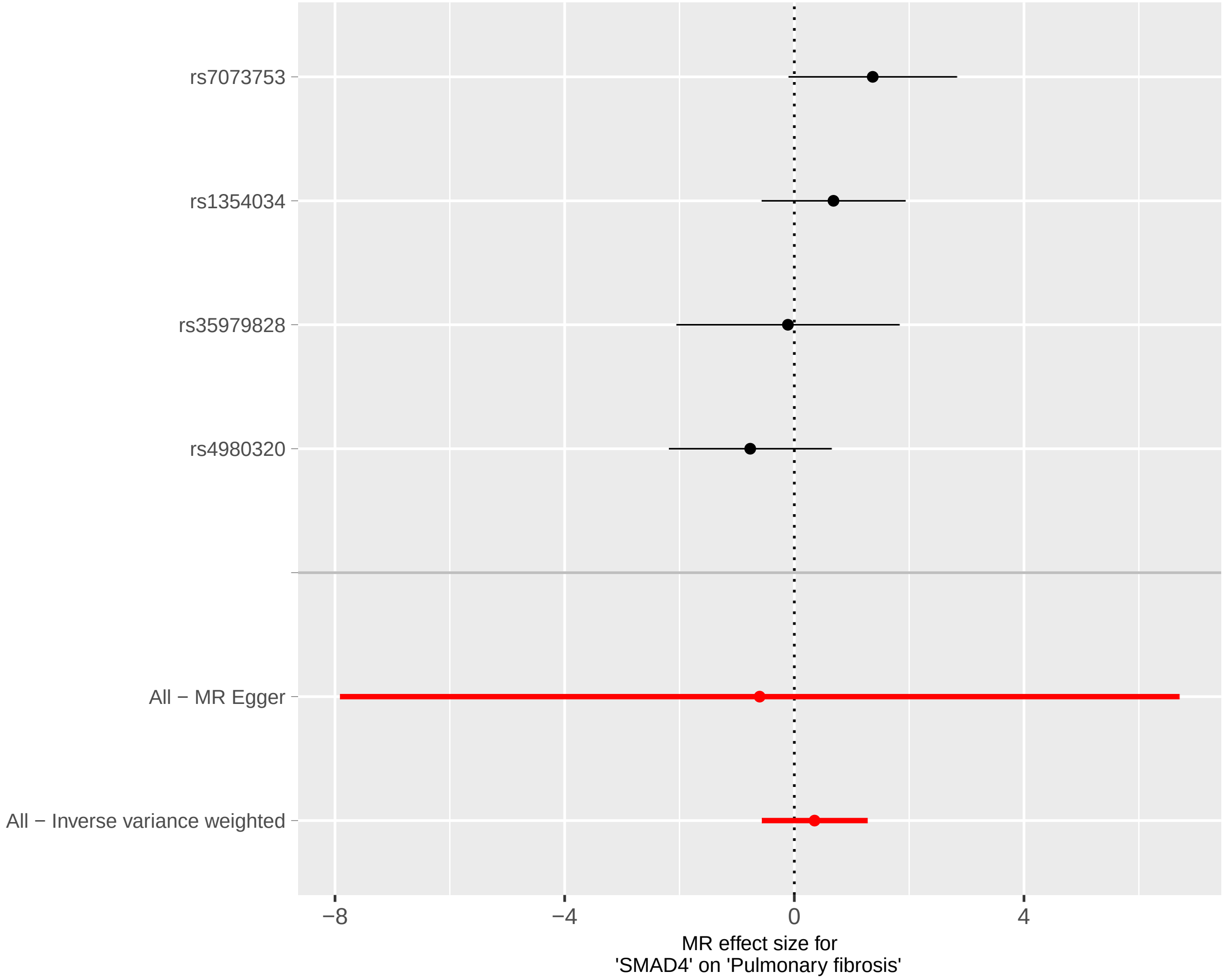
rs7073753
rs1354034
rs35979828
rs4980320
All − MR Egger
All − Inverse variance weighted
−8
−4
0
4
MR effect size for
'SMAD4' on 'Pulmonary fibrosis'

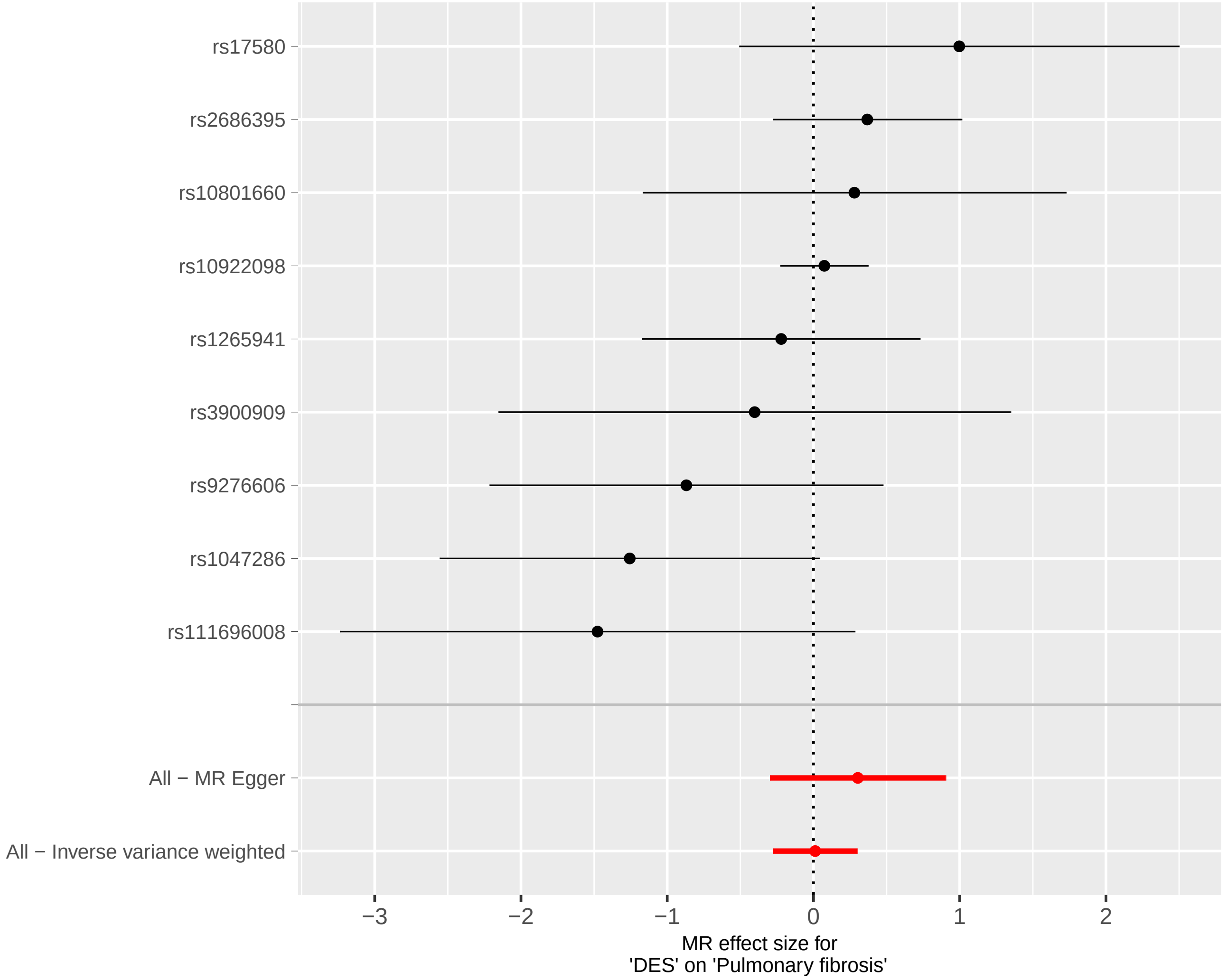
rs17580
rs2686395
rs10801660
rs10922098
rs1265941
rs3900909
rs9276606
rs1047286
rs111696008
All − MR Egger
All − Inverse variance weighted
−3
−2
−1
0
1
2
MR effect size for
'DES' on 'Pulmonary fibrosis'

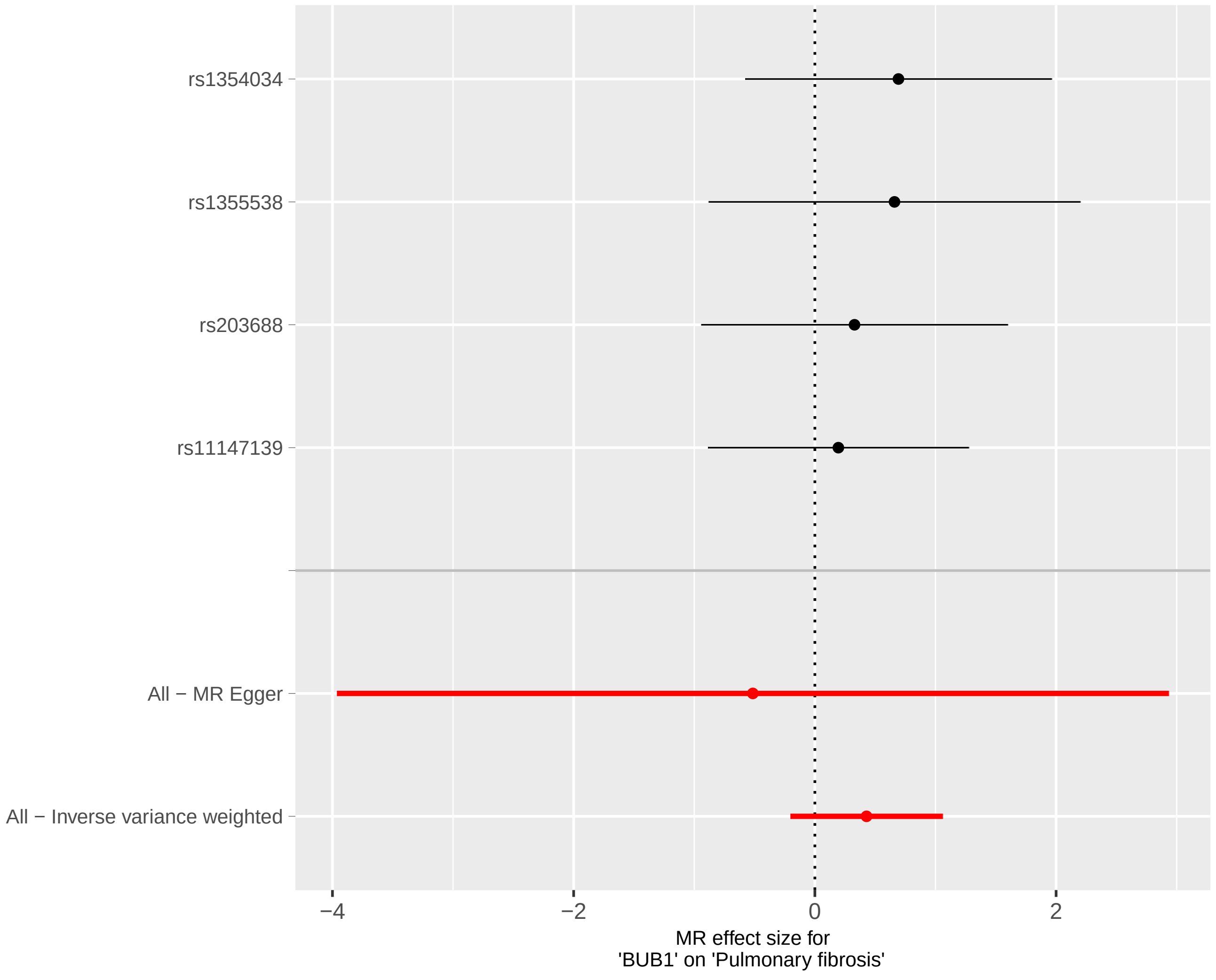
rs1354034
rs1355538
rs203688
rs11147139
All − MR Egger
All − Inverse variance weighted
−4
−2
0
2
MR effect size for
'BUB1' on 'Pulmonary fibrosis'

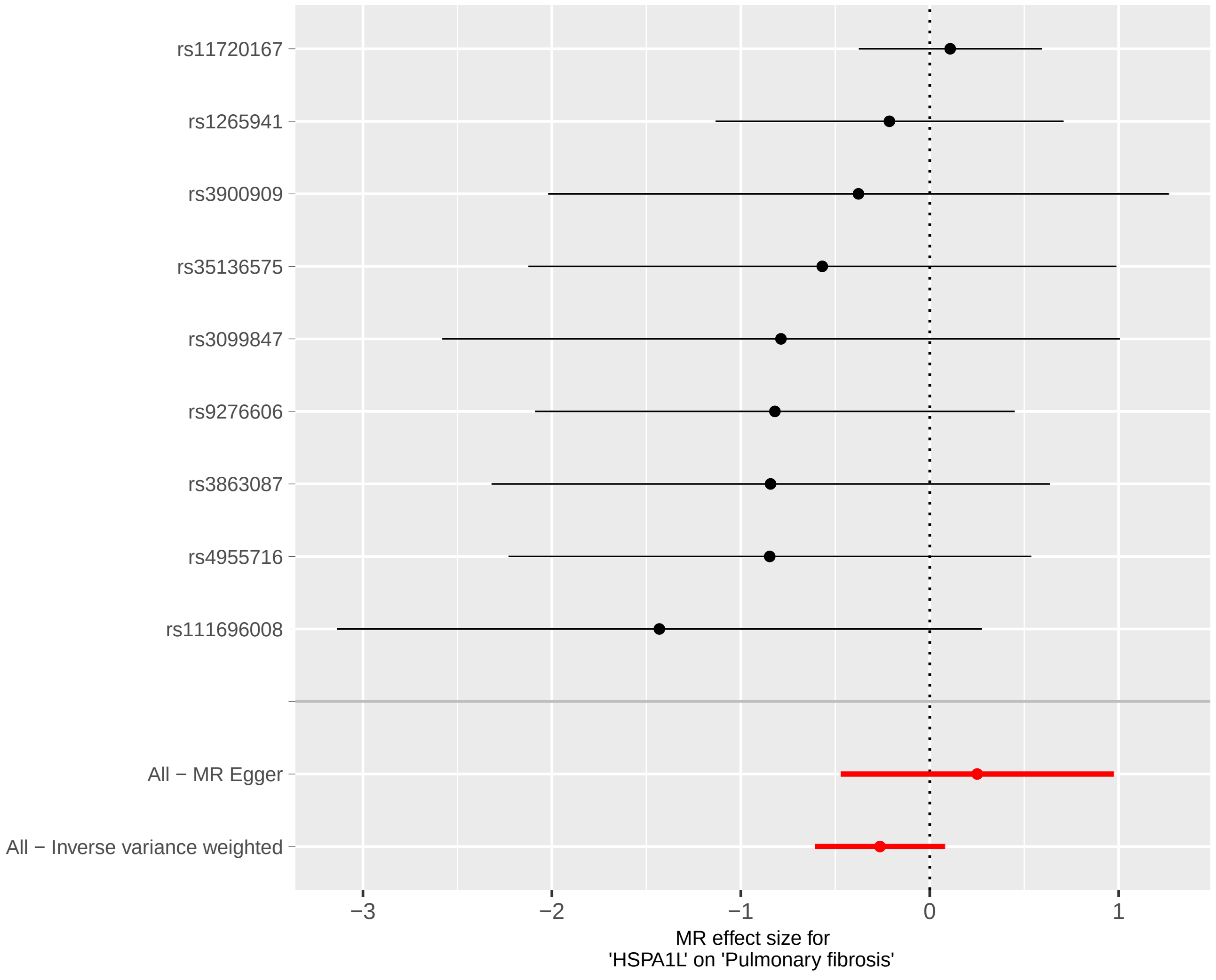

rs11720167
rs1265941
rs3900909
rs35136575
rs3099847
rs9276606
rs3863087
rs4955716
rs111696008
All − MR Egger
All − Inverse variance weighted
−3
−2
−1
0
1
MR effect size for
'HSPA1L' on 'Pulmonary fibrosis'

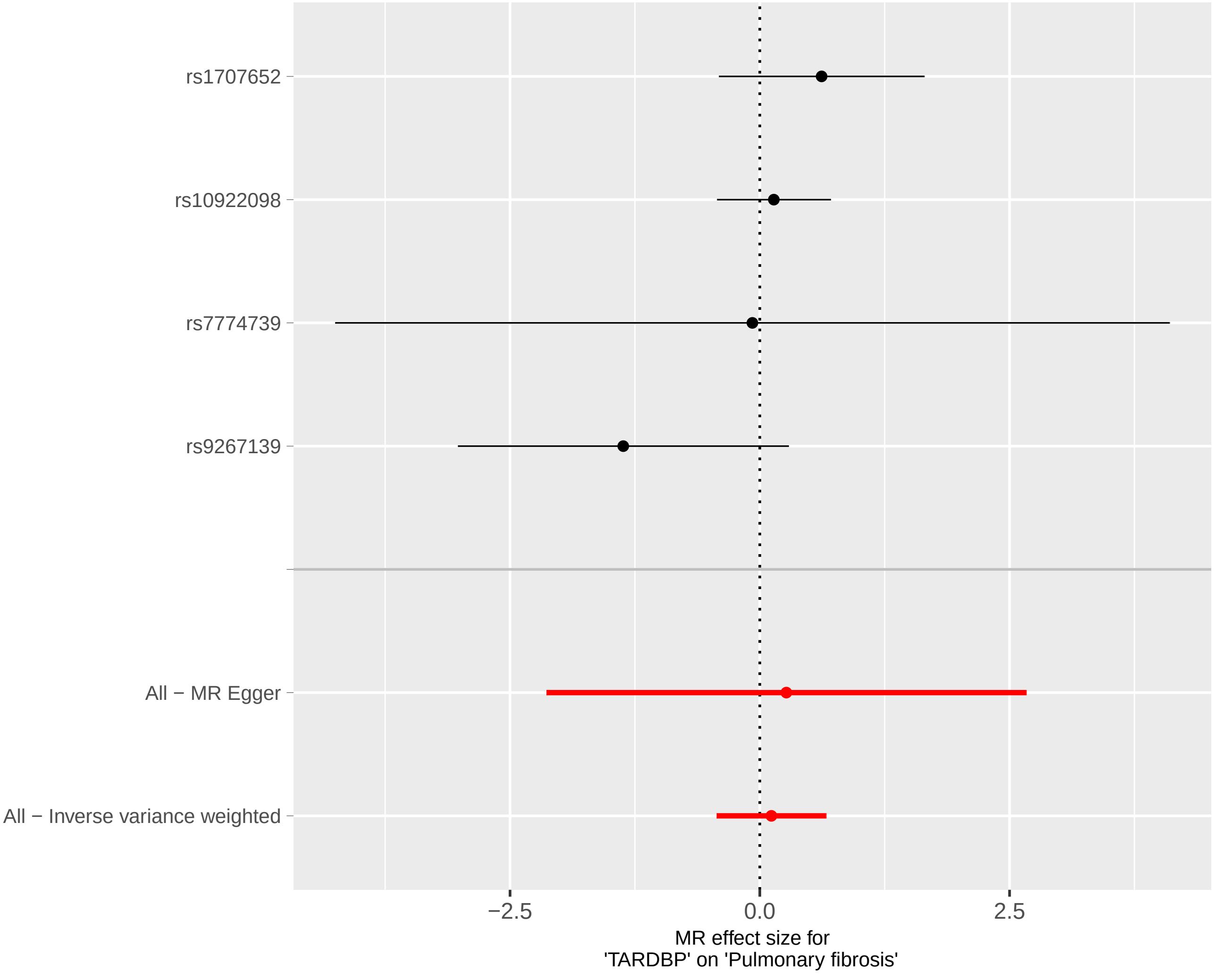

rs1707652
rs10922098
rs7774739
rs9267139
All − MR Egger
All − Inverse variance weighted
−2.5
0.0
2.5
MR effect size for
'TARDBP' on 'Pulmonary fibrosis'

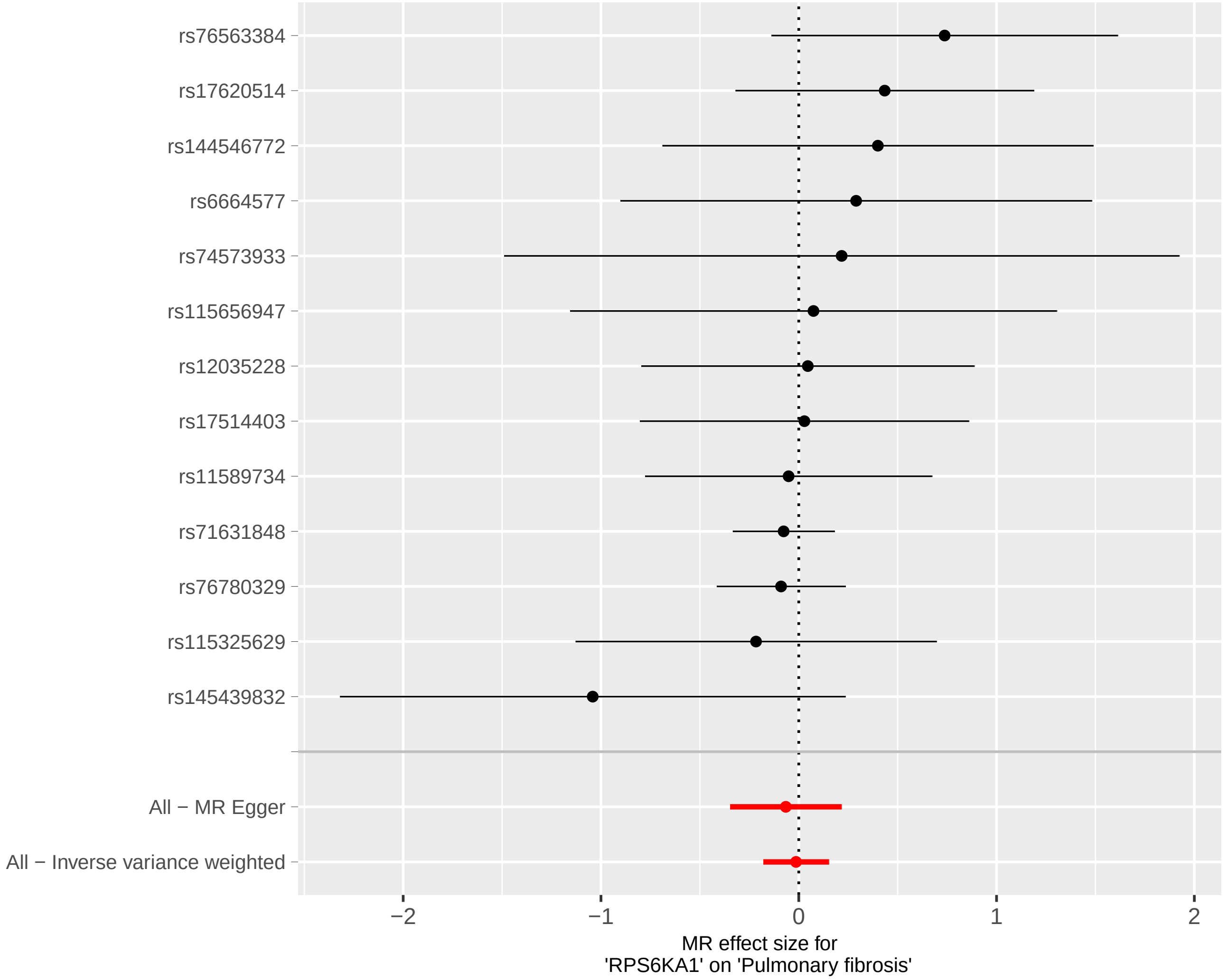
rs76563384
rs17620514
rs144546772
rs6664577
rs74573933
rs115656947
rs12035228
rs17514403
rs11589734
rs71631848
rs76780329
rs115325629
rs145439832
All − MR Egger
All − Inverse variance weighted
−2
−1
0
1
2
MR effect size for
'RPS6KA1' on 'Pulmonary fibrosis'

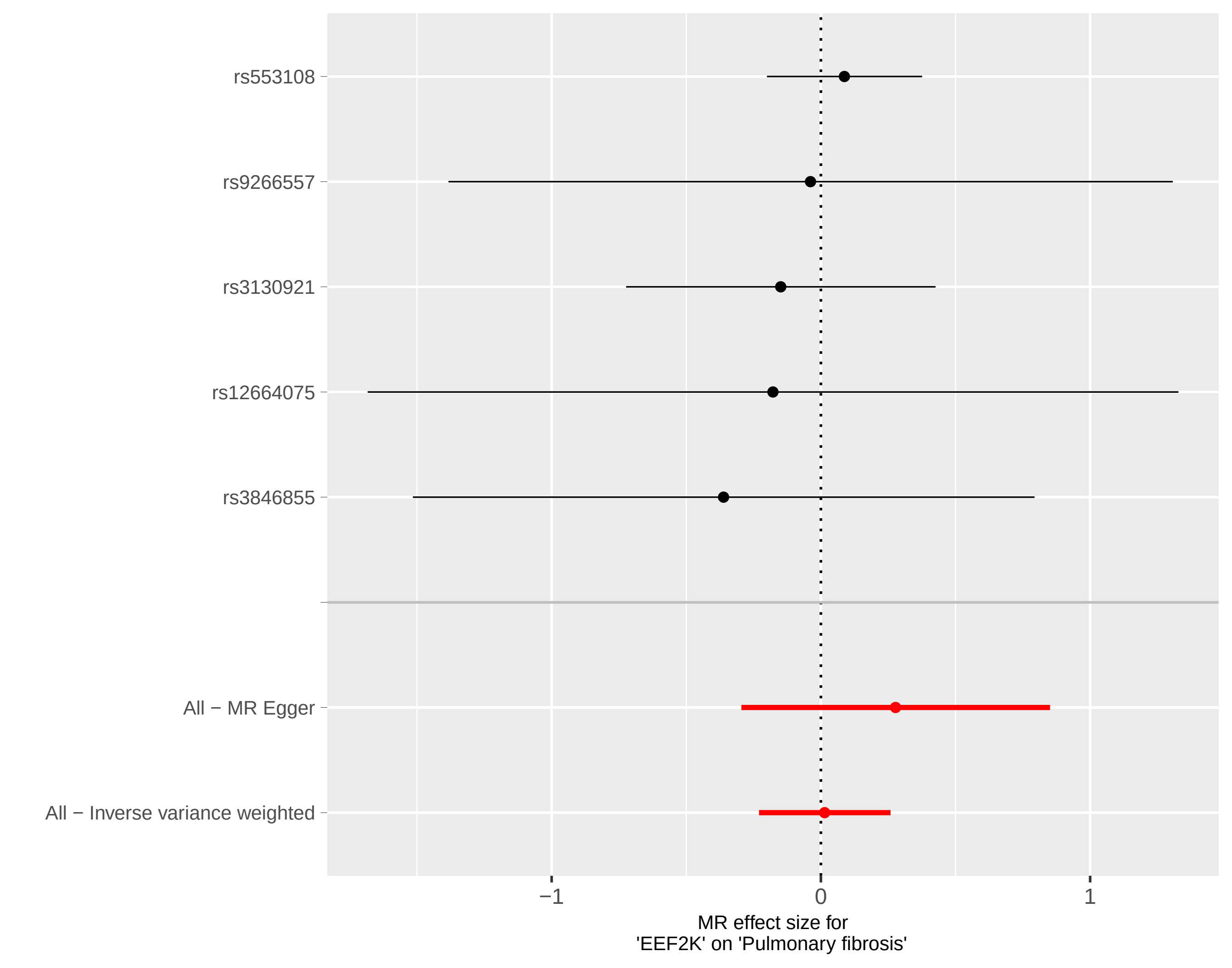

rs553108
rs9266557
rs3130921
rs12664075
rs3846855
All − MR Egger
All − Inverse variance weighted
−1
0
1
MR effect size for
'EEF2K' on 'Pulmonary fibrosis'

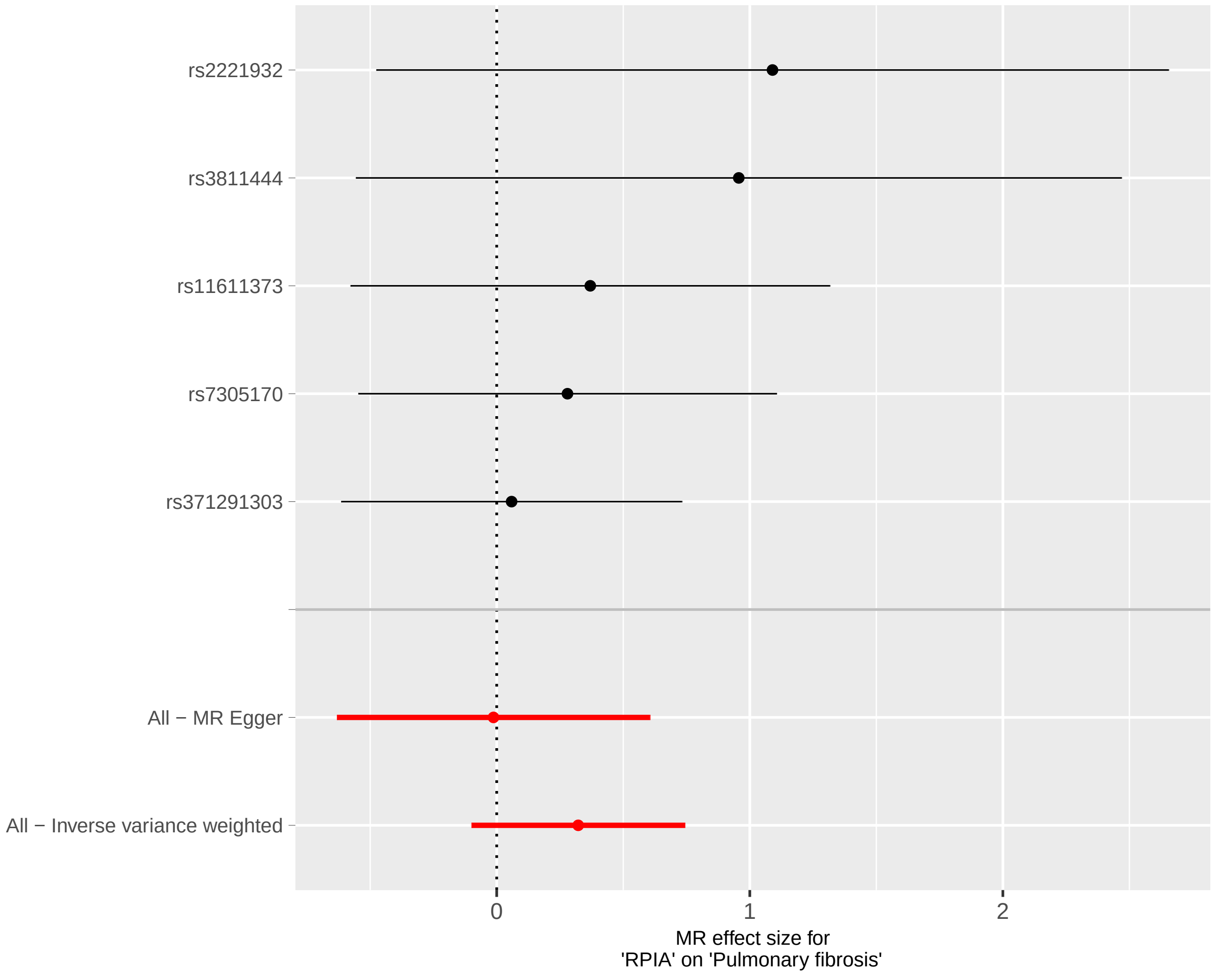
rs2221932
rs3811444
rs11611373
rs7305170
rs371291303
All − MR Egger
All − Inverse variance weighted
0
1
2
MR effect size for
'RPIA' on 'Pulmonary fibrosis'

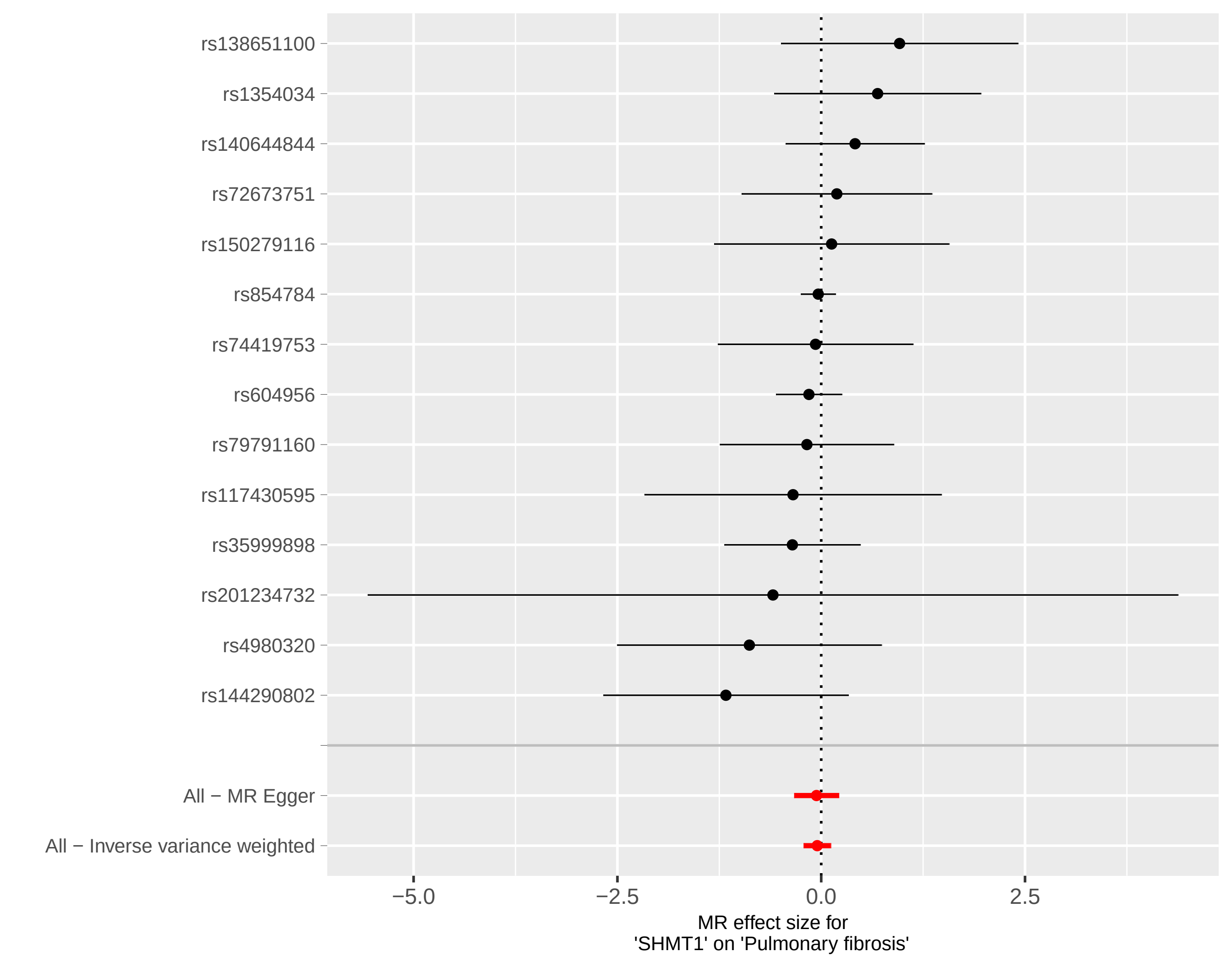

rs138651100
rs1354034
rs140644844
rs72673751
rs150279116
rs854784
rs74419753
rs604956
rs79791160
rs117430595
rs35999898
rs201234732
rs4980320
rs144290802
All − MR Egger
All − Inverse variance weighted
−5.0
−2.5
0.0
2.5
MR effect size for
'SHMT1' on 'Pulmonary fibrosis'

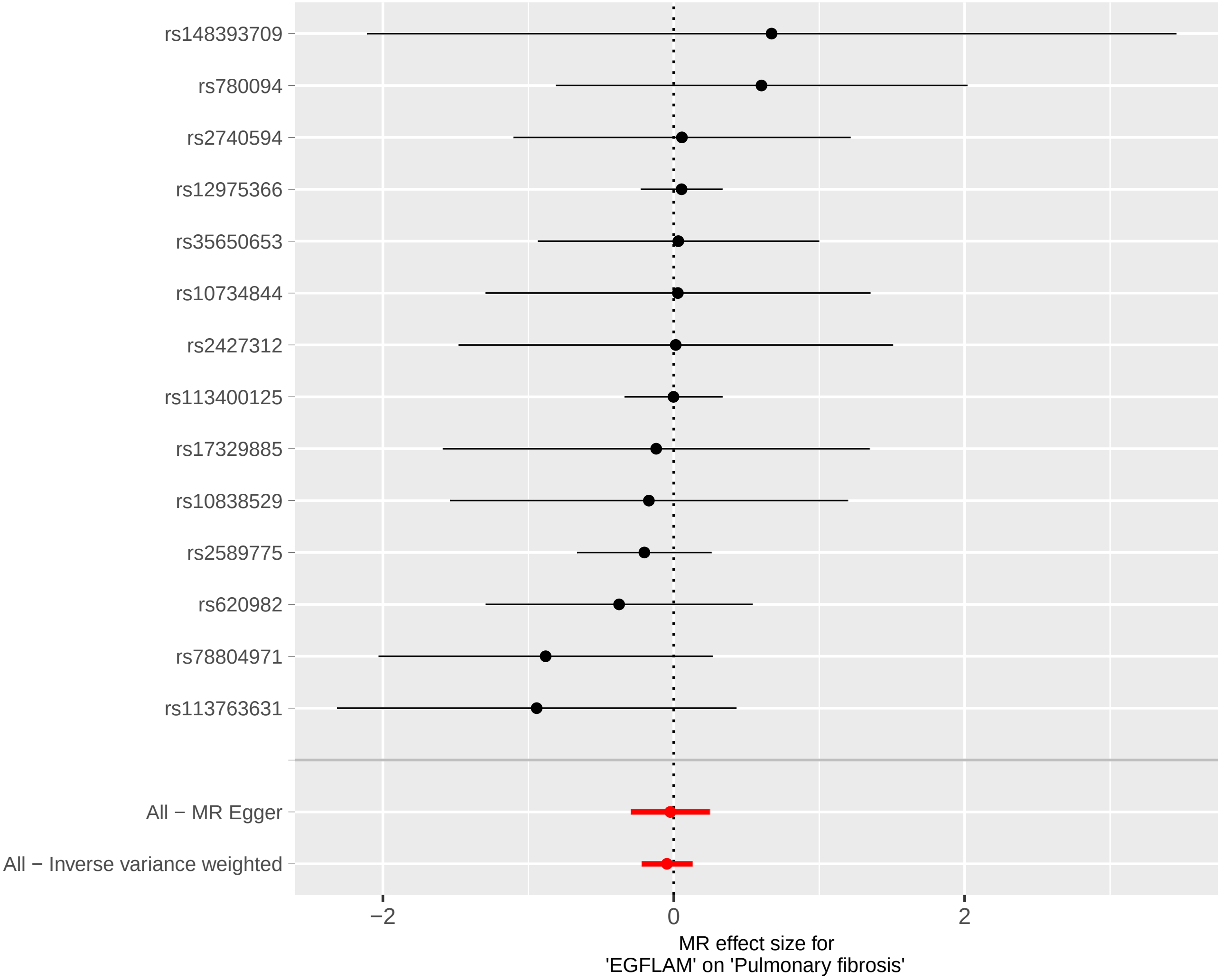
rs148393709
rs780094
rs2740594
rs12975366
rs35650653
rs10734844
rs2427312
rs113400125
rs17329885
rs10838529
rs2589775
rs620982
rs78804971
rs113763631
All − MR Egger
All − Inverse variance weighted
−2
0
2
MR effect size for
'EGFLAM' on 'Pulmonary fibrosis'

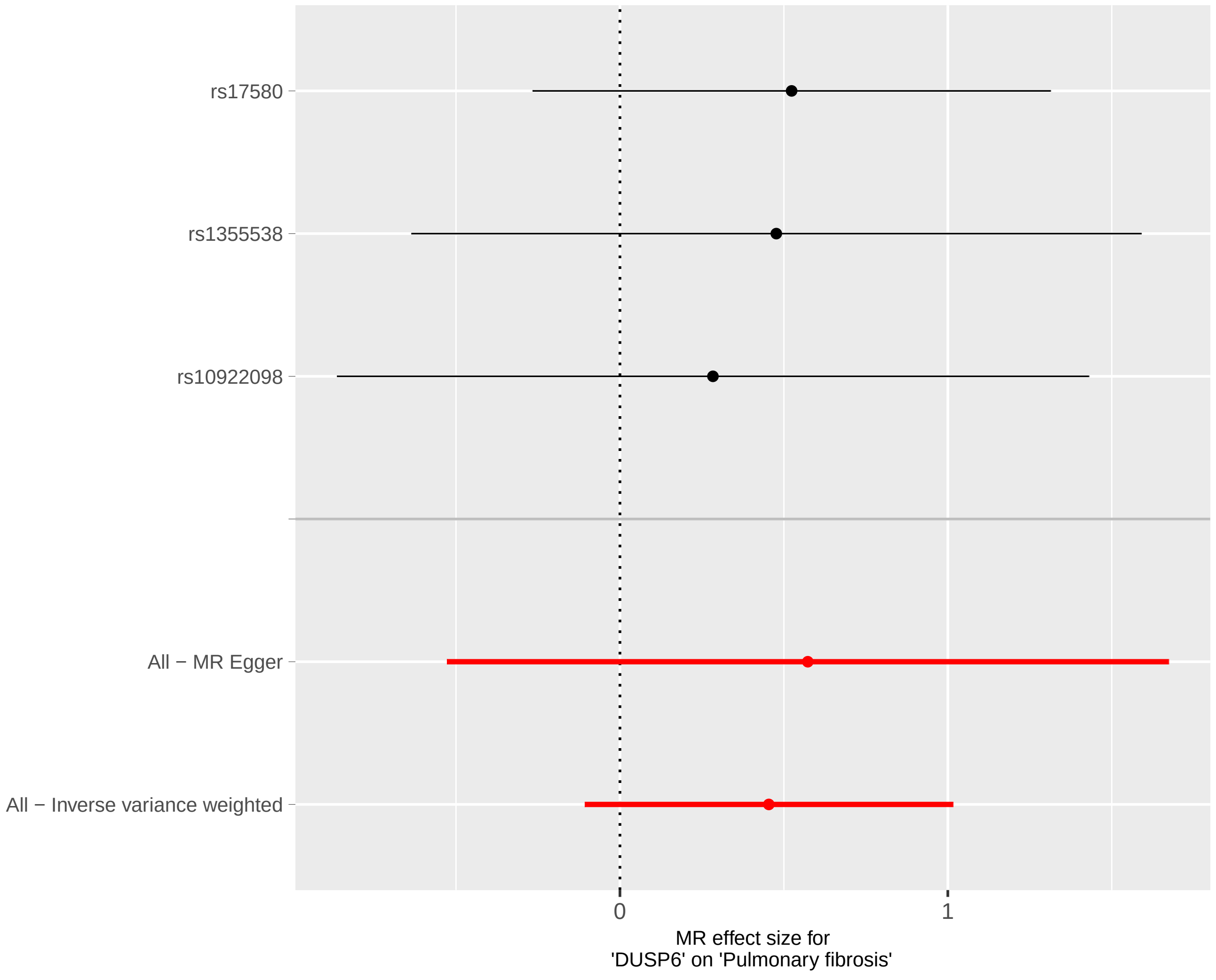
rs17580
rs1355538
rs10922098
All − MR Egger
All − Inverse variance weighted
0
1
MR effect size for
'DUSP6' on 'Pulmonary fibrosis'

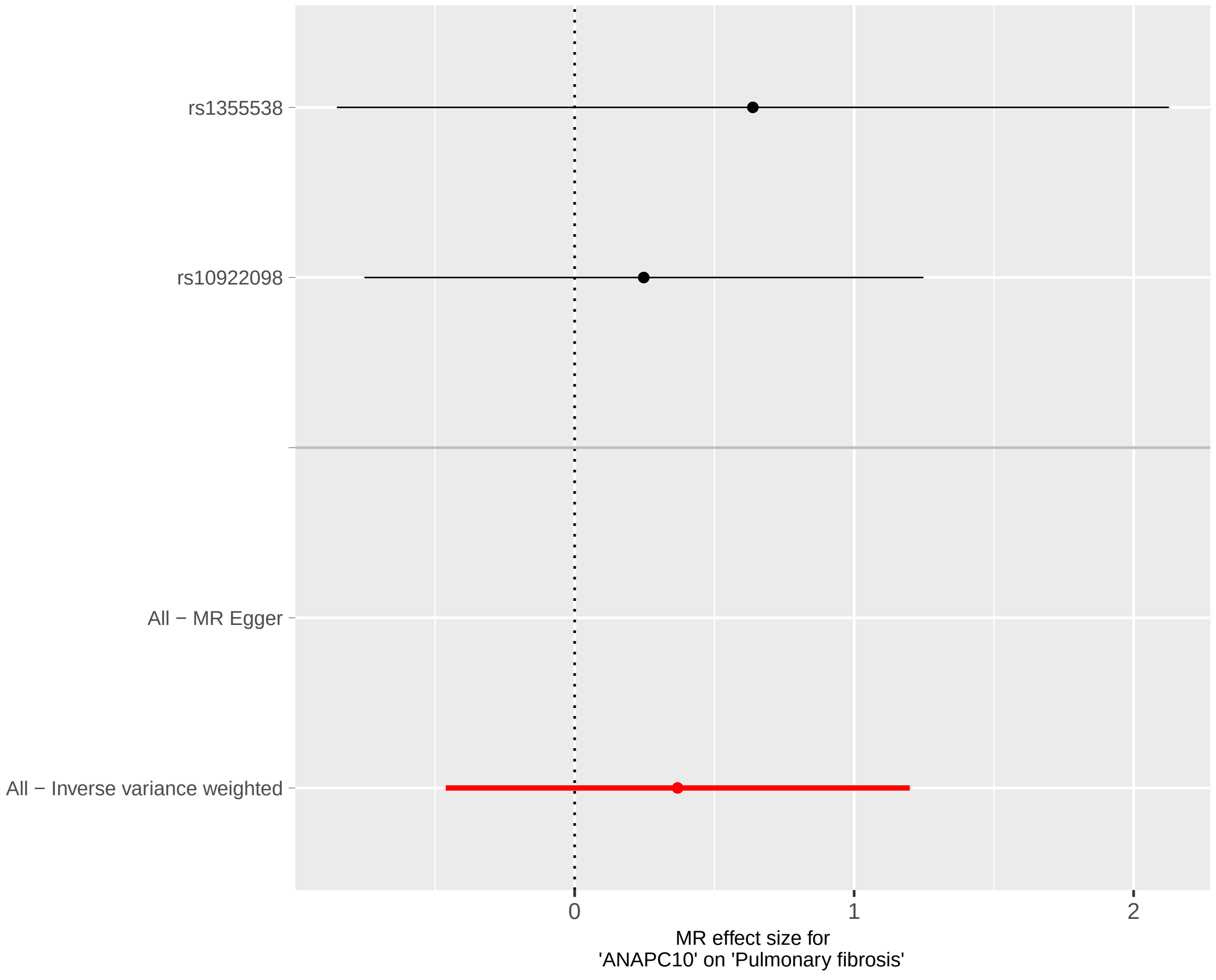
rs1355538
rs10922098
All − MR Egger
All − Inverse variance weighted
0
1
2
MR effect size for
'ANAPC10' on 'Pulmonary fibrosis'

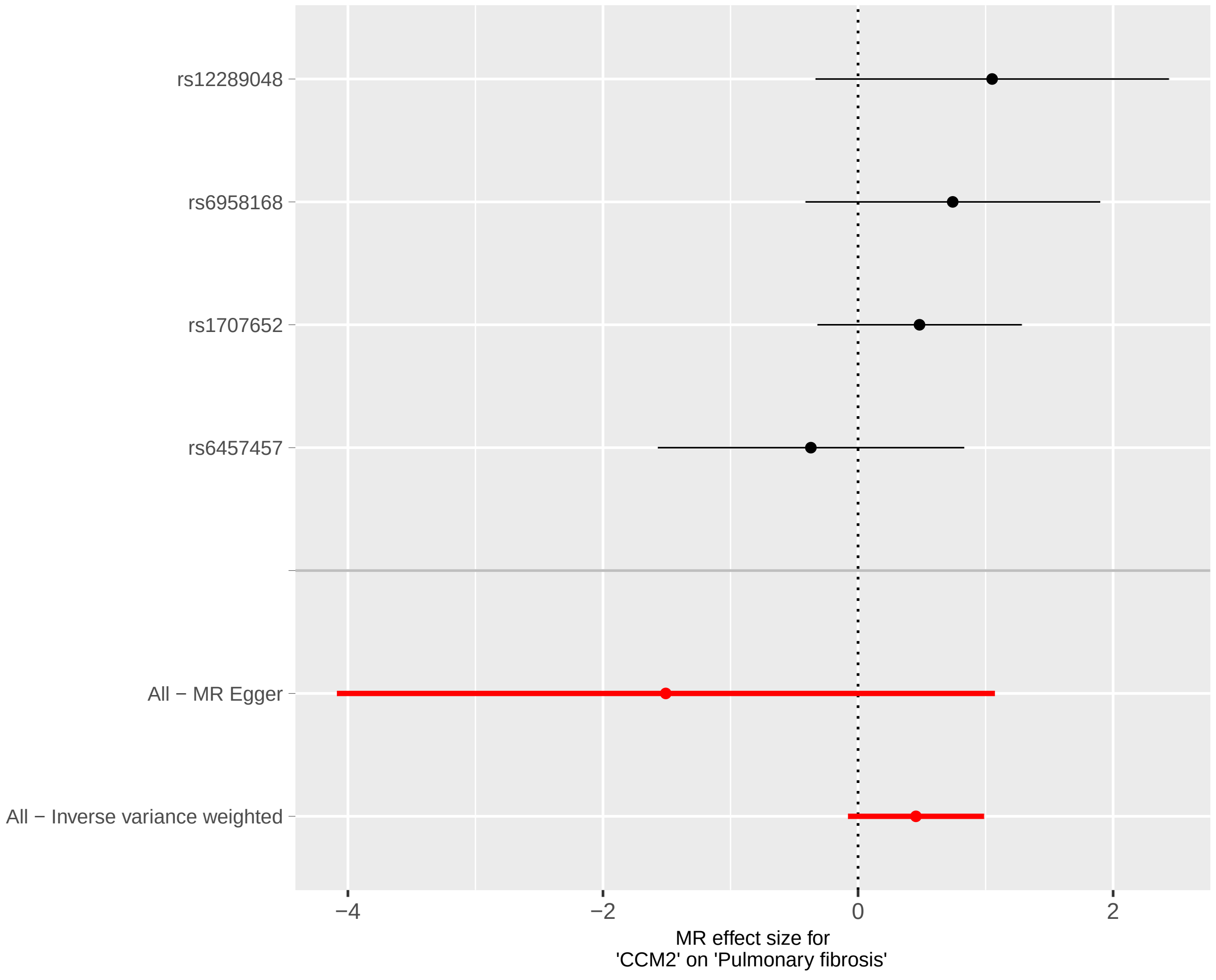
rs12289048
rs6958168
rs1707652
rs6457457
All − MR Egger
All − Inverse variance weighted
−4
−2
0
2
MR effect size for
'CCM2' on 'Pulmonary fibrosis'

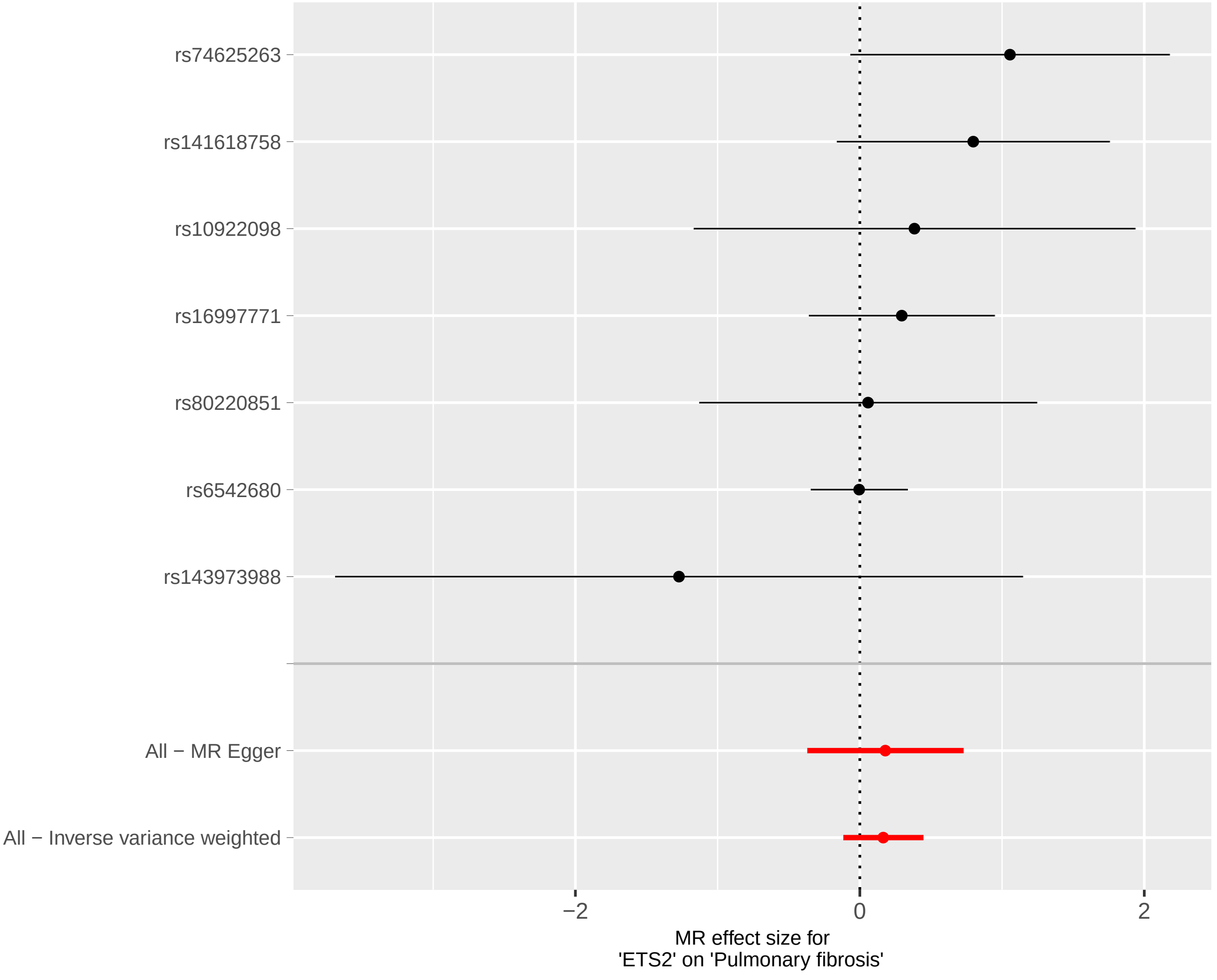
rs74625263
rs141618758
rs10922098
rs16997771
rs80220851
rs6542680
rs143973988
All − MR Egger
All − Inverse variance weighted
−2
0
2
MR effect size for
'ETS2' on 'Pulmonary fibrosis'

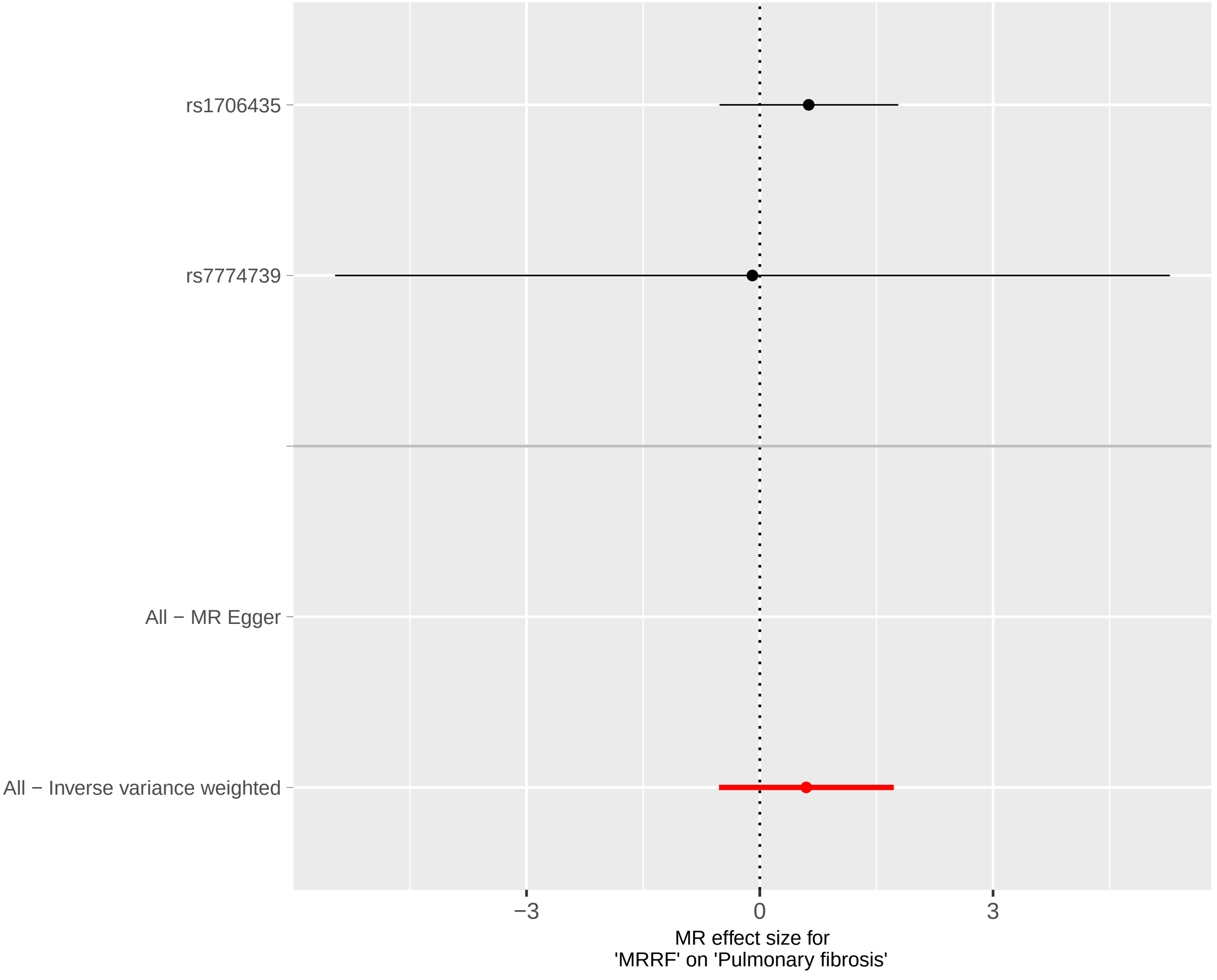
rs1706435
rs7774739
All − MR Egger
All − Inverse variance weighted
−3
0
3
MR effect size for
'MRRF' on 'Pulmonary fibrosis'

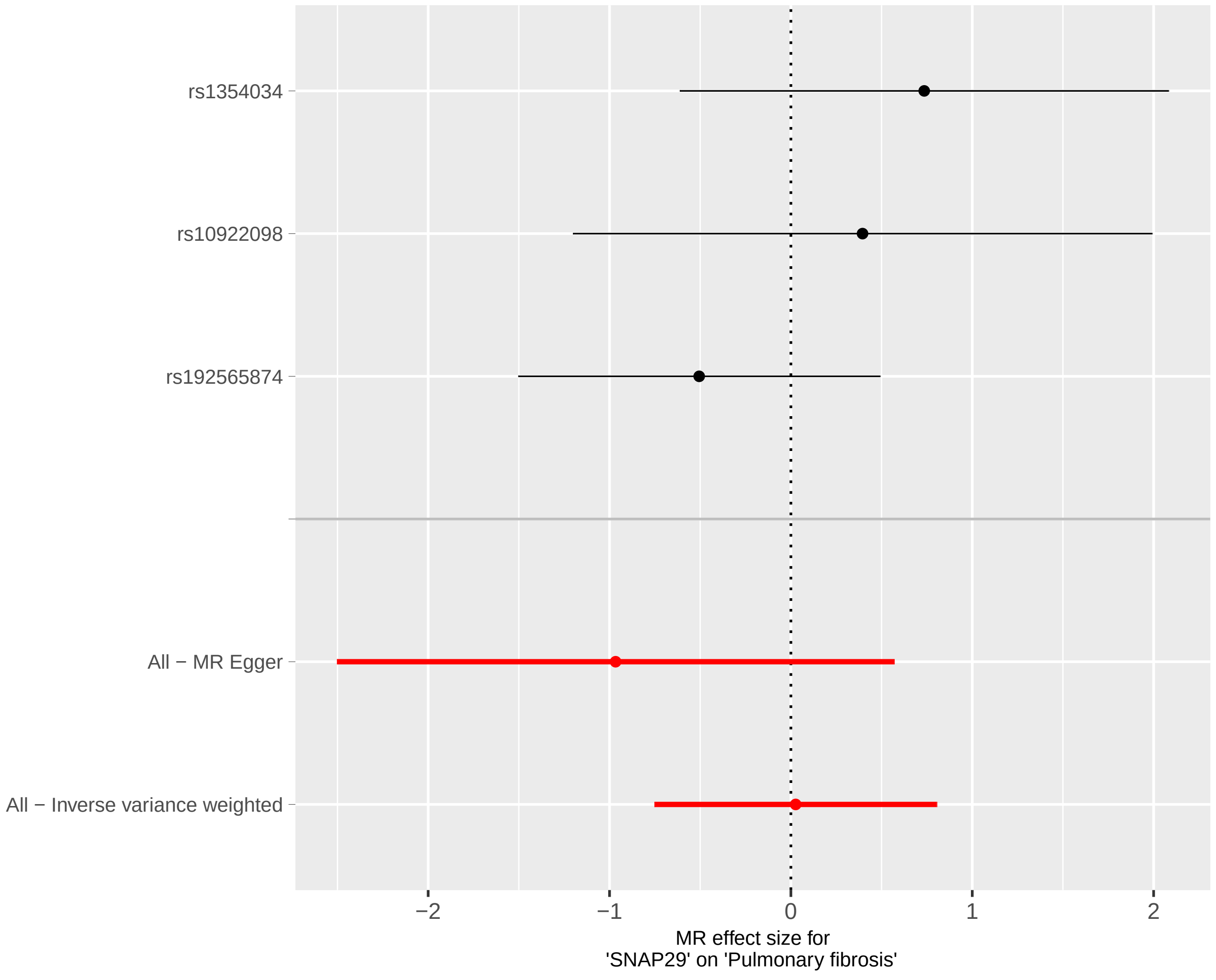
rs1354034
rs10922098
rs192565874
All − MR Egger
All − Inverse variance weighted
−2
−1
0
1
2
MR effect size for
'SNAP29' on 'Pulmonary fibrosis'

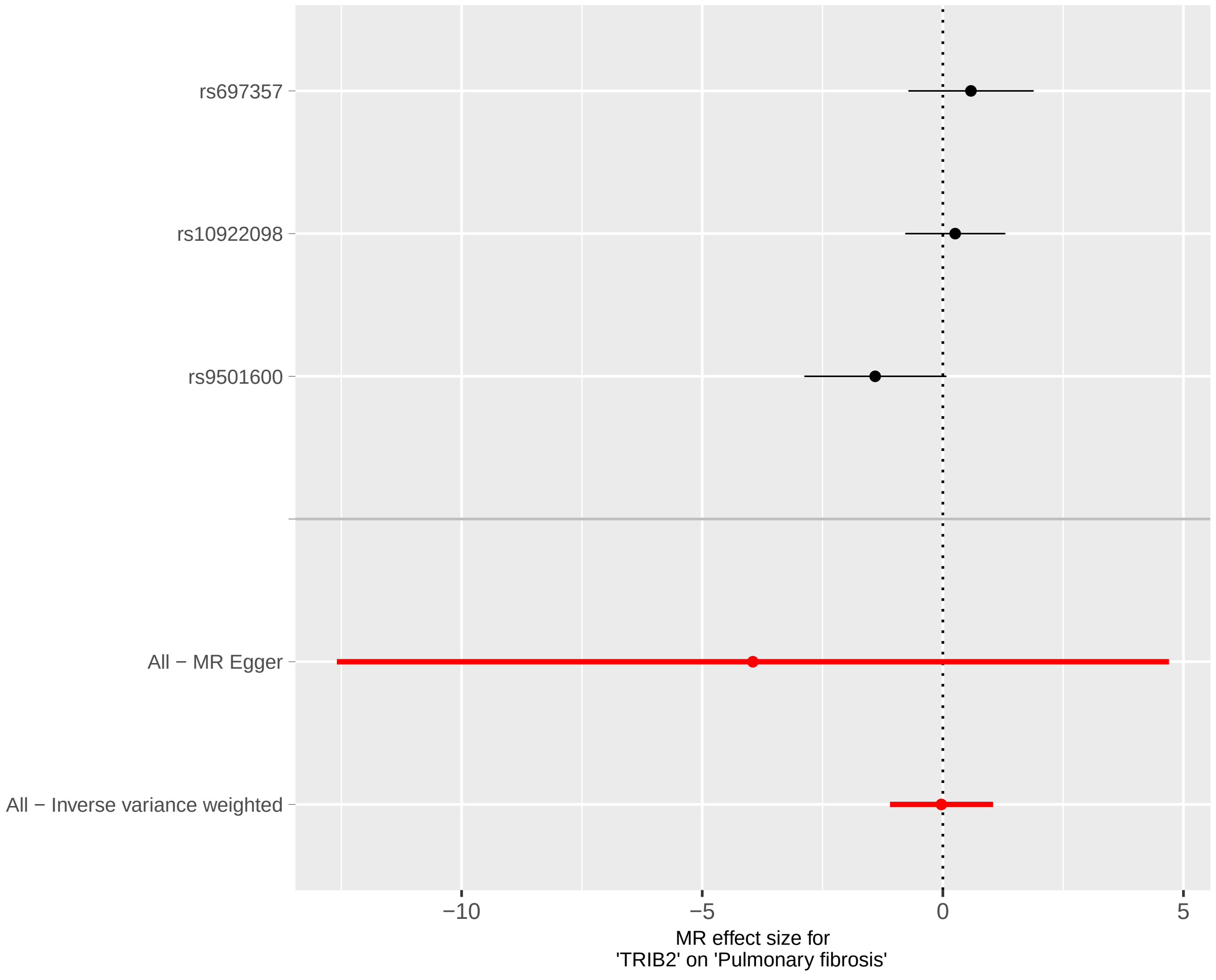

rs697357
rs10922098
rs9501600
All − MR Egger
All − Inverse variance weighted
−10
−5
0
5
MR effect size for
'TRIB2' on 'Pulmonary fibrosis'

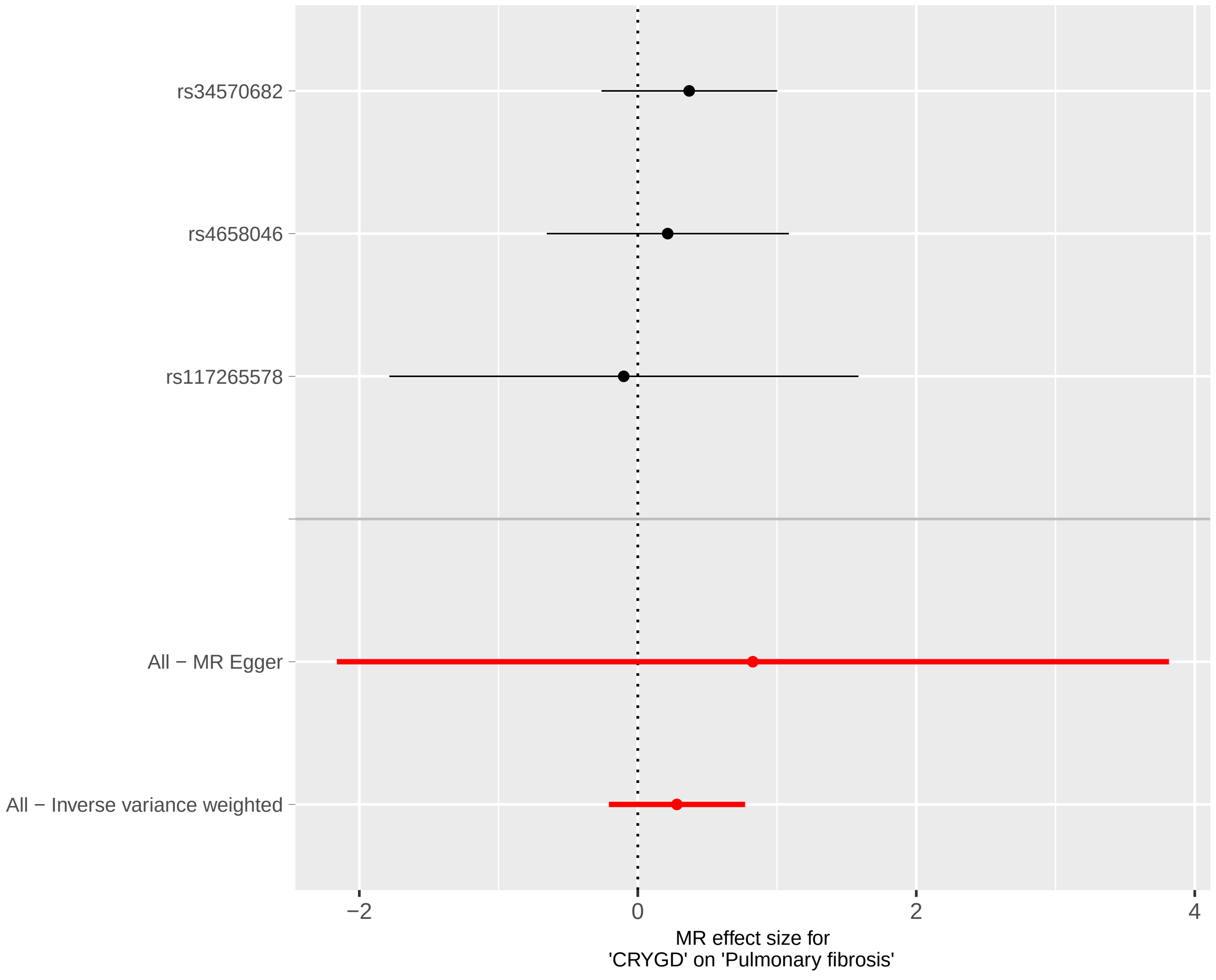
rs34570682
rs4658046
rs117265578
All − MR Egger
All − Inverse variance weighted
−2
0
2
4
MR effect size for
'CRYGD' on 'Pulmonary fibrosis'

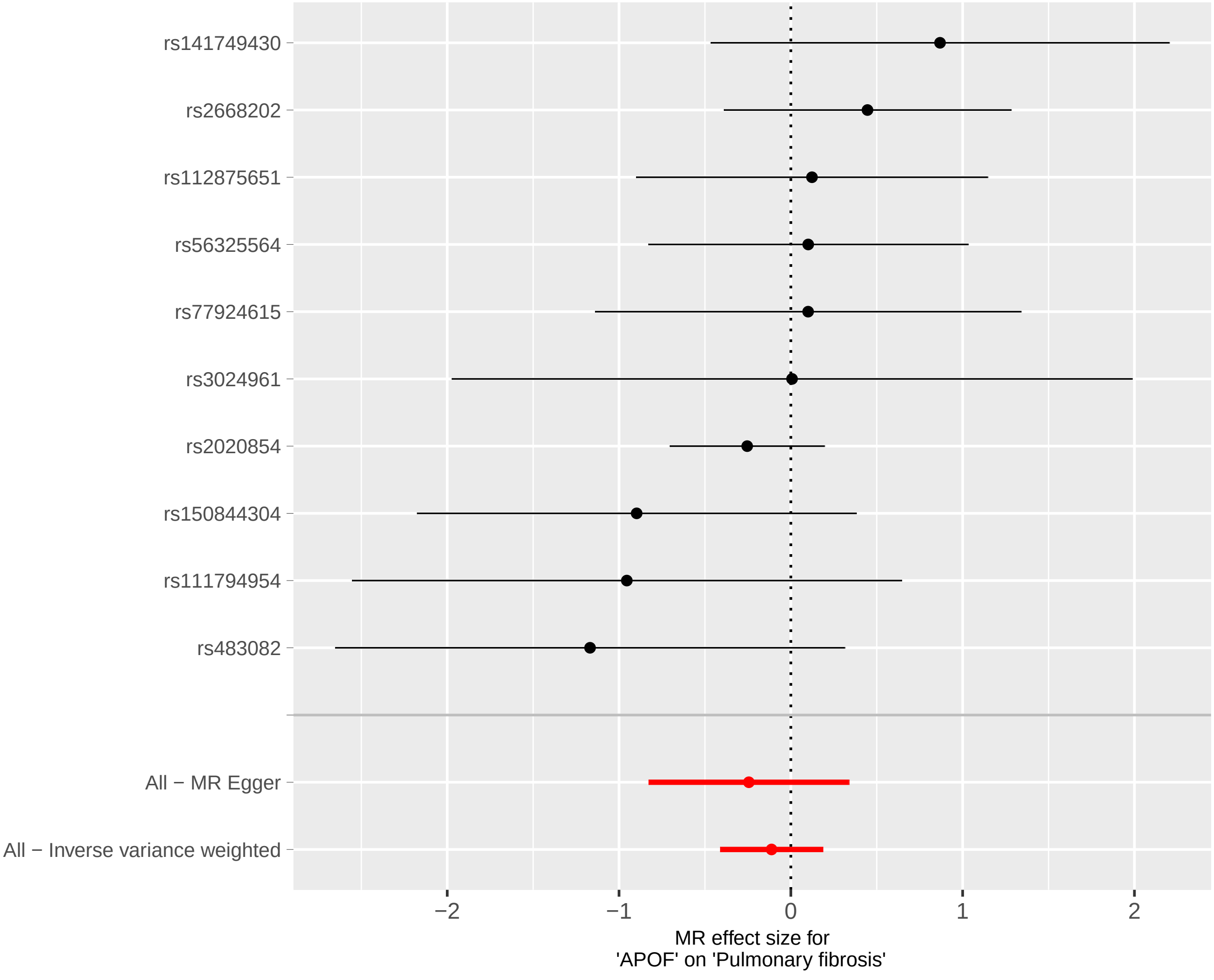
rs141749430
rs2668202
rs112875651
rs56325564
rs77924615
rs3024961
rs2020854
rs150844304
rs111794954
rs483082
All − MR Egger
All − Inverse variance weighted
−2
−1
0
1
2
MR effect size for
'APOF' on 'Pulmonary fibrosis'

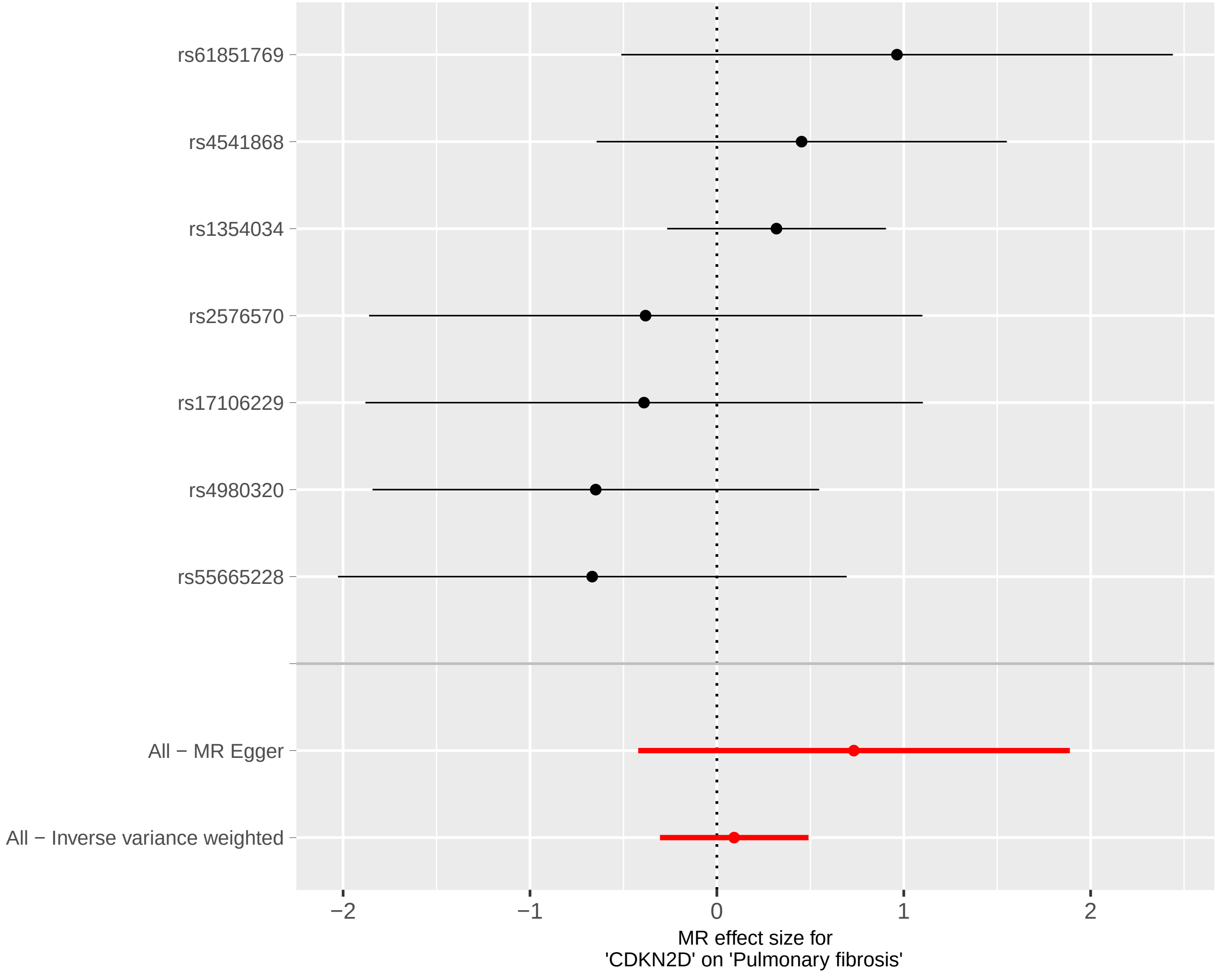

rs61851769
rs4541868
rs1354034
rs2576570
rs17106229
rs4980320
rs55665228
All − MR Egger
All − Inverse variance weighted
−2
−1
0
1
2
MR effect size for
'CDKN2D' on 'Pulmonary fibrosis'

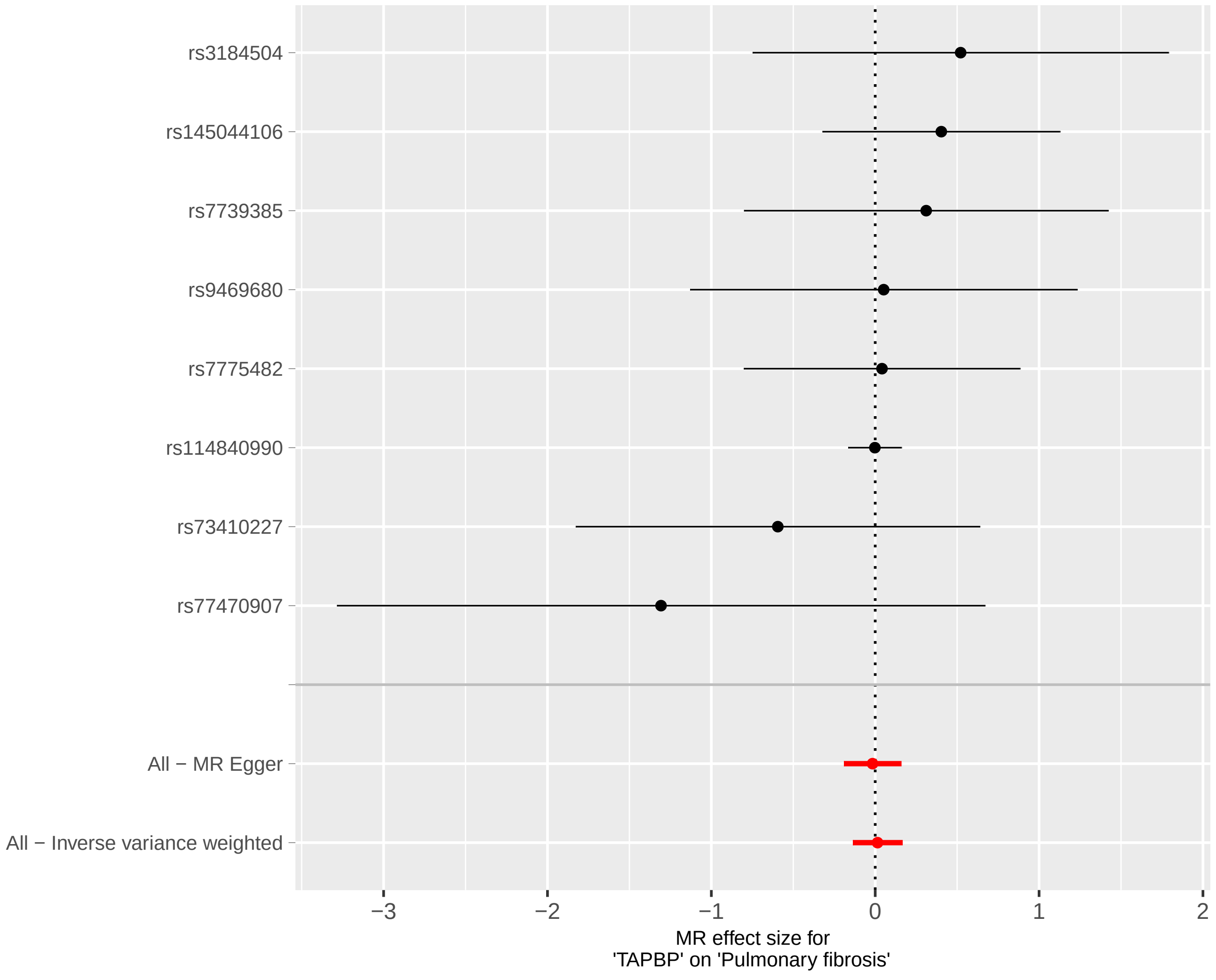
rs3184504
rs145044106
rs7739385
rs9469680
rs7775482
rs114840990
rs73410227
rs77470907
All − MR Egger
All − Inverse variance weighted
−3
−2
−1
0
1
2
MR effect size for
'TAPBP' on 'Pulmonary fibrosis'

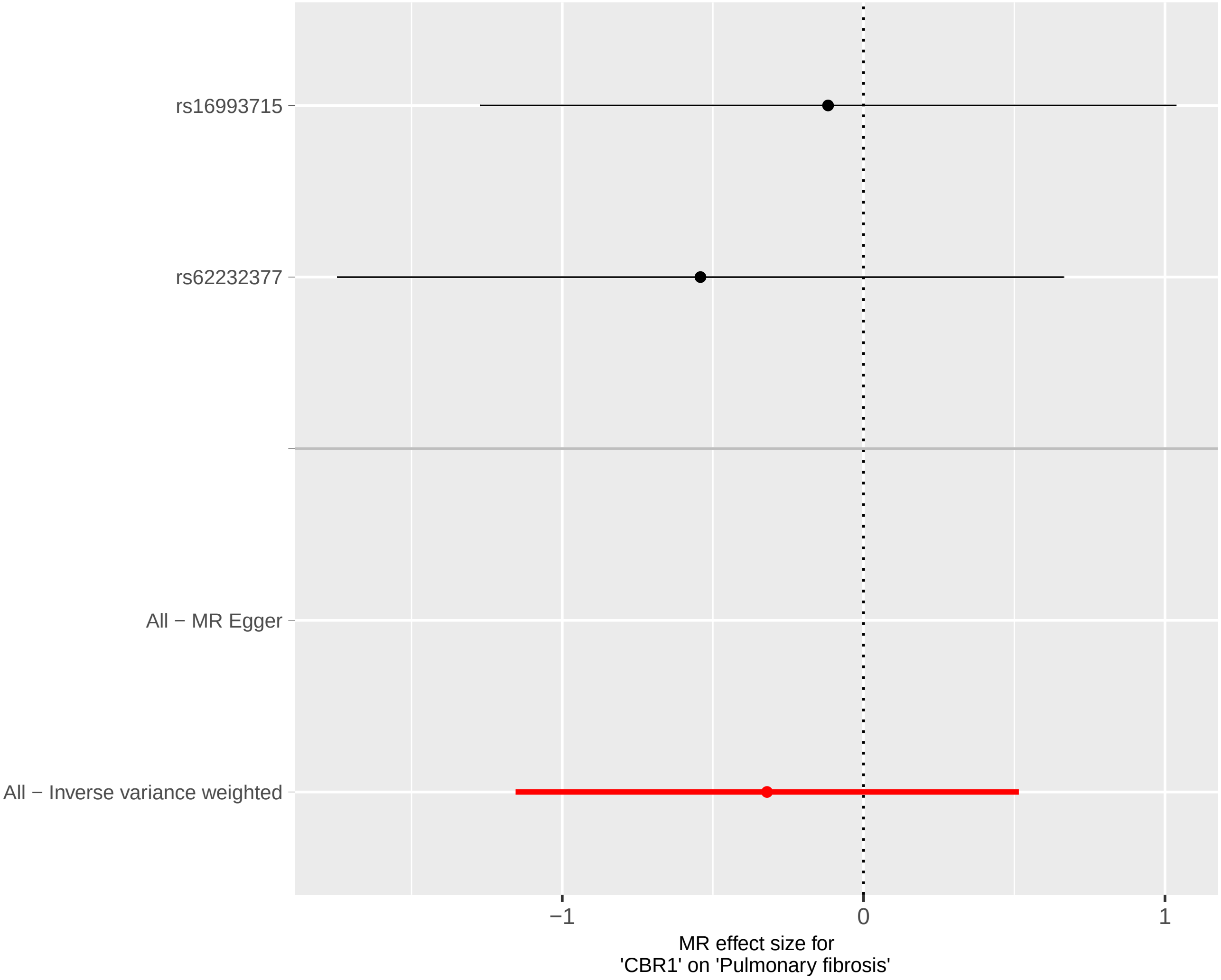
rs16993715
rs62232377
All − MR Egger
All − Inverse variance weighted
−1
0
1
MR effect size for
'CBR1' on 'Pulmonary fibrosis'

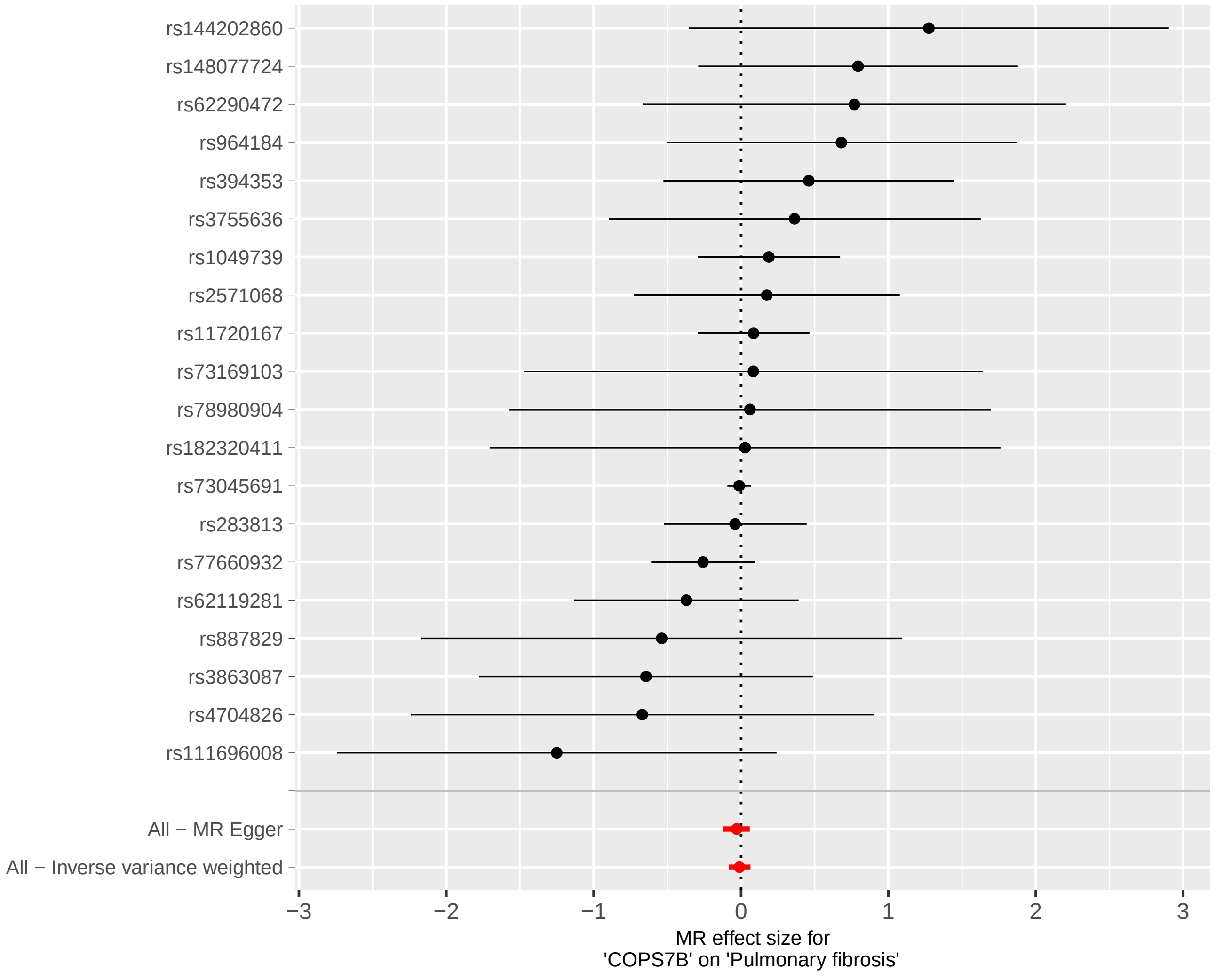
rs144202860
rs148077724
rs62290472
rs964184
rs394353
rs3755636
rs1049739
rs2571068
rs11720167
rs73169103
rs78980904
rs182320411
rs73045691
rs283813
rs77660932
rs62119281
rs887829
rs3863087
rs4704826
rs111696008
All − MR Egger
All − Inverse variance weighted
−3
−2
−1
0
1
2
3
MR effect size for
'COPS7B' on 'Pulmonary fibrosis'

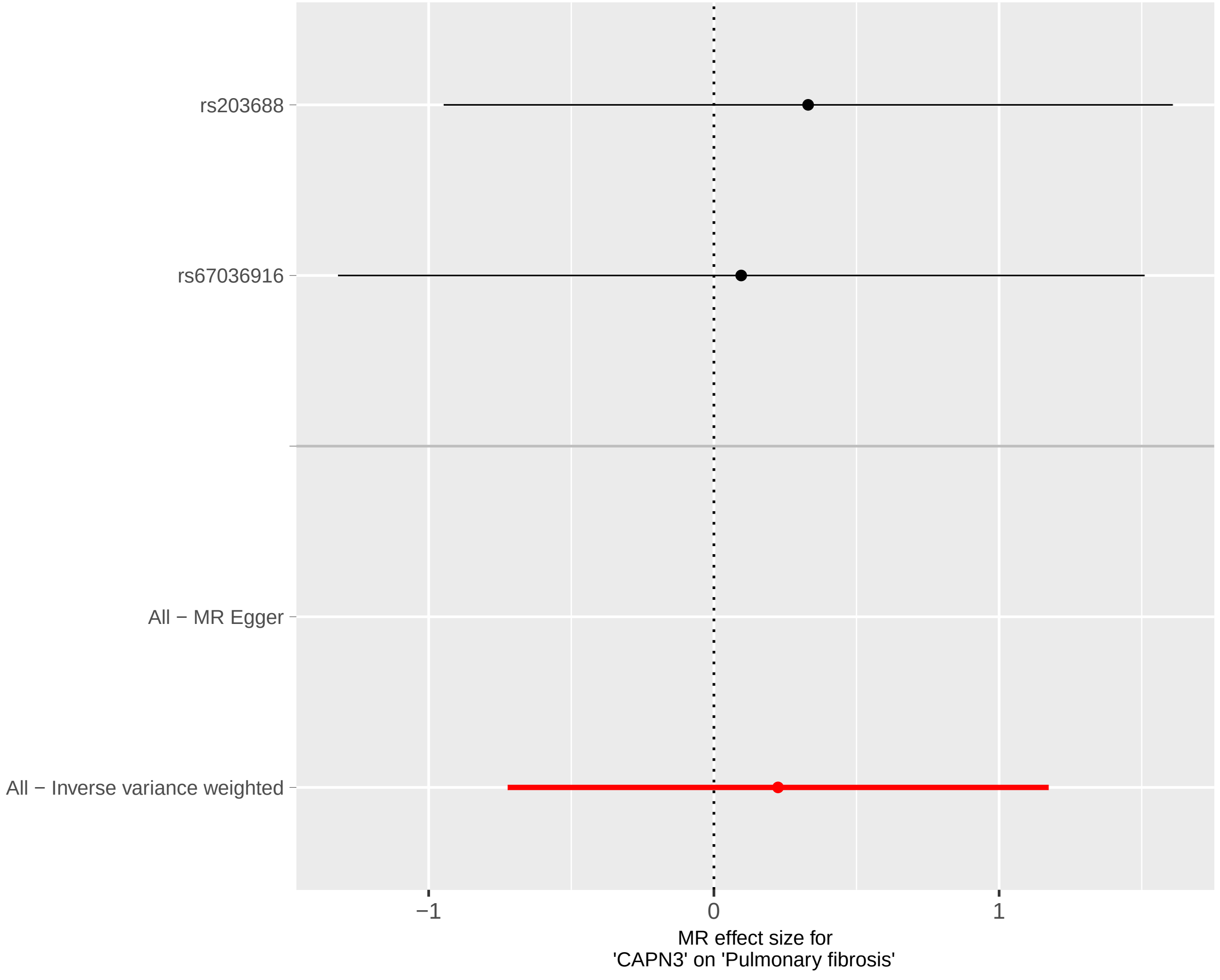

rs203688
rs67036916
All − MR Egger
All − Inverse variance weighted
−1
0
1
MR effect size for
'CAPN3' on 'Pulmonary fibrosis'

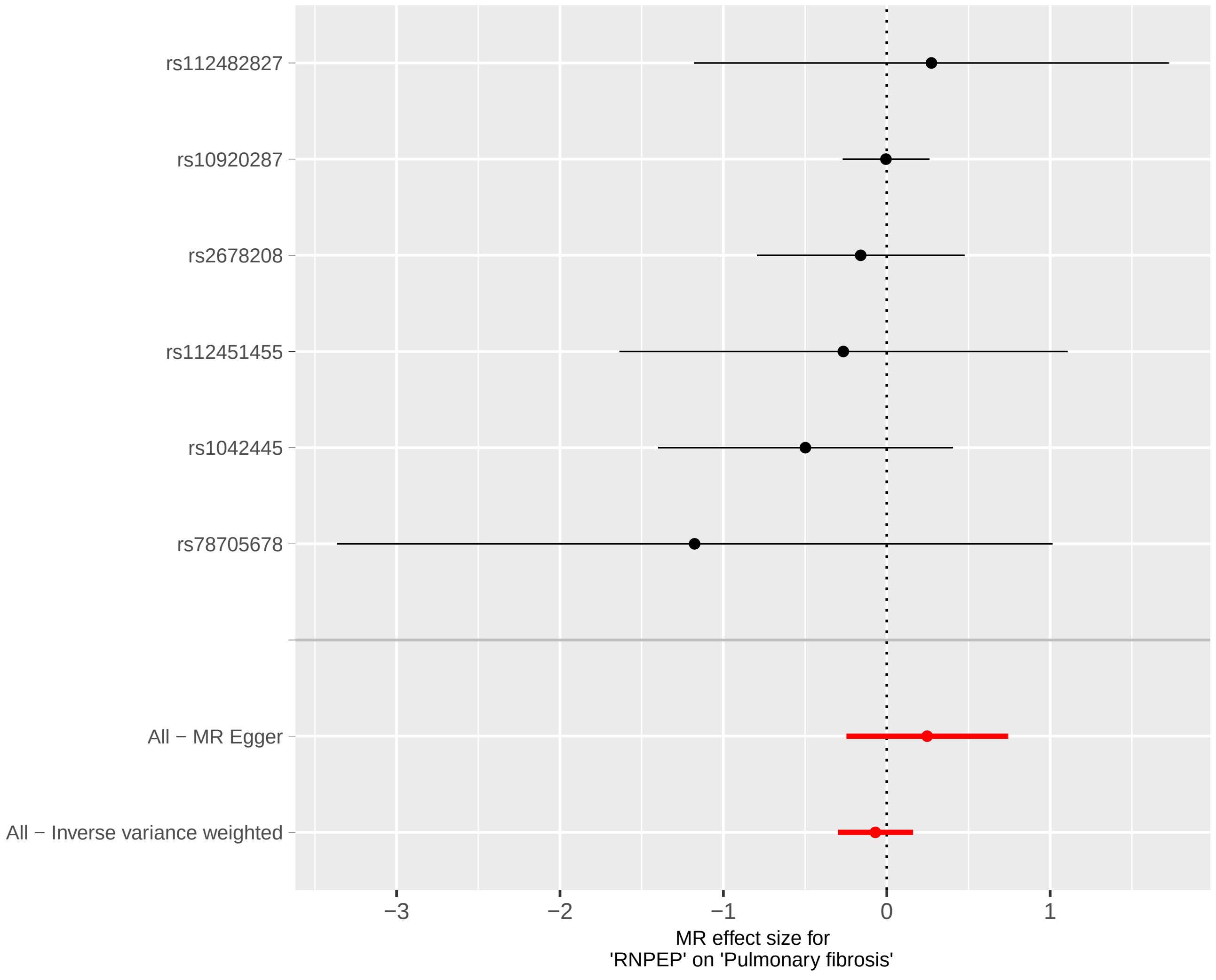

rs112482827
rs10920287
rs2678208
rs112451455
rs1042445
rs78705678
All − MR Egger
All − Inverse variance weighted
−3
−2
−1
0
1
MR effect size for
'RNPEP' on 'Pulmonary fibrosis'

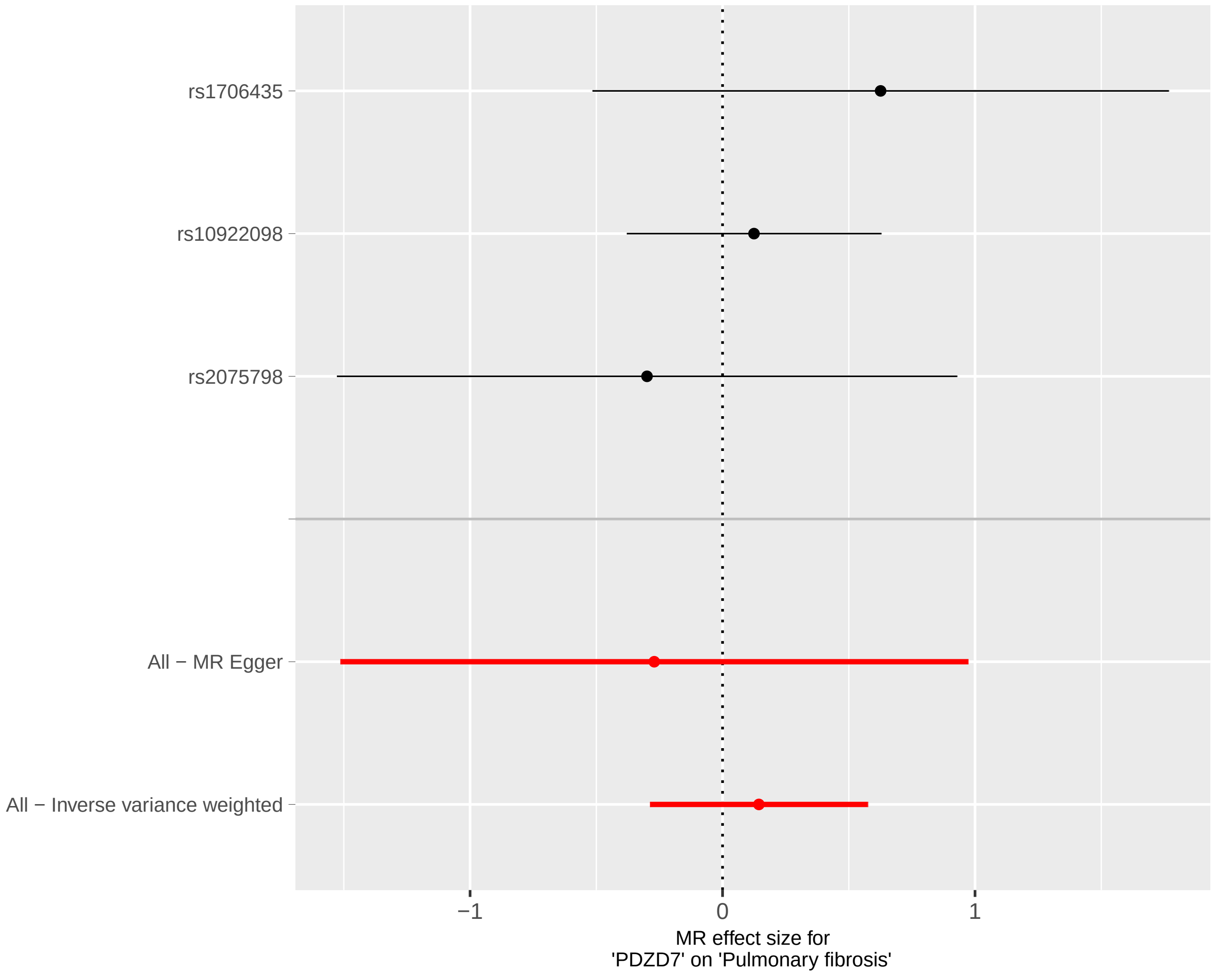
rs1706435
rs10922098
rs2075798
All − MR Egger
All − Inverse variance weighted
−1
0
1
MR effect size for
'PDZD7' on 'Pulmonary fibrosis'

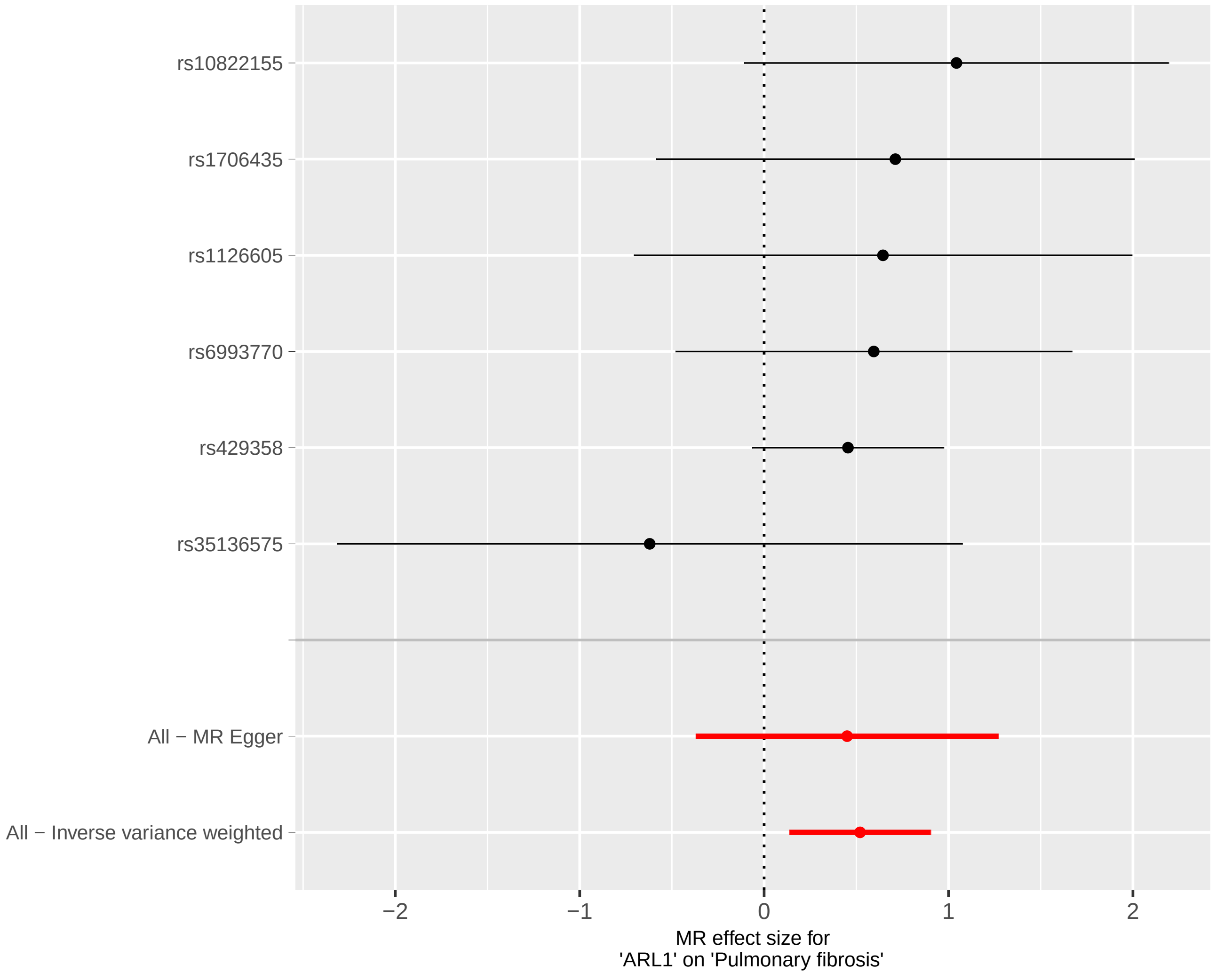

rs10822155
rs1706435
rs1126605
rs6993770
rs429358
rs35136575
All − MR Egger
All − Inverse variance weighted
−2
−1
0
1
2
MR effect size for
'ARL1' on 'Pulmonary fibrosis'

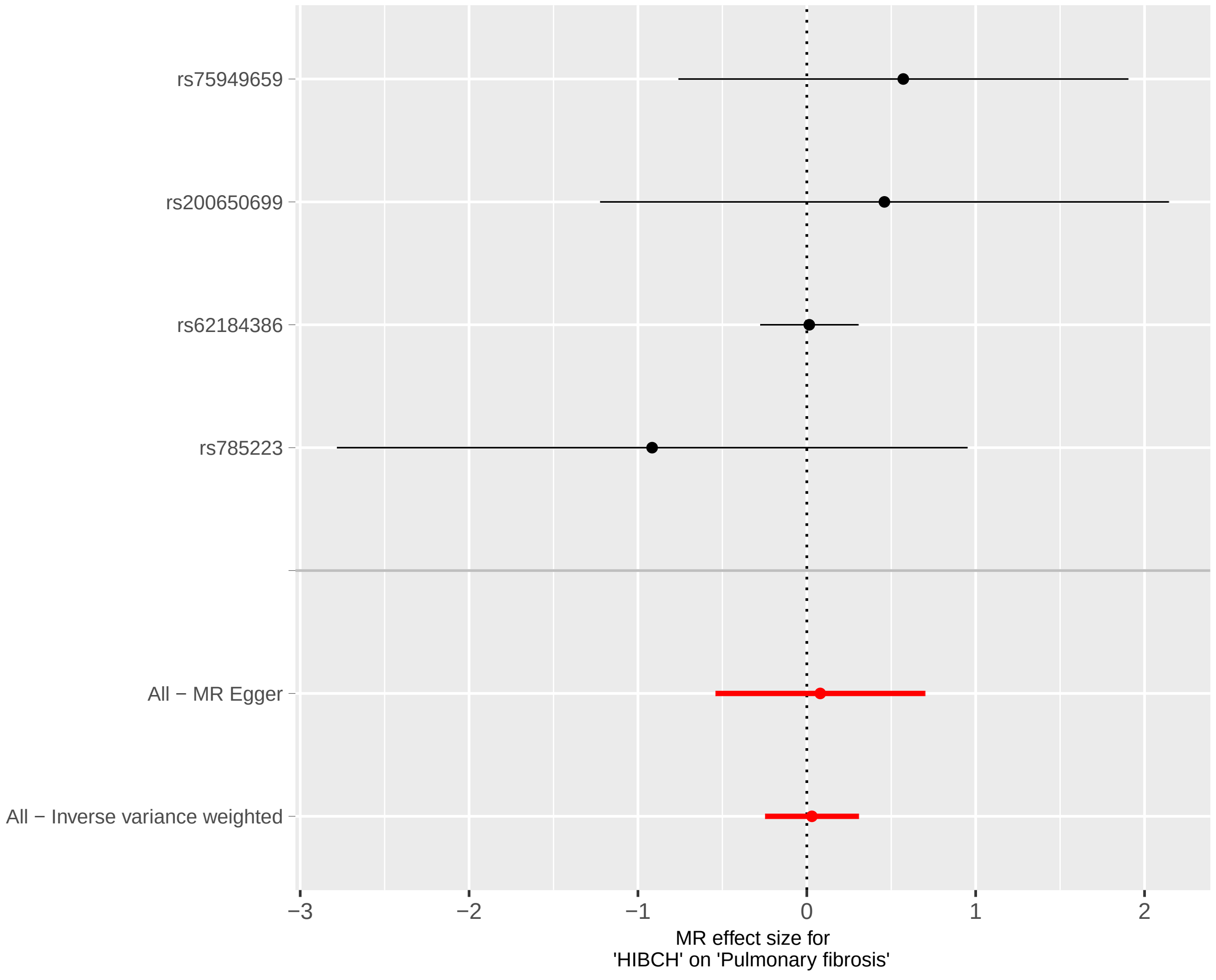

rs75949659
rs200650699
rs62184386
rs785223
All − MR Egger
All − Inverse variance weighted
−3
−2
−1
0
1
2
MR effect size for
'HIBCH' on 'Pulmonary fibrosis'

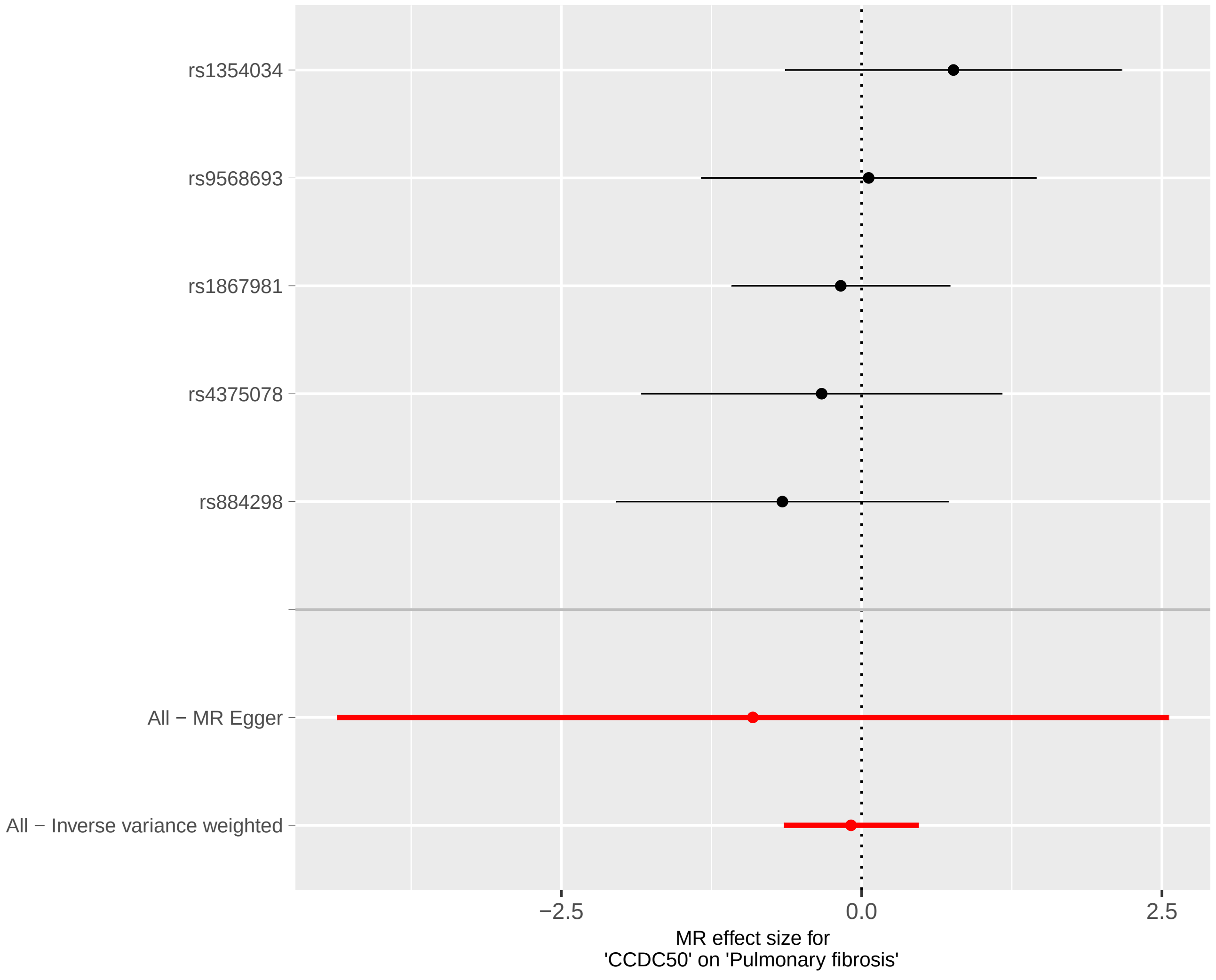
rs1354034
rs9568693
rs1867981
rs4375078
rs884298
All − MR Egger
All − Inverse variance weighted
−2.5
0.0
2.5
MR effect size for
'CCDC50' on 'Pulmonary fibrosis'

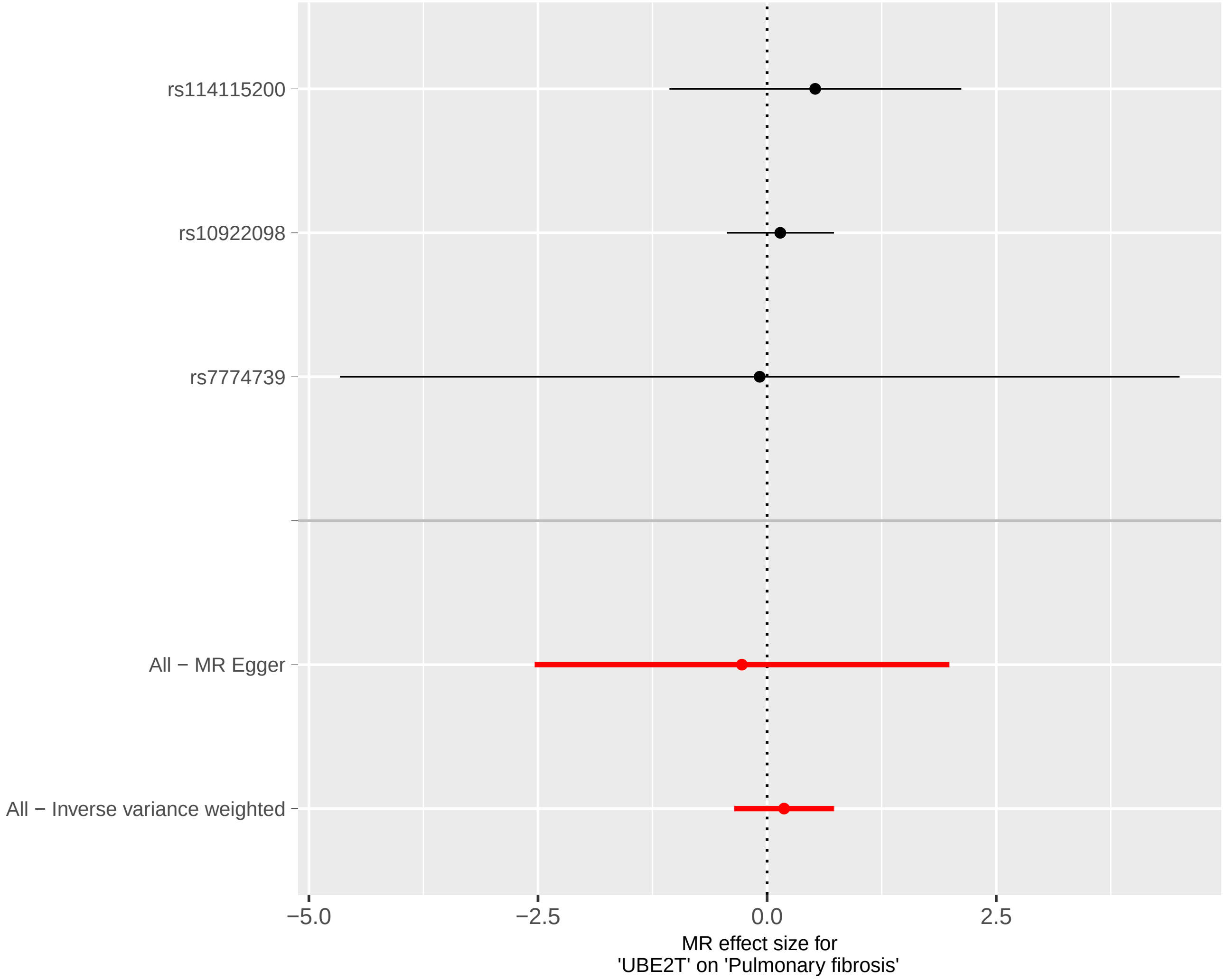
rs114115200
rs10922098
rs7774739
All − MR Egger
All − Inverse variance weighted
−5.0
−2.5
0.0
2.5
MR effect size for
'UBE2T' on 'Pulmonary fibrosis'

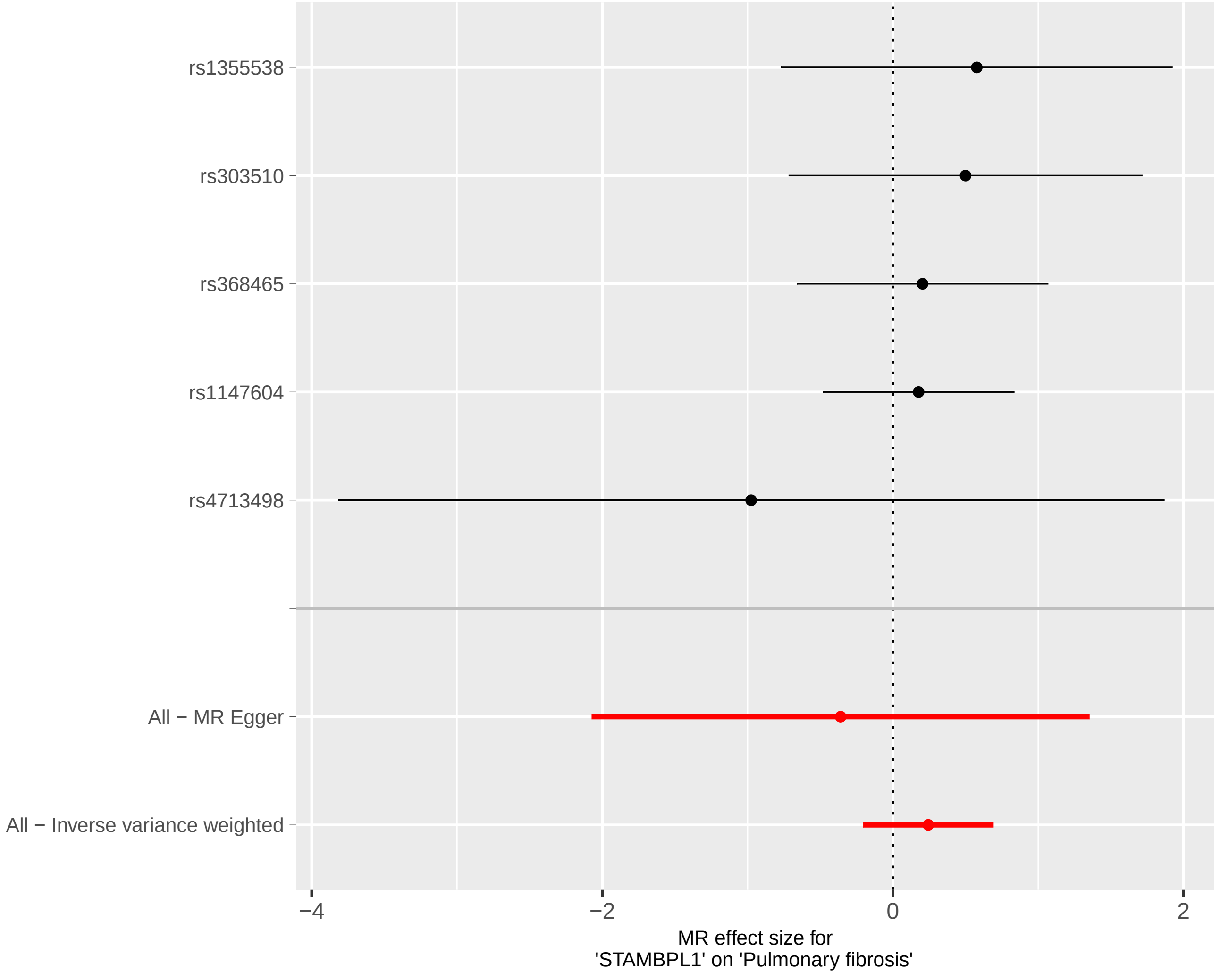
rs1355538
rs303510
rs368465
rs1147604
rs4713498
All − MR Egger
All − Inverse variance weighted
−4
−2
0
2
MR effect size for
'STAMBPL1' on 'Pulmonary fibrosis'

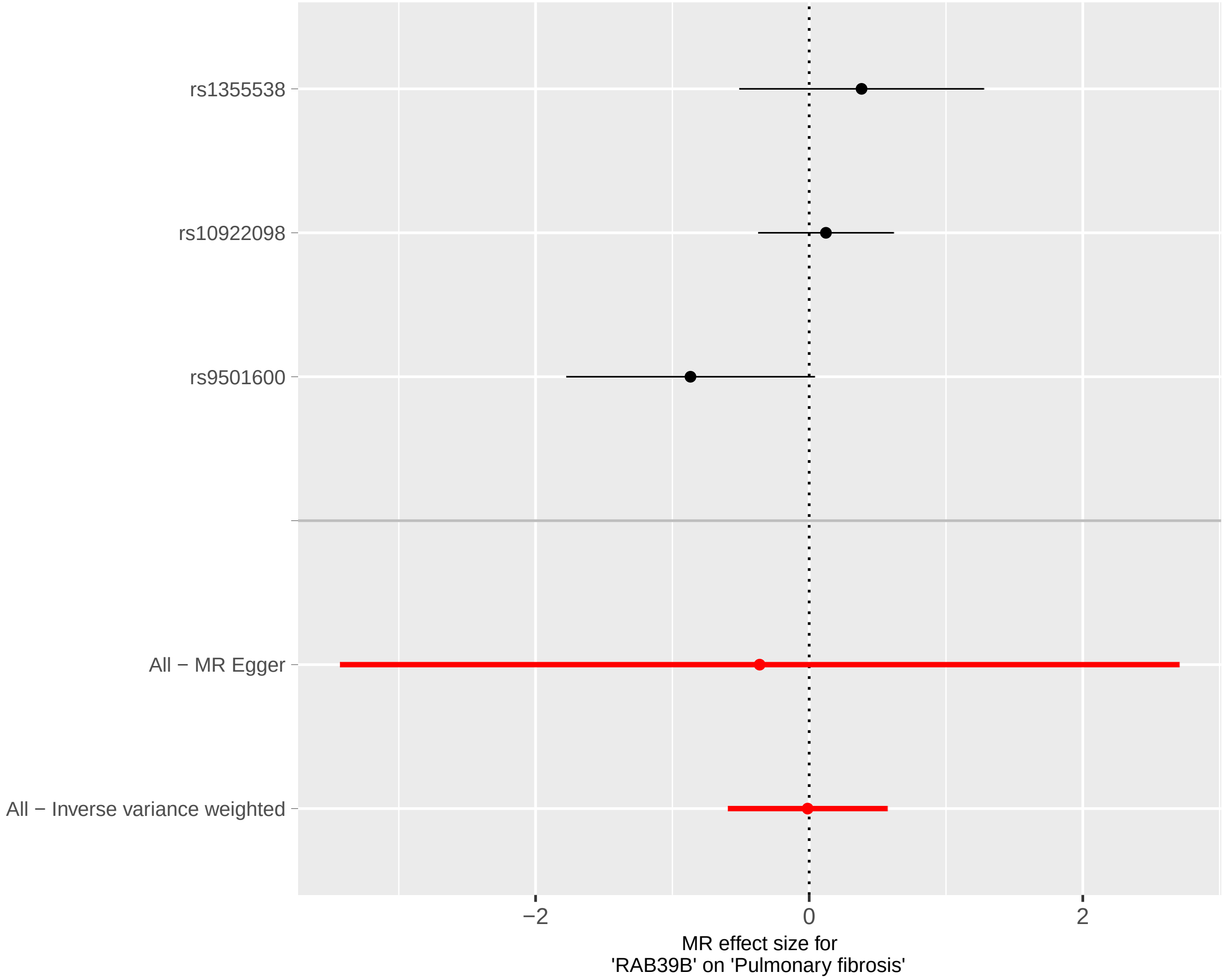

rs1355538
rs10922098
rs9501600
All − MR Egger
All − Inverse variance weighted
−2
0
2
MR effect size for
'RAB39B' on 'Pulmonary fibrosis'

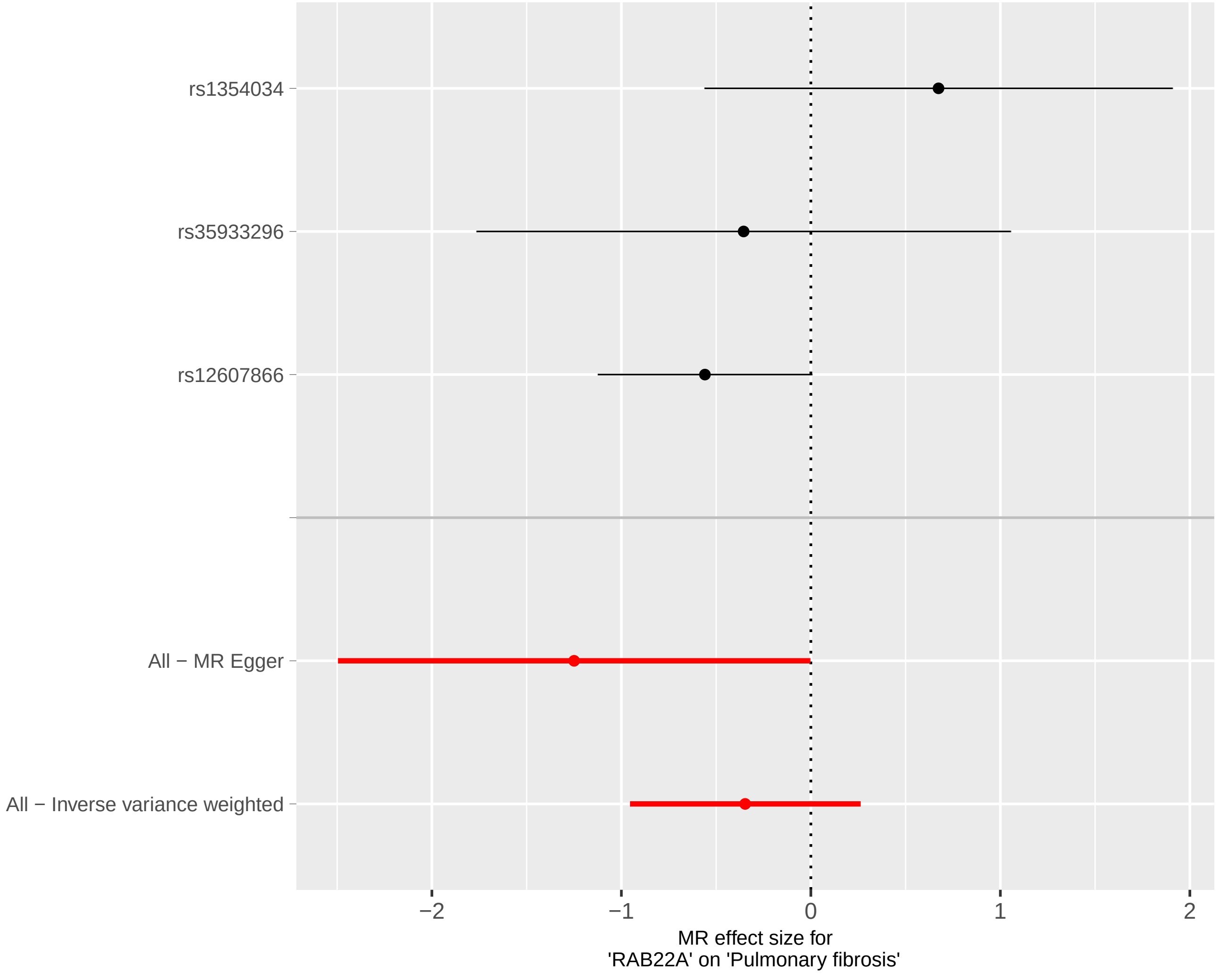
rs1354034
rs35933296
rs12607866
All − MR Egger
All − Inverse variance weighted
−2
−1
0
1
2
MR effect size for
'RAB22A' on 'Pulmonary fibrosis'

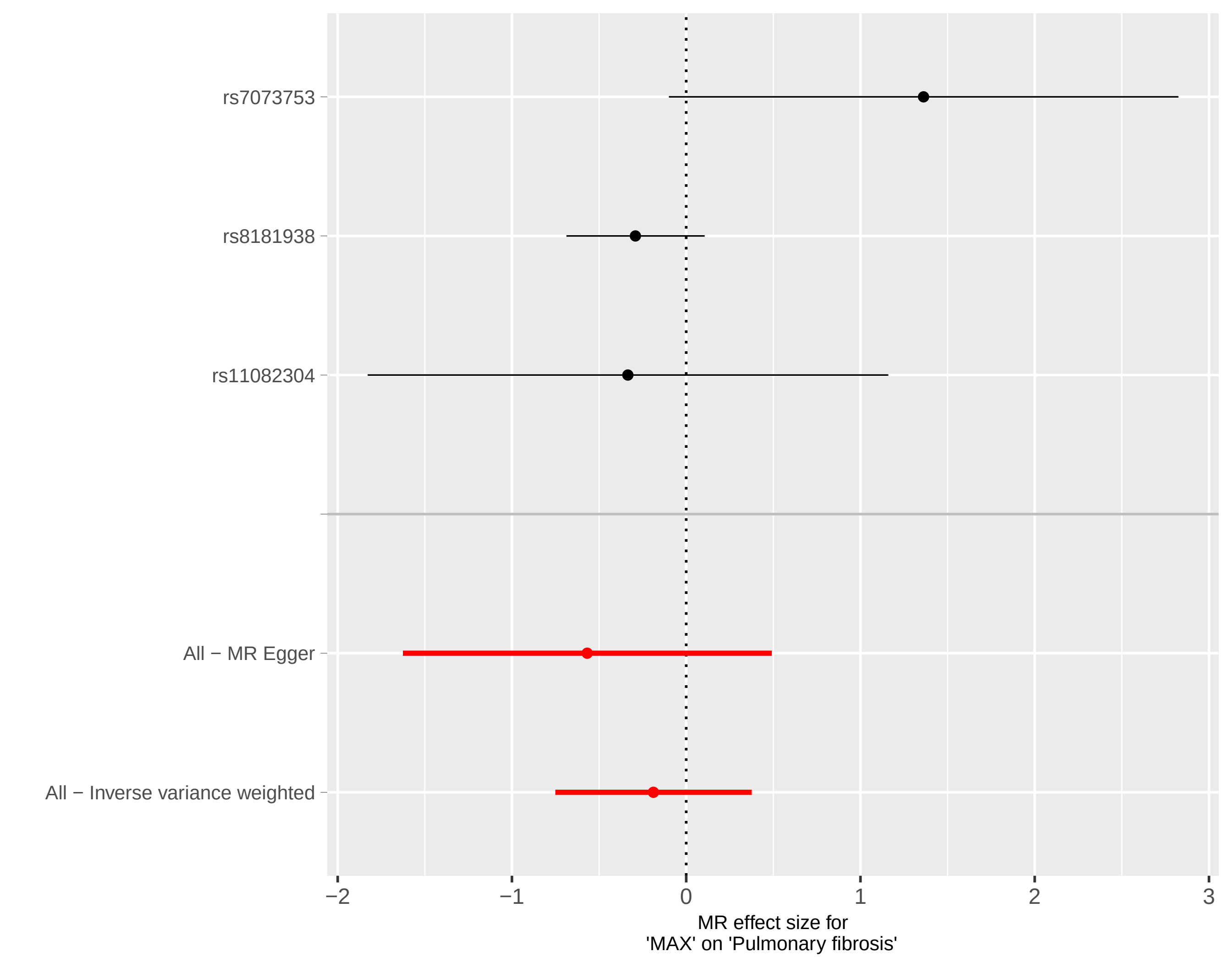

rs7073753
rs8181938
rs11082304
All − MR Egger
All − Inverse variance weighted
−2
−1
0
1
2
3
MR effect size for
'MAX' on 'Pulmonary fibrosis'

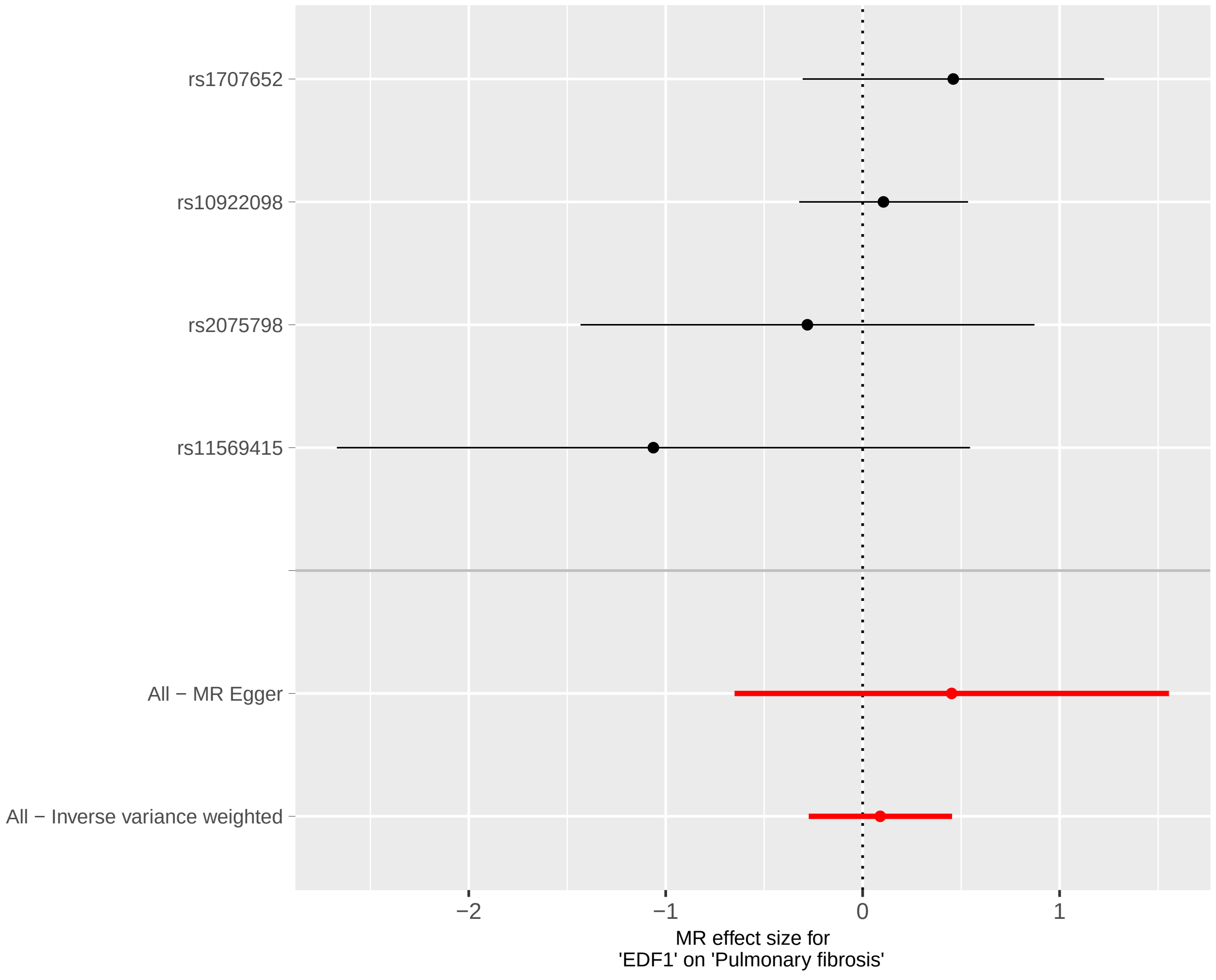
rs1707652
rs10922098
rs2075798
rs11569415
All − MR Egger
All − Inverse variance weighted
−2
−1
0
1
MR effect size for
'EDF1' on 'Pulmonary fibrosis'

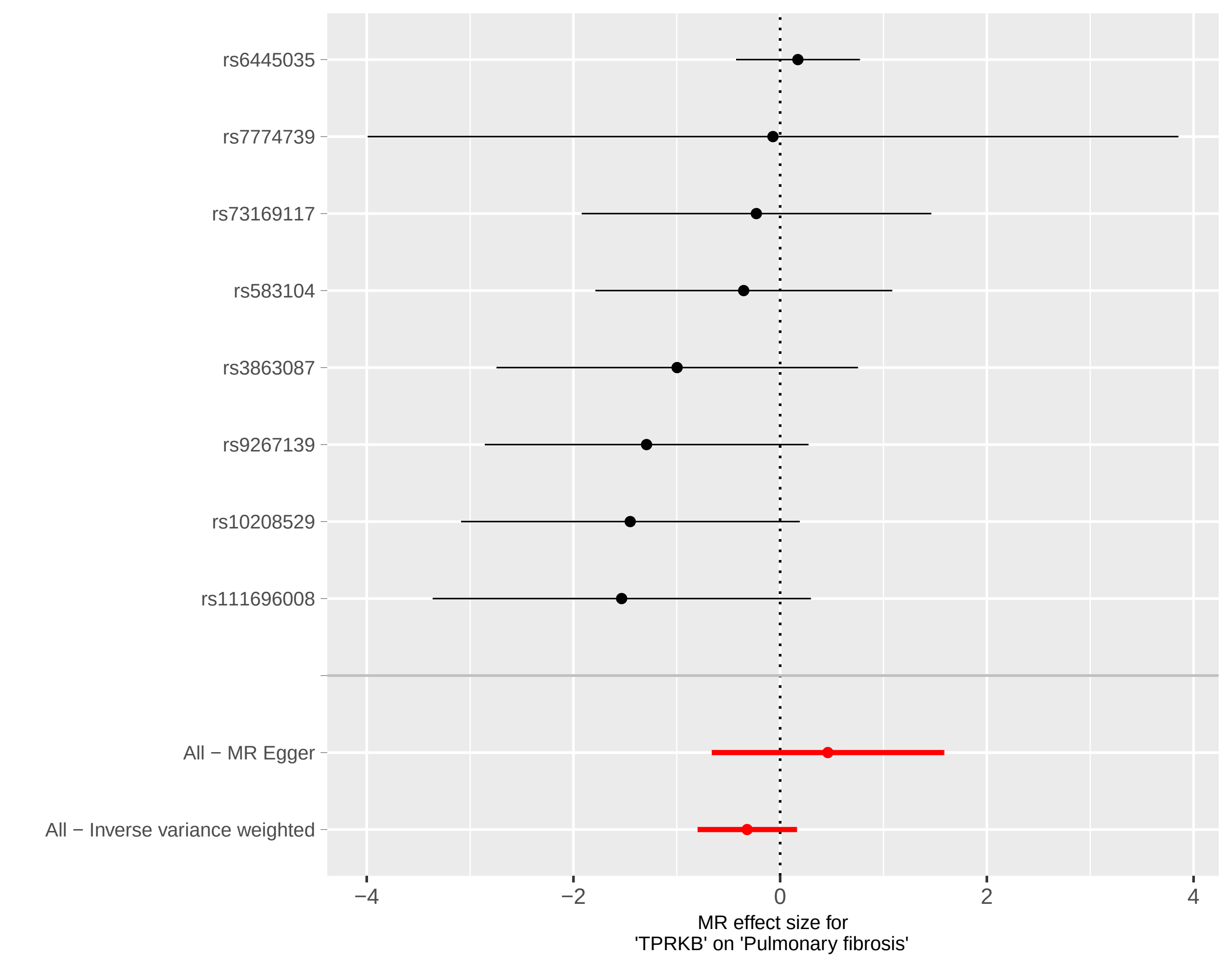
rs6445035
rs7774739
rs73169117
rs583104
rs3863087
rs9267139
rs10208529
rs111696008
All − MR Egger
All − Inverse variance weighted
−4
−2
0
2
4
MR effect size for
'TPRKB' on 'Pulmonary fibrosis'

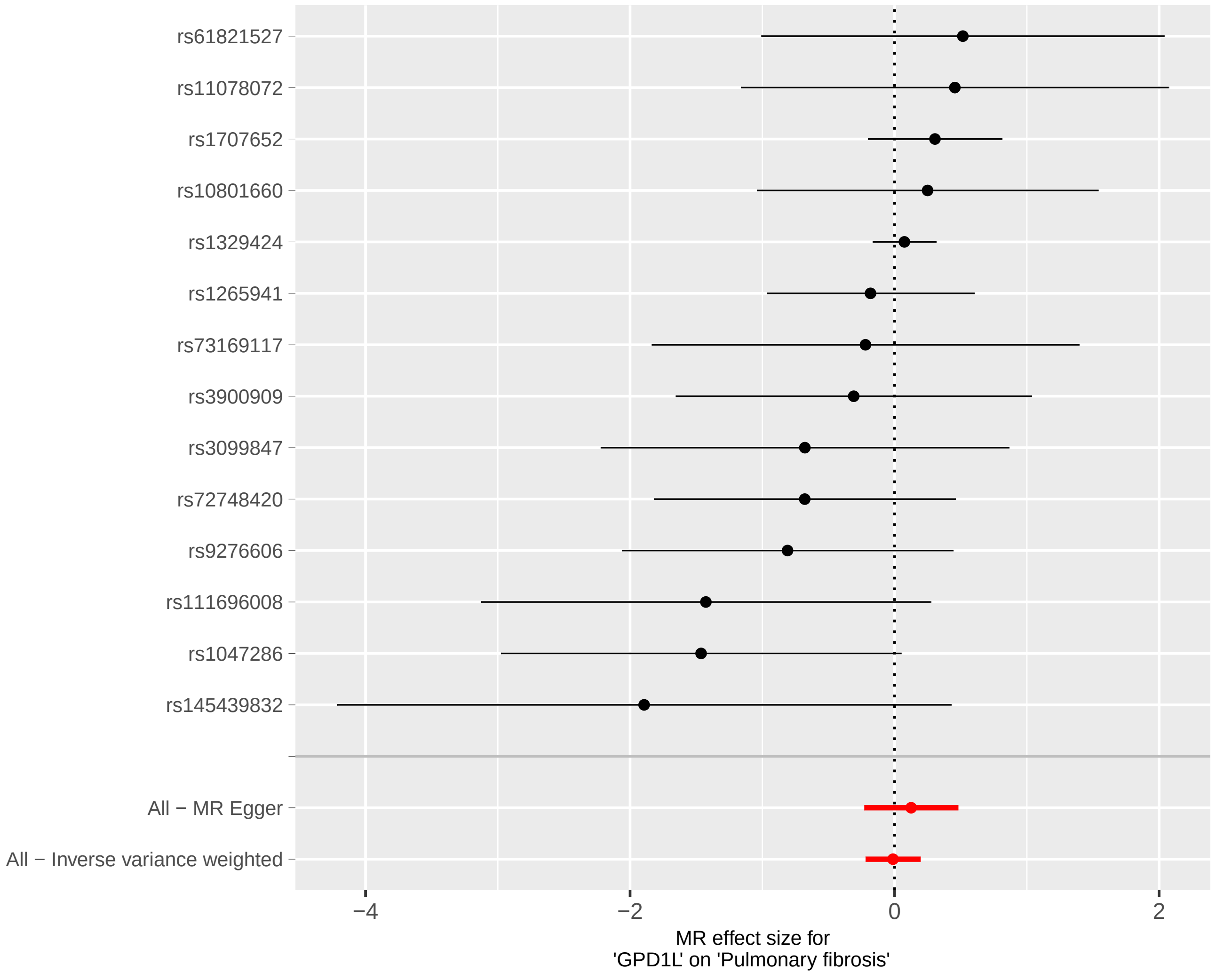
rs61821527
rs11078072
rs1707652
rs10801660
rs1329424
rs1265941
rs73169117
rs3900909
rs3099847
rs72748420
rs9276606
rs111696008
rs1047286
rs145439832
All − MR Egger
All − Inverse variance weighted
−4
−2
0
2
MR effect size for
'GPD1L' on 'Pulmonary fibrosis'

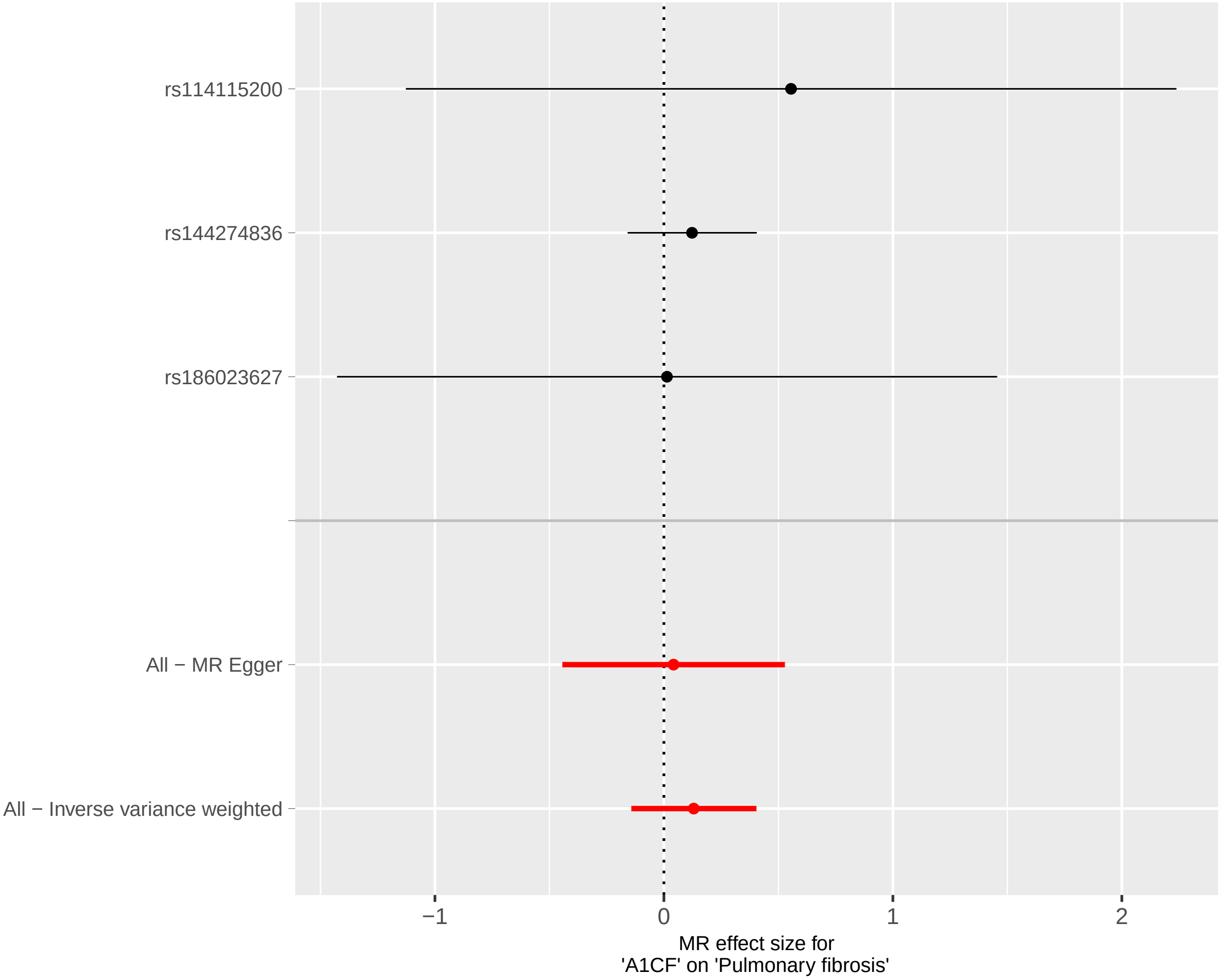
rs114115200
rs144274836
rs186023627
All − MR Egger
All − Inverse variance weighted
−1
0
1
2
MR effect size for
'A1CF' on 'Pulmonary fibrosis'

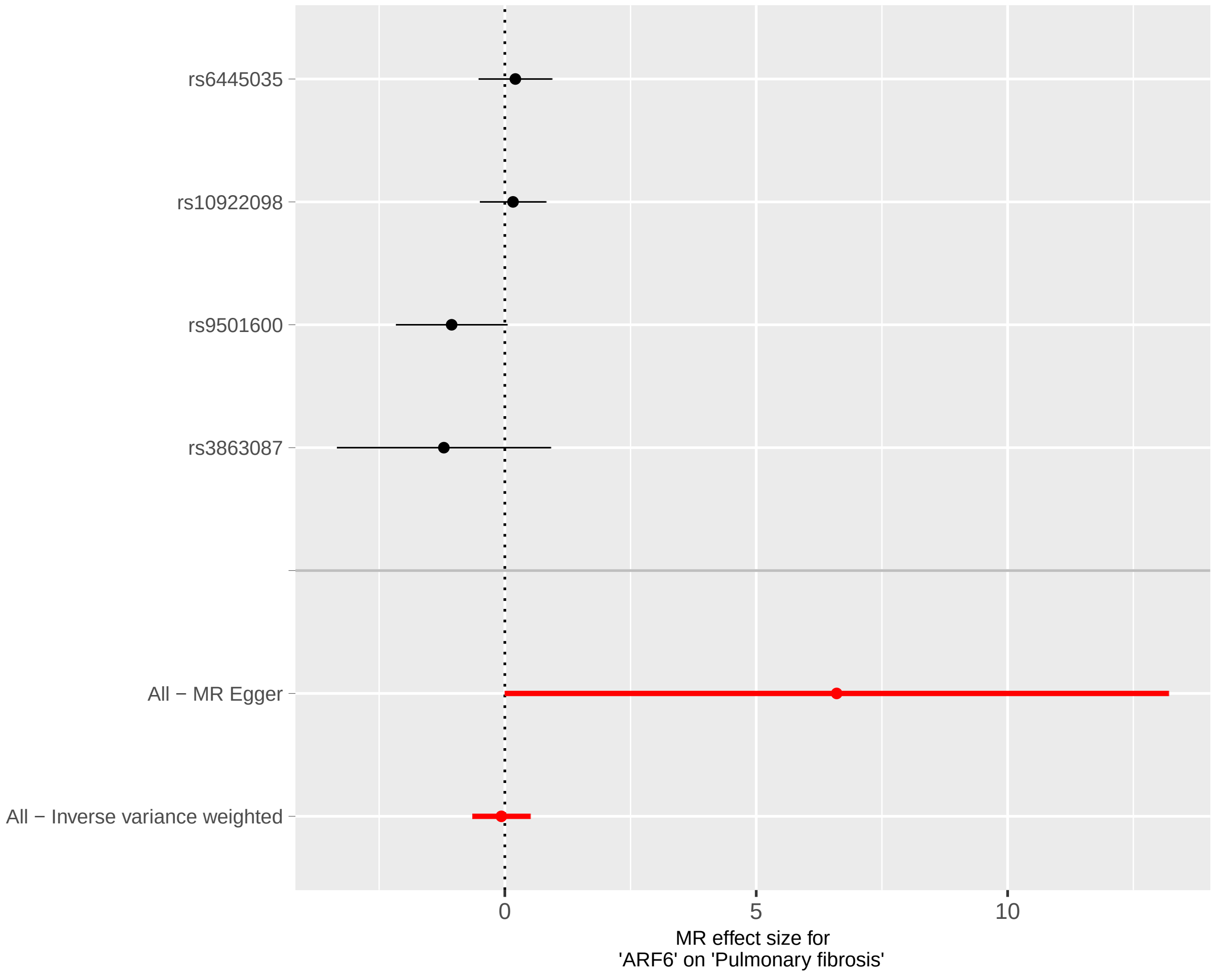
rs6445035
rs10922098
rs9501600
rs3863087
All − MR Egger
All − Inverse variance weighted
0
5
10
MR effect size for
'ARF6' on 'Pulmonary fibrosis'

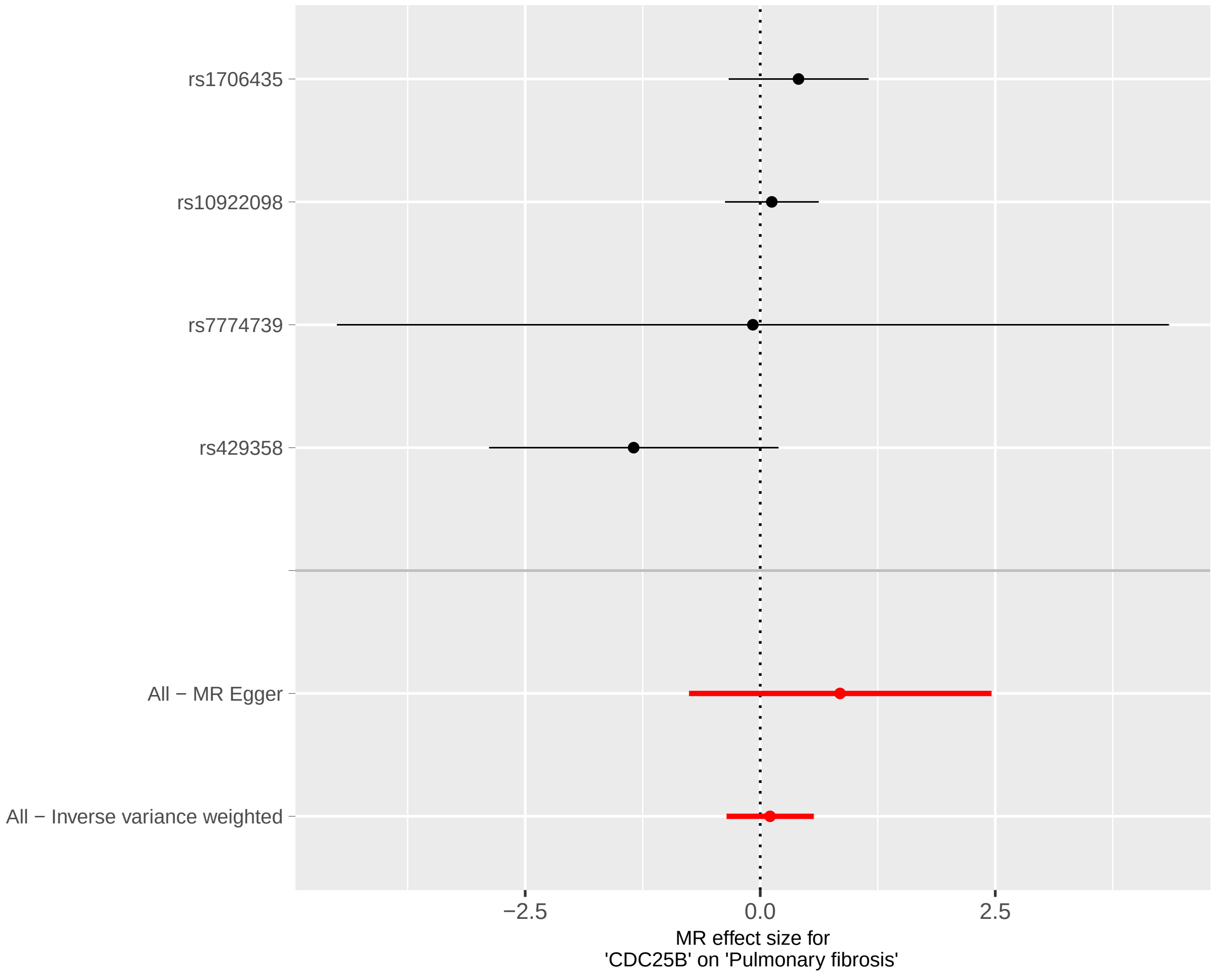

rs1706435
rs10922098
rs7774739
rs429358
All − MR Egger
All − Inverse variance weighted
−2.5
0.0
2.5
MR effect size for
'CDC25B' on 'Pulmonary fibrosis'

Insufficient number of SNPs

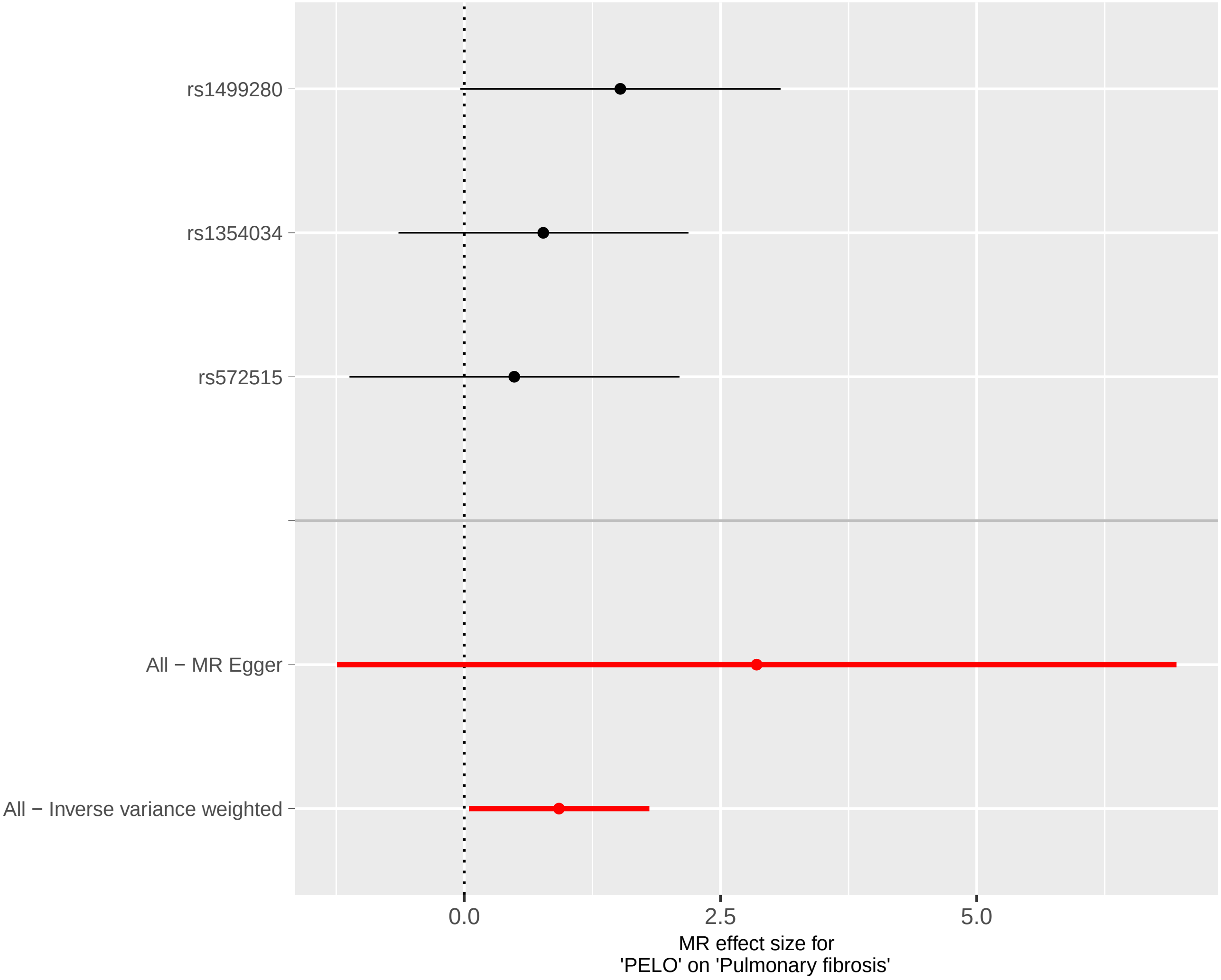

rs1499280
rs1354034
rs572515
All − MR Egger
All − Inverse variance weighted
0.0
2.5
5.0
MR effect size for
'PELO' on 'Pulmonary fibrosis'

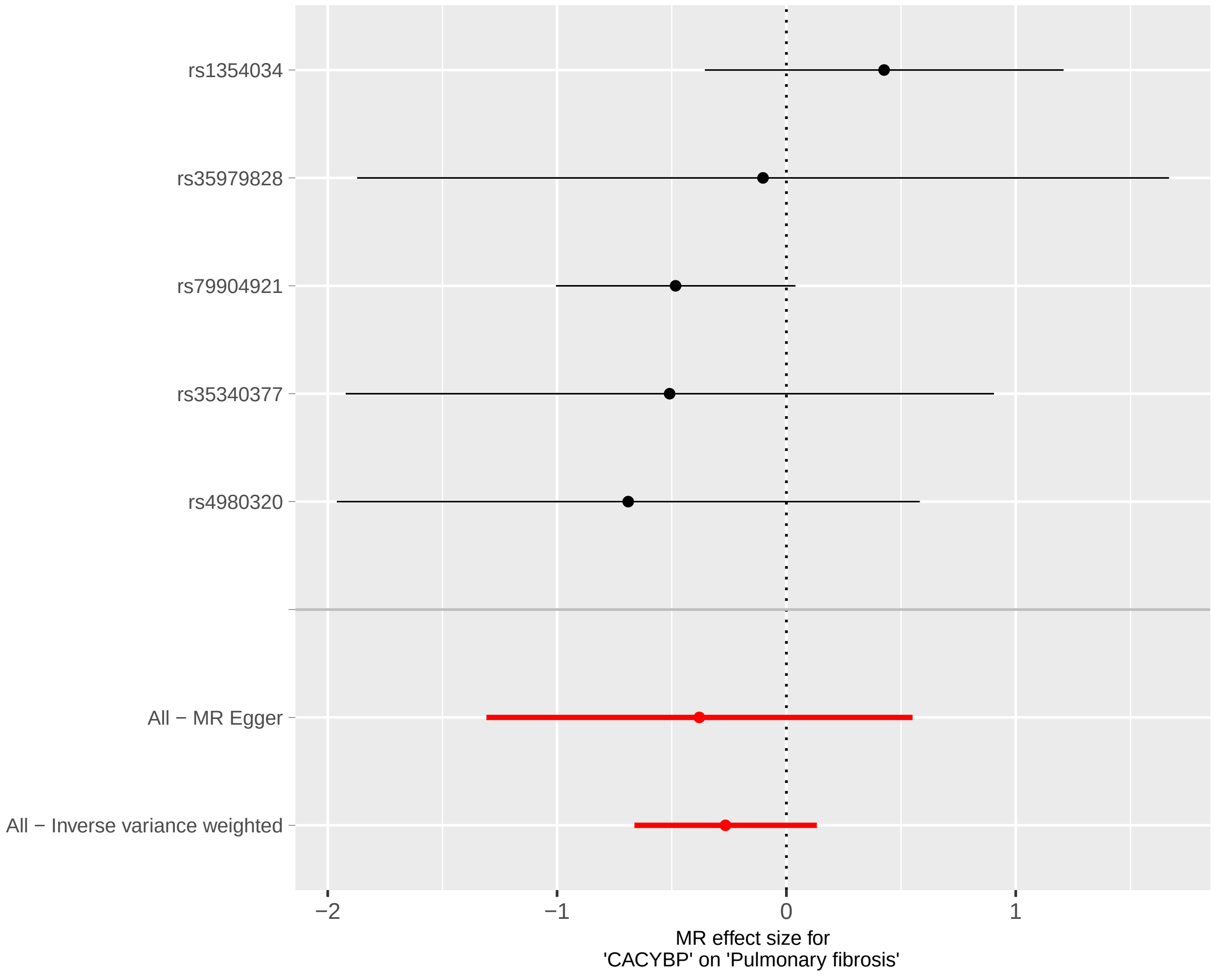
rs1354034
rs35979828
rs79904921
rs35340377
rs4980320
All − MR Egger
All − Inverse variance weighted
−2
−1
0
1
MR effect size for
'CACYBP' on 'Pulmonary fibrosis'

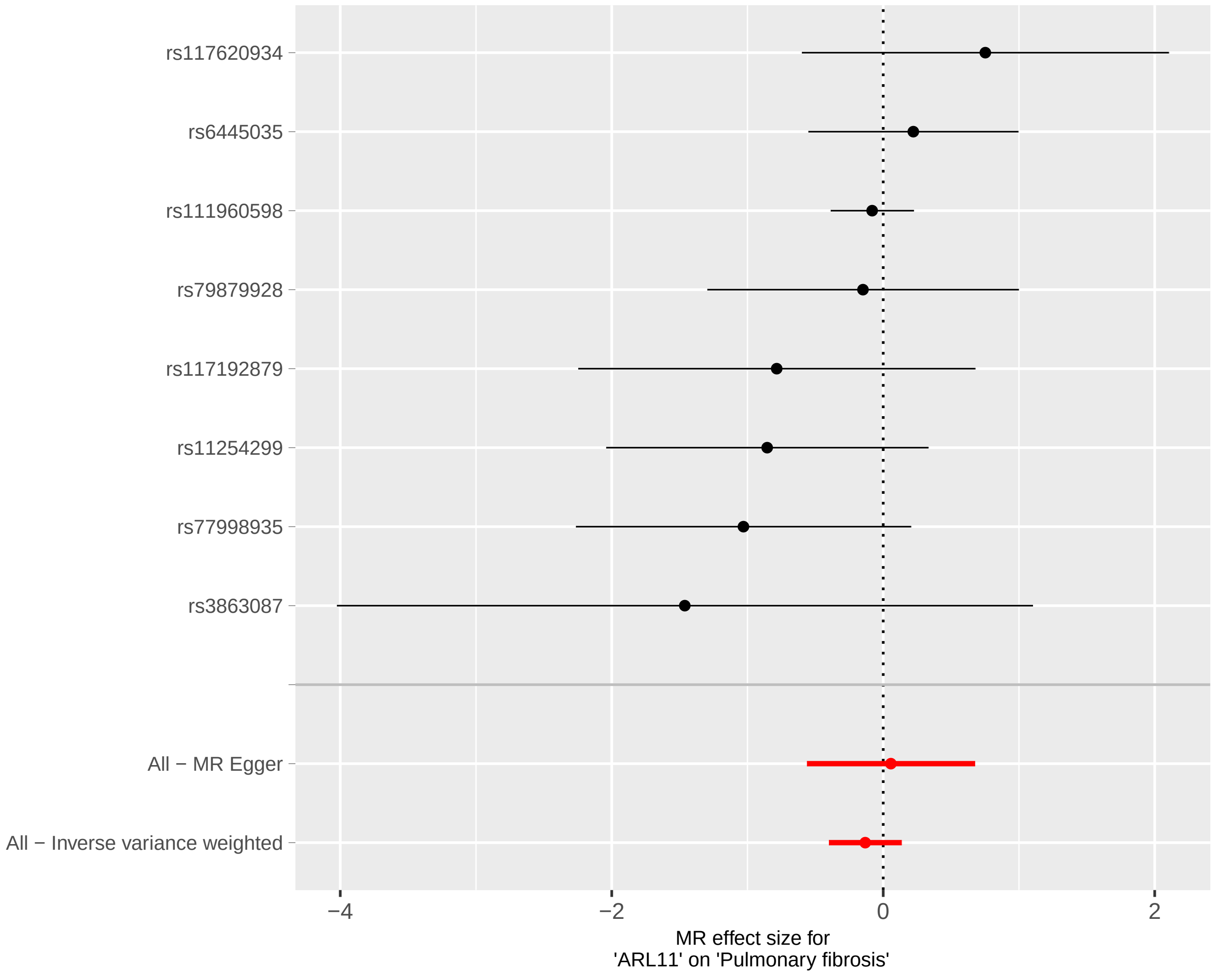
rs117620934
rs6445035
rs111960598
rs79879928
rs117192879
rs11254299
rs77998935
rs3863087
All − MR Egger
All − Inverse variance weighted
−4
−2
0
2
MR effect size for
'ARL11' on 'Pulmonary fibrosis'

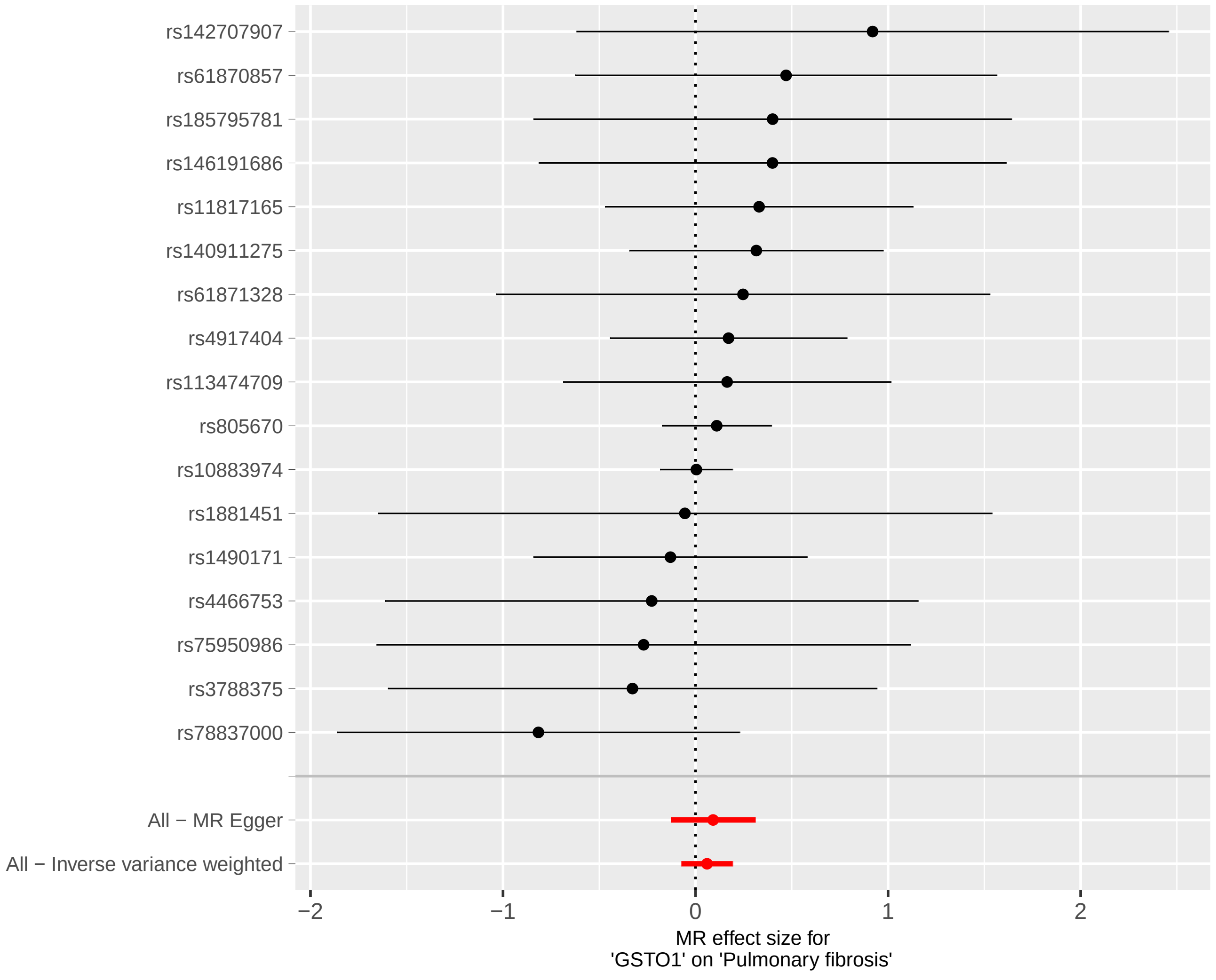

rs142707907
rs61870857
rs185795781
rs146191686
rs11817165
rs140911275
rs61871328
rs4917404
rs113474709
rs805670
rs10883974
rs1881451
rs1490171
rs4466753
rs75950986
rs3788375
rs78837000
All − MR Egger
All − Inverse variance weighted
−2
−1
0
1
2
MR effect size for
'GSTO1' on 'Pulmonary fibrosis'

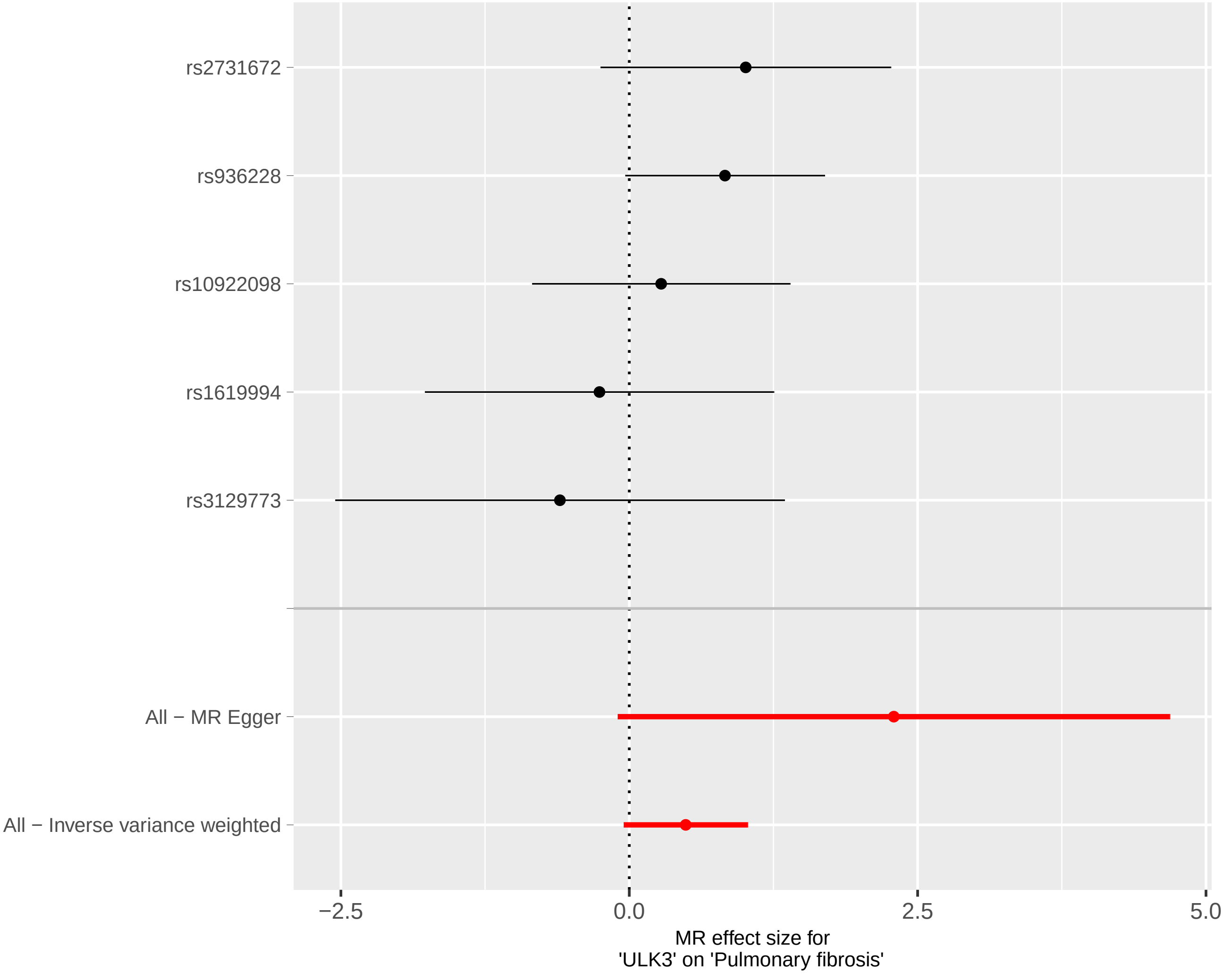

rs2731672
rs936228
rs10922098
rs1619994
rs3129773
All − MR Egger
All − Inverse variance weighted
−2.5
0.0
2.5
5.0
MR effect size for
'ULK3' on 'Pulmonary fibrosis'

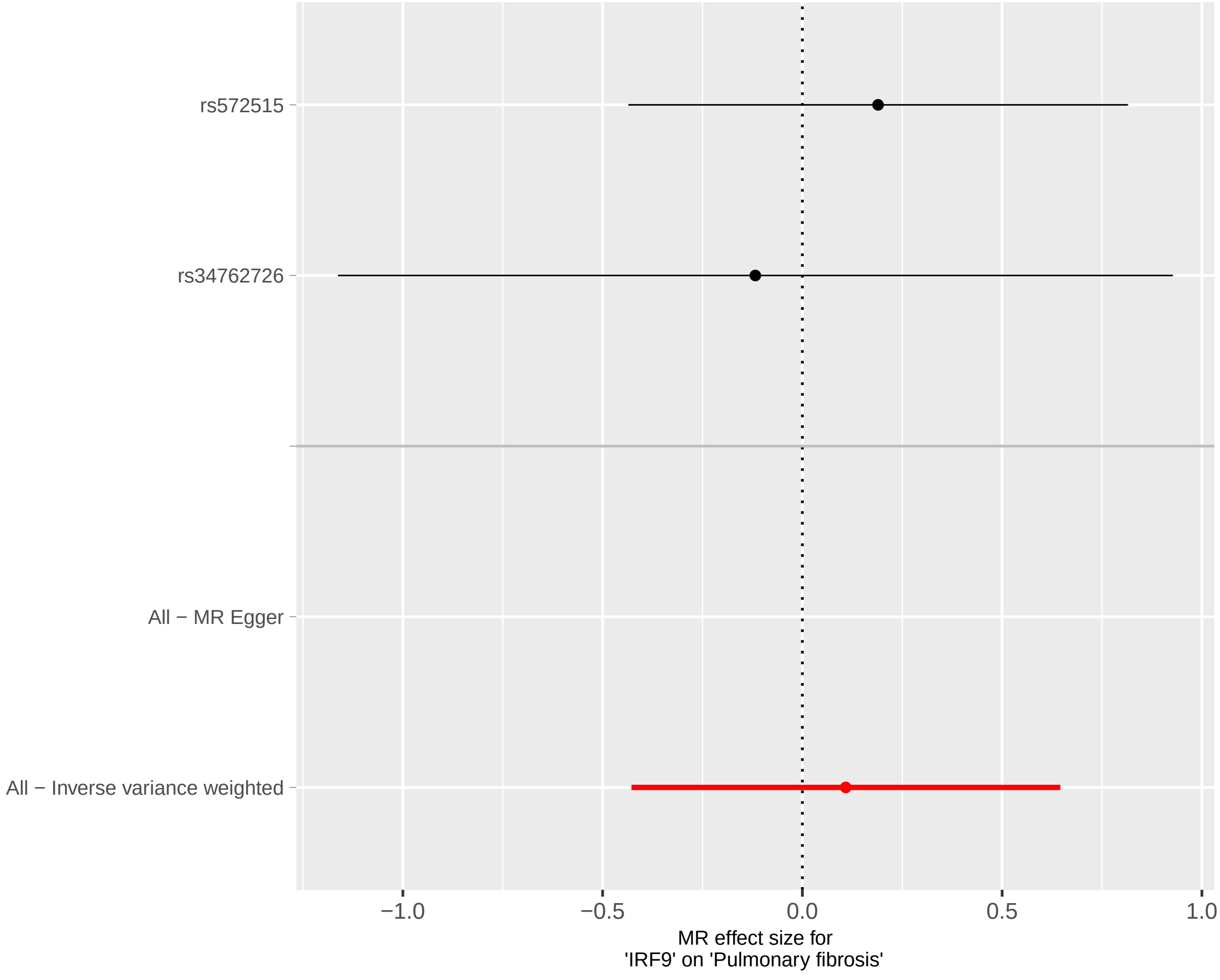
rs572515
rs34762726
All − MR Egger
All − Inverse variance weighted
−1.0
−0.5
0.0
0.5
1.0
MR effect size for
'IRF9' on 'Pulmonary fibrosis'

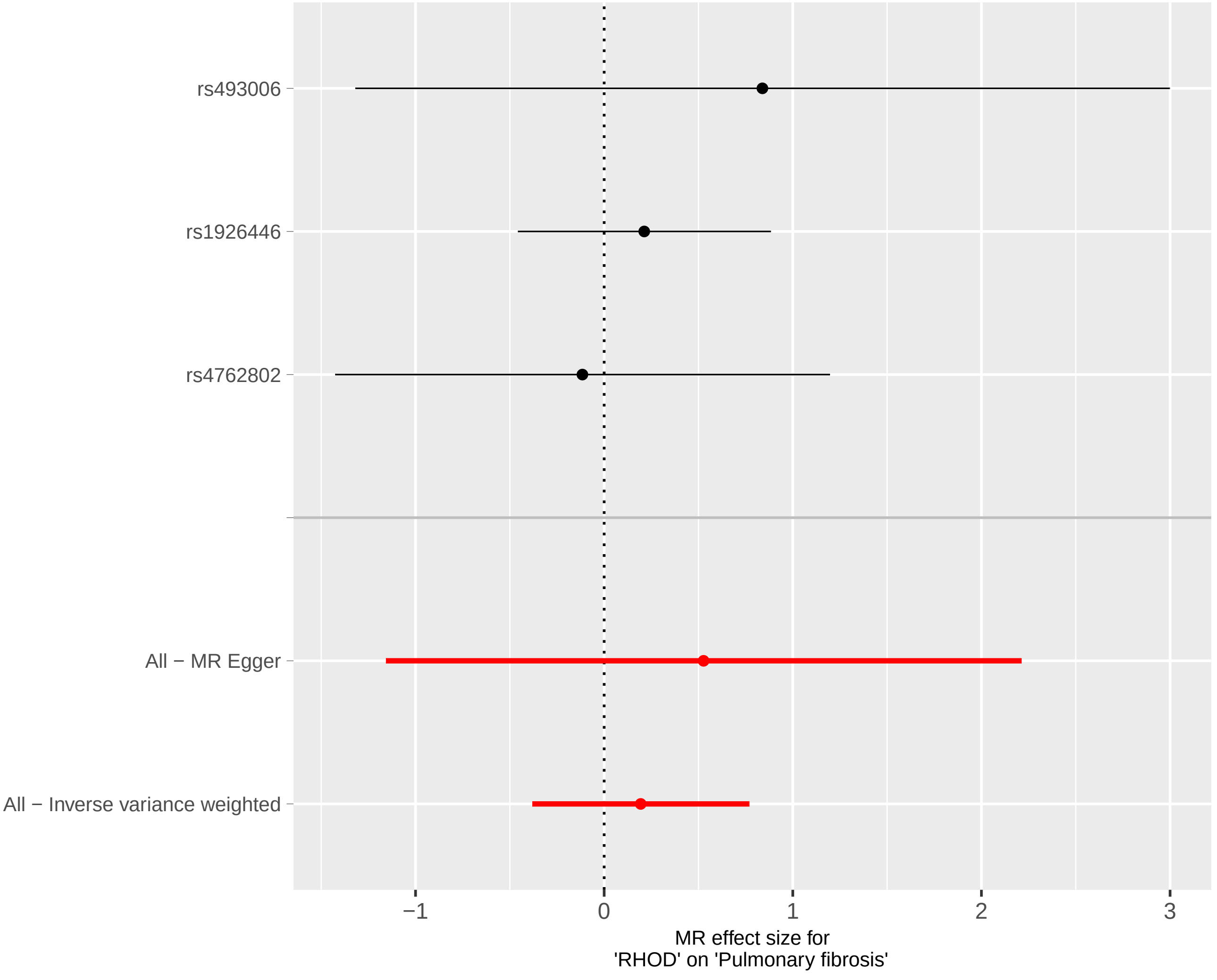

rs493006
rs1926446
rs4762802
All − MR Egger
All − Inverse variance weighted
−1
0
1
2
3
MR effect size for
'RHOD' on 'Pulmonary fibrosis'

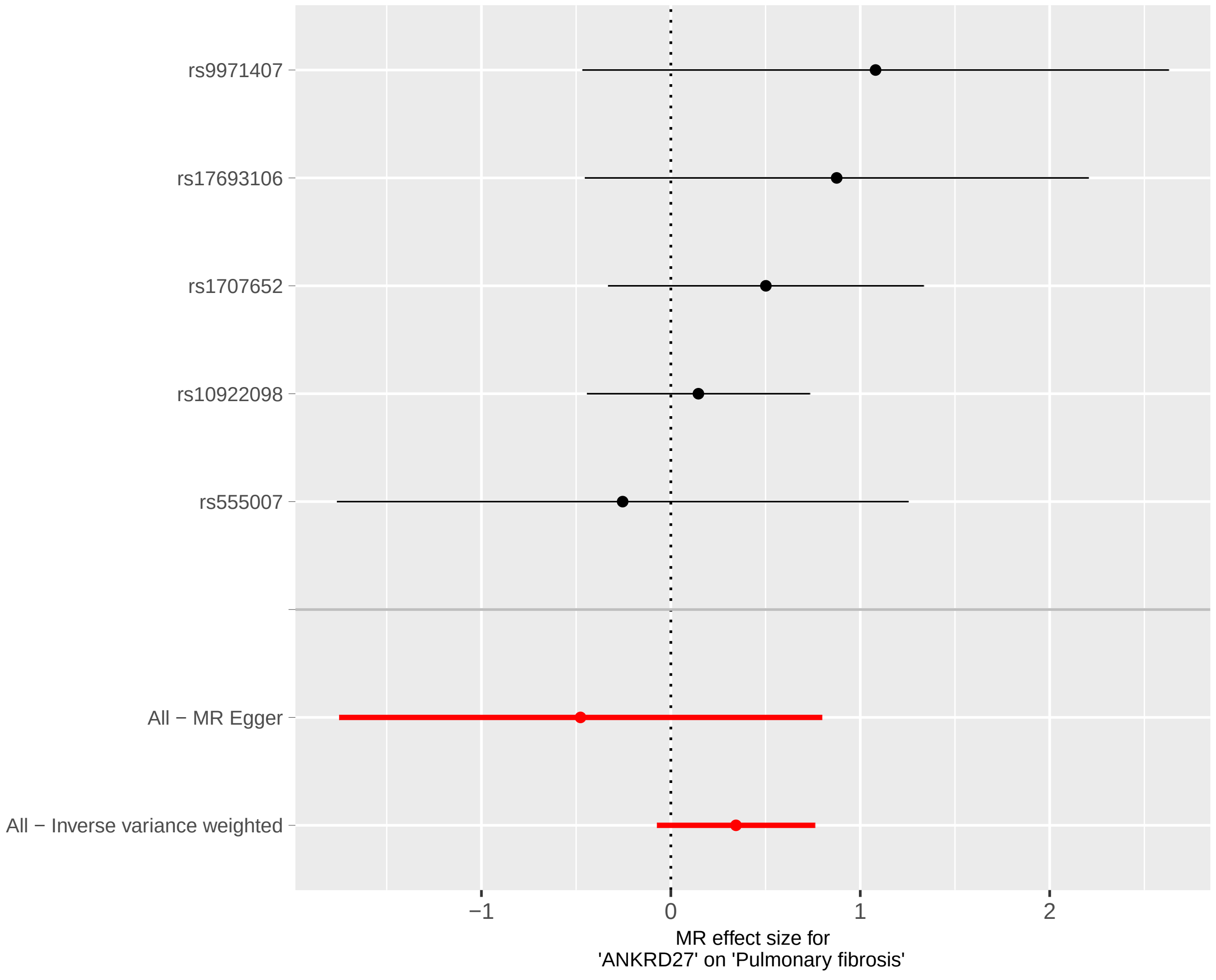

rs9971407
rs17693106
rs1707652
rs10922098
rs555007
All − MR Egger
All − Inverse variance weighted
−1
0
1
2
MR effect size for
'ANKRD27' on 'Pulmonary fibrosis'

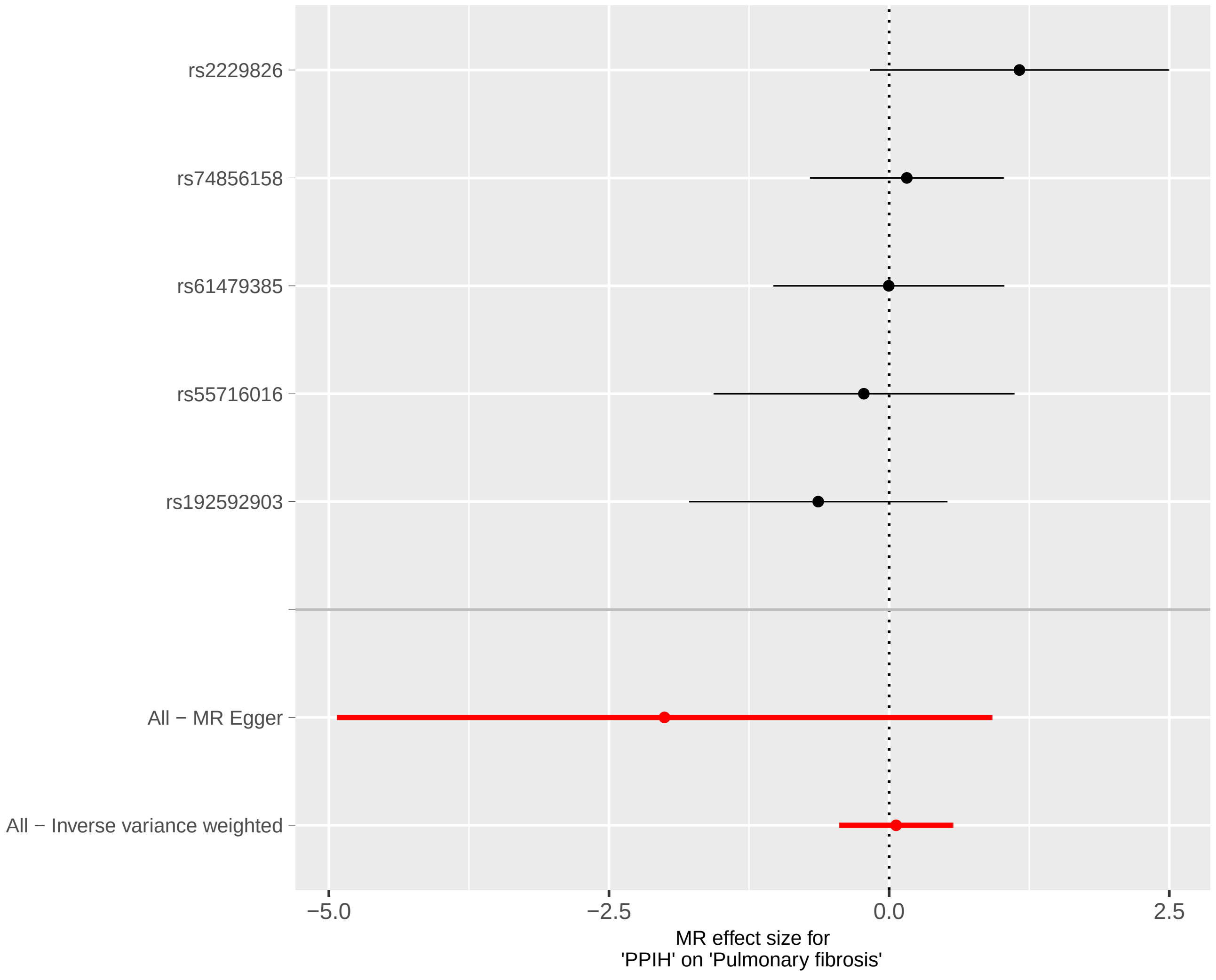
rs2229826
rs74856158
rs61479385
rs55716016
rs192592903
All − MR Egger
All − Inverse variance weighted
−5.0
−2.5
0.0
2.5
MR effect size for
'PPIH' on 'Pulmonary fibrosis'

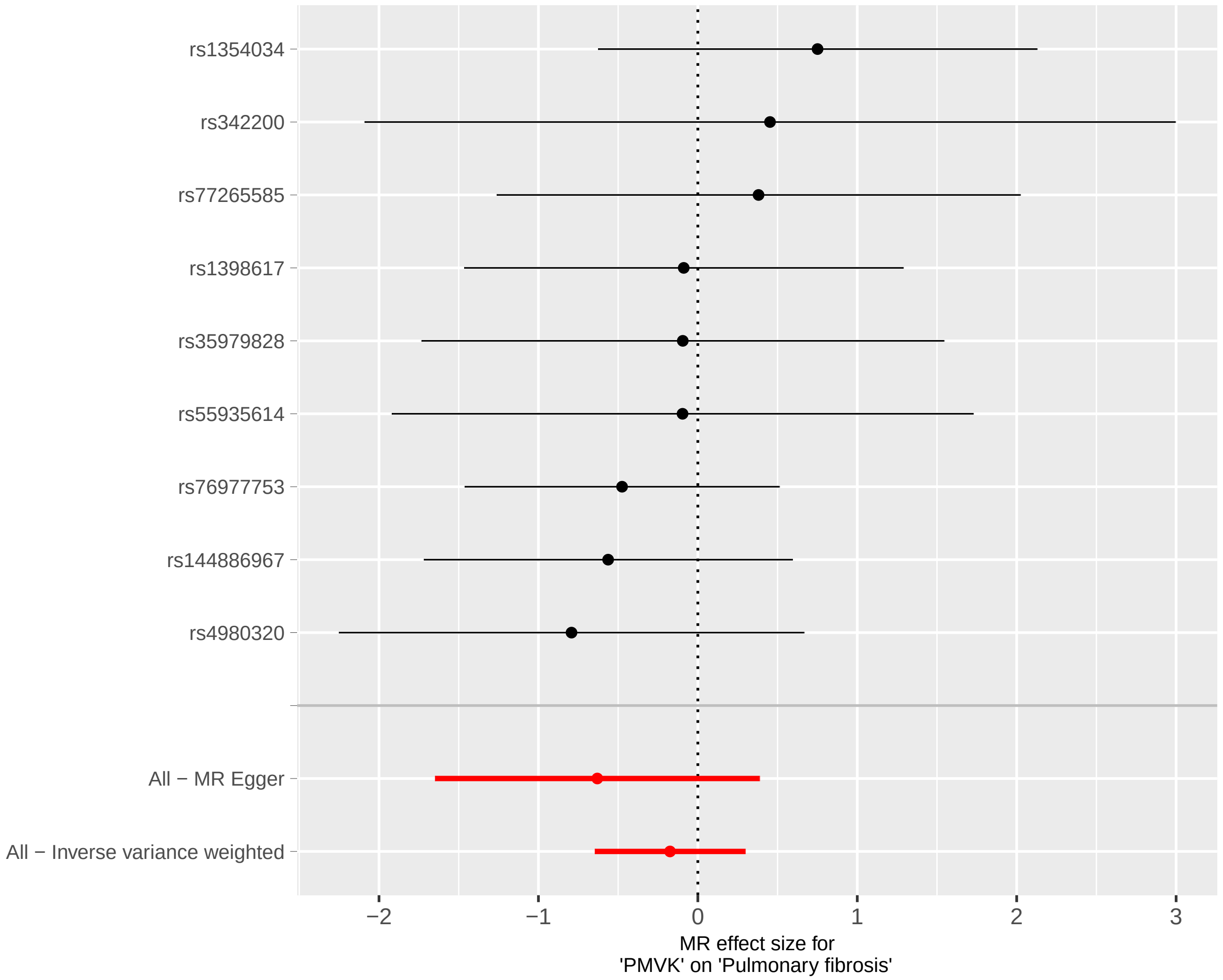
rs1354034
rs342200
rs77265585
rs1398617
rs35979828
rs55935614
rs76977753
rs144886967
rs4980320
All − MR Egger
All − Inverse variance weighted
−2
−1
0
1
2
3
MR effect size for
'PMVK' on 'Pulmonary fibrosis'

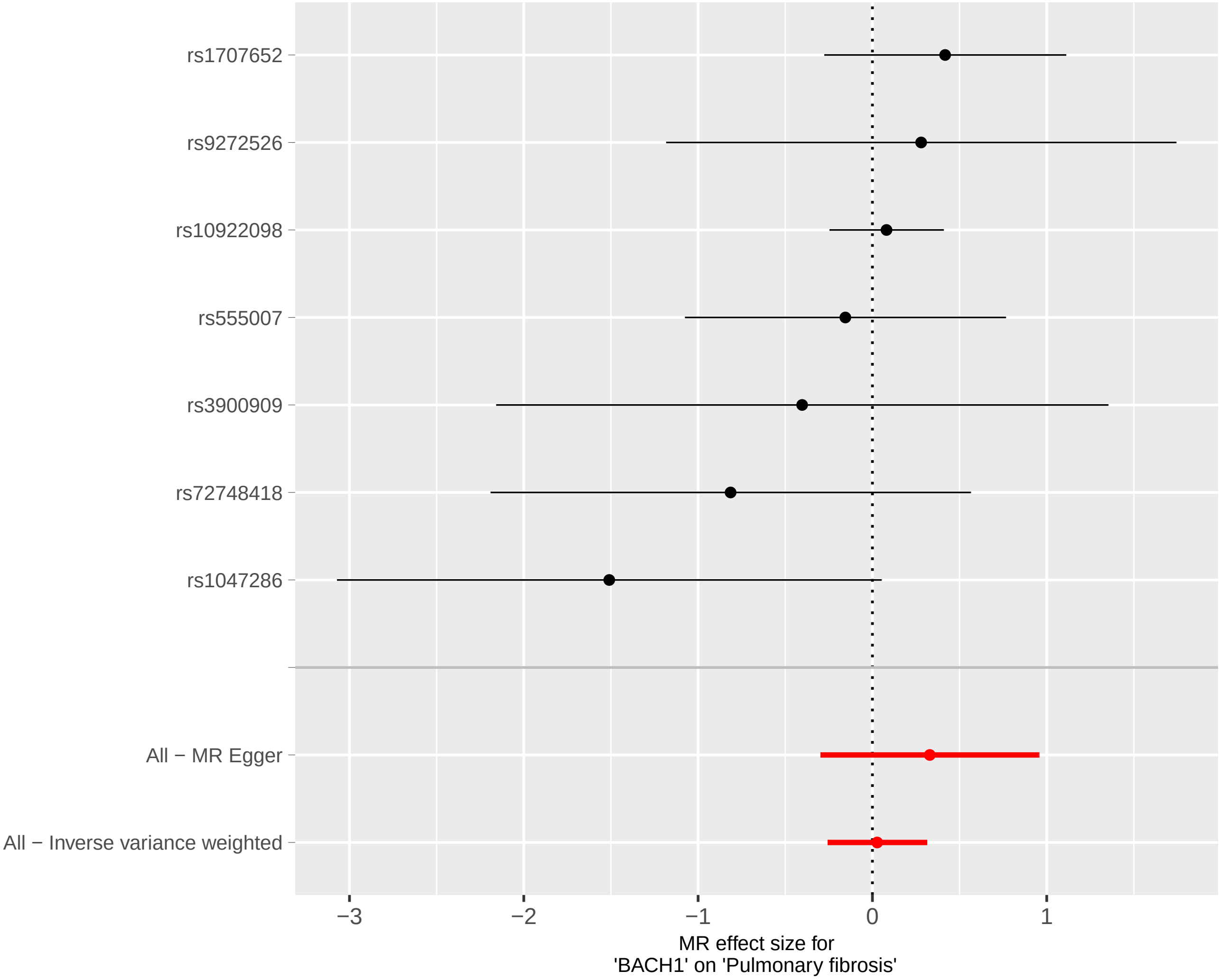

rs1707652
rs9272526
rs10922098
rs555007
rs3900909
rs72748418
rs1047286
All − MR Egger
All − Inverse variance weighted
−3
−2
−1
0
1
MR effect size for
'BACH1' on 'Pulmonary fibrosis'

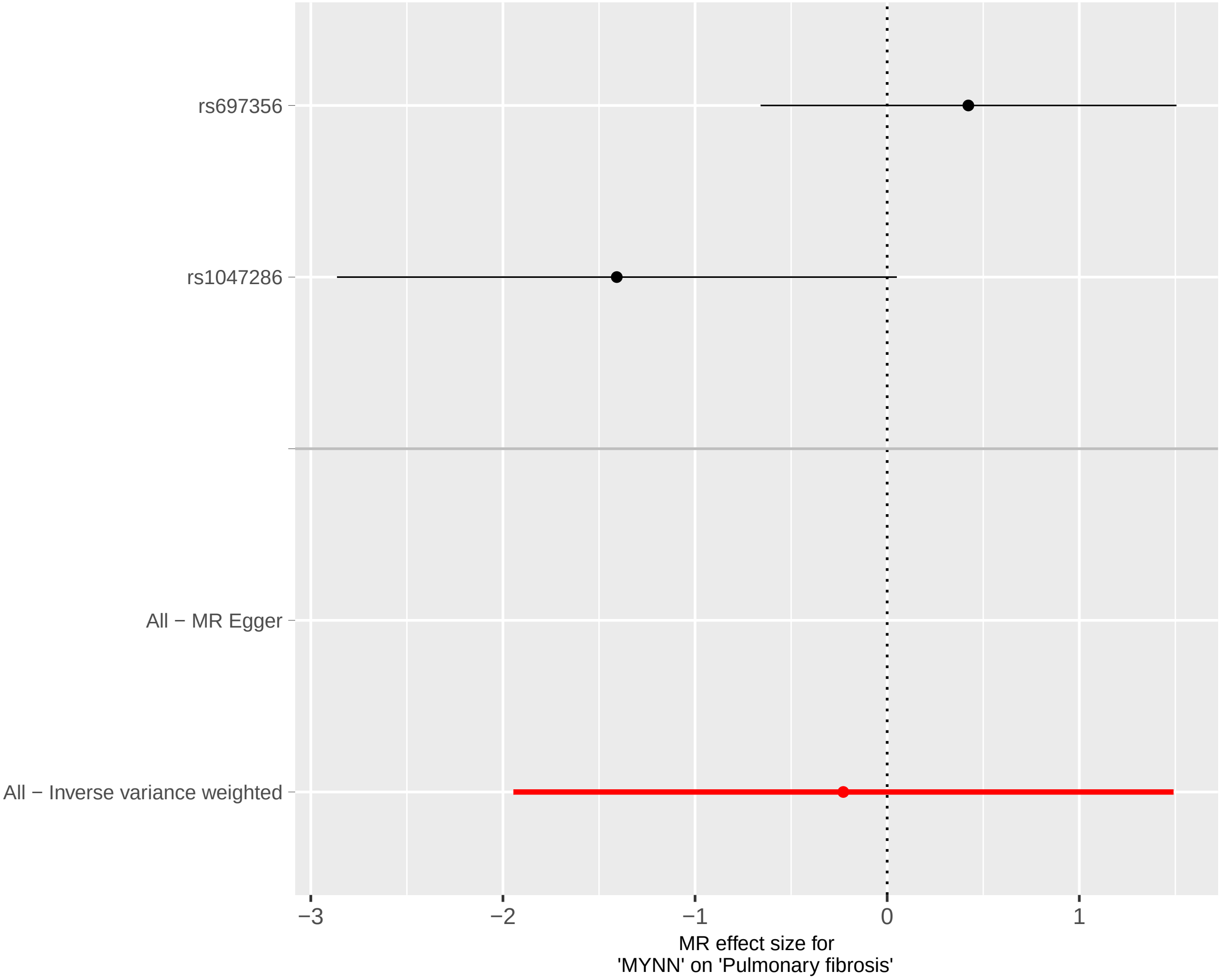
rs697356
rs1047286
All − MR Egger
All − Inverse variance weighted
−3
−2
−1
0
1
MR effect size for
'MYNN' on 'Pulmonary fibrosis'

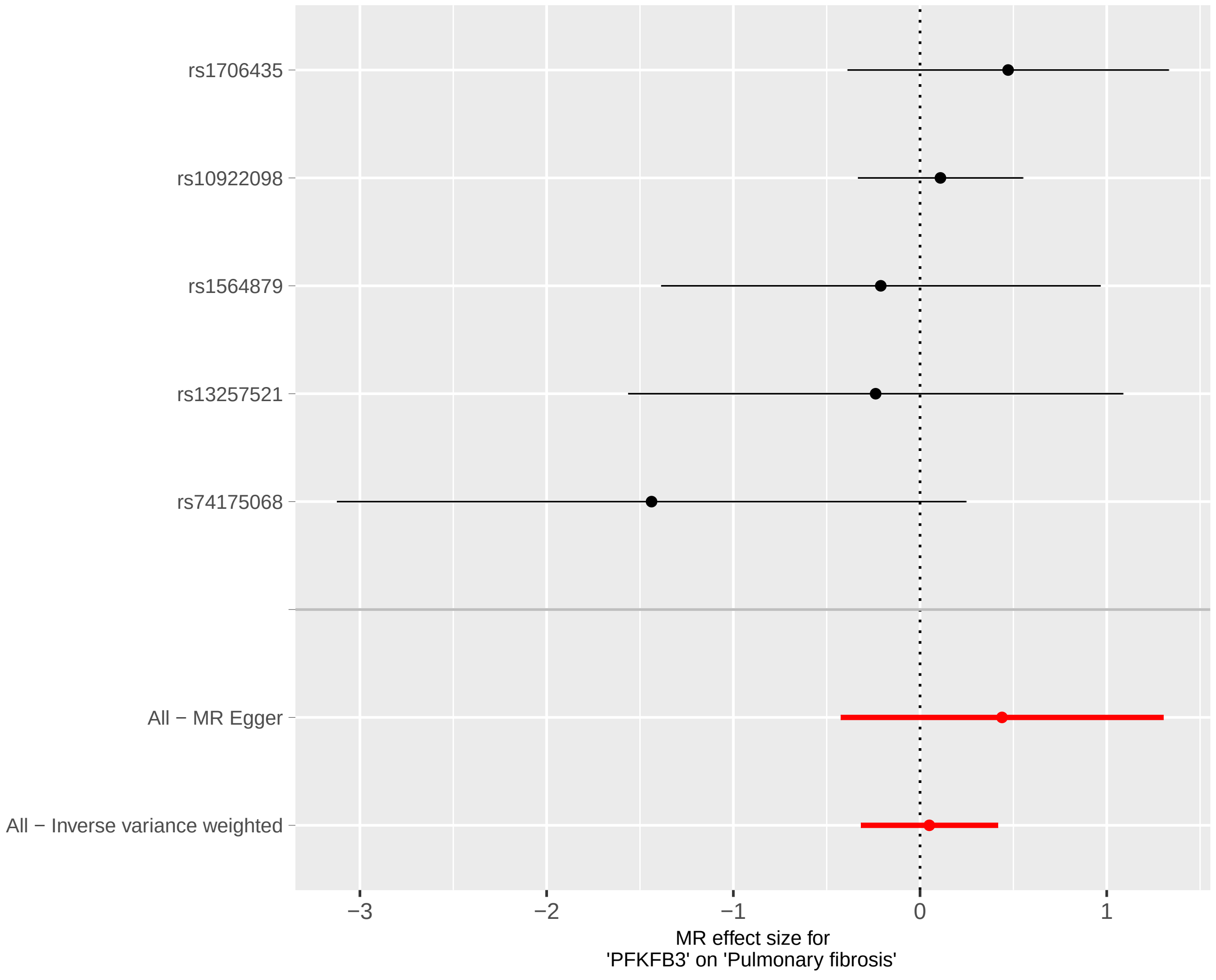
rs1706435
rs10922098
rs1564879
rs13257521
rs74175068
All − MR Egger
All − Inverse variance weighted
−3
−2
−1
0
1
MR effect size for
'PFKFB3' on 'Pulmonary fibrosis'

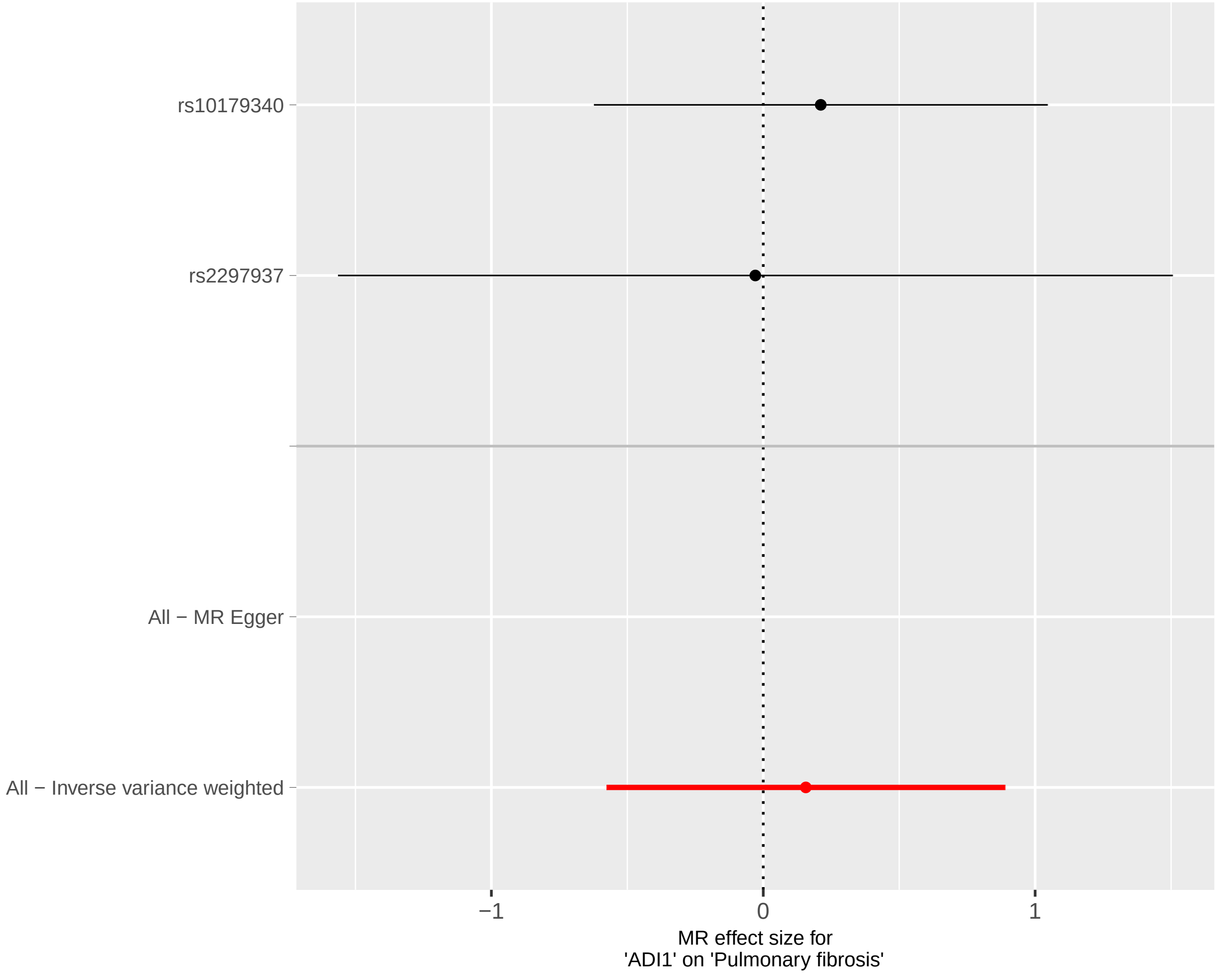
rs10179340
rs2297937
All − MR Egger
All − Inverse variance weighted
−1
0
1
MR effect size for
'ADI1' on 'Pulmonary fibrosis'

Insufficient number of SNPs

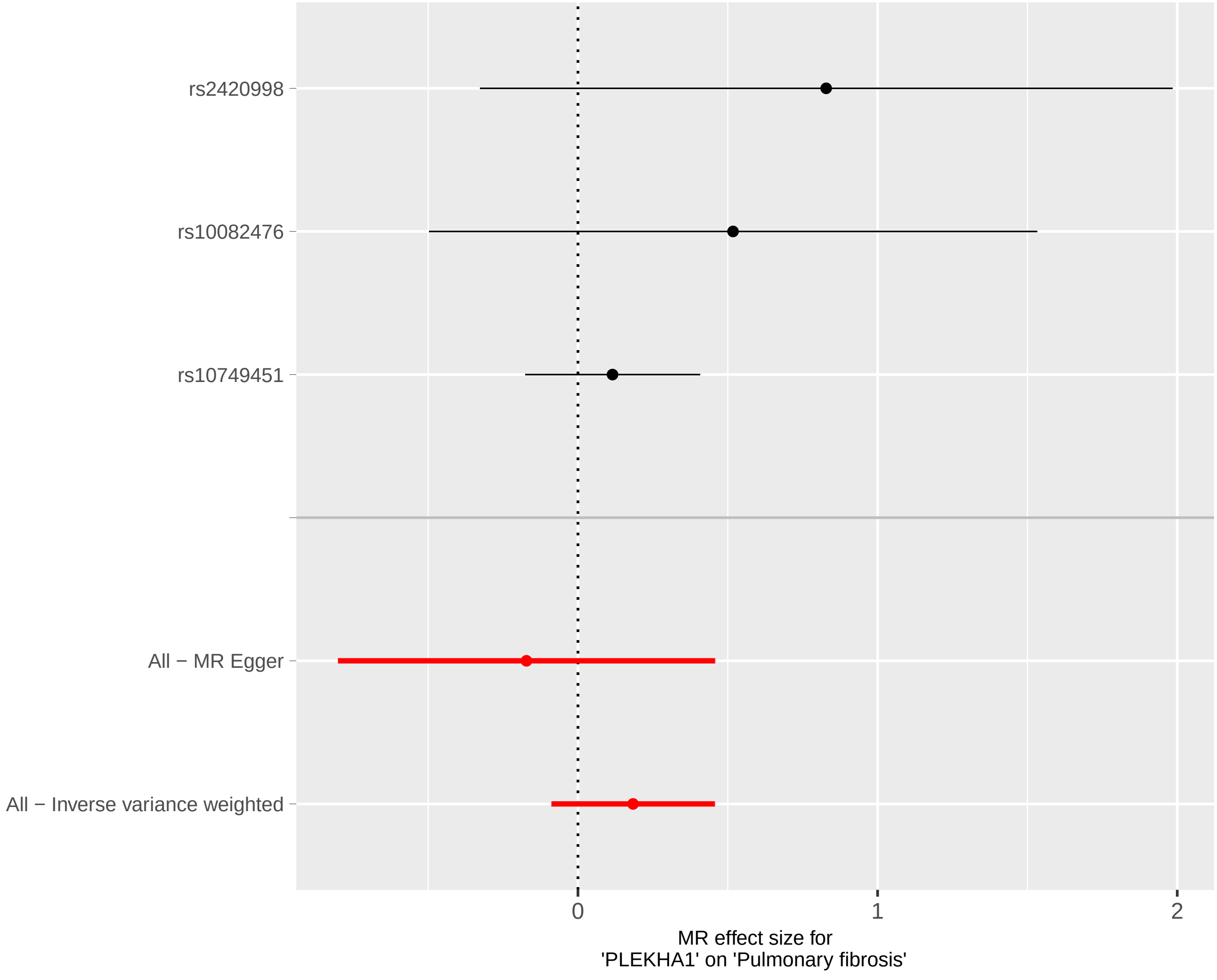
rs2420998
rs10082476
rs10749451
All − MR Egger
All − Inverse variance weighted
0
1
2
MR effect size for
'PLEKHA1' on 'Pulmonary fibrosis'

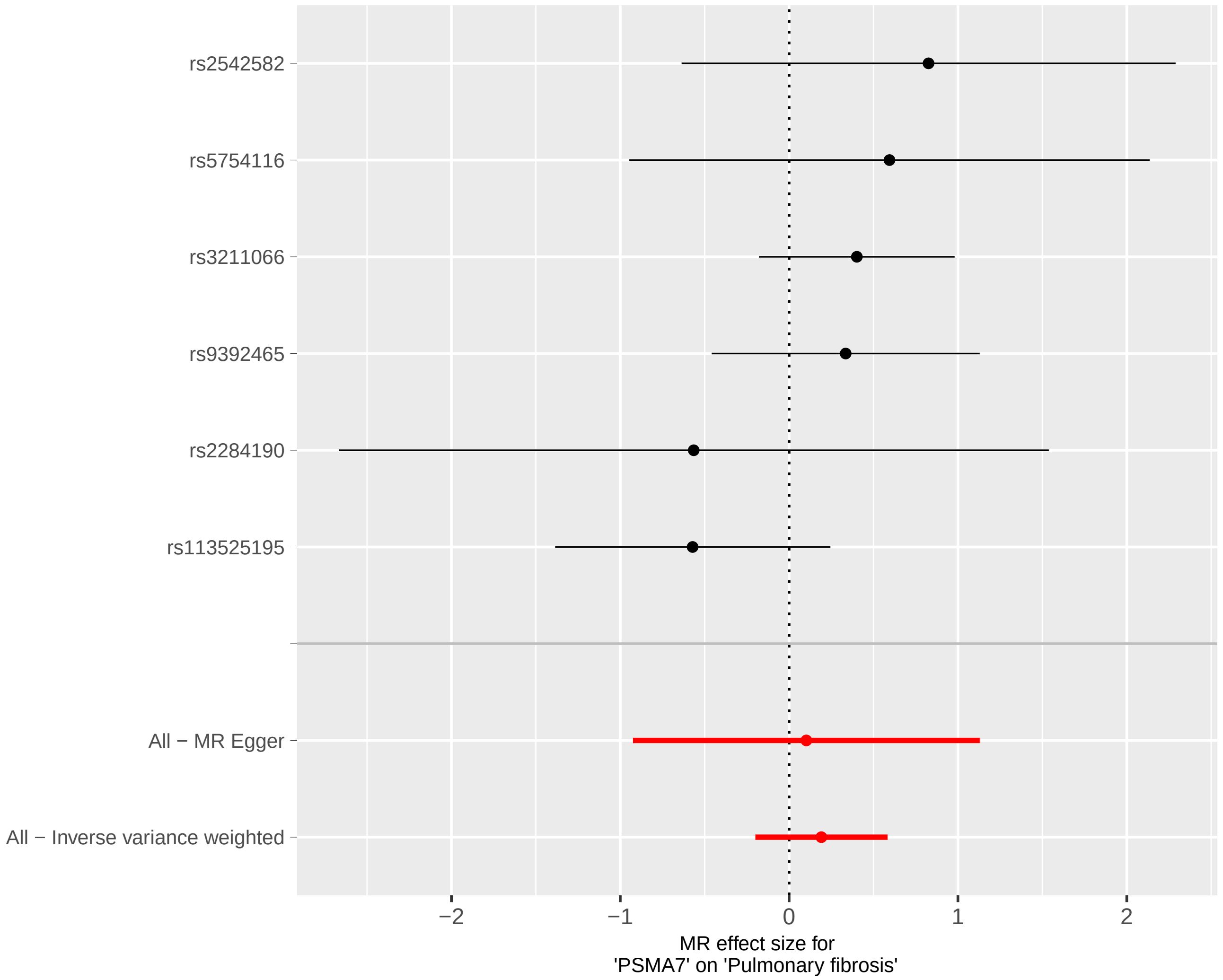
rs2542582
rs5754116
rs3211066
rs9392465
rs2284190
rs113525195
All − MR Egger
All − Inverse variance weighted
−2
−1
0
1
2
MR effect size for
'PSMA7' on 'Pulmonary fibrosis'

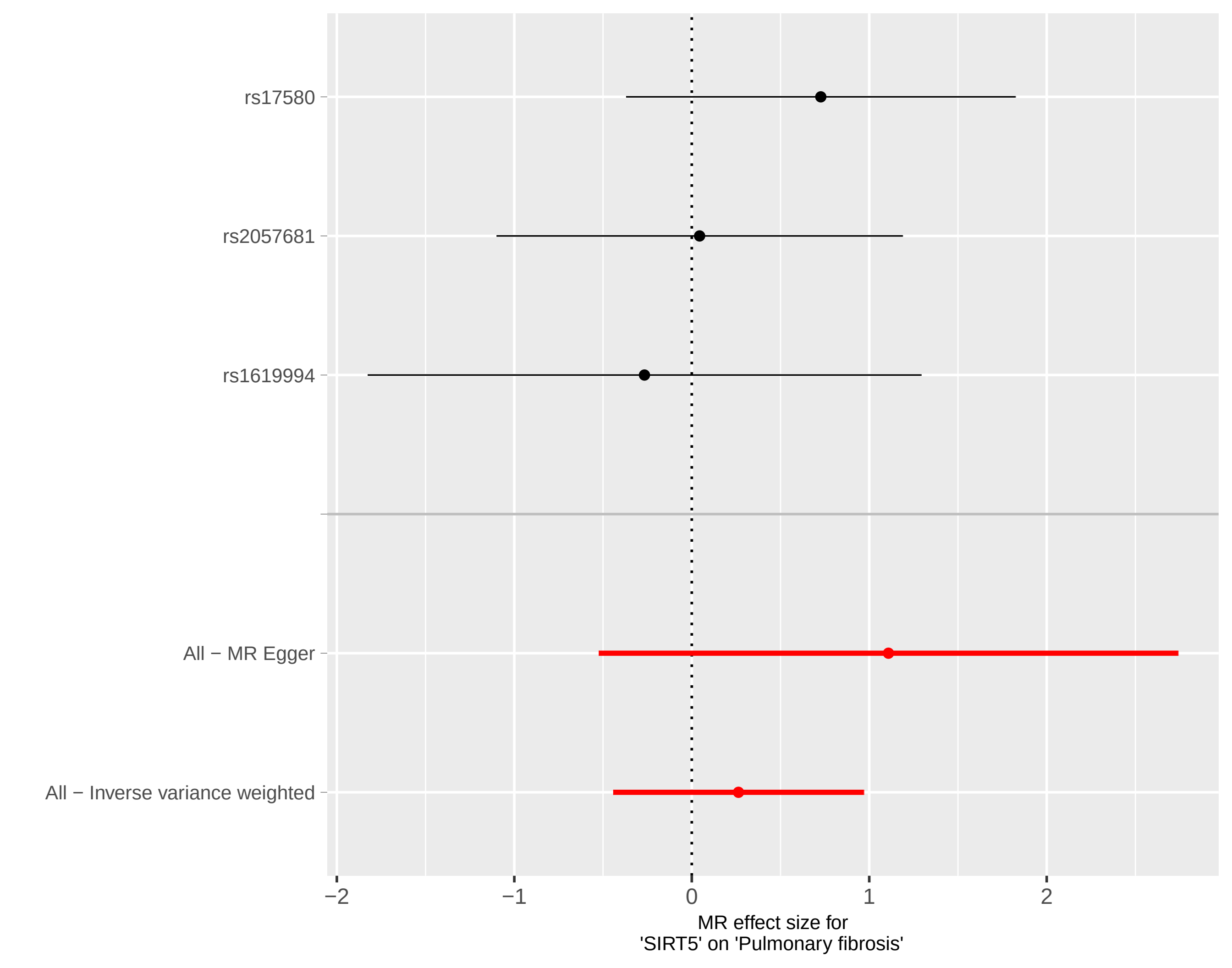
rs17580
rs2057681
rs1619994
All − MR Egger
All − Inverse variance weighted
−2
−1
0
1
2
MR effect size for
'SIRT5' on 'Pulmonary fibrosis'

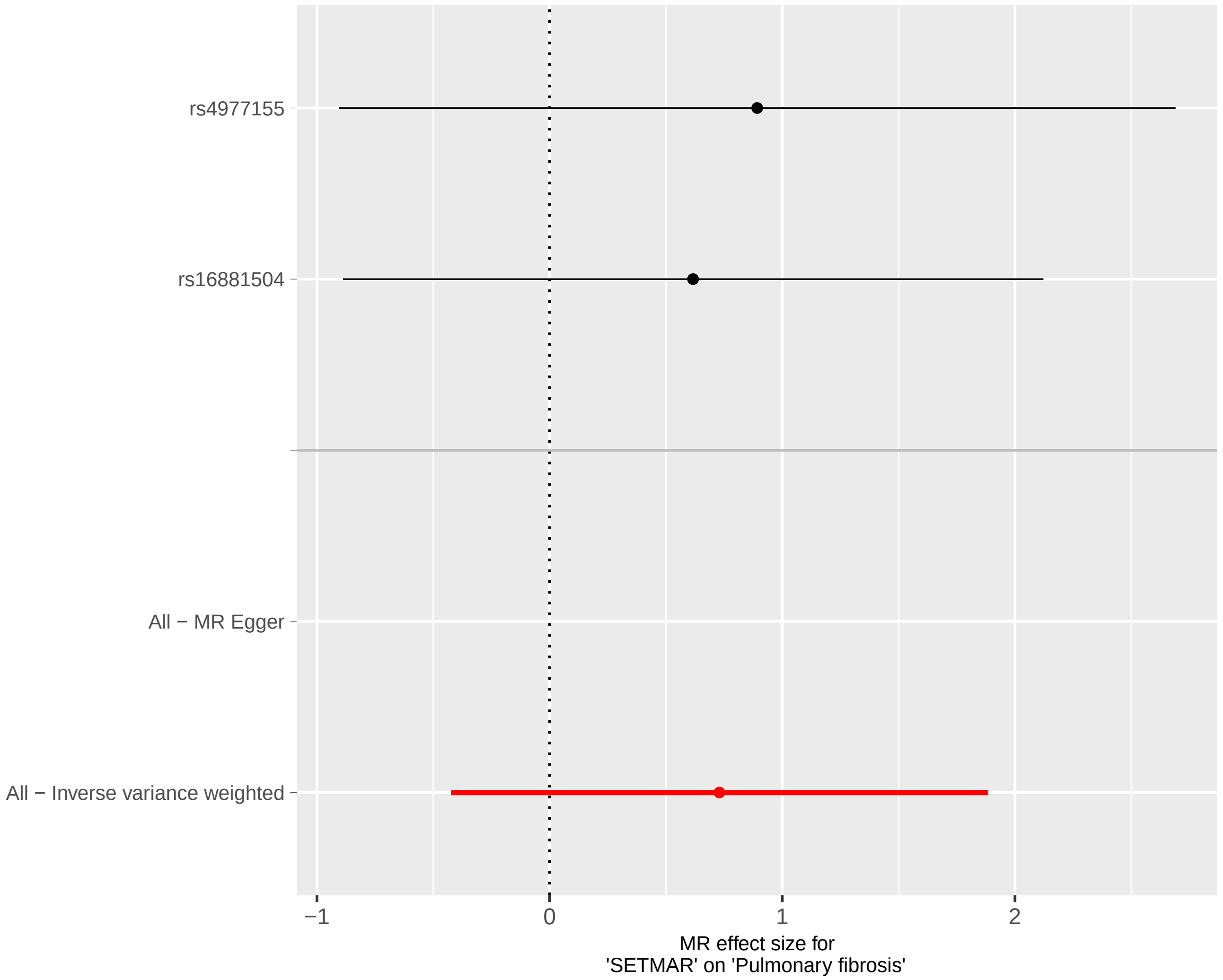
rs4977155
rs16881504
All − MR Egger
All − Inverse variance weighted
−1
0
1
2
MR effect size for
'SETMAR' on 'Pulmonary fibrosis'

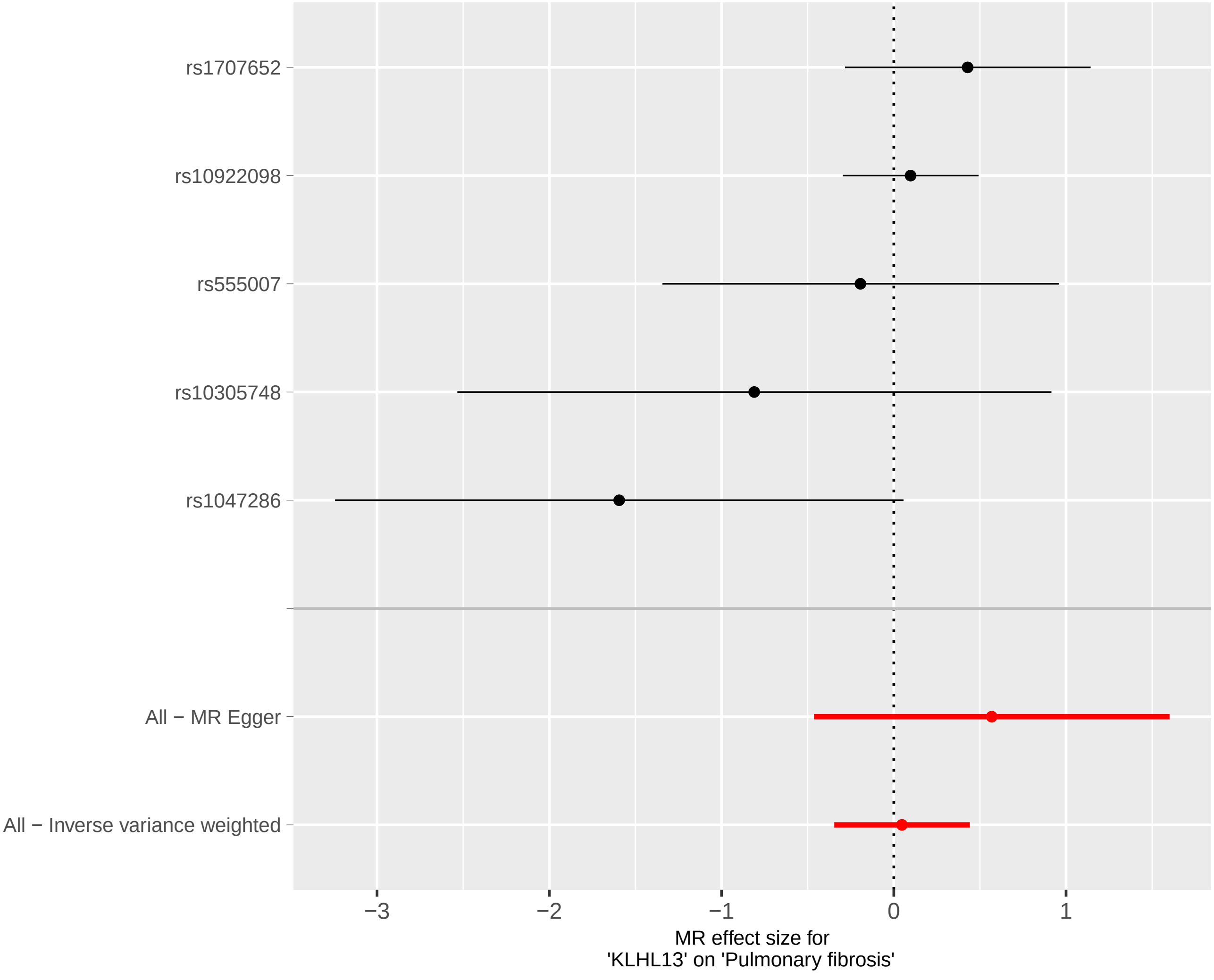
rs1707652
rs10922098
rs555007
rs10305748
rs1047286
All − MR Egger
All − Inverse variance weighted
−3
−2
−1
0
1
MR effect size for
'KLHL13' on 'Pulmonary fibrosis'

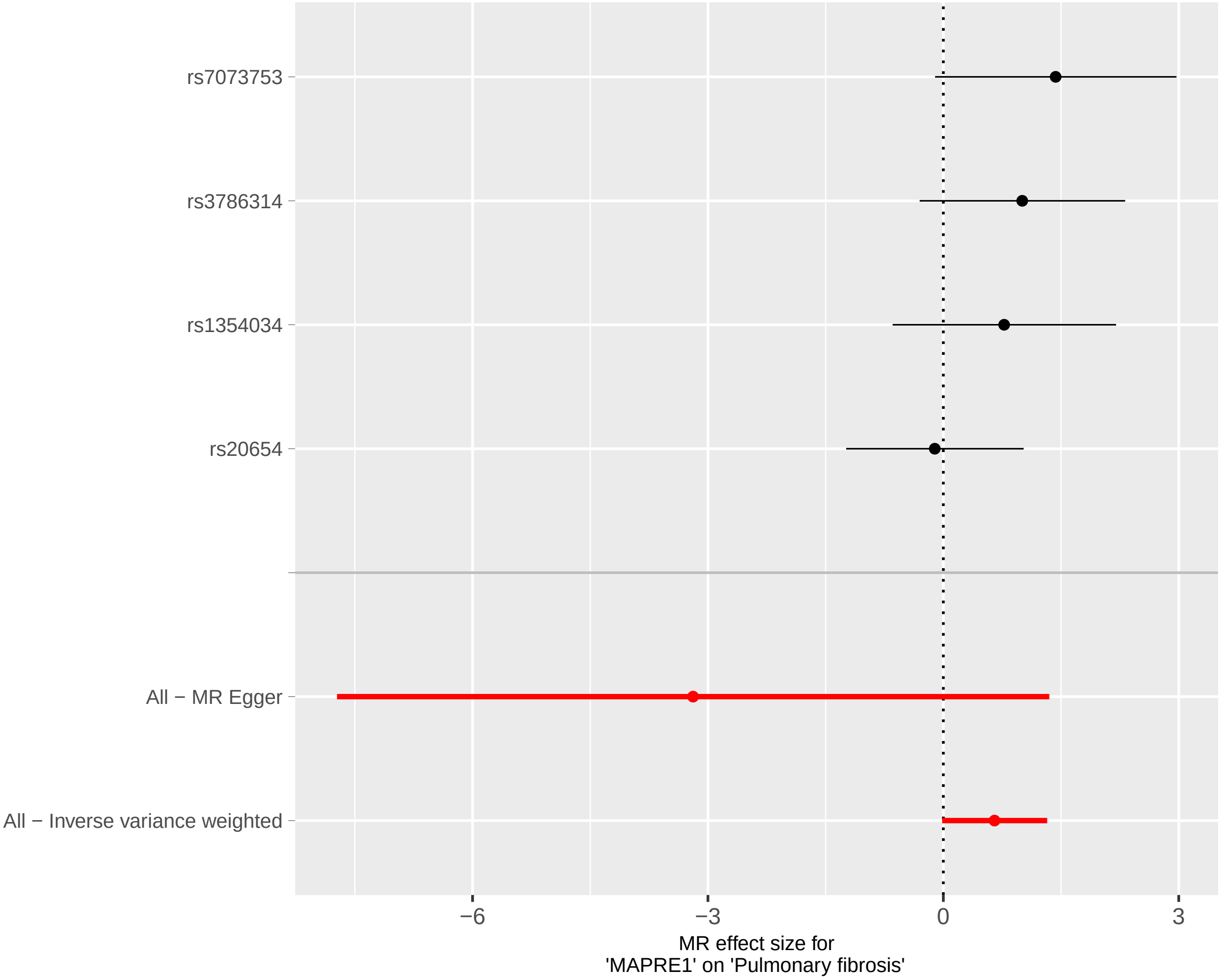

rs7073753
rs3786314
rs1354034
rs20654
All − MR Egger
All − Inverse variance weighted
−6
−3
0
3
MR effect size for
'MAPRE1' on 'Pulmonary fibrosis'

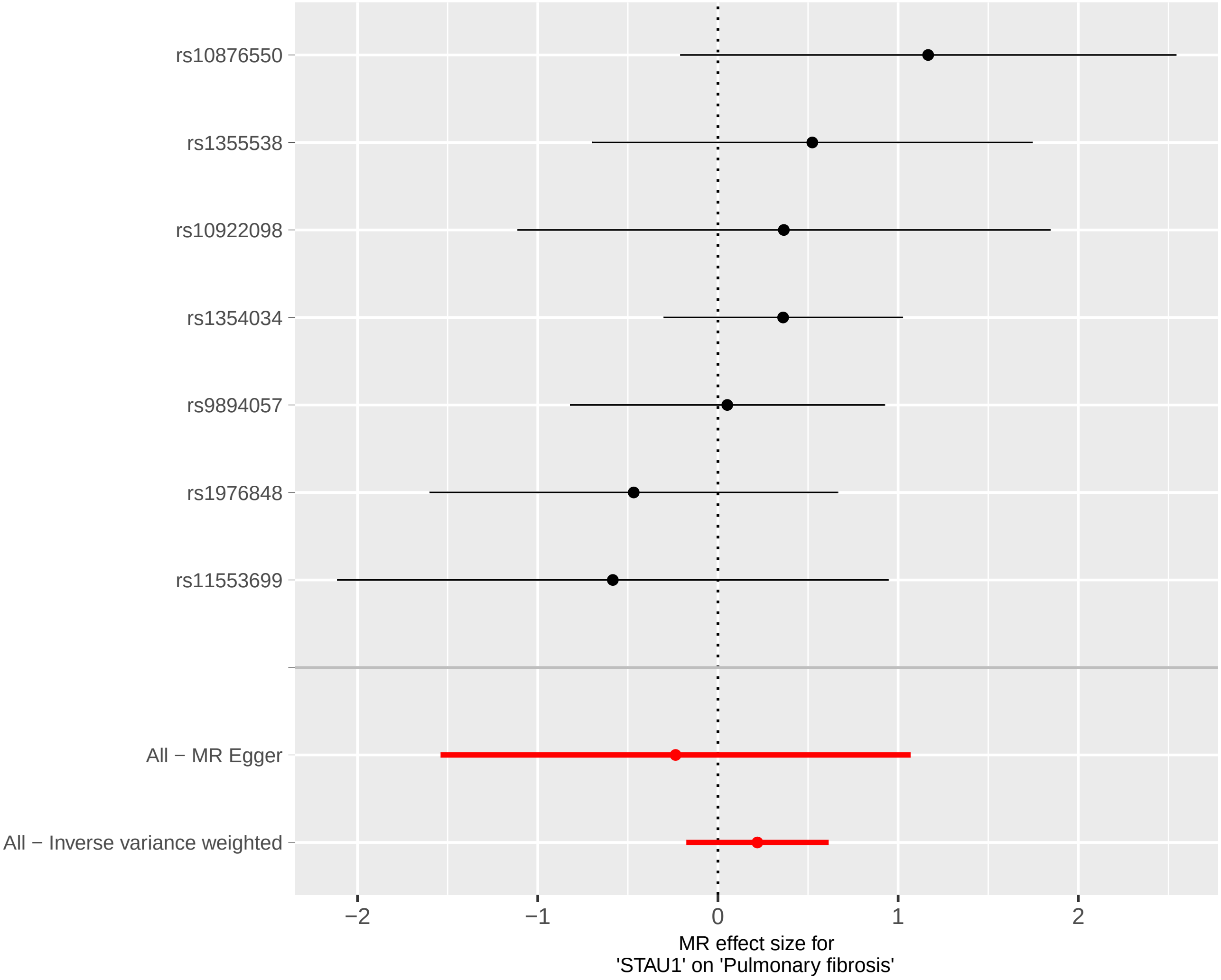
rs10876550
rs1355538
rs10922098
rs1354034
rs9894057
rs1976848
rs11553699
All − MR Egger
All − Inverse variance weighted
−2
−1
0
1
2
MR effect size for
'STAU1' on 'Pulmonary fibrosis'

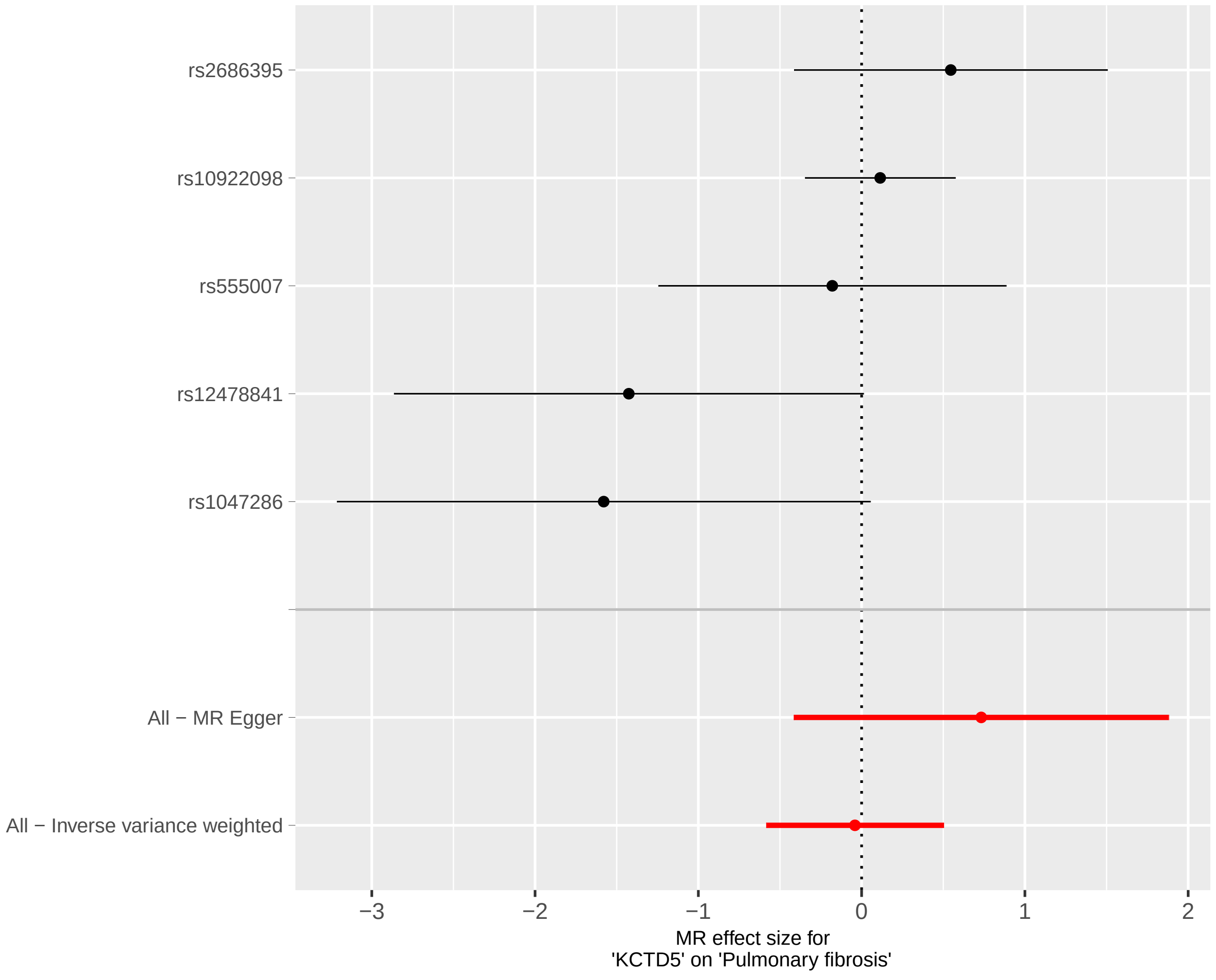
rs2686395
rs10922098
rs555007
rs12478841
rs1047286
All − MR Egger
All − Inverse variance weighted
−3
−2
−1
0
1
2
MR effect size for
'KCTD5' on 'Pulmonary fibrosis'

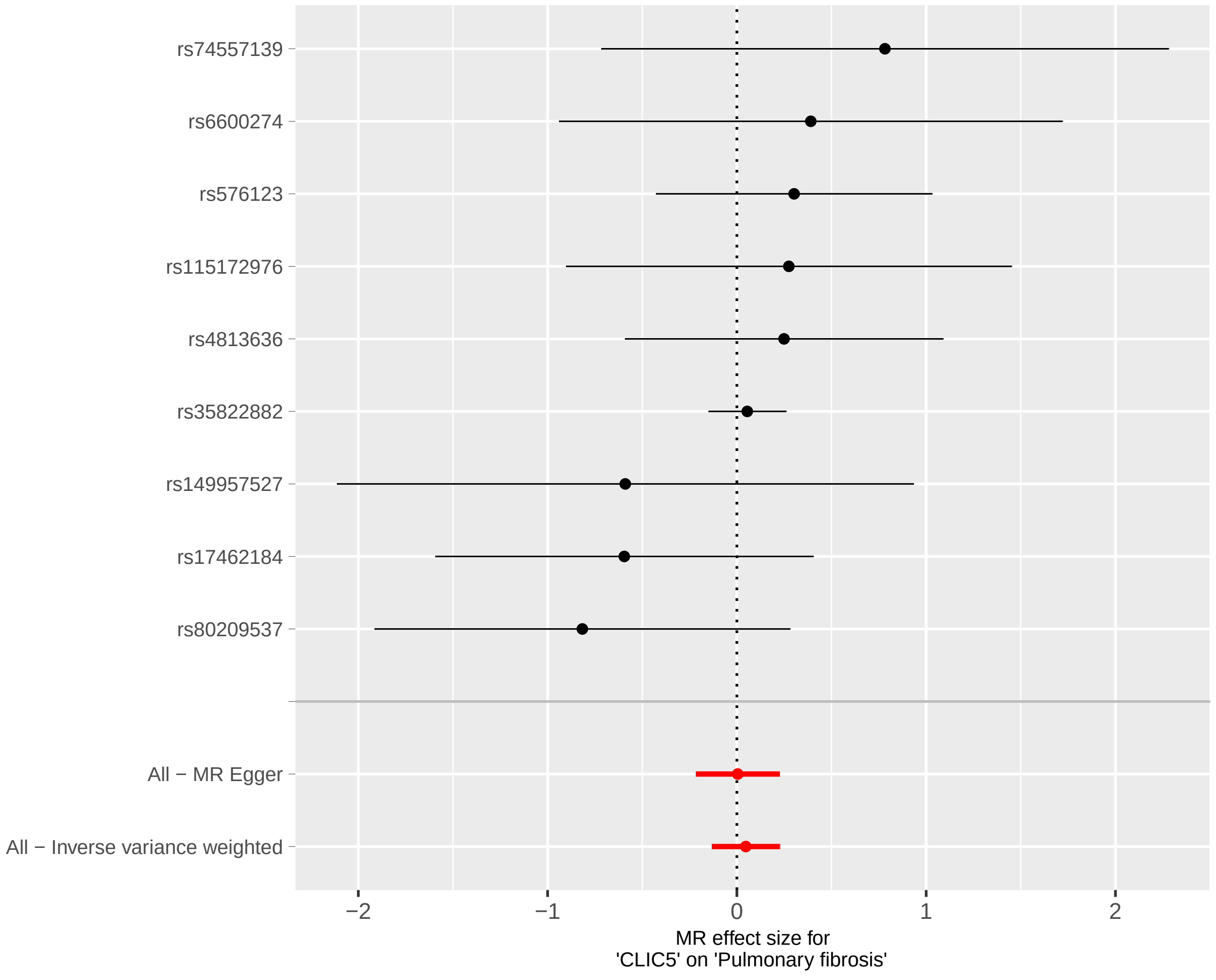
rs74557139
rs6600274
rs576123
rs115172976
rs4813636
rs35822882
rs149957527
rs17462184
rs80209537
All − MR Egger
All − Inverse variance weighted
−2
−1
0
1
2
MR effect size for
'CLIC5' on 'Pulmonary fibrosis'

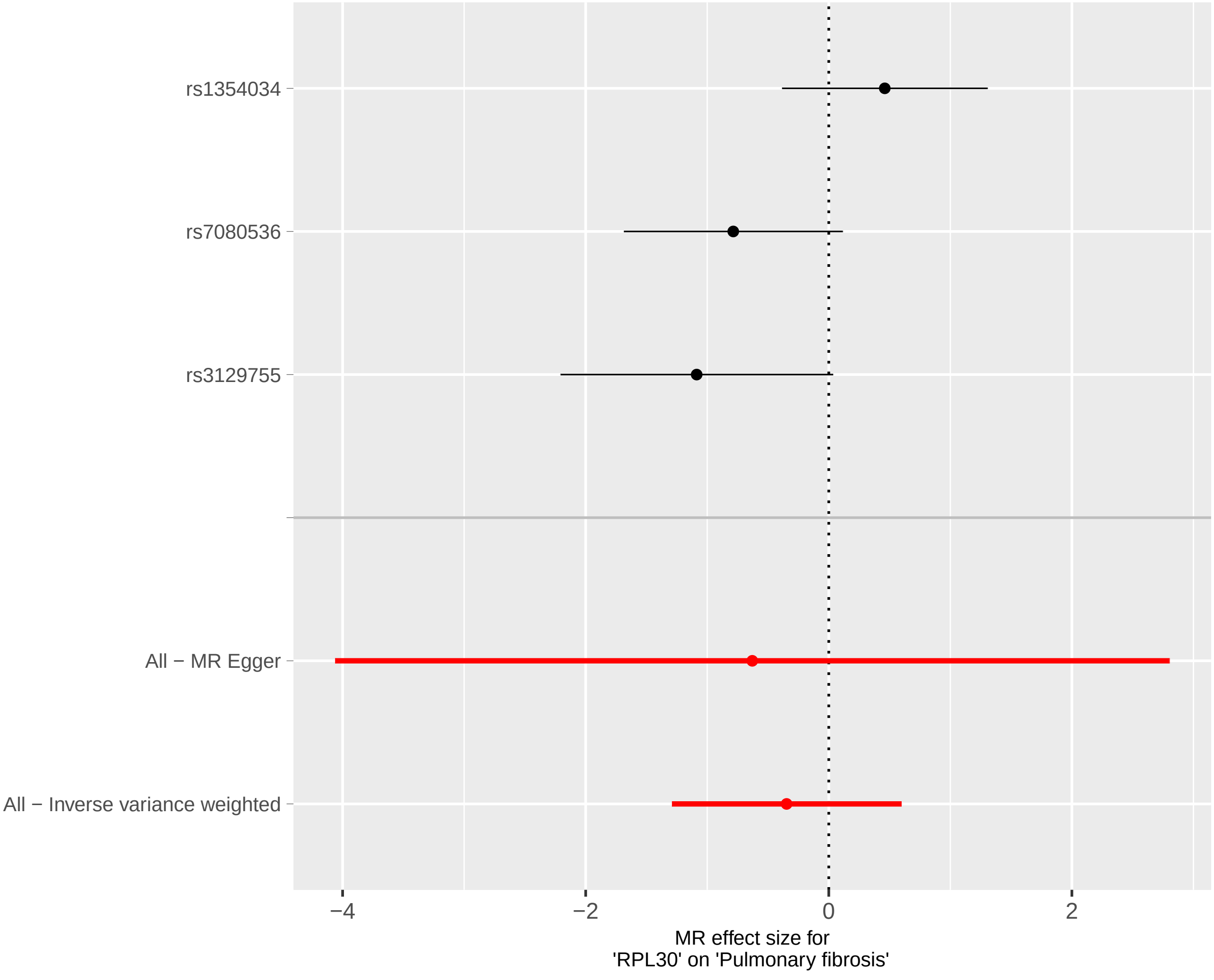
rs1354034
rs7080536
rs3129755
All − MR Egger
All − Inverse variance weighted
−4
−2
0
2
MR effect size for
'RPL30' on 'Pulmonary fibrosis'

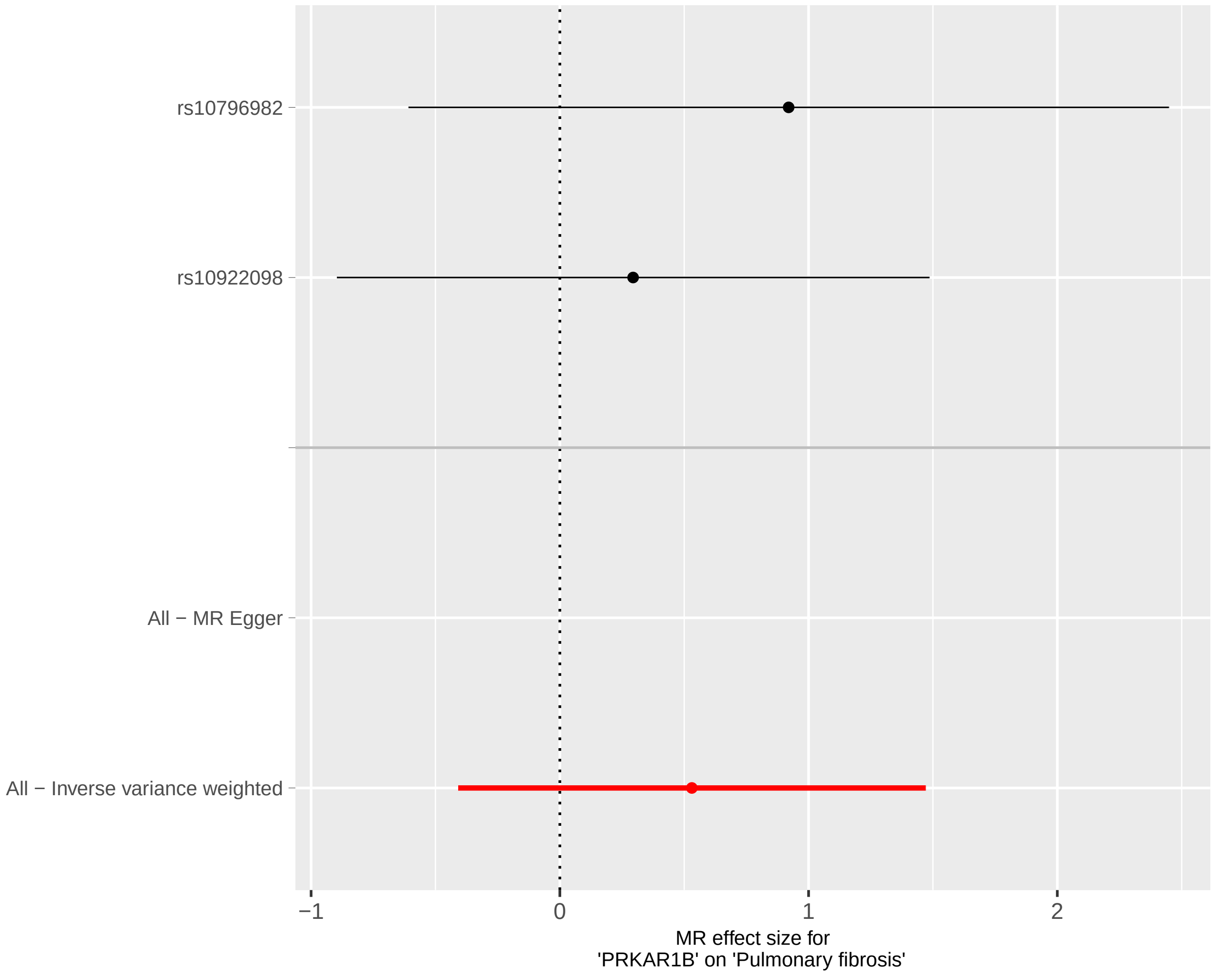

rs10796982
rs10922098
All − MR Egger
All − Inverse variance weighted
−1
0
1
2
MR effect size for
'PRKAR1B' on 'Pulmonary fibrosis'

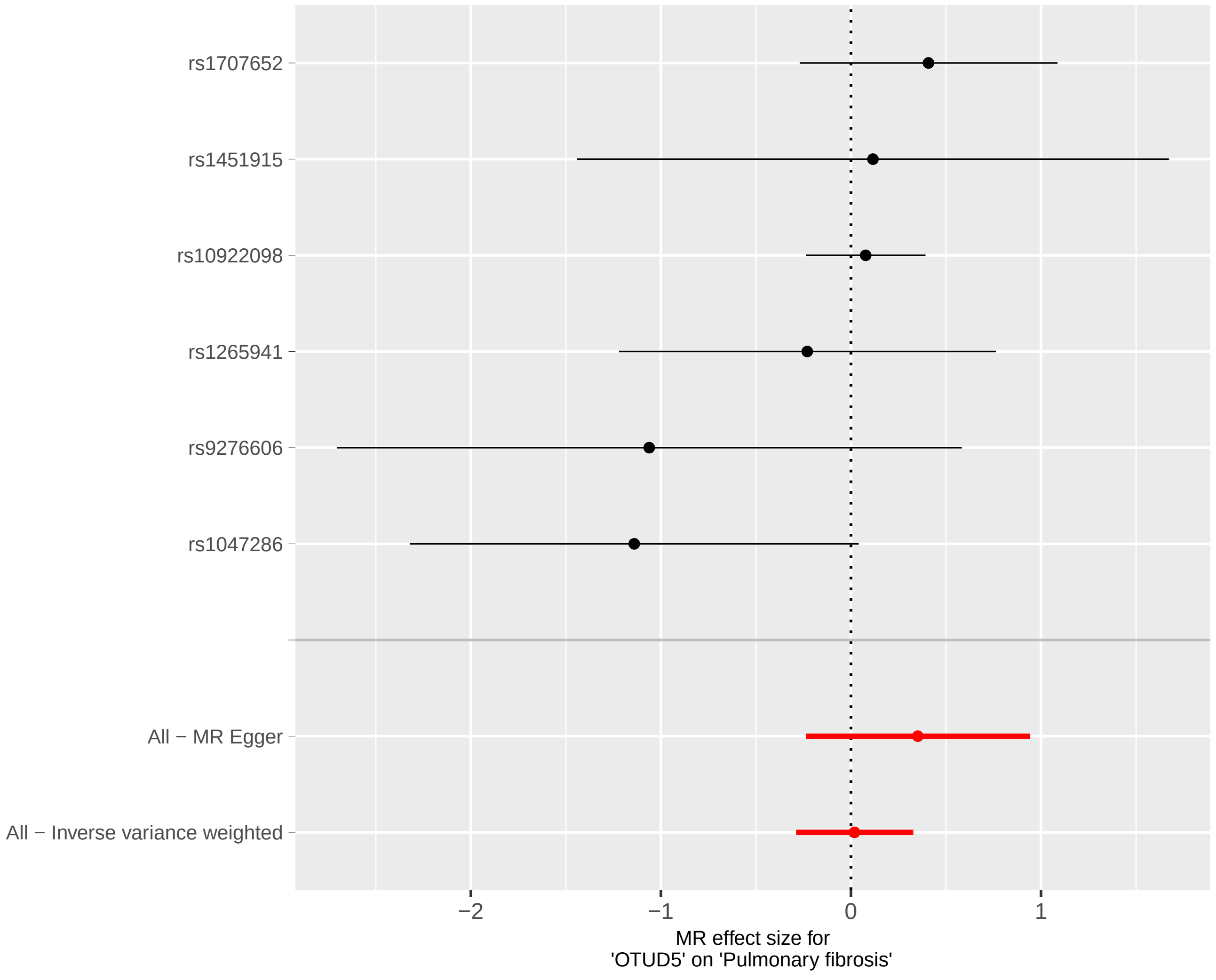
rs1707652
rs1451915
rs10922098
rs1265941
rs9276606
rs1047286
All − MR Egger
All − Inverse variance weighted
−2
−1
0
1
MR effect size for
'OTUD5' on 'Pulmonary fibrosis'

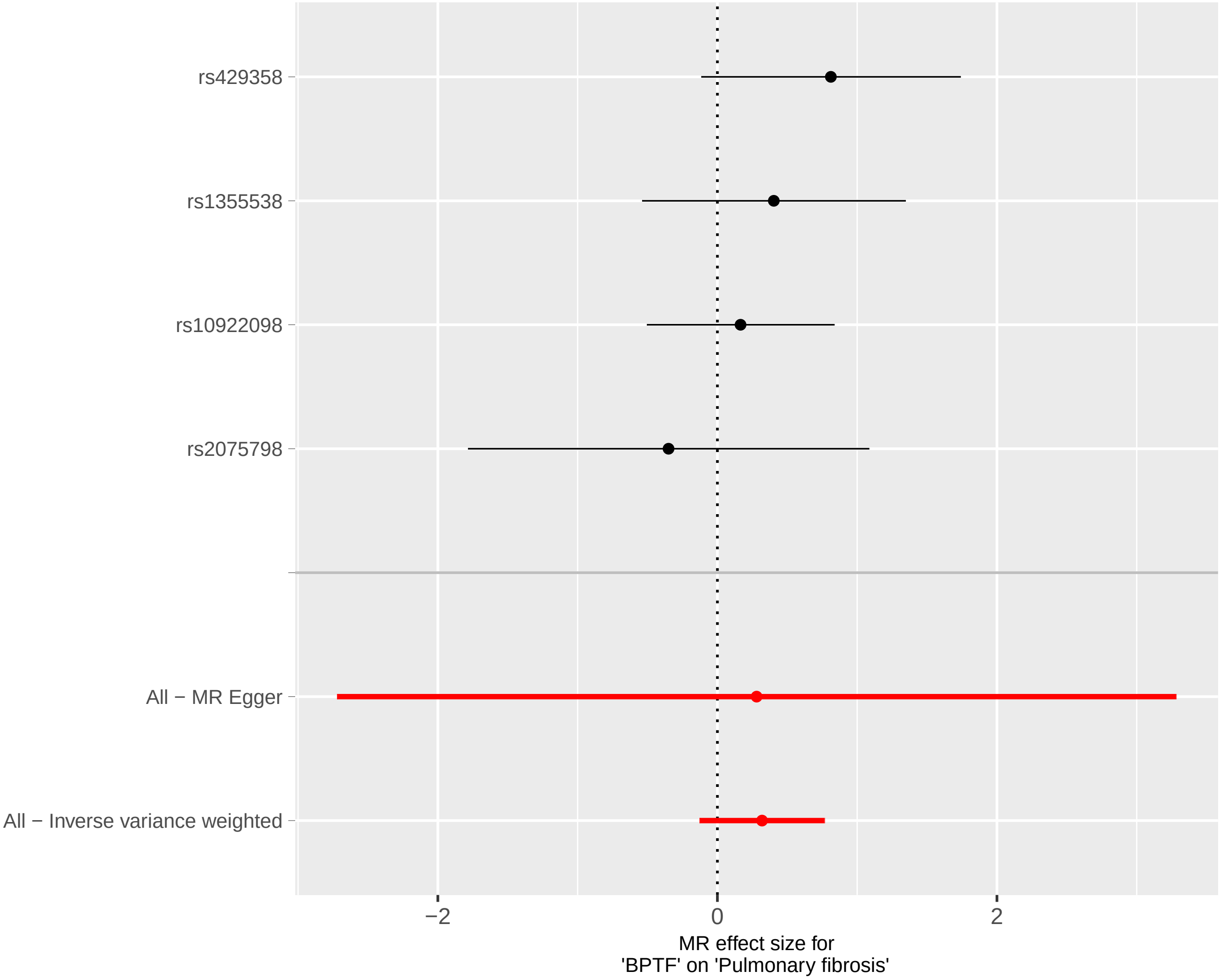
rs429358
rs1355538
rs10922098
rs2075798
All − MR Egger
All − Inverse variance weighted
−2
0
2
MR effect size for
'BPTF' on 'Pulmonary fibrosis'

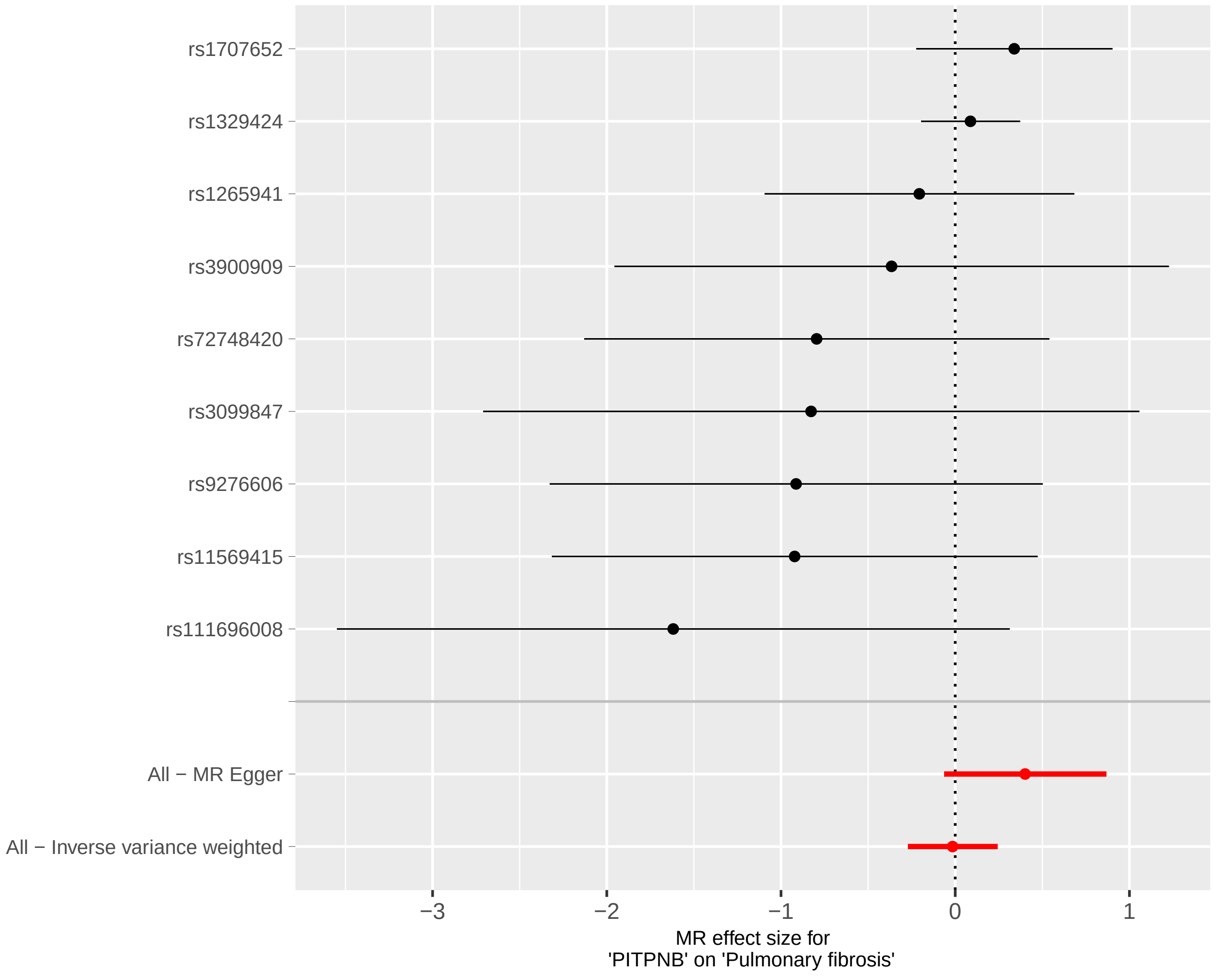

rs1707652
rs1329424
rs1265941
rs3900909
rs72748420
rs3099847
rs9276606
rs11569415
rs111696008
All − MR Egger
All − Inverse variance weighted
−3
−2
−1
0
1
MR effect size for
'PITPNB' on 'Pulmonary fibrosis'

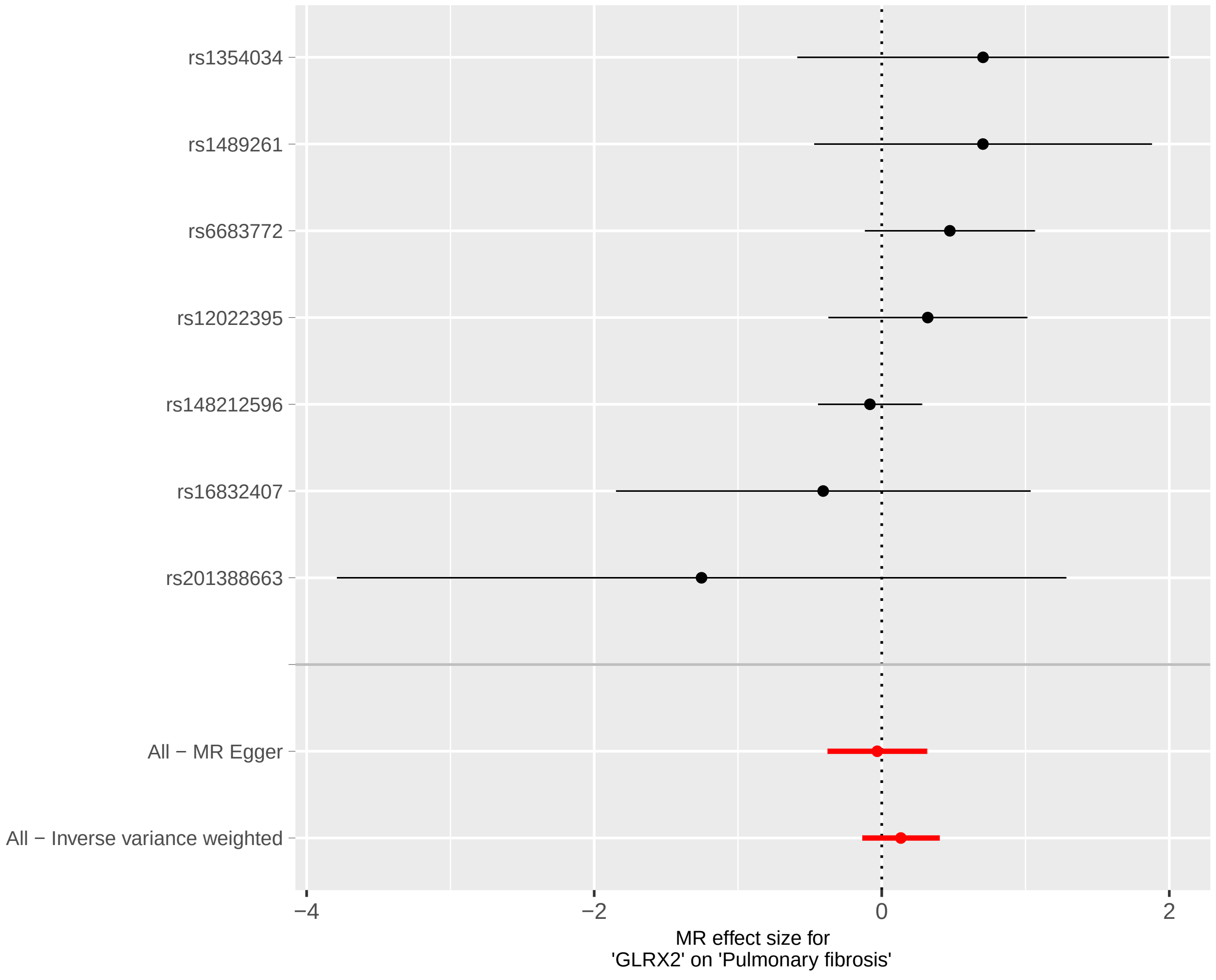
rs1354034
rs1489261
rs6683772
rs12022395
rs148212596
rs16832407
rs201388663
All − MR Egger
All − Inverse variance weighted
−4
−2
0
2
MR effect size for
'GLRX2' on 'Pulmonary fibrosis'

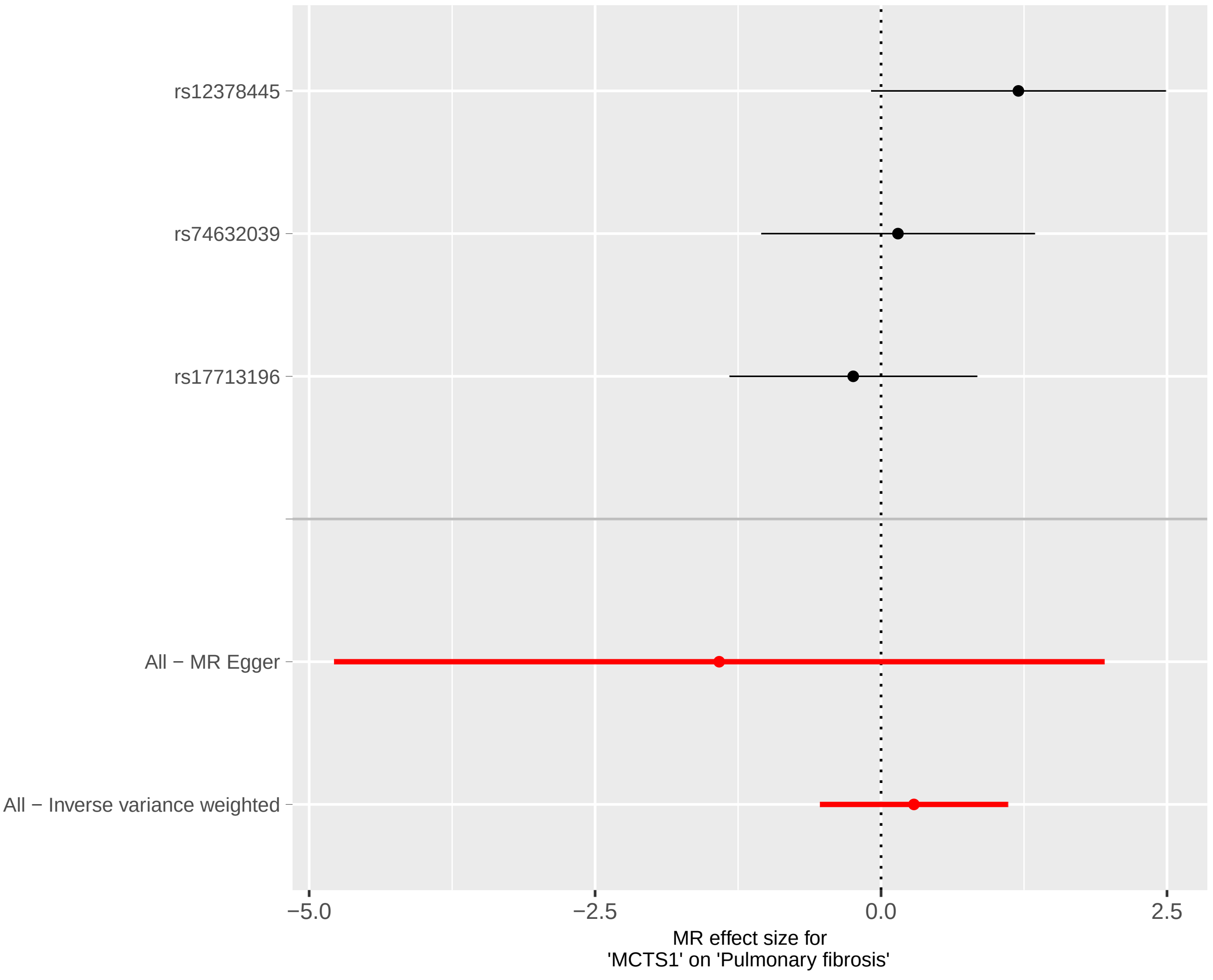
rs12378445
rs74632039
rs17713196
All − MR Egger
All − Inverse variance weighted
−5.0
−2.5
0.0
2.5
MR effect size for
'MCTS1' on 'Pulmonary fibrosis'

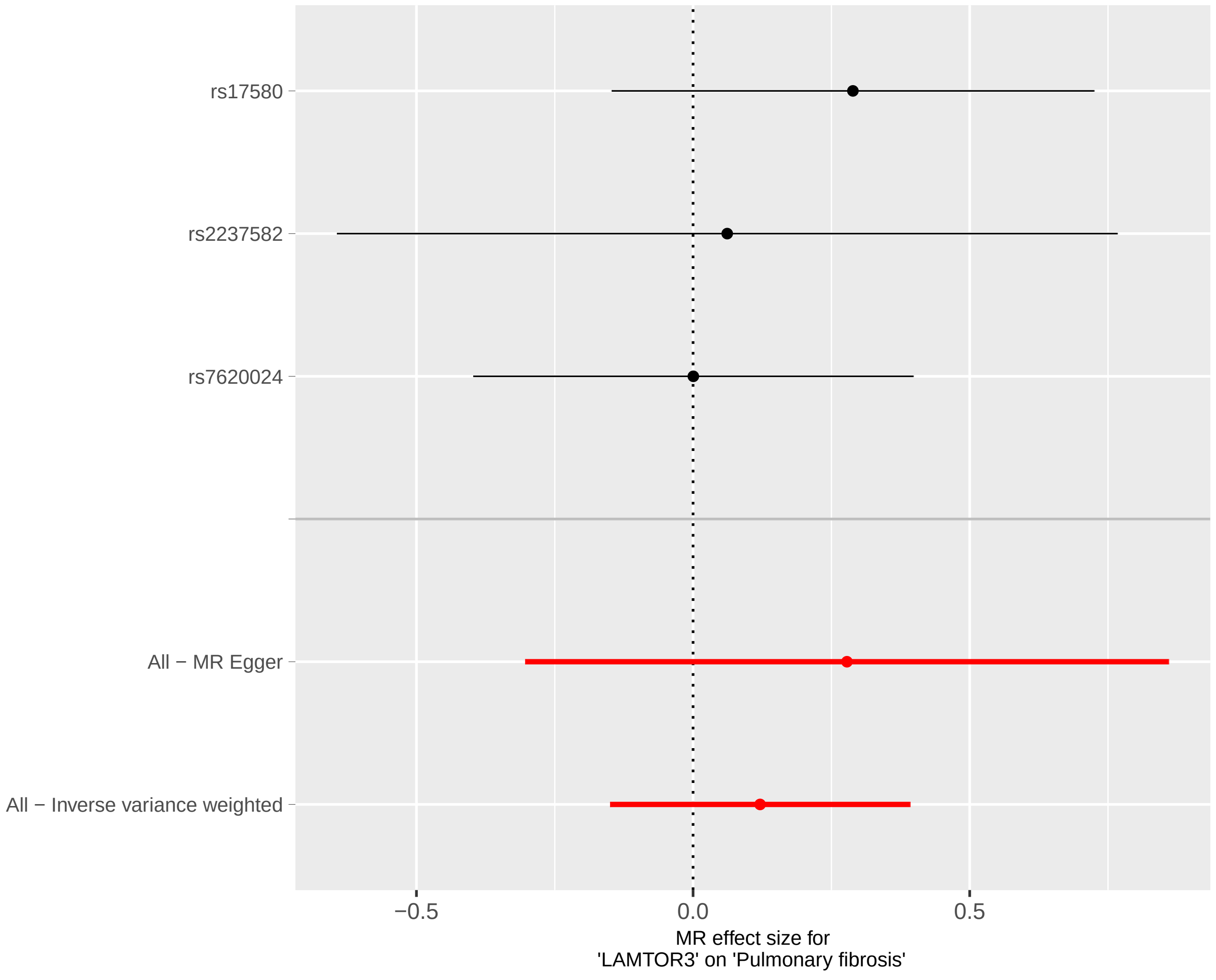
rs17580
rs2237582
rs7620024
All − MR Egger
All − Inverse variance weighted
−0.5
0.0
0.5
MR effect size for
'LAMTOR3' on 'Pulmonary fibrosis'

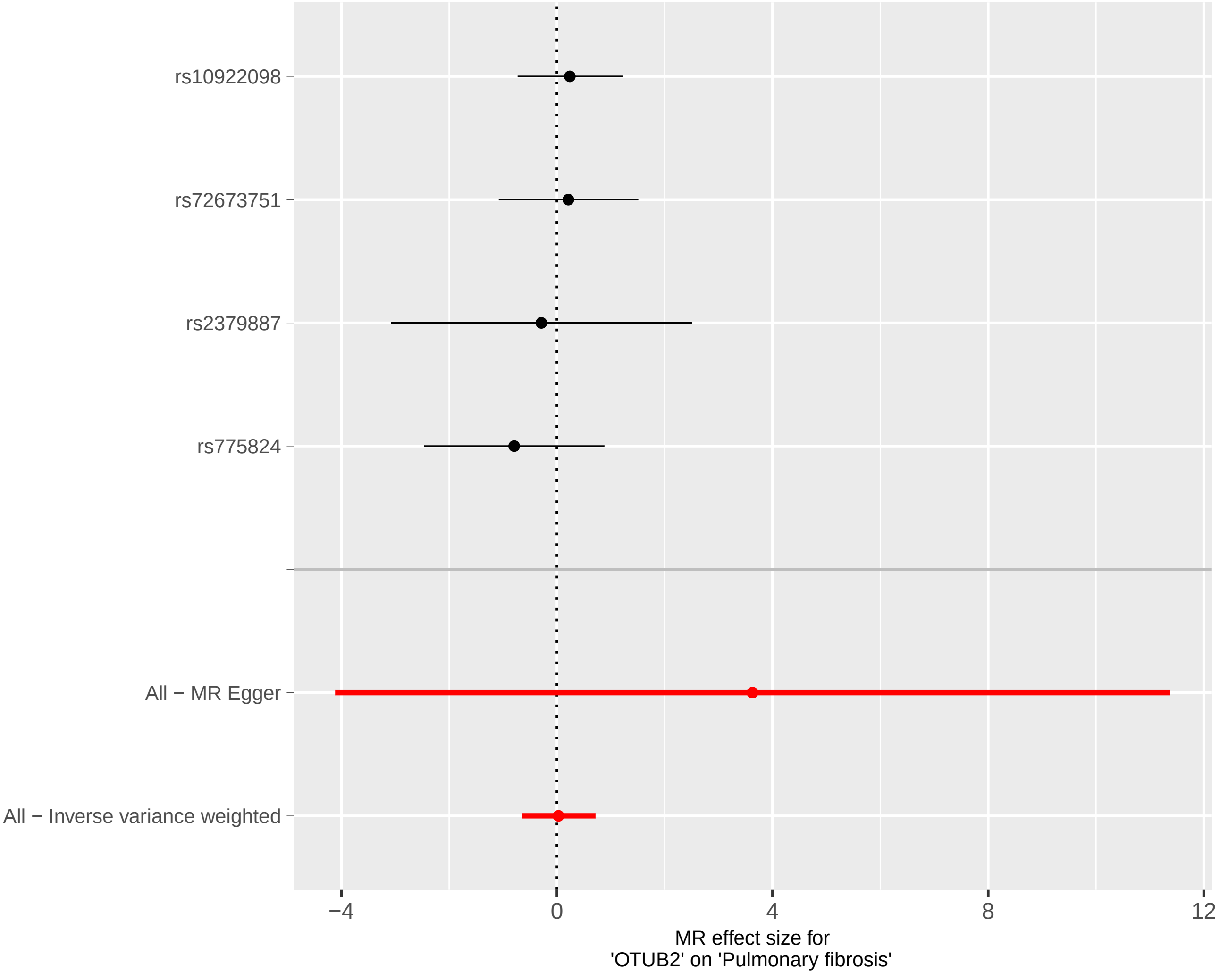
rs10922098
rs72673751
rs2379887
rs775824
All − MR Egger
All − Inverse variance weighted
−4
0
4
8
12
MR effect size for
'OTUB2' on 'Pulmonary fibrosis'

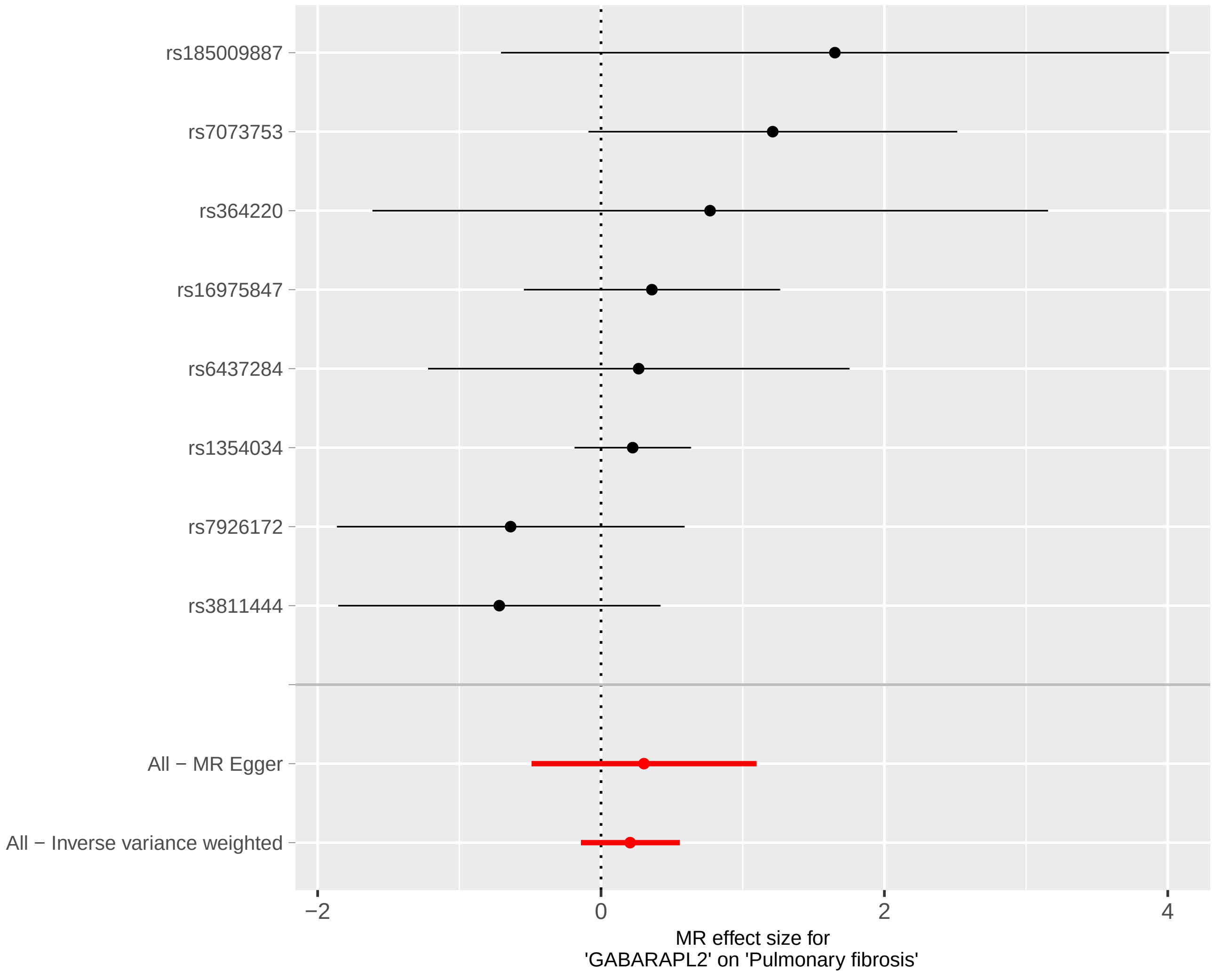
rs185009887
rs7073753
rs364220
rs16975847
rs6437284
rs1354034
rs7926172
rs3811444
All − MR Egger
All − Inverse variance weighted
−2
0
2
4
MR effect size for
'GABARAPL2' on 'Pulmonary fibrosis'

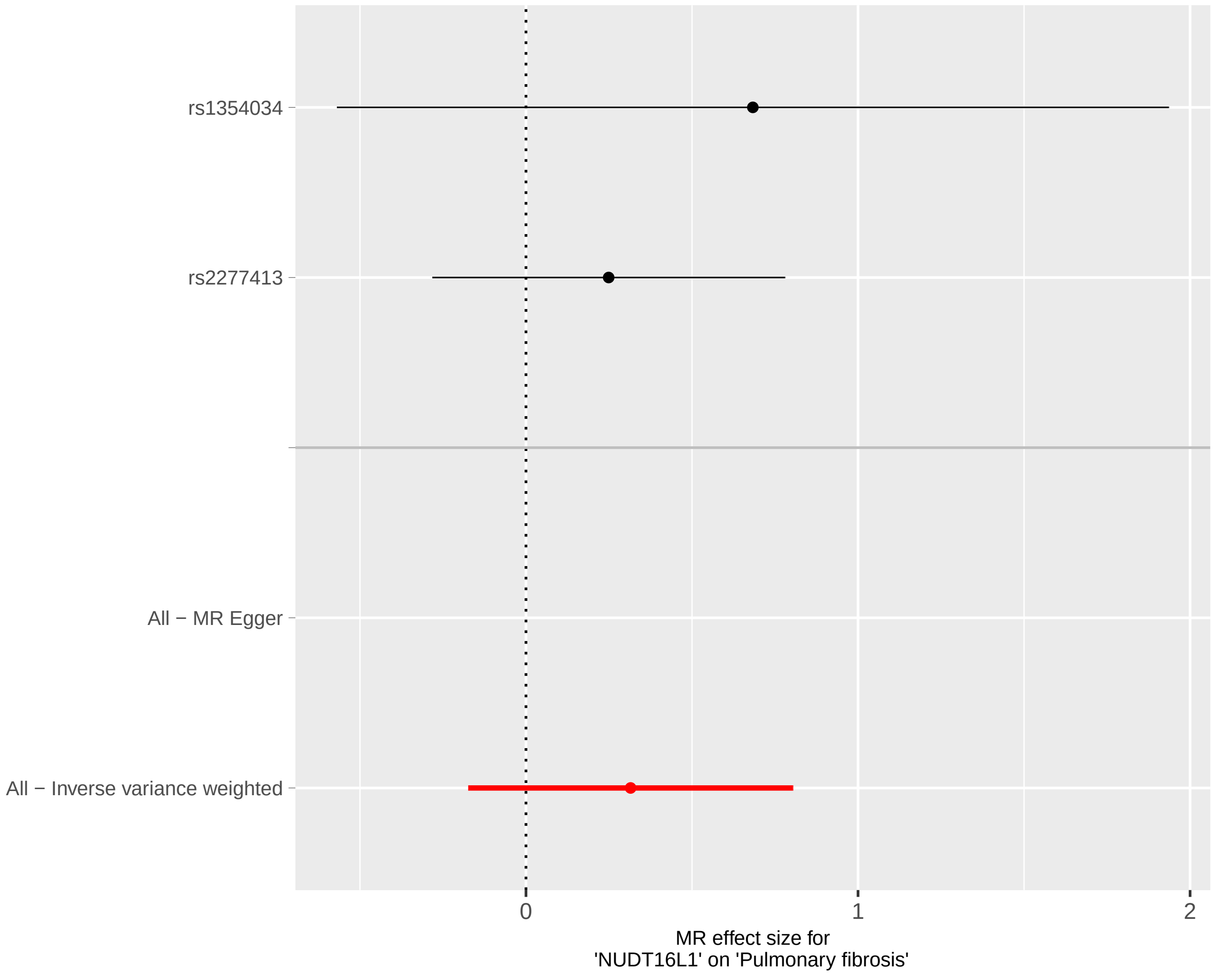
rs1354034
rs2277413
All − MR Egger
All − Inverse variance weighted
0
1
2
MR effect size for
'NUDT16L1' on 'Pulmonary fibrosis'

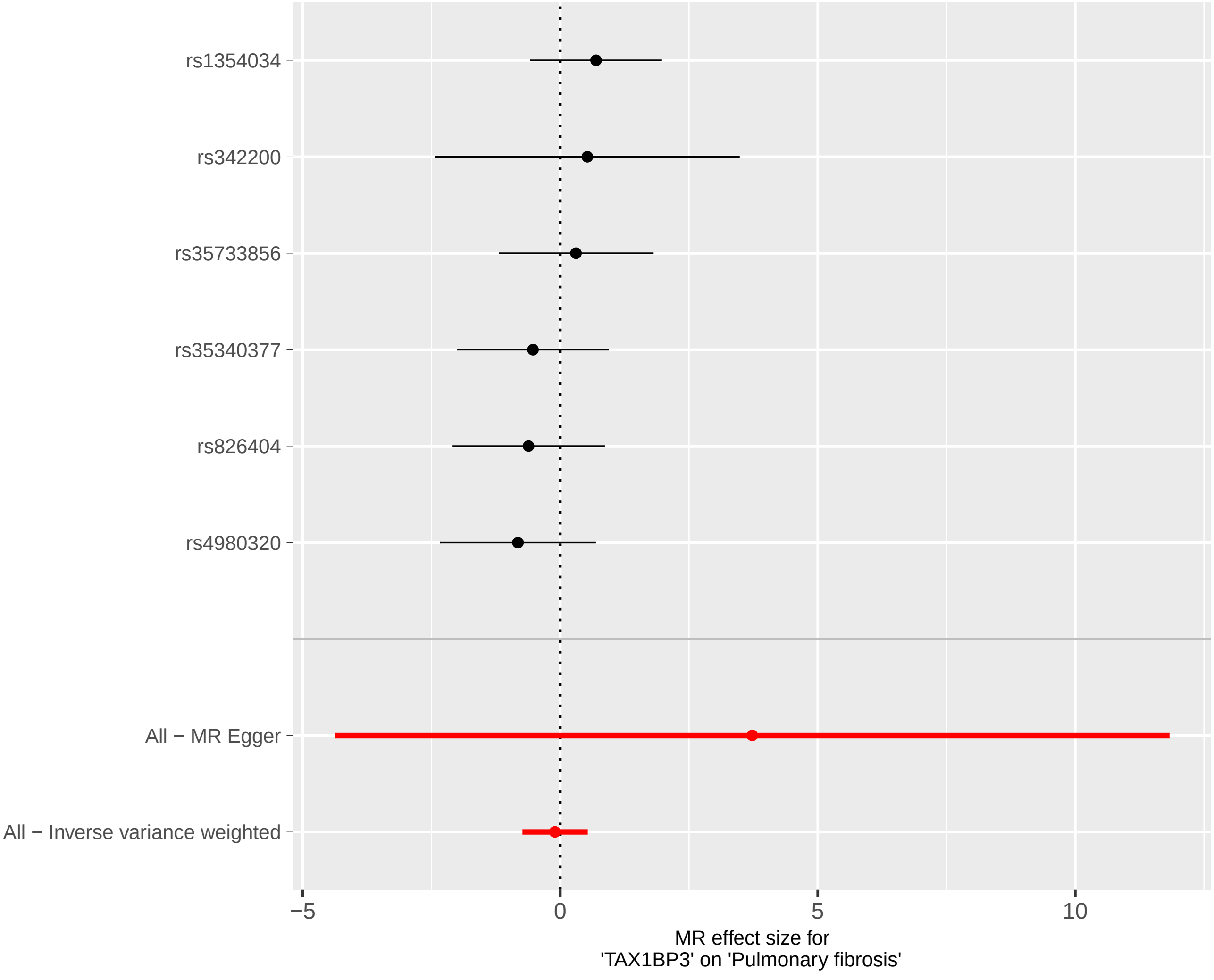

rs1354034
rs342200
rs35733856
rs35340377
rs826404
rs4980320
All − MR Egger
All − Inverse variance weighted
−5
0
5
10
MR effect size for
'TAX1BP3' on 'Pulmonary fibrosis'

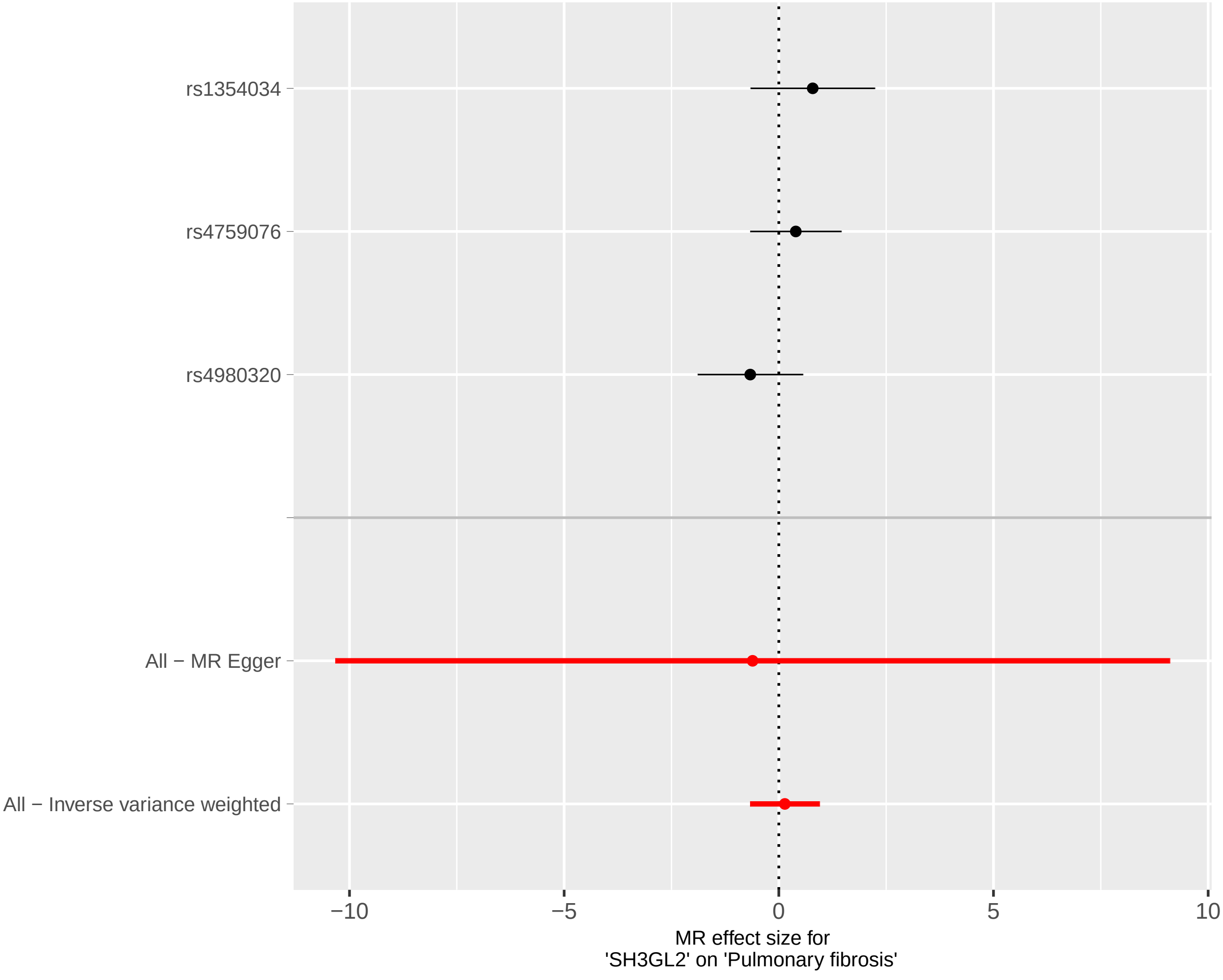
rs1354034
rs4759076
rs4980320
All − MR Egger
All − Inverse variance weighted
−10
−5
0
5
10
MR effect size for
'SH3GL2' on 'Pulmonary fibrosis'

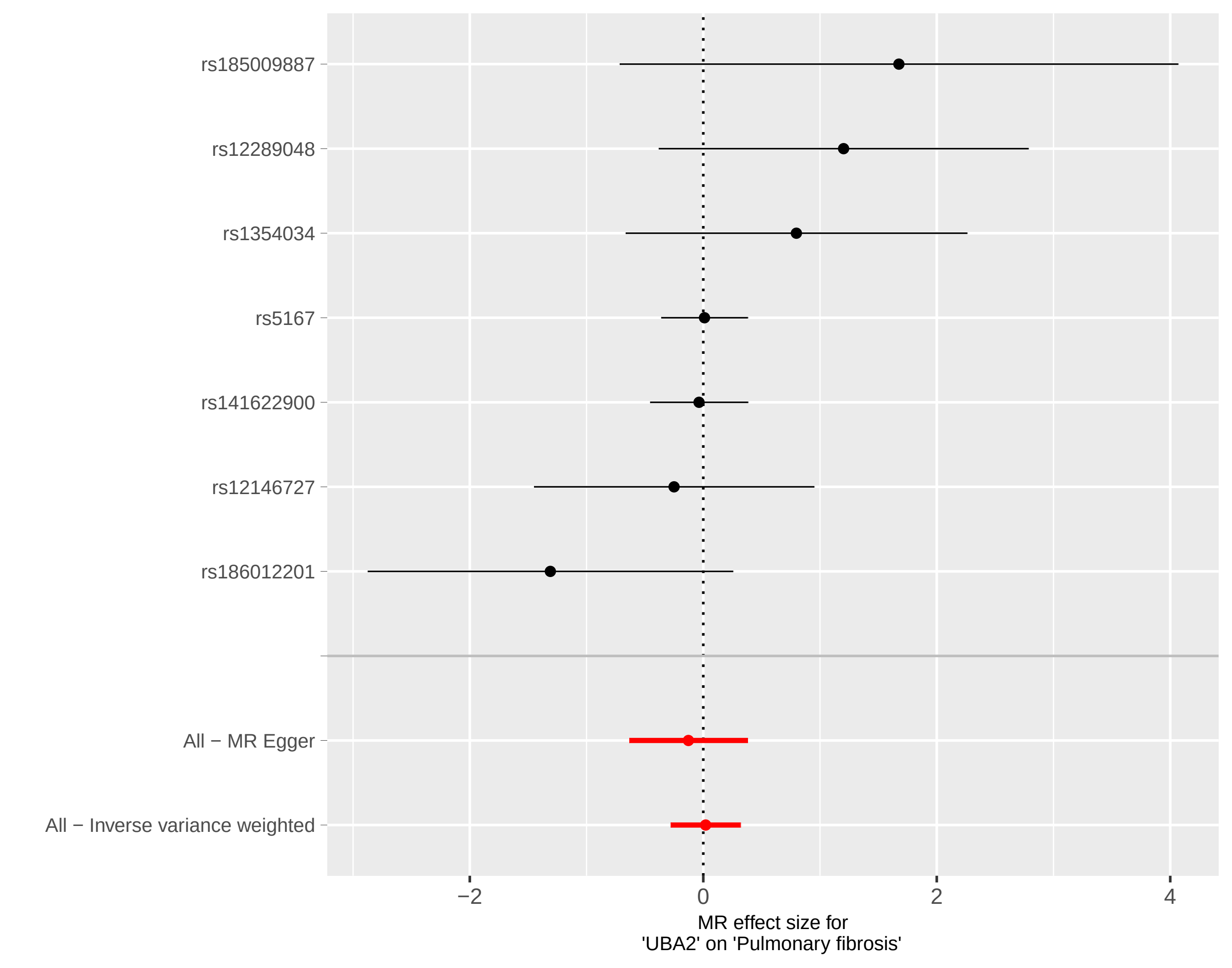
rs185009887
rs12289048
rs1354034
rs5167
rs141622900
rs12146727
rs186012201
All − MR Egger
All − Inverse variance weighted
−2
0
2
4
MR effect size for
'UBA2' on 'Pulmonary fibrosis'

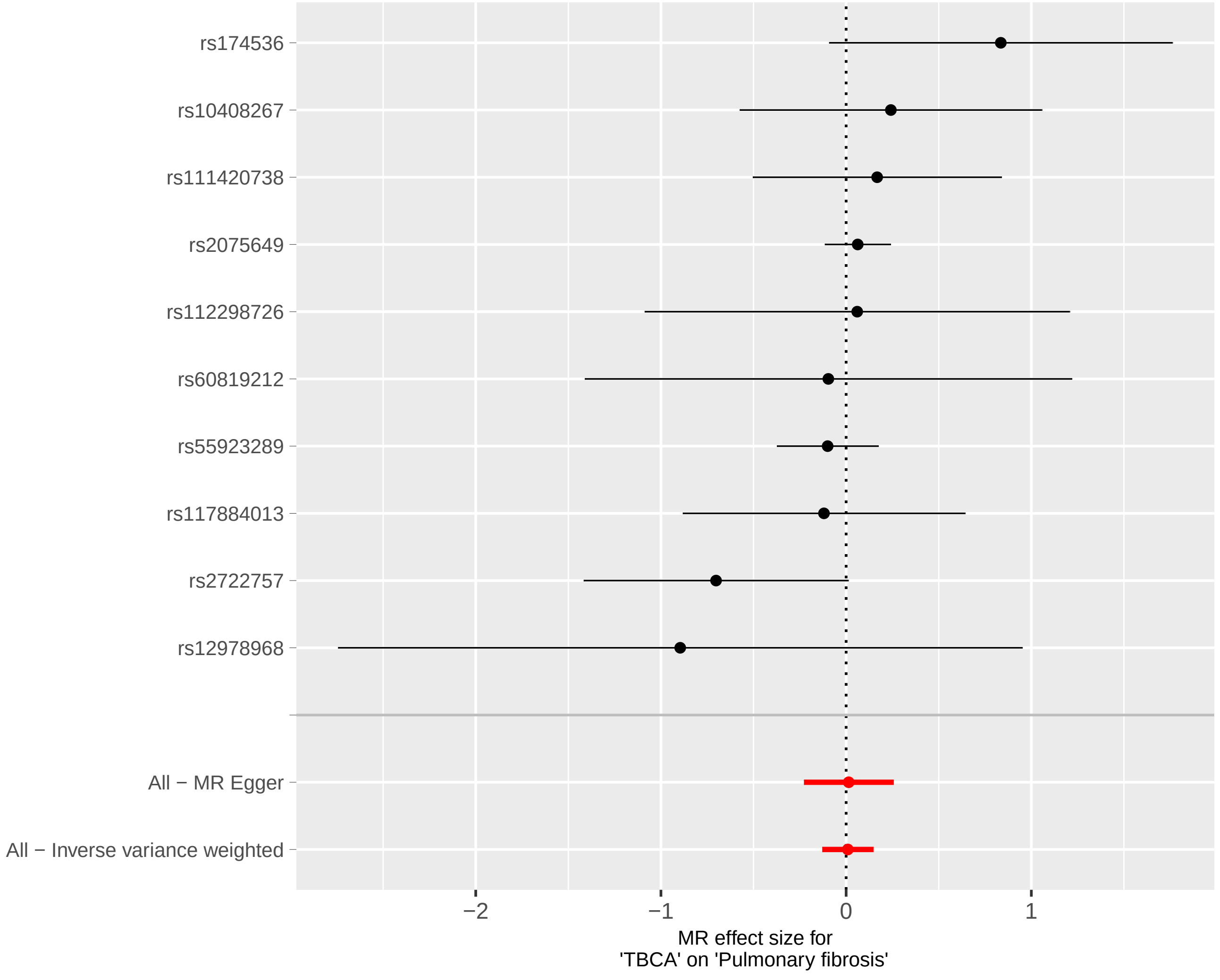
rs174536
rs10408267
rs111420738
rs2075649
rs112298726
rs60819212
rs55923289
rs117884013
rs2722757
rs12978968
All − MR Egger
All − Inverse variance weighted
−2
−1
0
1
MR effect size for
'TBCA' on 'Pulmonary fibrosis'

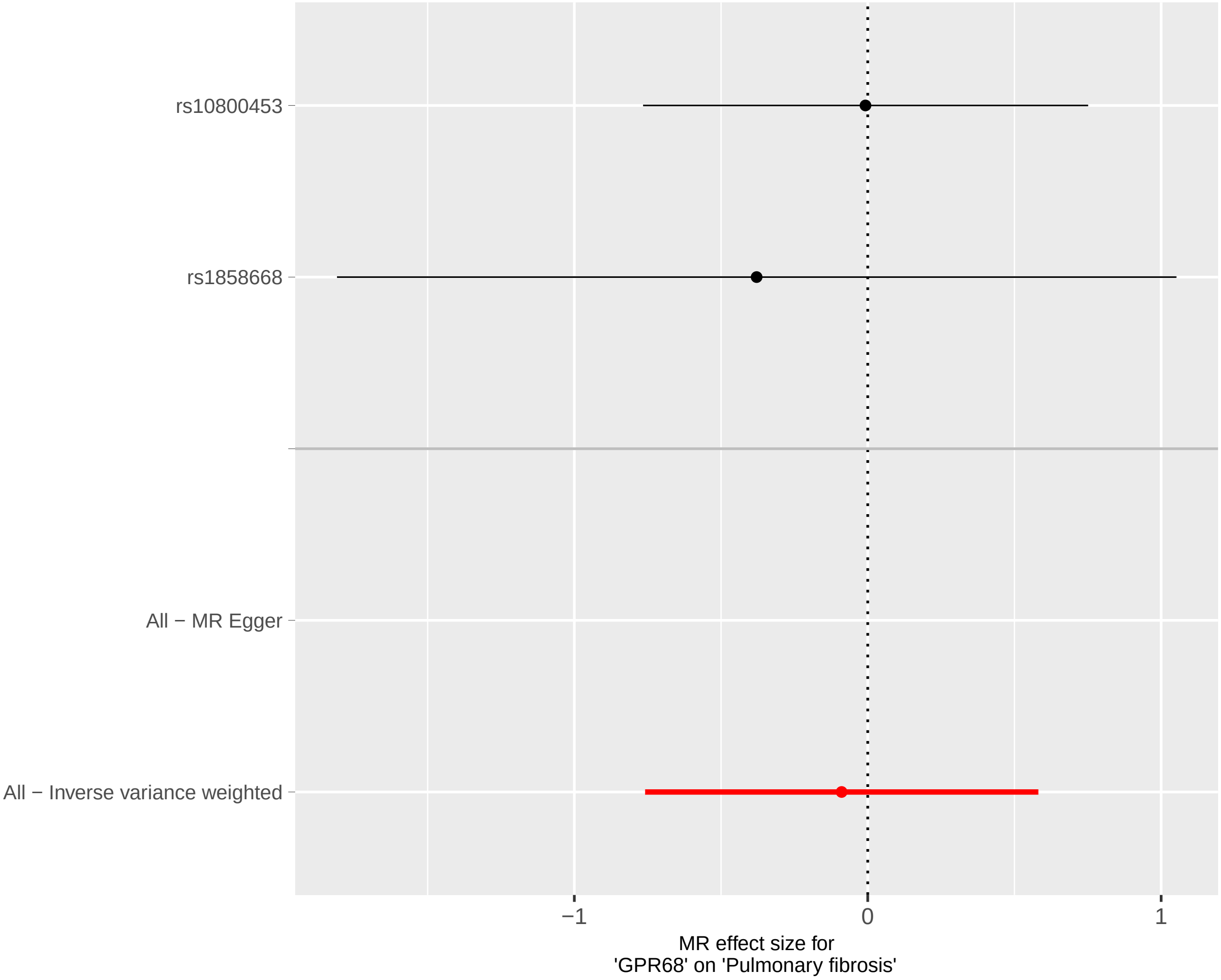
rs10800453
rs1858668
All − MR Egger
All − Inverse variance weighted
−1
0
1
MR effect size for
'GPR68' on 'Pulmonary fibrosis'

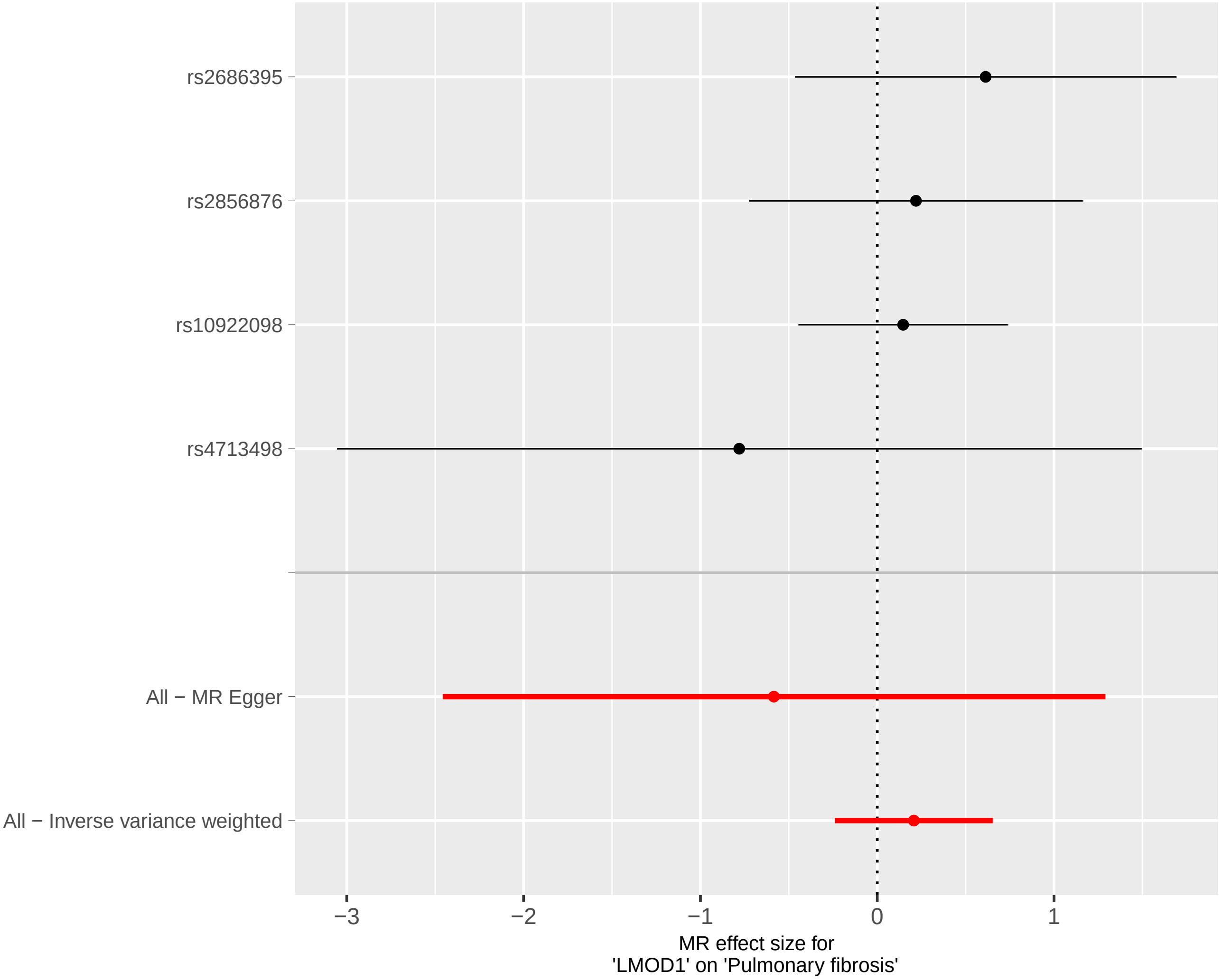

rs2686395
rs2856876
rs10922098
rs4713498
All − MR Egger
All − Inverse variance weighted
−3
−2
−1
0
1
MR effect size for
'LMOD1' on 'Pulmonary fibrosis'

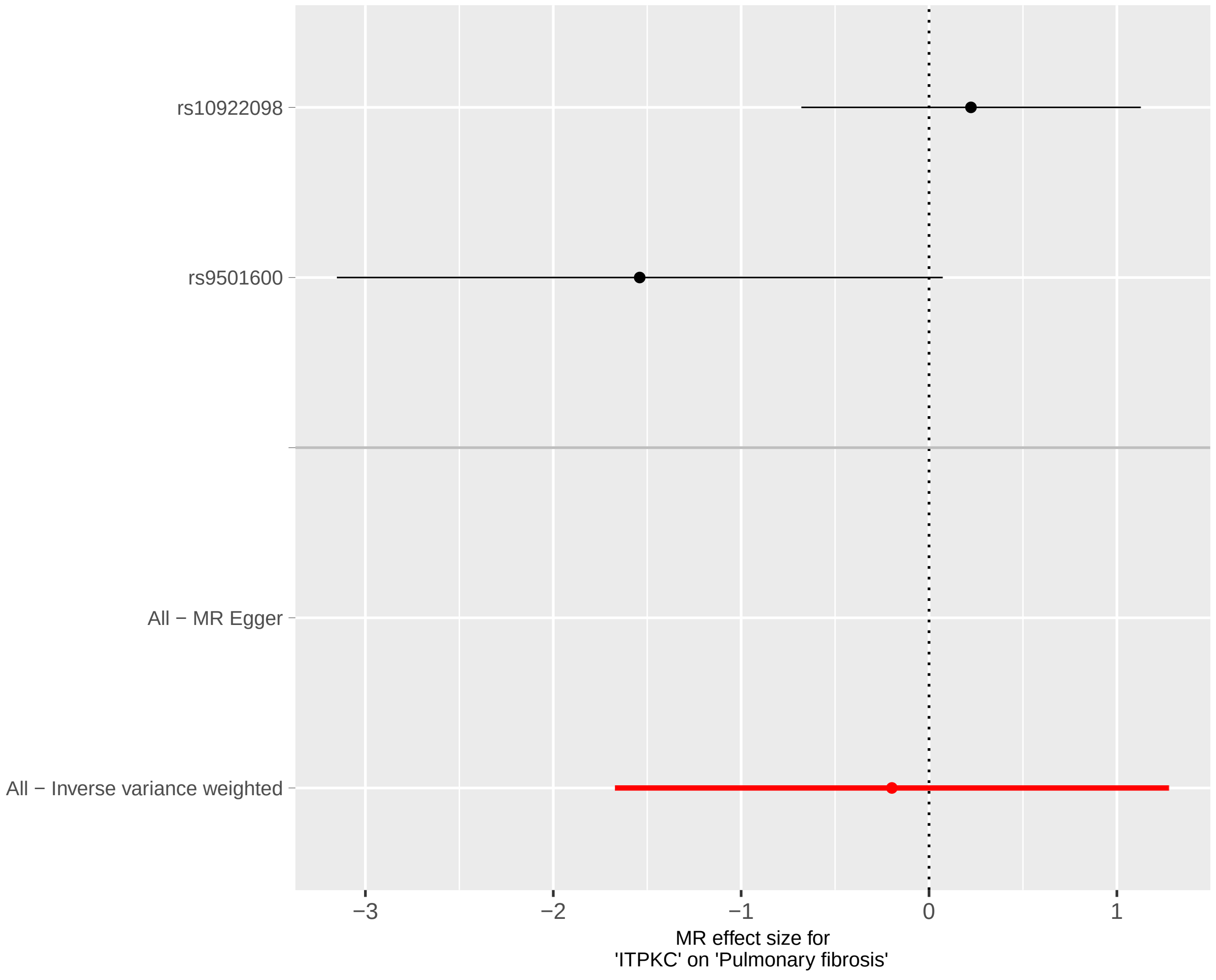
rs10922098
rs9501600
All − MR Egger
All − Inverse variance weighted
−3
−2
−1
0
1
MR effect size for
'ITPKC' on 'Pulmonary fibrosis'

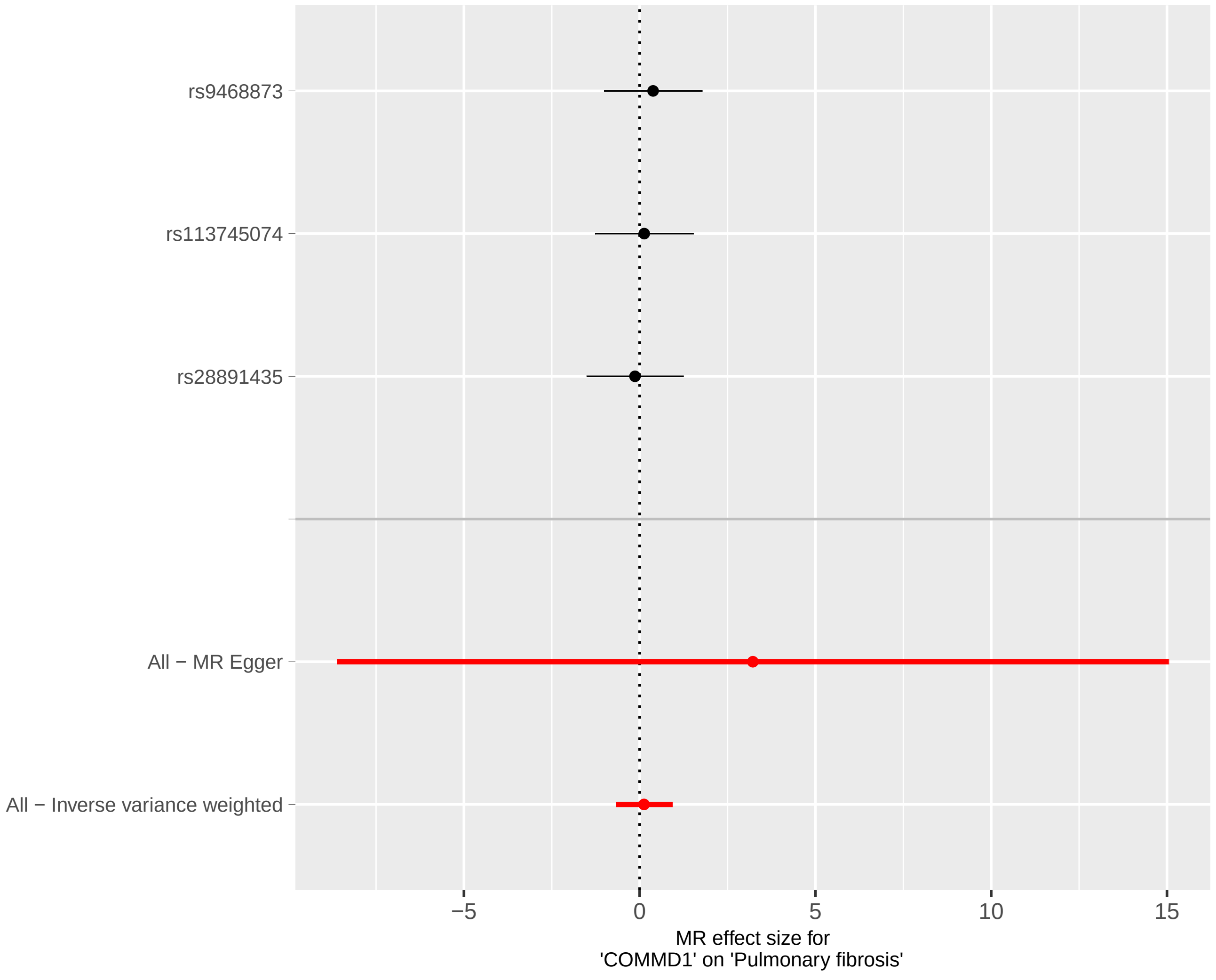
rs9468873
rs113745074
rs28891435
All − MR Egger
All − Inverse variance weighted
−5
0
5
10
15
MR effect size for
'COMMD1' on 'Pulmonary fibrosis'

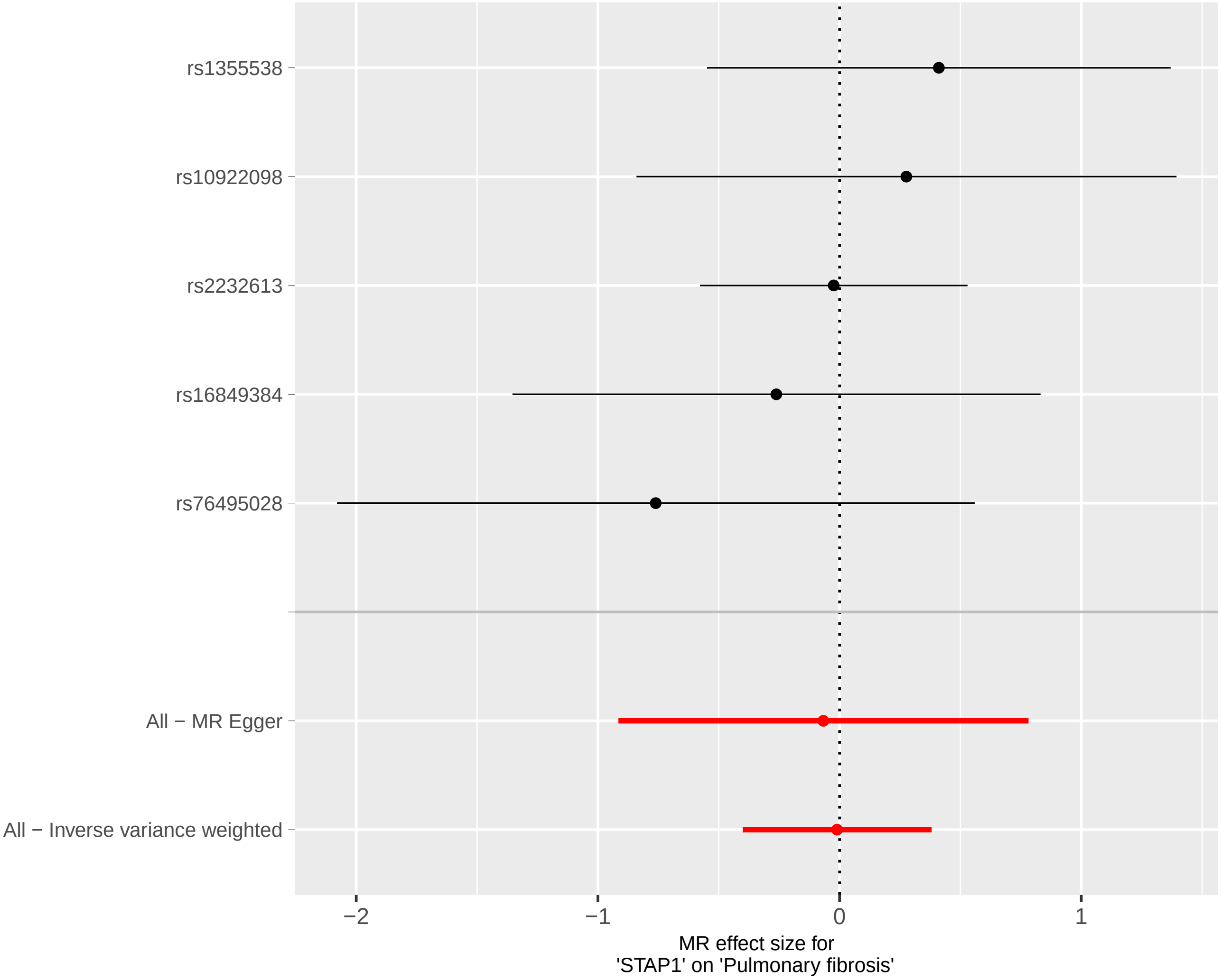
rs1355538
rs10922098
rs2232613
rs16849384
rs76495028
All − MR Egger
All − Inverse variance weighted
−2
−1
0
1
MR effect size for
'STAP1' on 'Pulmonary fibrosis'

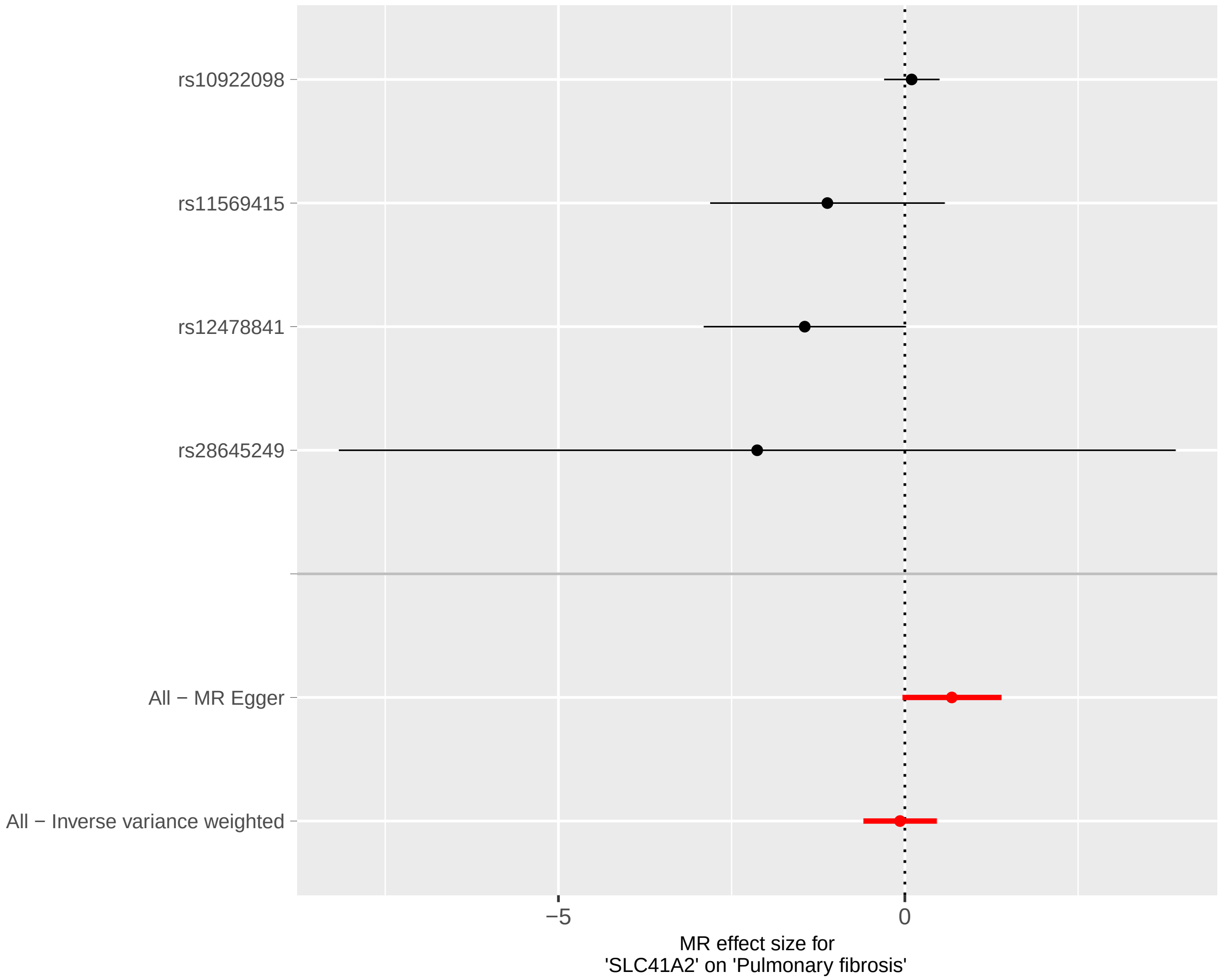
rs10922098
rs11569415
rs12478841
rs28645249
All − MR Egger
All − Inverse variance weighted
−5
0
MR effect size for
'SLC41A2' on 'Pulmonary fibrosis'

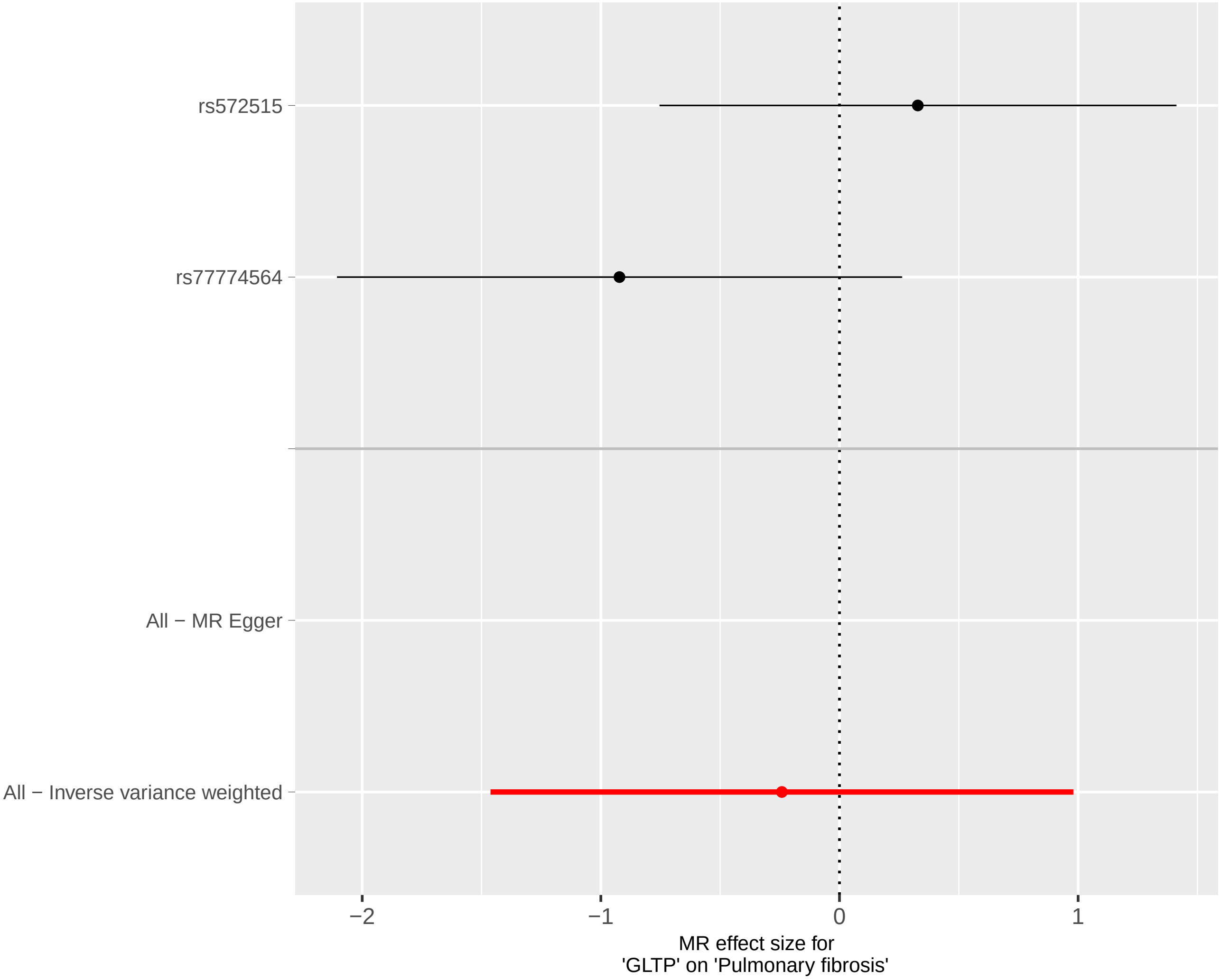

rs572515
rs77774564
All − MR Egger
All − Inverse variance weighted
−2
−1
0
1
MR effect size for
'GLTP' on 'Pulmonary fibrosis'

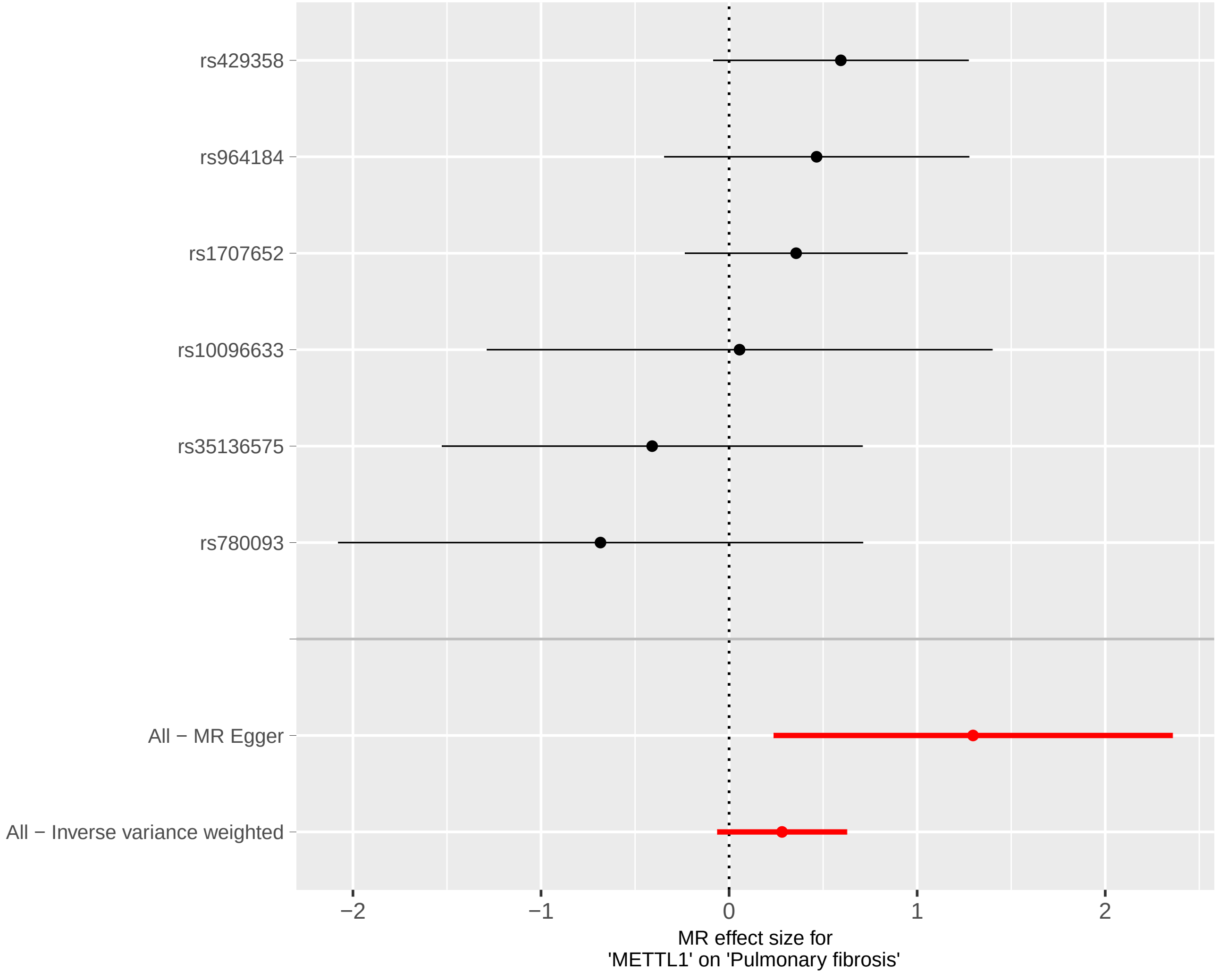
rs429358
rs964184
rs1707652
rs10096633
rs35136575
rs780093
All − MR Egger
All − Inverse variance weighted
−2
−1
0
1
2
MR effect size for
'METTL1' on 'Pulmonary fibrosis'

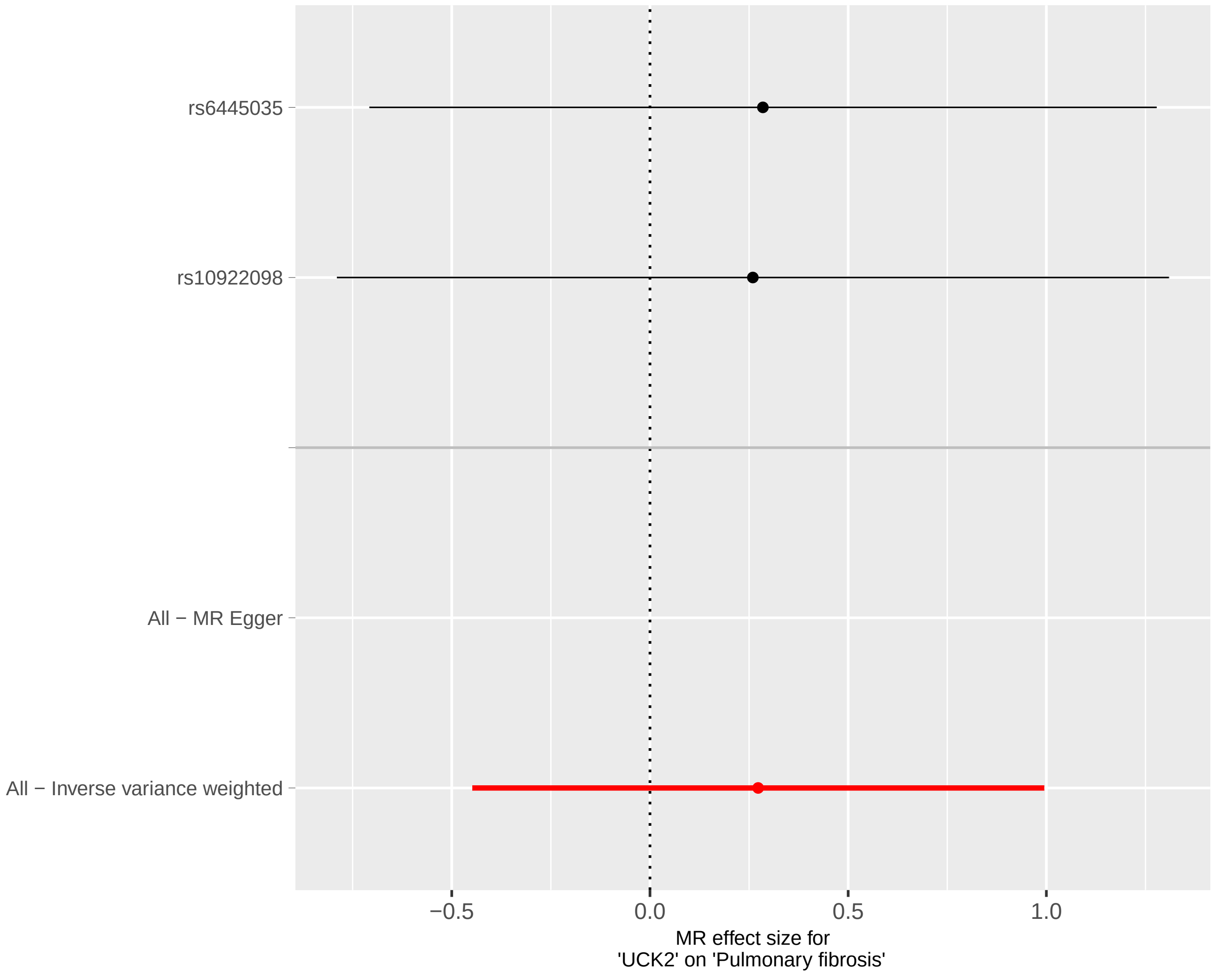
rs6445035
rs10922098
All − MR Egger
All − Inverse variance weighted
−0.5
0.0
0.5
1.0
MR effect size for
'UCK2' on 'Pulmonary fibrosis'

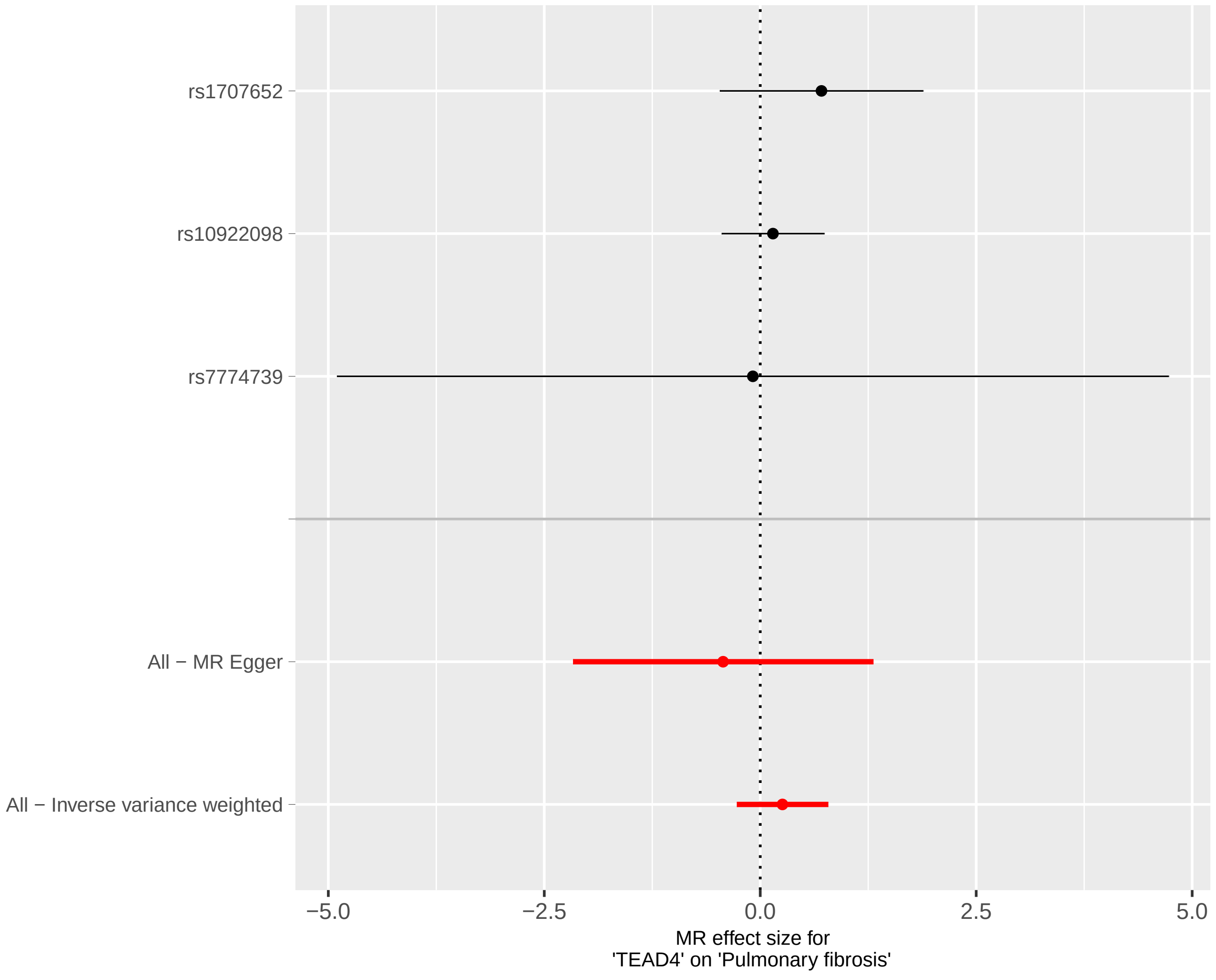
rs1707652
rs10922098
rs7774739
All − MR Egger
All − Inverse variance weighted
−5.0
−2.5
0.0
2.5
5.0
MR effect size for
'TEAD4' on 'Pulmonary fibrosis'

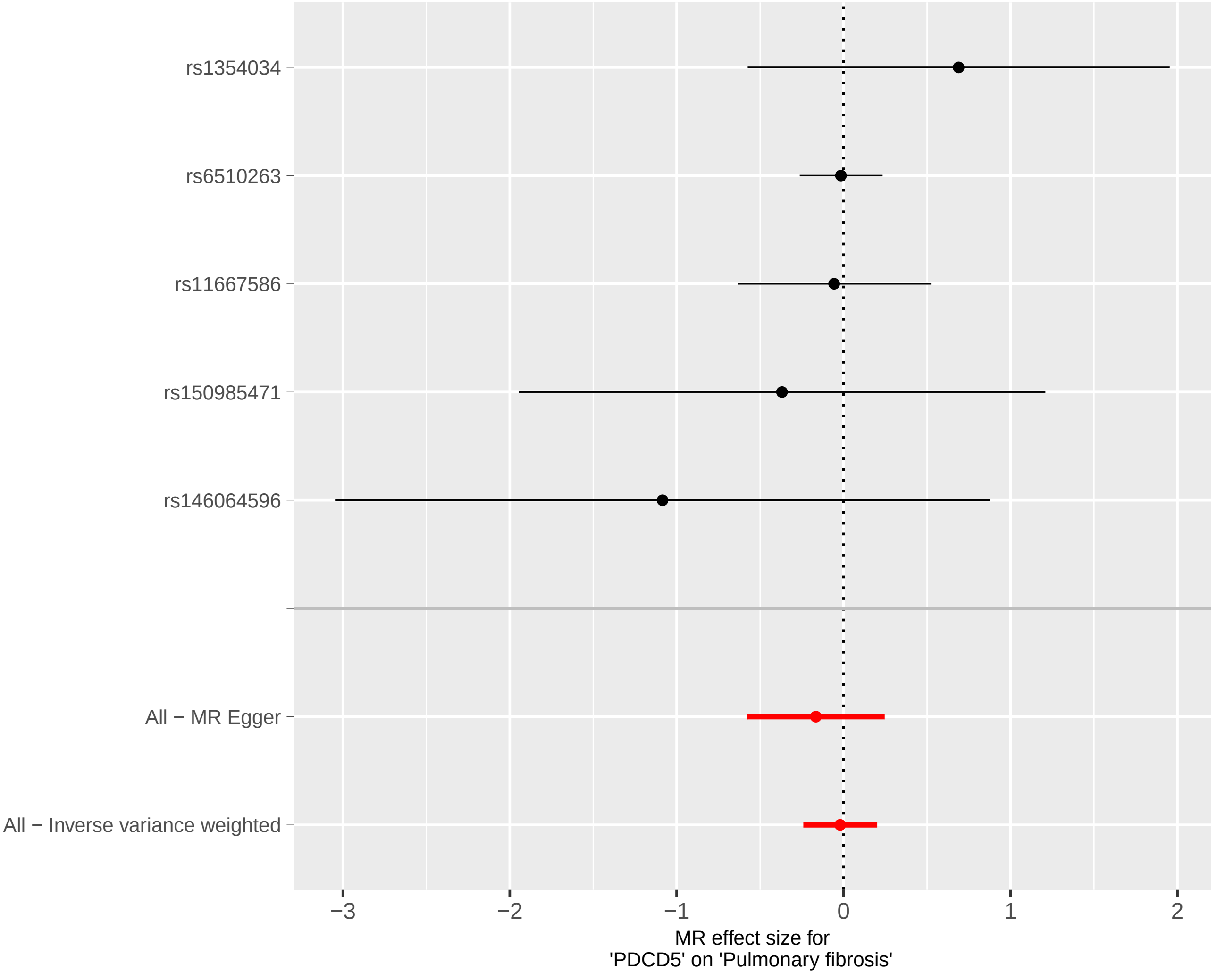
rs1354034
rs6510263
rs11667586
rs150985471
rs146064596
All − MR Egger
All − Inverse variance weighted
−3
−2
−1
0
1
2
MR effect size for
'PDCD5' on 'Pulmonary fibrosis'

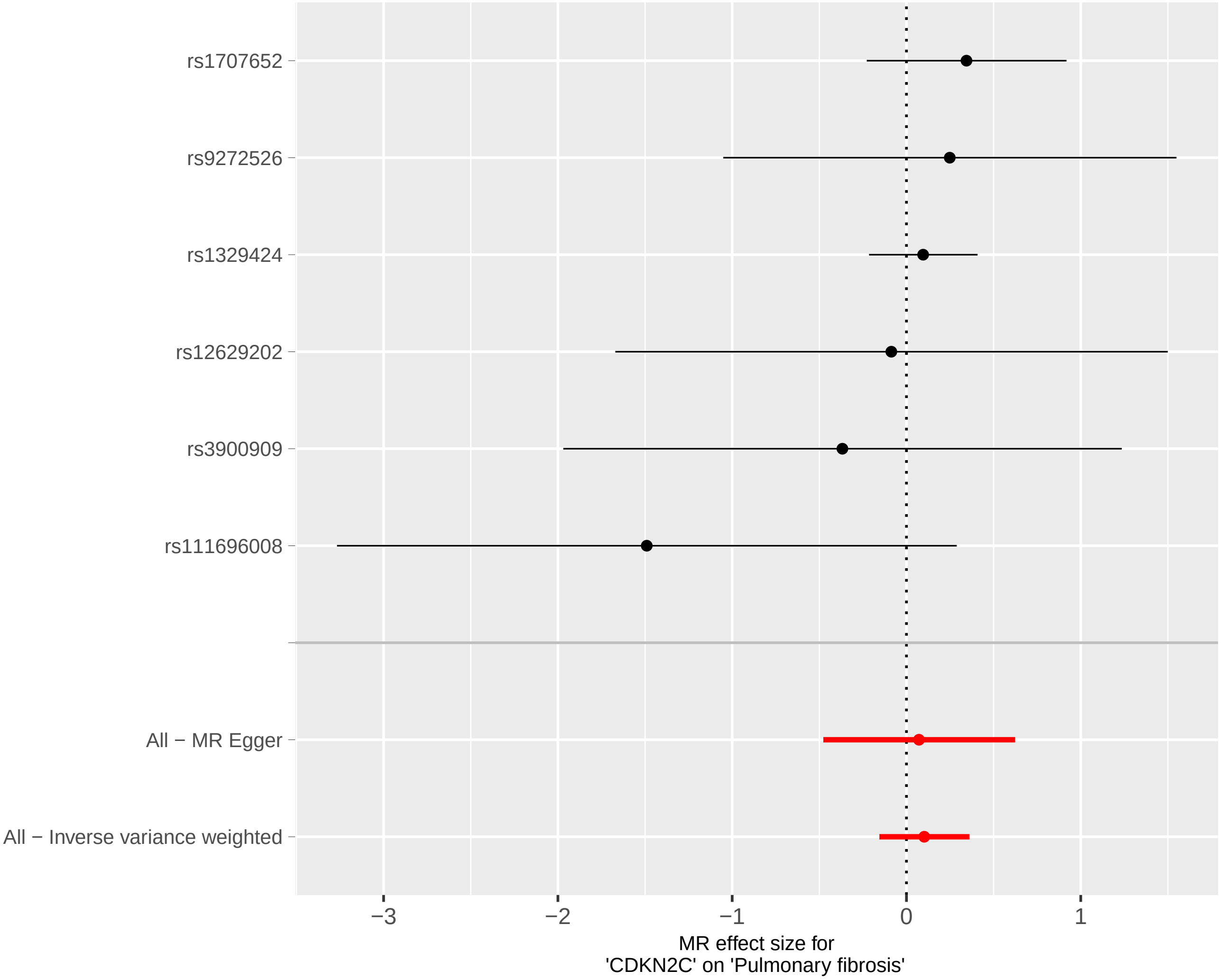

rs1707652
rs9272526
rs1329424
rs12629202
rs3900909
rs111696008
All − MR Egger
All − Inverse variance weighted
−3
−2
−1
0
1
MR effect size for
'CDKN2C' on 'Pulmonary fibrosis'

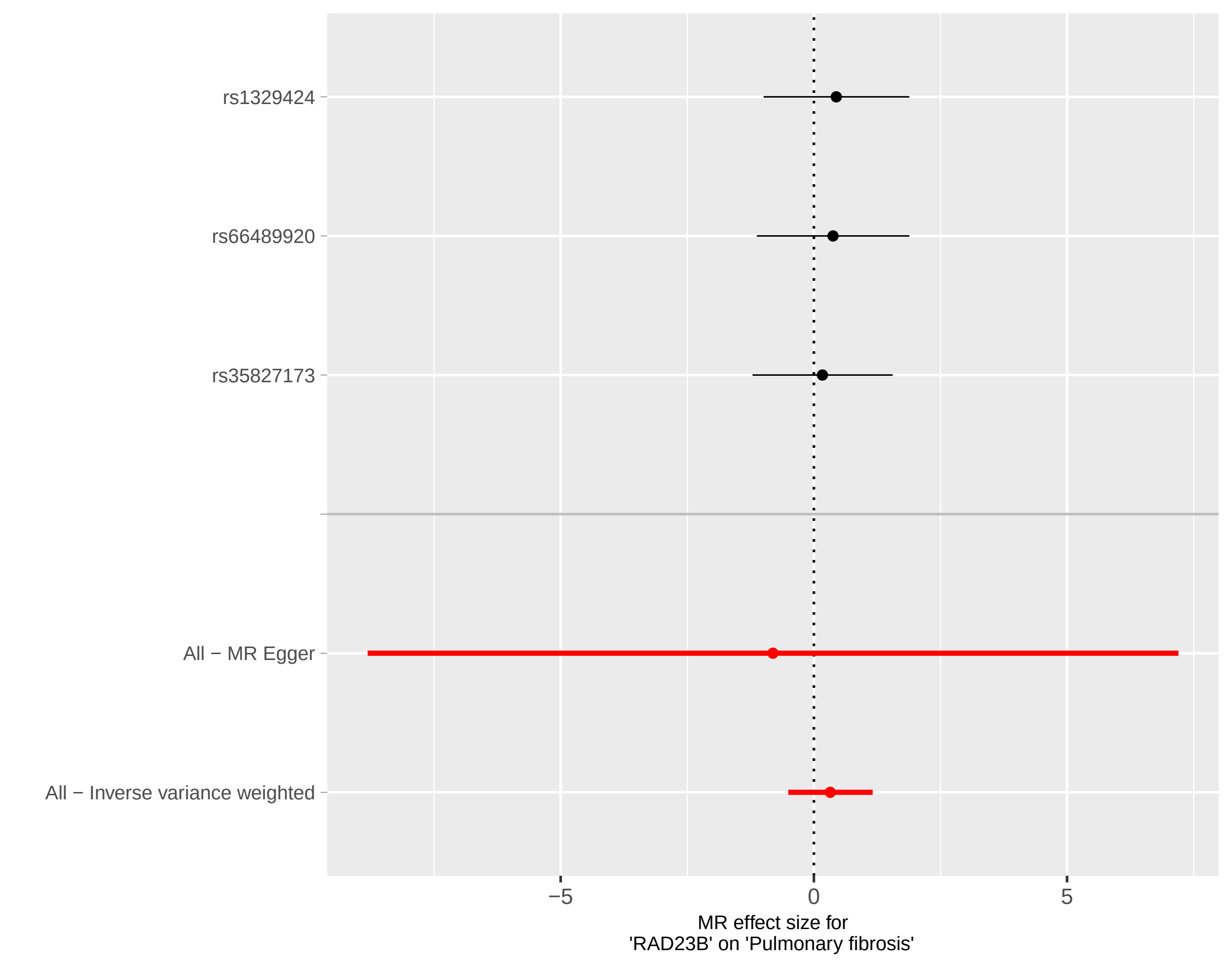
rs1329424
rs66489920
rs35827173
All − MR Egger
All − Inverse variance weighted
−5
0
5
MR effect size for
'RAD23B' on 'Pulmonary fibrosis'

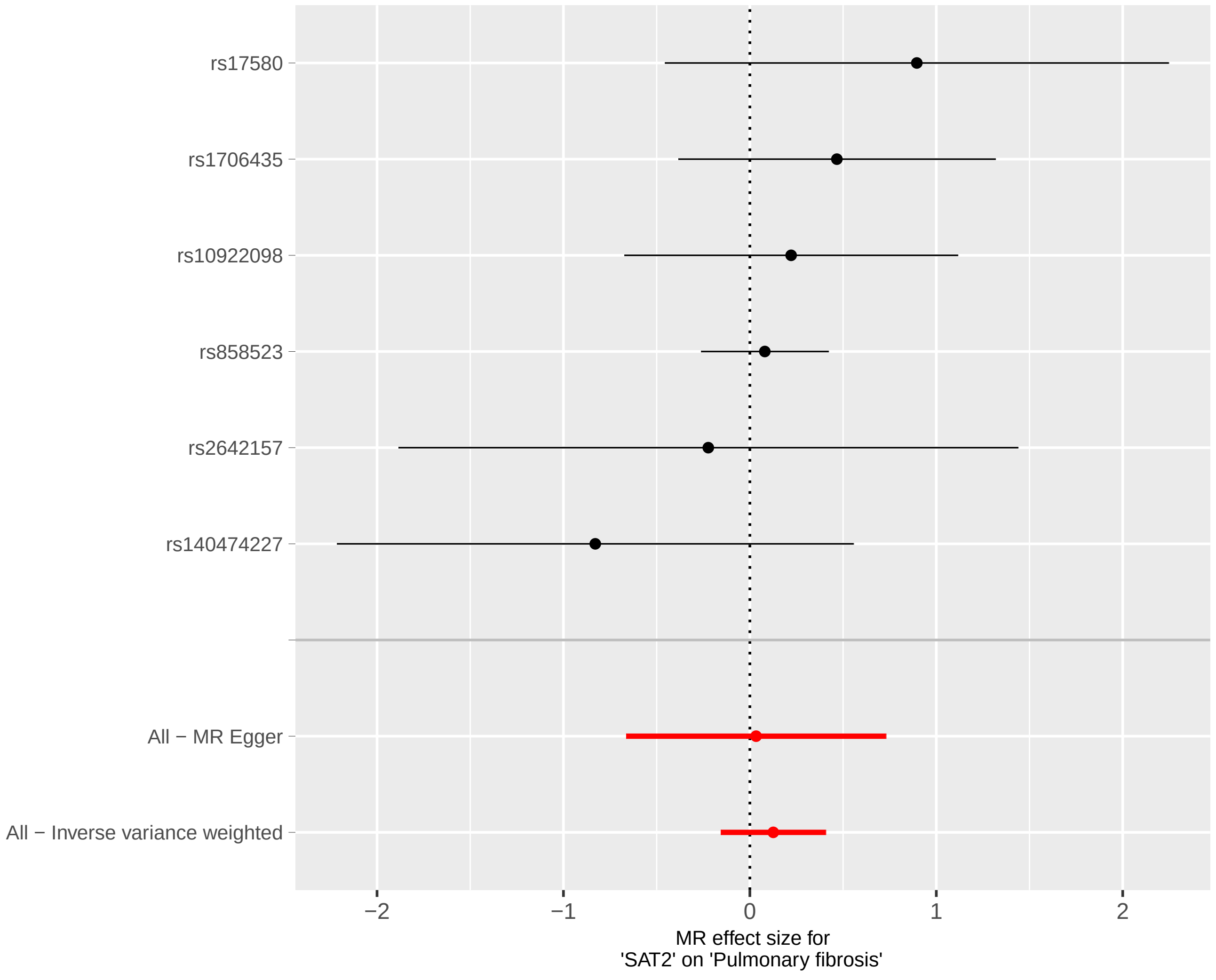
rs17580
rs1706435
rs10922098
rs858523
rs2642157
rs140474227
All − MR Egger
All − Inverse variance weighted
−2
−1
0
1
2
MR effect size for
'SAT2' on 'Pulmonary fibrosis'

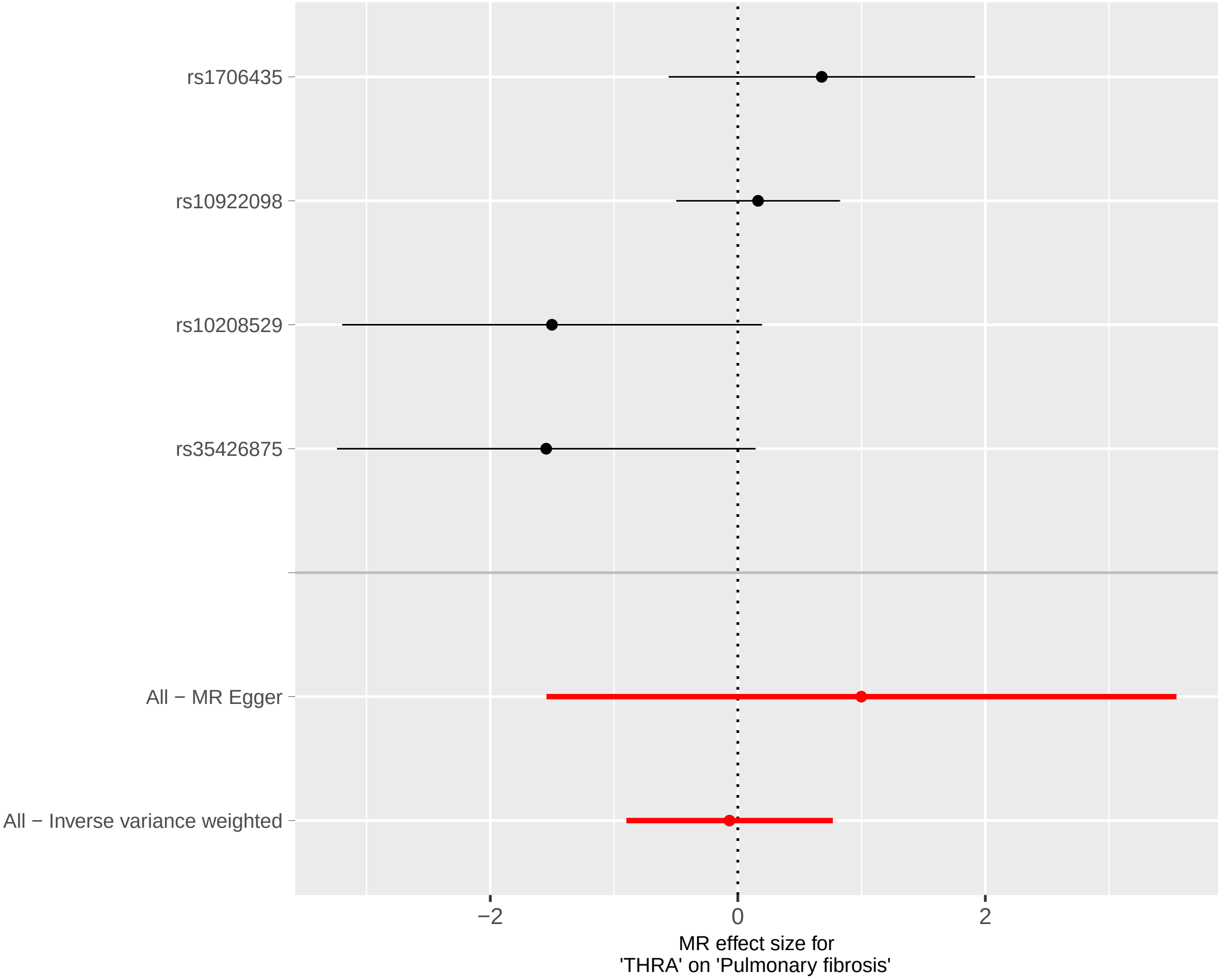
rs1706435
rs10922098
rs10208529
rs35426875
All − MR Egger
All − Inverse variance weighted
−2
0
2
MR effect size for
'THRA' on 'Pulmonary fibrosis'

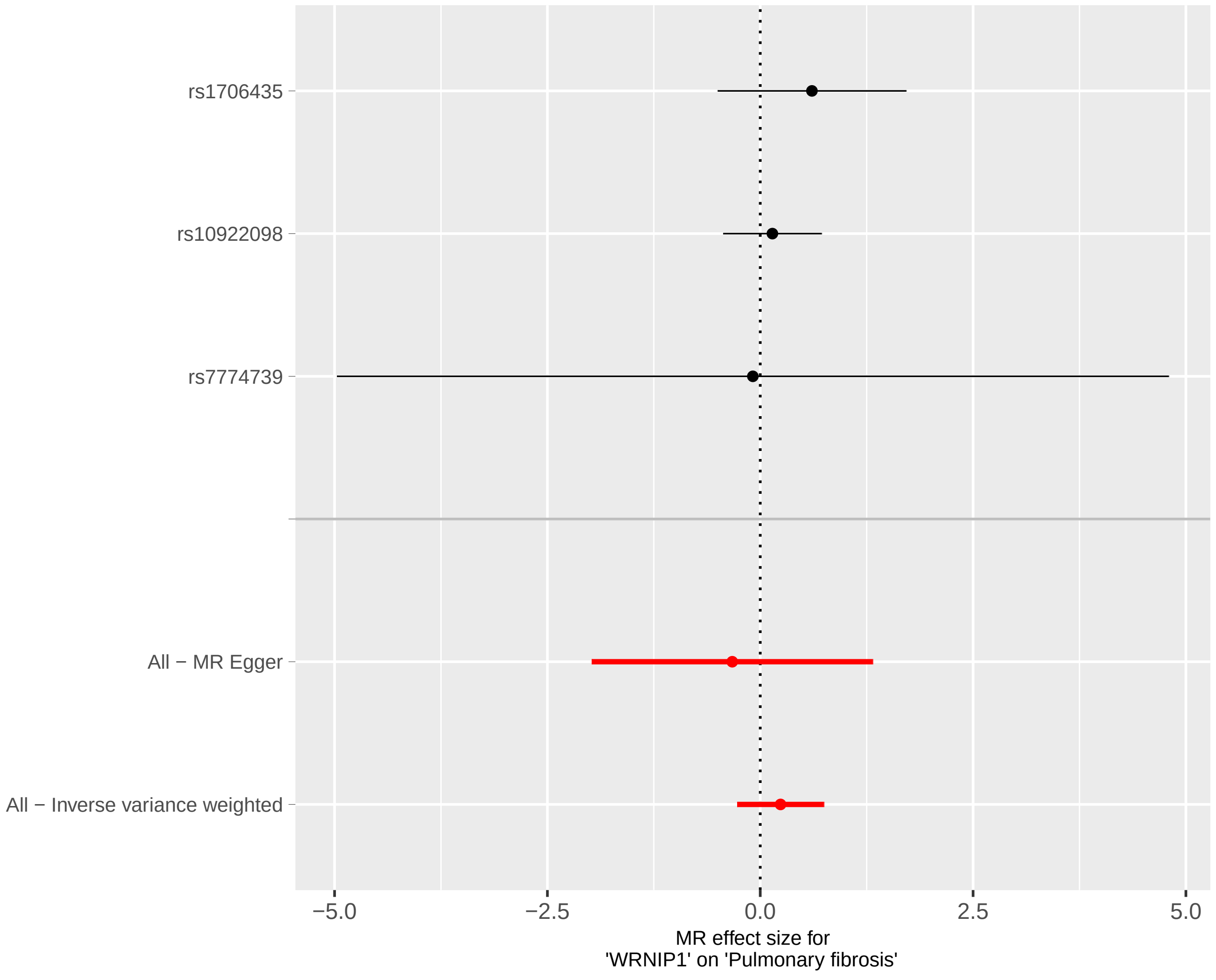
rs1706435
rs10922098
rs7774739
All − MR Egger
All − Inverse variance weighted
−5.0
−2.5
0.0
2.5
5.0
MR effect size for
'WRNIP1' on 'Pulmonary fibrosis'

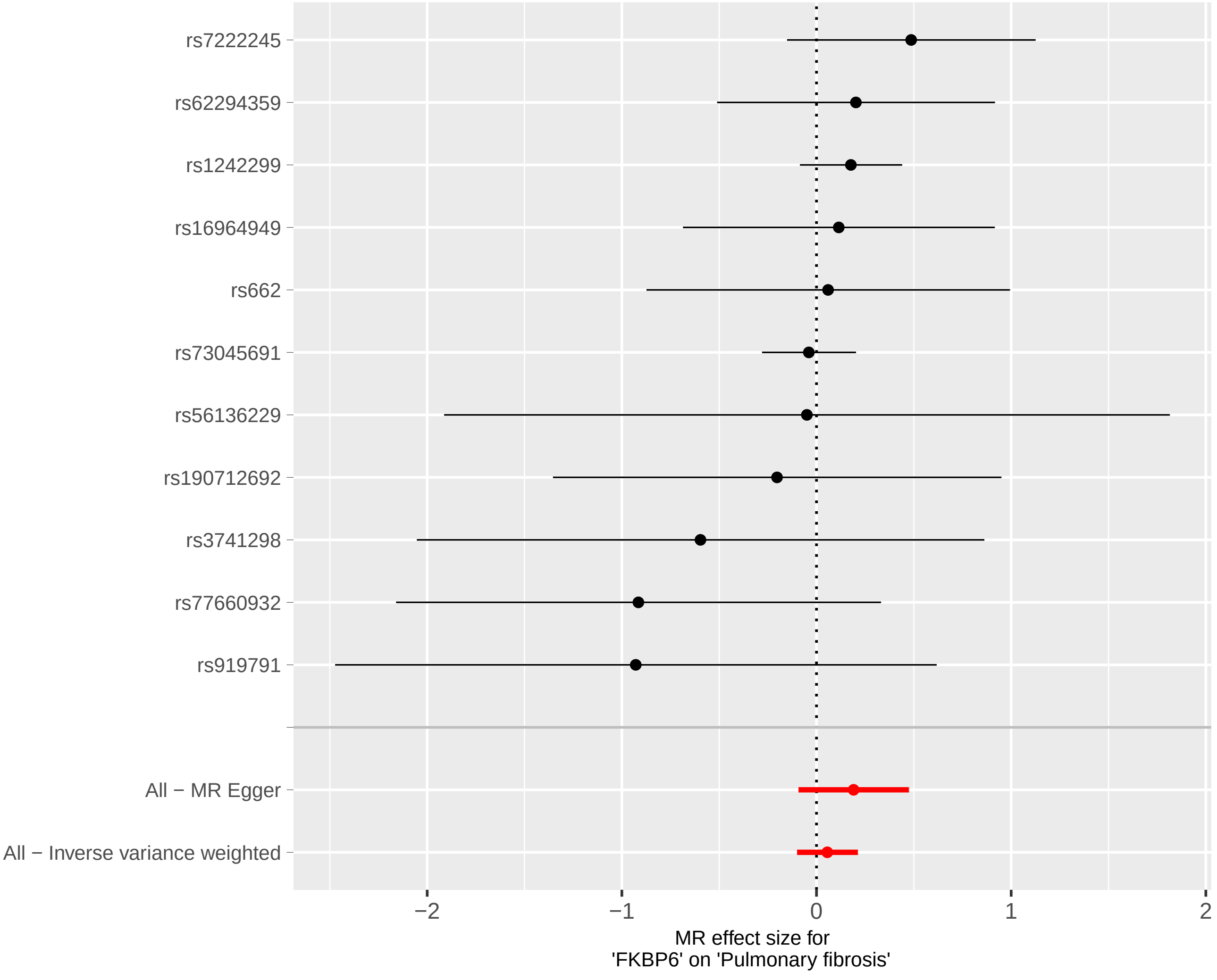

rs7222245
rs62294359
rs1242299
rs16964949
rs662
rs73045691
rs56136229
rs190712692
rs3741298
rs77660932
rs919791
All − MR Egger
All − Inverse variance weighted
−2
−1
0
1
2
MR effect size for
'FKBP6' on 'Pulmonary fibrosis'

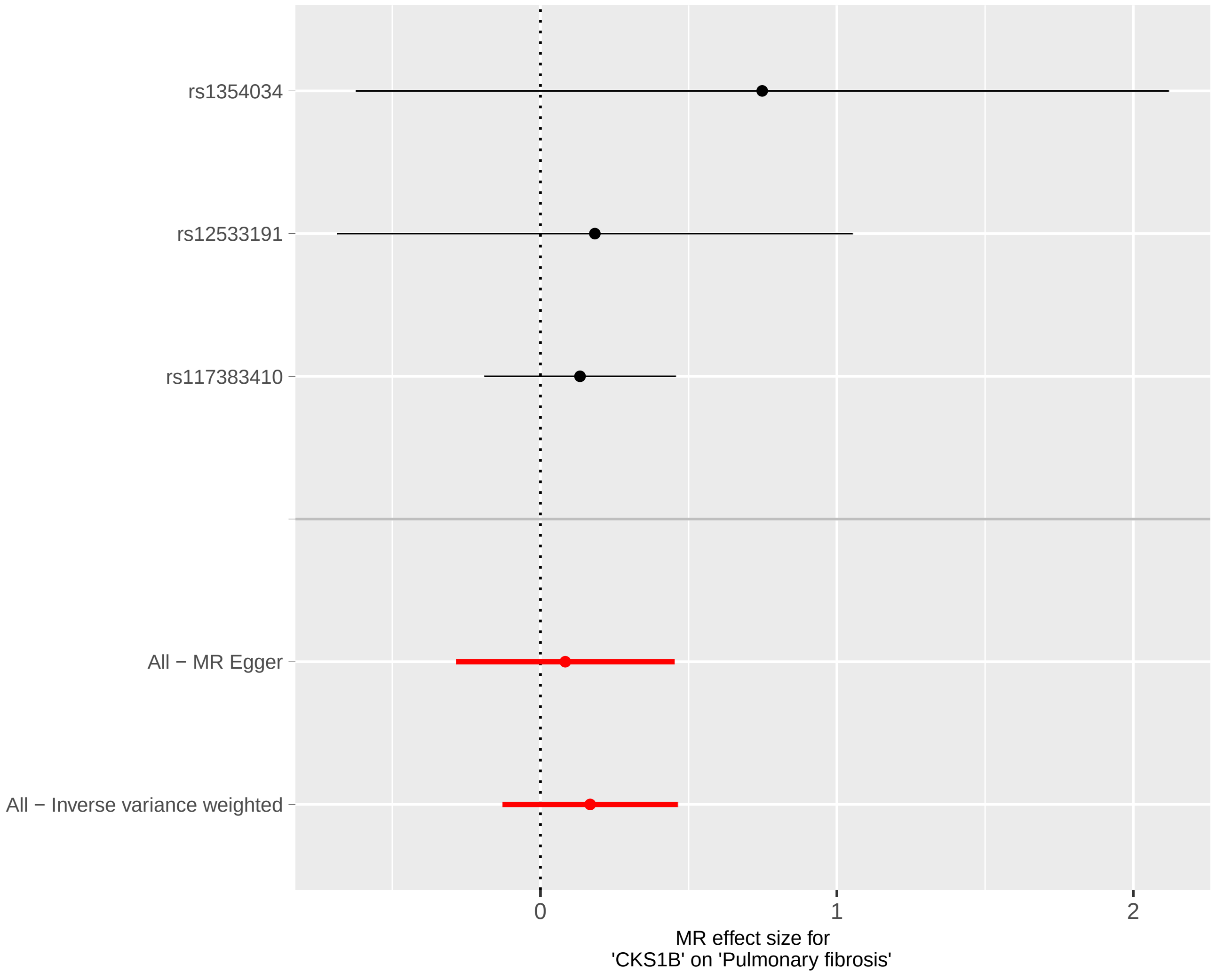

rs1354034
rs12533191
rs117383410
All − MR Egger
All − Inverse variance weighted
0
1
2
MR effect size for
'CKS1B' on 'Pulmonary fibrosis'

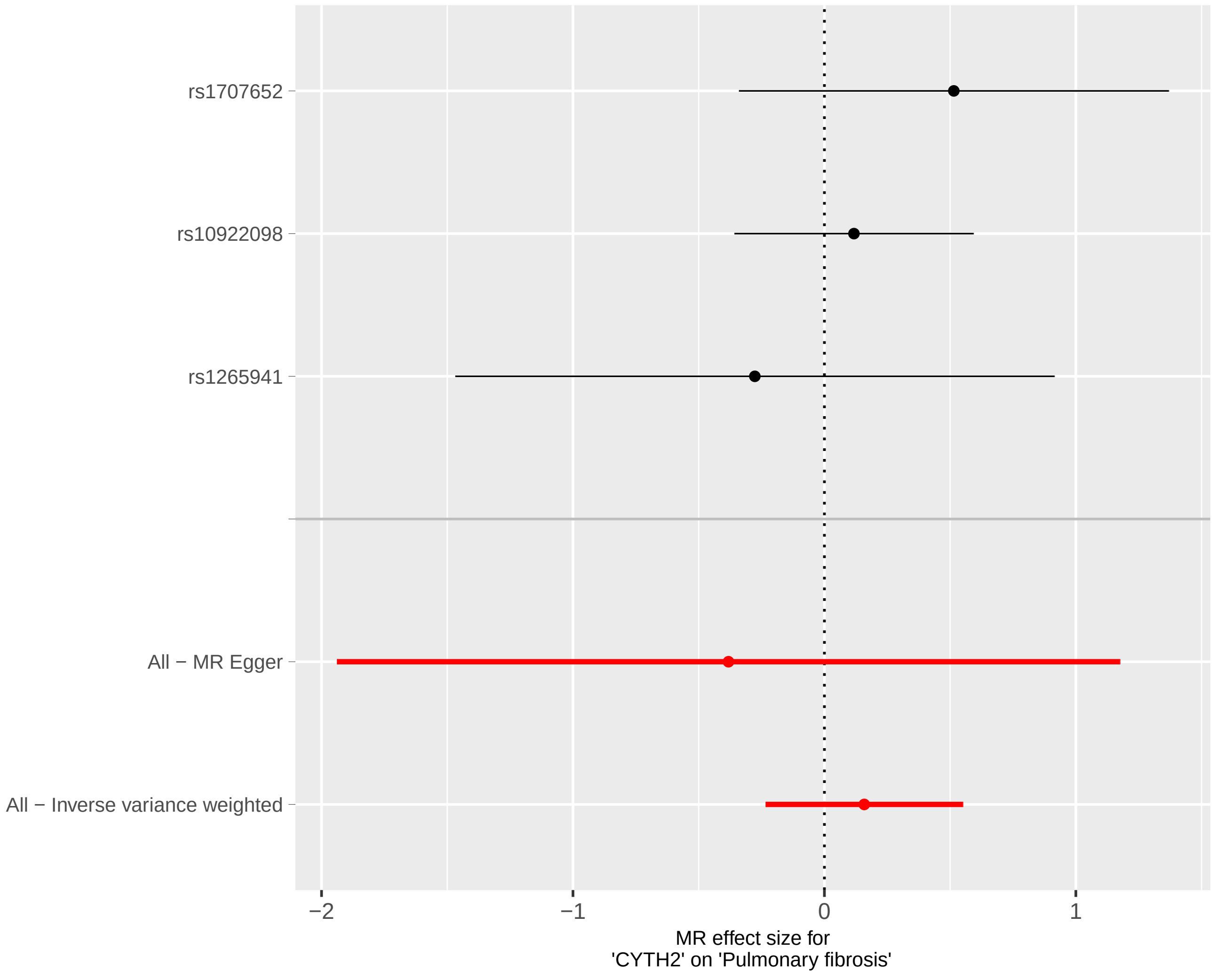
rs1707652
rs10922098
rs1265941
All − MR Egger
All − Inverse variance weighted
−2
−1
0
1
MR effect size for
'CYTH2' on 'Pulmonary fibrosis'

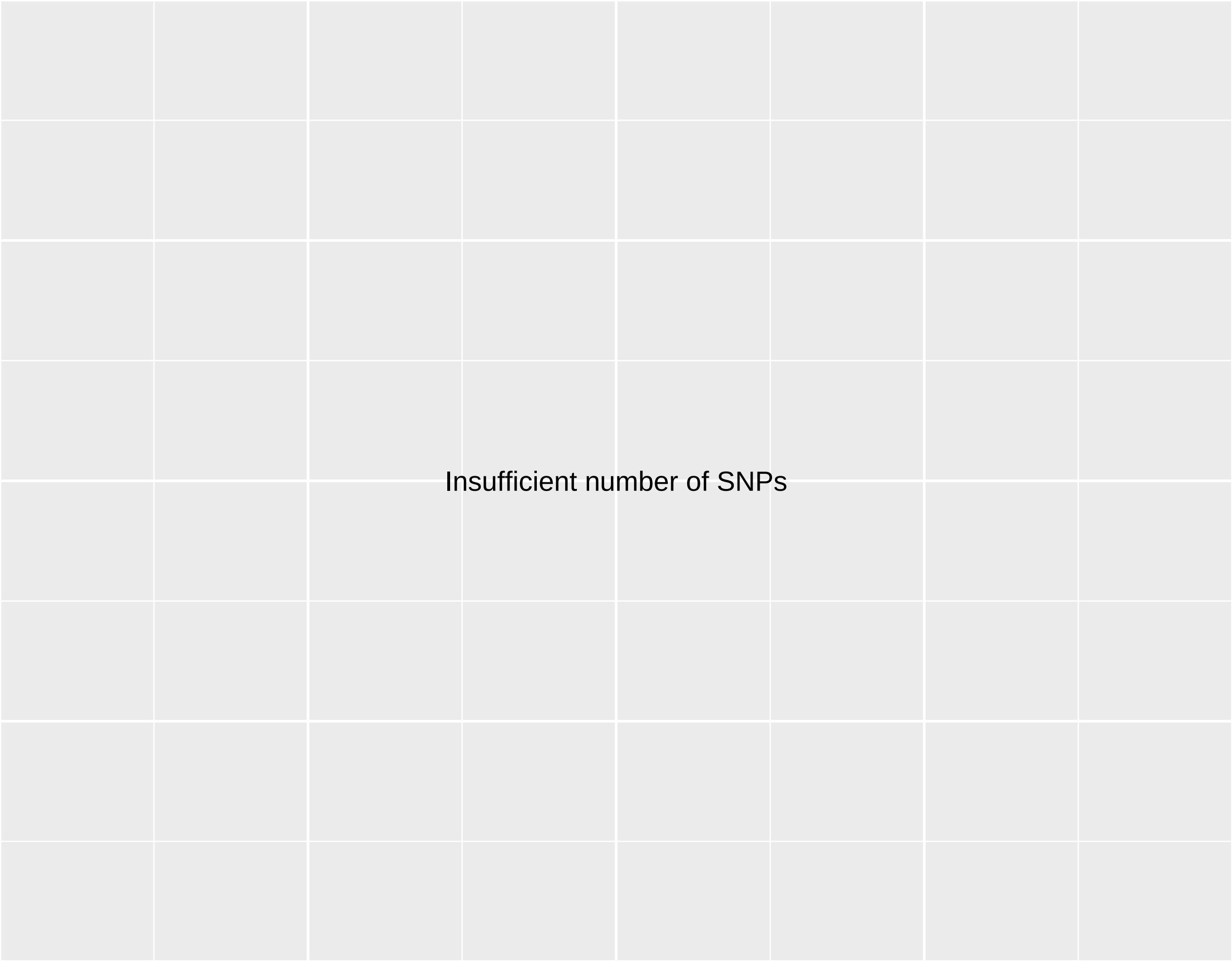
Insufficient number of SNPs

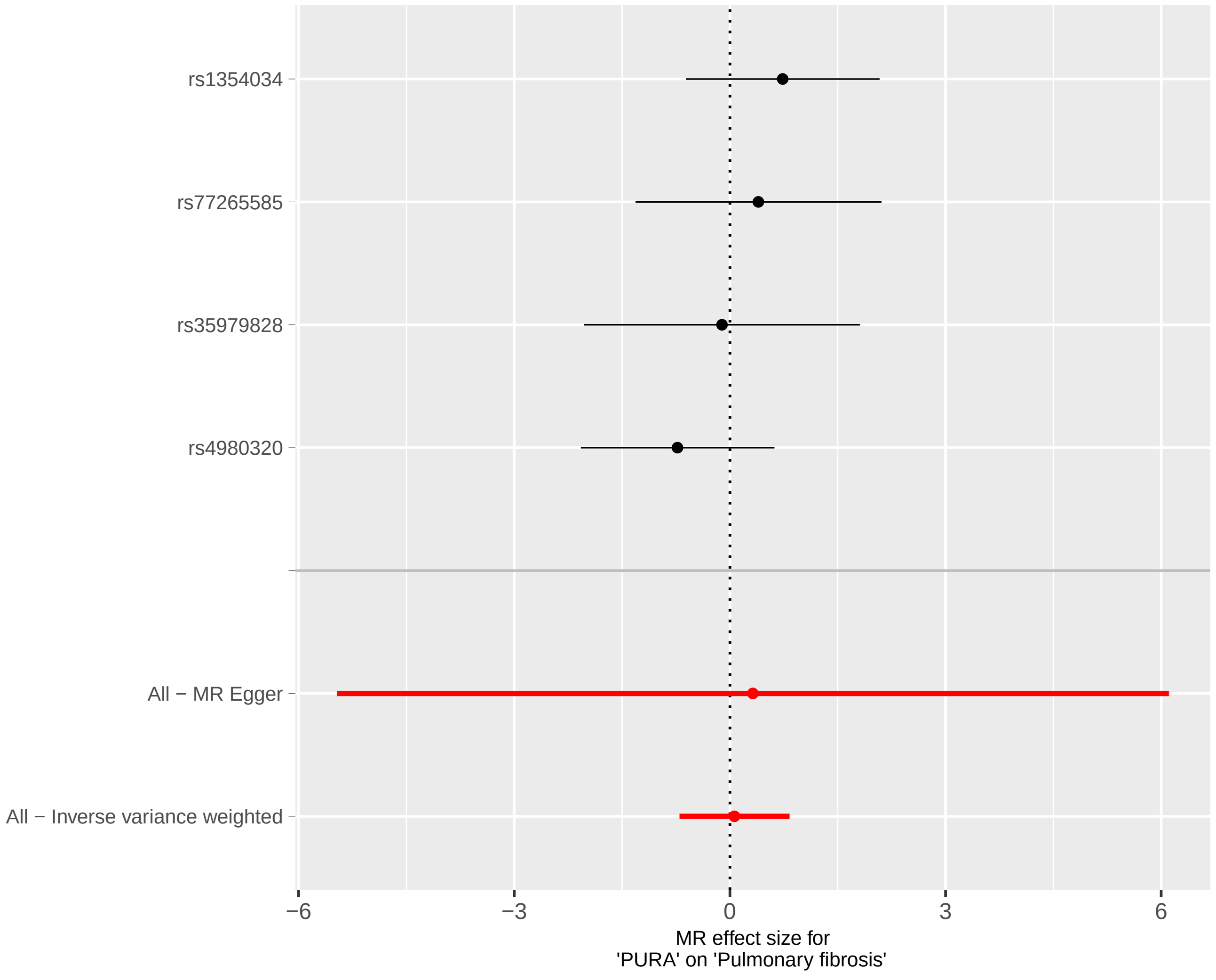
rs1354034
rs77265585
rs35979828
rs4980320
All − MR Egger
All − Inverse variance weighted
−6
−3
0
3
6
MR effect size for
'PURA' on 'Pulmonary fibrosis'

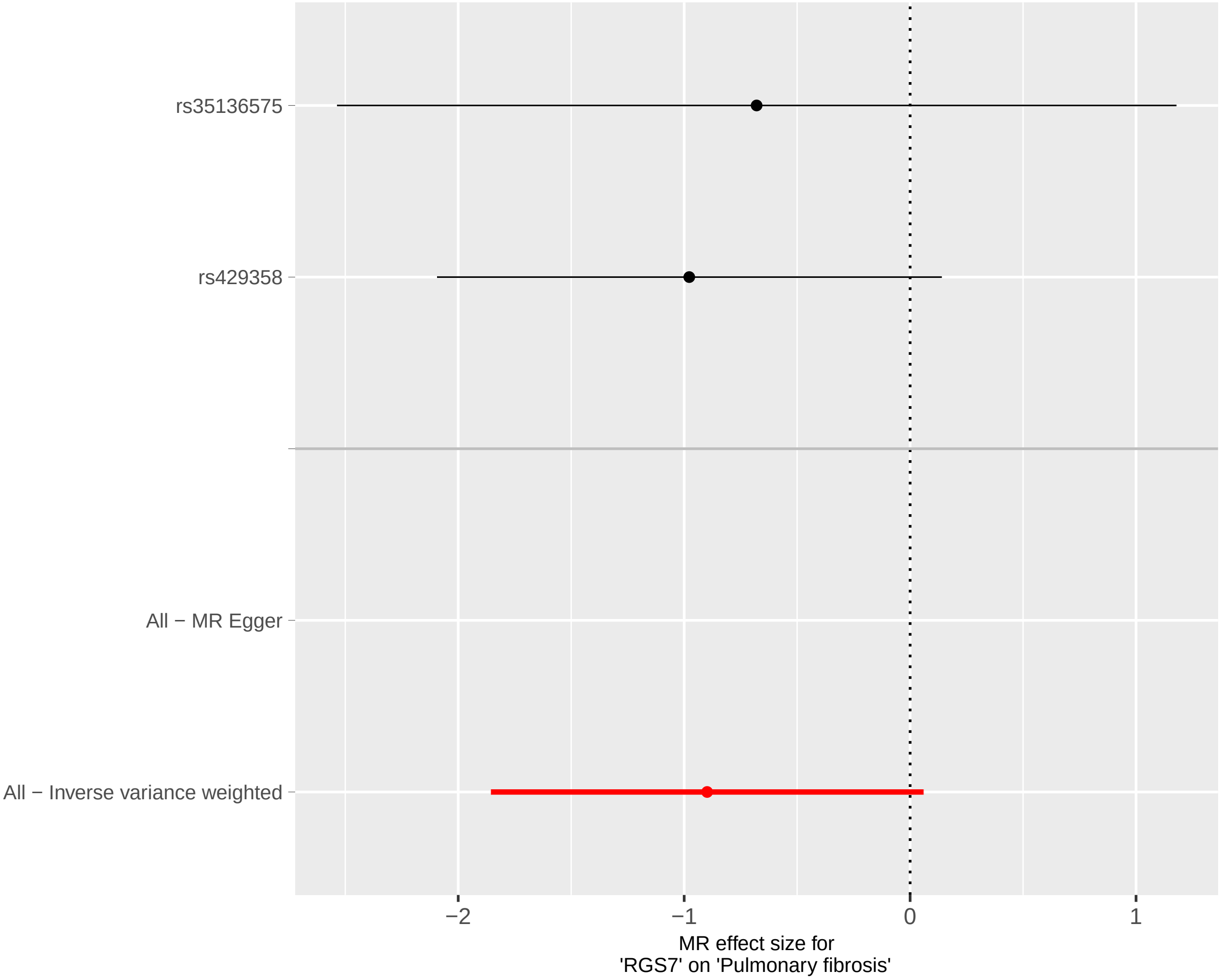
rs35136575
rs429358
All − MR Egger
All − Inverse variance weighted
−2
−1
0
1
MR effect size for
'RGS7' on 'Pulmonary fibrosis'

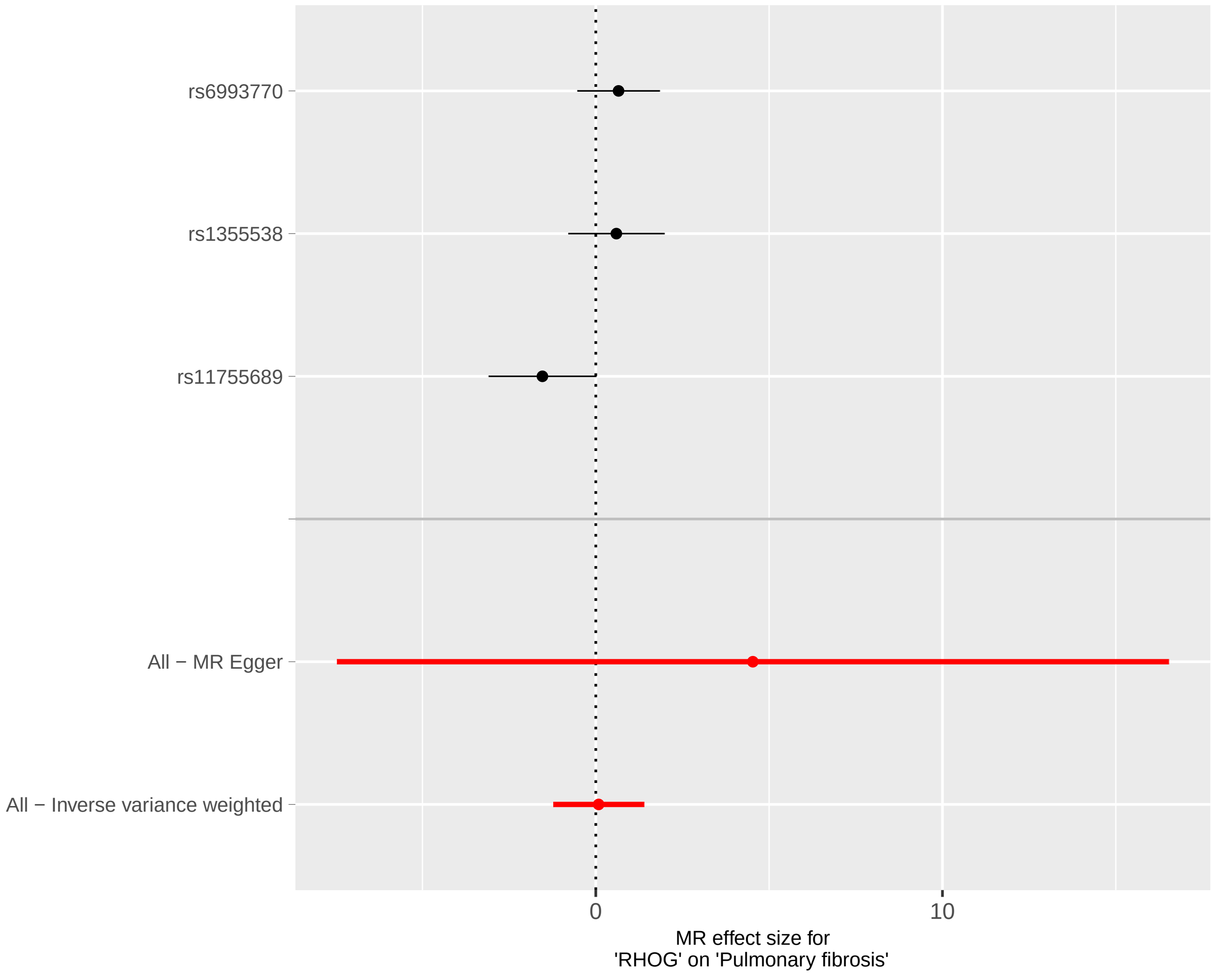

rs6993770
rs1355538
rs11755689
All − MR Egger
All − Inverse variance weighted
0
10
MR effect size for
'RHOG' on 'Pulmonary fibrosis'

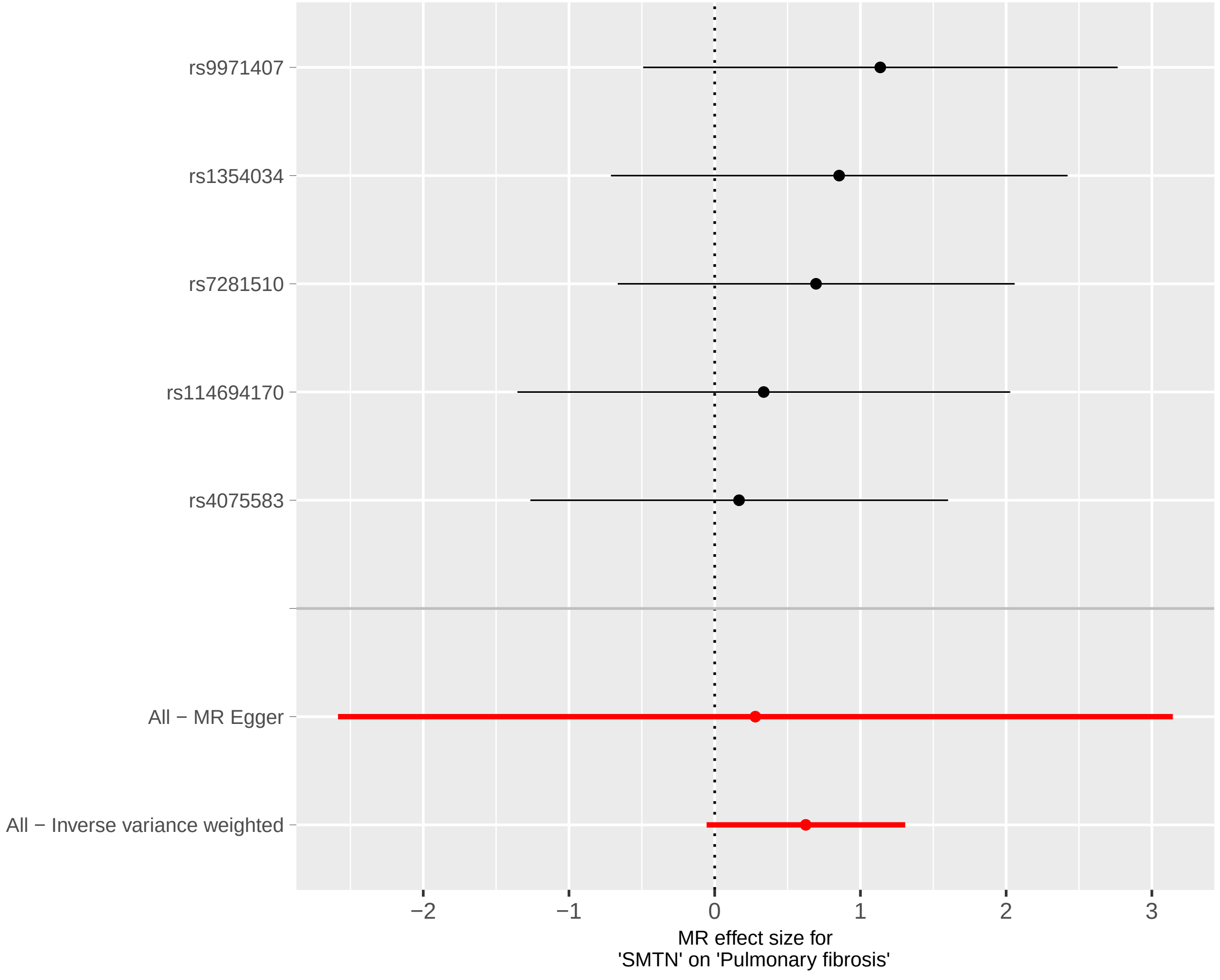

rs9971407
rs1354034
rs7281510
rs114694170
rs4075583
All − MR Egger
All − Inverse variance weighted
−2
−1
0
1
2
3
MR effect size for
'SMTN' on 'Pulmonary fibrosis'

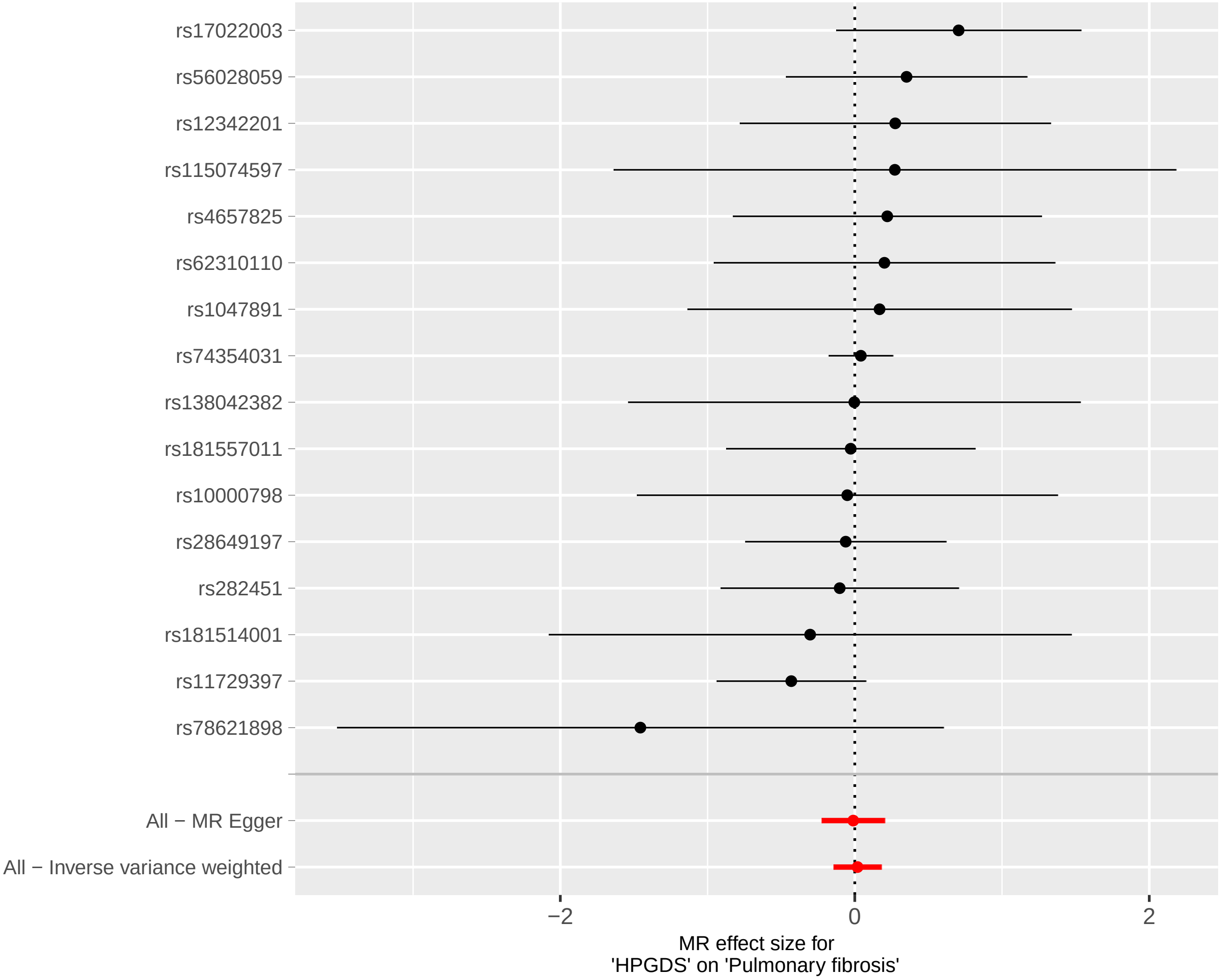
rs17022003
rs56028059
rs12342201
rs115074597
rs4657825
rs62310110
rs1047891
rs74354031
rs138042382
rs181557011
rs10000798
rs28649197
rs282451
rs181514001
rs11729397
rs78621898
All − MR Egger
All − Inverse variance weighted
−2
0
2
MR effect size for
'HPGDS' on 'Pulmonary fibrosis'

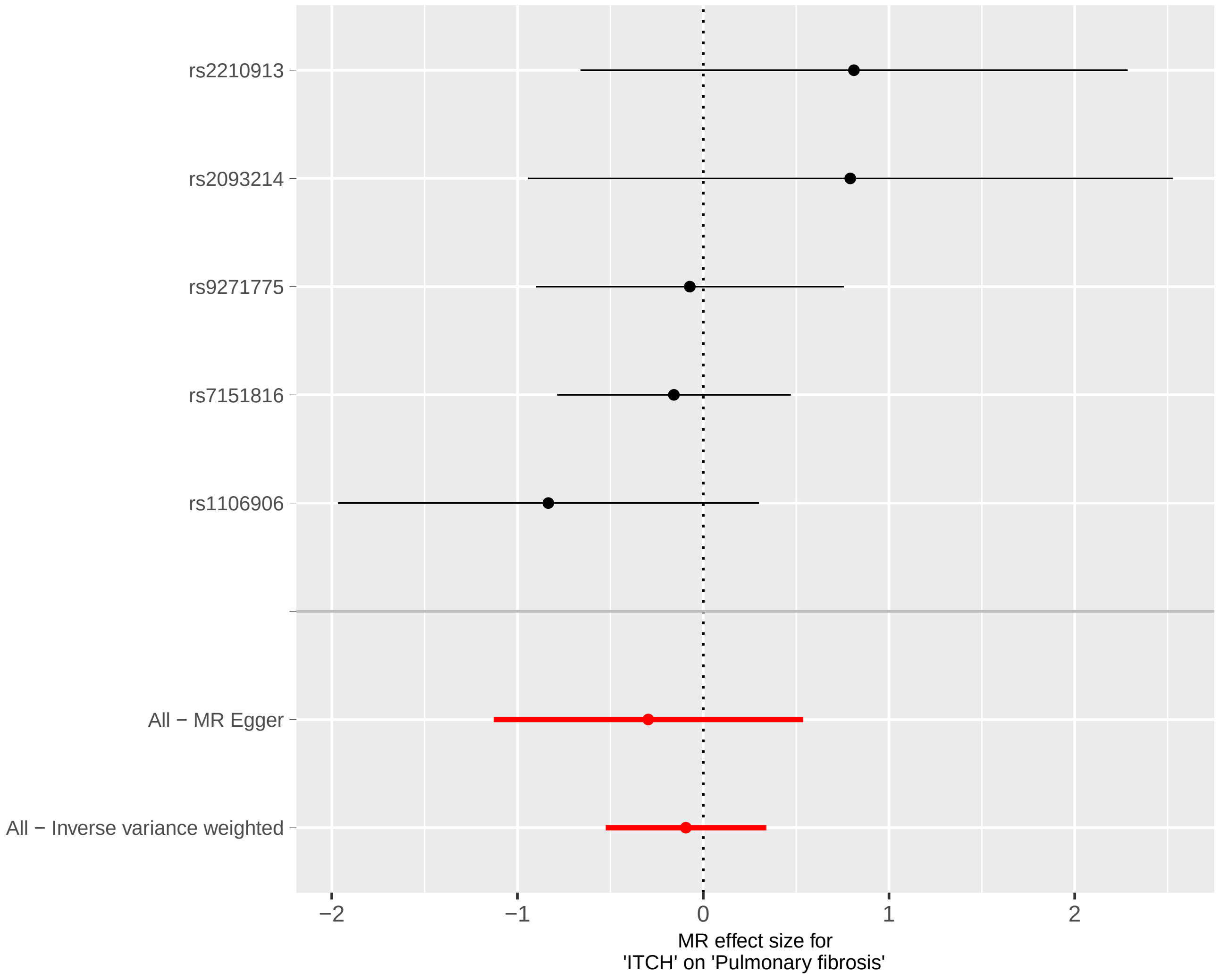
rs2210913
rs2093214
rs9271775
rs7151816
rs1106906
All − MR Egger
All − Inverse variance weighted
−2
−1
0
1
2
MR effect size for
'ITCH' on 'Pulmonary fibrosis'

Insufficient number of SNPs

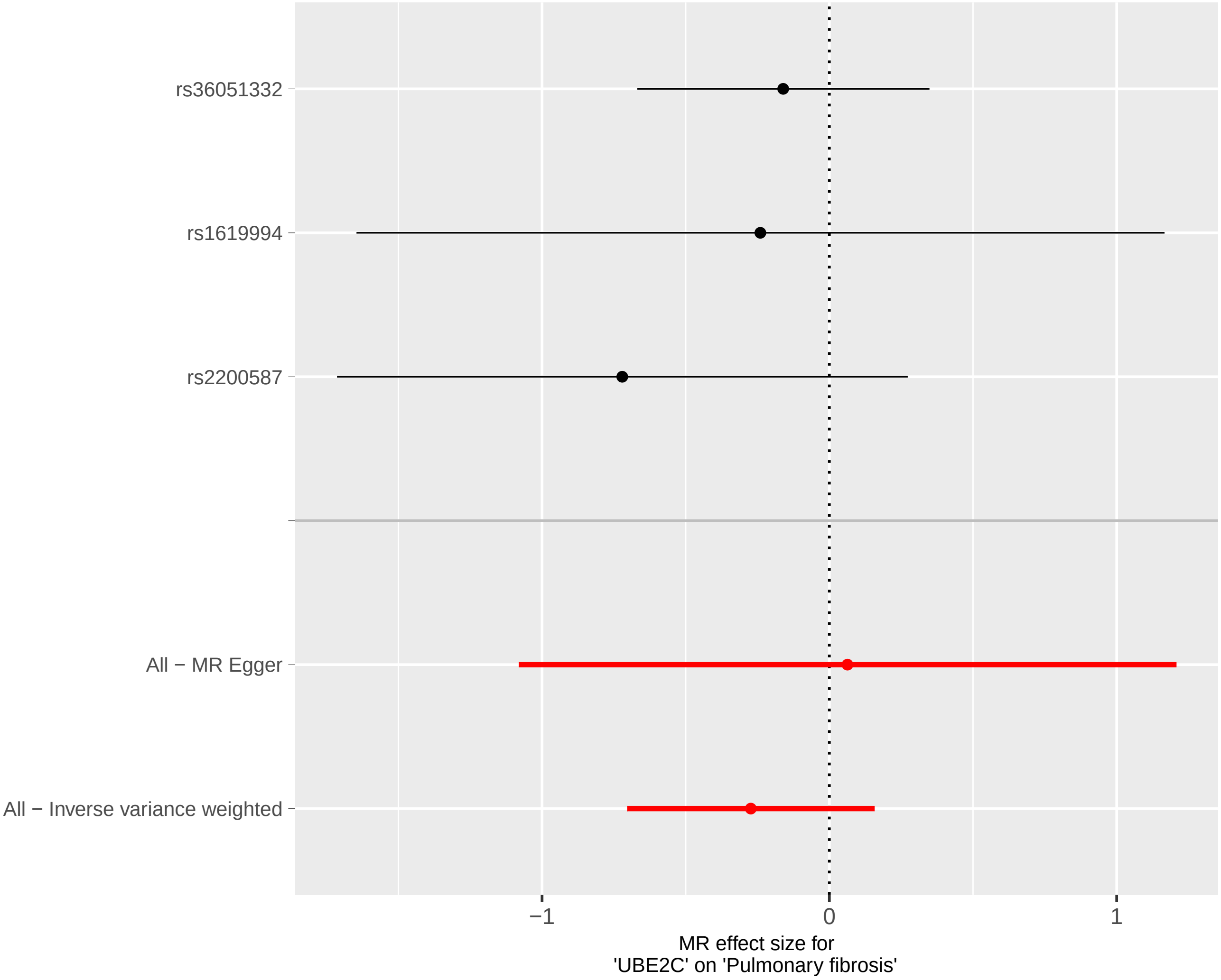

rs36051332
rs1619994
rs2200587
All − MR Egger
All − Inverse variance weighted
−1
0
1
MR effect size for
'UBE2C' on 'Pulmonary fibrosis'

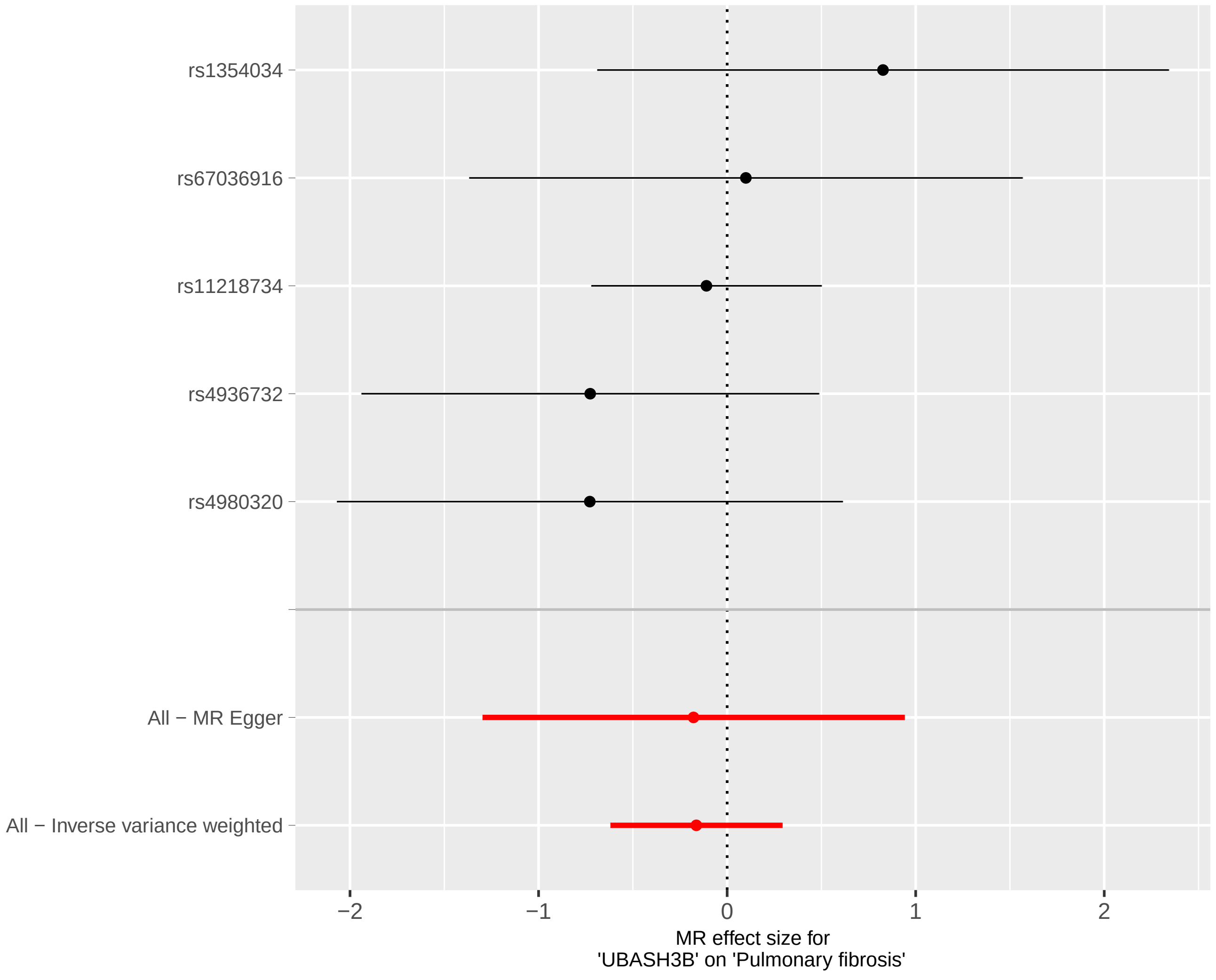

rs1354034
rs67036916
rs11218734
rs4936732
rs4980320
All − MR Egger
All − Inverse variance weighted
−2
−1
0
1
2
MR effect size for
'UBASH3B' on 'Pulmonary fibrosis'

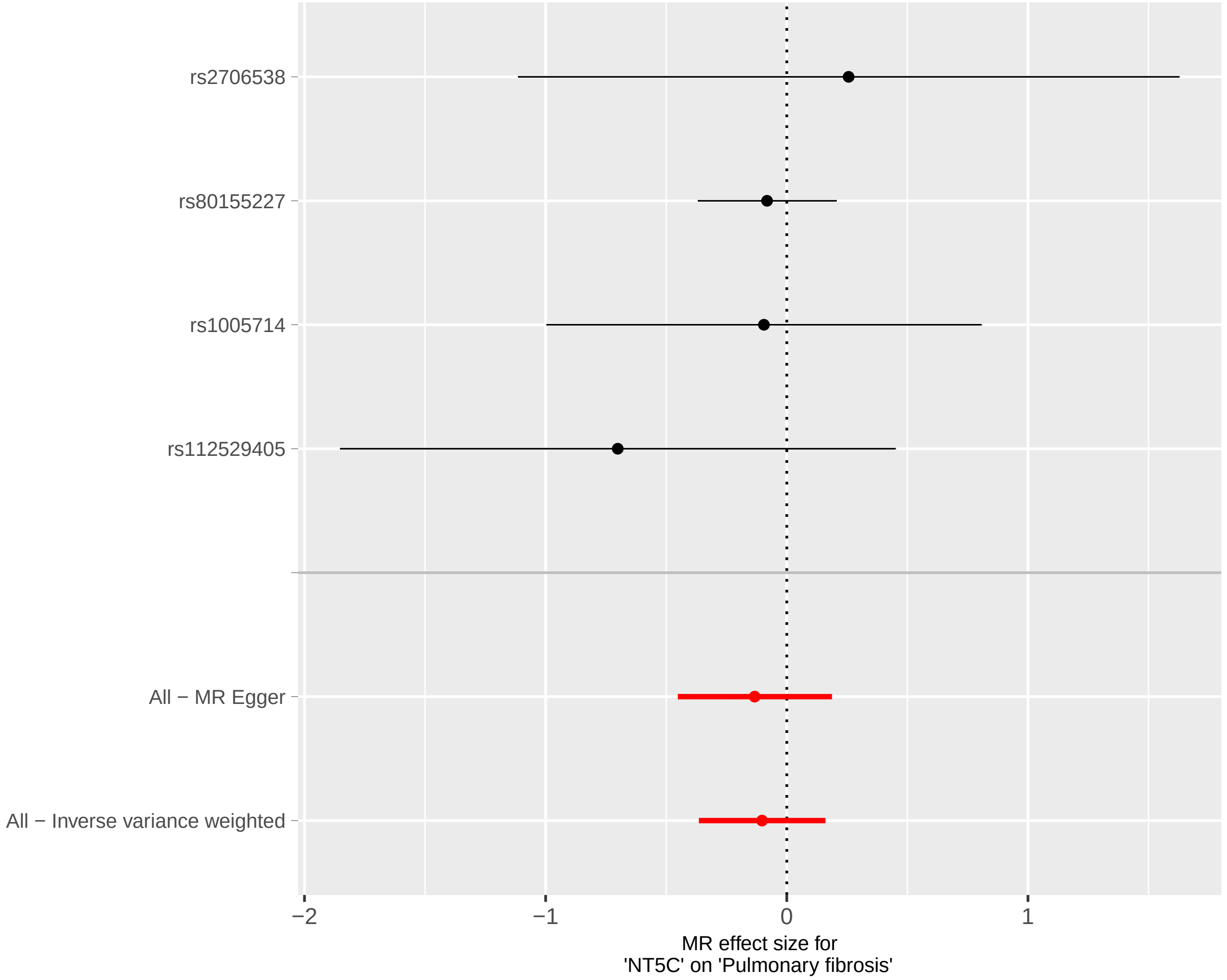
rs2706538
rs80155227
rs1005714
rs112529405
All − MR Egger
All − Inverse variance weighted
−2
−1
0
1
MR effect size for
'NT5C' on 'Pulmonary fibrosis'

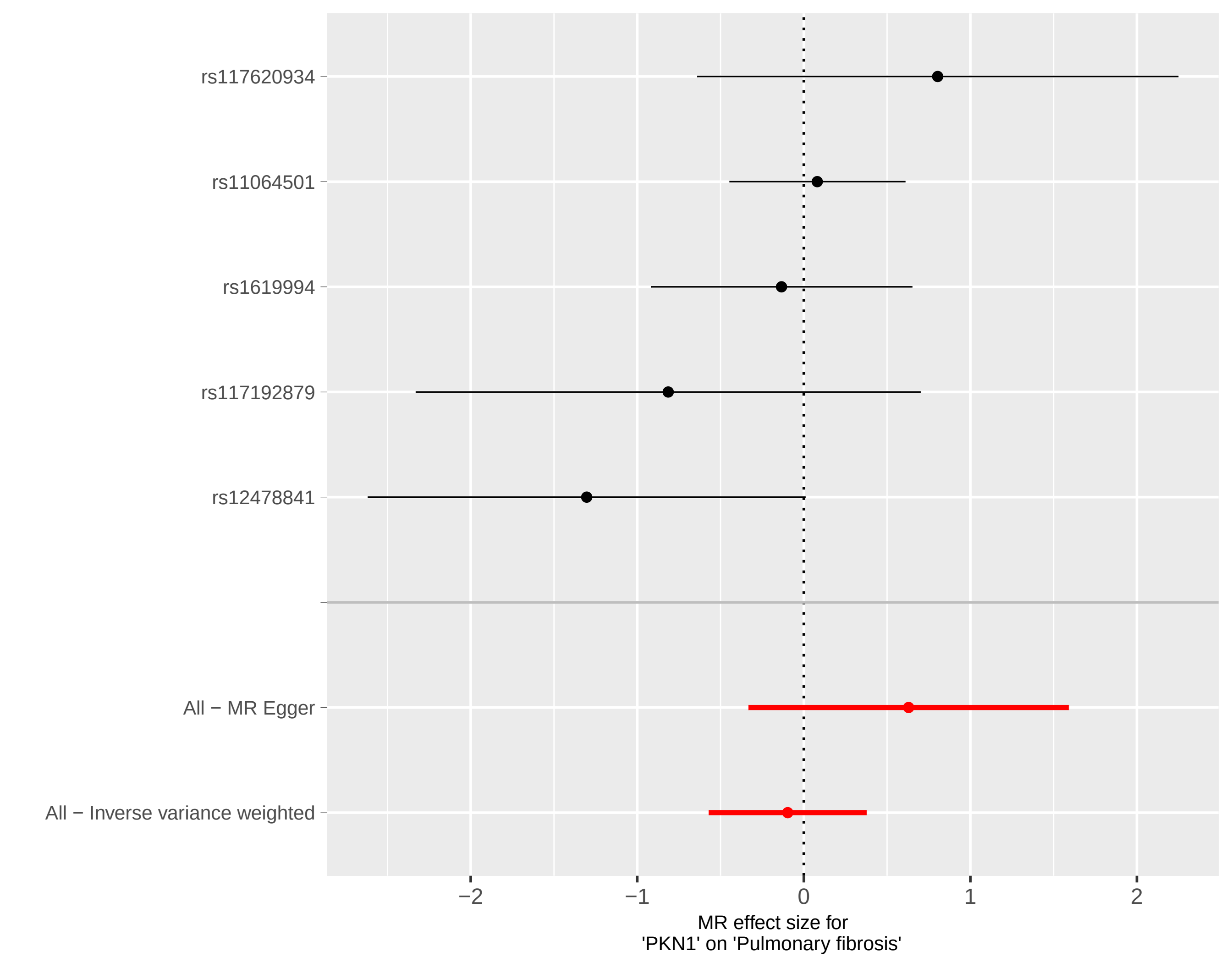
rs117620934
rs11064501
rs1619994
rs117192879
rs12478841
All − MR Egger
All − Inverse variance weighted
−2
−1
0
1
2
MR effect size for
'PKN1' on 'Pulmonary fibrosis'

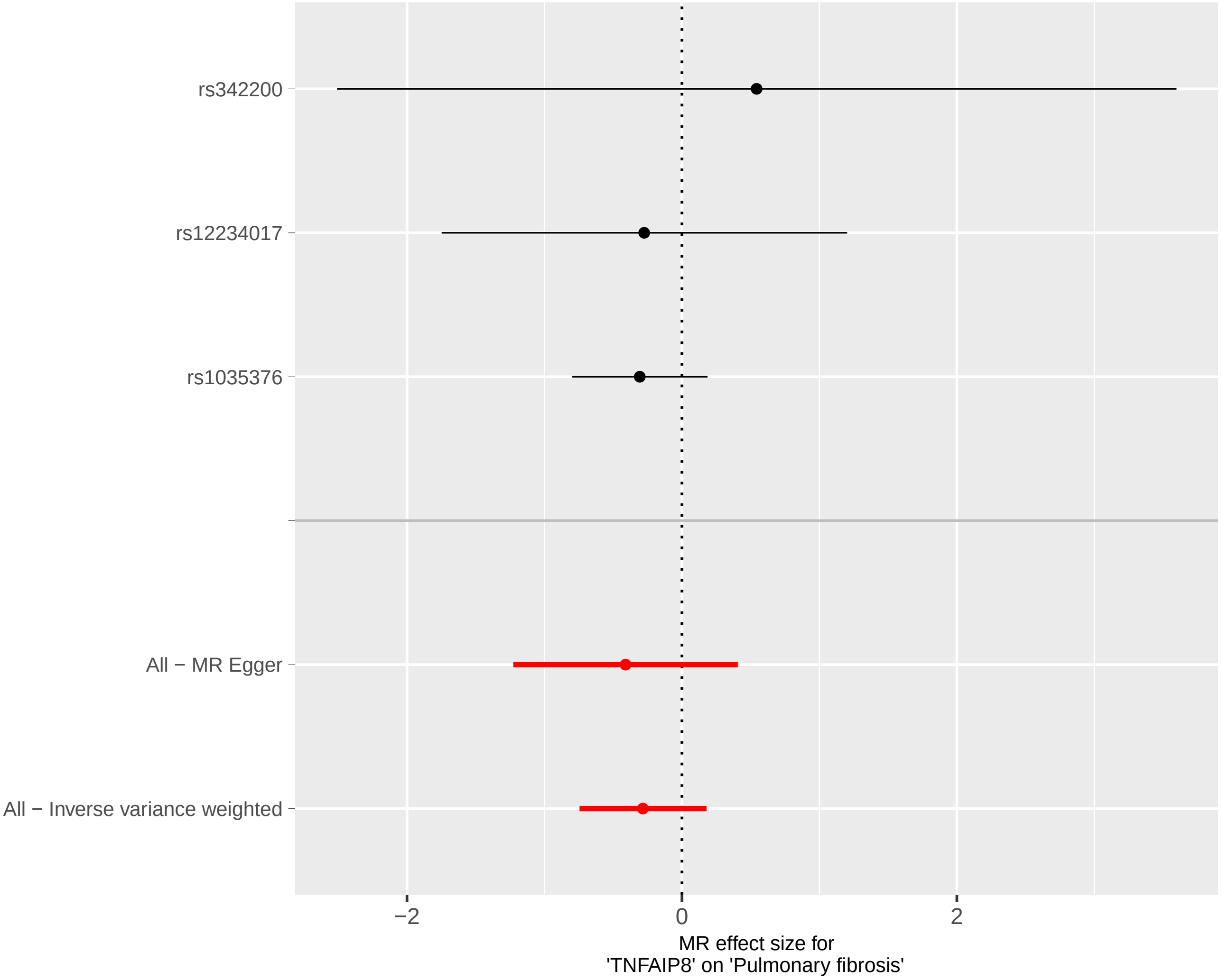

rs342200
rs12234017
rs1035376
All − MR Egger
All − Inverse variance weighted
−2
0
2
MR effect size for
'TNFAIP8' on 'Pulmonary fibrosis'

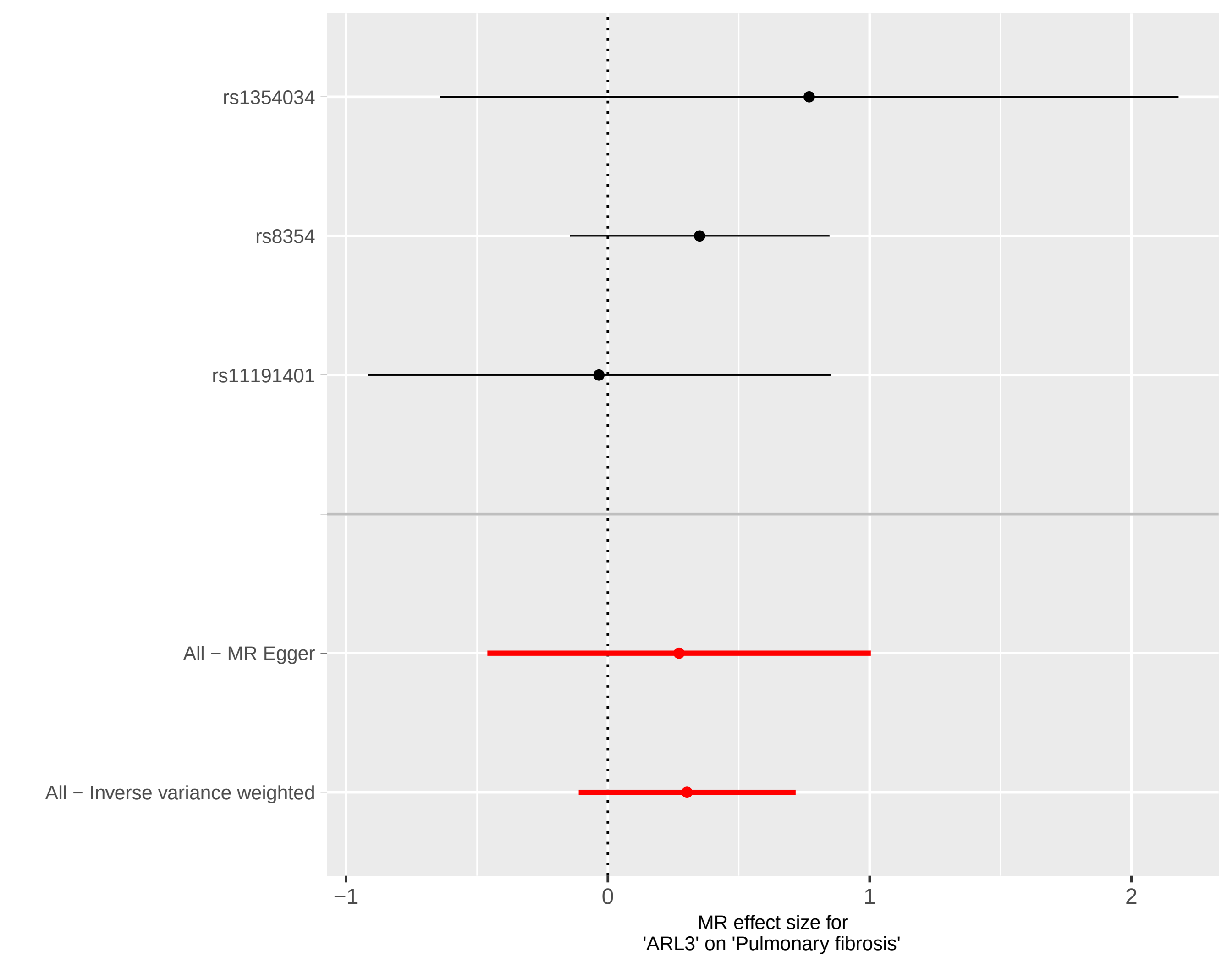
rs1354034
rs8354
rs11191401
All − MR Egger
All − Inverse variance weighted
−1
0
1
2
MR effect size for
'ARL3' on 'Pulmonary fibrosis'

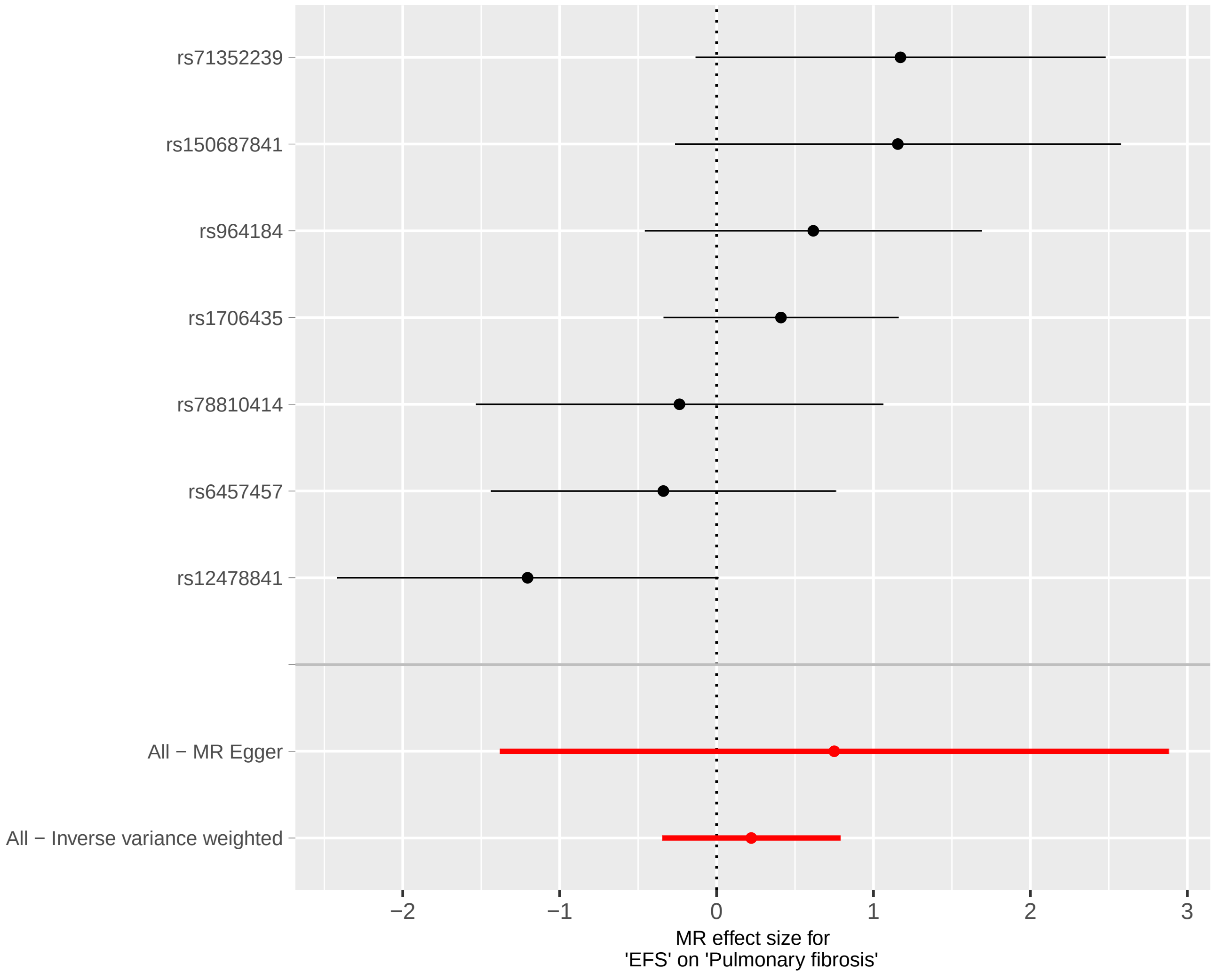

rs71352239
rs150687841
rs964184
rs1706435
rs78810414
rs6457457
rs12478841
All − MR Egger
All − Inverse variance weighted
−2
−1
0
1
2
3
MR effect size for
'EFS' on 'Pulmonary fibrosis'

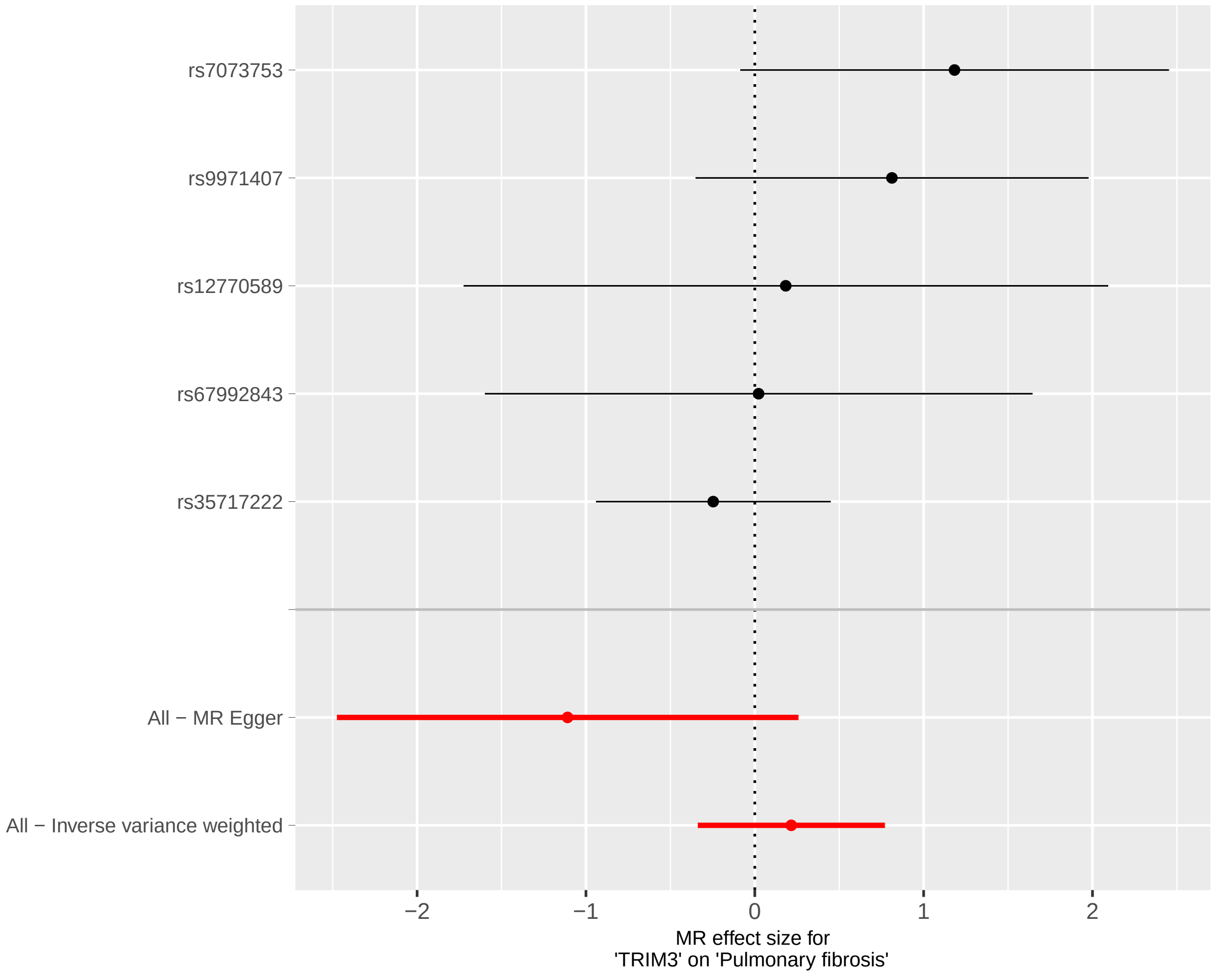
rs7073753
rs9971407
rs12770589
rs67992843
rs35717222
All − MR Egger
All − Inverse variance weighted
−2
−1
0
1
2
MR effect size for
'TRIM3' on 'Pulmonary fibrosis'

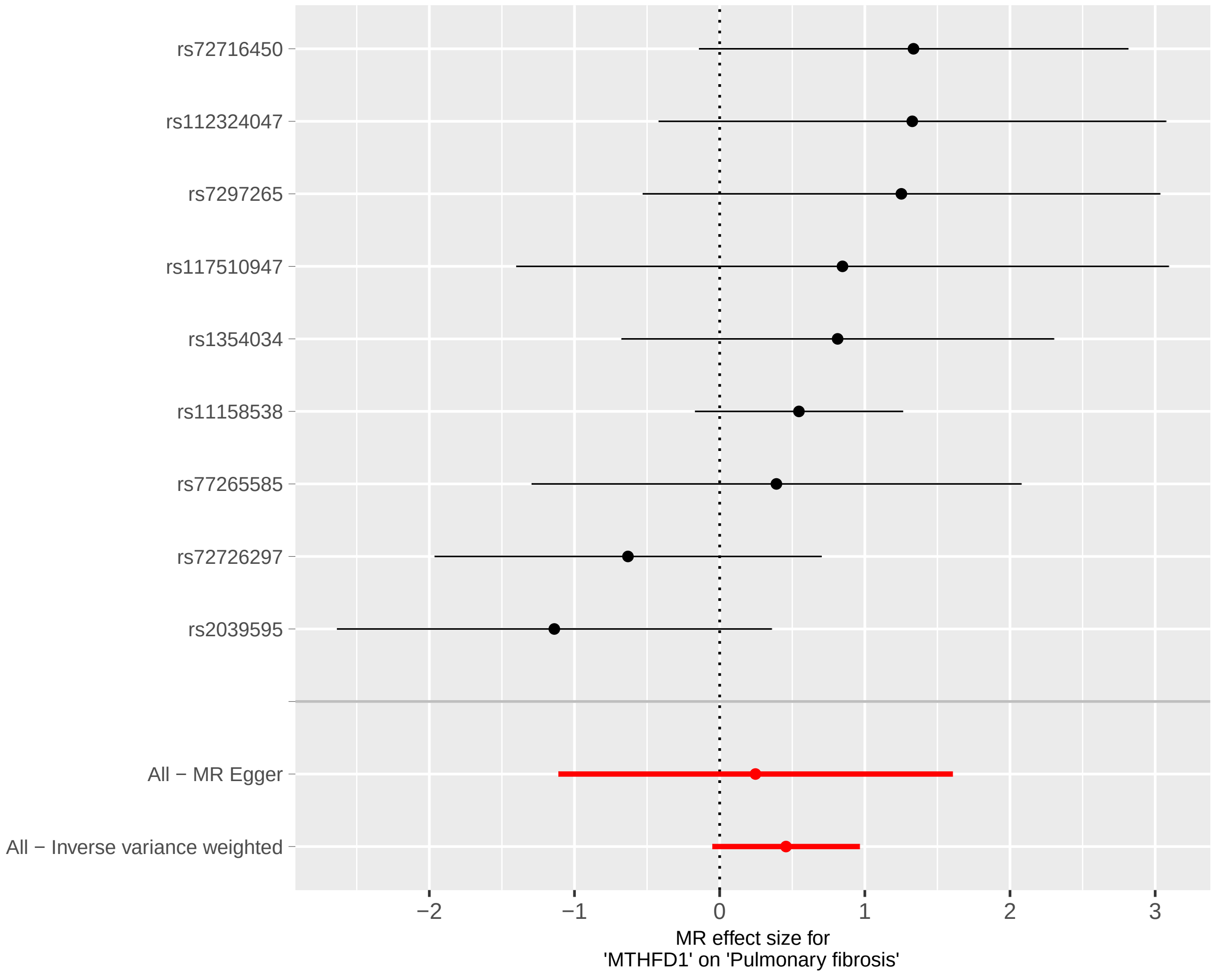
rs72716450
rs112324047
rs7297265
rs117510947
rs1354034
rs11158538
rs77265585
rs72726297
rs2039595
All − MR Egger
All − Inverse variance weighted
−2
−1
0
1
2
3
MR effect size for
'MTHFD1' on 'Pulmonary fibrosis'

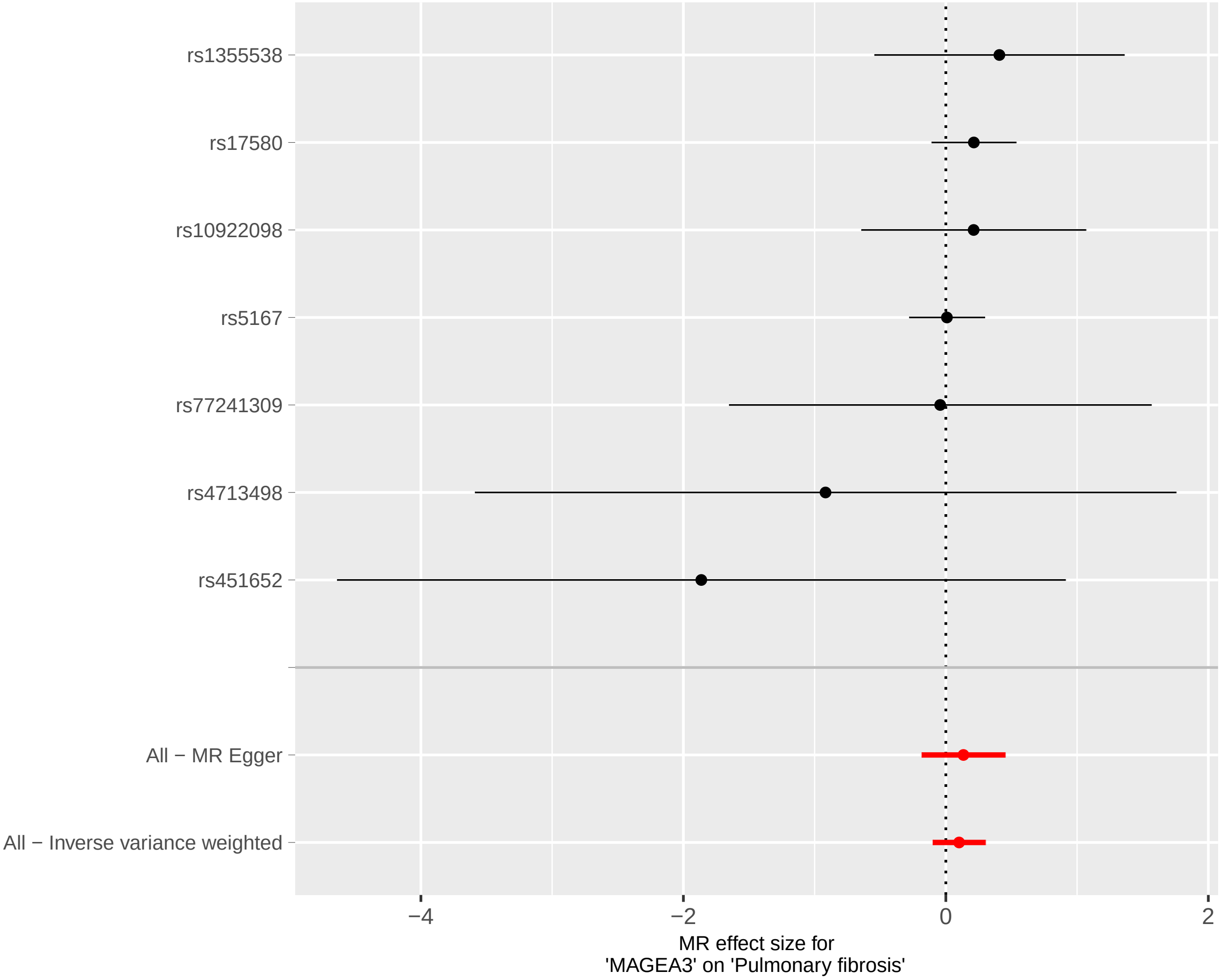
rs1355538
rs17580
rs10922098
rs5167
rs77241309
rs4713498
rs451652
All − MR Egger
All − Inverse variance weighted
−4
−2
0
2
MR effect size for
'MAGEA3' on 'Pulmonary fibrosis'

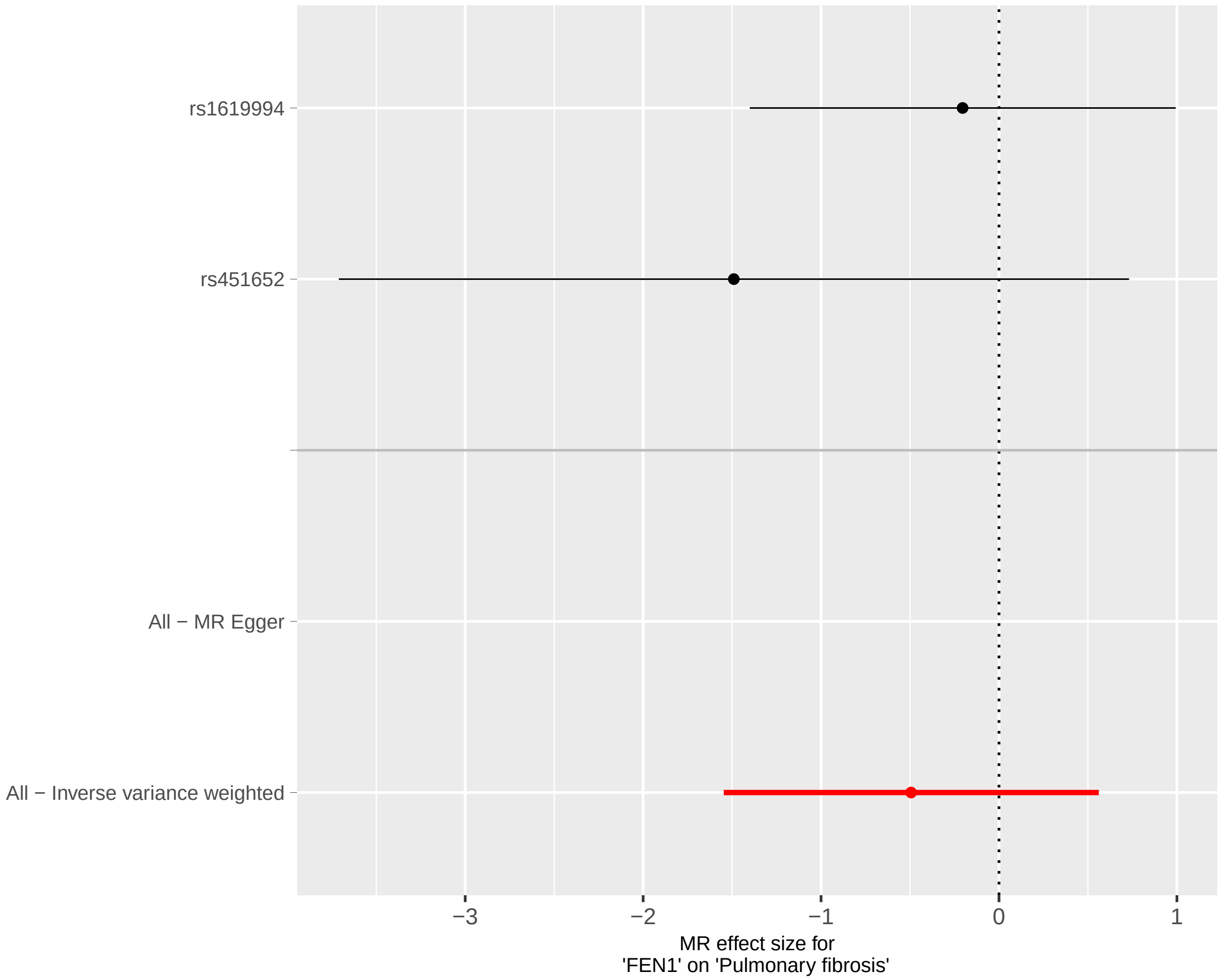
rs1619994
rs451652
All − MR Egger
All − Inverse variance weighted
−3
−2
−1
0
1
MR effect size for
'FEN1' on 'Pulmonary fibrosis'

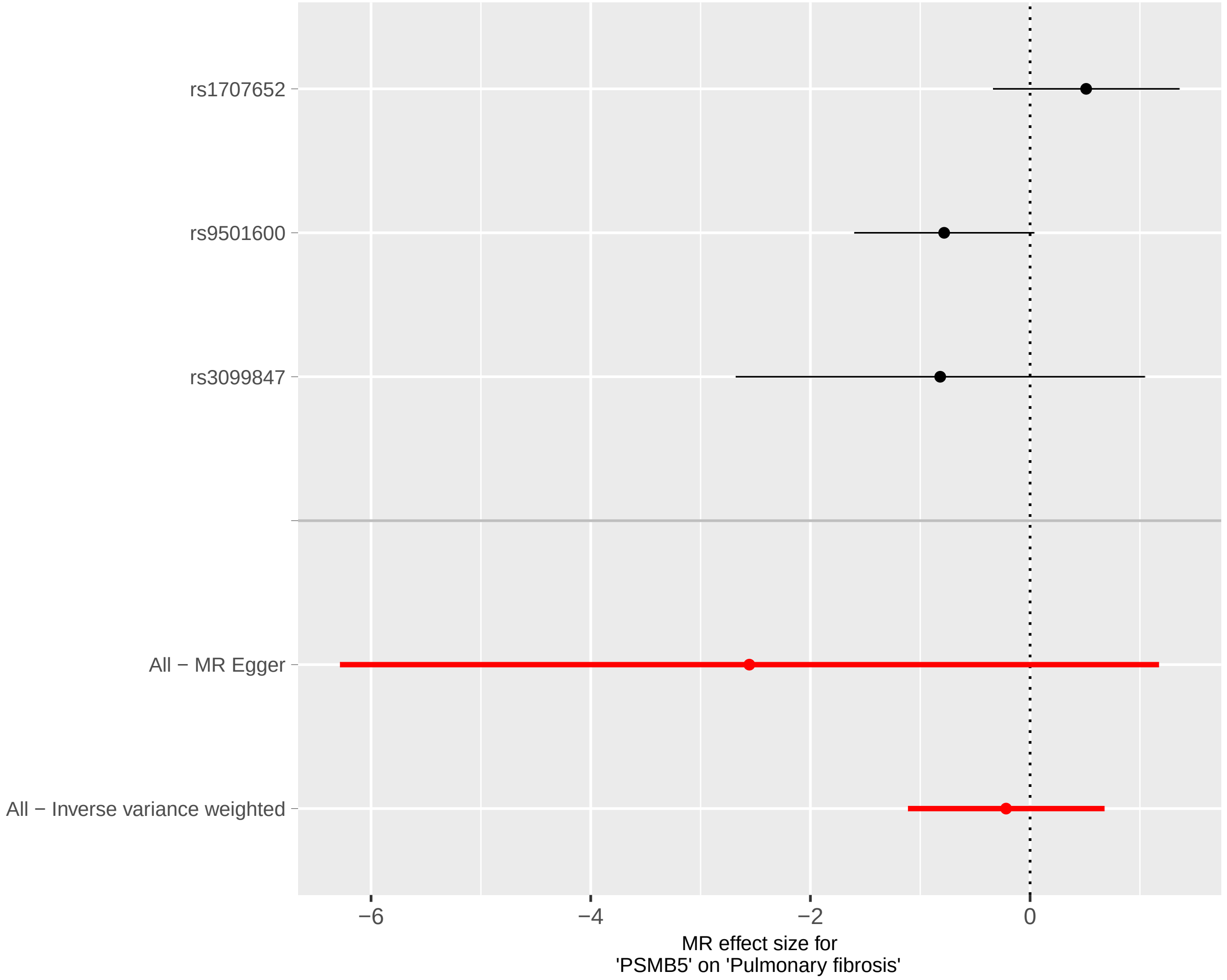
rs1707652
rs9501600
rs3099847
All − MR Egger
All − Inverse variance weighted
−6
−4
−2
0
MR effect size for
'PSMB5' on 'Pulmonary fibrosis'

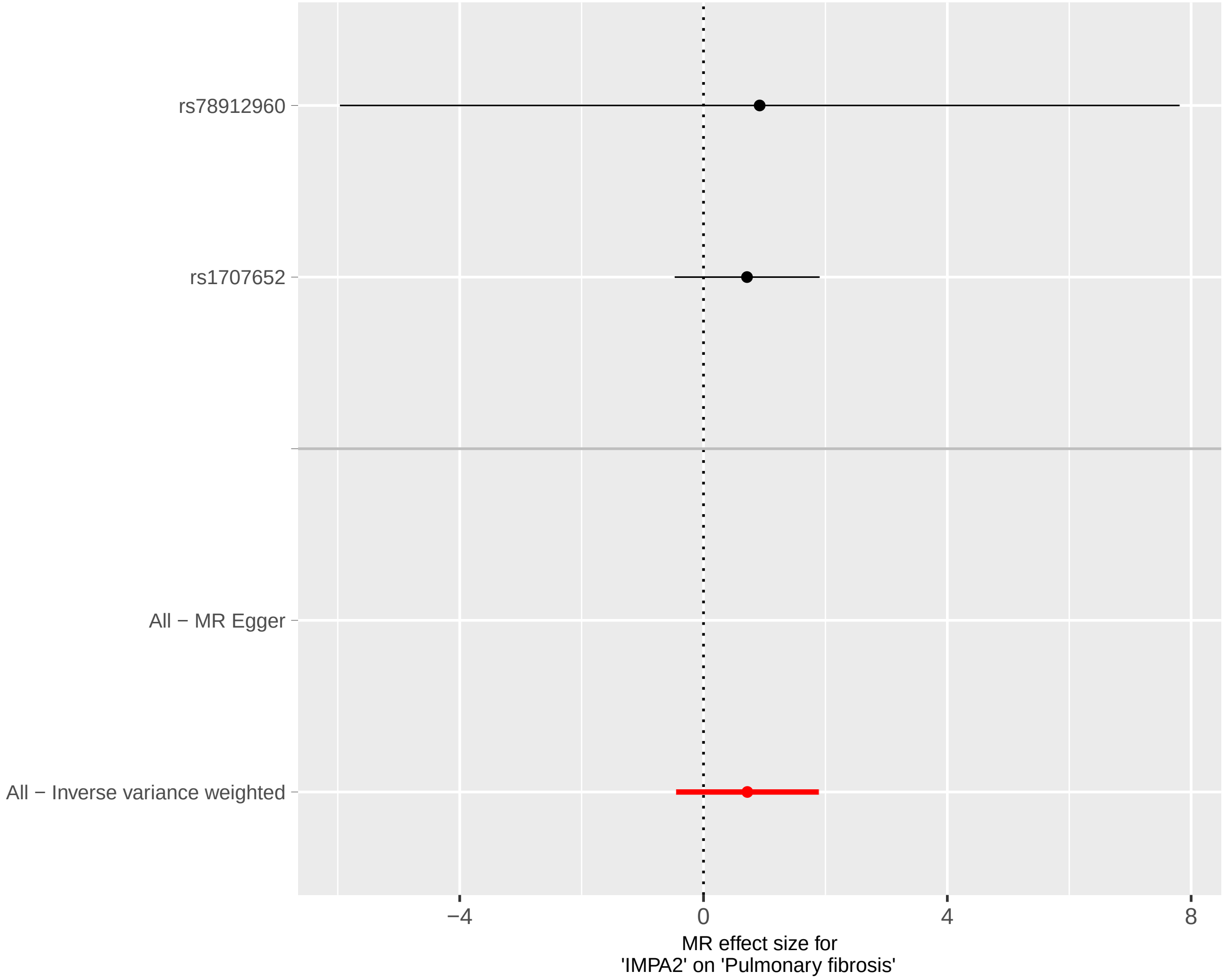

rs78912960
rs1707652
All − MR Egger
All − Inverse variance weighted
−4
0
4
8
MR effect size for
'IMPA2' on 'Pulmonary fibrosis'

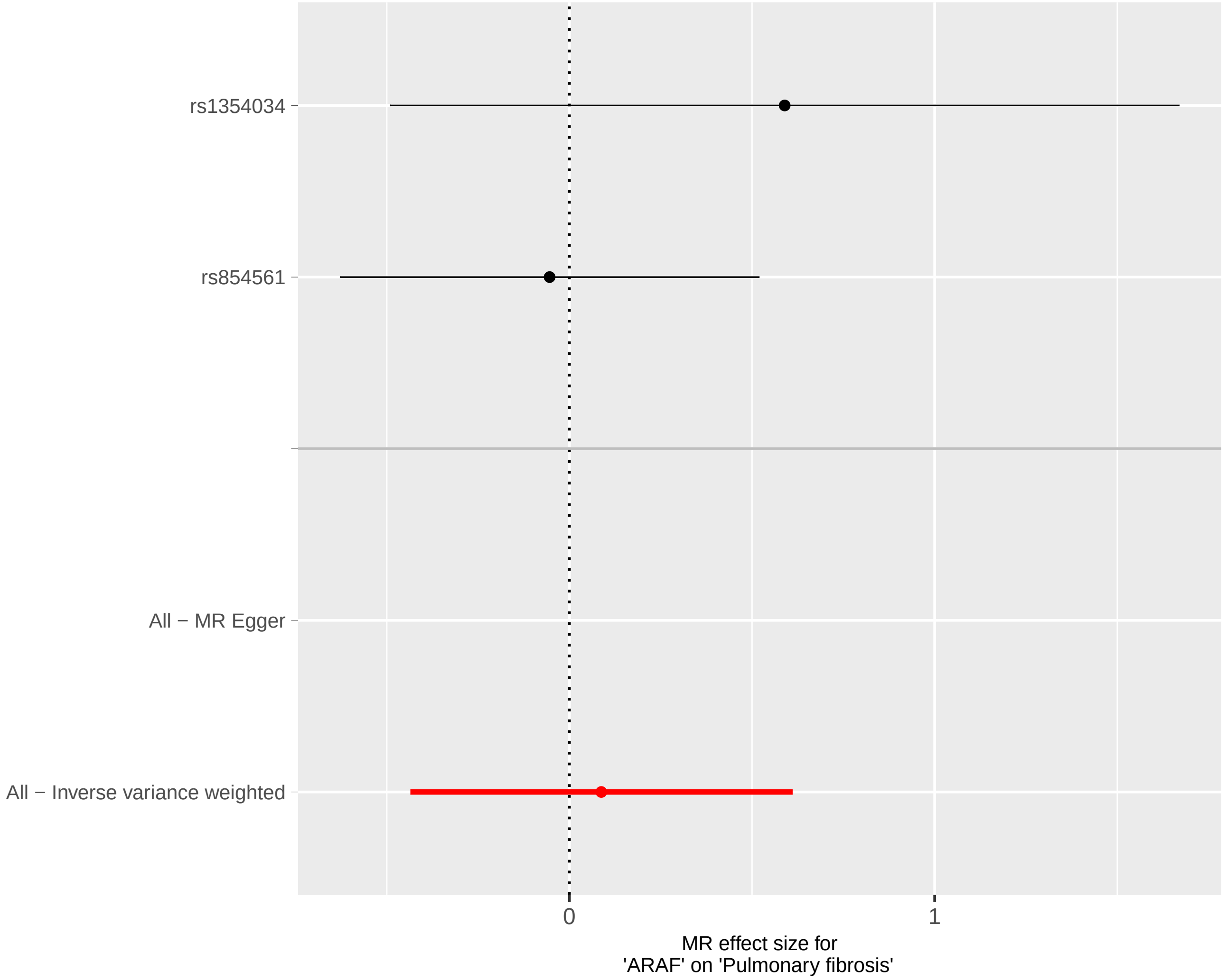
rs1354034
rs854561
All − MR Egger
All − Inverse variance weighted
0
1
MR effect size for
'ARAF' on 'Pulmonary fibrosis'

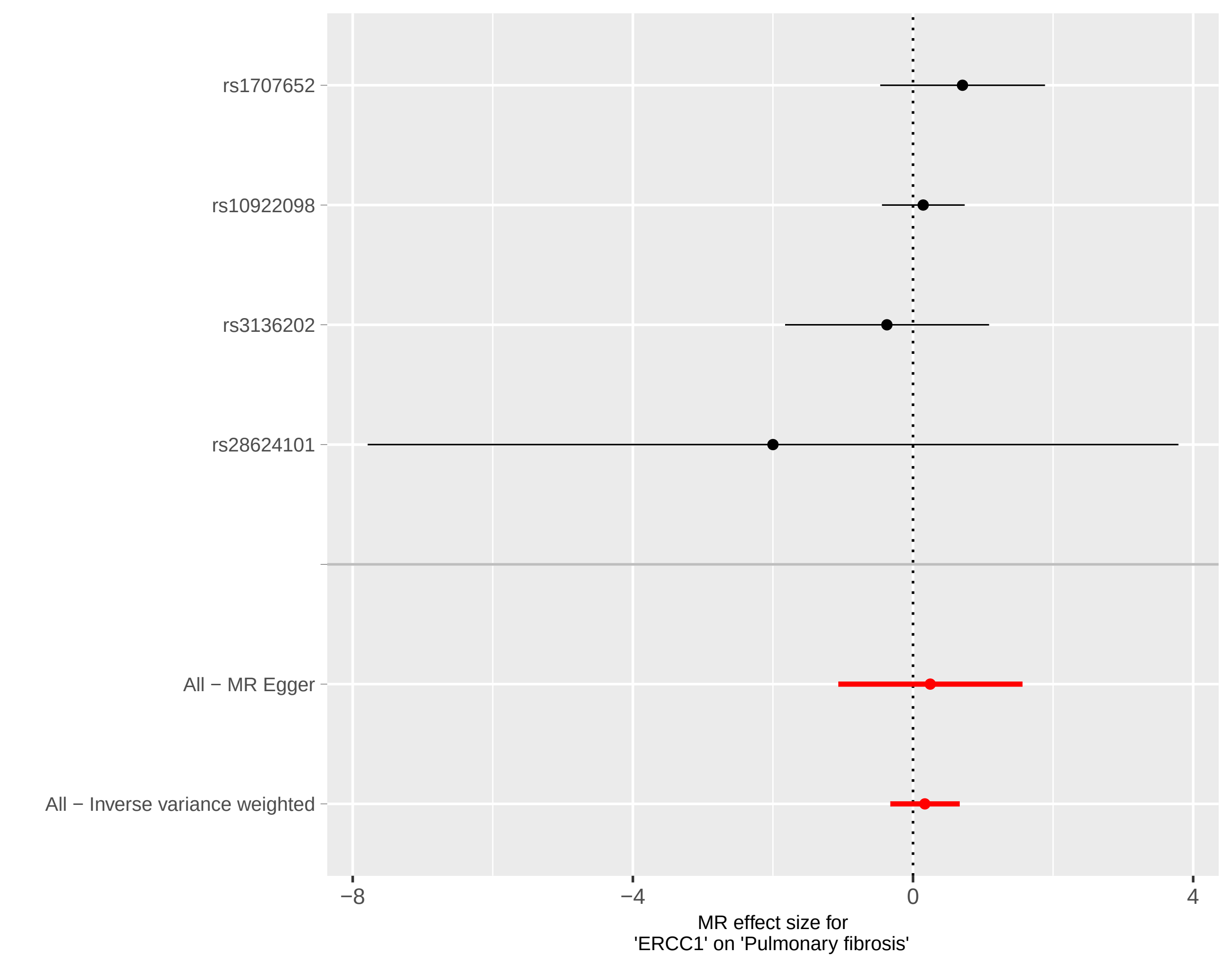

rs1707652
rs10922098
rs3136202
rs28624101
All − MR Egger
All − Inverse variance weighted
−8
−4
0
4
MR effect size for
'ERCC1' on 'Pulmonary fibrosis'

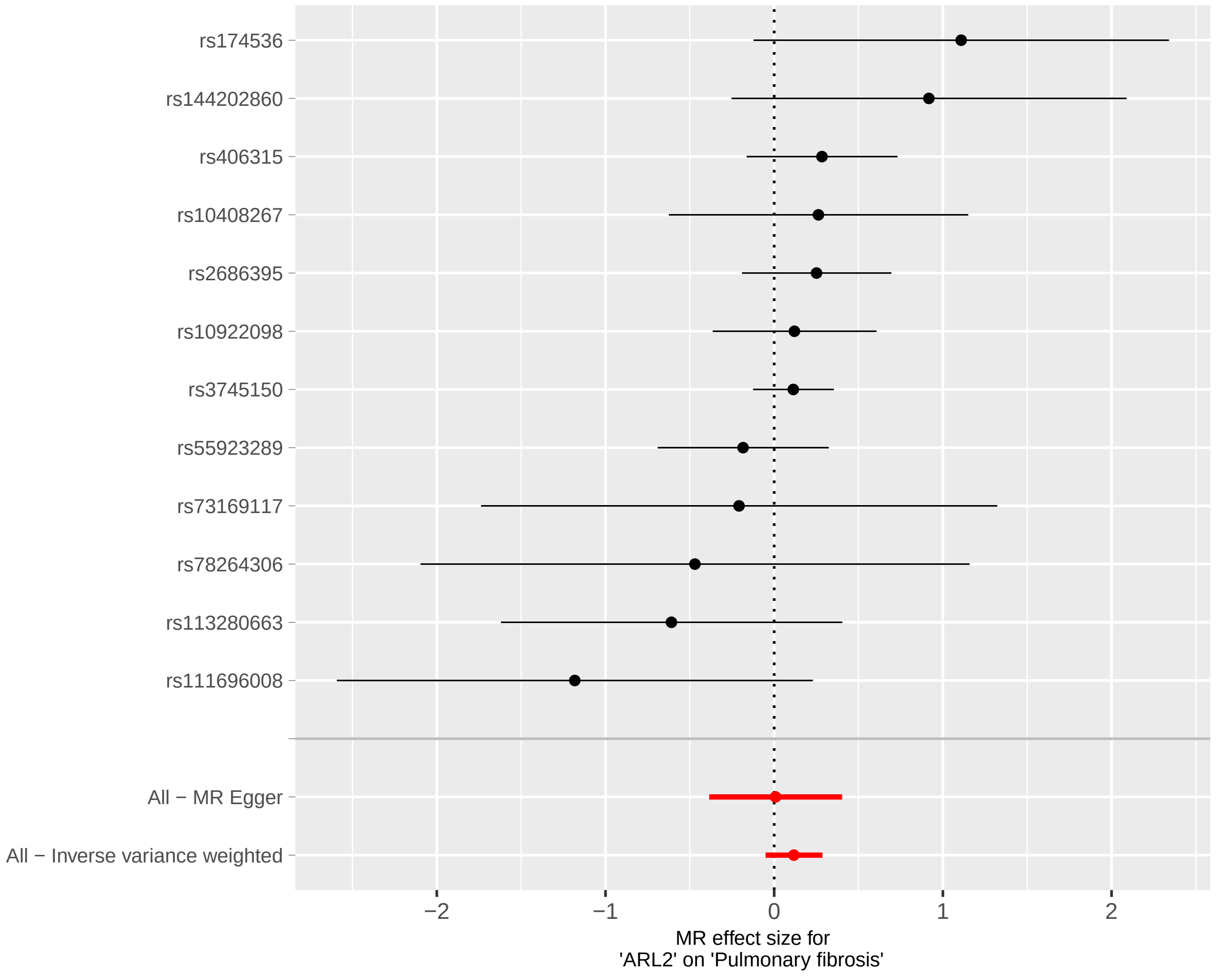
rs174536
rs144202860
rs406315
rs10408267
rs2686395
rs10922098
rs3745150
rs55923289
rs73169117
rs78264306
rs113280663
rs111696008
All − MR Egger
All − Inverse variance weighted
−2
−1
0
1
2
MR effect size for
'ARL2' on 'Pulmonary fibrosis'

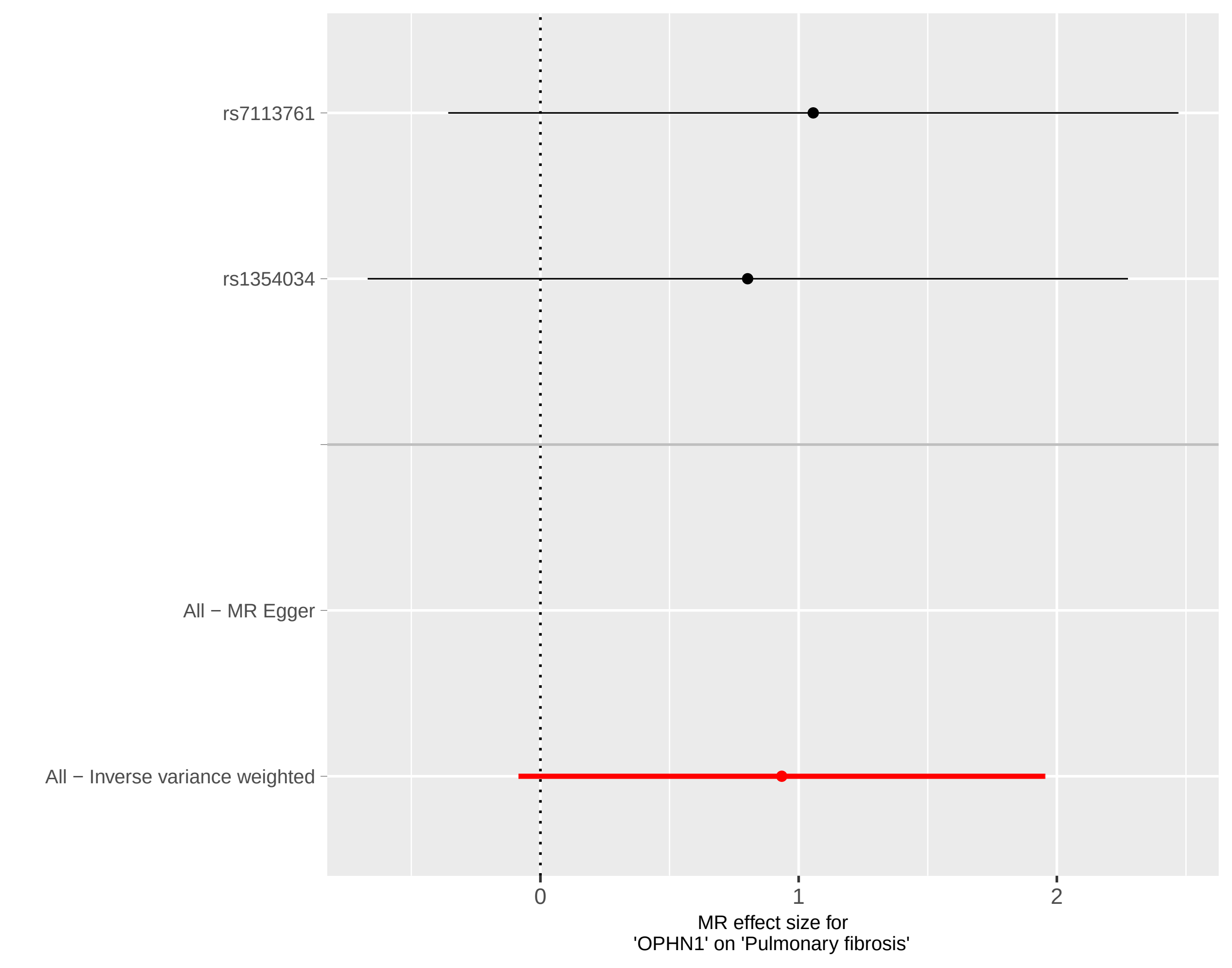
rs7113761
rs1354034
All − MR Egger
All − Inverse variance weighted
0
1
2
MR effect size for
'OPHN1' on 'Pulmonary fibrosis'

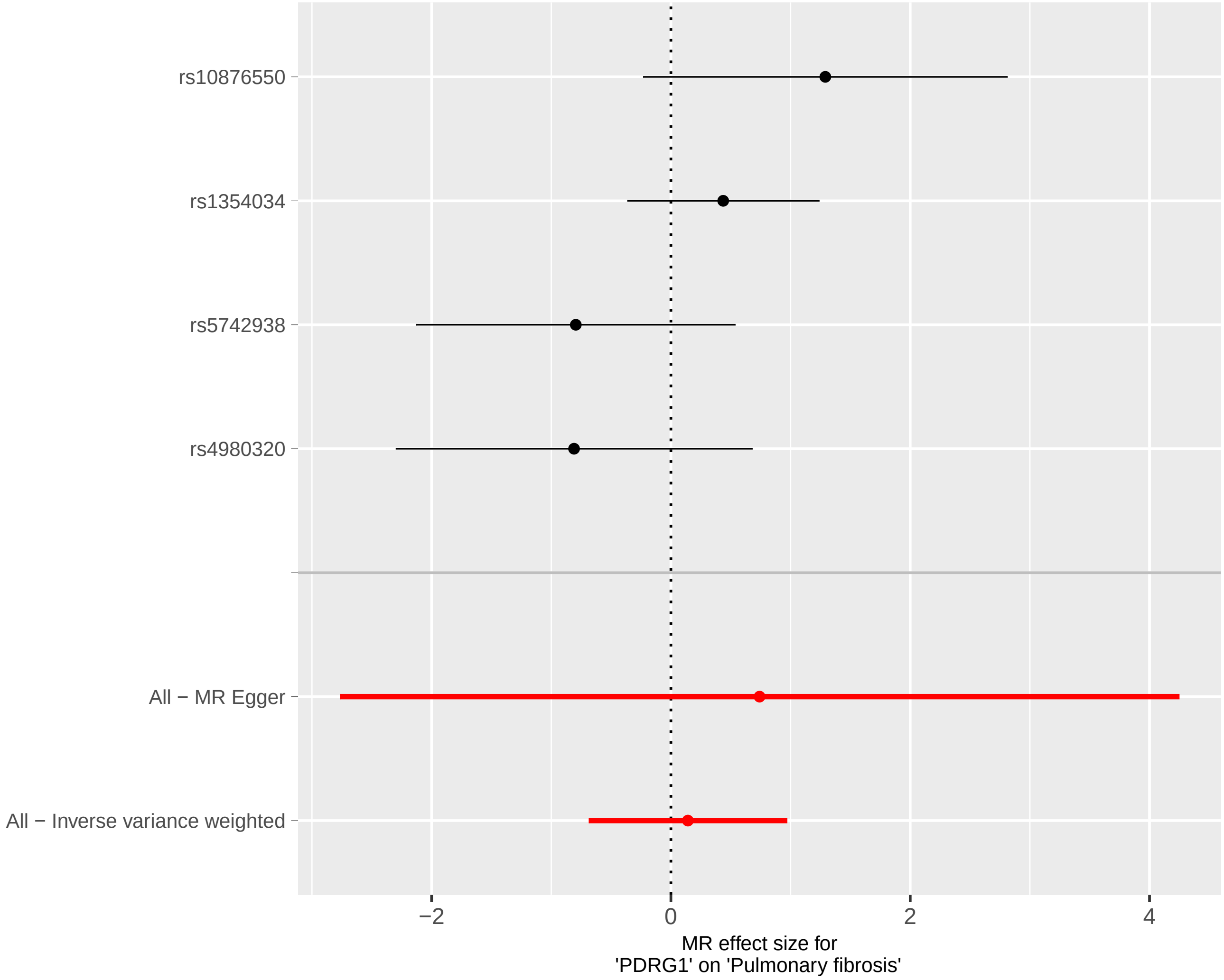

rs10876550
rs1354034
rs5742938
rs4980320
All − MR Egger
All − Inverse variance weighted
−2
0
2
4
MR effect size for
'PDRG1' on 'Pulmonary fibrosis'

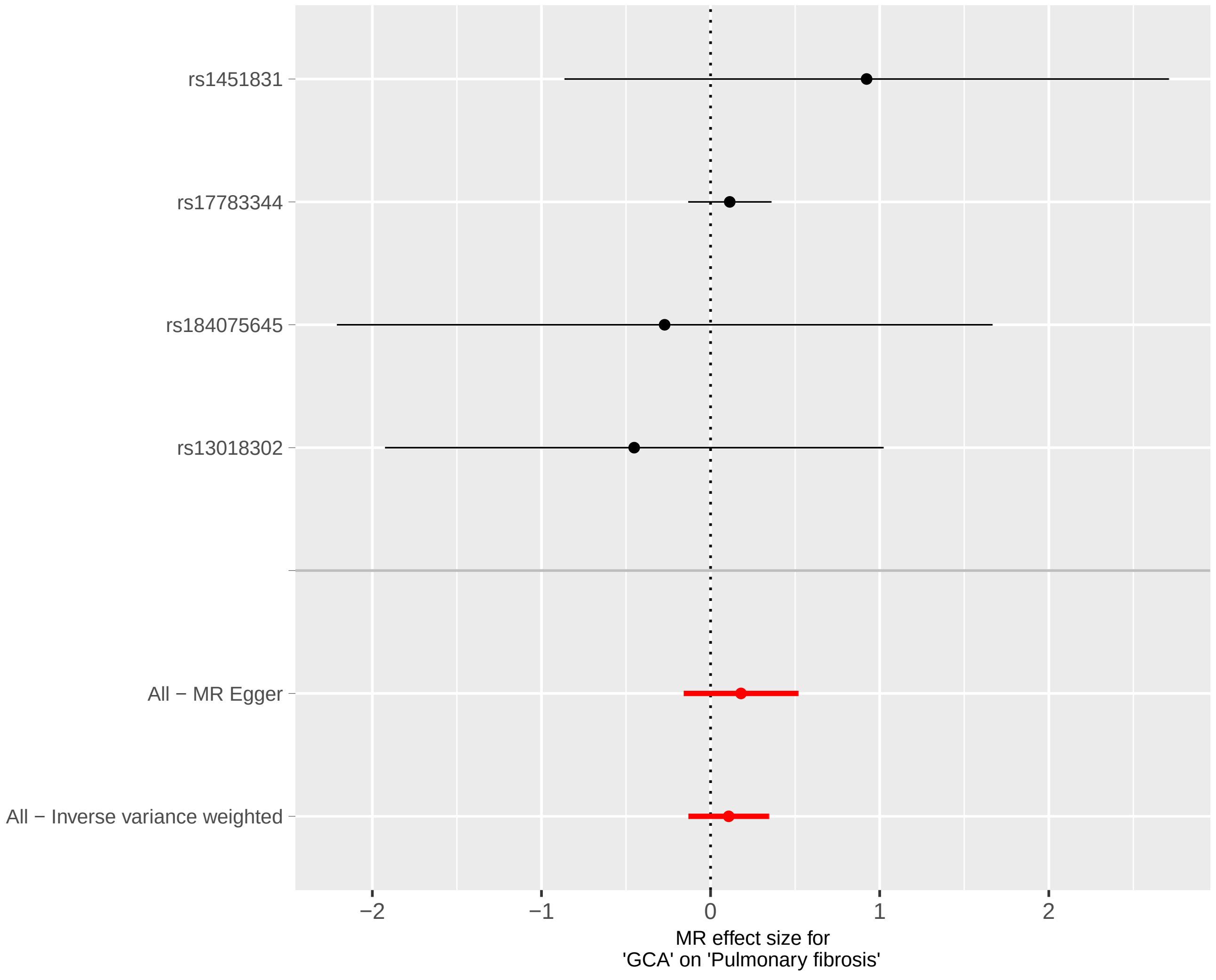
rs1451831
rs17783344
rs184075645
rs13018302
All − MR Egger
All − Inverse variance weighted
−2
−1
0
1
2
MR effect size for
'GCA' on 'Pulmonary fibrosis'

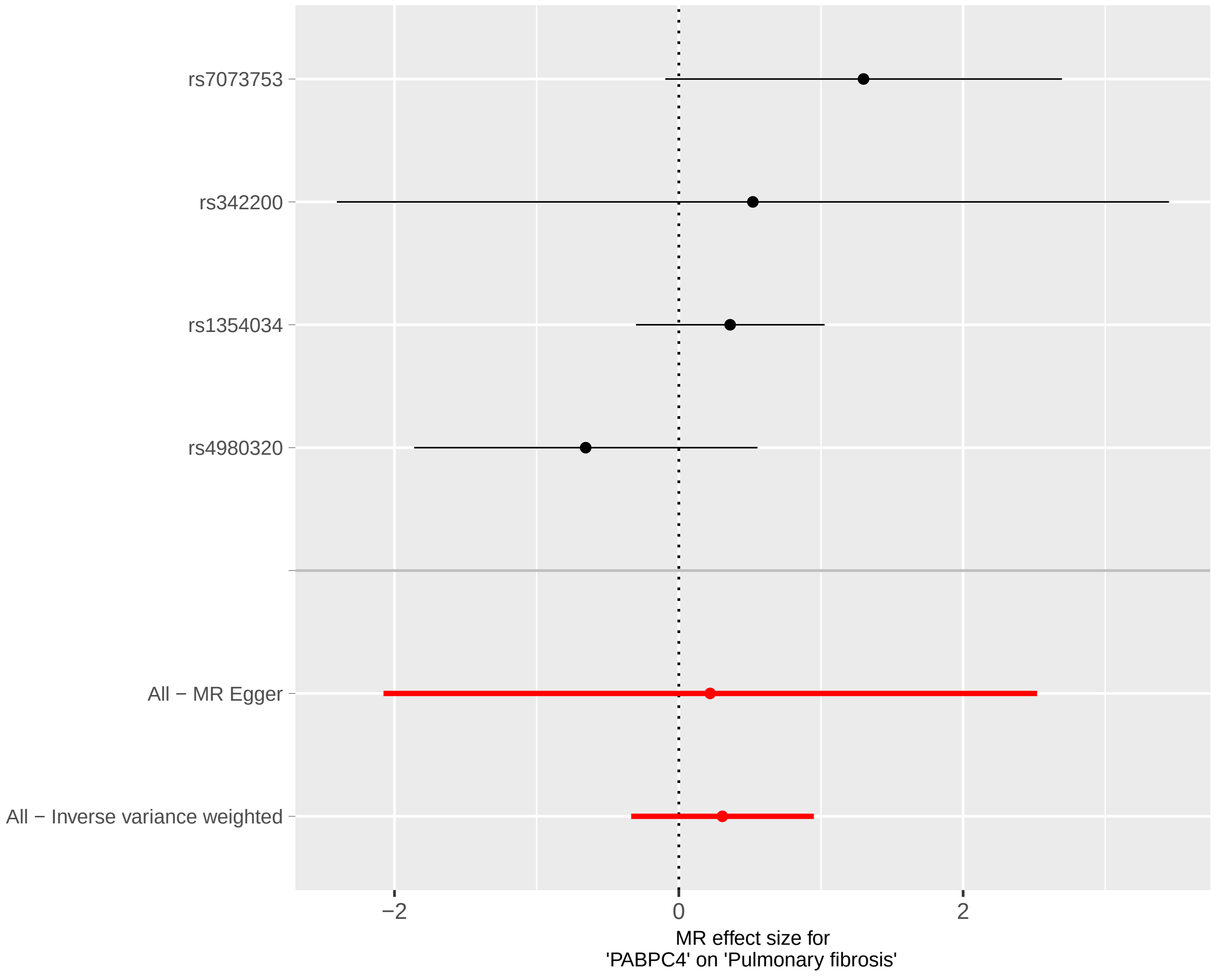
rs7073753
rs342200
rs1354034
rs4980320
All − MR Egger
All − Inverse variance weighted
−2
0
2
MR effect size for
'PABPC4' on 'Pulmonary fibrosis'

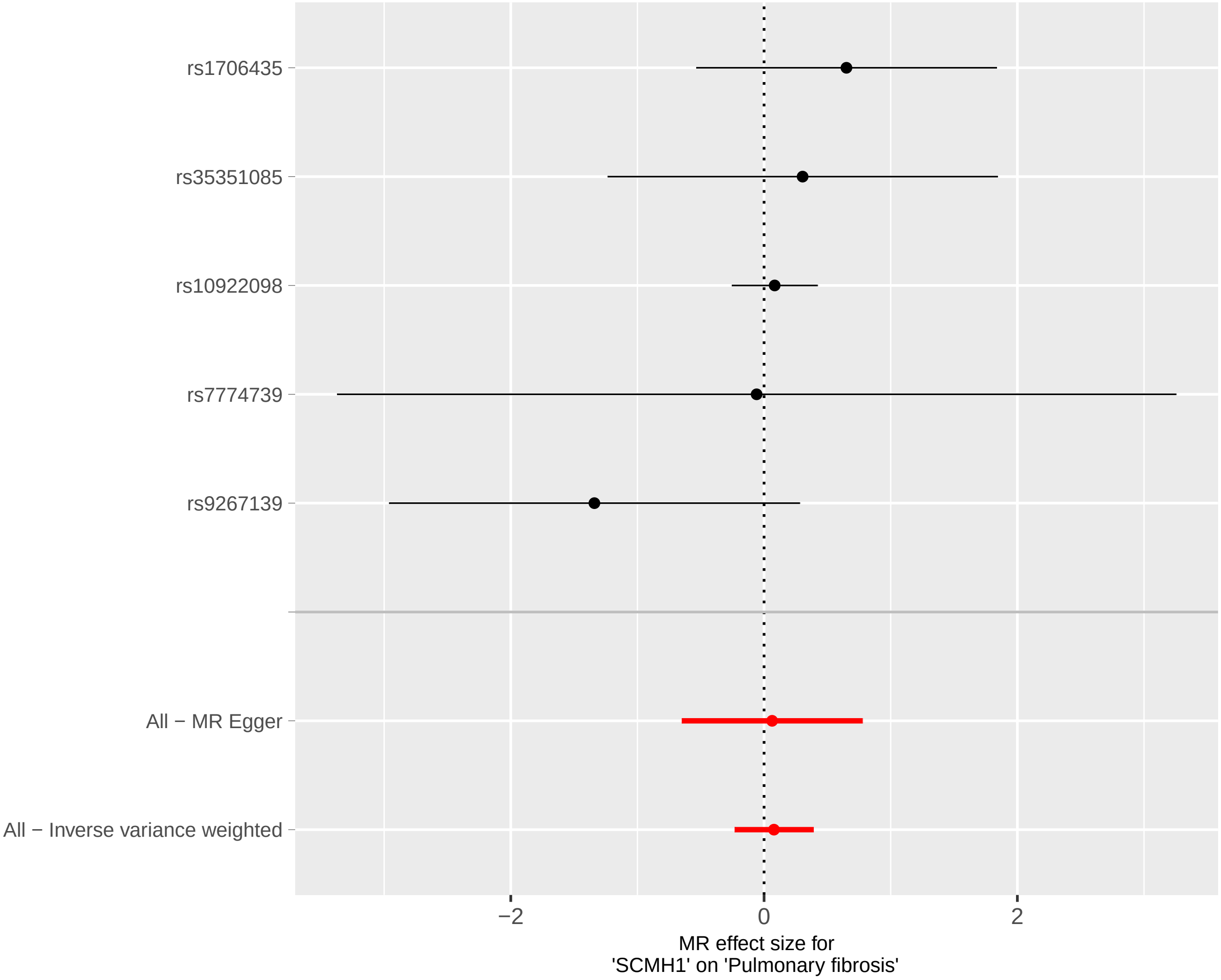
rs1706435
rs35351085
rs10922098
rs7774739
rs9267139
All − MR Egger
All − Inverse variance weighted
−2
0
2
MR effect size for
'SCMH1' on 'Pulmonary fibrosis'

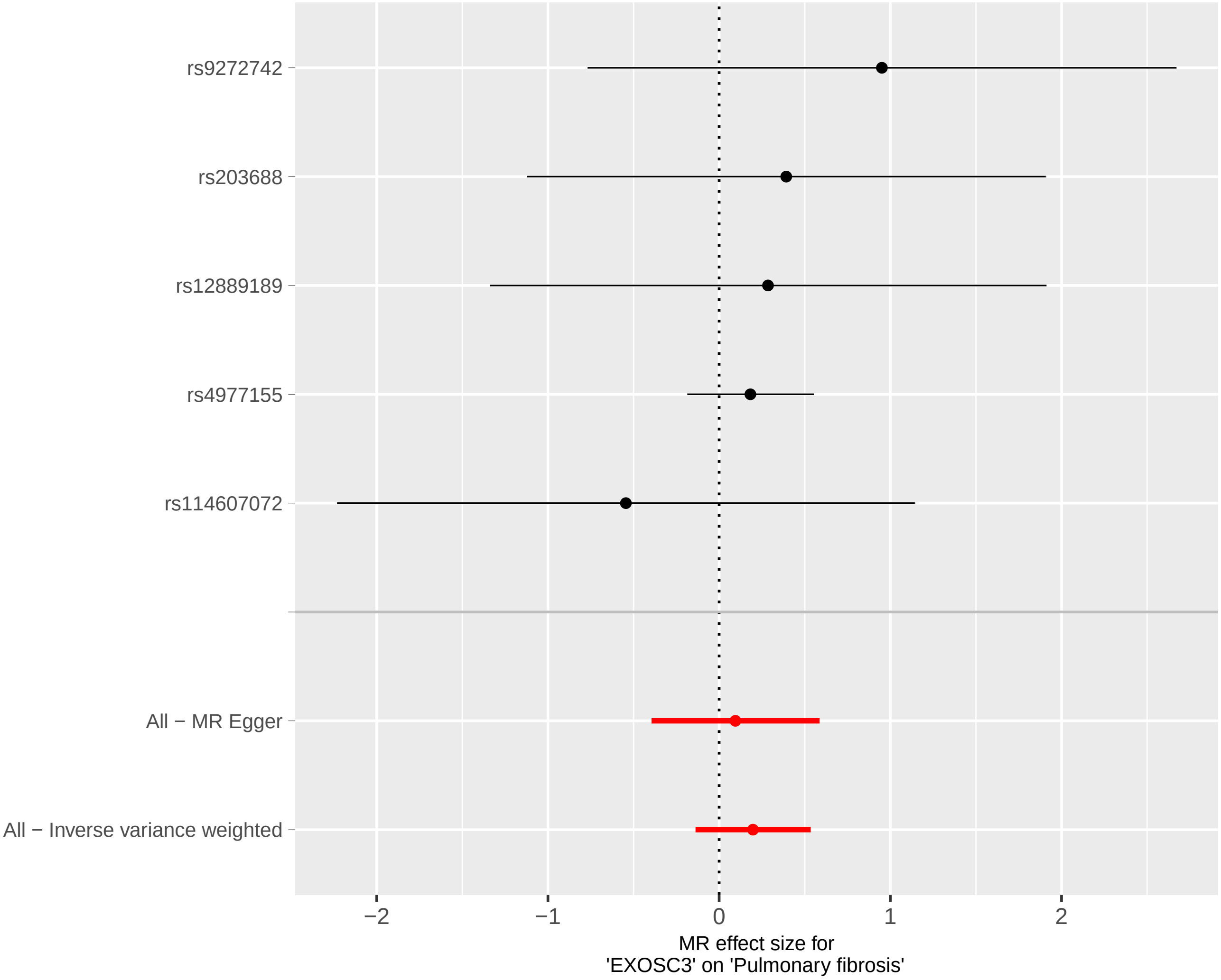
rs9272742
rs203688
rs12889189
rs4977155
rs114607072
All − MR Egger
All − Inverse variance weighted
−2
−1
0
1
2
MR effect size for
'EXOSC3' on 'Pulmonary fibrosis'

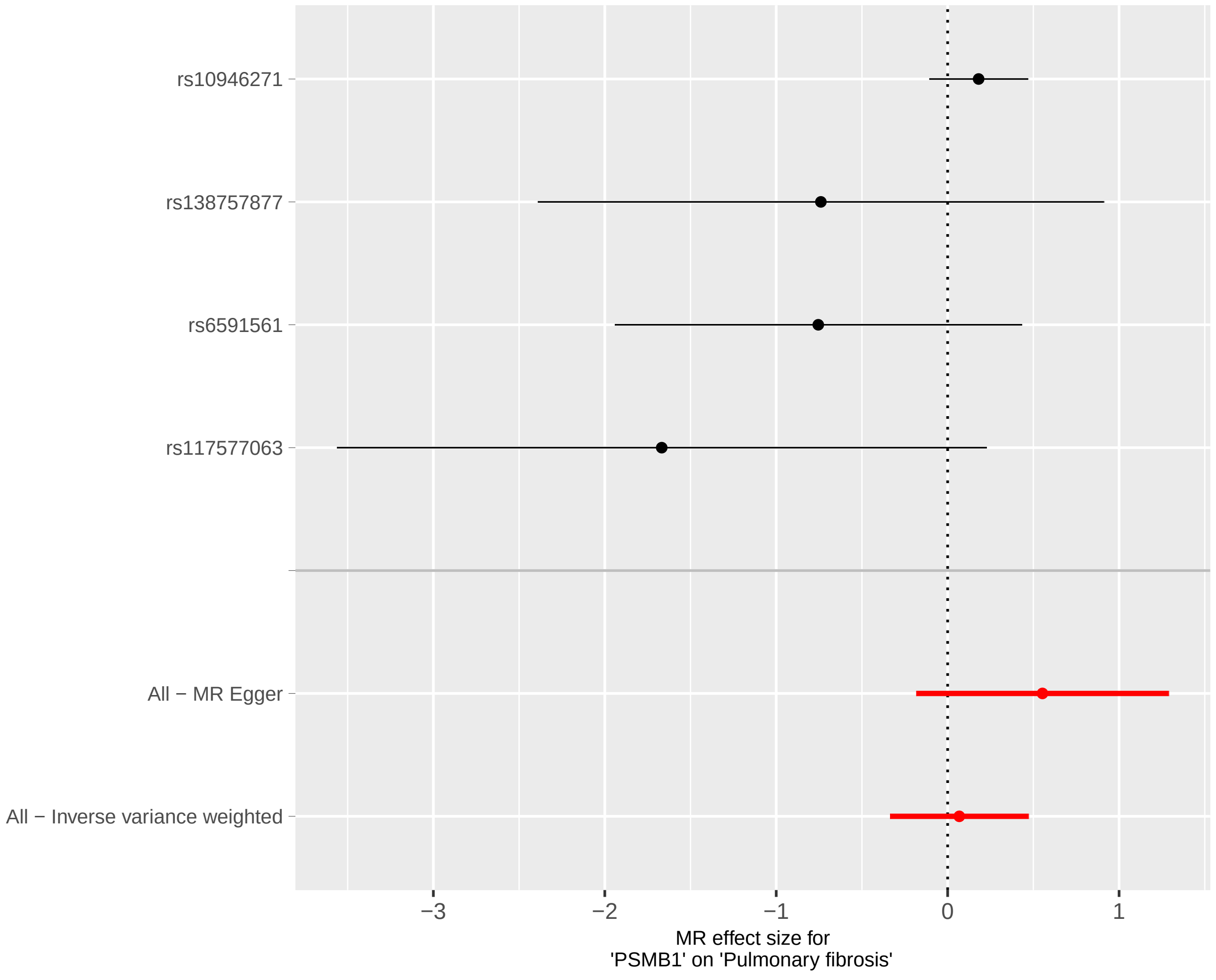
rs10946271
rs138757877
rs6591561
rs117577063
All − MR Egger
All − Inverse variance weighted
−3
−2
−1
0
1
MR effect size for
'PSMB1' on 'Pulmonary fibrosis'

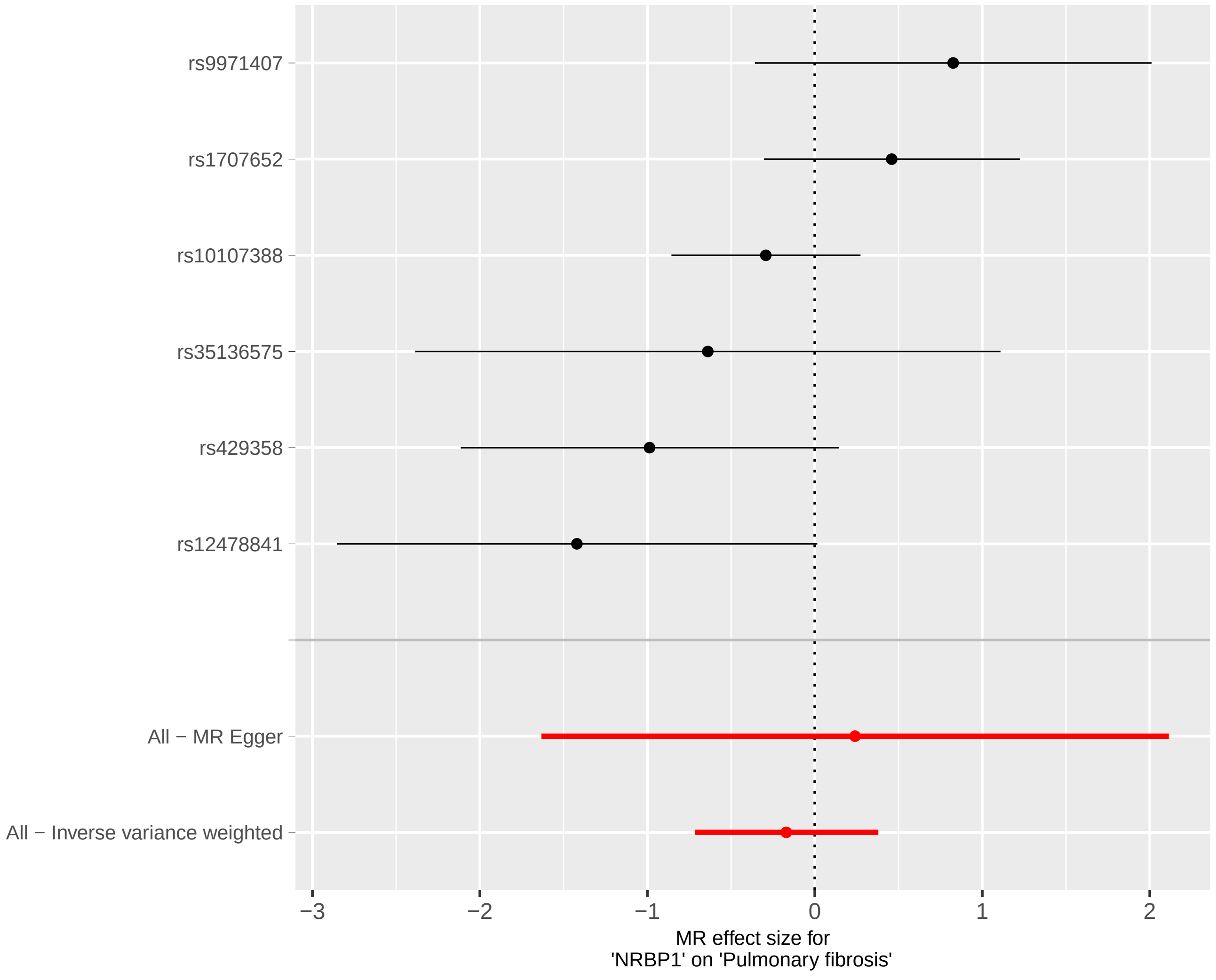
rs9971407
rs1707652
rs10107388
rs35136575
rs429358
rs12478841
All − MR Egger
All − Inverse variance weighted
−3
−2
−1
0
1
2
MR effect size for
'NRBP1' on 'Pulmonary fibrosis'

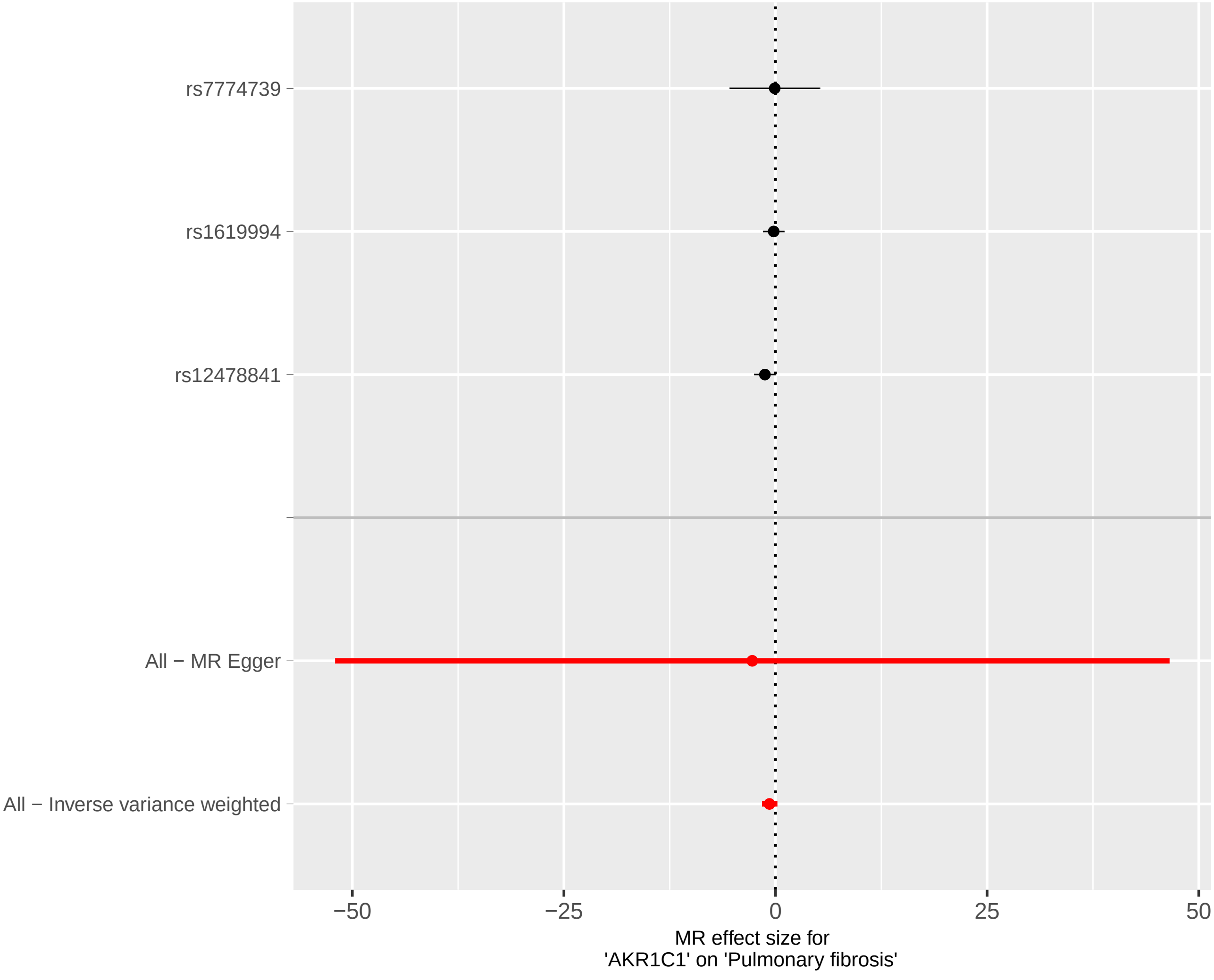

rs7774739
rs1619994
rs12478841
All − MR Egger
All − Inverse variance weighted
−50
−25
0
25
50
MR effect size for
'AKR1C1' on 'Pulmonary fibrosis'

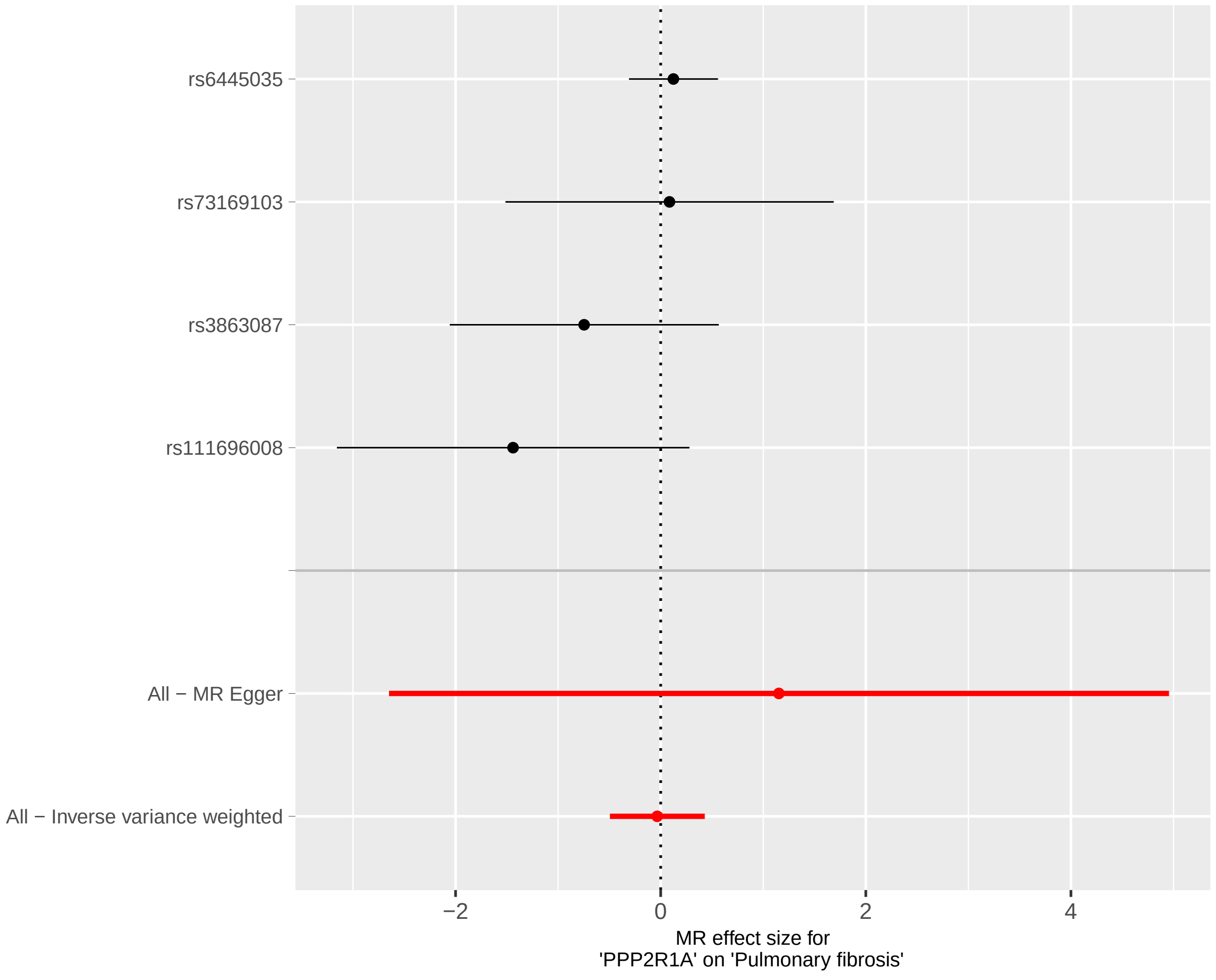
rs6445035
rs73169103
rs3863087
rs11696008
All − MR Egger
All − Inverse variance weighted
−2
0
2
4
MR effect size for
'PPP2R1A' on 'Pulmonary fibrosis'

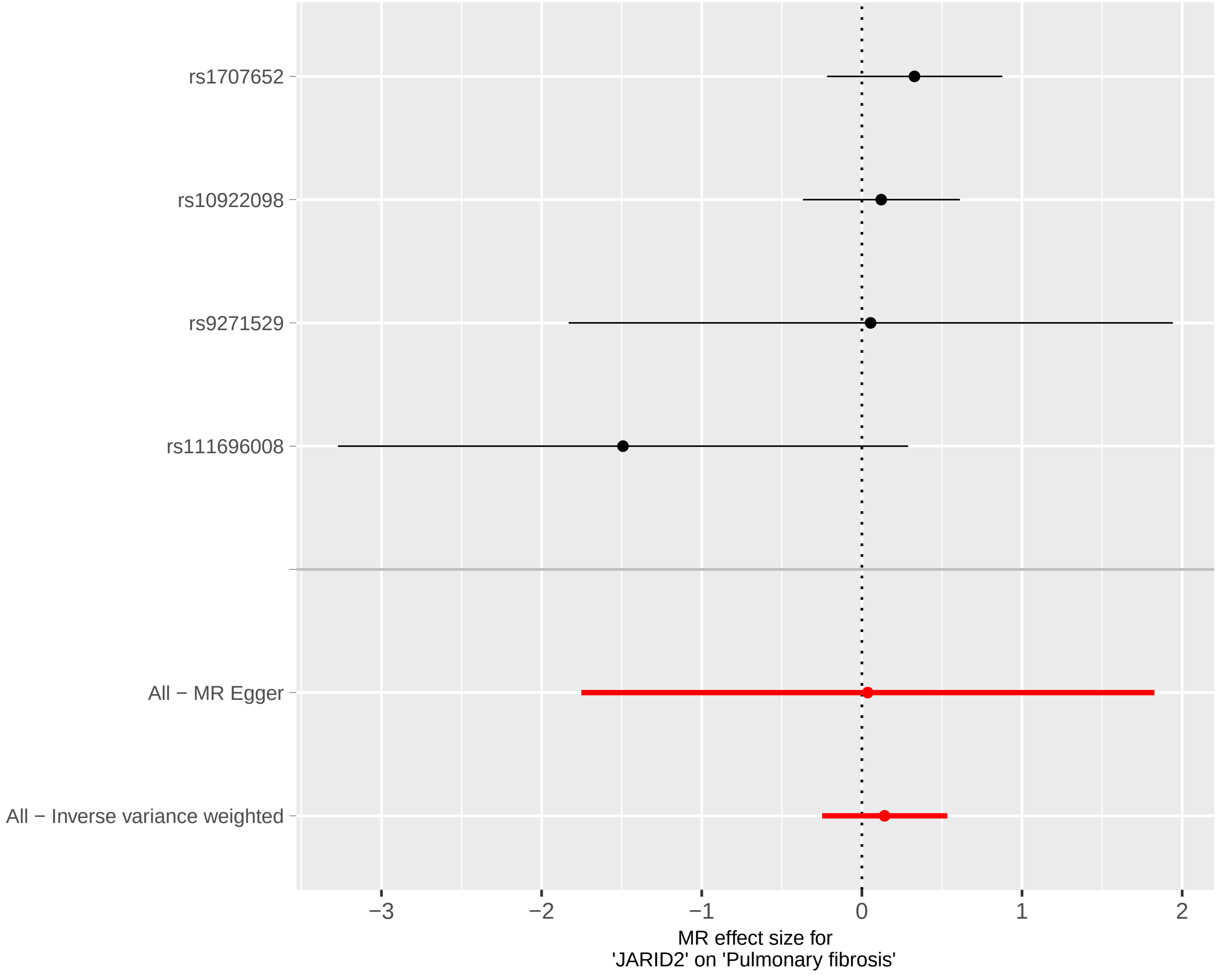

rs1707652
rs10922098
rs9271529
rs11696008
All − MR Egger
All − Inverse variance weighted
−3
−2
−1
0
1
2
MR effect size for
'JARID2' on 'Pulmonary fibrosis'

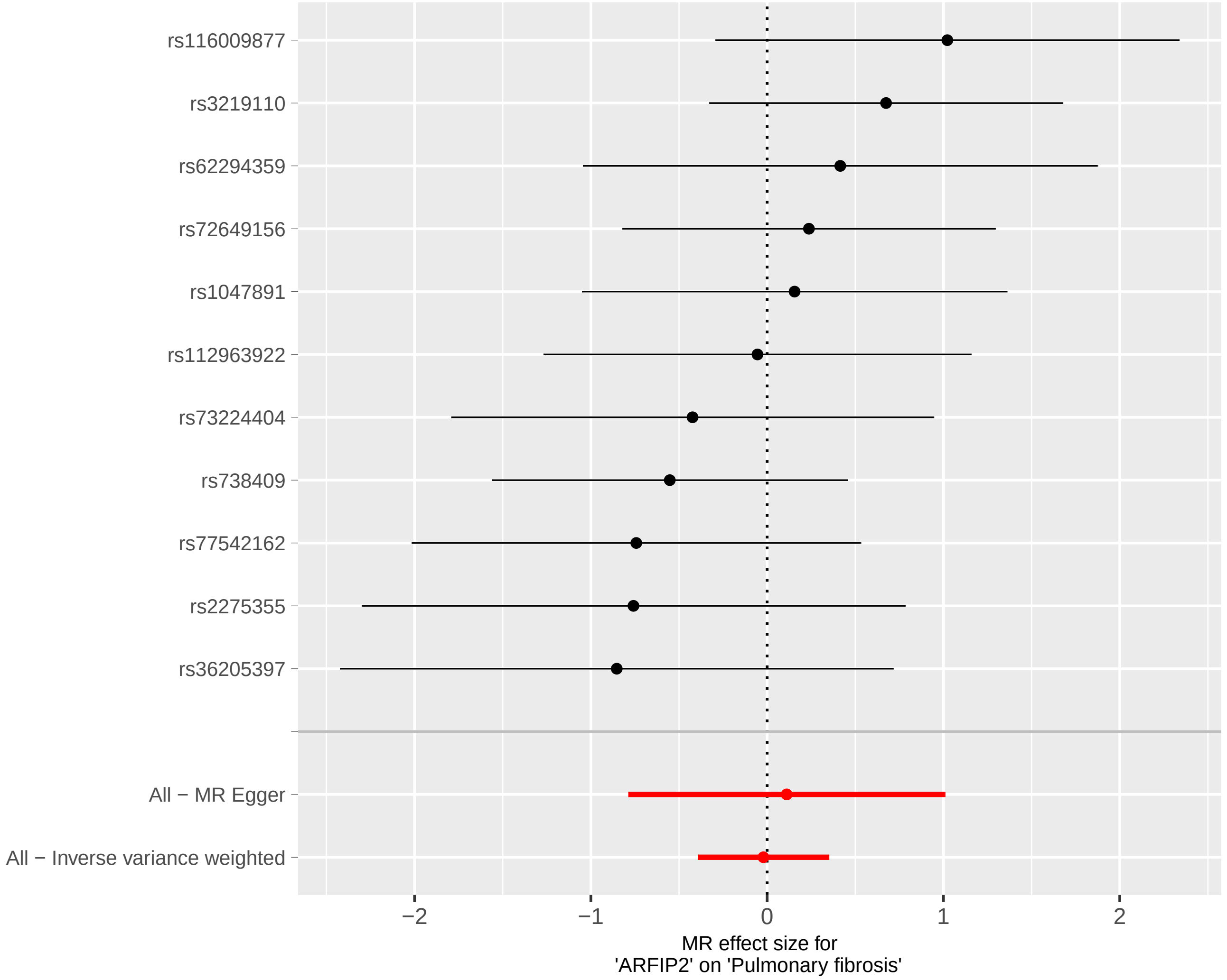
rs116009877
rs3219110
rs62294359
rs72649156
rs1047891
rs112963922
rs73224404
rs738409
rs77542162
rs2275355
rs36205397
All − MR Egger
All − Inverse variance weighted
−2
−1
0
1
2
MR effect size for
'ARFIP2' on 'Pulmonary fibrosis'

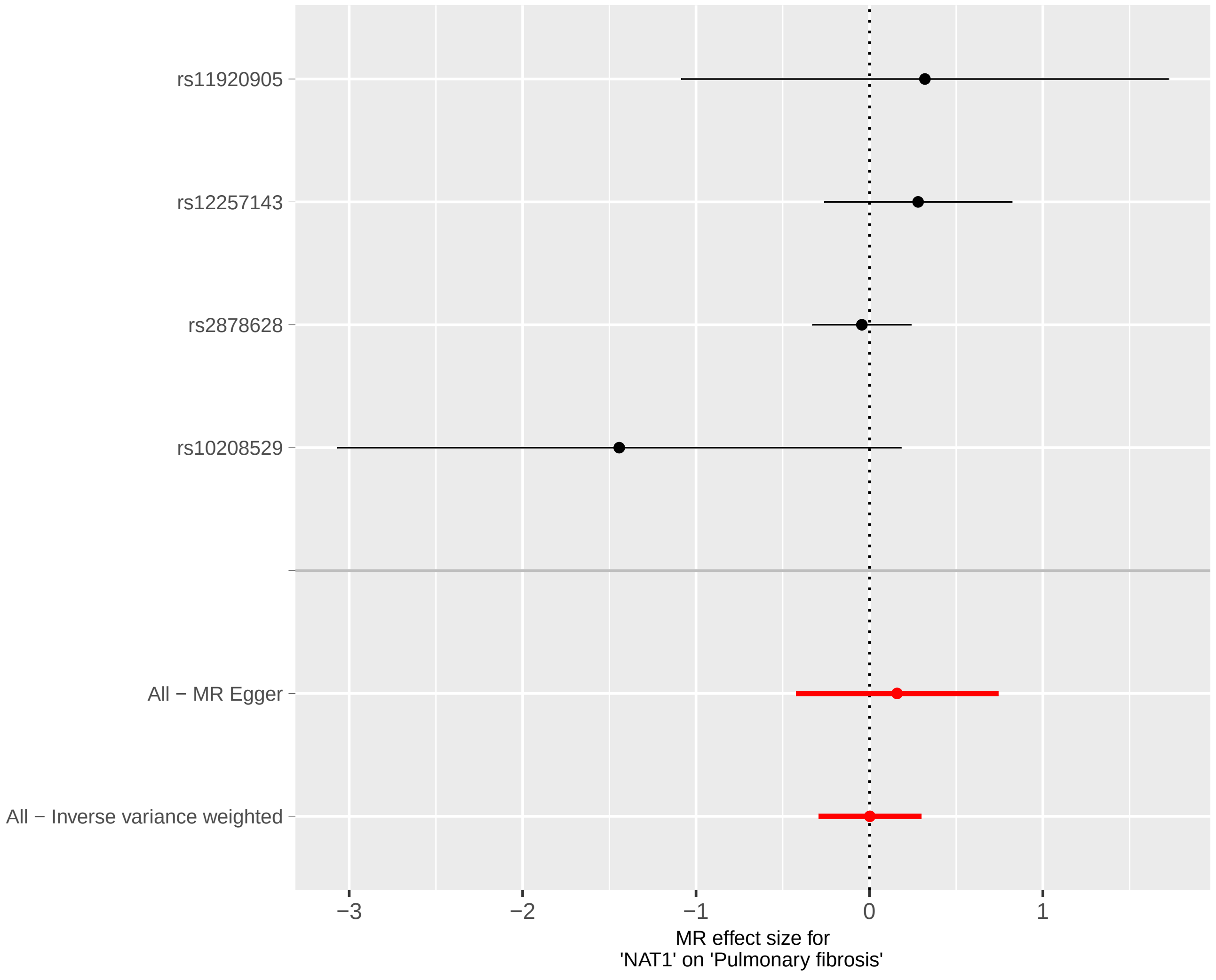
rs11920905
rs12257143
rs2878628
rs10208529
All − MR Egger
All − Inverse variance weighted
−3
−2
−1
0
1
MR effect size for
'NAT1' on 'Pulmonary fibrosis'

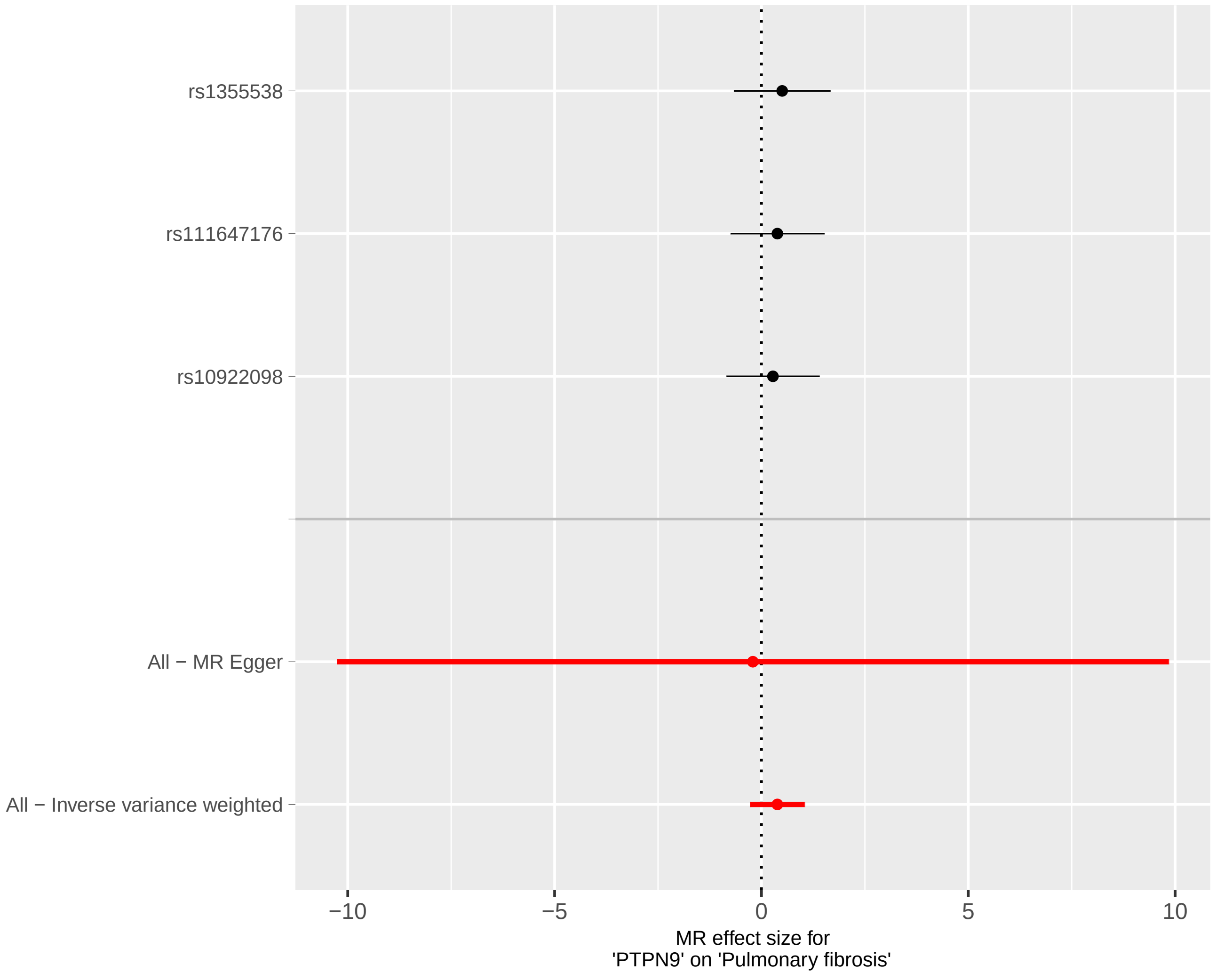

rs1355538
rs111647176
rs10922098
All − MR Egger
All − Inverse variance weighted
−10
−5
0
5
10
MR effect size for
'PTPN9' on 'Pulmonary fibrosis'

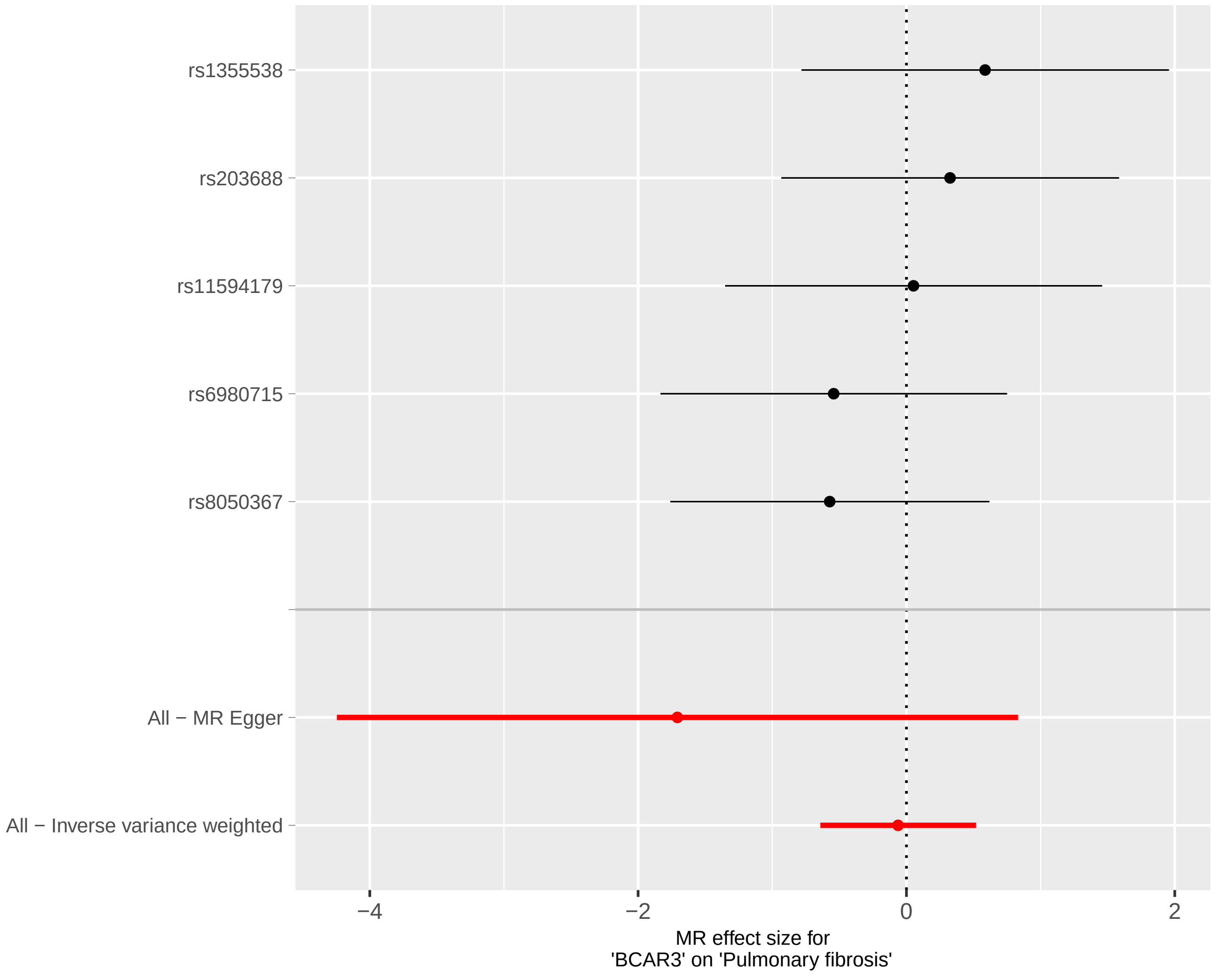
rs1355538
rs203688
rs11594179
rs6980715
rs8050367
All − MR Egger
All − Inverse variance weighted
−4
−2
0
2
MR effect size for
'BCAR3' on 'Pulmonary fibrosis'

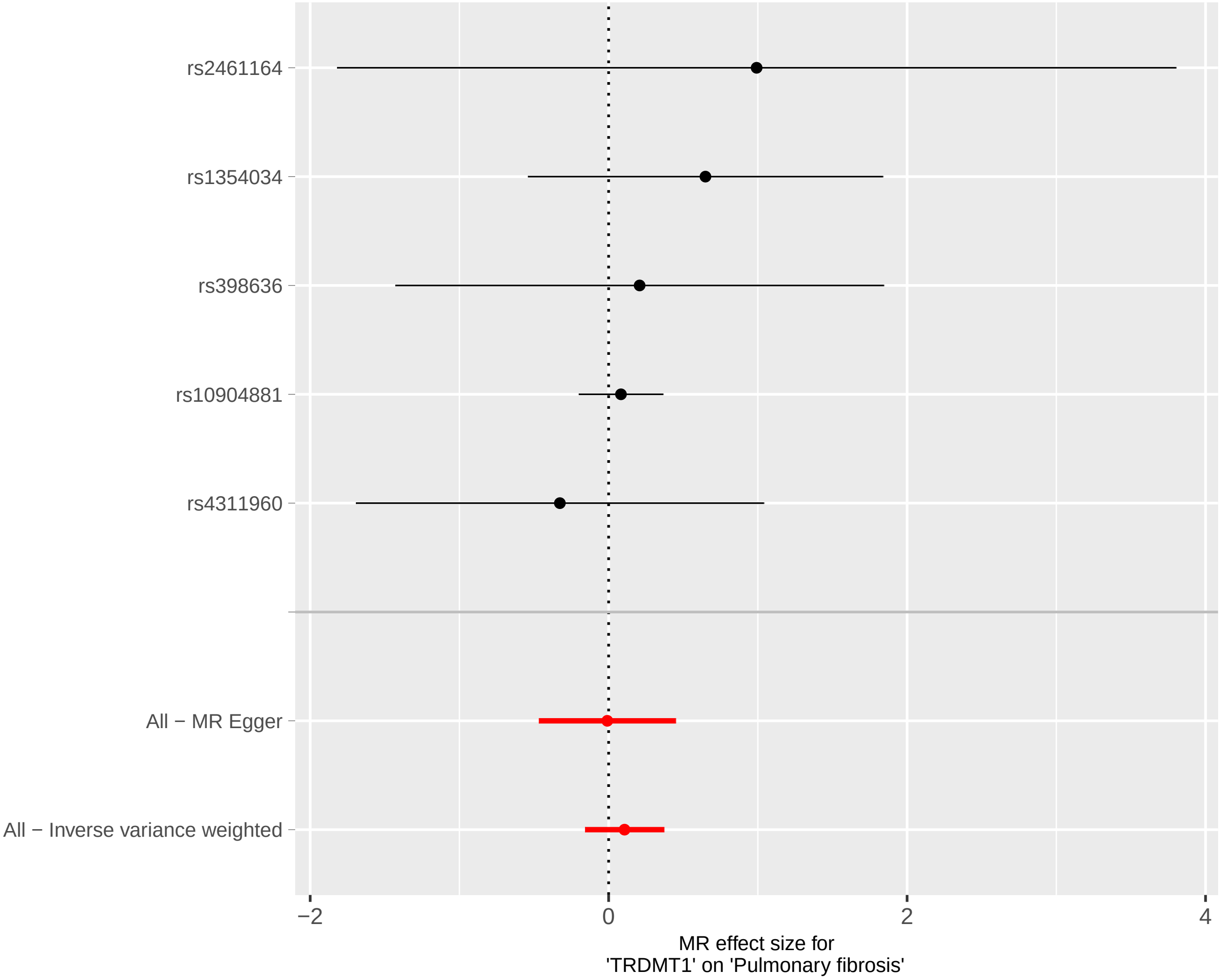
rs2461164
rs1354034
rs398636
rs10904881
rs4311960
All − MR Egger
All − Inverse variance weighted
−2
0
2
4
MR effect size for
'TRDMT1' on 'Pulmonary fibrosis'

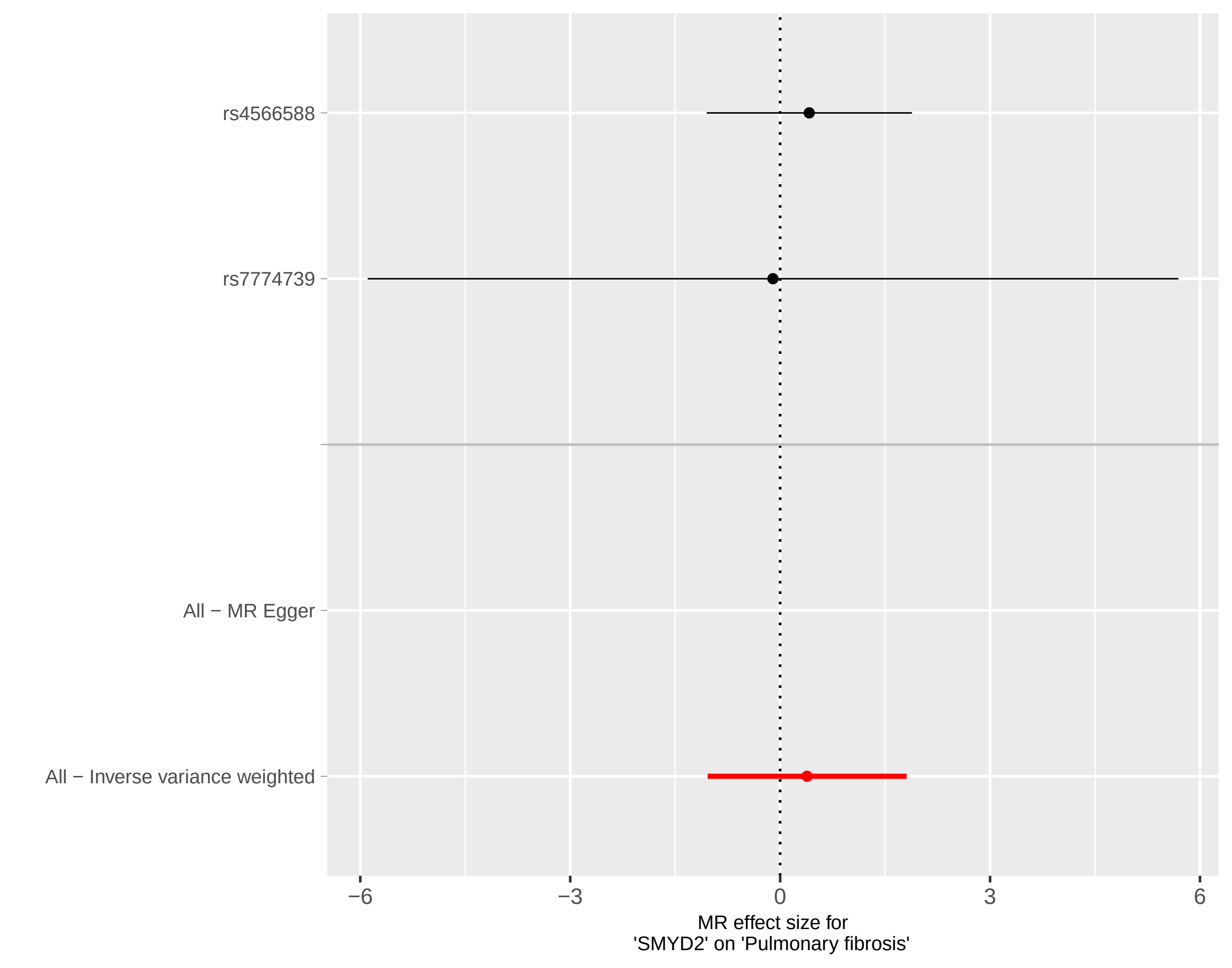
rs4566588
rs7774739
All − MR Egger
All − Inverse variance weighted
−6
−3
0
3
6
MR effect size for
'SMYD2' on 'Pulmonary fibrosis'

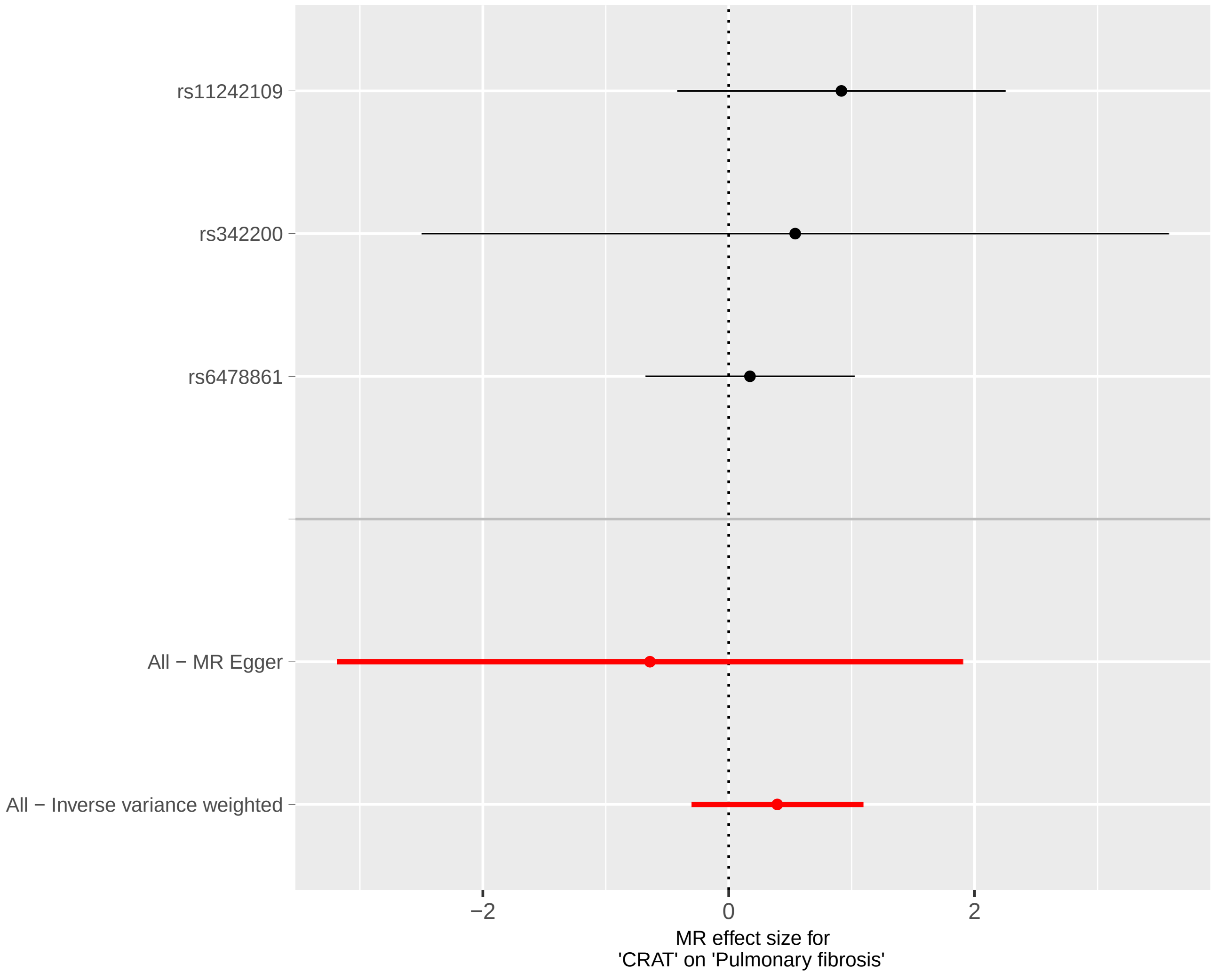
rs11242109
rs342200
rs6478861
All − MR Egger
All − Inverse variance weighted
−2
0
2
MR effect size for
'CRAT' on 'Pulmonary fibrosis'

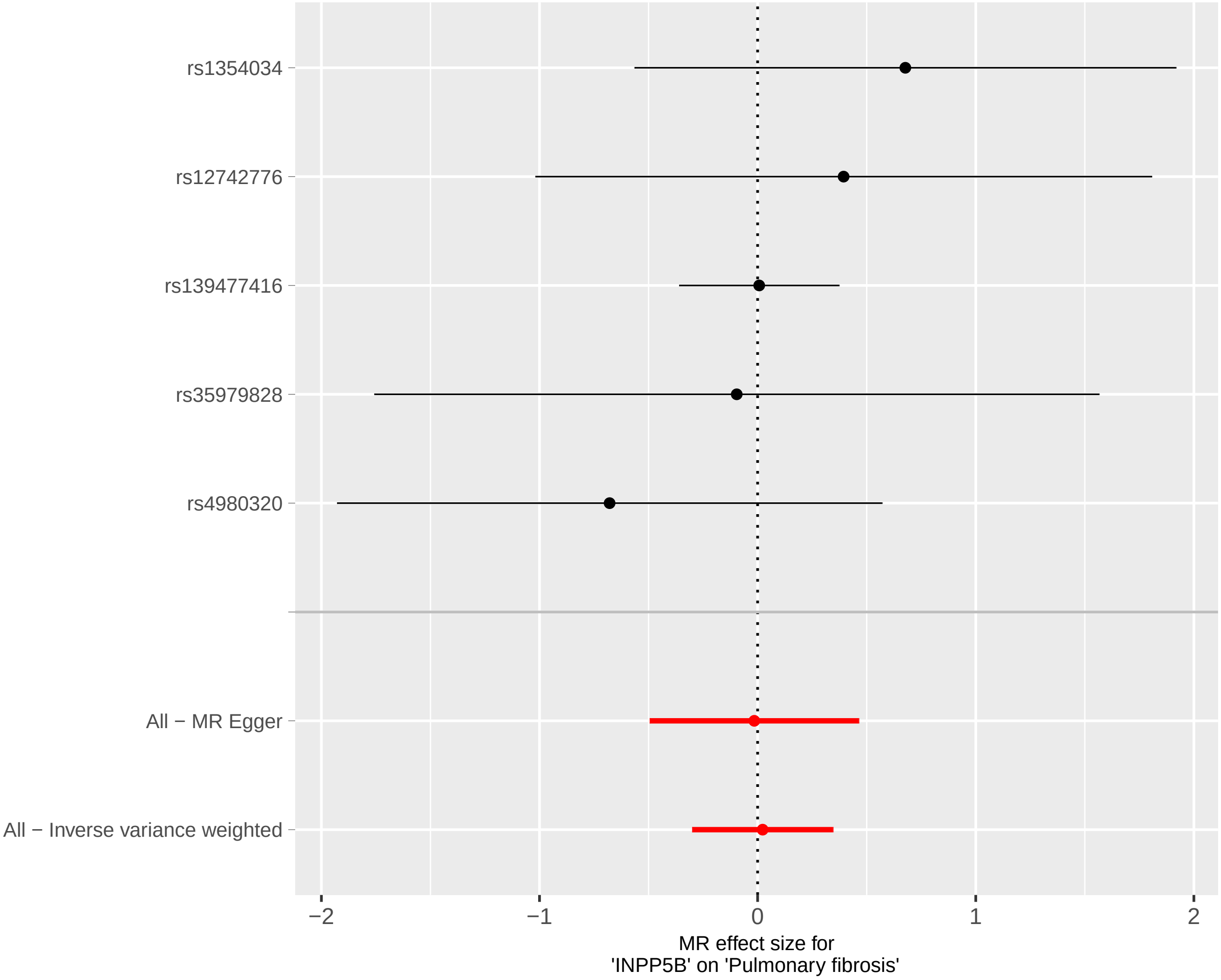
rs1354034
rs12742776
rs139477416
rs35979828
rs4980320
All − MR Egger
All − Inverse variance weighted
−2
−1
0
1
2
MR effect size for
'INPP5B' on 'Pulmonary fibrosis'

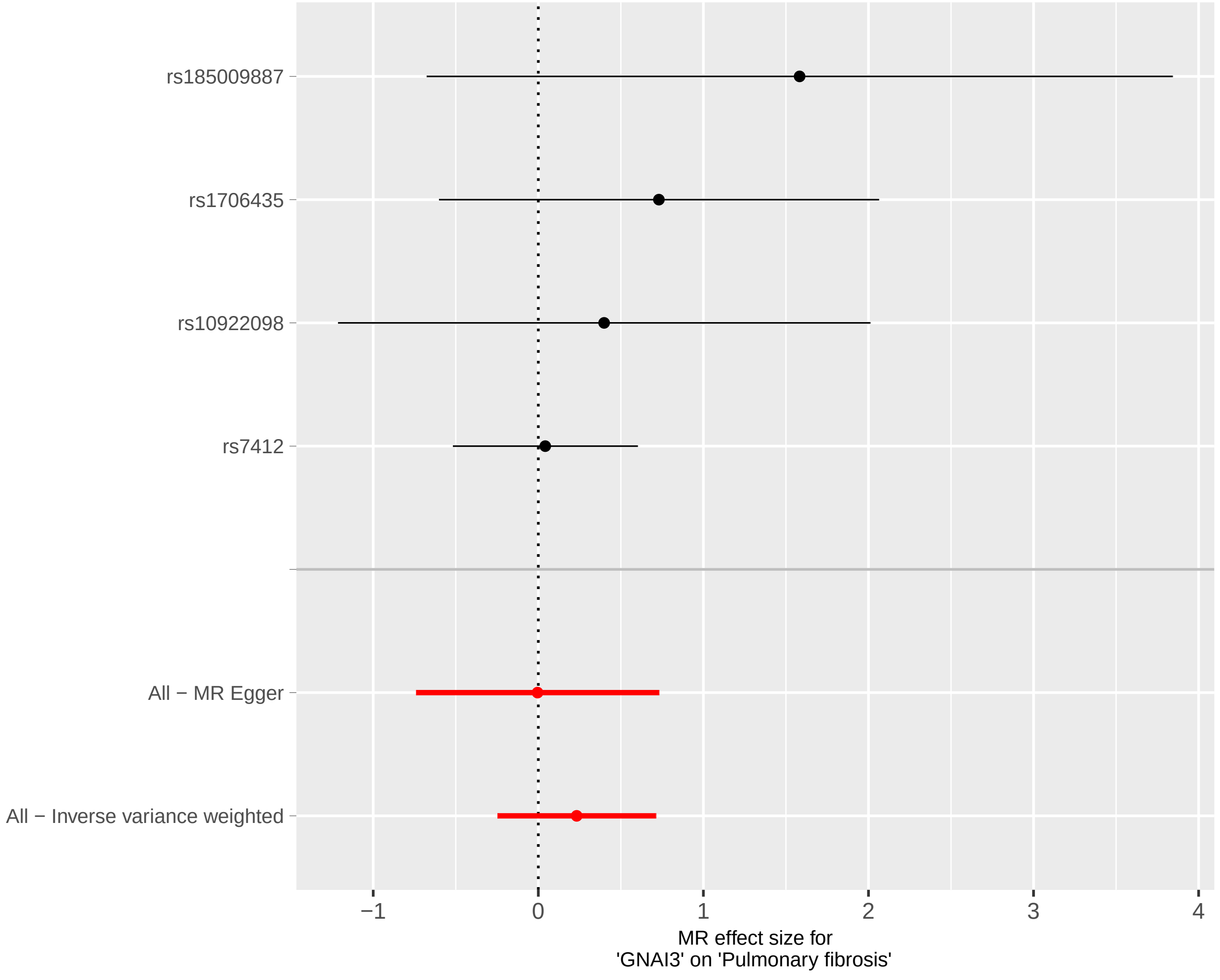

rs185009887
rs1706435
rs10922098
rs7412
All − MR Egger
All − Inverse variance weighted
−1
0
1
2
3
4
MR effect size for
'GNAI3' on 'Pulmonary fibrosis'

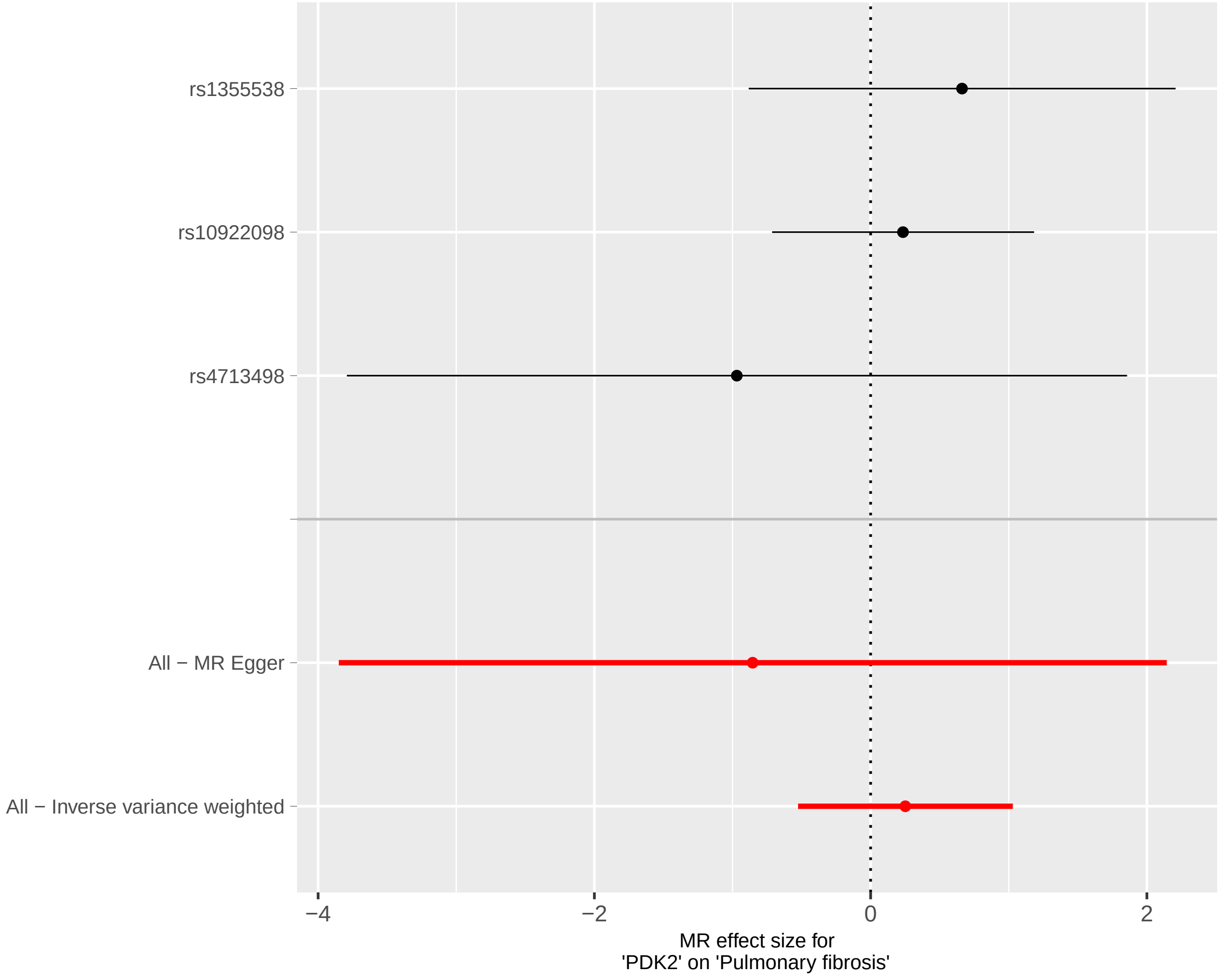

rs1355538
rs10922098
rs4713498
All − MR Egger
All − Inverse variance weighted
−4
−2
0
2
MR effect size for
'PDK2' on 'Pulmonary fibrosis'

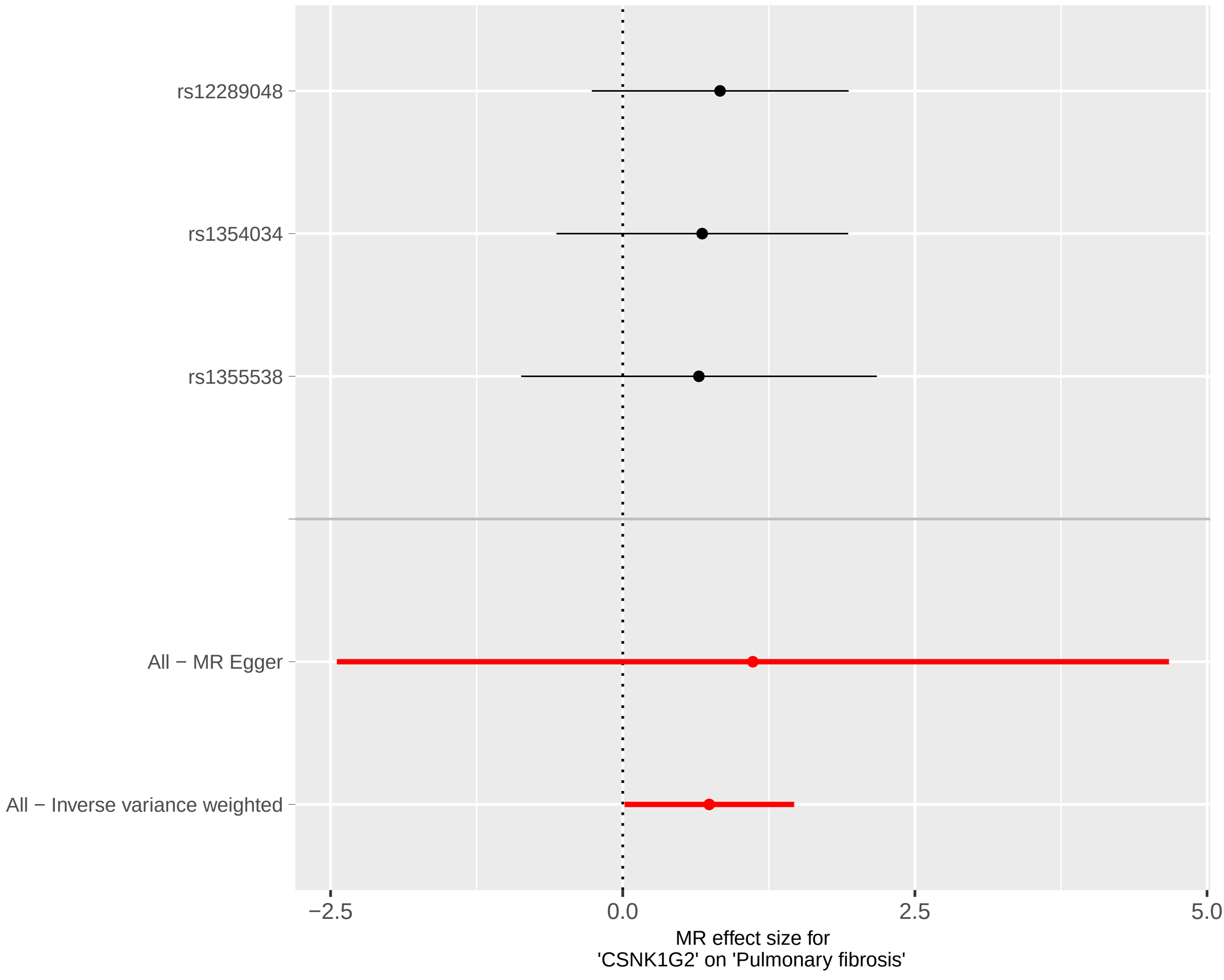
rs12289048
rs1354034
rs1355538
All − MR Egger
All − Inverse variance weighted
−2.5
0.0
2.5
5.0
MR effect size for
'CSNK1G2' on 'Pulmonary fibrosis'

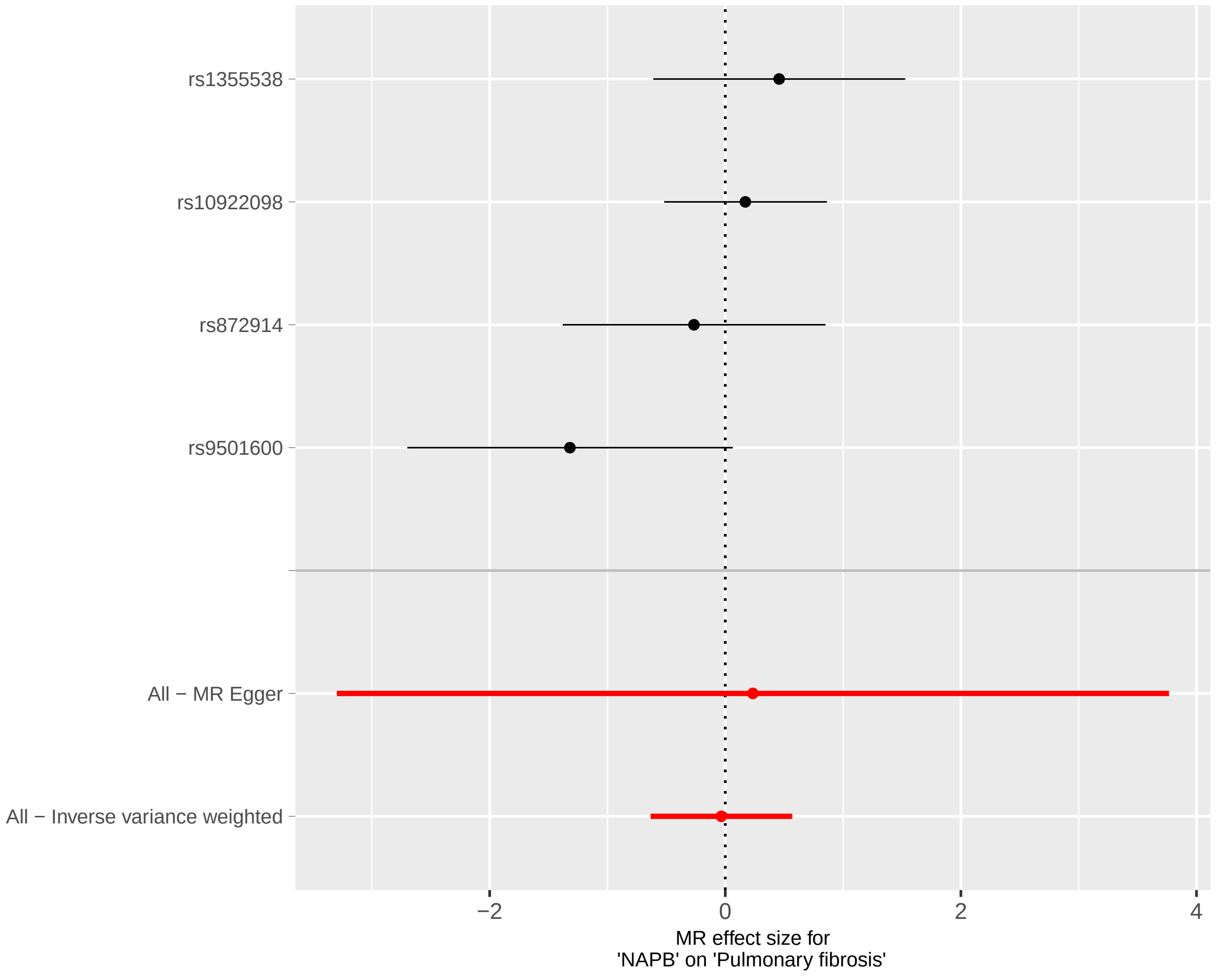
rs1355538
rs10922098
rs872914
rs9501600
All − MR Egger
All − Inverse variance weighted
−2
0
2
4
MR effect size for
'NAPB' on 'Pulmonary fibrosis'

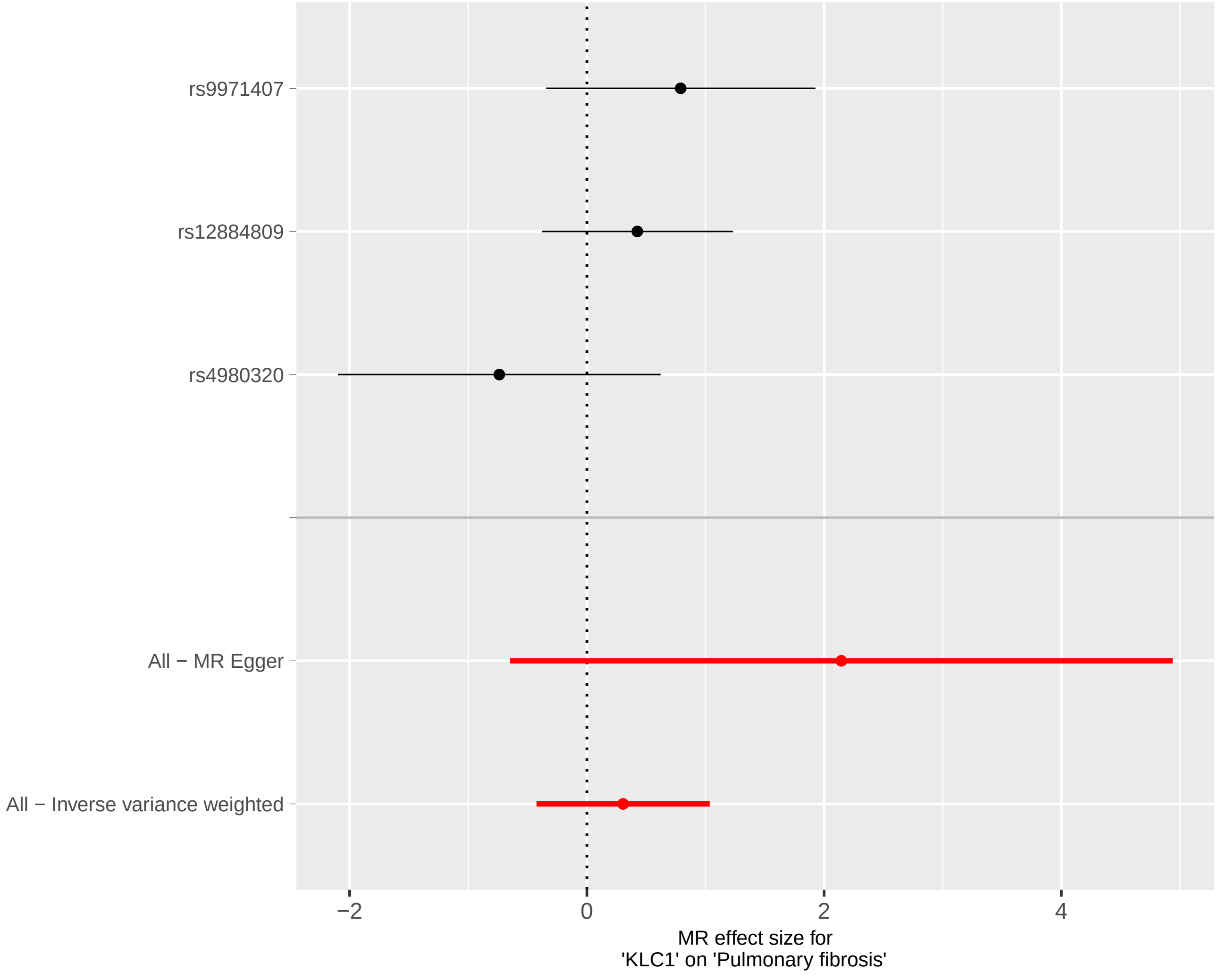
rs9971407
rs12884809
rs4980320
All − MR Egger
All − Inverse variance weighted
−2
0
2
4
MR effect size for
'KLC1' on 'Pulmonary fibrosis'

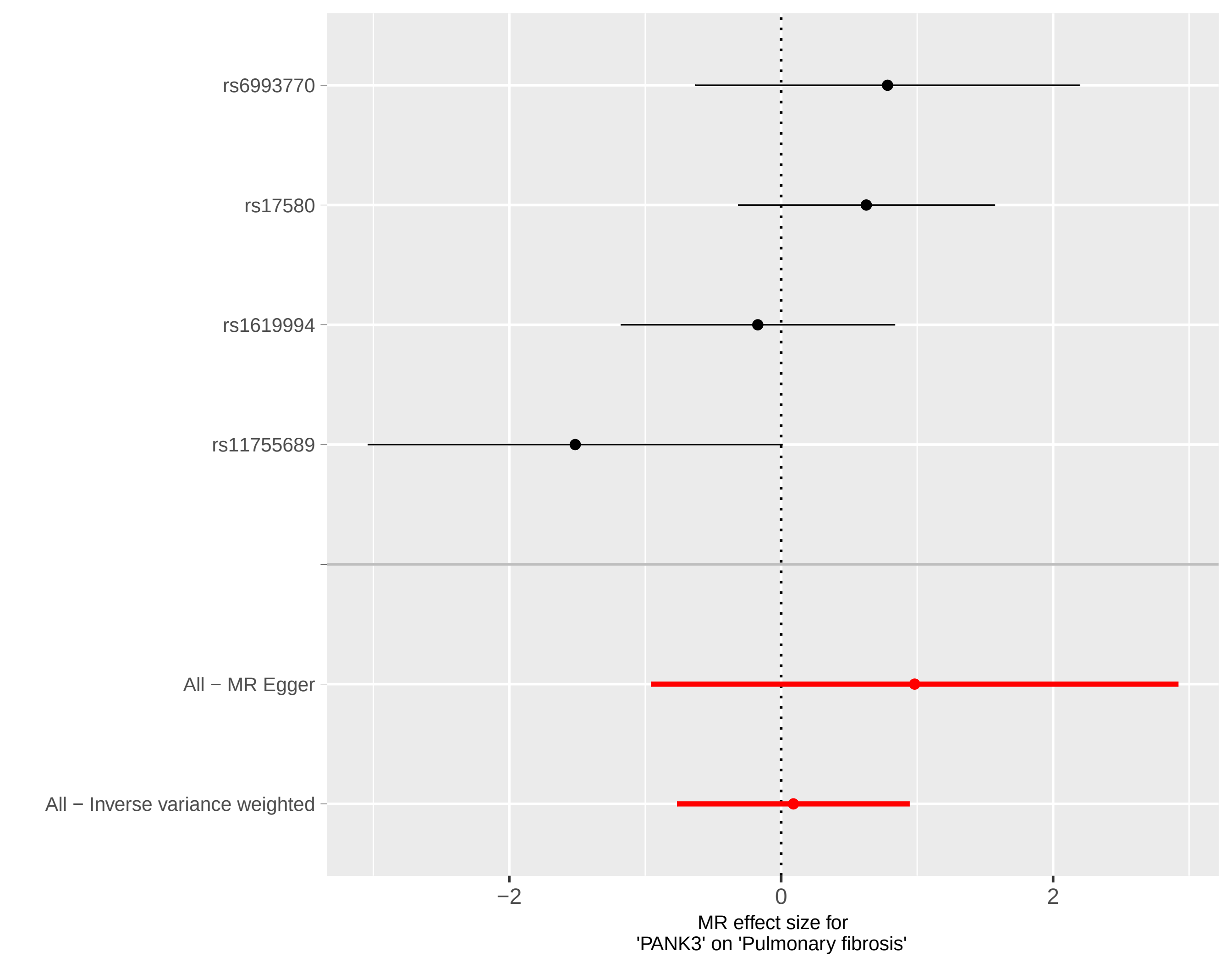
rs6993770
rs17580
rs1619994
rs11755689
All − MR Egger
All − Inverse variance weighted
−2
0
2
MR effect size for
'PANK3' on 'Pulmonary fibrosis'

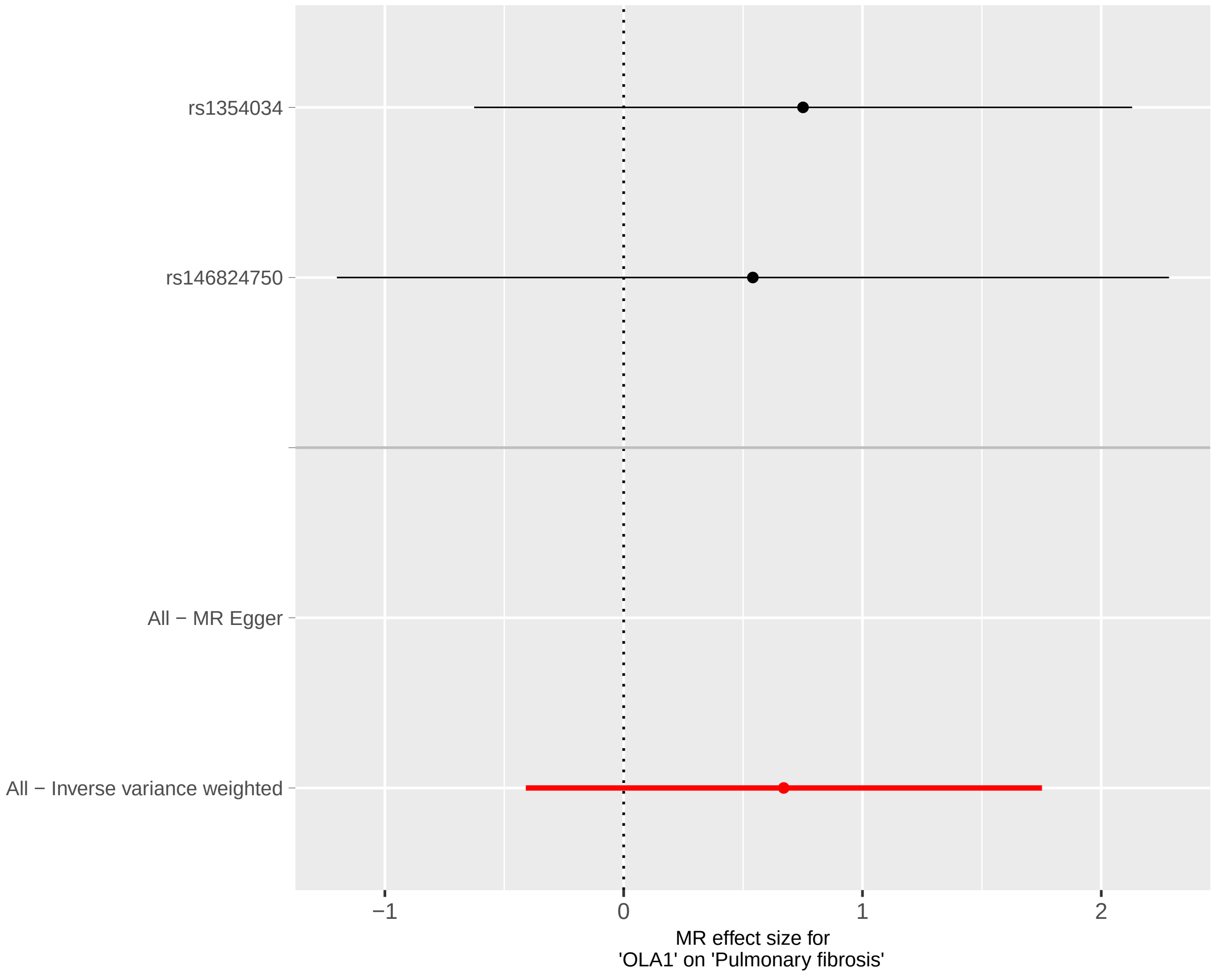

rs1354034
rs146824750
All − MR Egger
All − Inverse variance weighted
−1
0
1
2
MR effect size for
'OLA1' on 'Pulmonary fibrosis'

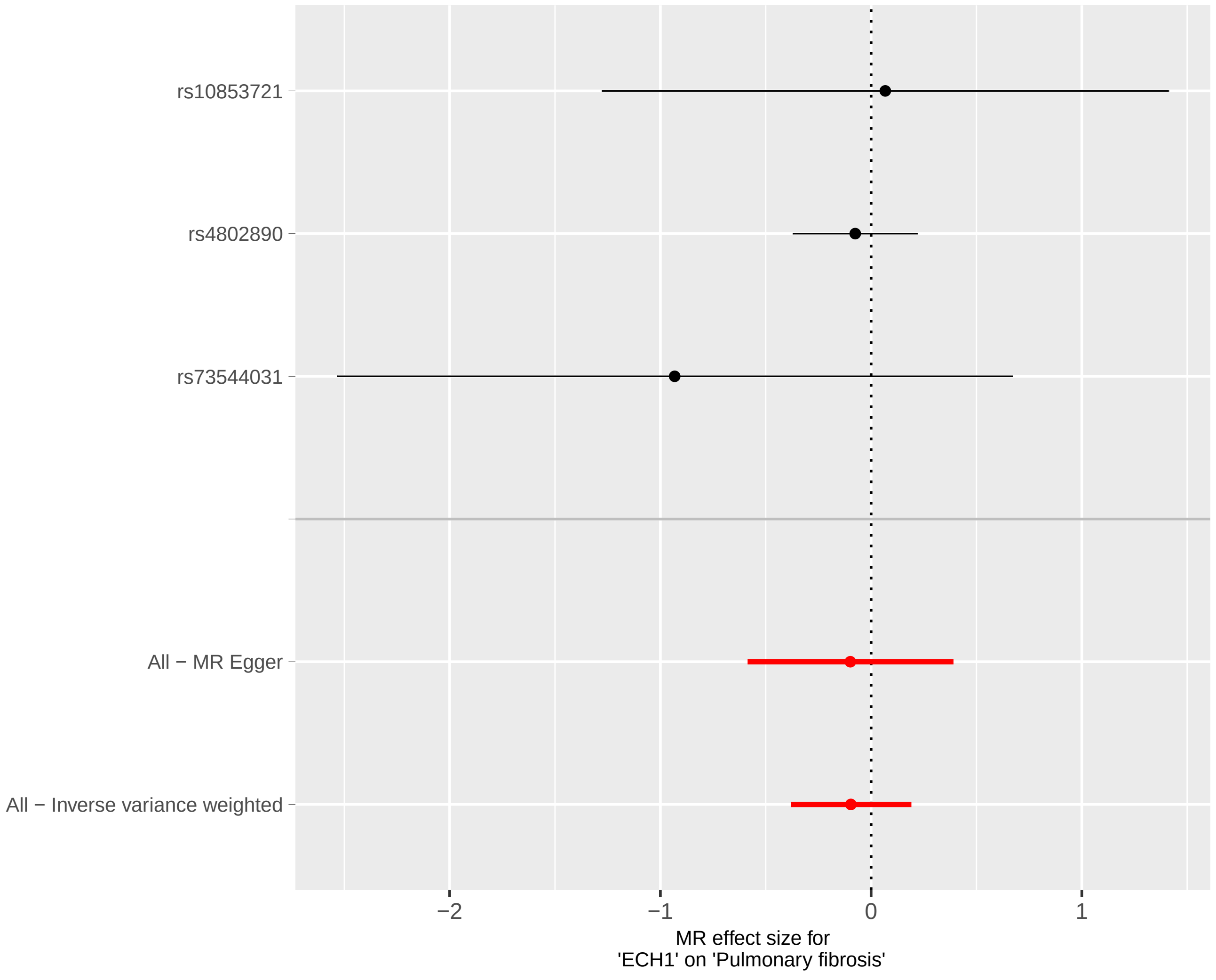
rs10853721
rs4802890
rs73544031
All − MR Egger
All − Inverse variance weighted
−2
−1
0
1
MR effect size for
'ECH1' on 'Pulmonary fibrosis'

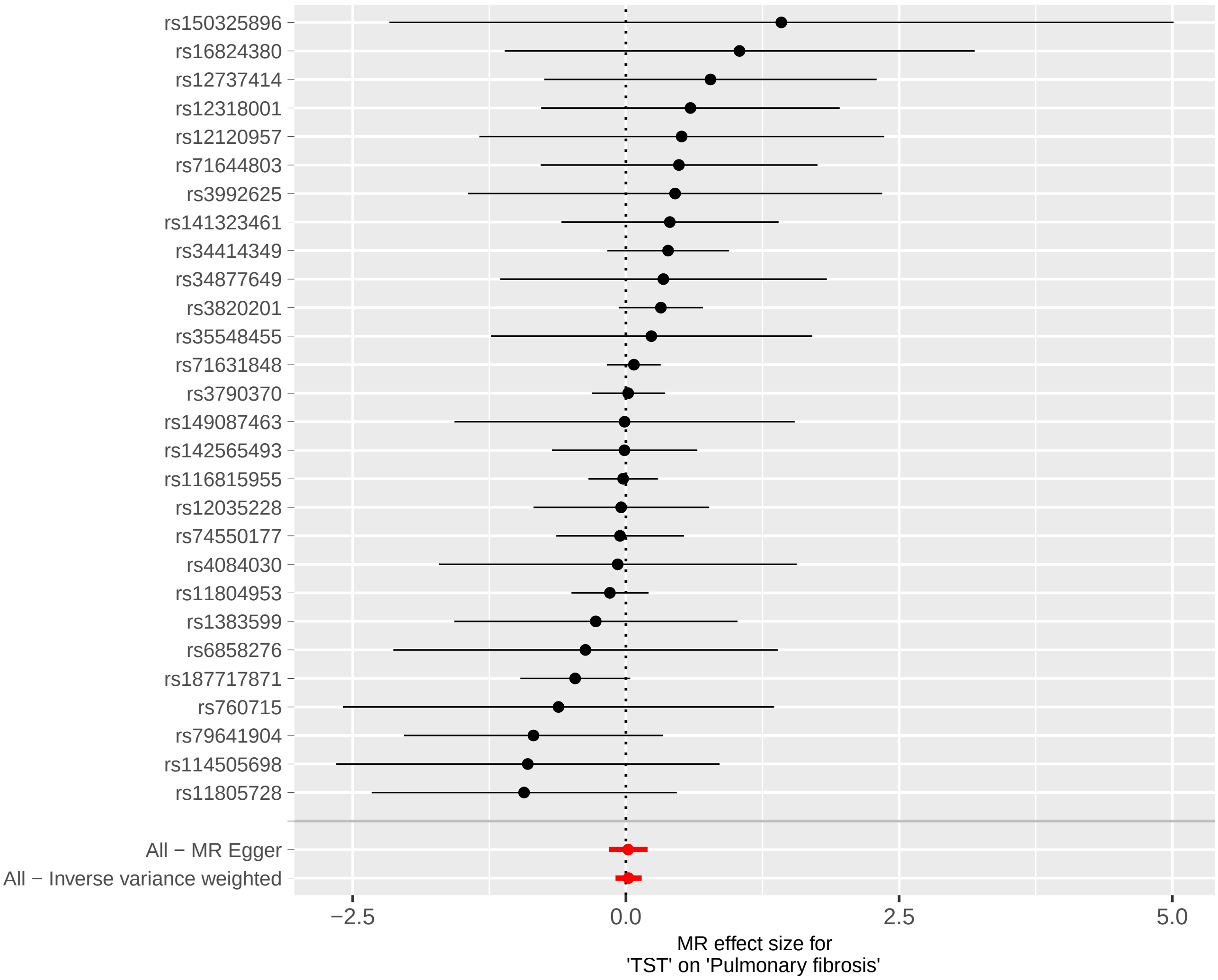
rs150325896
rs16824380
rs12737414
rs12318001
rs12120957
rs71644803
rs3992625
rs141323461
rs34414349
rs34877649
rs3820201
rs35548455
rs71631848
rs3790370
rs149087463
rs142565493
rs116815955
rs12035228
rs74550177
rs4084030
rs11804953
rs1383599
rs6858276
rs187717871
rs760715
rs79641904
rs114505698
rs11805728
All − MR Egger
All − Inverse variance weighted
−2.5
0.0
2.5
5.0
MR effect size for
'TST' on 'Pulmonary fibrosis'

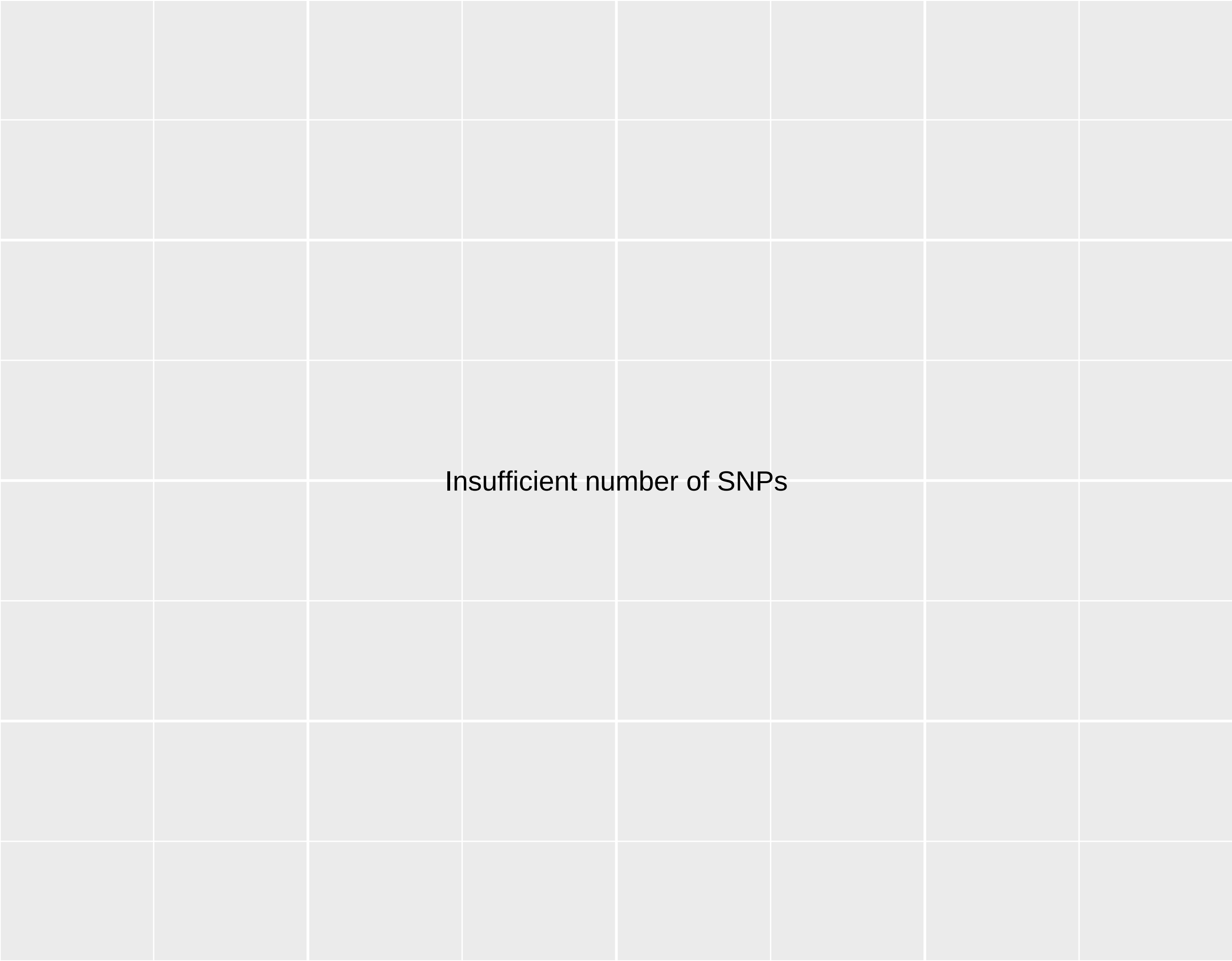
Insufficient number of SNPs

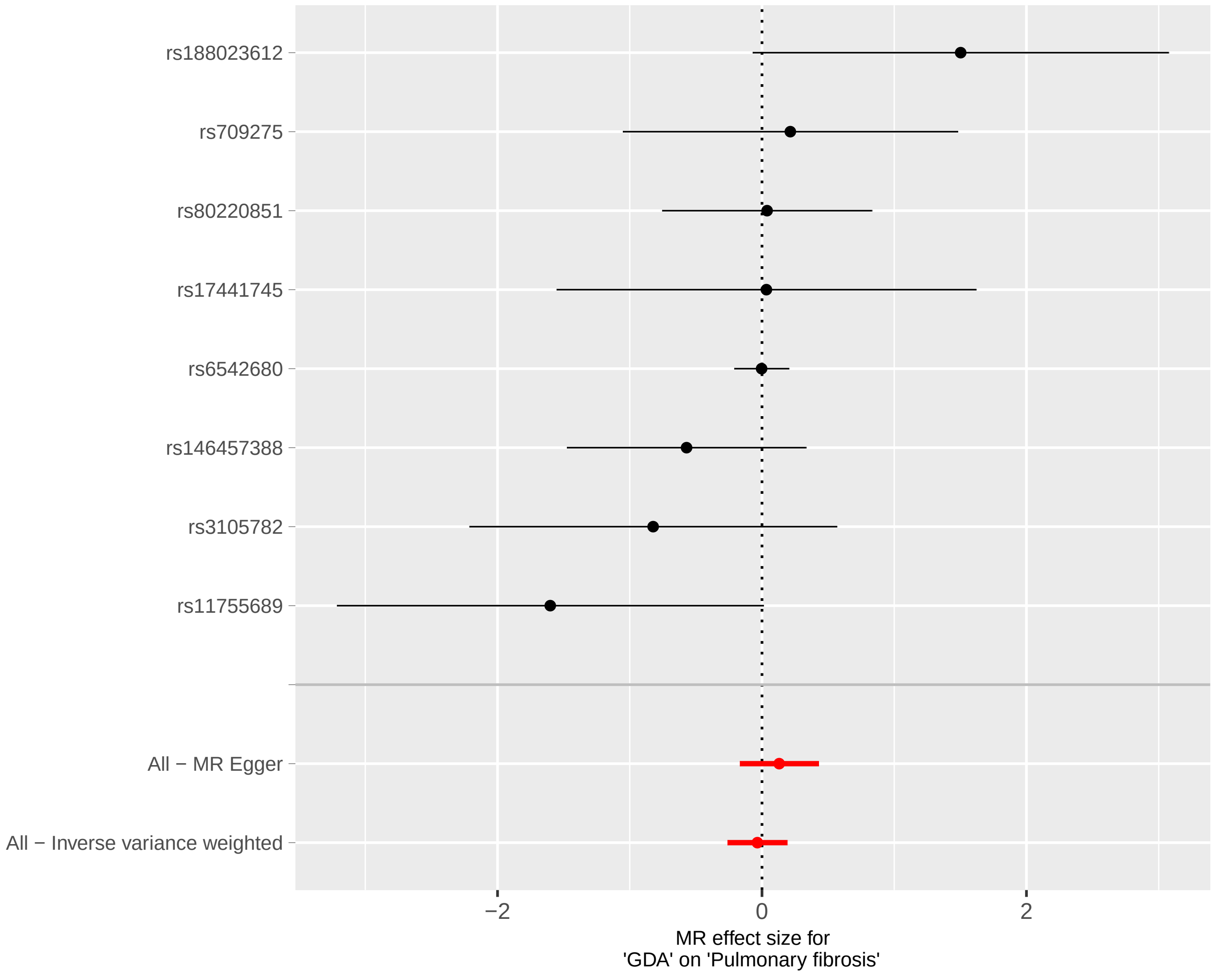
rs188023612
rs709275
rs80220851
rs17441745
rs6542680
rs146457388
rs3105782
rs11755689
All − MR Egger
All − Inverse variance weighted
−2
0
2
MR effect size for
'GDA' on 'Pulmonary fibrosis'

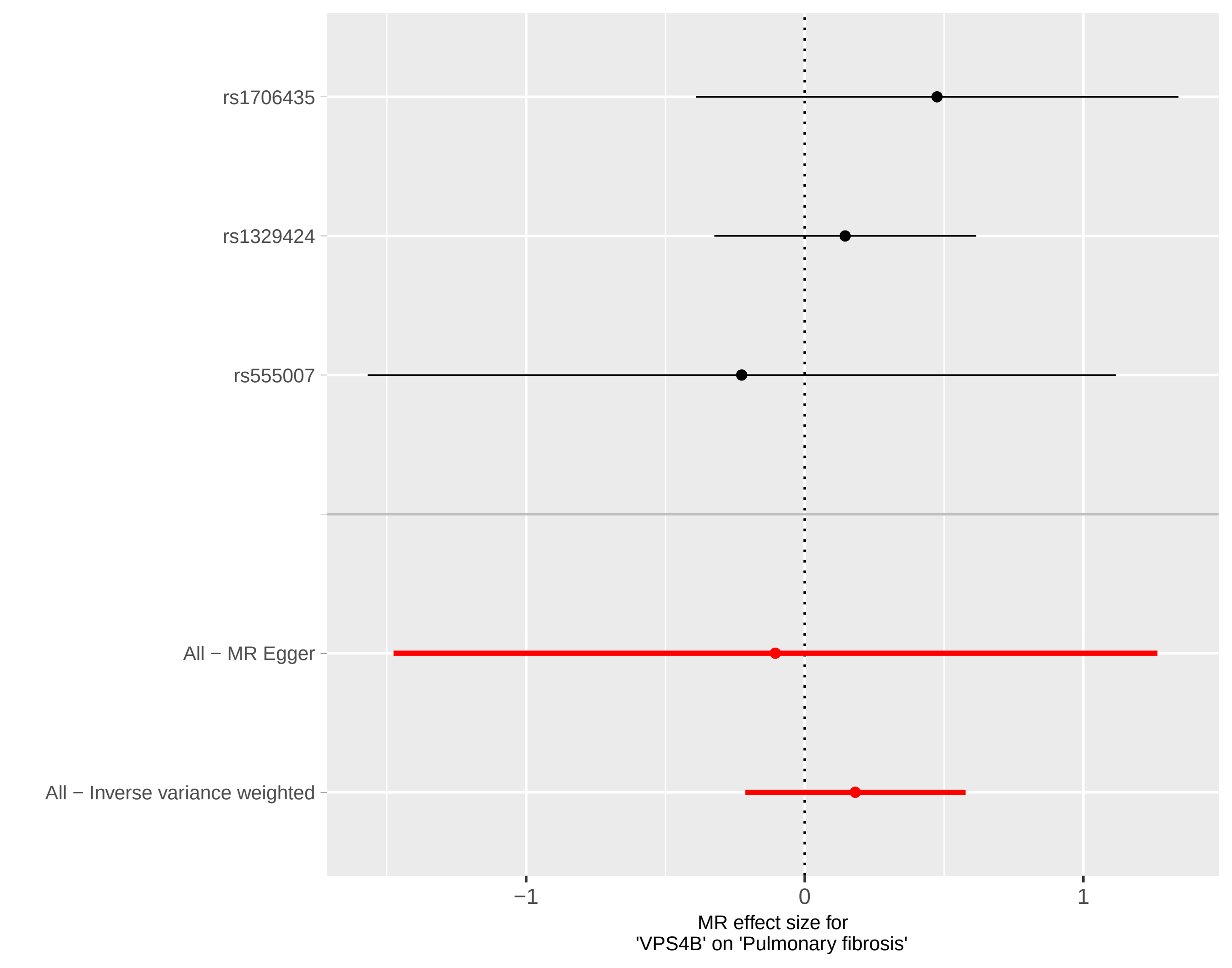

rs1706435
rs1329424
rs555007
All − MR Egger
All − Inverse variance weighted
−1
0
1
MR effect size for
'VPS4B' on 'Pulmonary fibrosis'

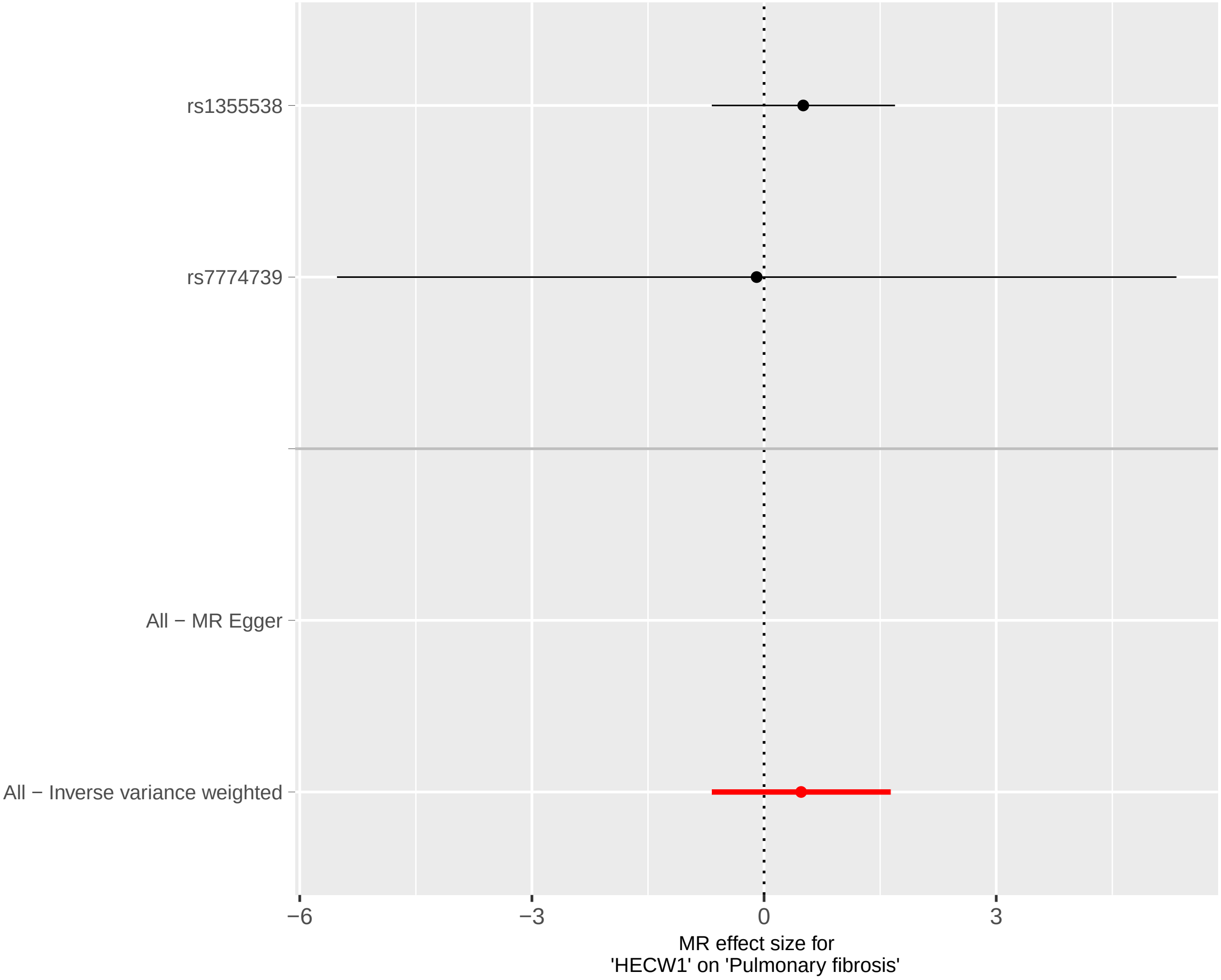

rs1355538
rs7774739
All − MR Egger
All − Inverse variance weighted
−6
−3
0
3
MR effect size for
'HECW1' on 'Pulmonary fibrosis'

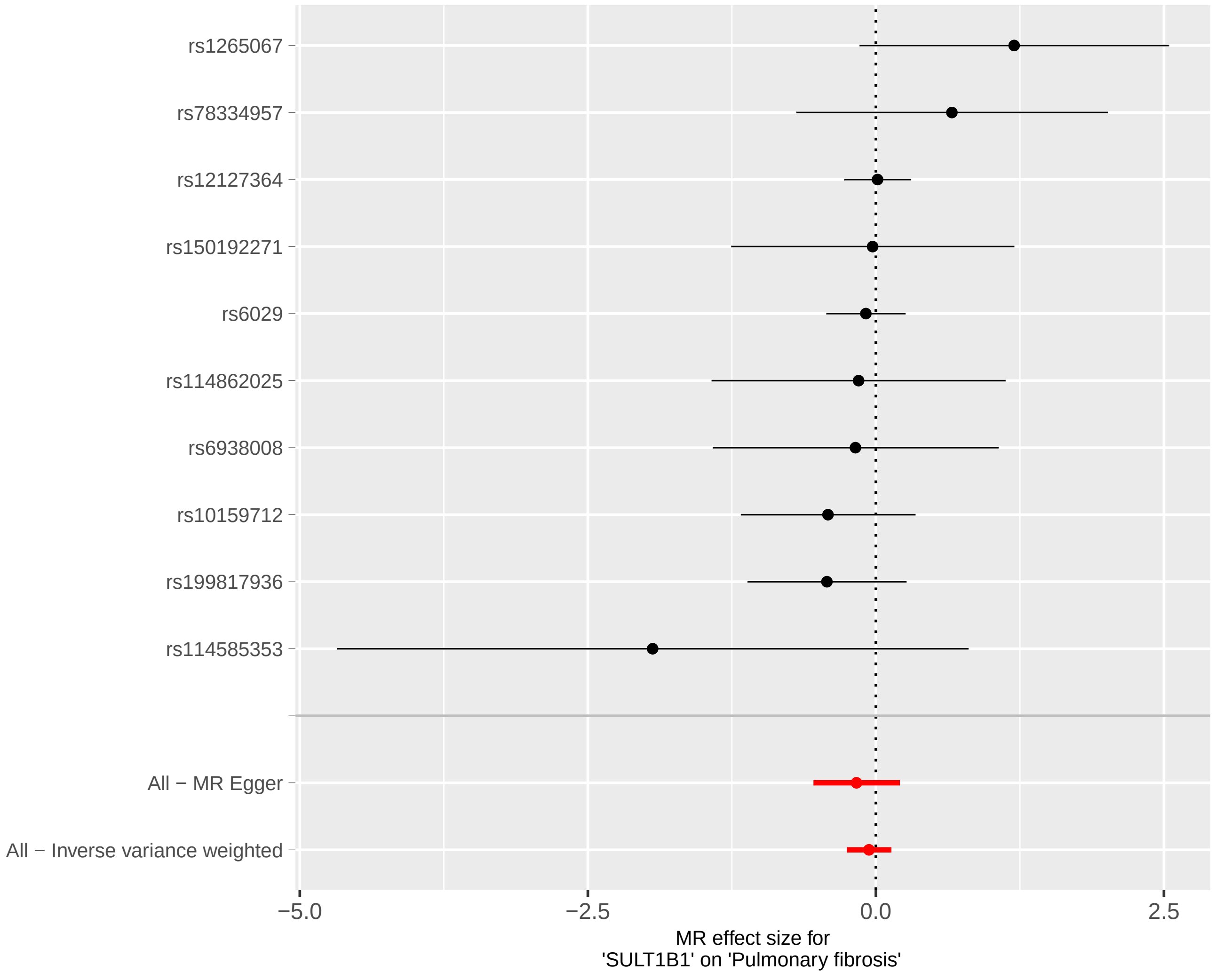
rs1265067
rs78334957
rs12127364
rs150192271
rs6029
rs114862025
rs6938008
rs10159712
rs199817936
rs114585353
All − MR Egger
All − Inverse variance weighted
−5.0
−2.5
0.0
2.5
MR effect size for
'SULT1B1' on 'Pulmonary fibrosis'

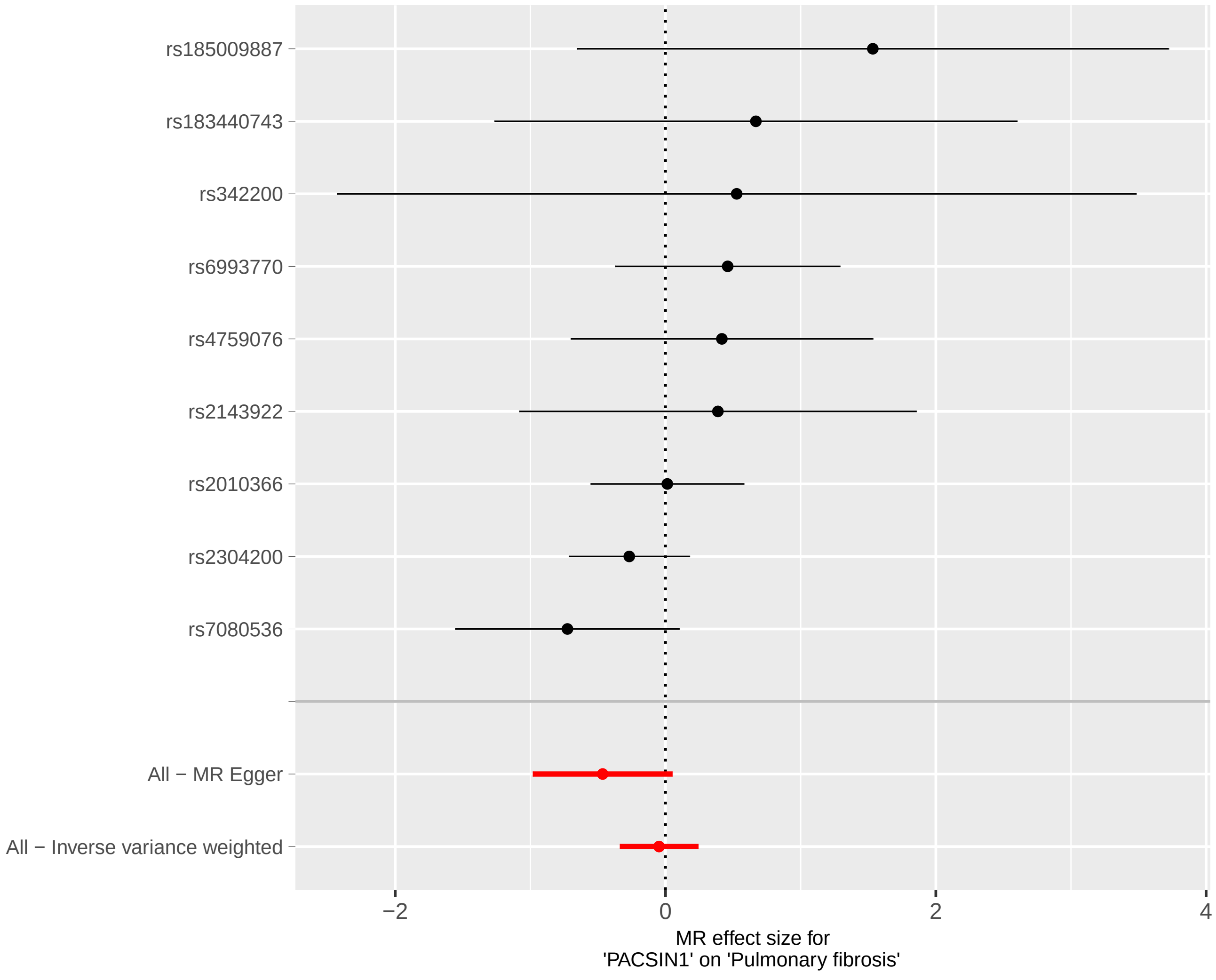
rs185009887
rs183440743
rs342200
rs6993770
rs4759076
rs2143922
rs2010366
rs2304200
rs7080536
All − MR Egger
All − Inverse variance weighted
−2
0
2
4
MR effect size for
'PACSIN1' on 'Pulmonary fibrosis'

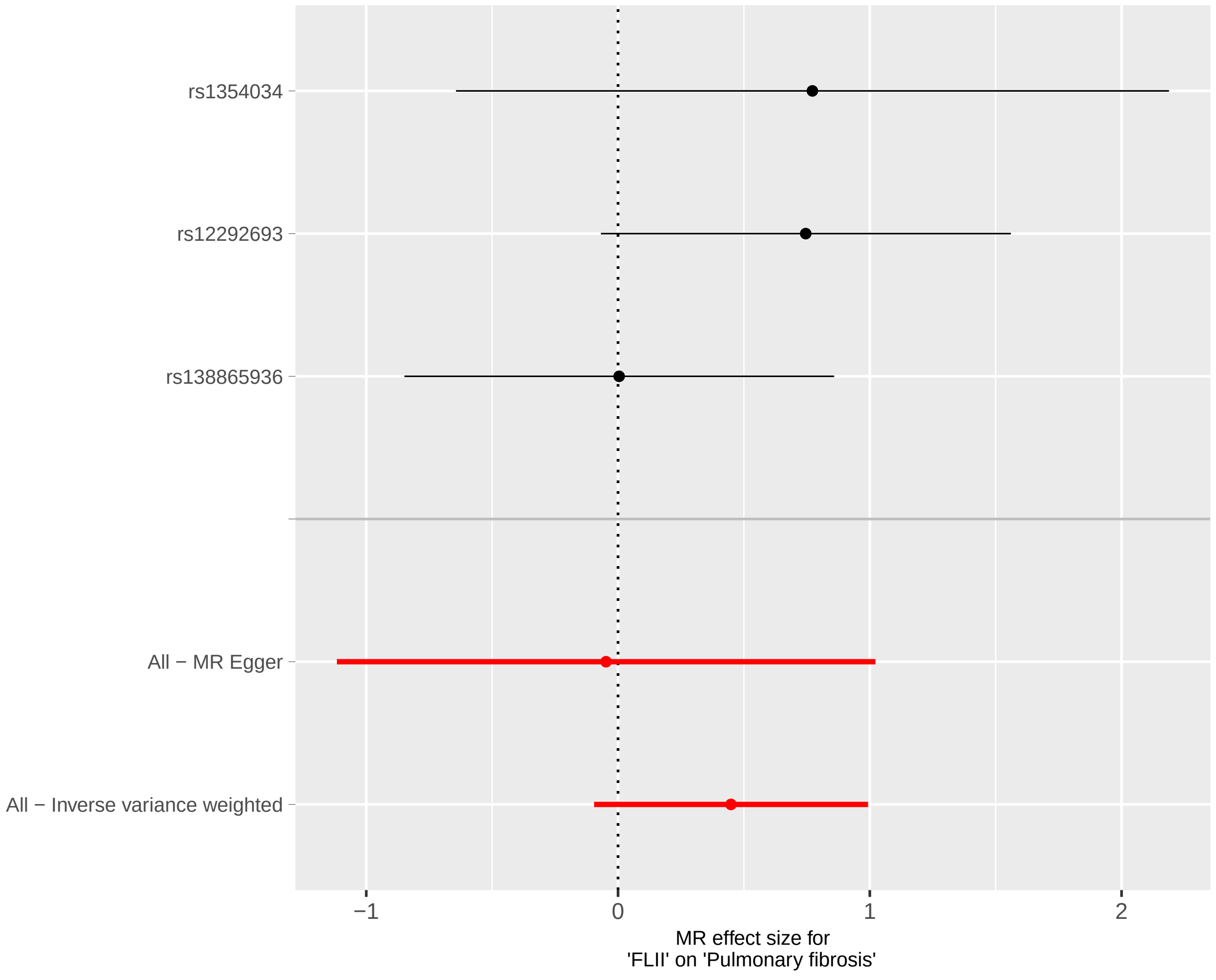
rs1354034
rs12292693
rs138865936
All − MR Egger
All − Inverse variance weighted
−1
0
1
2
MR effect size for
'FLII' on 'Pulmonary fibrosis'

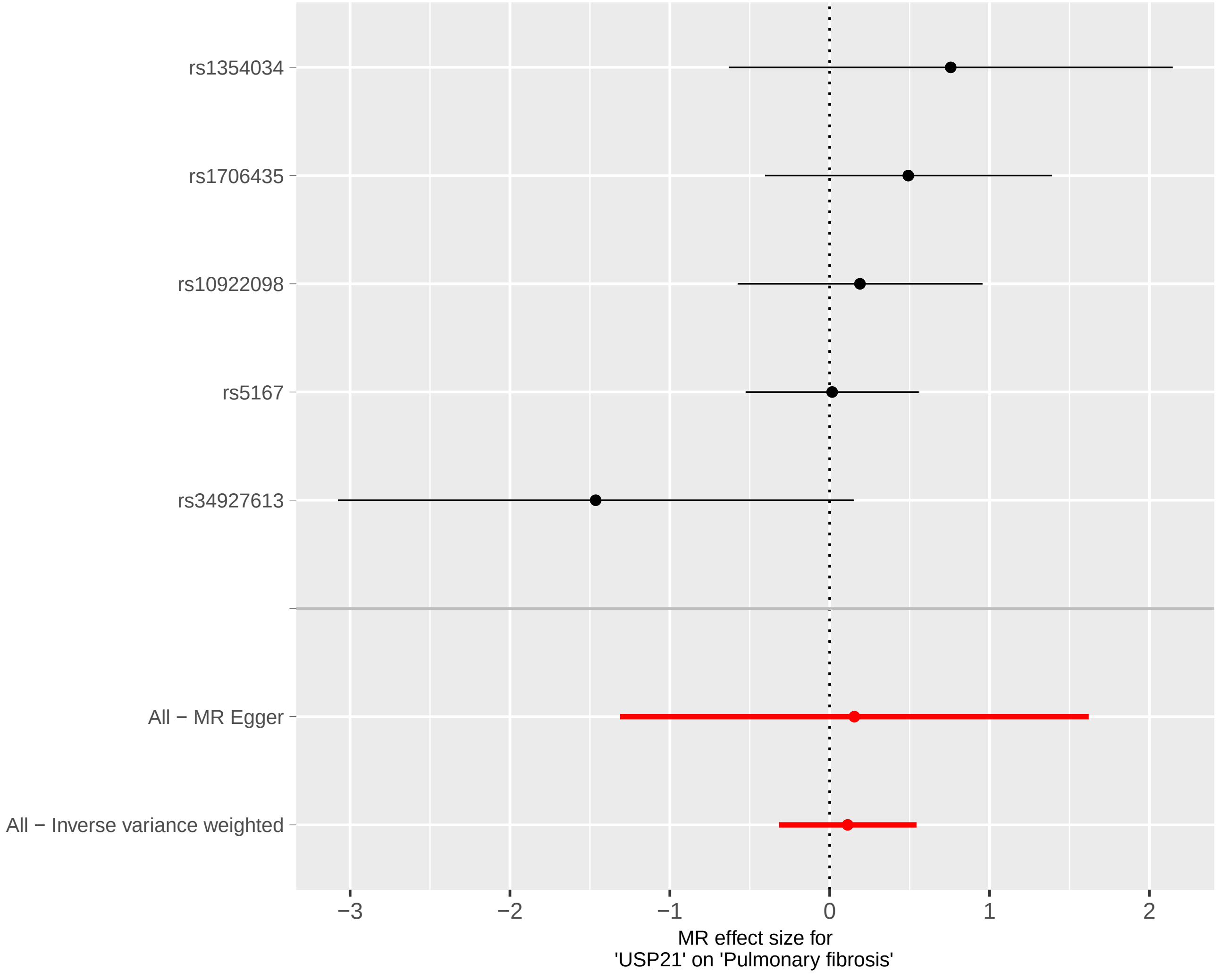
rs1354034
rs1706435
rs10922098
rs5167
rs34927613
All − MR Egger
All − Inverse variance weighted
−3
−2
−1
0
1
2
MR effect size for
'USP21' on 'Pulmonary fibrosis'

Insufficient number of SNPs

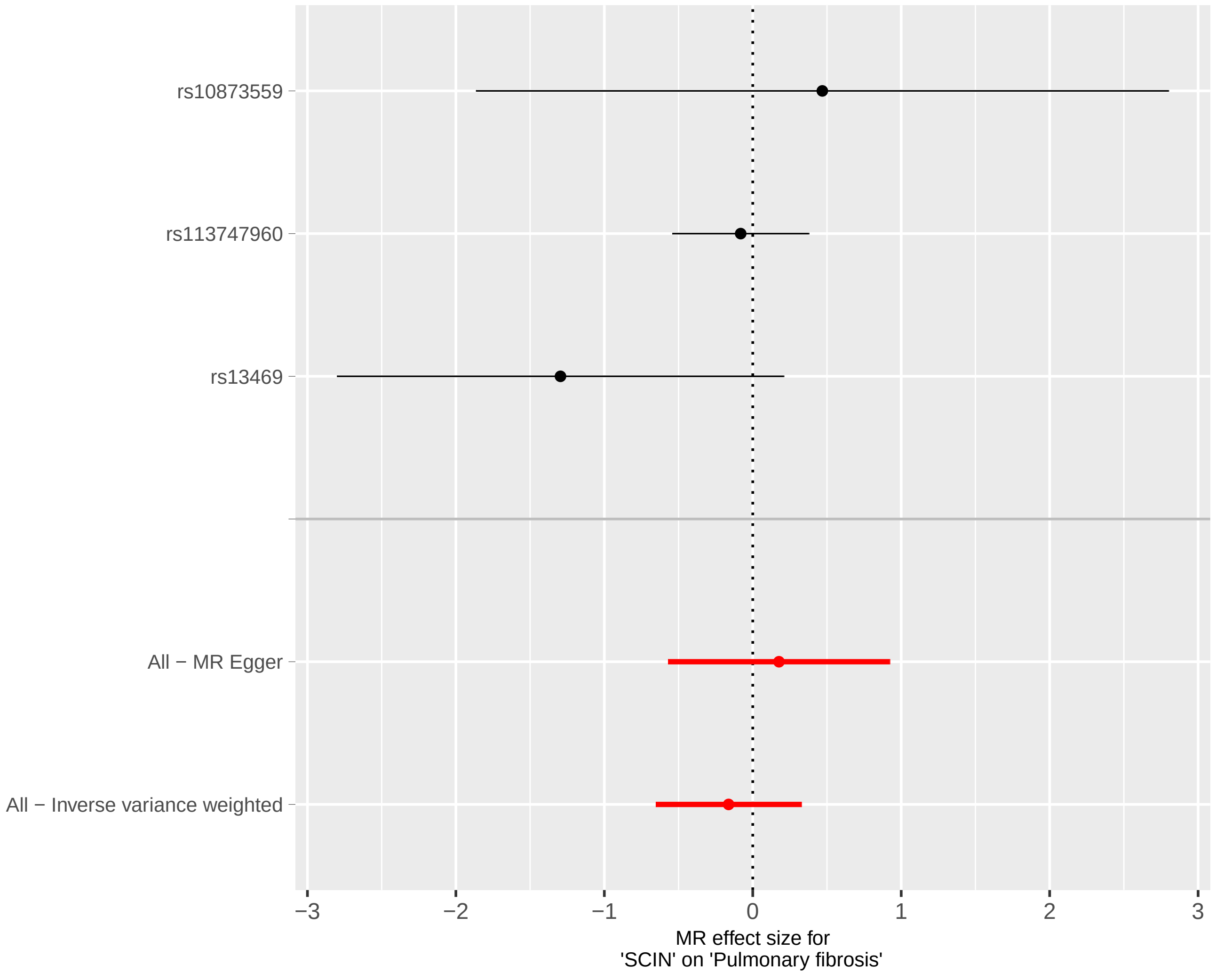
rs10873559
rs113747960
rs13469
All − MR Egger
All − Inverse variance weighted
−3
−2
−1
0
1
2
3
MR effect size for
'SCIN' on 'Pulmonary fibrosis'

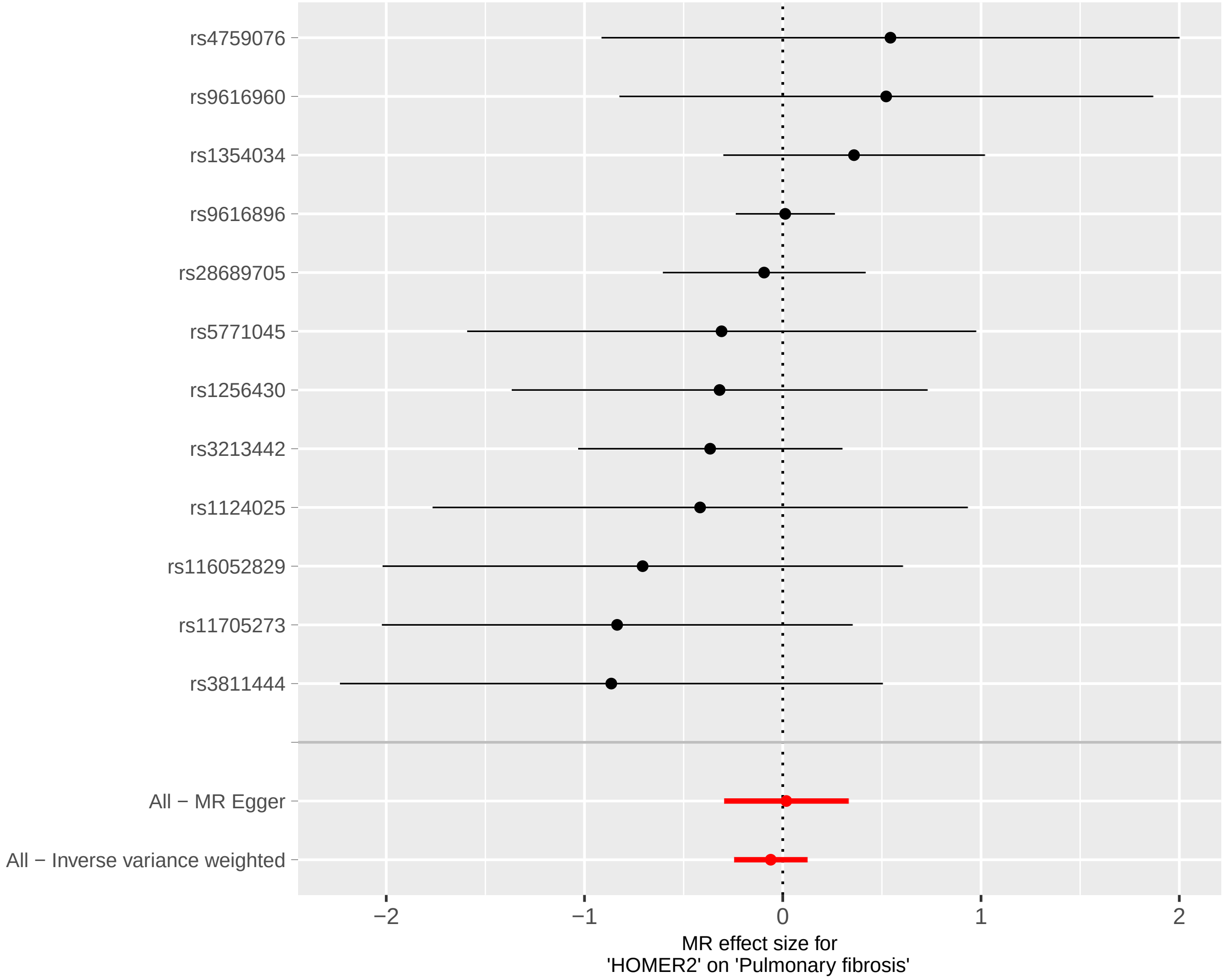

rs4759076
rs9616960
rs1354034
rs9616896
rs28689705
rs5771045
rs1256430
rs3213442
rs1124025
rs116052829
rs11705273
rs3811444
All − MR Egger
All − Inverse variance weighted
−2
−1
0
1
2
MR effect size for
'HOMER2' on 'Pulmonary fibrosis'

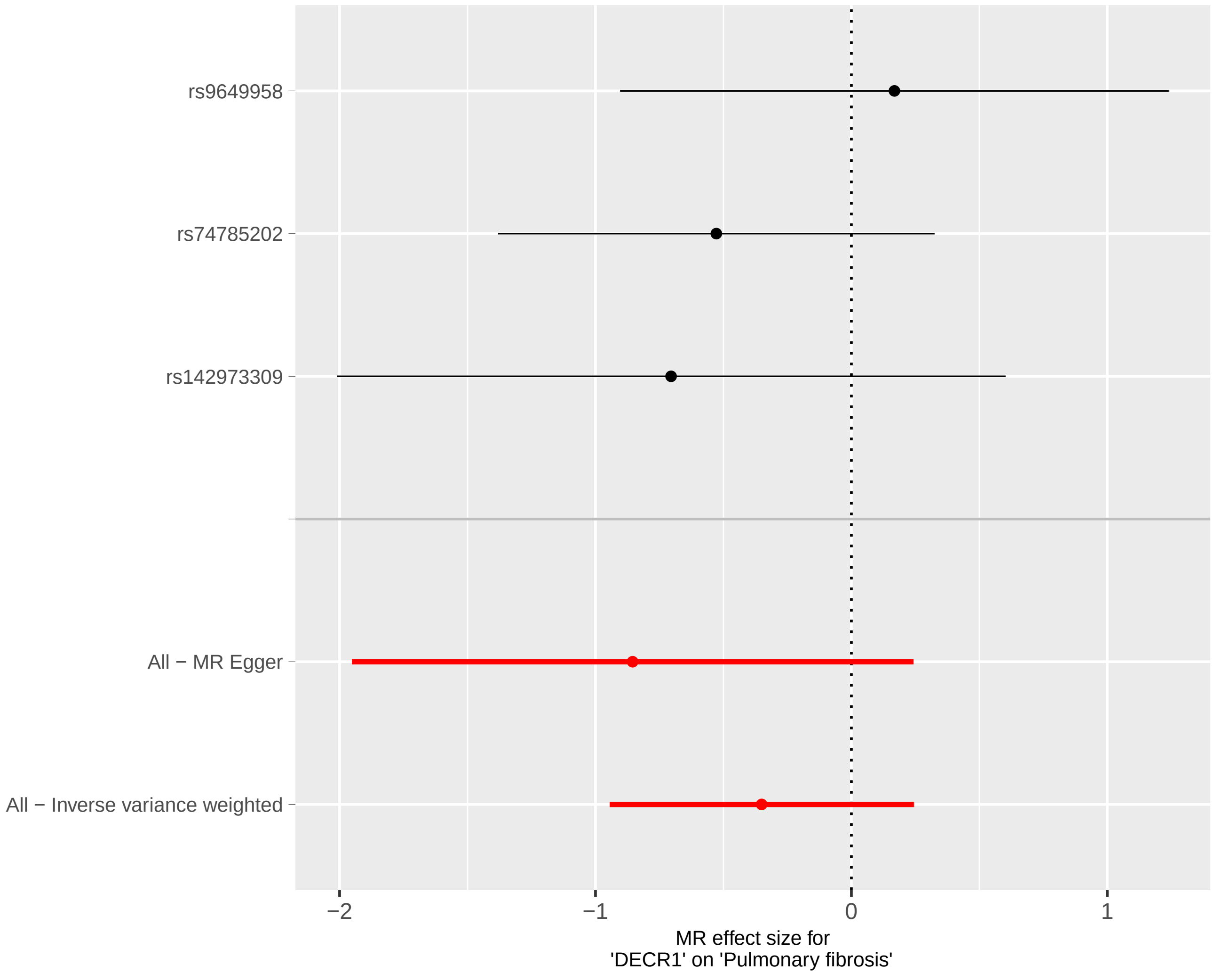
rs9649958
rs74785202
rs142973309
All − MR Egger
All − Inverse variance weighted
−2
−1
0
1
MR effect size for
'DECR1' on 'Pulmonary fibrosis'

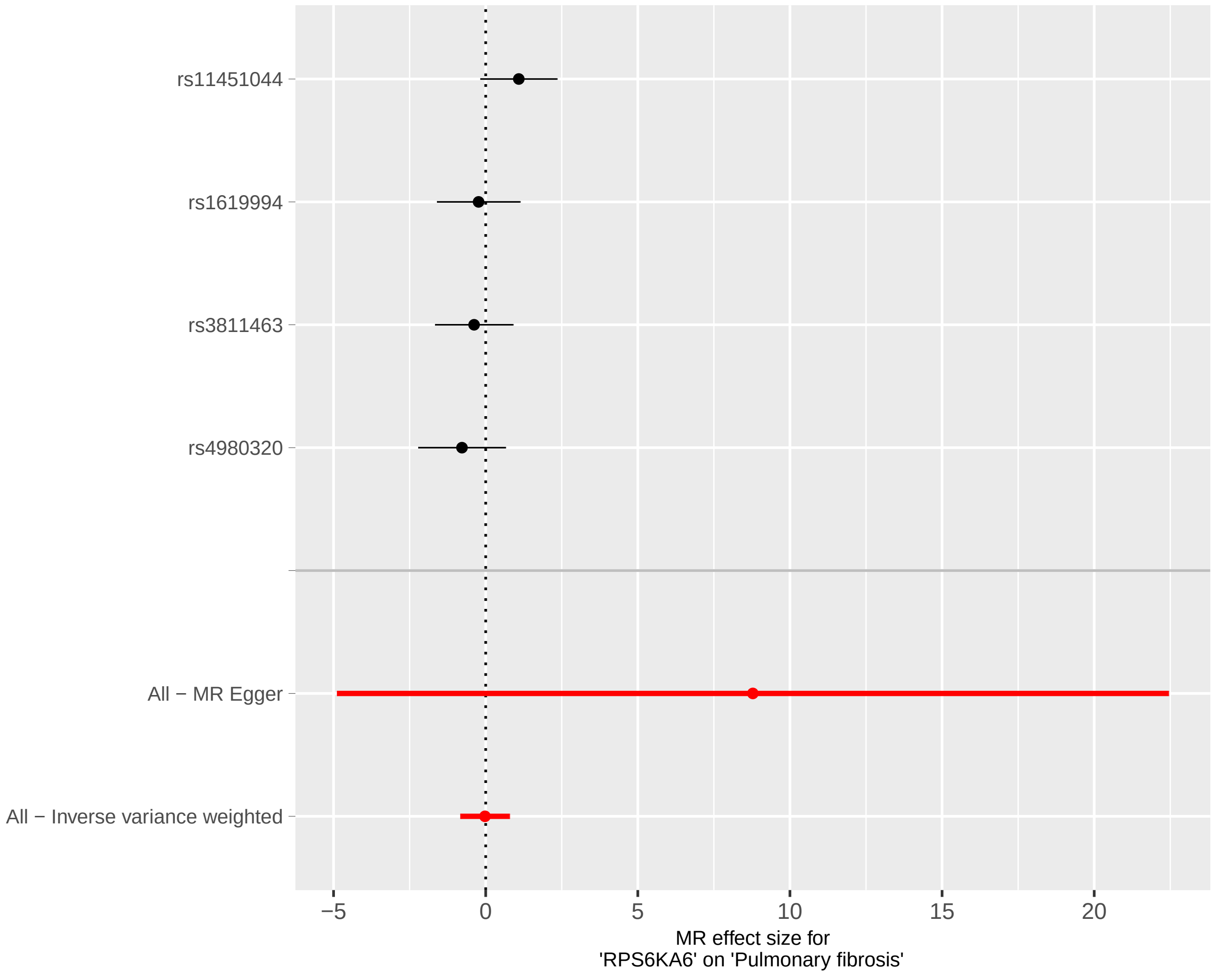
rs11451044
rs1619994
rs3811463
rs4980320
All − MR Egger
All − Inverse variance weighted
−5
0
5
10
15
20
MR effect size for
'RPS6KA6' on 'Pulmonary fibrosis'

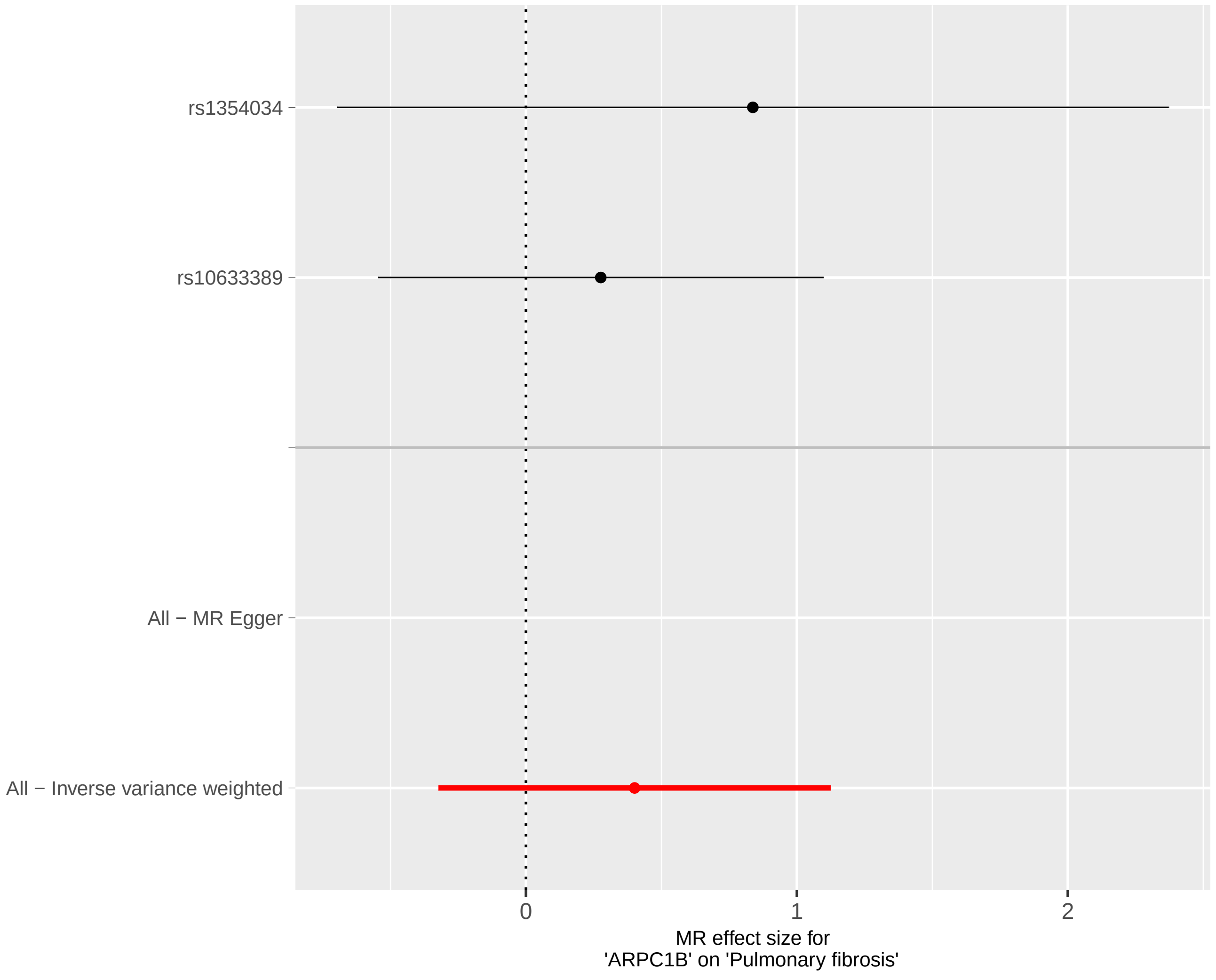

rs1354034
rs10633389
All − MR Egger
All − Inverse variance weighted
0
1
2
MR effect size for
'ARPC1B' on 'Pulmonary fibrosis'

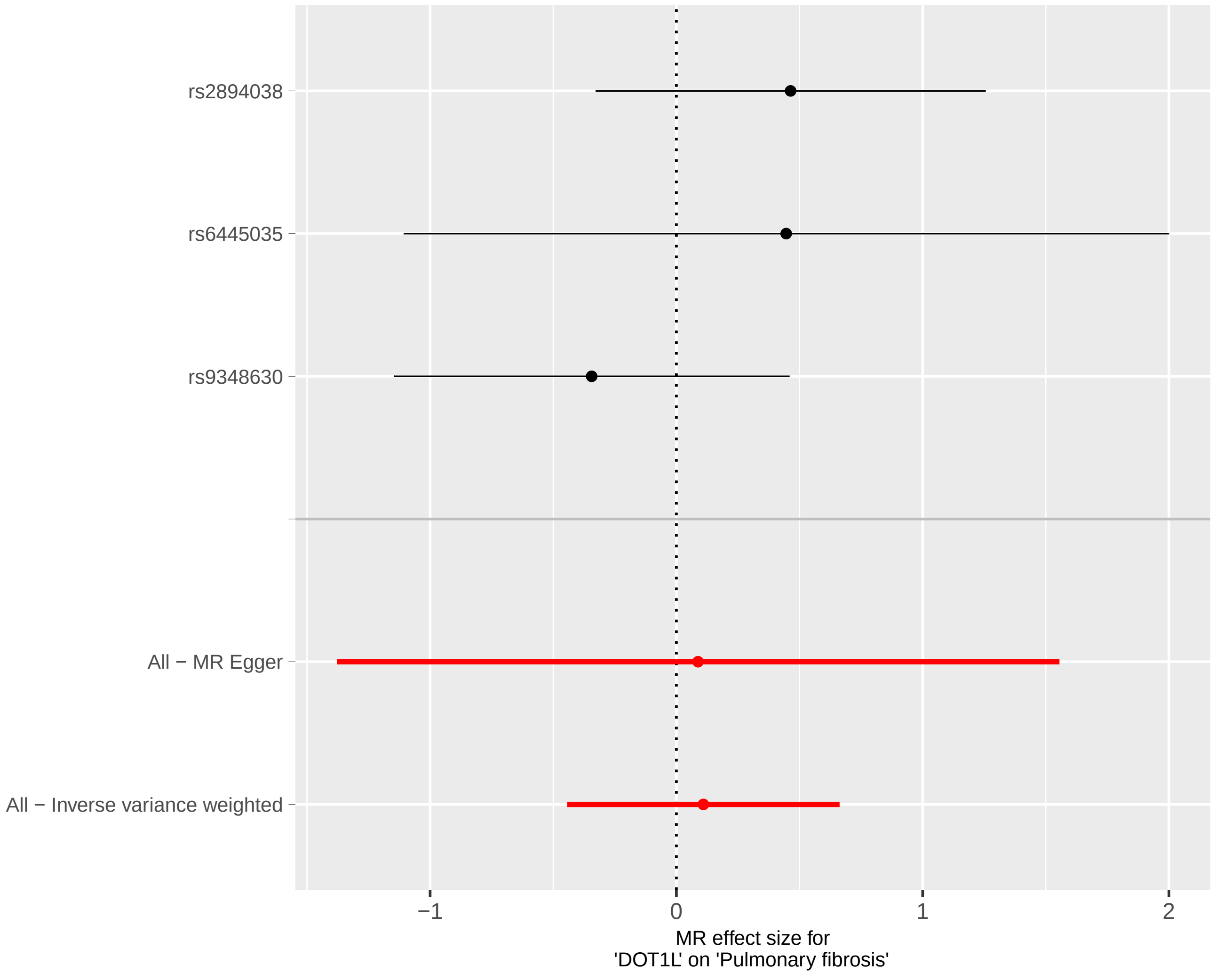
rs2894038
rs6445035
rs9348630
All − MR Egger
All − Inverse variance weighted
−1
0
1
2
MR effect size for
'DOT1L' on 'Pulmonary fibrosis'

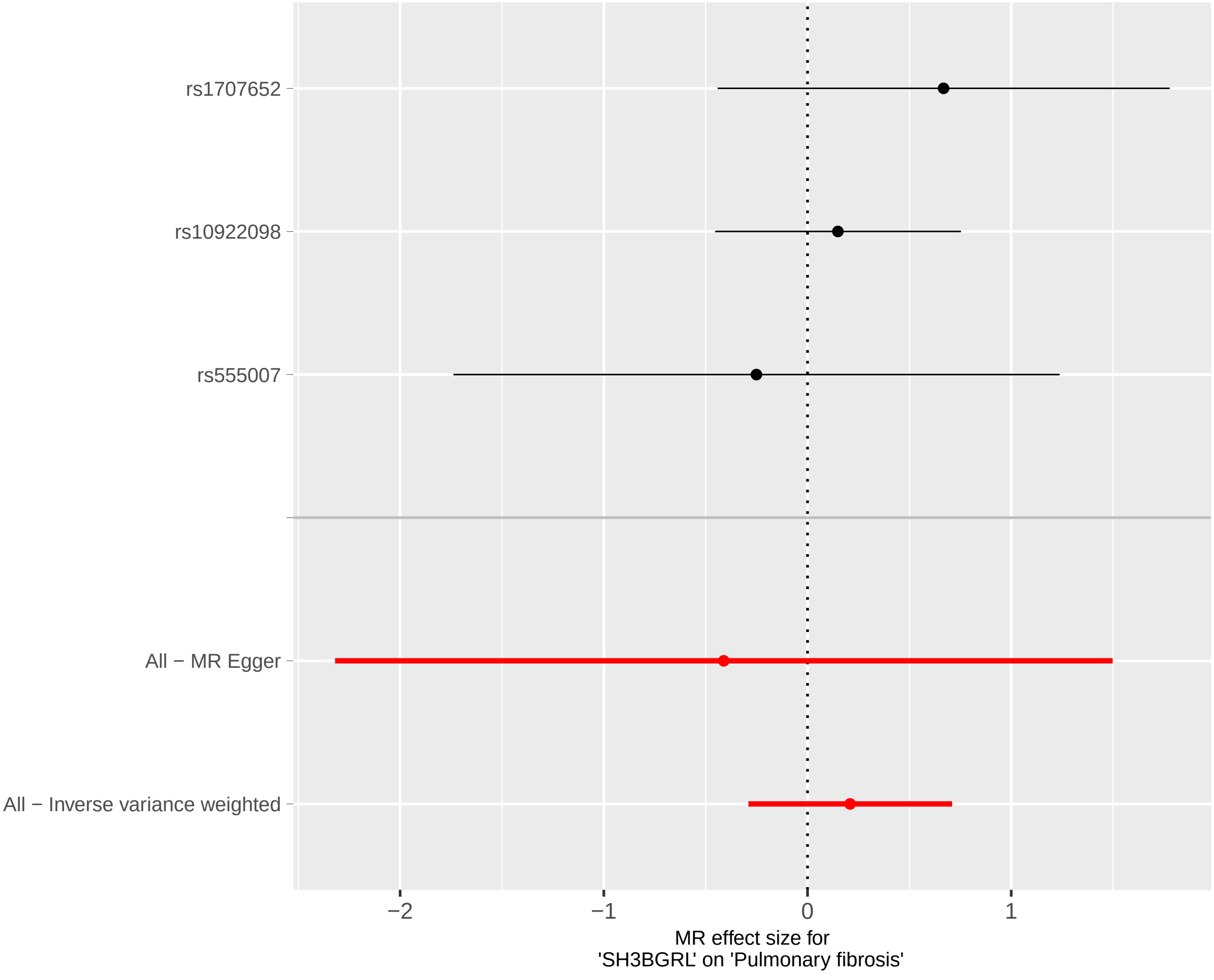

rs1707652
rs10922098
rs555007
All − MR Egger
All − Inverse variance weighted
−2
−1
0
1
MR effect size for
'SH3BGRL' on 'Pulmonary fibrosis'

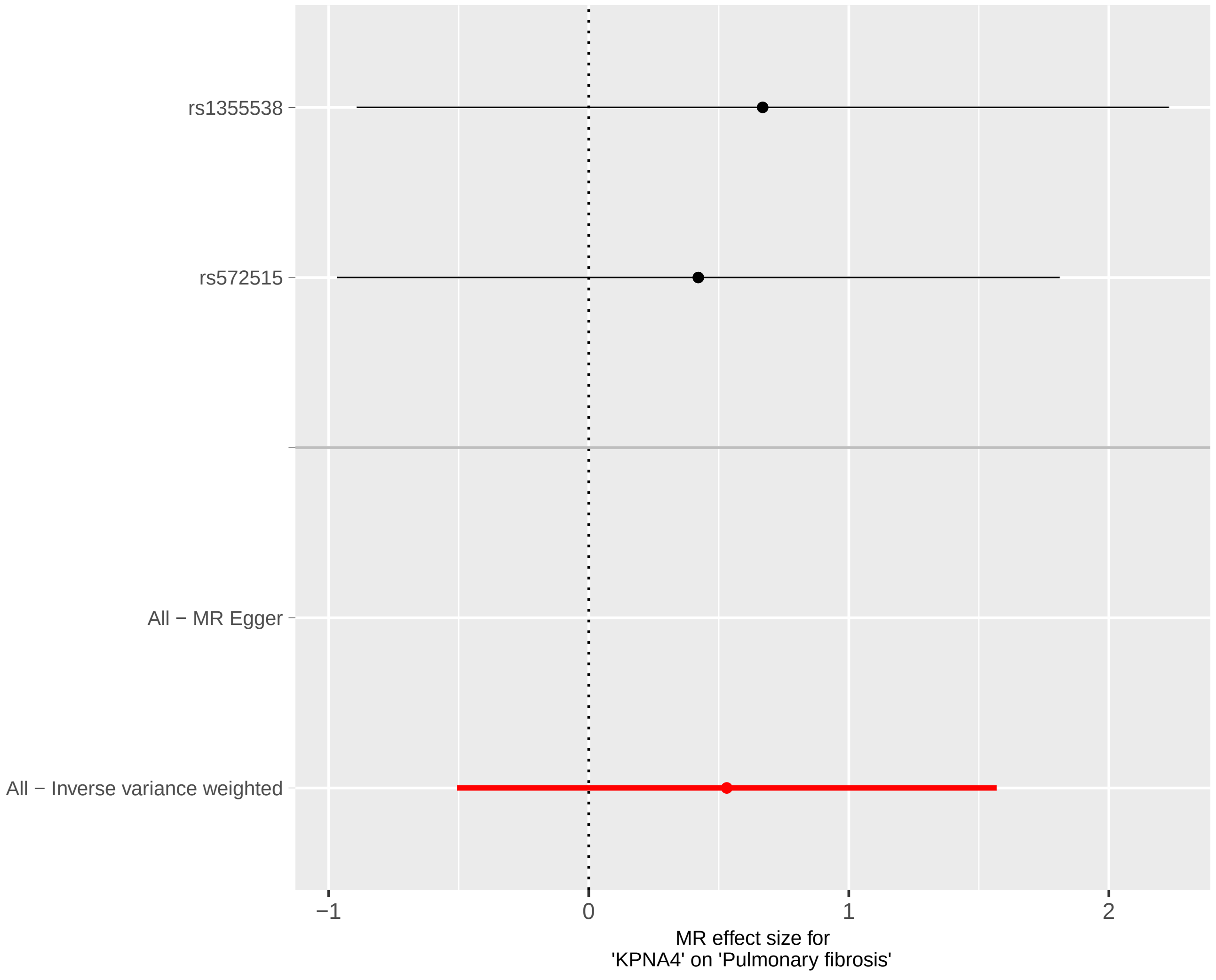
rs1355538
rs572515
All − MR Egger
All − Inverse variance weighted
−1
0
1
2
MR effect size for
'KPNA4' on 'Pulmonary fibrosis'

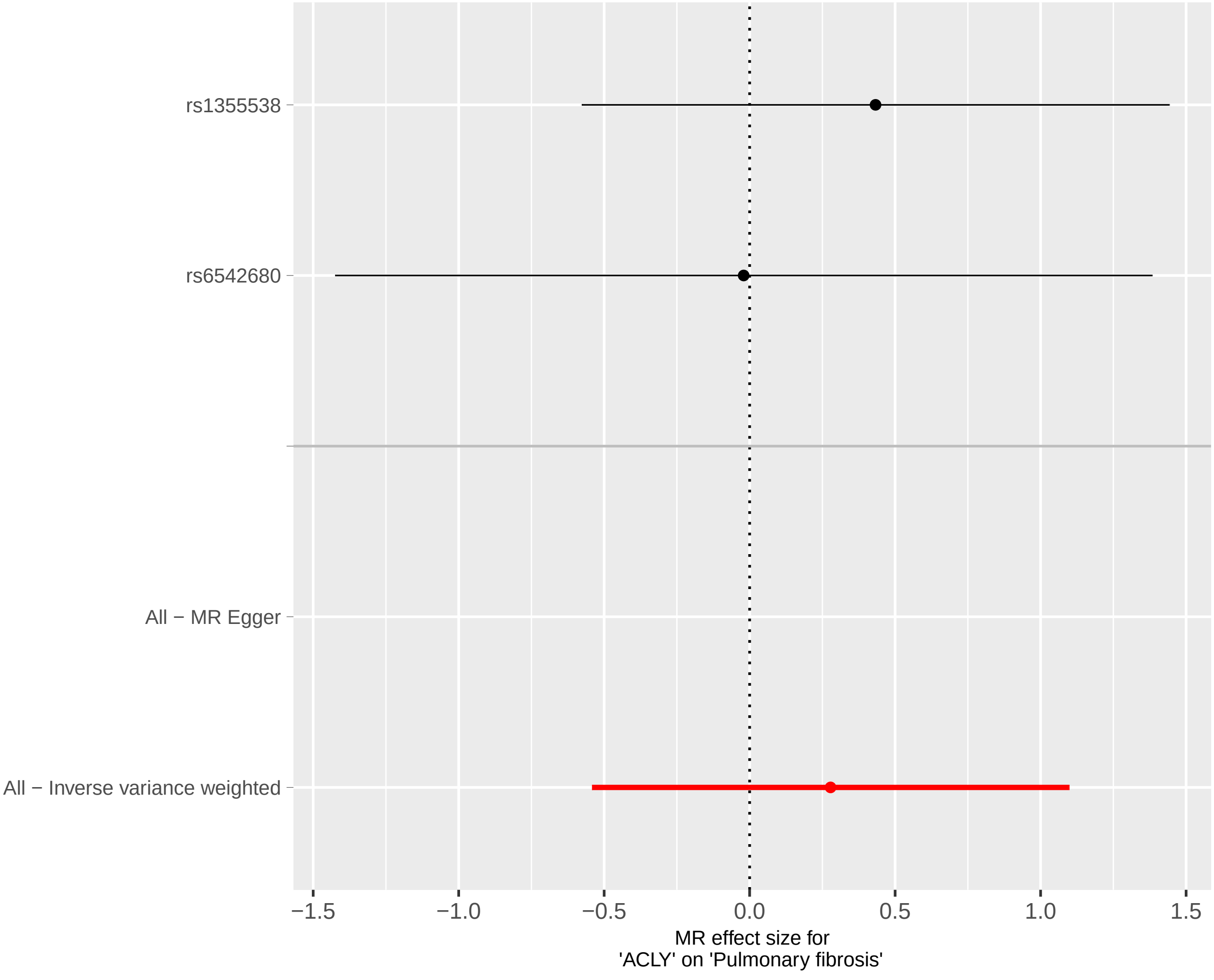
rs1355538
rs6542680
All − MR Egger
All − Inverse variance weighted
−1.5
−1.0
−0.5
0.0
0.5
1.0
1.5
MR effect size for
'ACLY' on 'Pulmonary fibrosis'

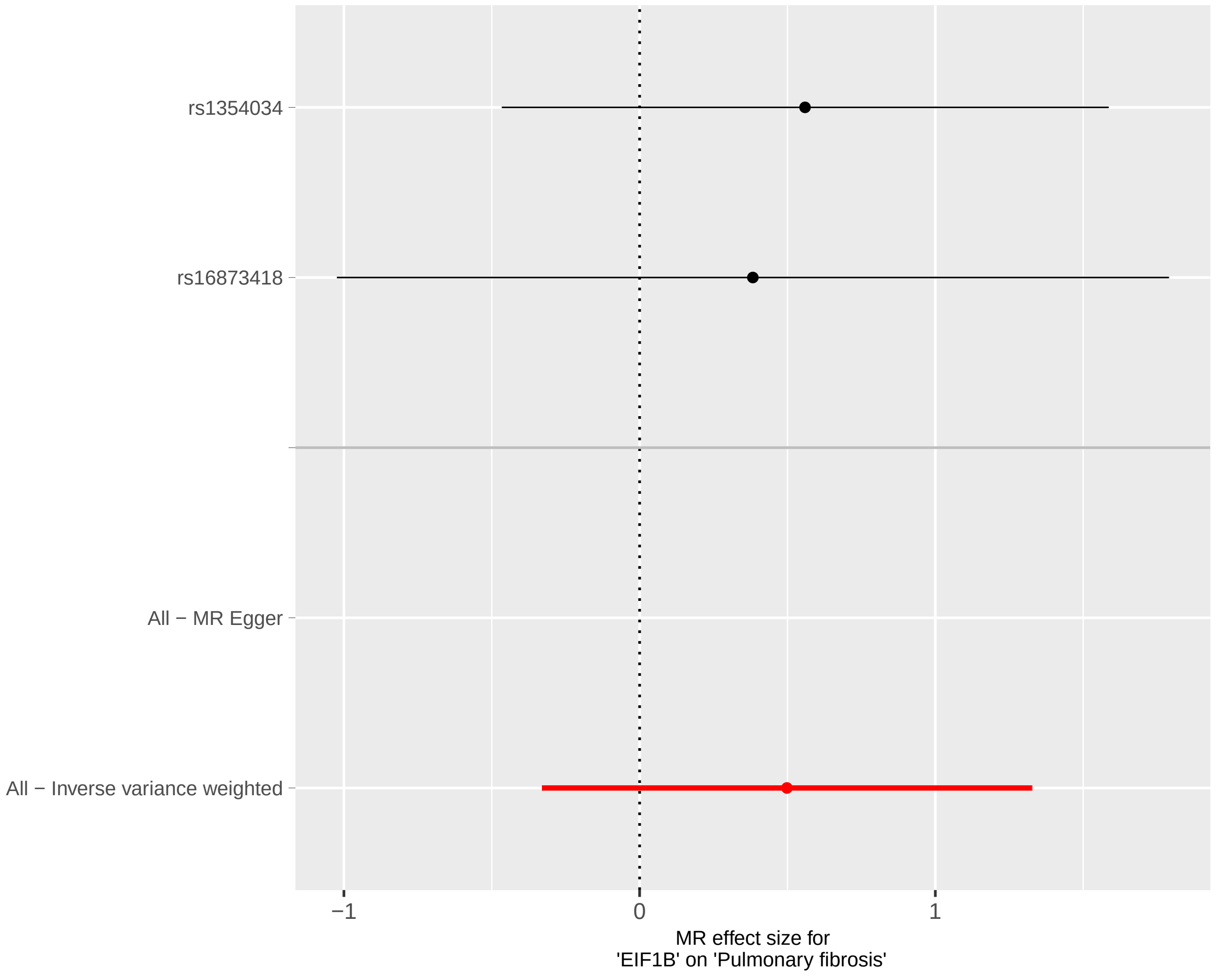

rs1354034
rs16873418
All − MR Egger
All − Inverse variance weighted
−1
0
1
MR effect size for
'EIF1B' on 'Pulmonary fibrosis'

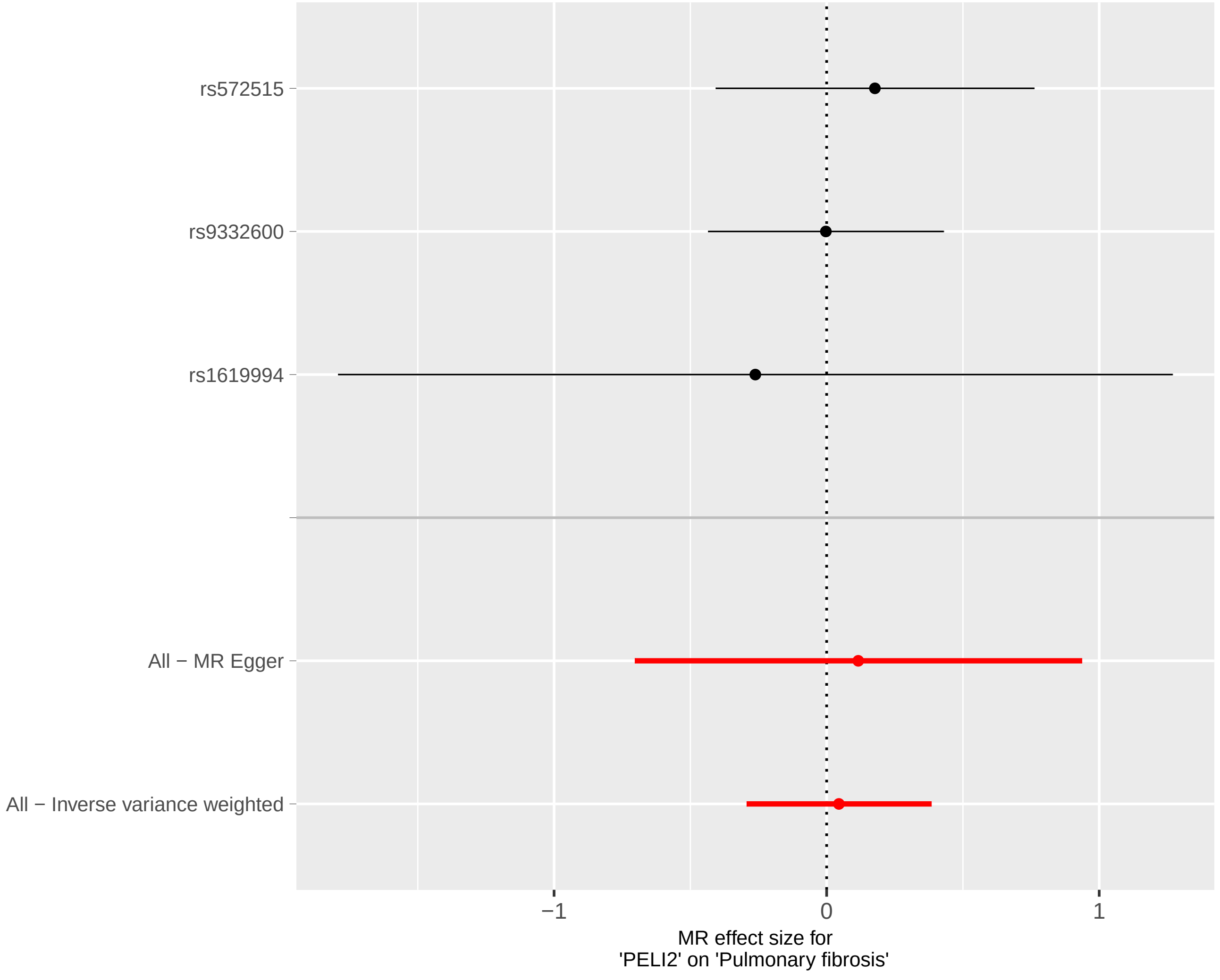
rs572515
rs9332600
rs1619994
All − MR Egger
All − Inverse variance weighted
−1
0
1
MR effect size for
'PELI2' on 'Pulmonary fibrosis'

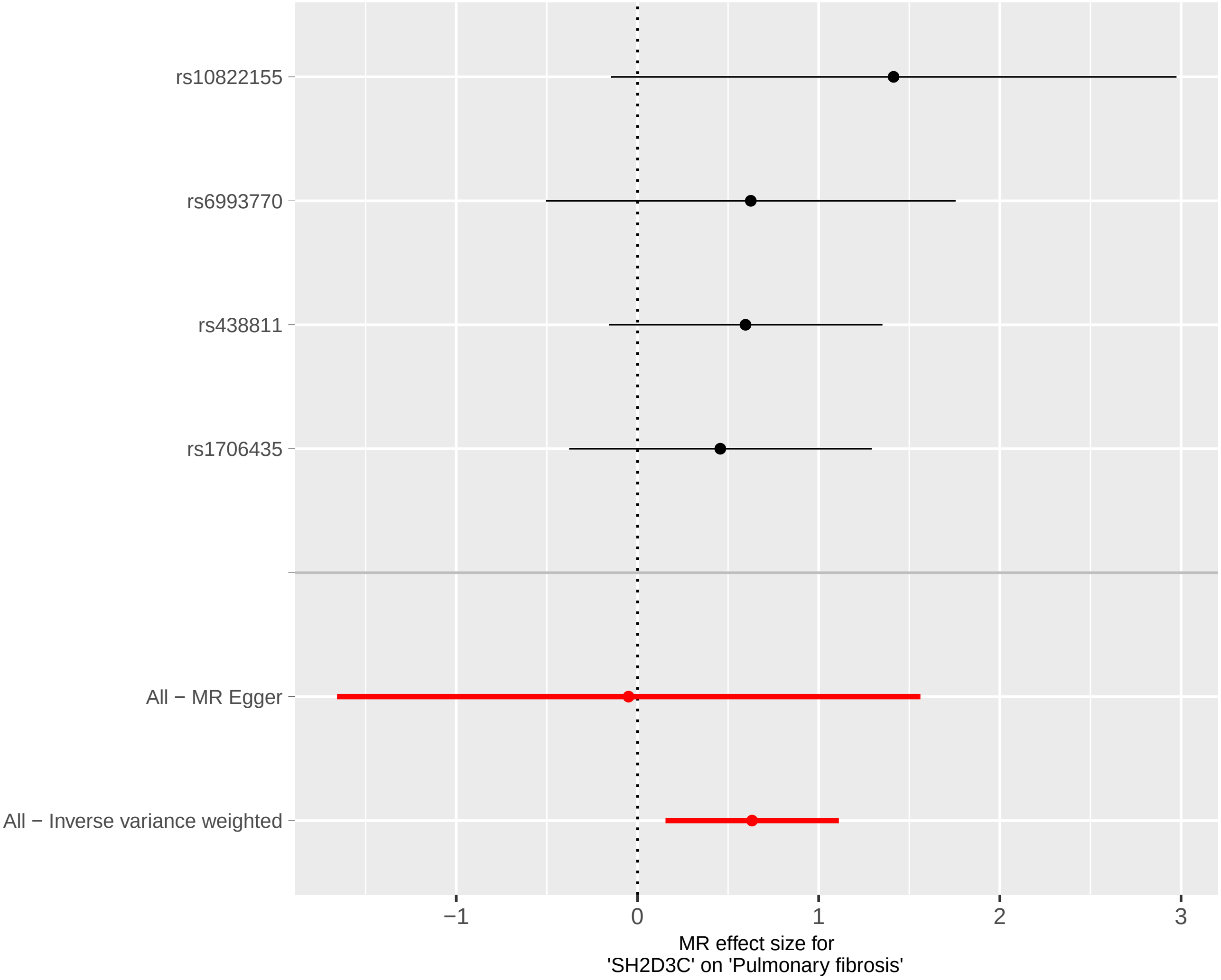
rs10822155
rs6993770
rs438811
rs1706435
All − MR Egger
All − Inverse variance weighted
−1
0
1
2
3
MR effect size for
'SH2D3C' on 'Pulmonary fibrosis'

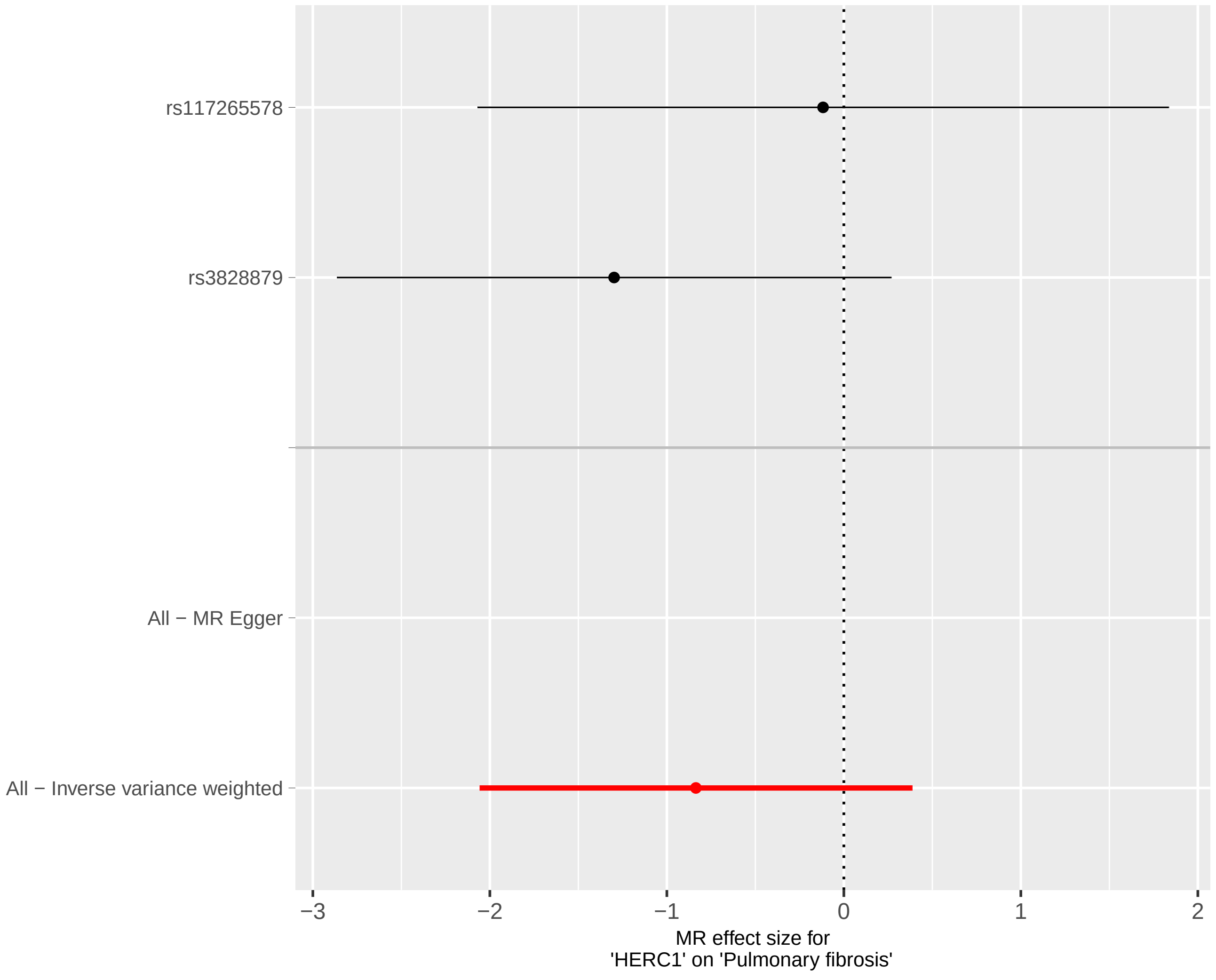
rs117265578
rs3828879
All − MR Egger
All − Inverse variance weighted
−3
−2
−1
0
1
2
MR effect size for
'HERC1' on 'Pulmonary fibrosis'

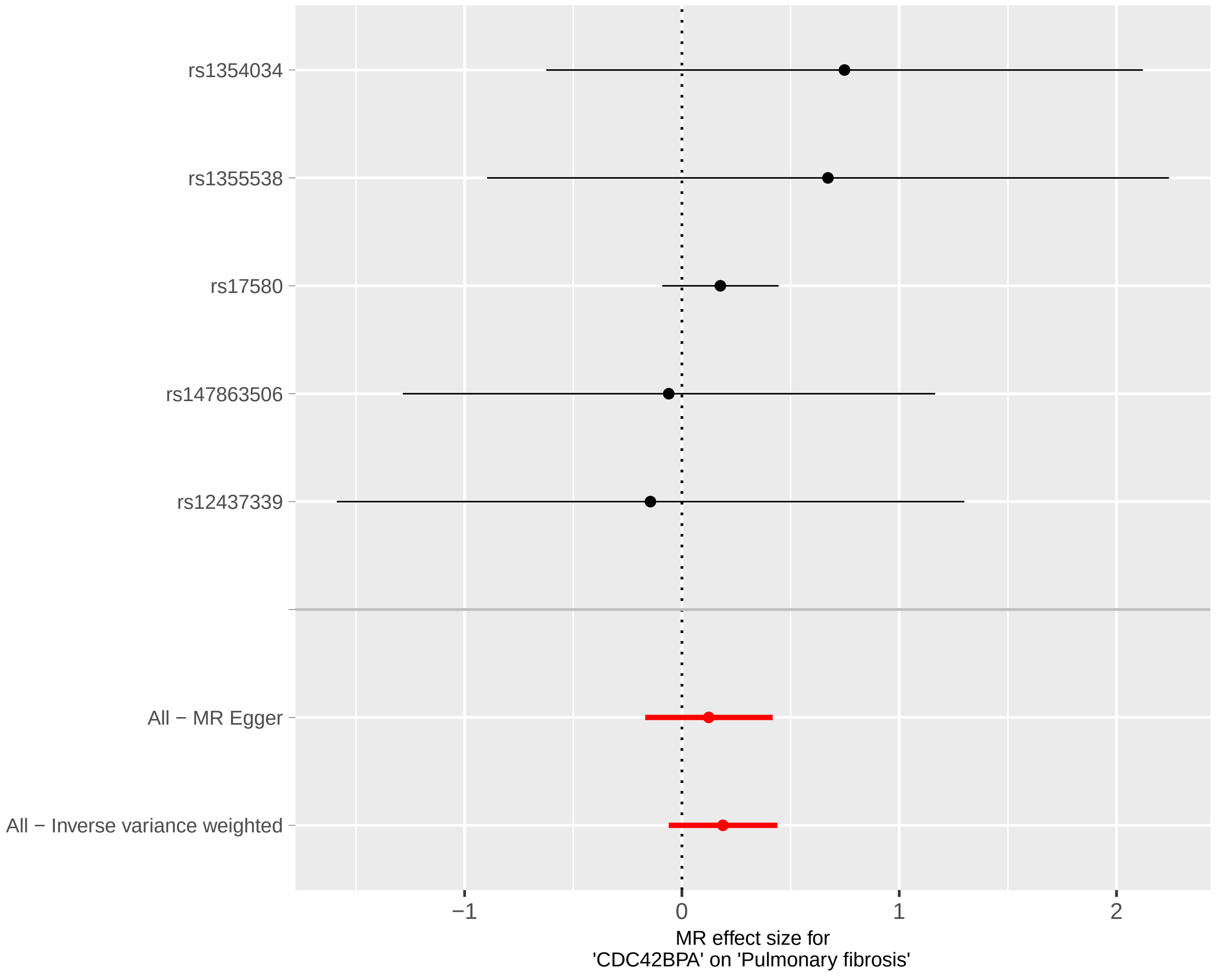
rs1354034
rs1355538
rs17580
rs147863506
rs12437339
All − MR Egger
All − Inverse variance weighted
−1
0
1
2
MR effect size for
'CDC42BPA' on 'Pulmonary fibrosis'

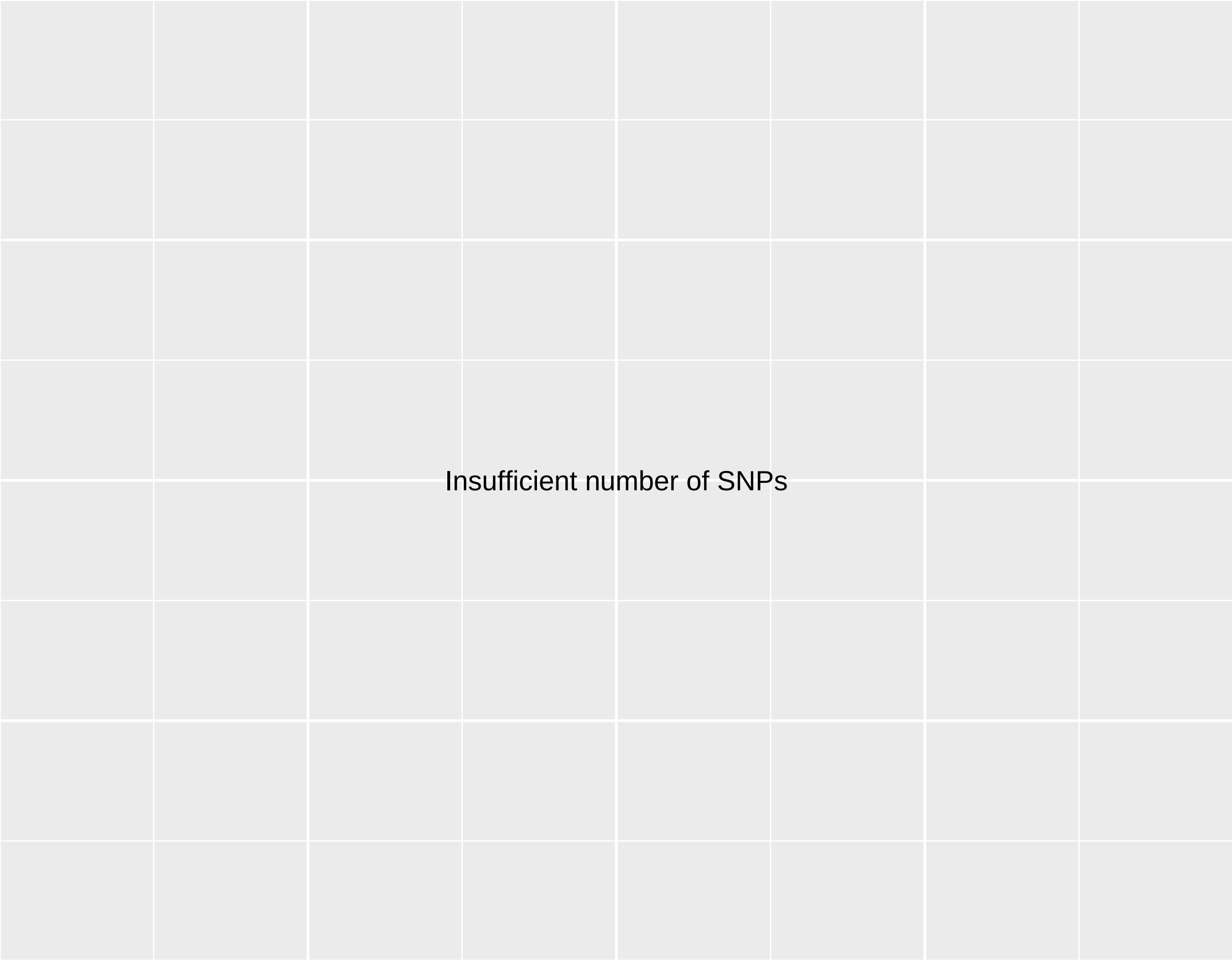
Insufficient number of SNPs

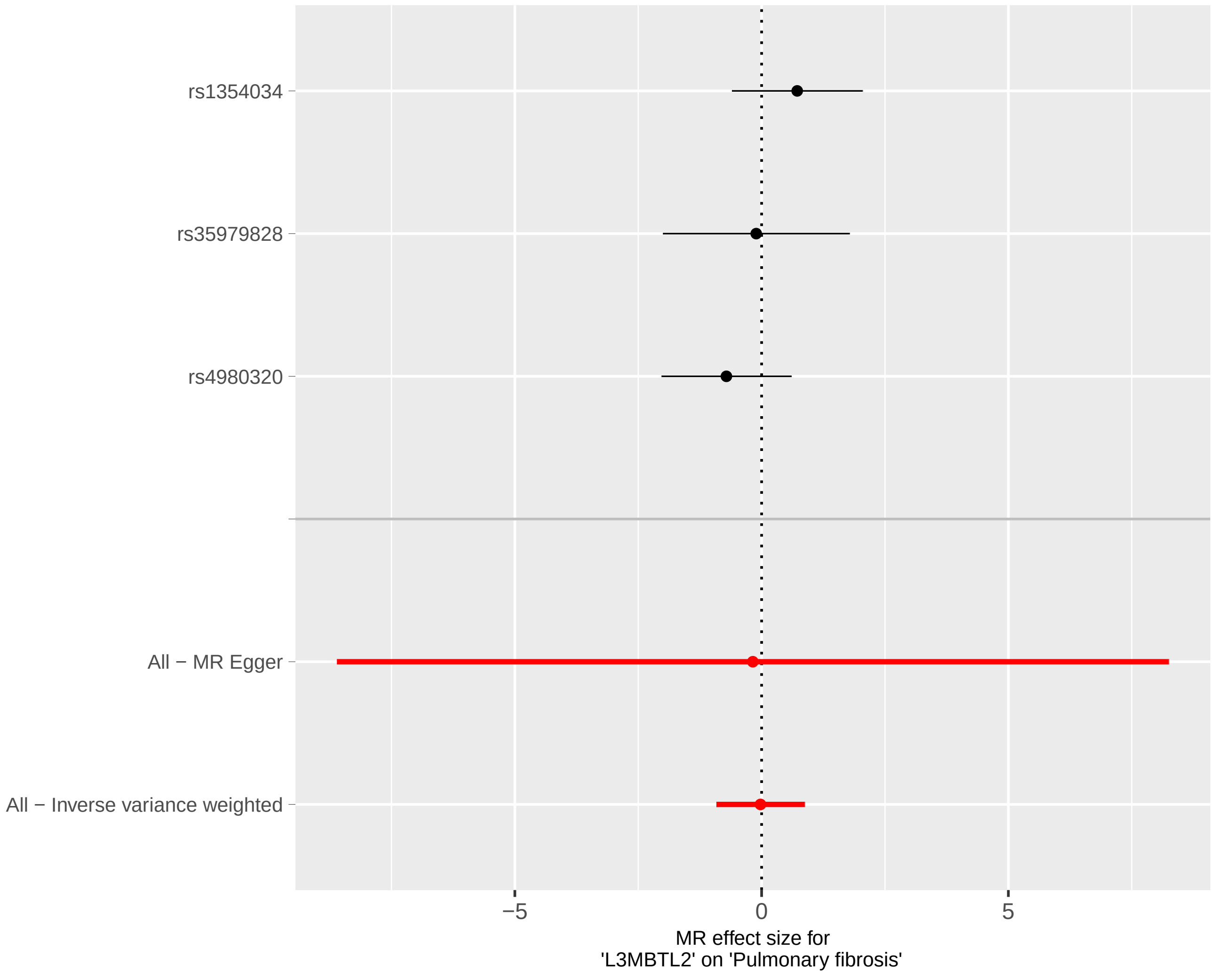
rs1354034
rs35979828
rs4980320
All − MR Egger
All − Inverse variance weighted
−5
0
5
MR effect size for
'L3MBTL2' on 'Pulmonary fibrosis'

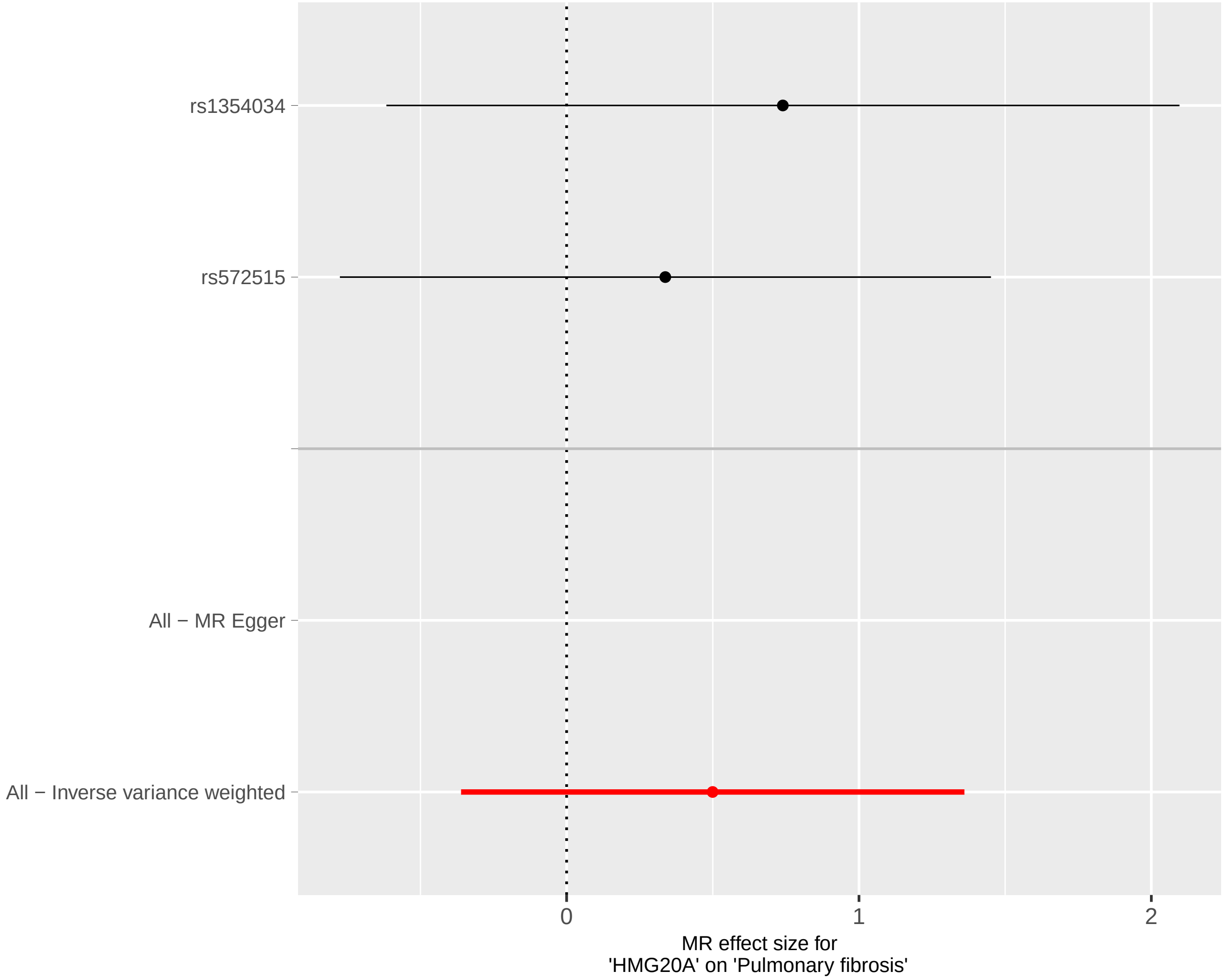
rs1354034
rs572515
All − MR Egger
All − Inverse variance weighted
0
1
2
MR effect size for
'HMG20A' on 'Pulmonary fibrosis'

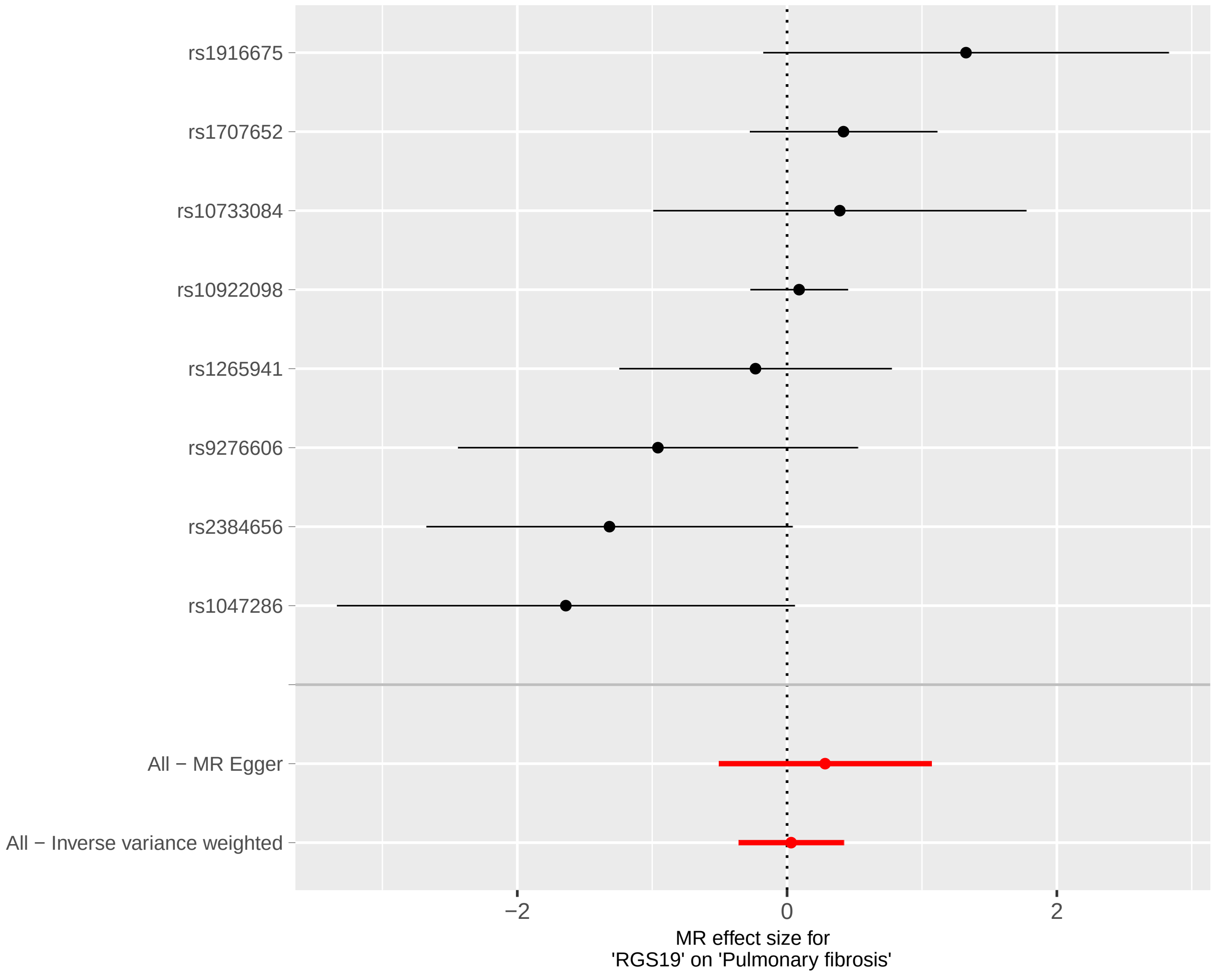
rs1916675
rs1707652
rs10733084
rs10922098
rs1265941
rs9276606
rs2384656
rs1047286
All − MR Egger
All − Inverse variance weighted
−2
0
2
MR effect size for
'RGS19' on 'Pulmonary fibrosis'

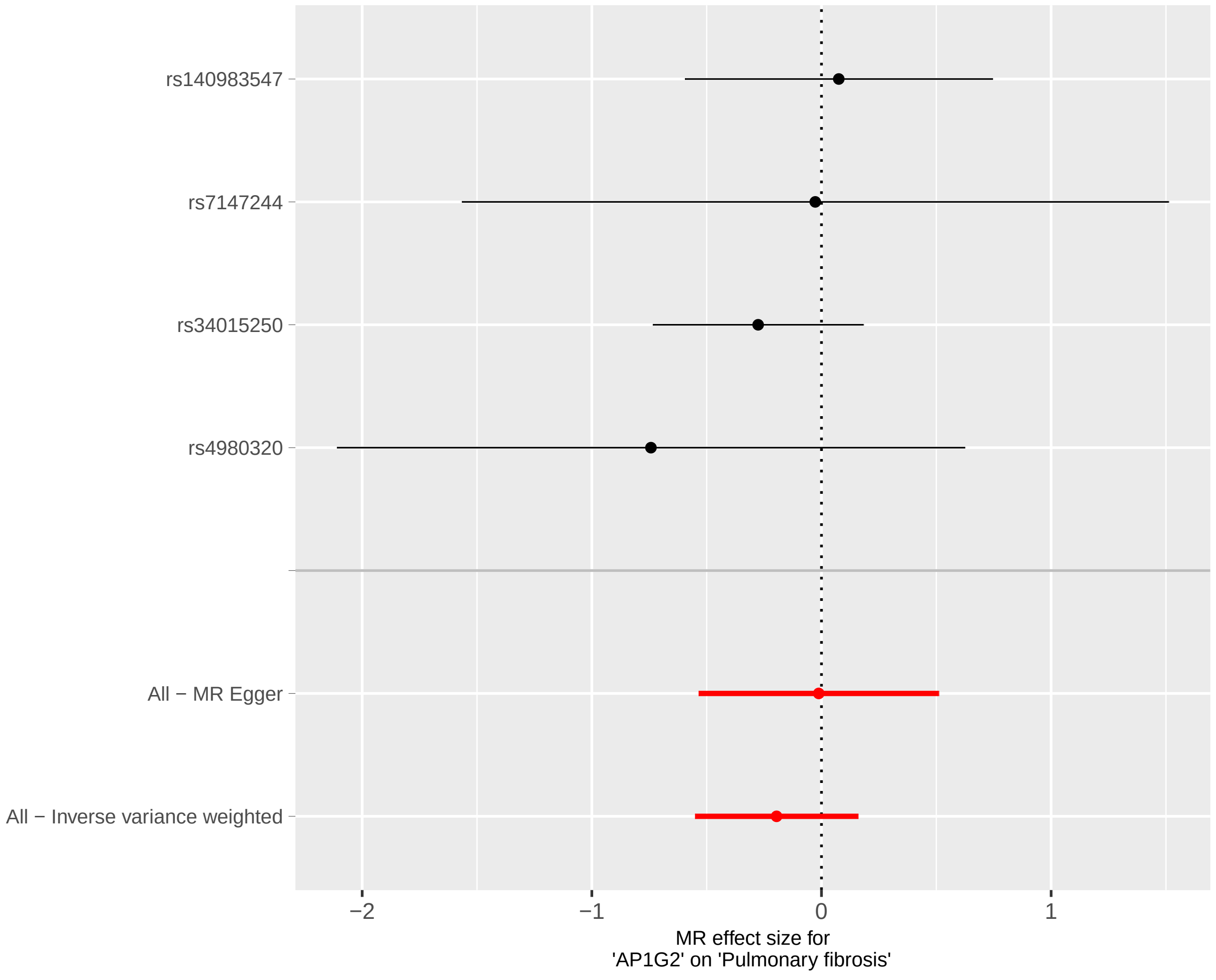

rs140983547
rs7147244
rs34015250
rs4980320
All − MR Egger
All − Inverse variance weighted
−2
−1
0
1
MR effect size for
'AP1G2' on 'Pulmonary fibrosis'

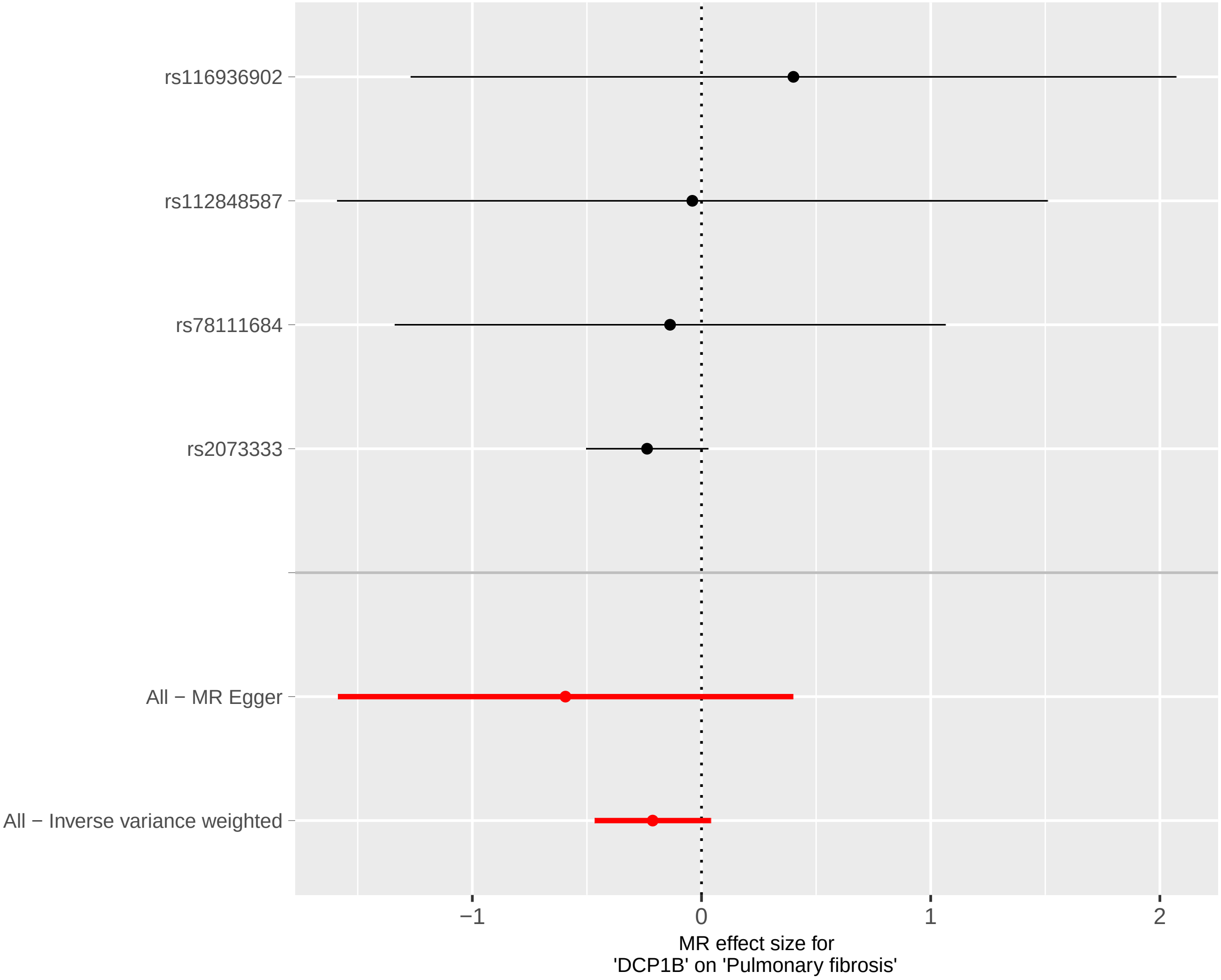
rs116936902
rs112848587
rs78111684
rs2073333
All − MR Egger
All − Inverse variance weighted
−1
0
1
2
MR effect size for
'DCP1B' on 'Pulmonary fibrosis'

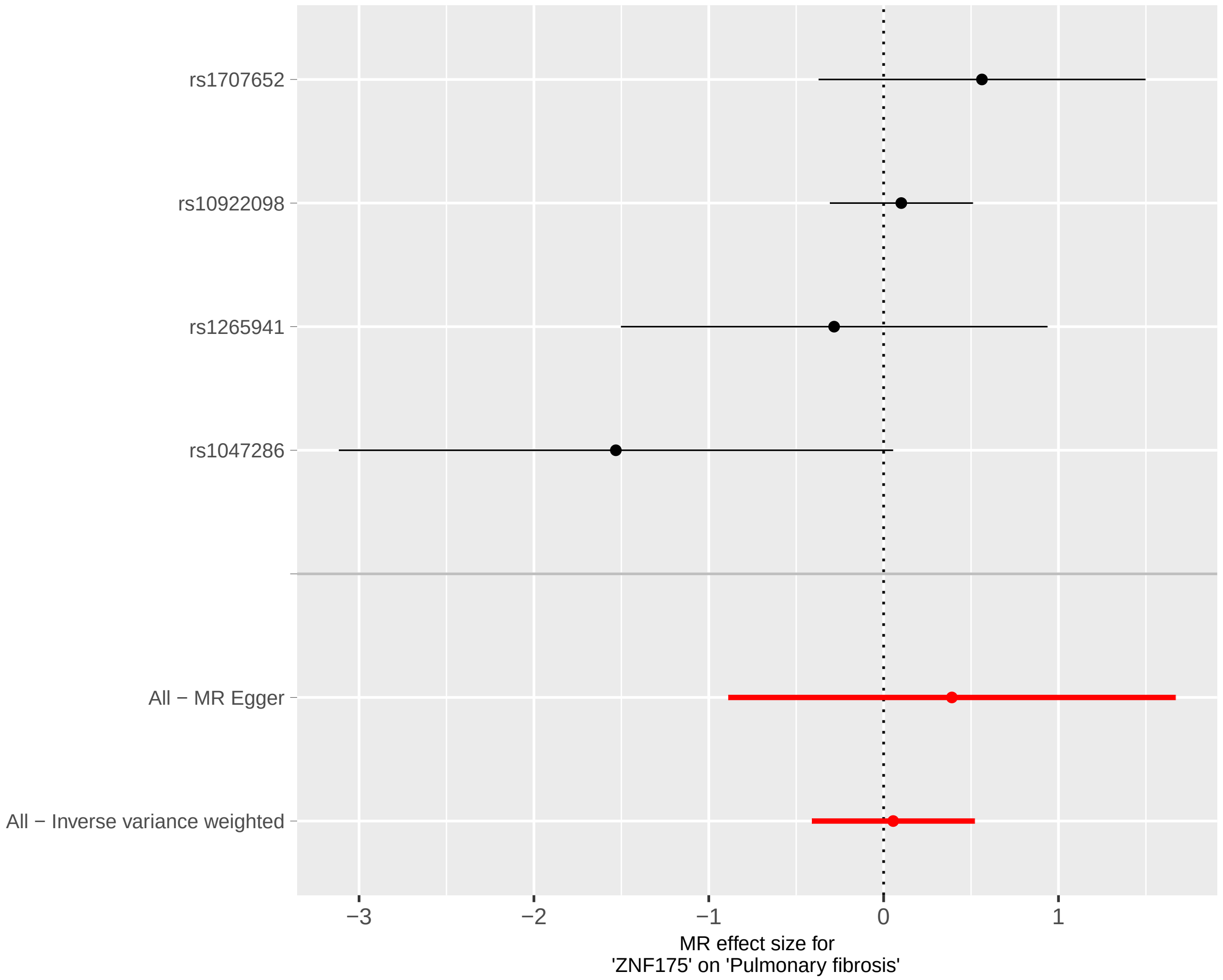

rs1707652
rs10922098
rs1265941
rs1047286
All − MR Egger
All − Inverse variance weighted
−3
−2
−1
0
1
MR effect size for
'ZNF175' on 'Pulmonary fibrosis'

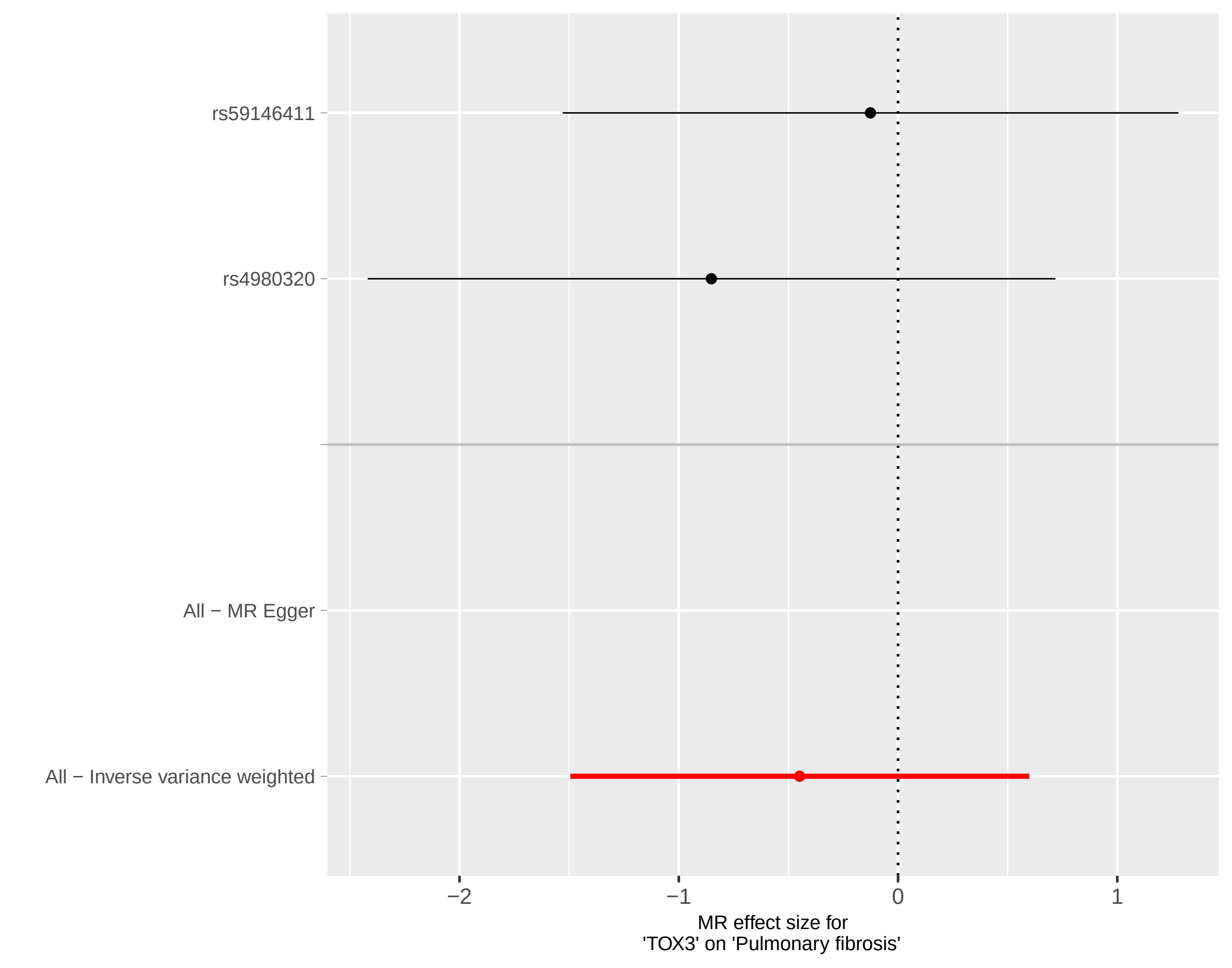
rs59146411
rs4980320
All − MR Egger
All − Inverse variance weighted
−2
−1
0
1
MR effect size for
'TOX3' on 'Pulmonary fibrosis'

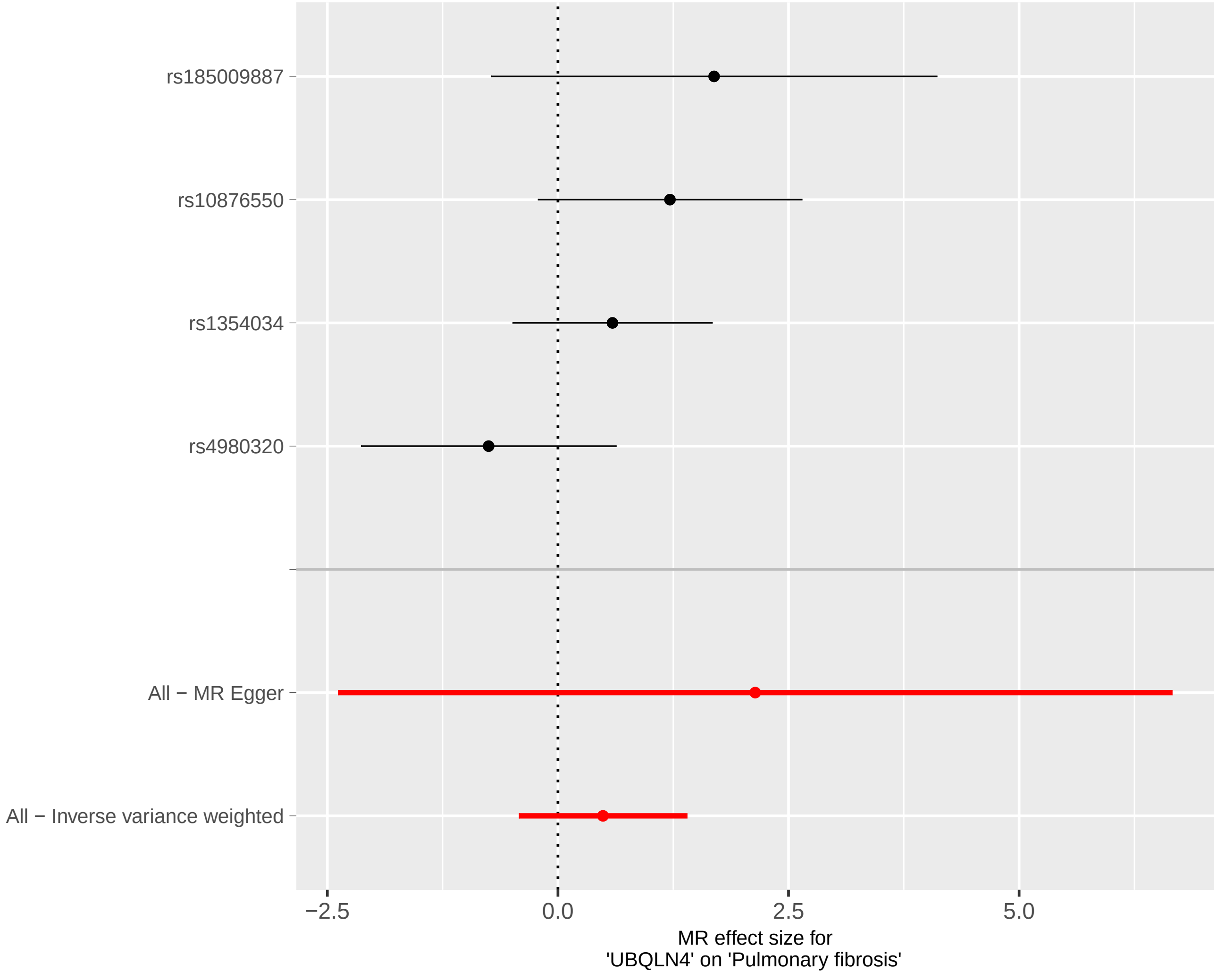
rs185009887
rs10876550
rs1354034
rs4980320
All − MR Egger
All − Inverse variance weighted
−2.5
0.0
2.5
5.0
MR effect size for
'UBQLN4' on 'Pulmonary fibrosis'

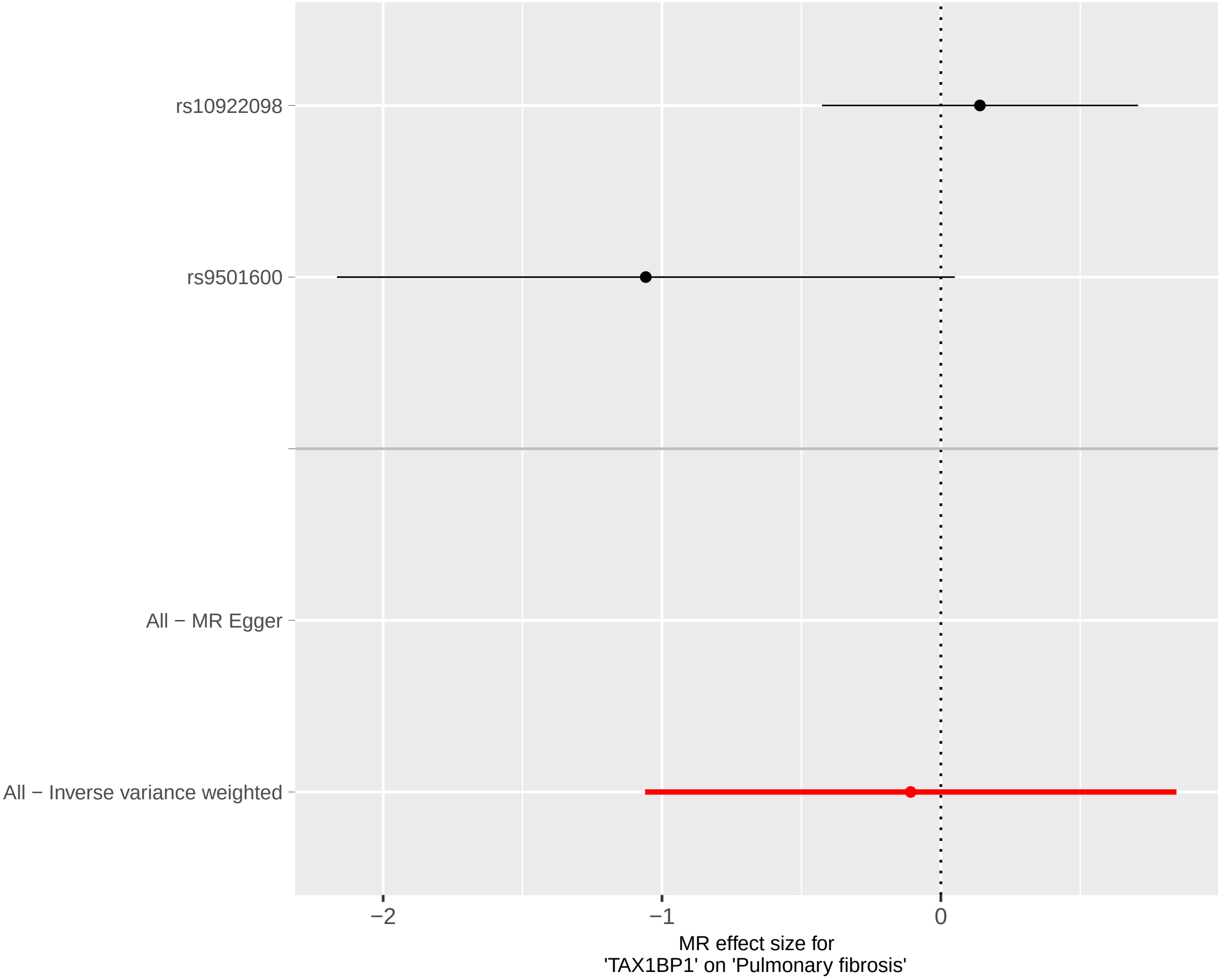
rs10922098
rs9501600
All − MR Egger
All − Inverse variance weighted
−2
−1
0
MR effect size for
'TAX1BP1' on 'Pulmonary fibrosis'

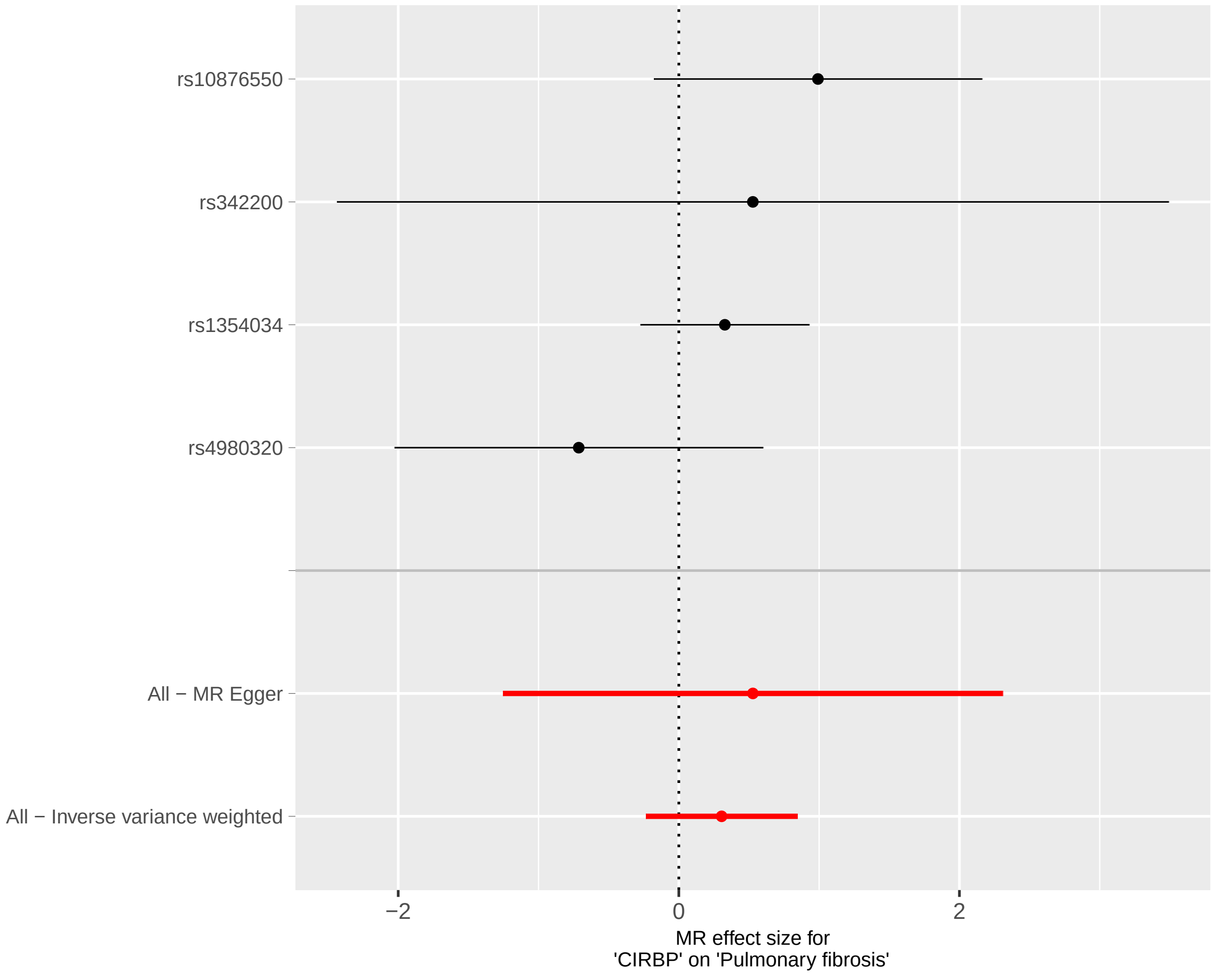

rs10876550
rs342200
rs1354034
rs4980320
All − MR Egger
All − Inverse variance weighted
−2
0
2
MR effect size for
'CIRBP' on 'Pulmonary fibrosis'

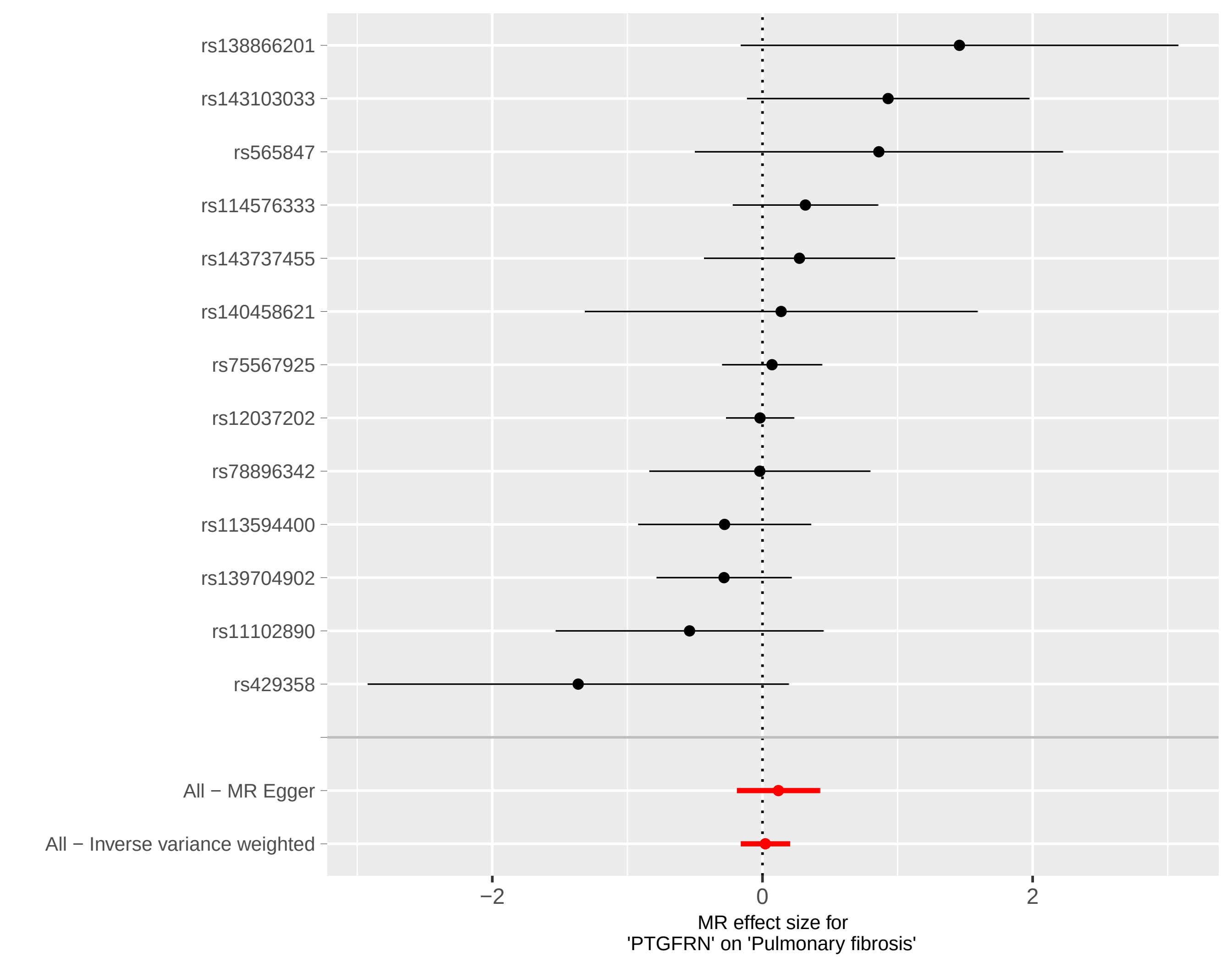
rs138866201
rs143103033
rs565847
rs114576333
rs143737455
rs140458621
rs75567925
rs12037202
rs78896342
rs113594400
rs139704902
rs11102890
rs429358
All − MR Egger
All − Inverse variance weighted
−2
0
2
MR effect size for
'PTGFRN' on 'Pulmonary fibrosis'

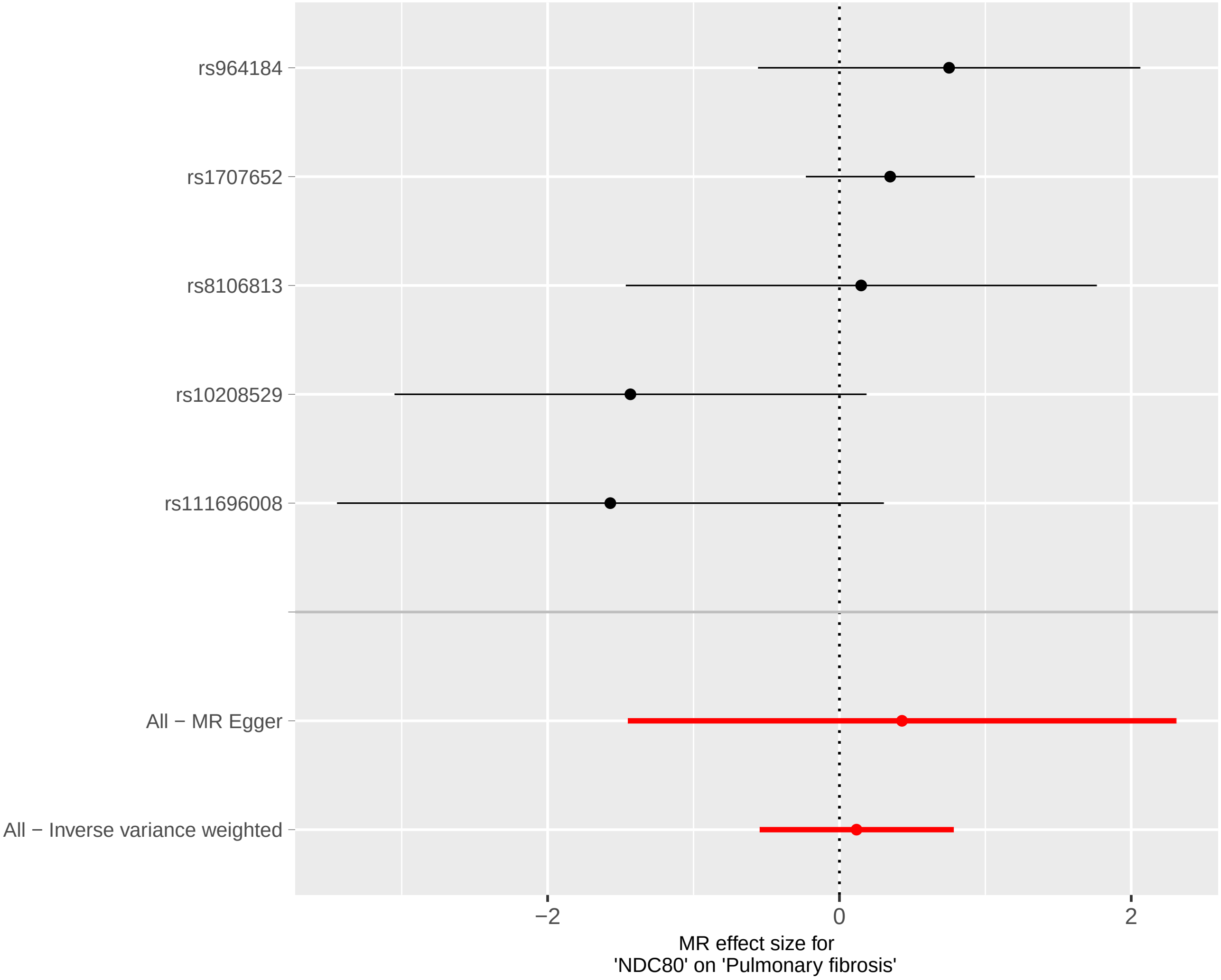
rs964184
rs1707652
rs8106813
rs10208529
rs111696008
All − MR Egger
All − Inverse variance weighted
−2
0
2
MR effect size for
'NDC80' on 'Pulmonary fibrosis'

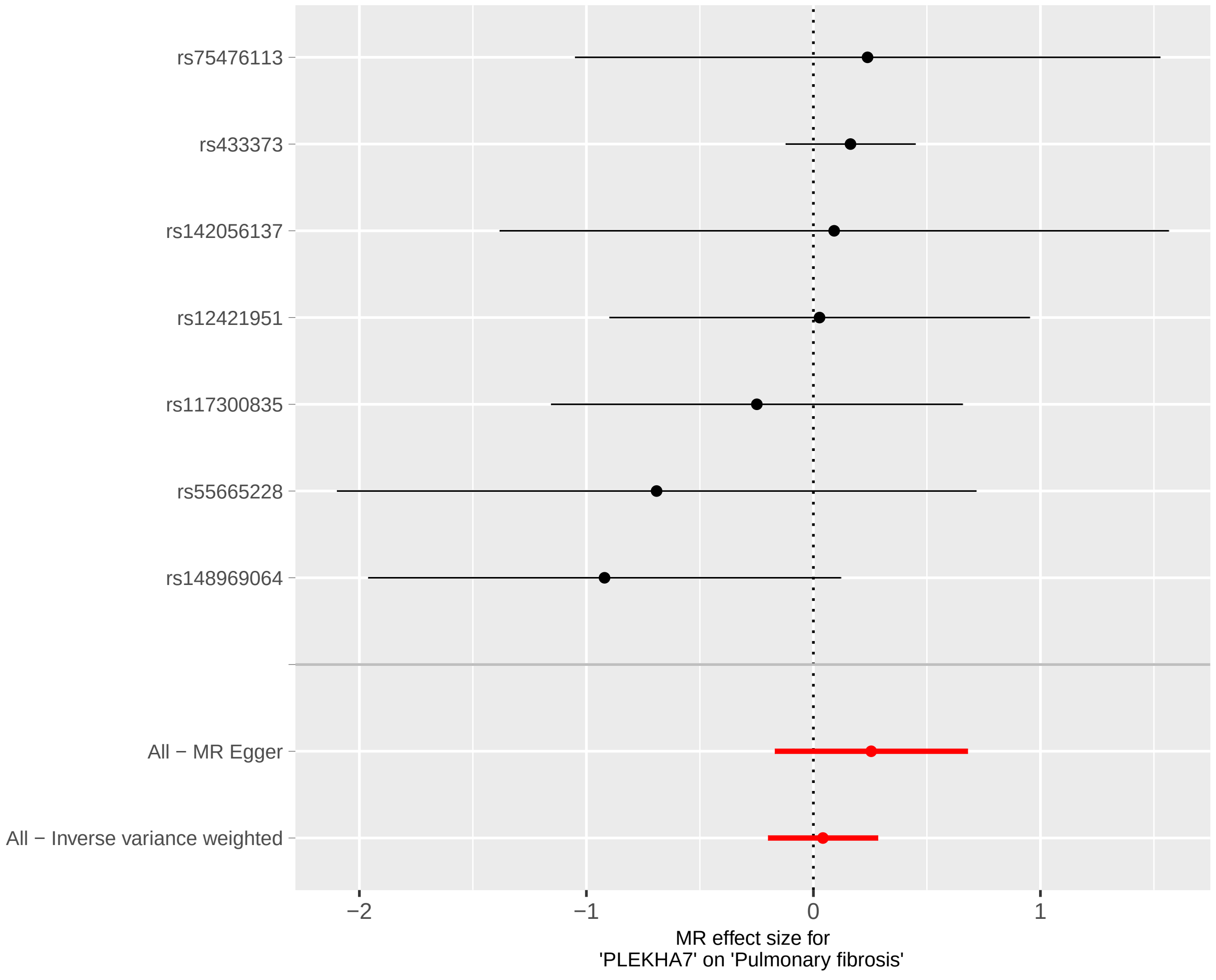

rs75476113
rs433373
rs142056137
rs12421951
rs117300835
rs55665228
rs148969064
All − MR Egger
All − Inverse variance weighted
−2
−1
0
1
MR effect size for
'PLEKHA7' on 'Pulmonary fibrosis'

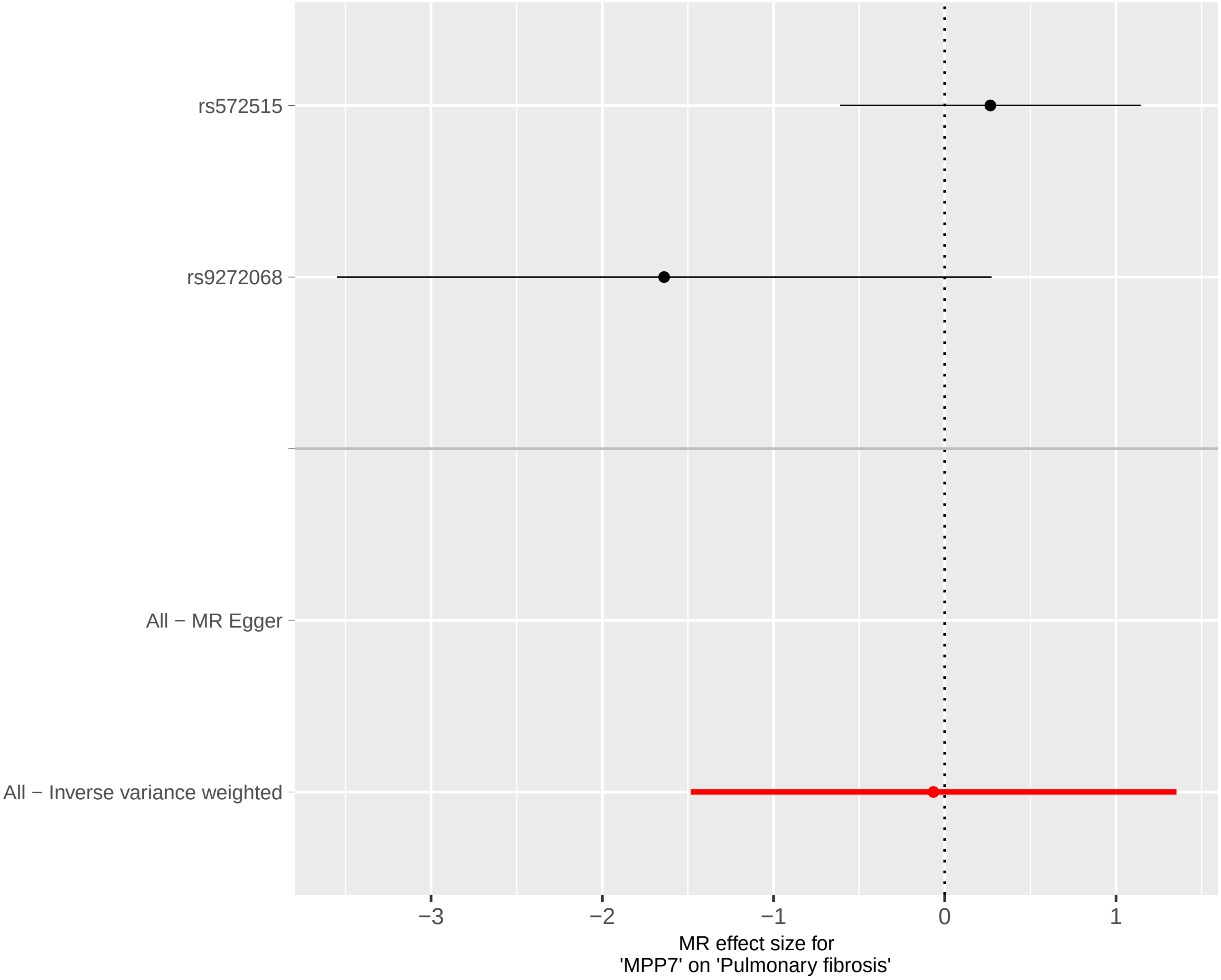

rs572515
rs9272068
All − MR Egger
All − Inverse variance weighted
−3
−2
−1
0
1
MR effect size for
'MPP7' on 'Pulmonary fibrosis'

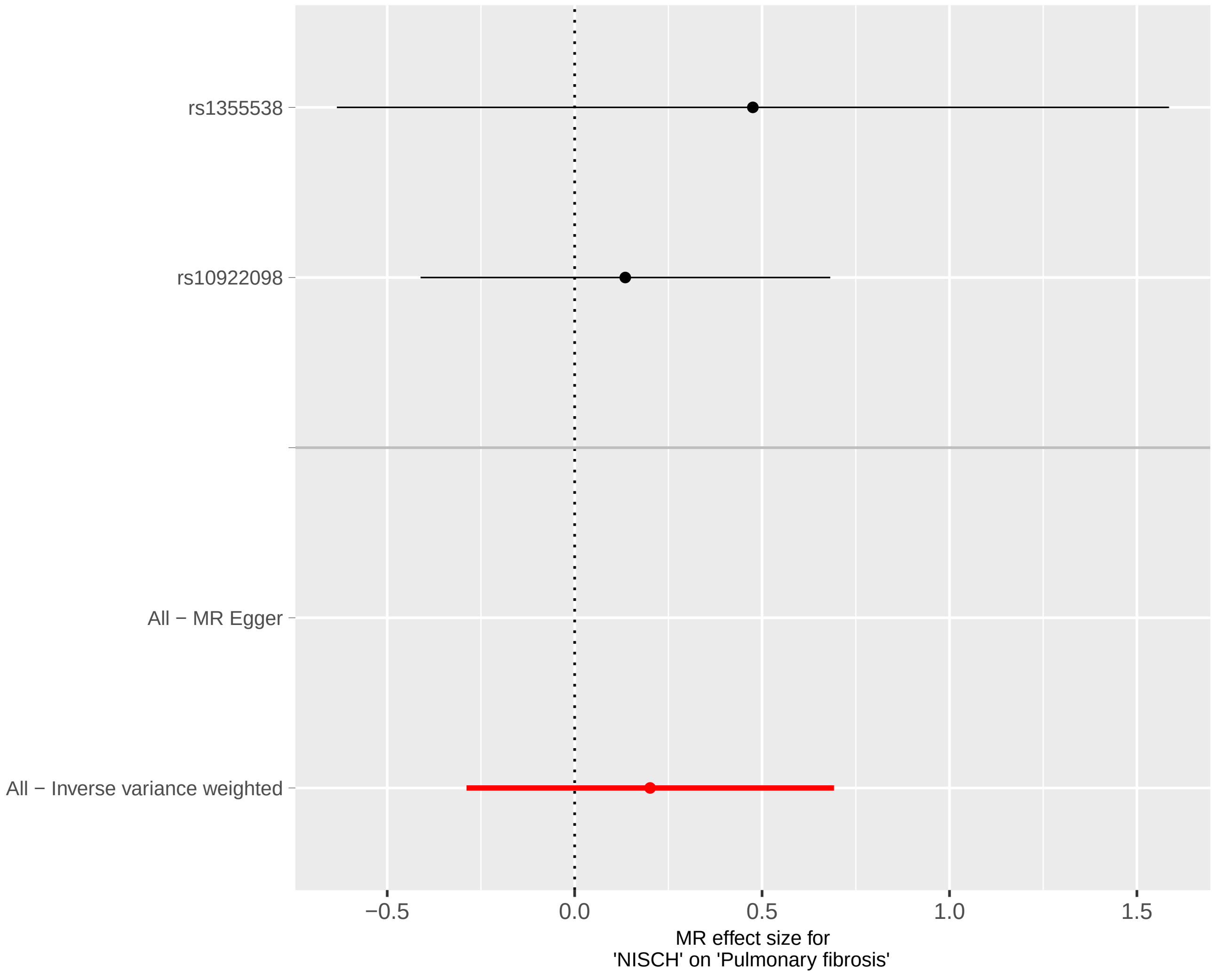
rs1355538
rs10922098
All − MR Egger
All − Inverse variance weighted
−0.5
0.0
0.5
1.0
1.5
MR effect size for
'NISCH' on 'Pulmonary fibrosis'

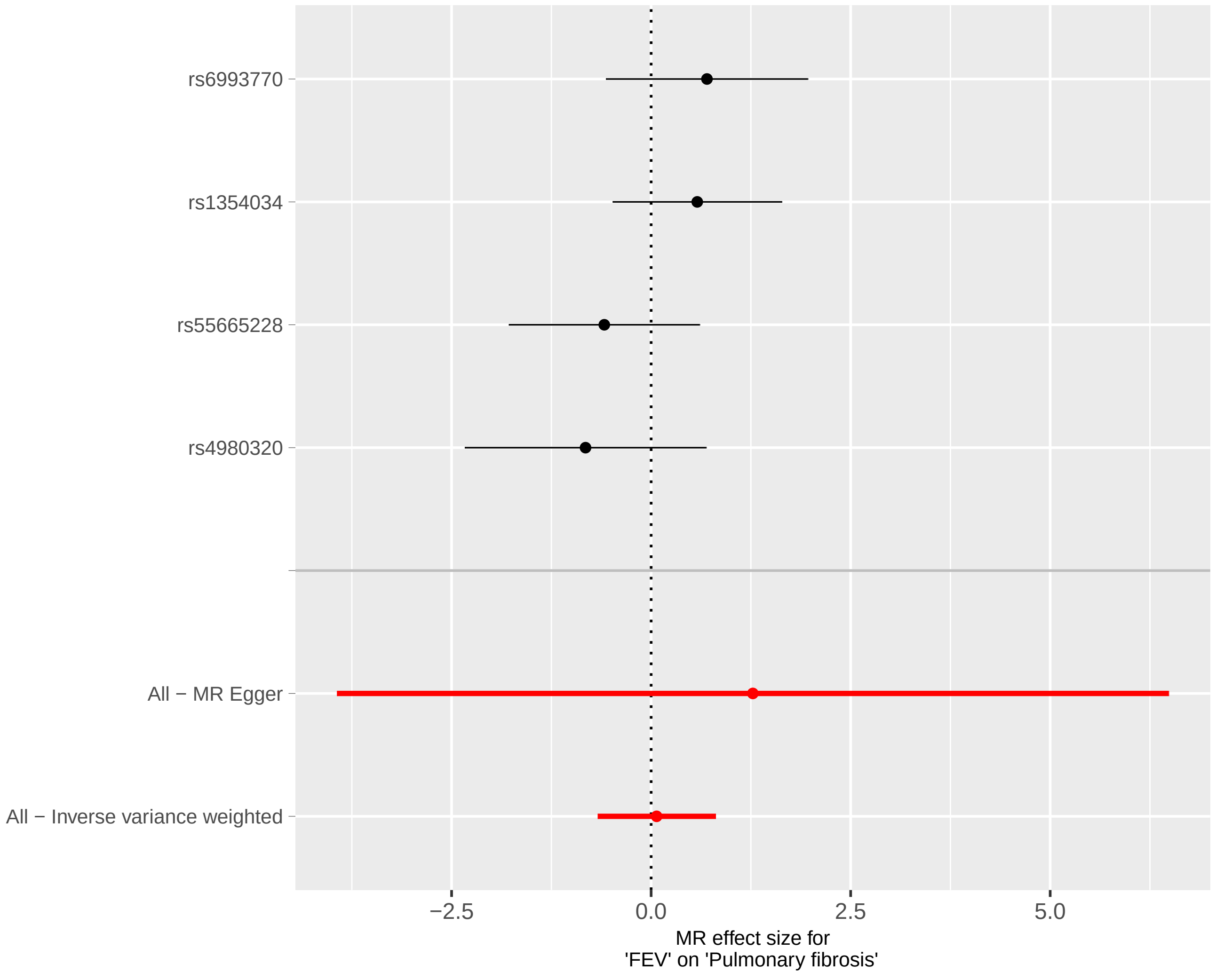

rs6993770
rs1354034
rs55665228
rs4980320
All − MR Egger
All − Inverse variance weighted
−2.5
0.0
2.5
5.0
MR effect size for
'FEV' on 'Pulmonary fibrosis'

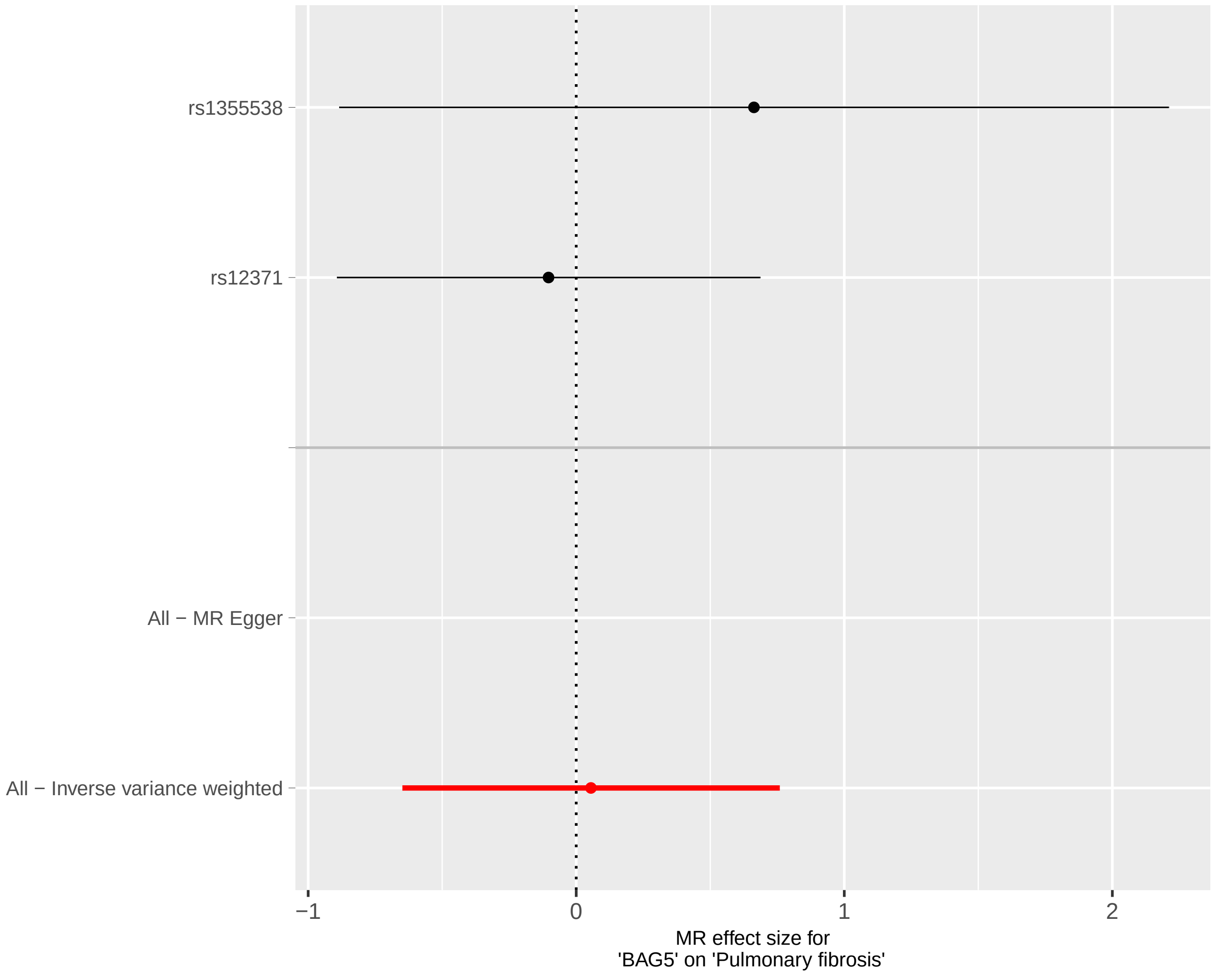
rs1355538
rs12371
All − MR Egger
All − Inverse variance weighted
−1
0
1
2
MR effect size for
'BAG5' on 'Pulmonary fibrosis'

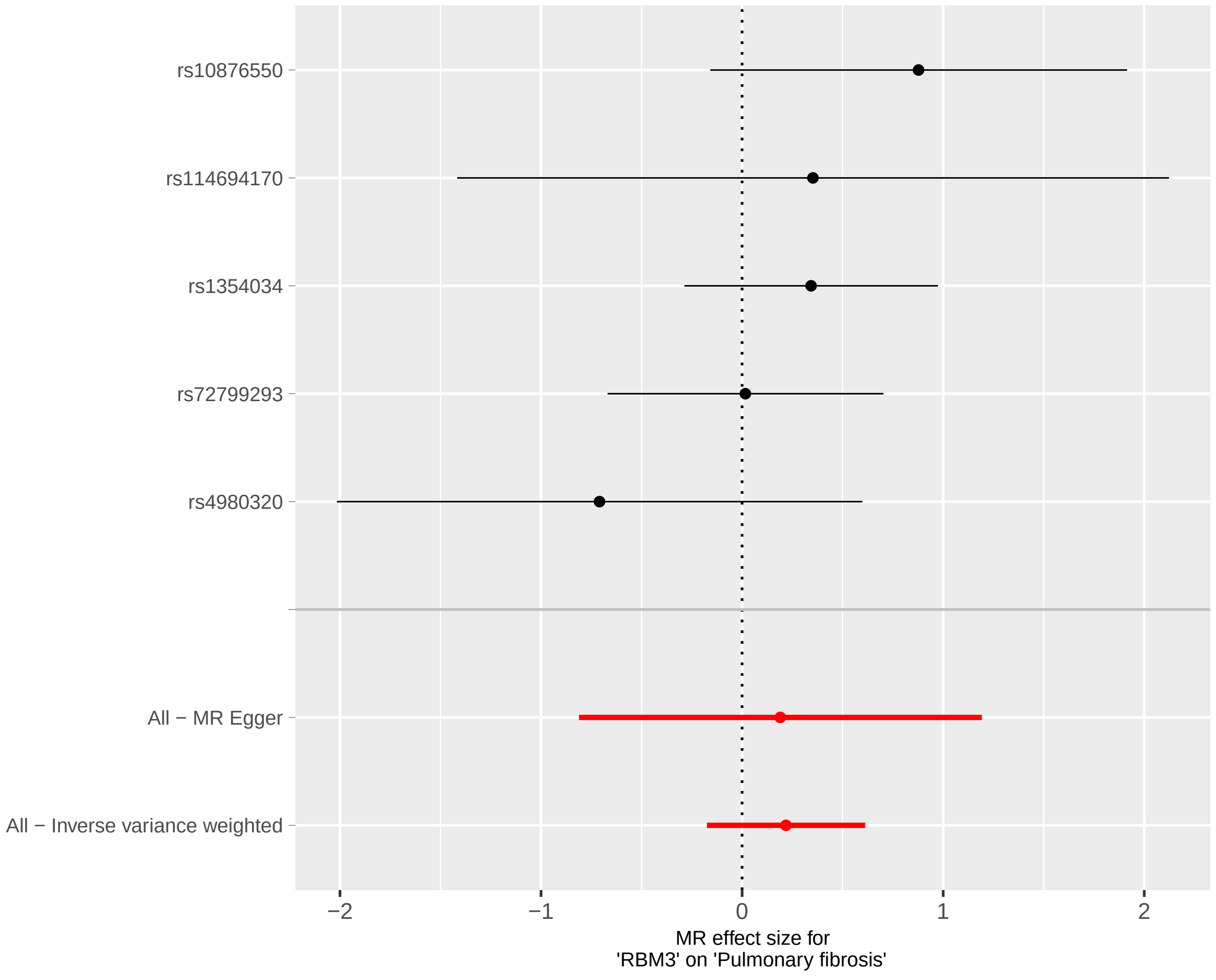

rs10876550
rs114694170
rs1354034
rs72799293
rs4980320
All − MR Egger
All − Inverse variance weighted
−2
−1
0
1
2
MR effect size for
'RBM3' on 'Pulmonary fibrosis'

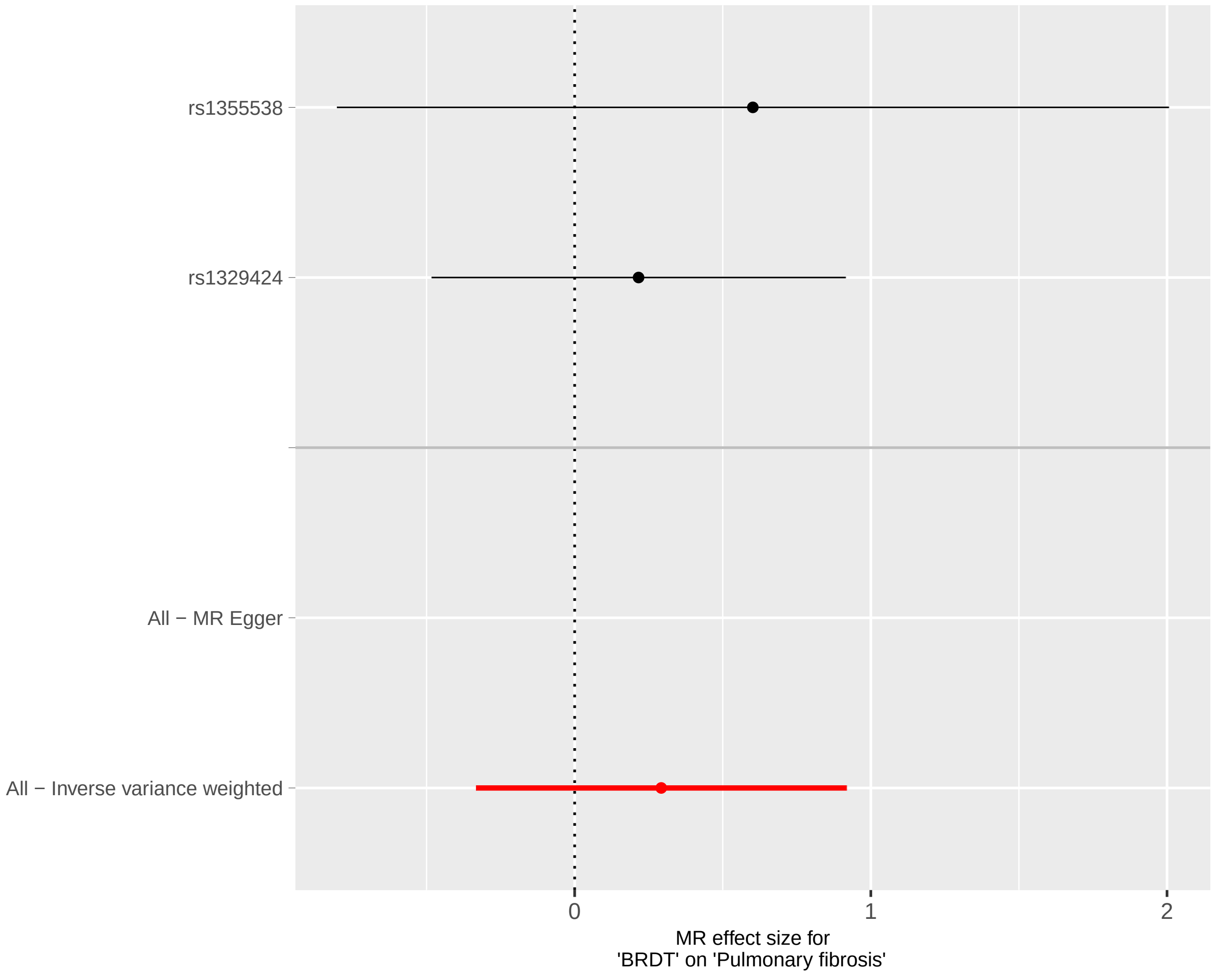

rs1355538
rs1329424
All − MR Egger
All − Inverse variance weighted
0
1
2
MR effect size for
'BRDT' on 'Pulmonary fibrosis'

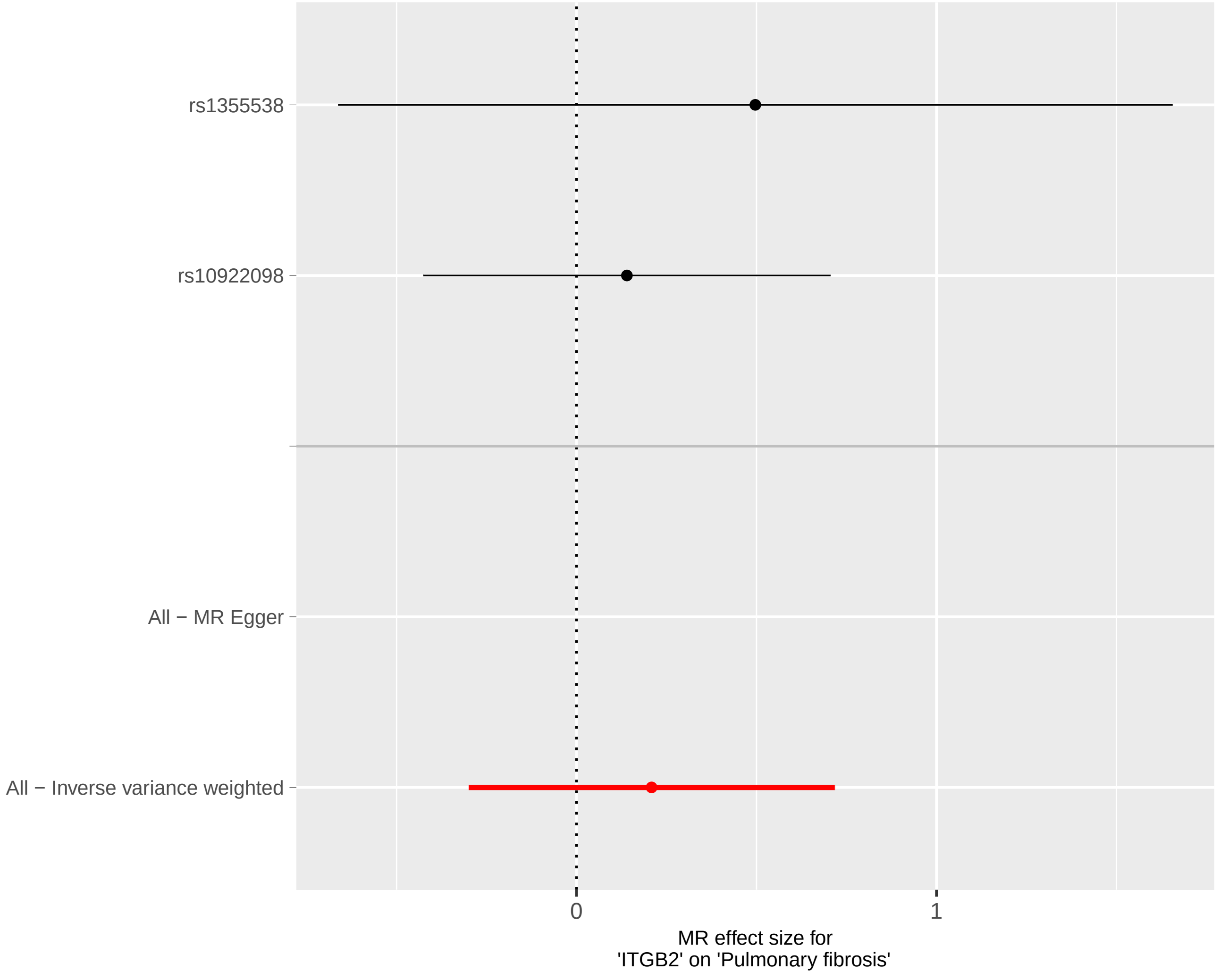

rs1355538
rs10922098
All − MR Egger
All − Inverse variance weighted
0
1
MR effect size for
'ITGB2' on 'Pulmonary fibrosis'

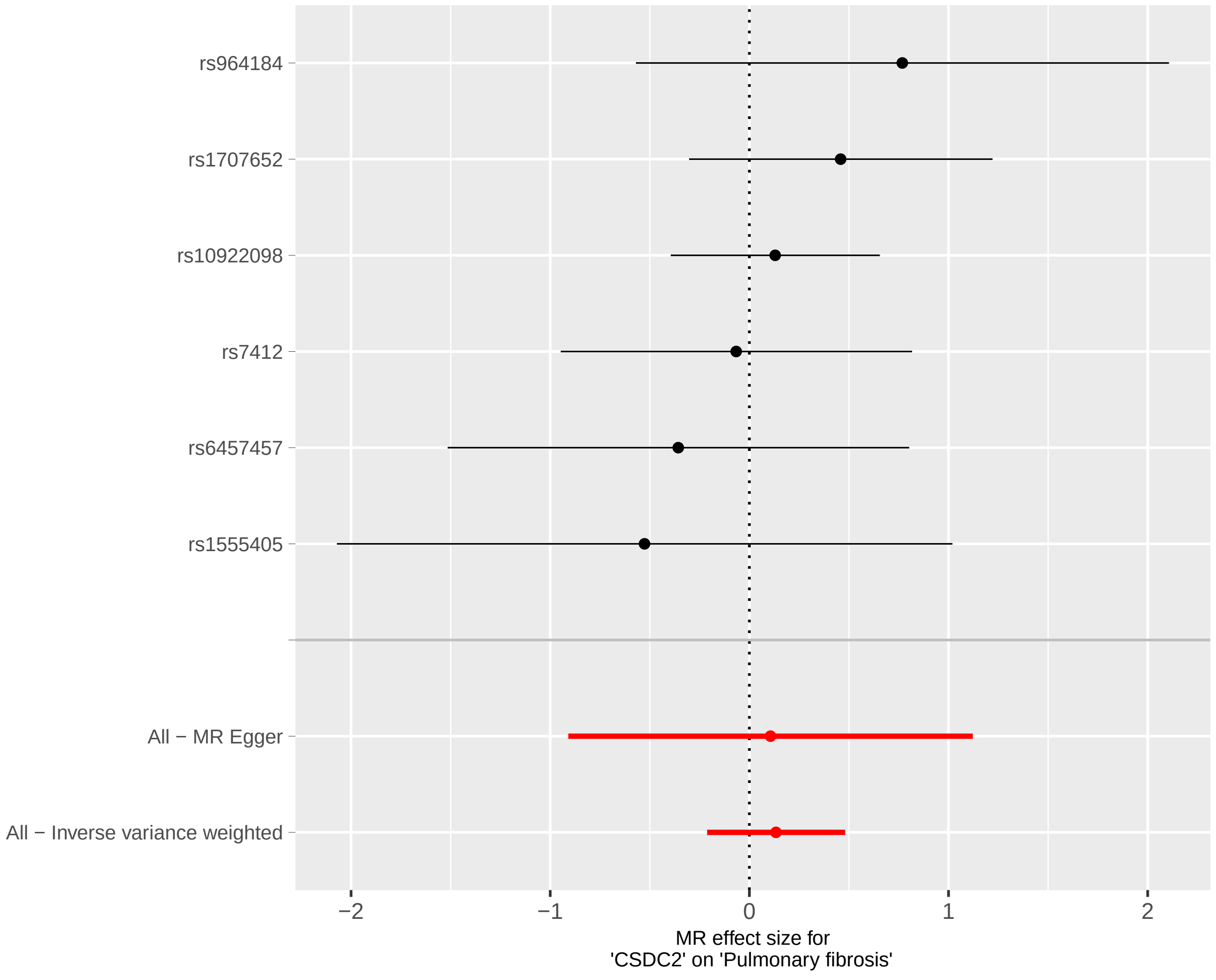

rs964184
rs1707652
rs10922098
rs7412
rs6457457
rs1555405
All − MR Egger
All − Inverse variance weighted
−2
−1
0
1
2
MR effect size for
'CSDC2' on 'Pulmonary fibrosis'

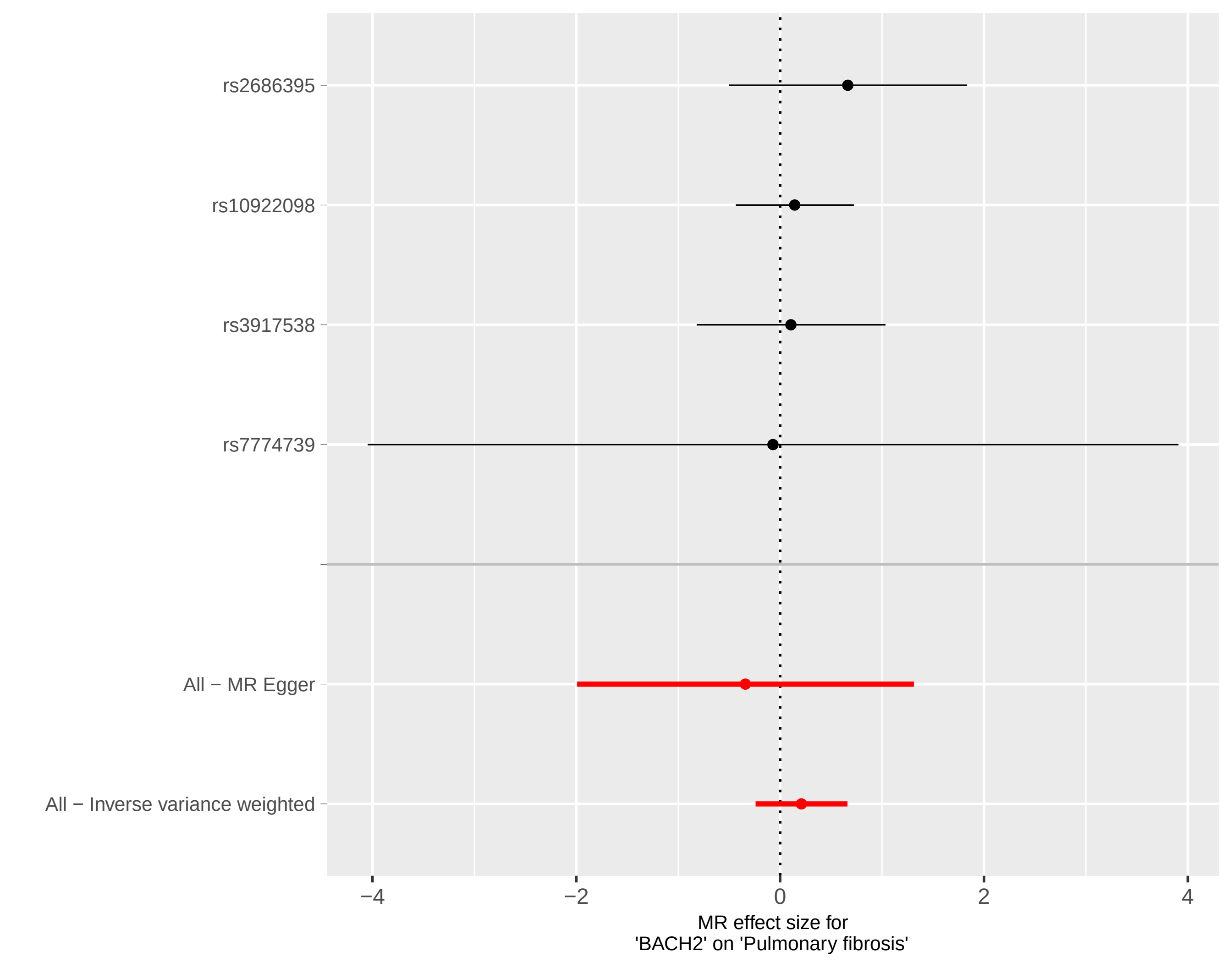
rs2686395
rs10922098
rs3917538
rs7774739
All − MR Egger
All − Inverse variance weighted
−4
−2
0
2
4
MR effect size for
'BACH2' on 'Pulmonary fibrosis'

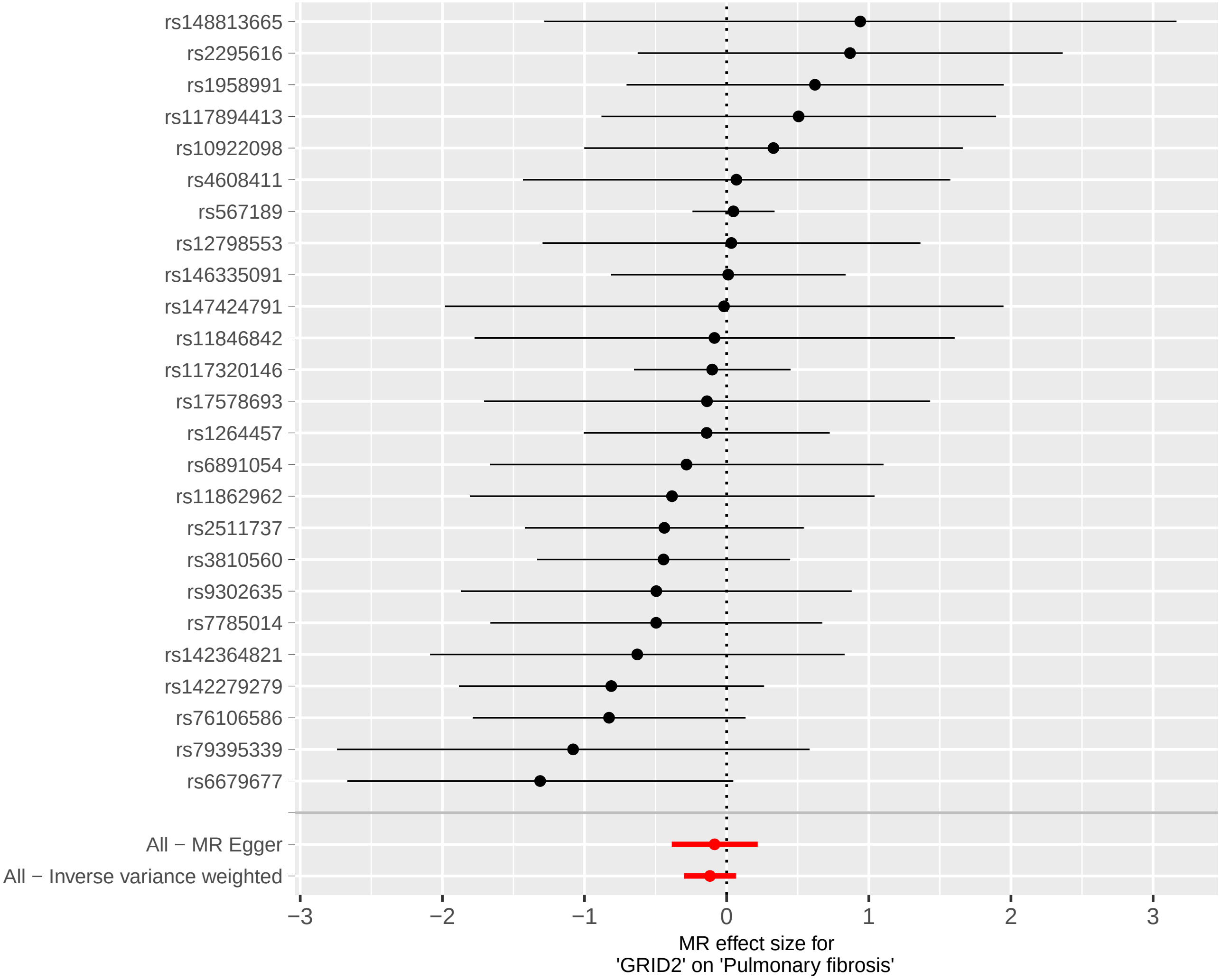
rs148813665
rs2295616
rs1958991
rs117894413
rs10922098
rs4608411
rs567189
rs12798553
rs146335091
rs147424791
rs11846842
rs117320146
rs17578693
rs1264457
rs6891054
rs11862962
rs2511737
rs3810560
rs9302635
rs7785014
rs142364821
rs142279279
rs76106586
rs79395339
rs6679677
All − MR Egger
All − Inverse variance weighted
−3
−2
−1
0
1
2
3
MR effect size for
'GRID2' on 'Pulmonary fibrosis'

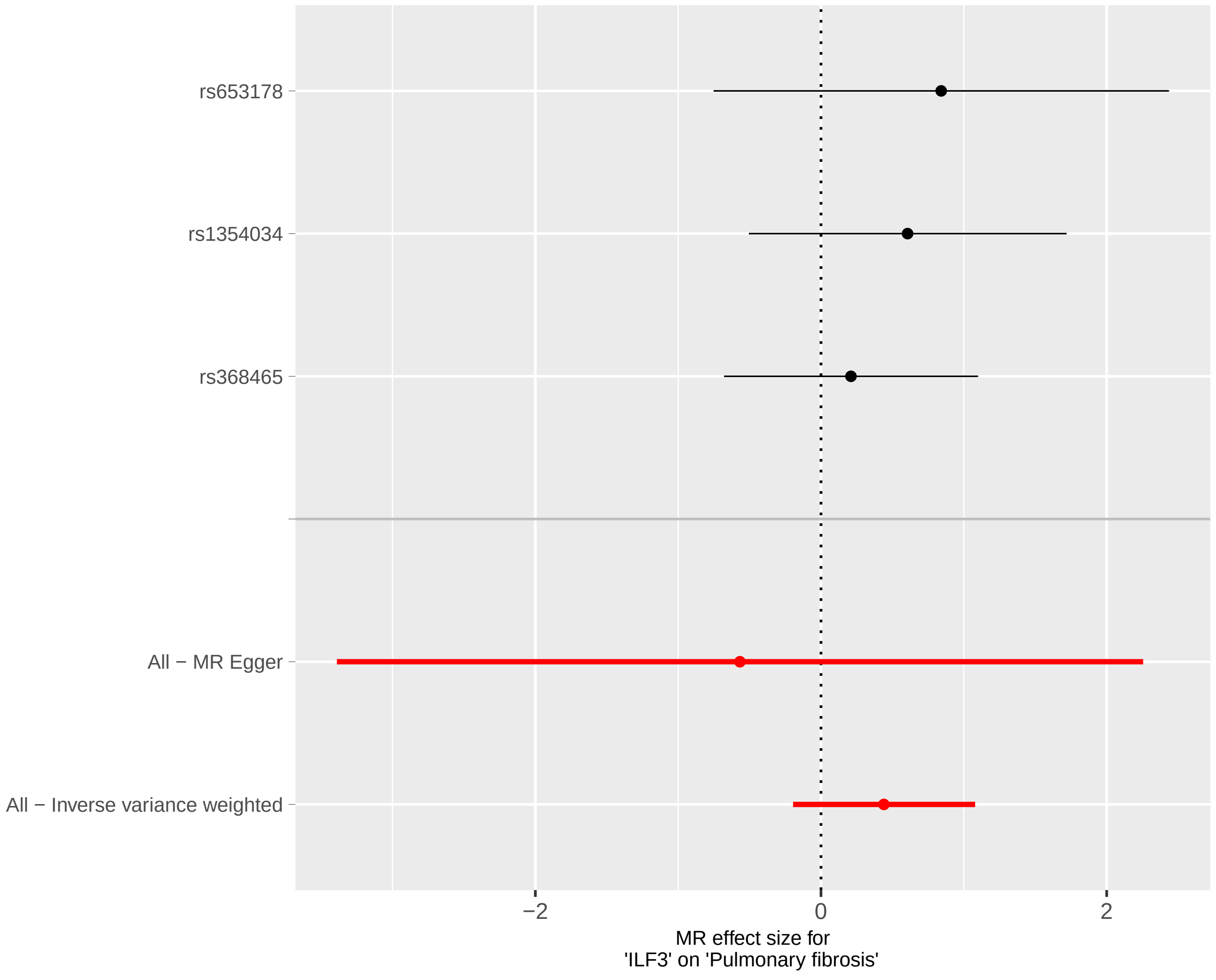

rs653178
rs1354034
rs368465
All − MR Egger
All − Inverse variance weighted
−2
0
2
MR effect size for
'ILF3' on 'Pulmonary fibrosis'

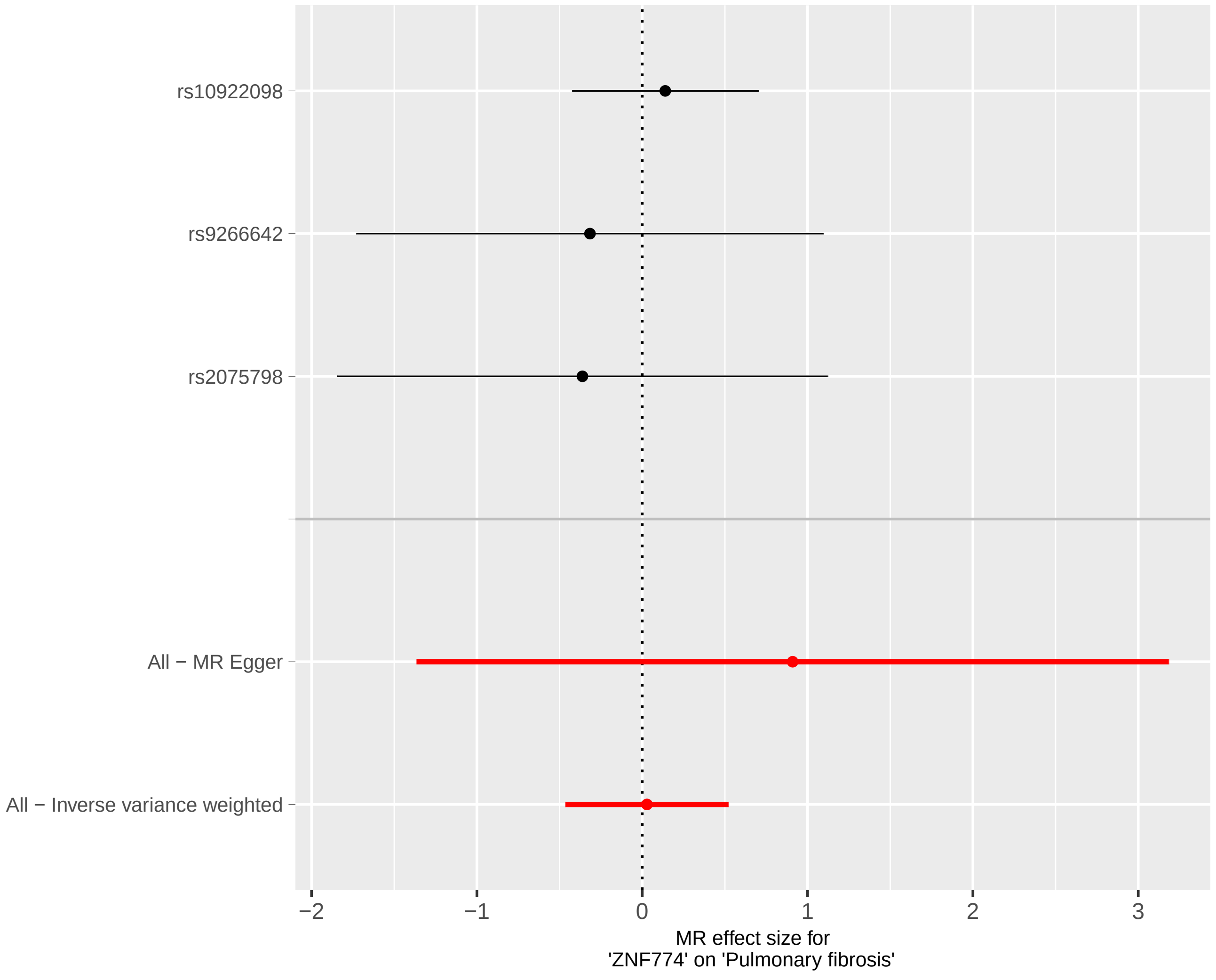
rs10922098
rs9266642
rs2075798
All − MR Egger
All − Inverse variance weighted
−2
−1
0
1
2
3
MR effect size for
'ZNF774' on 'Pulmonary fibrosis'

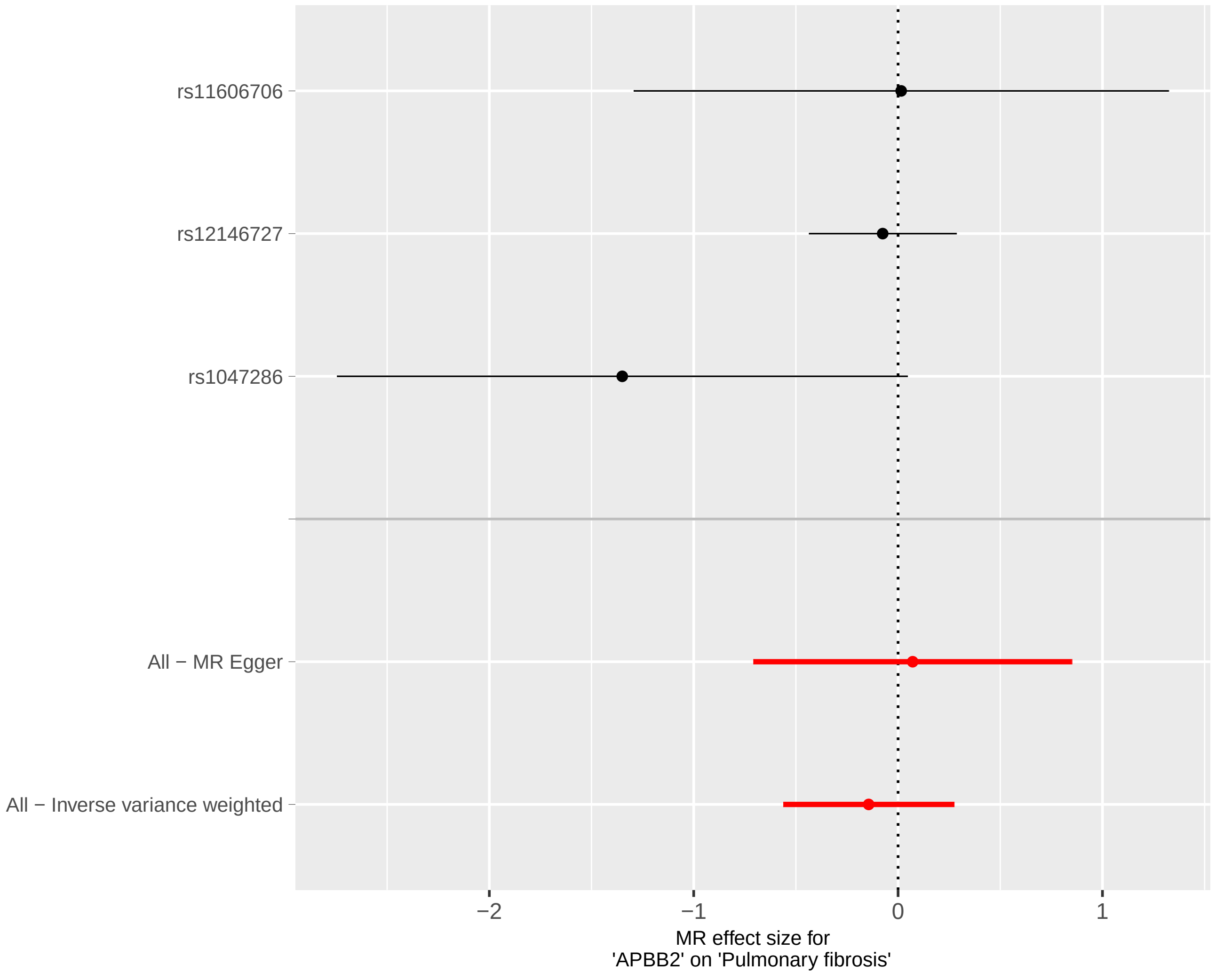

rs11606706
rs12146727
rs1047286
All − MR Egger
All − Inverse variance weighted
−2
−1
0
1
MR effect size for
'APBB2' on 'Pulmonary fibrosis'

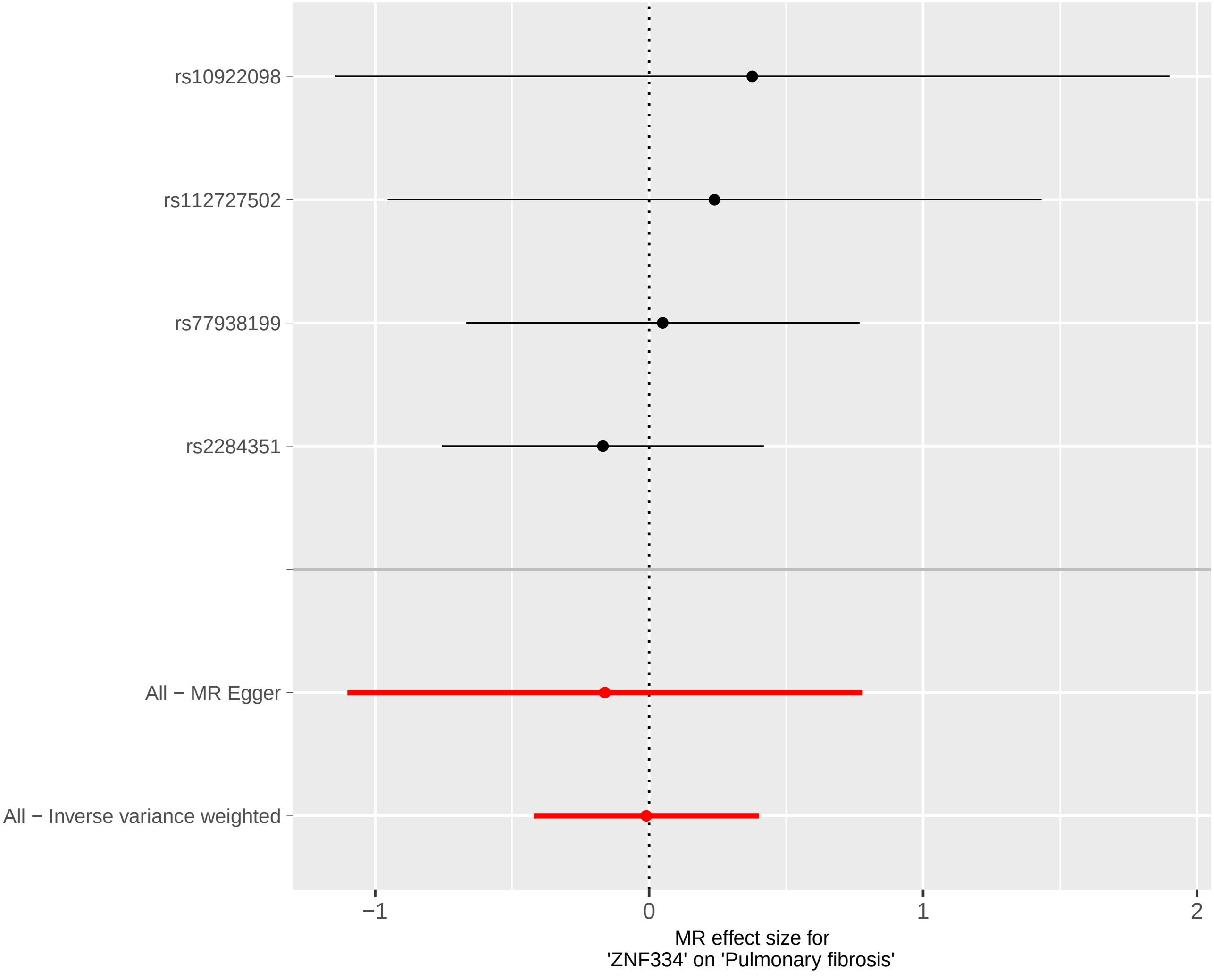
rs10922098
rs112727502
rs77938199
rs2284351
All − MR Egger
All − Inverse variance weighted
−1
0
1
2
MR effect size for
'ZNF334' on 'Pulmonary fibrosis'

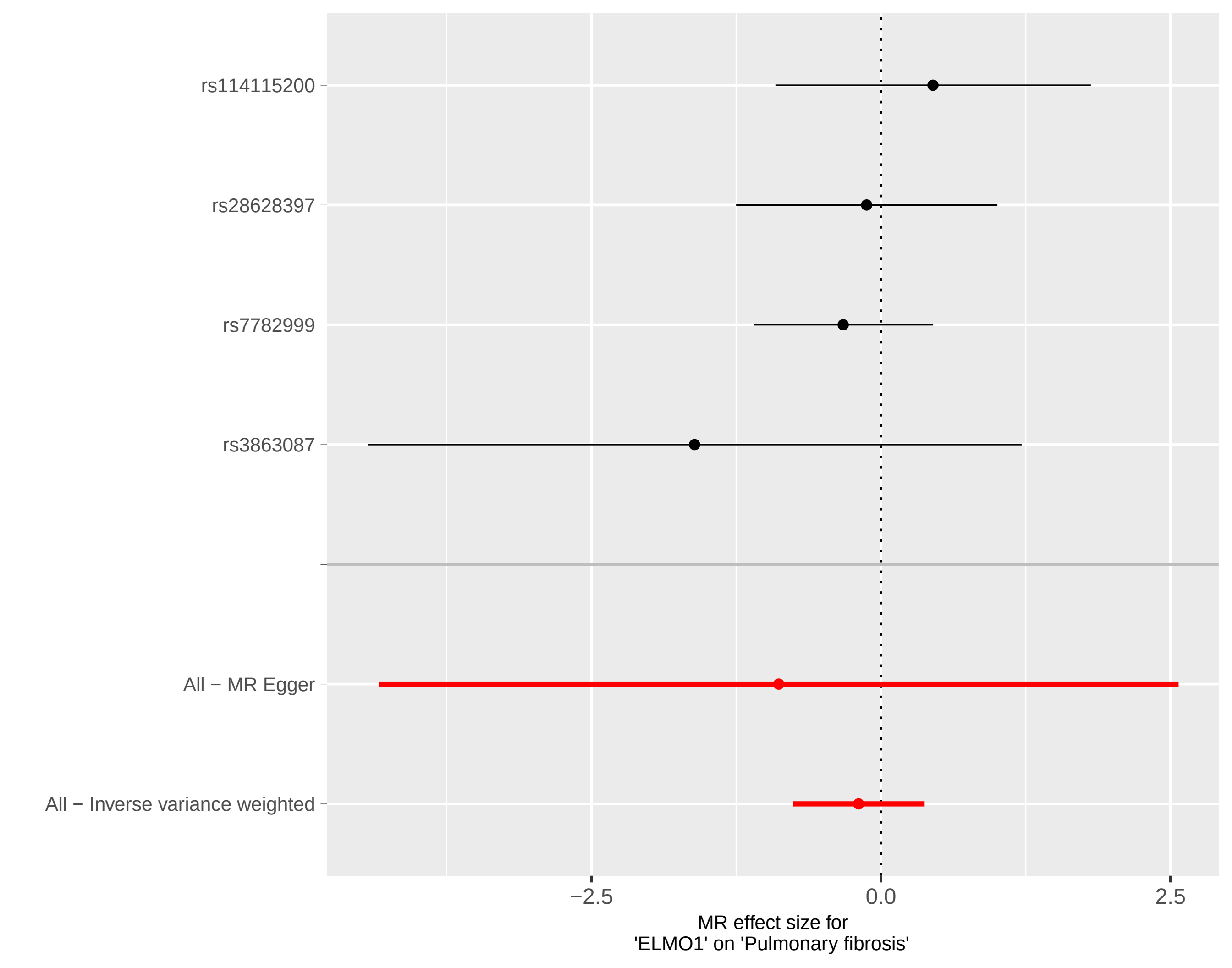

rs114115200
rs28628397
rs7782999
rs3863087
All − MR Egger
All − Inverse variance weighted
−2.5
0.0
2.5
MR effect size for
'ELMO1' on 'Pulmonary fibrosis'

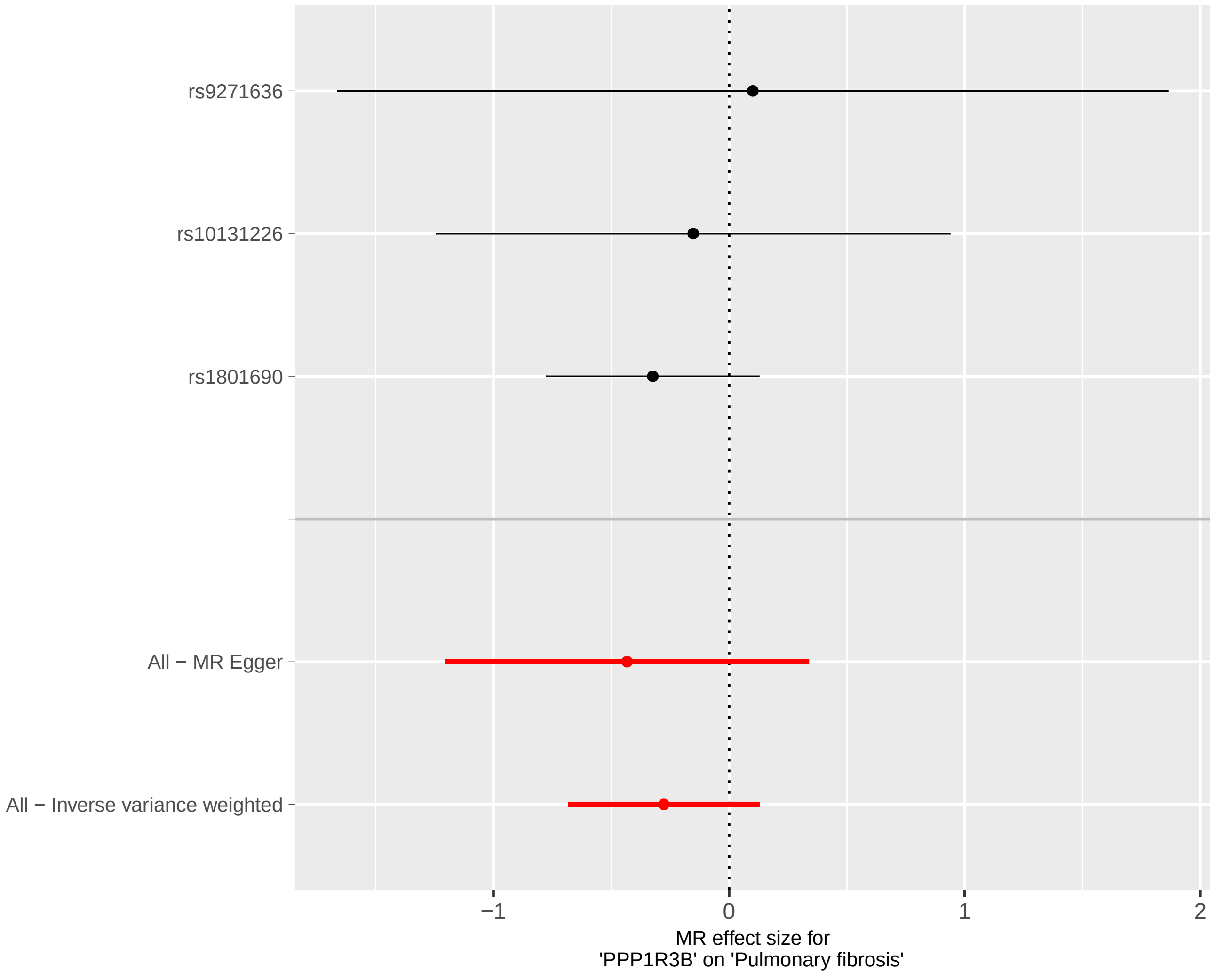
rs9271636
rs10131226
rs1801690
All − MR Egger
All − Inverse variance weighted
−1
0
1
2
MR effect size for
'PPP1R3B' on 'Pulmonary fibrosis'

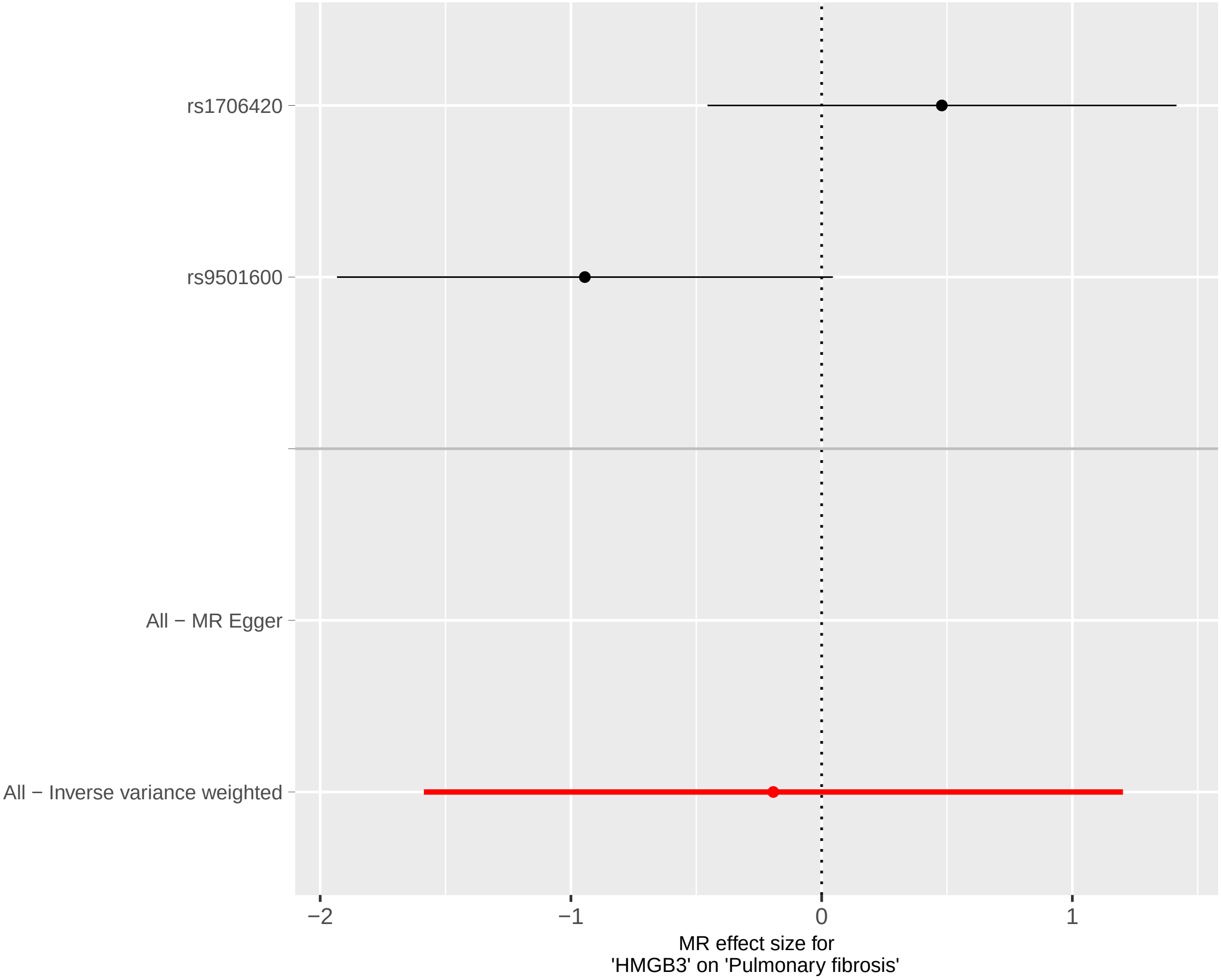
rs1706420
rs9501600
All − MR Egger
All − Inverse variance weighted
−2
−1
0
1
MR effect size for
'HMGB3' on 'Pulmonary fibrosis'

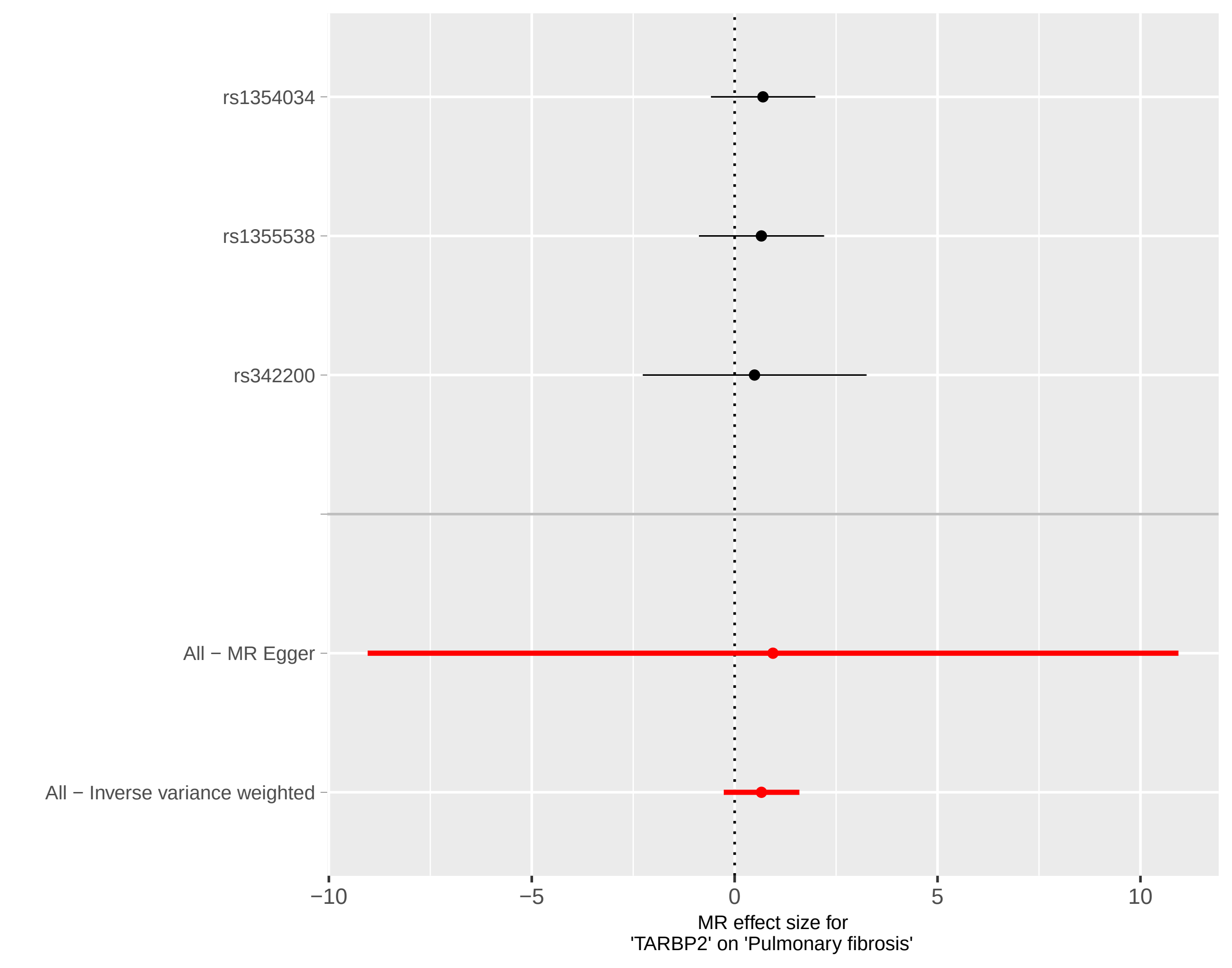
rs1354034
rs1355538
rs342200
All − MR Egger
All − Inverse variance weighted
−10
−5
0
5
10
MR effect size for
'TARBP2' on 'Pulmonary fibrosis'

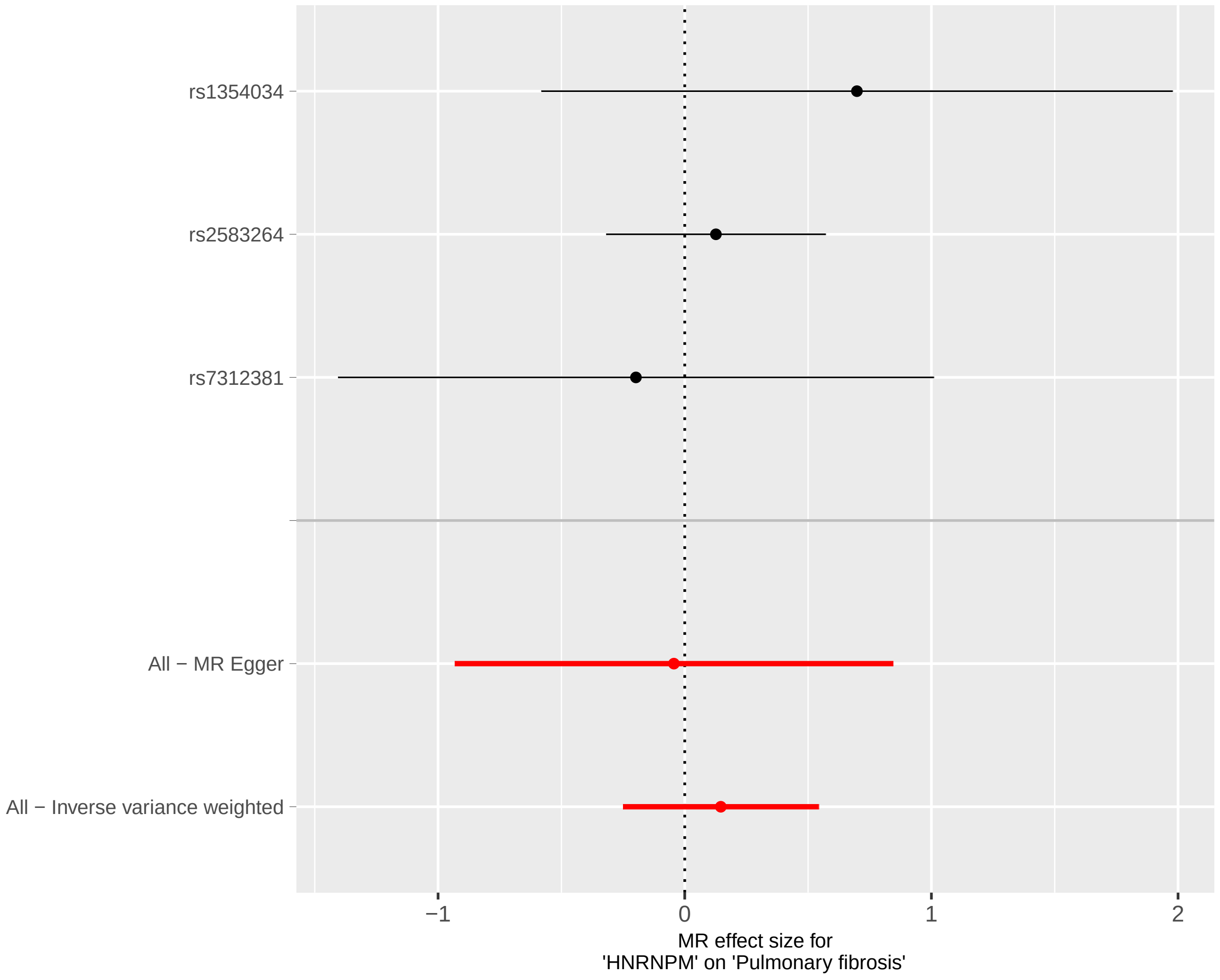
rs1354034
rs2583264
rs7312381
All − MR Egger
All − Inverse variance weighted
−1
0
1
2
MR effect size for
'HNRNPM' on 'Pulmonary fibrosis'

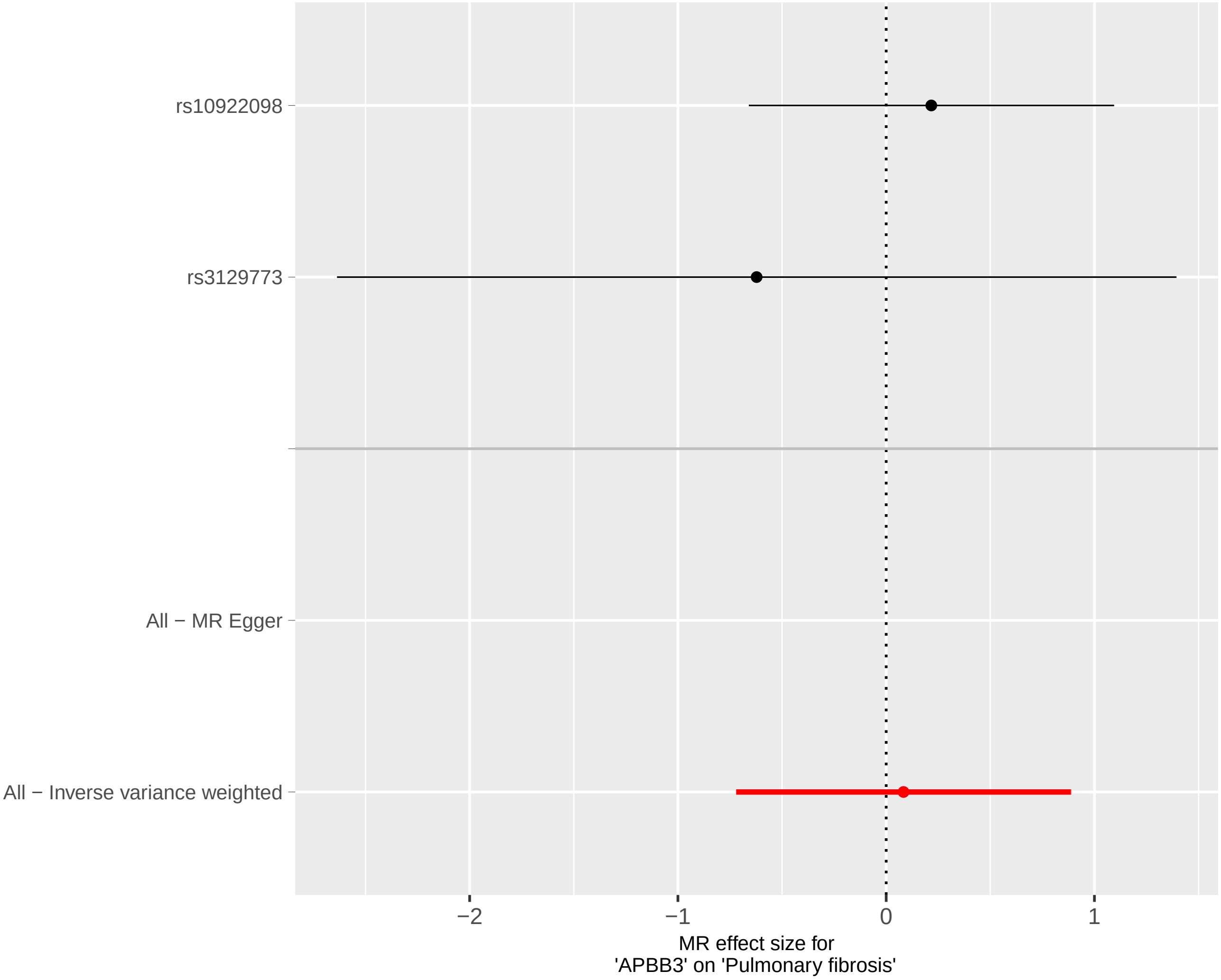

rs10922098
rs3129773
All − MR Egger
All − Inverse variance weighted
−2
−1
0
1
MR effect size for
'APBB3' on 'Pulmonary fibrosis'

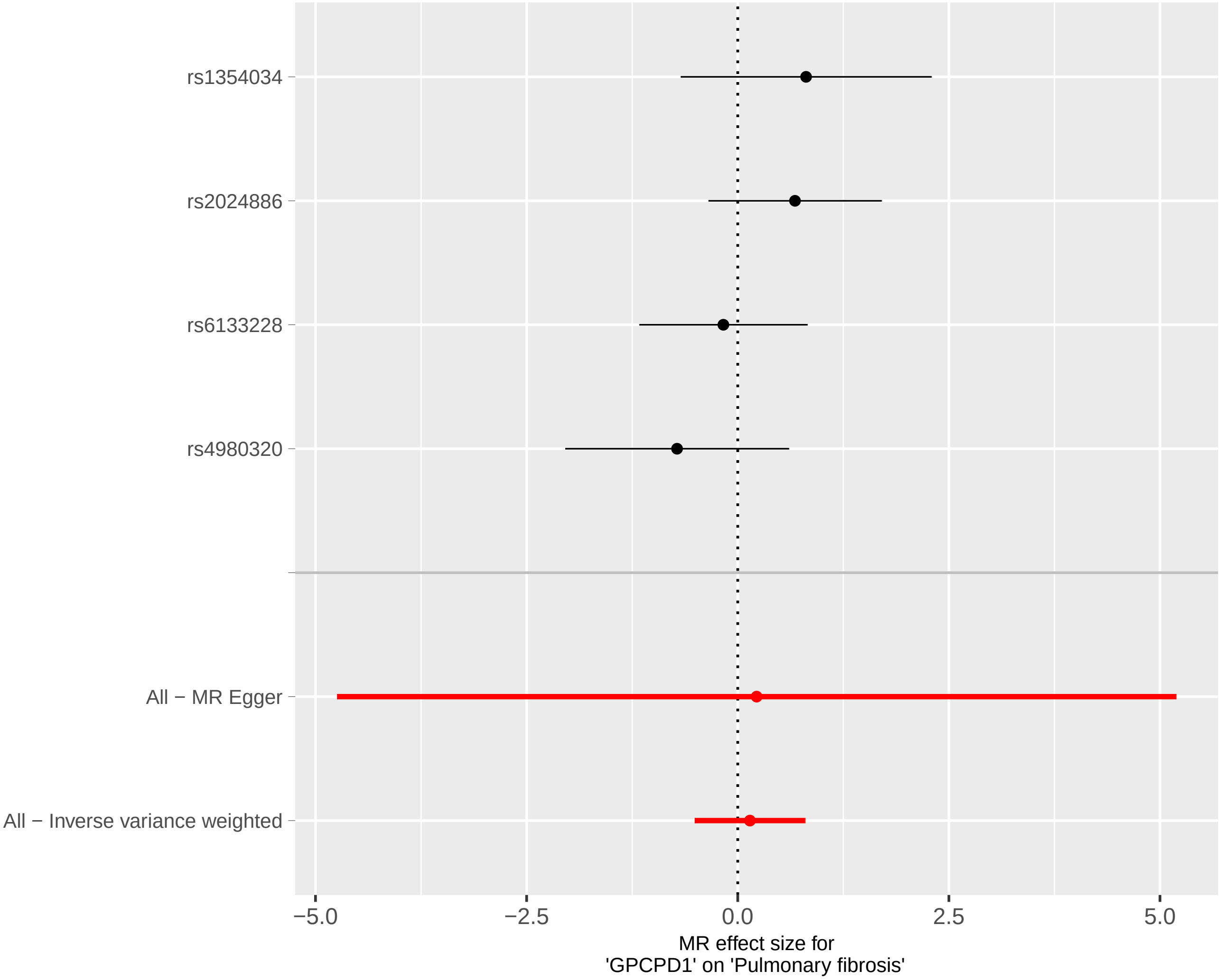

rs1354034
rs2024886
rs6133228
rs4980320
All − MR Egger
All − Inverse variance weighted
−5.0
−2.5
0.0
2.5
5.0
MR effect size for
'GPCPD1' on 'Pulmonary fibrosis'

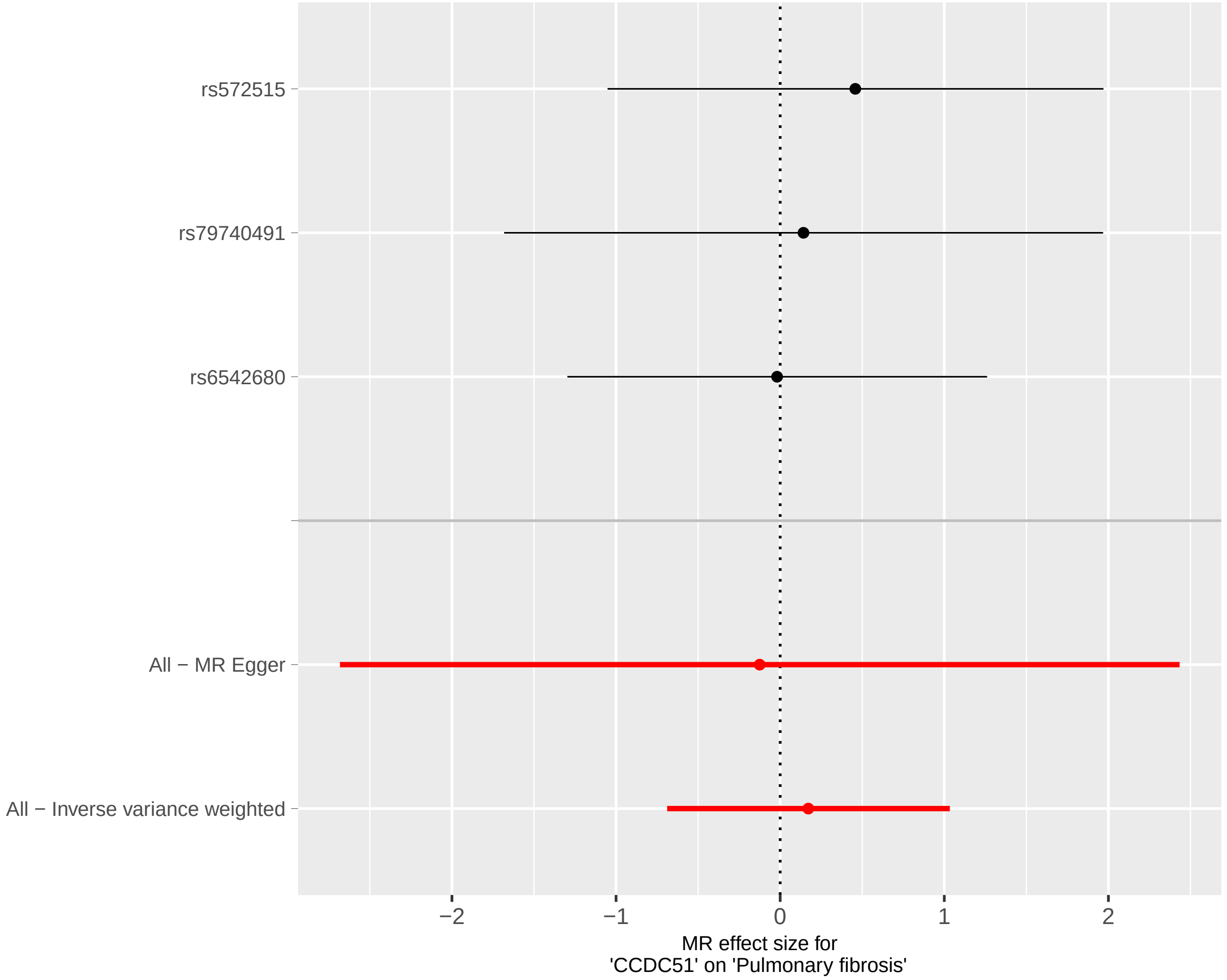
rs572515
rs79740491
rs6542680
All − MR Egger
All − Inverse variance weighted
−2
−1
0
1
2
MR effect size for
'CCDC51' on 'Pulmonary fibrosis'

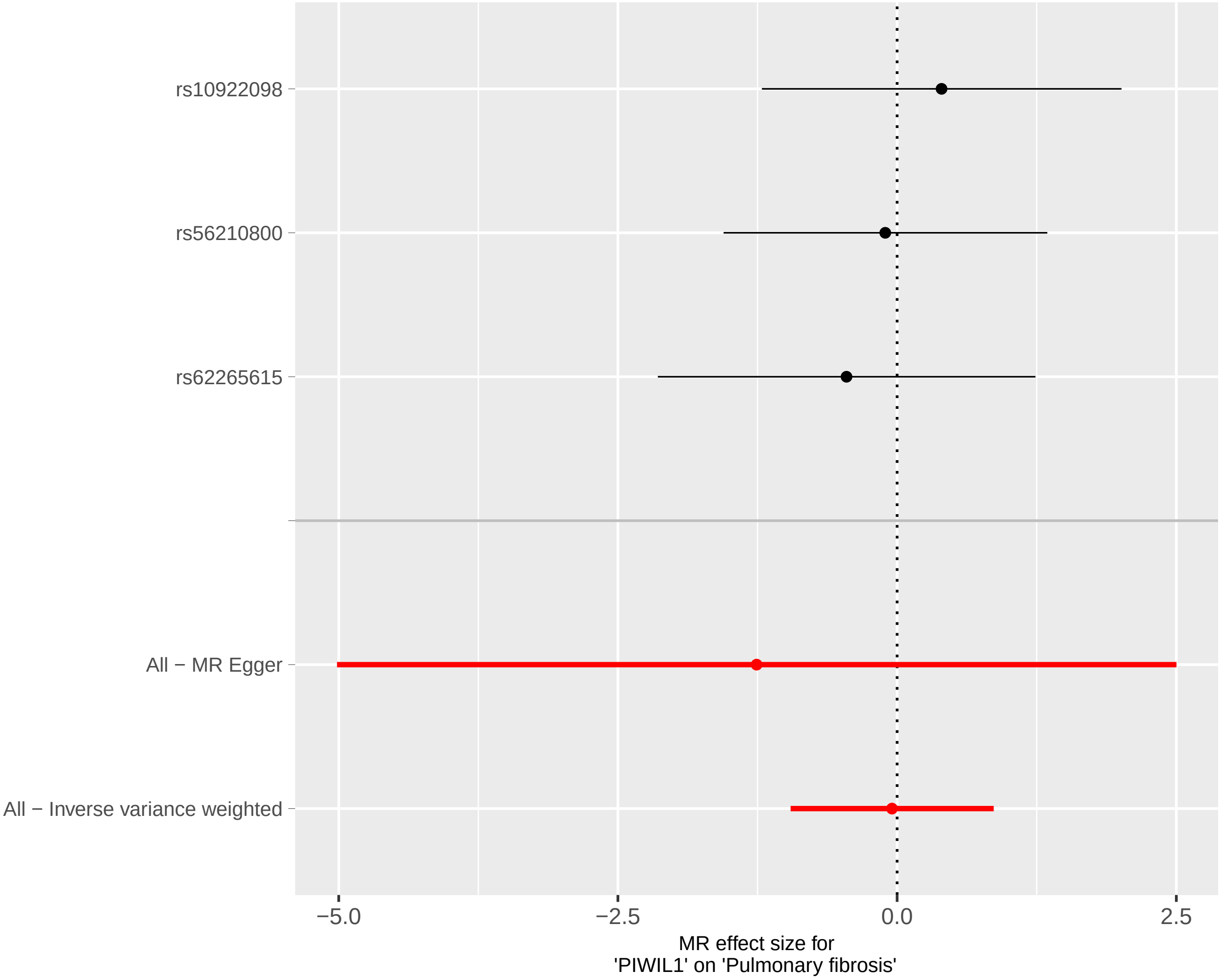
rs10922098
rs56210800
rs62265615
All − MR Egger
All − Inverse variance weighted
−5.0
−2.5
0.0
2.5
MR effect size for
'PIWIL1' on 'Pulmonary fibrosis'

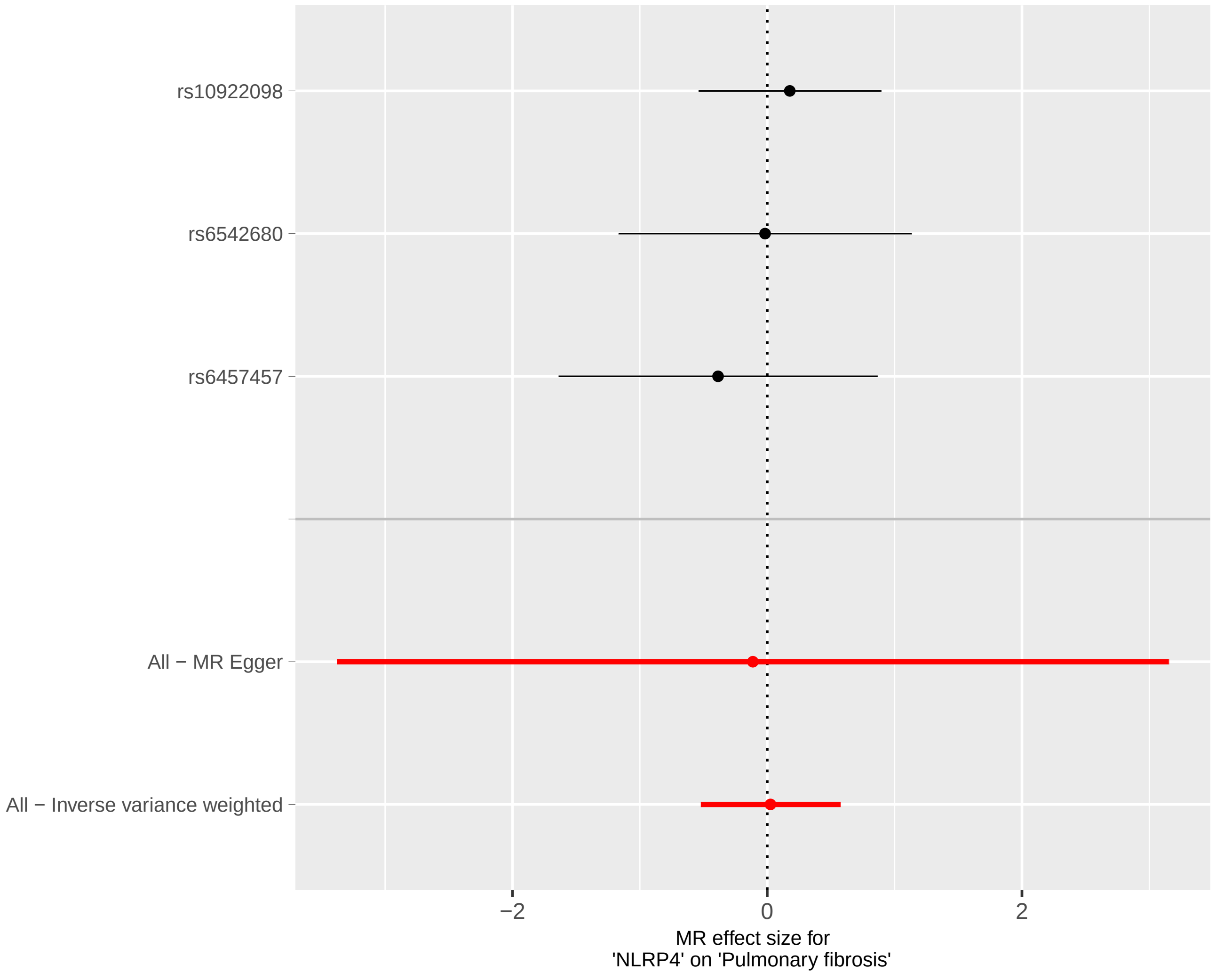
rs10922098
rs6542680
rs6457457
All − MR Egger
All − Inverse variance weighted
−2
0
2
MR effect size for
'NLRP4' on 'Pulmonary fibrosis'

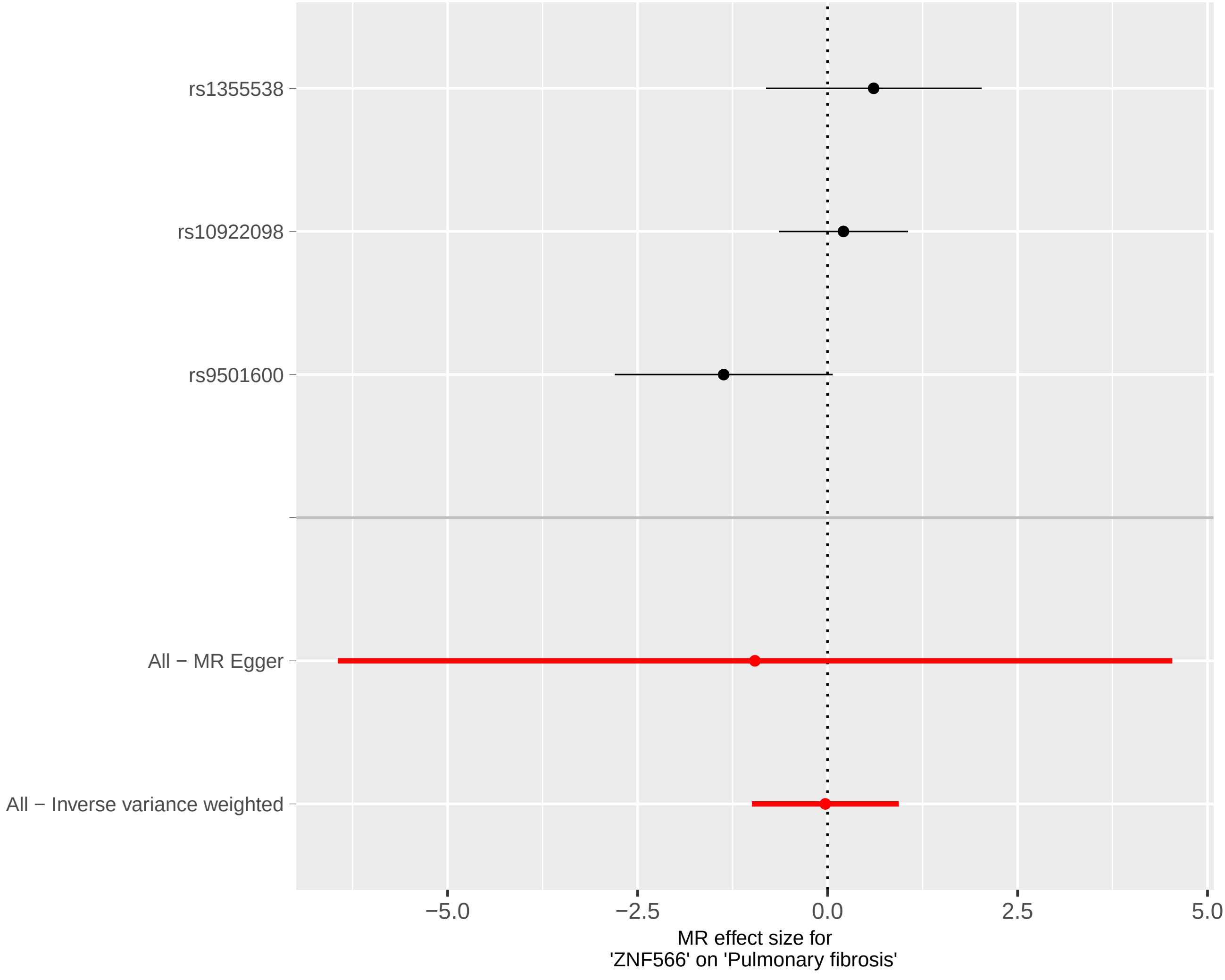

rs1355538
rs10922098
rs9501600
All − MR Egger
All − Inverse variance weighted
−5.0
−2.5
0.0
2.5
5.0
MR effect size for
'ZNF566' on 'Pulmonary fibrosis'

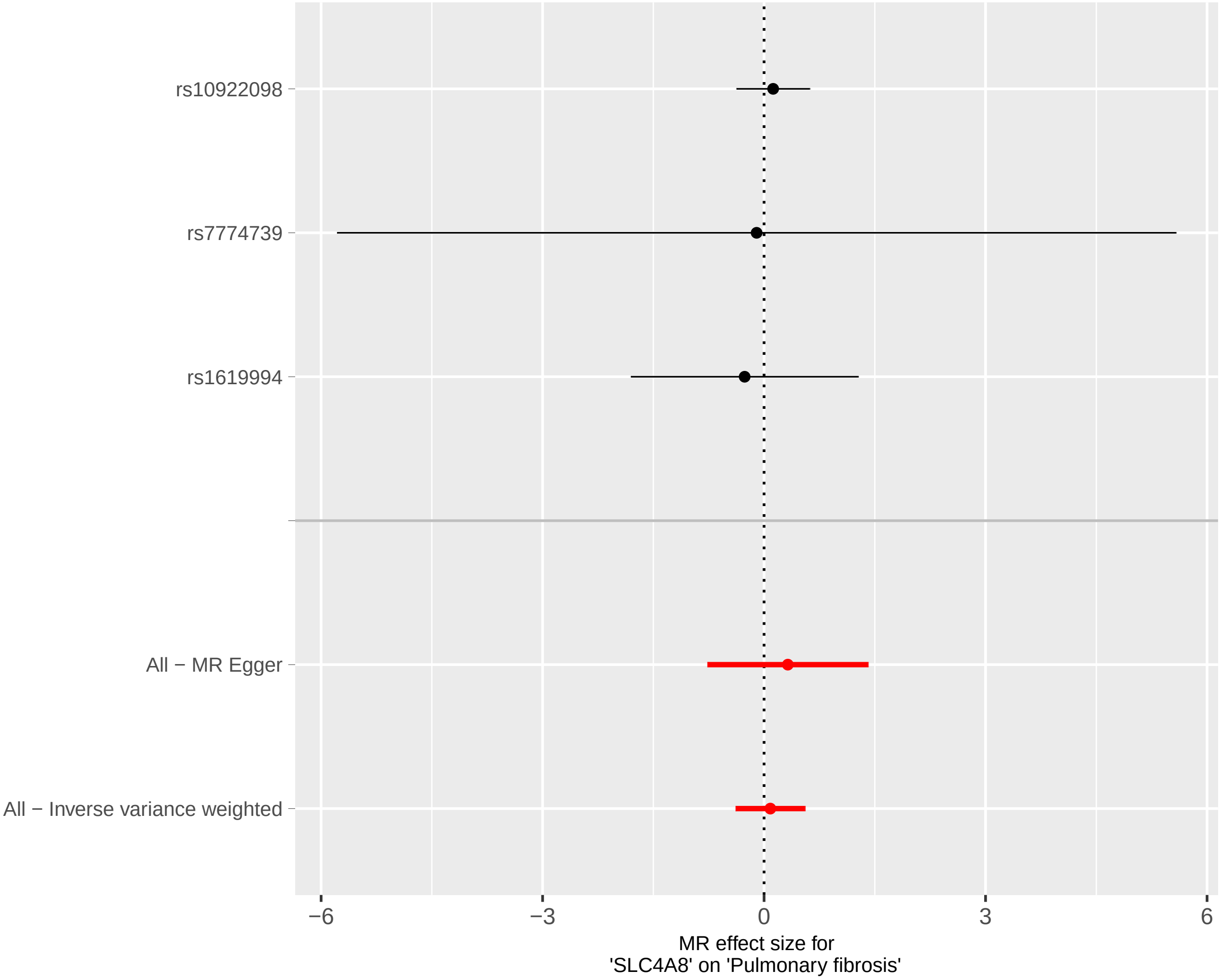
rs10922098
rs7774739
rs1619994
All − MR Egger
All − Inverse variance weighted
−6
−3
0
3
6
MR effect size for
'SLC4A8' on 'Pulmonary fibrosis'

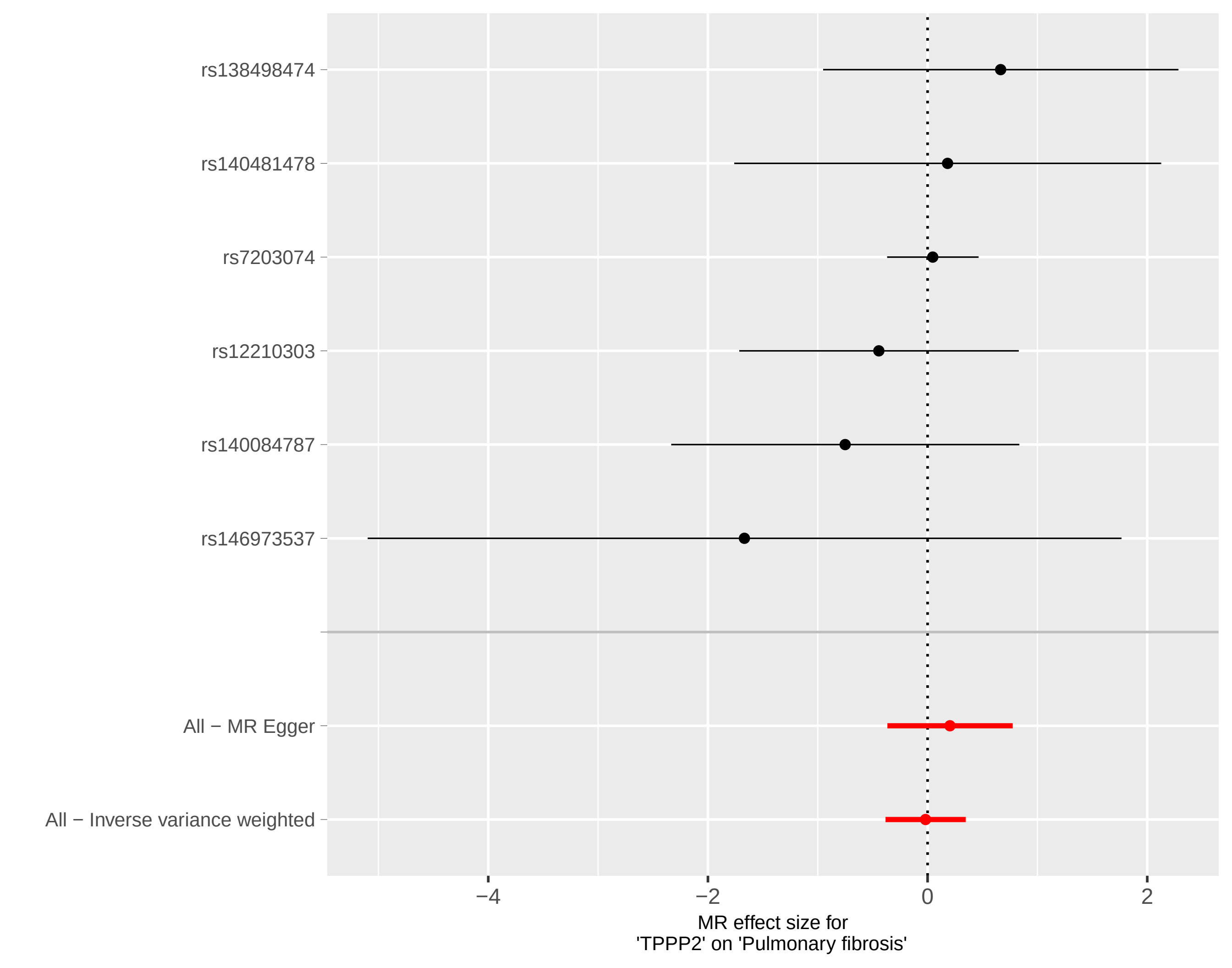
rs138498474
rs140481478
rs7203074
rs12210303
rs140084787
rs146973537
All − MR Egger
All − Inverse variance weighted
−4
−2
0
2
MR effect size for
'TPPP2' on 'Pulmonary fibrosis'

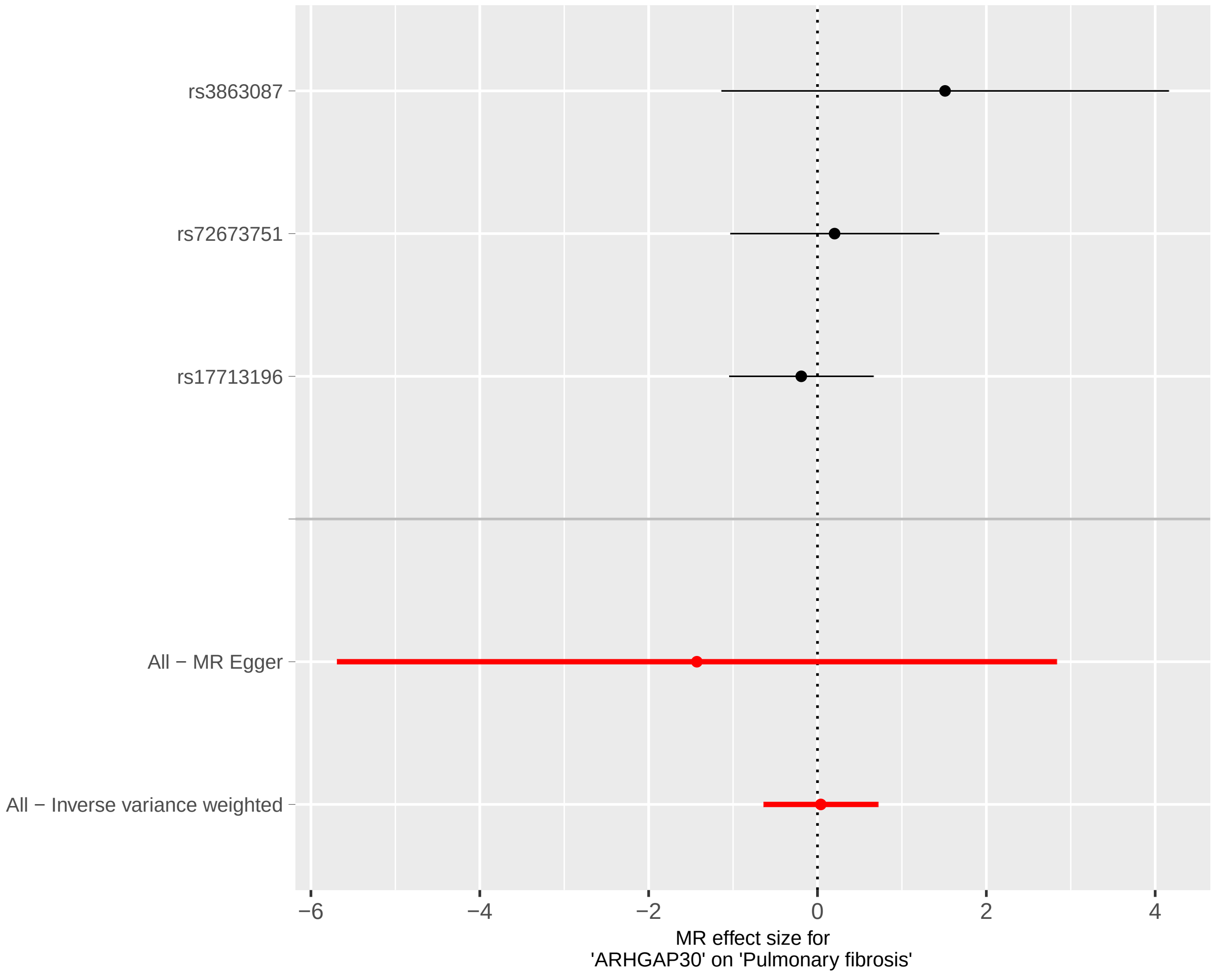

rs3863087
rs72673751
rs17713196
All − MR Egger
All − Inverse variance weighted
−6
−4
−2
0
2
4
MR effect size for
'ARHGAP30' on 'Pulmonary fibrosis'

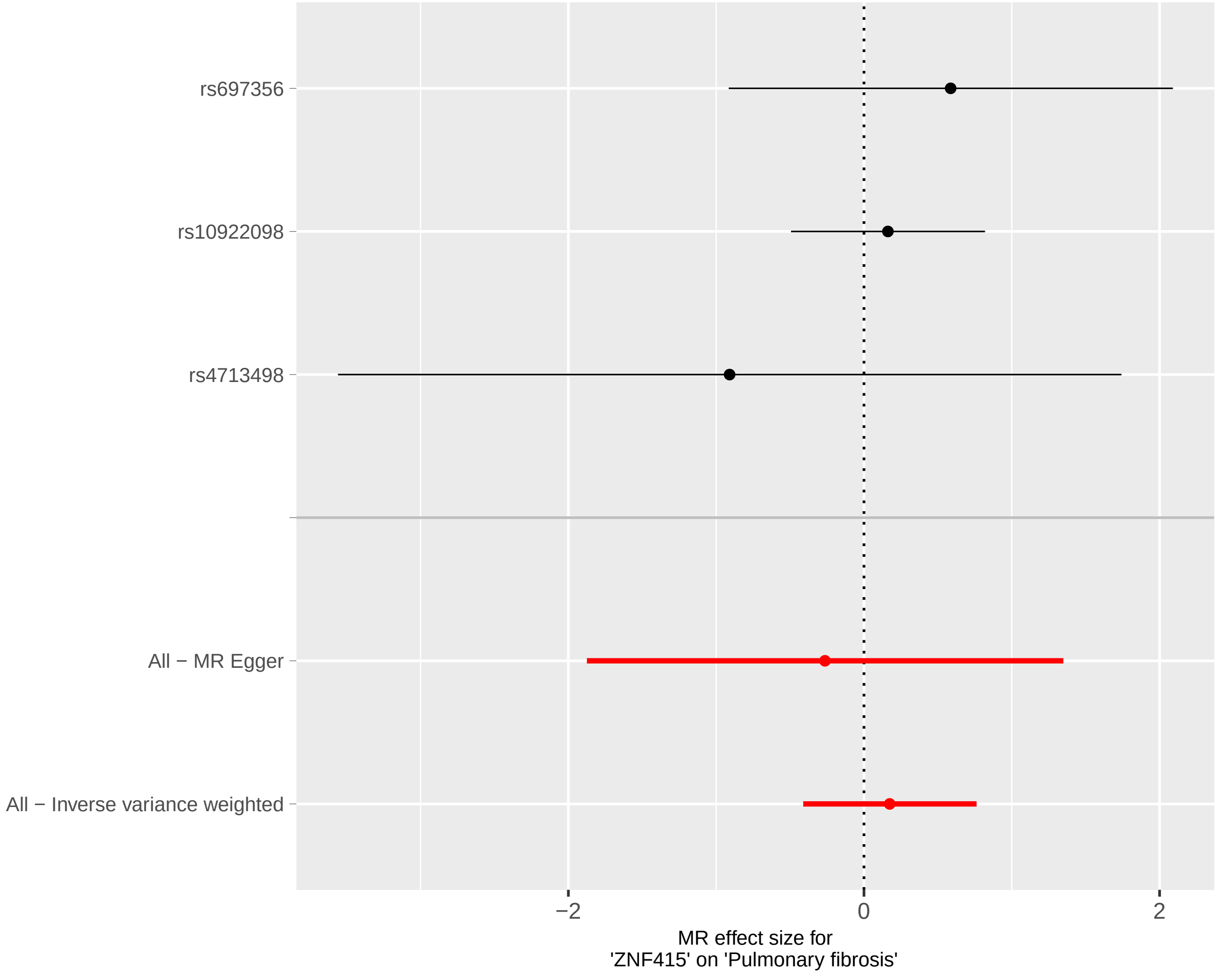
rs697356
rs10922098
rs4713498
All − MR Egger
All − Inverse variance weighted
−2
0
2
MR effect size for
'ZNF415' on 'Pulmonary fibrosis'

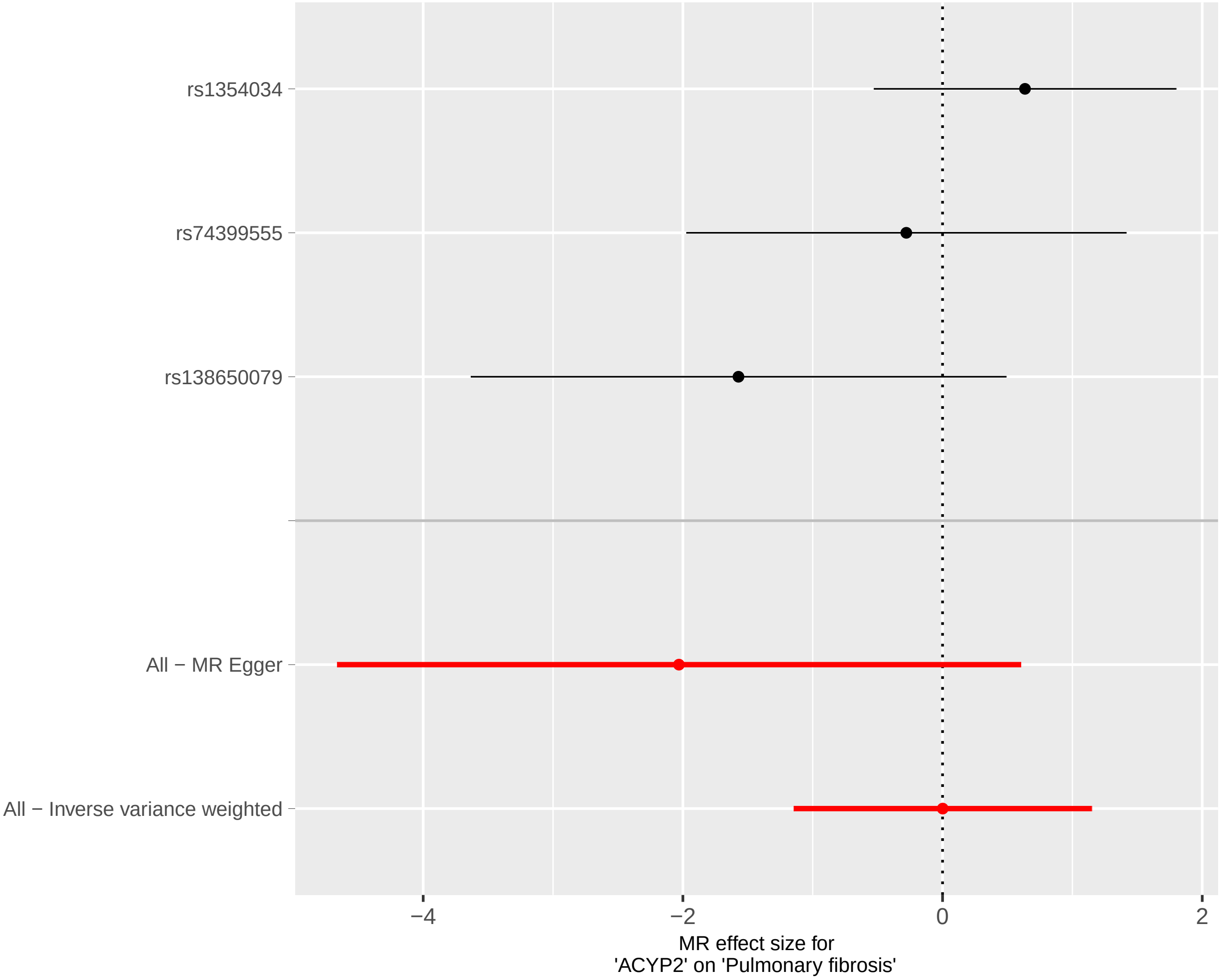

rs1354034
rs74399555
rs138650079
All − MR Egger
All − Inverse variance weighted
−4
−2
0
2
MR effect size for
'ACYP2' on 'Pulmonary fibrosis'

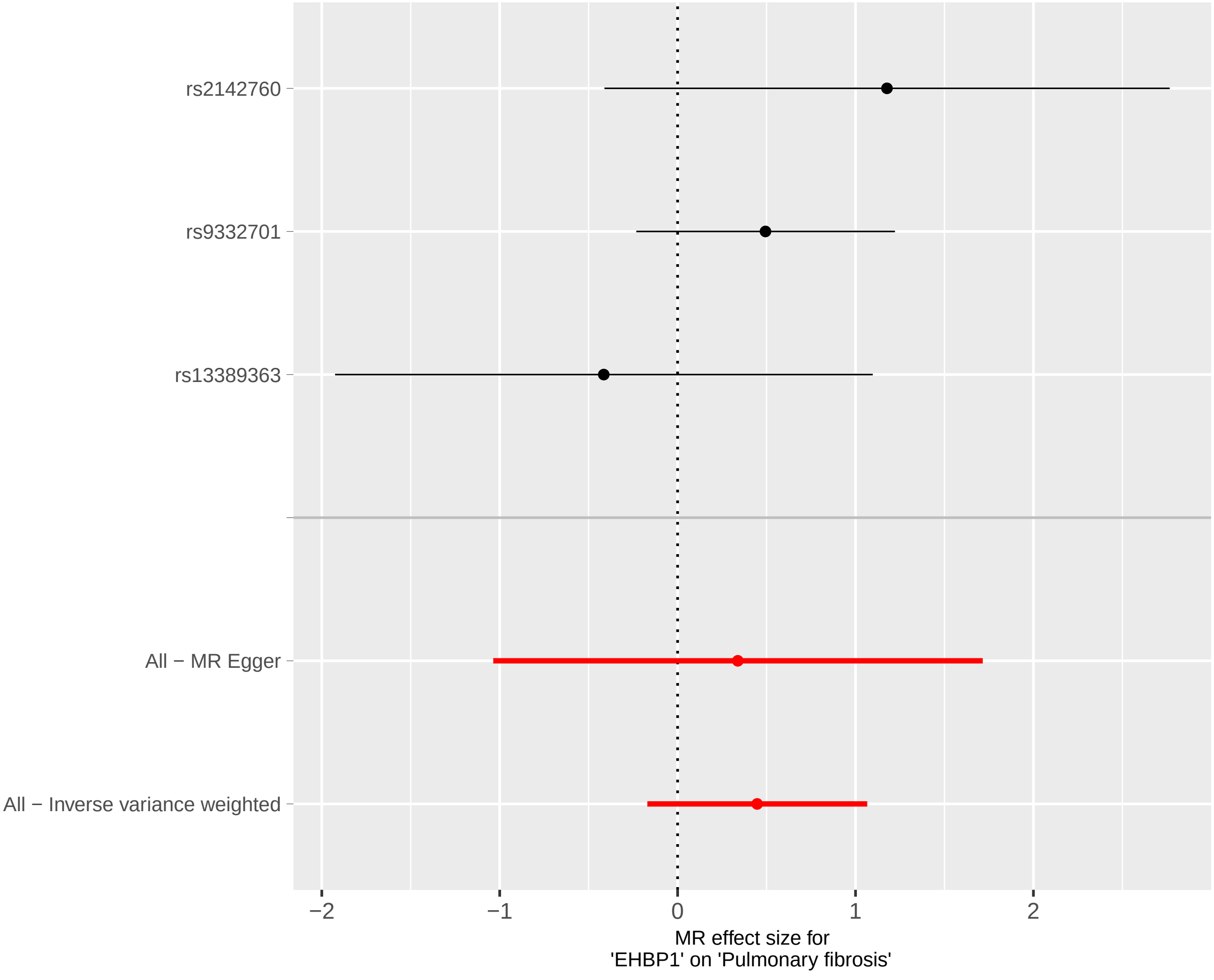

rs2142760
rs9332701
rs13389363
All − MR Egger
All − Inverse variance weighted
−2
−1
0
1
2
MR effect size for
'EHBP1' on 'Pulmonary fibrosis'

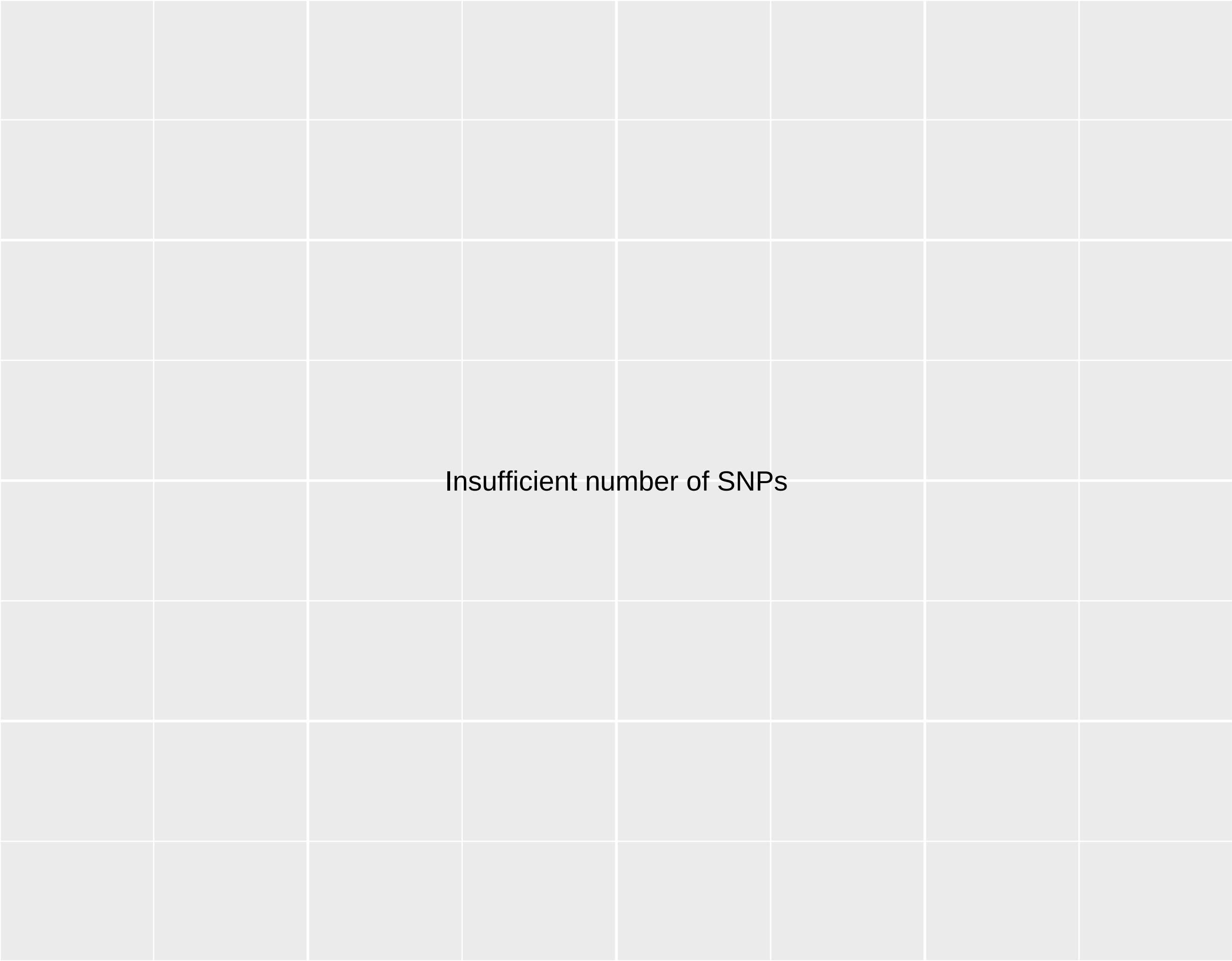
Insufficient number of SNPs

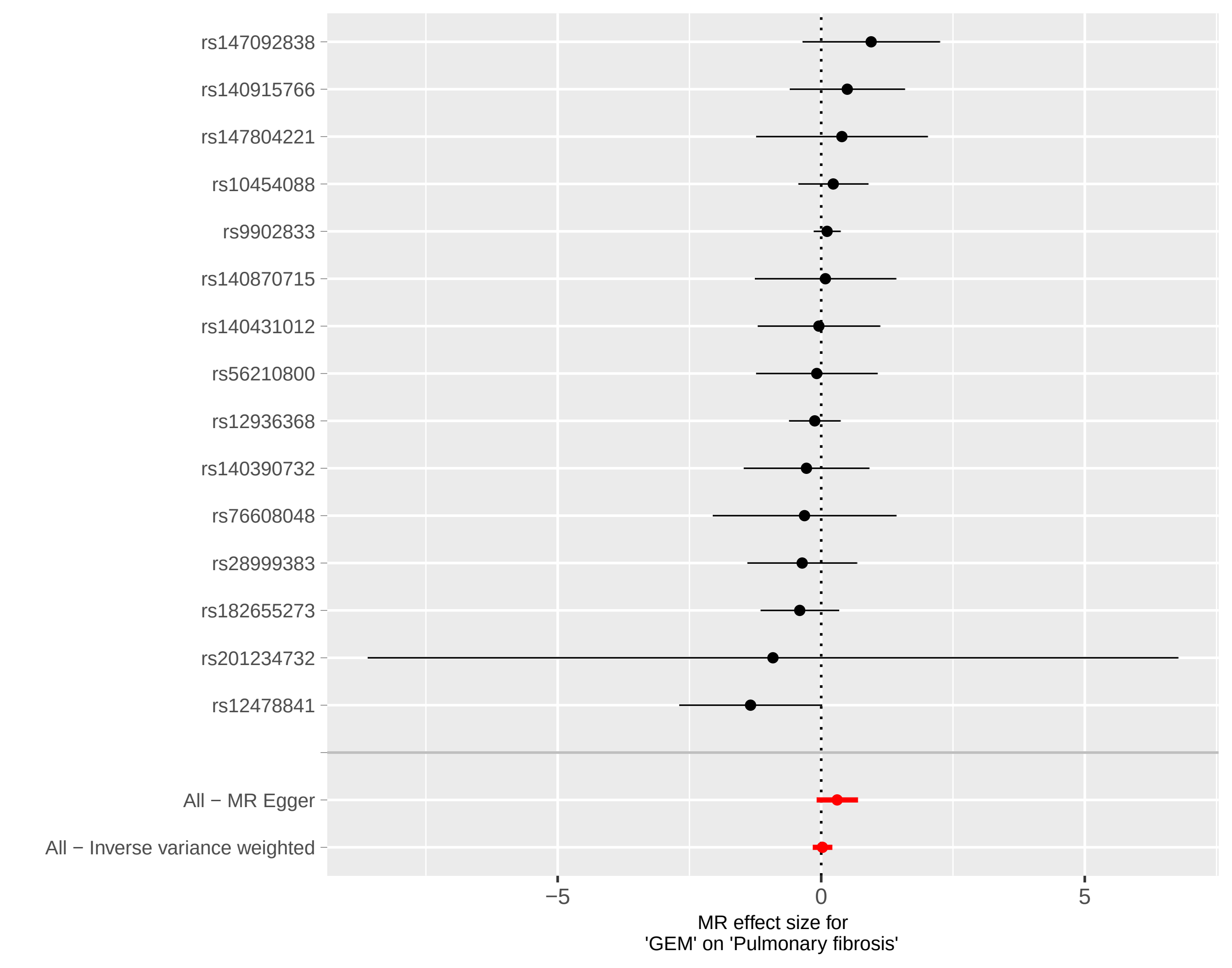
rs147092838
rs140915766
rs147804221
rs10454088
rs9902833
rs140870715
rs140431012
rs56210800
rs12936368
rs140390732
rs76608048
rs28999383
rs182655273
rs201234732
rs12478841
All − MR Egger
All − Inverse variance weighted
−5
0
5
MR effect size for
'GEM' on 'Pulmonary fibrosis'

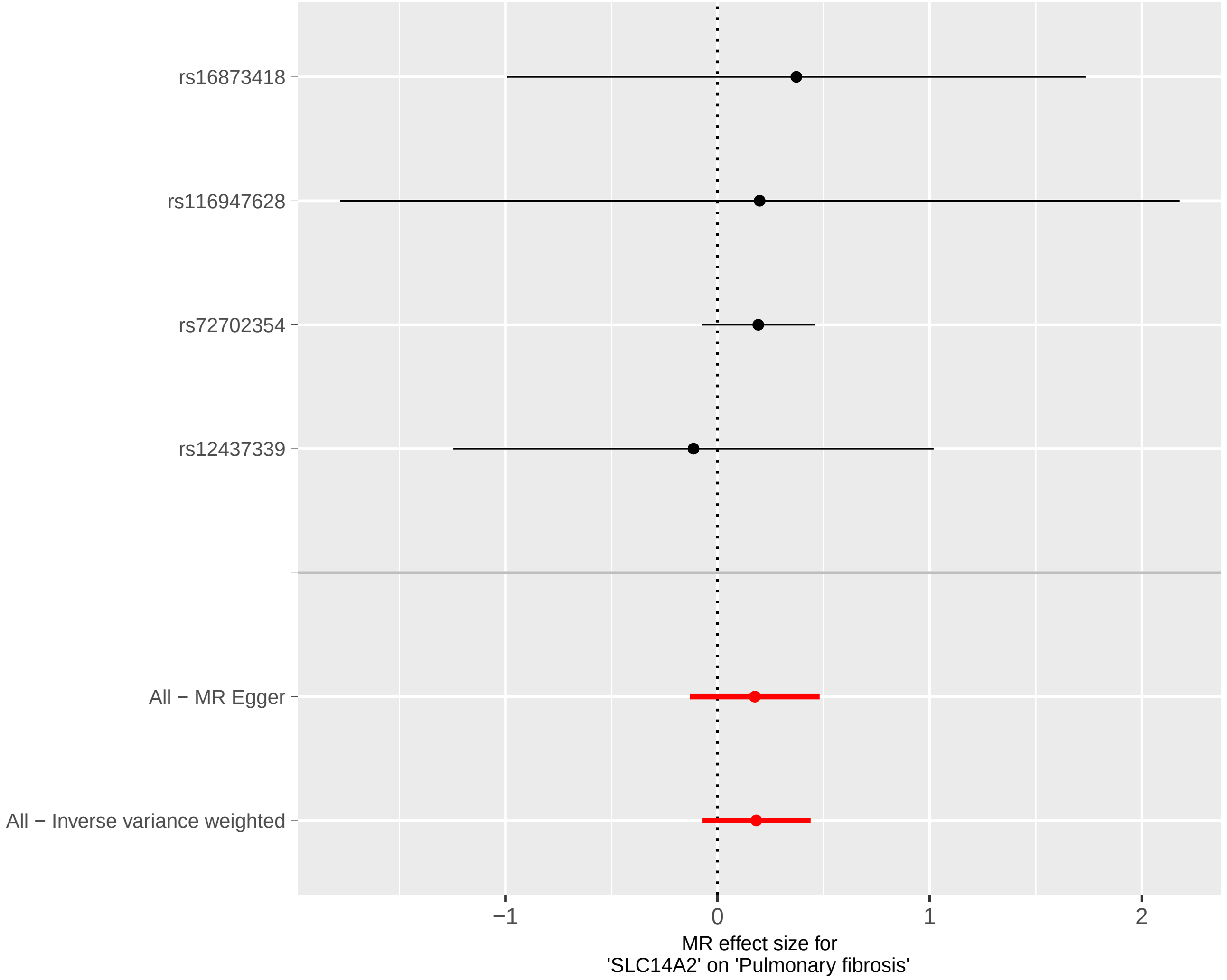

rs16873418
rs116947628
rs72702354
rs12437339
All − MR Egger
All − Inverse variance weighted
−1
0
1
2
MR effect size for
'SLC14A2' on 'Pulmonary fibrosis'

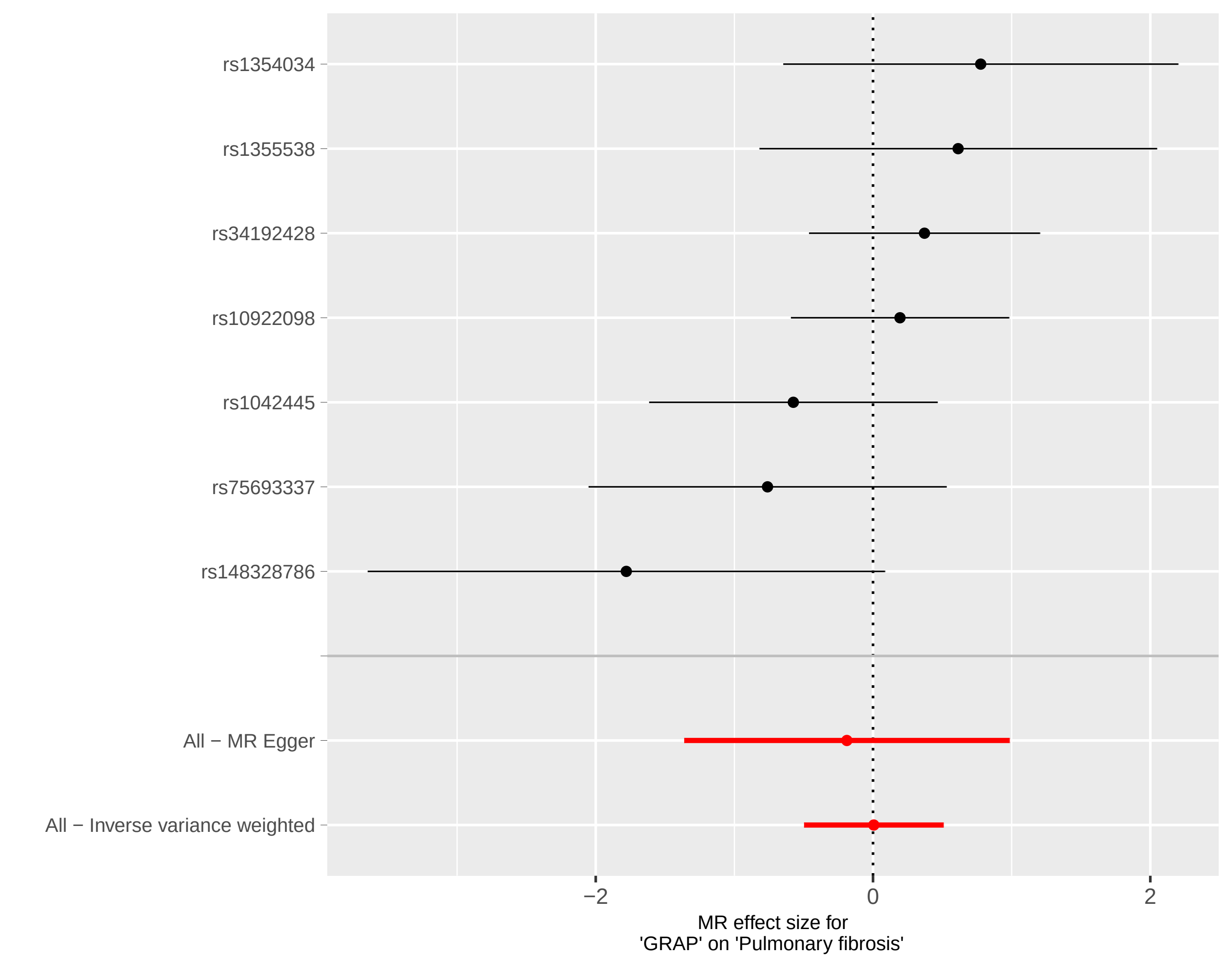
rs1354034
rs1355538
rs34192428
rs10922098
rs1042445
rs75693337
rs148328786
All − MR Egger
All − Inverse variance weighted
−2
0
2
MR effect size for
'GRAP' on 'Pulmonary fibrosis'

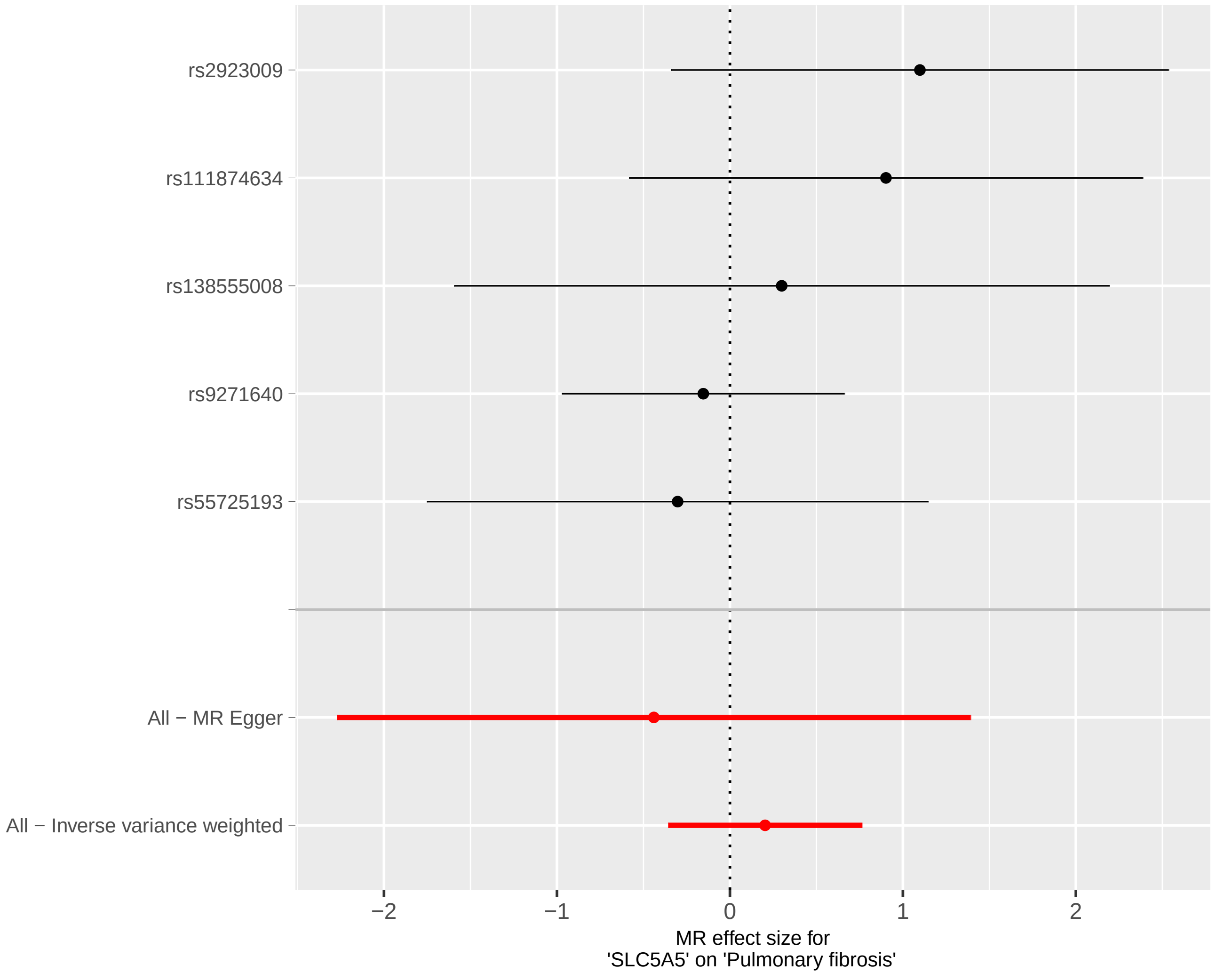
rs2923009
rs111874634
rs138555008
rs9271640
rs55725193
All − MR Egger
All − Inverse variance weighted
−2
−1
0
1
2
MR effect size for
'SLC5A5' on 'Pulmonary fibrosis'

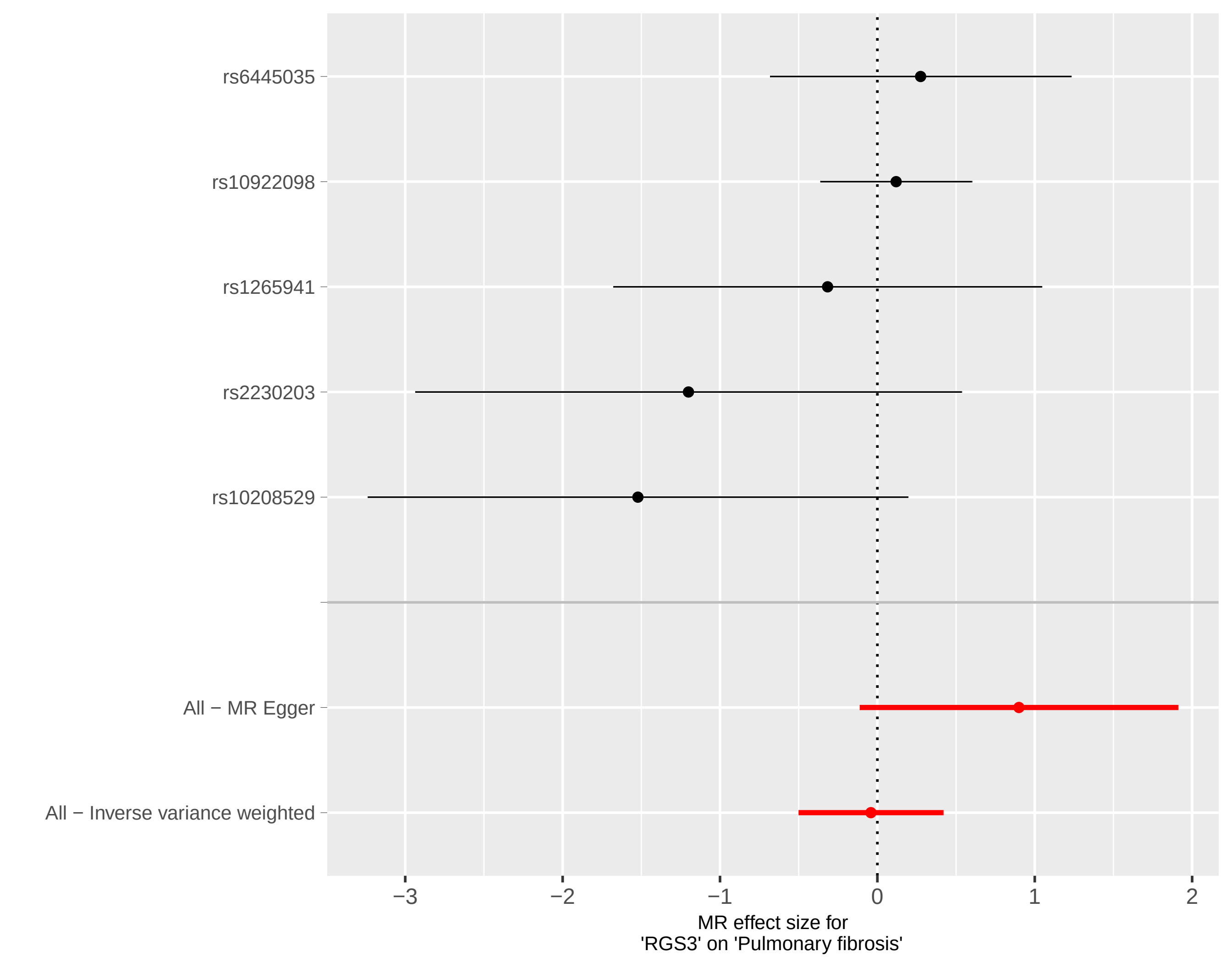
rs6445035
rs10922098
rs1265941
rs2230203
rs10208529
All − MR Egger
All − Inverse variance weighted
−3
−2
−1
0
1
2
MR effect size for
'RGS3' on 'Pulmonary fibrosis'

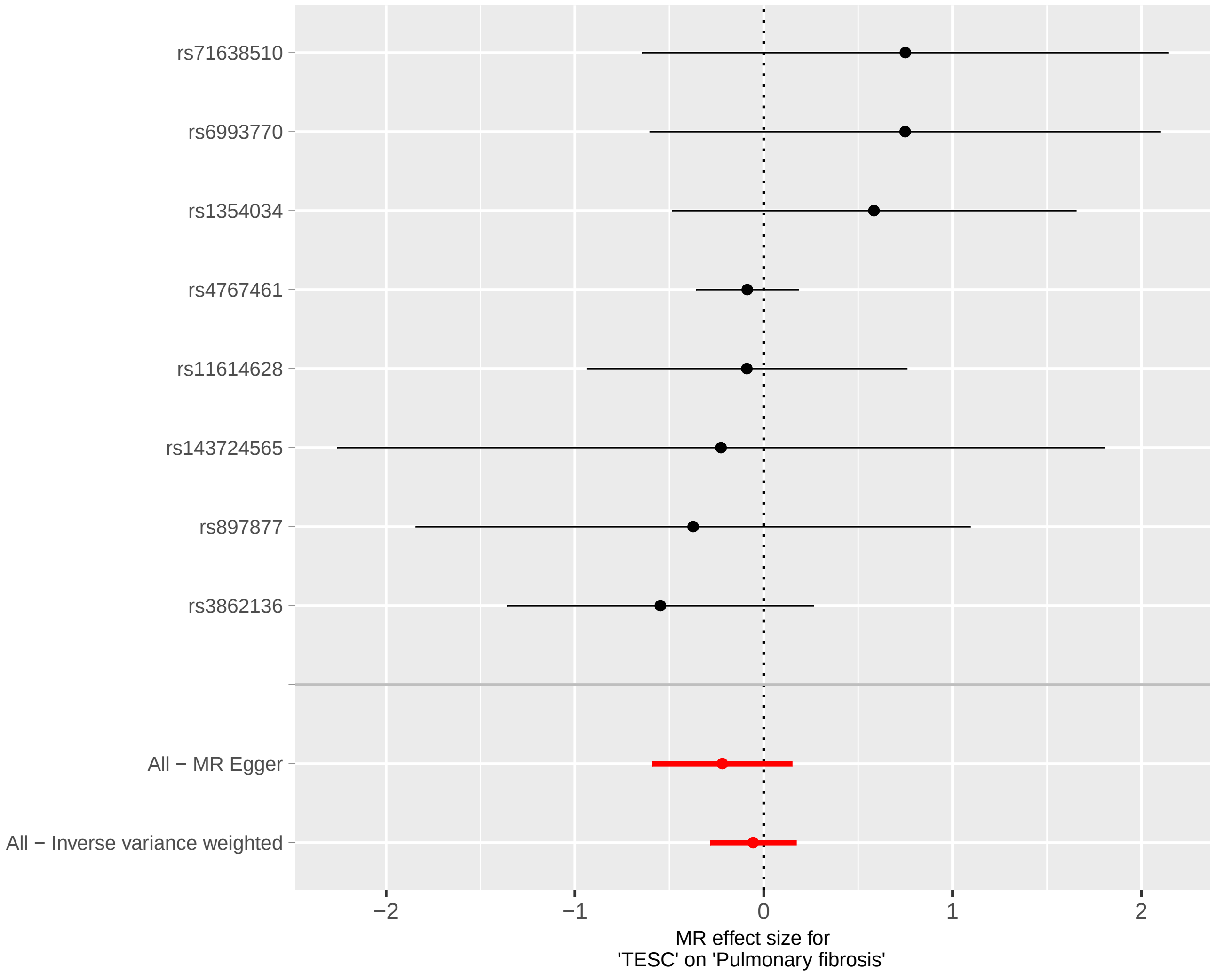

rs71638510
rs6993770
rs1354034
rs4767461
rs11614628
rs143724565
rs897877
rs3862136
All − MR Egger
All − Inverse variance weighted
−2
−1
0
1
2
MR effect size for
'TESC' on 'Pulmonary fibrosis'

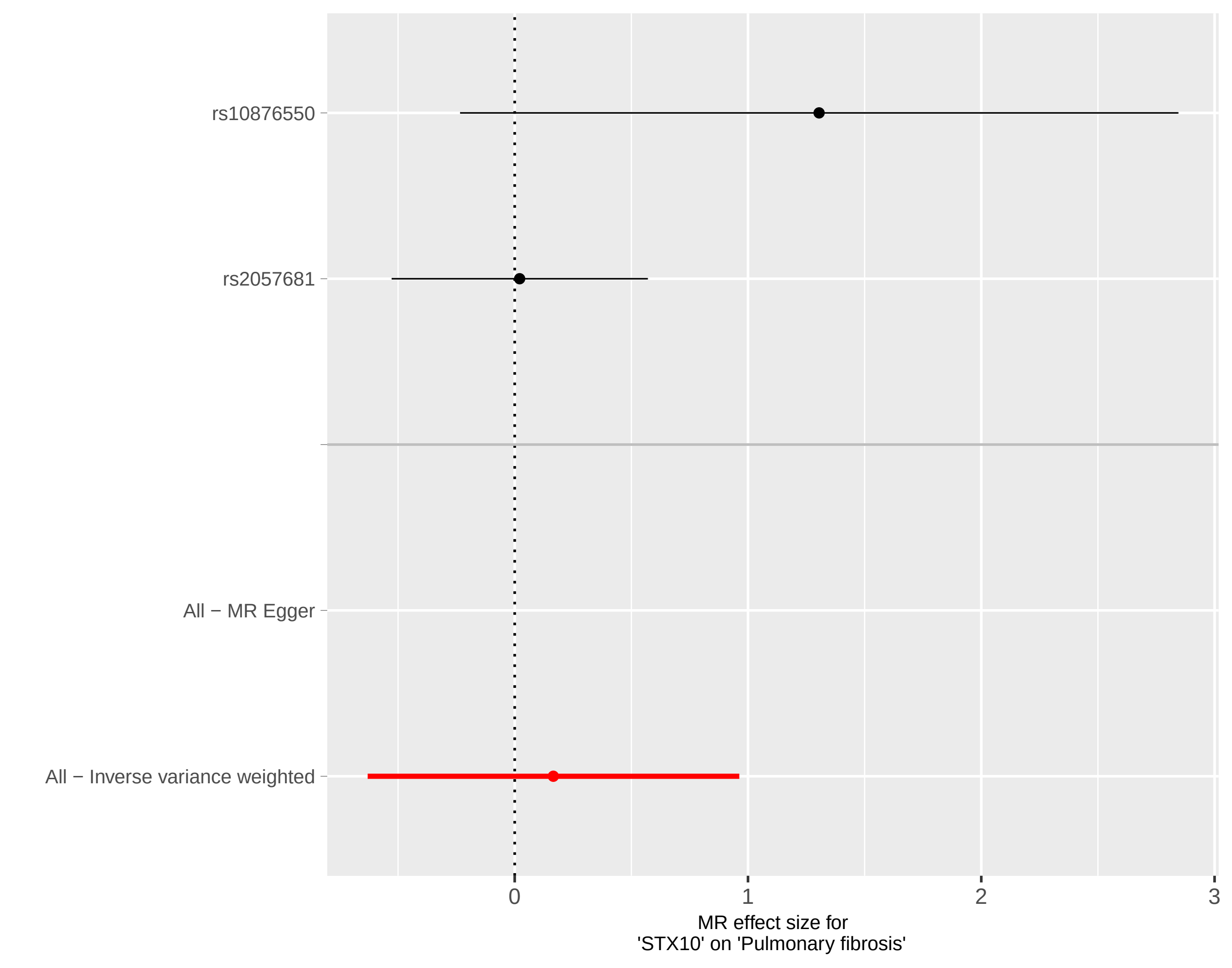
rs10876550
rs2057681
All − MR Egger
All − Inverse variance weighted
0
1
2
3
MR effect size for
'STX10' on 'Pulmonary fibrosis'

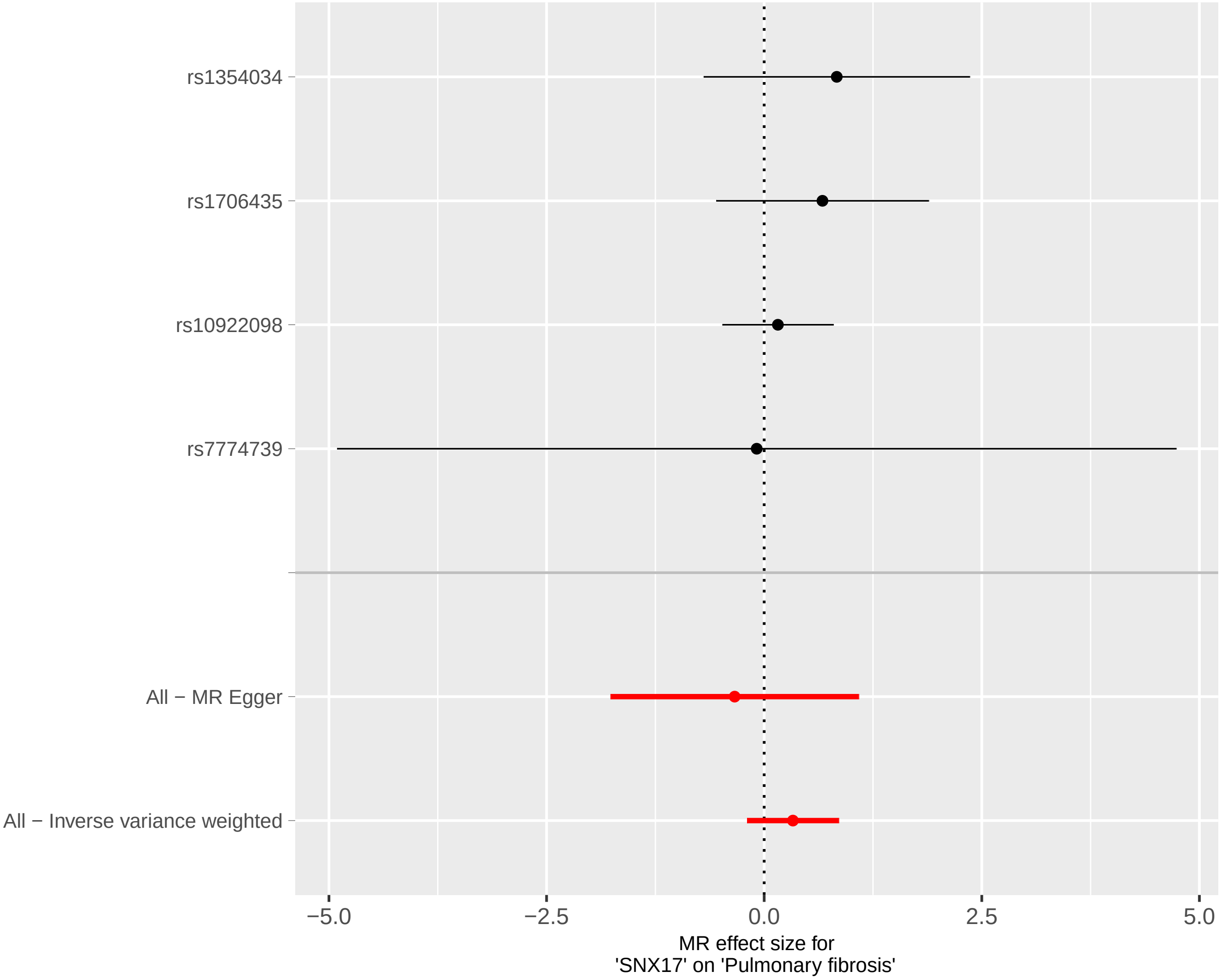
rs1354034
rs1706435
rs10922098
rs7774739
All − MR Egger
All − Inverse variance weighted
−5.0
−2.5
0.0
2.5
5.0
MR effect size for
'SNX17' on 'Pulmonary fibrosis'

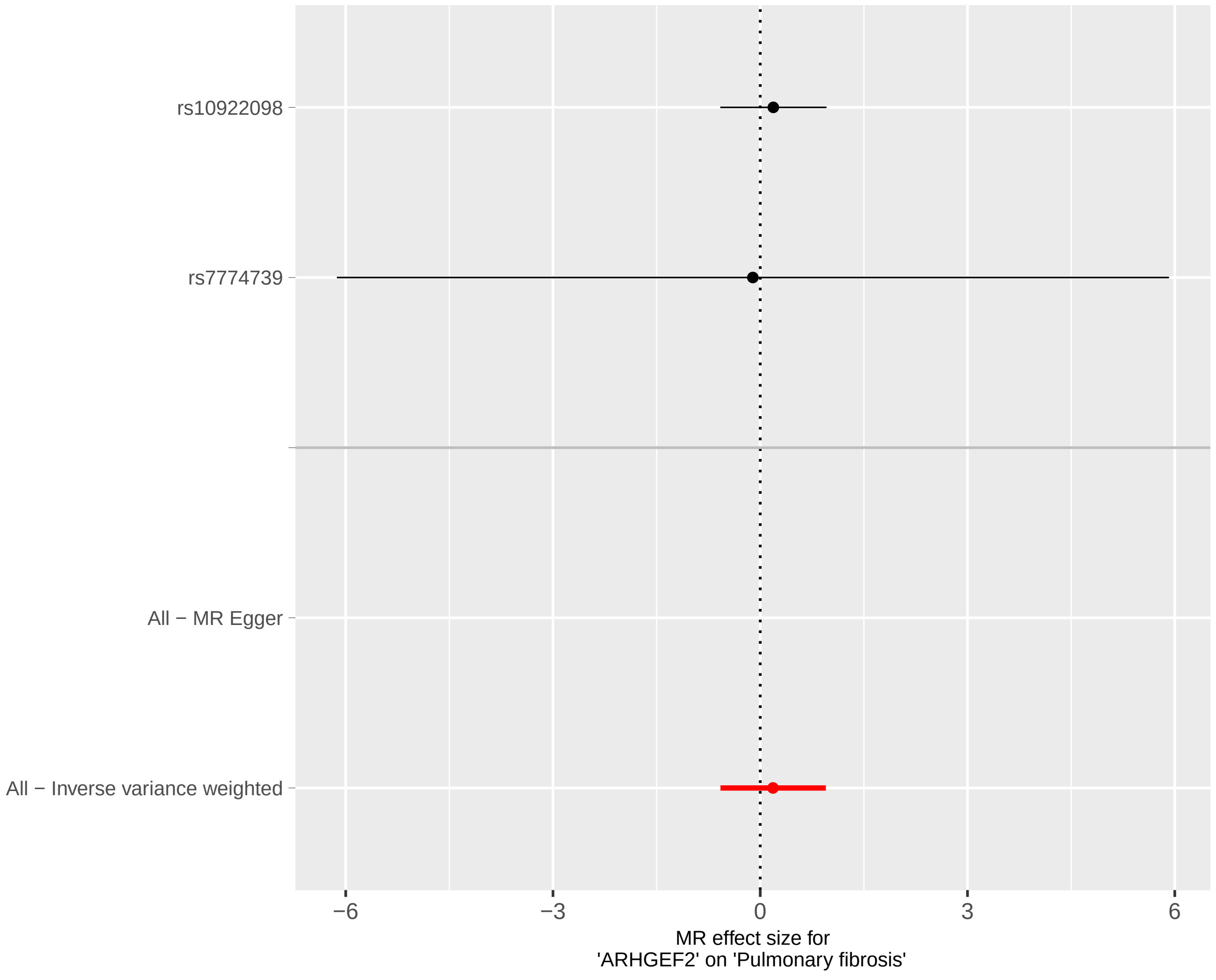

rs10922098
rs7774739
All − MR Egger
All − Inverse variance weighted
−6
−3
0
3
6
MR effect size for
'ARHGEF2' on 'Pulmonary fibrosis'

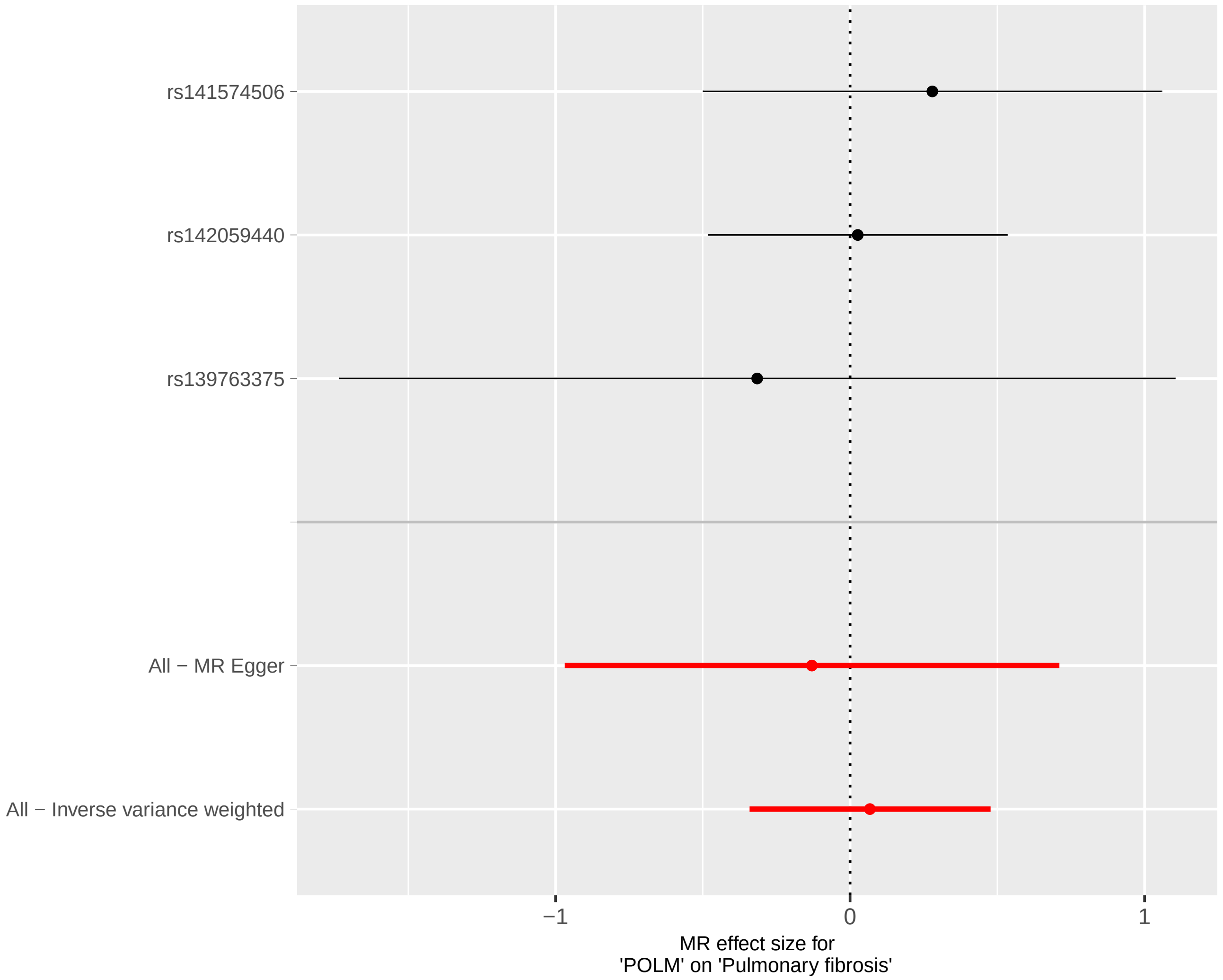
rs141574506
rs142059440
rs139763375
All − MR Egger
All − Inverse variance weighted
−1
0
1
MR effect size for
'POLM' on 'Pulmonary fibrosis'

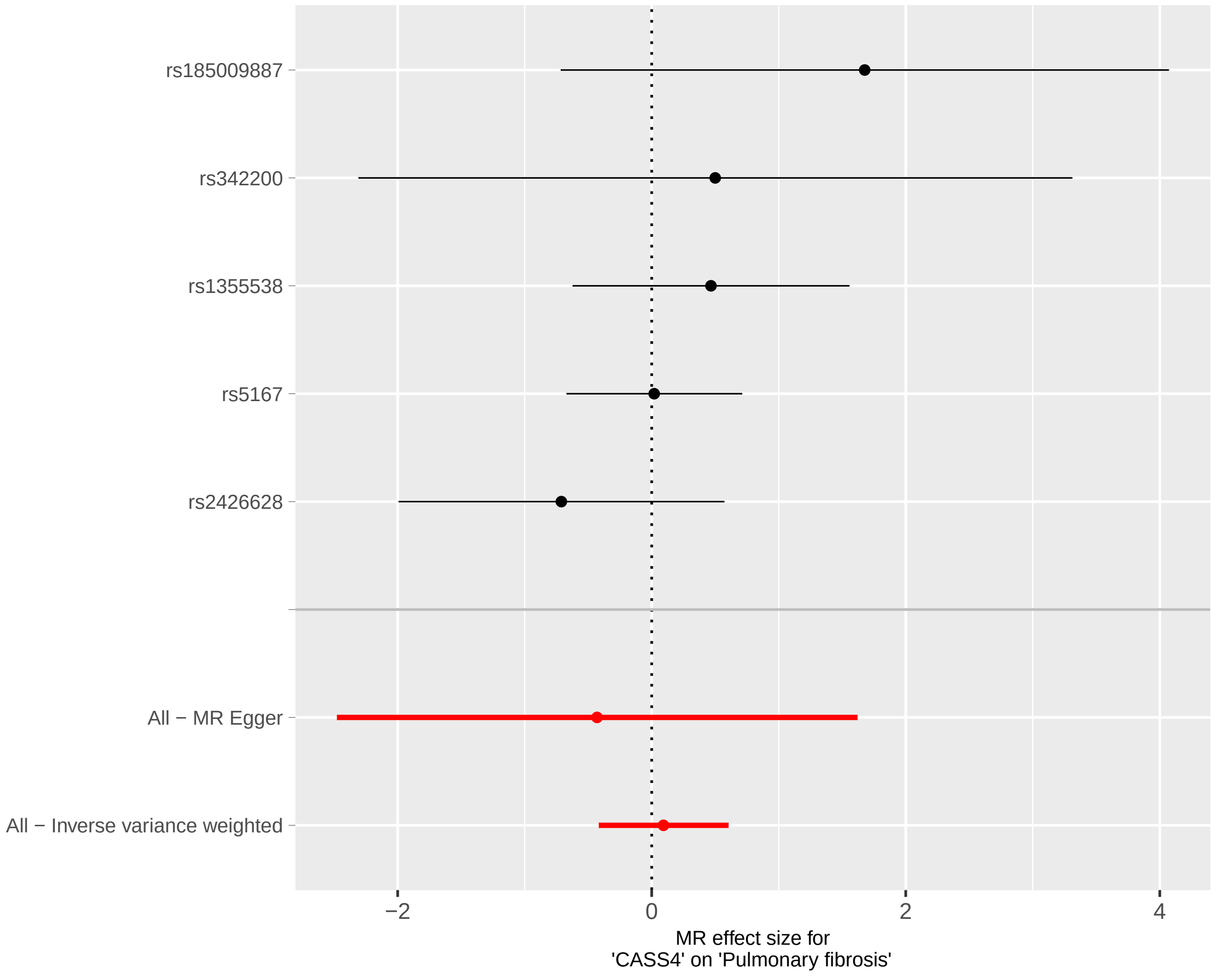

rs185009887
rs342200
rs1355538
rs5167
rs2426628
All − MR Egger
All − Inverse variance weighted
−2
0
2
4
MR effect size for
'CASS4' on 'Pulmonary fibrosis'

Insufficient number of SNPs

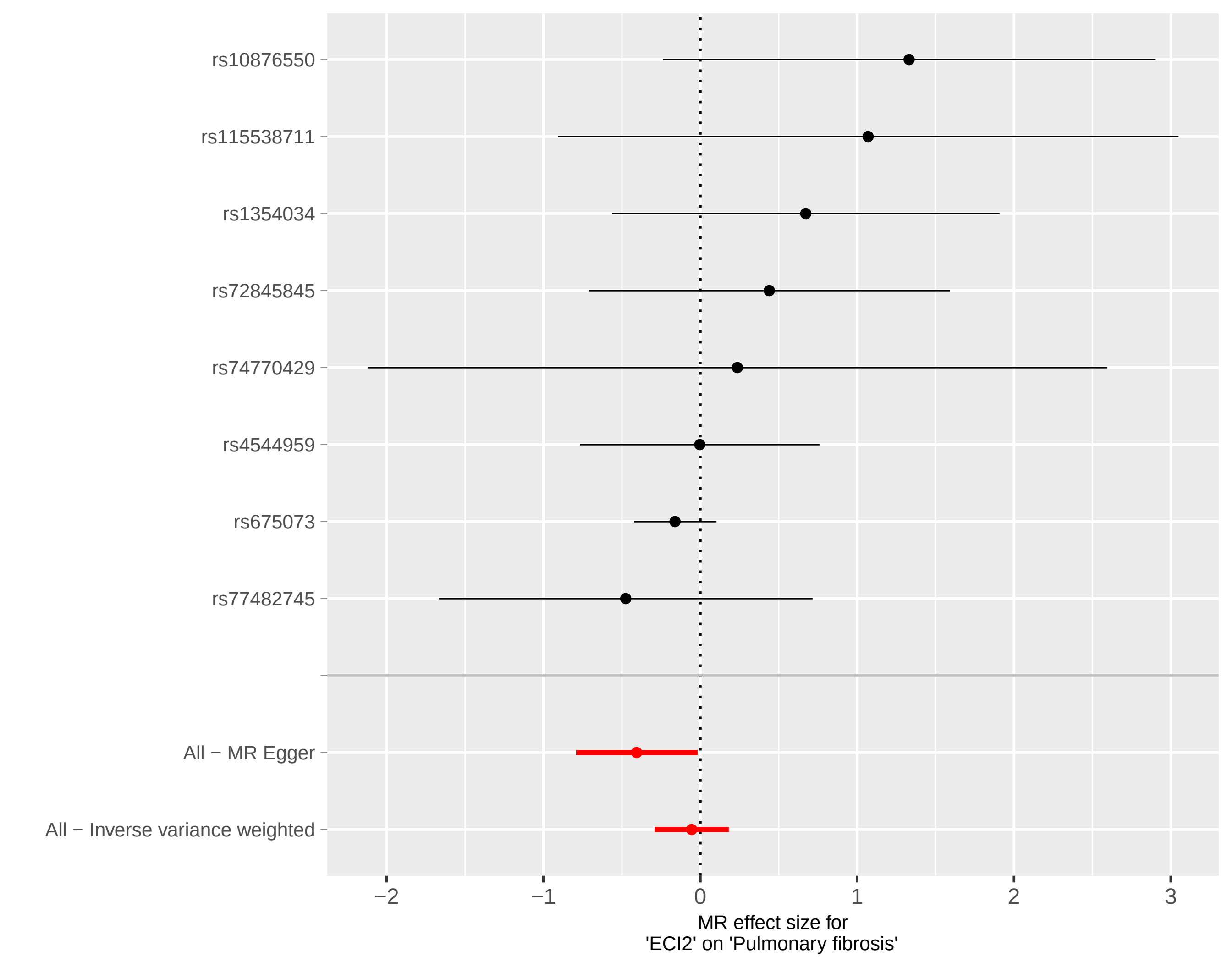
rs10876550
rs115538711
rs1354034
rs72845845
rs74770429
rs4544959
rs675073
rs77482745
All − MR Egger
All − Inverse variance weighted
−2
−1
0
1
2
3
MR effect size for
'ECI2' on 'Pulmonary fibrosis'

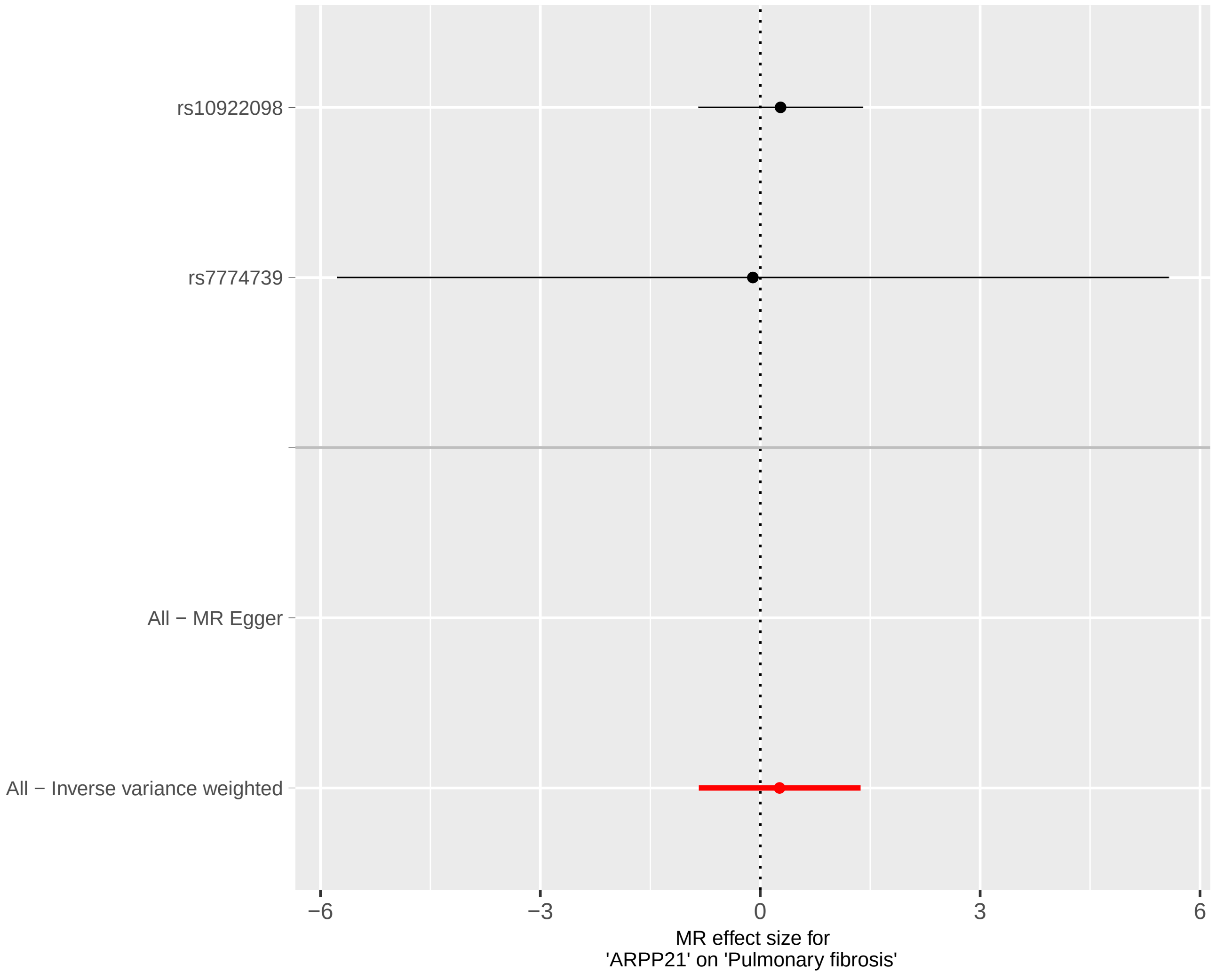
rs10922098
rs7774739
All − MR Egger
All − Inverse variance weighted
−6
−3
0
3
6
MR effect size for
'ARPP21' on 'Pulmonary fibrosis'

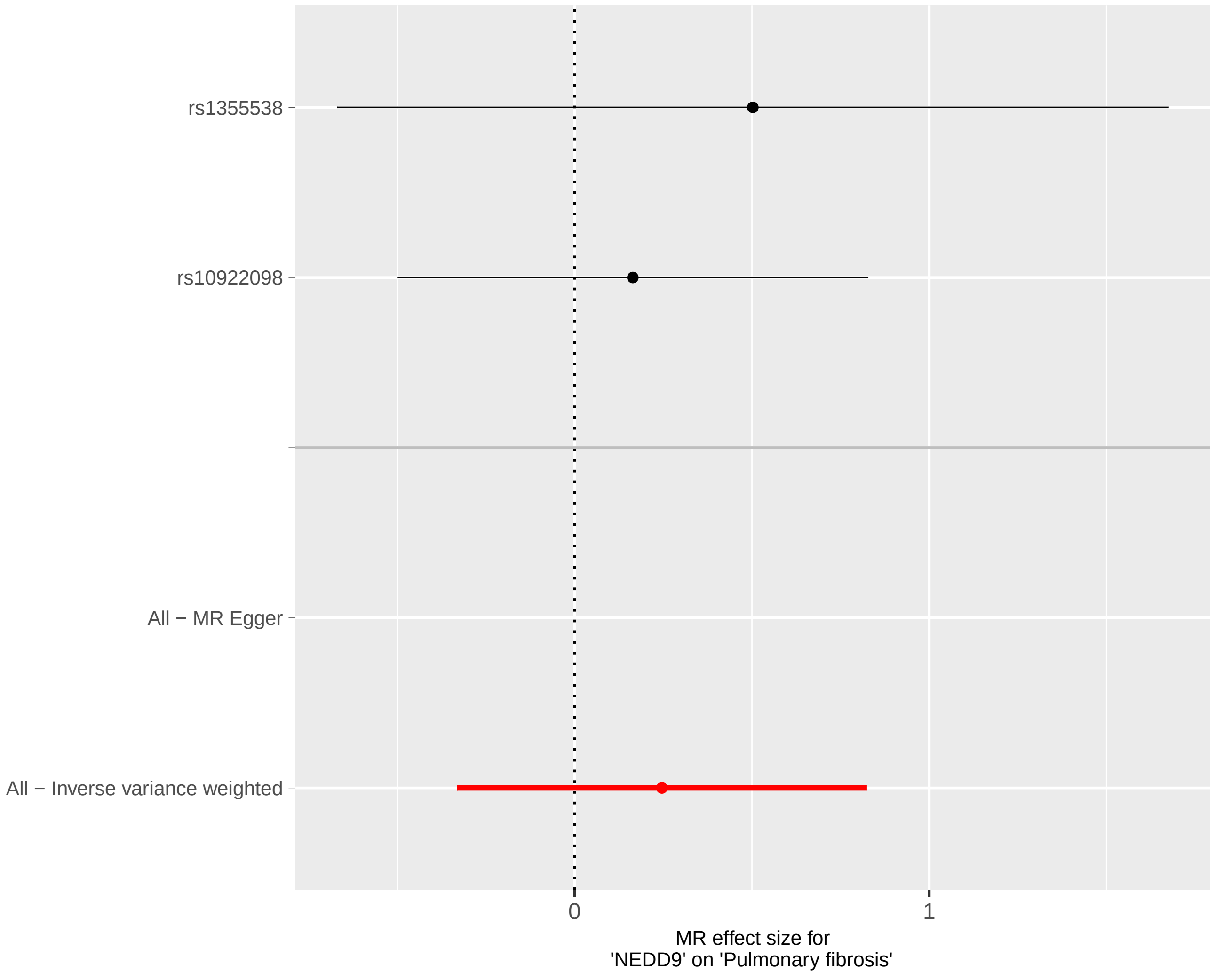
rs1355538
rs10922098
All − MR Egger
All − Inverse variance weighted
0
1
MR effect size for
'NEDD9' on 'Pulmonary fibrosis'

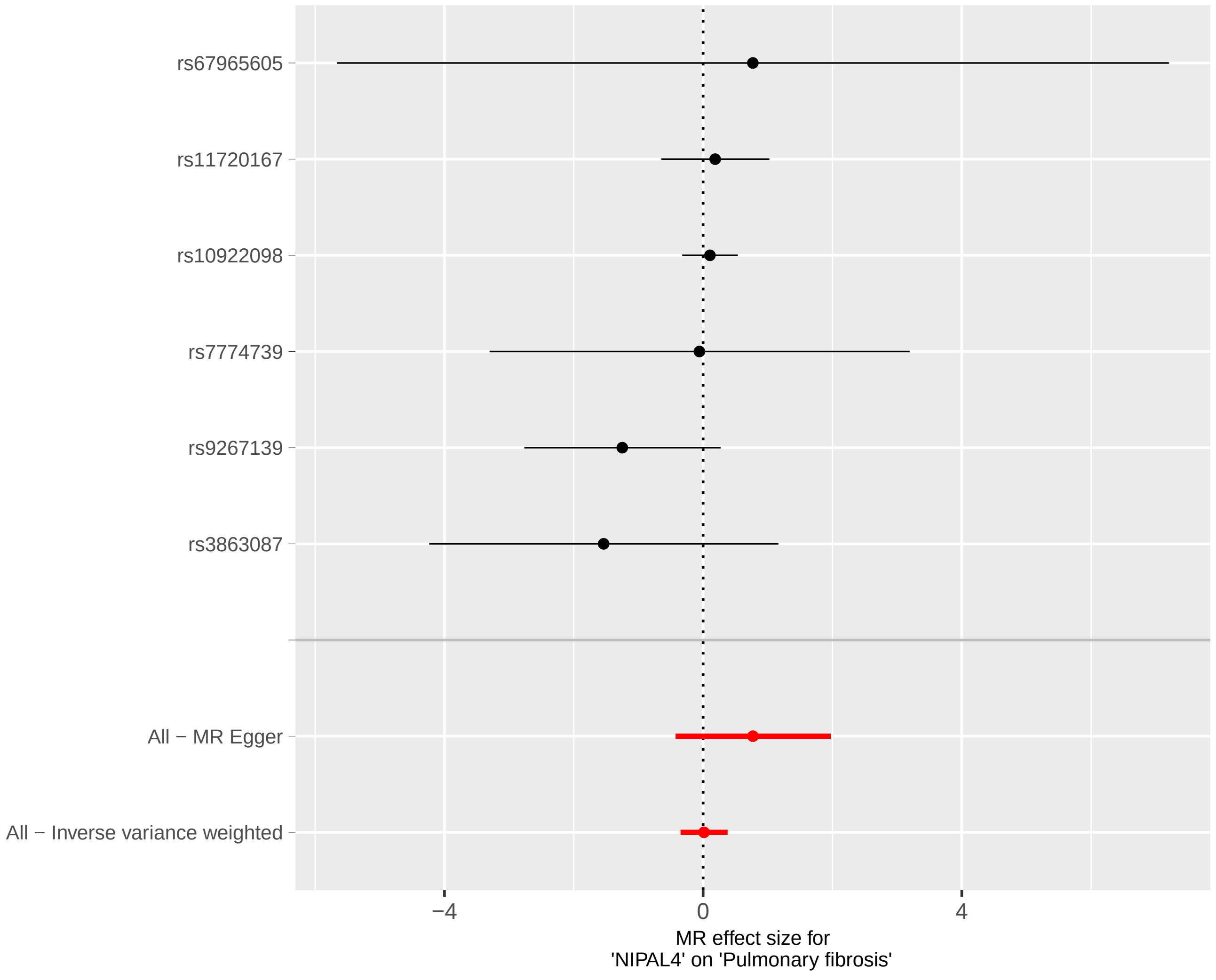

rs67965605
rs11720167
rs10922098
rs7774739
rs9267139
rs3863087
All − MR Egger
All − Inverse variance weighted
−4
0
4
MR effect size for
'NIPAL4' on 'Pulmonary fibrosis'

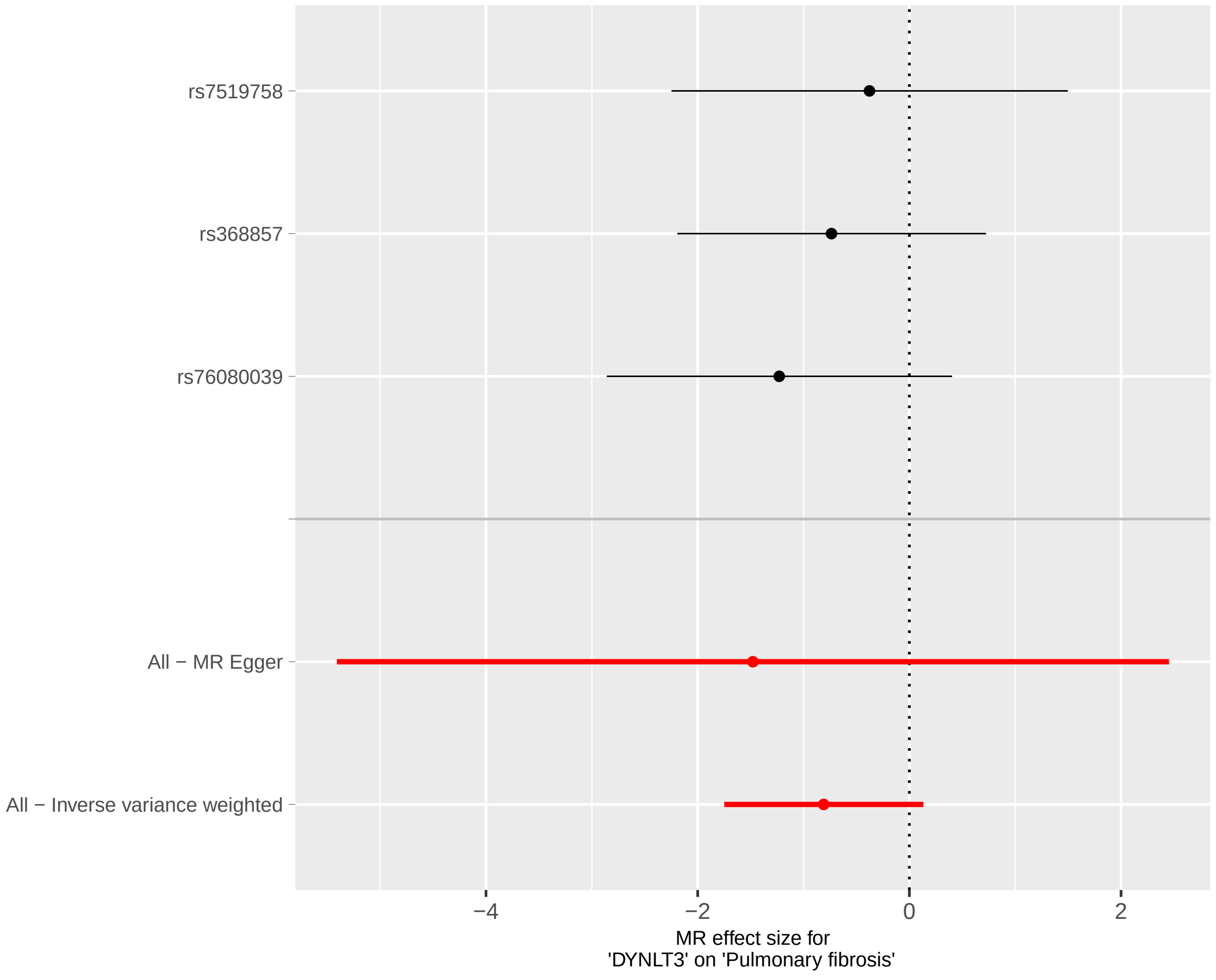
rs7519758
rs368857
rs76080039
All − MR Egger
All − Inverse variance weighted
−4
−2
0
2
MR effect size for
'DYNLT3' on 'Pulmonary fibrosis'

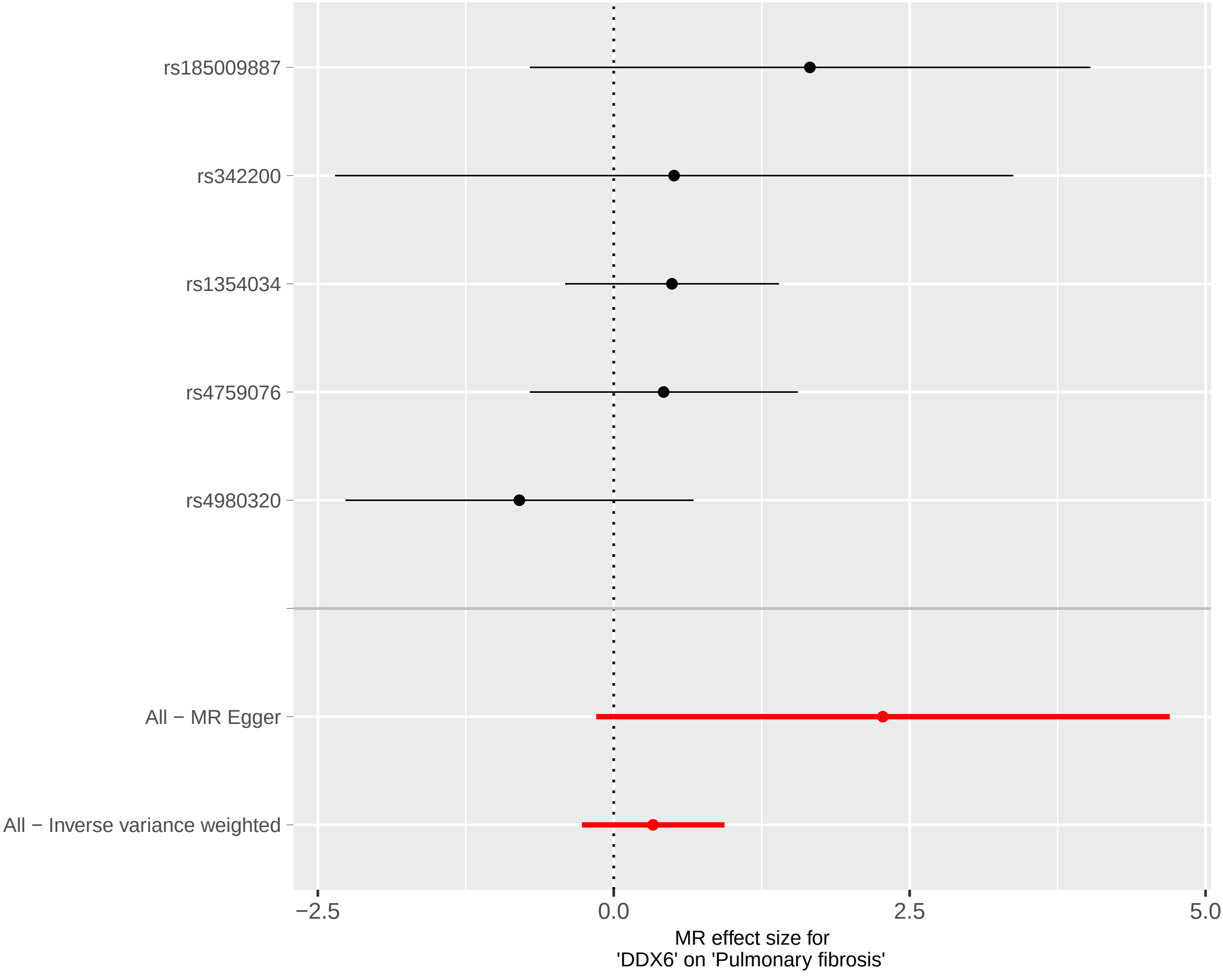

rs185009887
rs342200
rs1354034
rs4759076
rs4980320
All − MR Egger
All − Inverse variance weighted
−2.5
0.0
2.5
5.0
MR effect size for
'DDX6' on 'Pulmonary fibrosis'

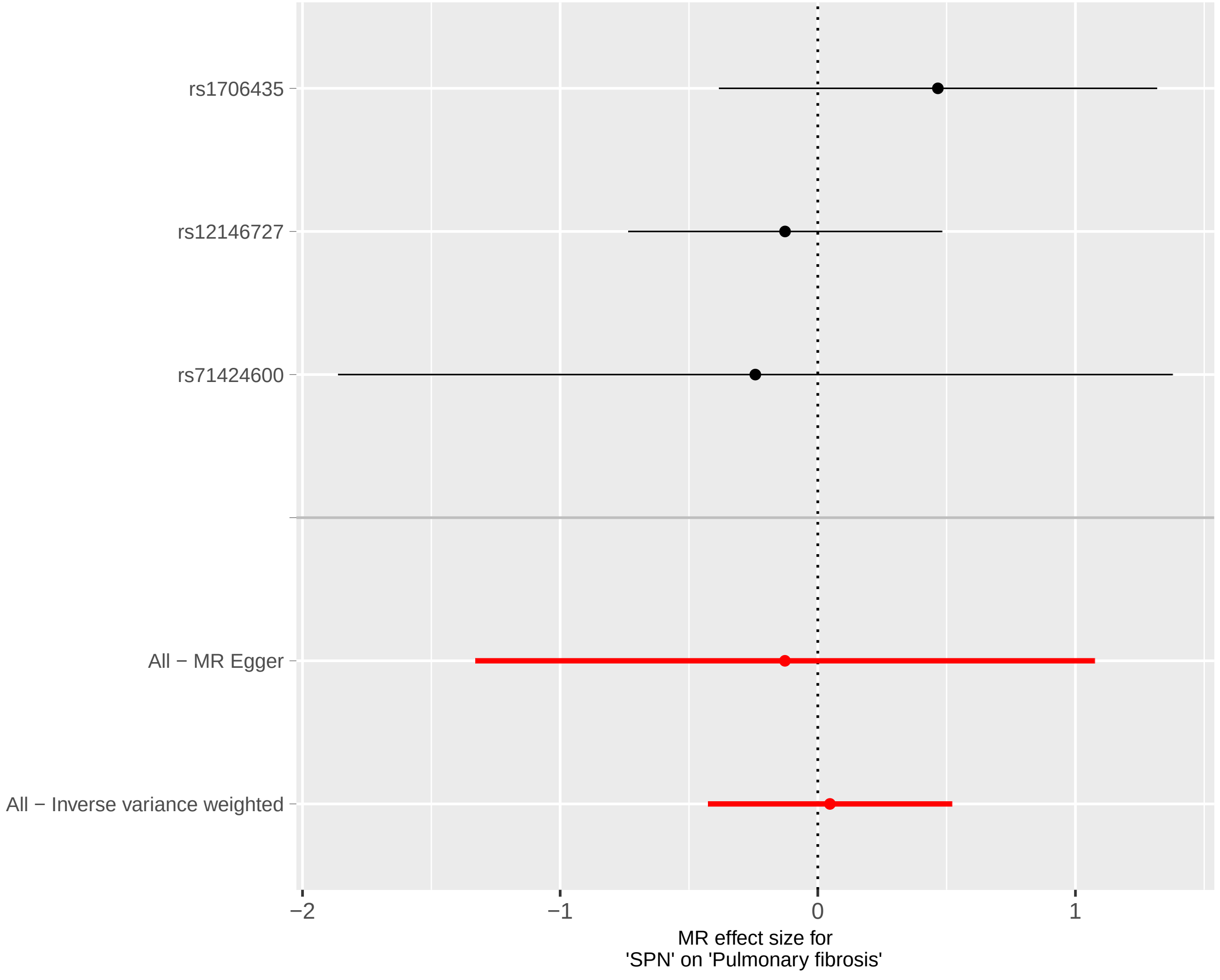

rs1706435
rs12146727
rs71424600
All − MR Egger
All − Inverse variance weighted
−2
−1
0
1
MR effect size for
'SPN' on 'Pulmonary fibrosis'

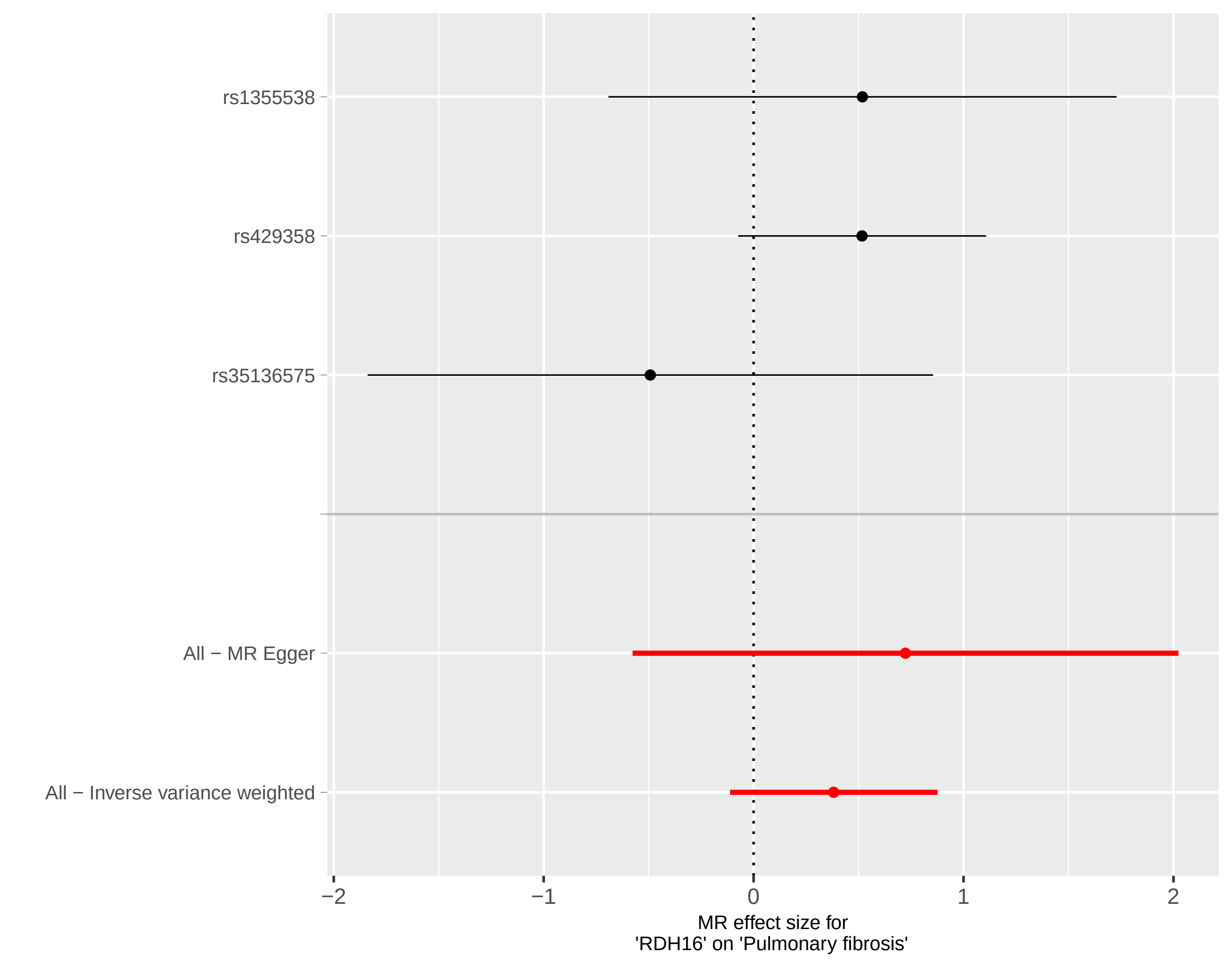

rs1355538
rs429358
rs35136575
All − MR Egger
All − Inverse variance weighted
−2
−1
0
1
2
MR effect size for
'RDH16' on 'Pulmonary fibrosis'

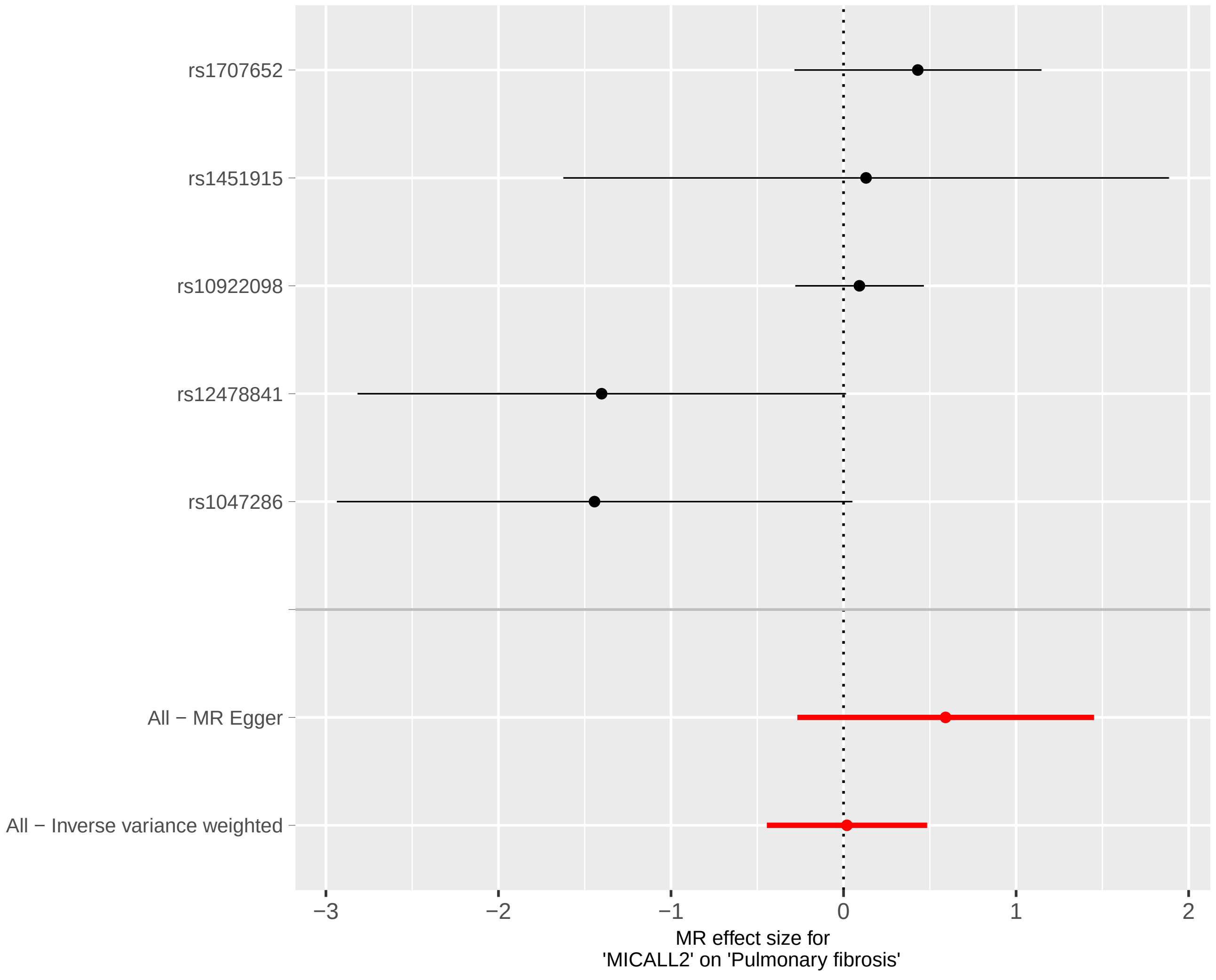
rs1707652
rs1451915
rs10922098
rs12478841
rs1047286
All − MR Egger
All − Inverse variance weighted
−3
−2
−1
0
1
2
MR effect size for
'MICALL2' on 'Pulmonary fibrosis'

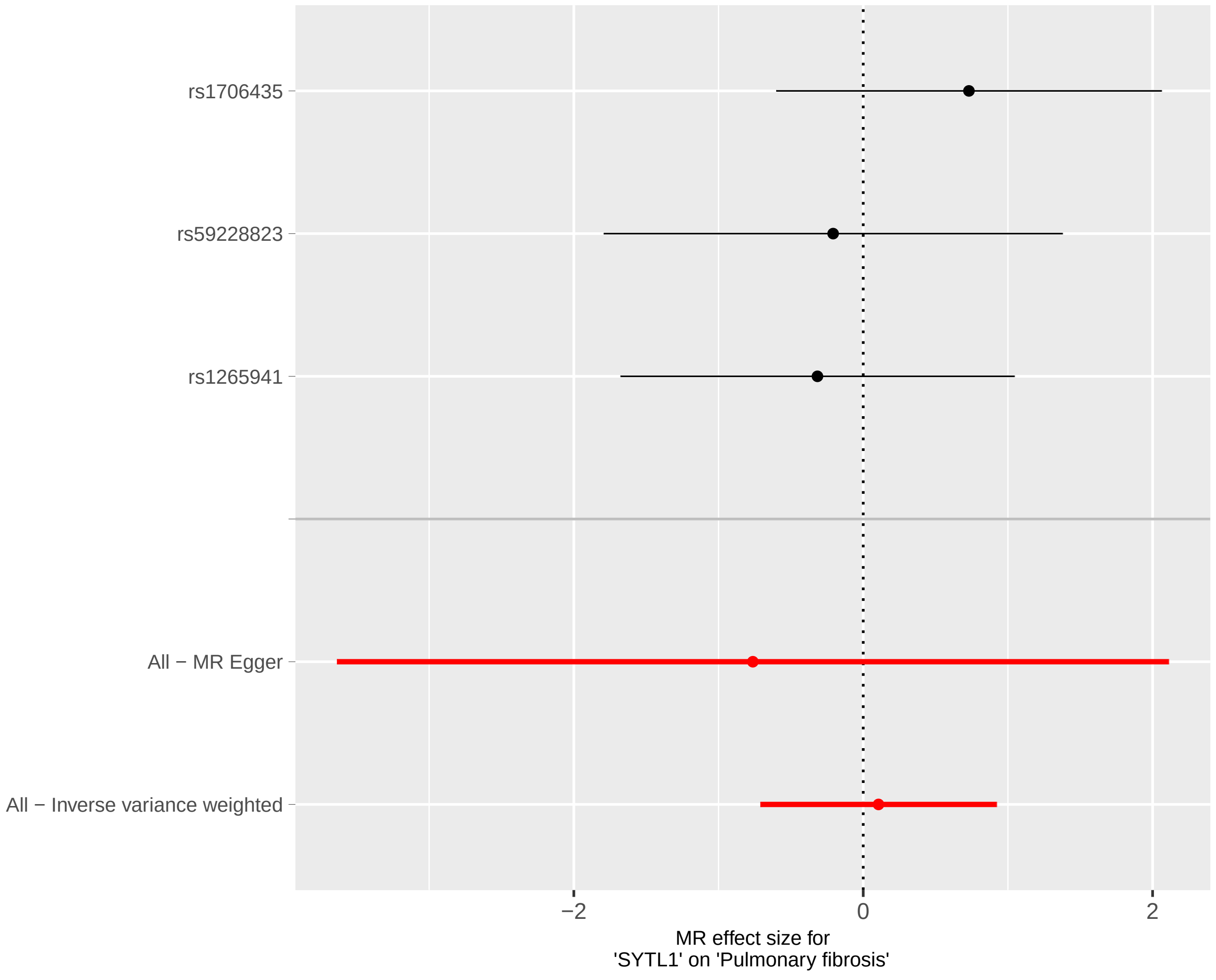

rs1706435
rs59228823
rs1265941
All − MR Egger
All − Inverse variance weighted
−2
0
2
MR effect size for
'SYTL1' on 'Pulmonary fibrosis'

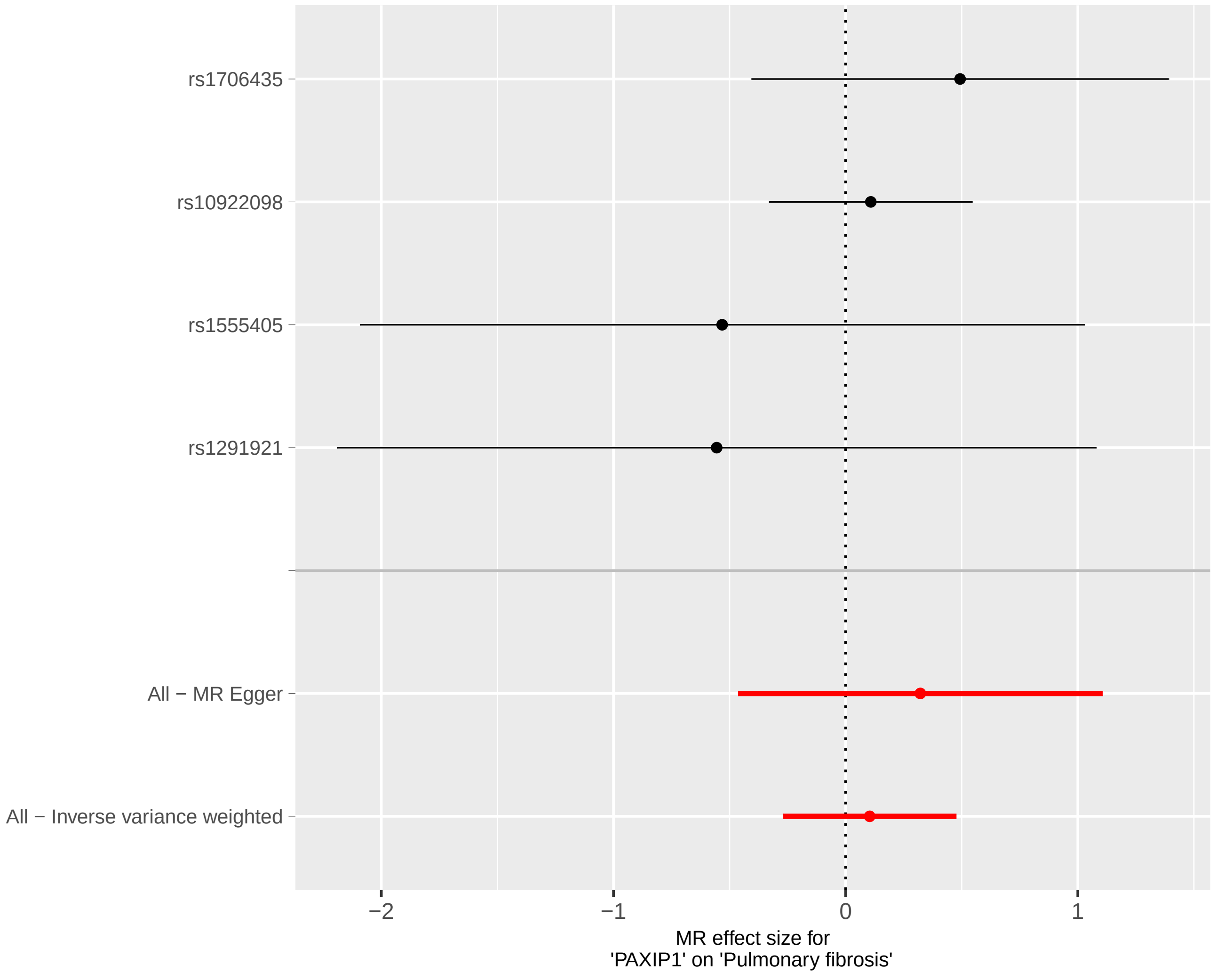
rs1706435
rs10922098
rs1555405
rs1291921
All − MR Egger
All − Inverse variance weighted
−2
−1
0
1
MR effect size for
'PAXIP1' on 'Pulmonary fibrosis'

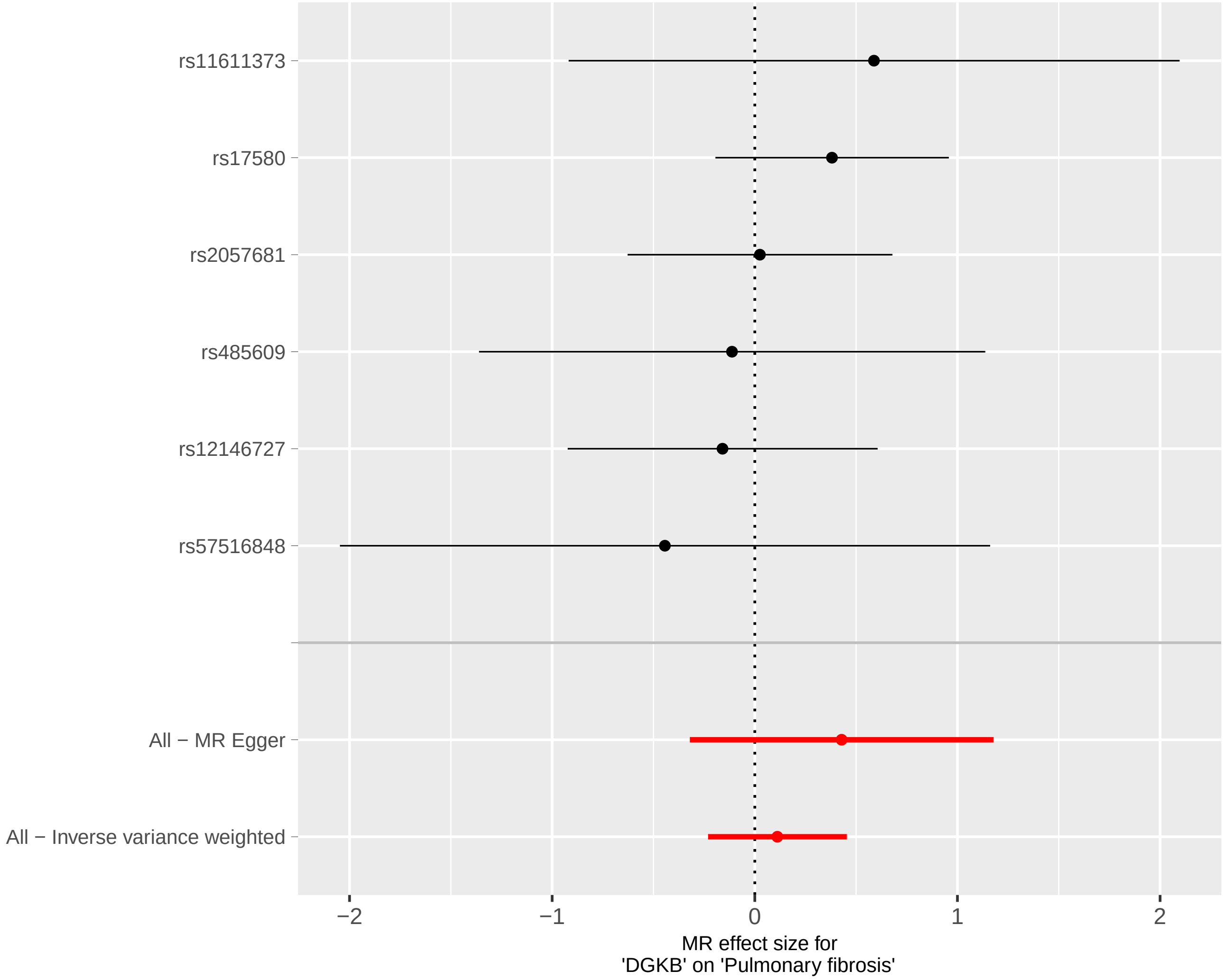

rs11611373
rs17580
rs2057681
rs485609
rs12146727
rs57516848
All − MR Egger
All − Inverse variance weighted
−2
−1
0
1
2
MR effect size for
'DGKB' on 'Pulmonary fibrosis'

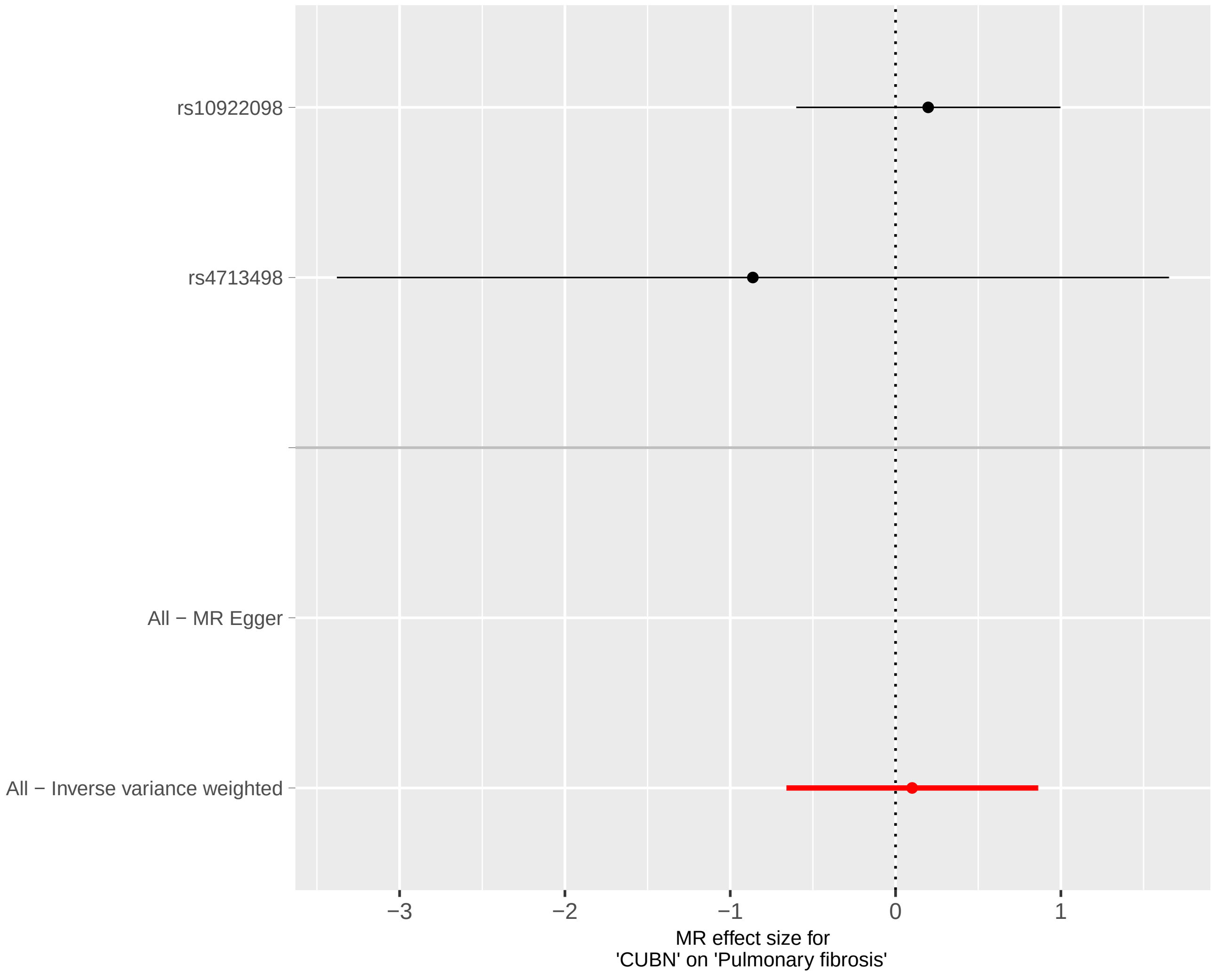
rs10922098
rs4713498
All − MR Egger
All − Inverse variance weighted
−3
−2
−1
0
1
MR effect size for
'CUBN' on 'Pulmonary fibrosis'

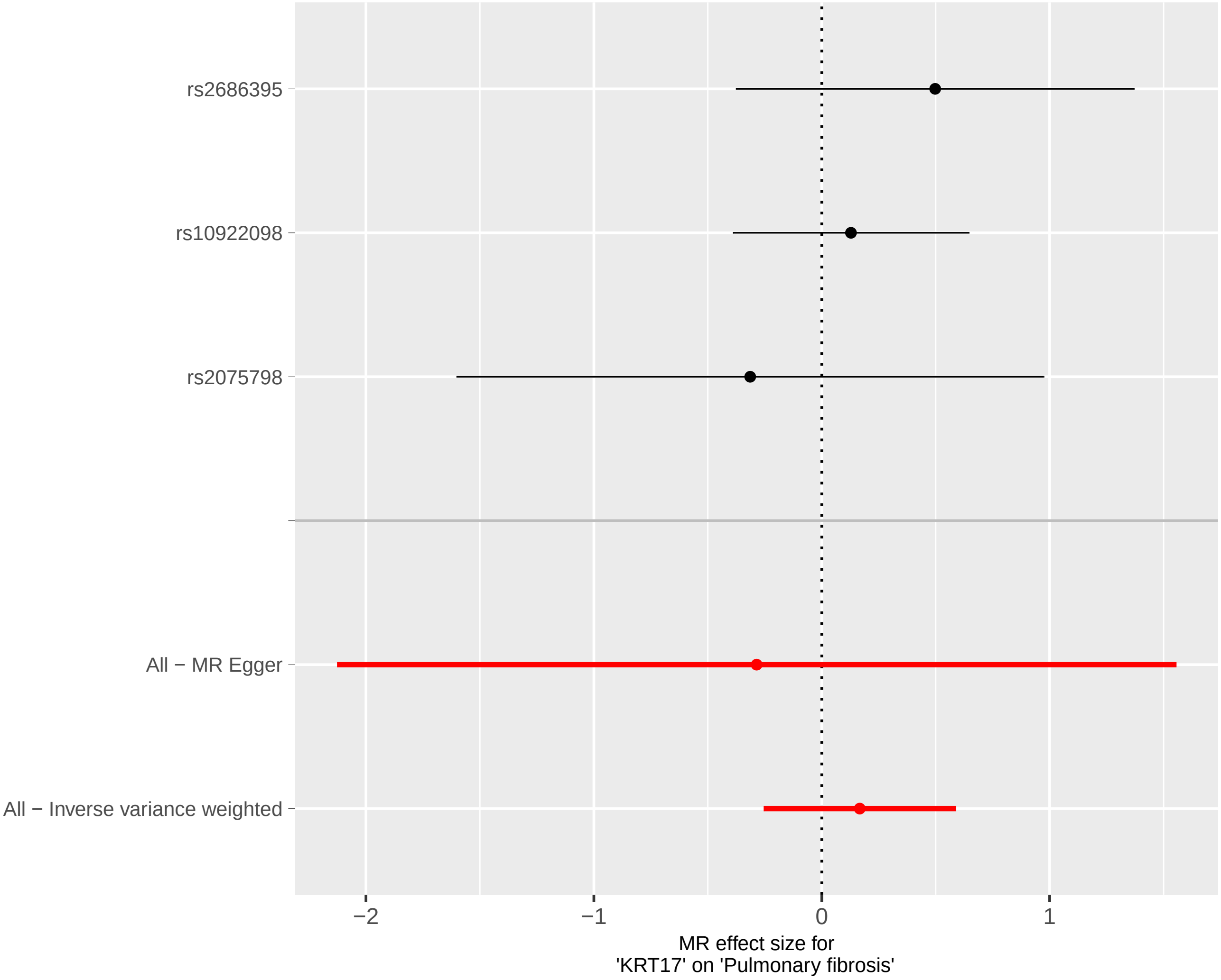

rs2686395
rs10922098
rs2075798
All − MR Egger
All − Inverse variance weighted
−2
−1
0
1
MR effect size for
'KRT17' on 'Pulmonary fibrosis'

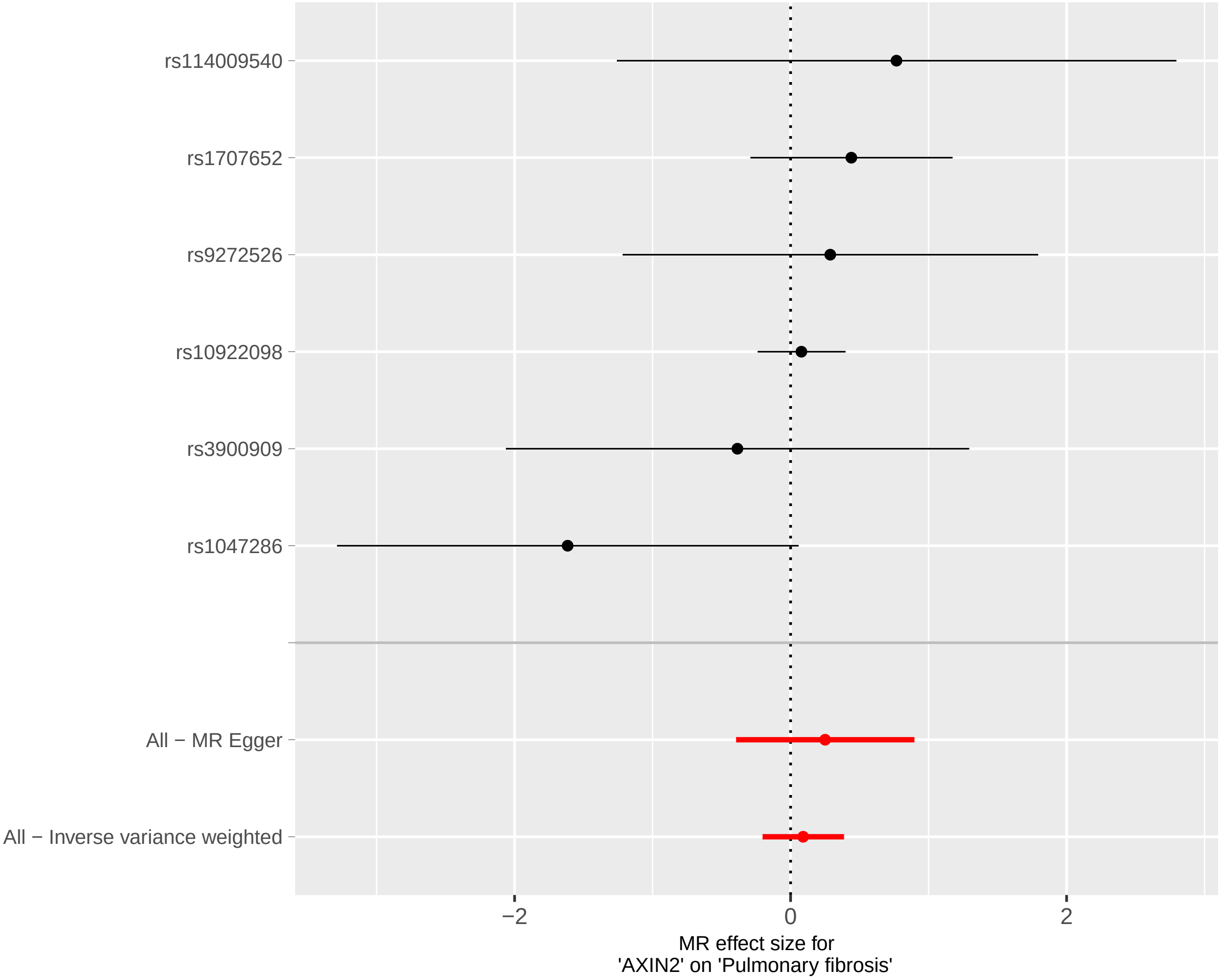
rs114009540
rs1707652
rs9272526
rs10922098
rs3900909
rs1047286
All − MR Egger
All − Inverse variance weighted
−2
0
2
MR effect size for
'AXIN2' on 'Pulmonary fibrosis'

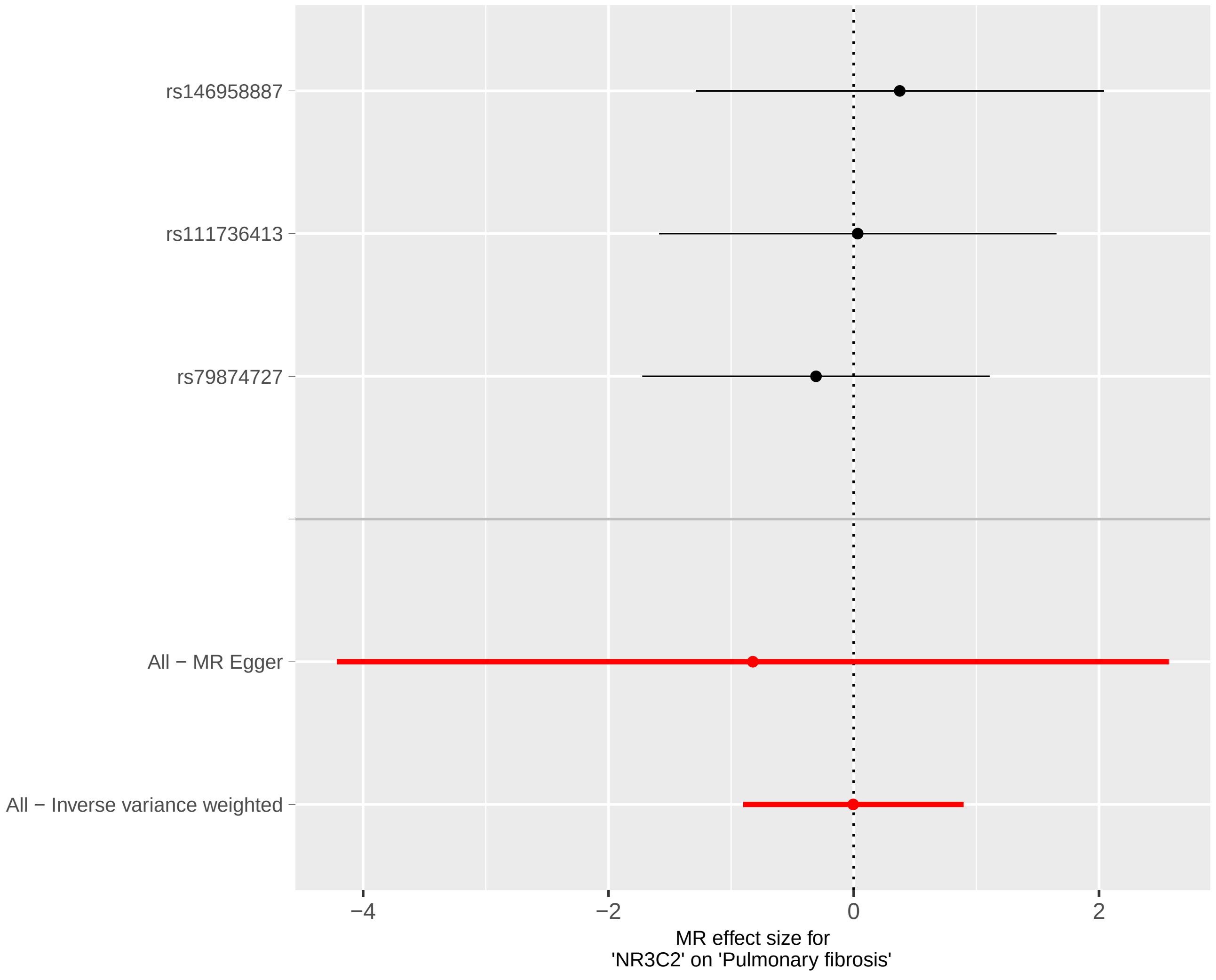
rs146958887
rs111736413
rs79874727
All − MR Egger
All − Inverse variance weighted
−4
−2
0
2
MR effect size for
'NR3C2' on 'Pulmonary fibrosis'

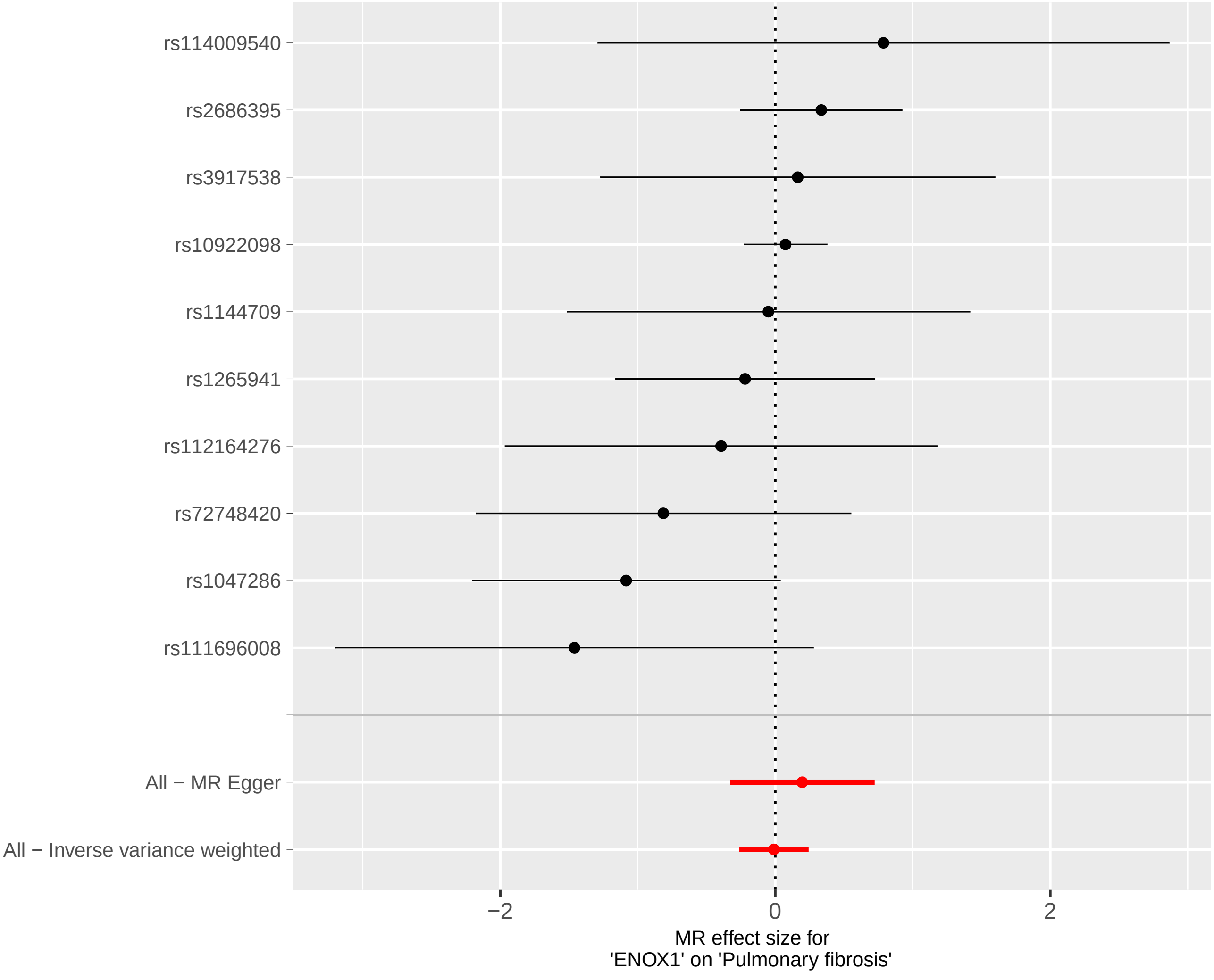

rs114009540
rs2686395
rs3917538
rs10922098
rs1144709
rs1265941
rs112164276
rs72748420
rs1047286
rs111696008
All − MR Egger
All − Inverse variance weighted
−2
0
2
MR effect size for
'ENOX1' on 'Pulmonary fibrosis'

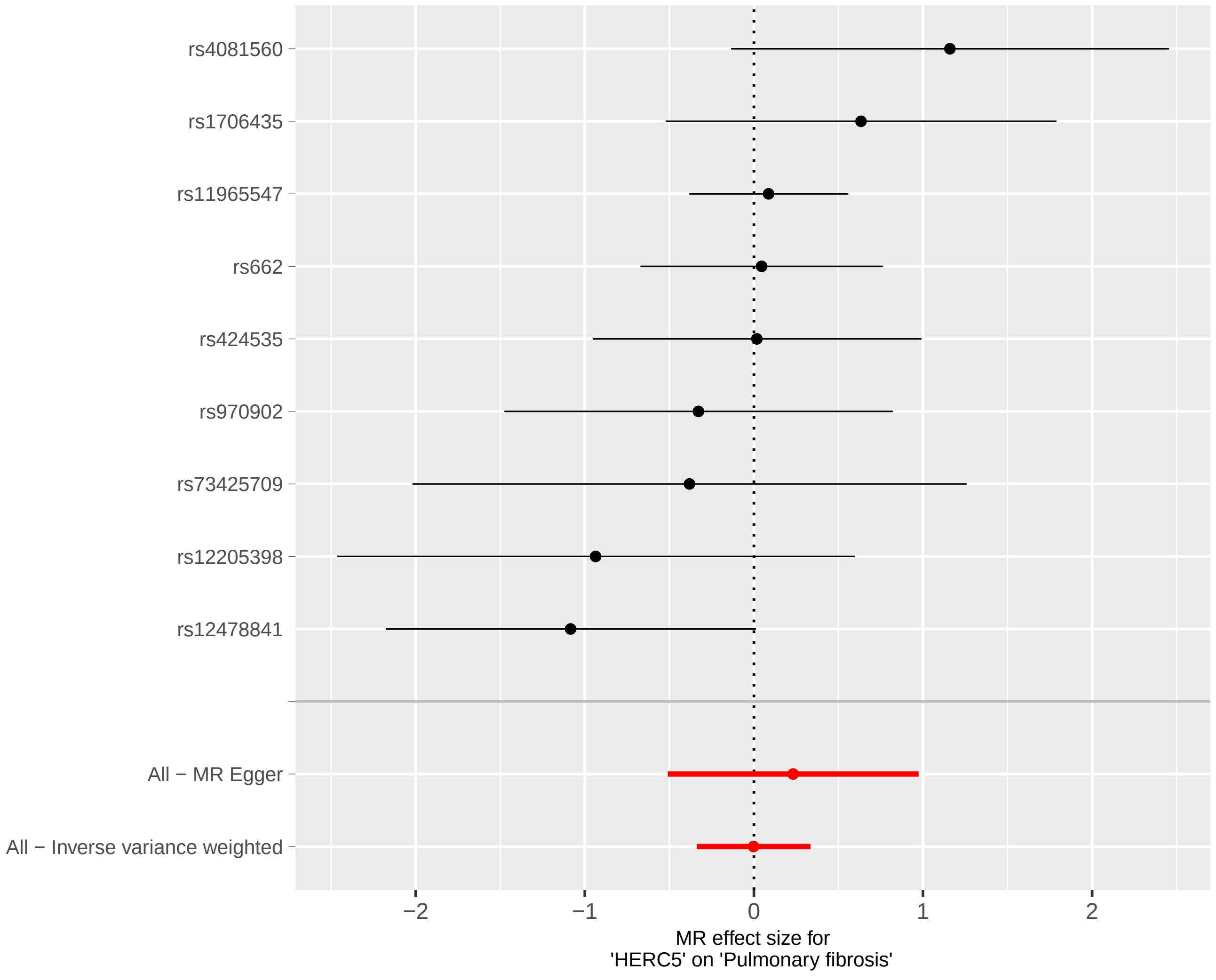
rs4081560
rs1706435
rs11965547
rs662
rs424535
rs970902
rs73425709
rs12205398
rs12478841
All − MR Egger
All − Inverse variance weighted
−2
−1
0
1
2
MR effect size for
'HERC5' on 'Pulmonary fibrosis'

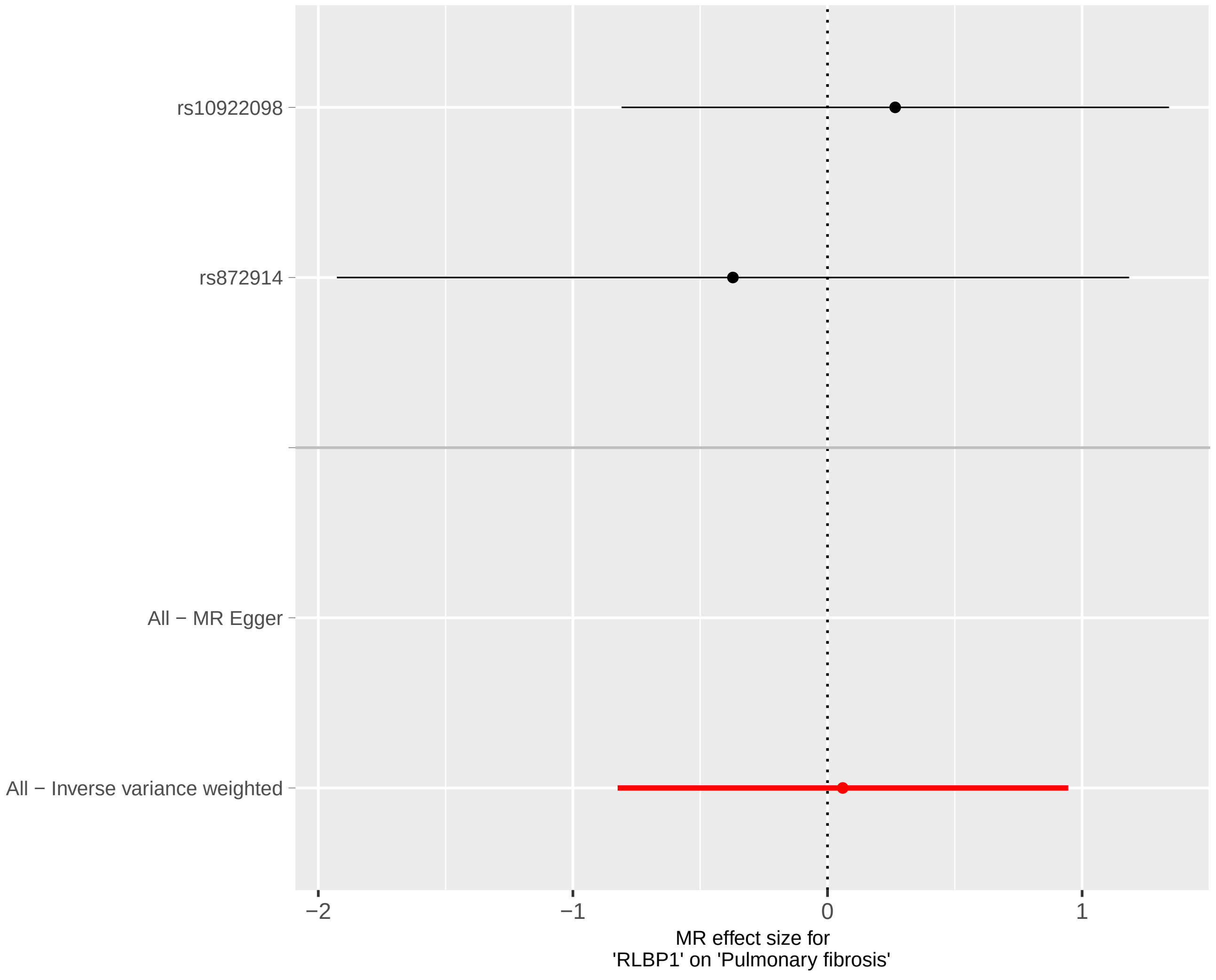
rs10922098
rs872914
All − MR Egger
All − Inverse variance weighted
−2
−1
0
1
MR effect size for
'RLBP1' on 'Pulmonary fibrosis'

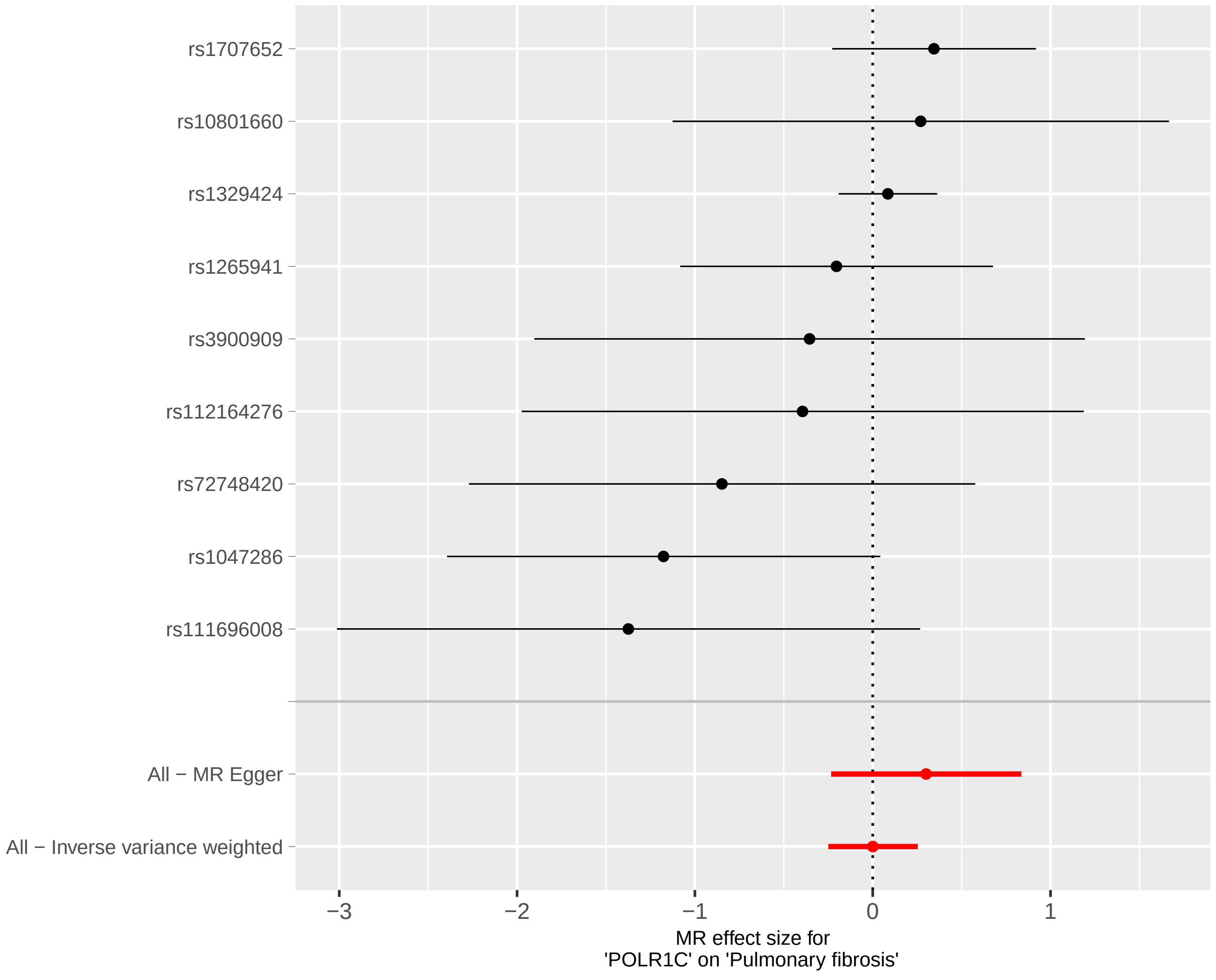

rs1707652
rs10801660
rs1329424
rs1265941
rs3900909
rs112164276
rs72748420
rs1047286
rs111696008
All − MR Egger
All − Inverse variance weighted
−3
−2
−1
0
1
MR effect size for
'POLR1C' on 'Pulmonary fibrosis'

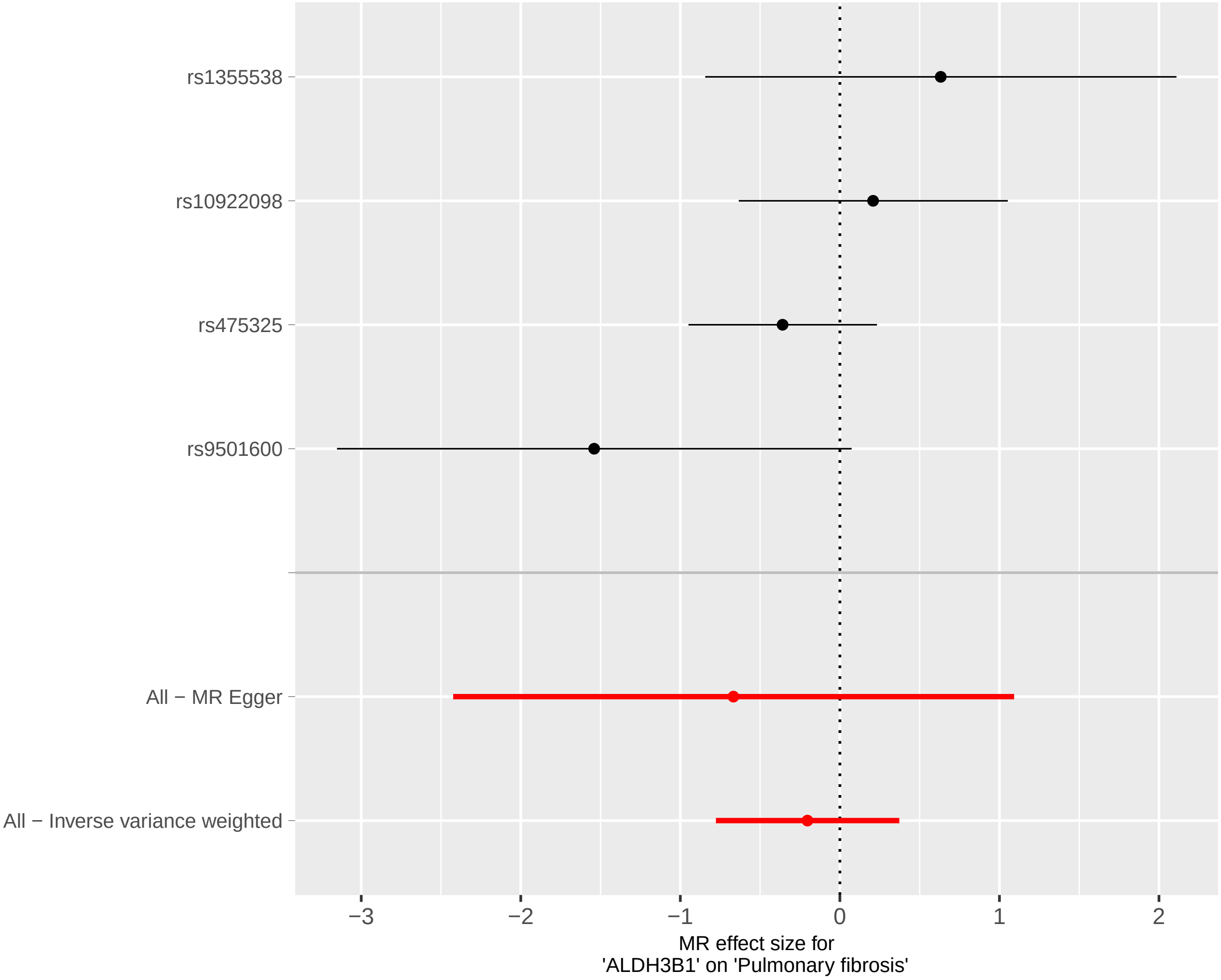

rs1355538
rs10922098
rs475325
rs9501600
All − MR Egger
All − Inverse variance weighted
−3
−2
−1
0
1
2
MR effect size for
'ALDH3B1' on 'Pulmonary fibrosis'

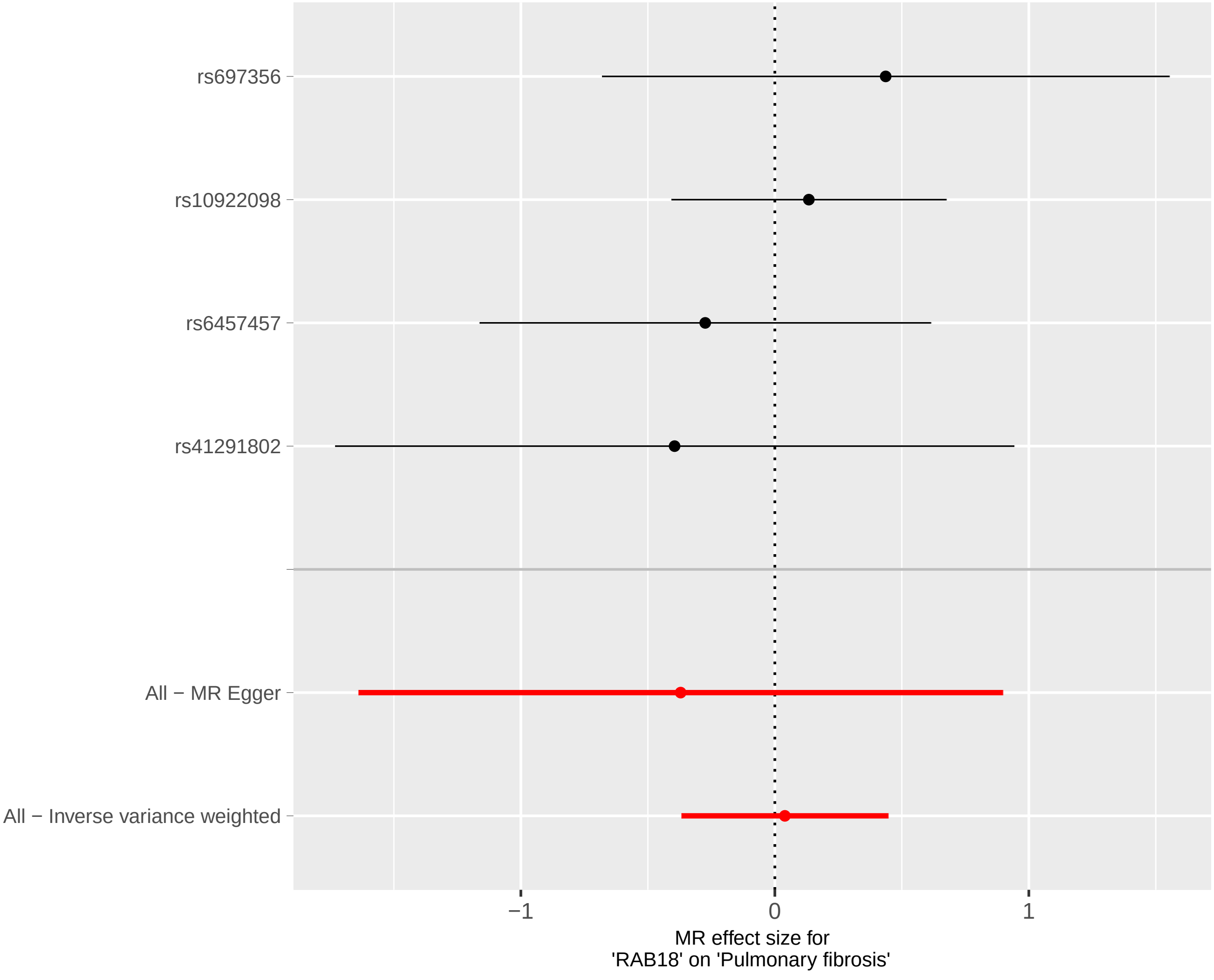
rs697356
rs10922098
rs6457457
rs41291802
All − MR Egger
All − Inverse variance weighted
−1
0
1
MR effect size for
'RAB18' on 'Pulmonary fibrosis'

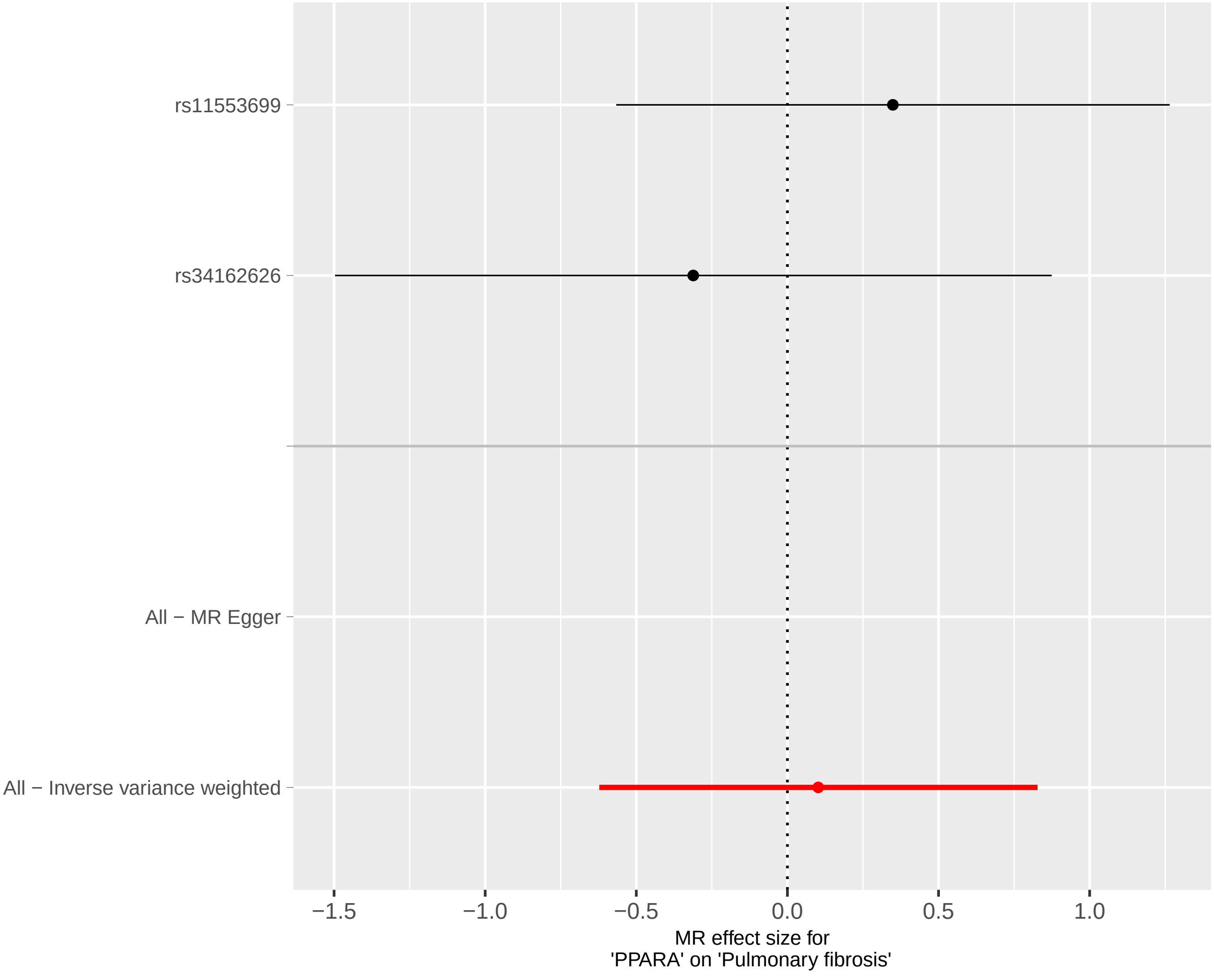
rs11553699
rs34162626
All − MR Egger
All − Inverse variance weighted
−1.5
−1.0
−0.5
0.0
0.5
1.0
MR effect size for
'PPARA' on 'Pulmonary fibrosis'

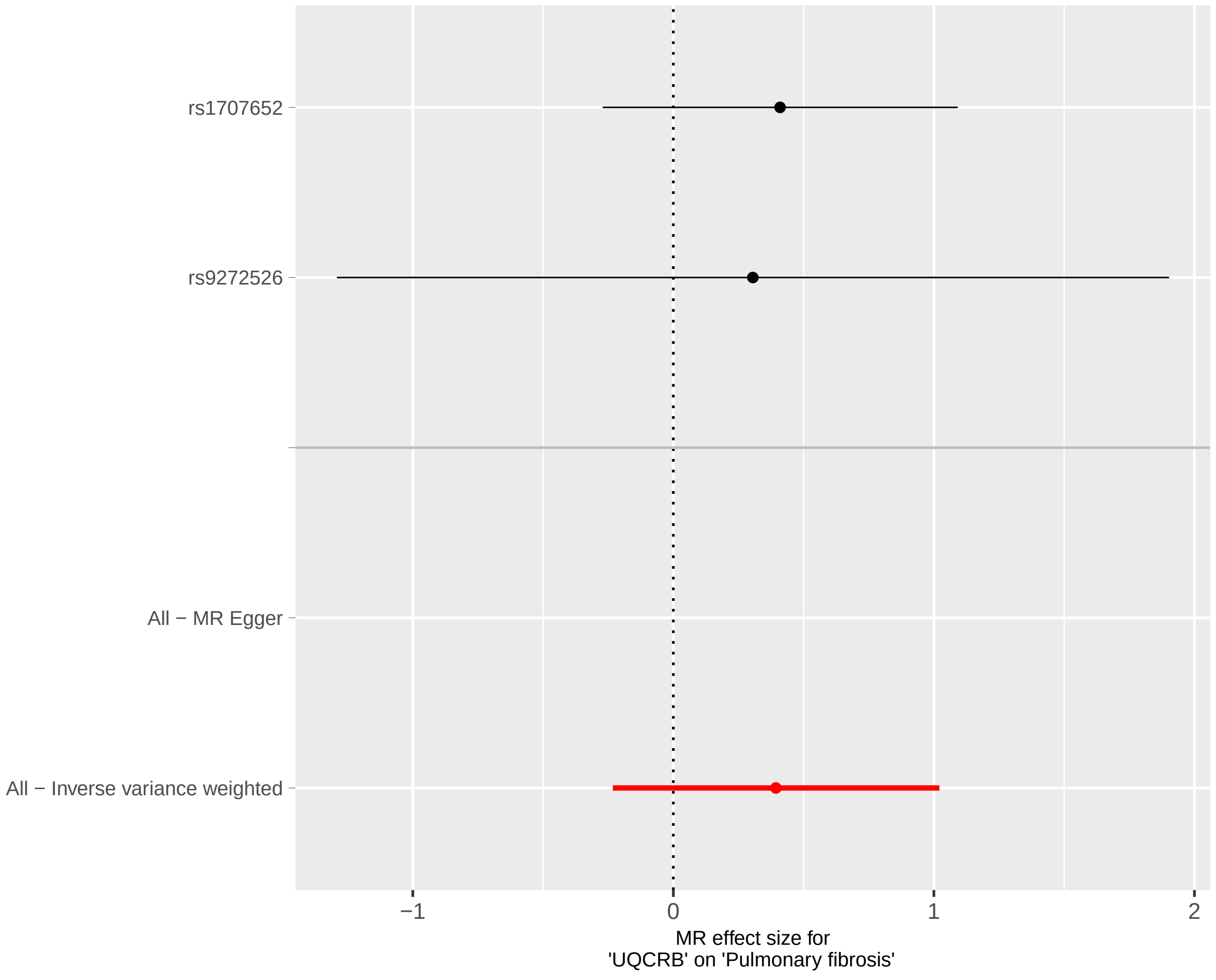
rs1707652
rs9272526
All − MR Egger
All − Inverse variance weighted
−1
0
1
2
MR effect size for
'UQCRB' on 'Pulmonary fibrosis'

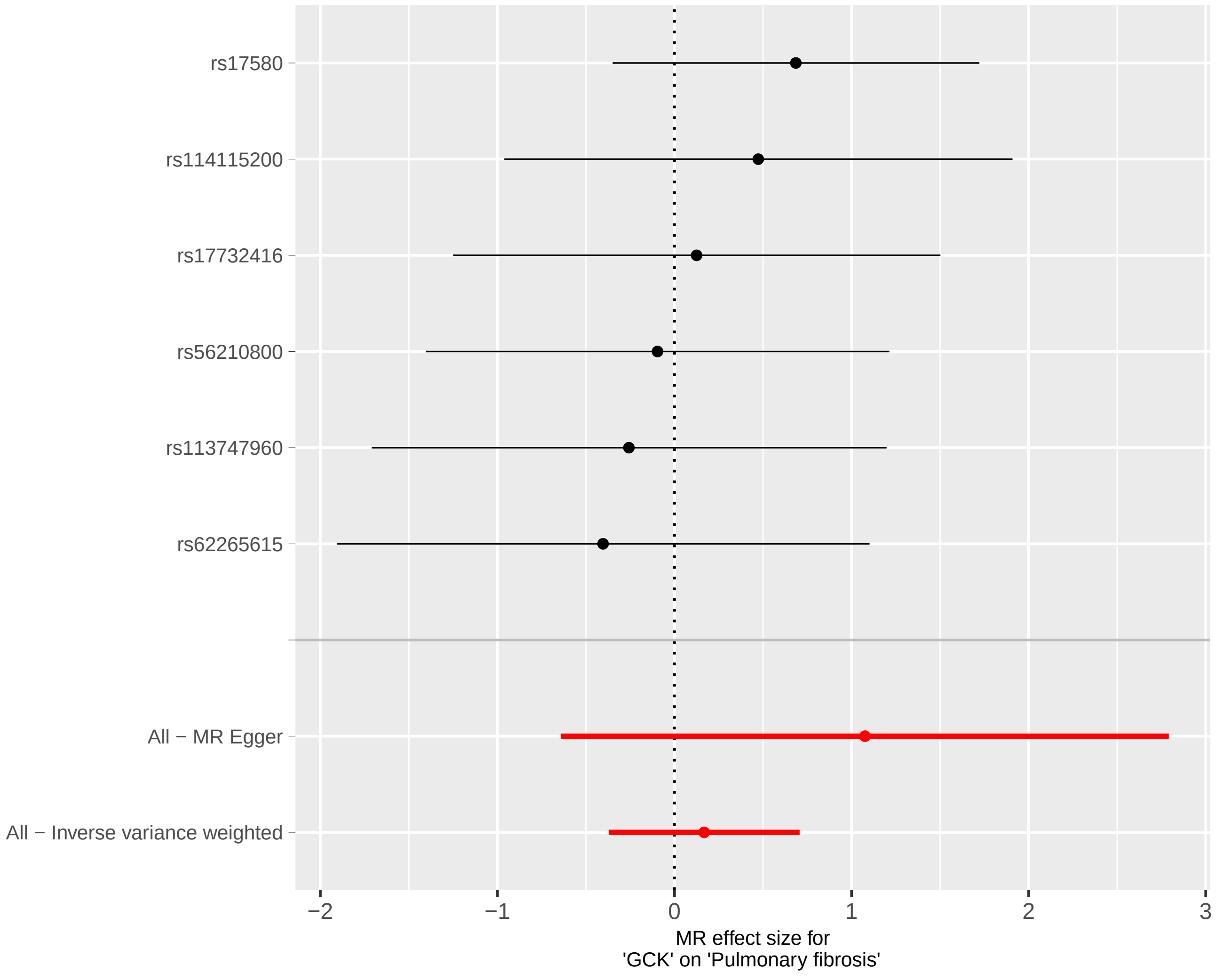
rs17580
rs114115200
rs17732416
rs56210800
rs113747960
rs62265615
All − MR Egger
All − Inverse variance weighted
−2
−1
0
1
2
3
MR effect size for
'GCK' on 'Pulmonary fibrosis'

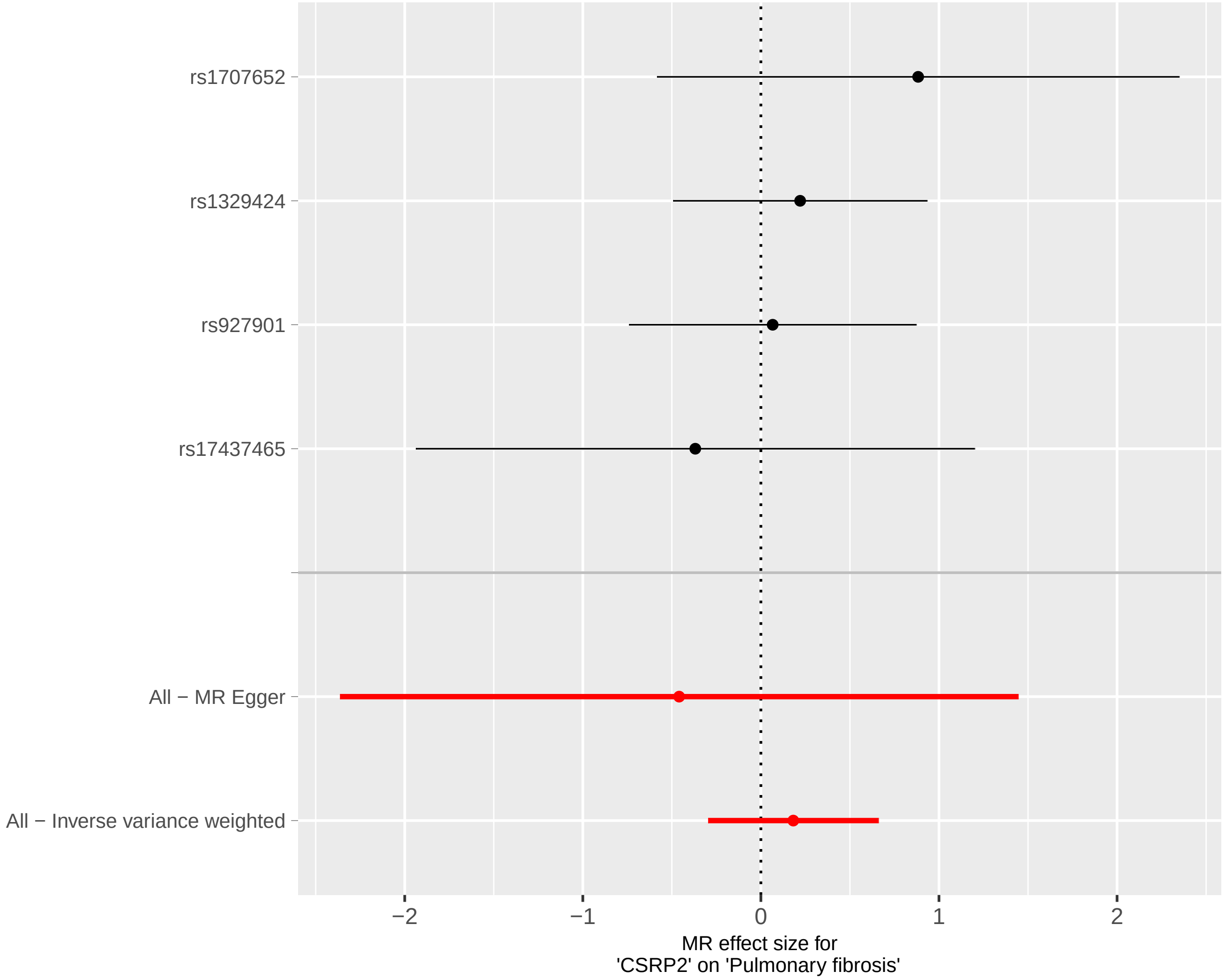
rs1707652
rs1329424
rs927901
rs17437465
All − MR Egger
All − Inverse variance weighted
−2
−1
0
1
2
MR effect size for
'CSRP2' on 'Pulmonary fibrosis'

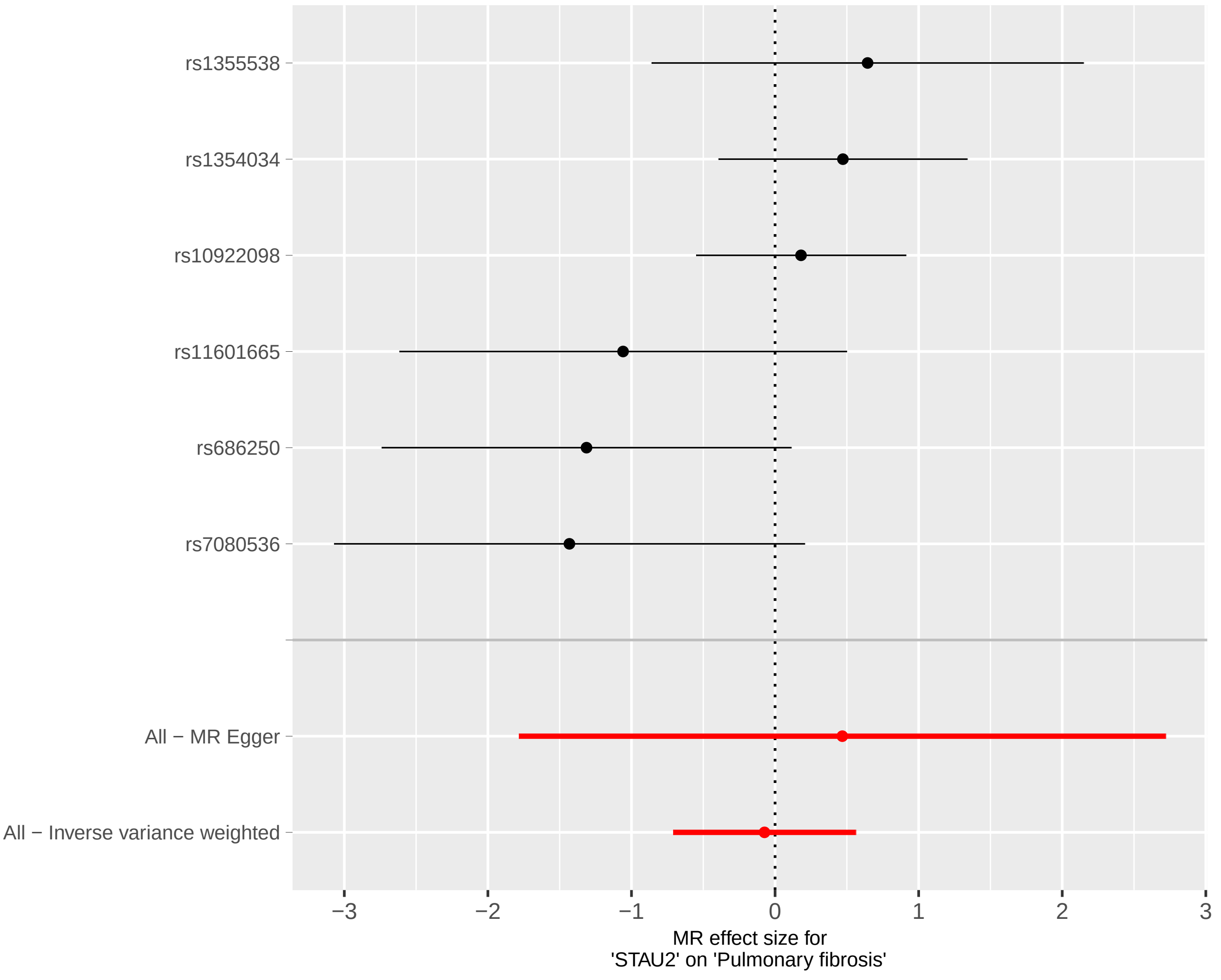
rs1355538
rs1354034
rs10922098
rs11601665
rs686250
rs7080536
All − MR Egger
All − Inverse variance weighted
−3
−2
−1
0
1
2
3
MR effect size for
'STAU2' on 'Pulmonary fibrosis'

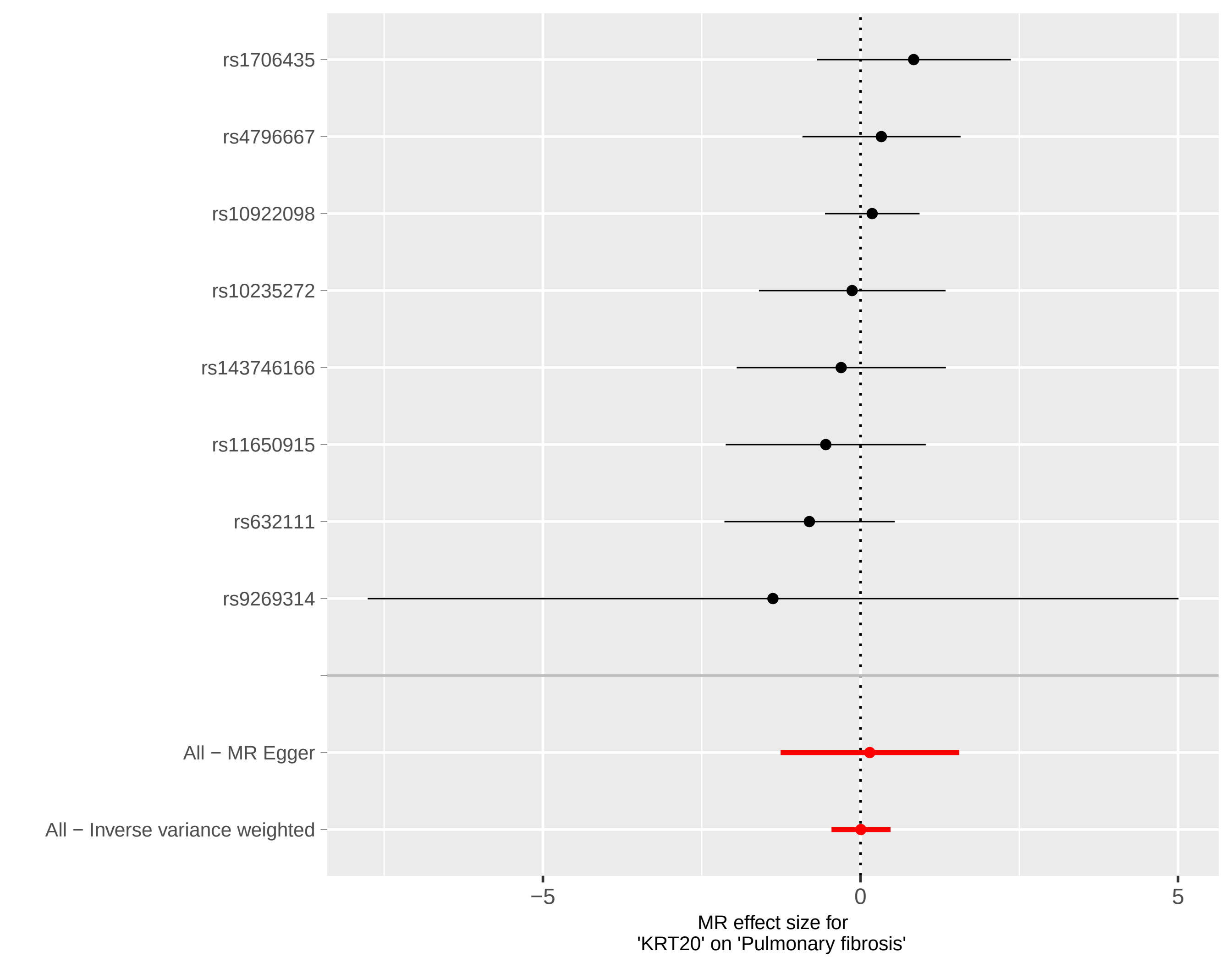
rs1706435
rs4796667
rs10922098
rs10235272
rs143746166
rs11650915
rs632111
rs9269314
All − MR Egger
All − Inverse variance weighted
−5
0
5
MR effect size for
'KRT20' on 'Pulmonary fibrosis'

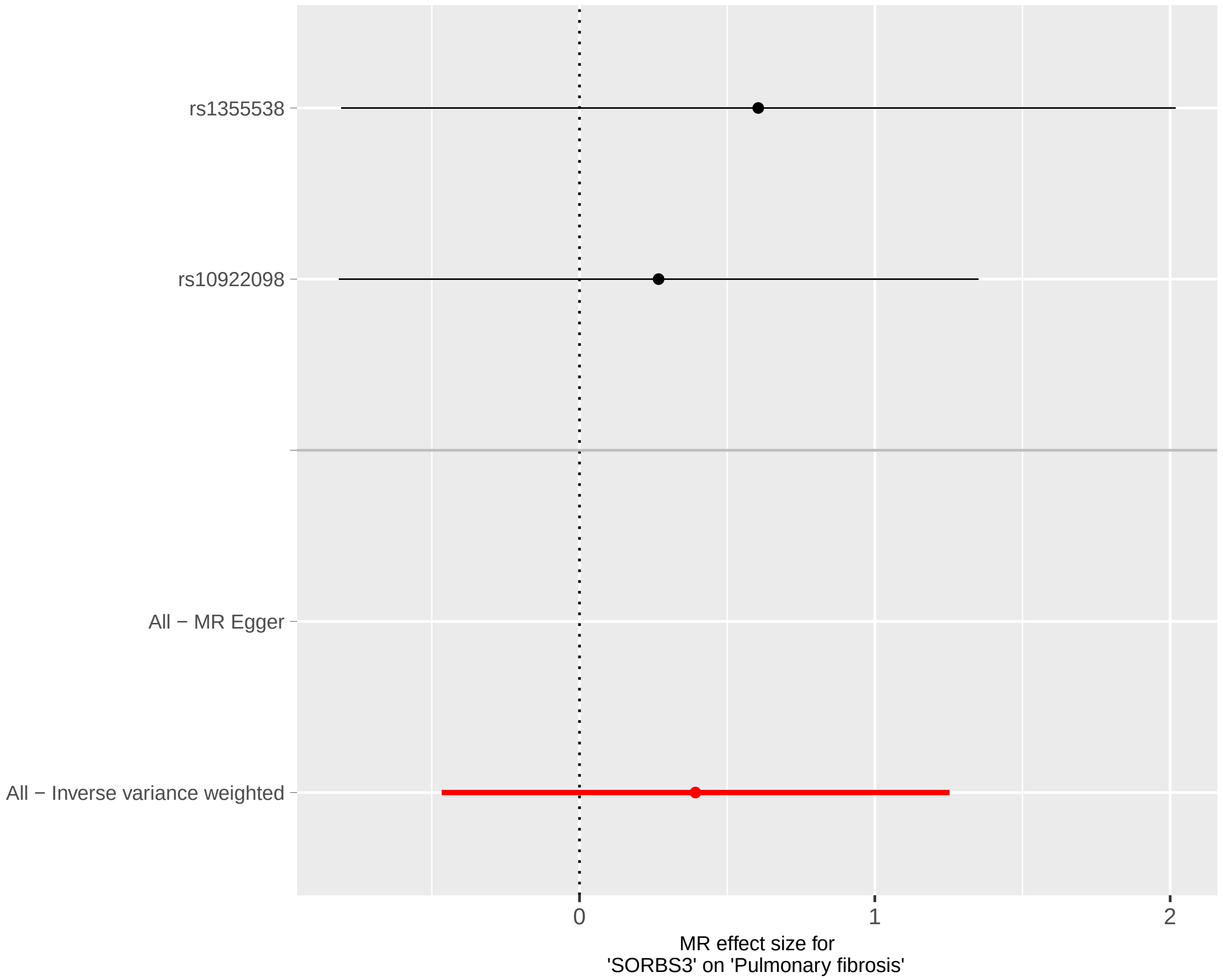

rs1355538
rs10922098
All − MR Egger
All − Inverse variance weighted
0
1
2
MR effect size for
'SORBS3' on 'Pulmonary fibrosis'

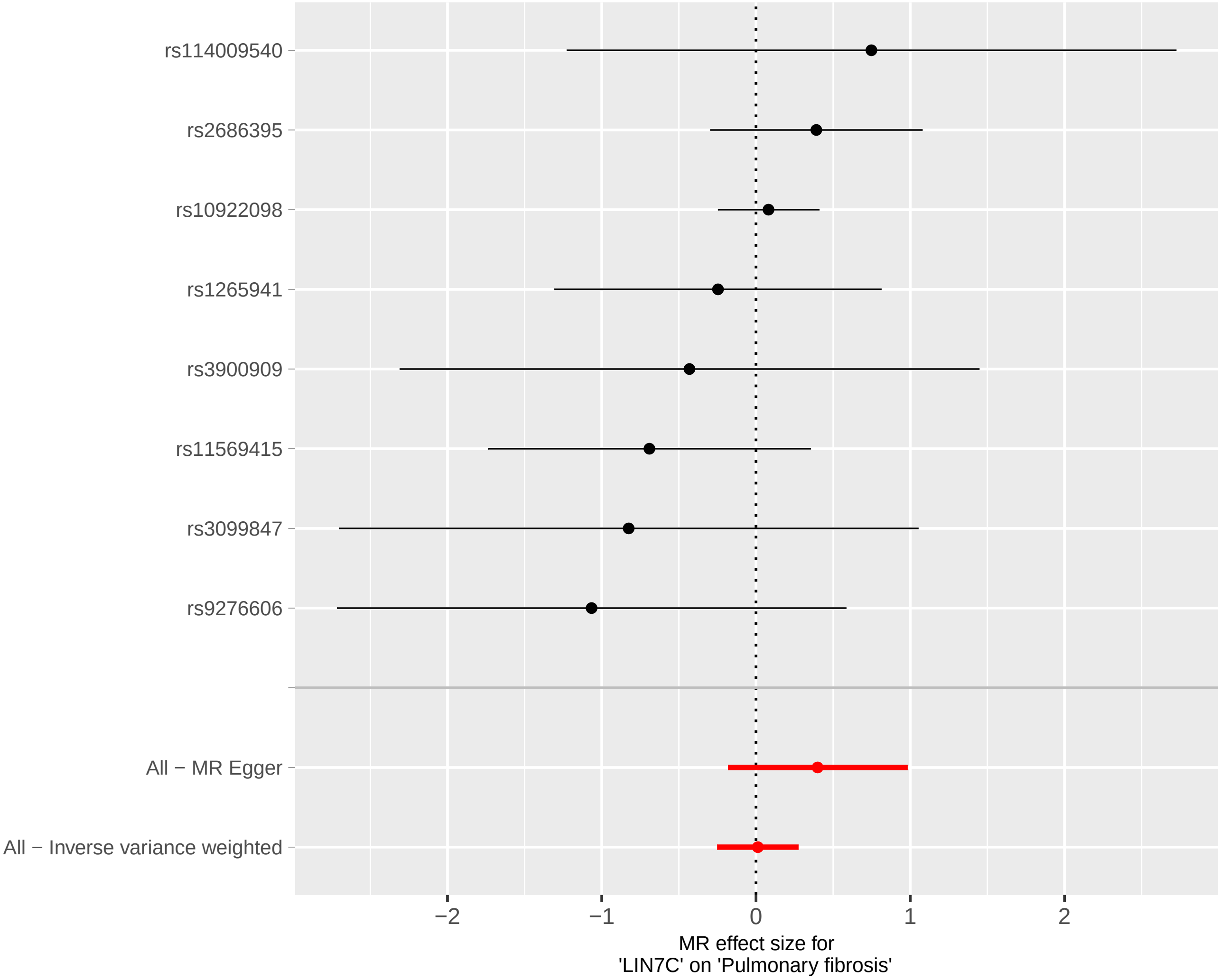
rs114009540
rs2686395
rs10922098
rs1265941
rs3900909
rs11569415
rs3099847
rs9276606
All − MR Egger
All − Inverse variance weighted
−2
−1
0
1
2
MR effect size for
'LIN7C' on 'Pulmonary fibrosis'

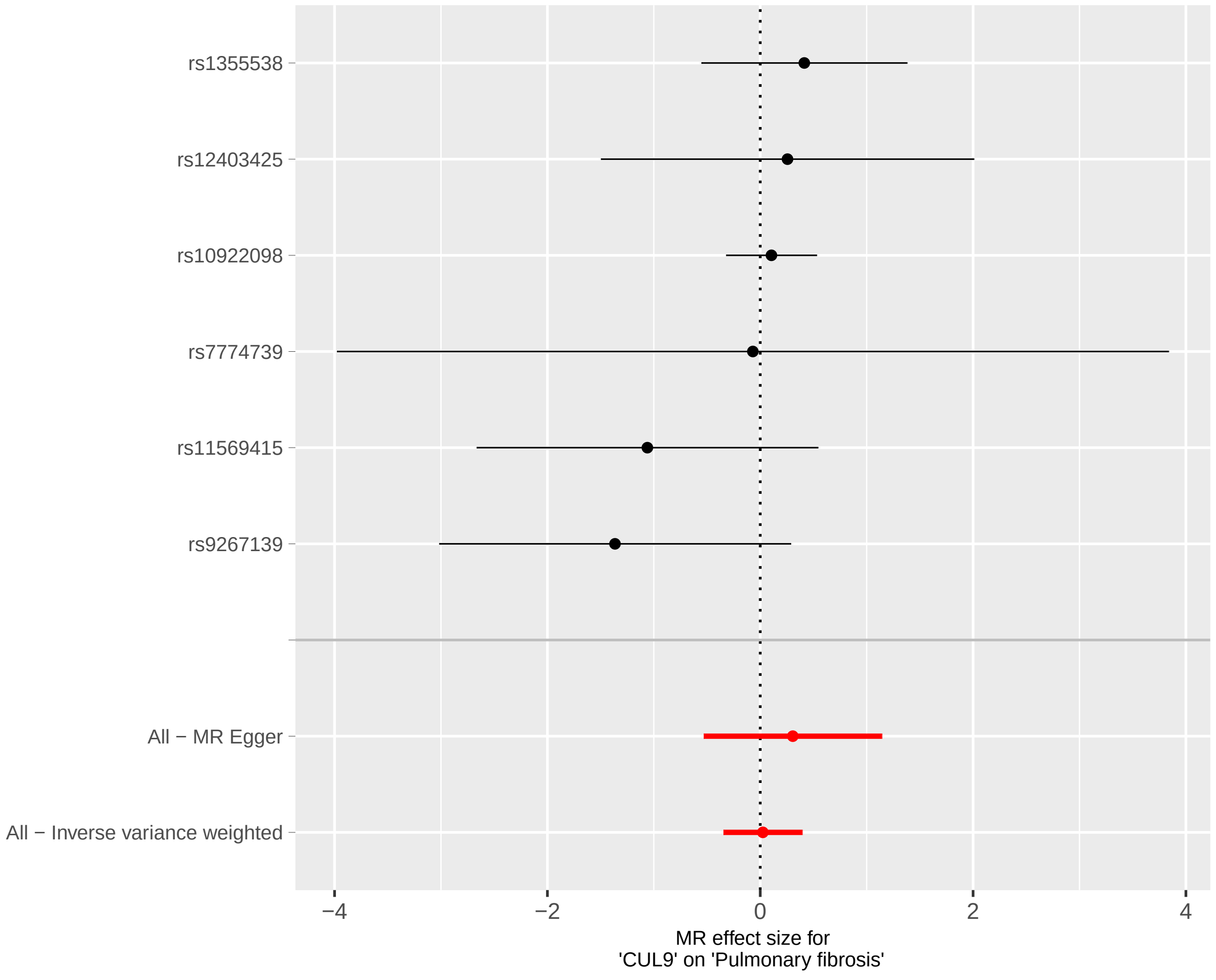
rs1355538
rs12403425
rs10922098
rs7774739
rs11569415
rs9267139
All − MR Egger
All − Inverse variance weighted
−4
−2
0
2
4
MR effect size for
'CUL9' on 'Pulmonary fibrosis'

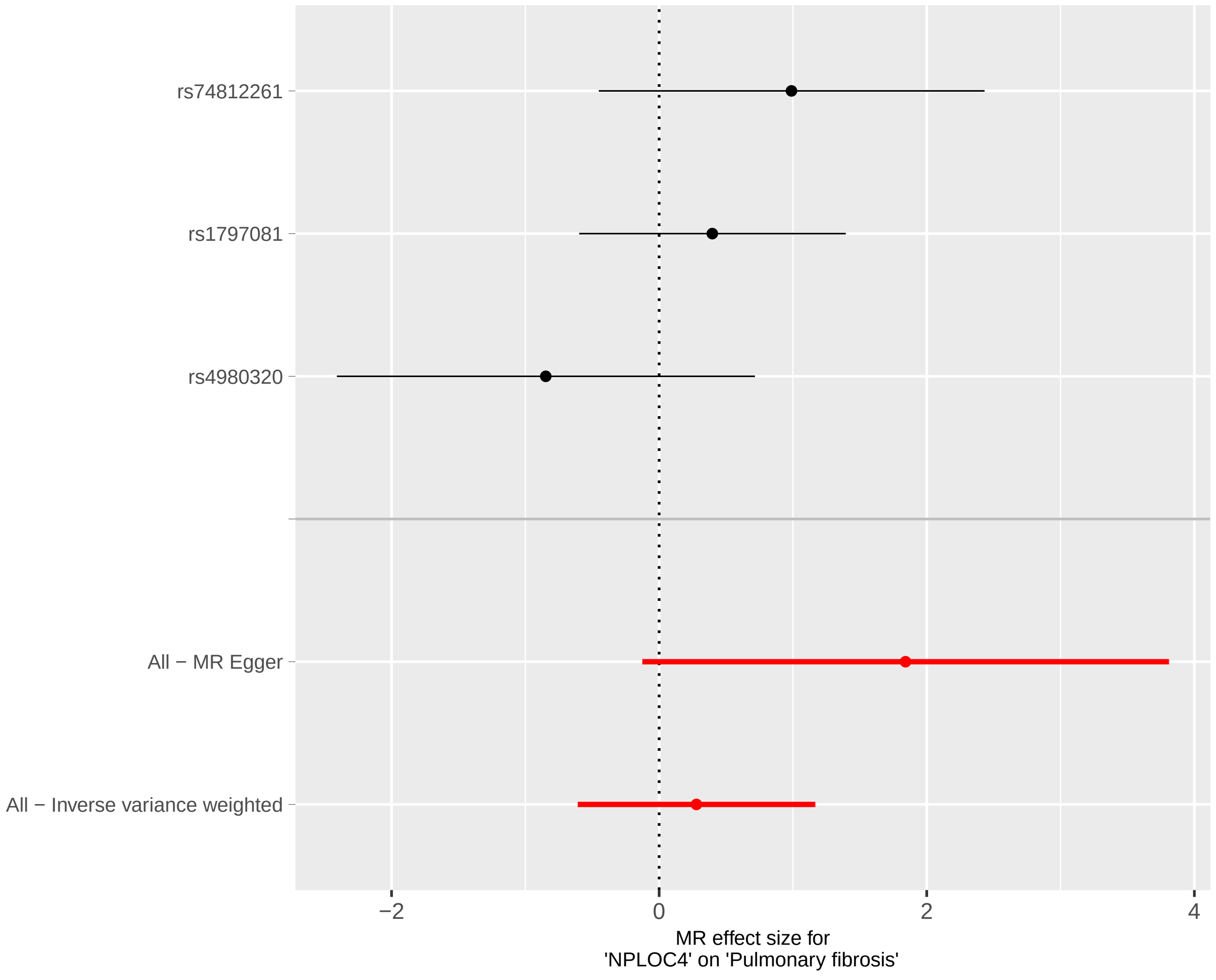
rs74812261
rs1797081
rs4980320
All − MR Egger
All − Inverse variance weighted
−2
0
2
4
MR effect size for
'NPLOC4' on 'Pulmonary fibrosis'

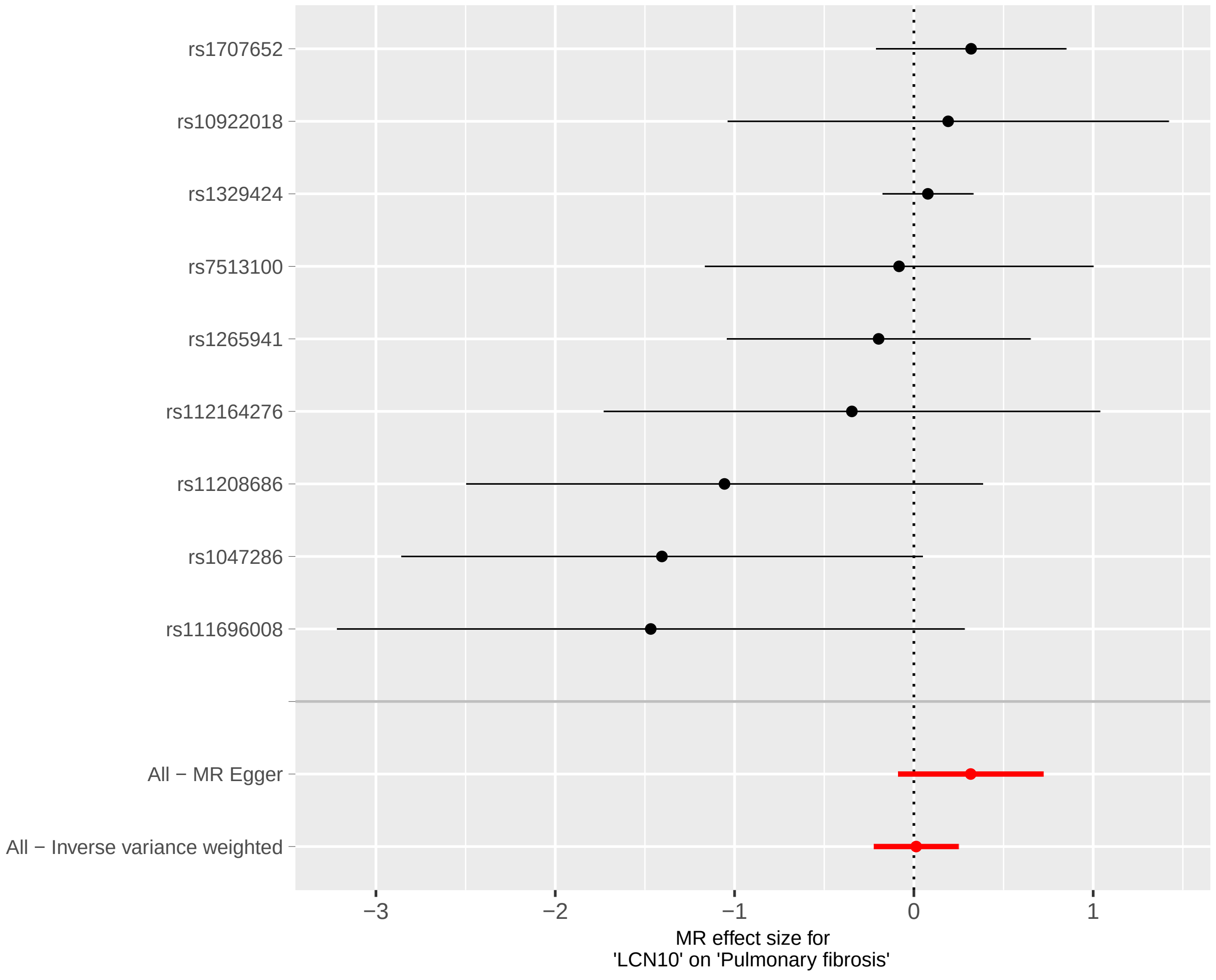
rs1707652
rs10922018
rs1329424
rs7513100
rs1265941
rs112164276
rs11208686
rs1047286
rs111696008
All − MR Egger
All − Inverse variance weighted
−3
−2
−1
0
1
MR effect size for
'LCN10' on 'Pulmonary fibrosis'

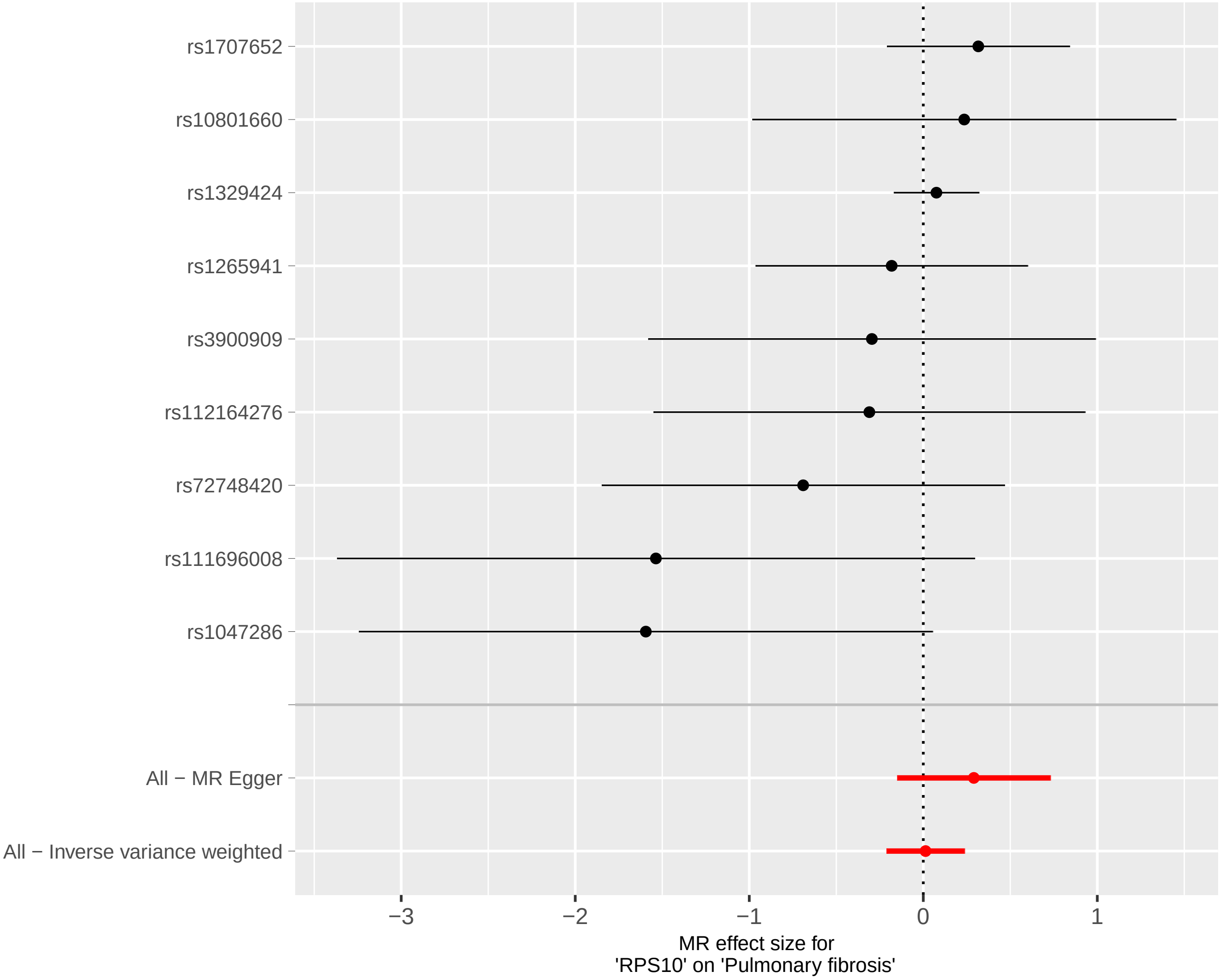
rs1707652
rs10801660
rs1329424
rs1265941
rs3900909
rs112164276
rs72748420
rs111696008
rs1047286
All − MR Egger
All − Inverse variance weighted
−3
−2
−1
0
1
MR effect size for
'RPS10' on 'Pulmonary fibrosis'

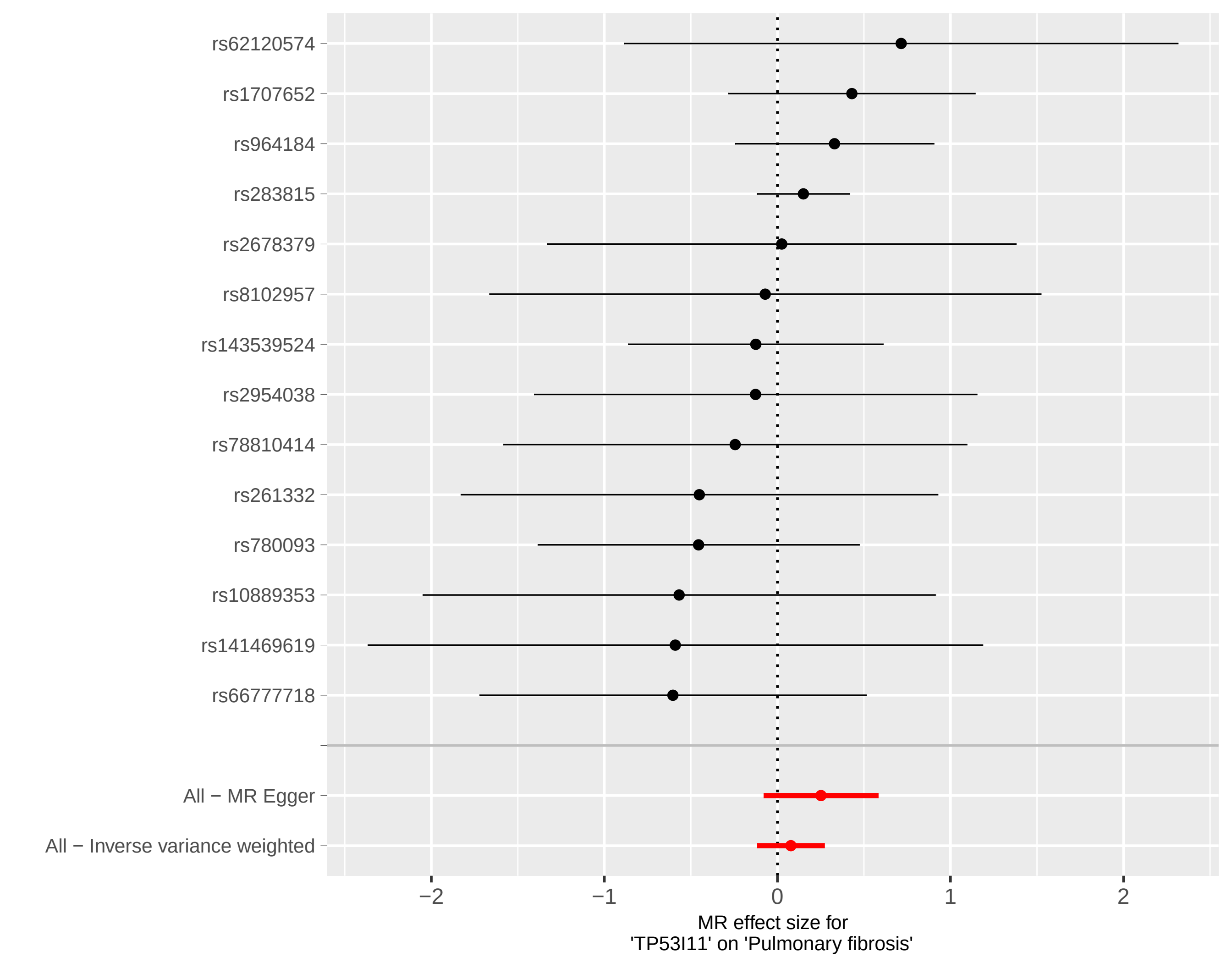

rs62120574
rs1707652
rs964184
rs283815
rs2678379
rs8102957
rs143539524
rs2954038
rs78810414
rs261332
rs780093
rs10889353
rs141469619
rs66777718
All − MR Egger
All − Inverse variance weighted
−2
−1
0
1
2
MR effect size for
'TP53I11' on 'Pulmonary fibrosis'

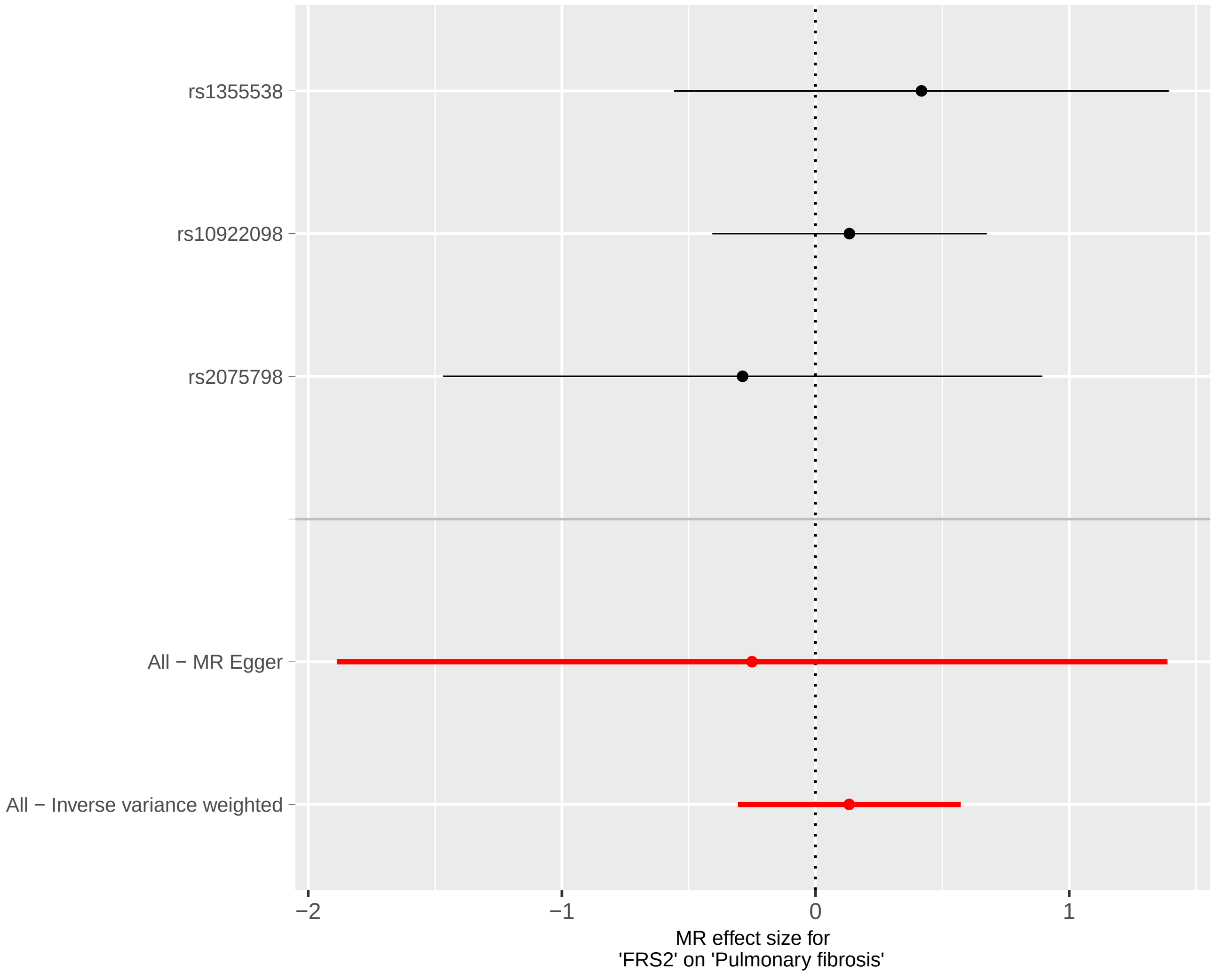

rs1355538
rs10922098
rs2075798
All − MR Egger
All − Inverse variance weighted
−2
−1
0
1
MR effect size for
'FRS2' on 'Pulmonary fibrosis'

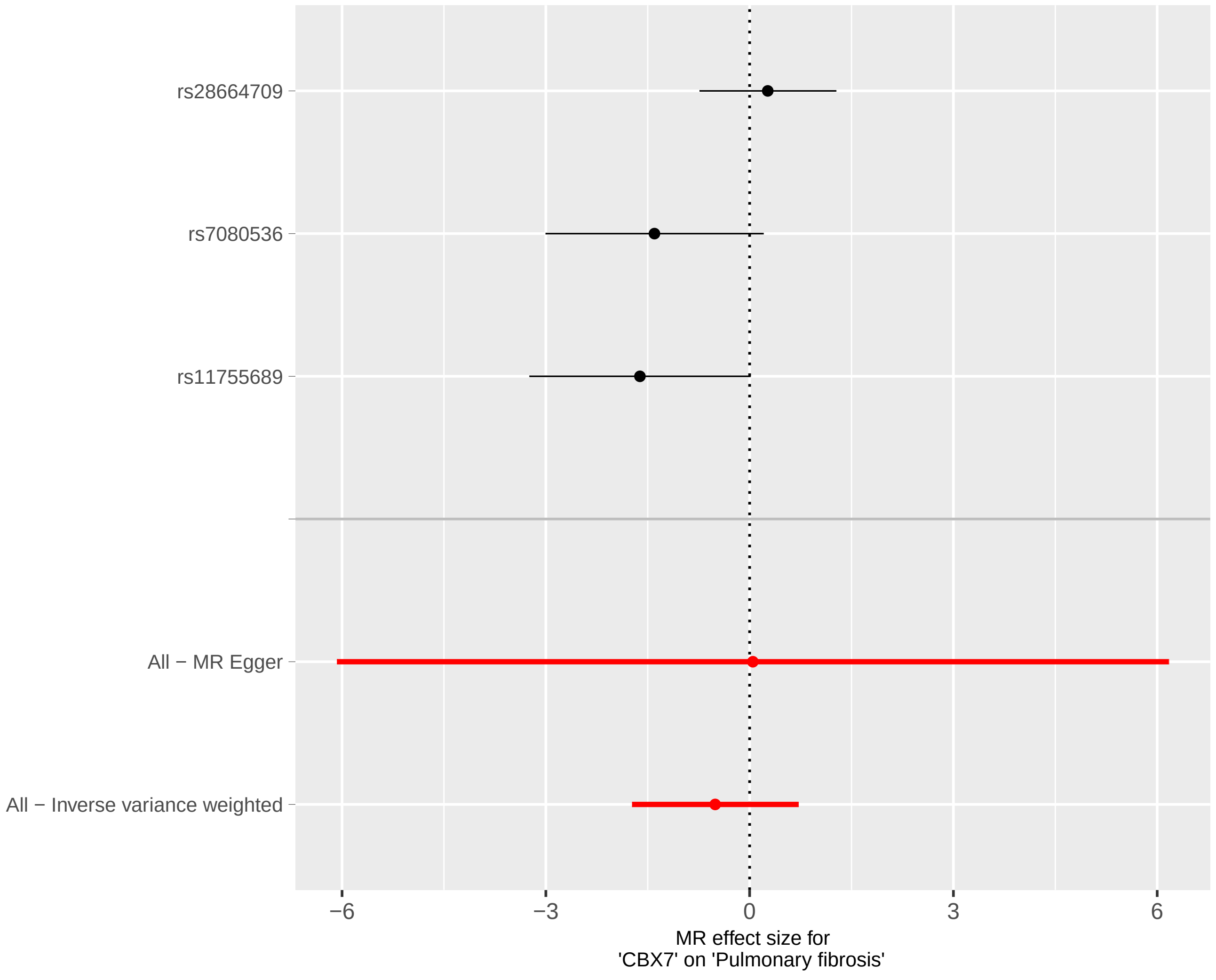
rs28664709
rs7080536
rs11755689
All − MR Egger
All − Inverse variance weighted
−6
−3
0
3
6
MR effect size for
'CBX7' on 'Pulmonary fibrosis'

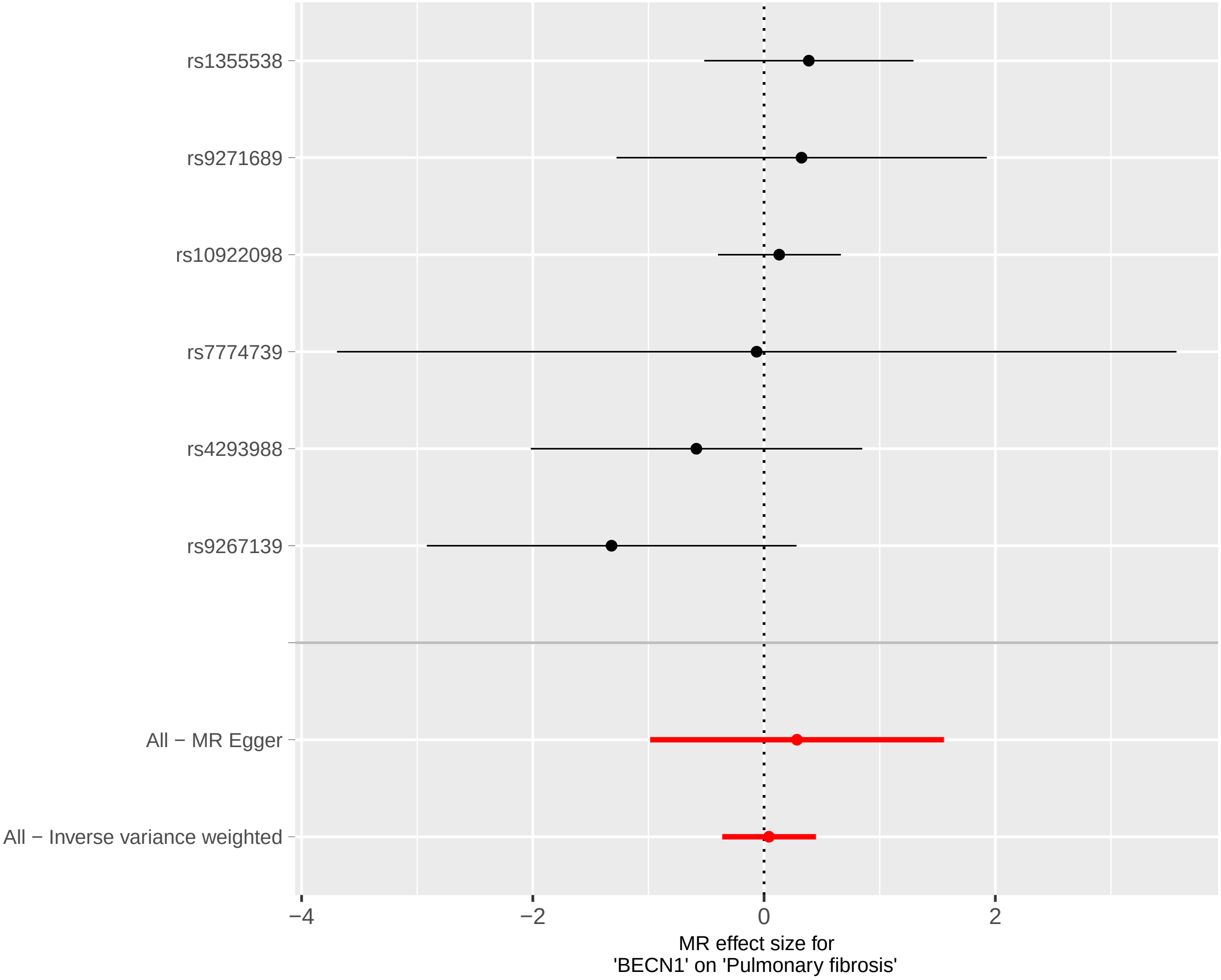

rs1355538
rs9271689
rs10922098
rs7774739
rs4293988
rs9267139
All − MR Egger
All − Inverse variance weighted
−4
−2
0
2
MR effect size for
'BECN1' on 'Pulmonary fibrosis'

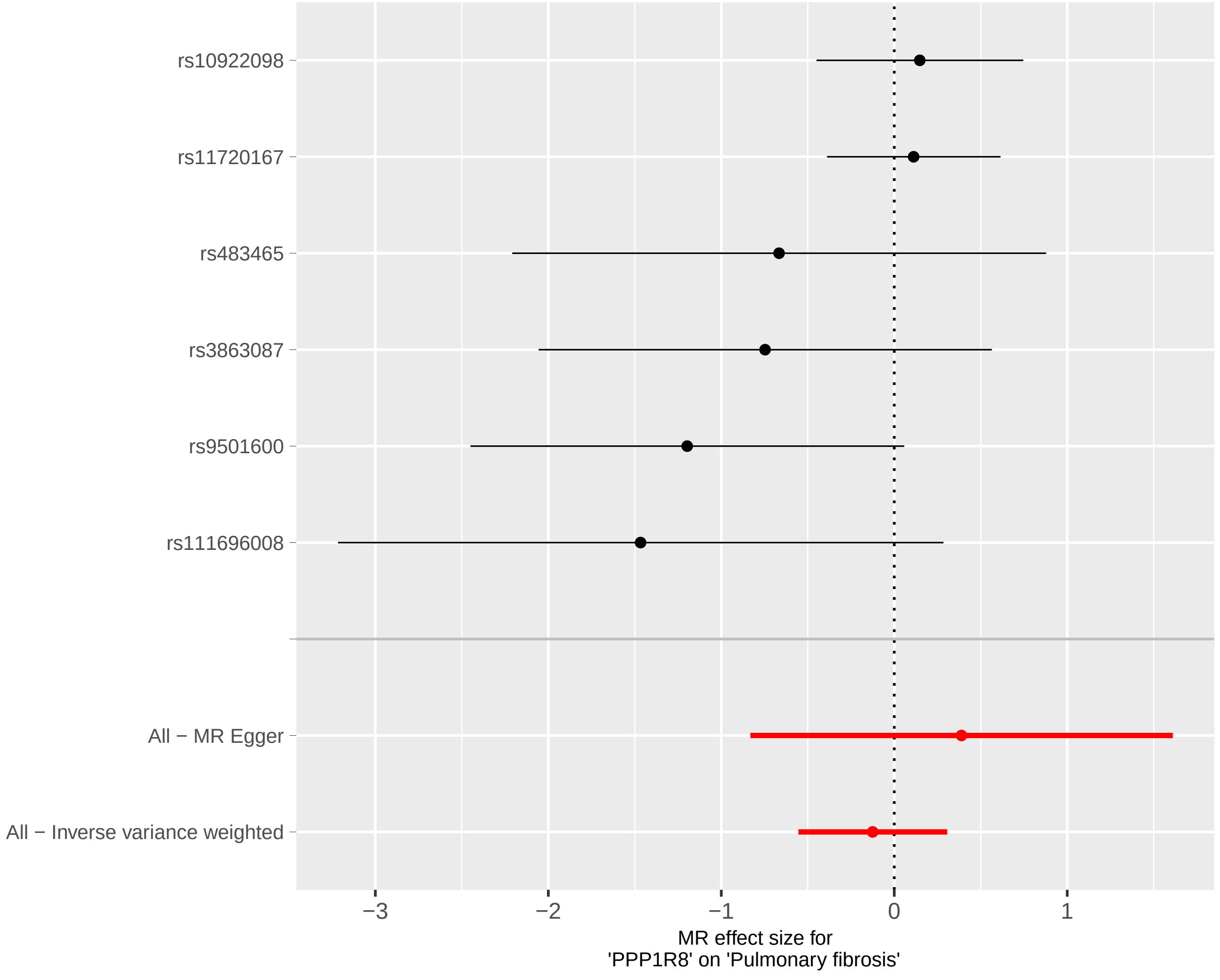

rs10922098
rs11720167
rs483465
rs3863087
rs9501600
rs111696008
All − MR Egger
All − Inverse variance weighted
−3
−2
−1
0
1
MR effect size for
'PPP1R8' on 'Pulmonary fibrosis'

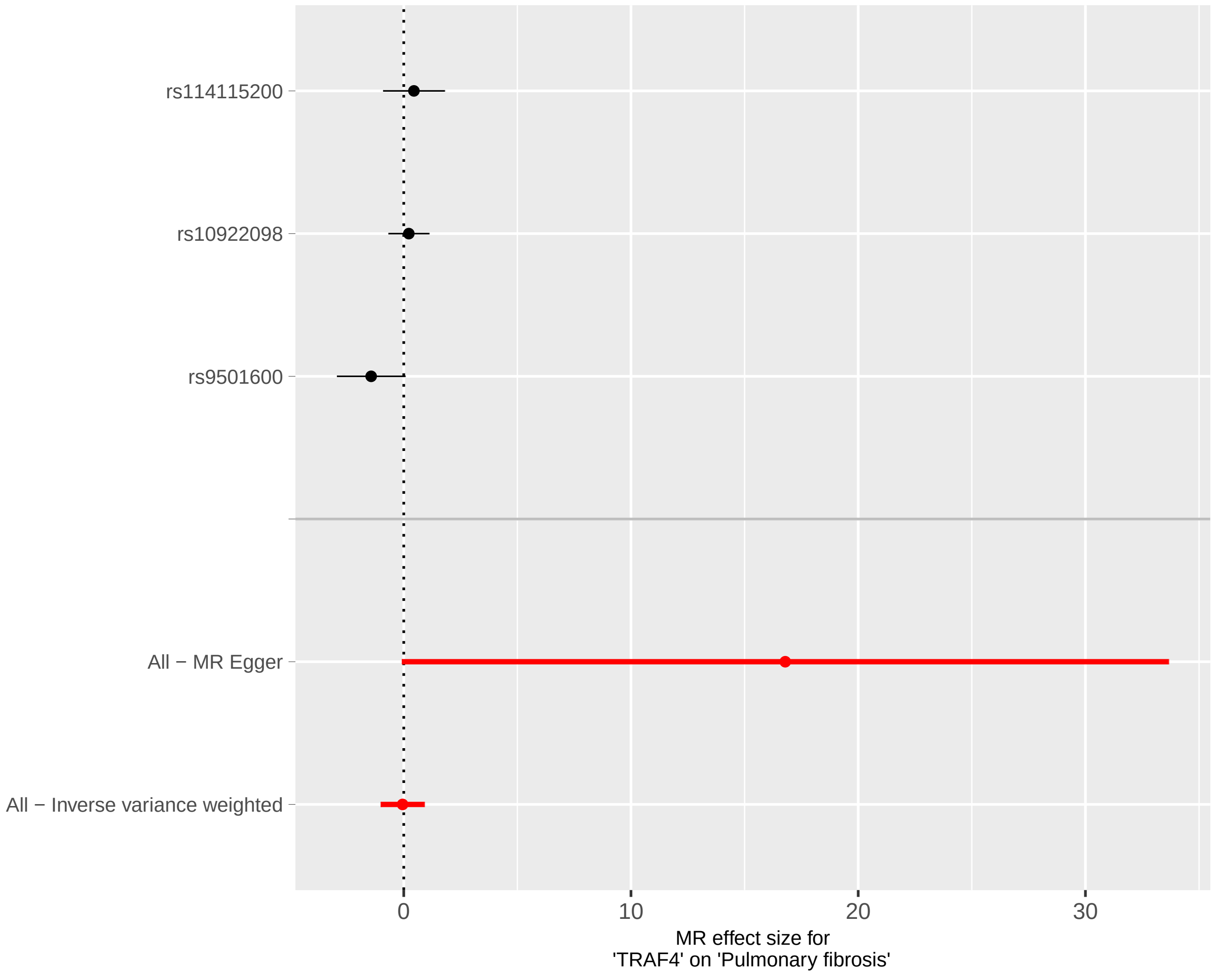

rs114115200
rs10922098
rs9501600
All − MR Egger
All − Inverse variance weighted
0
10
20
30
MR effect size for
'TRAF4' on 'Pulmonary fibrosis'

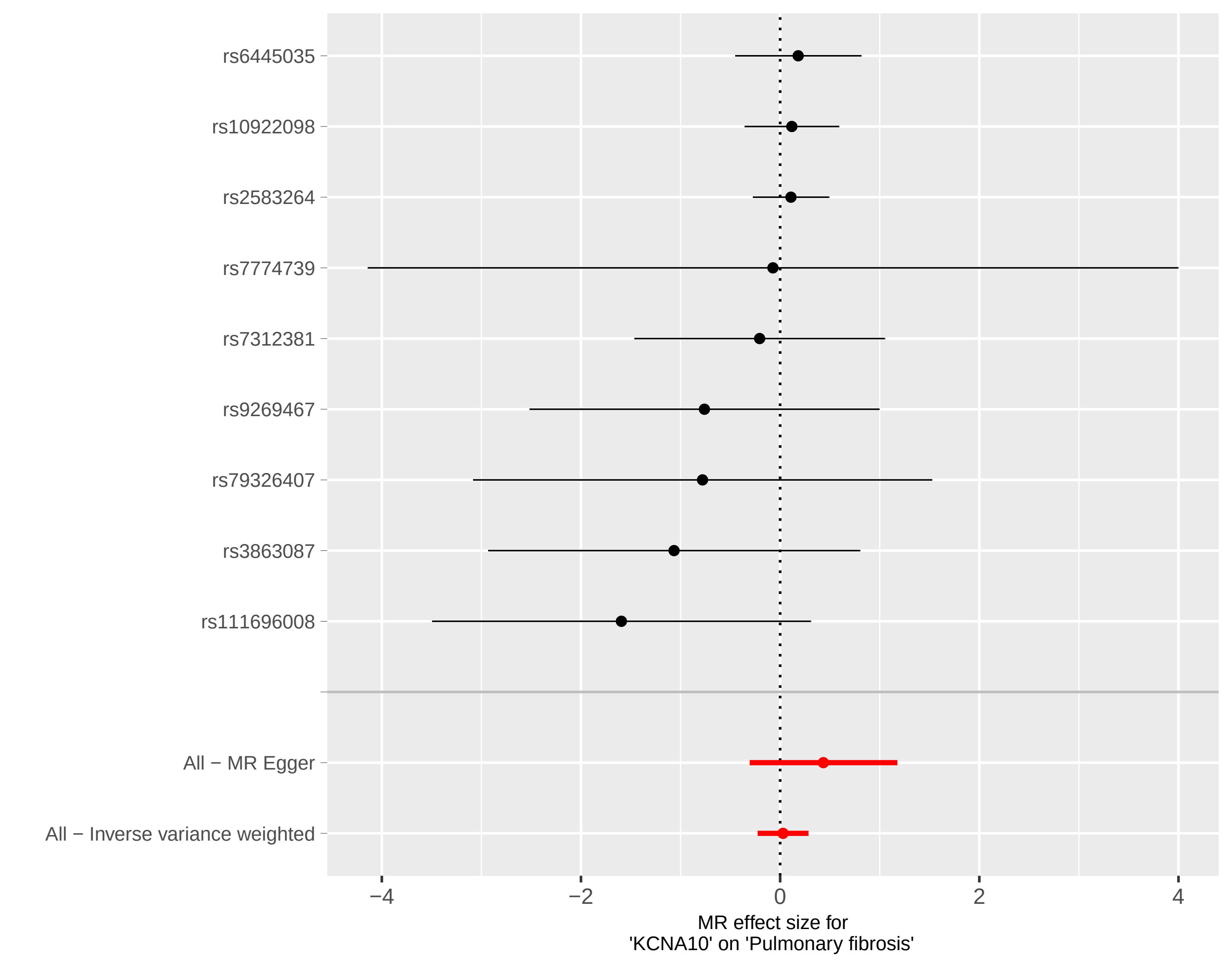
rs6445035
rs10922098
rs2583264
rs7774739
rs7312381
rs9269467
rs79326407
rs3863087
rs111696008
All − MR Egger
All − Inverse variance weighted
−4
−2
0
2
4
MR effect size for
'KCNA10' on 'Pulmonary fibrosis'

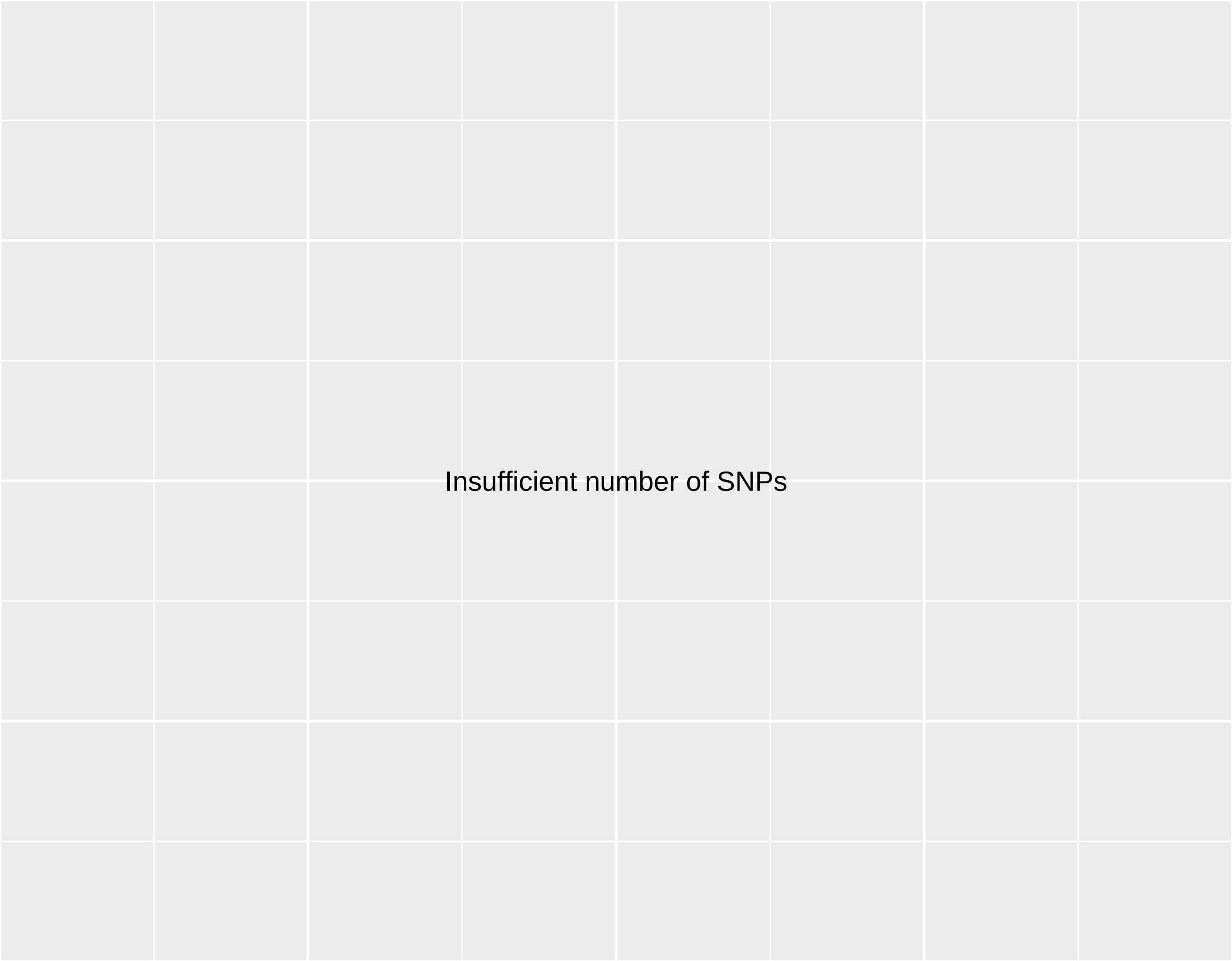
Insufficient number of SNPs

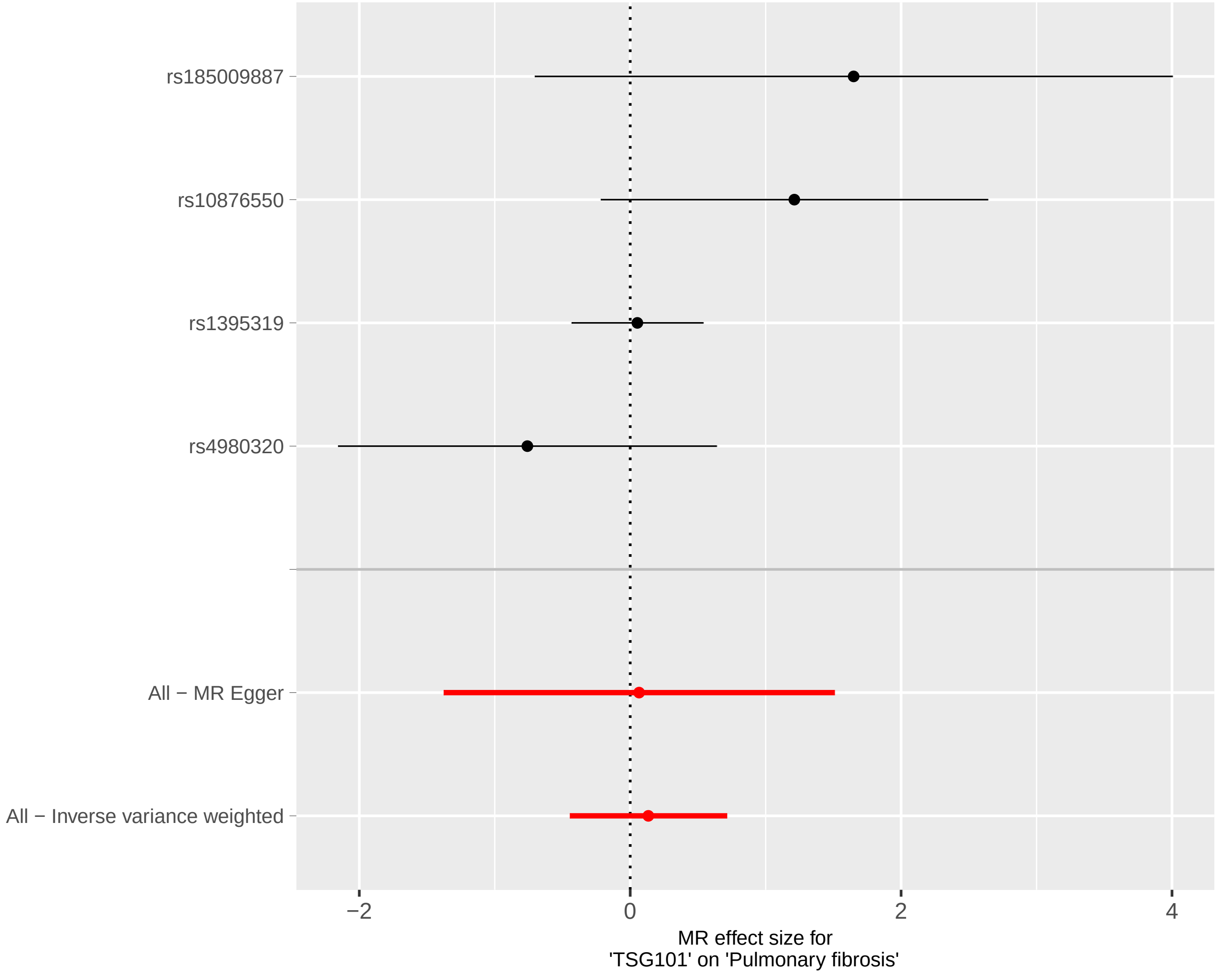
rs185009887
rs10876550
rs1395319
rs4980320
All − MR Egger
All − Inverse variance weighted
−2
0
2
4
MR effect size for
'TSG101' on 'Pulmonary fibrosis'

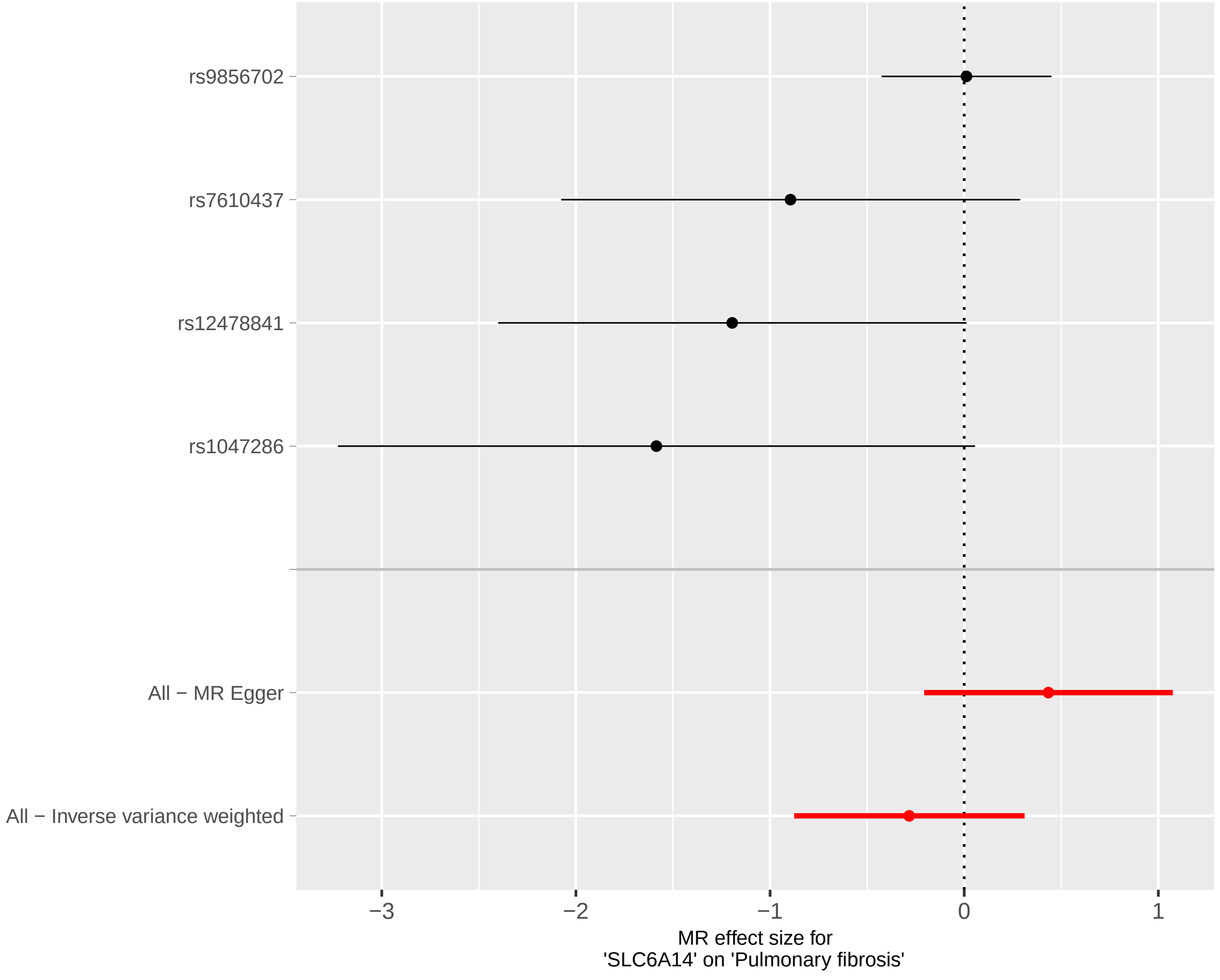
rs9856702
rs7610437
rs12478841
rs1047286
All − MR Egger
All − Inverse variance weighted
−3
−2
−1
0
1
MR effect size for
'SLC6A14' on 'Pulmonary fibrosis'

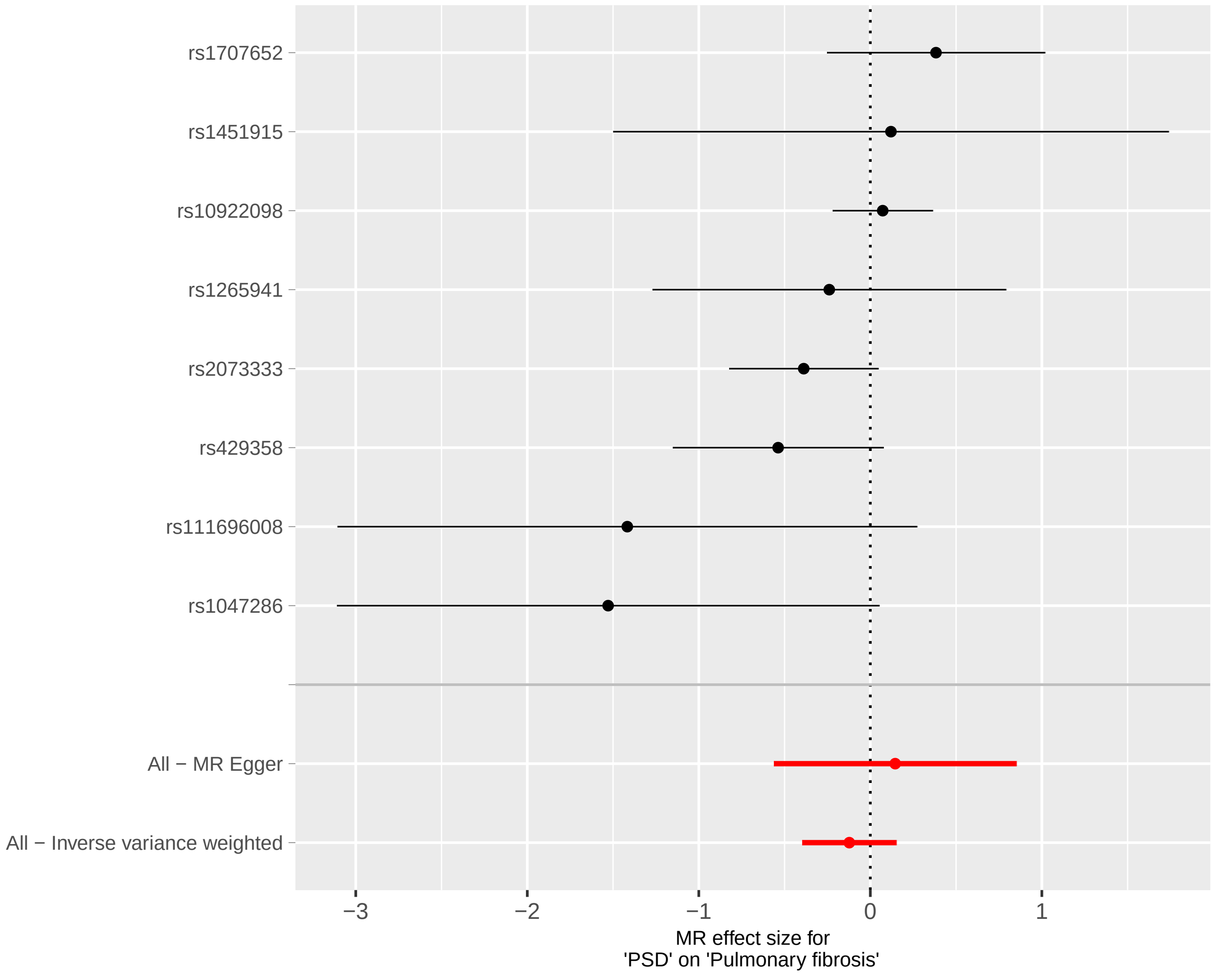

rs1707652
rs1451915
rs10922098
rs1265941
rs2073333
rs429358
rs111696008
rs1047286
All − MR Egger
All − Inverse variance weighted
−3
−2
−1
0
1
MR effect size for
'PSD' on 'Pulmonary fibrosis'

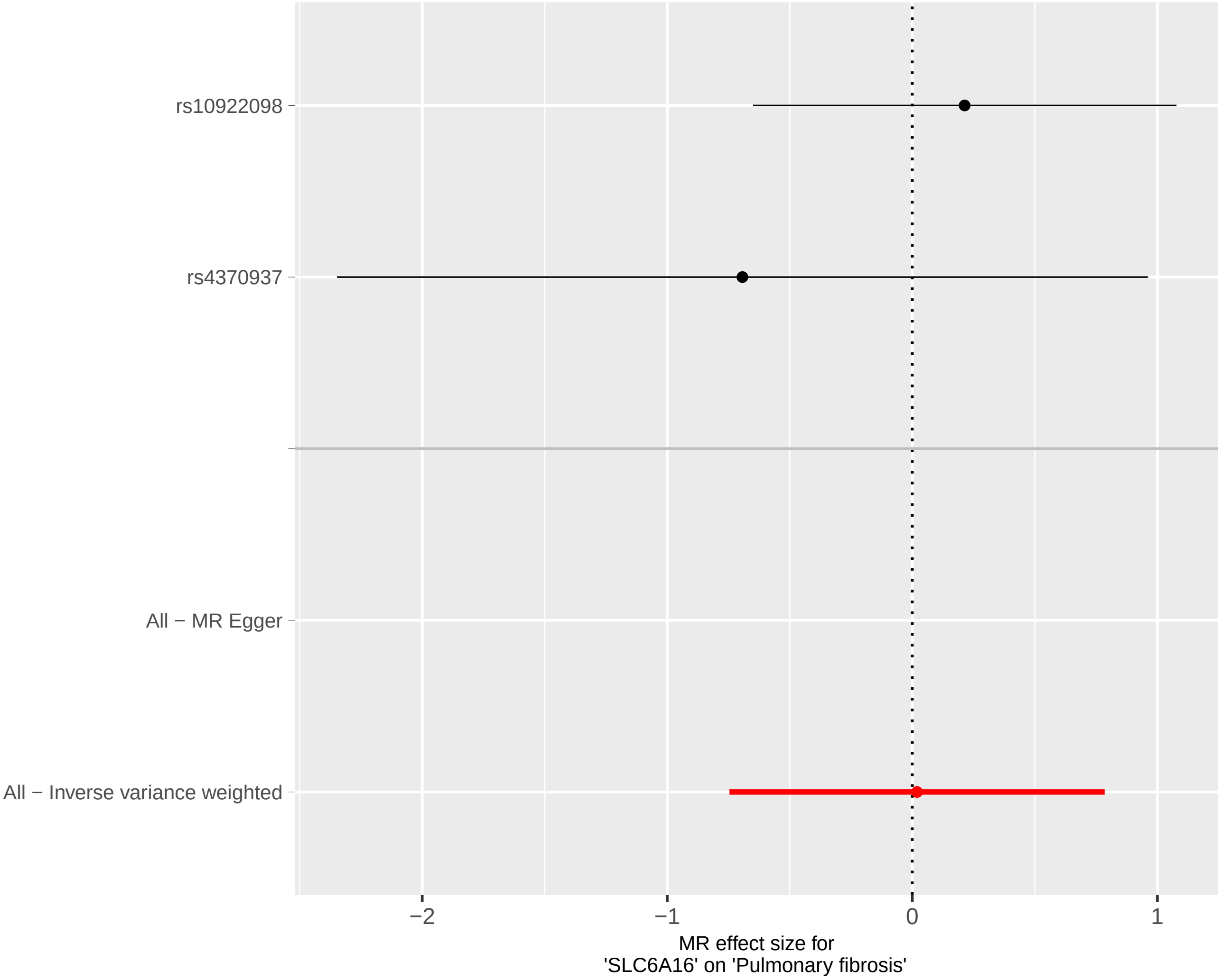
rs10922098
rs4370937
All − MR Egger
All − Inverse variance weighted
−2
−1
0
1
MR effect size for
'SLC6A16' on 'Pulmonary fibrosis'

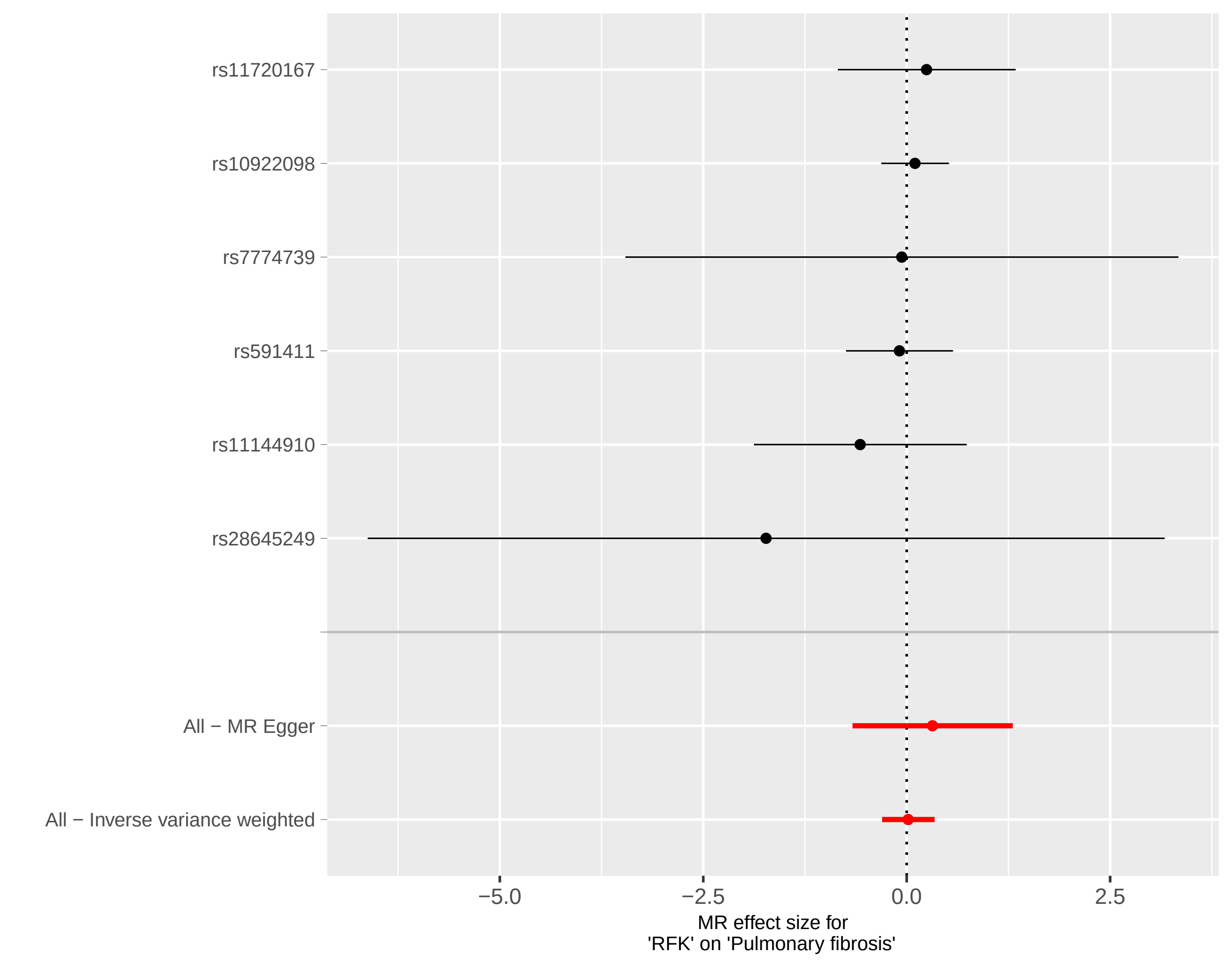
rs11720167
rs10922098
rs7774739
rs591411
rs11144910
rs28645249
All − MR Egger
All − Inverse variance weighted
−5.0
−2.5
0.0
2.5
MR effect size for
'RFK' on 'Pulmonary fibrosis'

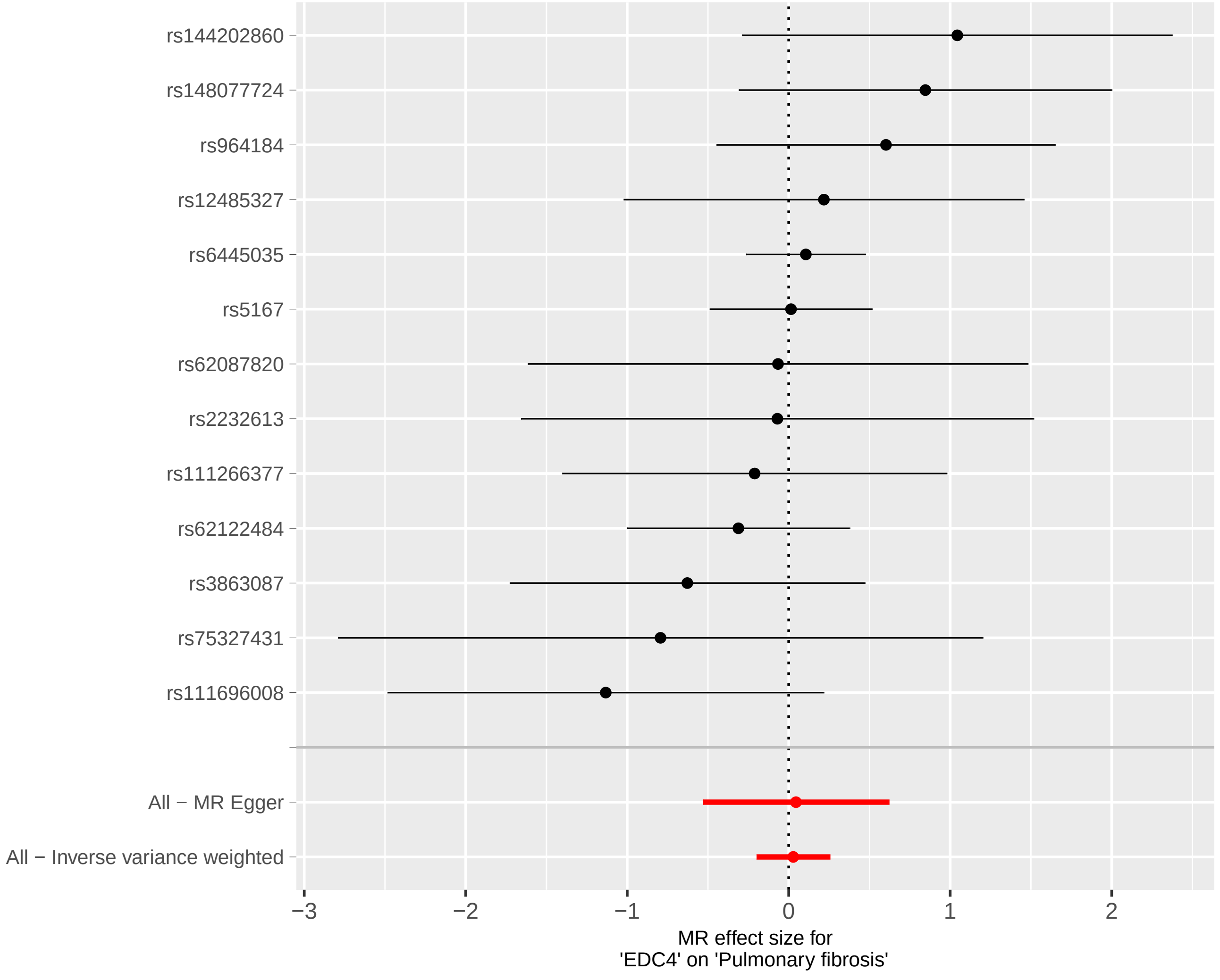
rs144202860
rs148077724
rs964184
rs12485327
rs6445035
rs5167
rs62087820
rs2232613
rs111266377
rs62122484
rs3863087
rs75327431
rs111696008
All − MR Egger
All − Inverse variance weighted
−3
−2
−1
0
1
2
MR effect size for
'EDC4' on 'Pulmonary fibrosis'

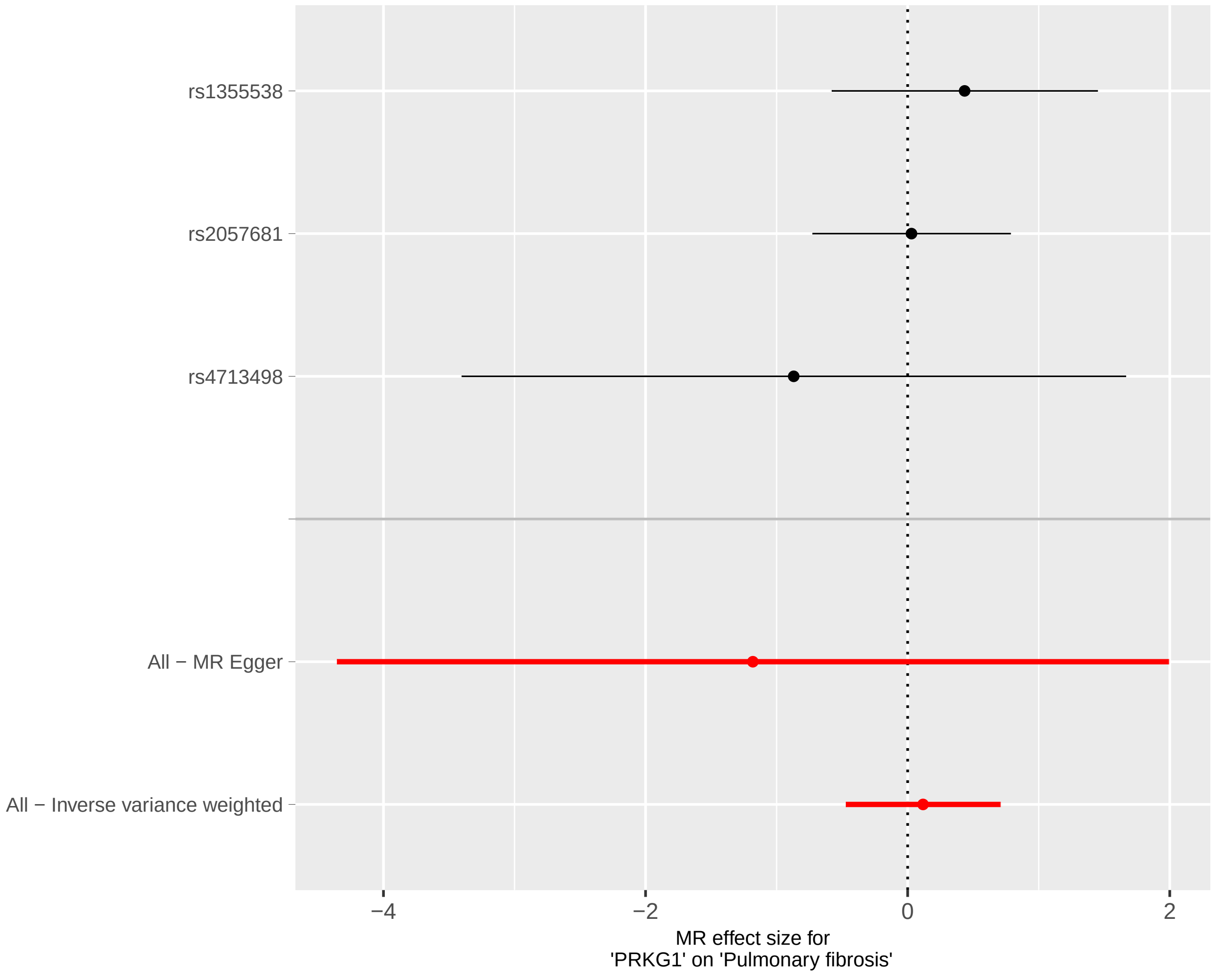
rs1355538
rs2057681
rs4713498
All − MR Egger
All − Inverse variance weighted
−4
−2
0
2
MR effect size for
'PRKG1' on 'Pulmonary fibrosis'

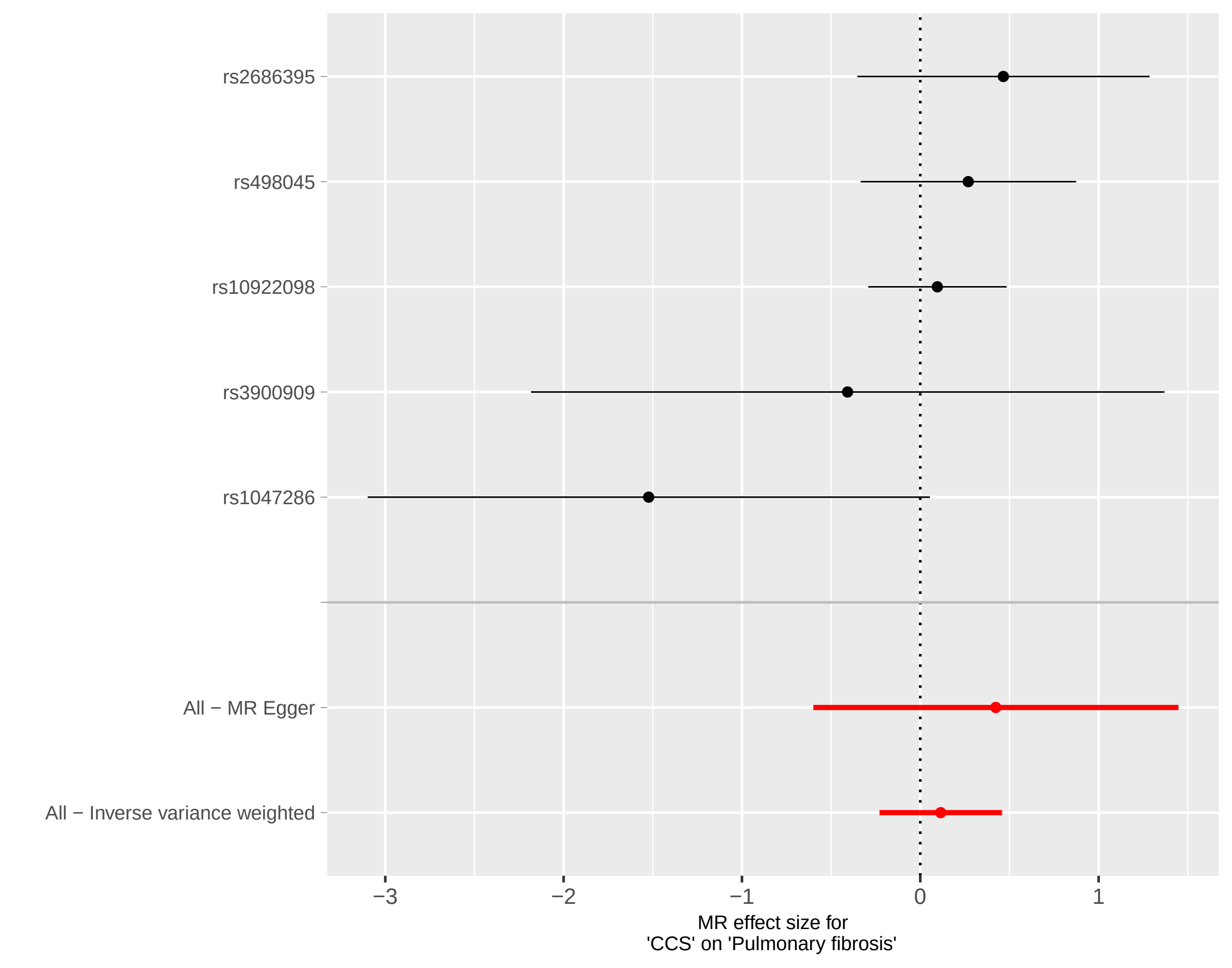

rs2686395
rs498045
rs10922098
rs3900909
rs1047286
All − MR Egger
All − Inverse variance weighted
−3
−2
−1
0
1
MR effect size for
'CCS' on 'Pulmonary fibrosis'

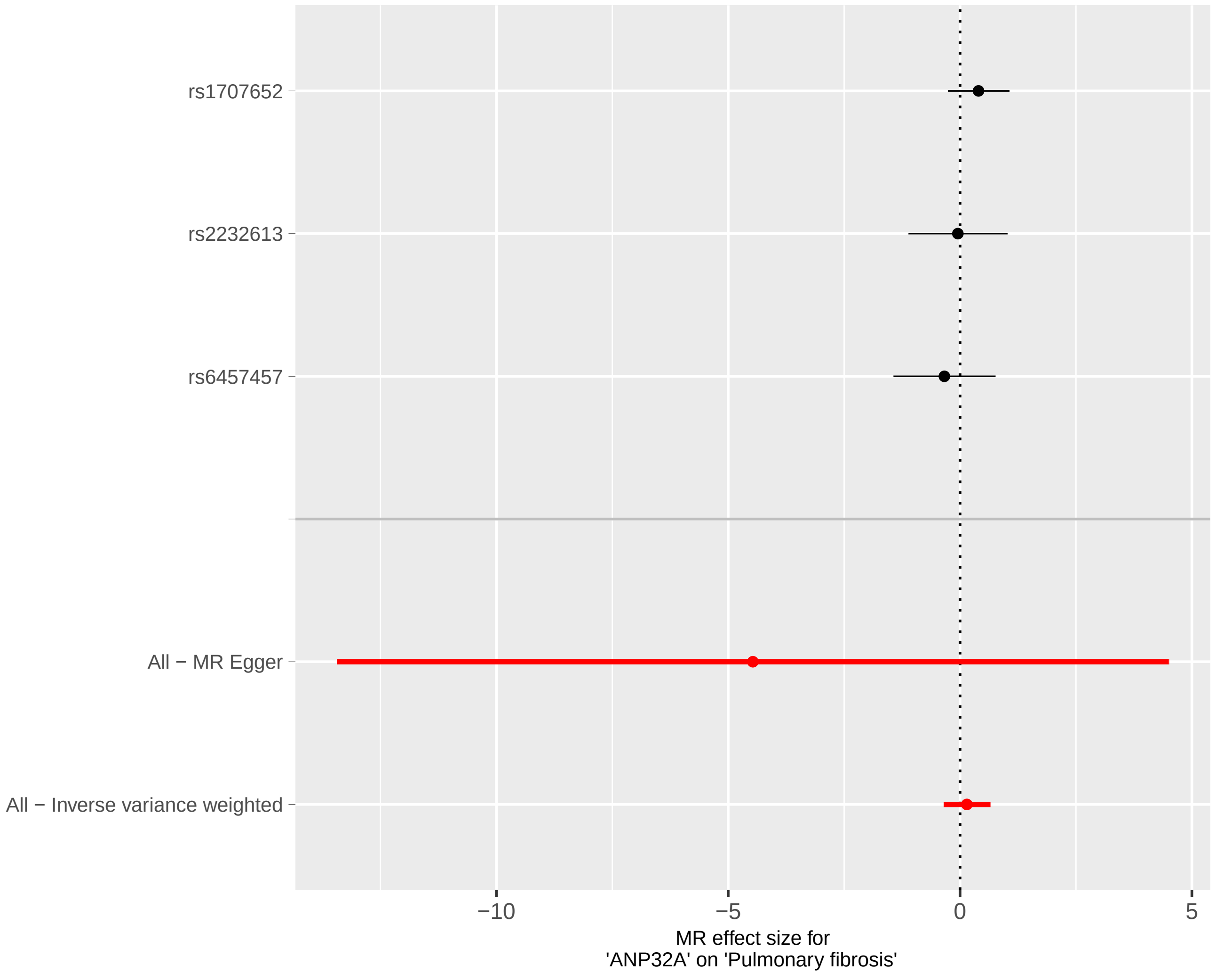

rs1707652
rs2232613
rs6457457
All − MR Egger
All − Inverse variance weighted
−10
−5
0
5
MR effect size for
'ANP32A' on 'Pulmonary fibrosis'

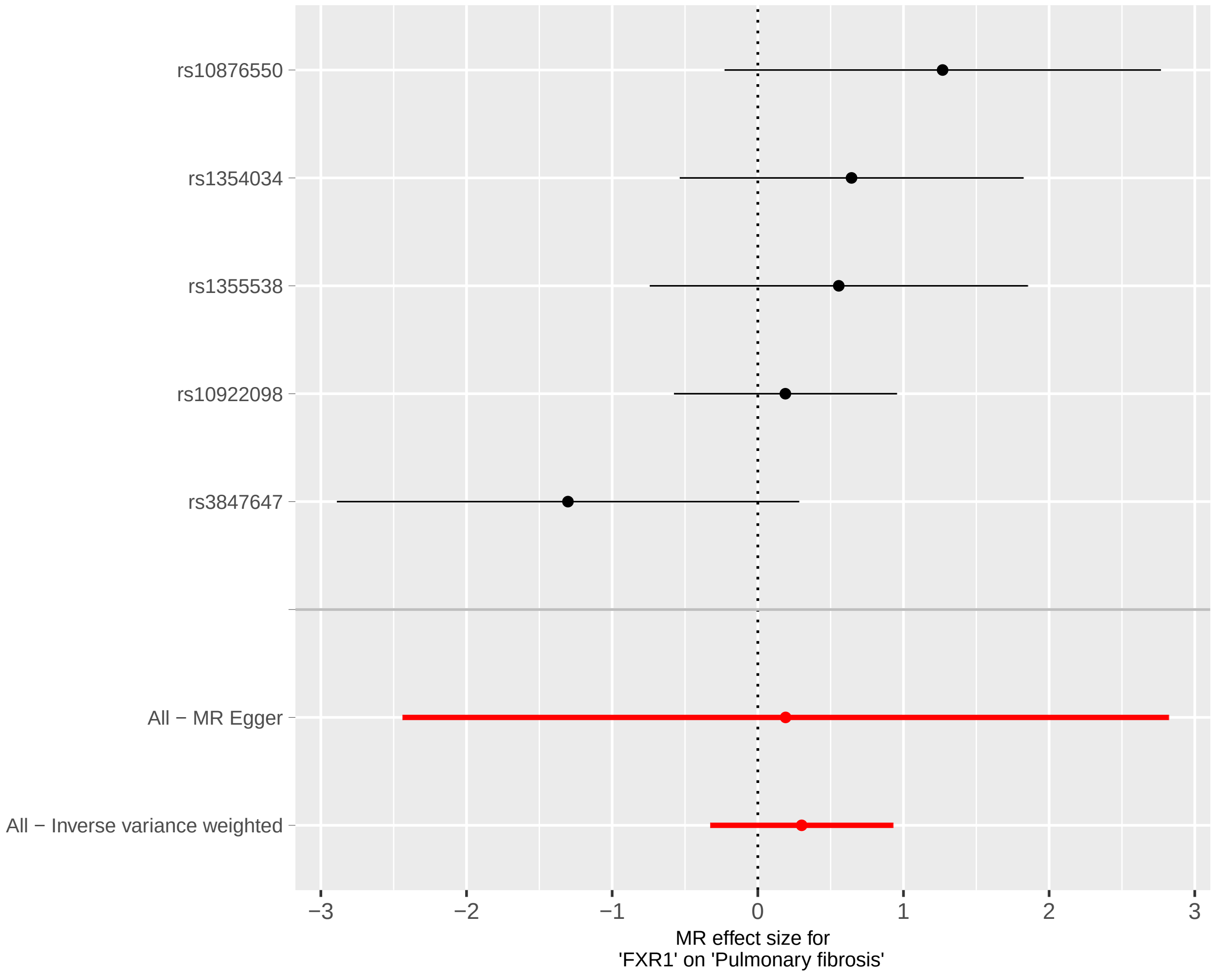

rs10876550
rs1354034
rs1355538
rs10922098
rs3847647
All − MR Egger
All − Inverse variance weighted
−3
−2
−1
0
1
2
3
MR effect size for
'FXR1' on 'Pulmonary fibrosis'

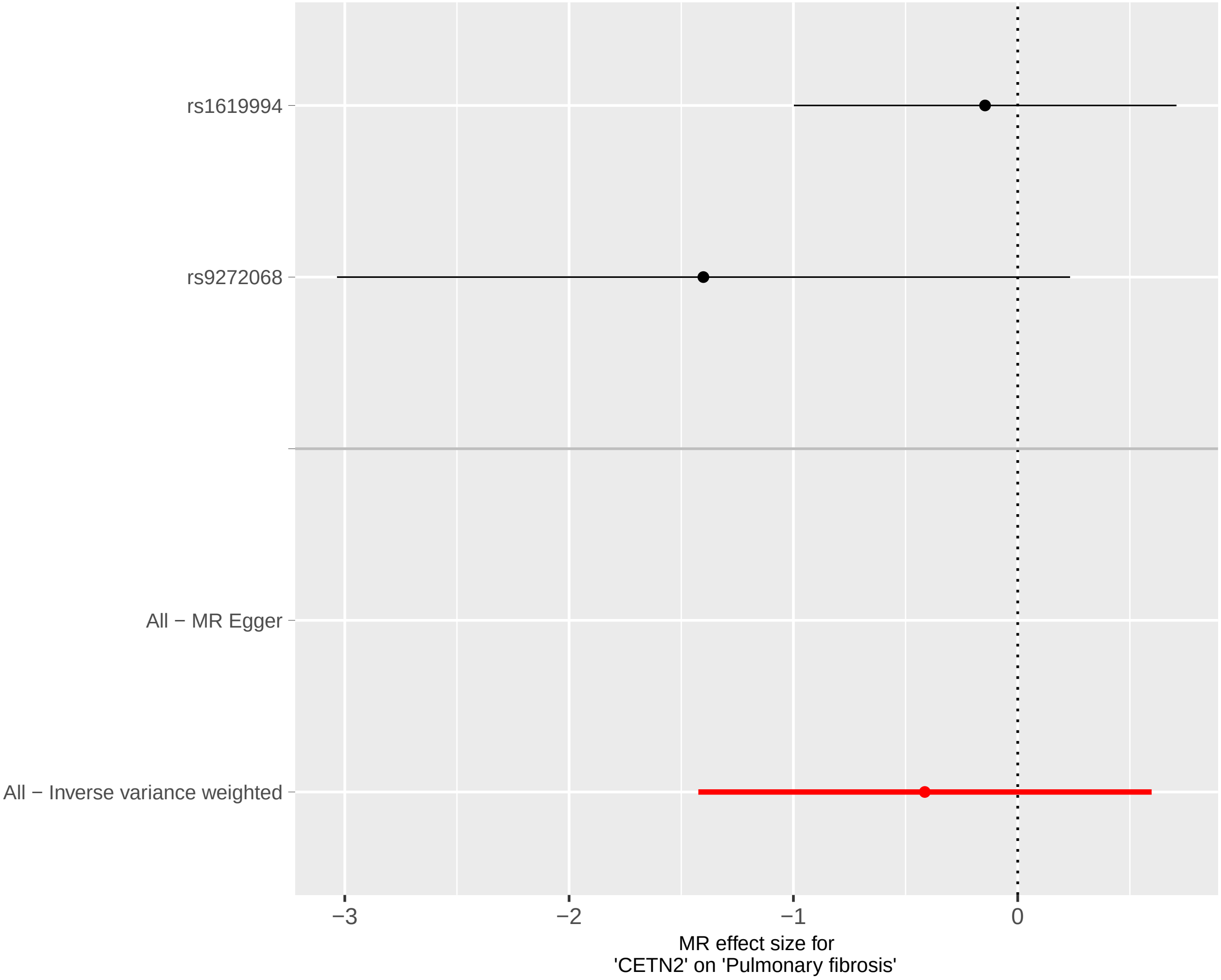
rs1619994
rs9272068
All − MR Egger
All − Inverse variance weighted
−3
−2
−1
0
MR effect size for
'CETN2' on 'Pulmonary fibrosis'

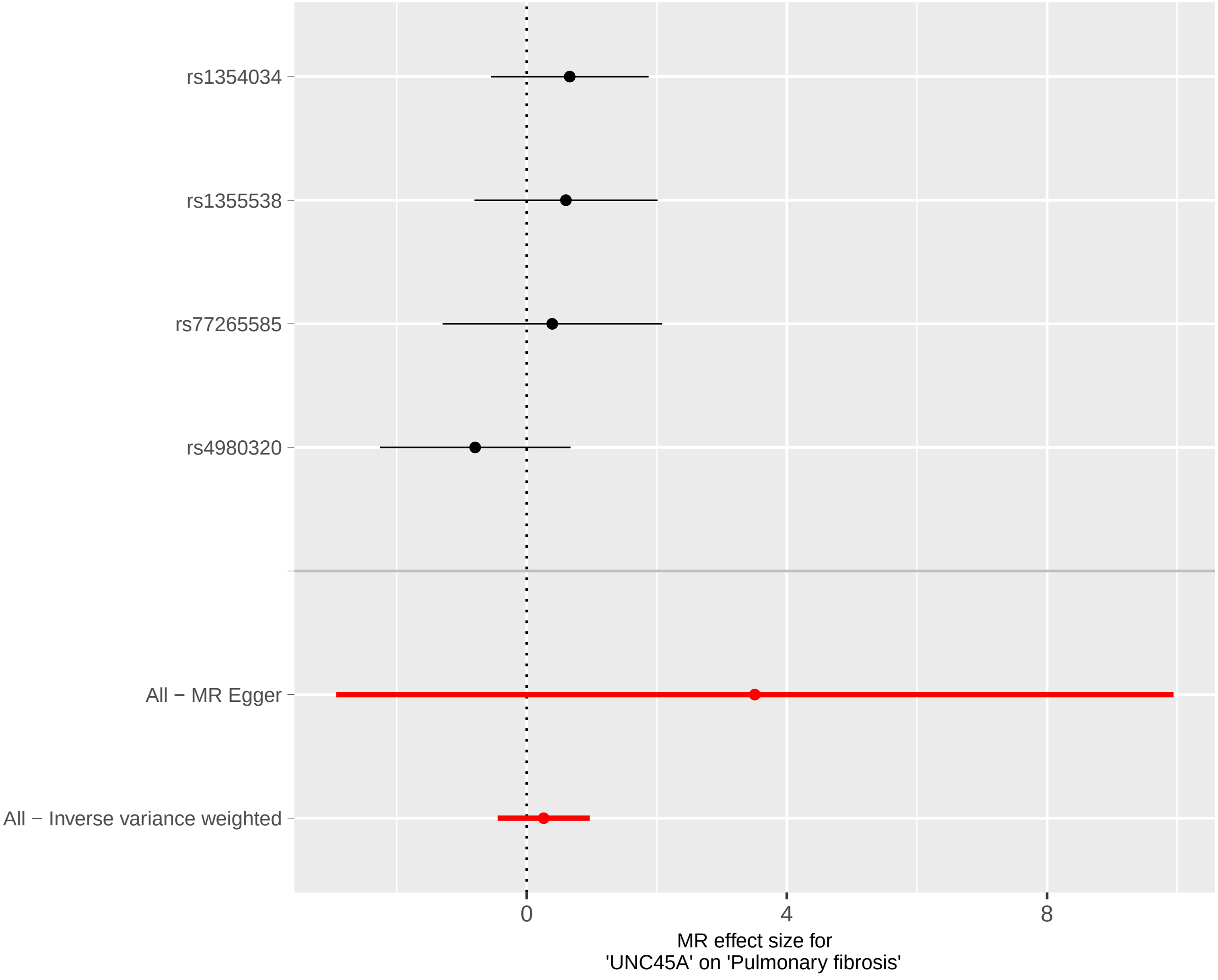

rs1354034
rs1355538
rs77265585
rs4980320
All − MR Egger
All − Inverse variance weighted
0
4
8
MR effect size for
'UNC45A' on 'Pulmonary fibrosis'

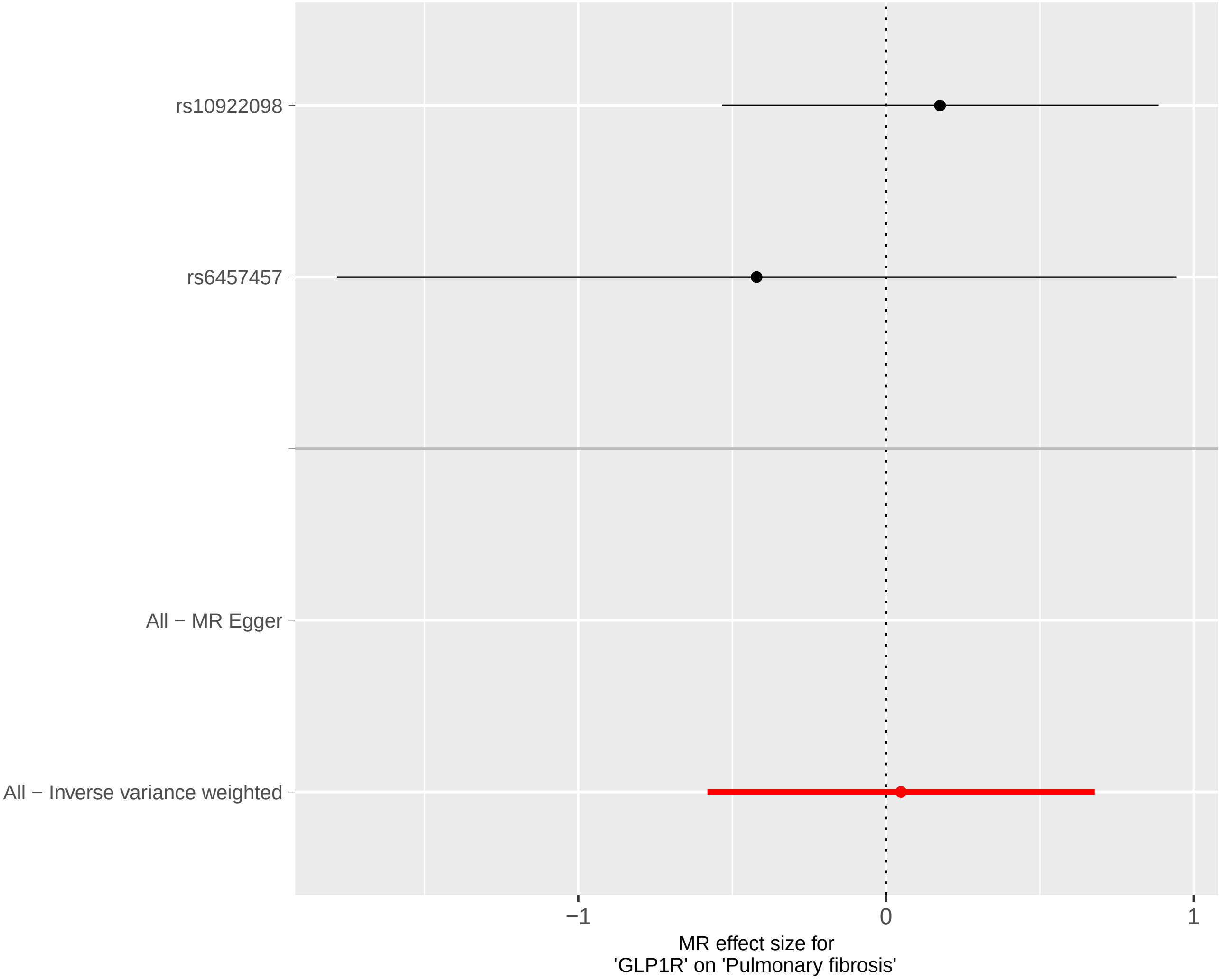
rs10922098
rs6457457
All − MR Egger
All − Inverse variance weighted
−1
0
1
MR effect size for
'GLP1R' on 'Pulmonary fibrosis'

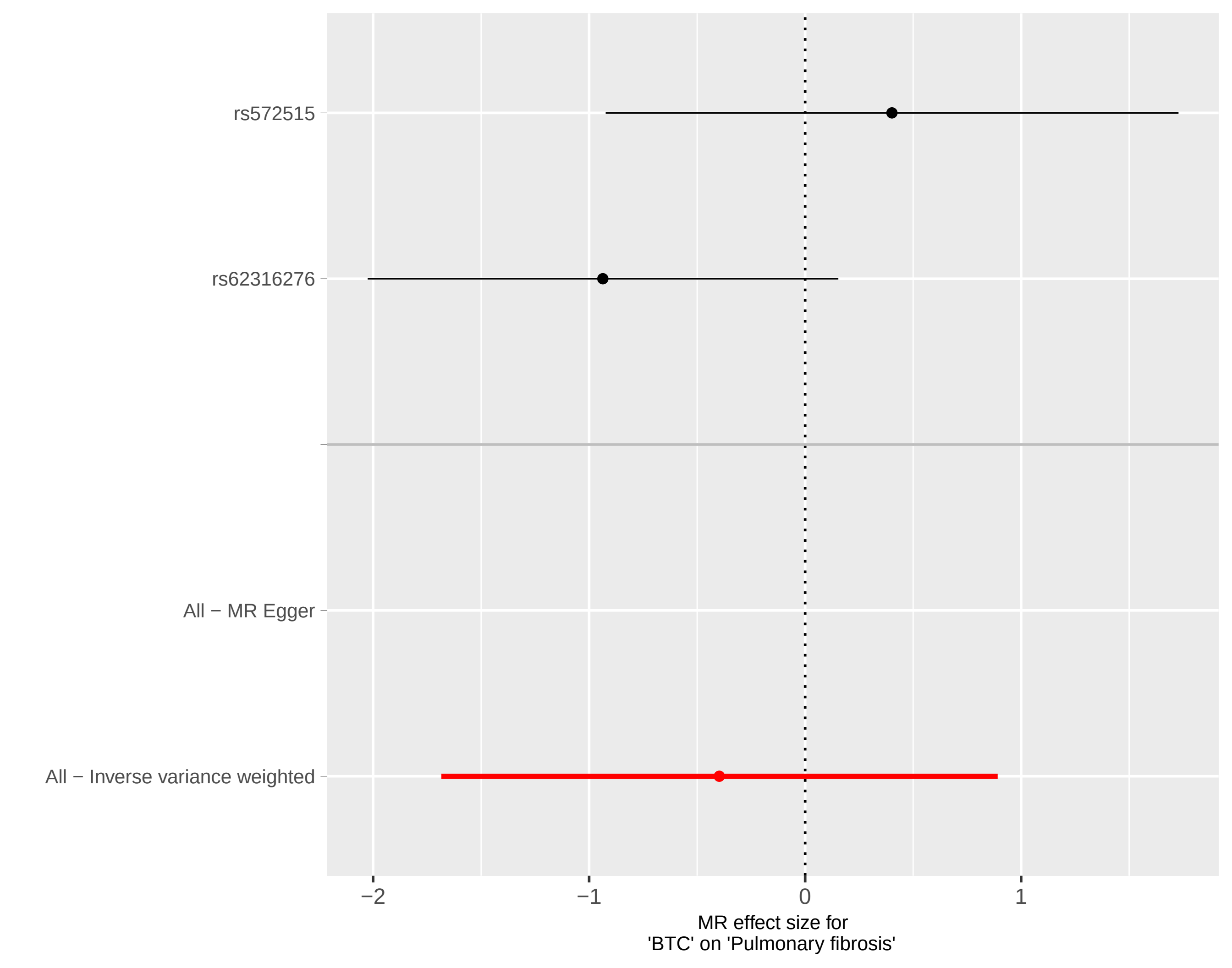
rs572515
rs62316276
All − MR Egger
All − Inverse variance weighted
−2
−1
0
1
MR effect size for
'BTC' on 'Pulmonary fibrosis'

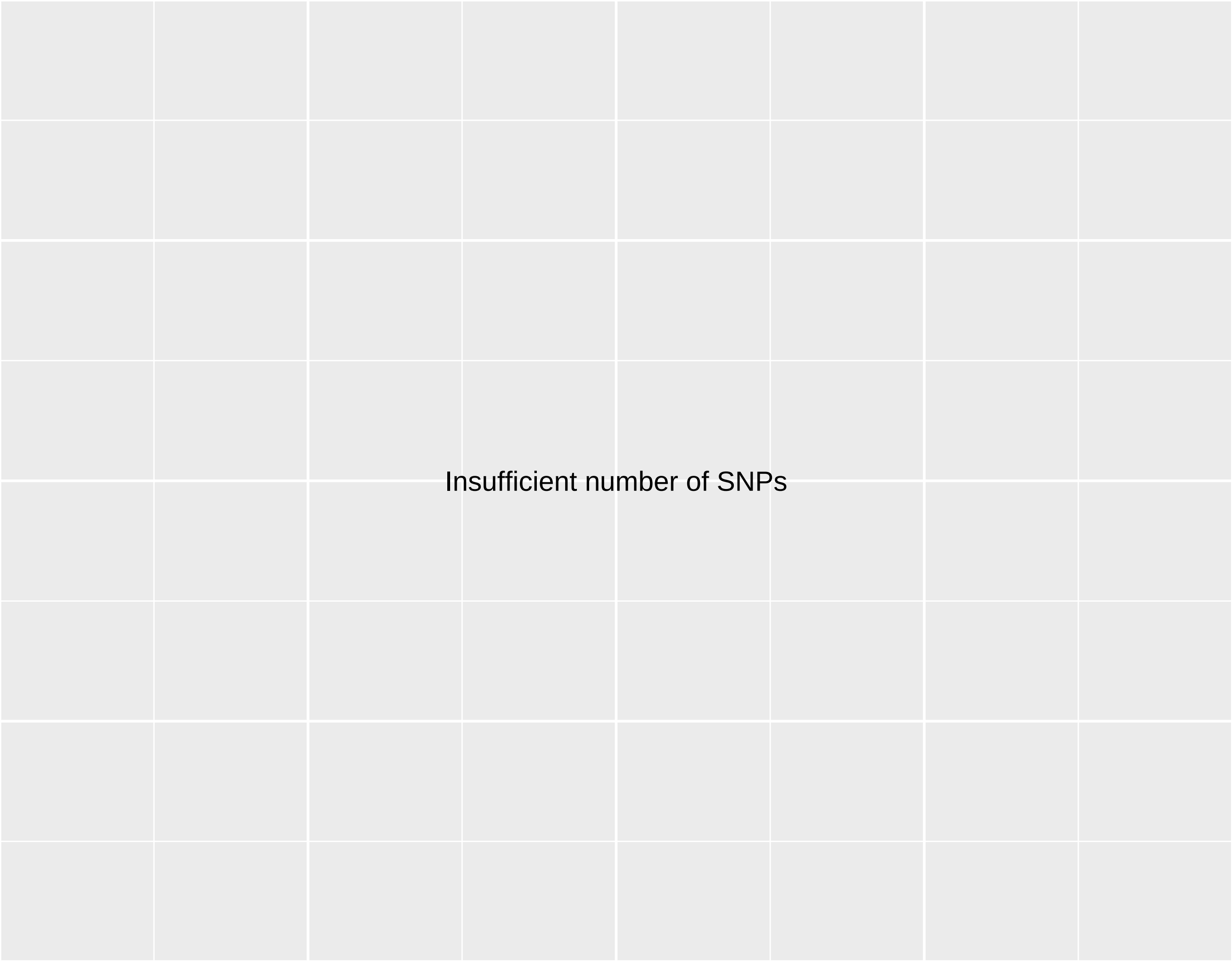
Insufficient number of SNPs

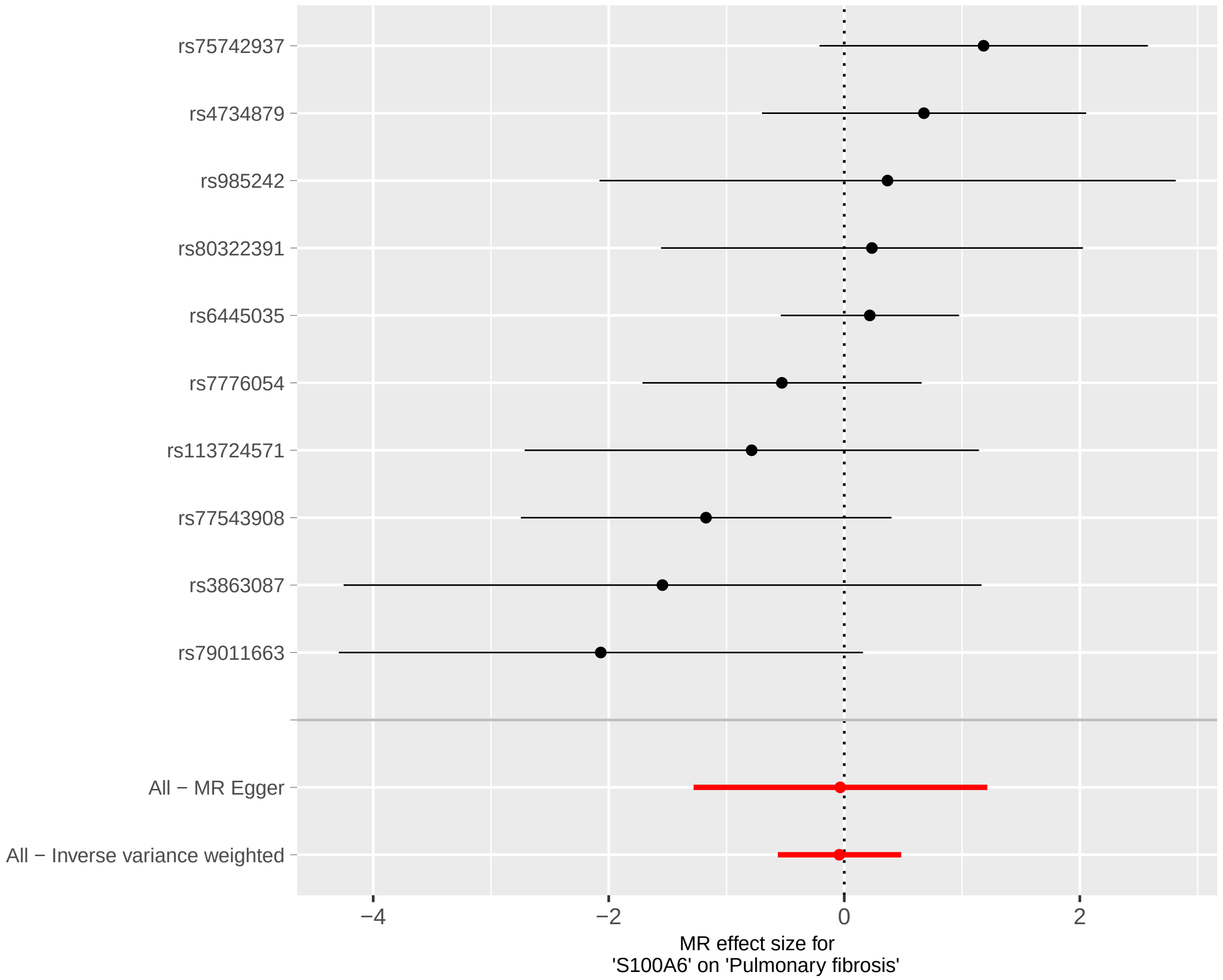
rs75742937
rs4734879
rs985242
rs80322391
rs6445035
rs7776054
rs113724571
rs77543908
rs3863087
rs79011663
All − MR Egger
All − Inverse variance weighted
−4
−2
0
2
MR effect size for
'S100A6' on 'Pulmonary fibrosis'

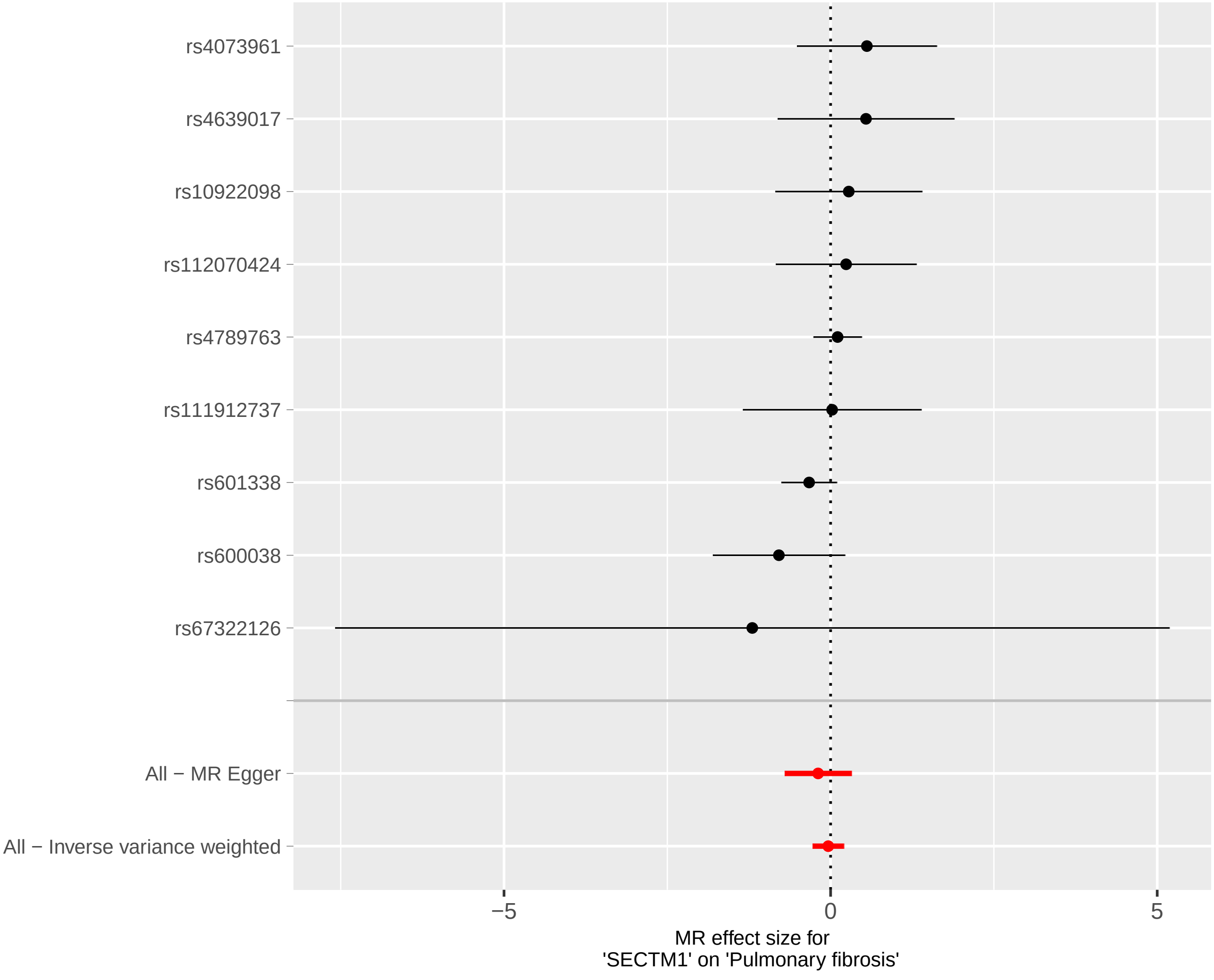
rs4073961
rs4639017
rs10922098
rs112070424
rs4789763
rs111912737
rs601338
rs600038
rs67322126
All − MR Egger
All − Inverse variance weighted
−5
0
5
MR effect size for
'SECTM1' on 'Pulmonary fibrosis'

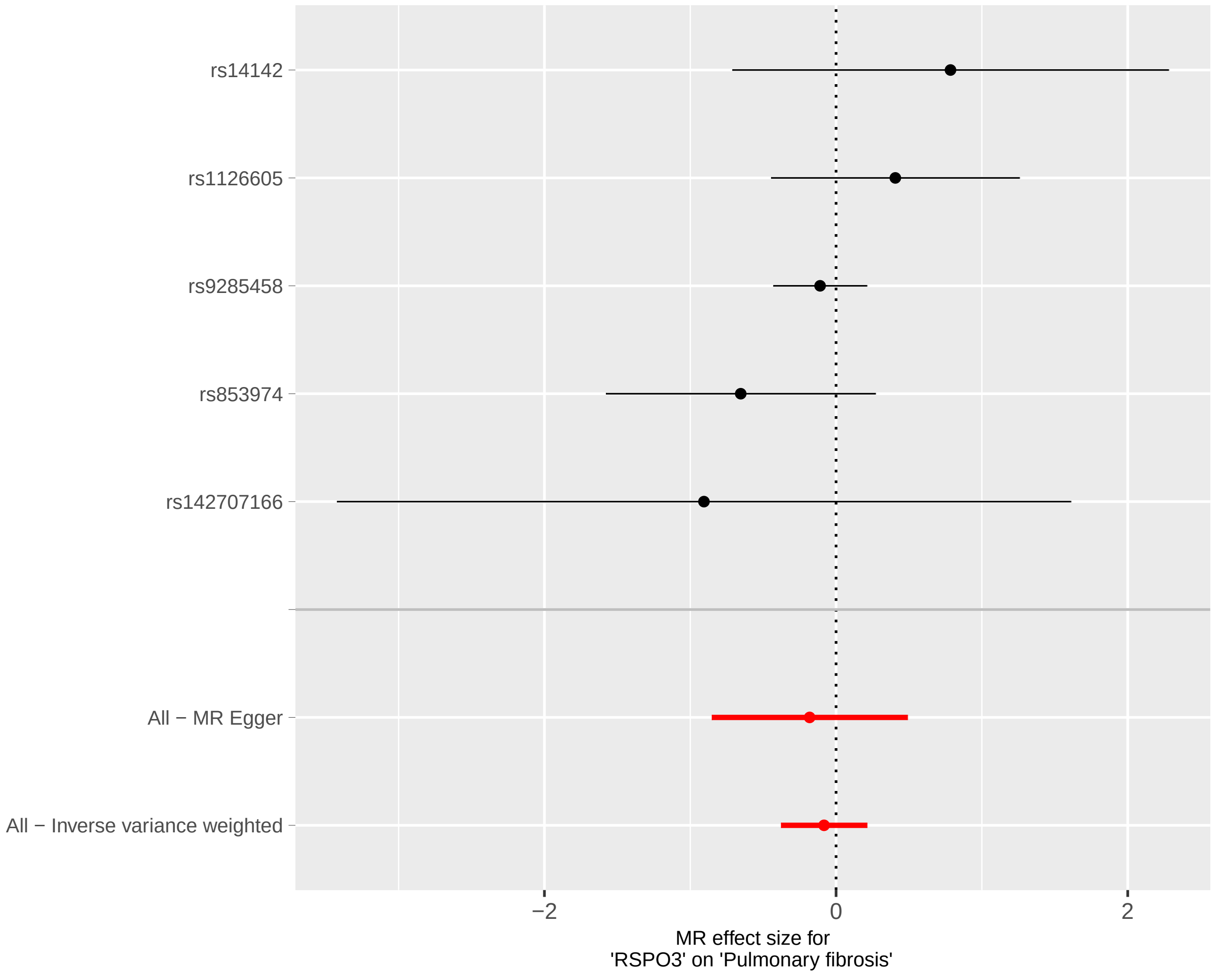
rs14142
rs1126605
rs9285458
rs853974
rs142707166
All − MR Egger
All − Inverse variance weighted
−2
0
2
MR effect size for
'RSPO3' on 'Pulmonary fibrosis'

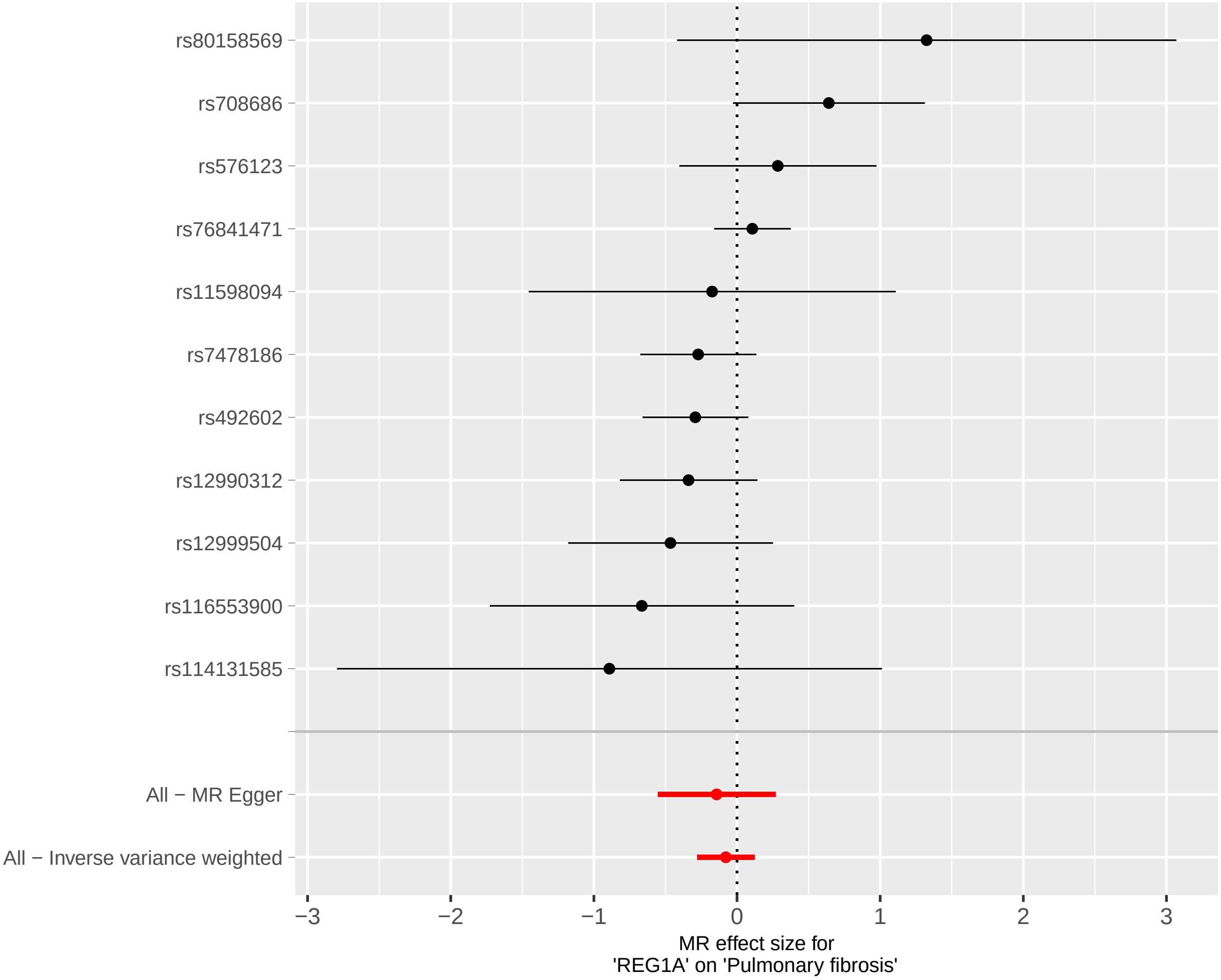
rs80158569
rs708686
rs576123
rs76841471
rs11598094
rs7478186
rs492602
rs12990312
rs12999504
rs116553900
rs114131585
All − MR Egger
All − Inverse variance weighted
−3
−2
−1
0
1
2
3
MR effect size for
'REG1A' on 'Pulmonary fibrosis'

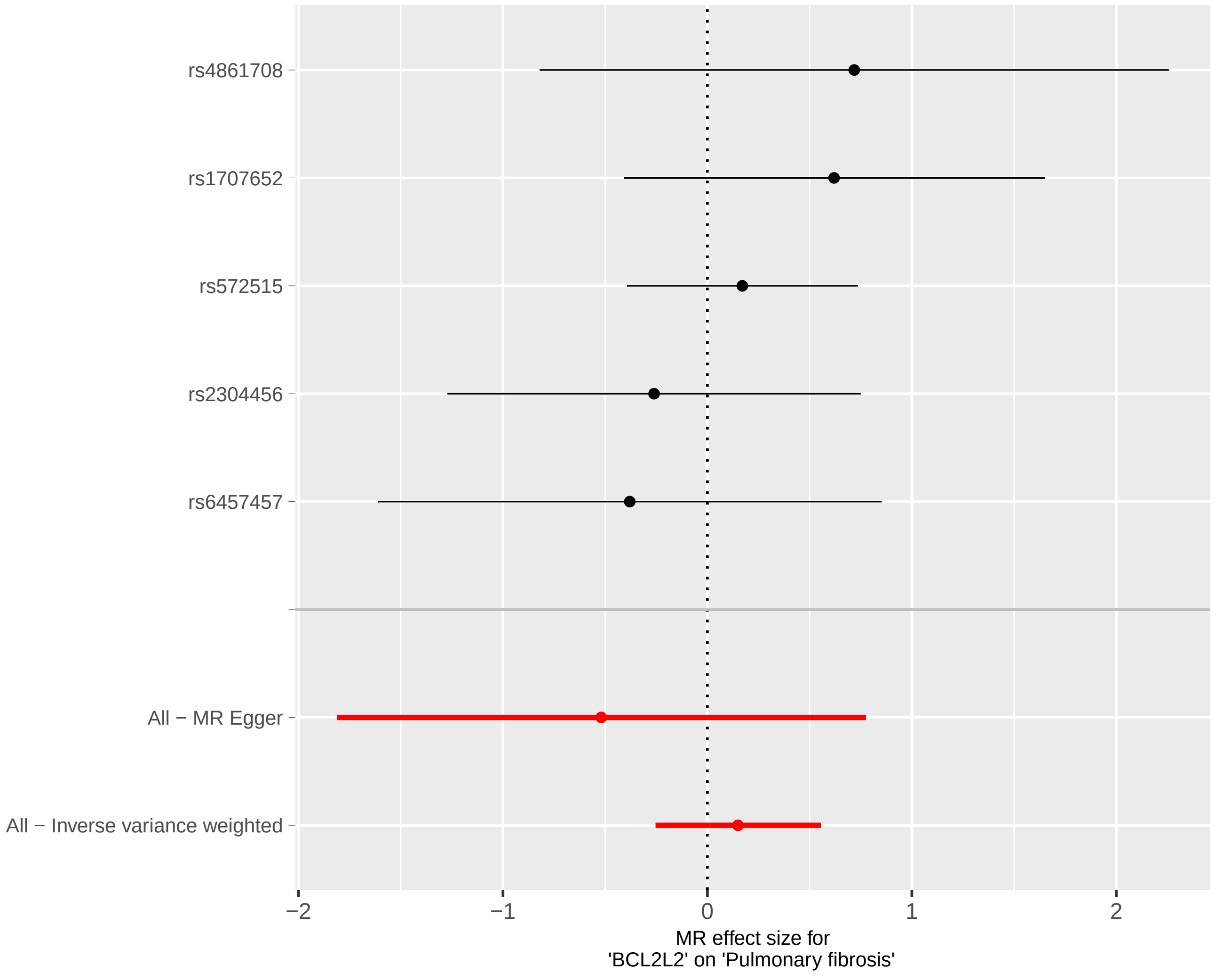

rs4861708
rs1707652
rs572515
rs2304456
rs6457457
All − MR Egger
All − Inverse variance weighted
−2
−1
0
1
2
MR effect size for
'BCL2L2' on 'Pulmonary fibrosis'

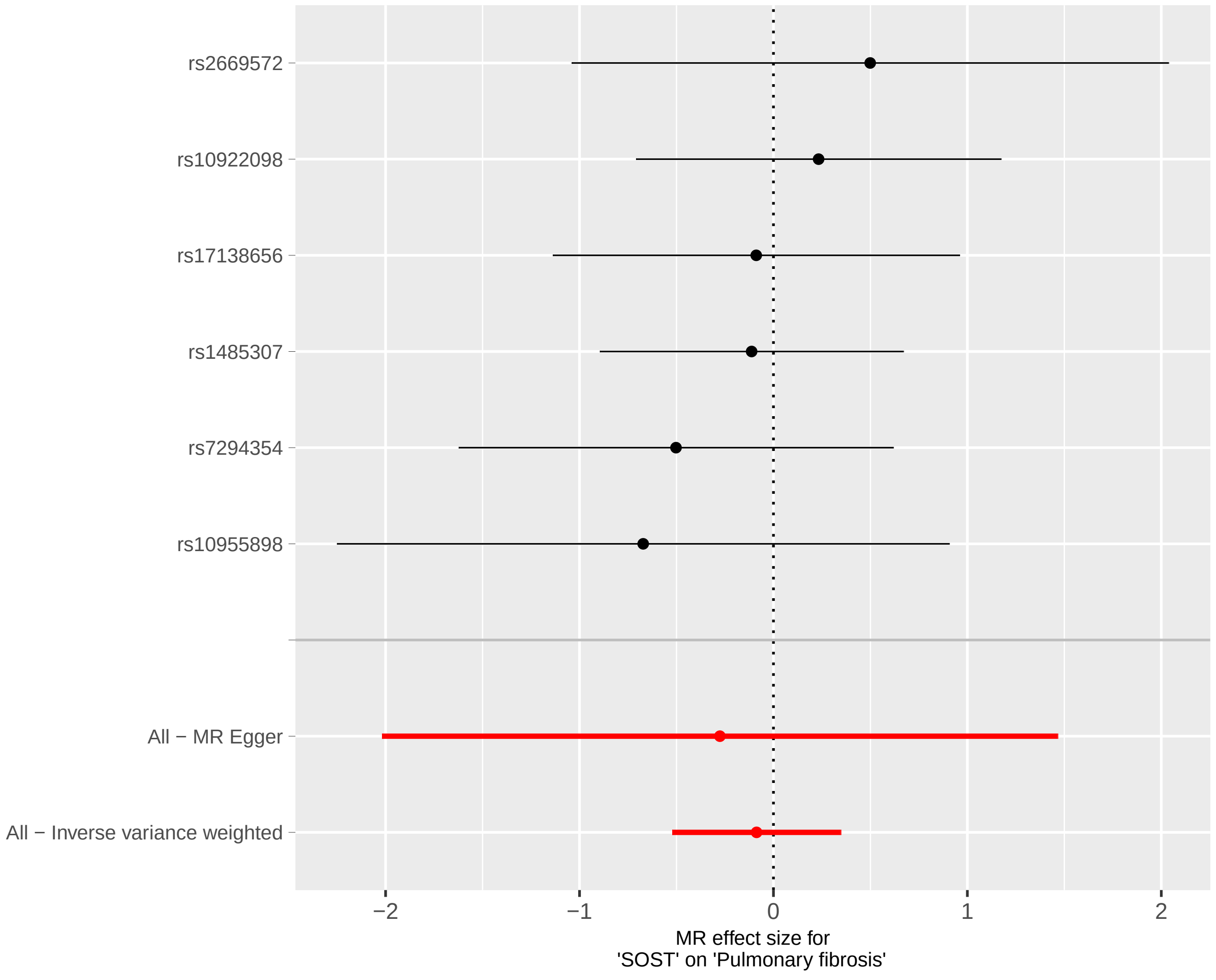
rs2669572
rs10922098
rs17138656
rs1485307
rs7294354
rs10955898
All − MR Egger
All − Inverse variance weighted
−2
−1
0
1
2
MR effect size for
'SOST' on 'Pulmonary fibrosis'

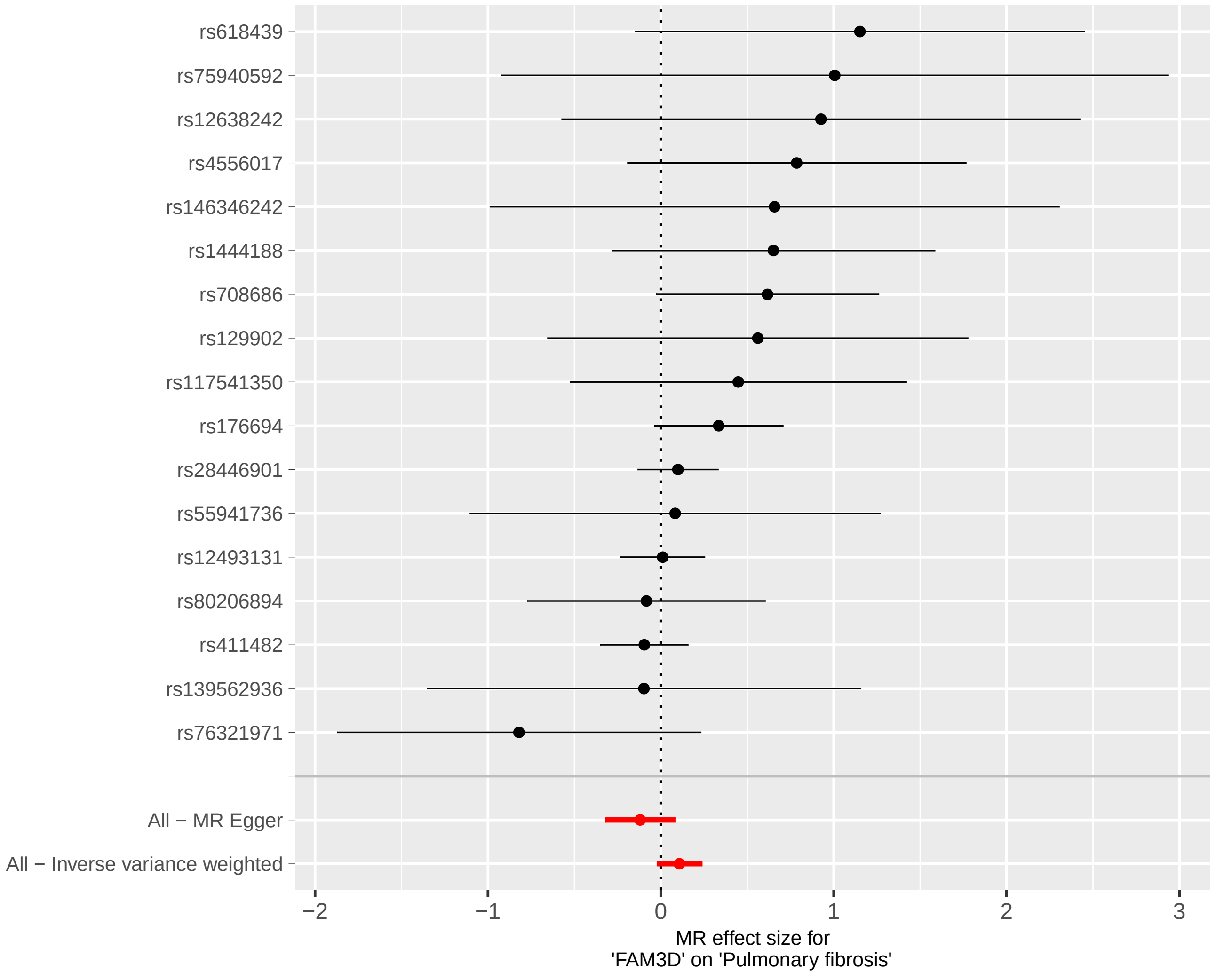
rs618439
rs75940592
rs12638242
rs4556017
rs146346242
rs1444188
rs708686
rs129902
rs117541350
rs176694
rs28446901
rs55941736
rs12493131
rs80206894
rs411482
rs139562936
rs76321971
All − MR Egger
All − Inverse variance weighted
−2
−1
0
1
2
3
MR effect size for
'FAM3D' on 'Pulmonary fibrosis'

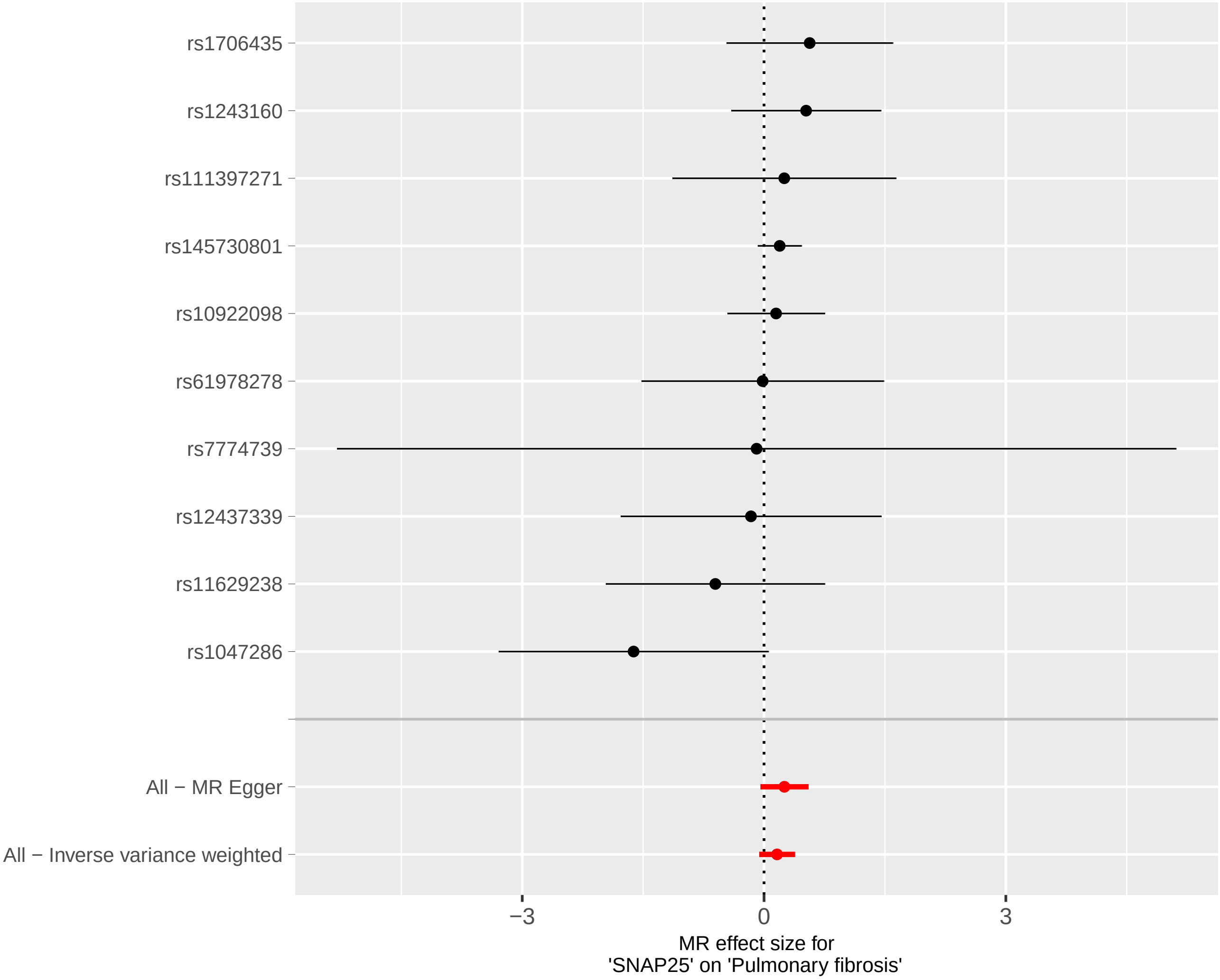
rs1706435
rs1243160
rs111397271
rs145730801
rs10922098
rs61978278
rs7774739
rs12437339
rs11629238
rs1047286
All − MR Egger
All − Inverse variance weighted
−3
0
3
MR effect size for
'SNAP25' on 'Pulmonary fibrosis'

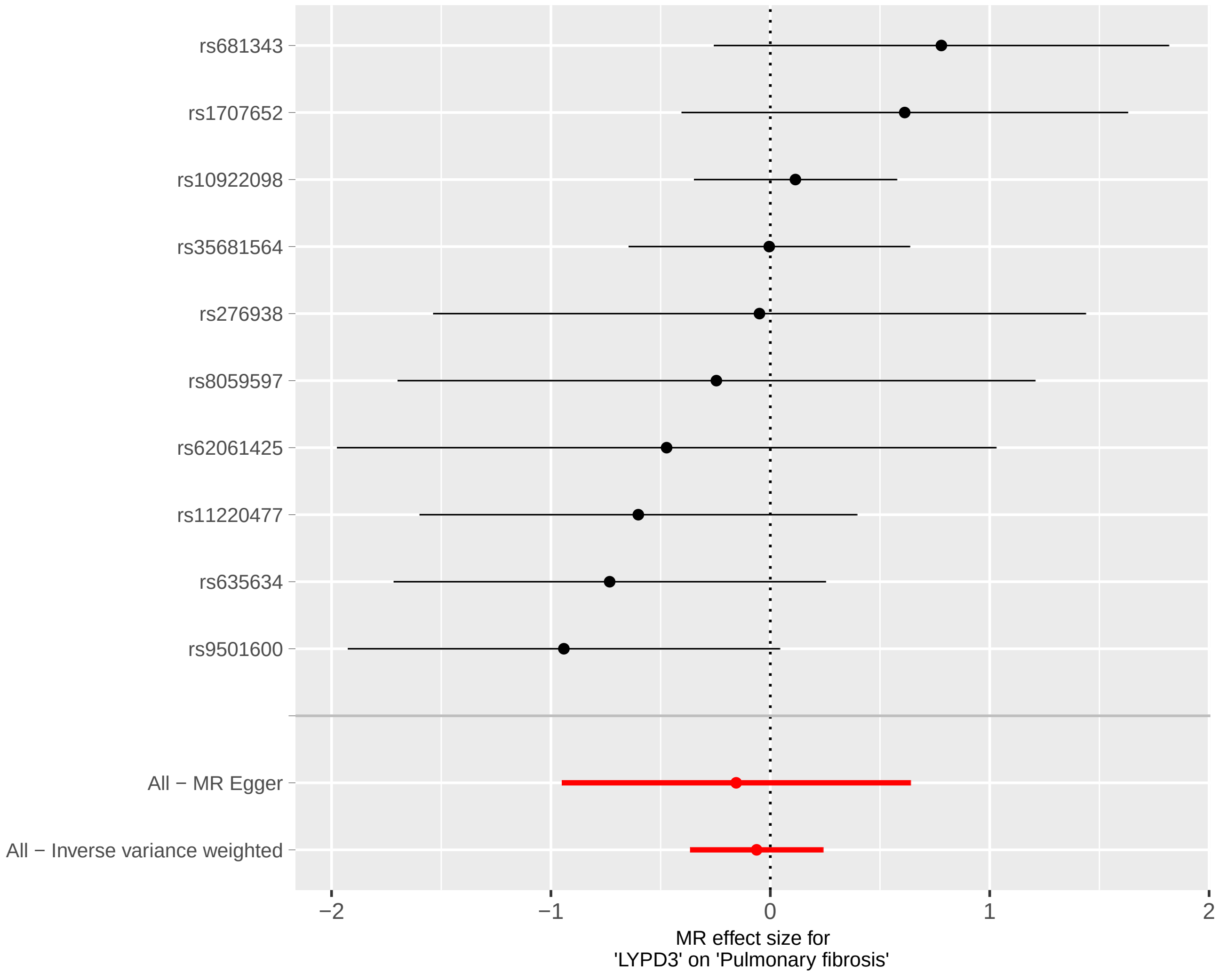
rs681343
rs1707652
rs10922098
rs35681564
rs276938
rs8059597
rs62061425
rs11220477
rs635634
rs9501600
All − MR Egger
All − Inverse variance weighted
−2
−1
0
1
2
MR effect size for
'LYPD3' on 'Pulmonary fibrosis'

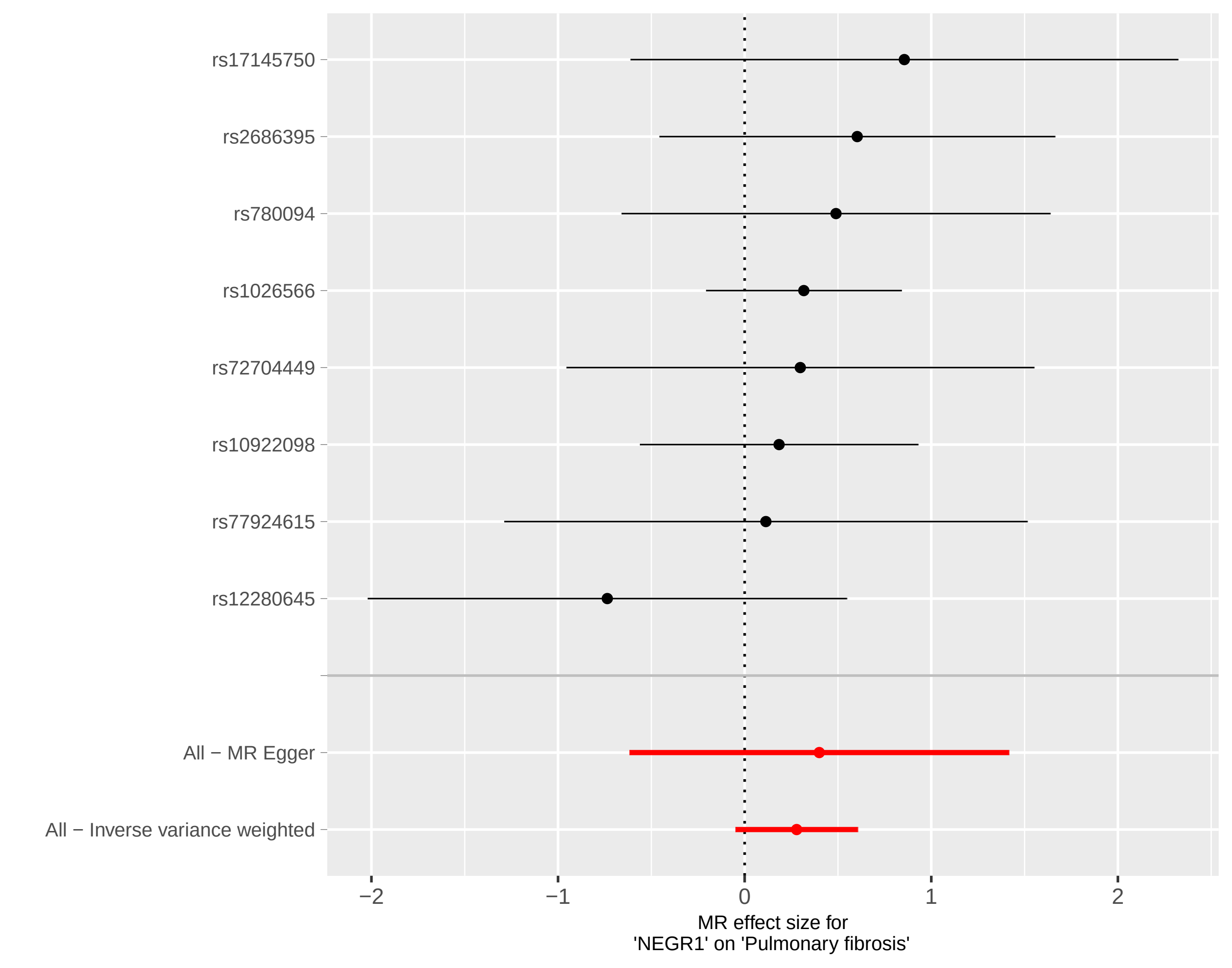

rs17145750
rs2686395
rs780094
rs1026566
rs72704449
rs10922098
rs77924615
rs12280645
All − MR Egger
All − Inverse variance weighted
−2
−1
0
1
2
MR effect size for
'NEGR1' on 'Pulmonary fibrosis'

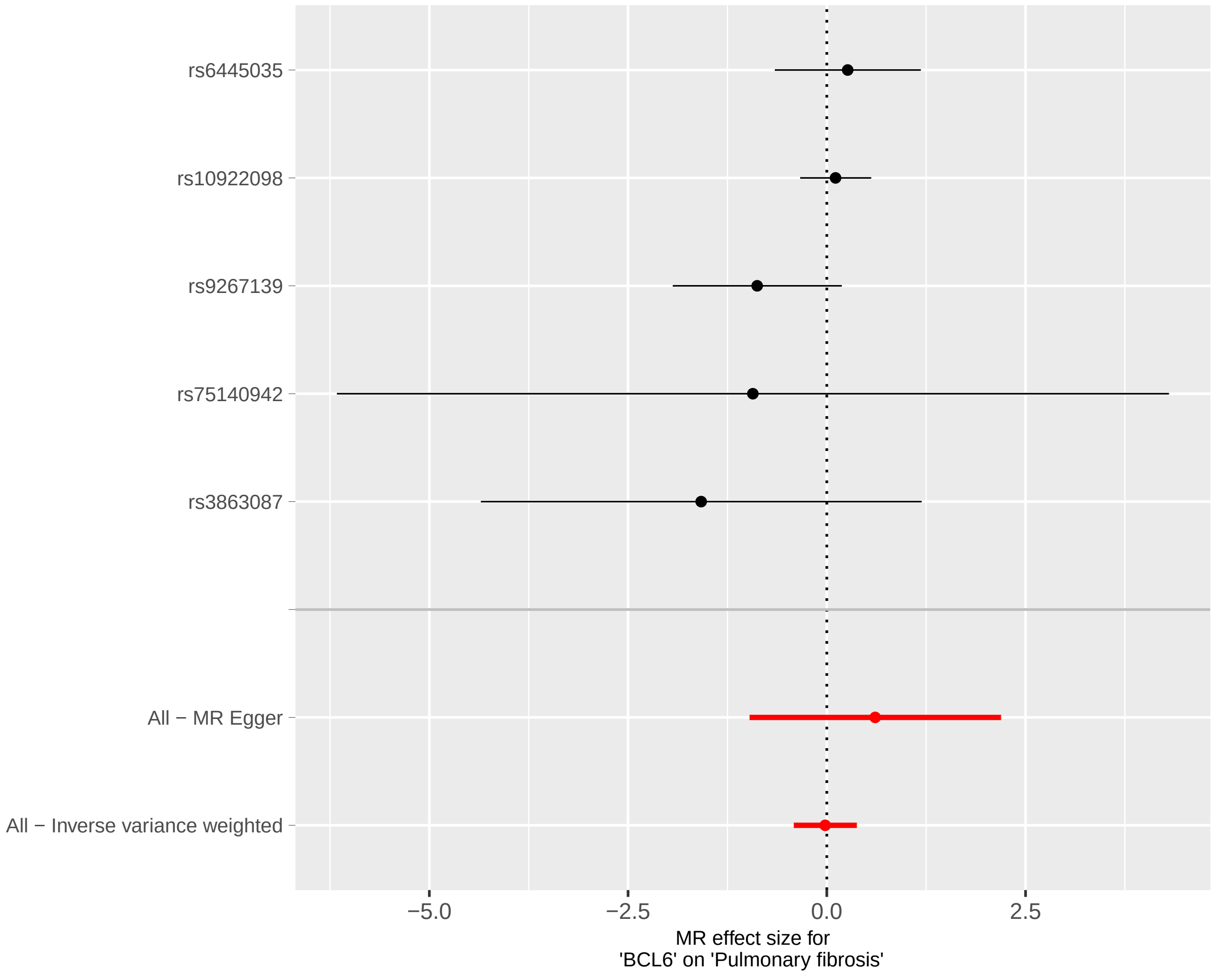

rs6445035
rs10922098
rs9267139
rs75140942
rs3863087
All − MR Egger
All − Inverse variance weighted
−5.0
−2.5
0.0
2.5
MR effect size for
'BCL6' on 'Pulmonary fibrosis'

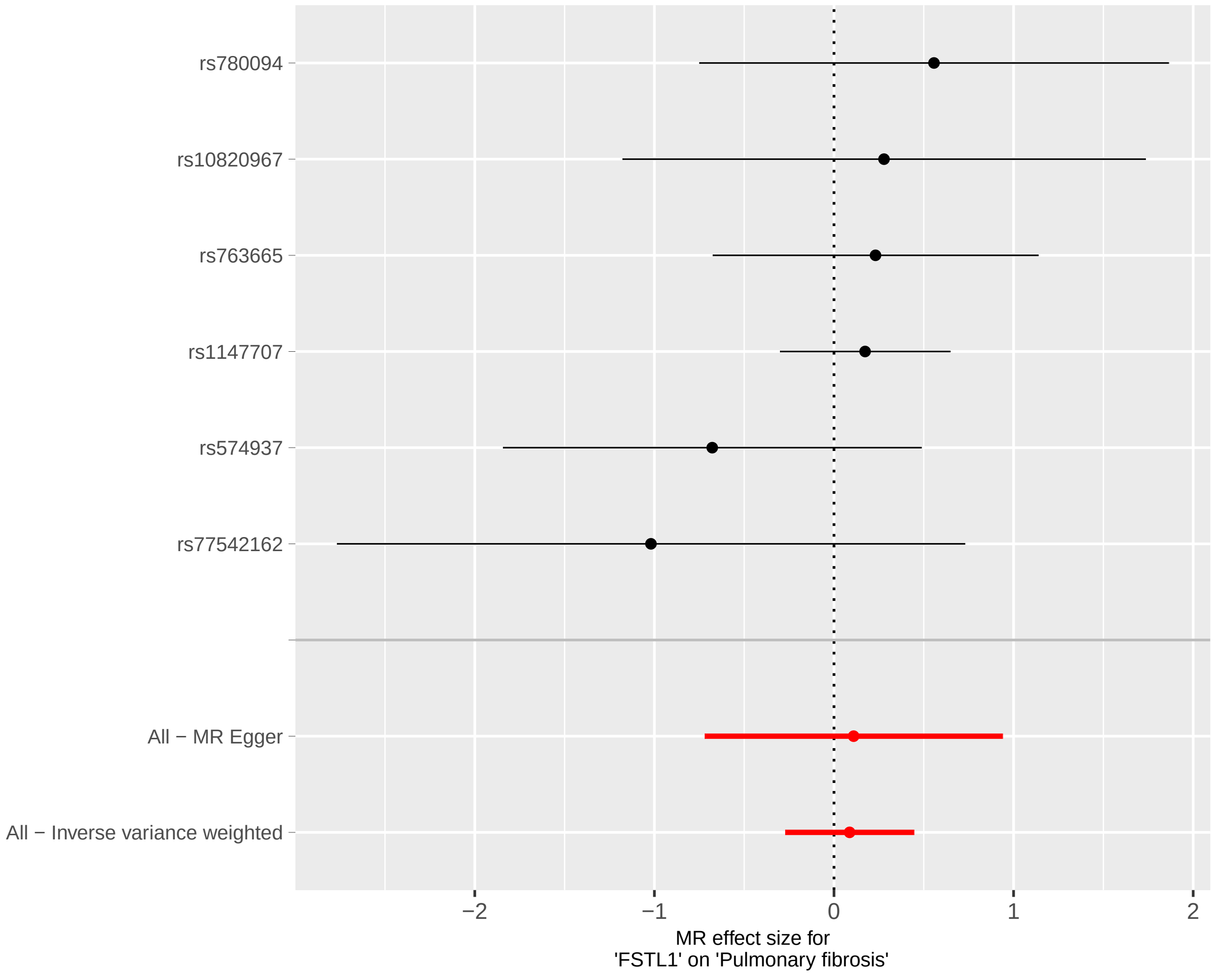
rs780094
rs10820967
rs763665
rs1147707
rs574937
rs77542162
All − MR Egger
All − Inverse variance weighted
−2
−1
0
1
2
MR effect size for
'FSTL1' on 'Pulmonary fibrosis'

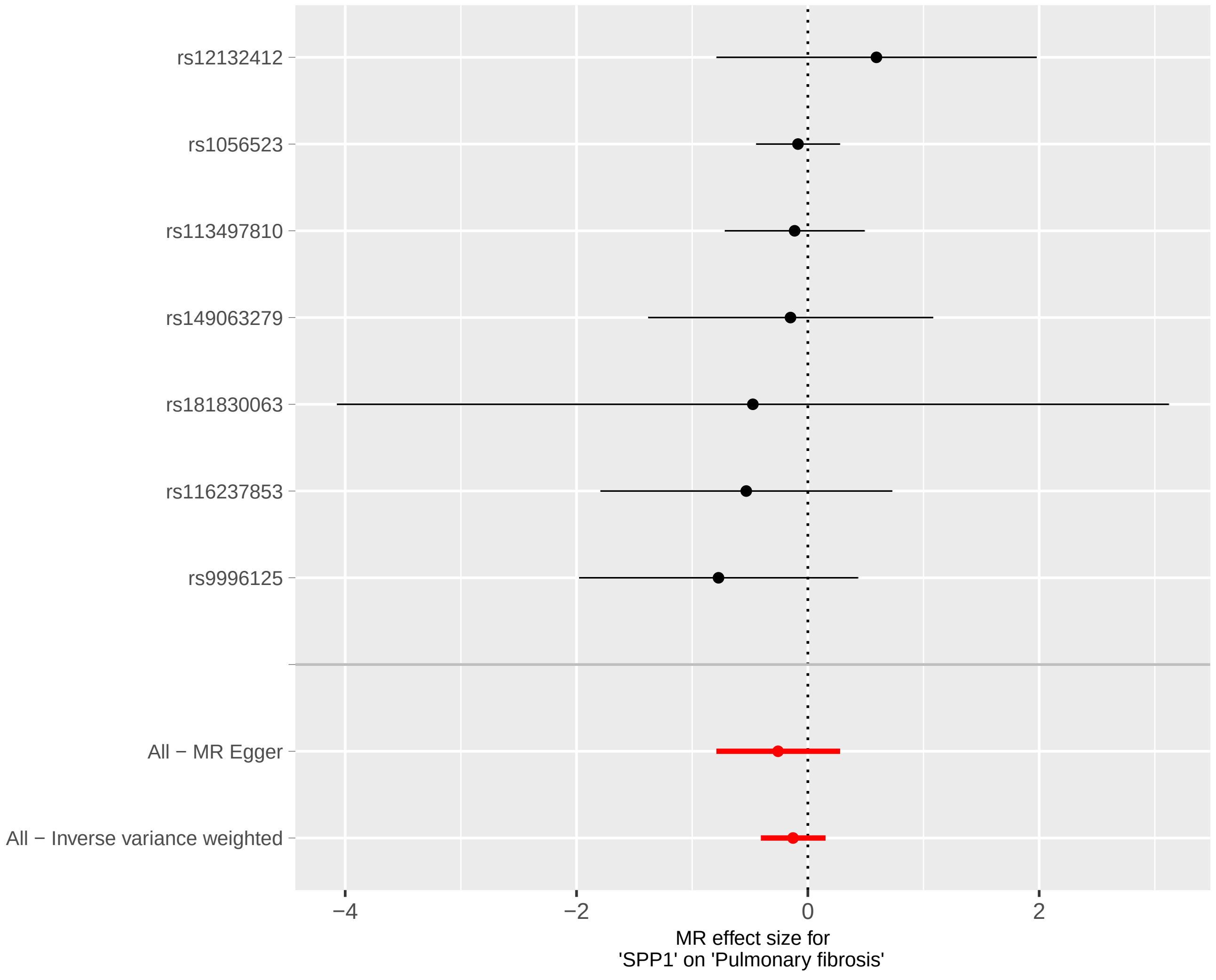

rs12132412
rs1056523
rs113497810
rs149063279
rs181830063
rs116237853
rs9996125
All − MR Egger
All − Inverse variance weighted
−4
−2
0
2
MR effect size for
'SPP1' on 'Pulmonary fibrosis'

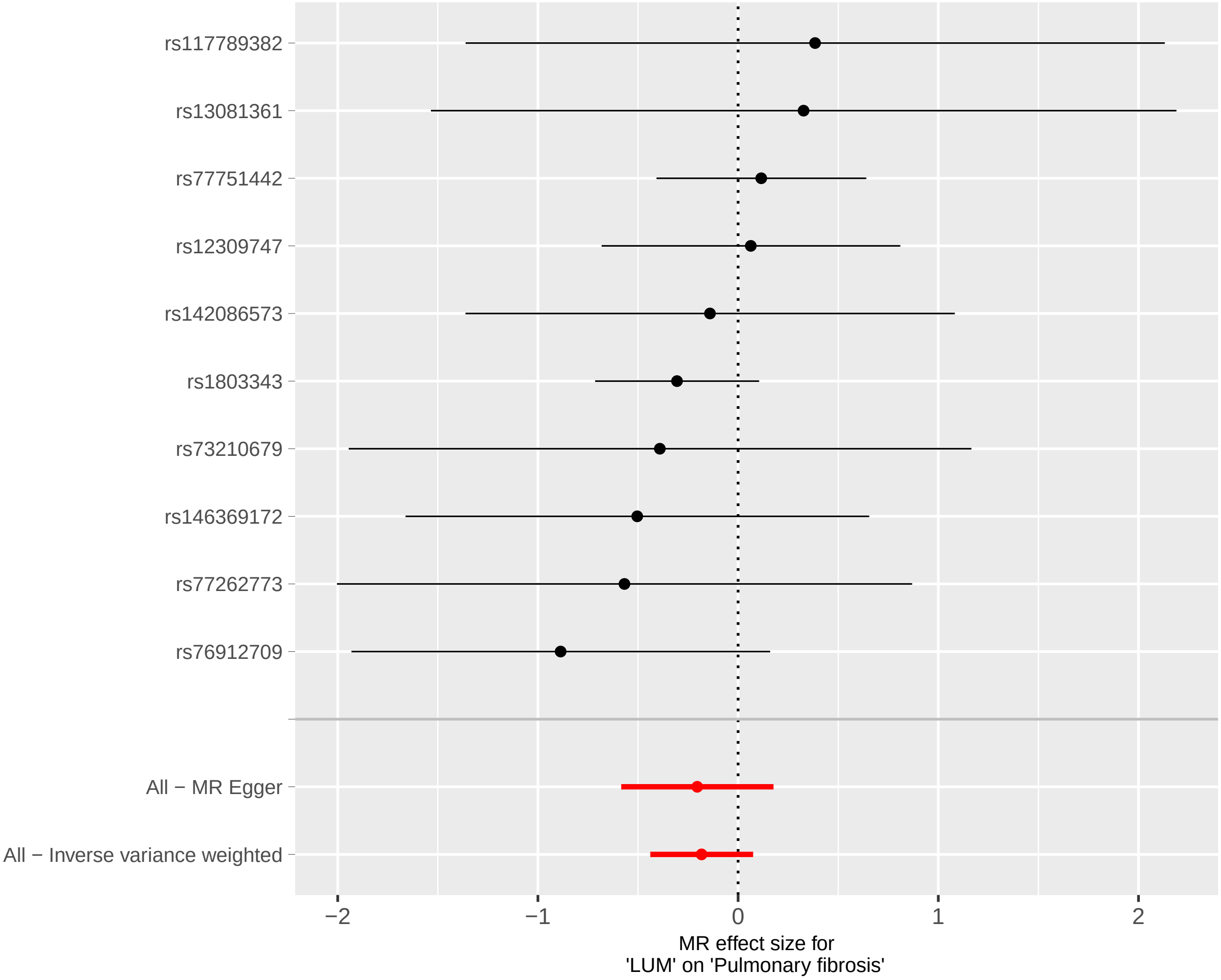

rs117789382
rs13081361
rs77751442
rs12309747
rs142086573
rs1803343
rs73210679
rs146369172
rs77262773
rs76912709
All − MR Egger
All − Inverse variance weighted
−2
−1
0
1
2
MR effect size for
'LUM' on 'Pulmonary fibrosis'

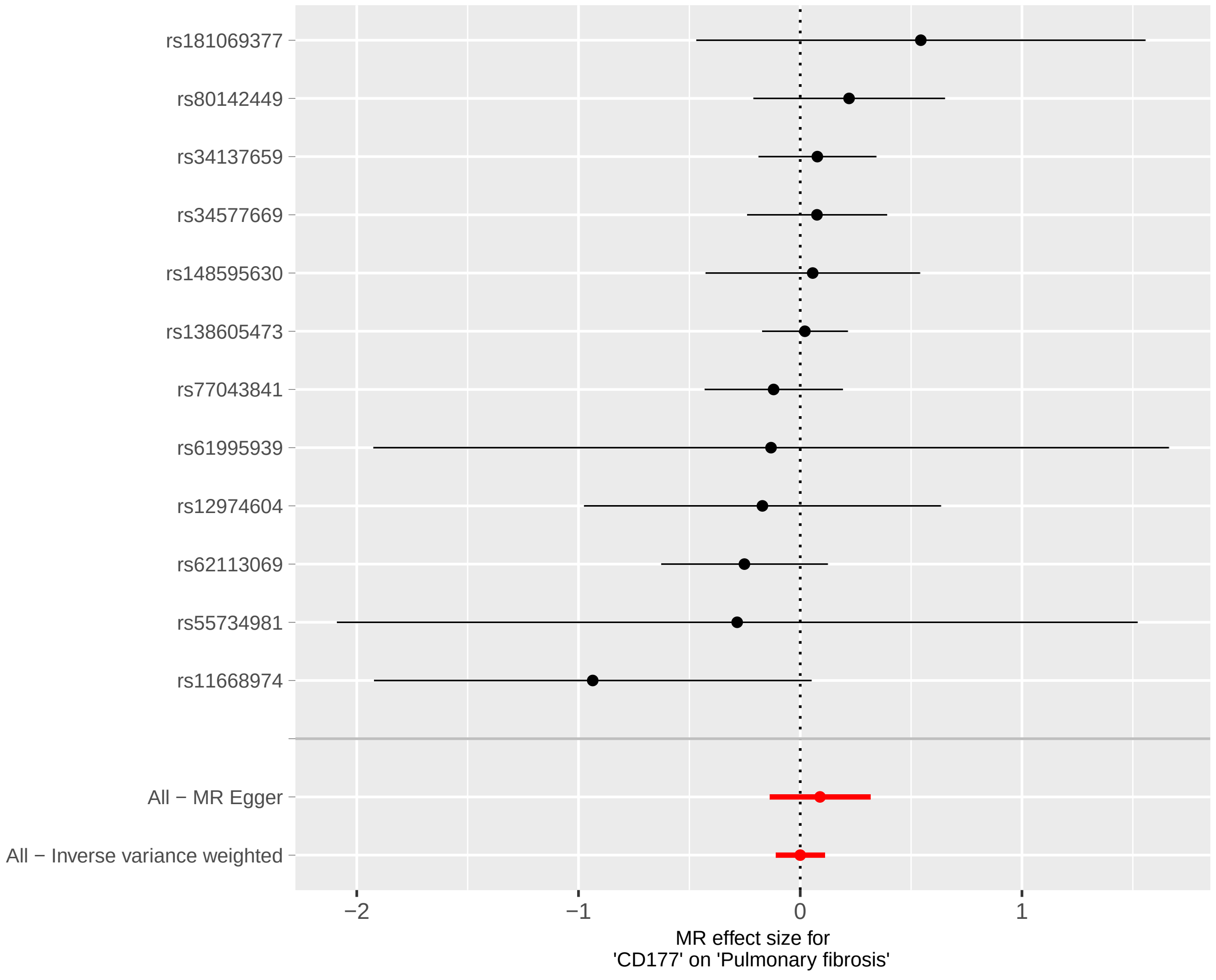

rs181069377
rs80142449
rs34137659
rs34577669
rs148595630
rs138605473
rs77043841
rs61995939
rs12974604
rs62113069
rs55734981
rs11668974
All − MR Egger
All − Inverse variance weighted
−2
−1
0
1
MR effect size for
'CD177' on 'Pulmonary fibrosis'

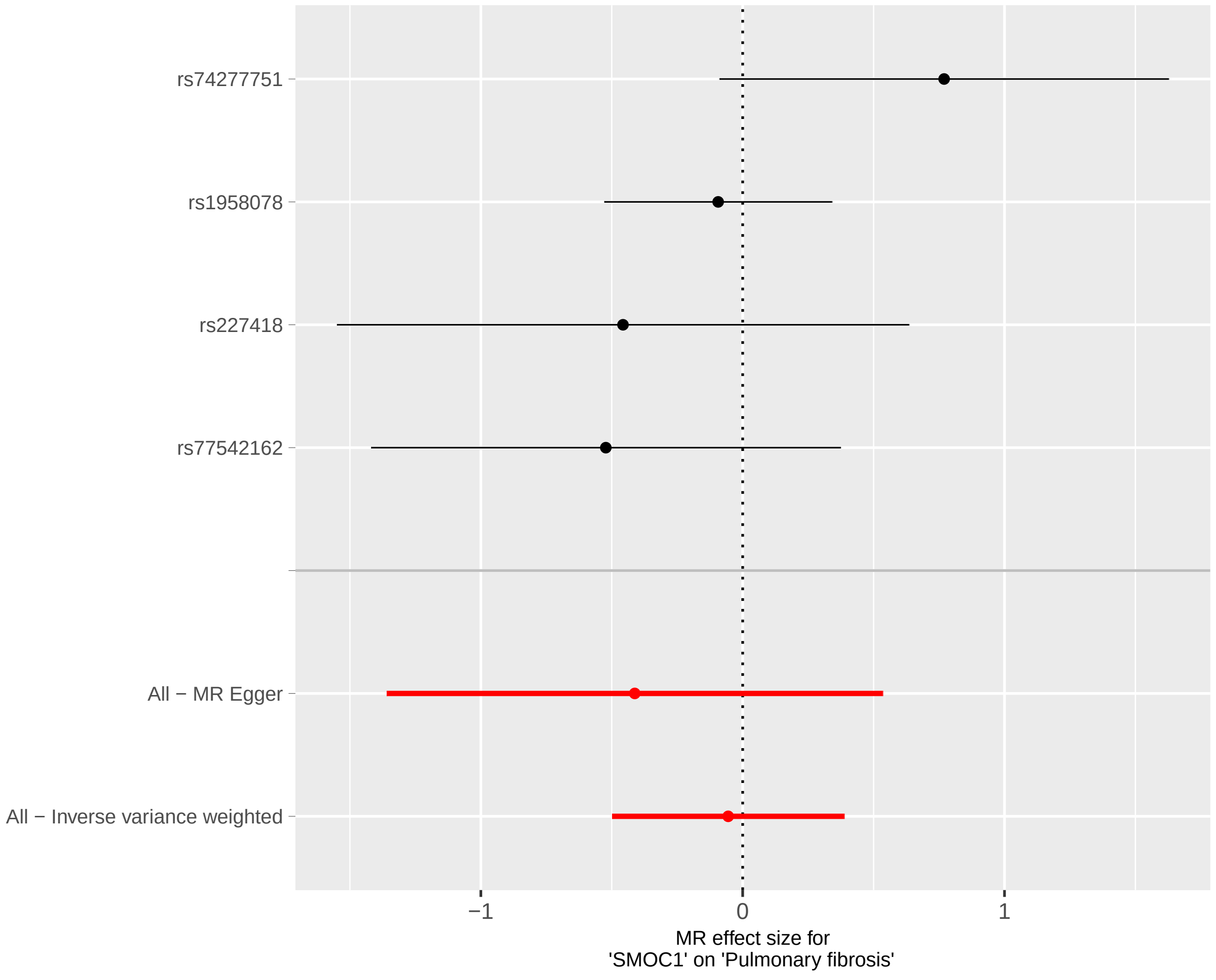
rs74277751
rs1958078
rs227418
rs77542162
All − MR Egger
All − Inverse variance weighted
−1
0
1
MR effect size for
'SMOC1' on 'Pulmonary fibrosis'

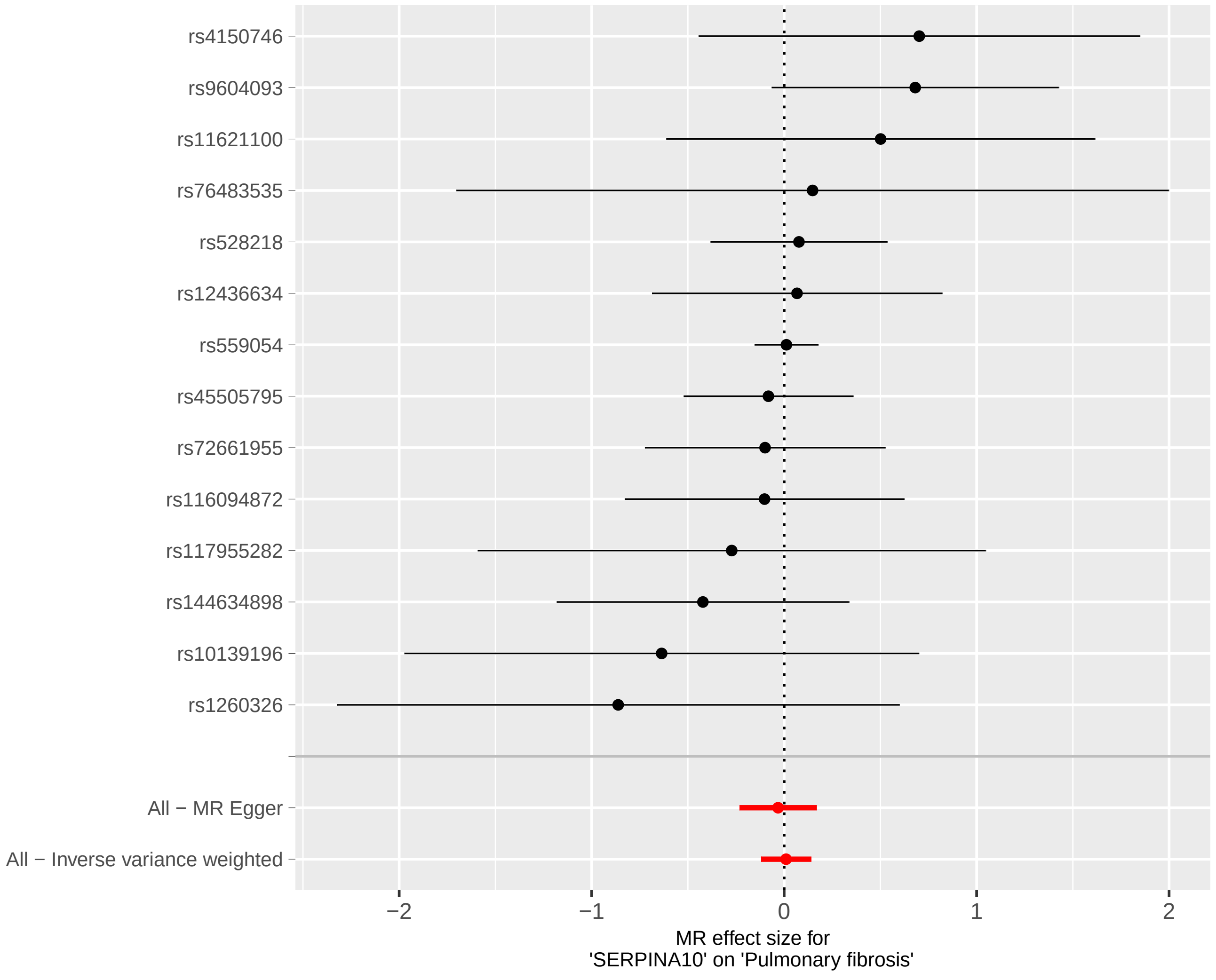
rs4150746
rs9604093
rs11621100
rs76483535
rs528218
rs12436634
rs559054
rs45505795
rs72661955
rs116094872
rs117955282
rs144634898
rs10139196
rs1260326
All − MR Egger
All − Inverse variance weighted
−2
−1
0
1
2
MR effect size for
'SERPINA10' on 'Pulmonary fibrosis'

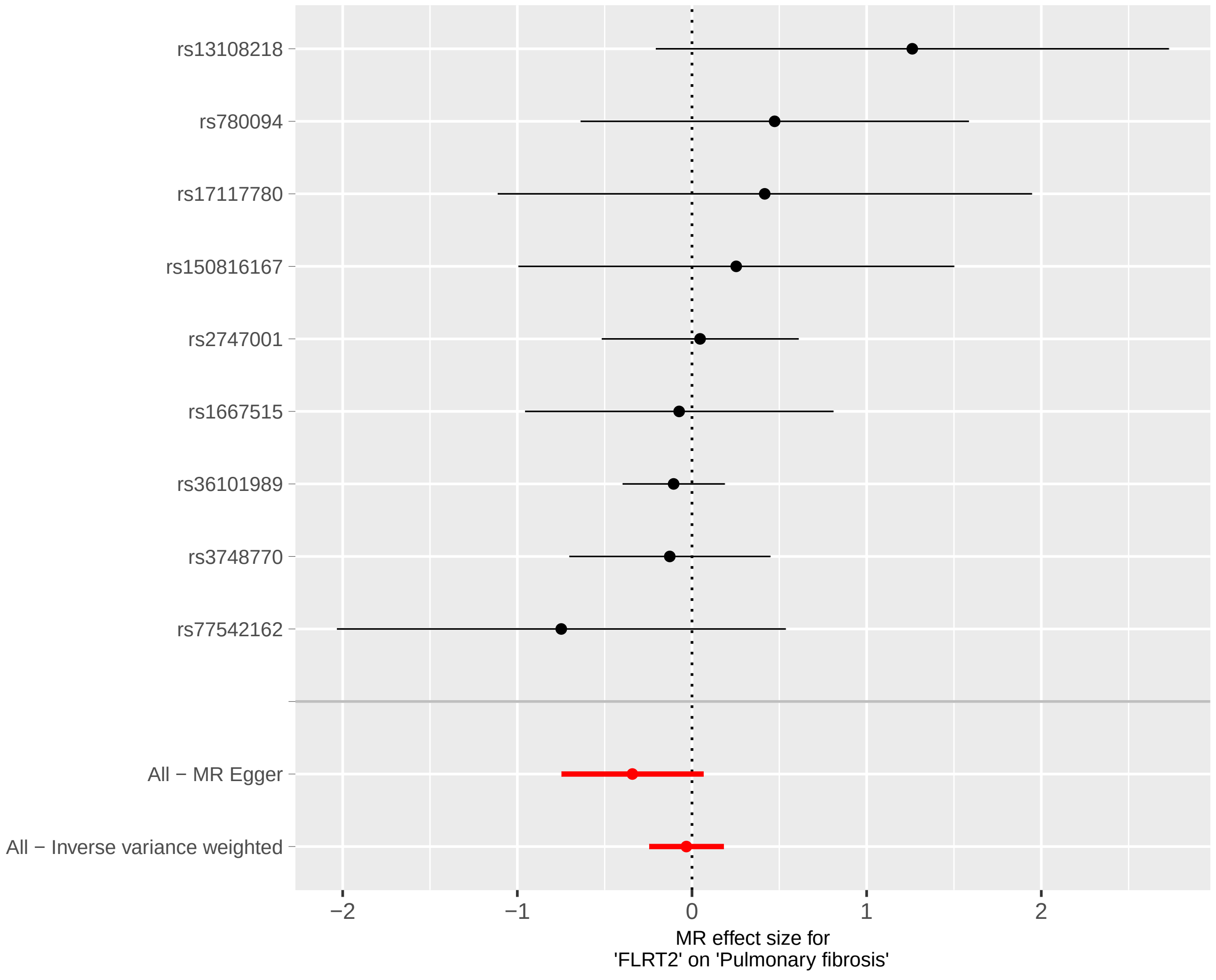

rs13108218
rs780094
rs17117780
rs150816167
rs2747001
rs1667515
rs36101989
rs3748770
rs77542162
All − MR Egger
All − Inverse variance weighted
−2
−1
0
1
2
MR effect size for
'FLRT2' on 'Pulmonary fibrosis'

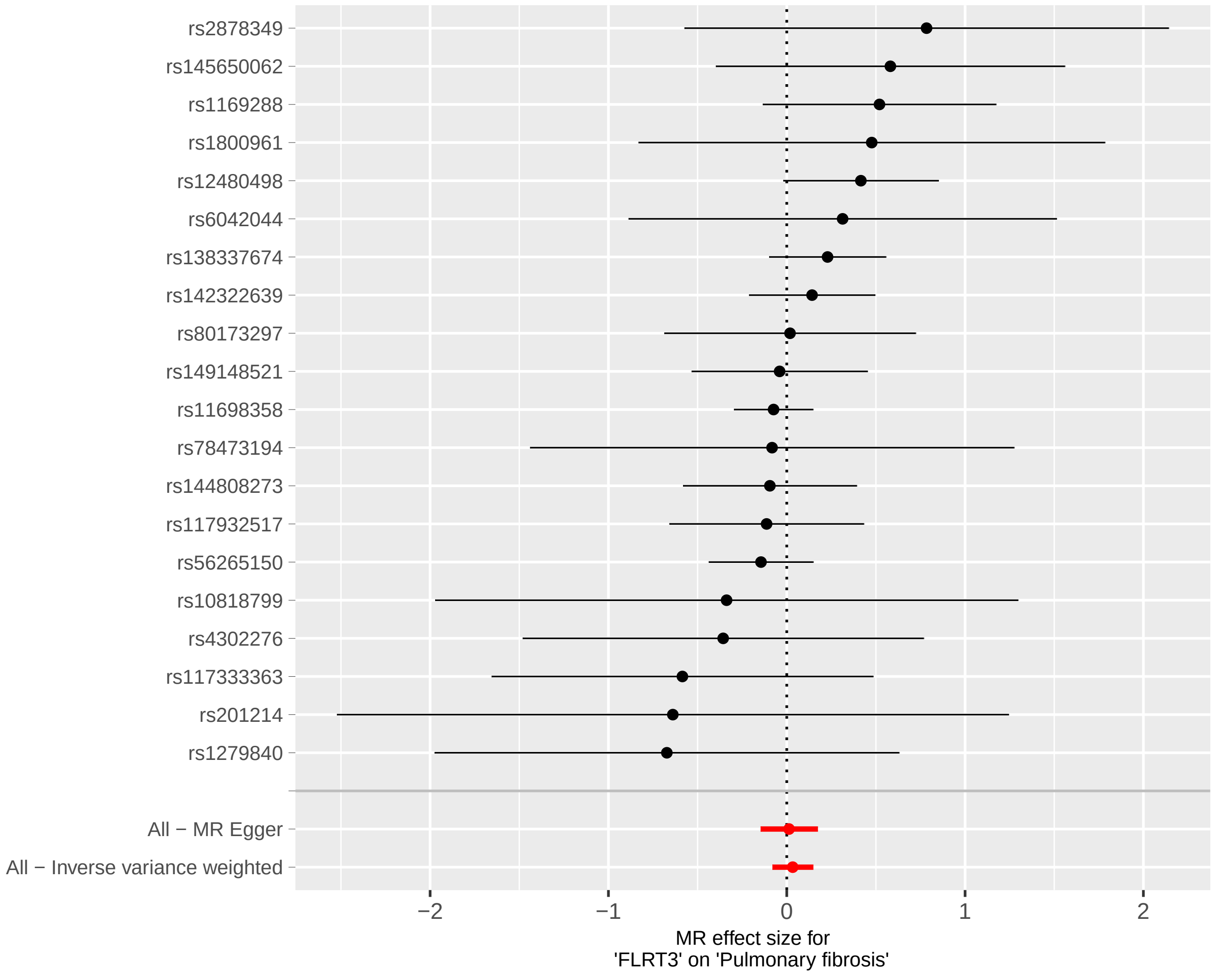
rs2878349
rs145650062
rs1169288
rs1800961
rs12480498
rs6042044
rs138337674
rs142322639
rs80173297
rs149148521
rs11698358
rs78473194
rs144808273
rs117932517
rs56265150
rs10818799
rs4302276
rs117333363
rs201214
rs1279840
All − MR Egger
All − Inverse variance weighted
−2
−1
0
1
2
MR effect size for
'FLRT3' on 'Pulmonary fibrosis'

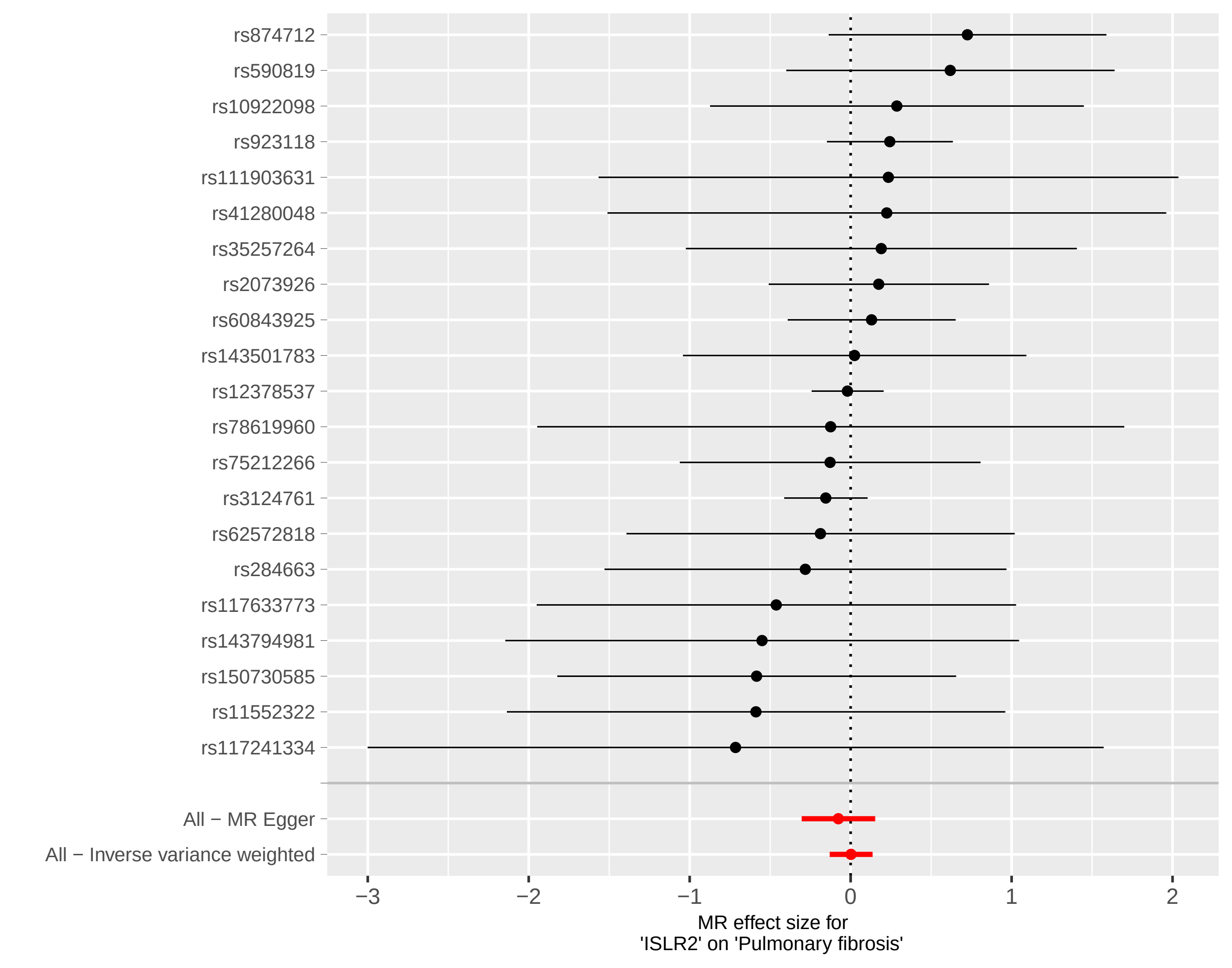
rs874712
rs590819
rs10922098
rs923118
rs111903631
rs41280048
rs35257264
rs2073926
rs60843925
rs143501783
rs12378537
rs78619960
rs75212266
rs3124761
rs62572818
rs284663
rs117633773
rs143794981
rs150730585
rs11552322
rs117241334
All − MR Egger
All − Inverse variance weighted
−3
−2
−1
0
1
2
MR effect size for
'ISLR2' on 'Pulmonary fibrosis'

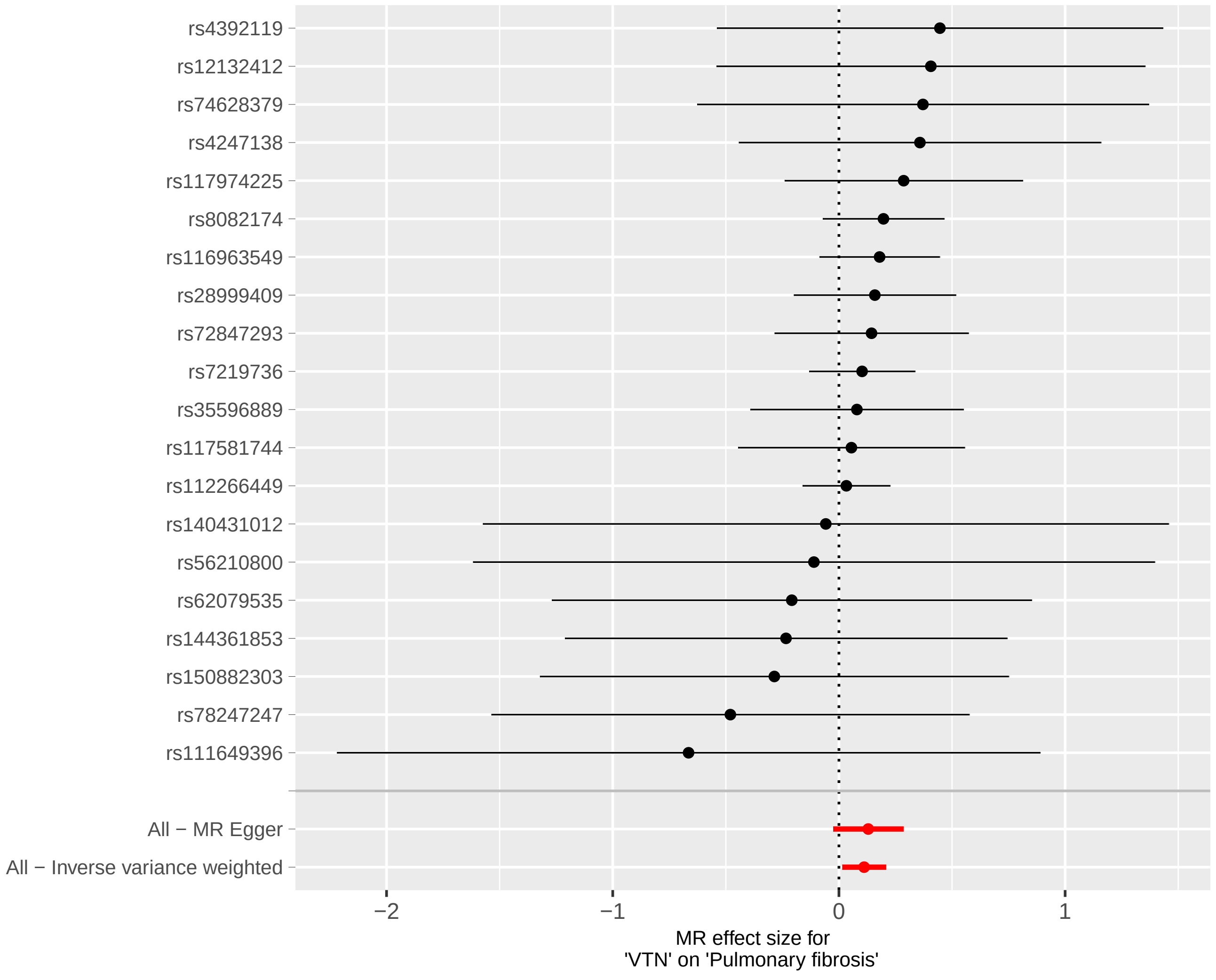

rs4392119
rs12132412
rs74628379
rs4247138
rs117974225
rs8082174
rs116963549
rs28999409
rs72847293
rs7219736
rs35596889
rs117581744
rs112266449
rs140431012
rs56210800
rs62079535
rs144361853
rs150882303
rs78247247
rs111649396
All − MR Egger
All − Inverse variance weighted
−2
−1
0
1
MR effect size for
'VTN' on 'Pulmonary fibrosis'

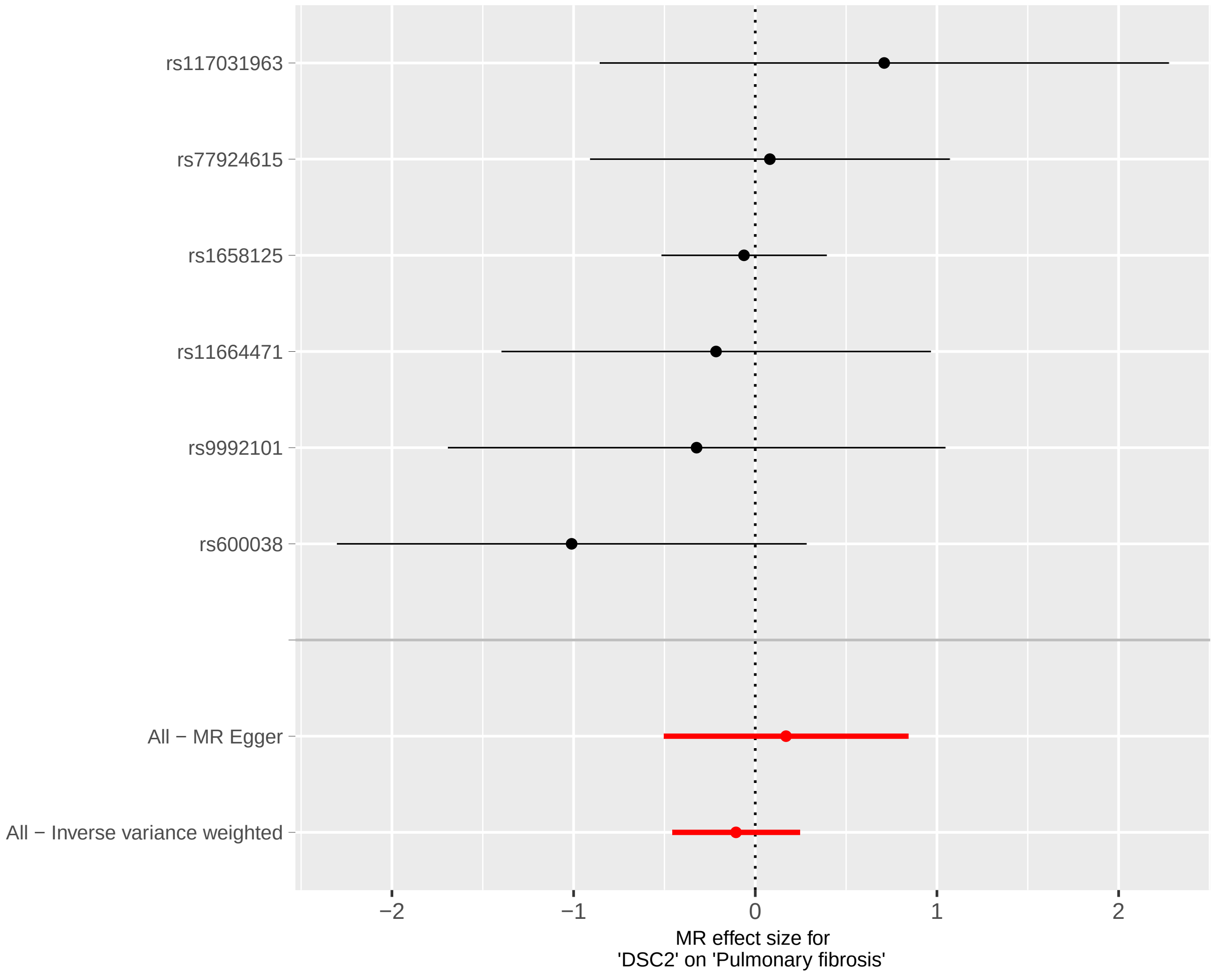

rs117031963
rs77924615
rs1658125
rs11664471
rs9992101
rs600038
All − MR Egger
All − Inverse variance weighted
−2
−1
0
1
2
MR effect size for
'DSC2' on 'Pulmonary fibrosis'

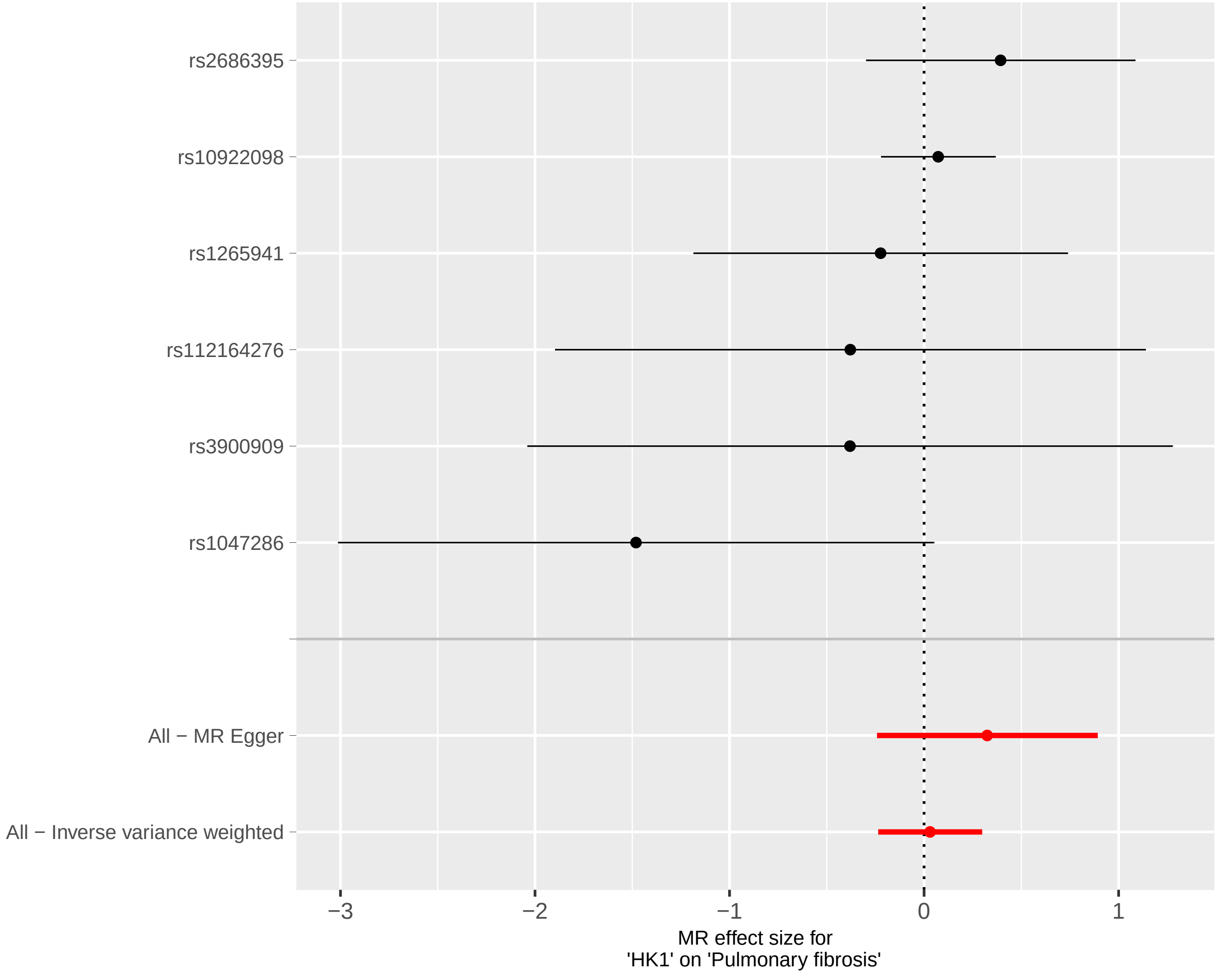
rs2686395
rs10922098
rs1265941
rs112164276
rs3900909
rs1047286
All − MR Egger
All − Inverse variance weighted
−3
−2
−1
0
1
MR effect size for
'HK1' on 'Pulmonary fibrosis'

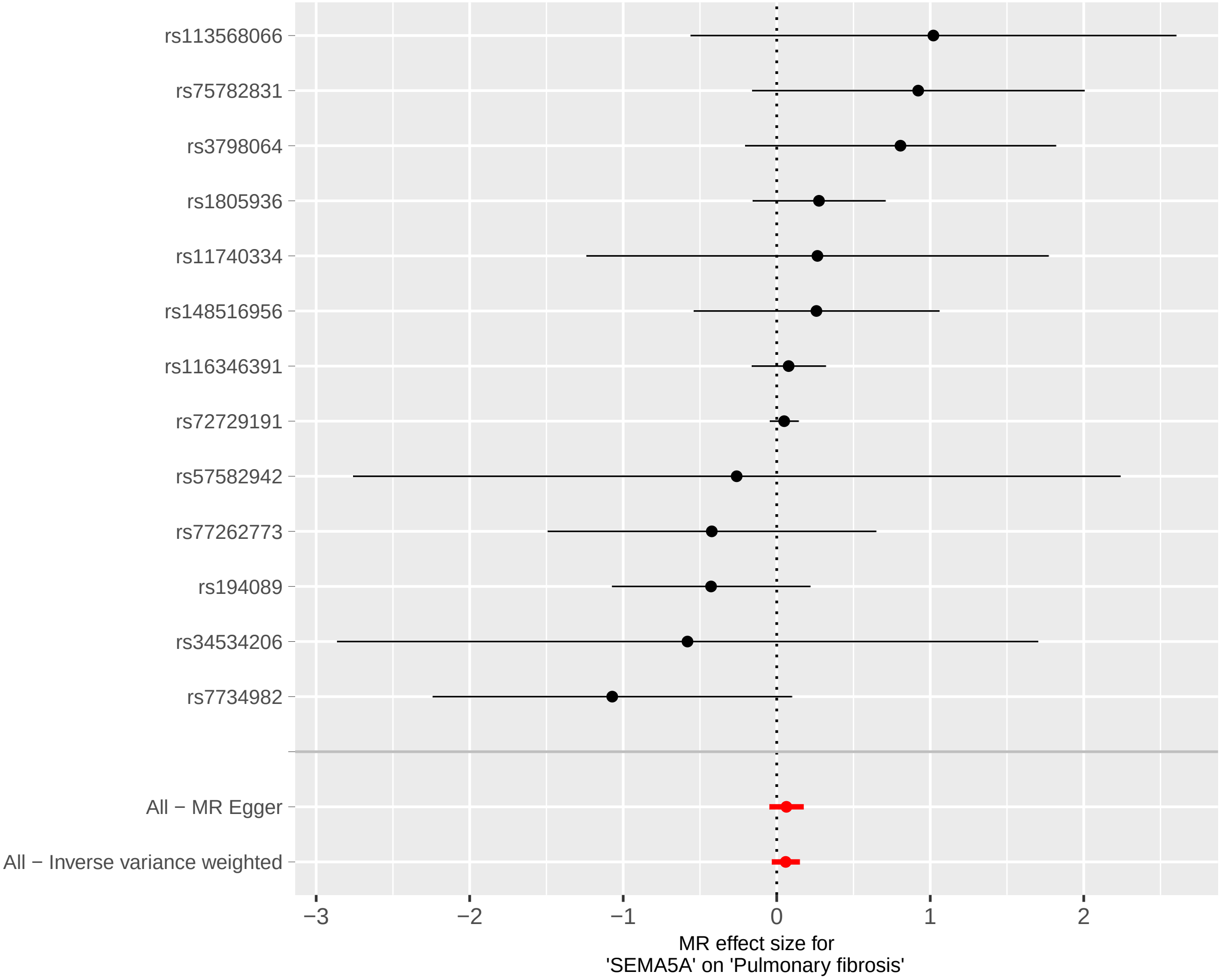

rs113568066
rs75782831
rs3798064
rs1805936
rs11740334
rs148516956
rs116346391
rs72729191
rs57582942
rs77262773
rs194089
rs34534206
rs7734982
All − MR Egger
All − Inverse variance weighted
−3
−2
−1
0
1
2
MR effect size for
'SEMA5A' on 'Pulmonary fibrosis'

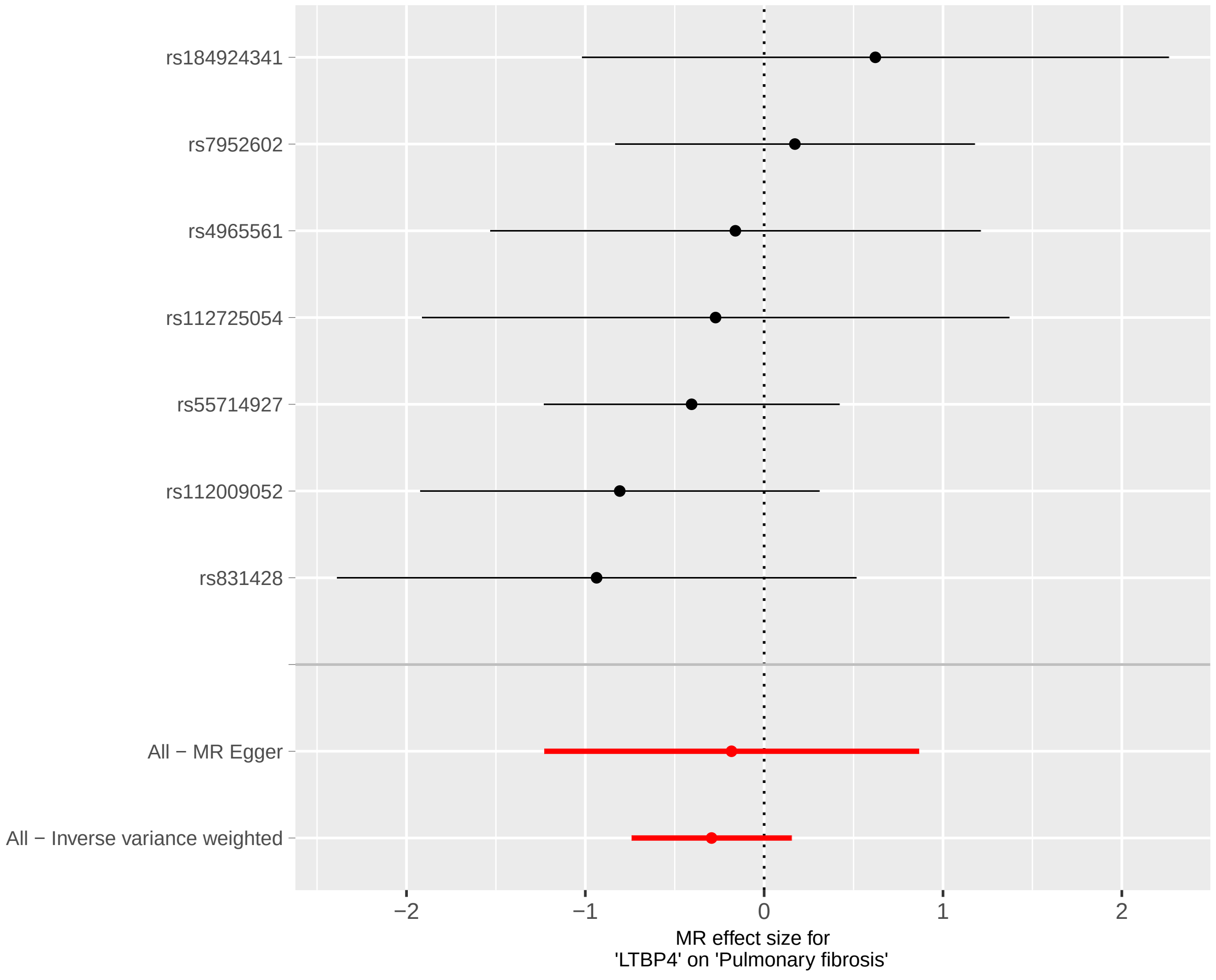
rs184924341
rs7952602
rs4965561
rs112725054
rs55714927
rs112009052
rs831428
All − MR Egger
All − Inverse variance weighted
−2
−1
0
1
2
MR effect size for
'LTBP4' on 'Pulmonary fibrosis'

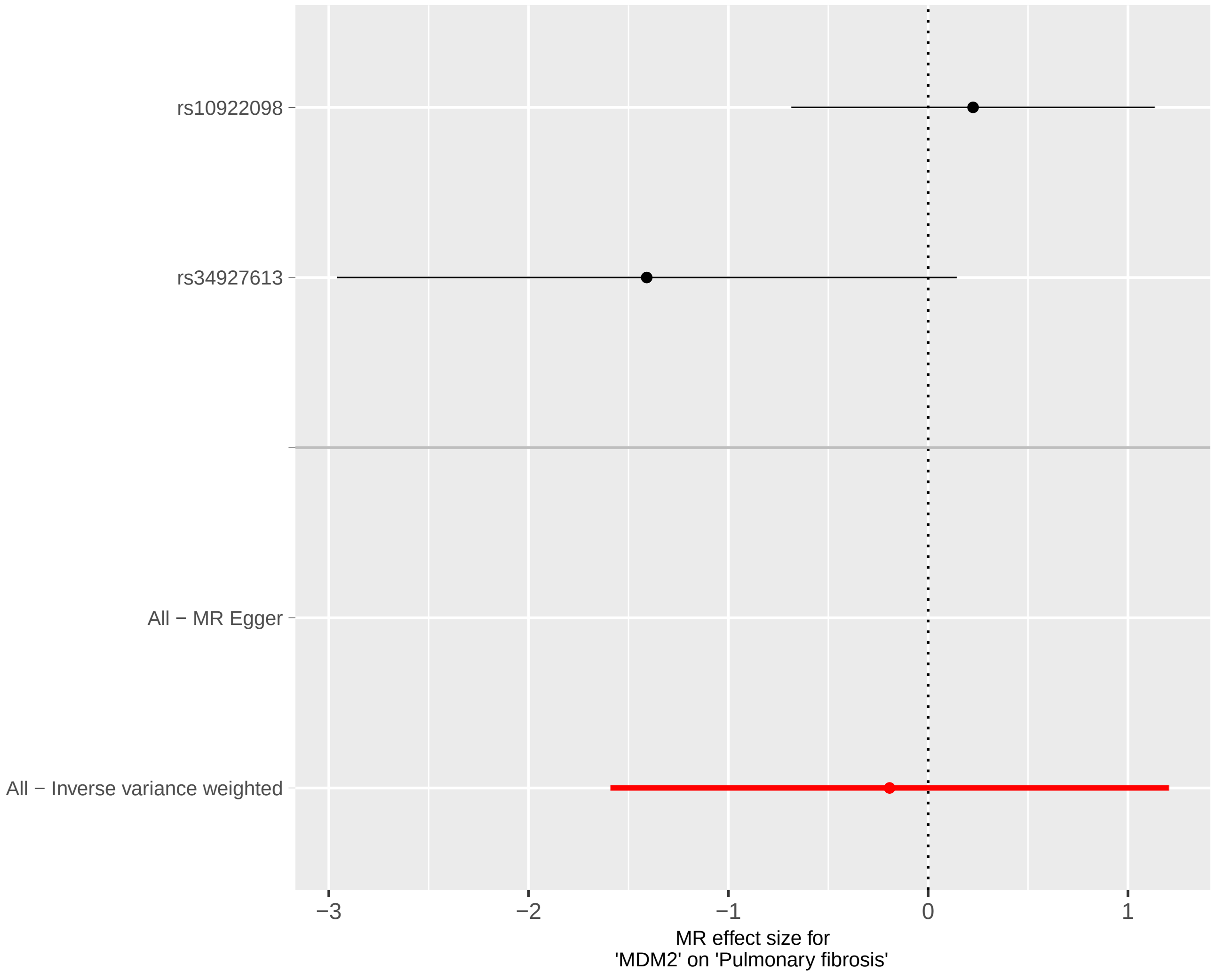
rs10922098
rs34927613
All − MR Egger
All − Inverse variance weighted
−3
−2
−1
0
1
MR effect size for
'MDM2' on 'Pulmonary fibrosis'

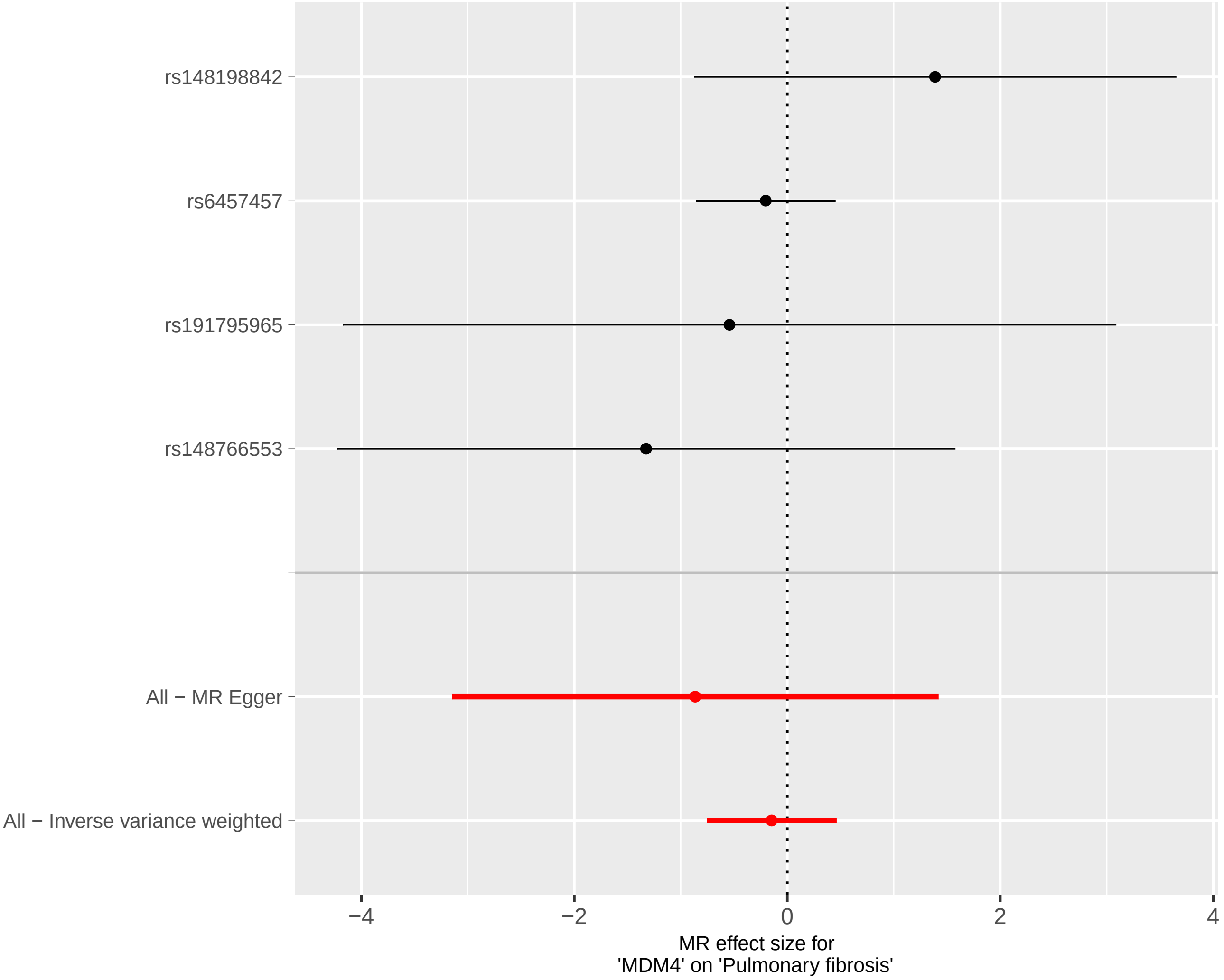
rs148198842
rs6457457
rs191795965
rs148766553
All − MR Egger
All − Inverse variance weighted
−4
−2
0
2
4
MR effect size for
'MDM4' on 'Pulmonary fibrosis'

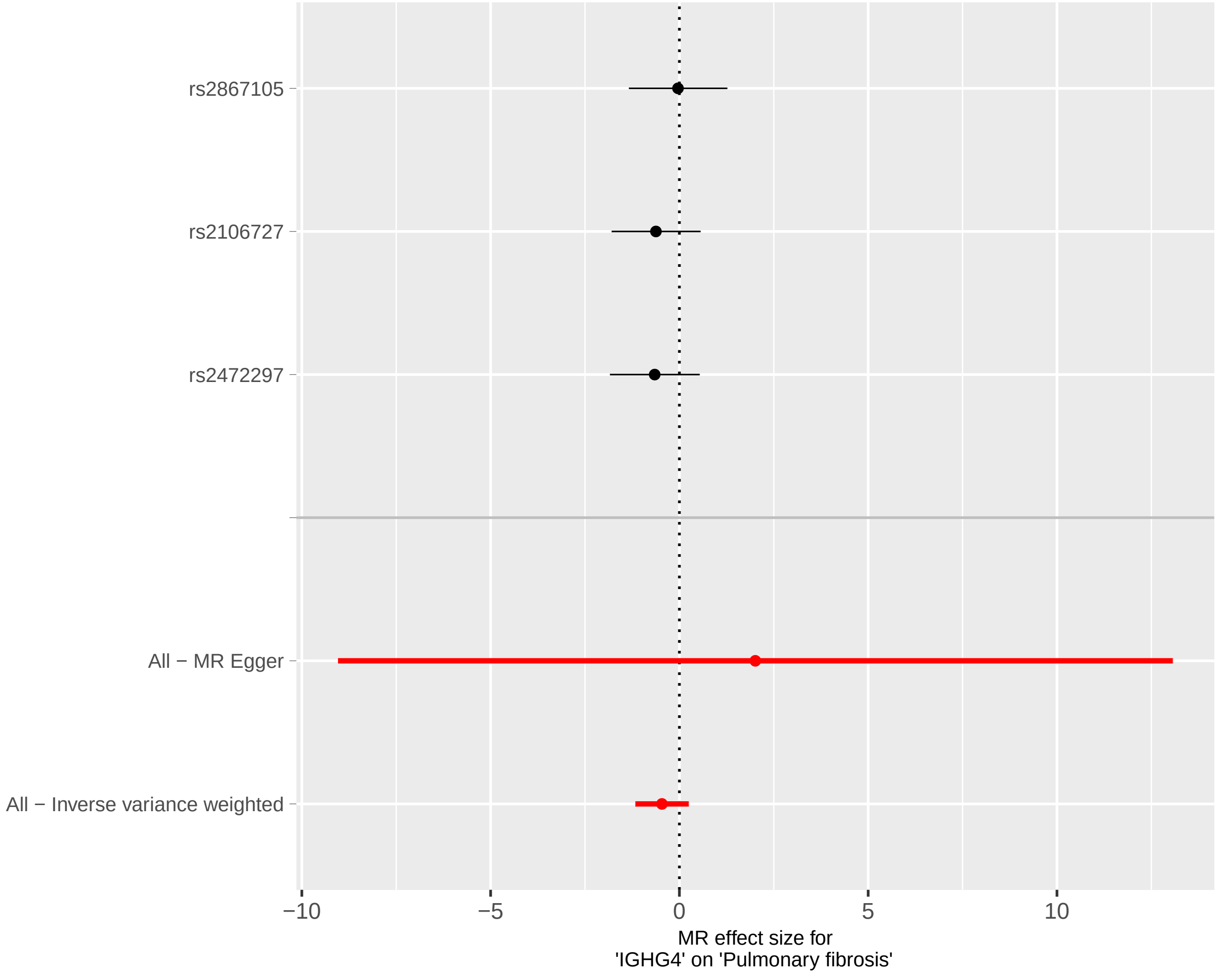

rs2867105
rs2106727
rs2472297
All − MR Egger
All − Inverse variance weighted
−10
−5
0
5
10
MR effect size for
'IGHG4' on 'Pulmonary fibrosis'

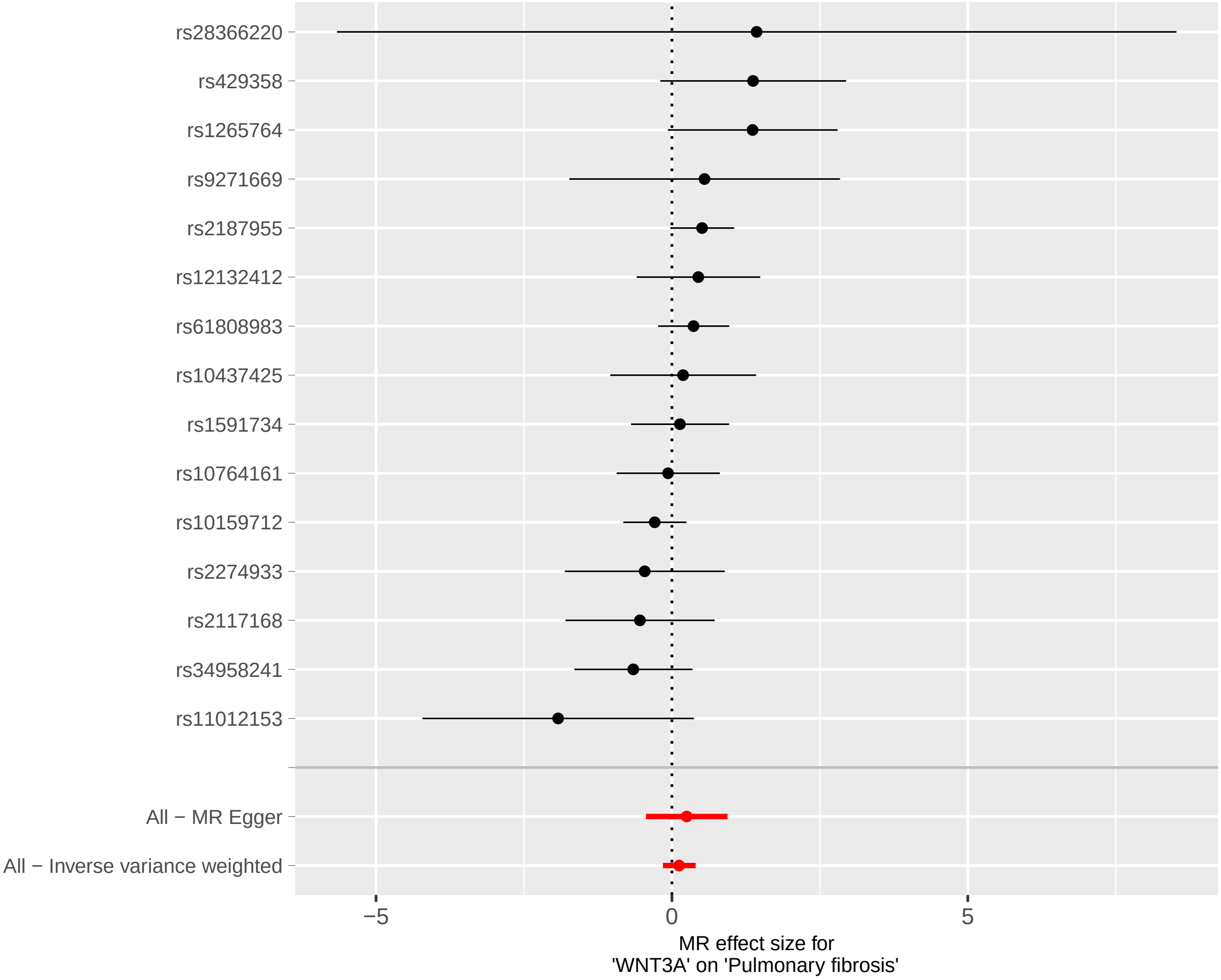
rs28366220
rs429358
rs1265764
rs9271669
rs2187955
rs12132412
rs61808983
rs10437425
rs1591734
rs10764161
rs10159712
rs2274933
rs2117168
rs34958241
rs11012153
All − MR Egger
All − Inverse variance weighted
−5
0
5
MR effect size for
'WNT3A' on 'Pulmonary fibrosis'

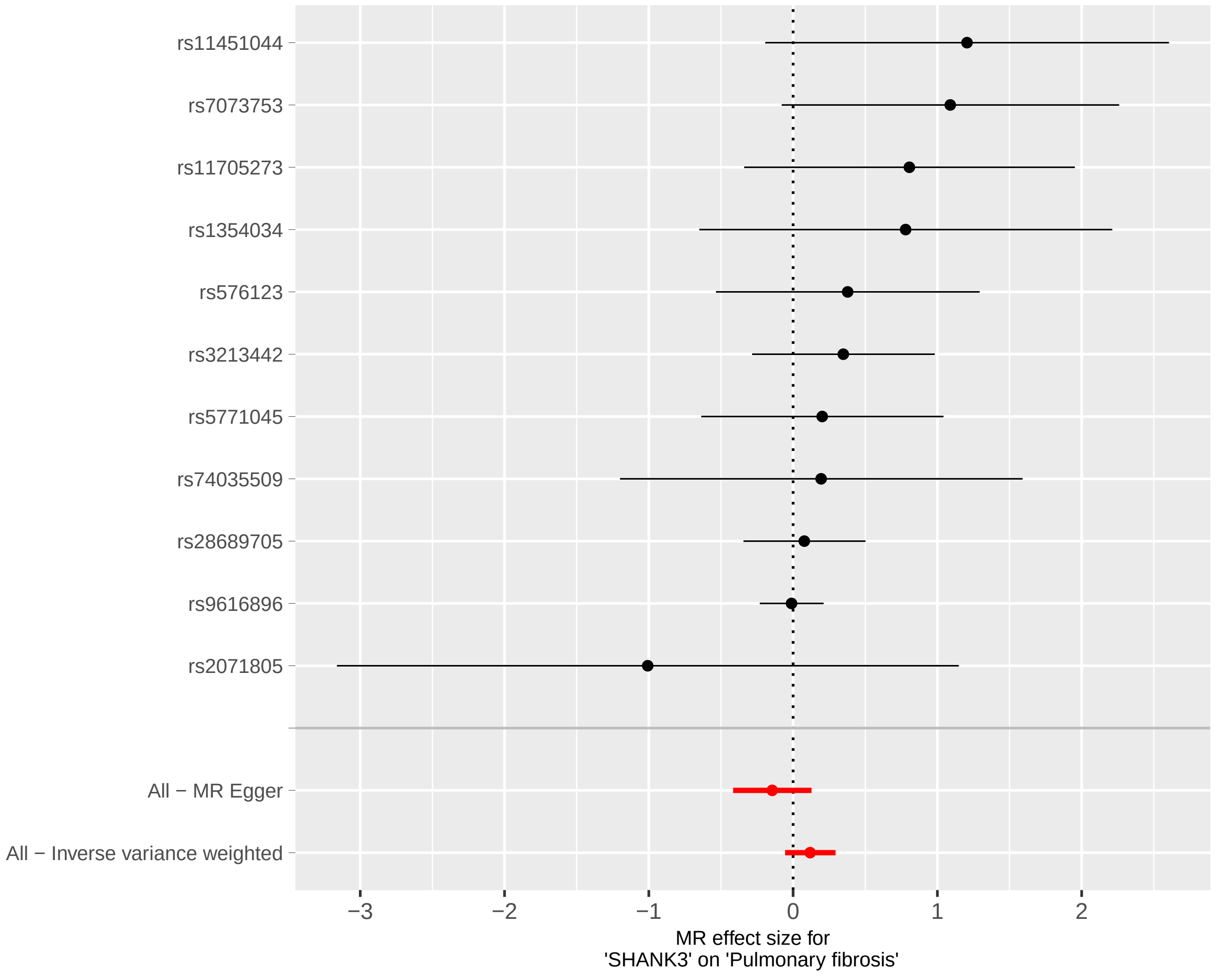

rs11451044
rs7073753
rs11705273
rs1354034
rs576123
rs3213442
rs5771045
rs74035509
rs28689705
rs9616896
rs2071805
All − MR Egger
All − Inverse variance weighted
−3
−2
−1
0
1
2
MR effect size for
'SHANK3' on 'Pulmonary fibrosis'

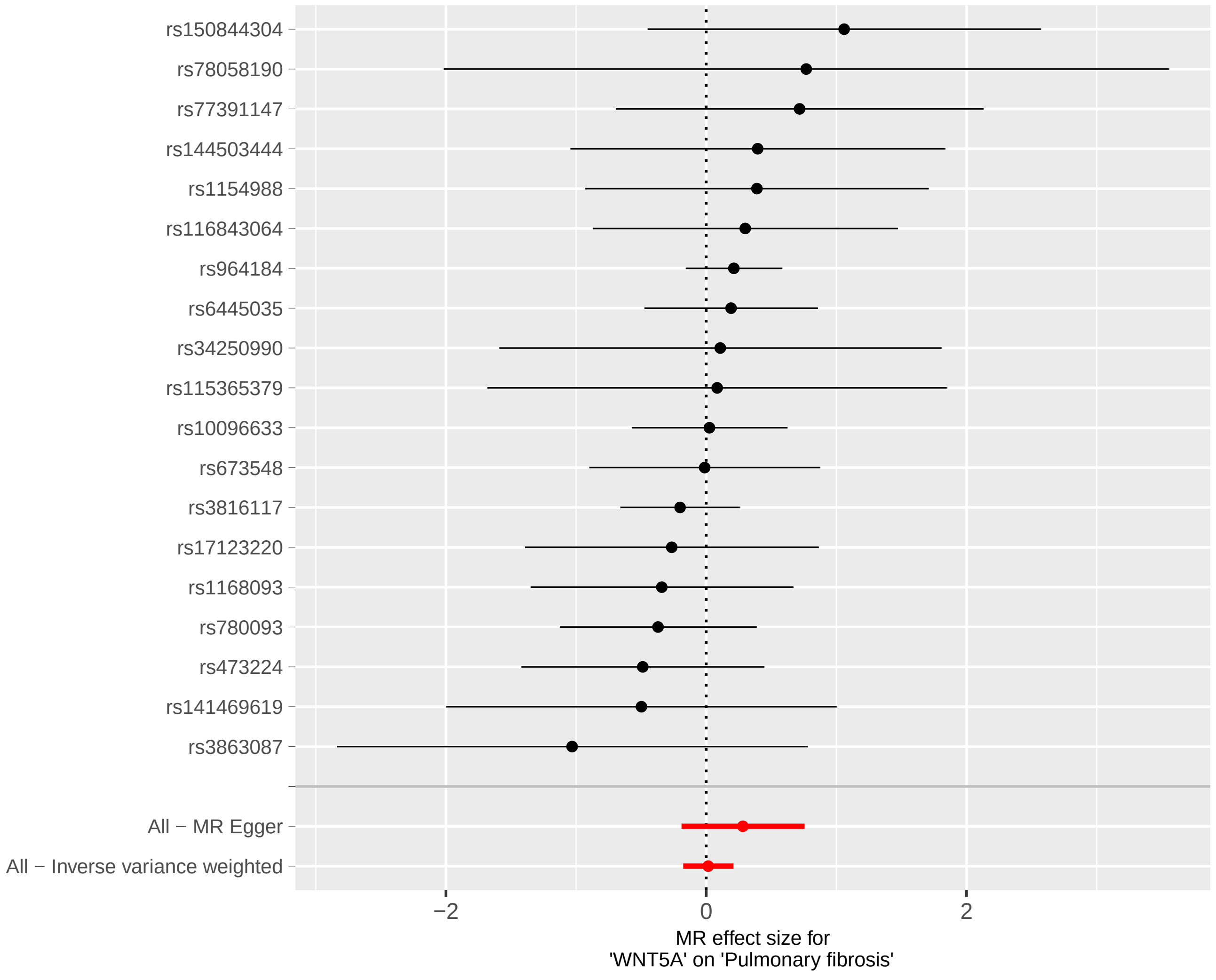
rs150844304
rs78058190
rs77391147
rs144503444
rs1154988
rs116843064
rs964184
rs6445035
rs34250990
rs115365379
rs10096633
rs673548
rs3816117
rs17123220
rs1168093
rs780093
rs473224
rs141469619
rs3863087
All − MR Egger
All − Inverse variance weighted
−2
0
2
MR effect size for
'WNT5A' on 'Pulmonary fibrosis'

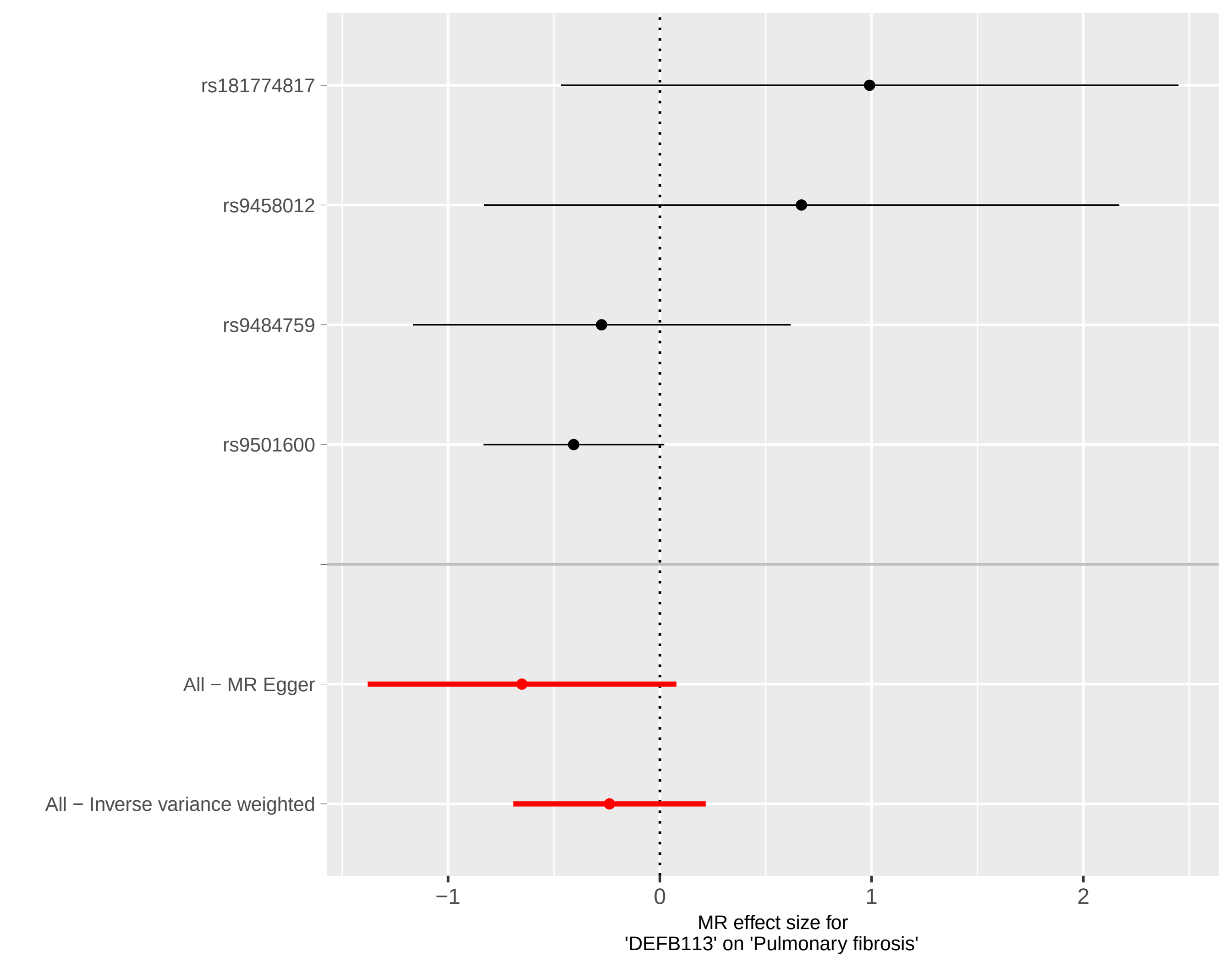
rs181774817
rs9458012
rs9484759
rs9501600
All − MR Egger
All − Inverse variance weighted
−1
0
1
2
MR effect size for
'DEFB113' on 'Pulmonary fibrosis'

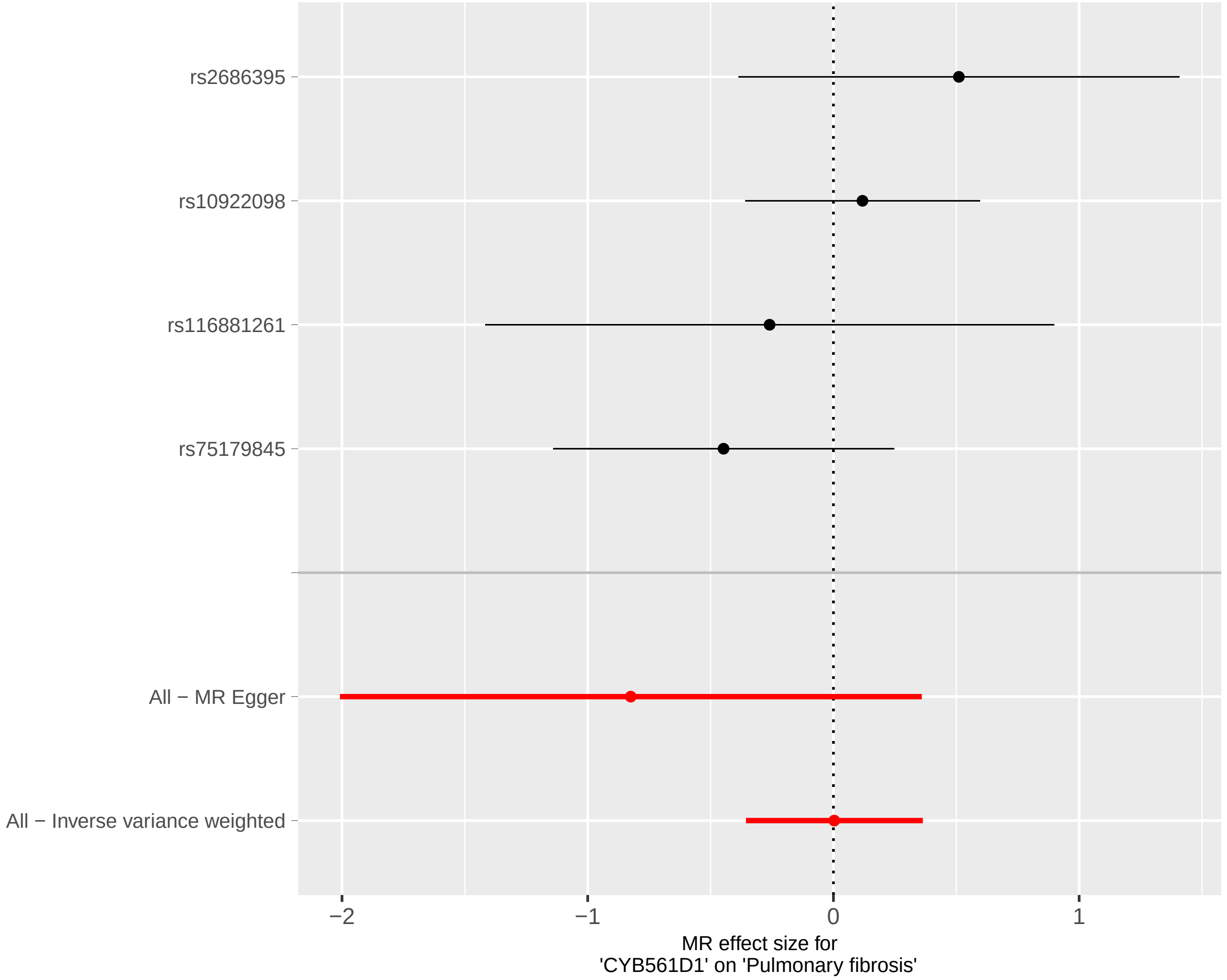
rs2686395
rs10922098
rs116881261
rs75179845
All − MR Egger
All − Inverse variance weighted
−2
−1
0
1
MR effect size for
'CYB561D1' on 'Pulmonary fibrosis'

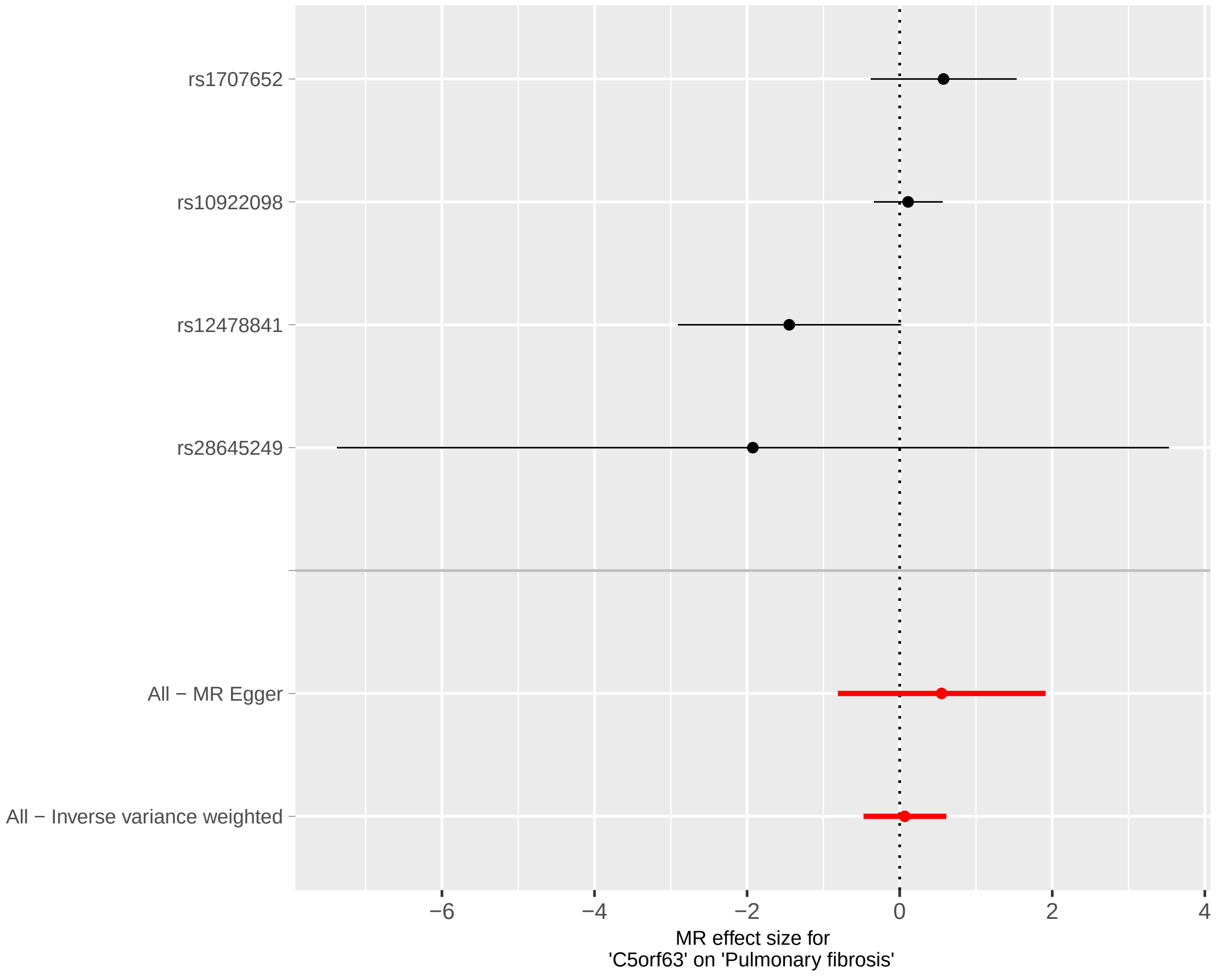
rs1707652
rs10922098
rs12478841
rs28645249
All − MR Egger
All − Inverse variance weighted
−6
−4
−2
0
2
4
MR effect size for
'C5orf63' on 'Pulmonary fibrosis'

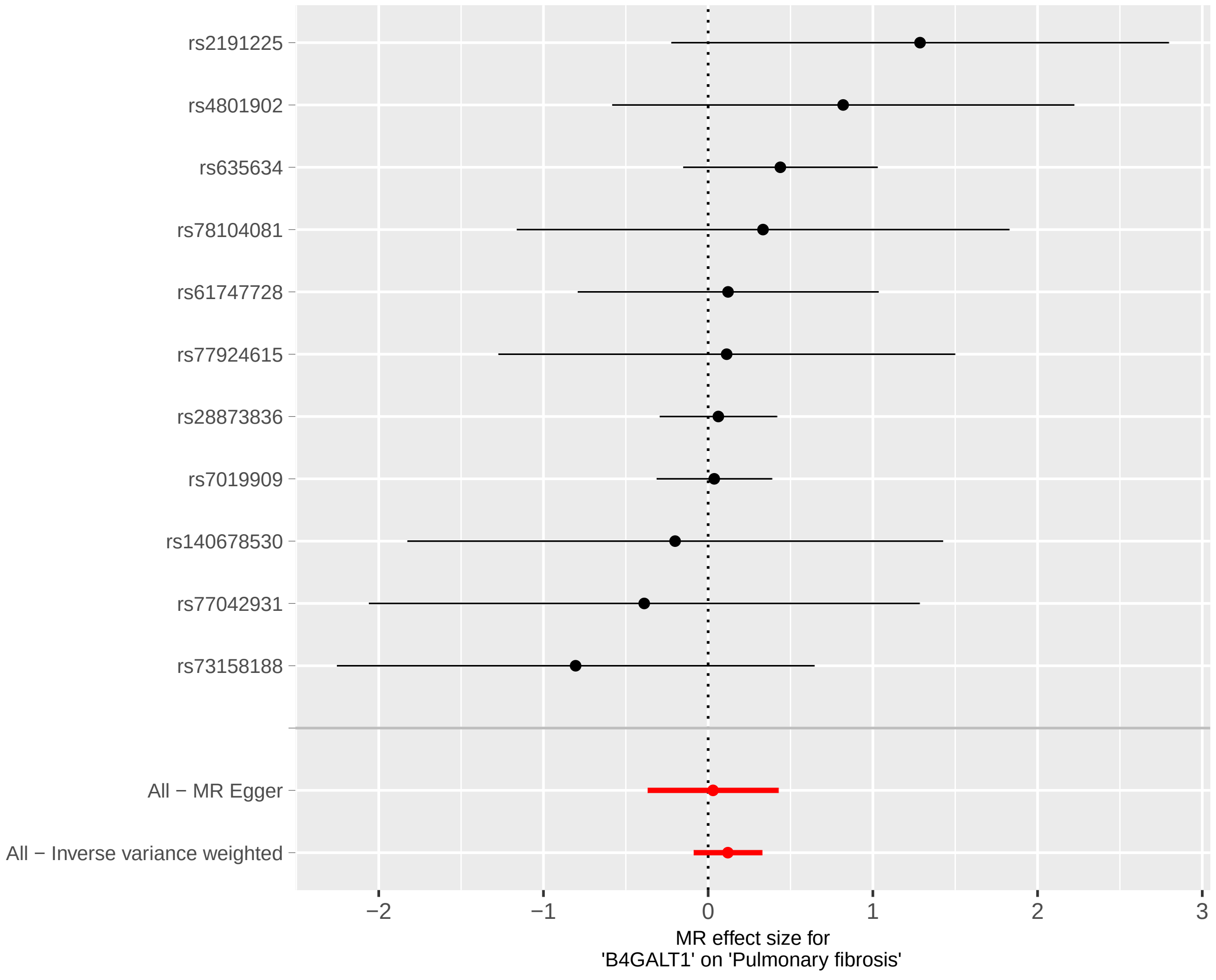
rs2191225
rs4801902
rs635634
rs78104081
rs61747728
rs77924615
rs28873836
rs7019909
rs140678530
rs77042931
rs73158188
All − MR Egger
All − Inverse variance weighted
−2
−1
0
1
2
3
MR effect size for
'B4GALT1' on 'Pulmonary fibrosis'

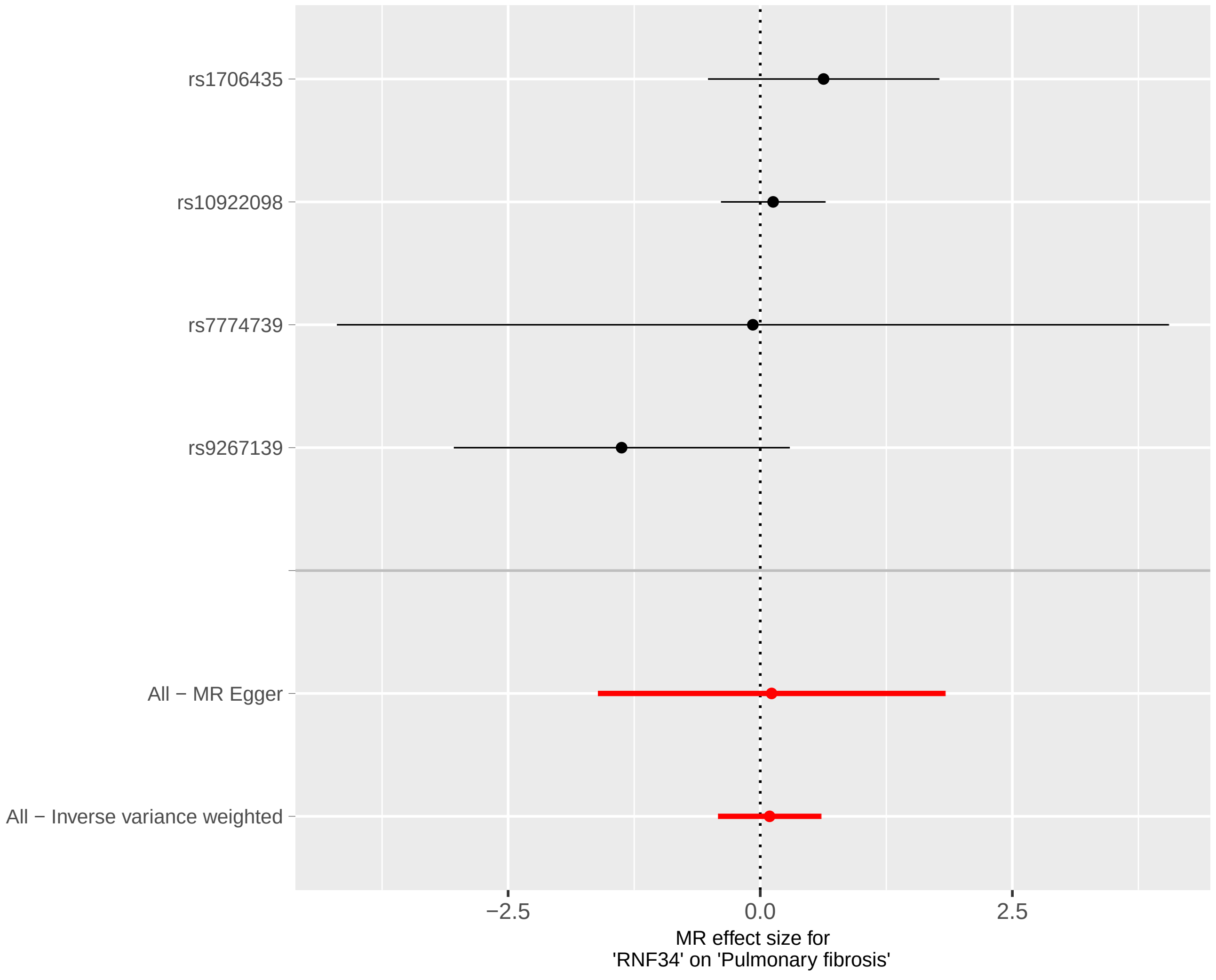
rs1706435
rs10922098
rs7774739
rs9267139
All − MR Egger
All − Inverse variance weighted
−2.5
0.0
2.5
MR effect size for
'RNF34' on 'Pulmonary fibrosis'

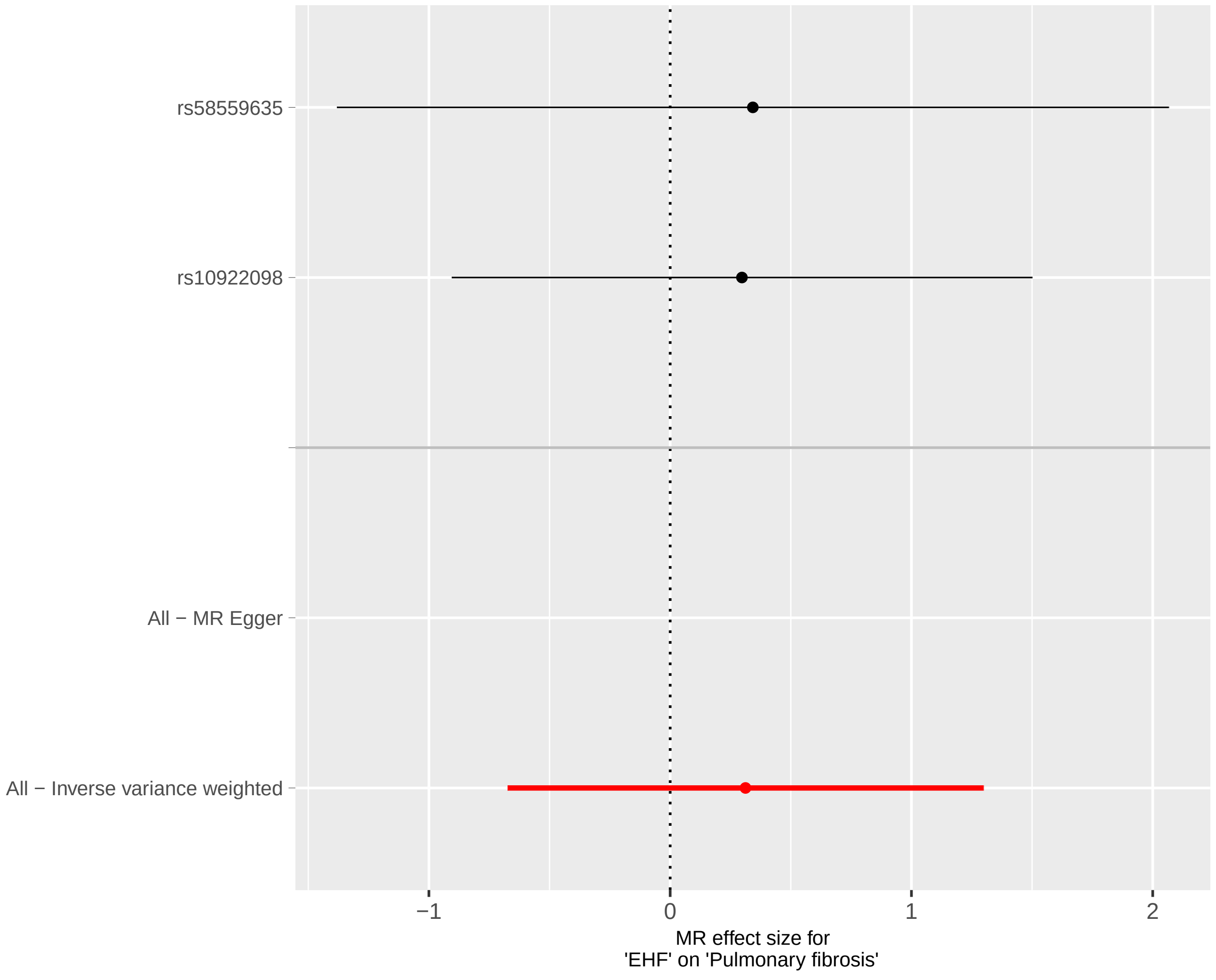
rs58559635
rs10922098
All − MR Egger
All − Inverse variance weighted
−1
0
1
2
MR effect size for
'EHF' on 'Pulmonary fibrosis'

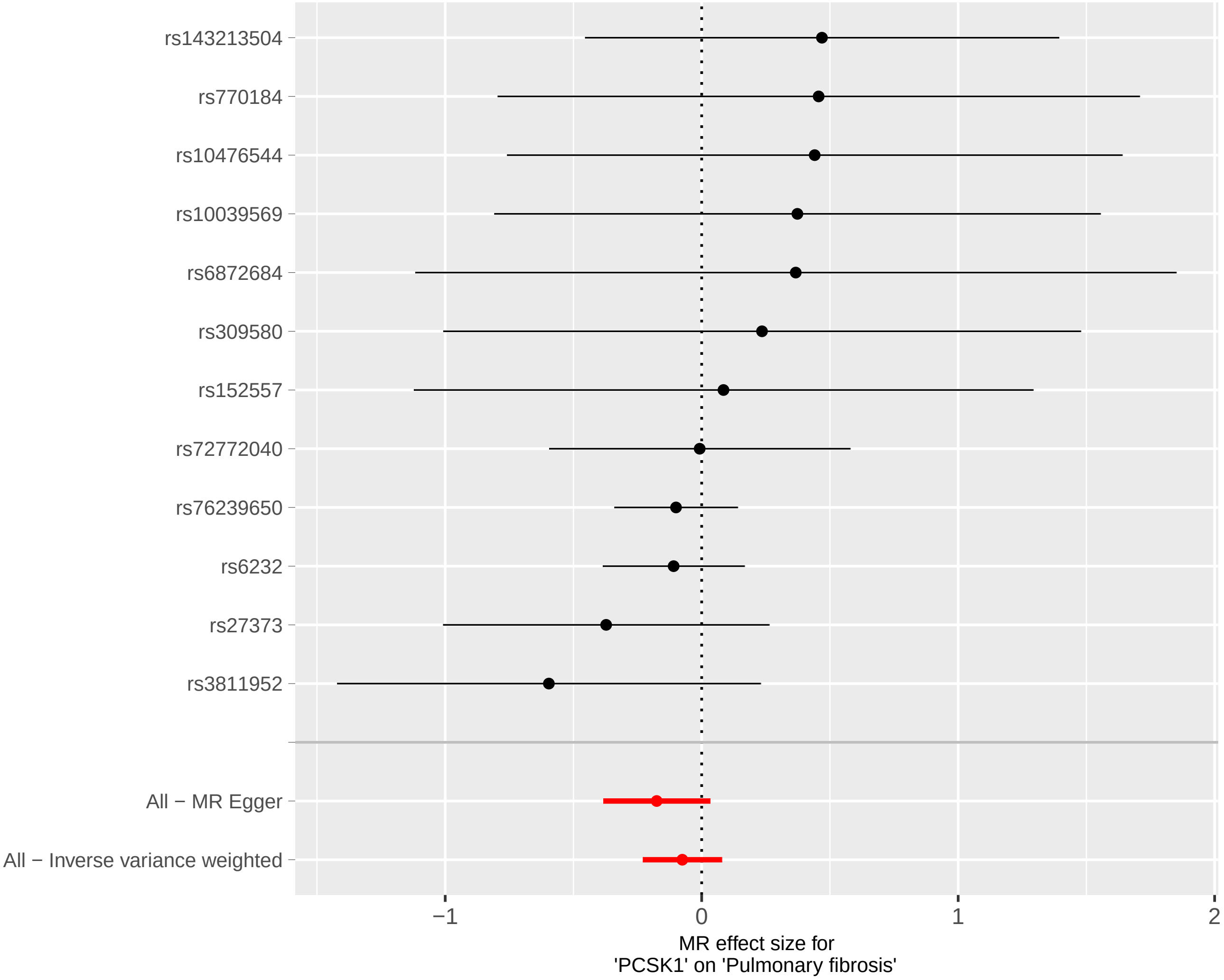
rs143213504
rs770184
rs10476544
rs10039569
rs6872684
rs309580
rs152557
rs72772040
rs76239650
rs6232
rs27373
rs3811952
All − MR Egger
All − Inverse variance weighted
−1
0
1
2
MR effect size for
'PCSK1' on 'Pulmonary fibrosis'

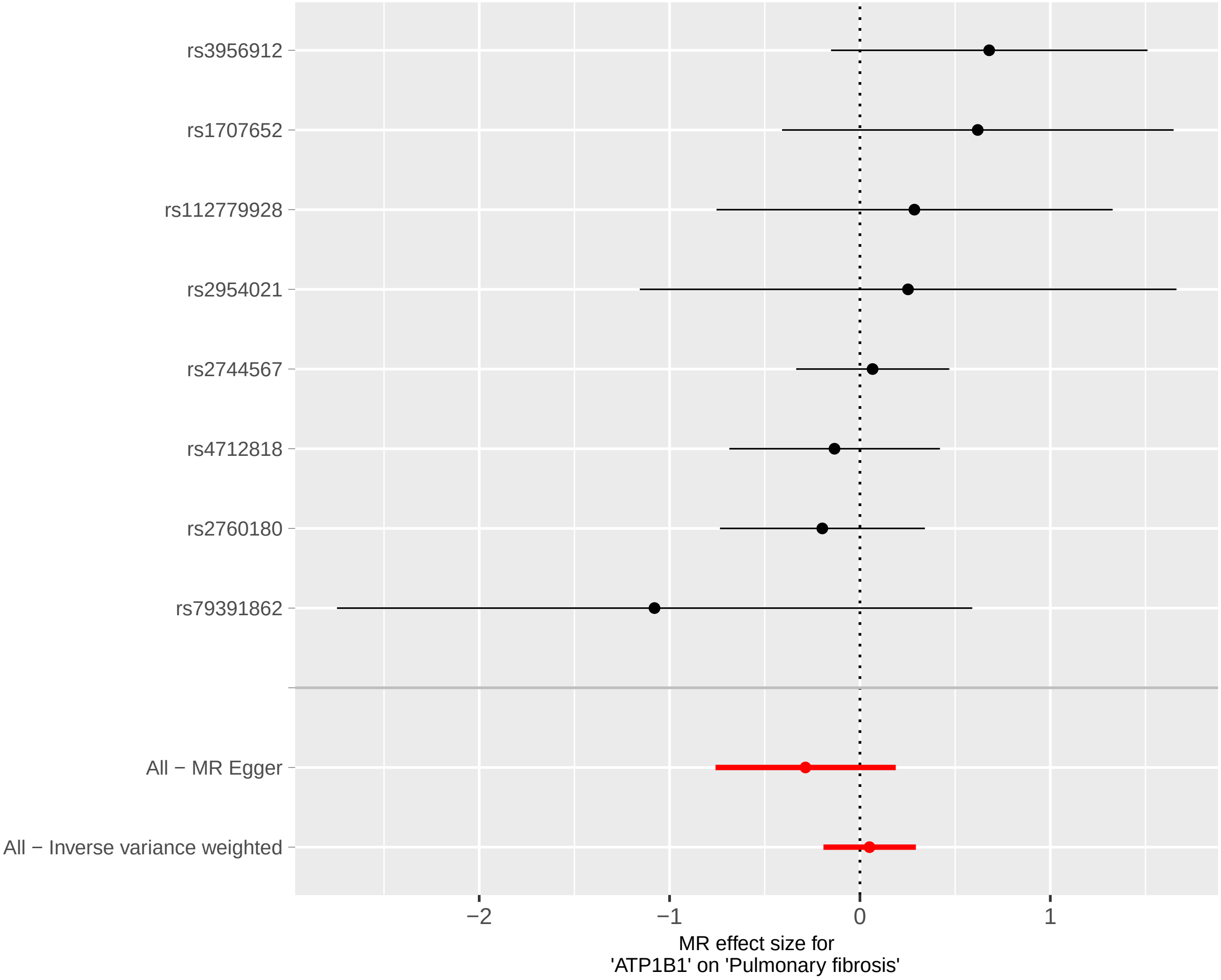
rs3956912
rs1707652
rs112779928
rs2954021
rs2744567
rs4712818
rs2760180
rs79391862
All − MR Egger
All − Inverse variance weighted
−2
−1
0
1
MR effect size for
'ATP1B1' on 'Pulmonary fibrosis'

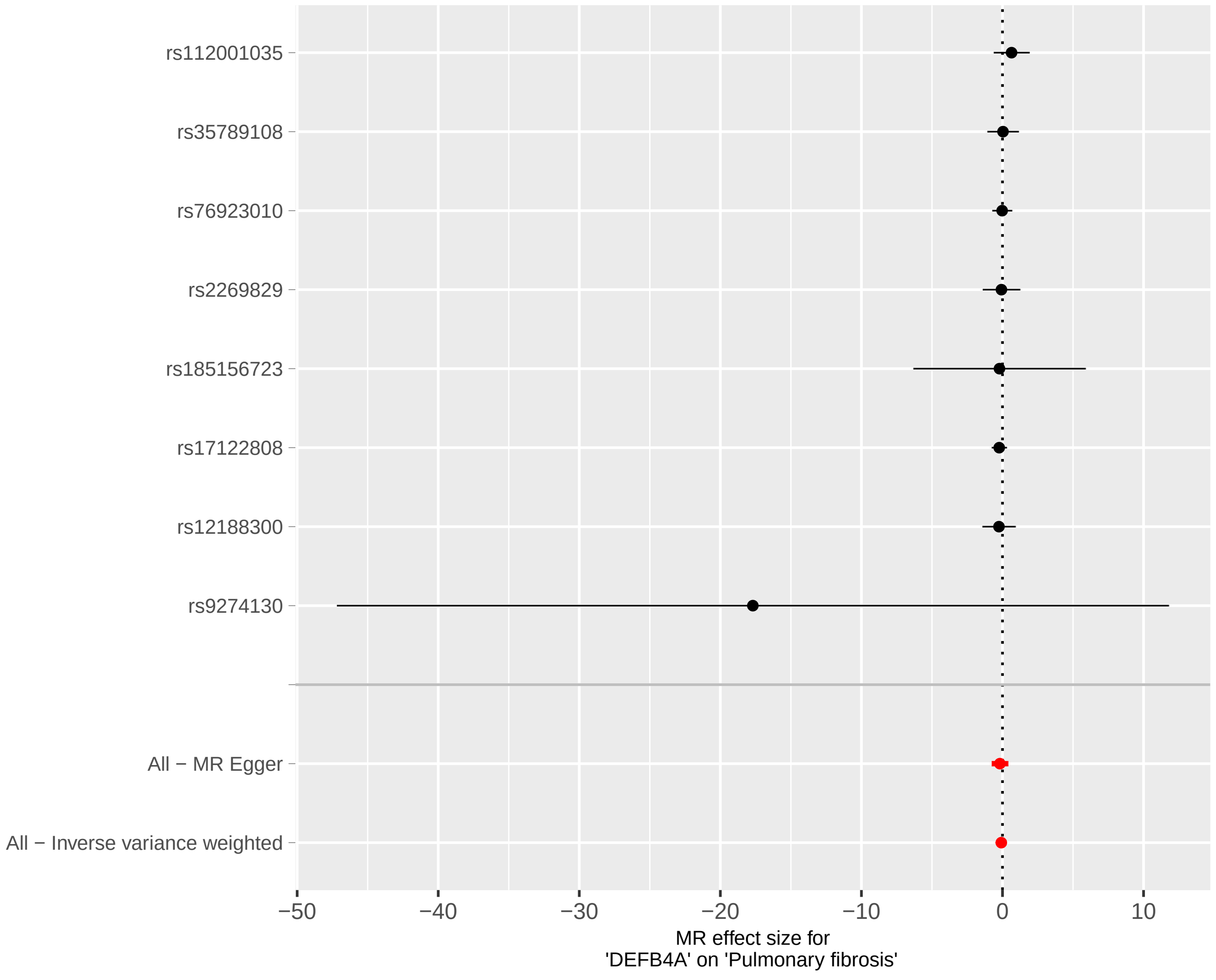
rs112001035
rs35789108
rs76923010
rs2269829
rs185156723
rs17122808
rs12188300
rs9274130
All − MR Egger
All − Inverse variance weighted
−50
−40
−30
−20
−10
0
10
MR effect size for
'DEFB4A' on 'Pulmonary fibrosis'

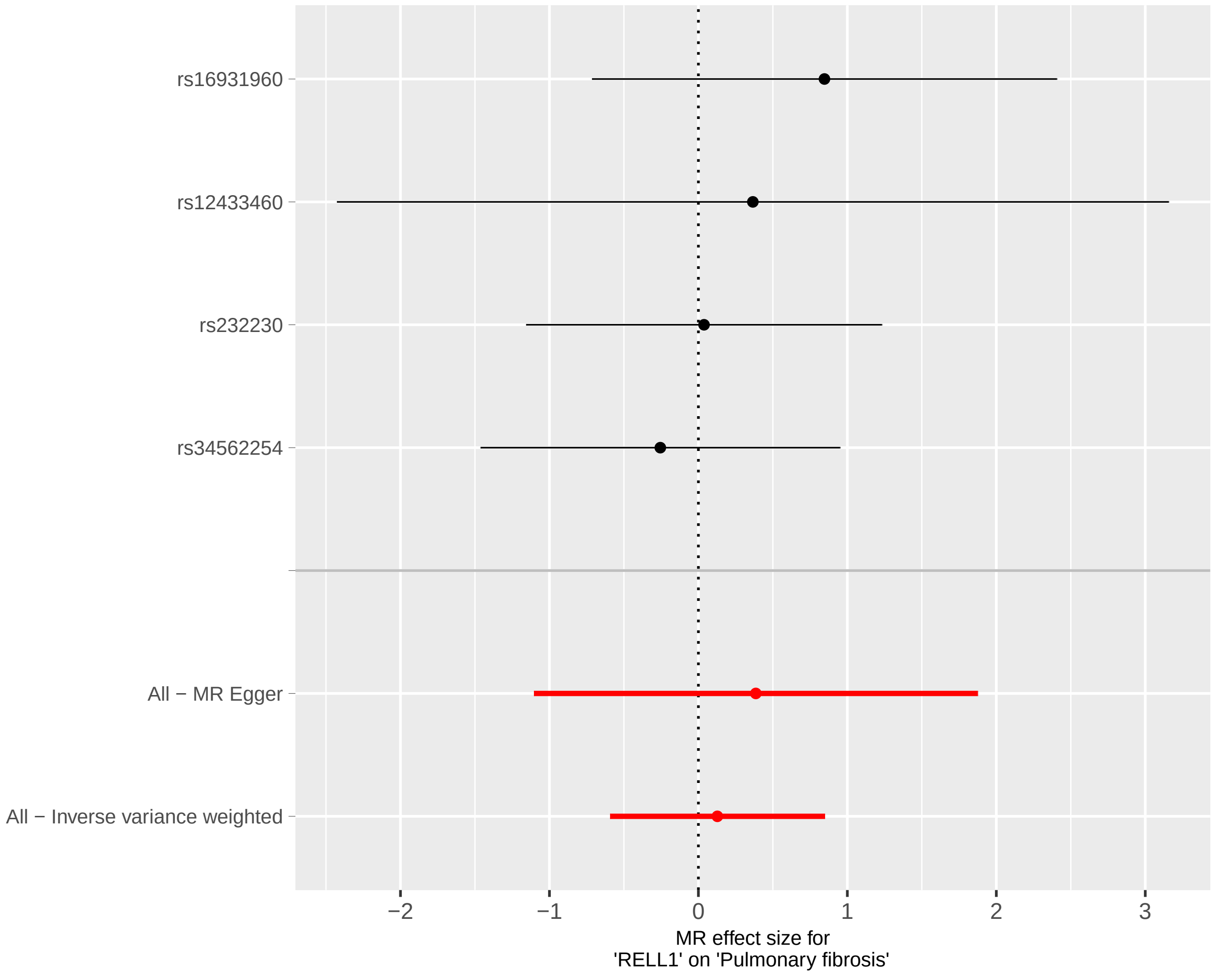
rs16931960
rs12433460
rs232230
rs34562254
All − MR Egger
All − Inverse variance weighted
−2
−1
0
1
2
3
MR effect size for
'RELL1' on 'Pulmonary fibrosis'

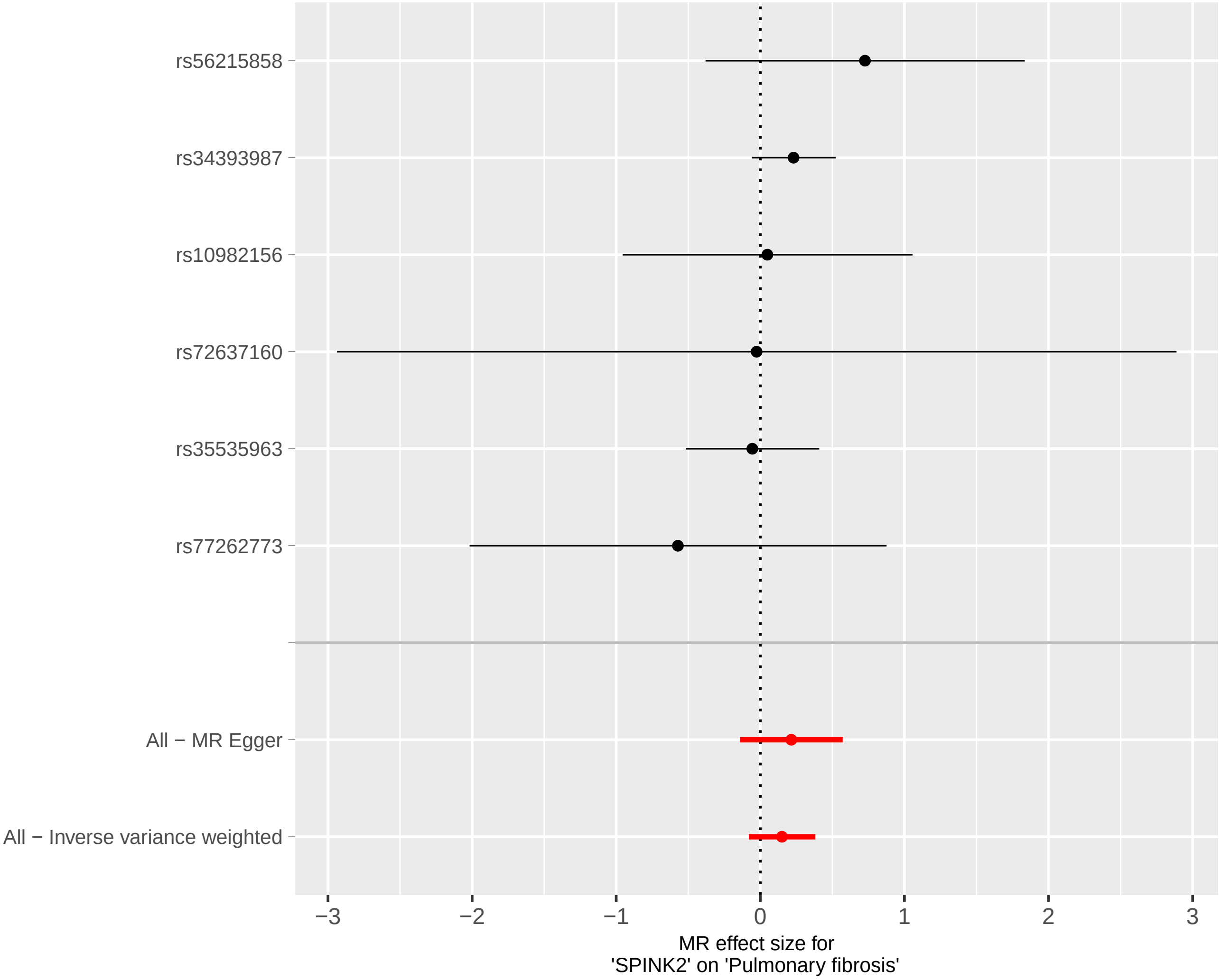
rs56215858
rs34393987
rs10982156
rs72637160
rs35535963
rs77262773
All − MR Egger
All − Inverse variance weighted
−3
−2
−1
0
1
2
3
MR effect size for
'SPINK2' on 'Pulmonary fibrosis'

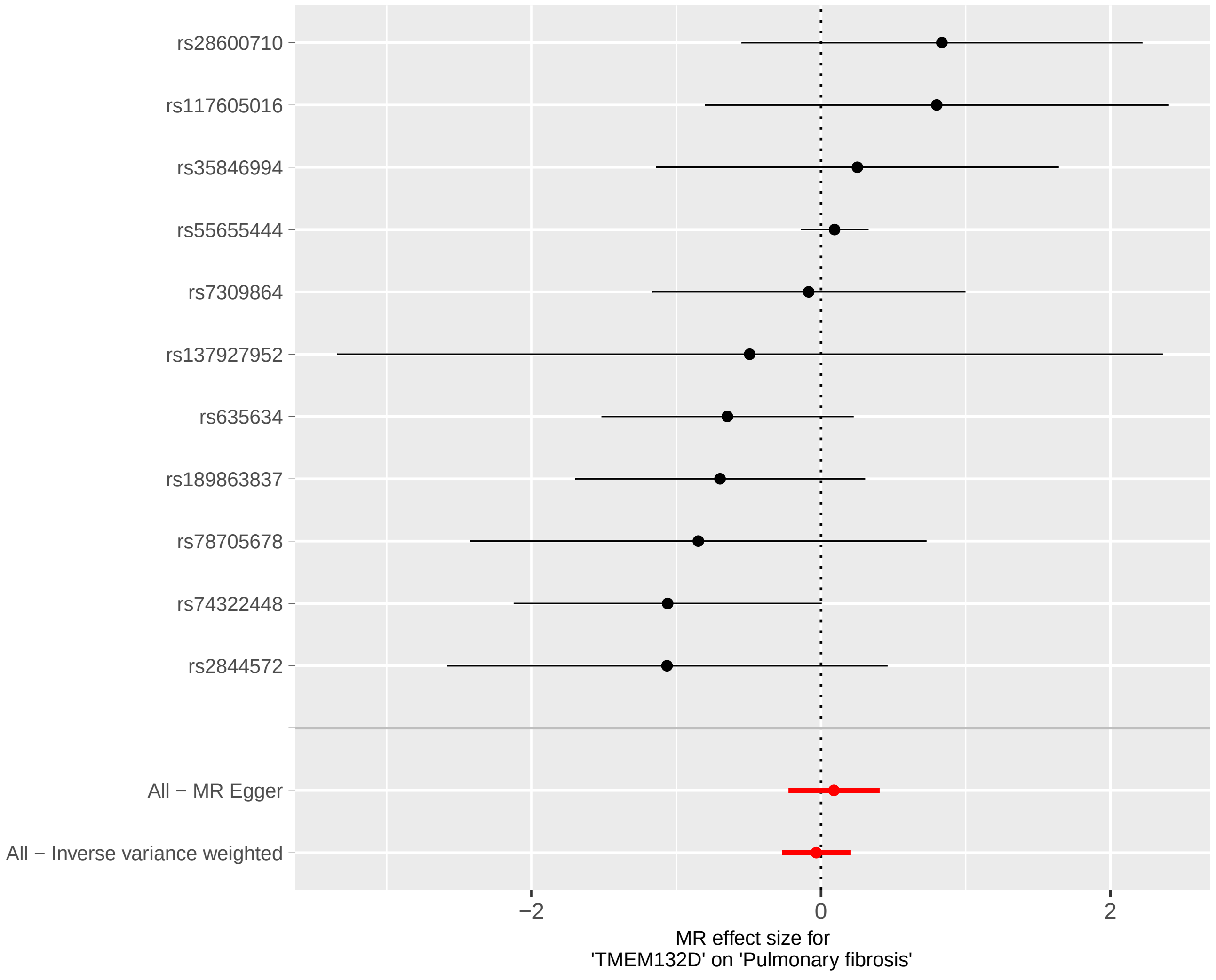
rs28600710
rs117605016
rs35846994
rs55655444
rs7309864
rs137927952
rs635634
rs189863837
rs78705678
rs74322448
rs2844572
All − MR Egger
All − Inverse variance weighted
−2
0
2
MR effect size for
'TMEM132D' on 'Pulmonary fibrosis'

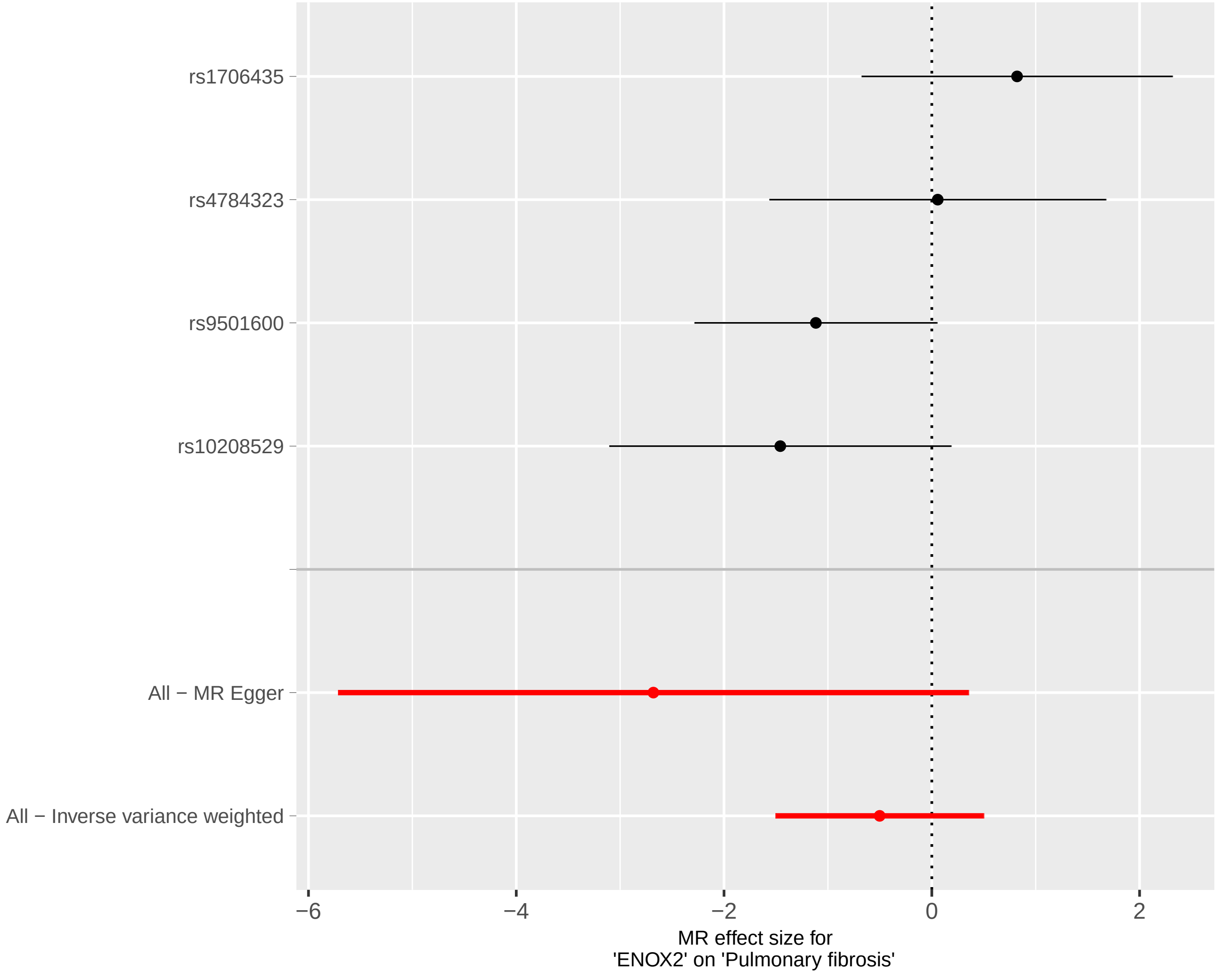
rs1706435
rs4784323
rs9501600
rs10208529
All − MR Egger
All − Inverse variance weighted
−6
−4
−2
0
2
MR effect size for
'ENOX2' on 'Pulmonary fibrosis'

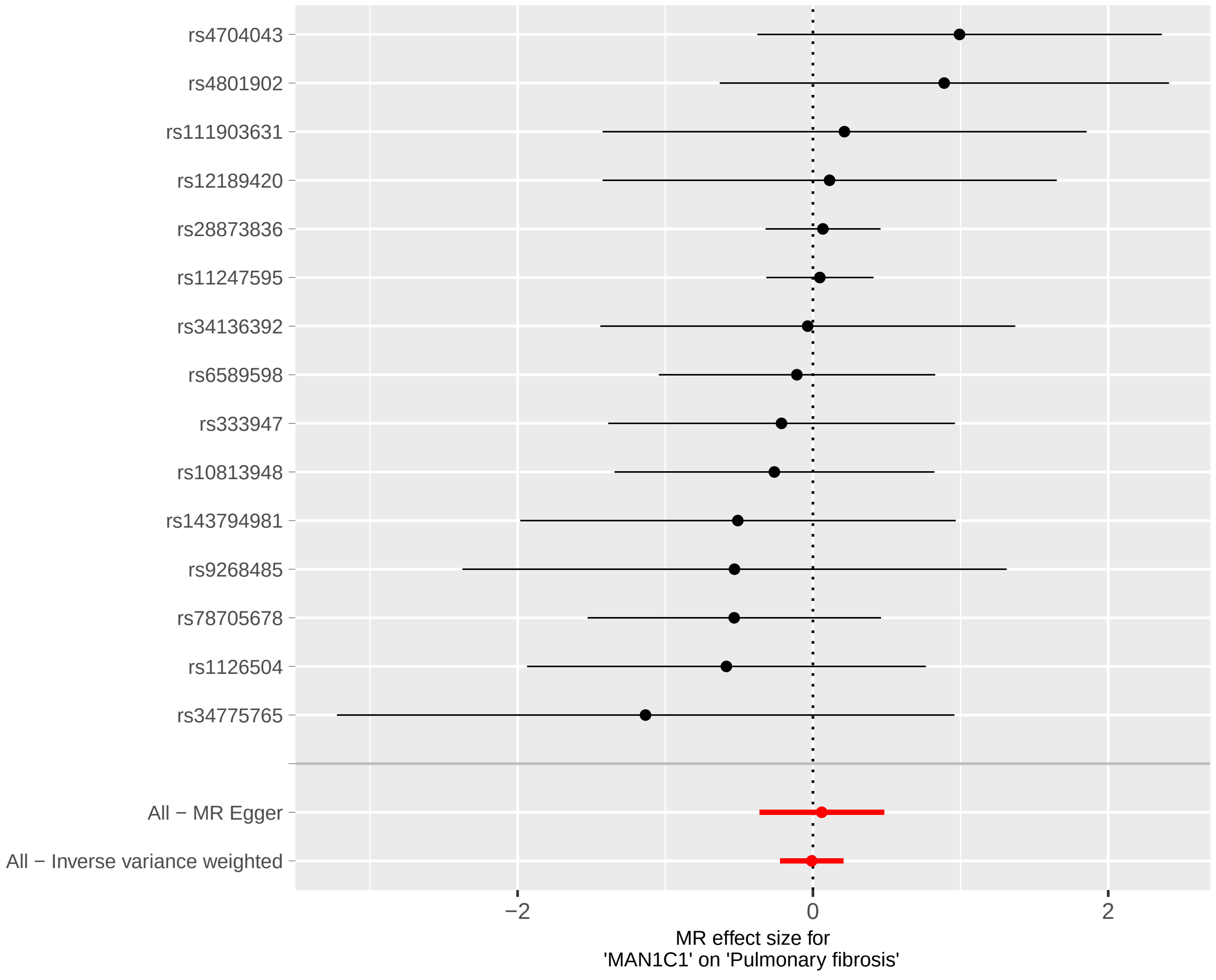
rs4704043
rs4801902
rs111903631
rs12189420
rs28873836
rs11247595
rs34136392
rs6589598
rs333947
rs10813948
rs143794981
rs9268485
rs78705678
rs1126504
rs34775765
All − MR Egger
All − Inverse variance weighted
−2
0
2
MR effect size for
'MAN1C1' on 'Pulmonary fibrosis'

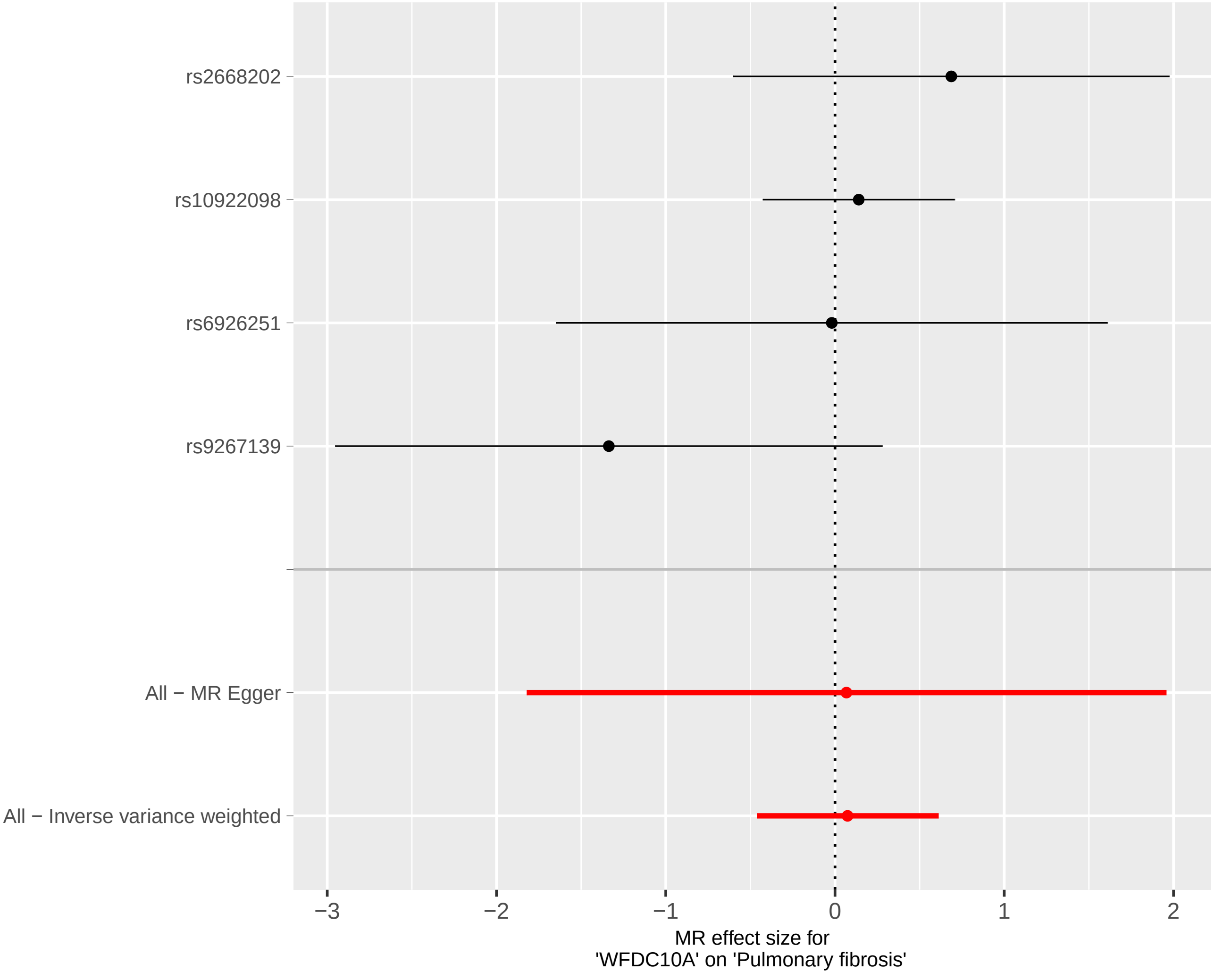
rs2668202
rs10922098
rs6926251
rs9267139
All − MR Egger
All − Inverse variance weighted
−3
−2
−1
0
1
2
MR effect size for
'WFDC10A' on 'Pulmonary fibrosis'

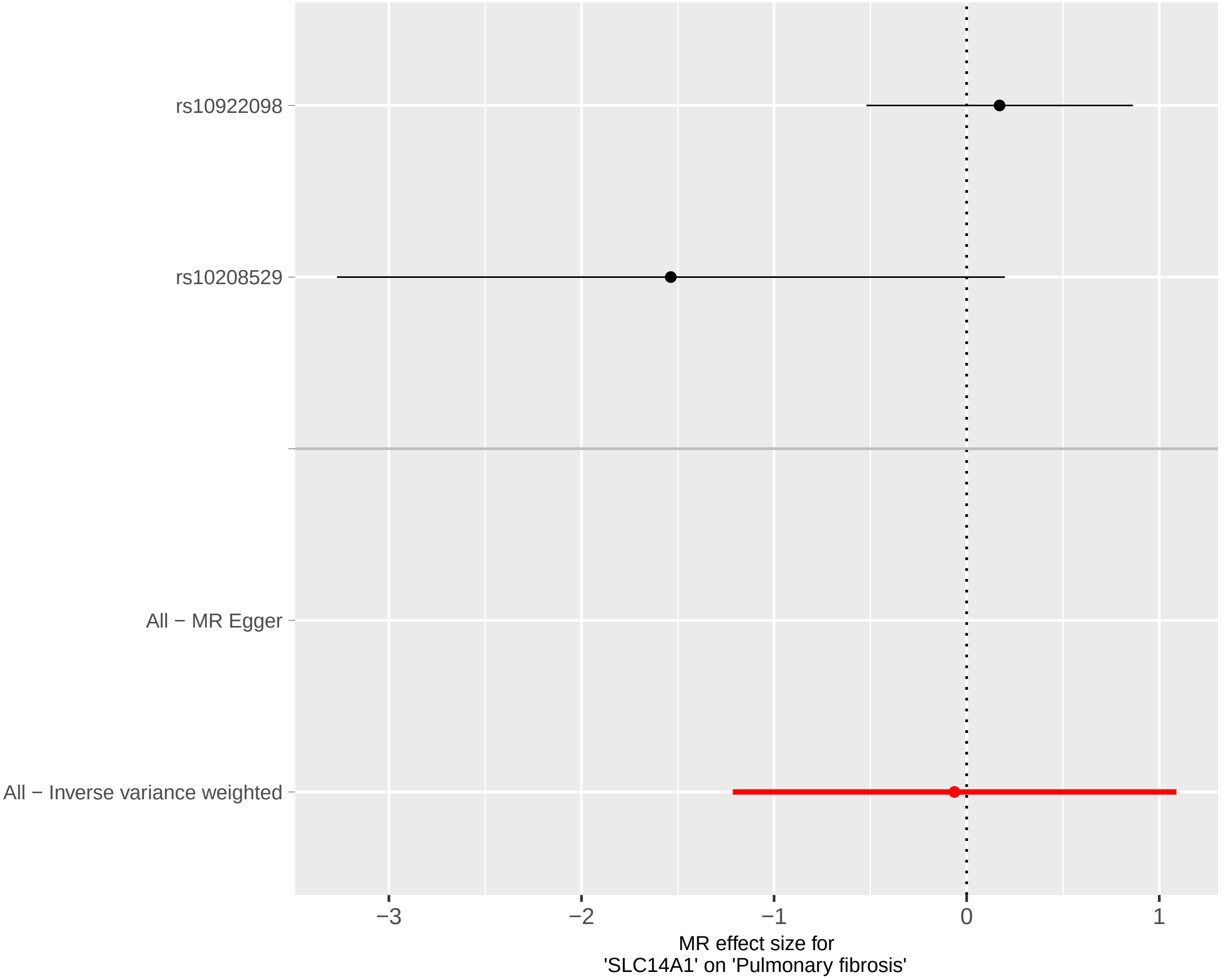
rs10922098
rs10208529
All − MR Egger
All − Inverse variance weighted
−3
−2
−1
0
1
MR effect size for
'SLC14A1' on 'Pulmonary fibrosis'

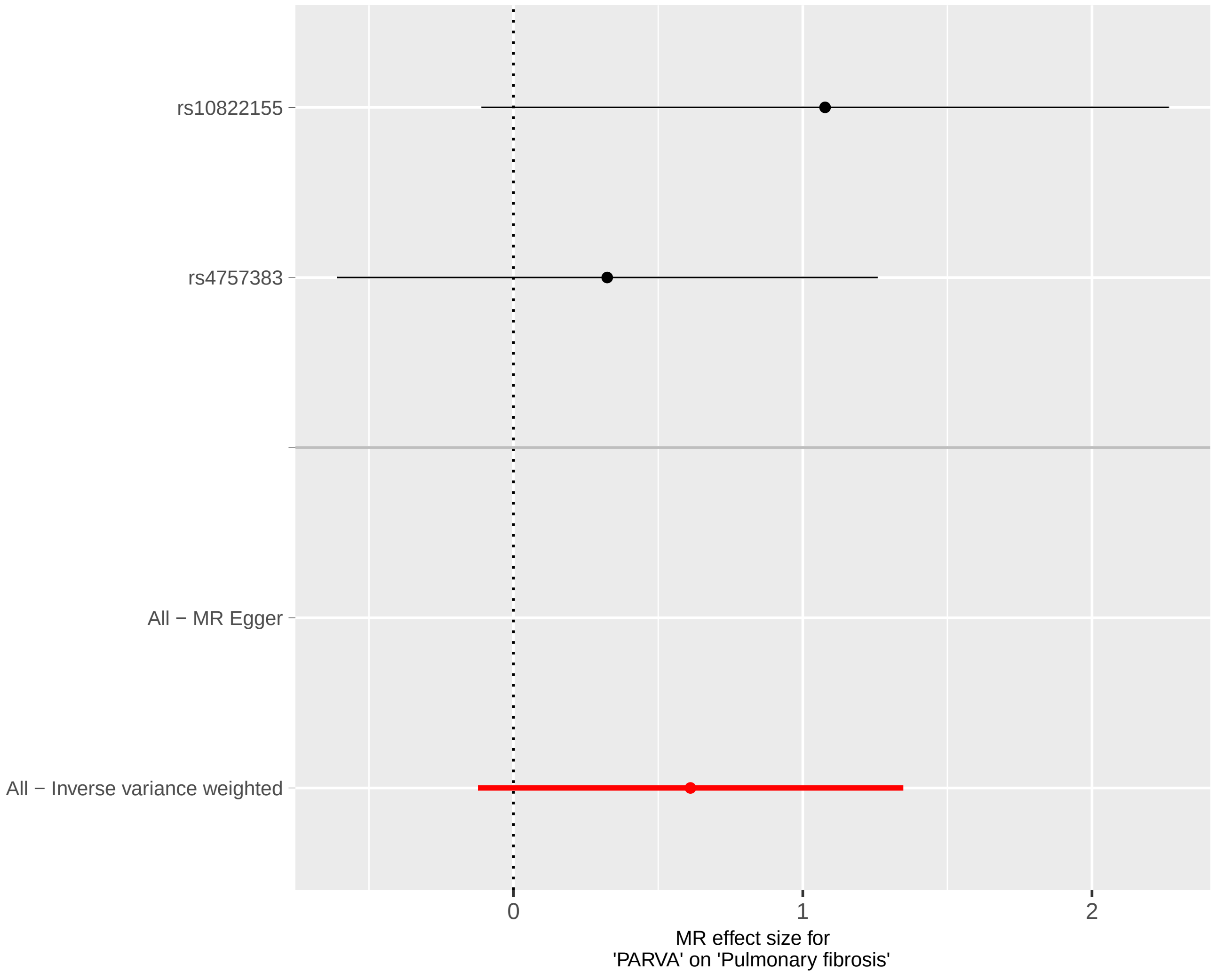

rs10822155
rs4757383
All − MR Egger
All − Inverse variance weighted
0
1
2
MR effect size for
'PARVA' on 'Pulmonary fibrosis'

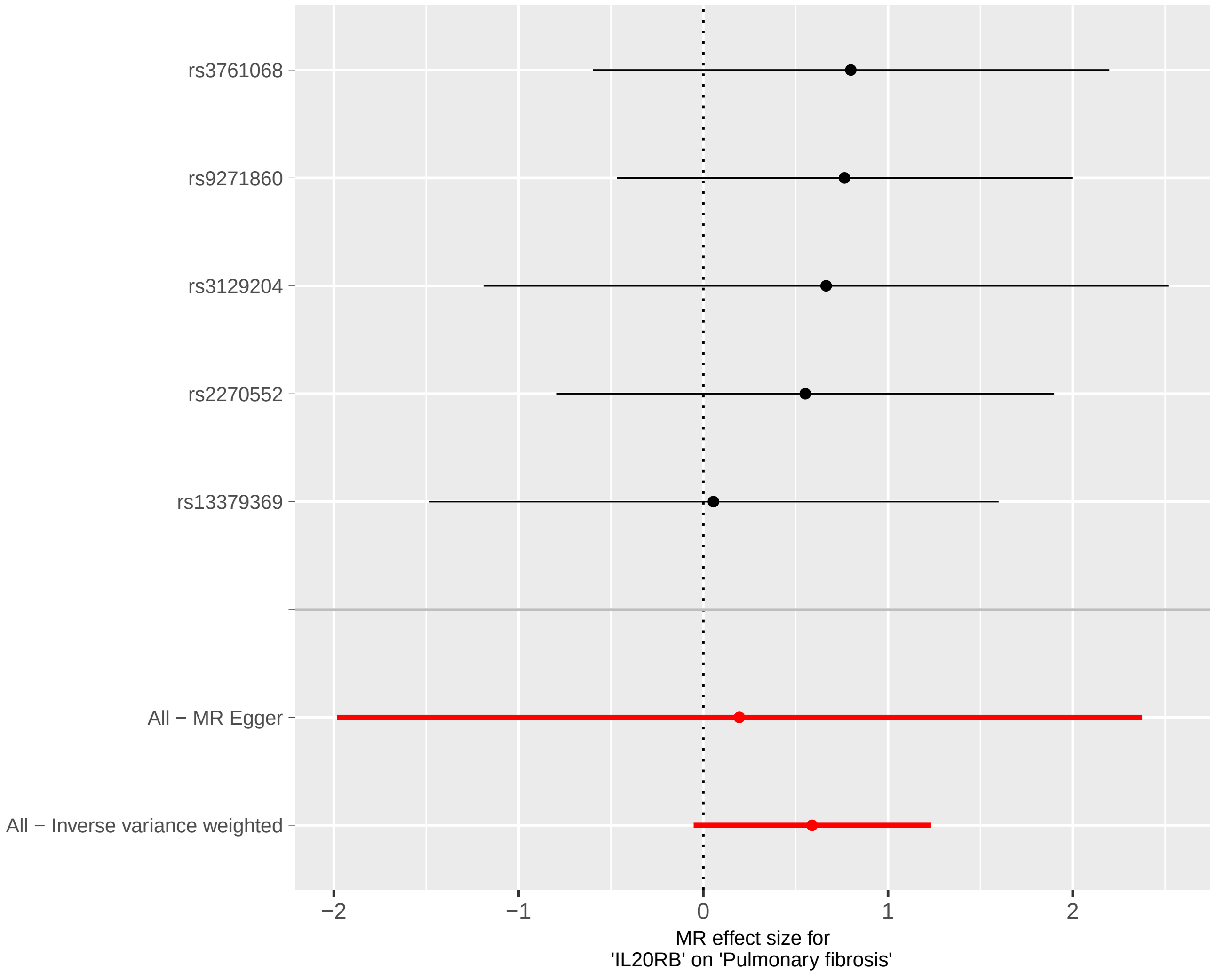
rs3761068
rs9271860
rs3129204
rs2270552
rs13379369
All − MR Egger
All − Inverse variance weighted
−2
−1
0
1
2
MR effect size for
'IL20RB' on 'Pulmonary fibrosis'

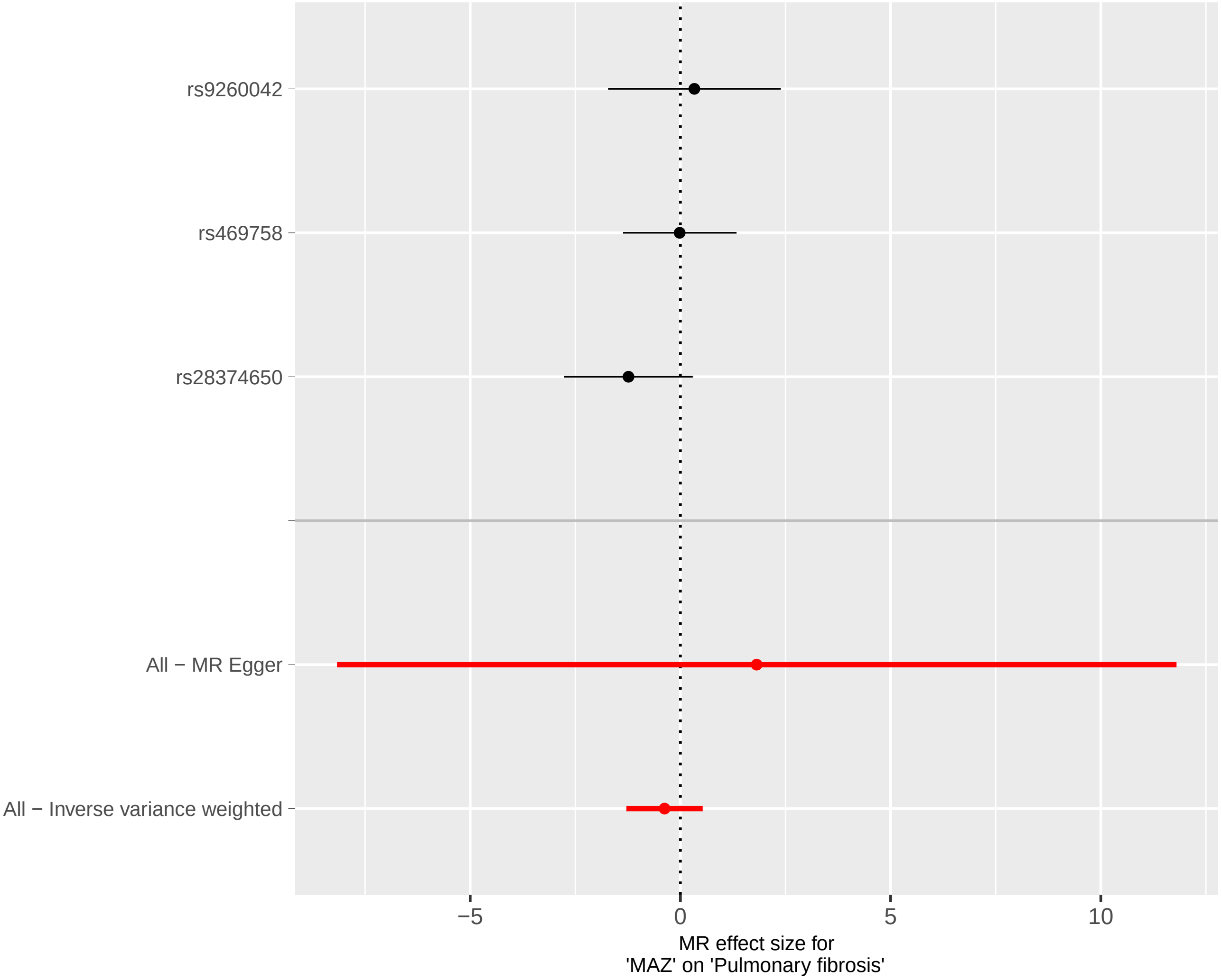

rs9260042
rs469758
rs28374650
All − MR Egger
All − Inverse variance weighted
−5
0
5
10
MR effect size for
'MAZ' on 'Pulmonary fibrosis'

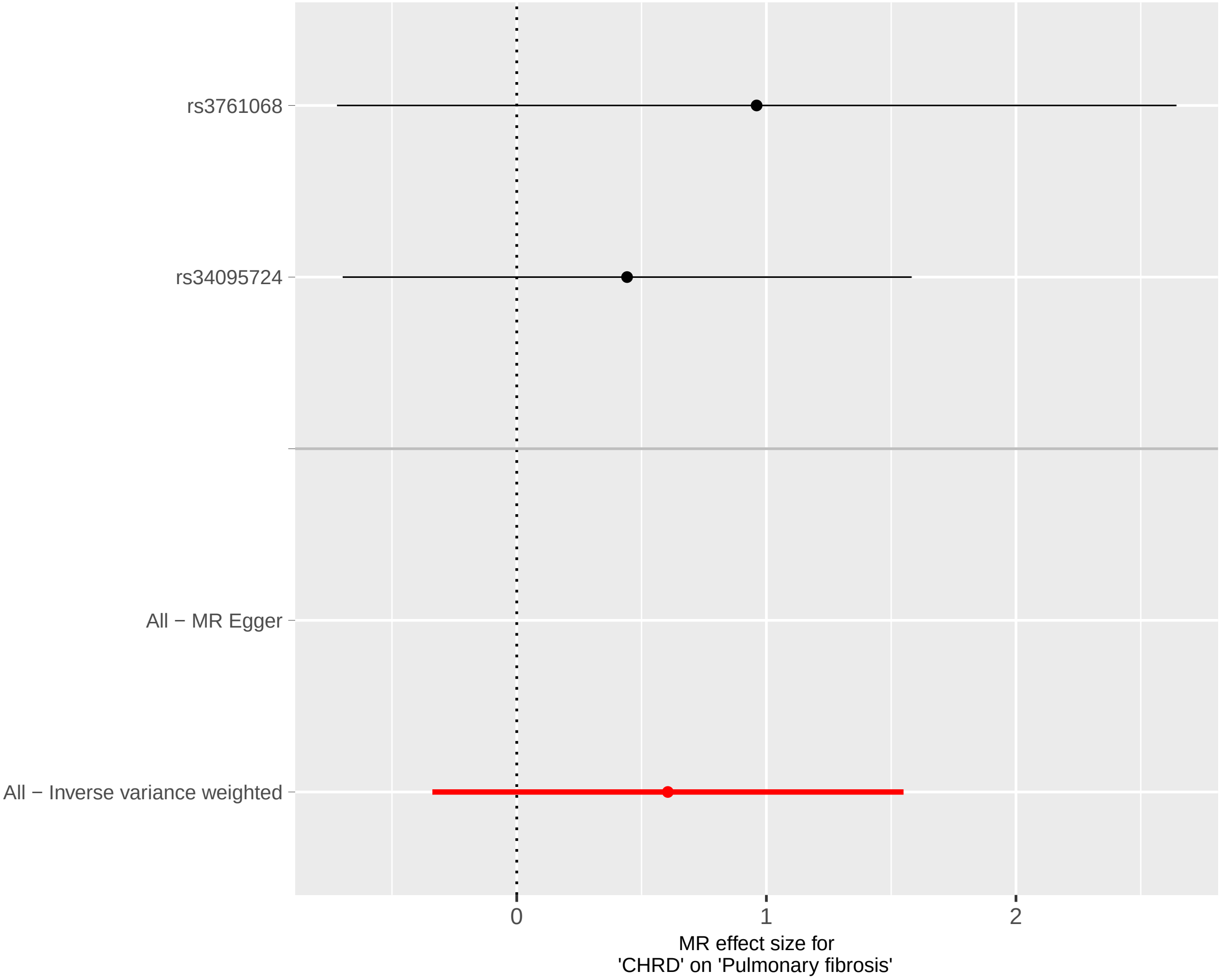
rs3761068
rs34095724
All − MR Egger
All − Inverse variance weighted
0
1
2
MR effect size for
'CHRD' on 'Pulmonary fibrosis'

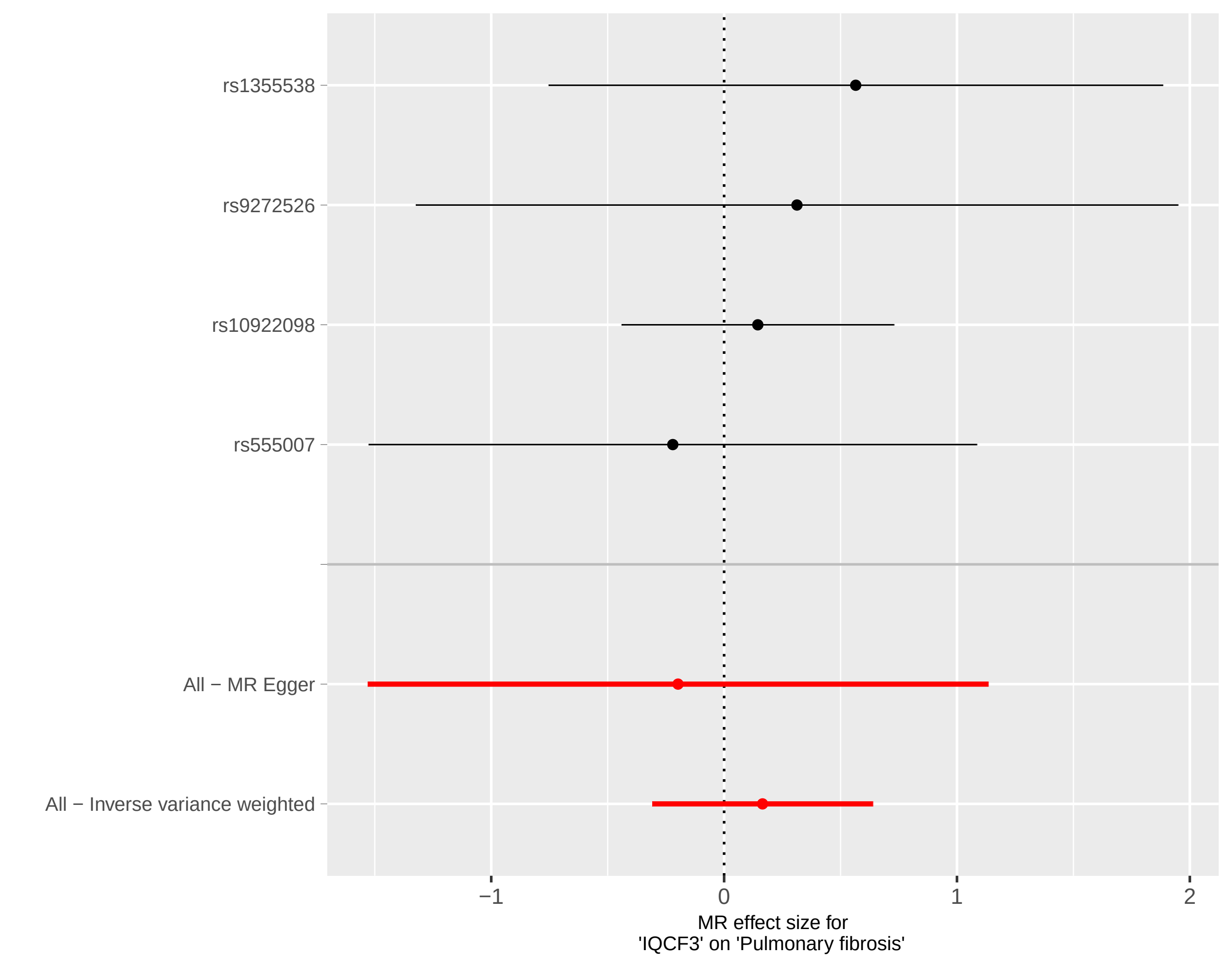
rs1355538
rs9272526
rs10922098
rs555007
All − MR Egger
All − Inverse variance weighted
−1
0
1
2
MR effect size for
'IQCF3' on 'Pulmonary fibrosis'

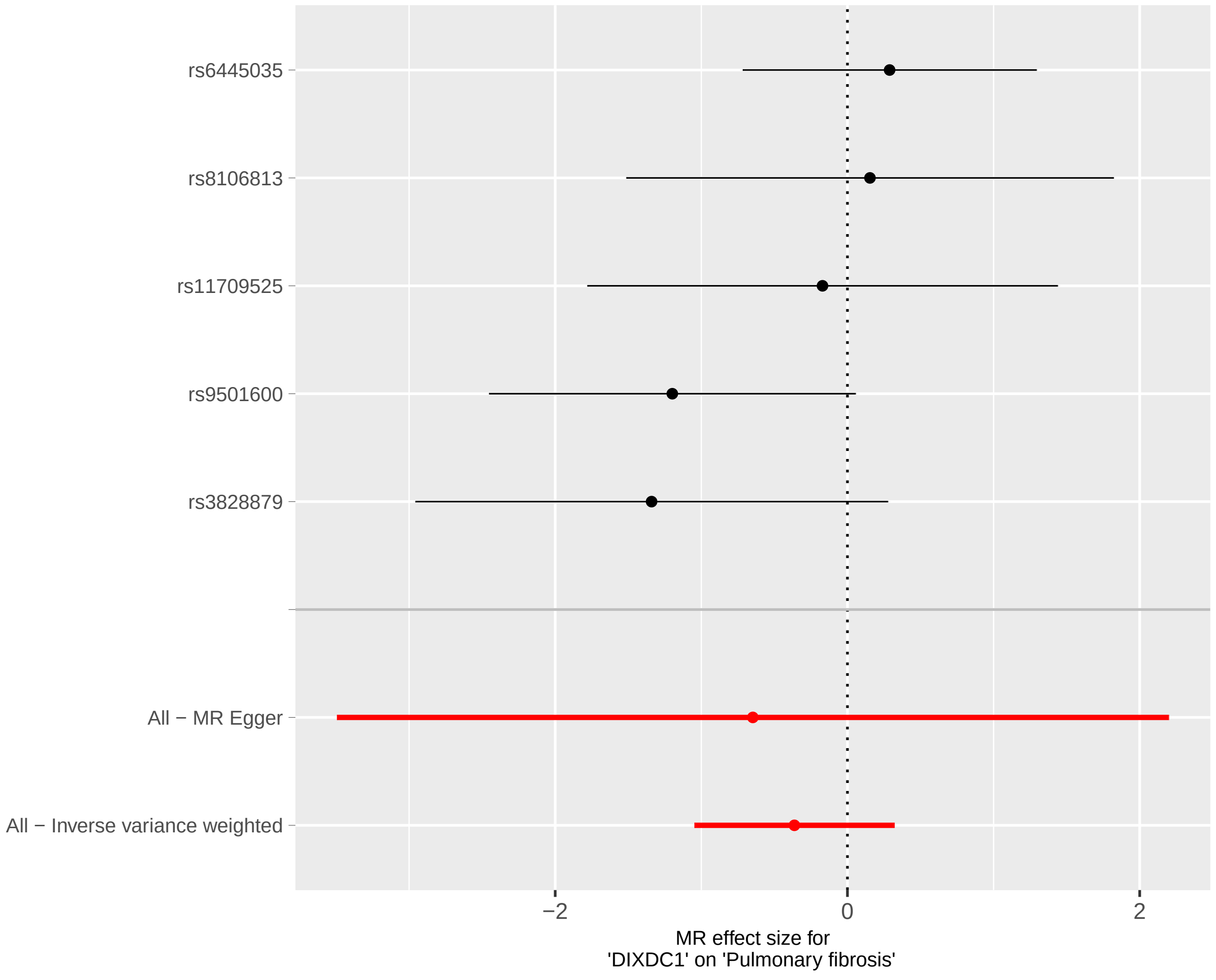

rs6445035
rs8106813
rs11709525
rs9501600
rs3828879
All − MR Egger
All − Inverse variance weighted
−2
0
2
MR effect size for
'DIXDC1' on 'Pulmonary fibrosis'

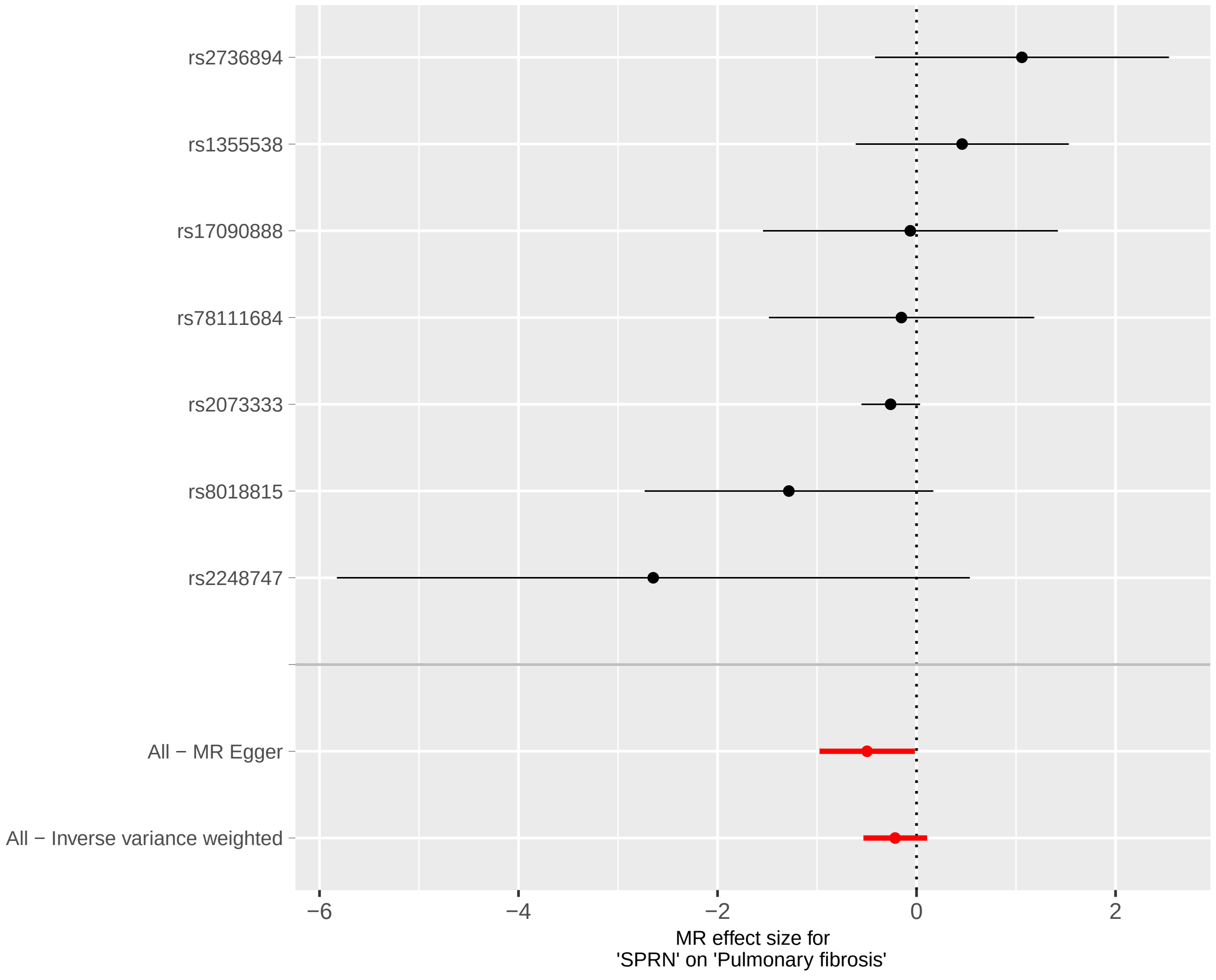
rs2736894
rs1355538
rs17090888
rs78111684
rs2073333
rs8018815
rs2248747
All − MR Egger
All − Inverse variance weighted
−6
−4
−2
0
2
MR effect size for
'SPRN' on 'Pulmonary fibrosis'

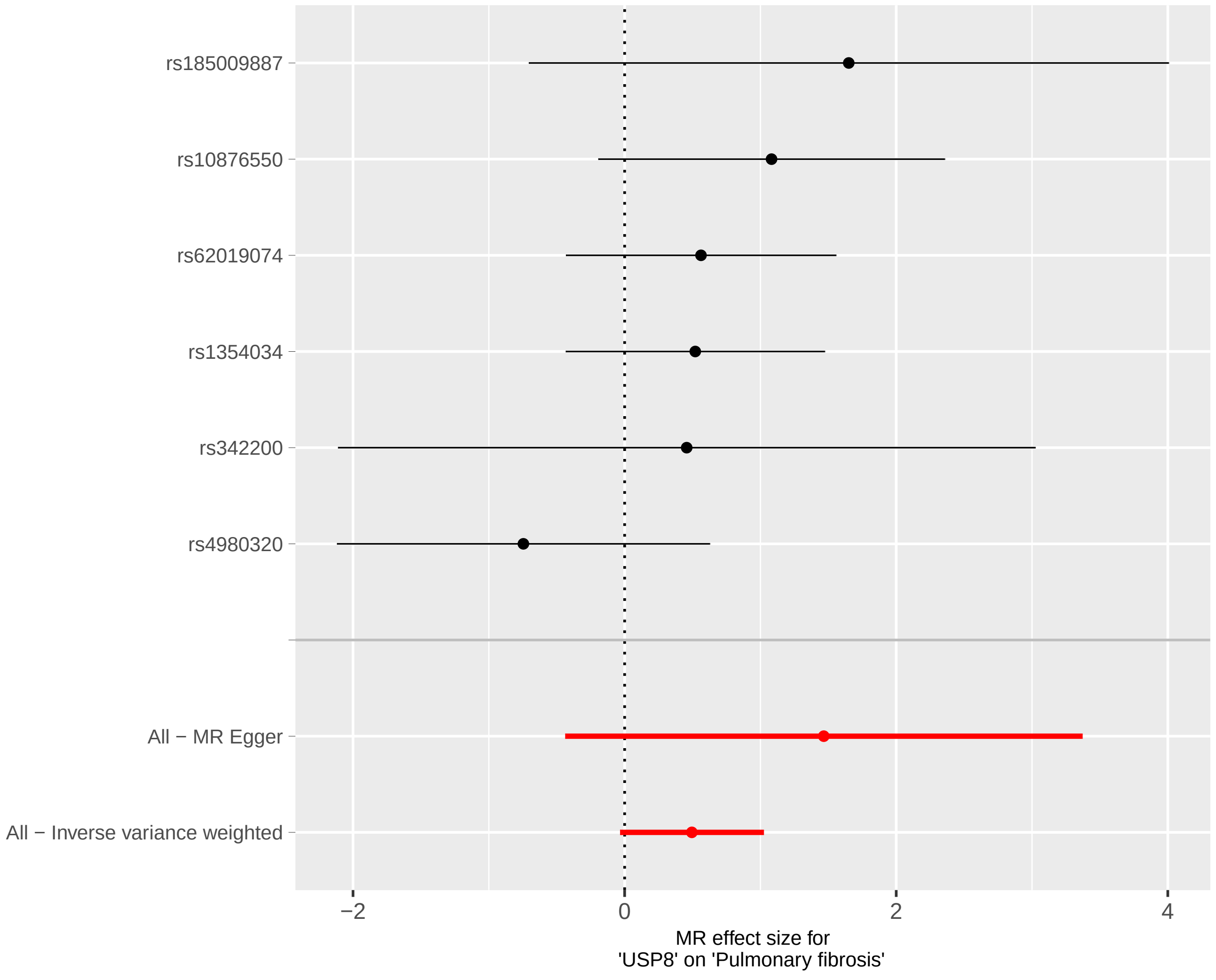
rs185009887
rs10876550
rs62019074
rs1354034
rs342200
rs4980320
All − MR Egger
All − Inverse variance weighted
−2
0
2
4
MR effect size for
'USP8' on 'Pulmonary fibrosis'

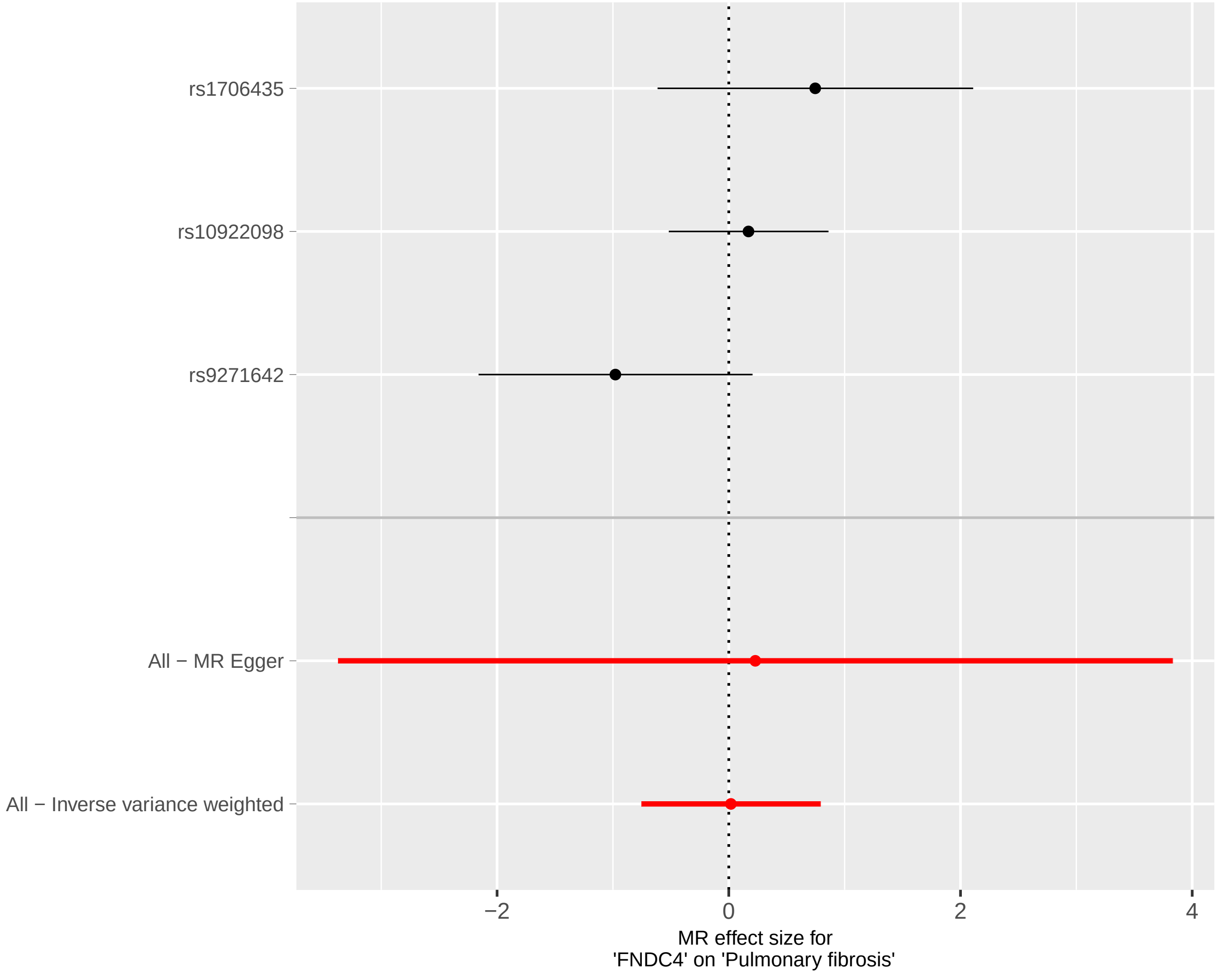
rs1706435
rs10922098
rs9271642
All − MR Egger
All − Inverse variance weighted
−2
0
2
4
MR effect size for
'FNDC4' on 'Pulmonary fibrosis'

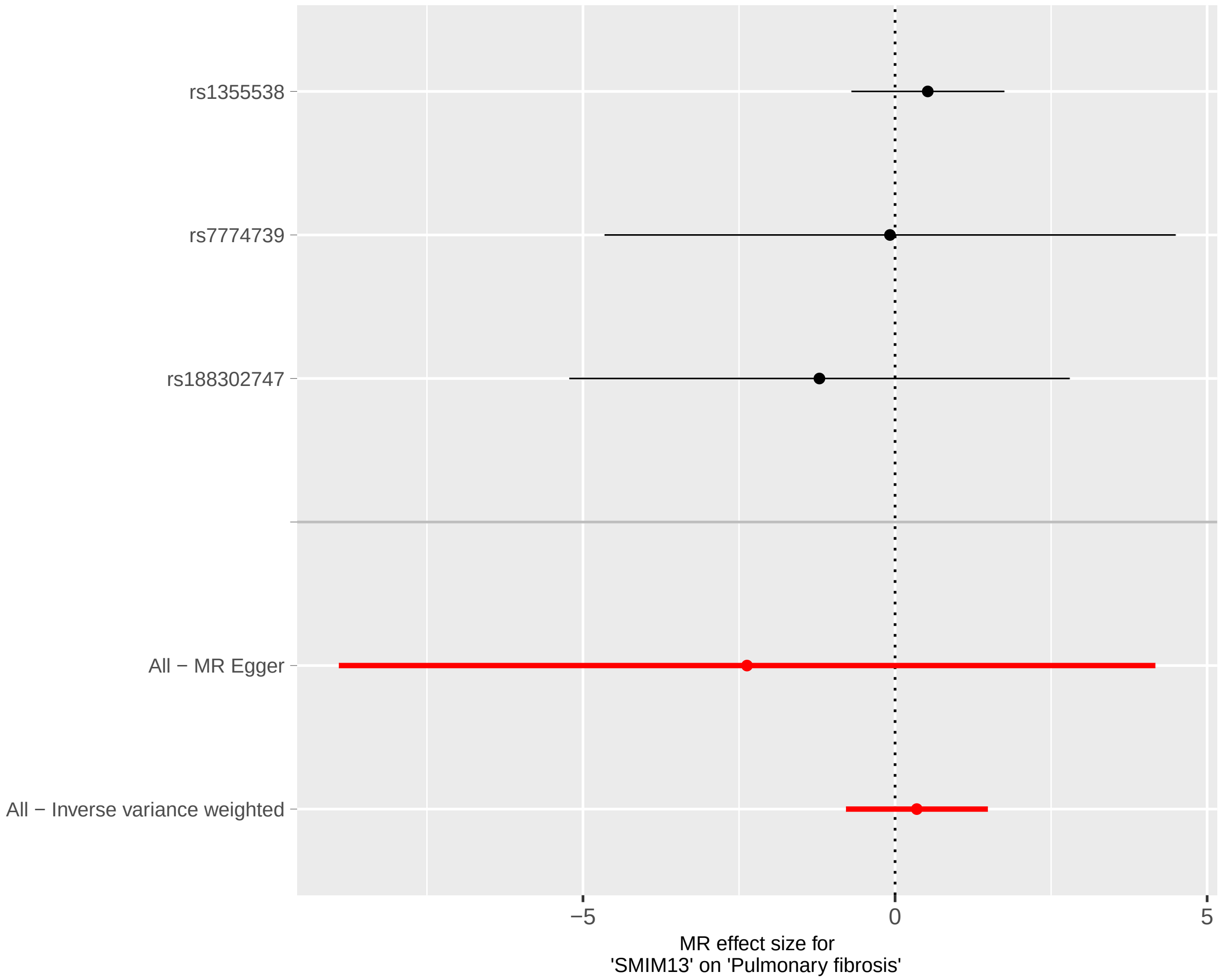
rs1355538
rs7774739
rs188302747
All − MR Egger
All − Inverse variance weighted
−5
0
5
MR effect size for
'SMIM13' on 'Pulmonary fibrosis'

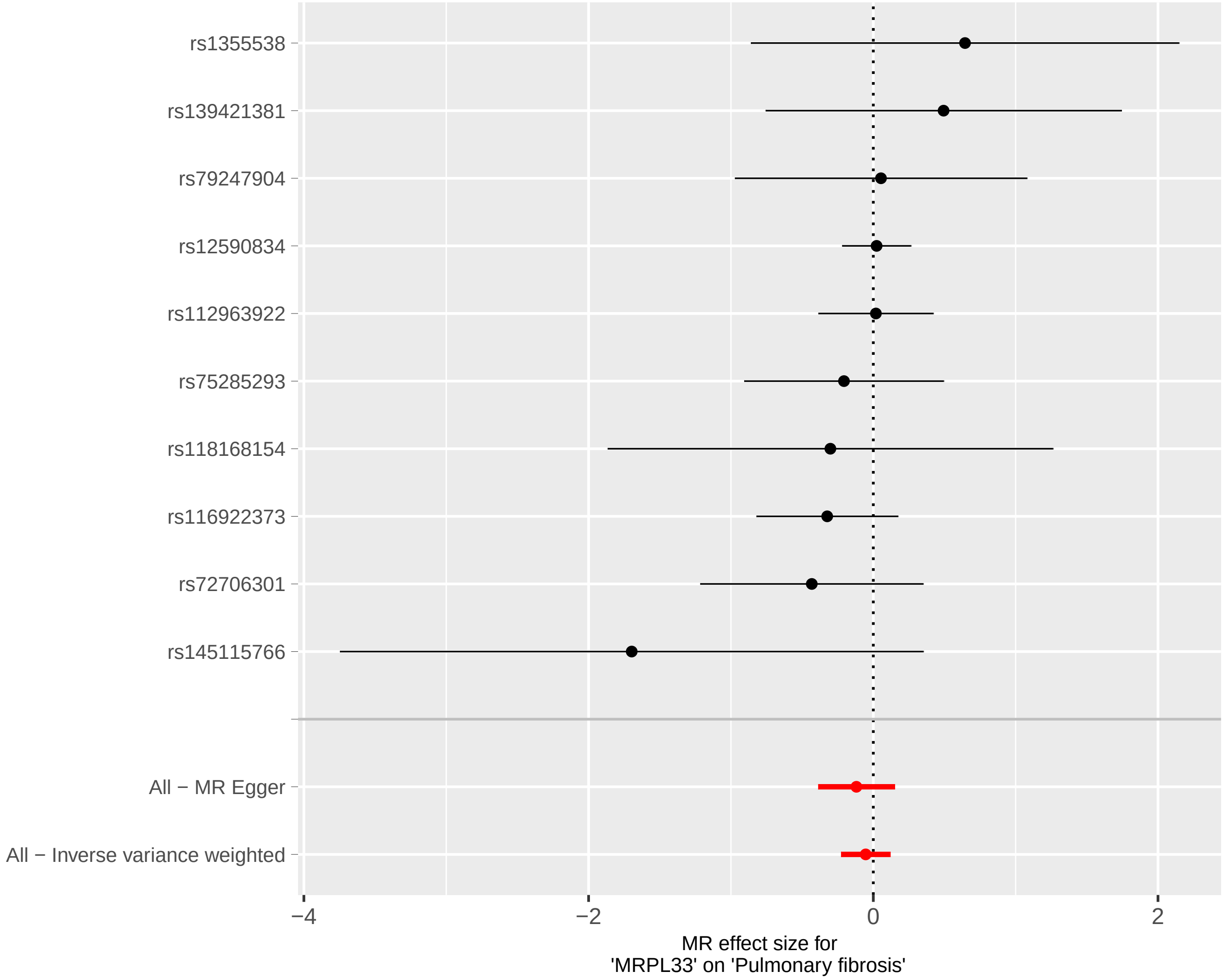
rs1355538
rs139421381
rs79247904
rs12590834
rs112963922
rs75285293
rs118168154
rs116922373
rs72706301
rs145115766
All − MR Egger
All − Inverse variance weighted
−4
−2
0
2
MR effect size for
'MRPL33' on 'Pulmonary fibrosis'

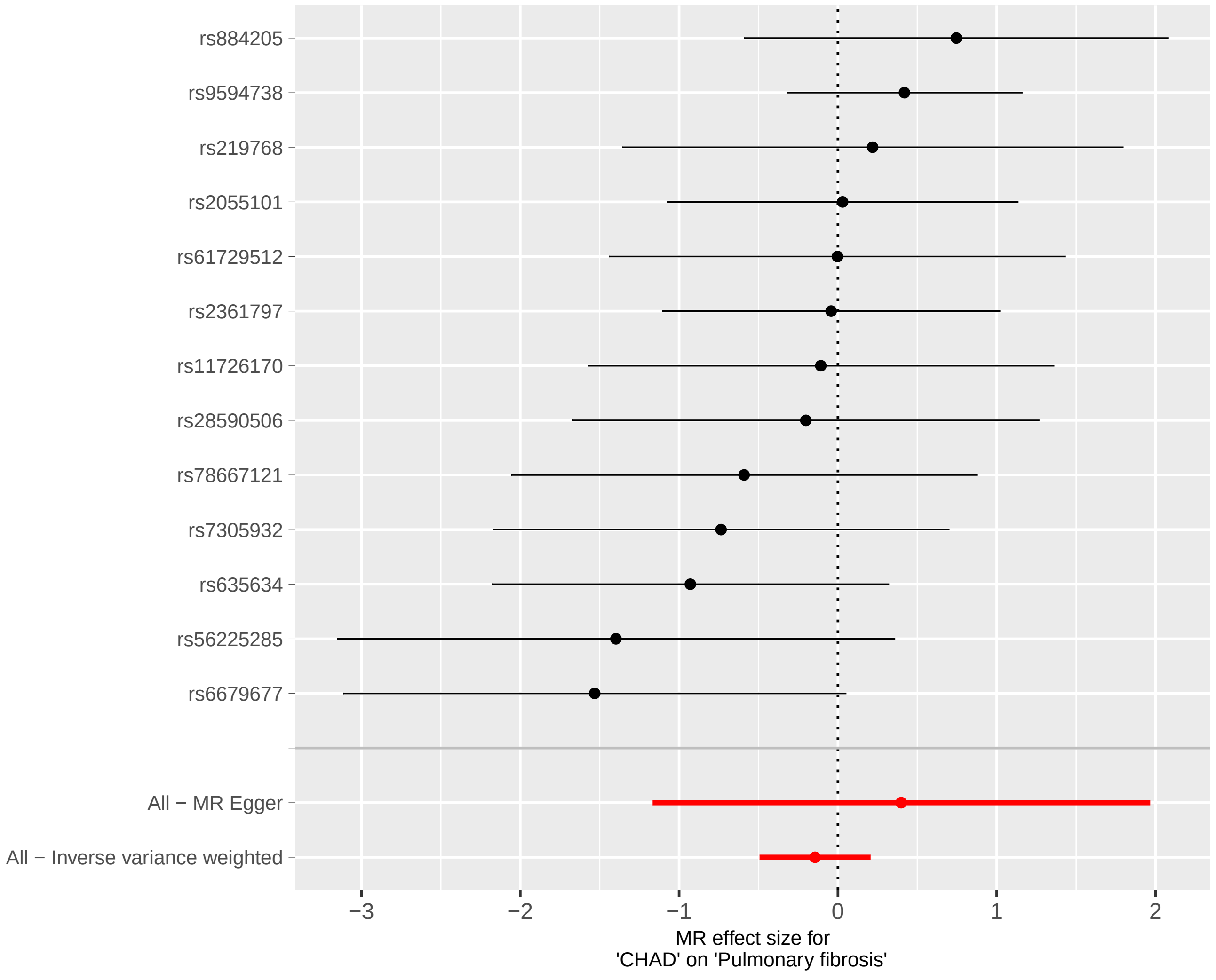

rs884205
rs9594738
rs219768
rs2055101
rs61729512
rs2361797
rs11726170
rs28590506
rs78667121
rs7305932
rs635634
rs56225285
rs6679677
All − MR Egger
All − Inverse variance weighted
−3
−2
−1
0
1
2
MR effect size for
'CHAD' on 'Pulmonary fibrosis'

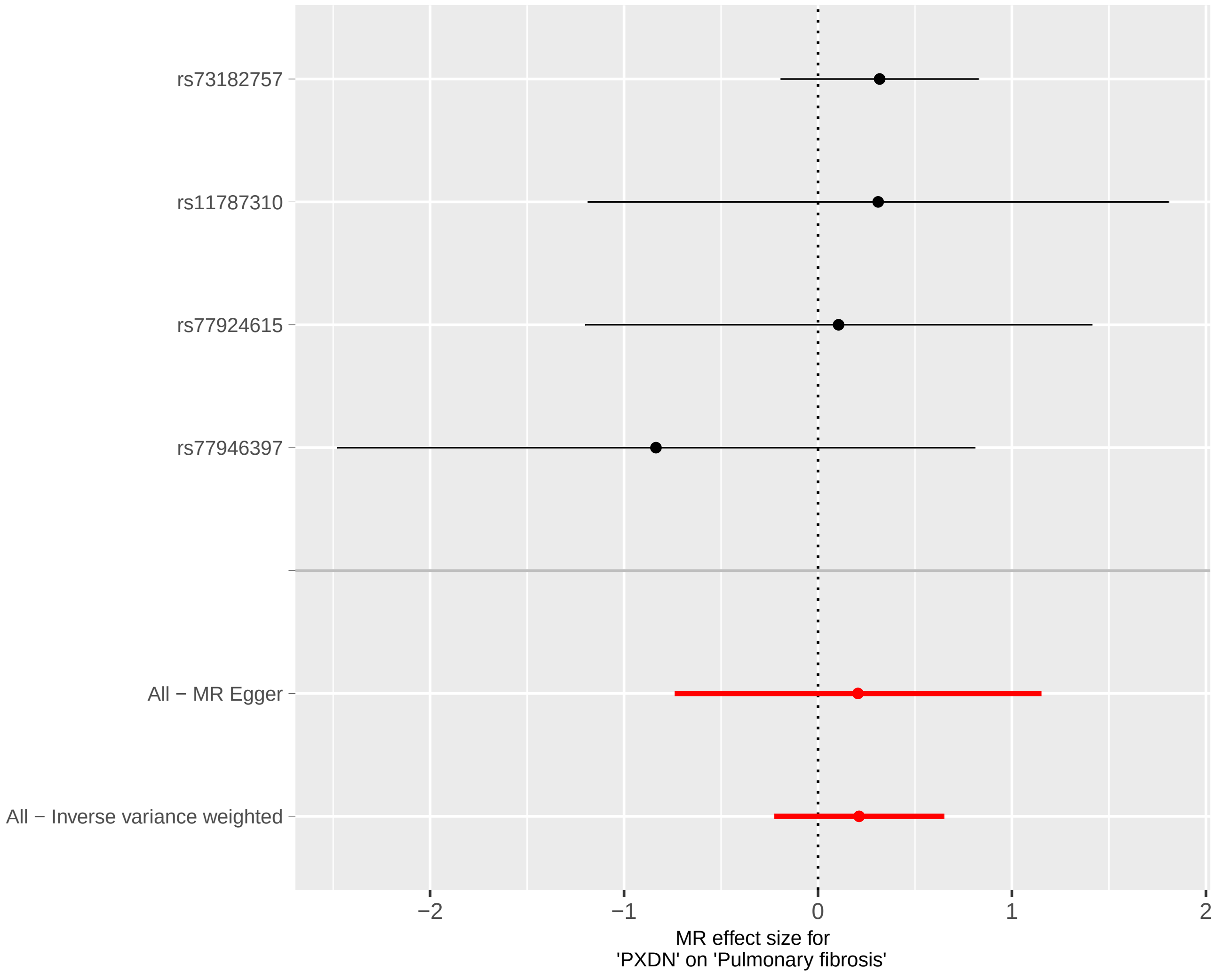
rs73182757
rs11787310
rs77924615
rs77946397
All − MR Egger
All − Inverse variance weighted
−2
−1
0
1
2
MR effect size for
'PXDN' on 'Pulmonary fibrosis'

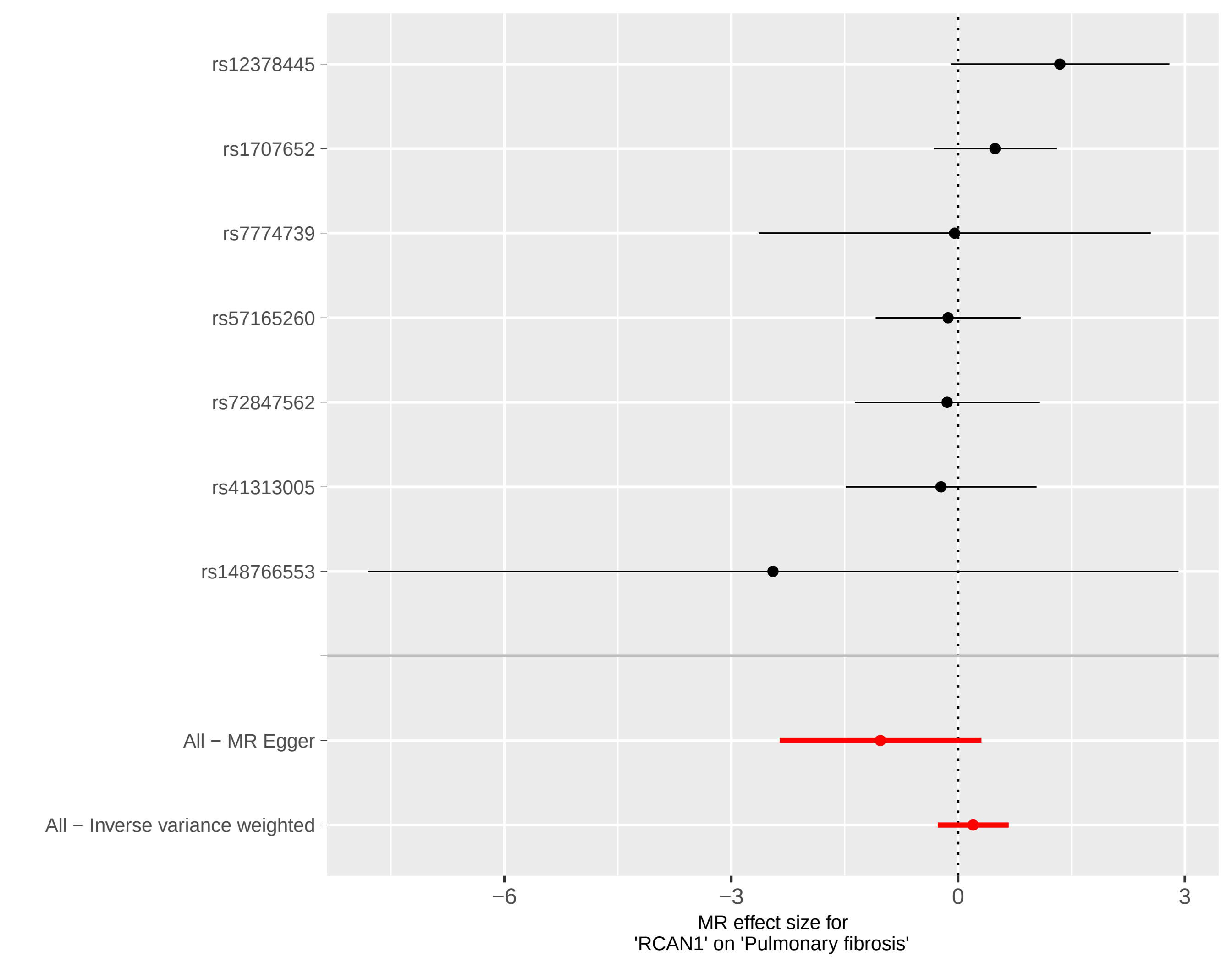

rs12378445
rs1707652
rs7774739
rs57165260
rs72847562
rs41313005
rs148766553
All − MR Egger
All − Inverse variance weighted
−6
−3
0
3
MR effect size for
'RCAN1' on 'Pulmonary fibrosis'

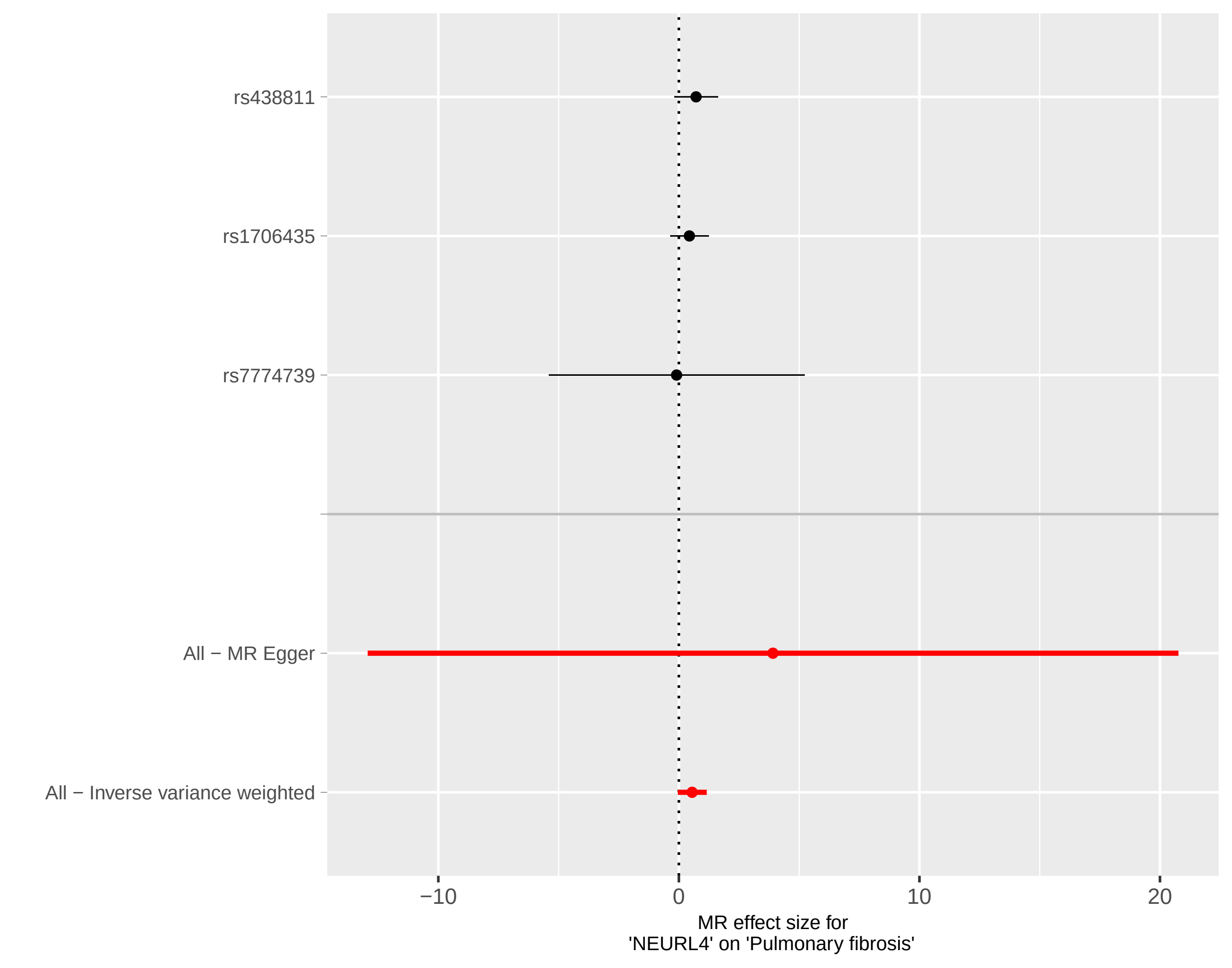
rs438811
rs1706435
rs7774739
All − MR Egger
All − Inverse variance weighted
−10
0
10
20
MR effect size for
'NEURL4' on 'Pulmonary fibrosis'

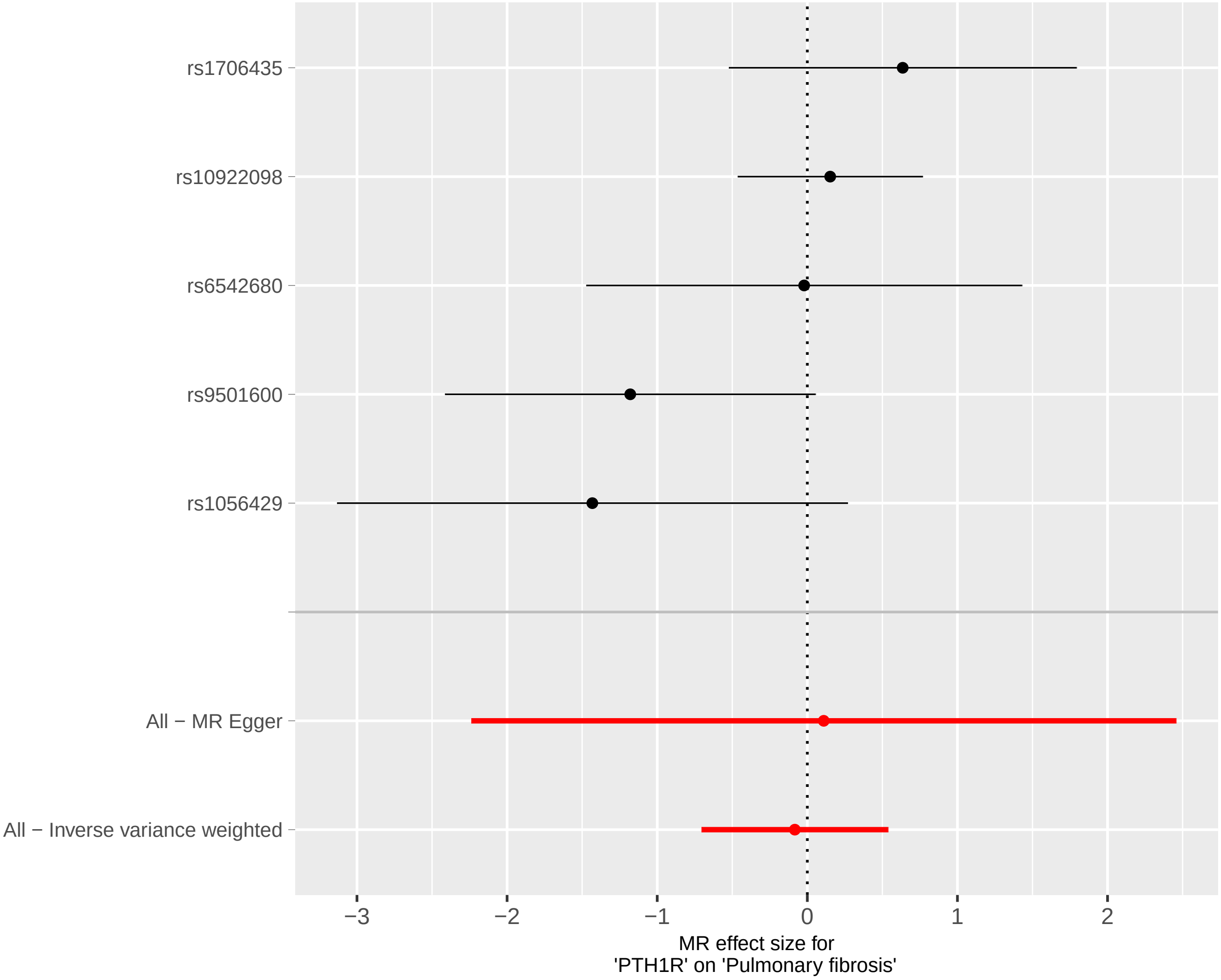
rs1706435
rs10922098
rs6542680
rs9501600
rs1056429
All − MR Egger
All − Inverse variance weighted
−3
−2
−1
0
1
2
MR effect size for
'PTH1R' on 'Pulmonary fibrosis'

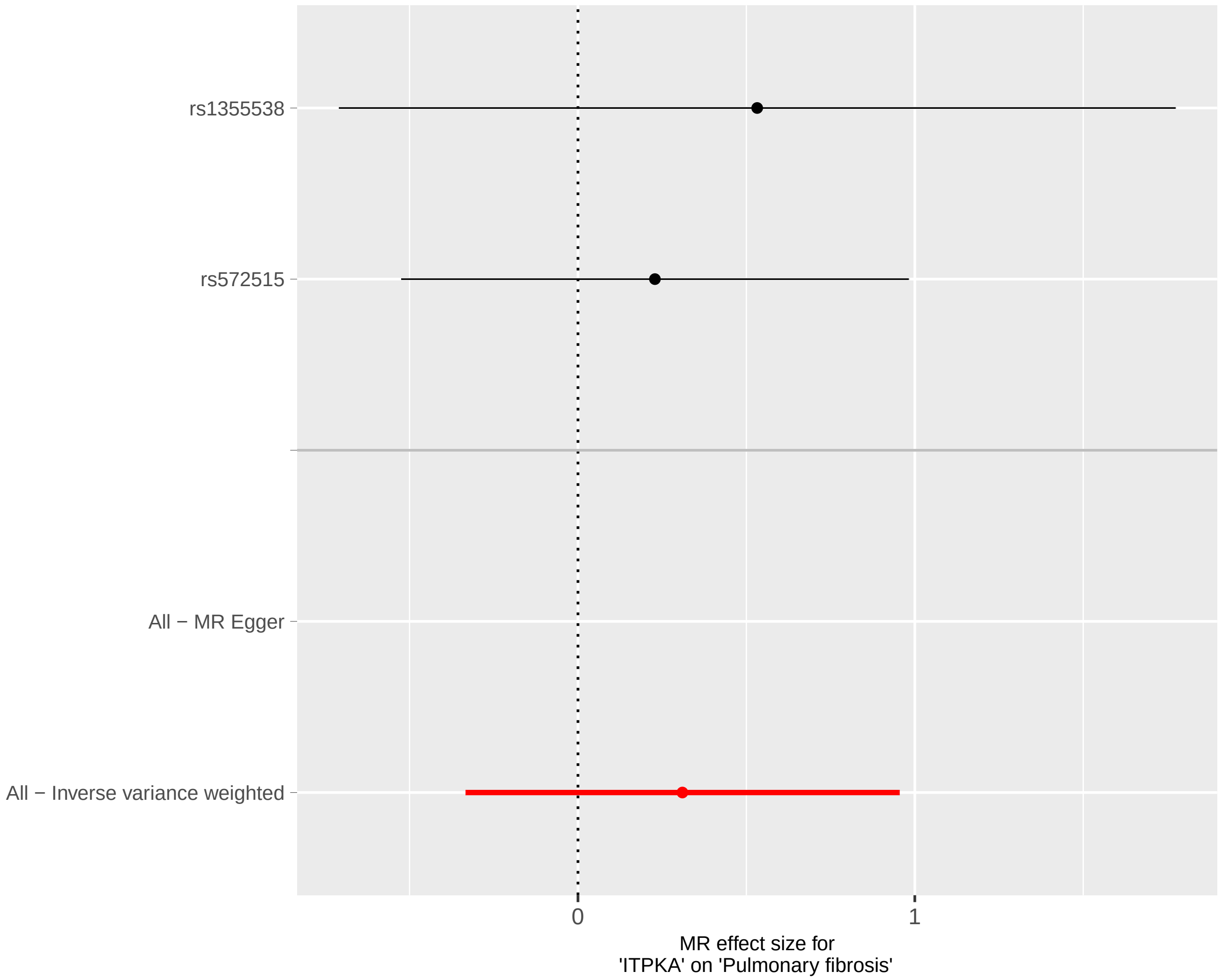

rs1355538
rs572515
All − MR Egger
All − Inverse variance weighted
0
1
MR effect size for
'ITPKA' on 'Pulmonary fibrosis'

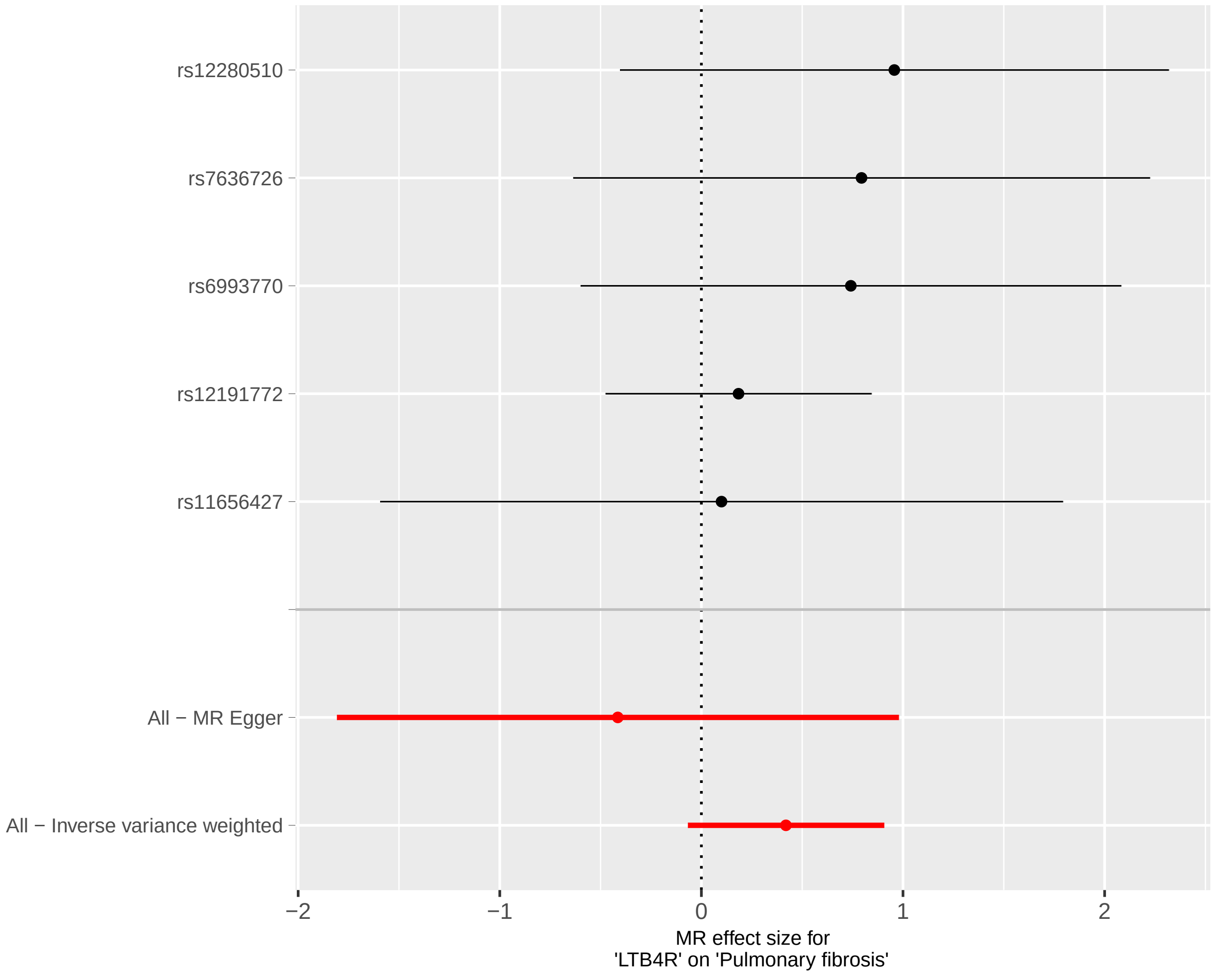
rs12280510
rs7636726
rs6993770
rs12191772
rs11656427
All − MR Egger
All − Inverse variance weighted
−2
−1
0
1
2
MR effect size for
'LTB4R' on 'Pulmonary fibrosis'

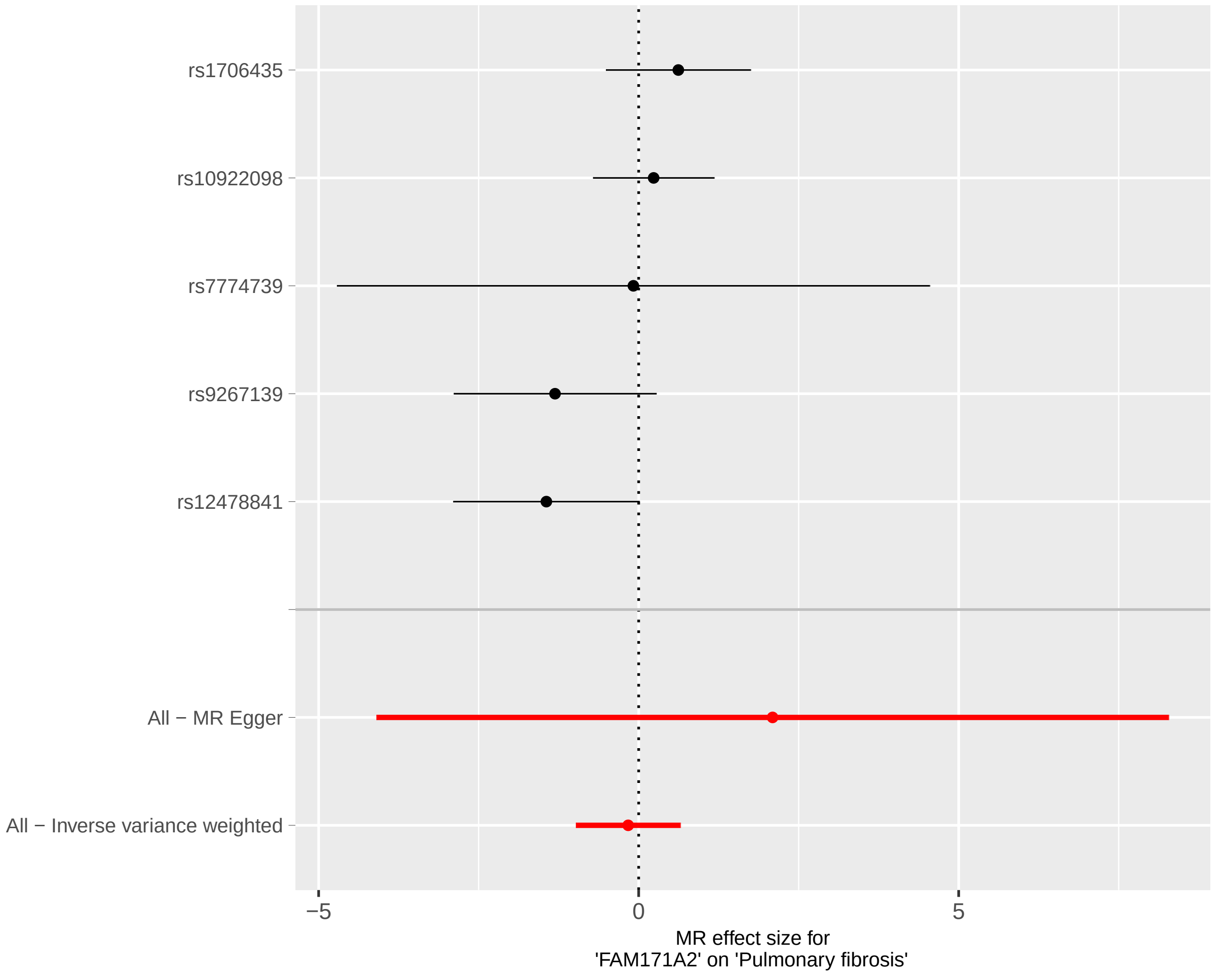
rs1706435
rs10922098
rs7774739
rs9267139
rs12478841
All − MR Egger
All − Inverse variance weighted
−5
0
5
MR effect size for
'FAM171A2' on 'Pulmonary fibrosis'

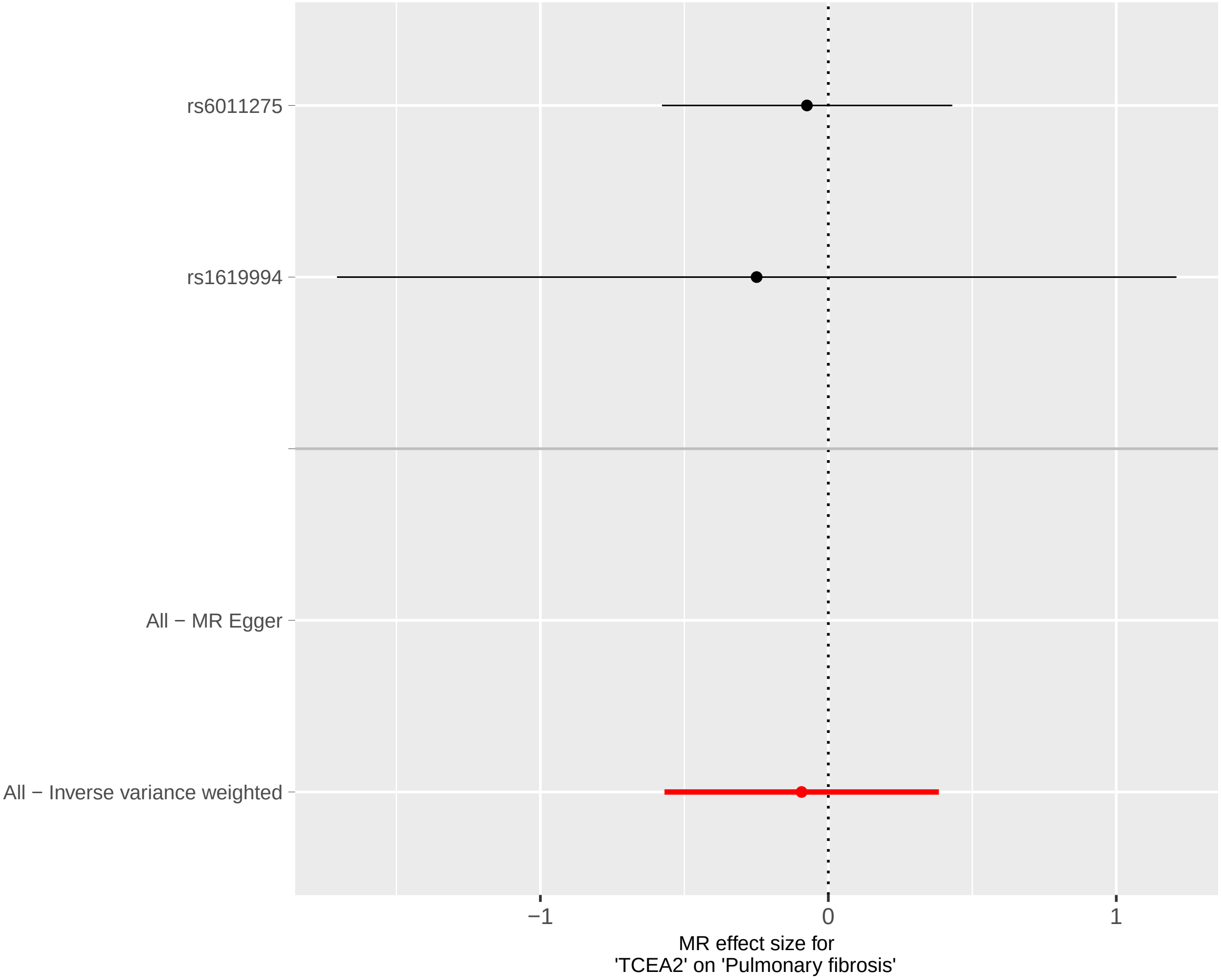
rs6011275
rs1619994
All − MR Egger
All − Inverse variance weighted
−1
0
1
MR effect size for
'TCEA2' on 'Pulmonary fibrosis'

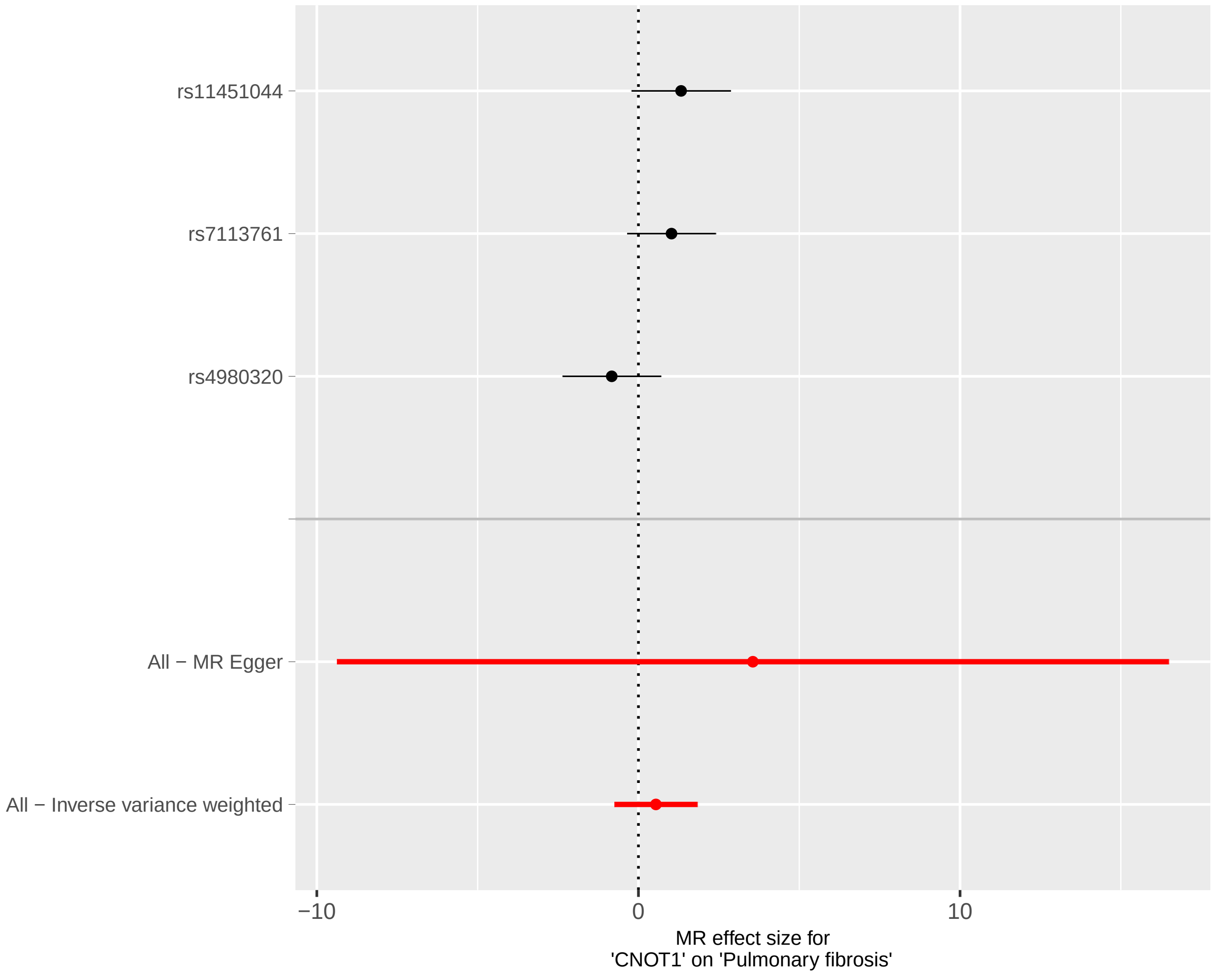
rs11451044
rs7113761
rs4980320
All − MR Egger
All − Inverse variance weighted
−10
0
10
MR effect size for
'CNOT1' on 'Pulmonary fibrosis'

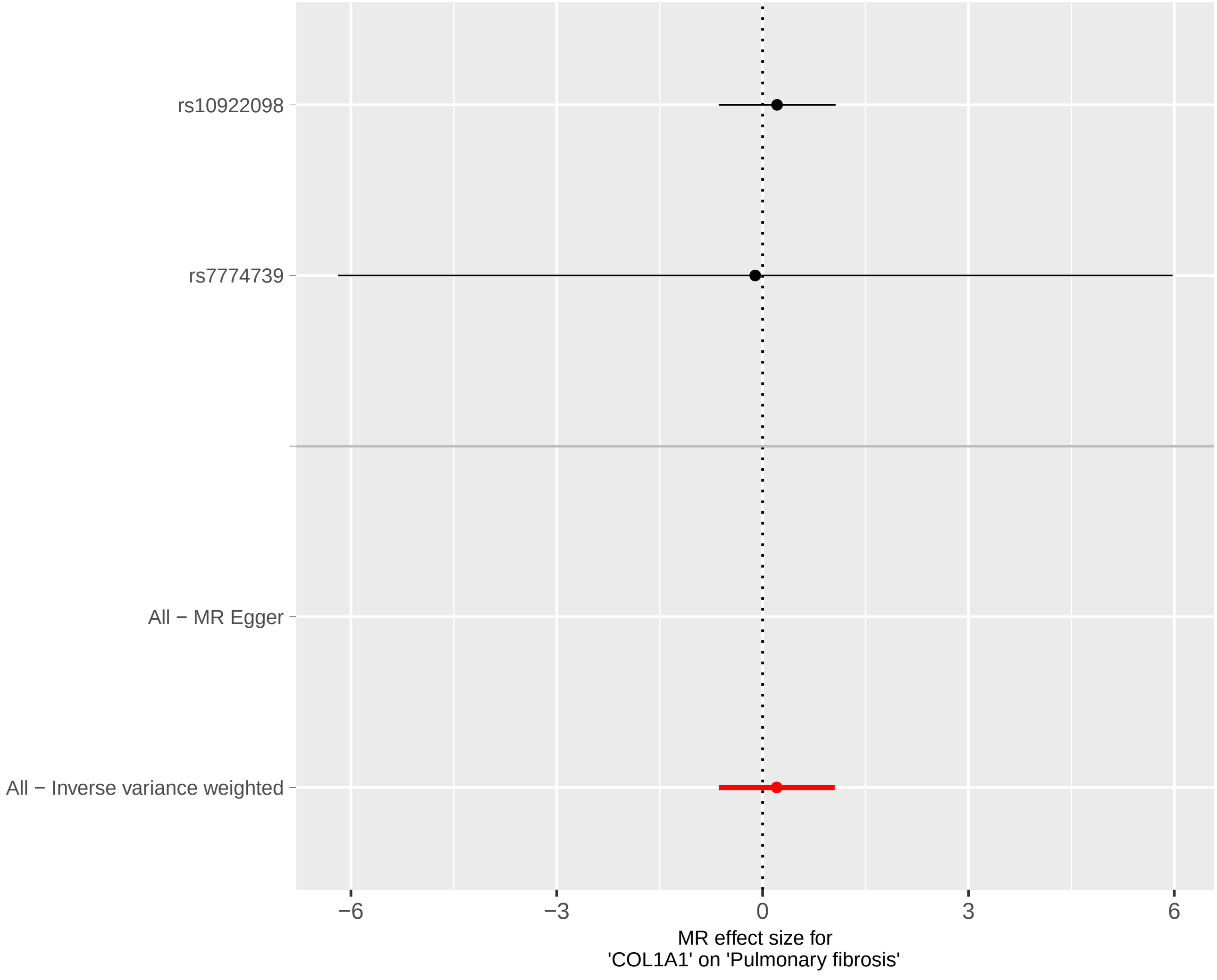
rs10922098
rs7774739
All − MR Egger
All − Inverse variance weighted
−6
−3
0
3
6
MR effect size for
'COL1A1' on 'Pulmonary fibrosis'

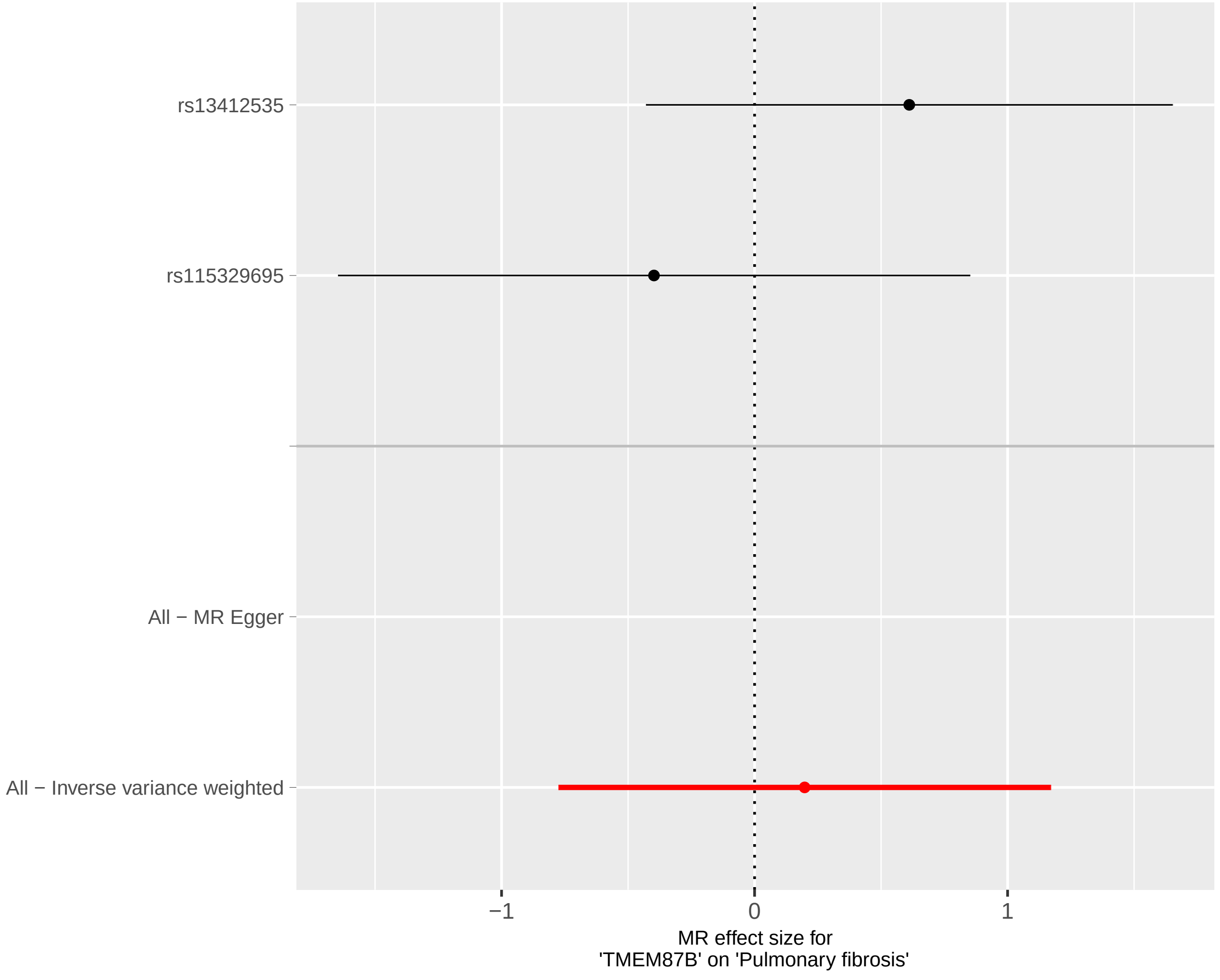
rs13412535
rs115329695
All − MR Egger
All − Inverse variance weighted
−1
0
1
MR effect size for
'TMEM87B' on 'Pulmonary fibrosis'

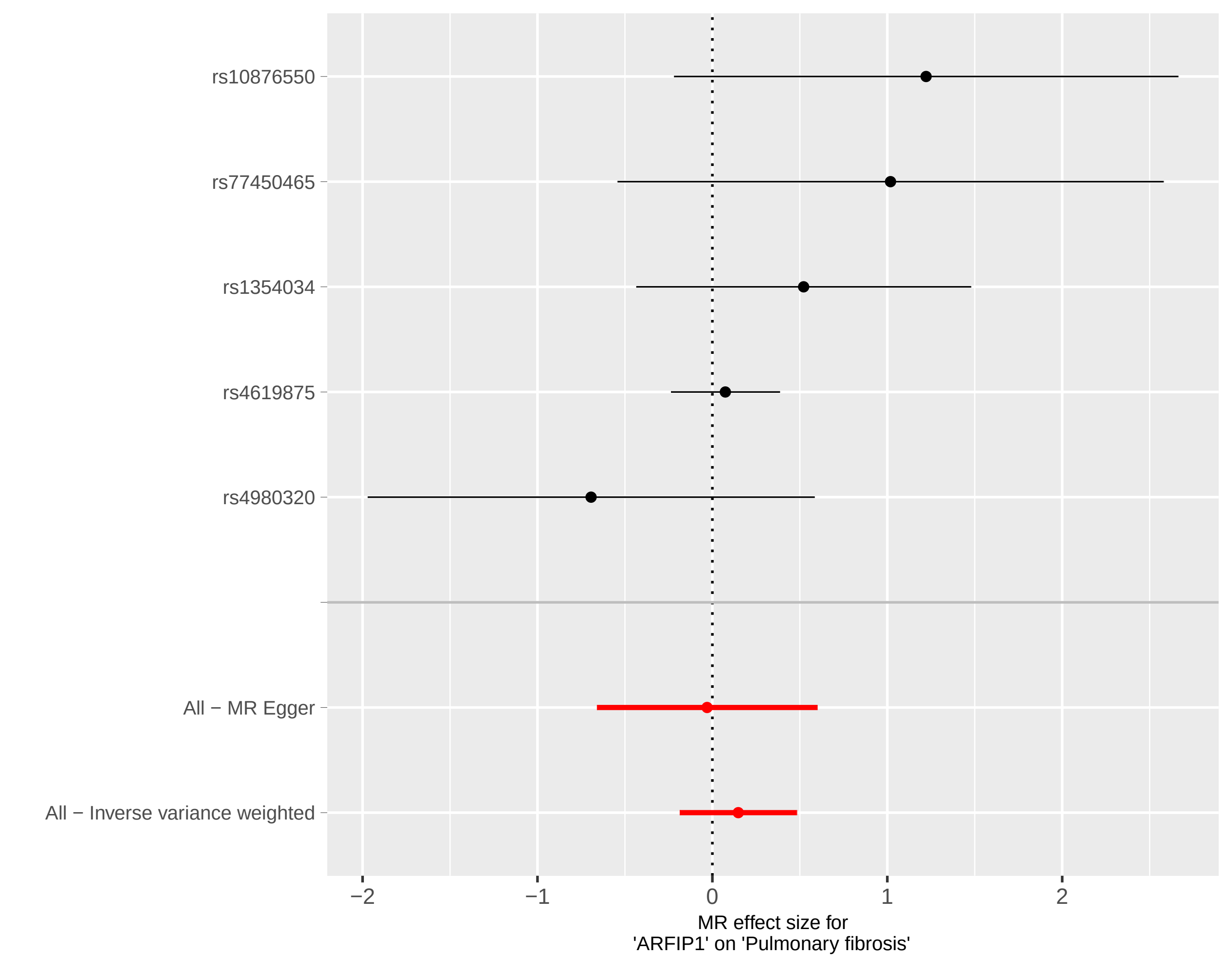
rs10876550
rs77450465
rs1354034
rs4619875
rs4980320
All − MR Egger
All − Inverse variance weighted
−2
−1
0
1
2
MR effect size for
'ARFIP1' on 'Pulmonary fibrosis'

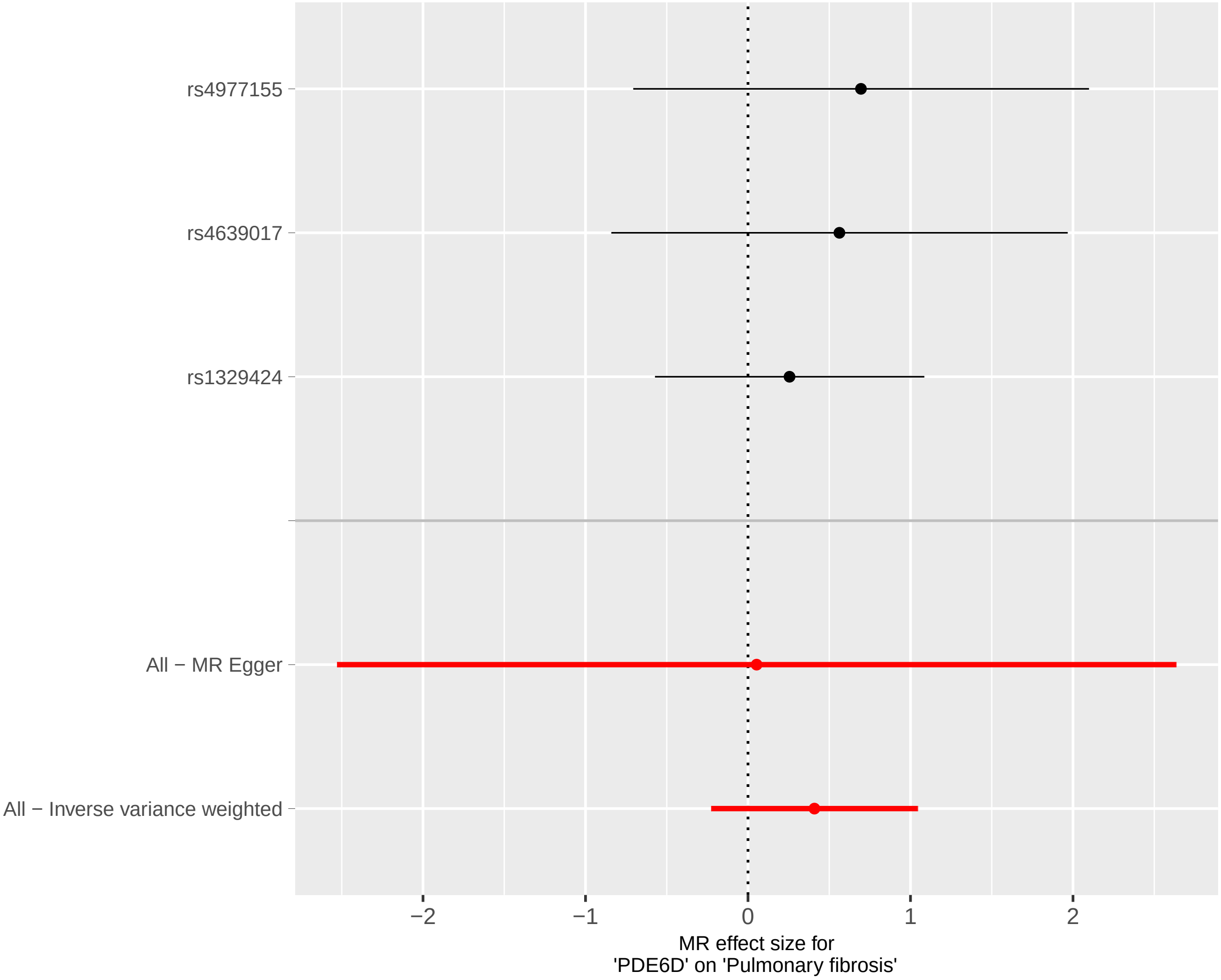
rs4977155
rs4639017
rs1329424
All − MR Egger
All − Inverse variance weighted
−2
−1
0
1
2
MR effect size for
'PDE6D' on 'Pulmonary fibrosis'

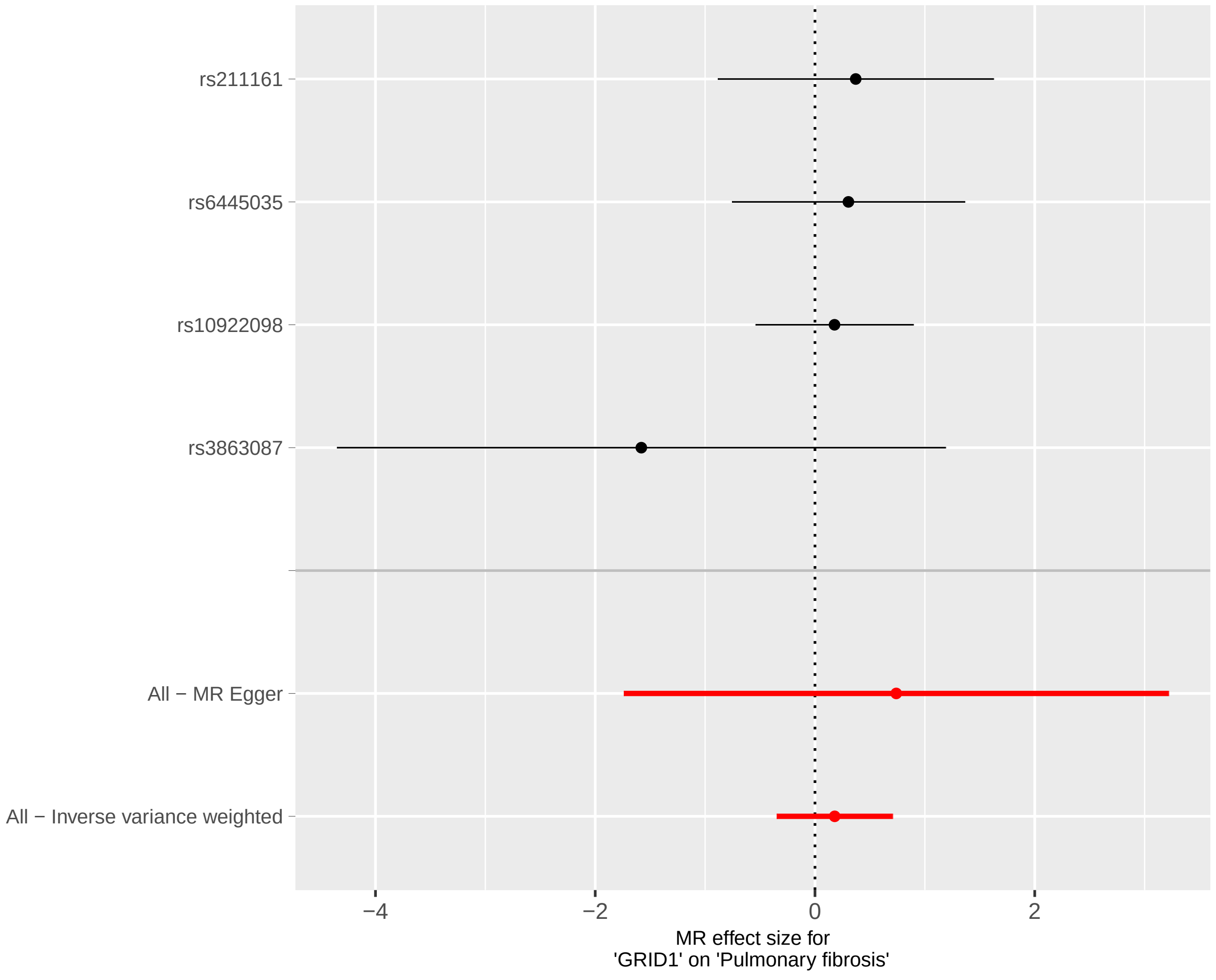

rs211161
rs6445035
rs10922098
rs3863087
All − MR Egger
All − Inverse variance weighted
−4
−2
0
2
MR effect size for
'GRID1' on 'Pulmonary fibrosis'

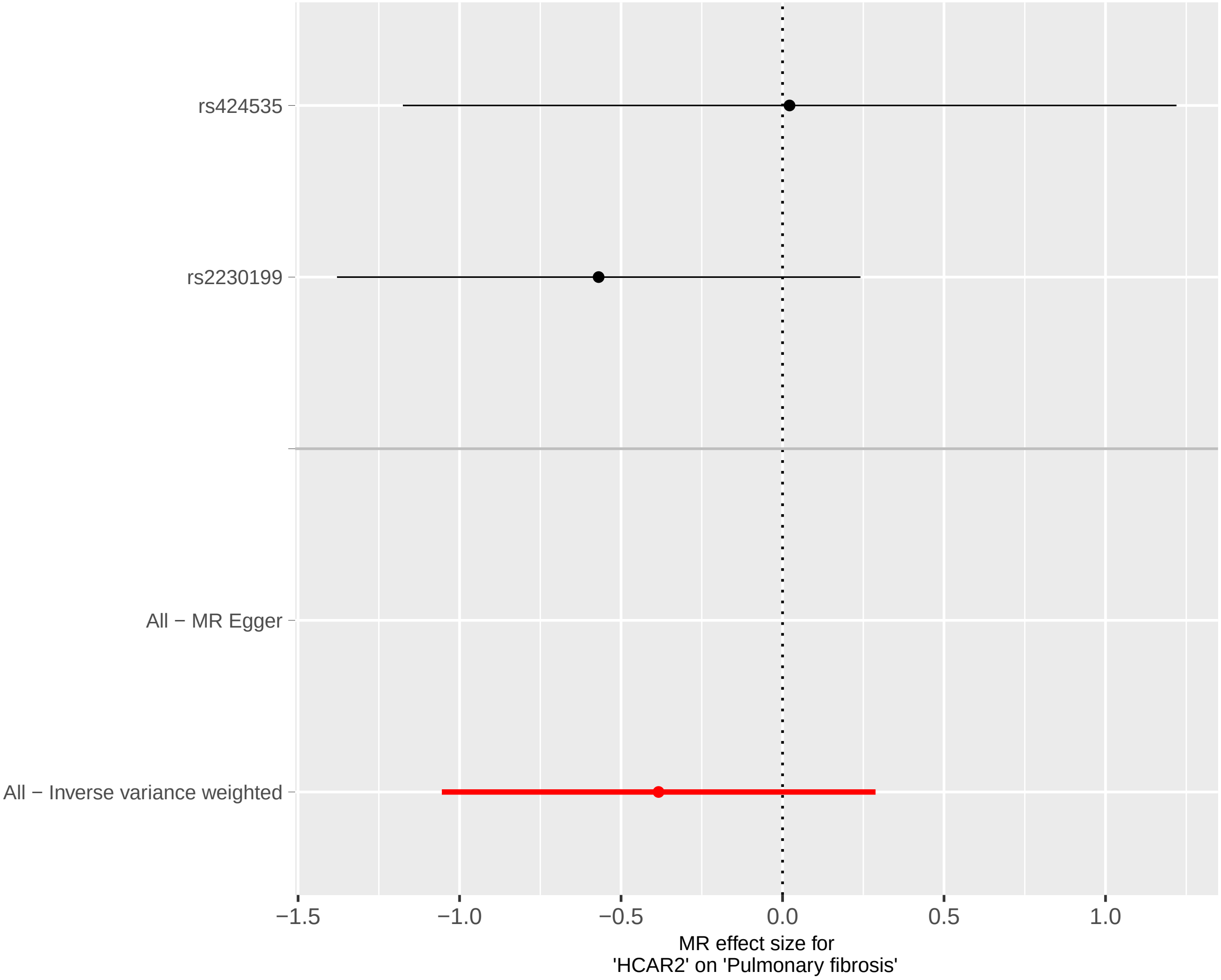
rs424535
rs2230199
All − MR Egger
All − Inverse variance weighted
−1.5
−1.0
−0.5
0.0
0.5
1.0
MR effect size for
'HCAR2' on 'Pulmonary fibrosis'

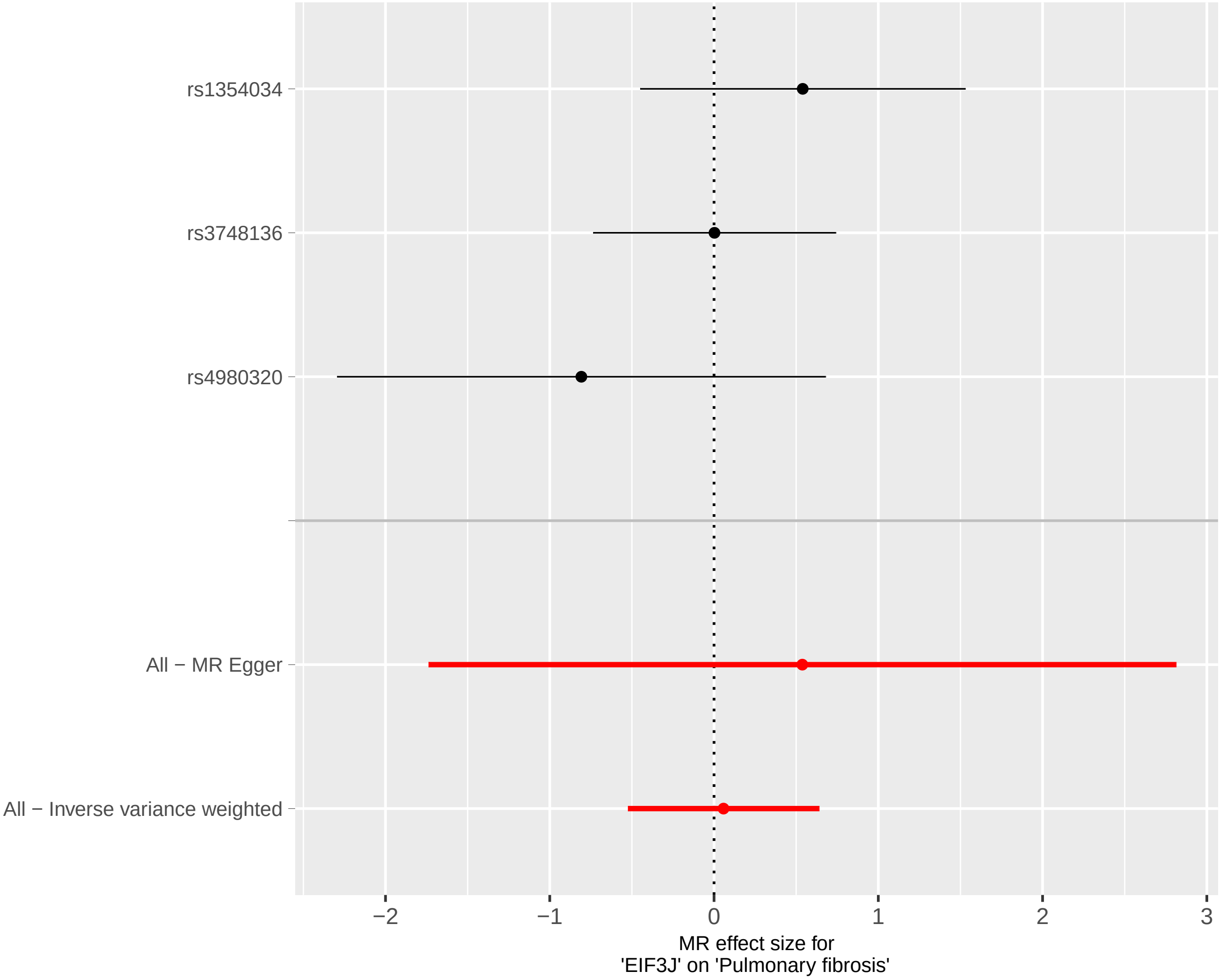

rs1354034
rs3748136
rs4980320
All − MR Egger
All − Inverse variance weighted
−2
−1
0
1
2
3
MR effect size for
'EIF3J' on 'Pulmonary fibrosis'

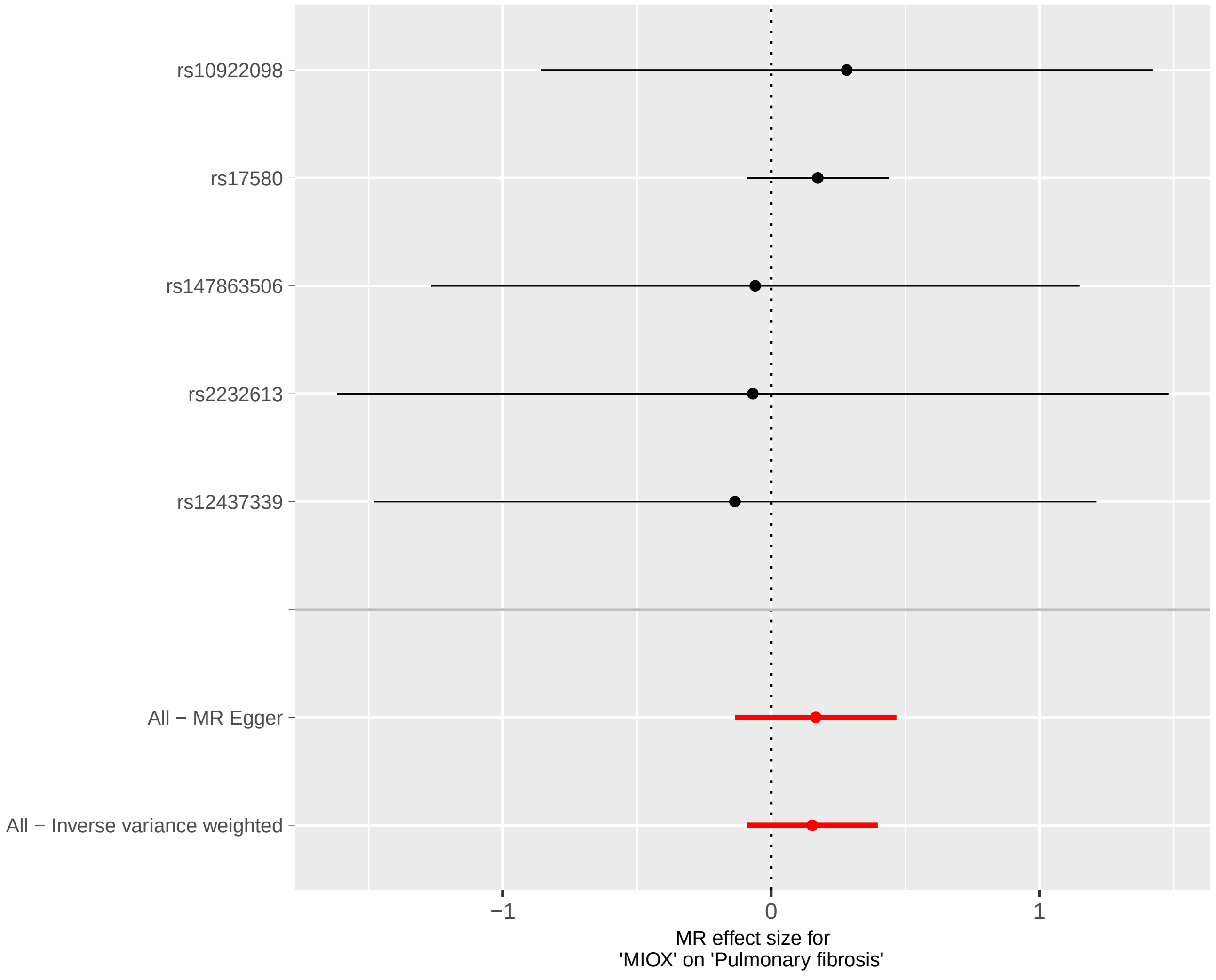
rs10922098
rs17580
rs147863506
rs2232613
rs12437339
All − MR Egger
All − Inverse variance weighted
−1
0
1
MR effect size for
'MIOX' on 'Pulmonary fibrosis'

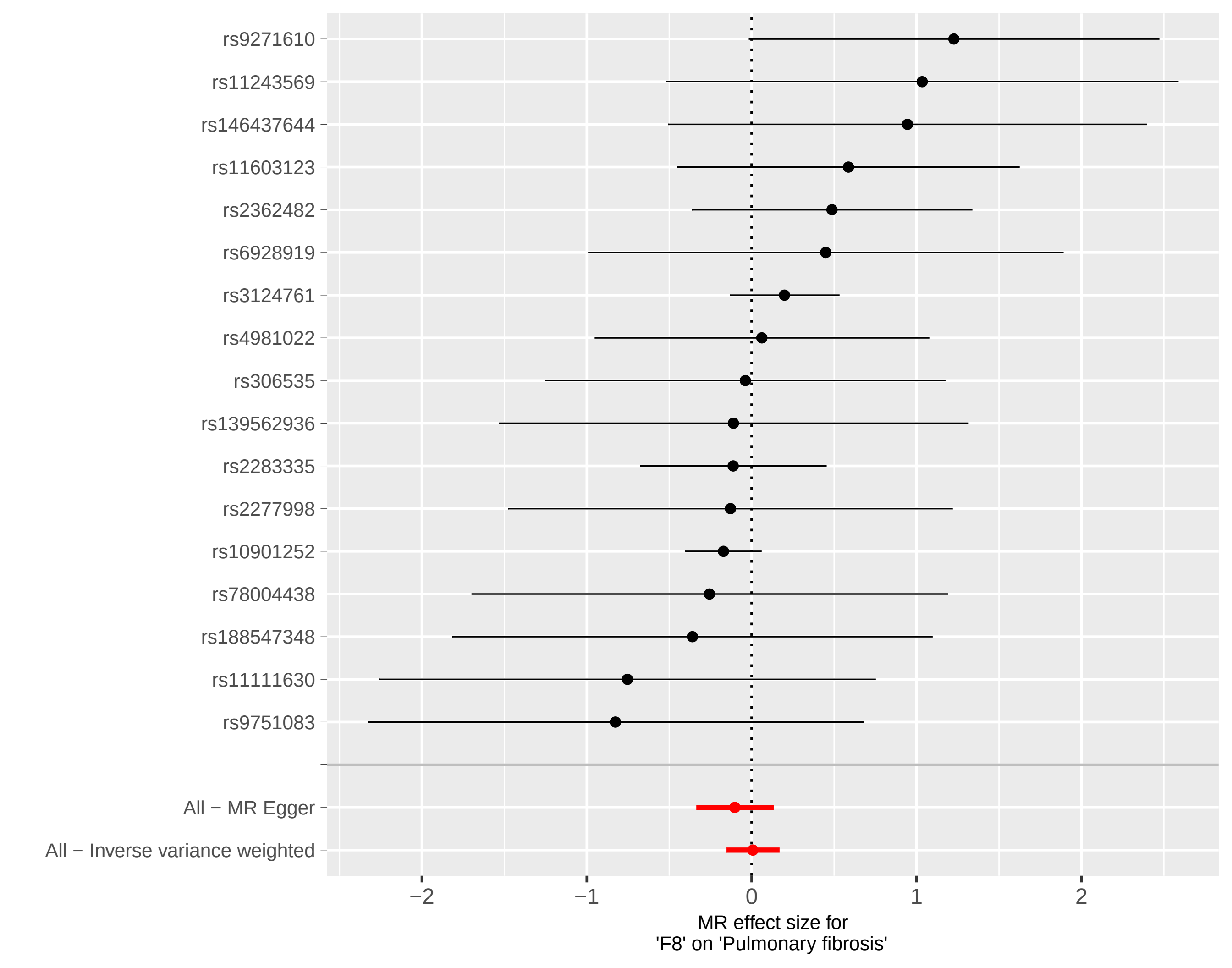
rs9271610
rs11243569
rs146437644
rs11603123
rs2362482
rs6928919
rs3124761
rs4981022
rs306535
rs139562936
rs2283335
rs2277998
rs10901252
rs78004438
rs188547348
rs11111630
rs9751083
All − MR Egger
All − Inverse variance weighted
−2
−1
0
1
2
MR effect size for
'F8' on 'Pulmonary fibrosis'

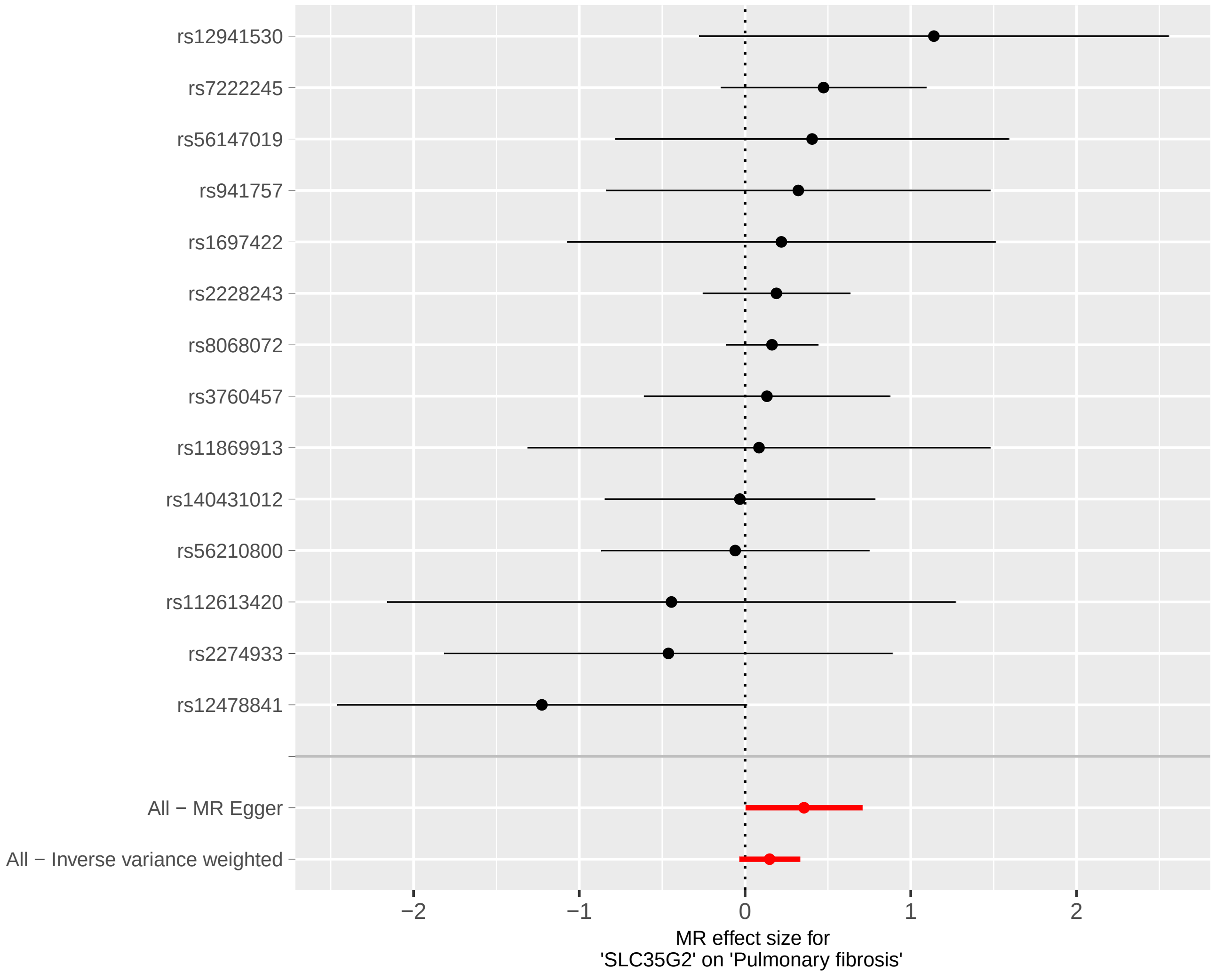
rs12941530
rs7222245
rs56147019
rs941757
rs1697422
rs2228243
rs8068072
rs3760457
rs11869913
rs140431012
rs56210800
rs112613420
rs2274933
rs12478841
All − MR Egger
All − Inverse variance weighted
−2
−1
0
1
2
MR effect size for
'SLC35G2' on 'Pulmonary fibrosis'

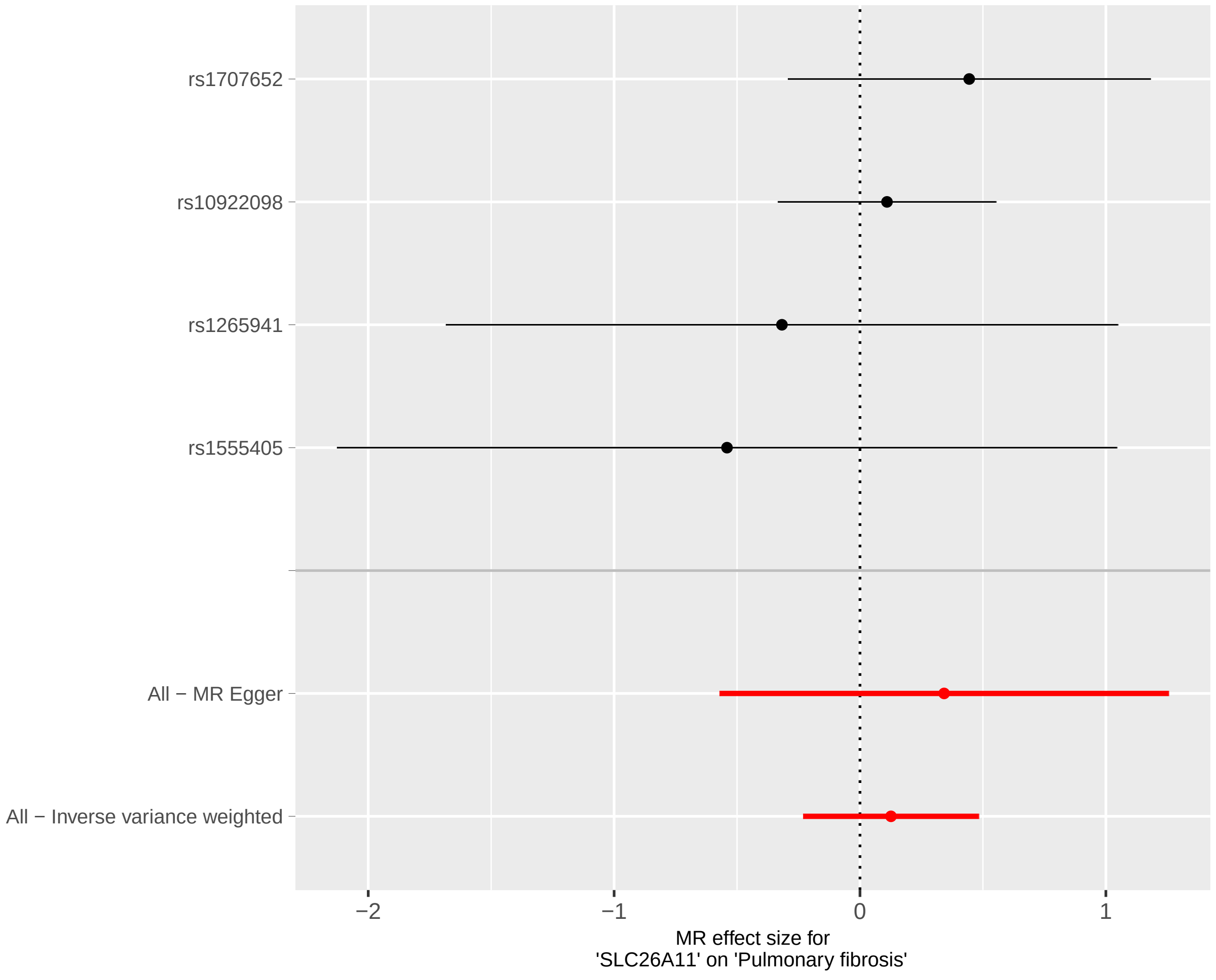
rs1707652
rs10922098
rs1265941
rs1555405
All − MR Egger
All − Inverse variance weighted
−2
−1
0
1
MR effect size for
'SLC26A11' on 'Pulmonary fibrosis'

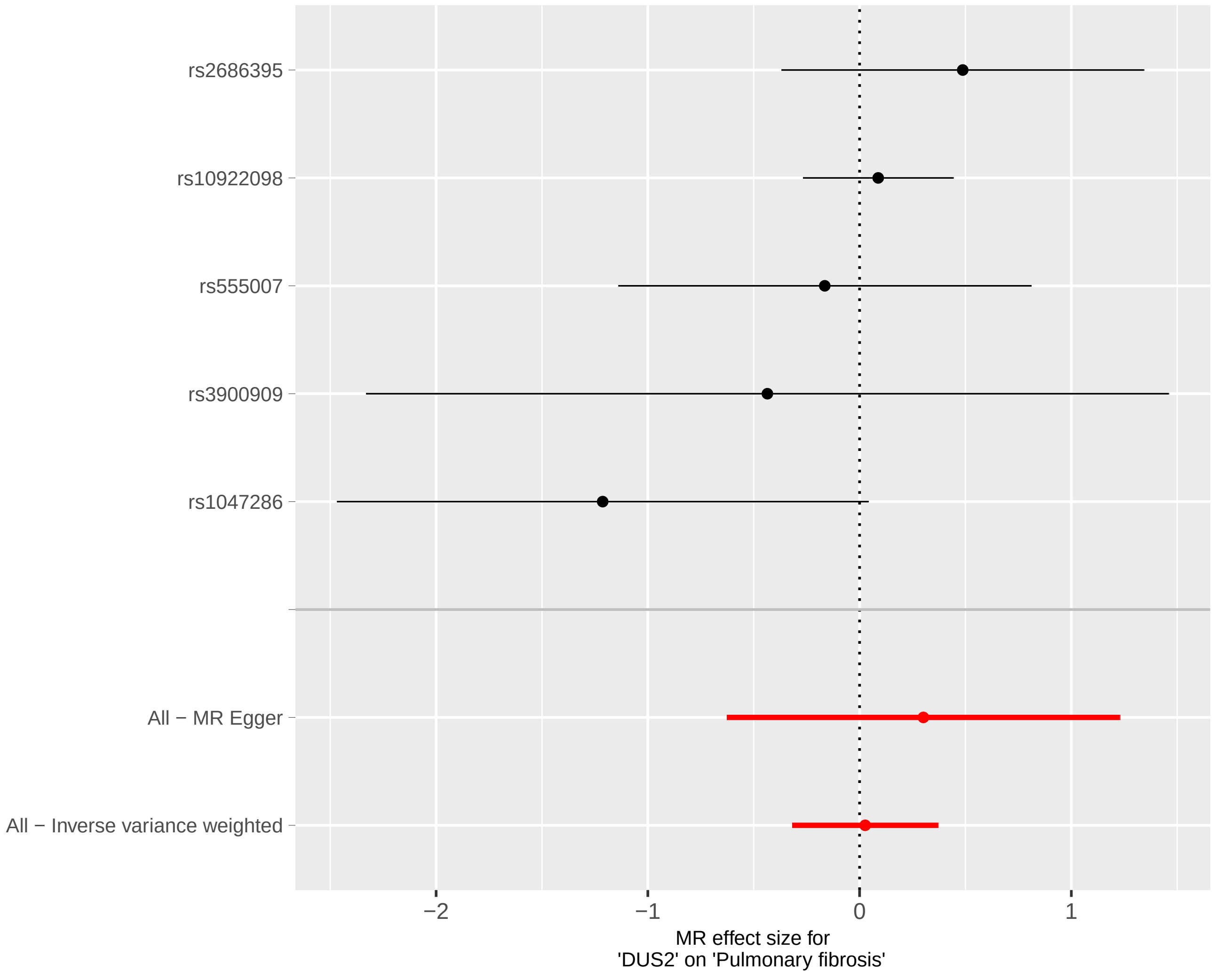

rs2686395
rs10922098
rs555007
rs3900909
rs1047286
All − MR Egger
All − Inverse variance weighted
−2
−1
0
1
MR effect size for
'DUS2' on 'Pulmonary fibrosis'

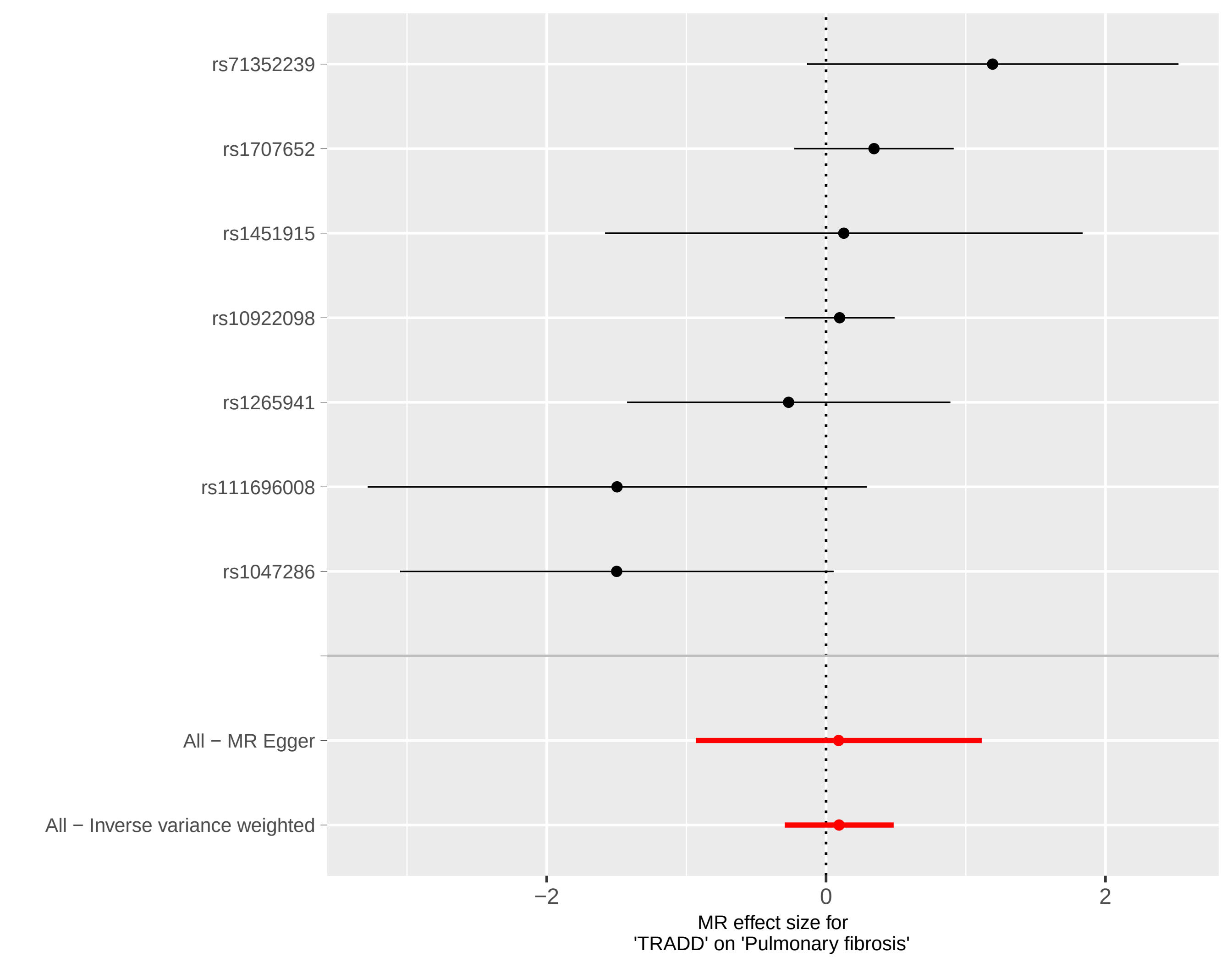

rs71352239
rs1707652
rs1451915
rs10922098
rs1265941
rs111696008
rs1047286
All − MR Egger
All − Inverse variance weighted
−2
0
2
MR effect size for
'TRADD' on 'Pulmonary fibrosis'

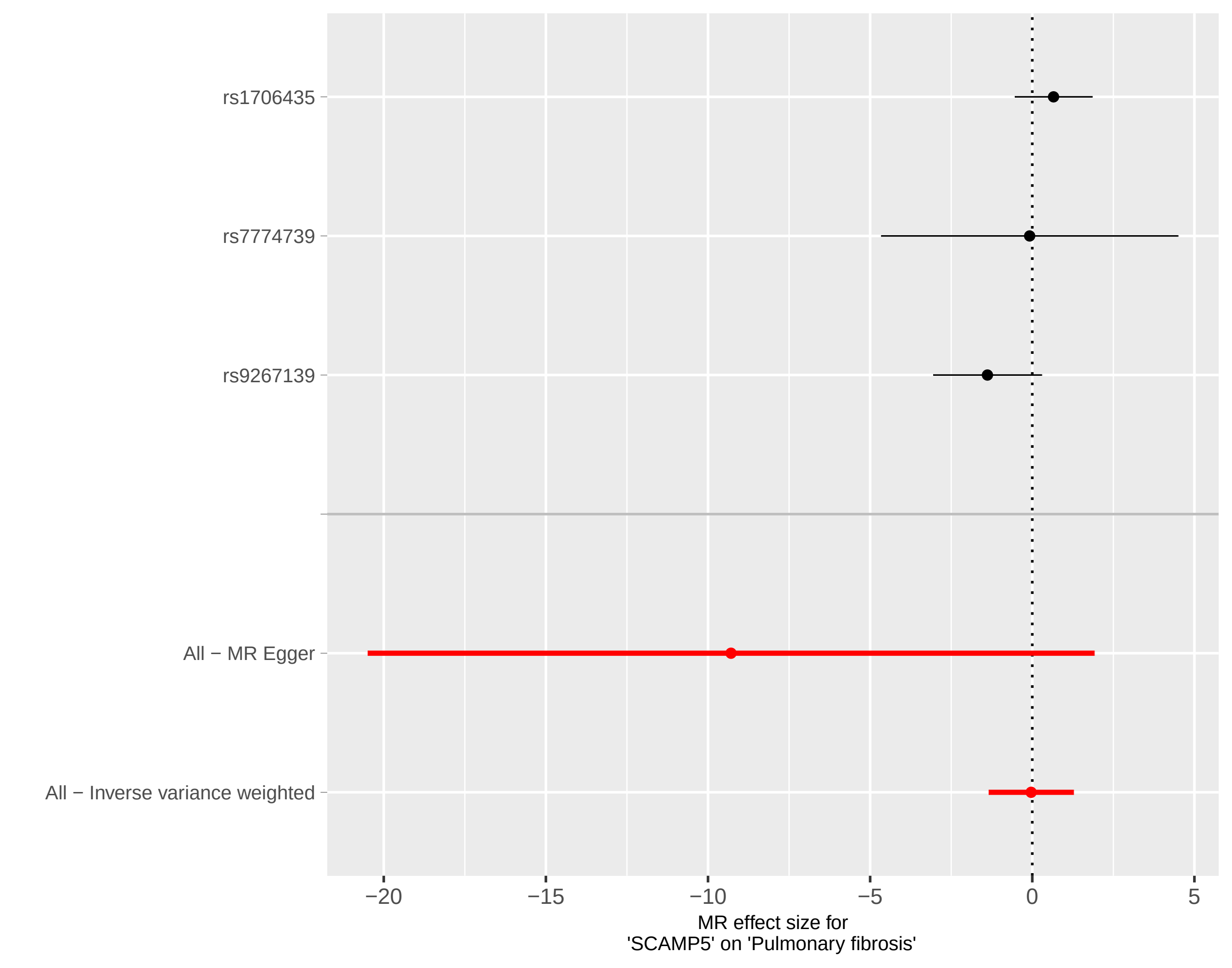

rs1706435
rs7774739
rs9267139
All − MR Egger
All − Inverse variance weighted
−20
−15
−10
−5
0
5
MR effect size for
'SCAMP5' on 'Pulmonary fibrosis'

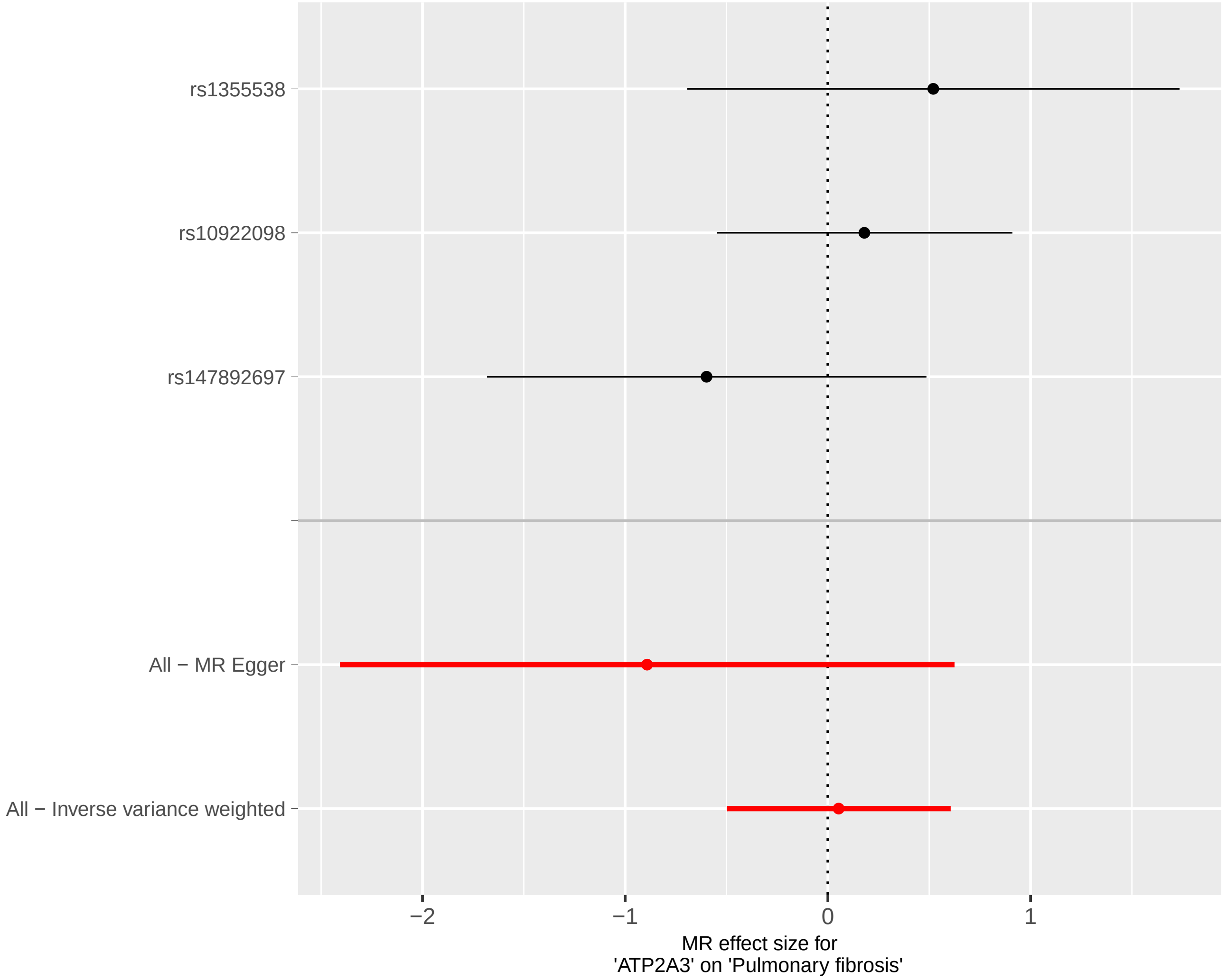
rs1355538
rs10922098
rs147892697
All − MR Egger
All − Inverse variance weighted
−2
−1
0
1
MR effect size for
'ATP2A3' on 'Pulmonary fibrosis'

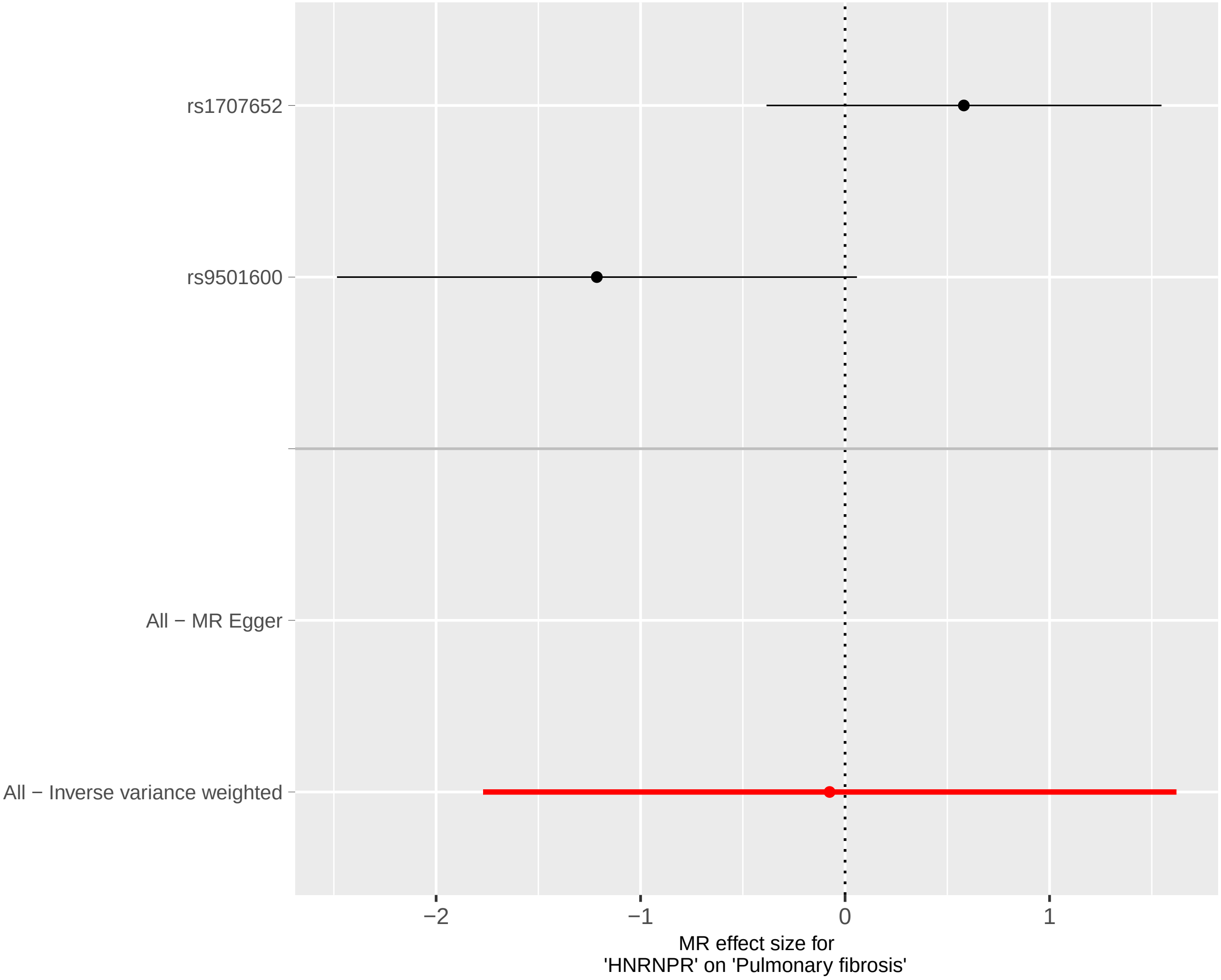

rs1707652
rs9501600
All − MR Egger
All − Inverse variance weighted
−2
−1
0
1
MR effect size for
'HNRNPR' on 'Pulmonary fibrosis'

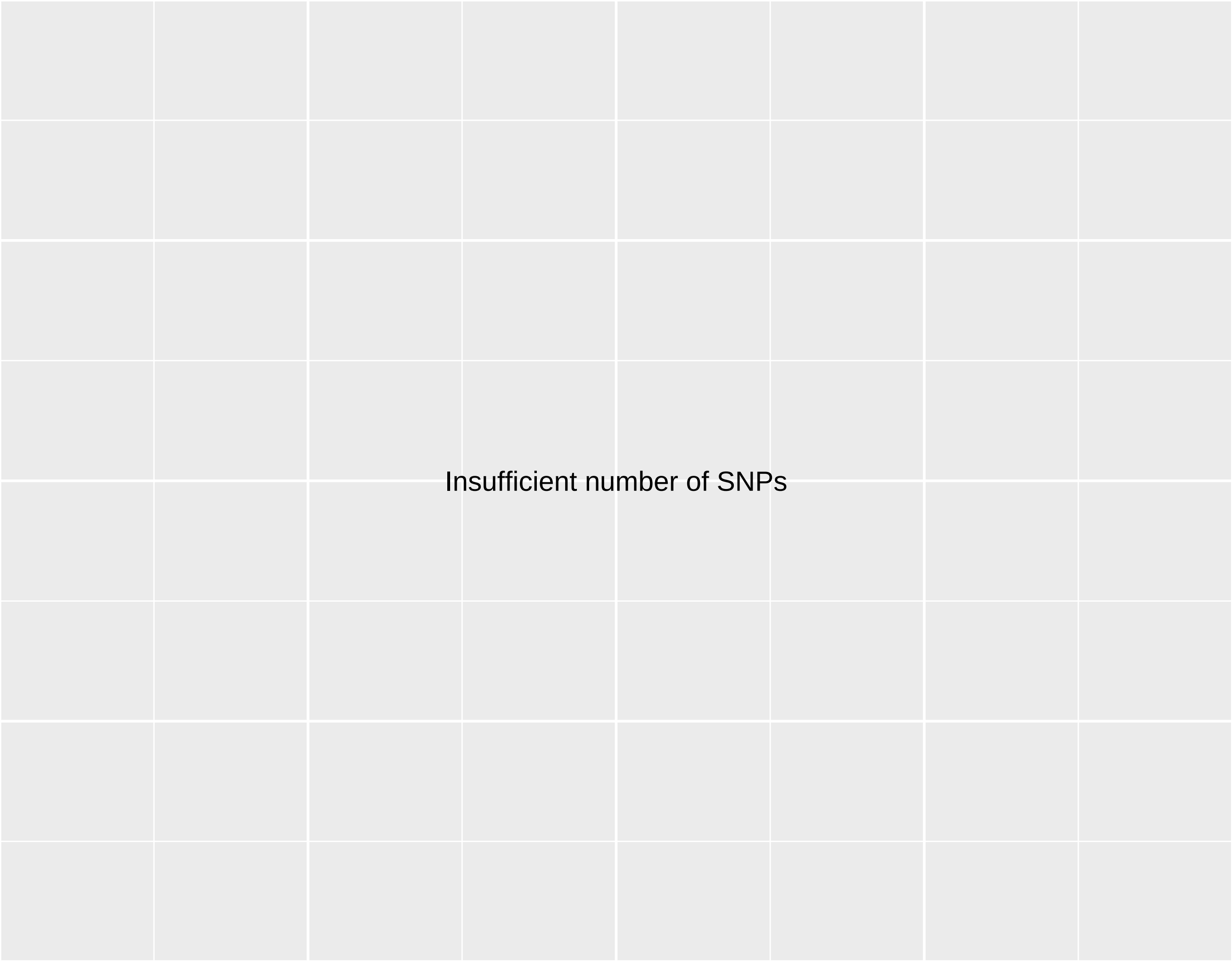
Insufficient number of SNPs

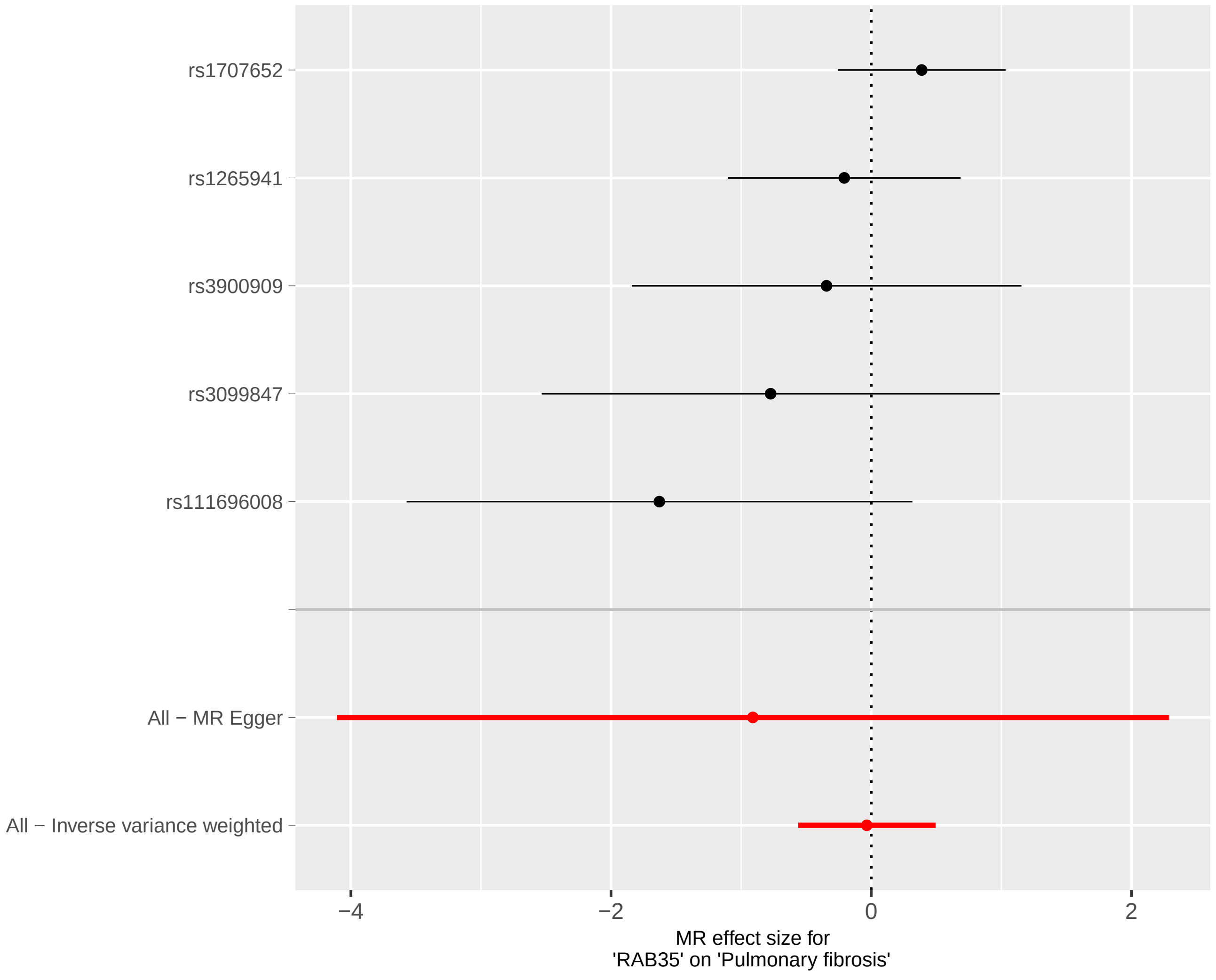

rs1707652
rs1265941
rs3900909
rs3099847
rs111696008
All − MR Egger
All − Inverse variance weighted
−4
−2
0
2
MR effect size for
'RAB35' on 'Pulmonary fibrosis'

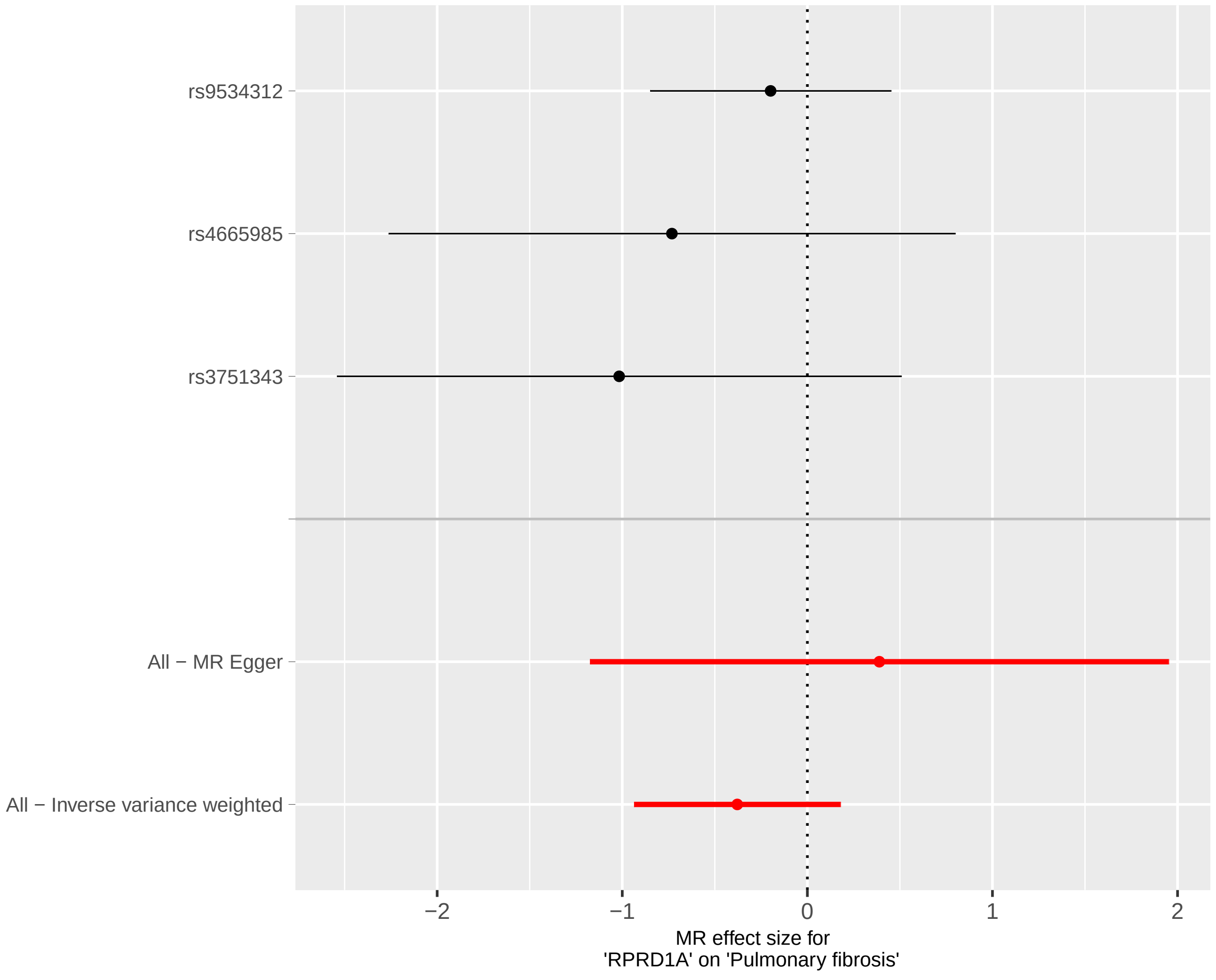

rs9534312
rs4665985
rs3751343
All − MR Egger
All − Inverse variance weighted
−2
−1
0
1
2
MR effect size for
'RPRD1A' on 'Pulmonary fibrosis'

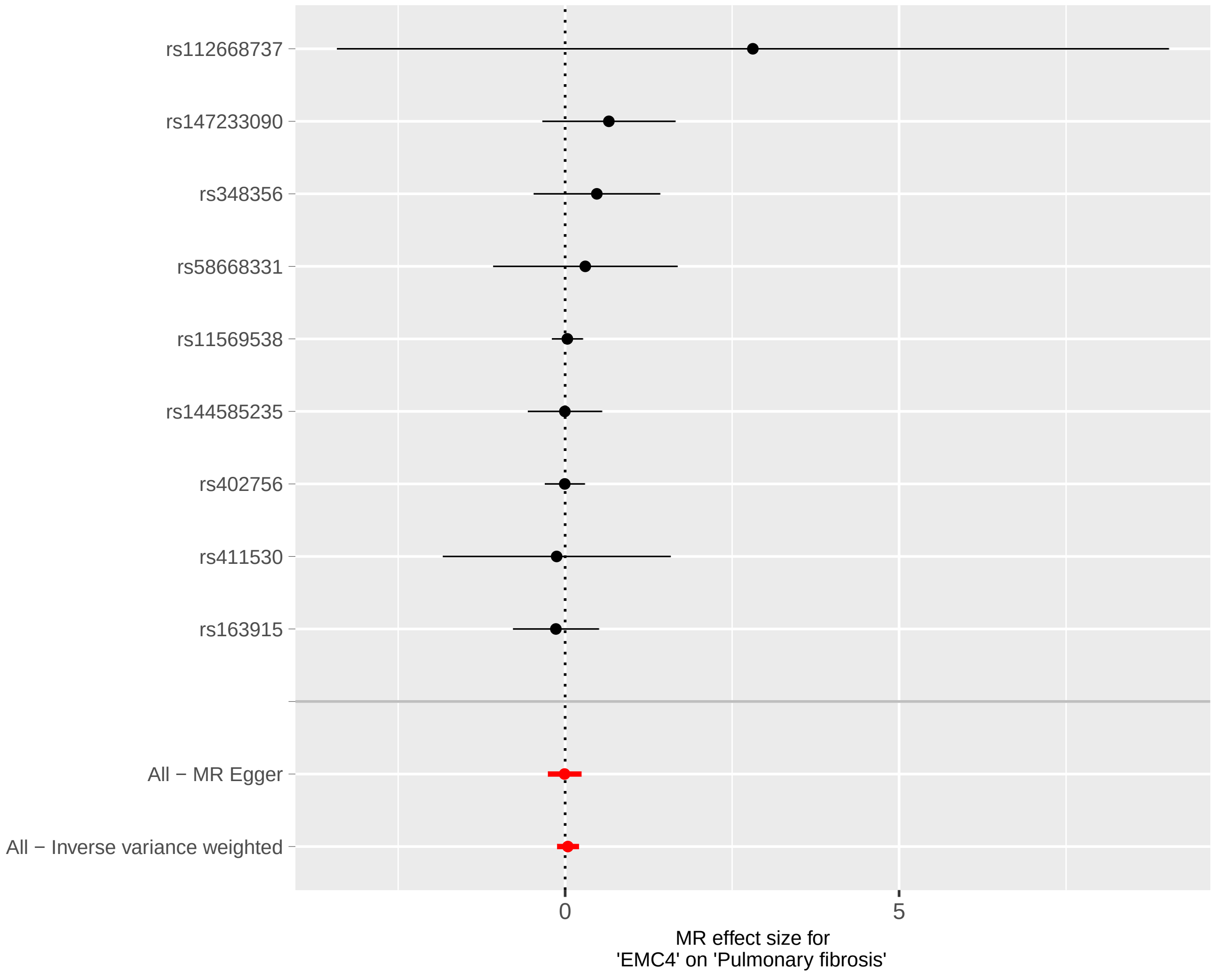
rs112668737
rs147233090
rs348356
rs58668331
rs11569538
rs144585235
rs402756
rs411530
rs163915
All − MR Egger
All − Inverse variance weighted
0
5
MR effect size for
'EMC4' on 'Pulmonary fibrosis'

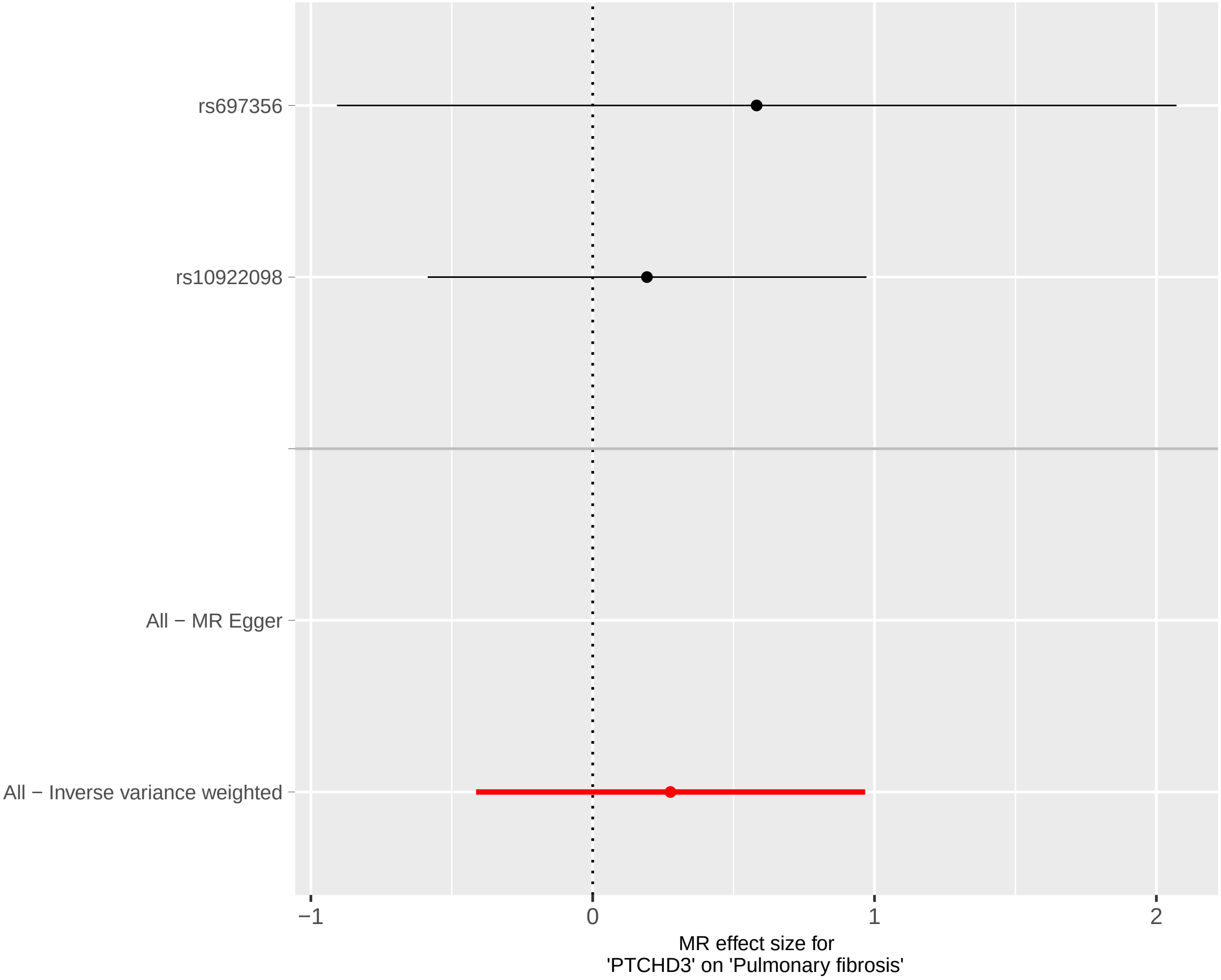

rs697356
rs10922098
All − MR Egger
All − Inverse variance weighted
−1
0
1
2
MR effect size for
'PTCHD3' on 'Pulmonary fibrosis'

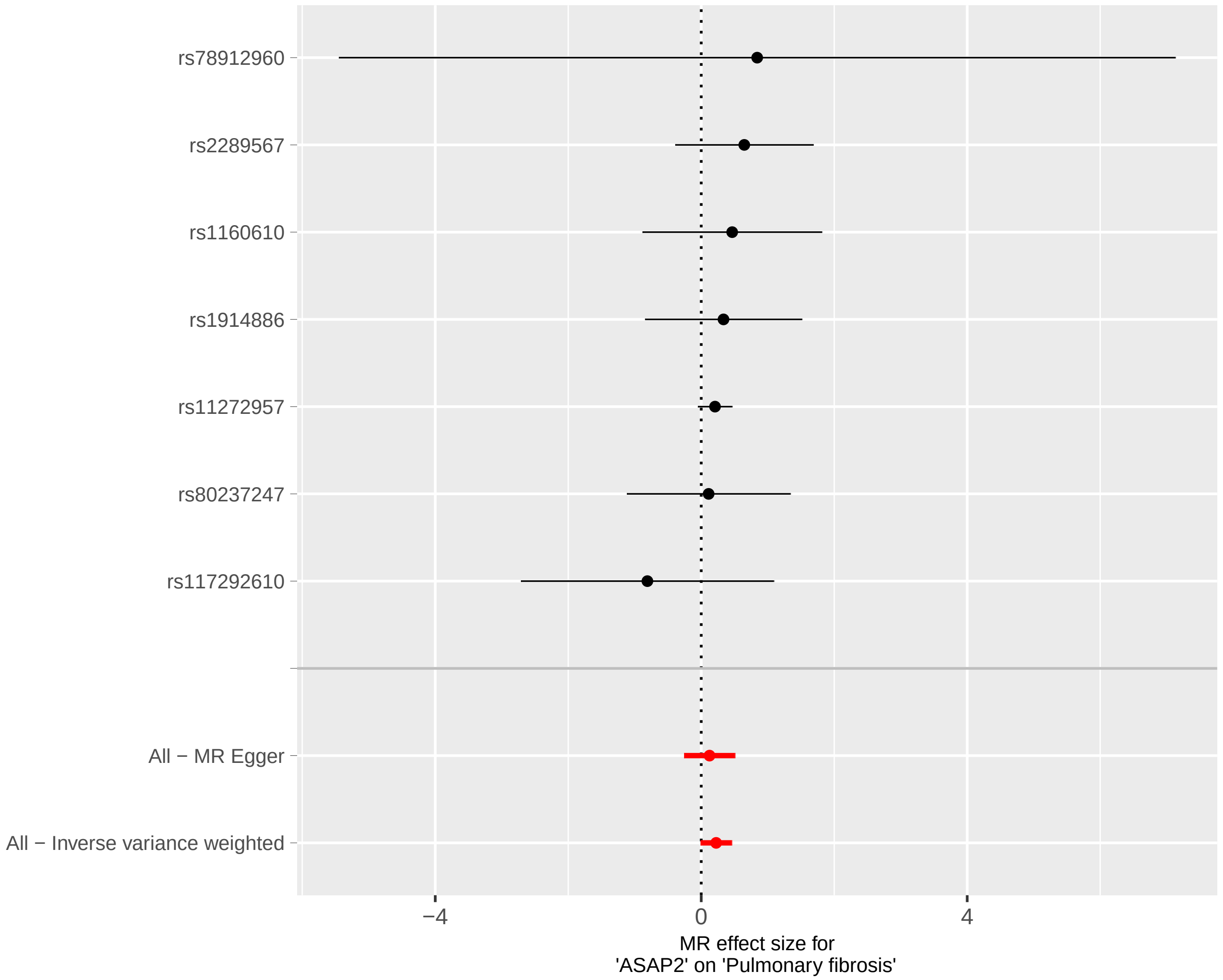

rs78912960
rs2289567
rs1160610
rs1914886
rs11272957
rs80237247
rs117292610
All − MR Egger
All − Inverse variance weighted
−4
0
4
MR effect size for
'ASAP2' on 'Pulmonary fibrosis'

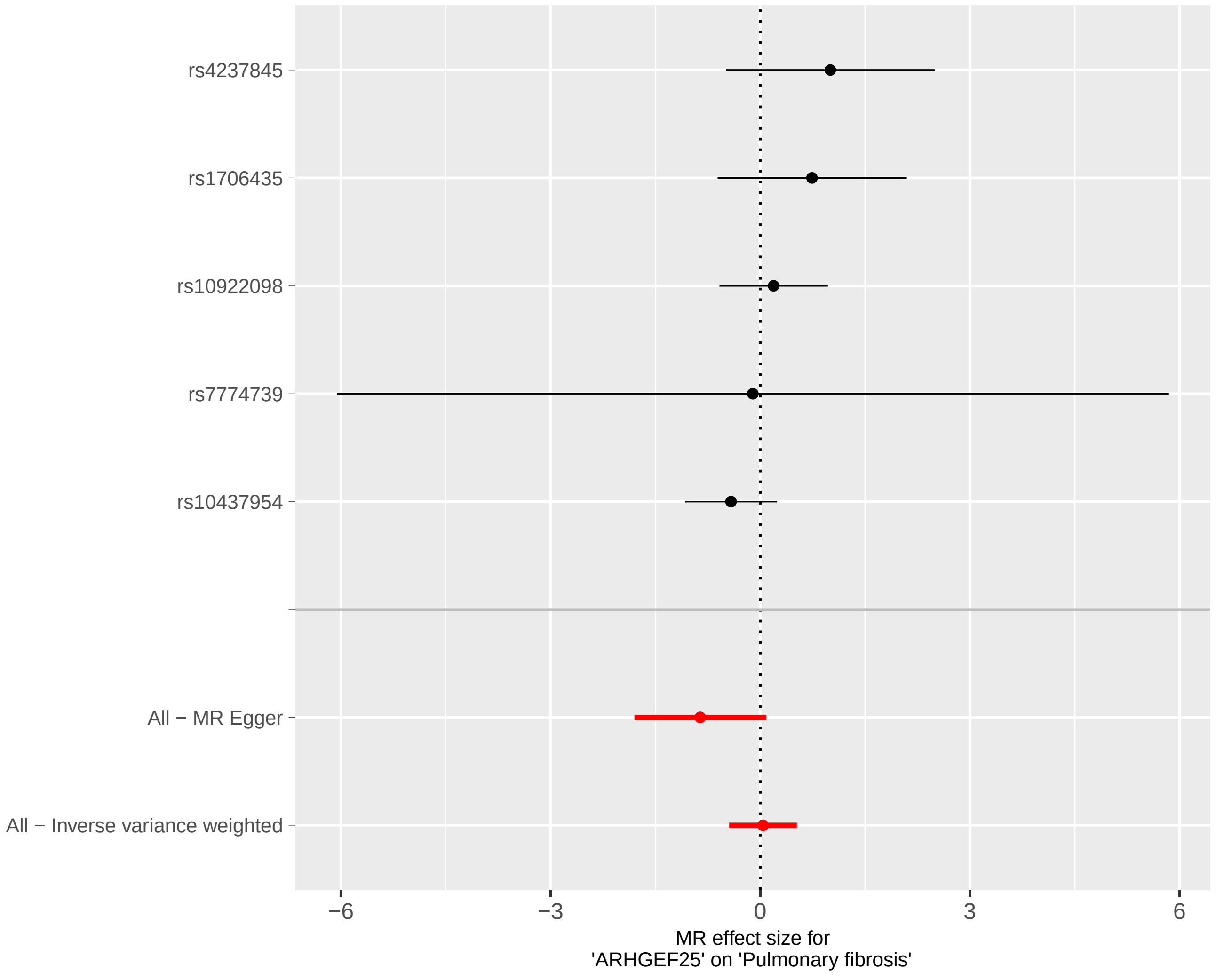

rs4237845
rs1706435
rs10922098
rs7774739
rs10437954
All − MR Egger
All − Inverse variance weighted
−6
−3
0
3
6
MR effect size for
'ARHGEF25' on 'Pulmonary fibrosis'

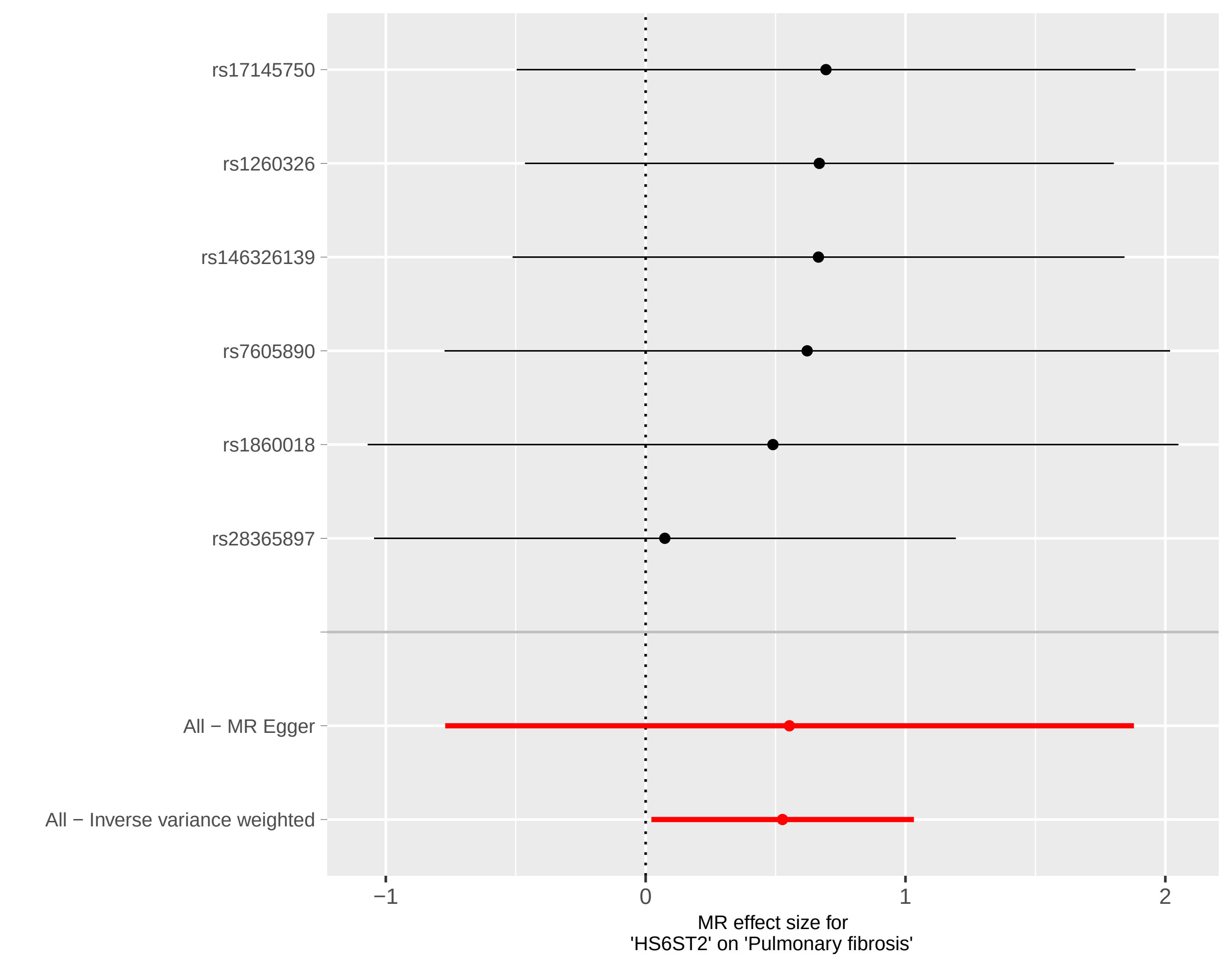
rs17145750
rs1260326
rs146326139
rs7605890
rs1860018
rs28365897
All − MR Egger
All − Inverse variance weighted
−1
0
1
2
MR effect size for
'HS6ST2' on 'Pulmonary fibrosis'

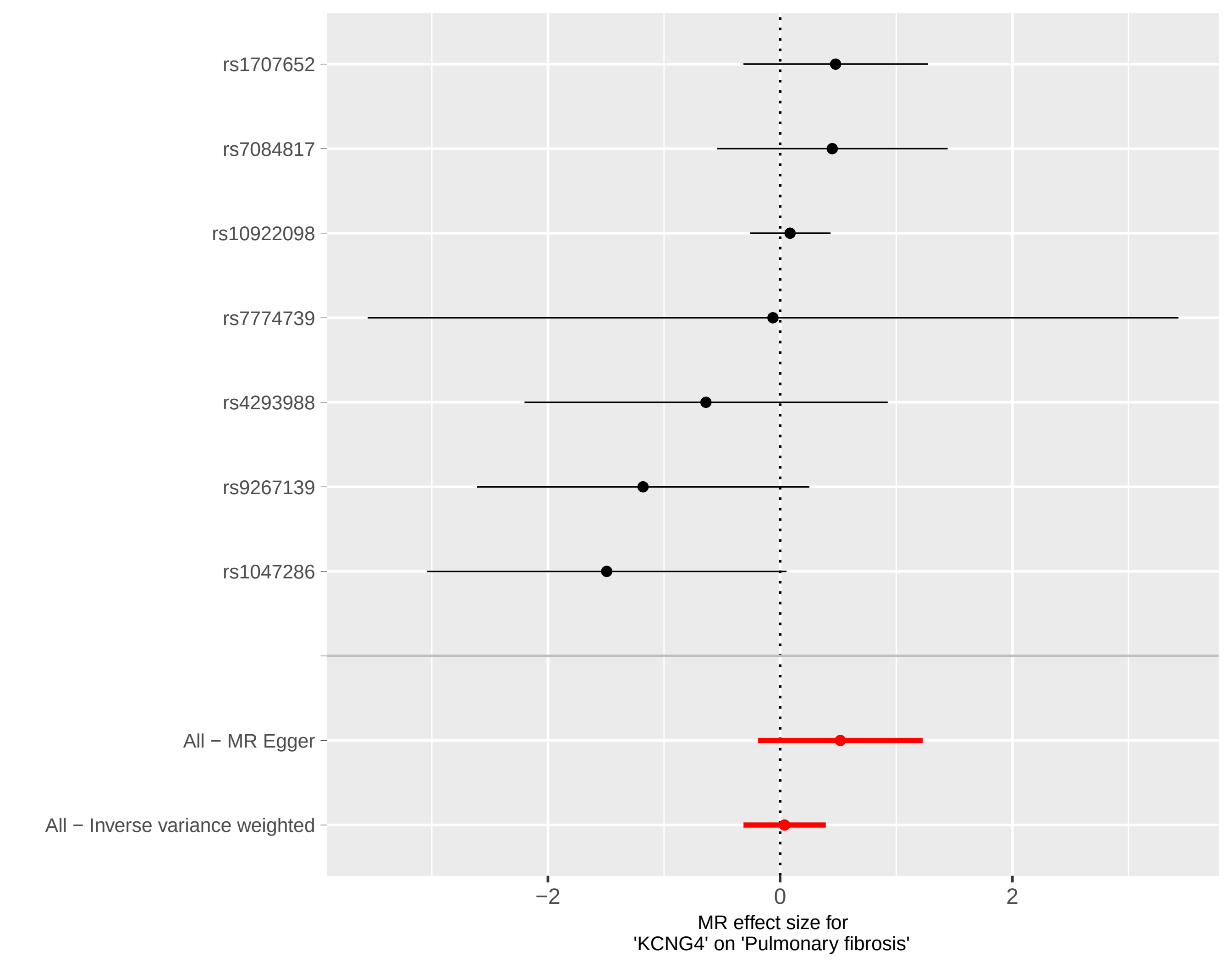
rs1707652
rs7084817
rs10922098
rs7774739
rs4293988
rs9267139
rs1047286
All − MR Egger
All − Inverse variance weighted
−2
0
2
MR effect size for
'KCNG4' on 'Pulmonary fibrosis'

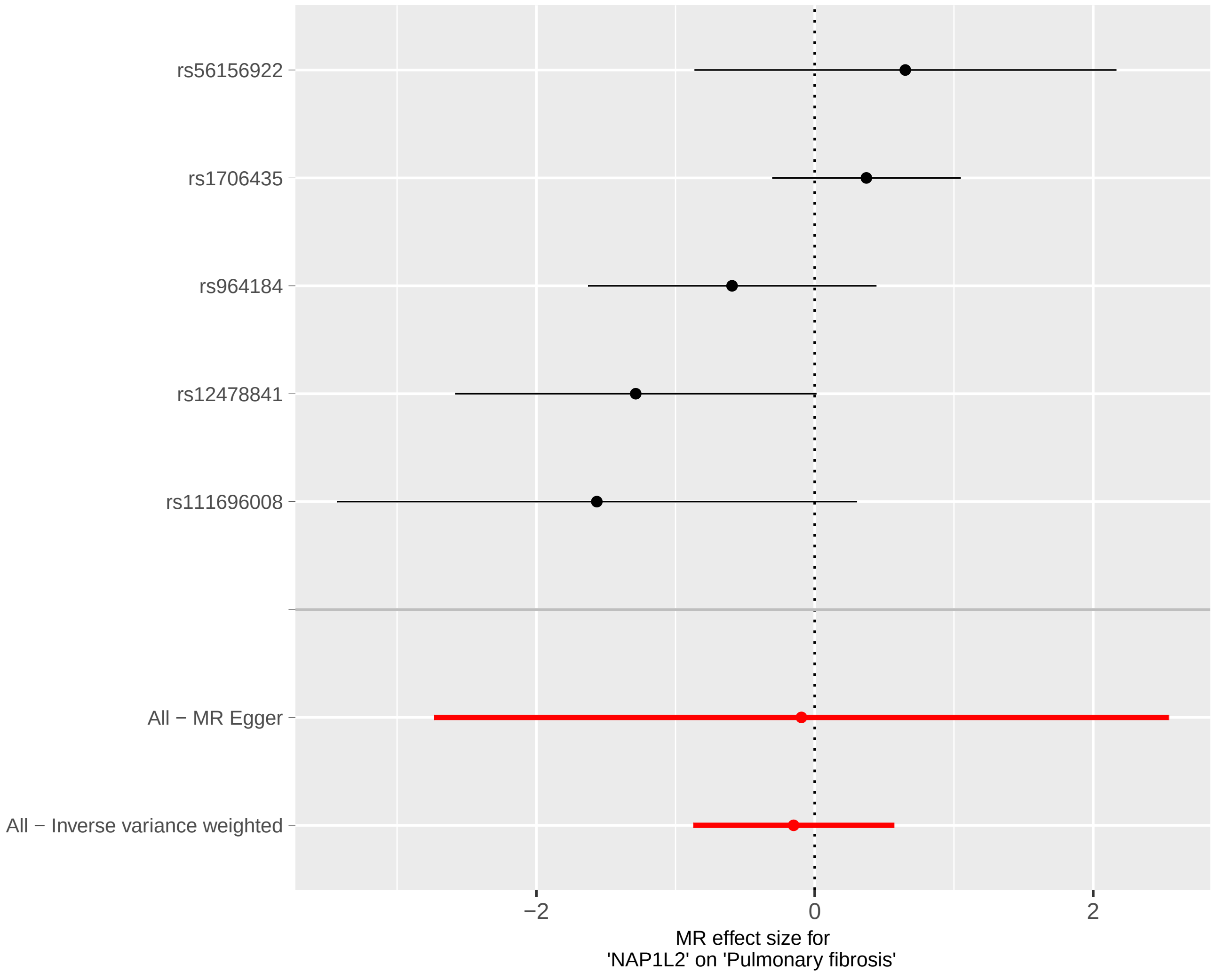
rs56156922
rs1706435
rs964184
rs12478841
rs111696008
All − MR Egger
All − Inverse variance weighted
−2
0
2
MR effect size for
'NAP1L2' on 'Pulmonary fibrosis'

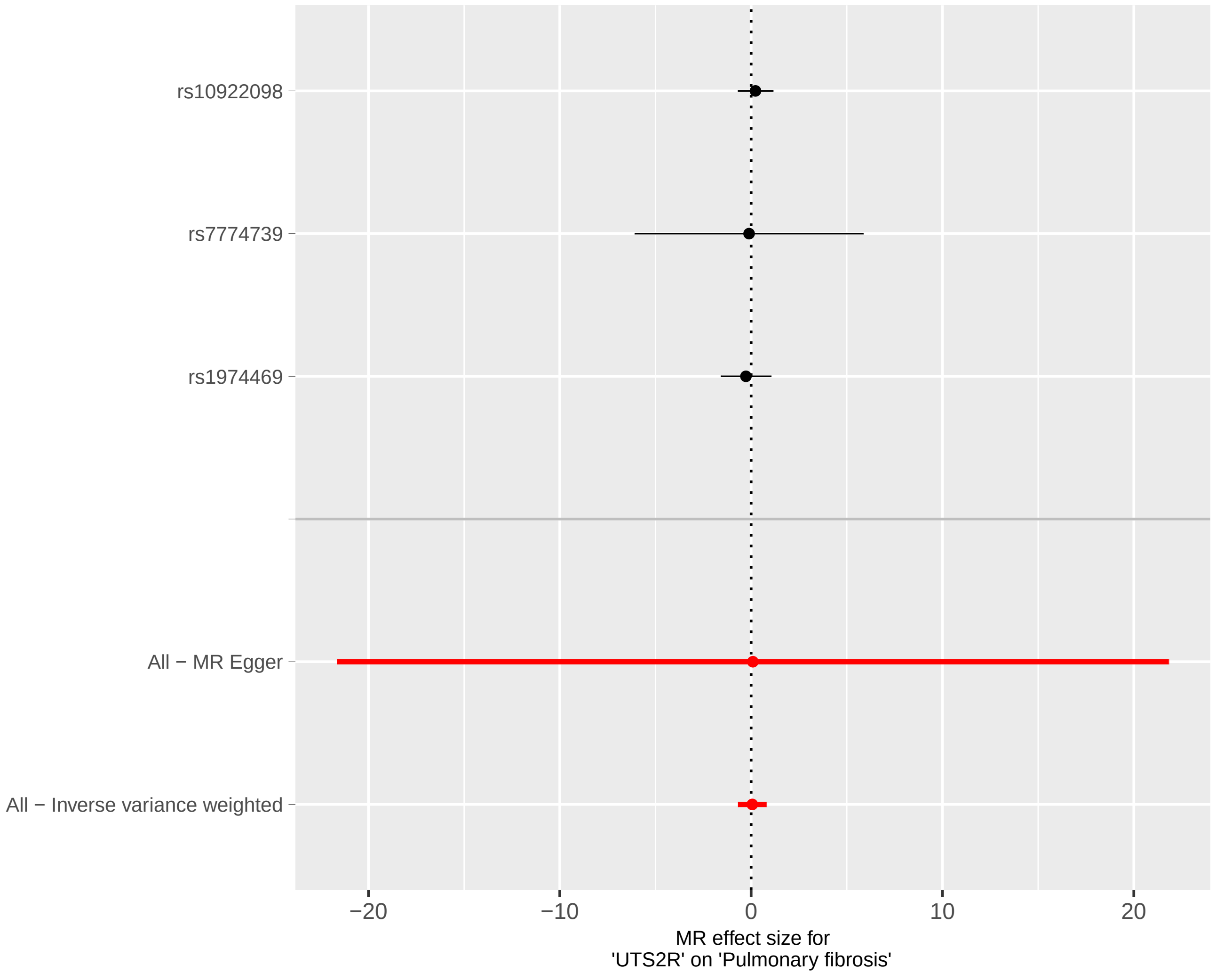

rs10922098
rs7774739
rs1974469
All − MR Egger
All − Inverse variance weighted
−20
−10
0
10
20
MR effect size for
'UTS2R' on 'Pulmonary fibrosis'

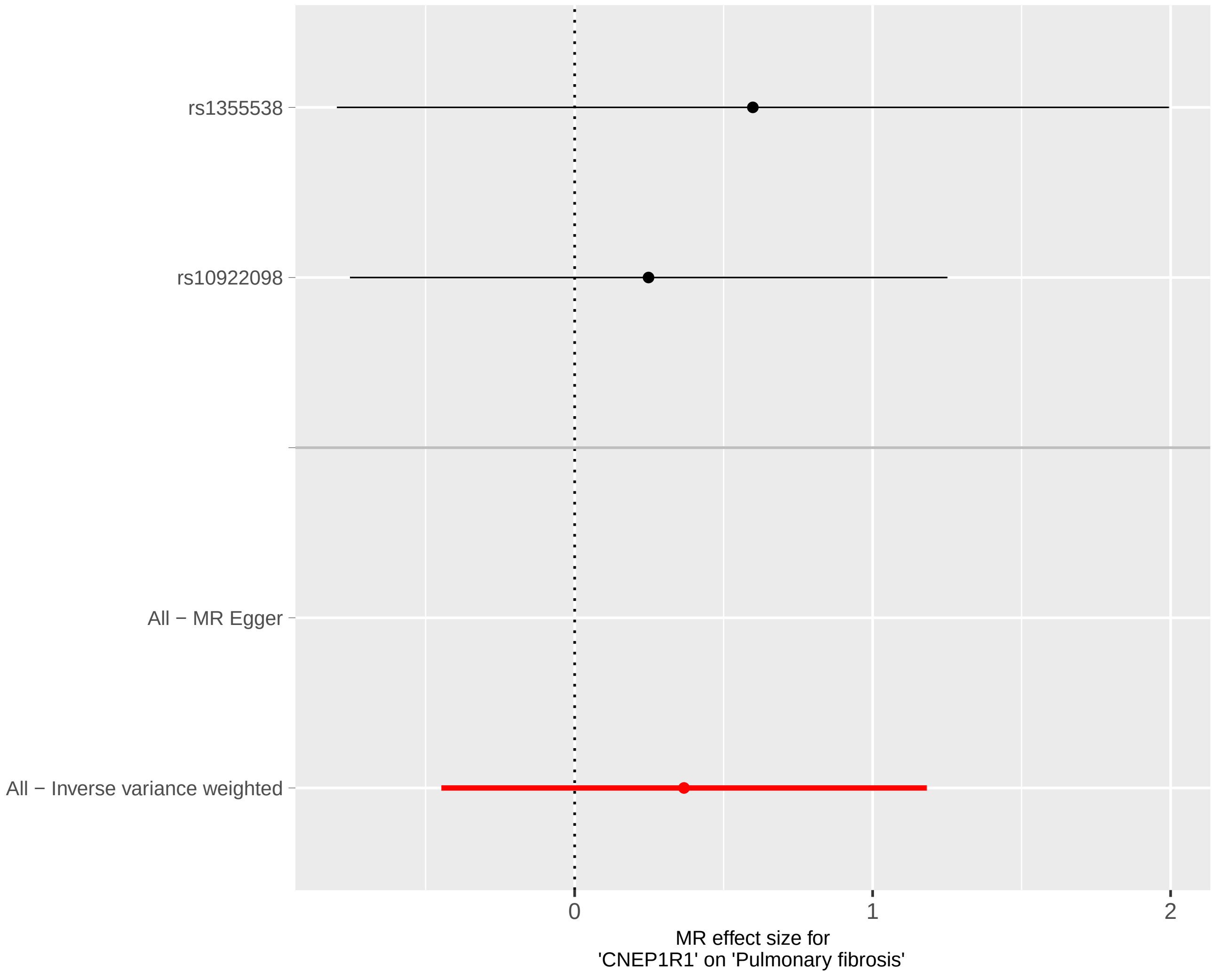
rs1355538
rs10922098
All − MR Egger
All − Inverse variance weighted
0
1
2
MR effect size for
'CNEP1R1' on 'Pulmonary fibrosis'

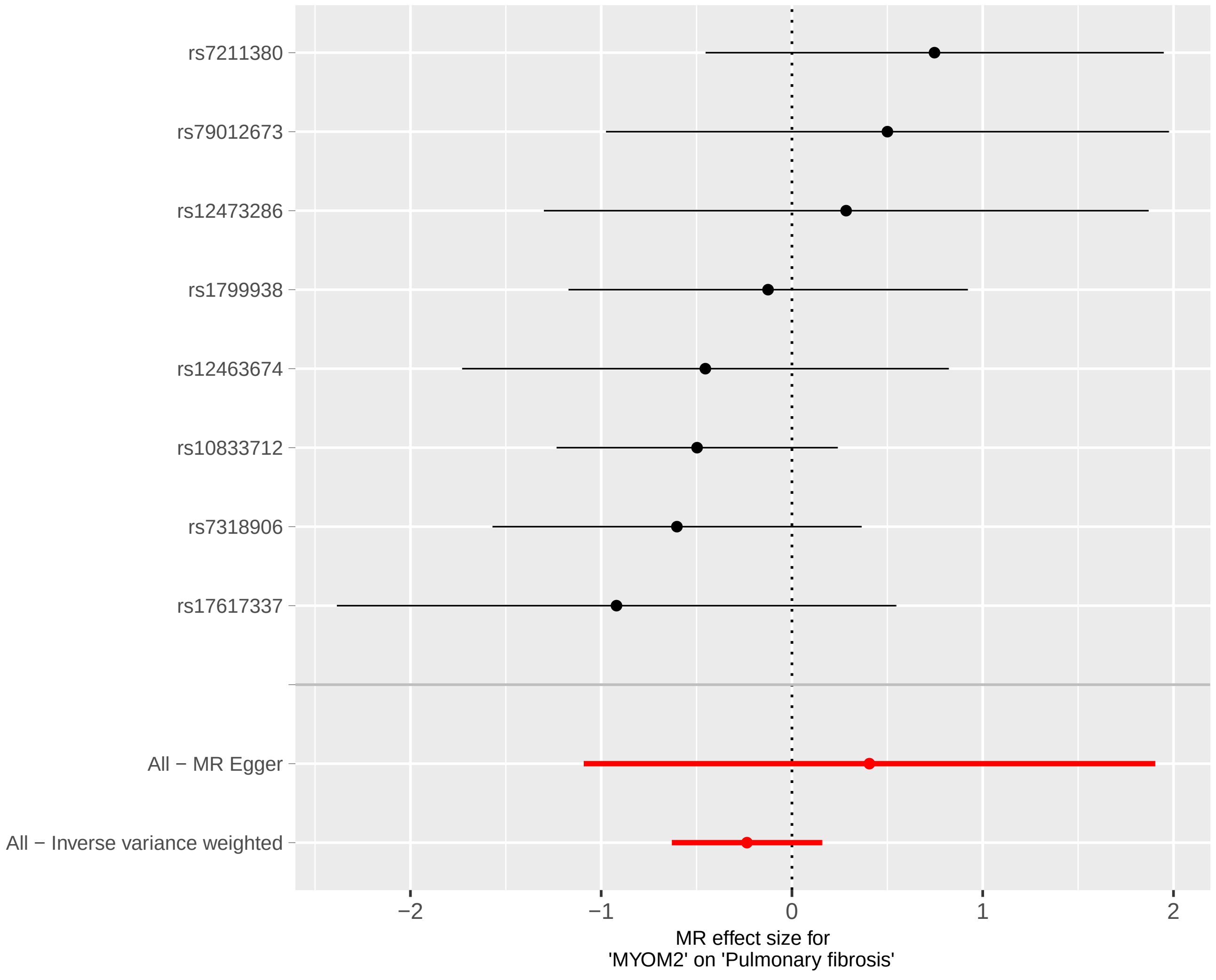
rs7211380
rs79012673
rs12473286
rs1799938
rs12463674
rs10833712
rs7318906
rs17617337
All − MR Egger
All − Inverse variance weighted
−2
−1
0
1
2
MR effect size for
'MYOM2' on 'Pulmonary fibrosis'

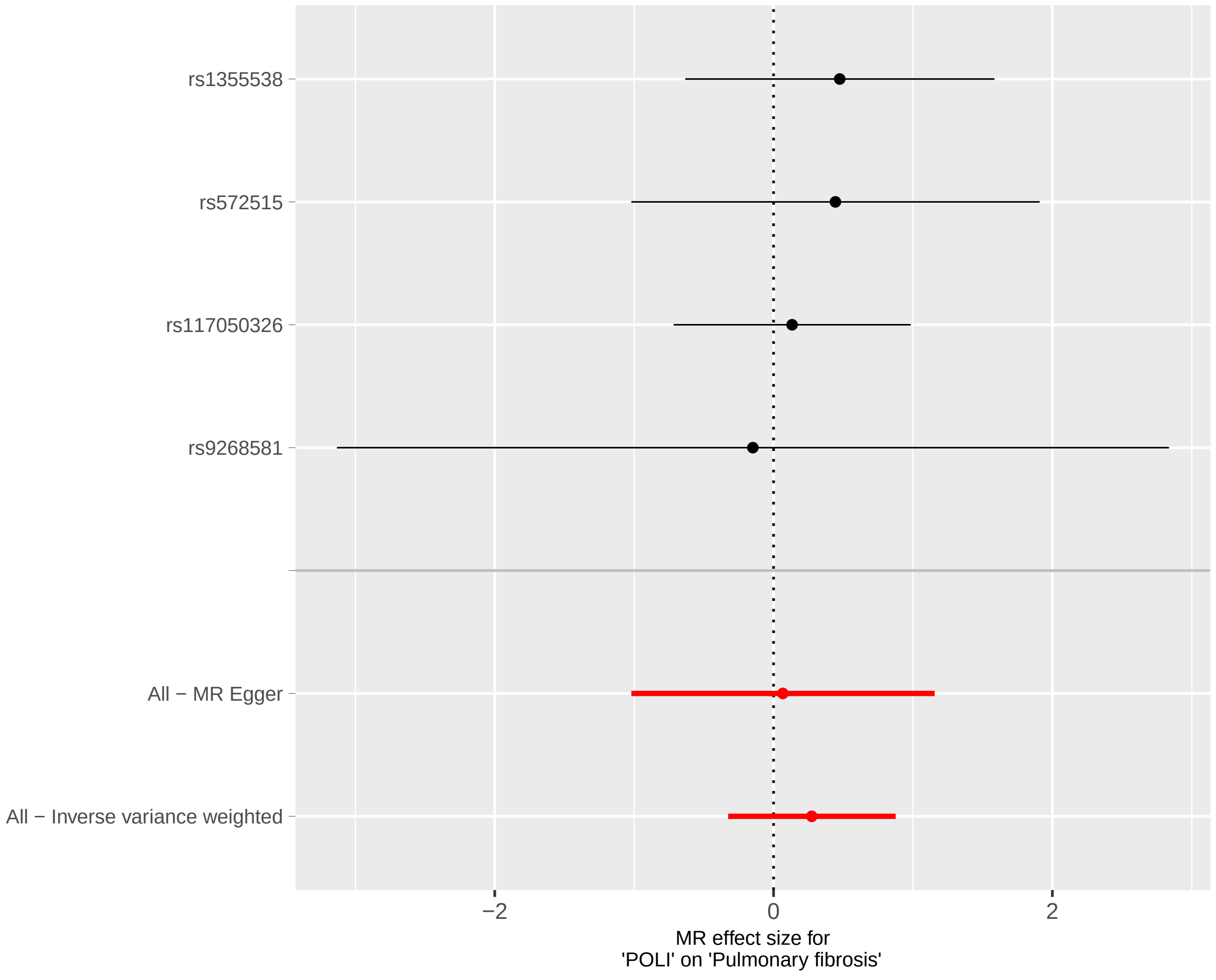
rs1355538
rs572515
rs117050326
rs9268581
All − MR Egger
All − Inverse variance weighted
−2
0
2
MR effect size for
'POLI' on 'Pulmonary fibrosis'

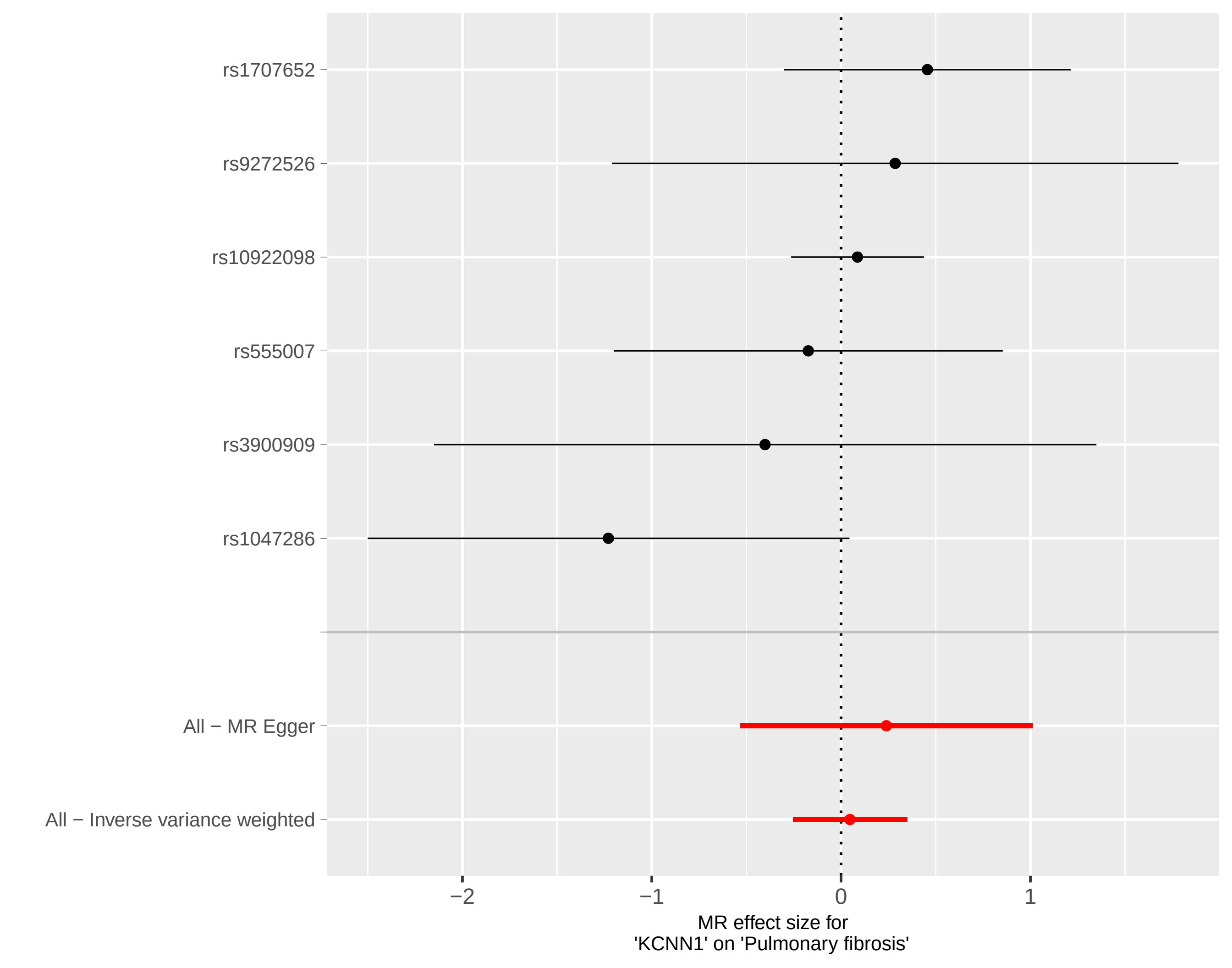
rs1707652
rs9272526
rs10922098
rs555007
rs3900909
rs1047286
All − MR Egger
All − Inverse variance weighted
−2
−1
0
1
MR effect size for
'KCNN1' on 'Pulmonary fibrosis'

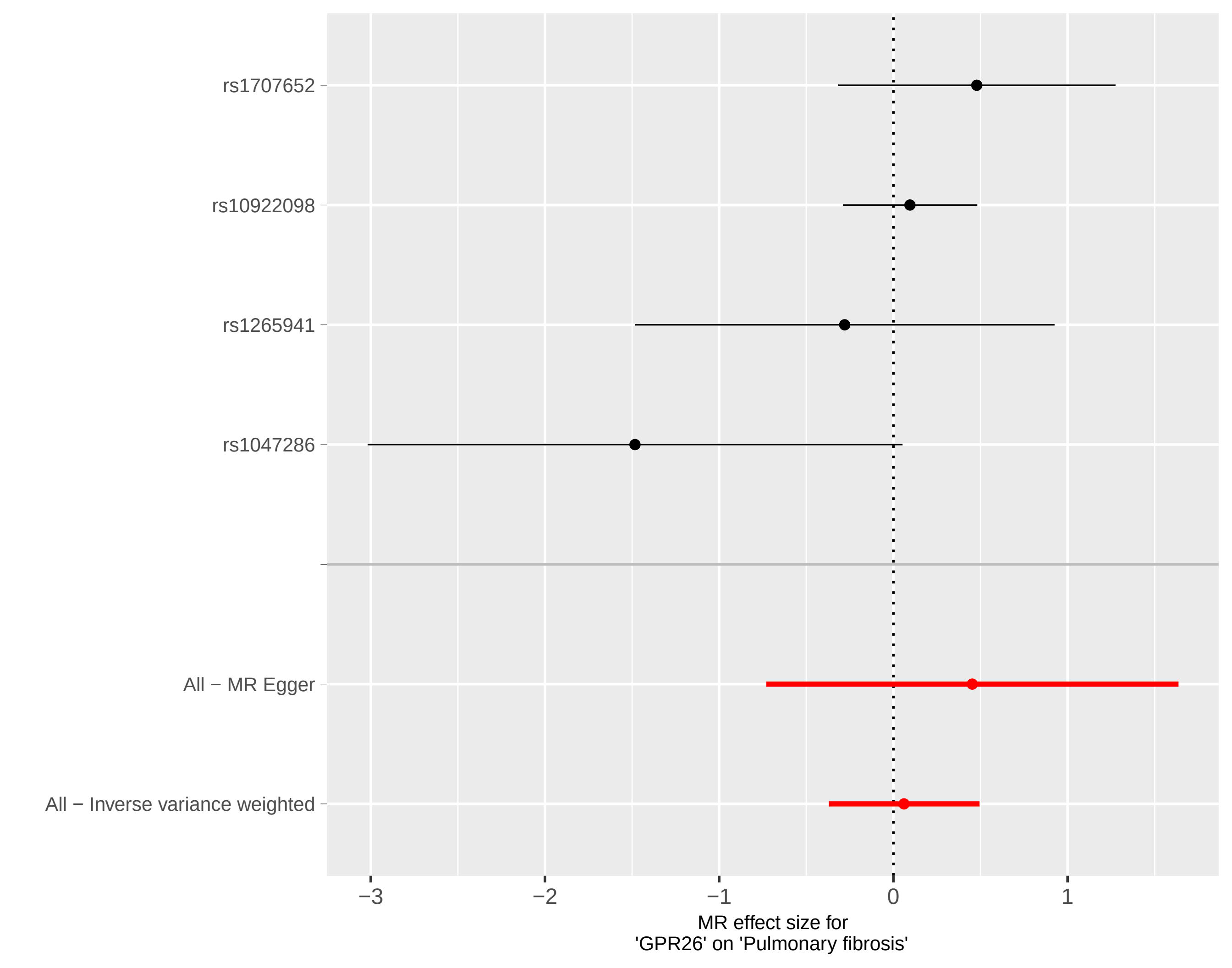
rs1707652
rs10922098
rs1265941
rs1047286
All − MR Egger
All − Inverse variance weighted
−3
−2
−1
0
1
MR effect size for
'GPR26' on 'Pulmonary fibrosis'

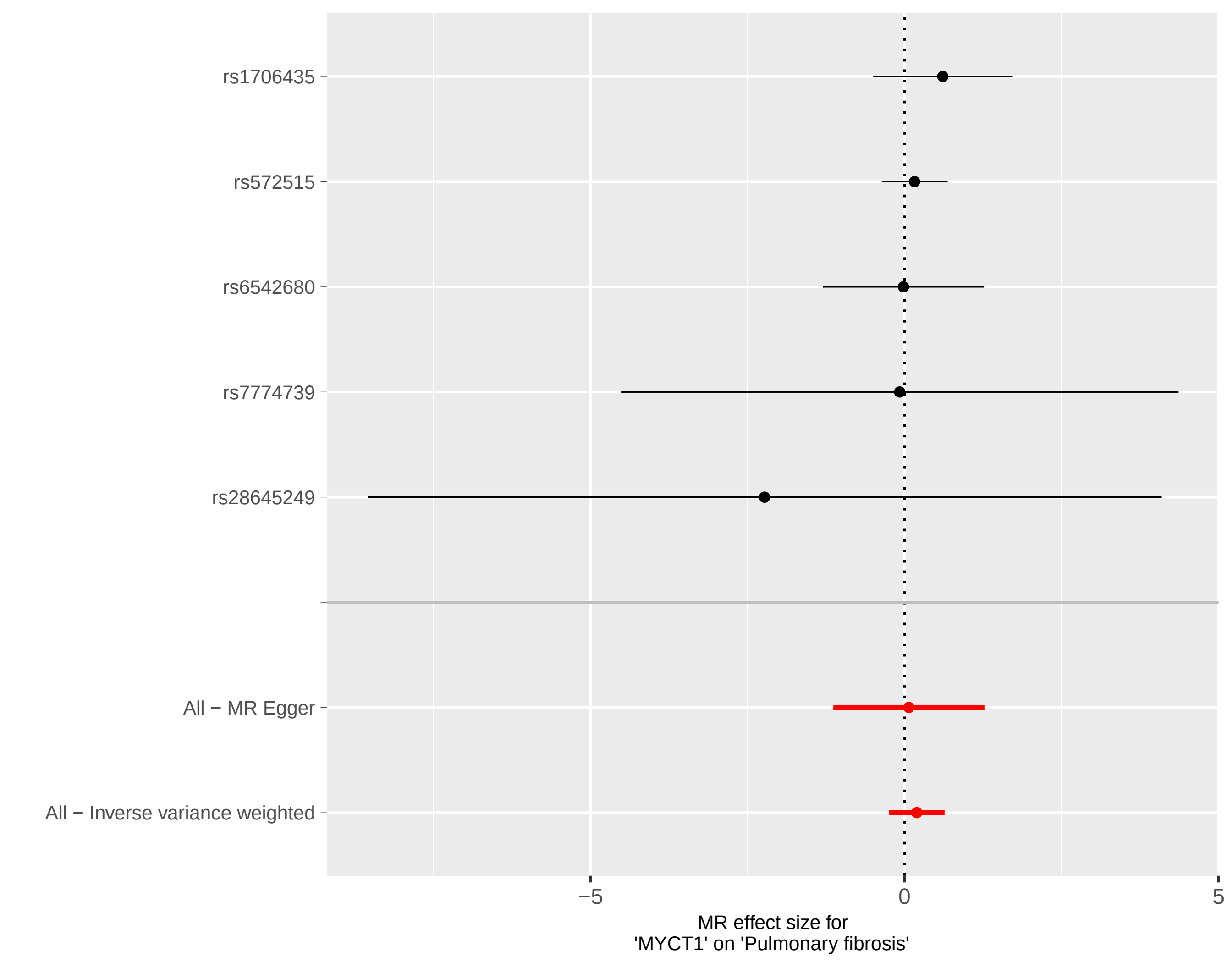

rs1706435
rs572515
rs6542680
rs7774739
rs28645249
All − MR Egger
All − Inverse variance weighted
−5
0
5
MR effect size for
'MYCT1' on 'Pulmonary fibrosis'

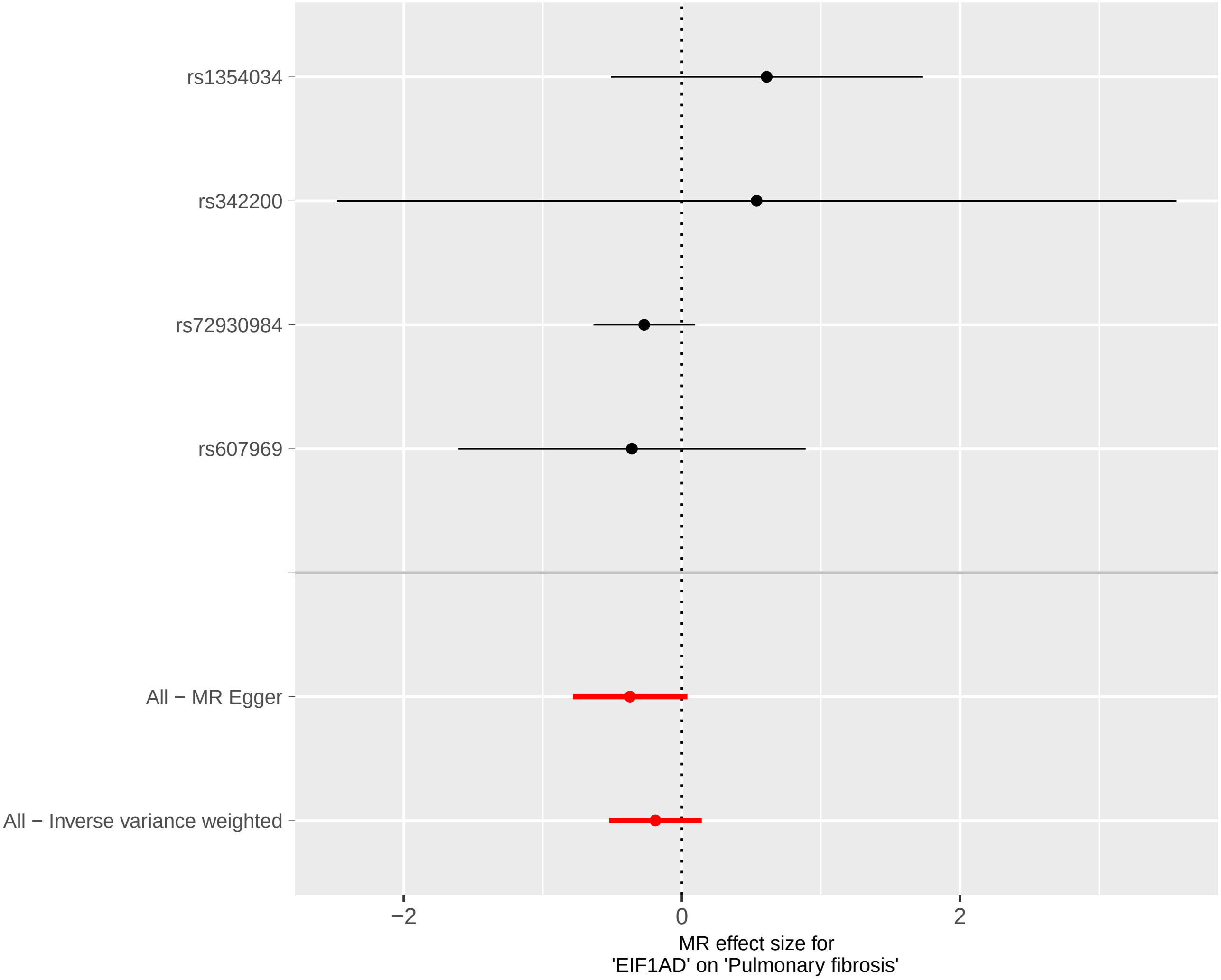

rs1354034
rs342200
rs72930984
rs607969
All − MR Egger
All − Inverse variance weighted
−2
0
2
MR effect size for
'EIF1AD' on 'Pulmonary fibrosis'

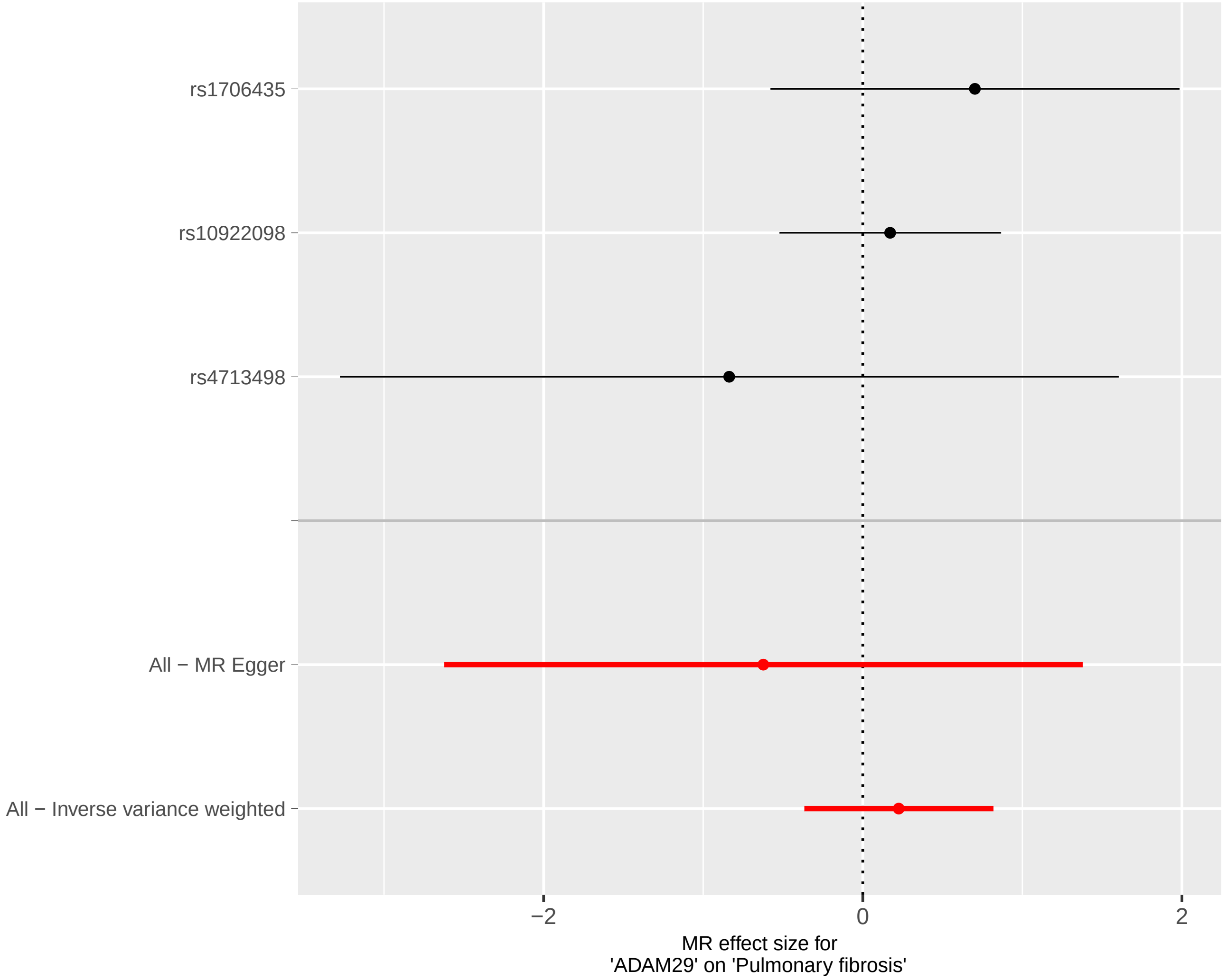
rs1706435
rs10922098
rs4713498
All − MR Egger
All − Inverse variance weighted
−2
0
2
MR effect size for
'ADAM29' on 'Pulmonary fibrosis'

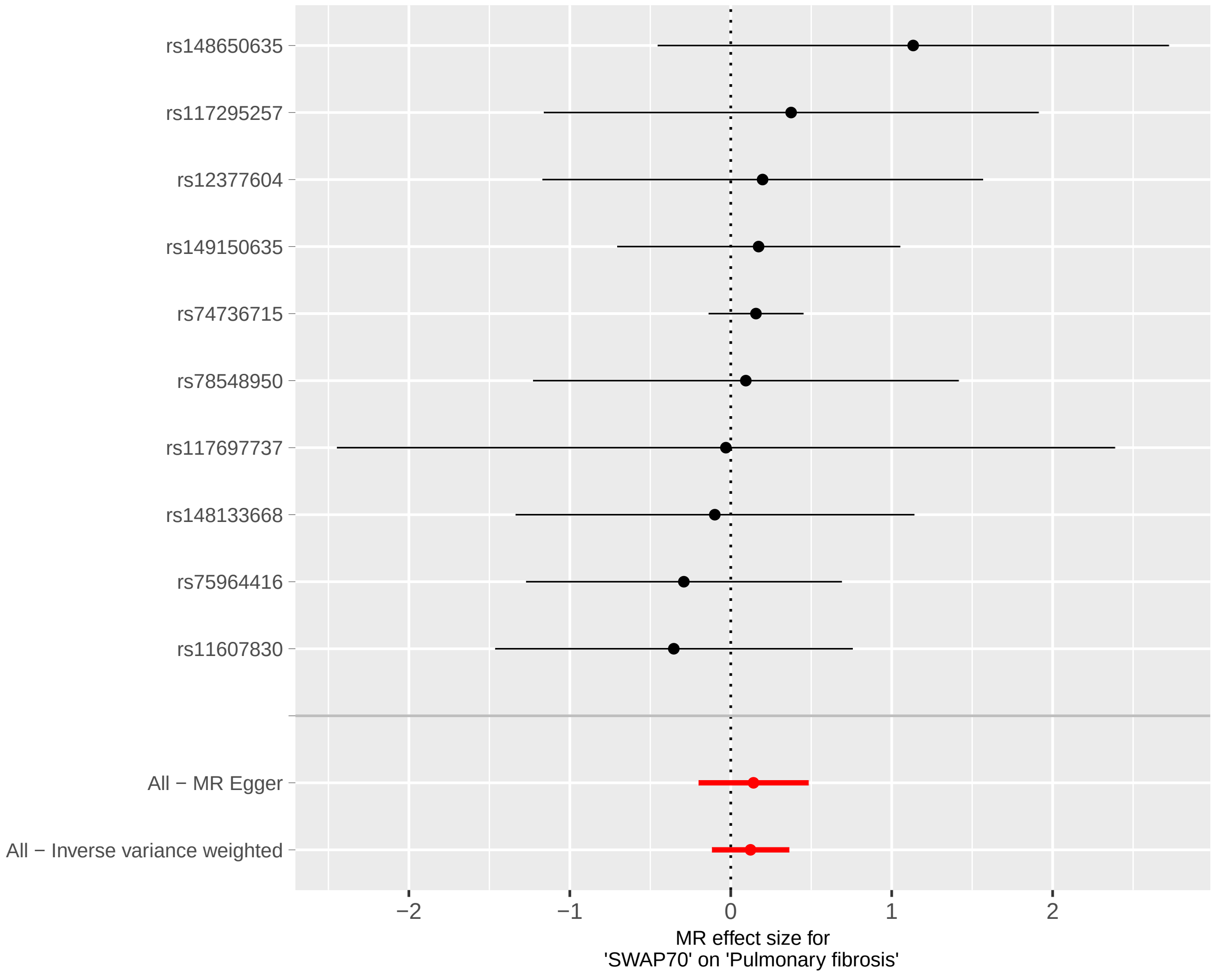
rs148650635
rs117295257
rs12377604
rs149150635
rs74736715
rs78548950
rs117697737
rs148133668
rs75964416
rs11607830
All − MR Egger
All − Inverse variance weighted
−2
−1
0
1
2
MR effect size for
'SWAP70' on 'Pulmonary fibrosis'

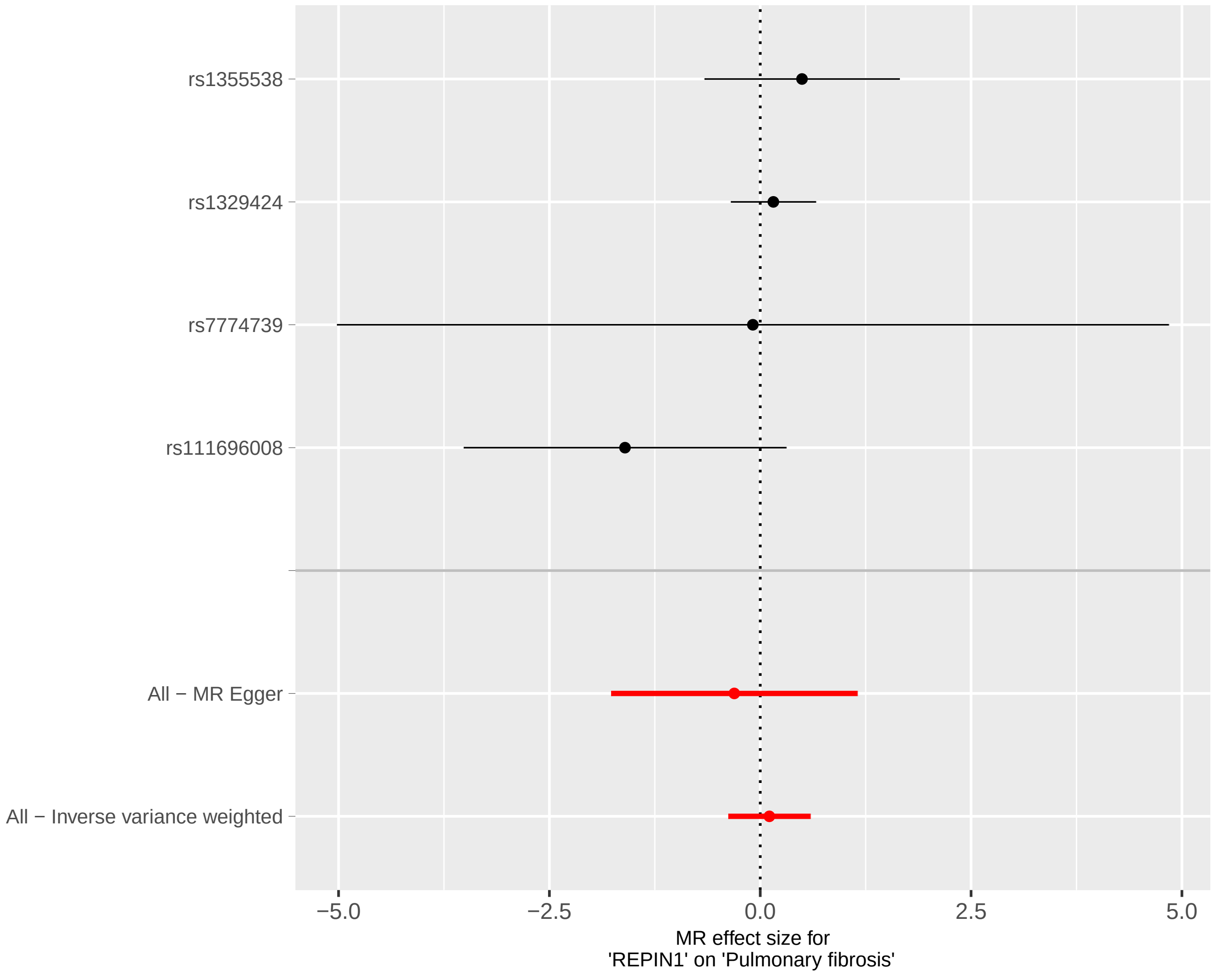

rs1355538
rs1329424
rs7774739
rs111696008
All − MR Egger
All − Inverse variance weighted
−5.0
−2.5
0.0
2.5
5.0
MR effect size for
'REPIN1' on 'Pulmonary fibrosis'

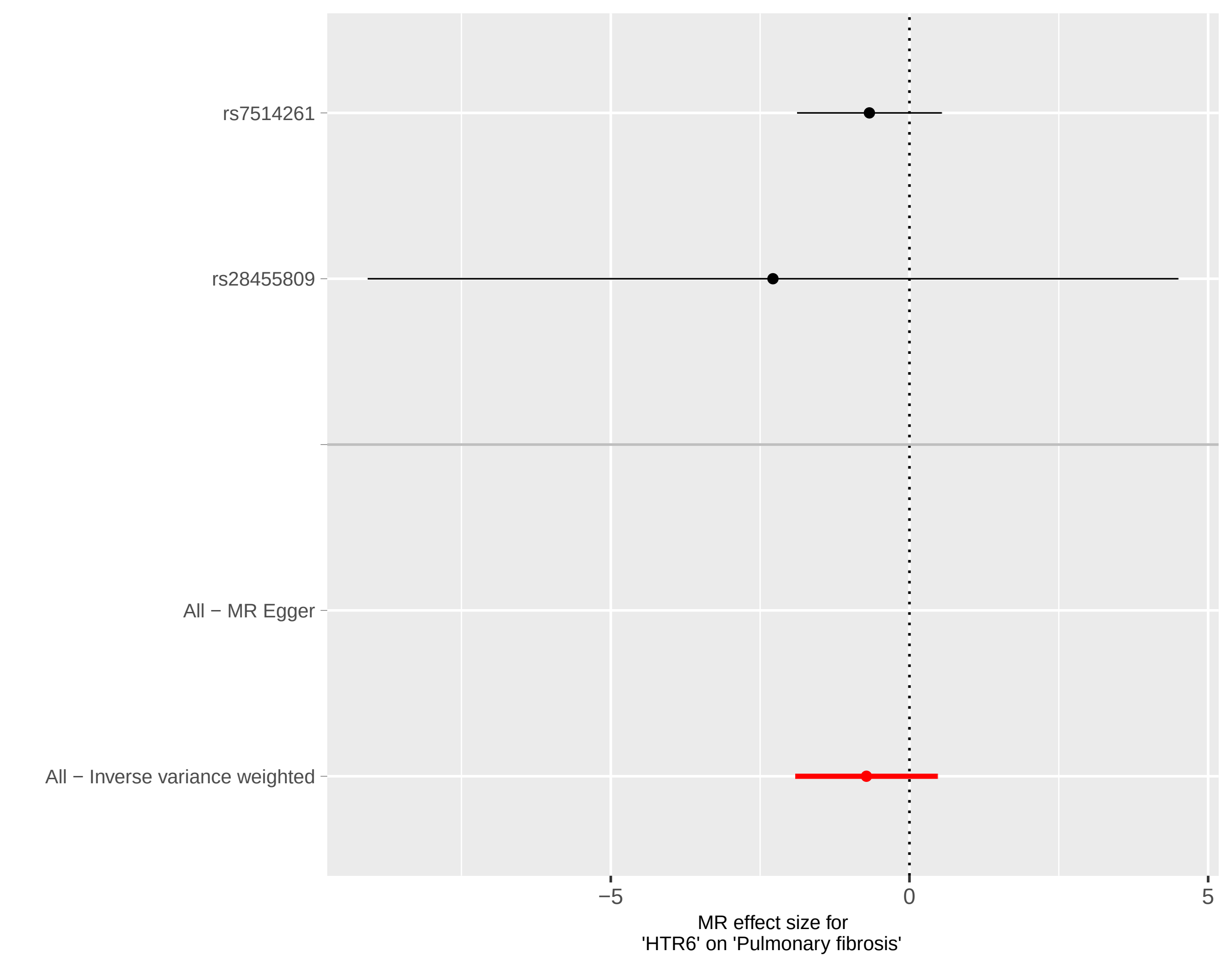
rs7514261
rs28455809
All − MR Egger
All − Inverse variance weighted
−5
0
5
MR effect size for
'HTR6' on 'Pulmonary fibrosis'

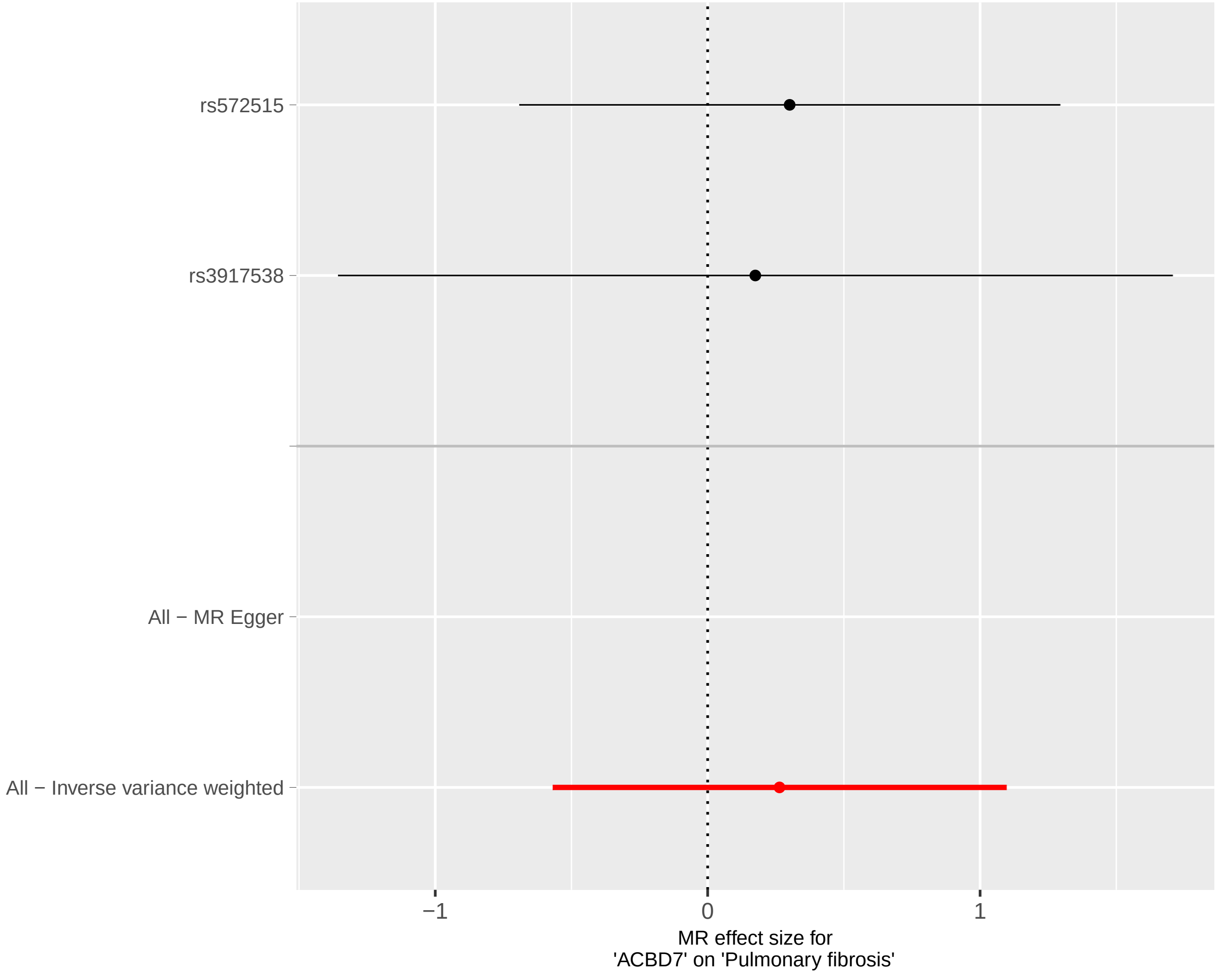
rs572515
rs3917538
All − MR Egger
All − Inverse variance weighted
−1
0
1
MR effect size for
'ACBD7' on 'Pulmonary fibrosis'

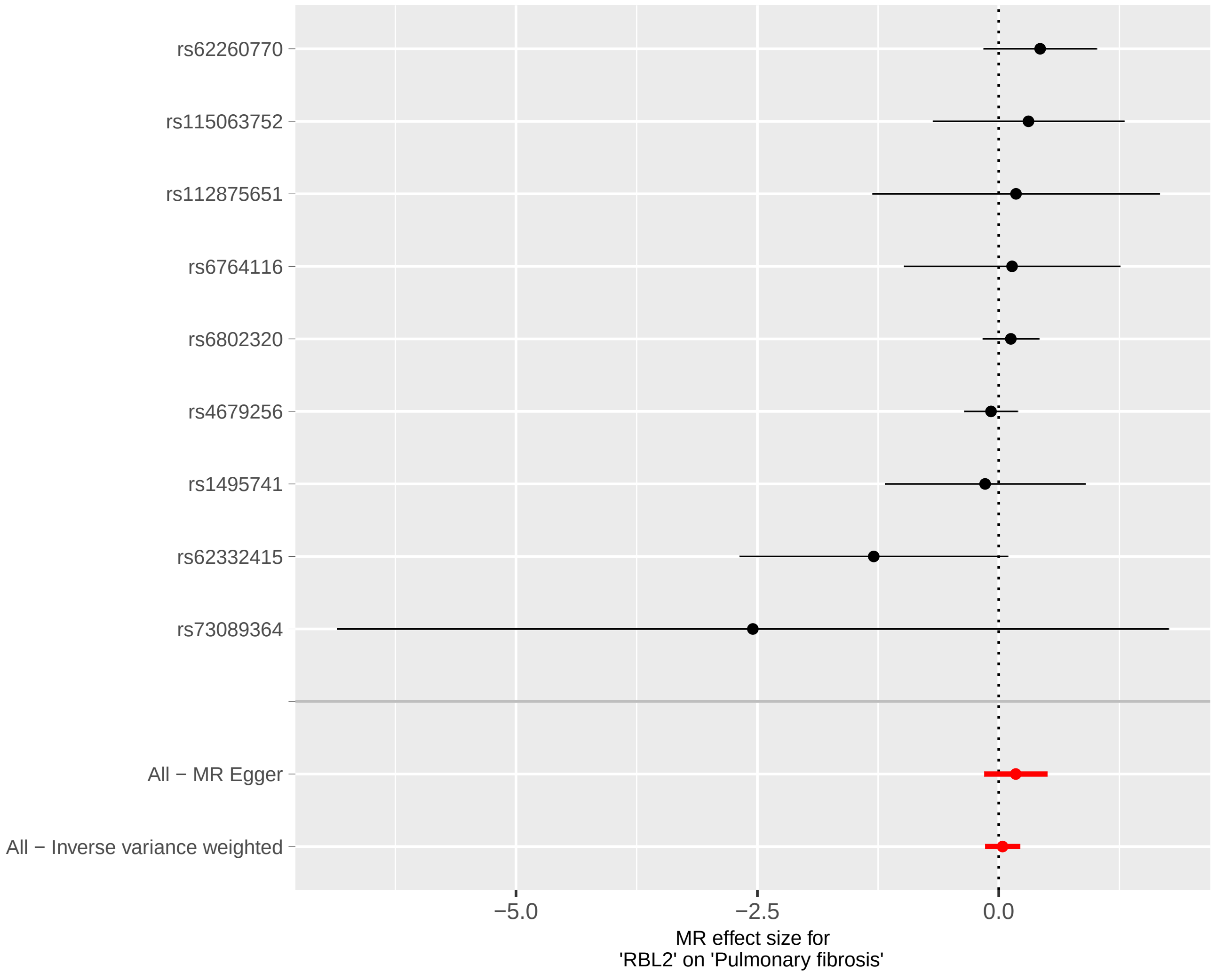
rs62260770
rs115063752
rs112875651
rs6764116
rs6802320
rs4679256
rs1495741
rs62332415
rs73089364
All − MR Egger
All − Inverse variance weighted
−5.0
−2.5
0.0
MR effect size for
'RBL2' on 'Pulmonary fibrosis'

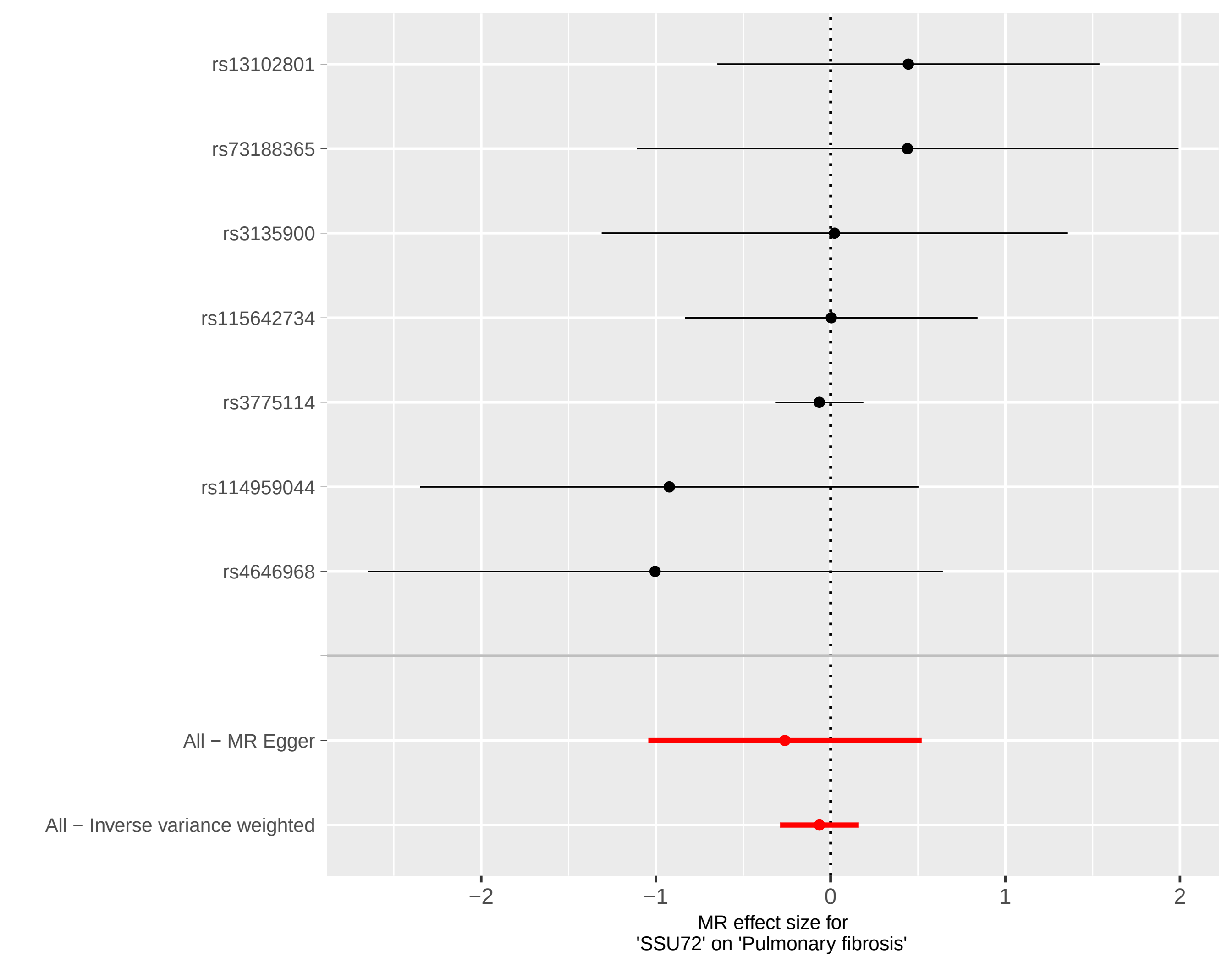
rs13102801
rs73188365
rs3135900
rs115642734
rs3775114
rs114959044
rs4646968
All − MR Egger
All − Inverse variance weighted
−2
−1
0
1
2
MR effect size for
'SSU72' on 'Pulmonary fibrosis'

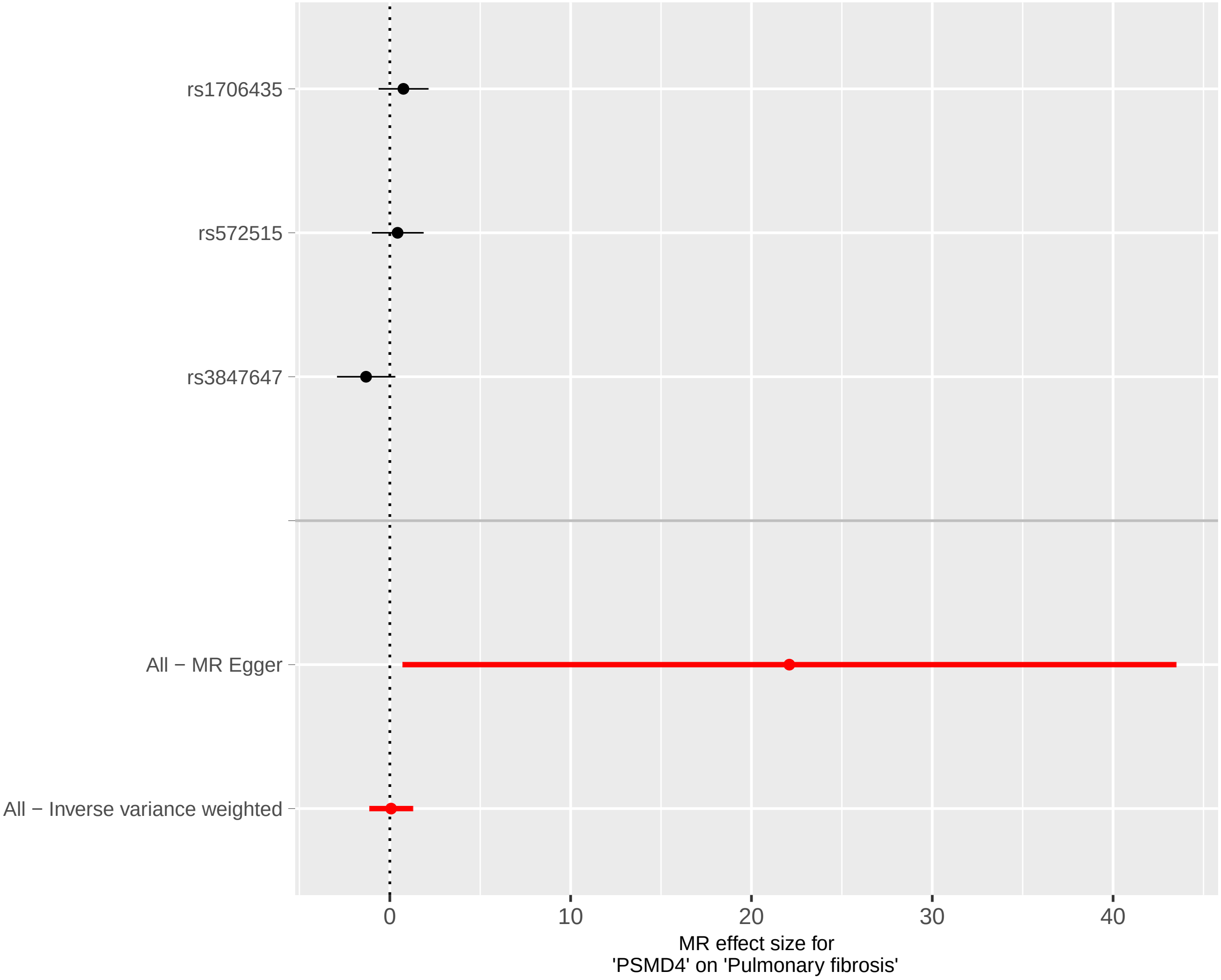
rs1706435
rs572515
rs3847647
All − MR Egger
All − Inverse variance weighted
0
10
20
30
40
MR effect size for
'PSMD4' on 'Pulmonary fibrosis'

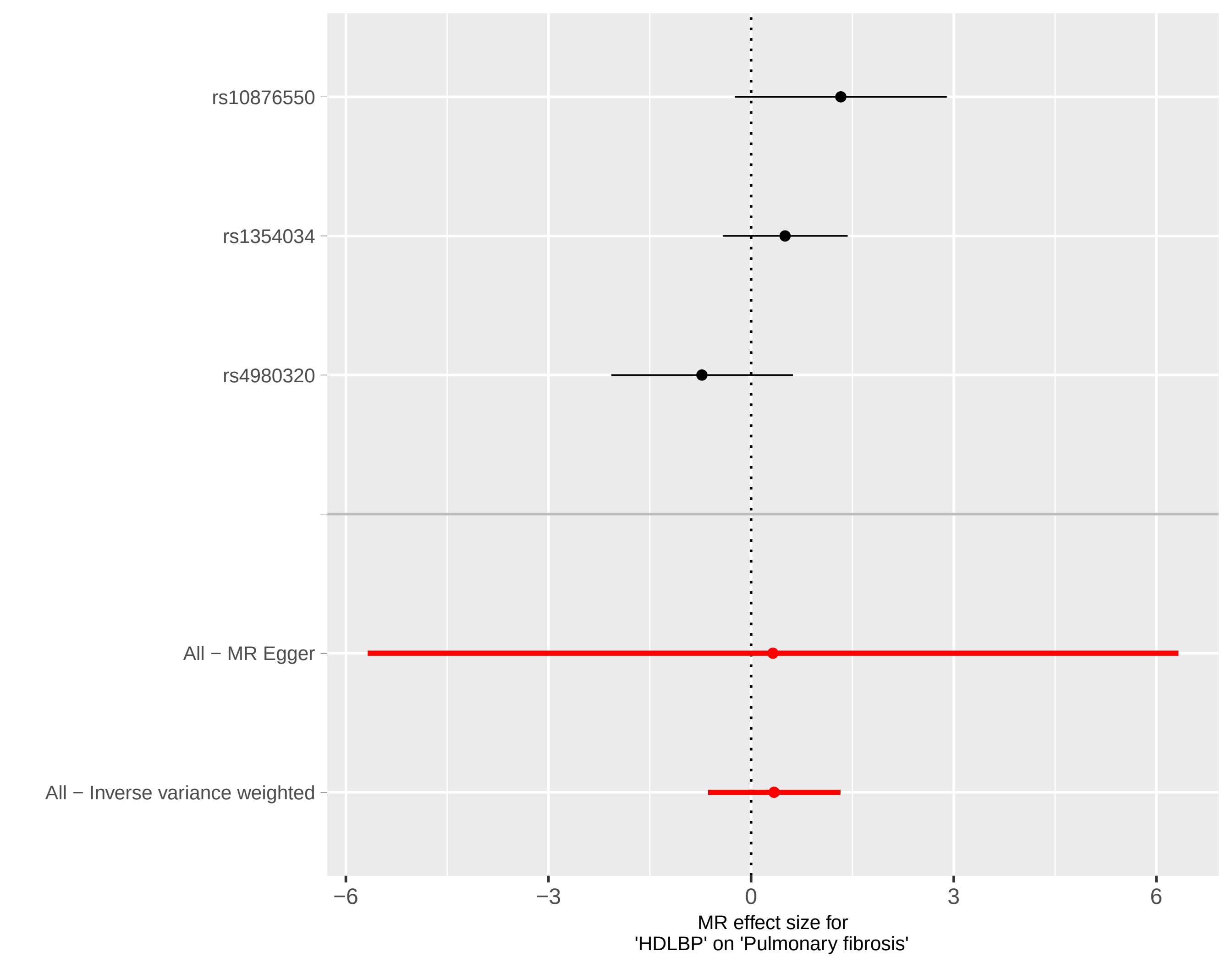
rs10876550
rs1354034
rs4980320
All − MR Egger
All − Inverse variance weighted
−6
−3
0
3
6
MR effect size for
'HDLBP' on 'Pulmonary fibrosis'

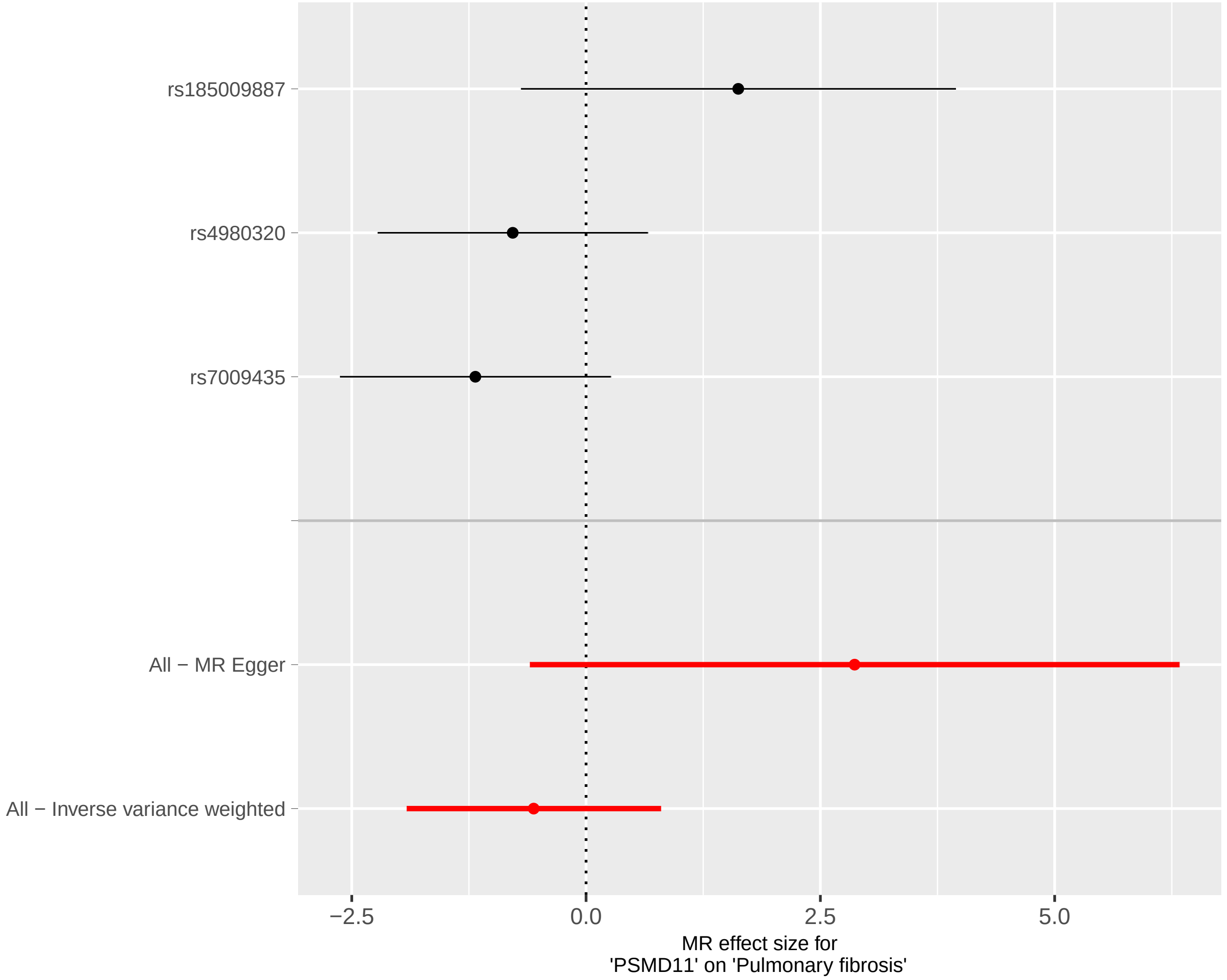
rs185009887
rs4980320
rs7009435
All − MR Egger
All − Inverse variance weighted
−2.5
0.0
2.5
5.0
MR effect size for
'PSMD11' on 'Pulmonary fibrosis'

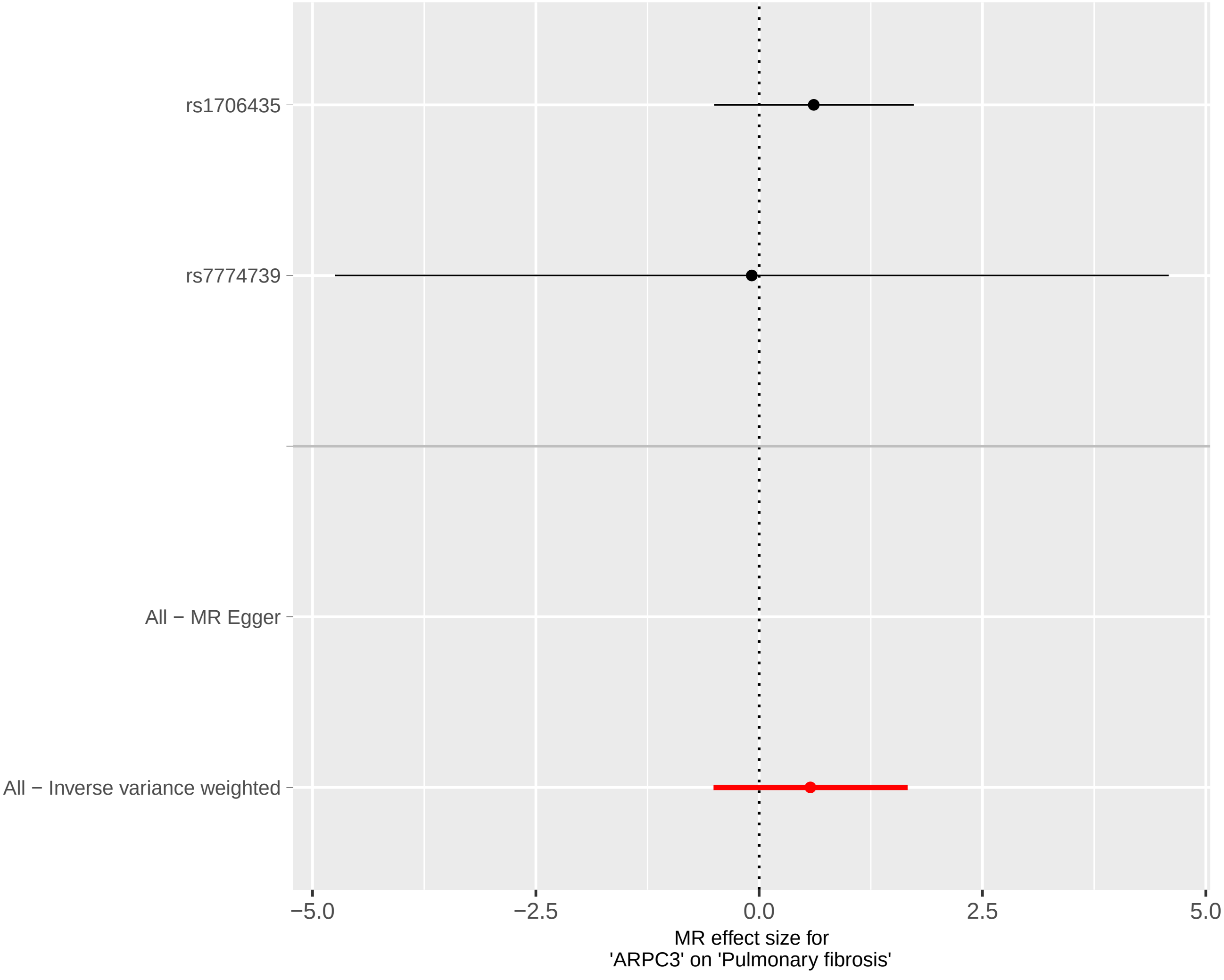

rs1706435
rs7774739
All − MR Egger
All − Inverse variance weighted
−5.0
−2.5
0.0
2.5
5.0
MR effect size for
'ARPC3' on 'Pulmonary fibrosis'

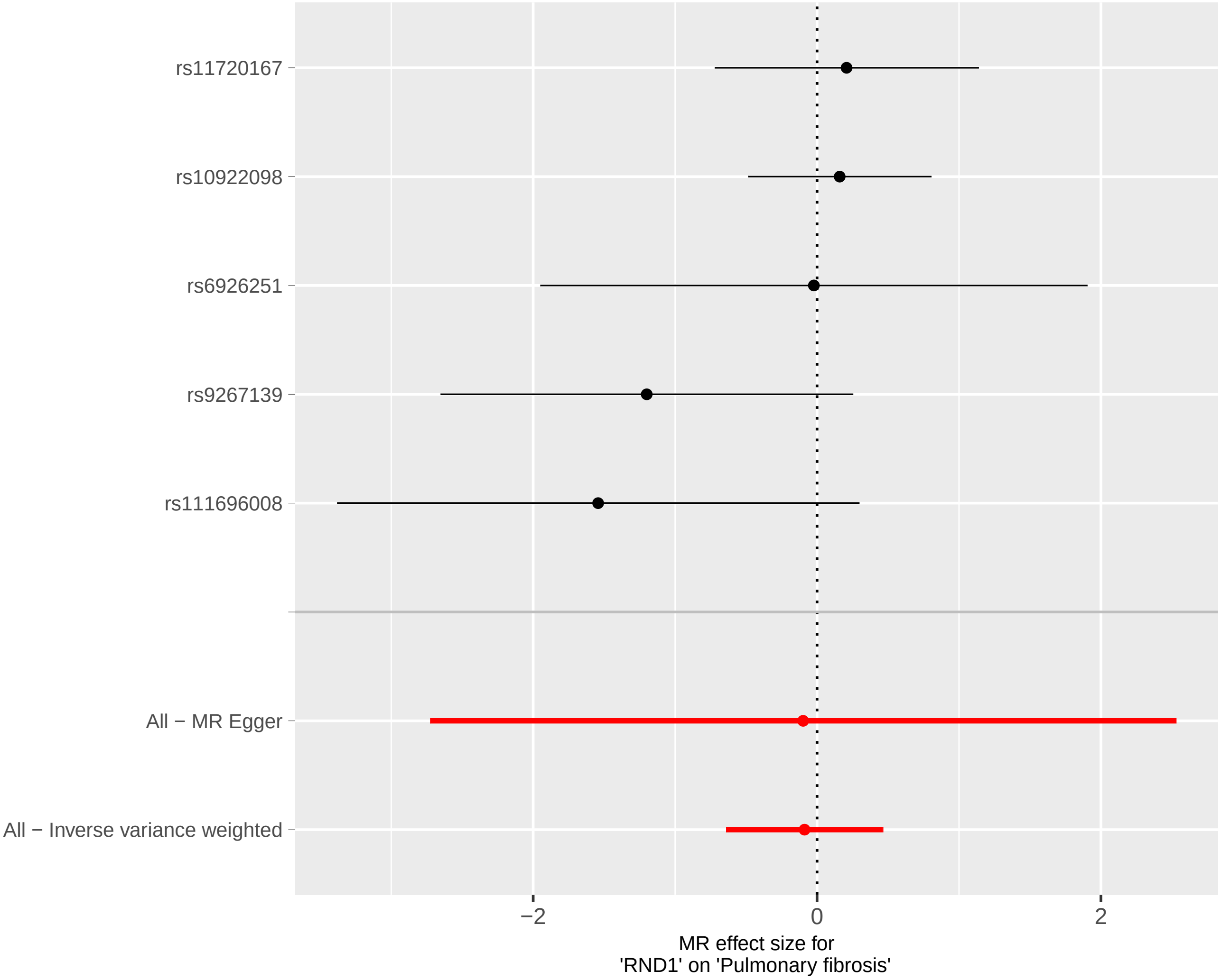
rs11720167
rs10922098
rs6926251
rs9267139
rs111696008
All − MR Egger
All − Inverse variance weighted
−2
0
2
MR effect size for
'RND1' on 'Pulmonary fibrosis'

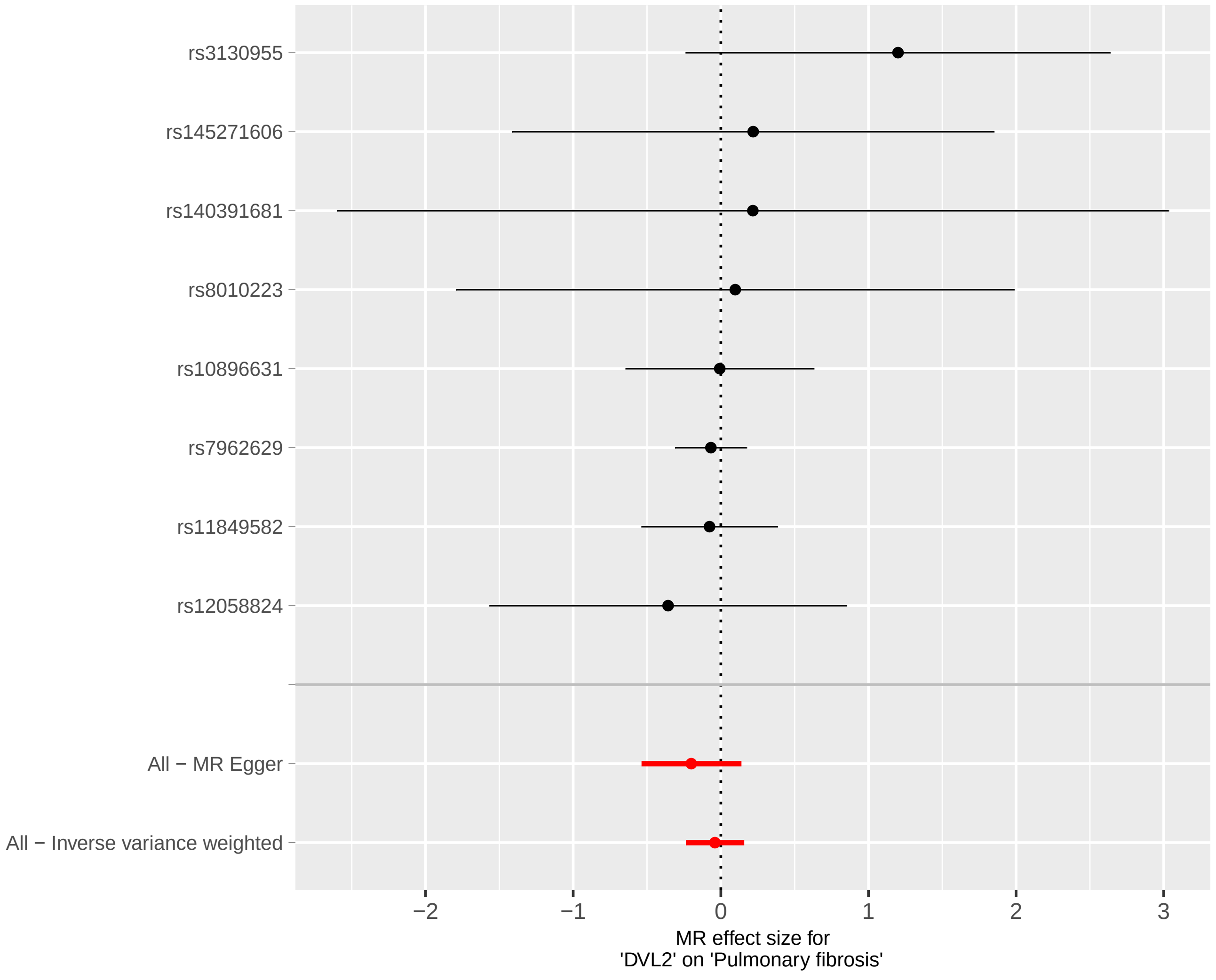
rs3130955
rs145271606
rs140391681
rs8010223
rs10896631
rs7962629
rs11849582
rs12058824
All − MR Egger
All − Inverse variance weighted
−2
−1
0
1
2
3
MR effect size for
'DVL2' on 'Pulmonary fibrosis'

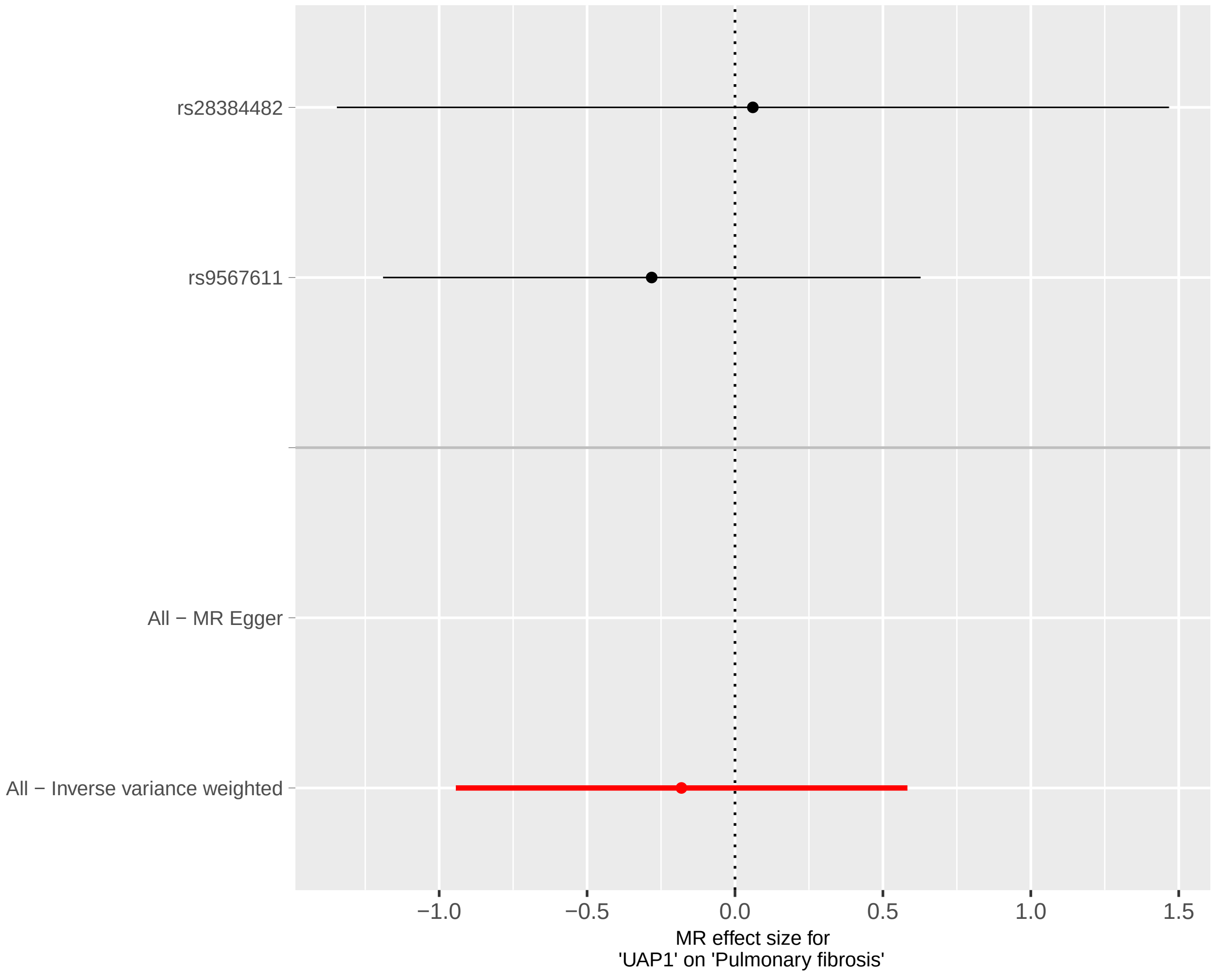
rs28384482
rs9567611
All − MR Egger
All − Inverse variance weighted
−1.0
−0.5
0.0
0.5
1.0
1.5
MR effect size for
'UAP1' on 'Pulmonary fibrosis'

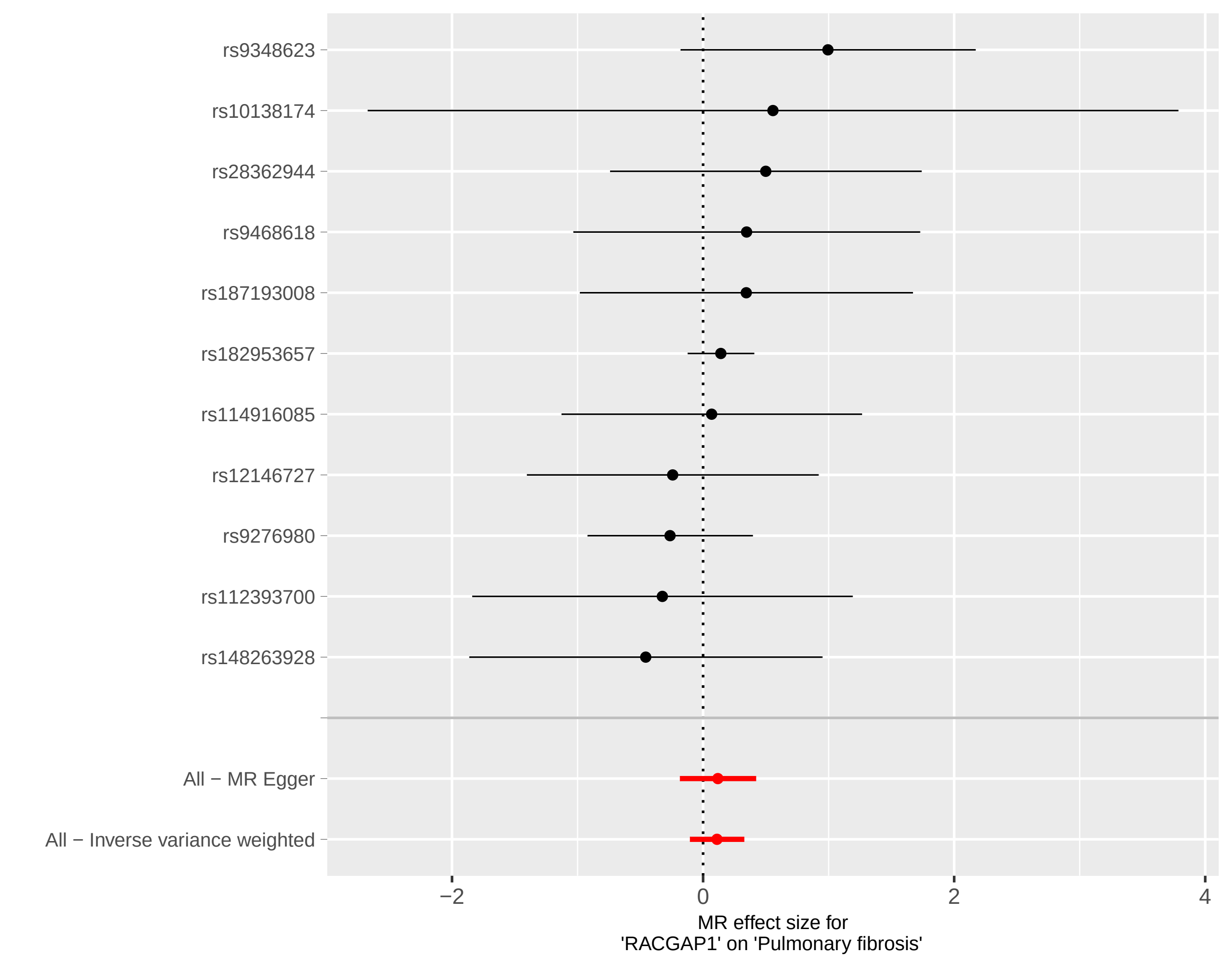
rs9348623
rs10138174
rs28362944
rs9468618
rs187193008
rs182953657
rs114916085
rs12146727
rs9276980
rs112393700
rs148263928
All − MR Egger
All − Inverse variance weighted
−2
0
2
4
MR effect size for
'RACGAP1' on 'Pulmonary fibrosis'

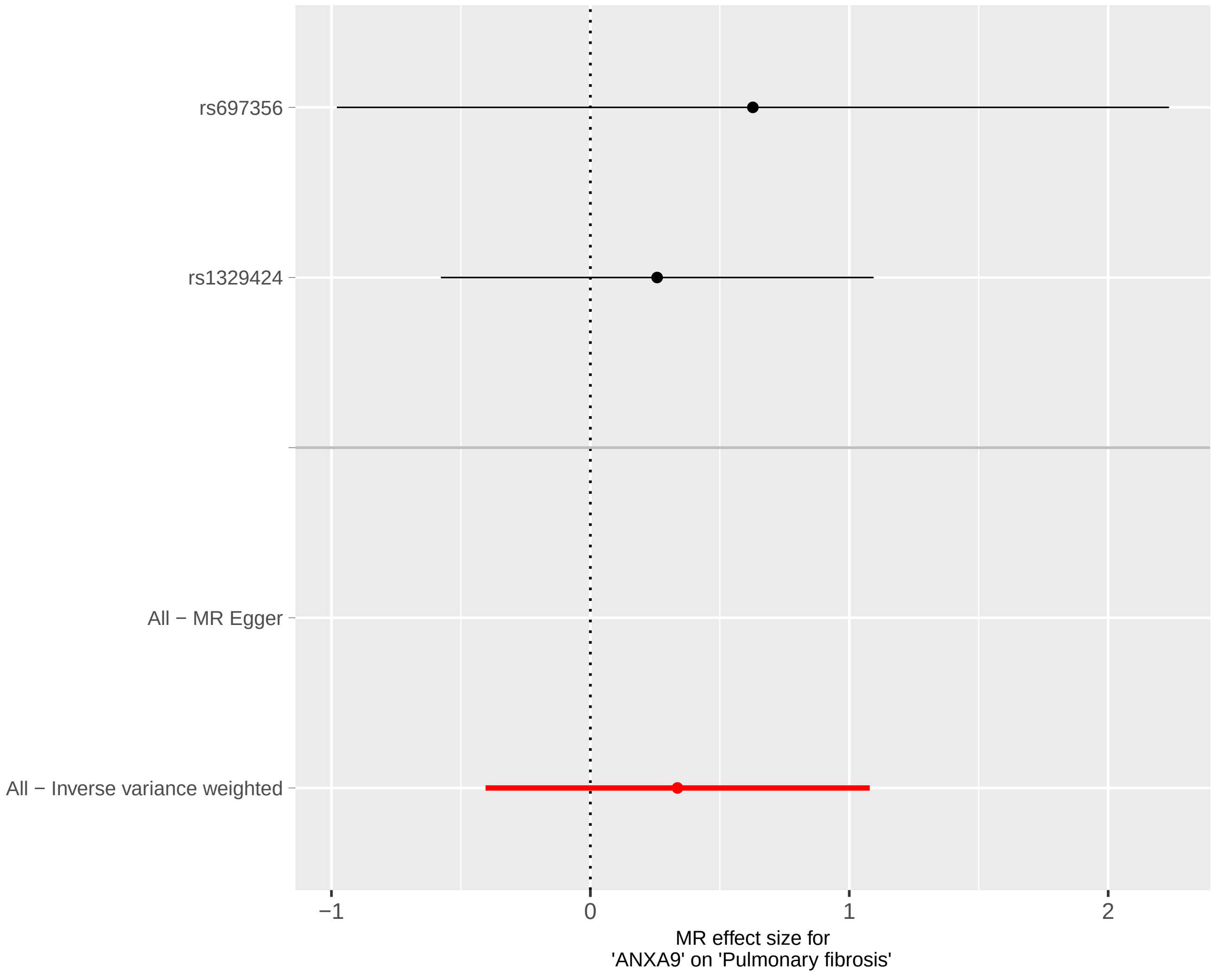
rs697356
rs1329424
All − MR Egger
All − Inverse variance weighted
−1
0
1
2
MR effect size for
'ANXA9' on 'Pulmonary fibrosis'

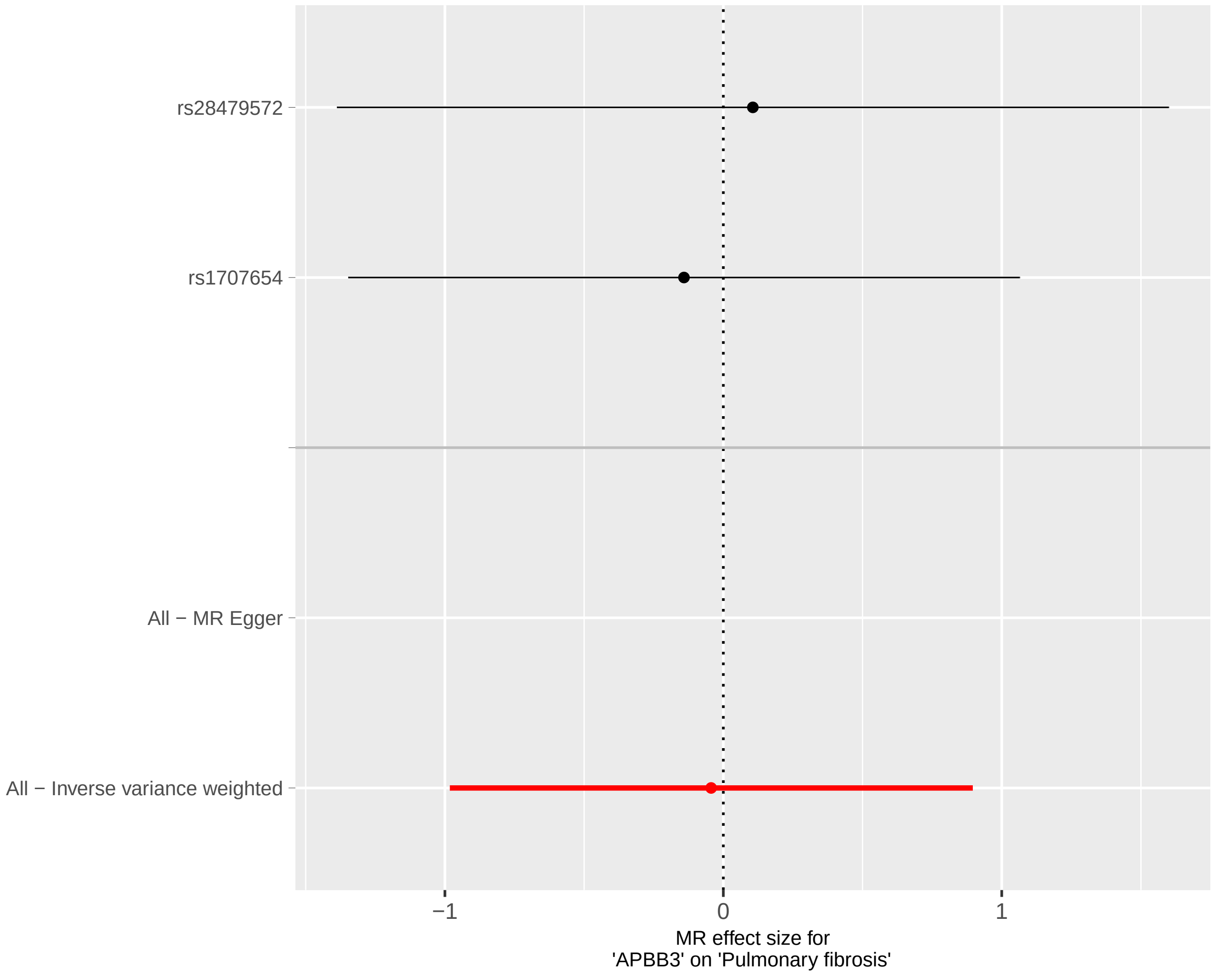
rs28479572
rs1707654
All − MR Egger
All − Inverse variance weighted
−1
0
1
MR effect size for
'APBB3' on 'Pulmonary fibrosis'

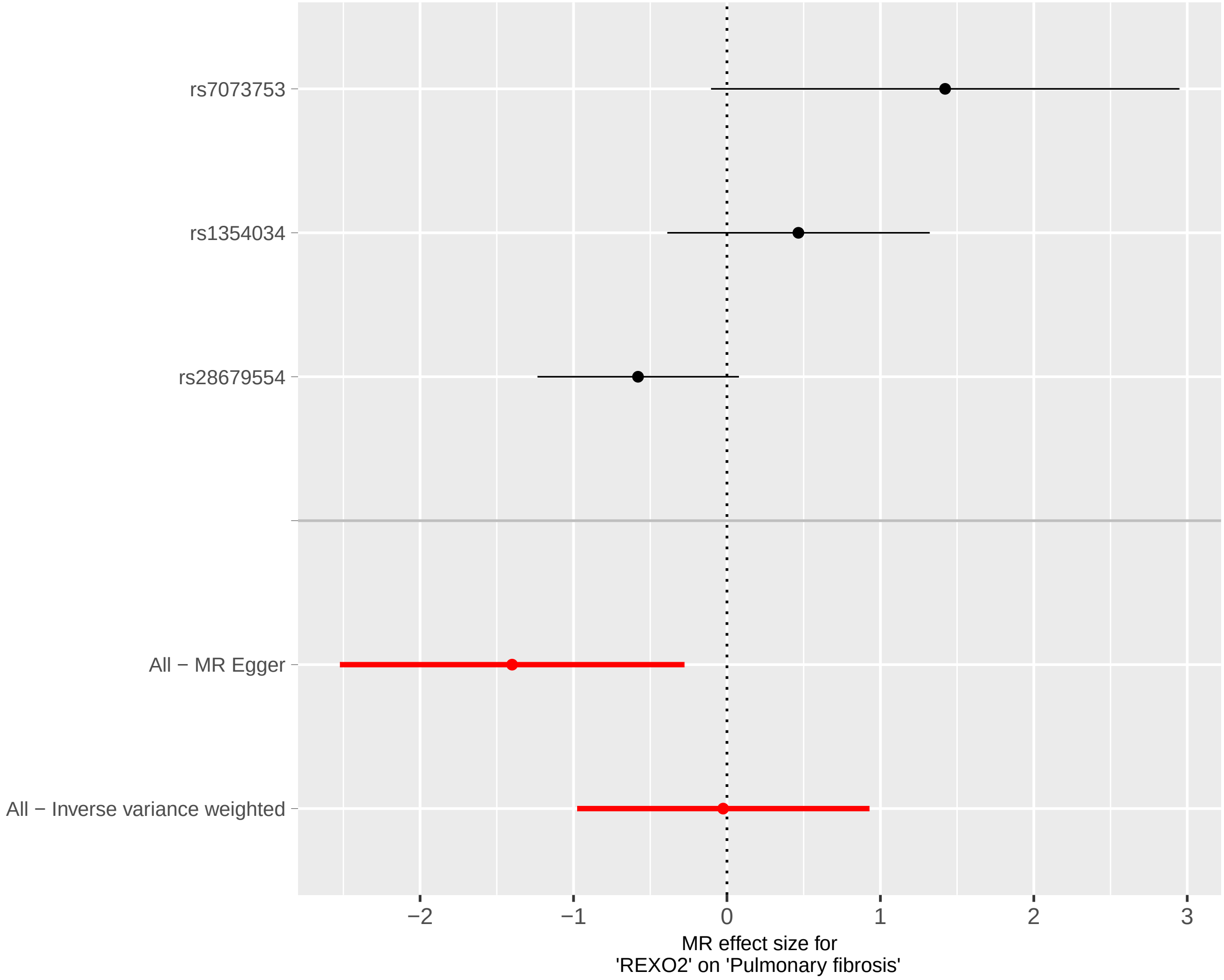

rs7073753
rs1354034
rs28679554
All − MR Egger
All − Inverse variance weighted
−2
−1
0
1
2
3
MR effect size for
'REXO2' on 'Pulmonary fibrosis'

Insufficient number of SNPs

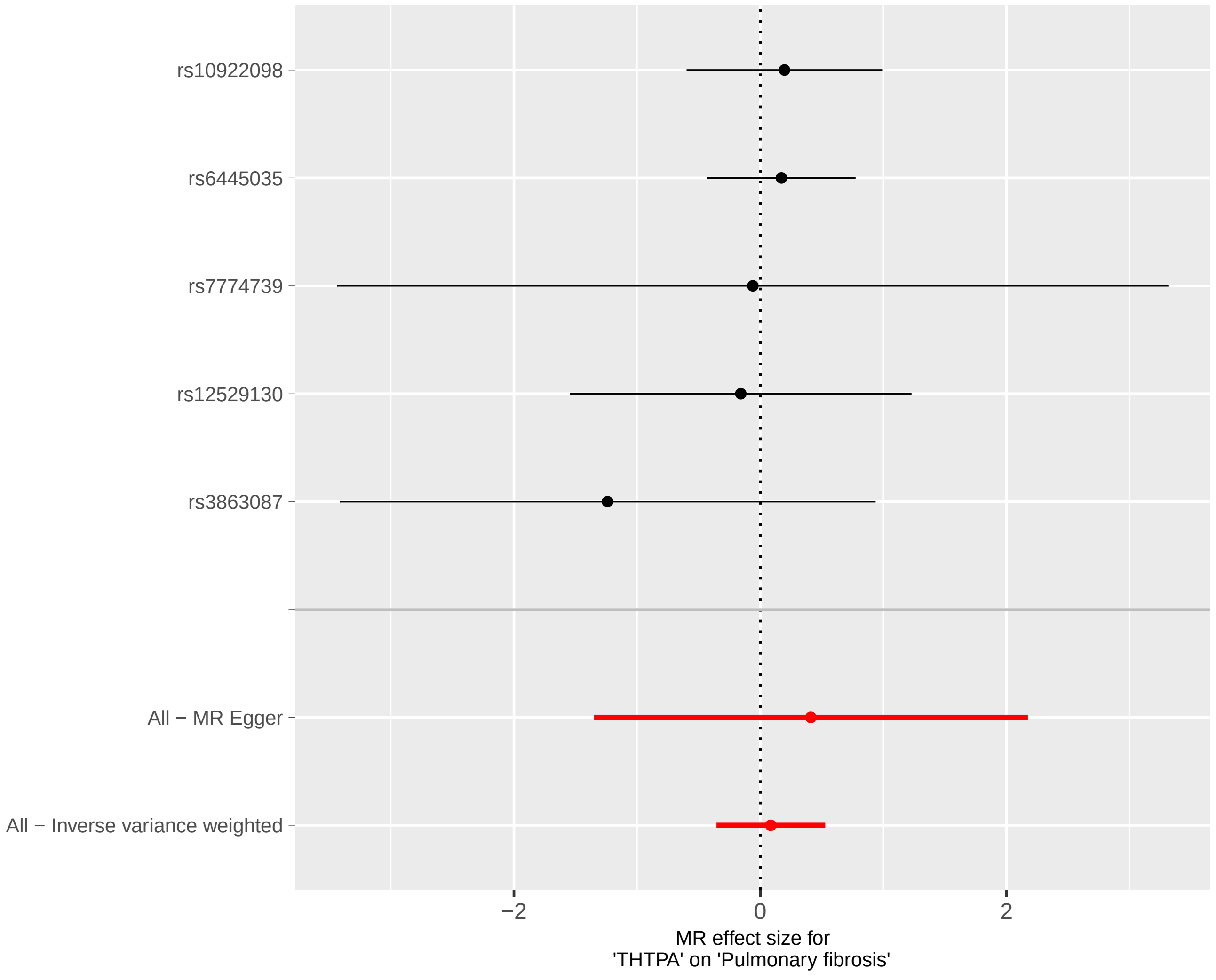
rs10922098
rs6445035
rs7774739
rs12529130
rs3863087
All − MR Egger
All − Inverse variance weighted
−2
0
2
MR effect size for
'THTPA' on 'Pulmonary fibrosis'

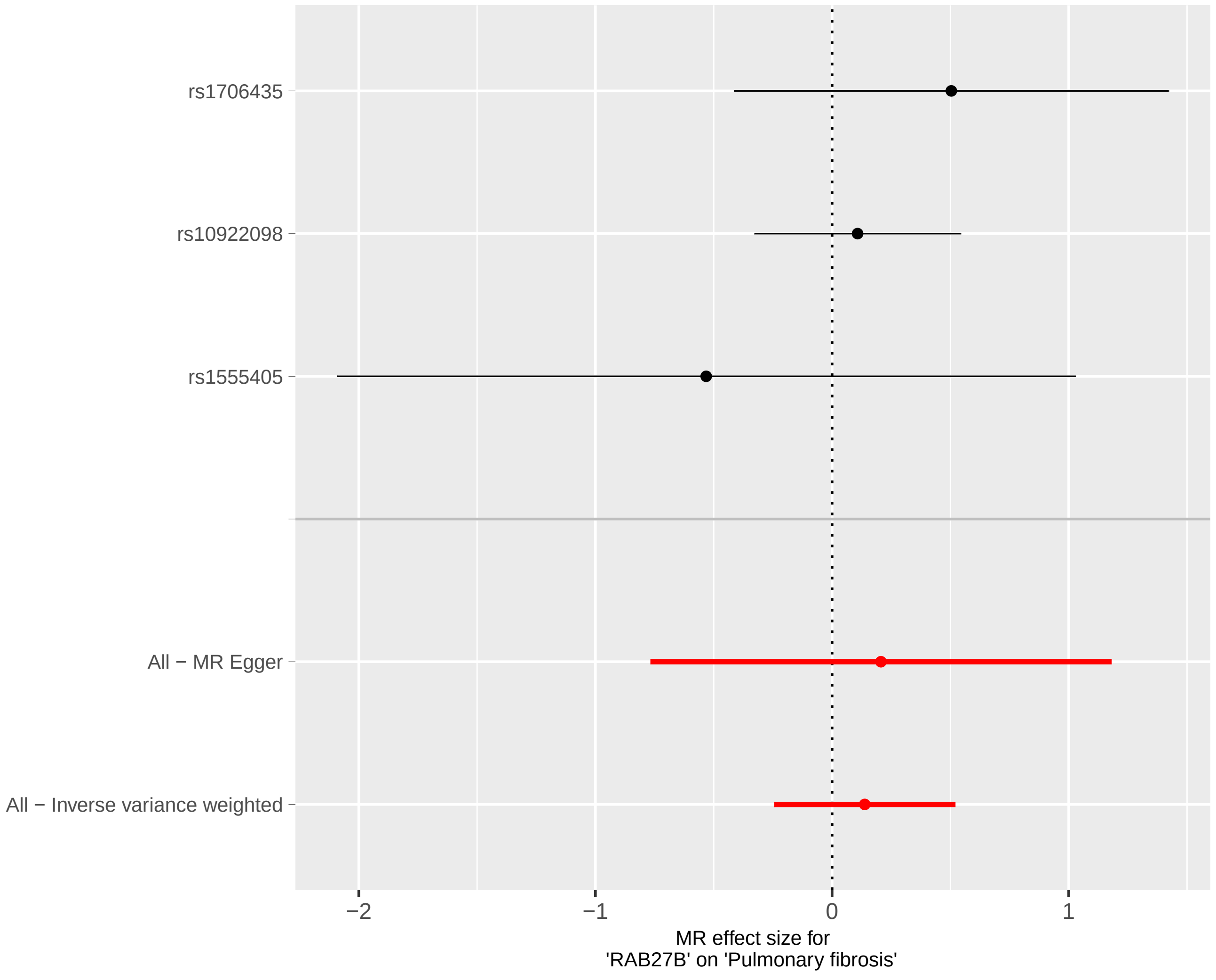

rs1706435
rs10922098
rs1555405
All − MR Egger
All − Inverse variance weighted
−2
−1
0
1
MR effect size for
'RAB27B' on 'Pulmonary fibrosis'

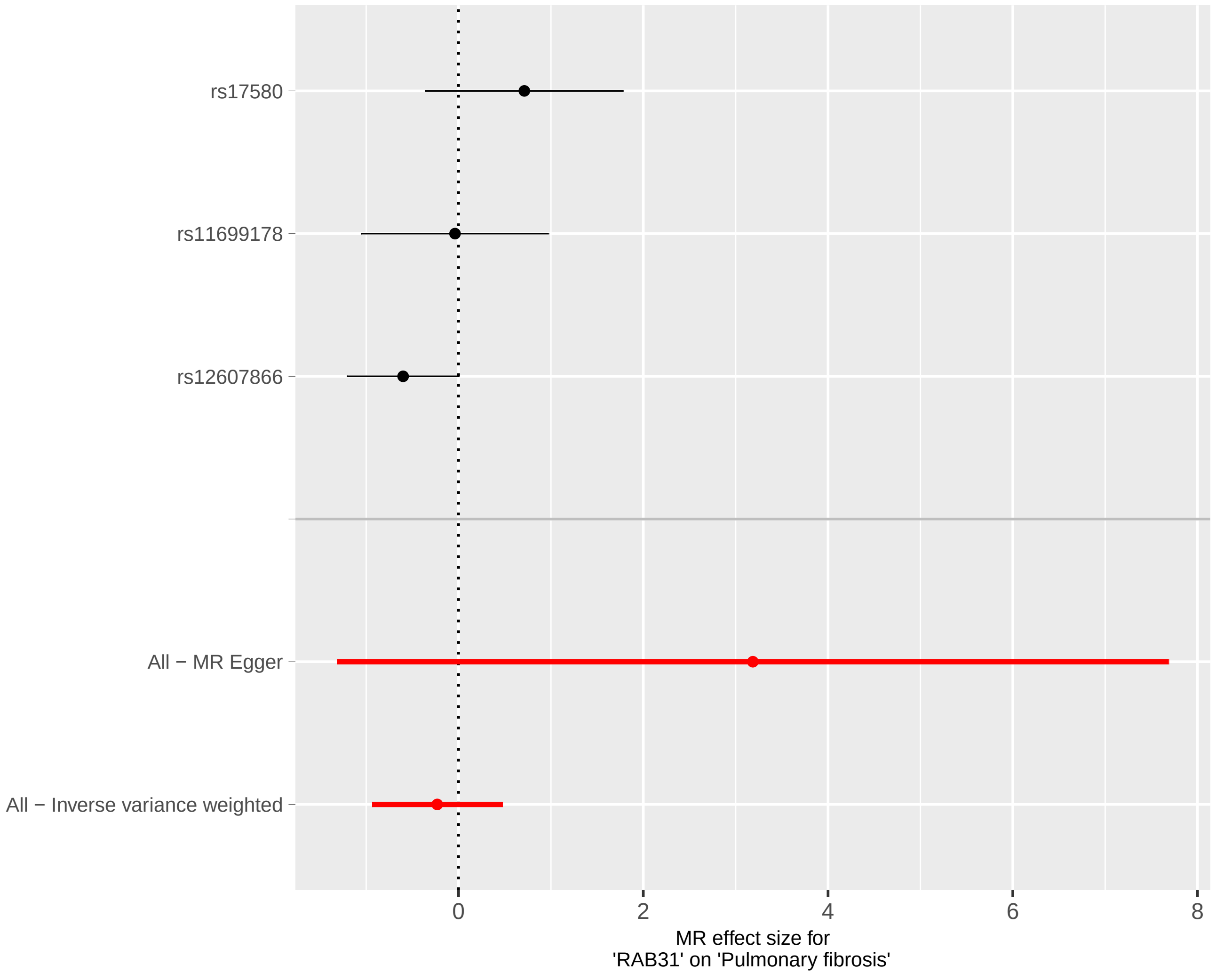
rs17580
rs11699178
rs12607866
All − MR Egger
All − Inverse variance weighted
0
2
4
6
8
MR effect size for
'RAB31' on 'Pulmonary fibrosis'

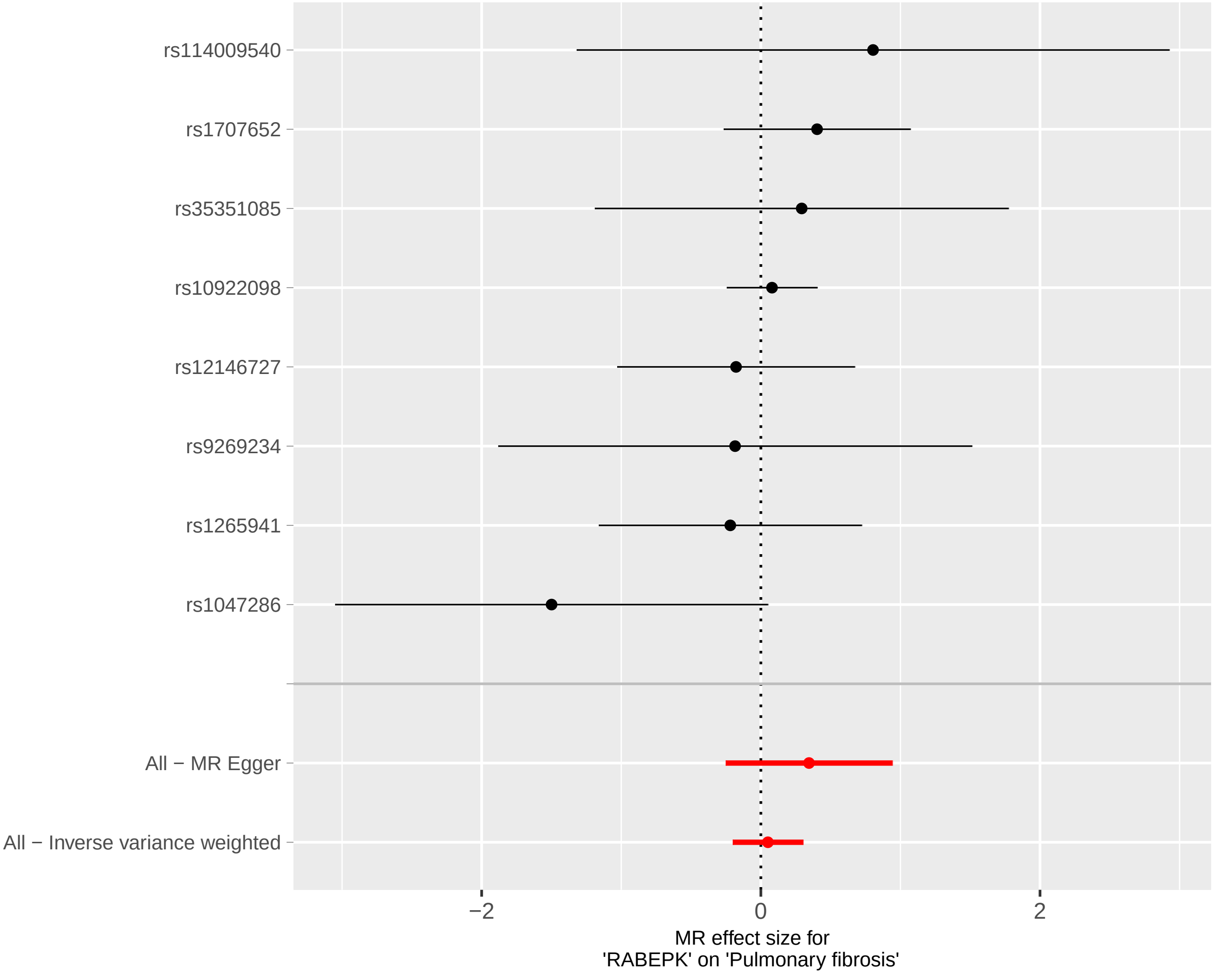

rs114009540
rs1707652
rs35351085
rs10922098
rs12146727
rs9269234
rs1265941
rs1047286
All − MR Egger
All − Inverse variance weighted
−2
0
2
MR effect size for
'RABEPK' on 'Pulmonary fibrosis'

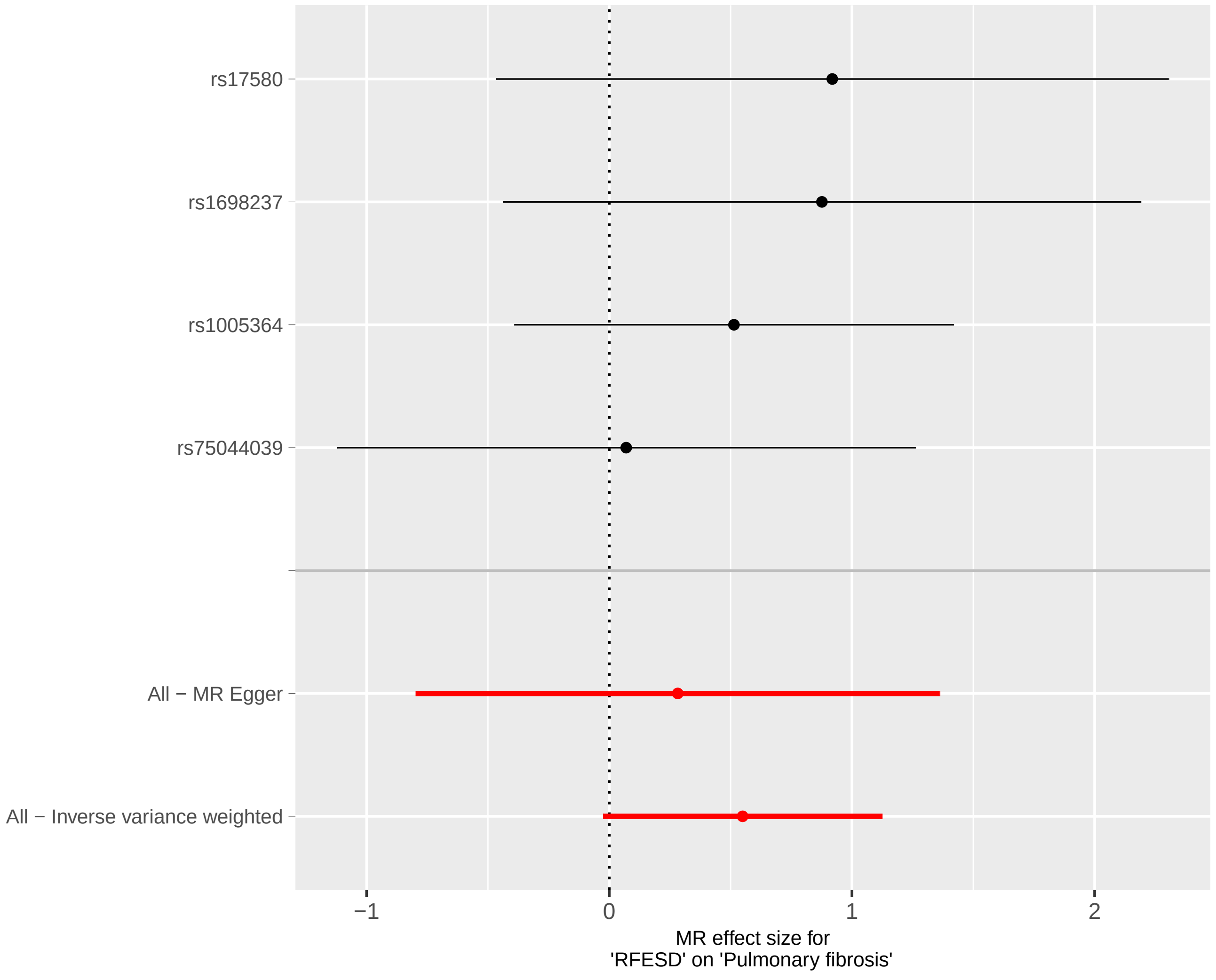
rs17580
rs1698237
rs1005364
rs75044039
All − MR Egger
All − Inverse variance weighted
−1
0
1
2
MR effect size for
'RFESD' on 'Pulmonary fibrosis'

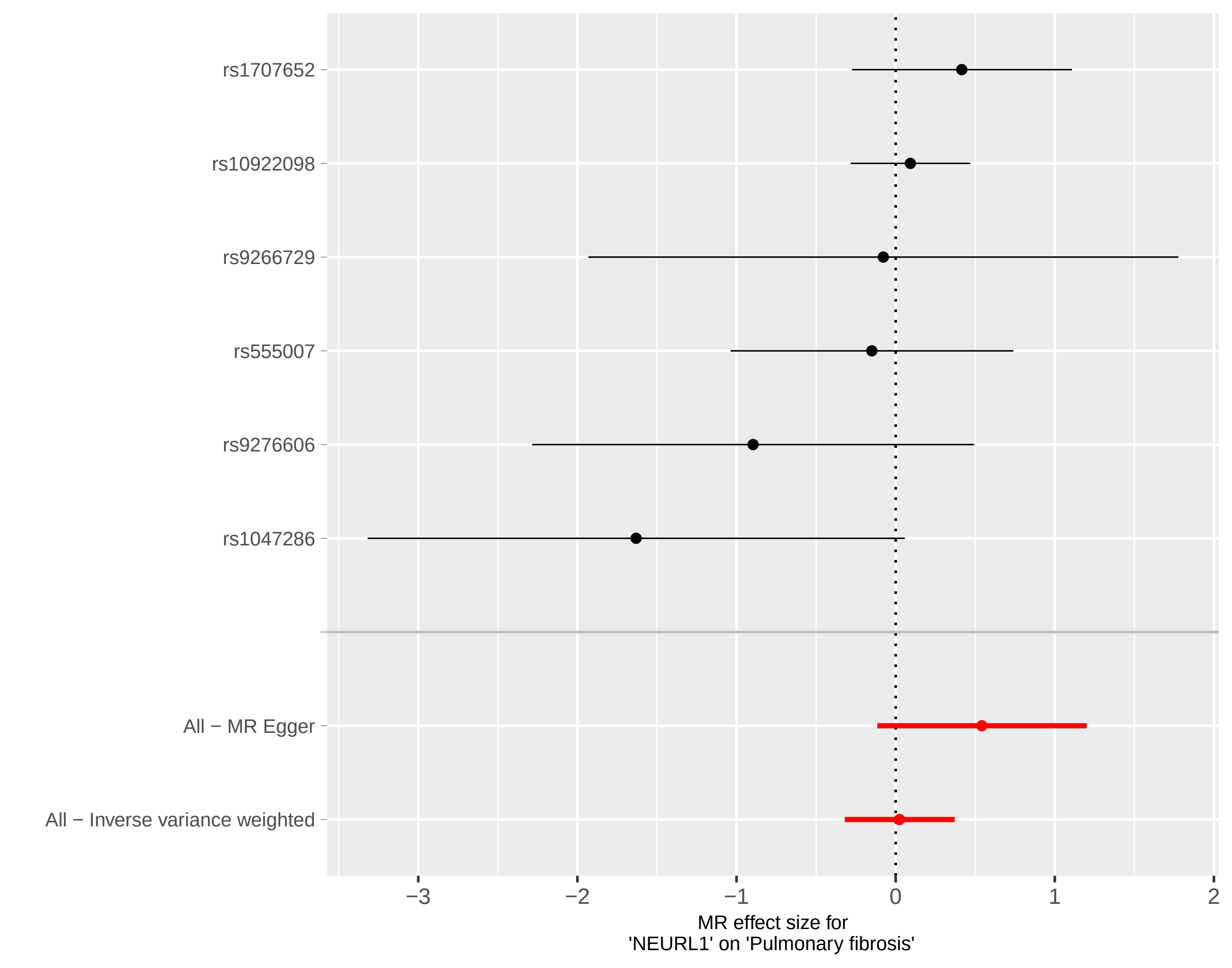

rs1707652
rs10922098
rs9266729
rs555007
rs9276606
rs1047286
All − MR Egger
All − Inverse variance weighted
−3
−2
−1
0
1
2
MR effect size for
'NEURL1' on 'Pulmonary fibrosis'

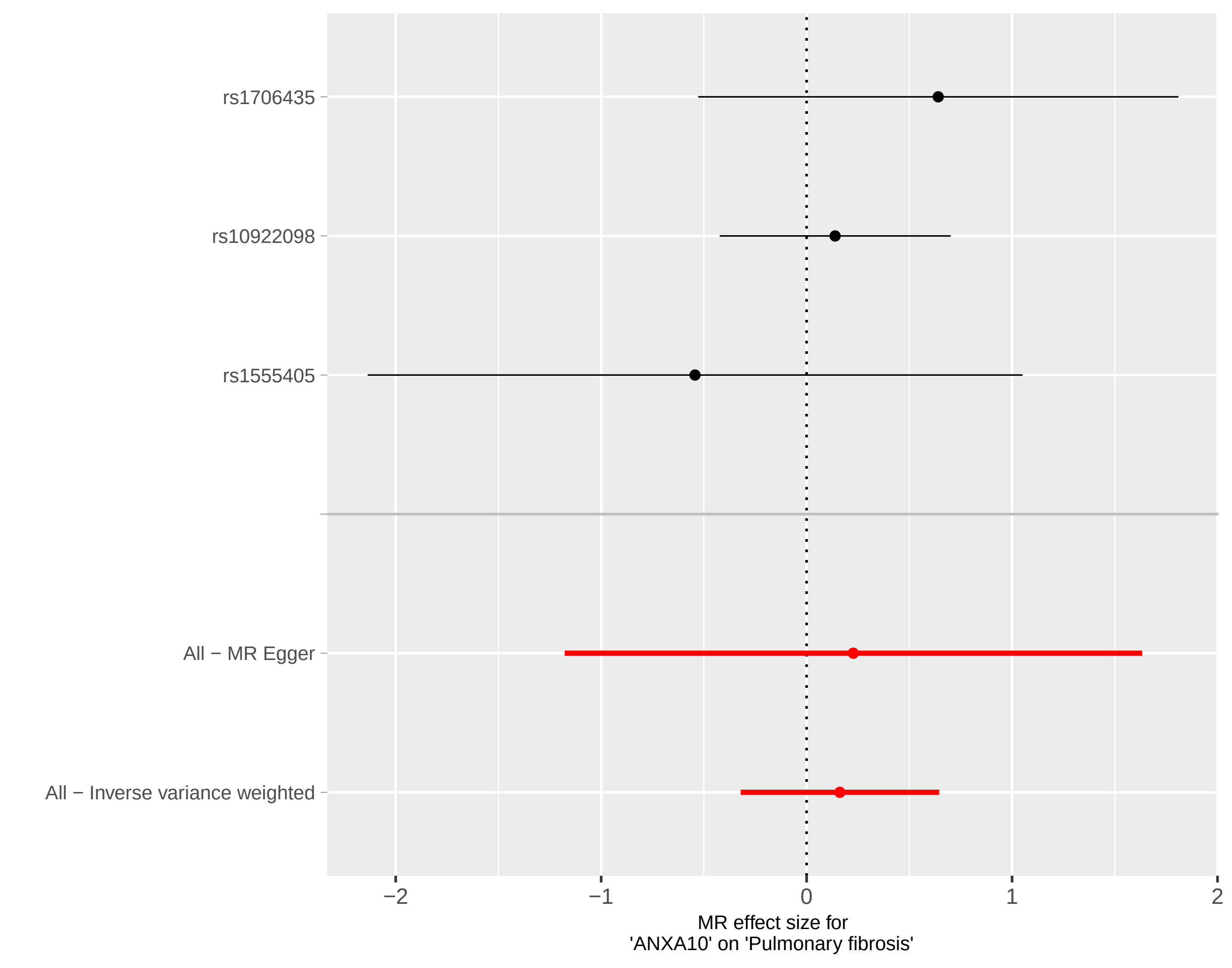

rs1706435
rs10922098
rs1555405
All − MR Egger
All − Inverse variance weighted
−2
−1
0
1
2
MR effect size for
'ANXA10' on 'Pulmonary fibrosis'

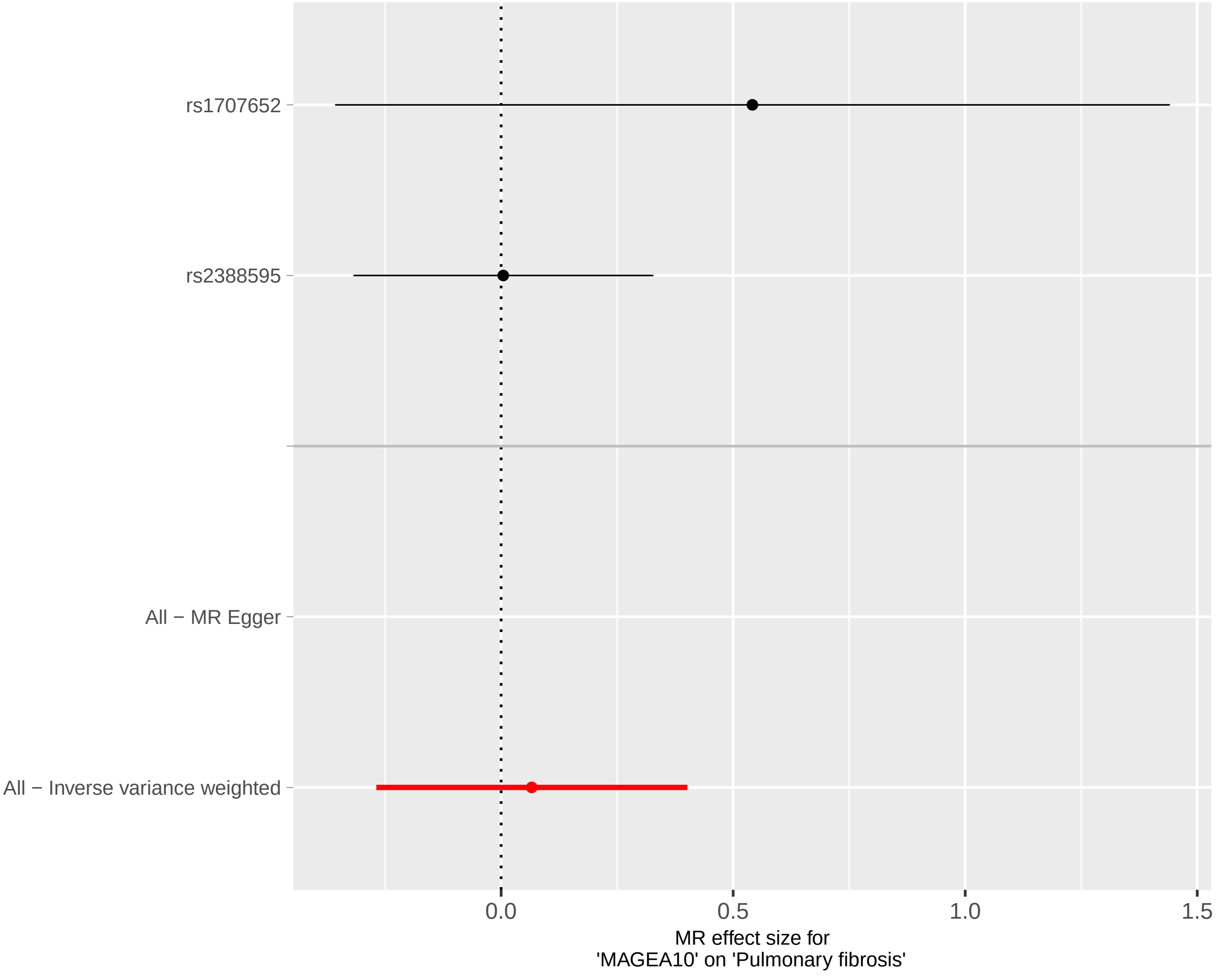
rs1707652
rs2388595
All − MR Egger
All − Inverse variance weighted
0.0
0.5
1.0
1.5
MR effect size for
'MAGEA10' on 'Pulmonary fibrosis'

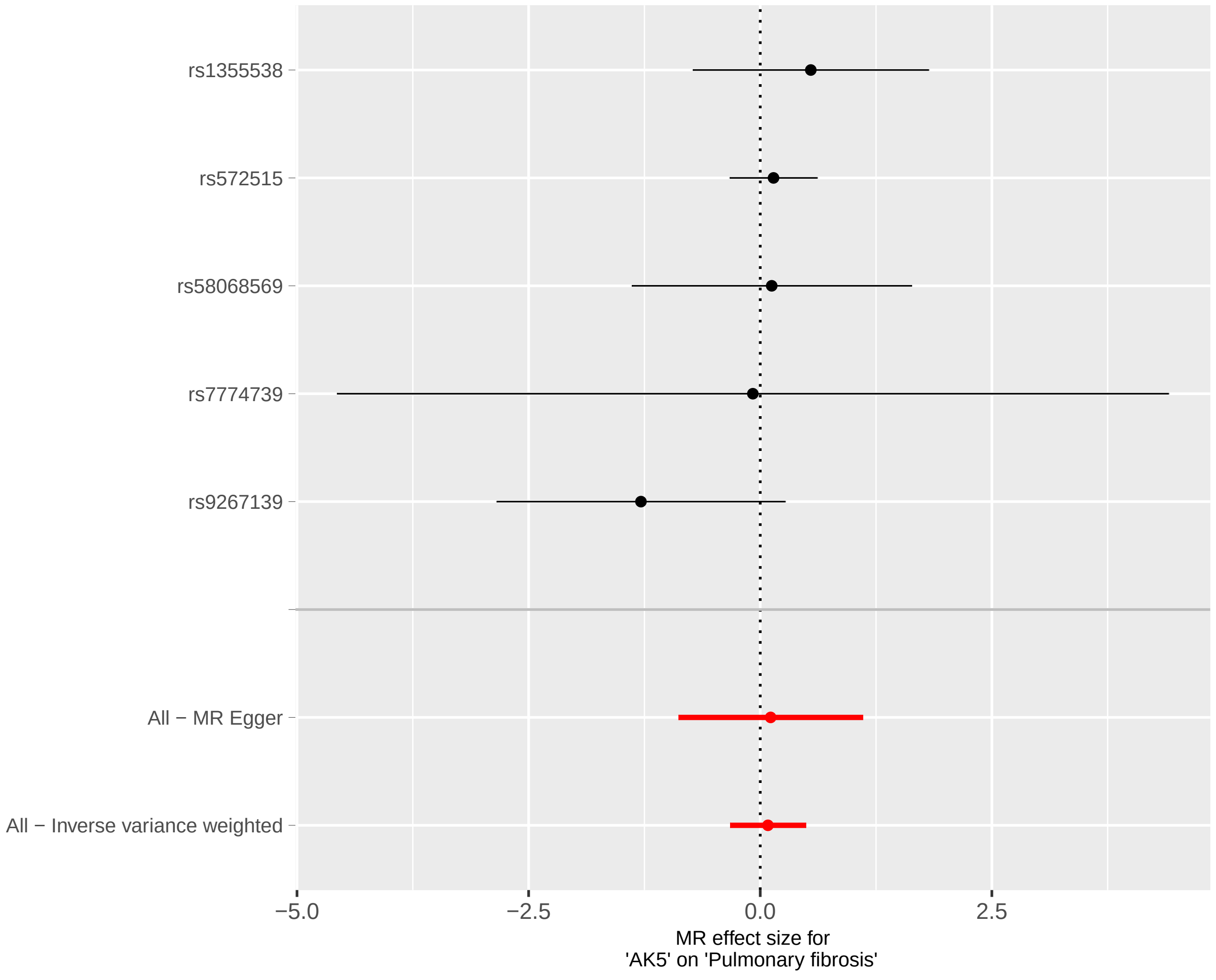
rs1355538
rs572515
rs58068569
rs7774739
rs9267139
All − MR Egger
All − Inverse variance weighted
−5.0
−2.5
0.0
2.5
MR effect size for
'AK5' on 'Pulmonary fibrosis'

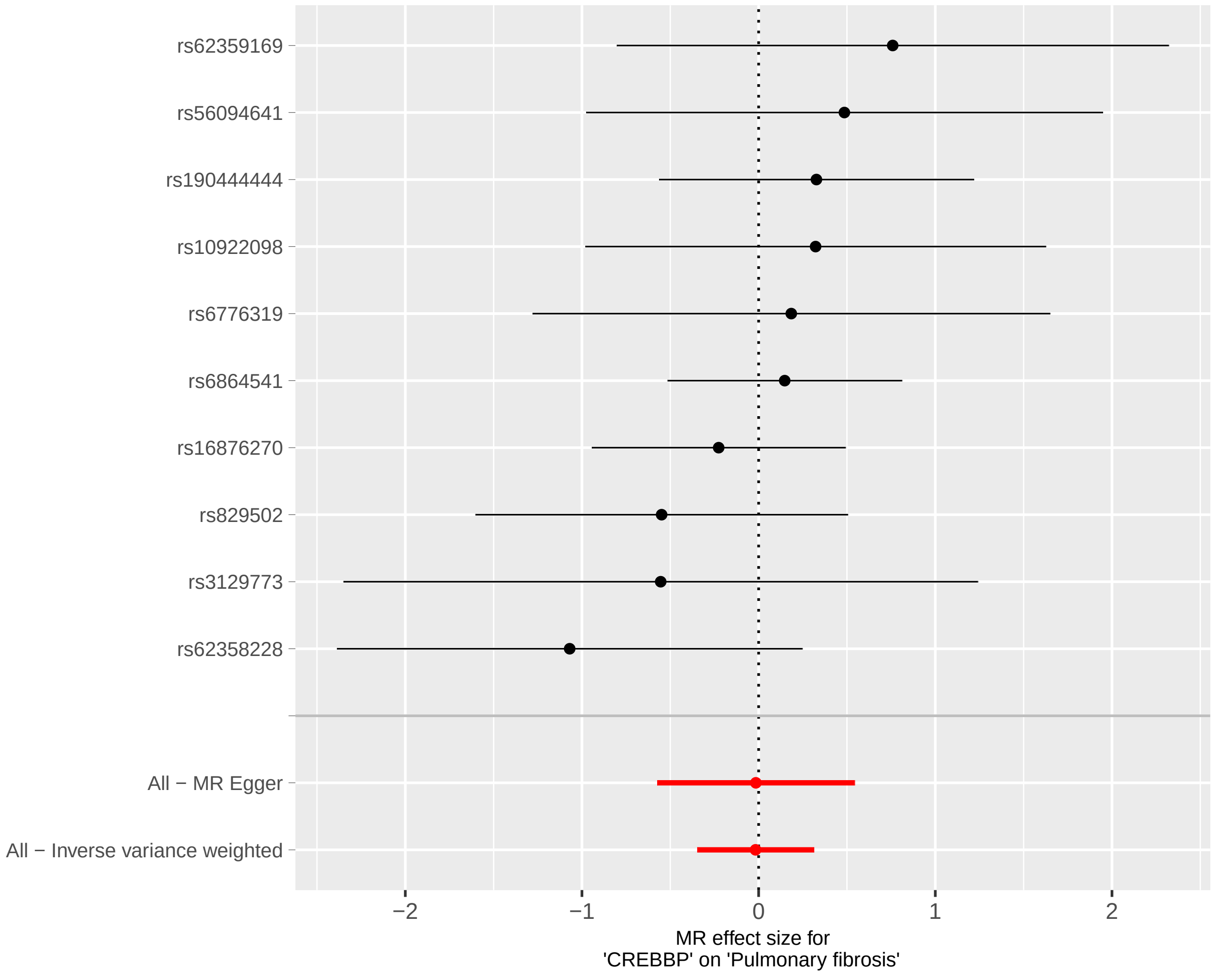

rs62359169
rs56094641
rs190444444
rs10922098
rs6776319
rs6864541
rs16876270
rs829502
rs3129773
rs62358228
All − MR Egger
All − Inverse variance weighted
−2
−1
0
1
2
MR effect size for
'CREBBP' on 'Pulmonary fibrosis'

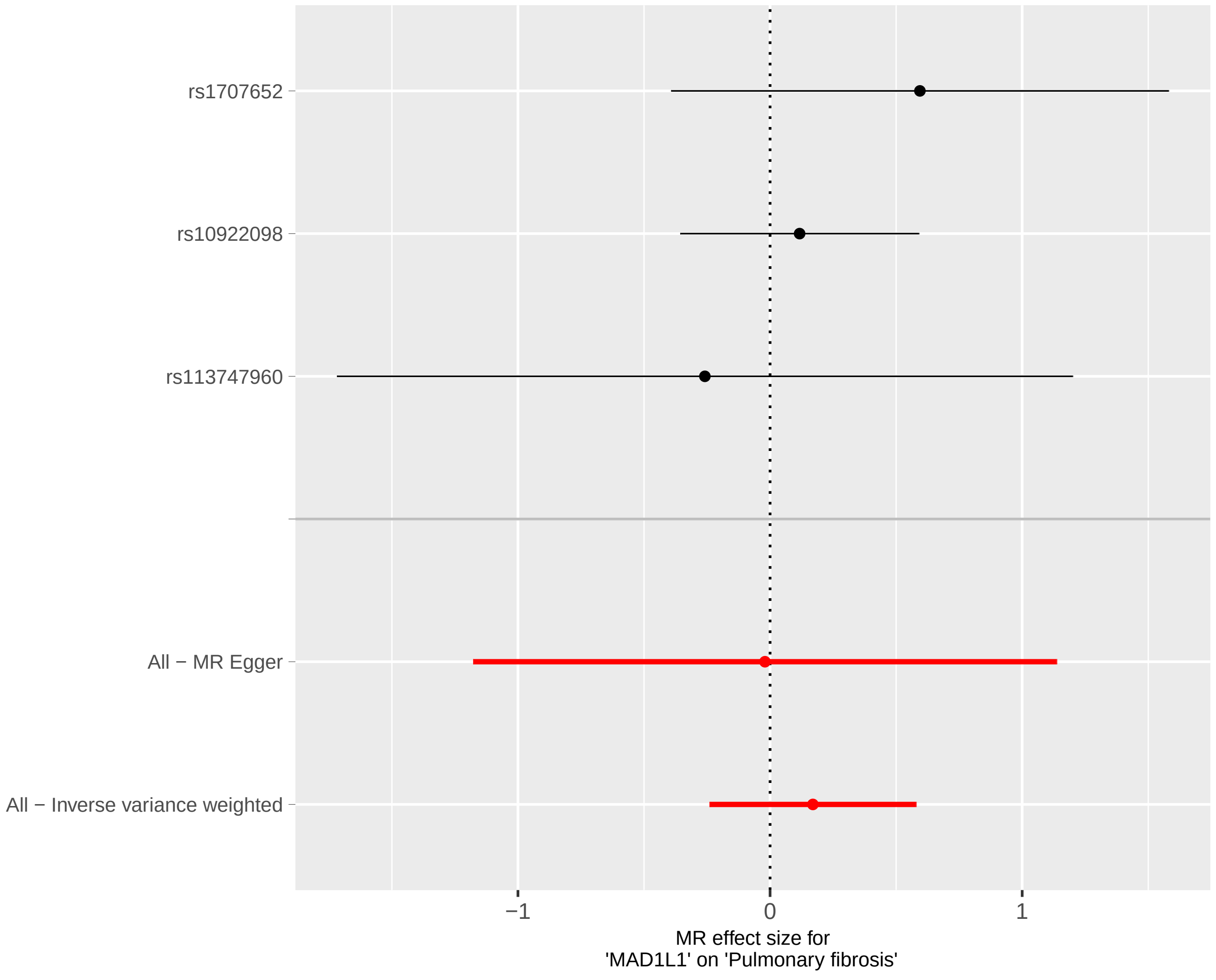
rs1707652
rs10922098
rs113747960
All − MR Egger
All − Inverse variance weighted
−1
0
1
MR effect size for
'MAD1L1' on 'Pulmonary fibrosis'

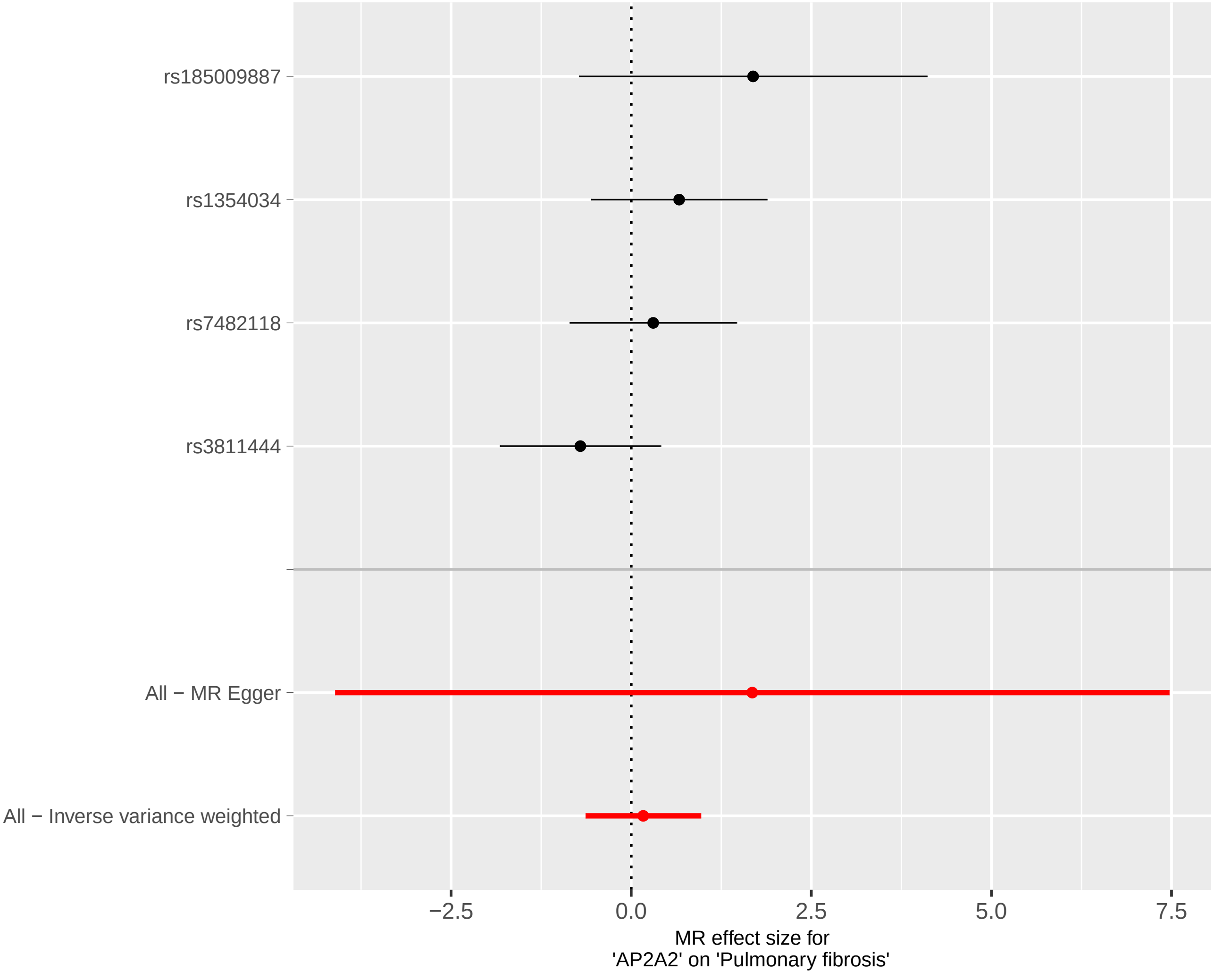

rs185009887
rs1354034
rs7482118
rs3811444
All − MR Egger
All − Inverse variance weighted
−2.5
0.0
2.5
5.0
7.5
MR effect size for
'AP2A2' on 'Pulmonary fibrosis'

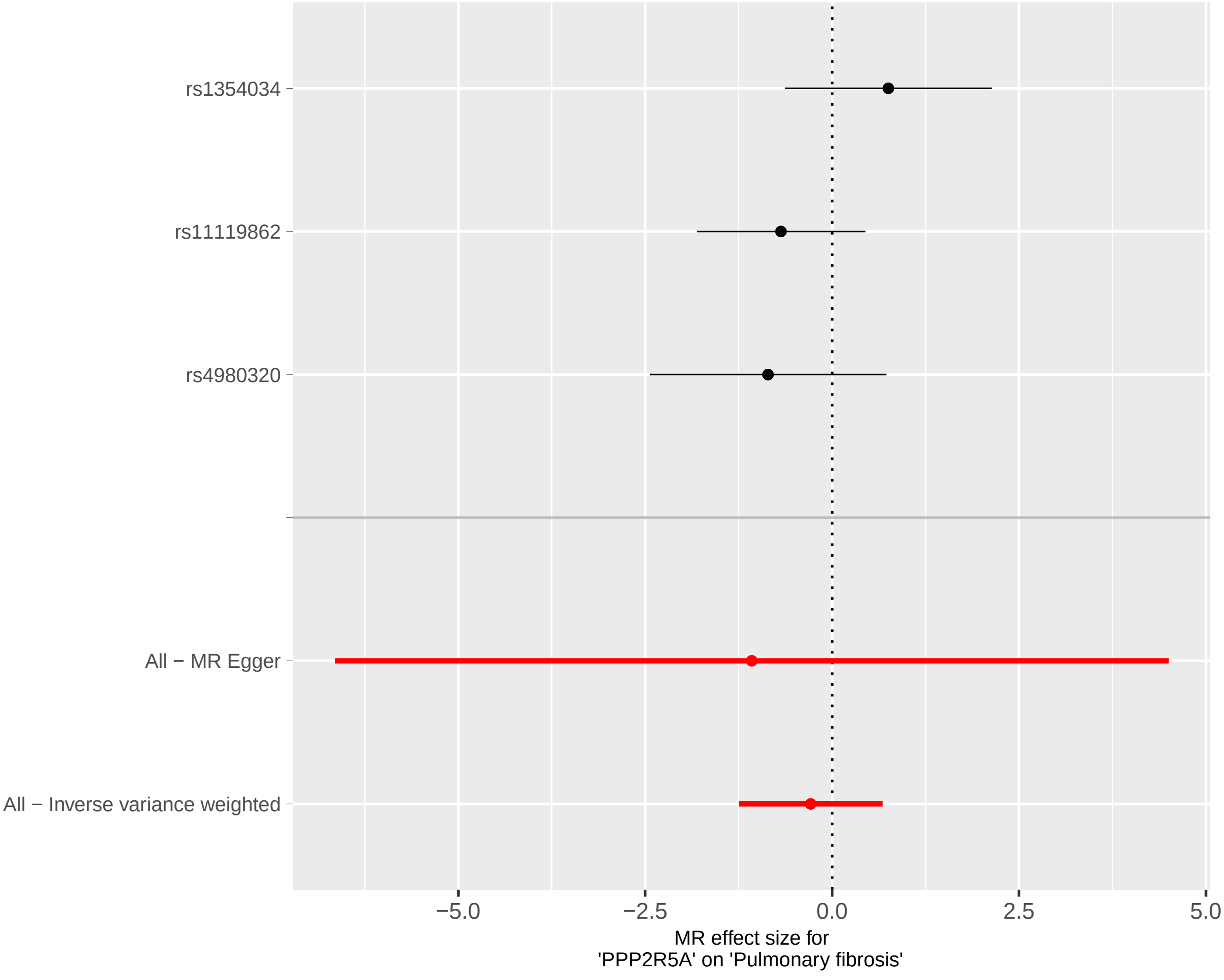
rs1354034
rs11119862
rs4980320
All − MR Egger
All − Inverse variance weighted
−5.0
−2.5
0.0
2.5
5.0
MR effect size for
'PPP2R5A' on 'Pulmonary fibrosis'

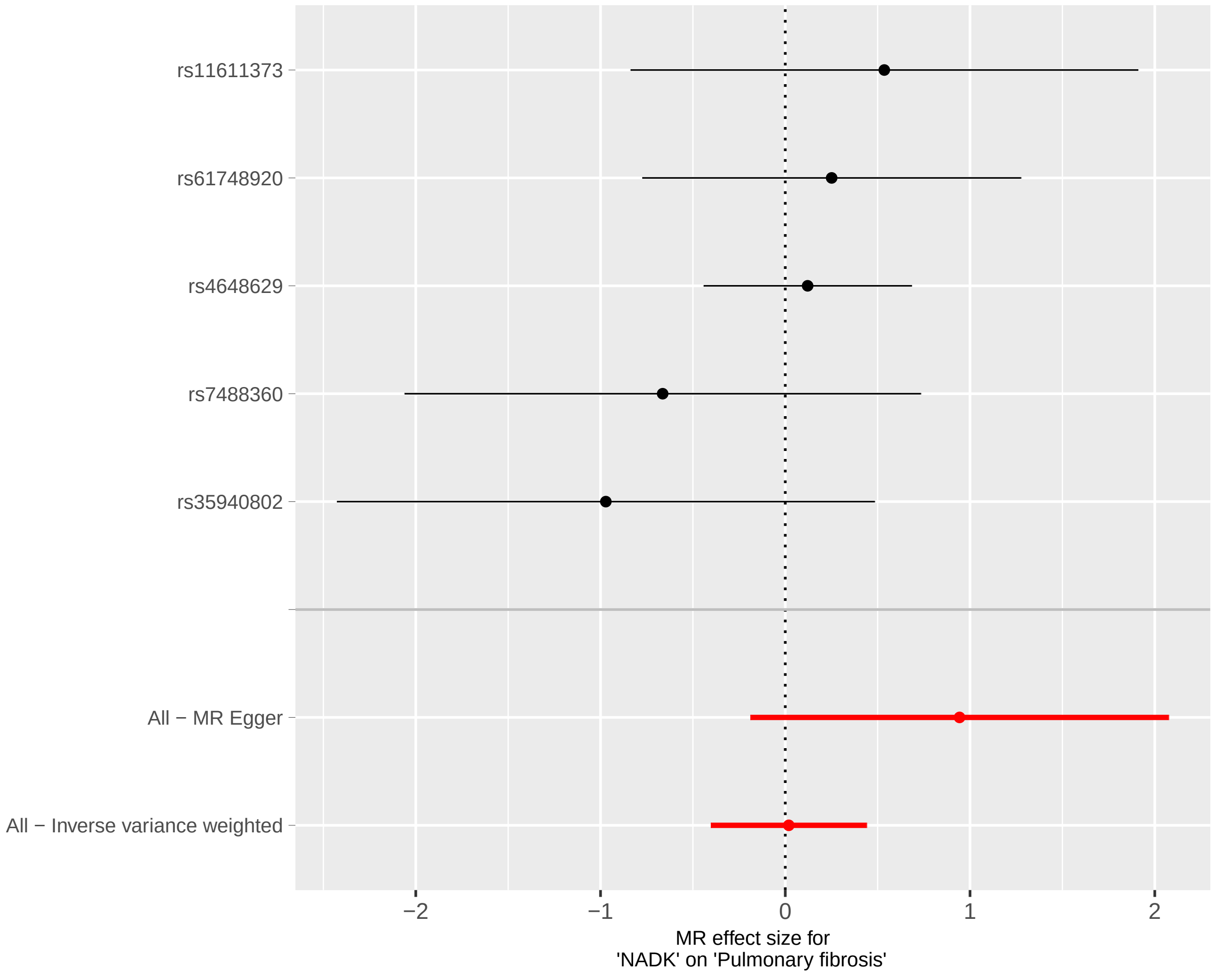
rs11611373
rs61748920
rs4648629
rs7488360
rs35940802
All − MR Egger
All − Inverse variance weighted
−2
−1
0
1
2
MR effect size for
'NADK' on 'Pulmonary fibrosis'

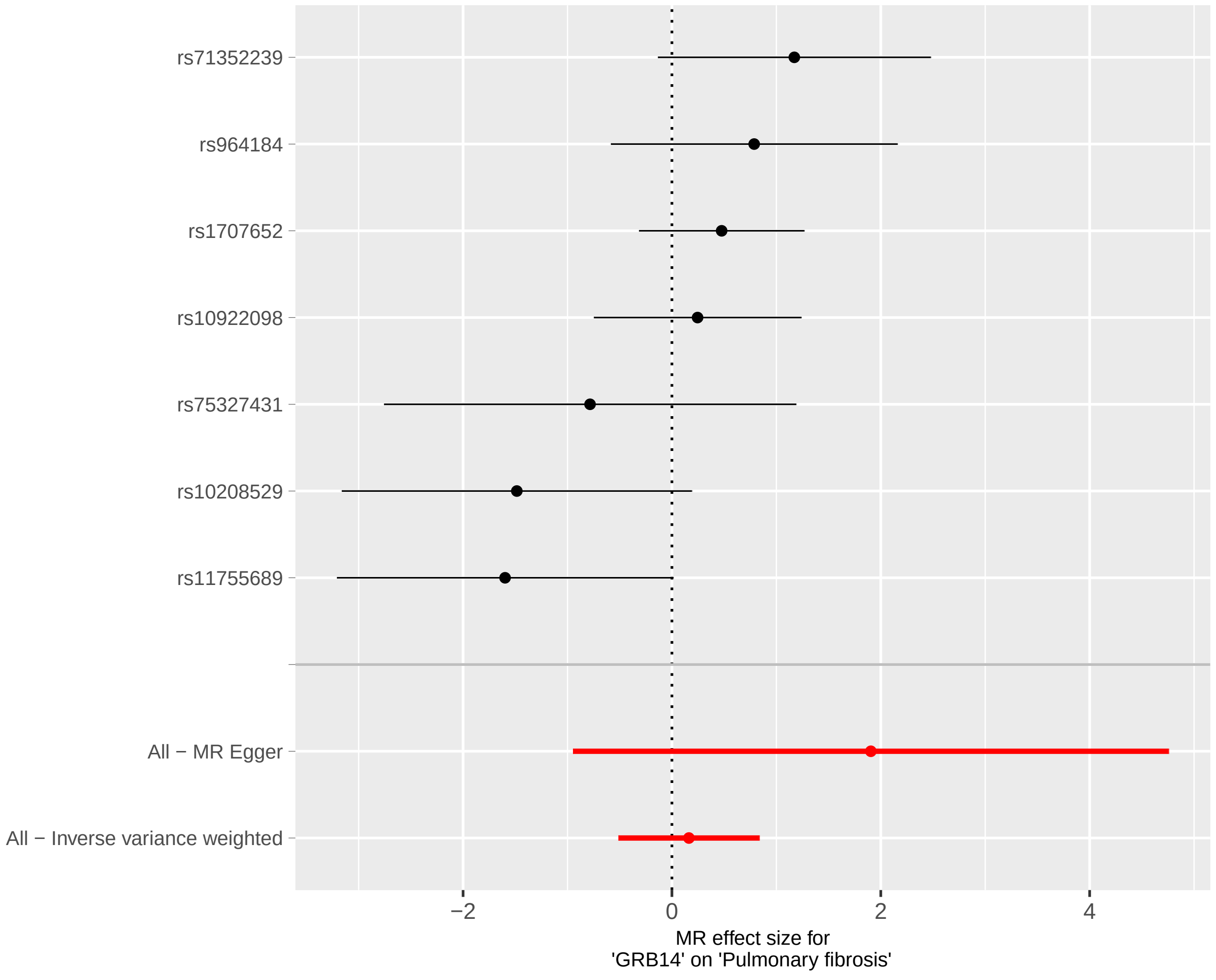
rs71352239
rs964184
rs1707652
rs10922098
rs75327431
rs10208529
rs11755689
All − MR Egger
All − Inverse variance weighted
−2
0
2
4
MR effect size for
'GRB14' on 'Pulmonary fibrosis'

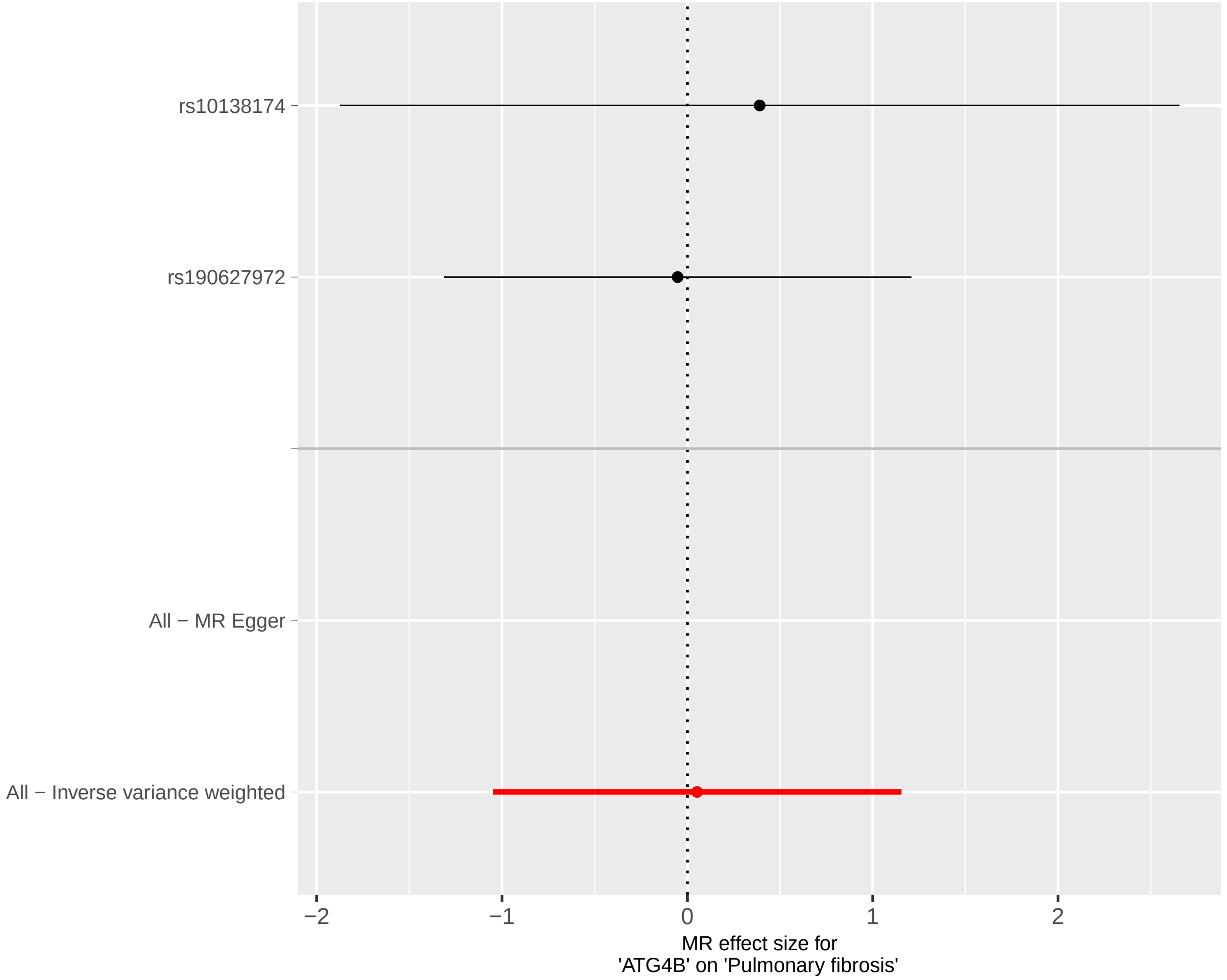

rs10138174
rs190627972
All − MR Egger
All − Inverse variance weighted
−2
−1
0
1
2
MR effect size for
'ATG4B' on 'Pulmonary fibrosis'

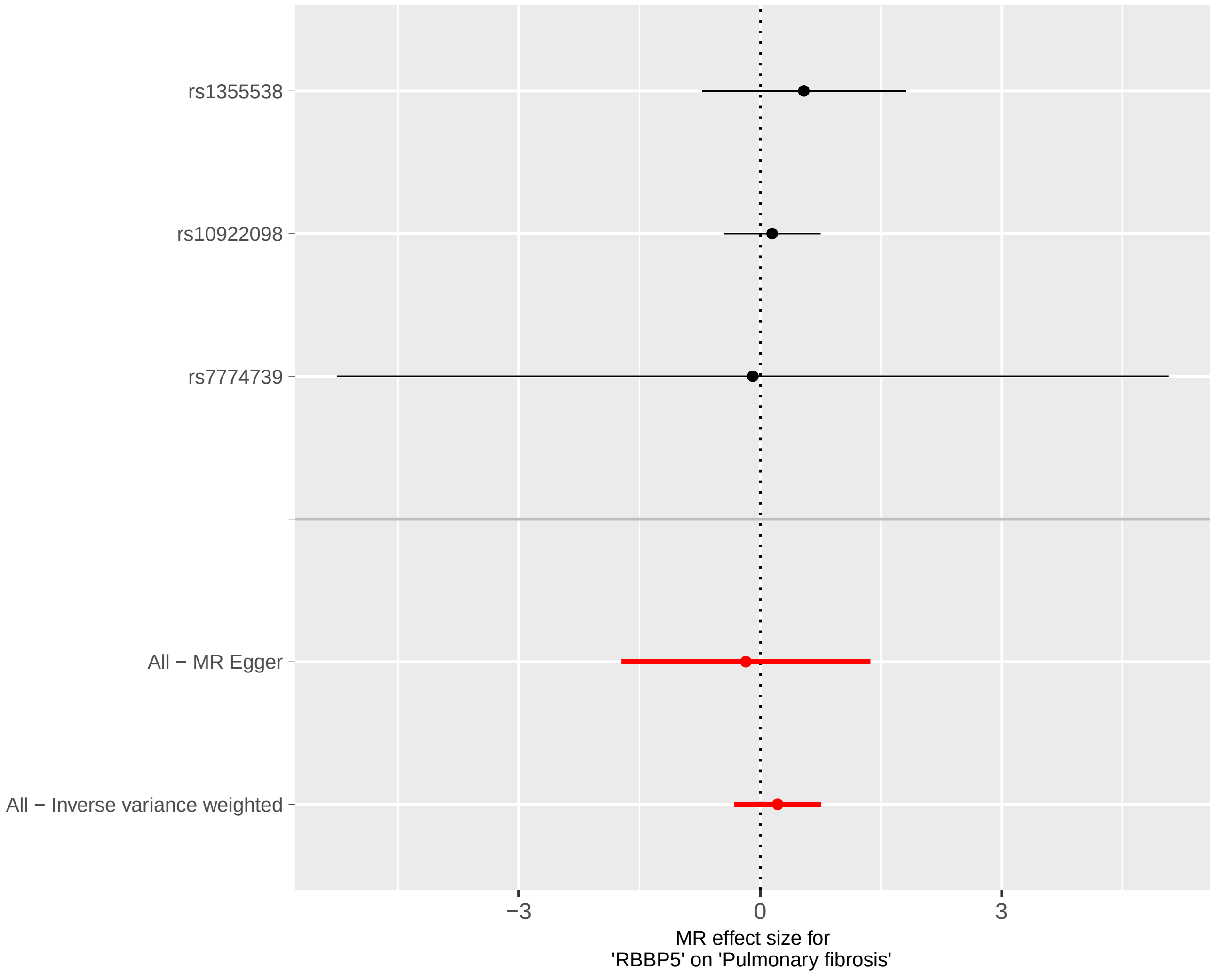
rs1355538
rs10922098
rs7774739
All − MR Egger
All − Inverse variance weighted
−3
0
3
MR effect size for
'RBBP5' on 'Pulmonary fibrosis'

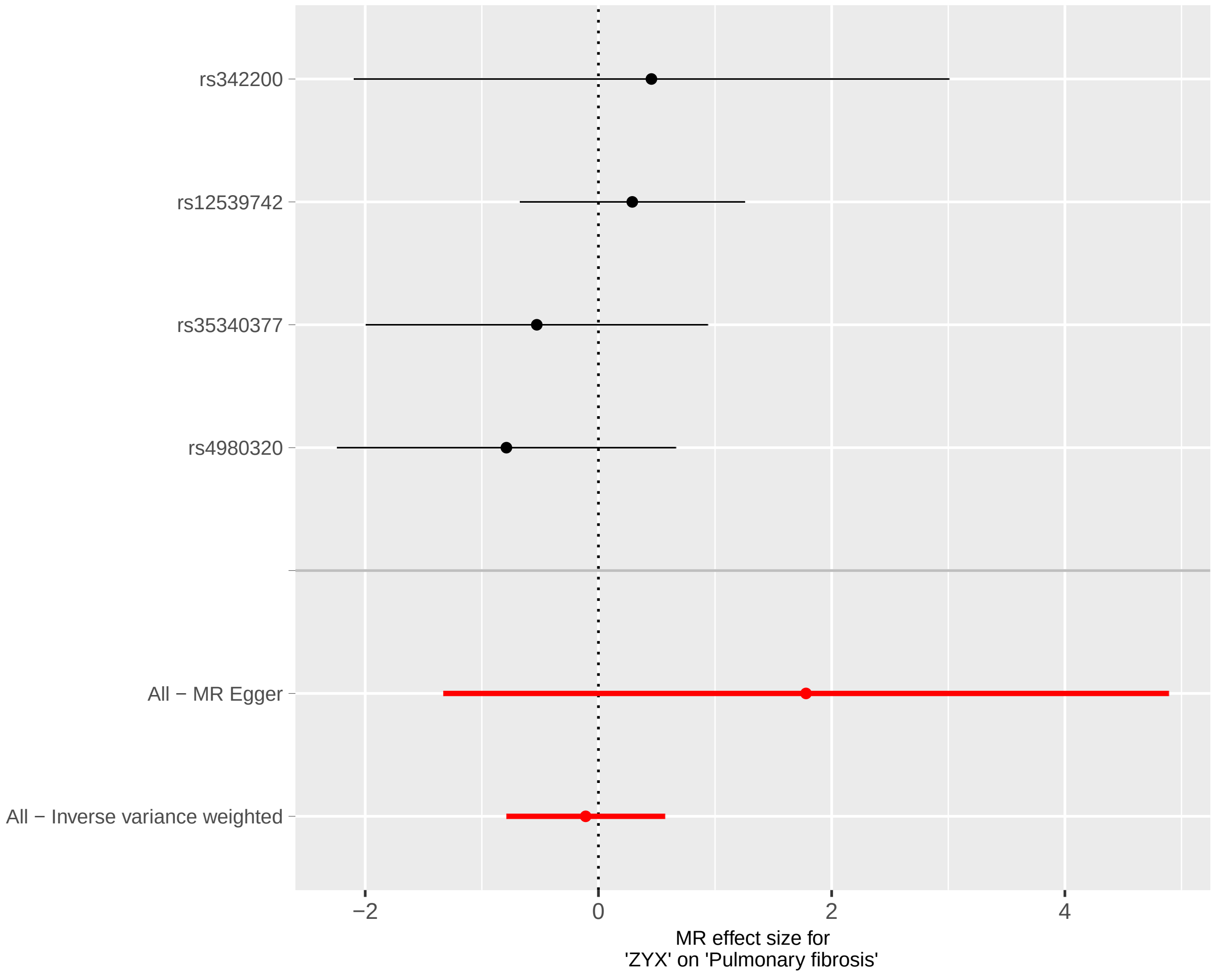

rs342200
rs12539742
rs35340377
rs4980320
All − MR Egger
All − Inverse variance weighted
−2
0
2
4
MR effect size for
'ZYX' on 'Pulmonary fibrosis'

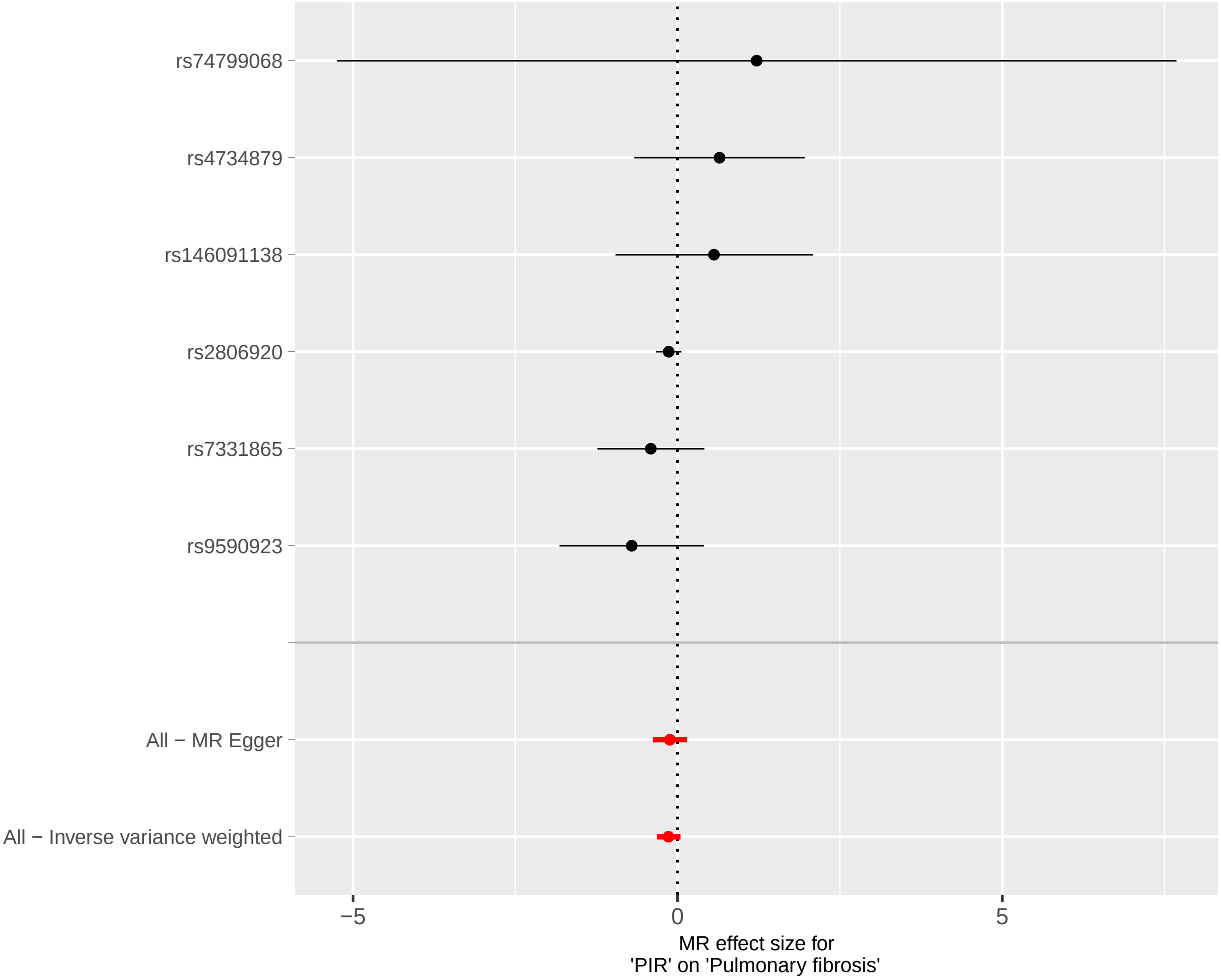
rs74799068
rs4734879
rs146091138
rs2806920
rs7331865
rs9590923
All − MR Egger
All − Inverse variance weighted
−5
0
5
MR effect size for
'PIR' on 'Pulmonary fibrosis'

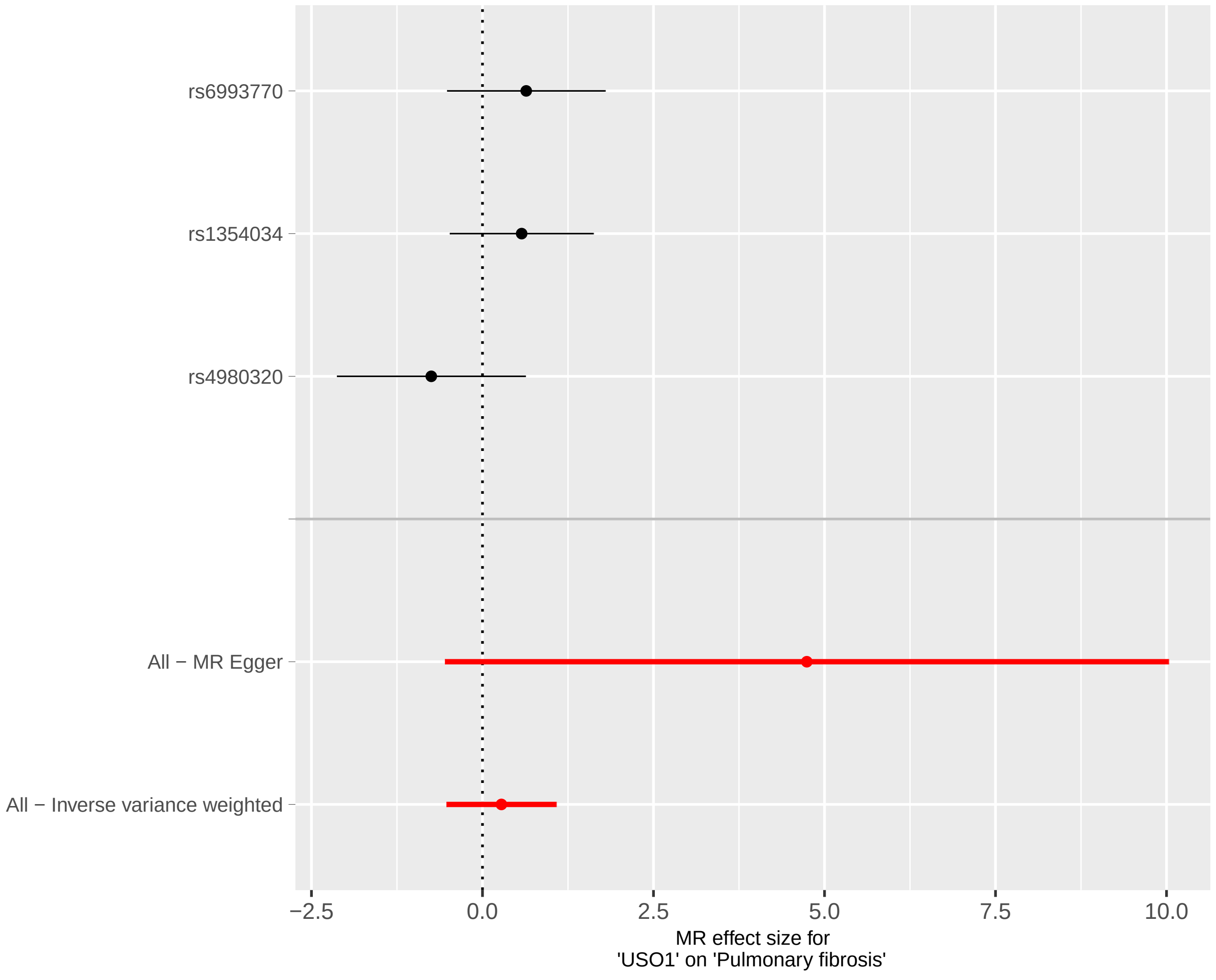

rs6993770
rs1354034
rs4980320
All − MR Egger
All − Inverse variance weighted
−2.5
0.0
2.5
5.0
7.5
10.0
MR effect size for
'USO1' on 'Pulmonary fibrosis'

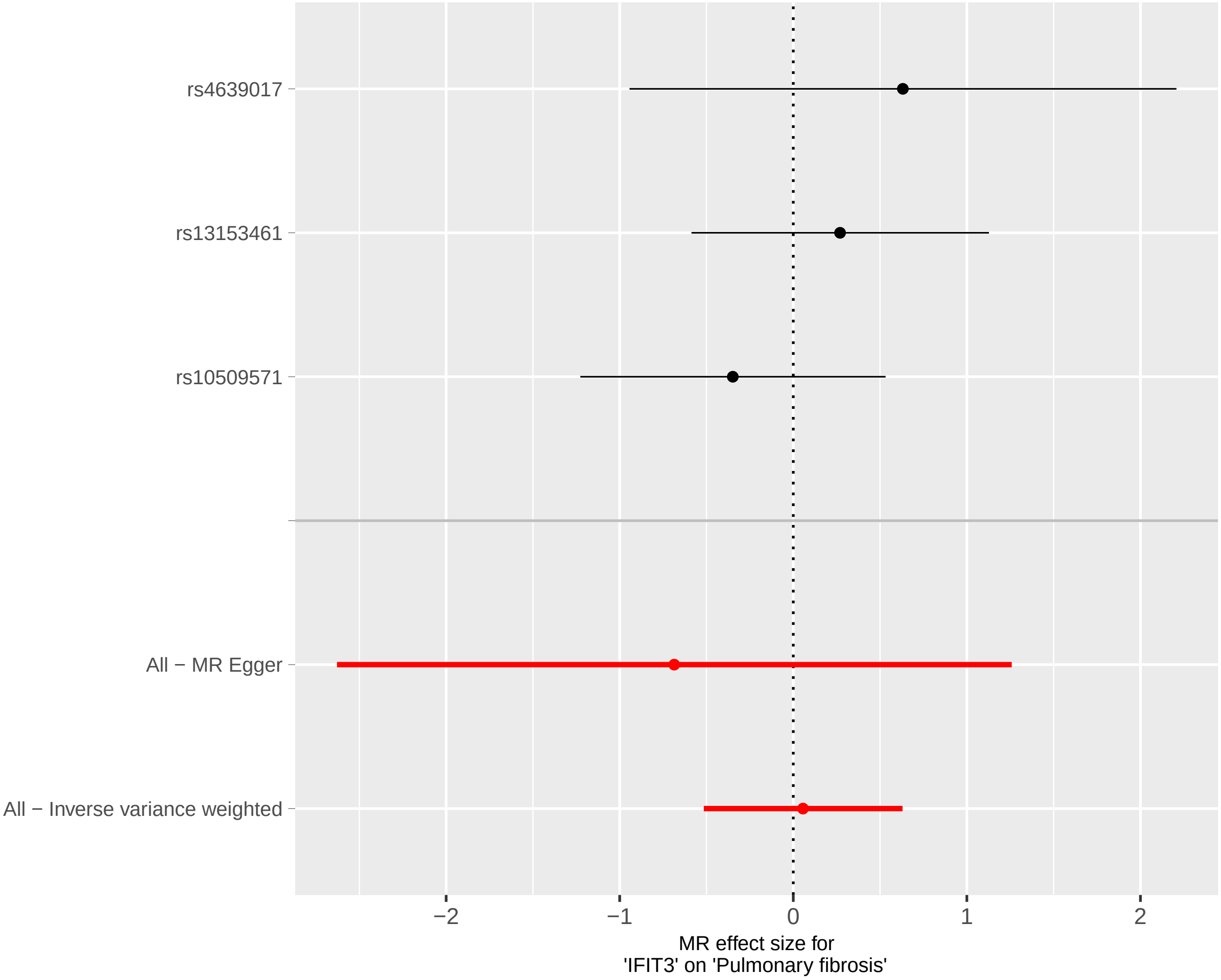
rs4639017
rs13153461
rs10509571
All − MR Egger
All − Inverse variance weighted
−2
−1
0
1
2
MR effect size for
'IFIT3' on 'Pulmonary fibrosis'

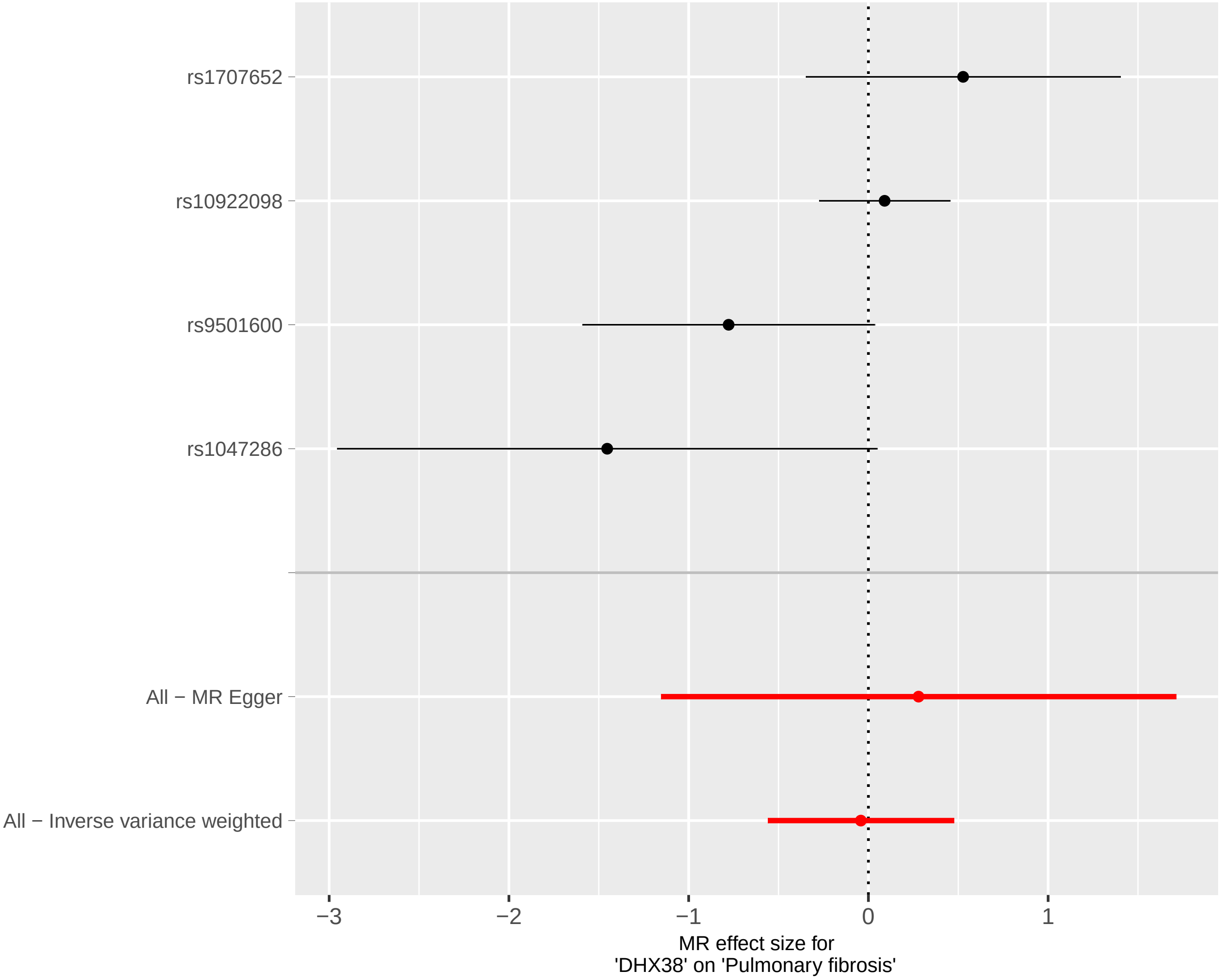

rs1707652
rs10922098
rs9501600
rs1047286
All − MR Egger
All − Inverse variance weighted
−3
−2
−1
0
1
MR effect size for
'DHX38' on 'Pulmonary fibrosis'

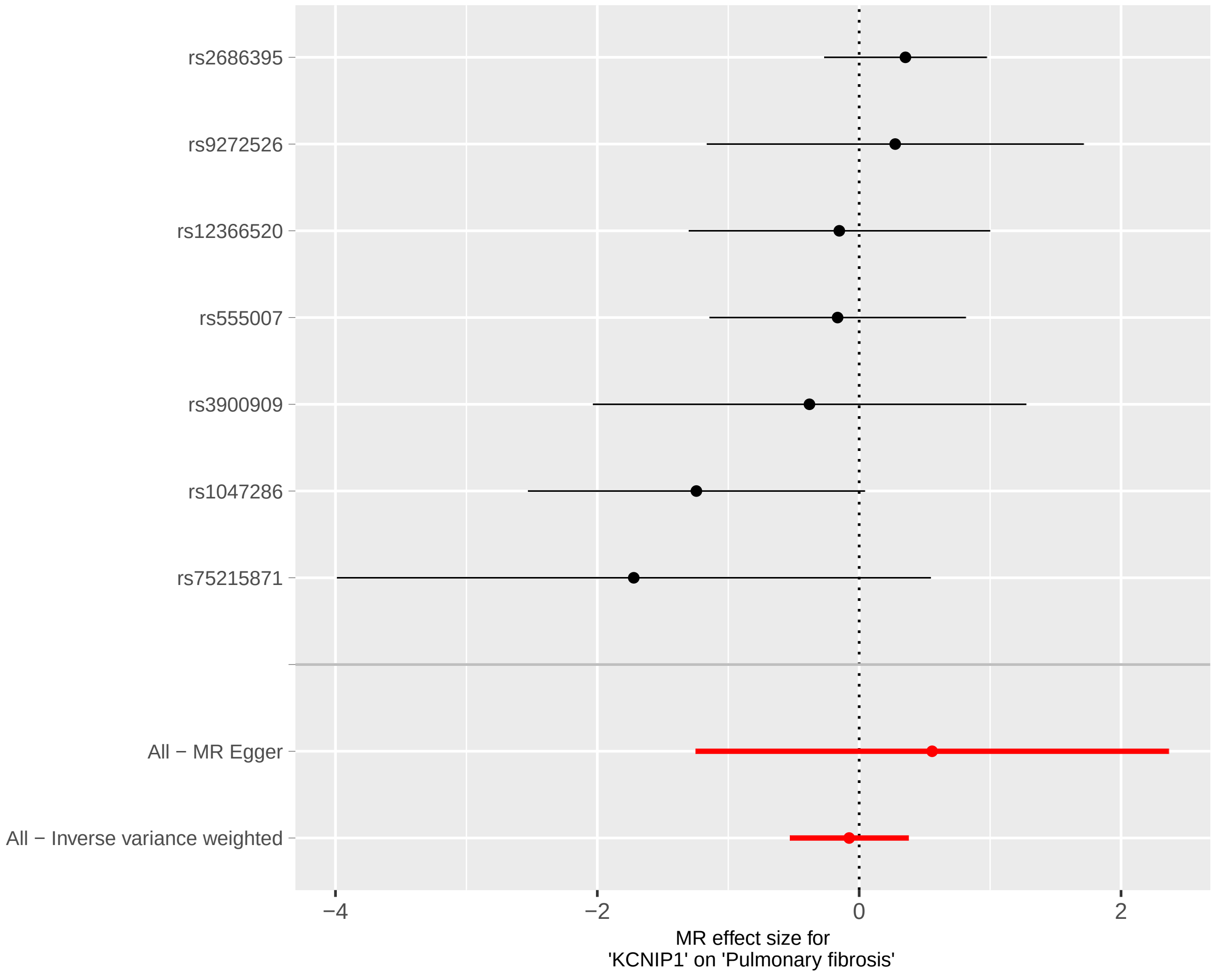
rs2686395
rs9272526
rs12366520
rs555007
rs3900909
rs1047286
rs75215871
All − MR Egger
All − Inverse variance weighted
−4
−2
0
2
MR effect size for
'KCNIP1' on 'Pulmonary fibrosis'

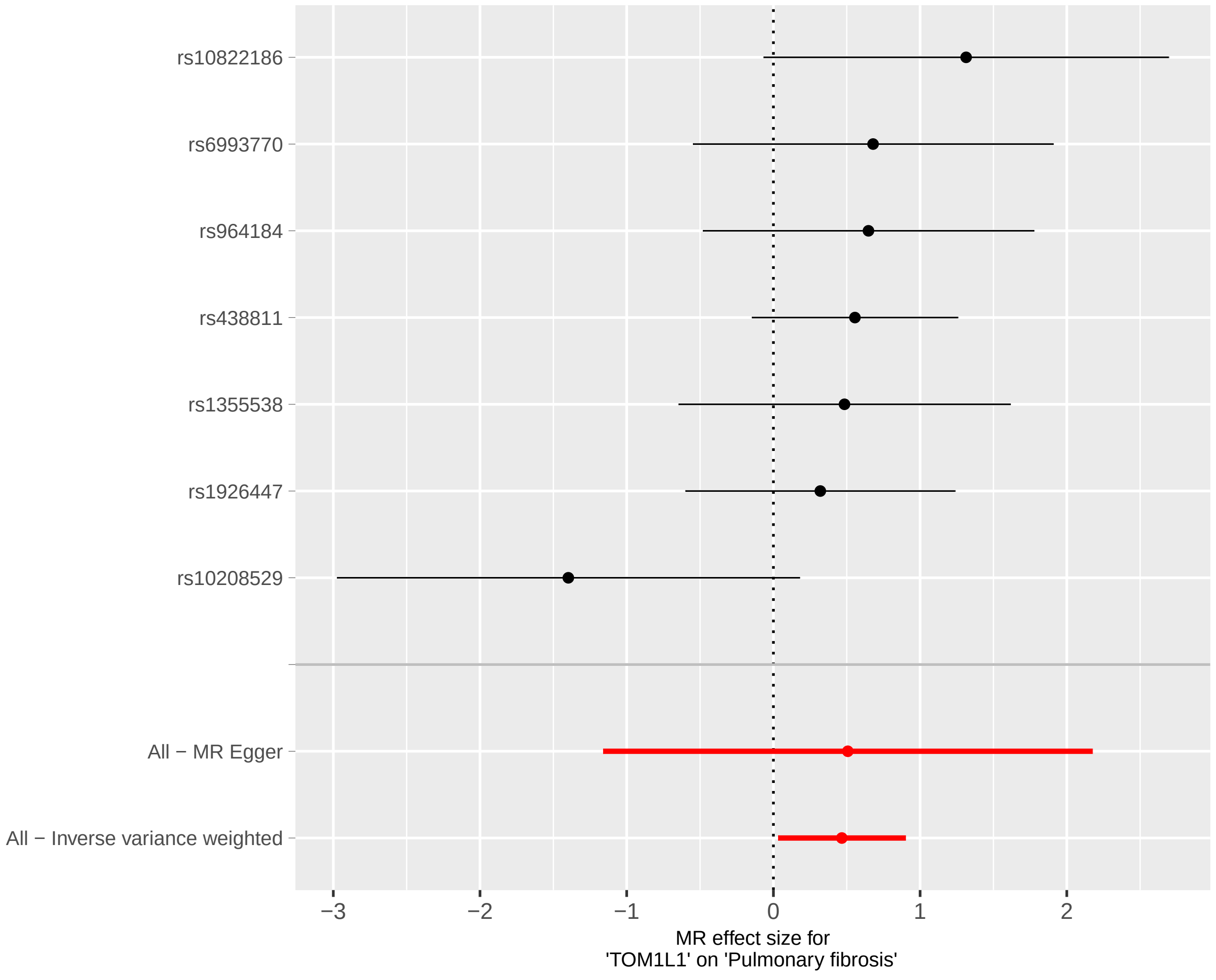

rs10822186
rs6993770
rs964184
rs438811
rs1355538
rs1926447
rs10208529
All − MR Egger
All − Inverse variance weighted
−3
−2
−1
0
1
2
MR effect size for
'TOM1L1' on 'Pulmonary fibrosis'

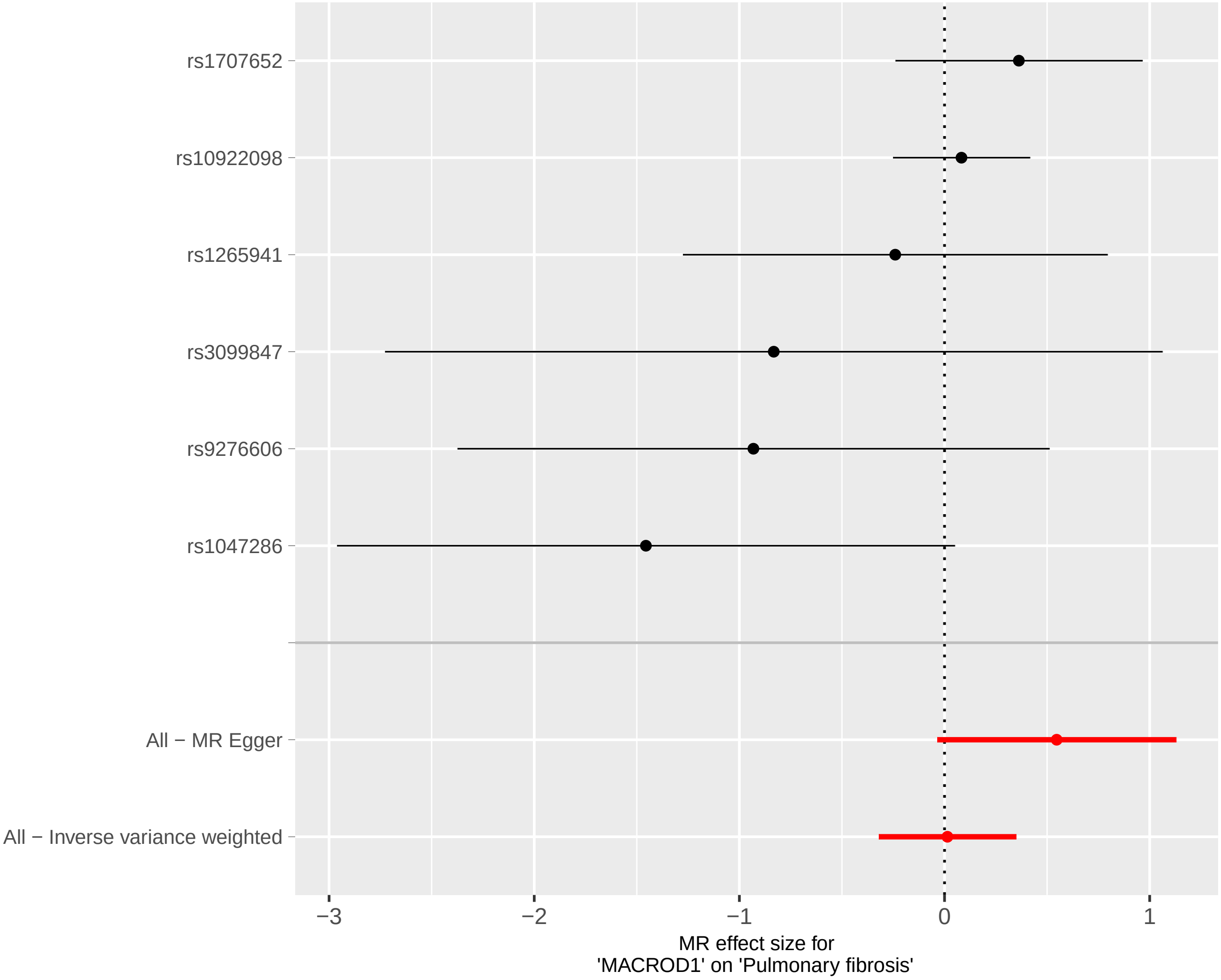
rs1707652
rs10922098
rs1265941
rs3099847
rs9276606
rs1047286
All − MR Egger
All − Inverse variance weighted
−3
−2
−1
0
1
MR effect size for
'MACROD1' on 'Pulmonary fibrosis'

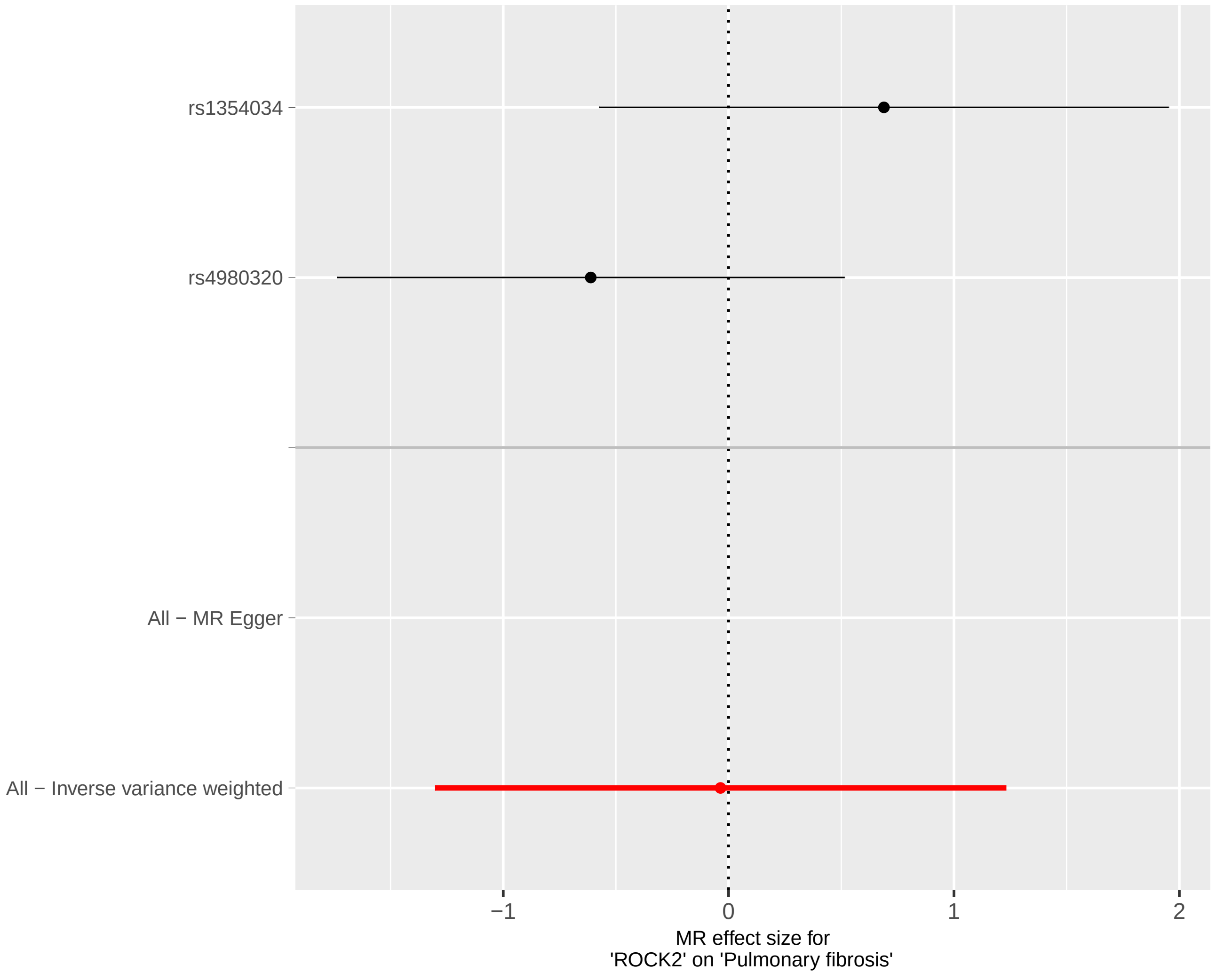
rs1354034
rs4980320
All − MR Egger
All − Inverse variance weighted
−1
0
1
2
MR effect size for
'ROCK2' on 'Pulmonary fibrosis'

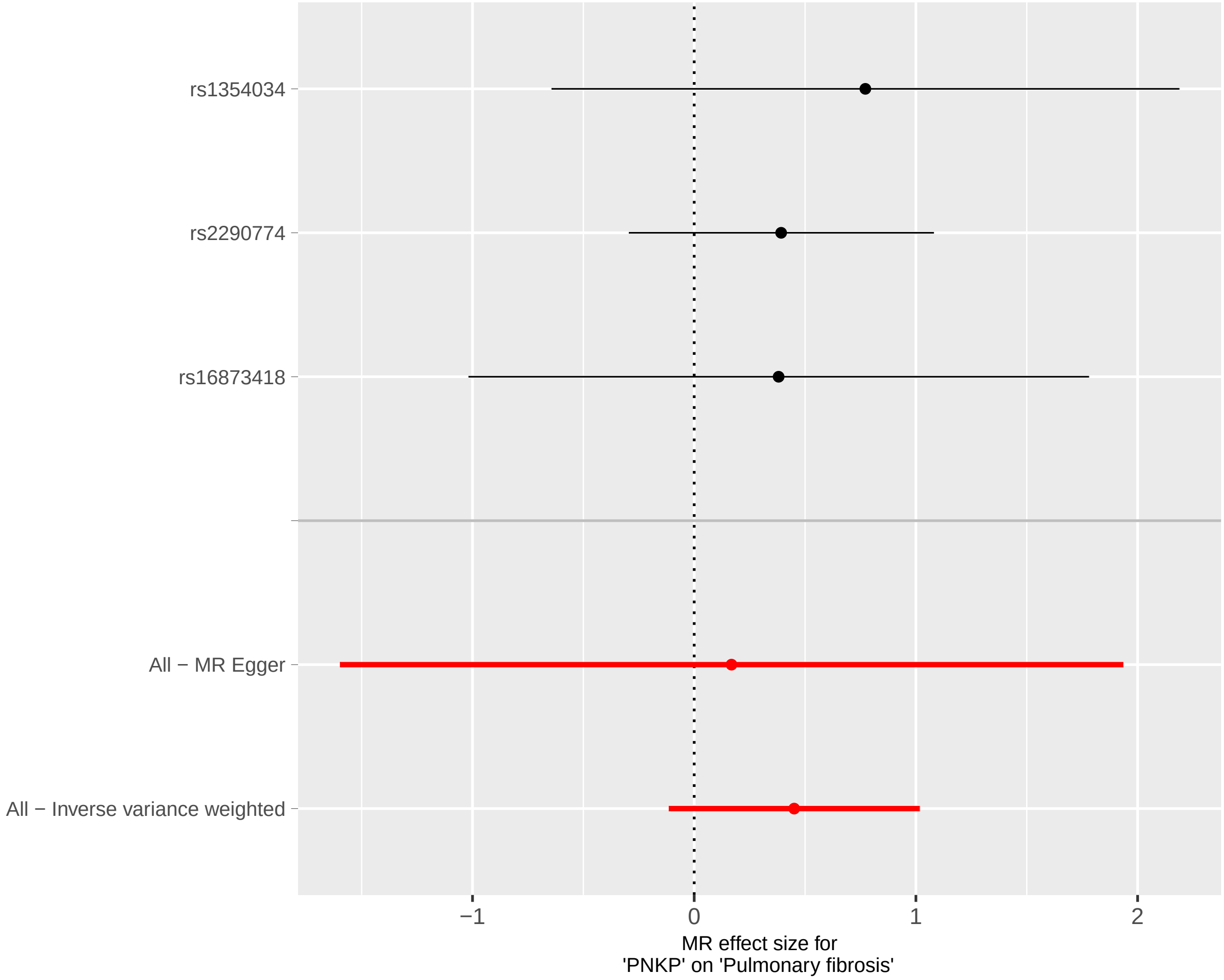
rs1354034
rs2290774
rs16873418
All − MR Egger
All − Inverse variance weighted
−1
0
1
2
MR effect size for
'PNKP' on 'Pulmonary fibrosis'

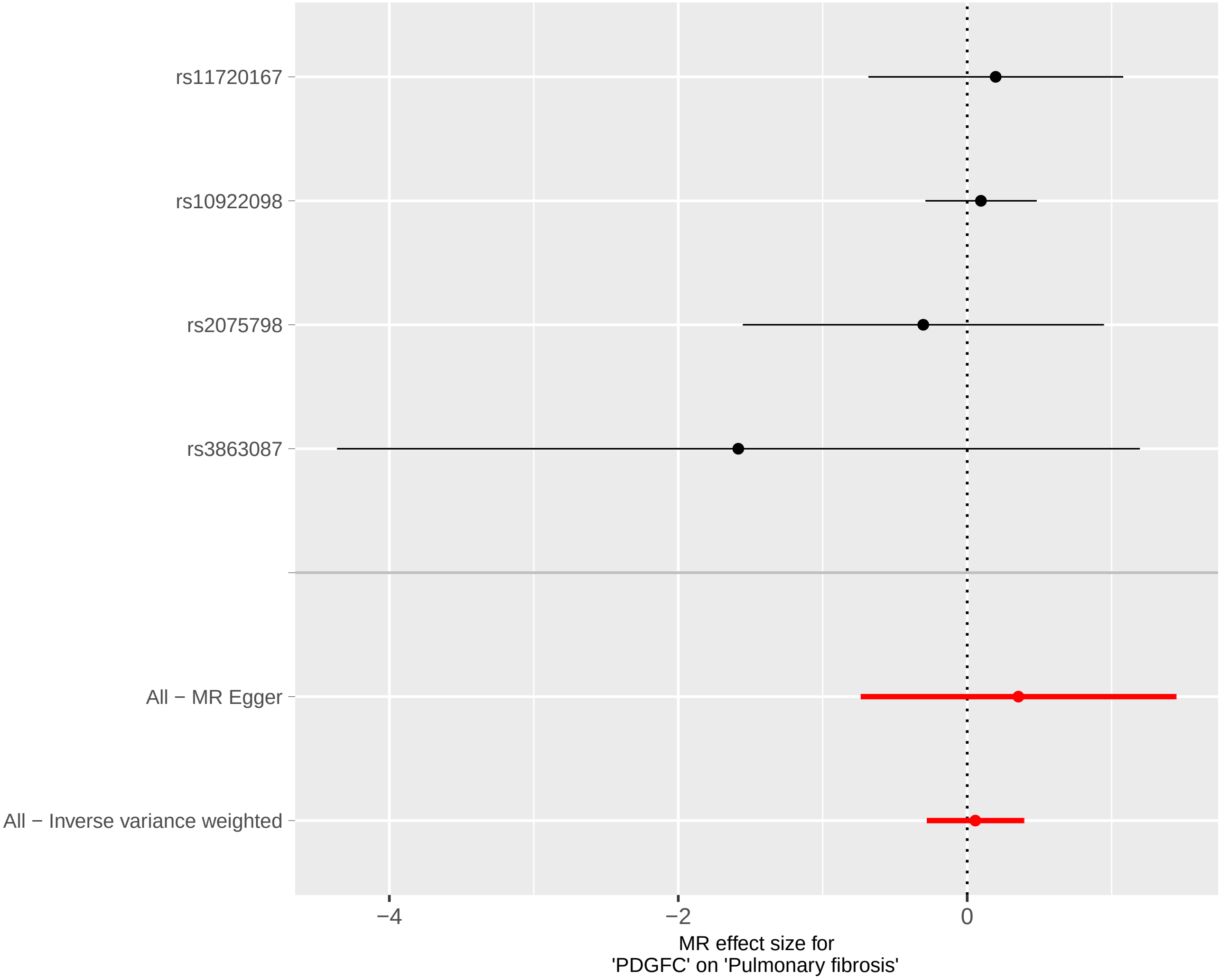

rs11720167
rs10922098
rs2075798
rs3863087
All − MR Egger
All − Inverse variance weighted
−4
−2
0
MR effect size for
'PDGFC' on 'Pulmonary fibrosis'

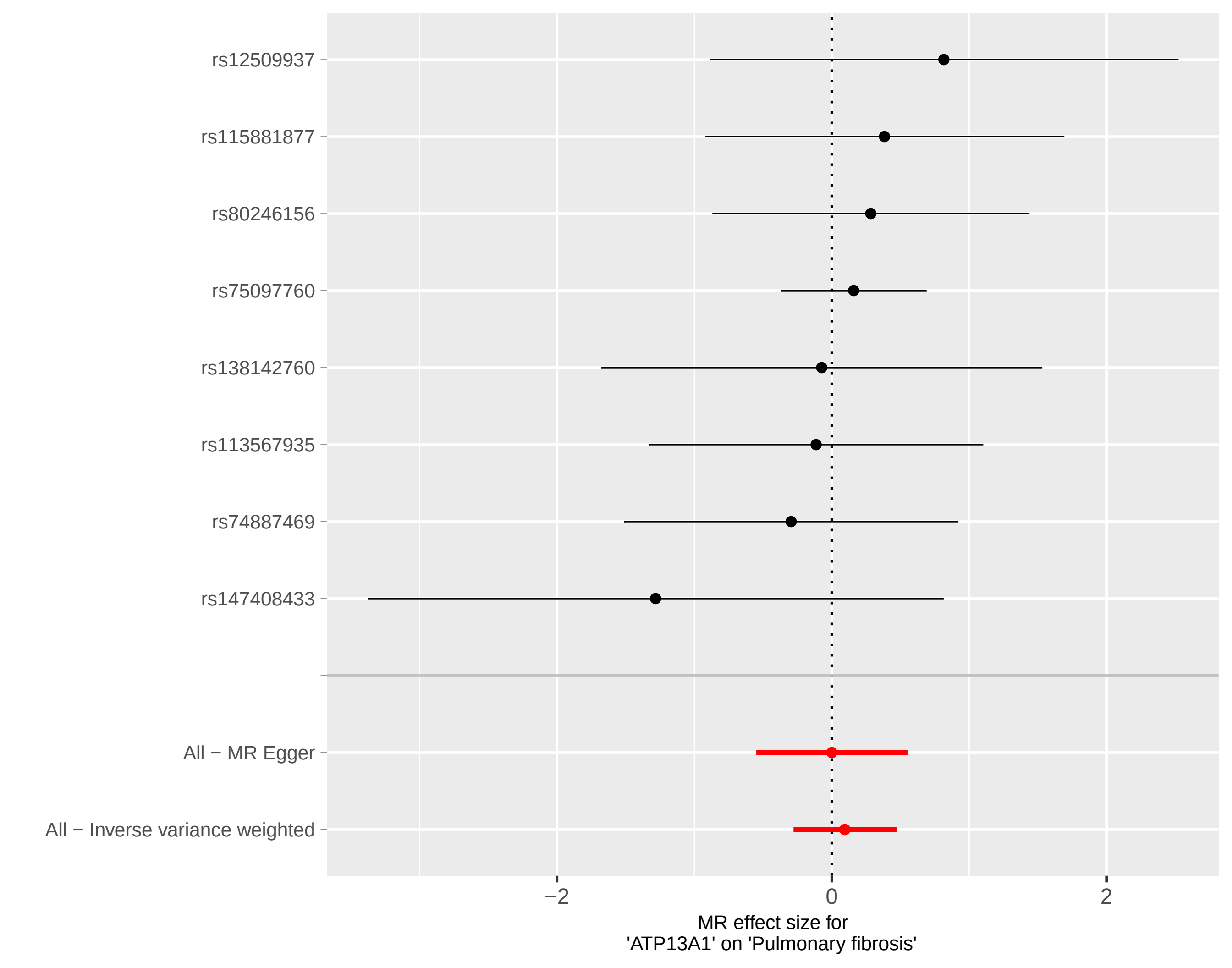
rs12509937
rs115881877
rs80246156
rs75097760
rs138142760
rs113567935
rs74887469
rs147408433
All − MR Egger
All − Inverse variance weighted
−2
0
2
MR effect size for
'ATP13A1' on 'Pulmonary fibrosis'

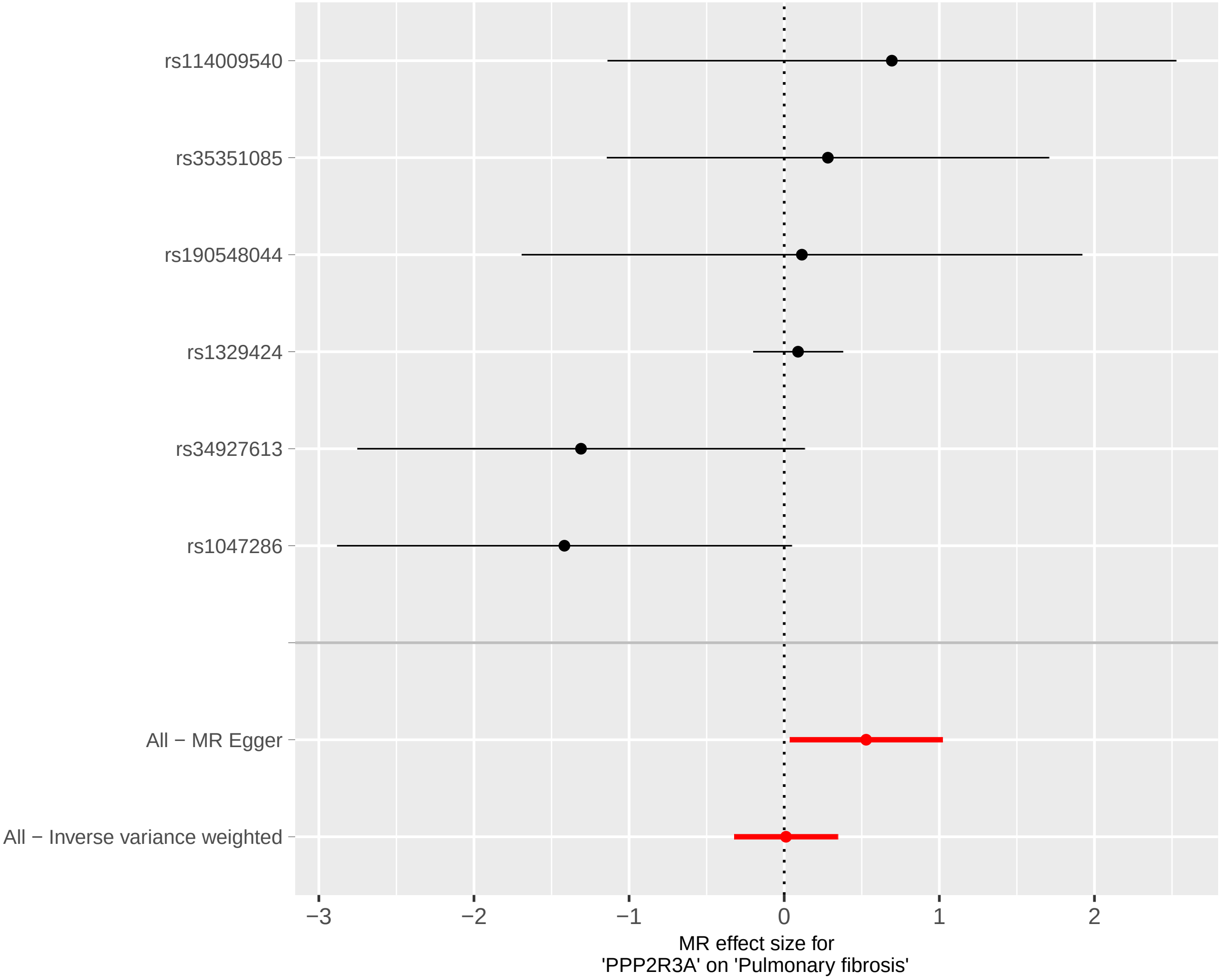

rs114009540
rs35351085
rs190548044
rs1329424
rs34927613
rs1047286
All − MR Egger
All − Inverse variance weighted
−3
−2
−1
0
1
2
MR effect size for
'PPP2R3A' on 'Pulmonary fibrosis'

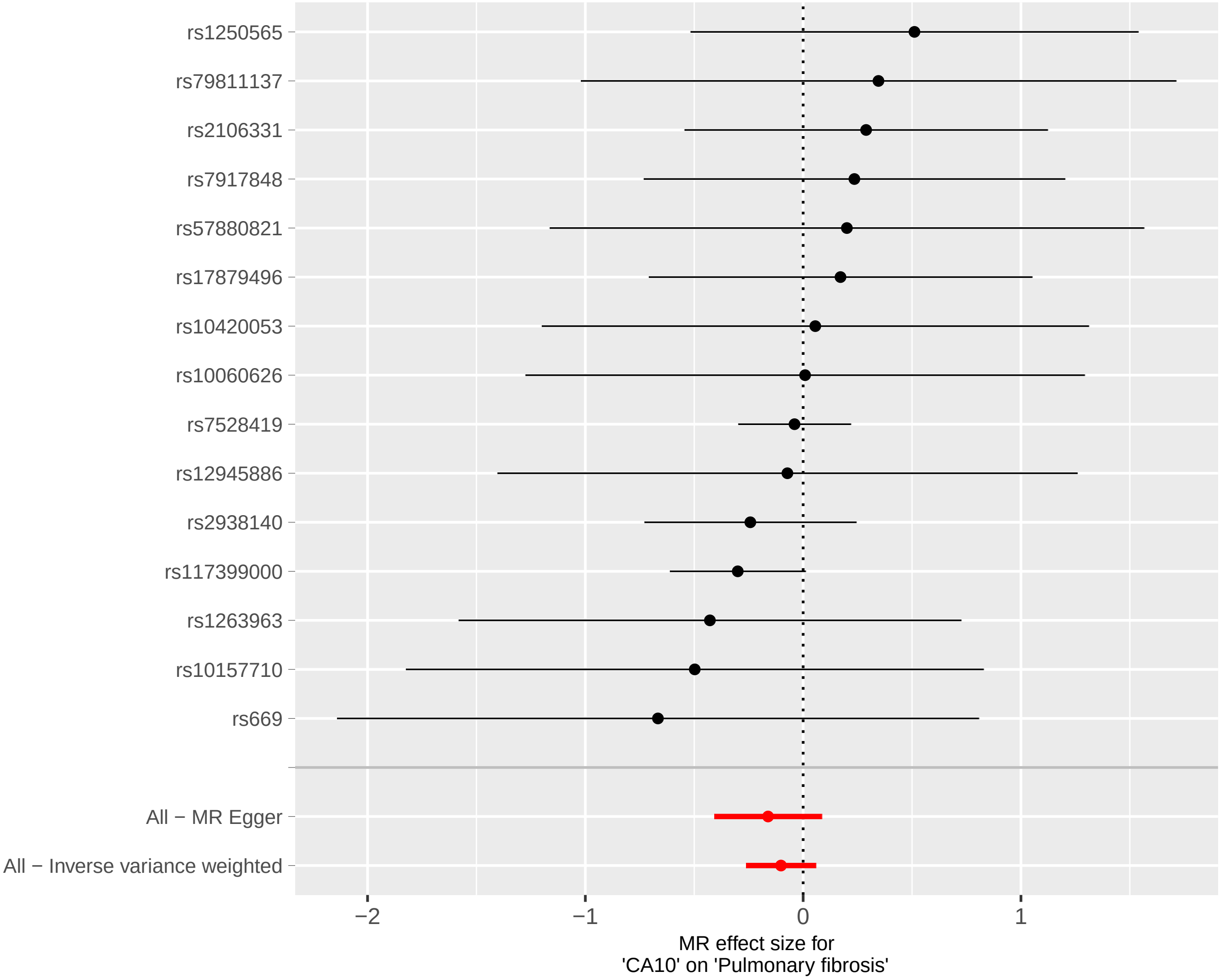

rs1250565
rs79811137
rs2106331
rs7917848
rs57880821
rs17879496
rs10420053
rs10060626
rs7528419
rs12945886
rs2938140
rs117399000
rs1263963
rs10157710
rs669
All − MR Egger
All − Inverse variance weighted
−2
−1
0
1
MR effect size for
'CA10' on 'Pulmonary fibrosis'

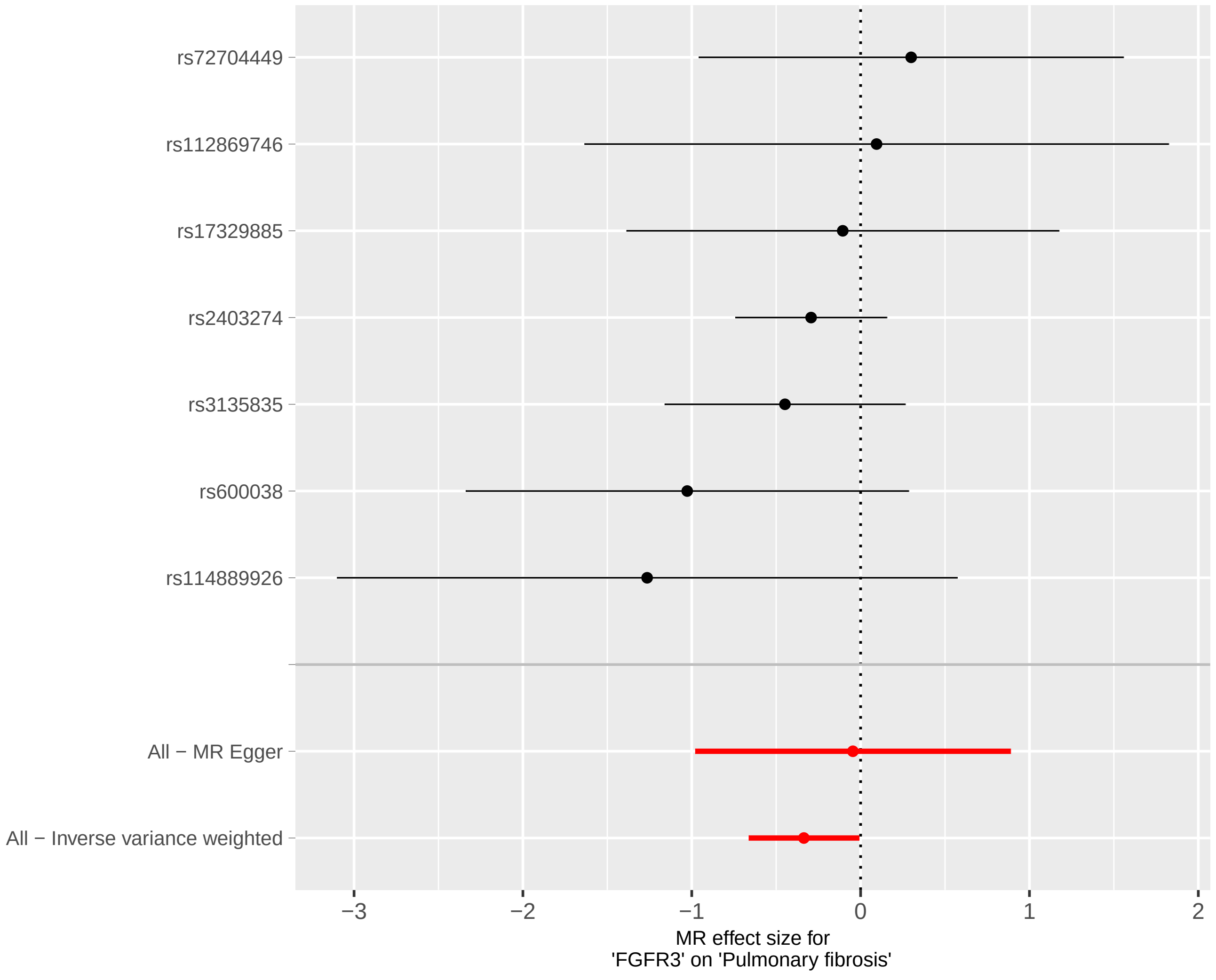

rs72704449
rs112869746
rs17329885
rs2403274
rs3135835
rs600038
rs114889926
All − MR Egger
All − Inverse variance weighted
−3
−2
−1
0
1
2
MR effect size for
'FGFR3' on 'Pulmonary fibrosis'

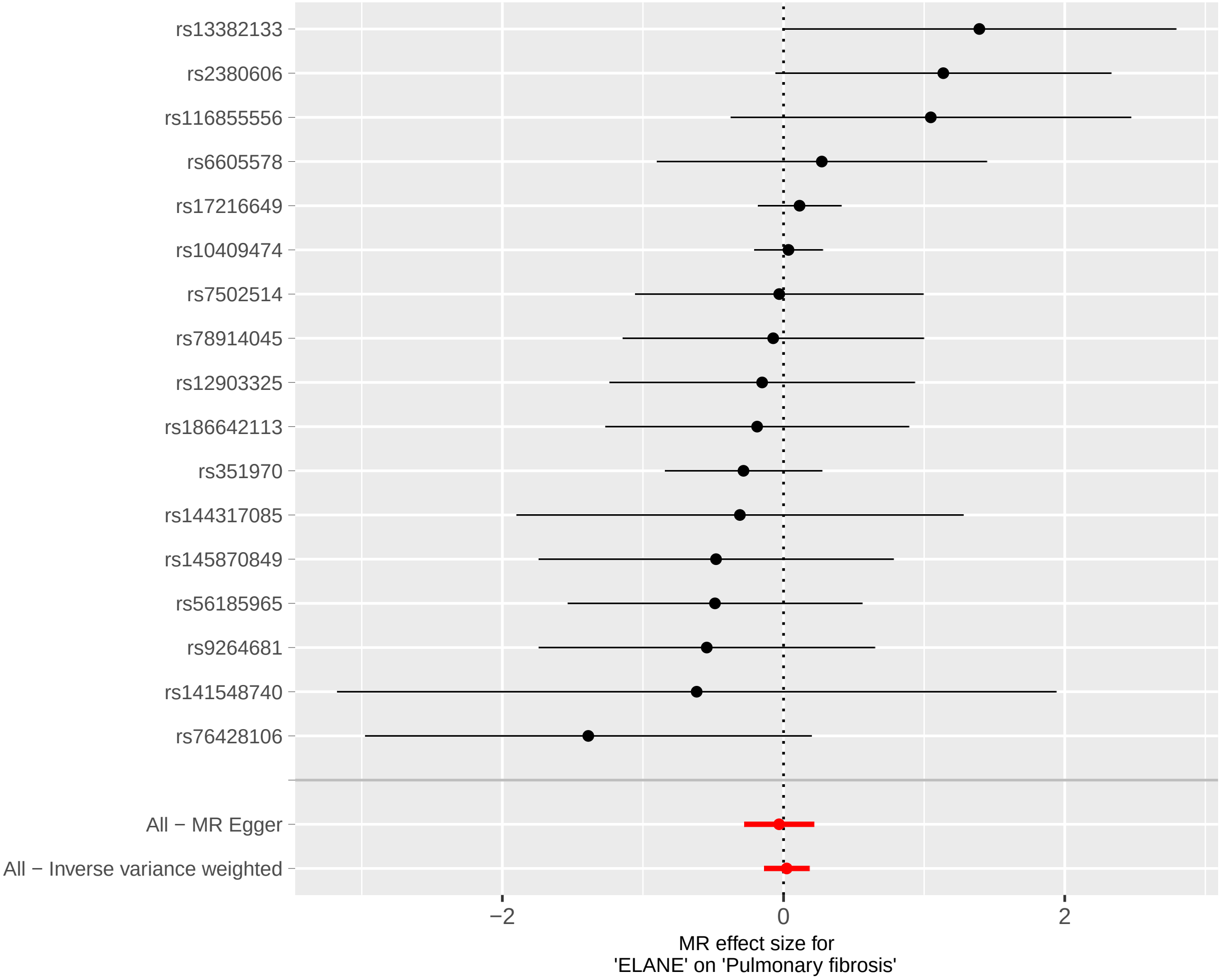

rs13382133
rs2380606
rs116855556
rs6605578
rs17216649
rs10409474
rs7502514
rs78914045
rs12903325
rs186642113
rs351970
rs144317085
rs145870849
rs56185965
rs9264681
rs141548740
rs76428106
All − MR Egger
All − Inverse variance weighted
−2
0
2
MR effect size for
'ELANE' on 'Pulmonary fibrosis'

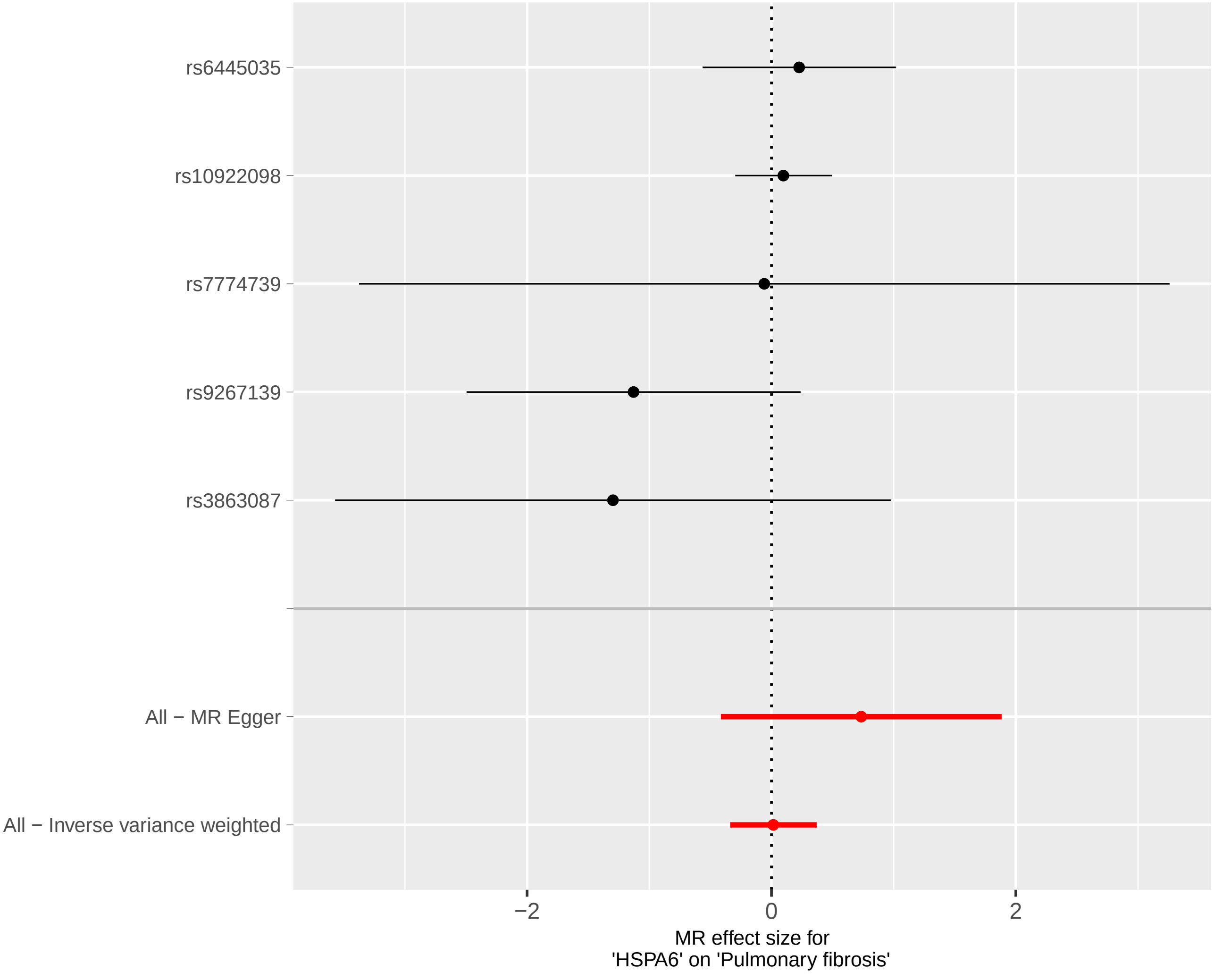
rs6445035
rs10922098
rs7774739
rs9267139
rs3863087
All − MR Egger
All − Inverse variance weighted
−2
0
2
MR effect size for
'HSPA6' on 'Pulmonary fibrosis'

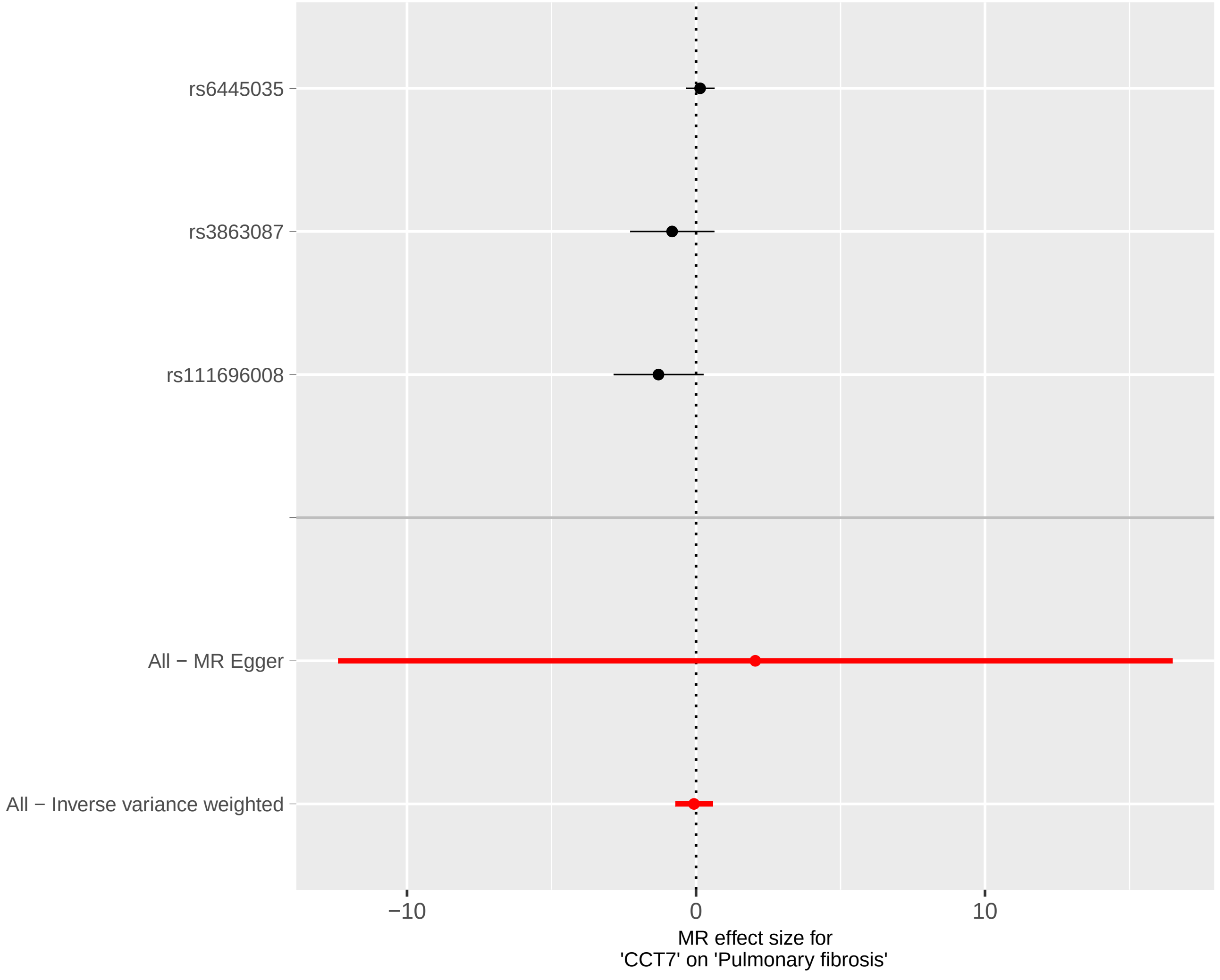

rs6445035
rs3863087
rs111696008
All − MR Egger
All − Inverse variance weighted
−10
0
10
MR effect size for
'CCT7' on 'Pulmonary fibrosis'

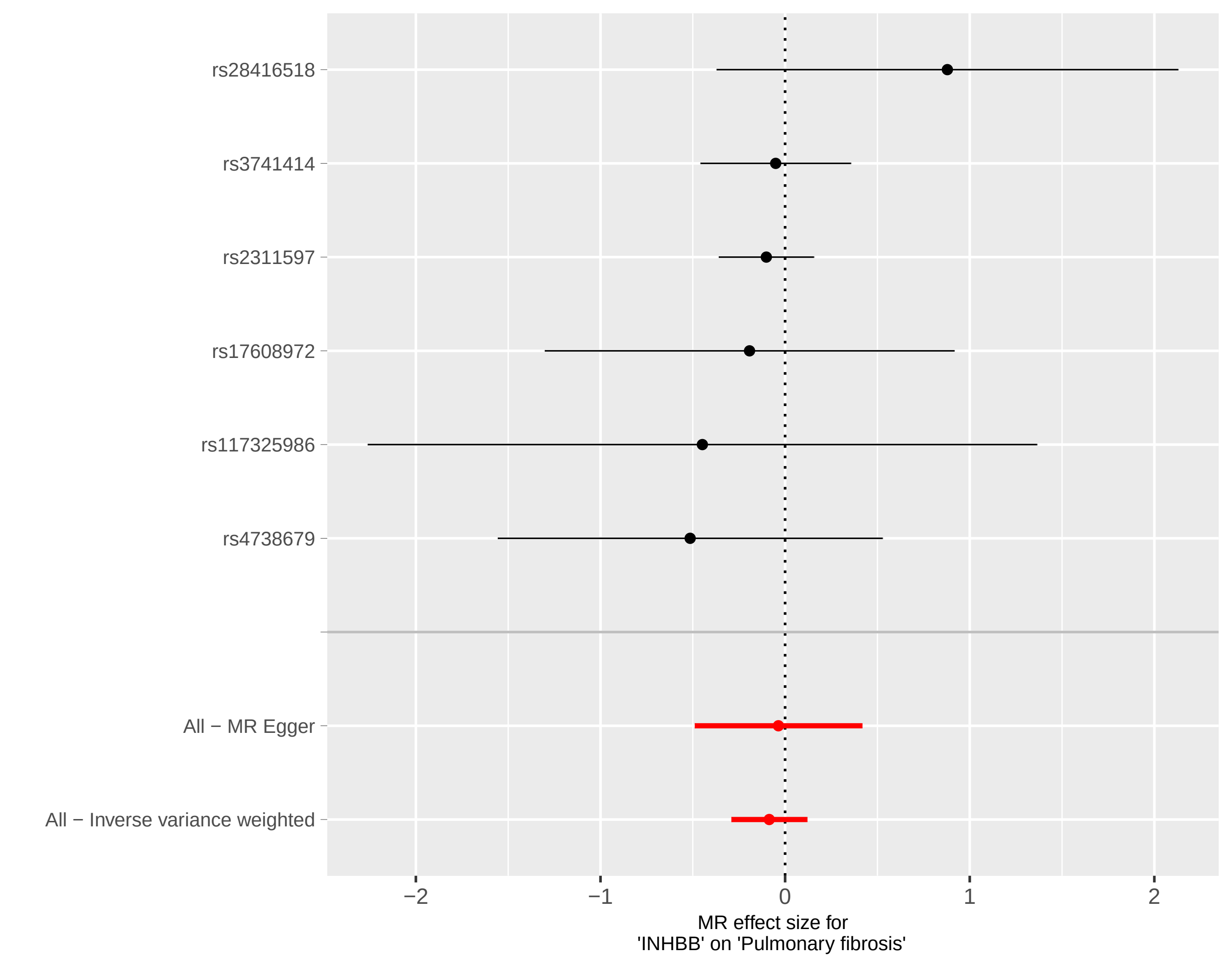
rs28416518
rs3741414
rs2311597
rs17608972
rs117325986
rs4738679
All − MR Egger
All − Inverse variance weighted
−2
−1
0
1
2
MR effect size for
'INHBB' on 'Pulmonary fibrosis'

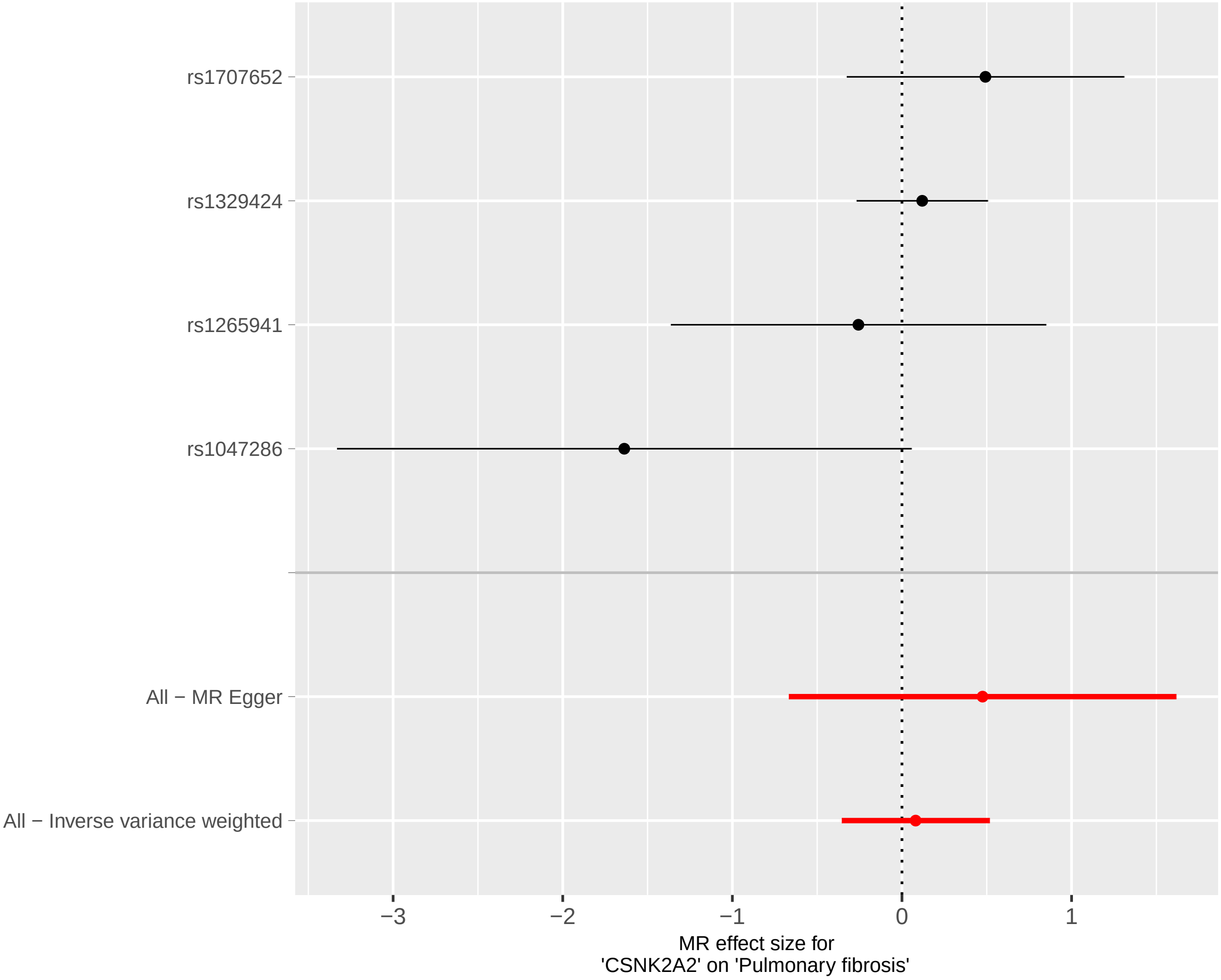
rs1707652
rs1329424
rs1265941
rs1047286
All − MR Egger
All − Inverse variance weighted
−3
−2
−1
0
1
MR effect size for
'CSNK2A2' on 'Pulmonary fibrosis'

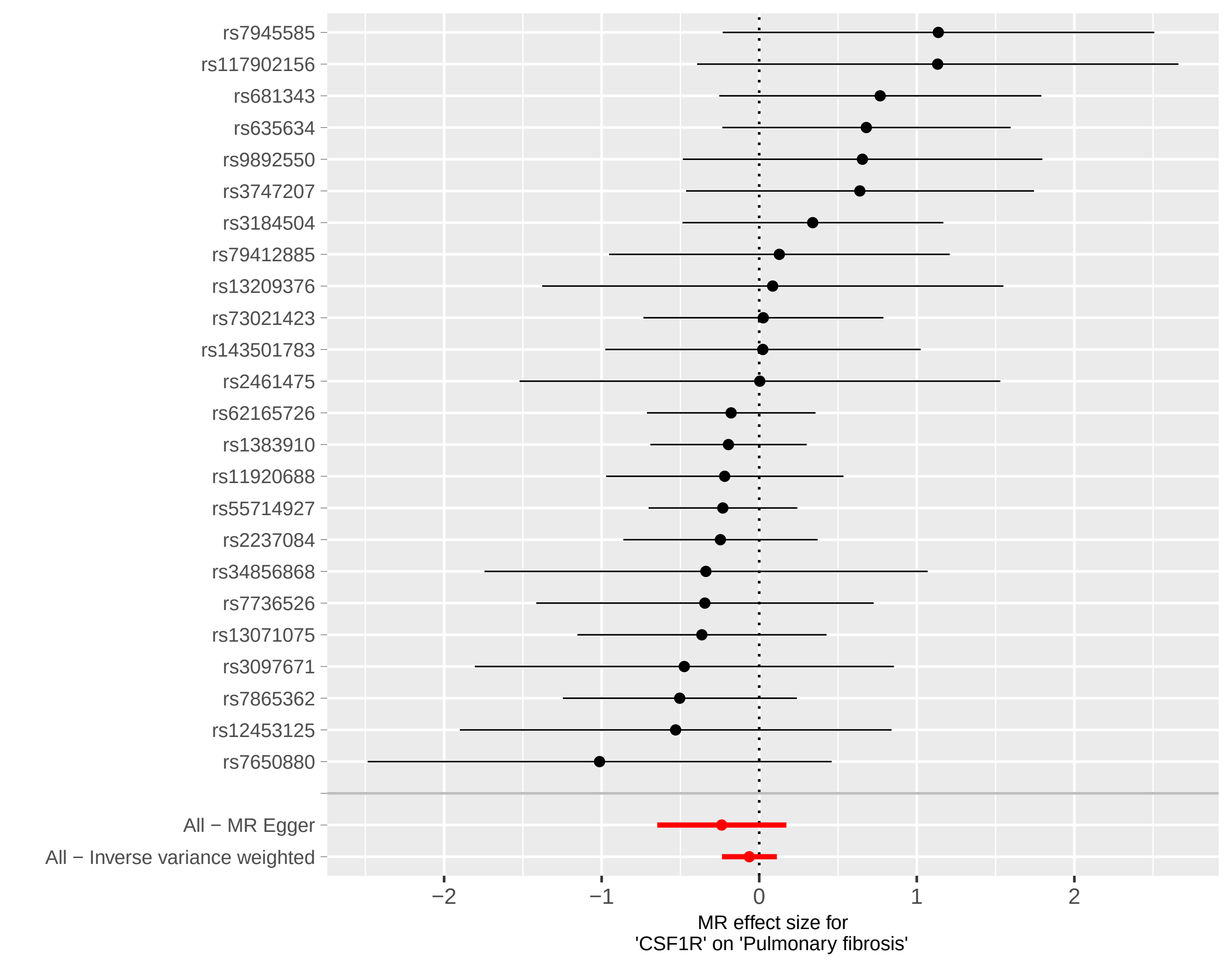
rs7945585
rs117902156
rs681343
rs635634
rs9892550
rs3747207
rs3184504
rs79412885
rs13209376
rs73021423
rs143501783
rs2461475
rs62165726
rs1383910
rs11920688
rs55714927
rs2237084
rs34856868
rs7736526
rs13071075
rs3097671
rs7865362
rs12453125
rs7650880
All − MR Egger
All − Inverse variance weighted
−2
−1
0
1
2
MR effect size for
'CSF1R' on 'Pulmonary fibrosis'

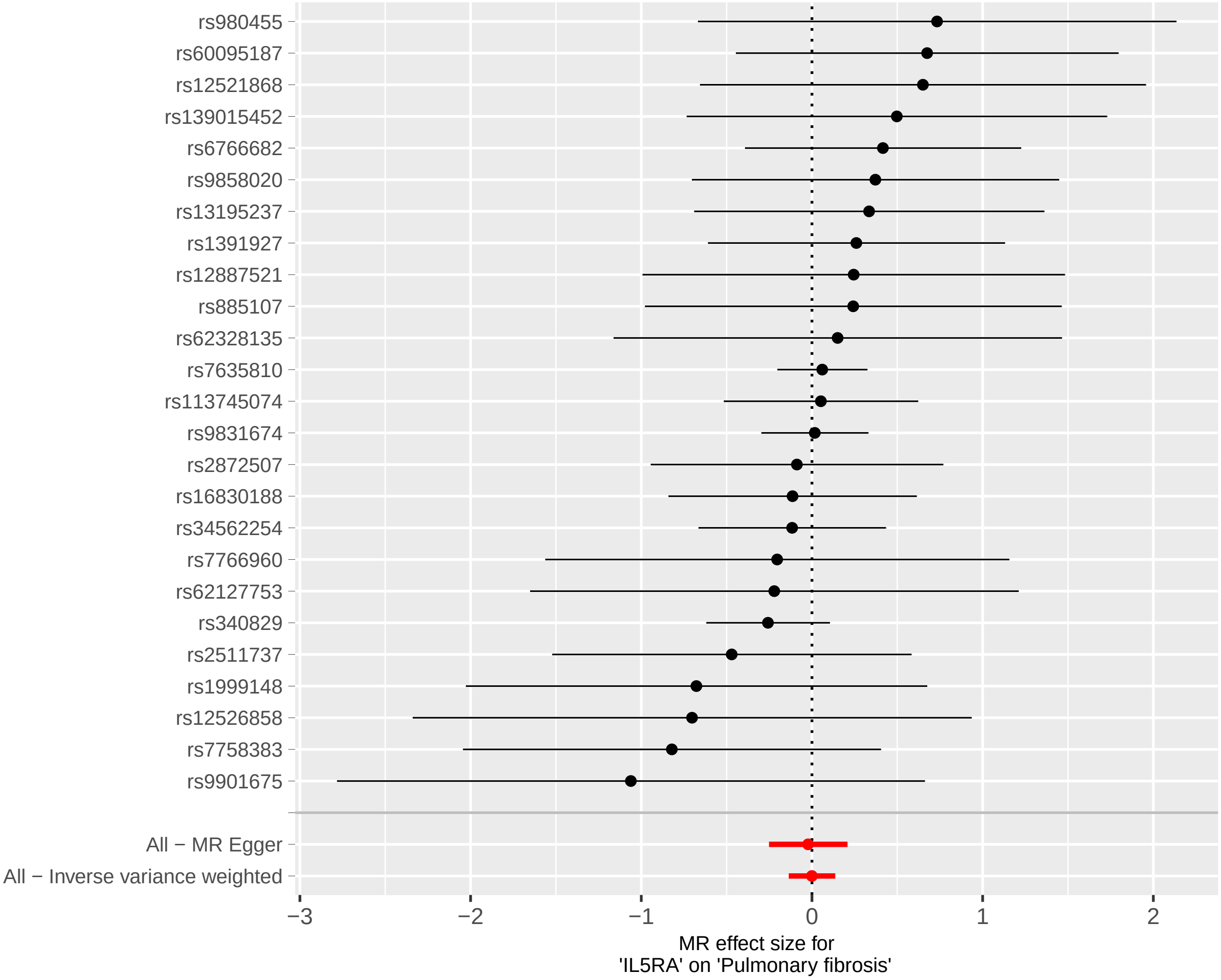

rs980455
rs60095187
rs12521868
rs139015452
rs6766682
rs9858020
rs13195237
rs1391927
rs12887521
rs885107
rs62328135
rs7635810
rs113745074
rs9831674
rs2872507
rs16830188
rs34562254
rs7766960
rs62127753
rs340829
rs2511737
rs1999148
rs12526858
rs7758383
rs9901675
All − MR Egger
All − Inverse variance weighted
−3
−2
−1
0
1
2
MR effect size for
'IL5RA' on 'Pulmonary fibrosis'

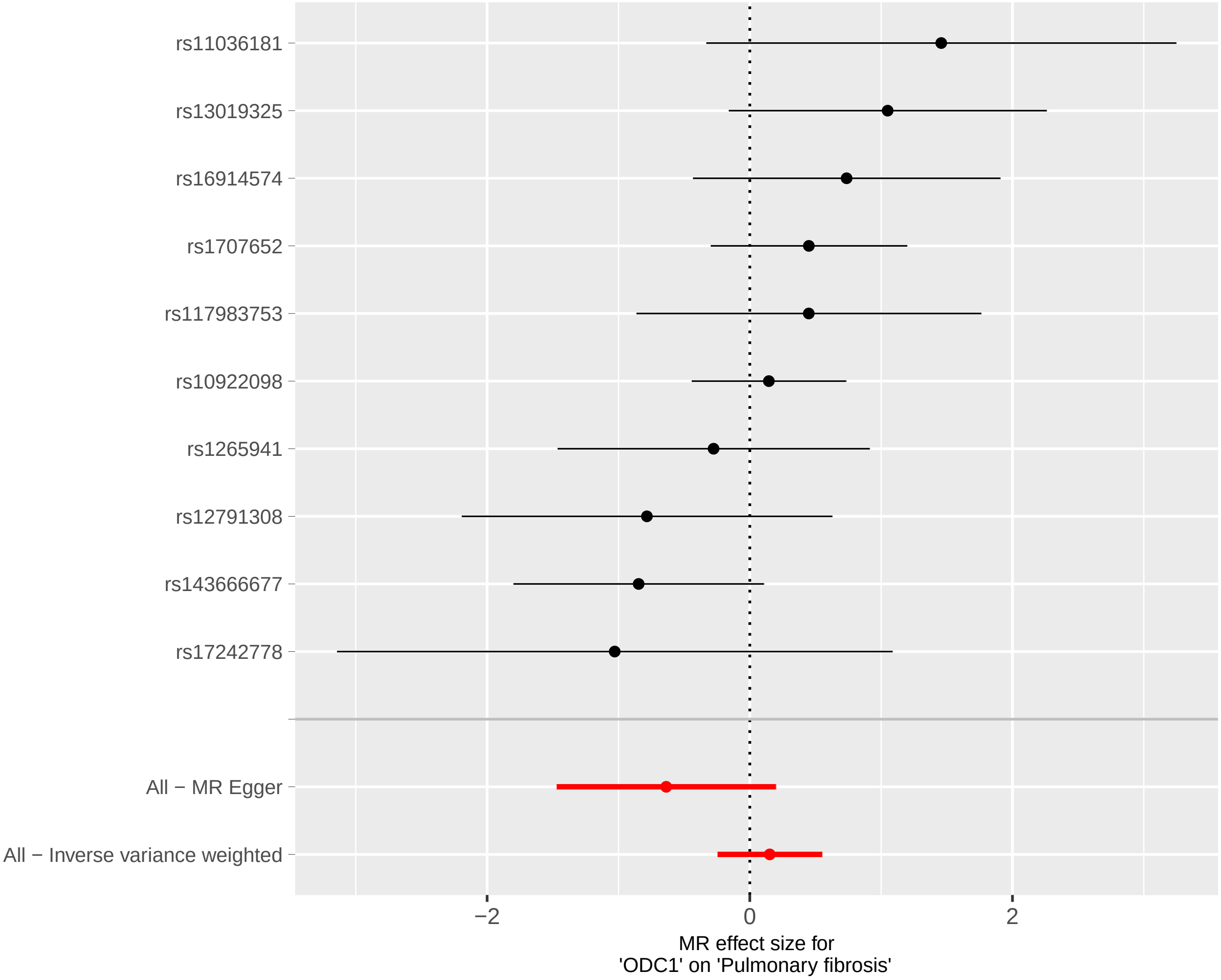
rs11036181
rs13019325
rs16914574
rs1707652
rs117983753
rs10922098
rs1265941
rs12791308
rs143666677
rs17242778
All − MR Egger
All − Inverse variance weighted
−2
0
2
MR effect size for
'ODC1' on 'Pulmonary fibrosis'

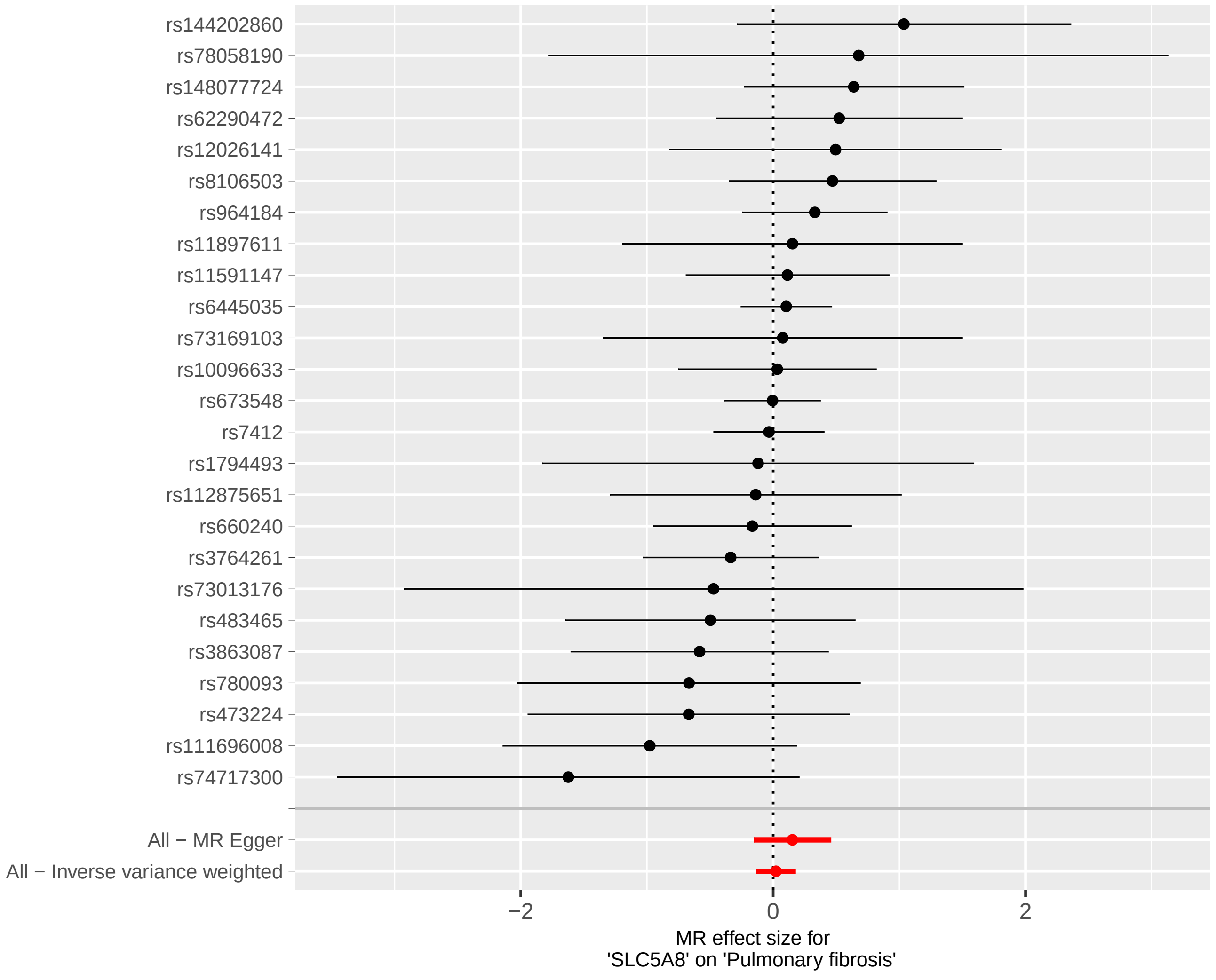
rs144202860
rs78058190
rs148077724
rs62290472
rs12026141
rs8106503
rs964184
rs11897611
rs11591147
rs6445035
rs73169103
rs10096633
rs673548
rs7412
rs1794493
rs112875651
rs660240
rs3764261
rs73013176
rs483465
rs3863087
rs780093
rs473224
rs111696008
rs74717300
All − MR Egger
All − Inverse variance weighted
−2
0
2
MR effect size for
'SLC5A8' on 'Pulmonary fibrosis'

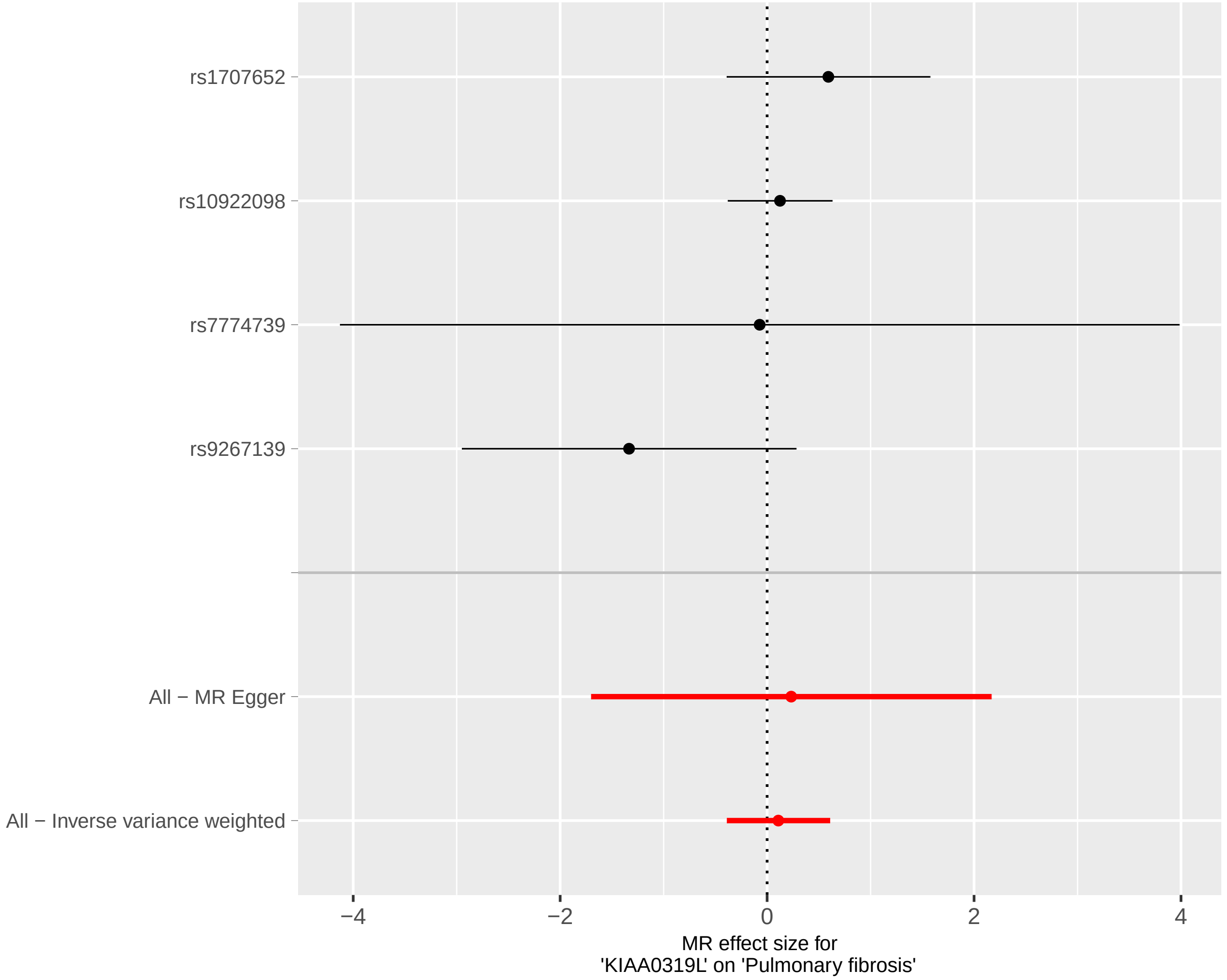
rs1707652
rs10922098
rs7774739
rs9267139
All − MR Egger
All − Inverse variance weighted
−4
−2
0
2
4
MR effect size for
'KIAA0319L' on 'Pulmonary fibrosis'

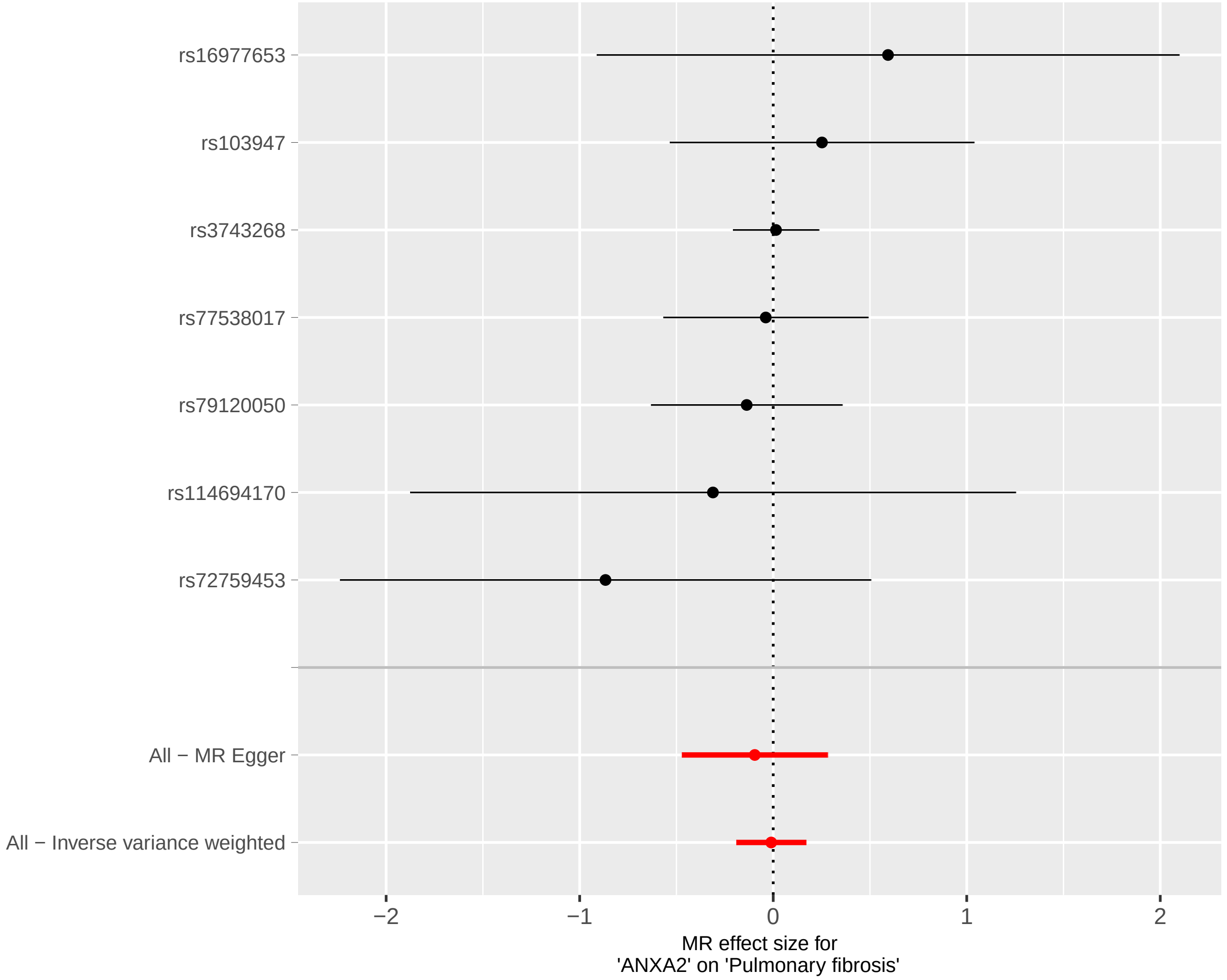
rs16977653
rs103947
rs3743268
rs77538017
rs79120050
rs114694170
rs72759453
All − MR Egger
All − Inverse variance weighted
−2
−1
0
1
2
MR effect size for
'ANXA2' on 'Pulmonary fibrosis'

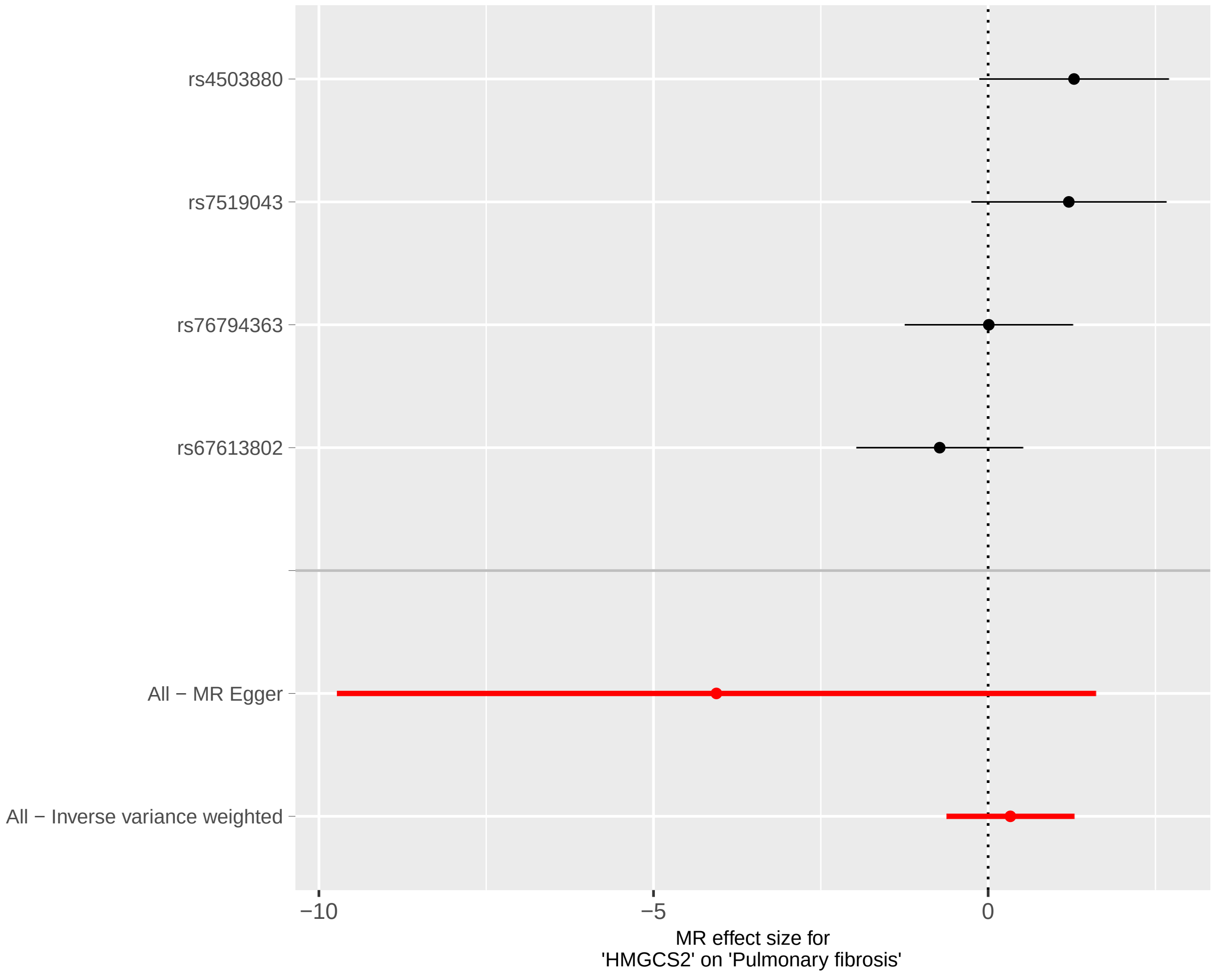
rs4503880
rs7519043
rs76794363
rs67613802
All − MR Egger
All − Inverse variance weighted
−10
−5
0
MR effect size for
'HMGCS2' on 'Pulmonary fibrosis'

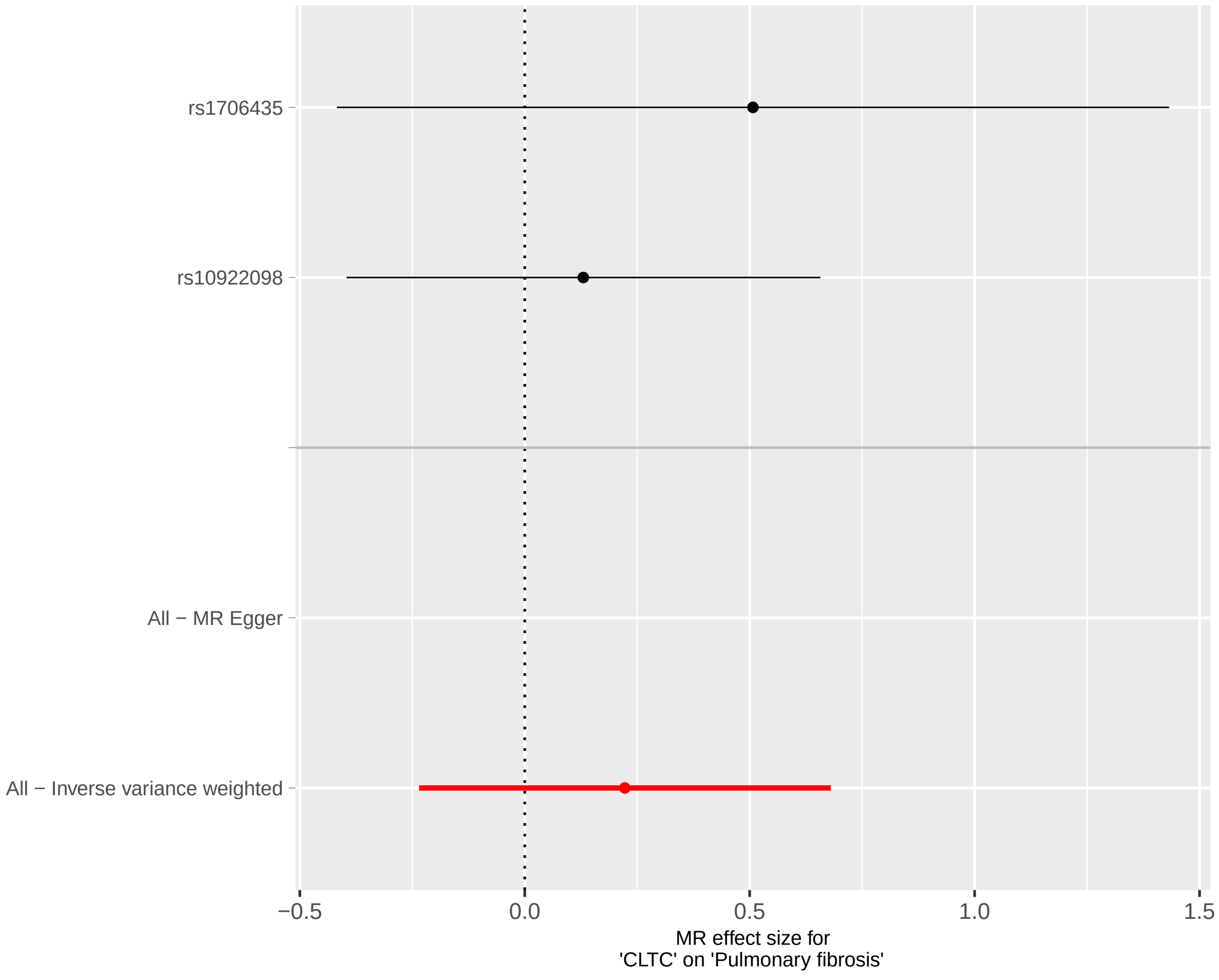

rs1706435
rs10922098
All − MR Egger
All − Inverse variance weighted
−0.5
0.0
0.5
1.0
1.5
MR effect size for
'CLTC' on 'Pulmonary fibrosis'

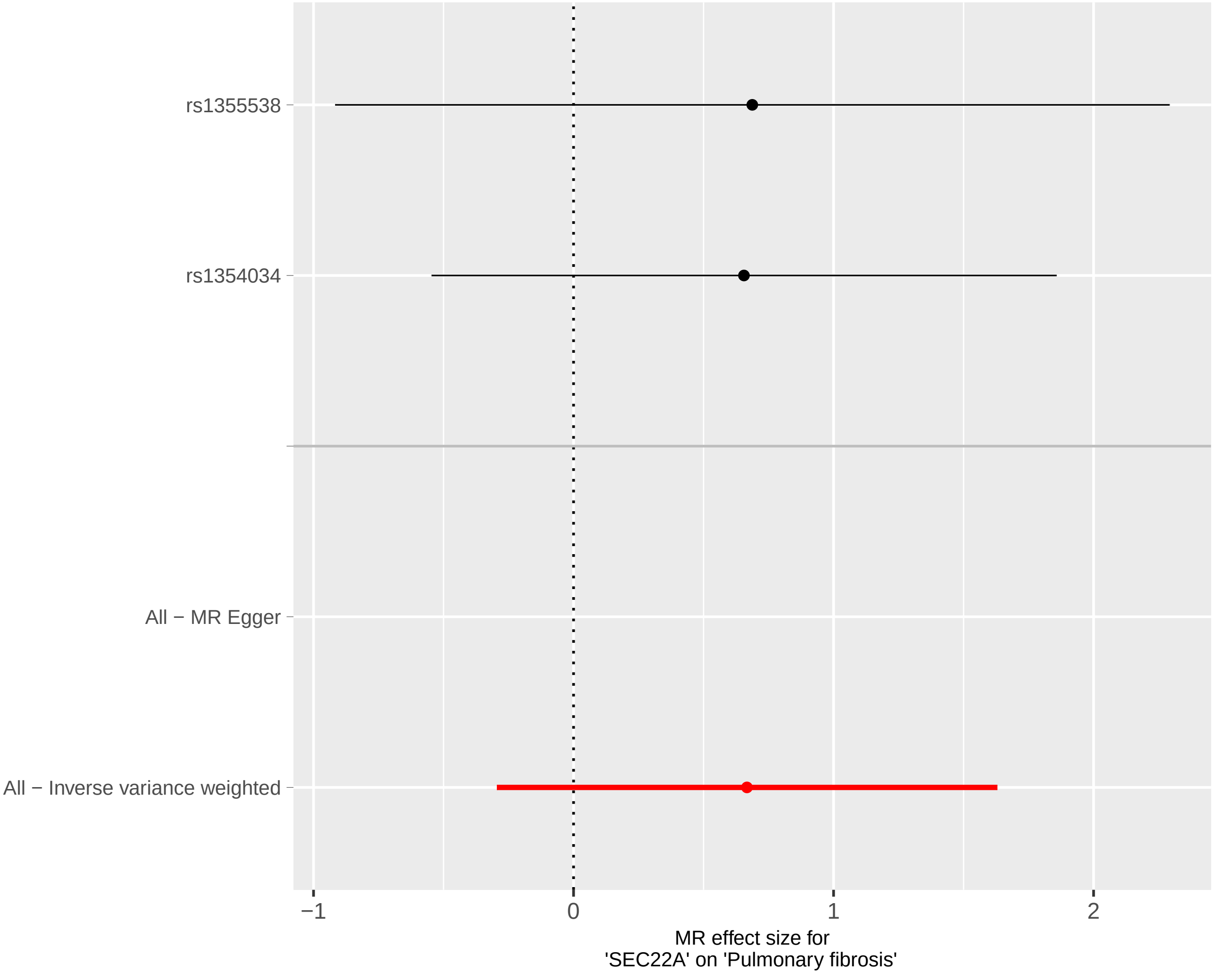

rs1355538
rs1354034
All − MR Egger
All − Inverse variance weighted
−1
0
1
2
MR effect size for
'SEC22A' on 'Pulmonary fibrosis'

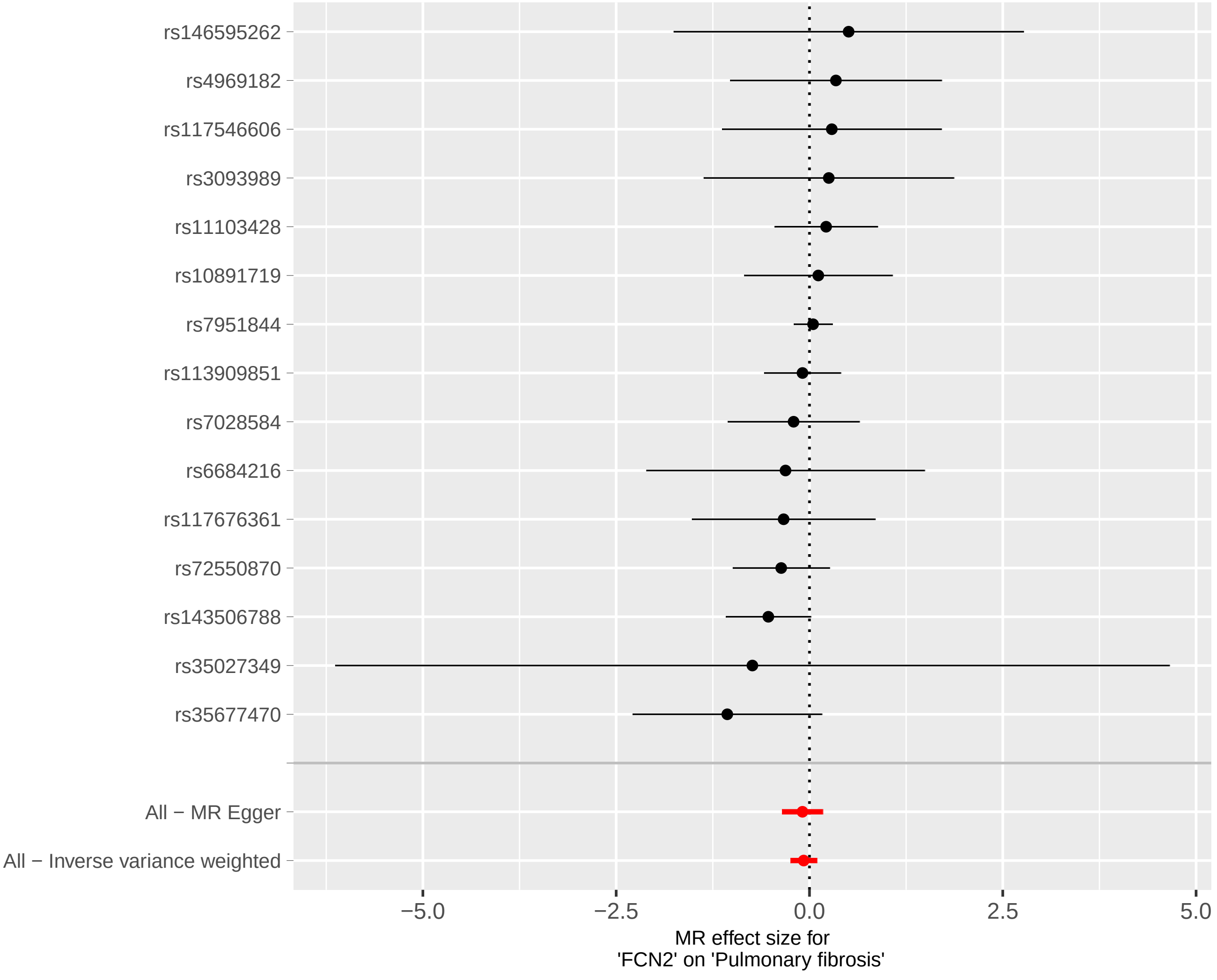
rs146595262
rs4969182
rs117546606
rs3093989
rs11103428
rs10891719
rs7951844
rs113909851
rs7028584
rs6684216
rs117676361
rs72550870
rs143506788
rs35027349
rs35677470
All − MR Egger
All − Inverse variance weighted
−5.0
−2.5
0.0
2.5
5.0
MR effect size for
'FCN2' on 'Pulmonary fibrosis'

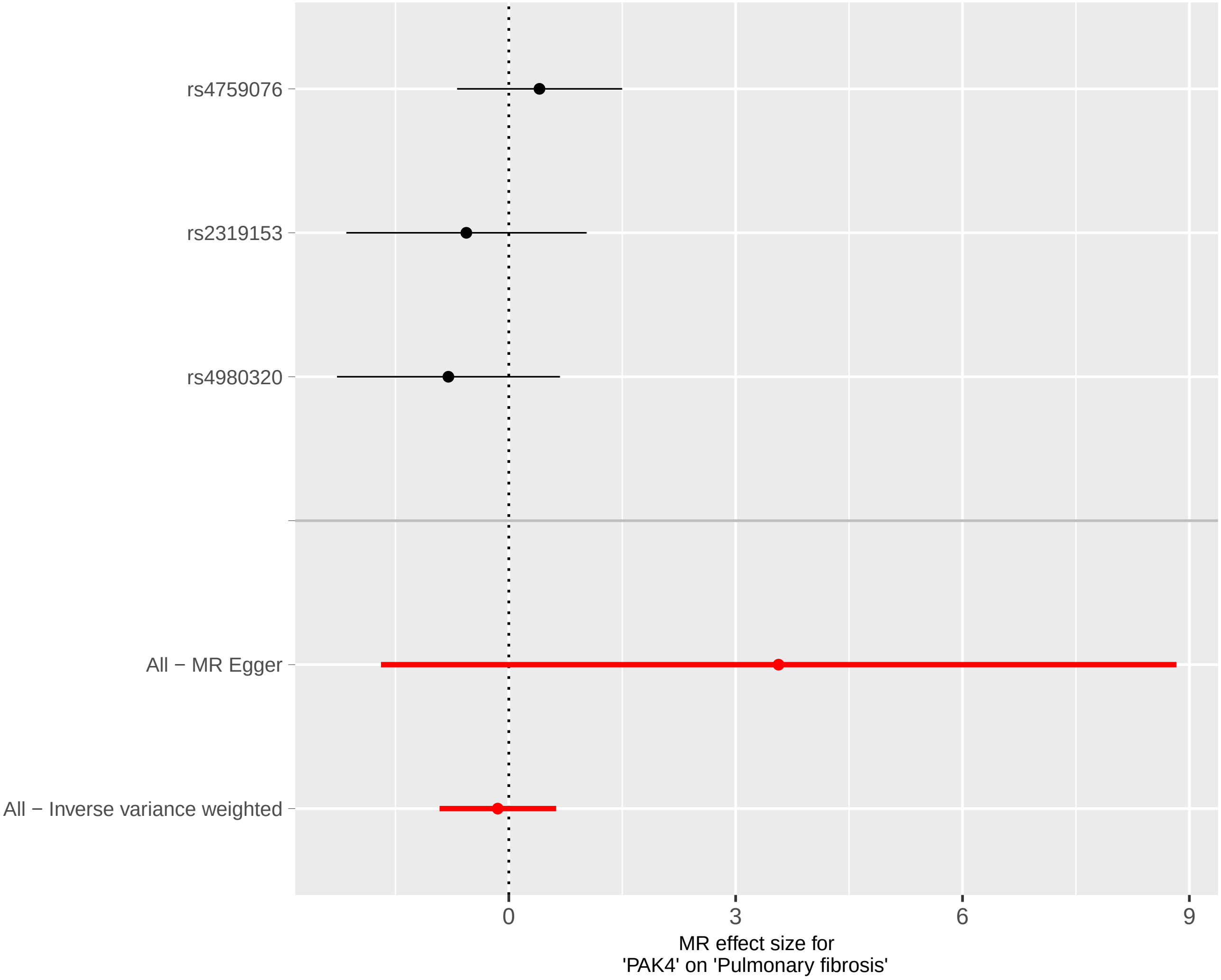

rs4759076
rs2319153
rs4980320
All − MR Egger
All − Inverse variance weighted
0
3
6
9
MR effect size for
'PAK4' on 'Pulmonary fibrosis'

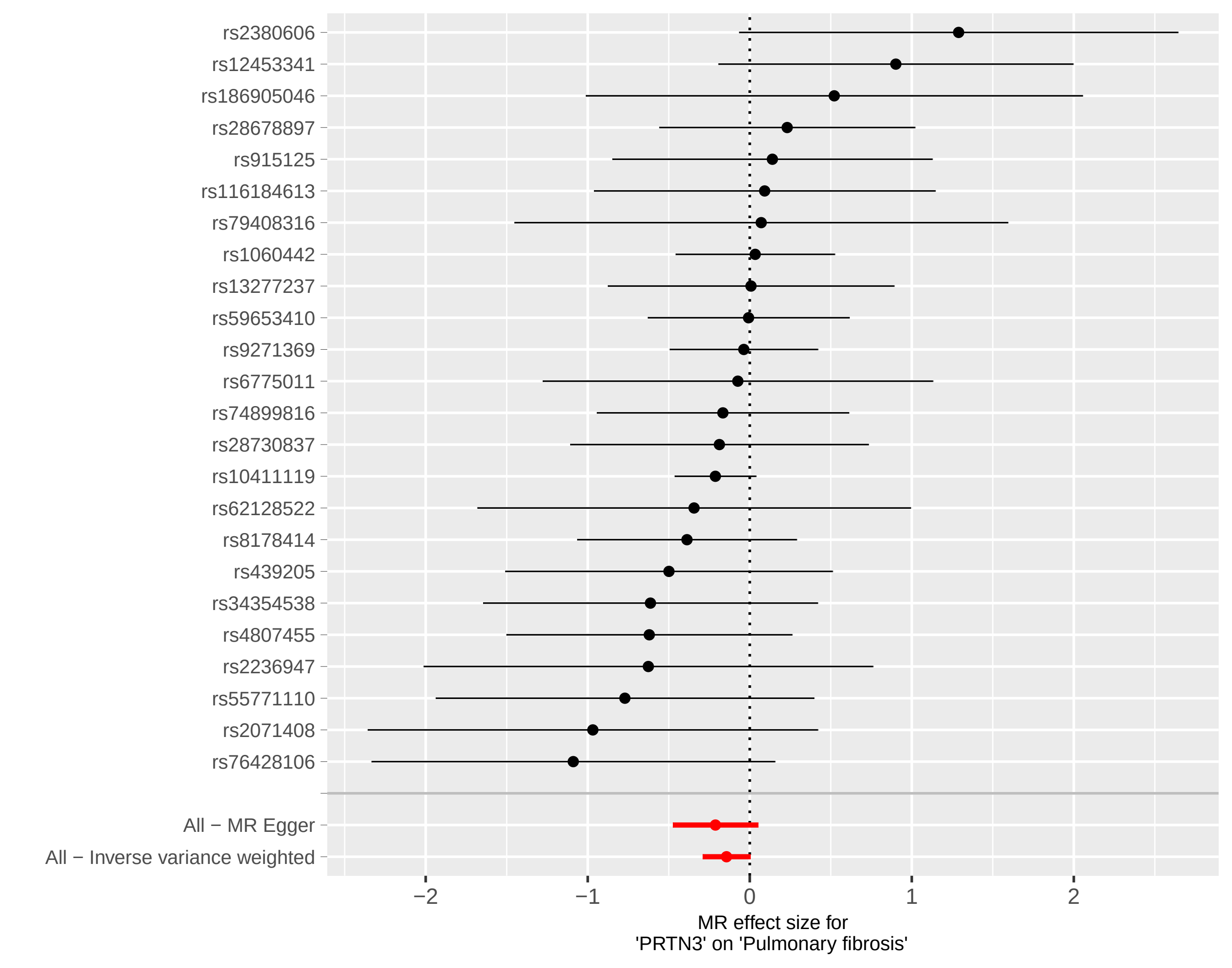
rs2380606
rs12453341
rs186905046
rs28678897
rs915125
rs116184613
rs79408316
rs1060442
rs13277237
rs59653410
rs9271369
rs6775011
rs74899816
rs28730837
rs10411119
rs62128522
rs8178414
rs439205
rs34354538
rs4807455
rs2236947
rs55771110
rs2071408
rs76428106
All − MR Egger
All − Inverse variance weighted
−2
−1
0
1
2
MR effect size for
'PRTN3' on 'Pulmonary fibrosis'

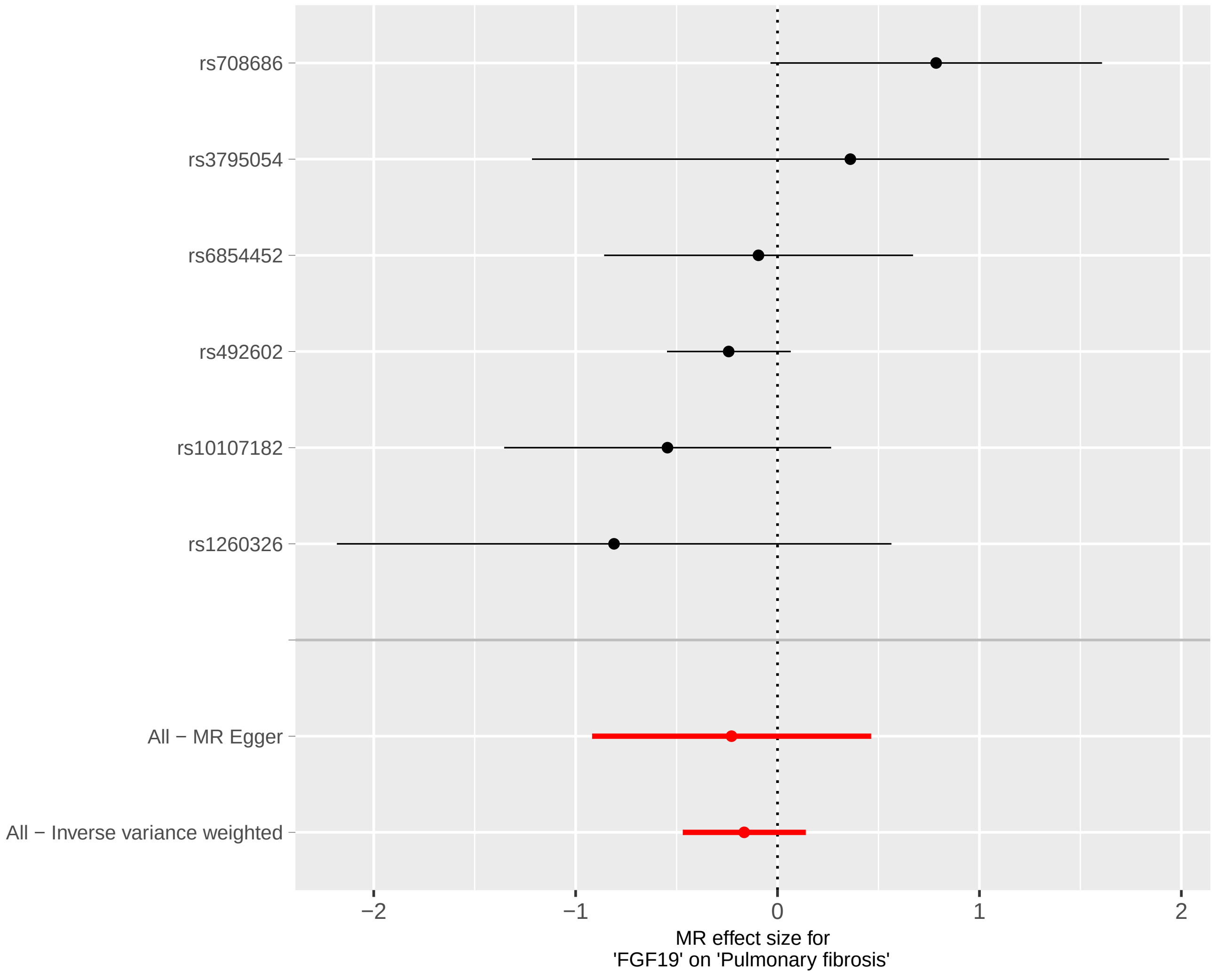
rs708686
rs3795054
rs6854452
rs492602
rs10107182
rs1260326
All − MR Egger
All − Inverse variance weighted
−2
−1
0
1
2
MR effect size for
'FGF19' on 'Pulmonary fibrosis'

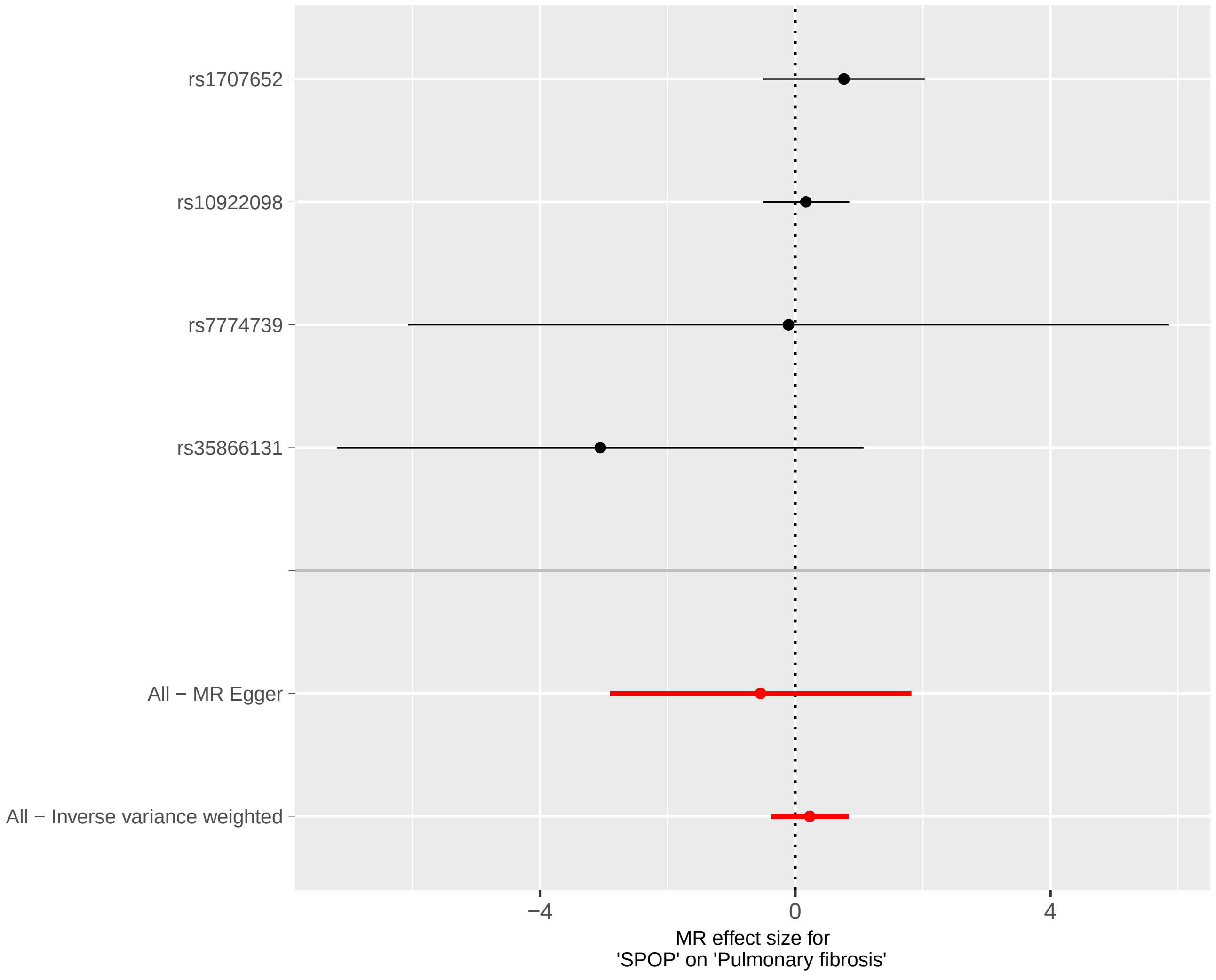

rs1707652
rs10922098
rs7774739
rs35866131
All − MR Egger
All − Inverse variance weighted
−4
0
4
MR effect size for
'SPOP' on 'Pulmonary fibrosis'

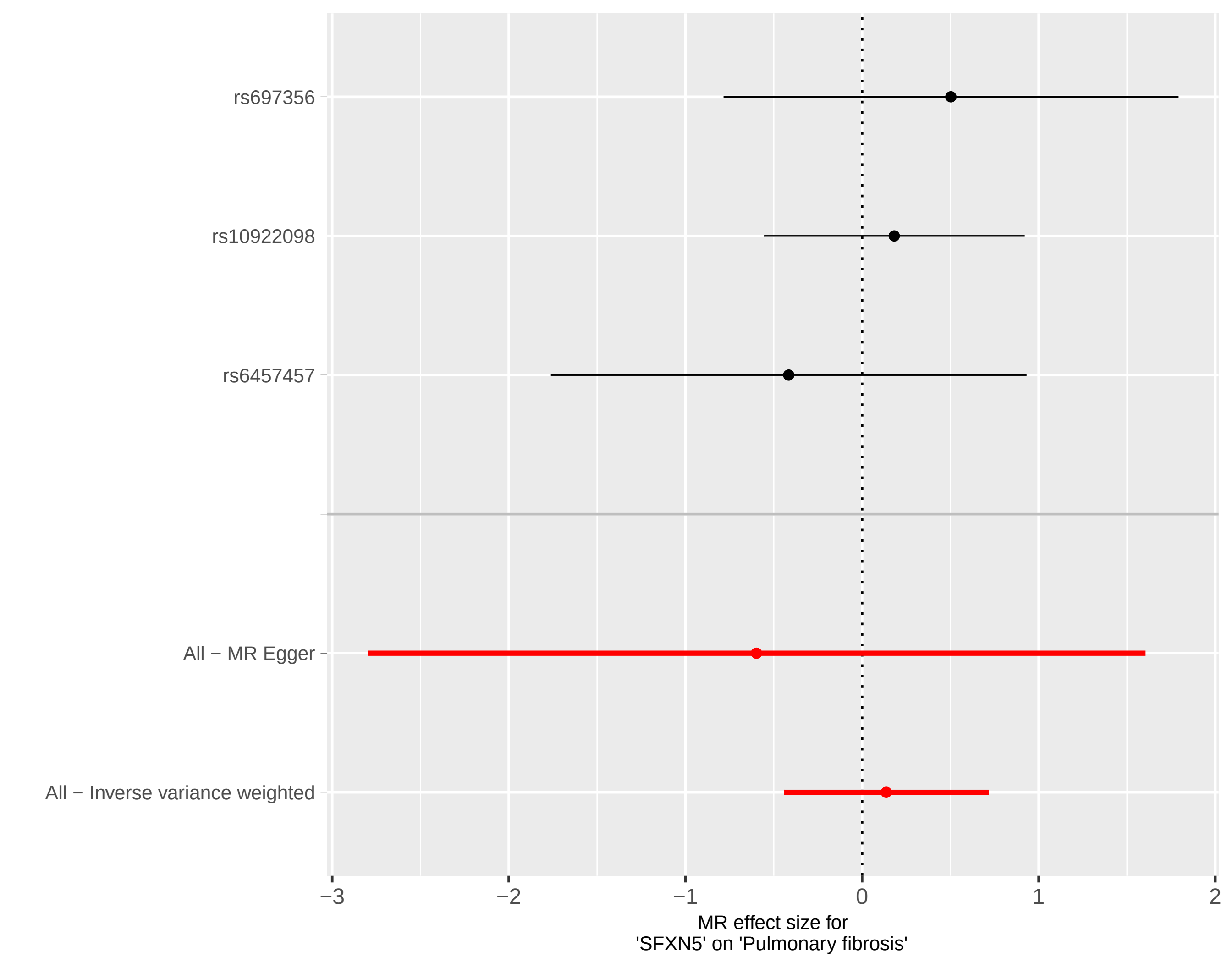
rs697356
rs10922098
rs6457457
All − MR Egger
All − Inverse variance weighted
−3
−2
−1
0
1
2
MR effect size for
'SFXN5' on 'Pulmonary fibrosis'

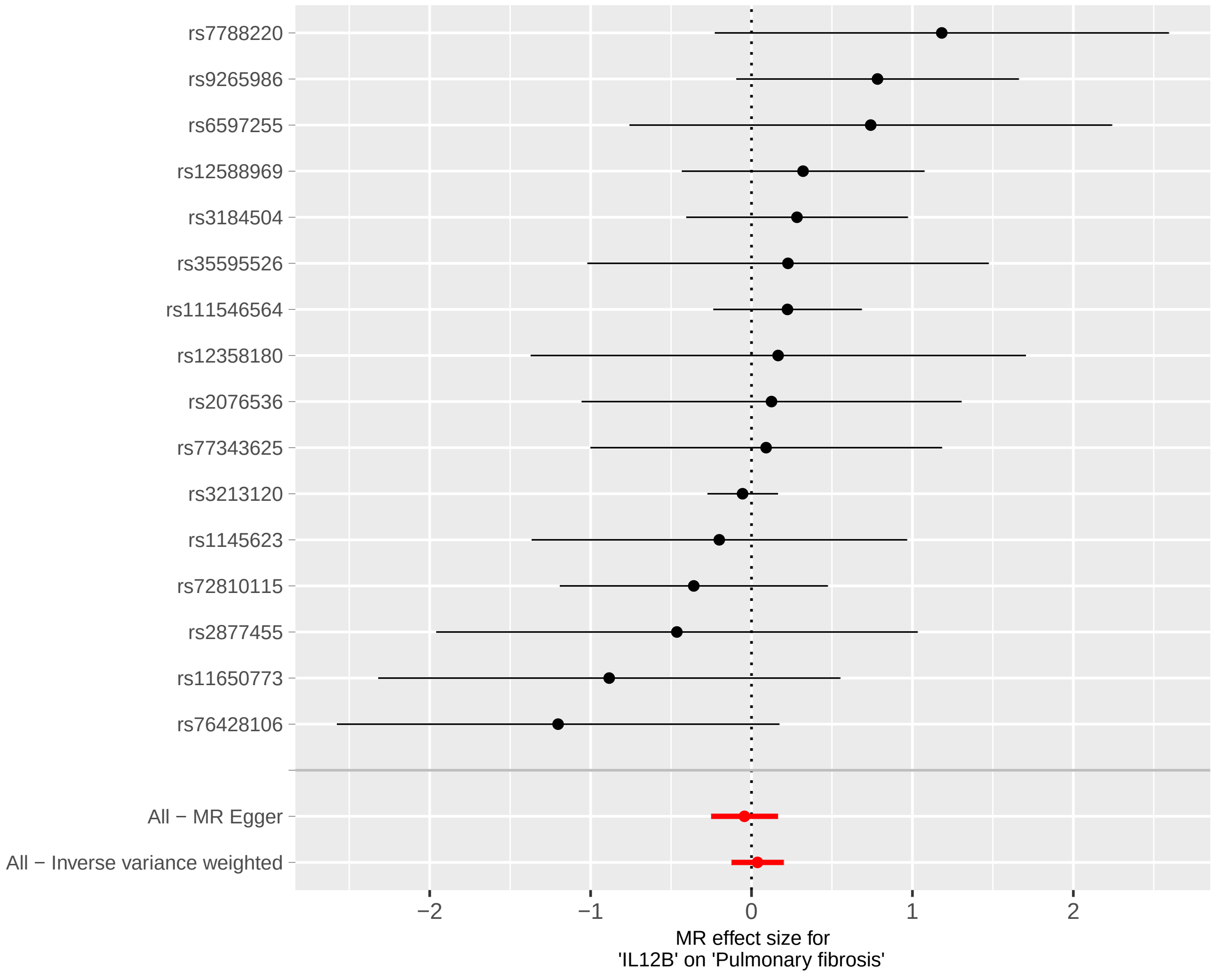

rs7788220
rs9265986
rs6597255
rs12588969
rs3184504
rs35595526
rs111546564
rs12358180
rs2076536
rs77343625
rs3213120
rs1145623
rs72810115
rs2877455
rs11650773
rs76428106
All − MR Egger
All − Inverse variance weighted
−2
−1
0
1
2
MR effect size for
'IL12B' on 'Pulmonary fibrosis'

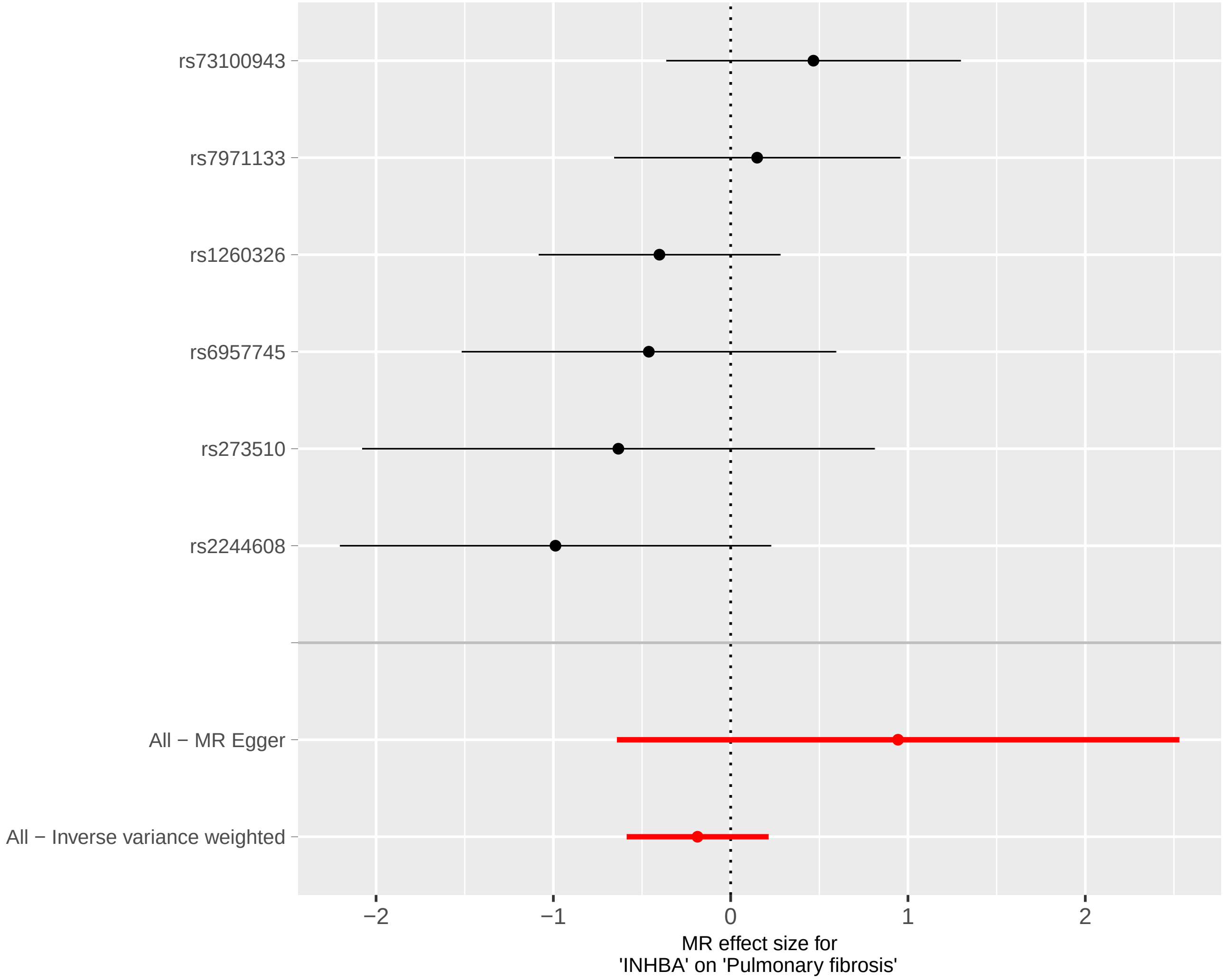
rs73100943
rs7971133
rs1260326
rs6957745
rs273510
rs2244608
All − MR Egger
All − Inverse variance weighted
−2
−1
0
1
2
MR effect size for
'INHBA' on 'Pulmonary fibrosis'

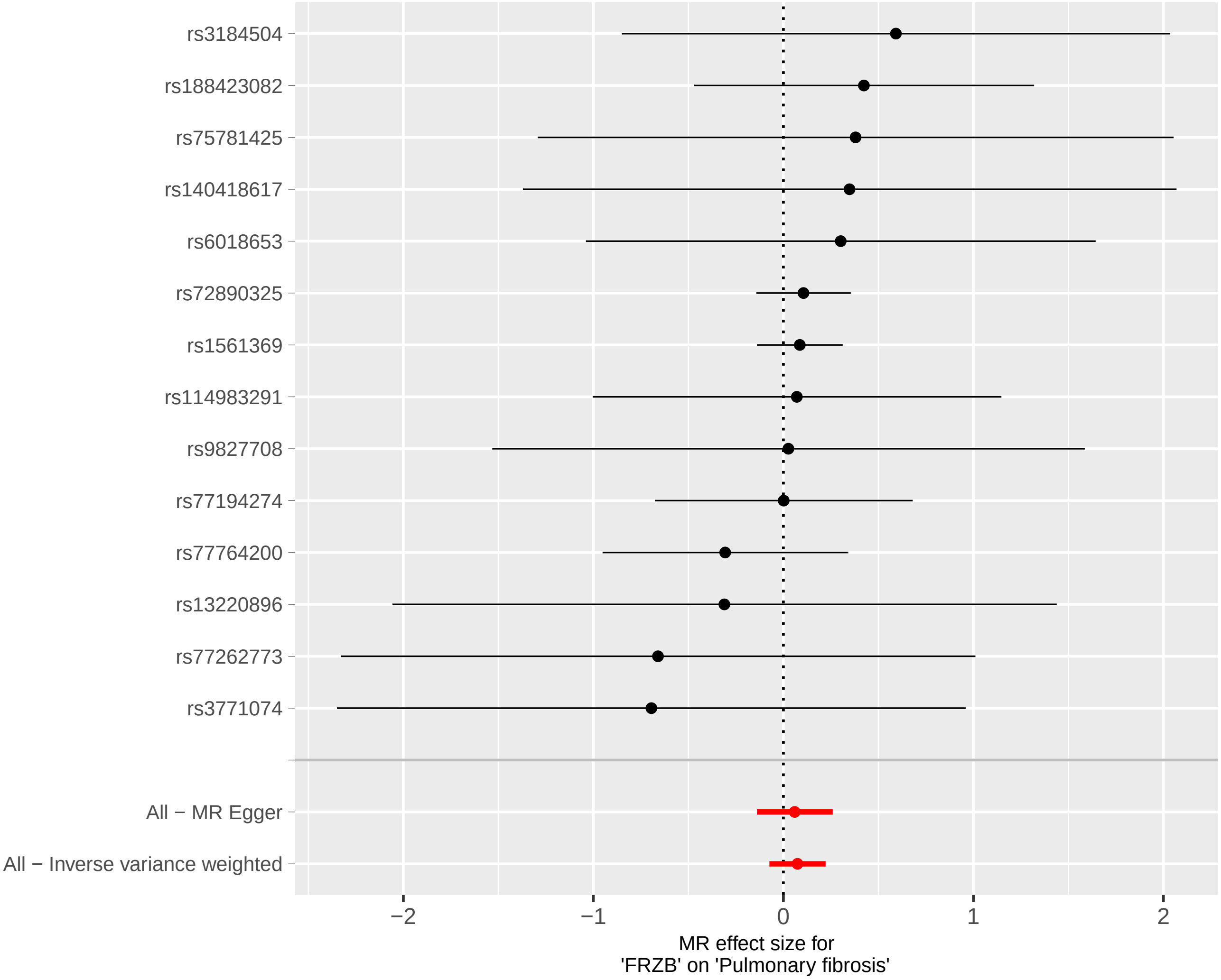
rs3184504
rs188423082
rs75781425
rs140418617
rs6018653
rs72890325
rs1561369
rs114983291
rs9827708
rs77194274
rs77764200
rs13220896
rs77262773
rs3771074
All − MR Egger
All − Inverse variance weighted
−2
−1
0
1
2
MR effect size for
'FRZB' on 'Pulmonary fibrosis'

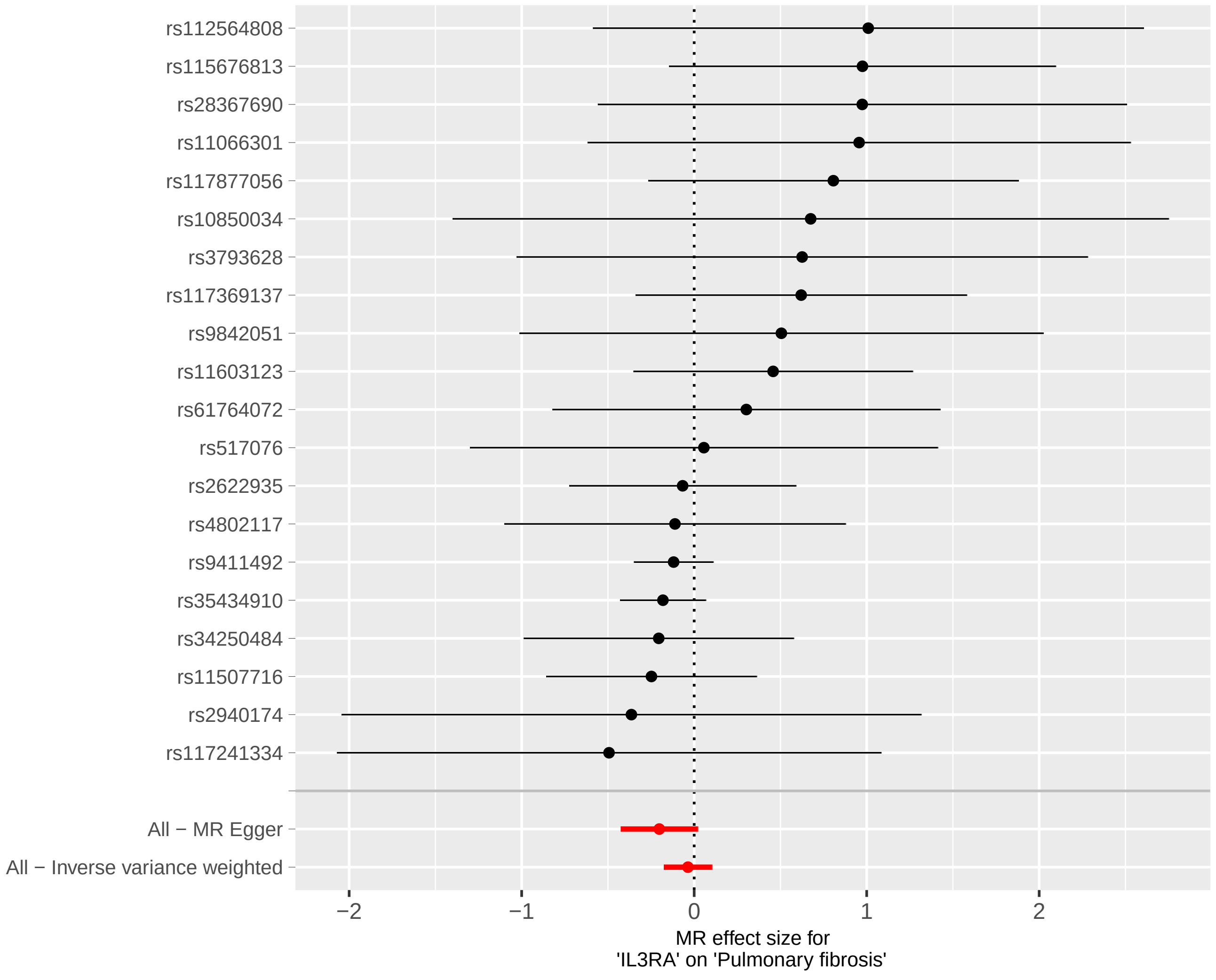
rs112564808
rs115676813
rs28367690
rs11066301
rs117877056
rs10850034
rs3793628
rs117369137
rs9842051
rs11603123
rs61764072
rs517076
rs2622935
rs4802117
rs9411492
rs35434910
rs34250484
rs11507716
rs2940174
rs117241334
All − MR Egger
All − Inverse variance weighted
−2
−1
0
1
2
MR effect size for
'IL3RA' on 'Pulmonary fibrosis'

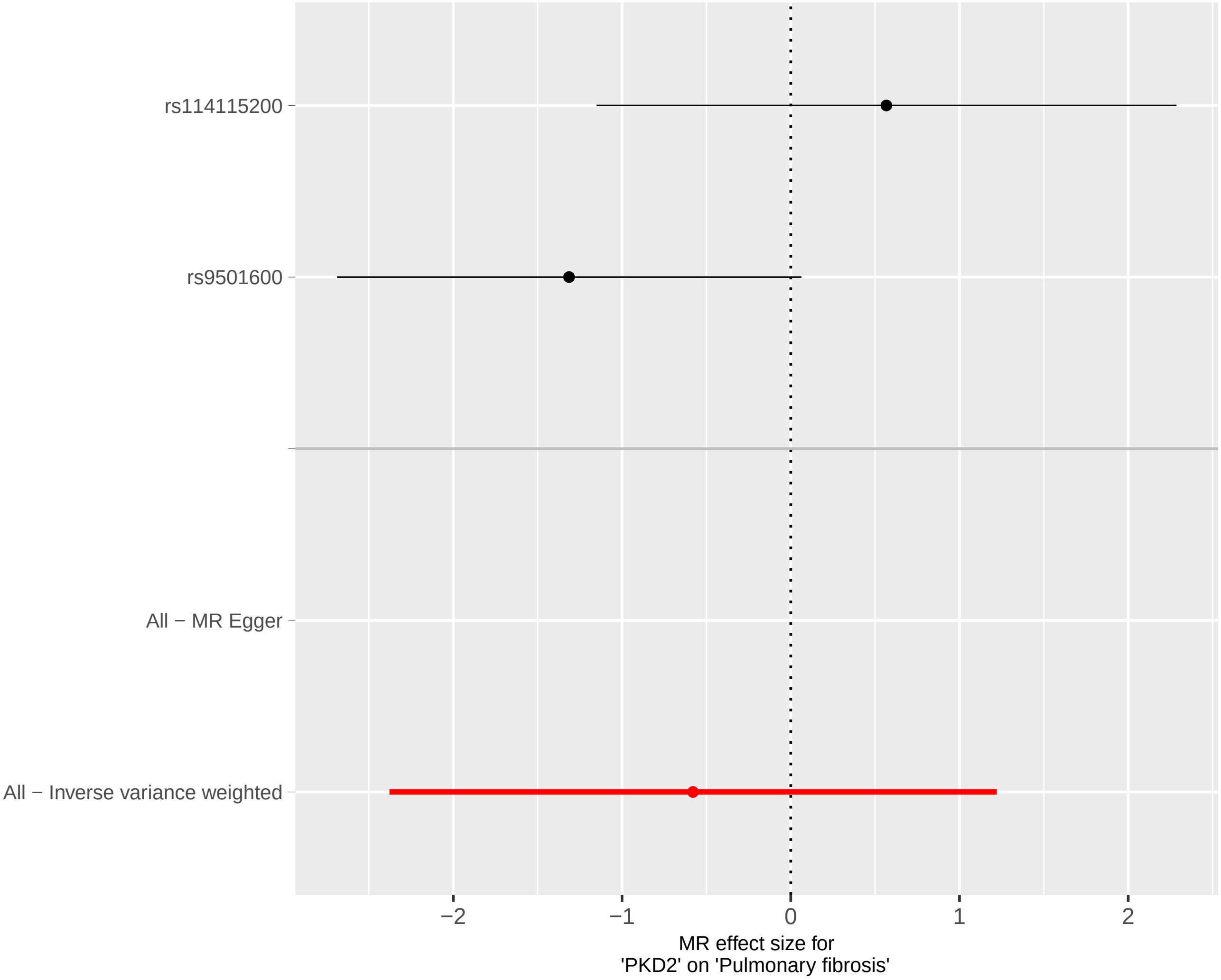
rs114115200
rs9501600
All − MR Egger
All − Inverse variance weighted
−2
−1
0
1
2
MR effect size for
'PKD2' on 'Pulmonary fibrosis'

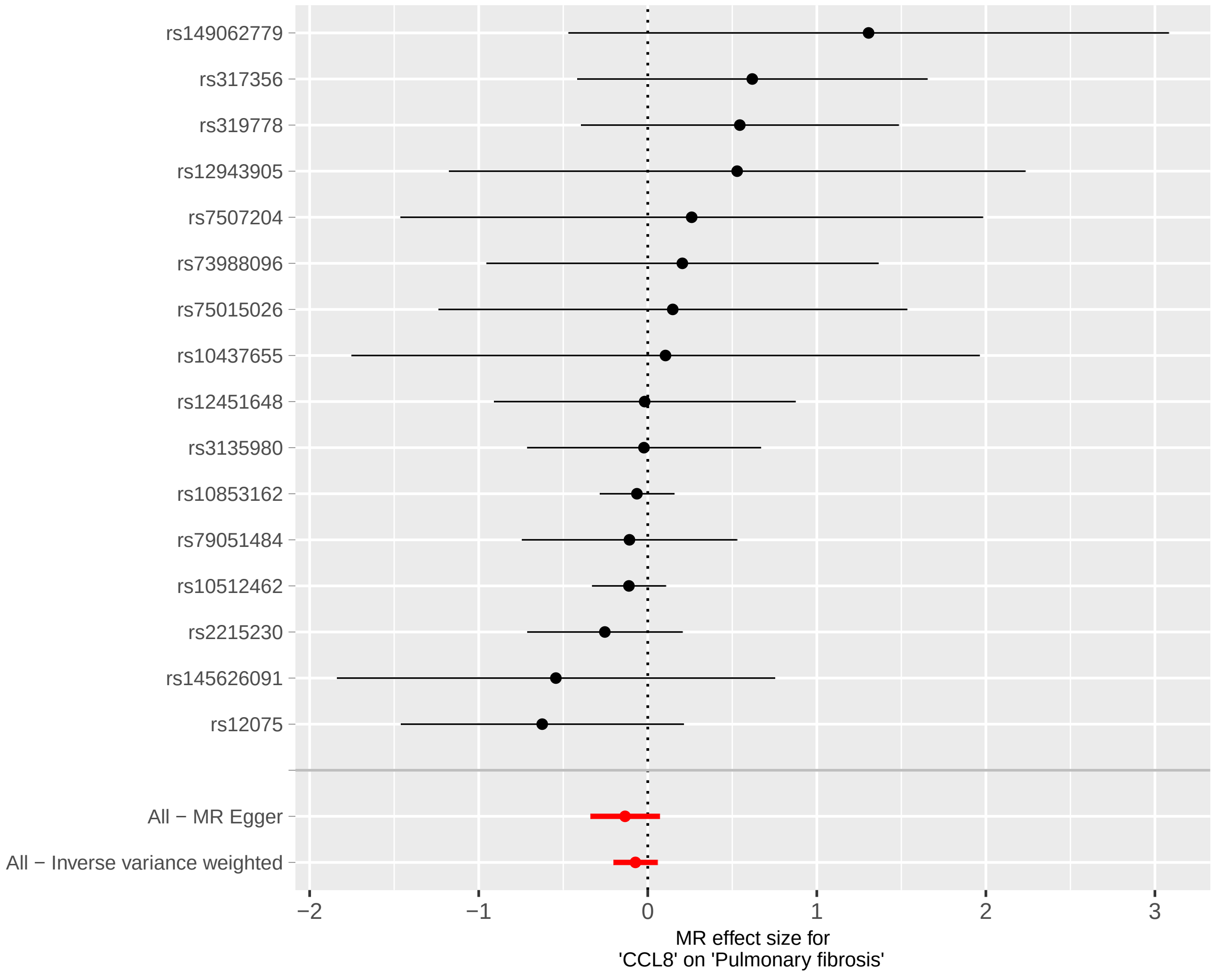

rs149062779
rs317356
rs319778
rs12943905
rs7507204
rs73988096
rs75015026
rs10437655
rs12451648
rs3135980
rs10853162
rs79051484
rs10512462
rs2215230
rs145626091
rs12075
All − MR Egger
All − Inverse variance weighted
−2
−1
0
1
2
3
MR effect size for
'CCL8' on 'Pulmonary fibrosis'

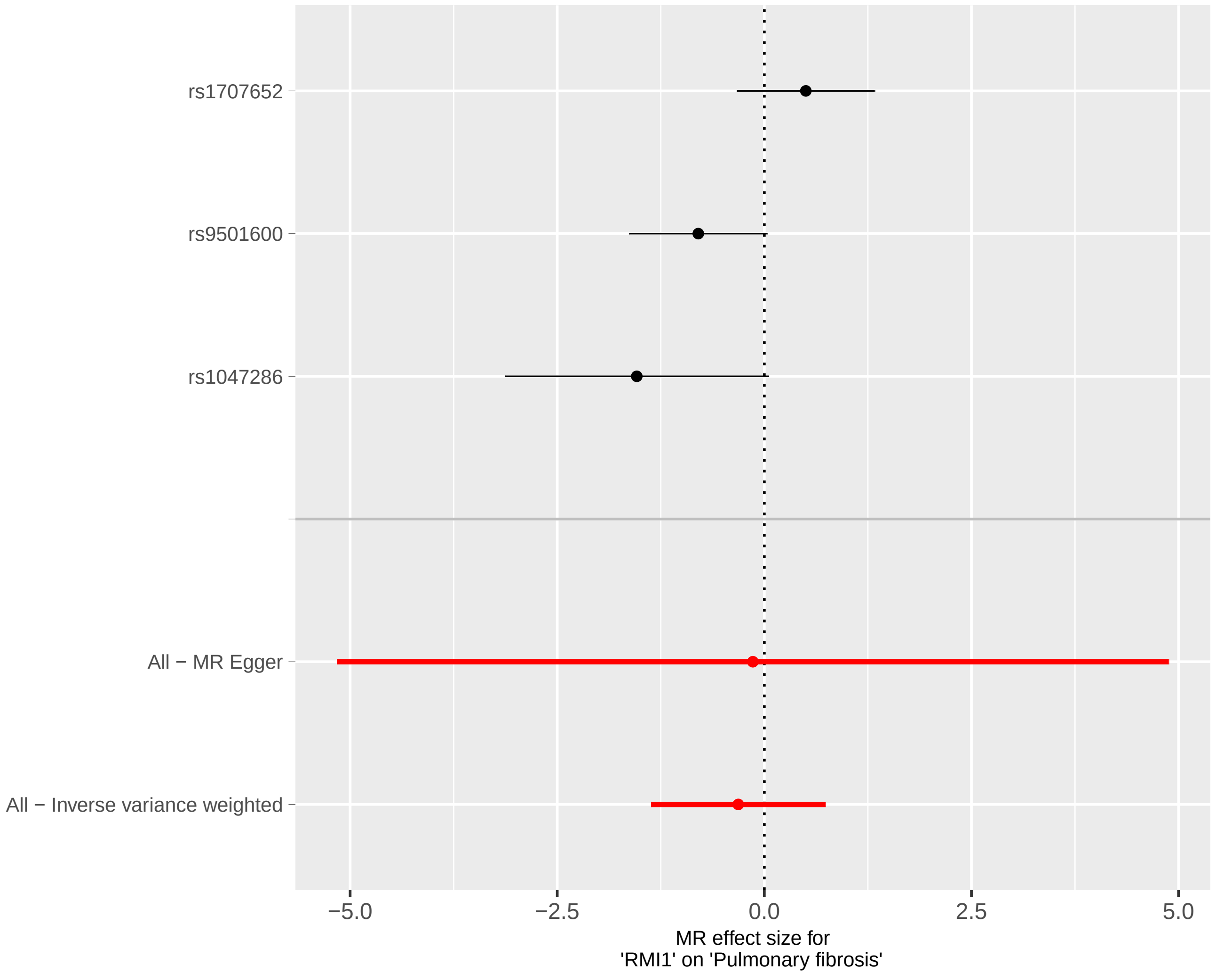
rs1707652
rs9501600
rs1047286
All − MR Egger
All − Inverse variance weighted
−5.0
−2.5
0.0
2.5
5.0
MR effect size for
'RMI1' on 'Pulmonary fibrosis'

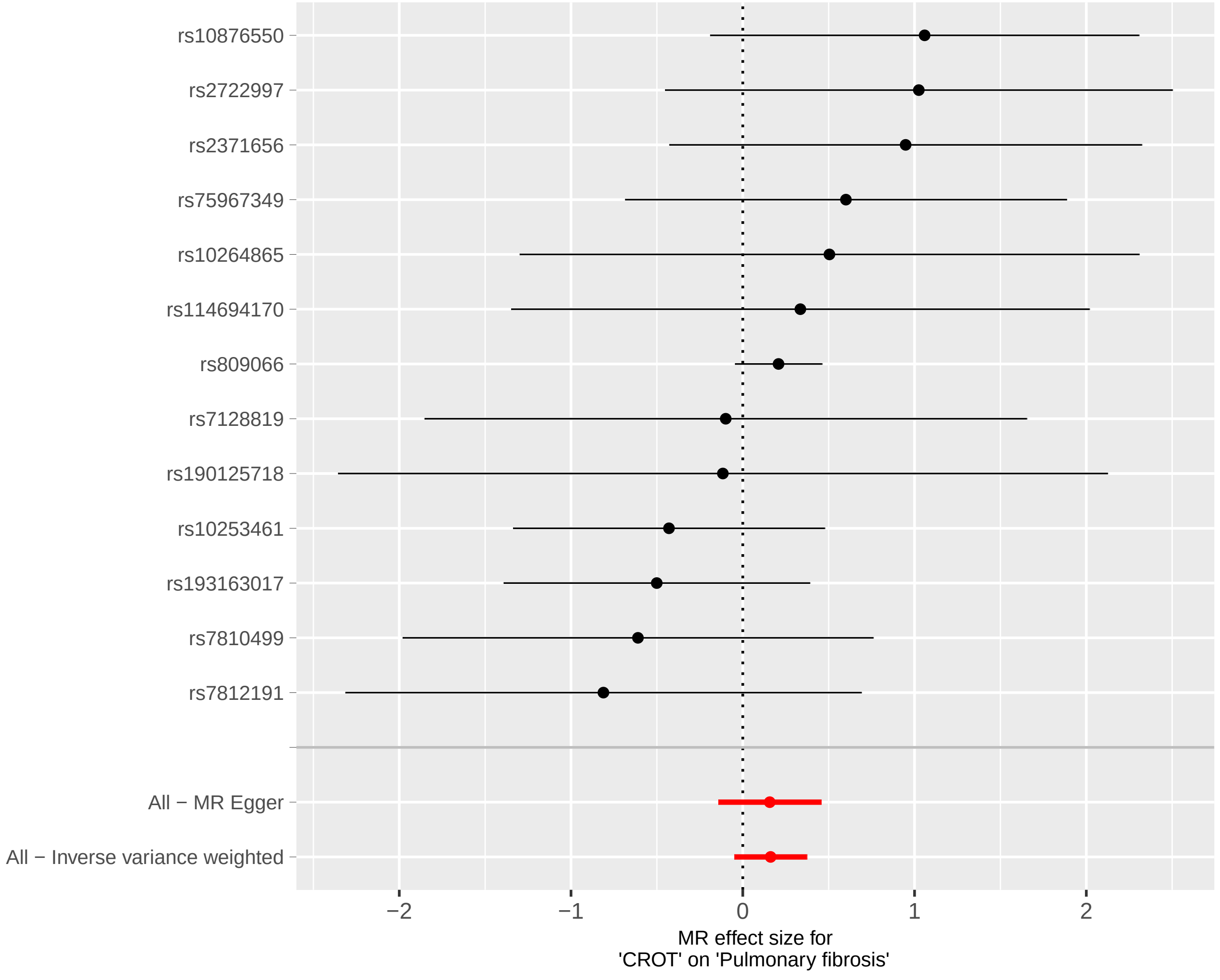
rs10876550
rs2722997
rs2371656
rs75967349
rs10264865
rs114694170
rs809066
rs7128819
rs190125718
rs10253461
rs193163017
rs7810499
rs7812191
All − MR Egger
All − Inverse variance weighted
−2
−1
0
1
2
MR effect size for
'CROT' on 'Pulmonary fibrosis'

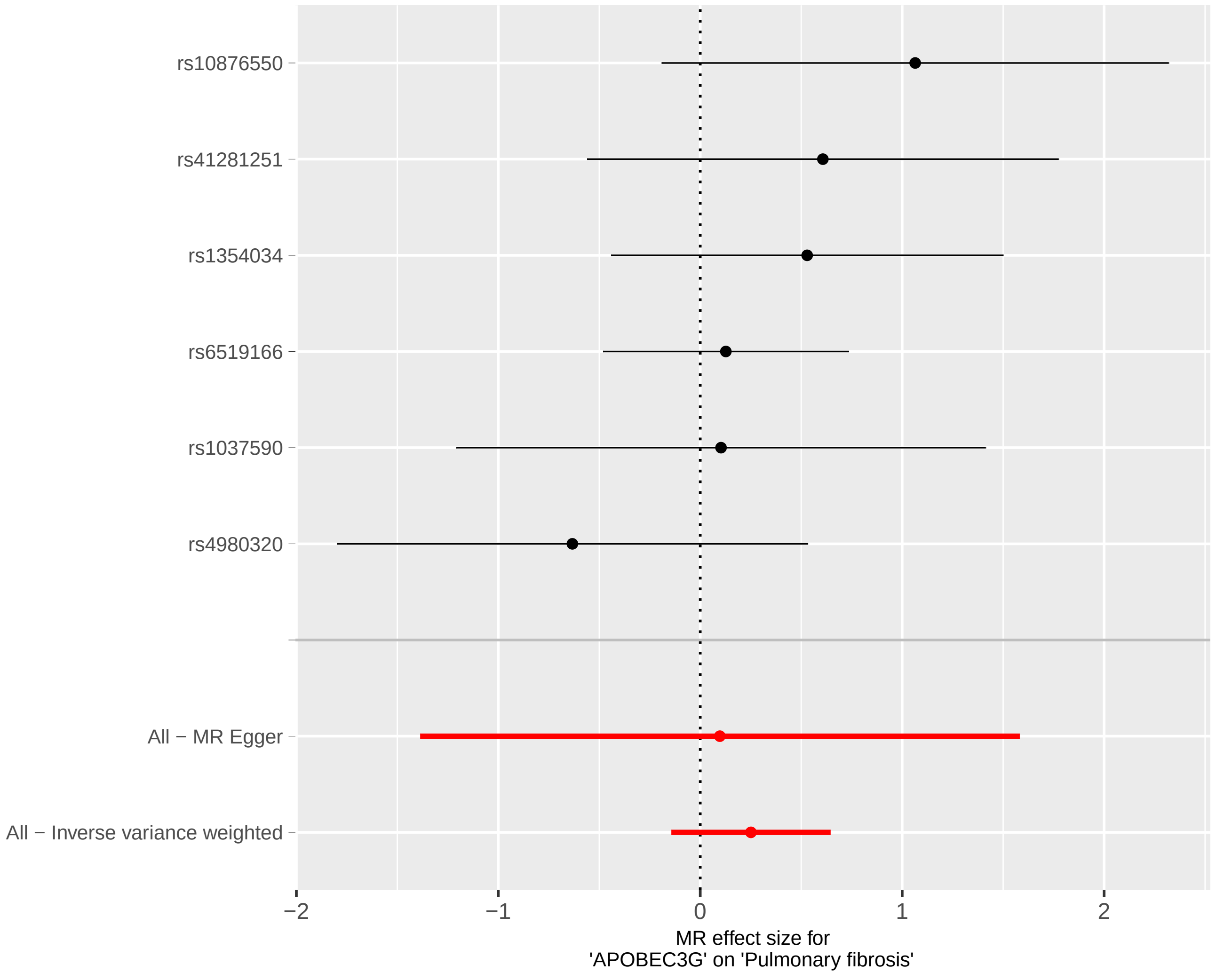
rs10876550
rs41281251
rs1354034
rs6519166
rs1037590
rs4980320
All − MR Egger
All − Inverse variance weighted
−2
−1
0
1
2
MR effect size for
'APOBEC3G' on 'Pulmonary fibrosis'

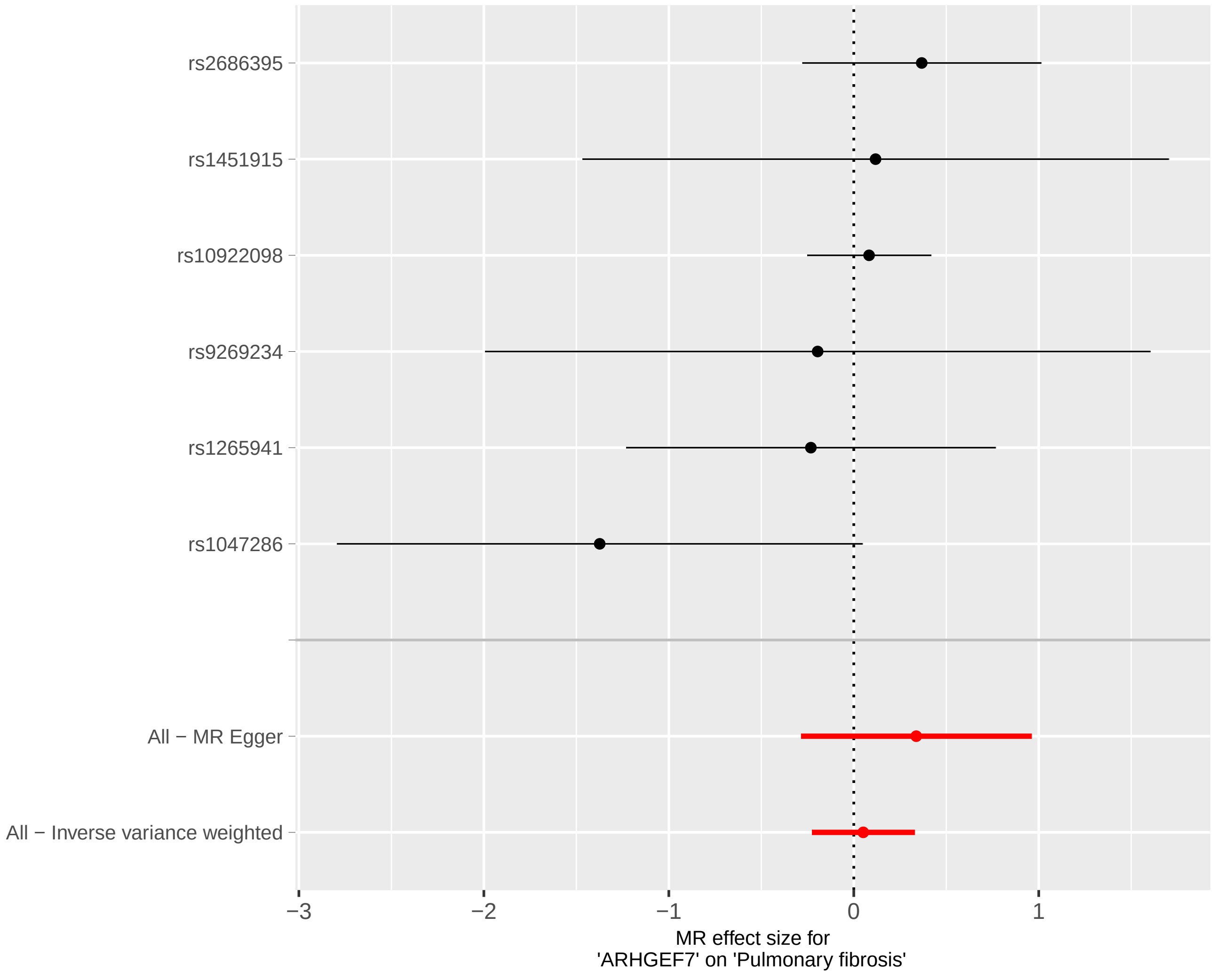
rs2686395
rs1451915
rs10922098
rs9269234
rs1265941
rs1047286
All − MR Egger
All − Inverse variance weighted
−3
−2
−1
0
1
MR effect size for
'ARHGEF7' on 'Pulmonary fibrosis'

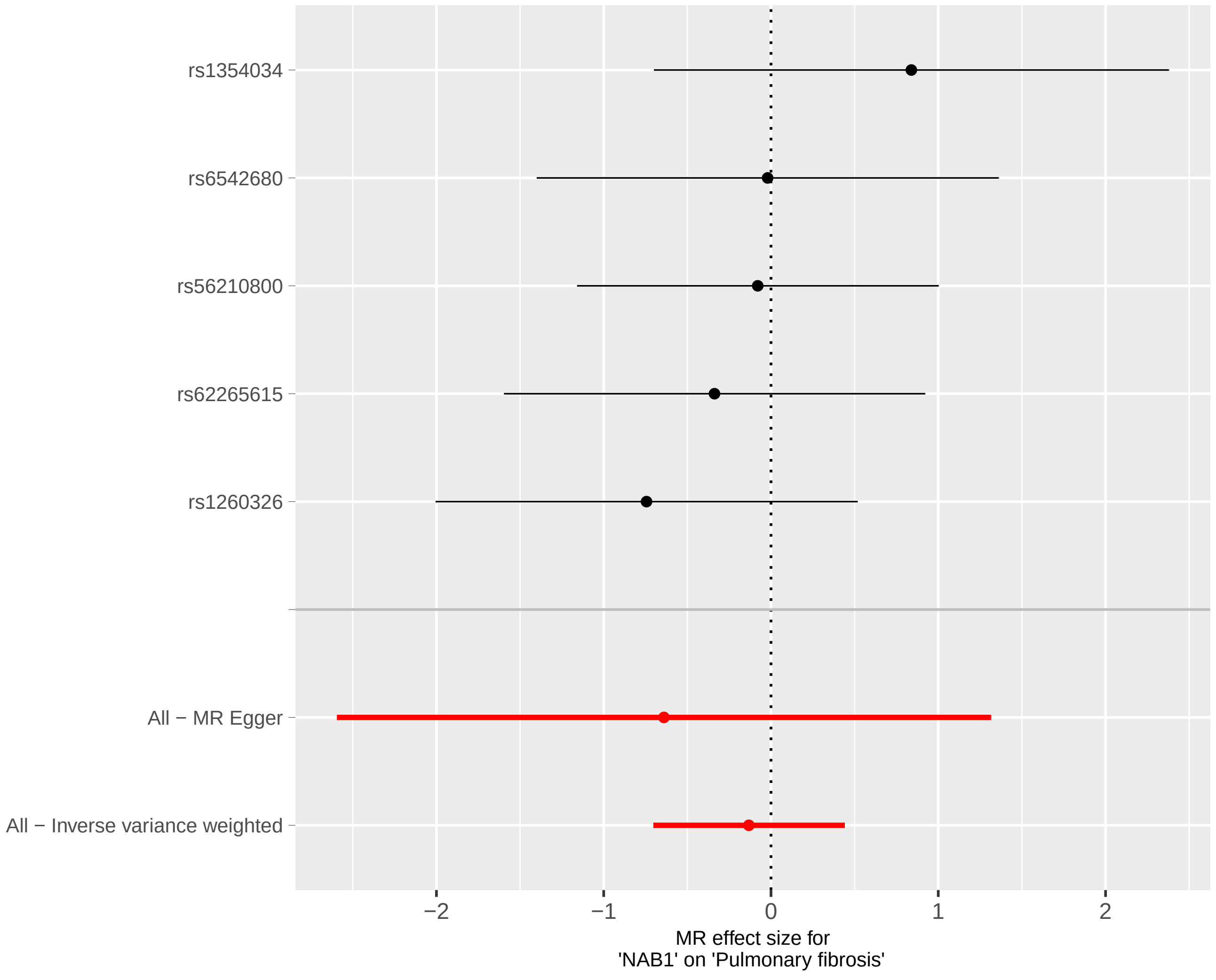
rs1354034
rs6542680
rs56210800
rs62265615
rs1260326
All − MR Egger
All − Inverse variance weighted
−2
−1
0
1
2
MR effect size for
'NAB1' on 'Pulmonary fibrosis'

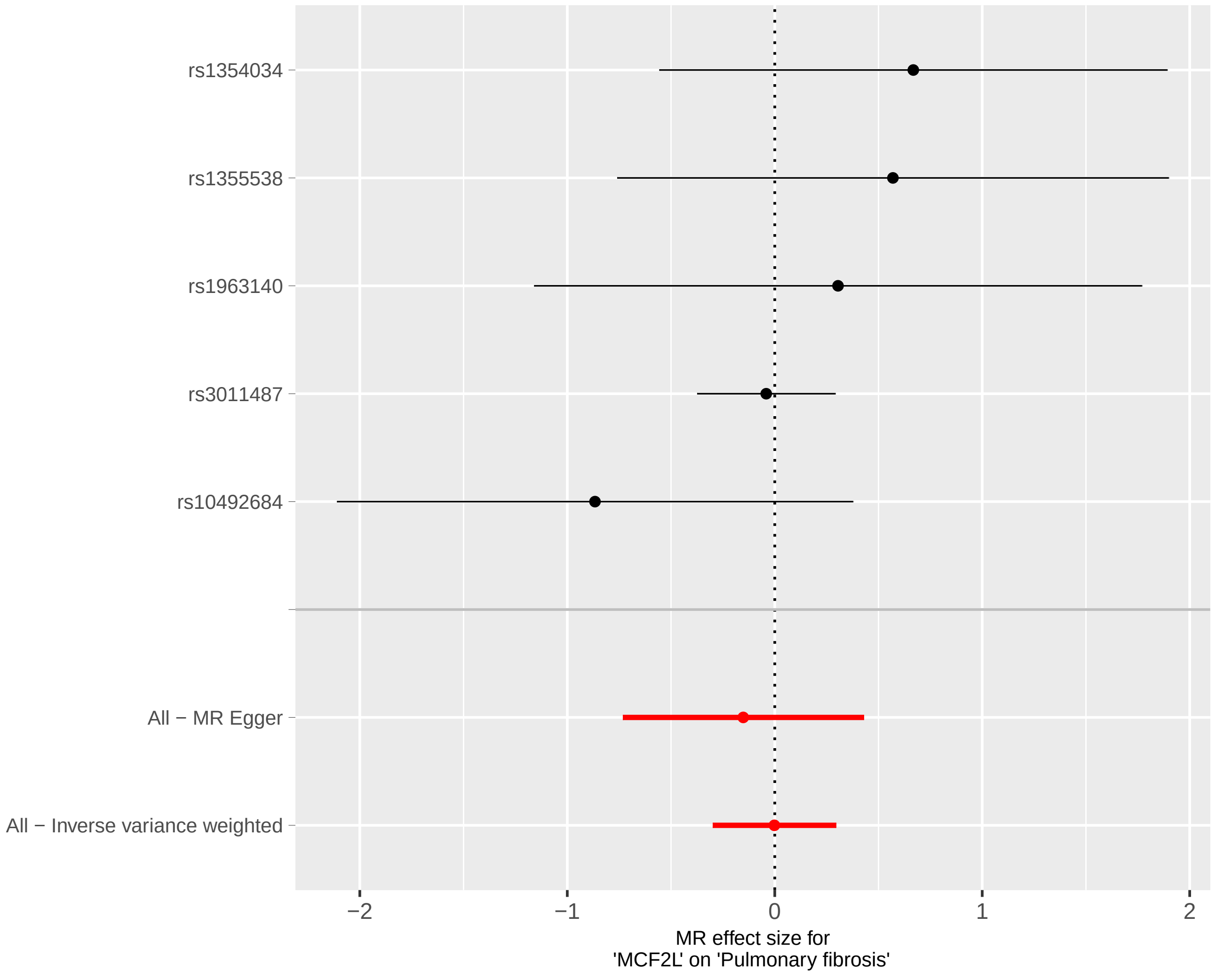

rs1354034
rs1355538
rs1963140
rs3011487
rs10492684
All − MR Egger
All − Inverse variance weighted
−2
−1
0
1
2
MR effect size for
'MCF2L' on 'Pulmonary fibrosis'

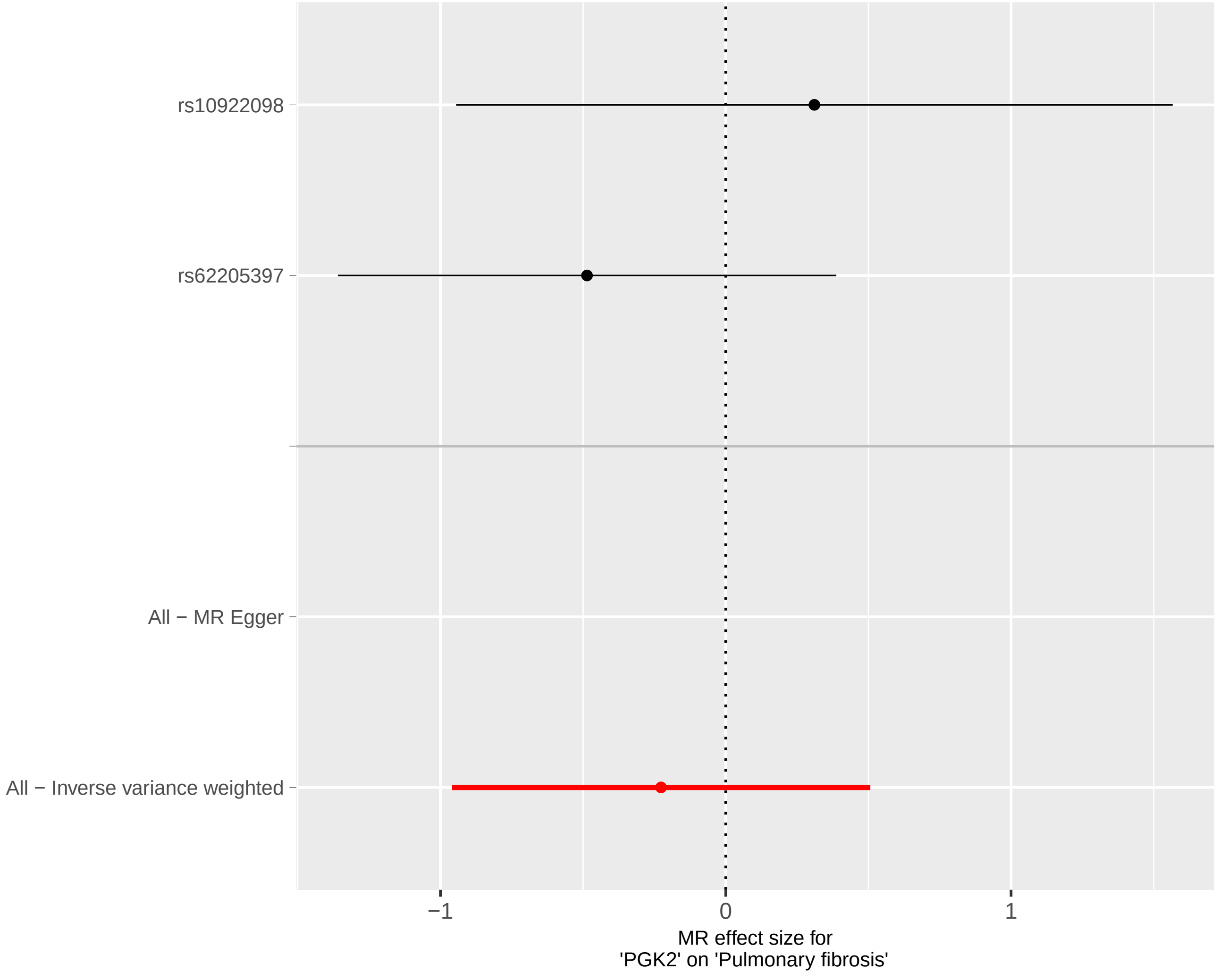
rs10922098
rs62205397
All − MR Egger
All − Inverse variance weighted
−1
0
1
MR effect size for
'PGK2' on 'Pulmonary fibrosis'

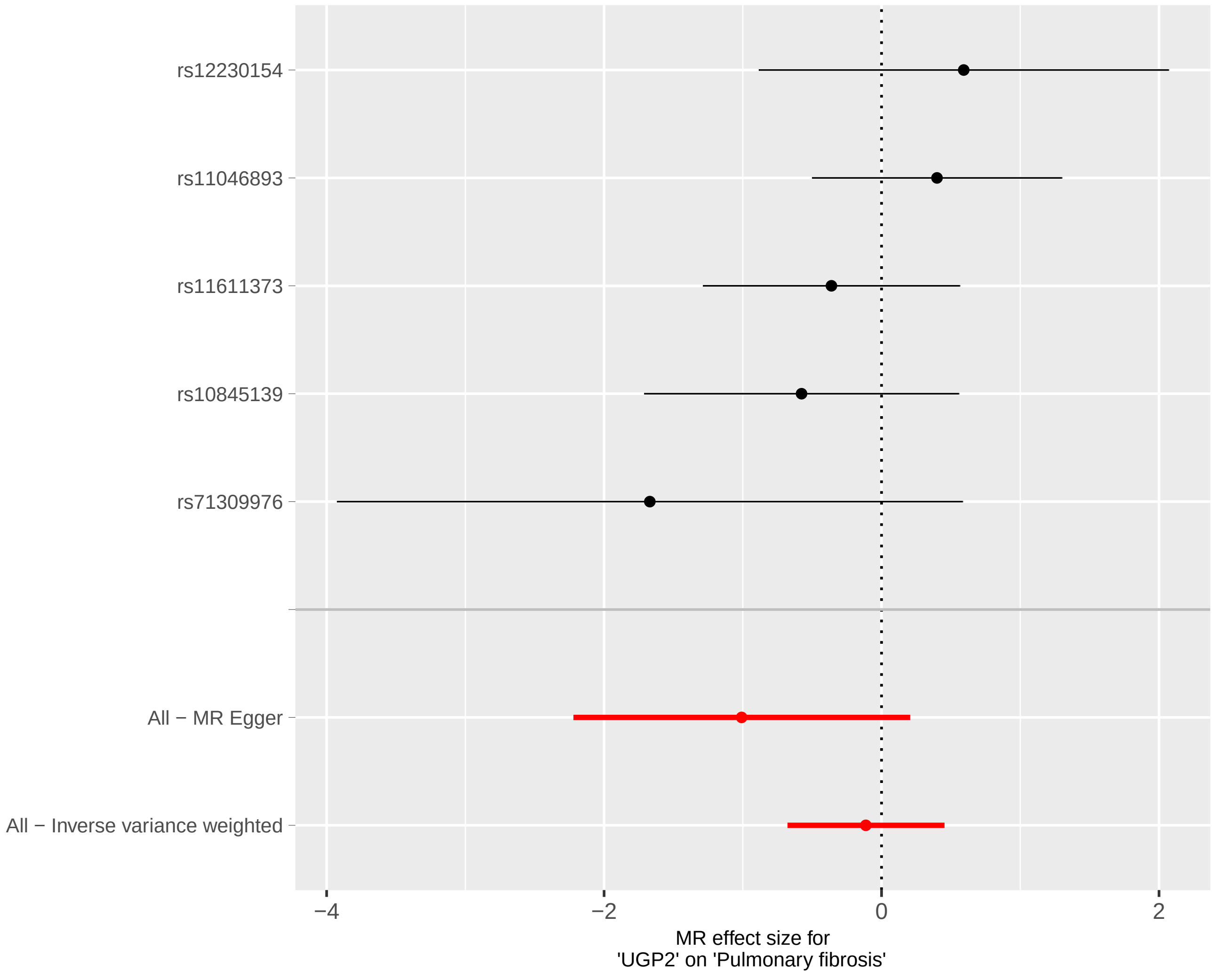
rs12230154
rs11046893
rs11611373
rs10845139
rs71309976
All − MR Egger
All − Inverse variance weighted
−4
−2
0
2
MR effect size for
'UGP2' on 'Pulmonary fibrosis'

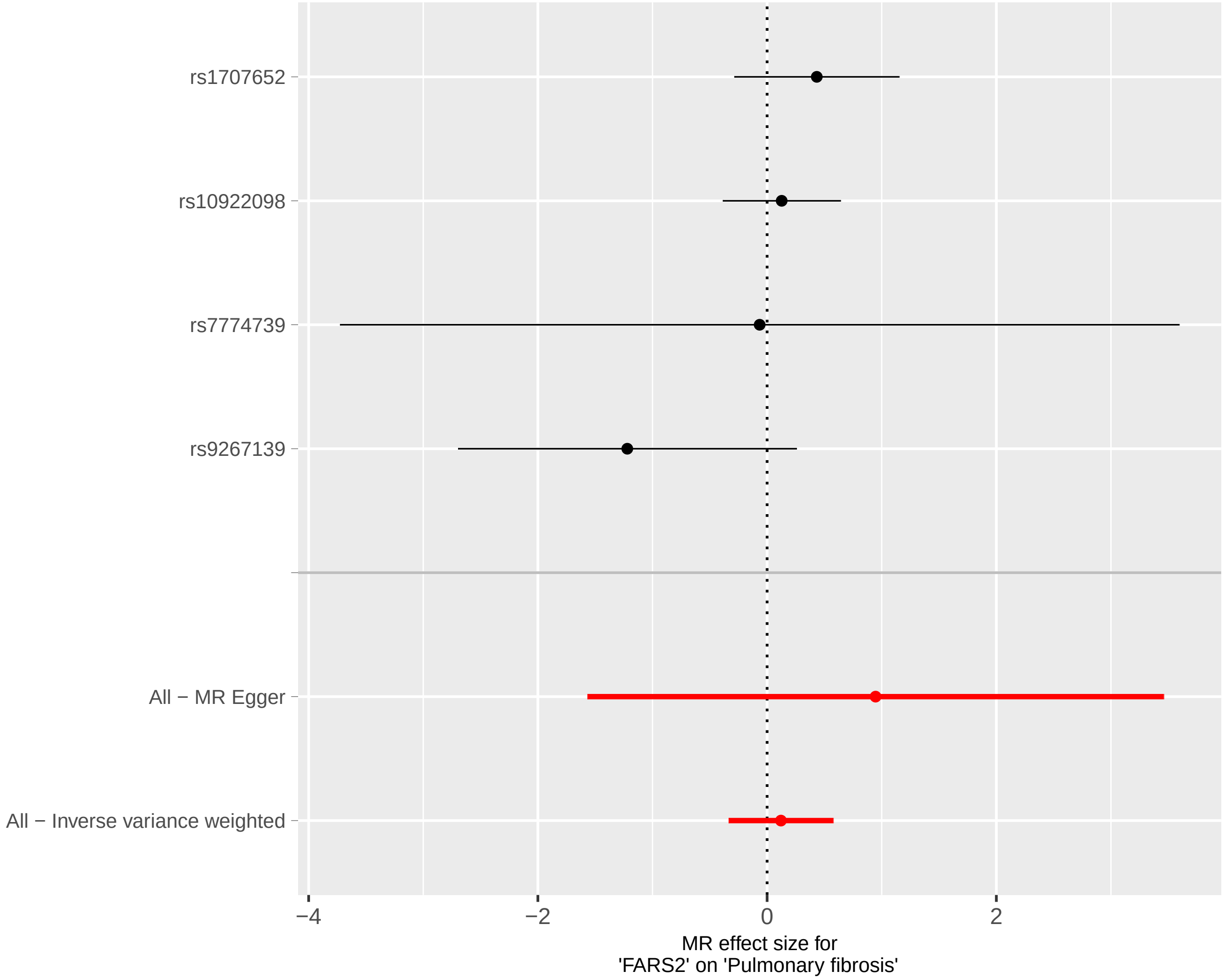

rs1707652
rs10922098
rs7774739
rs9267139
All − MR Egger
All − Inverse variance weighted
−4
−2
0
2
MR effect size for
'FARS2' on 'Pulmonary fibrosis'

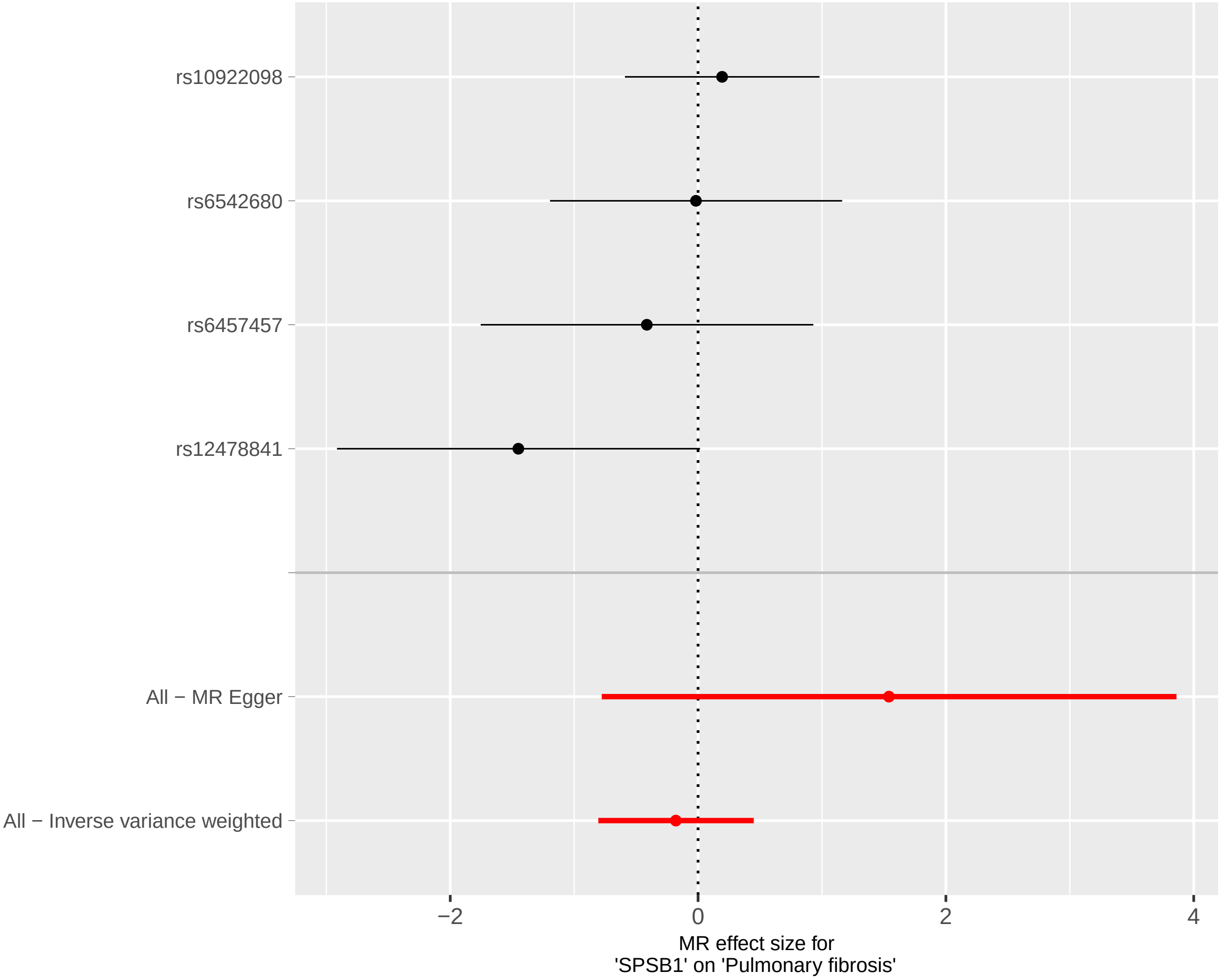

rs10922098
rs6542680
rs6457457
rs12478841
All − MR Egger
All − Inverse variance weighted
−2
0
2
4
MR effect size for
'SPSB1' on 'Pulmonary fibrosis'

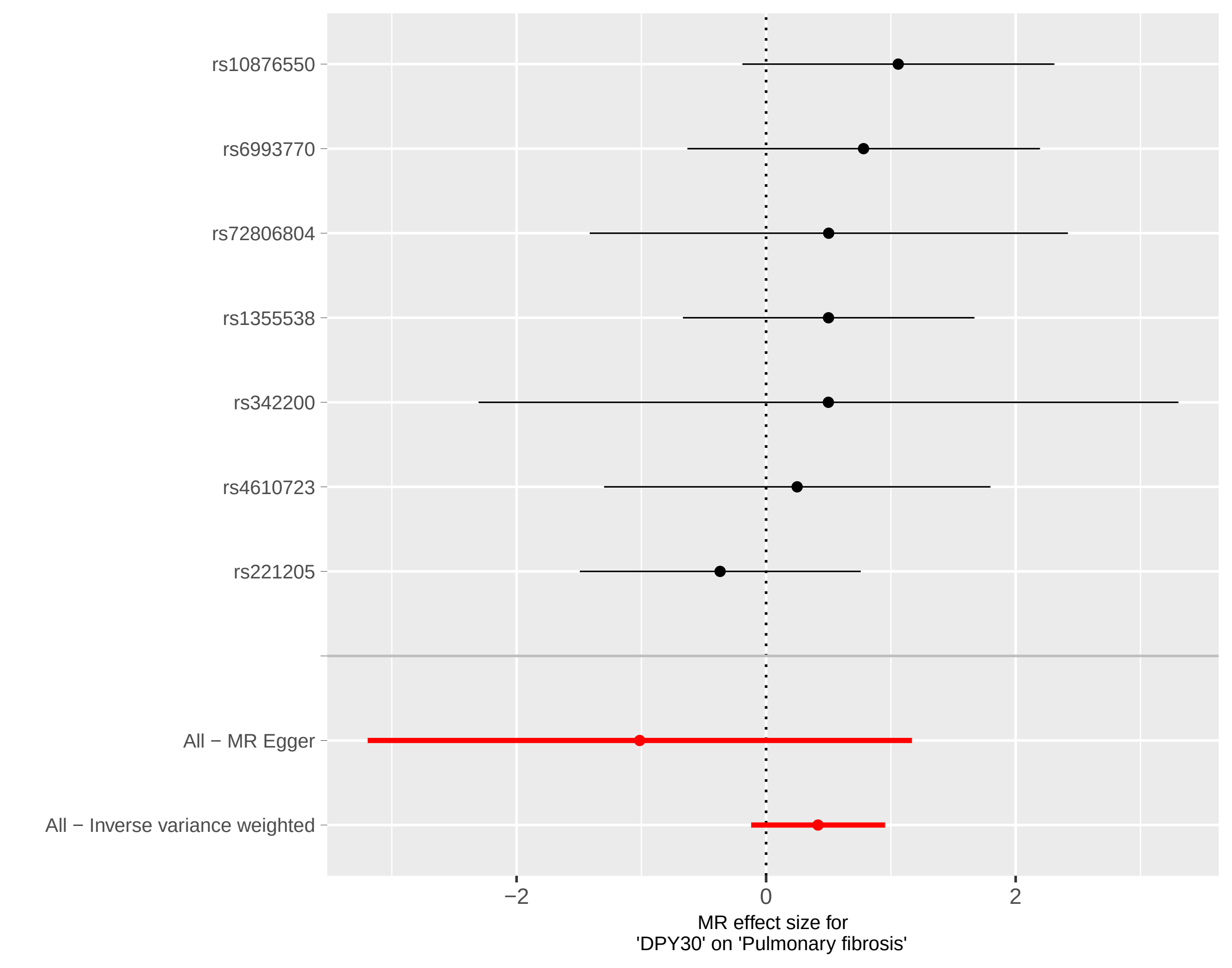
rs10876550
rs6993770
rs72806804
rs1355538
rs342200
rs4610723
rs221205
All − MR Egger
All − Inverse variance weighted
−2
0
2
MR effect size for
'DPY30' on 'Pulmonary fibrosis'

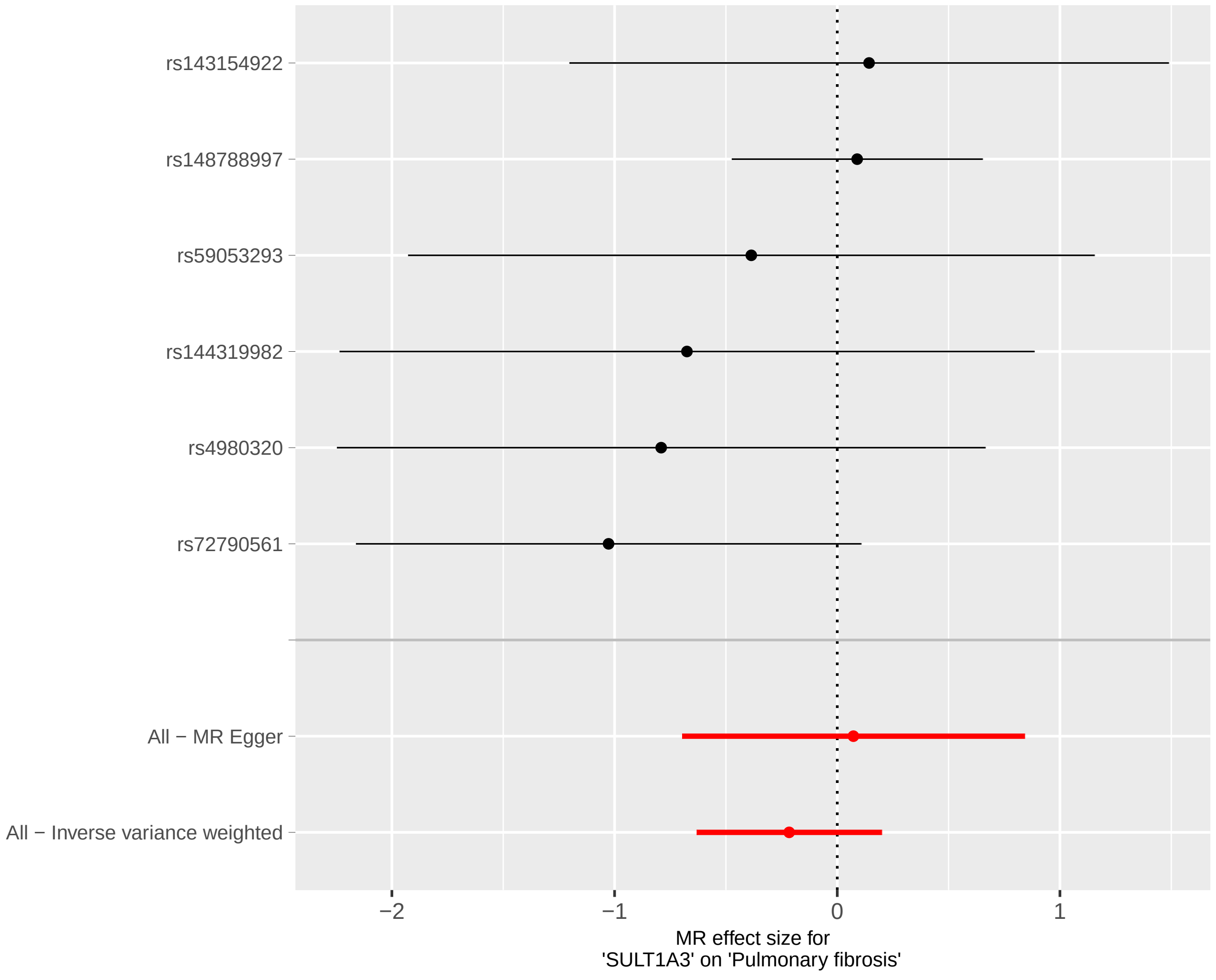
rs143154922
rs148788997
rs59053293
rs144319982
rs4980320
rs72790561
All − MR Egger
All − Inverse variance weighted
−2
−1
0
1
MR effect size for
'SULT1A3' on 'Pulmonary fibrosis'

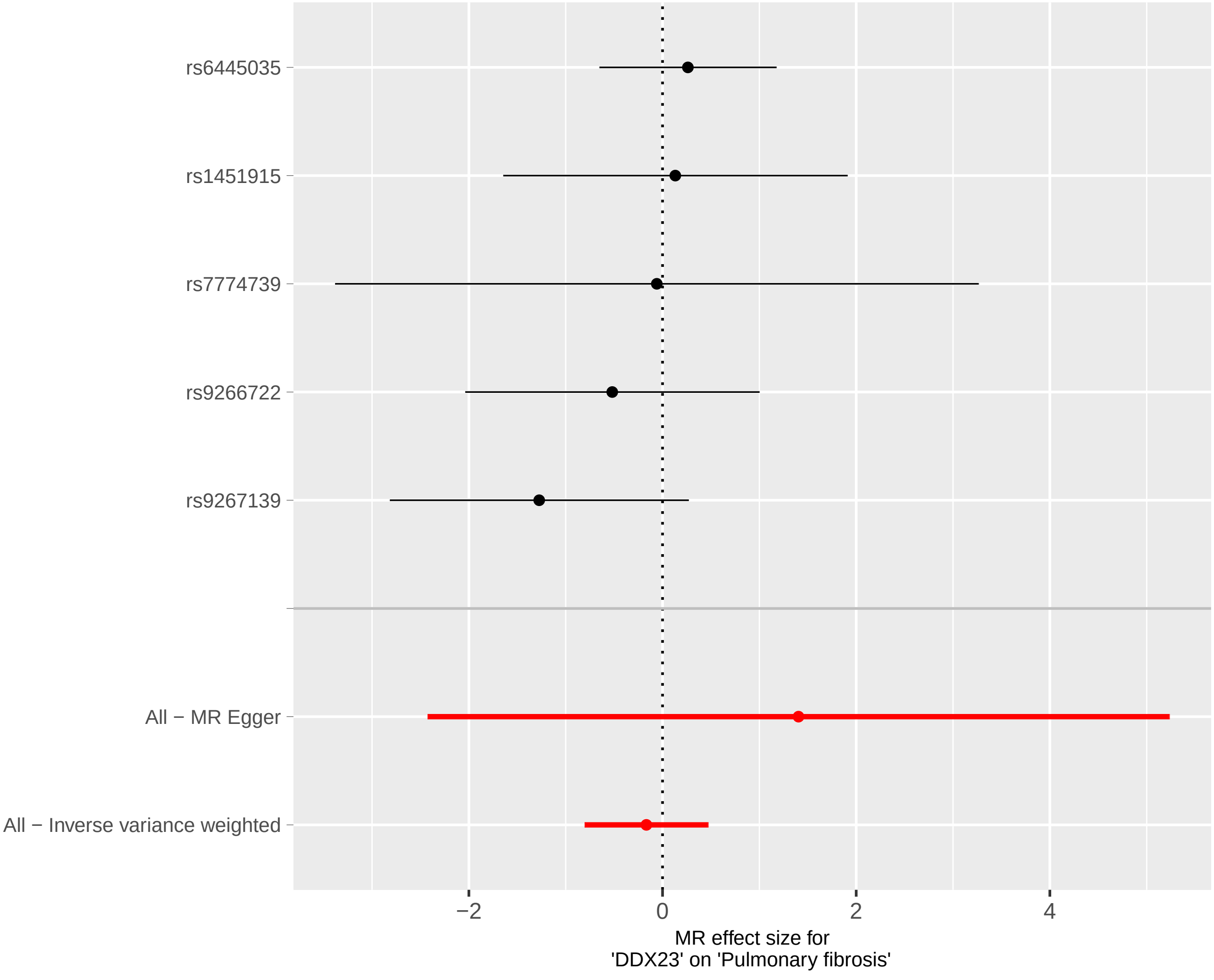
rs6445035
rs1451915
rs7774739
rs9266722
rs9267139
All − MR Egger
All − Inverse variance weighted
−2
0
2
4
MR effect size for
'DDX23' on 'Pulmonary fibrosis'

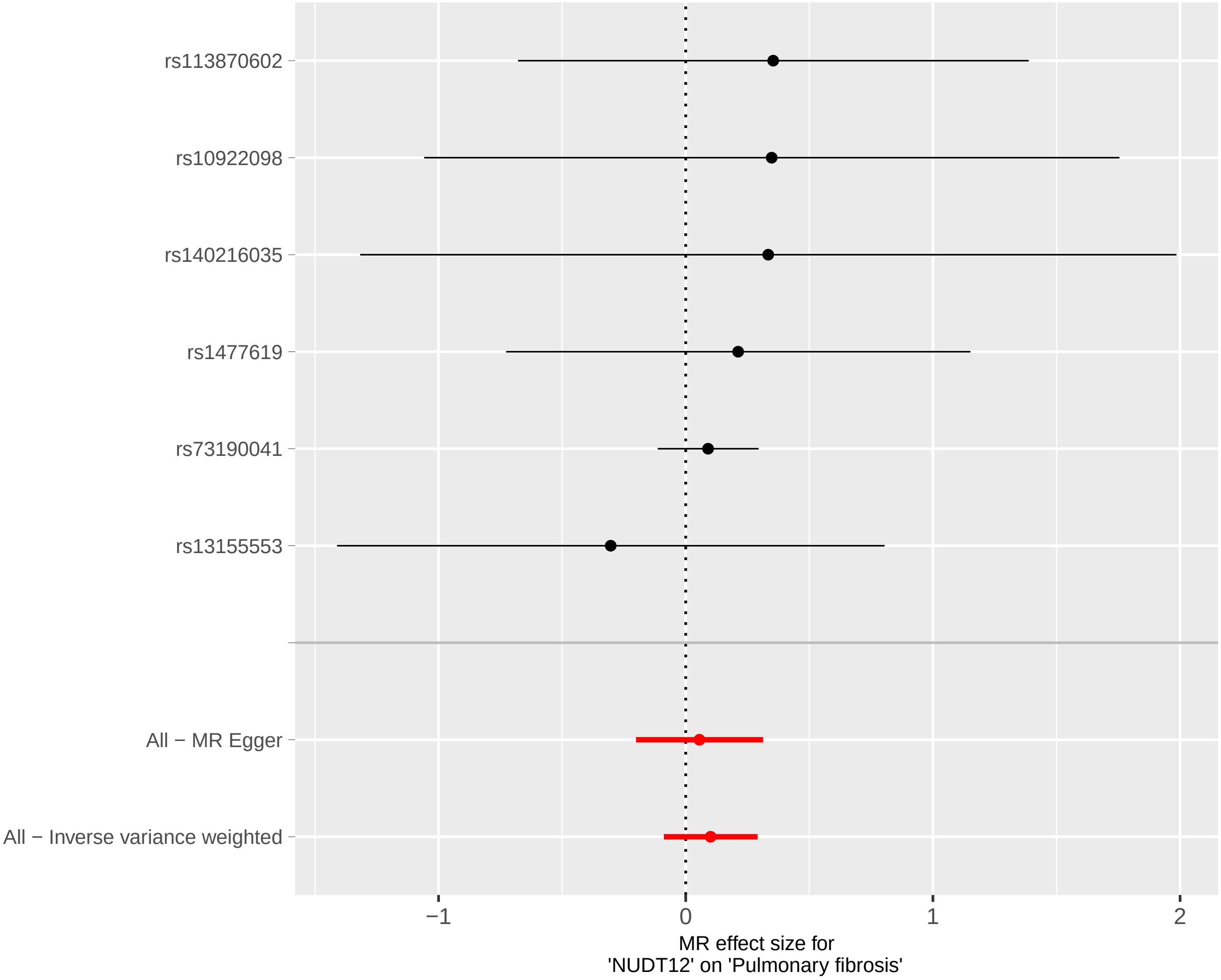
rs113870602
rs10922098
rs140216035
rs1477619
rs73190041
rs13155553
All − MR Egger
All − Inverse variance weighted
−1
0
1
2
MR effect size for
'NUDT12' on 'Pulmonary fibrosis'

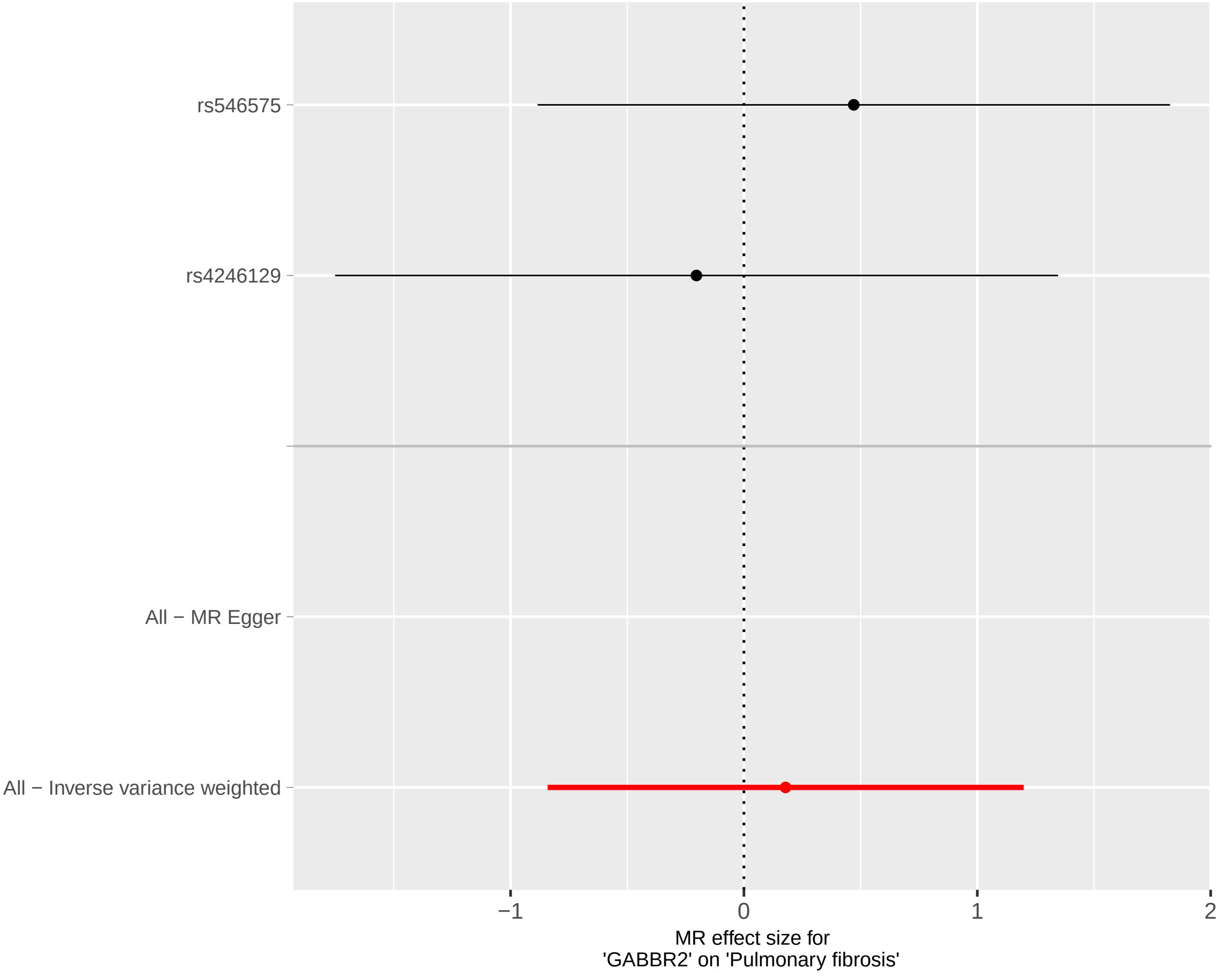
rs546575
rs4246129
All − MR Egger
All − Inverse variance weighted
−1
0
1
2
MR effect size for
'GABBR2' on 'Pulmonary fibrosis'

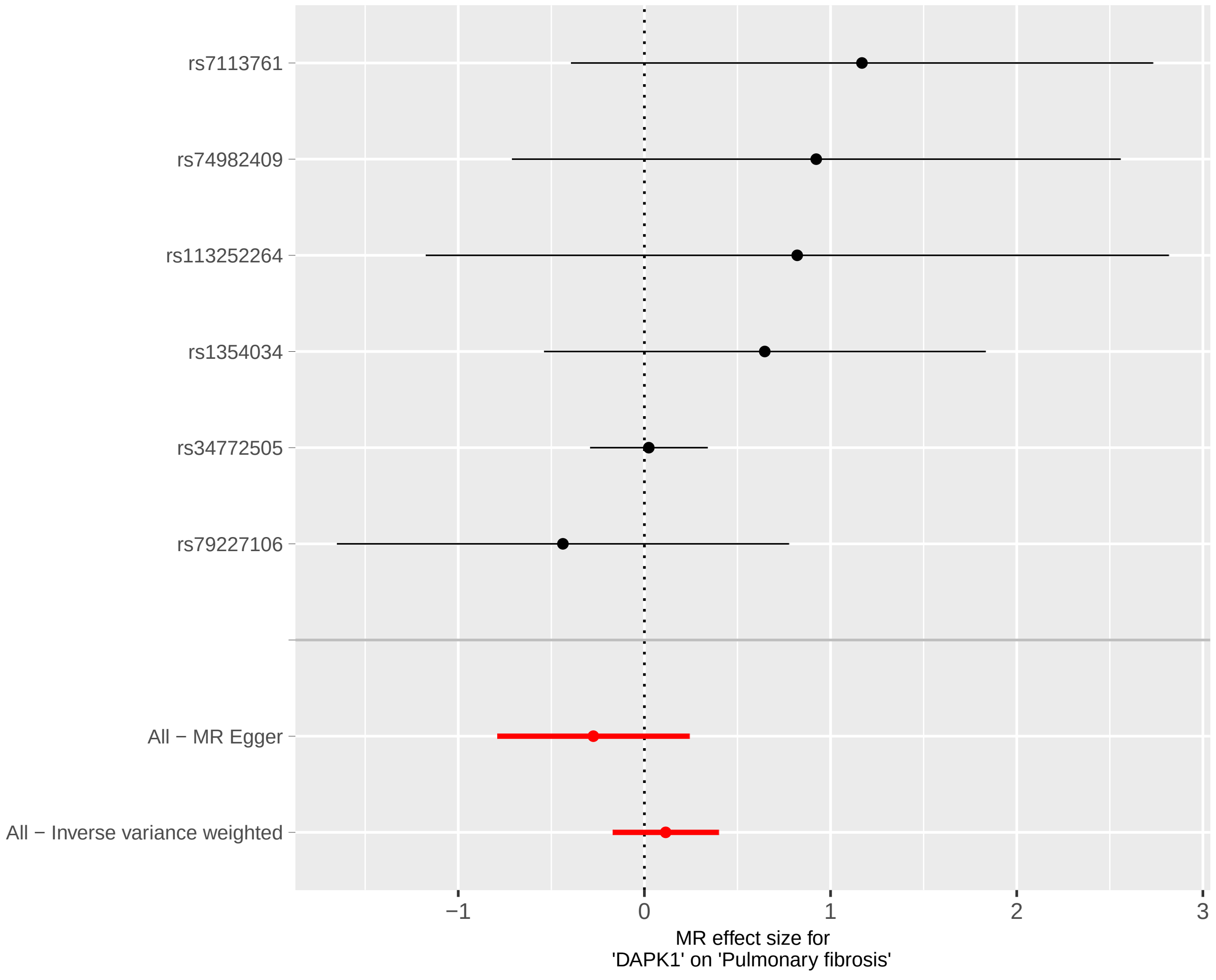

rs7113761
rs74982409
rs113252264
rs1354034
rs34772505
rs79227106
All − MR Egger
All − Inverse variance weighted
−1
0
1
2
3
MR effect size for
'DAPK1' on 'Pulmonary fibrosis'

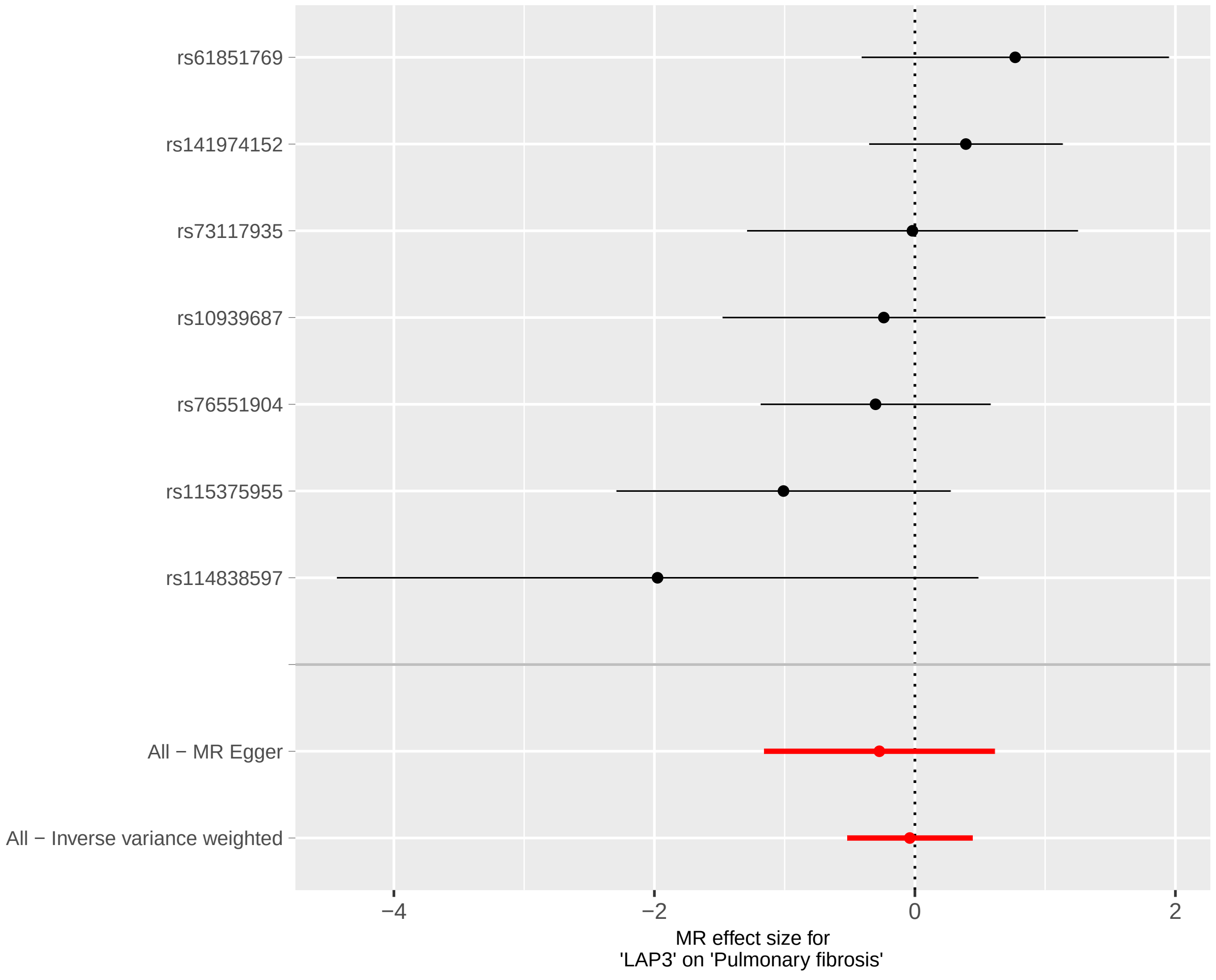
rs61851769
rs141974152
rs73117935
rs10939687
rs76551904
rs115375955
rs114838597
All − MR Egger
All − Inverse variance weighted
−4
−2
0
2
MR effect size for
'LAP3' on 'Pulmonary fibrosis'

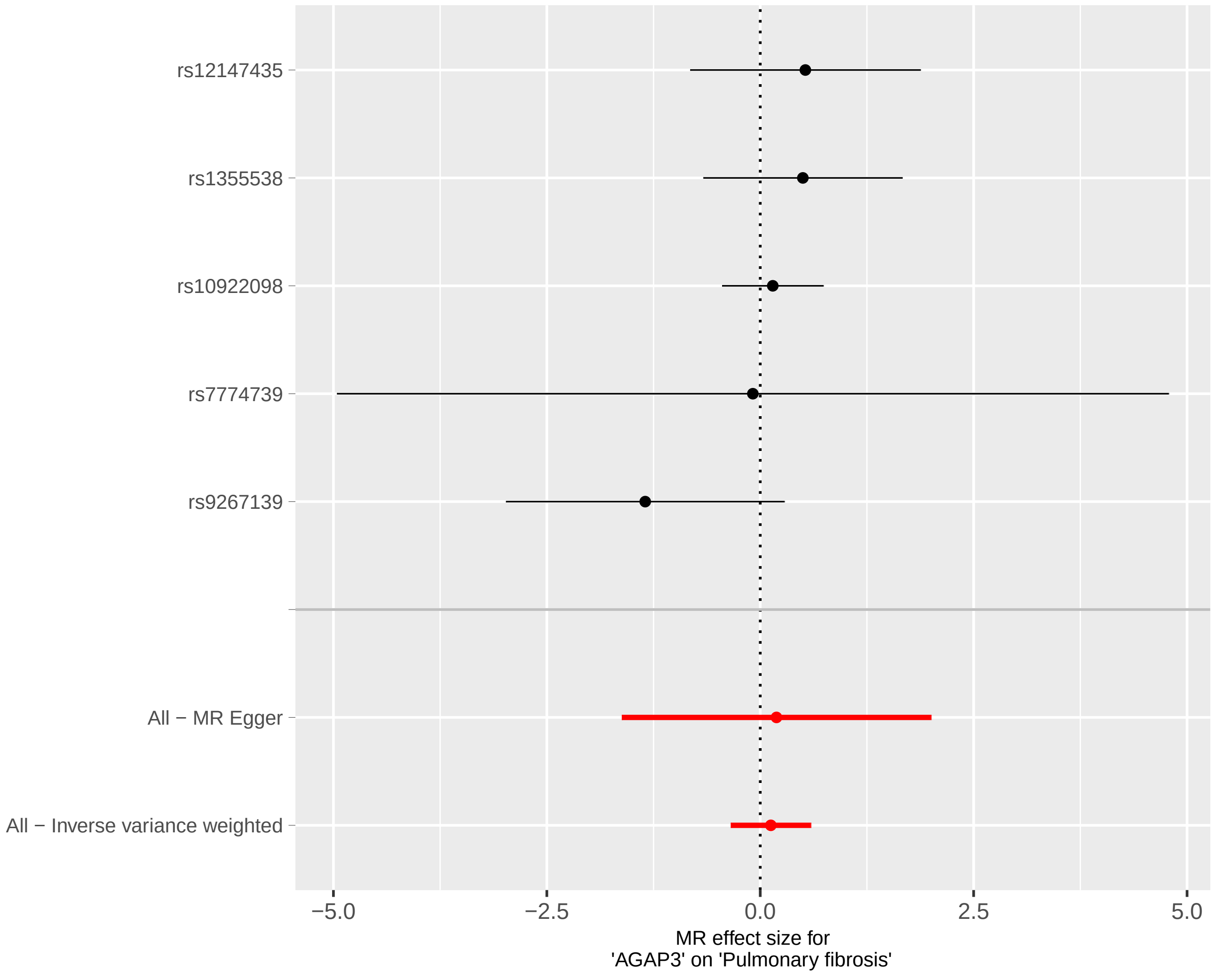
rs12147435
rs1355538
rs10922098
rs7774739
rs9267139
All − MR Egger
All − Inverse variance weighted
−5.0
−2.5
0.0
2.5
5.0
MR effect size for
'AGAP3' on 'Pulmonary fibrosis'

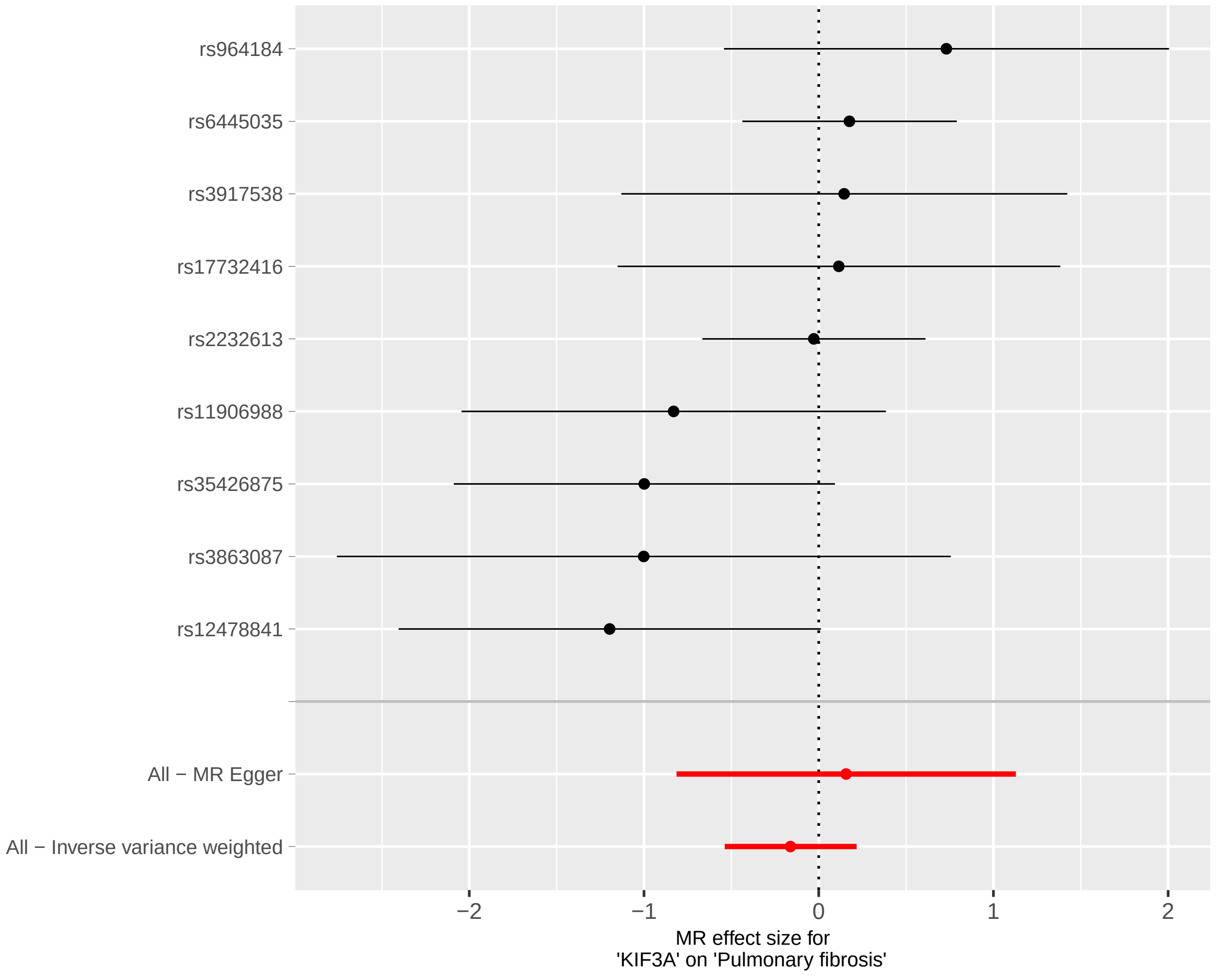
rs964184
rs6445035
rs3917538
rs17732416
rs2232613
rs11906988
rs35426875
rs3863087
rs12478841
All − MR Egger
All − Inverse variance weighted
−2
−1
0
1
2
MR effect size for
'KIF3A' on 'Pulmonary fibrosis'

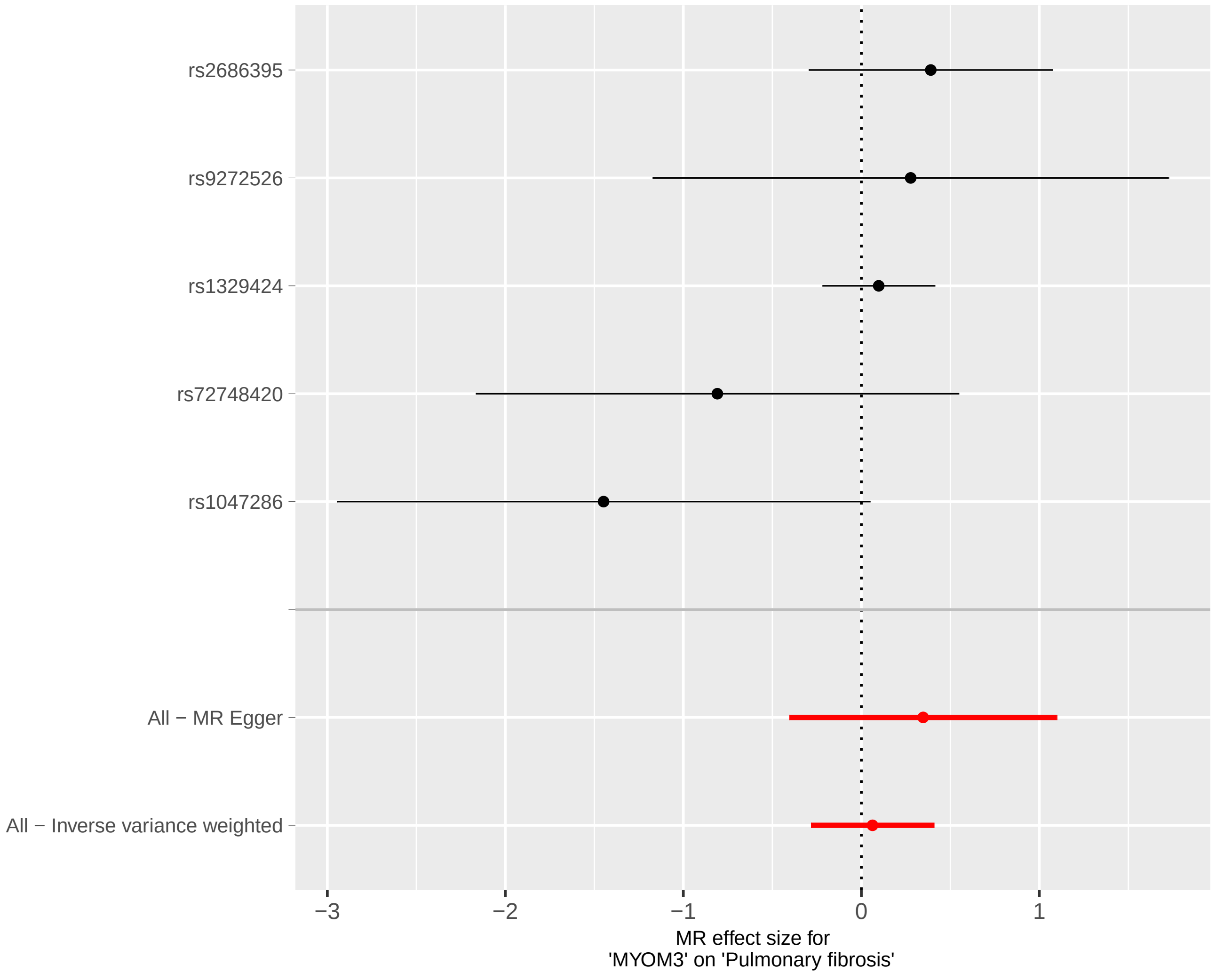
rs2686395
rs9272526
rs1329424
rs72748420
rs1047286
All − MR Egger
All − Inverse variance weighted
−3
−2
−1
0
1
MR effect size for
'MYOM3' on 'Pulmonary fibrosis'

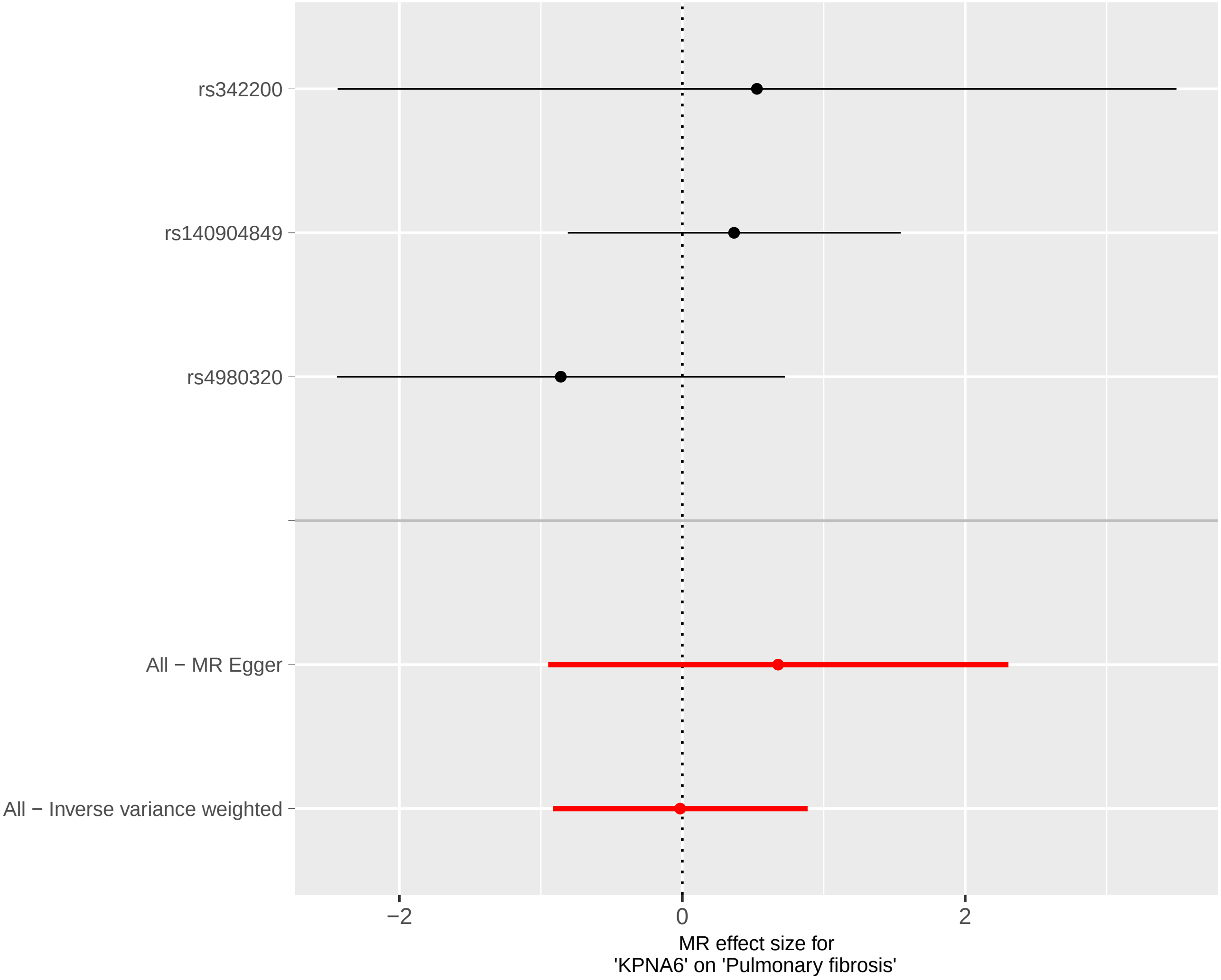
rs342200
rs140904849
rs4980320
All − MR Egger
All − Inverse variance weighted
−2
0
2
MR effect size for
'KPNA6' on 'Pulmonary fibrosis'

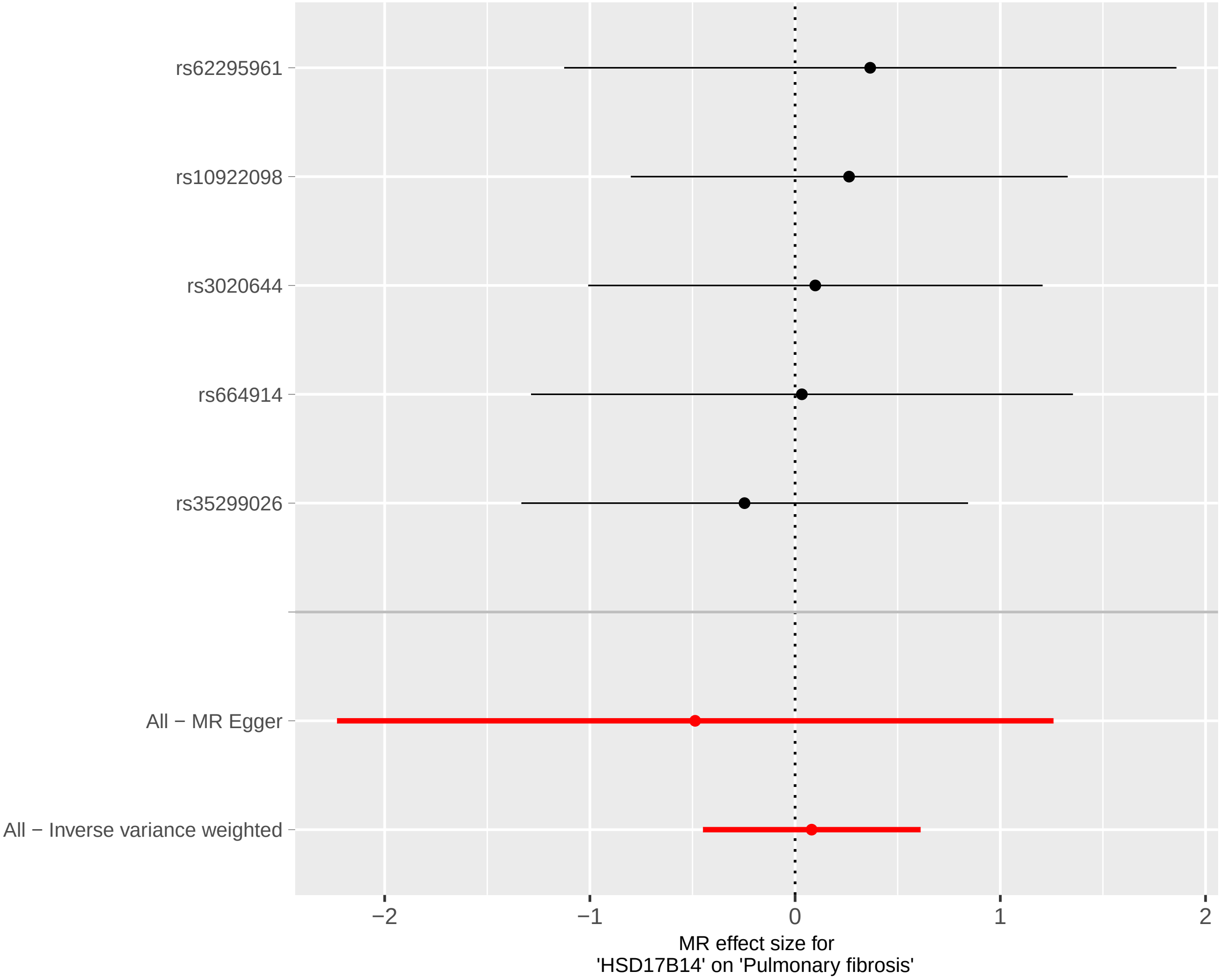
rs62295961
rs10922098
rs3020644
rs664914
rs35299026
All − MR Egger
All − Inverse variance weighted
−2
−1
0
1
2
MR effect size for
'HSD17B14' on 'Pulmonary fibrosis'

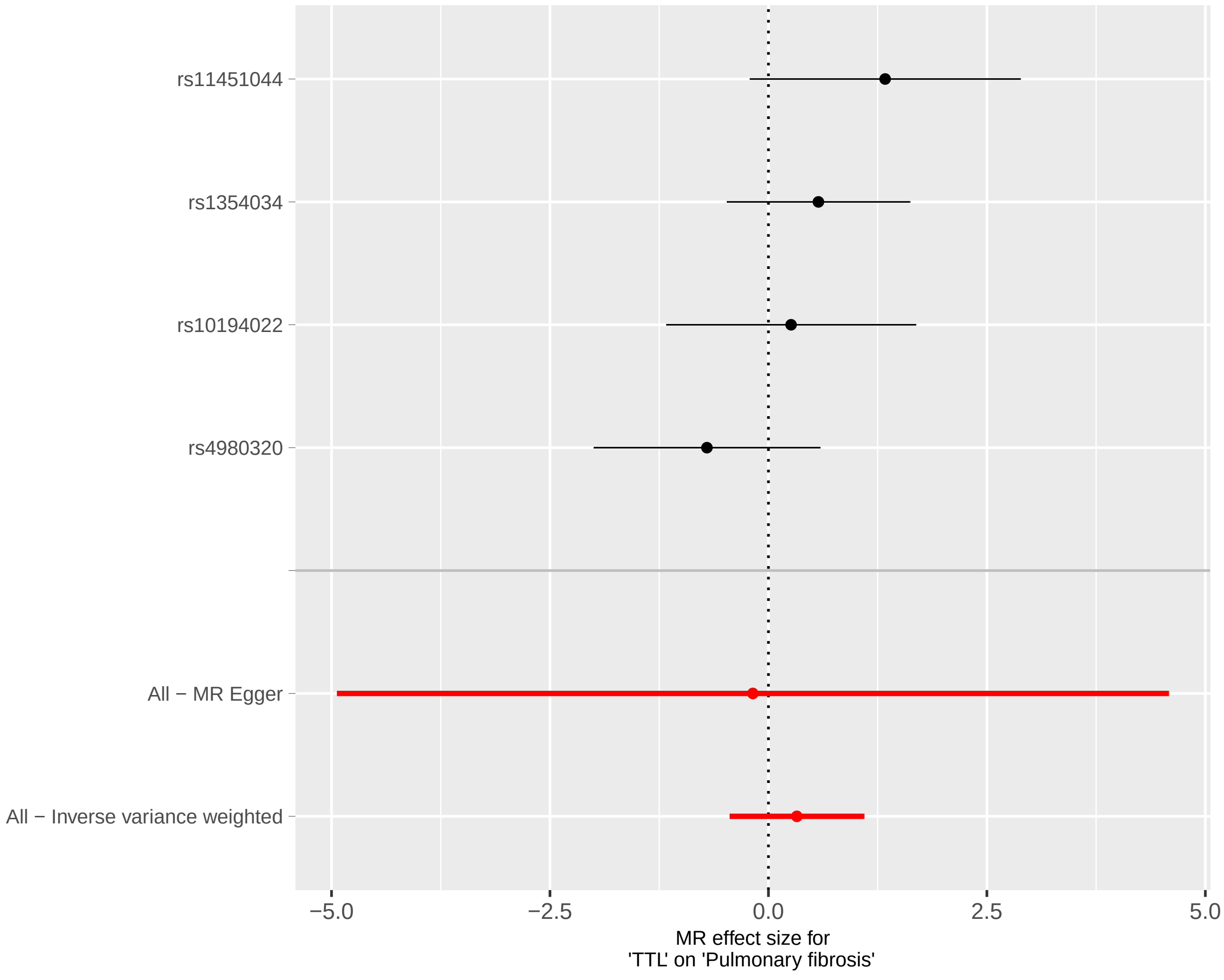
rs11451044
rs1354034
rs10194022
rs4980320
All − MR Egger
All − Inverse variance weighted
−5.0
−2.5
0.0
2.5
5.0
MR effect size for
'TTL' on 'Pulmonary fibrosis'

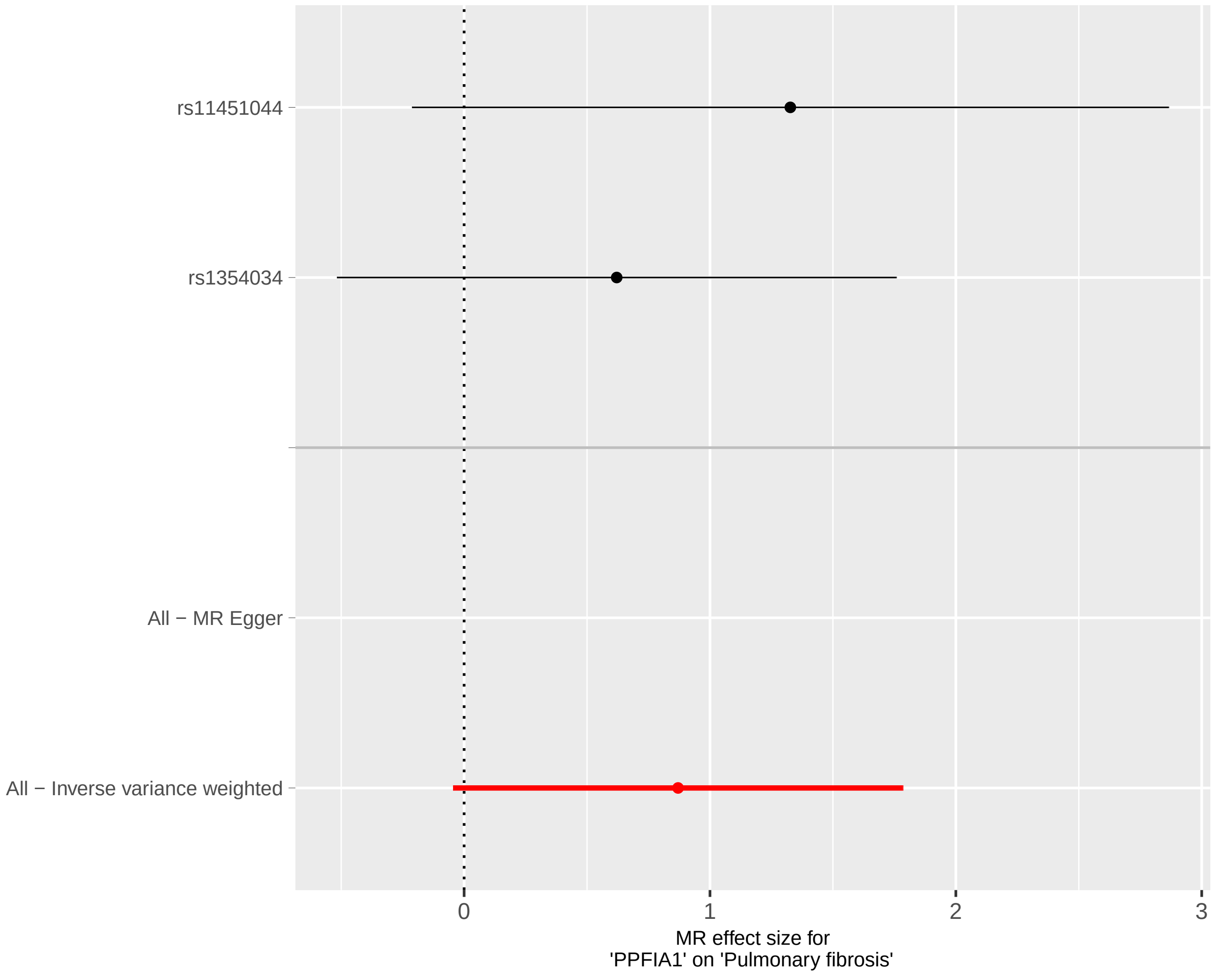
rs11451044
rs1354034
All − MR Egger
All − Inverse variance weighted
0
1
2
3
MR effect size for
'PPFIA1' on 'Pulmonary fibrosis'

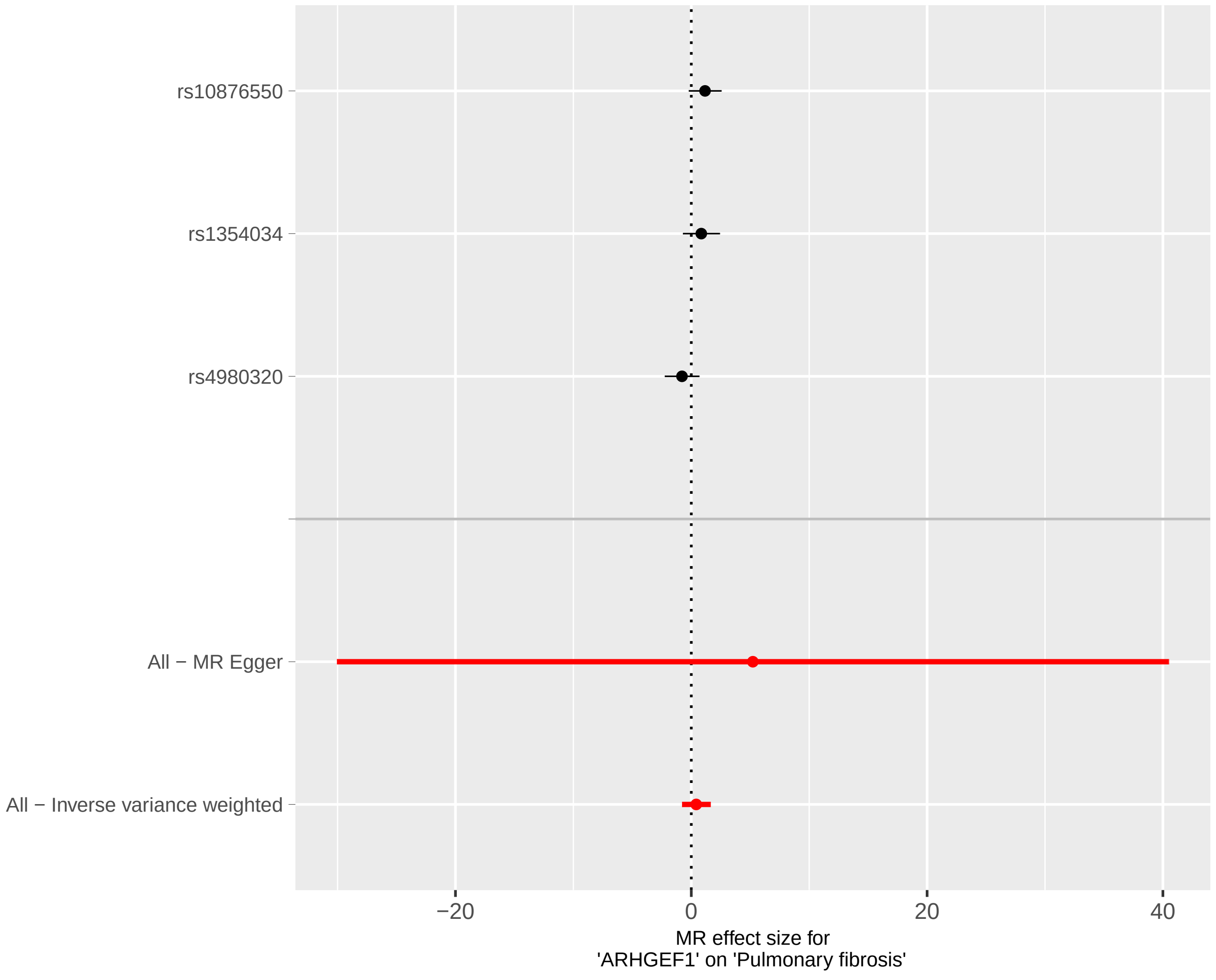

rs10876550
rs1354034
rs4980320
All − MR Egger
All − Inverse variance weighted
−20
0
20
40
MR effect size for
'ARHGEF1' on 'Pulmonary fibrosis'

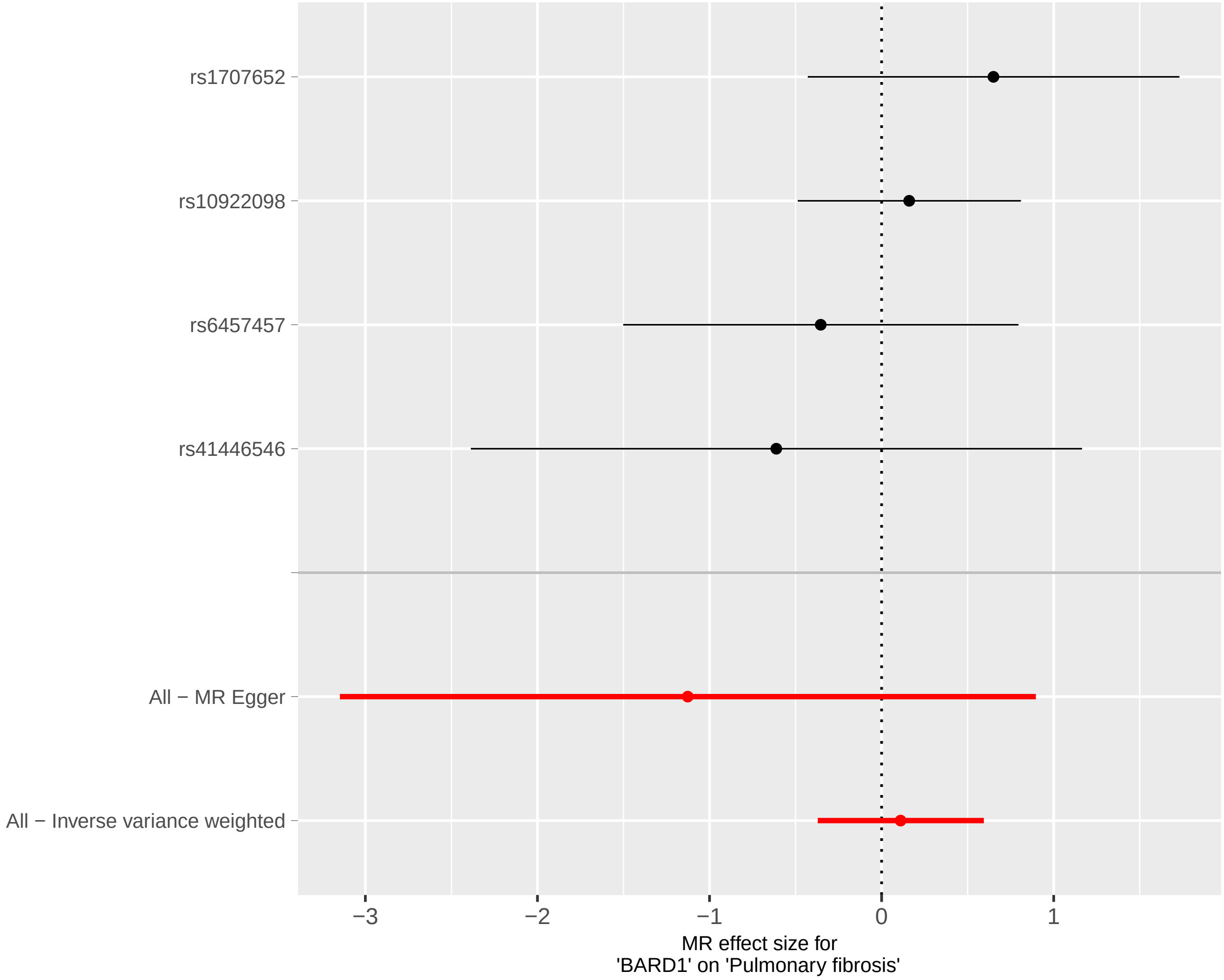

rs1707652
rs10922098
rs6457457
rs41446546
All − MR Egger
All − Inverse variance weighted
−3
−2
−1
0
1
MR effect size for
'BARD1' on 'Pulmonary fibrosis'

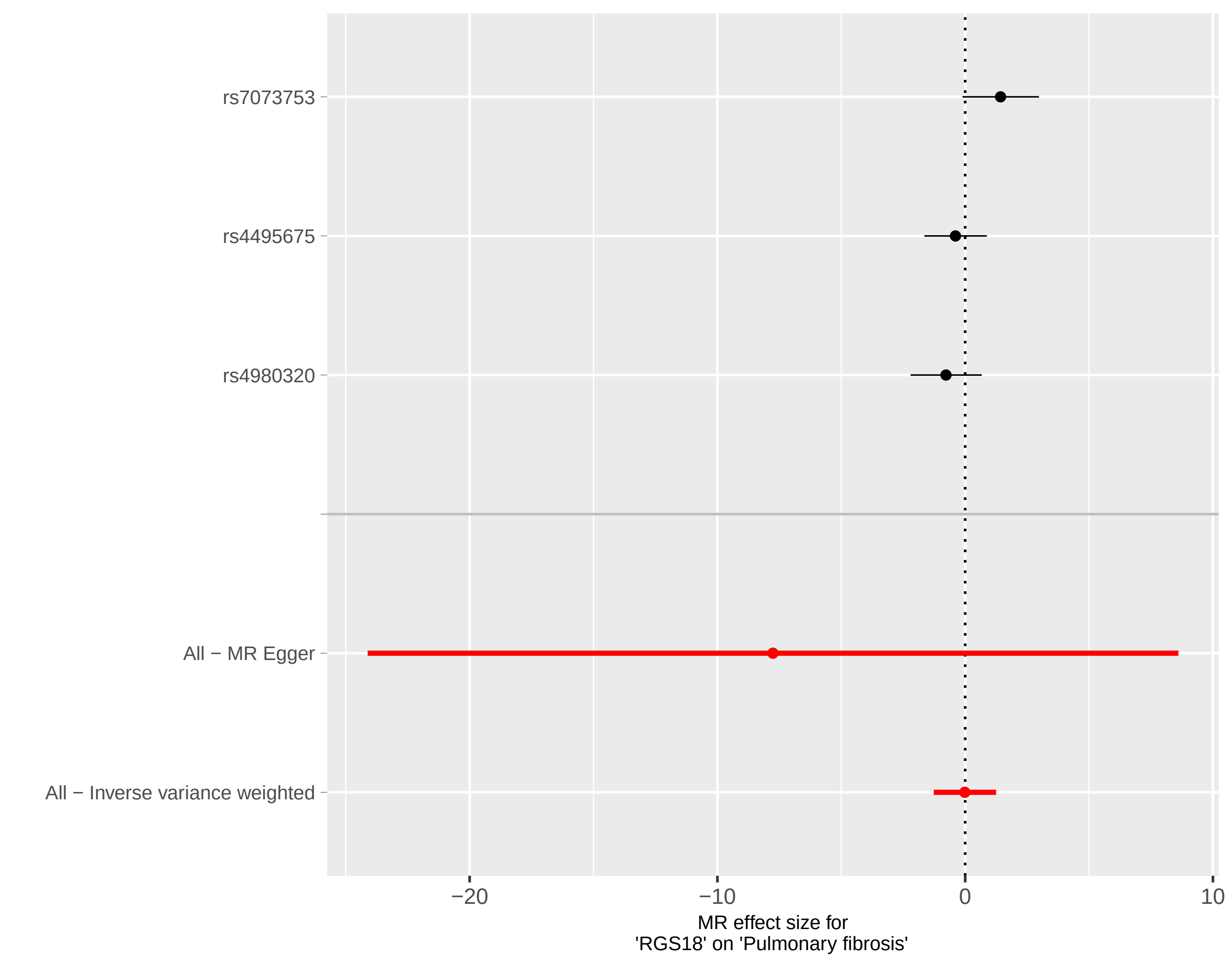
rs7073753
rs4495675
rs4980320
All − MR Egger
All − Inverse variance weighted
−20
−10
0
10
MR effect size for
'RGS18' on 'Pulmonary fibrosis'

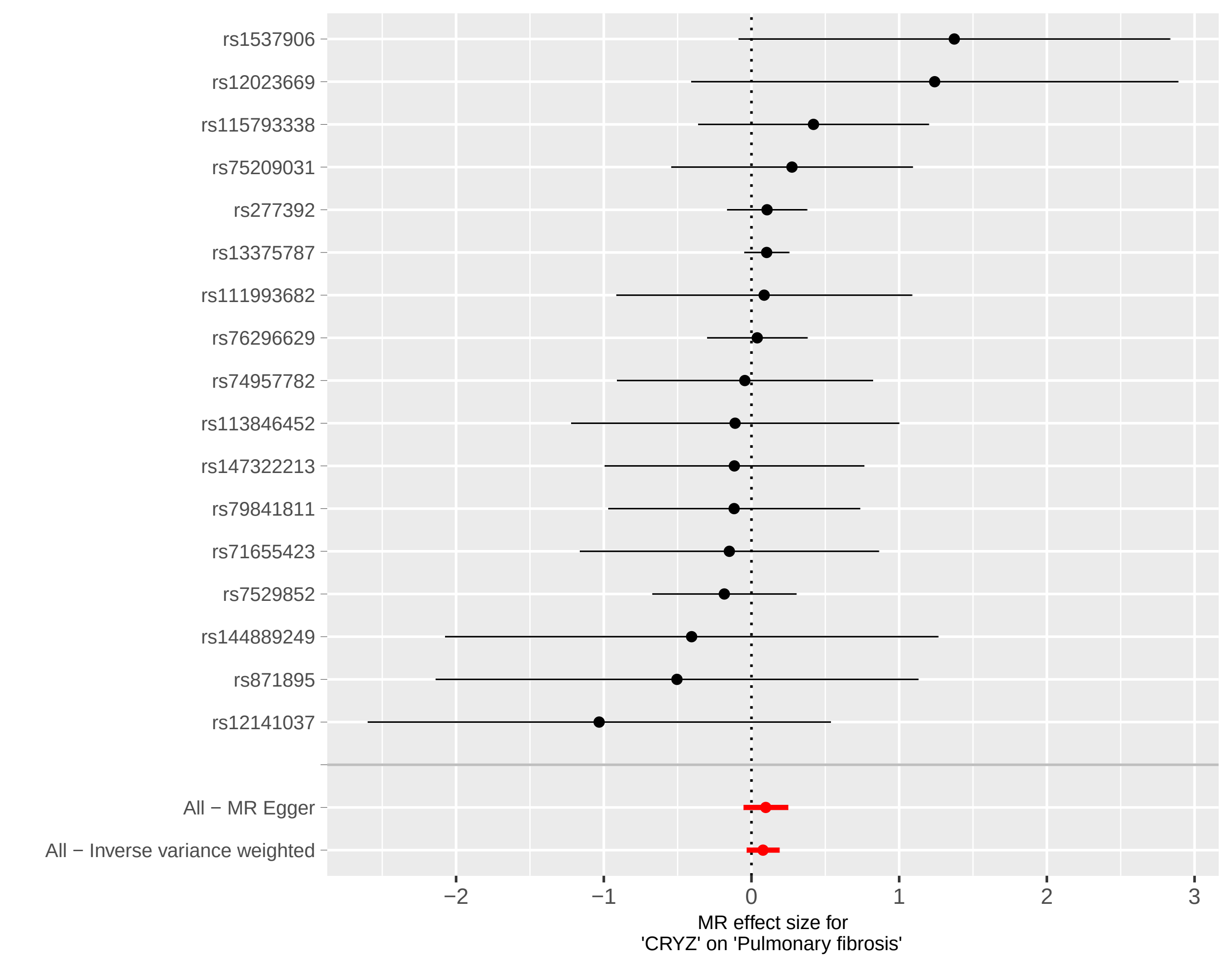

rs1537906
rs12023669
rs115793338
rs75209031
rs277392
rs13375787
rs111993682
rs76296629
rs74957782
rs113846452
rs147322213
rs79841811
rs71655423
rs7529852
rs144889249
rs871895
rs12141037
All − MR Egger
All − Inverse variance weighted
−2
−1
0
1
2
3
MR effect size for
'CRYZ' on 'Pulmonary fibrosis'

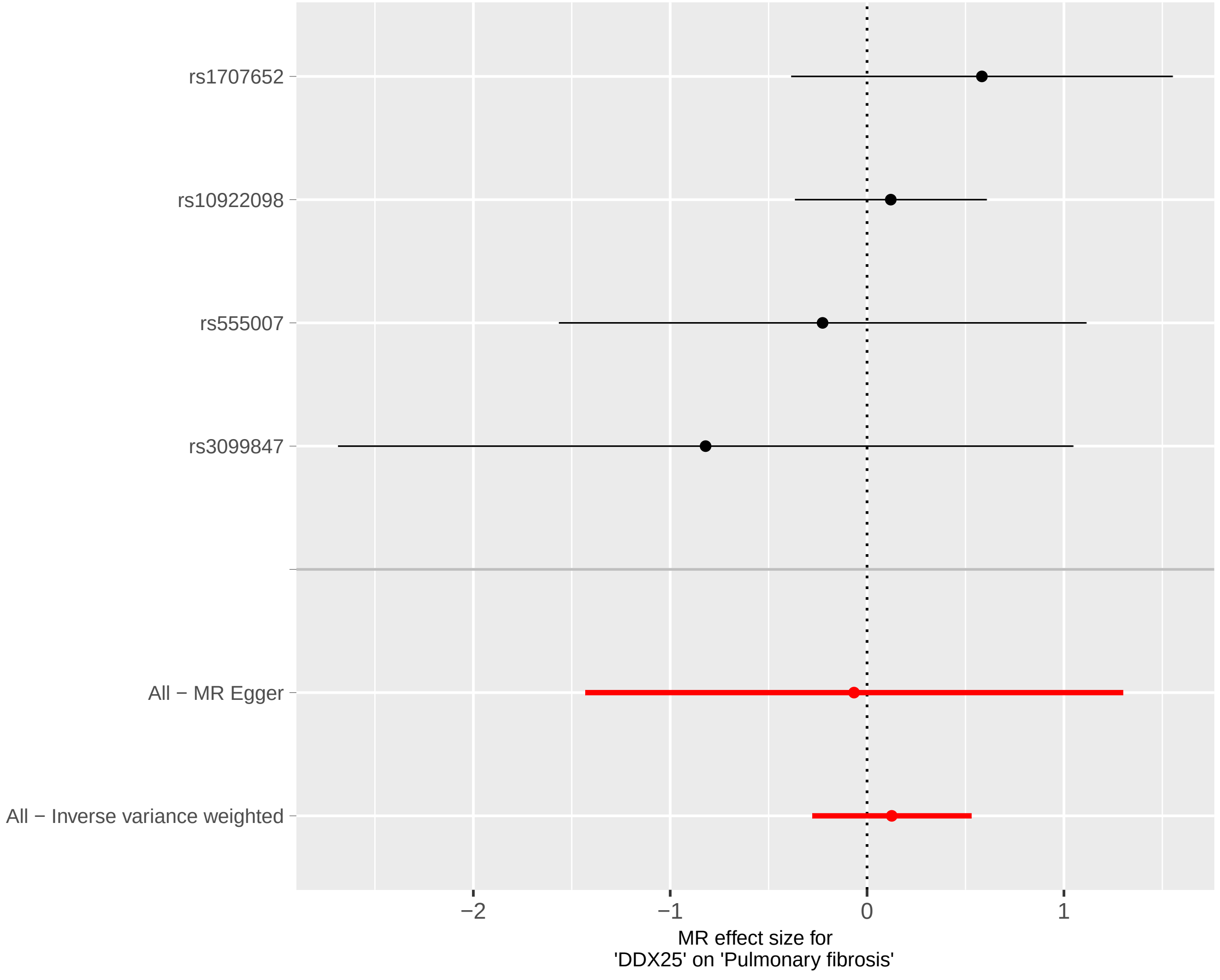
rs1707652
rs10922098
rs555007
rs3099847
All − MR Egger
All − Inverse variance weighted
−2
−1
0
1
MR effect size for
'DDX25' on 'Pulmonary fibrosis'

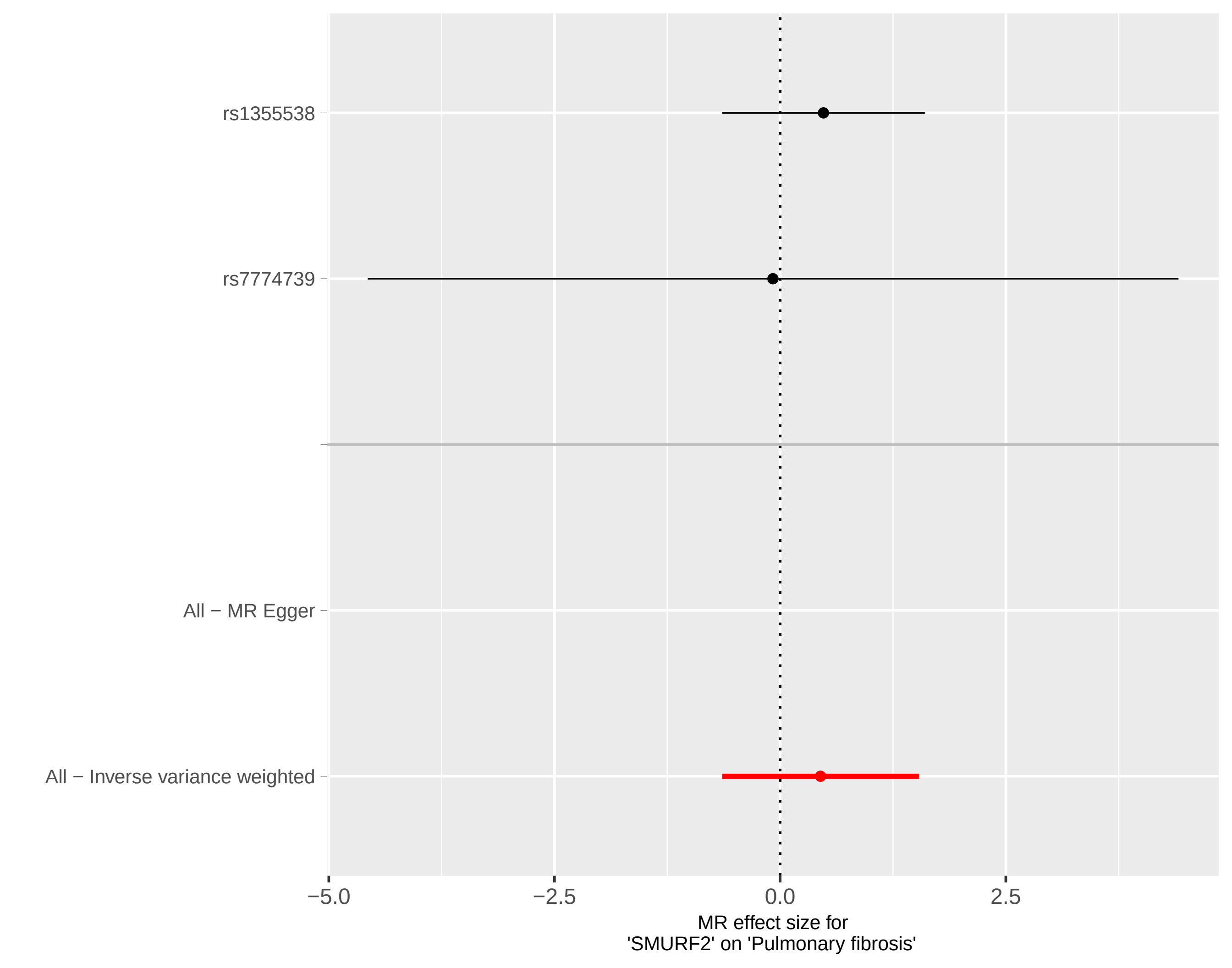

rs1355538
rs7774739
All − MR Egger
All − Inverse variance weighted
−5.0
−2.5
0.0
2.5
MR effect size for
'SMURF2' on 'Pulmonary fibrosis'

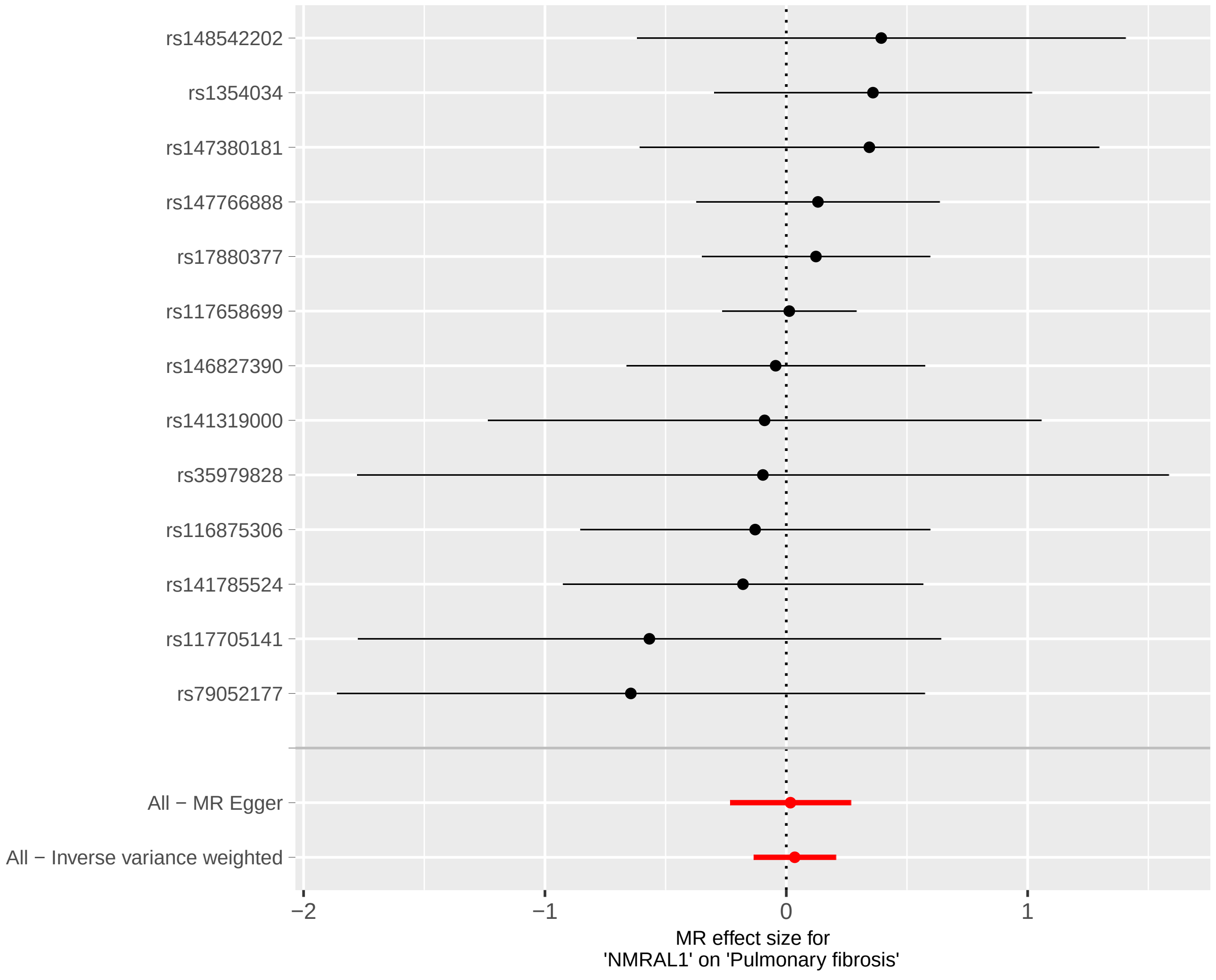
rs148542202
rs1354034
rs147380181
rs147766888
rs17880377
rs117658699
rs146827390
rs141319000
rs35979828
rs116875306
rs141785524
rs117705141
rs79052177
All − MR Egger
All − Inverse variance weighted
−2
−1
0
1
MR effect size for
'NMRAL1' on 'Pulmonary fibrosis'

Insufficient number of SNPs

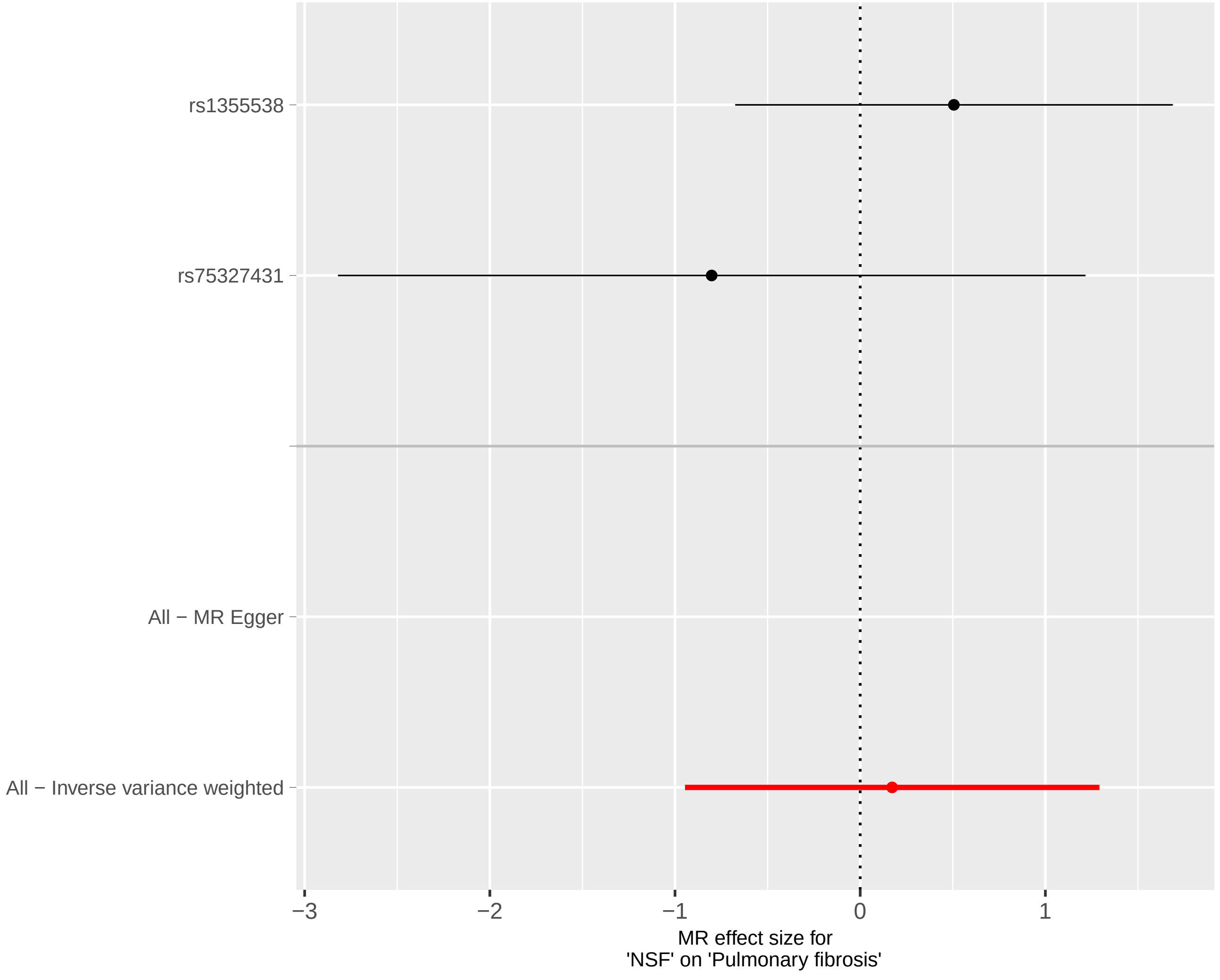
rs1355538
rs75327431
All − MR Egger
All − Inverse variance weighted
−3
−2
−1
0
1
MR effect size for
'NSF' on 'Pulmonary fibrosis'

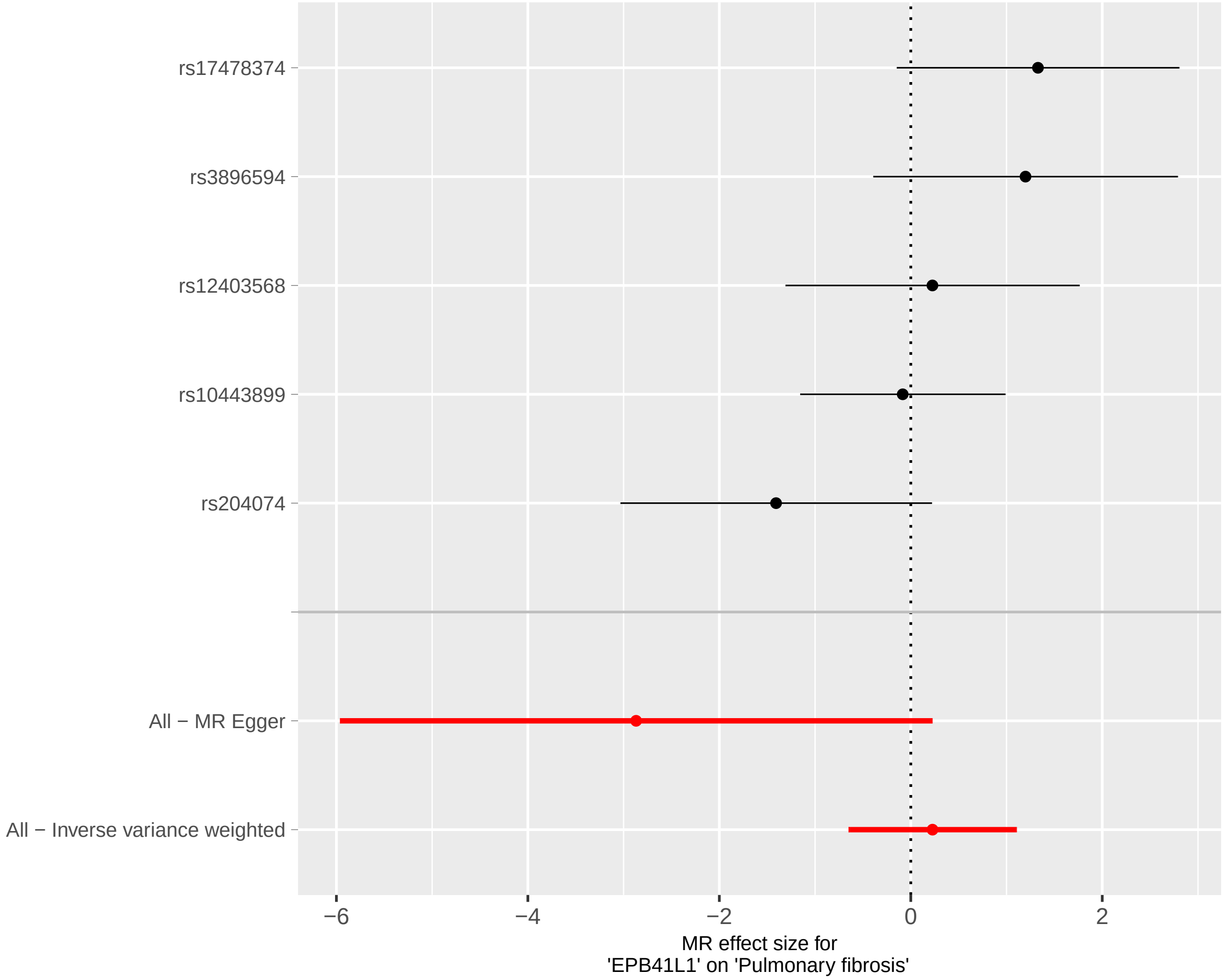

rs17478374
rs3896594
rs12403568
rs10443899
rs204074
All − MR Egger
All − Inverse variance weighted
−6
−4
−2
0
2
MR effect size for
'EPB41L1' on 'Pulmonary fibrosis'

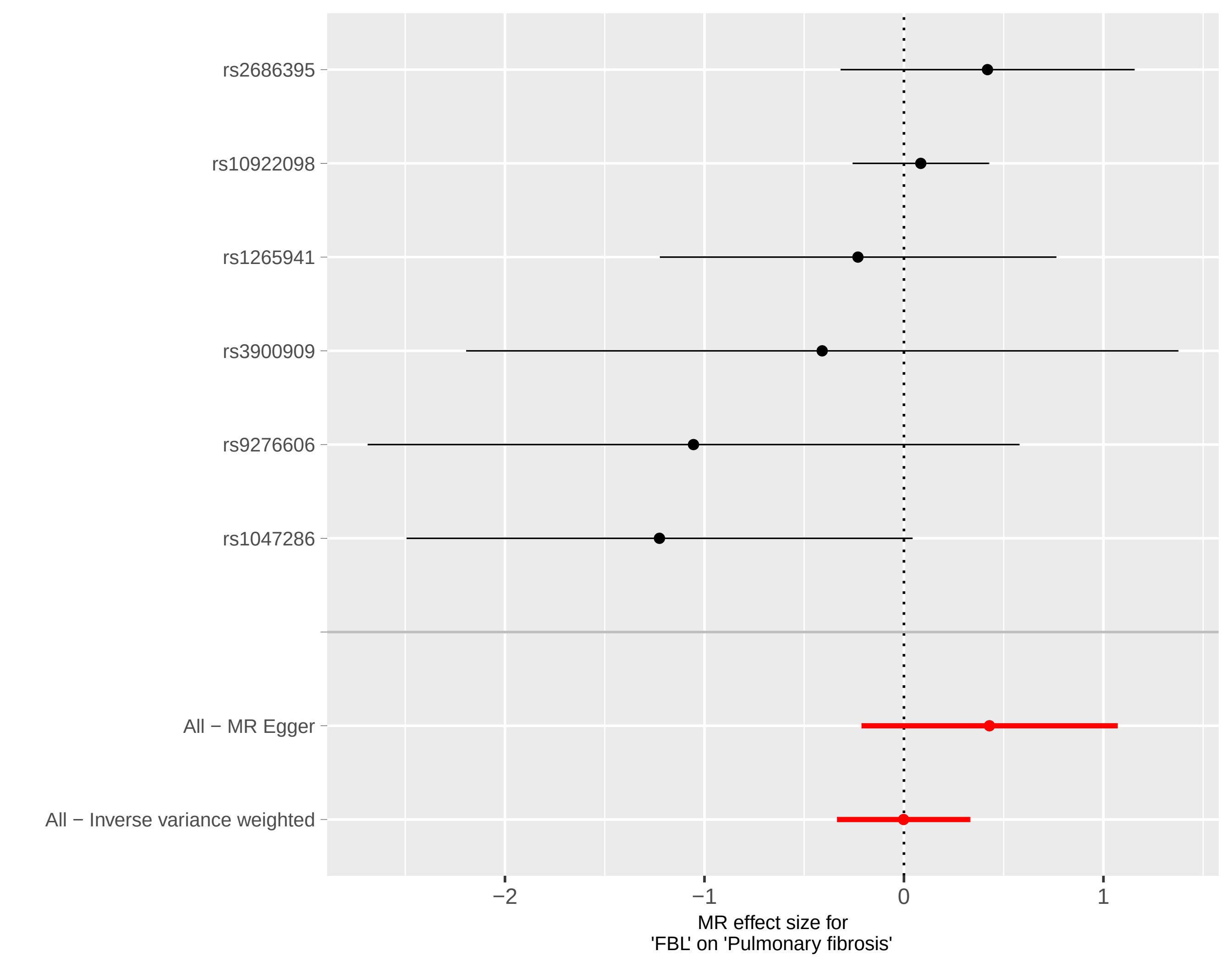
rs2686395
rs10922098
rs1265941
rs3900909
rs9276606
rs1047286
All − MR Egger
All − Inverse variance weighted
−2
−1
0
1
MR effect size for
'FBL' on 'Pulmonary fibrosis'

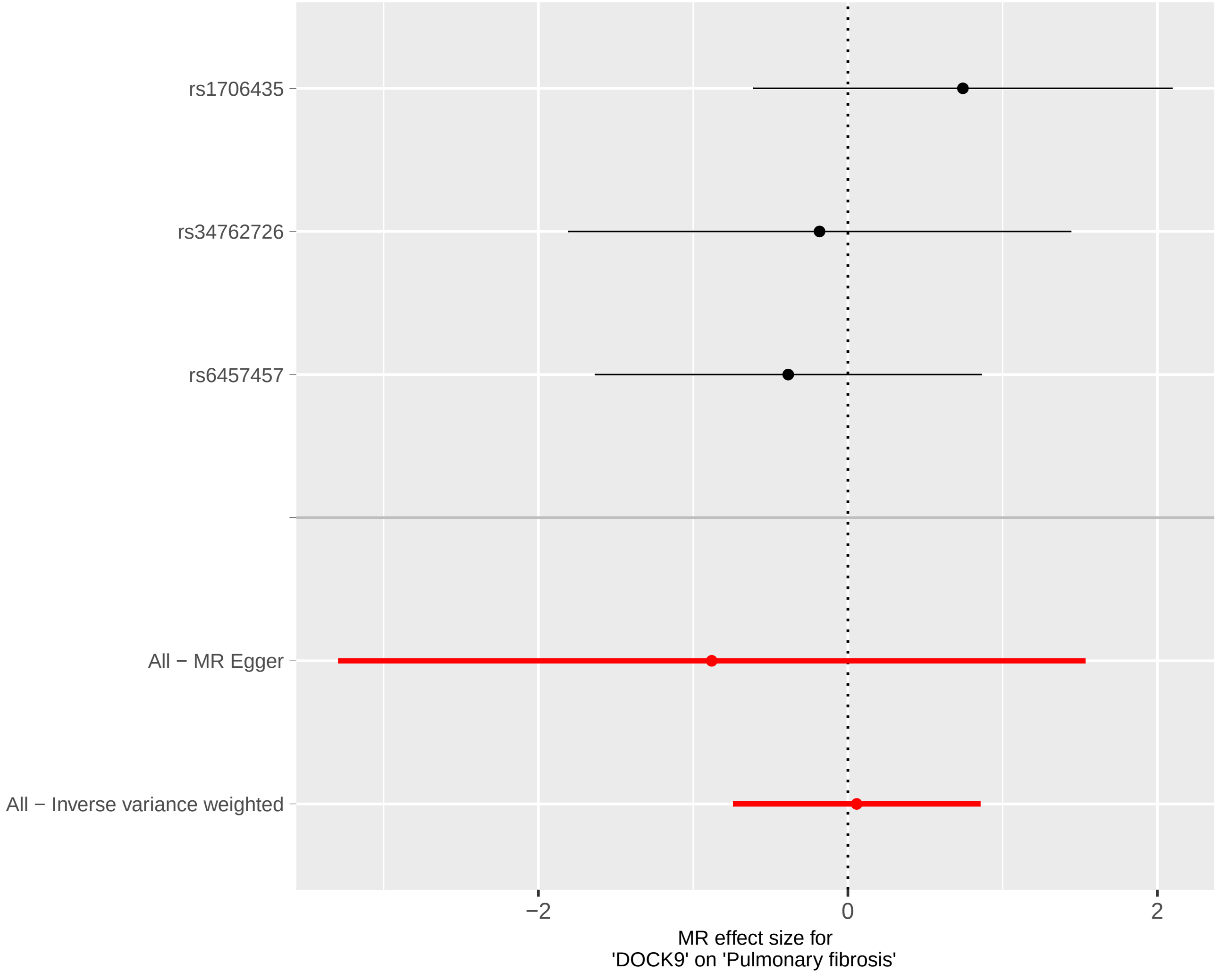

rs1706435
rs34762726
rs6457457
All − MR Egger
All − Inverse variance weighted
−2
0
2
MR effect size for
'DOCK9' on 'Pulmonary fibrosis'

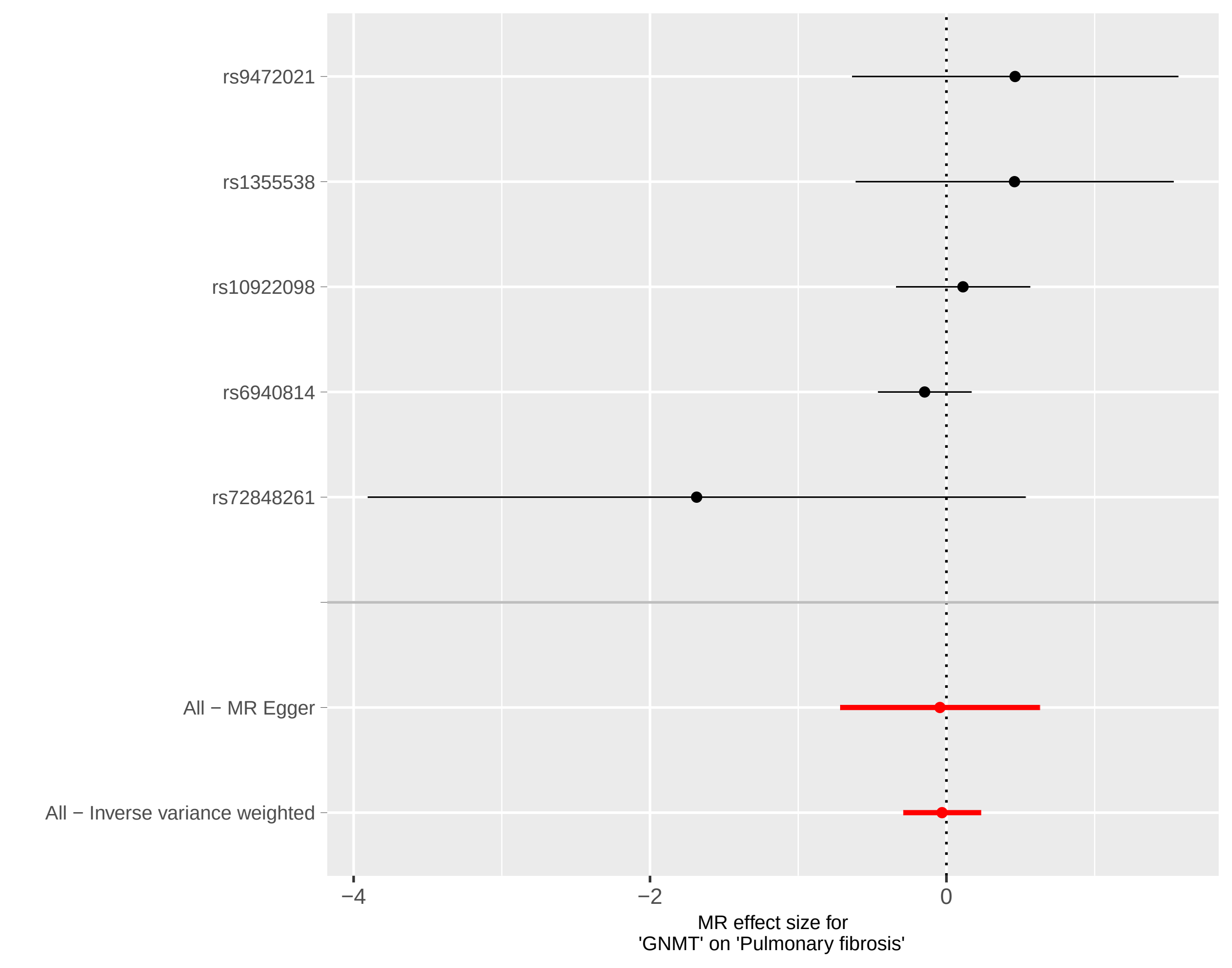

rs9472021
rs1355538
rs10922098
rs6940814
rs72848261
All − MR Egger
All − Inverse variance weighted
−4
−2
0
MR effect size for
'GNMT' on 'Pulmonary fibrosis'

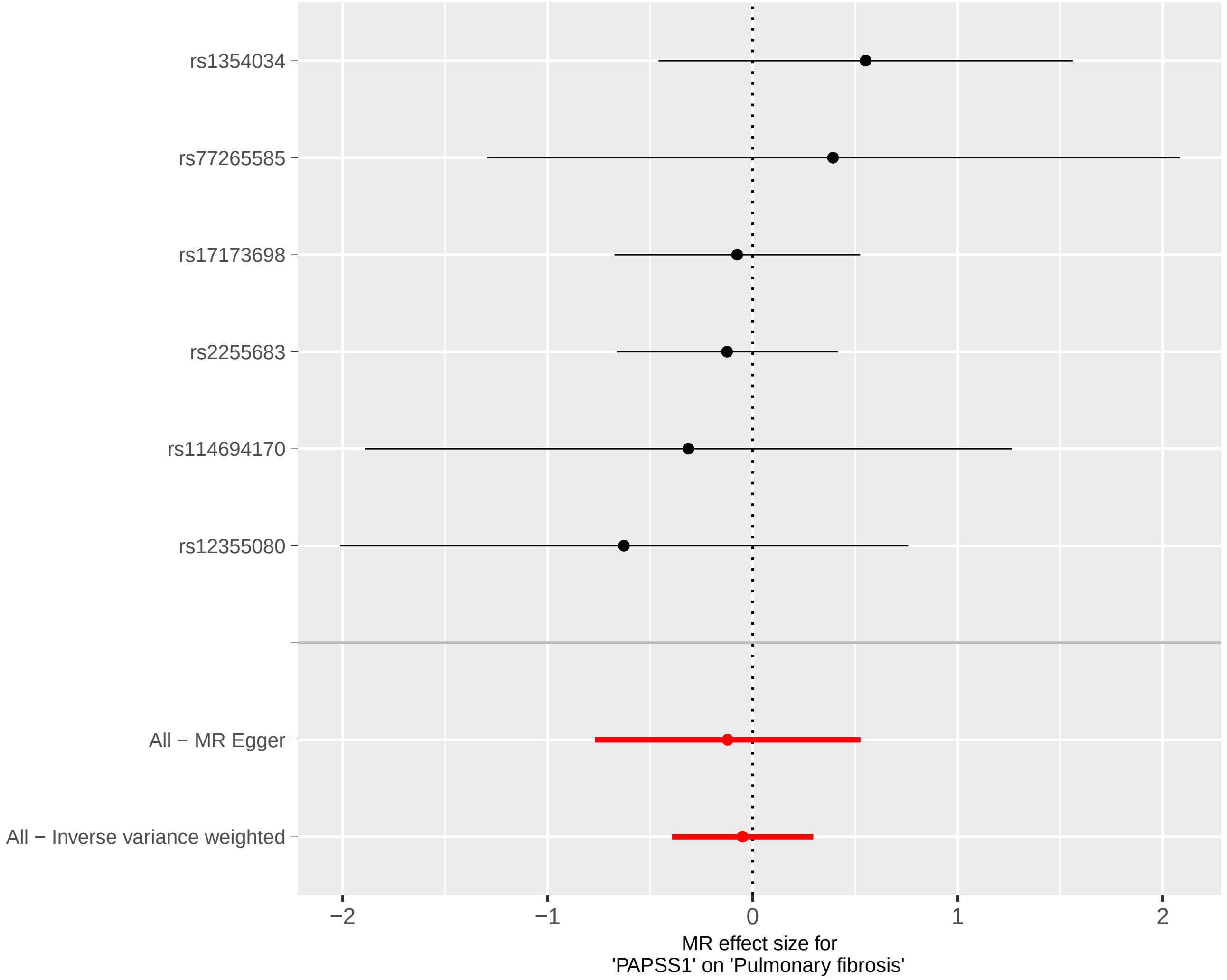
rs1354034
rs77265585
rs17173698
rs2255683
rs114694170
rs12355080
All − MR Egger
All − Inverse variance weighted
−2
−1
0
1
2
MR effect size for
'PAPSS1' on 'Pulmonary fibrosis'

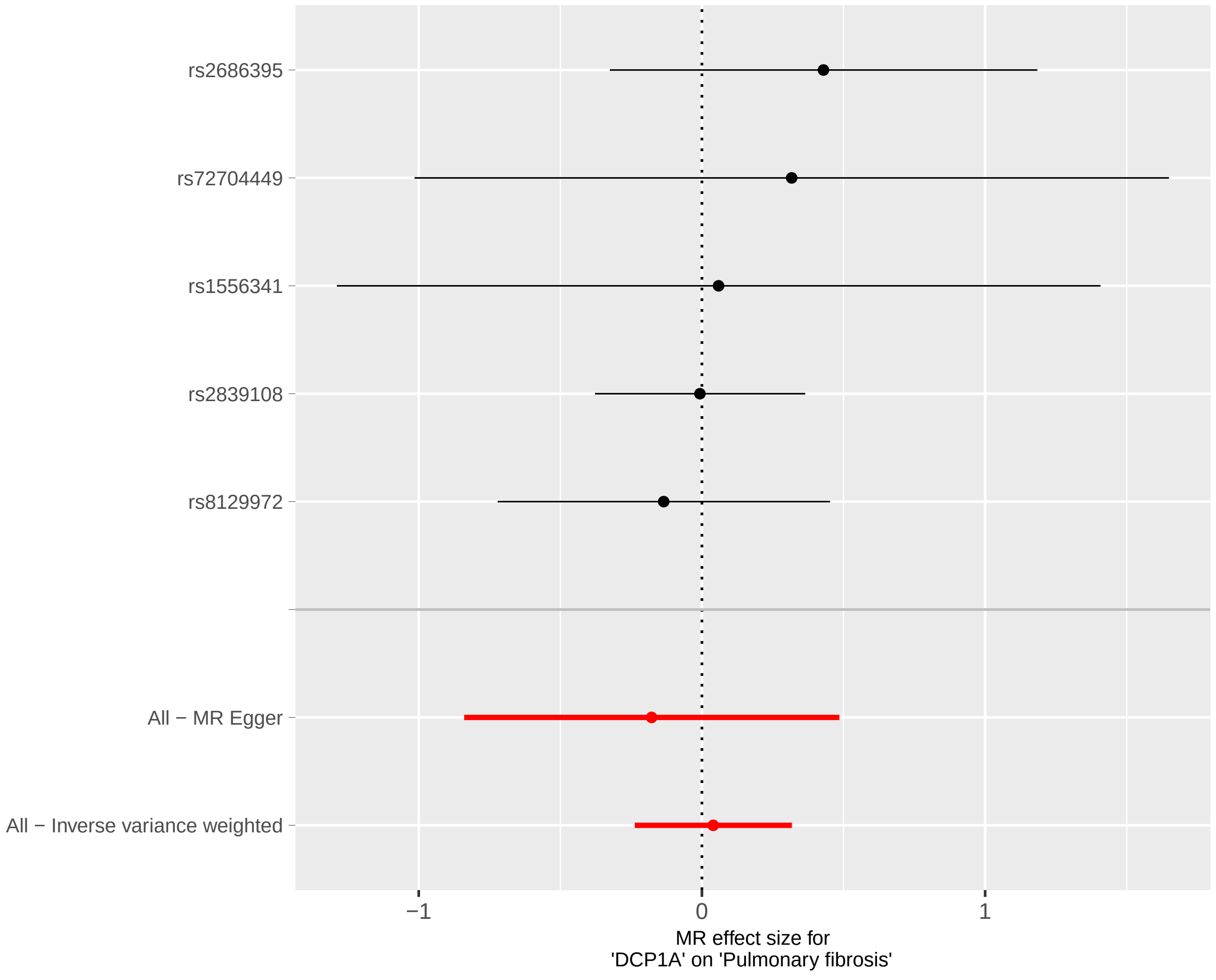
rs2686395
rs72704449
rs1556341
rs2839108
rs8129972
All − MR Egger
All − Inverse variance weighted
−1
0
1
MR effect size for
'DCP1A' on 'Pulmonary fibrosis'

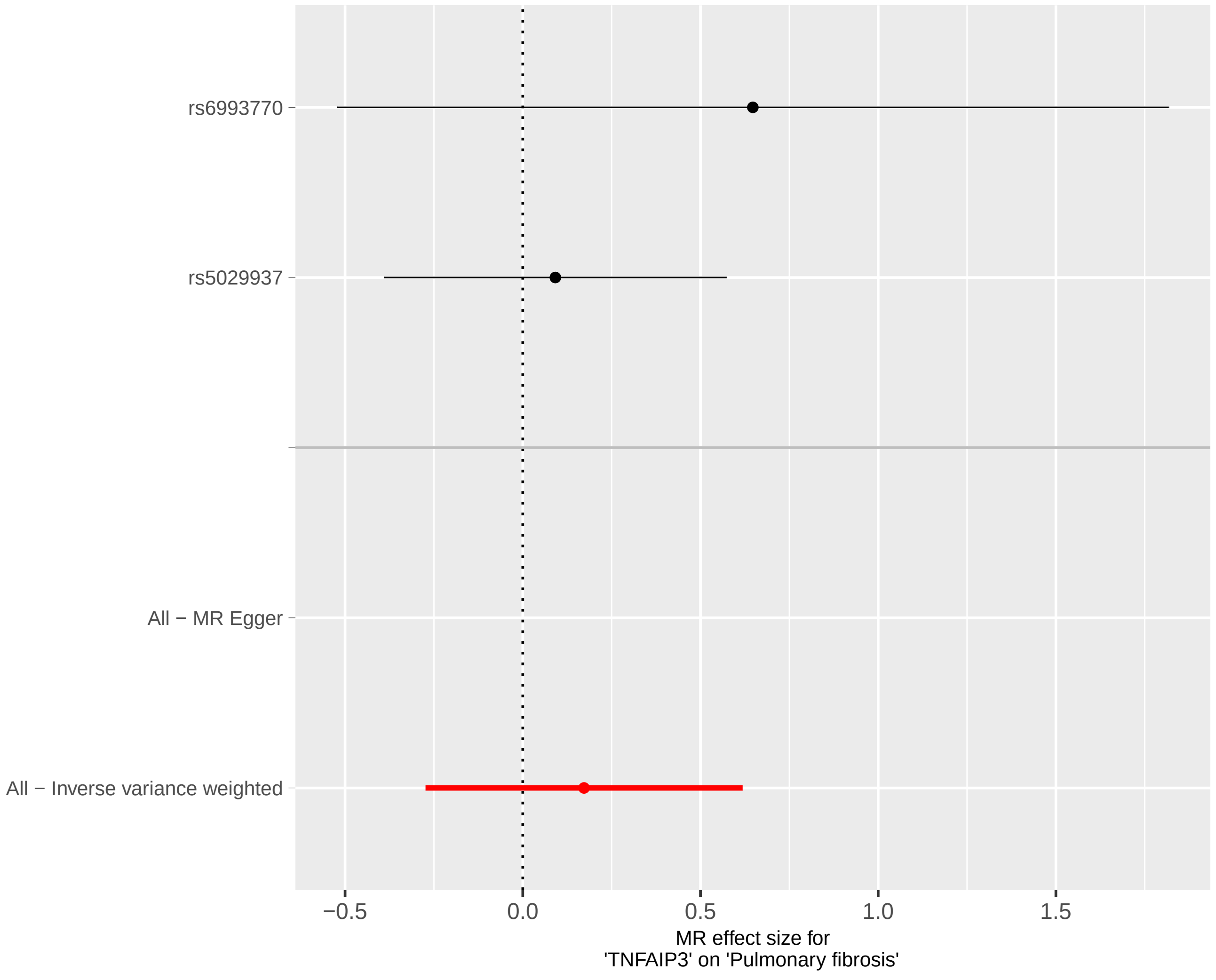
rs6993770
rs5029937
All − MR Egger
All − Inverse variance weighted
−0.5
0.0
0.5
1.0
1.5
MR effect size for
'TNFAIP3' on 'Pulmonary fibrosis'

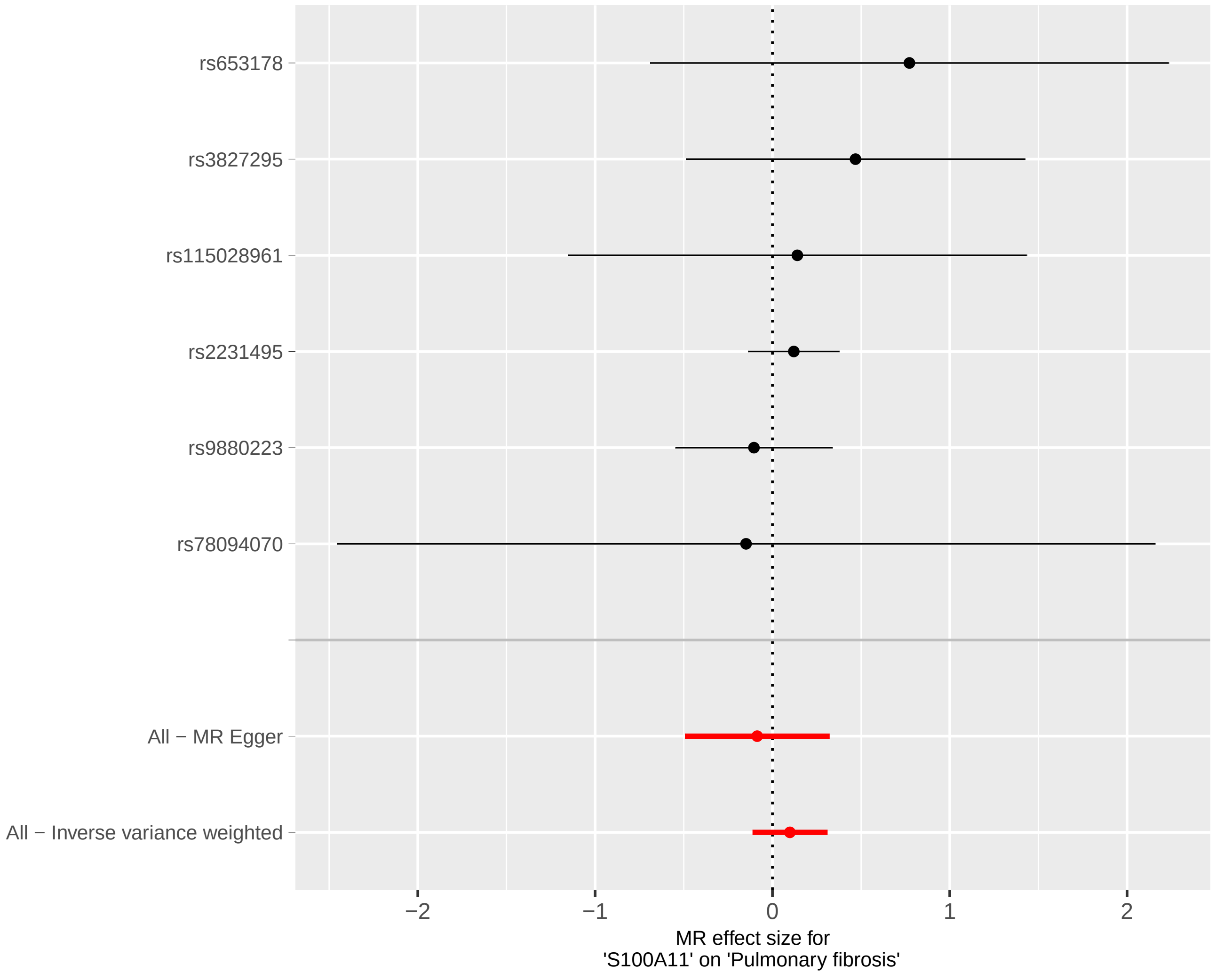
rs653178
rs3827295
rs115028961
rs2231495
rs9880223
rs78094070
All − MR Egger
All − Inverse variance weighted
−2
−1
0
1
2
MR effect size for
'S100A11' on 'Pulmonary fibrosis'

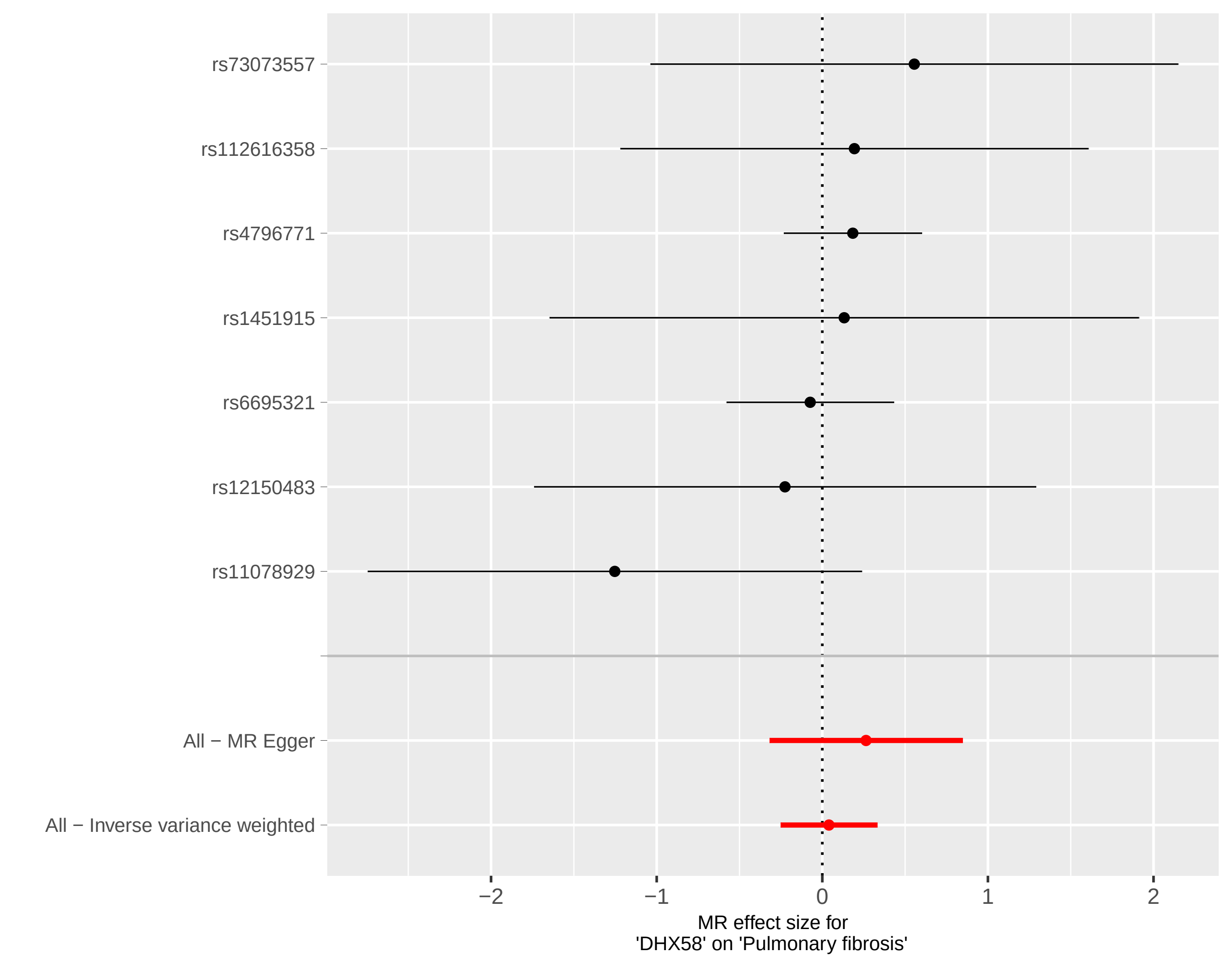

rs73073557
rs112616358
rs4796771
rs1451915
rs6695321
rs12150483
rs11078929
All − MR Egger
All − Inverse variance weighted
−2
−1
0
1
2
MR effect size for
'DHX58' on 'Pulmonary fibrosis'

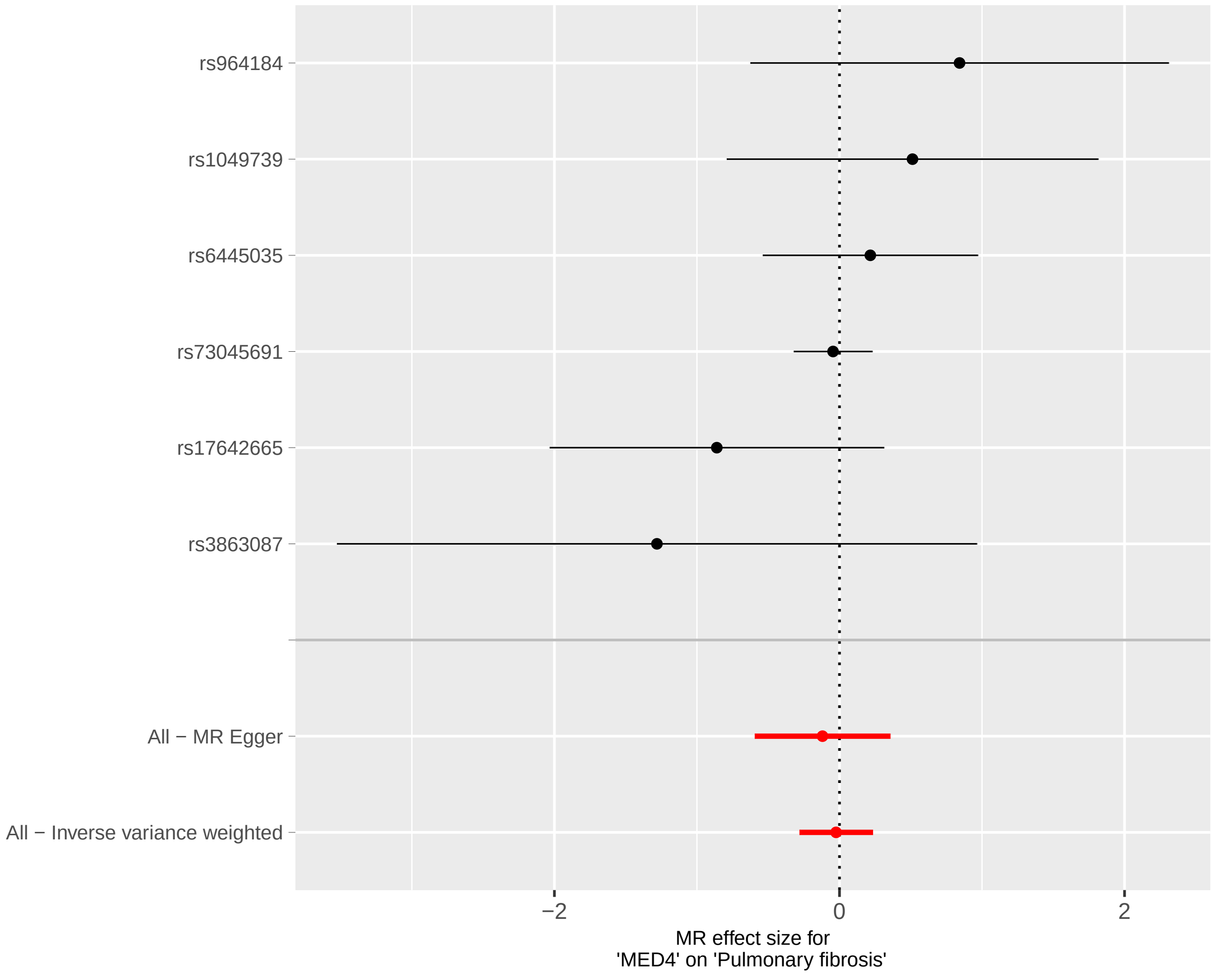
rs964184
rs1049739
rs6445035
rs73045691
rs17642665
rs3863087
All − MR Egger
All − Inverse variance weighted
−2
0
2
MR effect size for
'MED4' on 'Pulmonary fibrosis'

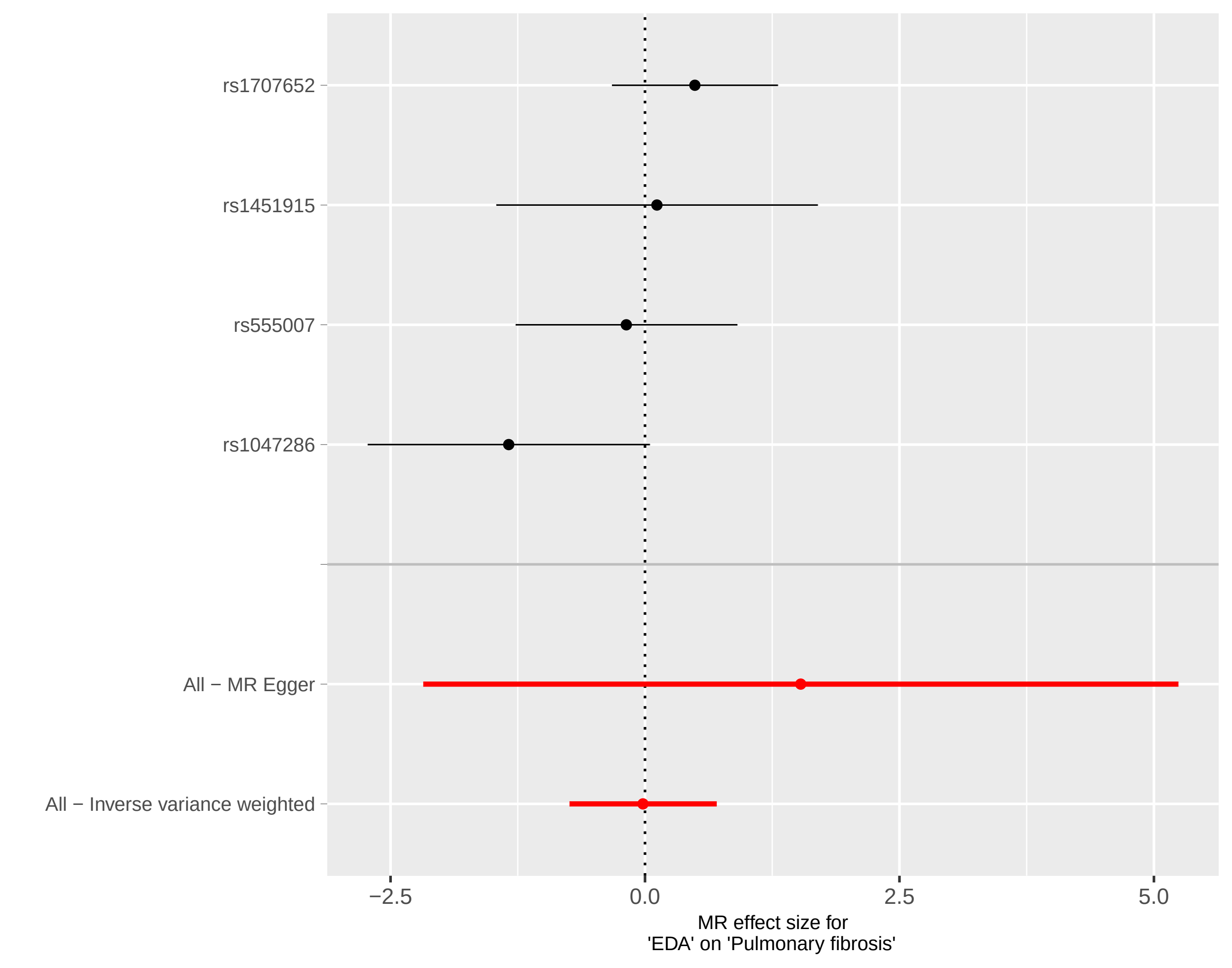

rs1707652
rs1451915
rs555007
rs1047286
All − MR Egger
All − Inverse variance weighted
−2.5
0.0
2.5
5.0
MR effect size for
'EDA' on 'Pulmonary fibrosis'

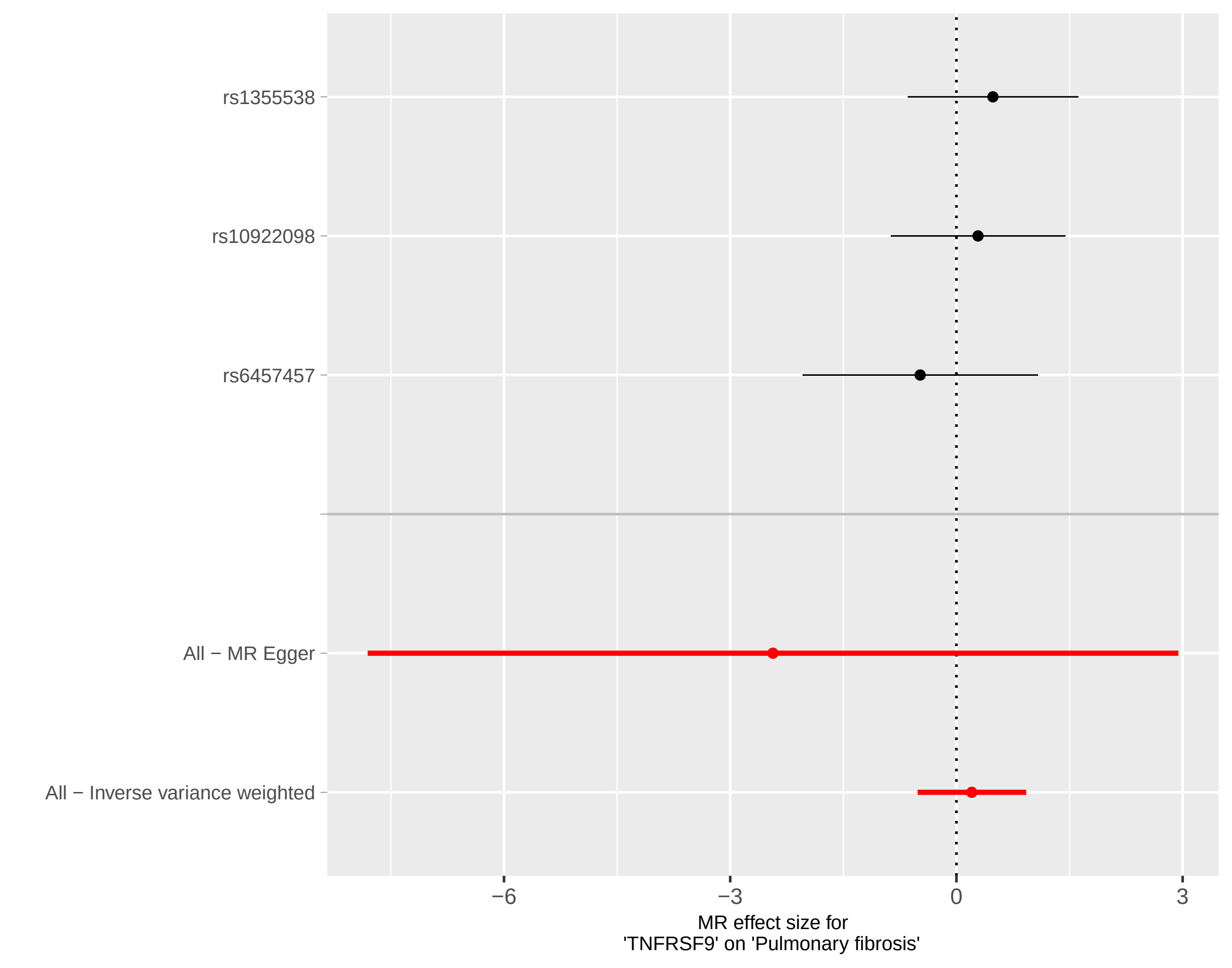

rs1355538
rs10922098
rs6457457
All − MR Egger
All − Inverse variance weighted
−6
−3
0
3
MR effect size for
'TNFRSF9' on 'Pulmonary fibrosis'

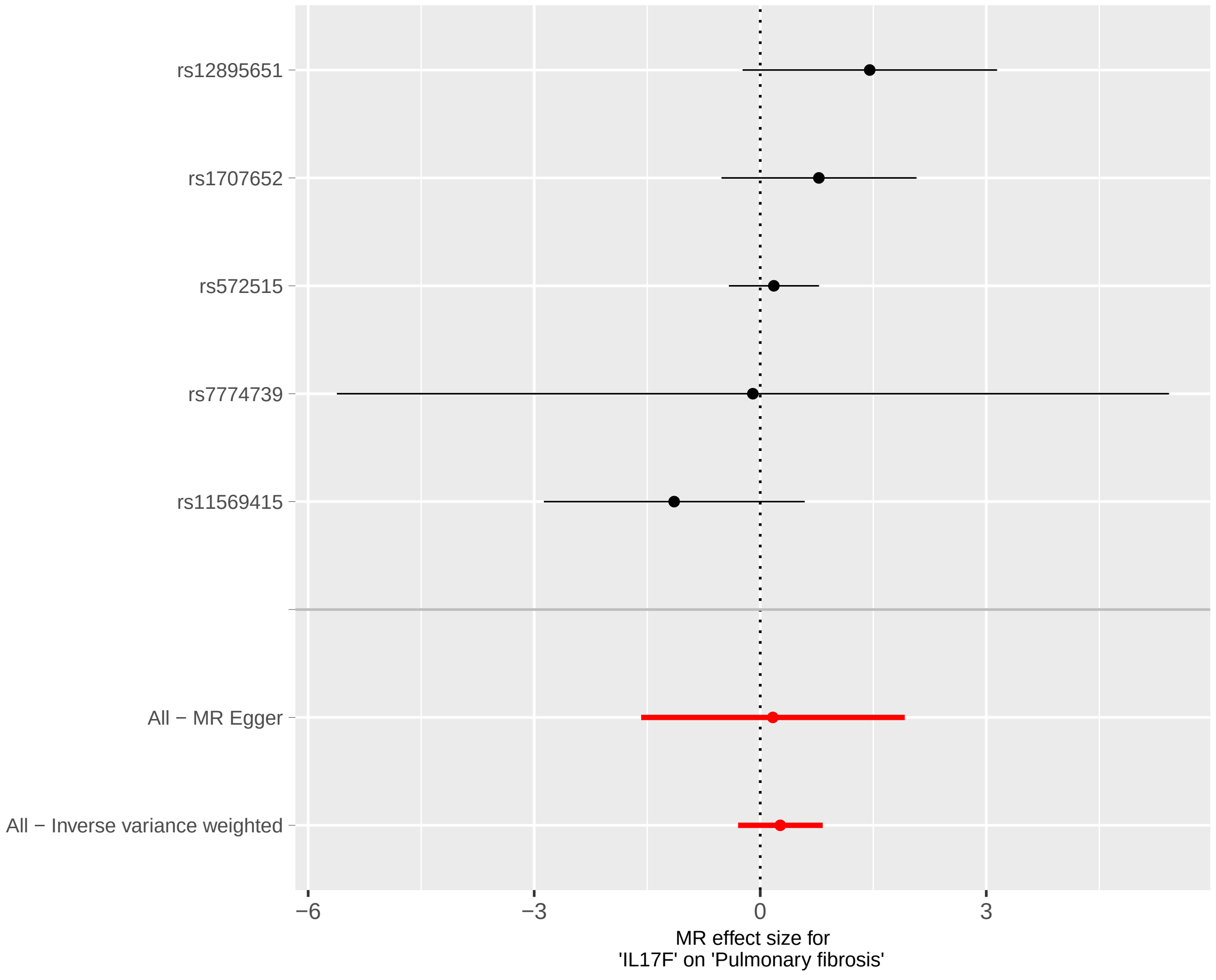

rs12895651
rs1707652
rs572515
rs7774739
rs11569415
All − MR Egger
All − Inverse variance weighted
−6
−3
0
3
MR effect size for
'IL17F' on 'Pulmonary fibrosis'

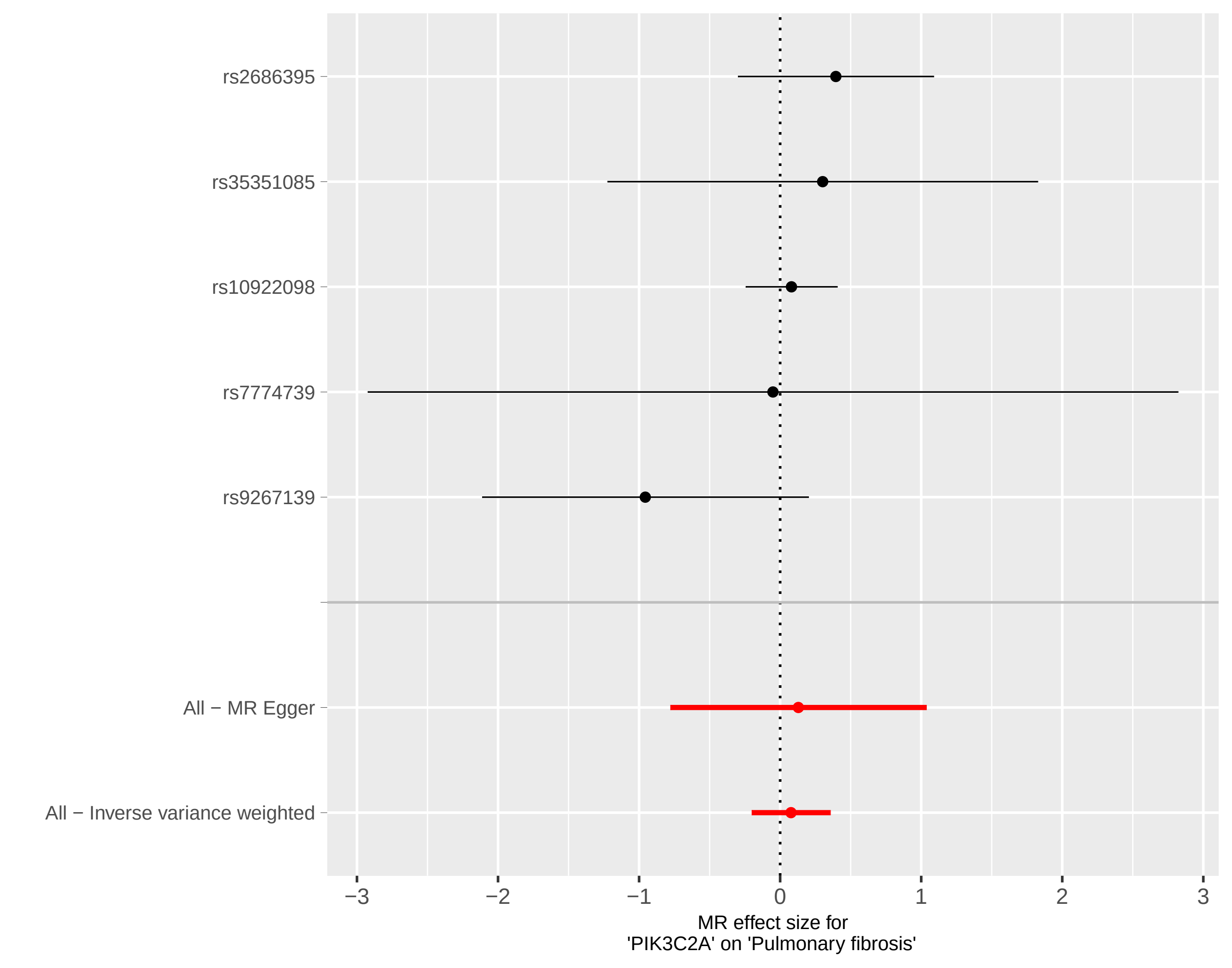
rs2686395
rs35351085
rs10922098
rs7774739
rs9267139
All − MR Egger
All − Inverse variance weighted
−3
−2
−1
0
1
2
3
MR effect size for
'PIK3C2A' on 'Pulmonary fibrosis'

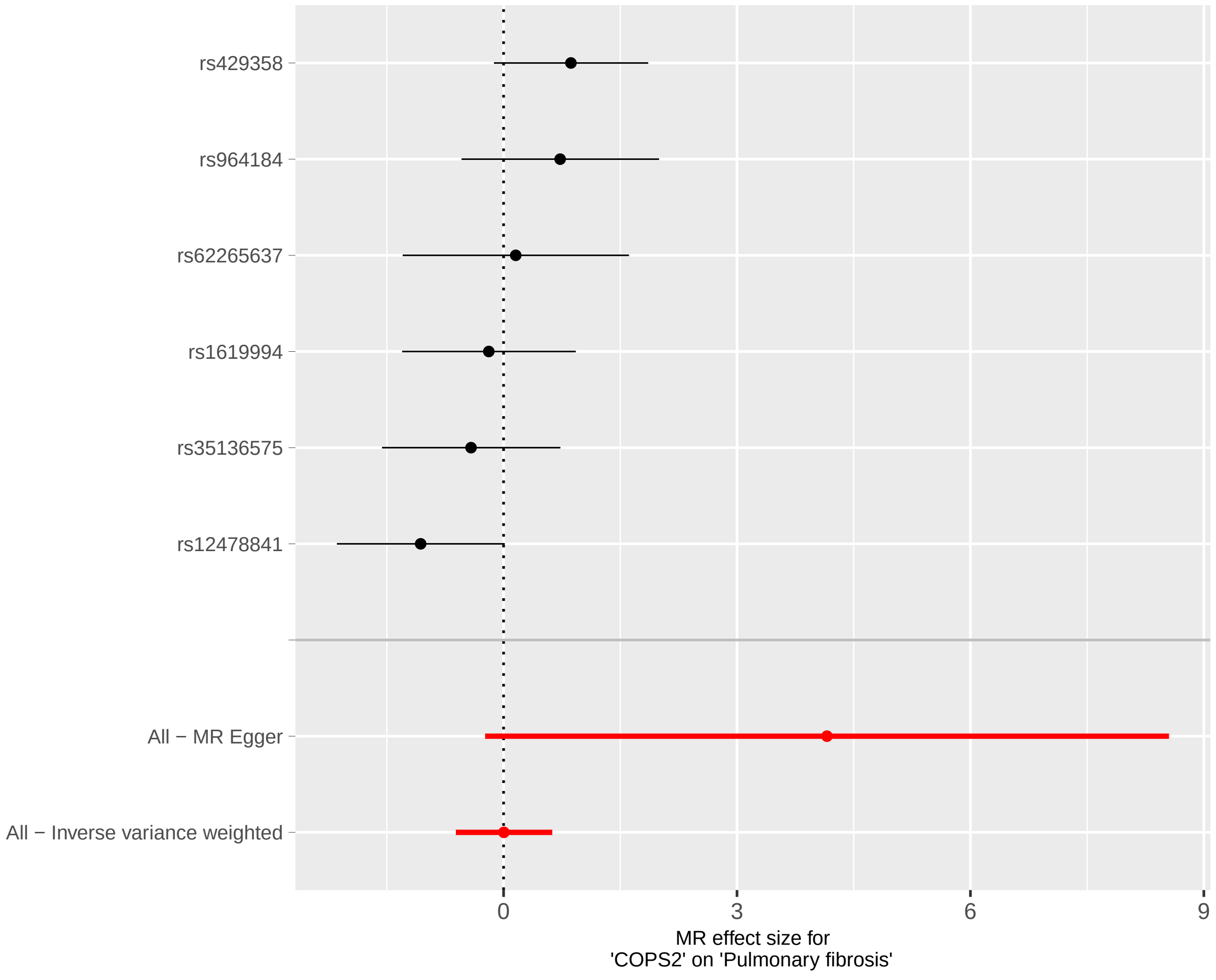
rs429358
rs964184
rs62265637
rs1619994
rs35136575
rs12478841
All − MR Egger
All − Inverse variance weighted
0
3
6
9
MR effect size for
'COPS2' on 'Pulmonary fibrosis'

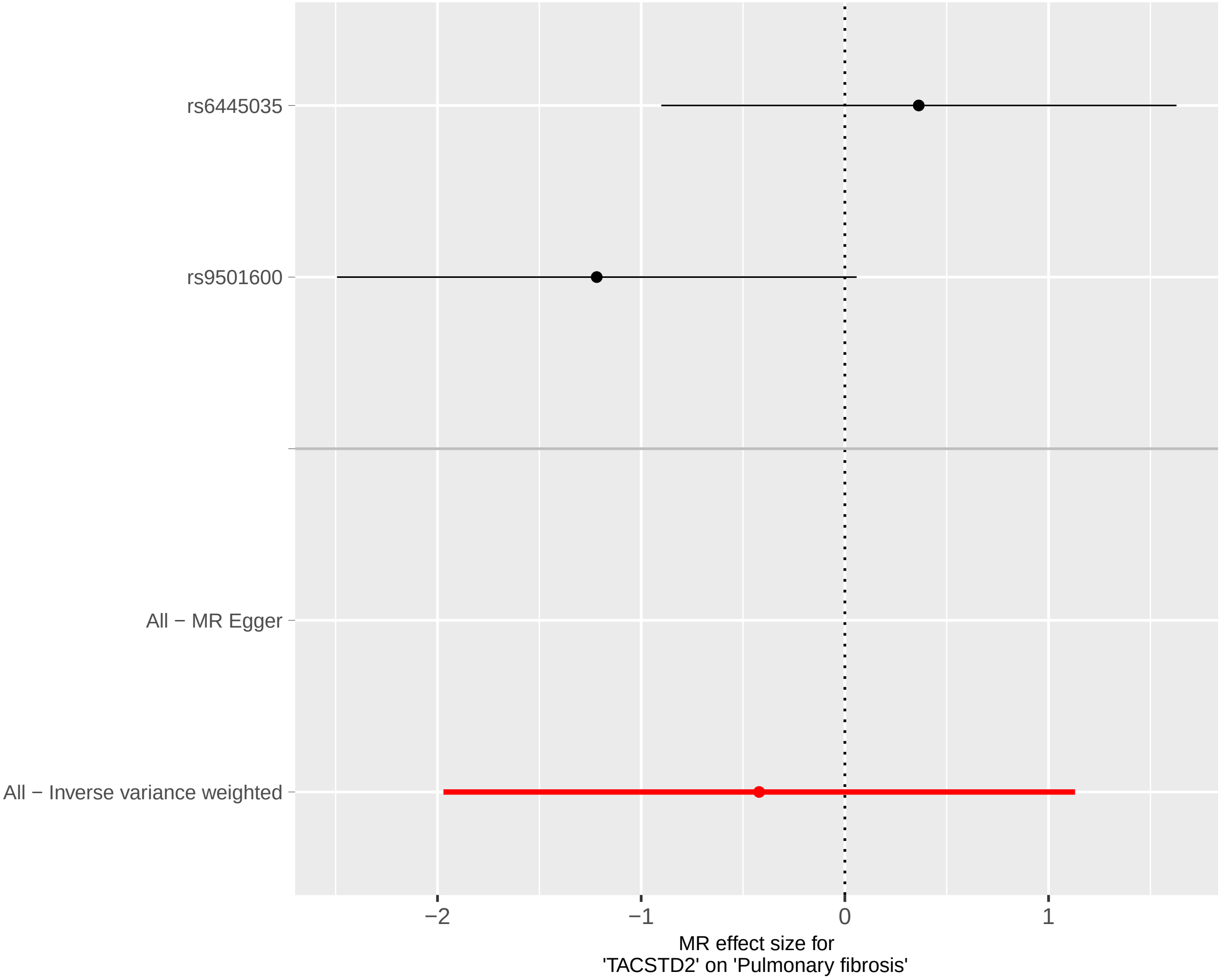
rs6445035
rs9501600
All − MR Egger
All − Inverse variance weighted
−2
−1
0
1
MR effect size for
'TACSTD2' on 'Pulmonary fibrosis'

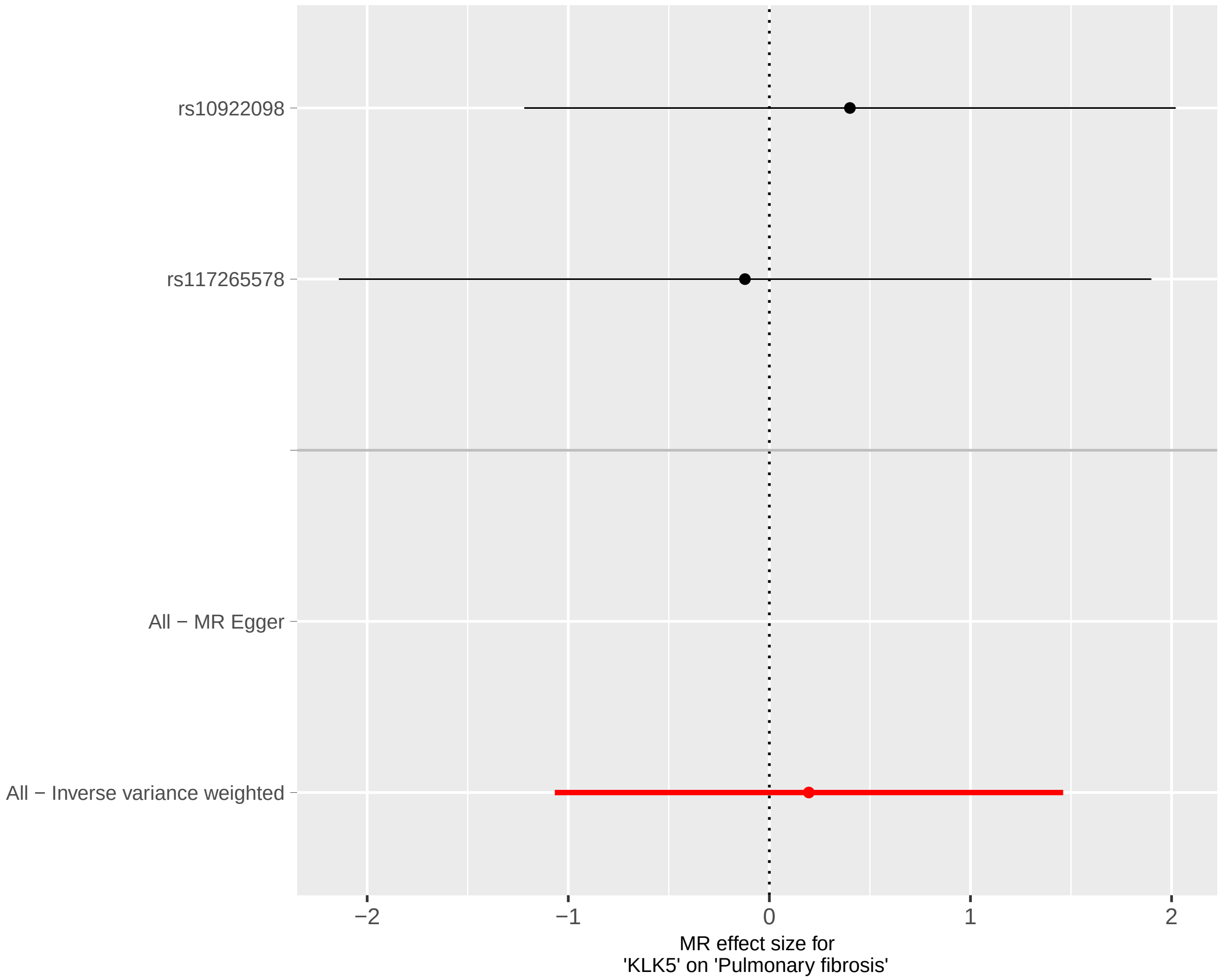
rs10922098
rs117265578
All − MR Egger
All − Inverse variance weighted
−2
−1
0
1
2
MR effect size for
'KLK5' on 'Pulmonary fibrosis'

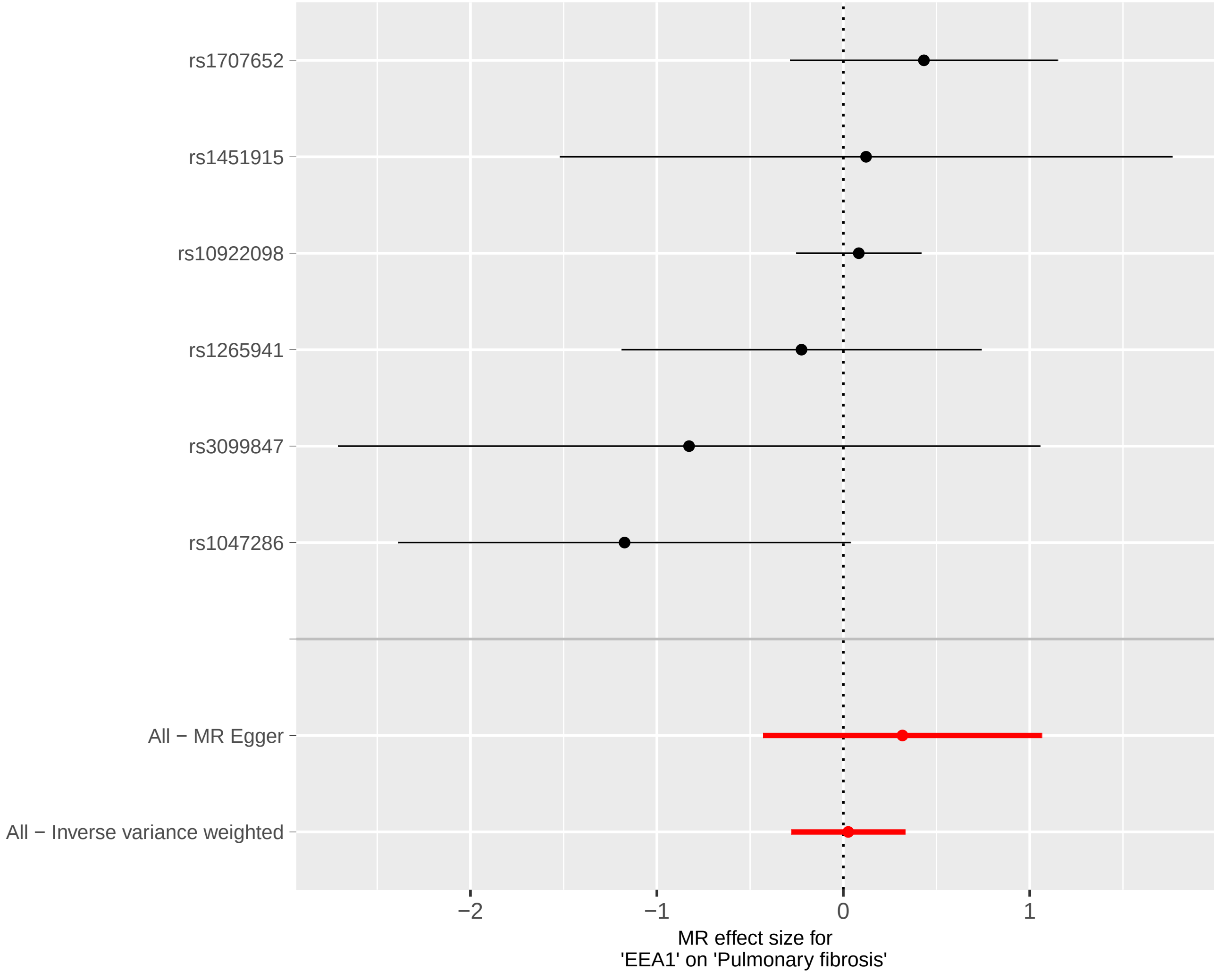

rs1707652
rs1451915
rs10922098
rs1265941
rs3099847
rs1047286
All − MR Egger
All − Inverse variance weighted
−2
−1
0
1
MR effect size for
'EEA1' on 'Pulmonary fibrosis'

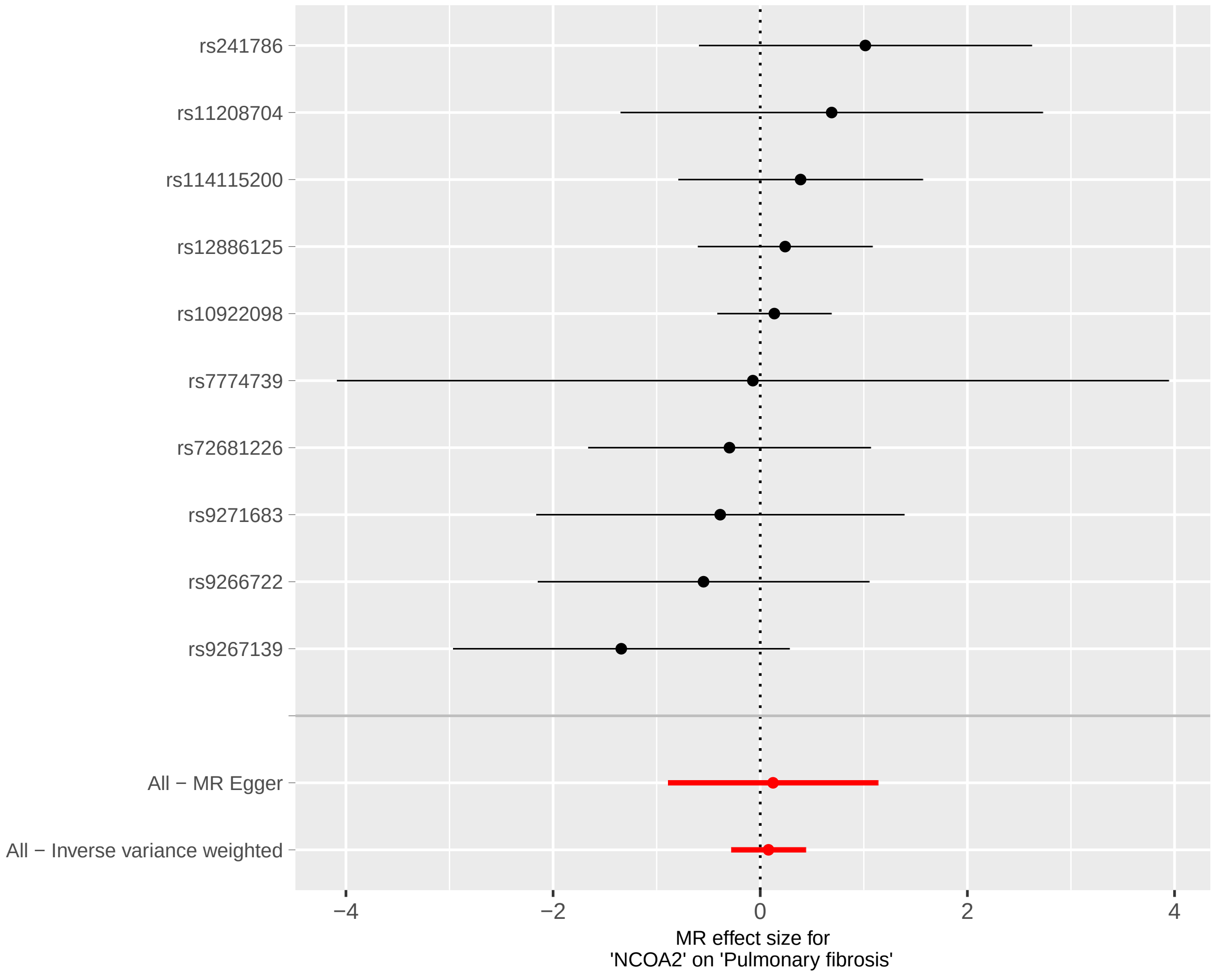
rs241786
rs11208704
rs114115200
rs12886125
rs10922098
rs7774739
rs72681226
rs9271683
rs9266722
rs9267139
All − MR Egger
All − Inverse variance weighted
−4
−2
0
2
4
MR effect size for
'NCOA2' on 'Pulmonary fibrosis'

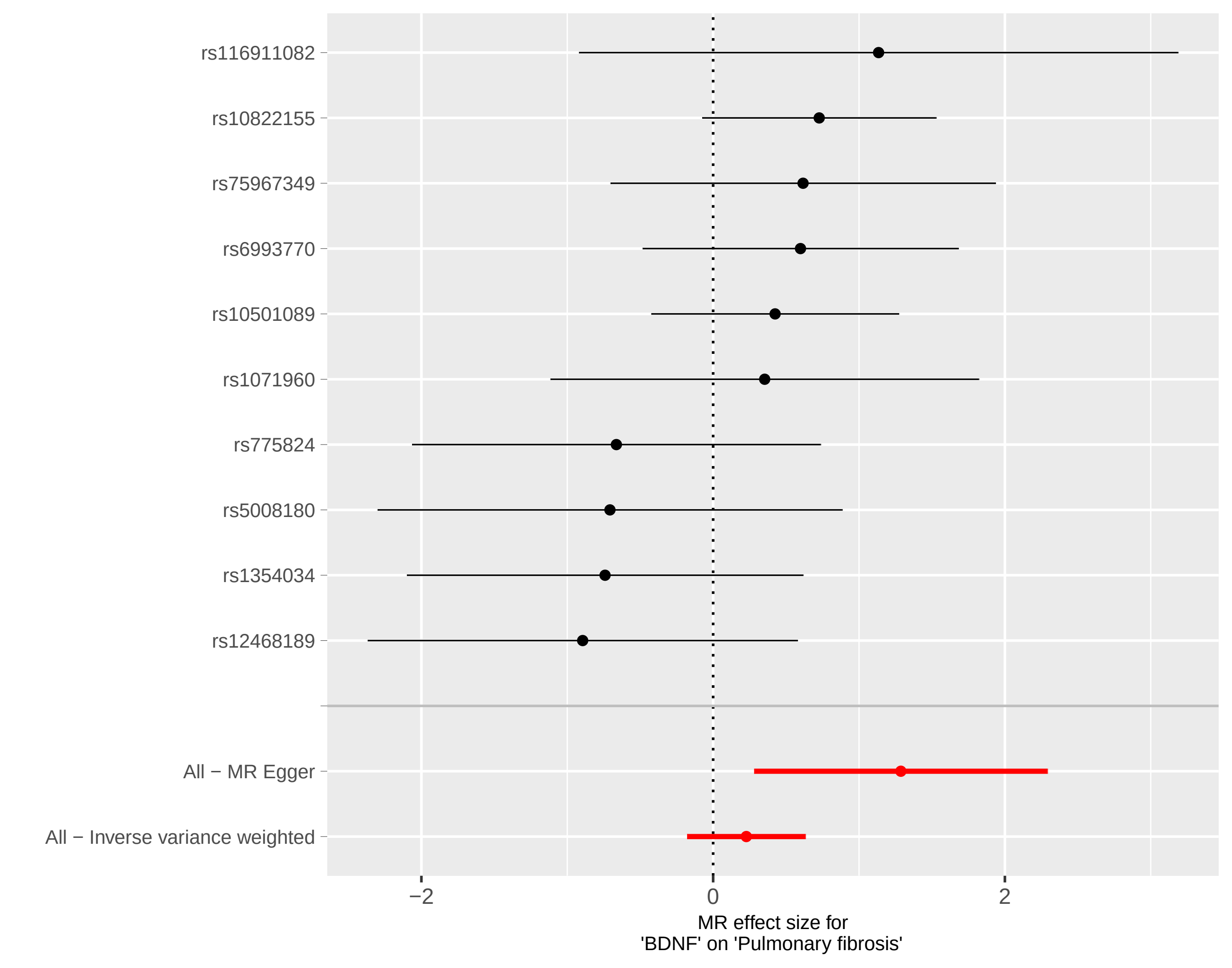
rs116911082
rs10822155
rs75967349
rs6993770
rs10501089
rs1071960
rs775824
rs5008180
rs1354034
rs12468189
All − MR Egger
All − Inverse variance weighted
−2
0
2
MR effect size for
'BDNF' on 'Pulmonary fibrosis'

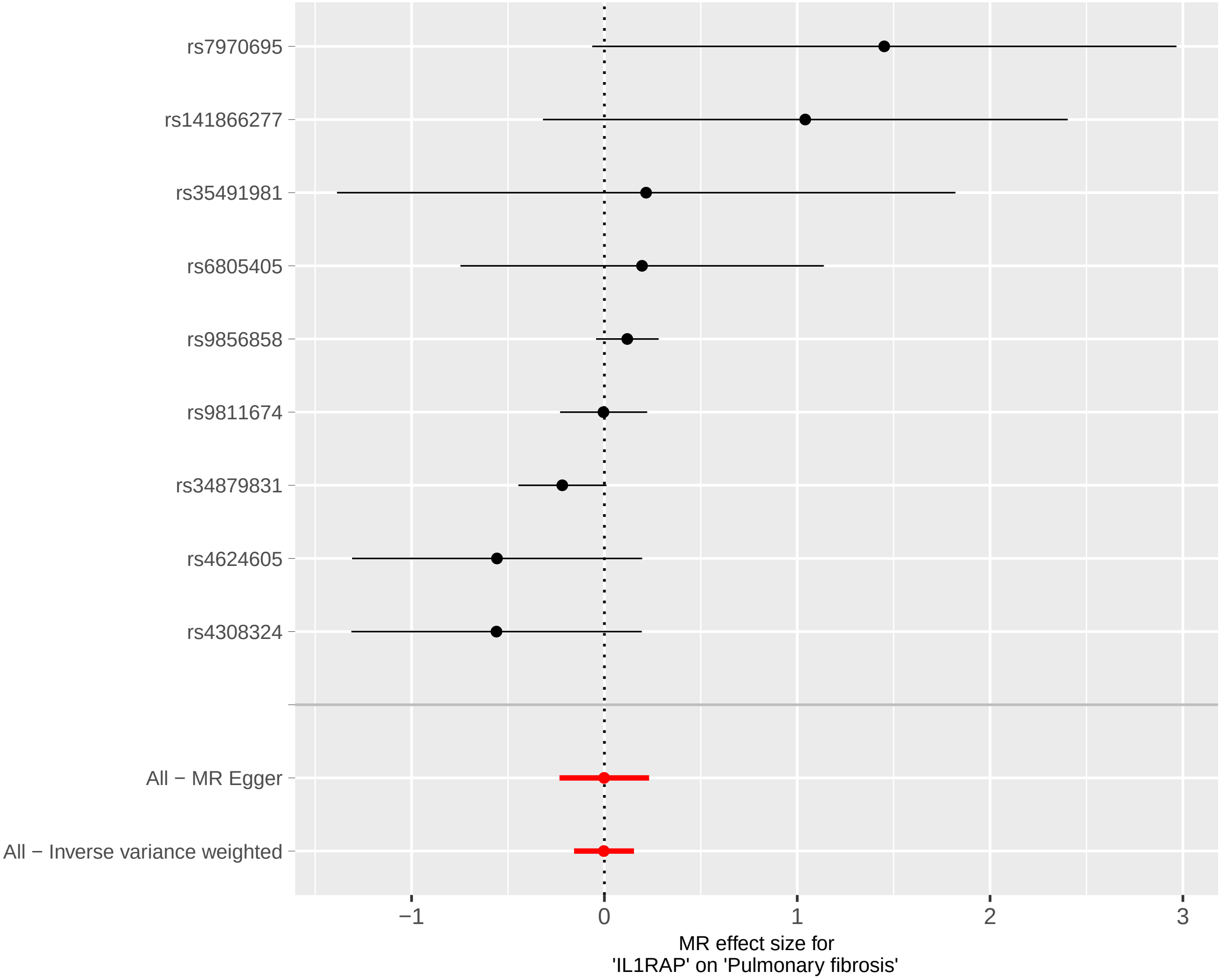

rs7970695
rs141866277
rs35491981
rs6805405
rs9856858
rs9811674
rs34879831
rs4624605
rs4308324
All − MR Egger
All − Inverse variance weighted
−1
0
1
2
3
MR effect size for
'IL1RAP' on 'Pulmonary fibrosis'

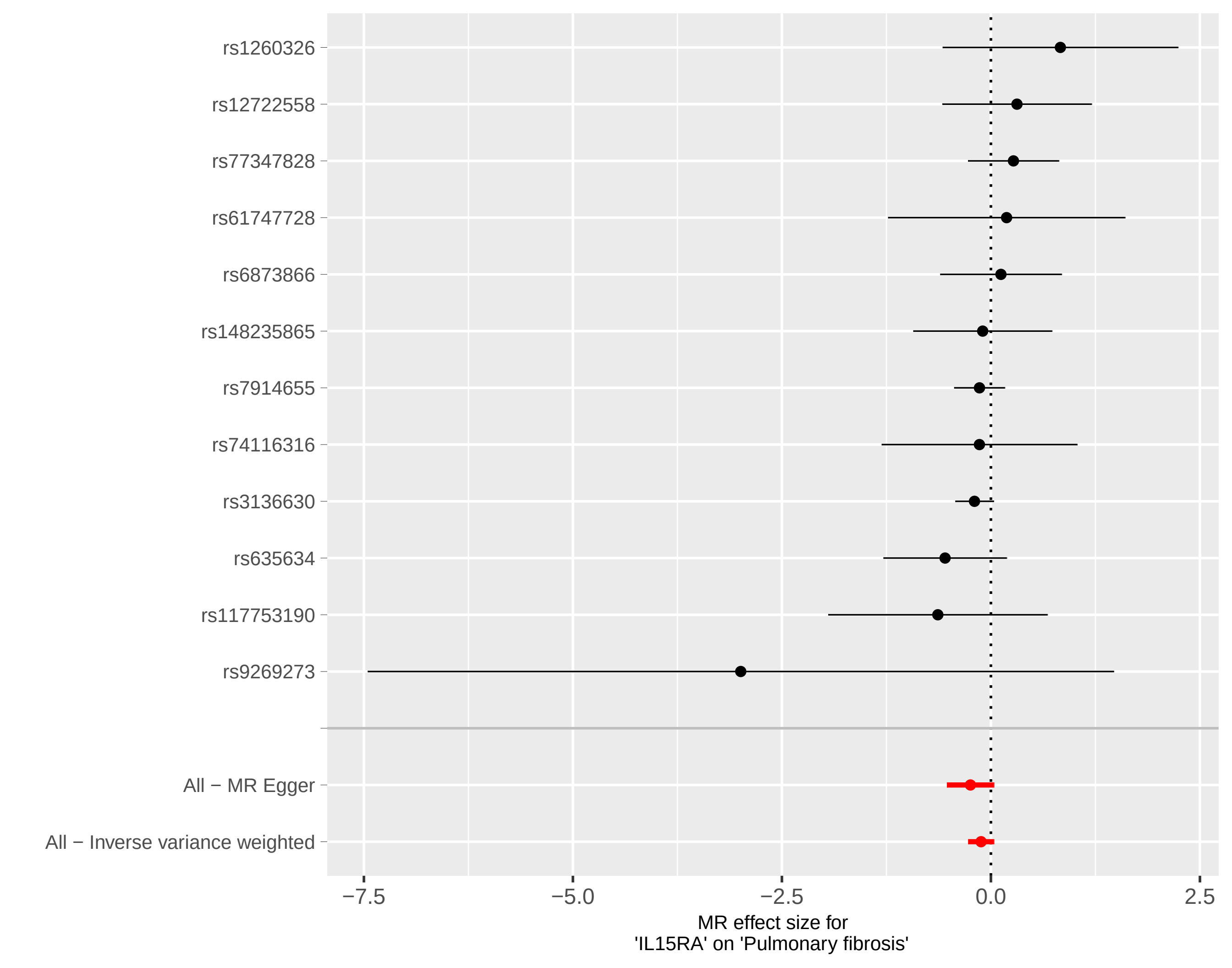

rs1260326
rs12722558
rs77347828
rs61747728
rs6873866
rs148235865
rs7914655
rs74116316
rs3136630
rs635634
rs117753190
rs9269273
All − MR Egger
All − Inverse variance weighted
−7.5
−5.0
−2.5
0.0
2.5
MR effect size for
'IL15RA' on 'Pulmonary fibrosis'

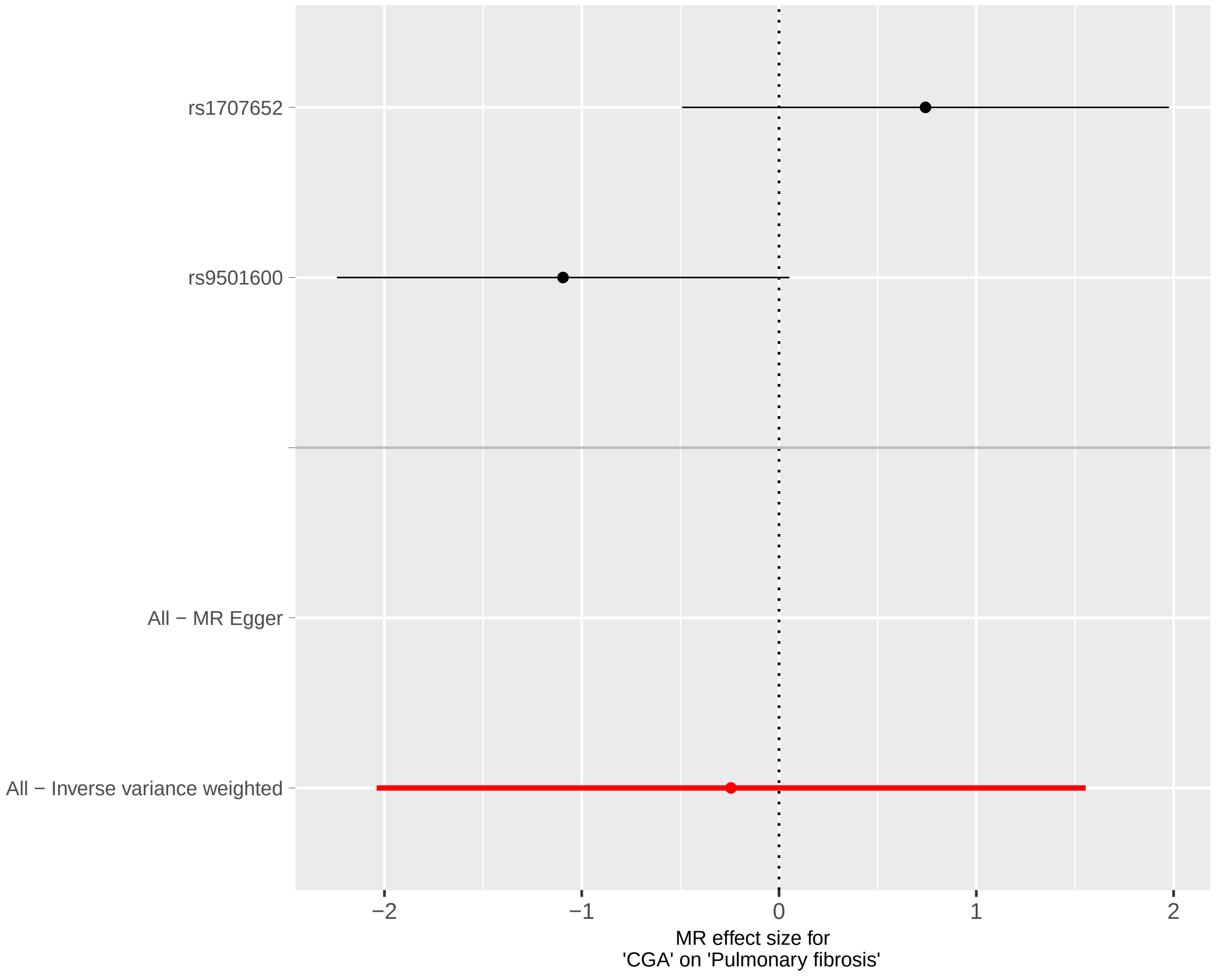
rs1707652
rs9501600
All − MR Egger
All − Inverse variance weighted
−2
−1
0
1
2
MR effect size for
'CGA' on 'Pulmonary fibrosis'

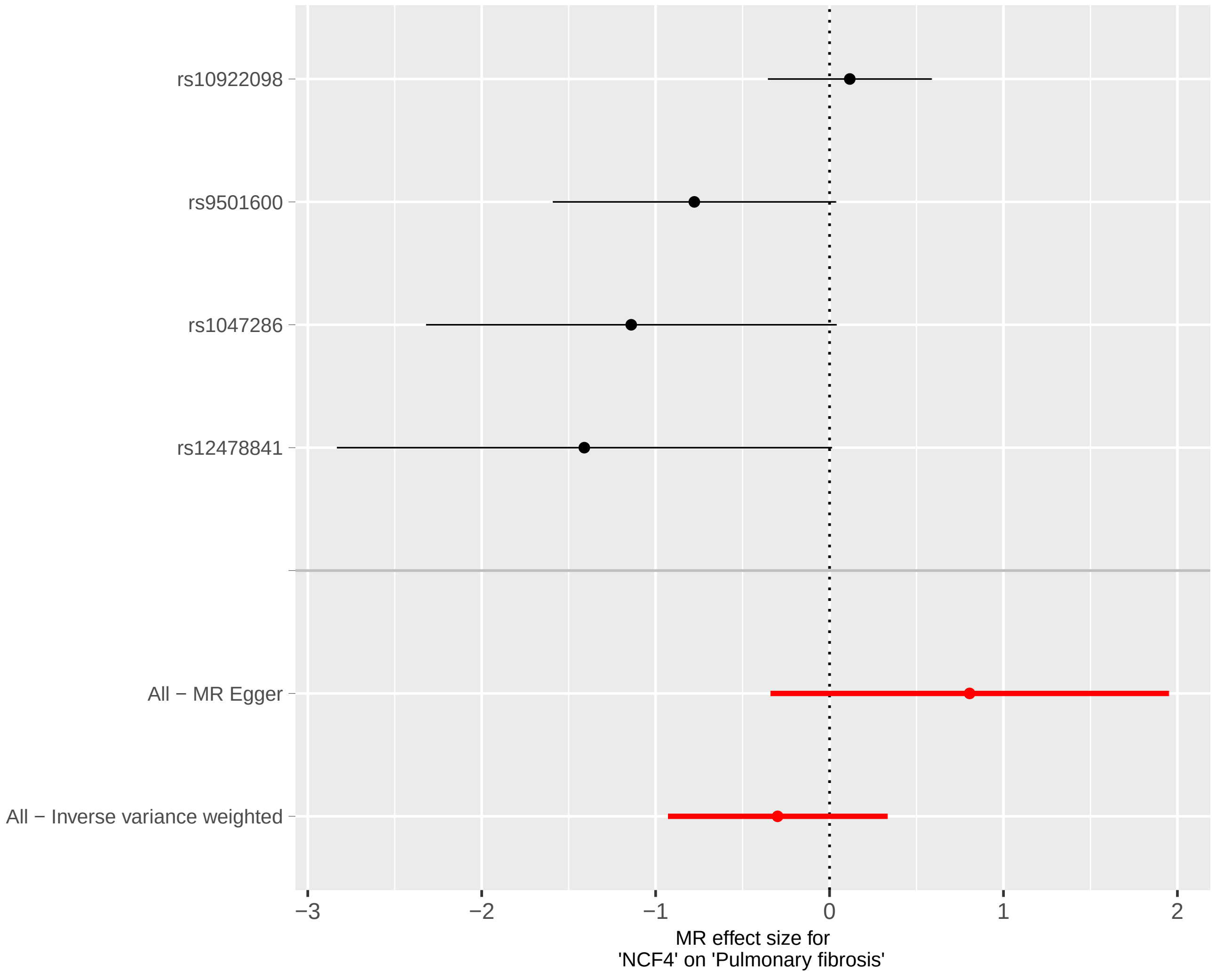
rs10922098
rs9501600
rs1047286
rs12478841
All − MR Egger
All − Inverse variance weighted
−3
−2
−1
0
1
2
MR effect size for
'NCF4' on 'Pulmonary fibrosis'

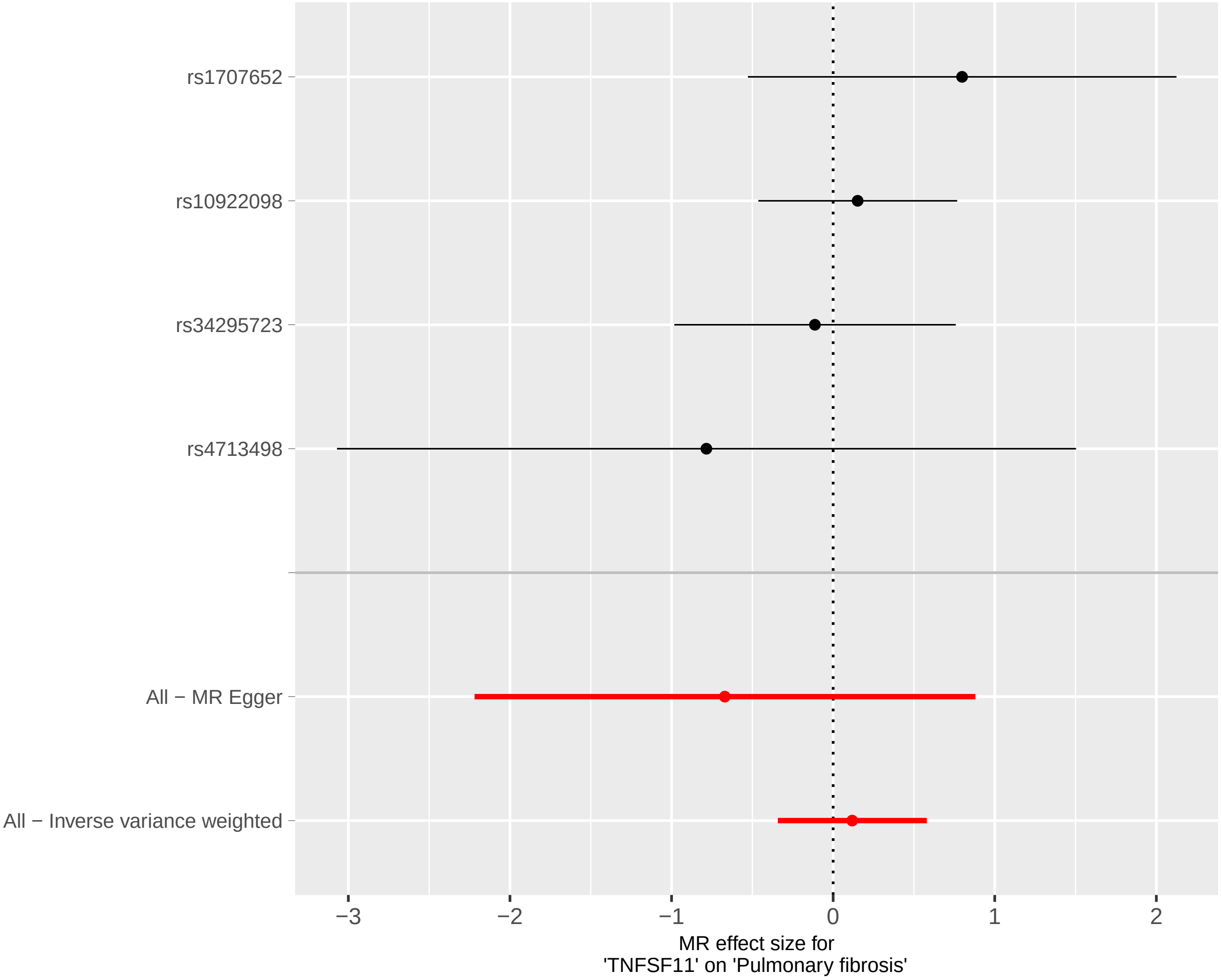

rs1707652
rs10922098
rs34295723
rs4713498
All − MR Egger
All − Inverse variance weighted
−3
−2
−1
0
1
2
MR effect size for
'TNFSF11' on 'Pulmonary fibrosis'

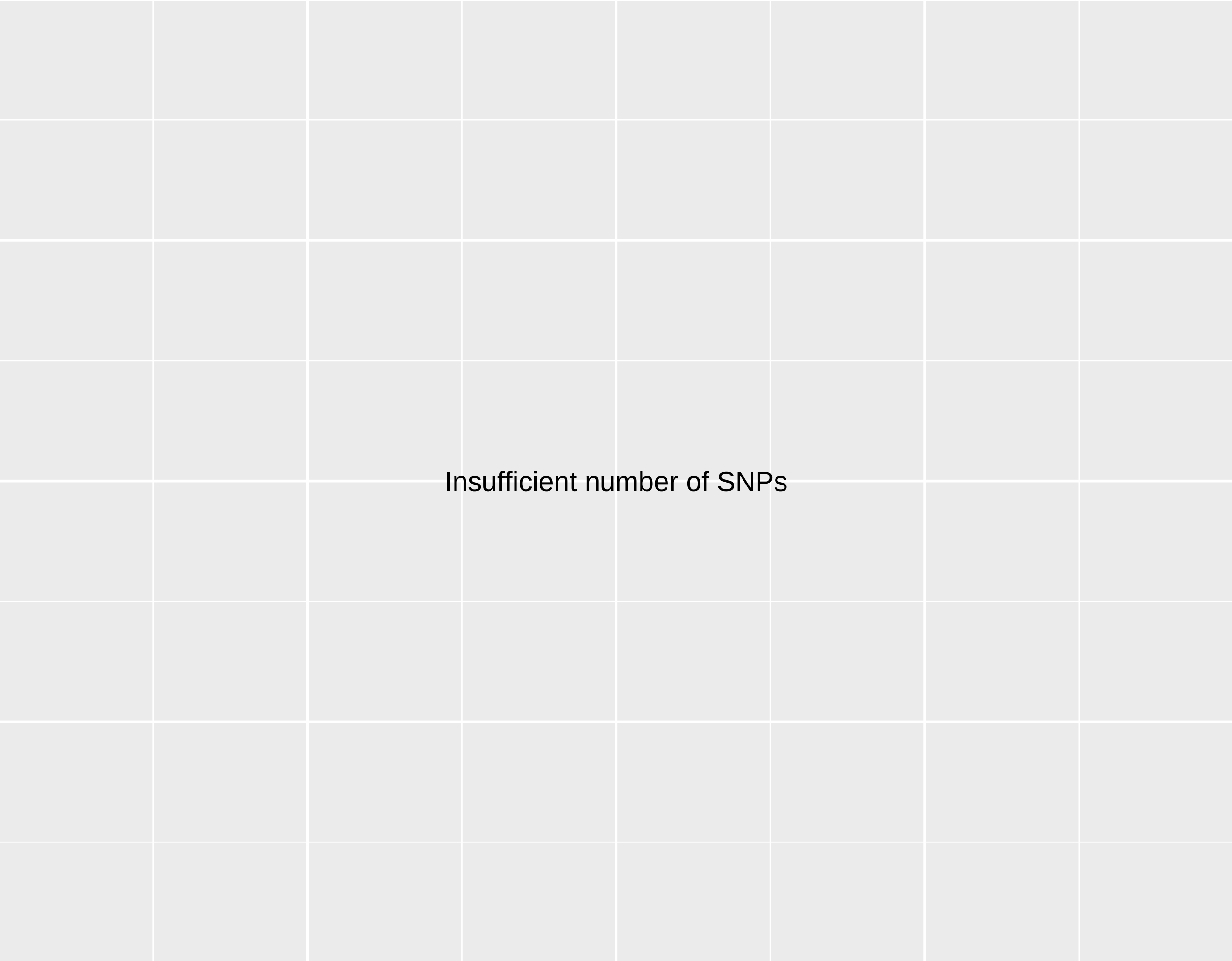
Insufficient number of SNPs

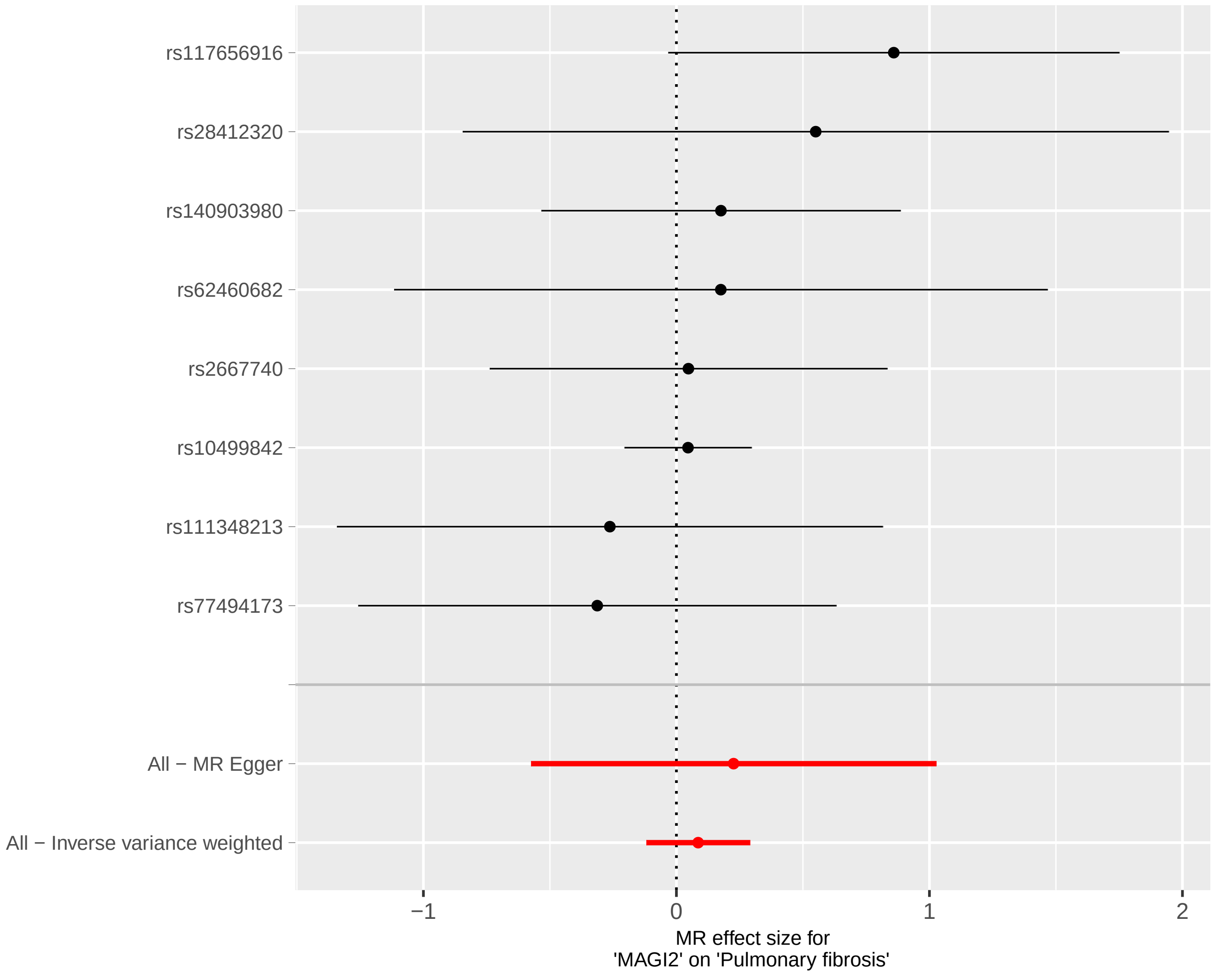
rs117656916
rs28412320
rs140903980
rs62460682
rs2667740
rs10499842
rs111348213
rs77494173
All − MR Egger
All − Inverse variance weighted
−1
0
1
2
MR effect size for
'MAGI2' on 'Pulmonary fibrosis'

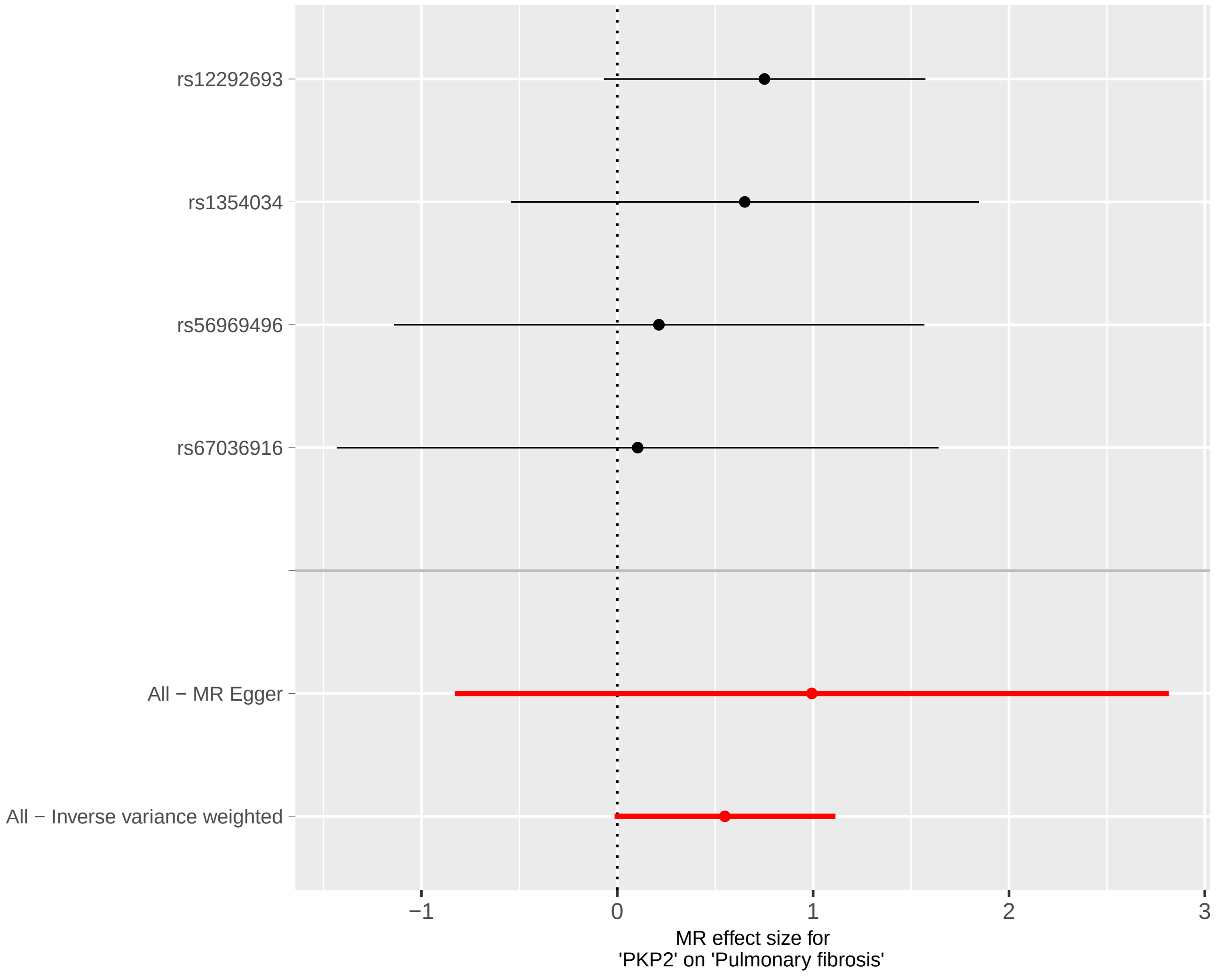

rs12292693
rs1354034
rs56969496
rs67036916
All − MR Egger
All − Inverse variance weighted
−1
0
1
2
3
MR effect size for
'PKP2' on 'Pulmonary fibrosis'

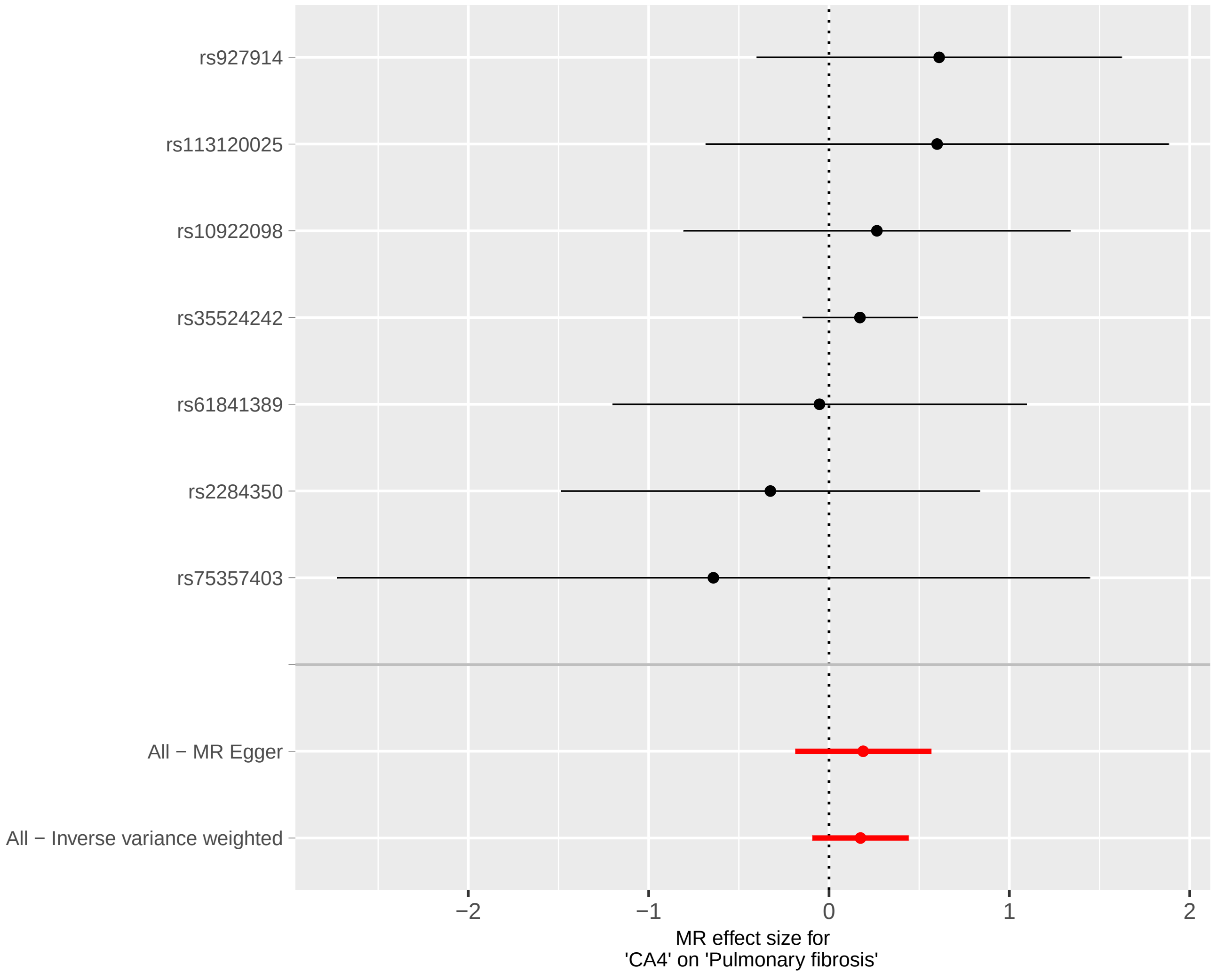

rs927914
rs113120025
rs10922098
rs35524242
rs61841389
rs2284350
rs75357403
All − MR Egger
All − Inverse variance weighted
−2
−1
0
1
2
MR effect size for
'CA4' on 'Pulmonary fibrosis'

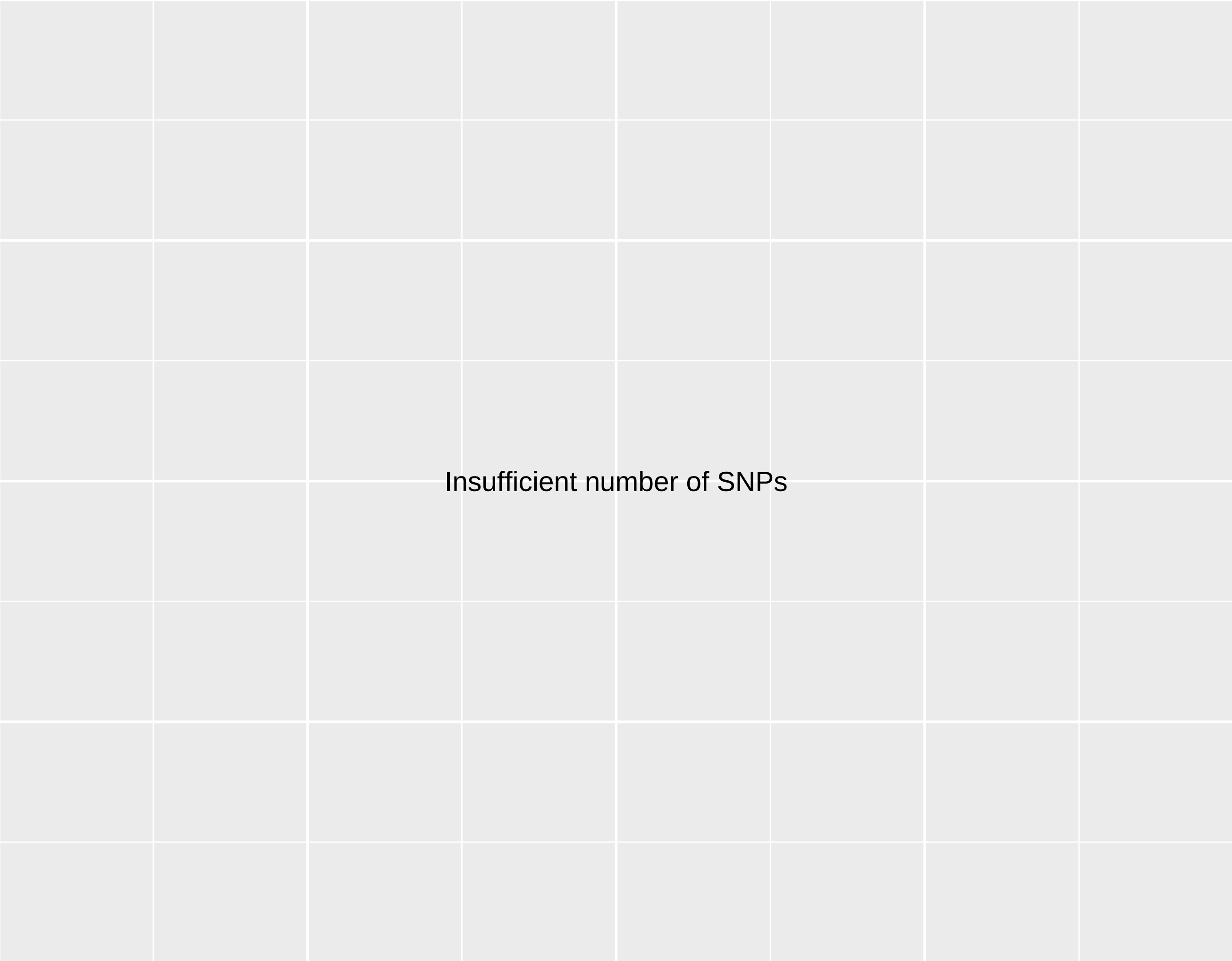
Insufficient number of SNPs

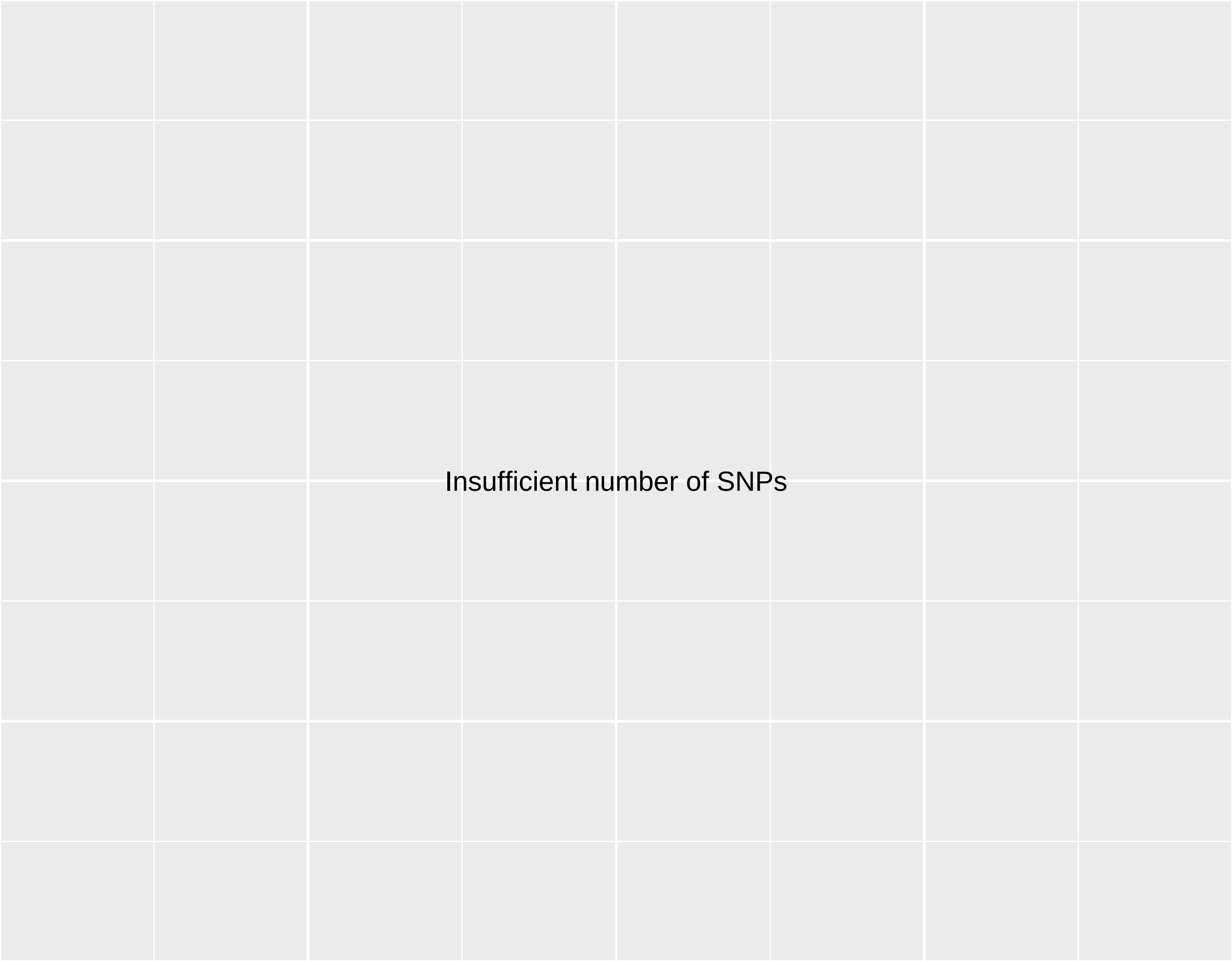
Insufficient number of SNPs

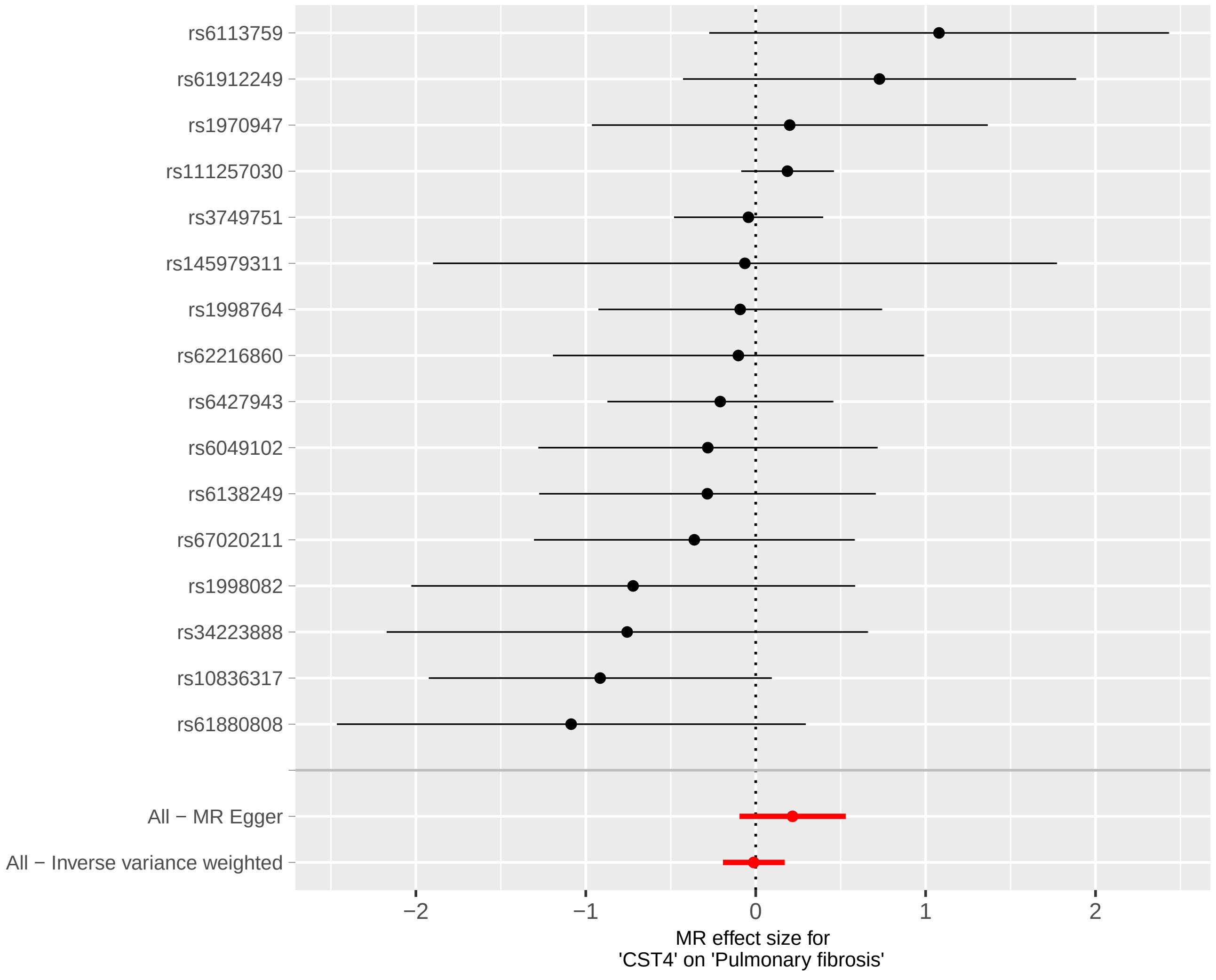
rs6113759
rs61912249
rs1970947
rs111257030
rs3749751
rs145979311
rs1998764
rs62216860
rs6427943
rs6049102
rs6138249
rs67020211
rs1998082
rs34223888
rs10836317
rs61880808
All − MR Egger
All − Inverse variance weighted
−2
−1
0
1
2
MR effect size for
'CST4' on 'Pulmonary fibrosis'

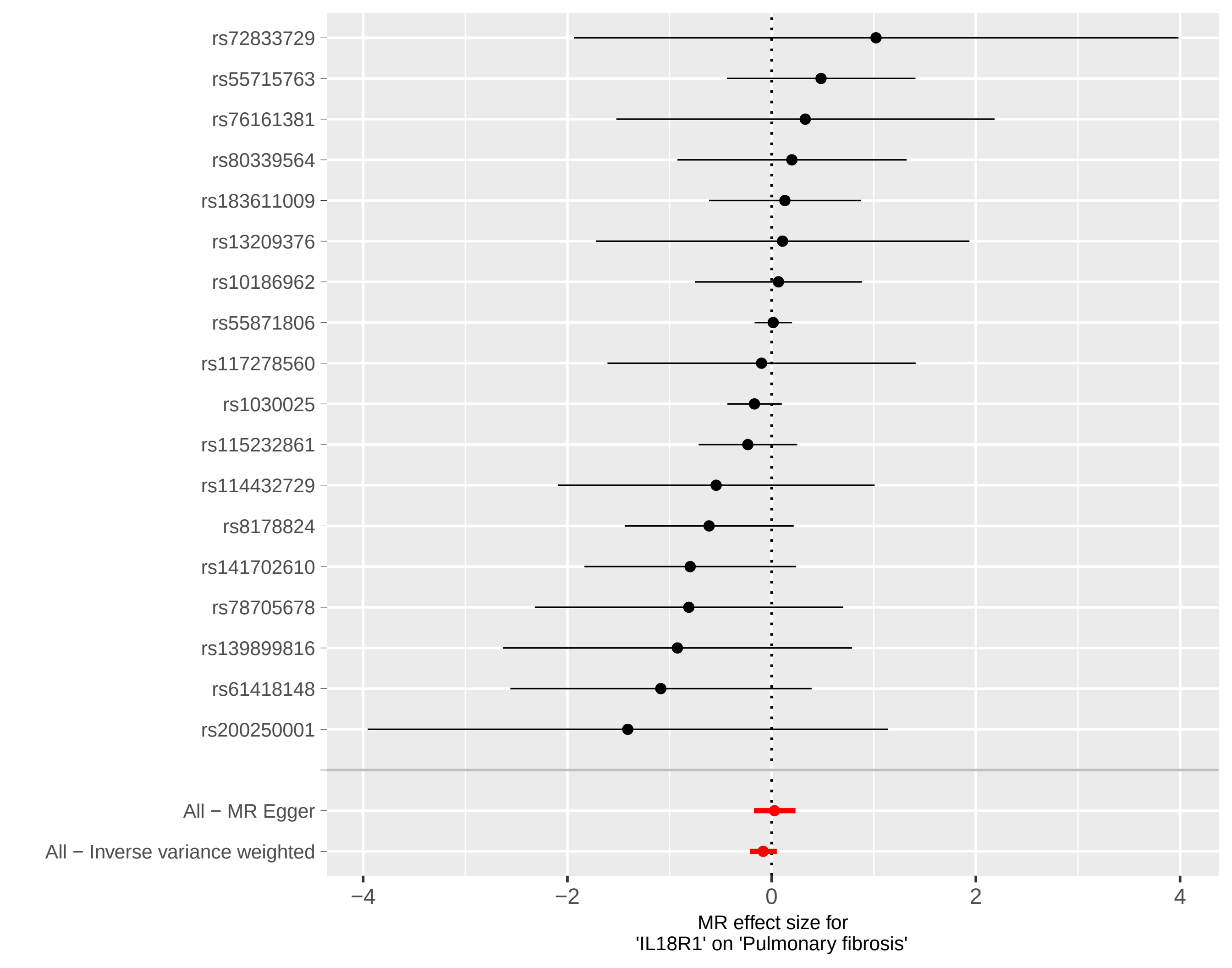
rs72833729
rs55715763
rs76161381
rs80339564
rs183611009
rs13209376
rs10186962
rs55871806
rs117278560
rs1030025
rs115232861
rs114432729
rs8178824
rs141702610
rs78705678
rs139899816
rs61418148
rs200250001
All − MR Egger
All − Inverse variance weighted
−4
−2
0
2
4
MR effect size for
'IL18R1' on 'Pulmonary fibrosis'

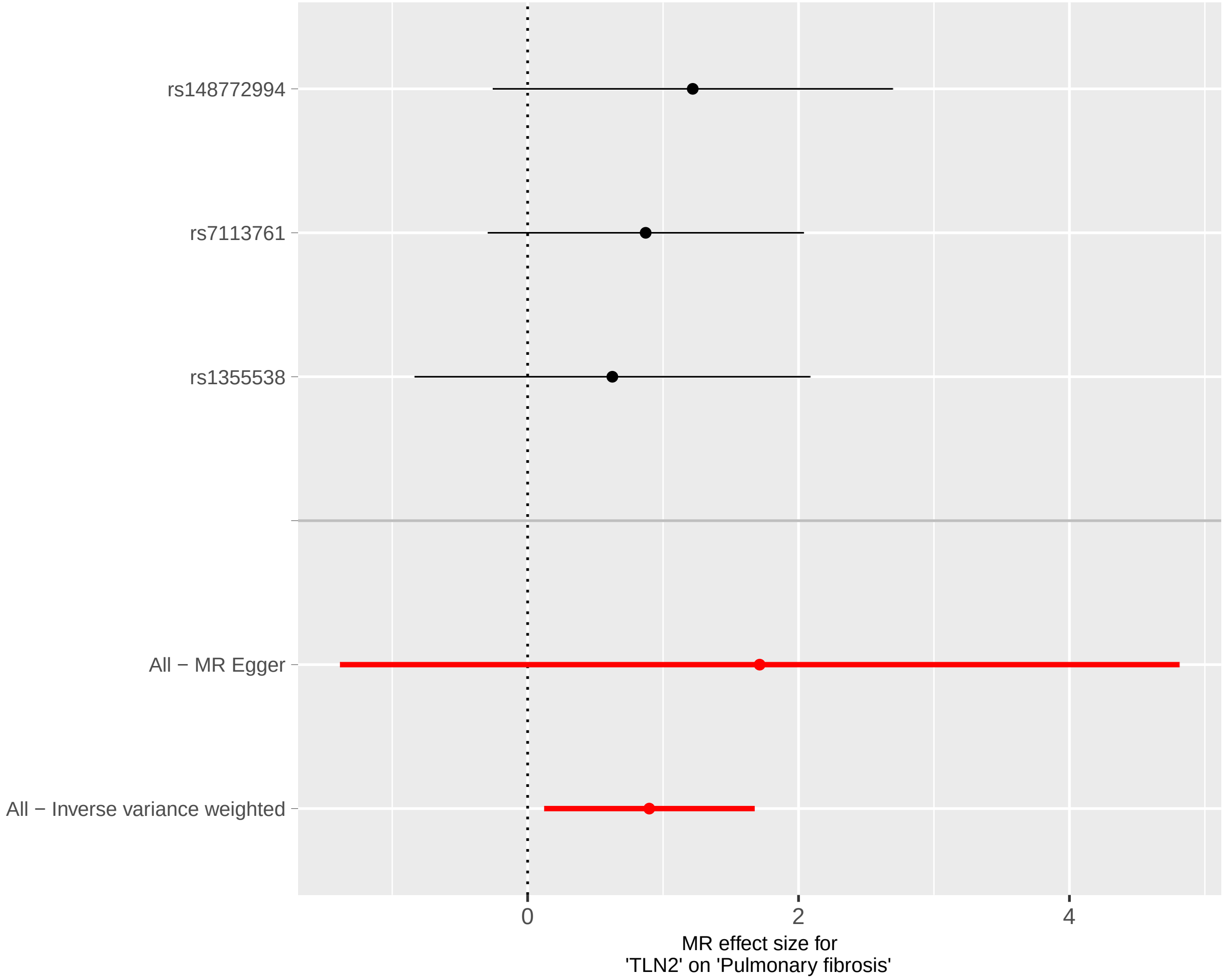
rs148772994
rs7113761
rs1355538
All − MR Egger
All − Inverse variance weighted
0
2
4
MR effect size for
'TLN2' on 'Pulmonary fibrosis'

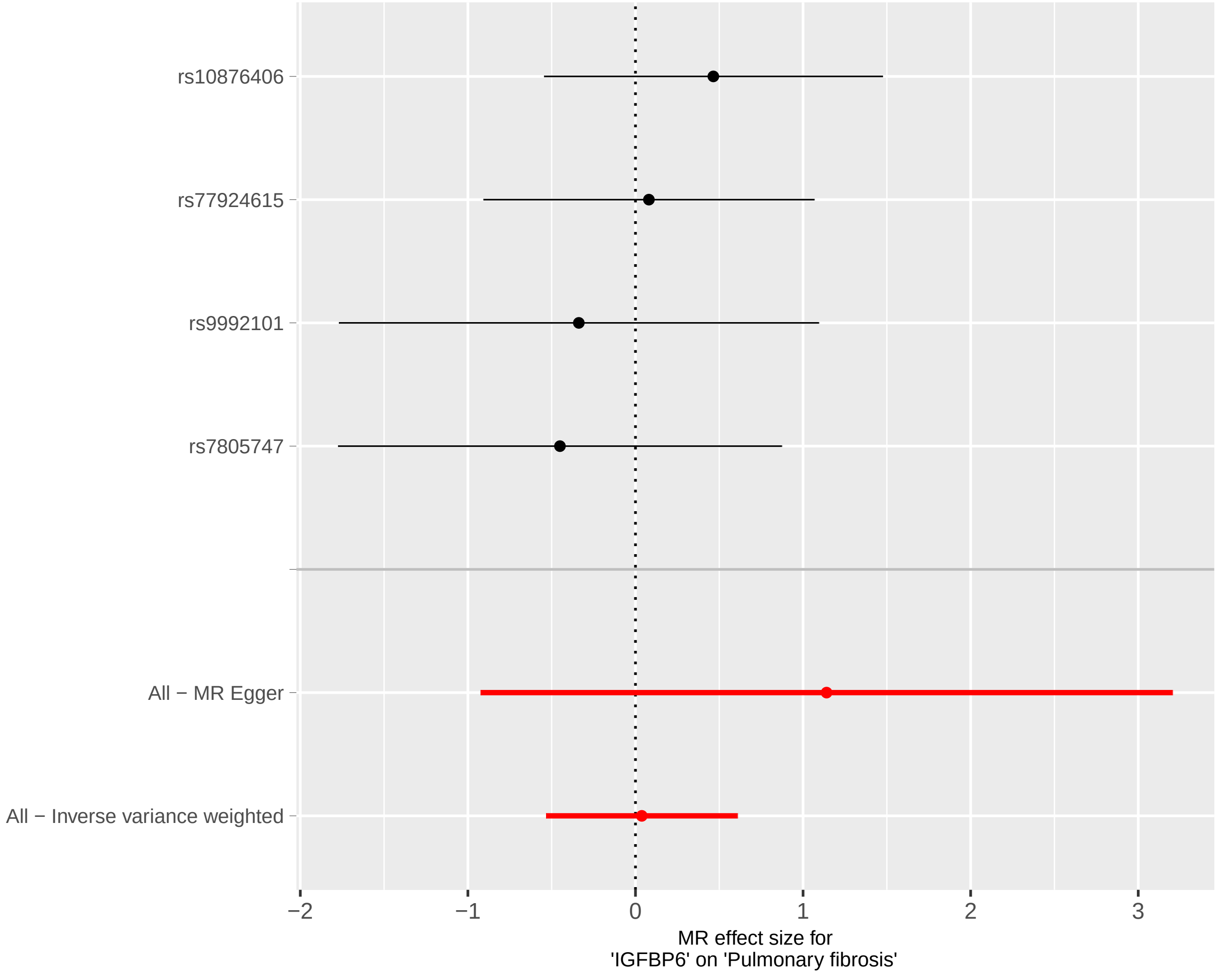
rs10876406
rs77924615
rs9992101
rs7805747
All − MR Egger
All − Inverse variance weighted
−2
−1
0
1
2
3
MR effect size for
'IGFBP6' on 'Pulmonary fibrosis'

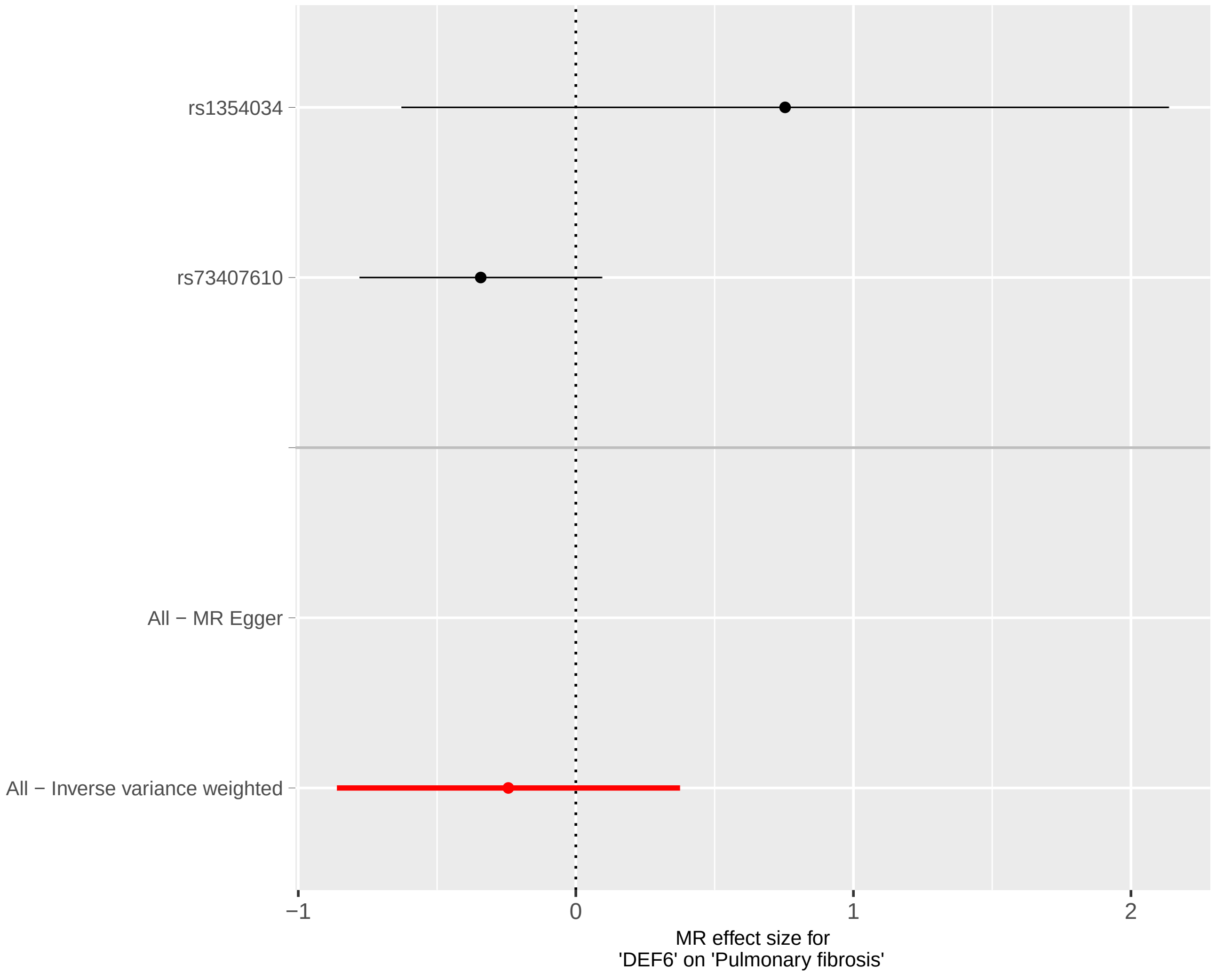

rs1354034
rs73407610
All − MR Egger
All − Inverse variance weighted
−1
0
1
2
MR effect size for
'DEF6' on 'Pulmonary fibrosis'

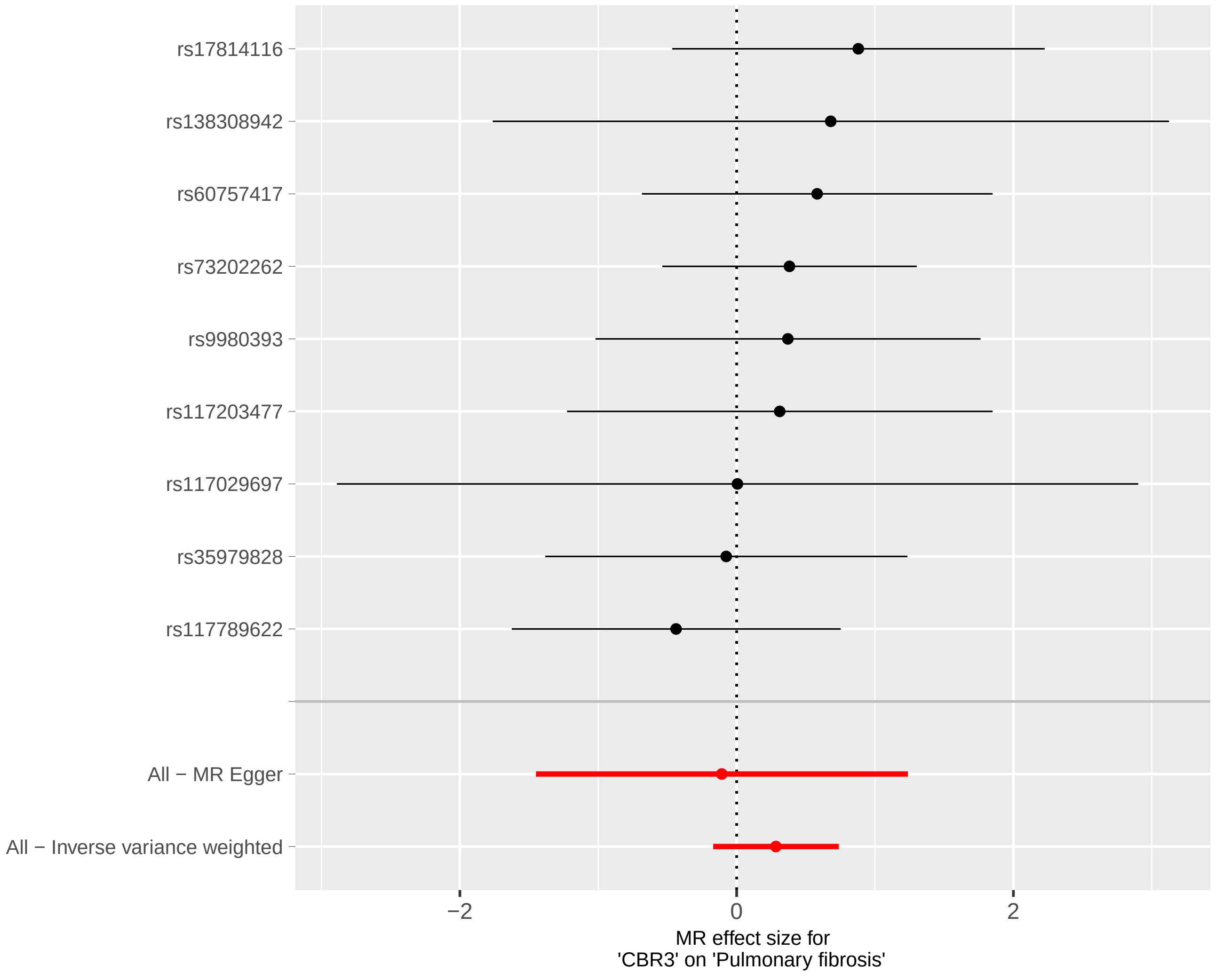
rs17814116
rs138308942
rs60757417
rs73202262
rs9980393
rs117203477
rs117029697
rs35979828
rs117789622
All − MR Egger
All − Inverse variance weighted
−2
0
2
MR effect size for
'CBR3' on 'Pulmonary fibrosis'

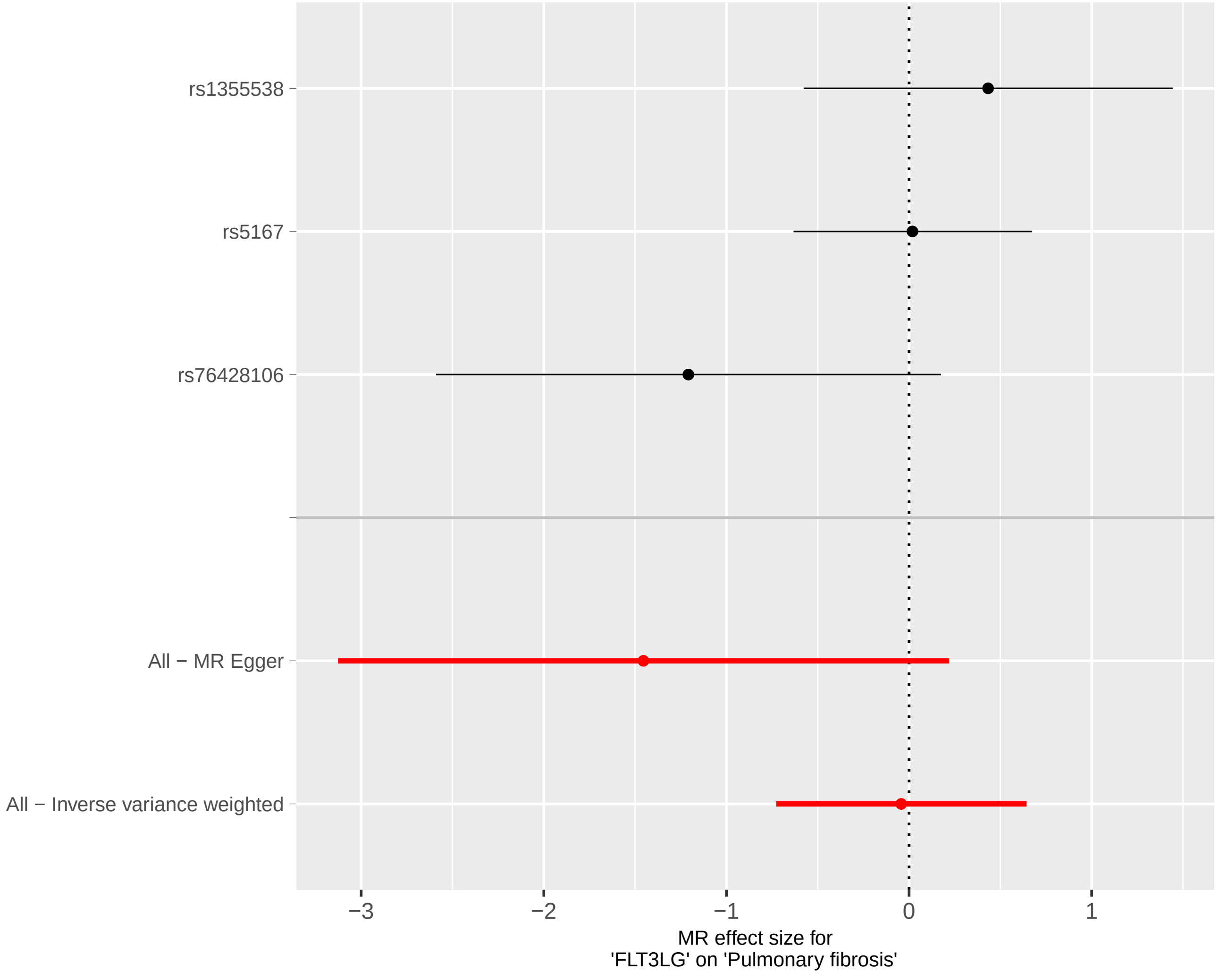

rs1355538
rs5167
rs76428106
All − MR Egger
All − Inverse variance weighted
−3
−2
−1
0
1
MR effect size for
'FLT3LG' on 'Pulmonary fibrosis'

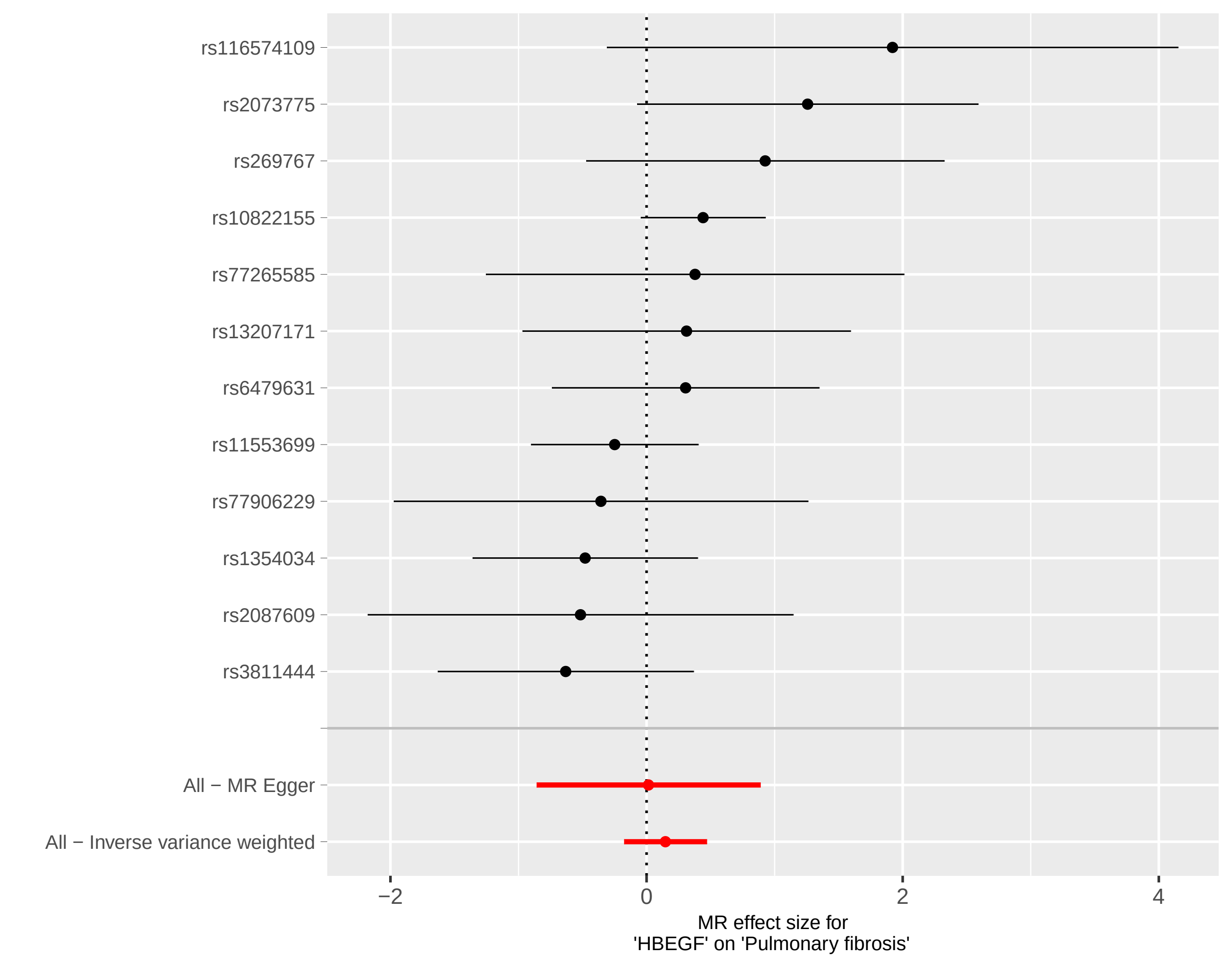
rs116574109
rs2073775
rs269767
rs10822155
rs77265585
rs13207171
rs6479631
rs11553699
rs77906229
rs1354034
rs2087609
rs3811444
All − MR Egger
All − Inverse variance weighted
−2
0
2
4
MR effect size for
'HBEGF' on 'Pulmonary fibrosis'

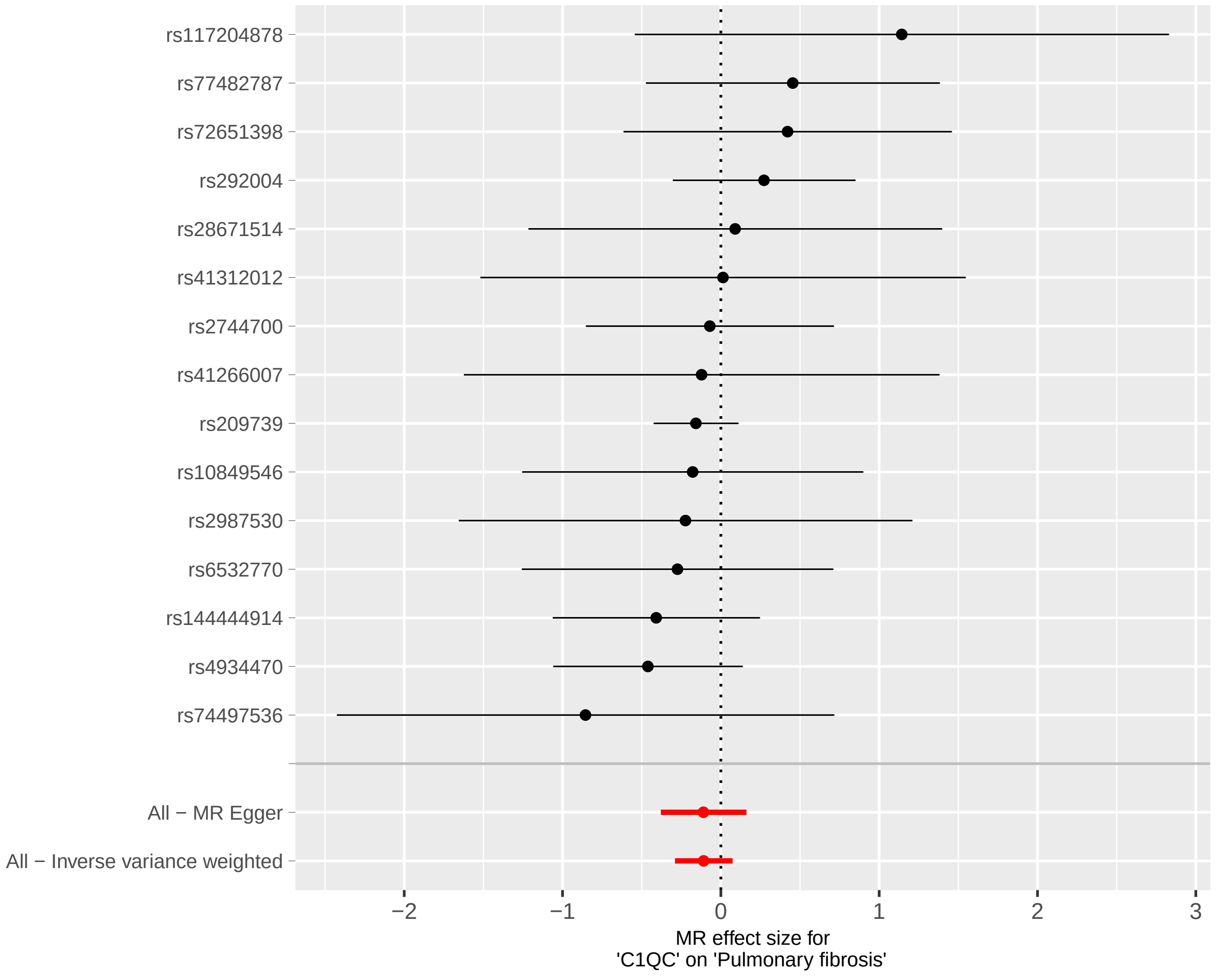
rs117204878
rs77482787
rs72651398
rs292004
rs28671514
rs41312012
rs2744700
rs41266007
rs209739
rs10849546
rs2987530
rs6532770
rs144444914
rs4934470
rs74497536
All − MR Egger
All − Inverse variance weighted
−2
−1
0
1
2
3
MR effect size for
'C1QC' on 'Pulmonary fibrosis'

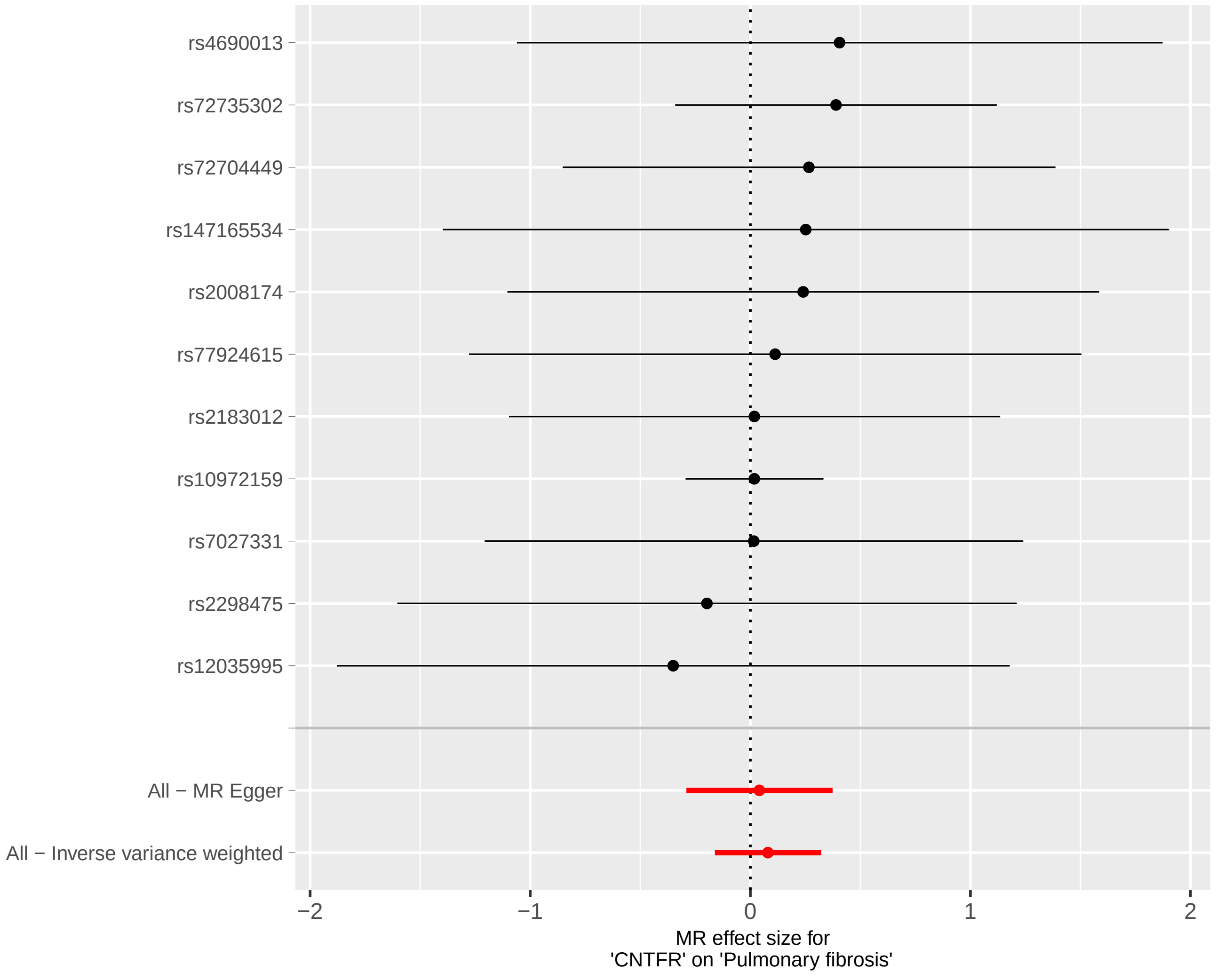
rs4690013
rs72735302
rs72704449
rs147165534
rs2008174
rs77924615
rs2183012
rs10972159
rs7027331
rs2298475
rs12035995
All − MR Egger
All − Inverse variance weighted
−2
−1
0
1
2
MR effect size for
'CNTFR' on 'Pulmonary fibrosis'

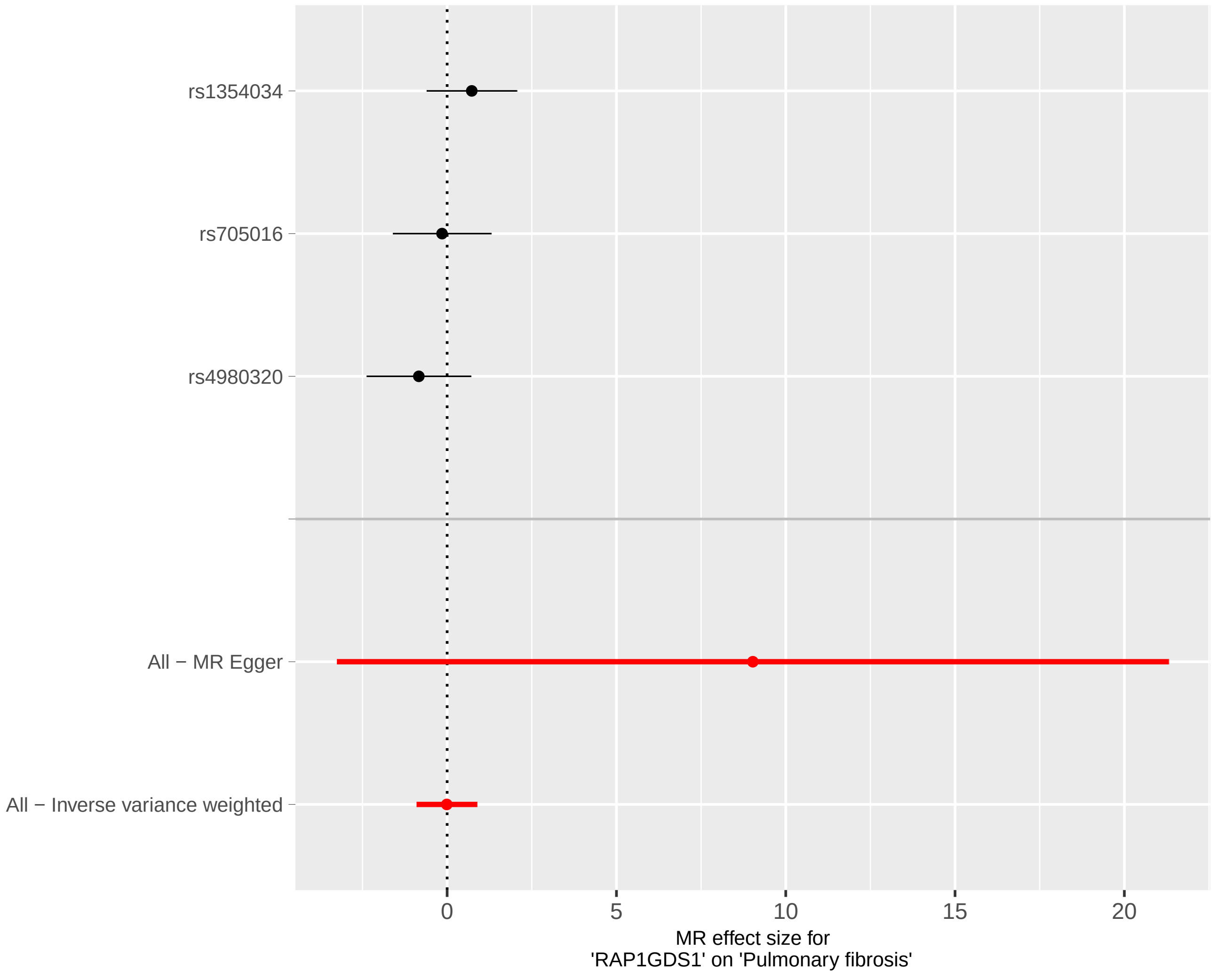
rs1354034
rs705016
rs4980320
All − MR Egger
All − Inverse variance weighted
0
5
10
15
20
MR effect size for
'RAP1GDS1' on 'Pulmonary fibrosis'

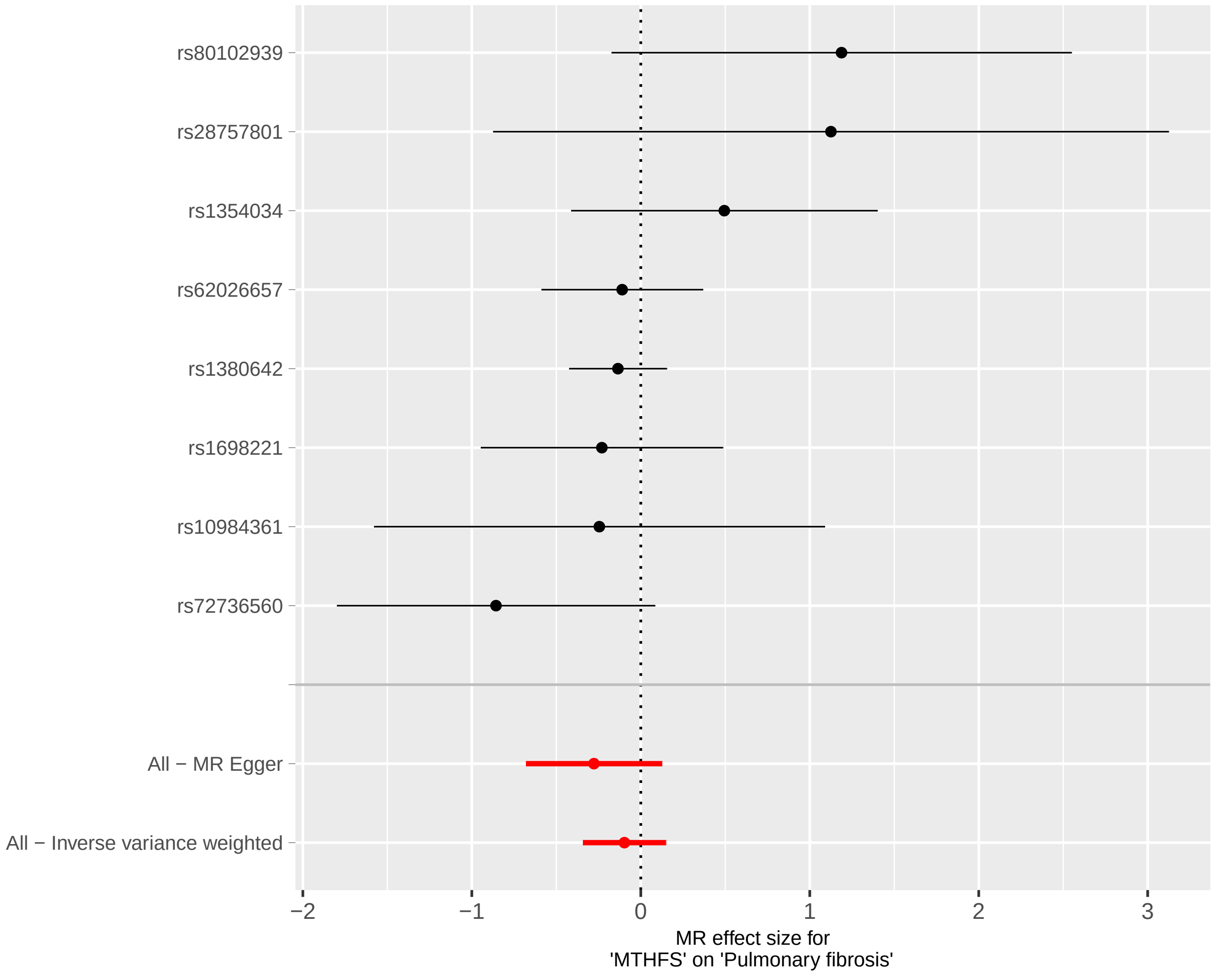
rs80102939
rs28757801
rs1354034
rs62026657
rs1380642
rs1698221
rs10984361
rs72736560
All − MR Egger
All − Inverse variance weighted
−2
−1
0
1
2
3
MR effect size for
'MTHFS' on 'Pulmonary fibrosis'

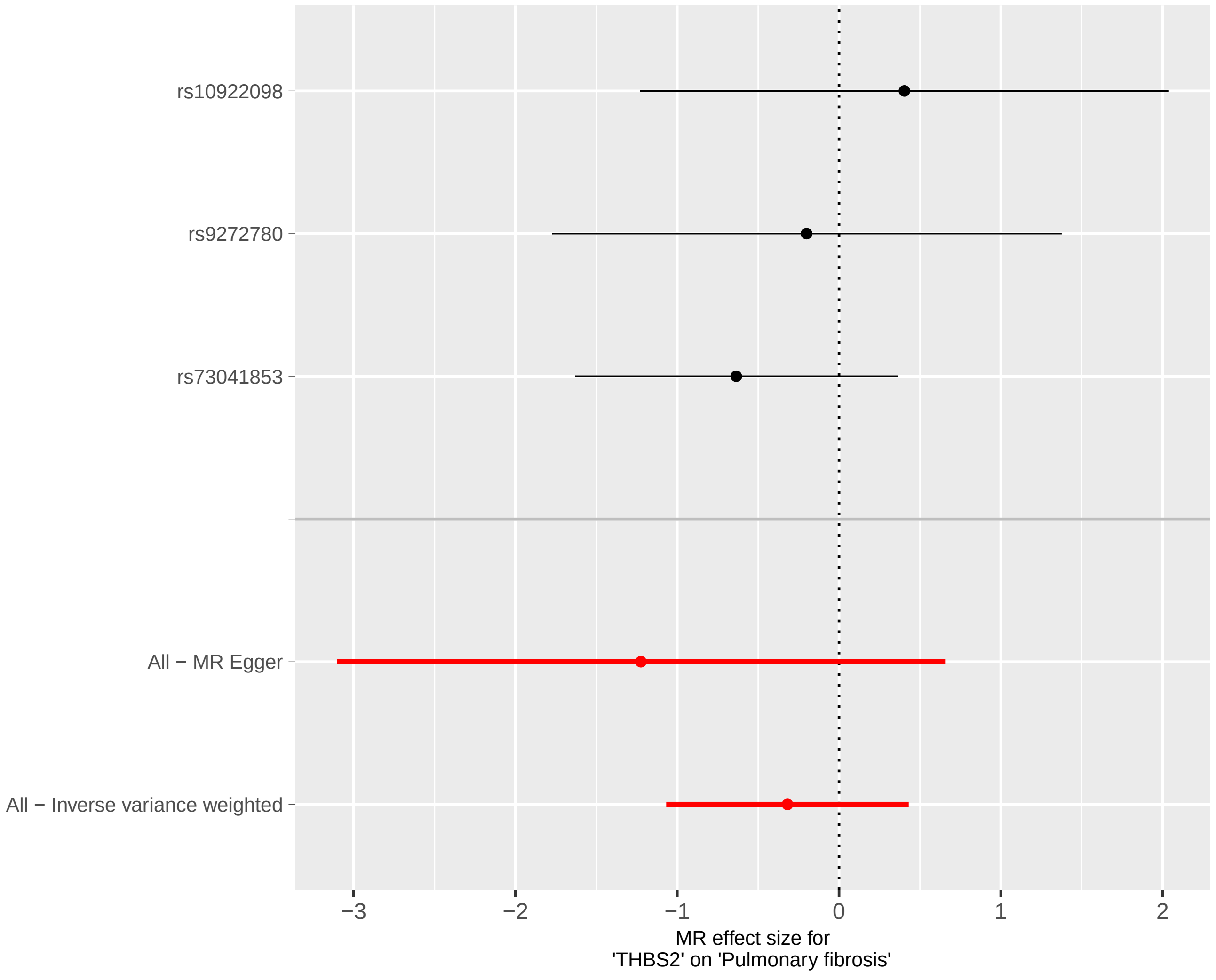
rs10922098
rs9272780
rs73041853
All − MR Egger
All − Inverse variance weighted
−3
−2
−1
0
1
2
MR effect size for
'THBS2' on 'Pulmonary fibrosis'

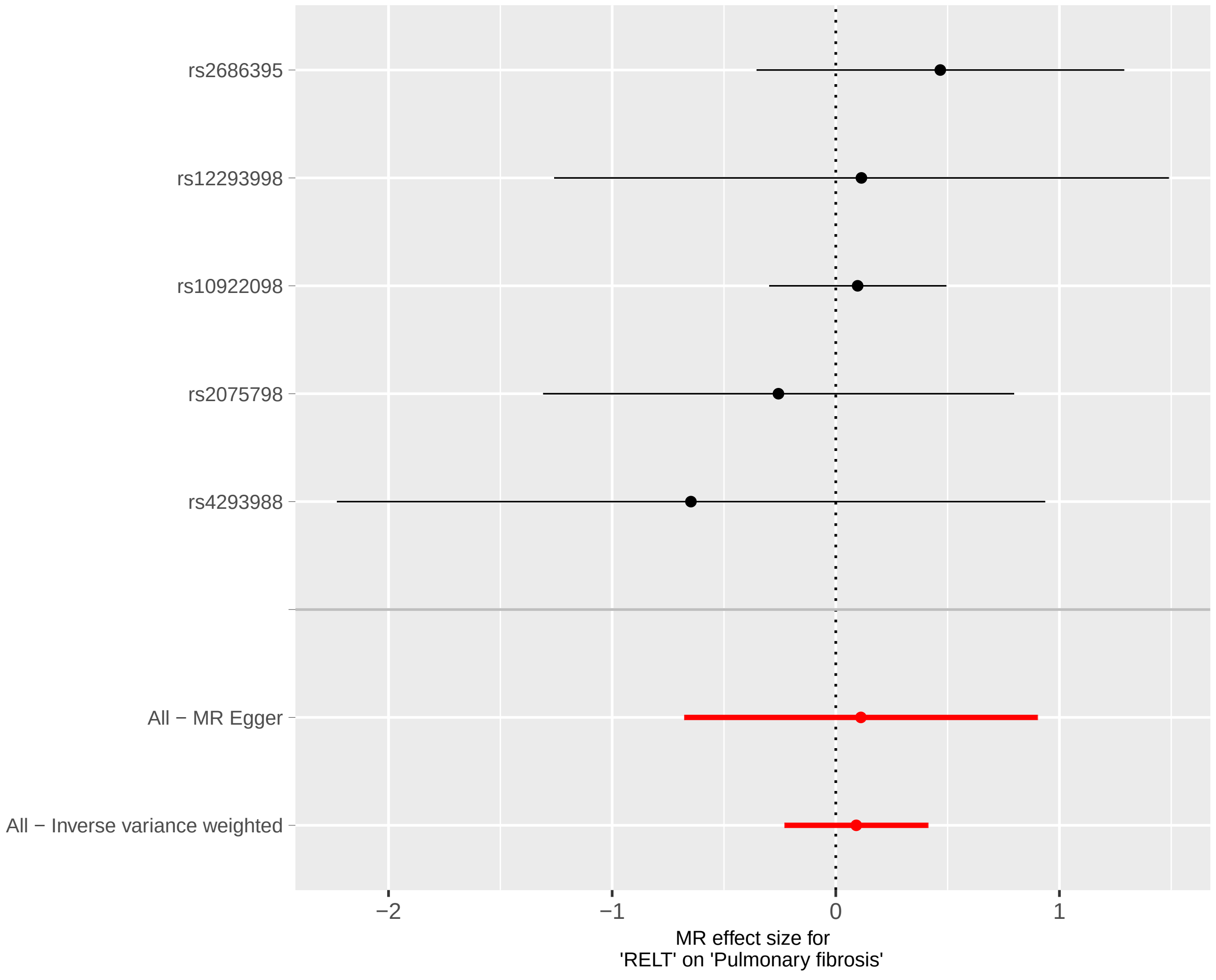
rs2686395
rs12293998
rs10922098
rs2075798
rs4293988
All − MR Egger
All − Inverse variance weighted
−2
−1
0
1
MR effect size for
'RELT' on 'Pulmonary fibrosis'

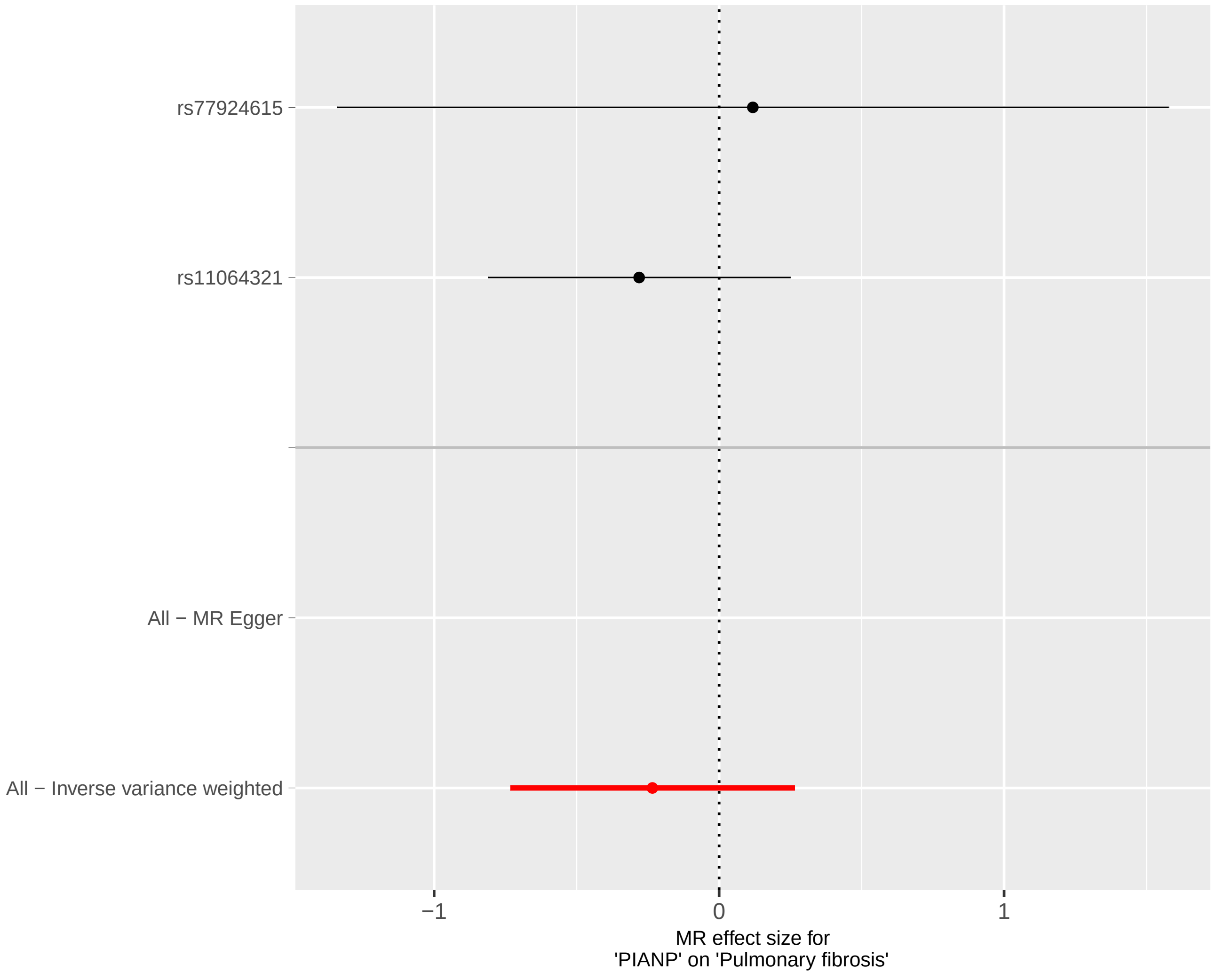
rs77924615
rs11064321
All − MR Egger
All − Inverse variance weighted
−1
0
1
MR effect size for
'PIANP' on 'Pulmonary fibrosis'

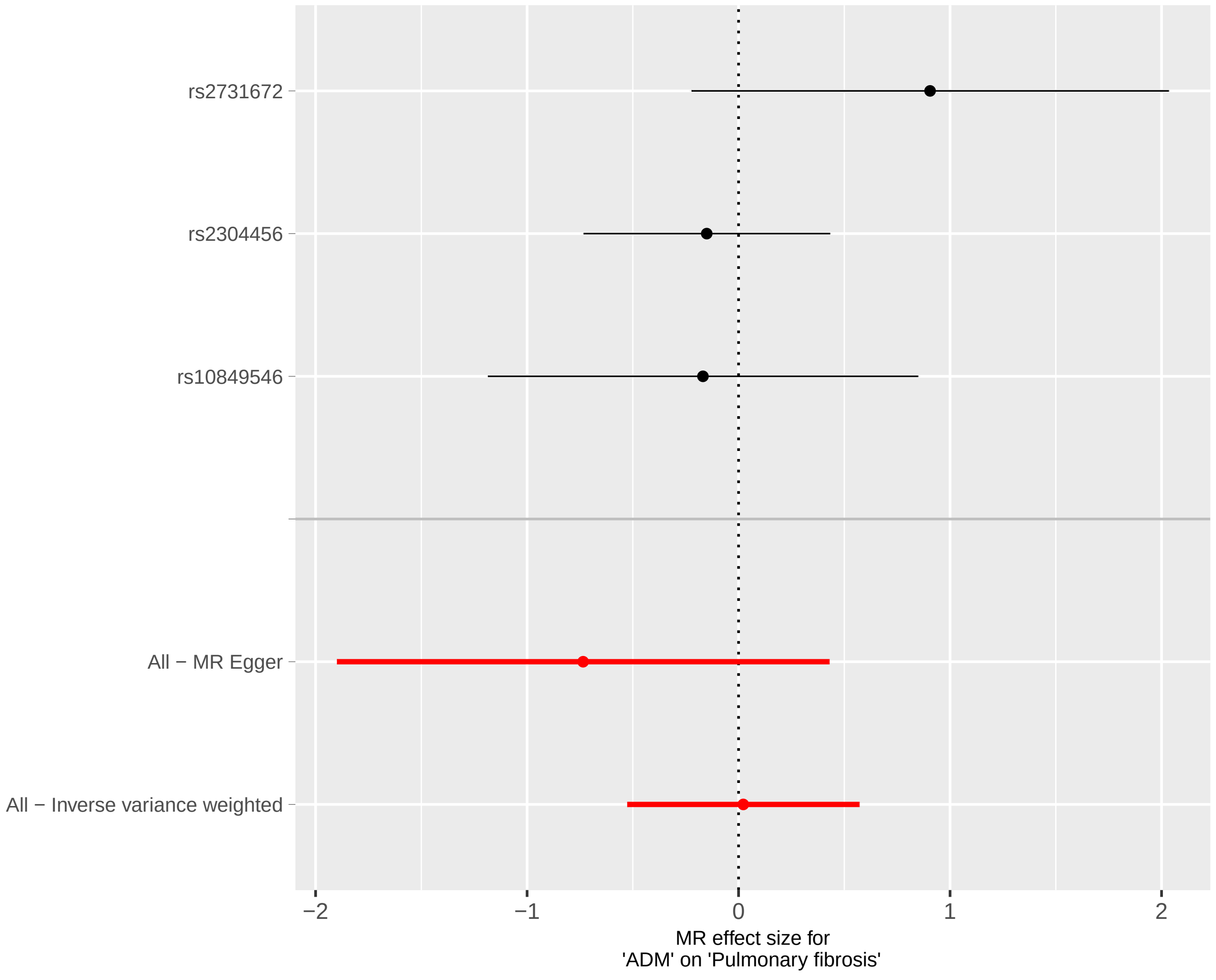
rs2731672
rs2304456
rs10849546
All − MR Egger
All − Inverse variance weighted
−2
−1
0
1
2
MR effect size for
'ADM' on 'Pulmonary fibrosis'

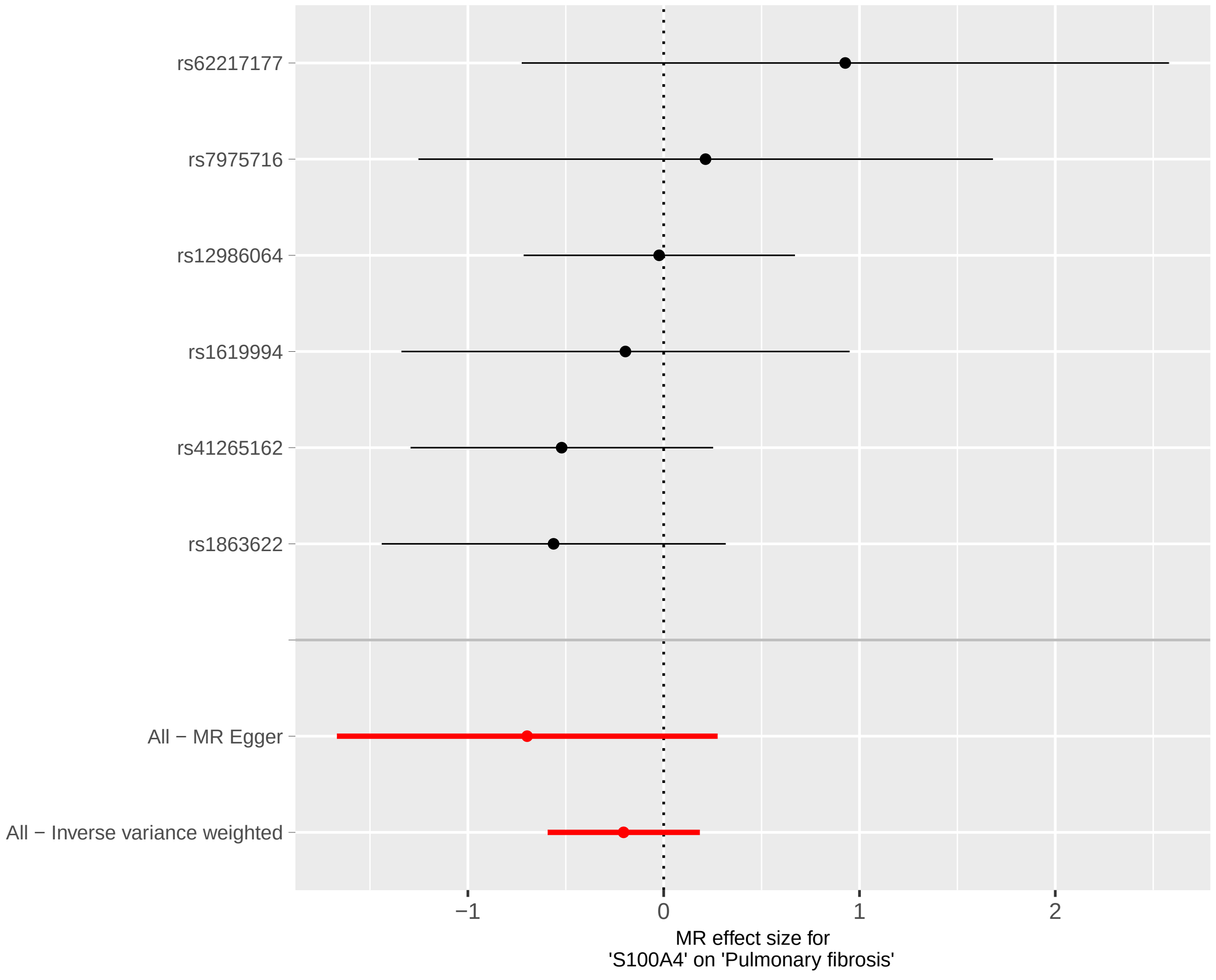

rs62217177
rs7975716
rs12986064
rs1619994
rs41265162
rs1863622
All − MR Egger
All − Inverse variance weighted
−1
0
1
2
MR effect size for
'S100A4' on 'Pulmonary fibrosis'

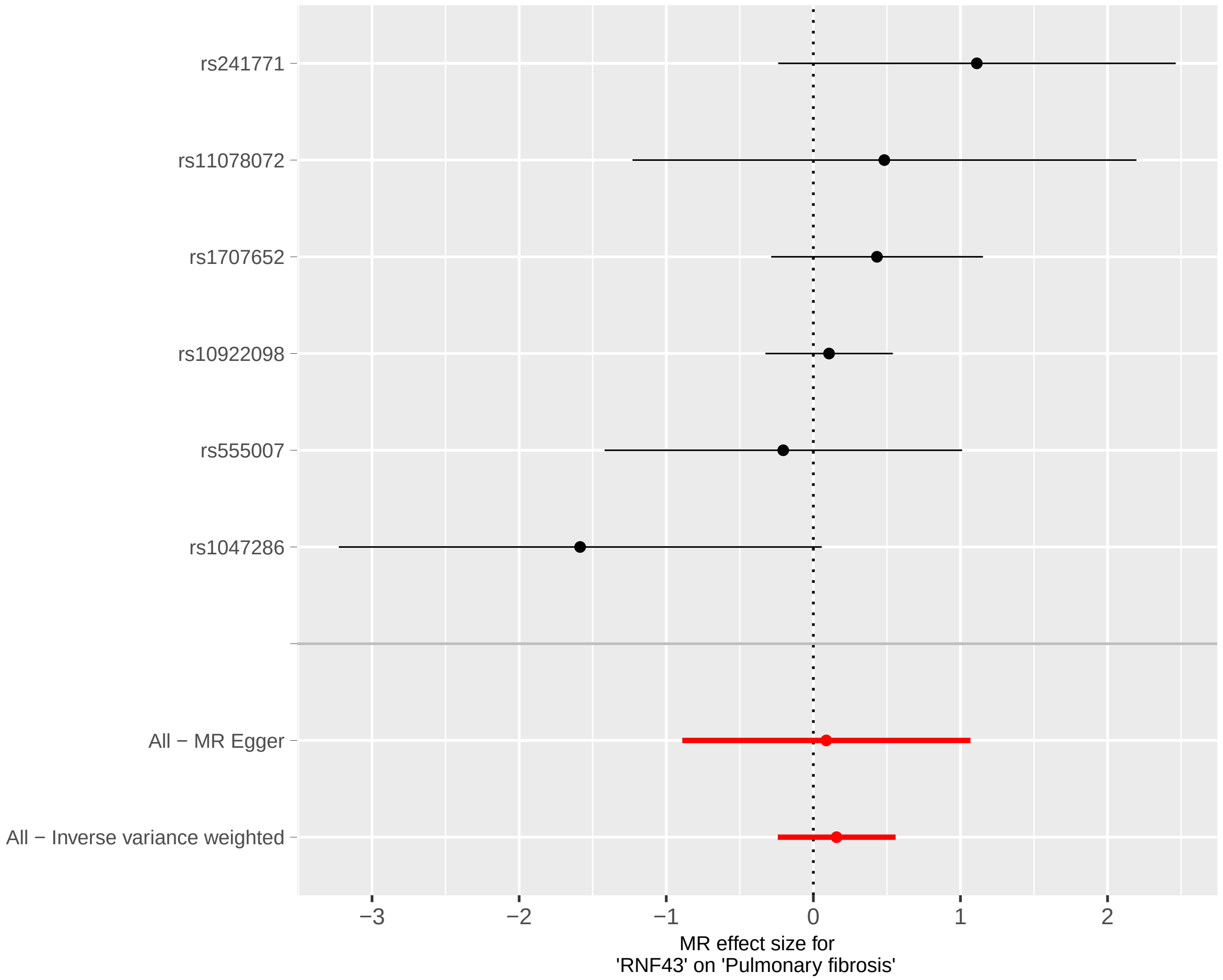
rs241771
rs11078072
rs1707652
rs10922098
rs555007
rs1047286
All − MR Egger
All − Inverse variance weighted
−3
−2
−1
0
1
2
MR effect size for
'RNF43' on 'Pulmonary fibrosis'

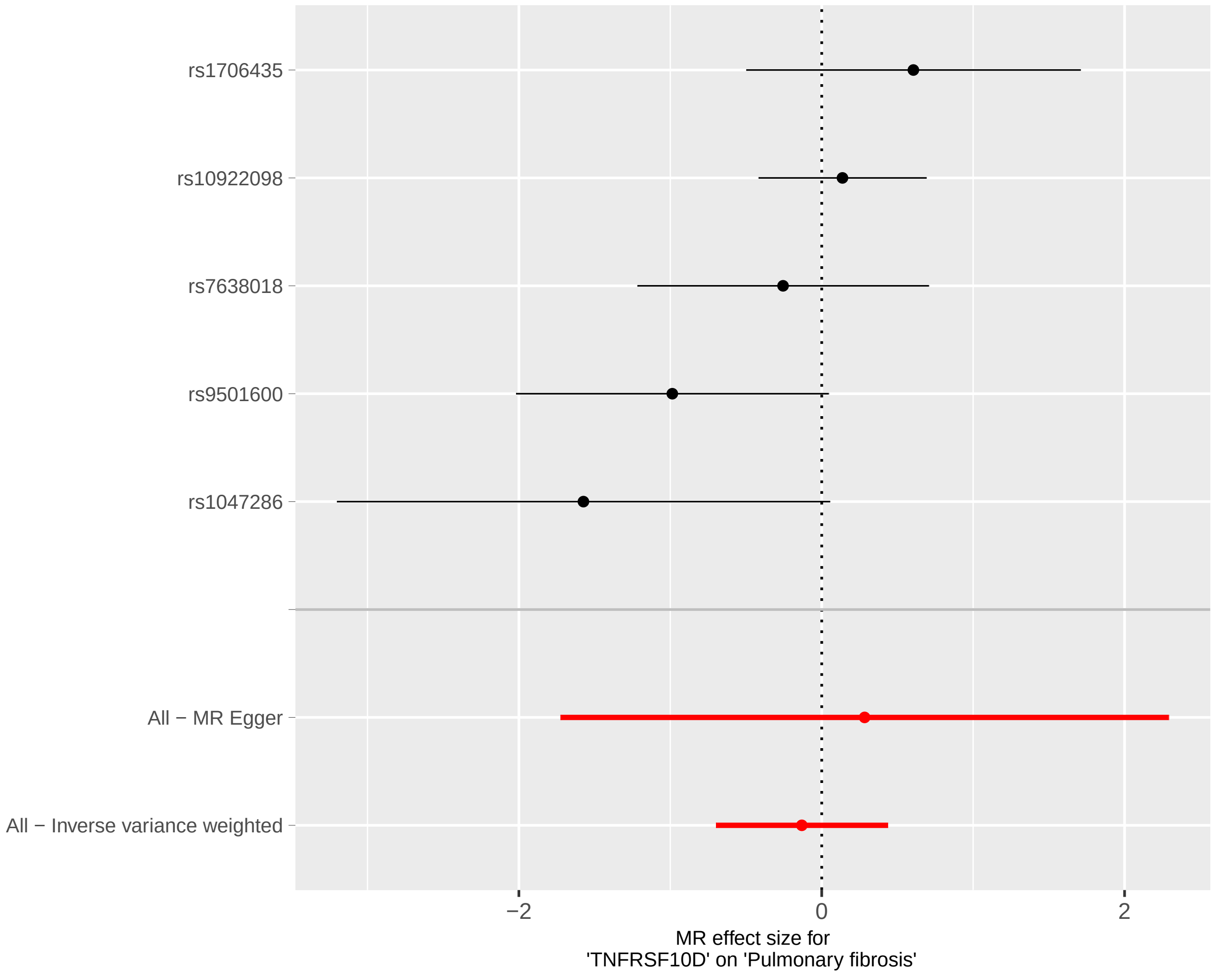
rs1706435
rs10922098
rs7638018
rs9501600
rs1047286
All − MR Egger
All − Inverse variance weighted
−2
0
2
MR effect size for
'TNFRSF10D' on 'Pulmonary fibrosis'

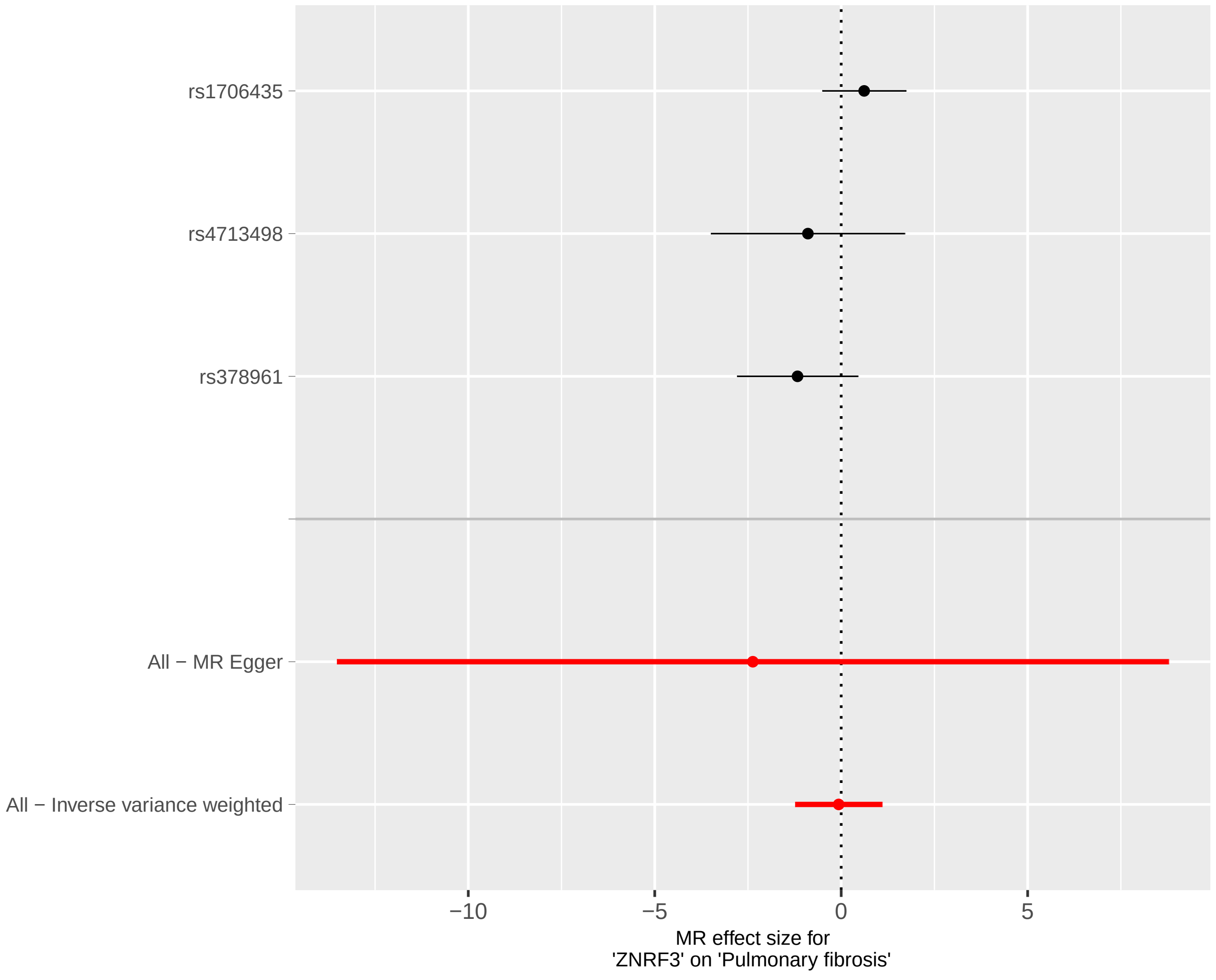
rs1706435
rs4713498
rs378961
All − MR Egger
All − Inverse variance weighted
−10
−5
0
5
MR effect size for
'ZNRF3' on 'Pulmonary fibrosis'

Insufficient number of SNPs

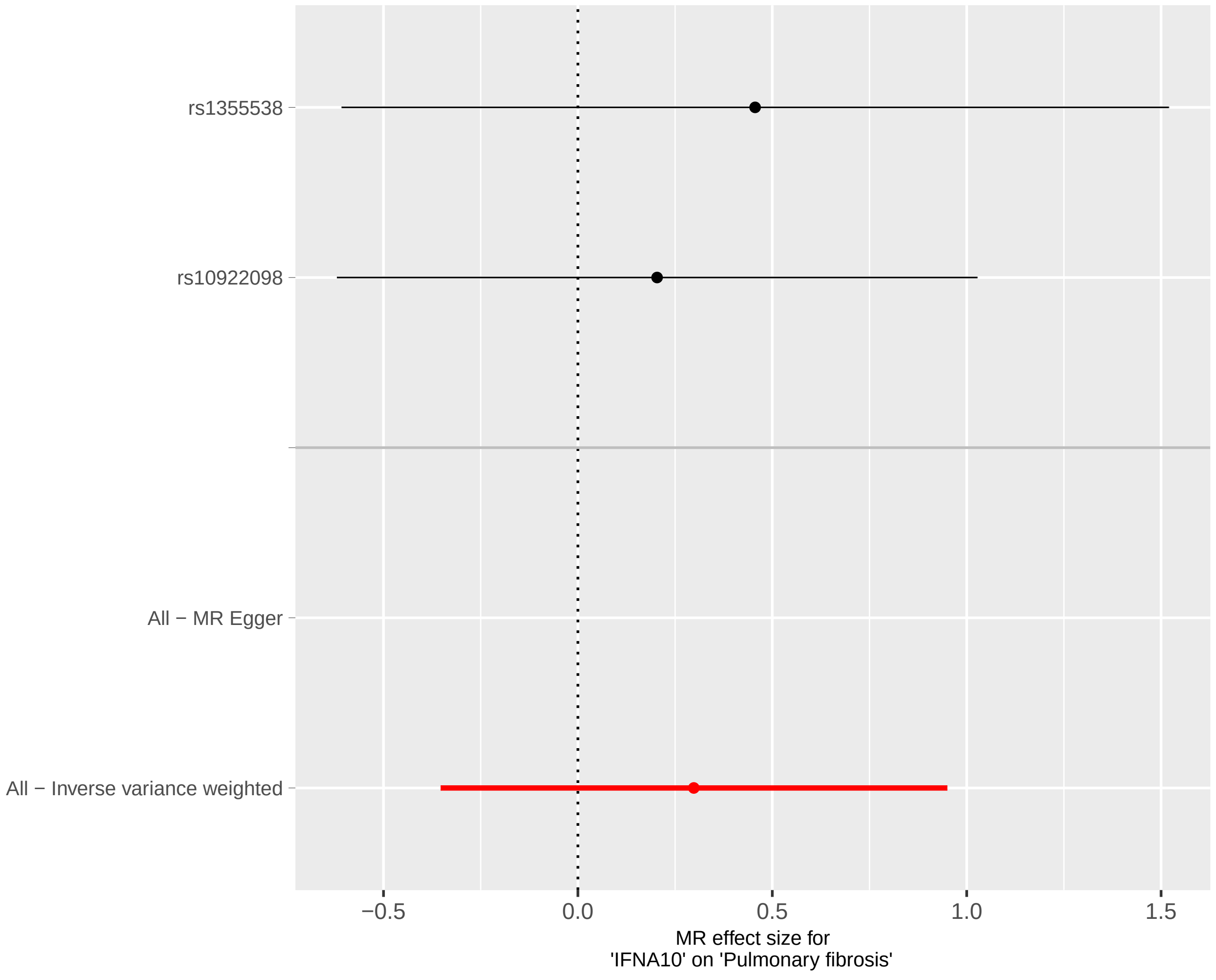
rs1355538
rs10922098
All − MR Egger
All − Inverse variance weighted
−0.5
0.0
0.5
1.0
1.5
MR effect size for
'IFNA10' on 'Pulmonary fibrosis'

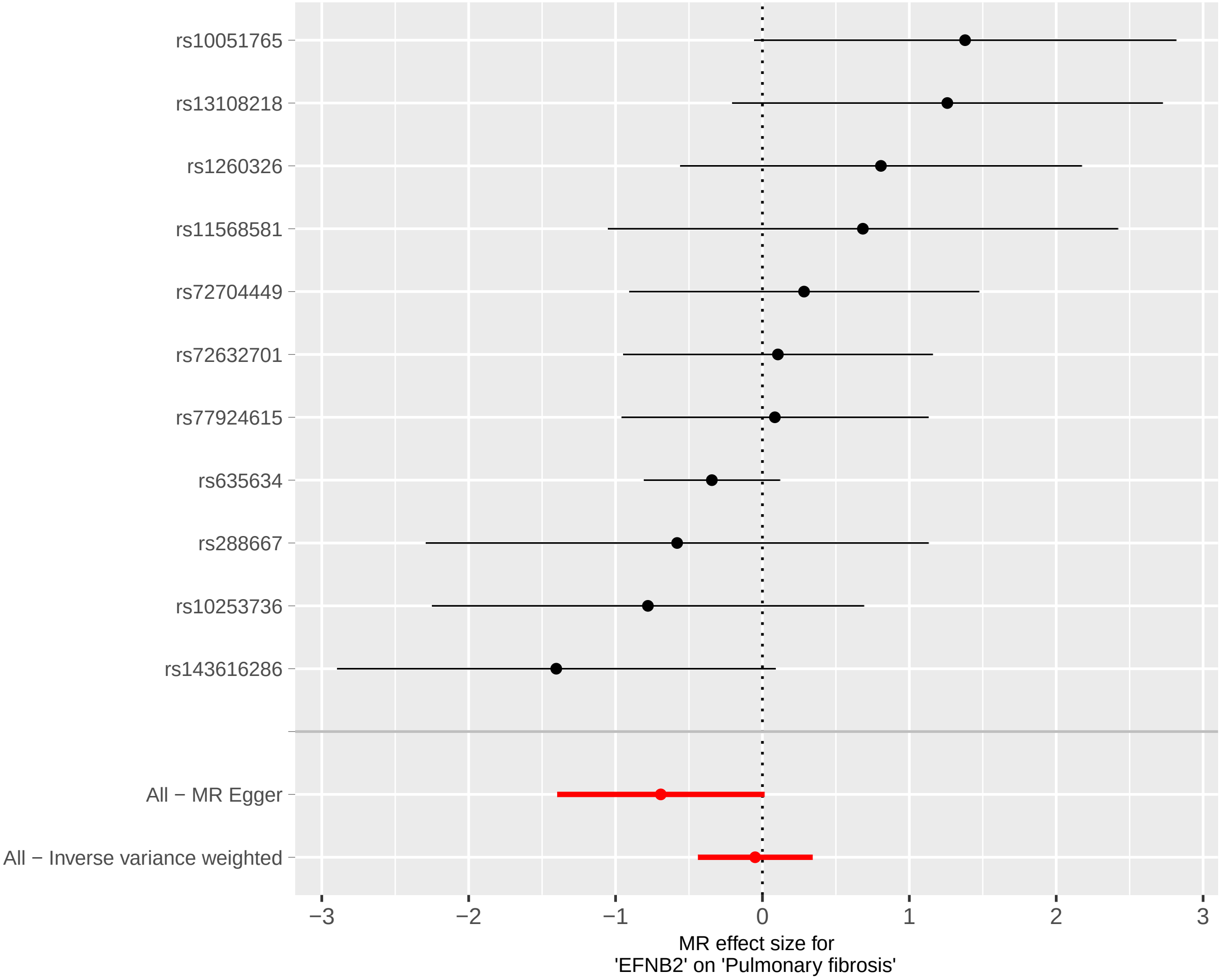

rs10051765
rs13108218
rs1260326
rs11568581
rs72704449
rs72632701
rs77924615
rs635634
rs288667
rs10253736
rs143616286
All − MR Egger
All − Inverse variance weighted
−3
−2
−1
0
1
2
3
MR effect size for
'EFNB2' on 'Pulmonary fibrosis'

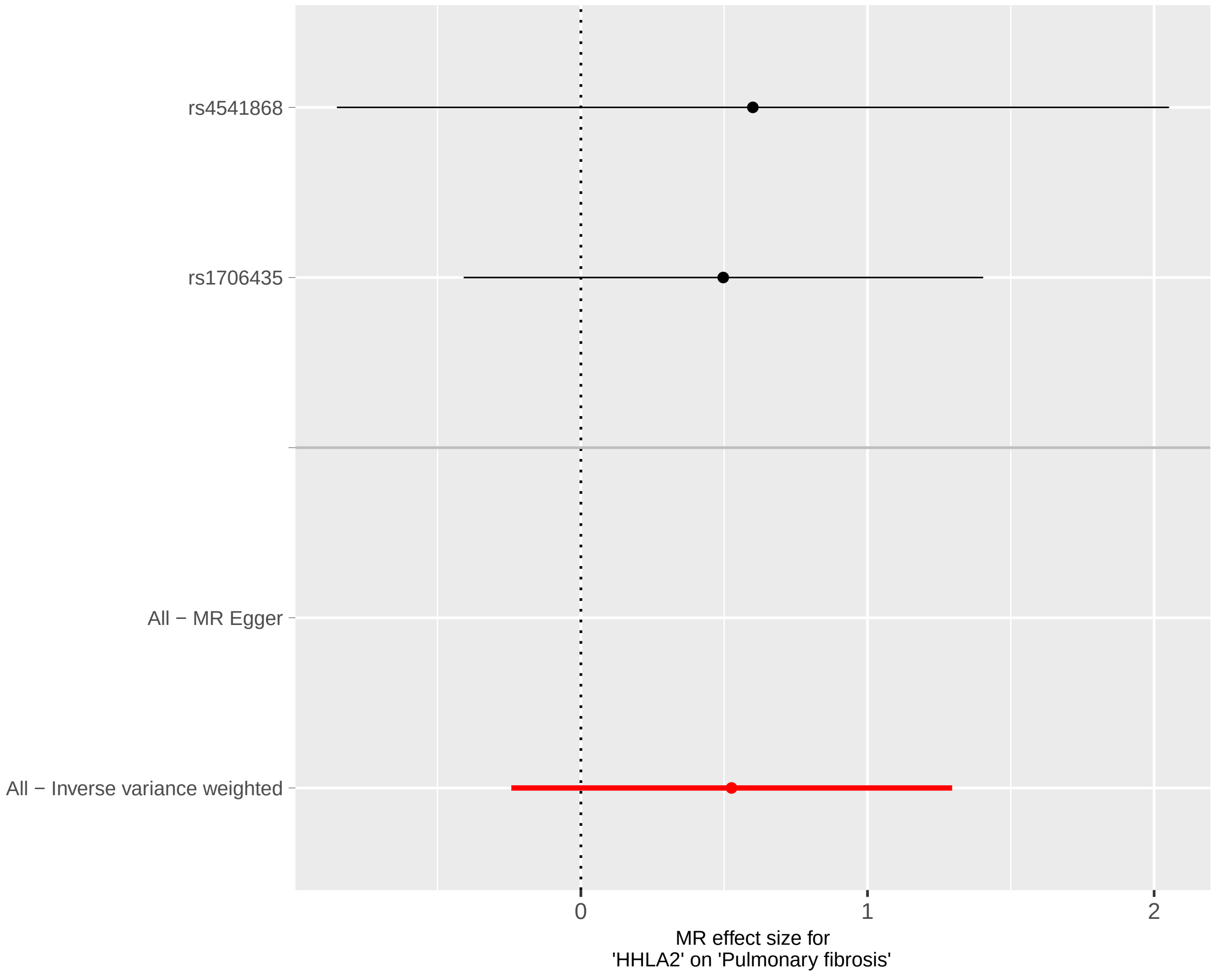
rs4541868
rs1706435
All − MR Egger
All − Inverse variance weighted
0
1
2
MR effect size for
'HHLA2' on 'Pulmonary fibrosis'

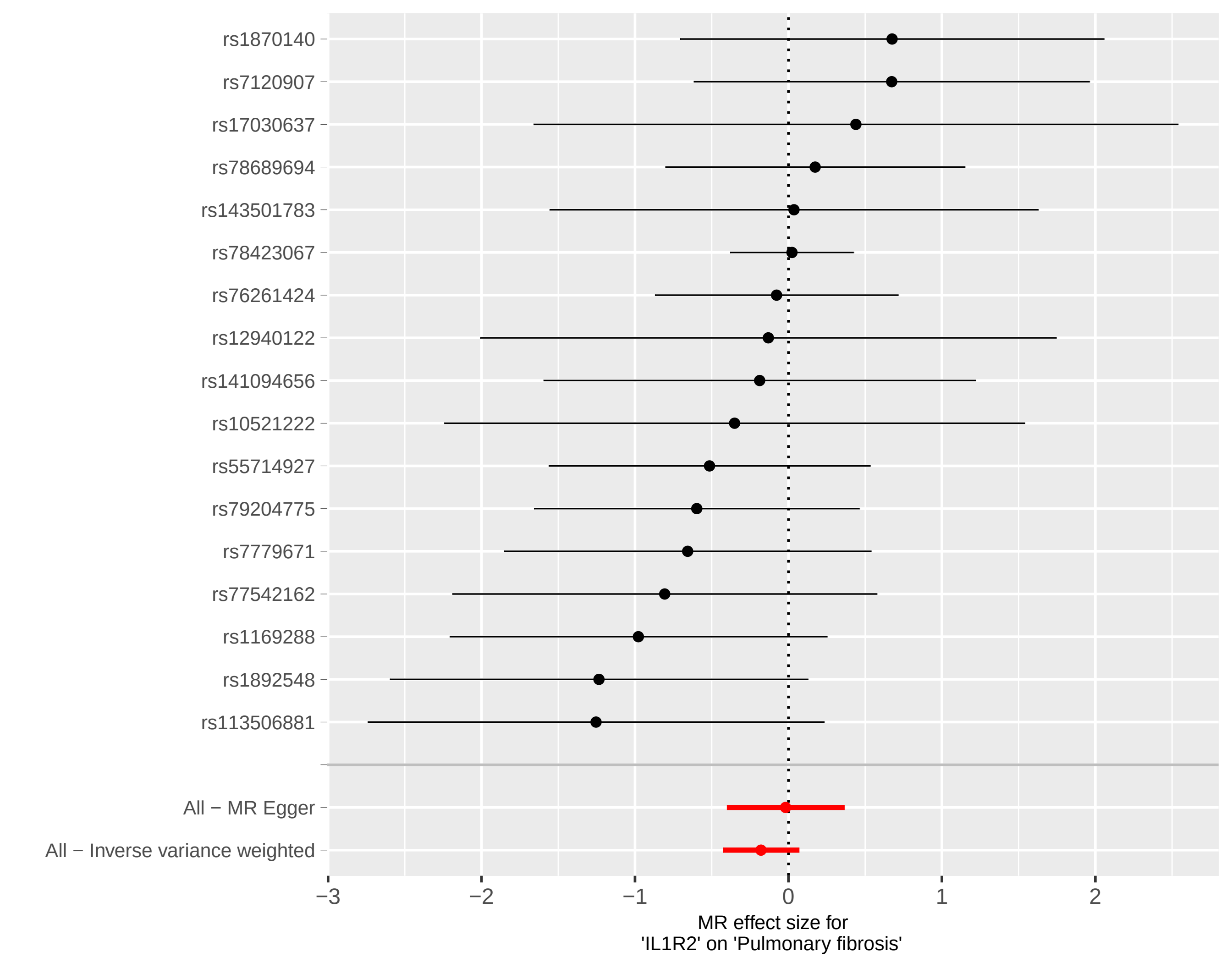

rs1870140
rs7120907
rs17030637
rs78689694
rs143501783
rs78423067
rs76261424
rs12940122
rs141094656
rs10521222
rs55714927
rs79204775
rs7779671
rs77542162
rs1169288
rs1892548
rs113506881
All − MR Egger
All − Inverse variance weighted
−3
−2
−1
0
1
2
MR effect size for
'IL1R2' on 'Pulmonary fibrosis'

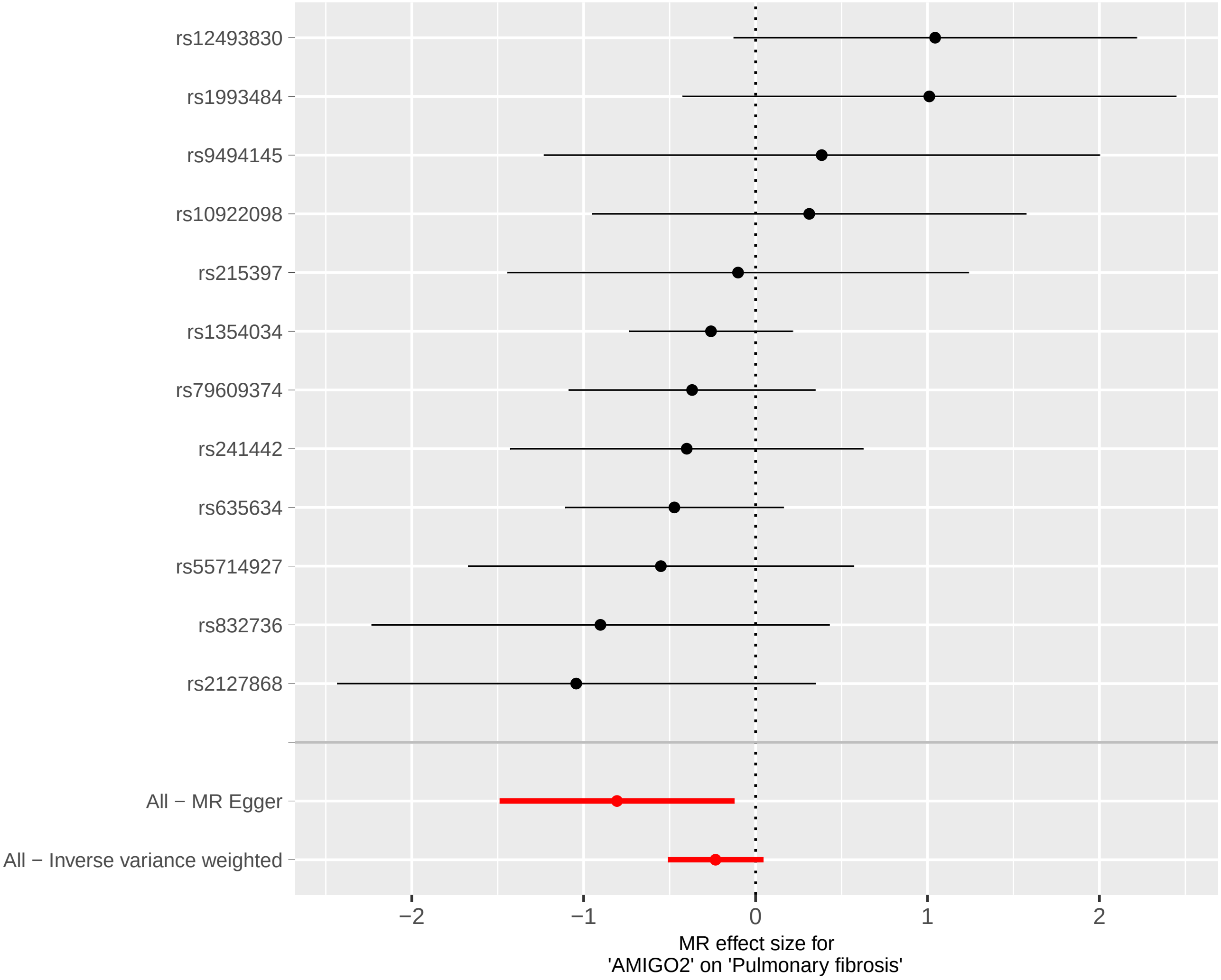

rs12493830
rs1993484
rs9494145
rs10922098
rs215397
rs1354034
rs79609374
rs241442
rs635634
rs55714927
rs832736
rs2127868
All − MR Egger
All − Inverse variance weighted
−2
−1
0
1
2
MR effect size for
'AMIGO2' on 'Pulmonary fibrosis'

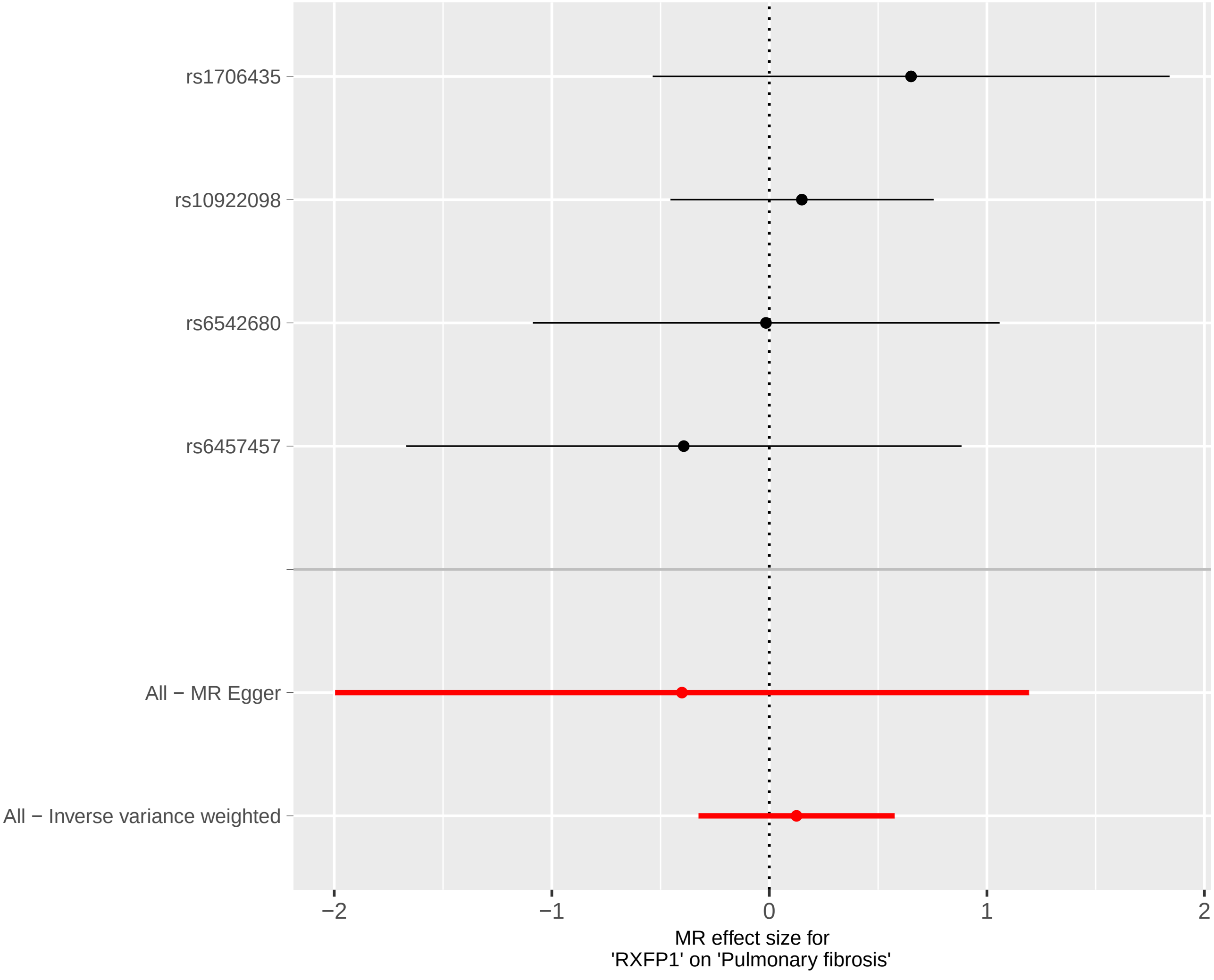
rs1706435
rs10922098
rs6542680
rs6457457
All − MR Egger
All − Inverse variance weighted
−2
−1
0
1
2
MR effect size for
'RXFP1' on 'Pulmonary fibrosis'

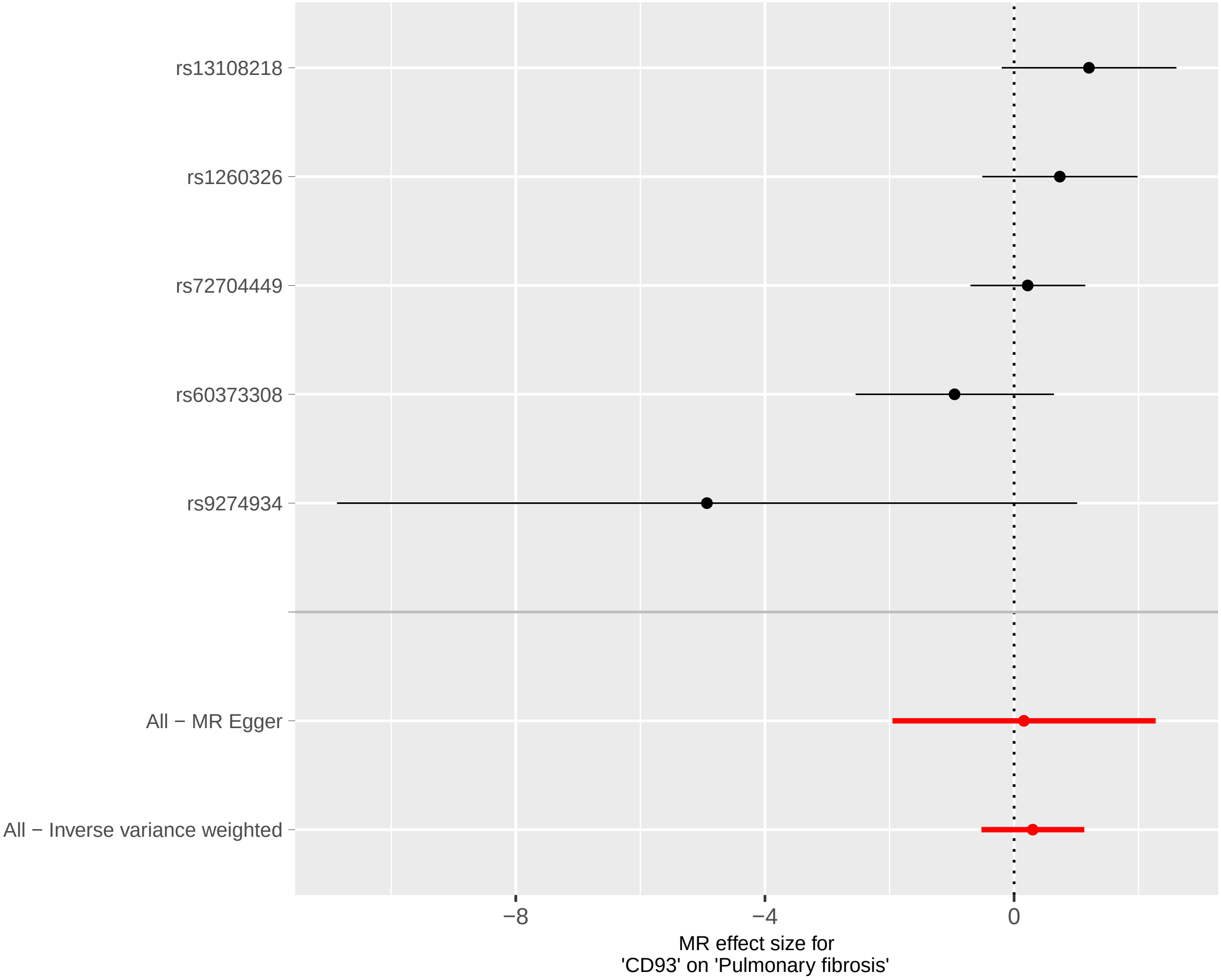

rs13108218
rs1260326
rs72704449
rs60373308
rs9274934
All − MR Egger
All − Inverse variance weighted
−8
−4
0
MR effect size for
'CD93' on 'Pulmonary fibrosis'

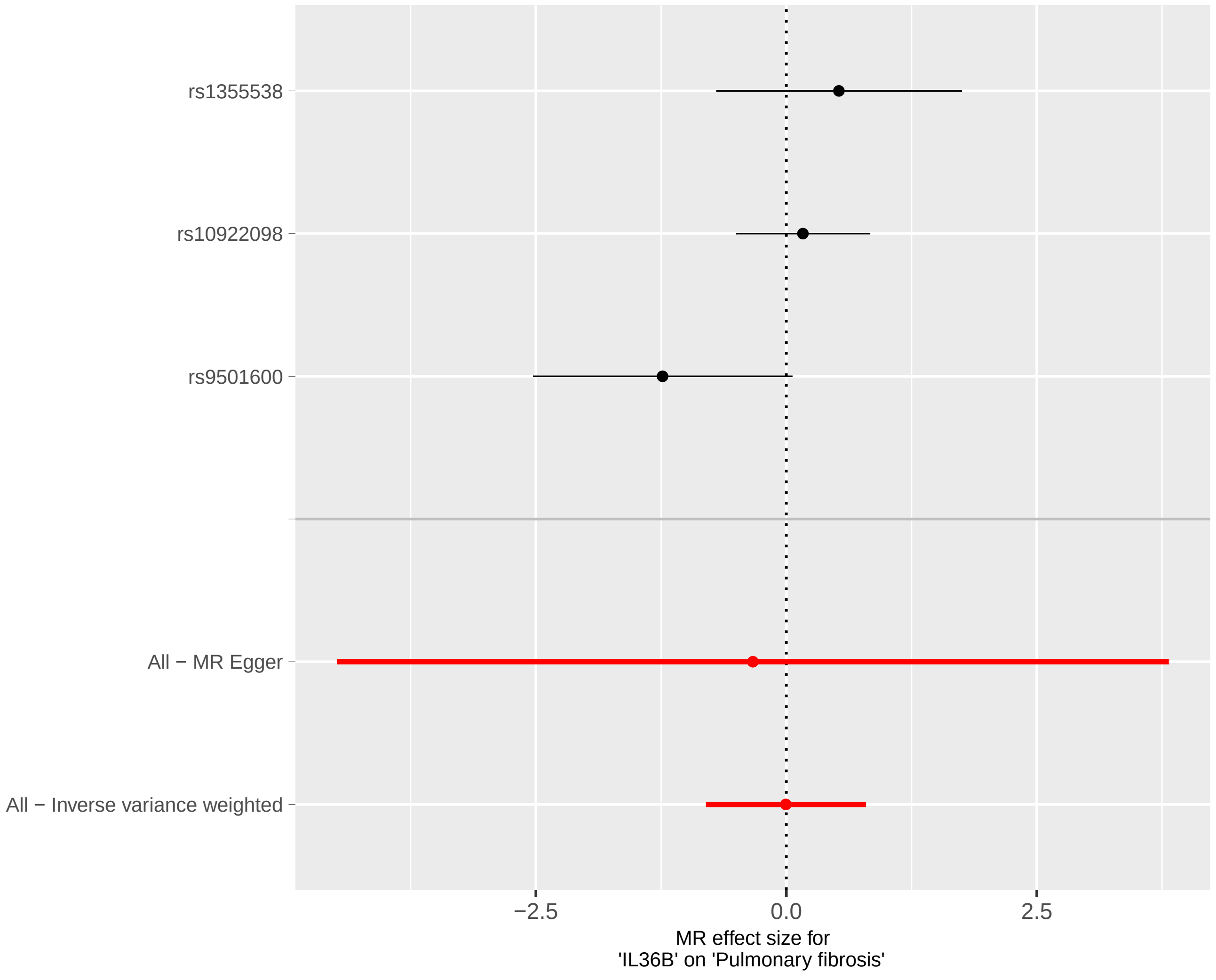

rs1355538
rs10922098
rs9501600
All − MR Egger
All − Inverse variance weighted
−2.5
0.0
2.5
MR effect size for
'IL36B' on 'Pulmonary fibrosis'

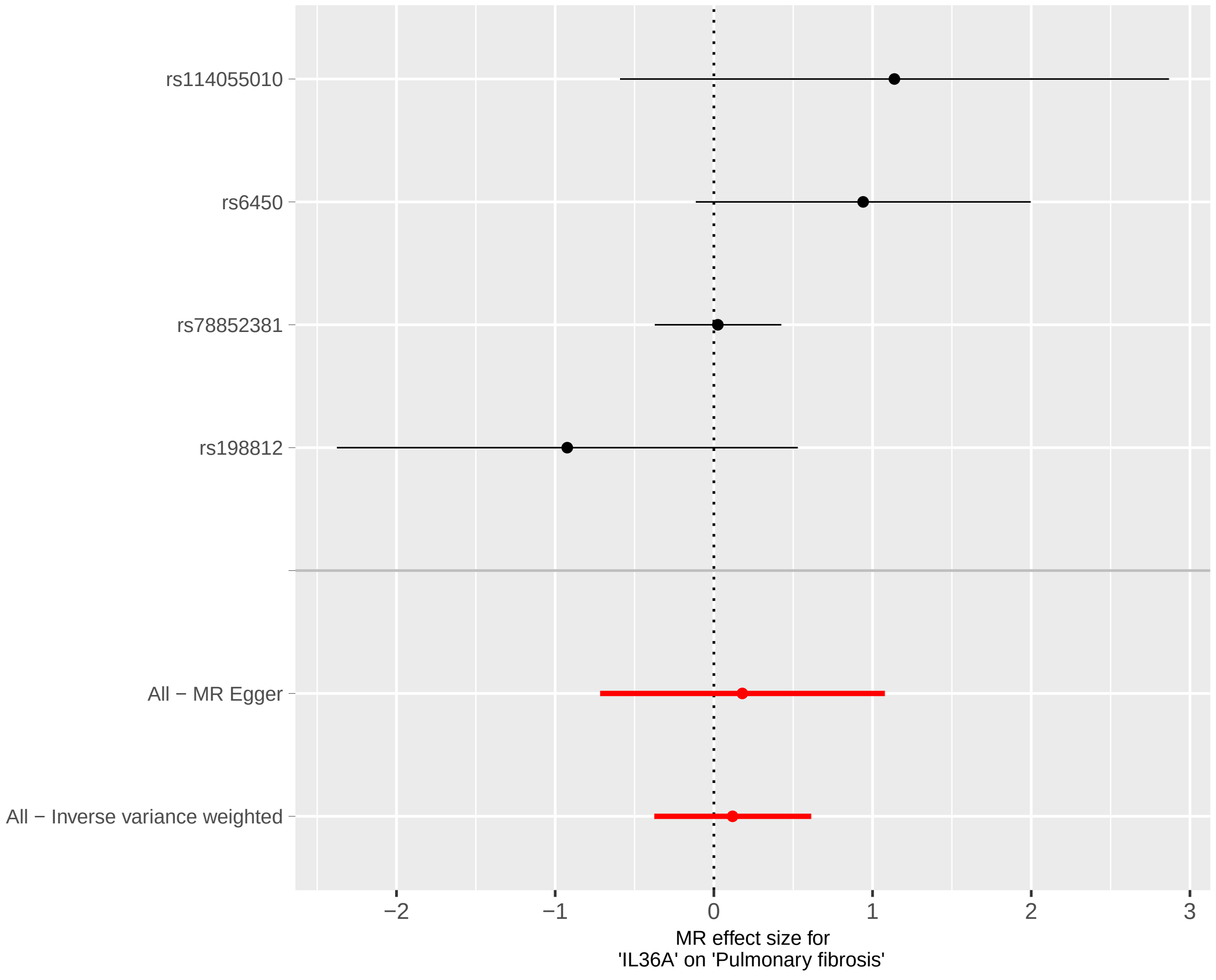

rs114055010
rs6450
rs78852381
rs198812
All − MR Egger
All − Inverse variance weighted
−2
−1
0
1
2
3
MR effect size for
'IL36A' on 'Pulmonary fibrosis'

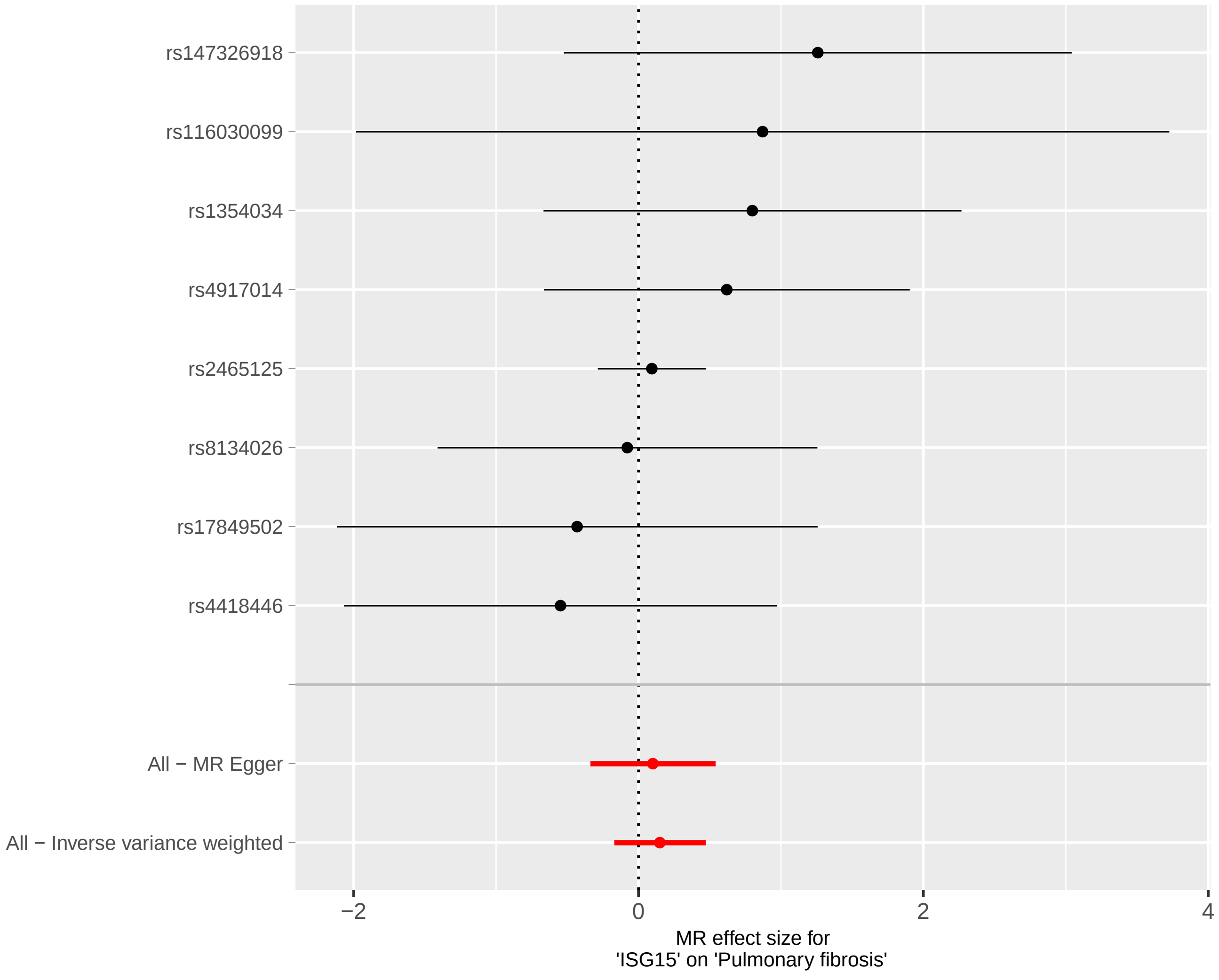
rs147326918
rs116030099
rs1354034
rs4917014
rs2465125
rs8134026
rs17849502
rs4418446
All − MR Egger
All − Inverse variance weighted
−2
0
2
4
MR effect size for
'ISG15' on 'Pulmonary fibrosis'

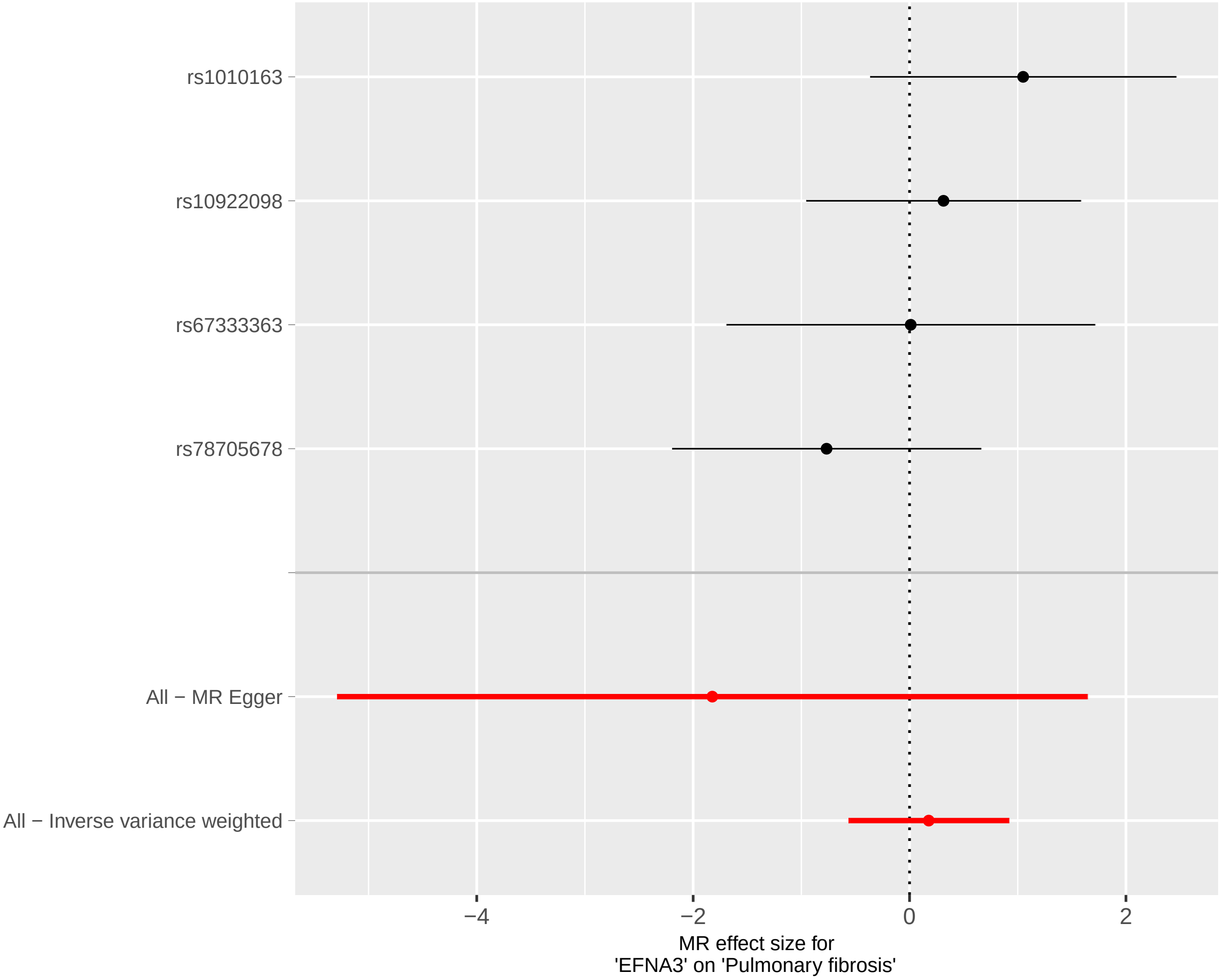

rs1010163
rs10922098
rs67333363
rs78705678
All − MR Egger
All − Inverse variance weighted
−4
−2
0
2
MR effect size for
'EFNA3' on 'Pulmonary fibrosis'

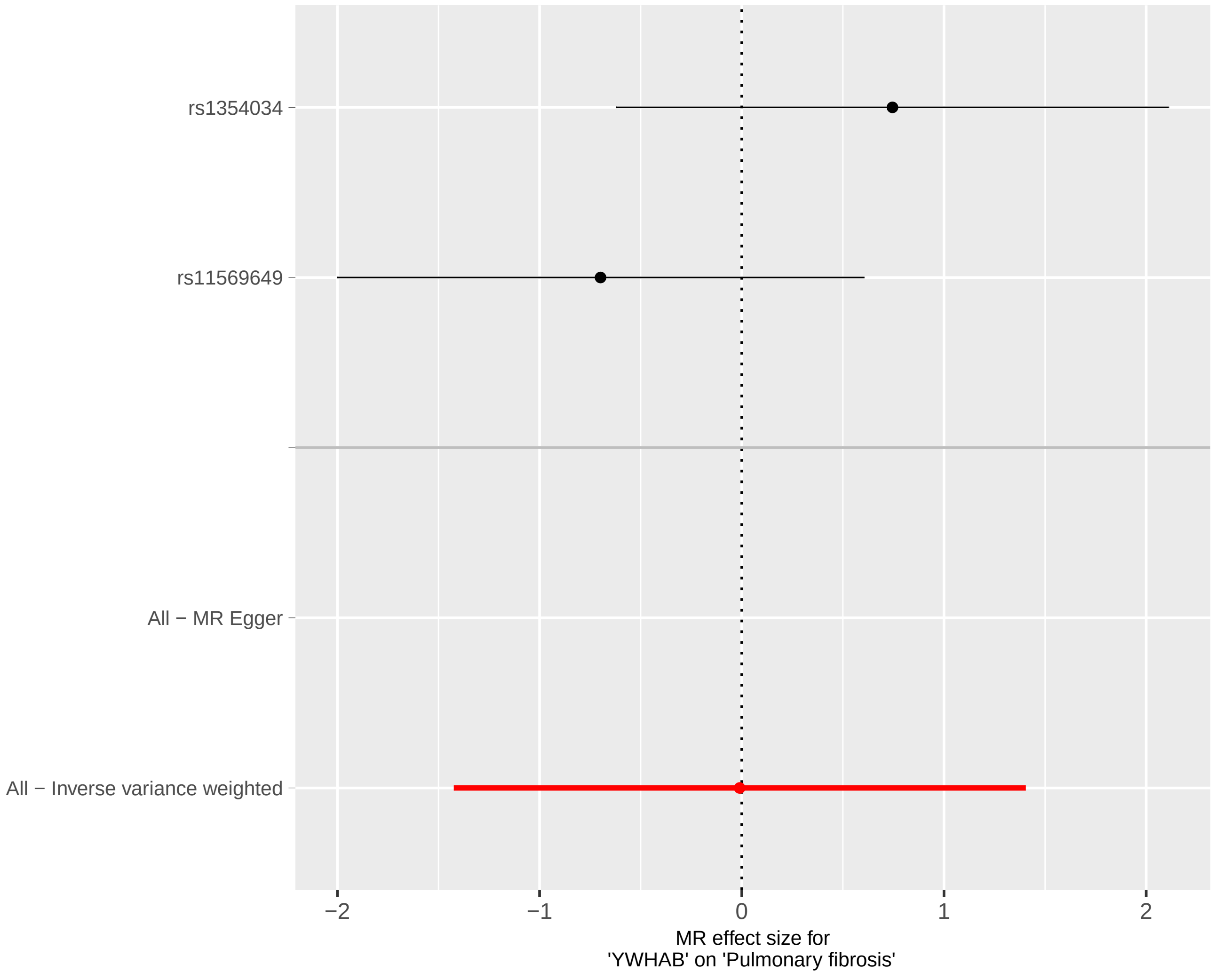
rs1354034
rs11569649
All − MR Egger
All − Inverse variance weighted
−2
−1
0
1
2
MR effect size for
'YWHAB' on 'Pulmonary fibrosis'

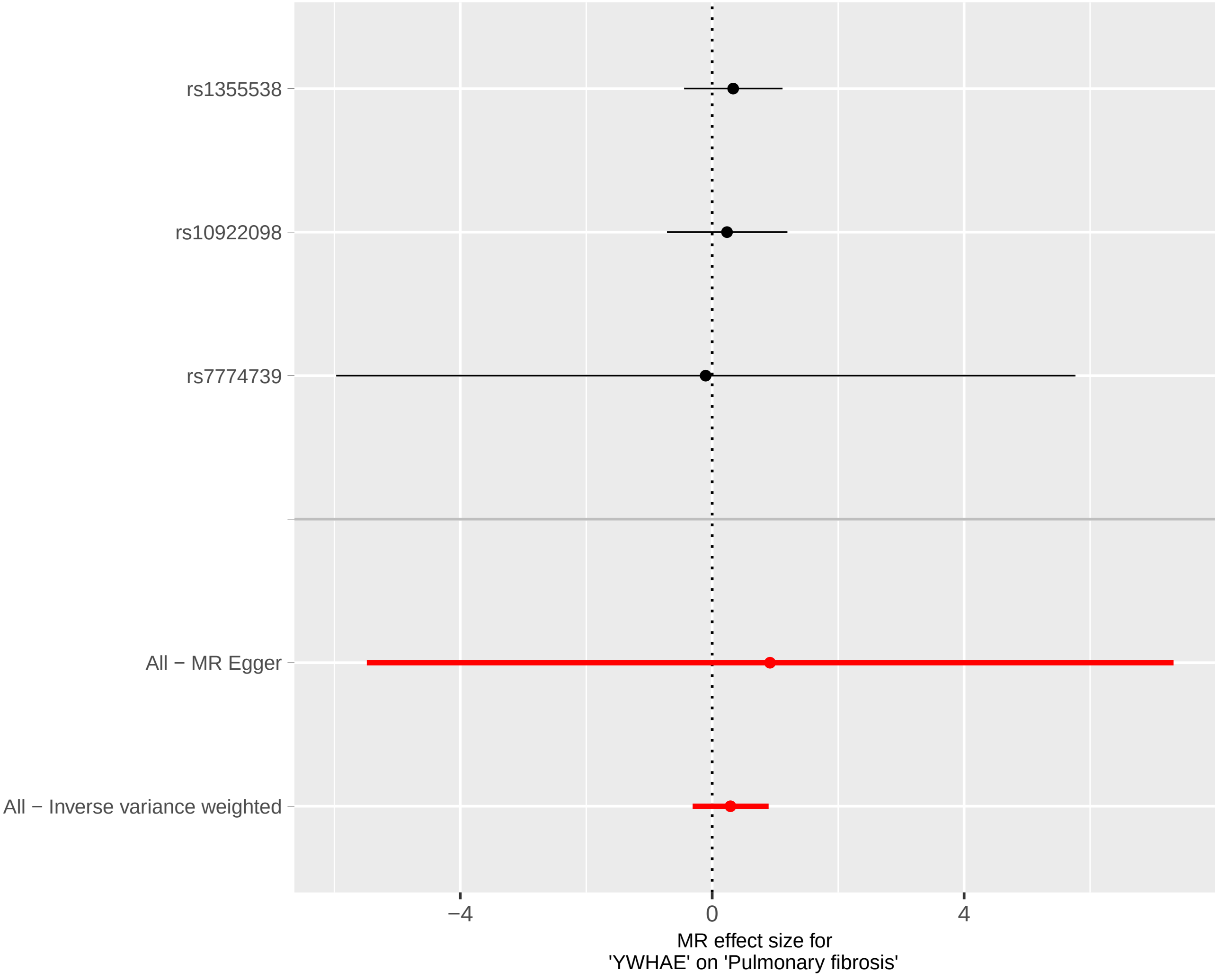
rs1355538
rs10922098
rs7774739
All − MR Egger
All − Inverse variance weighted
−4
0
4
MR effect size for
'YWHAE' on 'Pulmonary fibrosis'

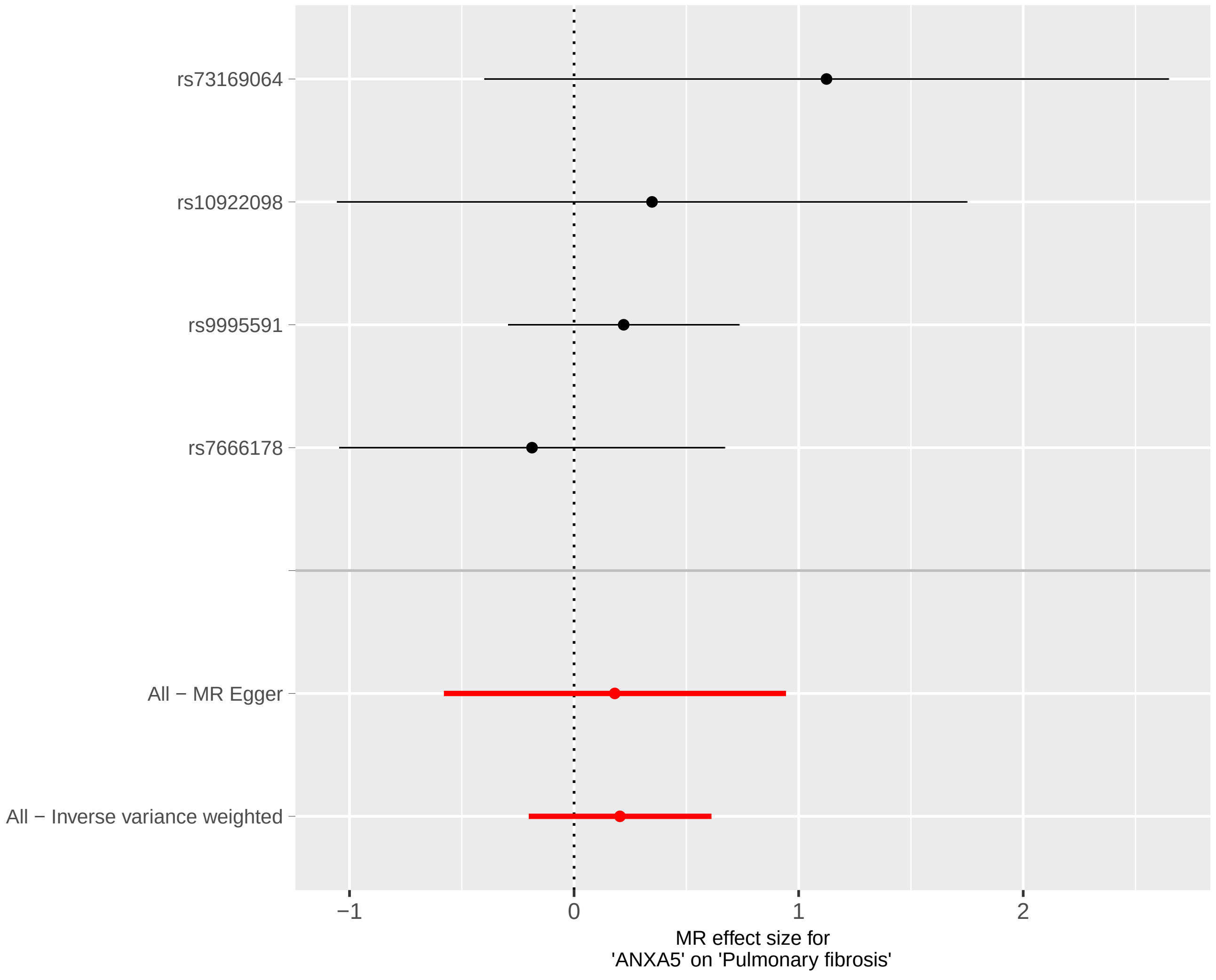
rs73169064
rs10922098
rs9995591
rs7666178
All − MR Egger
All − Inverse variance weighted
−1
0
1
2
MR effect size for
'ANXA5' on 'Pulmonary fibrosis'

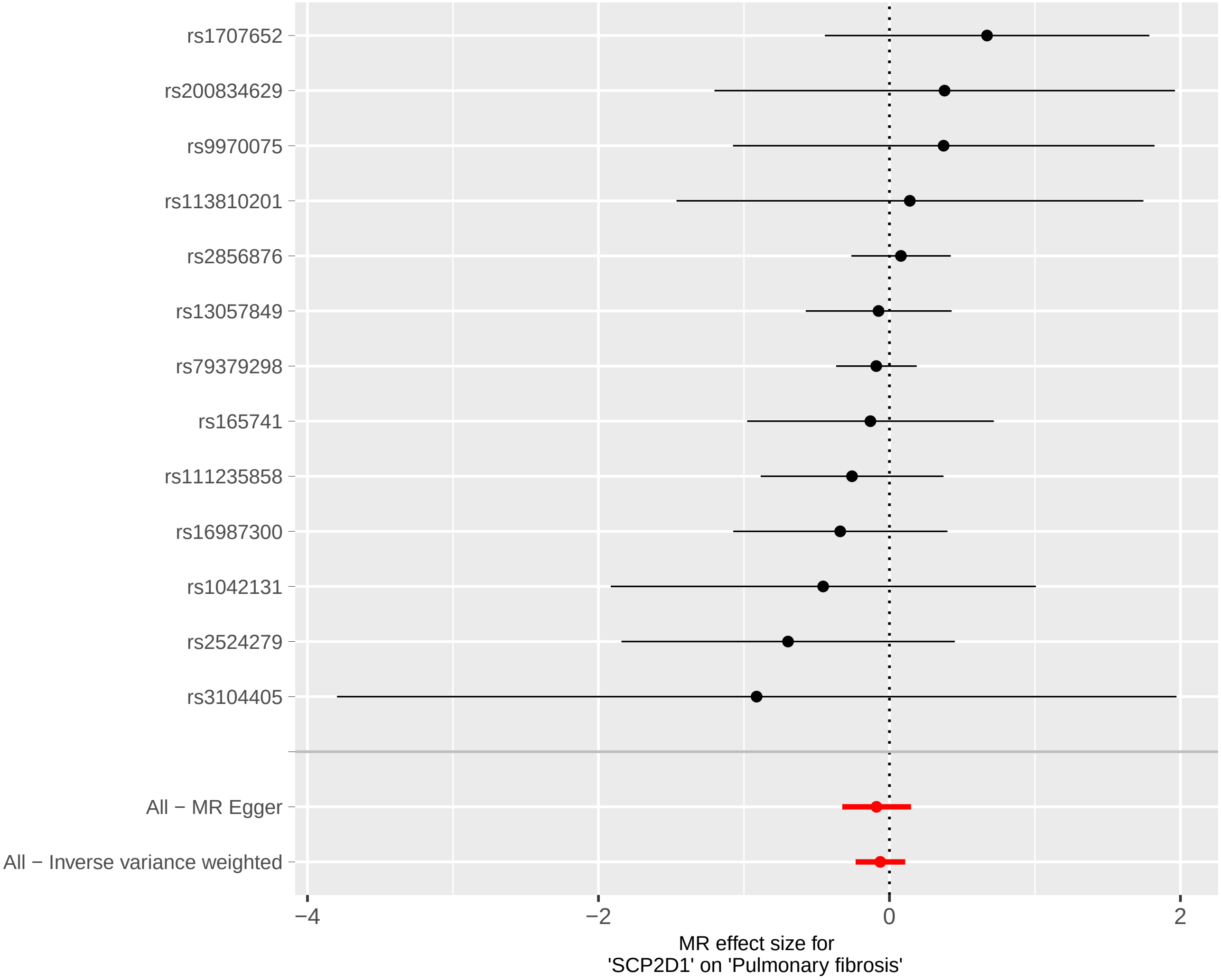
rs1707652
rs200834629
rs9970075
rs113810201
rs2856876
rs13057849
rs79379298
rs165741
rs111235858
rs16987300
rs1042131
rs2524279
rs3104405
All − MR Egger
All − Inverse variance weighted
−4
−2
0
2
MR effect size for
'SCP2D1' on 'Pulmonary fibrosis'

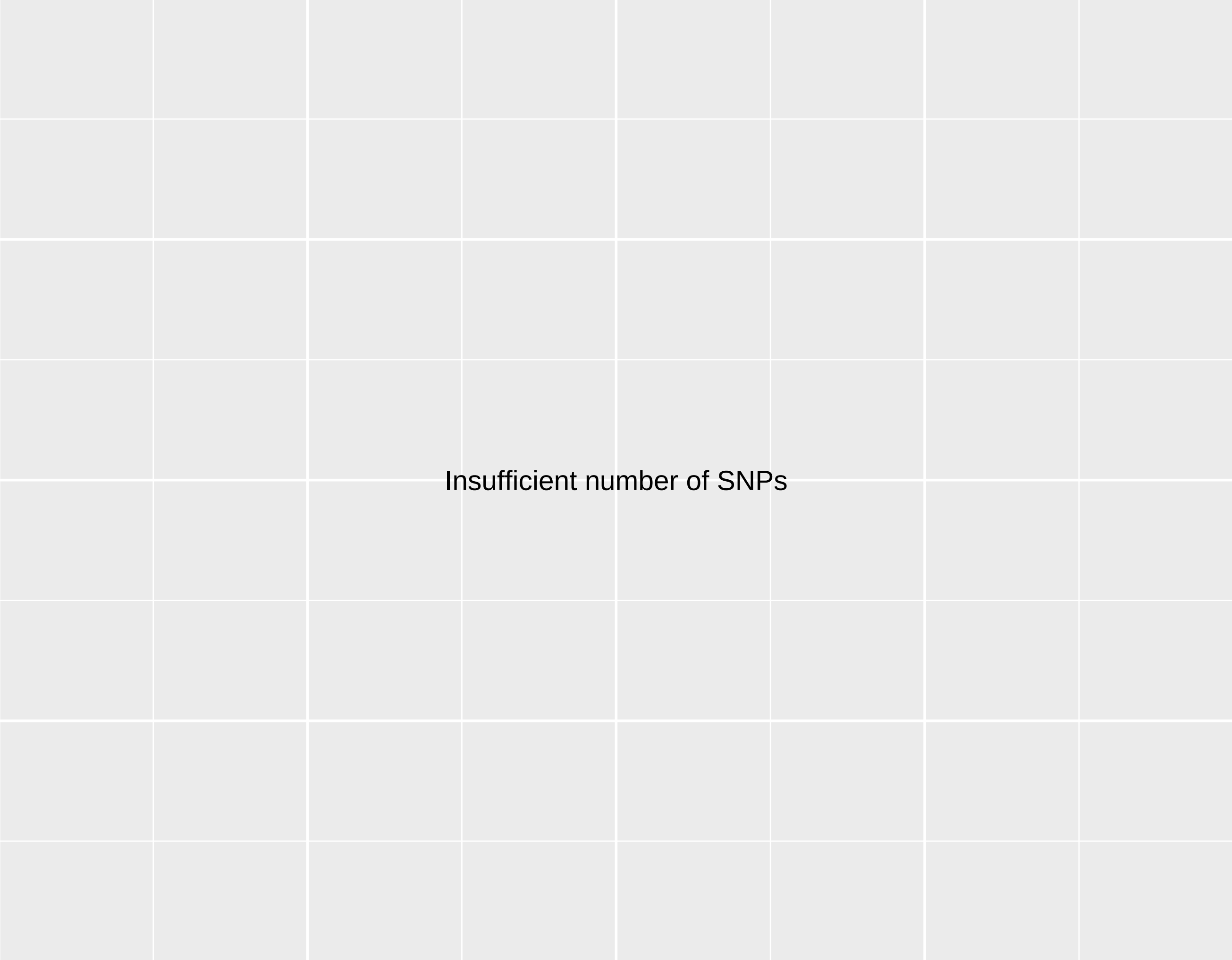
Insufficient number of SNPs

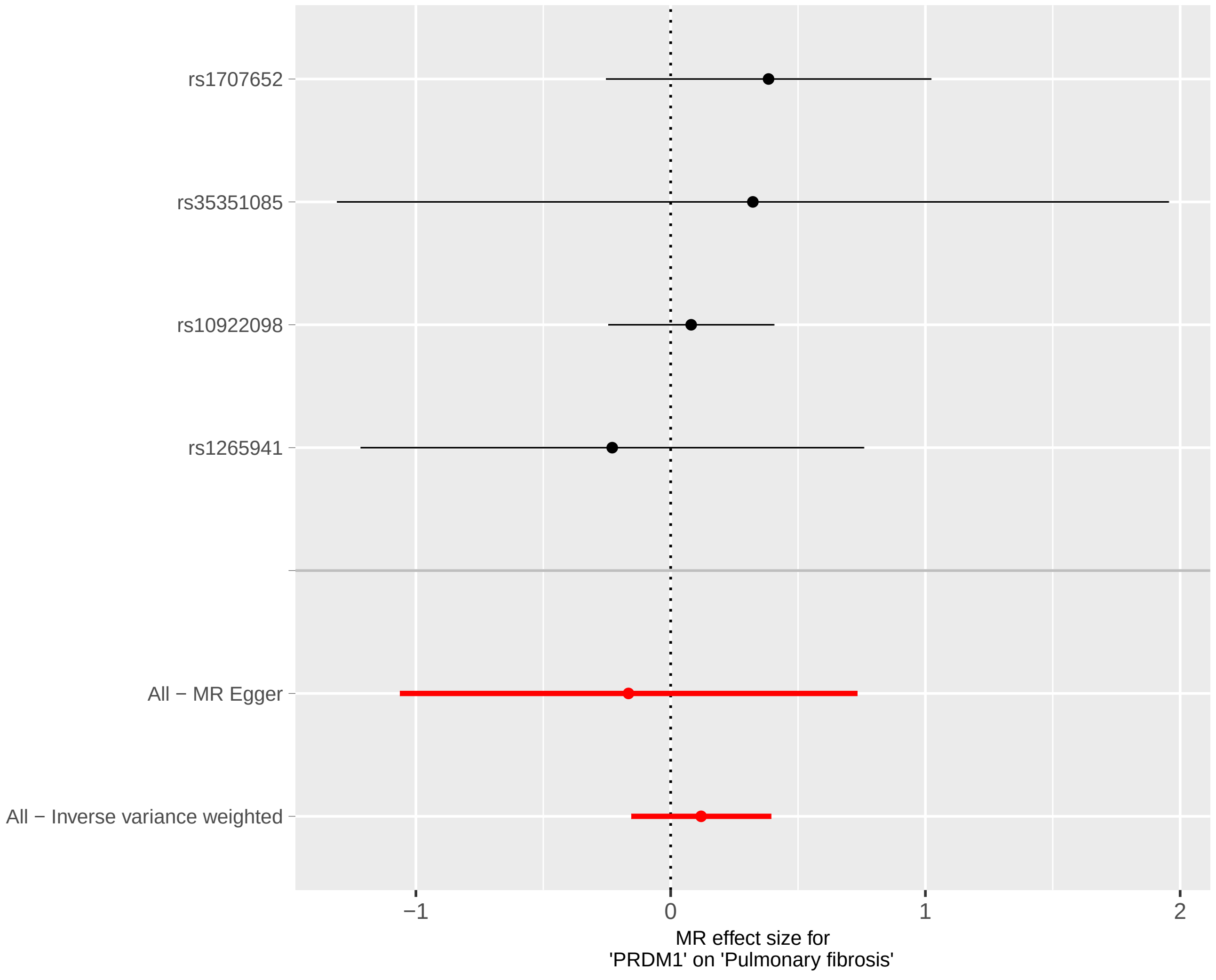
rs1707652
rs35351085
rs10922098
rs1265941
All − MR Egger
All − Inverse variance weighted
−1
0
1
2
MR effect size for
'PRDM1' on 'Pulmonary fibrosis'

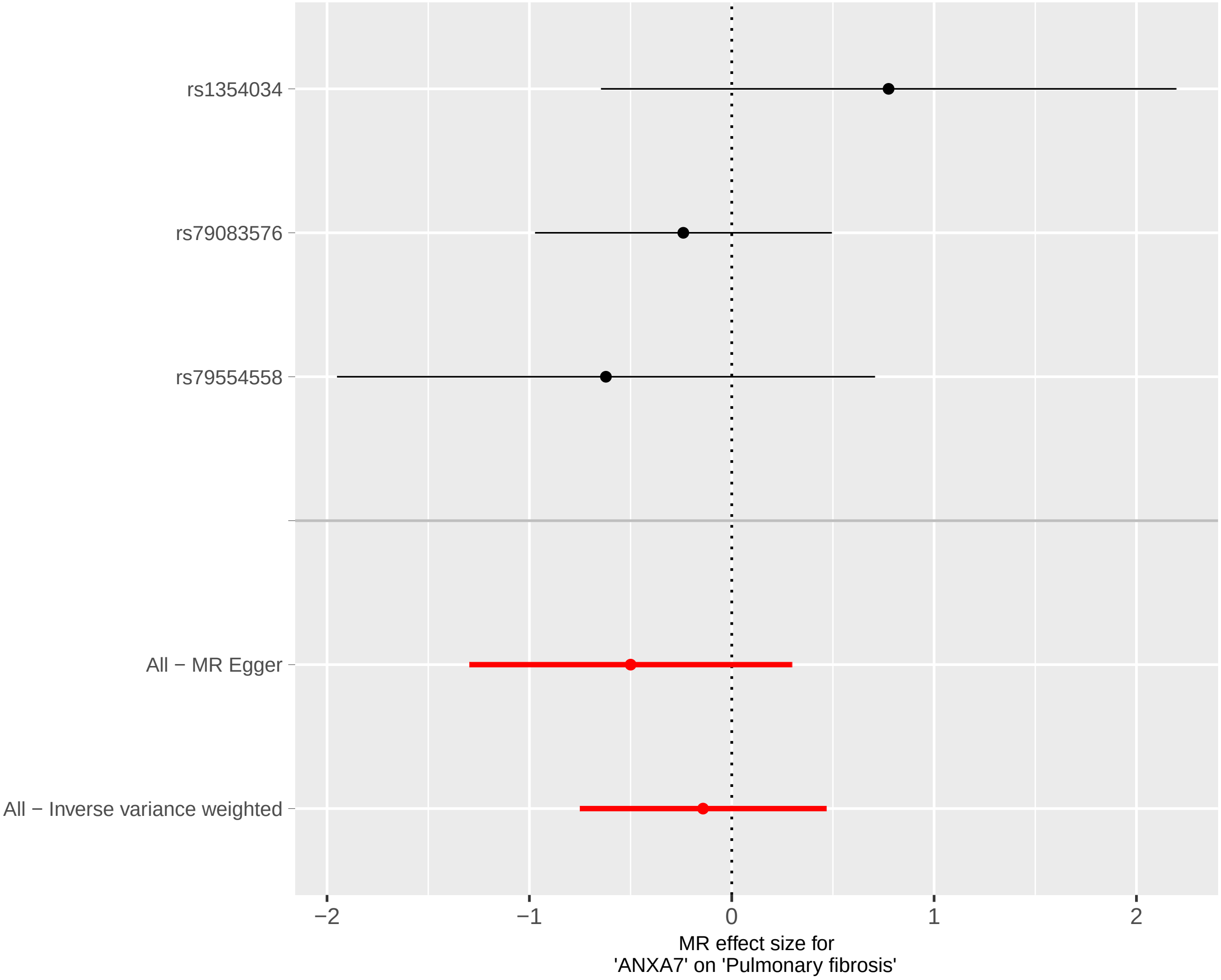
rs1354034
rs79083576
rs79554558
All − MR Egger
All − Inverse variance weighted
−2
−1
0
1
2
MR effect size for
'ANXA7' on 'Pulmonary fibrosis'

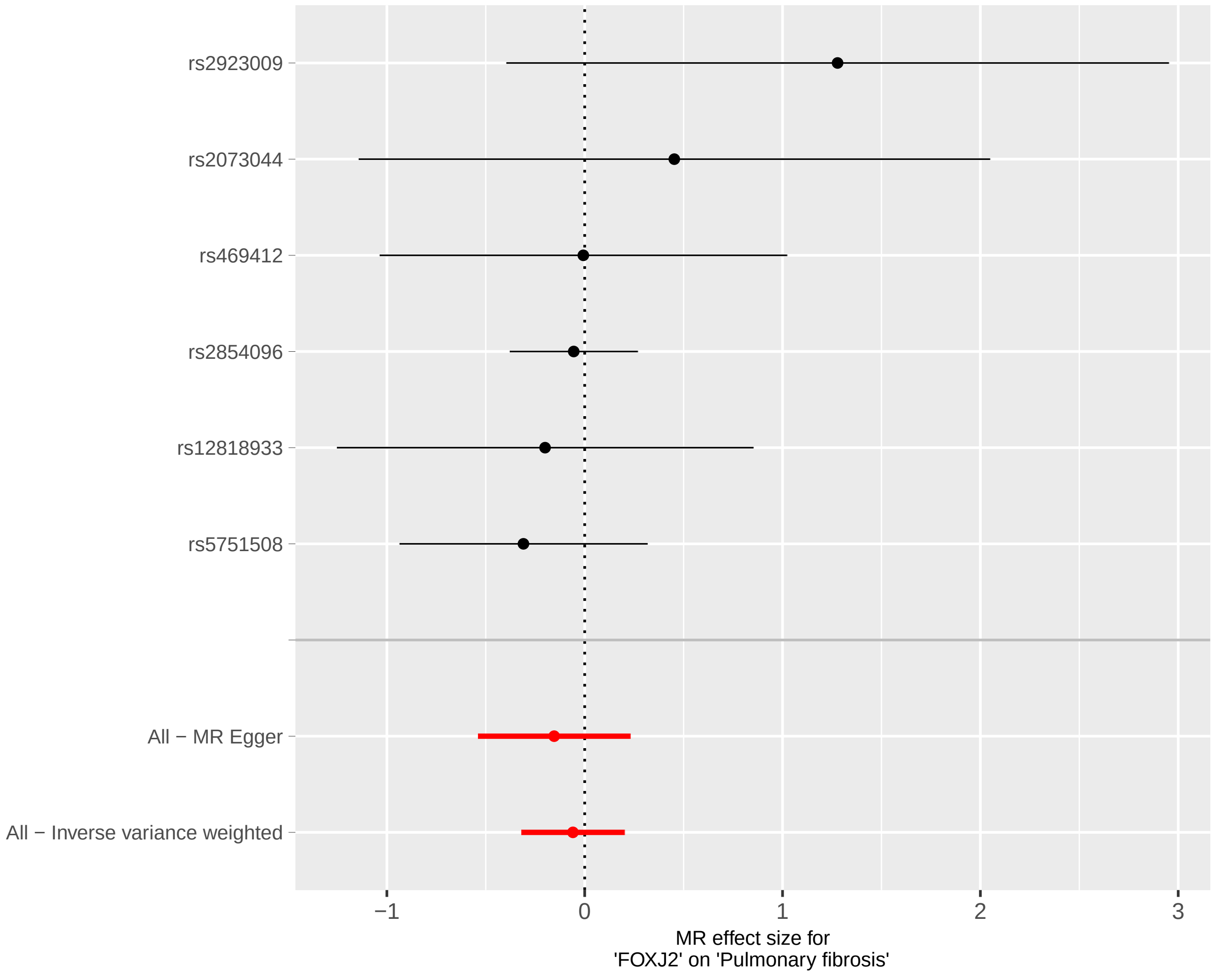
rs2923009
rs2073044
rs469412
rs2854096
rs12818933
rs5751508
All − MR Egger
All − Inverse variance weighted
−1
0
1
2
3
MR effect size for
'FOXJ2' on 'Pulmonary fibrosis'

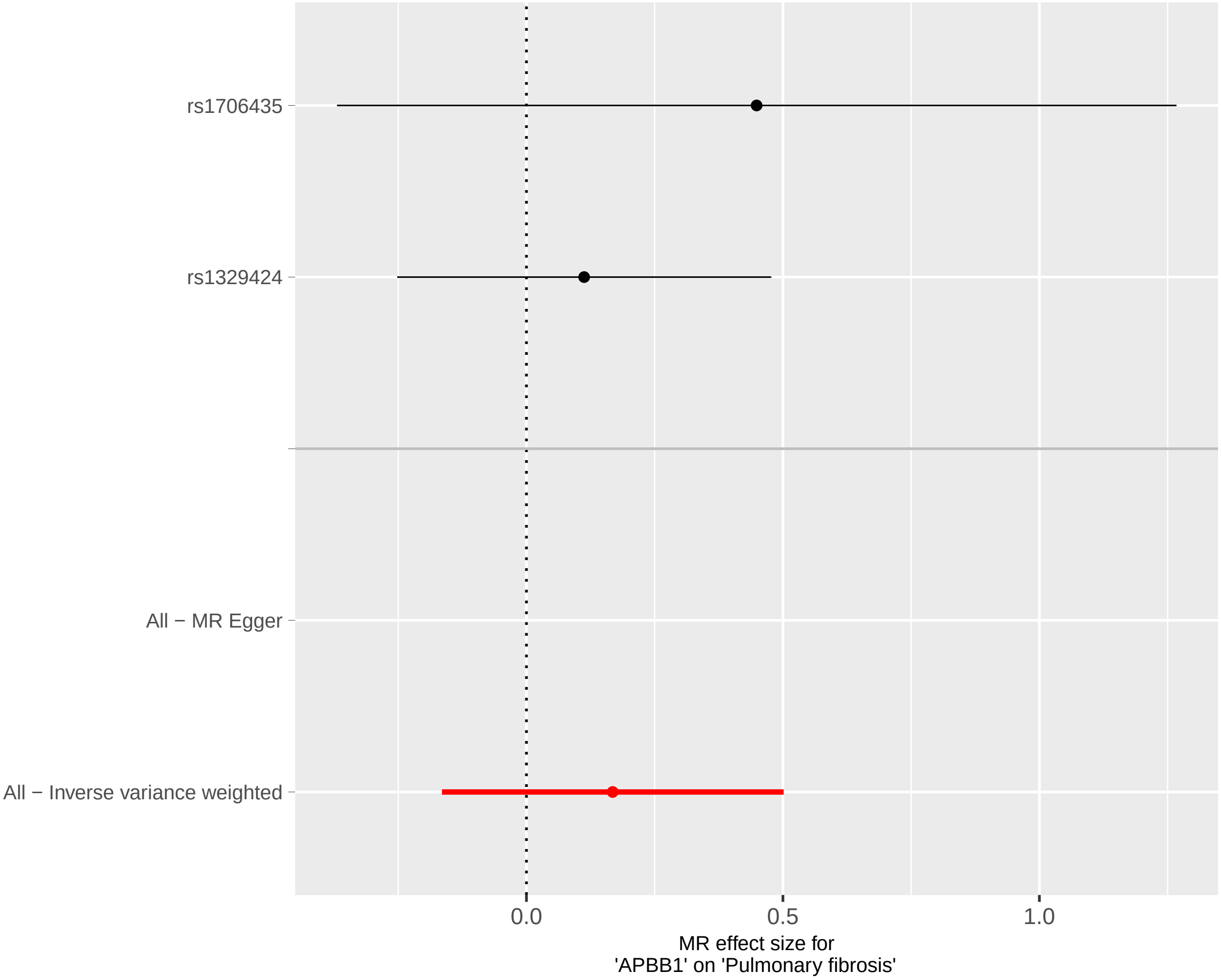
rs1706435
rs1329424
All − MR Egger
All − Inverse variance weighted
0.0
0.5
1.0
MR effect size for
'APBB1' on 'Pulmonary fibrosis'

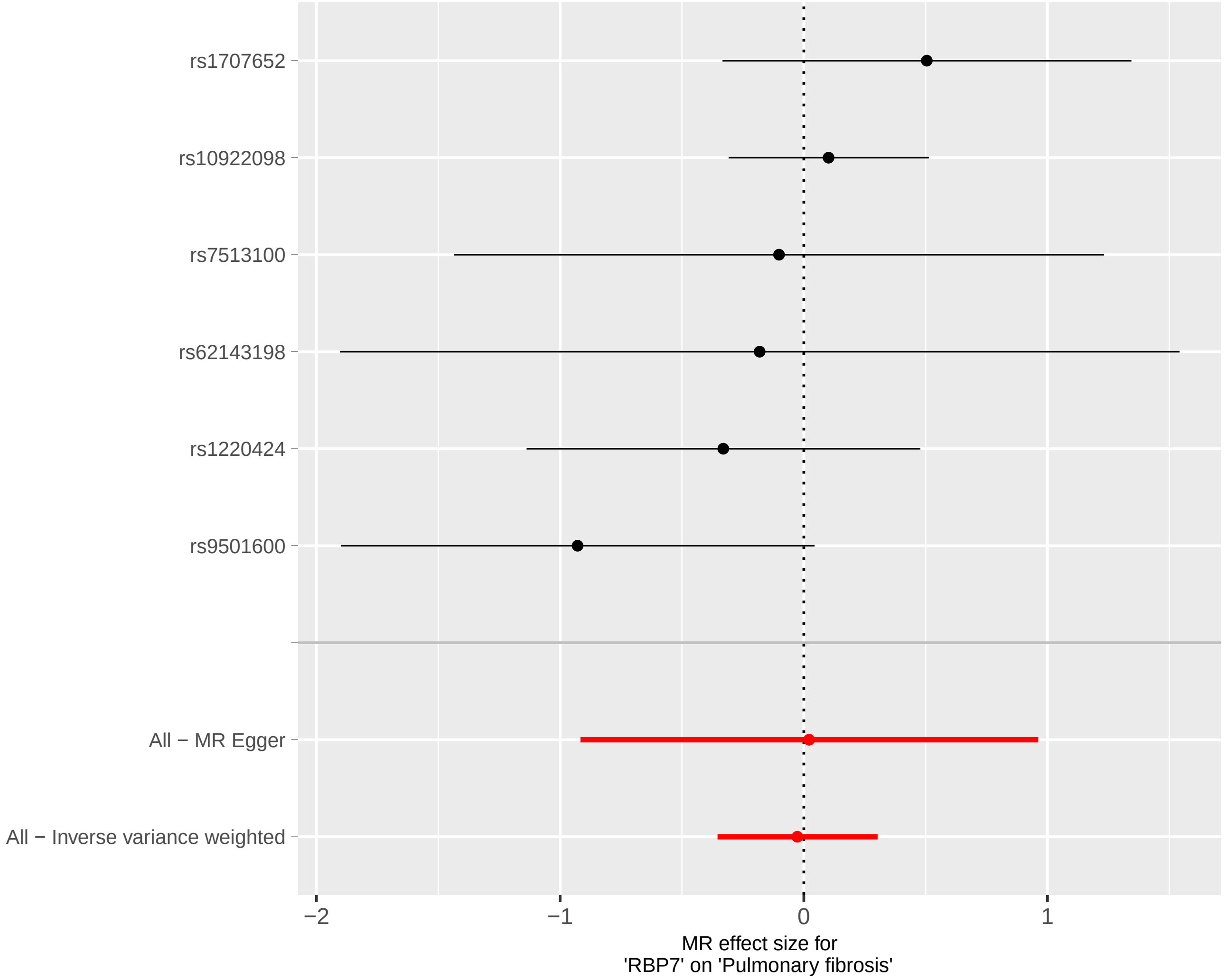
rs1707652
rs10922098
rs7513100
rs62143198
rs1220424
rs9501600
All − MR Egger
All − Inverse variance weighted
−2
−1
0
1
MR effect size for
'RBP7' on 'Pulmonary fibrosis'

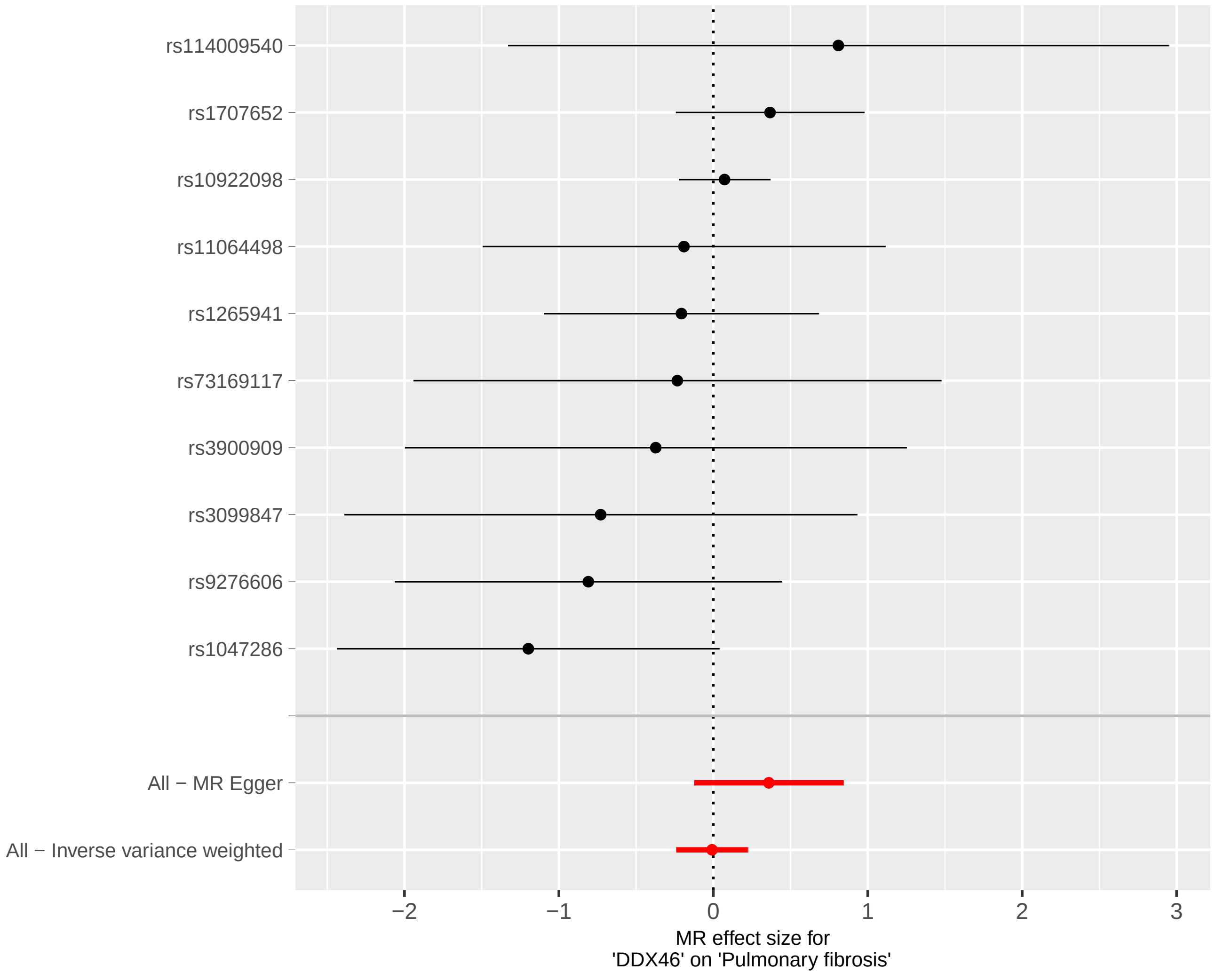
rs114009540
rs1707652
rs10922098
rs11064498
rs1265941
rs73169117
rs3900909
rs3099847
rs9276606
rs1047286
All − MR Egger
All − Inverse variance weighted
−2
−1
0
1
2
3
MR effect size for
'DDX46' on 'Pulmonary fibrosis'

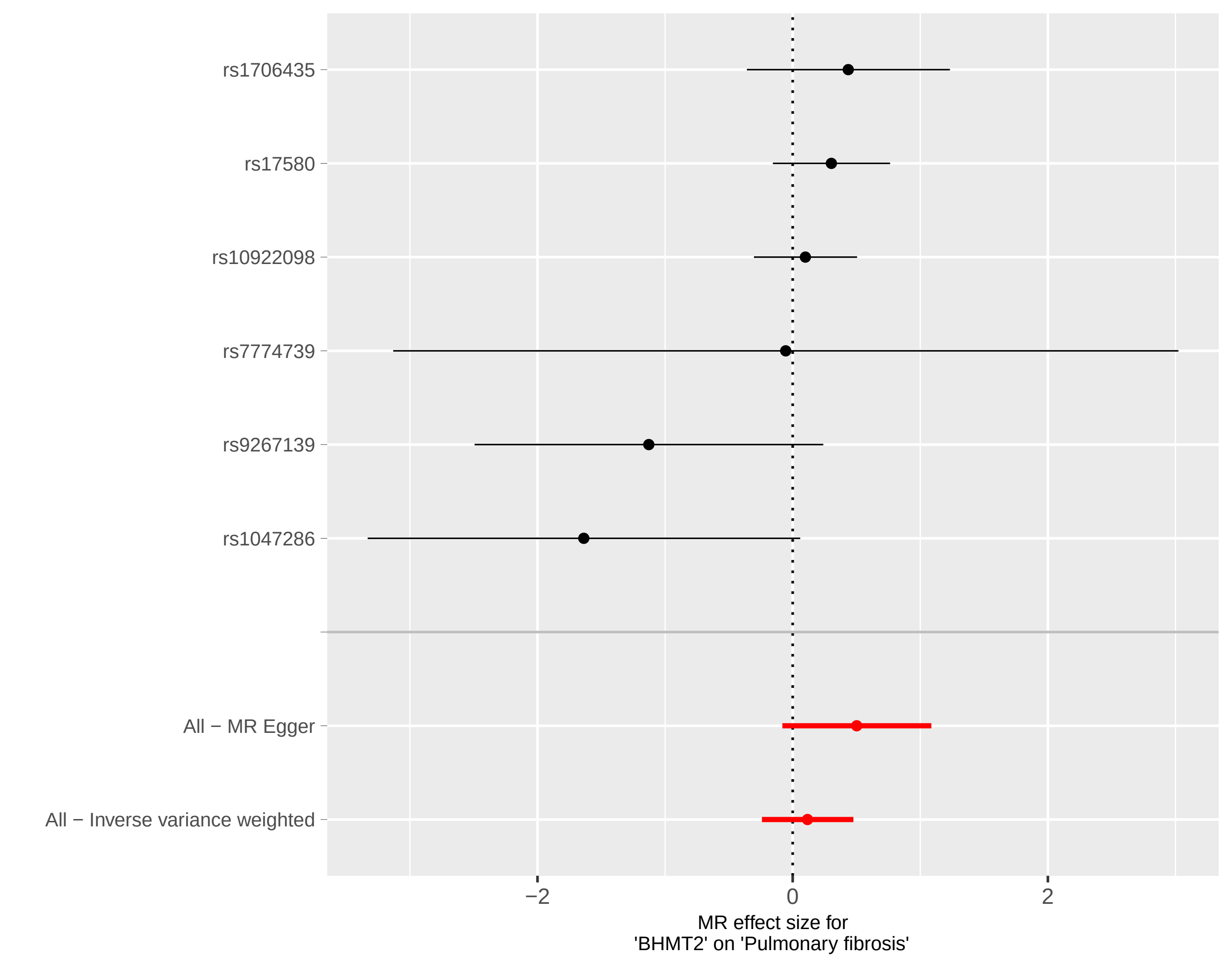
rs1706435
rs17580
rs10922098
rs7774739
rs9267139
rs1047286
All − MR Egger
All − Inverse variance weighted
−2
0
2
MR effect size for
'BHMT2' on 'Pulmonary fibrosis'

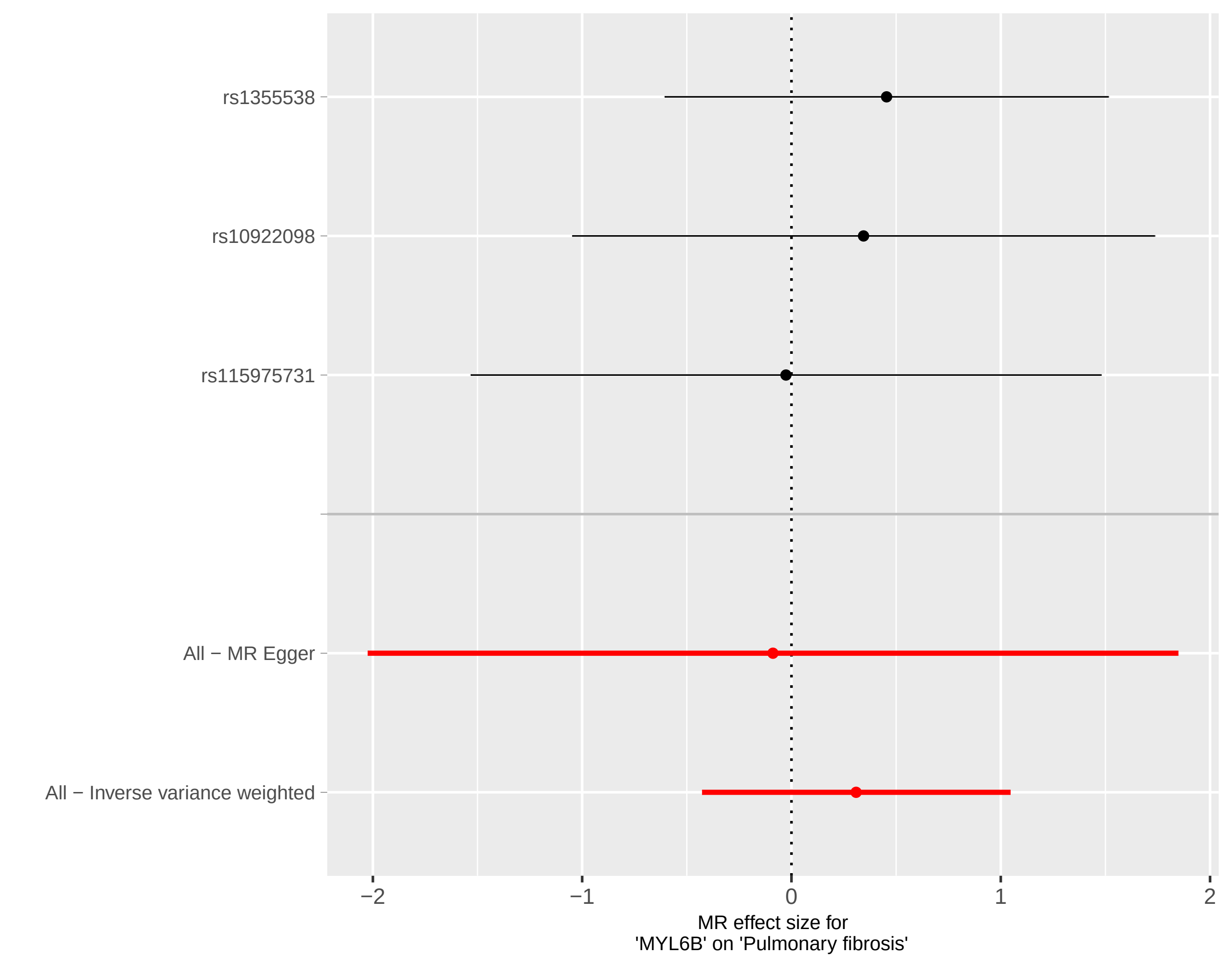

rs1355538
rs10922098
rs115975731
All − MR Egger
All − Inverse variance weighted
−2
−1
0
1
2
MR effect size for
'MYL6B' on 'Pulmonary fibrosis'

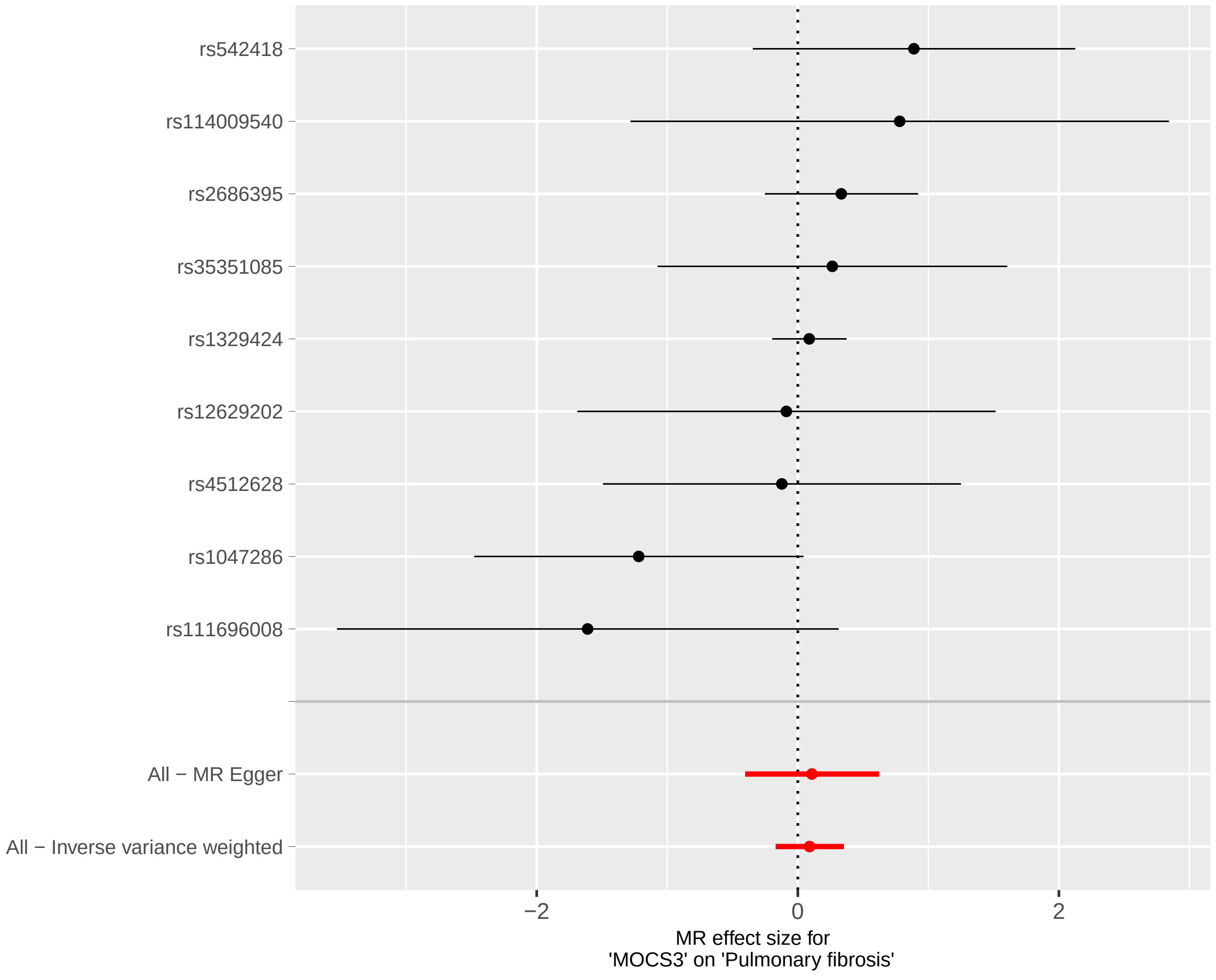

rs542418
rs114009540
rs2686395
rs35351085
rs1329424
rs12629202
rs4512628
rs1047286
rs111696008
All − MR Egger
All − Inverse variance weighted
−2
0
2
MR effect size for
'MOCS3' on 'Pulmonary fibrosis'

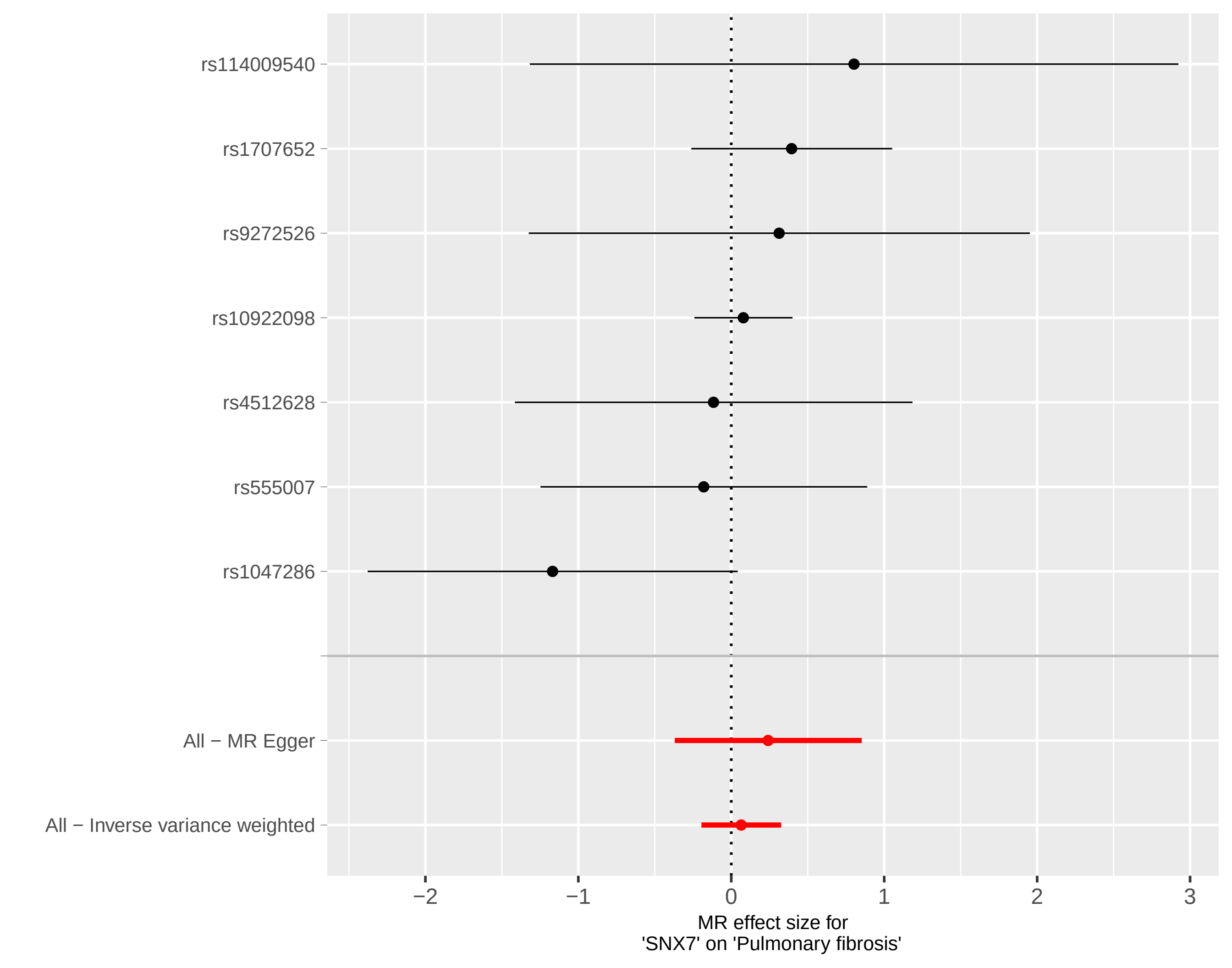
rs114009540
rs1707652
rs9272526
rs10922098
rs4512628
rs555007
rs1047286
All − MR Egger
All − Inverse variance weighted
−2
−1
0
1
2
3
MR effect size for
'SNX7' on 'Pulmonary fibrosis'

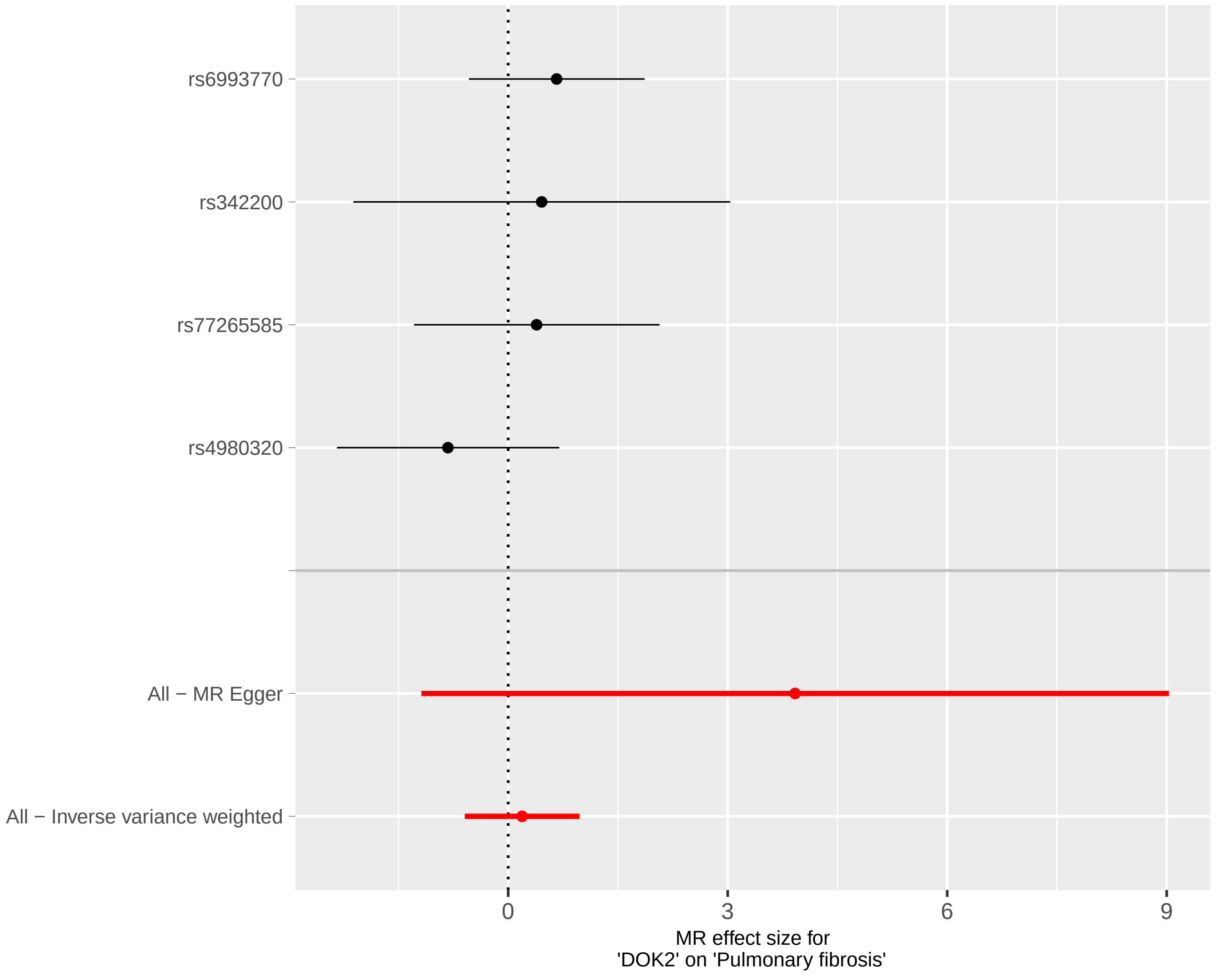
rs6993770
rs342200
rs77265585
rs4980320
All − MR Egger
All − Inverse variance weighted
0
3
6
9
MR effect size for
'DOK2' on 'Pulmonary fibrosis'

Insufficient number of SNPs

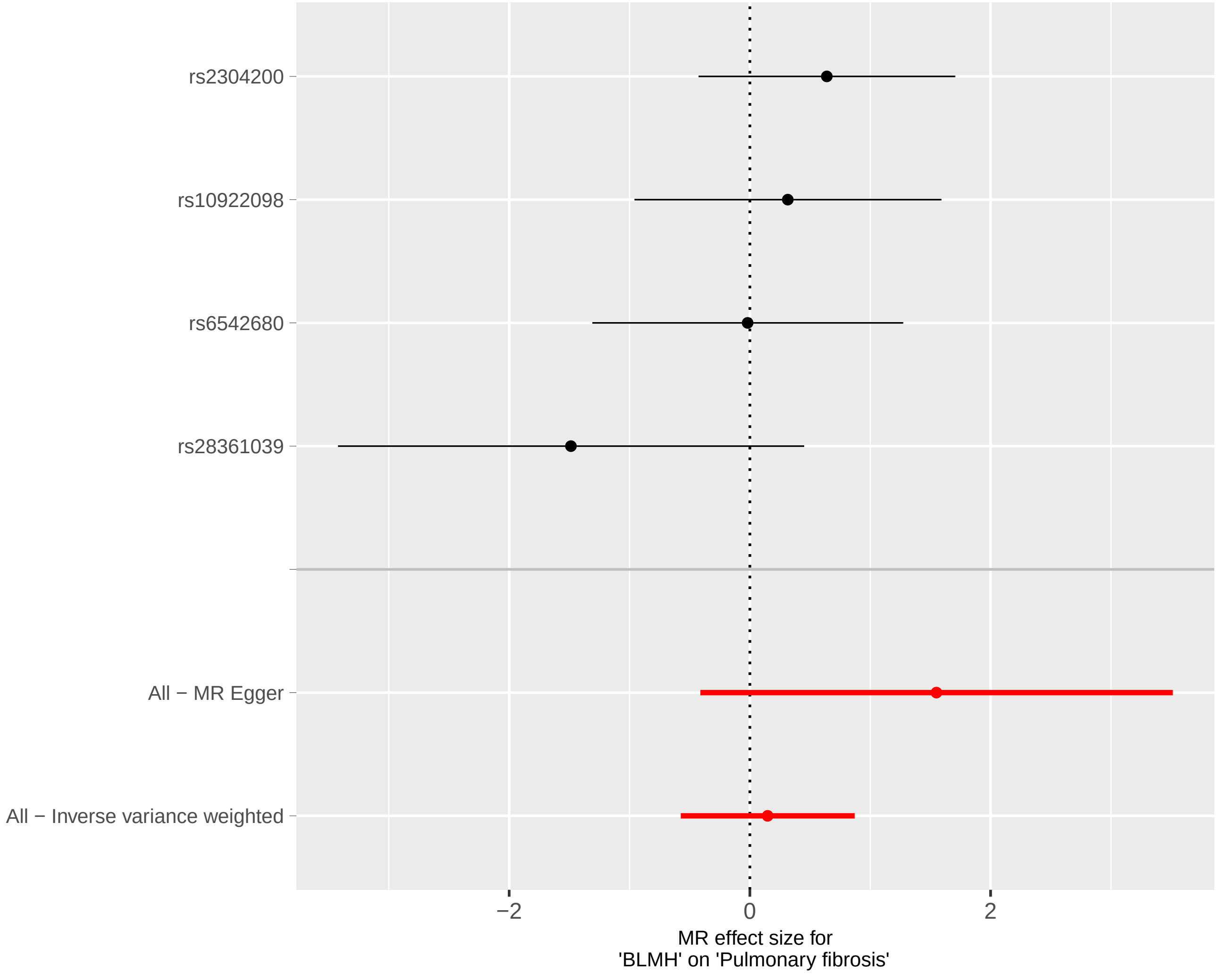
rs2304200
rs10922098
rs6542680
rs28361039
All − MR Egger
All − Inverse variance weighted
−2
0
2
MR effect size for
'BLMH' on 'Pulmonary fibrosis'

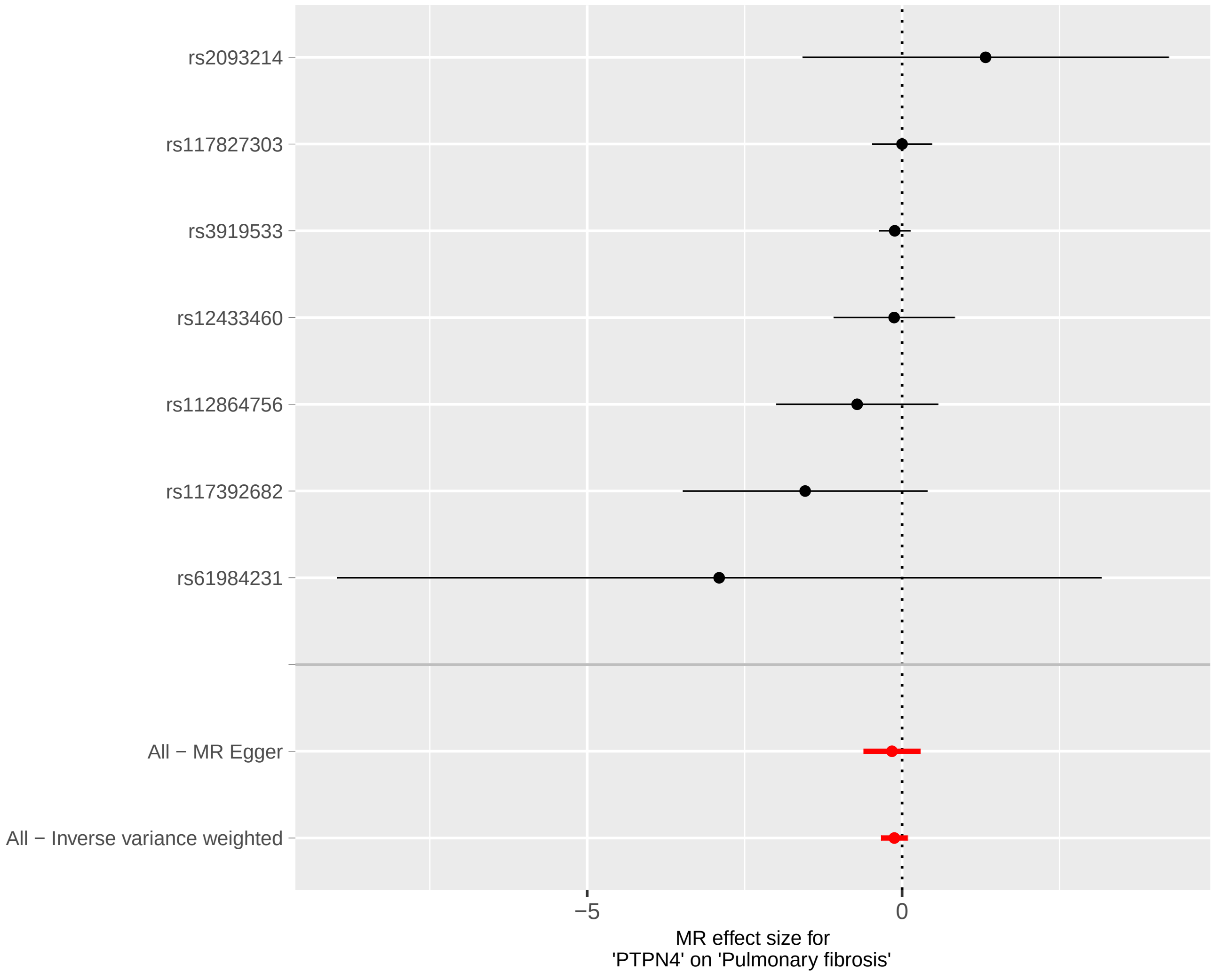
rs2093214
rs117827303
rs3919533
rs12433460
rs112864756
rs117392682
rs61984231
All − MR Egger
All − Inverse variance weighted
−5
0
MR effect size for
'PTPN4' on 'Pulmonary fibrosis'

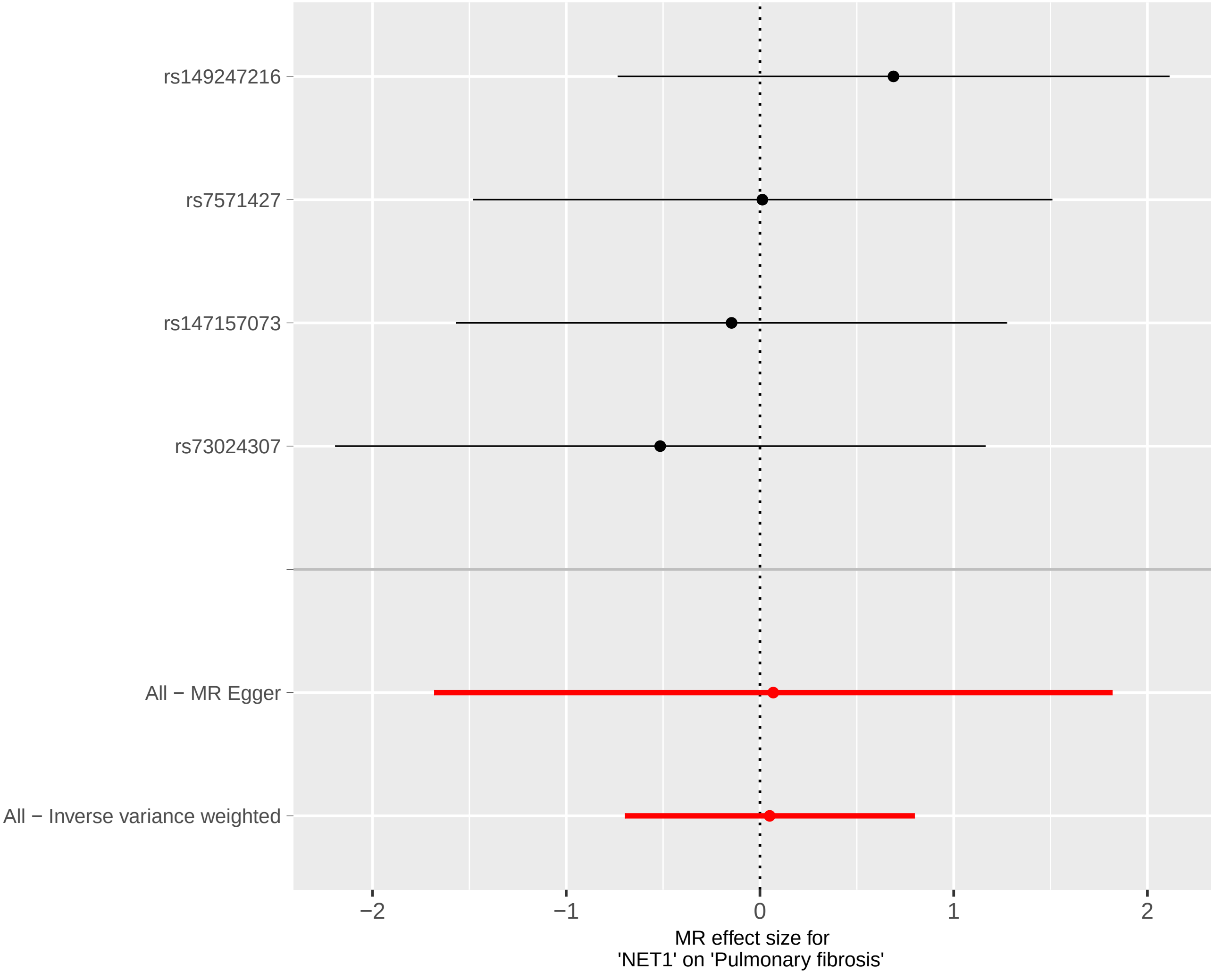

rs149247216
rs7571427
rs147157073
rs73024307
All − MR Egger
All − Inverse variance weighted
−2
−1
0
1
2
MR effect size for
'NET1' on 'Pulmonary fibrosis'

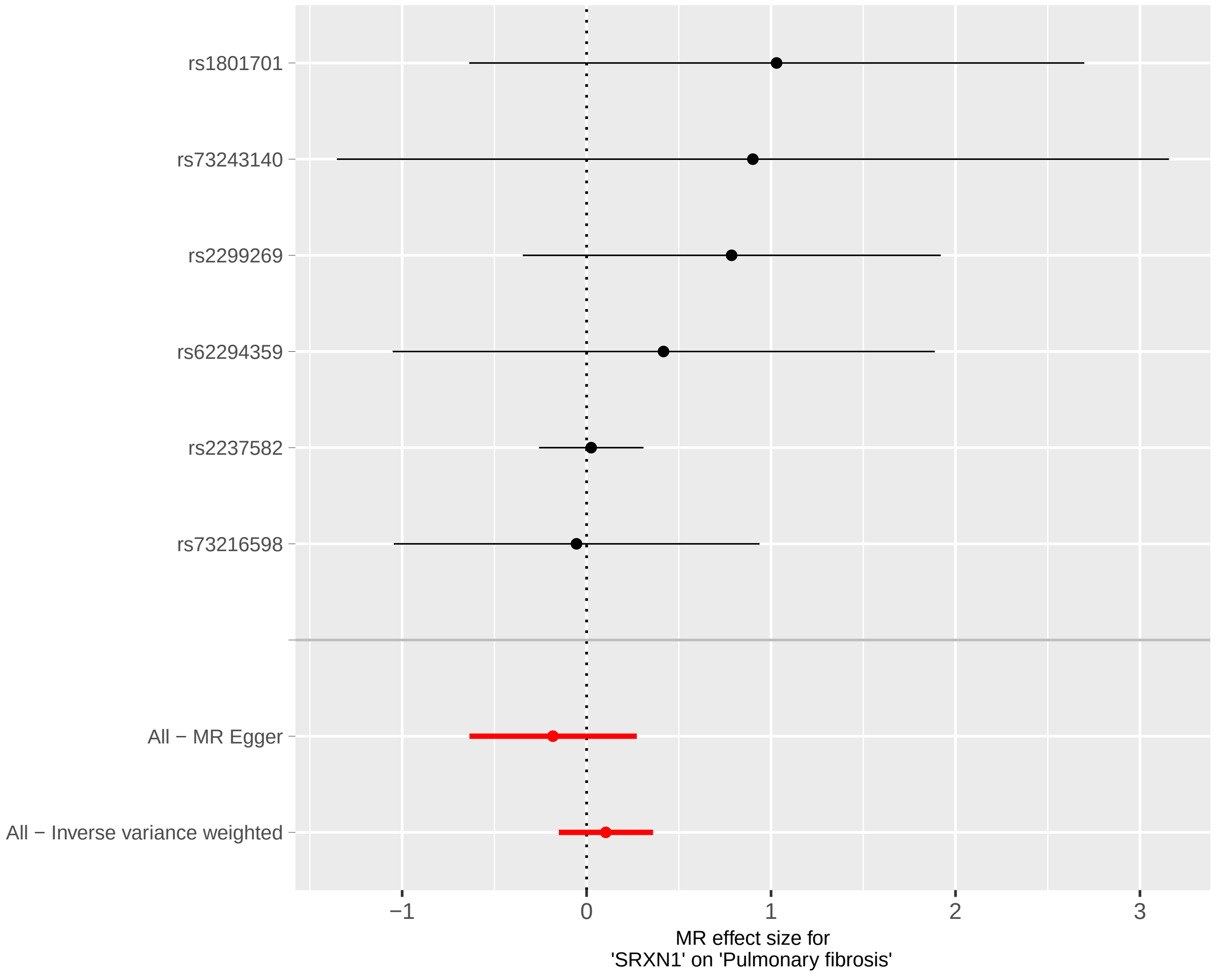
rs1801701
rs73243140
rs2299269
rs62294359
rs2237582
rs73216598
All − MR Egger
All − Inverse variance weighted
−1
0
1
2
3
MR effect size for
'SRXN1' on 'Pulmonary fibrosis'

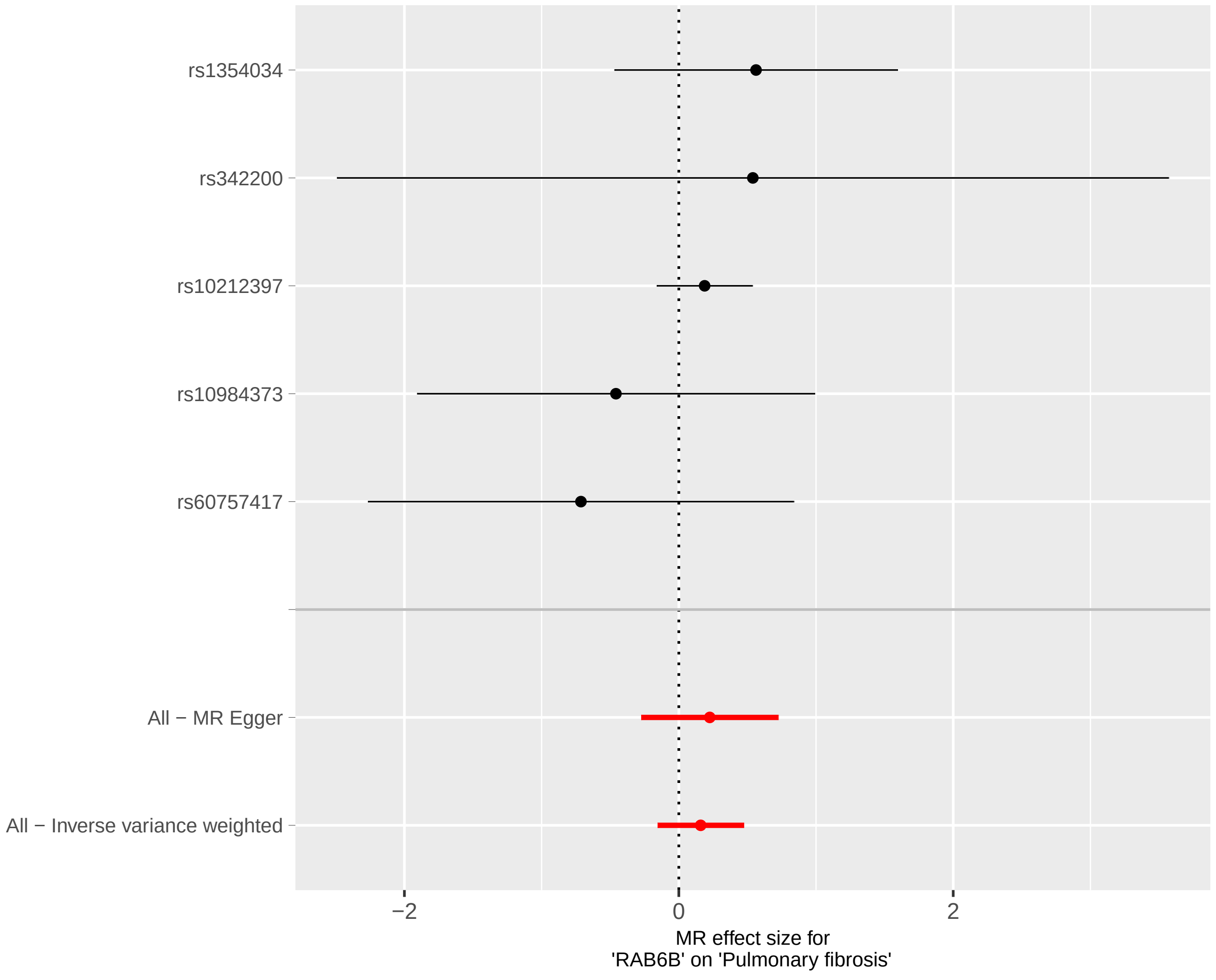
rs1354034
rs342200
rs10212397
rs10984373
rs60757417
All − MR Egger
All − Inverse variance weighted
−2
0
2
MR effect size for
'RAB6B' on 'Pulmonary fibrosis'

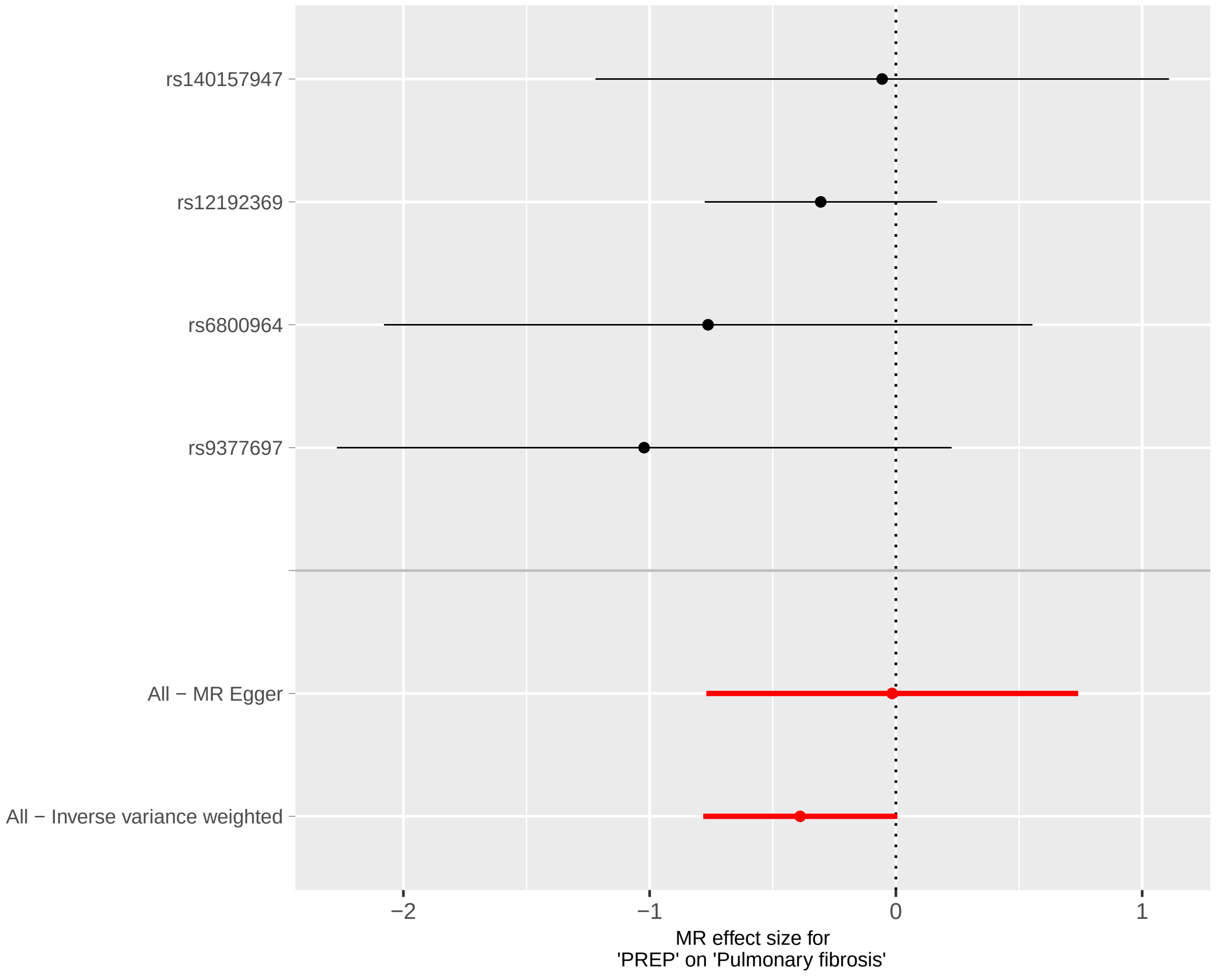
rs140157947
rs12192369
rs6800964
rs9377697
All − MR Egger
All − Inverse variance weighted
−2
−1
0
1
MR effect size for
'PREP' on 'Pulmonary fibrosis'

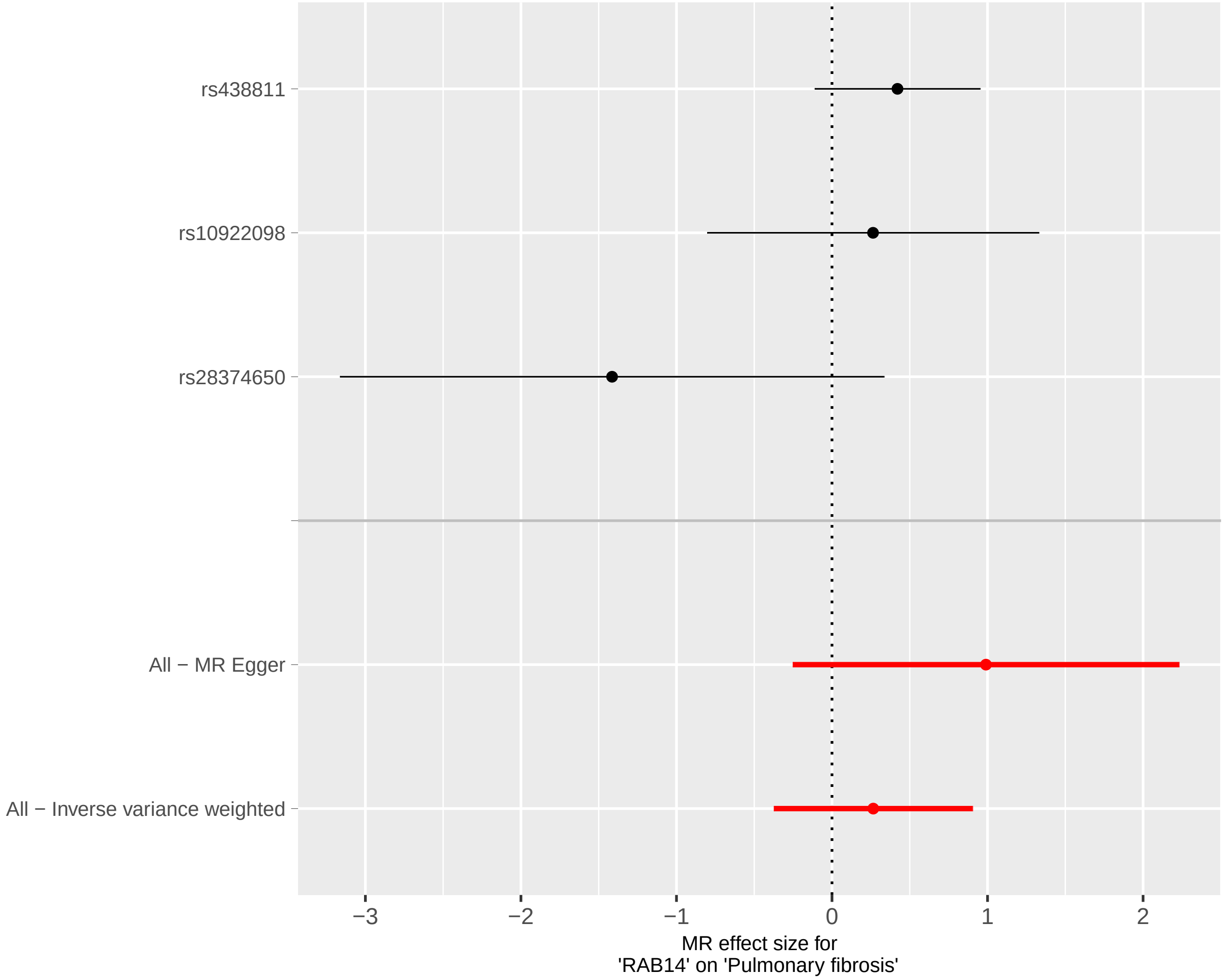

rs438811
rs10922098
rs28374650
All − MR Egger
All − Inverse variance weighted
−3
−2
−1
0
1
2
MR effect size for
'RAB14' on 'Pulmonary fibrosis'

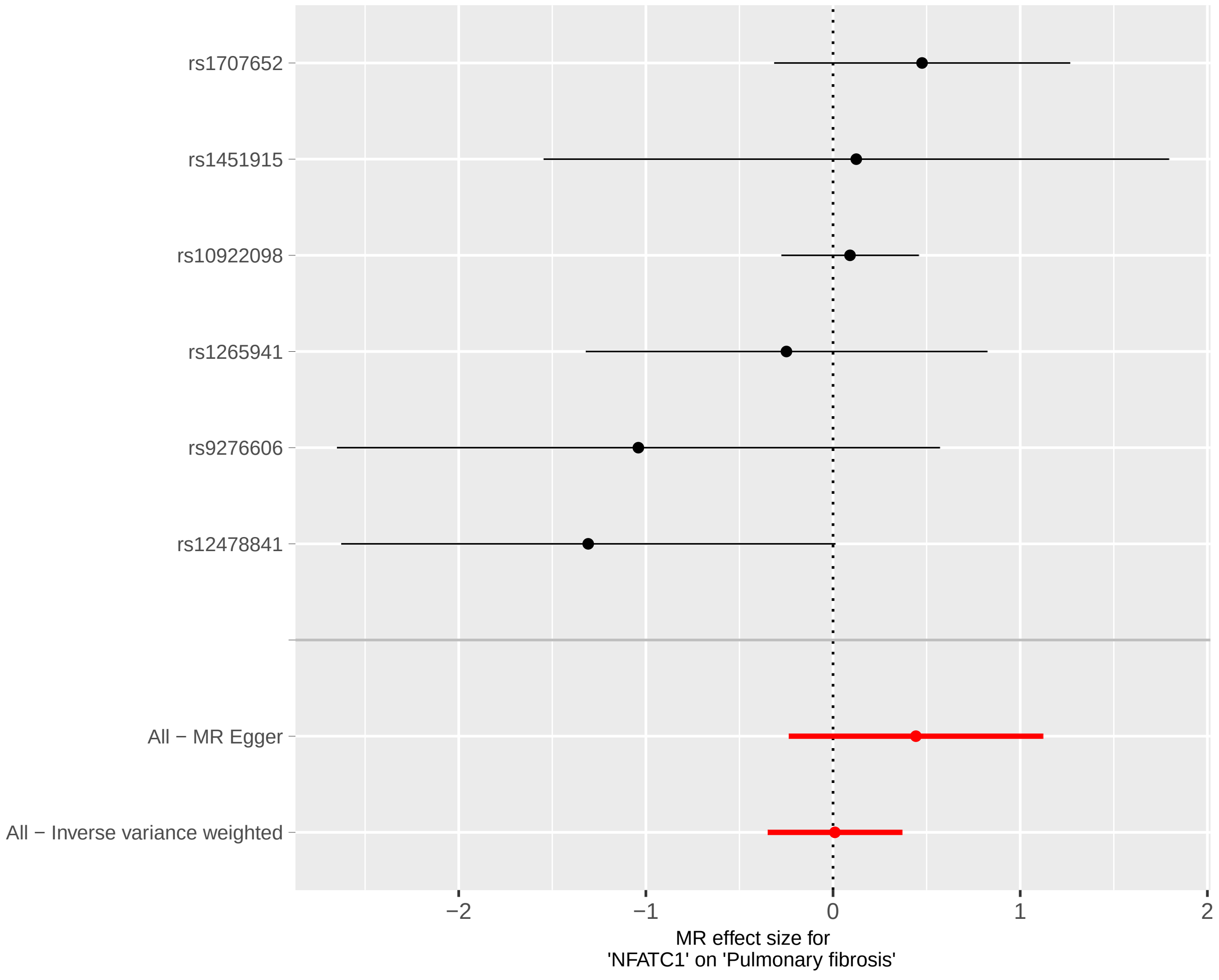
rs1707652
rs1451915
rs10922098
rs1265941
rs9276606
rs12478841
All − MR Egger
All − Inverse variance weighted
−2
−1
0
1
2
MR effect size for
'NFATC1' on 'Pulmonary fibrosis'

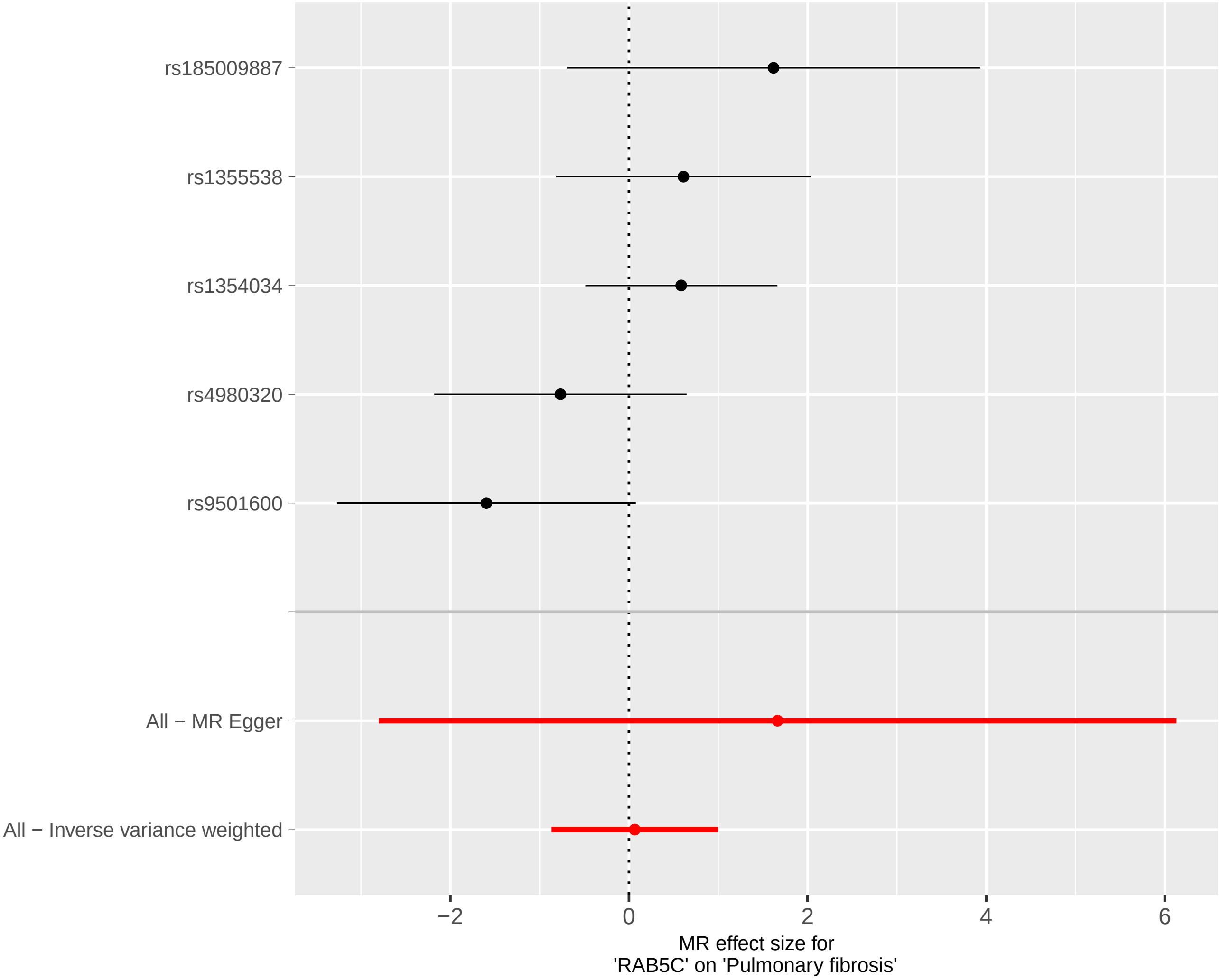
rs185009887
rs1355538
rs1354034
rs4980320
rs9501600
All − MR Egger
All − Inverse variance weighted
−2
0
2
4
6
MR effect size for
'RAB5C' on 'Pulmonary fibrosis'

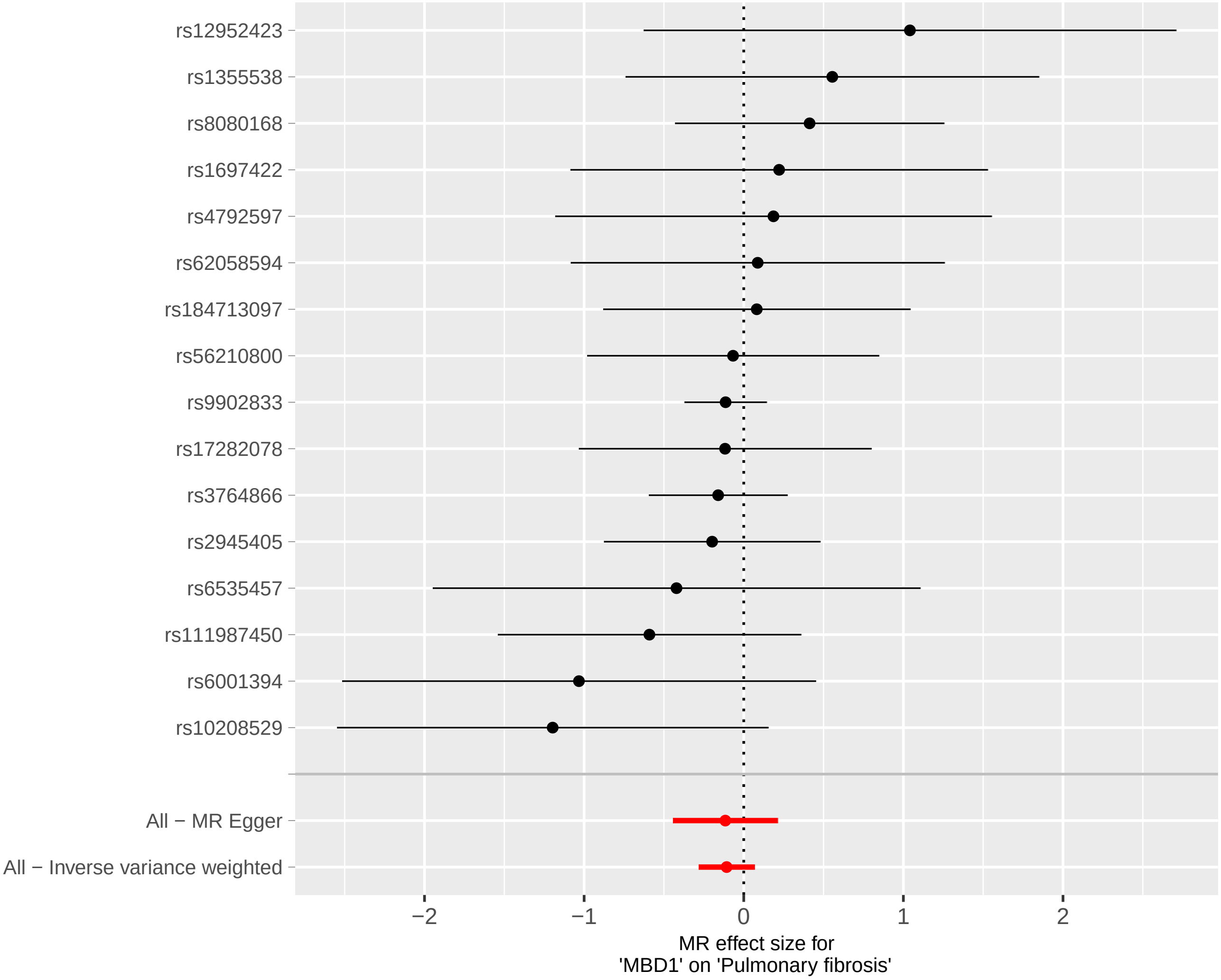
rs12952423
rs1355538
rs8080168
rs1697422
rs4792597
rs62058594
rs184713097
rs56210800
rs9902833
rs17282078
rs3764866
rs2945405
rs6535457
rs111987450
rs6001394
rs10208529
All − MR Egger
All − Inverse variance weighted
−2
−1
0
1
2
MR effect size for
'MBD1' on 'Pulmonary fibrosis'

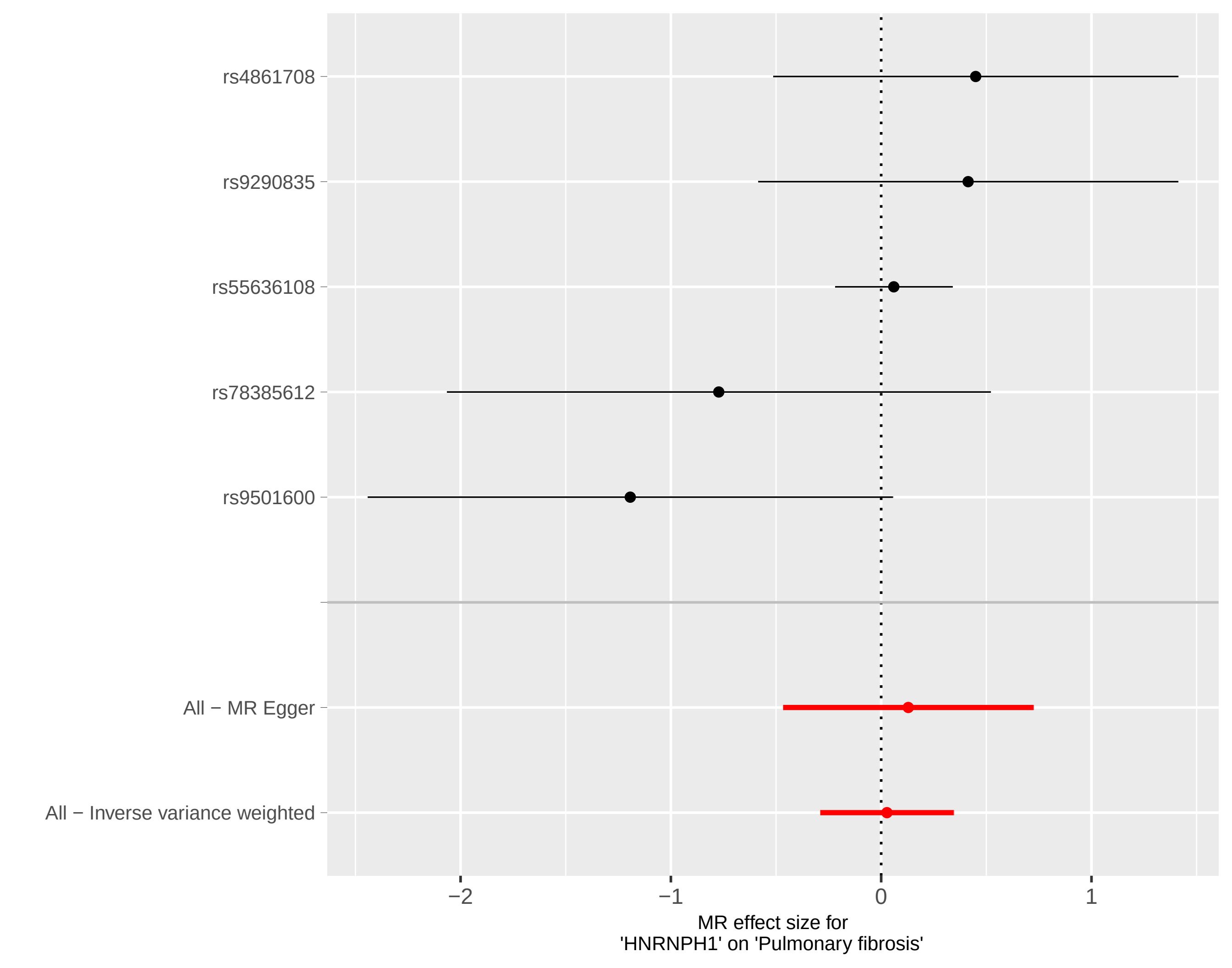
rs4861708
rs9290835
rs55636108
rs78385612
rs9501600
All − MR Egger
All − Inverse variance weighted
−2
−1
0
1
MR effect size for
'HNRNPH1' on 'Pulmonary fibrosis'

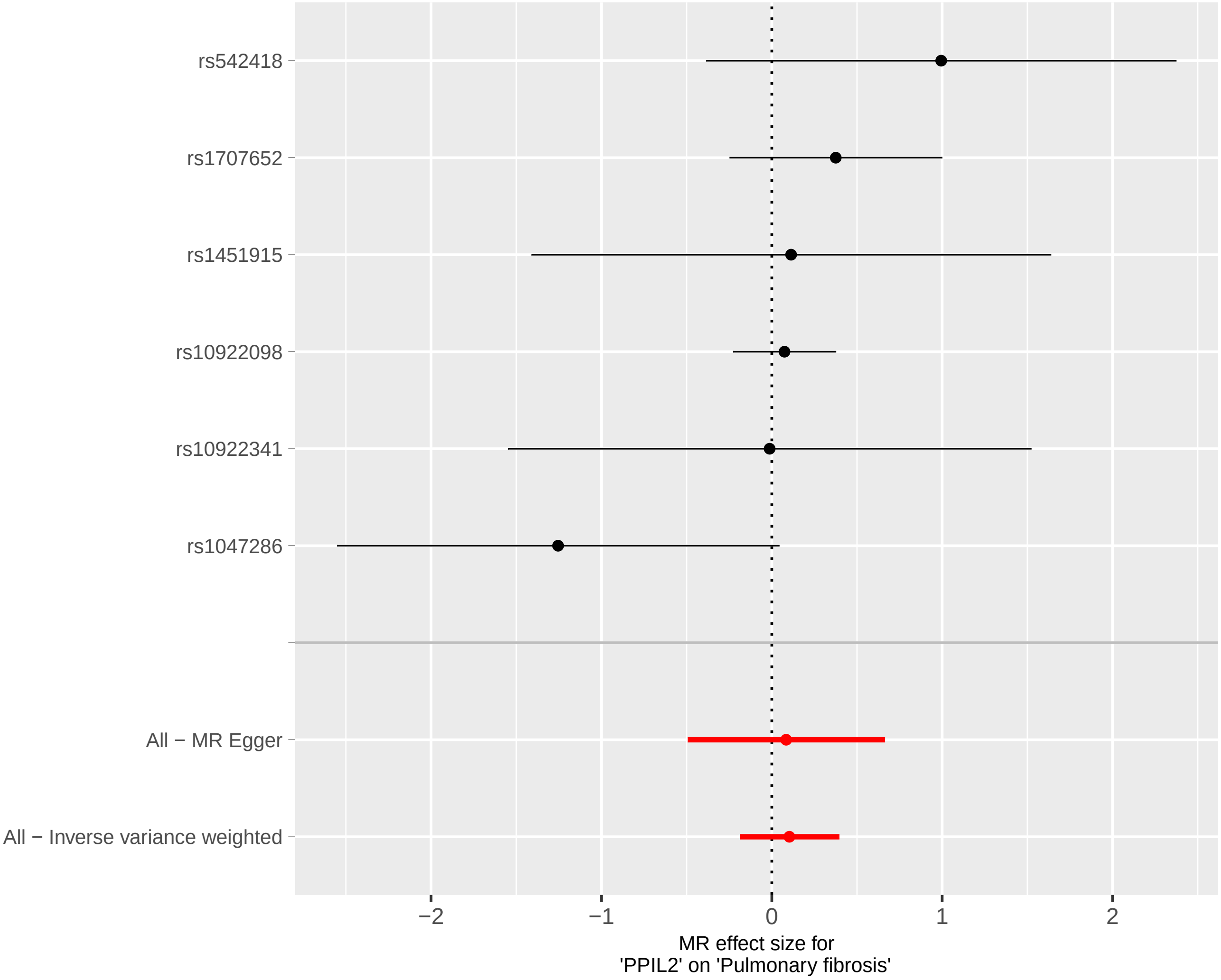

rs542418
rs1707652
rs1451915
rs10922098
rs10922341
rs1047286
All − MR Egger
All − Inverse variance weighted
−2
−1
0
1
2
MR effect size for
'PPIL2' on 'Pulmonary fibrosis'

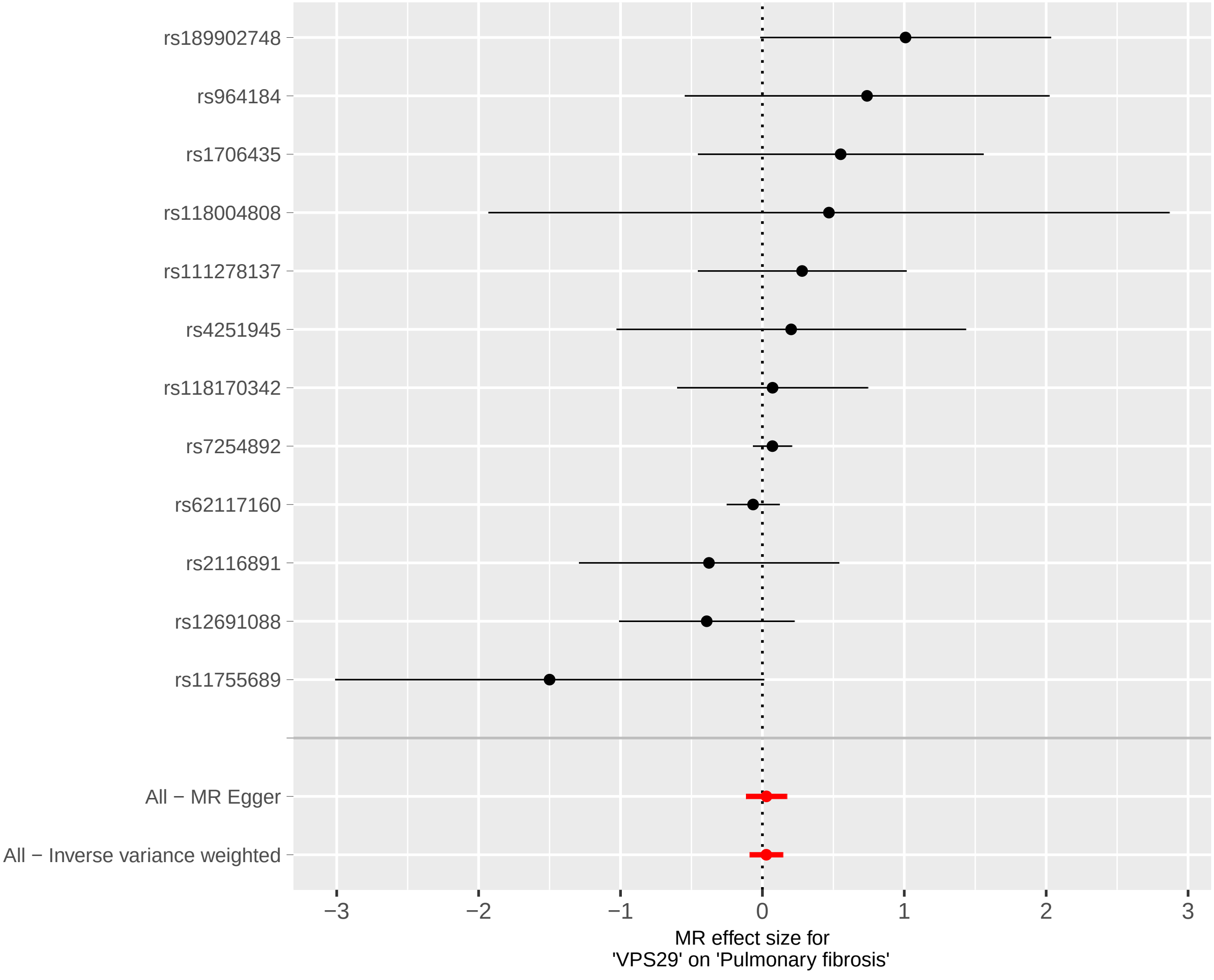

rs189902748
rs964184
rs1706435
rs118004808
rs111278137
rs4251945
rs118170342
rs7254892
rs62117160
rs2116891
rs12691088
rs11755689
All − MR Egger
All − Inverse variance weighted
−3
−2
−1
0
1
2
3
MR effect size for
'VPS29' on 'Pulmonary fibrosis'

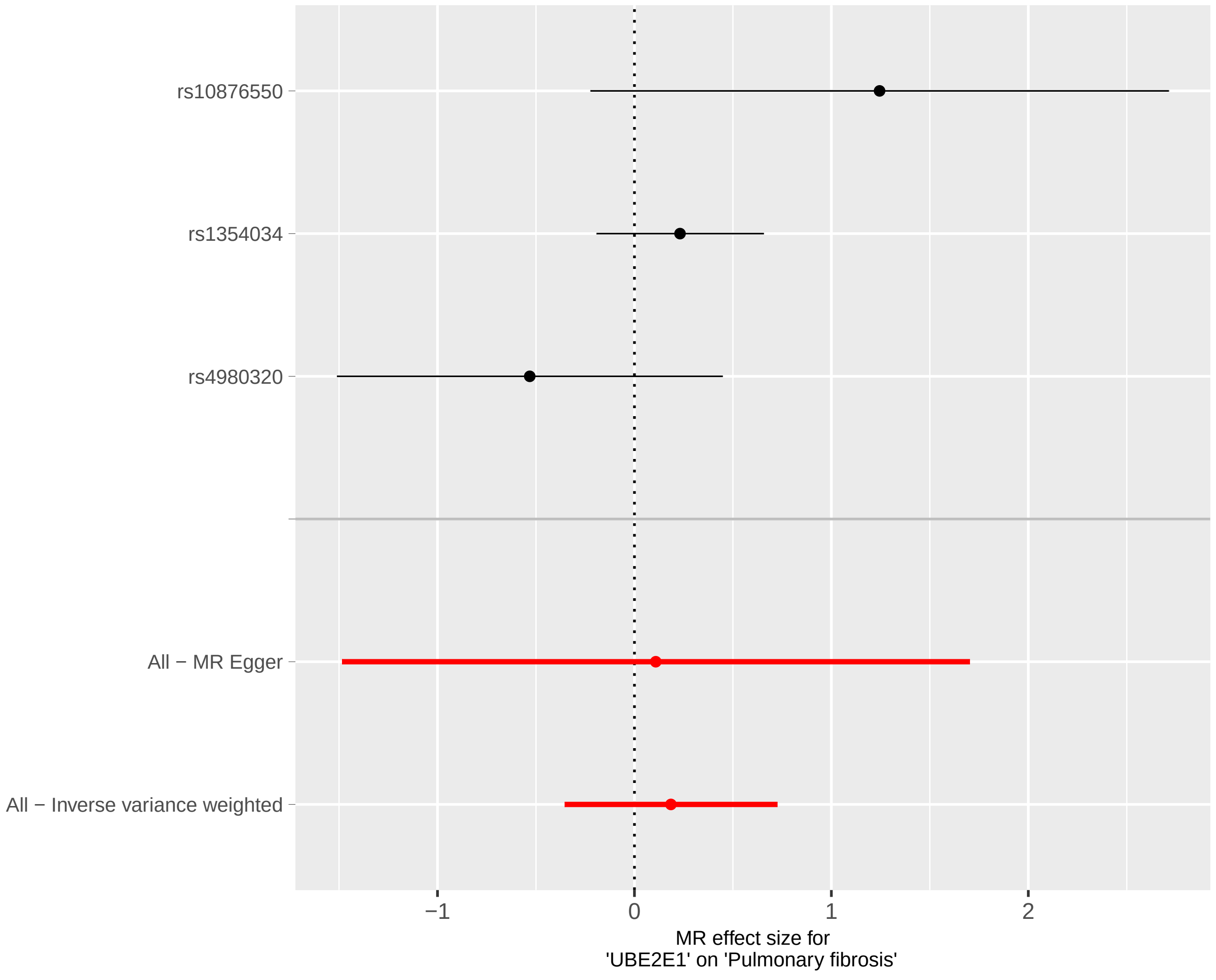

rs10876550
rs1354034
rs4980320
All − MR Egger
All − Inverse variance weighted
−1
0
1
2
MR effect size for
'UBE2E1' on 'Pulmonary fibrosis'

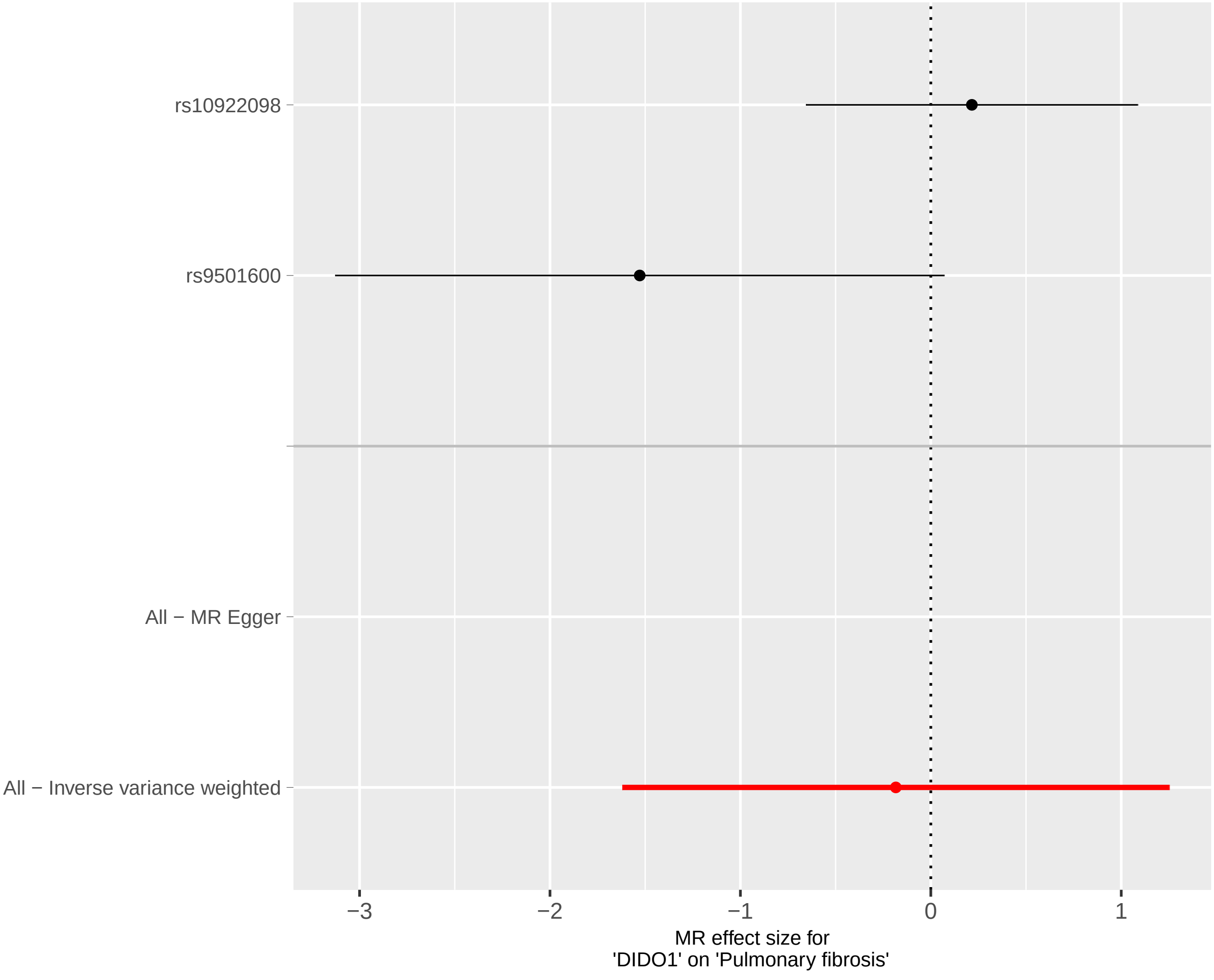

rs10922098
rs9501600
All − MR Egger
All − Inverse variance weighted
−3
−2
−1
0
1
MR effect size for
'DIDO1' on 'Pulmonary fibrosis'

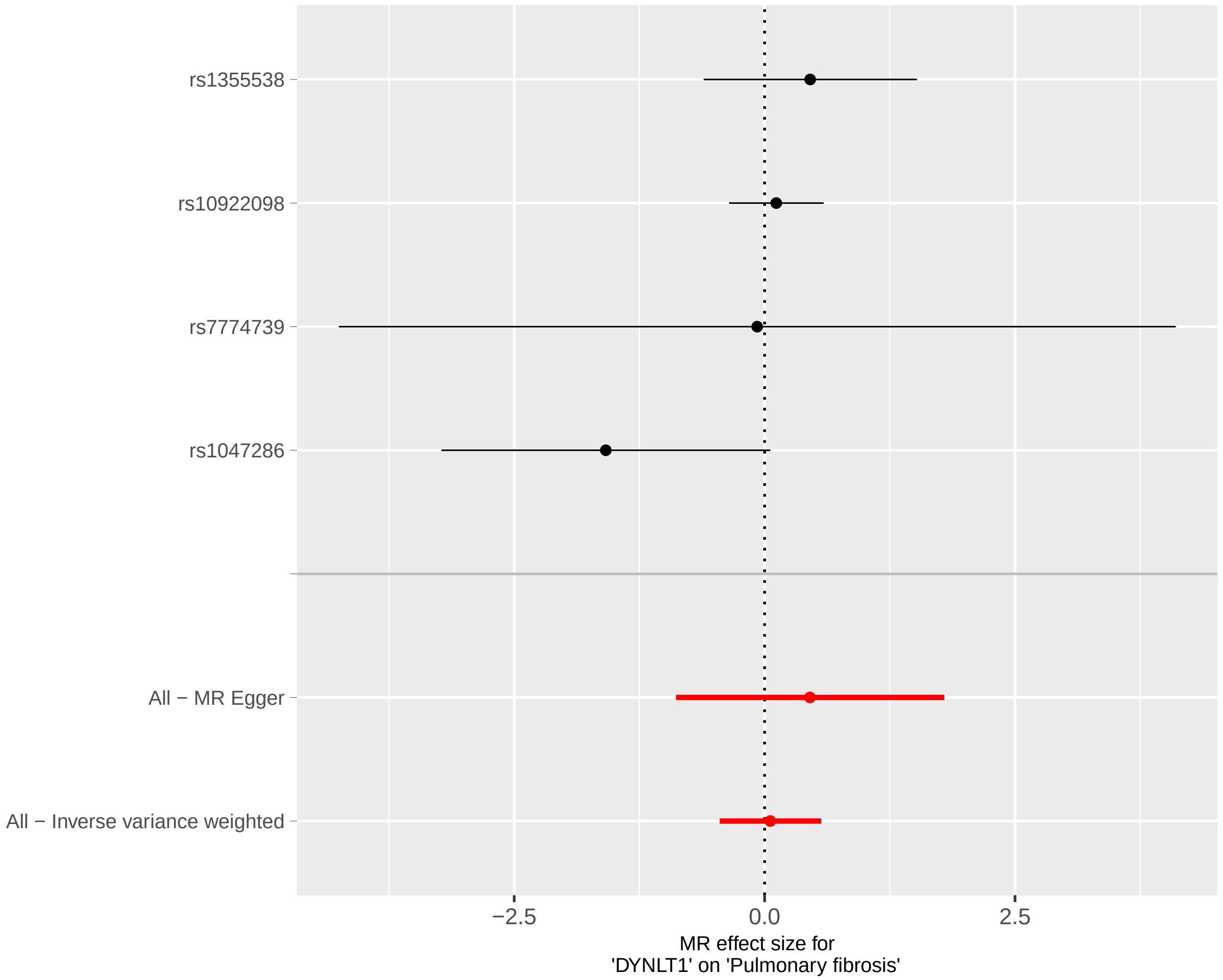

rs1355538
rs10922098
rs7774739
rs1047286
All − MR Egger
All − Inverse variance weighted
−2.5
0.0
2.5
MR effect size for
'DYNLT1' on 'Pulmonary fibrosis'

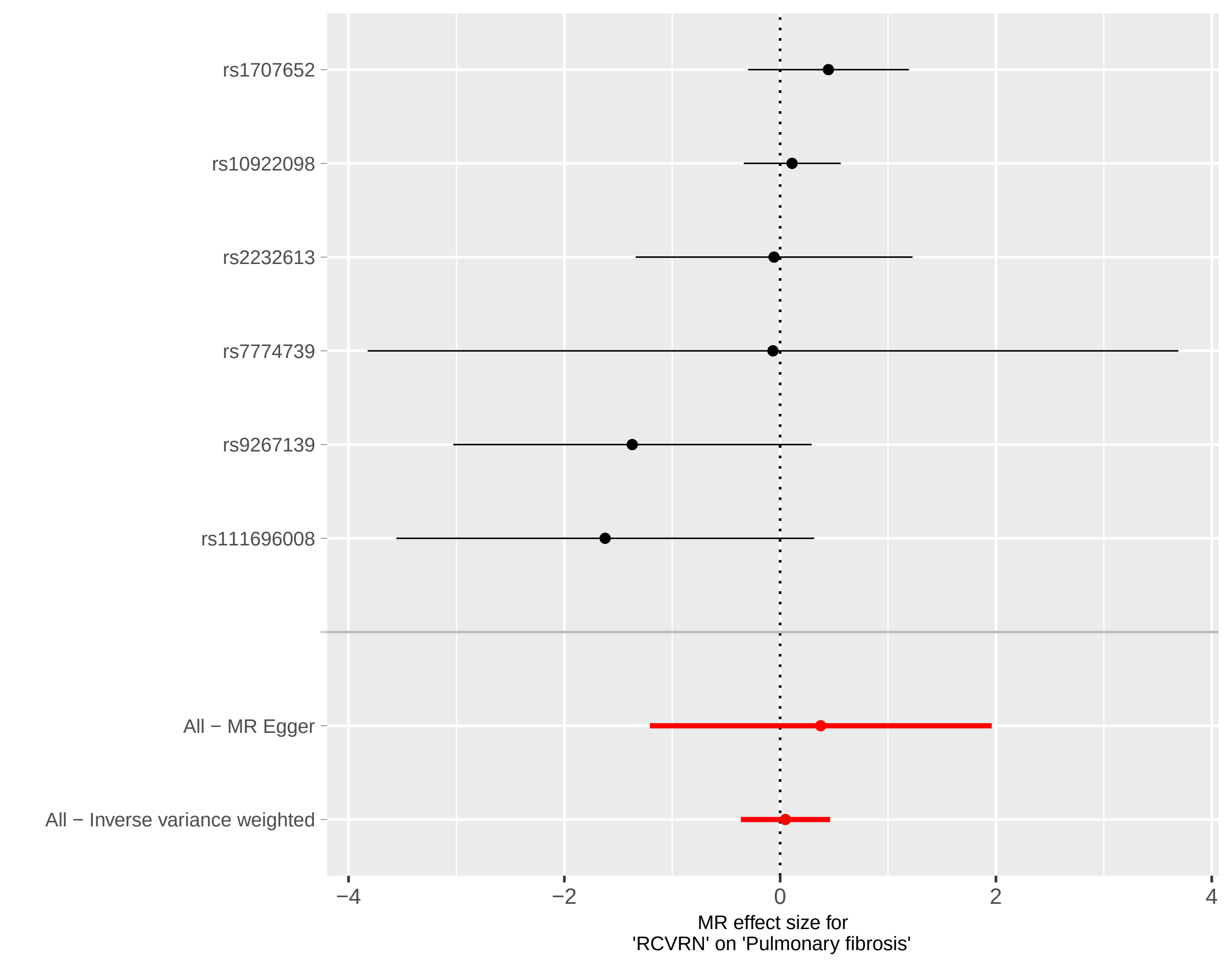
rs1707652
rs10922098
rs2232613
rs7774739
rs9267139
rs111696008
All − MR Egger
All − Inverse variance weighted
−4
−2
0
2
4
MR effect size for
'RCVRN' on 'Pulmonary fibrosis'

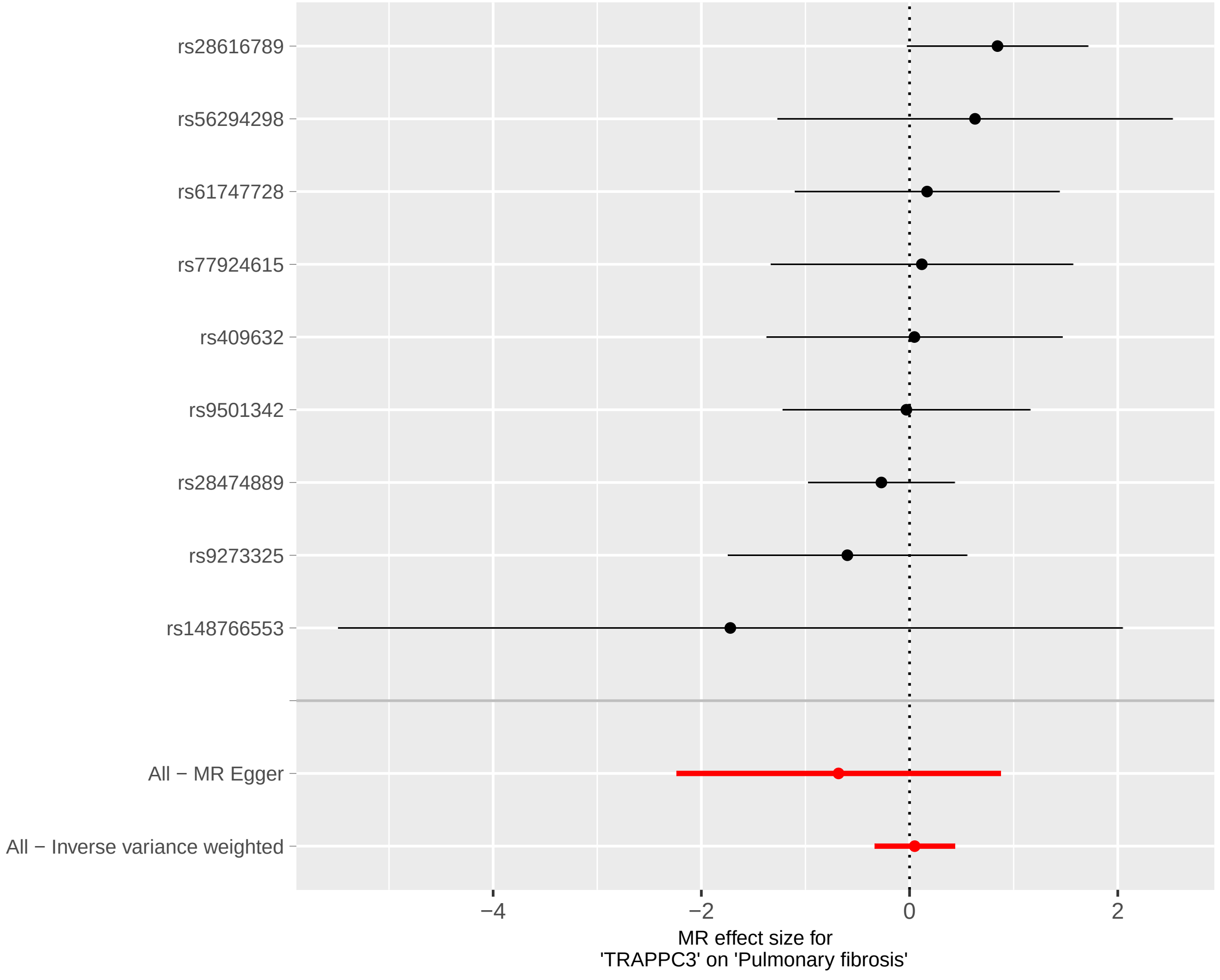

rs28616789
rs56294298
rs61747728
rs77924615
rs409632
rs9501342
rs28474889
rs9273325
rs148766553
All − MR Egger
All − Inverse variance weighted
−4
−2
0
2
MR effect size for
'TRAPPC3' on 'Pulmonary fibrosis'

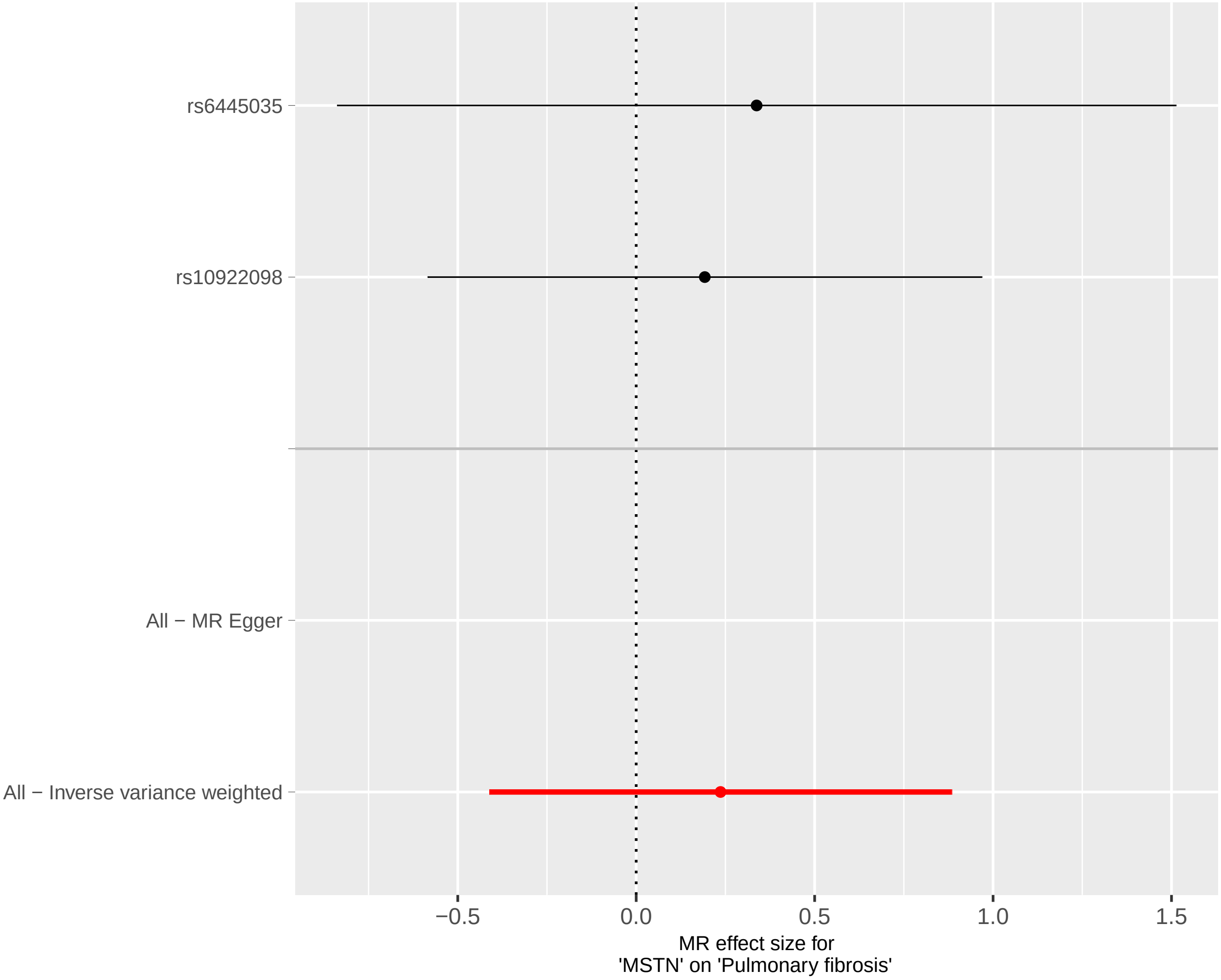
rs6445035
rs10922098
All − MR Egger
All − Inverse variance weighted
−0.5
0.0
0.5
1.0
1.5
MR effect size for
'MSTN' on 'Pulmonary fibrosis'

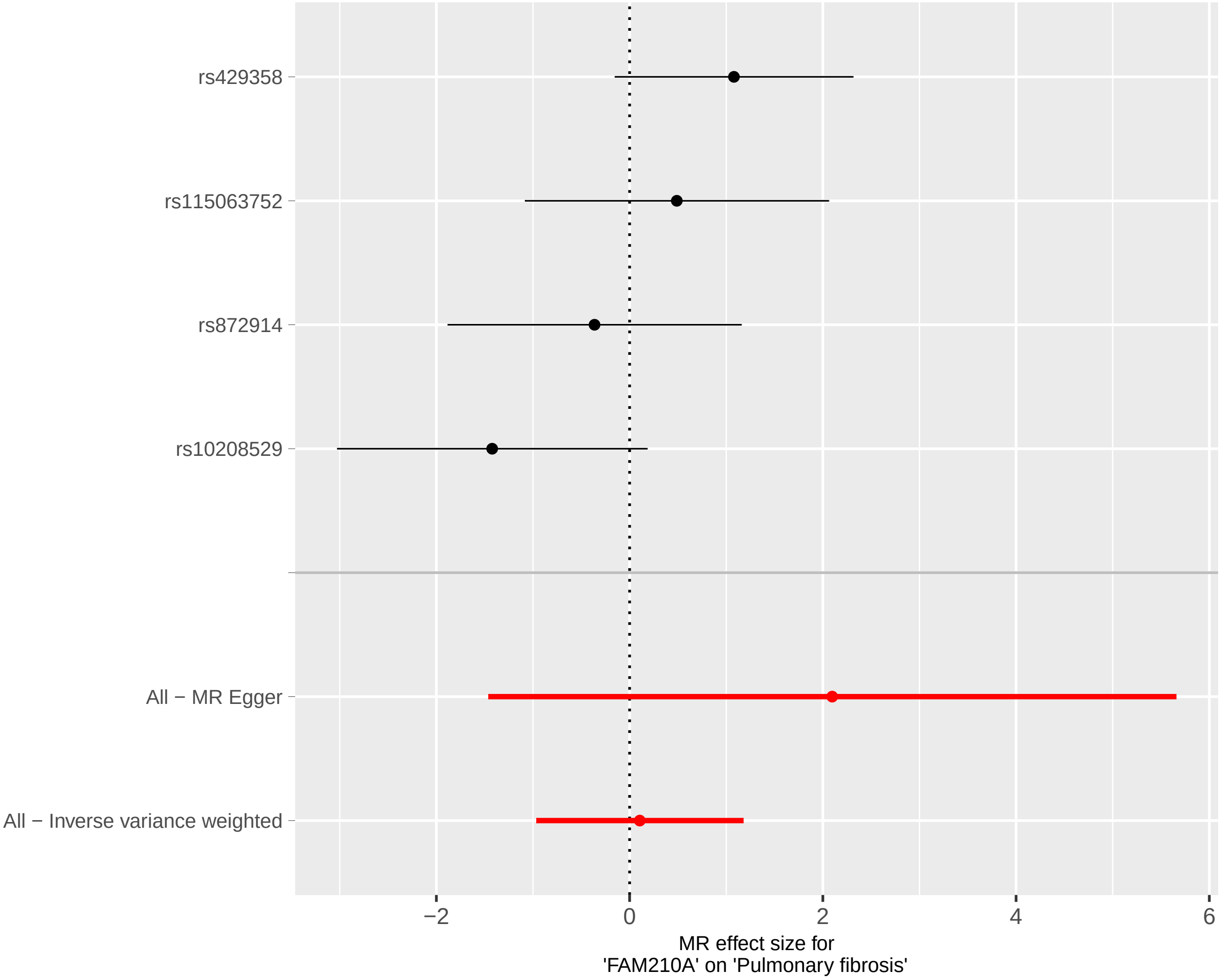
rs429358
rs115063752
rs872914
rs10208529
All − MR Egger
All − Inverse variance weighted
−2
0
2
4
6
MR effect size for
'FAM210A' on 'Pulmonary fibrosis'

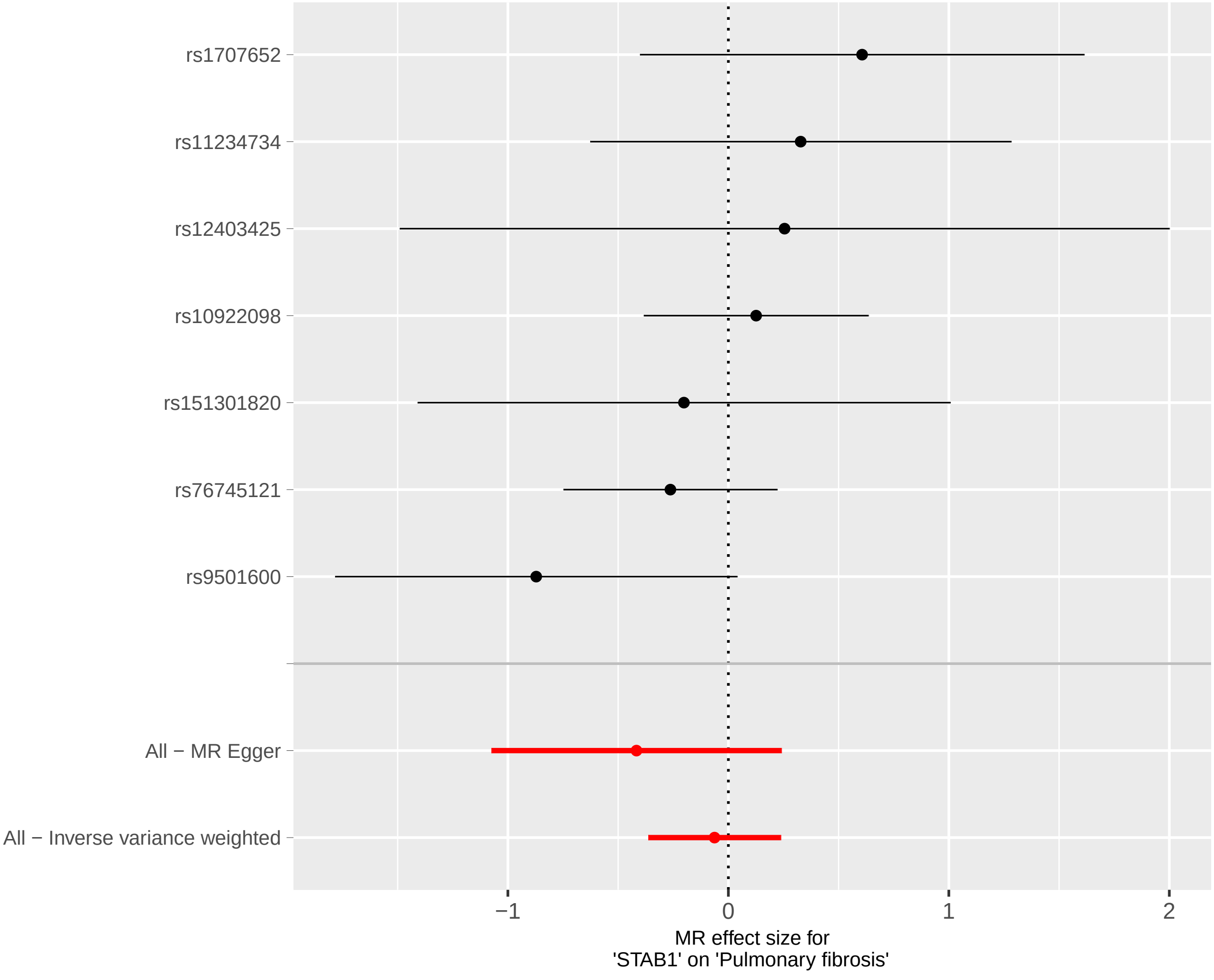

rs1707652
rs11234734
rs12403425
rs10922098
rs151301820
rs76745121
rs9501600
All − MR Egger
All − Inverse variance weighted
−1
0
1
2
MR effect size for
'STAB1' on 'Pulmonary fibrosis'

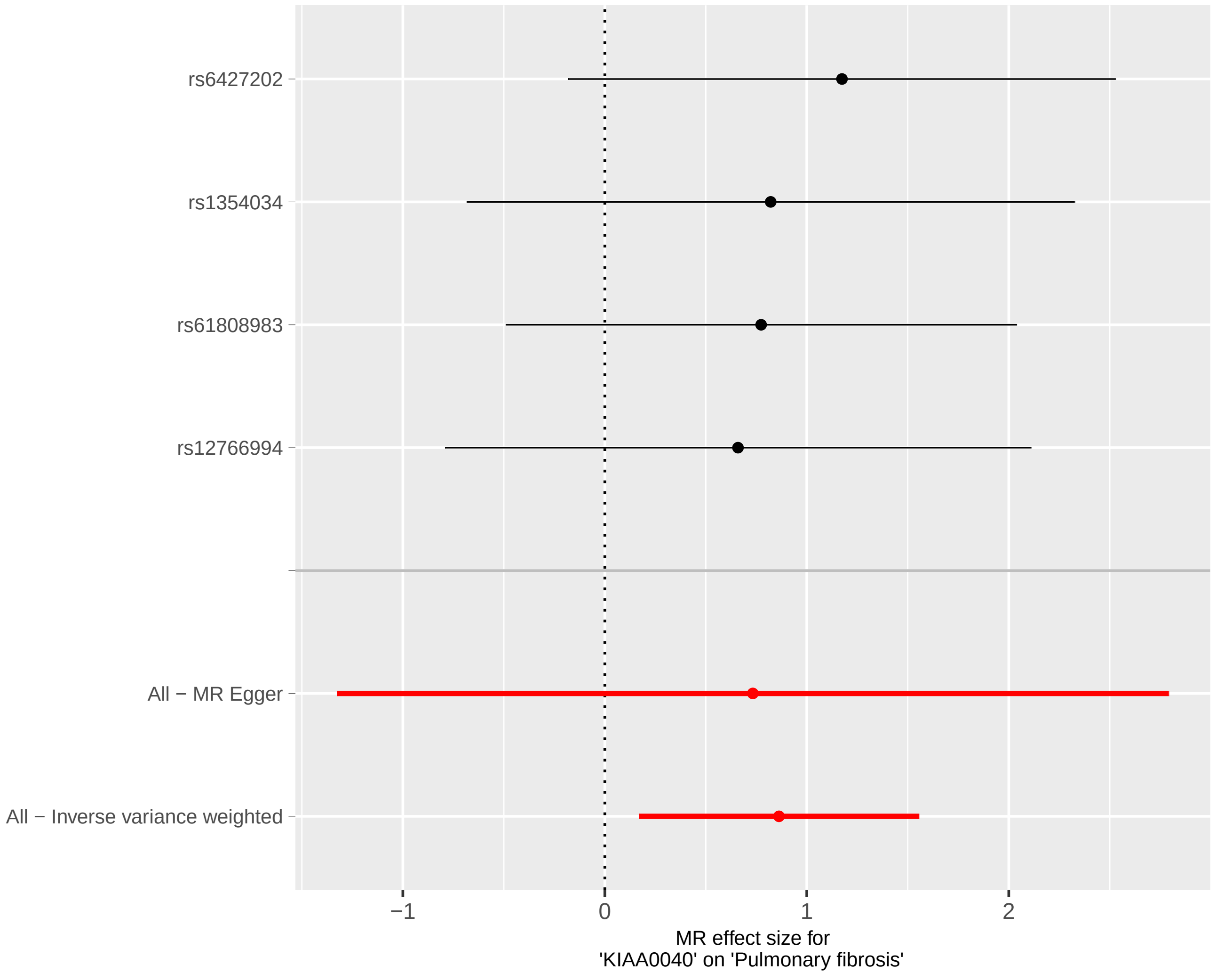

rs6427202
rs1354034
rs61808983
rs12766994
All − MR Egger
All − Inverse variance weighted
−1
0
1
2
MR effect size for
'KIAA0040' on 'Pulmonary fibrosis'

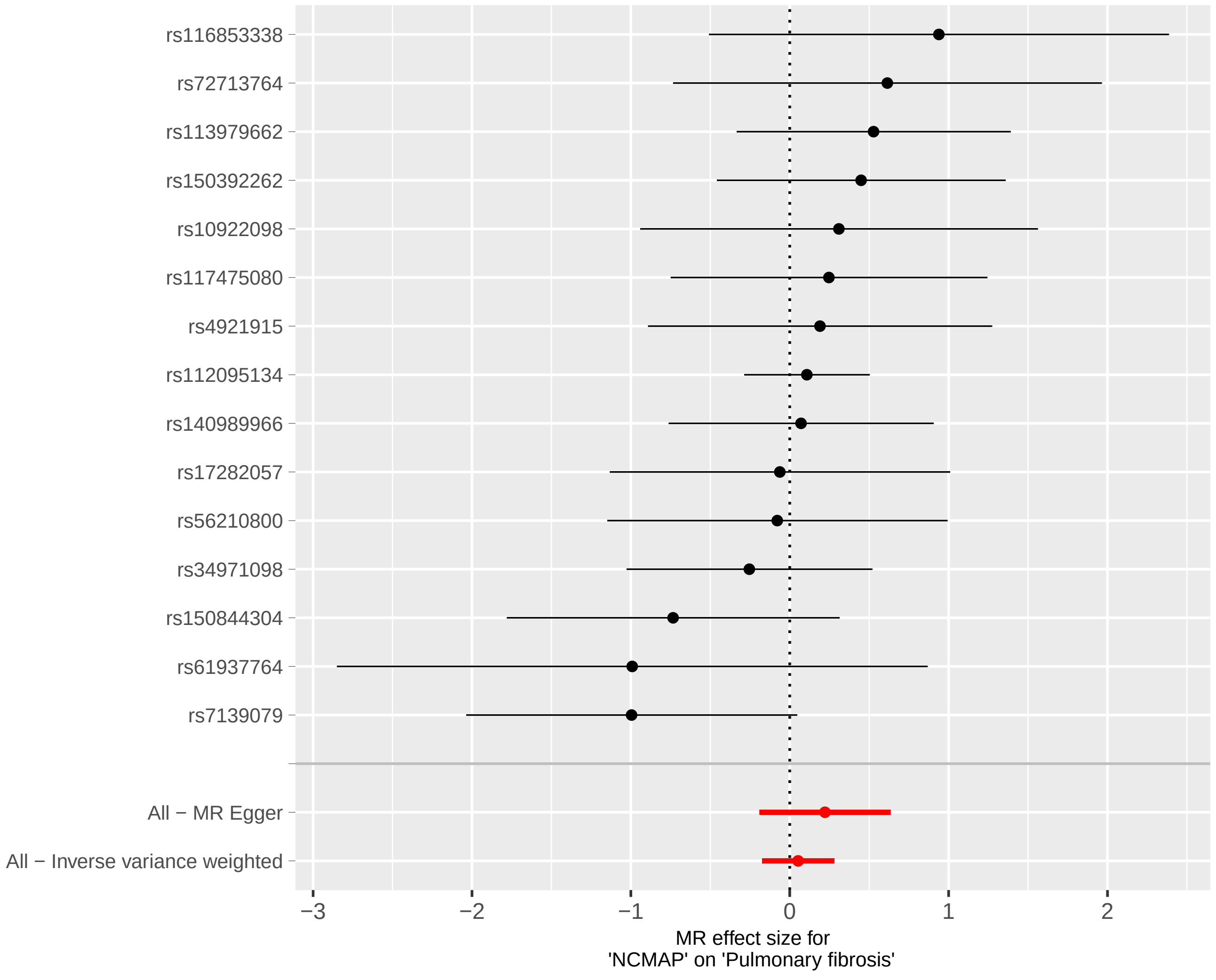
rs116853338
rs72713764
rs113979662
rs150392262
rs10922098
rs117475080
rs4921915
rs112095134
rs140989966
rs17282057
rs56210800
rs34971098
rs150844304
rs61937764
rs7139079
All − MR Egger
All − Inverse variance weighted
−3
−2
−1
0
1
2
MR effect size for
'NCMAP' on 'Pulmonary fibrosis'

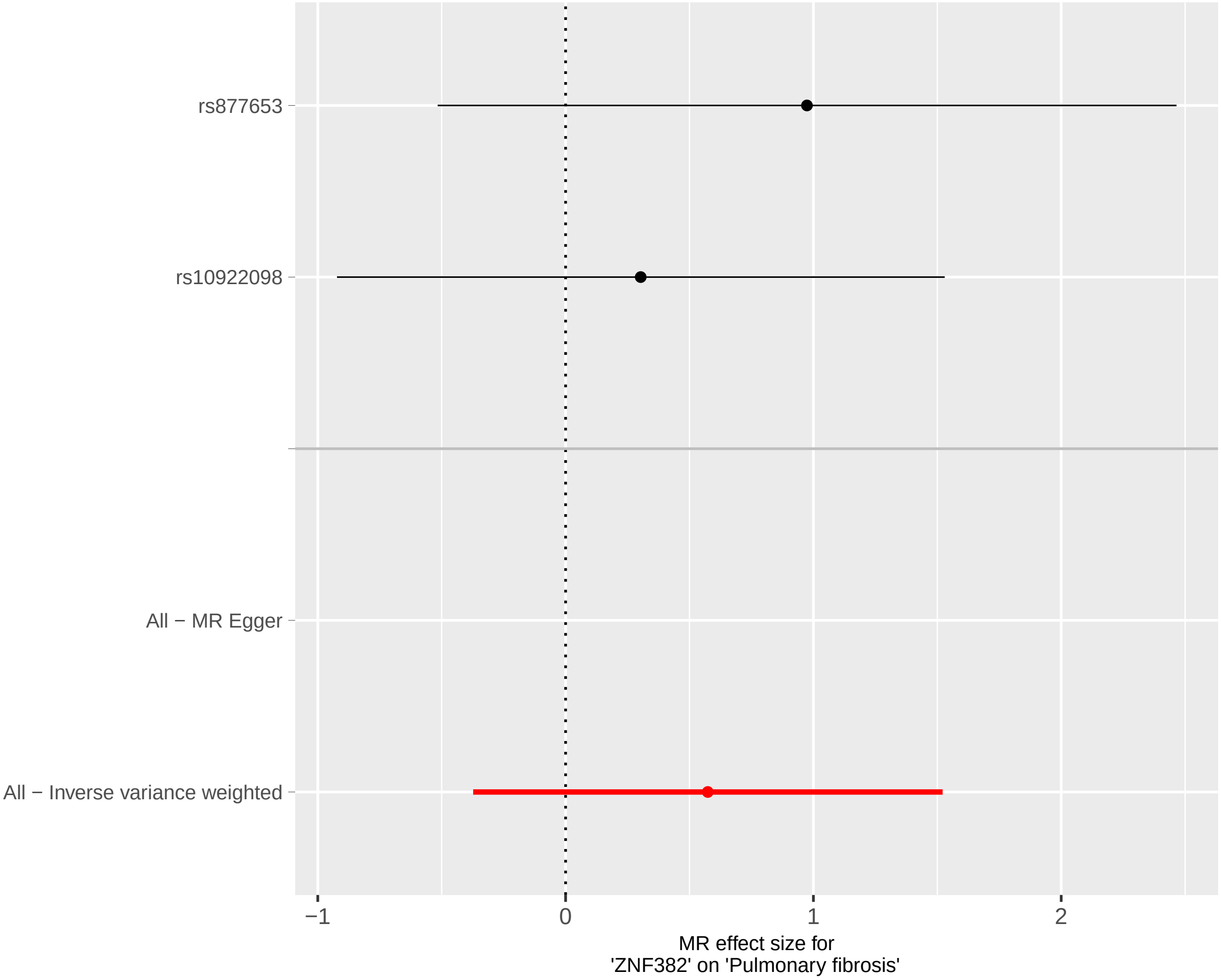
rs877653
rs10922098
All − MR Egger
All − Inverse variance weighted
−1
0
1
2
MR effect size for
'ZNF382' on 'Pulmonary fibrosis'

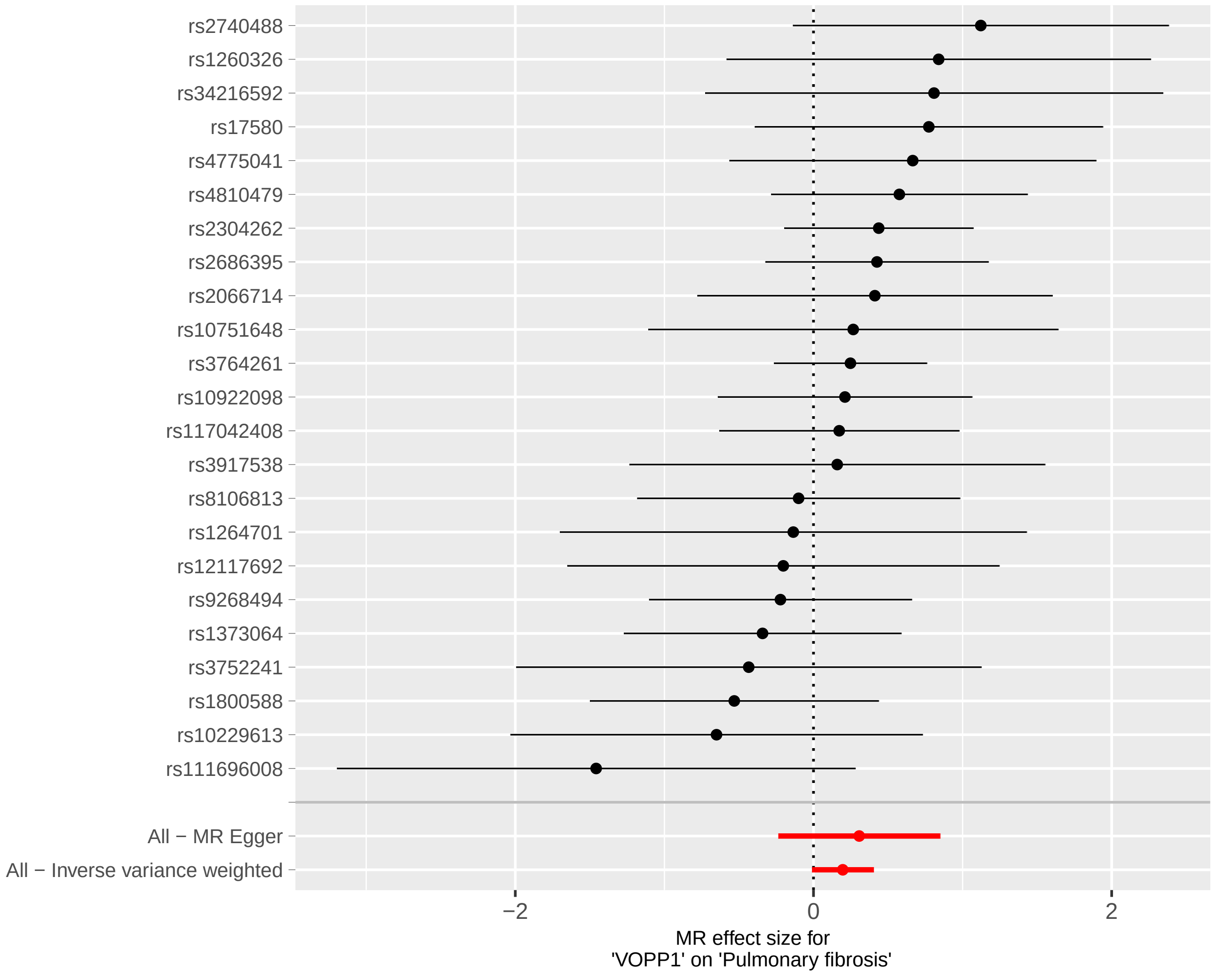

rs2740488
rs1260326
rs34216592
rs17580
rs4775041
rs4810479
rs2304262
rs2686395
rs2066714
rs10751648
rs3764261
rs10922098
rs117042408
rs3917538
rs8106813
rs1264701
rs12117692
rs9268494
rs1373064
rs3752241
rs1800588
rs10229613
rs111696008
All − MR Egger
All − Inverse variance weighted
−2
0
2
MR effect size for
'VOPP1' on 'Pulmonary fibrosis'

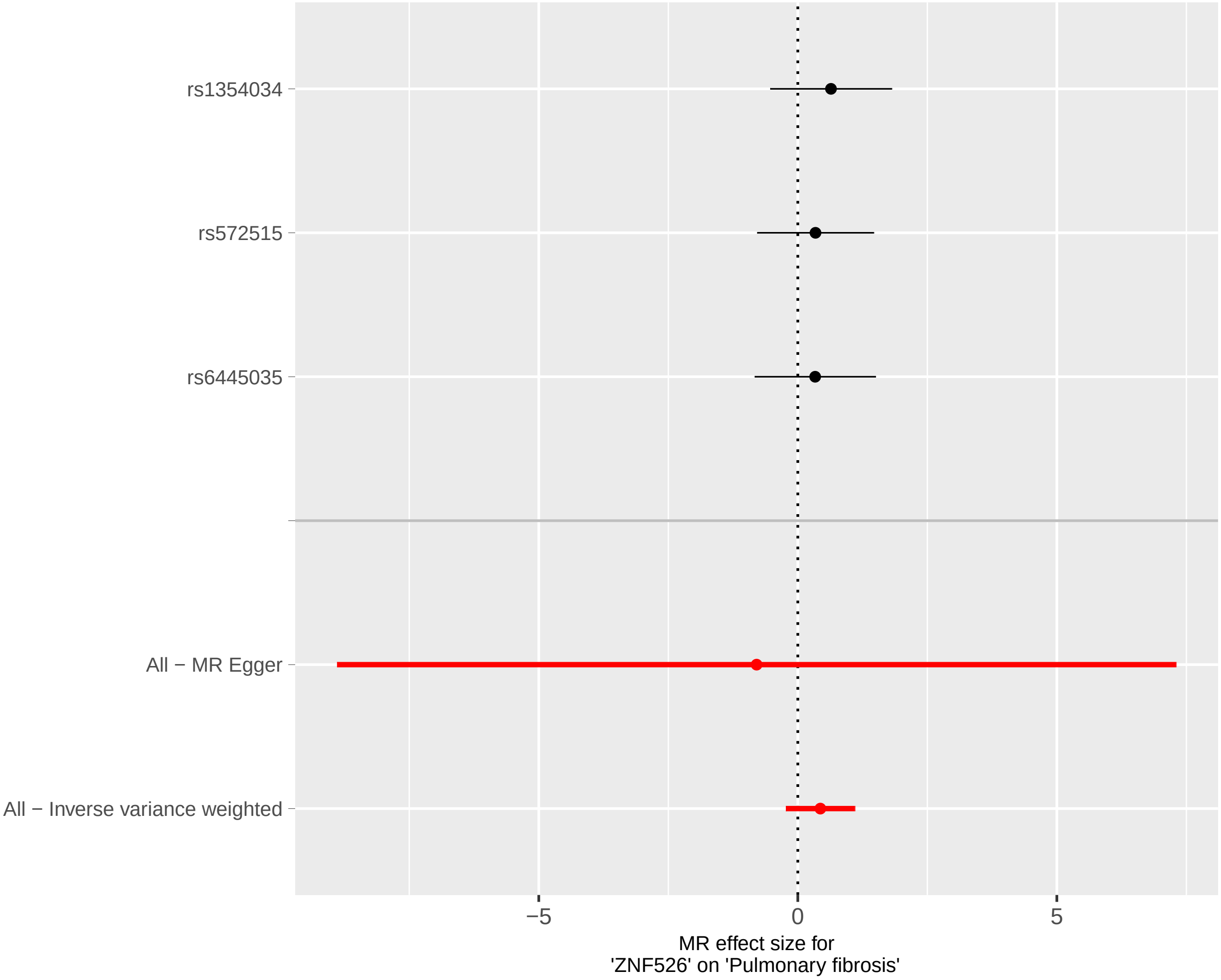
rs1354034
rs572515
rs6445035
All − MR Egger
All − Inverse variance weighted
−5
0
5
MR effect size for
'ZNF526' on 'Pulmonary fibrosis'

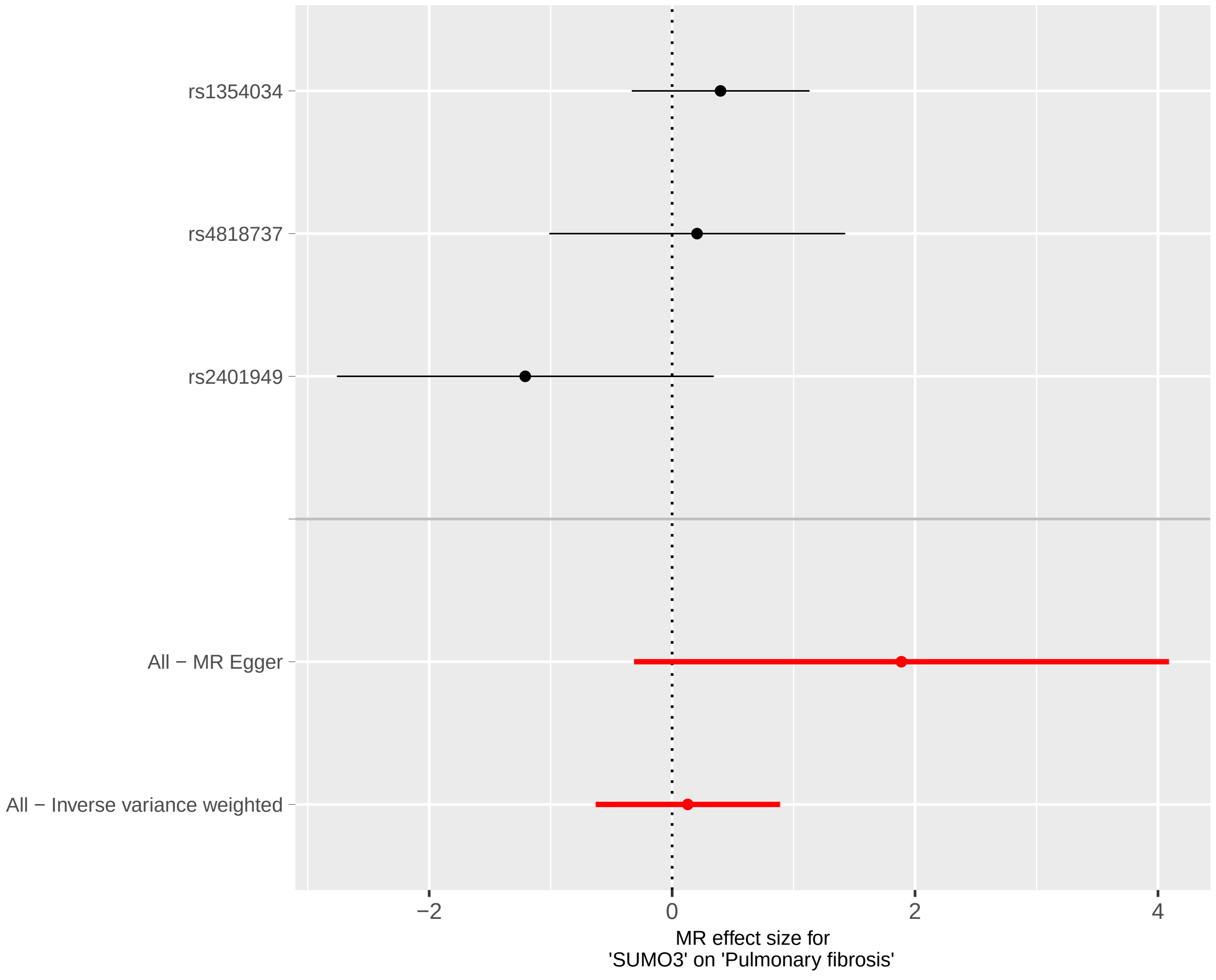
rs1354034
rs4818737
rs2401949
All − MR Egger
All − Inverse variance weighted
−2
0
2
4
MR effect size for
'SUMO3' on 'Pulmonary fibrosis'

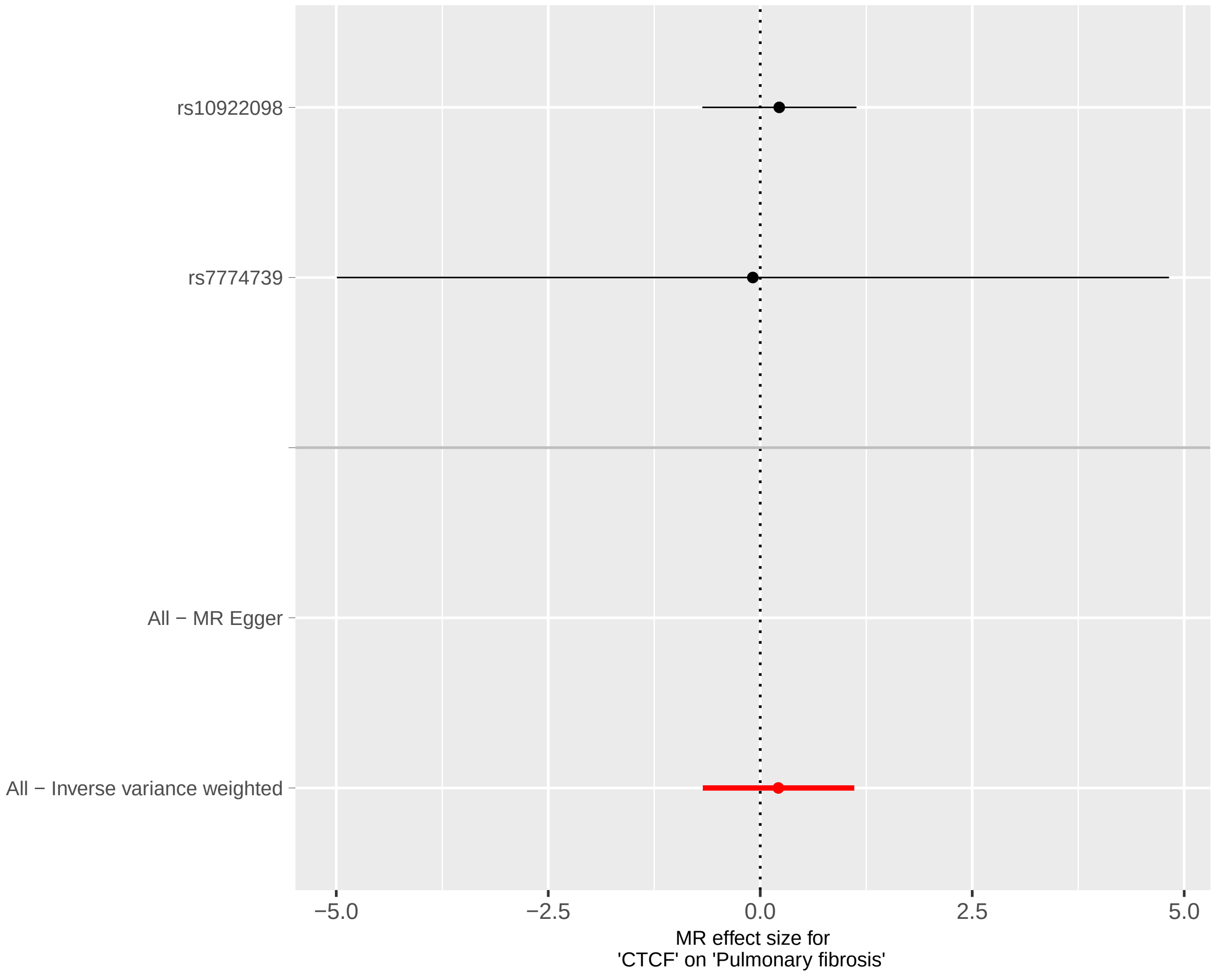
rs10922098
rs7774739
All − MR Egger
All − Inverse variance weighted
−5.0
−2.5
0.0
2.5
5.0
MR effect size for
'CTCF' on 'Pulmonary fibrosis'

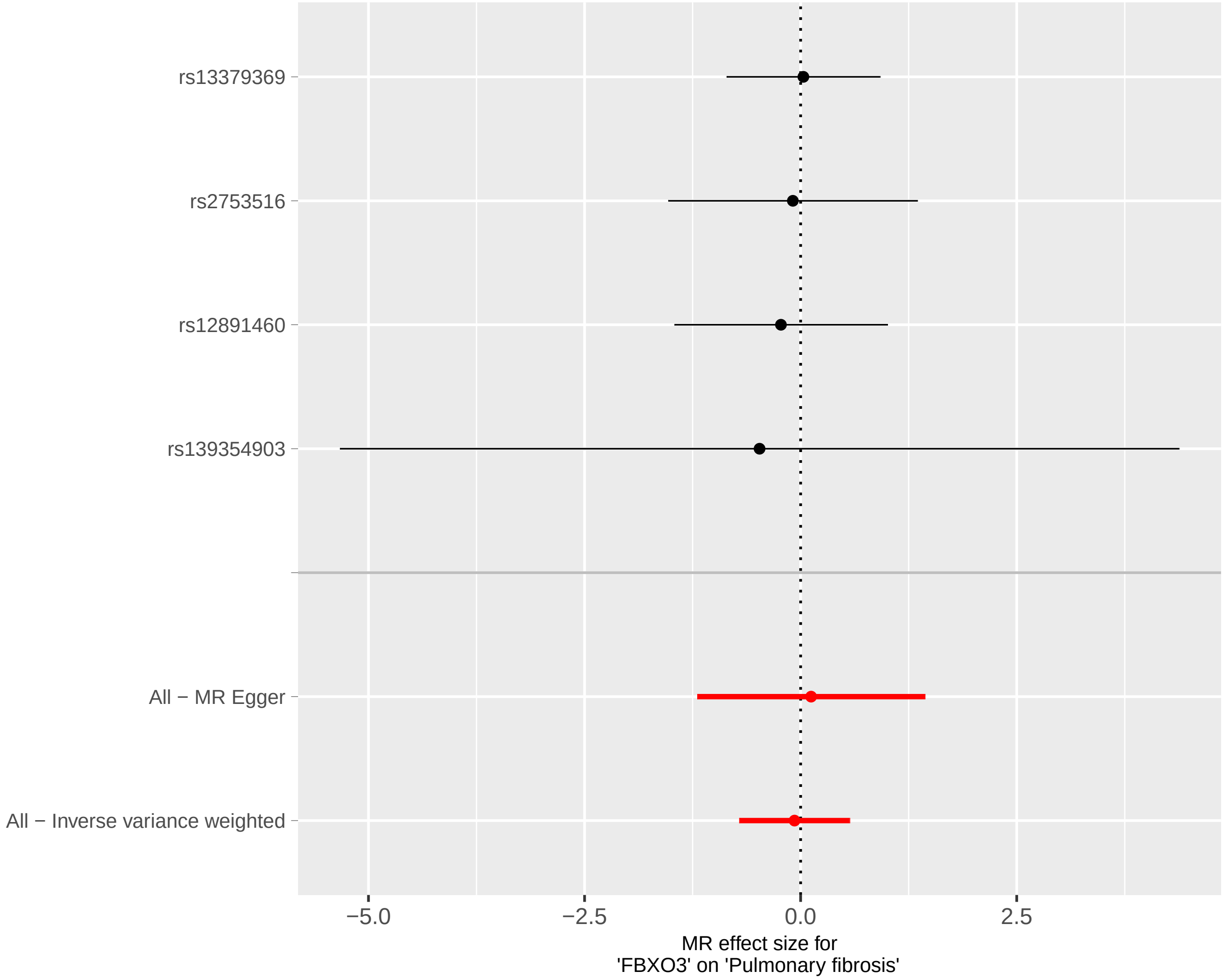
rs13379369
rs2753516
rs12891460
rs139354903
All − MR Egger
All − Inverse variance weighted
−5.0
−2.5
0.0
2.5
MR effect size for
'FBXO3' on 'Pulmonary fibrosis'

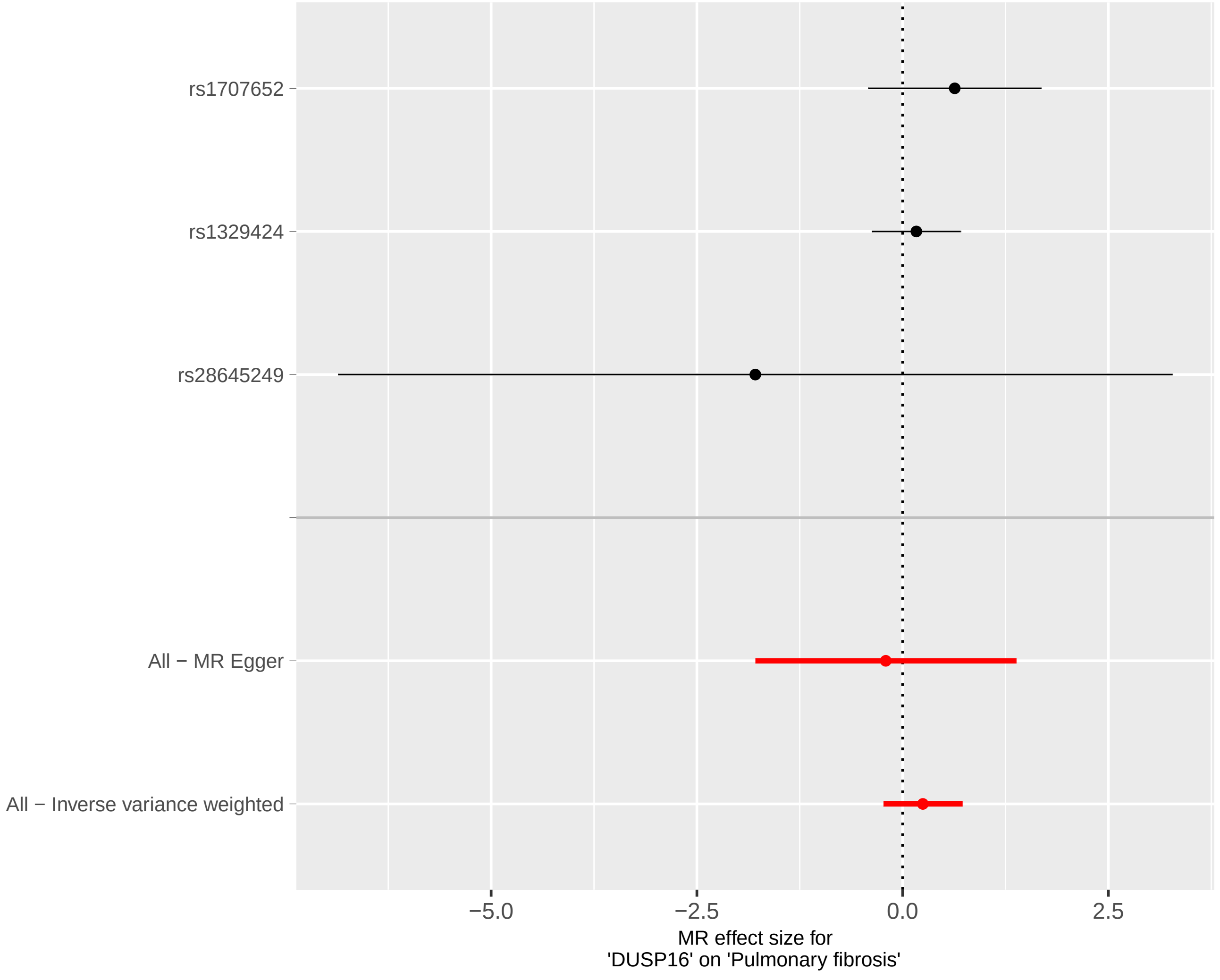

rs1707652
rs1329424
rs28645249
All − MR Egger
All − Inverse variance weighted
−5.0
−2.5
0.0
2.5
MR effect size for
'DUSP16' on 'Pulmonary fibrosis'

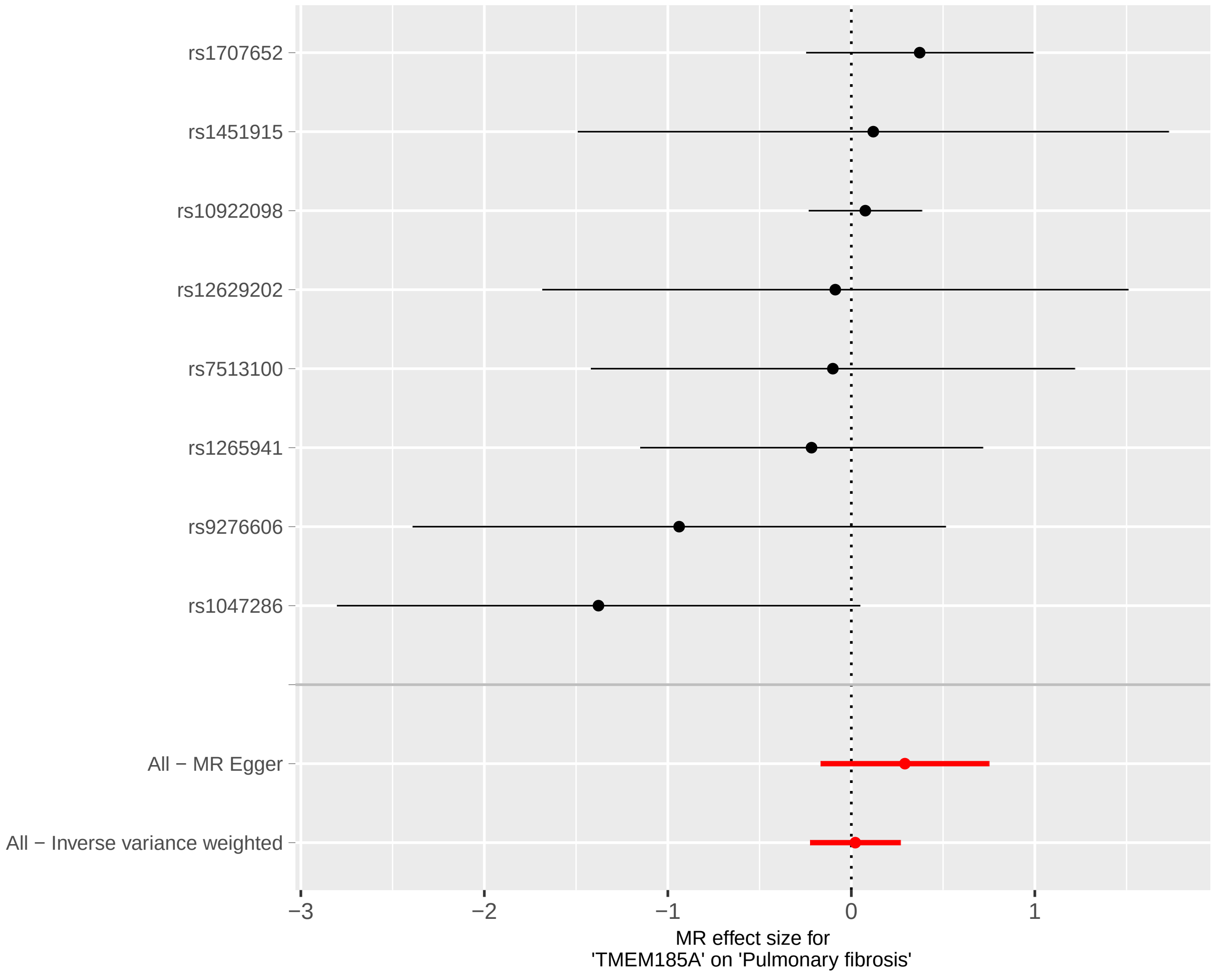
rs1707652
rs1451915
rs10922098
rs12629202
rs7513100
rs1265941
rs9276606
rs1047286
All − MR Egger
All − Inverse variance weighted
−3
−2
−1
0
1
MR effect size for
'TMEM185A' on 'Pulmonary fibrosis'

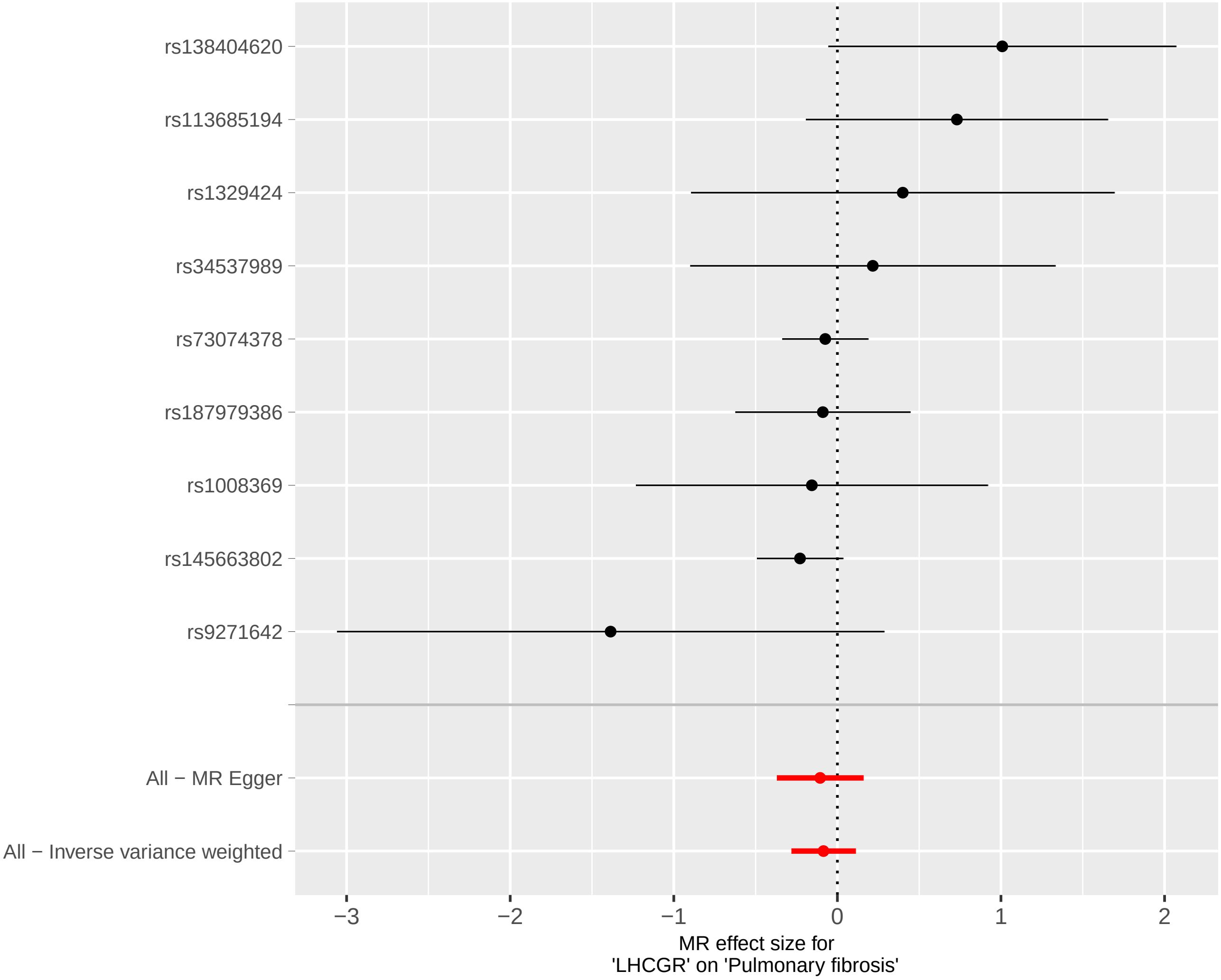
rs138404620
rs113685194
rs1329424
rs34537989
rs73074378
rs187979386
rs1008369
rs145663802
rs9271642
All − MR Egger
All − Inverse variance weighted
−3
−2
−1
0
1
2
MR effect size for
'LHCGR' on 'Pulmonary fibrosis'

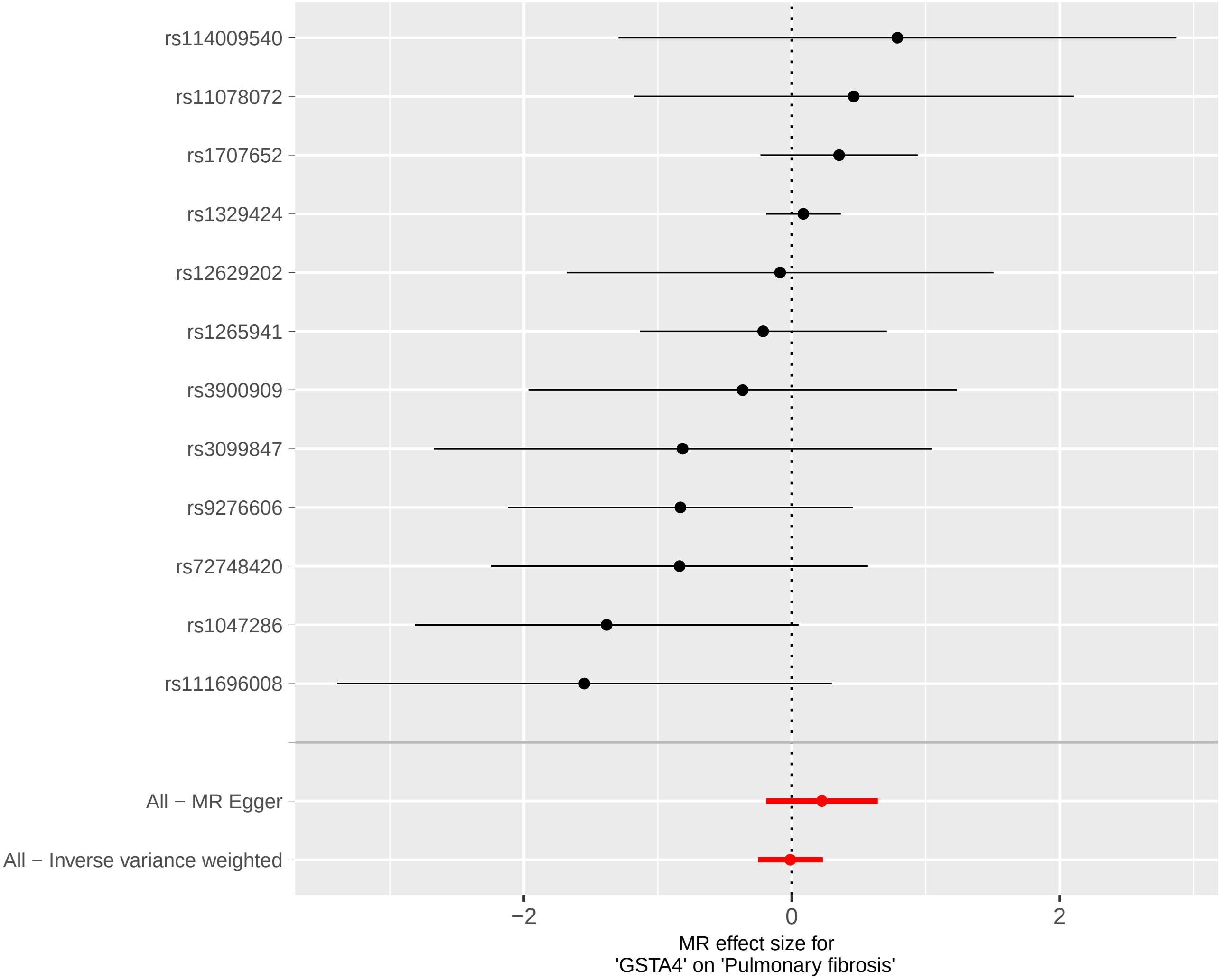
rs114009540
rs11078072
rs1707652
rs1329424
rs12629202
rs1265941
rs3900909
rs3099847
rs9276606
rs72748420
rs1047286
rs111696008
All − MR Egger
All − Inverse variance weighted
−2
0
2
MR effect size for
'GSTA4' on 'Pulmonary fibrosis'

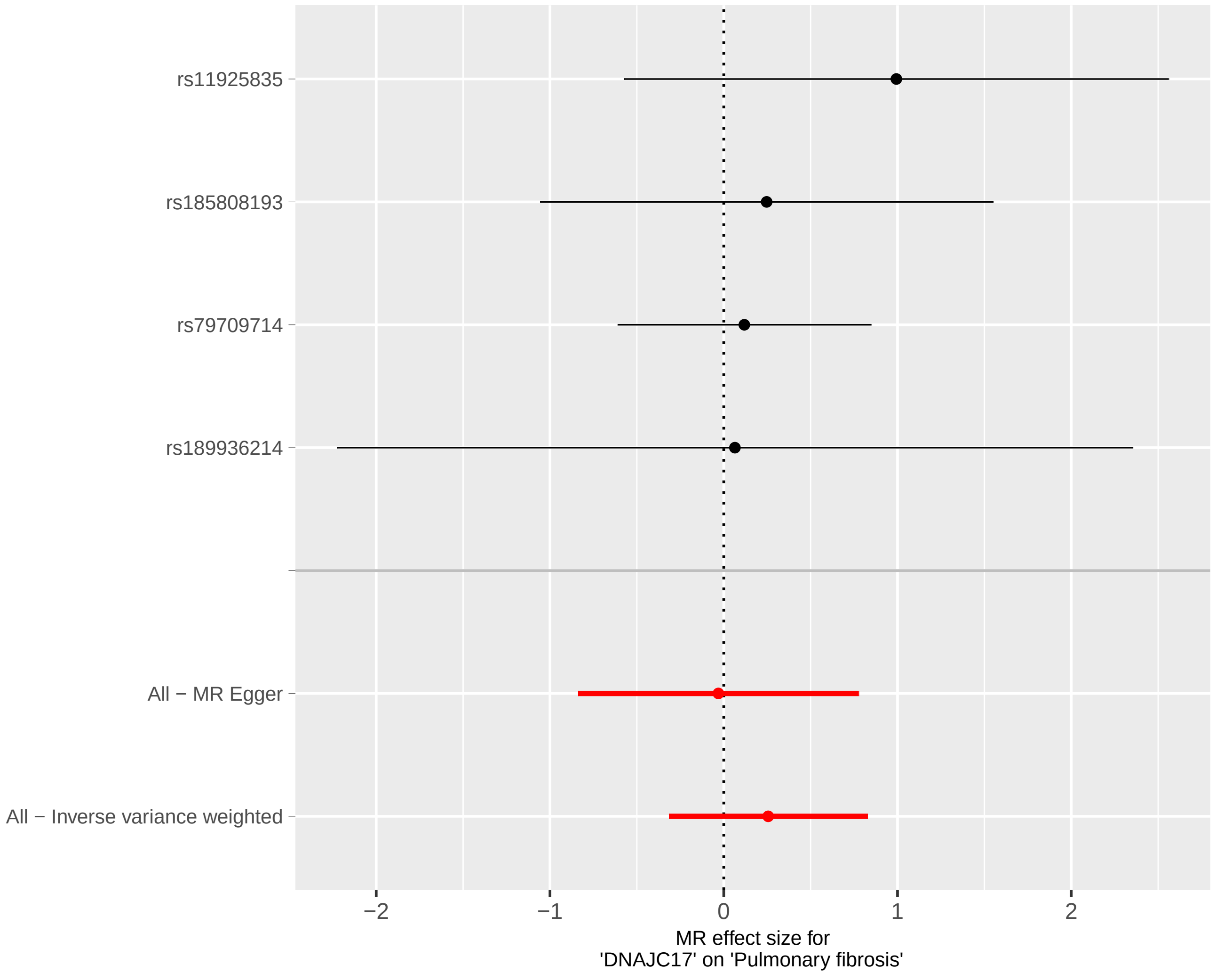

rs11925835
rs185808193
rs79709714
rs189936214
All − MR Egger
All − Inverse variance weighted
−2
−1
0
1
2
MR effect size for
'DNAJC17' on 'Pulmonary fibrosis'

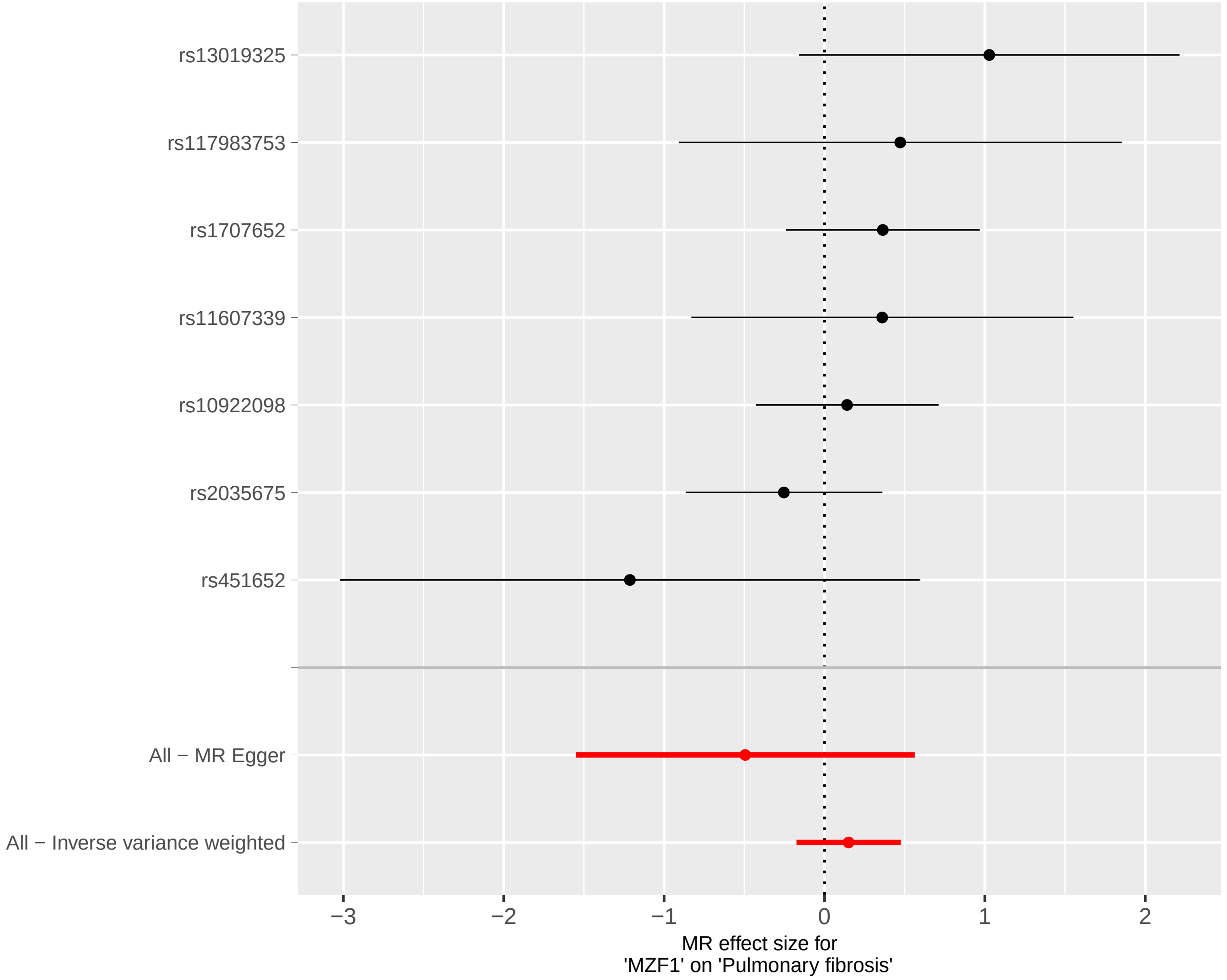

rs13019325
rs117983753
rs1707652
rs11607339
rs10922098
rs2035675
rs451652
All − MR Egger
All − Inverse variance weighted
−3
−2
−1
0
1
2
MR effect size for
'MZF1' on 'Pulmonary fibrosis'

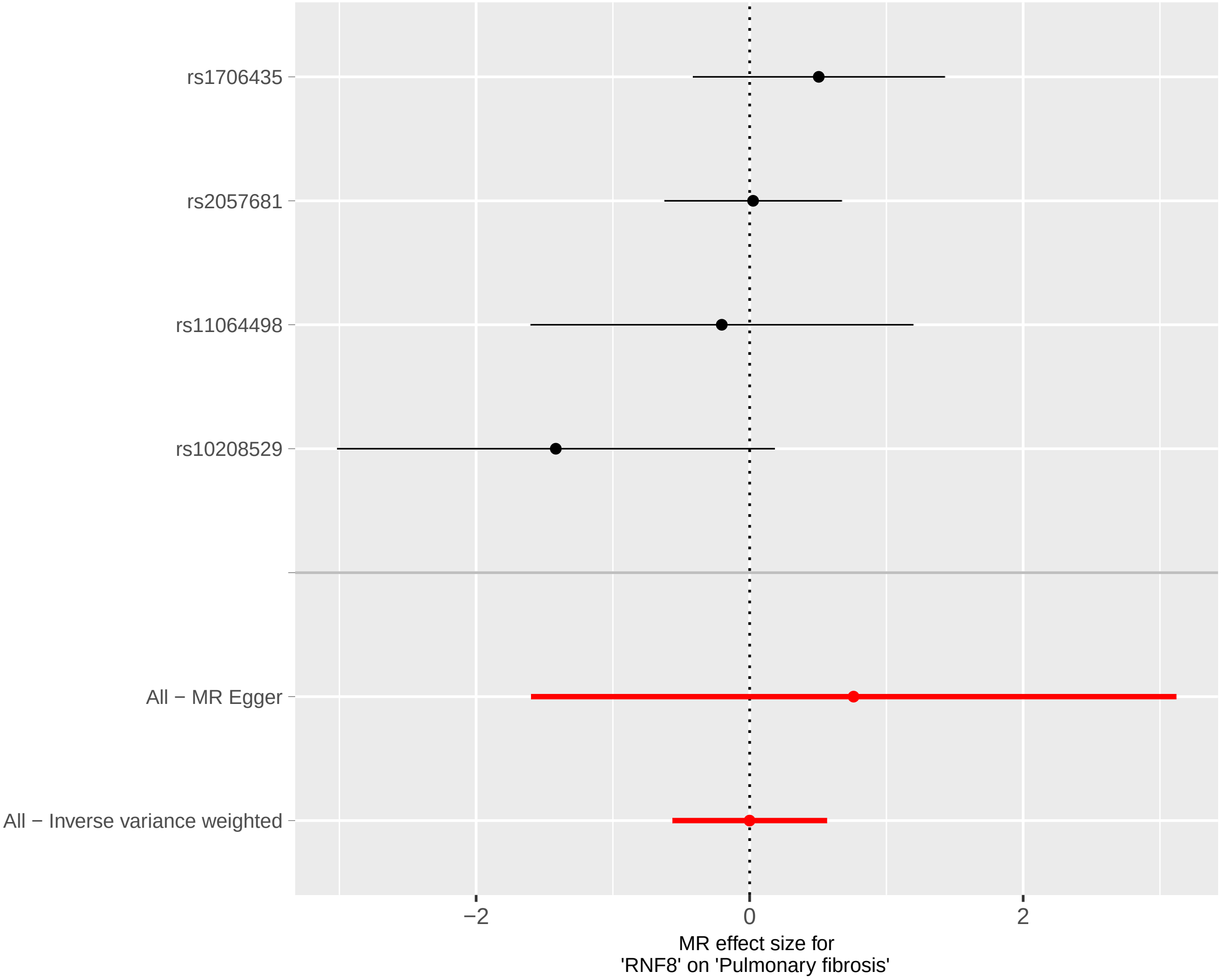
rs1706435
rs2057681
rs11064498
rs10208529
All − MR Egger
All − Inverse variance weighted
−2
0
2
MR effect size for
'RNF8' on 'Pulmonary fibrosis'

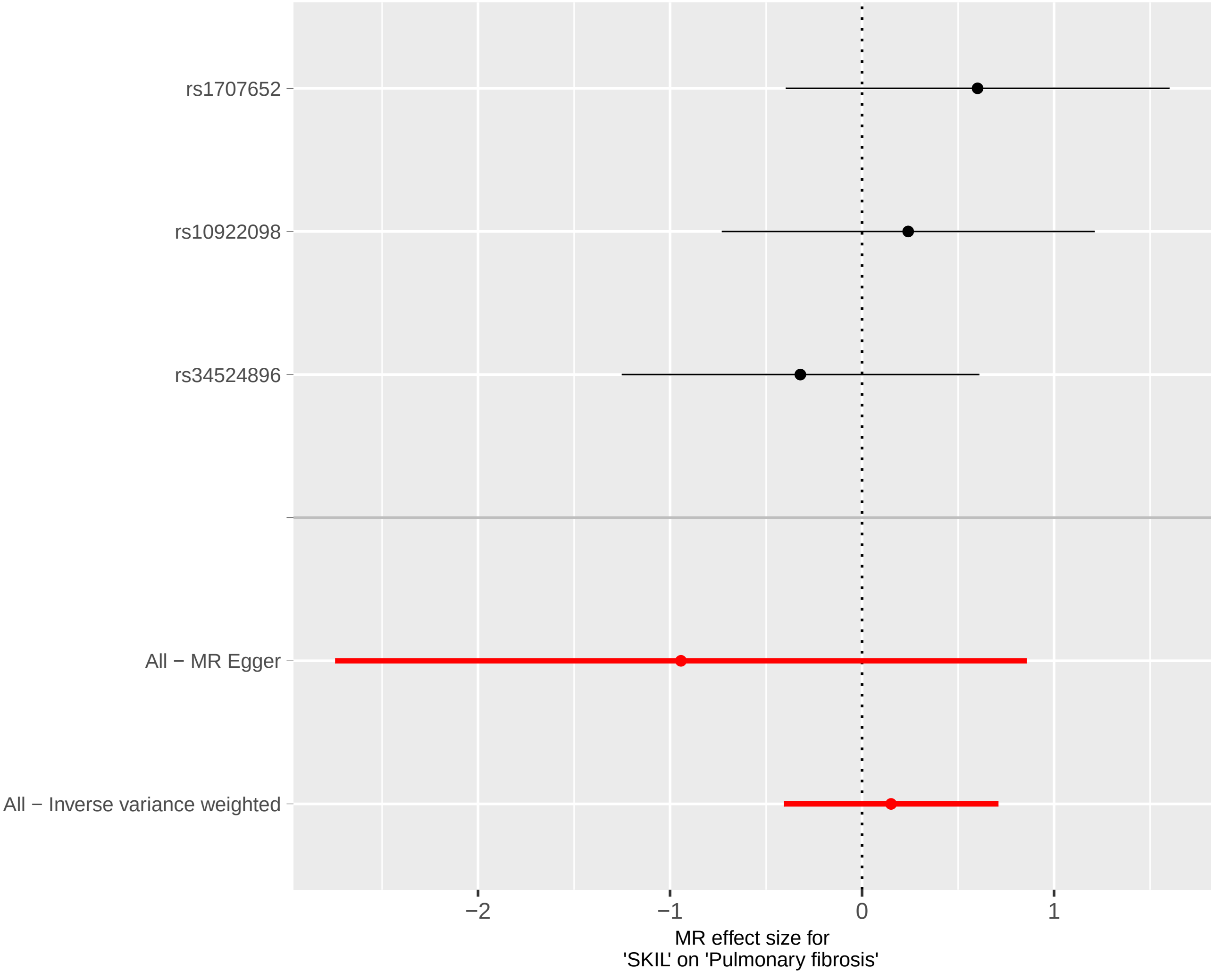

rs1707652
rs10922098
rs34524896
All − MR Egger
All − Inverse variance weighted
−2
−1
0
1
MR effect size for
'SKIL' on 'Pulmonary fibrosis'

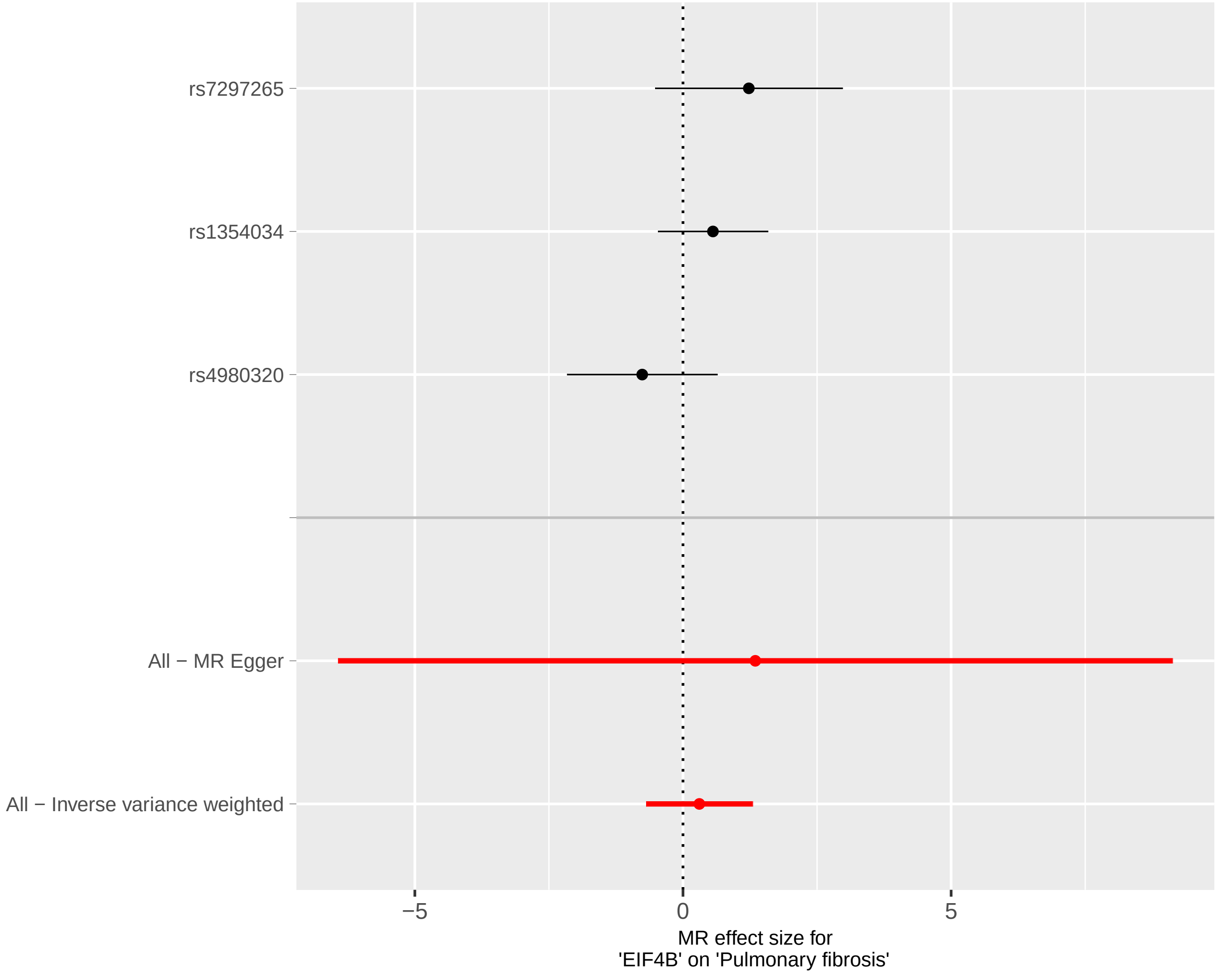
rs7297265
rs1354034
rs4980320
All − MR Egger
All − Inverse variance weighted
−5
0
5
MR effect size for
'EIF4B' on 'Pulmonary fibrosis'

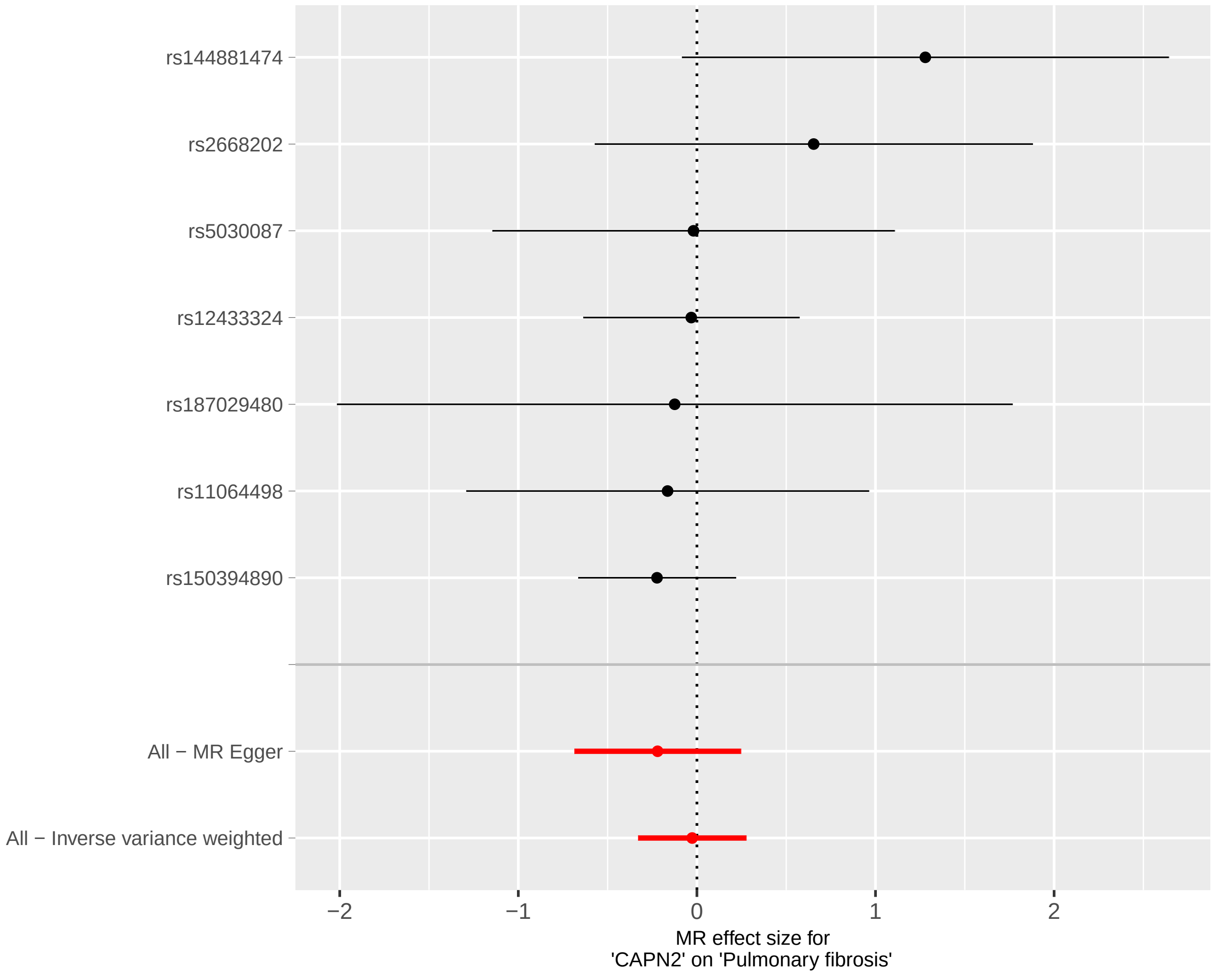

rs144881474
rs2668202
rs5030087
rs12433324
rs187029480
rs11064498
rs150394890
All − MR Egger
All − Inverse variance weighted
−2
−1
0
1
2
MR effect size for
'CAPN2' on 'Pulmonary fibrosis'

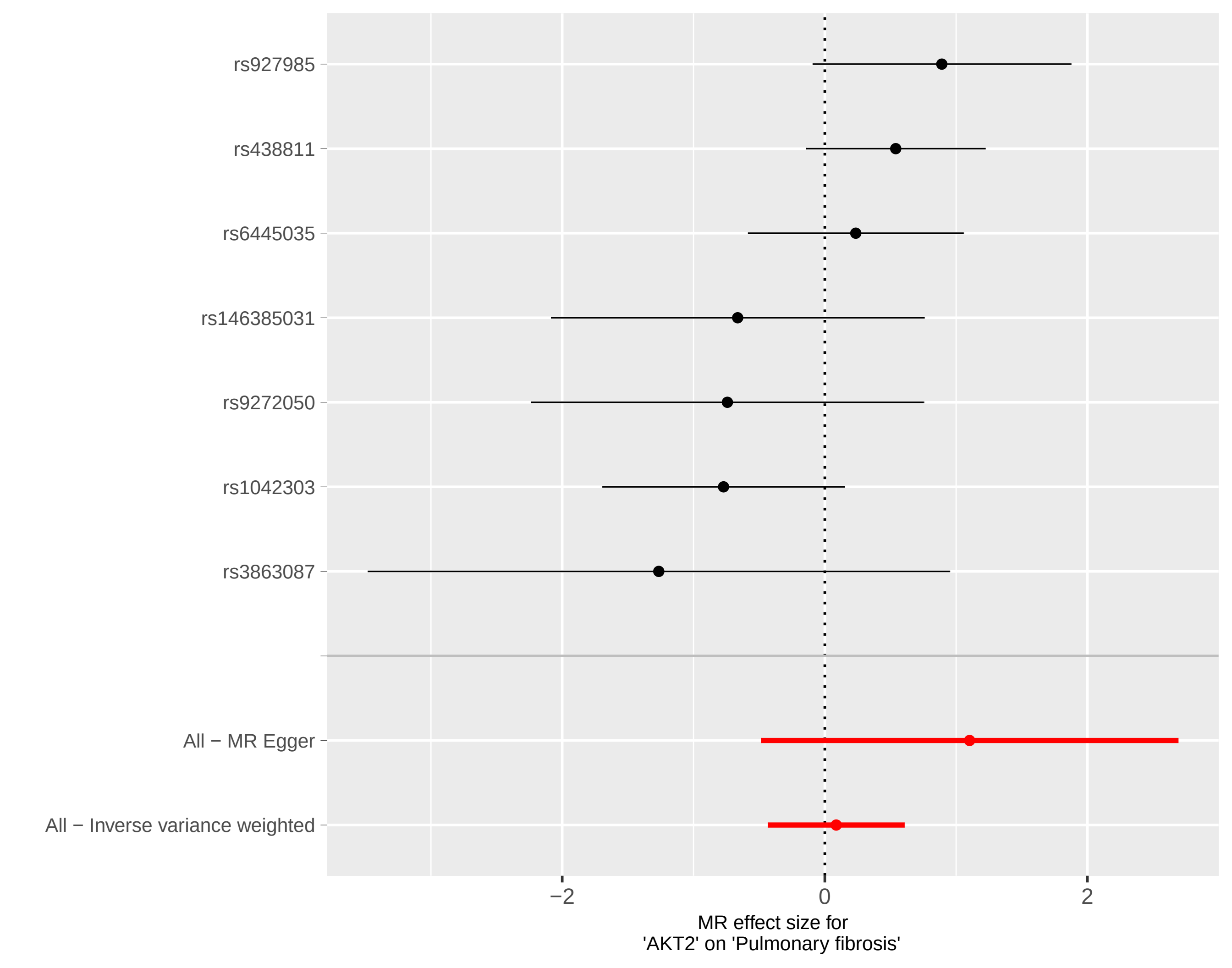
rs927985
rs438811
rs6445035
rs146385031
rs9272050
rs1042303
rs3863087
All − MR Egger
All − Inverse variance weighted
−2
0
2
MR effect size for
'AKT2' on 'Pulmonary fibrosis'

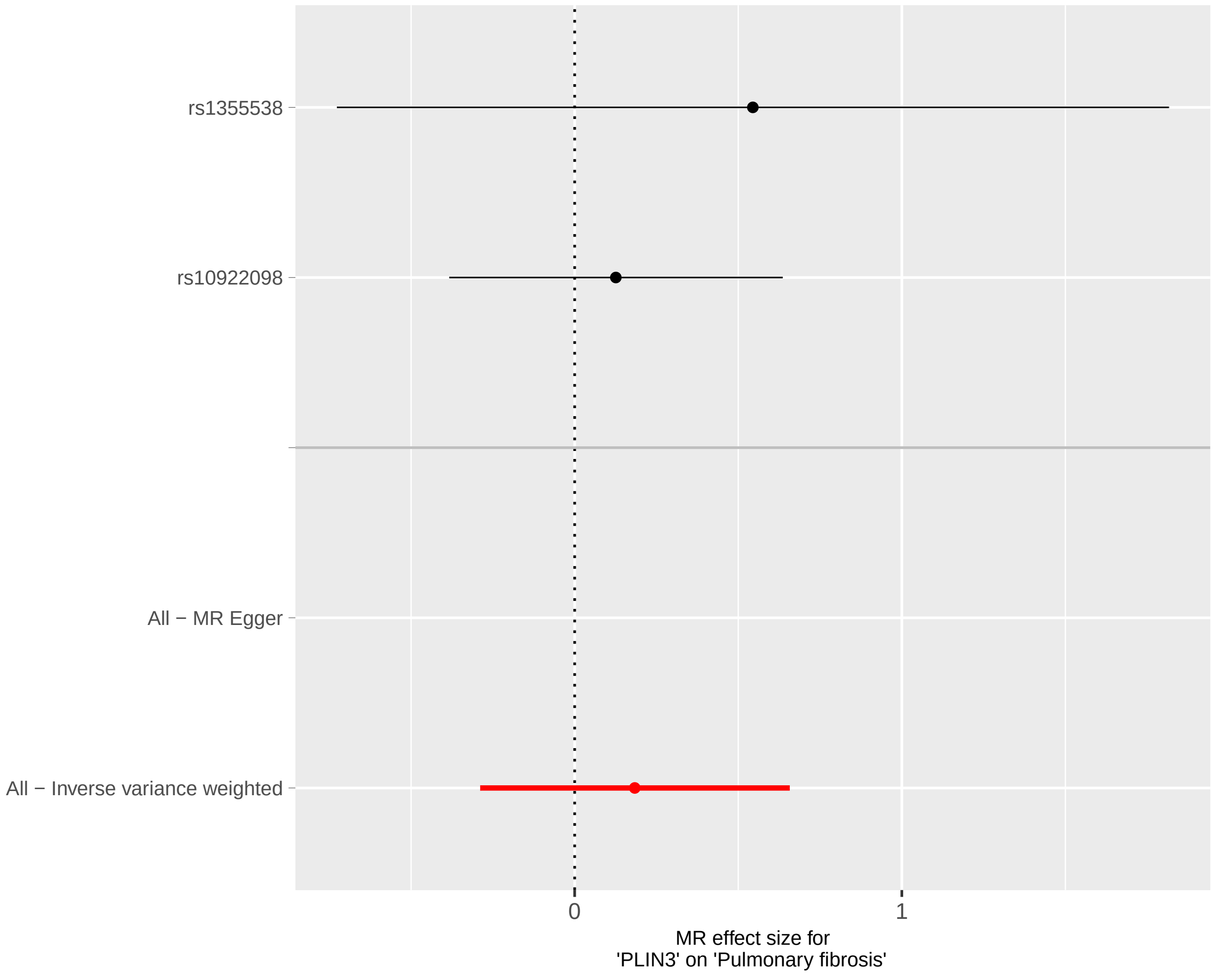

rs1355538
rs10922098
All − MR Egger
All − Inverse variance weighted
0
1
MR effect size for
'PLIN3' on 'Pulmonary fibrosis'

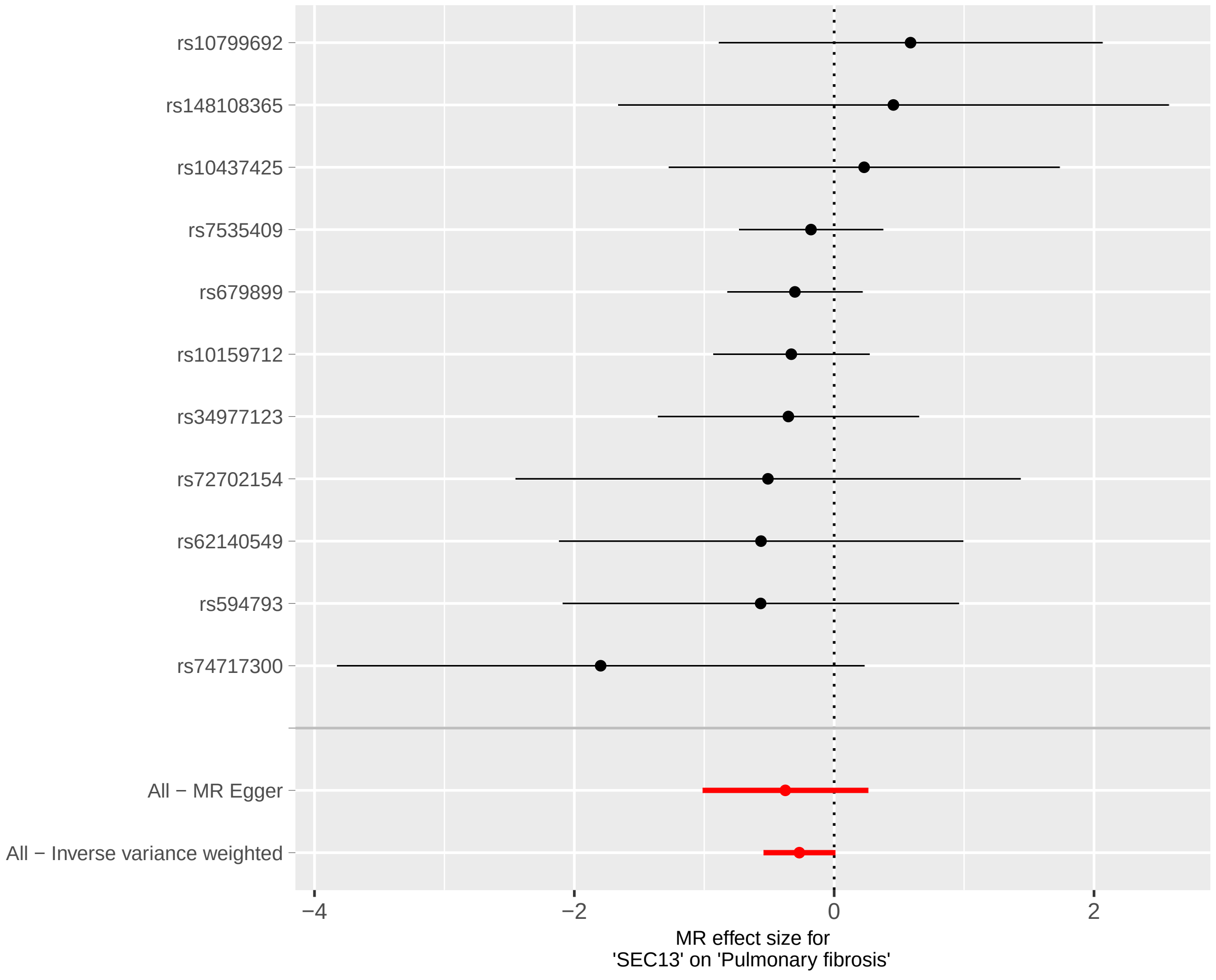
rs10799692
rs148108365
rs10437425
rs7535409
rs679899
rs10159712
rs34977123
rs72702154
rs62140549
rs594793
rs74717300
All − MR Egger
All − Inverse variance weighted
−4
−2
0
2
MR effect size for
'SEC13' on 'Pulmonary fibrosis'

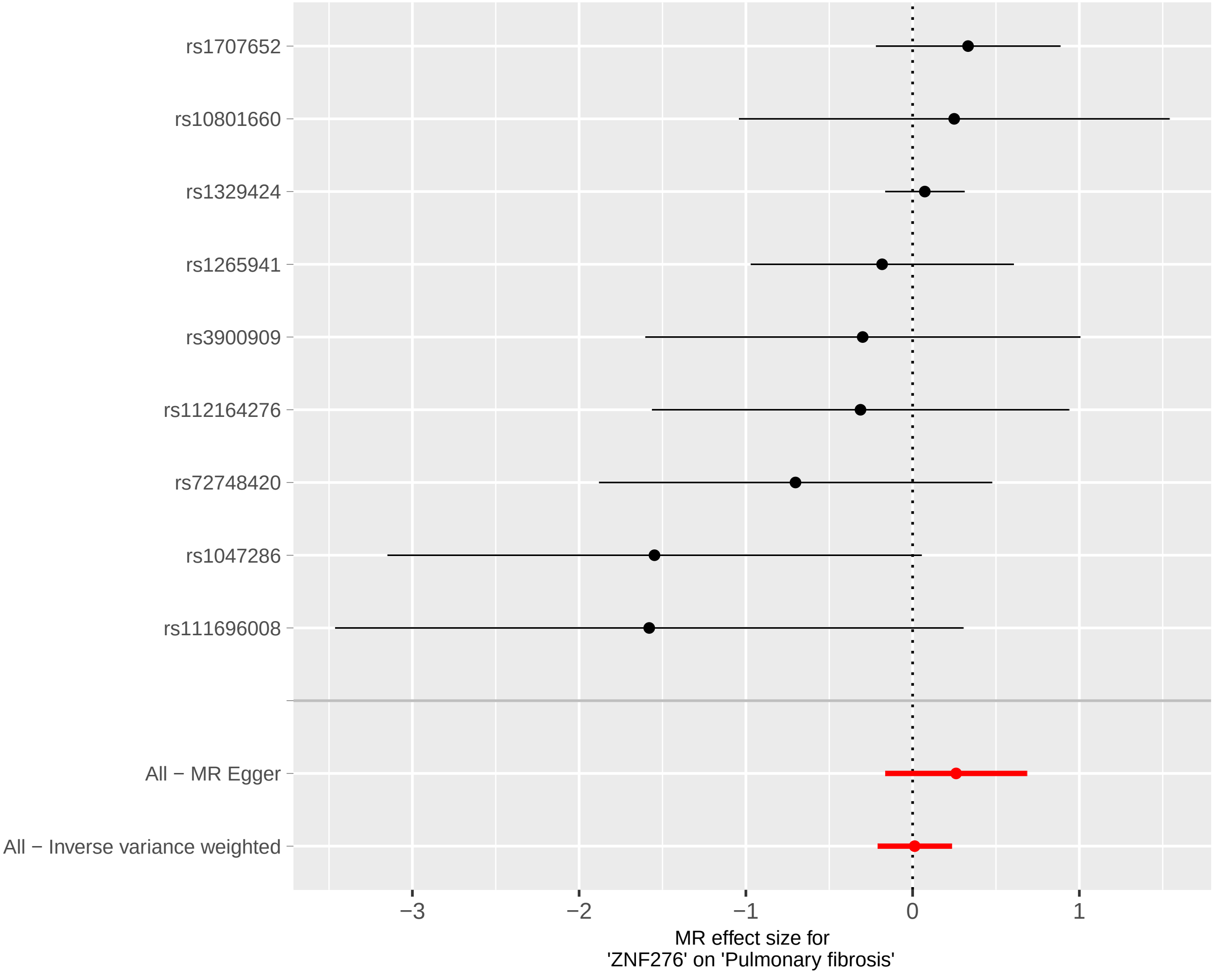
rs1707652
rs10801660
rs1329424
rs1265941
rs3900909
rs112164276
rs72748420
rs1047286
rs111696008
All − MR Egger
All − Inverse variance weighted
−3
−2
−1
0
1
MR effect size for
'ZNF276' on 'Pulmonary fibrosis'

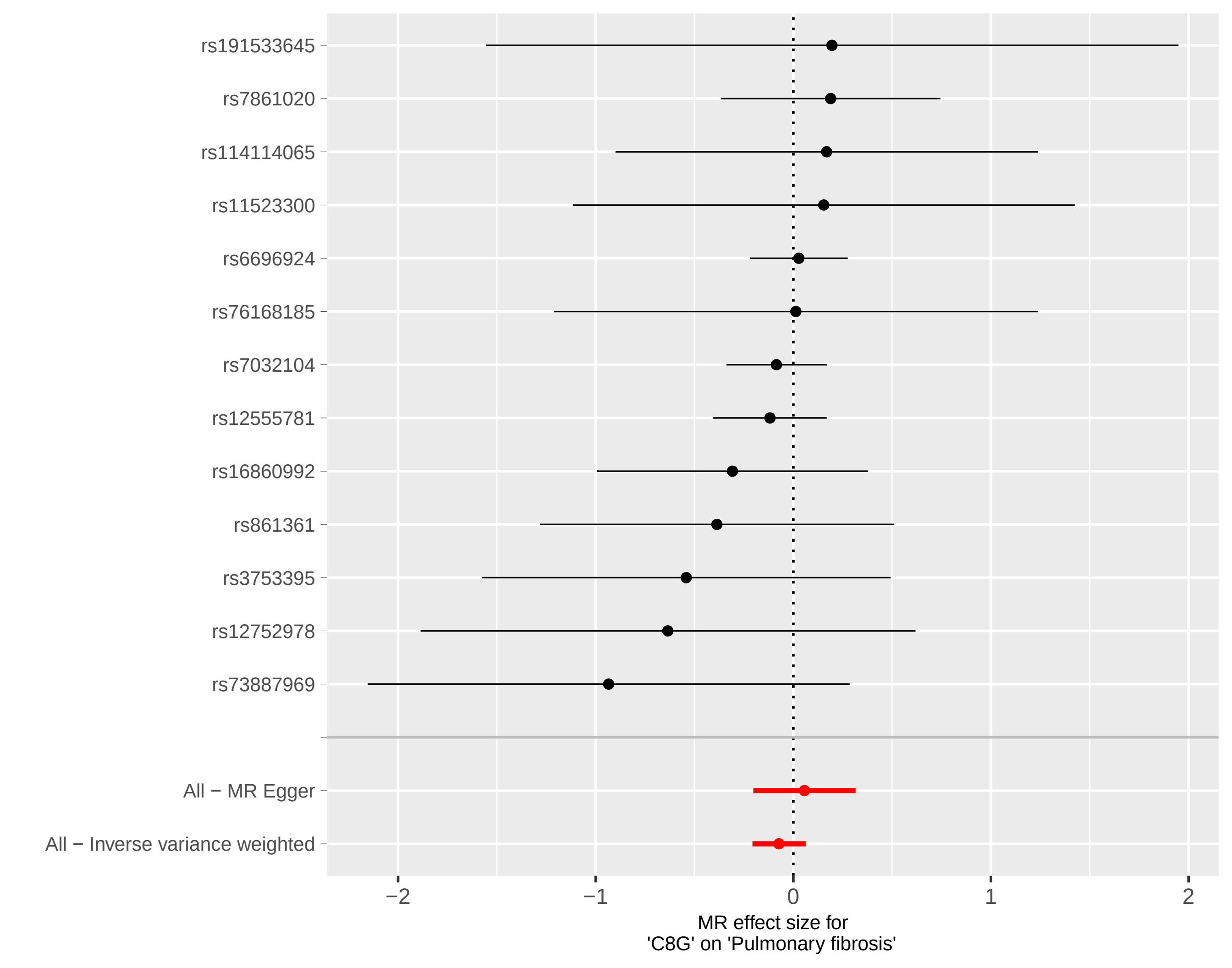
rs191533645
rs7861020
rs114114065
rs11523300
rs6696924
rs76168185
rs7032104
rs12555781
rs16860992
rs861361
rs3753395
rs12752978
rs73887969
All − MR Egger
All − Inverse variance weighted
−2
−1
0
1
2
MR effect size for
'C8G' on 'Pulmonary fibrosis'

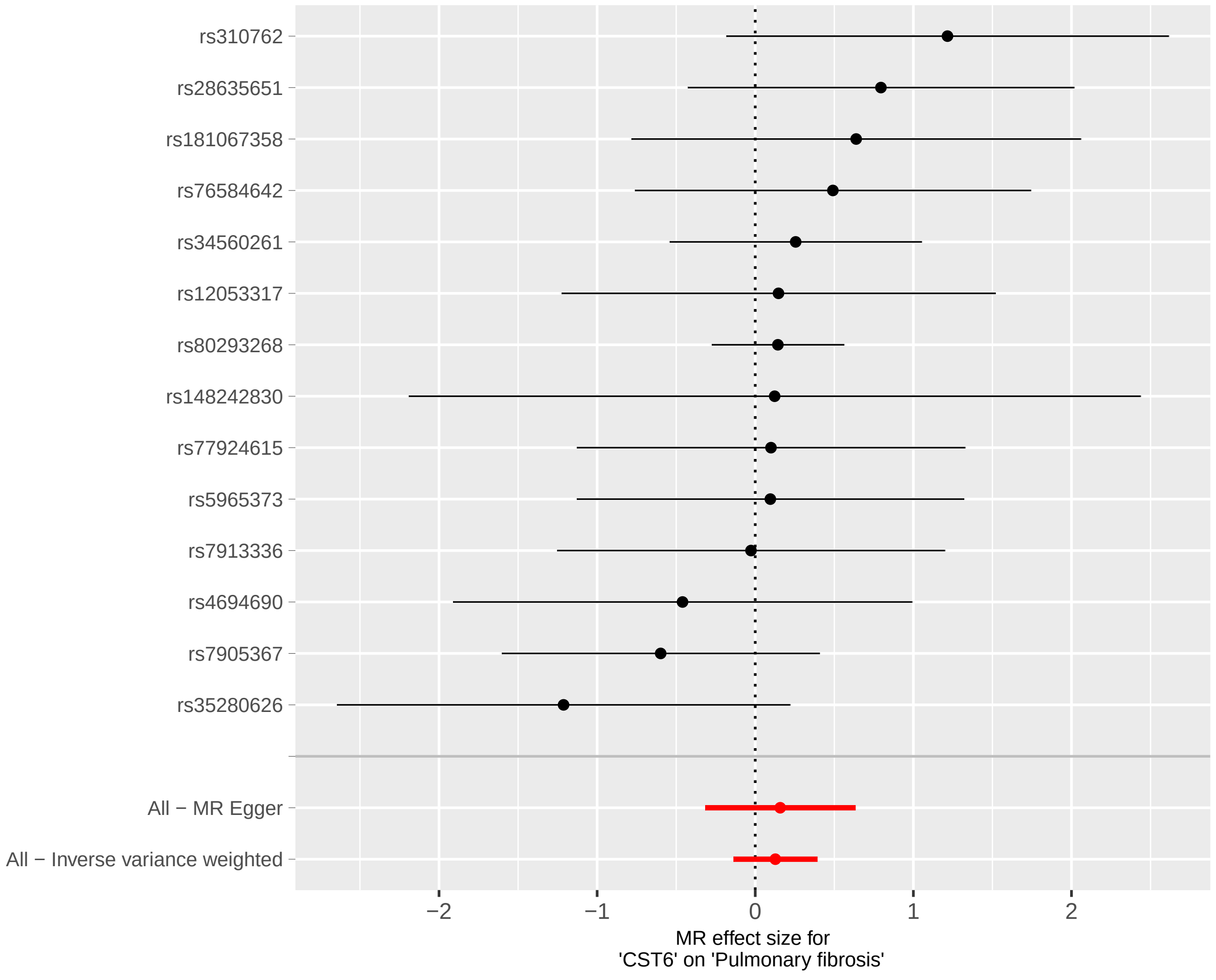

rs310762
rs28635651
rs181067358
rs76584642
rs34560261
rs12053317
rs80293268
rs148242830
rs77924615
rs5965373
rs7913336
rs4694690
rs7905367
rs35280626
All − MR Egger
All − Inverse variance weighted
−2
−1
0
1
2
MR effect size for
'CST6' on 'Pulmonary fibrosis'

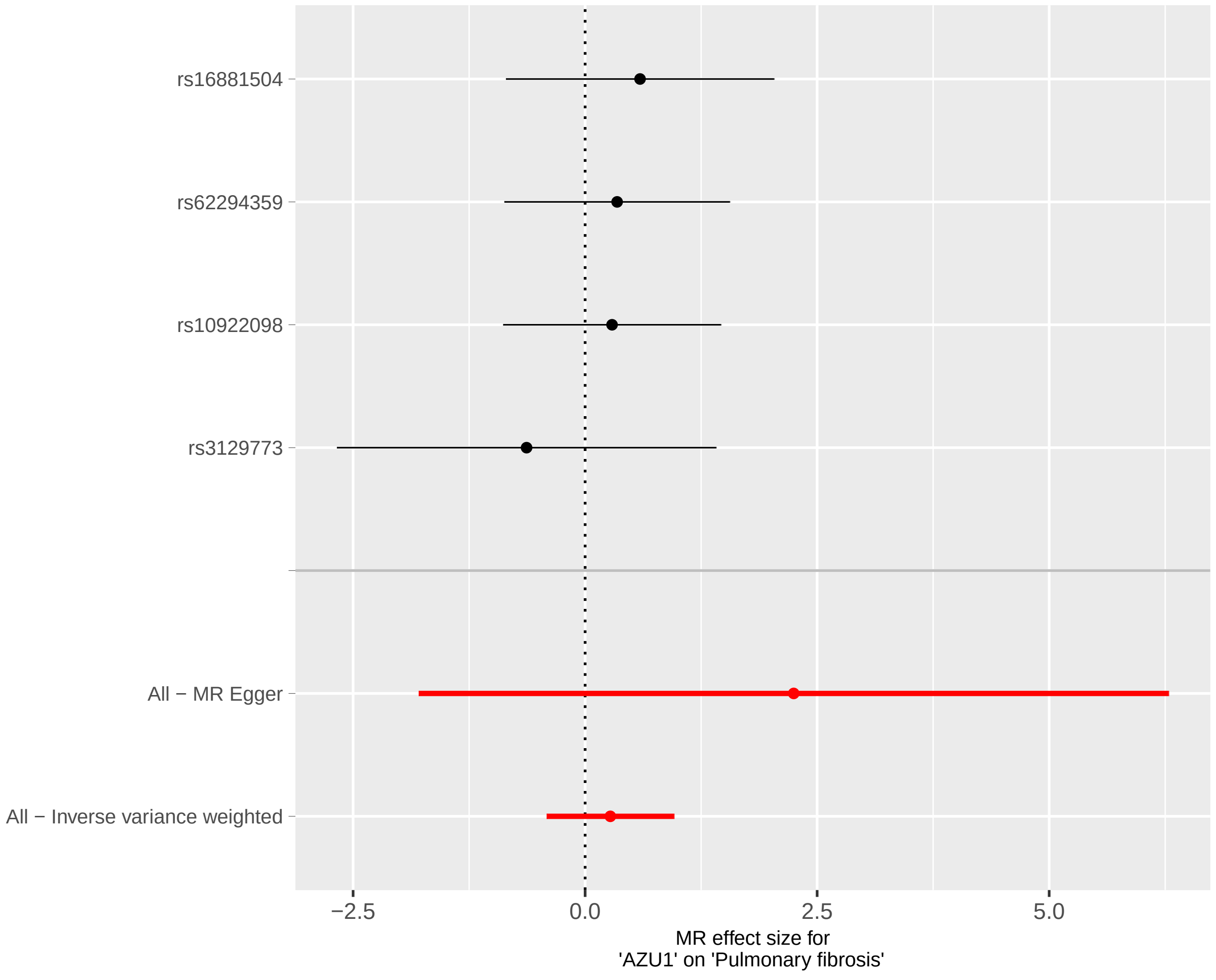
rs16881504
rs62294359
rs10922098
rs3129773
All − MR Egger
All − Inverse variance weighted
−2.5
0.0
2.5
5.0
MR effect size for
'AZU1' on 'Pulmonary fibrosis'

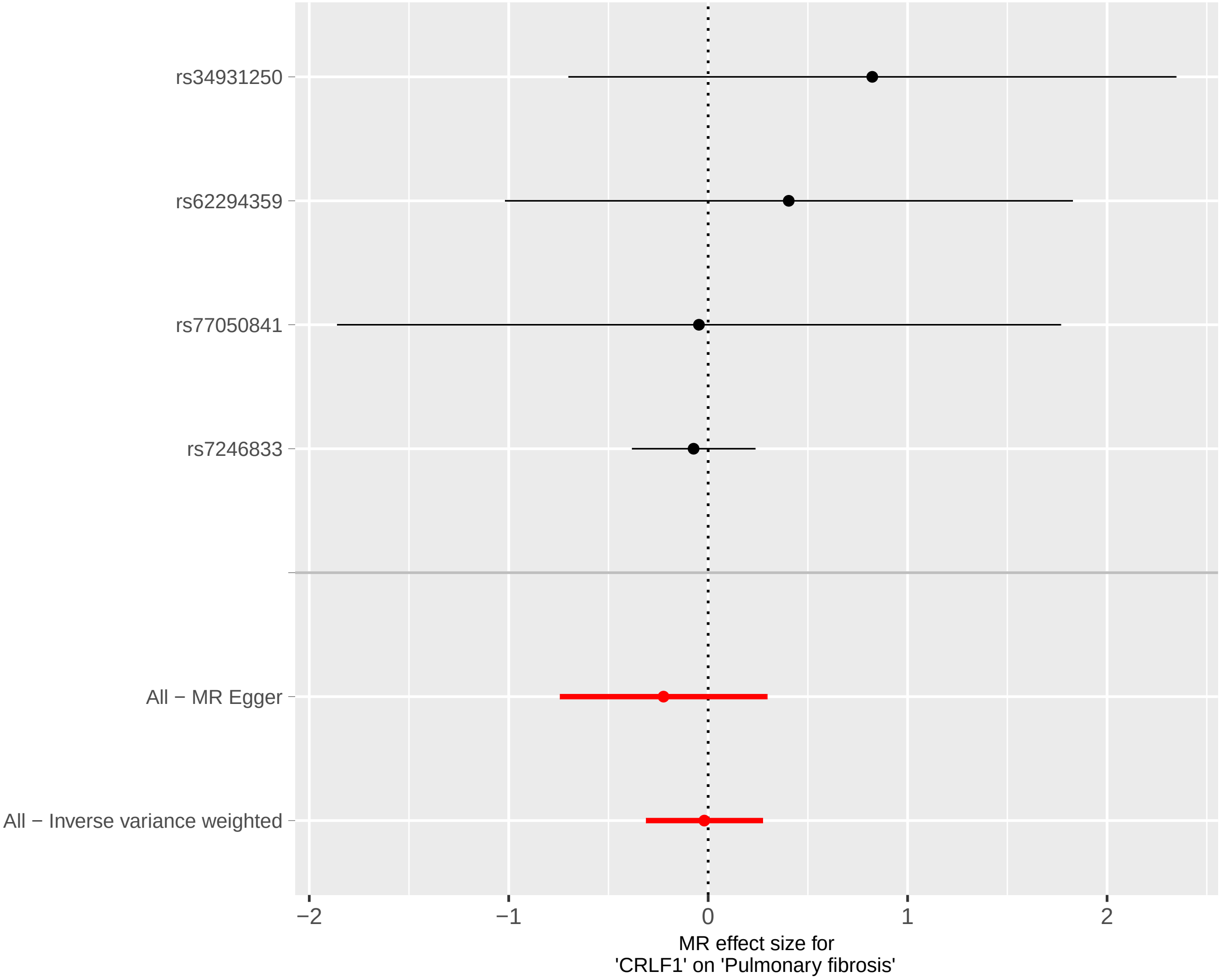

rs34931250
rs62294359
rs77050841
rs7246833
All − MR Egger
All − Inverse variance weighted
−2
−1
0
1
2
MR effect size for
'CRLF1' on 'Pulmonary fibrosis'

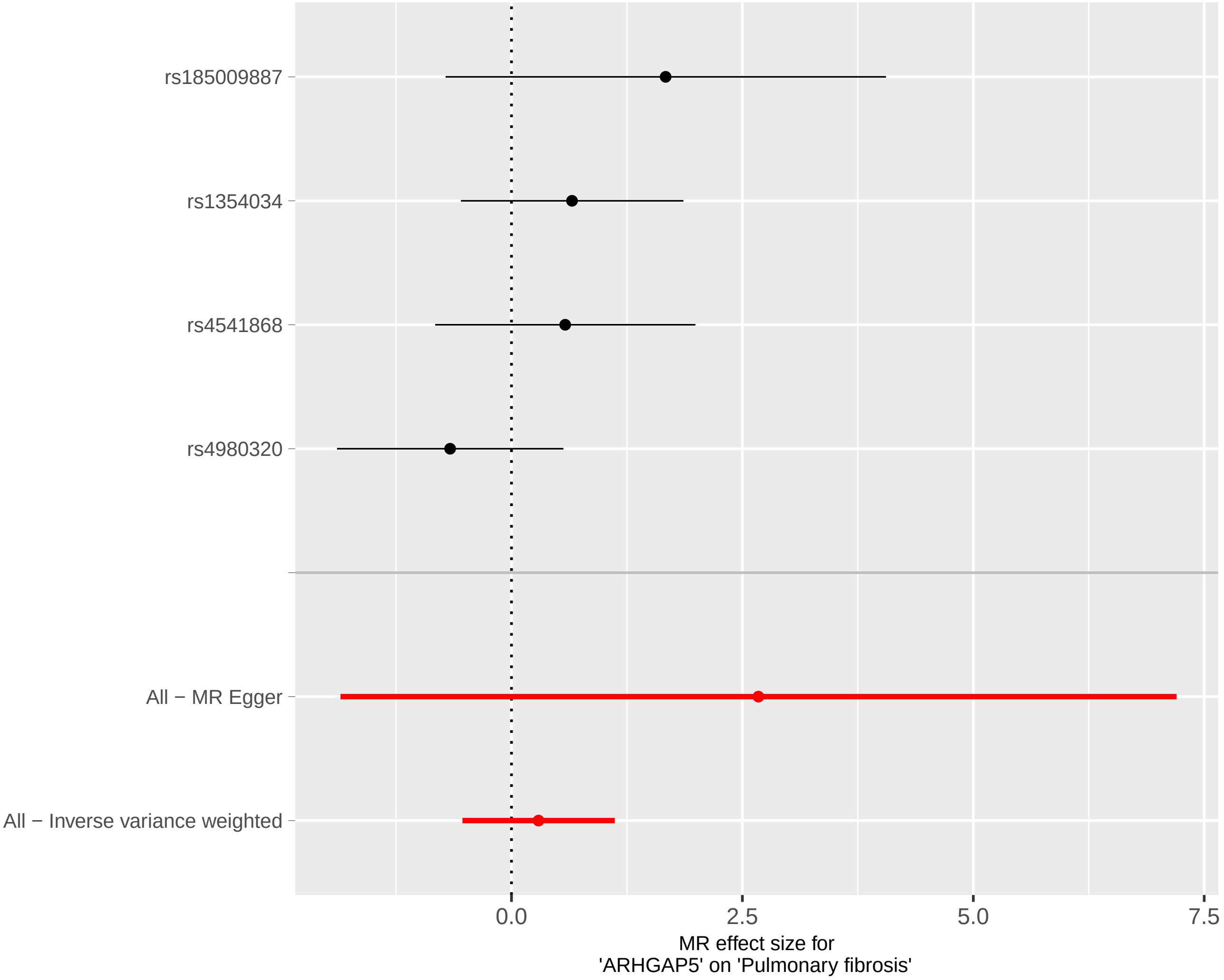

rs185009887
rs1354034
rs4541868
rs4980320
All − MR Egger
All − Inverse variance weighted
0.0
2.5
5.0
7.5
MR effect size for
'ARHGAP5' on 'Pulmonary fibrosis'

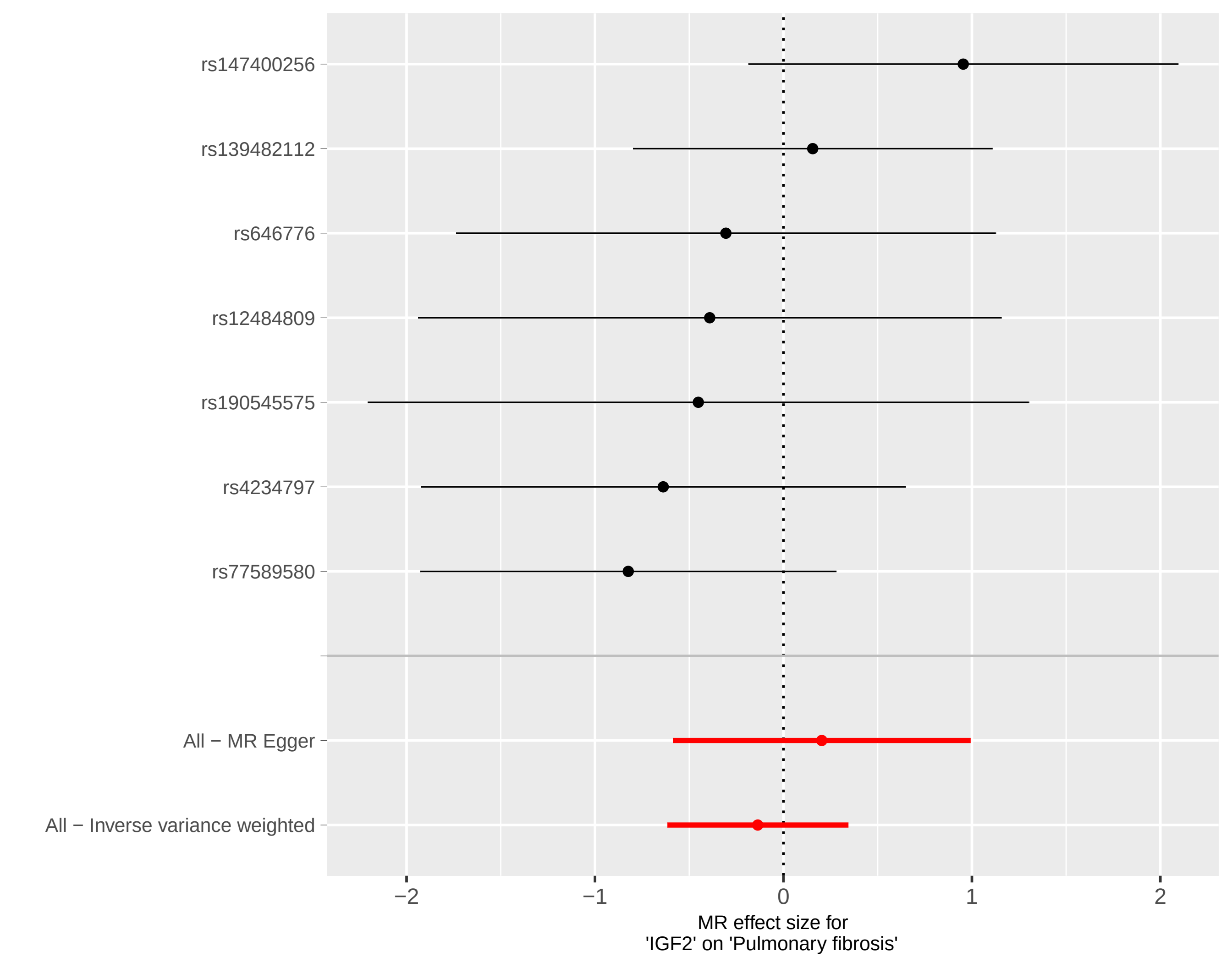

rs147400256
rs139482112
rs646776
rs12484809
rs190545575
rs4234797
rs77589580
All − MR Egger
All − Inverse variance weighted
−2
−1
0
1
2
MR effect size for
'IGF2' on 'Pulmonary fibrosis'

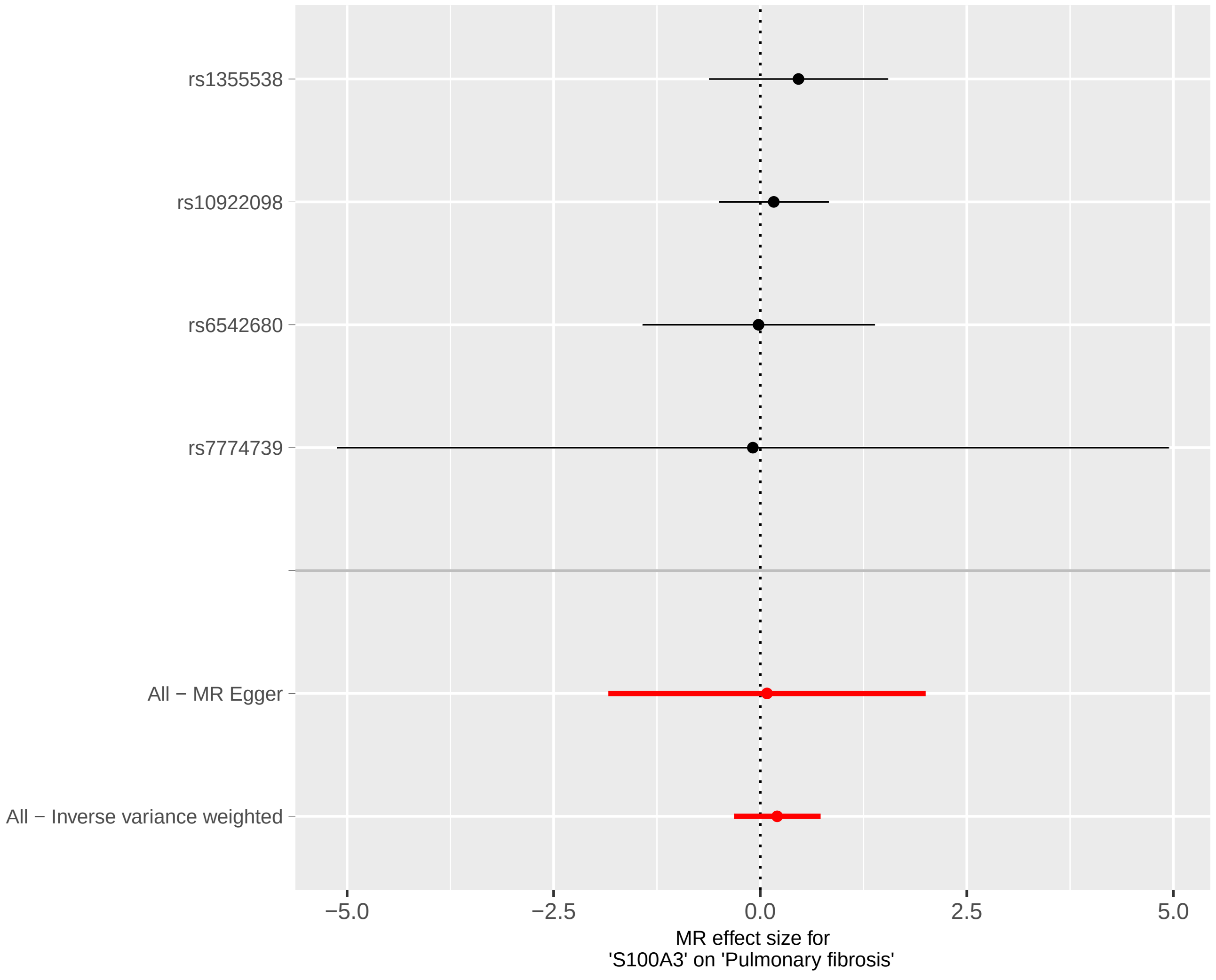
rs1355538
rs10922098
rs6542680
rs7774739
All − MR Egger
All − Inverse variance weighted
−5.0
−2.5
0.0
2.5
5.0
MR effect size for
'S100A3' on 'Pulmonary fibrosis'

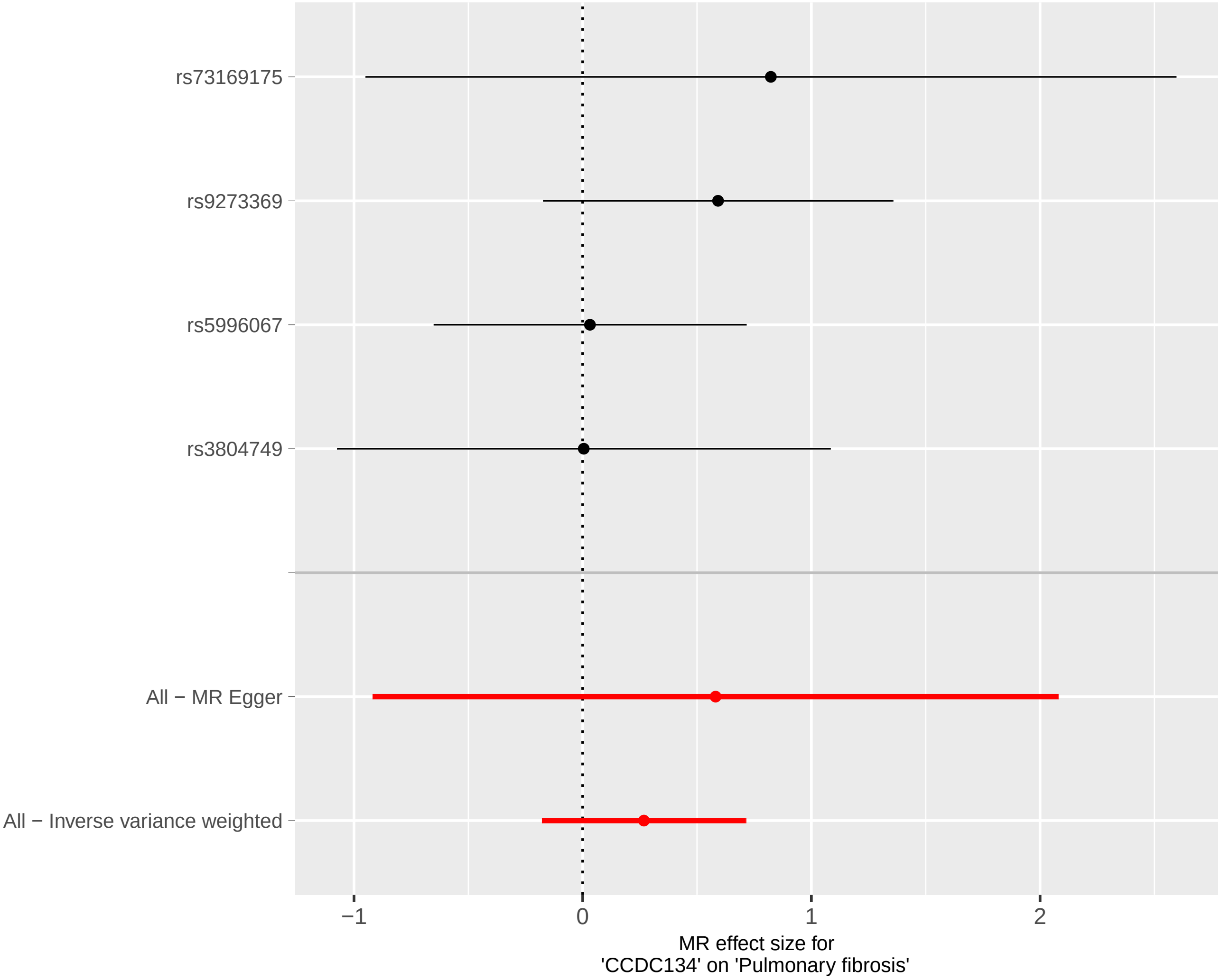

rs73169175
rs9273369
rs5996067
rs3804749
All − MR Egger
All − Inverse variance weighted
−1
0
1
2
MR effect size for
'CCDC134' on 'Pulmonary fibrosis'

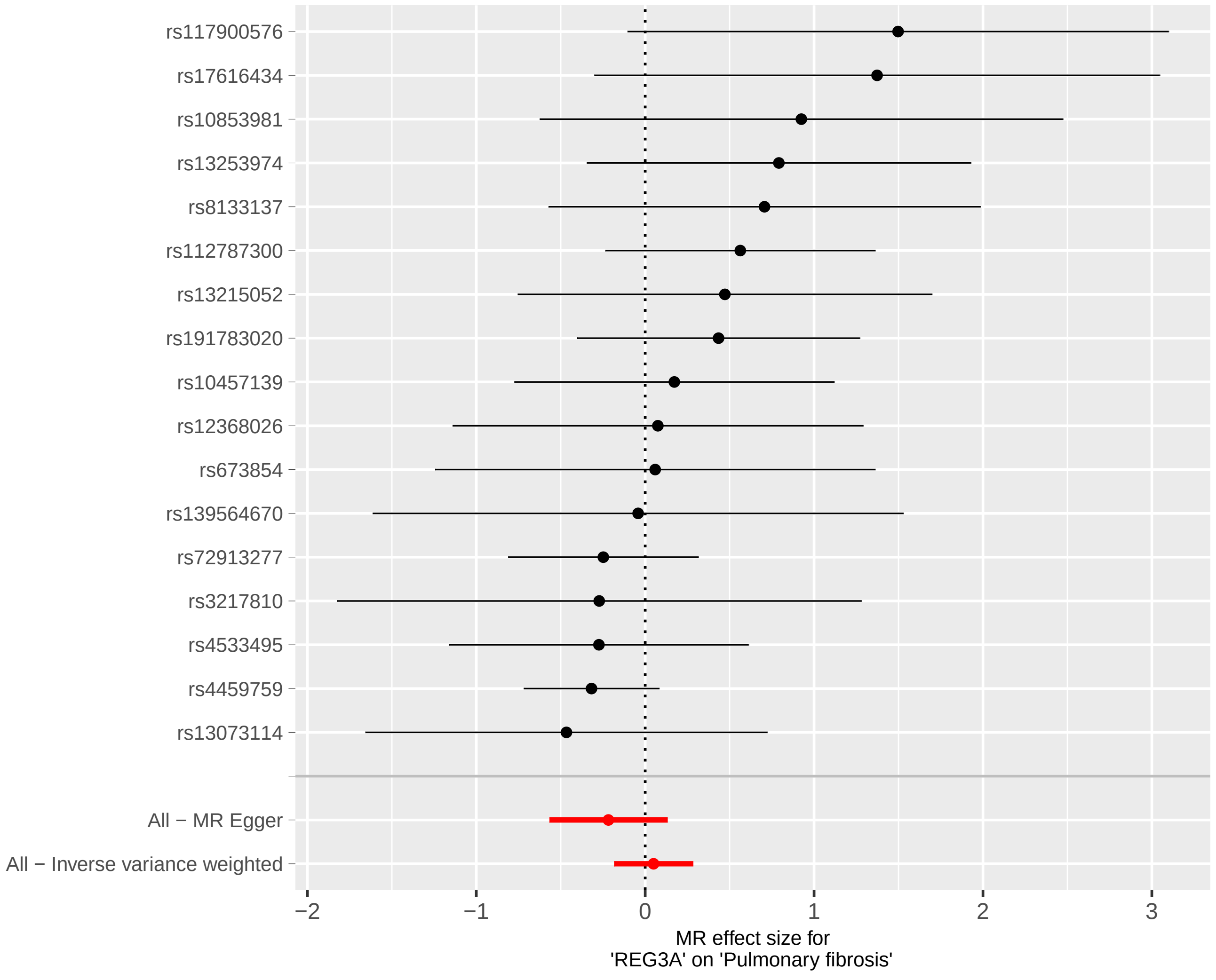

rs117900576
rs17616434
rs10853981
rs13253974
rs8133137
rs112787300
rs13215052
rs191783020
rs10457139
rs12368026
rs673854
rs139564670
rs72913277
rs3217810
rs4533495
rs4459759
rs13073114
All − MR Egger
All − Inverse variance weighted
−2
−1
0
1
2
3
MR effect size for
'REG3A' on 'Pulmonary fibrosis'

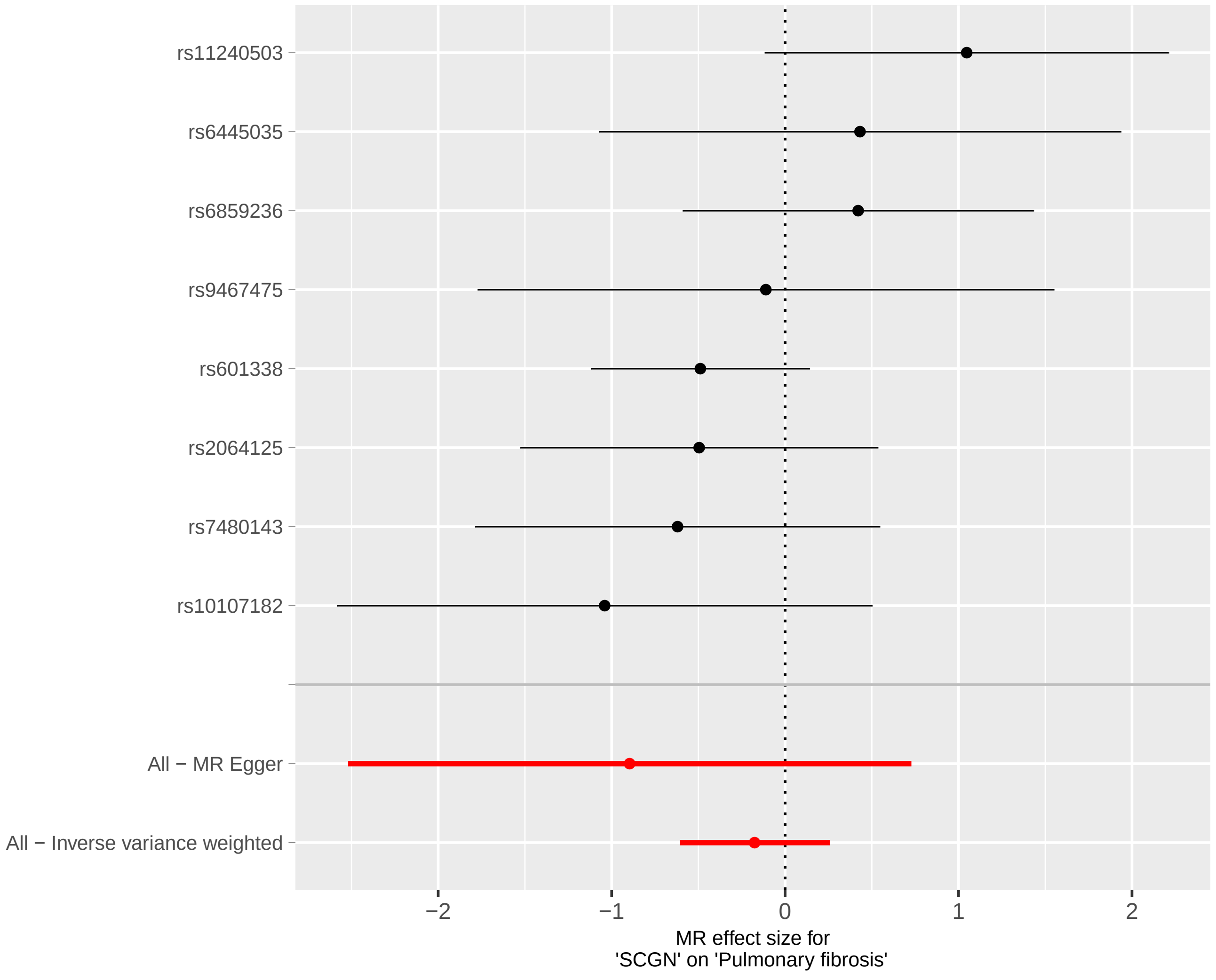
rs11240503
rs6445035
rs6859236
rs9467475
rs601338
rs2064125
rs7480143
rs10107182
All − MR Egger
All − Inverse variance weighted
−2
−1
0
1
2
MR effect size for
'SCGN' on 'Pulmonary fibrosis'

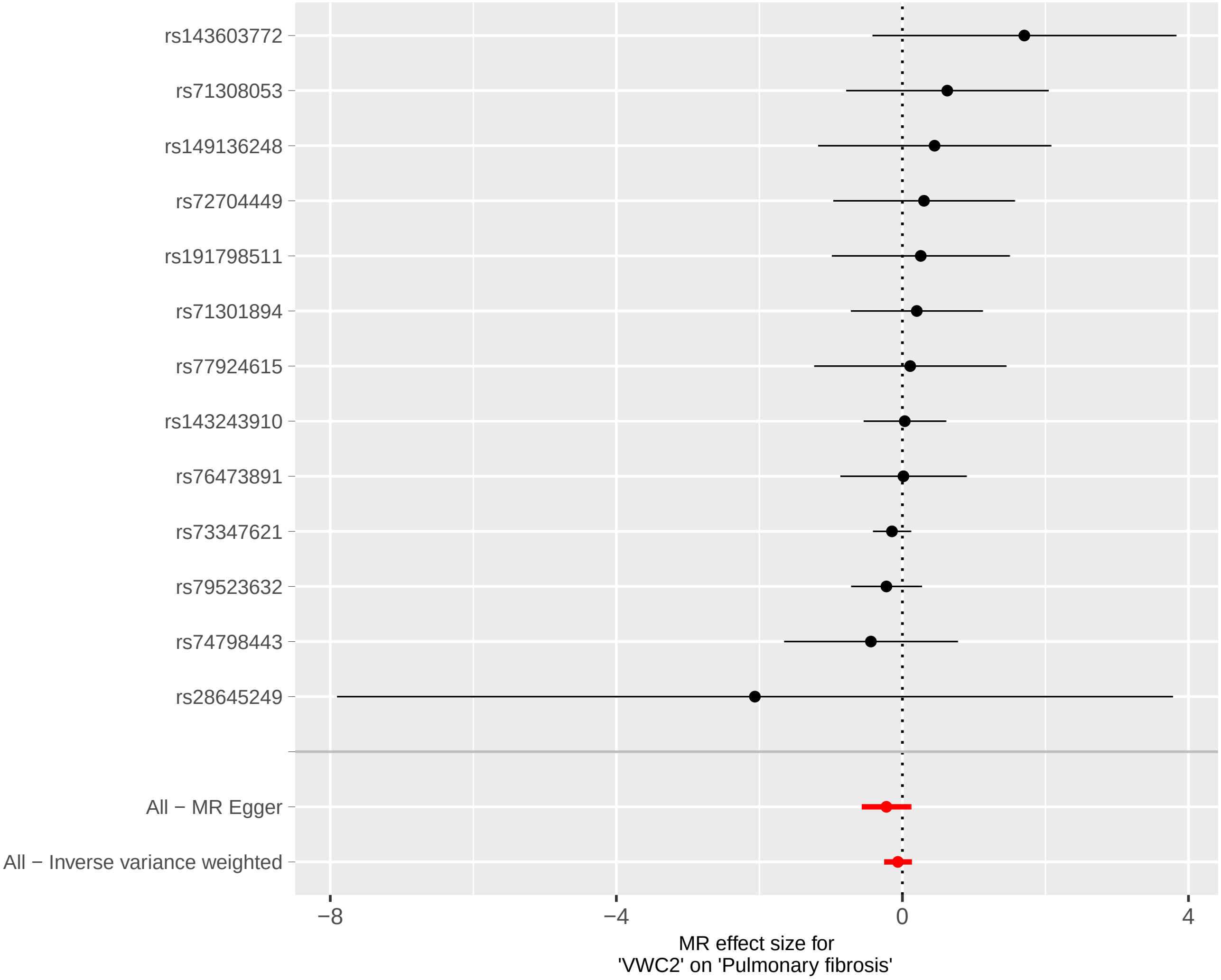

rs143603772
rs71308053
rs149136248
rs72704449
rs191798511
rs71301894
rs77924615
rs143243910
rs76473891
rs73347621
rs79523632
rs74798443
rs28645249
All − MR Egger
All − Inverse variance weighted
−8
−4
0
4
MR effect size for
'VWC2' on 'Pulmonary fibrosis'

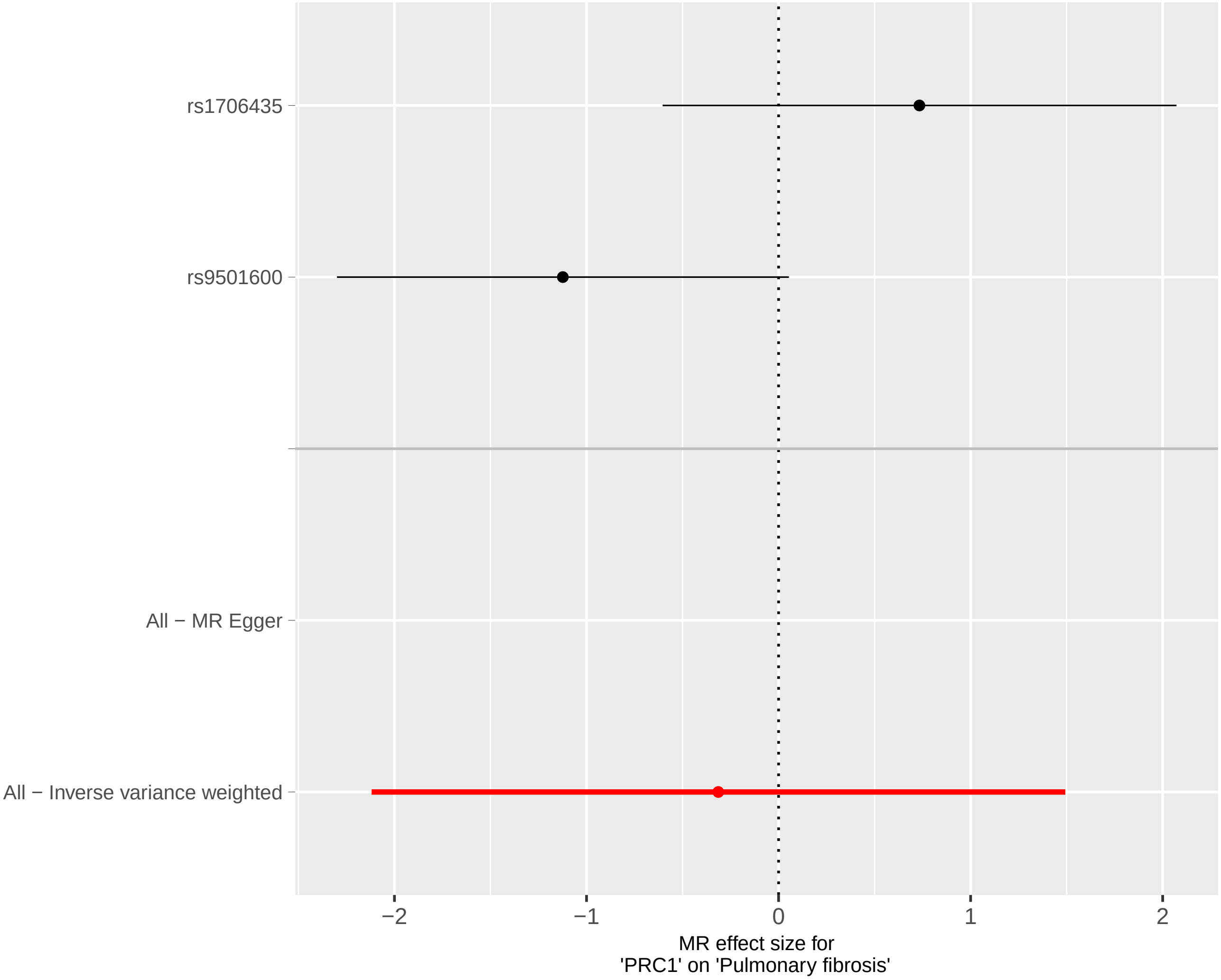

rs1706435
rs9501600
All − MR Egger
All − Inverse variance weighted
−2
−1
0
1
2
MR effect size for
'PRC1' on 'Pulmonary fibrosis'

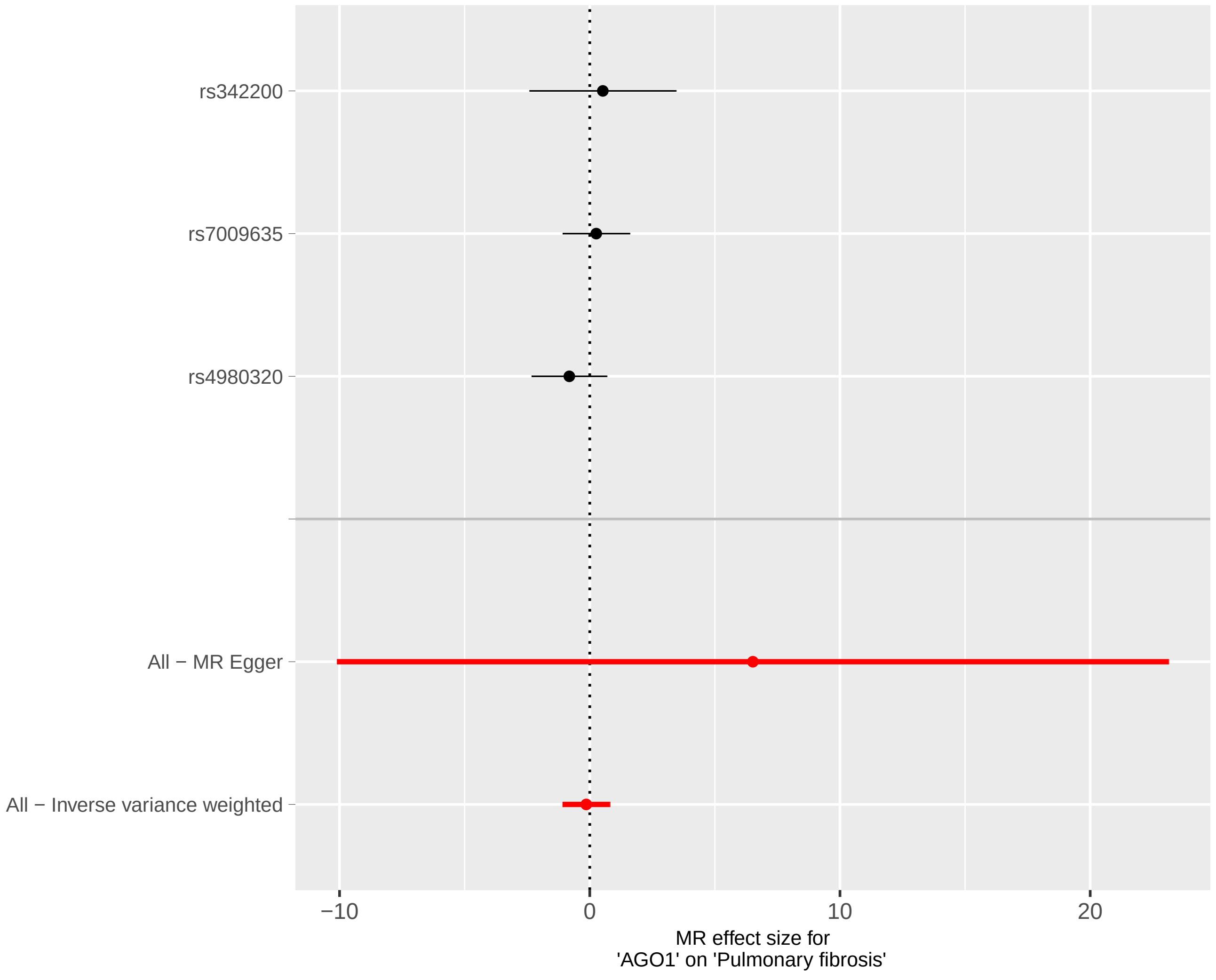

rs342200
rs7009635
rs4980320
All − MR Egger
All − Inverse variance weighted
−10
0
10
20
MR effect size for
'AGO1' on 'Pulmonary fibrosis'

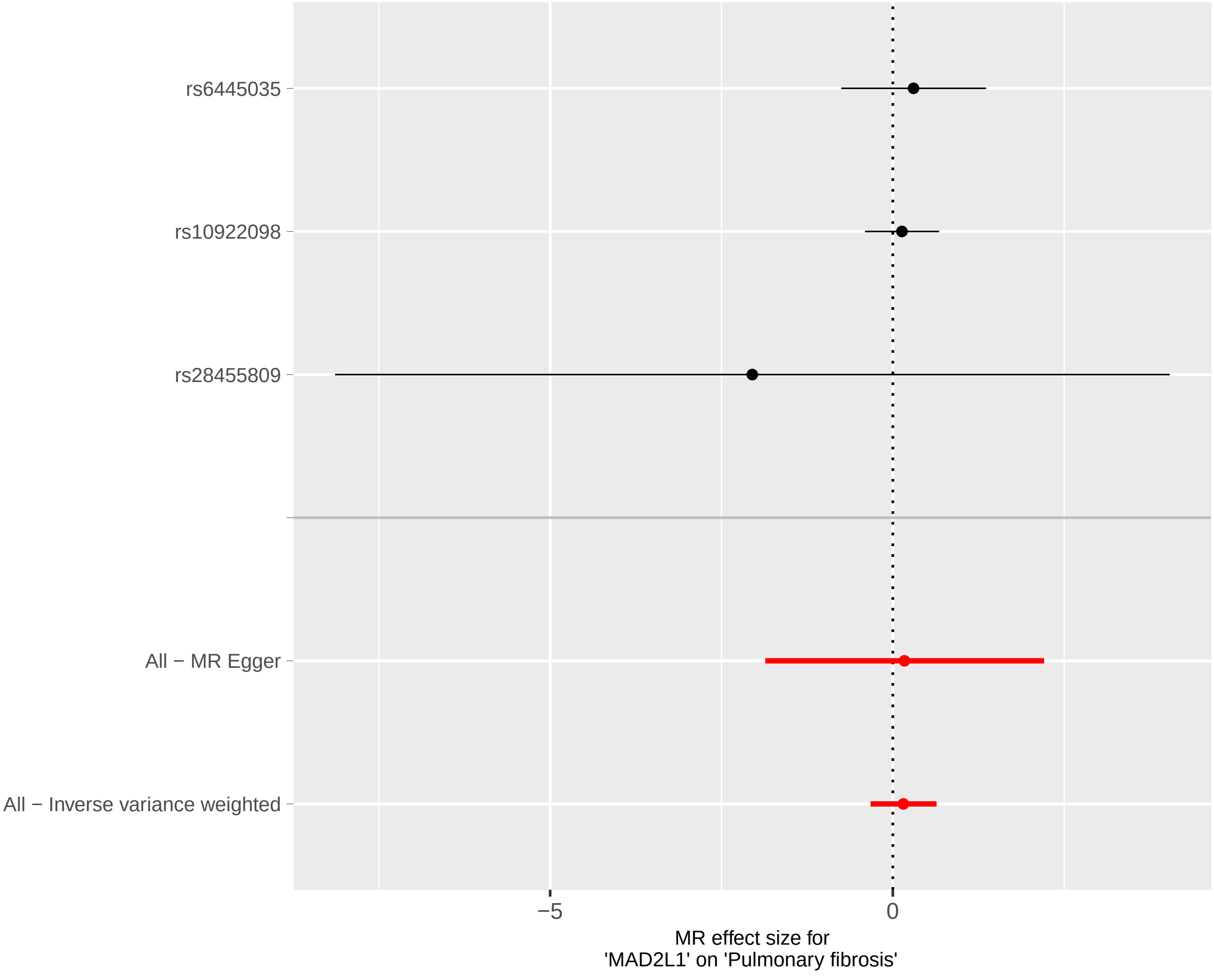
rs6445035
rs10922098
rs28455809
All − MR Egger
All − Inverse variance weighted
−5
0
MR effect size for
'MAD2L1' on 'Pulmonary fibrosis'

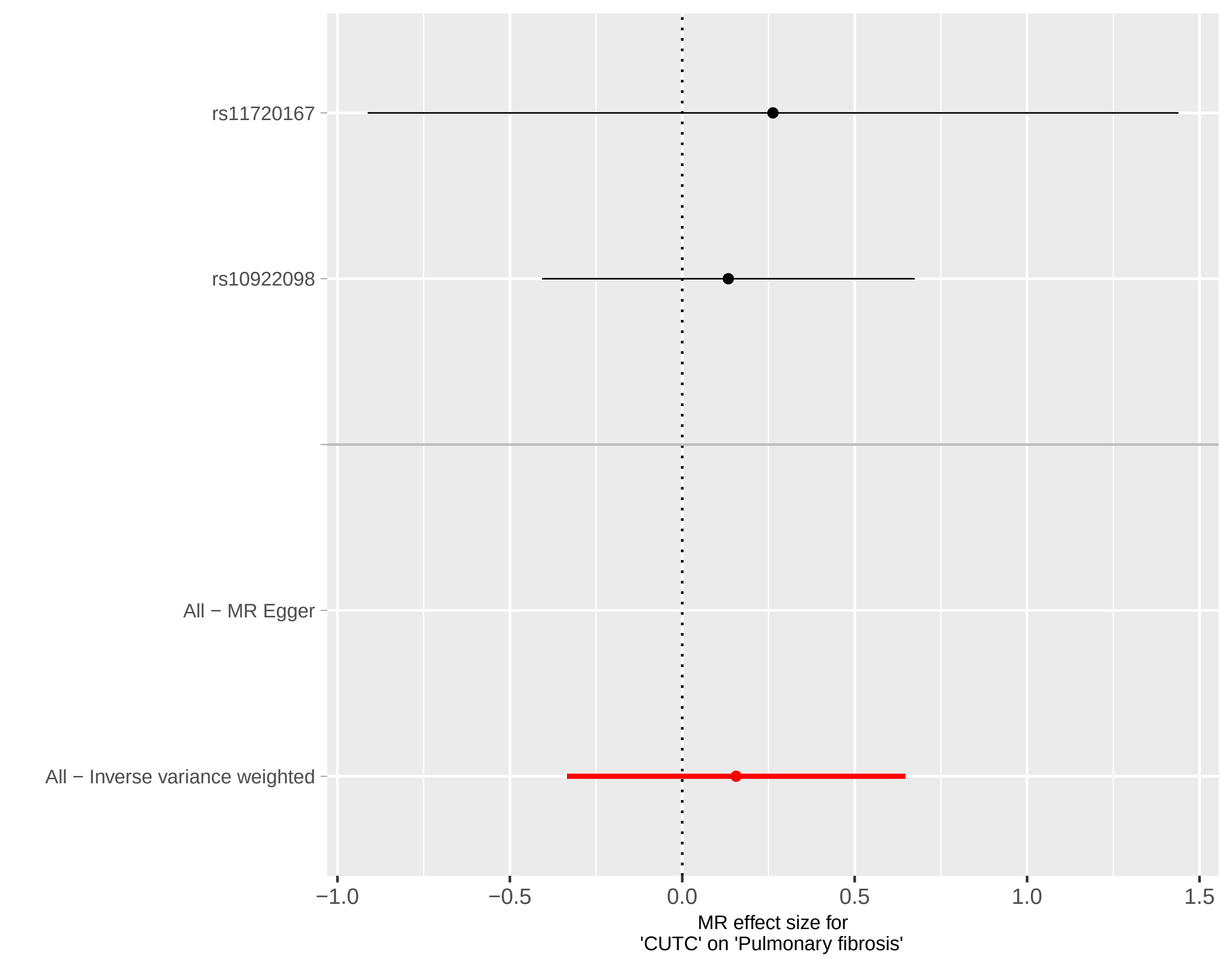

rs11720167
rs10922098
All − MR Egger
All − Inverse variance weighted
−1.0
−0.5
0.0
0.5
1.0
1.5
MR effect size for
'CUTC' on 'Pulmonary fibrosis'

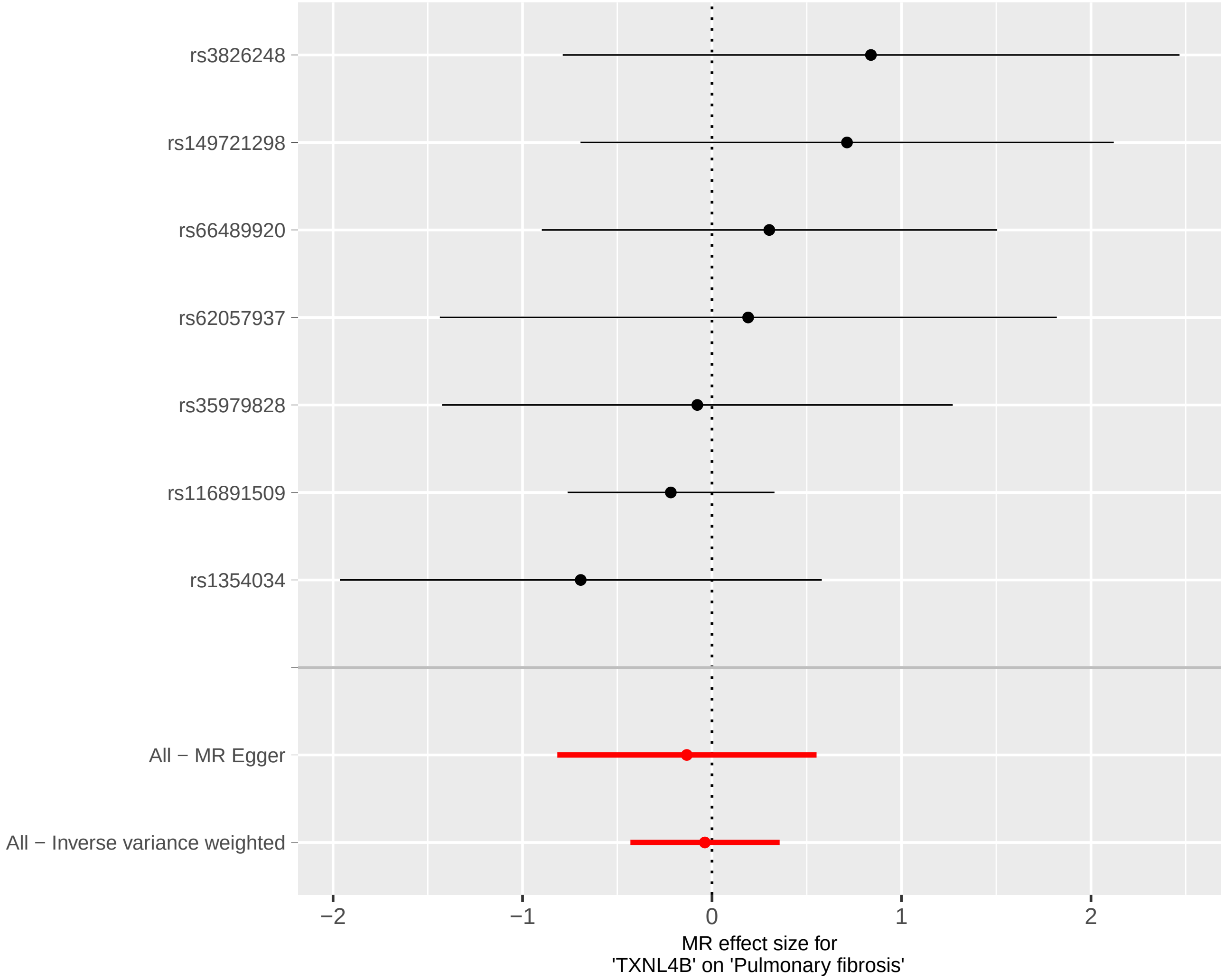
rs3826248
rs149721298
rs66489920
rs62057937
rs35979828
rs116891509
rs1354034
All − MR Egger
All − Inverse variance weighted
−2
−1
0
1
2
MR effect size for
'TXNL4B' on 'Pulmonary fibrosis'

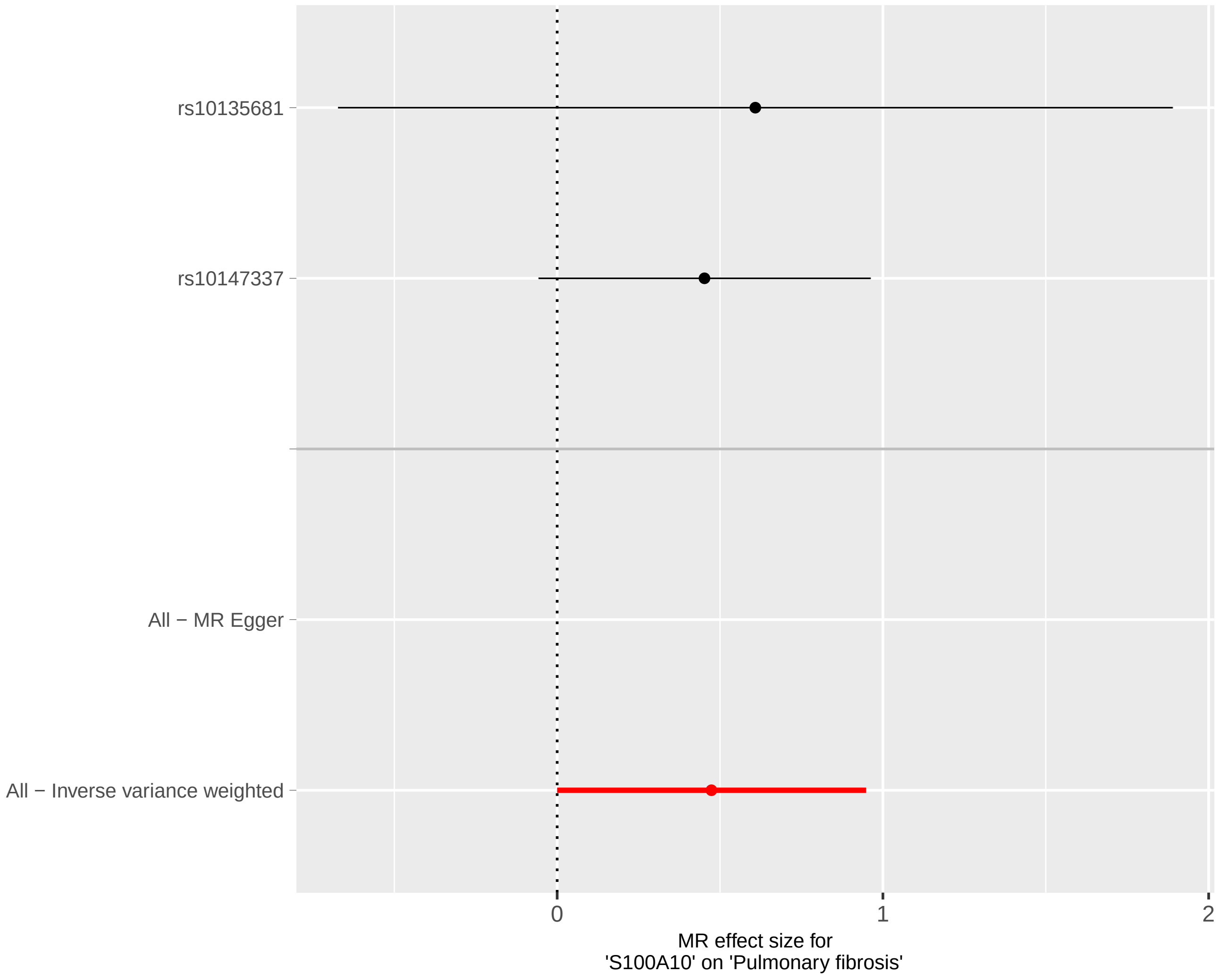
rs10135681
rs10147337
All − MR Egger
All − Inverse variance weighted
0
1
2
MR effect size for
'S100A10' on 'Pulmonary fibrosis'

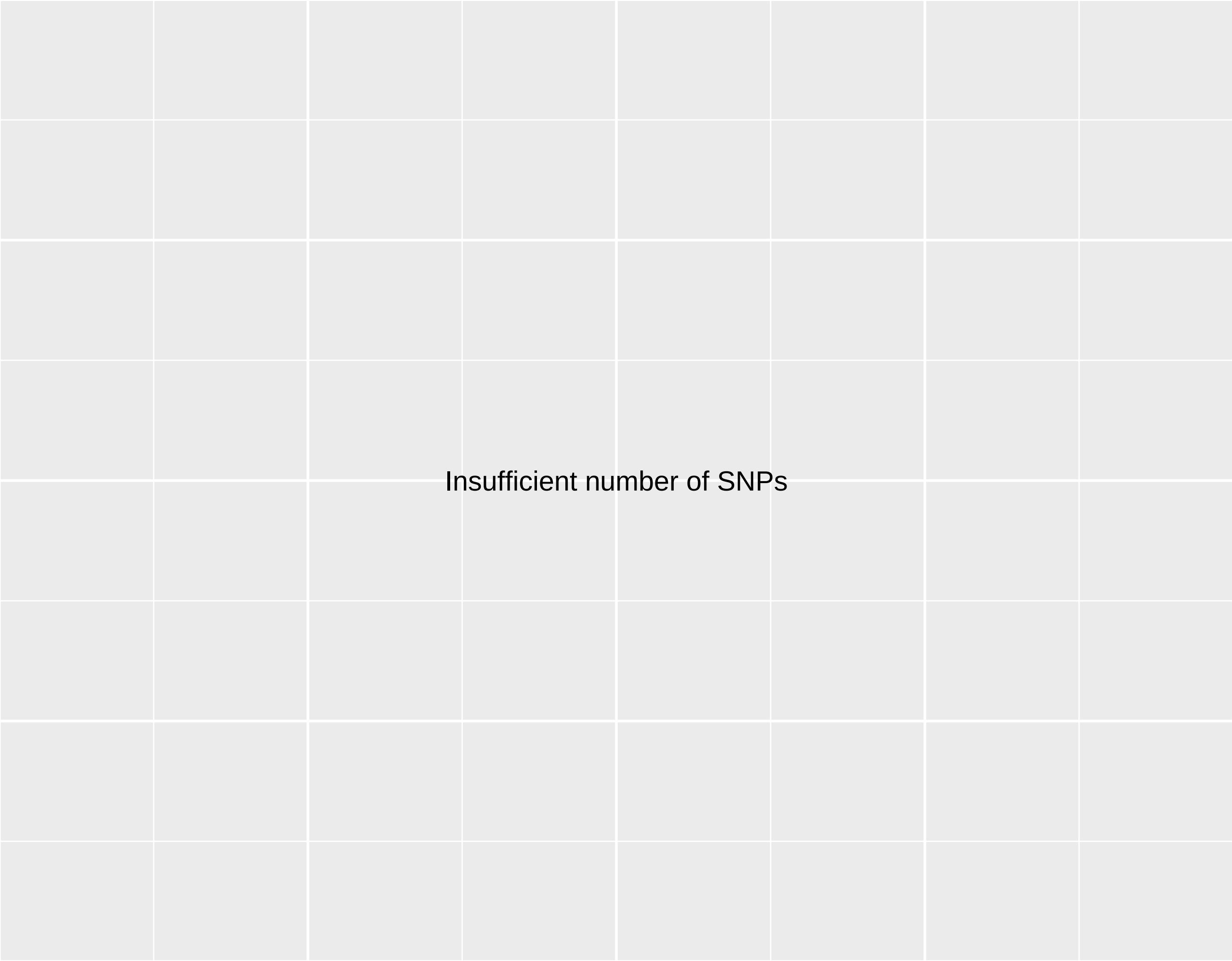
Insufficient number of SNPs

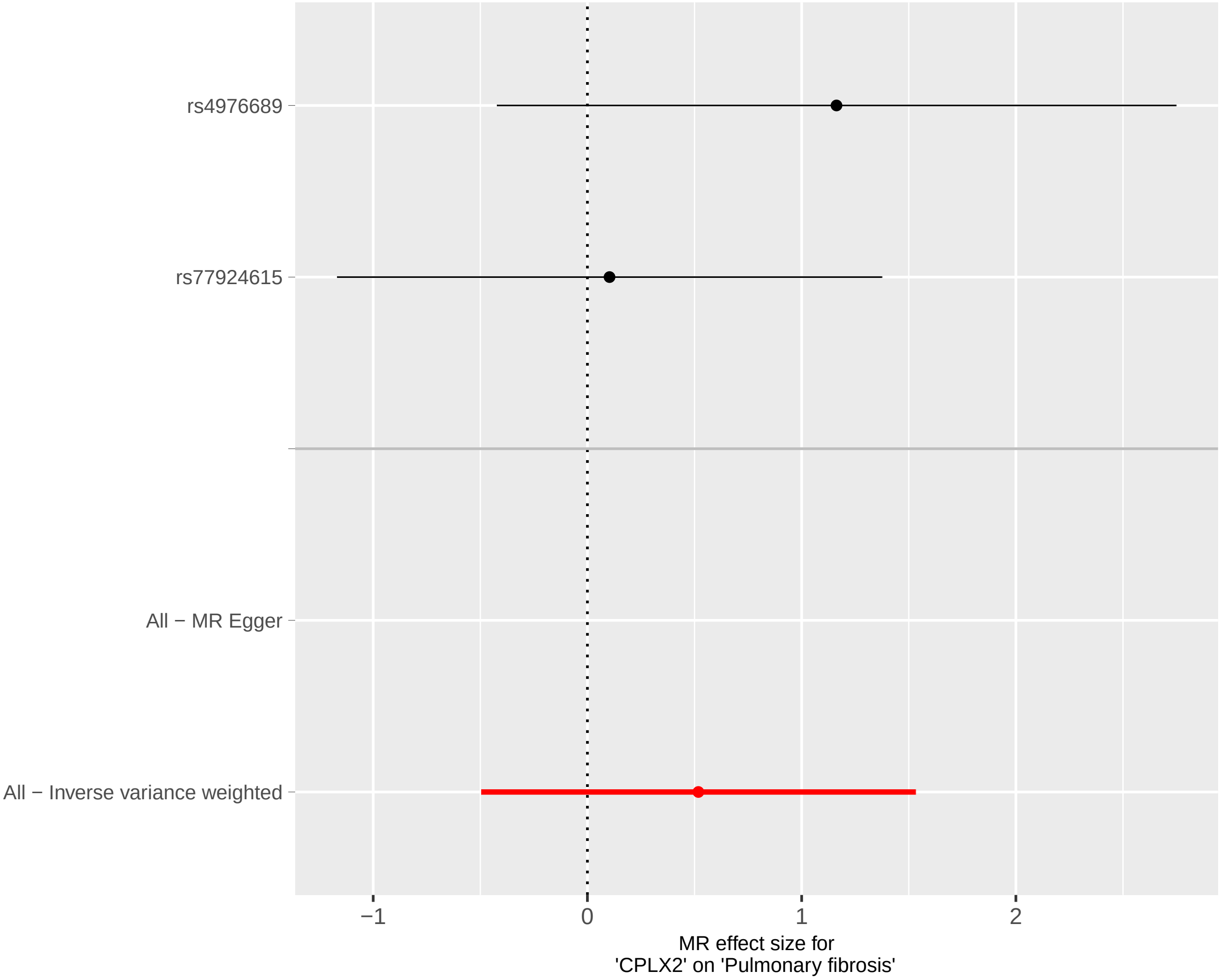

rs4976689
rs77924615
All − MR Egger
All − Inverse variance weighted
−1
0
1
2
MR effect size for
'CPLX2' on 'Pulmonary fibrosis'

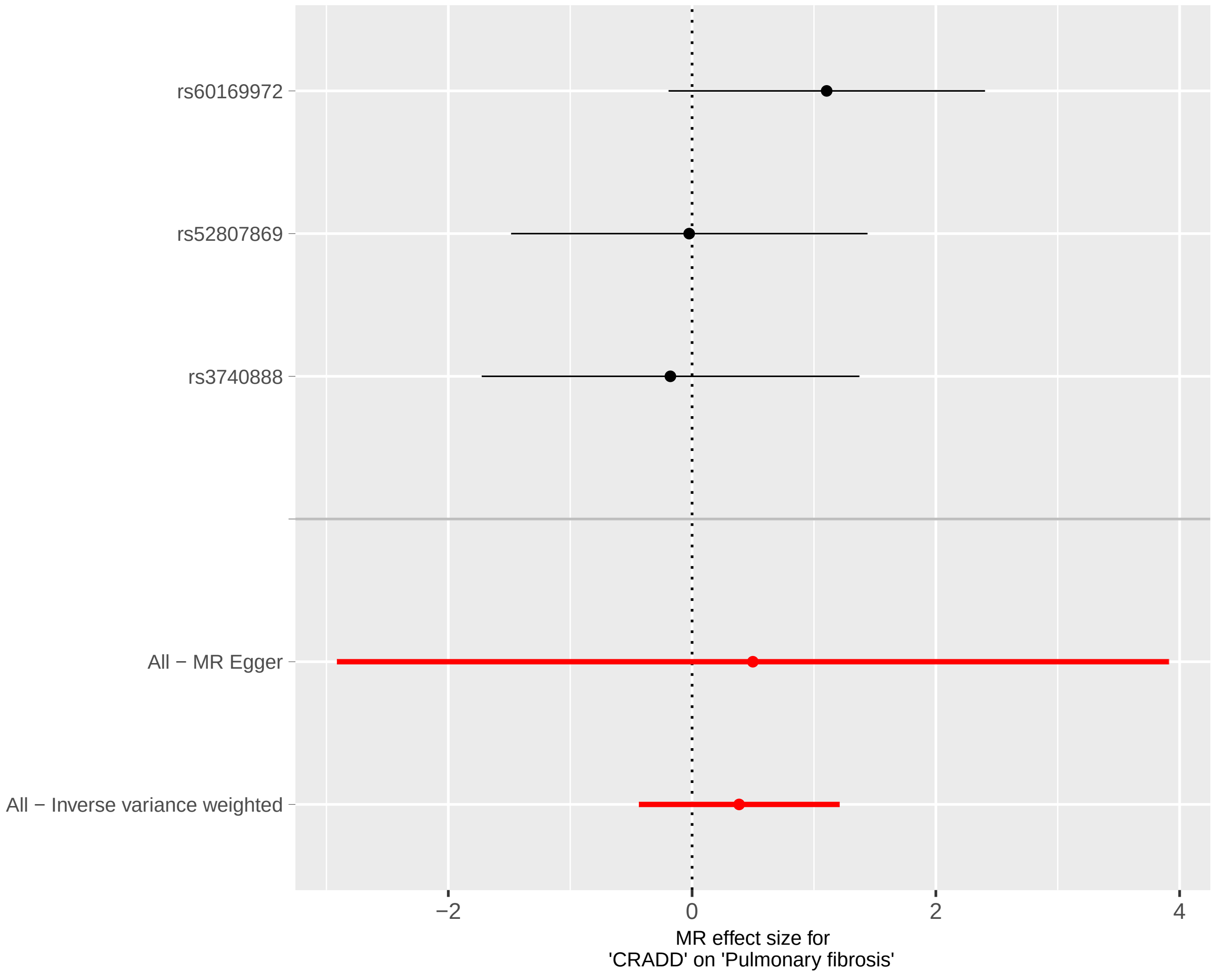
rs60169972
rs52807869
rs3740888
All − MR Egger
All − Inverse variance weighted
−2
0
2
4
MR effect size for
'CRADD' on 'Pulmonary fibrosis'

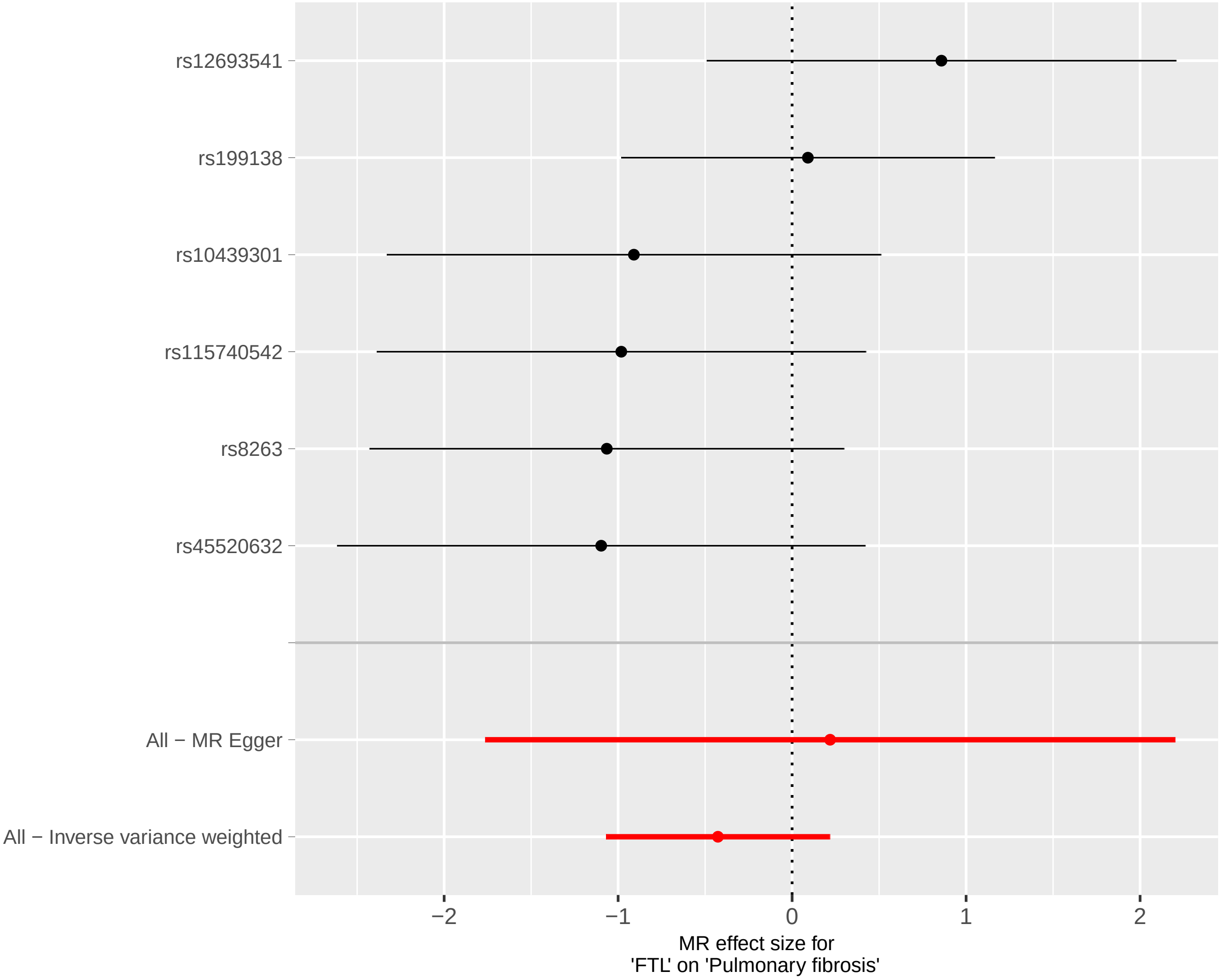

rs12693541
rs199138
rs10439301
rs115740542
rs8263
rs45520632
All − MR Egger
All − Inverse variance weighted
−2
−1
0
1
2
MR effect size for
'FTL' on 'Pulmonary fibrosis'

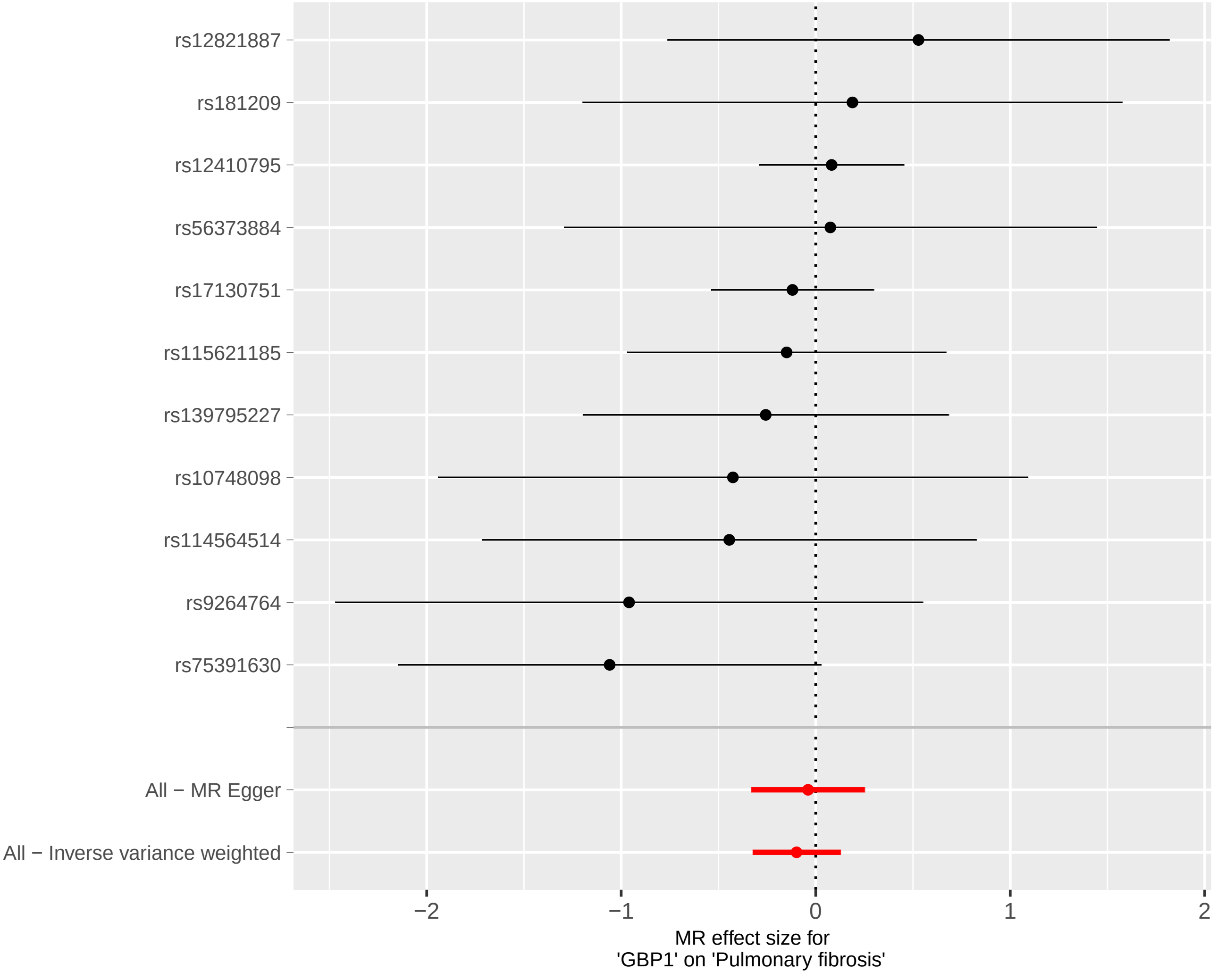

rs12821887
rs181209
rs12410795
rs56373884
rs17130751
rs115621185
rs139795227
rs10748098
rs114564514
rs9264764
rs75391630
All − MR Egger
All − Inverse variance weighted
−2
−1
0
1
2
MR effect size for
'GBP1' on 'Pulmonary fibrosis'

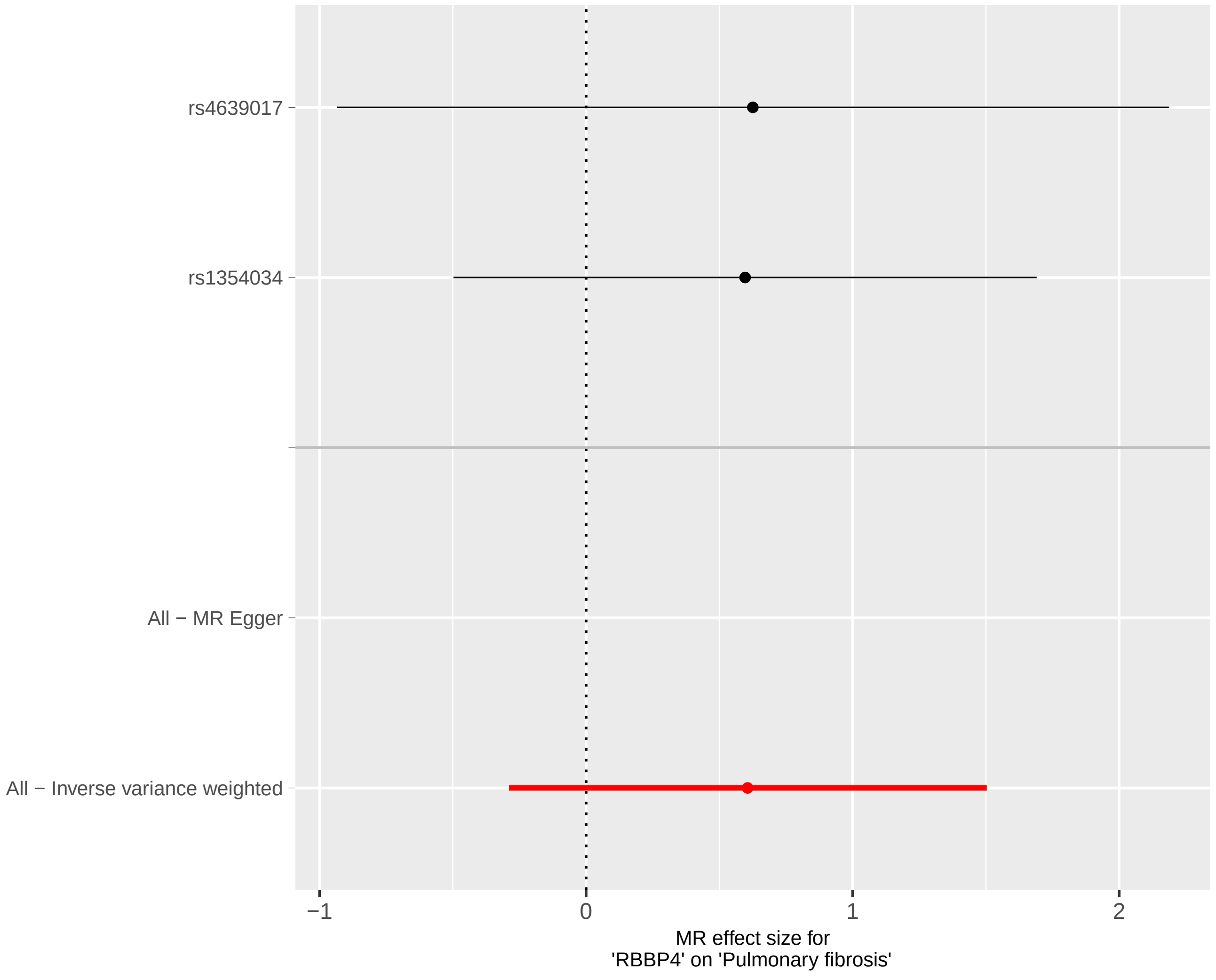
rs4639017
rs1354034
All − MR Egger
All − Inverse variance weighted
−1
0
1
2
MR effect size for
'RBBP4' on 'Pulmonary fibrosis'

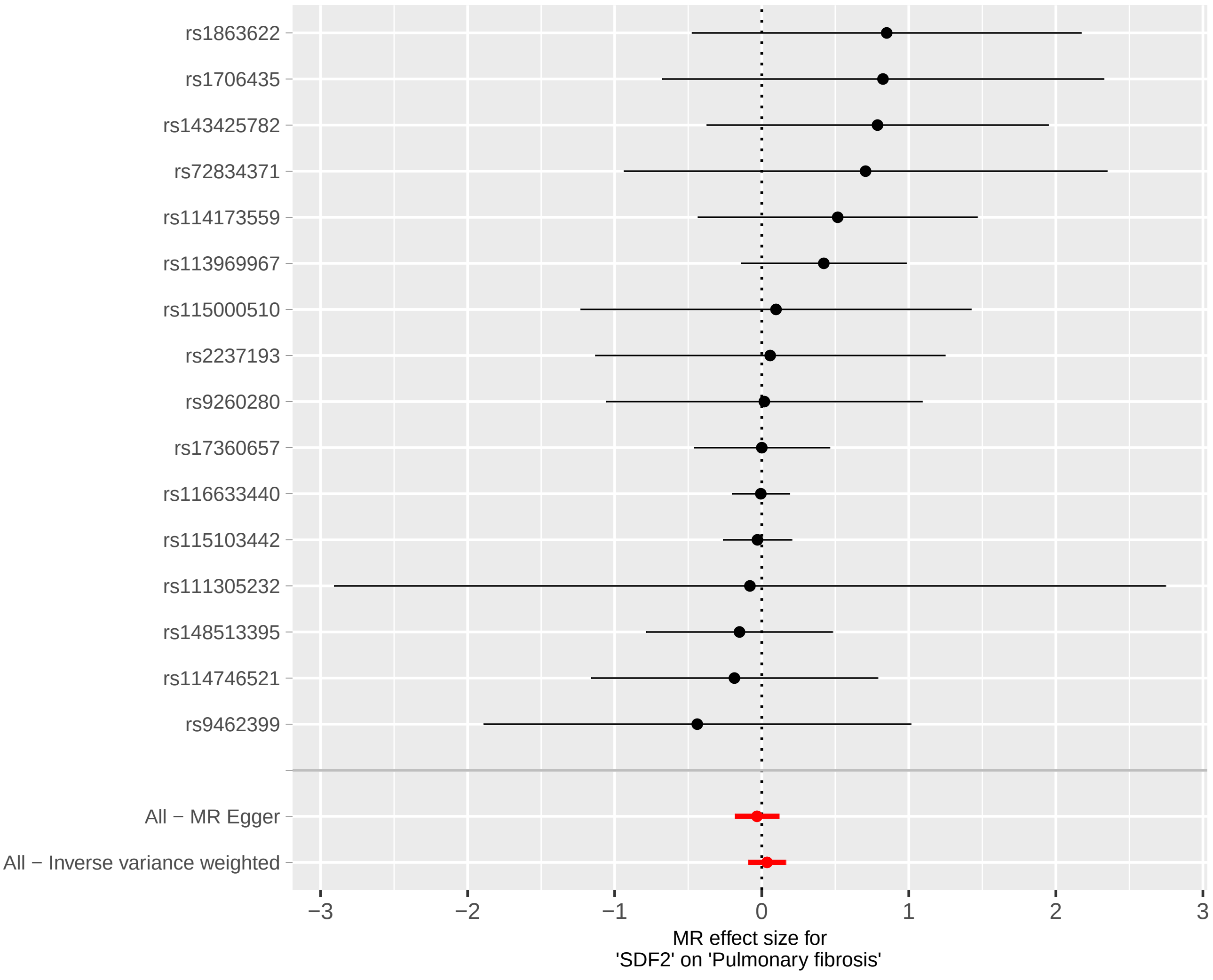

rs1863622
rs1706435
rs143425782
rs72834371
rs114173559
rs113969967
rs115000510
rs2237193
rs9260280
rs17360657
rs116633440
rs115103442
rs111305232
rs148513395
rs114746521
rs9462399
All − MR Egger
All − Inverse variance weighted
−3
−2
−1
0
1
2
3
MR effect size for
'SDF2' on 'Pulmonary fibrosis'

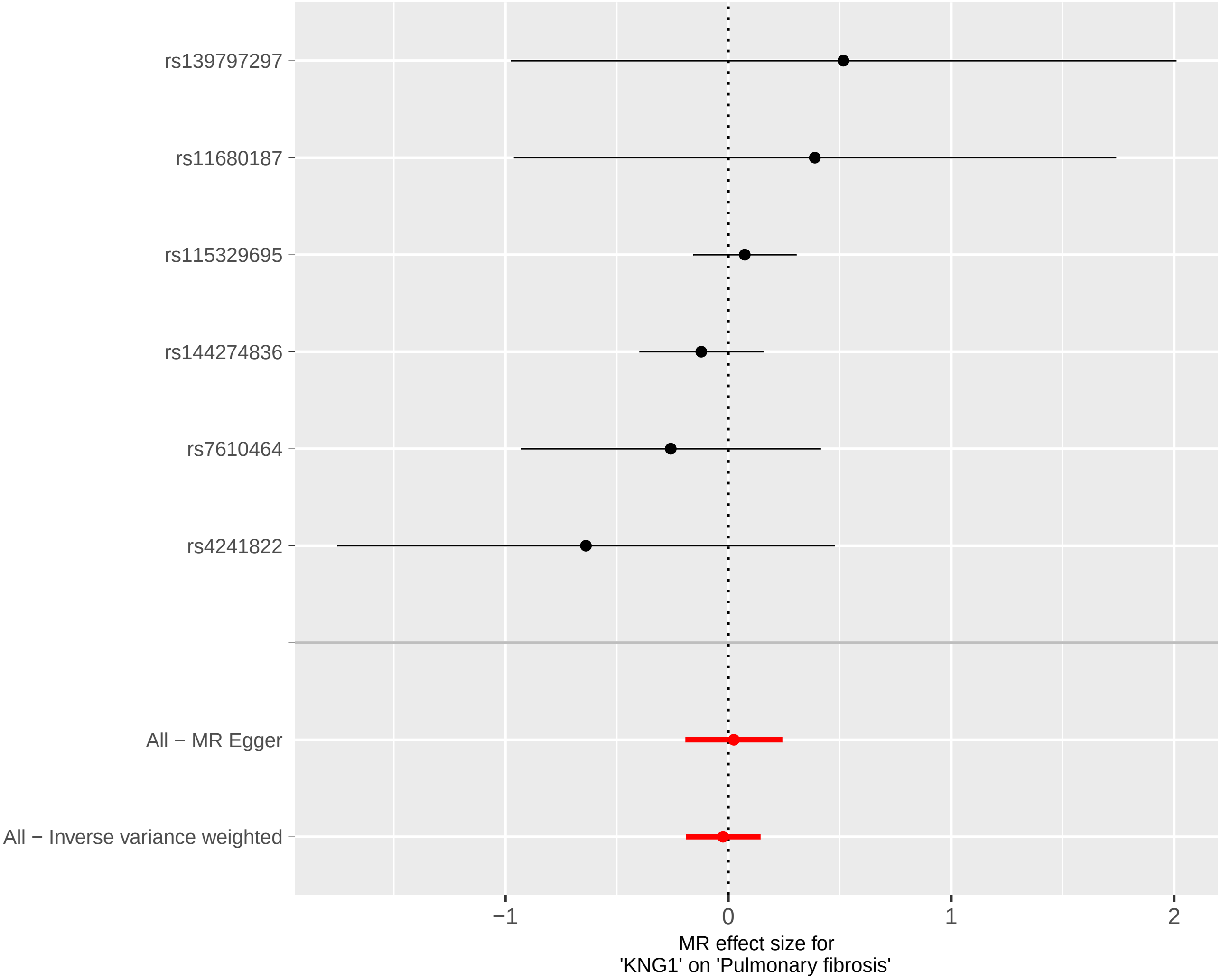

rs139797297
rs11680187
rs115329695
rs144274836
rs7610464
rs4241822
All − MR Egger
All − Inverse variance weighted
−1
0
1
2
MR effect size for
'KNG1' on 'Pulmonary fibrosis'

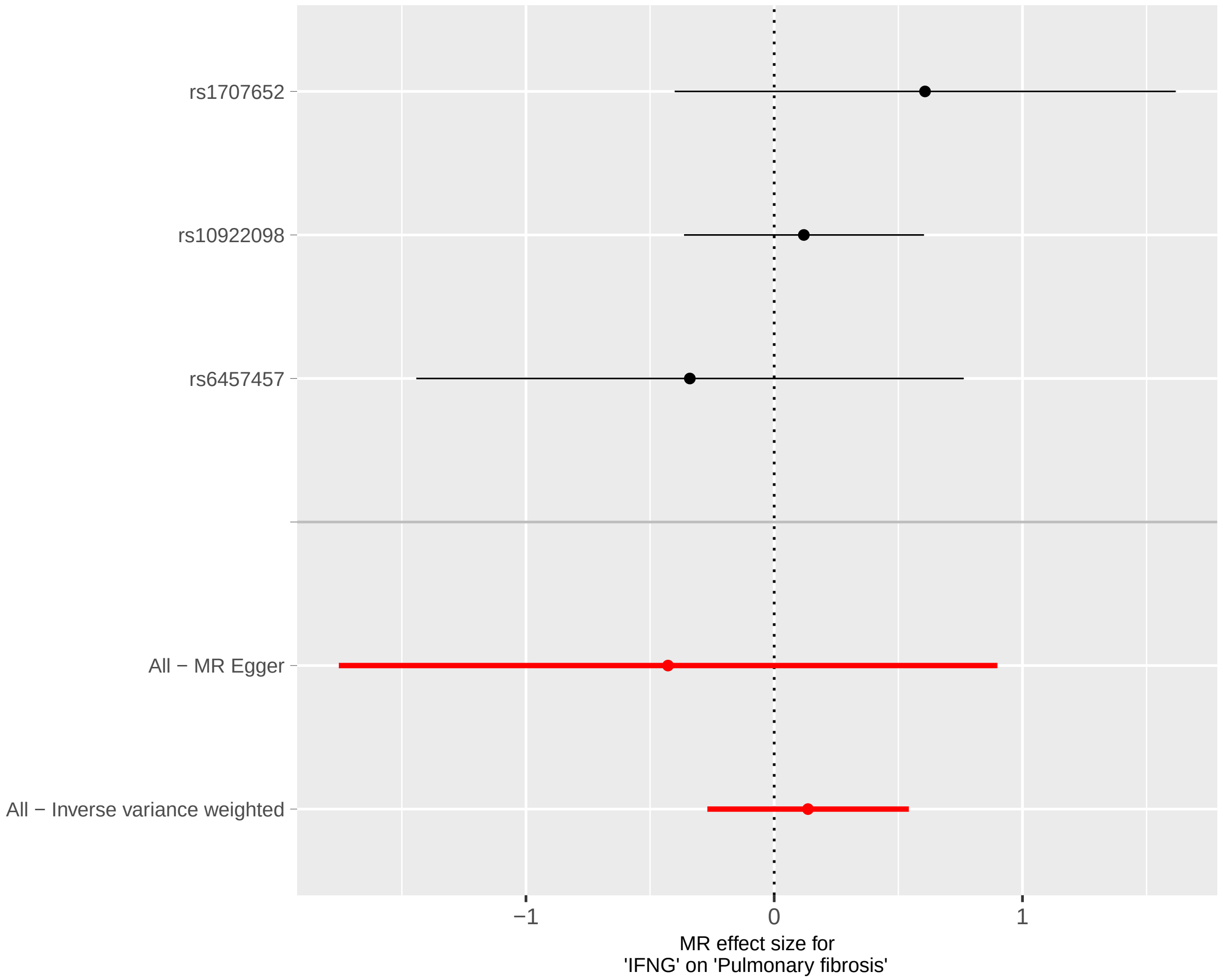
rs1707652
rs10922098
rs6457457
All − MR Egger
All − Inverse variance weighted
−1
0
1
MR effect size for
'IFNG' on 'Pulmonary fibrosis'

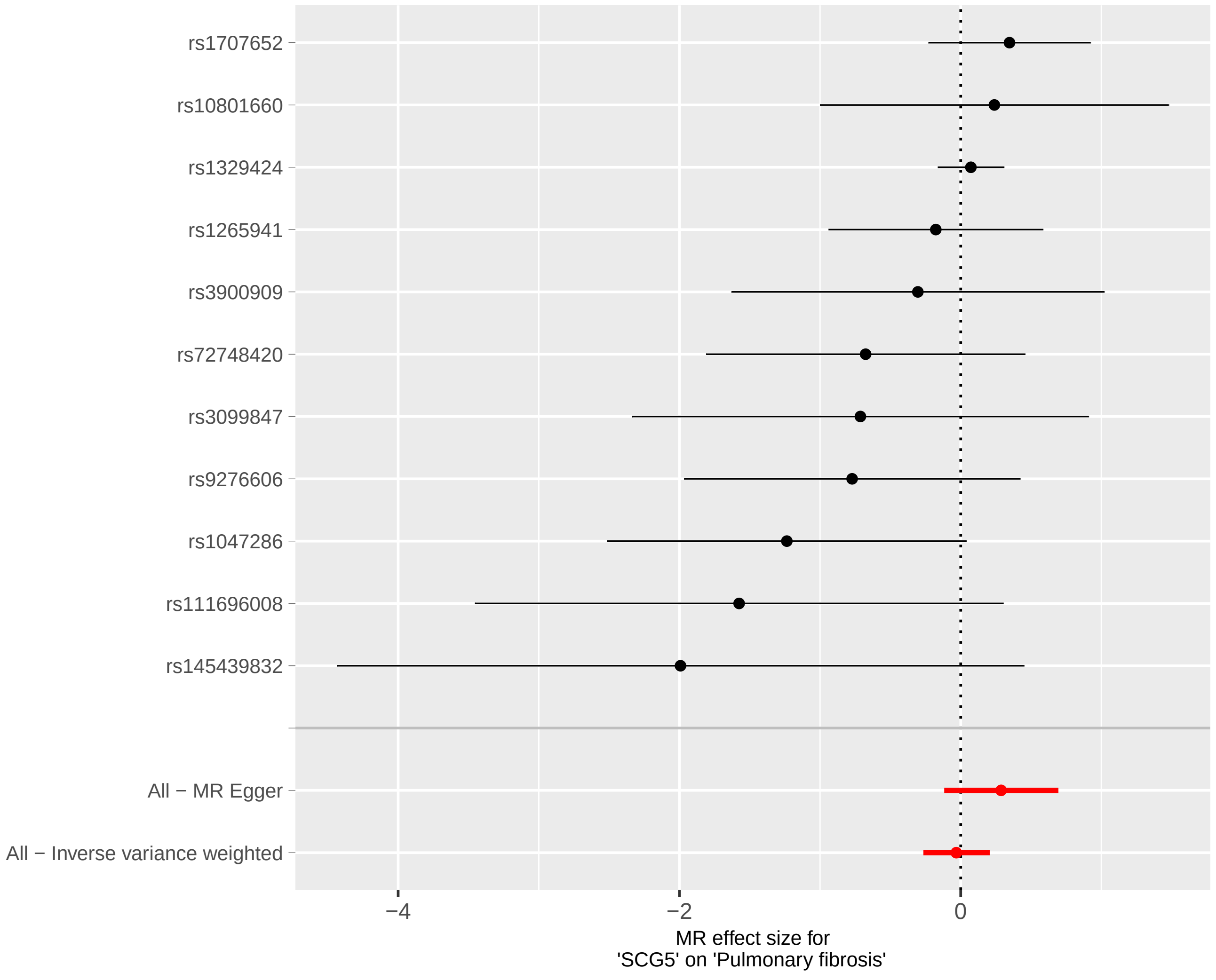

rs1707652
rs10801660
rs1329424
rs1265941
rs3900909
rs72748420
rs3099847
rs9276606
rs1047286
rs111696008
rs145439832
All − MR Egger
All − Inverse variance weighted
−4
−2
0
MR effect size for
'SCG5' on 'Pulmonary fibrosis'

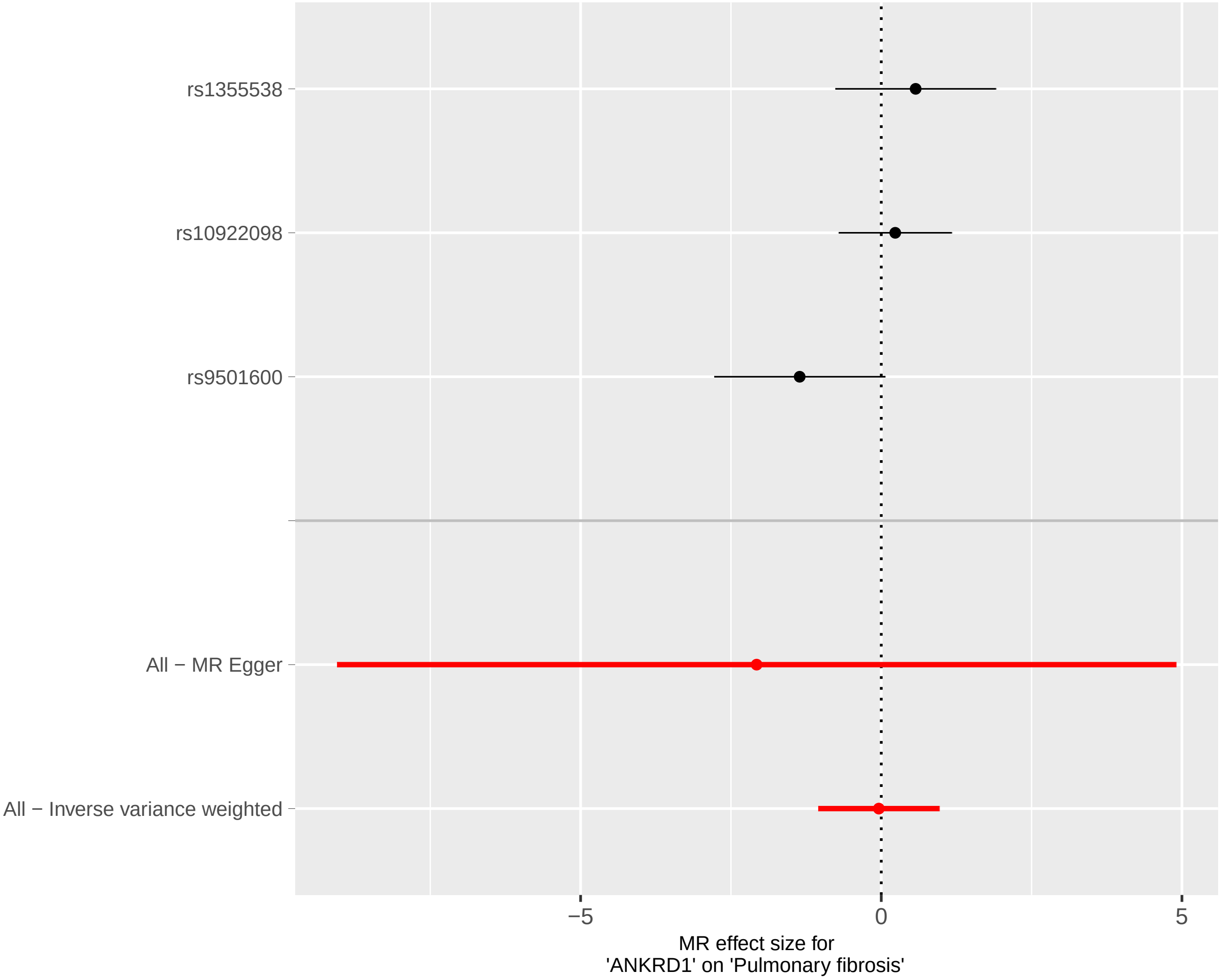
rs1355538
rs10922098
rs9501600
All − MR Egger
All − Inverse variance weighted
−5
0
5
MR effect size for
'ANKRD1' on 'Pulmonary fibrosis'

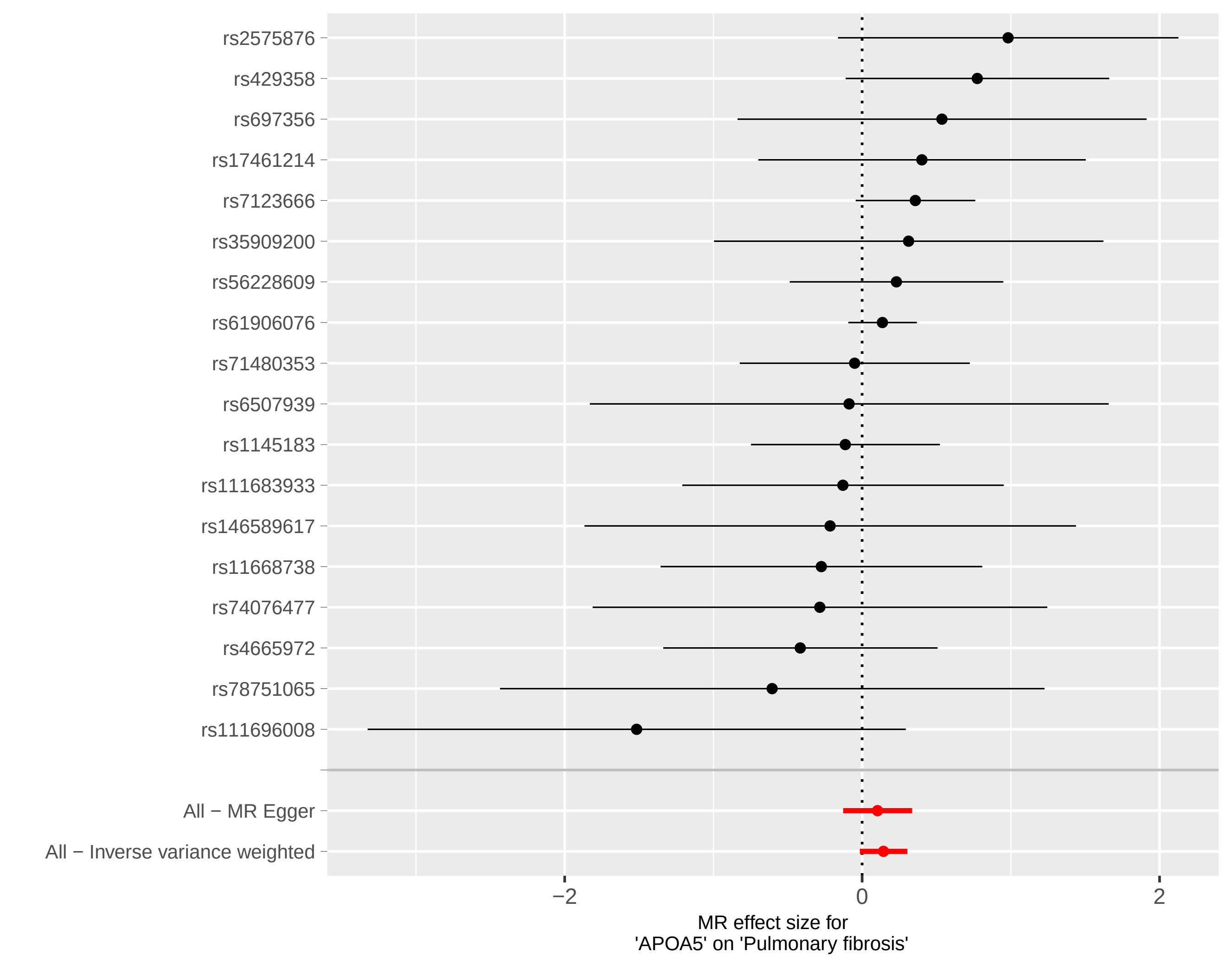
rs2575876
rs429358
rs697356
rs17461214
rs7123666
rs35909200
rs56228609
rs61906076
rs71480353
rs6507939
rs1145183
rs111683933
rs146589617
rs11668738
rs74076477
rs4665972
rs78751065
rs111696008
All − MR Egger
All − Inverse variance weighted
−2
0
2
MR effect size for
'APOA5' on 'Pulmonary fibrosis'

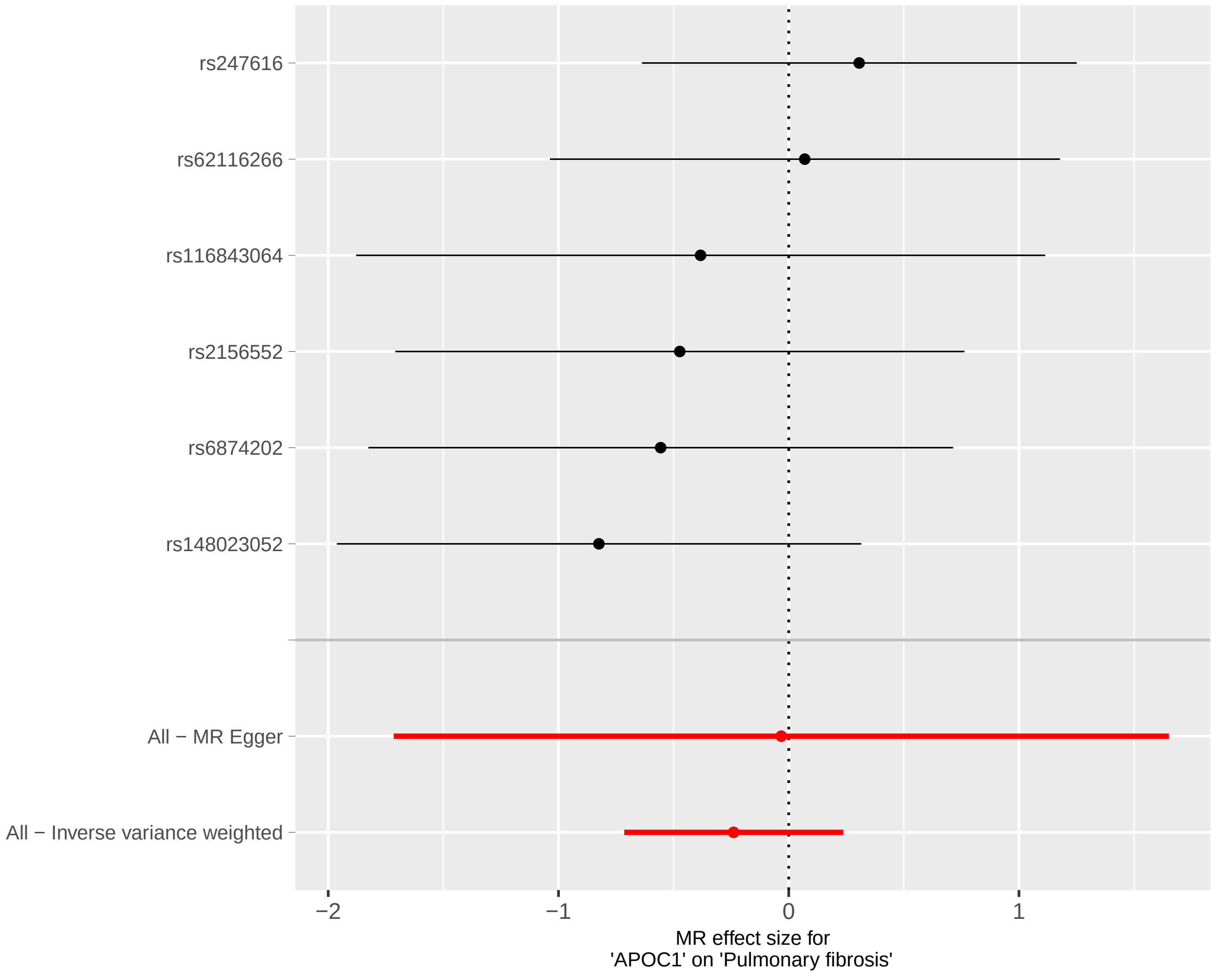
rs247616
rs62116266
rs116843064
rs2156552
rs6874202
rs148023052
All − MR Egger
All − Inverse variance weighted
−2
−1
0
1
MR effect size for
'APOC1' on 'Pulmonary fibrosis'

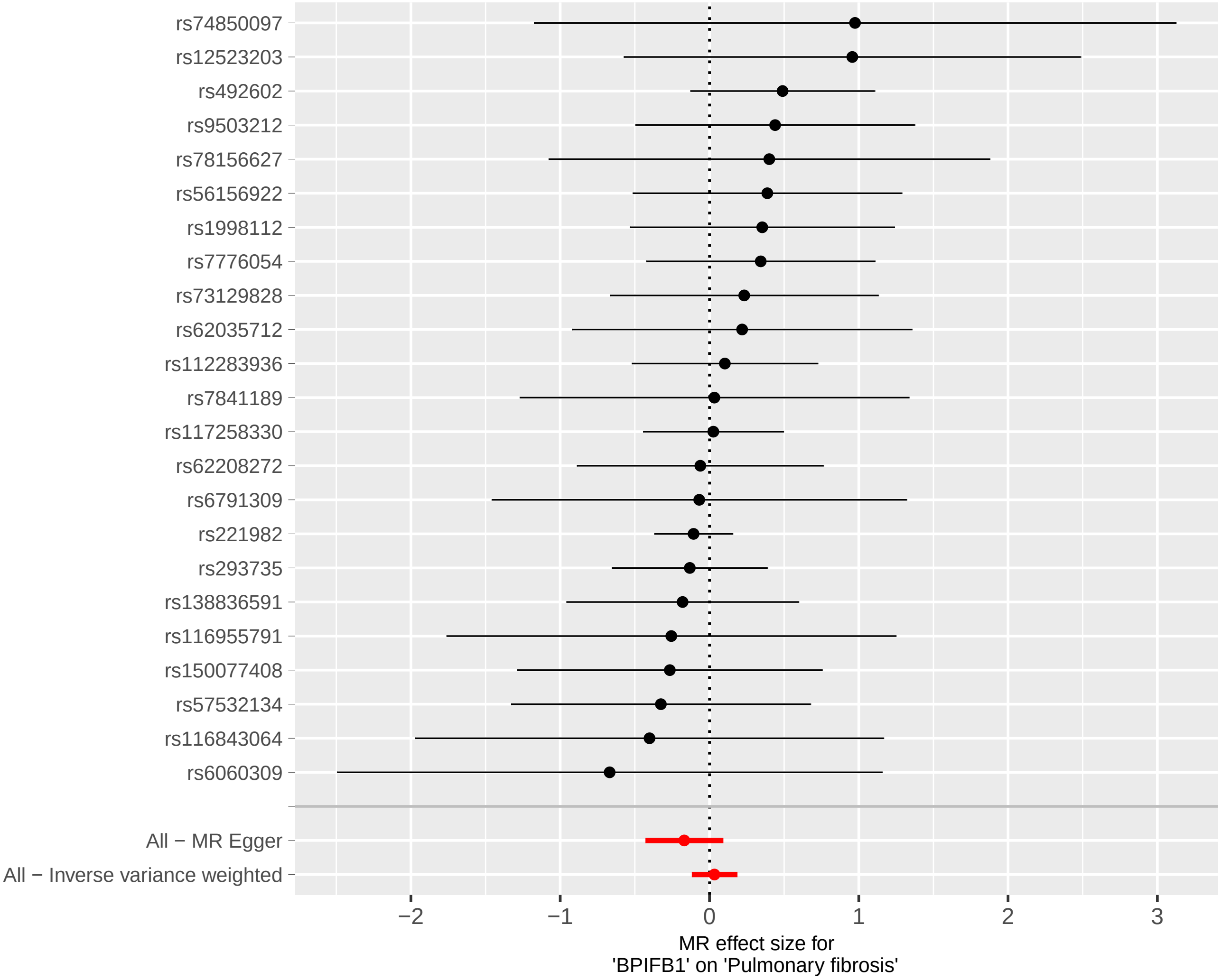

rs74850097
rs12523203
rs492602
rs9503212
rs78156627
rs56156922
rs1998112
rs7776054
rs73129828
rs62035712
rs112283936
rs7841189
rs117258330
rs62208272
rs6791309
rs221982
rs293735
rs138836591
rs116955791
rs150077408
rs57532134
rs116843064
rs6060309
All − MR Egger
All − Inverse variance weighted
−2
−1
0
1
2
3
MR effect size for
'BPIFB1' on 'Pulmonary fibrosis'

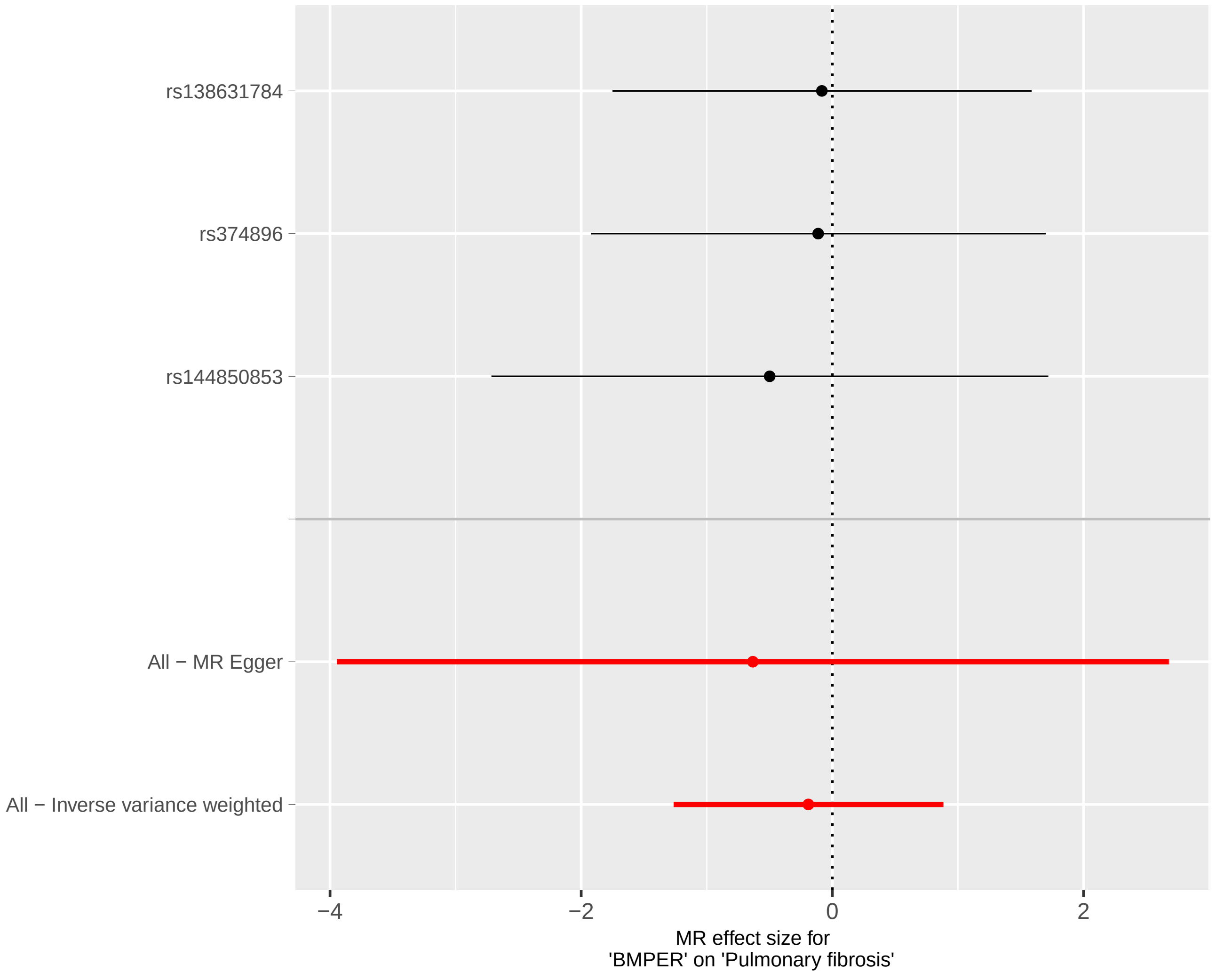

rs138631784
rs374896
rs144850853
All − MR Egger
All − Inverse variance weighted
−4
−2
0
2
MR effect size for
'BMPER' on 'Pulmonary fibrosis'

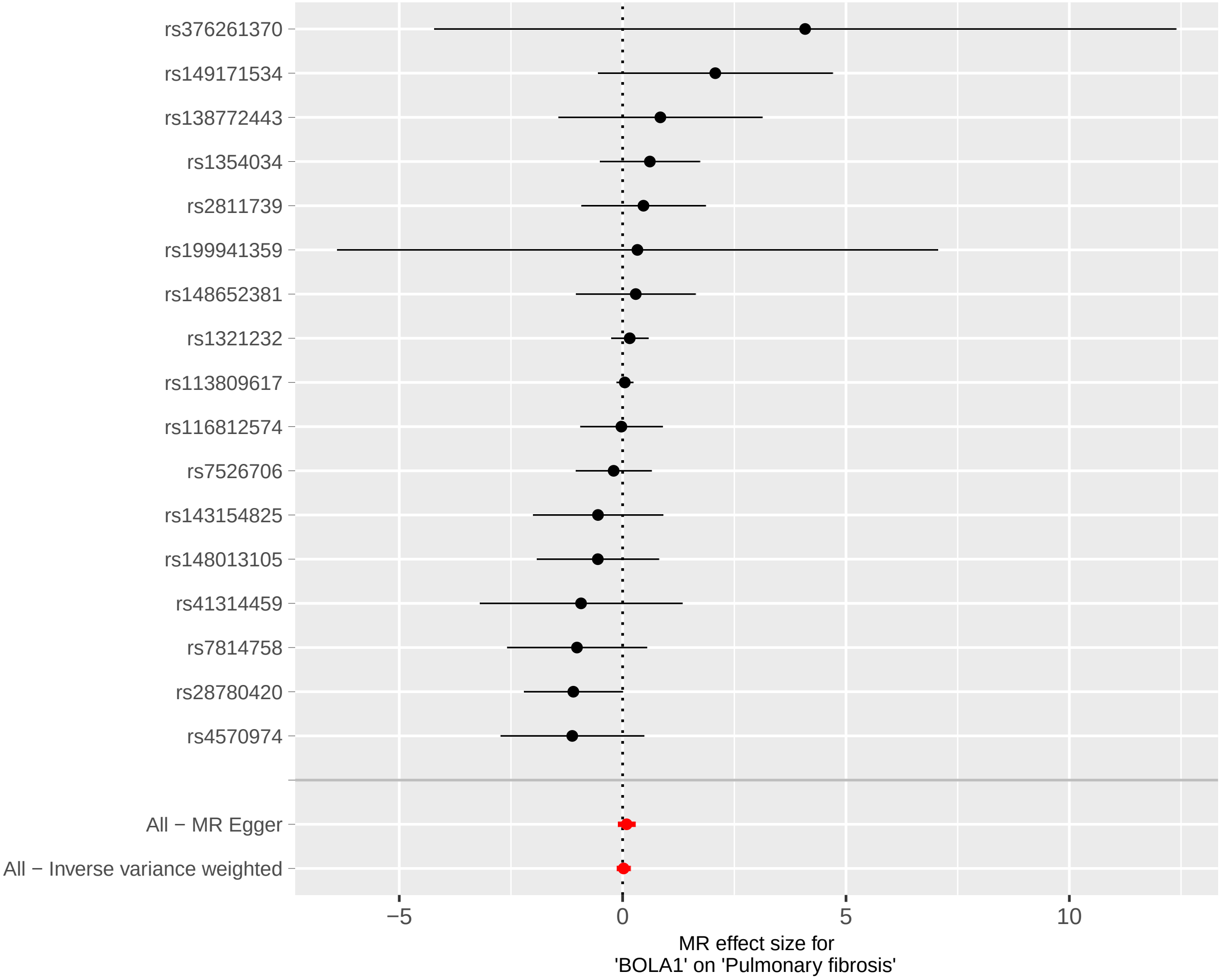

rs376261370
rs149171534
rs138772443
rs1354034
rs2811739
rs199941359
rs148652381
rs1321232
rs113809617
rs116812574
rs7526706
rs143154825
rs148013105
rs41314459
rs7814758
rs28780420
rs4570974
All − MR Egger
All − Inverse variance weighted
−5
0
5
10
MR effect size for
'BOLA1' on 'Pulmonary fibrosis'

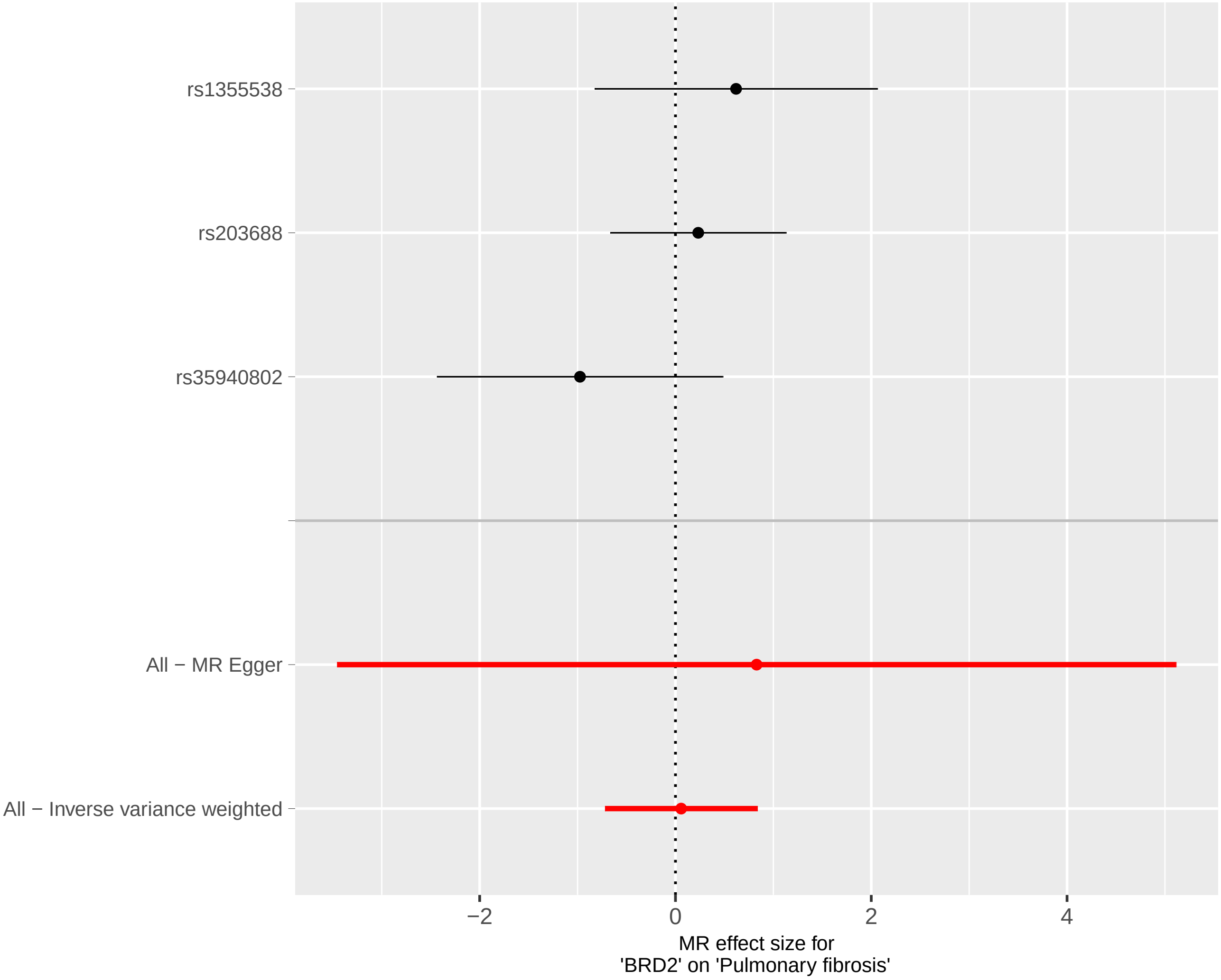

rs1355538
rs203688
rs35940802
All − MR Egger
All − Inverse variance weighted
−2
0
2
4
MR effect size for
'BRD2' on 'Pulmonary fibrosis'

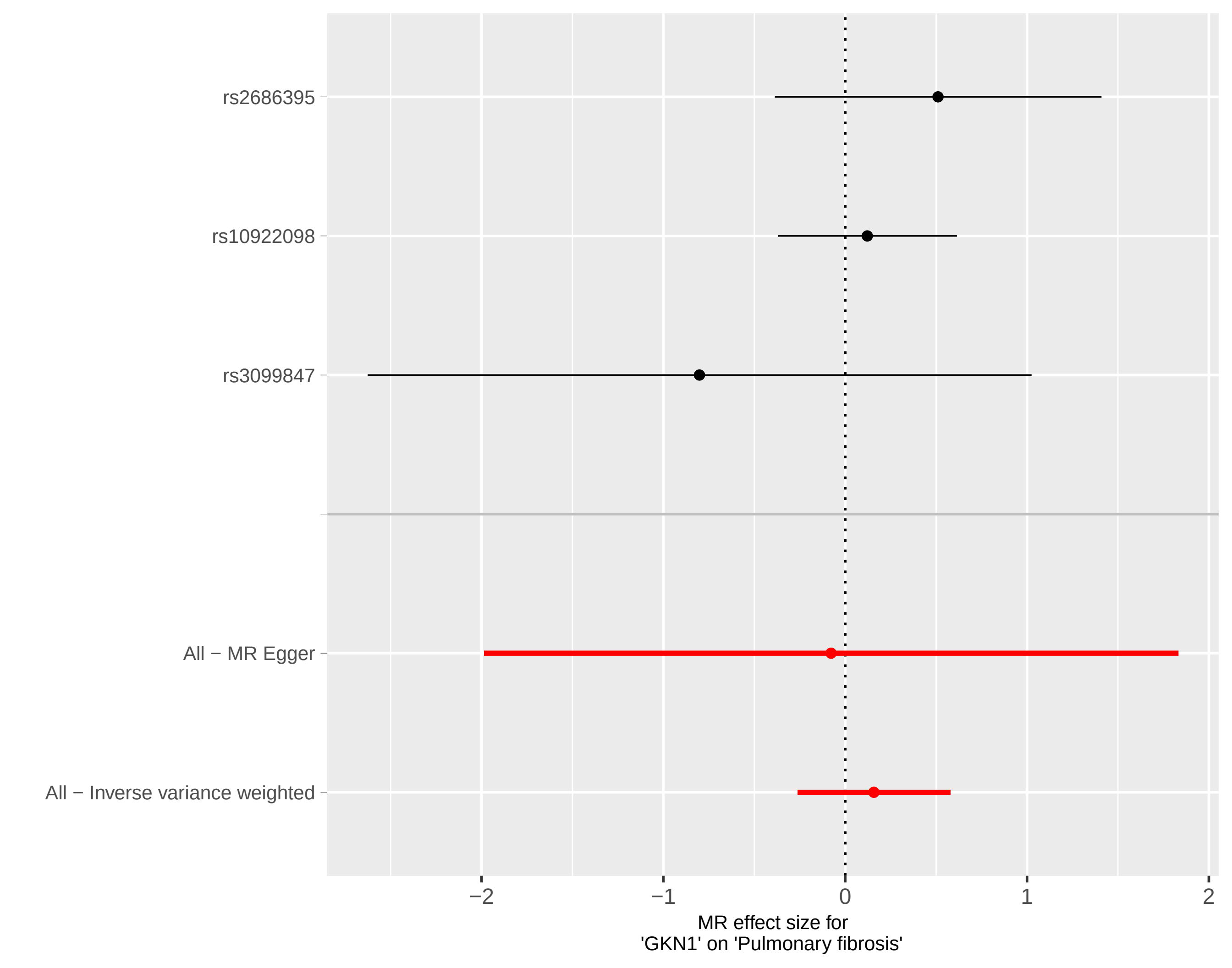

rs2686395
rs10922098
rs3099847
All − MR Egger
All − Inverse variance weighted
−2
−1
0
1
2
MR effect size for
'GKN1' on 'Pulmonary fibrosis'

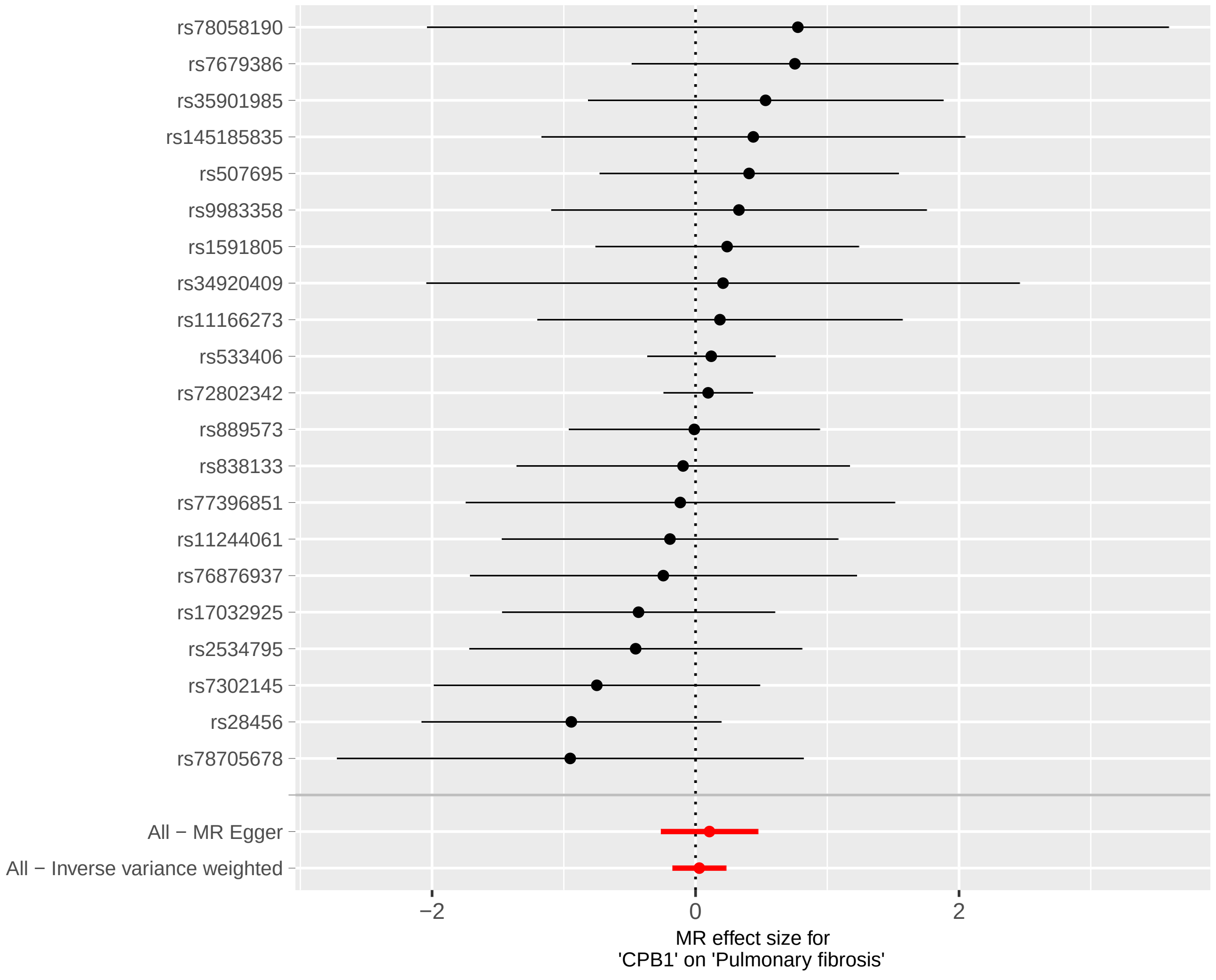

rs78058190
rs7679386
rs35901985
rs145185835
rs507695
rs9983358
rs1591805
rs34920409
rs11166273
rs533406
rs72802342
rs889573
rs838133
rs77396851
rs11244061
rs76876937
rs17032925
rs2534795
rs7302145
rs28456
rs78705678
All − MR Egger
All − Inverse variance weighted
−2
0
2
MR effect size for
'CPB1' on 'Pulmonary fibrosis'

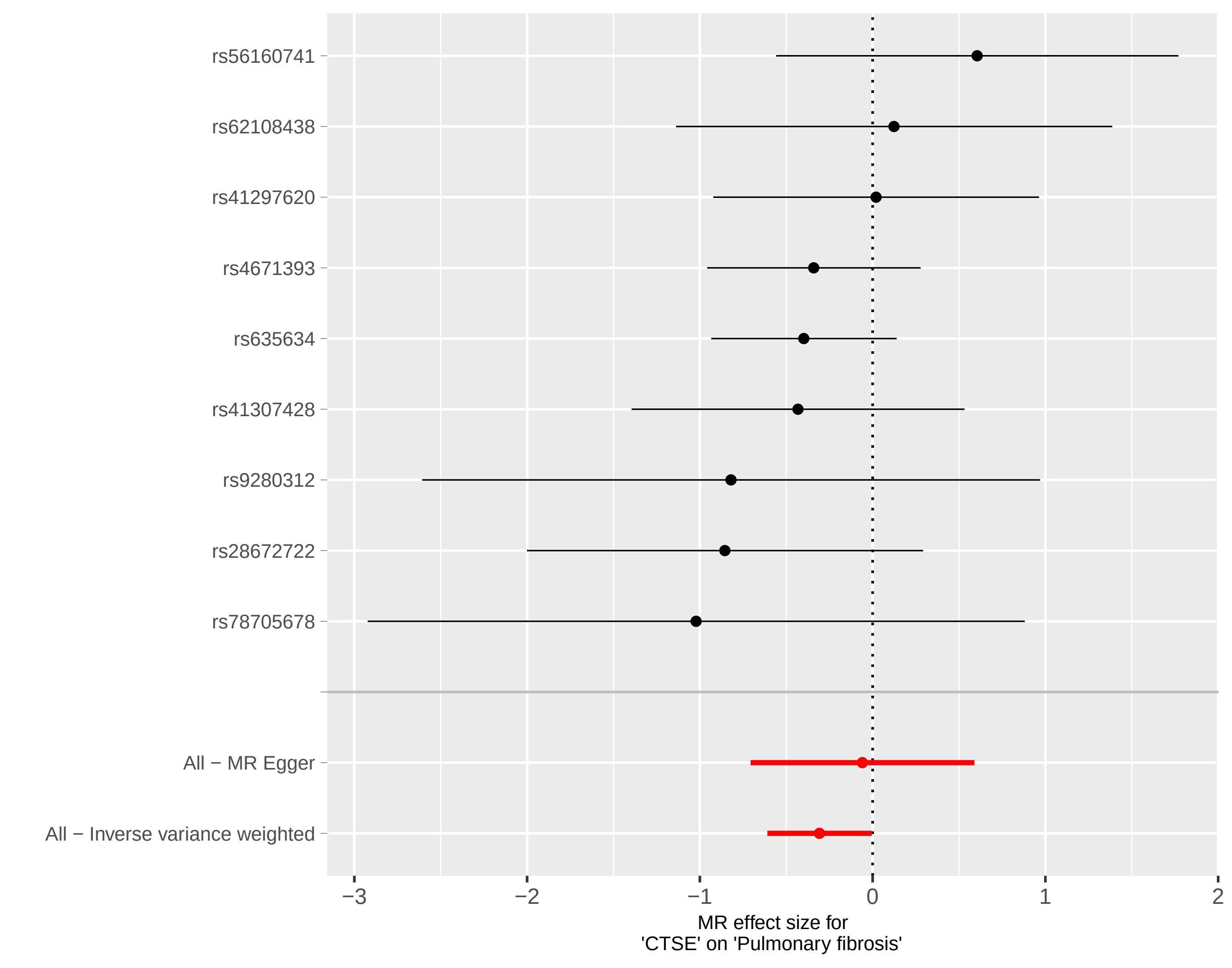
rs56160741
rs62108438
rs41297620
rs4671393
rs635634
rs41307428
rs9280312
rs28672722
rs78705678
All − MR Egger
All − Inverse variance weighted
−3
−2
−1
0
1
2
MR effect size for
'CTSE' on 'Pulmonary fibrosis'

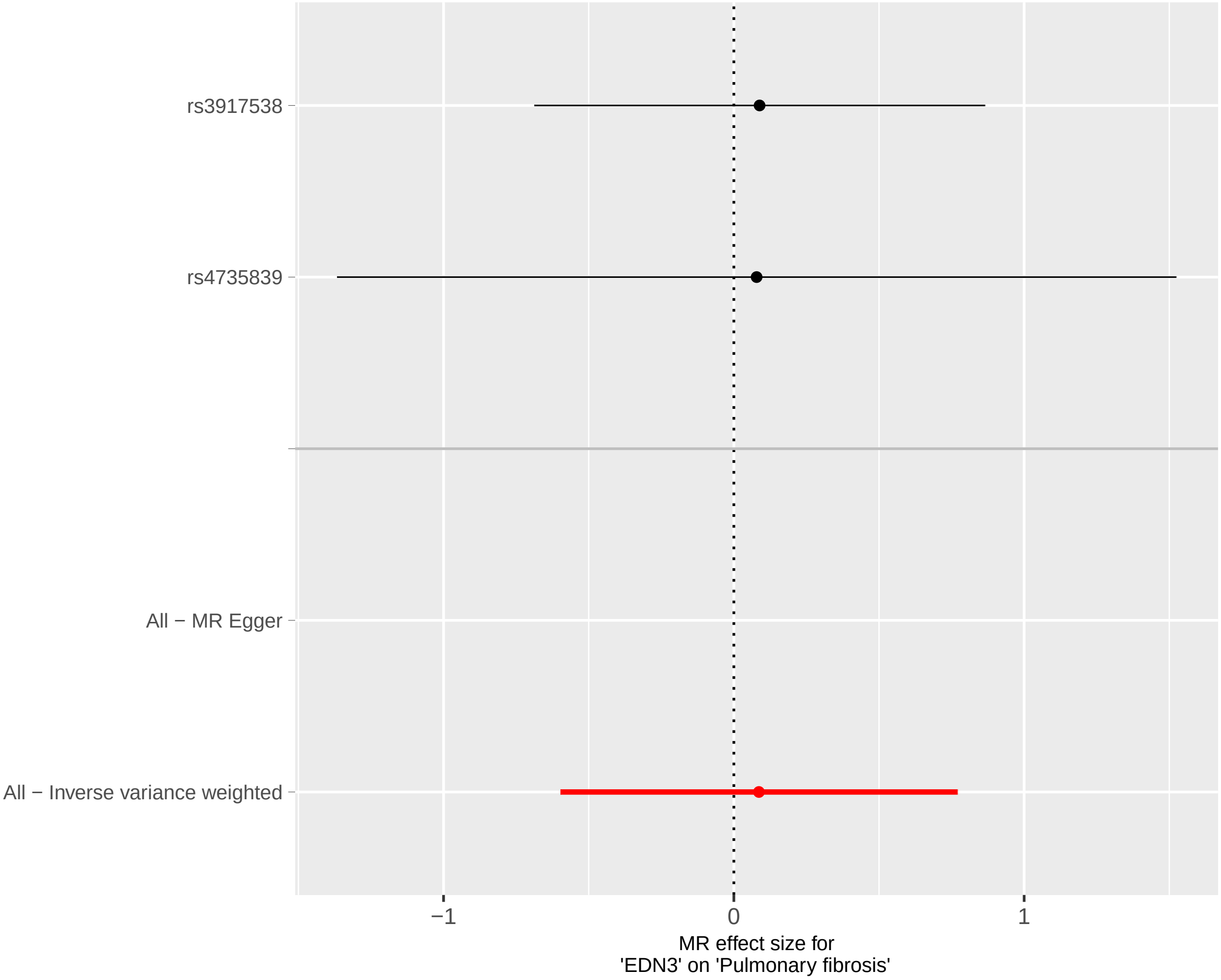
rs3917538
rs4735839
All − MR Egger
All − Inverse variance weighted
−1
0
1
MR effect size for
'EDN3' on 'Pulmonary fibrosis'

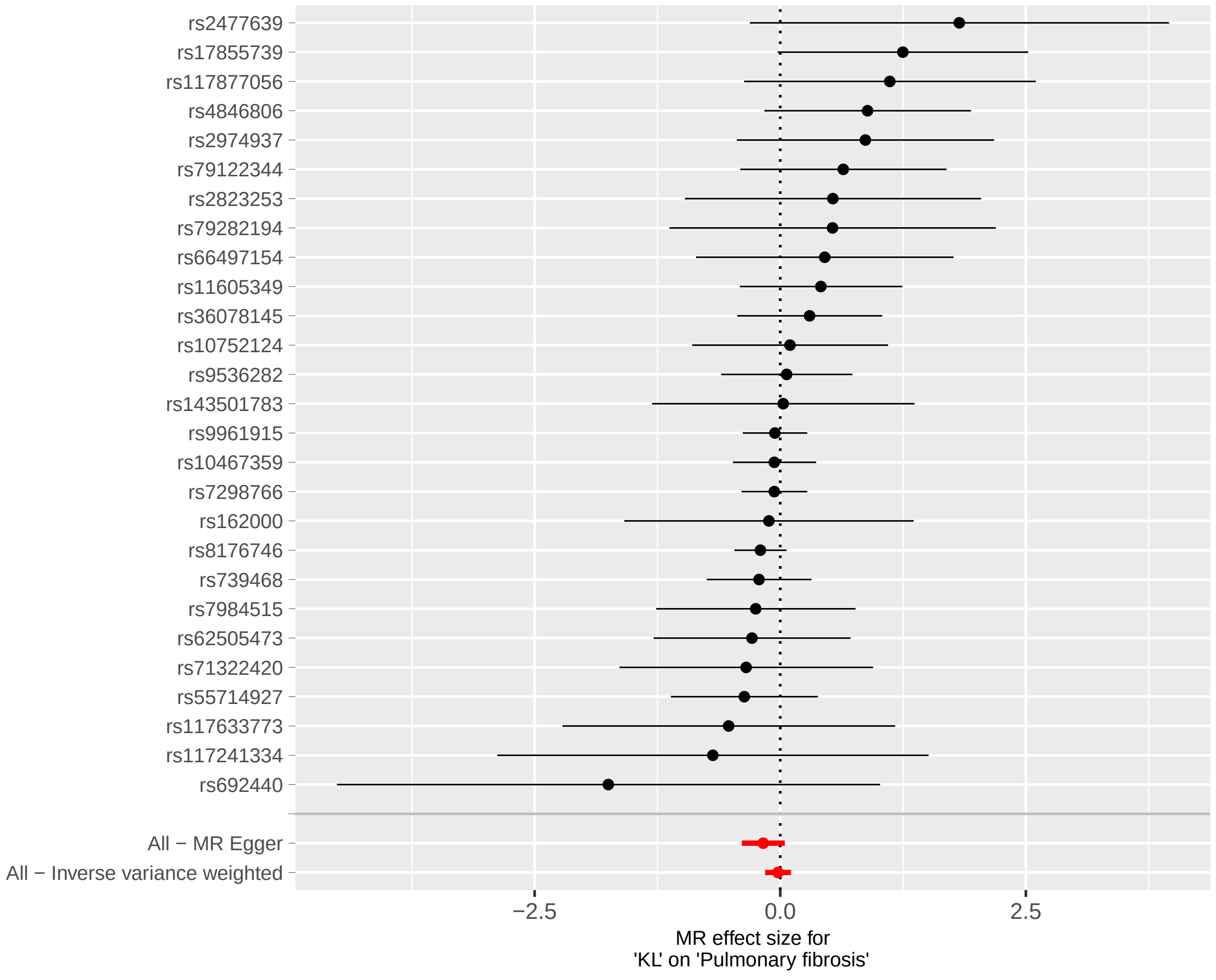
rs2477639
rs17855739
rs117877056
rs4846806
rs2974937
rs79122344
rs2823253
rs79282194
rs66497154
rs11605349
rs36078145
rs10752124
rs9536282
rs143501783
rs9961915
rs10467359
rs7298766
rs162000
rs8176746
rs739468
rs7984515
rs62505473
rs71322420
rs55714927
rs117633773
rs117241334
rs692440
All − MR Egger
All − Inverse variance weighted
−2.5
0.0
2.5
MR effect size for
'KL' on 'Pulmonary fibrosis'

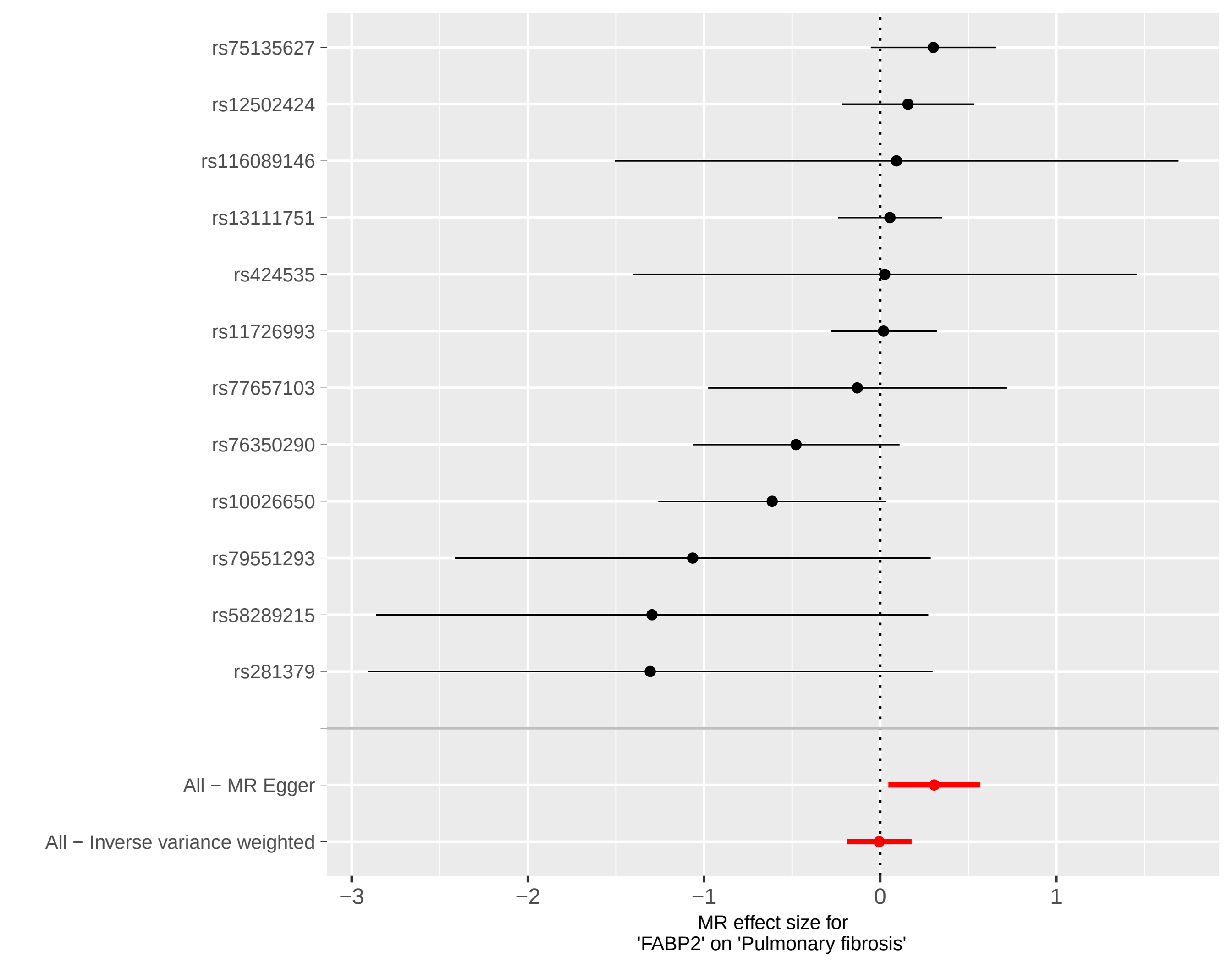
rs75135627
rs12502424
rs116089146
rs13111751
rs424535
rs11726993
rs77657103
rs76350290
rs10026650
rs79551293
rs58289215
rs281379
All − MR Egger
All − Inverse variance weighted
−3
−2
−1
0
1
MR effect size for
'FABP2' on 'Pulmonary fibrosis'

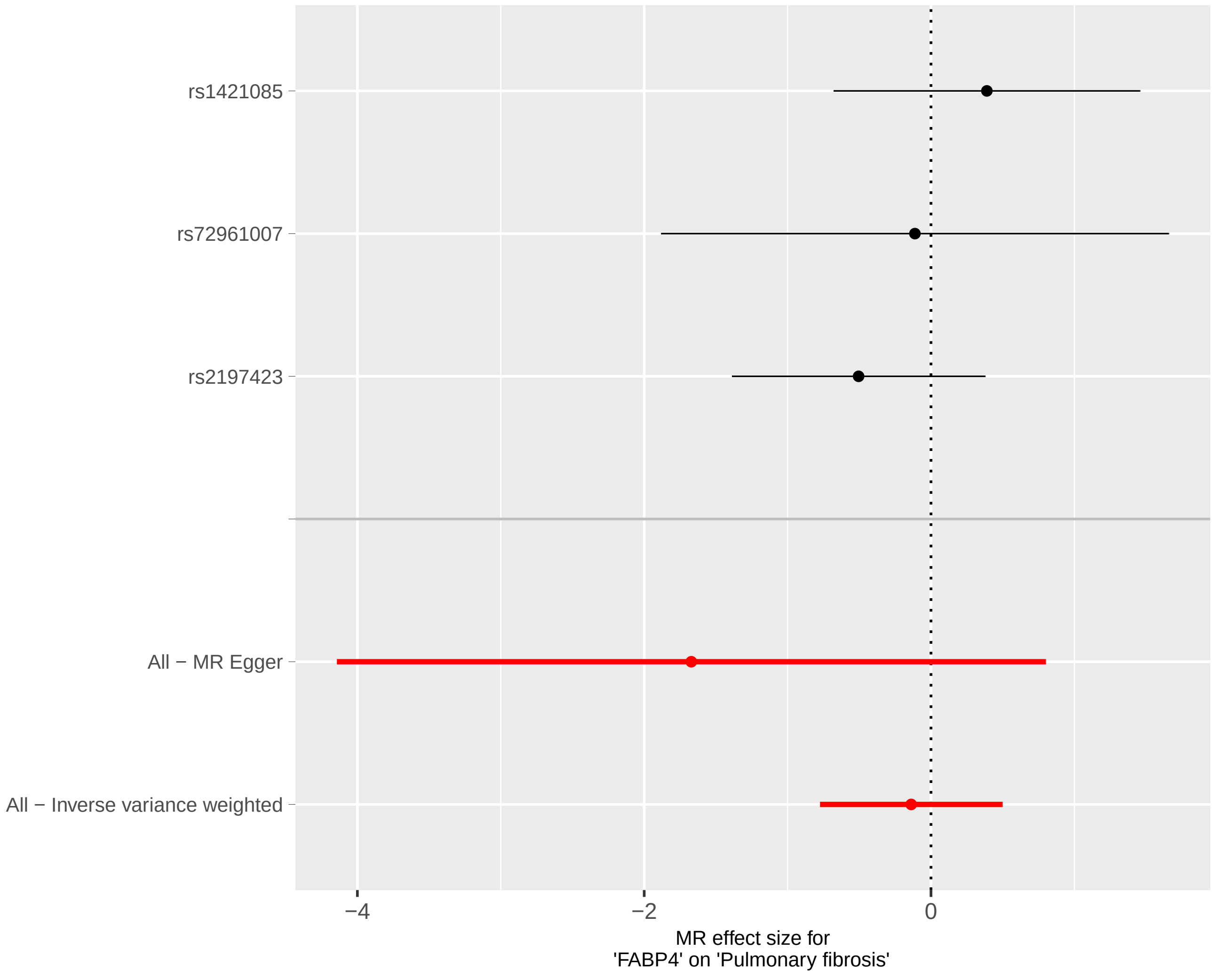
rs1421085
rs72961007
rs2197423
All − MR Egger
All − Inverse variance weighted
−4
−2
0
MR effect size for
'FABP4' on 'Pulmonary fibrosis'

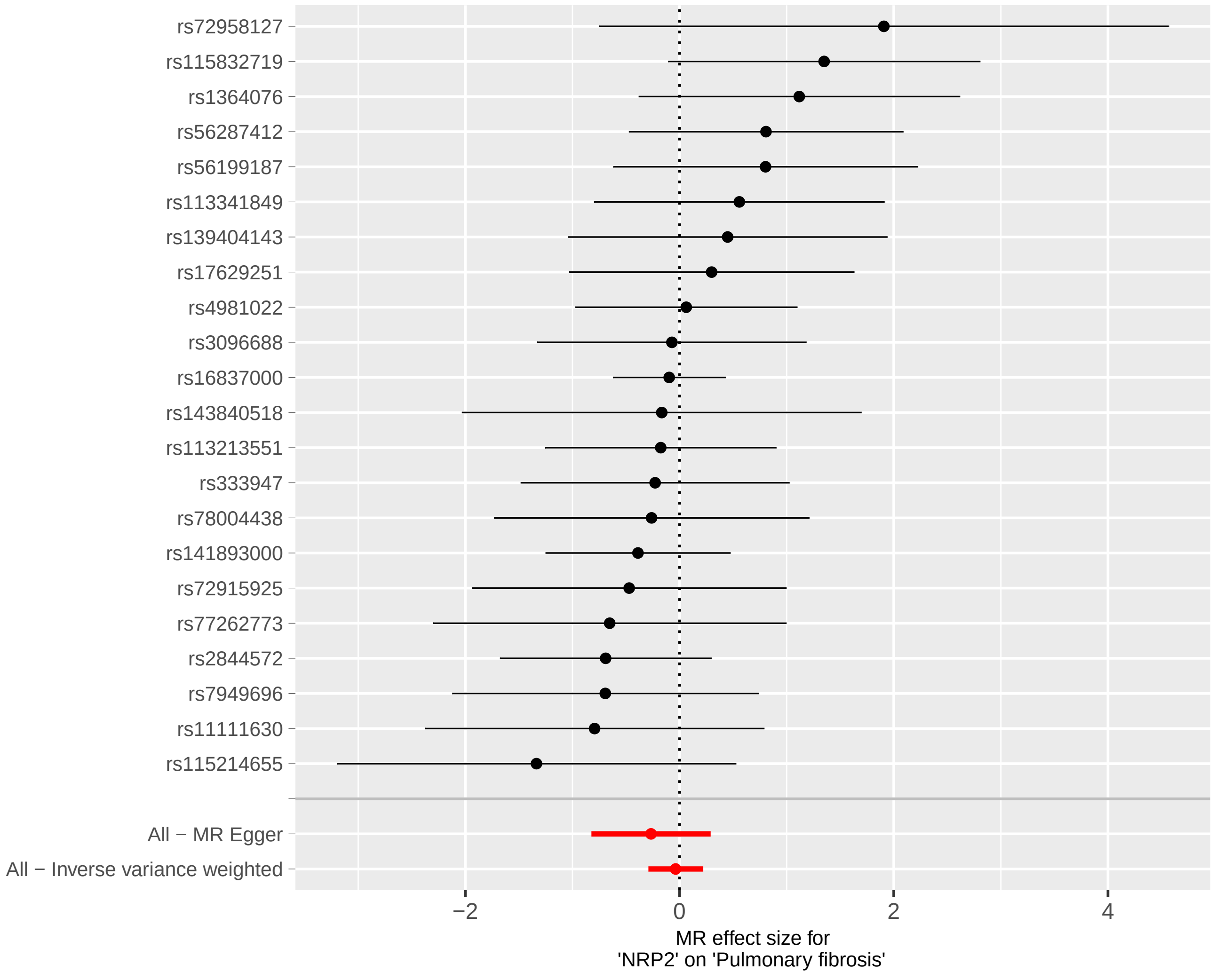

rs72958127
rs115832719
rs1364076
rs56287412
rs56199187
rs113341849
rs139404143
rs17629251
rs4981022
rs3096688
rs16837000
rs143840518
rs113213551
rs333947
rs78004438
rs141893000
rs72915925
rs77262773
rs2844572
rs7949696
rs11111630
rs115214655
All − MR Egger
All − Inverse variance weighted
−2
0
2
4
MR effect size for
'NRP2' on 'Pulmonary fibrosis'

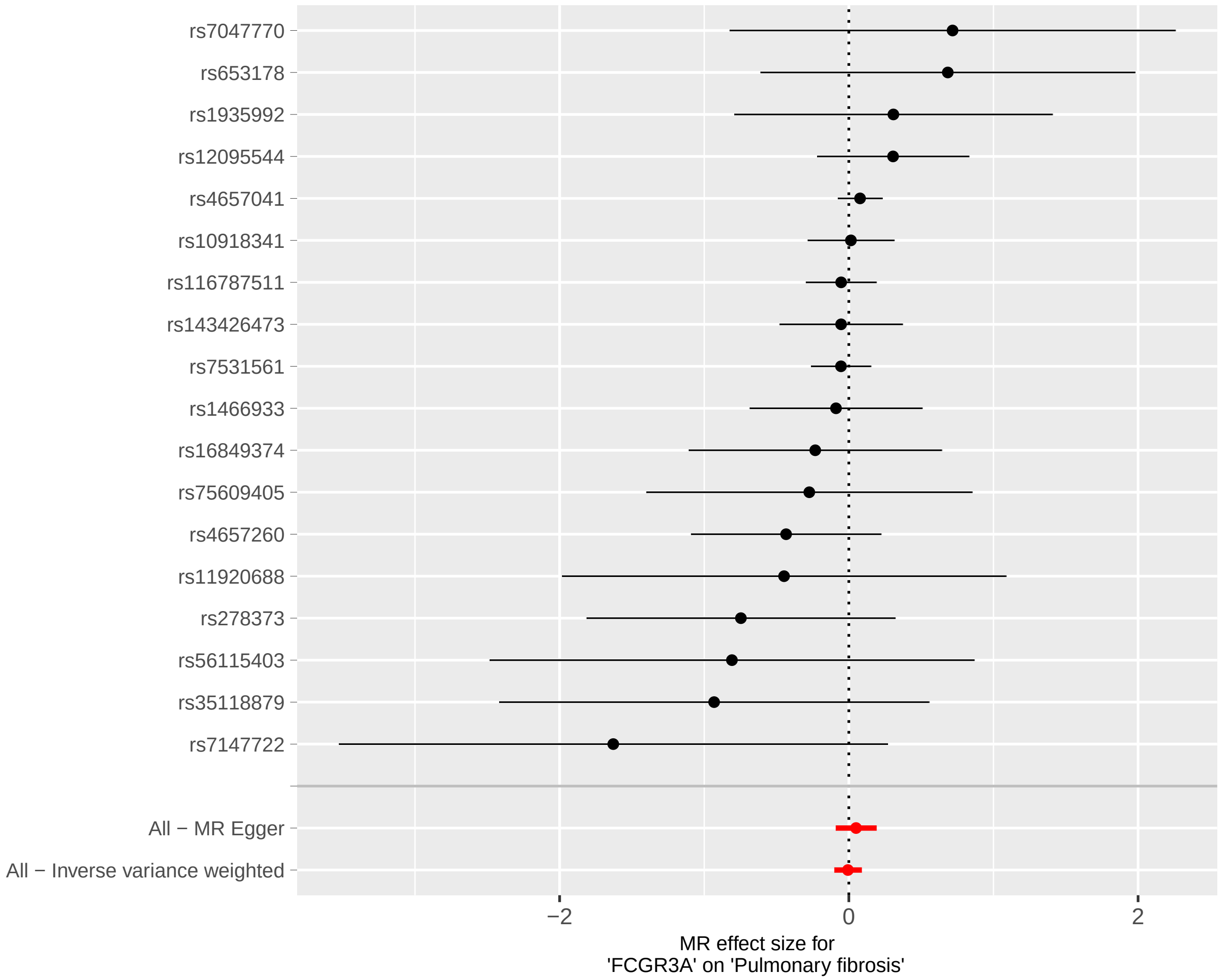
rs7047770
rs653178
rs1935992
rs12095544
rs4657041
rs10918341
rs116787511
rs143426473
rs7531561
rs1466933
rs16849374
rs75609405
rs4657260
rs11920688
rs278373
rs56115403
rs35118879
rs7147722
All − MR Egger
All − Inverse variance weighted
−2
0
2
MR effect size for
'FCGR3A' on 'Pulmonary fibrosis'

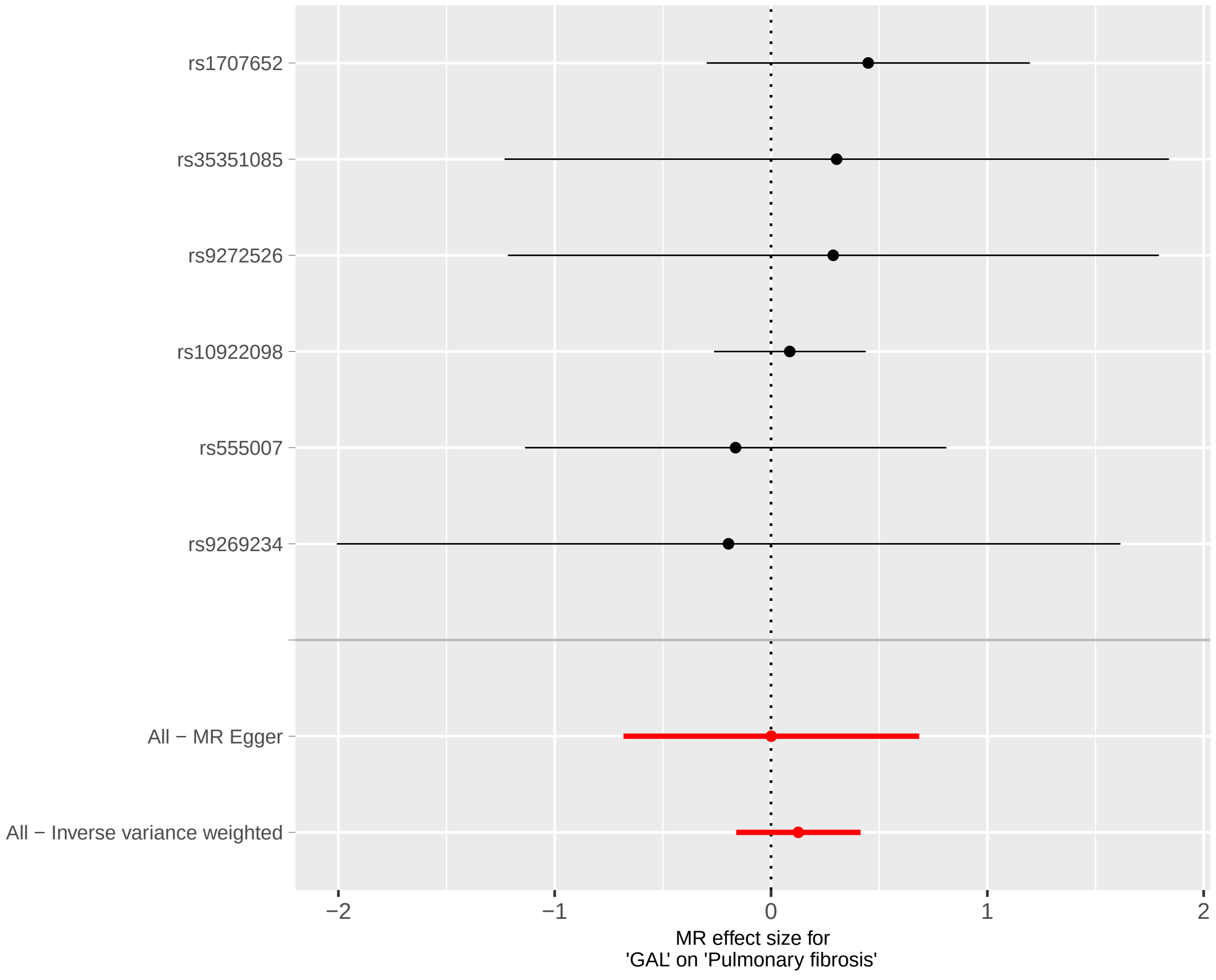
rs1707652
rs35351085
rs9272526
rs10922098
rs555007
rs9269234
All − MR Egger
All − Inverse variance weighted
−2
−1
0
1
2
MR effect size for
'GAL' on 'Pulmonary fibrosis'

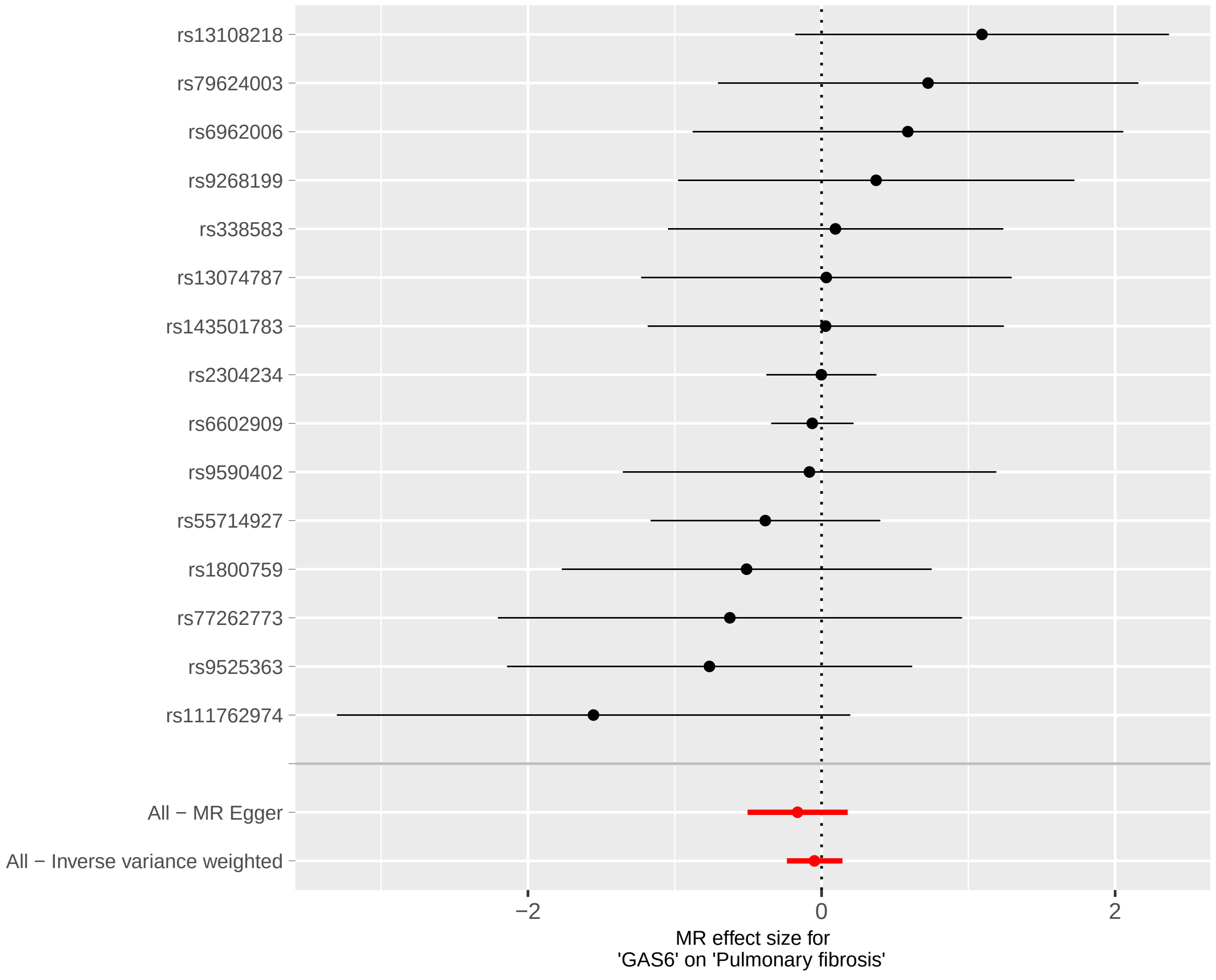

rs13108218
rs79624003
rs6962006
rs9268199
rs338583
rs13074787
rs143501783
rs2304234
rs6602909
rs9590402
rs55714927
rs1800759
rs77262773
rs9525363
rs111762974
All − MR Egger
All − Inverse variance weighted
−2
0
2
MR effect size for
'GAS6' on 'Pulmonary fibrosis'

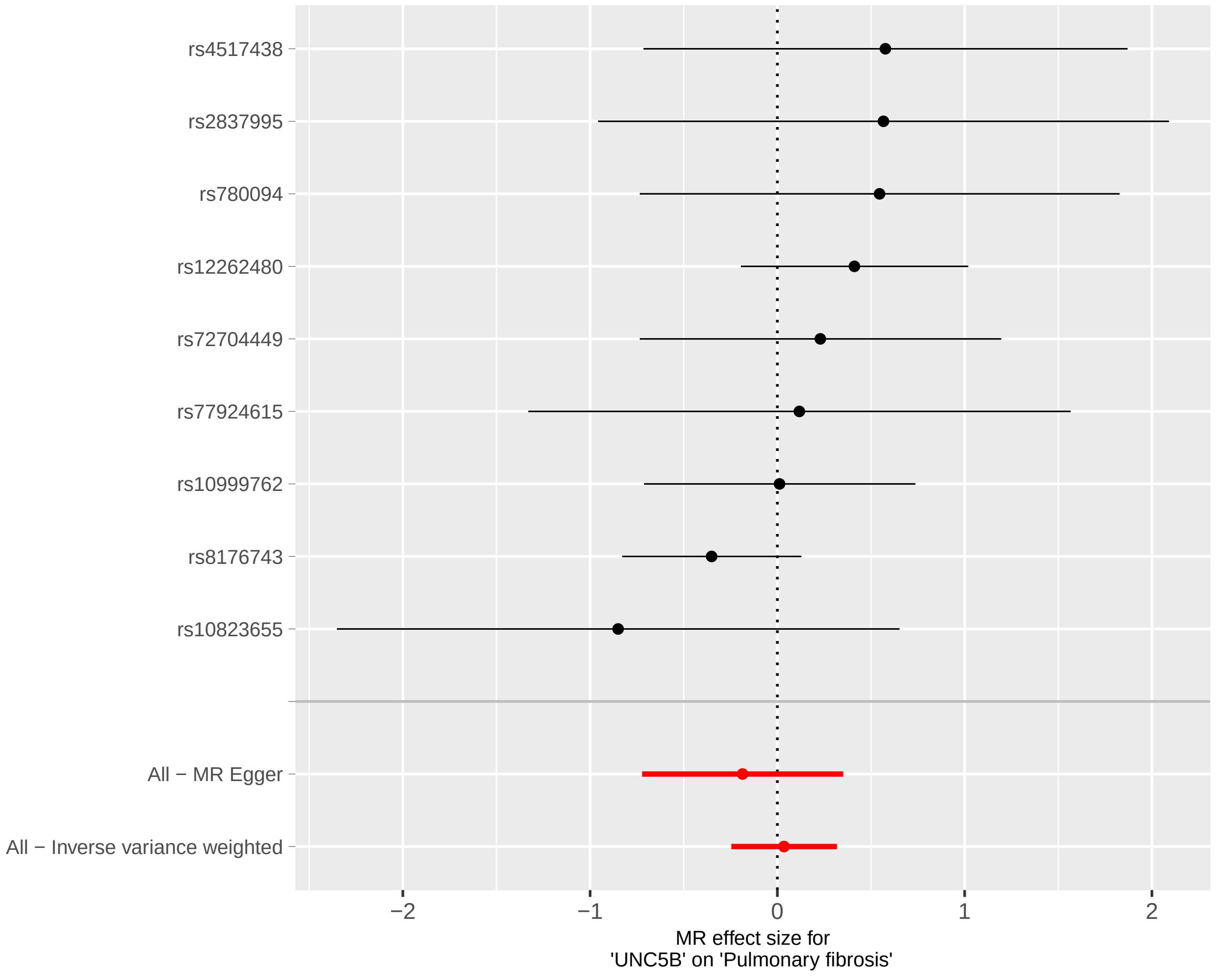

rs4517438
rs2837995
rs780094
rs12262480
rs72704449
rs77924615
rs10999762
rs8176743
rs10823655
All − MR Egger
All − Inverse variance weighted
−2
−1
0
1
2
MR effect size for
'UNC5B' on 'Pulmonary fibrosis'

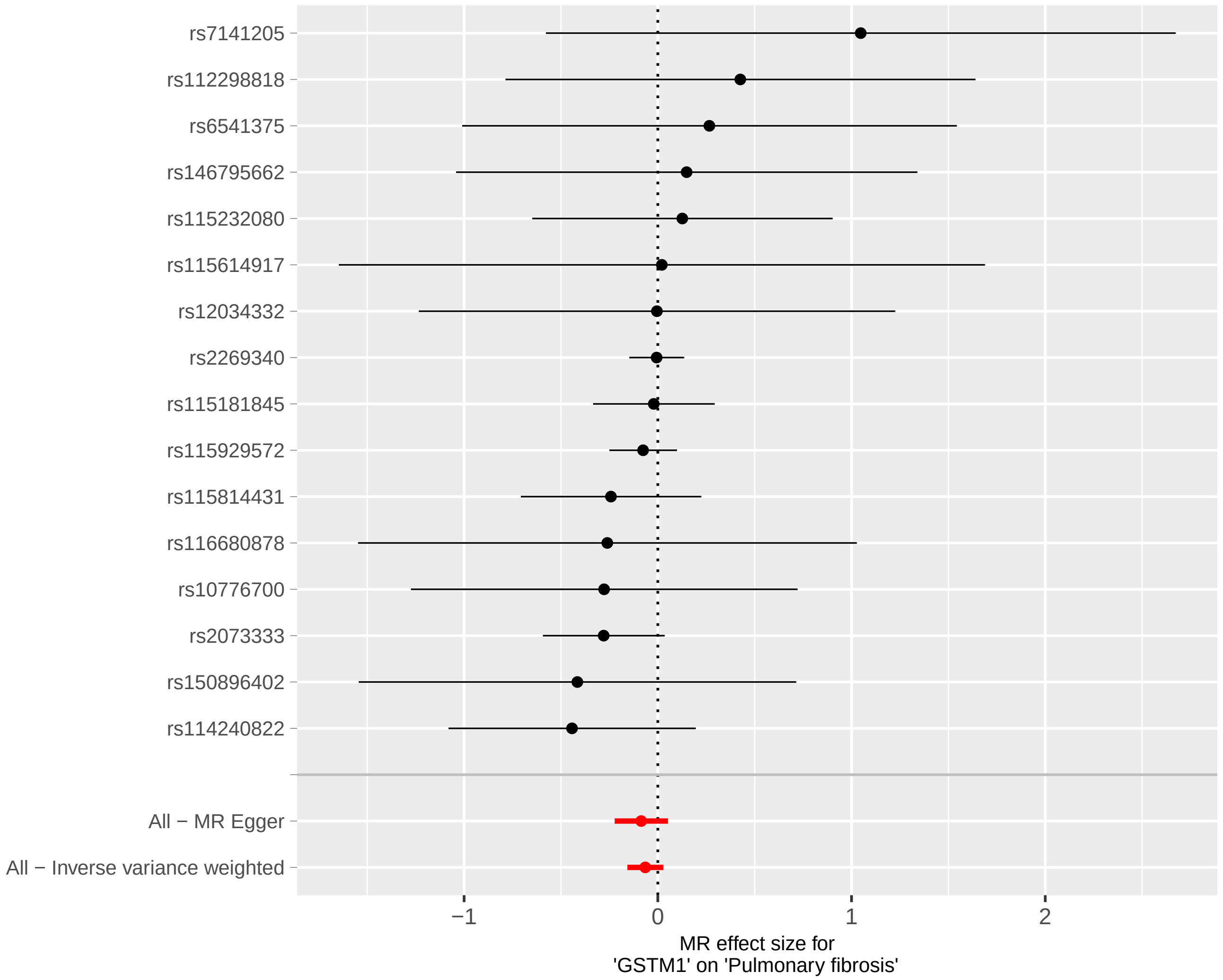

rs7141205
rs112298818
rs6541375
rs146795662
rs115232080
rs115614917
rs12034332
rs2269340
rs115181845
rs115929572
rs115814431
rs116680878
rs10776700
rs2073333
rs150896402
rs114240822
All − MR Egger
All − Inverse variance weighted
−1
0
1
2
MR effect size for
'GSTM1' on 'Pulmonary fibrosis'

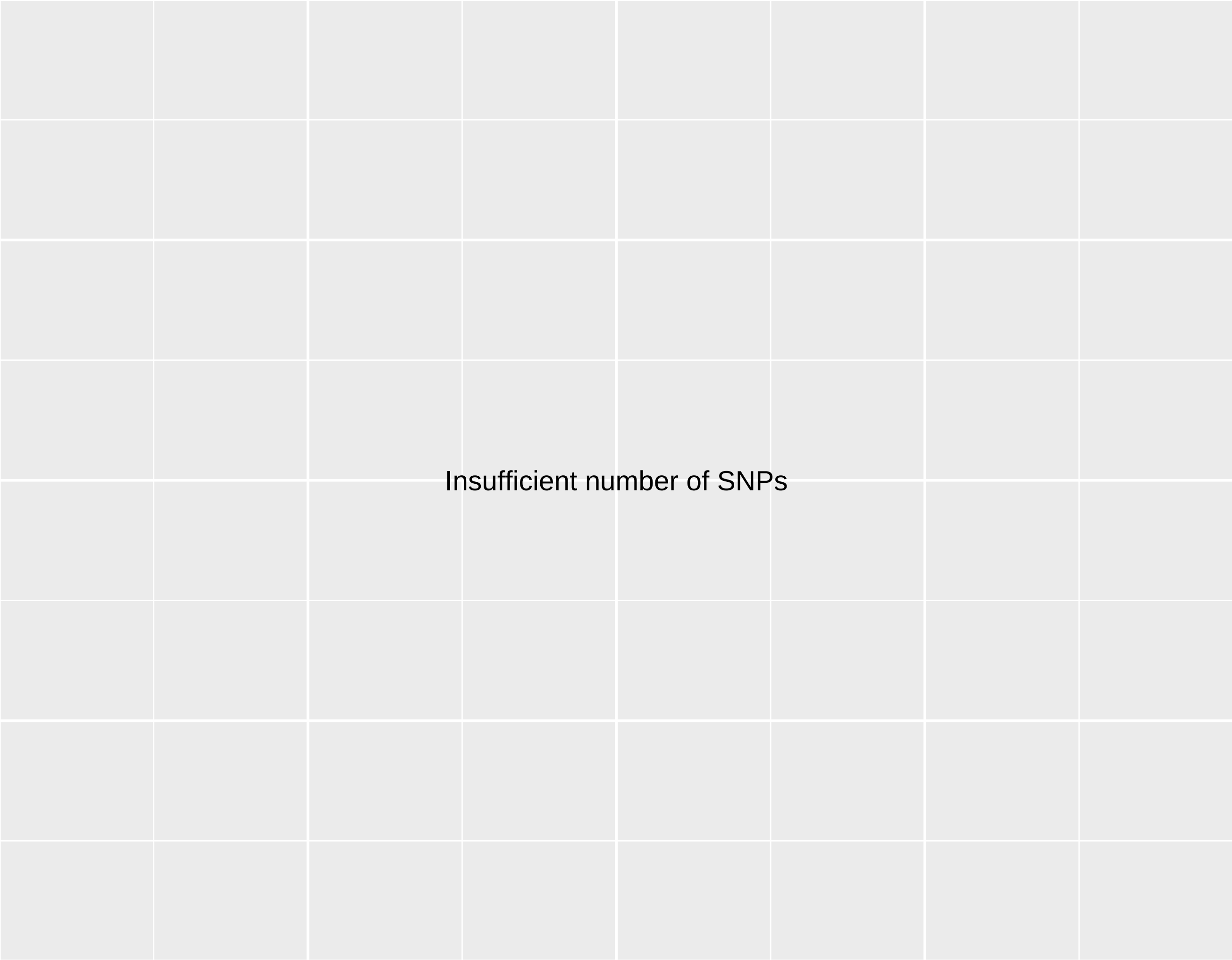
Insufficient number of SNPs

Insufficient number of SNPs

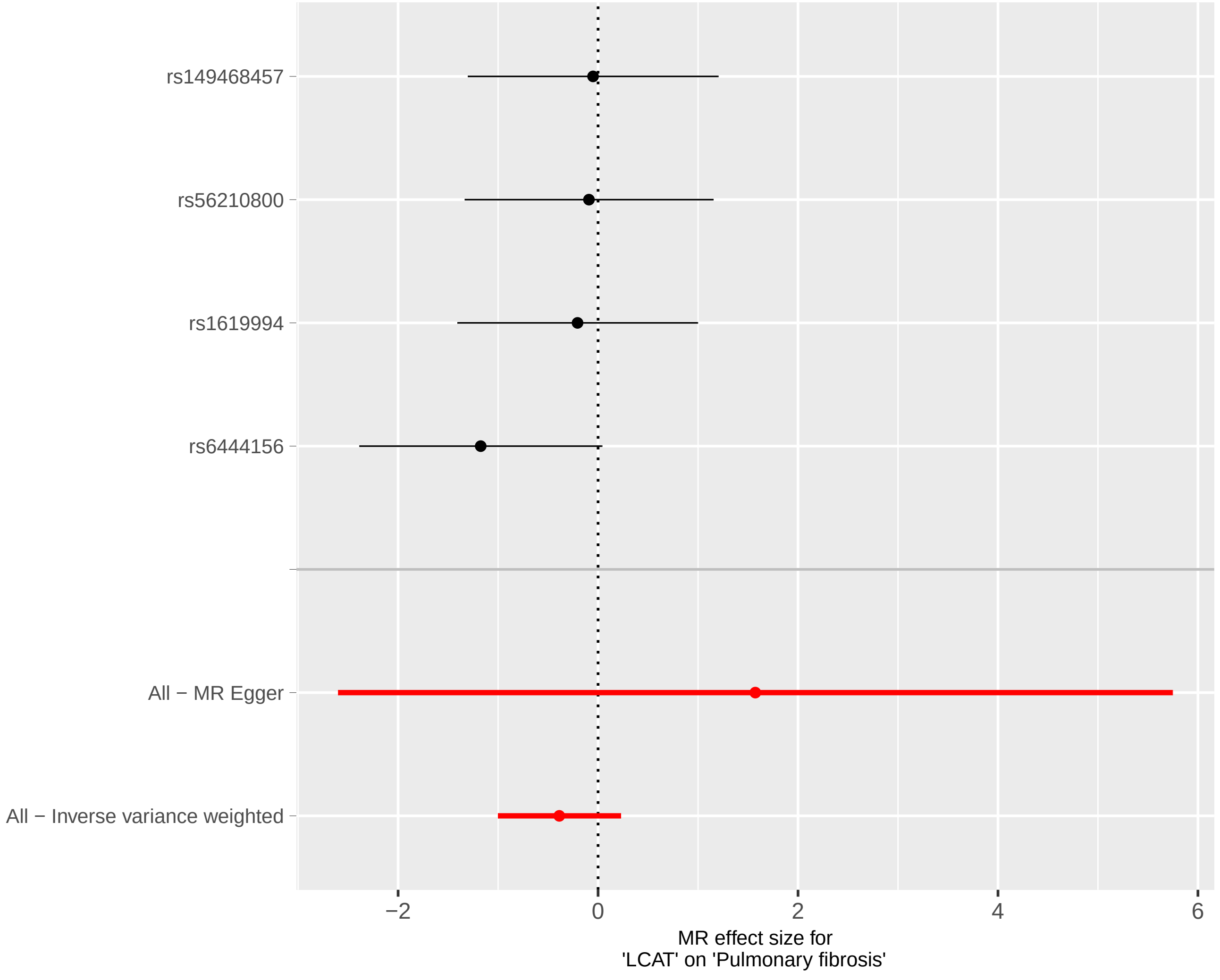

rs149468457
rs56210800
rs1619994
rs6444156
All − MR Egger
All − Inverse variance weighted
−2
0
2
4
6
MR effect size for
'LCAT' on 'Pulmonary fibrosis'

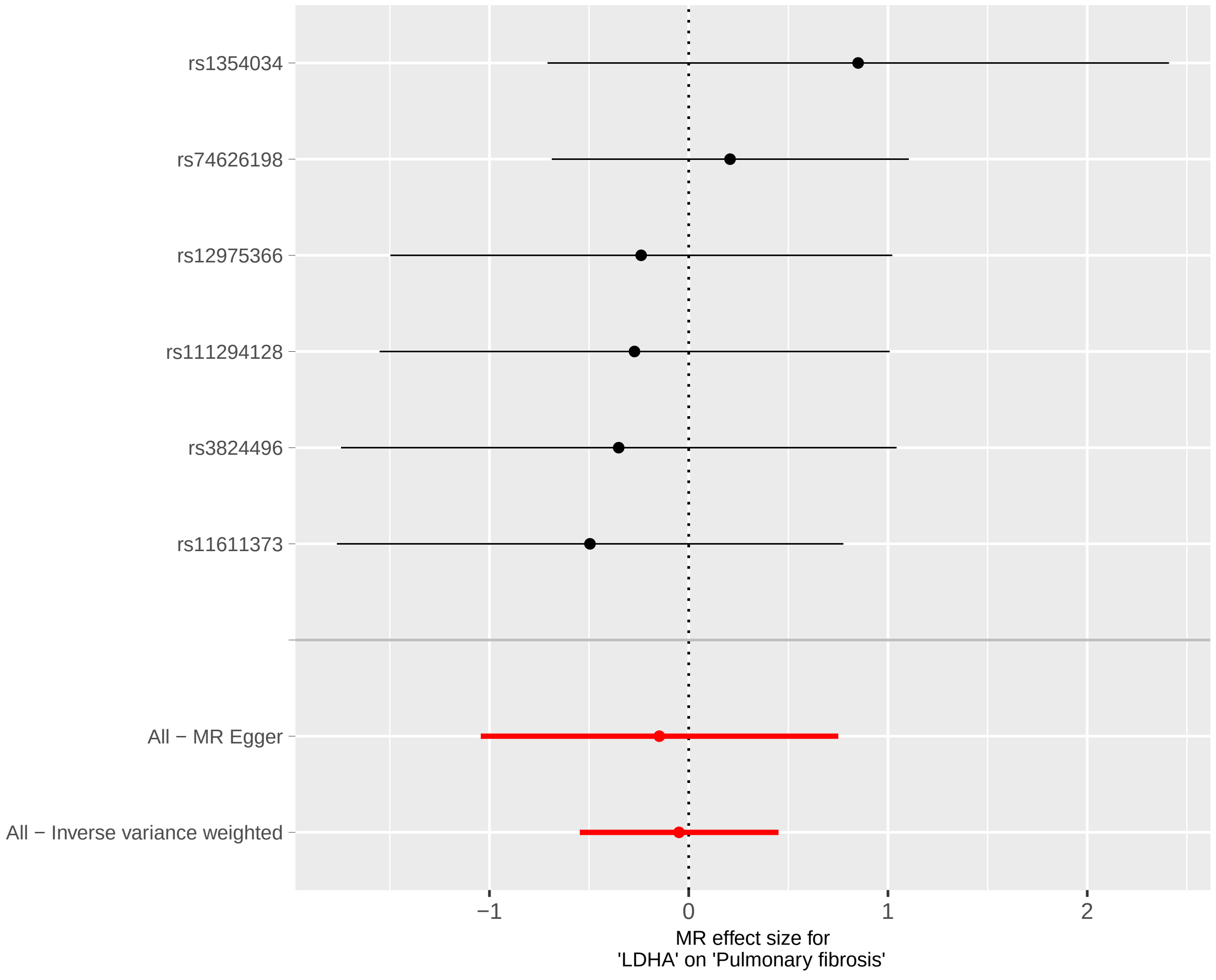

rs1354034
rs74626198
rs12975366
rs111294128
rs3824496
rs11611373
All − MR Egger
All − Inverse variance weighted
−1
0
1
2
MR effect size for
'LDHA' on 'Pulmonary fibrosis'

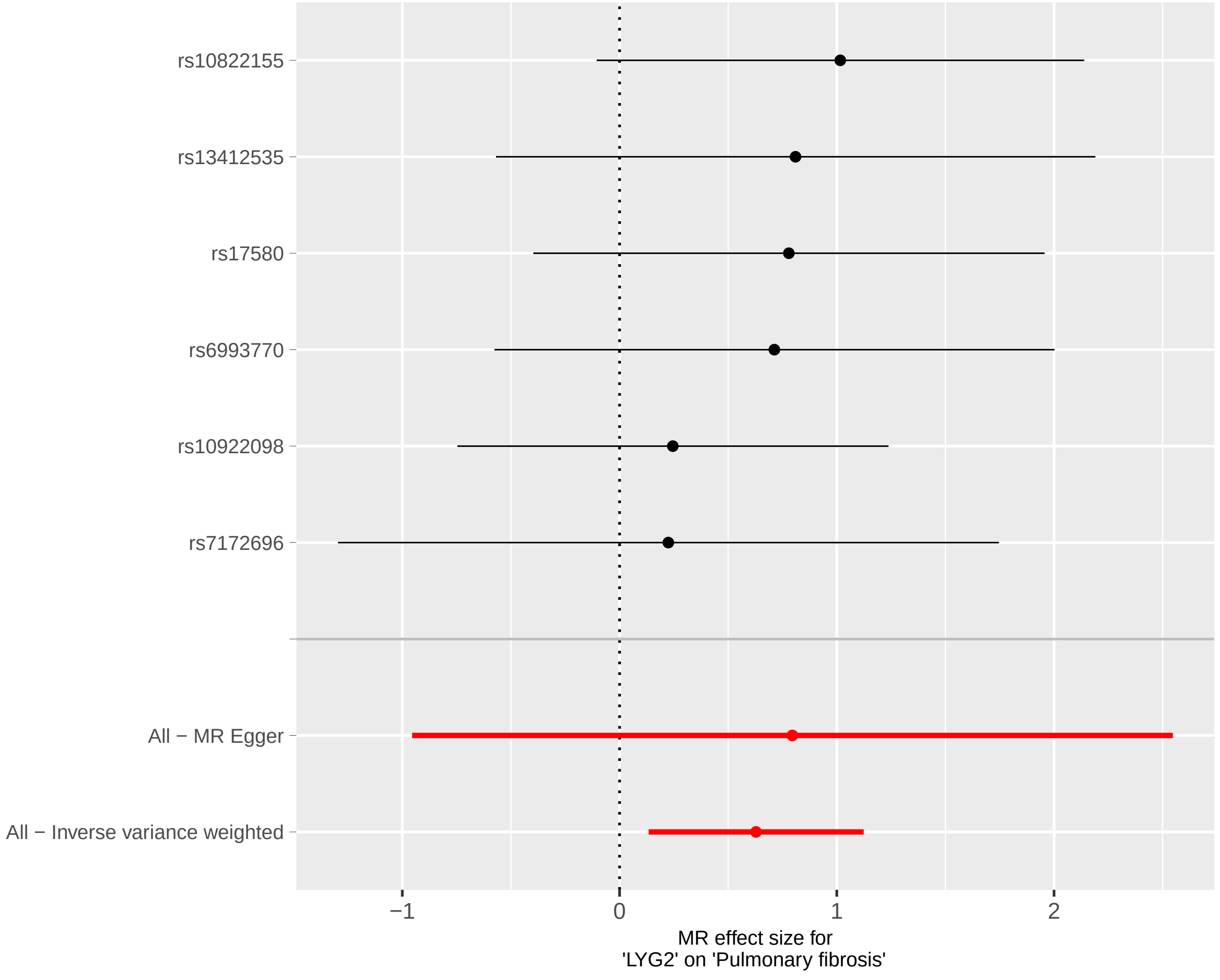
rs10822155
rs13412535
rs17580
rs6993770
rs10922098
rs7172696
All − MR Egger
All − Inverse variance weighted
−1
0
1
2
MR effect size for
'LYG2' on 'Pulmonary fibrosis'

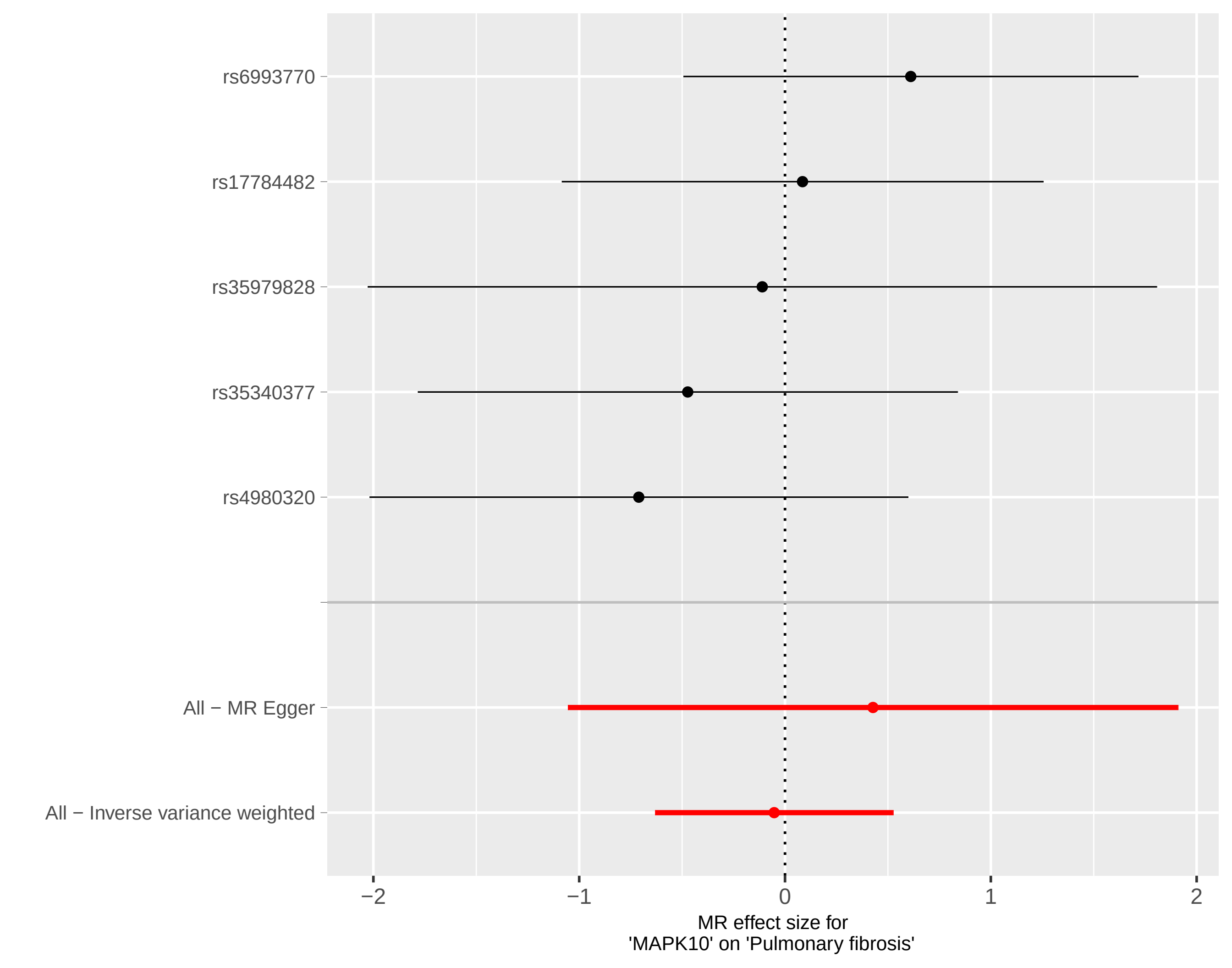

rs6993770
rs17784482
rs35979828
rs35340377
rs4980320
All − MR Egger
All − Inverse variance weighted
−2
−1
0
1
2
MR effect size for
'MAPK10' on 'Pulmonary fibrosis'

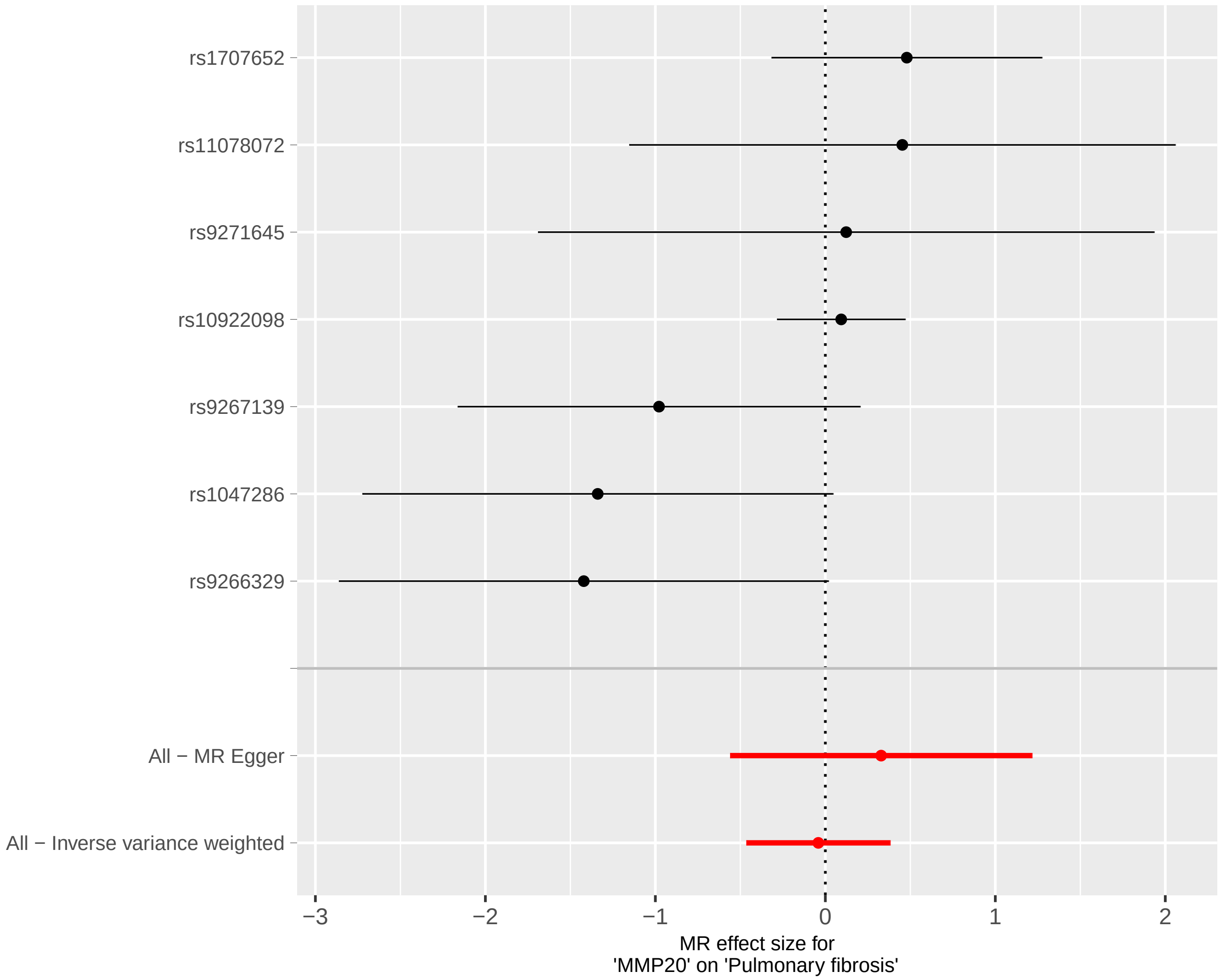
rs1707652
rs11078072
rs9271645
rs10922098
rs9267139
rs1047286
rs9266329
All − MR Egger
All − Inverse variance weighted
−3
−2
−1
0
1
2
MR effect size for
'MMP20' on 'Pulmonary fibrosis'

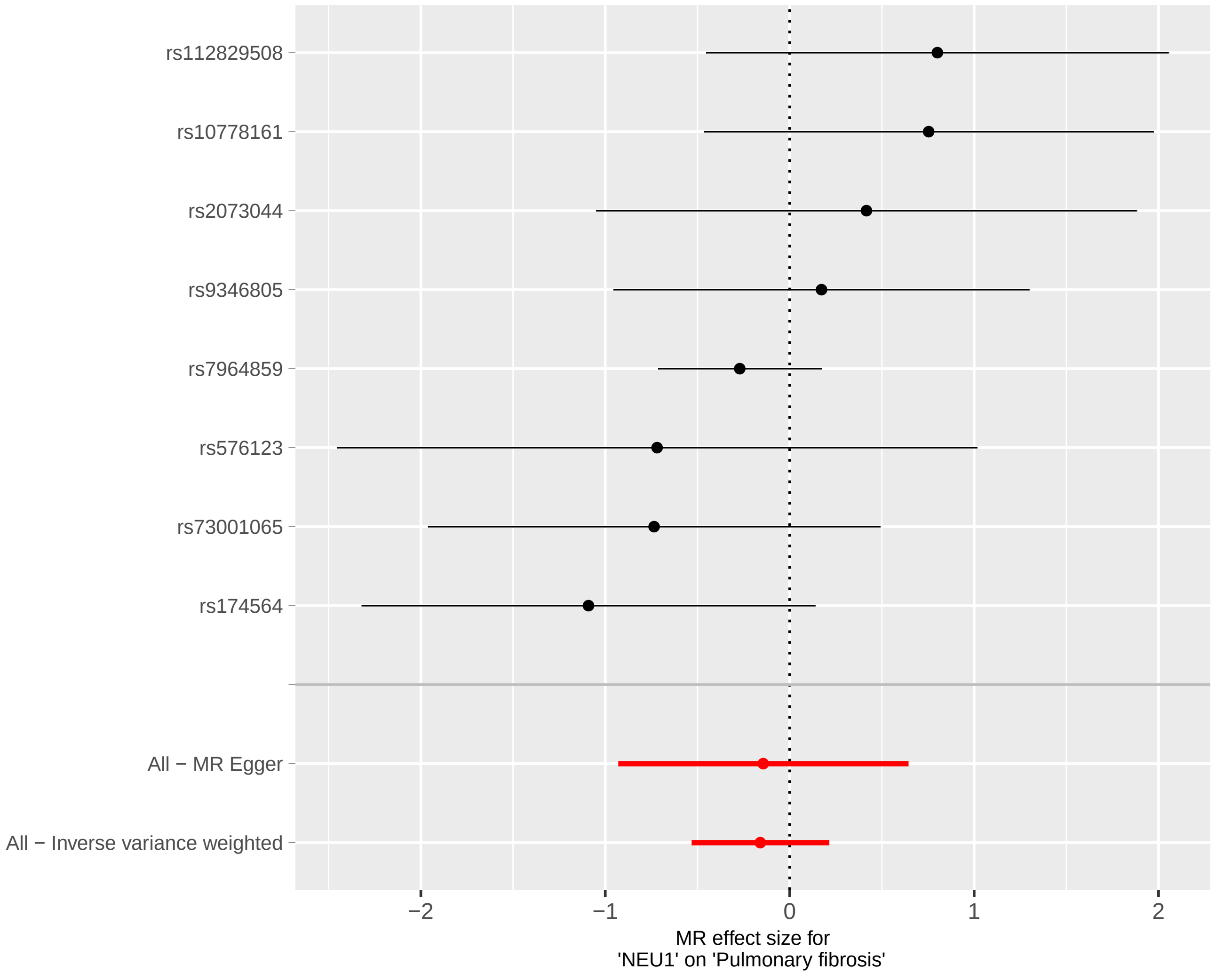
rs112829508
rs10778161
rs2073044
rs9346805
rs7964859
rs576123
rs73001065
rs174564
All − MR Egger
All − Inverse variance weighted
−2
−1
0
1
2
MR effect size for
'NEU1' on 'Pulmonary fibrosis'

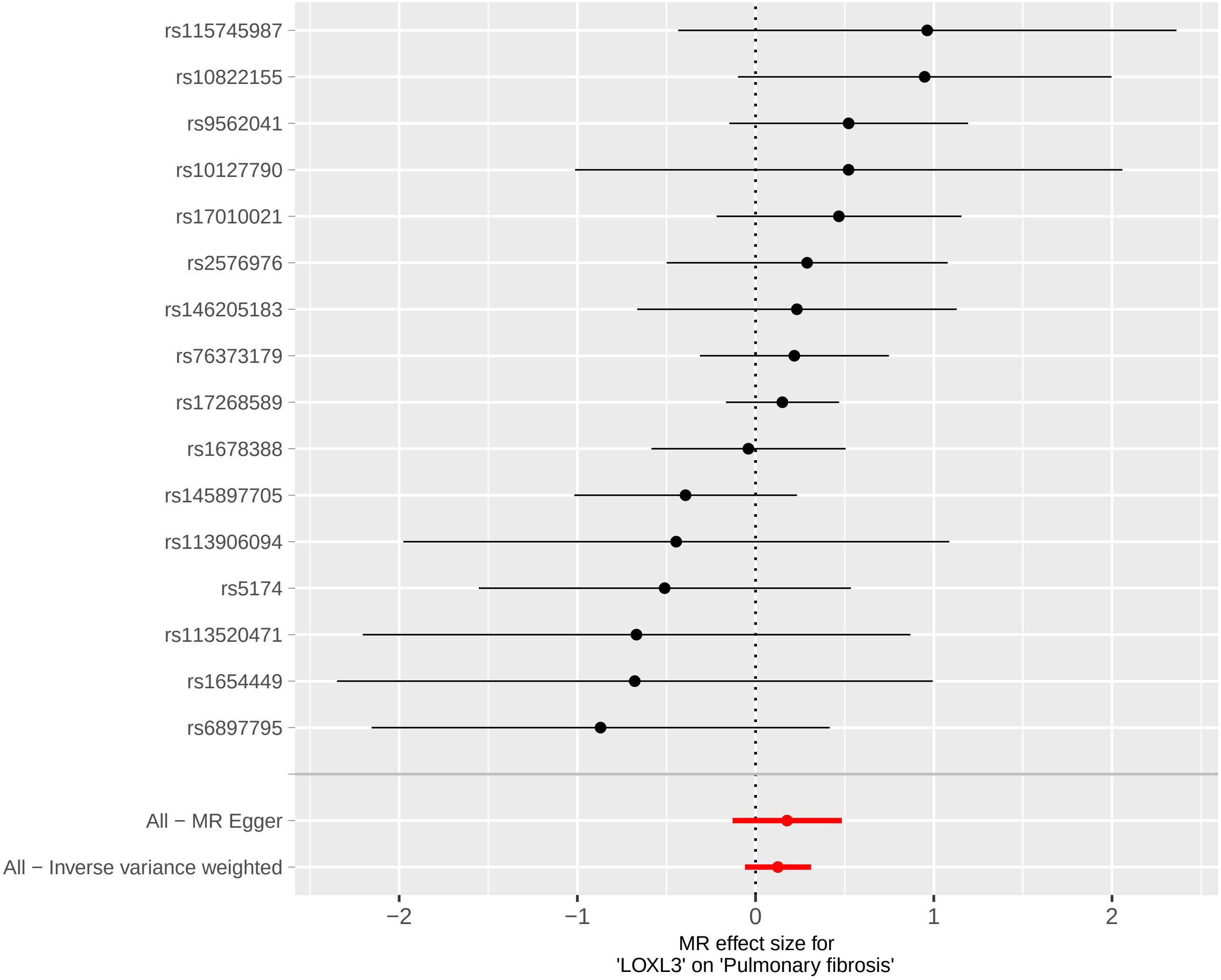

rs115745987
rs10822155
rs9562041
rs10127790
rs17010021
rs2576976
rs146205183
rs76373179
rs17268589
rs1678388
rs145897705
rs113906094
rs5174
rs113520471
rs1654449
rs6897795
All − MR Egger
All − Inverse variance weighted
−2
−1
0
1
2
MR effect size for
'LOXL3' on 'Pulmonary fibrosis'

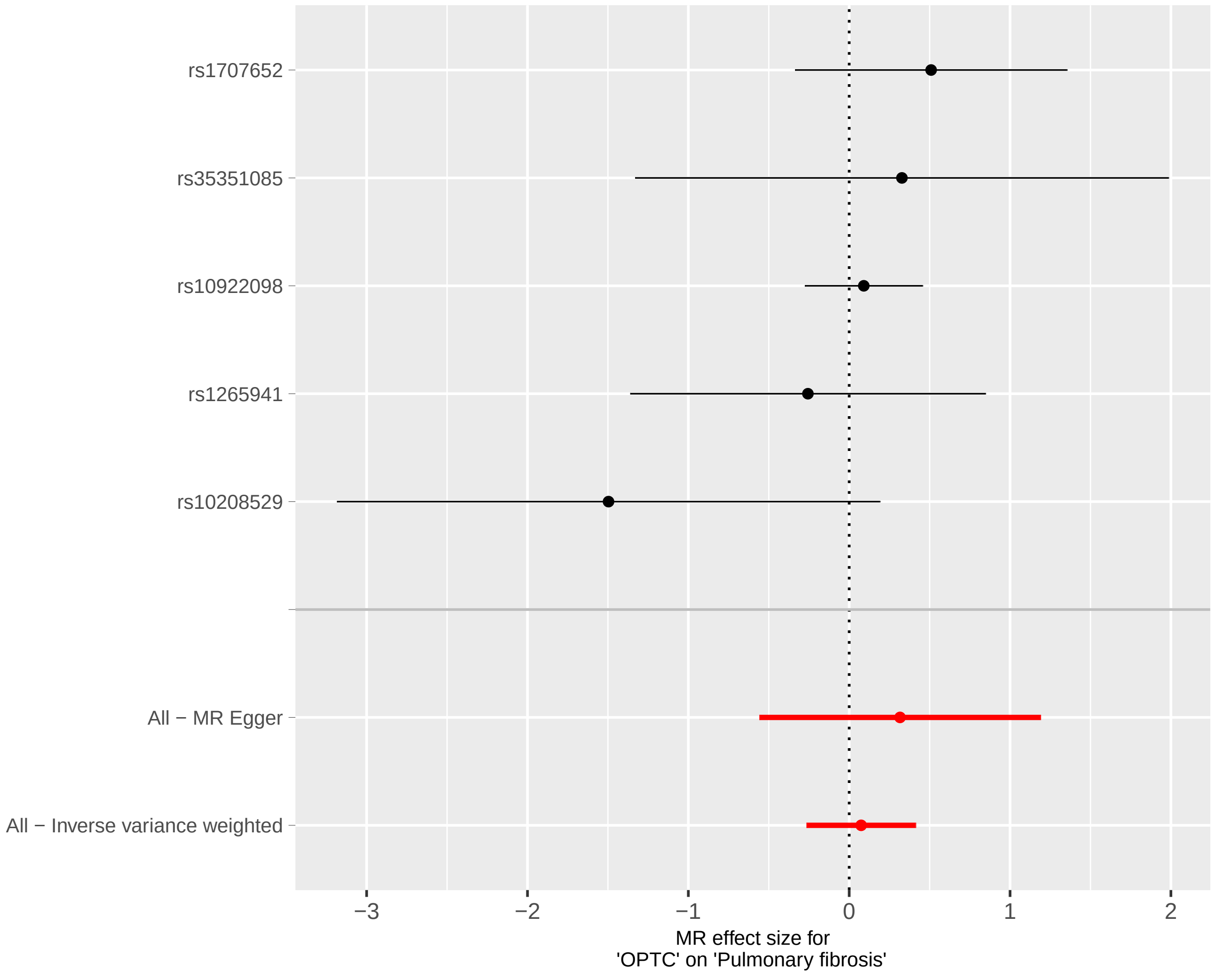

rs1707652
rs35351085
rs10922098
rs1265941
rs10208529
All − MR Egger
All − Inverse variance weighted
−3
−2
−1
0
1
2
MR effect size for
'OPTC' on 'Pulmonary fibrosis'

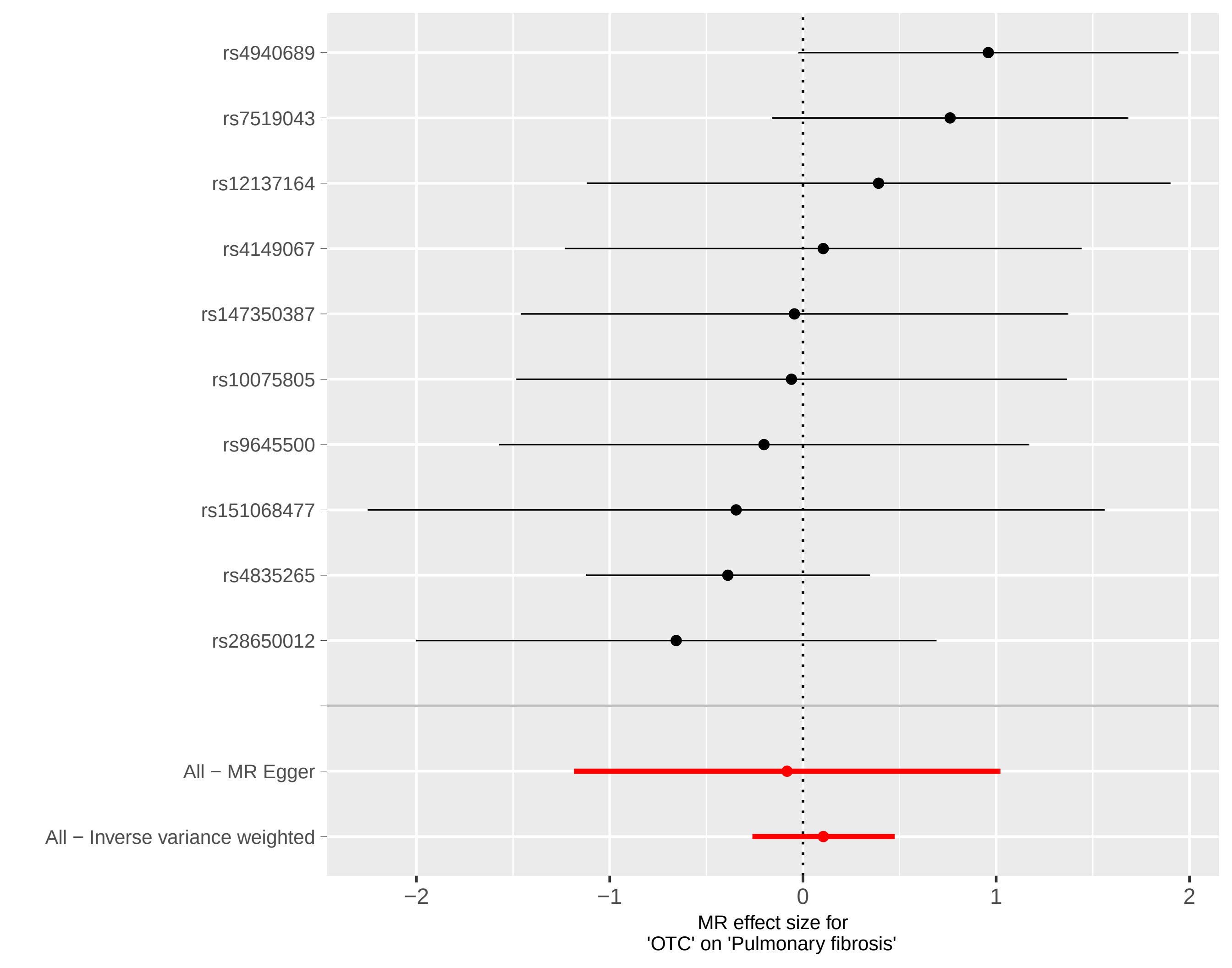
rs4940689
rs7519043
rs12137164
rs4149067
rs147350387
rs10075805
rs9645500
rs151068477
rs4835265
rs28650012
All − MR Egger
All − Inverse variance weighted
−2
−1
0
1
2
MR effect size for
'OTC' on 'Pulmonary fibrosis'

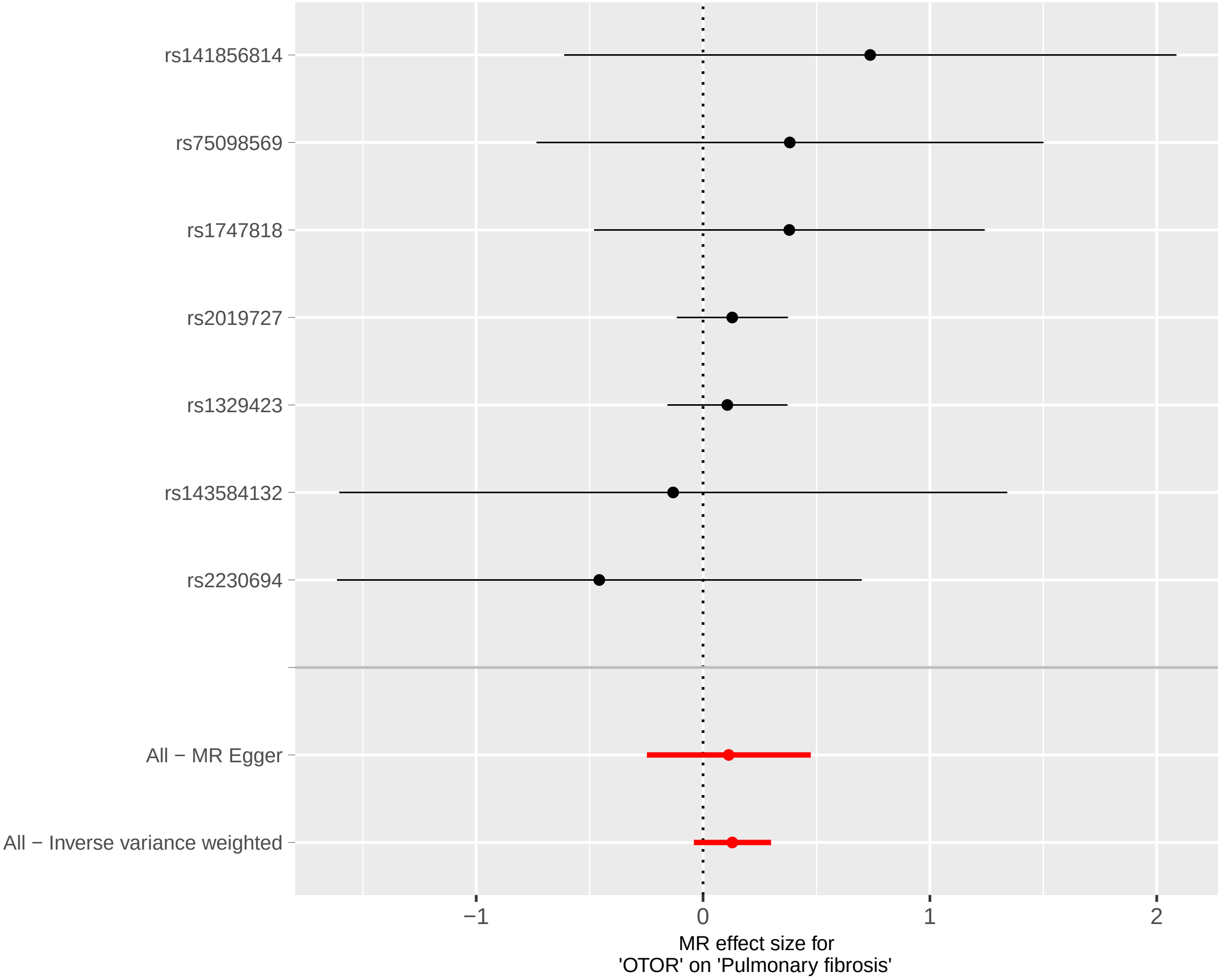
rs141856814
rs75098569
rs1747818
rs2019727
rs1329423
rs143584132
rs2230694
All − MR Egger
All − Inverse variance weighted
−1
0
1
2
MR effect size for
'OTOR' on 'Pulmonary fibrosis'

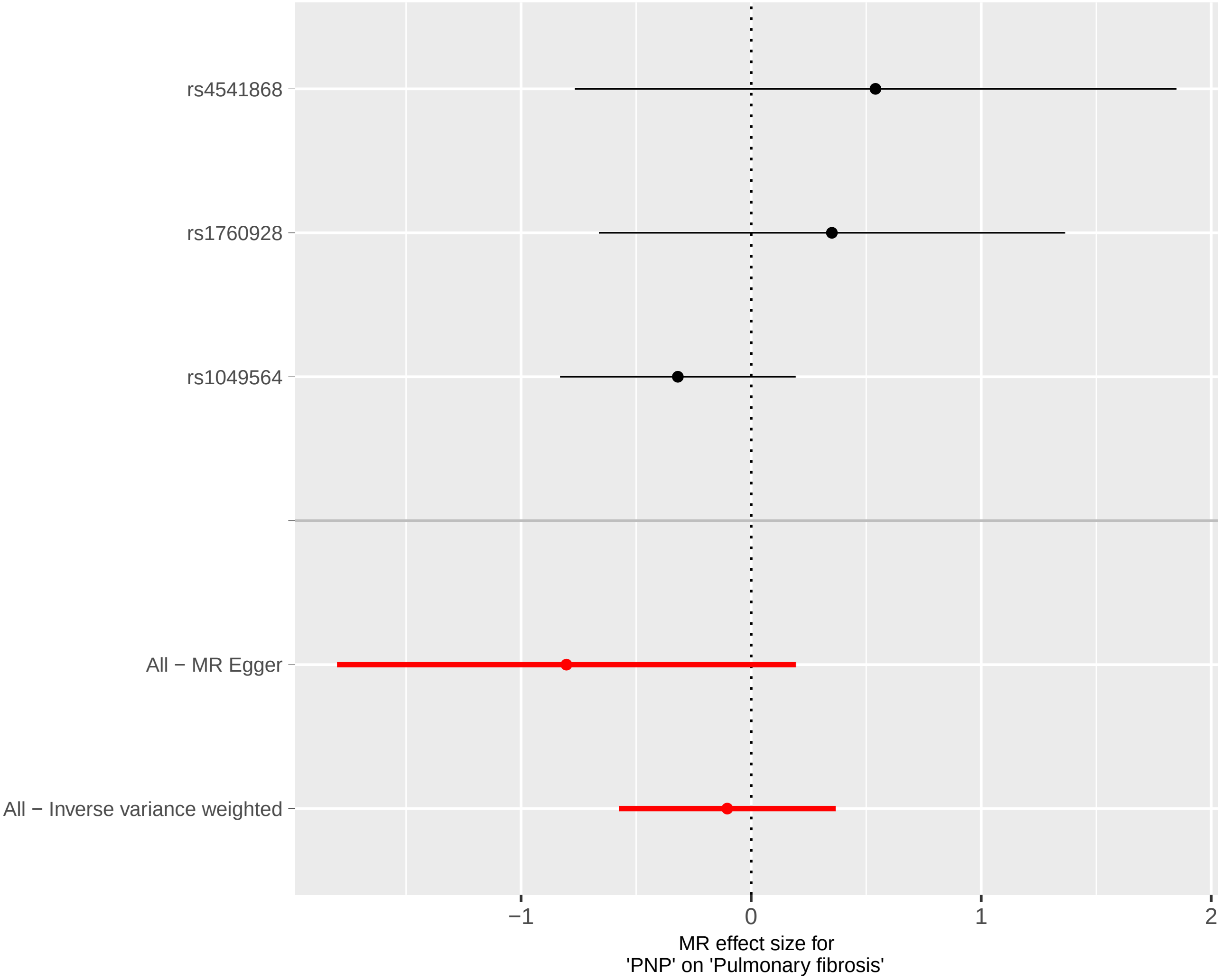
rs4541868
rs1760928
rs1049564
All − MR Egger
All − Inverse variance weighted
−1
0
1
2
MR effect size for
'PNP' on 'Pulmonary fibrosis'

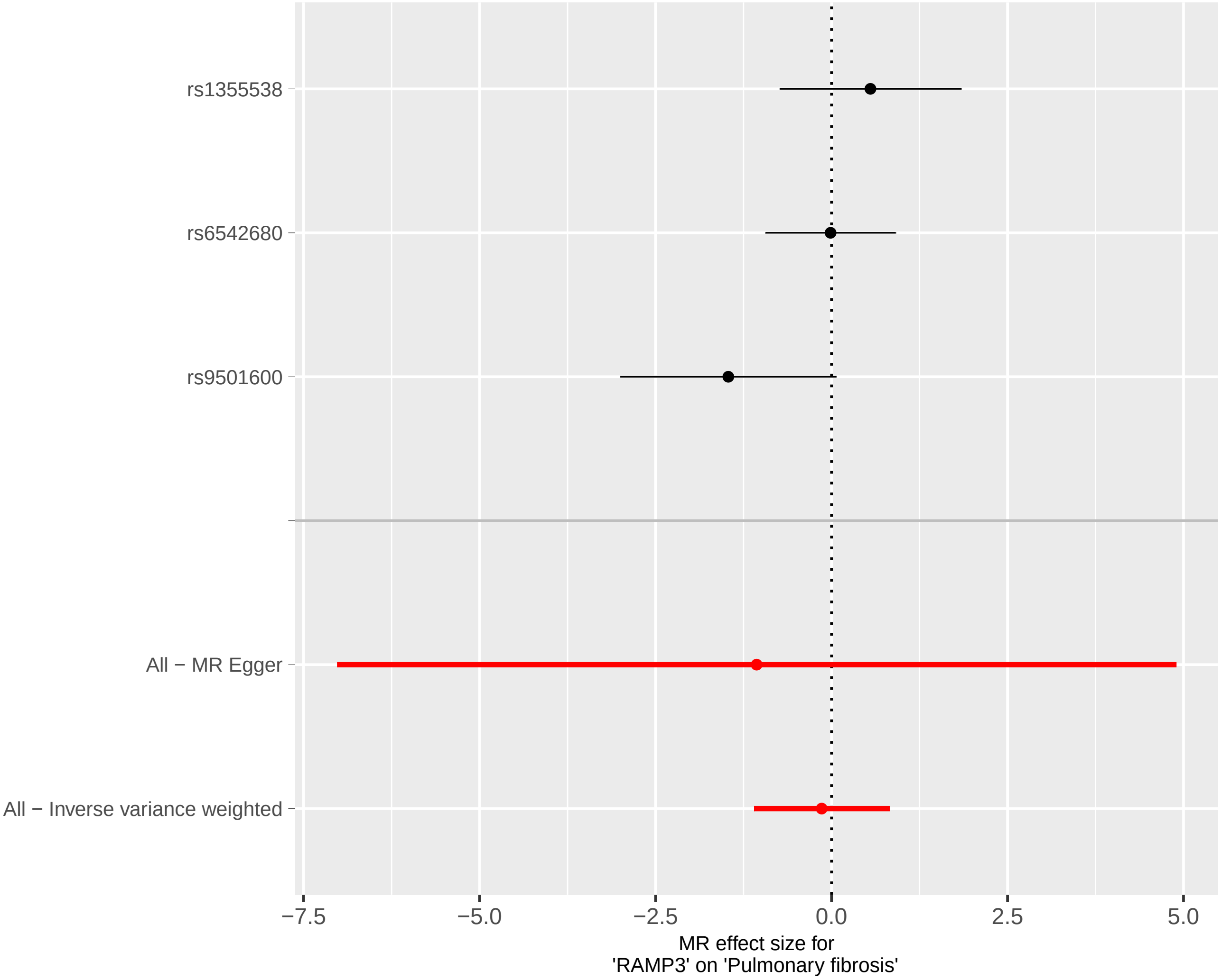
rs1355538
rs6542680
rs9501600
All − MR Egger
All − Inverse variance weighted
−7.5
−5.0
−2.5
0.0
2.5
5.0
MR effect size for
'RAMP3' on 'Pulmonary fibrosis'

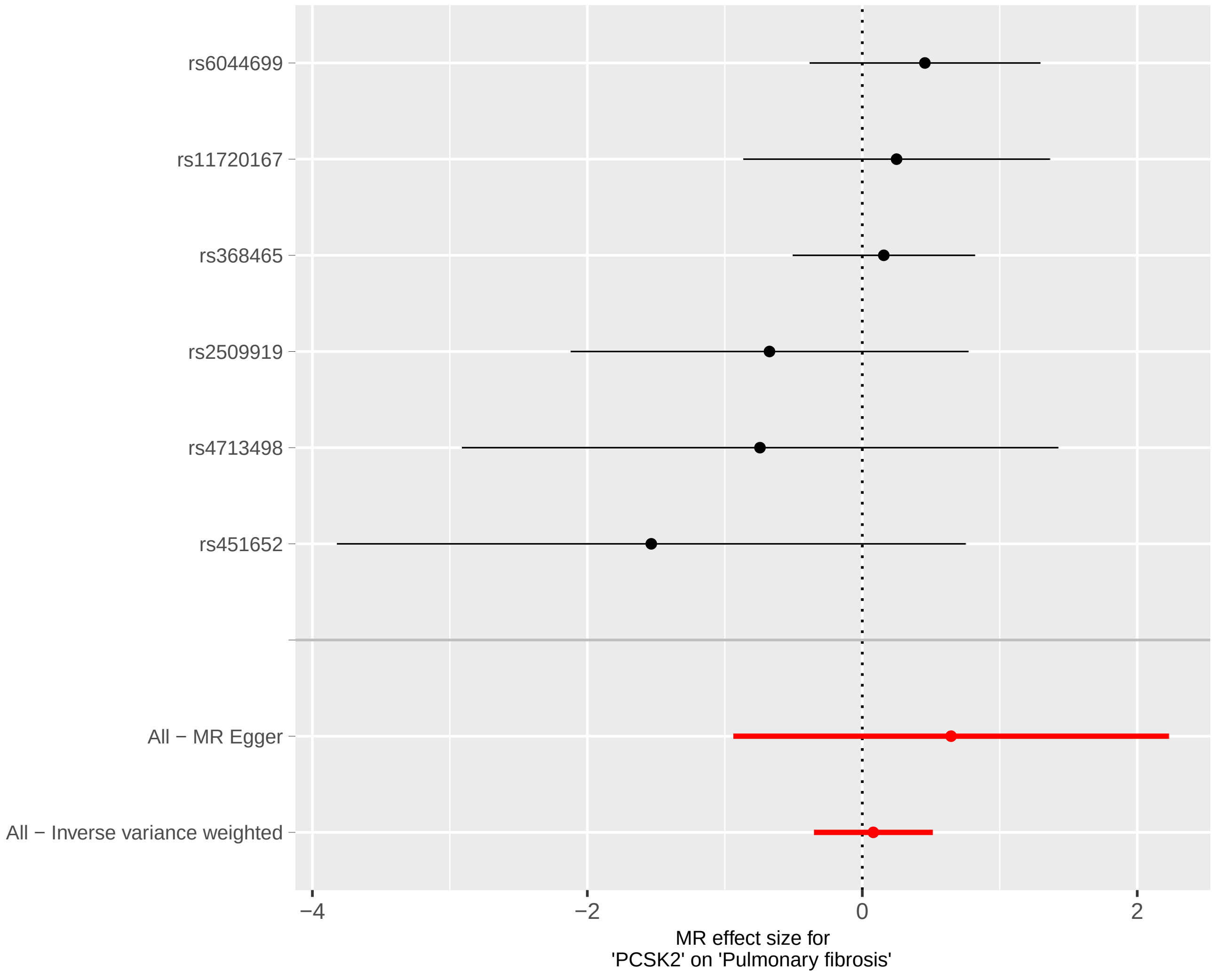
rs6044699
rs11720167
rs368465
rs2509919
rs4713498
rs451652
All − MR Egger
All − Inverse variance weighted
−4
−2
0
2
MR effect size for
'PCSK2' on 'Pulmonary fibrosis'

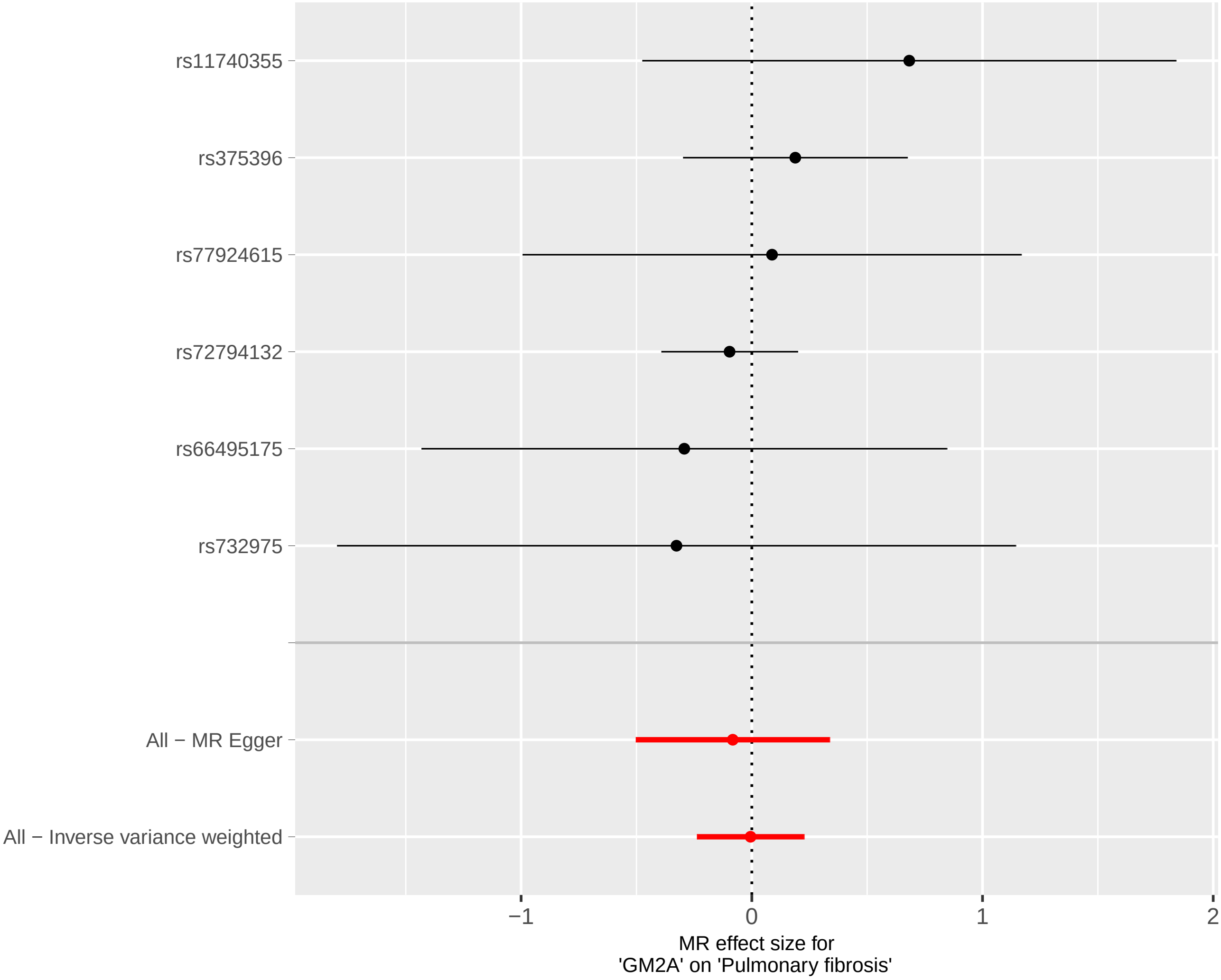

rs11740355
rs375396
rs77924615
rs72794132
rs66495175
rs732975
All − MR Egger
All − Inverse variance weighted
−1
0
1
2
MR effect size for
'GM2A' on 'Pulmonary fibrosis'

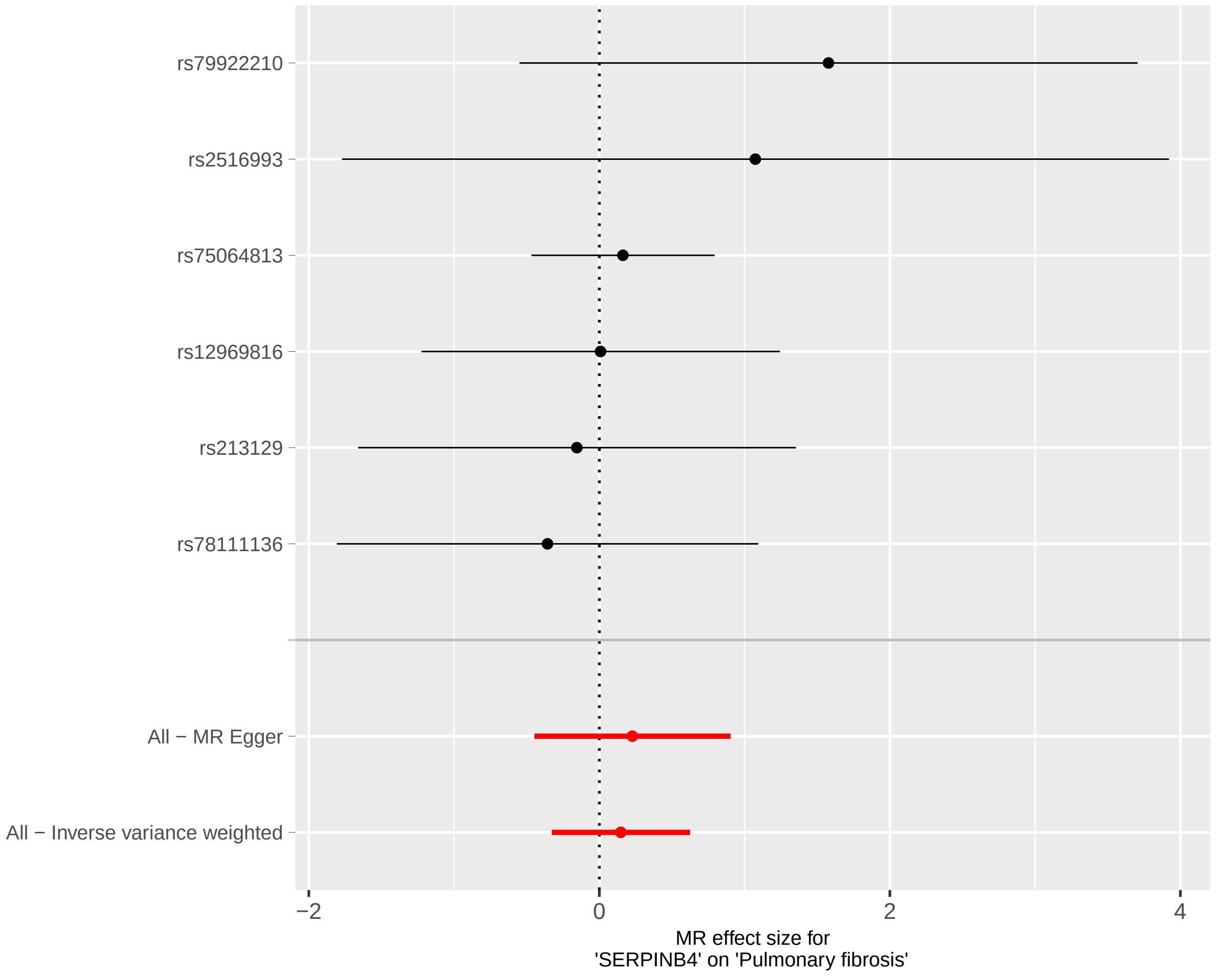
rs79922210
rs2516993
rs75064813
rs12969816
rs213129
rs78111136
All − MR Egger
All − Inverse variance weighted
−2
0
2
4
MR effect size for
'SERPINB4' on 'Pulmonary fibrosis'

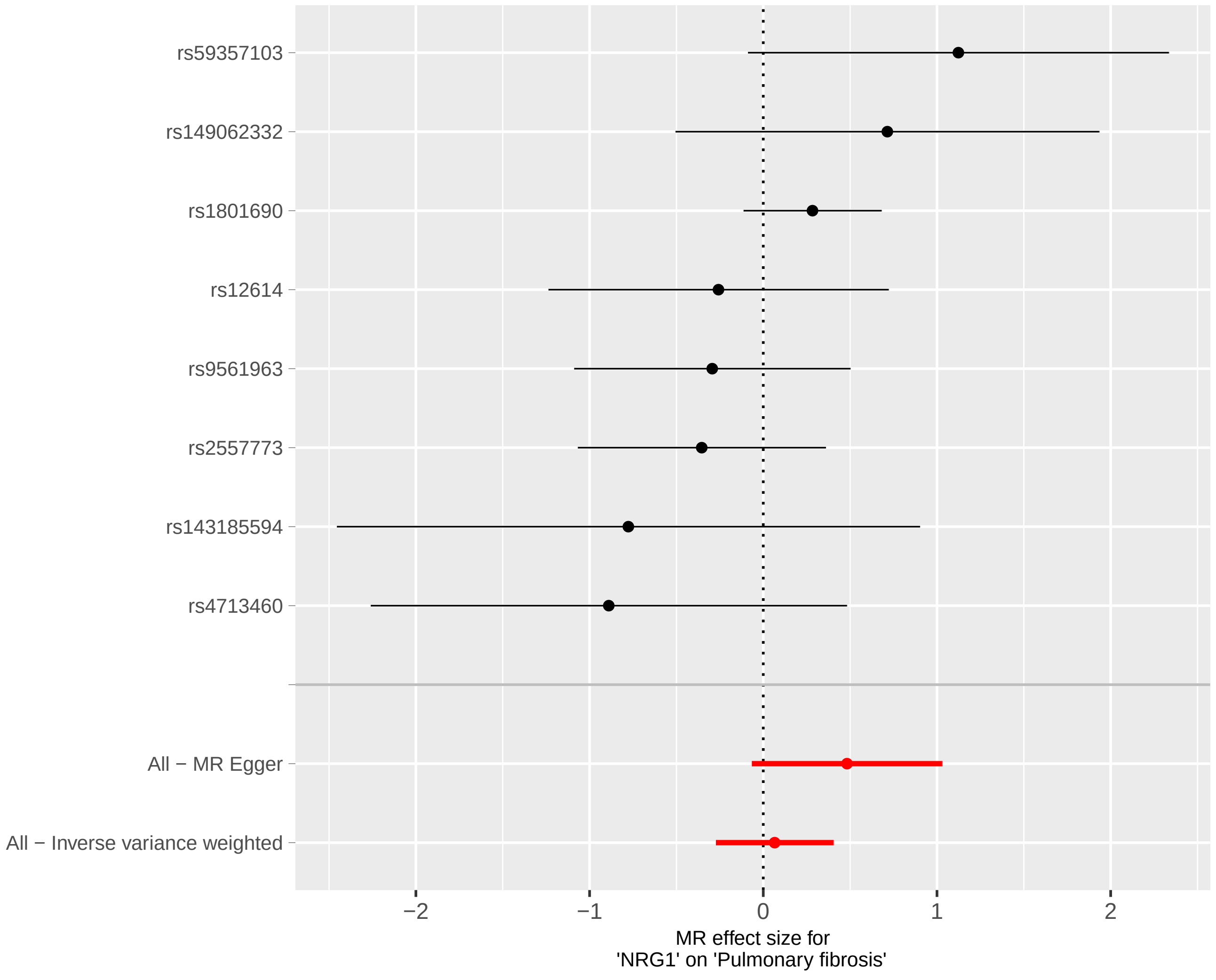
rs59357103
rs149062332
rs1801690
rs12614
rs9561963
rs2557773
rs143185594
rs4713460
All − MR Egger
All − Inverse variance weighted
−2
−1
0
1
2
MR effect size for
'NRG1' on 'Pulmonary fibrosis'

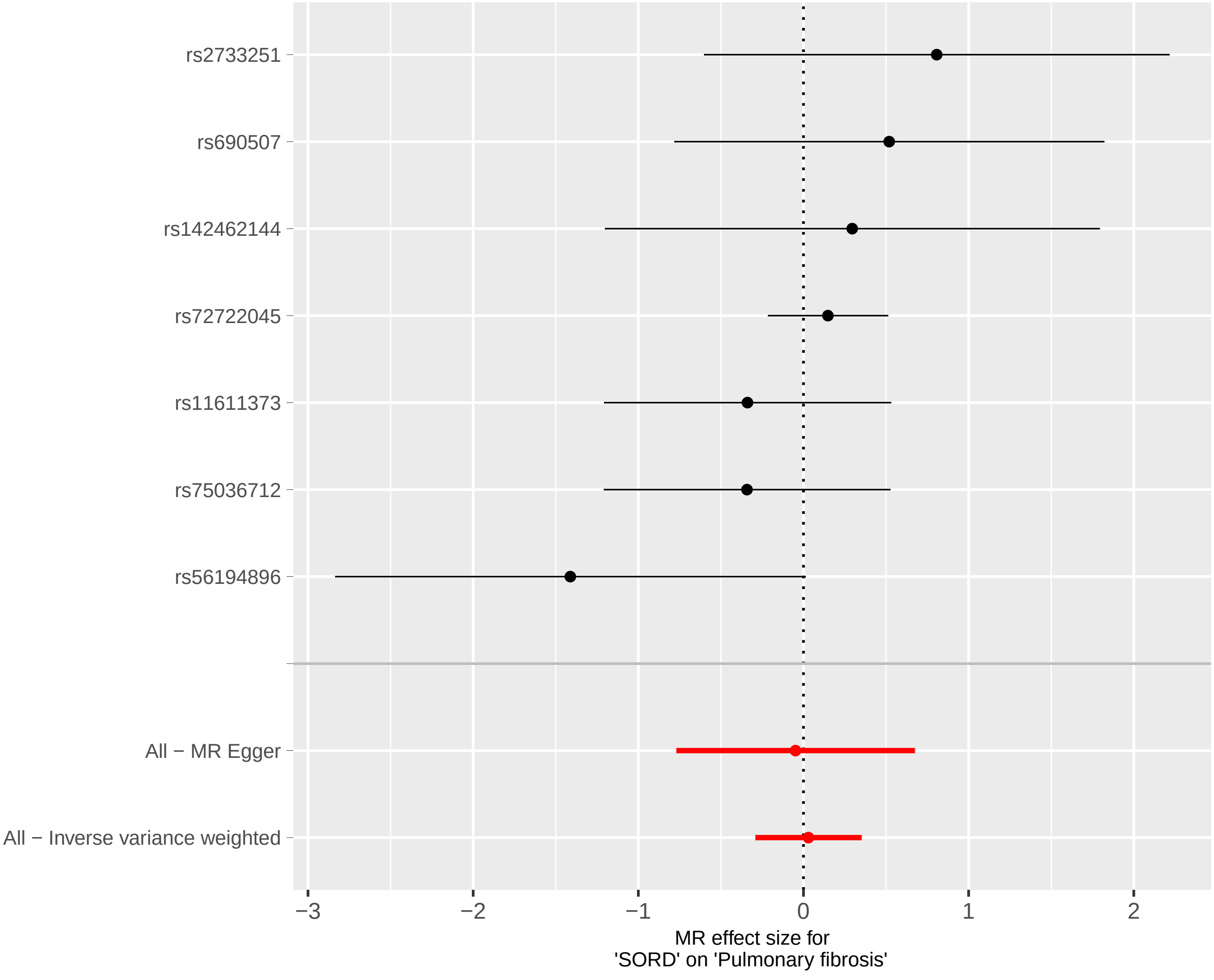

rs2733251
rs690507
rs142462144
rs72722045
rs11611373
rs75036712
rs56194896
All − MR Egger
All − Inverse variance weighted
−3
−2
−1
0
1
2
MR effect size for
'SORD' on 'Pulmonary fibrosis'

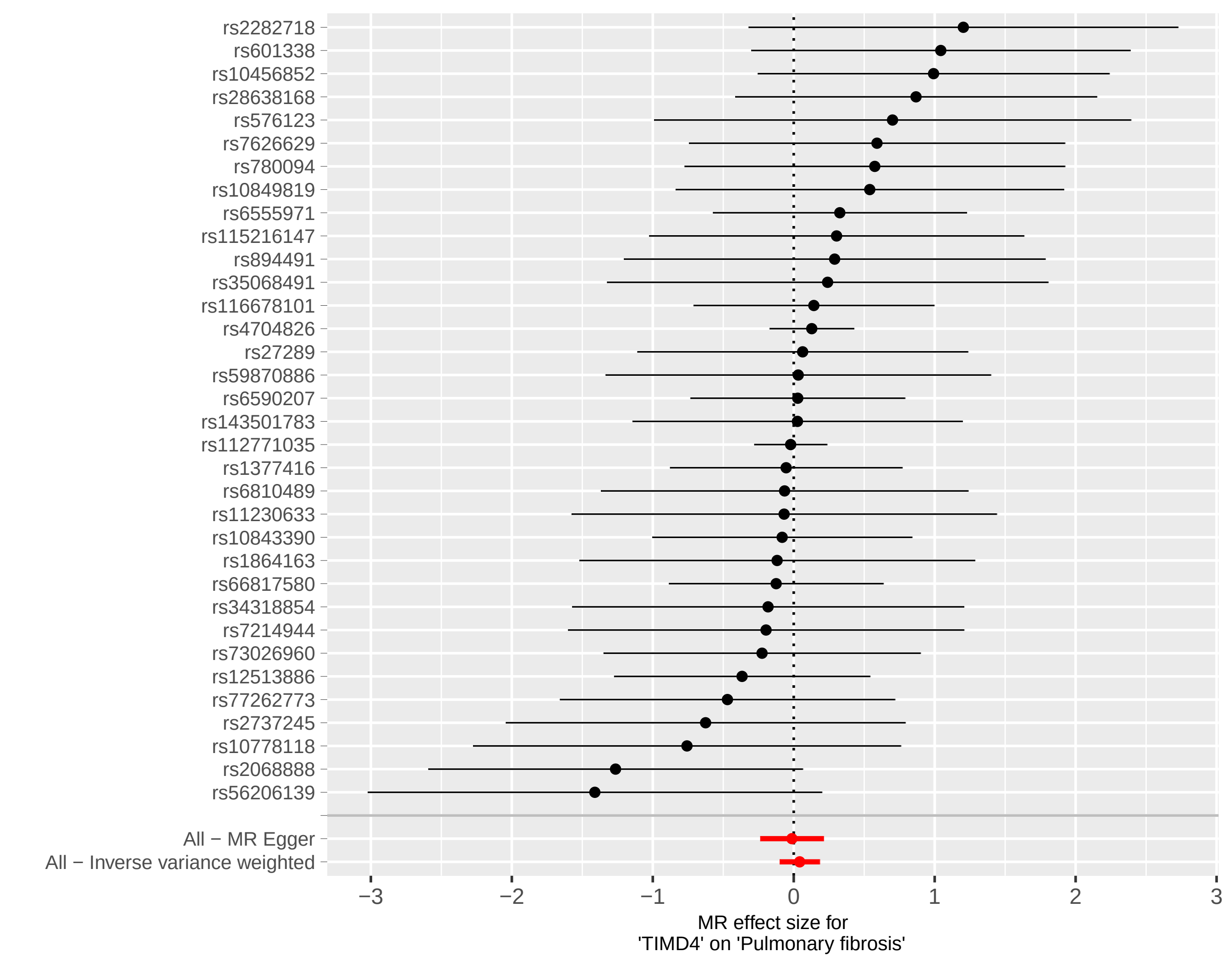
rs2282718
rs601338
rs10456852
rs28638168
rs576123
rs7626629
rs780094
rs10849819
rs6555971
rs115216147
rs894491
rs35068491
rs116678101
rs4704826
rs27289
rs59870886
rs6590207
rs143501783
rs112771035
rs1377416
rs6810489
rs11230633
rs10843390
rs1864163
rs66817580
rs34318854
rs7214944
rs73026960
rs12513886
rs77262773
rs2737245
rs10778118
rs2068888
rs56206139
All − MR Egger
All − Inverse variance weighted
−3
−2
−1
0
1
2
3
MR effect size for
'TIMD4' on 'Pulmonary fibrosis'

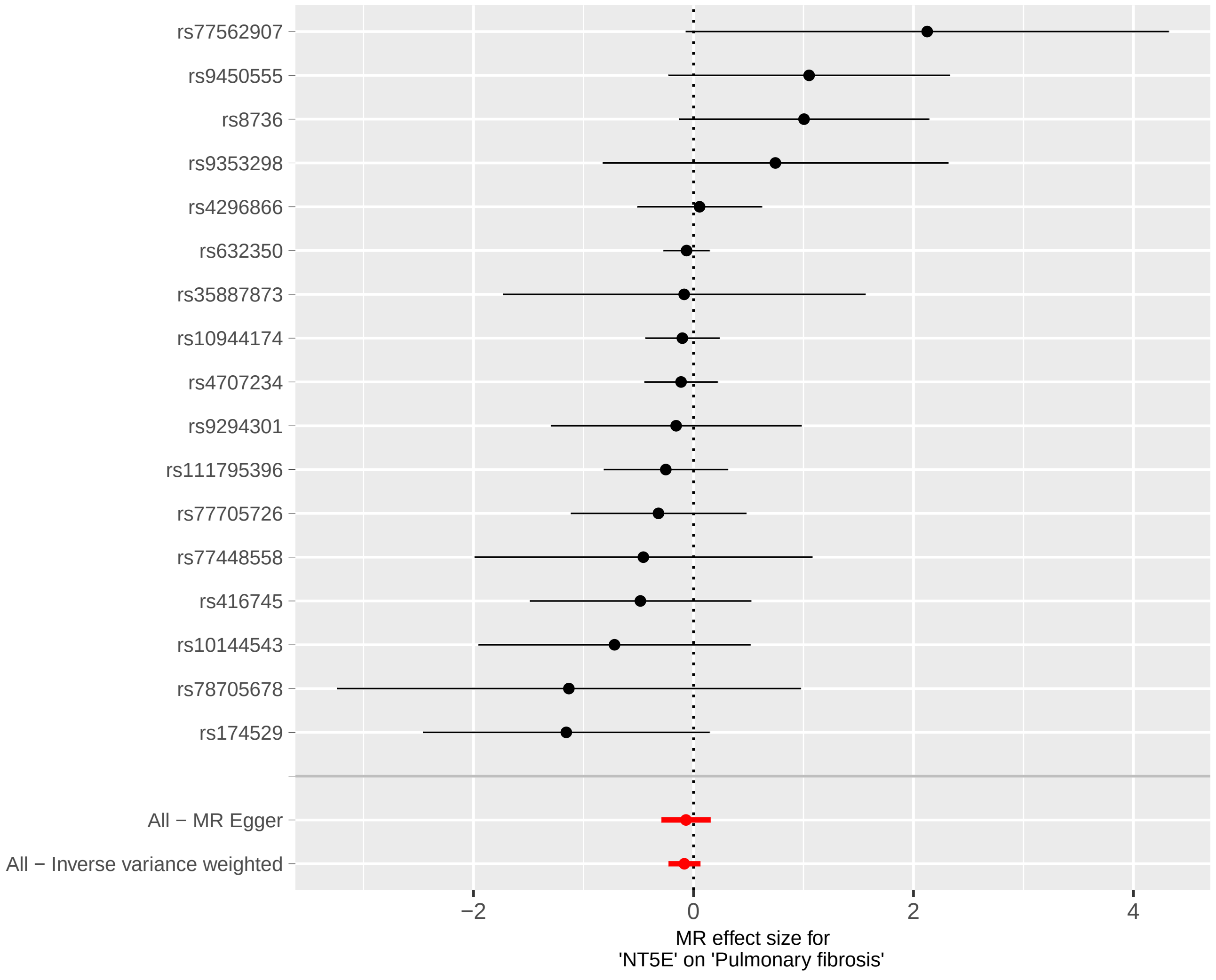

rs77562907
rs9450555
rs8736
rs9353298
rs4296866
rs632350
rs35887873
rs10944174
rs4707234
rs9294301
rs111795396
rs77705726
rs77448558
rs416745
rs10144543
rs78705678
rs174529
All − MR Egger
All − Inverse variance weighted
−2
0
2
4
MR effect size for
'NT5E' on 'Pulmonary fibrosis'

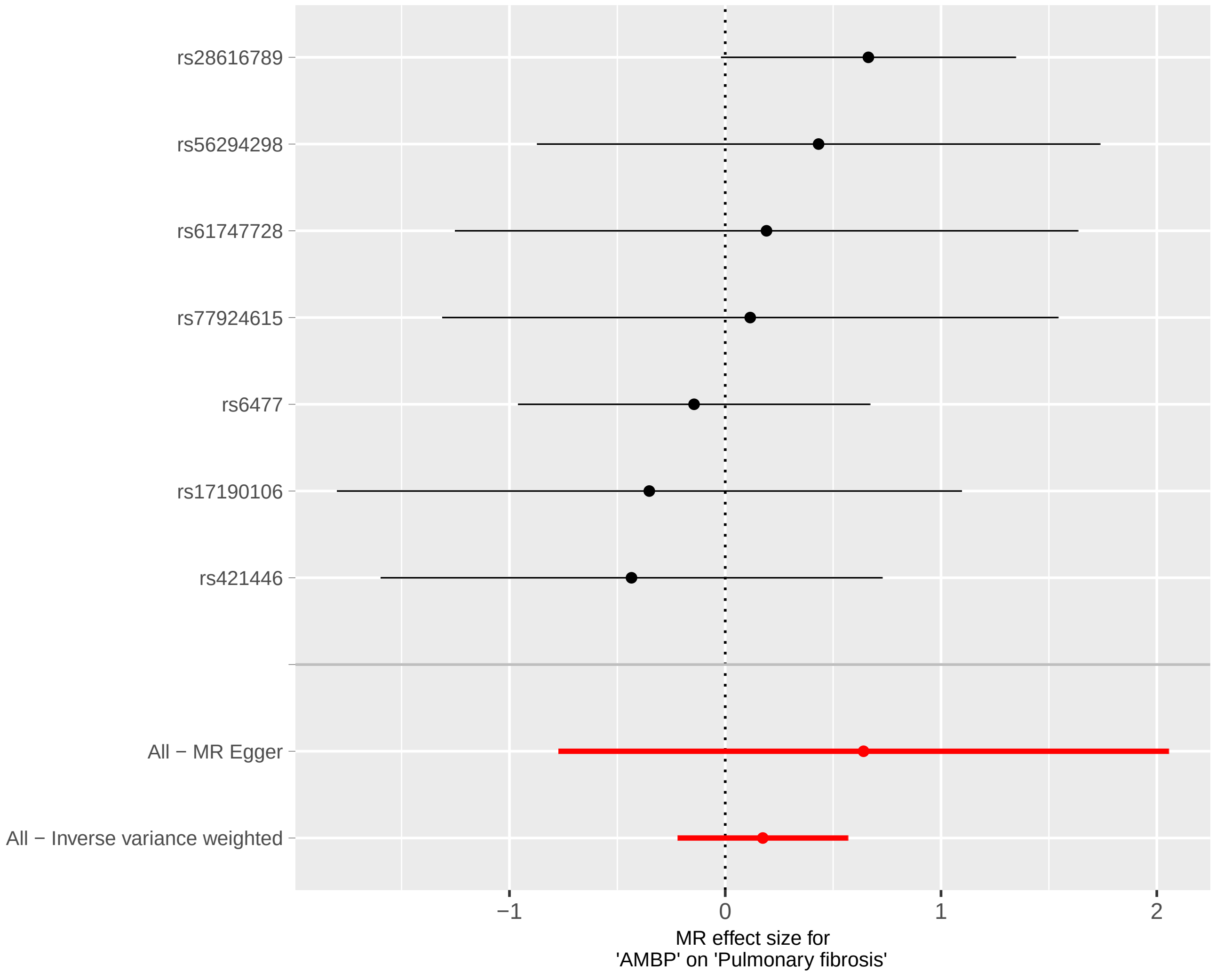
rs28616789
rs56294298
rs61747728
rs77924615
rs6477
rs17190106
rs421446
All − MR Egger
All − Inverse variance weighted
−1
0
1
2
MR effect size for
'AMBP' on 'Pulmonary fibrosis'

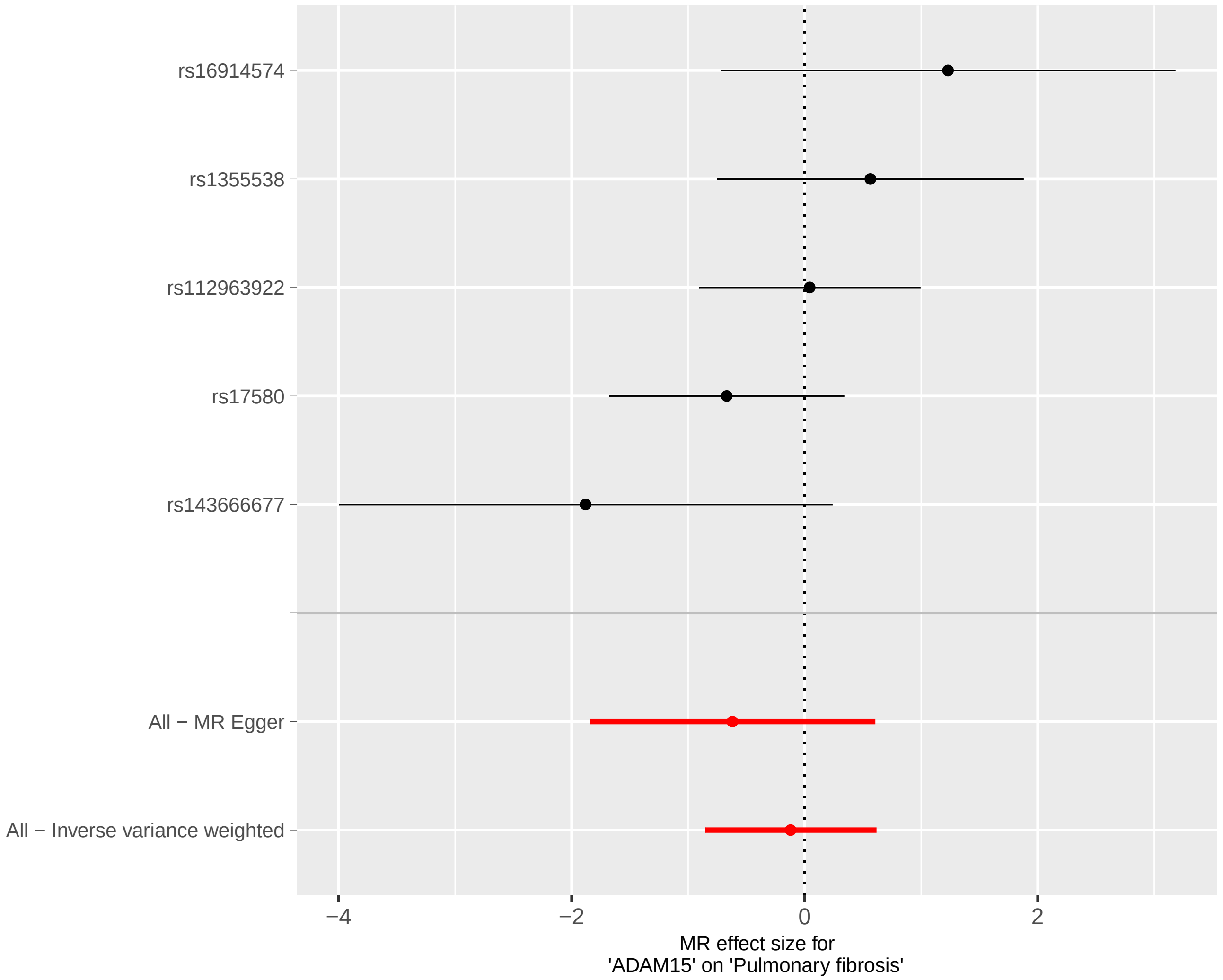

rs16914574
rs1355538
rs112963922
rs17580
rs143666677
All − MR Egger
All − Inverse variance weighted
−4
−2
0
2
MR effect size for
'ADAM15' on 'Pulmonary fibrosis'

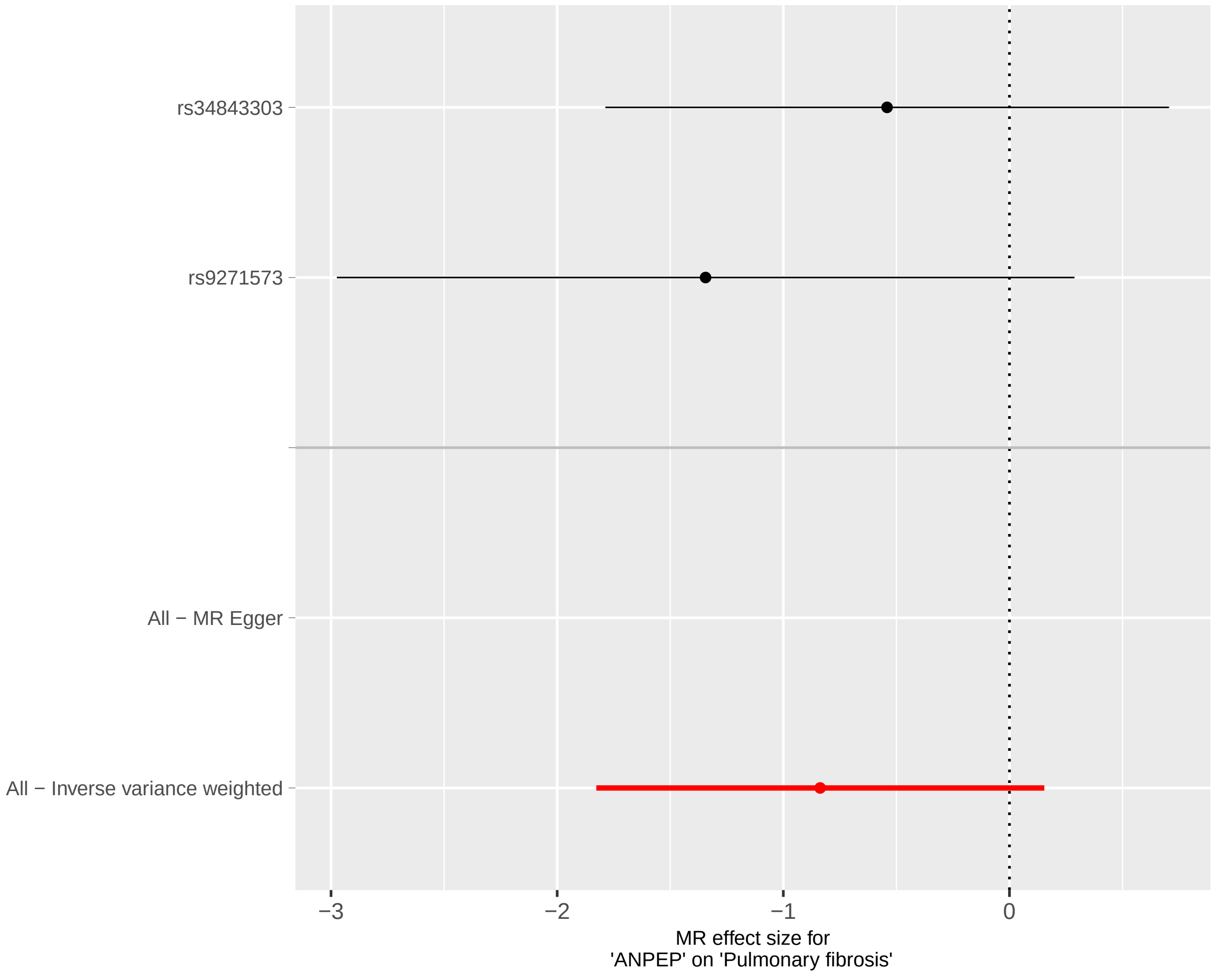
rs34843303
rs9271573
All − MR Egger
All − Inverse variance weighted
−3
−2
−1
0
MR effect size for
'ANPEP' on 'Pulmonary fibrosis'

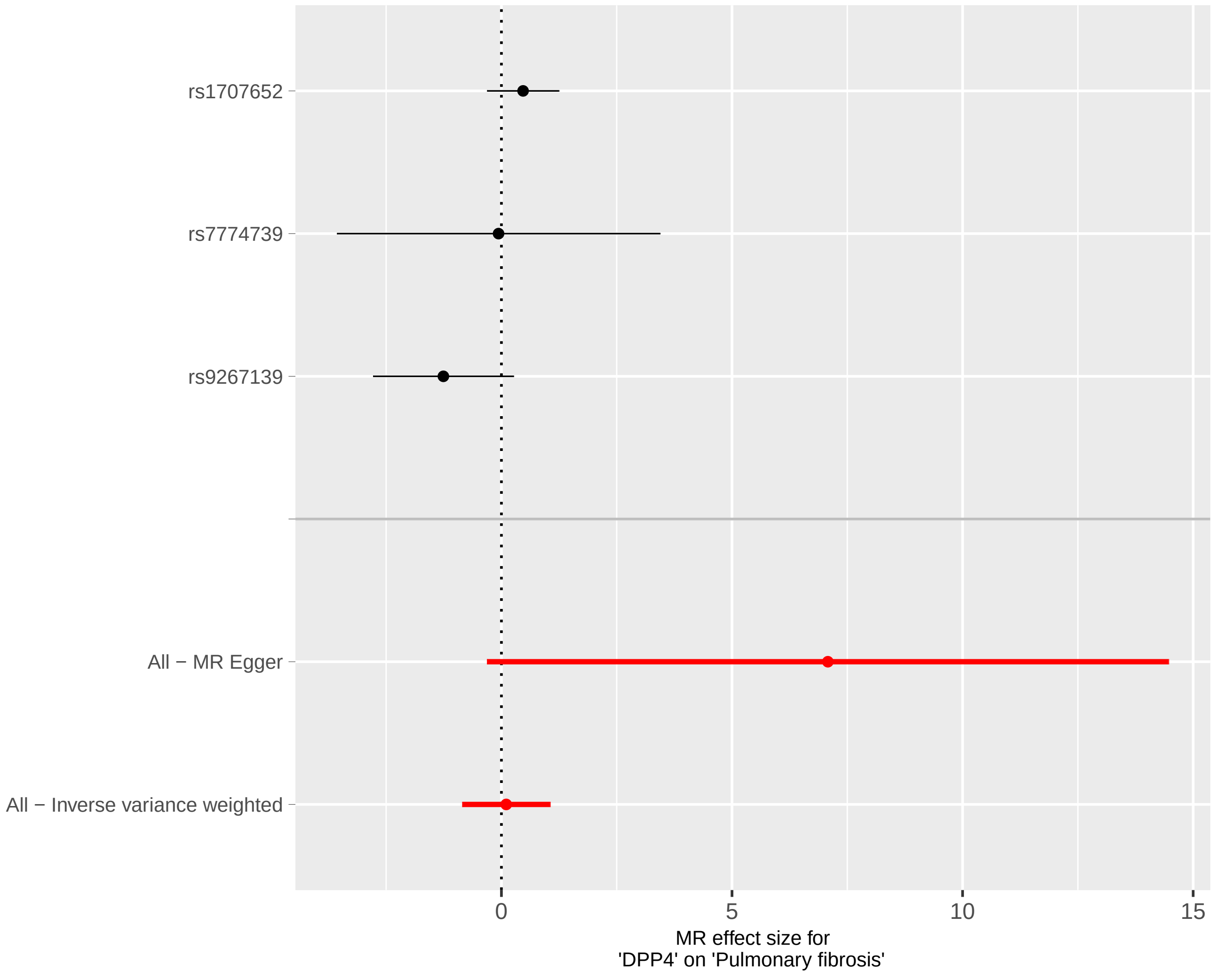

rs1707652
rs7774739
rs9267139
All − MR Egger
All − Inverse variance weighted
0
5
10
15
MR effect size for
'DPP4' on 'Pulmonary fibrosis'

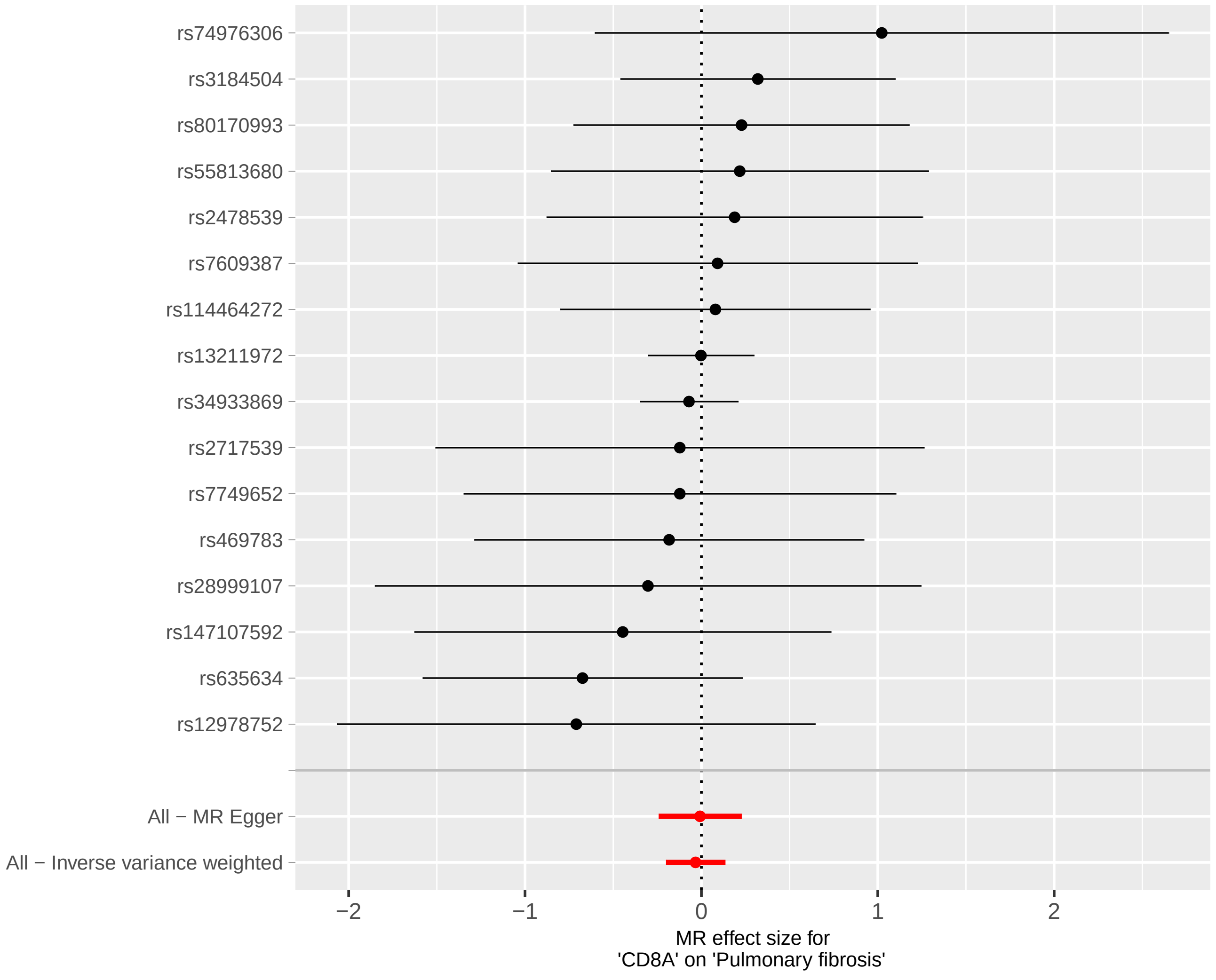

rs74976306
rs3184504
rs80170993
rs55813680
rs2478539
rs7609387
rs114464272
rs13211972
rs34933869
rs2717539
rs7749652
rs469783
rs28999107
rs147107592
rs635634
rs12978752
All − MR Egger
All − Inverse variance weighted
−2
−1
0
1
2
MR effect size for
'CD8A' on 'Pulmonary fibrosis'

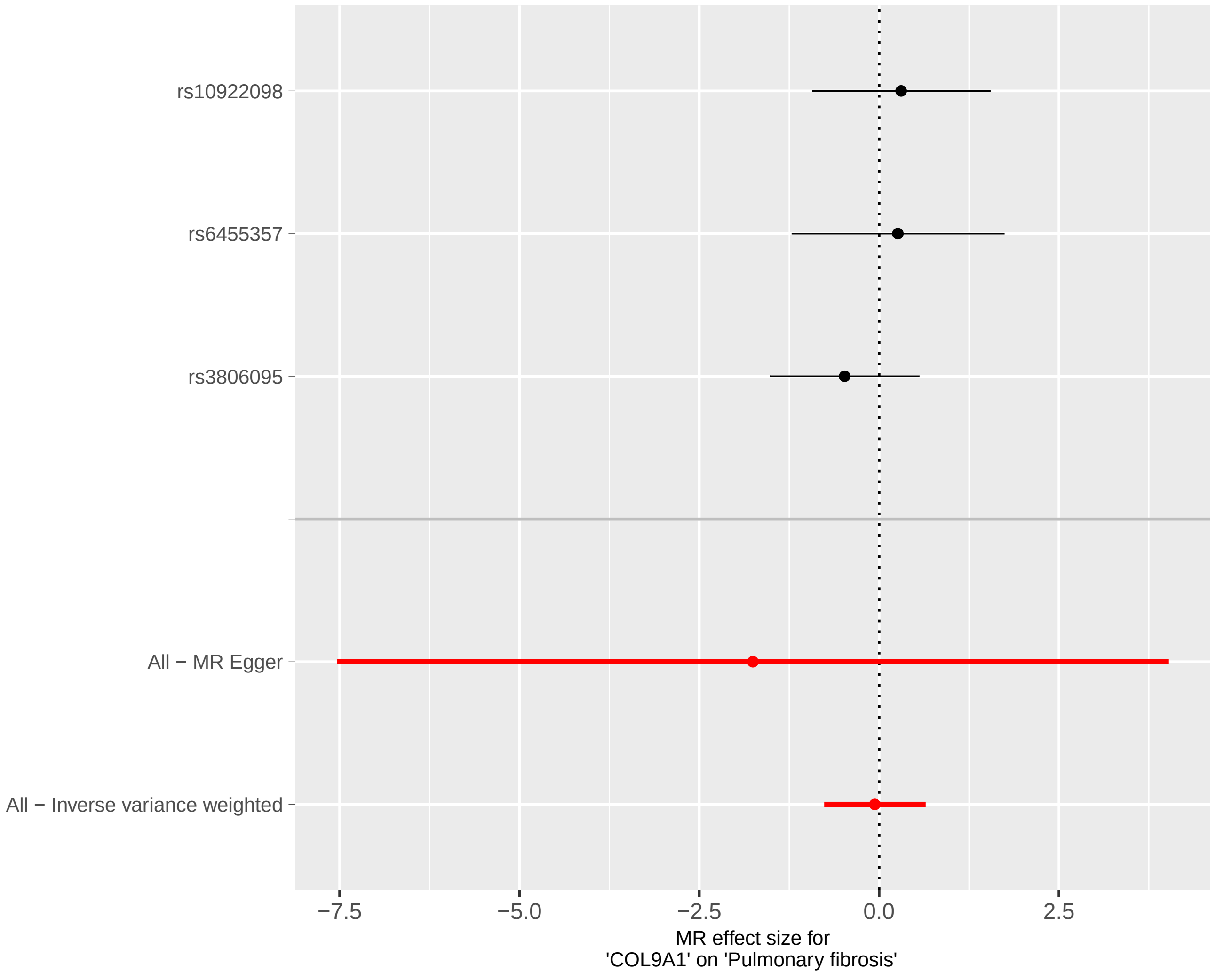

rs10922098
rs6455357
rs3806095
All − MR Egger
All − Inverse variance weighted
−7.5
−5.0
−2.5
0.0
2.5
MR effect size for
'COL9A1' on 'Pulmonary fibrosis'

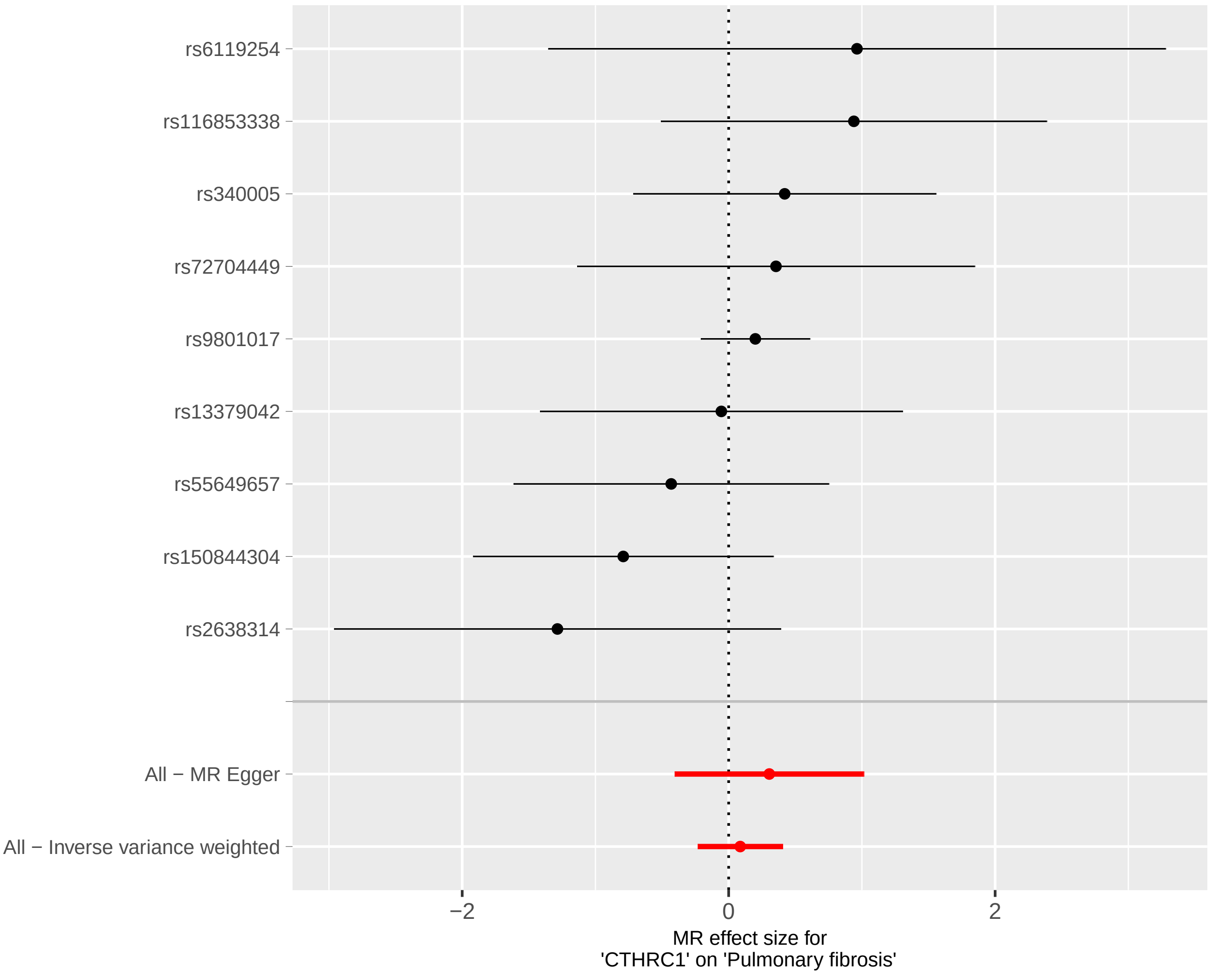
rs6119254
rs116853338
rs340005
rs72704449
rs9801017
rs13379042
rs55649657
rs150844304
rs2638314
All − MR Egger
All − Inverse variance weighted
−2
0
2
MR effect size for
'CTHRC1' on 'Pulmonary fibrosis'

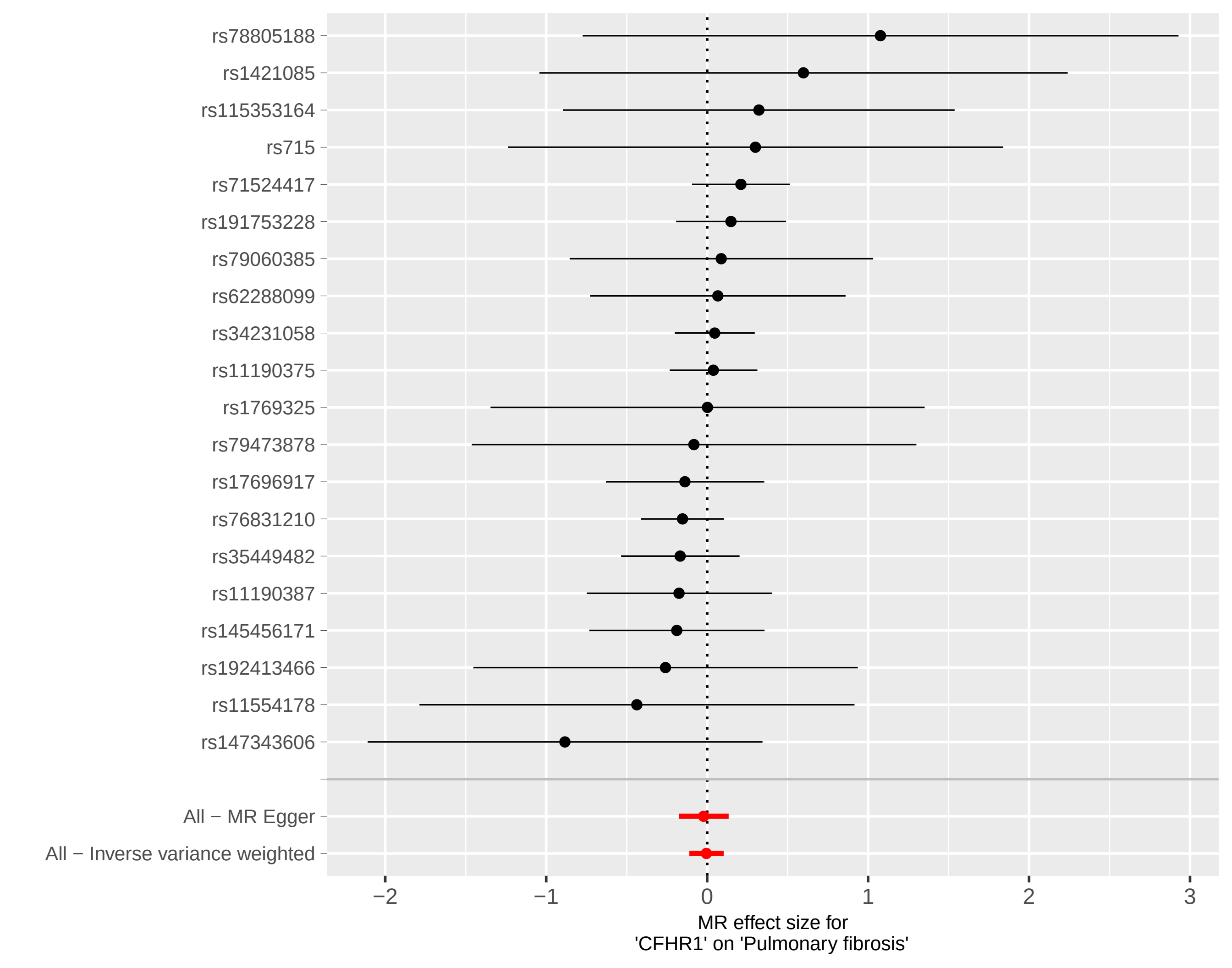
rs78805188
rs1421085
rs115353164
rs715
rs71524417
rs191753228
rs79060385
rs62288099
rs34231058
rs11190375
rs1769325
rs79473878
rs17696917
rs76831210
rs35449482
rs11190387
rs145456171
rs192413466
rs11554178
rs147343606
All − MR Egger
All − Inverse variance weighted
−2
−1
0
1
2
3
MR effect size for
'CFHR1' on 'Pulmonary fibrosis'

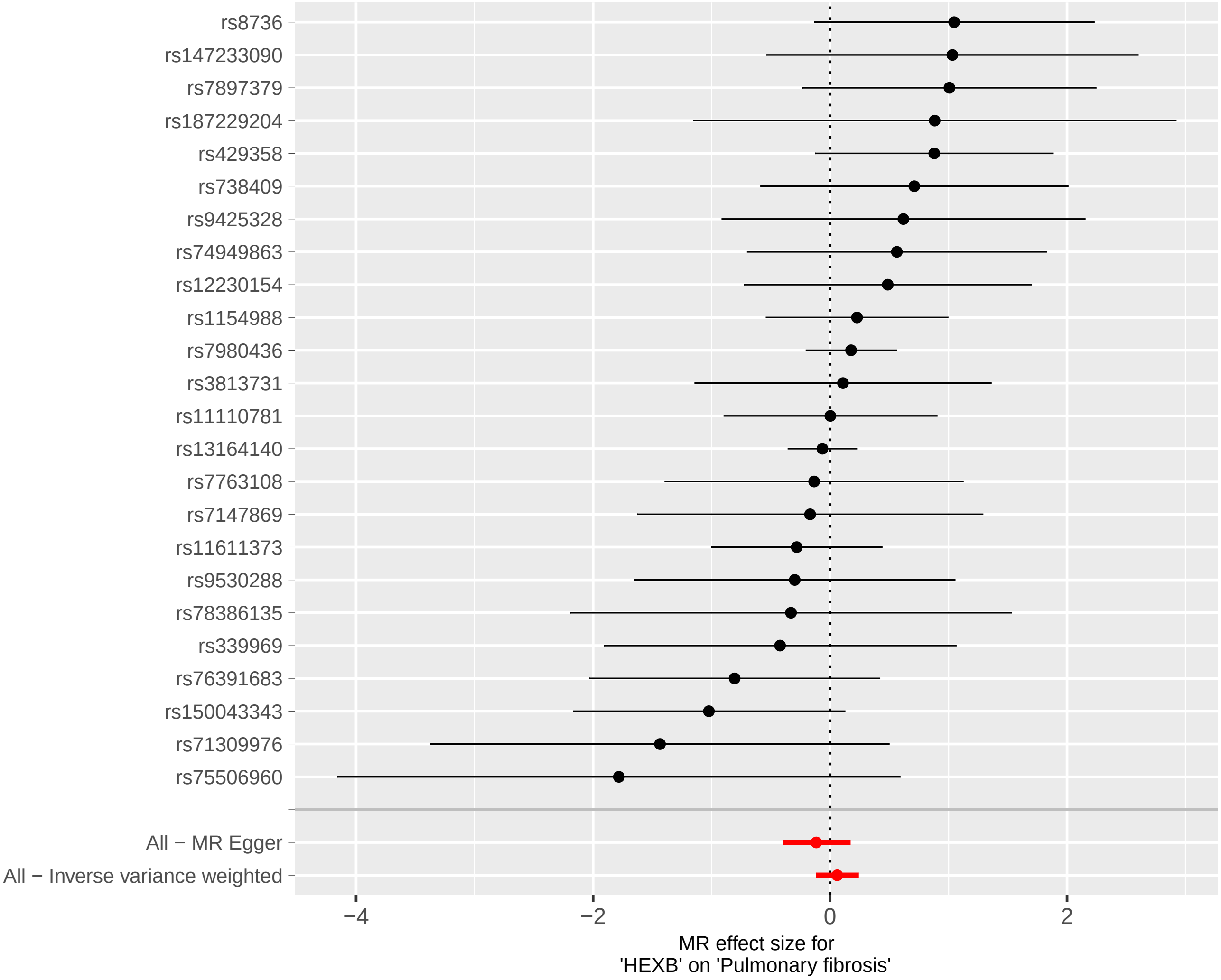
rs8736
rs147233090
rs7897379
rs187229204
rs429358
rs738409
rs9425328
rs74949863
rs12230154
rs1154988
rs7980436
rs3813731
rs11110781
rs13164140
rs7763108
rs7147869
rs11611373
rs9530288
rs78386135
rs339969
rs76391683
rs150043343
rs71309976
rs75506960
All − MR Egger
All − Inverse variance weighted
−4
−2
0
2
MR effect size for
'HEXB' on 'Pulmonary fibrosis'

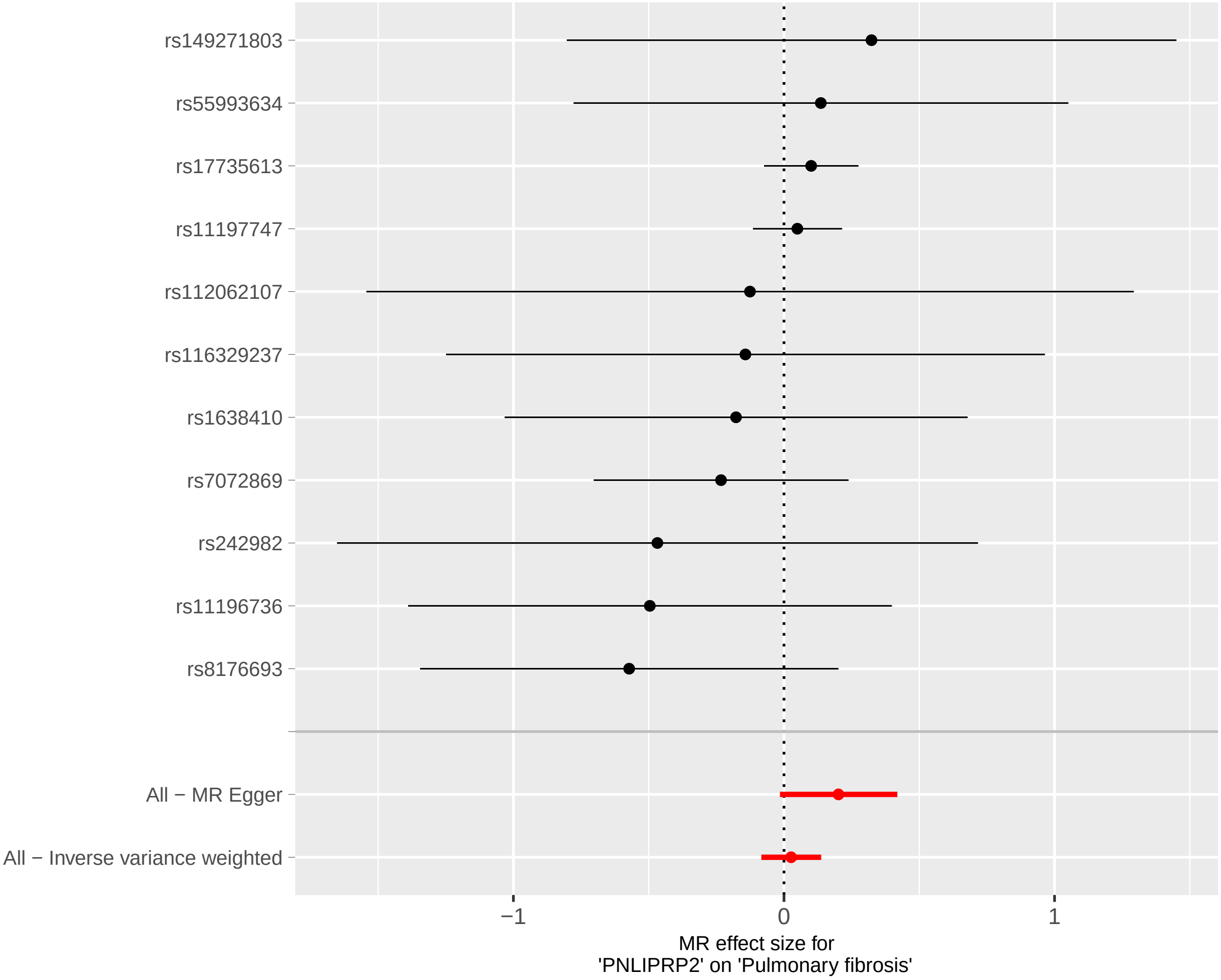

rs149271803
rs55993634
rs17735613
rs11197747
rs112062107
rs116329237
rs1638410
rs7072869
rs242982
rs11196736
rs8176693
All − MR Egger
All − Inverse variance weighted
−1
0
1
MR effect size for
'PNLIPRP2' on 'Pulmonary fibrosis'

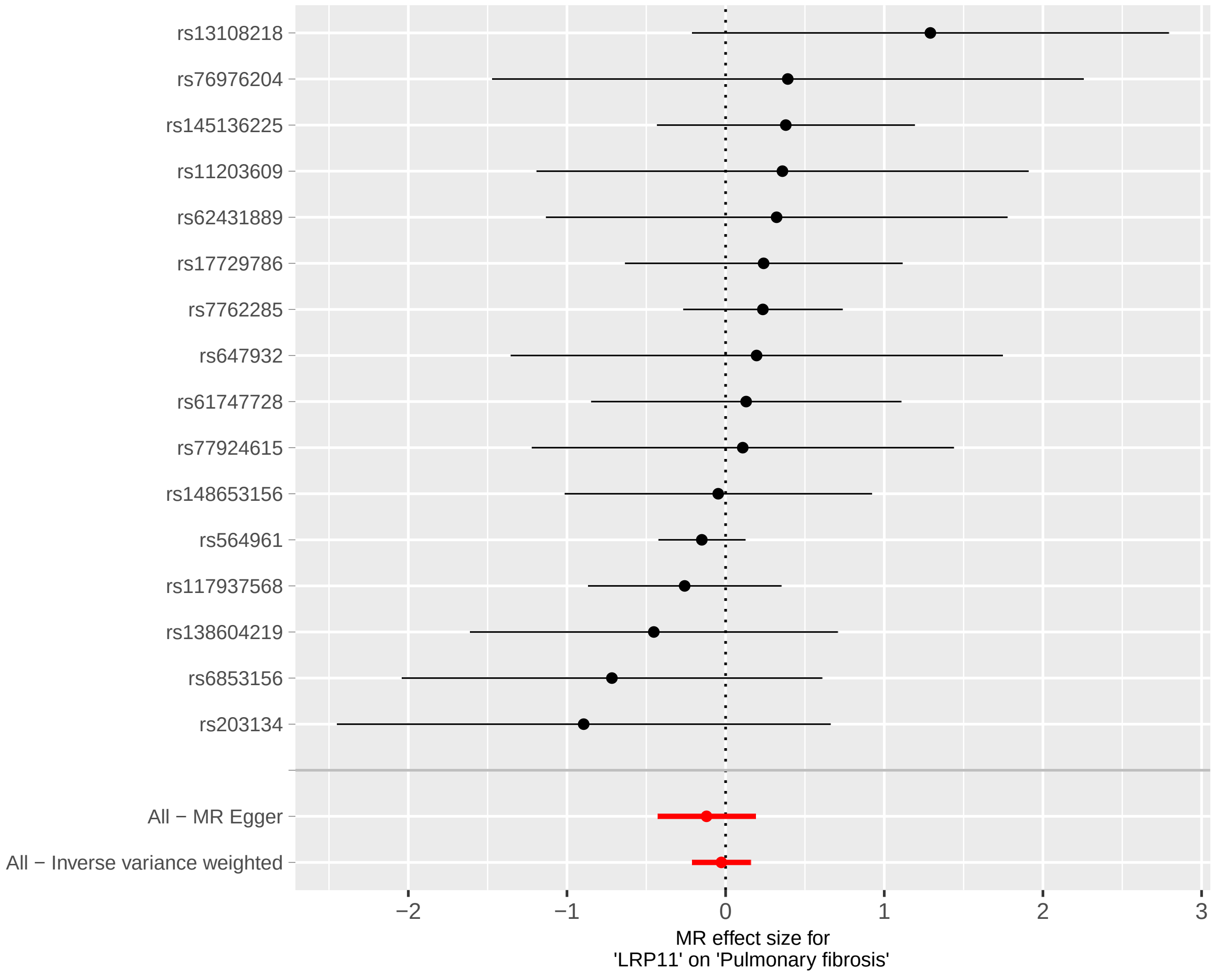
rs13108218
rs76976204
rs145136225
rs11203609
rs62431889
rs17729786
rs7762285
rs647932
rs61747728
rs77924615
rs148653156
rs564961
rs117937568
rs138604219
rs6853156
rs203134
All − MR Egger
All − Inverse variance weighted
−2
−1
0
1
2
3
MR effect size for
'LRP11' on 'Pulmonary fibrosis'

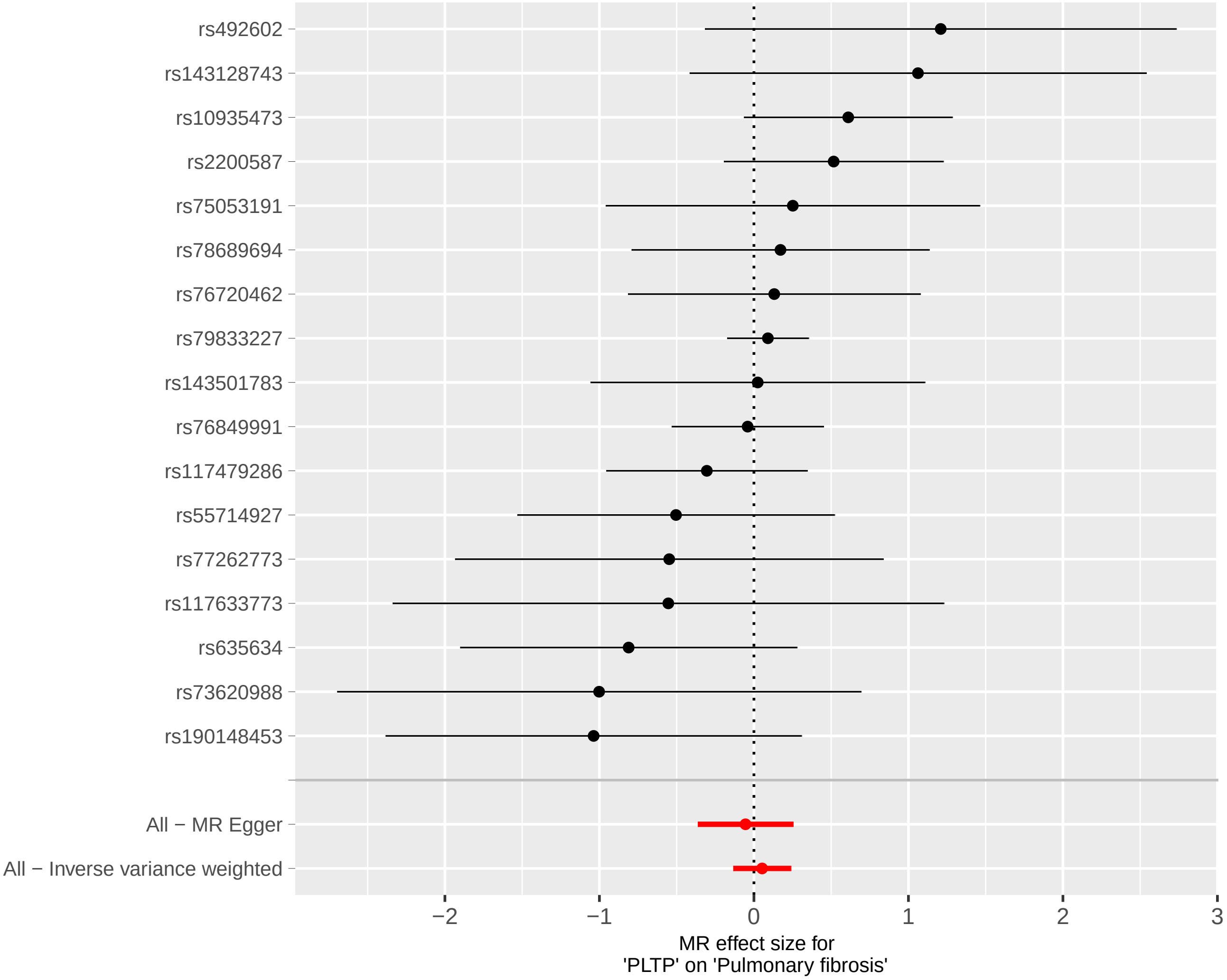

rs492602
rs143128743
rs10935473
rs2200587
rs75053191
rs78689694
rs76720462
rs79833227
rs143501783
rs76849991
rs117479286
rs55714927
rs77262773
rs117633773
rs635634
rs73620988
rs190148453
All − MR Egger
All − Inverse variance weighted
−2
−1
0
1
2
3
MR effect size for
'PLTP' on 'Pulmonary fibrosis'

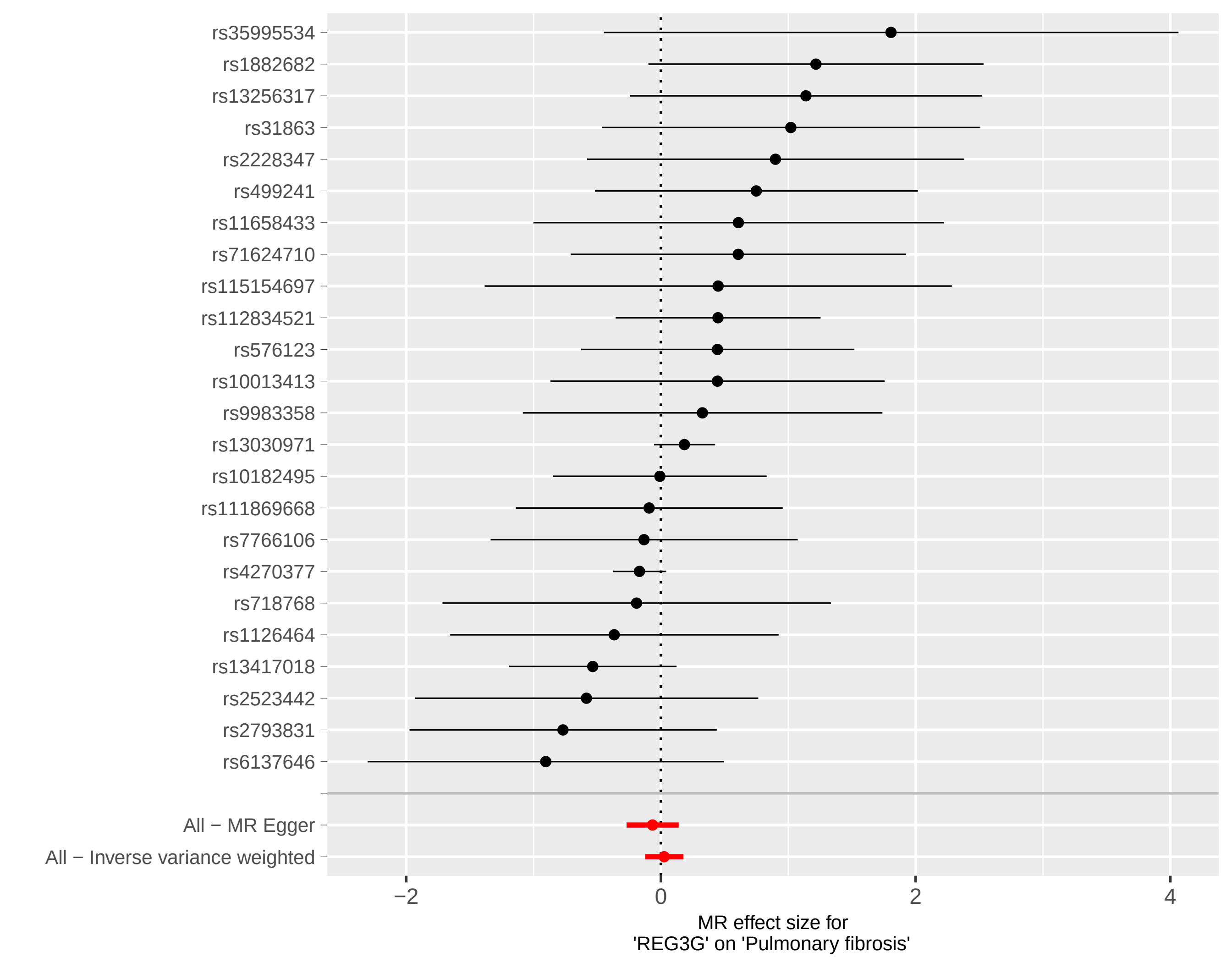
rs35995534
rs1882682
rs13256317
rs31863
rs2228347
rs499241
rs11658433
rs71624710
rs115154697
rs112834521
rs576123
rs10013413
rs9983358
rs13030971
rs10182495
rs111869668
rs7766106
rs4270377
rs718768
rs1126464
rs13417018
rs2523442
rs2793831
rs6137646
All − MR Egger
All − Inverse variance weighted
−2
0
2
4
MR effect size for
'REG3G' on 'Pulmonary fibrosis'

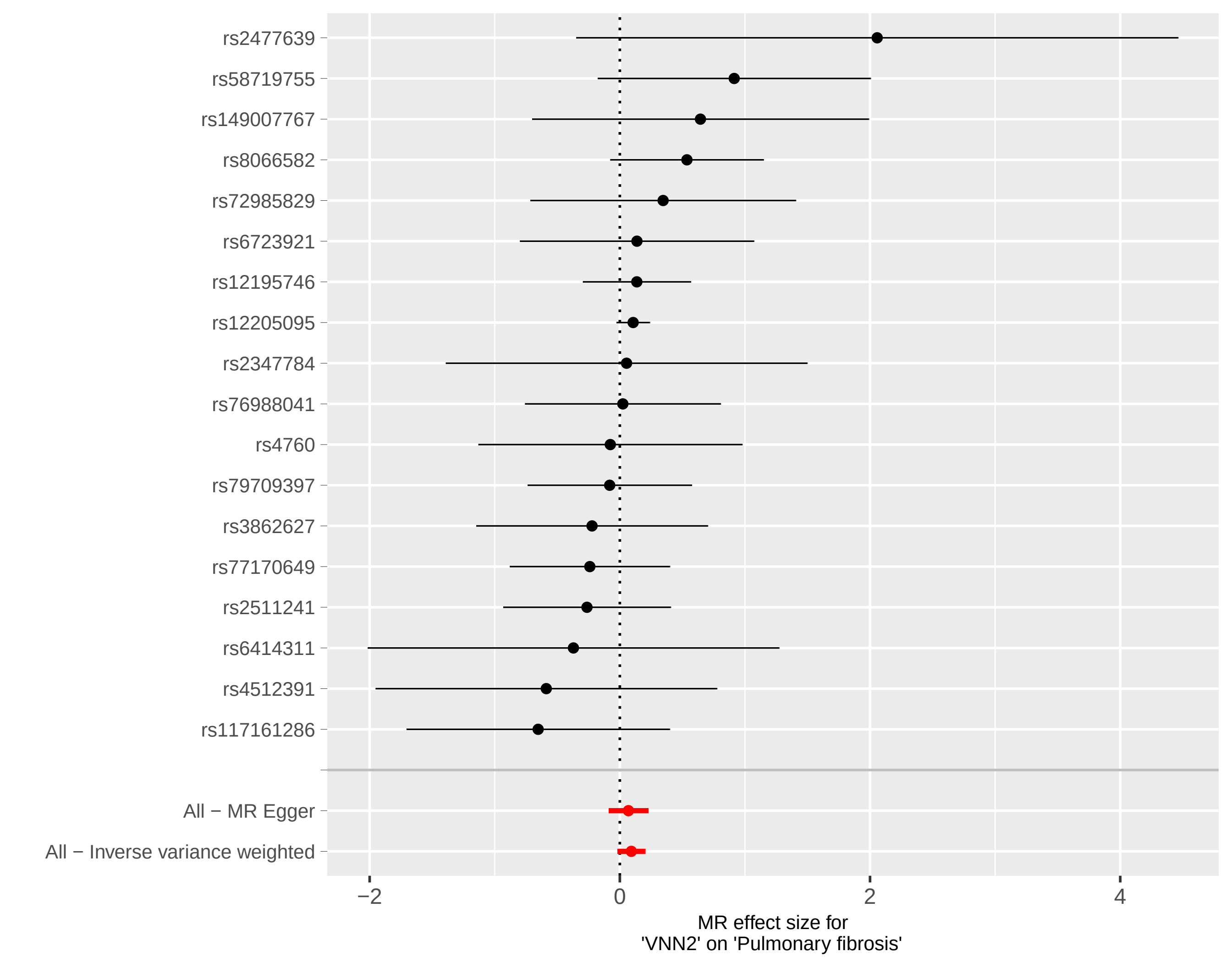

rs2477639
rs58719755
rs149007767
rs8066582
rs72985829
rs6723921
rs12195746
rs12205095
rs2347784
rs76988041
rs4760
rs79709397
rs3862627
rs77170649
rs2511241
rs6414311
rs4512391
rs117161286
All − MR Egger
All − Inverse variance weighted
−2
0
2
4
MR effect size for
'VNN2' on 'Pulmonary fibrosis'

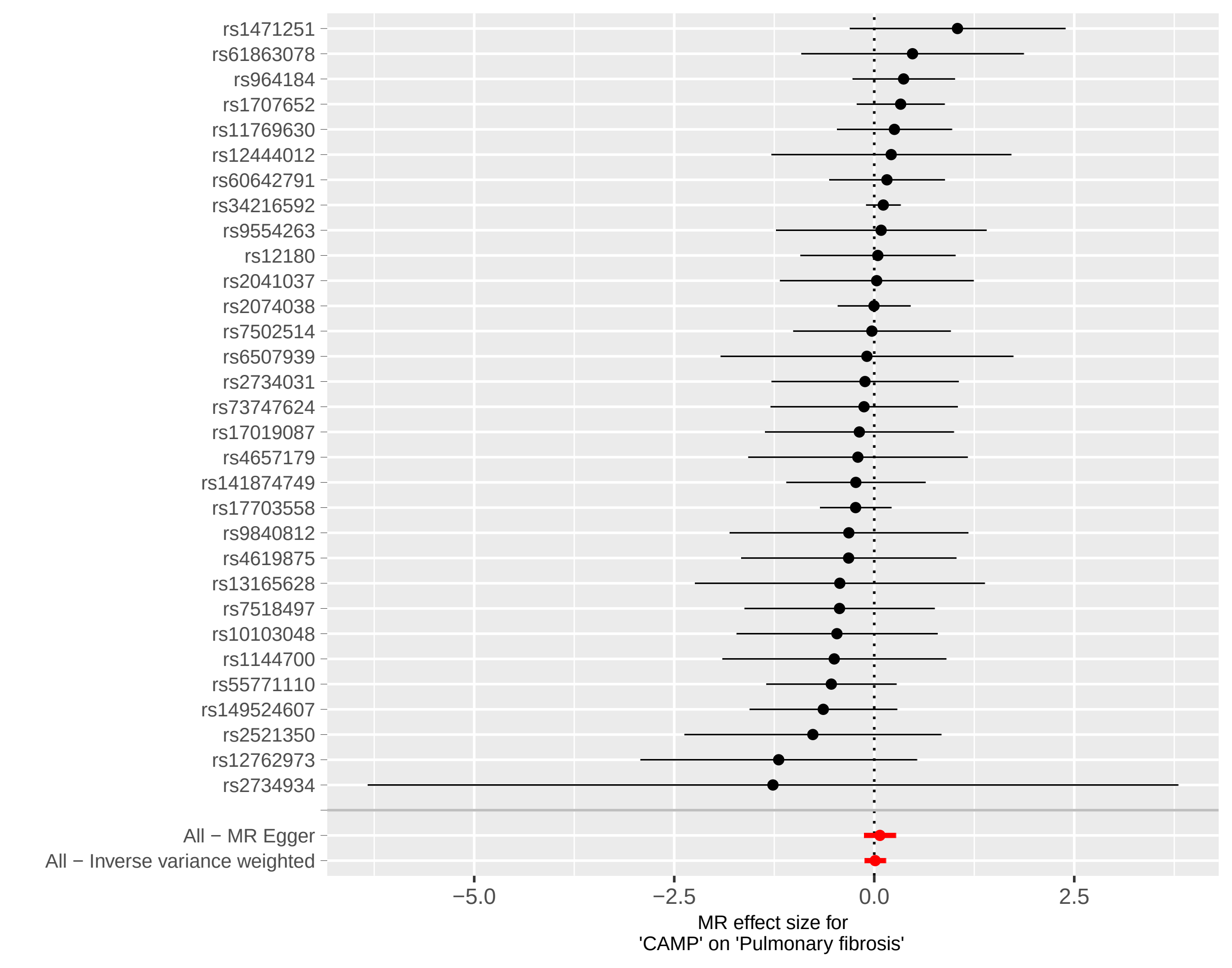
rs1471251
rs61863078
rs964184
rs1707652
rs11769630
rs12444012
rs60642791
rs34216592
rs9554263
rs12180
rs2041037
rs2074038
rs7502514
rs6507939
rs2734031
rs73747624
rs17019087
rs4657179
rs141874749
rs17703558
rs9840812
rs4619875
rs13165628
rs7518497
rs10103048
rs1144700
rs55771110
rs149524607
rs2521350
rs12762973
rs2734934
All − MR Egger
All − Inverse variance weighted
−5.0
−2.5
0.0
2.5
MR effect size for
'CAMP' on 'Pulmonary fibrosis'

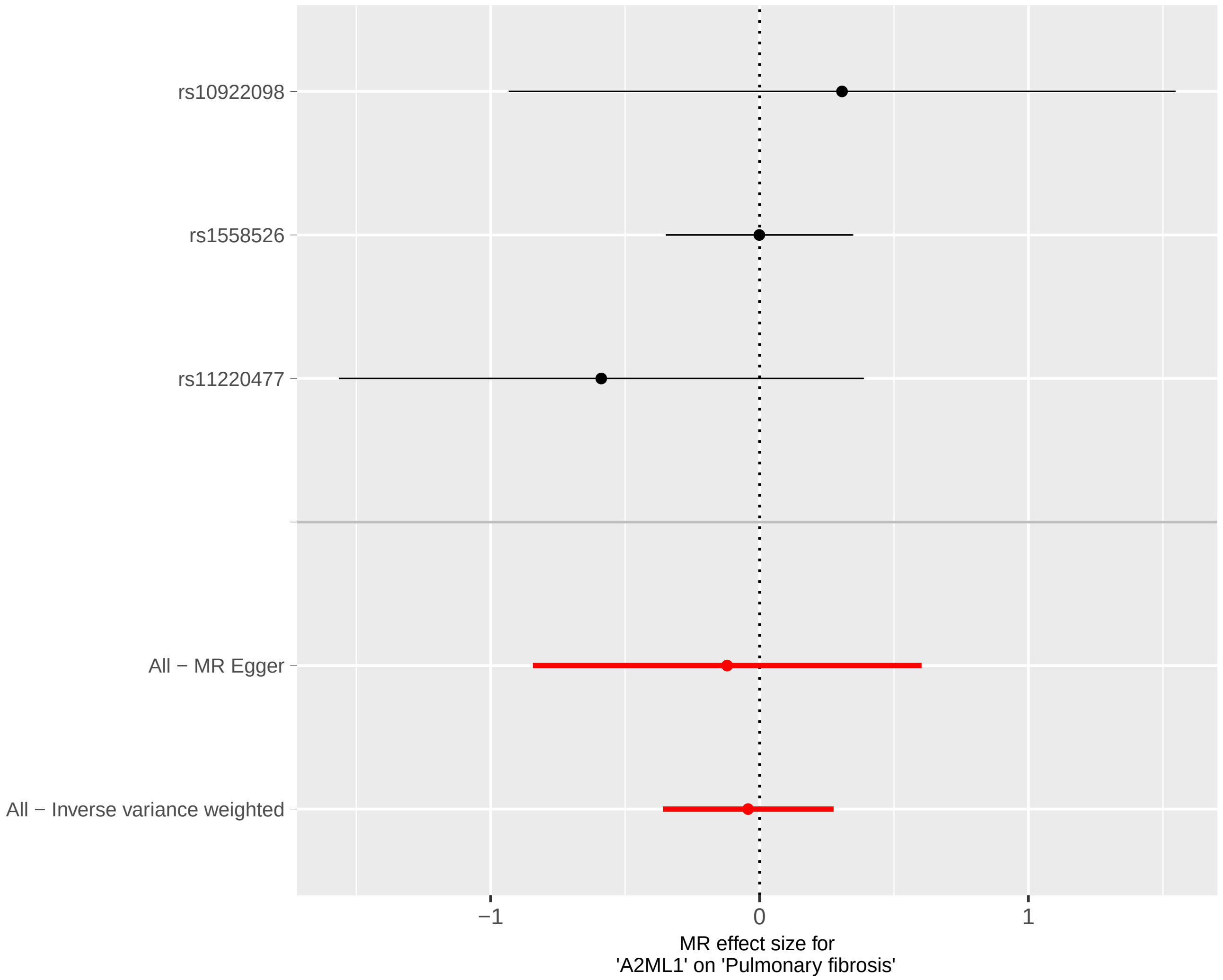
rs10922098
rs1558526
rs11220477
All − MR Egger
All − Inverse variance weighted
−1
0
1
MR effect size for
'A2ML1' on 'Pulmonary fibrosis'

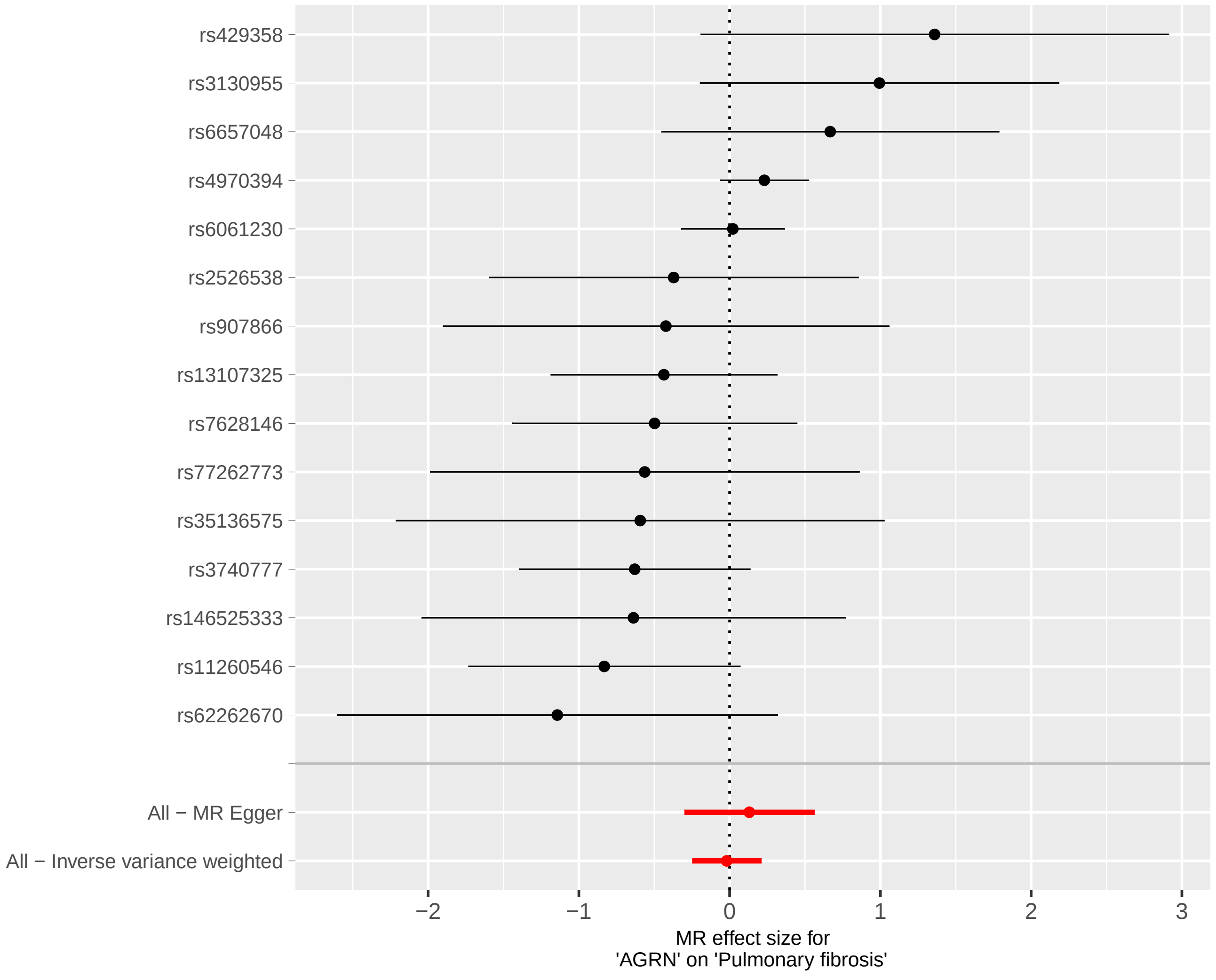
rs429358
rs3130955
rs6657048
rs4970394
rs6061230
rs2526538
rs907866
rs13107325
rs7628146
rs77262773
rs35136575
rs3740777
rs146525333
rs11260546
rs62262670
All − MR Egger
All − Inverse variance weighted
−2
−1
0
1
2
3
MR effect size for
'AGRN' on 'Pulmonary fibrosis'

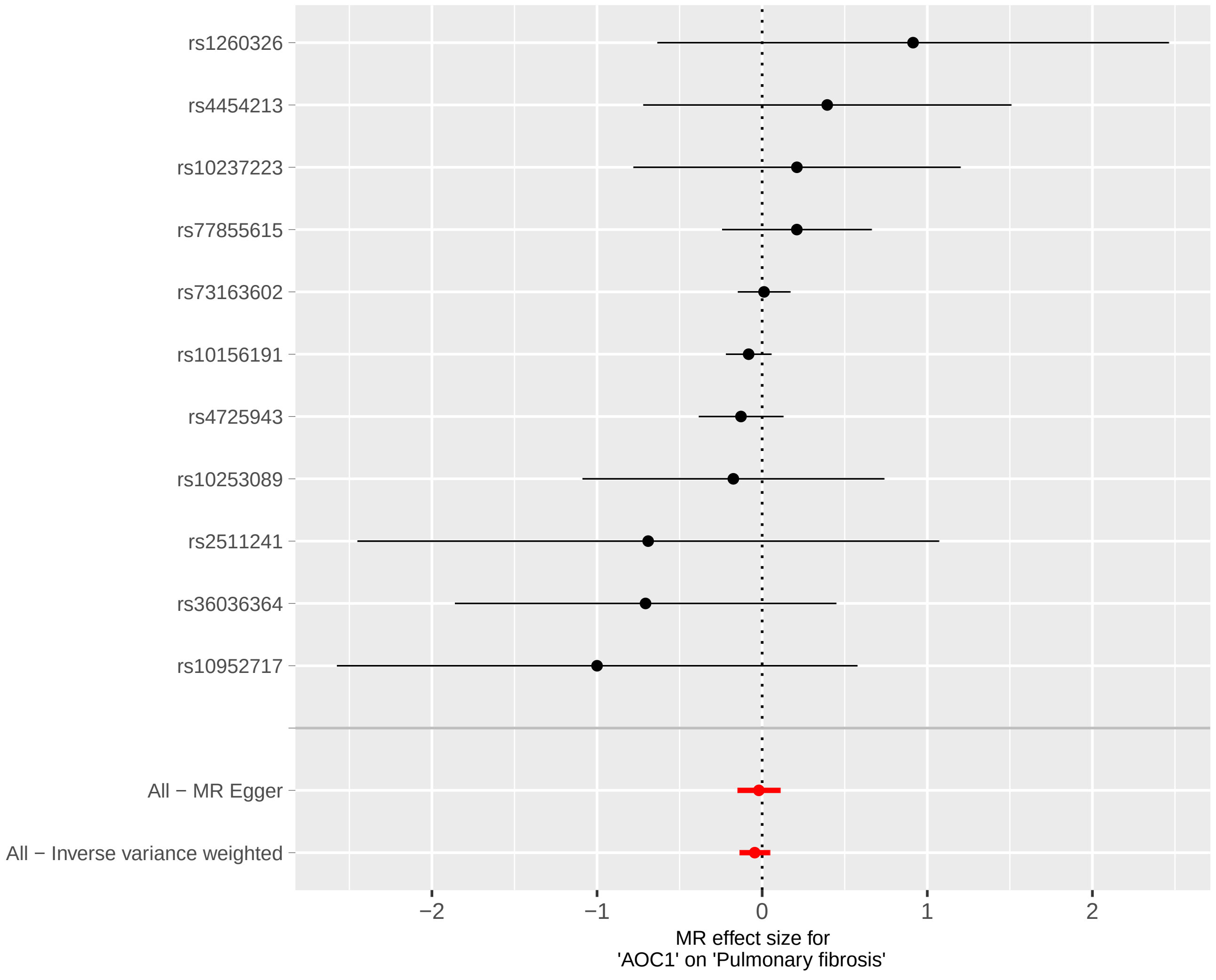

rs1260326
rs4454213
rs10237223
rs77855615
rs73163602
rs10156191
rs4725943
rs10253089
rs2511241
rs36036364
rs10952717
All − MR Egger
All − Inverse variance weighted
−2
−1
0
1
2
MR effect size for
'AOC1' on 'Pulmonary fibrosis'

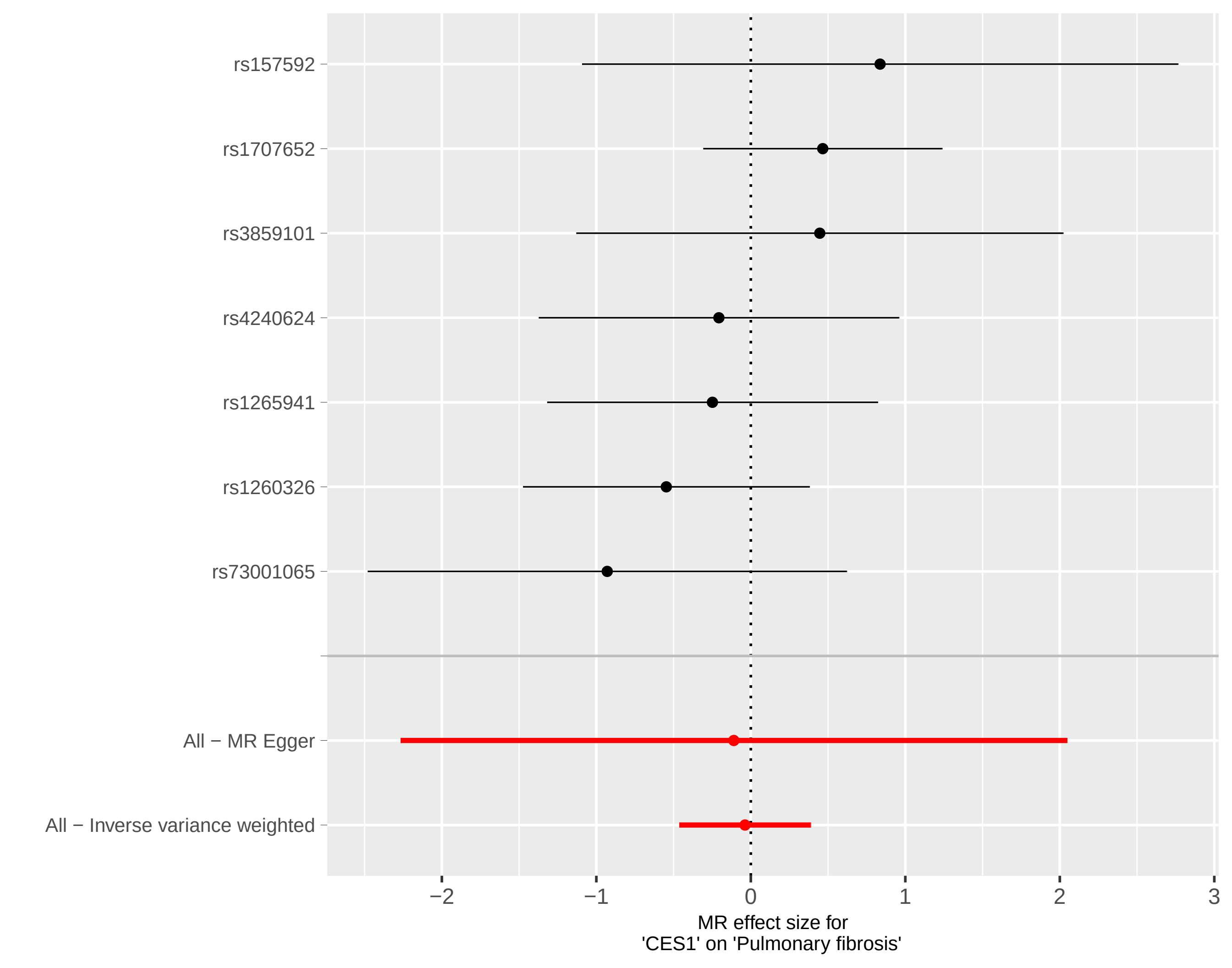
rs157592
rs1707652
rs3859101
rs4240624
rs1265941
rs1260326
rs73001065
All − MR Egger
All − Inverse variance weighted
−2
−1
0
1
2
3
MR effect size for
'CES1' on 'Pulmonary fibrosis'

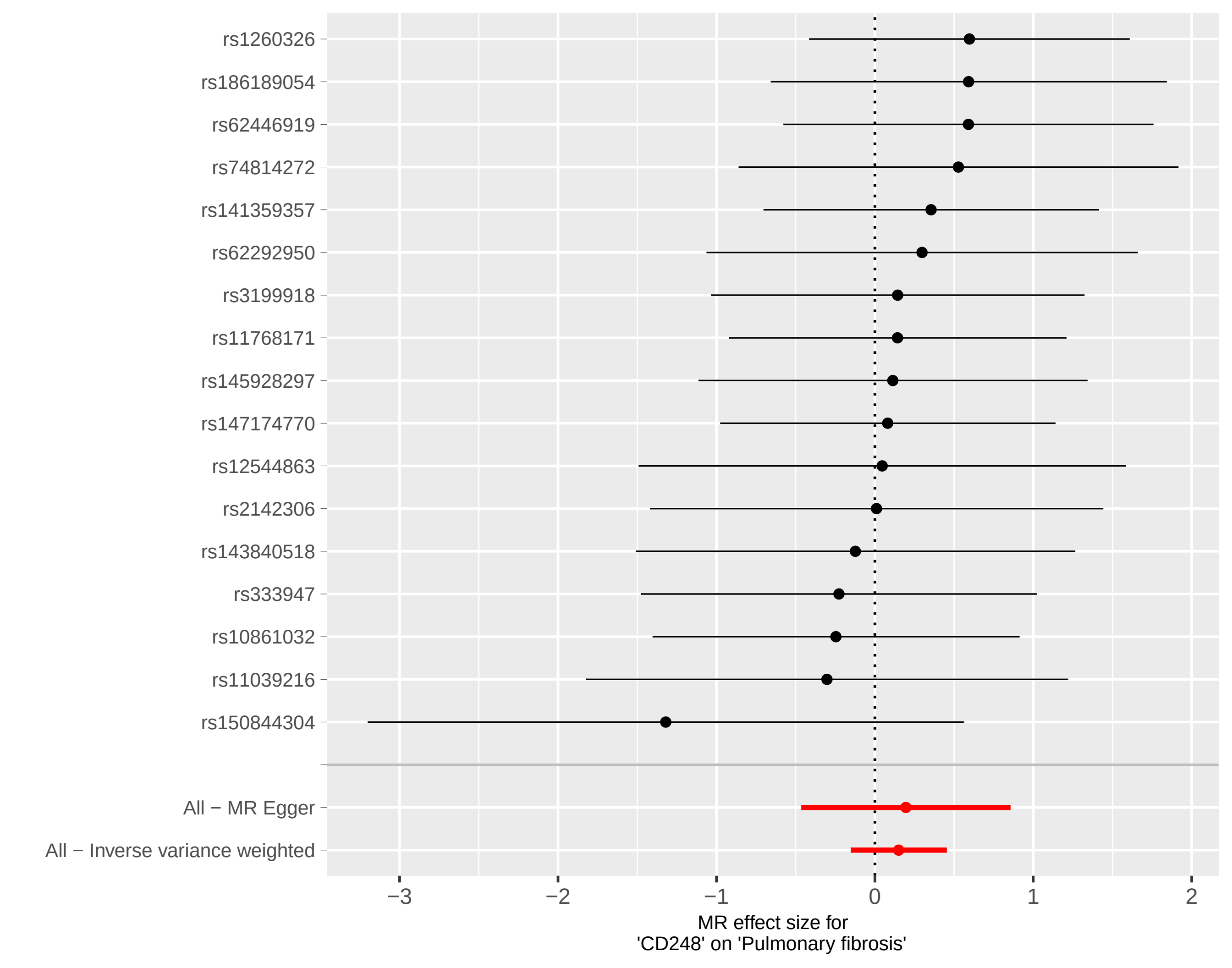
rs1260326
rs186189054
rs62446919
rs74814272
rs141359357
rs62292950
rs3199918
rs11768171
rs145928297
rs147174770
rs12544863
rs2142306
rs143840518
rs333947
rs10861032
rs11039216
rs150844304
All − MR Egger
All − Inverse variance weighted
−3
−2
−1
0
1
2
MR effect size for
'CD248' on 'Pulmonary fibrosis'

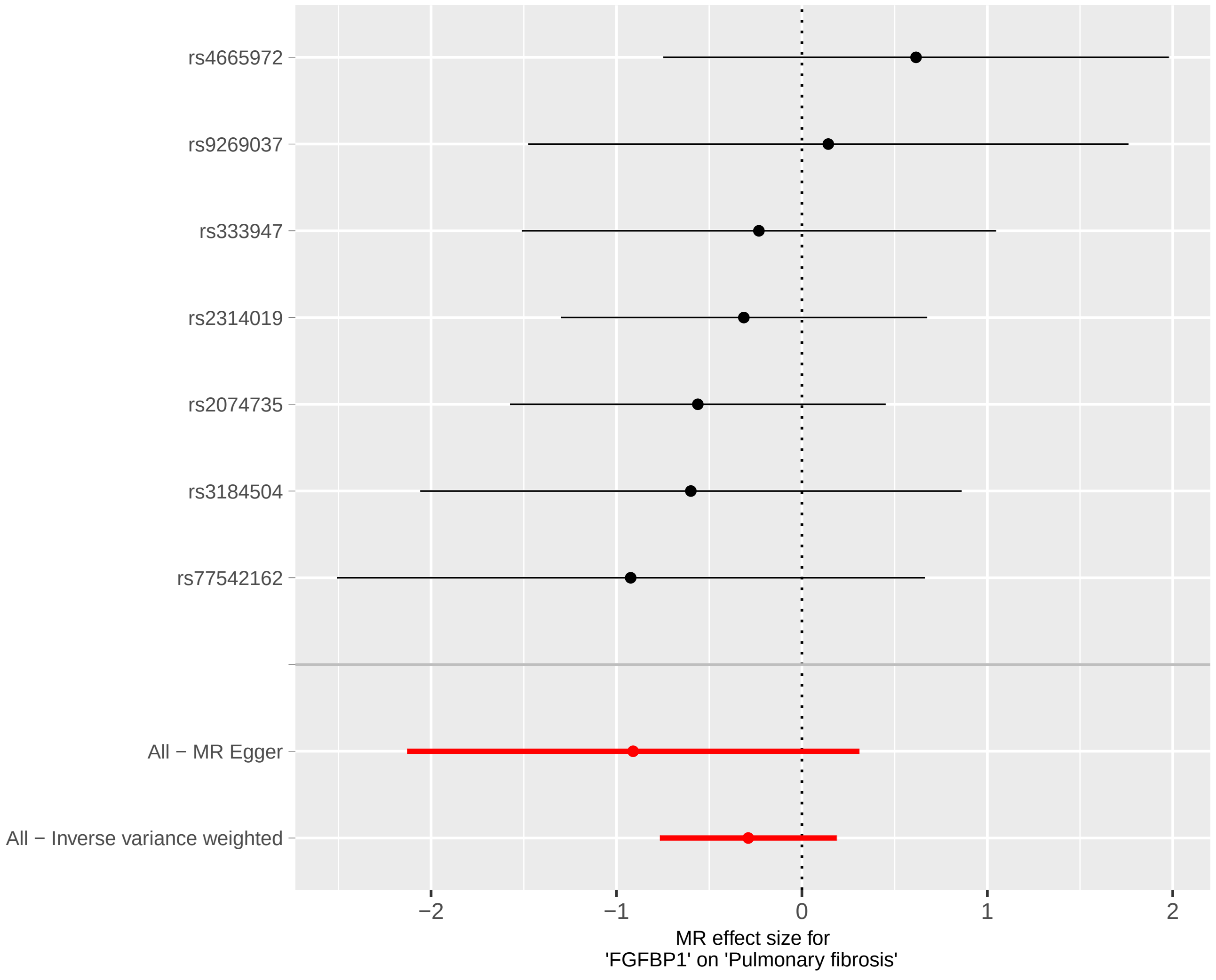
rs4665972
rs9269037
rs333947
rs2314019
rs2074735
rs3184504
rs77542162
All − MR Egger
All − Inverse variance weighted
−2
−1
0
1
2
MR effect size for
'FGFBP1' on 'Pulmonary fibrosis'

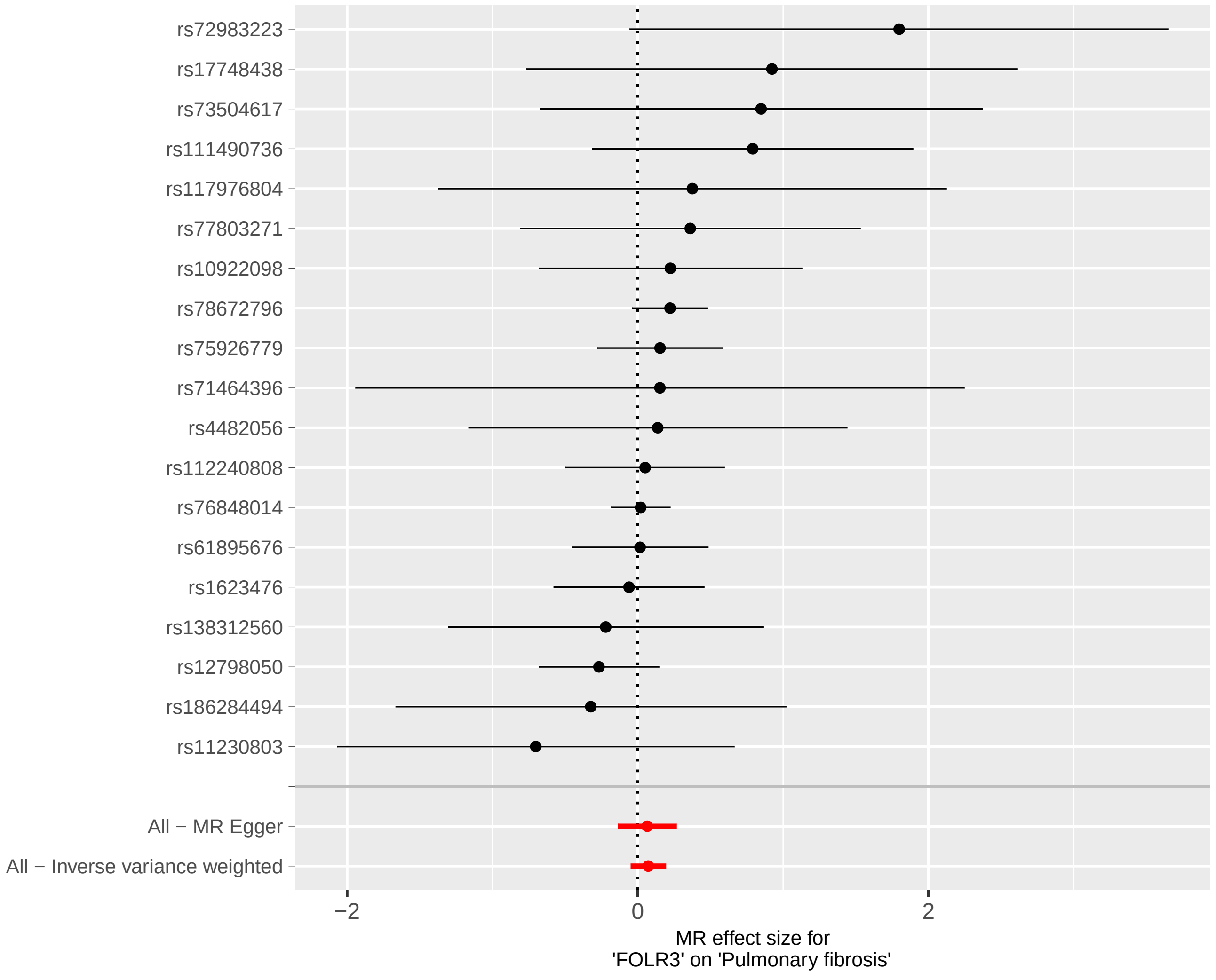
rs72983223
rs17748438
rs73504617
rs111490736
rs117976804
rs77803271
rs10922098
rs78672796
rs75926779
rs71464396
rs4482056
rs112240808
rs76848014
rs61895676
rs1623476
rs138312560
rs12798050
rs186284494
rs11230803
All − MR Egger
All − Inverse variance weighted
−2
0
2
MR effect size for
'FOLR3' on 'Pulmonary fibrosis'

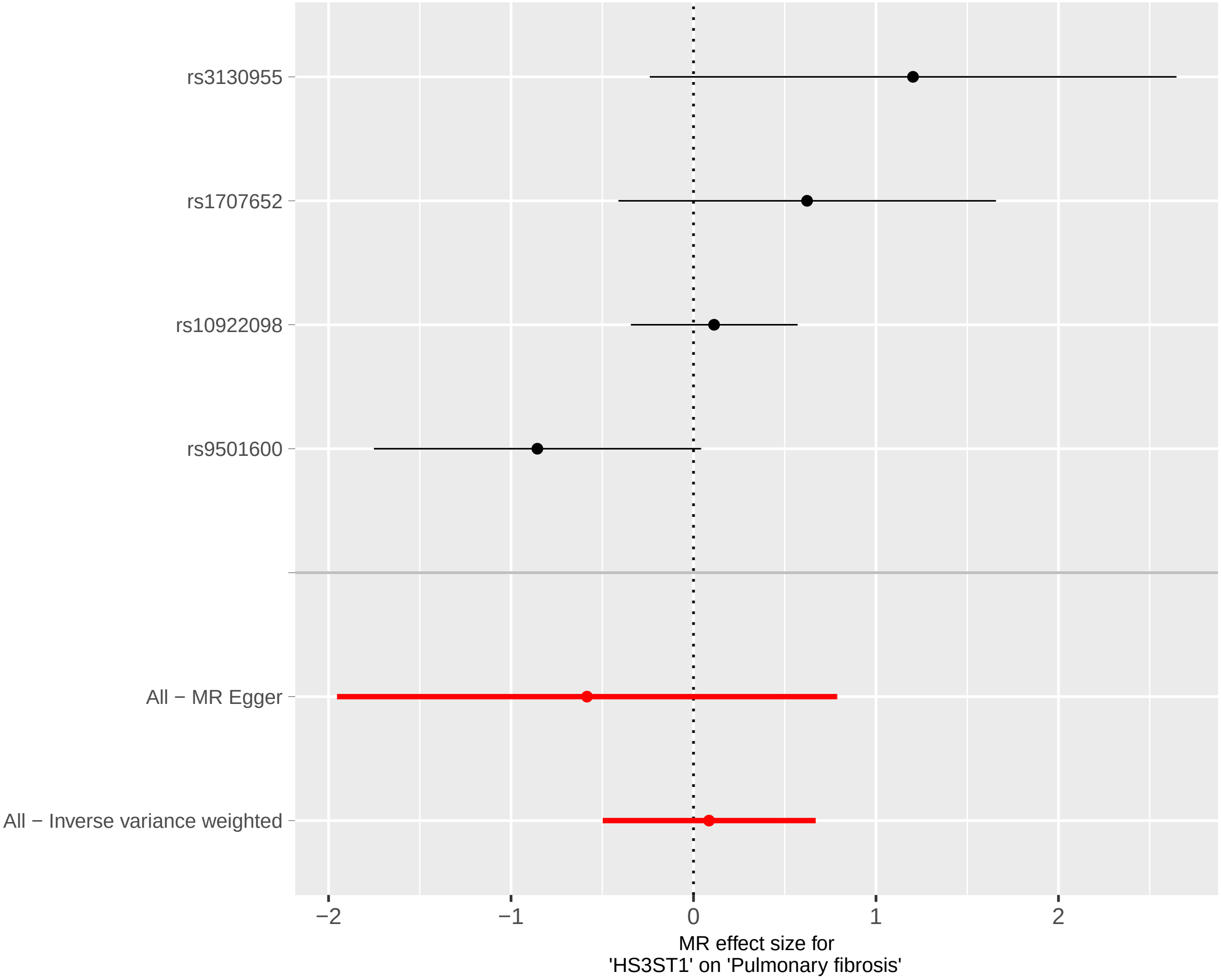
rs3130955
rs1707652
rs10922098
rs9501600
All − MR Egger
All − Inverse variance weighted
−2
−1
0
1
2
MR effect size for
'HS3ST1' on 'Pulmonary fibrosis'

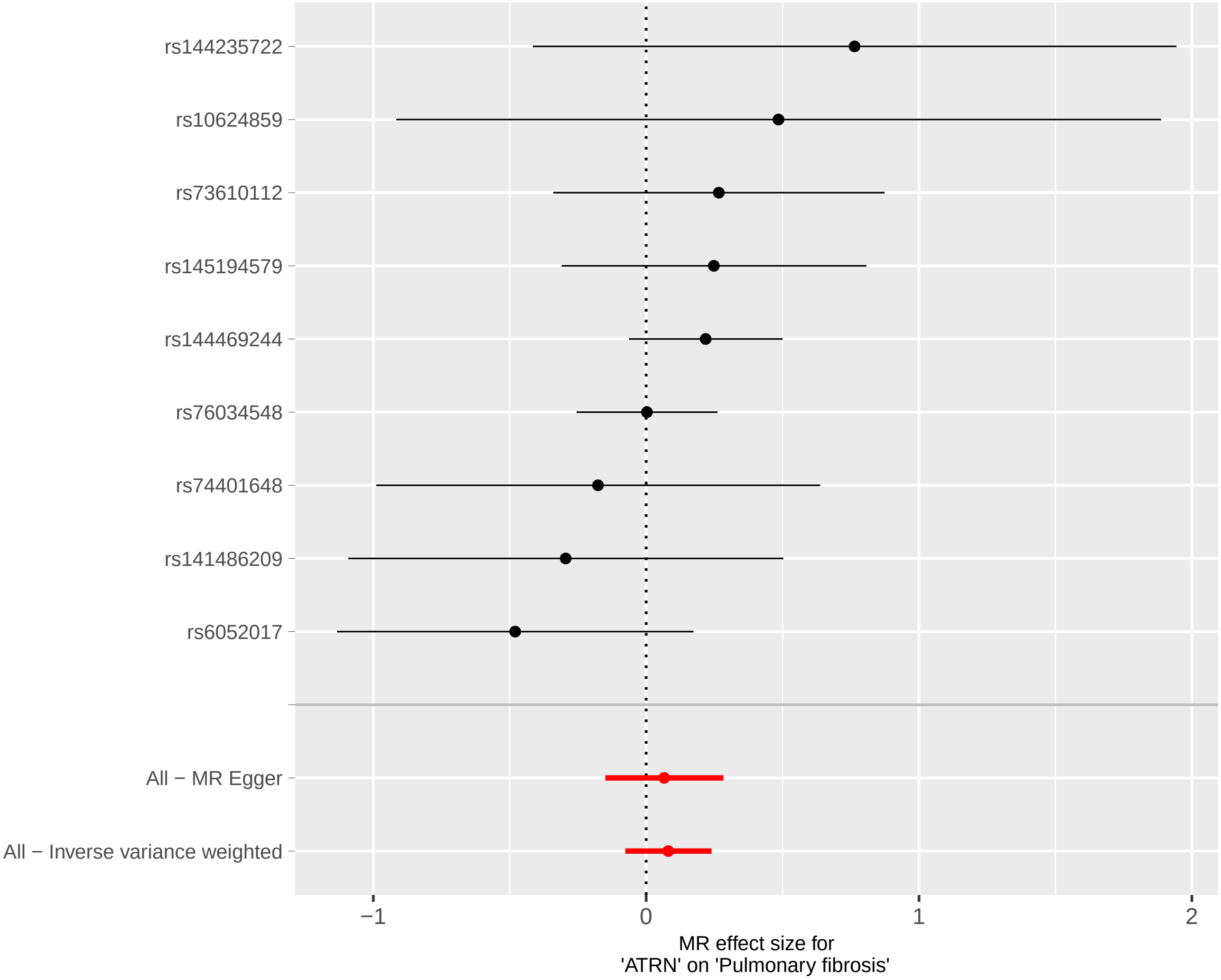

rs144235722
rs10624859
rs73610112
rs145194579
rs144469244
rs76034548
rs74401648
rs141486209
rs6052017
All − MR Egger
All − Inverse variance weighted
−1
0
1
2
MR effect size for
'ATRN' on 'Pulmonary fibrosis'

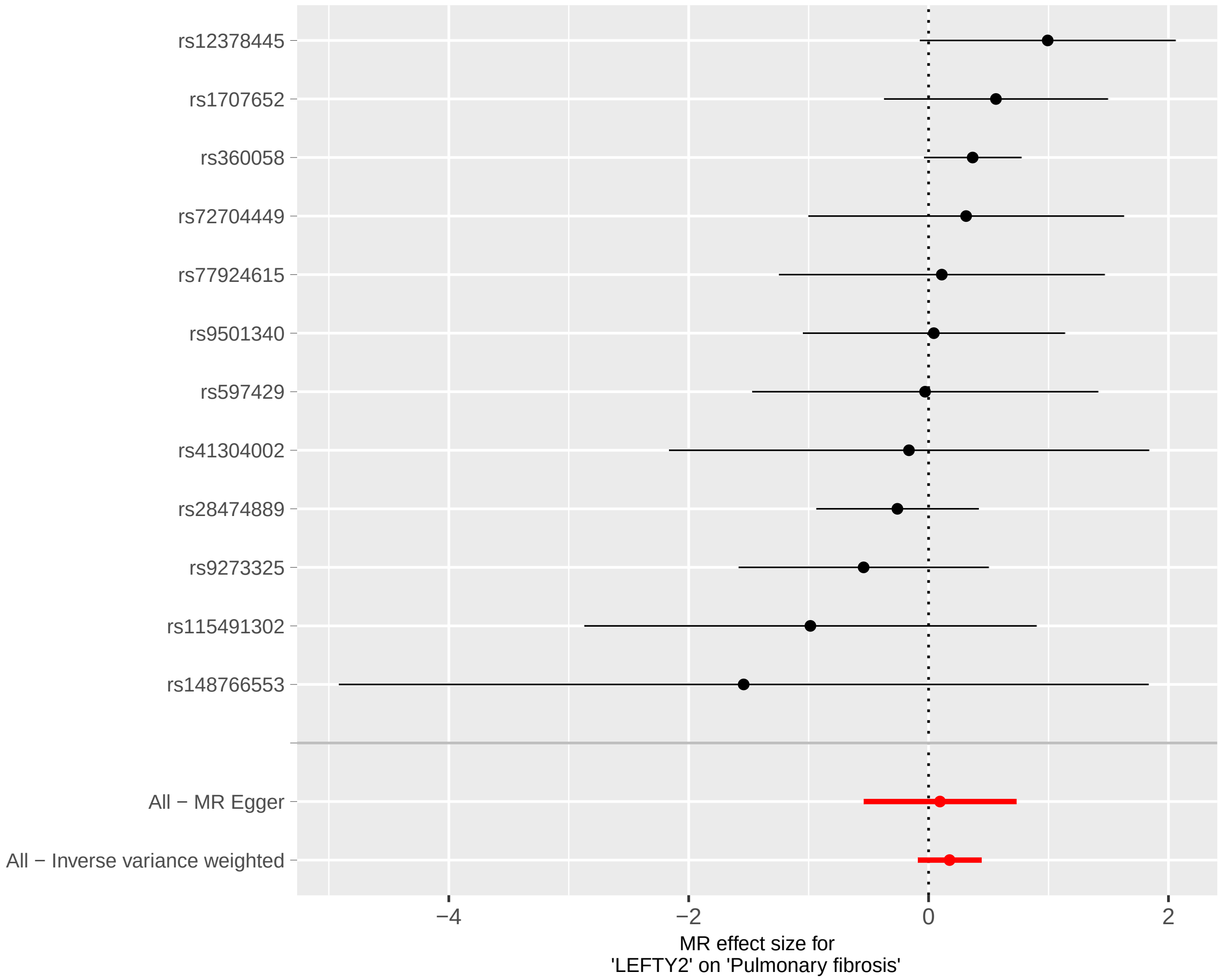

rs12378445
rs1707652
rs360058
rs72704449
rs77924615
rs9501340
rs597429
rs41304002
rs28474889
rs9273325
rs115491302
rs148766553
All − MR Egger
All − Inverse variance weighted
−4
−2
0
2
MR effect size for
'LEFTY2' on 'Pulmonary fibrosis'

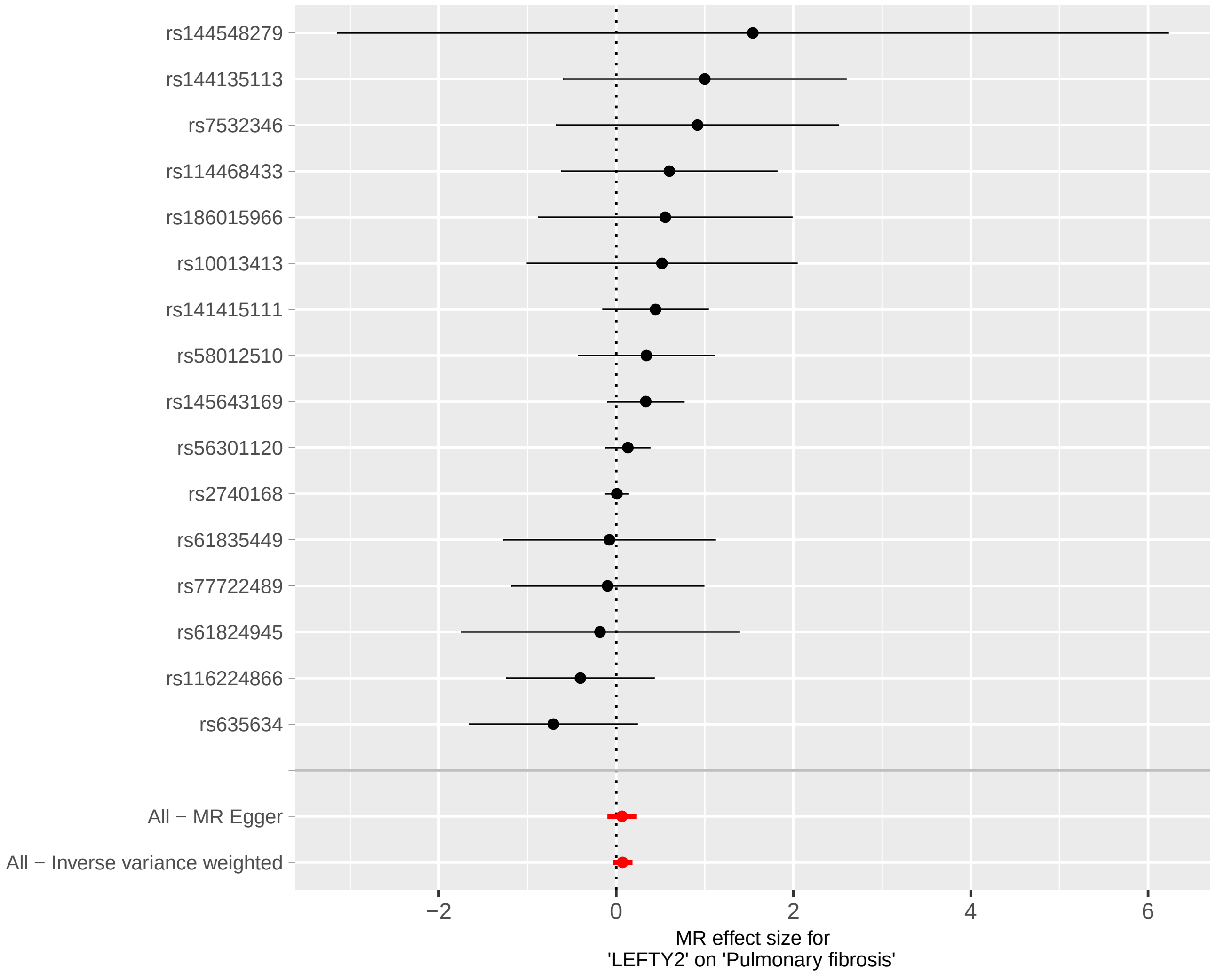
rs144548279
rs144135113
rs7532346
rs114468433
rs186015966
rs10013413
rs141415111
rs58012510
rs145643169
rs56301120
rs2740168
rs61835449
rs77722489
rs61824945
rs116224866
rs635634
All − MR Egger
All − Inverse variance weighted
−2
0
2
4
6
MR effect size for
'LEFTY2' on 'Pulmonary fibrosis'

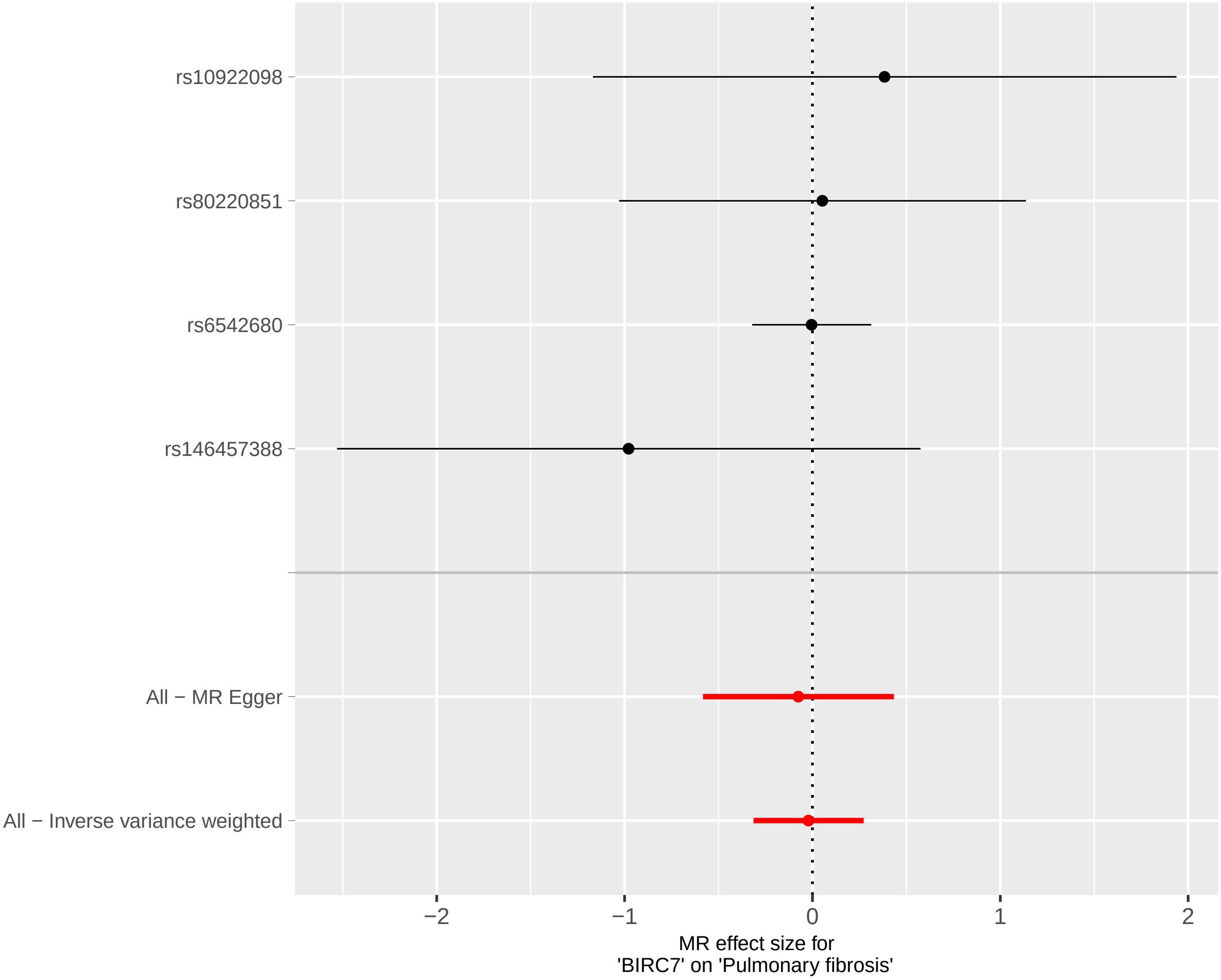

rs10922098
rs80220851
rs6542680
rs146457388
All − MR Egger
All − Inverse variance weighted
−2
−1
0
1
2
MR effect size for
'BIRC7' on 'Pulmonary fibrosis'

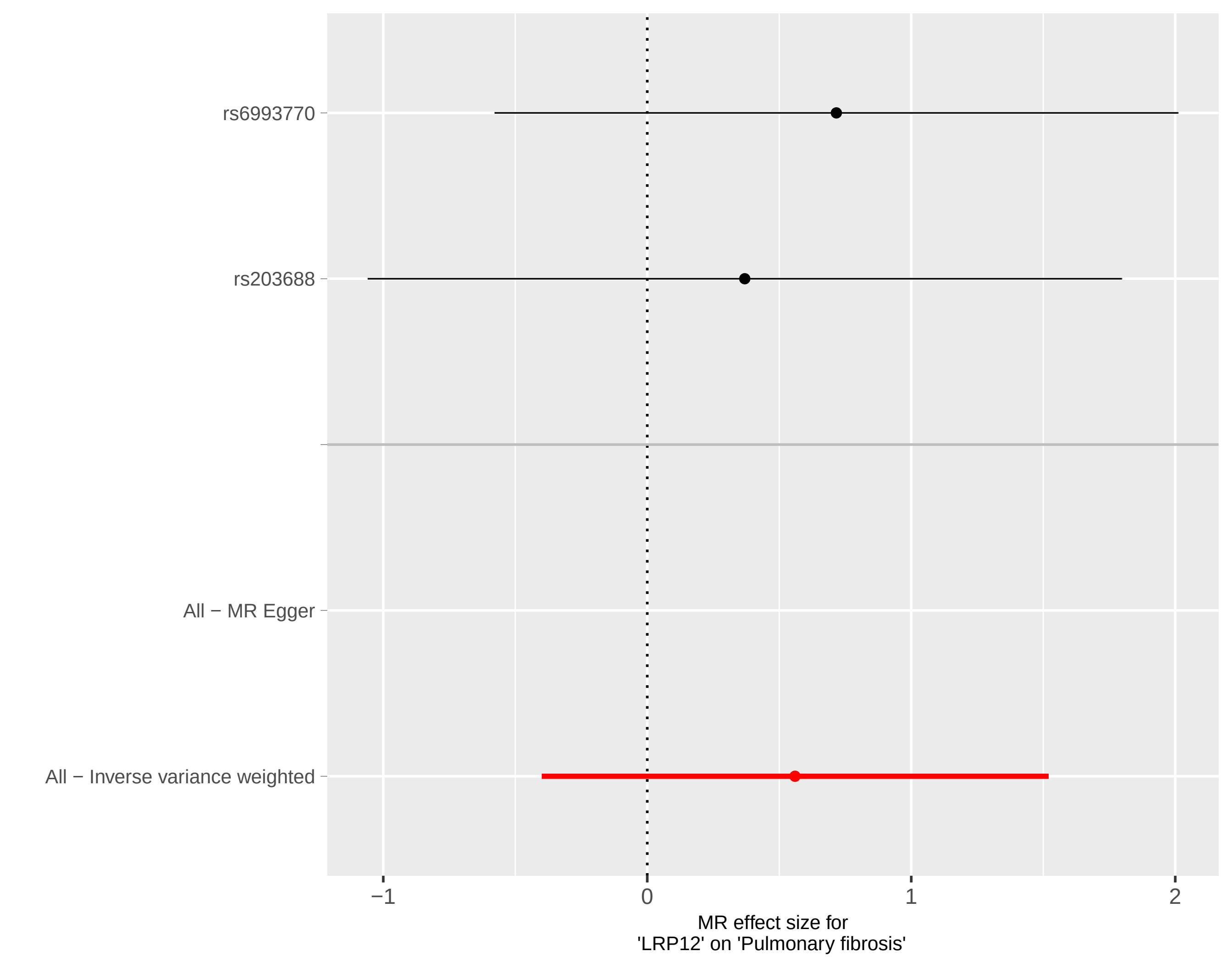

rs6993770
rs203688
All − MR Egger
All − Inverse variance weighted
−1
0
1
2
MR effect size for
'LRP12' on 'Pulmonary fibrosis'

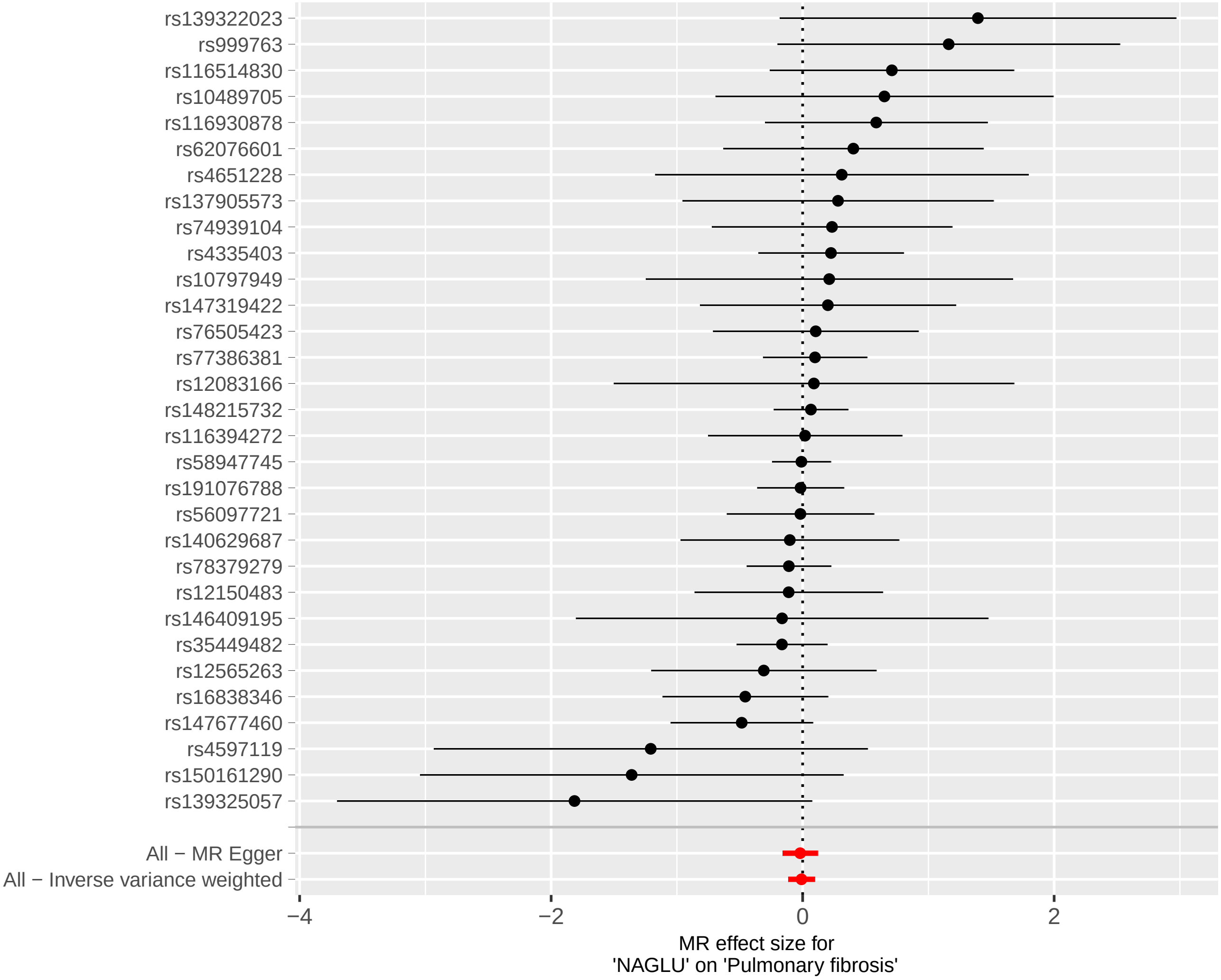
rs139322023
rs999763
rs116514830
rs10489705
rs116930878
rs62076601
rs4651228
rs137905573
rs74939104
rs4335403
rs10797949
rs147319422
rs76505423
rs77386381
rs12083166
rs148215732
rs116394272
rs58947745
rs191076788
rs56097721
rs140629687
rs78379279
rs12150483
rs146409195
rs35449482
rs12565263
rs16838346
rs147677460
rs4597119
rs150161290
rs139325057
All − MR Egger
All − Inverse variance weighted
−4
−2
0
2
MR effect size for
'NAGLU' on 'Pulmonary fibrosis'

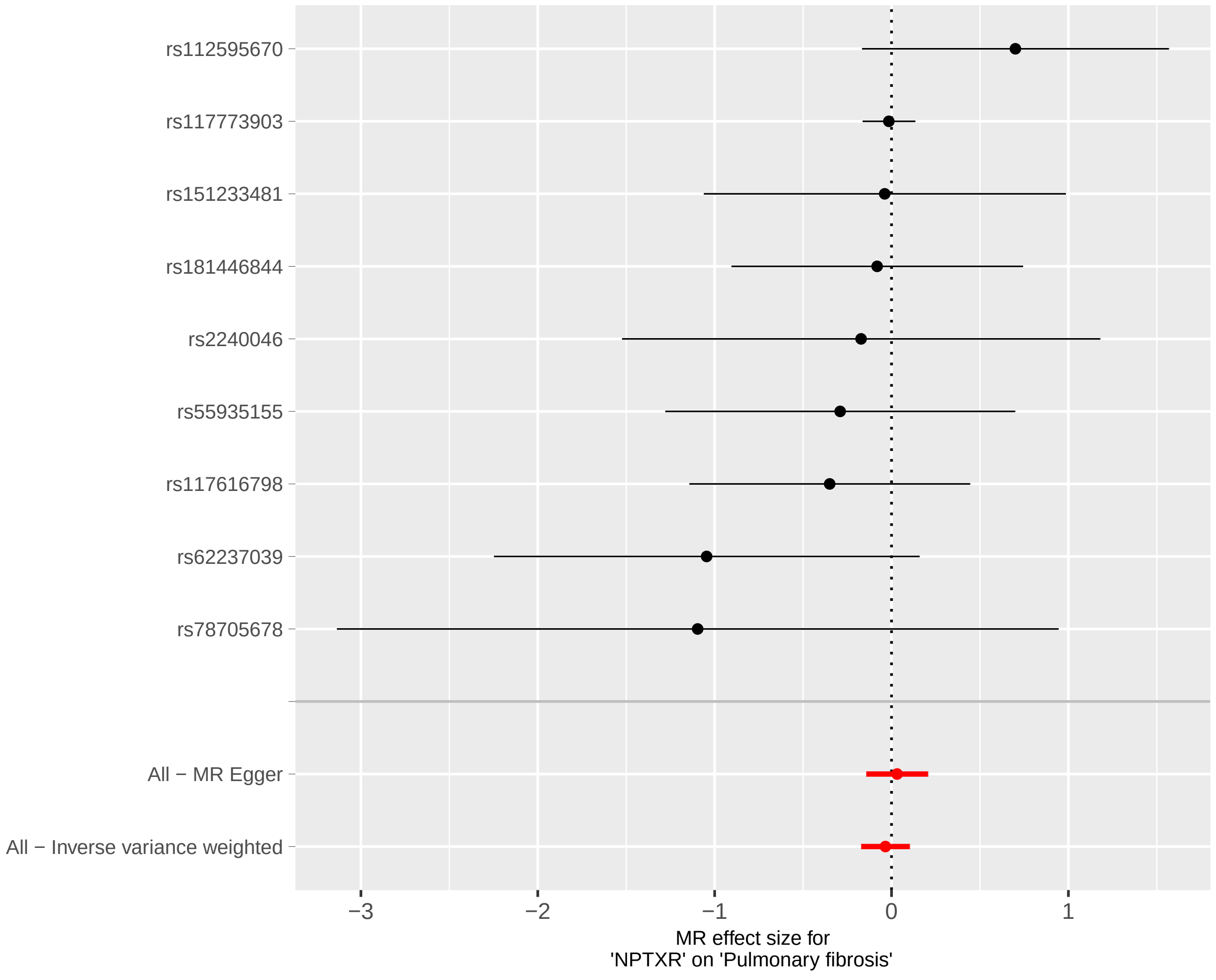
rs112595670
rs117773903
rs151233481
rs181446844
rs2240046
rs55935155
rs117616798
rs62237039
rs78705678
All − MR Egger
All − Inverse variance weighted
−3
−2
−1
0
1
MR effect size for
'NPTXR' on 'Pulmonary fibrosis'

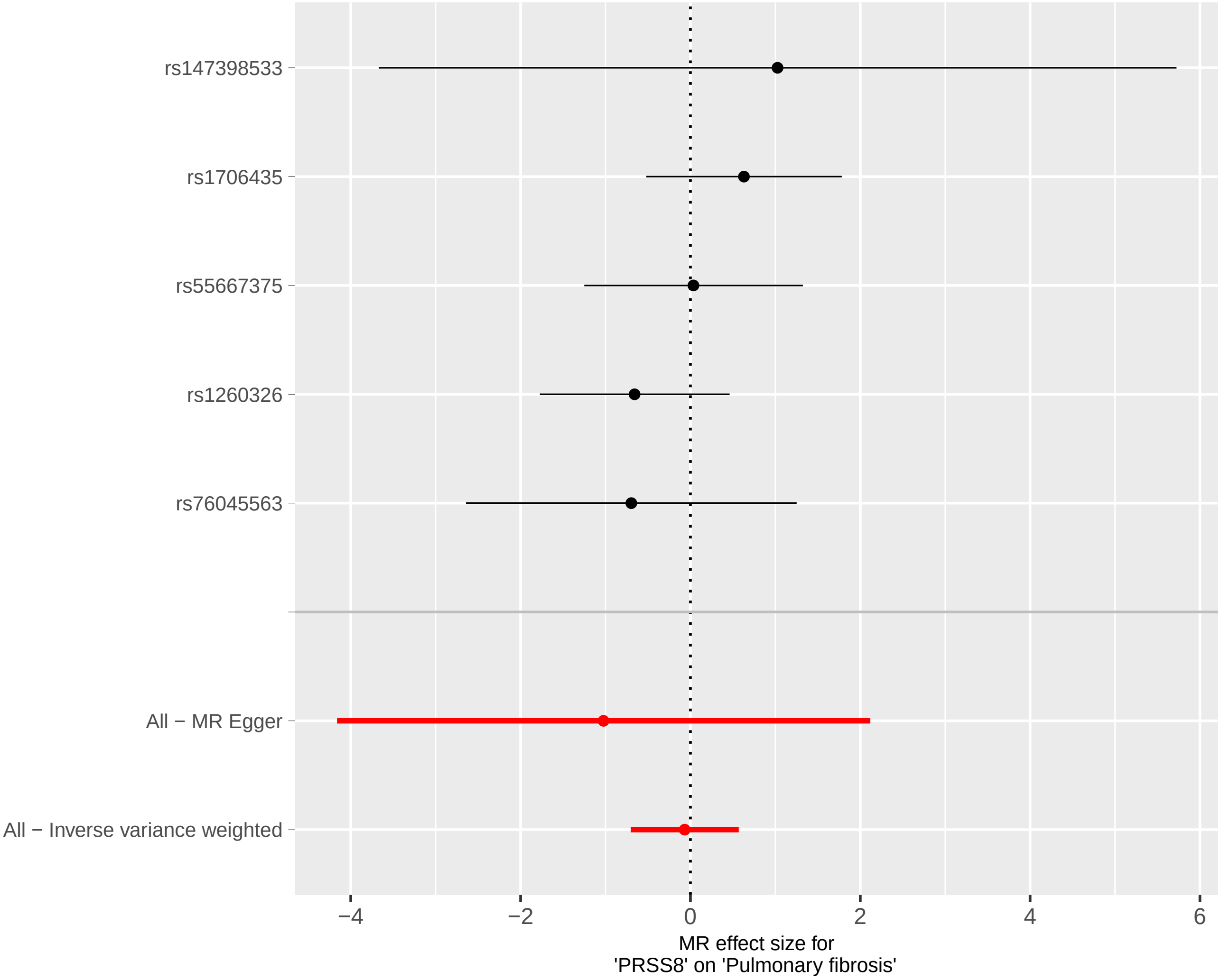

rs147398533
rs1706435
rs55667375
rs1260326
rs76045563
All − MR Egger
All − Inverse variance weighted
−4
−2
0
2
4
6
MR effect size for
'PRSS8' on 'Pulmonary fibrosis'

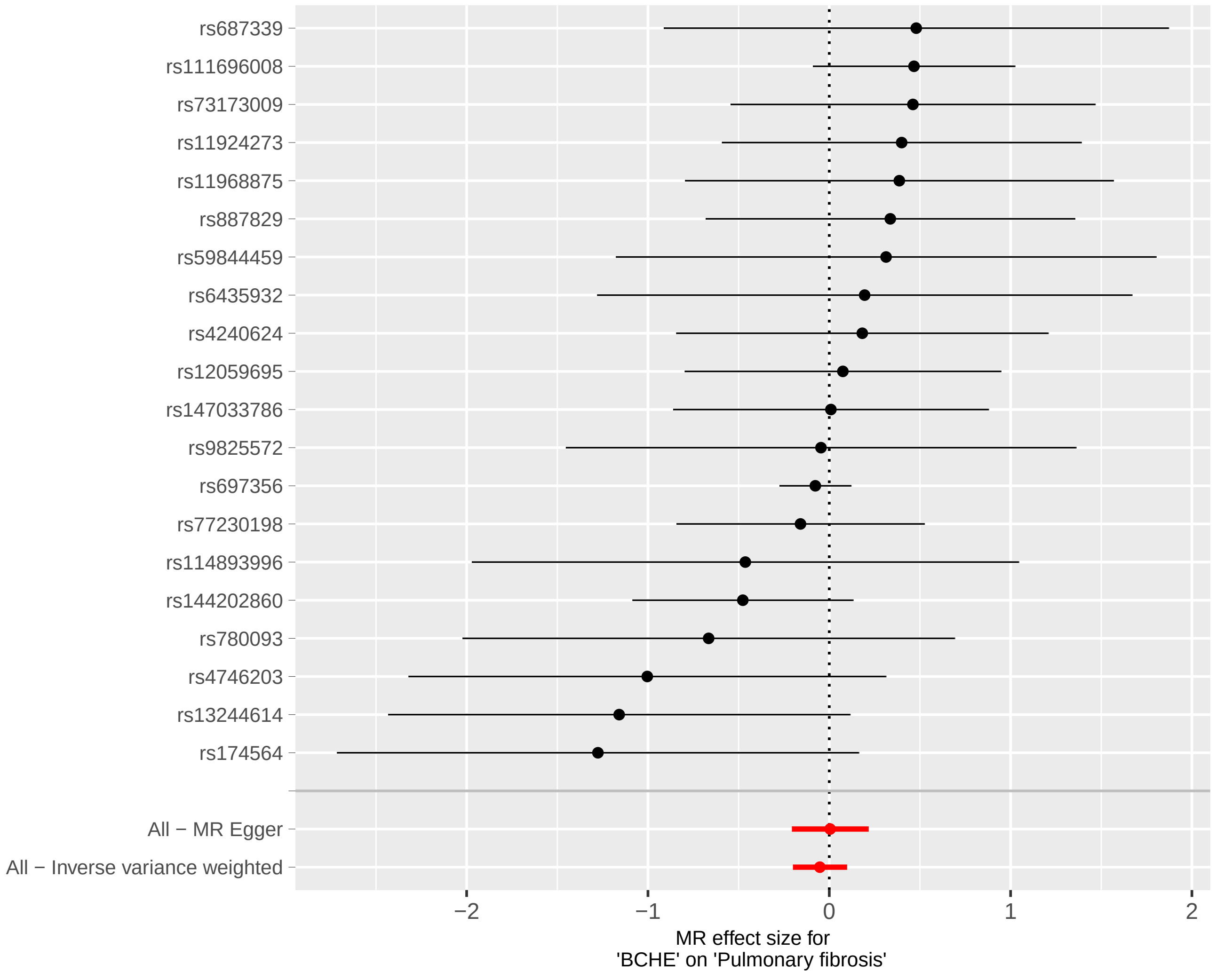

rs687339
rs111696008
rs73173009
rs11924273
rs11968875
rs887829
rs59844459
rs6435932
rs4240624
rs12059695
rs147033786
rs9825572
rs697356
rs77230198
rs114893996
rs144202860
rs780093
rs4746203
rs13244614
rs174564
All − MR Egger
All − Inverse variance weighted
−2
−1
0
1
2
MR effect size for
'BCHE' on 'Pulmonary fibrosis'

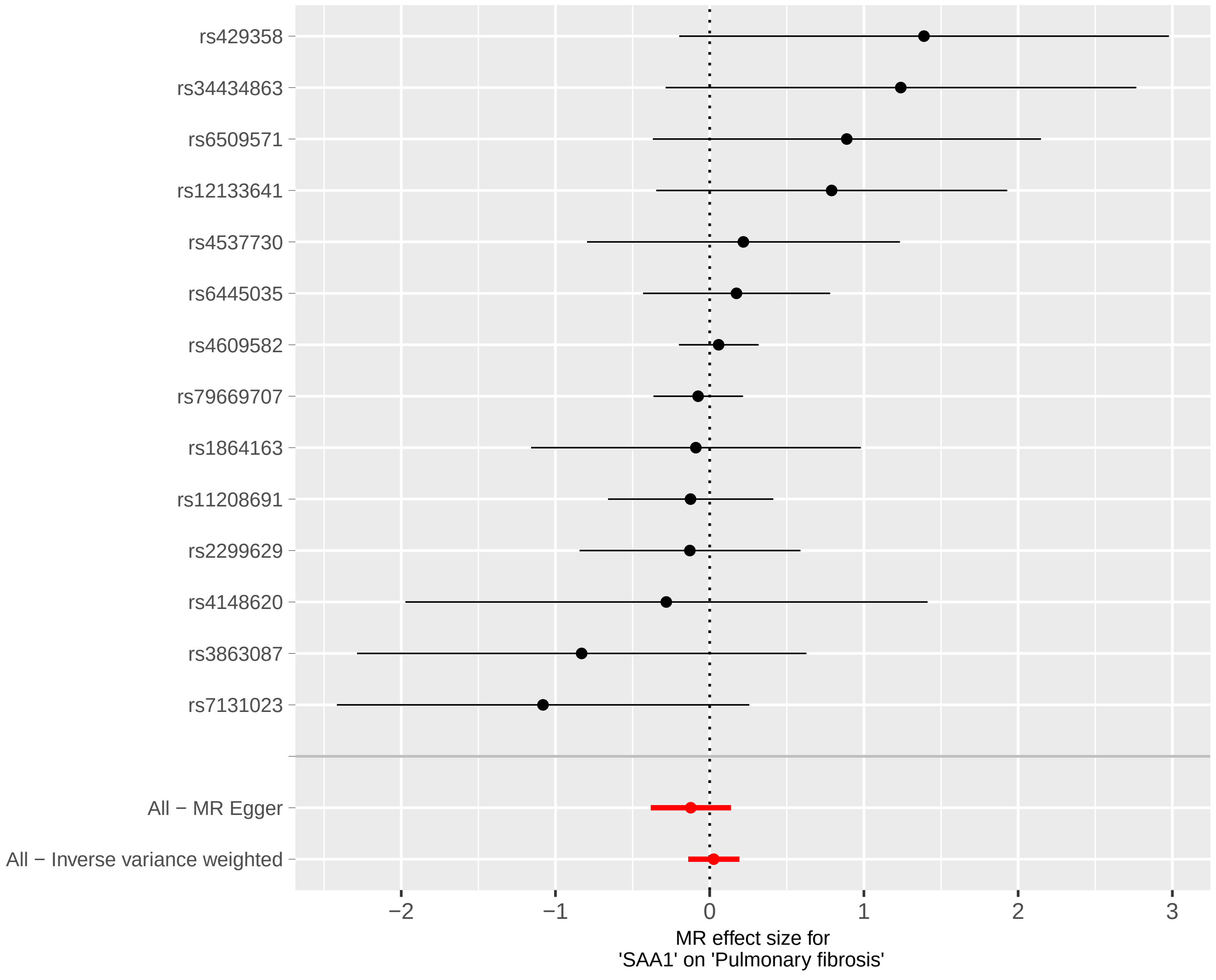

rs429358
rs34434863
rs6509571
rs12133641
rs4537730
rs6445035
rs4609582
rs79669707
rs1864163
rs11208691
rs2299629
rs4148620
rs3863087
rs7131023
All − MR Egger
All − Inverse variance weighted
−2
−1
0
1
2
3
MR effect size for
'SAA1' on 'Pulmonary fibrosis'

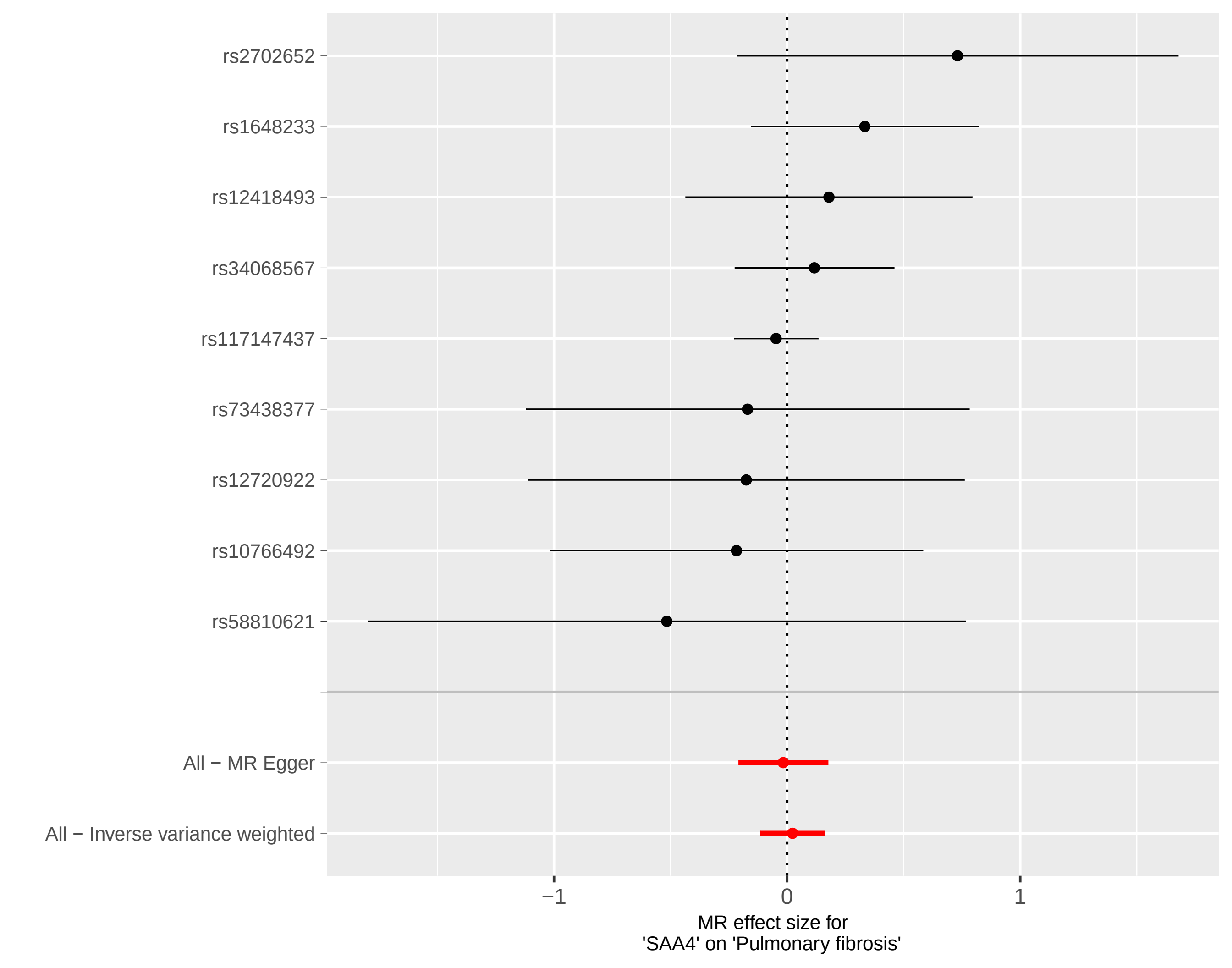

rs2702652
rs1648233
rs12418493
rs34068567
rs117147437
rs73438377
rs12720922
rs10766492
rs58810621
All − MR Egger
All − Inverse variance weighted
−1
0
1
MR effect size for
'SAA4' on 'Pulmonary fibrosis'

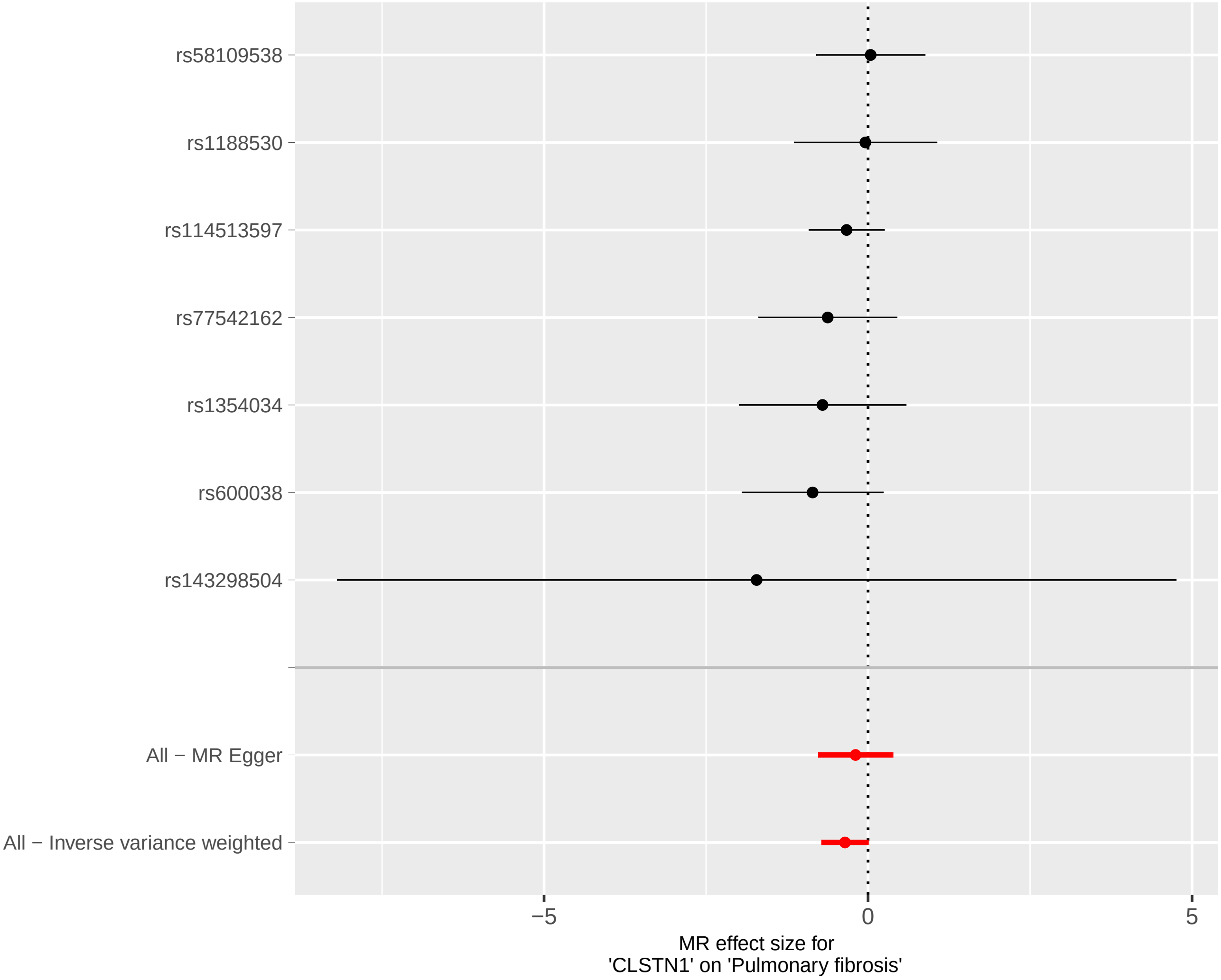
rs58109538
rs1188530
rs114513597
rs77542162
rs1354034
rs600038
rs143298504
All − MR Egger
All − Inverse variance weighted
−5
0
5
MR effect size for
'CLSTN1' on 'Pulmonary fibrosis'

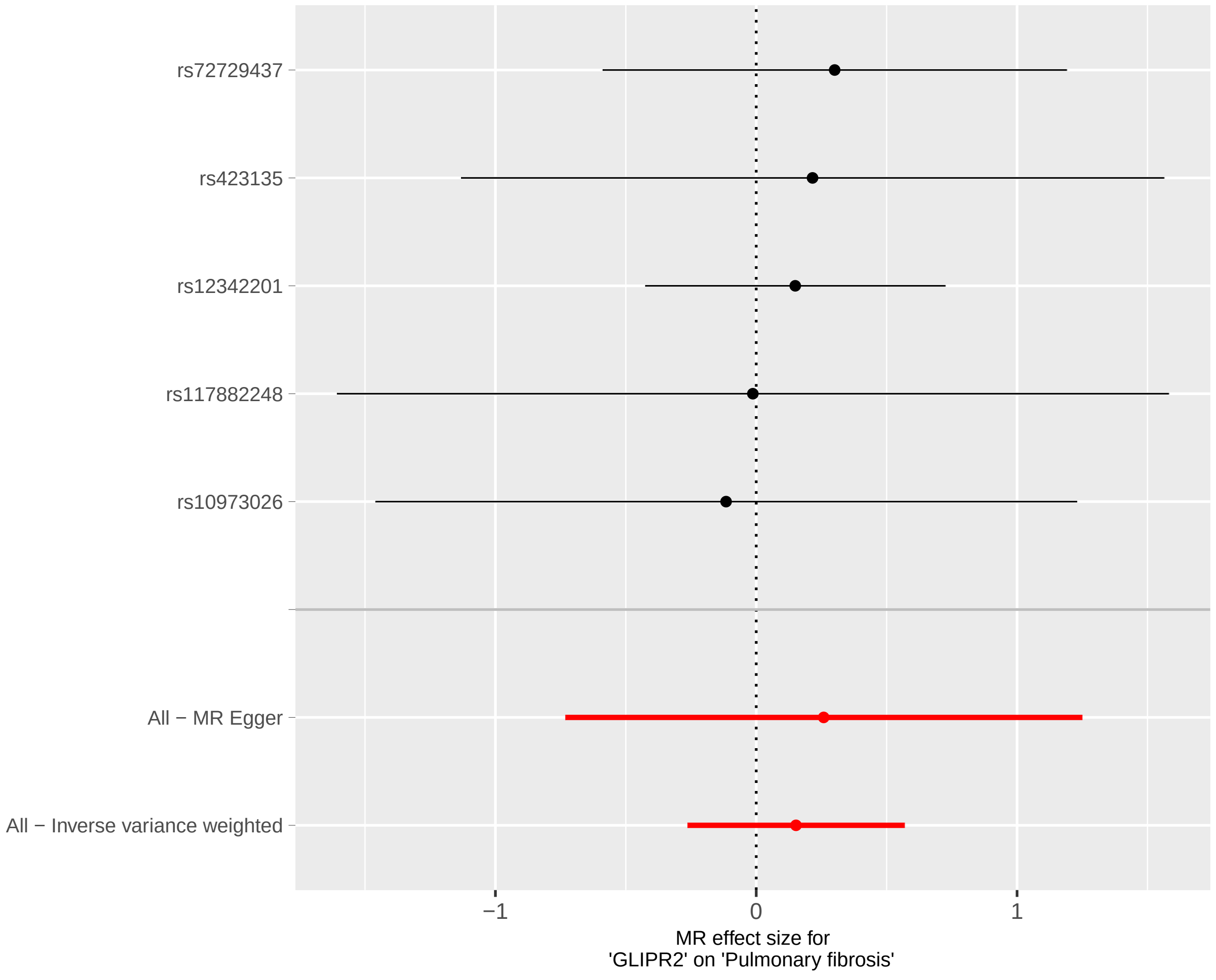

rs72729437
rs423135
rs12342201
rs117882248
rs10973026
All − MR Egger
All − Inverse variance weighted
−1
0
1
MR effect size for
'GLIPR2' on 'Pulmonary fibrosis'

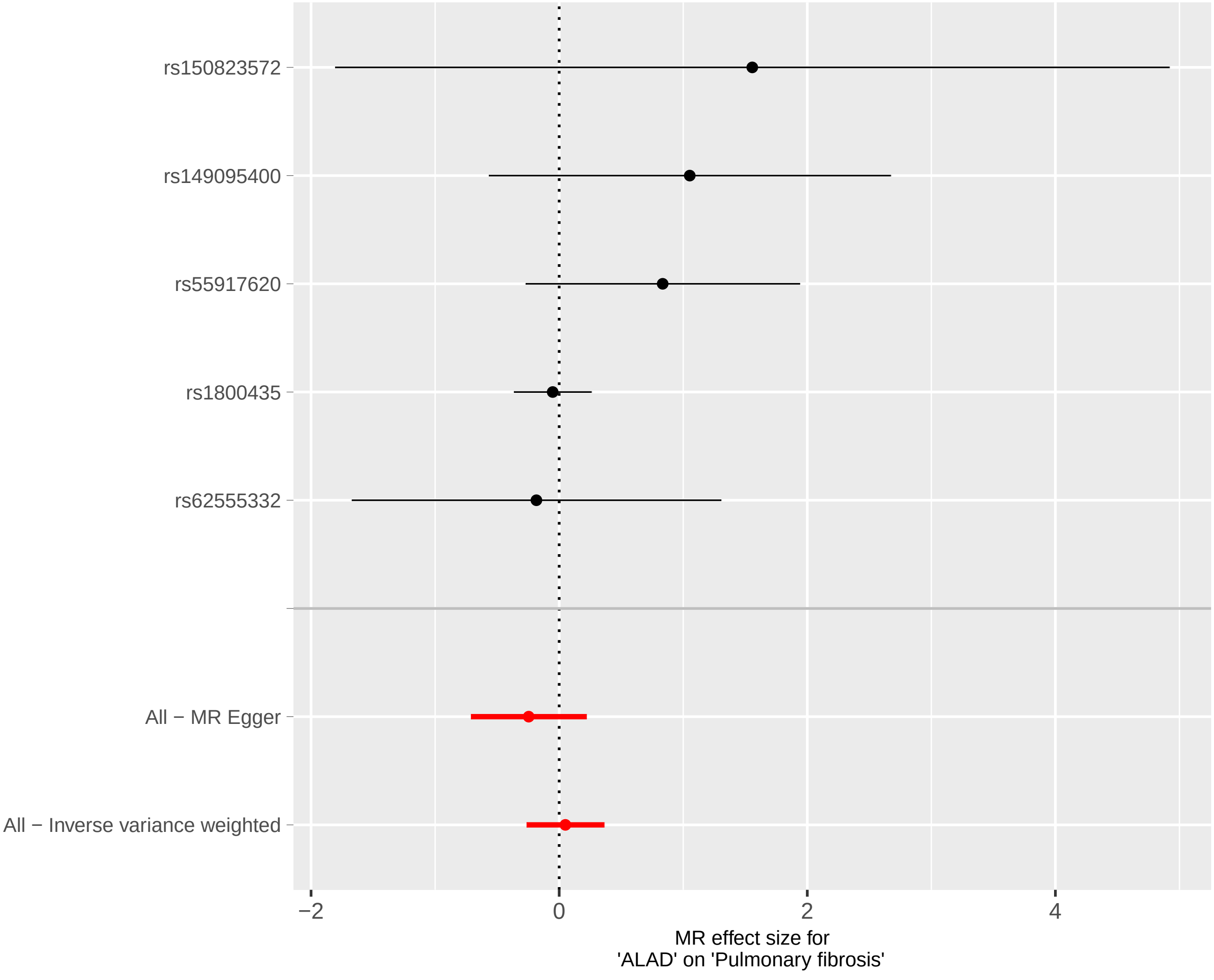

rs150823572
rs149095400
rs55917620
rs1800435
rs62555332
All − MR Egger
All − Inverse variance weighted
−2
0
2
4
MR effect size for
'ALAD' on 'Pulmonary fibrosis'

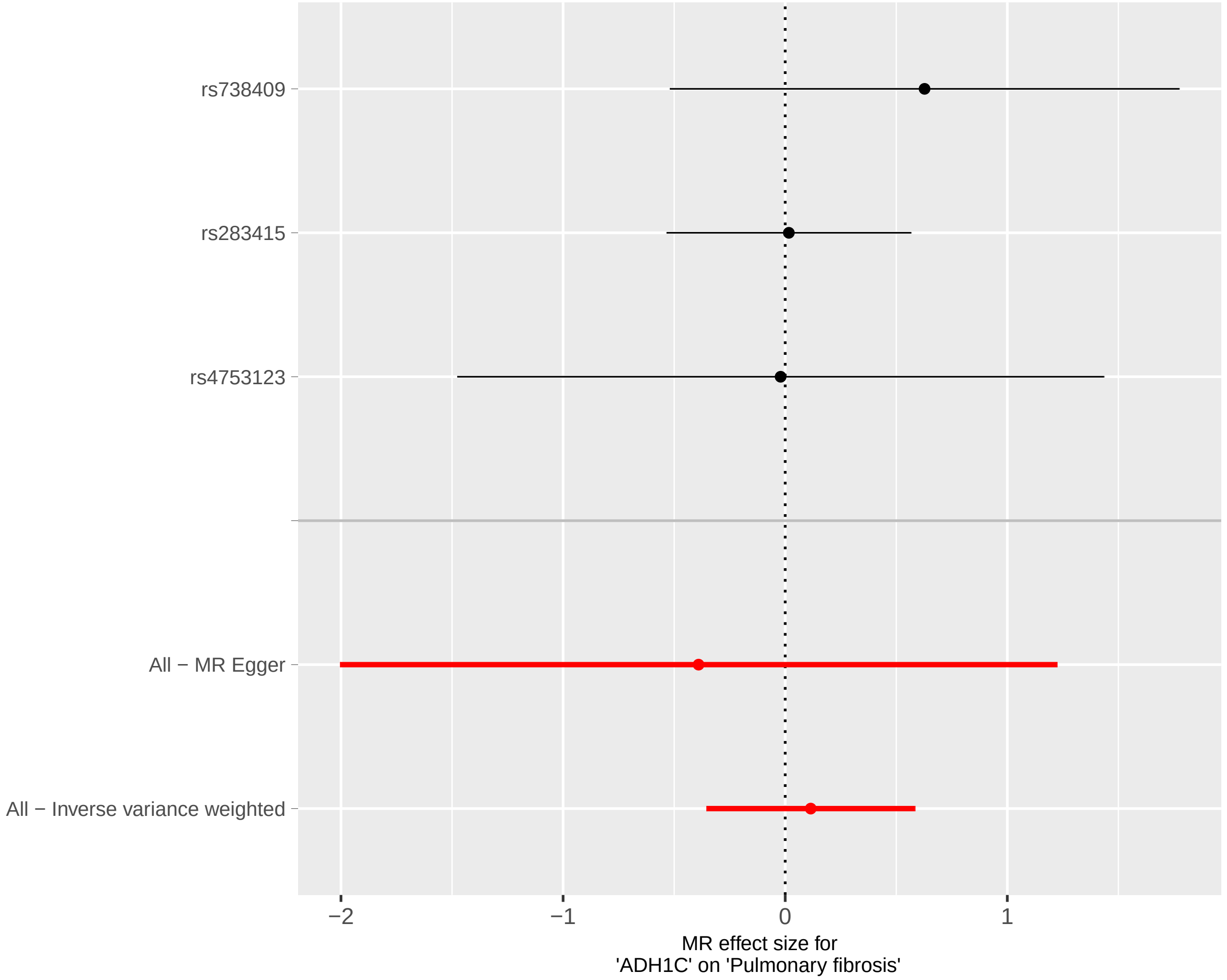

rs738409
rs283415
rs4753123
All − MR Egger
All − Inverse variance weighted
−2
−1
0
1
MR effect size for
'ADH1C' on 'Pulmonary fibrosis'

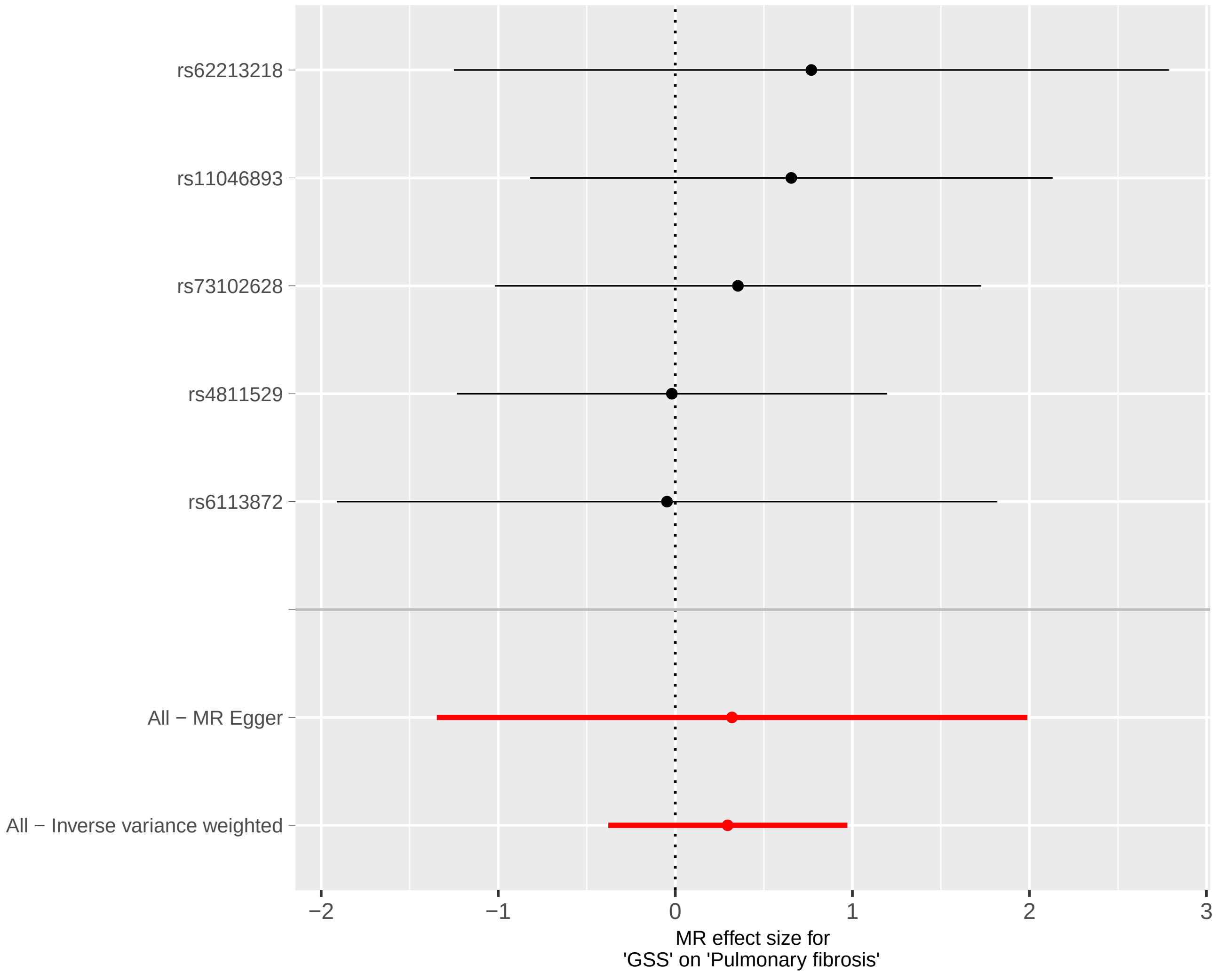

rs62213218
rs11046893
rs73102628
rs4811529
rs6113872
All − MR Egger
All − Inverse variance weighted
−2
−1
0
1
2
3
MR effect size for
'GSS' on 'Pulmonary fibrosis'

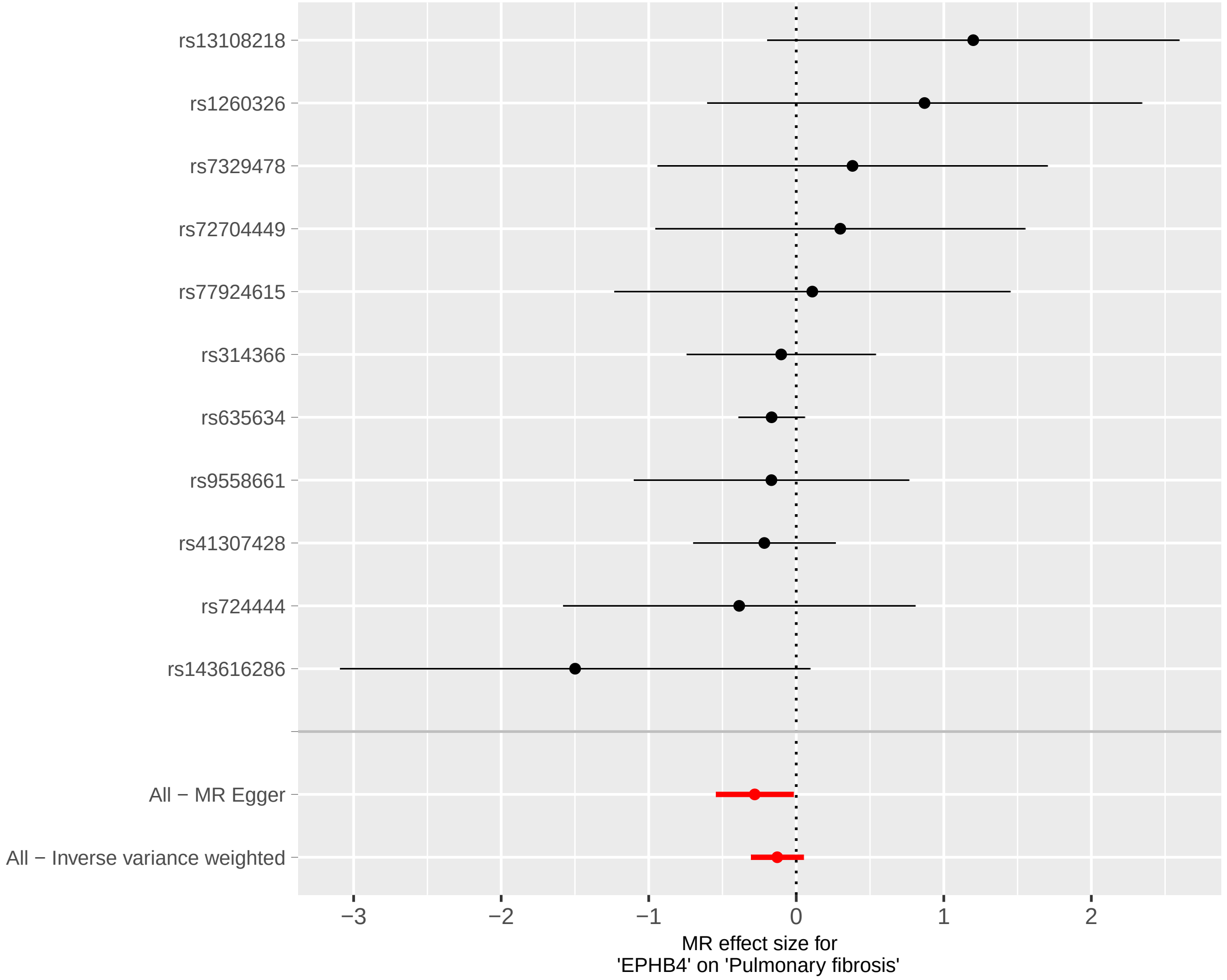
rs13108218
rs1260326
rs7329478
rs72704449
rs77924615
rs314366
rs635634
rs9558661
rs41307428
rs724444
rs143616286
All − MR Egger
All − Inverse variance weighted
−3
−2
−1
0
1
2
MR effect size for
'EPHB4' on 'Pulmonary fibrosis'

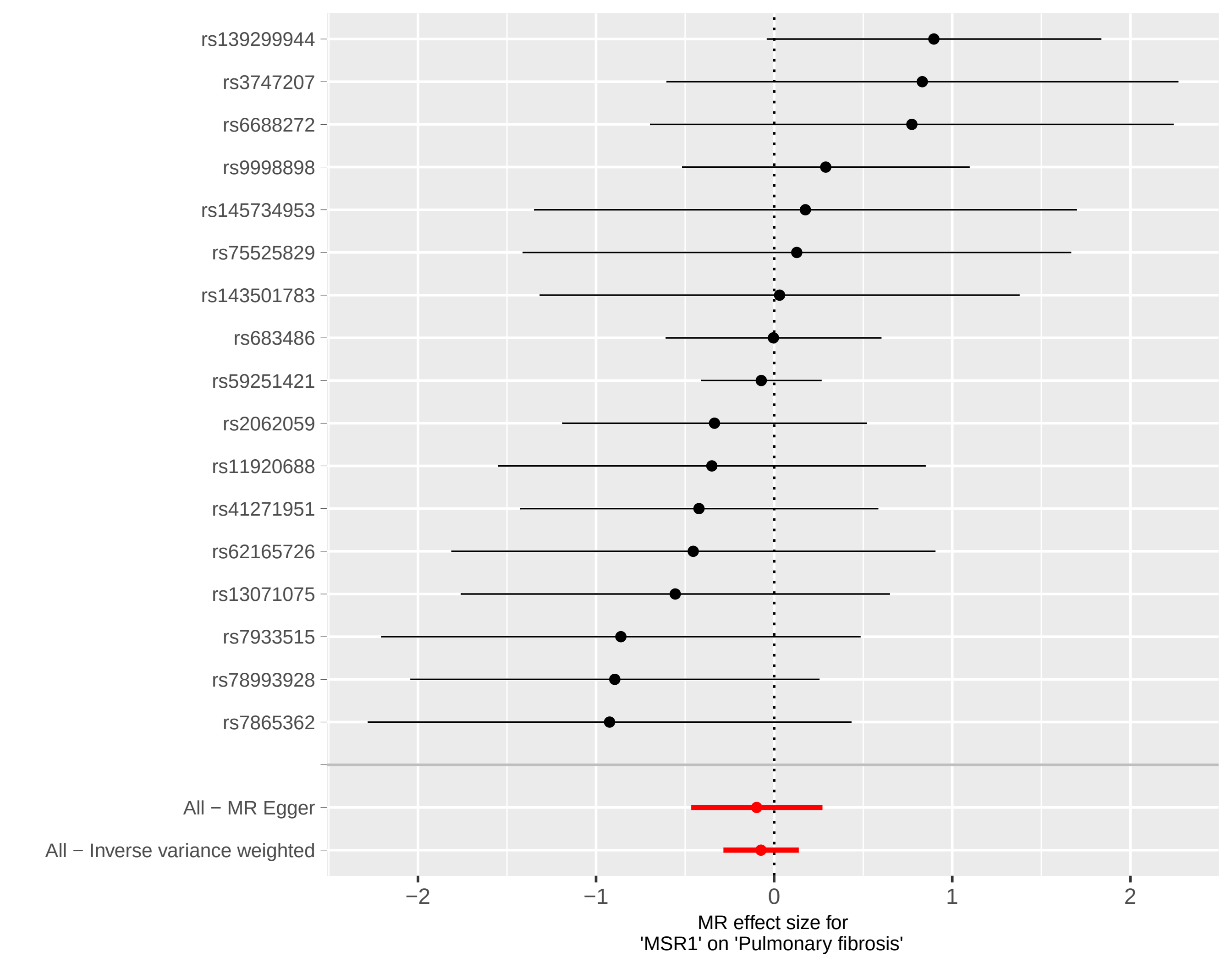
rs139299944
rs3747207
rs6688272
rs9998898
rs145734953
rs75525829
rs143501783
rs683486
rs59251421
rs2062059
rs11920688
rs41271951
rs62165726
rs13071075
rs7933515
rs78993928
rs7865362
All − MR Egger
All − Inverse variance weighted
−2
−1
0
1
2
MR effect size for
'MSR1' on 'Pulmonary fibrosis'

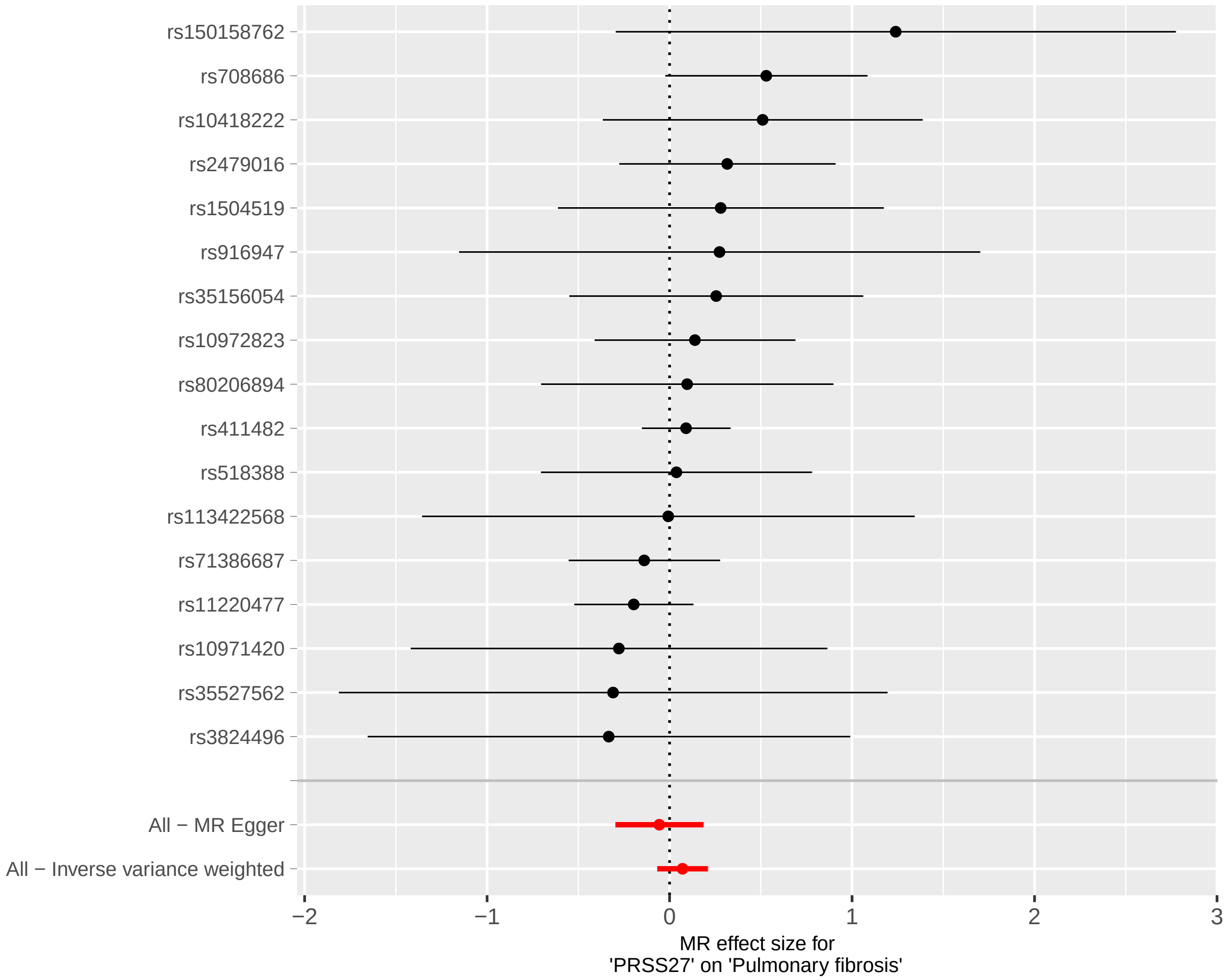

rs150158762
rs708686
rs10418222
rs2479016
rs1504519
rs916947
rs35156054
rs10972823
rs80206894
rs411482
rs518388
rs113422568
rs71386687
rs11220477
rs10971420
rs35527562
rs3824496
All − MR Egger
All − Inverse variance weighted
−2
−1
0
1
2
3
MR effect size for
'PRSS27' on 'Pulmonary fibrosis'

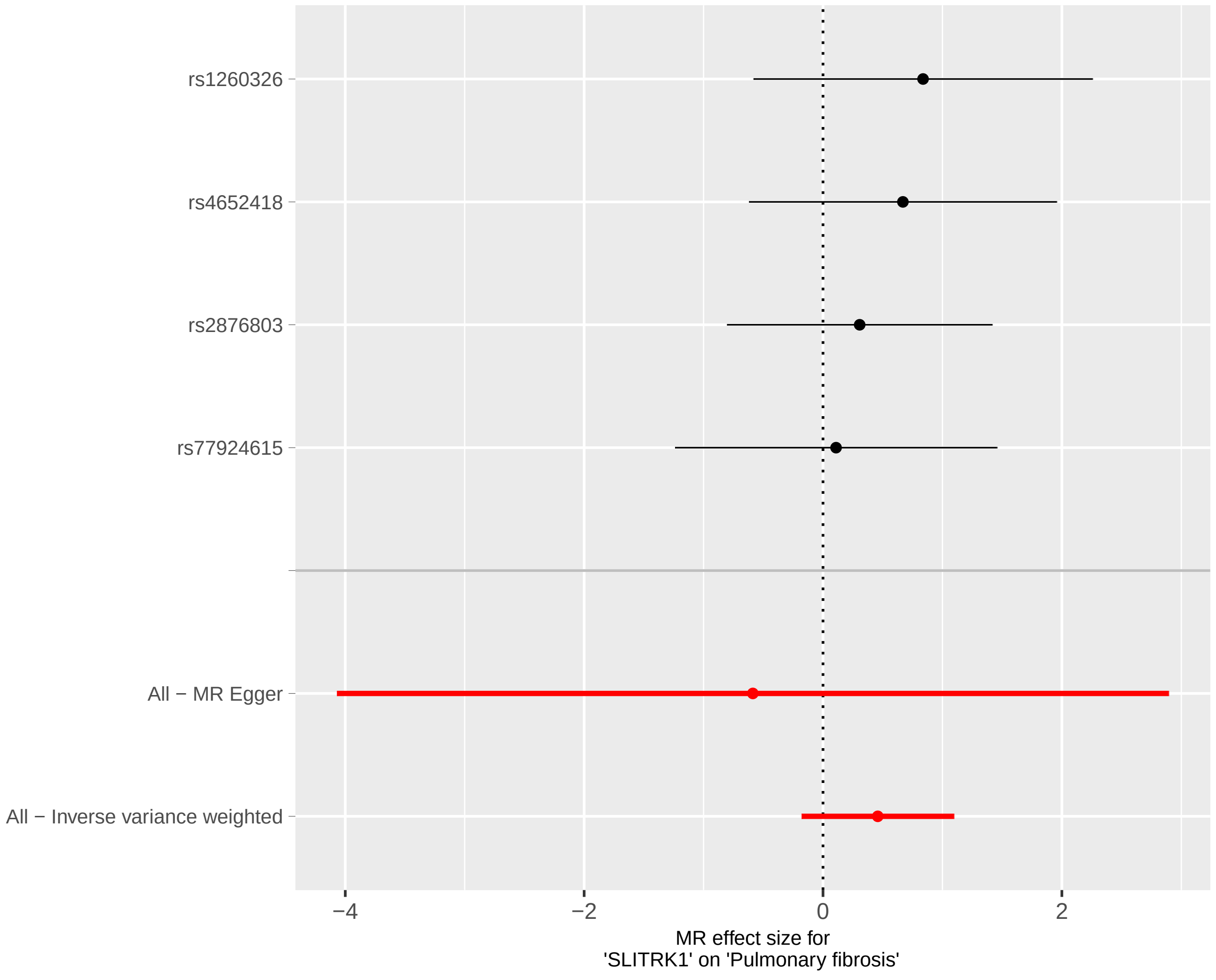
rs1260326
rs4652418
rs2876803
rs77924615
All − MR Egger
All − Inverse variance weighted
−4
−2
0
2
MR effect size for
'SLITRK1' on 'Pulmonary fibrosis'

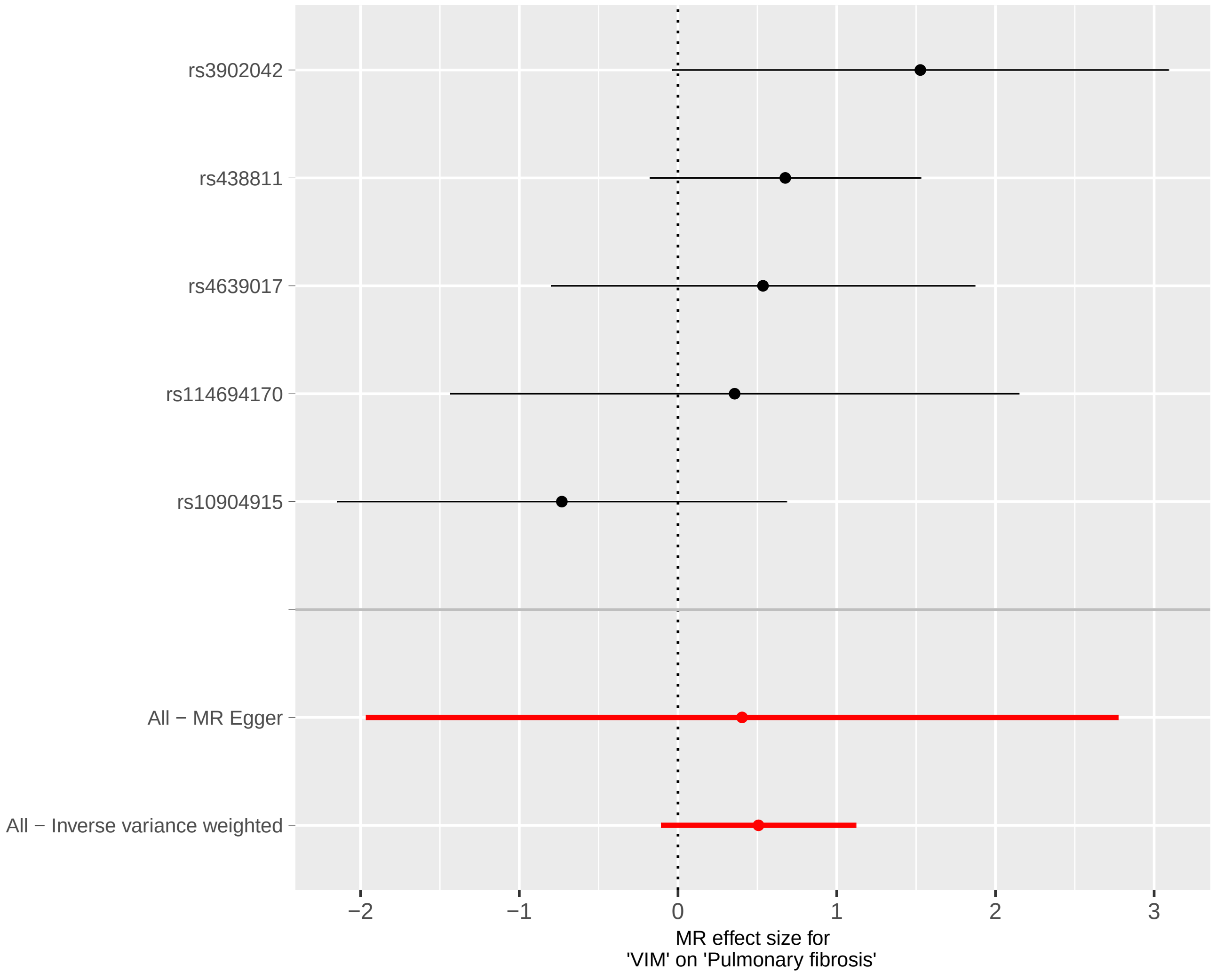

rs3902042
rs438811
rs4639017
rs114694170
rs10904915
All − MR Egger
All − Inverse variance weighted
−2
−1
0
1
2
3
MR effect size for
'VIM' on 'Pulmonary fibrosis'

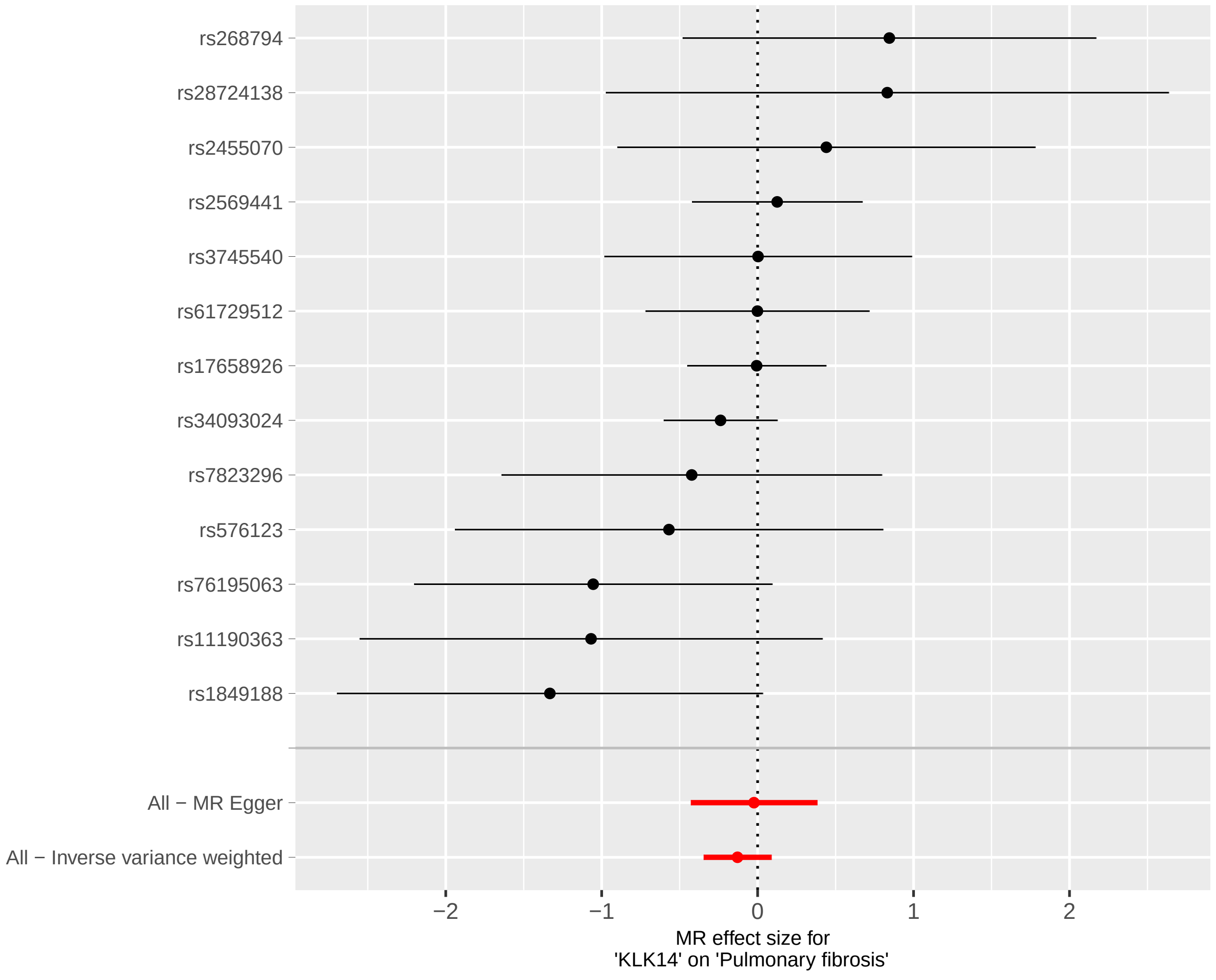
rs268794
rs28724138
rs2455070
rs2569441
rs3745540
rs61729512
rs17658926
rs34093024
rs7823296
rs576123
rs76195063
rs11190363
rs1849188
All − MR Egger
All − Inverse variance weighted
−2
−1
0
1
2
MR effect size for
'KLK14' on 'Pulmonary fibrosis'

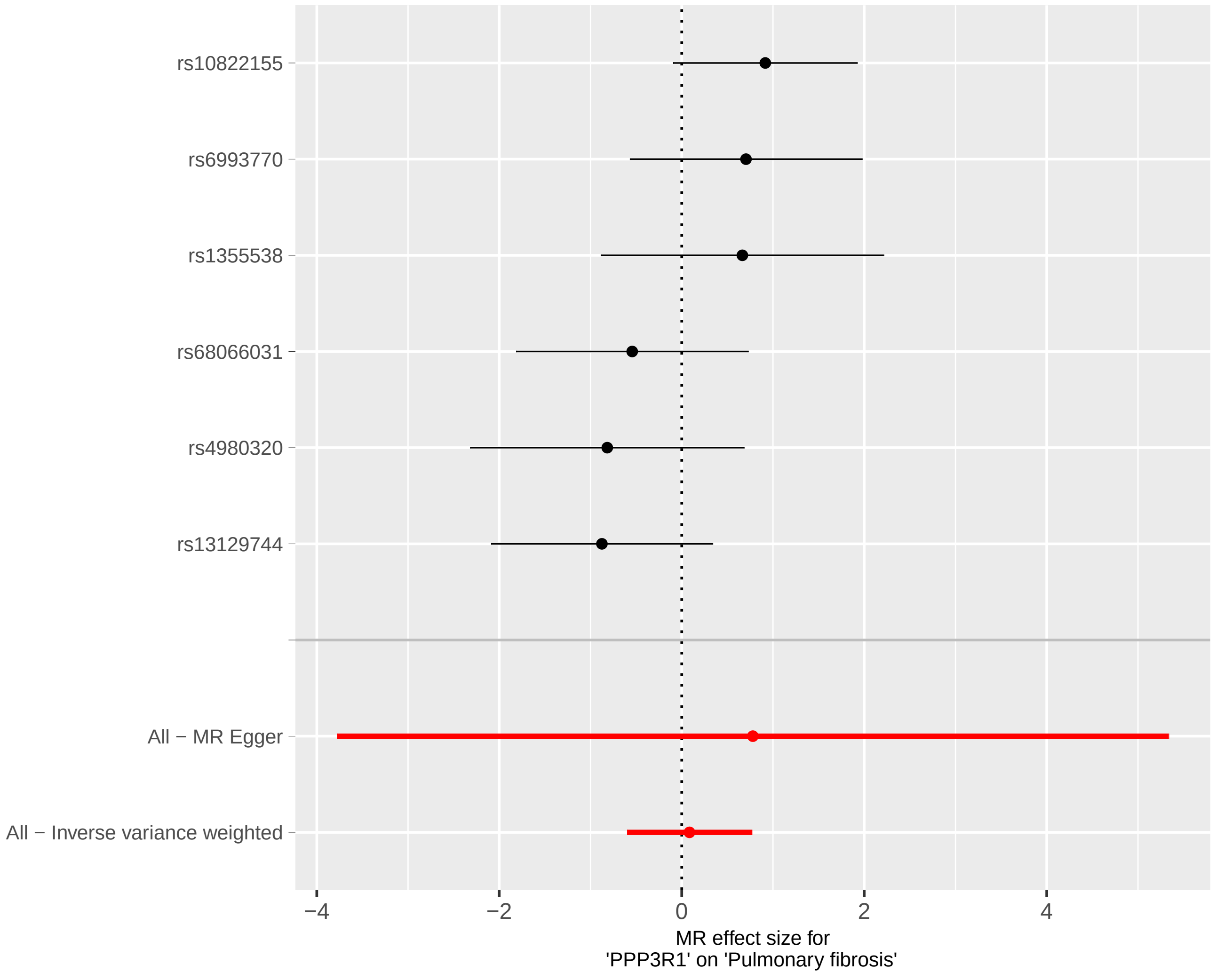
rs10822155
rs6993770
rs1355538
rs68066031
rs4980320
rs13129744
All − MR Egger
All − Inverse variance weighted
−4
−2
0
2
4
MR effect size for
'PPP3R1' on 'Pulmonary fibrosis'

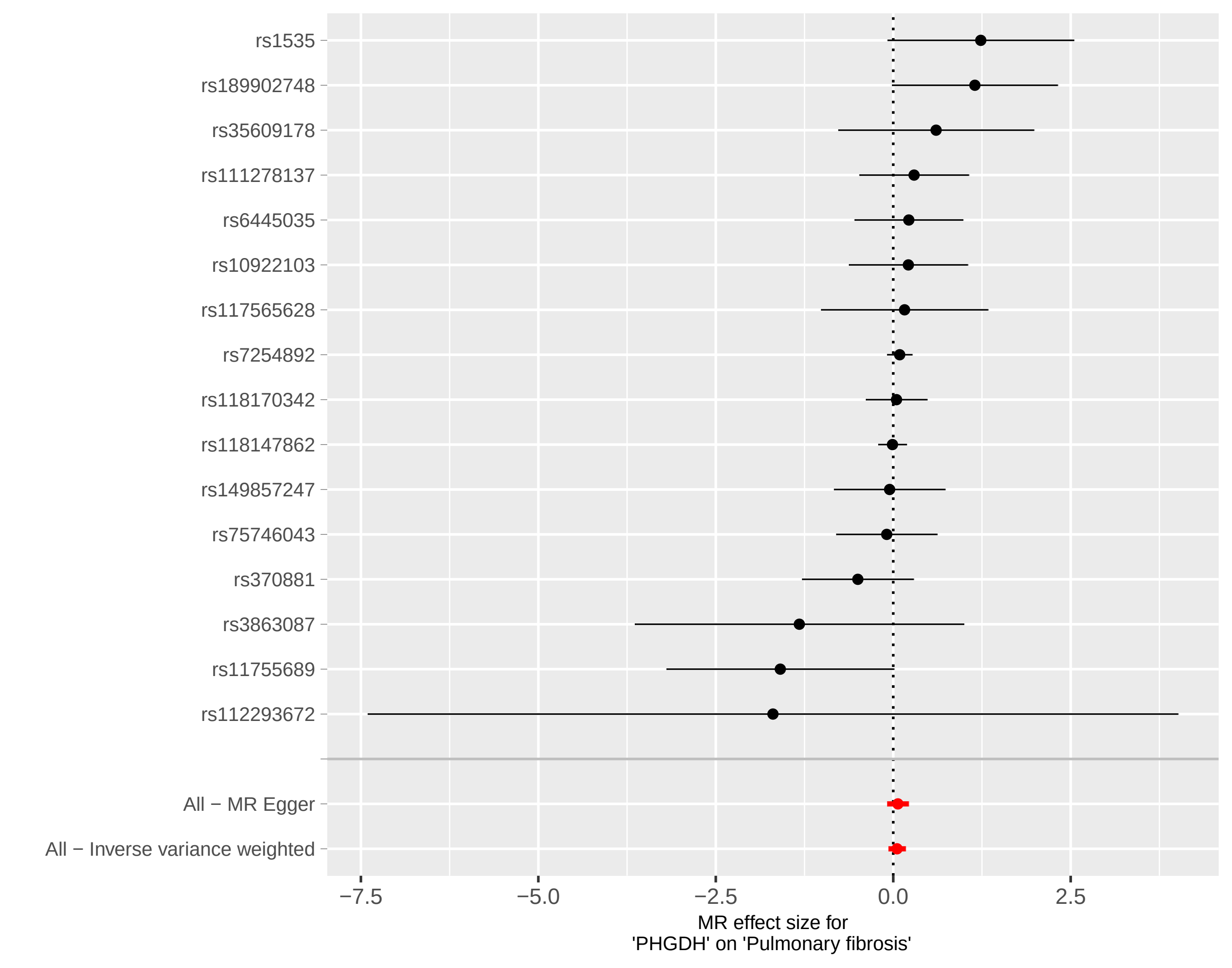

rs1535
rs189902748
rs35609178
rs111278137
rs6445035
rs10922103
rs117565628
rs7254892
rs118170342
rs118147862
rs149857247
rs75746043
rs370881
rs3863087
rs11755689
rs112293672
All − MR Egger
All − Inverse variance weighted
−7.5
−5.0
−2.5
0.0
2.5
MR effect size for
'PHGDH' on 'Pulmonary fibrosis'

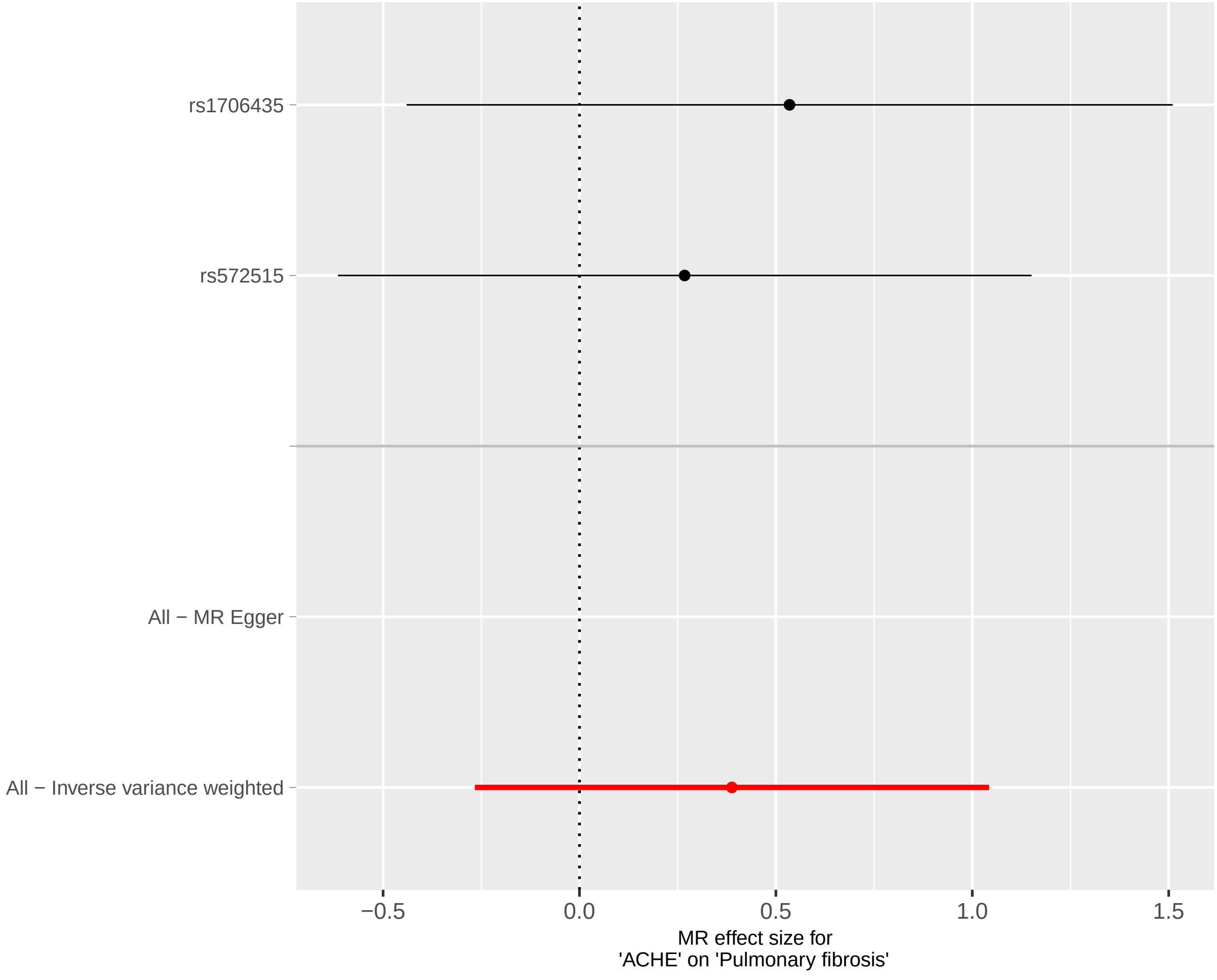
rs1706435
rs572515
All − MR Egger
All − Inverse variance weighted
−0.5
0.0
0.5
1.0
1.5
MR effect size for
'ACHE' on 'Pulmonary fibrosis'

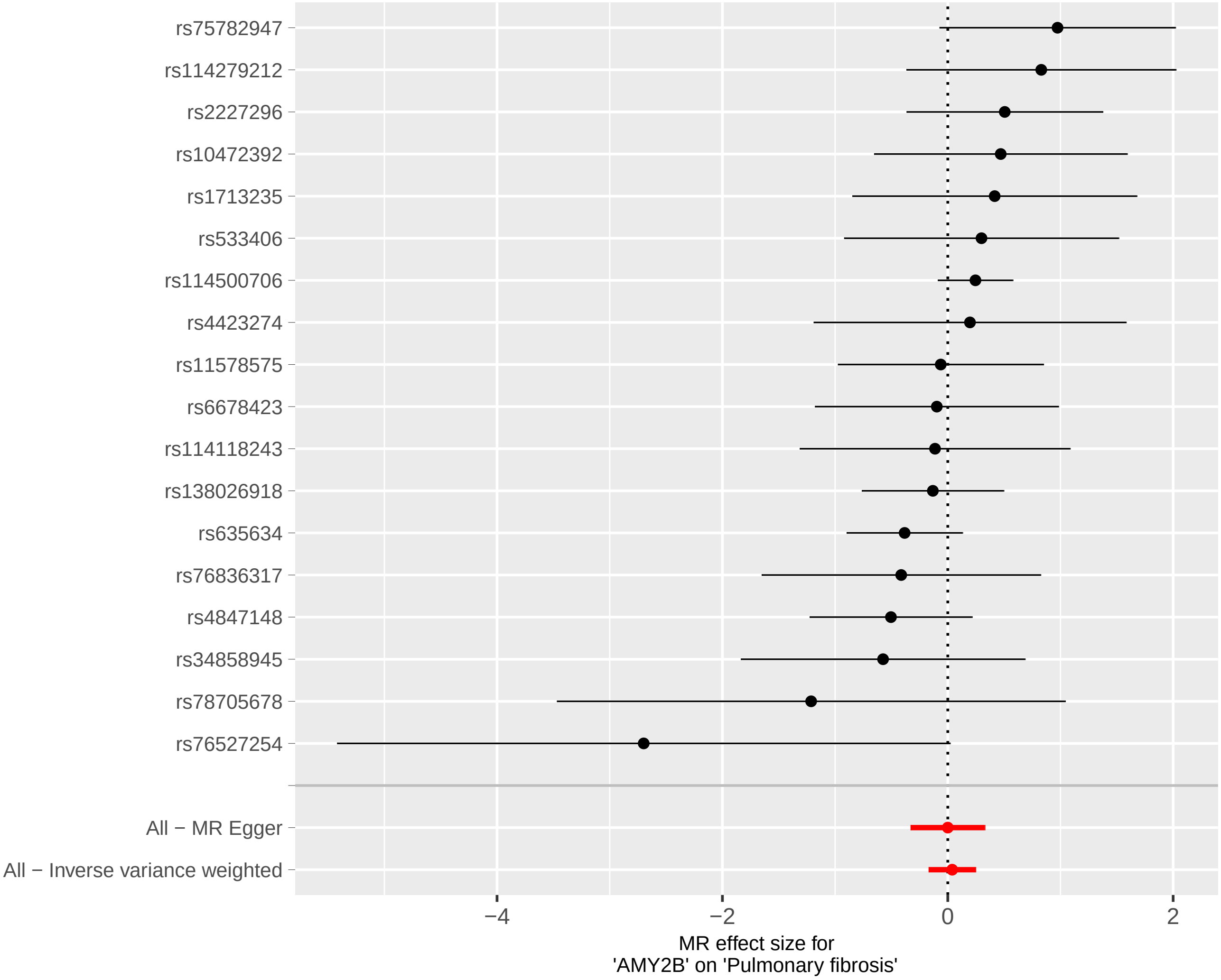

rs75782947
rs114279212
rs2227296
rs10472392
rs1713235
rs533406
rs114500706
rs4423274
rs11578575
rs6678423
rs114118243
rs138026918
rs635634
rs76836317
rs4847148
rs34858945
rs78705678
rs76527254
All − MR Egger
All − Inverse variance weighted
−4
−2
0
2
MR effect size for
'AMY2B' on 'Pulmonary fibrosis'

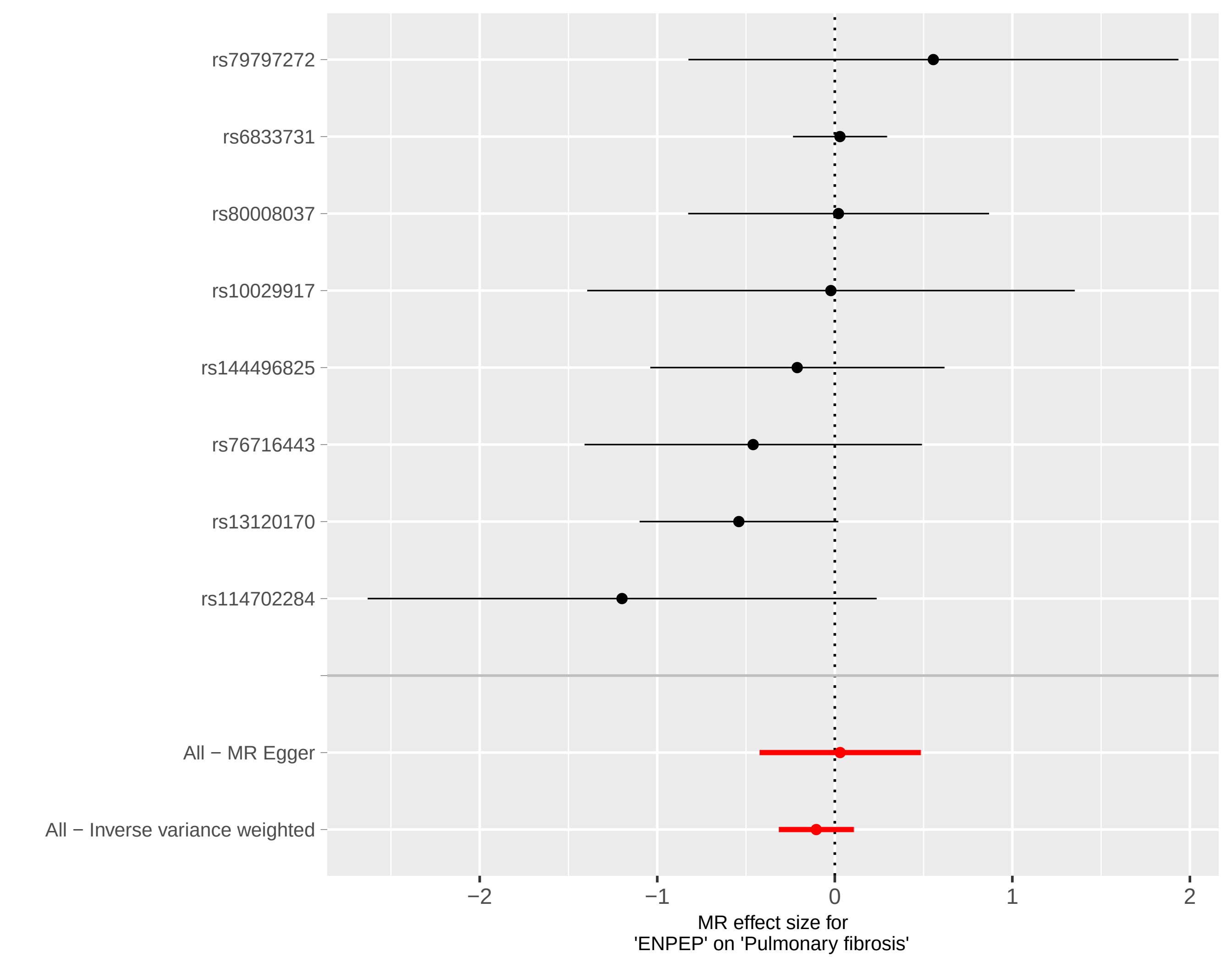
rs79797272
rs6833731
rs80008037
rs10029917
rs144496825
rs76716443
rs13120170
rs114702284
All − MR Egger
All − Inverse variance weighted
−2
−1
0
1
2
MR effect size for
'ENPEP' on 'Pulmonary fibrosis'

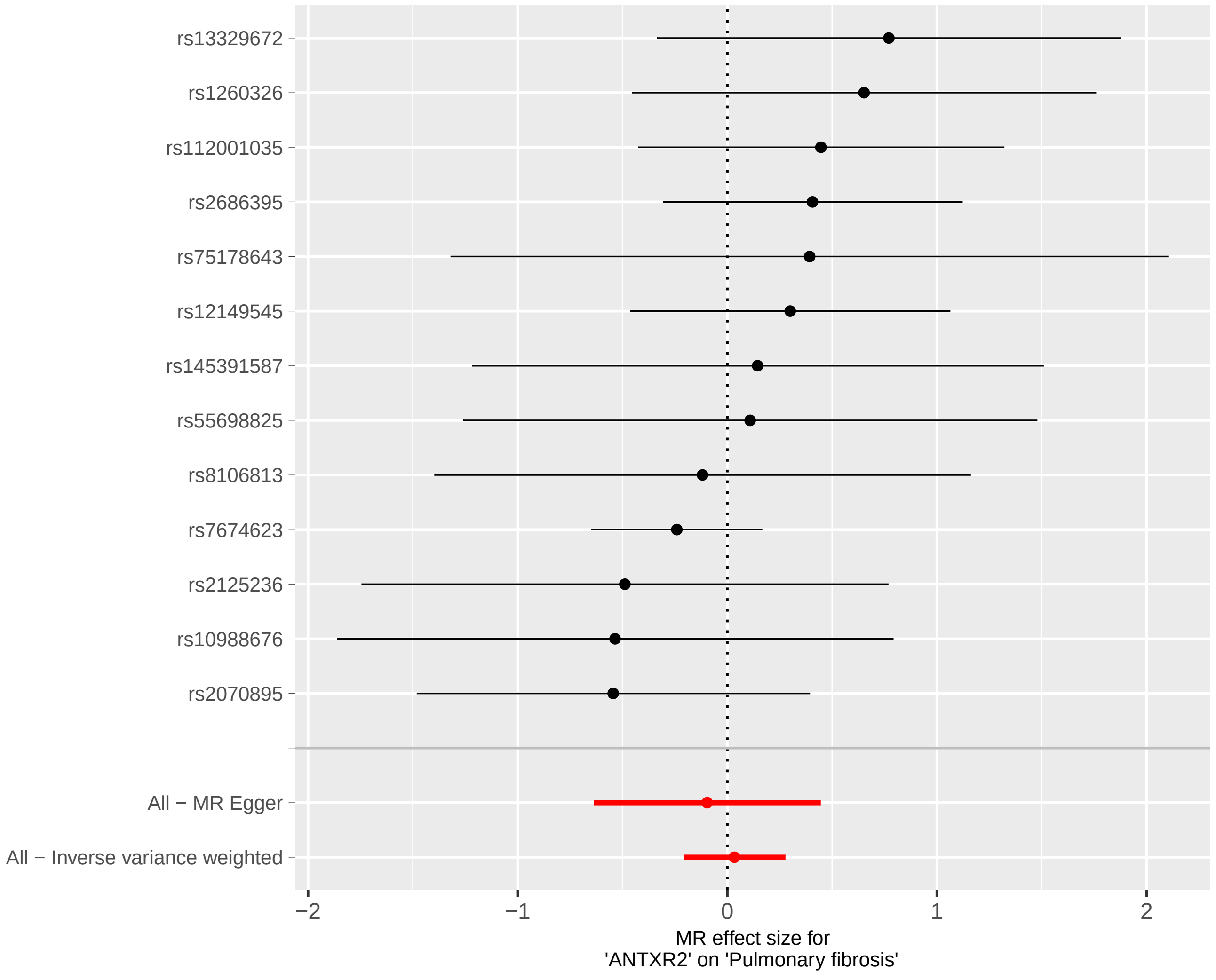
rs13329672
rs1260326
rs112001035
rs2686395
rs75178643
rs12149545
rs145391587
rs55698825
rs8106813
rs7674623
rs2125236
rs10988676
rs2070895
All − MR Egger
All − Inverse variance weighted
−2
−1
0
1
2
MR effect size for
'ANTXR2' on 'Pulmonary fibrosis'

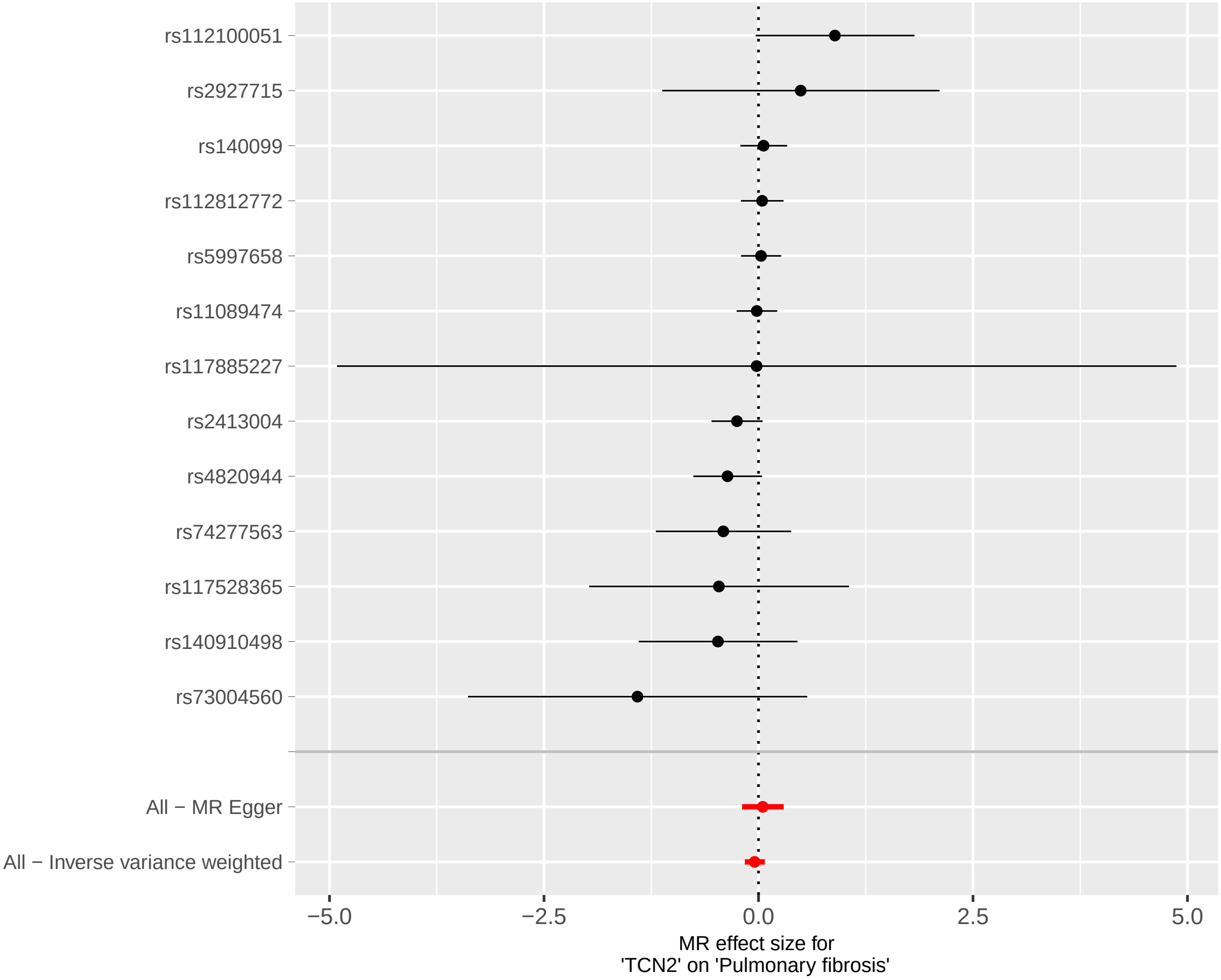

rs112100051
rs2927715
rs140099
rs112812772
rs5997658
rs11089474
rs117885227
rs2413004
rs4820944
rs74277563
rs117528365
rs140910498
rs73004560
All − MR Egger
All − Inverse variance weighted
−5.0
−2.5
0.0
2.5
5.0
MR effect size for
'TCN2' on 'Pulmonary fibrosis'

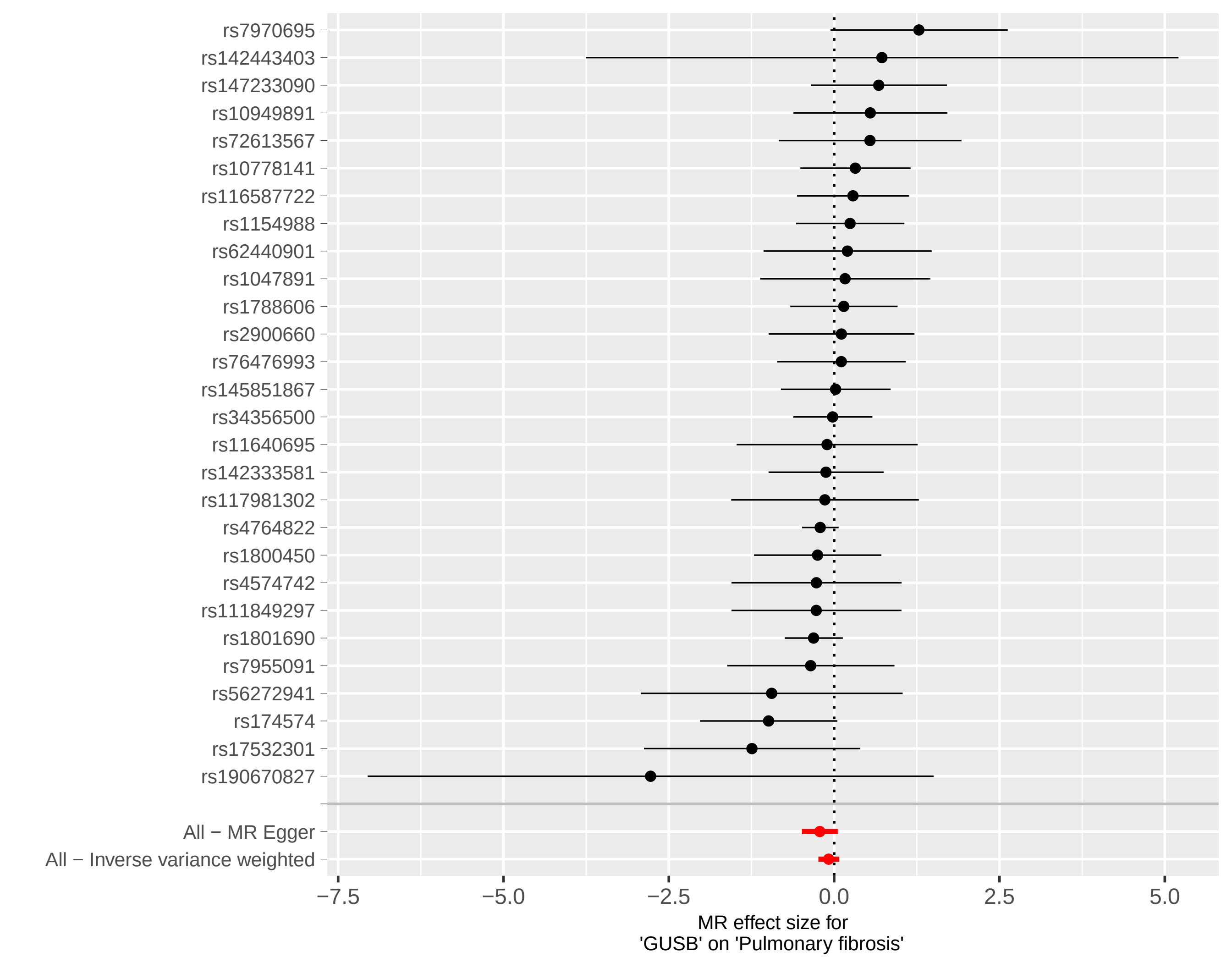
rs7970695
rs142443403
rs147233090
rs10949891
rs72613567
rs10778141
rs116587722
rs1154988
rs62440901
rs1047891
rs1788606
rs2900660
rs76476993
rs145851867
rs34356500
rs11640695
rs142333581
rs117981302
rs4764822
rs1800450
rs4574742
rs111849297
rs1801690
rs7955091
rs56272941
rs174574
rs17532301
rs190670827
All − MR Egger
All − Inverse variance weighted
−7.5
−5.0
−2.5
0.0
2.5
5.0
MR effect size for
'GUSB' on 'Pulmonary fibrosis'

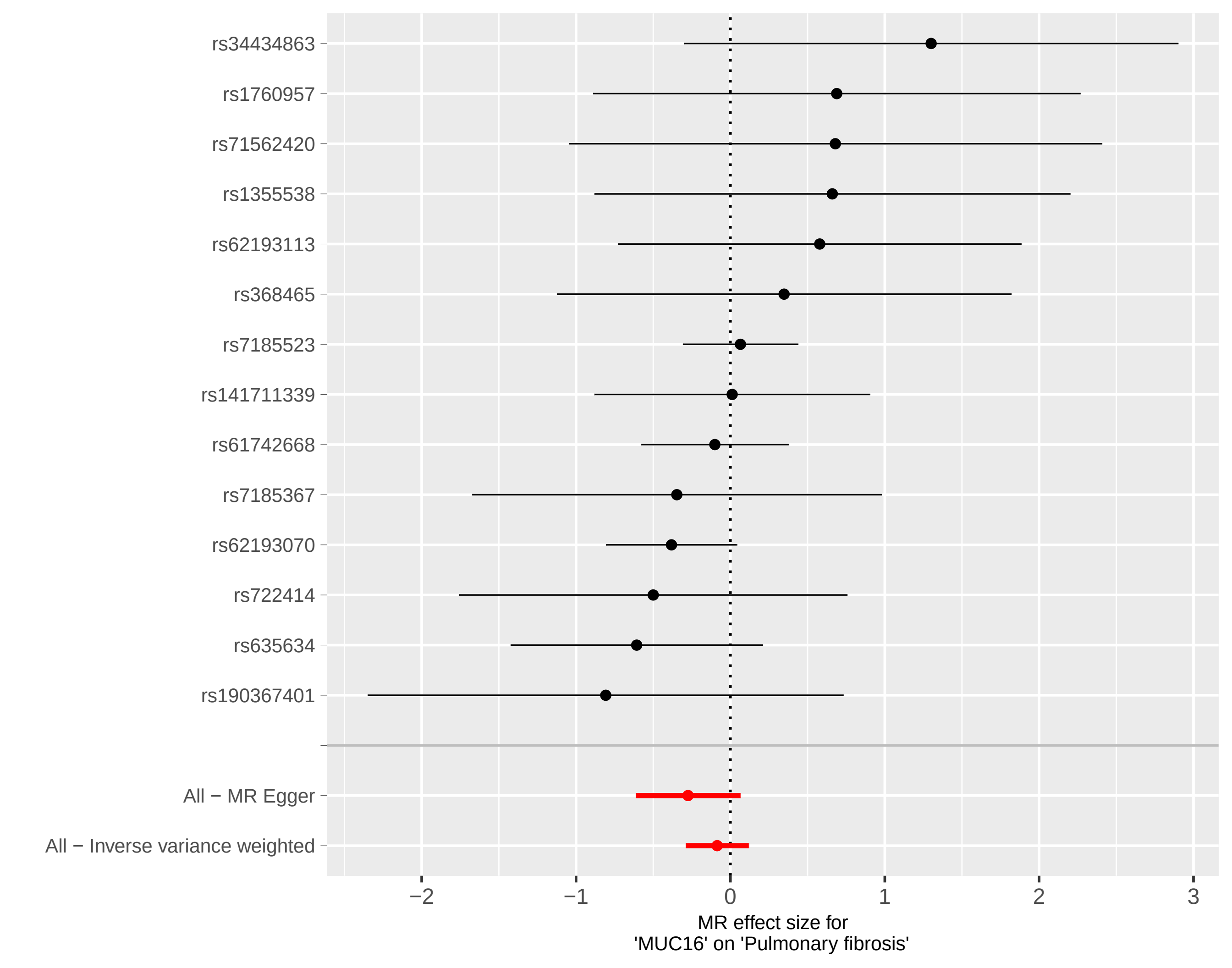
rs34434863
rs1760957
rs71562420
rs1355538
rs62193113
rs368465
rs7185523
rs141711339
rs61742668
rs7185367
rs62193070
rs722414
rs635634
rs190367401
All − MR Egger
All − Inverse variance weighted
−2
−1
0
1
2
3
MR effect size for
'MUC16' on 'Pulmonary fibrosis'

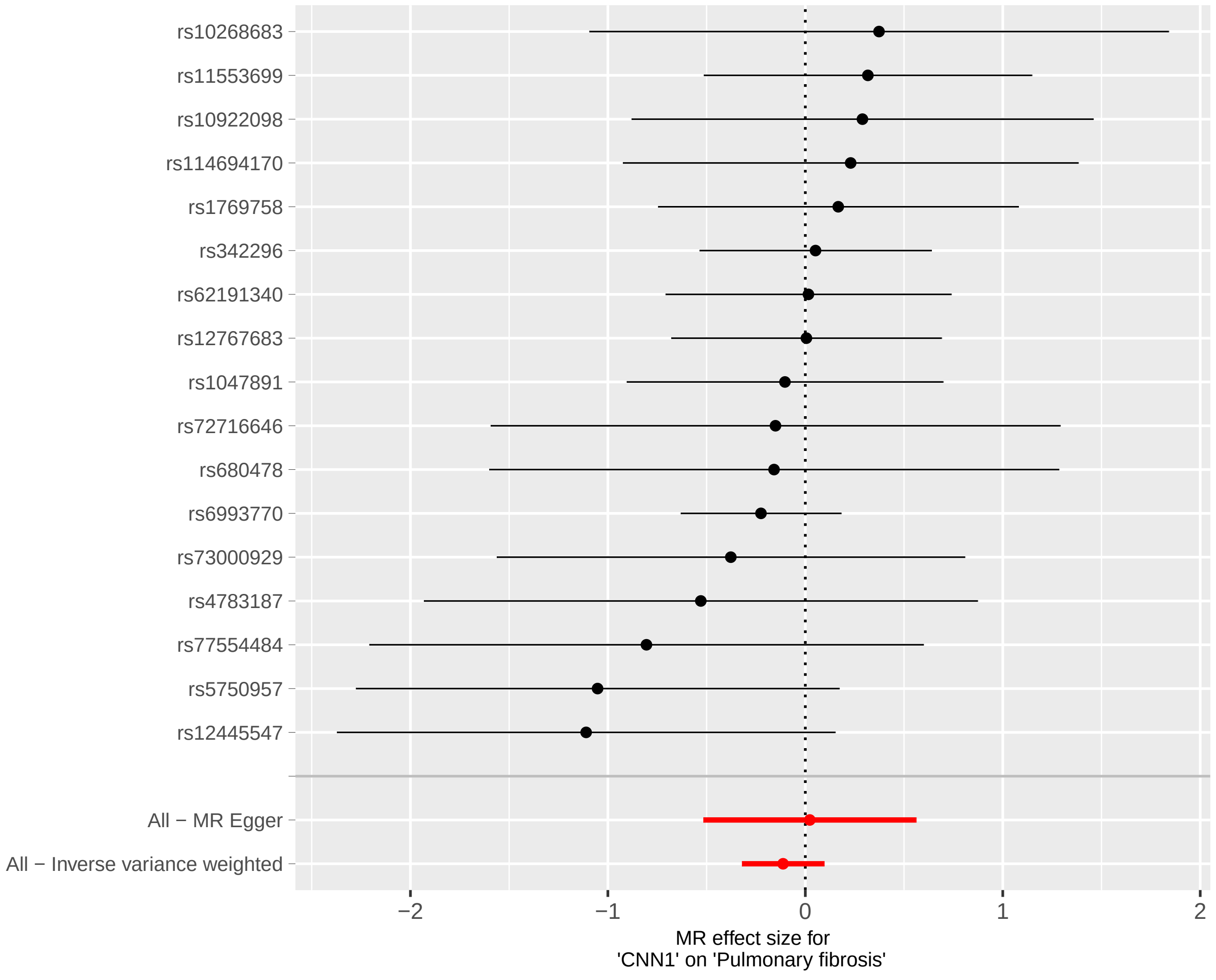
rs10268683
rs11553699
rs10922098
rs114694170
rs1769758
rs342296
rs62191340
rs12767683
rs1047891
rs72716646
rs680478
rs6993770
rs73000929
rs4783187
rs77554484
rs5750957
rs12445547
All − MR Egger
All − Inverse variance weighted
−2
−1
0
1
2
MR effect size for
'CNN1' on 'Pulmonary fibrosis'

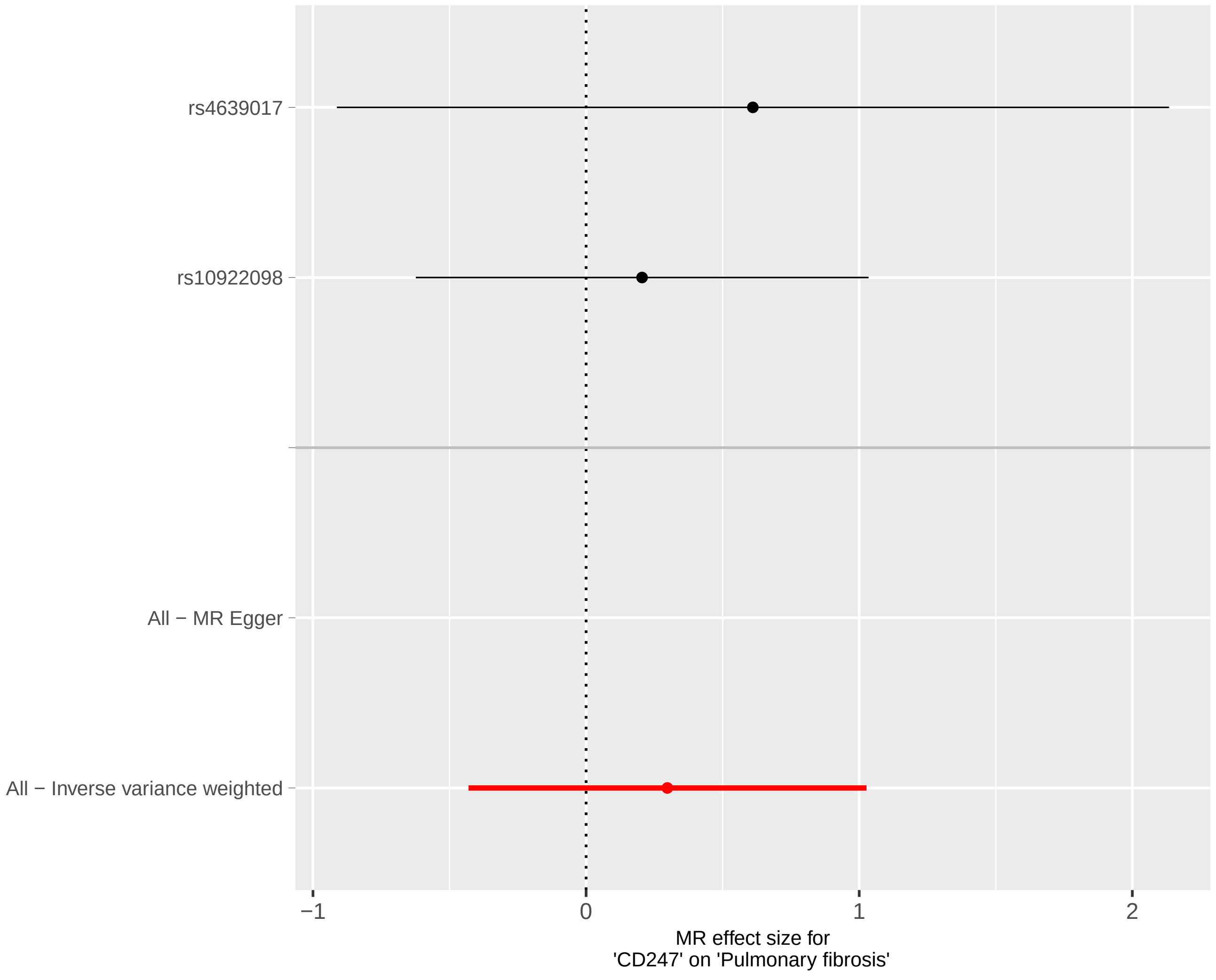

rs4639017
rs10922098
All − MR Egger
All − Inverse variance weighted
−1
0
1
2
MR effect size for
'CD247' on 'Pulmonary fibrosis'

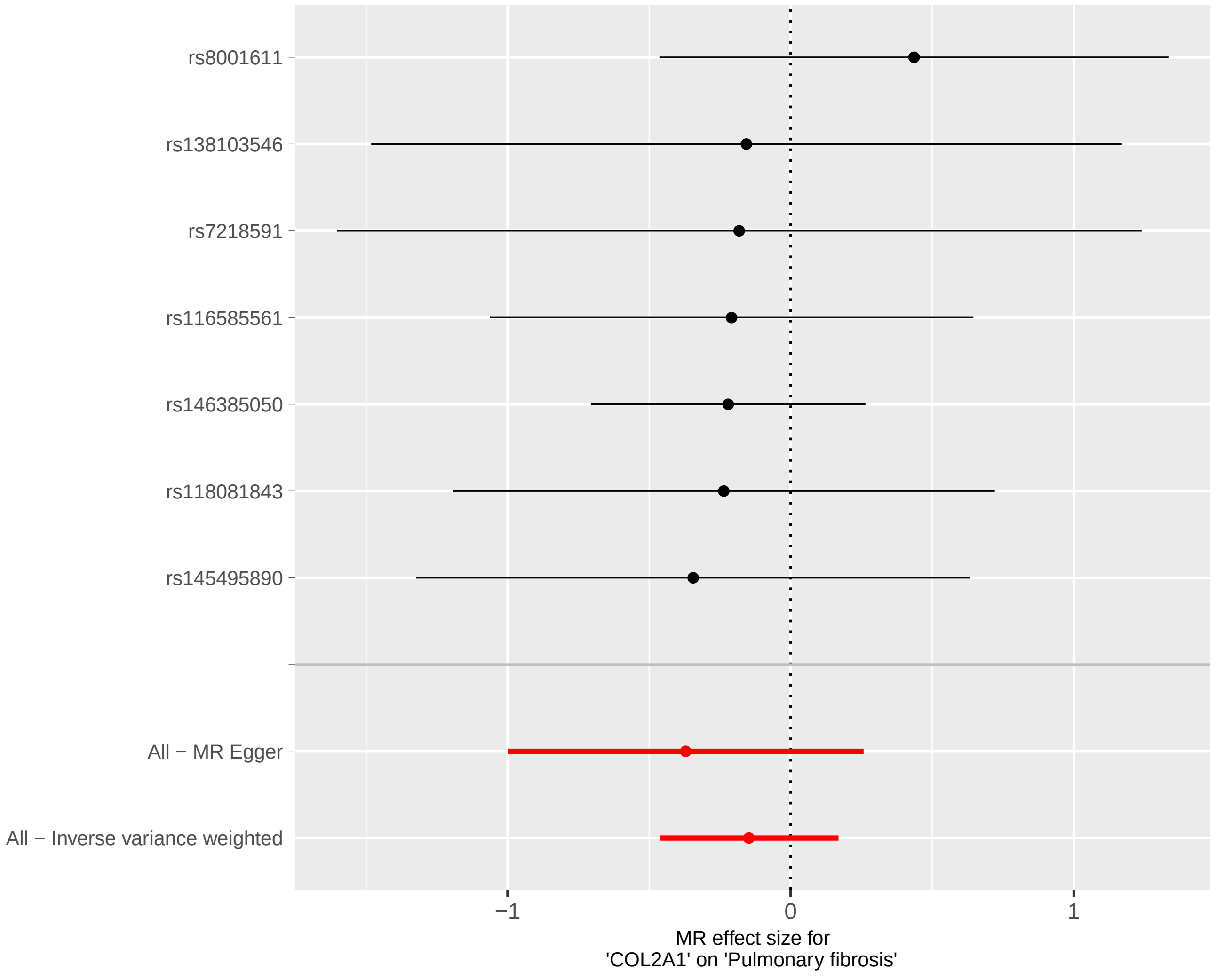
rs8001611
rs138103546
rs7218591
rs116585561
rs146385050
rs118081843
rs145495890
All − MR Egger
All − Inverse variance weighted
−1
0
1
MR effect size for
'COL2A1' on 'Pulmonary fibrosis'

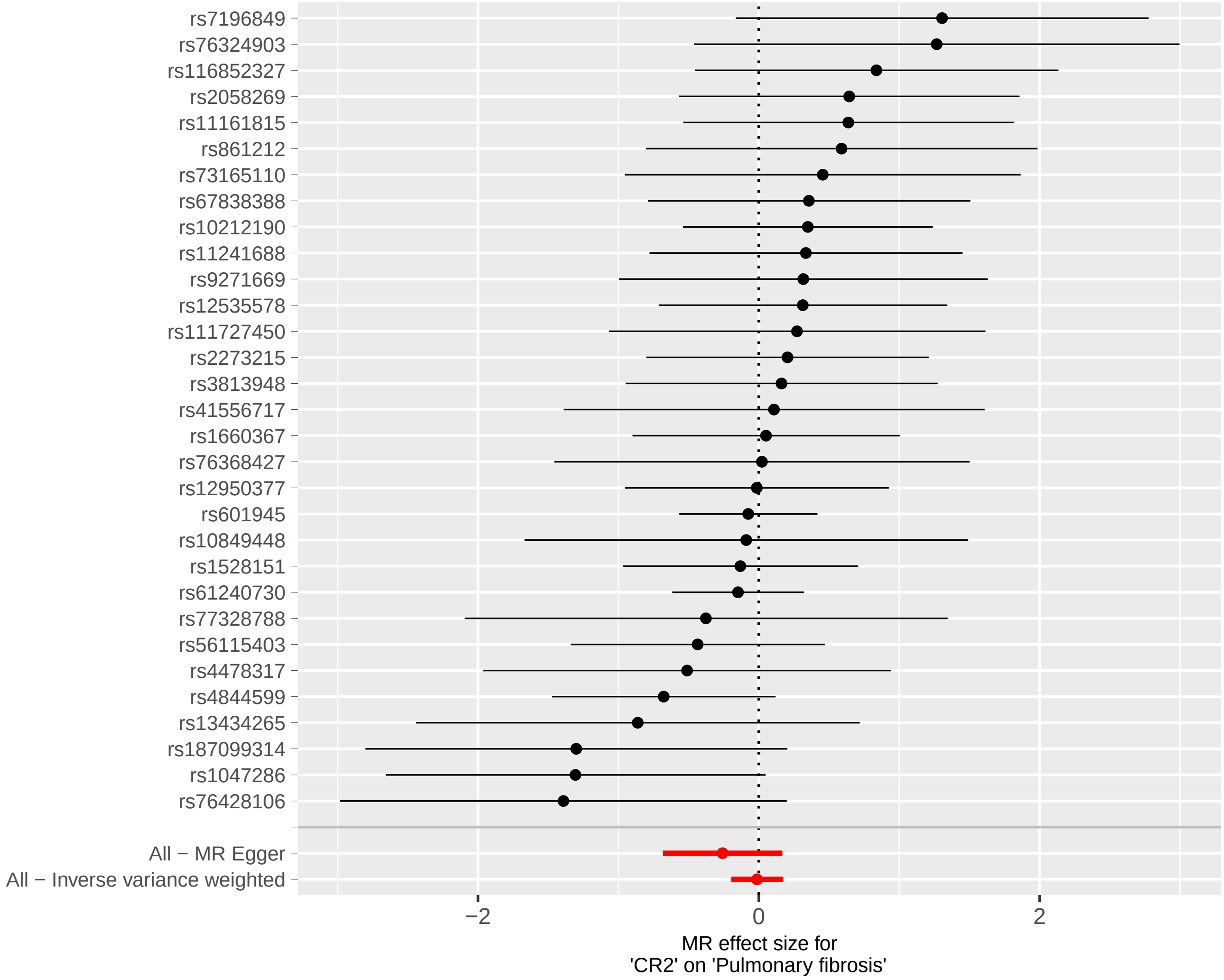
rs7196849
rs76324903
rs116852327
rs2058269
rs11161815
rs861212
rs73165110
rs67838388
rs10212190
rs11241688
rs9271669
rs12535578
rs111727450
rs2273215
rs3813948
rs41556717
rs1660367
rs76368427
rs12950377
rs601945
rs10849448
rs1528151
rs61240730
rs77328788
rs56115403
rs4478317
rs4844599
rs13434265
rs187099314
rs1047286
rs76428106
All − MR Egger
All − Inverse variance weighted
−2
0
2
MR effect size for
'CR2' on 'Pulmonary fibrosis'

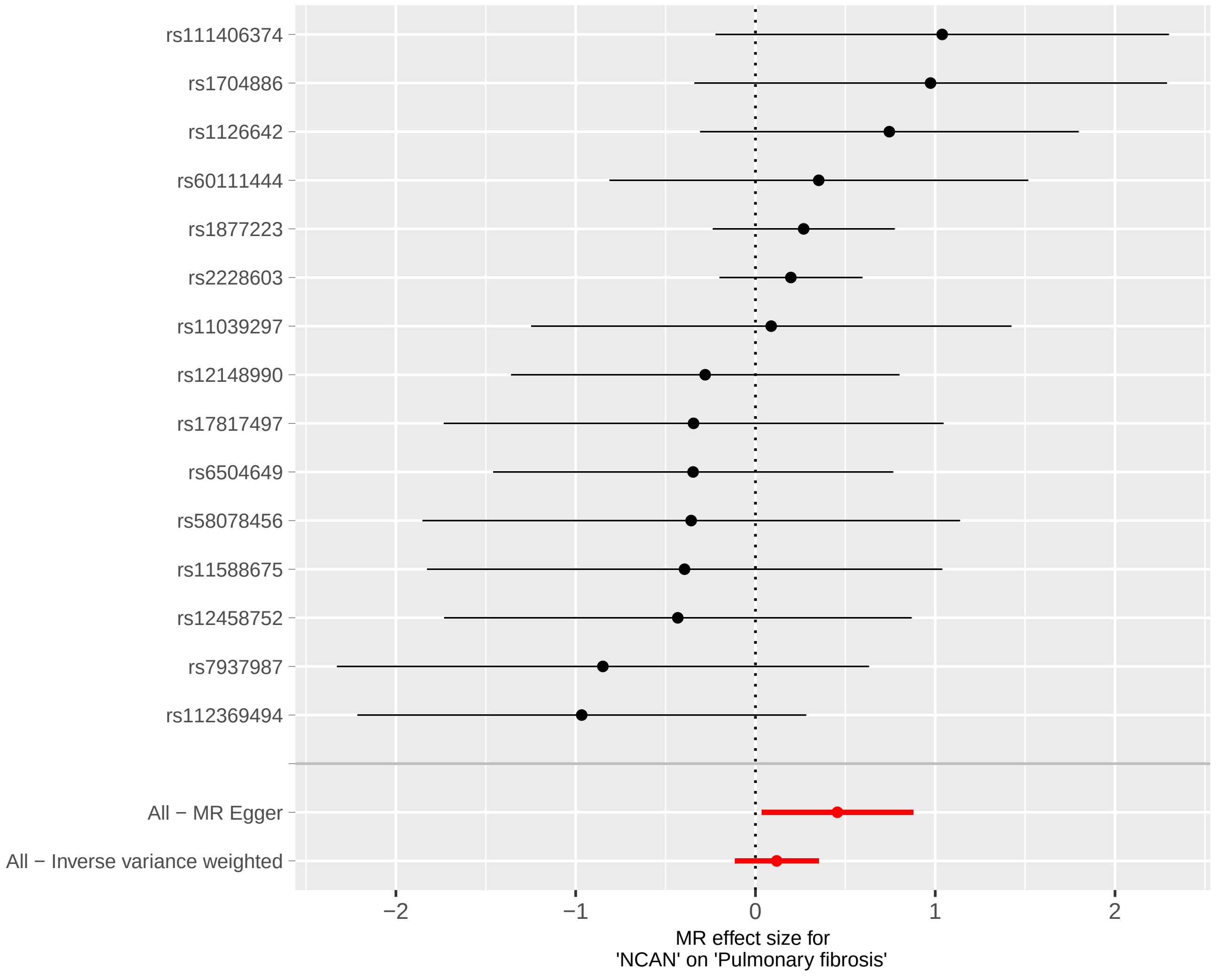

rs111406374
rs1704886
rs1126642
rs60111444
rs1877223
rs2228603
rs11039297
rs12148990
rs17817497
rs6504649
rs58078456
rs11588675
rs12458752
rs7937987
rs112369494
All − MR Egger
All − Inverse variance weighted
−2
−1
0
1
2
MR effect size for
'NCAN' on 'Pulmonary fibrosis'

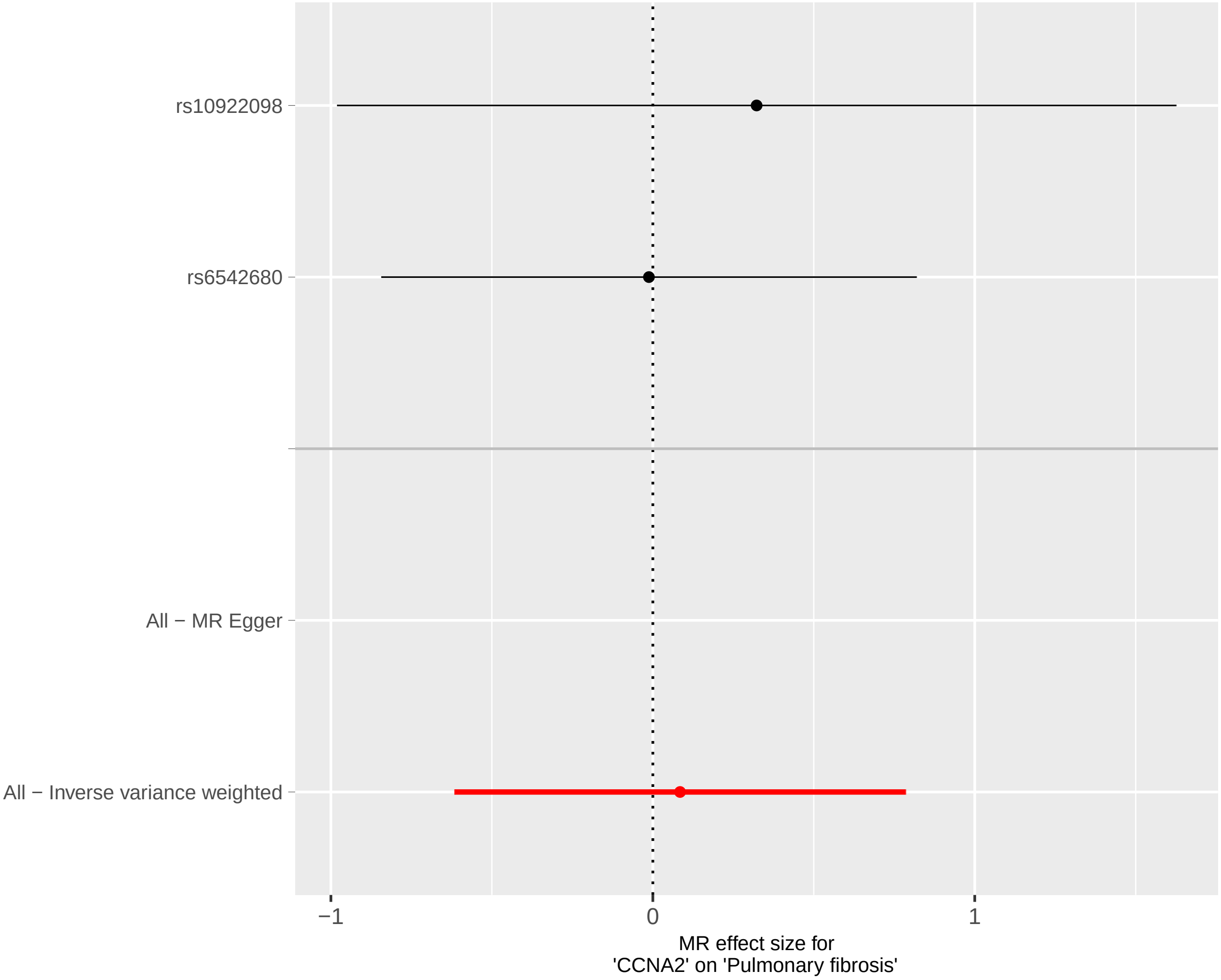
rs10922098
rs6542680
All − MR Egger
All − Inverse variance weighted
−1
0
1
MR effect size for
'CCNA2' on 'Pulmonary fibrosis'

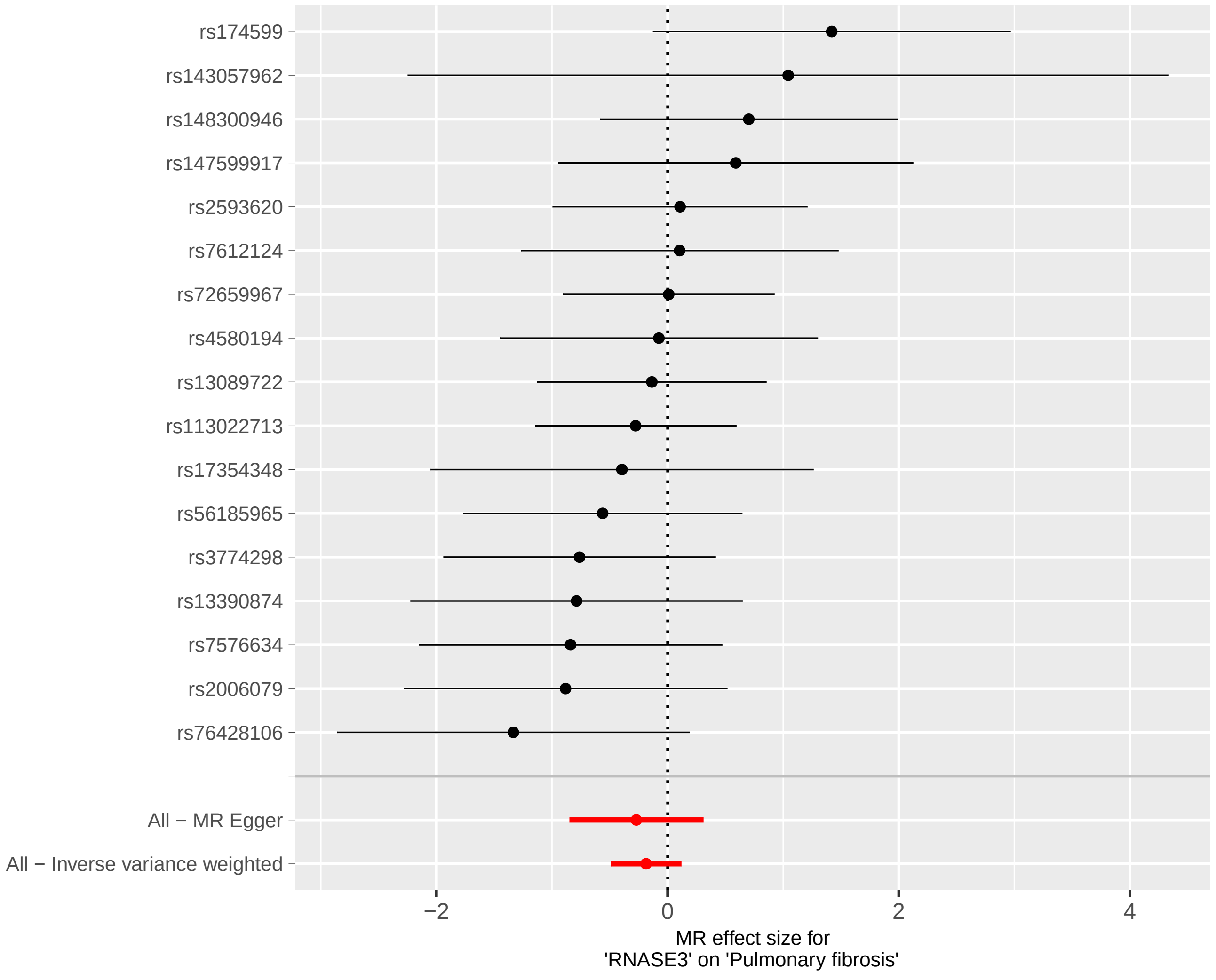

rs174599
rs143057962
rs148300946
rs147599917
rs2593620
rs7612124
rs72659967
rs4580194
rs13089722
rs113022713
rs17354348
rs56185965
rs3774298
rs13390874
rs7576634
rs2006079
rs76428106
All − MR Egger
All − Inverse variance weighted
−2
0
2
4
MR effect size for
'RNASE3' on 'Pulmonary fibrosis'

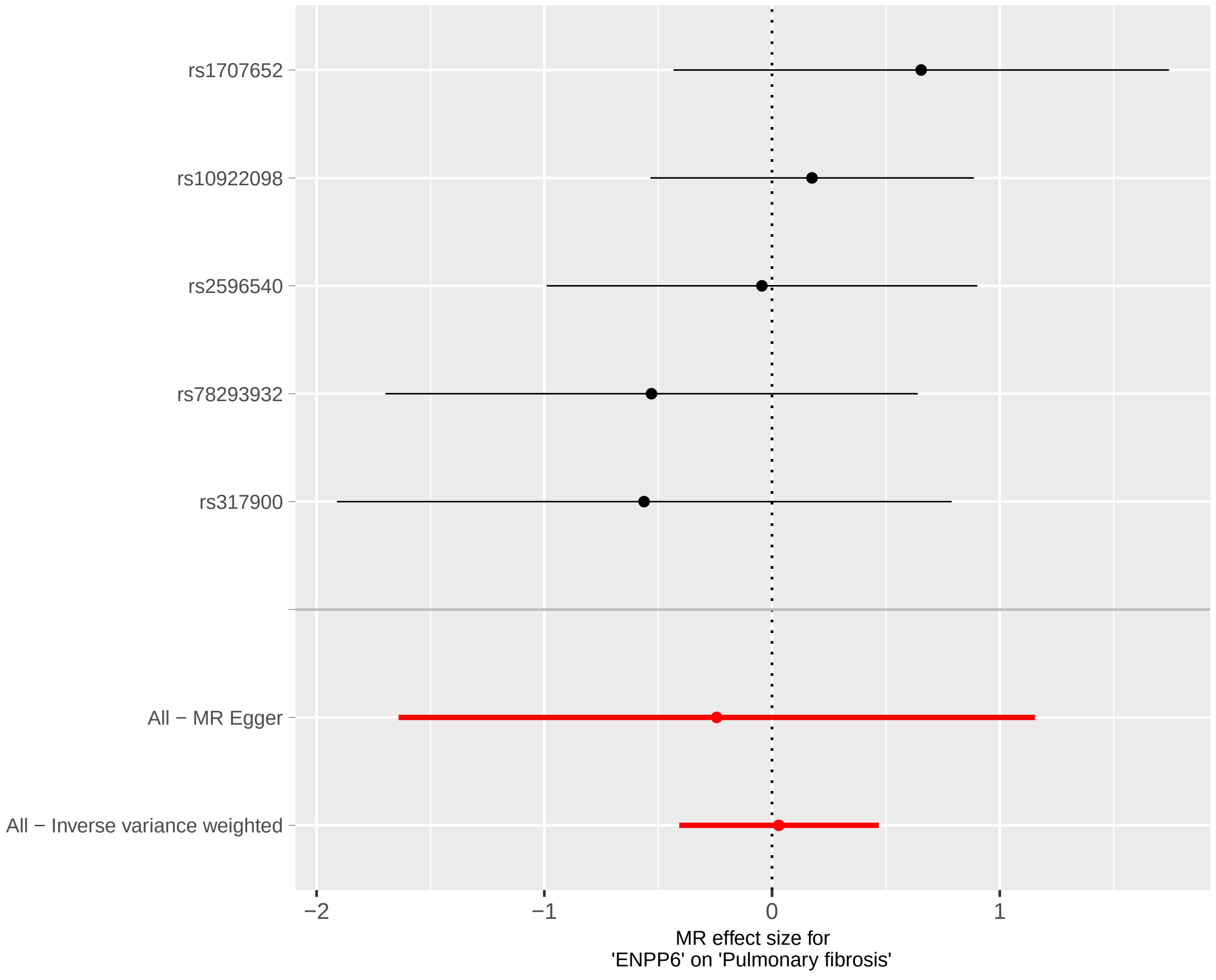

rs1707652
rs10922098
rs2596540
rs78293932
rs317900
All − MR Egger
All − Inverse variance weighted
−2
−1
0
1
MR effect size for
'ENPP6' on 'Pulmonary fibrosis'

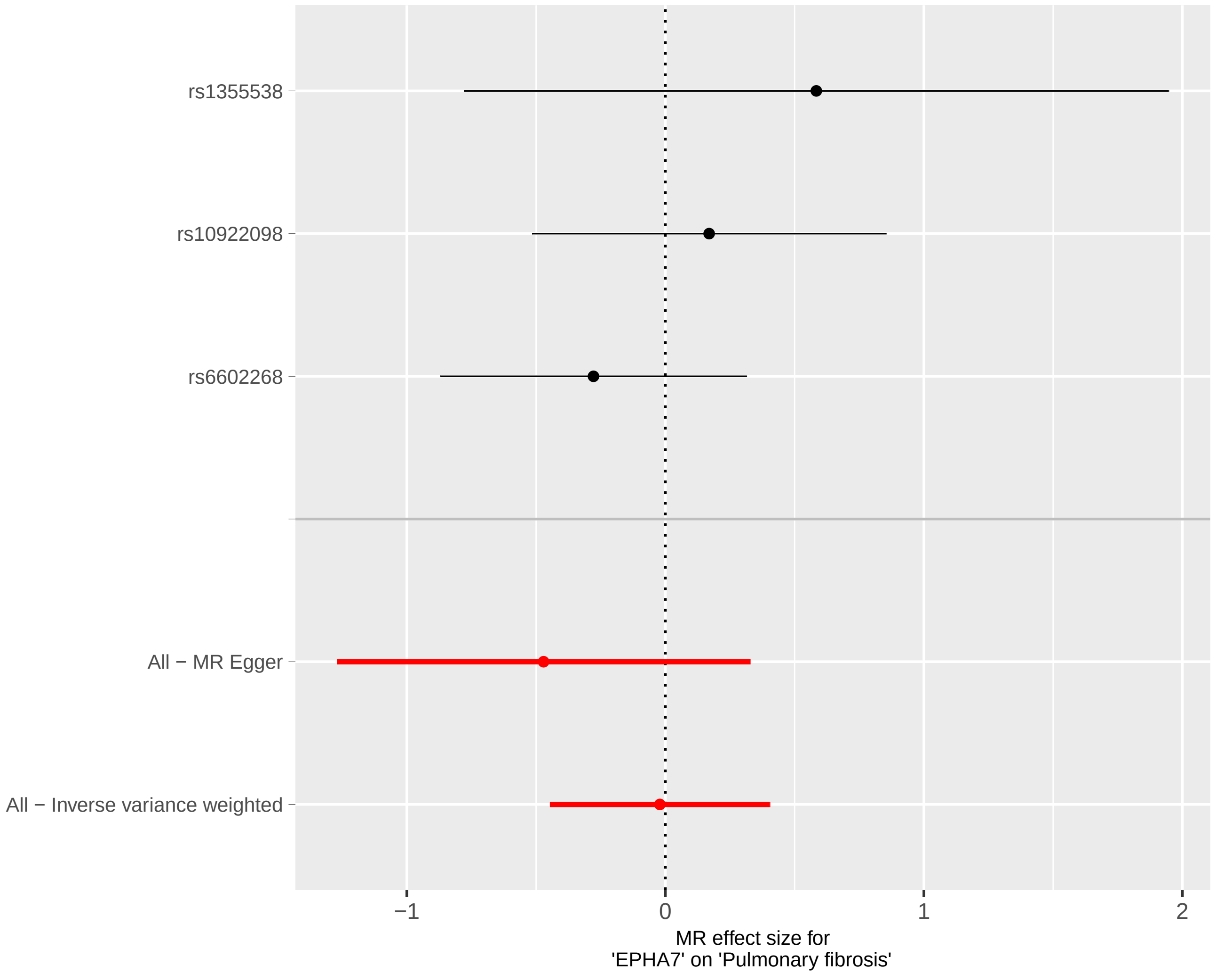

rs1355538
rs10922098
rs6602268
All − MR Egger
All − Inverse variance weighted
−1
0
1
2
MR effect size for
'EPHA7' on 'Pulmonary fibrosis'

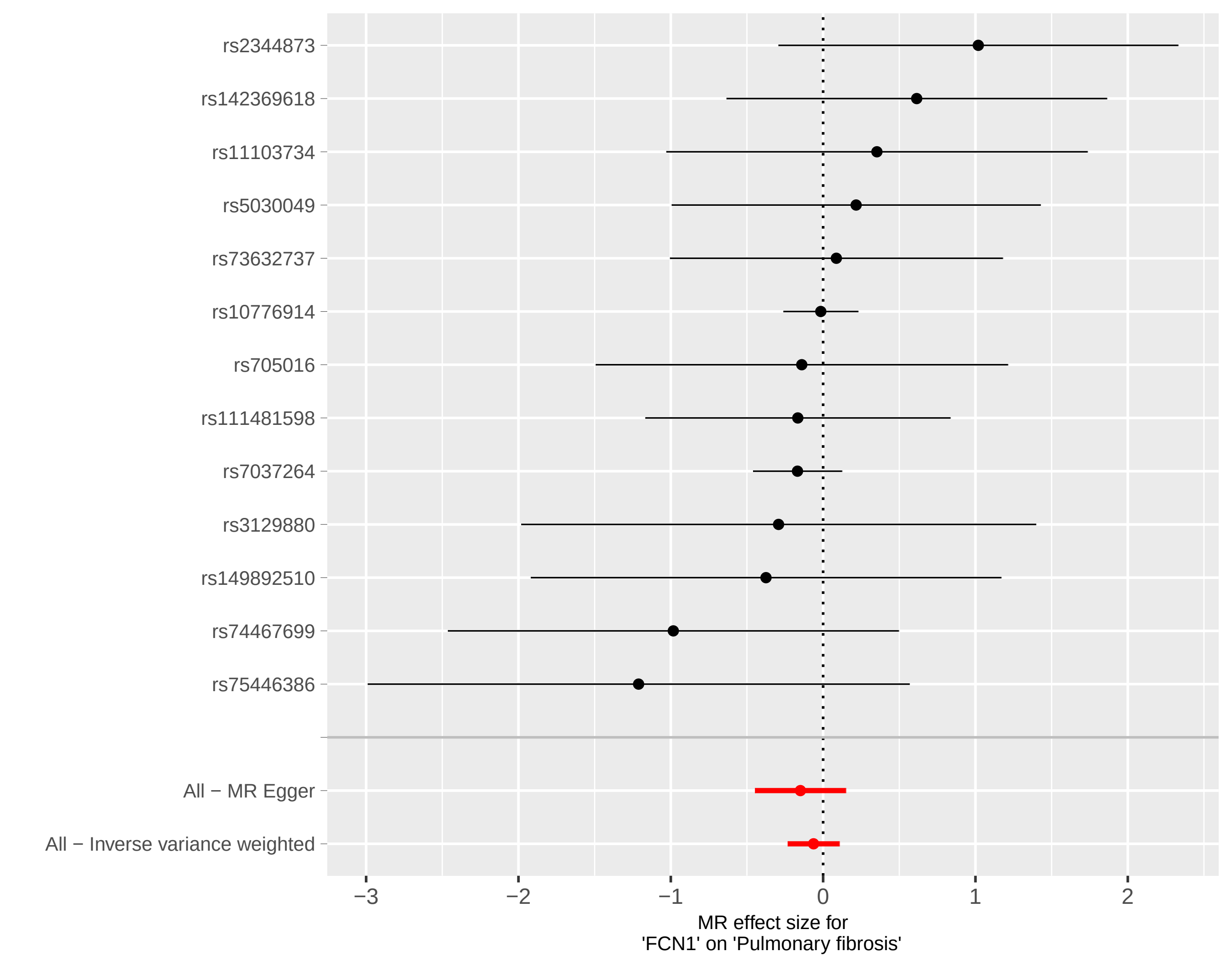

rs2344873
rs142369618
rs11103734
rs5030049
rs73632737
rs10776914
rs705016
rs111481598
rs7037264
rs3129880
rs149892510
rs74467699
rs75446386
All − MR Egger
All − Inverse variance weighted
−3
−2
−1
0
1
2
MR effect size for
'FCN1' on 'Pulmonary fibrosis'

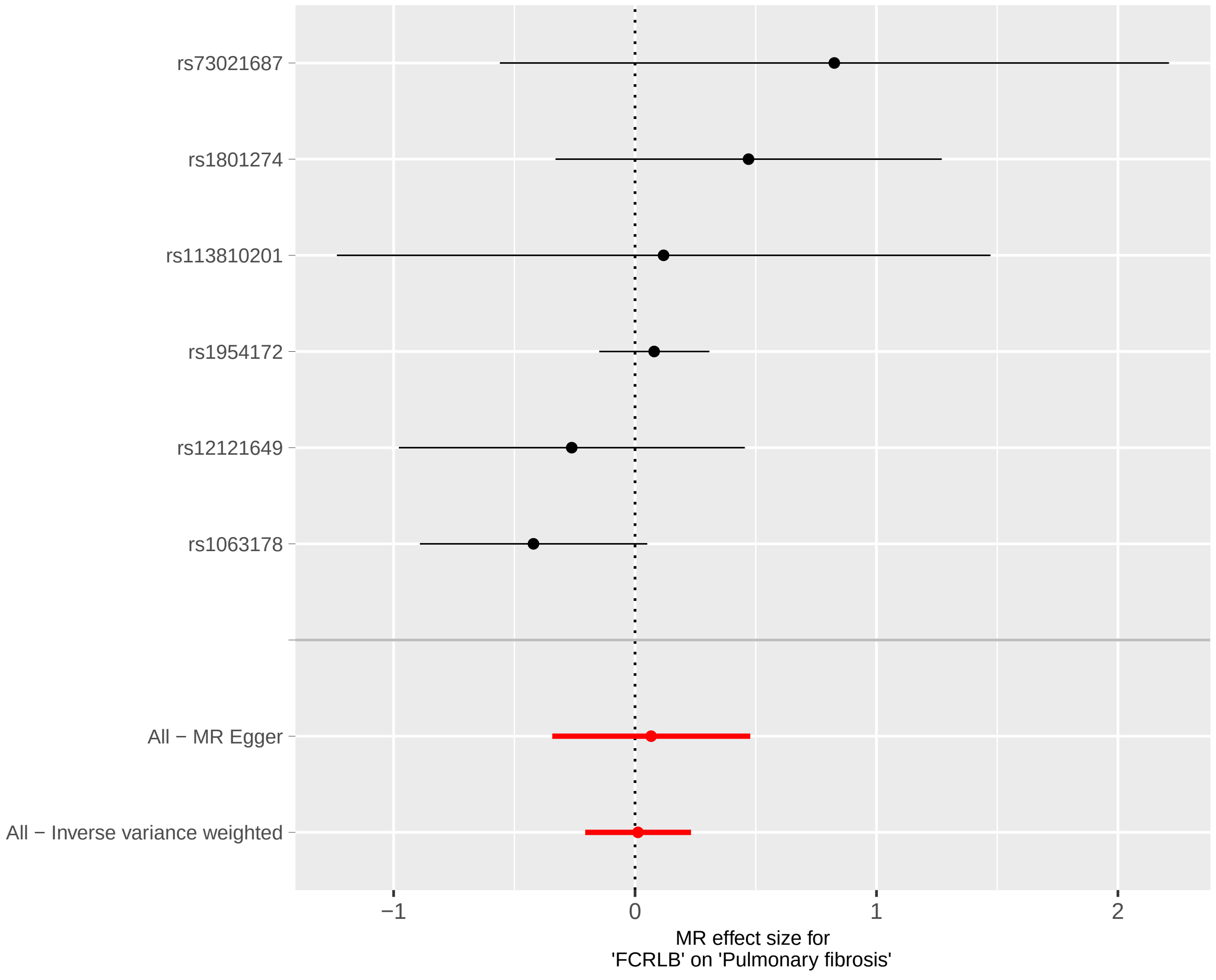
rs73021687
rs1801274
rs113810201
rs1954172
rs12121649
rs1063178
All − MR Egger
All − Inverse variance weighted
−1
0
1
2
MR effect size for
'FCRLB' on 'Pulmonary fibrosis'

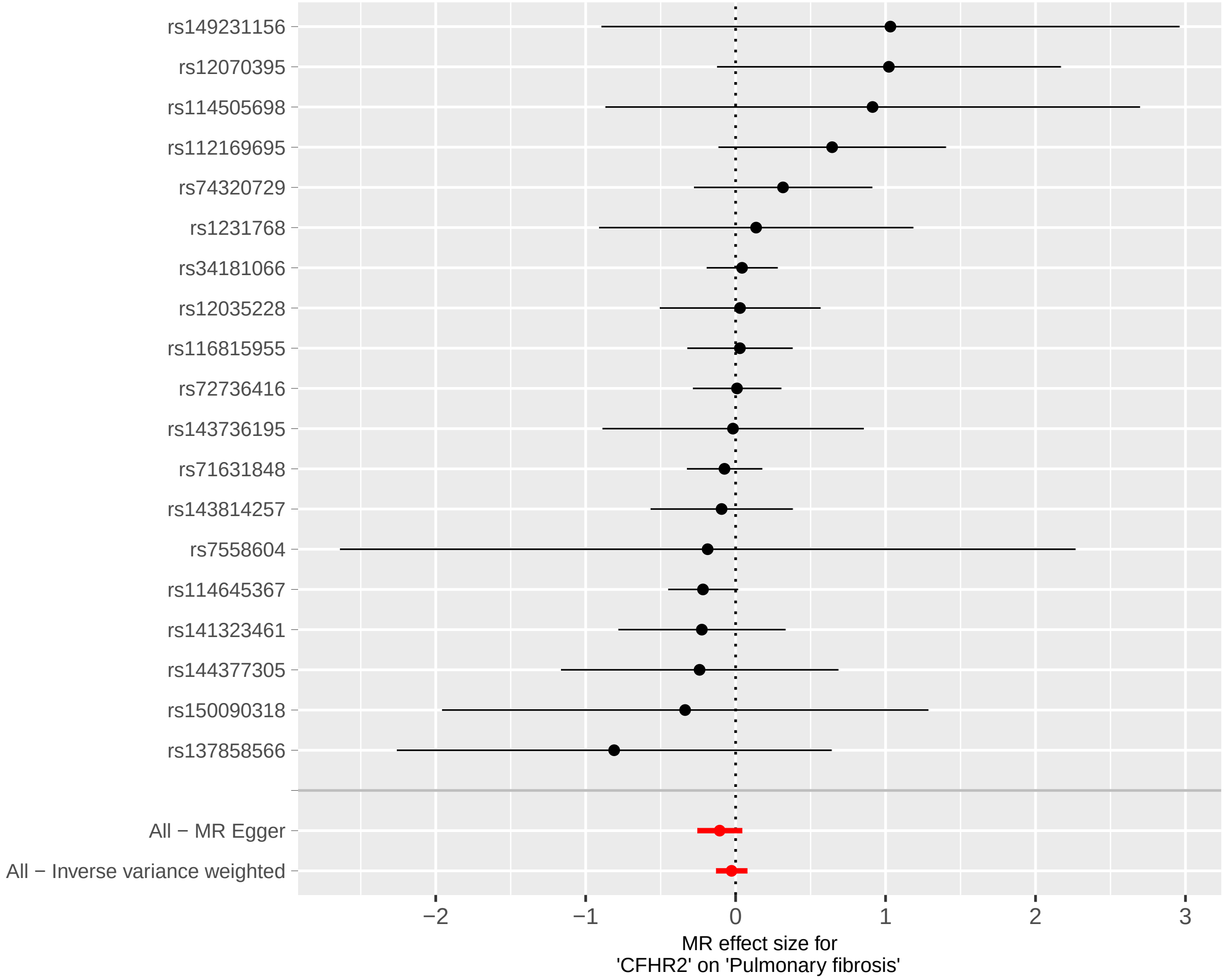
rs149231156
rs12070395
rs114505698
rs112169695
rs74320729
rs1231768
rs34181066
rs12035228
rs116815955
rs72736416
rs143736195
rs71631848
rs143814257
rs7558604
rs114645367
rs141323461
rs144377305
rs150090318
rs137858566
All − MR Egger
All − Inverse variance weighted
−2
−1
0
1
2
3
MR effect size for
'CFHR2' on 'Pulmonary fibrosis'

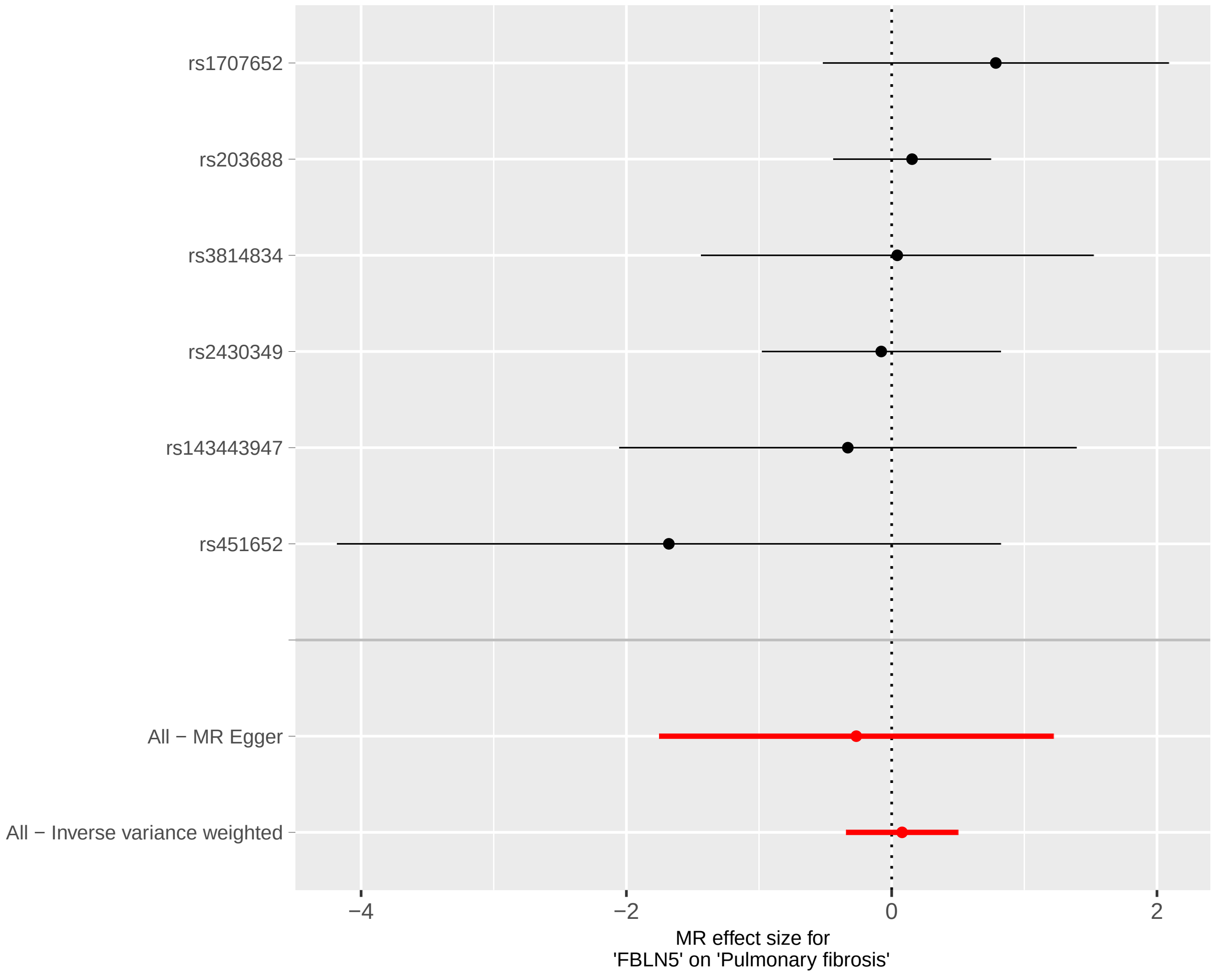

rs1707652
rs203688
rs3814834
rs2430349
rs143443947
rs451652
All − MR Egger
All − Inverse variance weighted
−4
−2
0
2
MR effect size for
'FBLN5' on 'Pulmonary fibrosis'

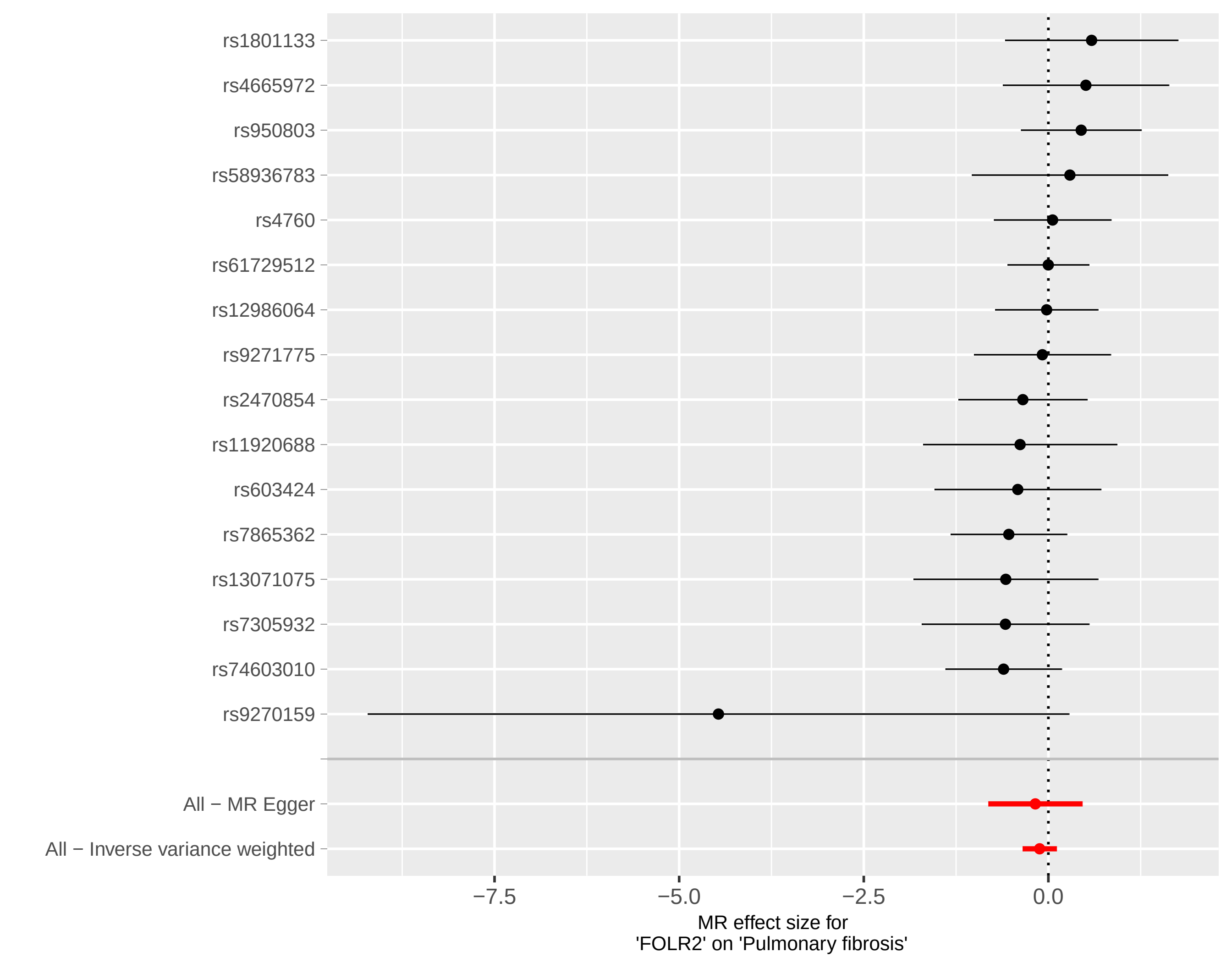
rs1801133
rs4665972
rs950803
rs58936783
rs4760
rs61729512
rs12986064
rs9271775
rs2470854
rs11920688
rs603424
rs7865362
rs13071075
rs7305932
rs74603010
rs9270159
All − MR Egger
All − Inverse variance weighted
−7.5
−5.0
−2.5
0.0
MR effect size for
'FOLR2' on 'Pulmonary fibrosis'

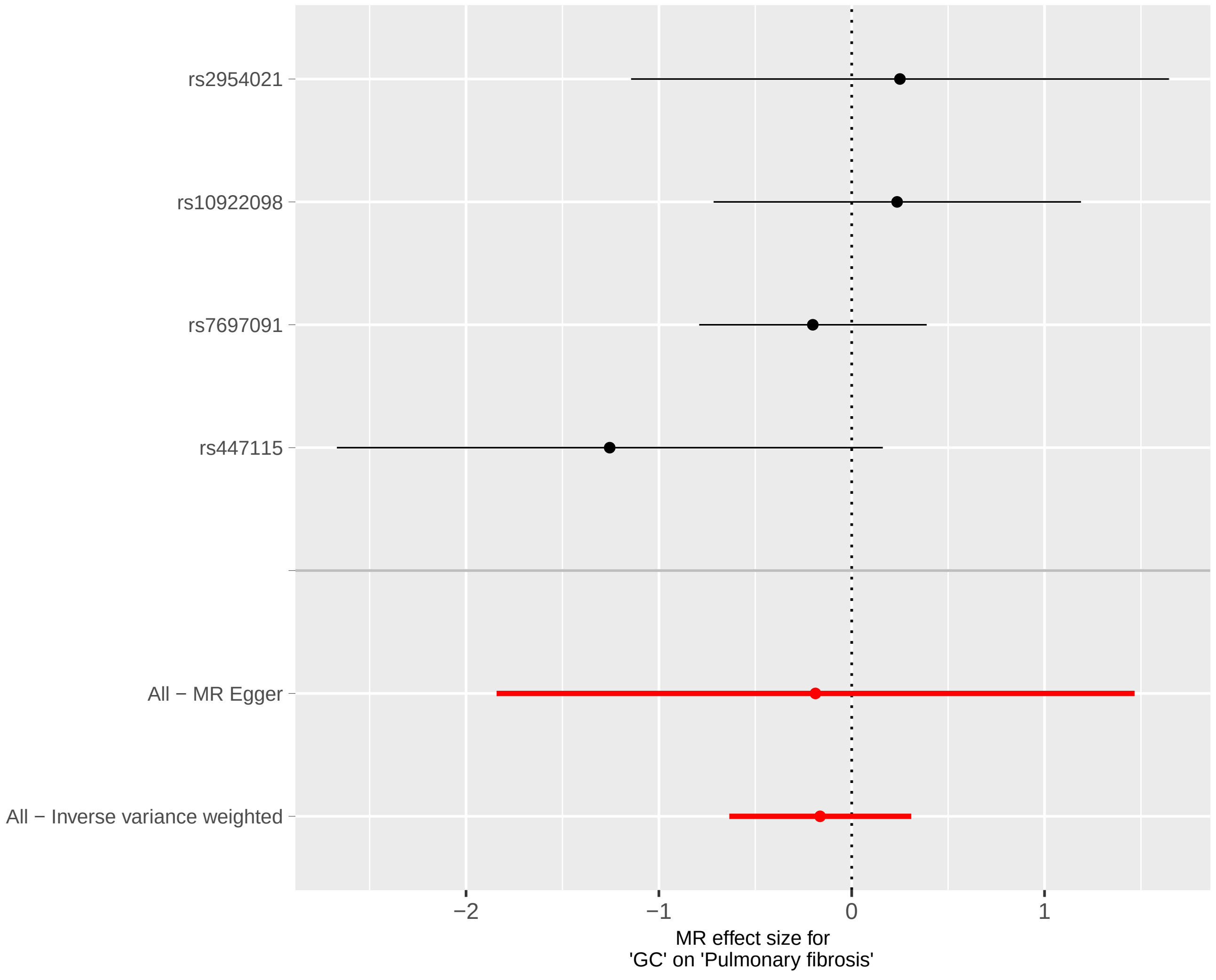
rs2954021
rs10922098
rs7697091
rs447115
All − MR Egger
All − Inverse variance weighted
−2
−1
0
1
MR effect size for
'GC' on 'Pulmonary fibrosis'

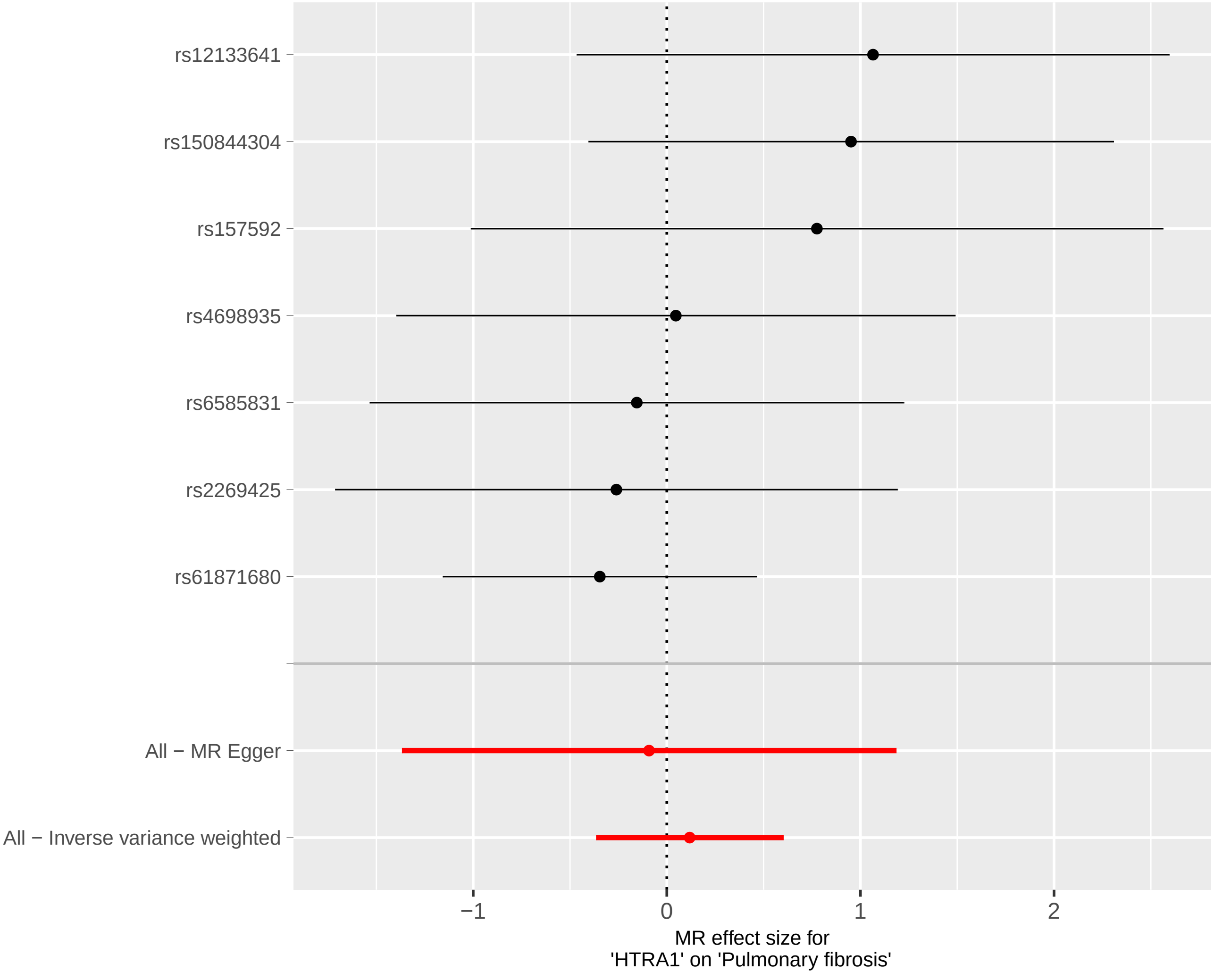

rs12133641
rs150844304
rs157592
rs4698935
rs6585831
rs2269425
rs61871680
All − MR Egger
All − Inverse variance weighted
−1
0
1
2
MR effect size for
'HTRA1' on 'Pulmonary fibrosis'

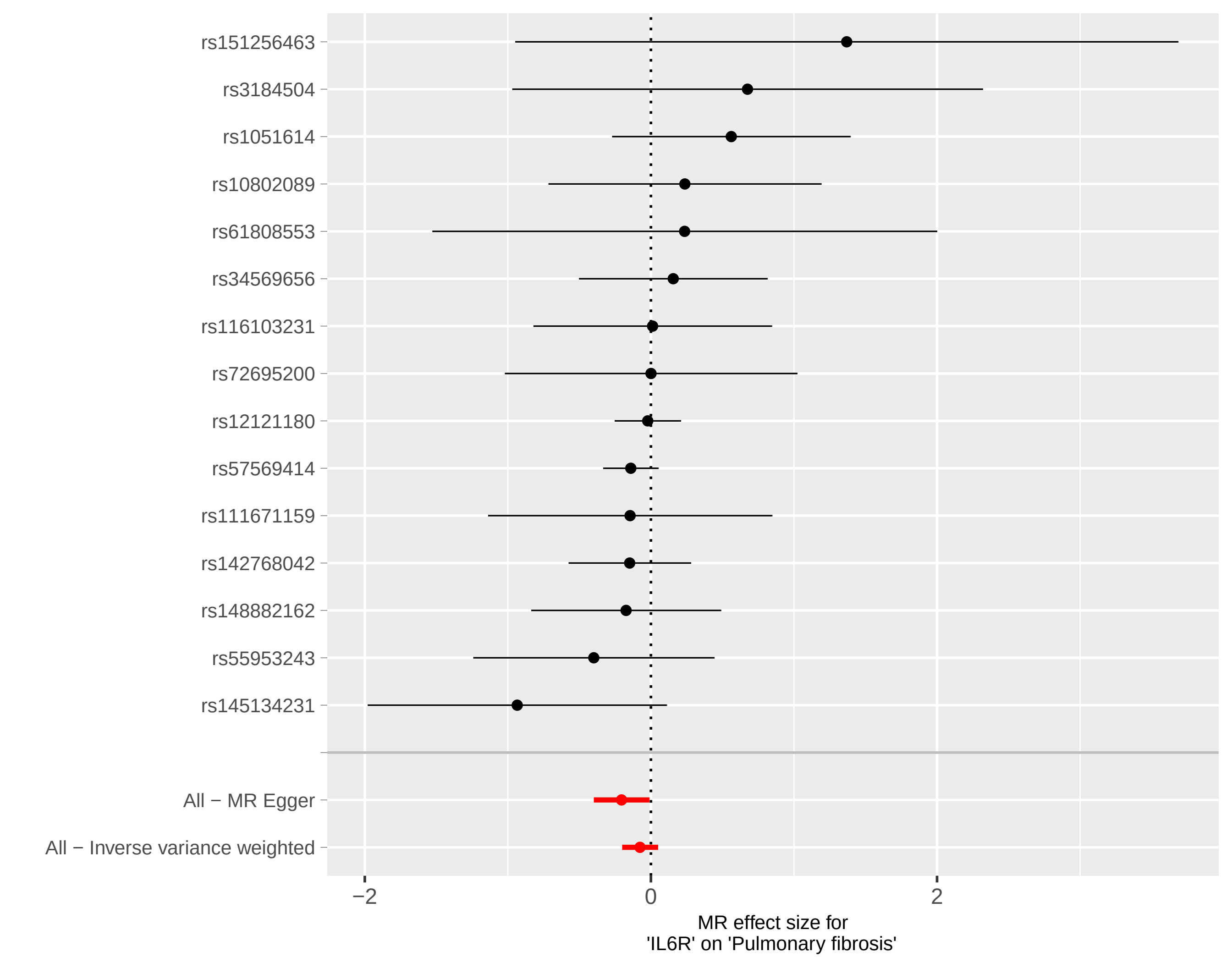
rs151256463
rs3184504
rs1051614
rs10802089
rs61808553
rs34569656
rs116103231
rs72695200
rs12121180
rs57569414
rs111671159
rs142768042
rs148882162
rs55953243
rs145134231
All − MR Egger
All − Inverse variance weighted
−2
0
2
MR effect size for
'IL6R' on 'Pulmonary fibrosis'

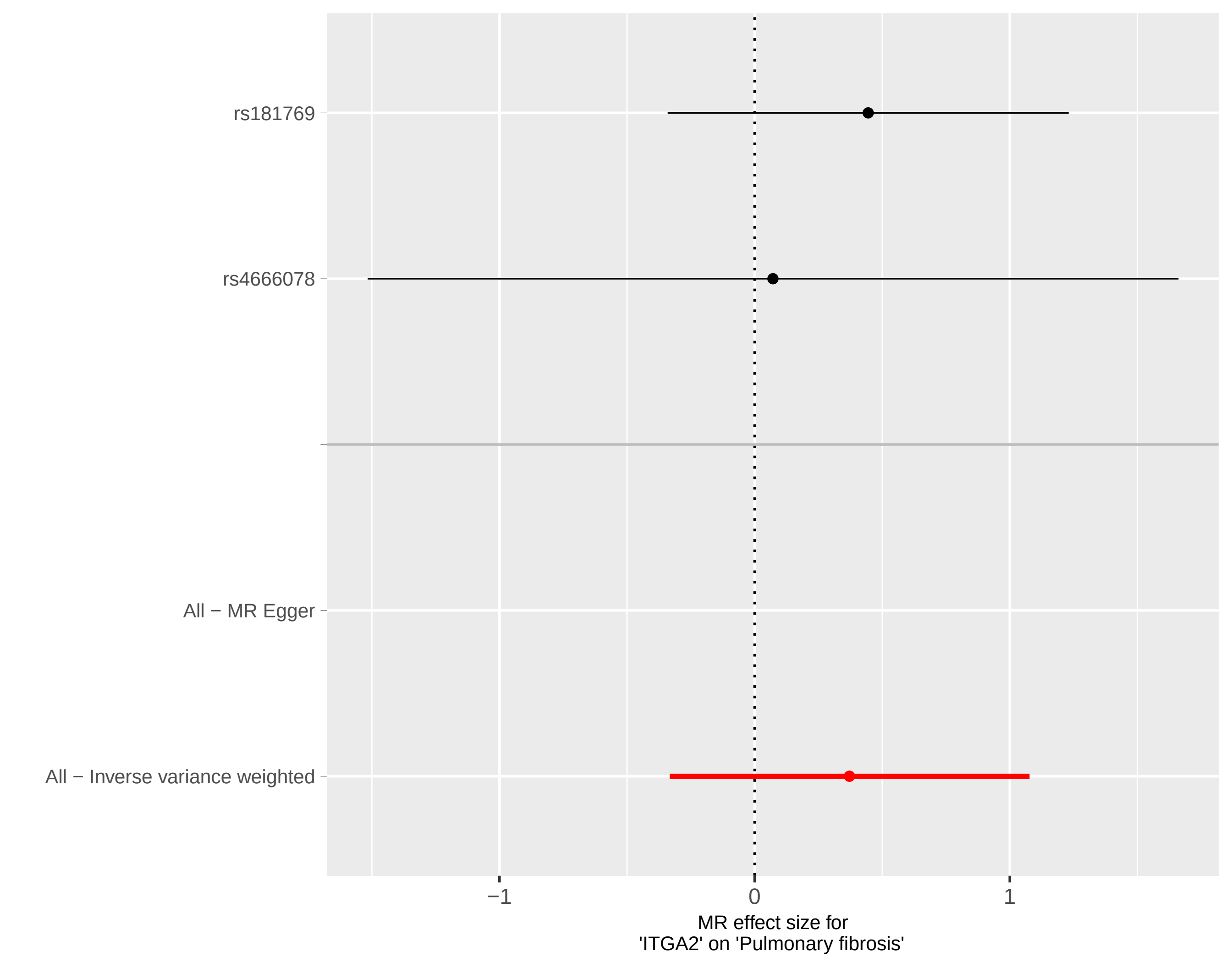
rs181769
rs4666078
All − MR Egger
All − Inverse variance weighted
−1
0
1
MR effect size for
'ITGA2' on 'Pulmonary fibrosis'

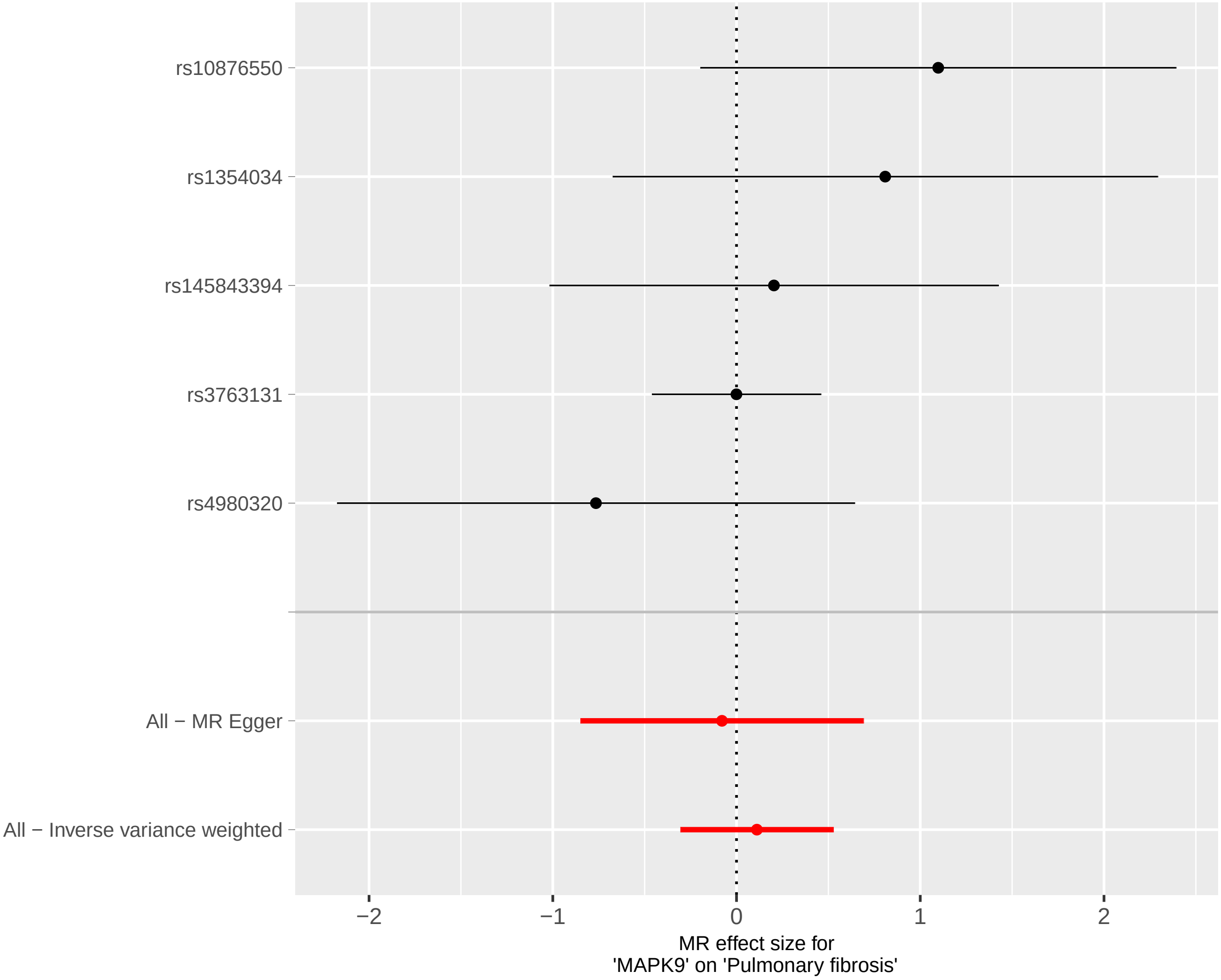

rs10876550
rs1354034
rs145843394
rs3763131
rs4980320
All − MR Egger
All − Inverse variance weighted
−2
−1
0
1
2
MR effect size for
'MAPK9' on 'Pulmonary fibrosis'

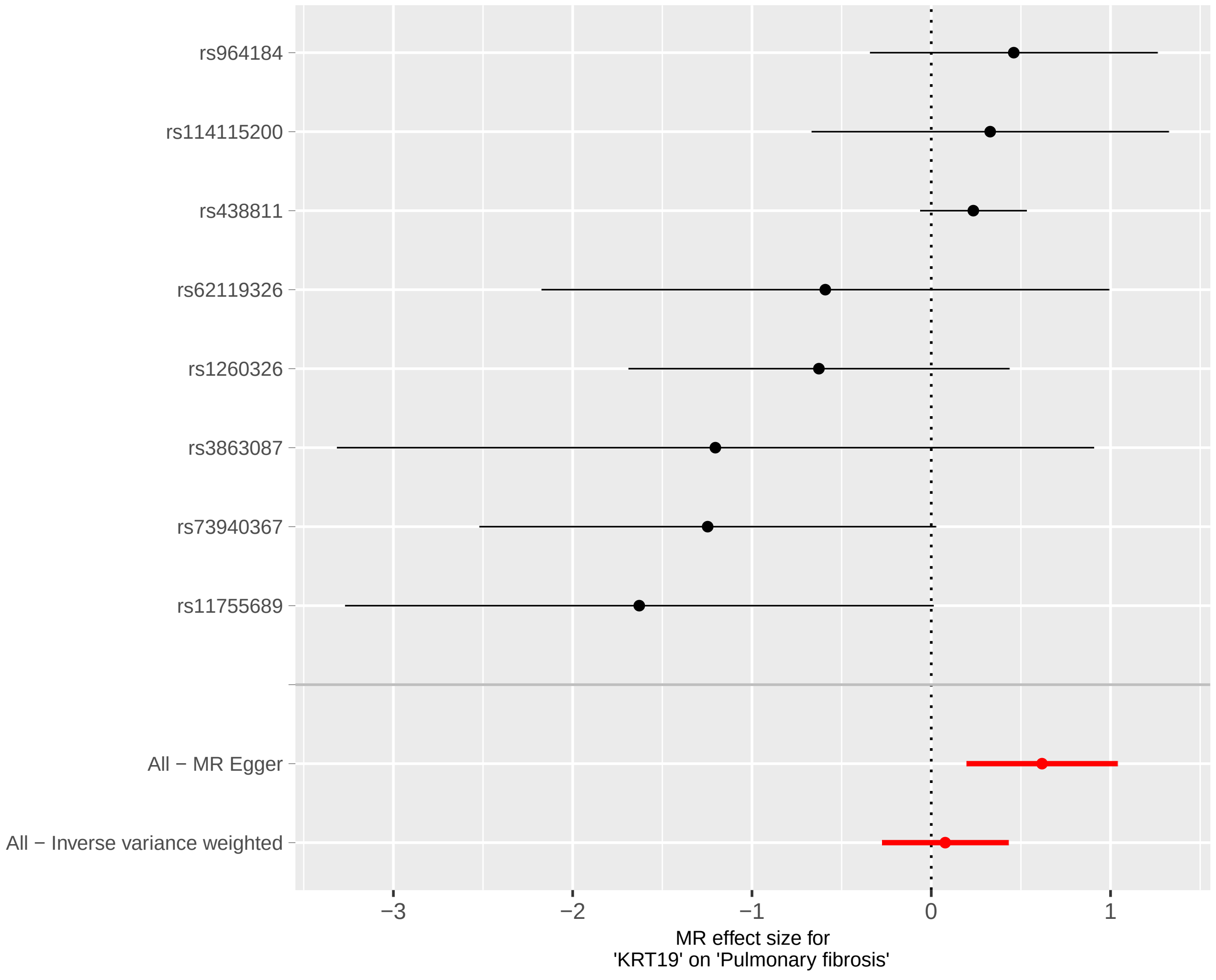

rs964184
rs114115200
rs438811
rs62119326
rs1260326
rs3863087
rs73940367
rs11755689
All − MR Egger
All − Inverse variance weighted
−3
−2
−1
0
1
MR effect size for
'KRT19' on 'Pulmonary fibrosis'

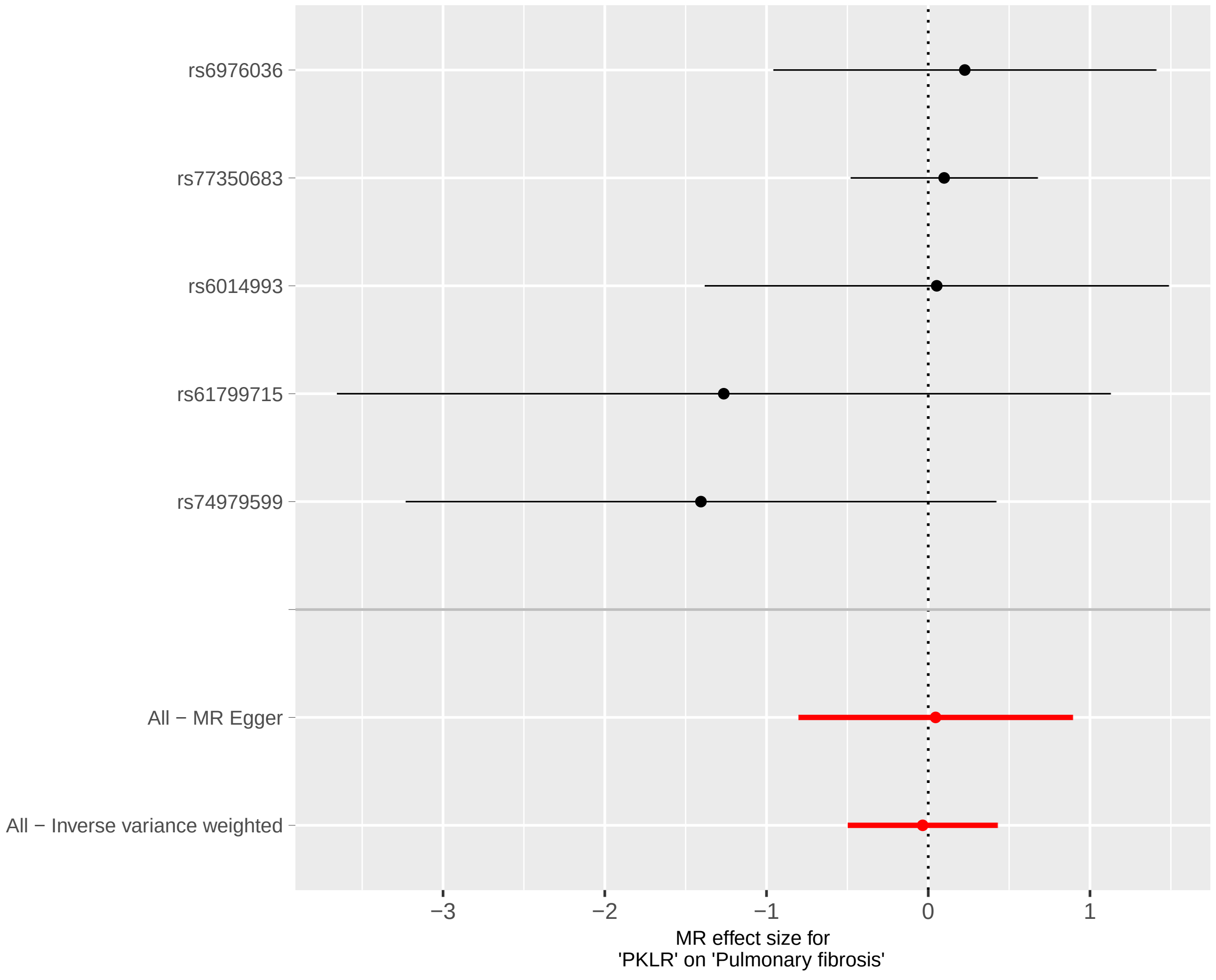

rs6976036
rs77350683
rs6014993
rs61799715
rs74979599
All − MR Egger
All − Inverse variance weighted
−3
−2
−1
0
1
MR effect size for
'PKLR' on 'Pulmonary fibrosis'

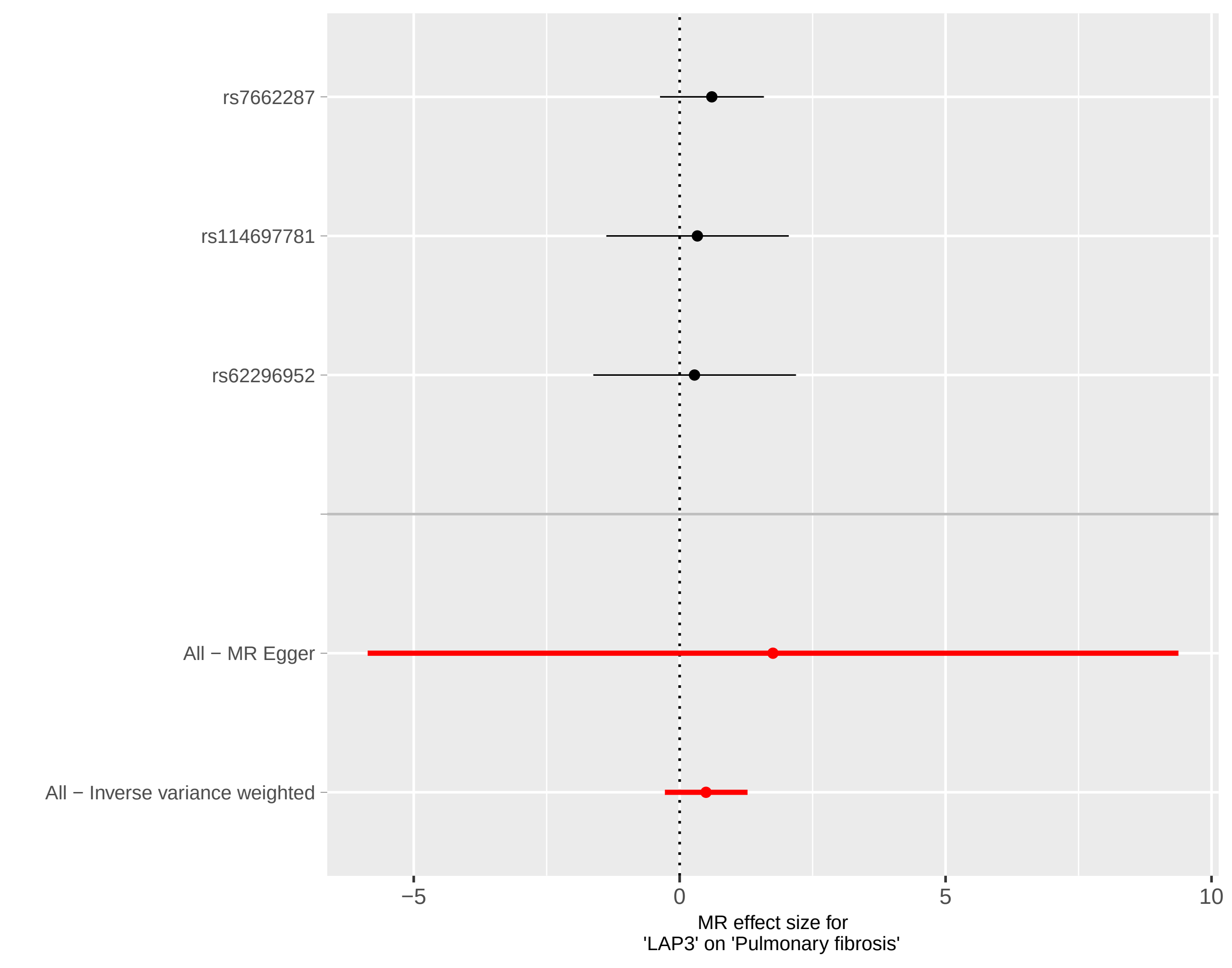
rs7662287
rs114697781
rs62296952
All − MR Egger
All − Inverse variance weighted
−5
0
5
10
MR effect size for
'LAP3' on 'Pulmonary fibrosis'

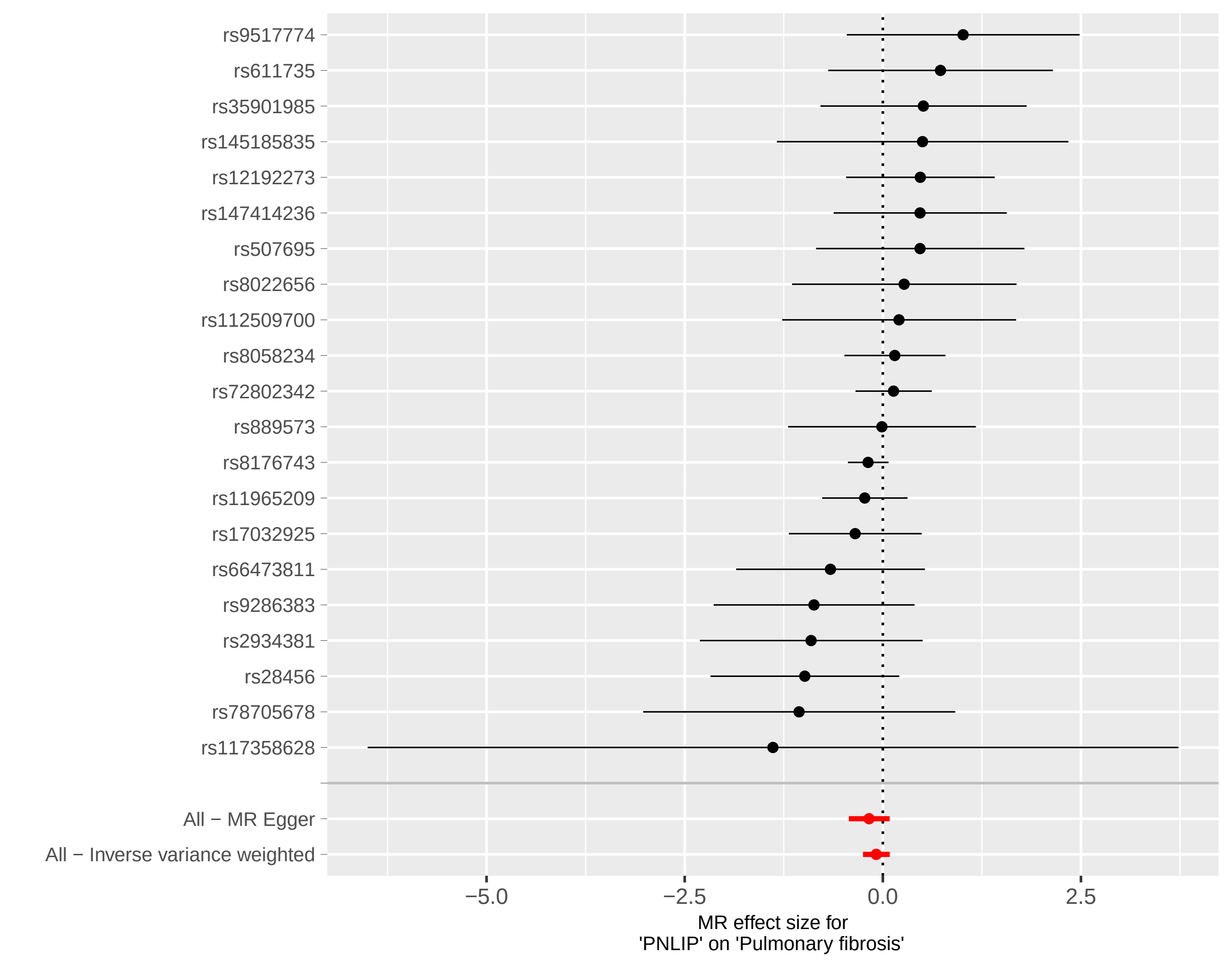

rs9517774
rs611735
rs35901985
rs145185835
rs12192273
rs147414236
rs507695
rs8022656
rs112509700
rs8058234
rs72802342
rs889573
rs8176743
rs11965209
rs17032925
rs66473811
rs9286383
rs2934381
rs28456
rs78705678
rs117358628
All − MR Egger
All − Inverse variance weighted
−5.0
−2.5
0.0
2.5
MR effect size for
'PNLIP' on 'Pulmonary fibrosis'

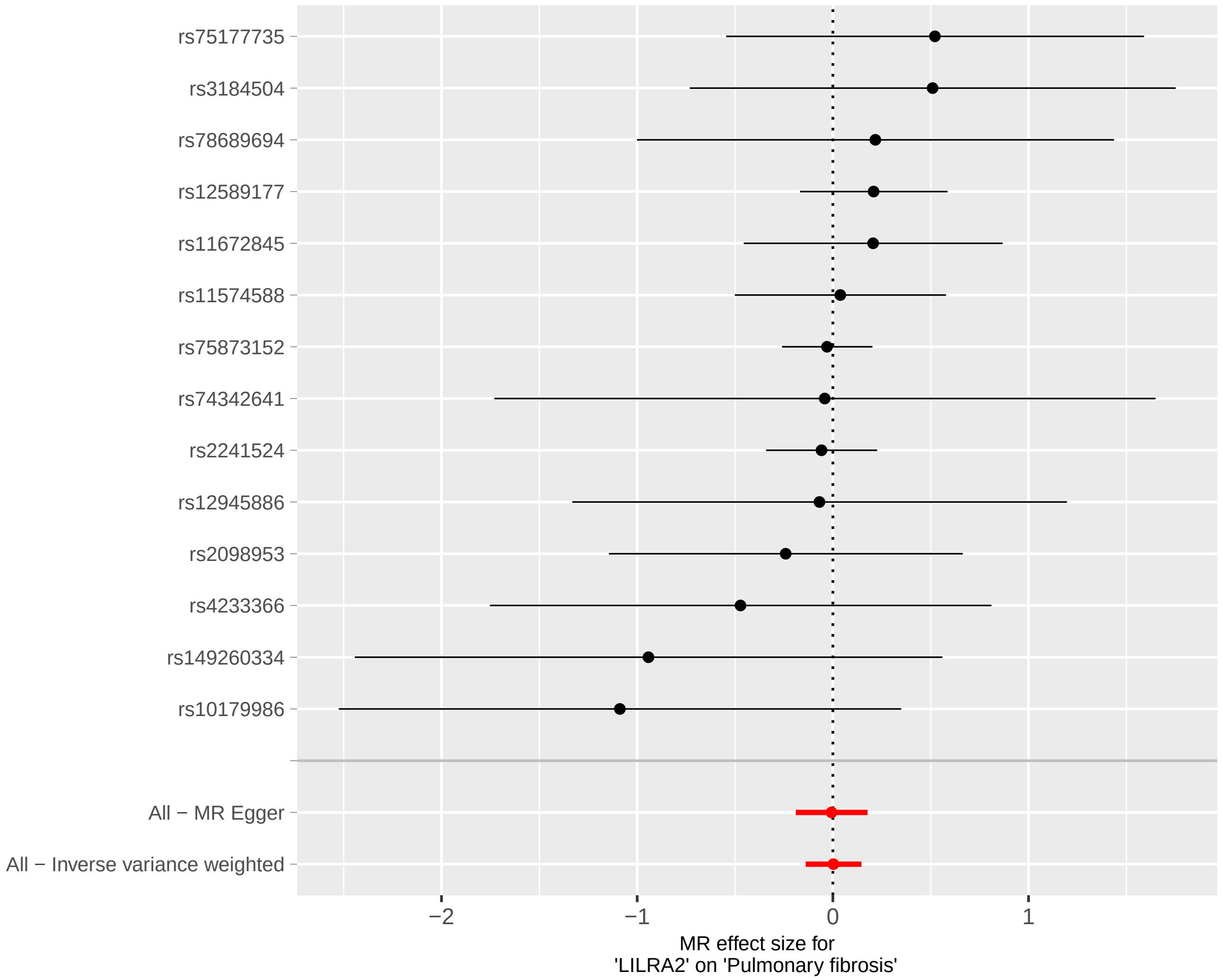

rs75177735
rs3184504
rs78689694
rs12589177
rs11672845
rs11574588
rs75873152
rs74342641
rs2241524
rs12945886
rs2098953
rs4233366
rs149260334
rs10179986
All − MR Egger
All − Inverse variance weighted
−2
−1
0
1
MR effect size for
'LILRA2' on 'Pulmonary fibrosis'

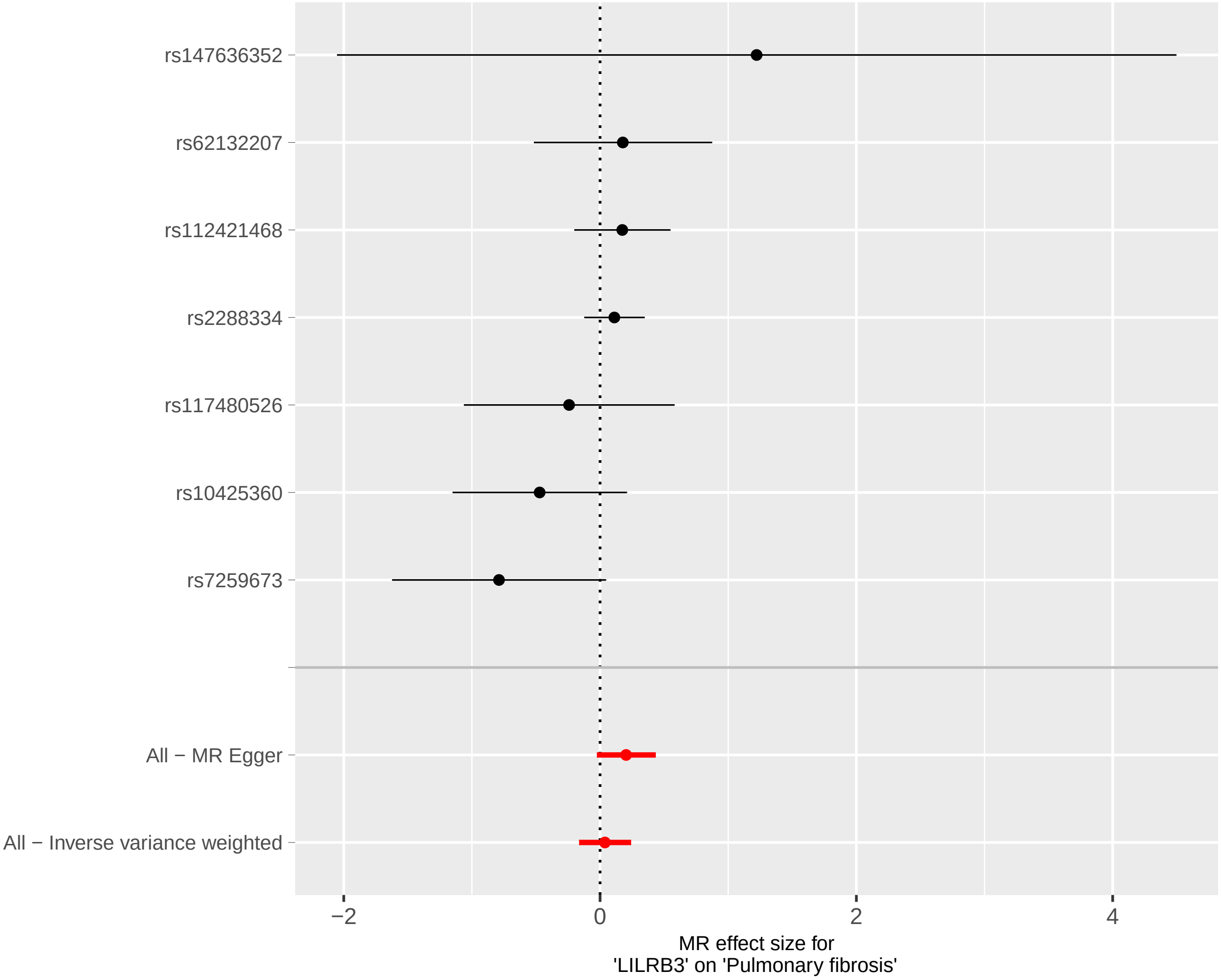

rs147636352
rs62132207
rs112421468
rs2288334
rs117480526
rs10425360
rs7259673
All − MR Egger
All − Inverse variance weighted
−2
0
2
4
MR effect size for
'LILRB3' on 'Pulmonary fibrosis'

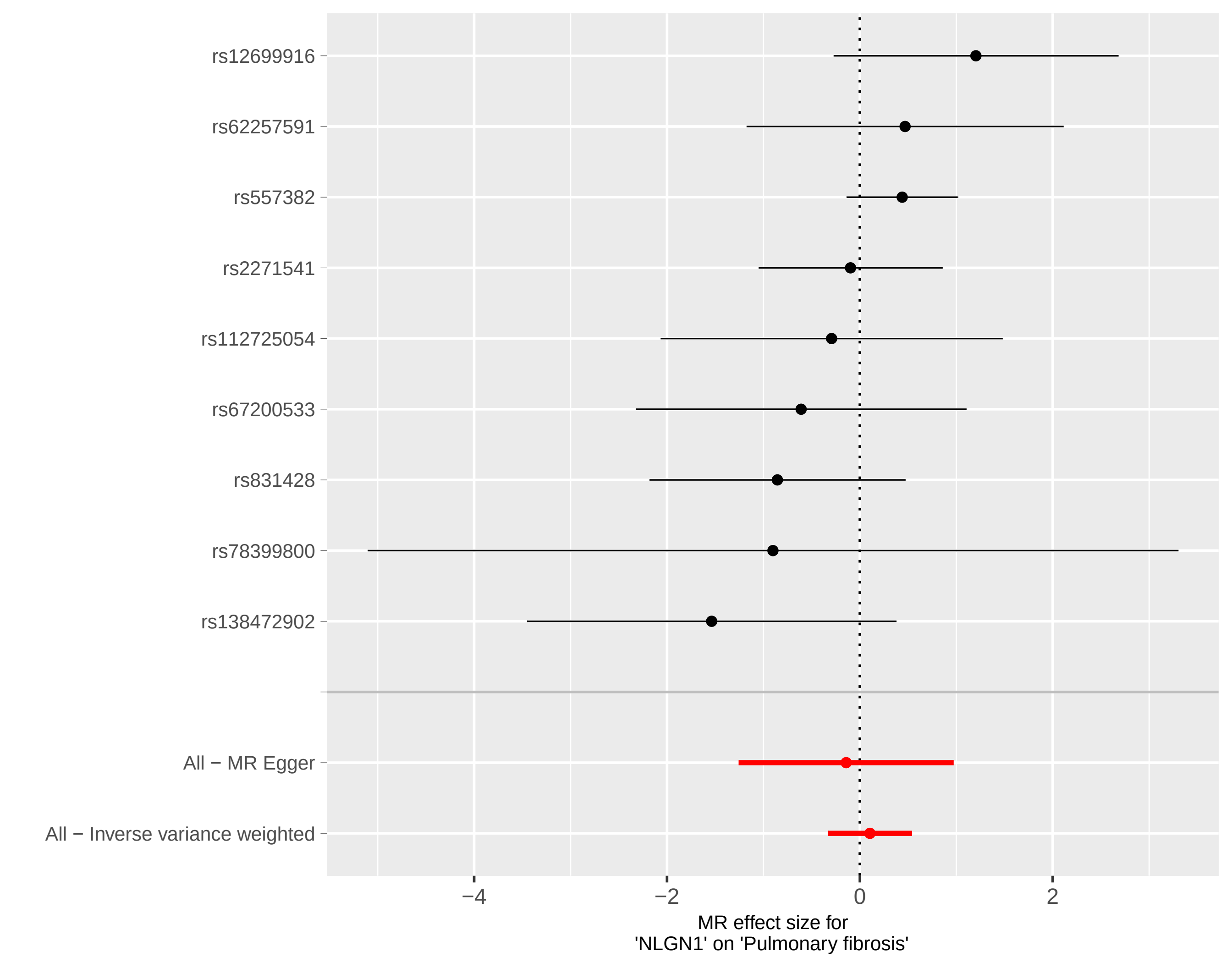

rs12699916
rs62257591
rs557382
rs2271541
rs112725054
rs67200533
rs831428
rs78399800
rs138472902
All − MR Egger
All − Inverse variance weighted
−4
−2
0
2
MR effect size for
'NLGN1' on 'Pulmonary fibrosis'

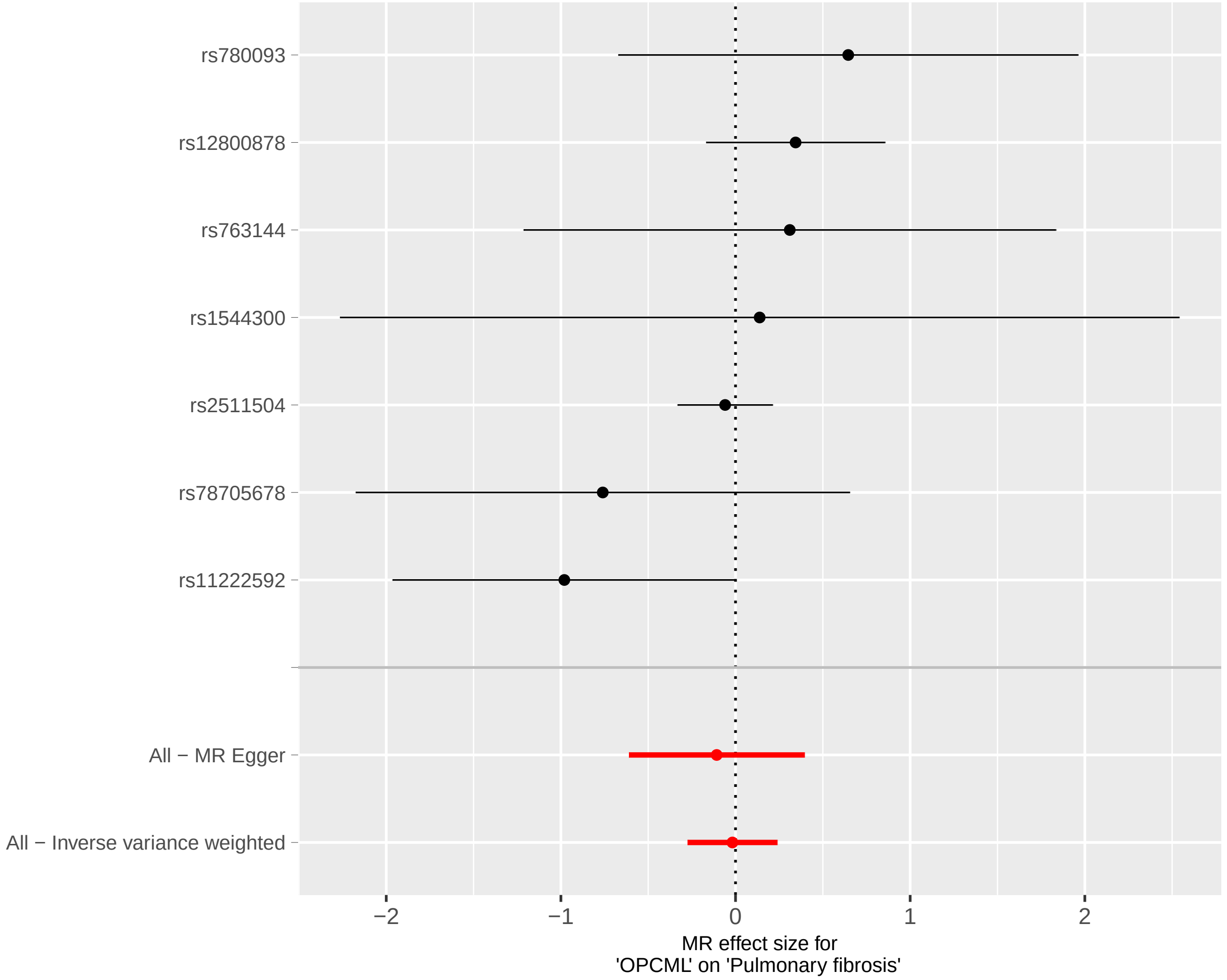

rs780093
rs12800878
rs763144
rs1544300
rs2511504
rs78705678
rs11222592
All − MR Egger
All − Inverse variance weighted
−2
−1
0
1
2
MR effect size for
'OPCML' on 'Pulmonary fibrosis'

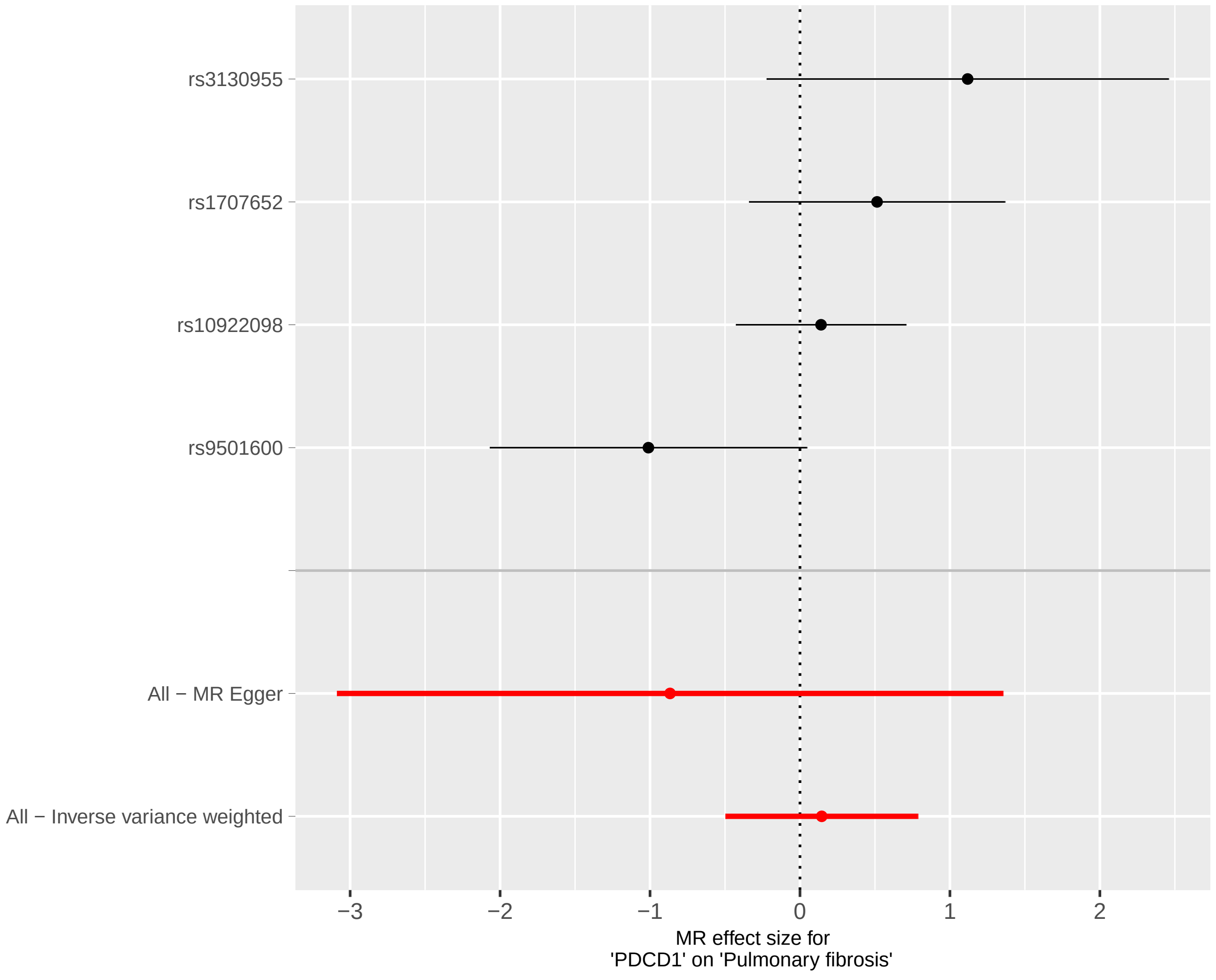
rs3130955
rs1707652
rs10922098
rs9501600
All − MR Egger
All − Inverse variance weighted
−3
−2
−1
0
1
2
MR effect size for
'PDCD1' on 'Pulmonary fibrosis'

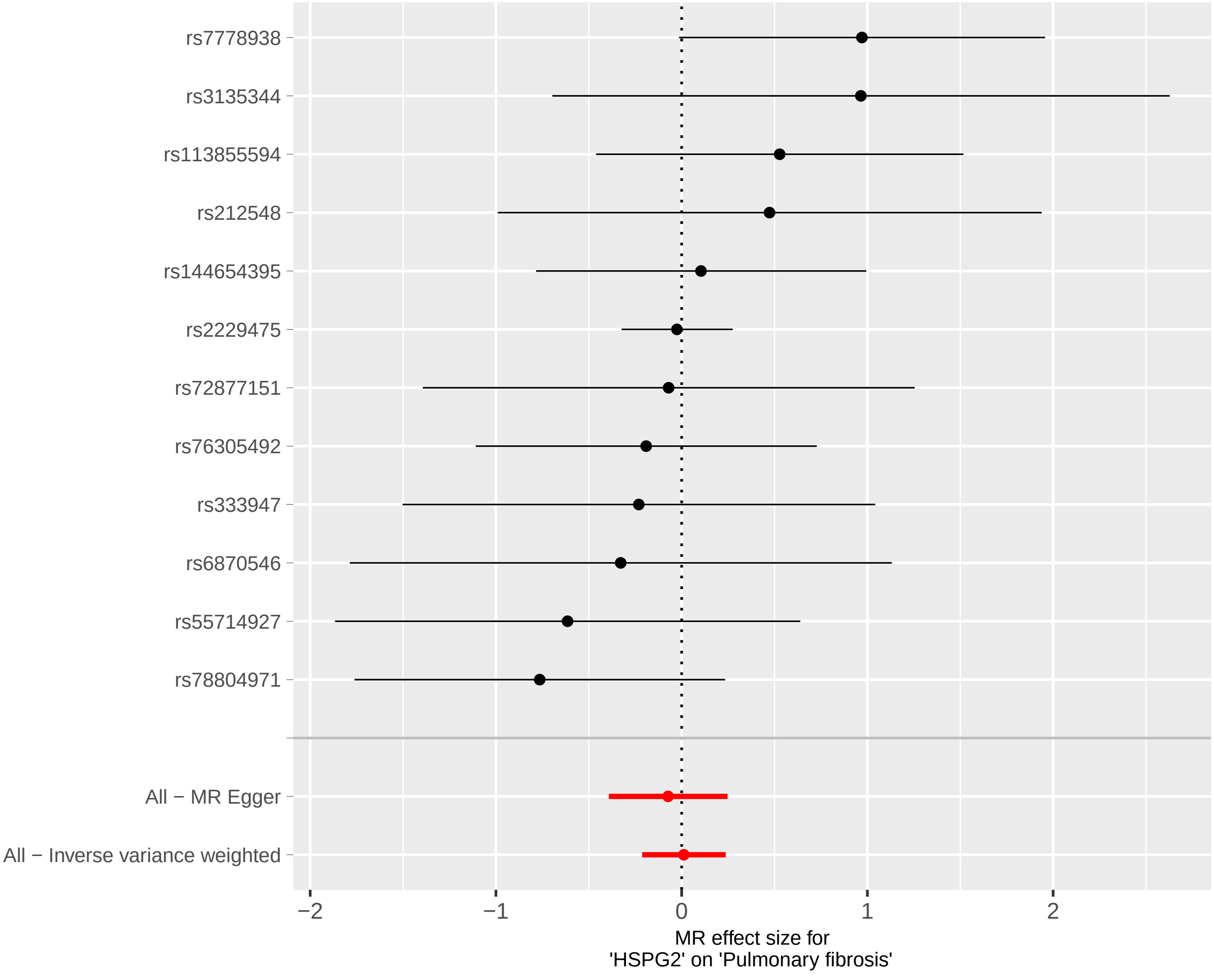
rs7778938
rs3135344
rs113855594
rs212548
rs144654395
rs2229475
rs72877151
rs76305492
rs333947
rs6870546
rs55714927
rs78804971
All − MR Egger
All − Inverse variance weighted
−2
−1
0
1
2
MR effect size for
'HSPG2' on 'Pulmonary fibrosis'

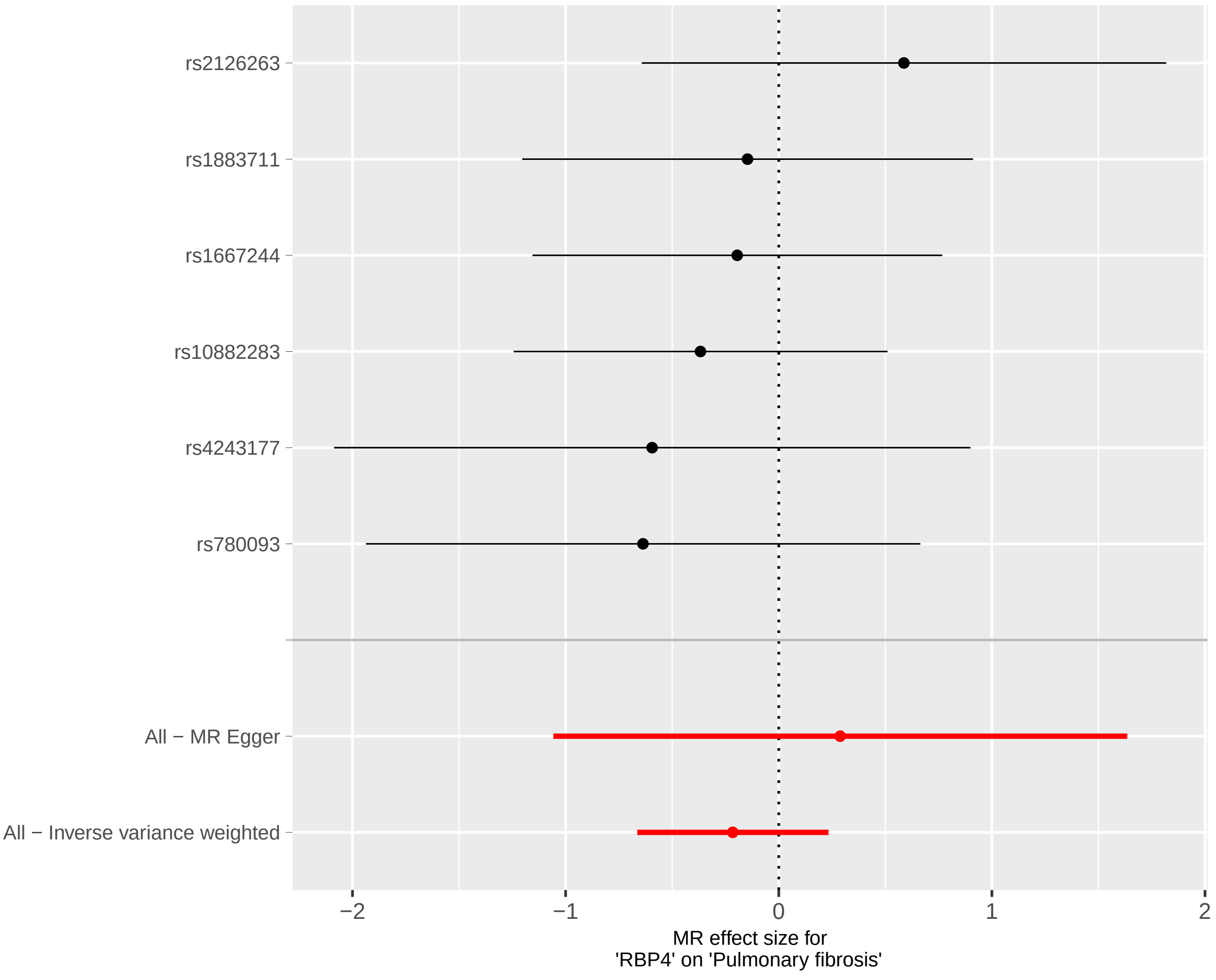

rs2126263
rs1883711
rs1667244
rs10882283
rs4243177
rs780093
All − MR Egger
All − Inverse variance weighted
−2
−1
0
1
2
MR effect size for
'RBP4' on 'Pulmonary fibrosis'

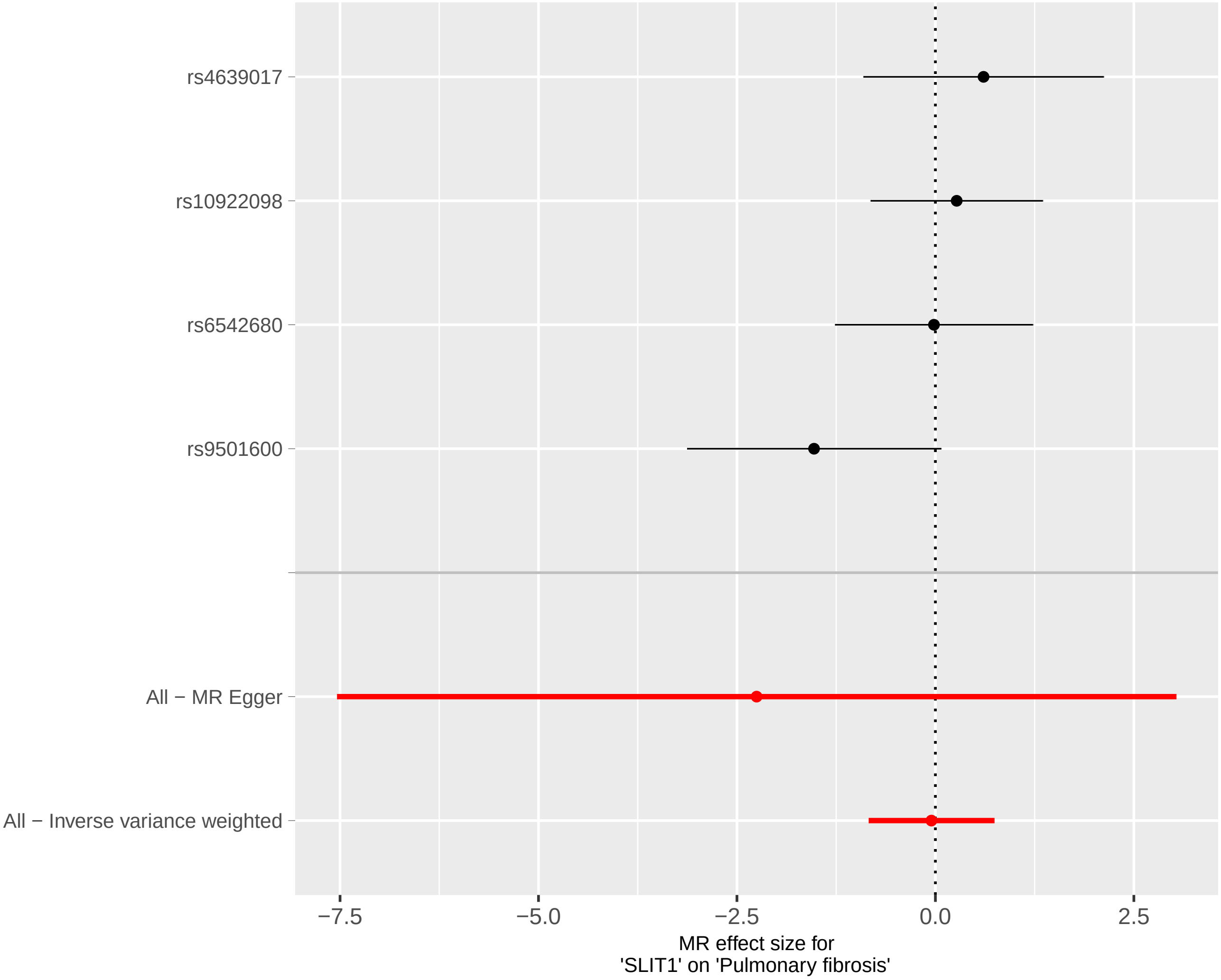
rs4639017
rs10922098
rs6542680
rs9501600
All − MR Egger
All − Inverse variance weighted
−7.5
−5.0
−2.5
0.0
2.5
MR effect size for
'SLIT1' on 'Pulmonary fibrosis'

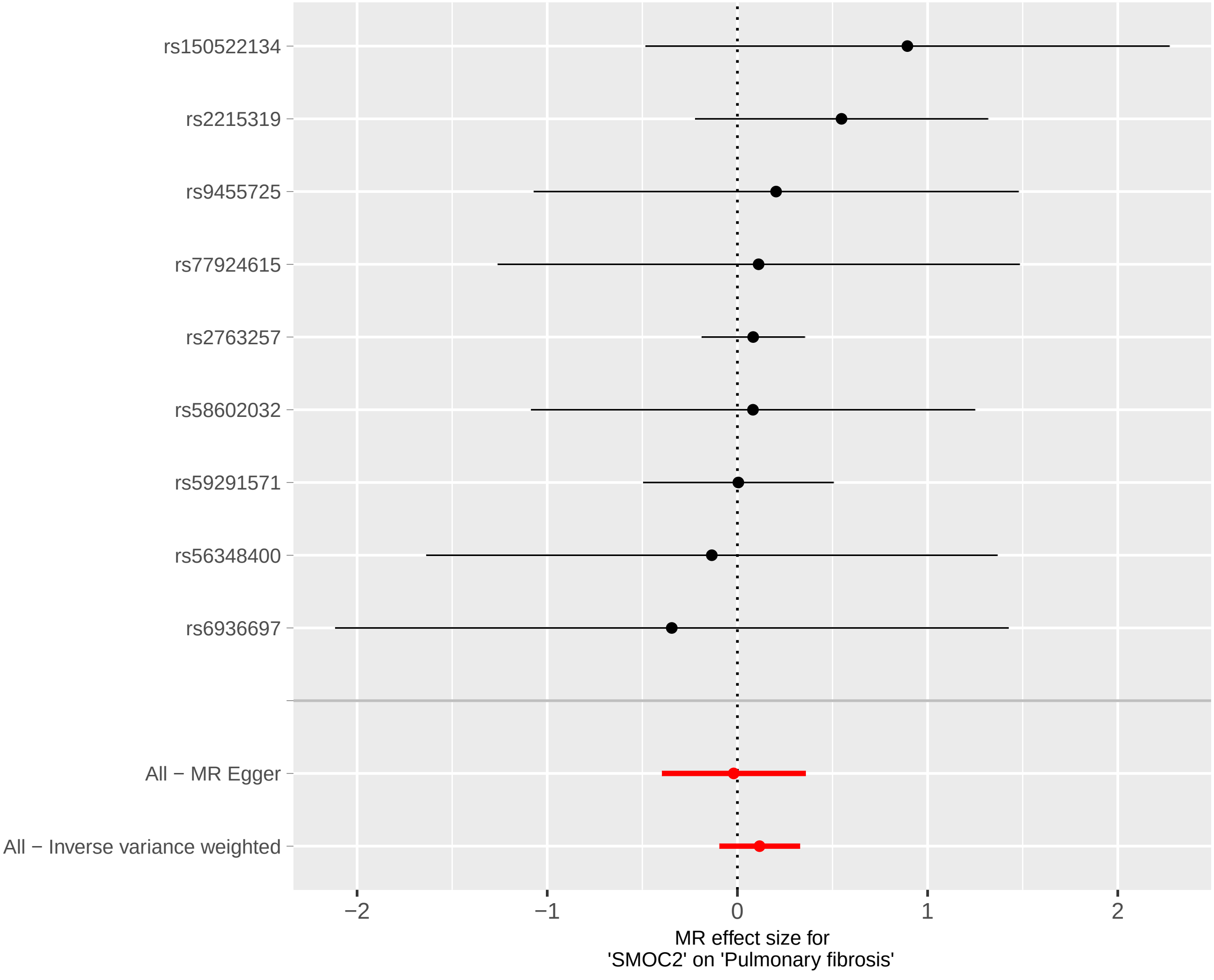

rs150522134
rs2215319
rs9455725
rs77924615
rs2763257
rs58602032
rs59291571
rs56348400
rs6936697
All − MR Egger
All − Inverse variance weighted
−2
−1
0
1
2
MR effect size for
'SMOC2' on 'Pulmonary fibrosis'

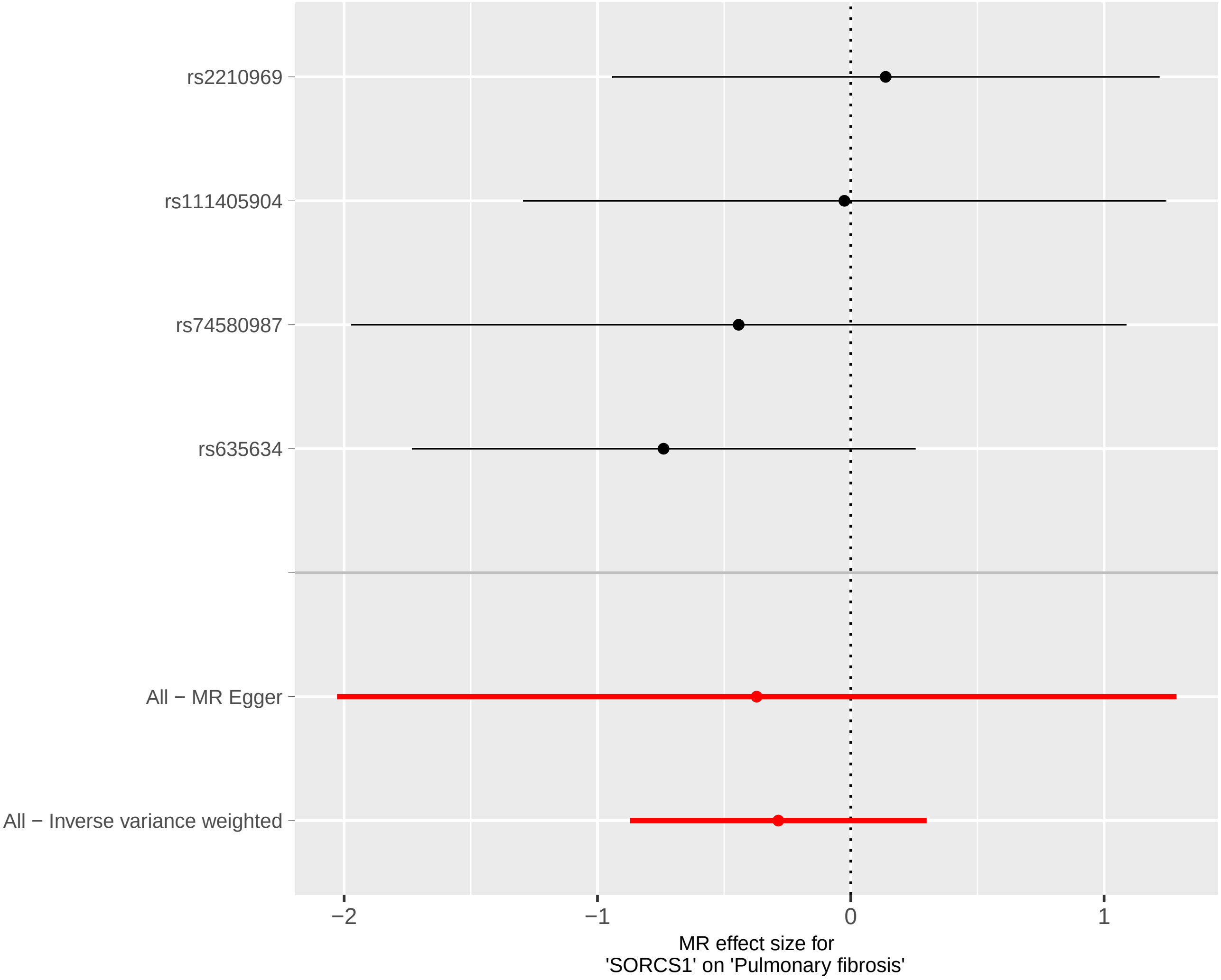
rs2210969
rs111405904
rs74580987
rs635634
All − MR Egger
All − Inverse variance weighted
−2
−1
0
1
MR effect size for
'SORCS1' on 'Pulmonary fibrosis'

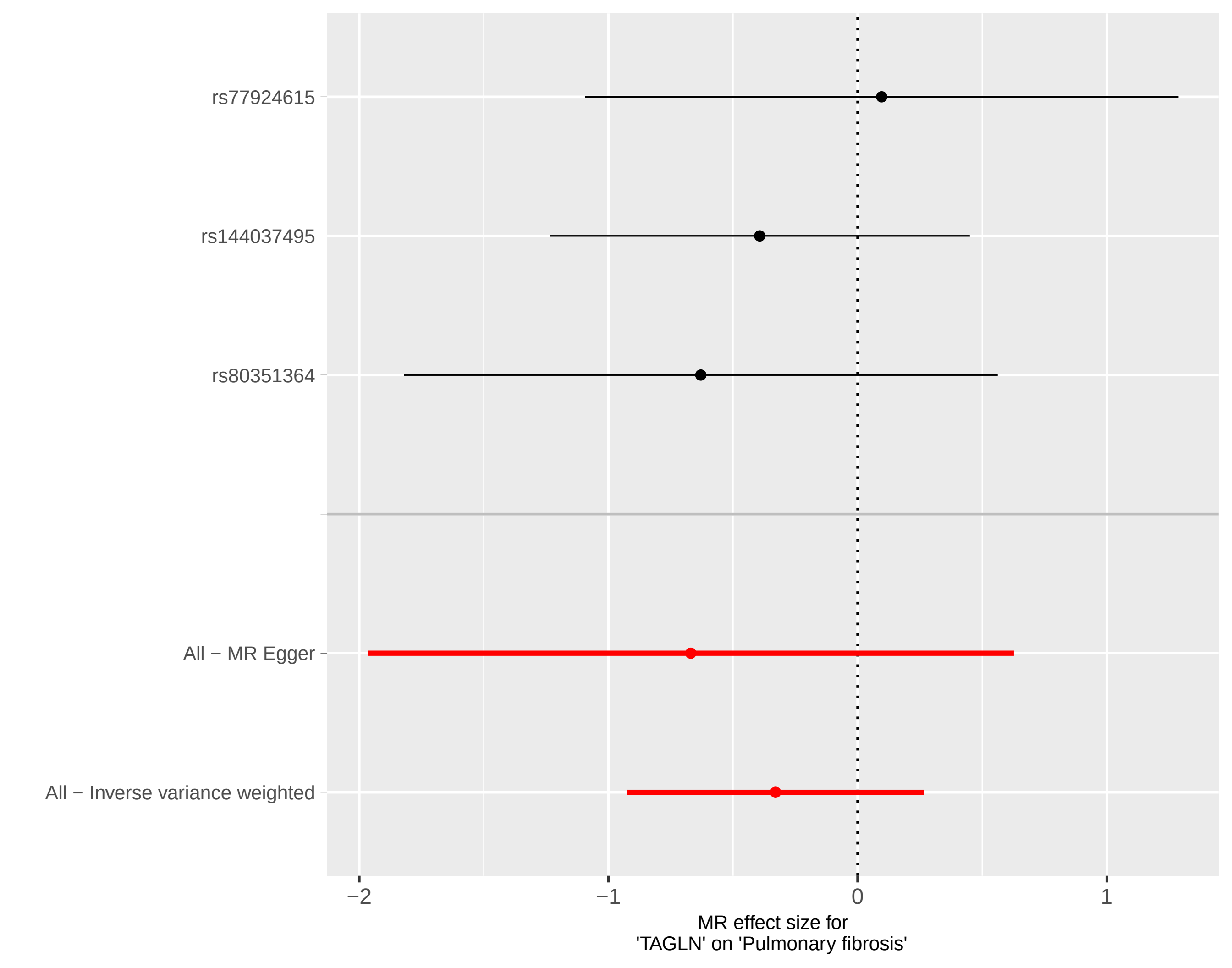
rs77924615
rs144037495
rs80351364
All − MR Egger
All − Inverse variance weighted
−2
−1
0
1
MR effect size for
'TAGLN' on 'Pulmonary fibrosis'

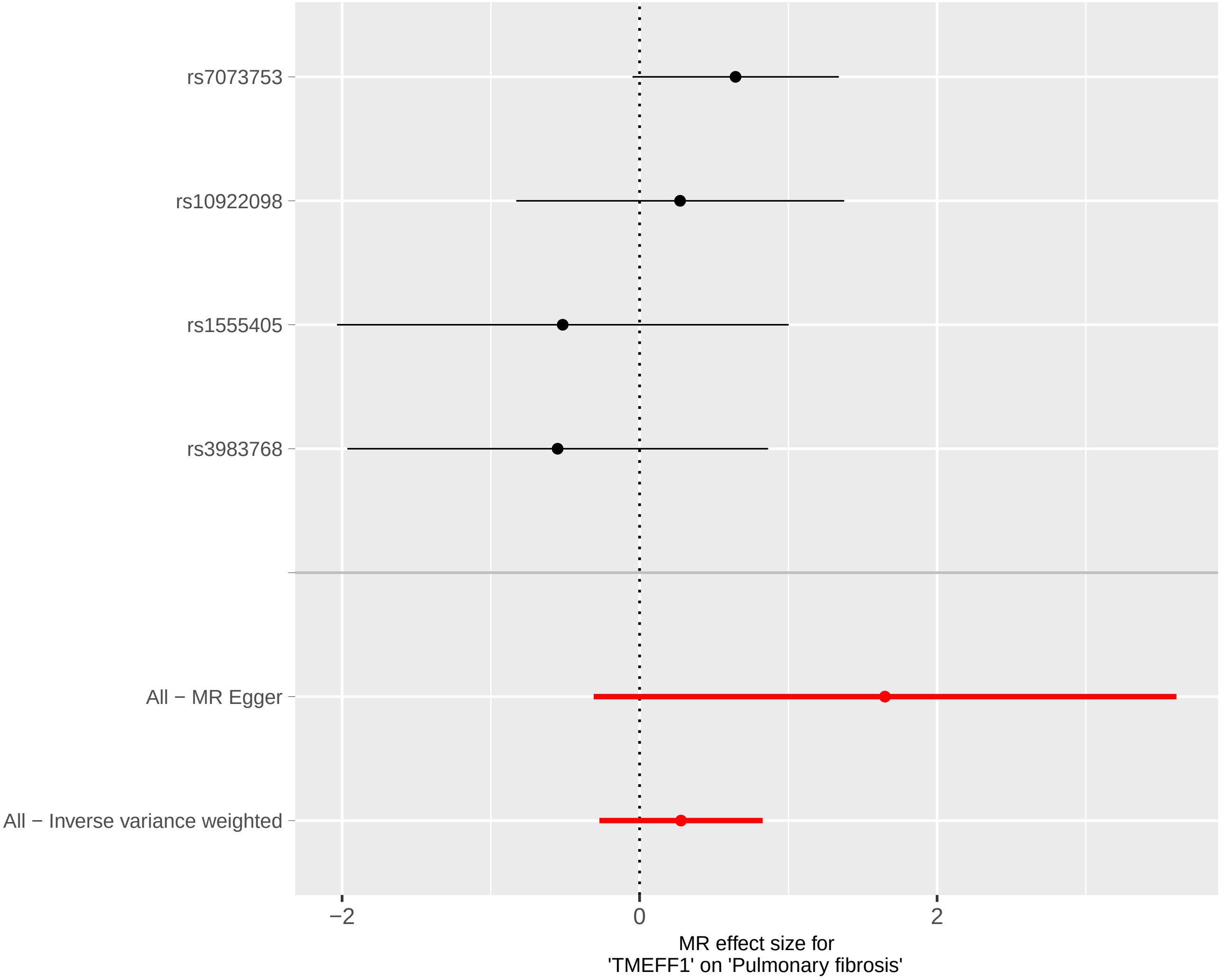
rs7073753
rs10922098
rs1555405
rs3983768
All − MR Egger
All − Inverse variance weighted
−2
0
2
MR effect size for
'TMEFF1' on 'Pulmonary fibrosis'

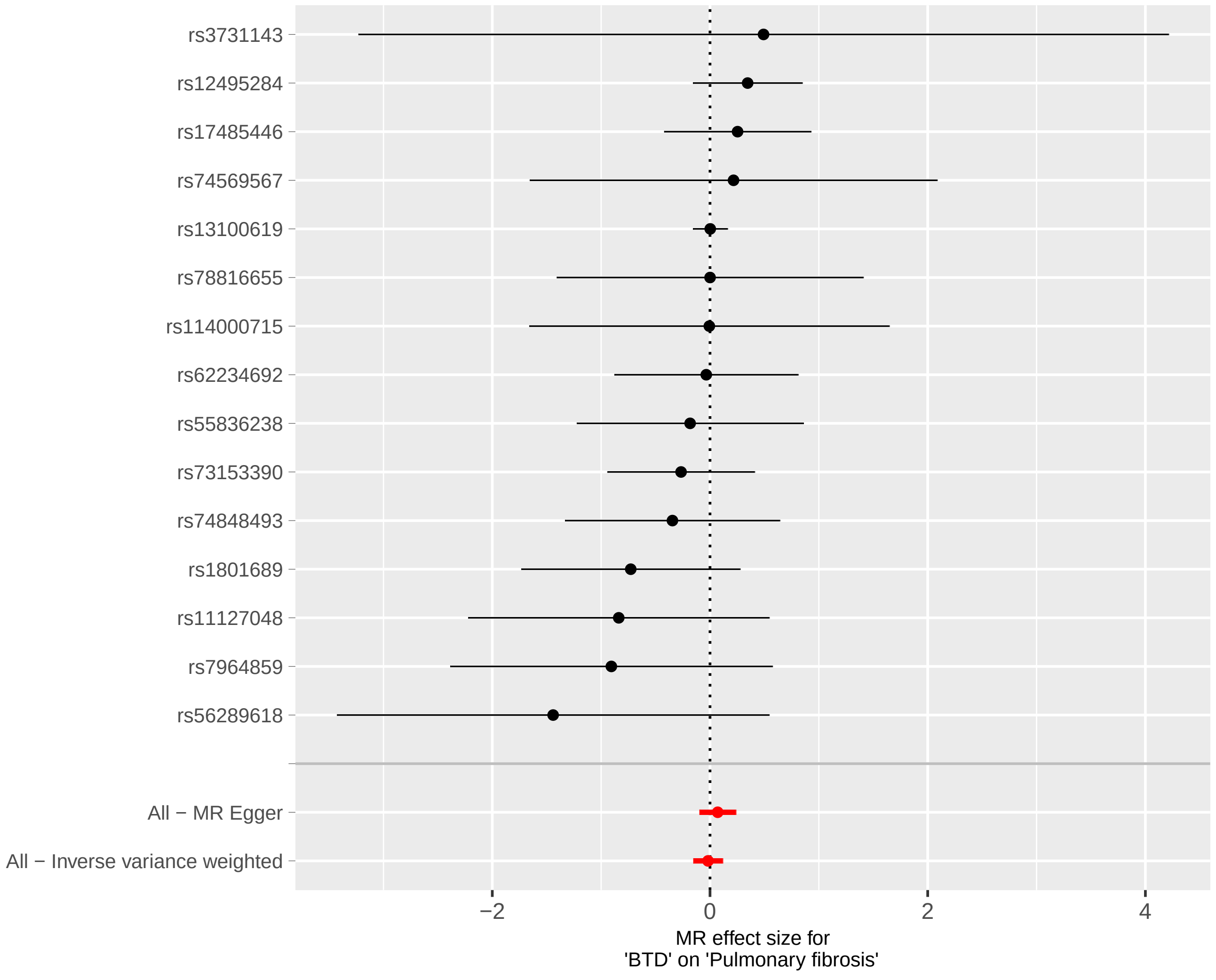
rs3731143
rs12495284
rs17485446
rs74569567
rs13100619
rs78816655
rs114000715
rs62234692
rs55836238
rs73153390
rs74848493
rs1801689
rs11127048
rs7964859
rs56289618
All − MR Egger
All − Inverse variance weighted
−2
0
2
4
MR effect size for
'BTD' on 'Pulmonary fibrosis'

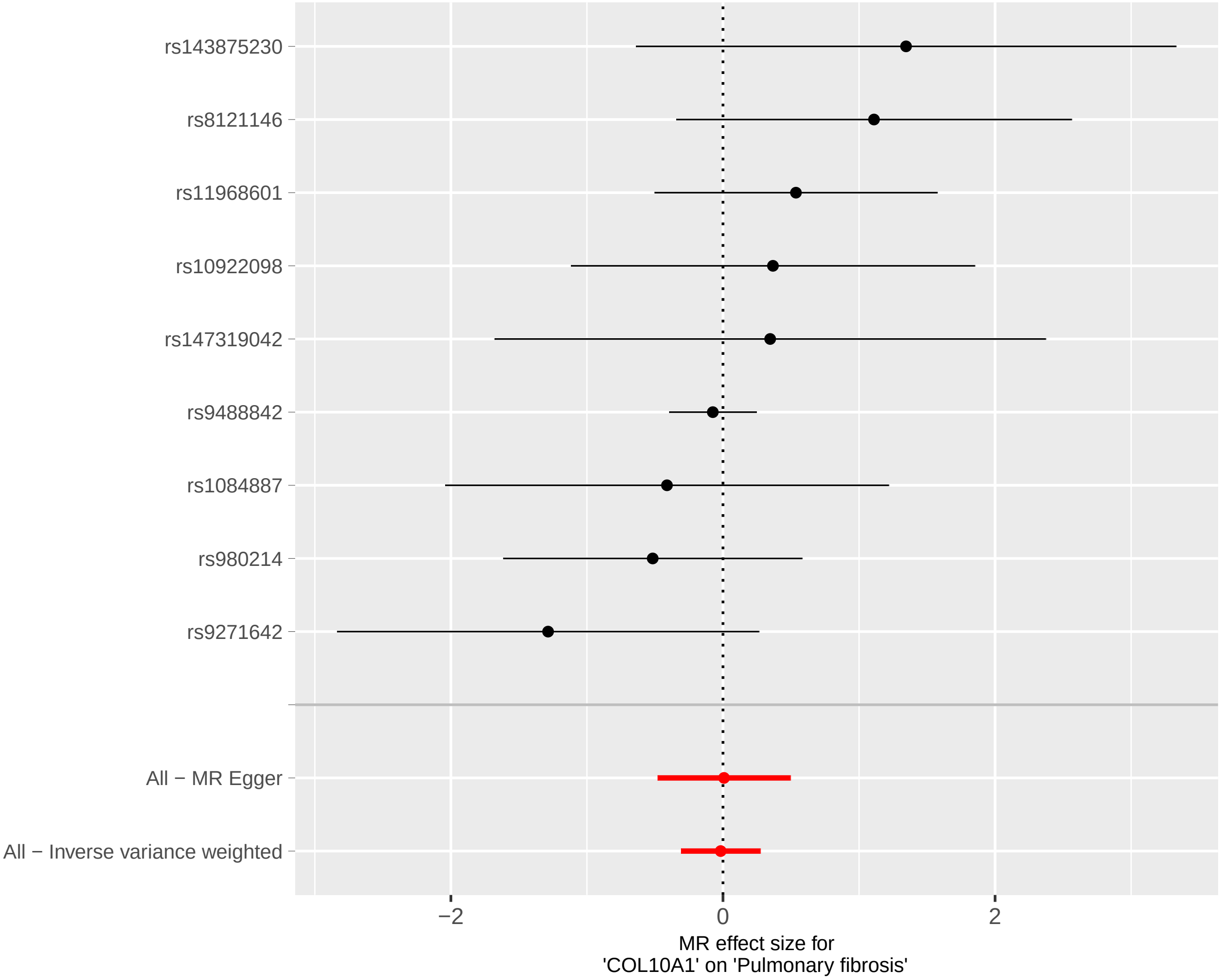

rs143875230
rs8121146
rs11968601
rs10922098
rs147319042
rs9488842
rs1084887
rs980214
rs9271642
All − MR Egger
All − Inverse variance weighted
−2
0
2
MR effect size for
'COL10A1' on 'Pulmonary fibrosis'

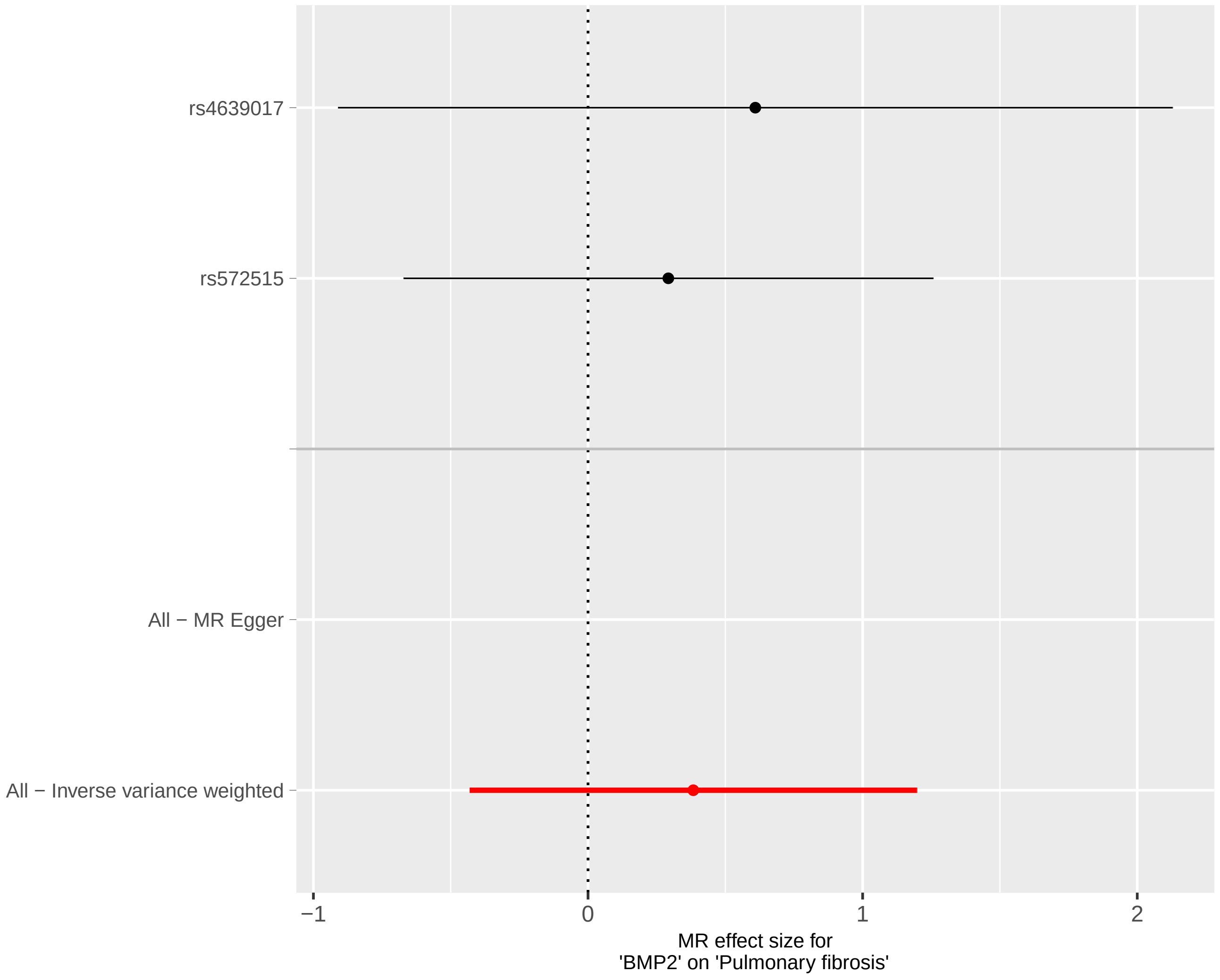

rs4639017
rs572515
All − MR Egger
All − Inverse variance weighted
−1
0
1
2
MR effect size for
'BMP2' on 'Pulmonary fibrosis'

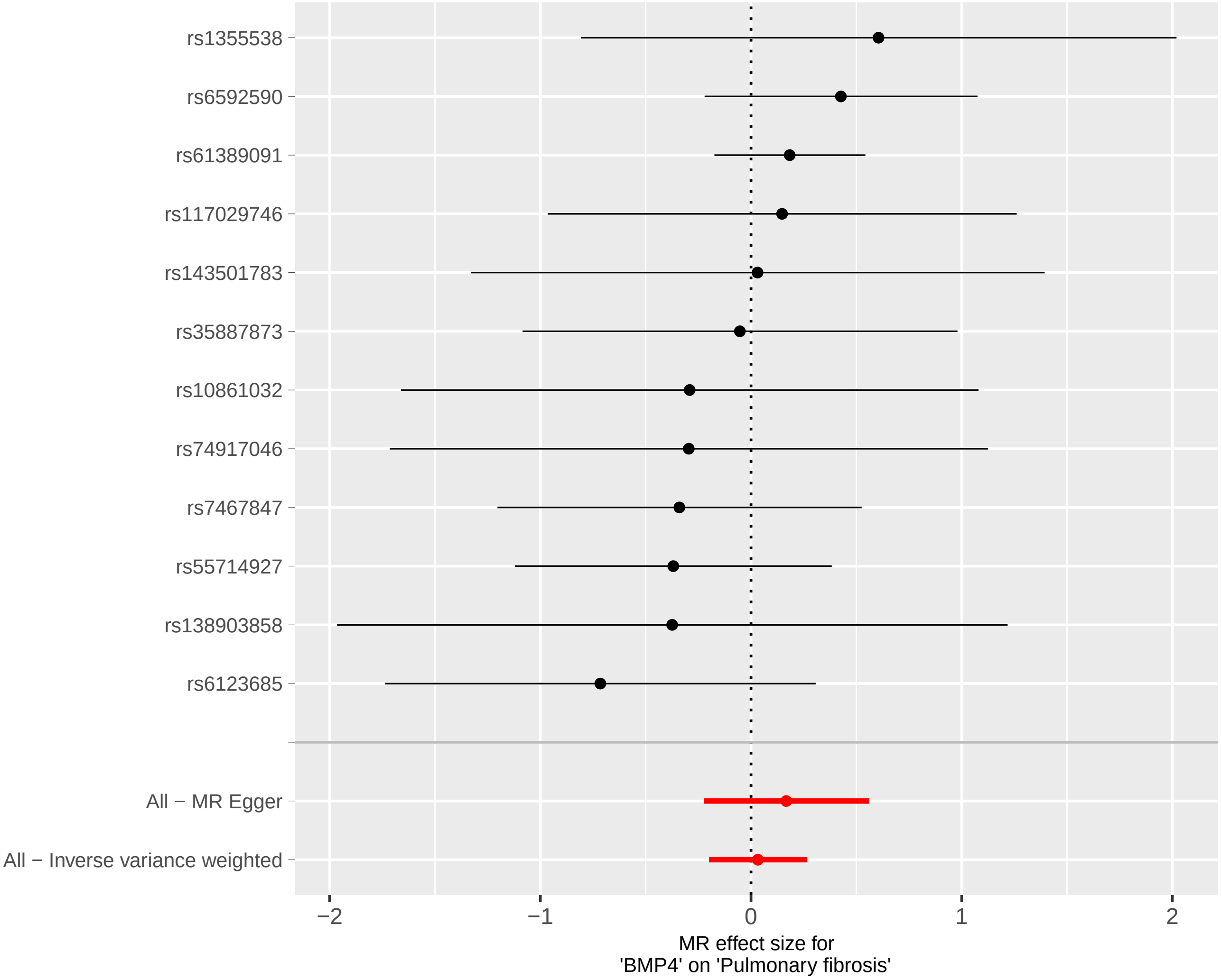
rs1355538
rs6592590
rs61389091
rs117029746
rs143501783
rs35887873
rs10861032
rs74917046
rs7467847
rs55714927
rs138903858
rs6123685
All − MR Egger
All − Inverse variance weighted
−2
−1
0
1
2
MR effect size for
'BMP4' on 'Pulmonary fibrosis'

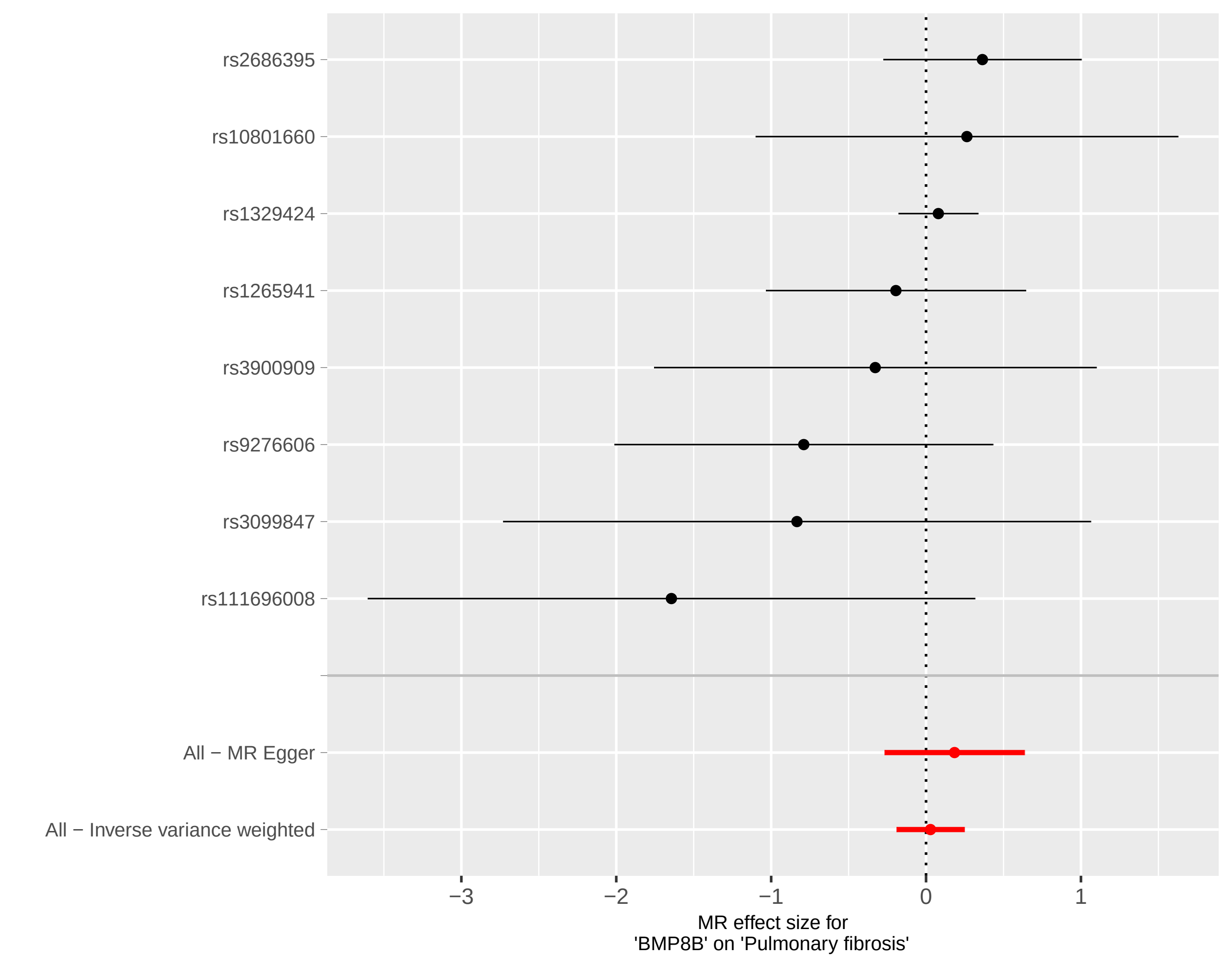
rs2686395
rs10801660
rs1329424
rs1265941
rs3900909
rs9276606
rs3099847
rs111696008
All − MR Egger
All − Inverse variance weighted
−3
−2
−1
0
1
MR effect size for
'BMP8B' on 'Pulmonary fibrosis'

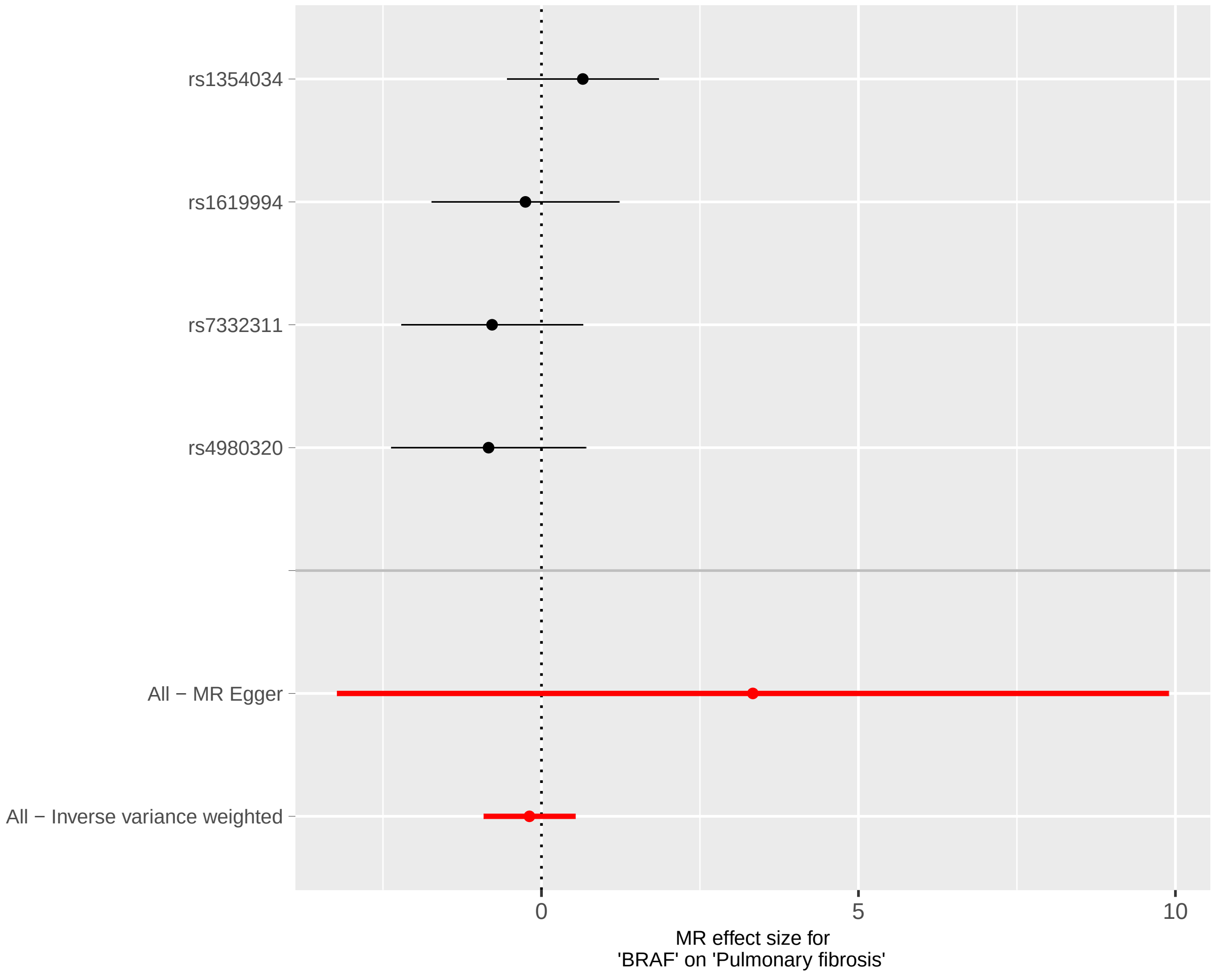

rs1354034
rs1619994
rs7332311
rs4980320
All − MR Egger
All − Inverse variance weighted
0
5
10
MR effect size for
'BRAF' on 'Pulmonary fibrosis'

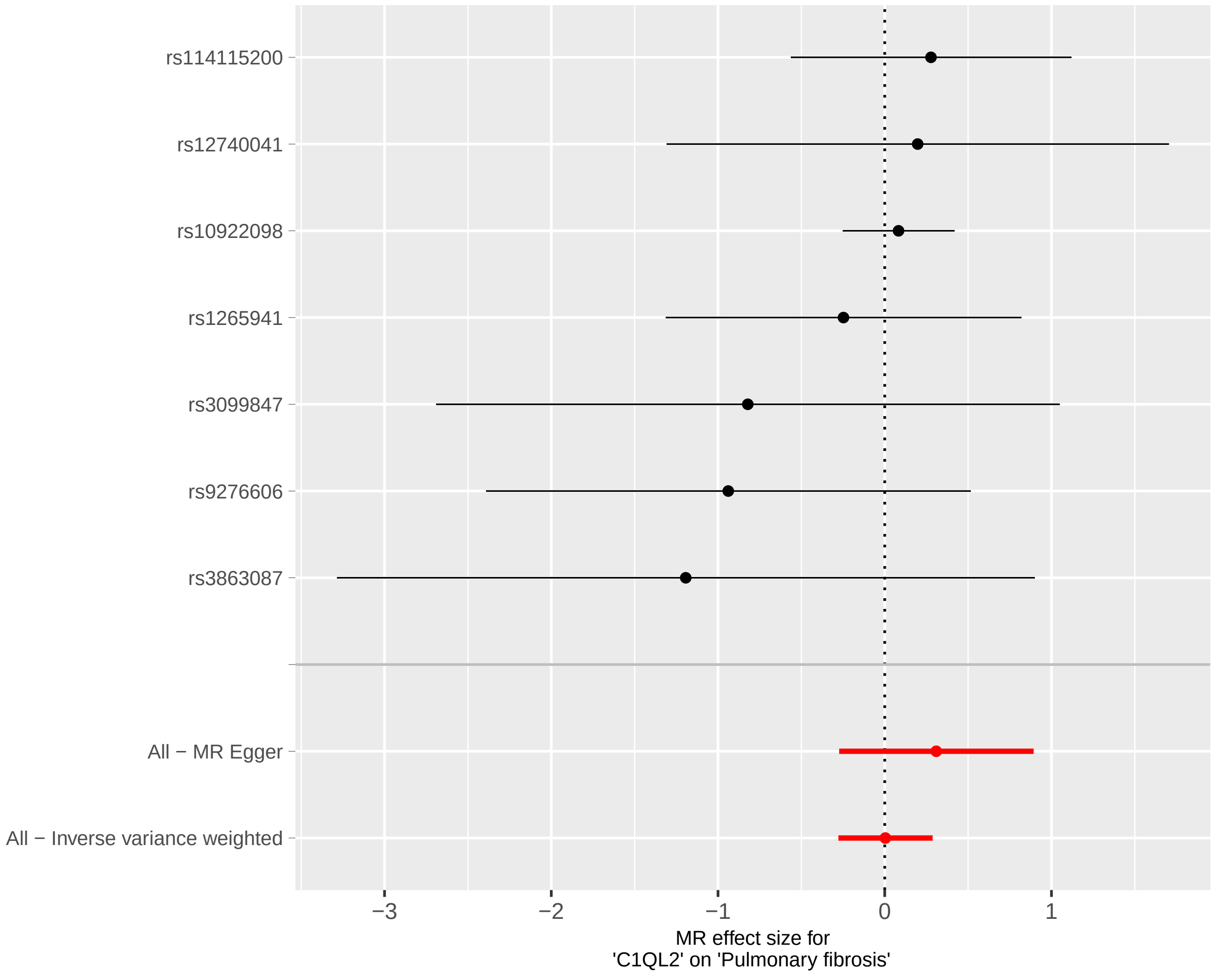

rs114115200
rs12740041
rs10922098
rs1265941
rs3099847
rs9276606
rs3863087
All − MR Egger
All − Inverse variance weighted
−3
−2
−1
0
1
MR effect size for
'C1QL2' on 'Pulmonary fibrosis'

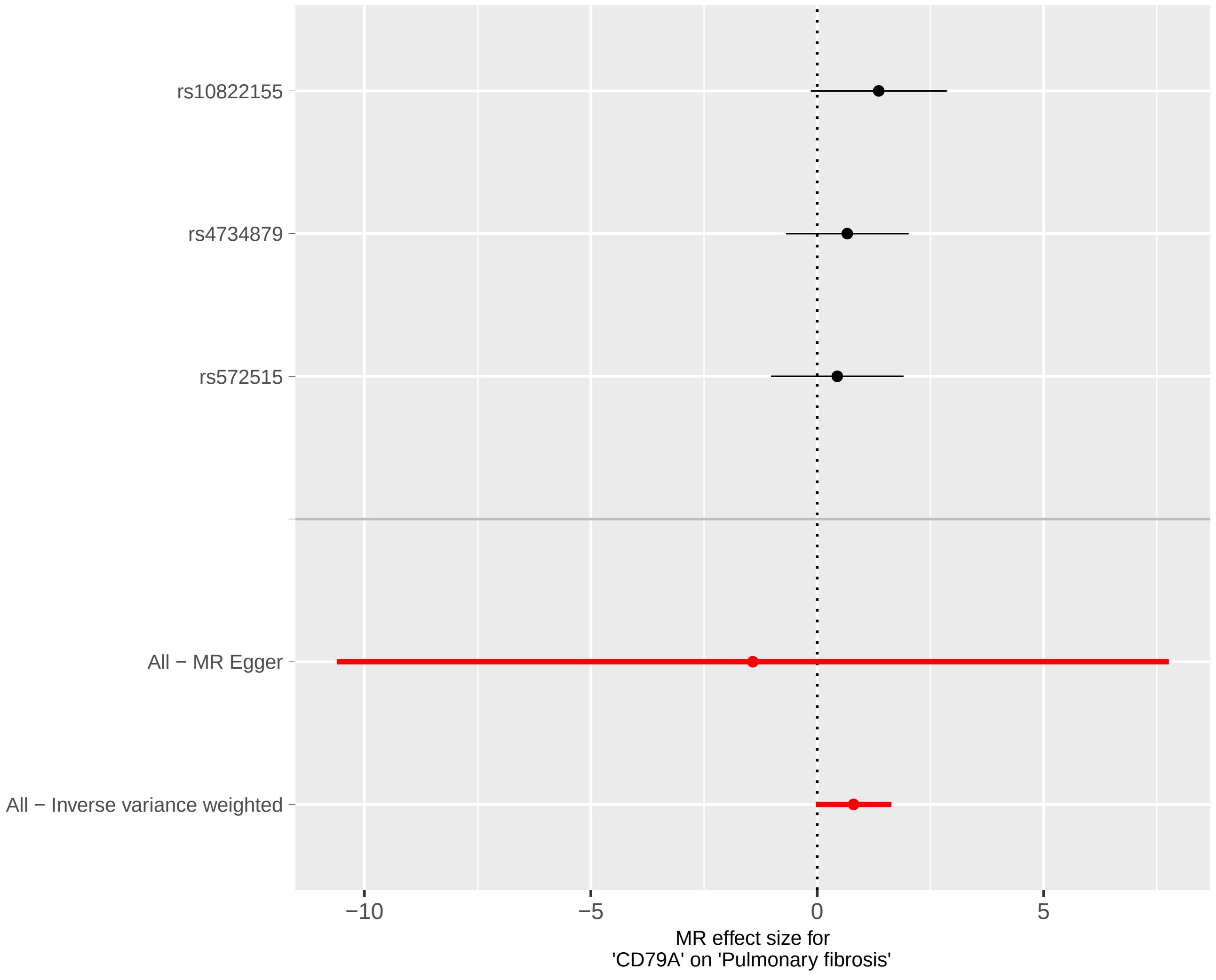

rs10822155
rs4734879
rs572515
All − MR Egger
All − Inverse variance weighted
−10
−5
0
5
MR effect size for
'CD79A' on 'Pulmonary fibrosis'

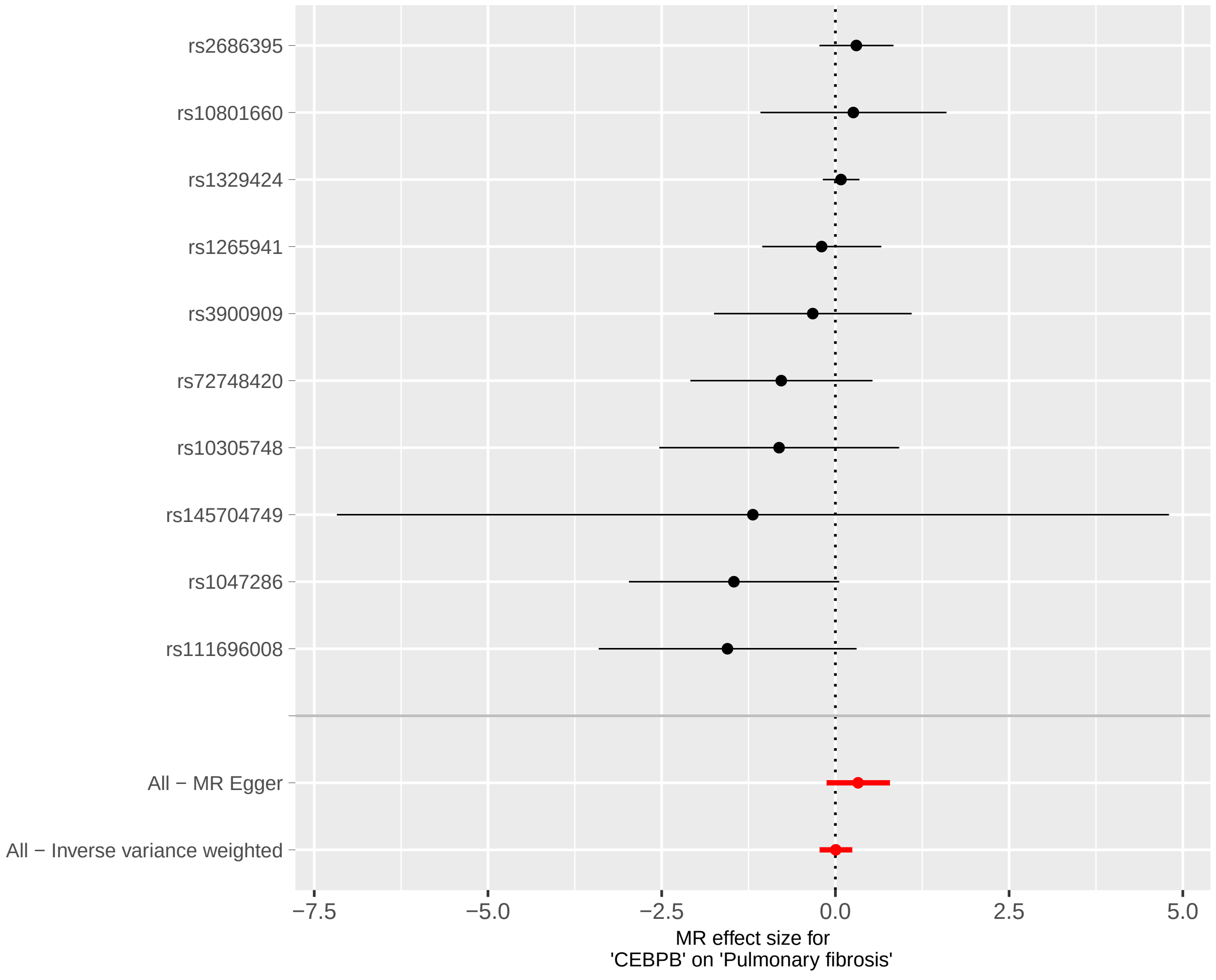
rs2686395
rs10801660
rs1329424
rs1265941
rs3900909
rs72748420
rs10305748
rs145704749
rs1047286
rs111696008
All − MR Egger
All − Inverse variance weighted
−7.5
−5.0
−2.5
0.0
2.5
5.0
MR effect size for
'CEBPB' on 'Pulmonary fibrosis'

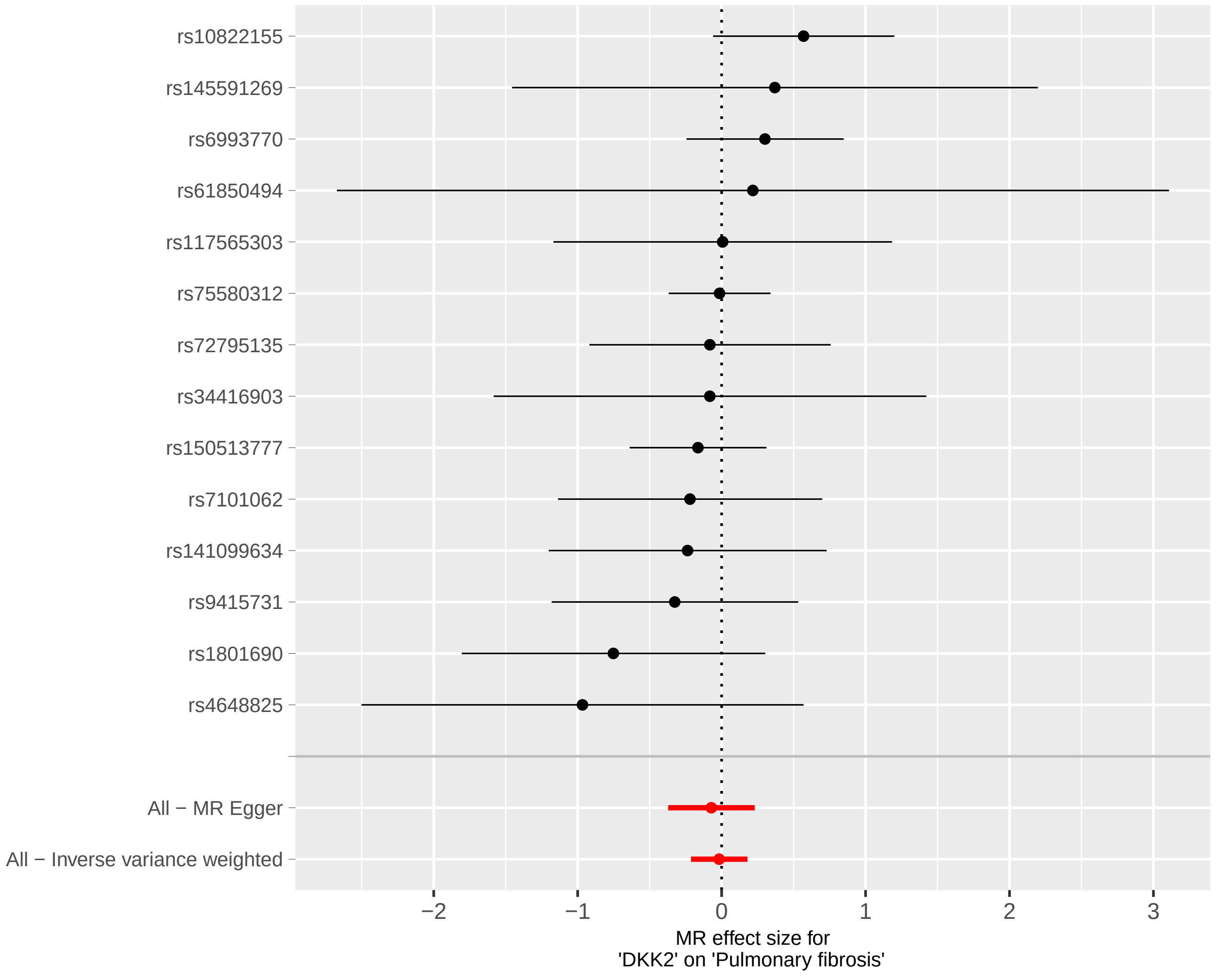

rs10822155
rs145591269
rs6993770
rs61850494
rs117565303
rs75580312
rs72795135
rs34416903
rs150513777
rs7101062
rs141099634
rs9415731
rs1801690
rs4648825
All − MR Egger
All − Inverse variance weighted
−2
−1
0
1
2
3
MR effect size for
'DKK2' on 'Pulmonary fibrosis'

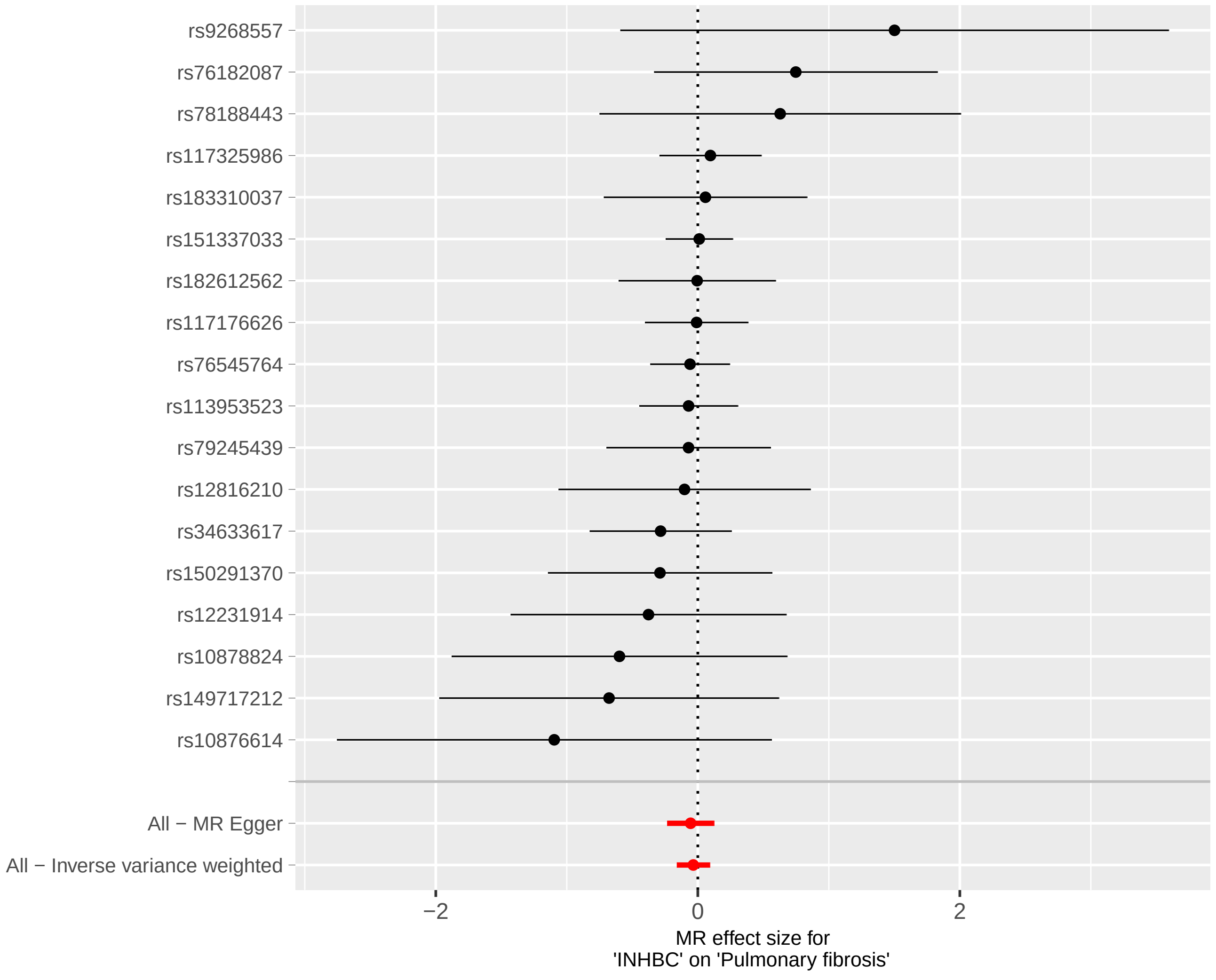

rs9268557
rs76182087
rs78188443
rs117325986
rs183310037
rs151337033
rs182612562
rs117176626
rs76545764
rs113953523
rs79245439
rs12816210
rs34633617
rs150291370
rs12231914
rs10878824
rs149717212
rs10876614
All − MR Egger
All − Inverse variance weighted
−2
0
2
MR effect size for
'INHBC' on 'Pulmonary fibrosis'

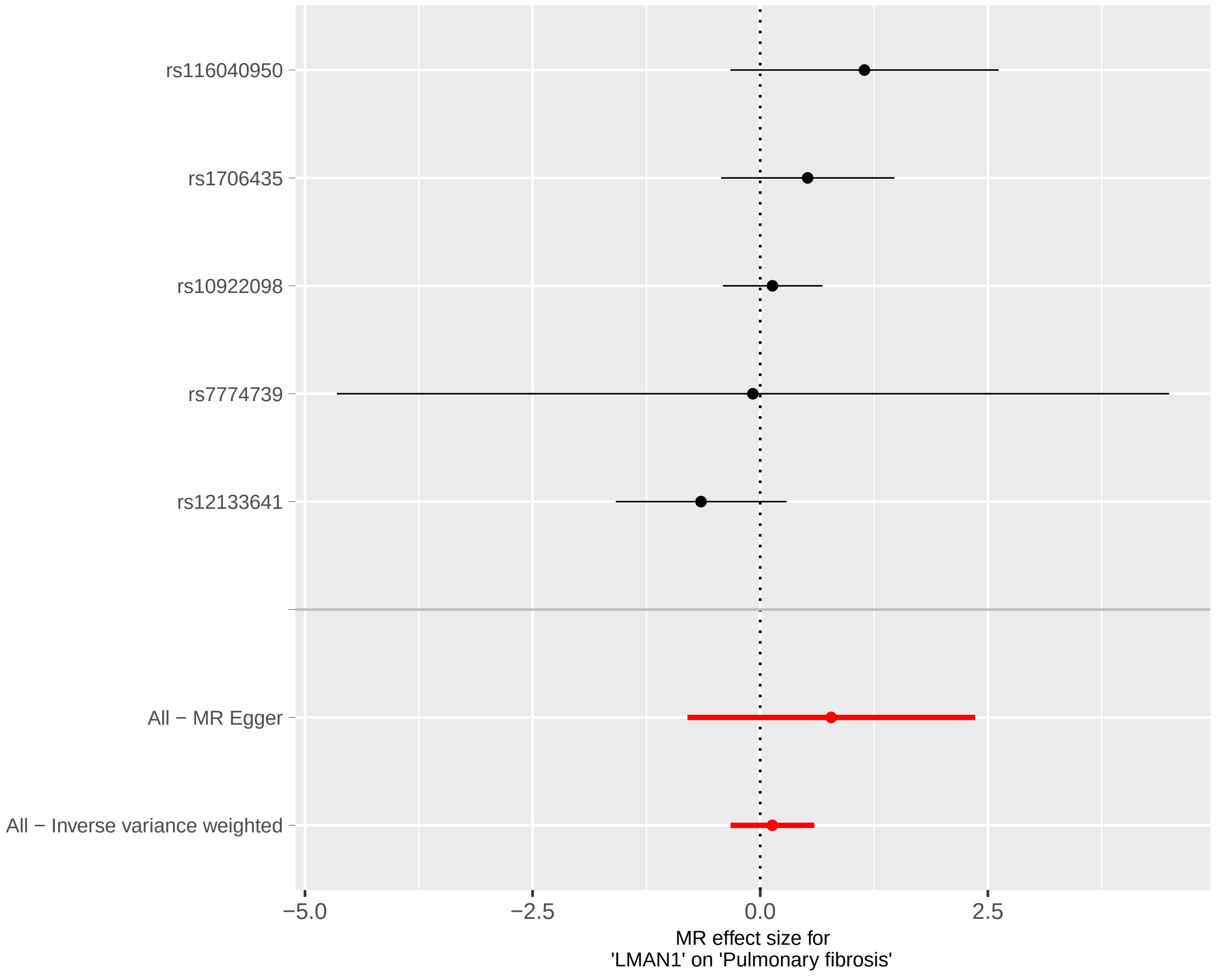
rs116040950
rs1706435
rs10922098
rs7774739
rs12133641
All − MR Egger
All − Inverse variance weighted
−5.0
−2.5
0.0
2.5
MR effect size for
'LMAN1' on 'Pulmonary fibrosis'

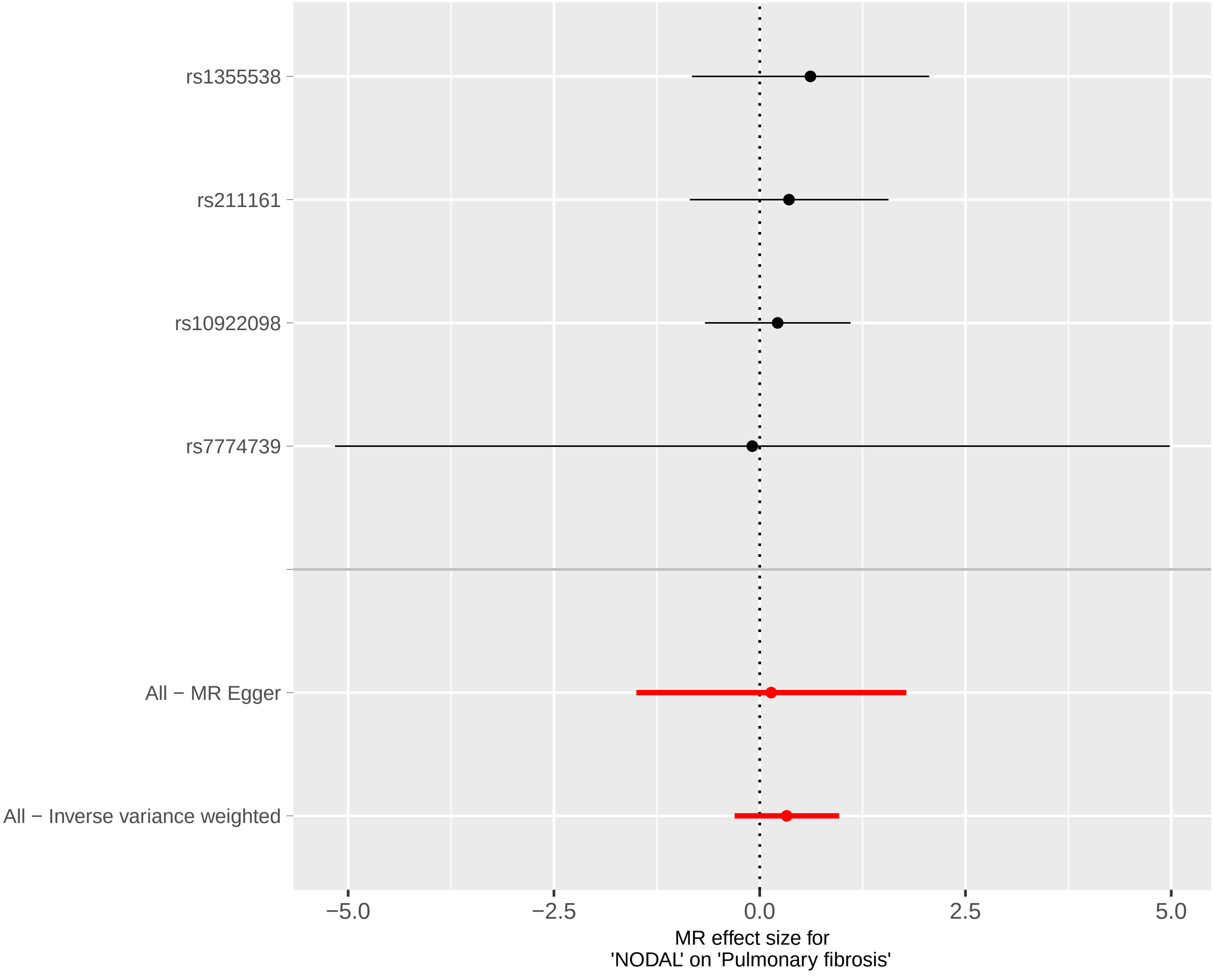

rs1355538
rs211161
rs10922098
rs7774739
All − MR Egger
All − Inverse variance weighted
−5.0
−2.5
0.0
2.5
5.0
MR effect size for
'NODAL' on 'Pulmonary fibrosis'

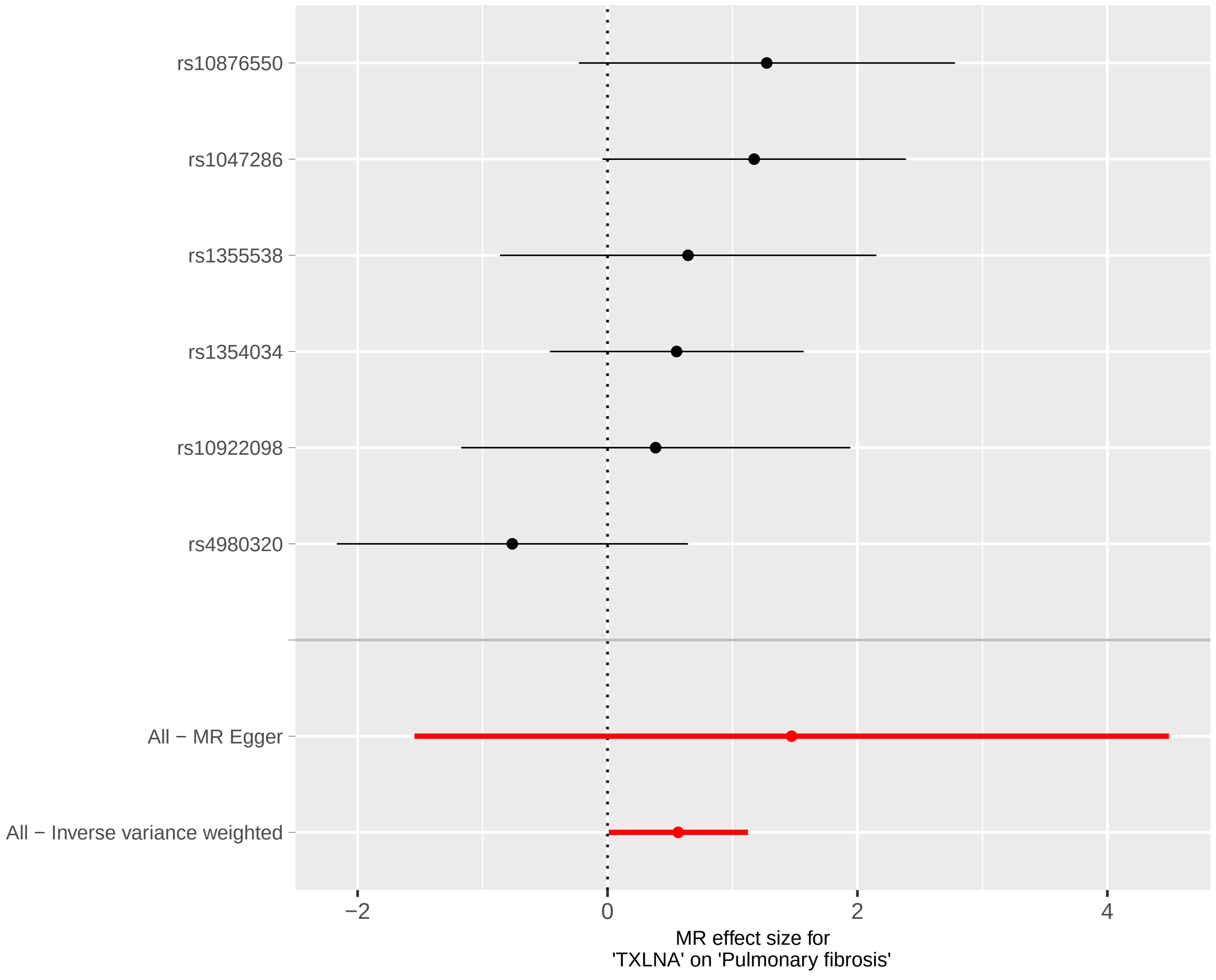
rs10876550
rs1047286
rs1355538
rs1354034
rs10922098
rs4980320
All − MR Egger
All − Inverse variance weighted
−2
0
2
4
MR effect size for
'TXLNA' on 'Pulmonary fibrosis'

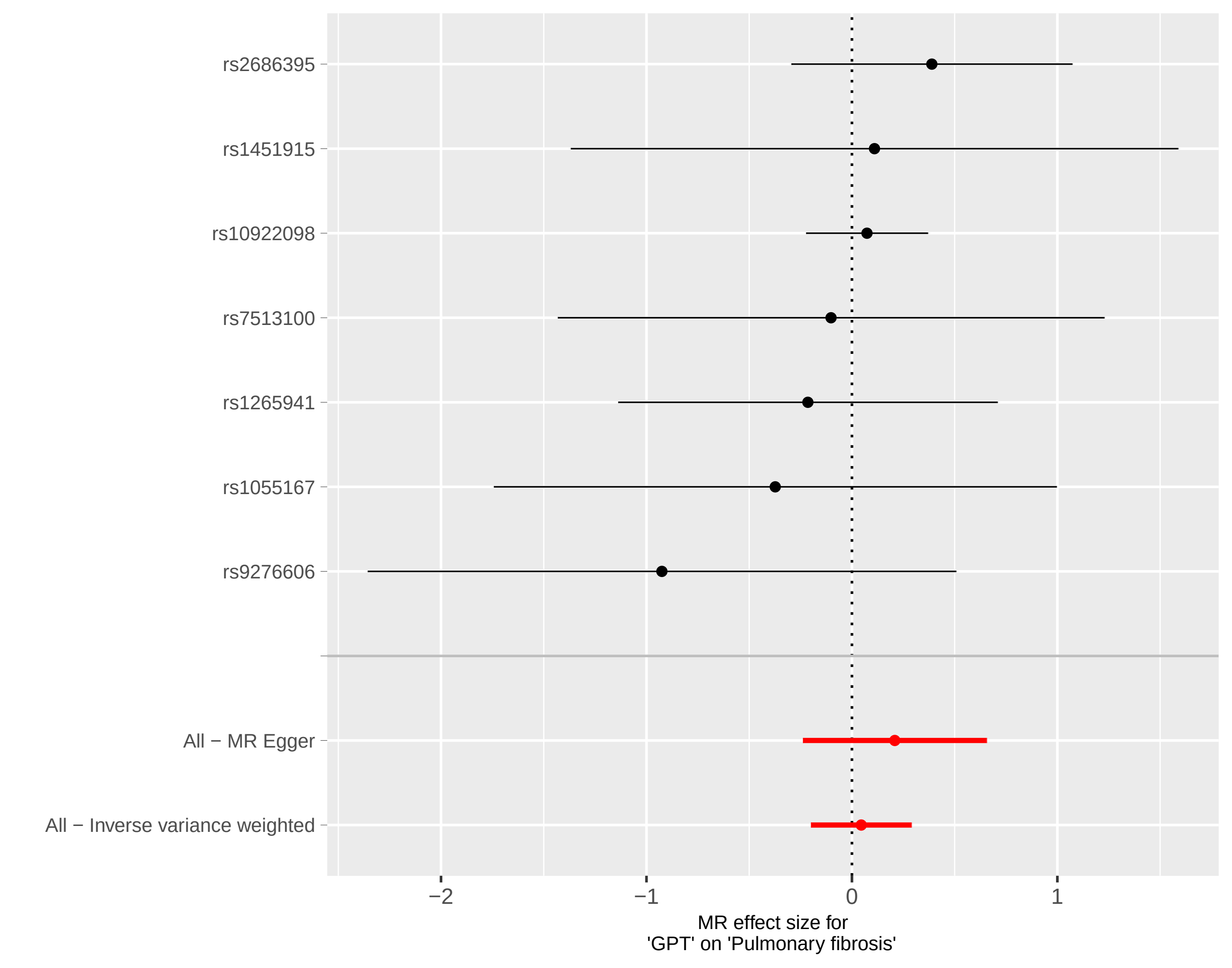

rs2686395
rs1451915
rs10922098
rs7513100
rs1265941
rs1055167
rs9276606
All − MR Egger
All − Inverse variance weighted
−2
−1
0
1
MR effect size for
'GPT' on 'Pulmonary fibrosis'

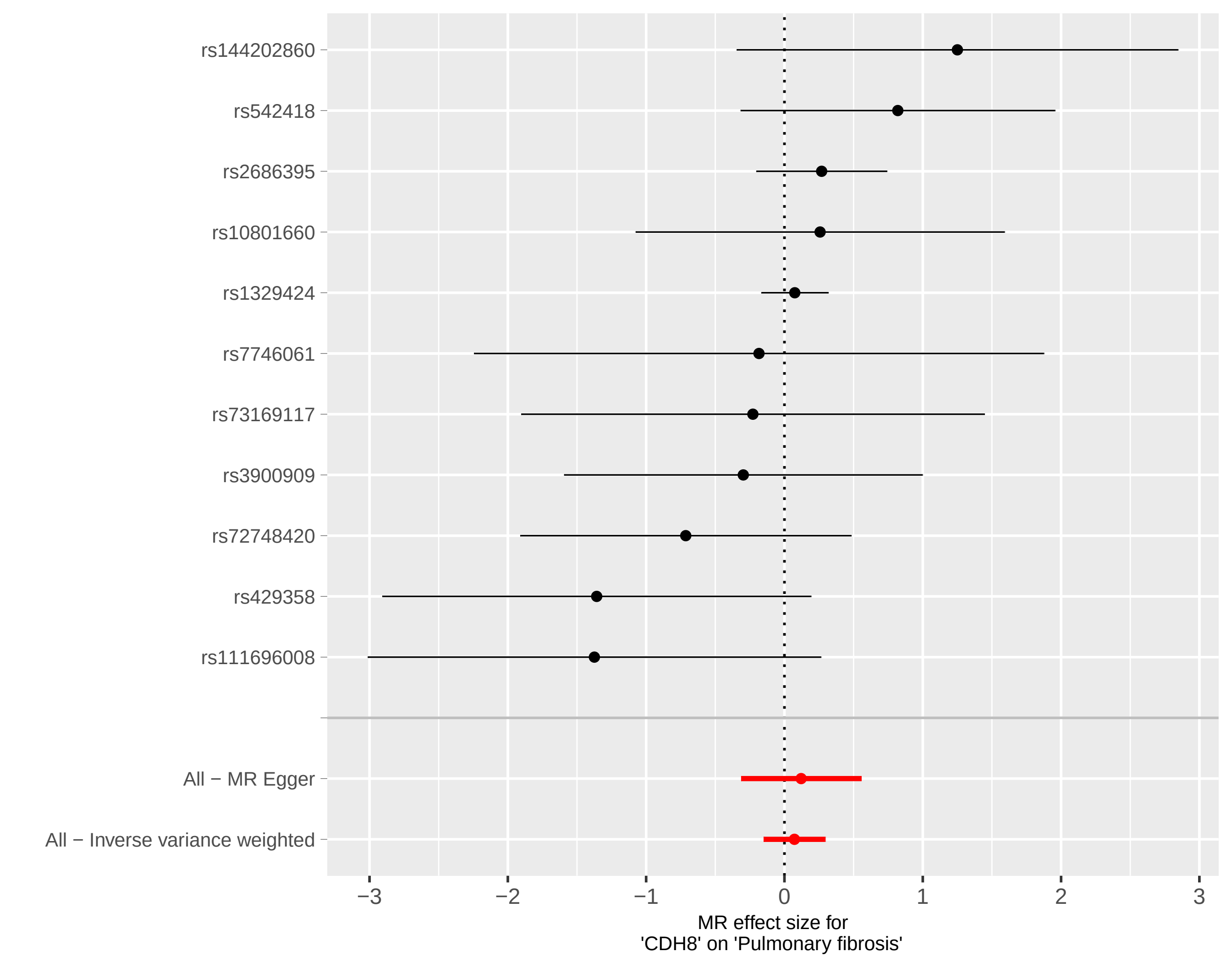
rs144202860
rs542418
rs2686395
rs10801660
rs1329424
rs7746061
rs73169117
rs3900909
rs72748420
rs429358
rs111696008
All − MR Egger
All − Inverse variance weighted
−3
−2
−1
0
1
2
3
MR effect size for
'CDH8' on 'Pulmonary fibrosis'

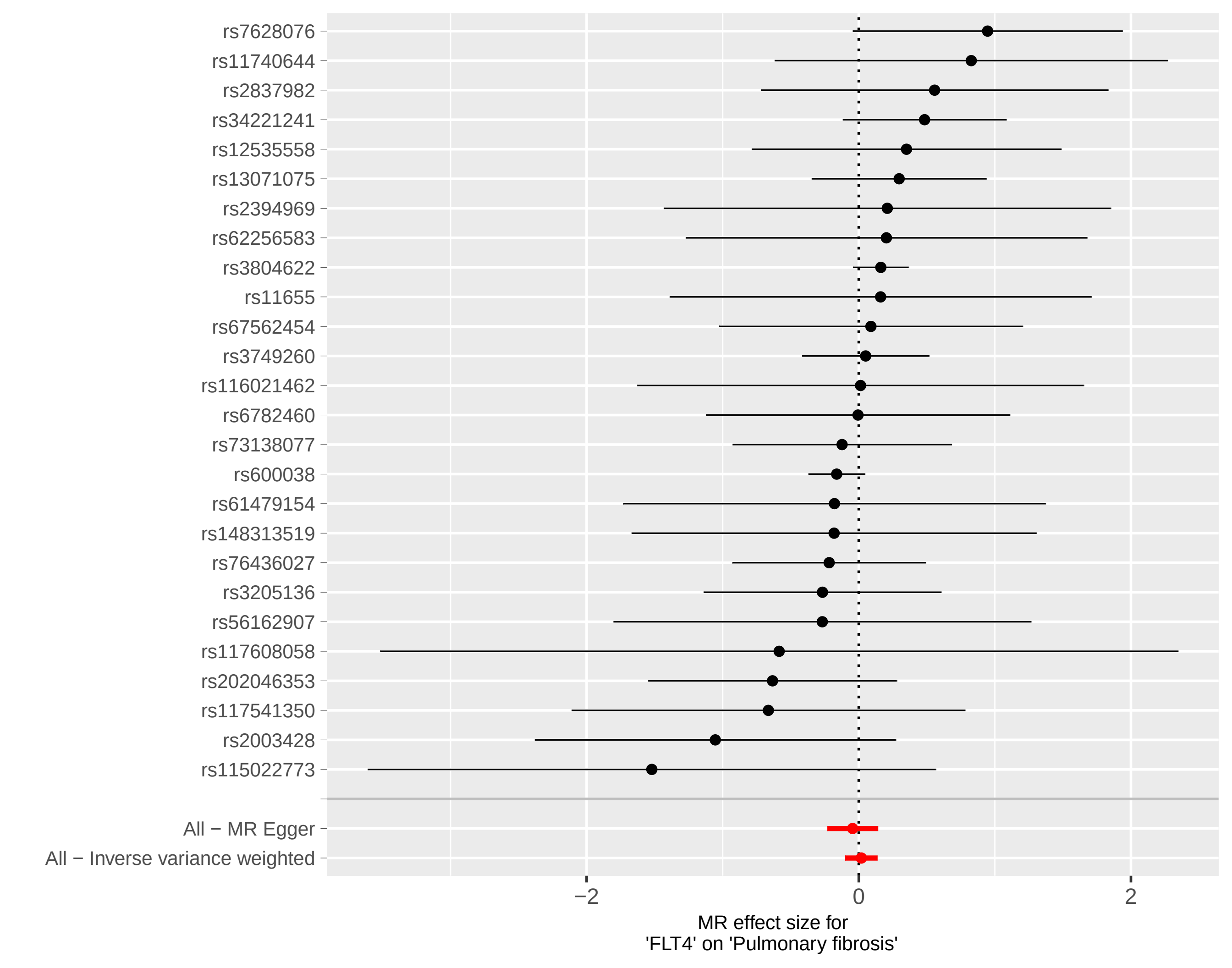

rs7628076
rs11740644
rs2837982
rs34221241
rs12535558
rs13071075
rs2394969
rs62256583
rs3804622
rs11655
rs67562454
rs3749260
rs116021462
rs6782460
rs73138077
rs600038
rs61479154
rs148313519
rs76436027
rs3205136
rs56162907
rs117608058
rs202046353
rs117541350
rs2003428
rs115022773
All − MR Egger
All − Inverse variance weighted
−2
0
2
MR effect size for
'FLT4' on 'Pulmonary fibrosis'

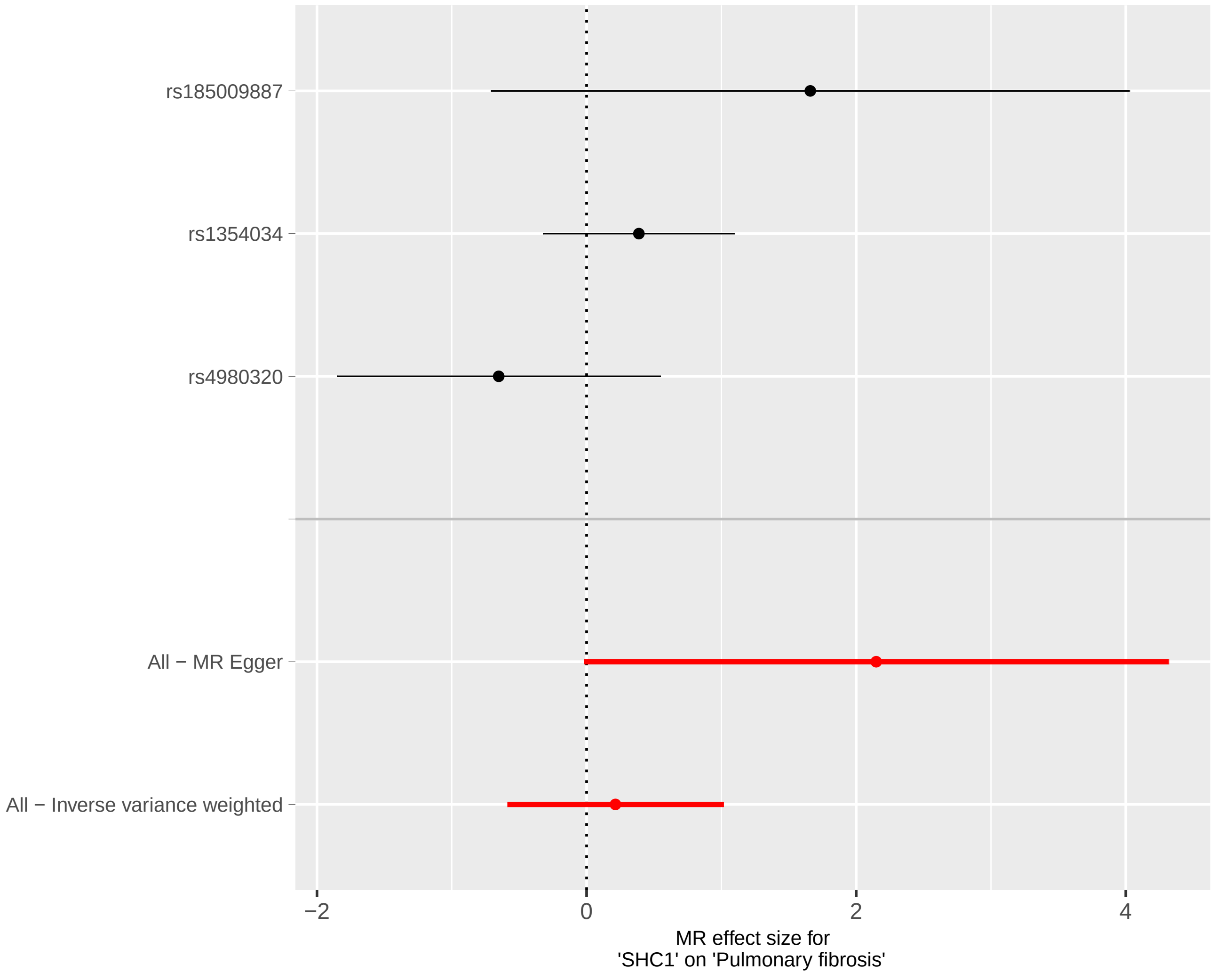

rs185009887
rs1354034
rs4980320
All − MR Egger
All − Inverse variance weighted
−2
0
2
4
MR effect size for
'SHC1' on 'Pulmonary fibrosis'

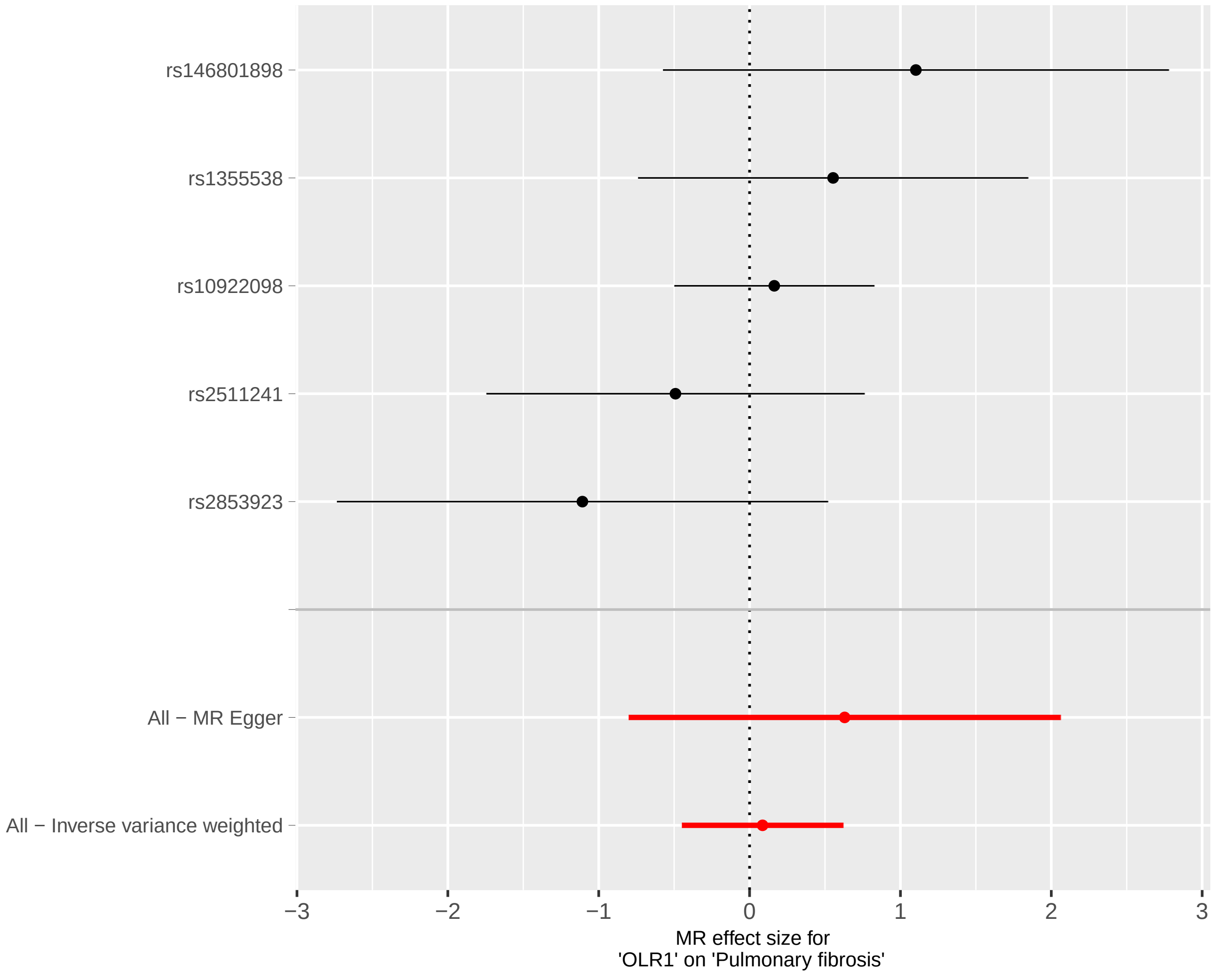

rs146801898
rs1355538
rs10922098
rs2511241
rs2853923
All − MR Egger
All − Inverse variance weighted
−3
−2
−1
0
1
2
3
MR effect size for
'OLR1' on 'Pulmonary fibrosis'

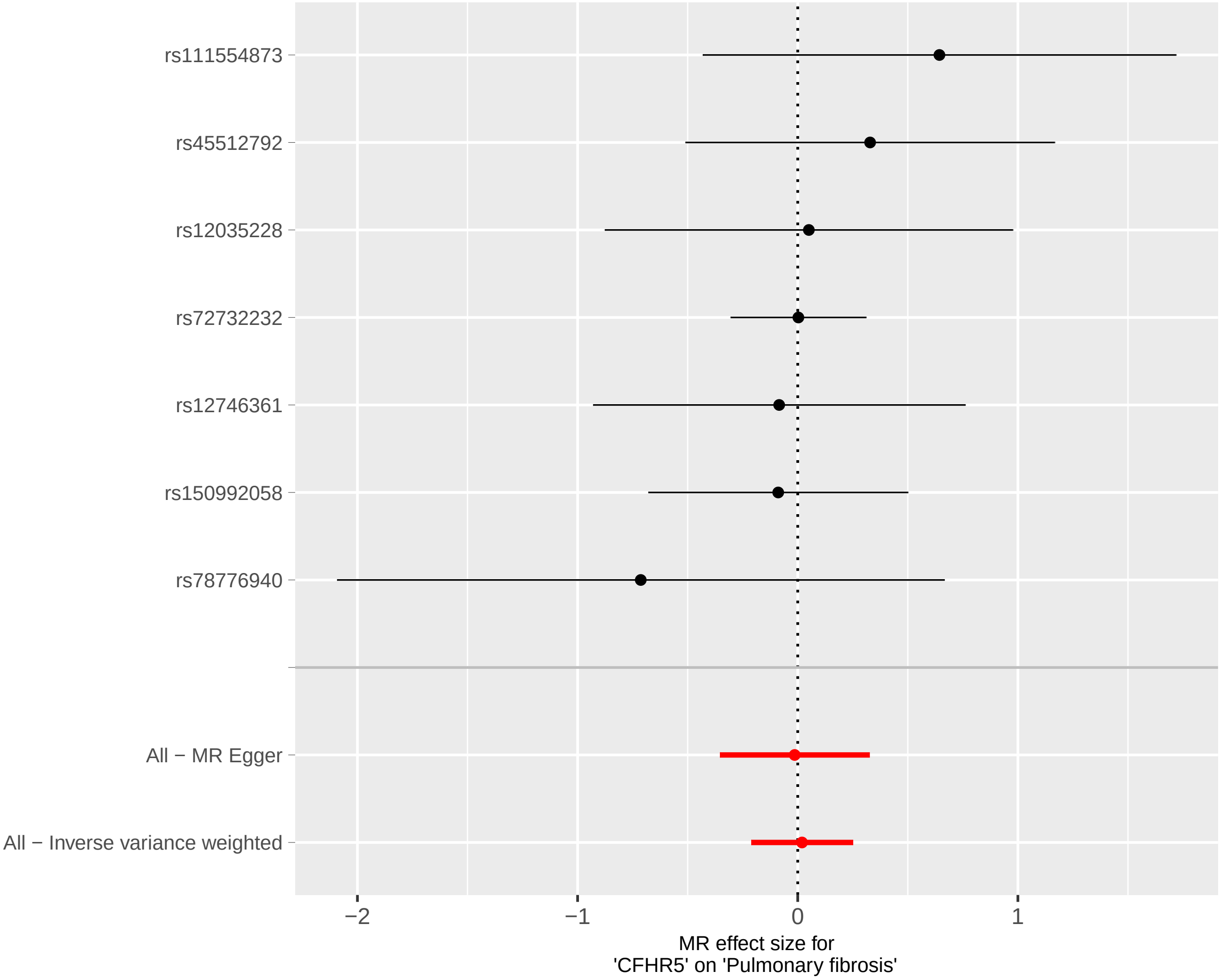

rs111554873
rs45512792
rs12035228
rs72732232
rs12746361
rs150992058
rs78776940
All − MR Egger
All − Inverse variance weighted
−2
−1
0
1
MR effect size for
'CFHR5' on 'Pulmonary fibrosis'

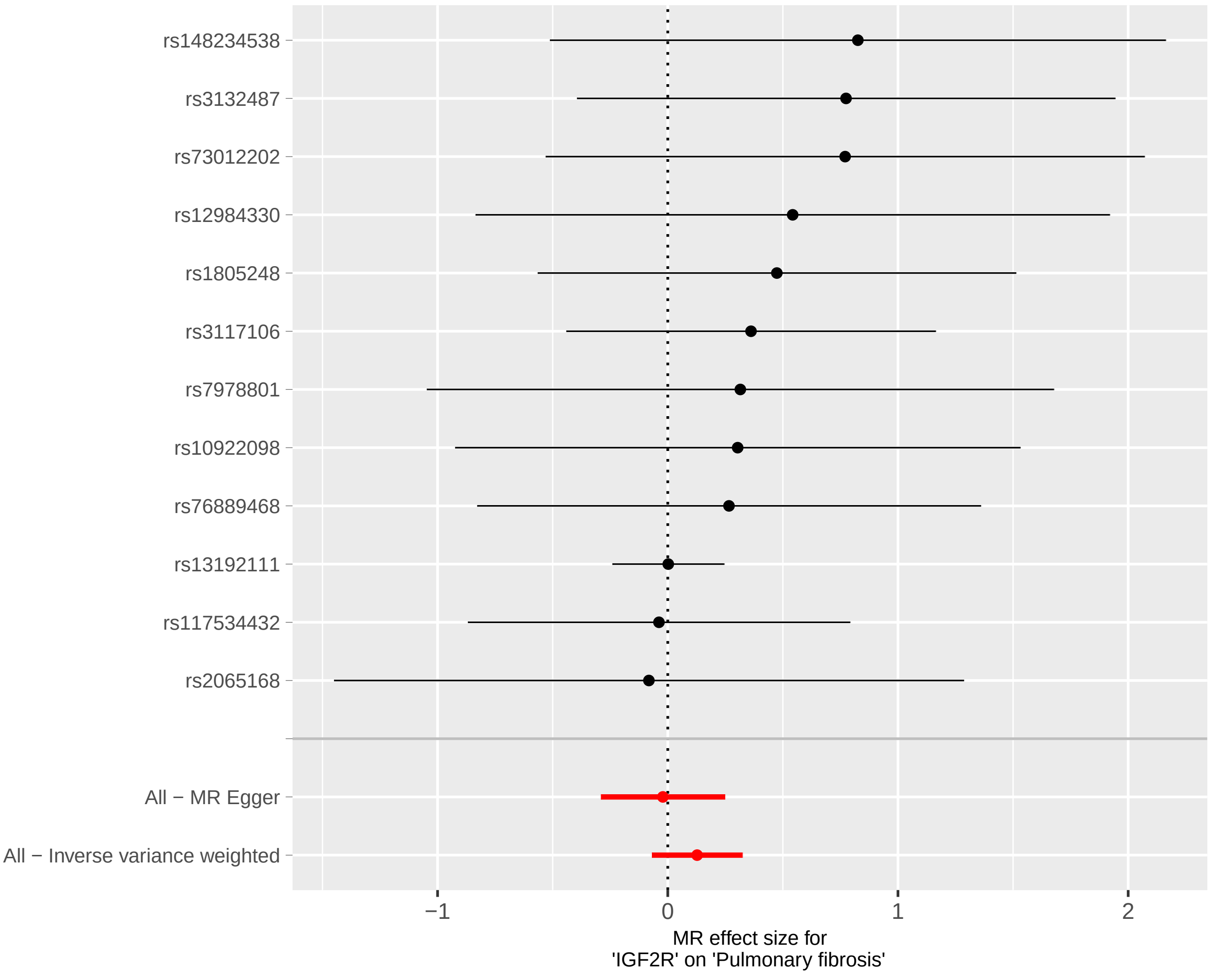

rs148234538
rs3132487
rs73012202
rs12984330
rs1805248
rs3117106
rs7978801
rs10922098
rs76889468
rs13192111
rs117534432
rs2065168
All − MR Egger
All − Inverse variance weighted
−1
0
1
2
MR effect size for
'IGF2R' on 'Pulmonary fibrosis'

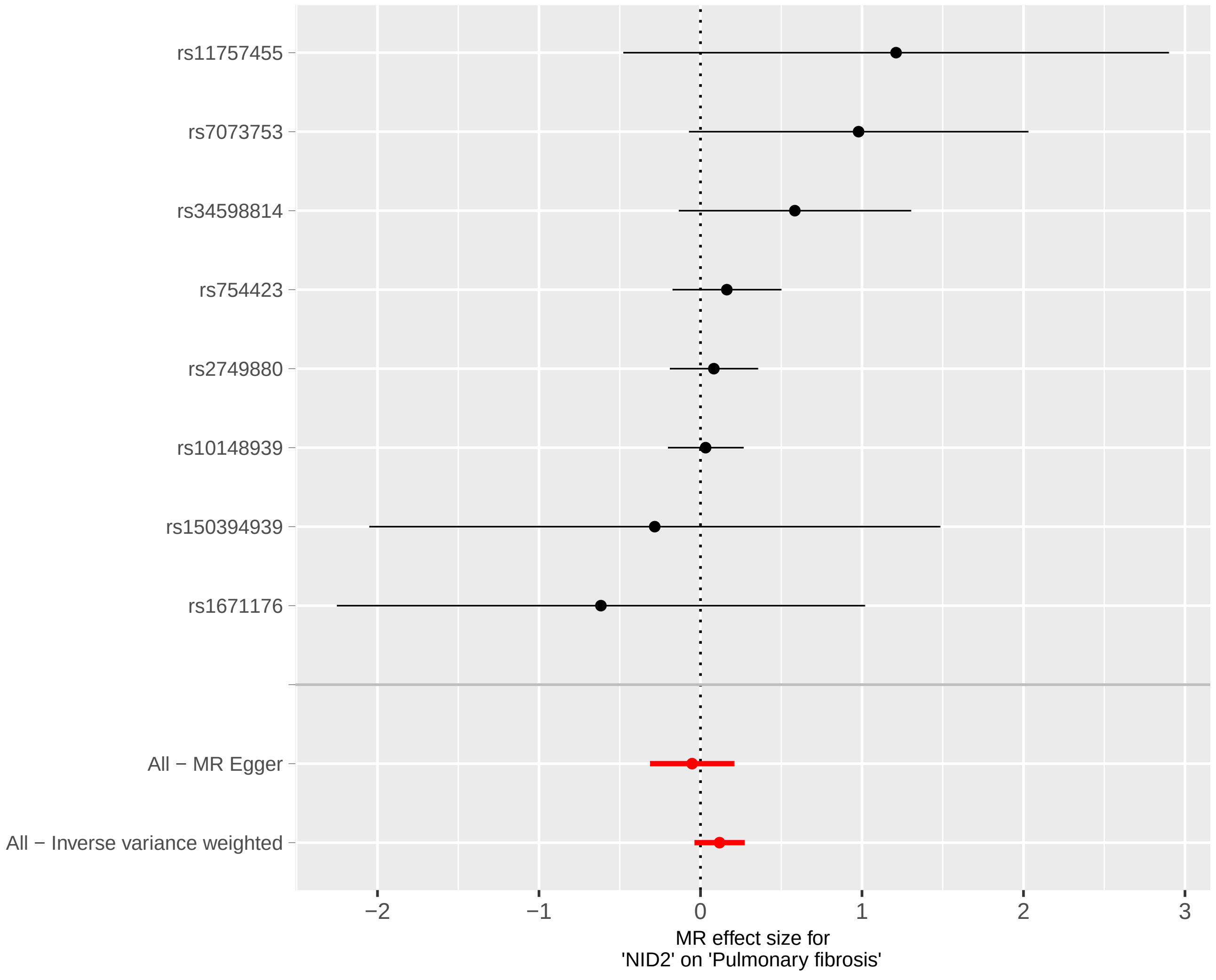

rs11757455
rs7073753
rs34598814
rs754423
rs2749880
rs10148939
rs150394939
rs1671176
All − MR Egger
All − Inverse variance weighted
−2
−1
0
1
2
3
MR effect size for
'NID2' on 'Pulmonary fibrosis'

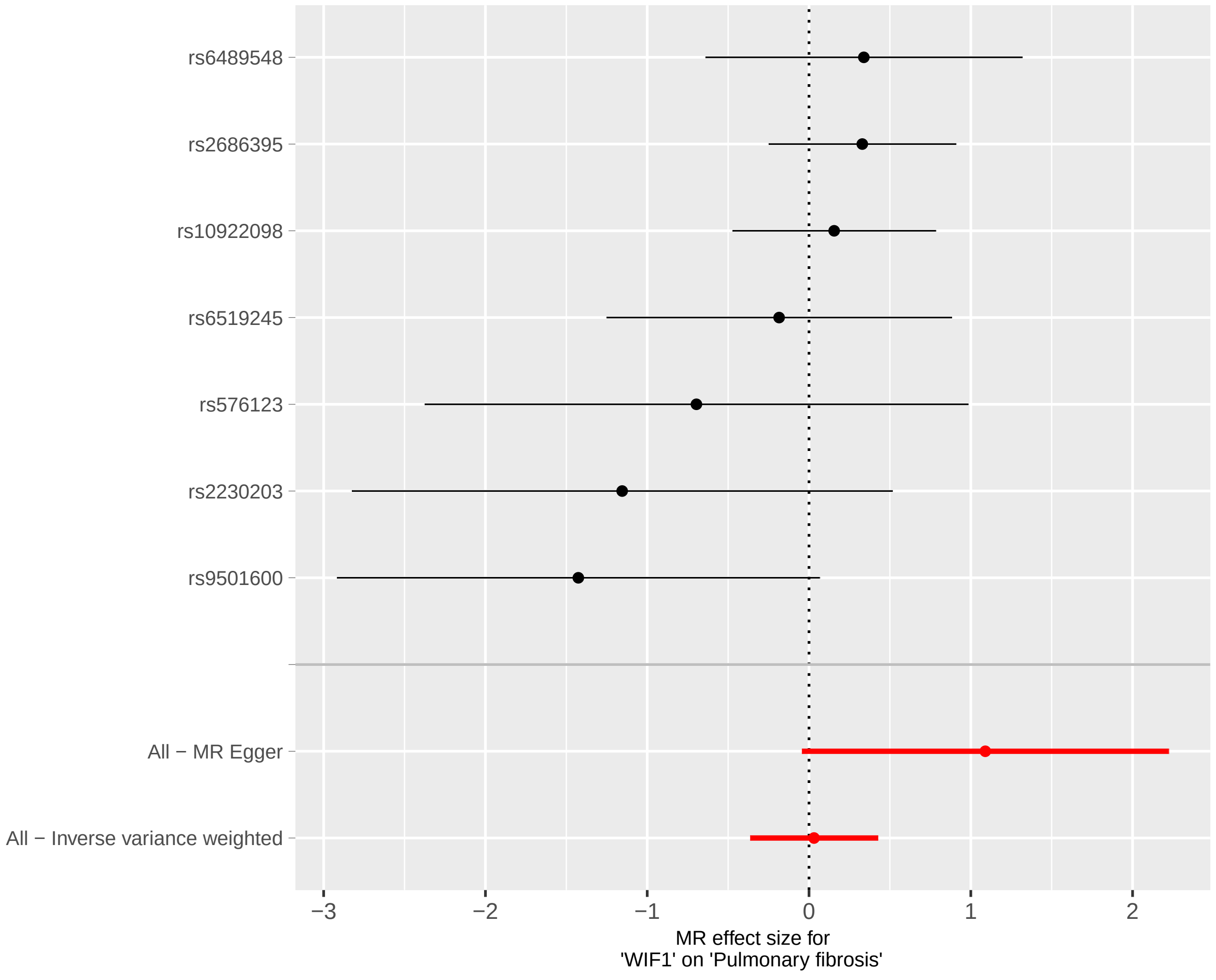

rs6489548
rs2686395
rs10922098
rs6519245
rs576123
rs2230203
rs9501600
All − MR Egger
All − Inverse variance weighted
−3
−2
−1
0
1
2
MR effect size for
'WIF1' on 'Pulmonary fibrosis'

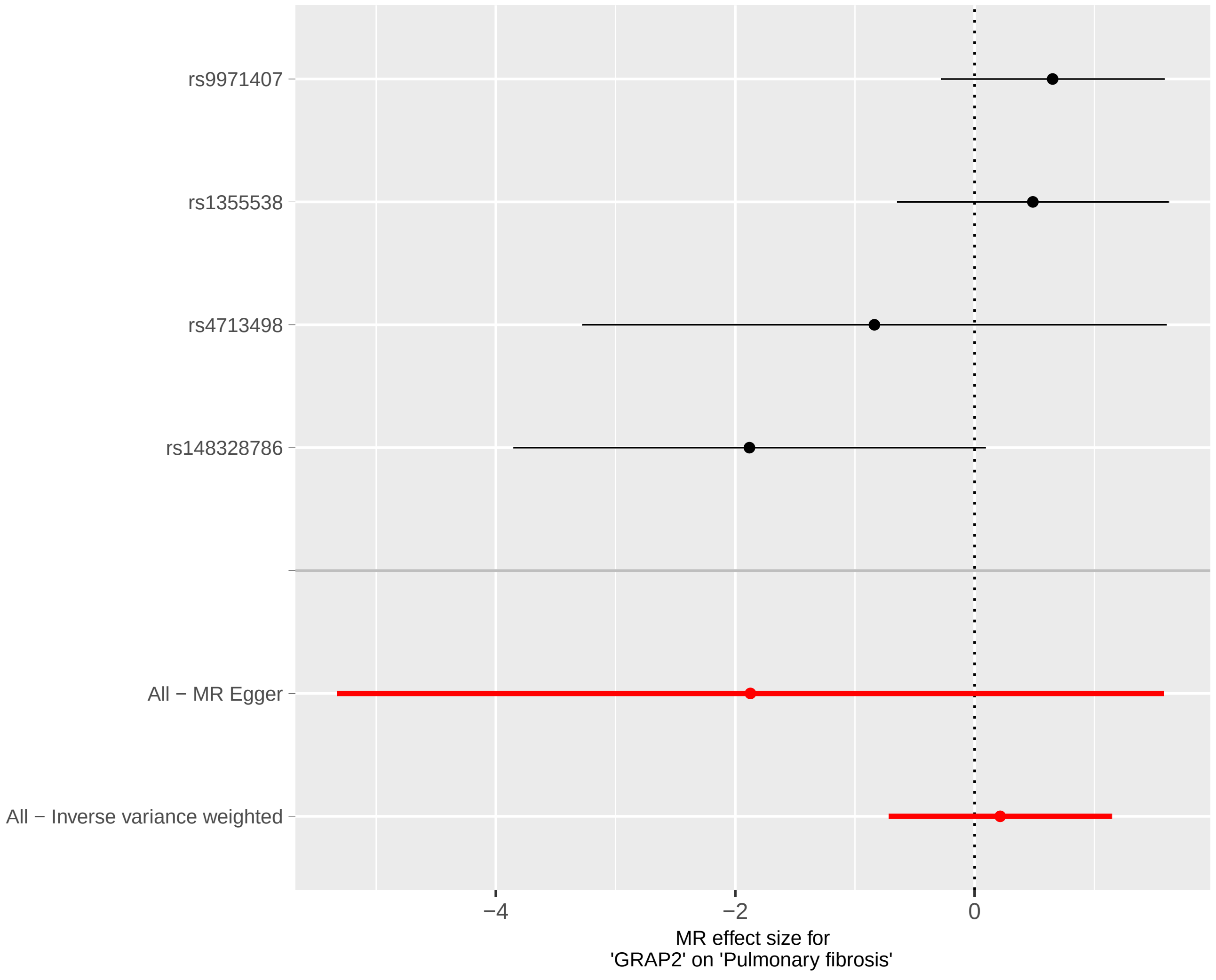
rs9971407
rs1355538
rs4713498
rs148328786
All − MR Egger
All − Inverse variance weighted
−4
−2
0
MR effect size for
'GRAP2' on 'Pulmonary fibrosis'

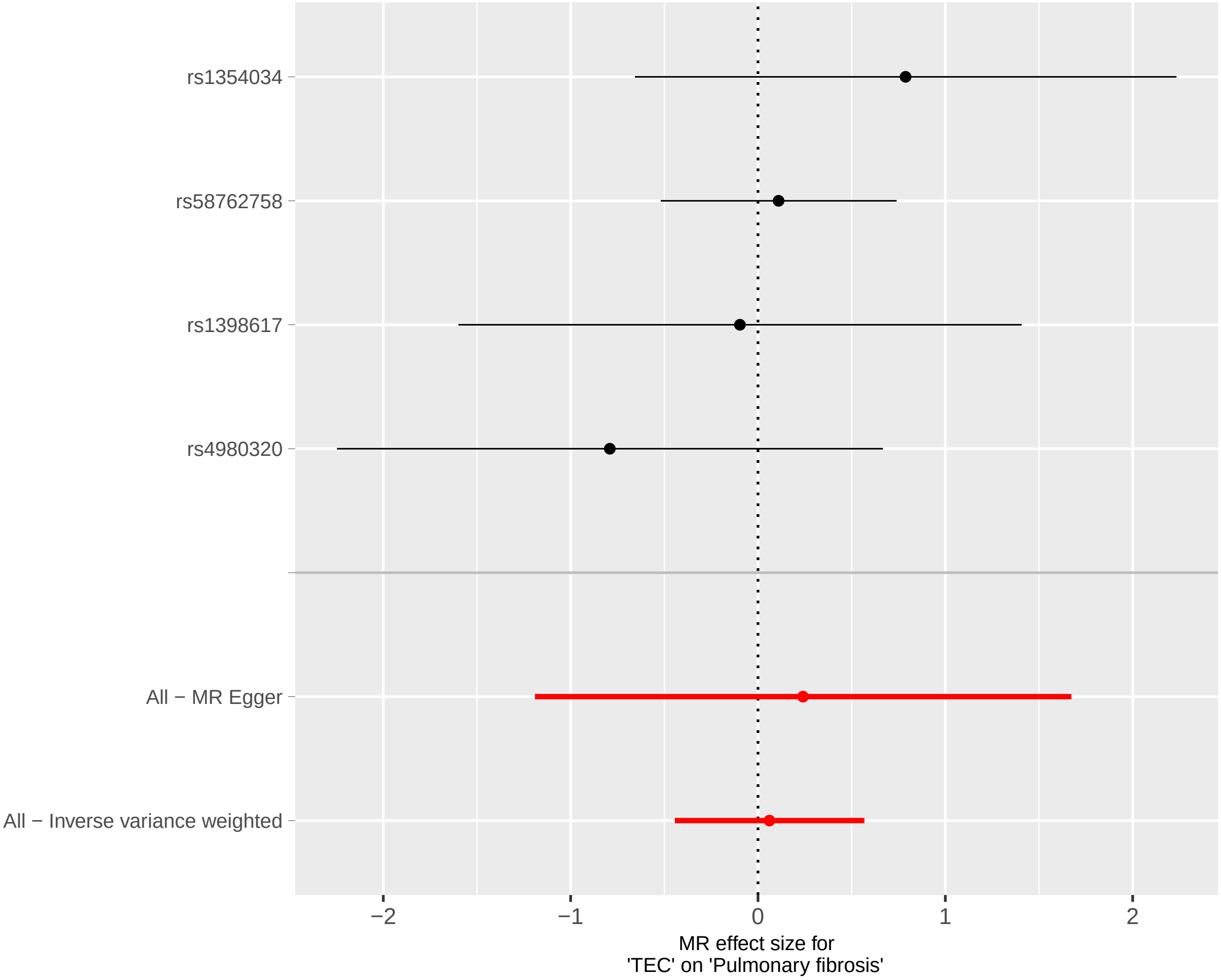
rs1354034
rs58762758
rs1398617
rs4980320
All − MR Egger
All − Inverse variance weighted
−2
−1
0
1
2
MR effect size for
'TEC' on 'Pulmonary fibrosis'

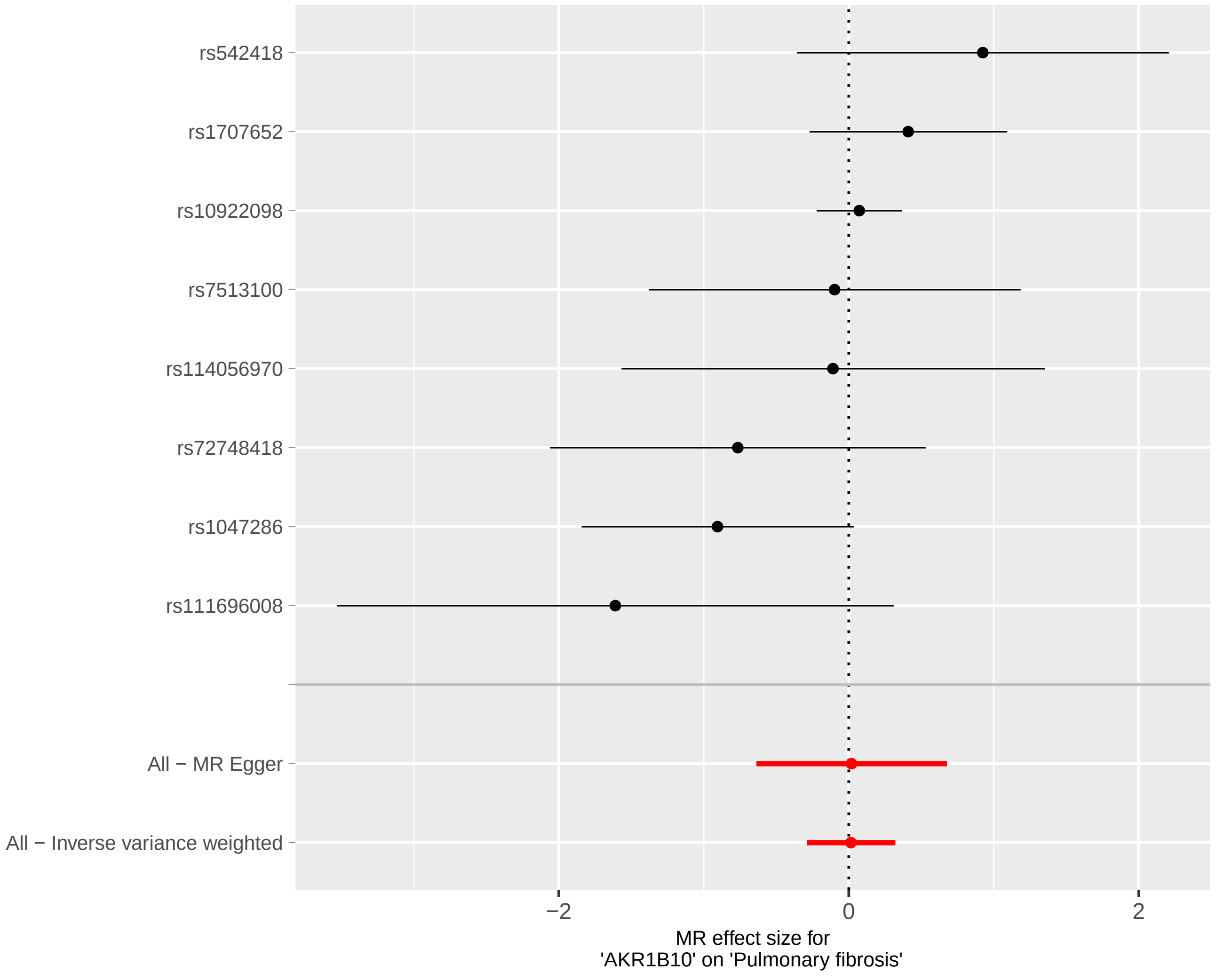

rs542418
rs1707652
rs10922098
rs7513100
rs114056970
rs72748418
rs1047286
rs111696008
All − MR Egger
All − Inverse variance weighted
−2
0
2
MR effect size for
'AKR1B10' on 'Pulmonary fibrosis'

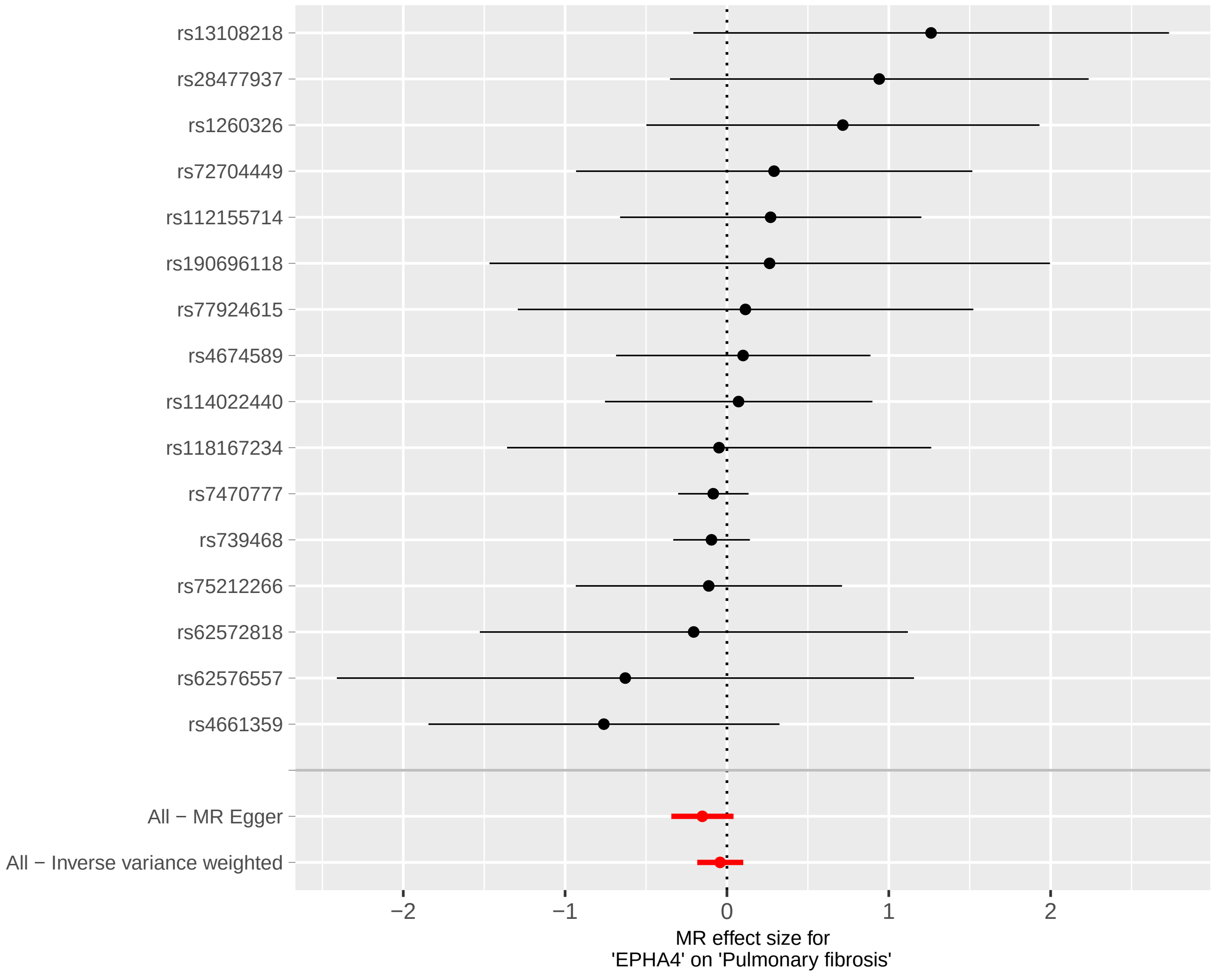
rs13108218
rs28477937
rs1260326
rs72704449
rs112155714
rs190696118
rs77924615
rs4674589
rs114022440
rs118167234
rs7470777
rs739468
rs75212266
rs62572818
rs62576557
rs4661359
All − MR Egger
All − Inverse variance weighted
−2
−1
0
1
2
MR effect size for
'EPHA4' on 'Pulmonary fibrosis'

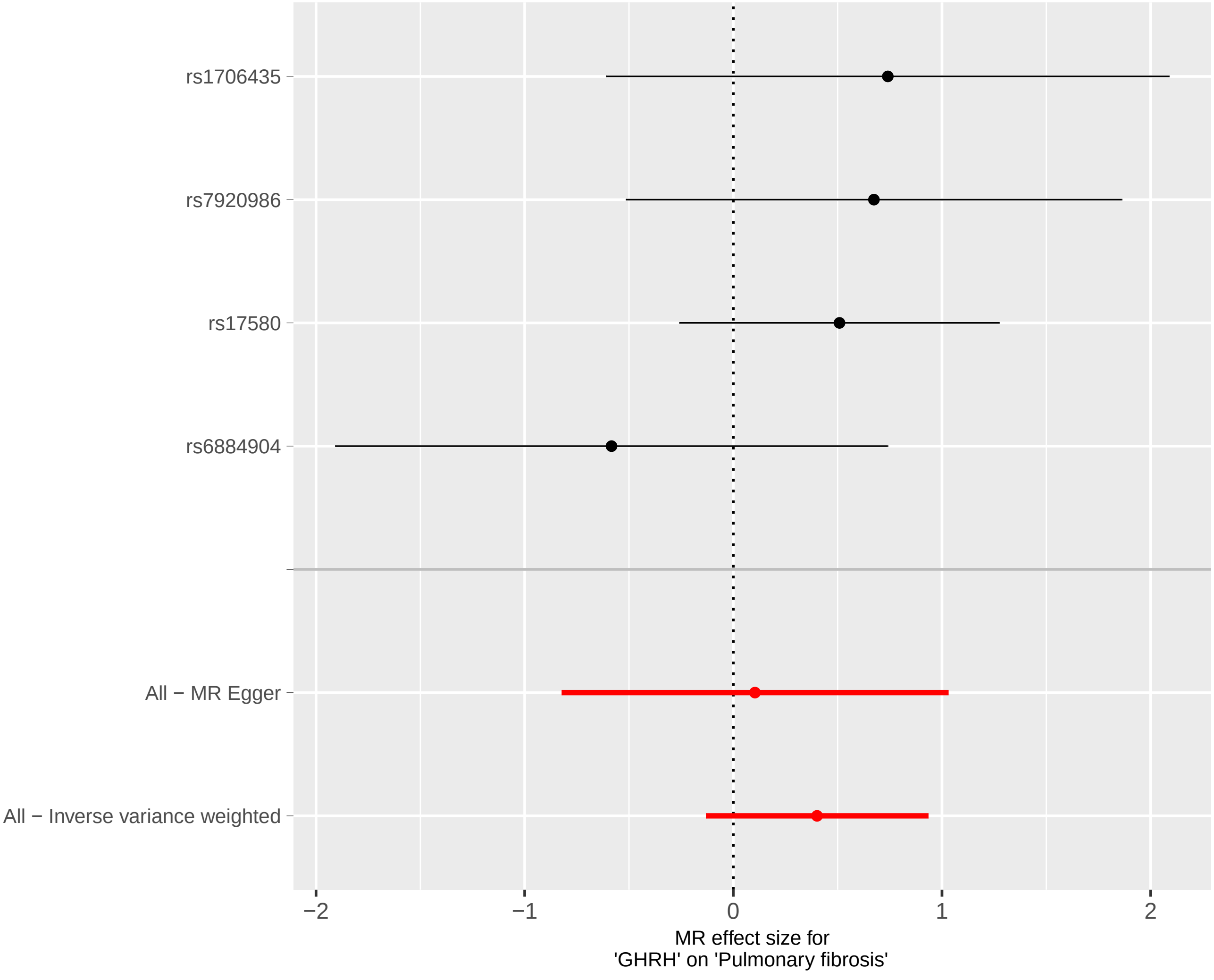

rs1706435
rs7920986
rs17580
rs6884904
All − MR Egger
All − Inverse variance weighted
−2
−1
0
1
2
MR effect size for
'GHRH' on 'Pulmonary fibrosis'

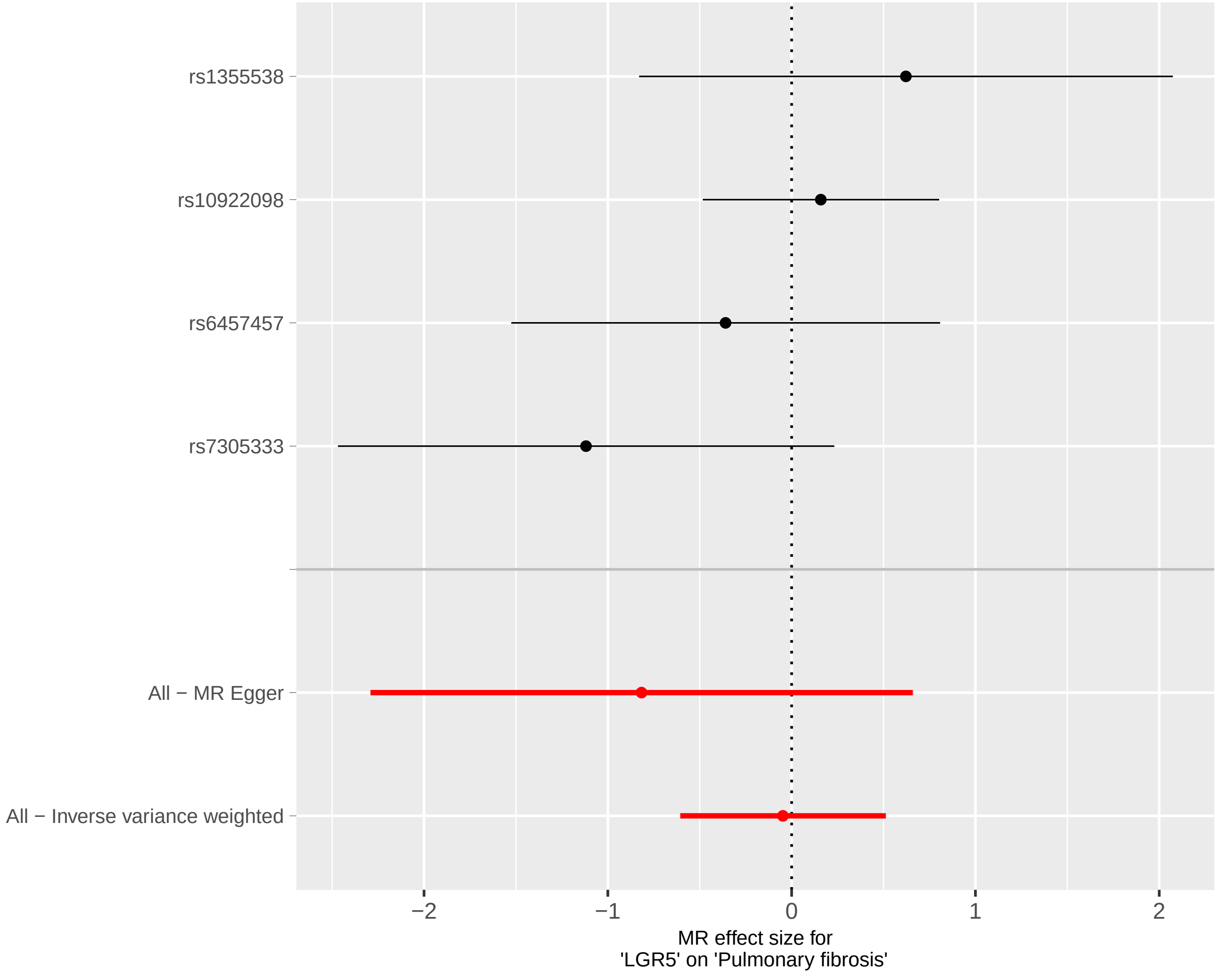
rs1355538
rs10922098
rs6457457
rs7305333
All − MR Egger
All − Inverse variance weighted
−2
−1
0
1
2
MR effect size for
'LGR5' on 'Pulmonary fibrosis'

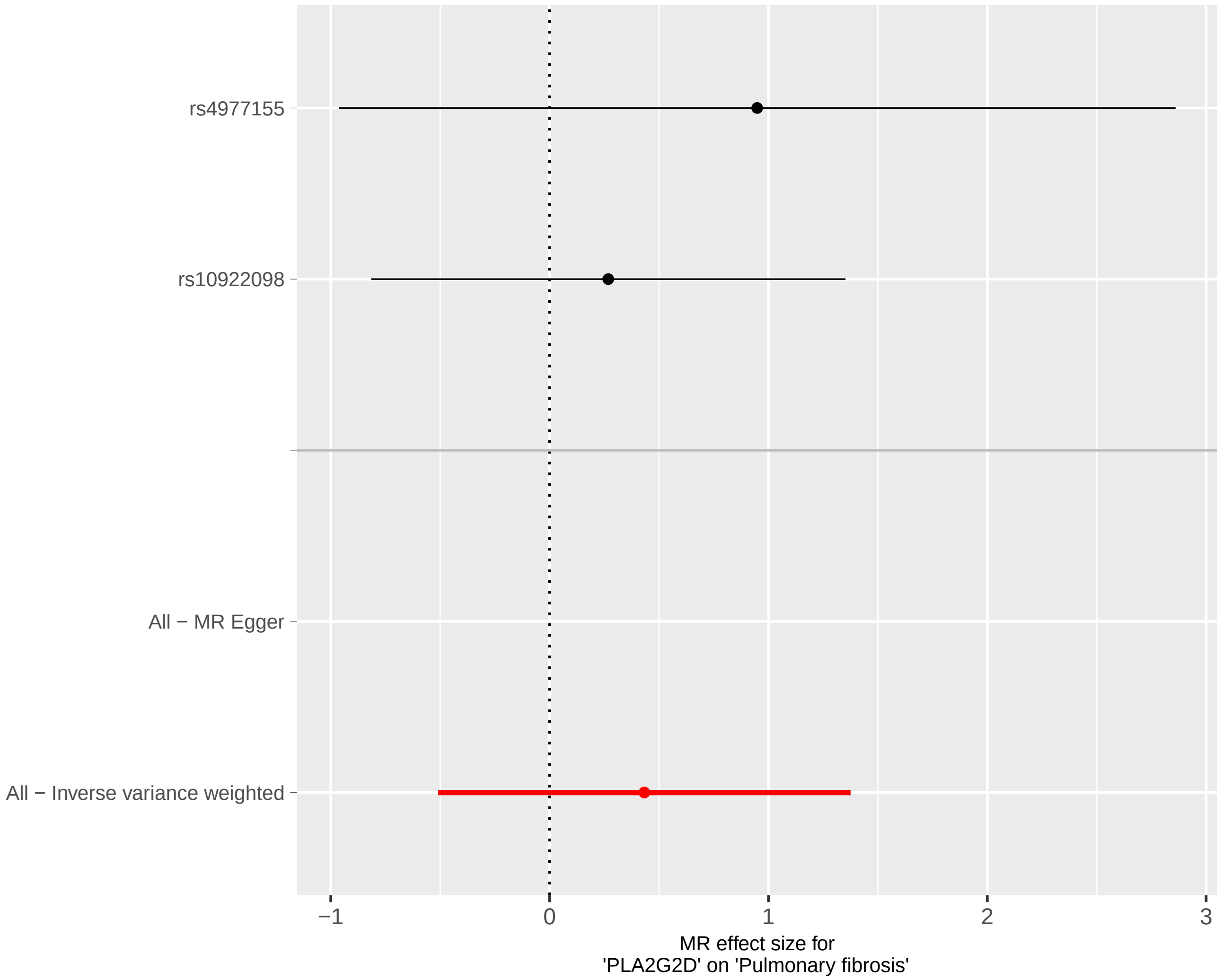
rs4977155
rs10922098
All − MR Egger
All − Inverse variance weighted
−1
0
1
2
3
MR effect size for
'PLA2G2D' on 'Pulmonary fibrosis'

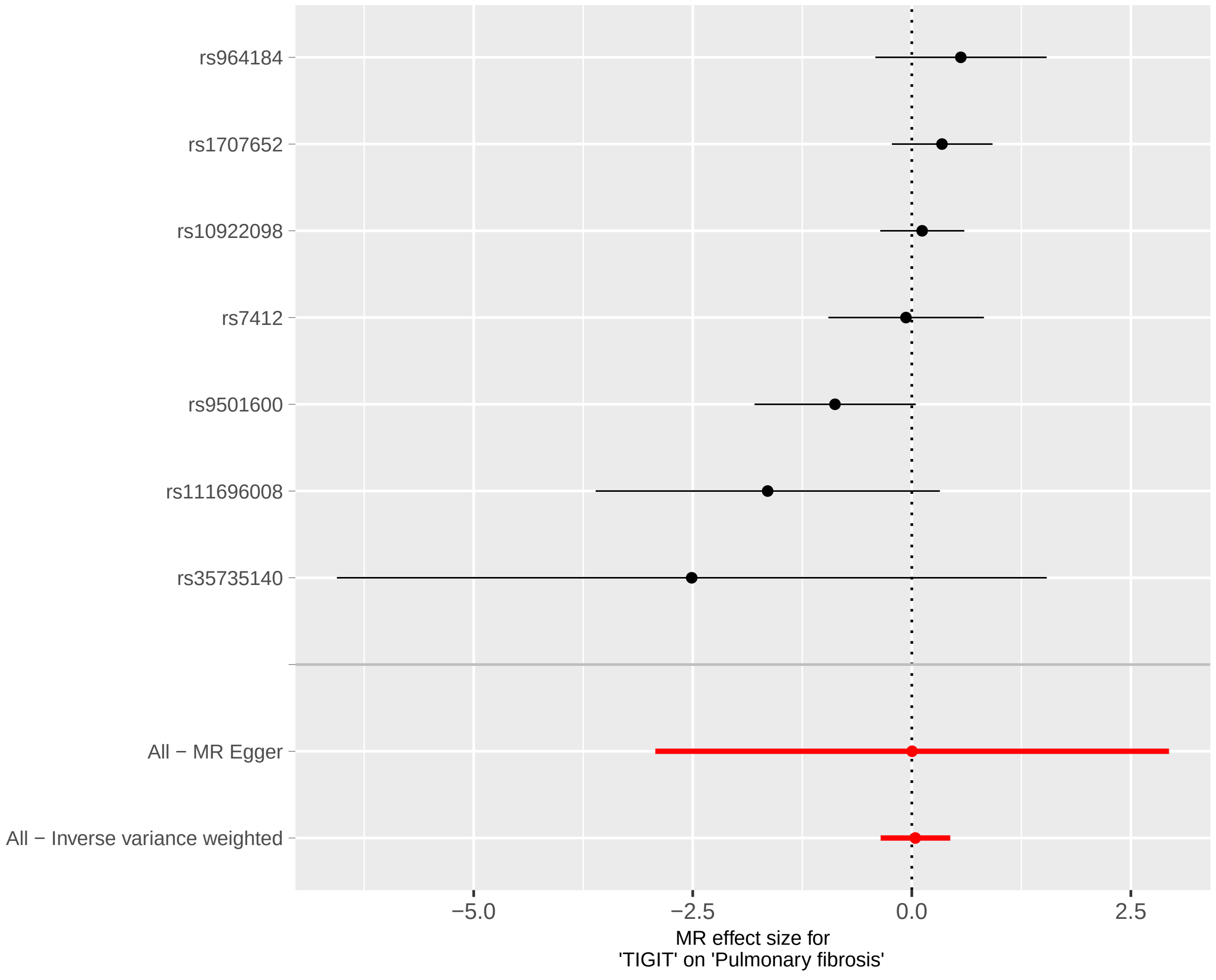

rs964184
rs1707652
rs10922098
rs7412
rs9501600
rs111696008
rs35735140
All − MR Egger
All − Inverse variance weighted
−5.0
−2.5
0.0
2.5
MR effect size for
'TIGIT' on 'Pulmonary fibrosis'

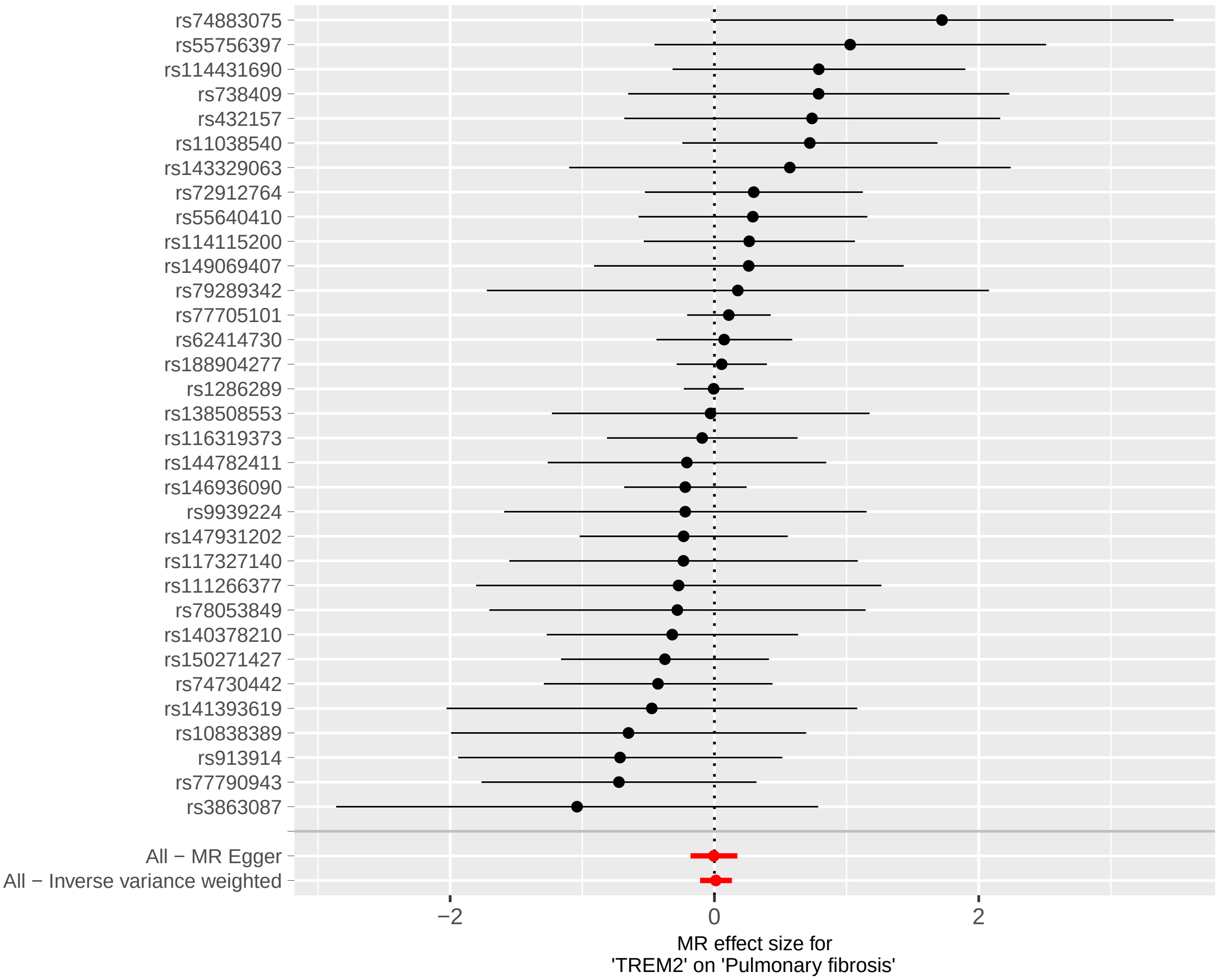

rs74883075
rs55756397
rs114431690
rs738409
rs432157
rs11038540
rs143329063
rs72912764
rs55640410
rs114115200
rs149069407
rs79289342
rs77705101
rs62414730
rs188904277
rs1286289
rs138508553
rs116319373
rs144782411
rs146936090
rs9939224
rs147931202
rs117327140
rs111266377
rs78053849
rs140378210
rs150271427
rs74730442
rs141393619
rs10838389
rs913914
rs77790943
rs3863087
All − MR Egger
All − Inverse variance weighted
−2
0
2
MR effect size for
'TREM2' on 'Pulmonary fibrosis'

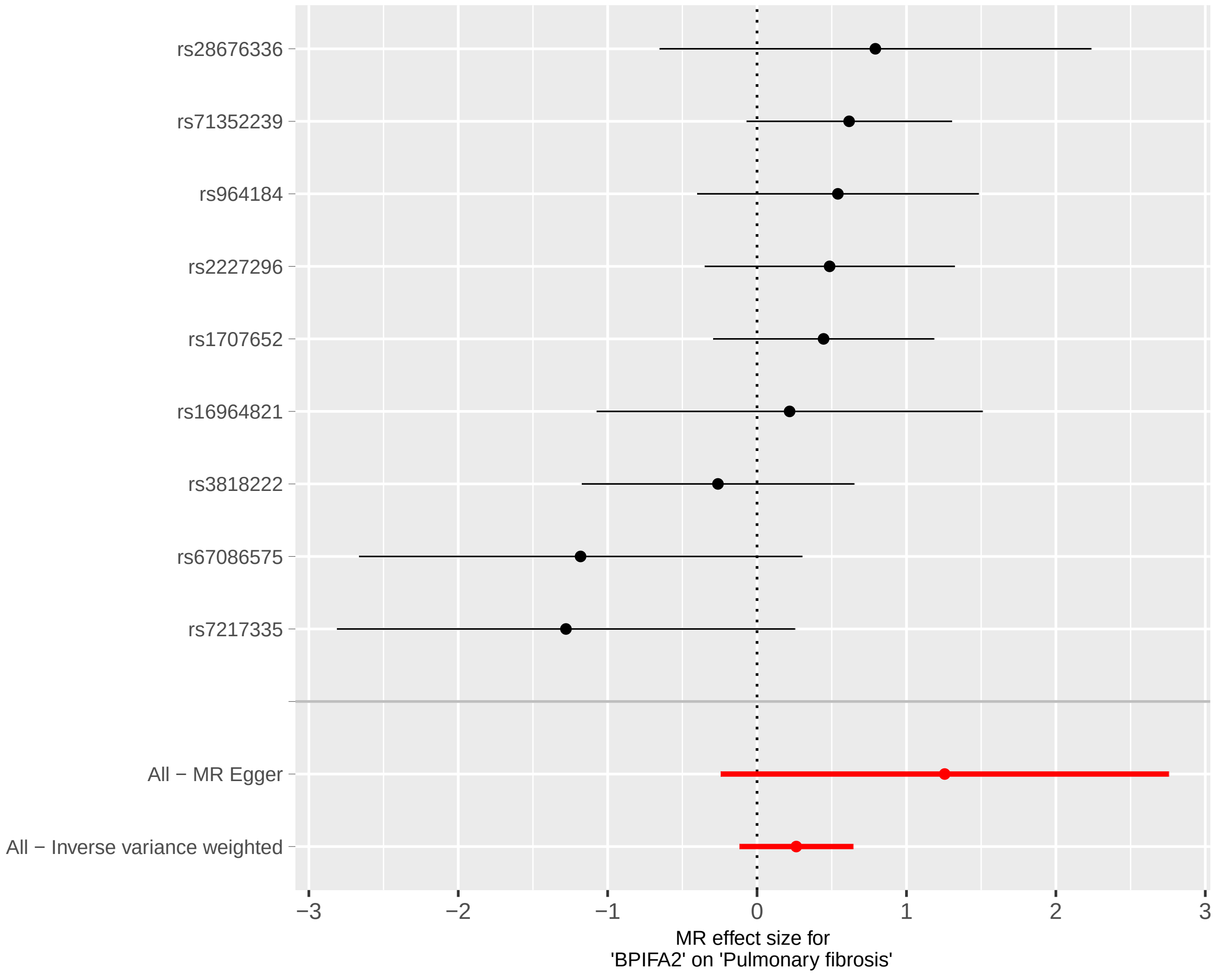
rs28676336
rs71352239
rs964184
rs2227296
rs1707652
rs16964821
rs3818222
rs67086575
rs7217335
All − MR Egger
All − Inverse variance weighted
−3
−2
−1
0
1
2
3
MR effect size for
'BPIFA2' on 'Pulmonary fibrosis'

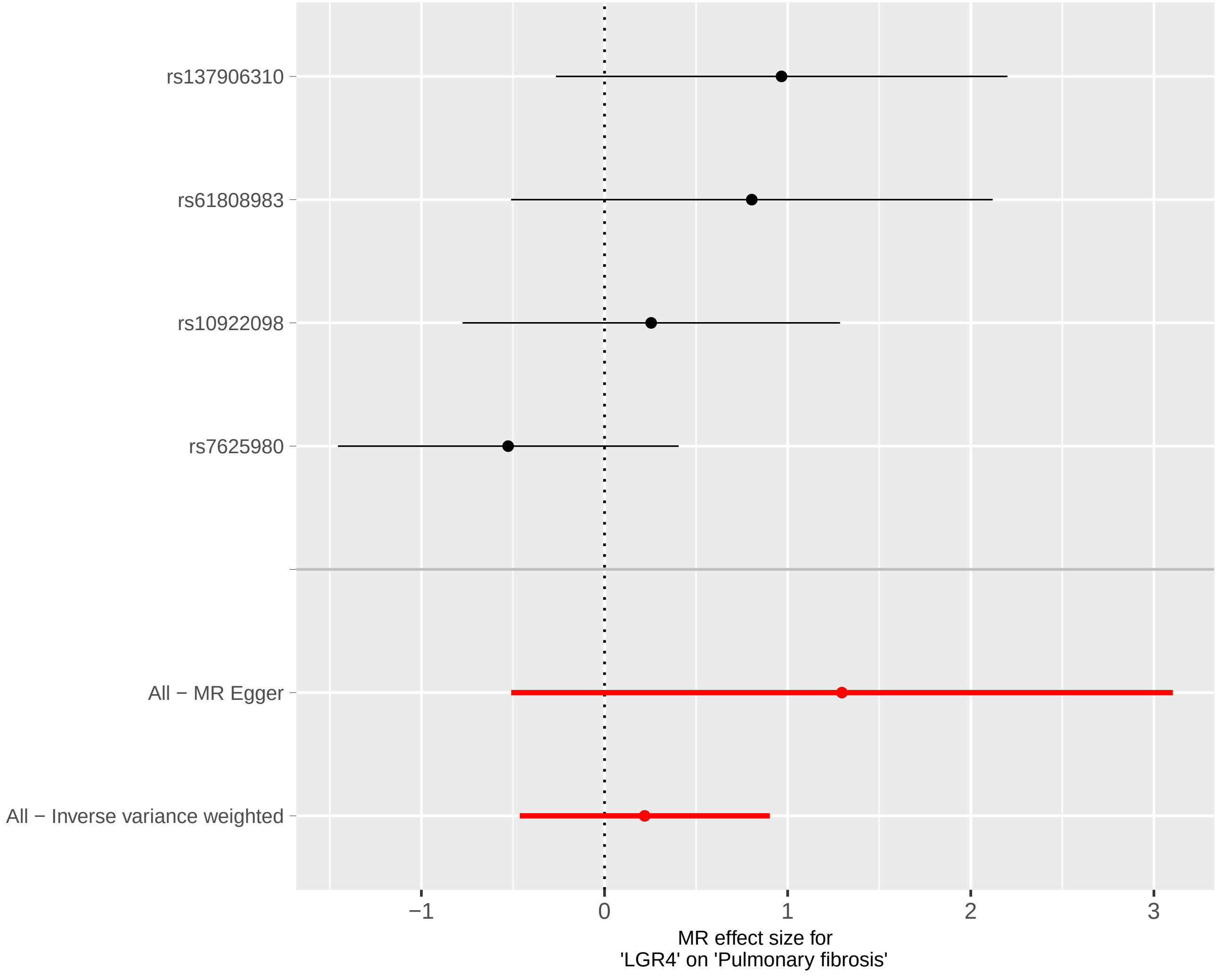

rs137906310
rs61808983
rs10922098
rs7625980
All − MR Egger
All − Inverse variance weighted
−1
0
1
2
3
MR effect size for
'LGR4' on 'Pulmonary fibrosis'

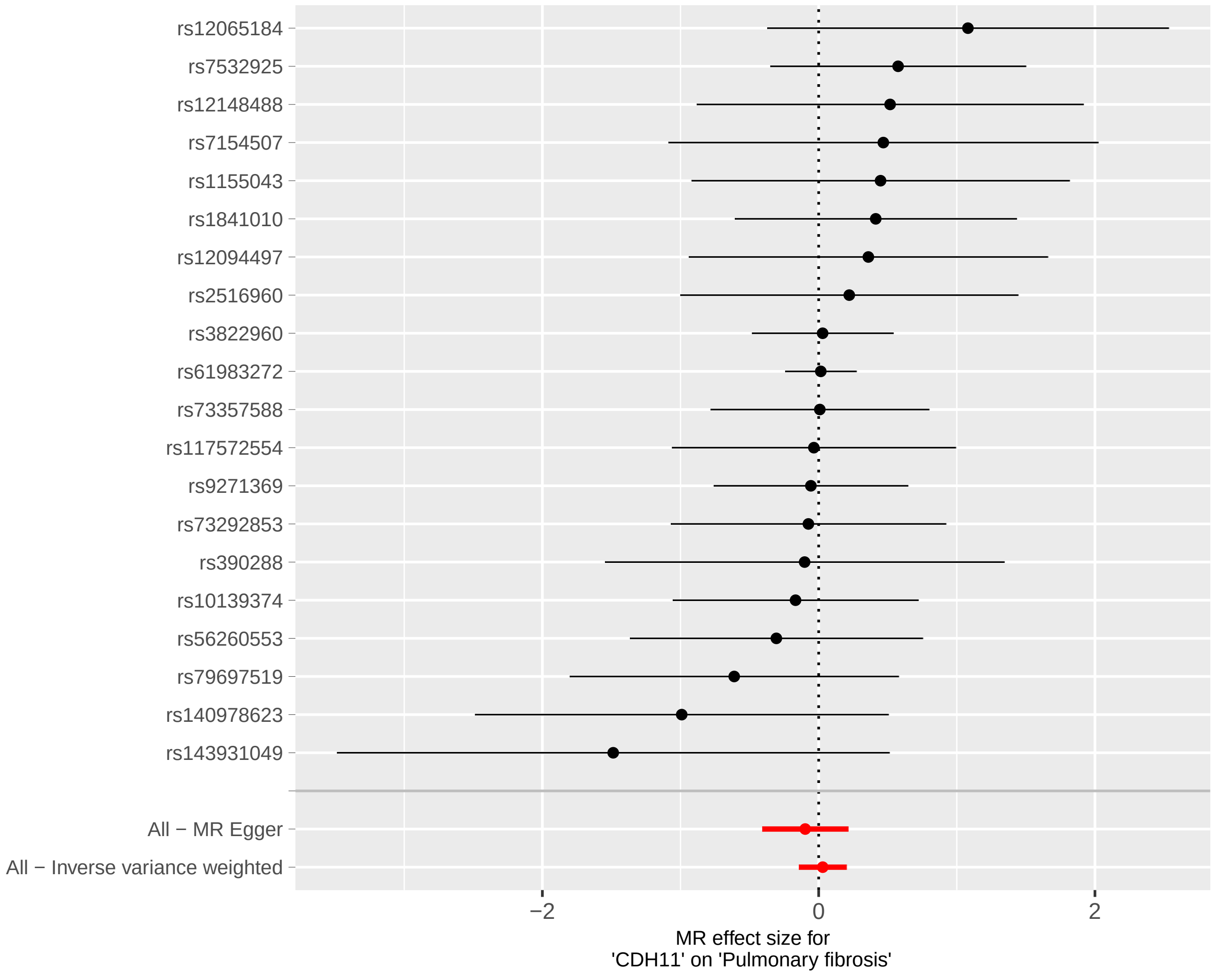
rs12065184
rs7532925
rs12148488
rs7154507
rs1155043
rs1841010
rs12094497
rs2516960
rs3822960
rs61983272
rs73357588
rs117572554
rs9271369
rs73292853
rs390288
rs10139374
rs56260553
rs79697519
rs140978623
rs143931049
All − MR Egger
All − Inverse variance weighted
−2
0
2
MR effect size for
'CDH11' on 'Pulmonary fibrosis'

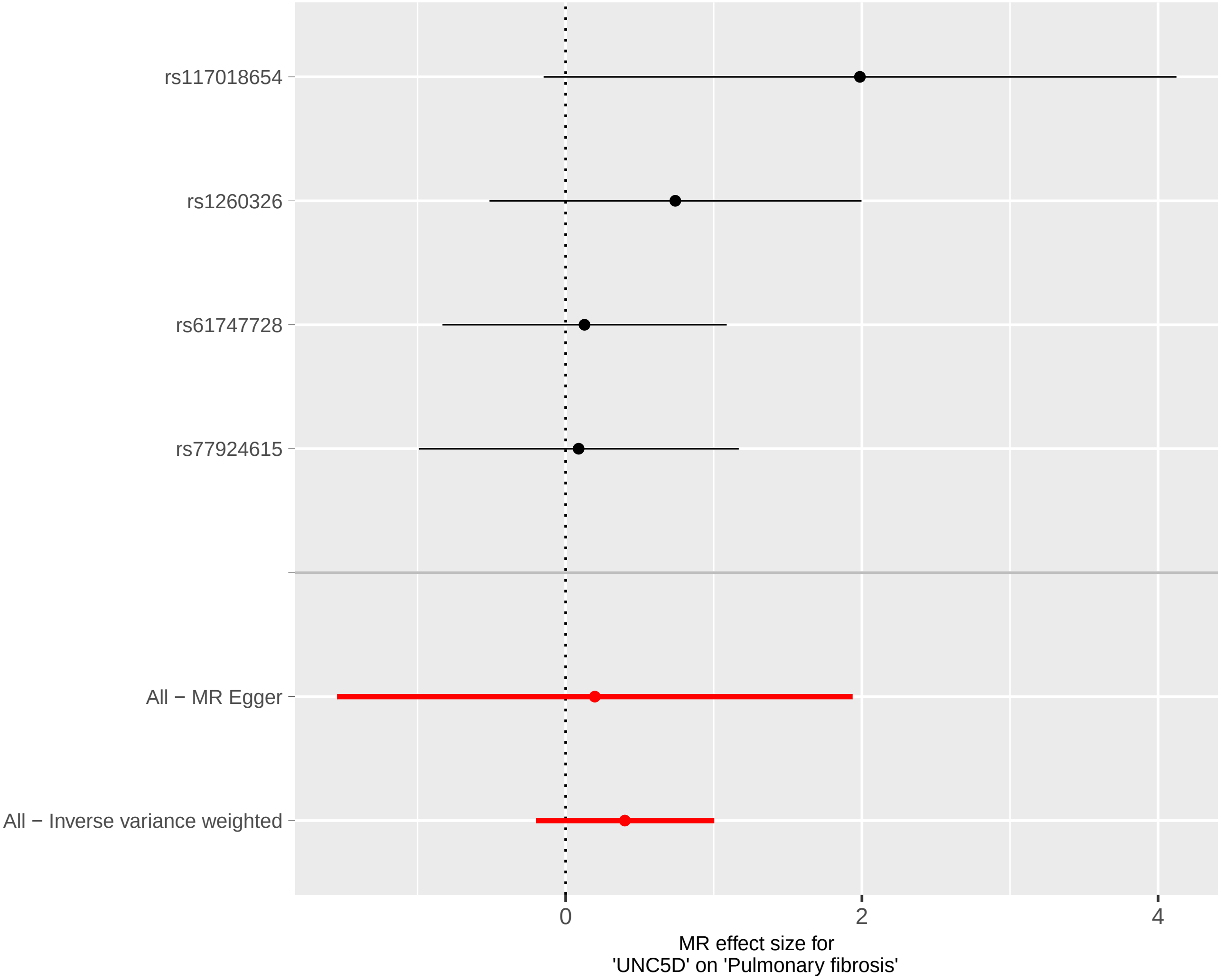

rs117018654
rs1260326
rs61747728
rs77924615
All − MR Egger
All − Inverse variance weighted
0
2
4
MR effect size for
'UNC5D' on 'Pulmonary fibrosis'

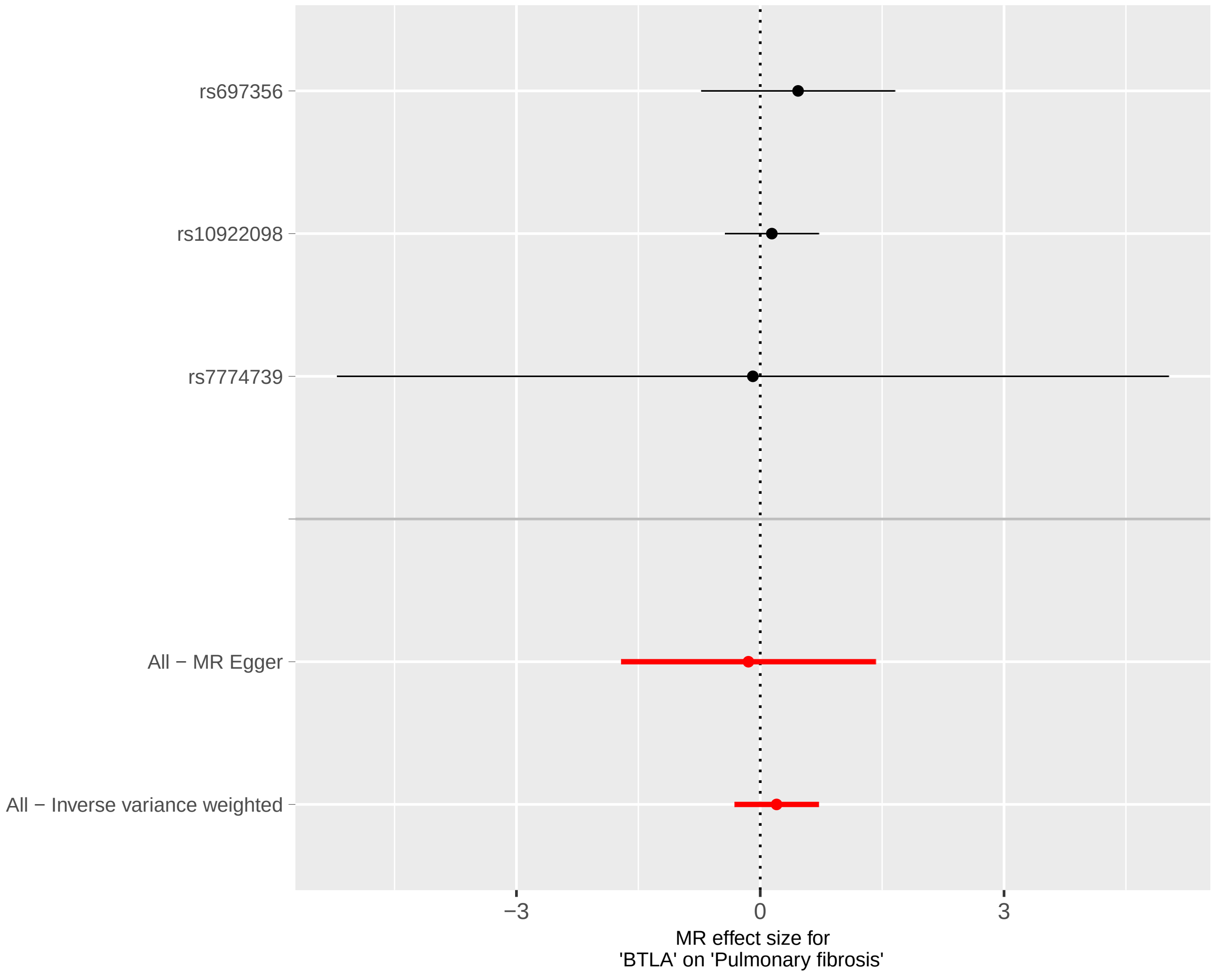

rs697356
rs10922098
rs7774739
All − MR Egger
All − Inverse variance weighted
−3
0
3
MR effect size for
'BTLA' on 'Pulmonary fibrosis'

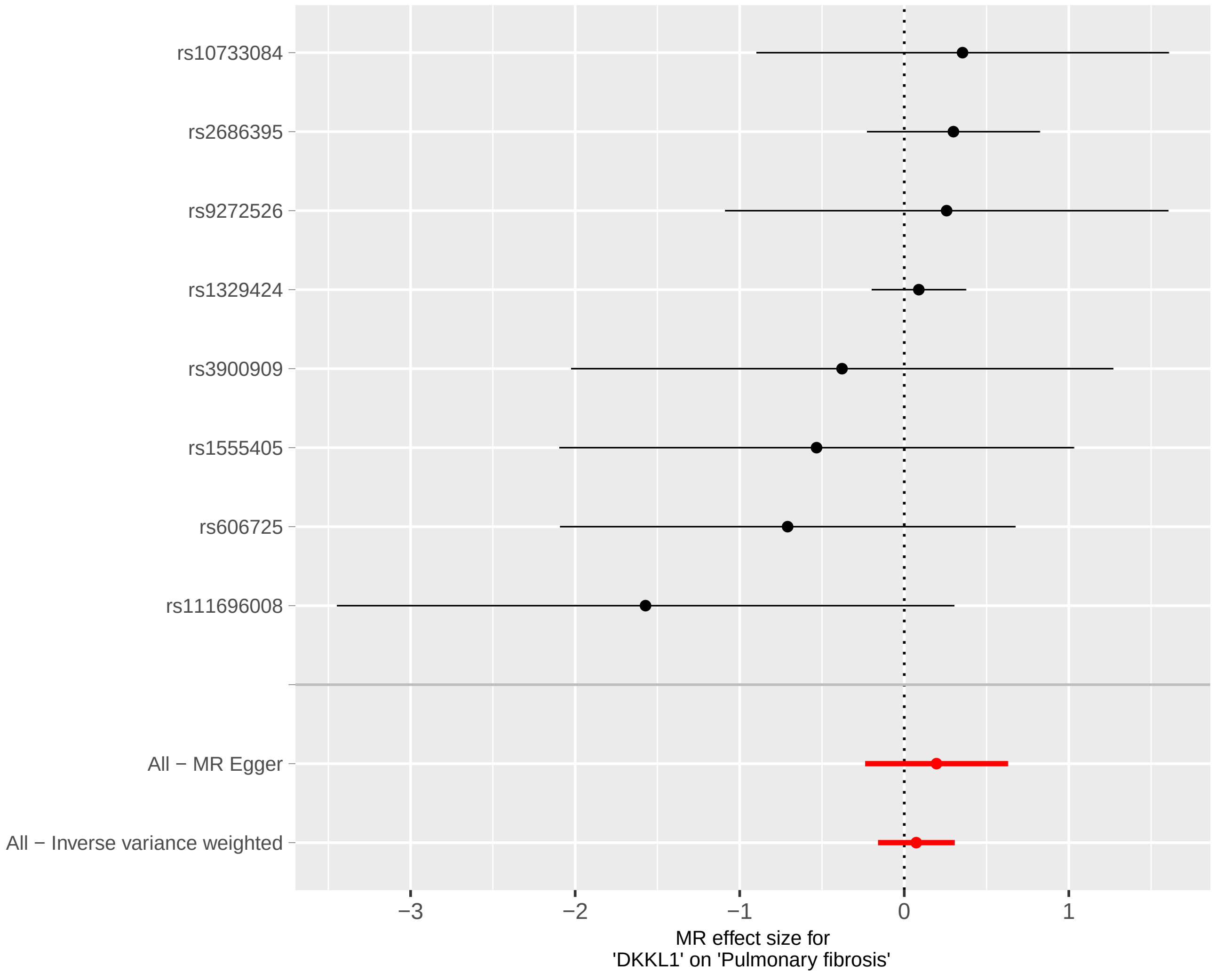

rs10733084
rs2686395
rs9272526
rs1329424
rs3900909
rs1555405
rs606725
rs111696008
All − MR Egger
All − Inverse variance weighted
−3
−2
−1
0
1
MR effect size for
'DKKL1' on 'Pulmonary fibrosis'

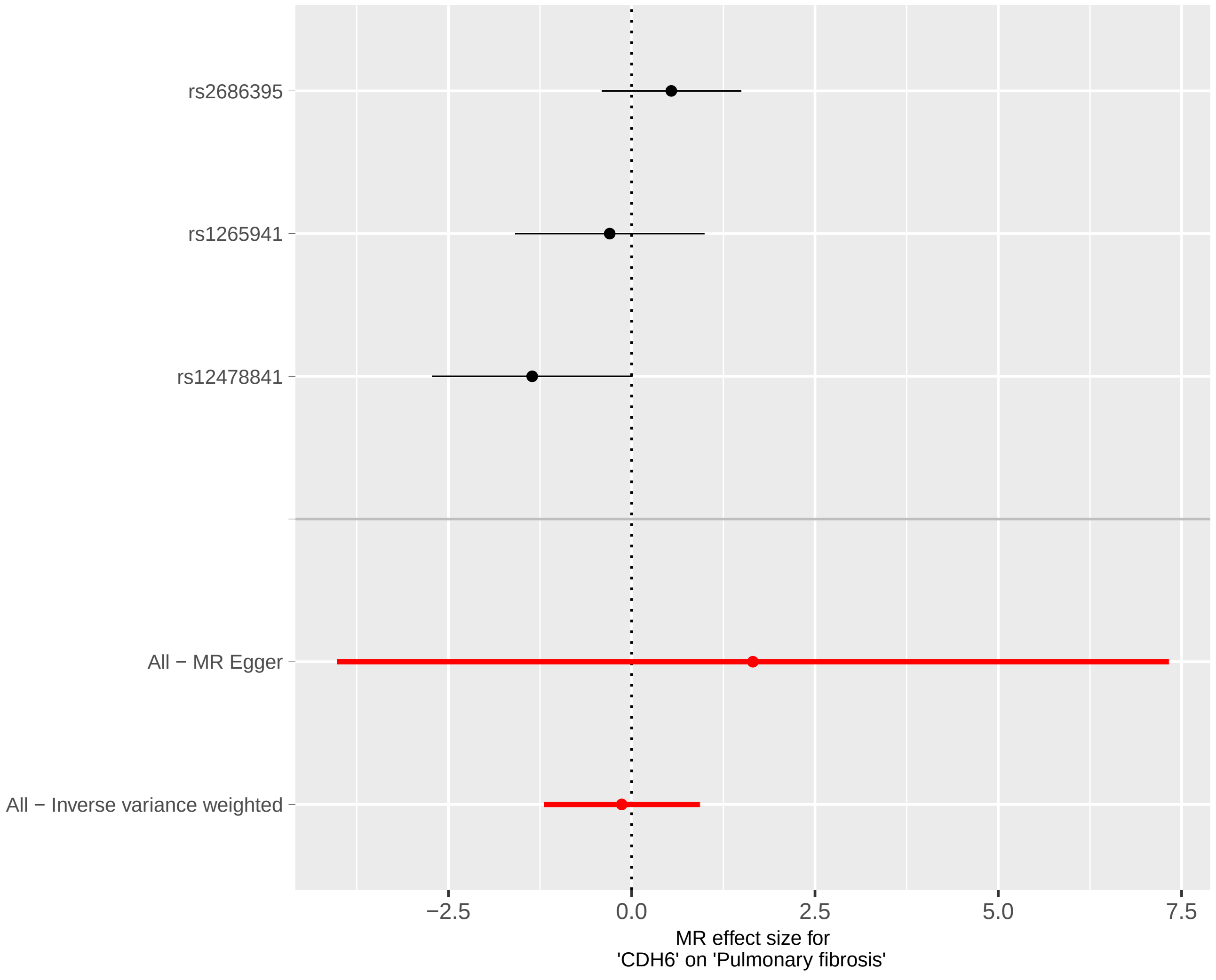

rs2686395
rs1265941
rs12478841
All − MR Egger
All − Inverse variance weighted
−2.5
0.0
2.5
5.0
7.5
MR effect size for
'CDH6' on 'Pulmonary fibrosis'

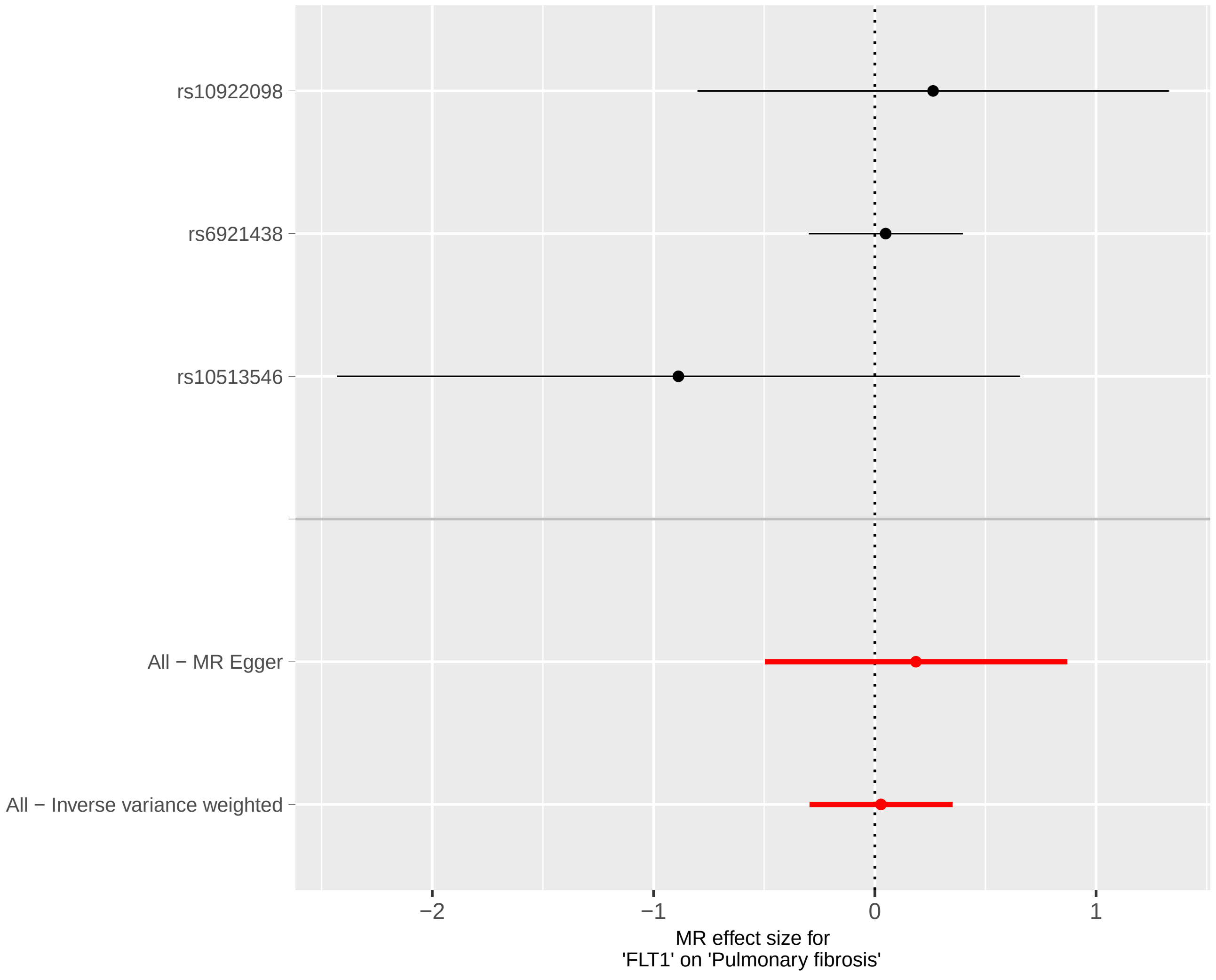

rs10922098
rs6921438
rs10513546
All − MR Egger
All − Inverse variance weighted
−2
−1
0
1
MR effect size for
'FLT1' on 'Pulmonary fibrosis'

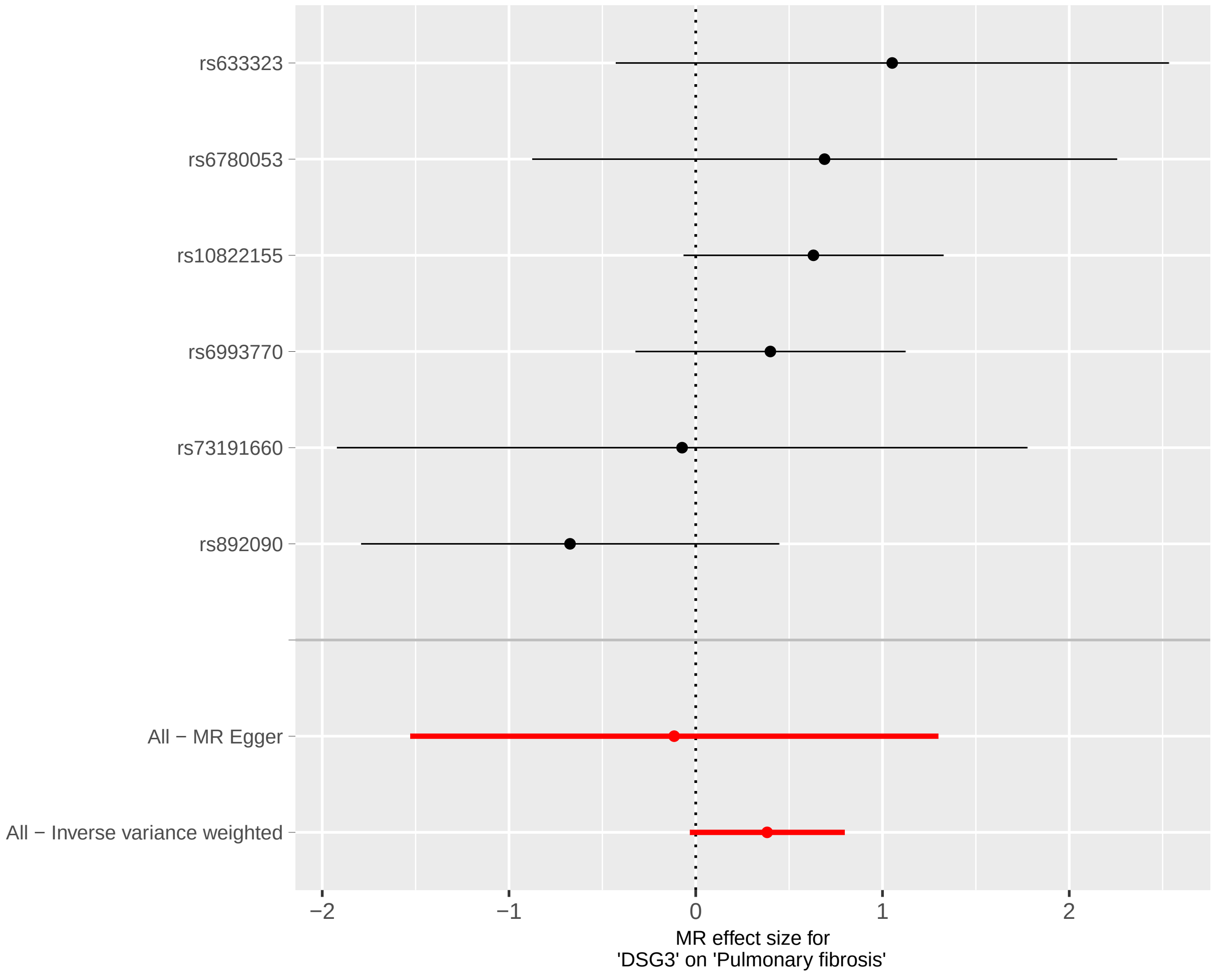

rs633323
rs6780053
rs10822155
rs6993770
rs73191660
rs892090
All − MR Egger
All − Inverse variance weighted
−2
−1
0
1
2
MR effect size for
'DSG3' on 'Pulmonary fibrosis'

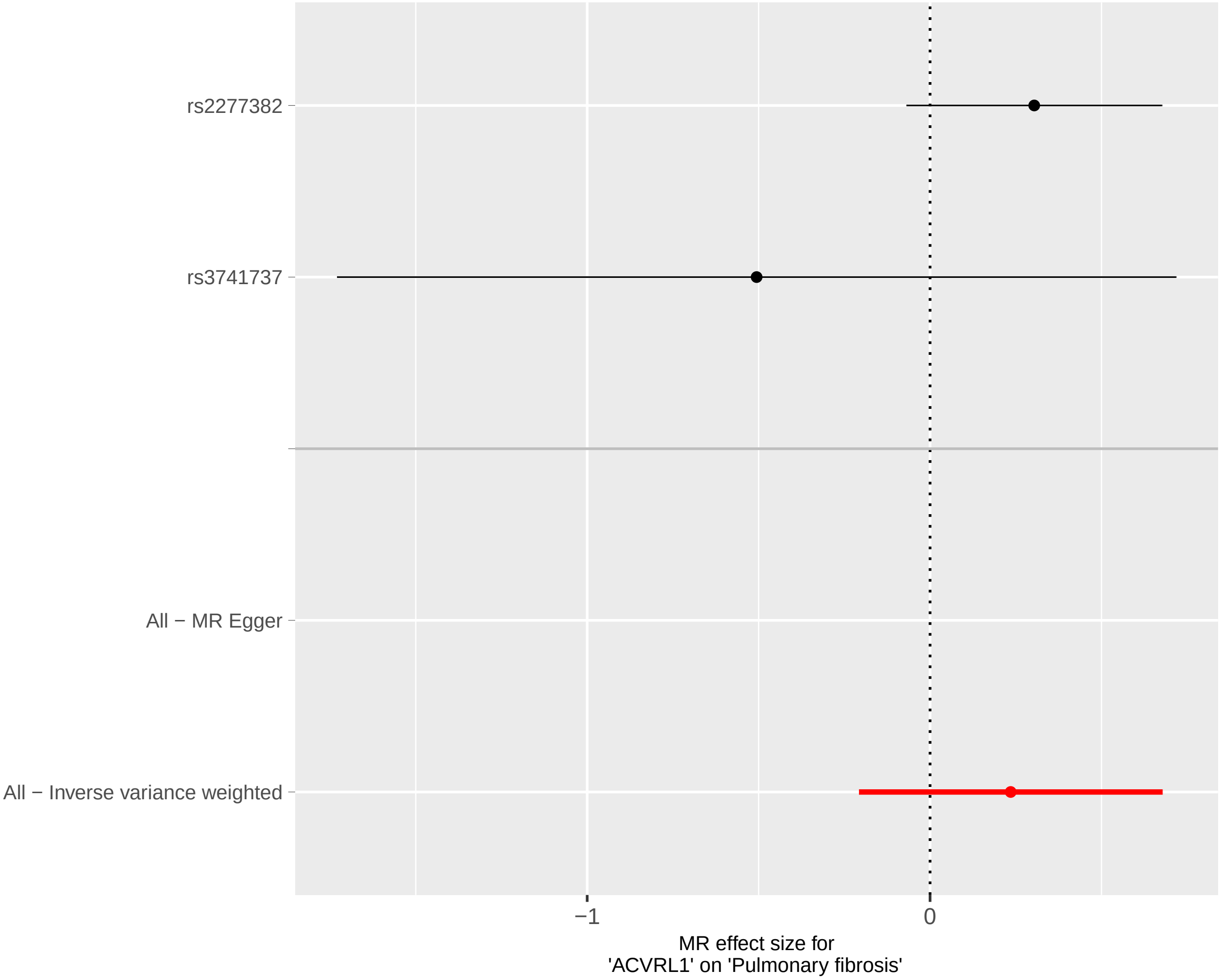
rs2277382
rs3741737
All − MR Egger
All − Inverse variance weighted
−1
0
MR effect size for
'ACVRL1' on 'Pulmonary fibrosis'

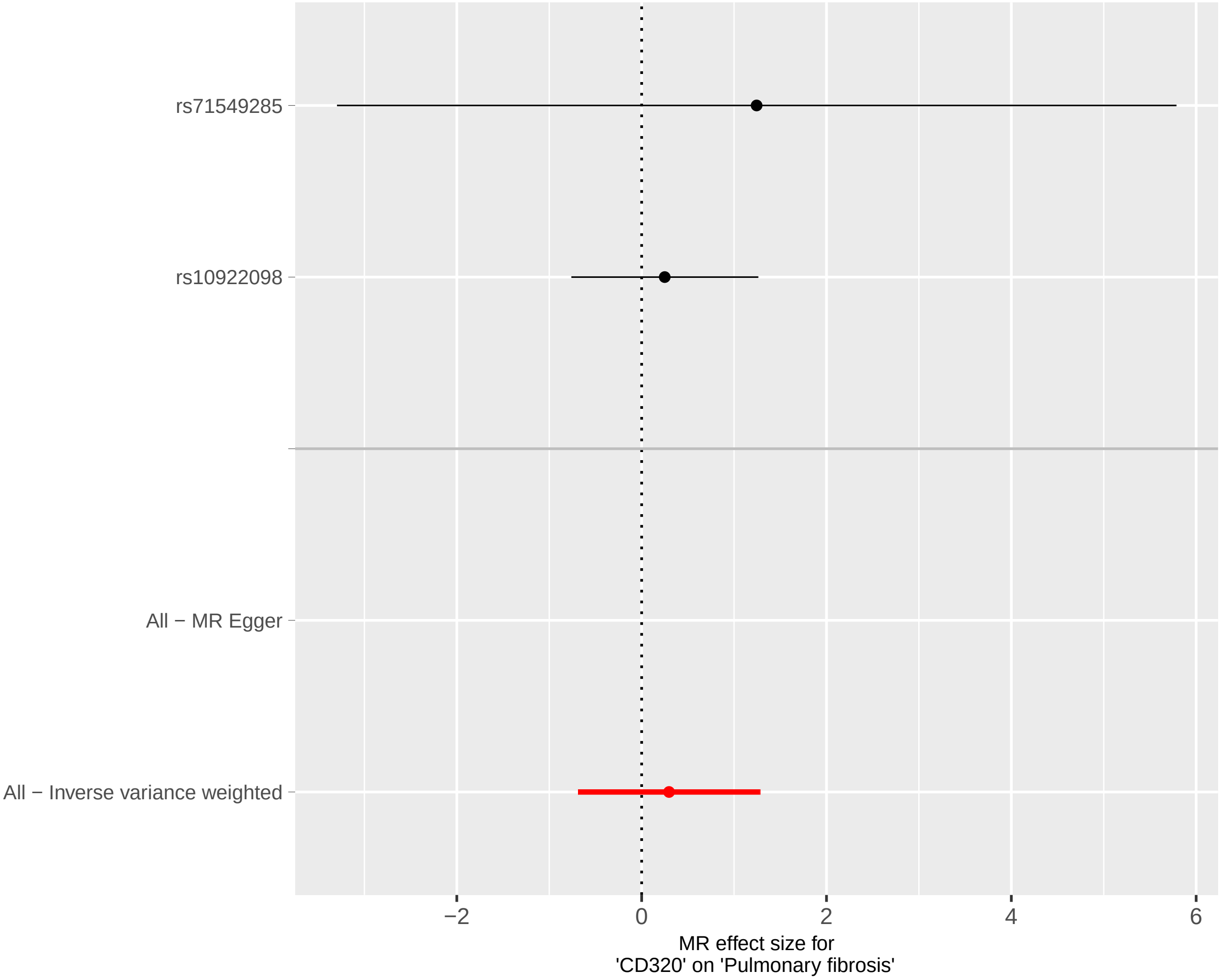
rs71549285
rs10922098
All − MR Egger
All − Inverse variance weighted
−2
0
2
4
6
MR effect size for
'CD320' on 'Pulmonary fibrosis'

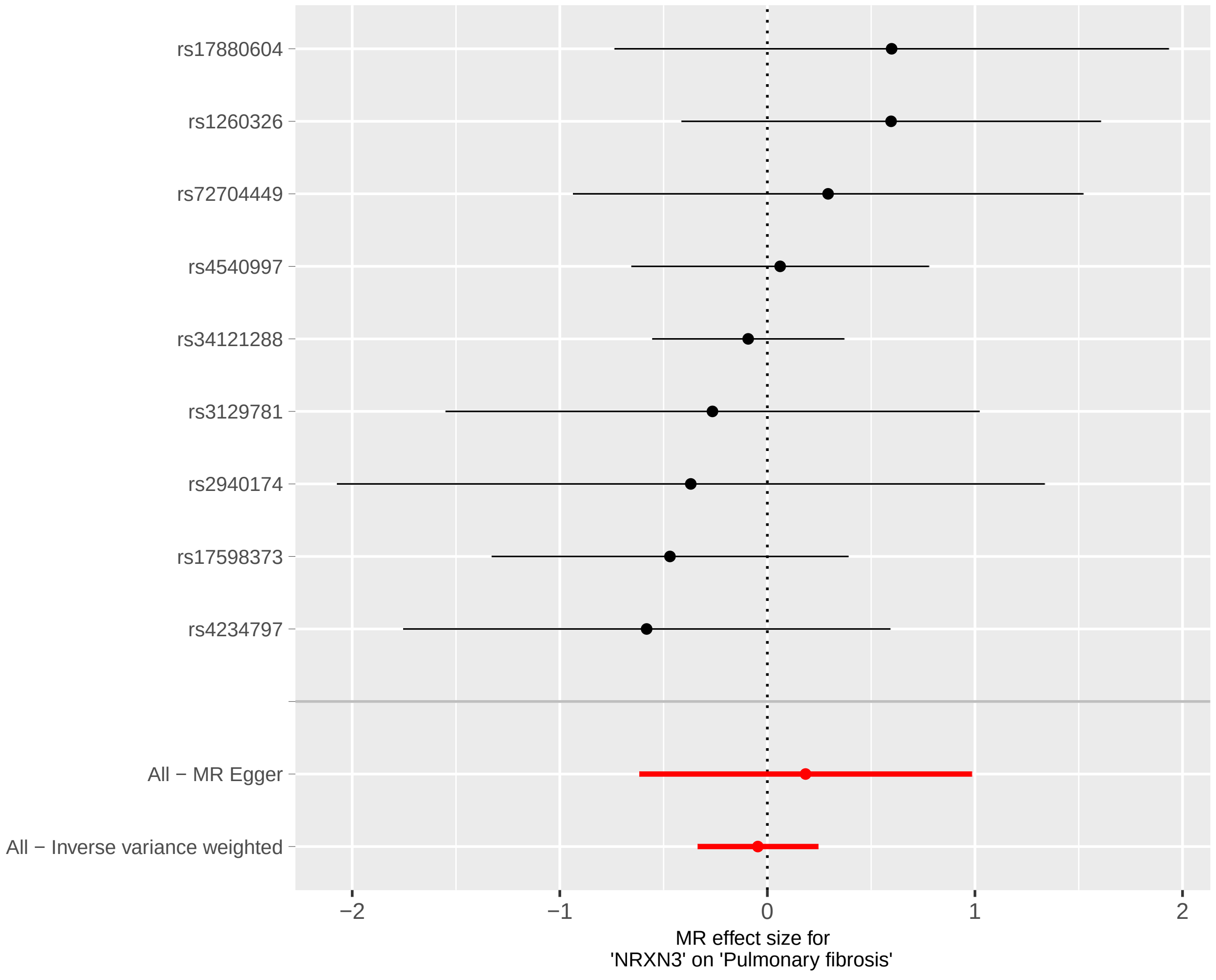
rs17880604
rs1260326
rs72704449
rs4540997
rs34121288
rs3129781
rs2940174
rs17598373
rs4234797
All − MR Egger
All − Inverse variance weighted
−2
−1
0
1
2
MR effect size for
'NRXN3' on 'Pulmonary fibrosis'

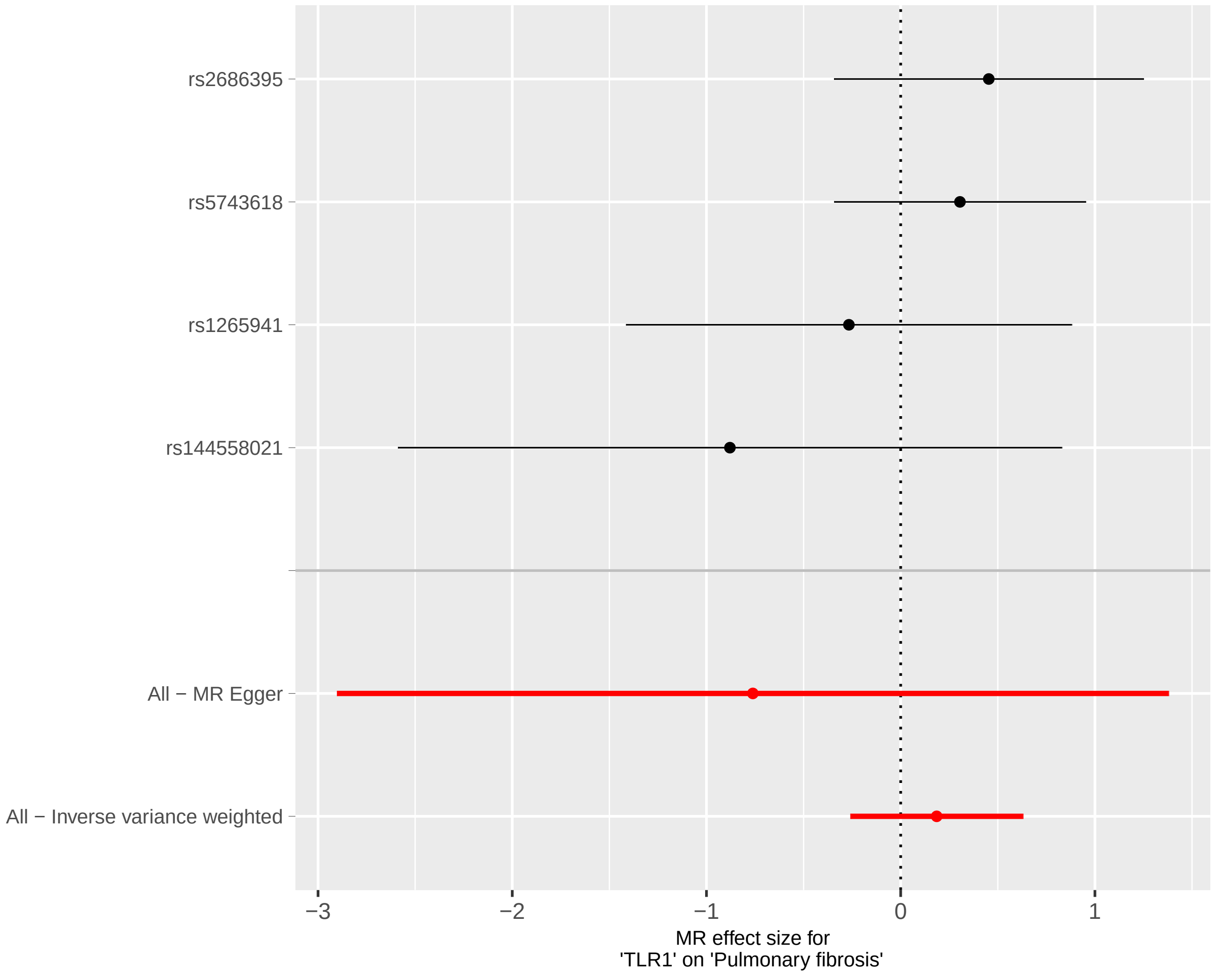
rs2686395
rs5743618
rs1265941
rs144558021
All − MR Egger
All − Inverse variance weighted
−3
−2
−1
0
1
MR effect size for
'TLR1' on 'Pulmonary fibrosis'

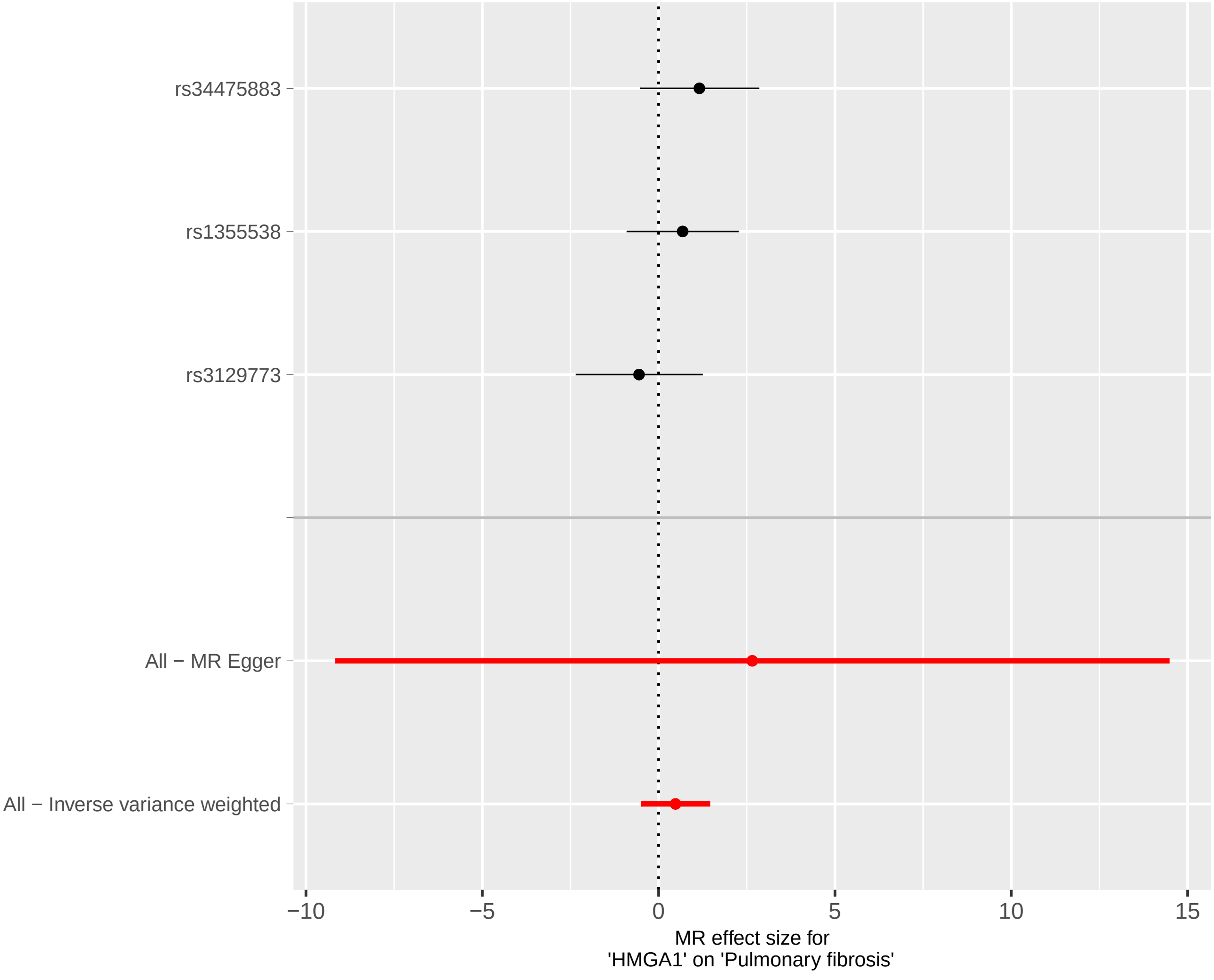

rs34475883
rs1355538
rs3129773
All − MR Egger
All − Inverse variance weighted
−10
−5
0
5
10
15
MR effect size for
'HMGA1' on 'Pulmonary fibrosis'

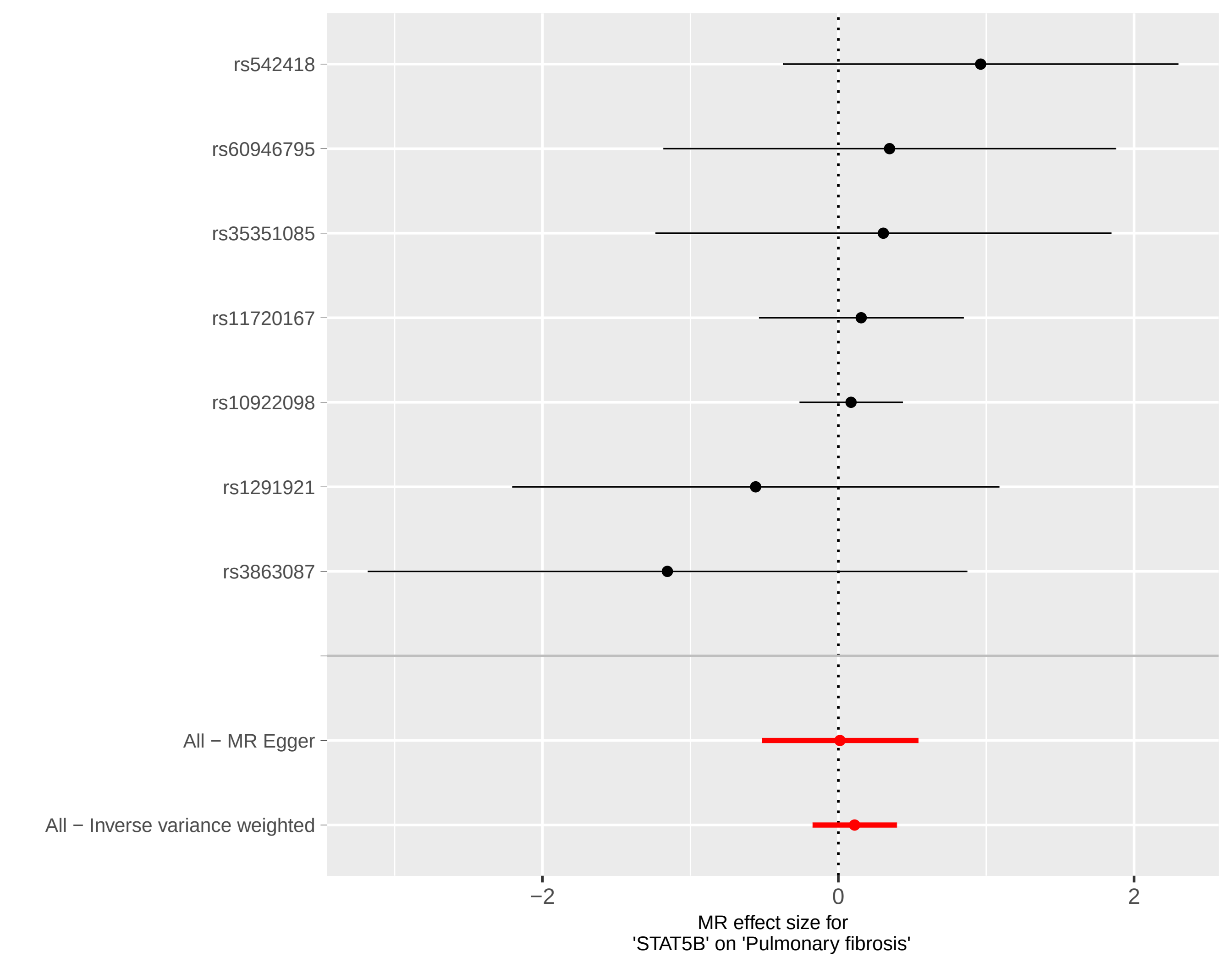
rs542418
rs60946795
rs35351085
rs11720167
rs10922098
rs1291921
rs3863087
All − MR Egger
All − Inverse variance weighted
−2
0
2
MR effect size for
'STAT5B' on 'Pulmonary fibrosis'

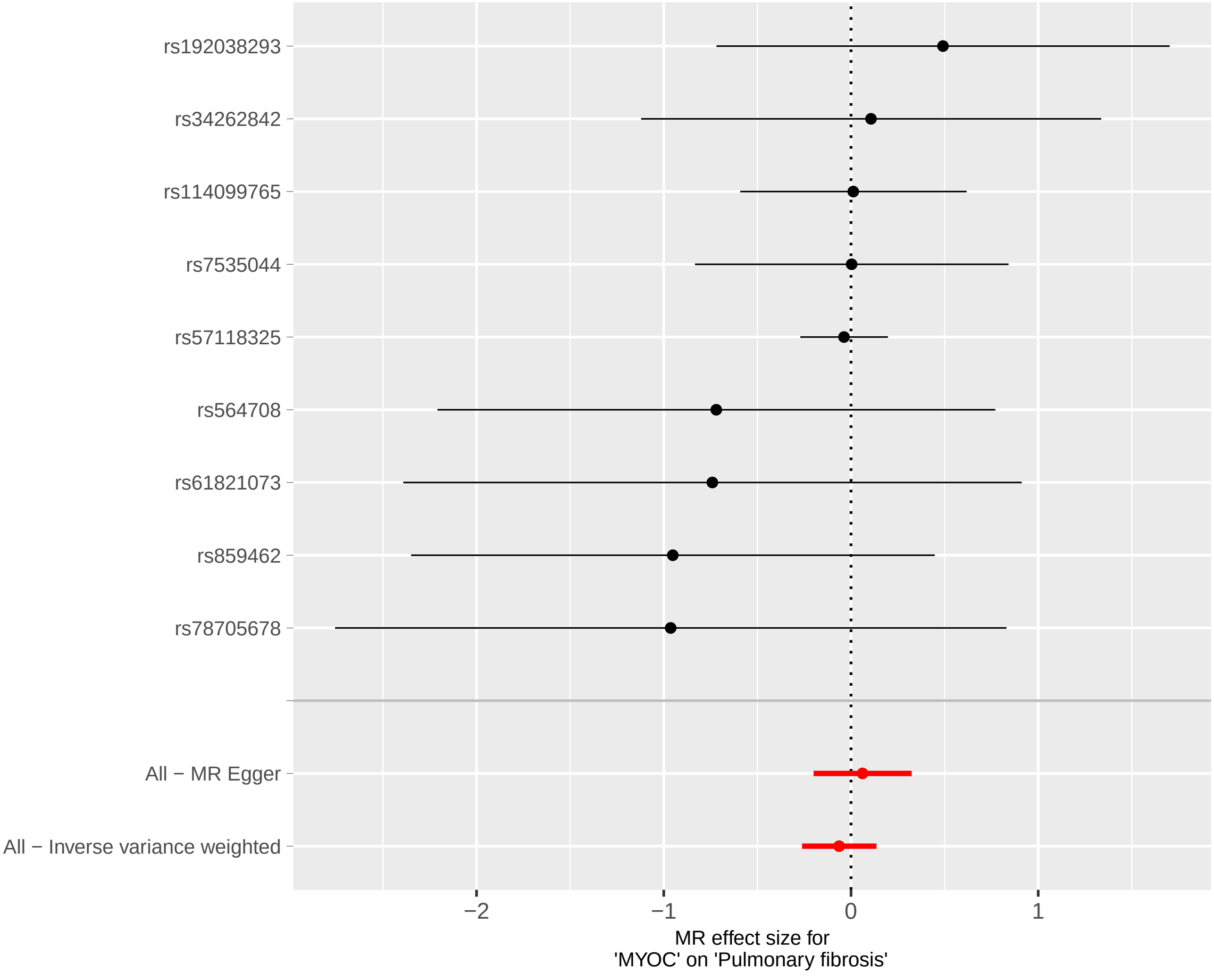
rs192038293
rs34262842
rs114099765
rs7535044
rs57118325
rs564708
rs61821073
rs859462
rs78705678
All − MR Egger
All − Inverse variance weighted
−2
−1
0
1
MR effect size for
'MYOC' on 'Pulmonary fibrosis'

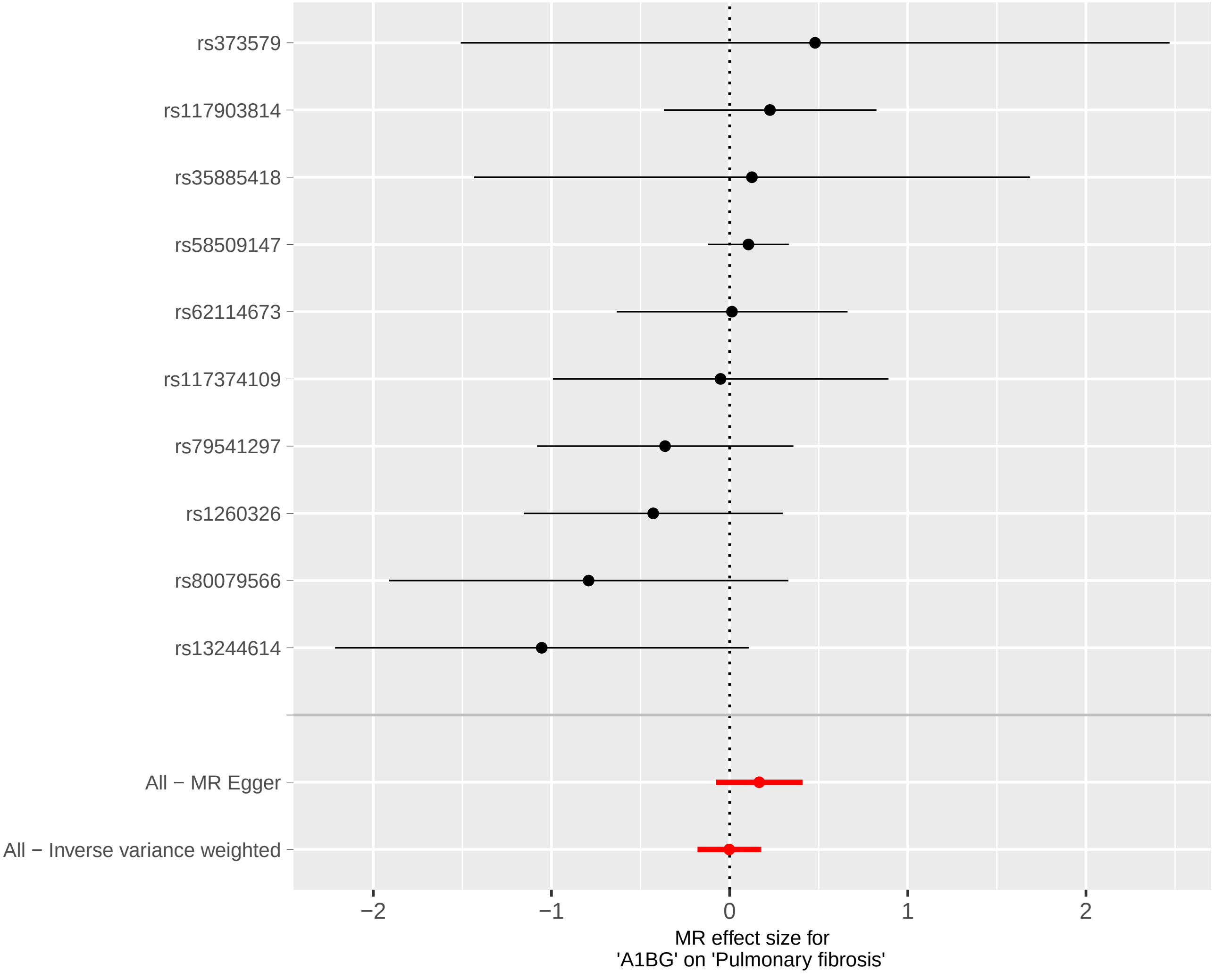
rs373579
rs117903814
rs35885418
rs58509147
rs62114673
rs117374109
rs79541297
rs1260326
rs80079566
rs13244614
All − MR Egger
All − Inverse variance weighted
−2
−1
0
1
2
MR effect size for
'A1BG' on 'Pulmonary fibrosis'

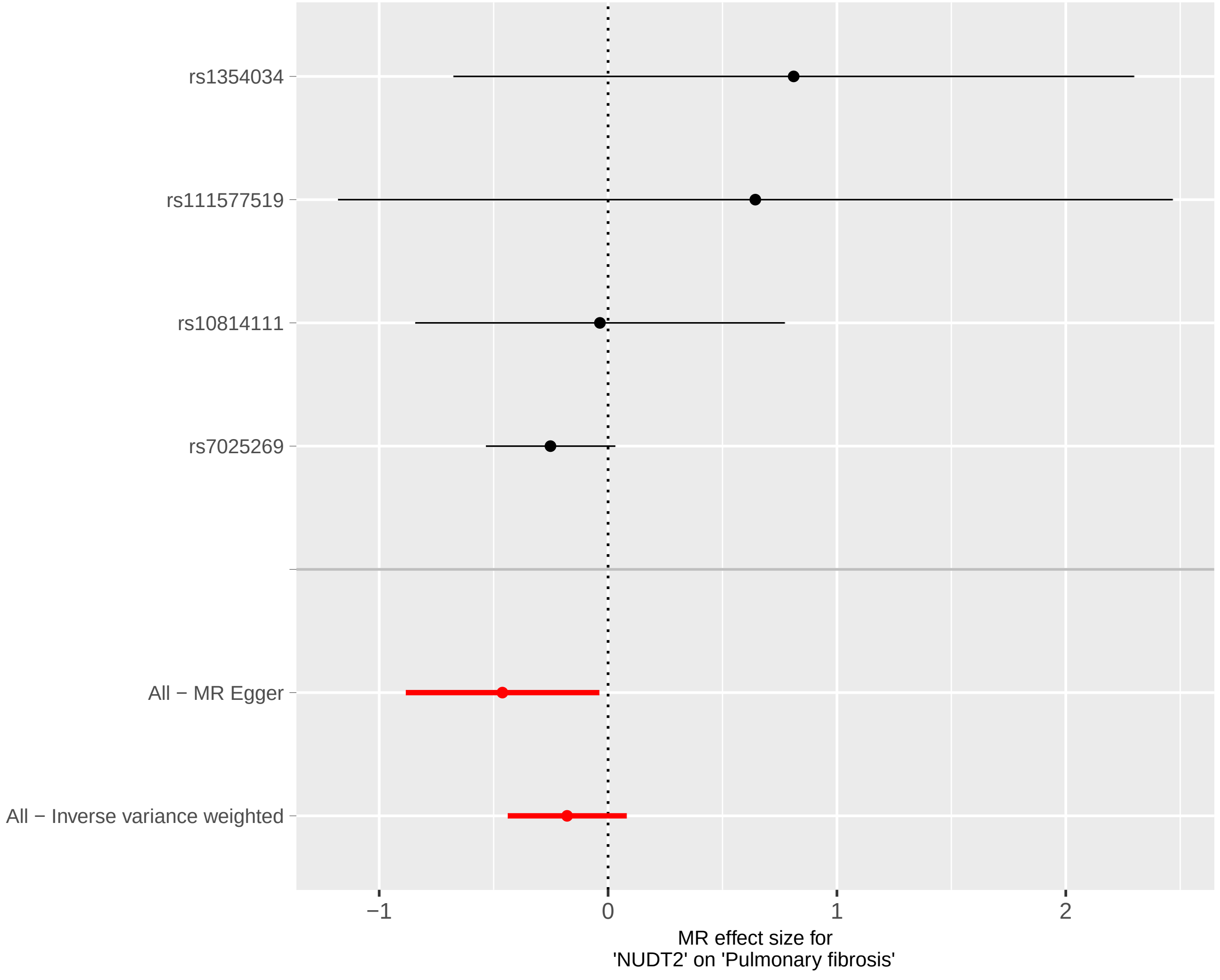

rs1354034
rs111577519
rs10814111
rs7025269
All − MR Egger
All − Inverse variance weighted
−1
0
1
2
MR effect size for
'NUDT2' on 'Pulmonary fibrosis'

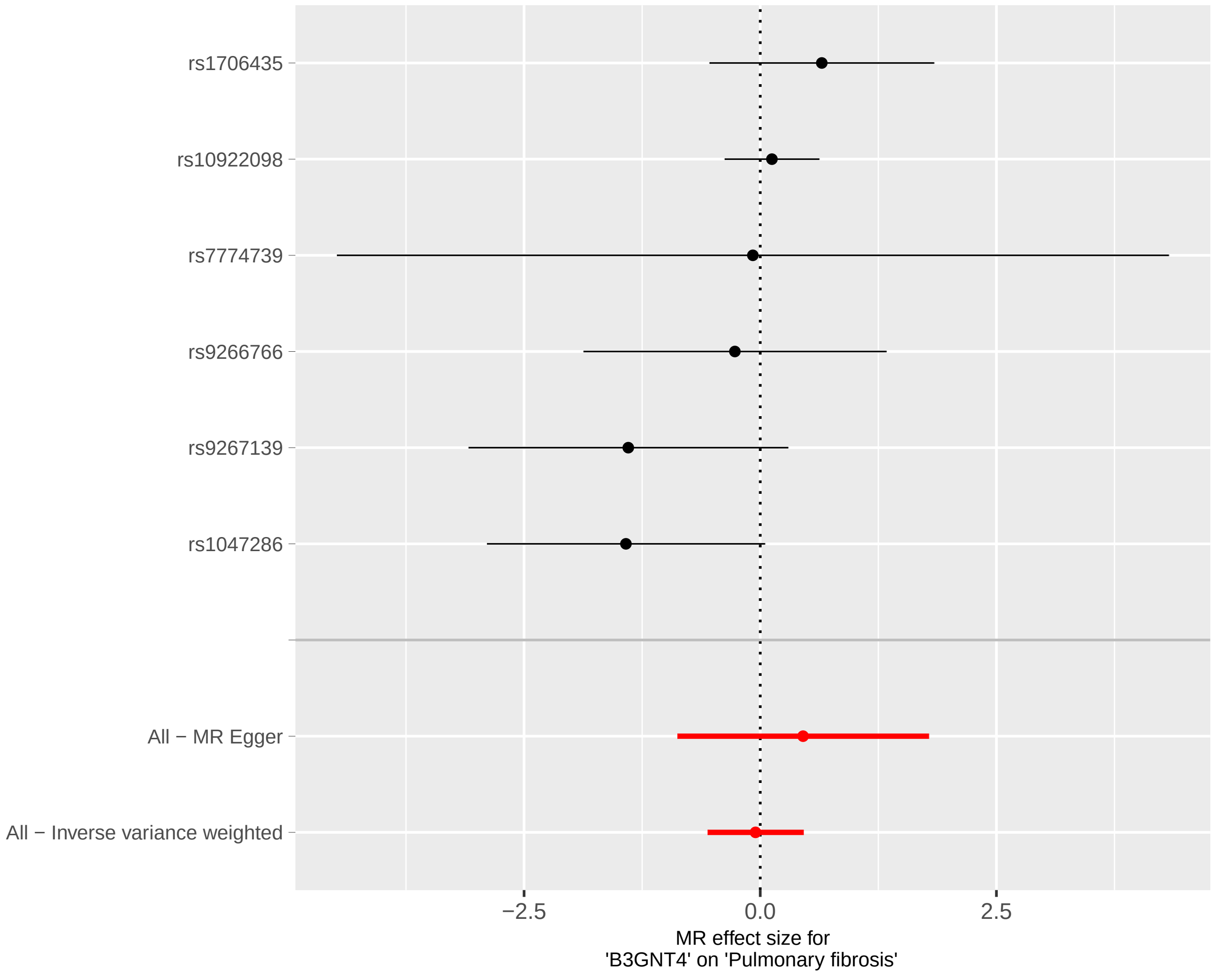

rs1706435
rs10922098
rs7774739
rs9266766
rs9267139
rs1047286
All − MR Egger
All − Inverse variance weighted
−2.5
0.0
2.5
MR effect size for
'B3GNT4' on 'Pulmonary fibrosis'

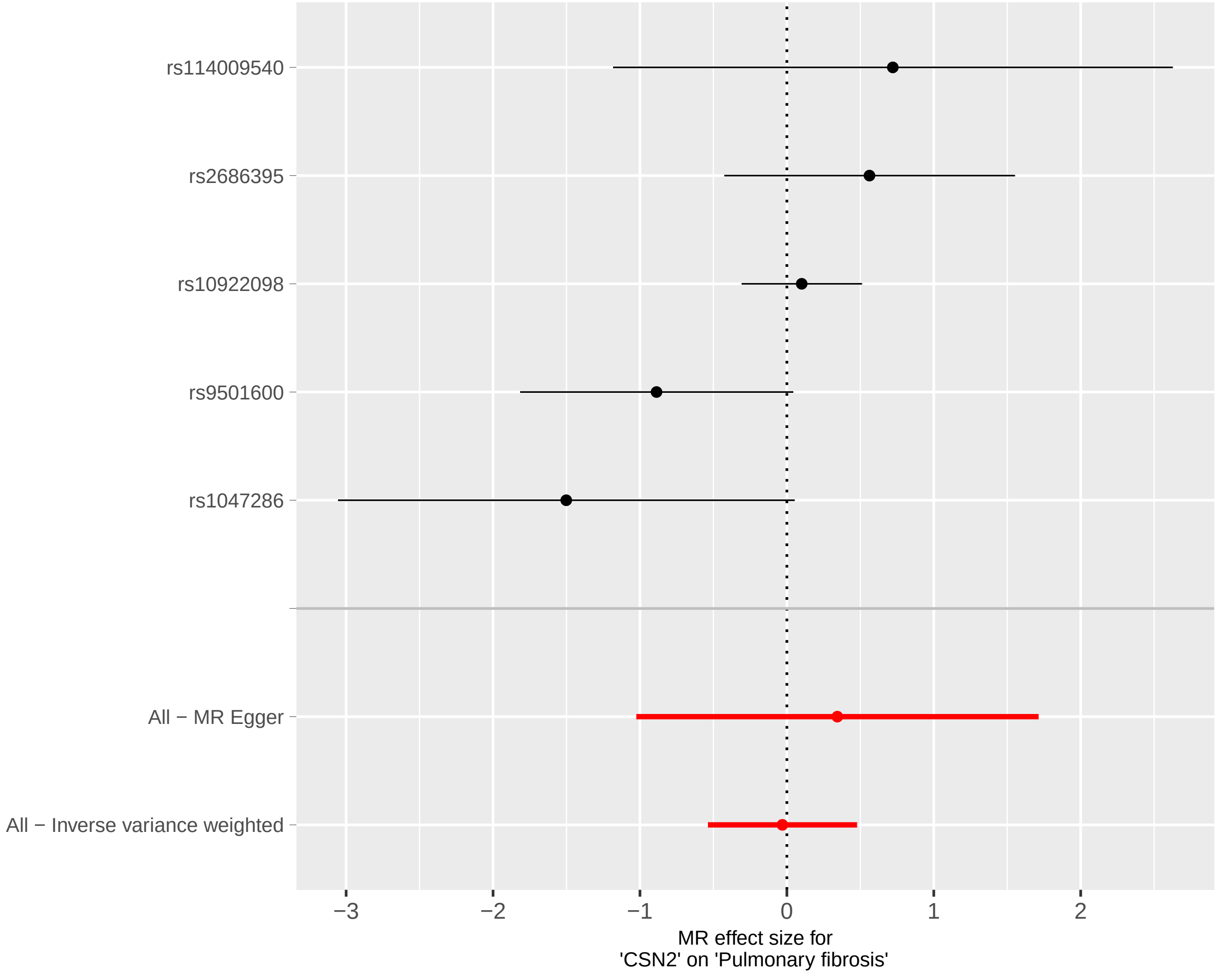

rs114009540
rs2686395
rs10922098
rs9501600
rs1047286
All − MR Egger
All − Inverse variance weighted
−3
−2
−1
0
1
2
MR effect size for
'CSN2' on 'Pulmonary fibrosis'

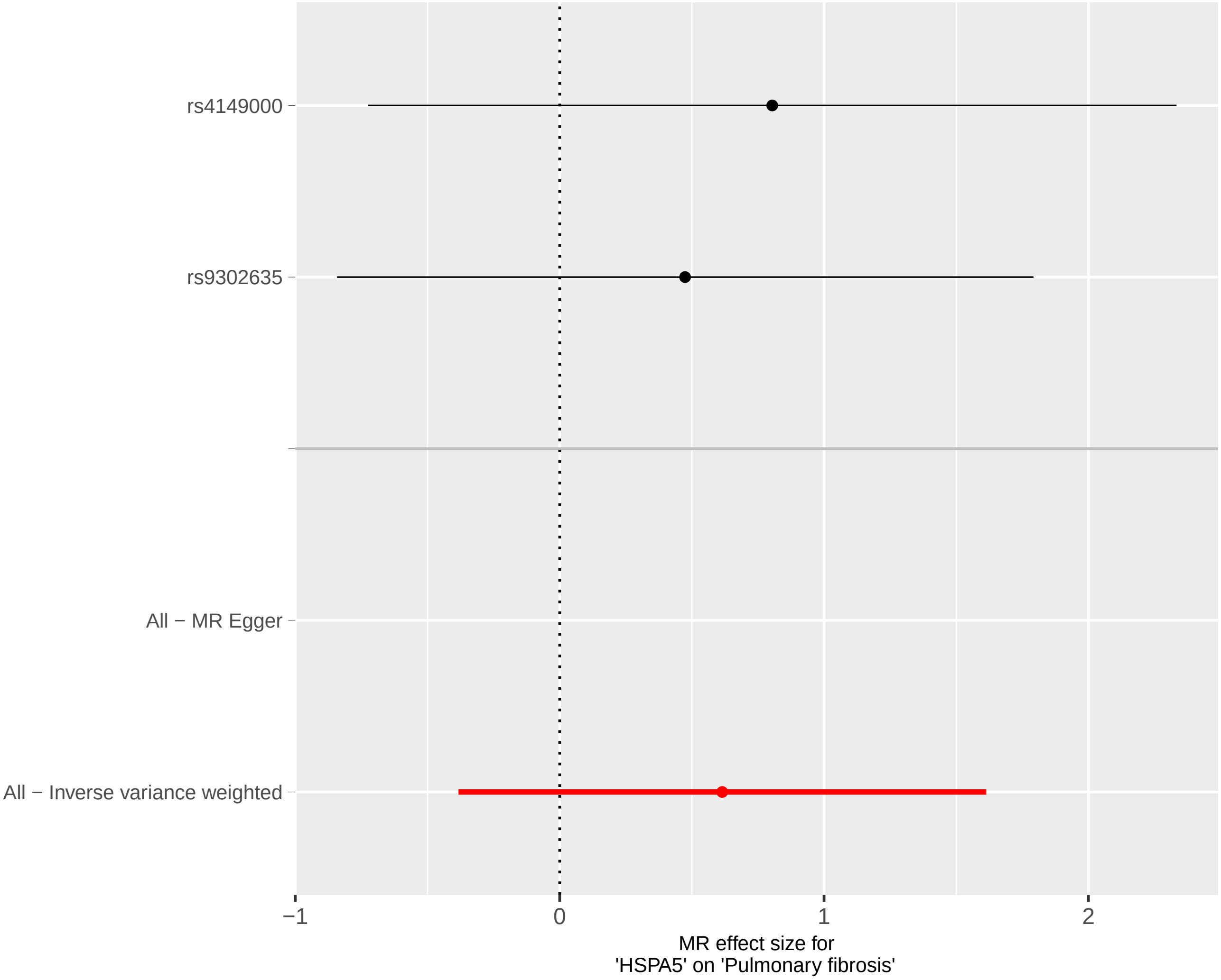

rs4149000
rs9302635
All − MR Egger
All − Inverse variance weighted
−1
0
1
2
MR effect size for
'HSPA5' on 'Pulmonary fibrosis'

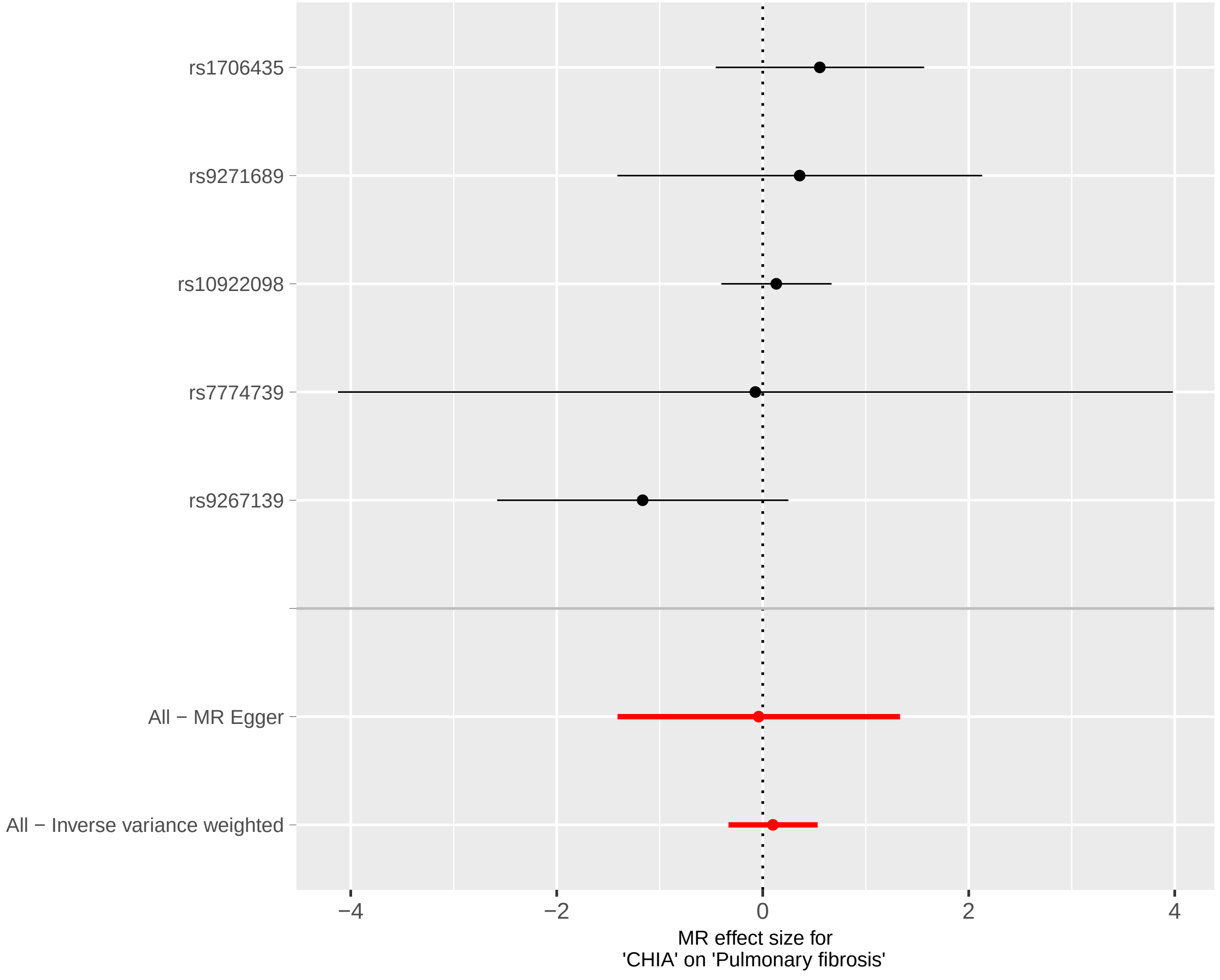
rs1706435
rs9271689
rs10922098
rs7774739
rs9267139
All − MR Egger
All − Inverse variance weighted
−4
−2
0
2
4
MR effect size for
'CHIA' on 'Pulmonary fibrosis'

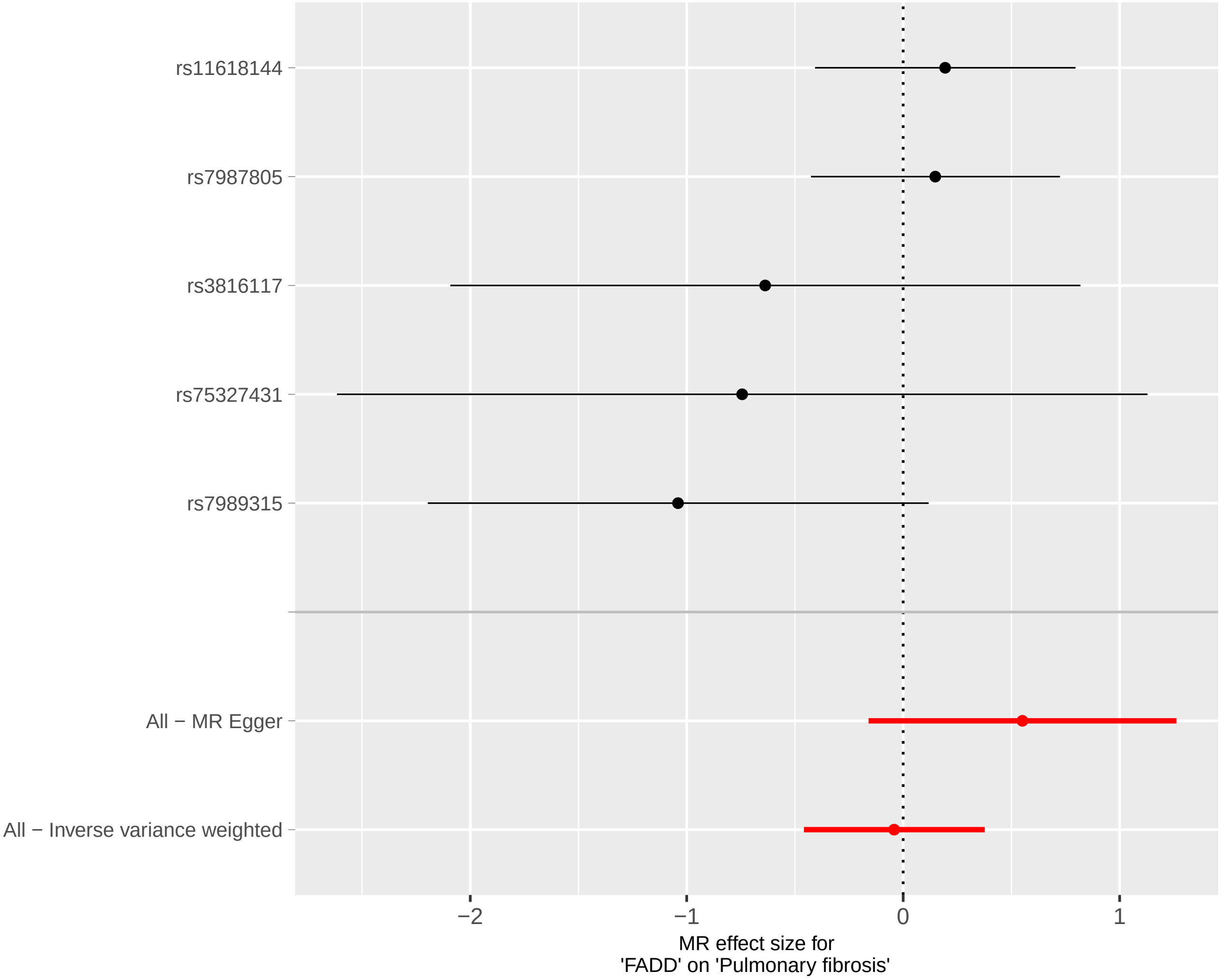
rs11618144
rs7987805
rs3816117
rs75327431
rs7989315
All − MR Egger
All − Inverse variance weighted
−2
−1
0
1
MR effect size for
'FADD' on 'Pulmonary fibrosis'

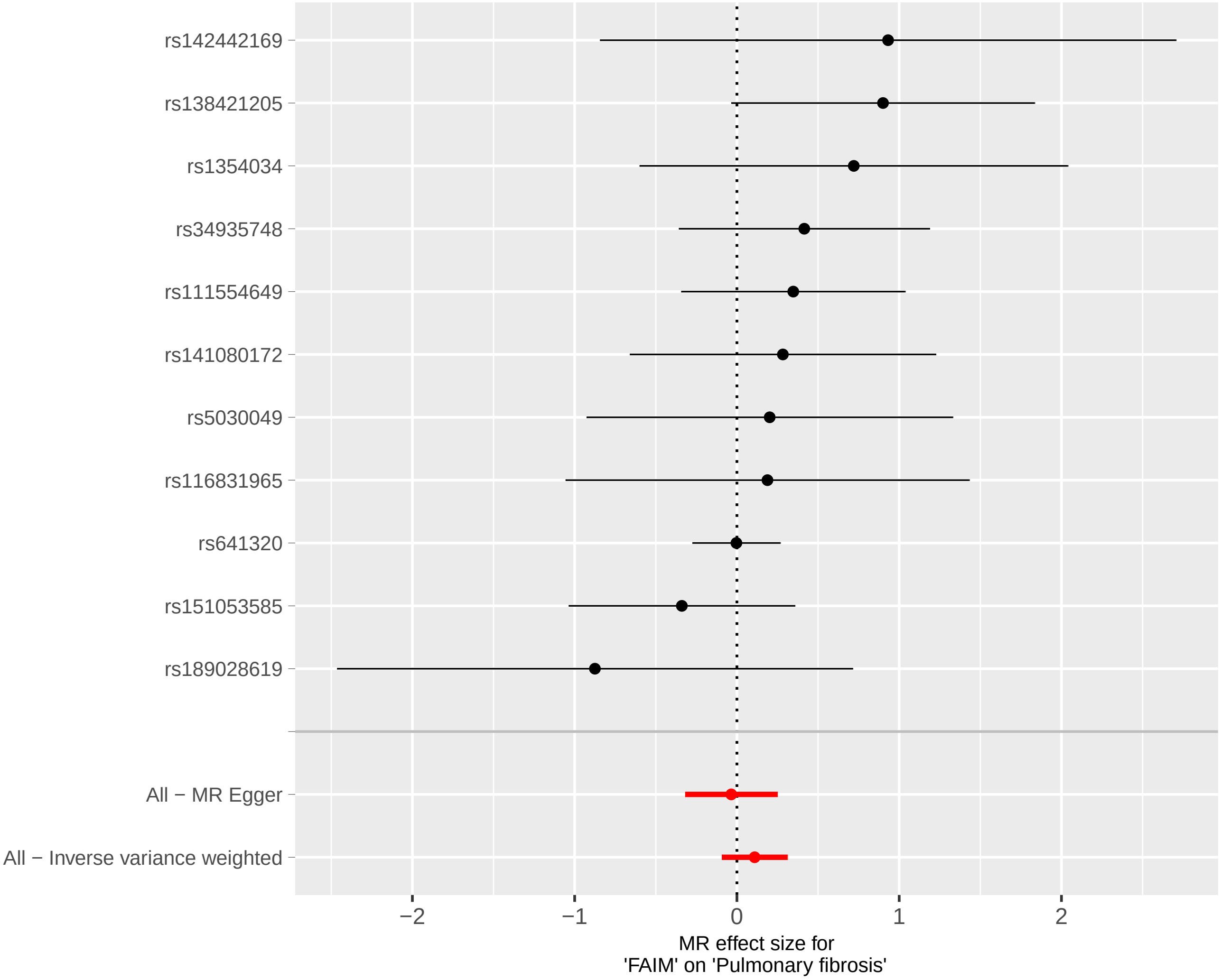
rs142442169
rs138421205
rs1354034
rs34935748
rs111554649
rs141080172
rs5030049
rs116831965
rs641320
rs151053585
rs189028619
All − MR Egger
All − Inverse variance weighted
−2
−1
0
1
2
MR effect size for
'FAIM' on 'Pulmonary fibrosis'

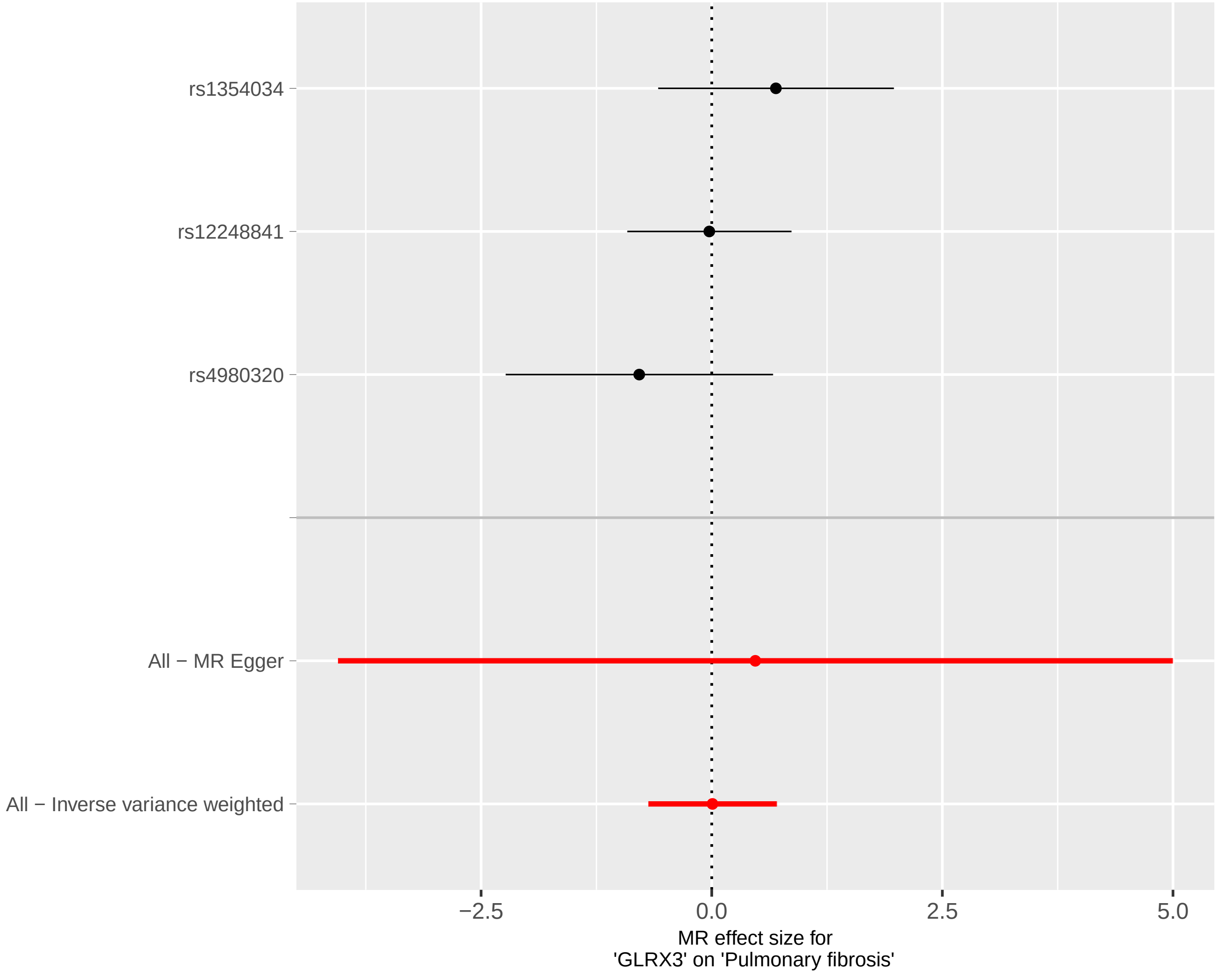
rs1354034
rs12248841
rs4980320
All − MR Egger
All − Inverse variance weighted
−2.5
0.0
2.5
5.0
MR effect size for
'GLRX3' on 'Pulmonary fibrosis'

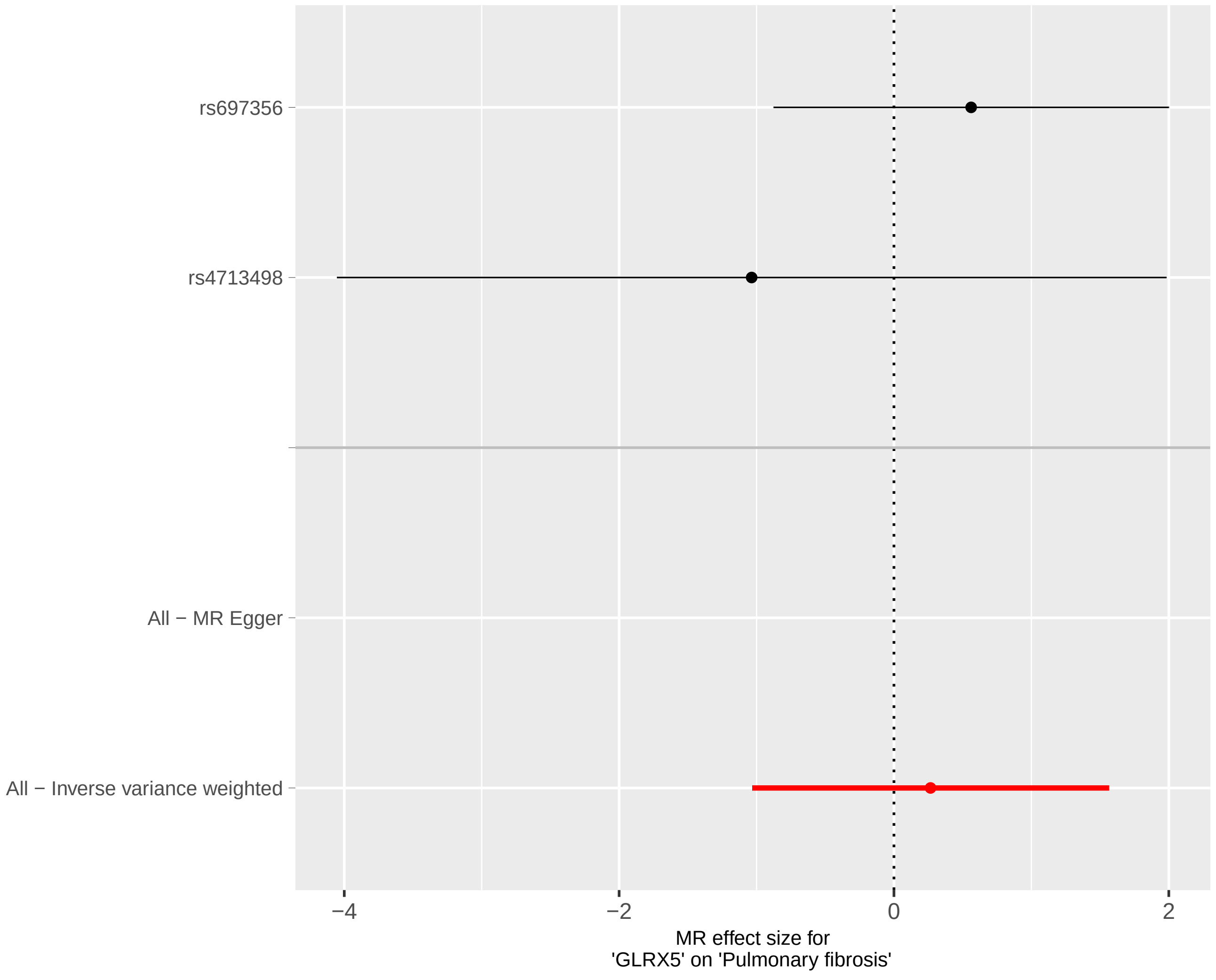

rs697356
rs4713498
All − MR Egger
All − Inverse variance weighted
−4
−2
0
2
MR effect size for
'GLRX5' on 'Pulmonary fibrosis'

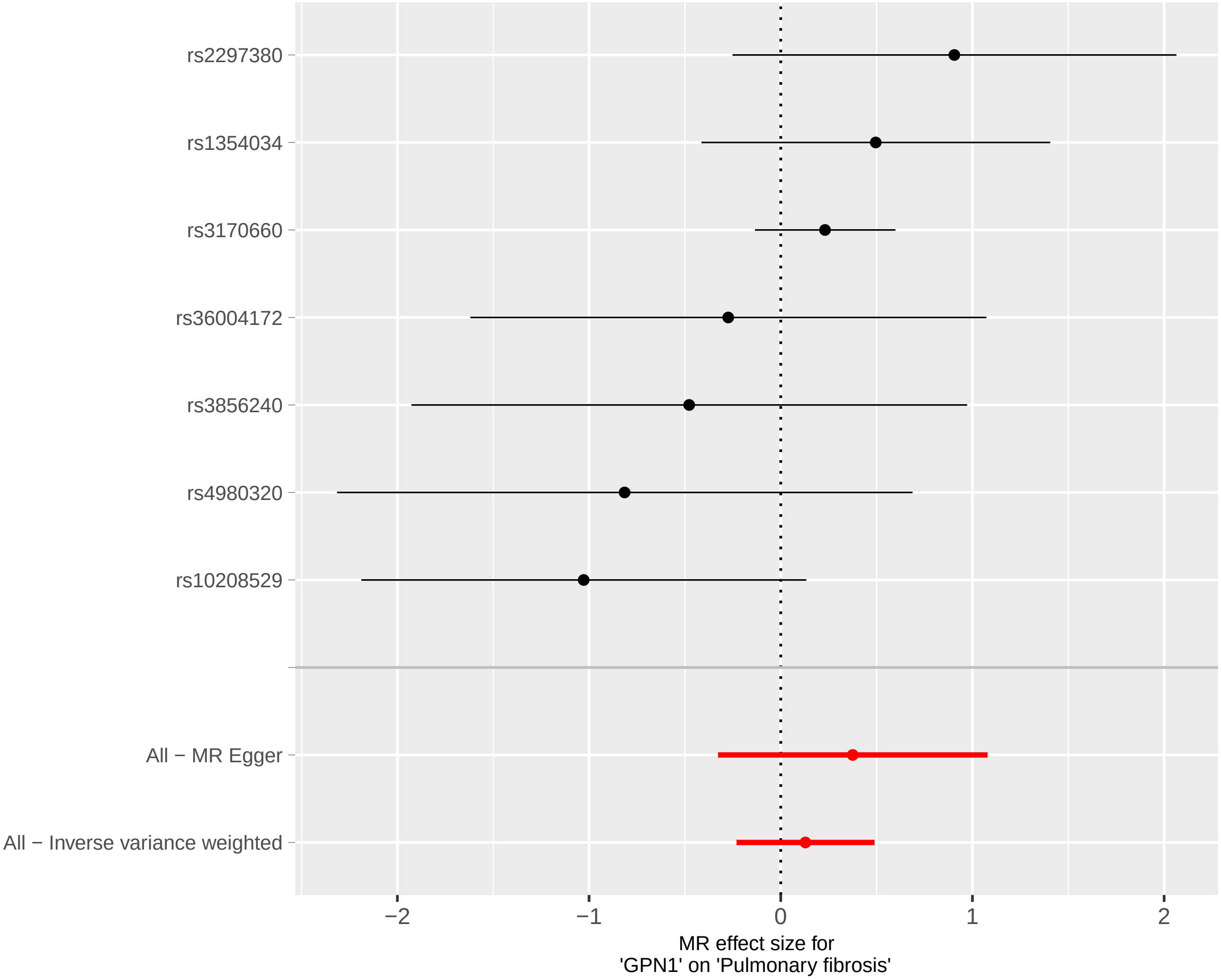

rs2297380
rs1354034
rs3170660
rs36004172
rs3856240
rs4980320
rs10208529
All − MR Egger
All − Inverse variance weighted
−2
−1
0
1
2
MR effect size for
'GPN1' on 'Pulmonary fibrosis'

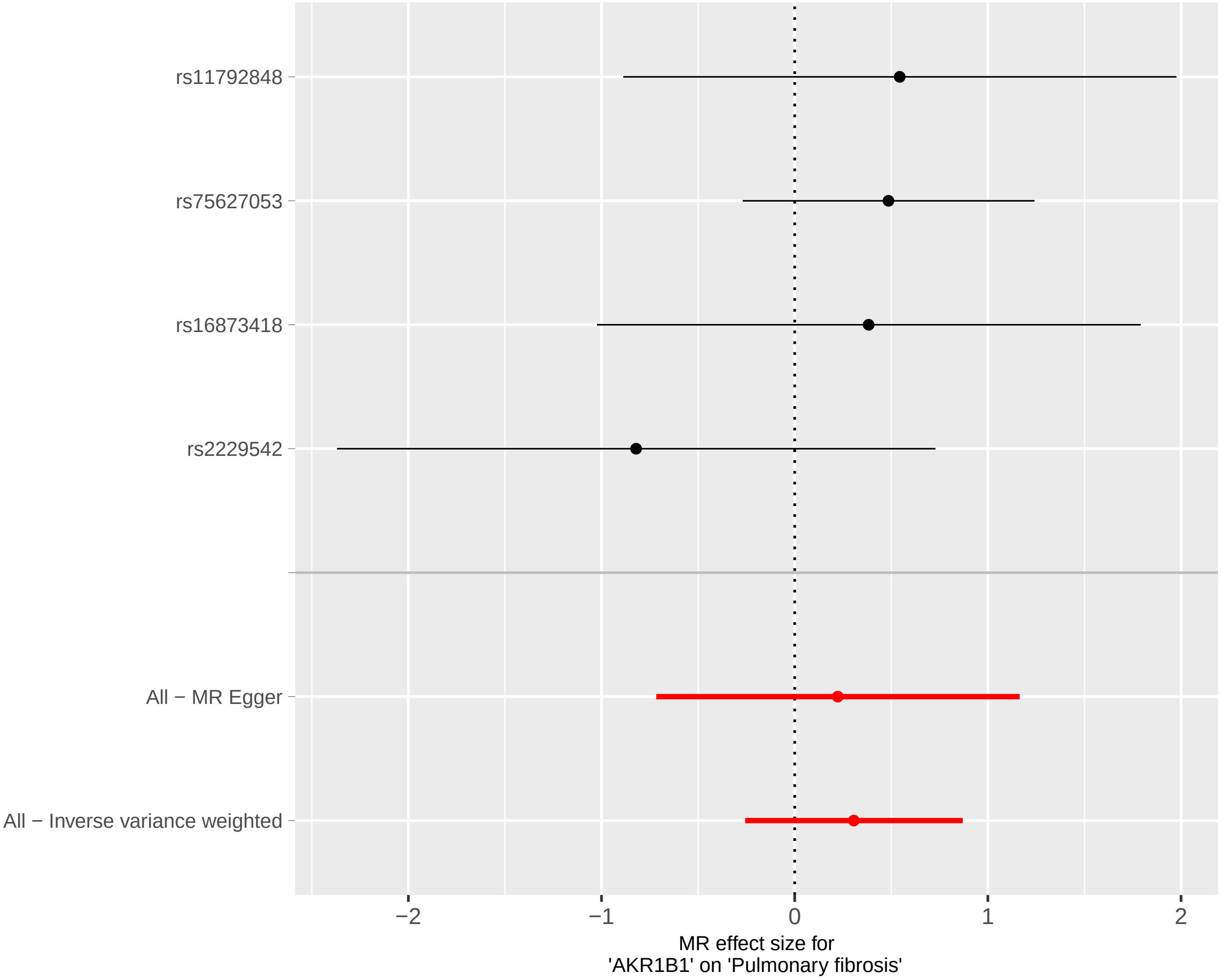
rs11792848
rs75627053
rs16873418
rs2229542
All − MR Egger
All − Inverse variance weighted
−2
−1
0
1
2
MR effect size for
'AKR1B1' on 'Pulmonary fibrosis'

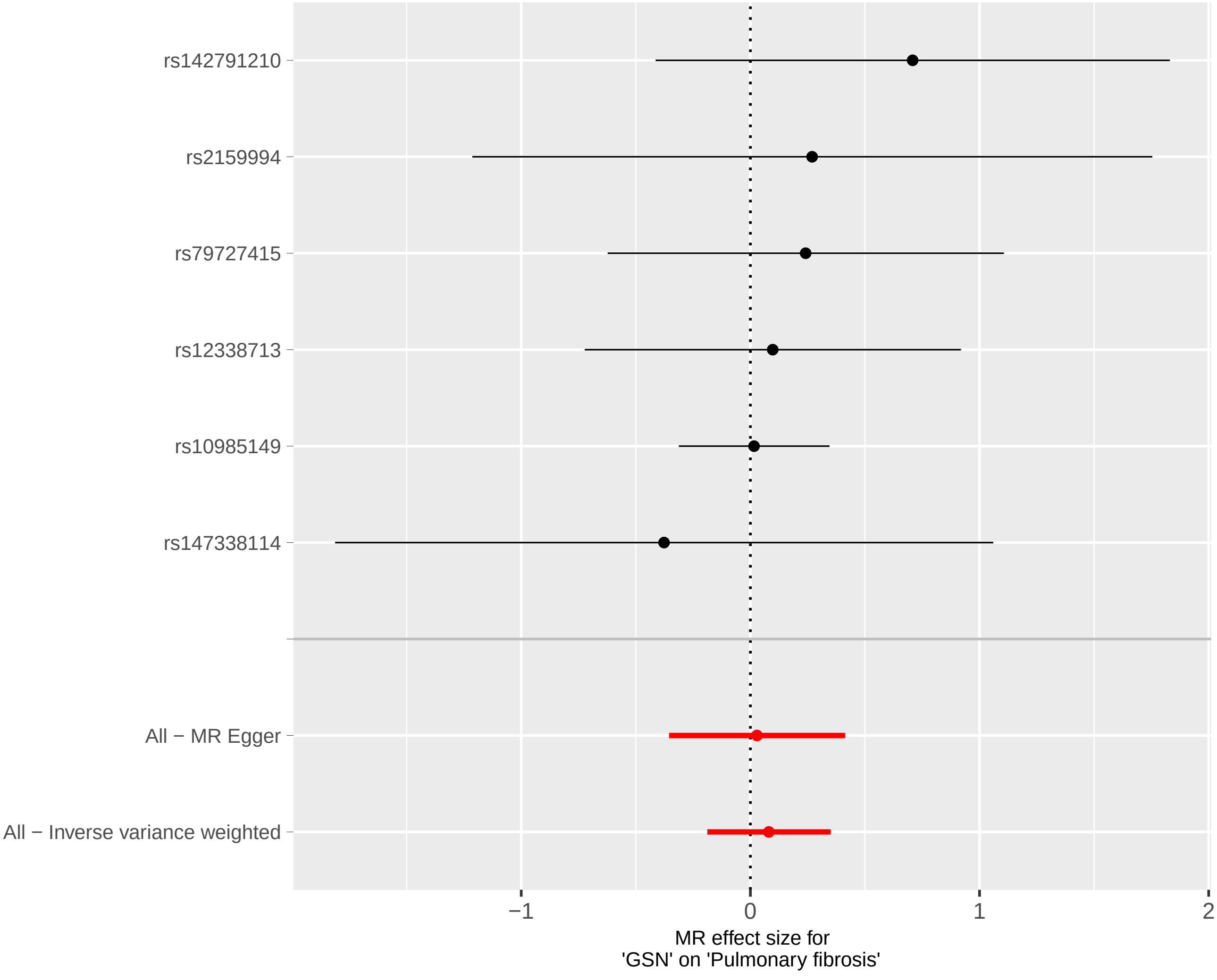
rs142791210
rs2159994
rs79727415
rs12338713
rs10985149
rs147338114
All − MR Egger
All − Inverse variance weighted
−1
0
1
2
MR effect size for
'GSN' on 'Pulmonary fibrosis'

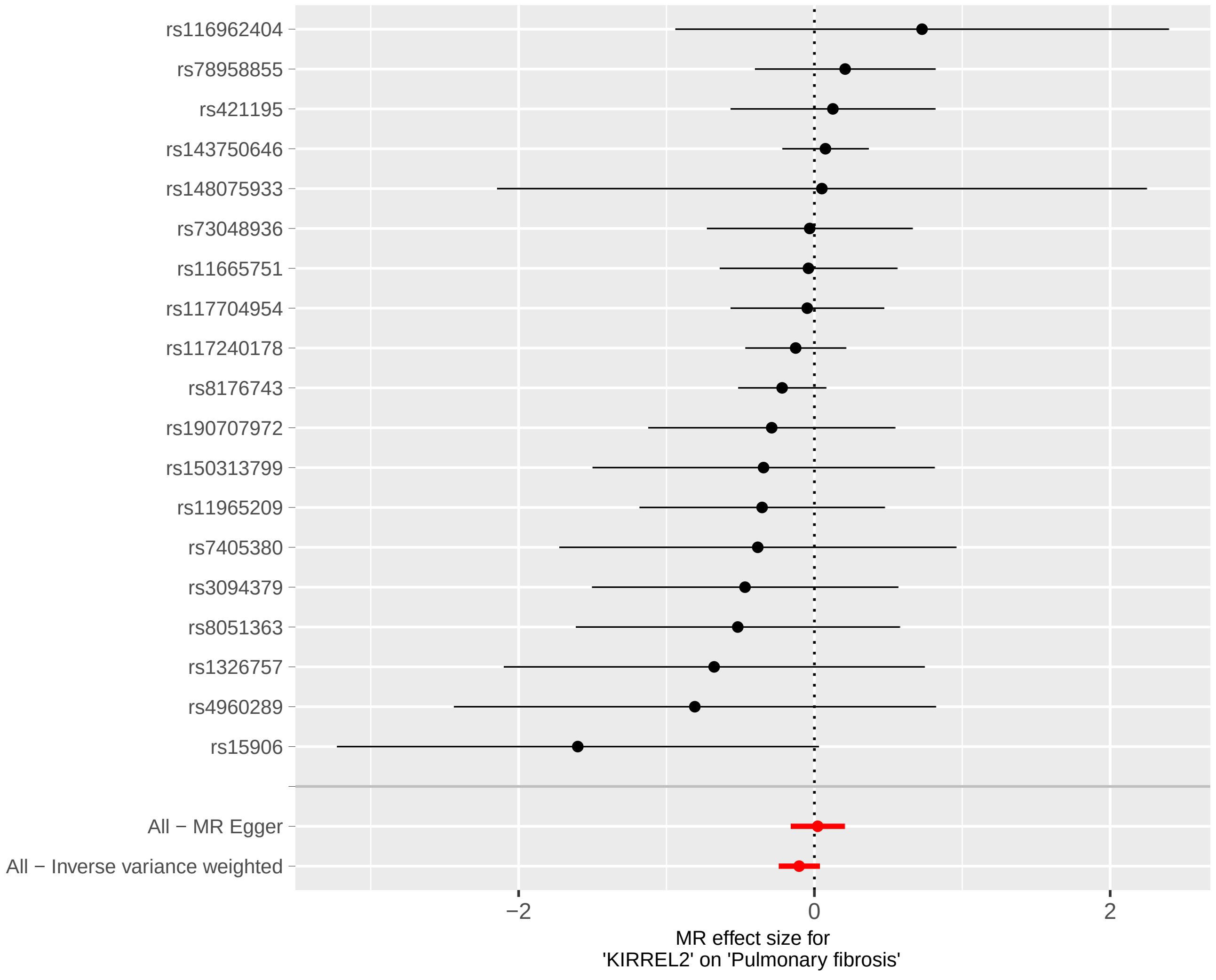

rs116962404
rs78958855
rs421195
rs143750646
rs148075933
rs73048936
rs11665751
rs117704954
rs117240178
rs8176743
rs190707972
rs150313799
rs11965209
rs7405380
rs3094379
rs8051363
rs1326757
rs4960289
rs15906
All − MR Egger
All − Inverse variance weighted
−2
0
2
MR effect size for
'KIRREL2' on 'Pulmonary fibrosis'

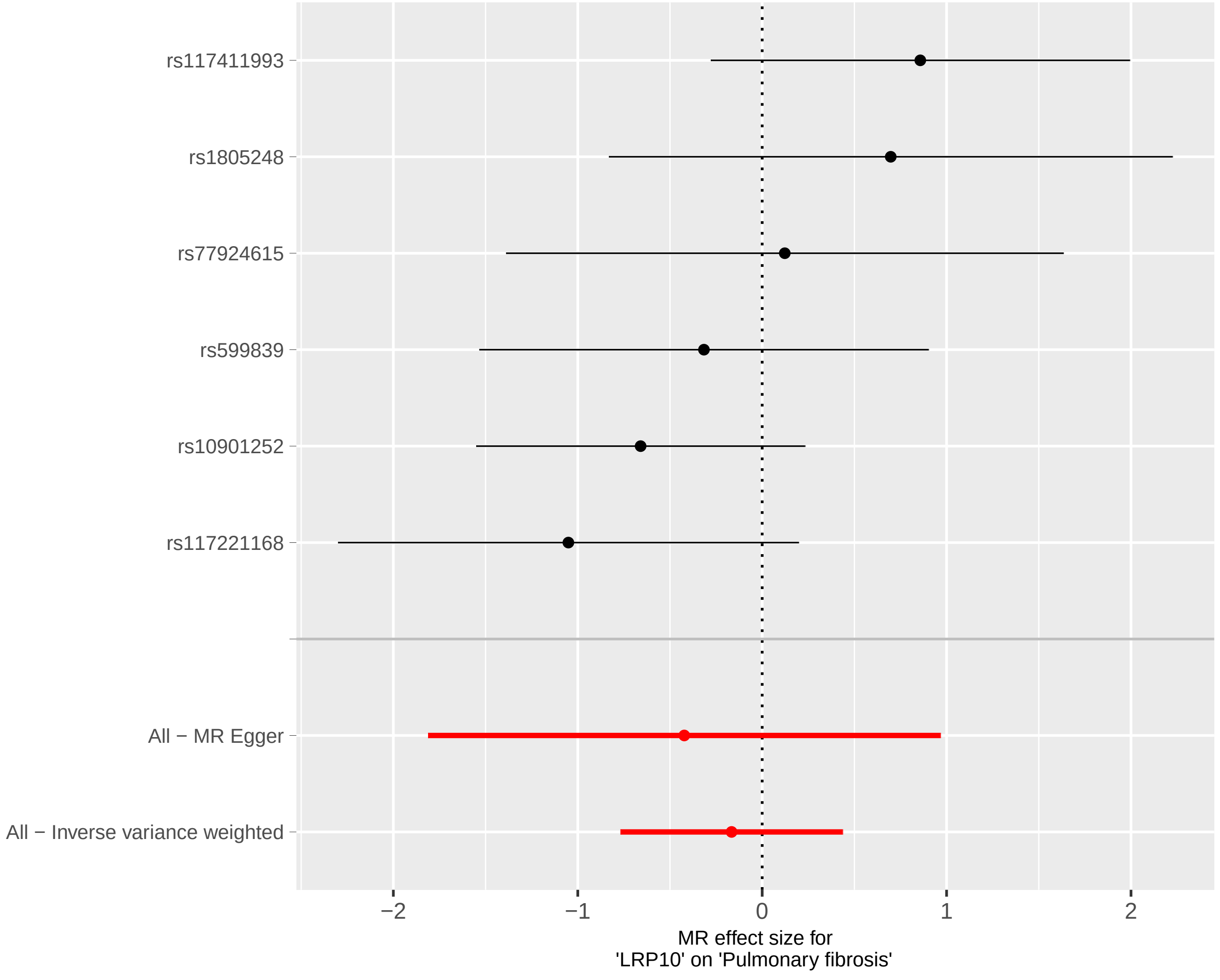
rs117411993
rs1805248
rs77924615
rs599839
rs10901252
rs117221168
All − MR Egger
All − Inverse variance weighted
−2
−1
0
1
2
MR effect size for
'LRP10' on 'Pulmonary fibrosis'

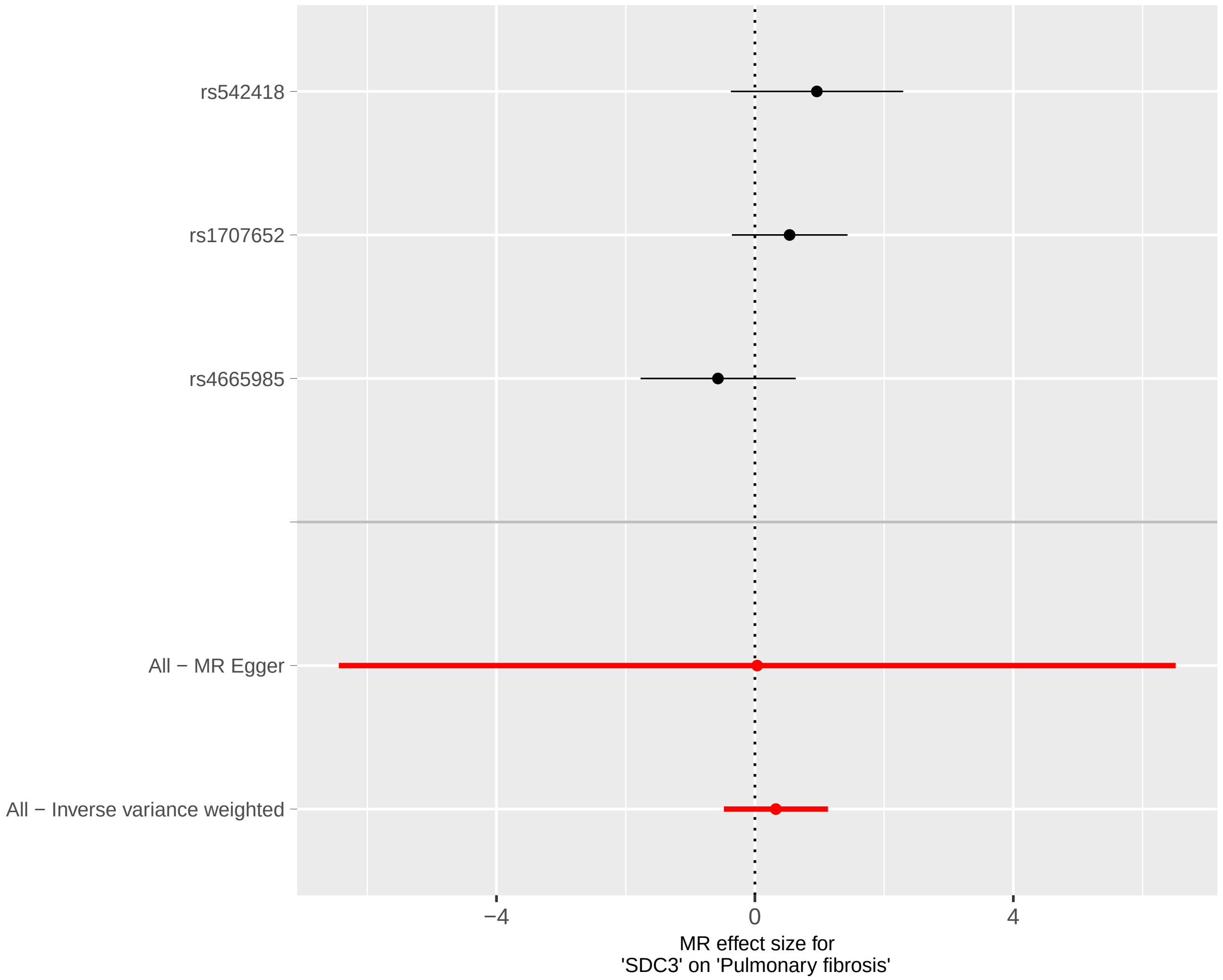
rs542418
rs1707652
rs4665985
All − MR Egger
All − Inverse variance weighted
−4
0
4
MR effect size for
'SDC3' on 'Pulmonary fibrosis'

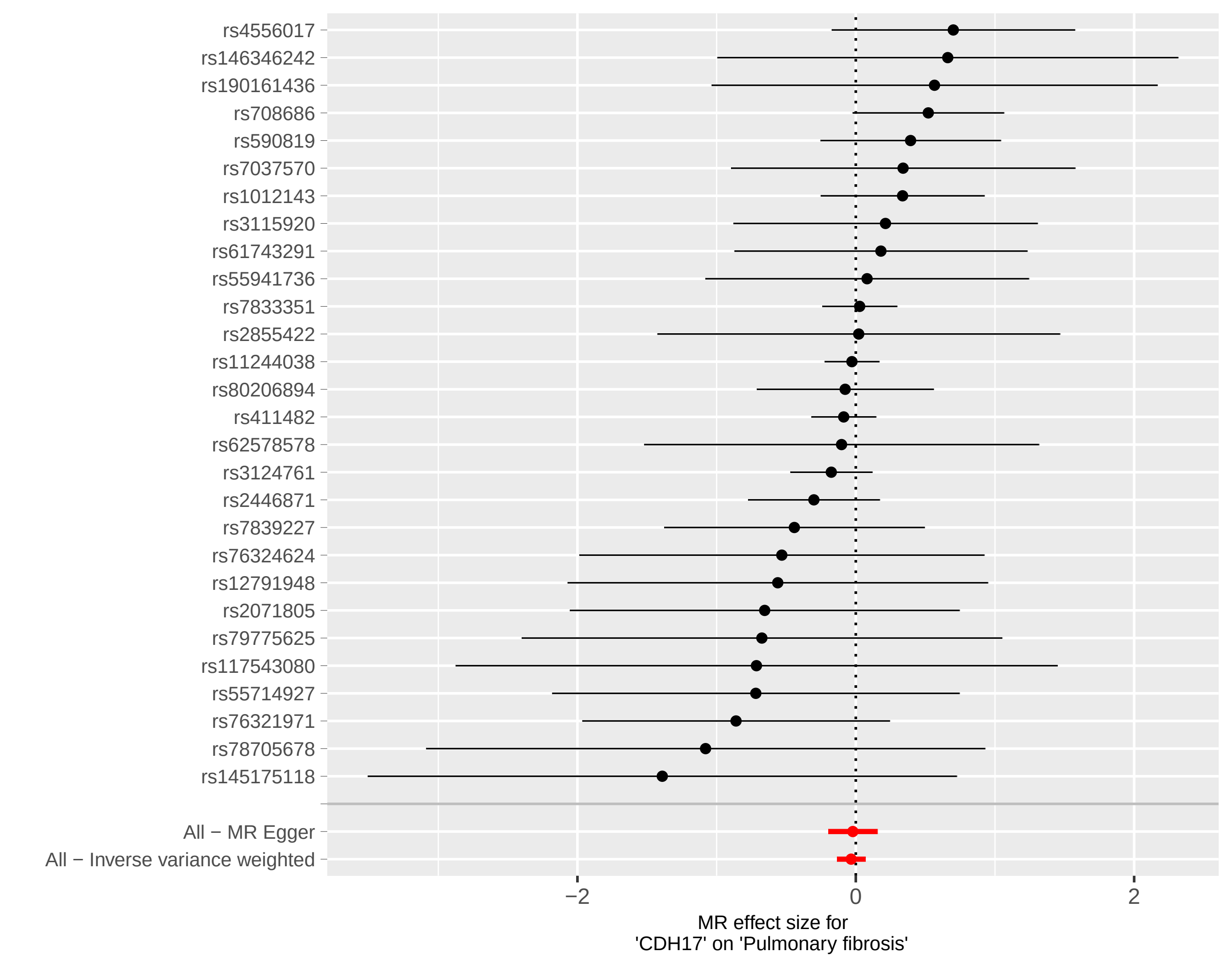

rs4556017
rs146346242
rs190161436
rs708686
rs590819
rs7037570
rs1012143
rs3115920
rs61743291
rs55941736
rs7833351
rs2855422
rs11244038
rs80206894
rs411482
rs62578578
rs3124761
rs2446871
rs7839227
rs76324624
rs12791948
rs2071805
rs79775625
rs117543080
rs55714927
rs76321971
rs78705678
rs145175118
All − MR Egger
All − Inverse variance weighted
−2
0
2
MR effect size for
'CDH17' on 'Pulmonary fibrosis'

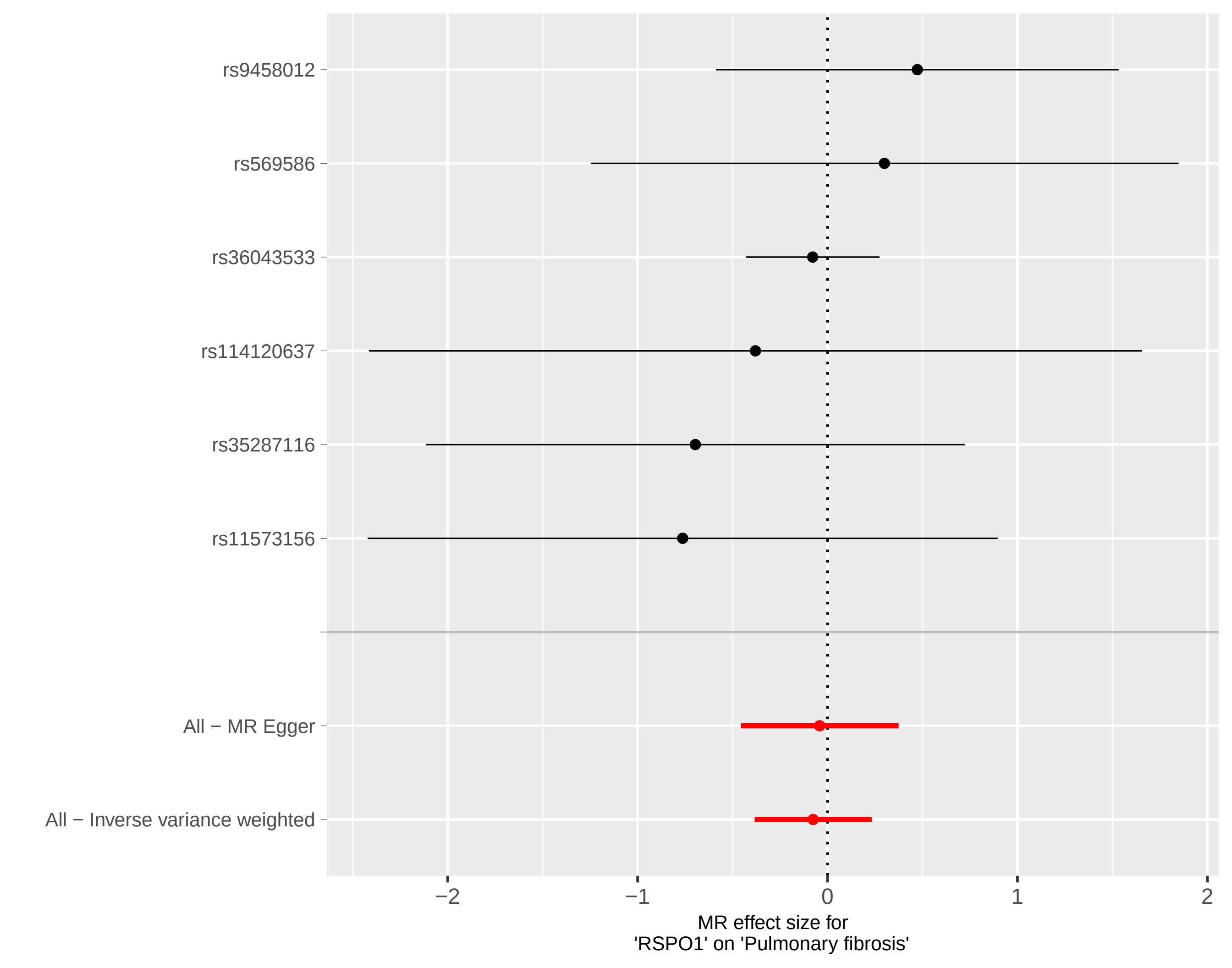
rs9458012
rs569586
rs36043533
rs114120637
rs35287116
rs11573156
All − MR Egger
All − Inverse variance weighted
−2
−1
0
1
2
MR effect size for
'RSPO1' on 'Pulmonary fibrosis'

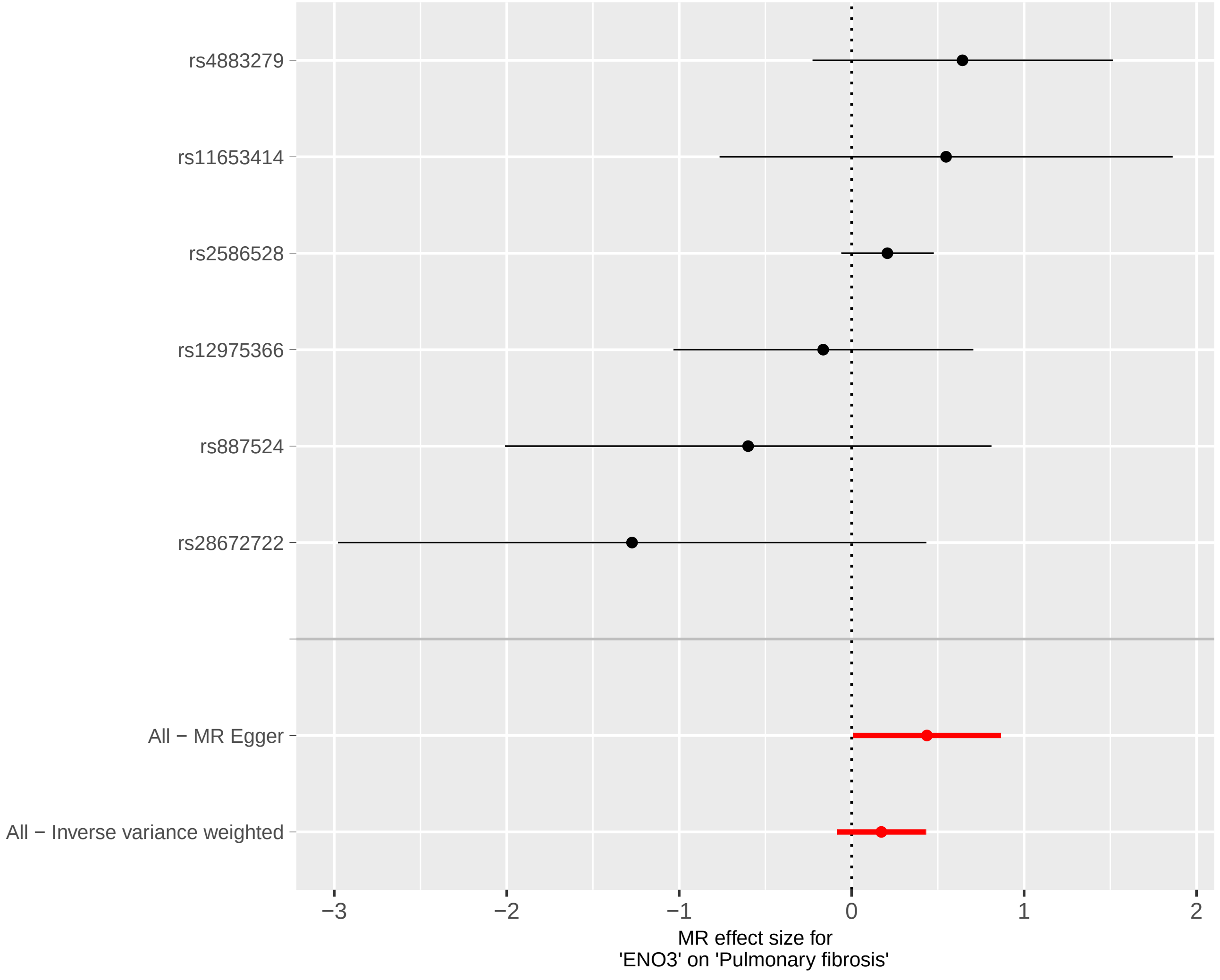
rs4883279
rs11653414
rs2586528
rs12975366
rs887524
rs28672722
All − MR Egger
All − Inverse variance weighted
−3
−2
−1
0
1
2
MR effect size for
'ENO3' on 'Pulmonary fibrosis'

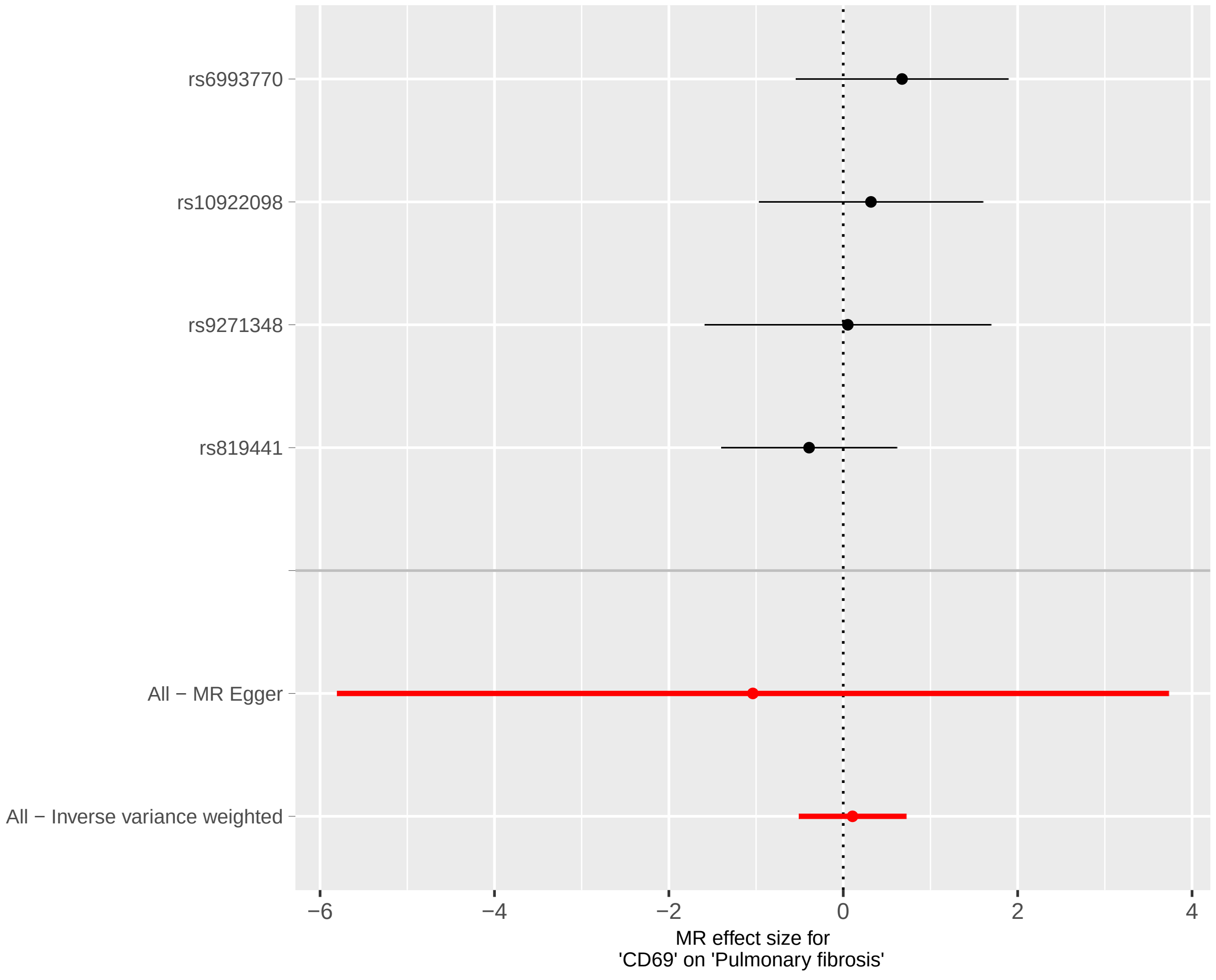

rs6993770
rs10922098
rs9271348
rs819441
All − MR Egger
All − Inverse variance weighted
−6
−4
−2
0
2
4
MR effect size for
'CD69' on 'Pulmonary fibrosis'

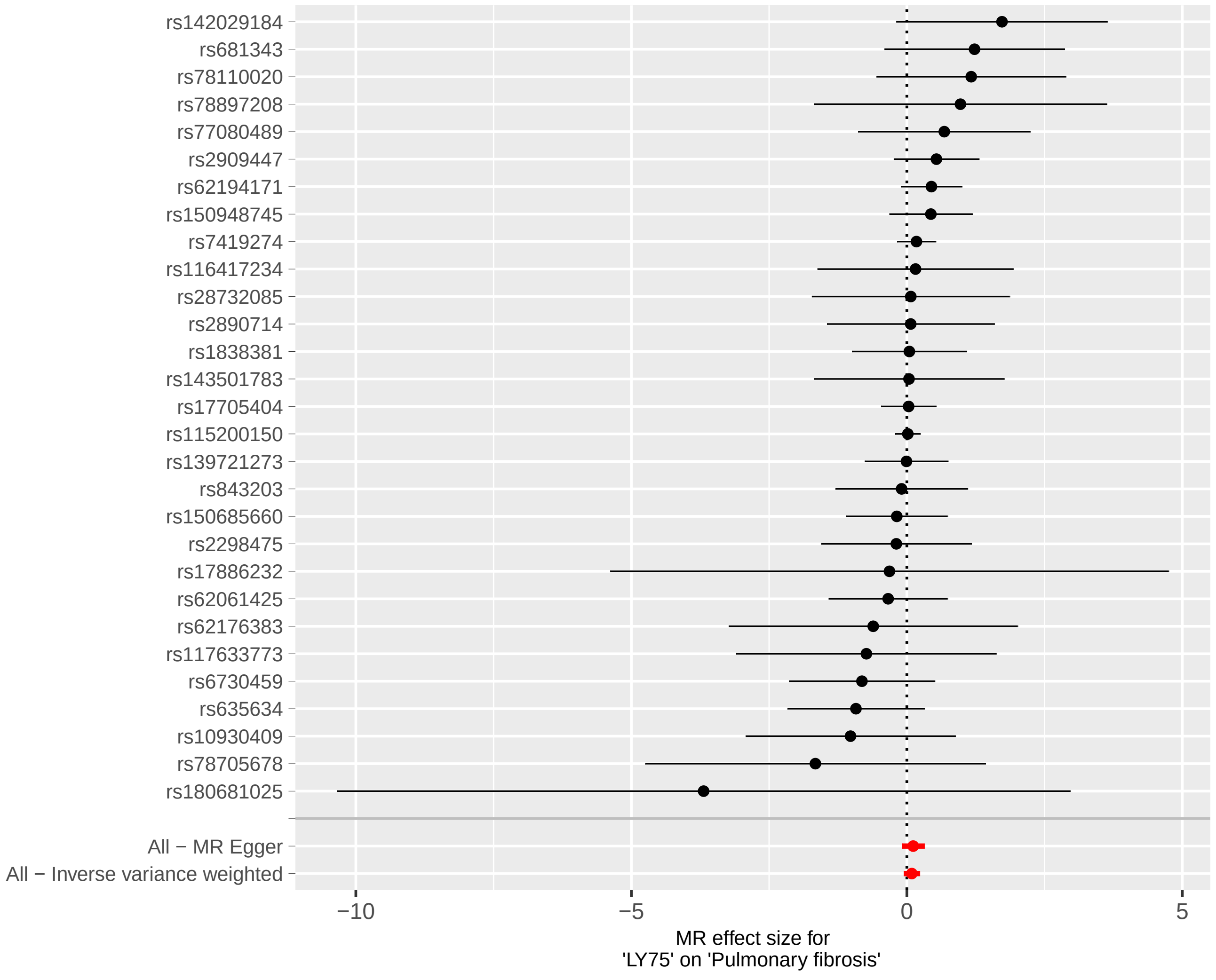

rs142029184
rs681343
rs78110020
rs78897208
rs77080489
rs2909447
rs62194171
rs150948745
rs7419274
rs116417234
rs28732085
rs2890714
rs1838381
rs143501783
rs17705404
rs115200150
rs139721273
rs843203
rs150685660
rs2298475
rs17886232
rs62061425
rs62176383
rs117633773
rs6730459
rs635634
rs10930409
rs78705678
rs180681025
All − MR Egger
All − Inverse variance weighted
−10
−5
0
5
MR effect size for
'LY75' on 'Pulmonary fibrosis'

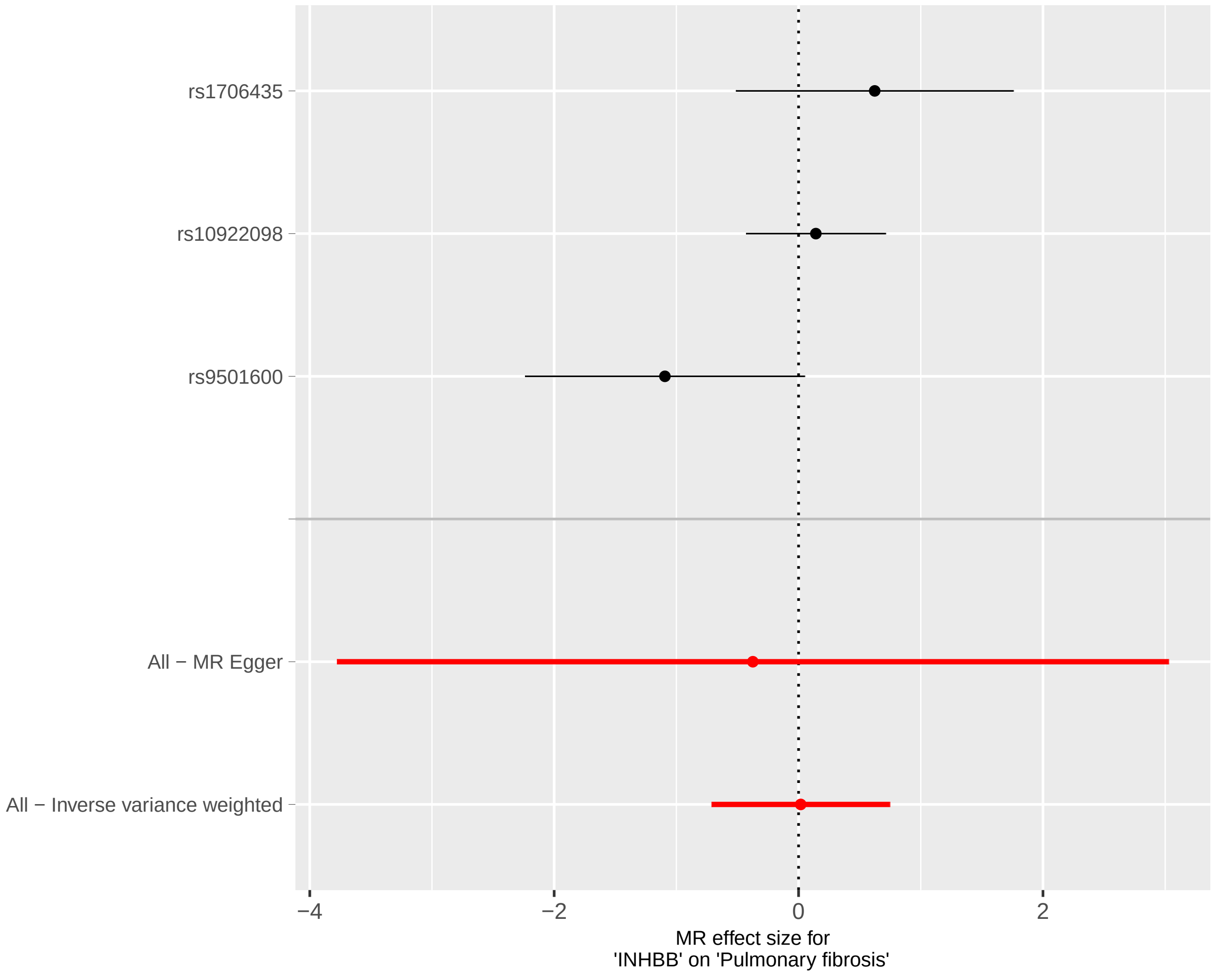

rs1706435
rs10922098
rs9501600
All − MR Egger
All − Inverse variance weighted
−4
−2
0
2
MR effect size for
'INHBB' on 'Pulmonary fibrosis'

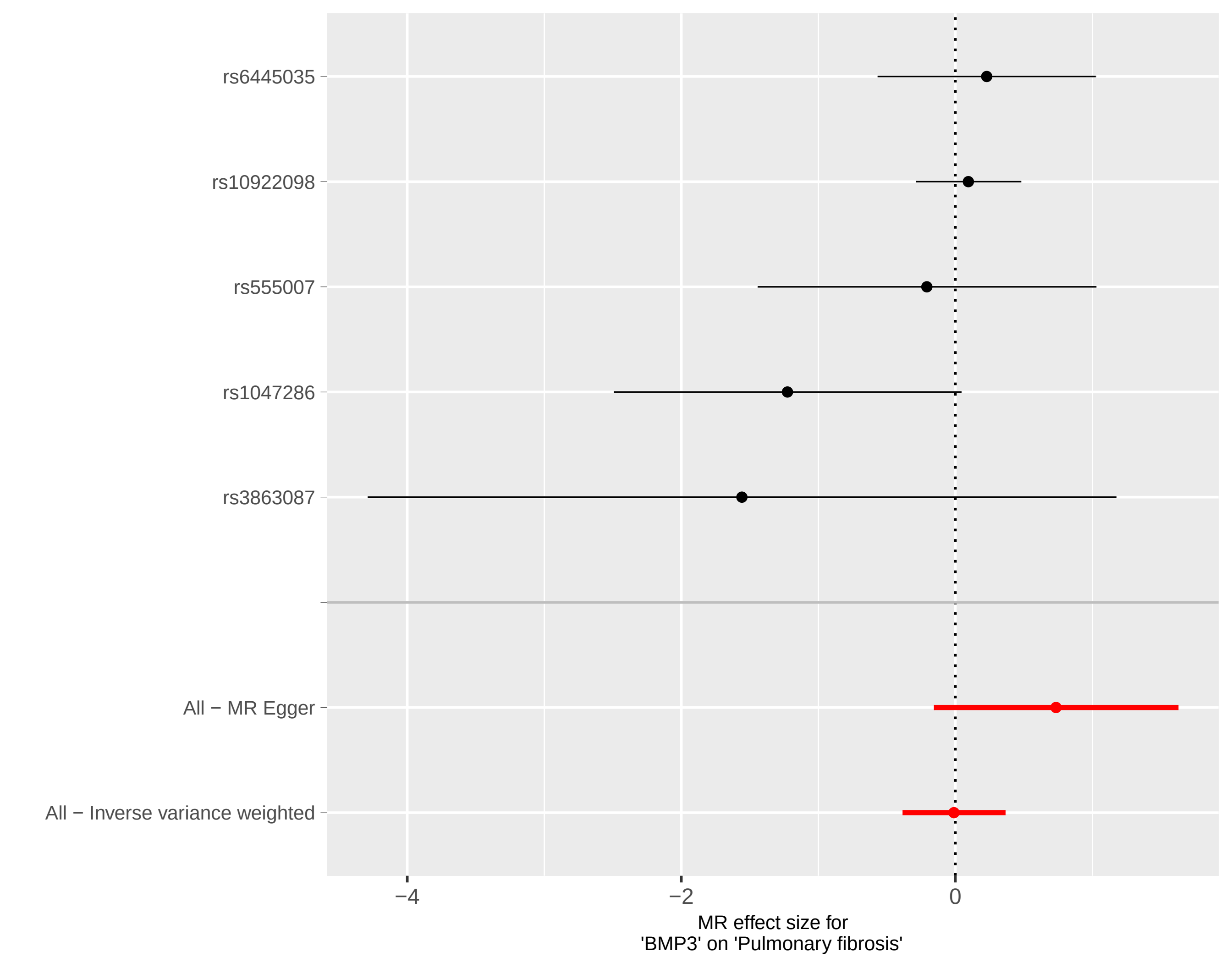

rs6445035
rs10922098
rs555007
rs1047286
rs3863087
All − MR Egger
All − Inverse variance weighted
−4
−2
0
MR effect size for
'BMP3' on 'Pulmonary fibrosis'

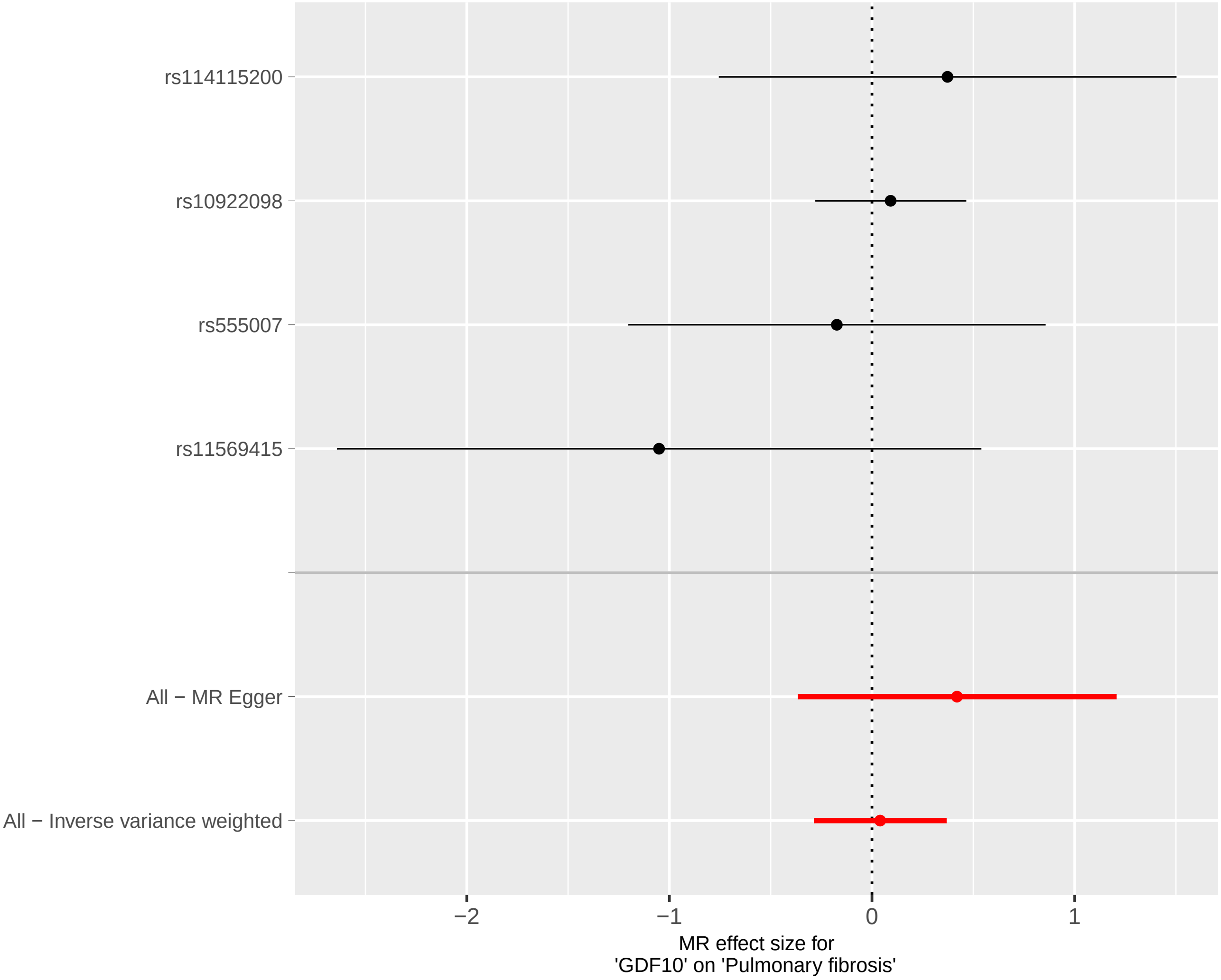
rs114115200
rs10922098
rs555007
rs11569415
All − MR Egger
All − Inverse variance weighted
−2
−1
0
1
MR effect size for
'GDF10' on 'Pulmonary fibrosis'

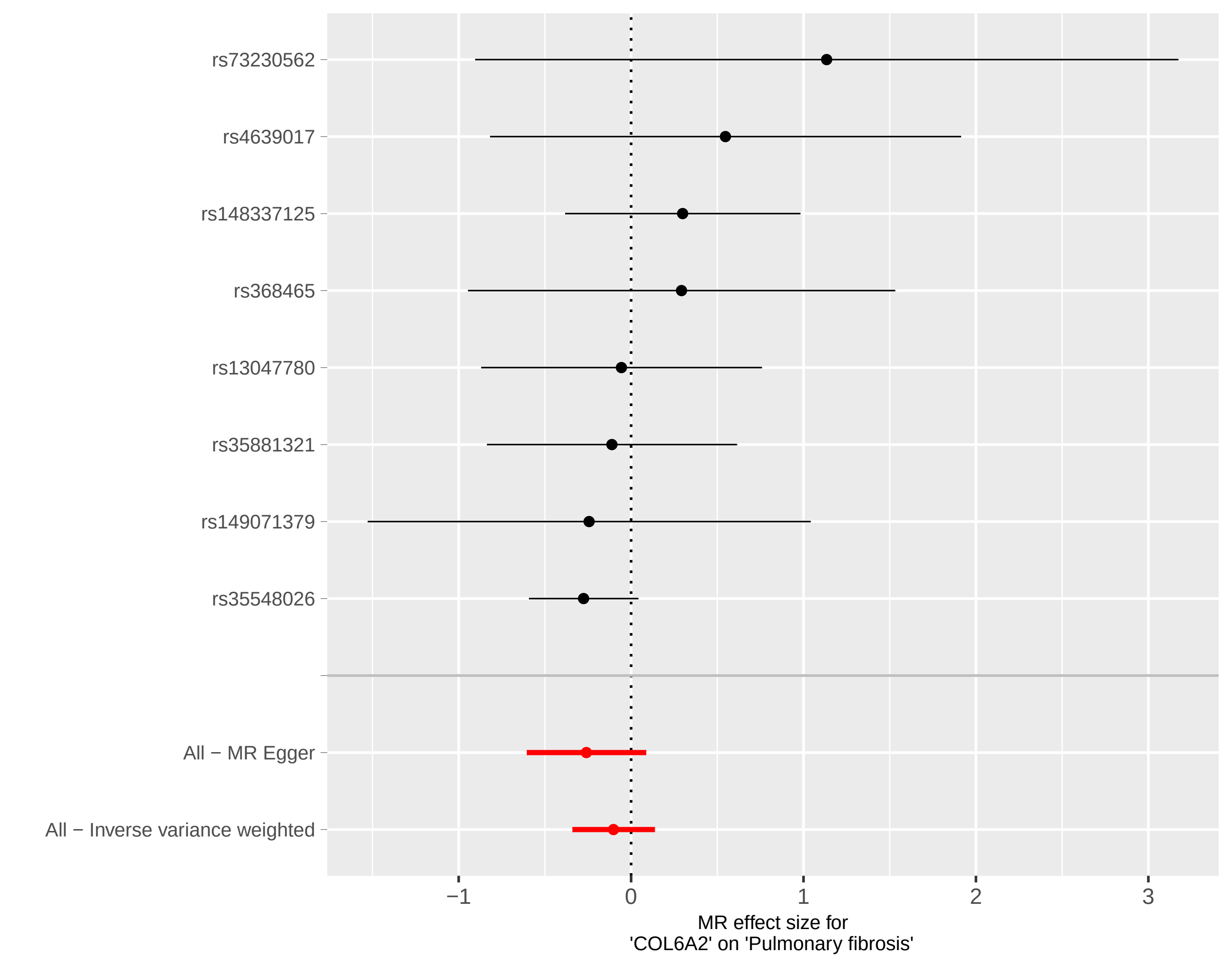

rs73230562
rs4639017
rs148337125
rs368465
rs13047780
rs35881321
rs149071379
rs35548026
All − MR Egger
All − Inverse variance weighted
−1
0
1
2
3
MR effect size for
'COL6A2' on 'Pulmonary fibrosis'

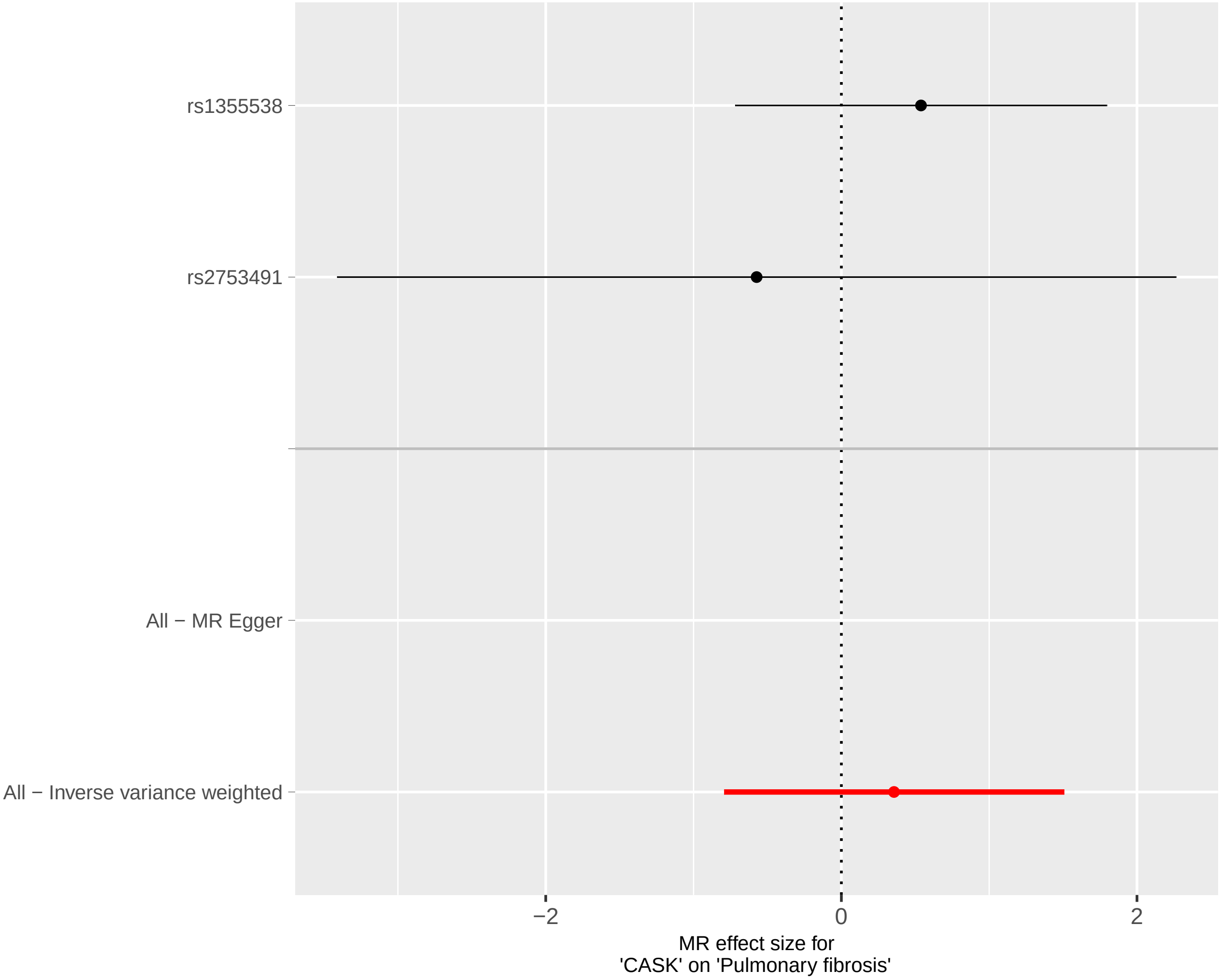
rs1355538
rs2753491
All − MR Egger
All − Inverse variance weighted
−2
0
2
MR effect size for
'CASK' on 'Pulmonary fibrosis'

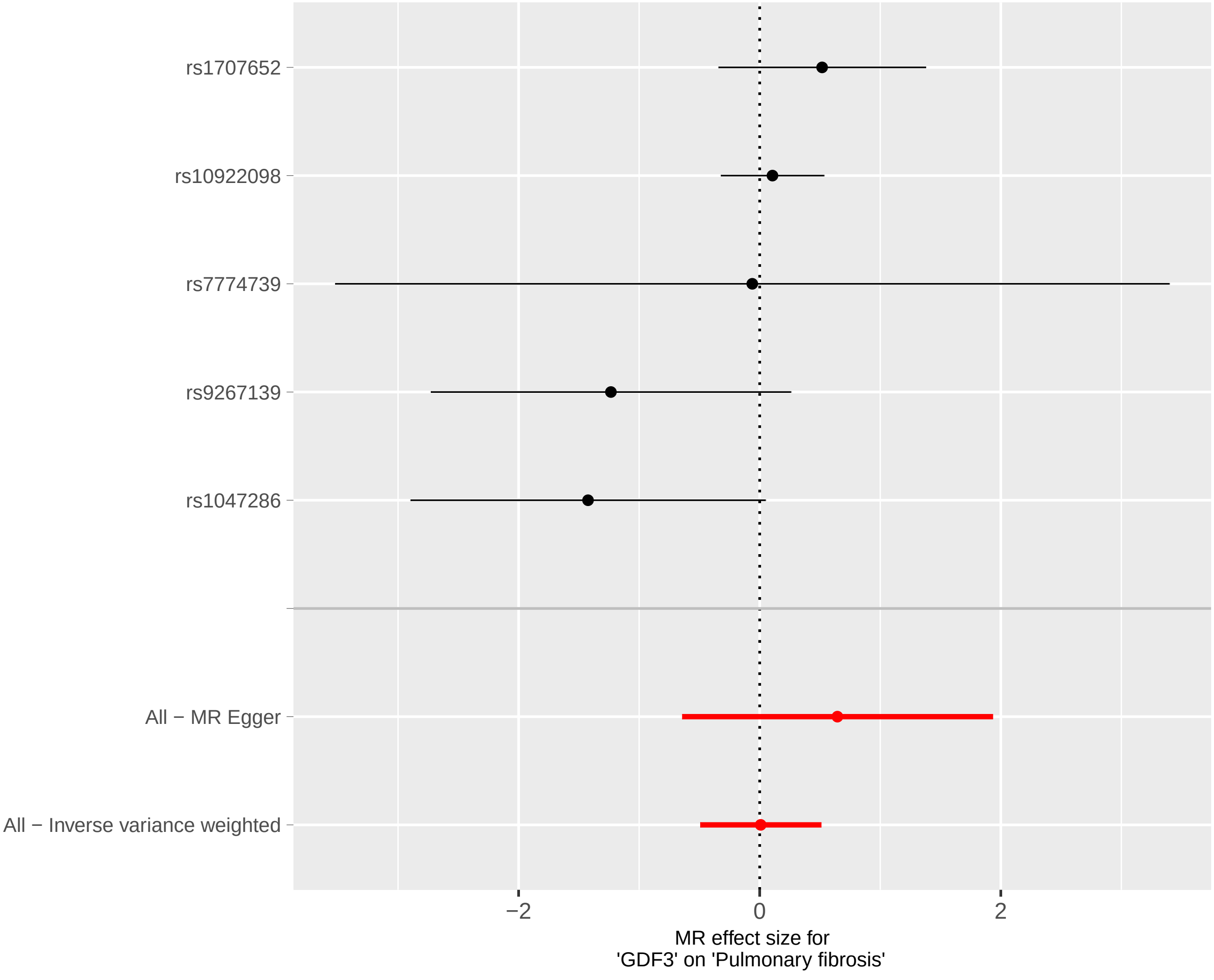
rs1707652
rs10922098
rs7774739
rs9267139
rs1047286
All − MR Egger
All − Inverse variance weighted
−2
0
2
MR effect size for
'GDF3' on 'Pulmonary fibrosis'

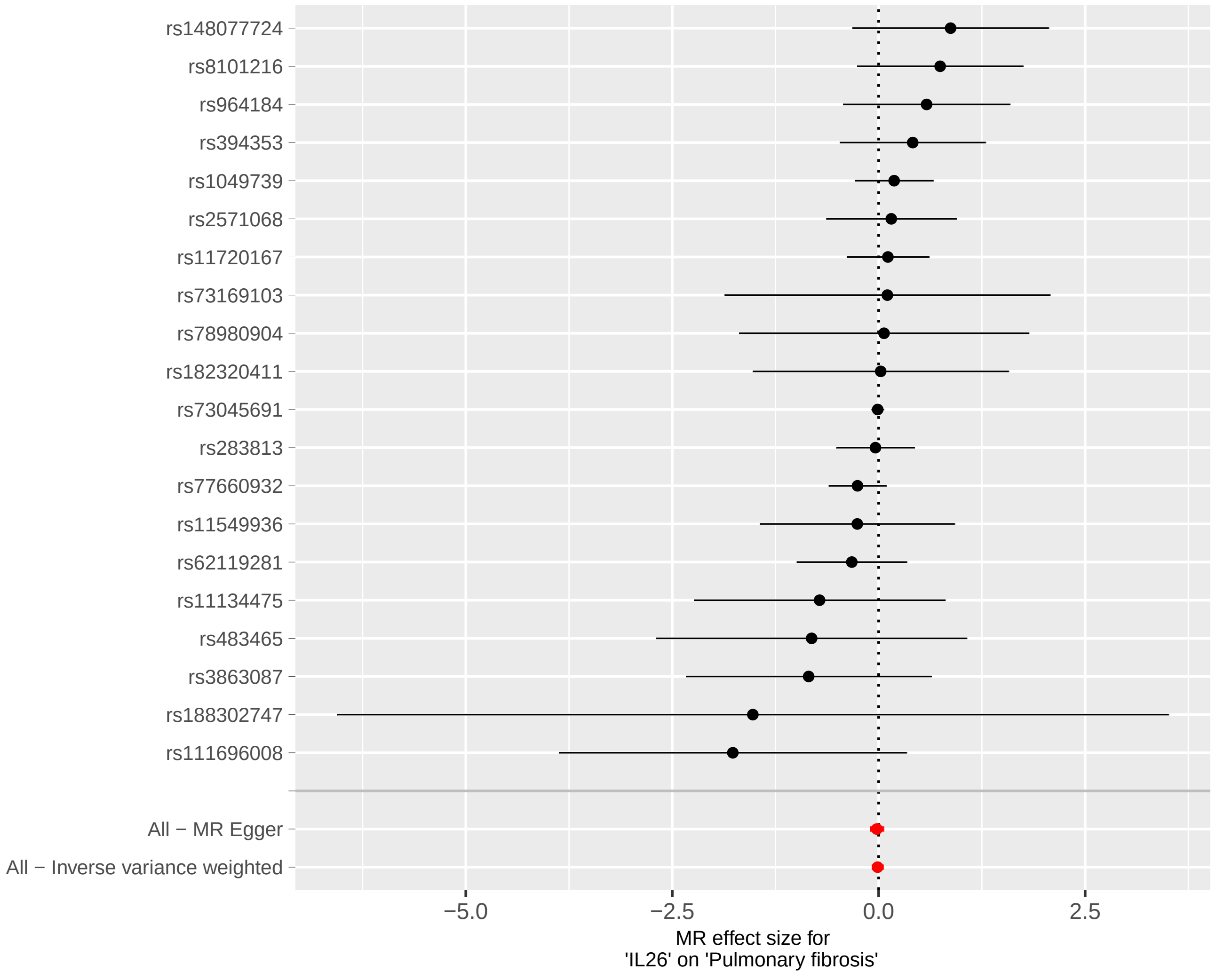

rs148077724
rs8101216
rs964184
rs394353
rs1049739
rs2571068
rs11720167
rs73169103
rs78980904
rs182320411
rs73045691
rs283813
rs77660932
rs11549936
rs62119281
rs11134475
rs483465
rs3863087
rs188302747
rs111696008
All − MR Egger
All − Inverse variance weighted
−5.0
−2.5
0.0
2.5
MR effect size for
'IL26' on 'Pulmonary fibrosis'

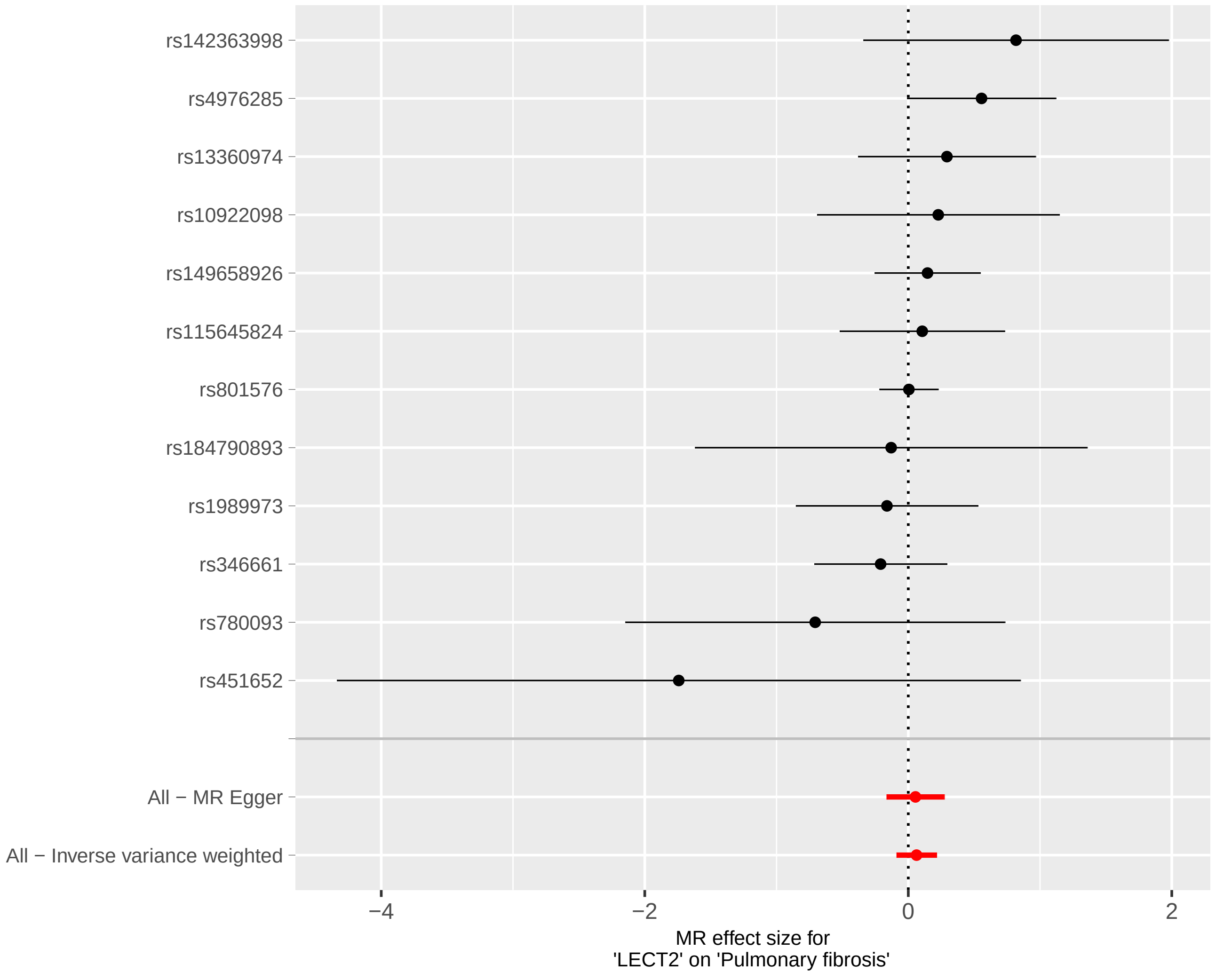
rs142363998
rs4976285
rs13360974
rs10922098
rs149658926
rs115645824
rs801576
rs184790893
rs1989973
rs346661
rs780093
rs451652
All − MR Egger
All − Inverse variance weighted
−4
−2
0
2
MR effect size for
'LECT2' on 'Pulmonary fibrosis'

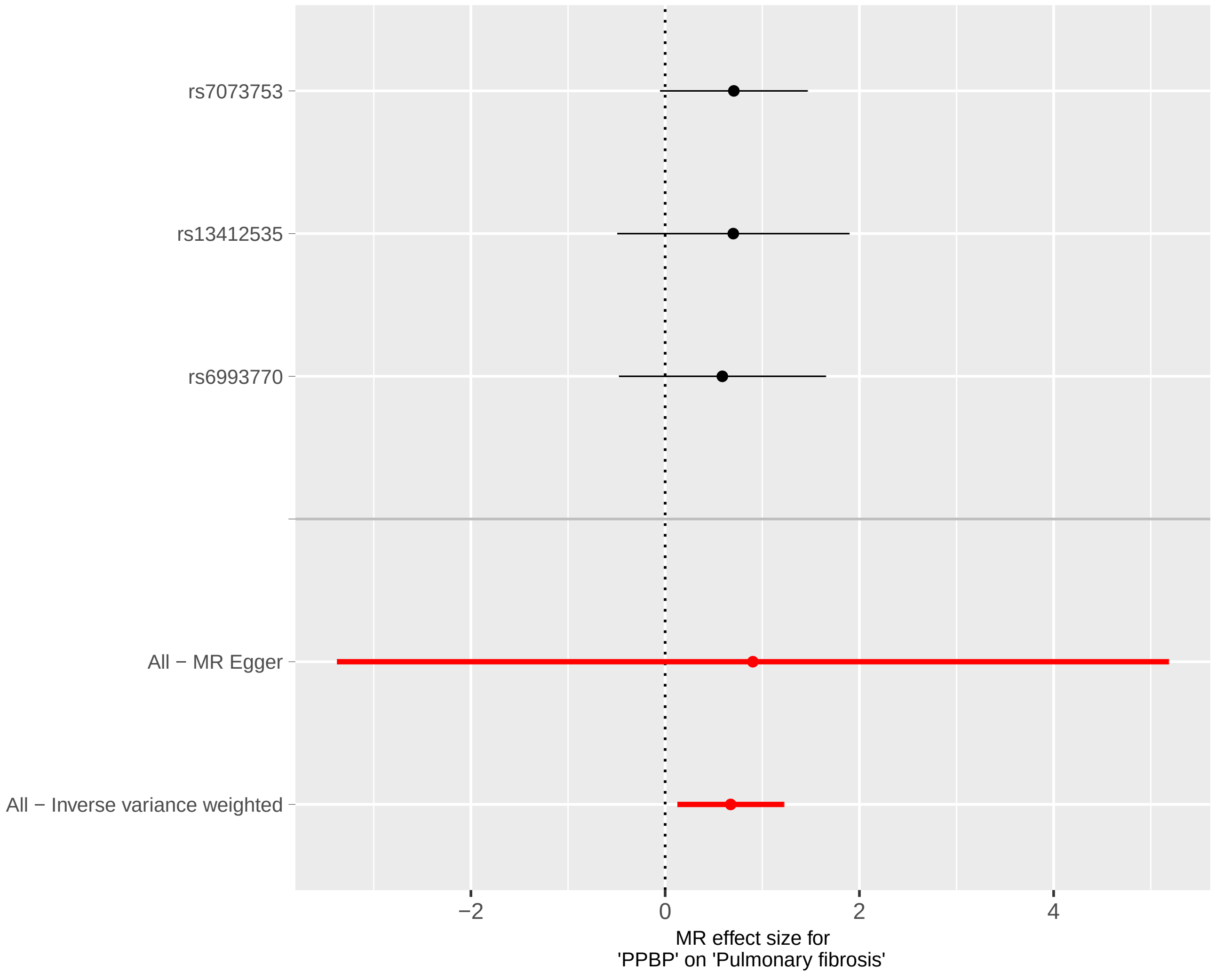

rs7073753
rs13412535
rs6993770
All − MR Egger
All − Inverse variance weighted
−2
0
2
4
MR effect size for
'PPBP' on 'Pulmonary fibrosis'

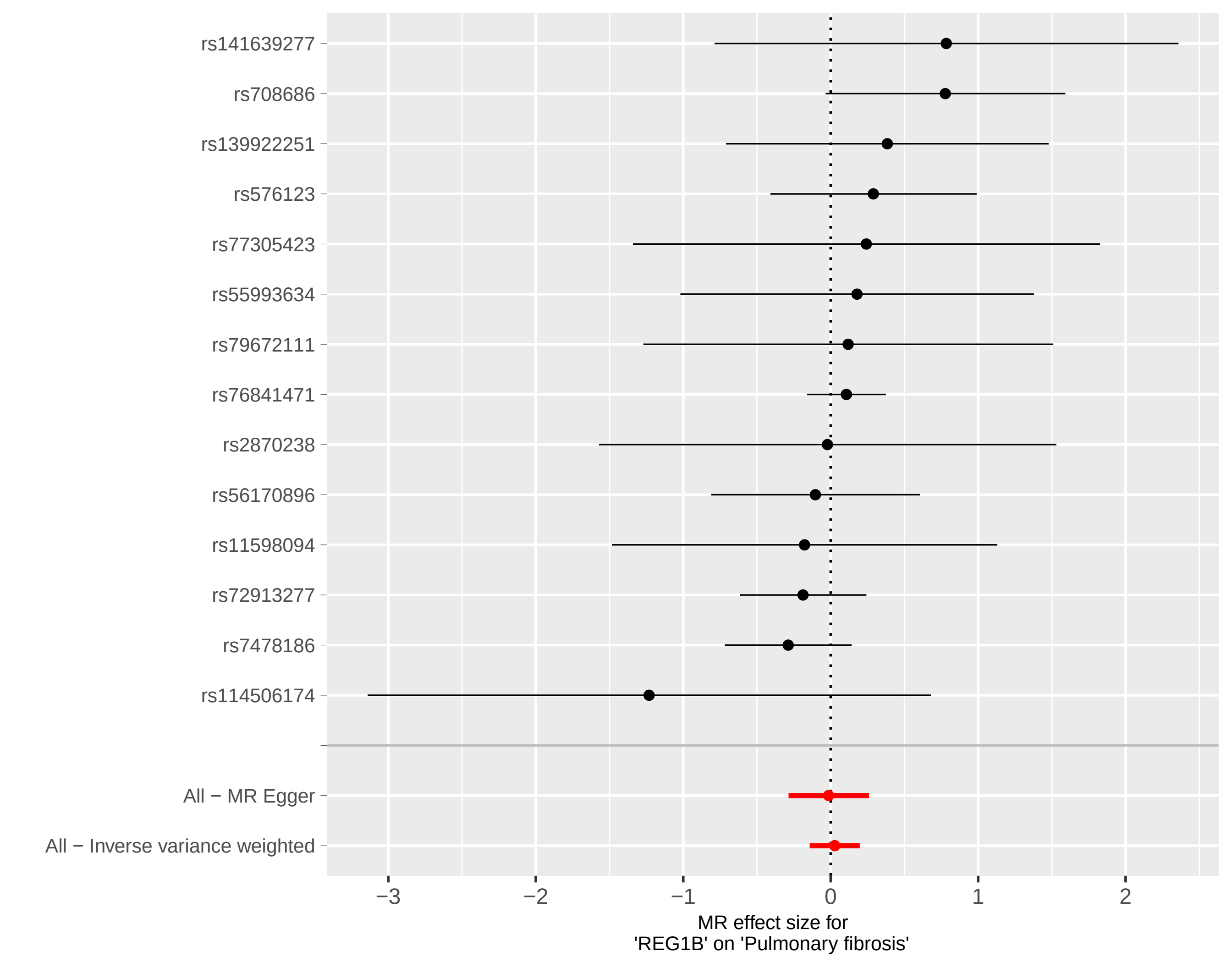

rs141639277
rs708686
rs139922251
rs576123
rs77305423
rs55993634
rs79672111
rs76841471
rs2870238
rs56170896
rs11598094
rs72913277
rs7478186
rs114506174
All − MR Egger
All − Inverse variance weighted
−3
−2
−1
0
1
2
MR effect size for
'REG1B' on 'Pulmonary fibrosis'

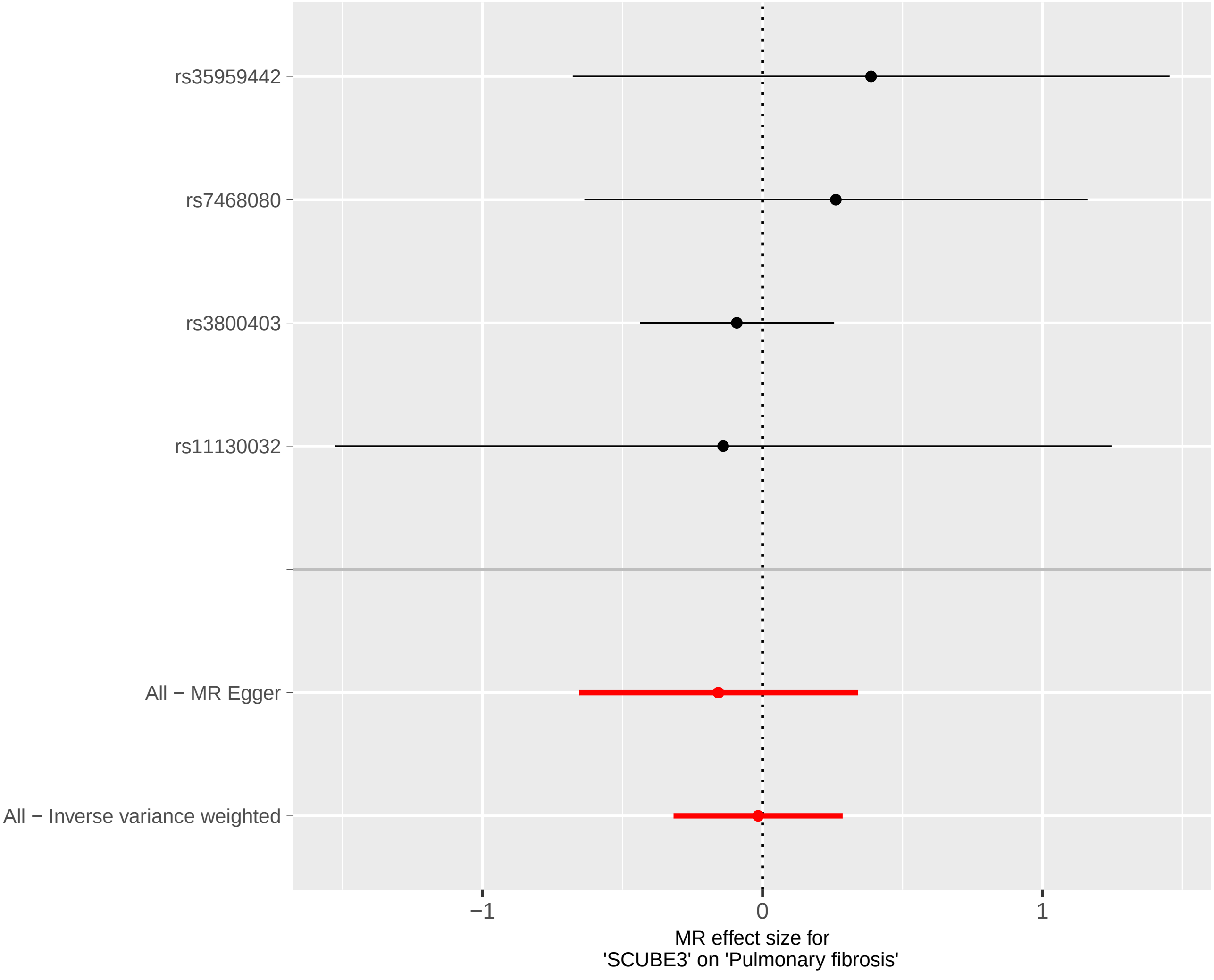

rs35959442
rs7468080
rs3800403
rs11130032
All − MR Egger
All − Inverse variance weighted
−1
0
1
MR effect size for
'SCUBE3' on 'Pulmonary fibrosis'

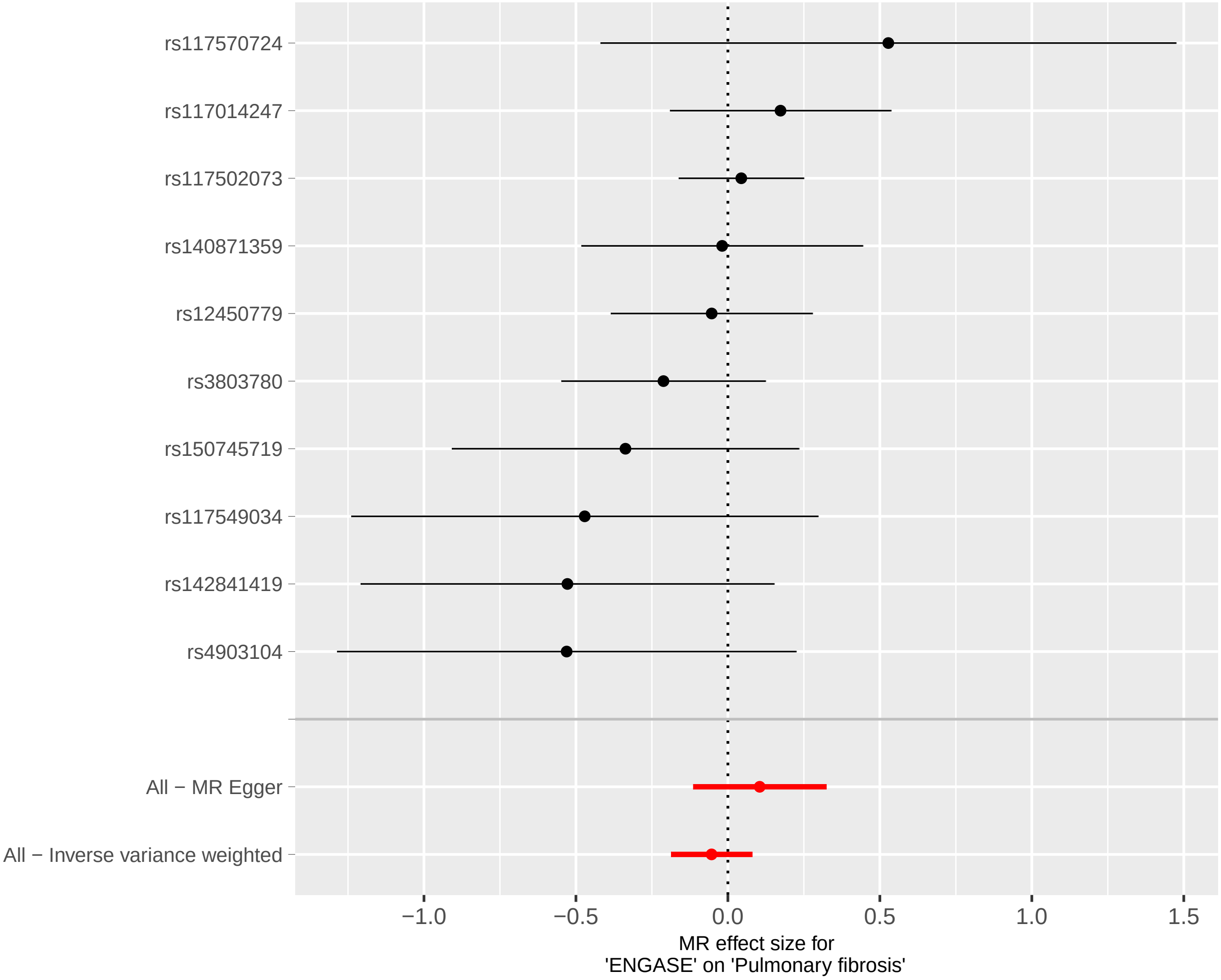
rs117570724
rs117014247
rs117502073
rs140871359
rs12450779
rs3803780
rs150745719
rs117549034
rs142841419
rs4903104
All − MR Egger
All − Inverse variance weighted
−1.0
−0.5
0.0
0.5
1.0
1.5
MR effect size for
'ENGASE' on 'Pulmonary fibrosis'

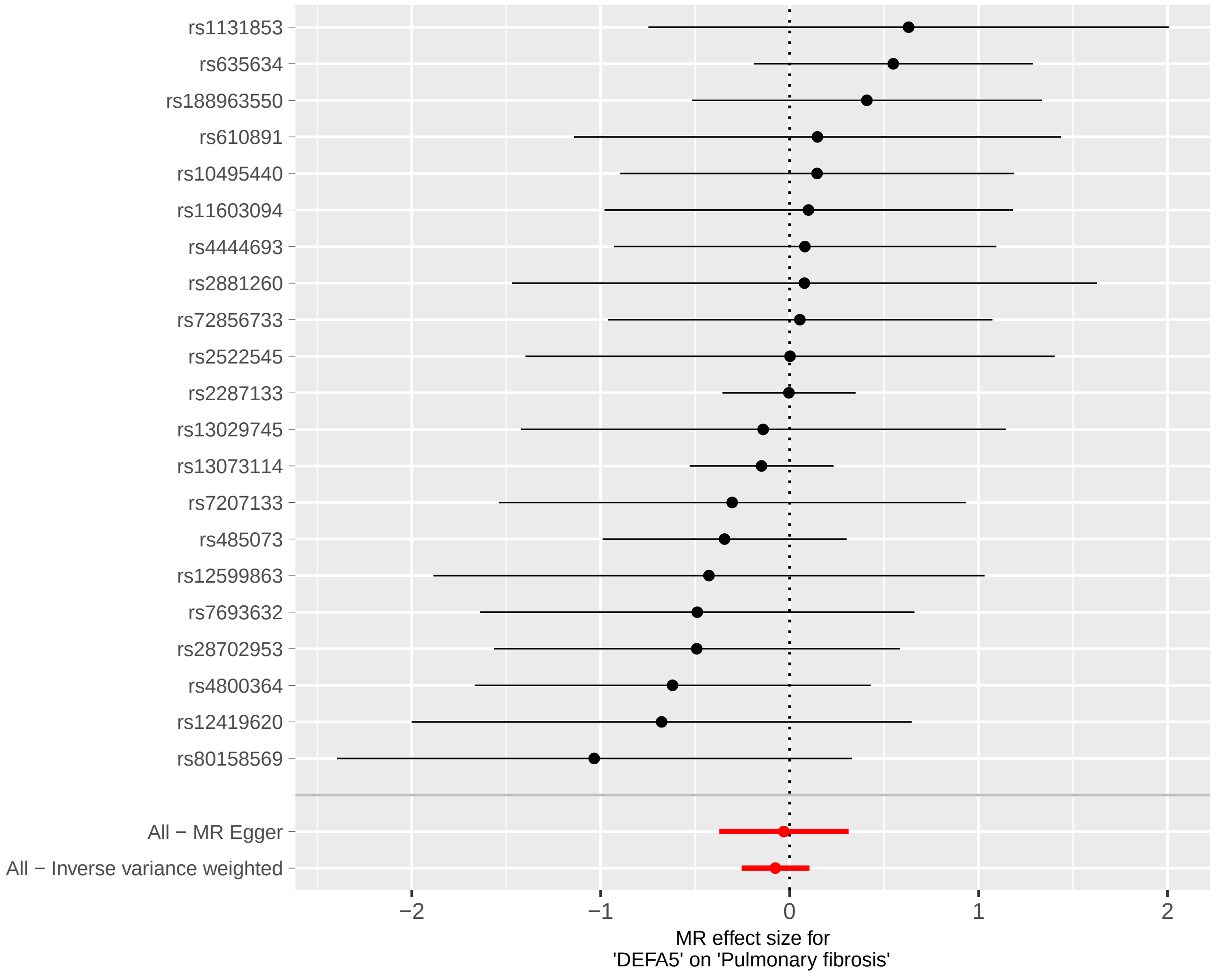

rs1131853
rs635634
rs188963550
rs610891
rs10495440
rs11603094
rs4444693
rs2881260
rs72856733
rs2522545
rs2287133
rs13029745
rs13073114
rs7207133
rs485073
rs12599863
rs7693632
rs28702953
rs4800364
rs12419620
rs80158569
All − MR Egger
All − Inverse variance weighted
−2
−1
0
1
2
MR effect size for
'DEFA5' on 'Pulmonary fibrosis'

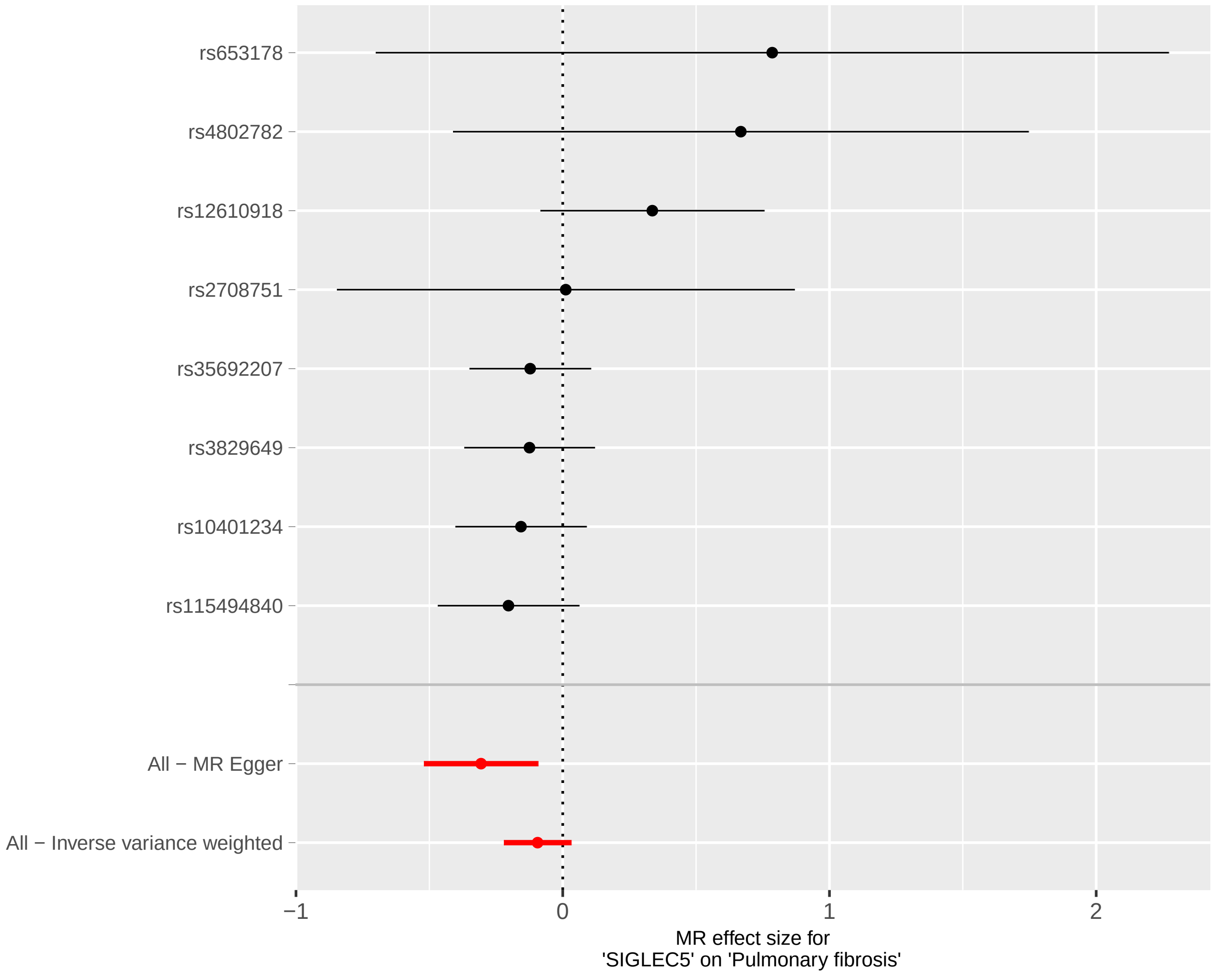

rs653178
rs4802782
rs12610918
rs2708751
rs35692207
rs3829649
rs10401234
rs115494840
All − MR Egger
All − Inverse variance weighted
−1
0
1
2
MR effect size for
'SIGLEC5' on 'Pulmonary fibrosis'

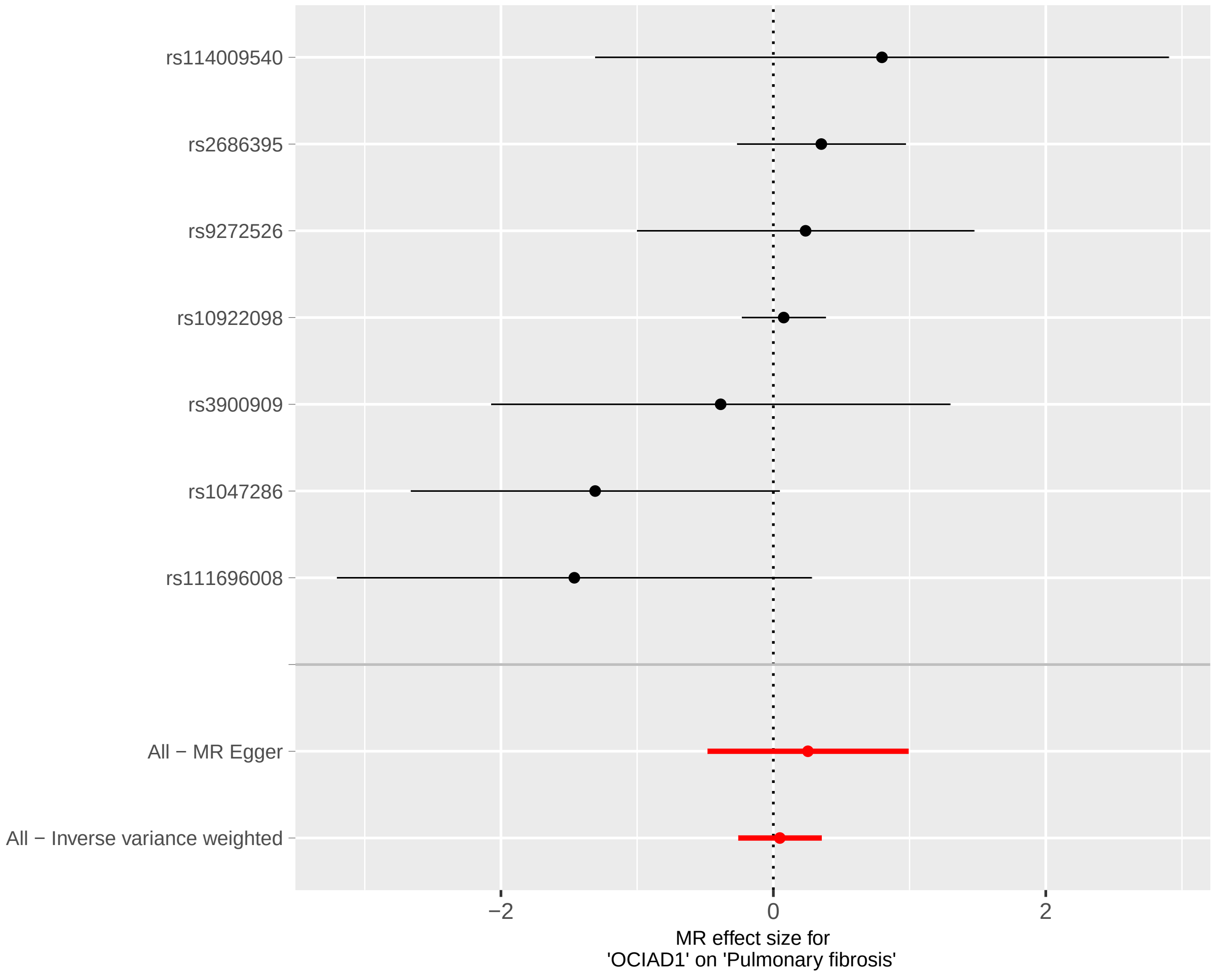

rs114009540
rs2686395
rs9272526
rs10922098
rs3900909
rs1047286
rs111696008
All − MR Egger
All − Inverse variance weighted
−2
0
2
MR effect size for
'OCIAD1' on 'Pulmonary fibrosis'

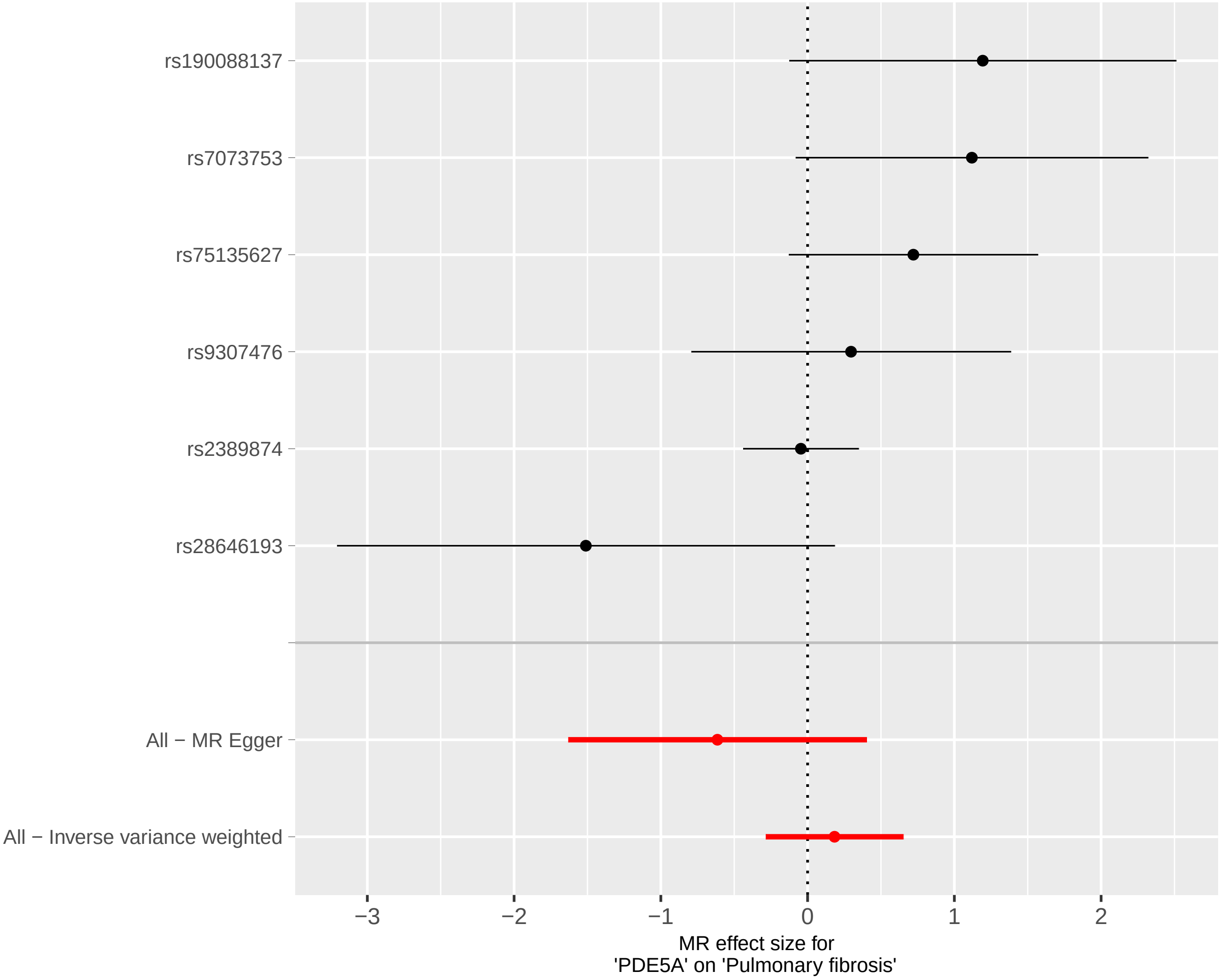
rs190088137
rs7073753
rs75135627
rs9307476
rs2389874
rs28646193
All − MR Egger
All − Inverse variance weighted
−3
−2
−1
0
1
2
MR effect size for
'PDE5A' on 'Pulmonary fibrosis'

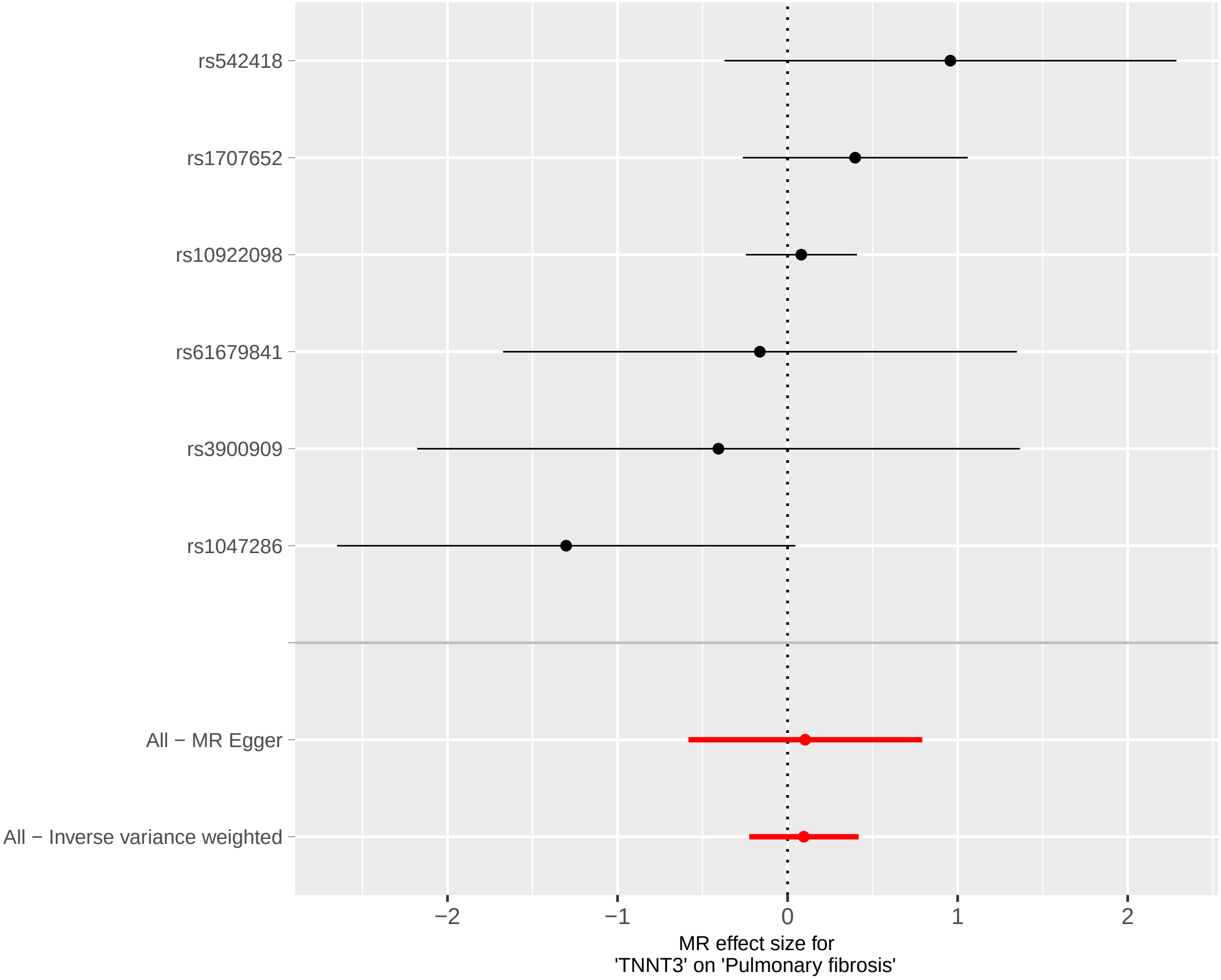

rs542418
rs1707652
rs10922098
rs61679841
rs3900909
rs1047286
All − MR Egger
All − Inverse variance weighted
−2
−1
0
1
2
MR effect size for
'TNNT3' on 'Pulmonary fibrosis'

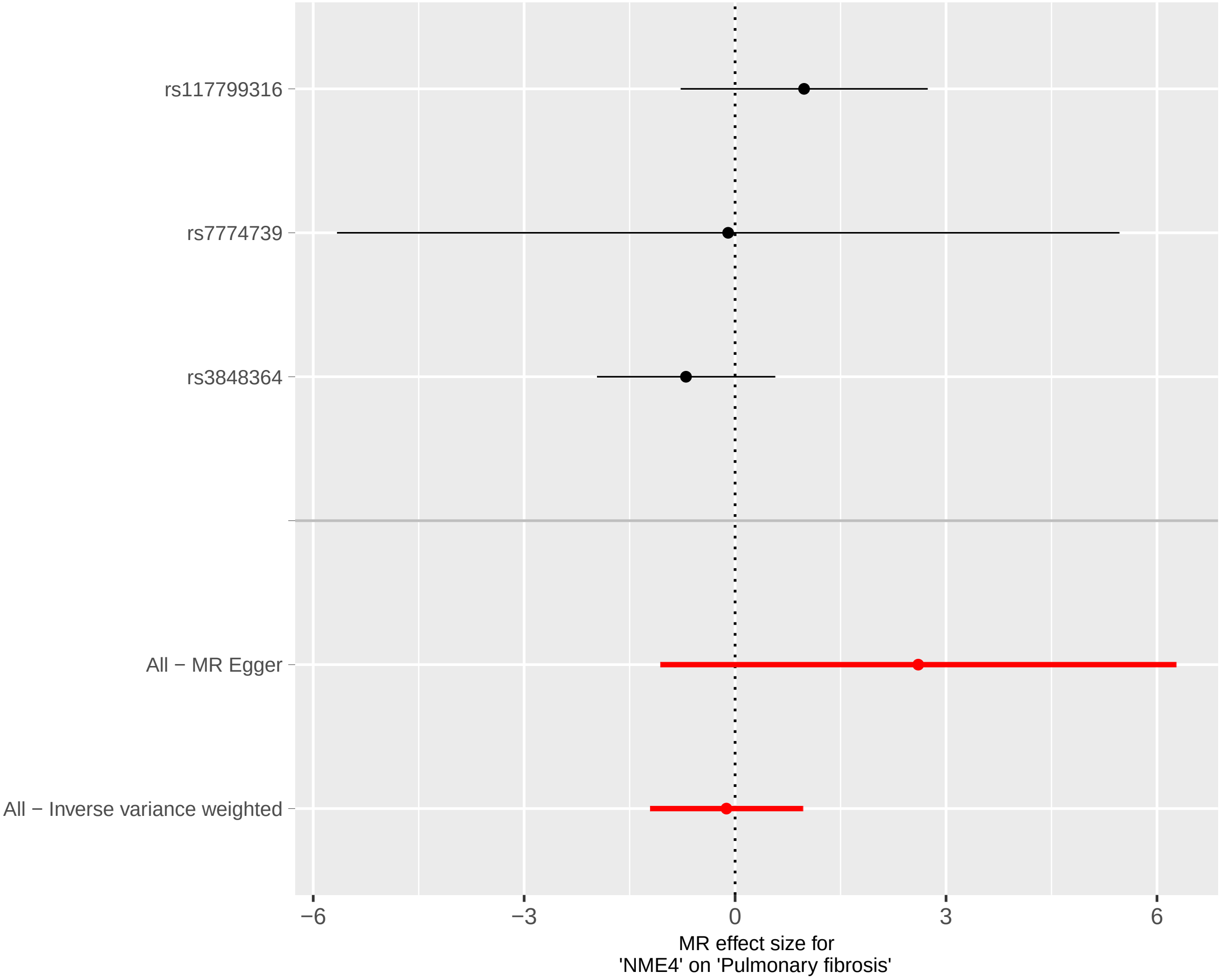

rs117799316
rs7774739
rs3848364
All − MR Egger
All − Inverse variance weighted
−6
−3
0
3
6
MR effect size for
'NME4' on 'Pulmonary fibrosis'

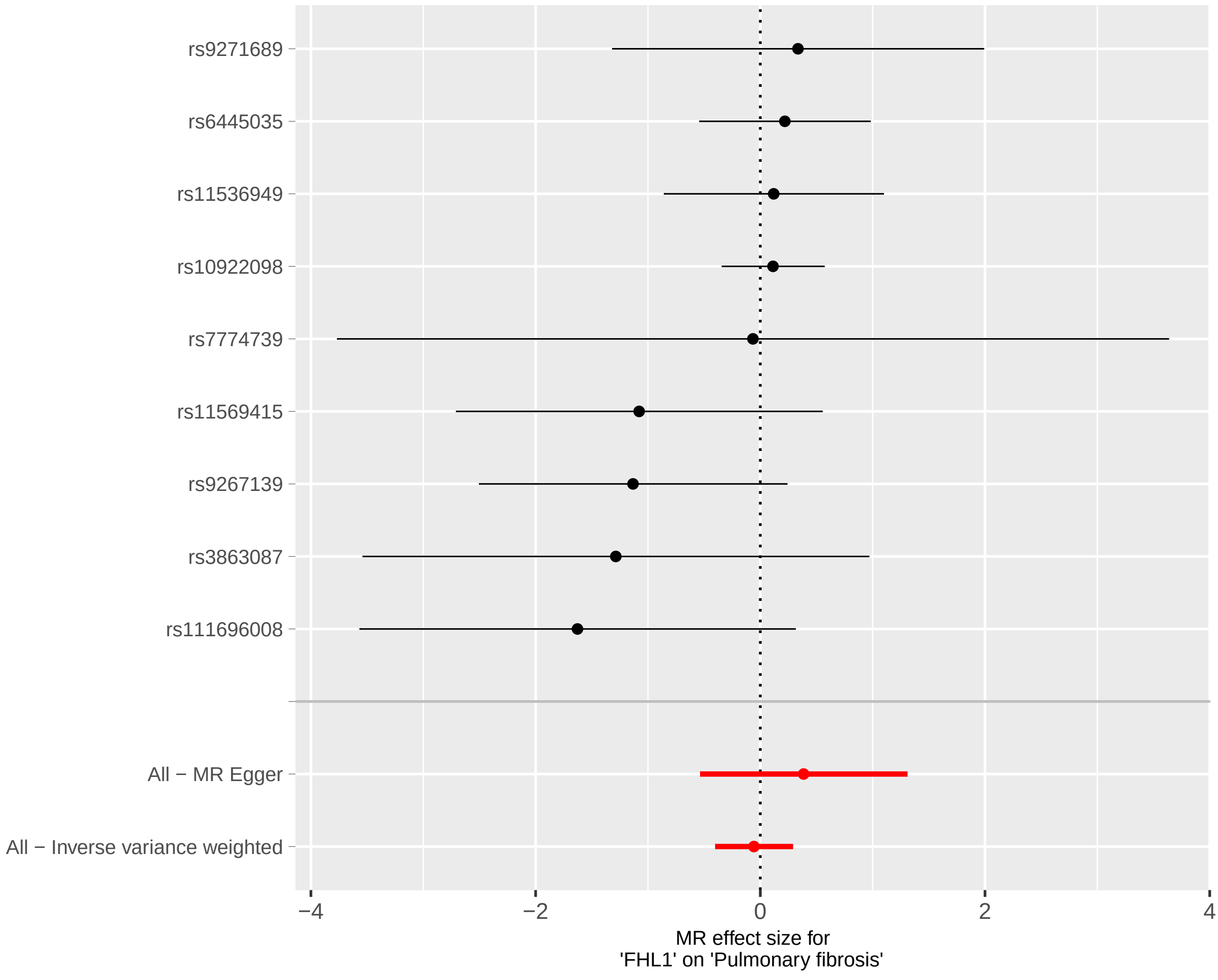
rs9271689
rs6445035
rs11536949
rs10922098
rs7774739
rs11569415
rs9267139
rs3863087
rs111696008
All − MR Egger
All − Inverse variance weighted
−4
−2
0
2
4
MR effect size for
'FHL1' on 'Pulmonary fibrosis'

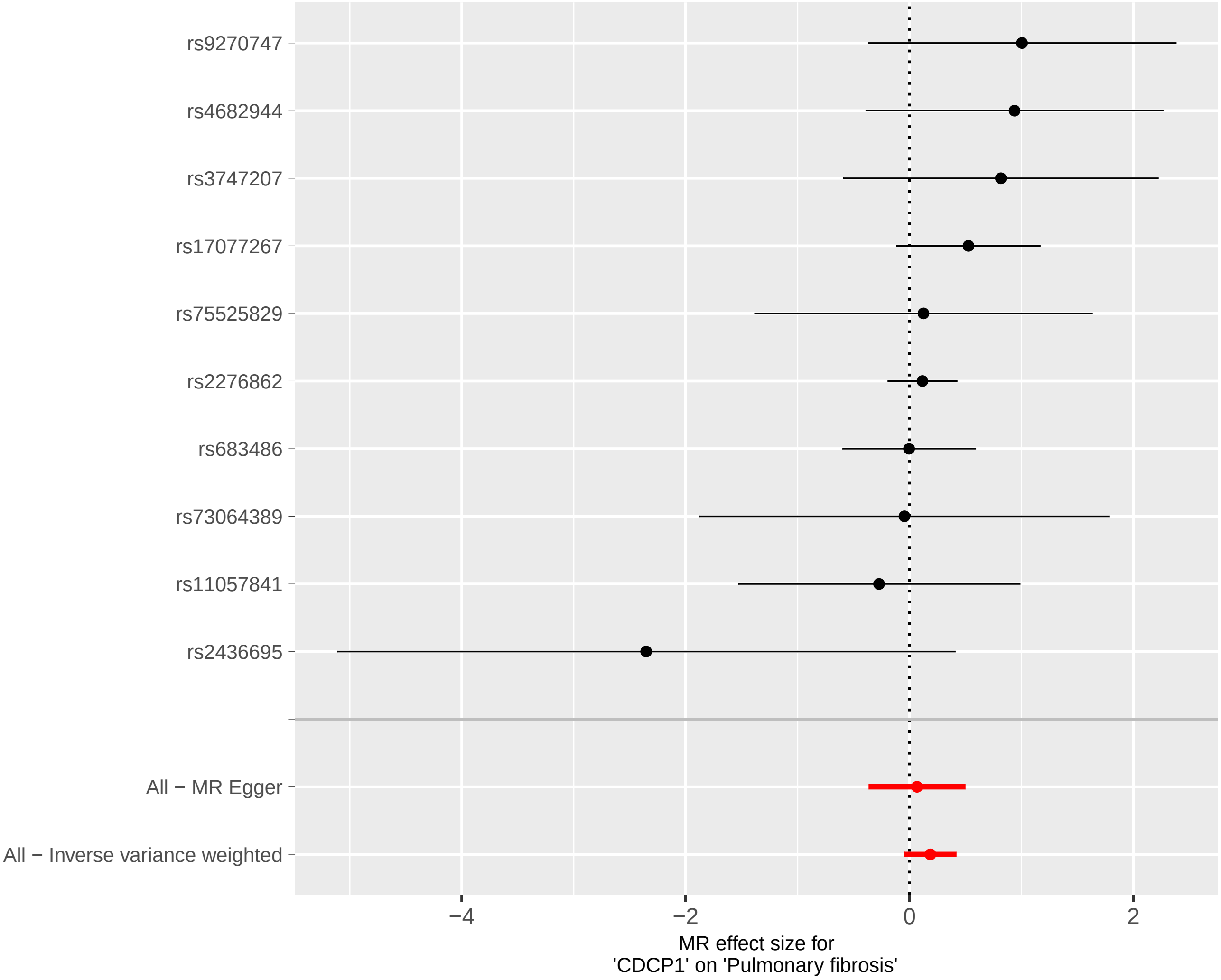
rs9270747
rs4682944
rs3747207
rs17077267
rs75525829
rs2276862
rs683486
rs73064389
rs11057841
rs2436695
All − MR Egger
All − Inverse variance weighted
−4
−2
0
2
MR effect size for
'CDCP1' on 'Pulmonary fibrosis'

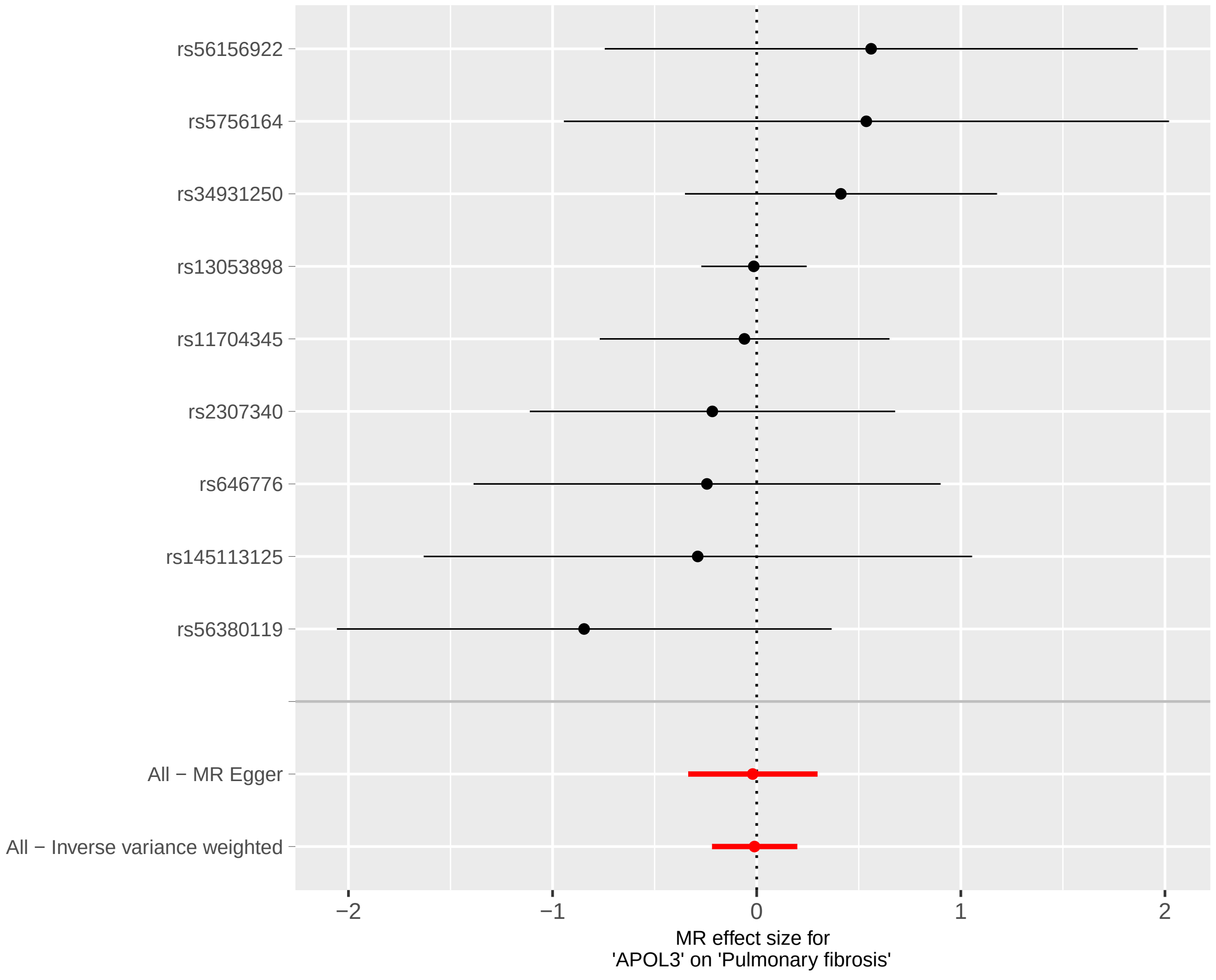

rs56156922
rs5756164
rs34931250
rs13053898
rs11704345
rs2307340
rs646776
rs145113125
rs56380119
All − MR Egger
All − Inverse variance weighted
−2
−1
0
1
2
MR effect size for
'APOL3' on 'Pulmonary fibrosis'

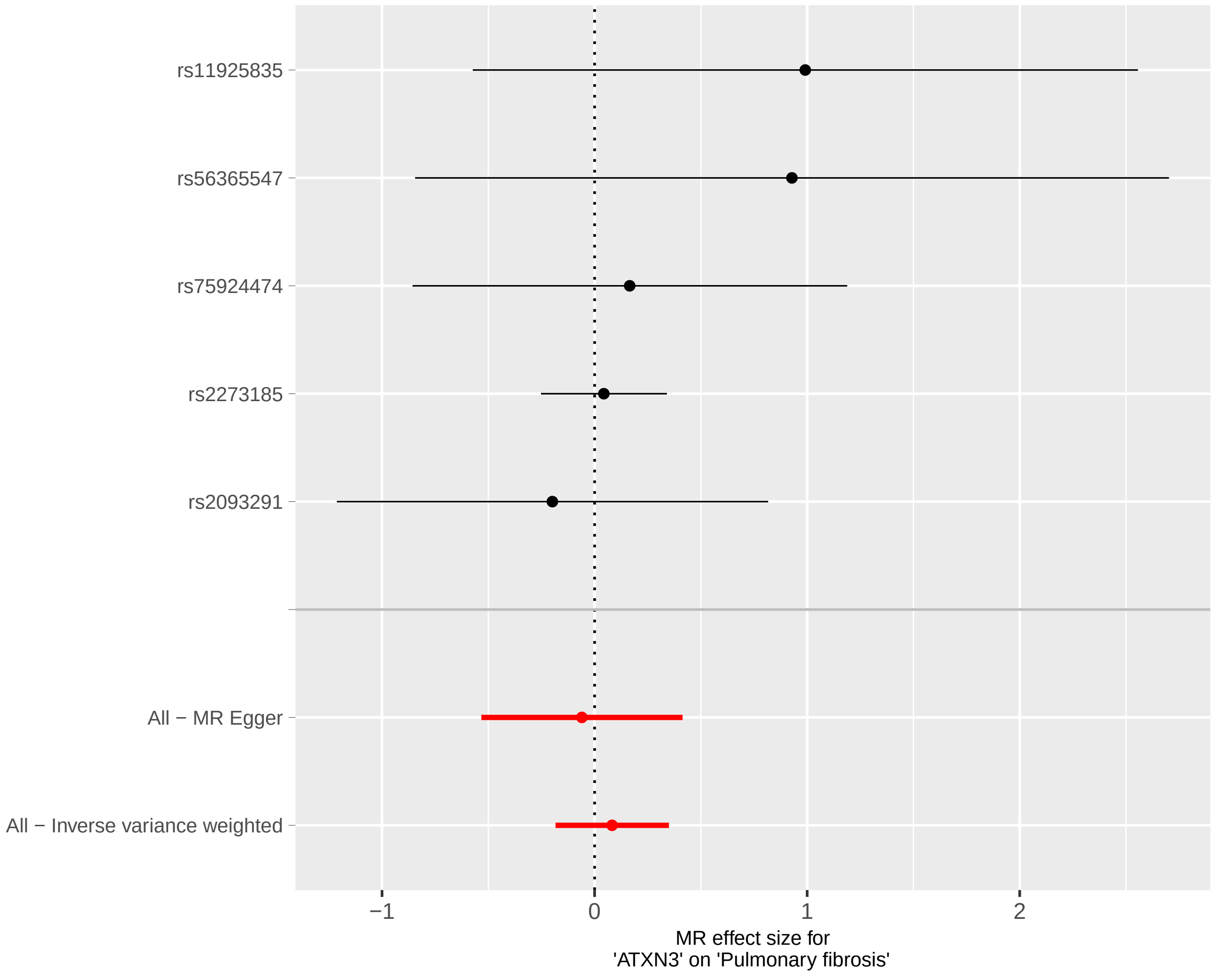

rs11925835
rs56365547
rs75924474
rs2273185
rs2093291
All − MR Egger
All − Inverse variance weighted
−1
0
1
2
MR effect size for
'ATXN3' on 'Pulmonary fibrosis'

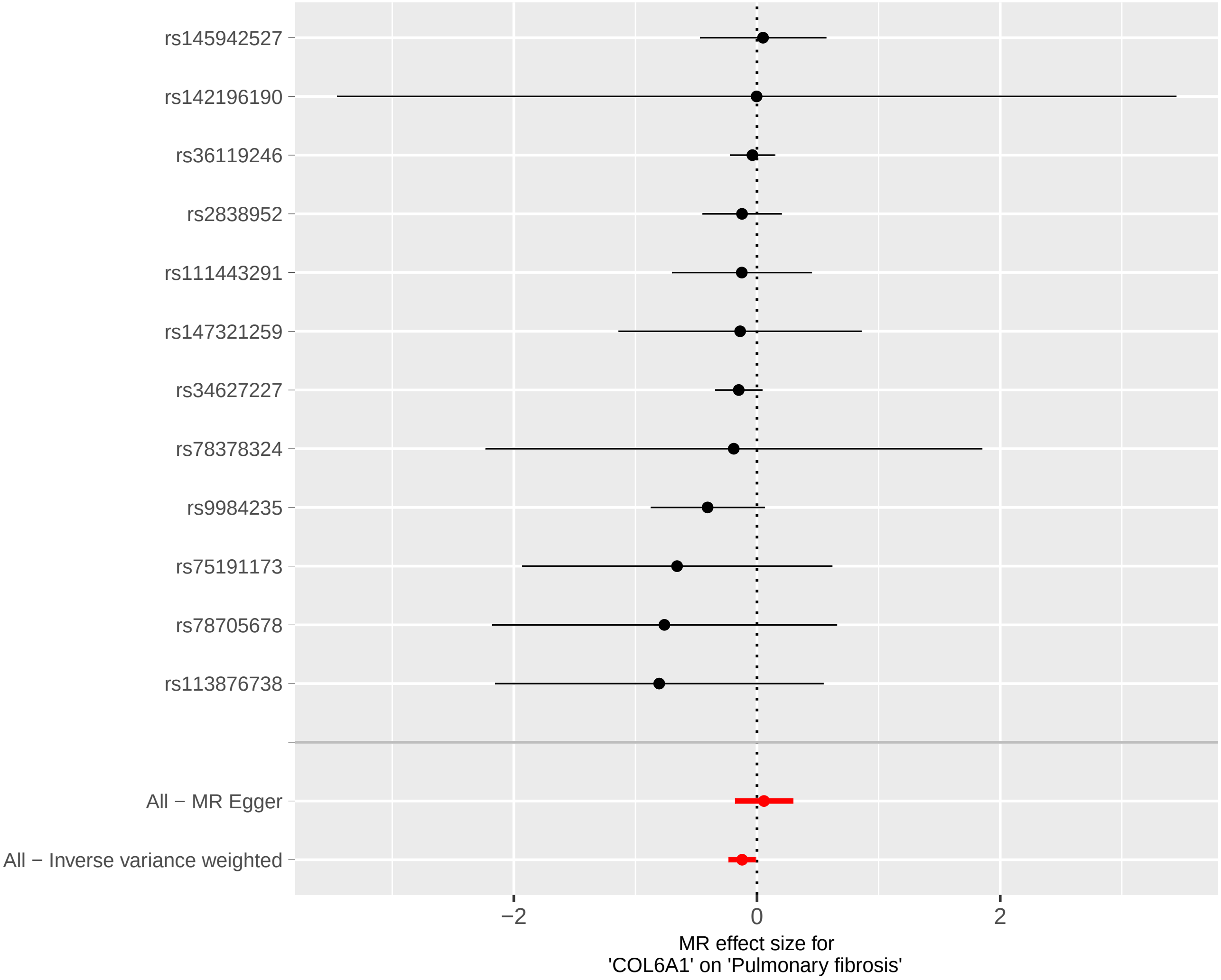

rs145942527
rs142196190
rs36119246
rs2838952
rs111443291
rs147321259
rs34627227
rs78378324
rs9984235
rs75191173
rs78705678
rs113876738
All − MR Egger
All − Inverse variance weighted
−2
0
2
MR effect size for
'COL6A1' on 'Pulmonary fibrosis'

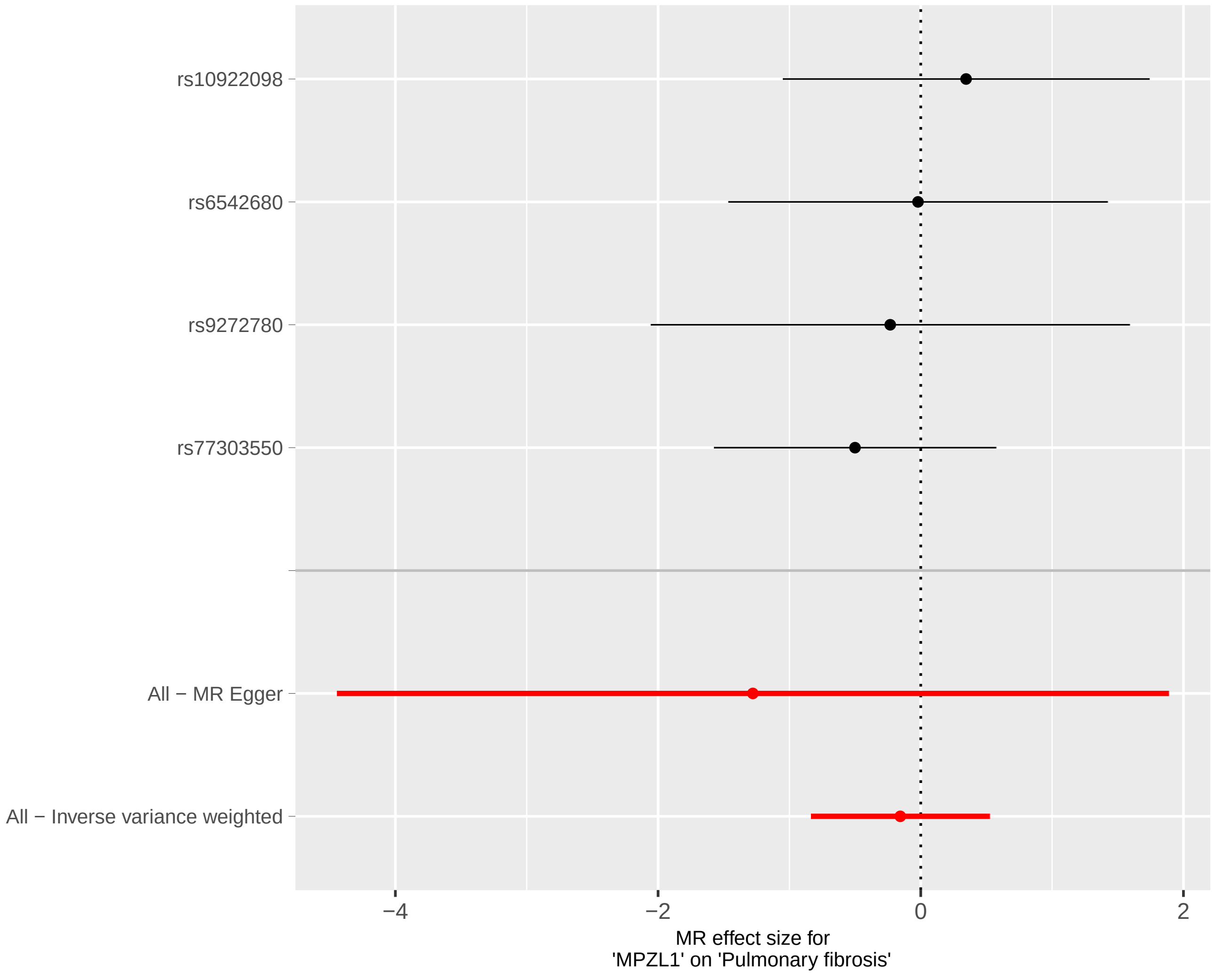

rs10922098
rs6542680
rs9272780
rs77303550
All − MR Egger
All − Inverse variance weighted
−4
−2
0
2
MR effect size for
'MPZL1' on 'Pulmonary fibrosis'

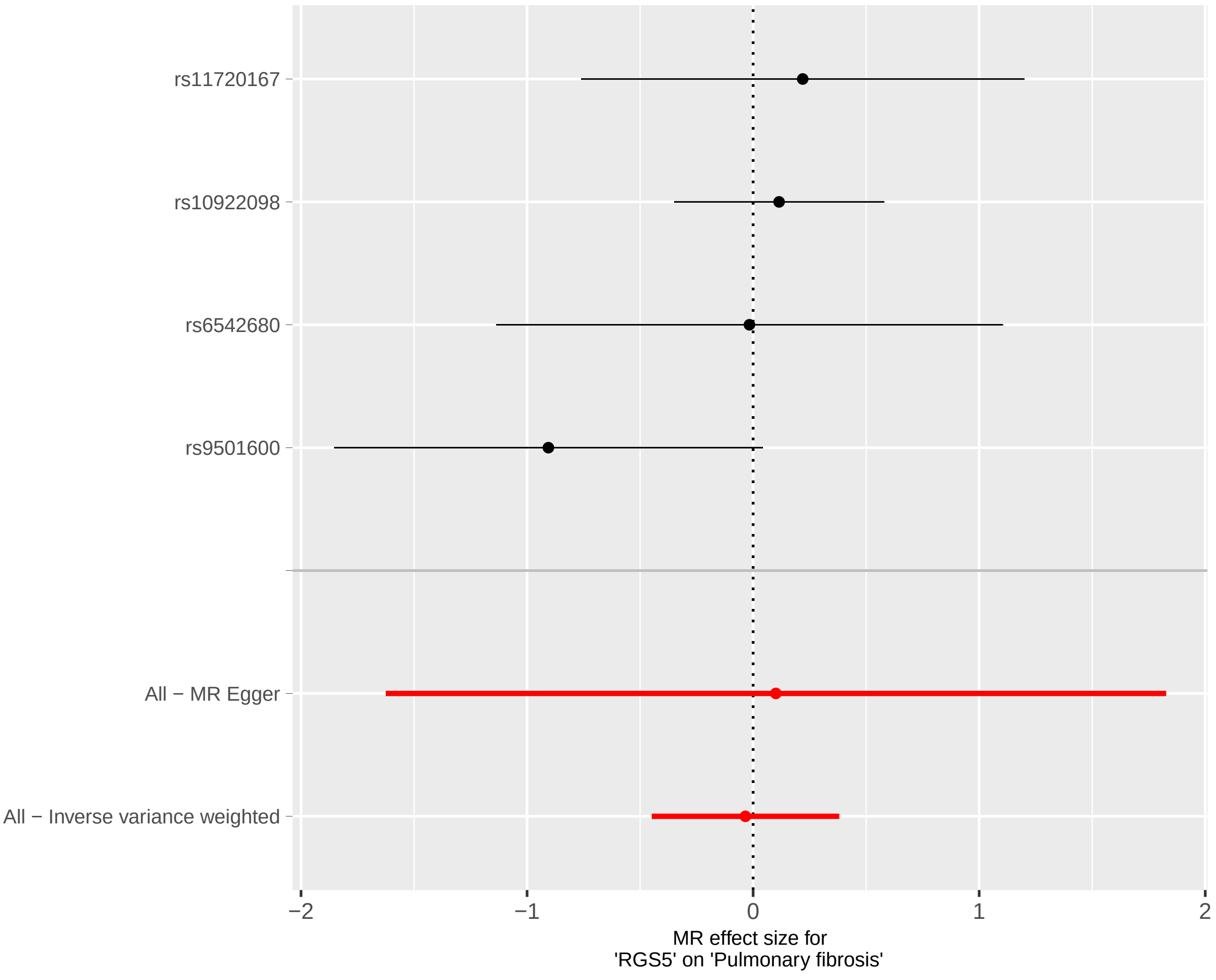

rs11720167
rs10922098
rs6542680
rs9501600
All − MR Egger
All − Inverse variance weighted
−2
−1
0
1
2
MR effect size for
'RGS5' on 'Pulmonary fibrosis'

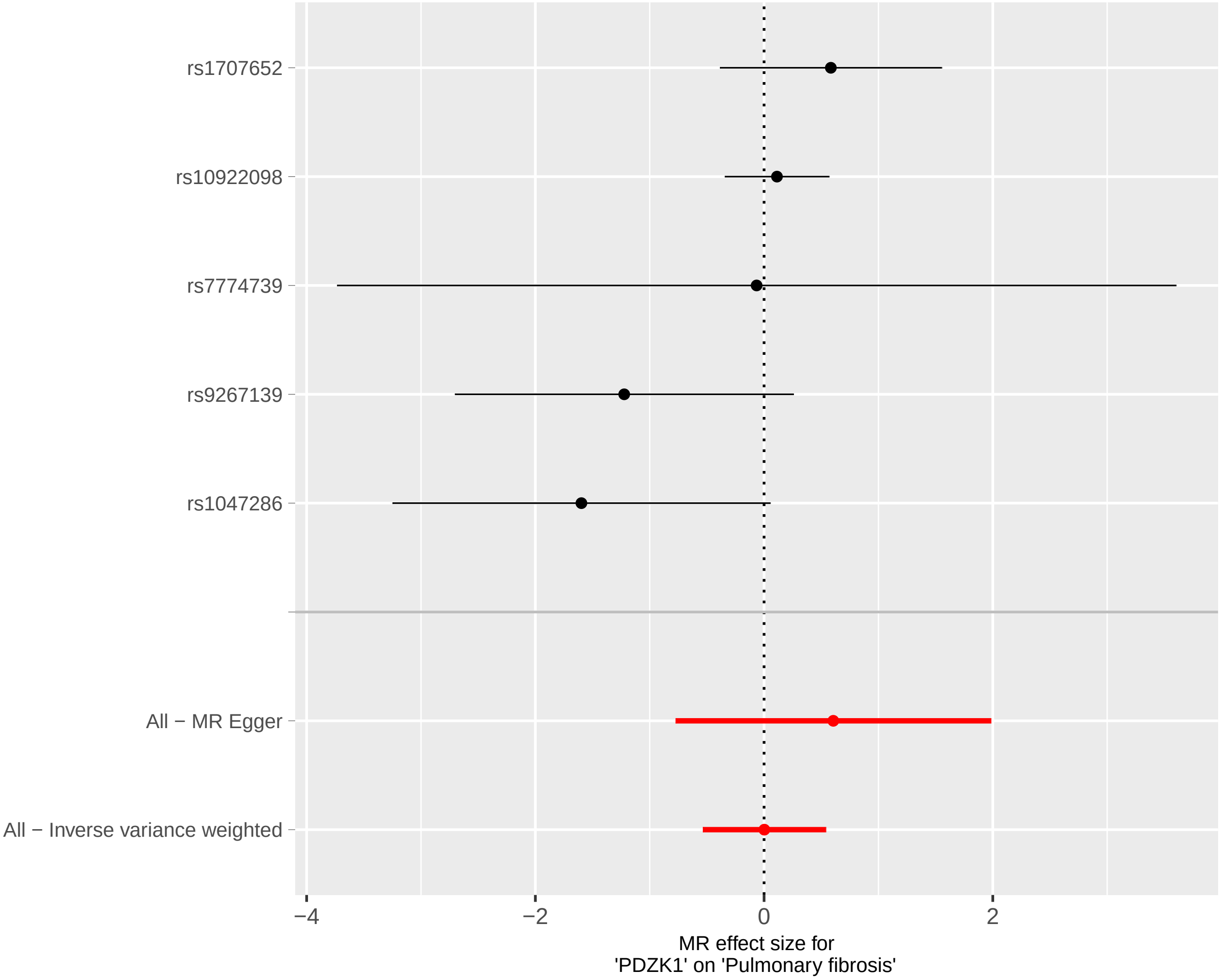

rs1707652
rs10922098
rs7774739
rs9267139
rs1047286
All − MR Egger
All − Inverse variance weighted
−4
−2
0
2
MR effect size for
'PDZK1' on 'Pulmonary fibrosis'

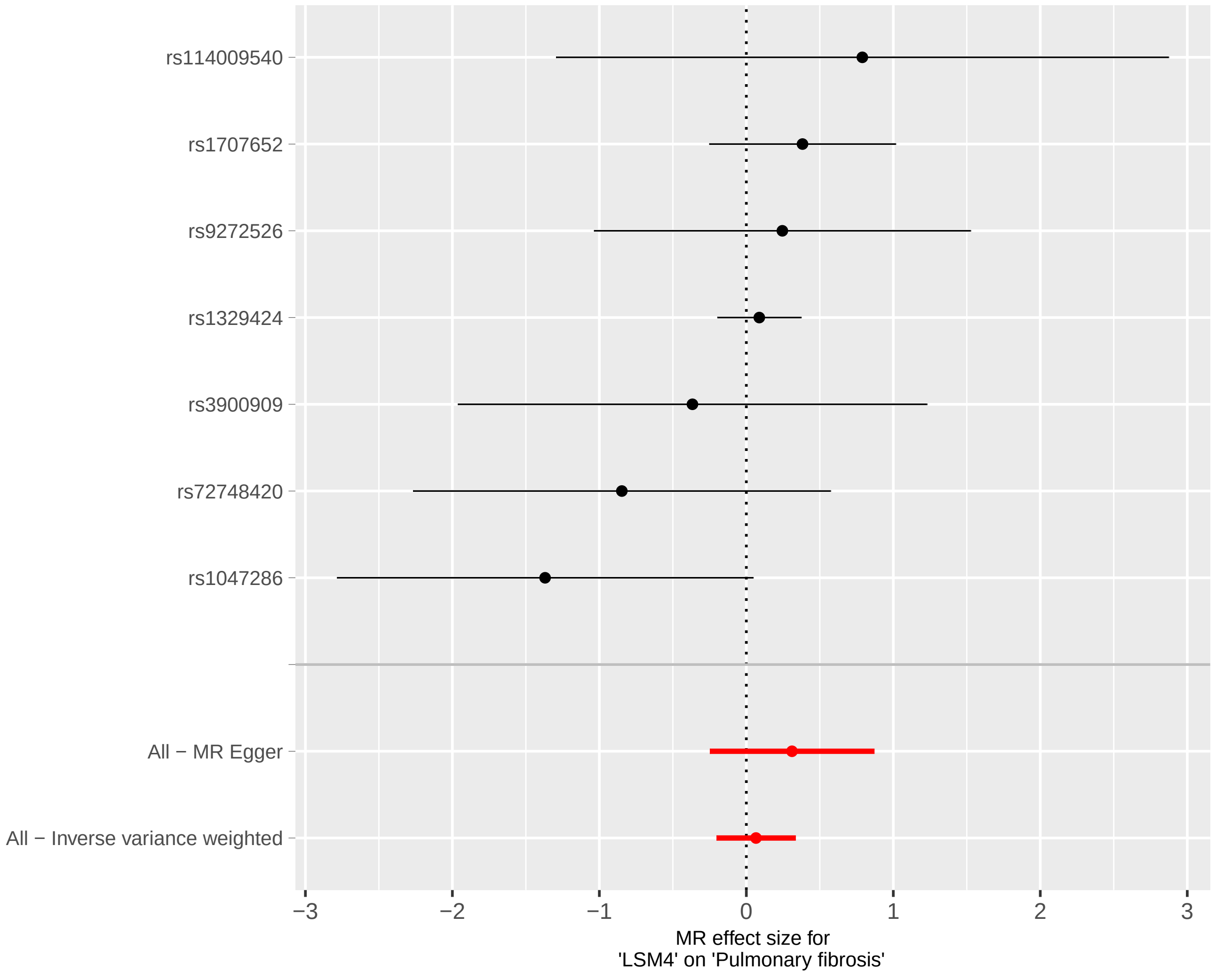

rs114009540
rs1707652
rs9272526
rs1329424
rs3900909
rs72748420
rs1047286
All − MR Egger
All − Inverse variance weighted
−3
−2
−1
0
1
2
3
MR effect size for
'LSM4' on 'Pulmonary fibrosis'

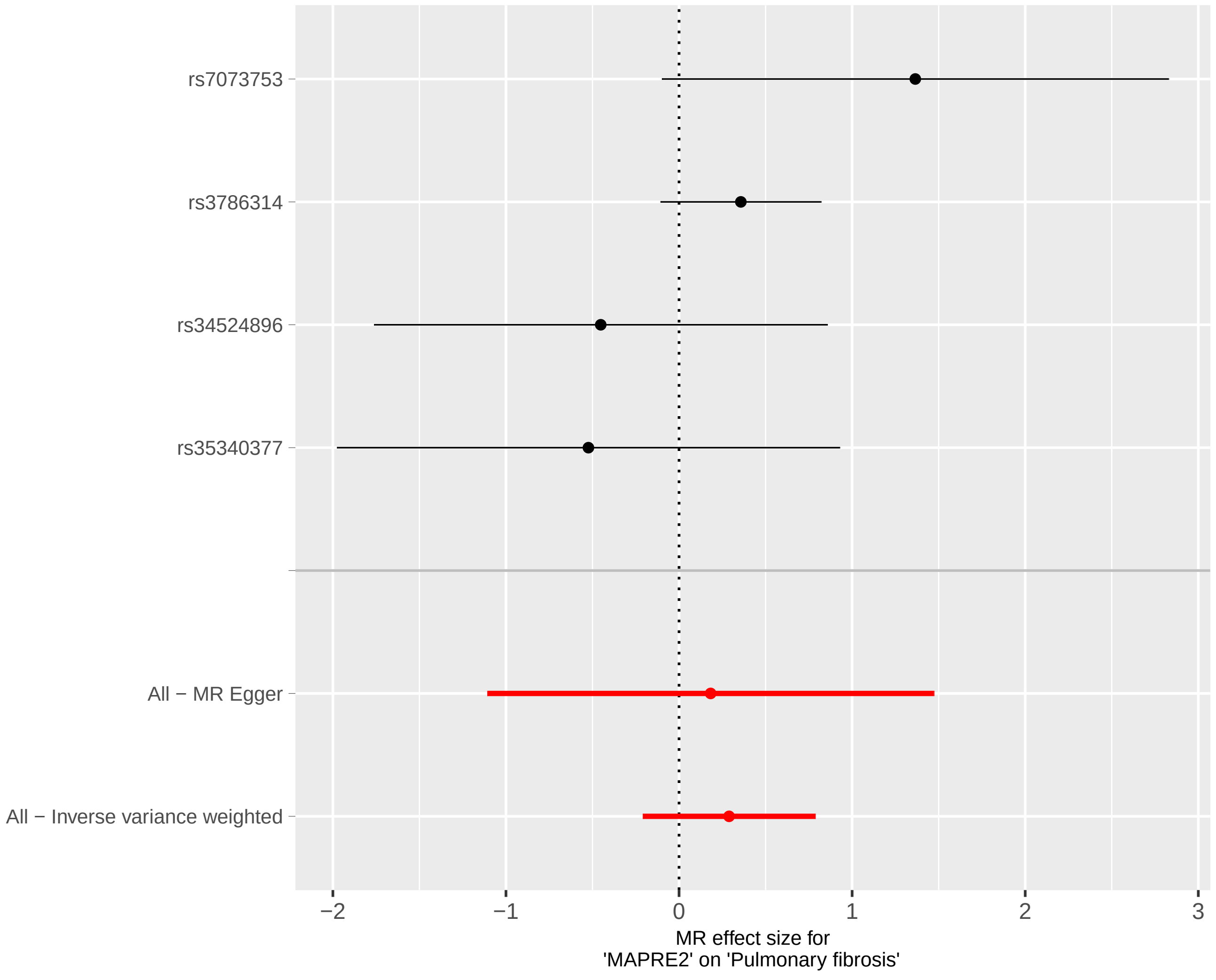

rs7073753
rs3786314
rs34524896
rs35340377
All − MR Egger
All − Inverse variance weighted
−2
−1
0
1
2
3
MR effect size for
'MAPRE2' on 'Pulmonary fibrosis'

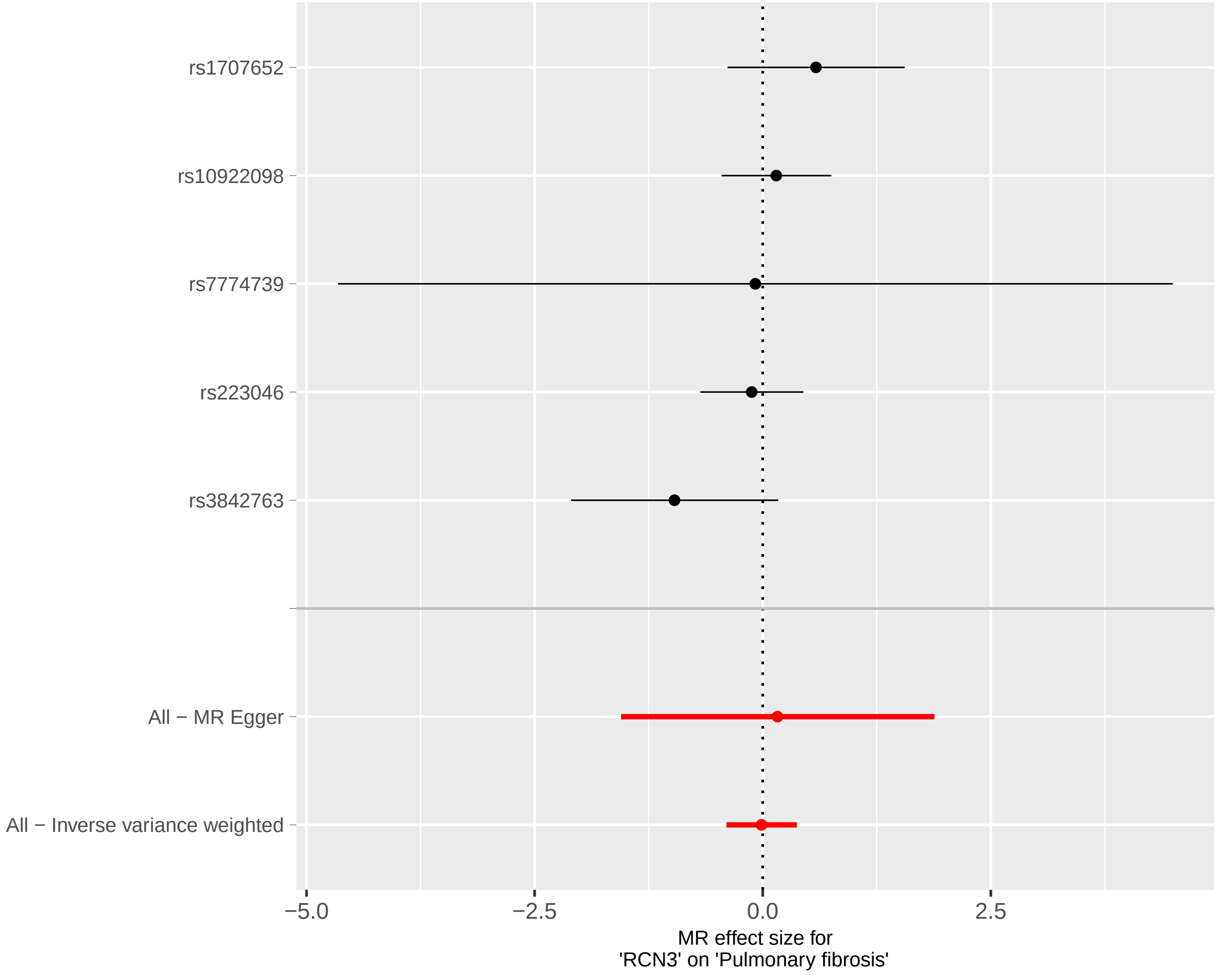

rs1707652
rs10922098
rs7774739
rs223046
rs3842763
All − MR Egger
All − Inverse variance weighted
−5.0
−2.5
0.0
2.5
MR effect size for
'RCN3' on 'Pulmonary fibrosis'

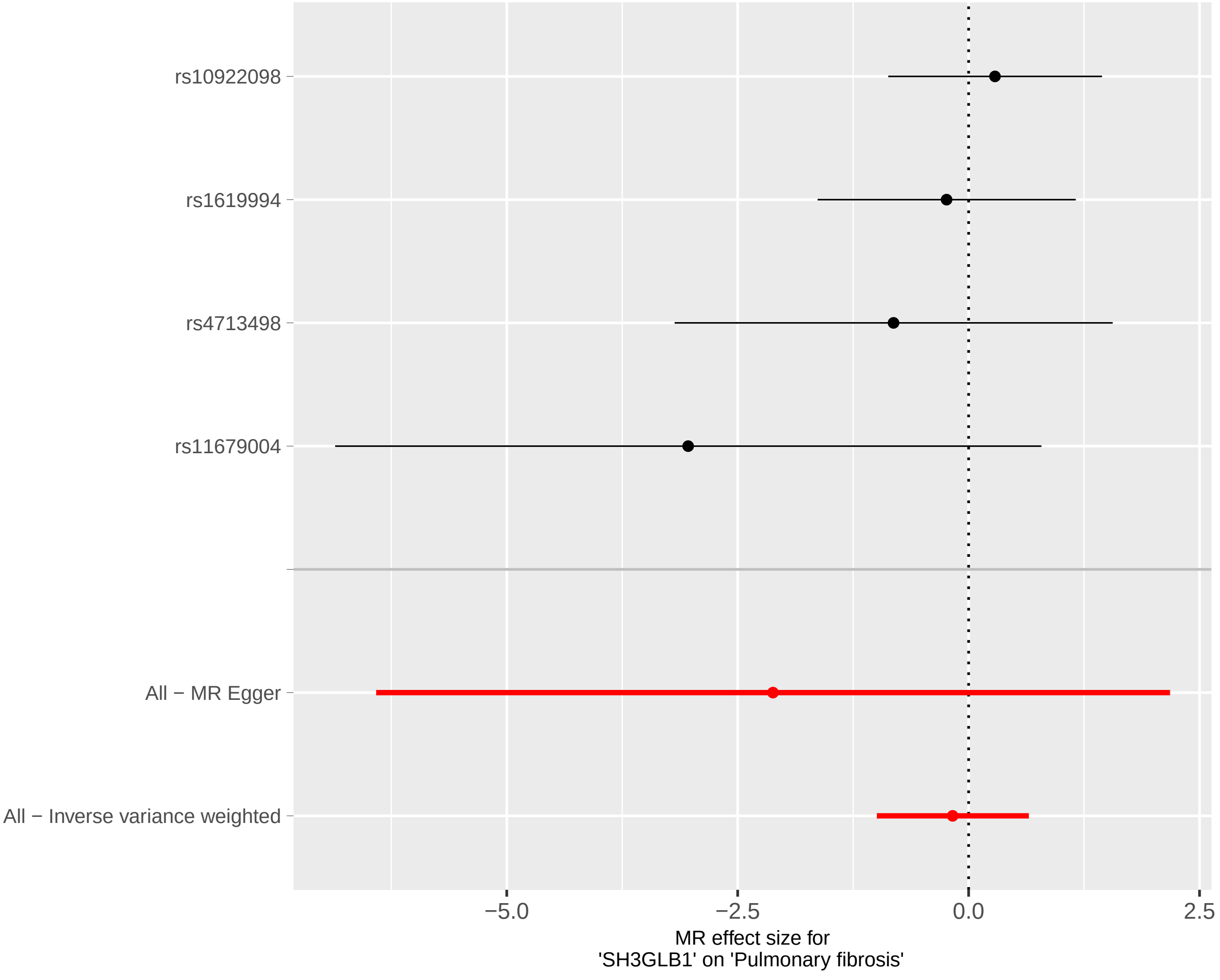

rs10922098
rs1619994
rs4713498
rs11679004
All − MR Egger
All − Inverse variance weighted
−5.0
−2.5
0.0
2.5
MR effect size for
'SH3GLB1' on 'Pulmonary fibrosis'

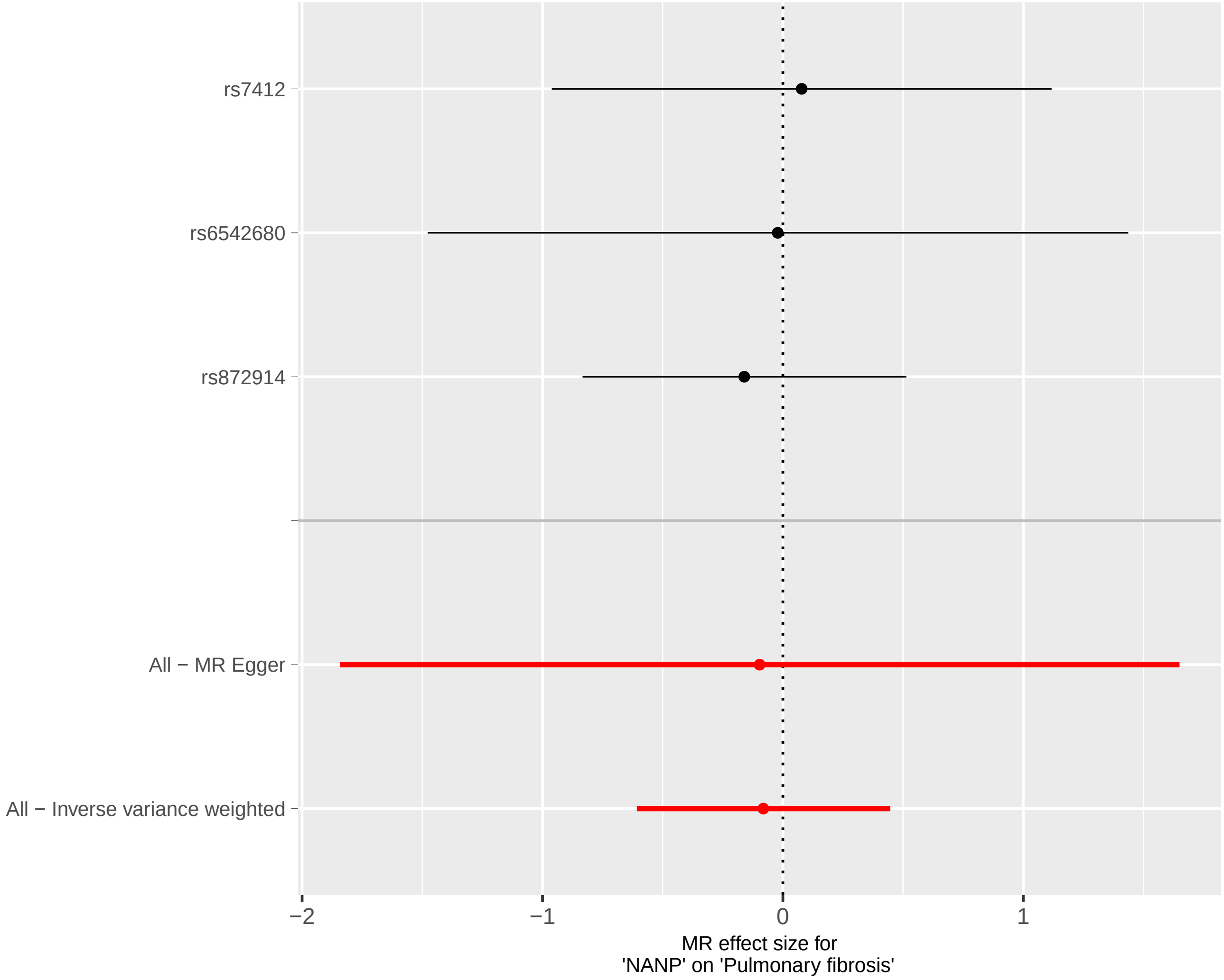

rs7412
rs6542680
rs872914
All − MR Egger
All − Inverse variance weighted
−2
−1
0
1
MR effect size for
'NANP' on 'Pulmonary fibrosis'

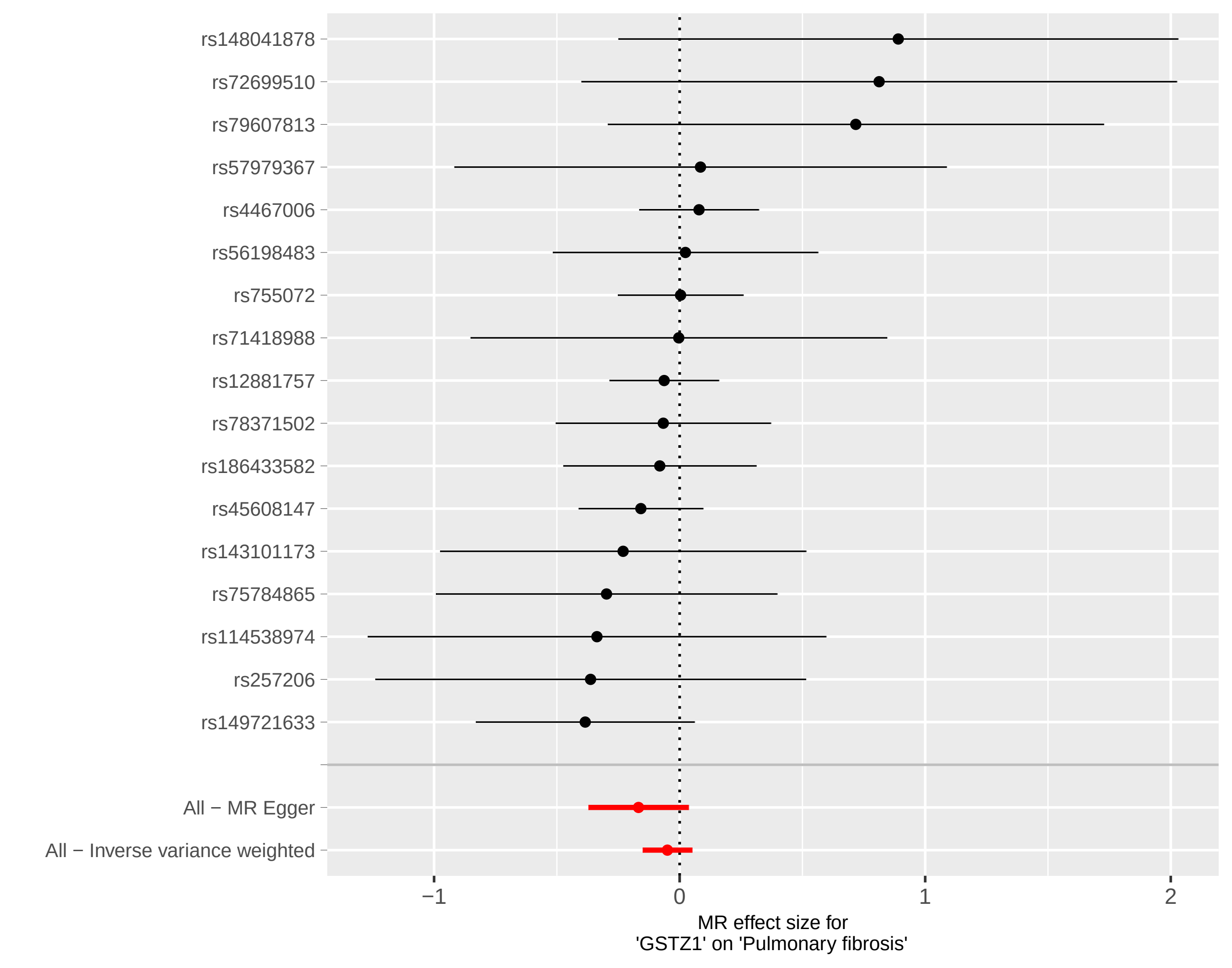

rs148041878
rs72699510
rs79607813
rs57979367
rs4467006
rs56198483
rs755072
rs71418988
rs12881757
rs78371502
rs186433582
rs45608147
rs143101173
rs75784865
rs114538974
rs257206
rs149721633
All − MR Egger
All − Inverse variance weighted
−1
0
1
2
MR effect size for
'GSTZ1' on 'Pulmonary fibrosis'

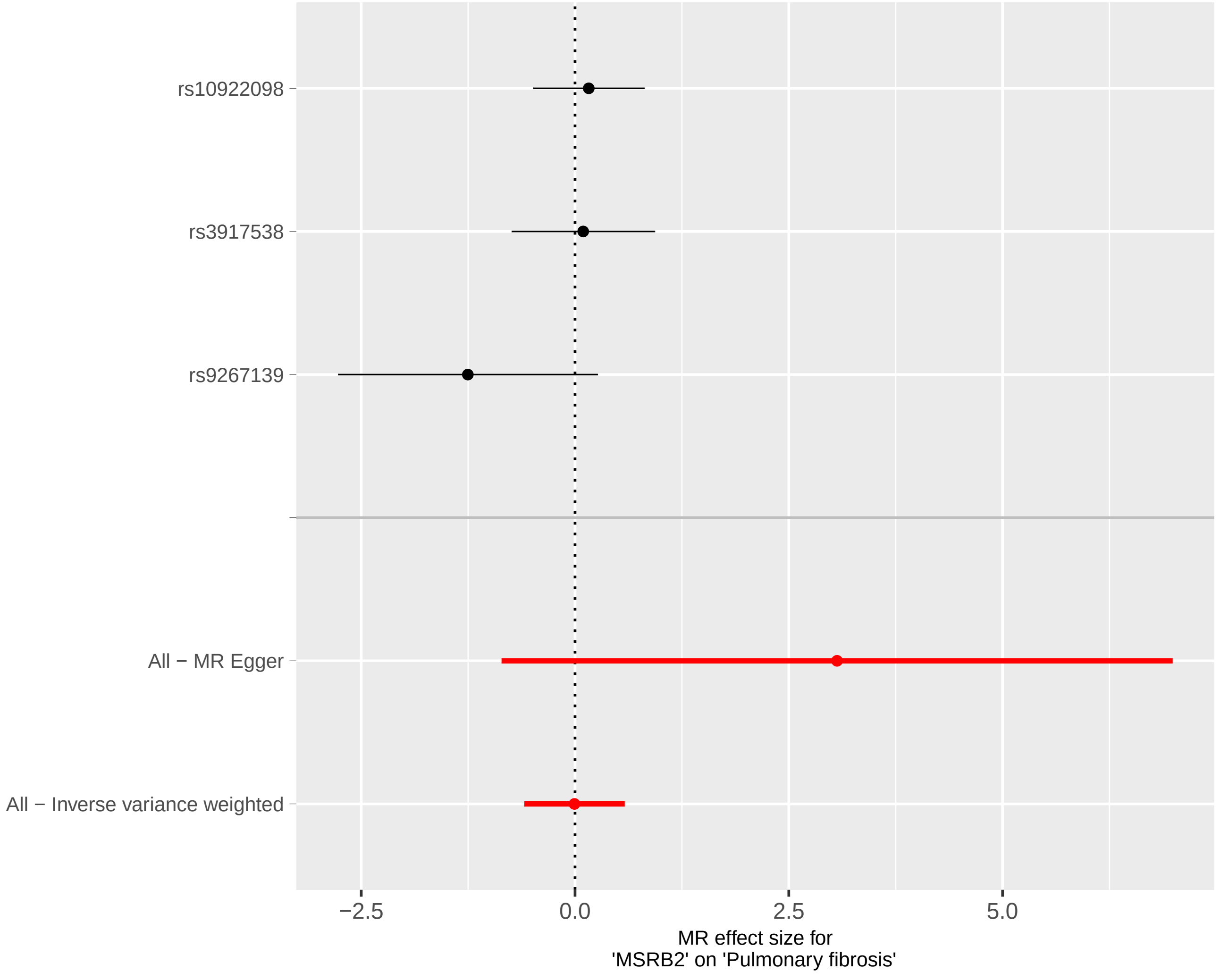

rs10922098
rs3917538
rs9267139
All − MR Egger
All − Inverse variance weighted
−2.5
0.0
2.5
5.0
MR effect size for
'MSRB2' on 'Pulmonary fibrosis'

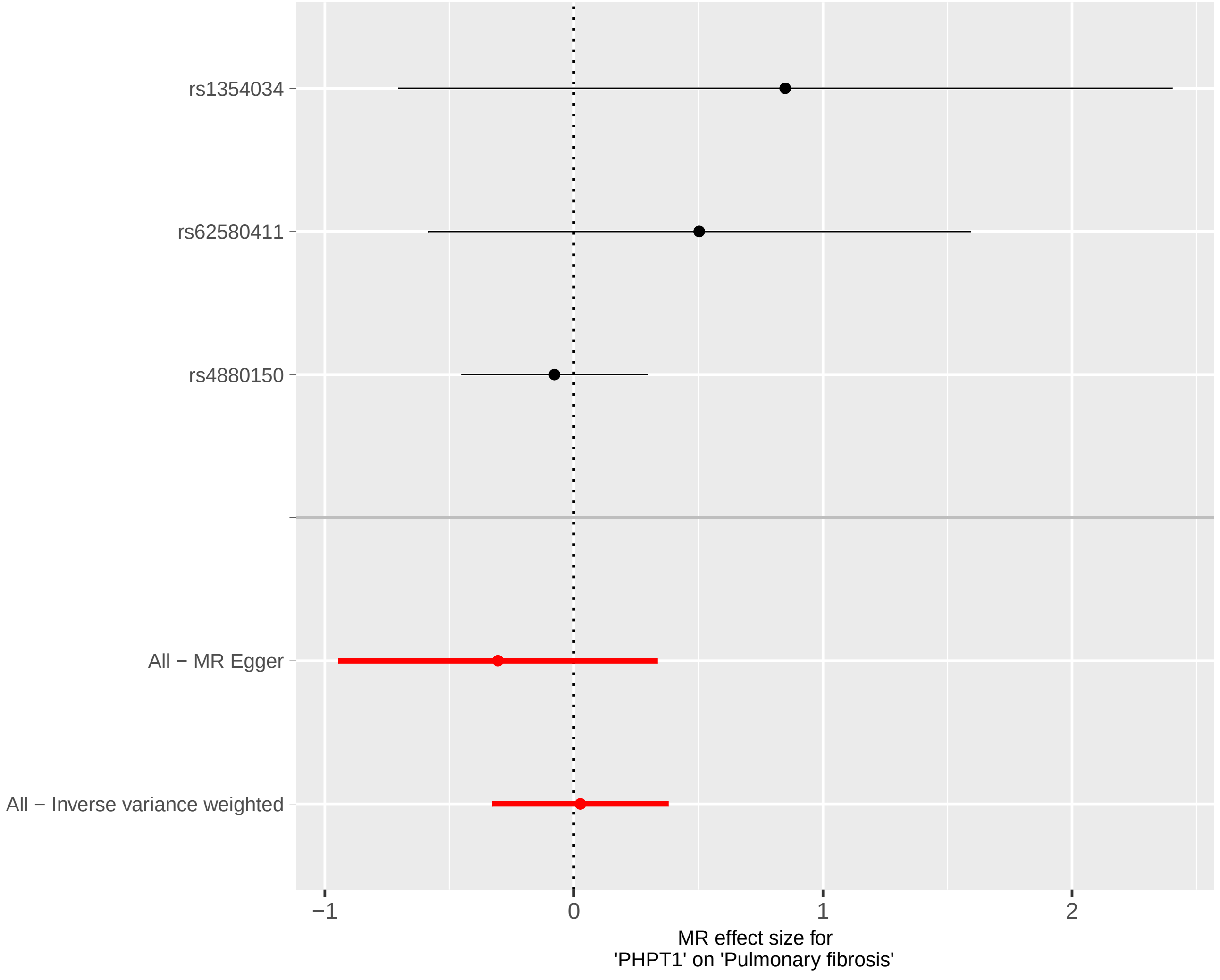

rs1354034
rs62580411
rs4880150
All − MR Egger
All − Inverse variance weighted
−1
0
1
2
MR effect size for
'PHPT1' on 'Pulmonary fibrosis'

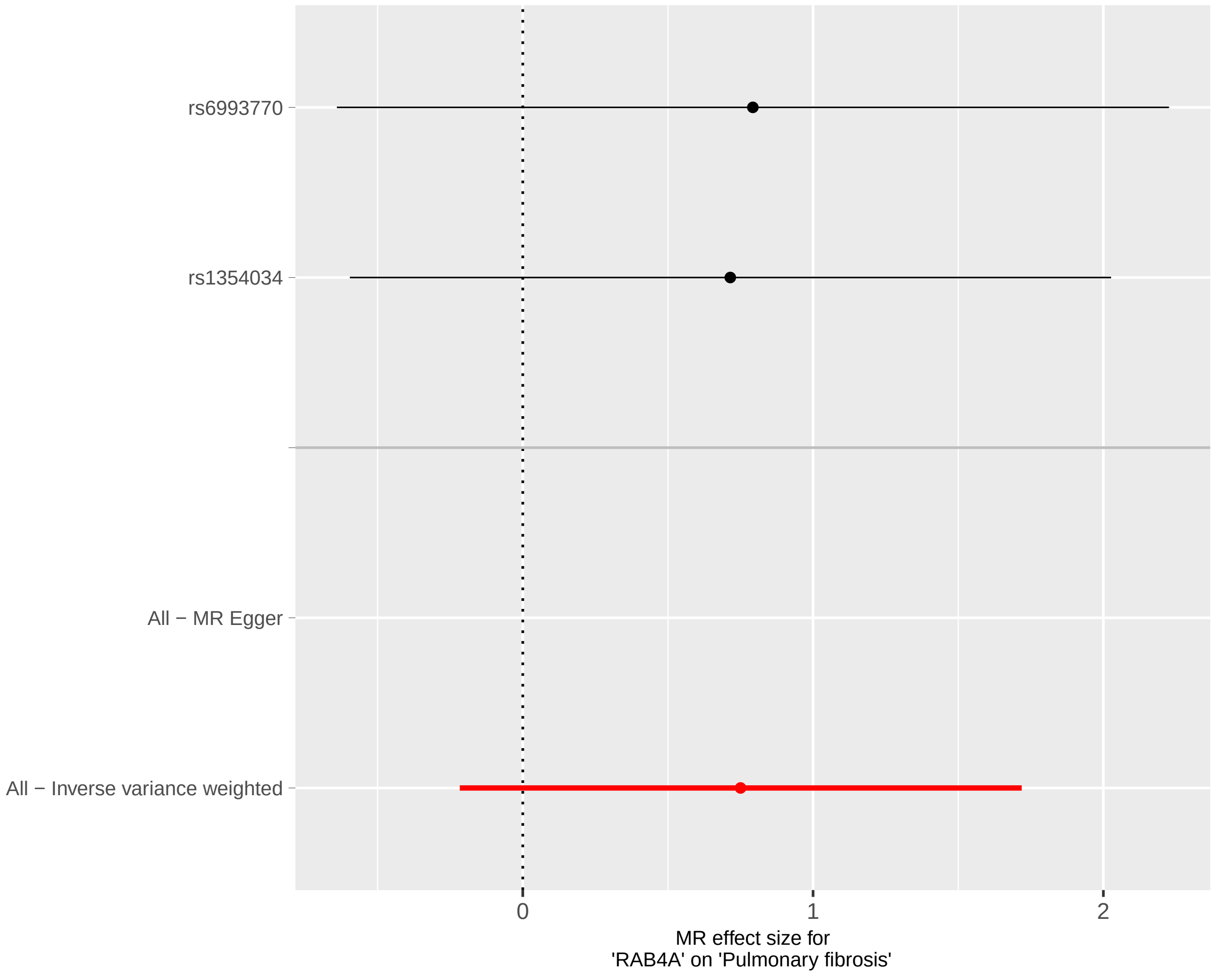
rs6993770
rs1354034
All − MR Egger
All − Inverse variance weighted
0
1
2
MR effect size for
'RAB4A' on 'Pulmonary fibrosis'

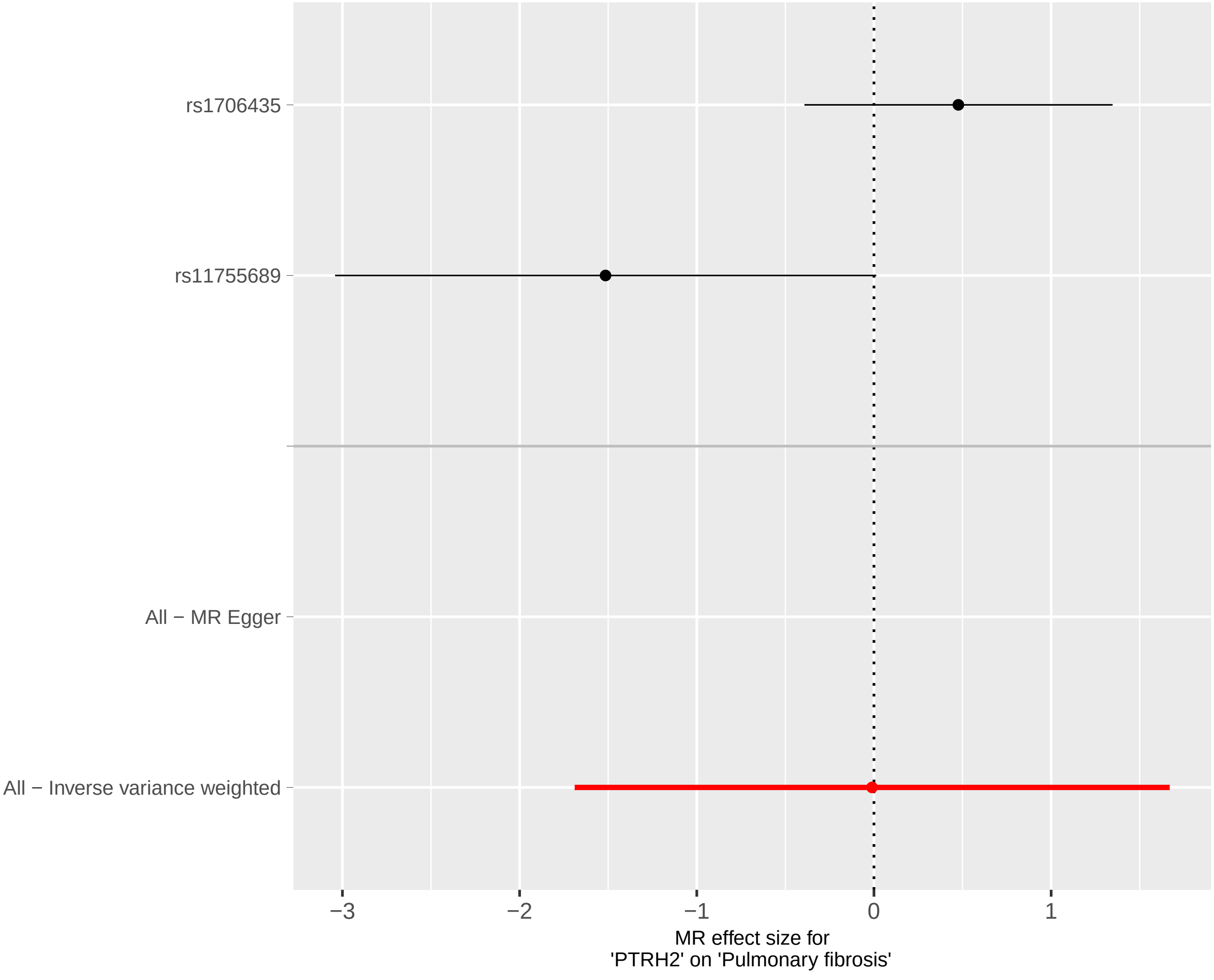
rs1706435
rs11755689
All − MR Egger
All − Inverse variance weighted
−3
−2
−1
0
1
MR effect size for
'PTRH2' on 'Pulmonary fibrosis'

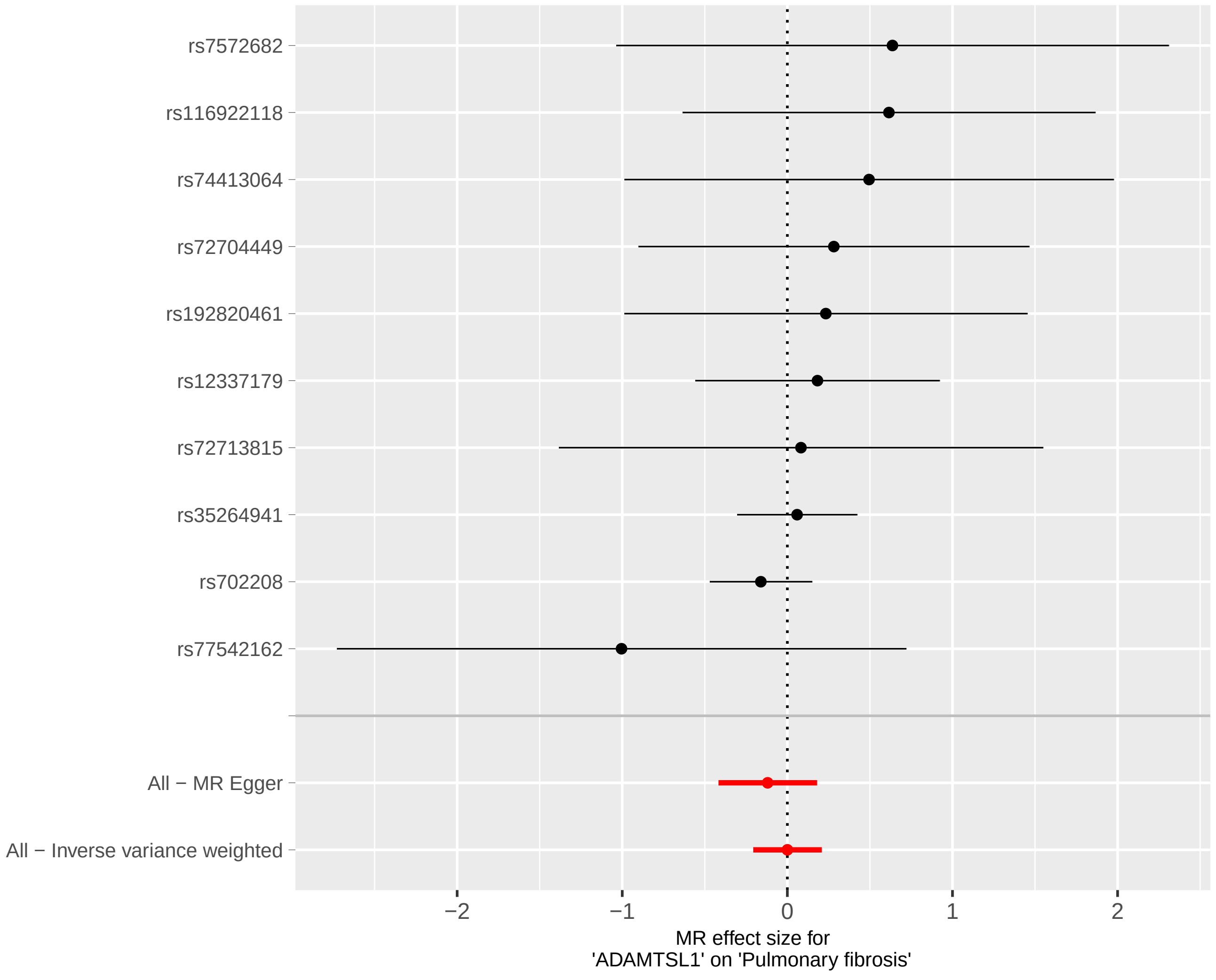

rs7572682
rs116922118
rs74413064
rs72704449
rs192820461
rs12337179
rs72713815
rs35264941
rs702208
rs77542162
All − MR Egger
All − Inverse variance weighted
−2
−1
0
1
2
MR effect size for
'ADAMTSL1' on 'Pulmonary fibrosis'

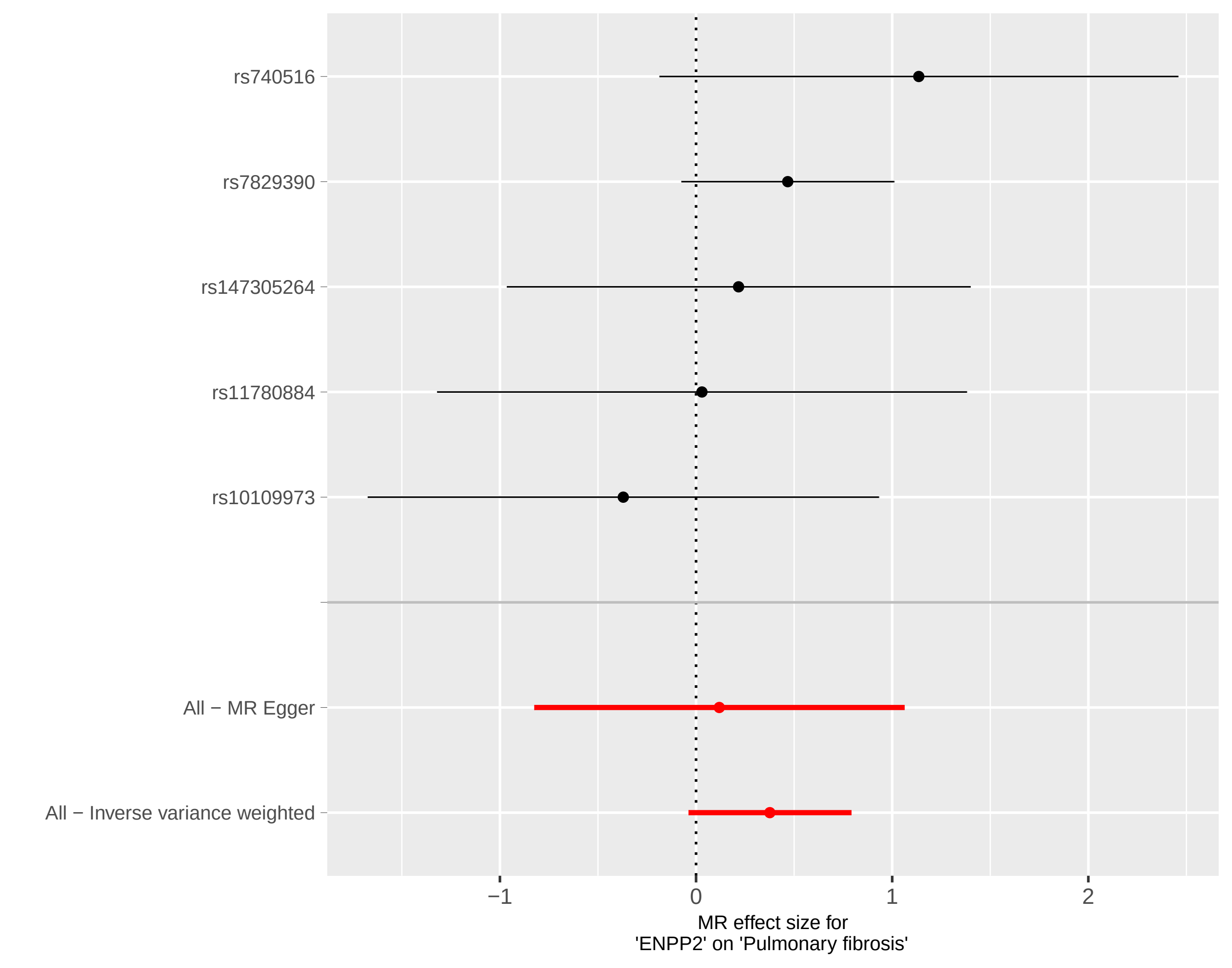
rs740516
rs7829390
rs147305264
rs11780884
rs10109973
All − MR Egger
All − Inverse variance weighted
−1
0
1
2
MR effect size for
'ENPP2' on 'Pulmonary fibrosis'

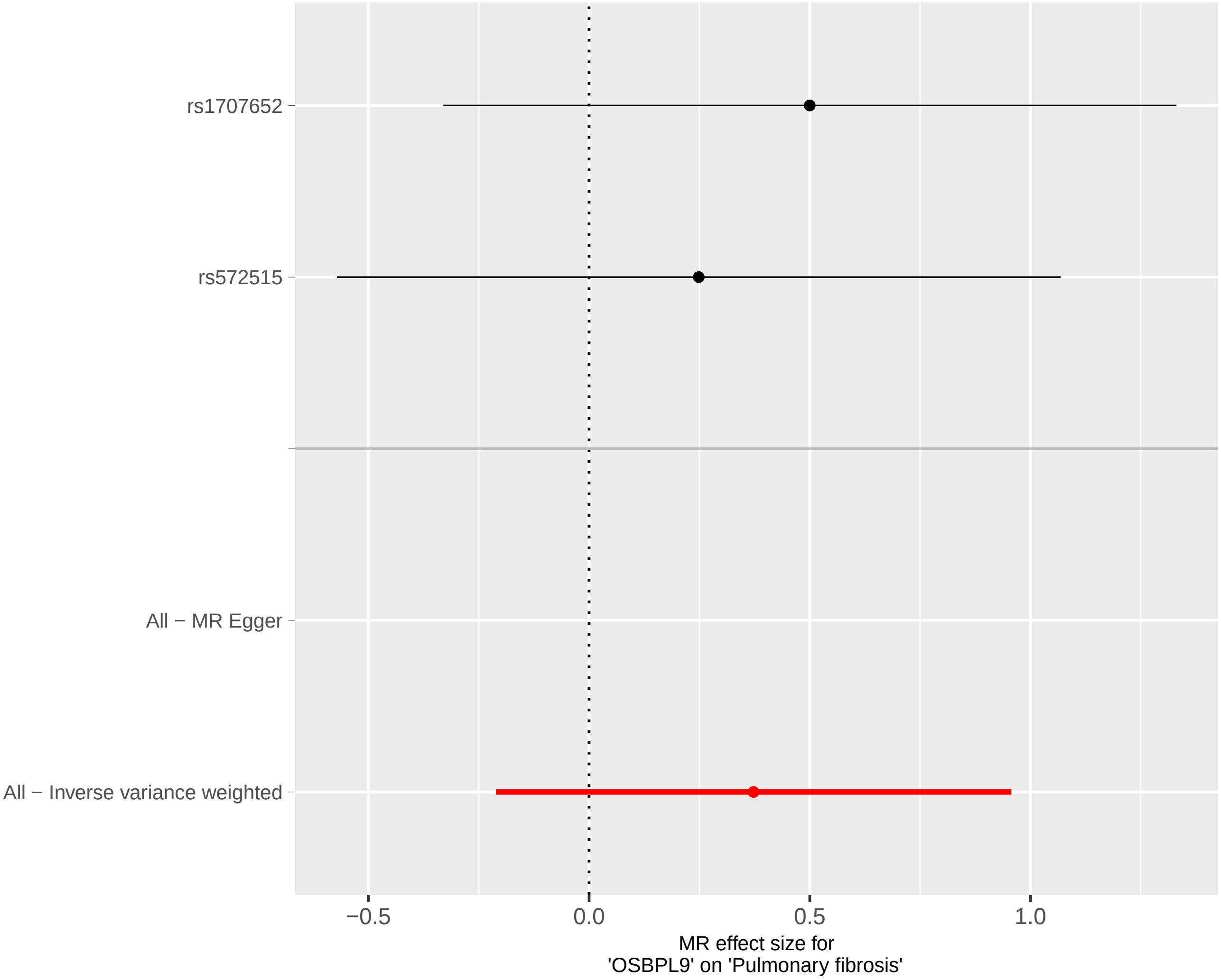

rs1707652
rs572515
All − MR Egger
All − Inverse variance weighted
−0.5
0.0
0.5
1.0
MR effect size for
'OSBPL9' on 'Pulmonary fibrosis'

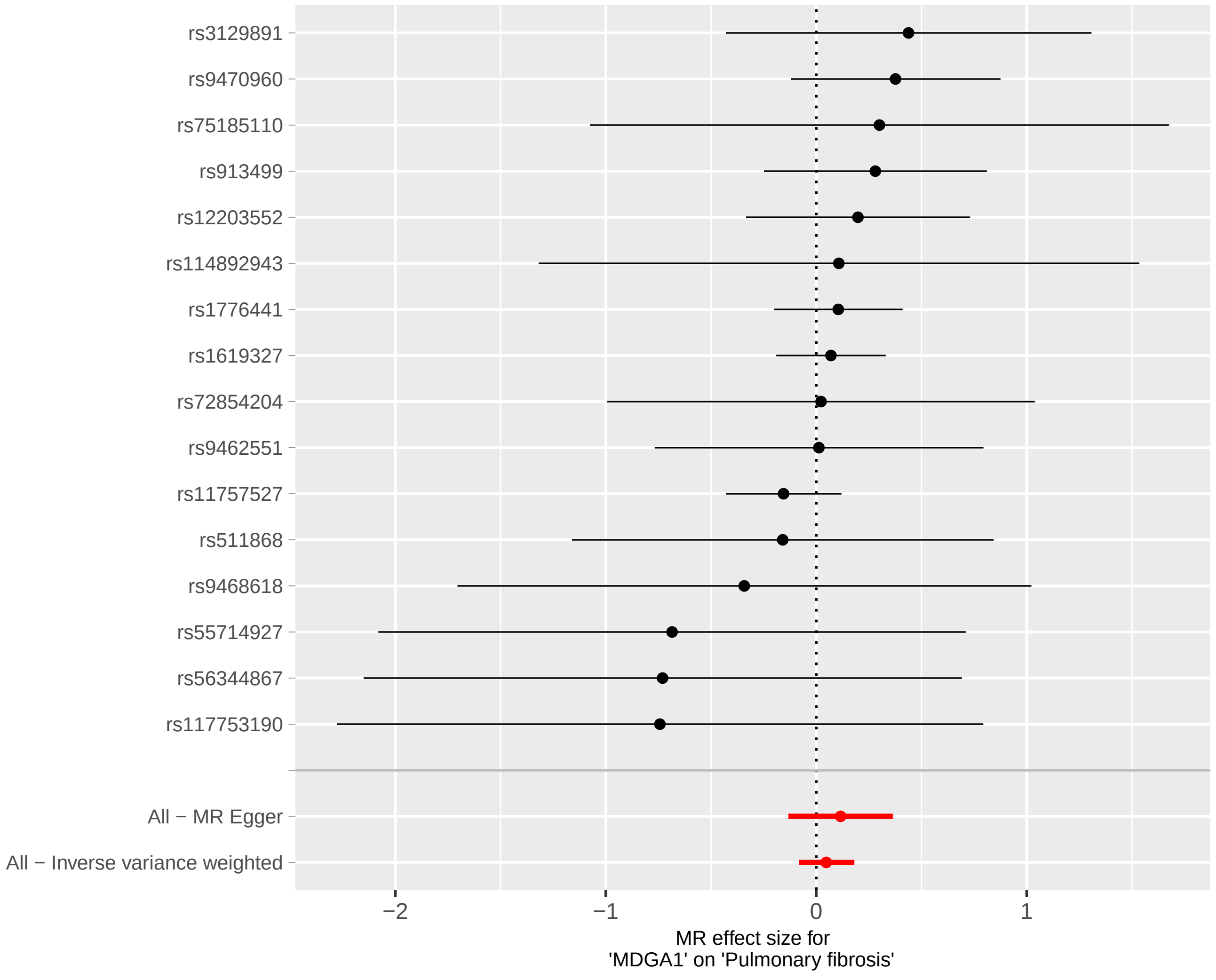

rs3129891
rs9470960
rs75185110
rs913499
rs12203552
rs114892943
rs1776441
rs1619327
rs72854204
rs9462551
rs11757527
rs511868
rs9468618
rs55714927
rs56344867
rs117753190
All − MR Egger
All − Inverse variance weighted
−2
−1
0
1
MR effect size for
'MDGA1' on 'Pulmonary fibrosis'

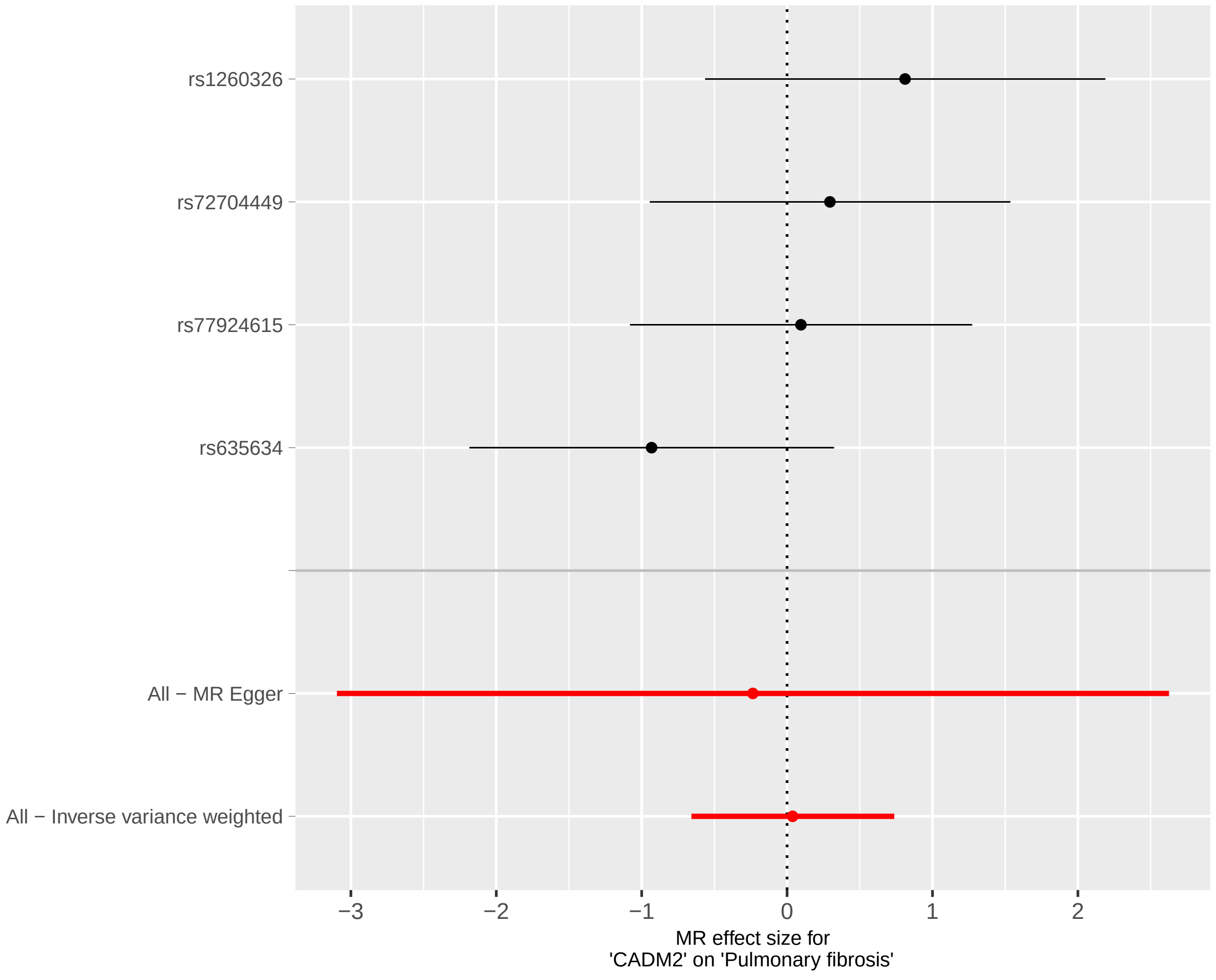

rs1260326
rs72704449
rs77924615
rs635634
All − MR Egger
All − Inverse variance weighted
−3
−2
−1
0
1
2
MR effect size for
'CADM2' on 'Pulmonary fibrosis'

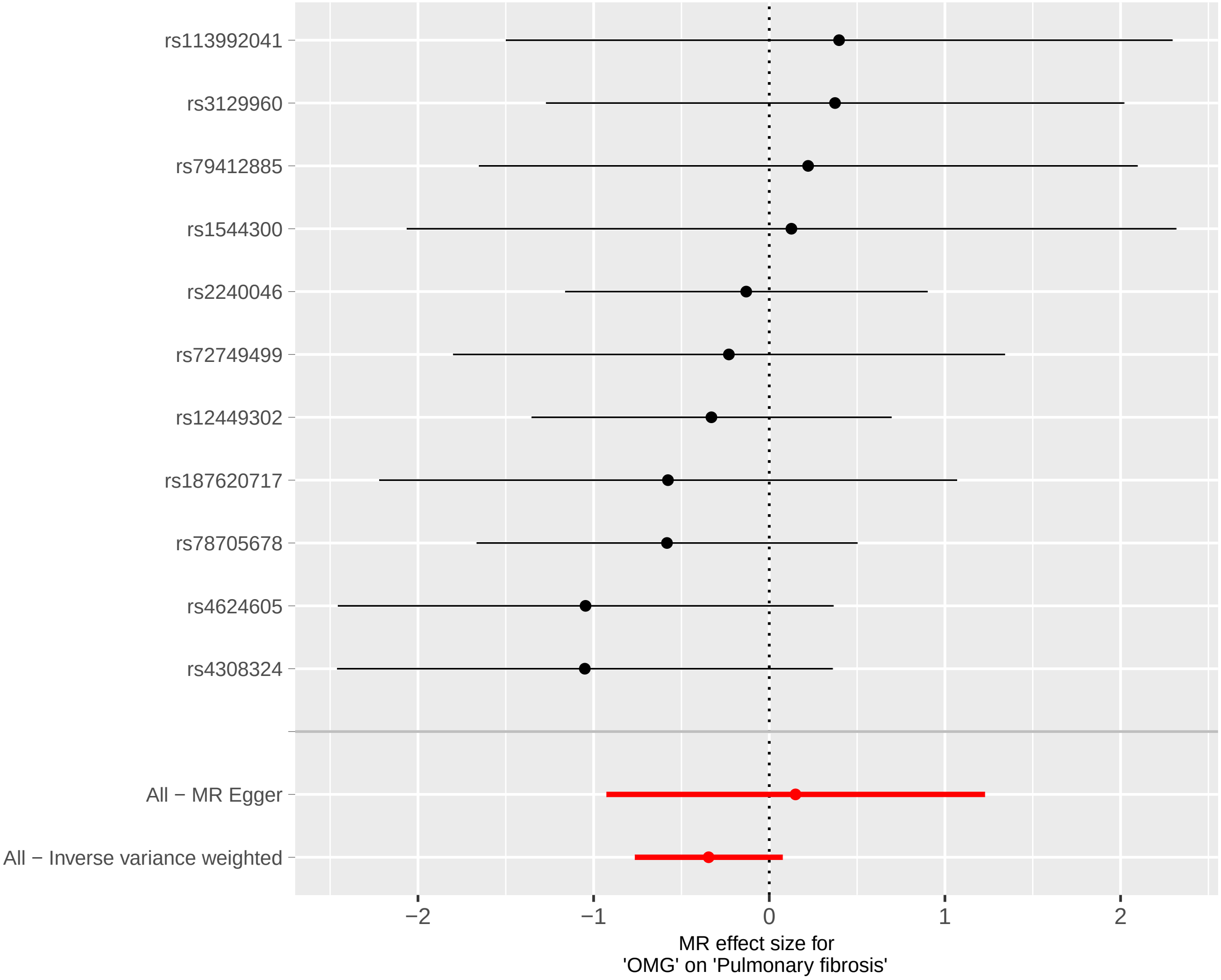

rs113992041
rs3129960
rs79412885
rs1544300
rs2240046
rs72749499
rs12449302
rs187620717
rs78705678
rs4624605
rs4308324
All − MR Egger
All − Inverse variance weighted
−2
−1
0
1
2
MR effect size for
'OMG' on 'Pulmonary fibrosis'

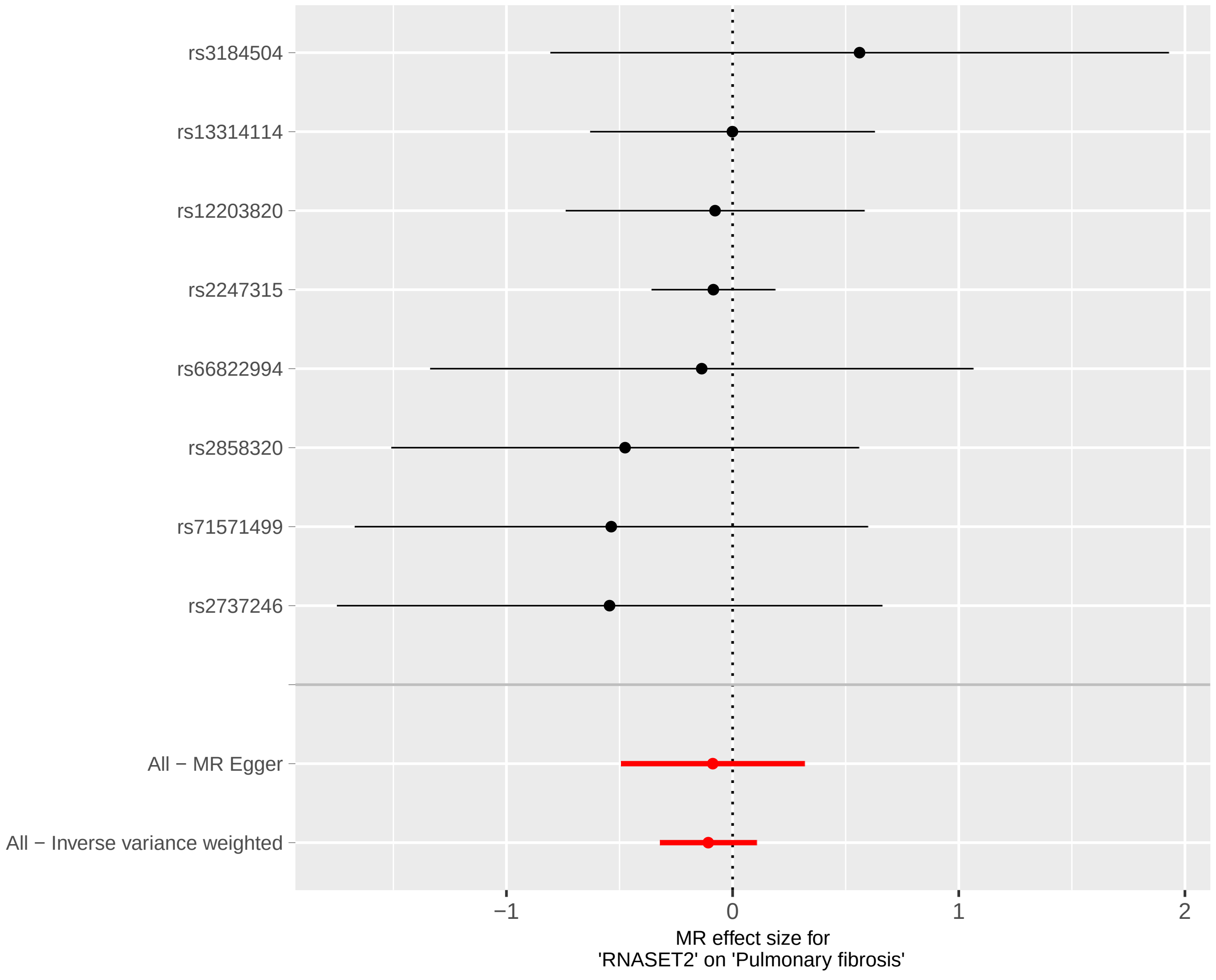

rs3184504
rs13314114
rs12203820
rs2247315
rs66822994
rs2858320
rs71571499
rs2737246
All − MR Egger
All − Inverse variance weighted
−1
0
1
2
MR effect size for
'RNASET2' on 'Pulmonary fibrosis'

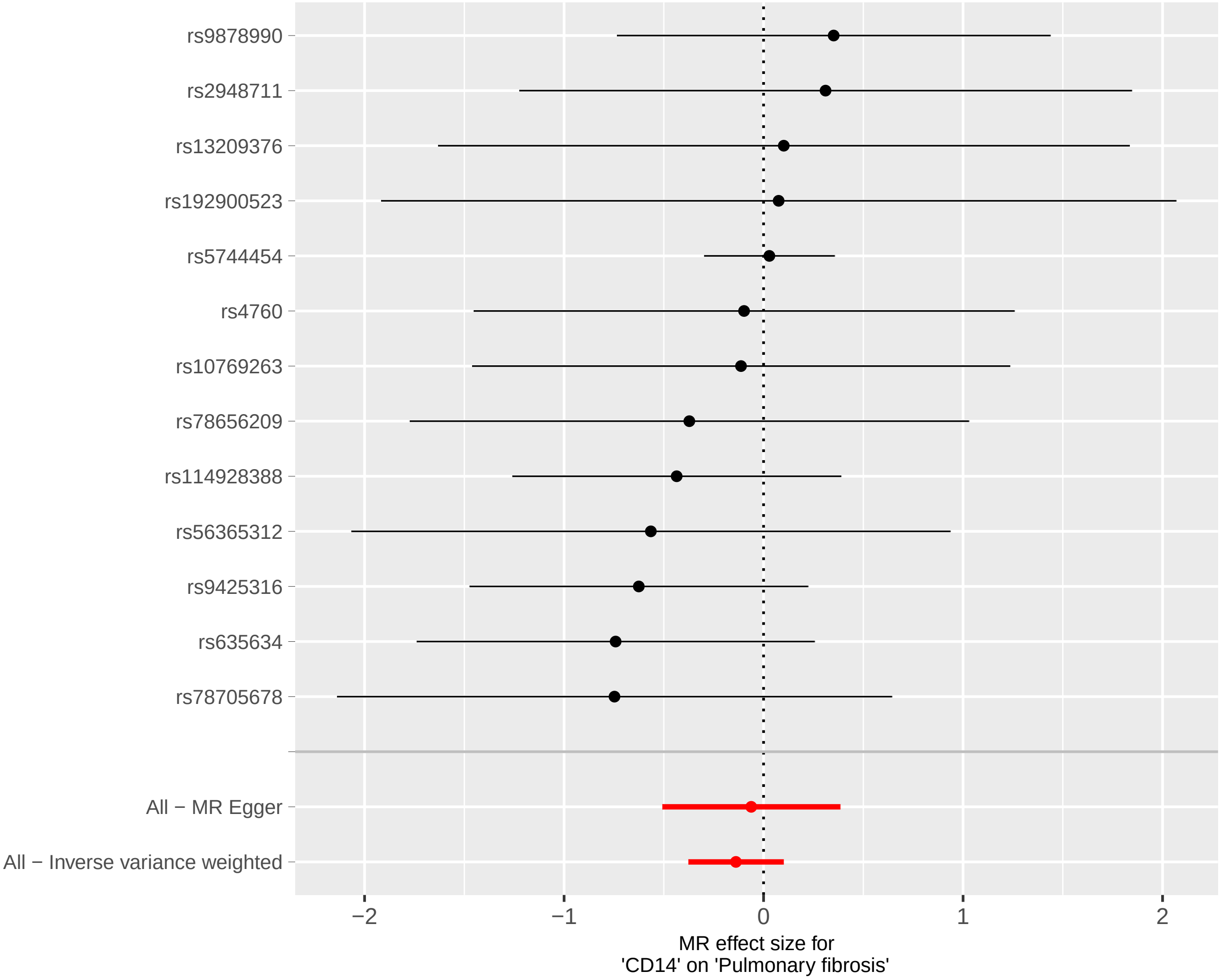
rs9878990
rs2948711
rs13209376
rs192900523
rs5744454
rs4760
rs10769263
rs78656209
rs114928388
rs56365312
rs9425316
rs635634
rs78705678
All − MR Egger
All − Inverse variance weighted
−2
−1
0
1
2
MR effect size for
'CD14' on 'Pulmonary fibrosis'

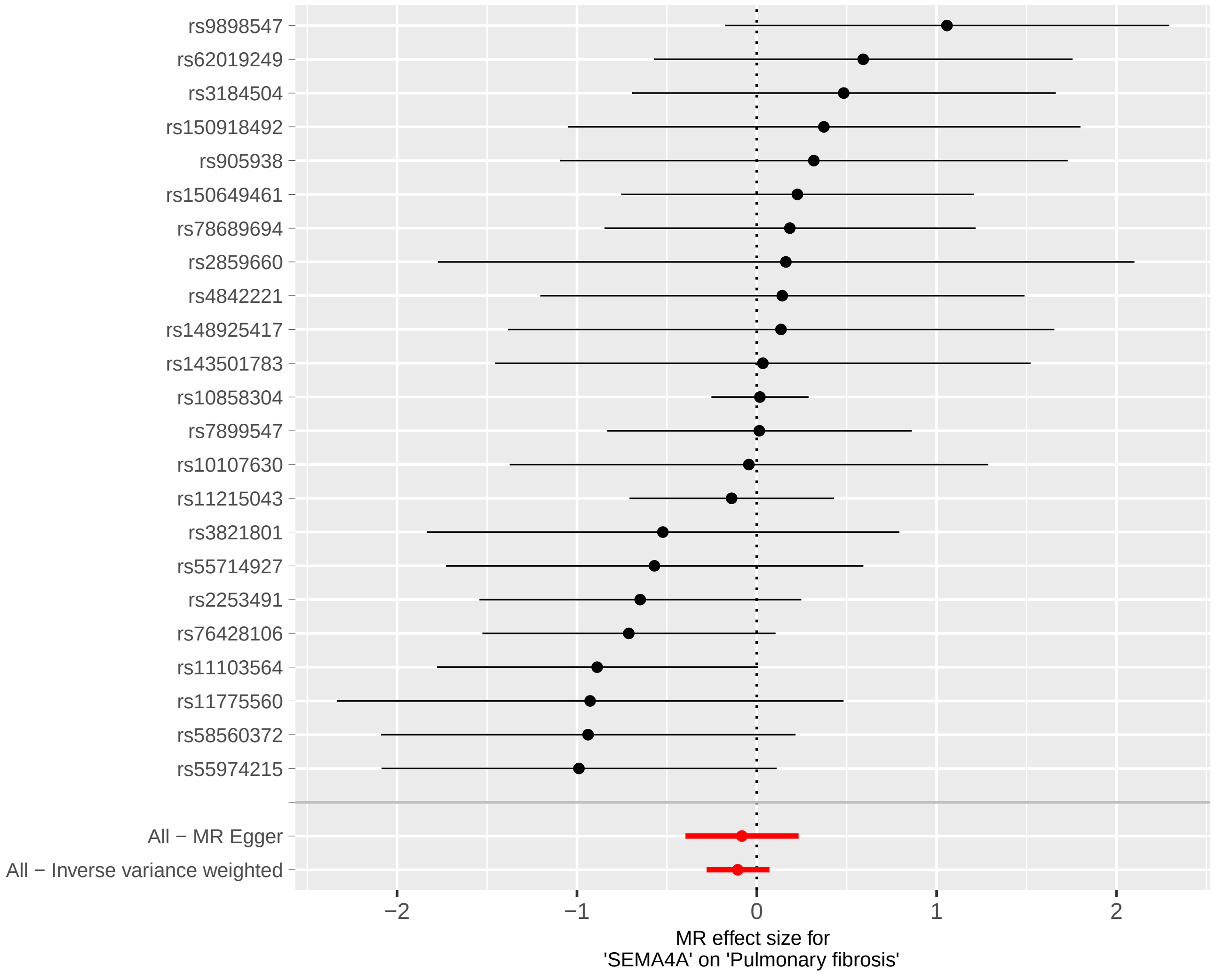
rs9898547
rs62019249
rs3184504
rs150918492
rs905938
rs150649461
rs78689694
rs2859660
rs4842221
rs148925417
rs143501783
rs10858304
rs7899547
rs10107630
rs11215043
rs3821801
rs55714927
rs2253491
rs76428106
rs11103564
rs11775560
rs58560372
rs55974215
All − MR Egger
All − Inverse variance weighted
−2
−1
0
1
2
MR effect size for
'SEMA4A' on 'Pulmonary fibrosis'

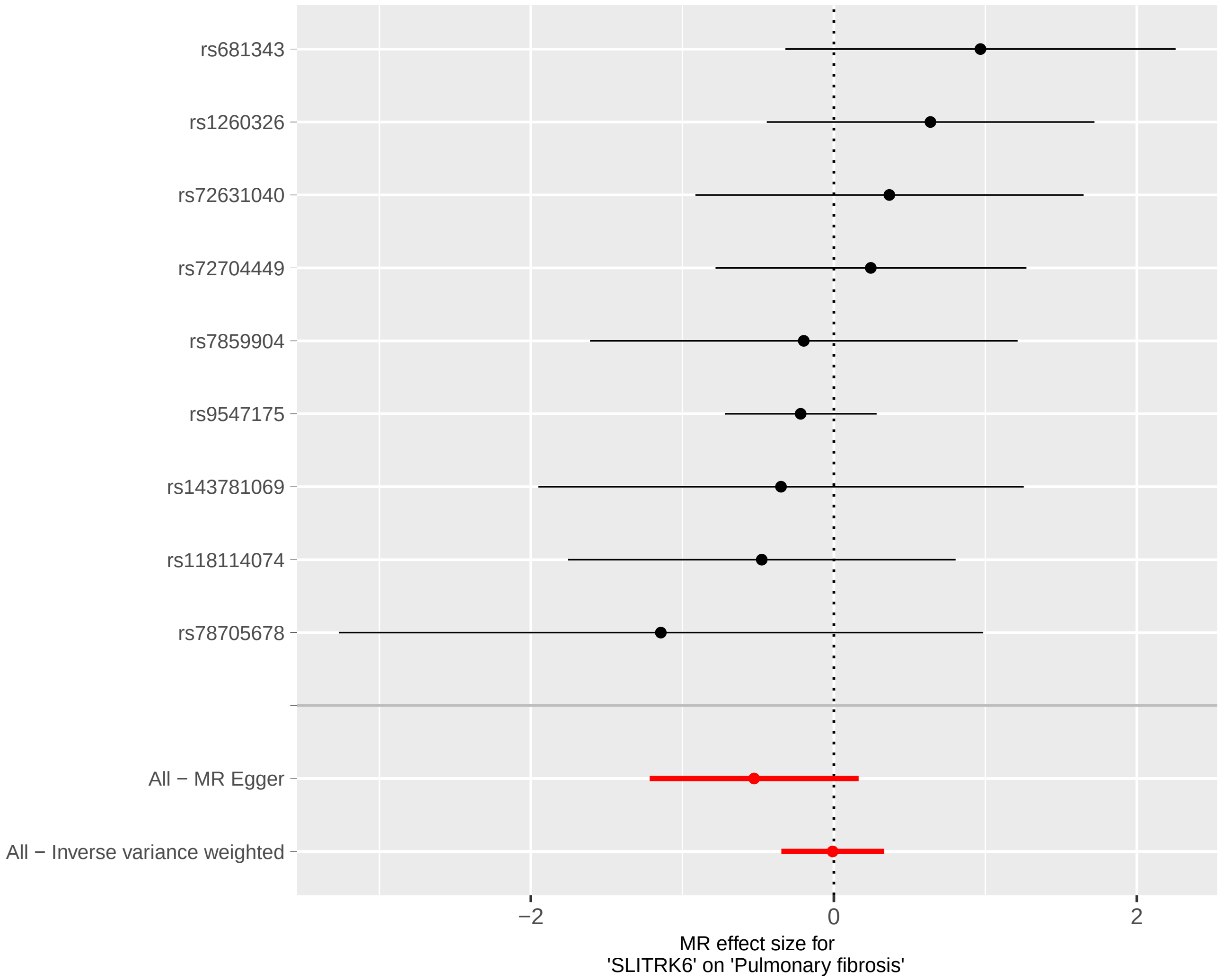

rs681343
rs1260326
rs72631040
rs72704449
rs7859904
rs9547175
rs143781069
rs118114074
rs78705678
All − MR Egger
All − Inverse variance weighted
−2
0
2
MR effect size for
'SLITRK6' on 'Pulmonary fibrosis'

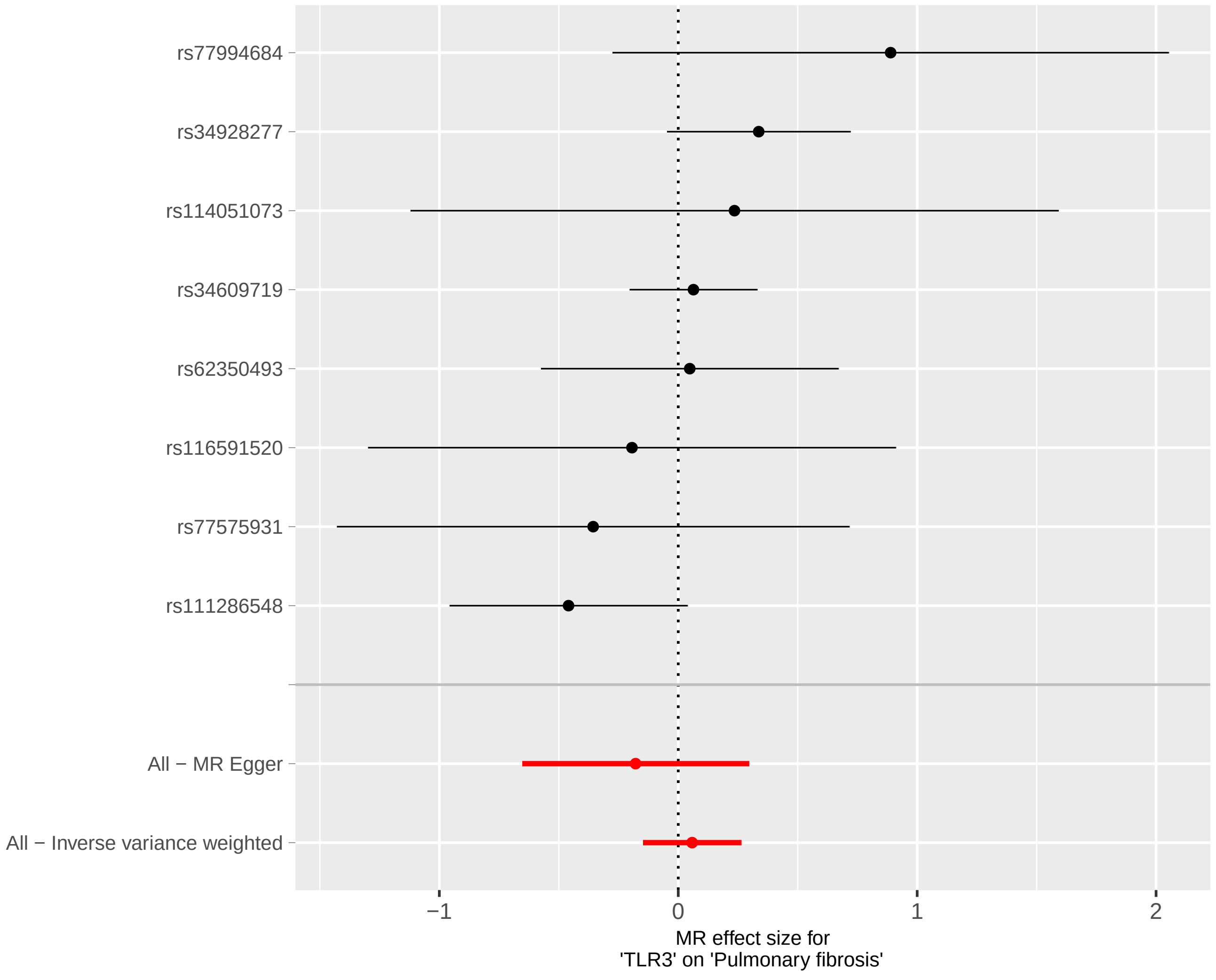
rs77994684
rs34928277
rs114051073
rs34609719
rs62350493
rs116591520
rs77575931
rs111286548
All − MR Egger
All − Inverse variance weighted
−1
0
1
2
MR effect size for
'TLR3' on 'Pulmonary fibrosis'

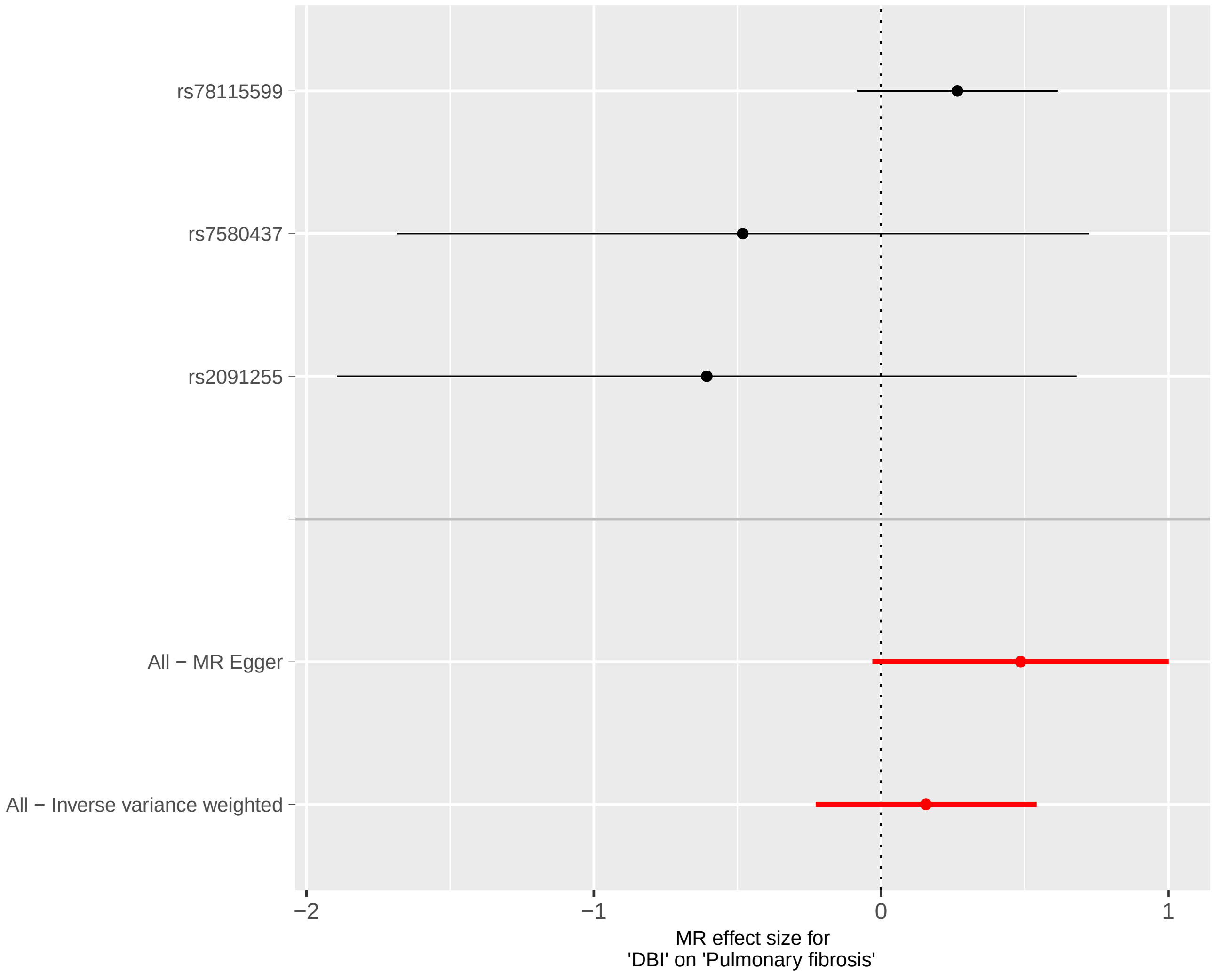

rs78115599
rs7580437
rs2091255
All − MR Egger
All − Inverse variance weighted
−2
−1
0
1
MR effect size for
'DBI' on 'Pulmonary fibrosis'

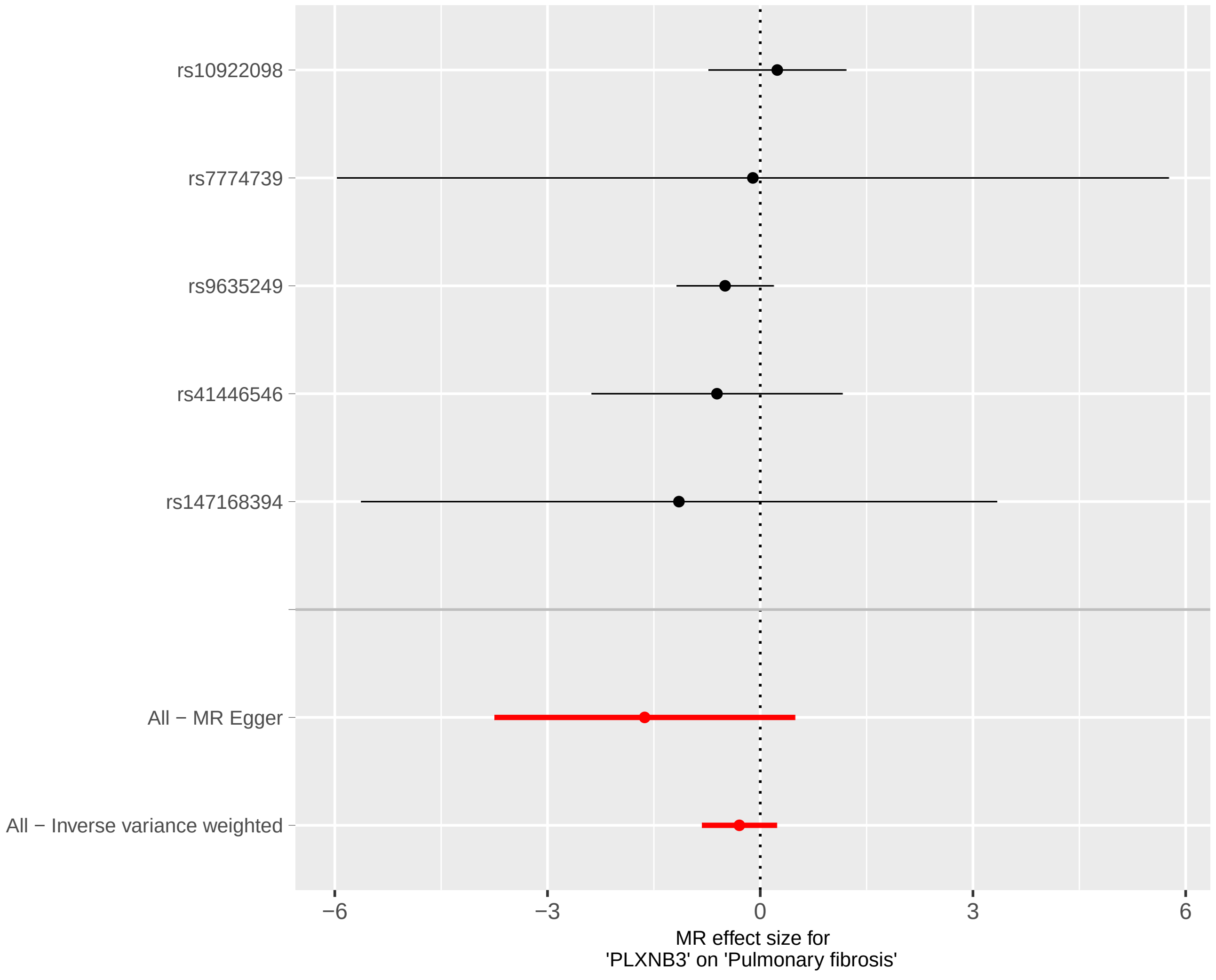
rs10922098
rs7774739
rs9635249
rs41446546
rs147168394
All − MR Egger
All − Inverse variance weighted
−6
−3
0
3
6
MR effect size for
'PLXNB3' on 'Pulmonary fibrosis'

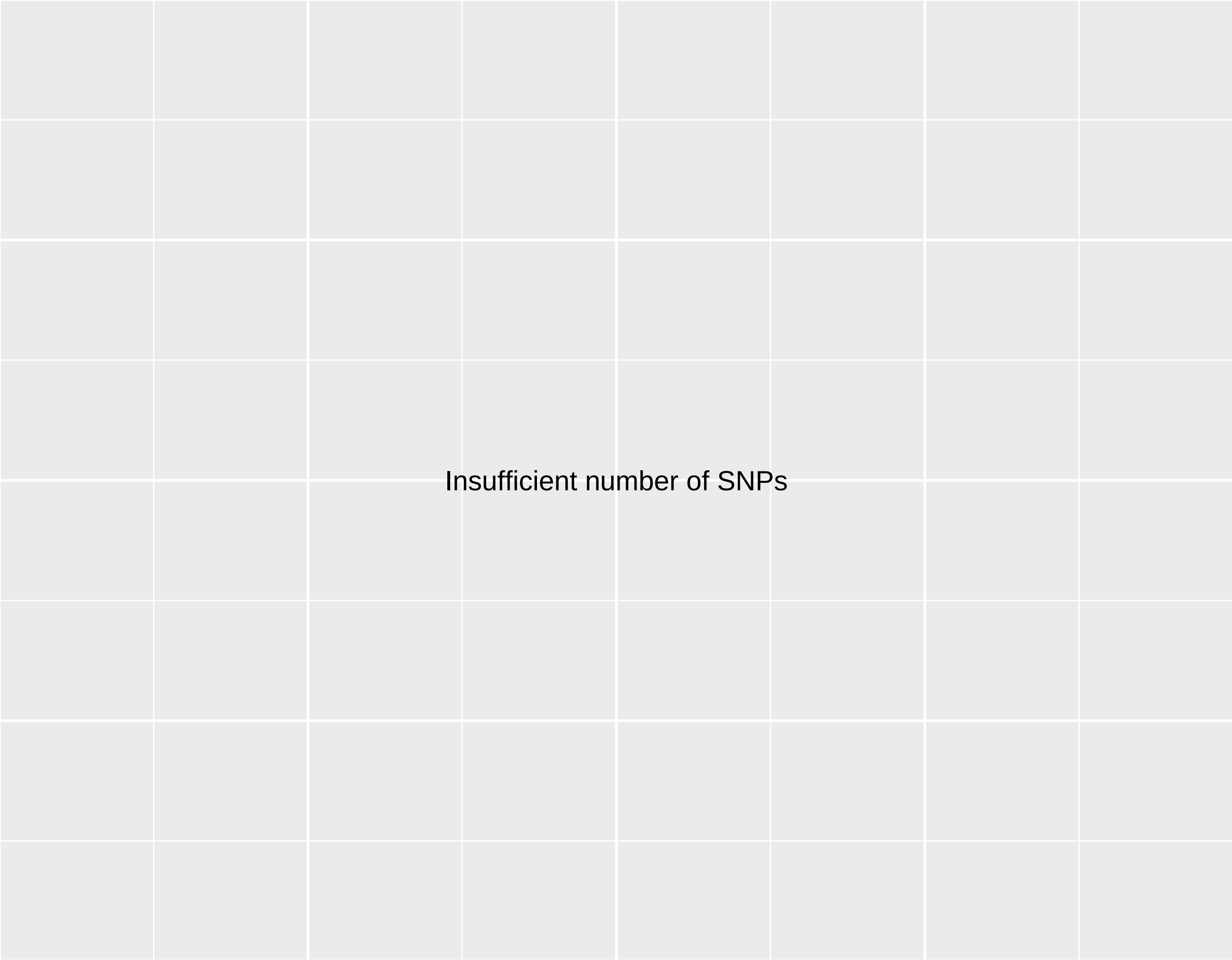
Insufficient number of SNPs

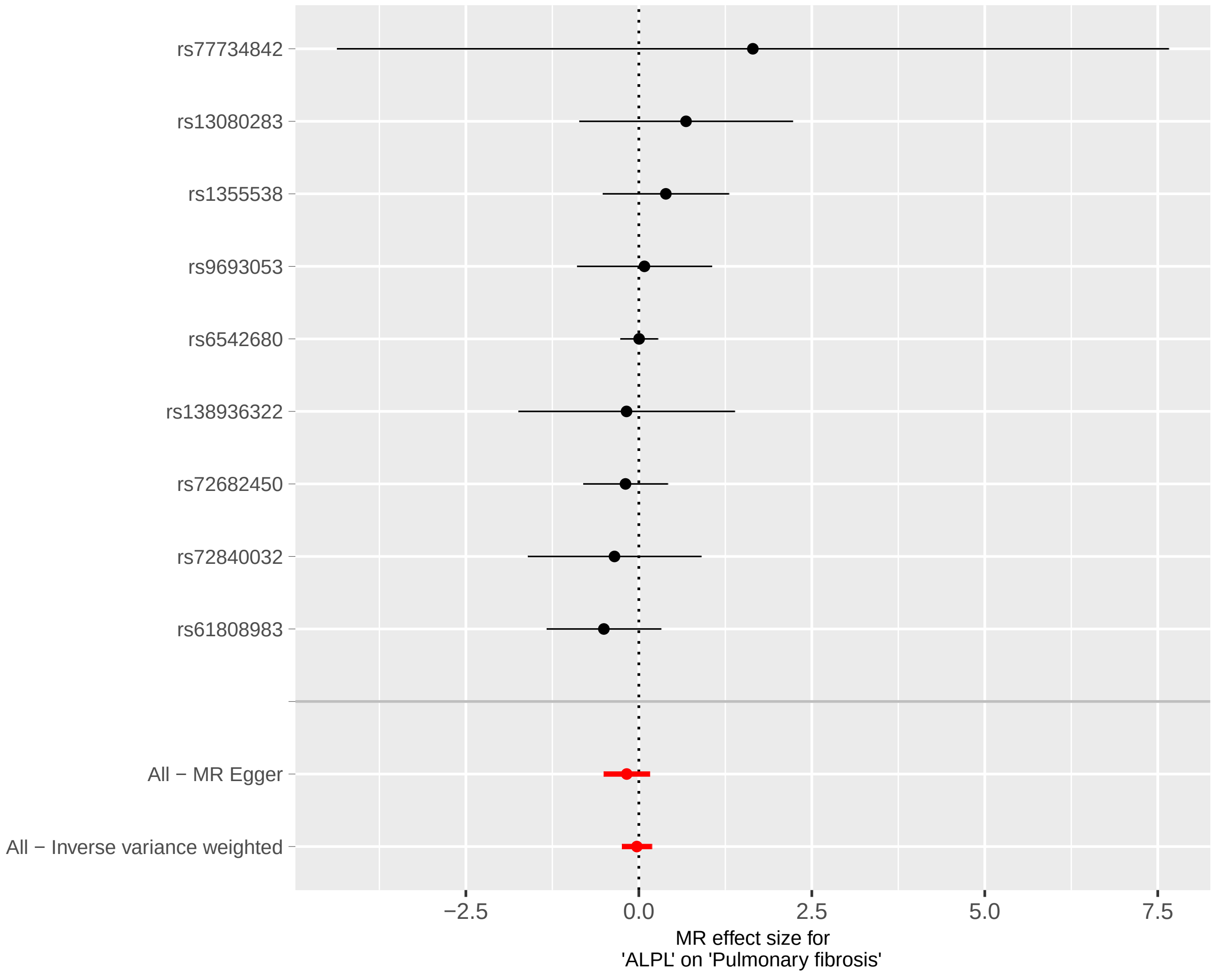
rs77734842
rs13080283
rs1355538
rs9693053
rs6542680
rs138936322
rs72682450
rs72840032
rs61808983
All − MR Egger
All − Inverse variance weighted
−2.5
0.0
2.5
5.0
7.5
MR effect size for
'ALPL' on 'Pulmonary fibrosis'

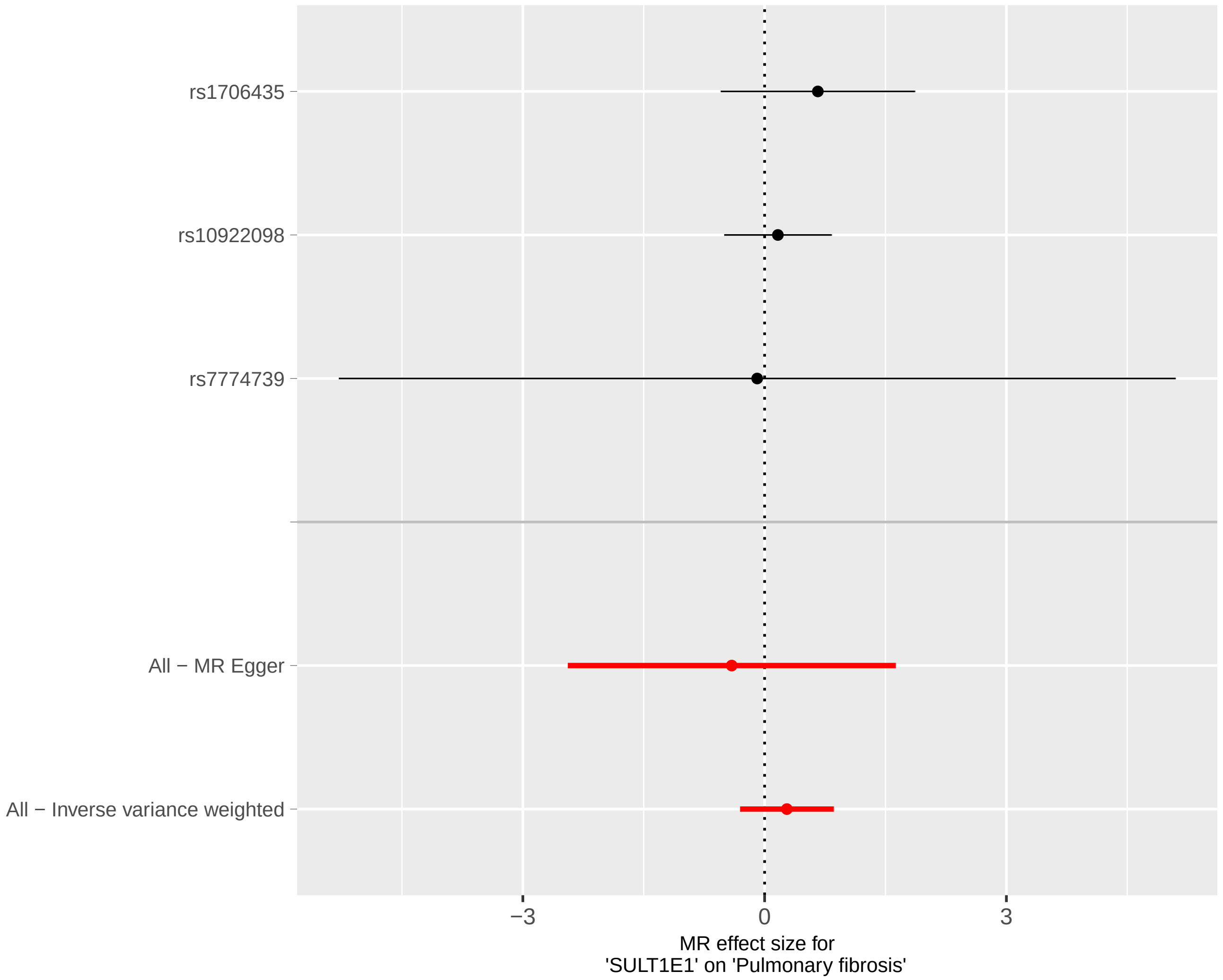

rs1706435
rs10922098
rs7774739
All − MR Egger
All − Inverse variance weighted
−3
0
3
MR effect size for
'SULT1E1' on 'Pulmonary fibrosis'

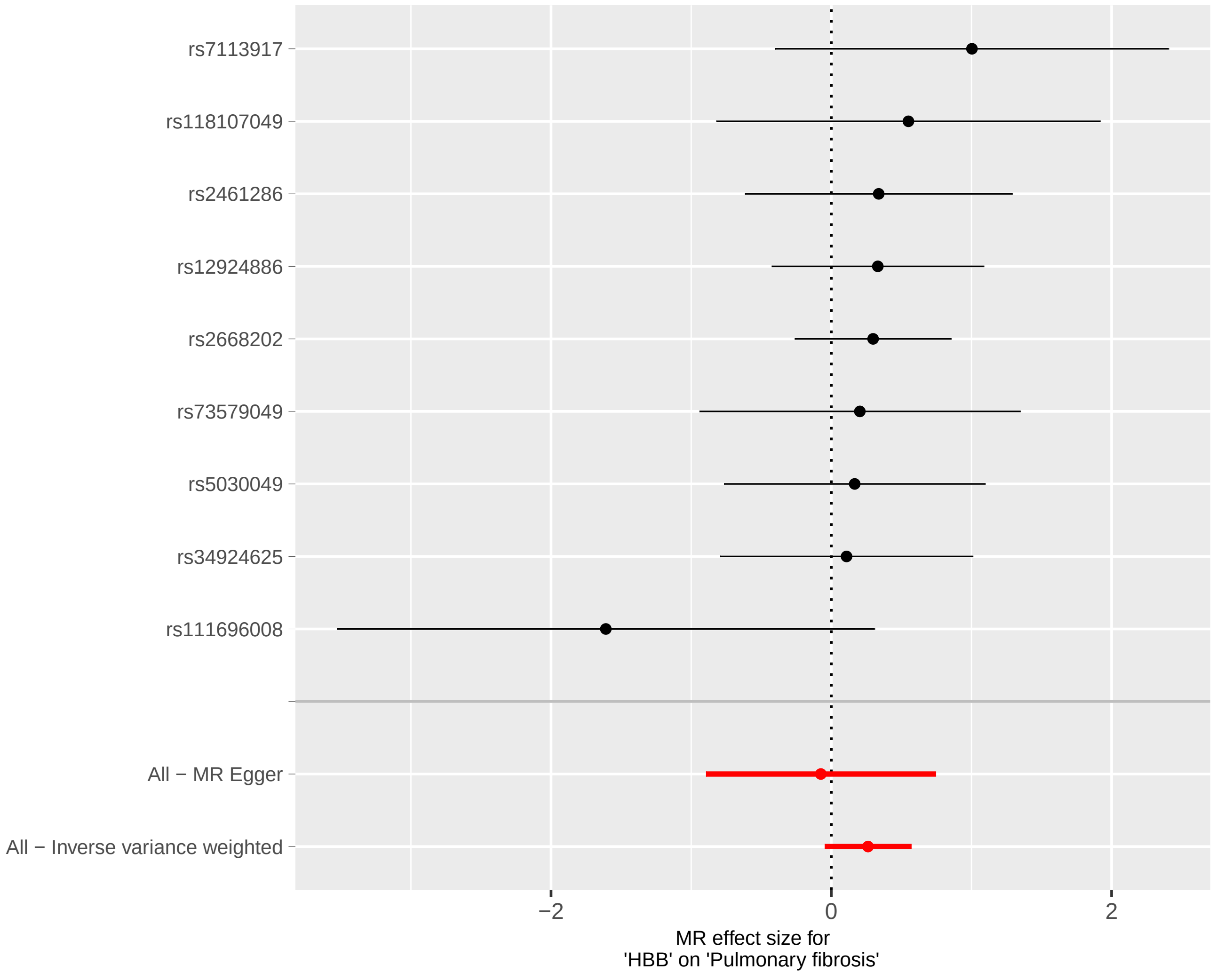
rs7113917
rs118107049
rs2461286
rs12924886
rs2668202
rs73579049
rs5030049
rs34924625
rs111696008
All − MR Egger
All − Inverse variance weighted
−2
0
2
MR effect size for
'HBB' on 'Pulmonary fibrosis'

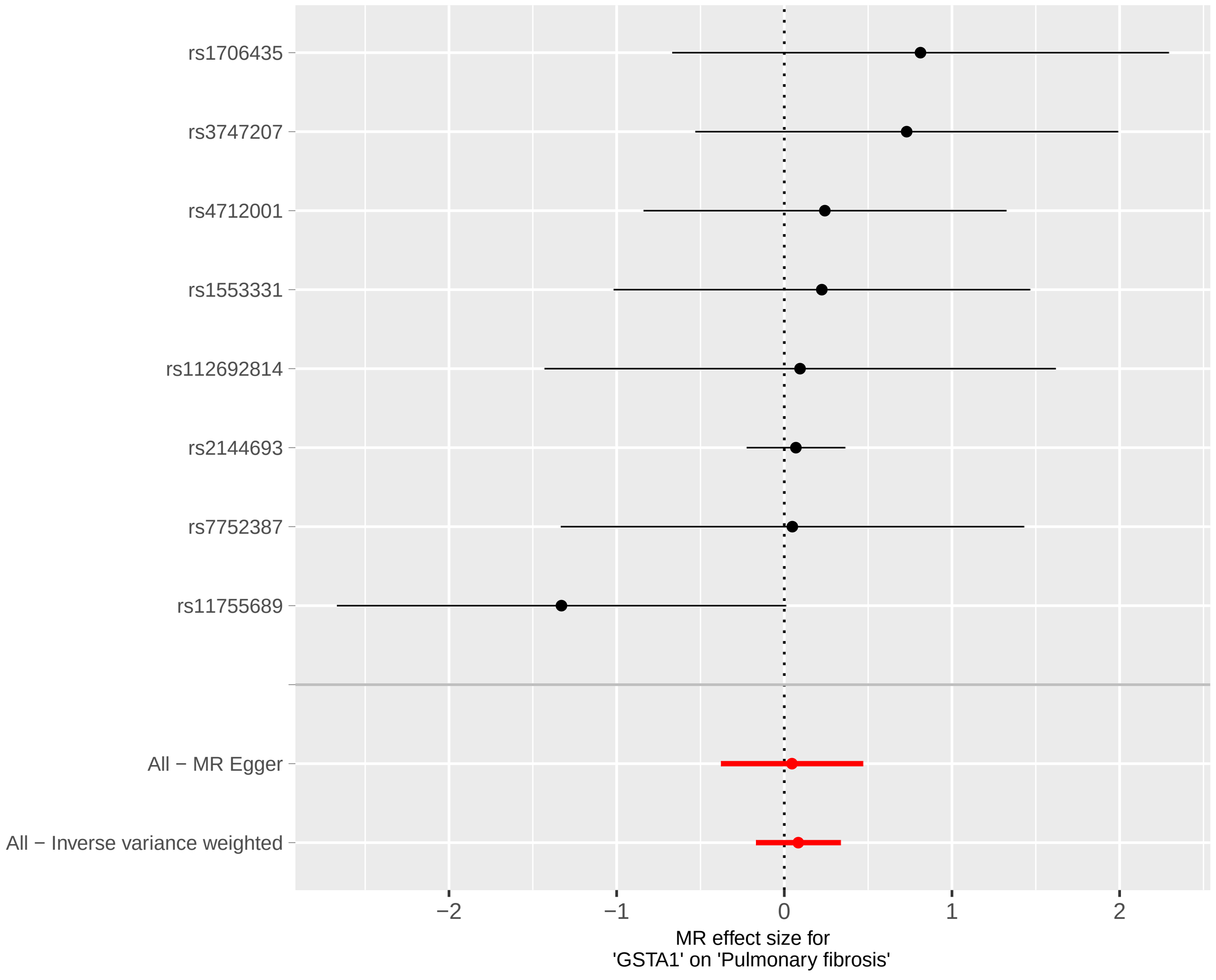
rs1706435
rs3747207
rs4712001
rs1553331
rs112692814
rs2144693
rs7752387
rs11755689
All − MR Egger
All − Inverse variance weighted
−2
−1
0
1
2
MR effect size for
'GSTA1' on 'Pulmonary fibrosis'

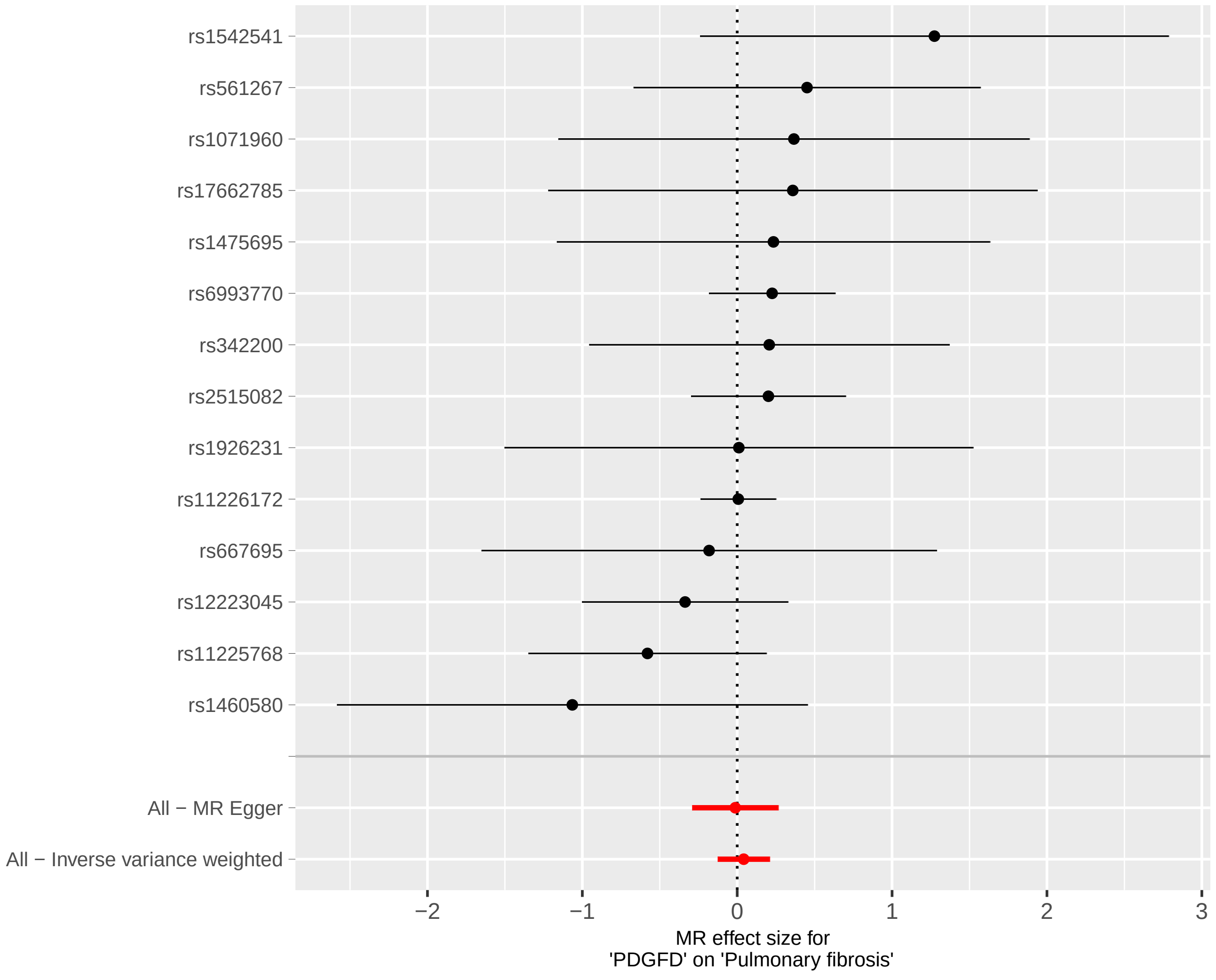

rs1542541
rs561267
rs1071960
rs17662785
rs1475695
rs6993770
rs342200
rs2515082
rs1926231
rs11226172
rs667695
rs12223045
rs11225768
rs1460580
All − MR Egger
All − Inverse variance weighted
−2
−1
0
1
2
3
MR effect size for
'PDGFD' on 'Pulmonary fibrosis'

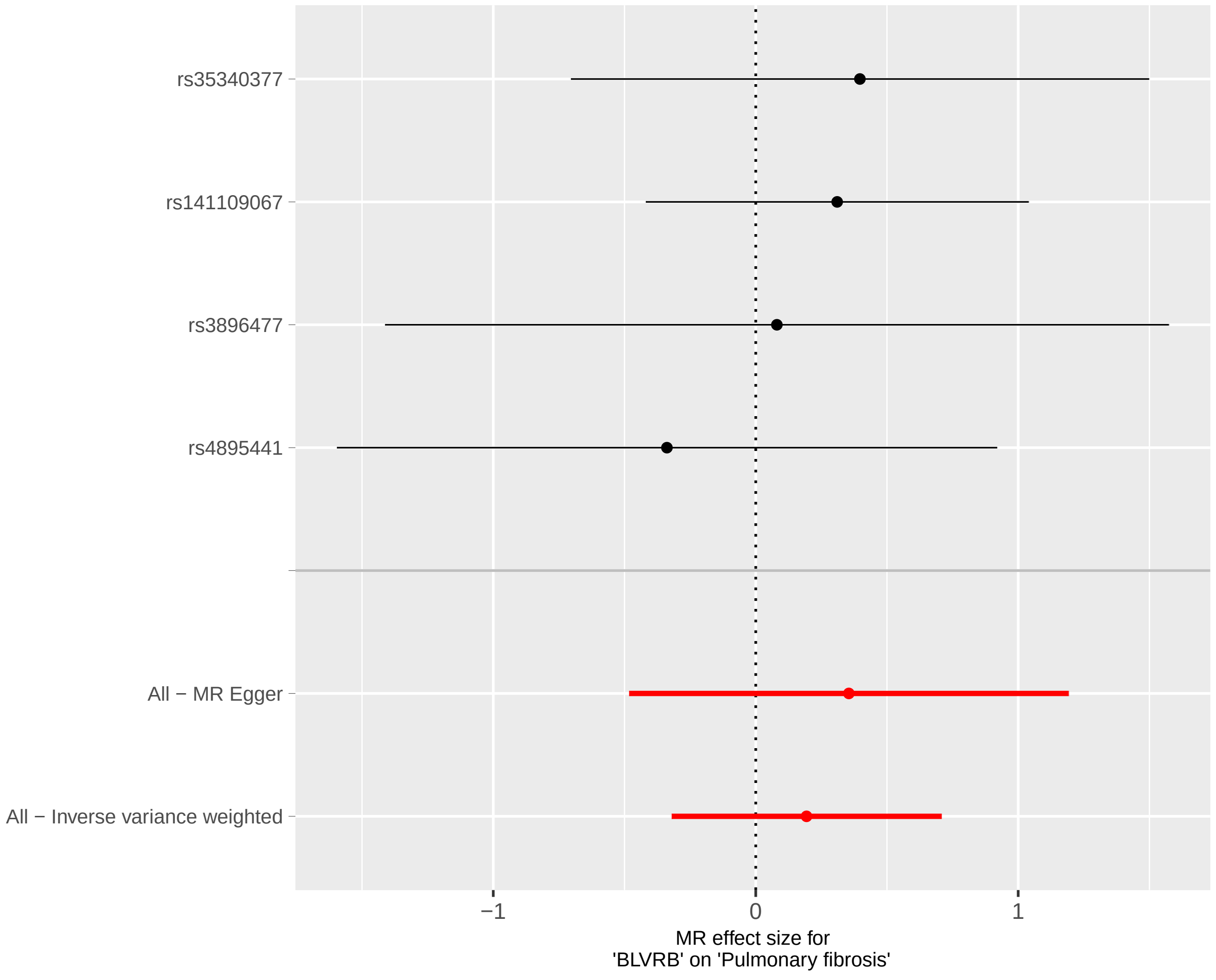

rs35340377
rs141109067
rs3896477
rs4895441
All − MR Egger
All − Inverse variance weighted
−1
0
1
MR effect size for
'BLVRB' on 'Pulmonary fibrosis'

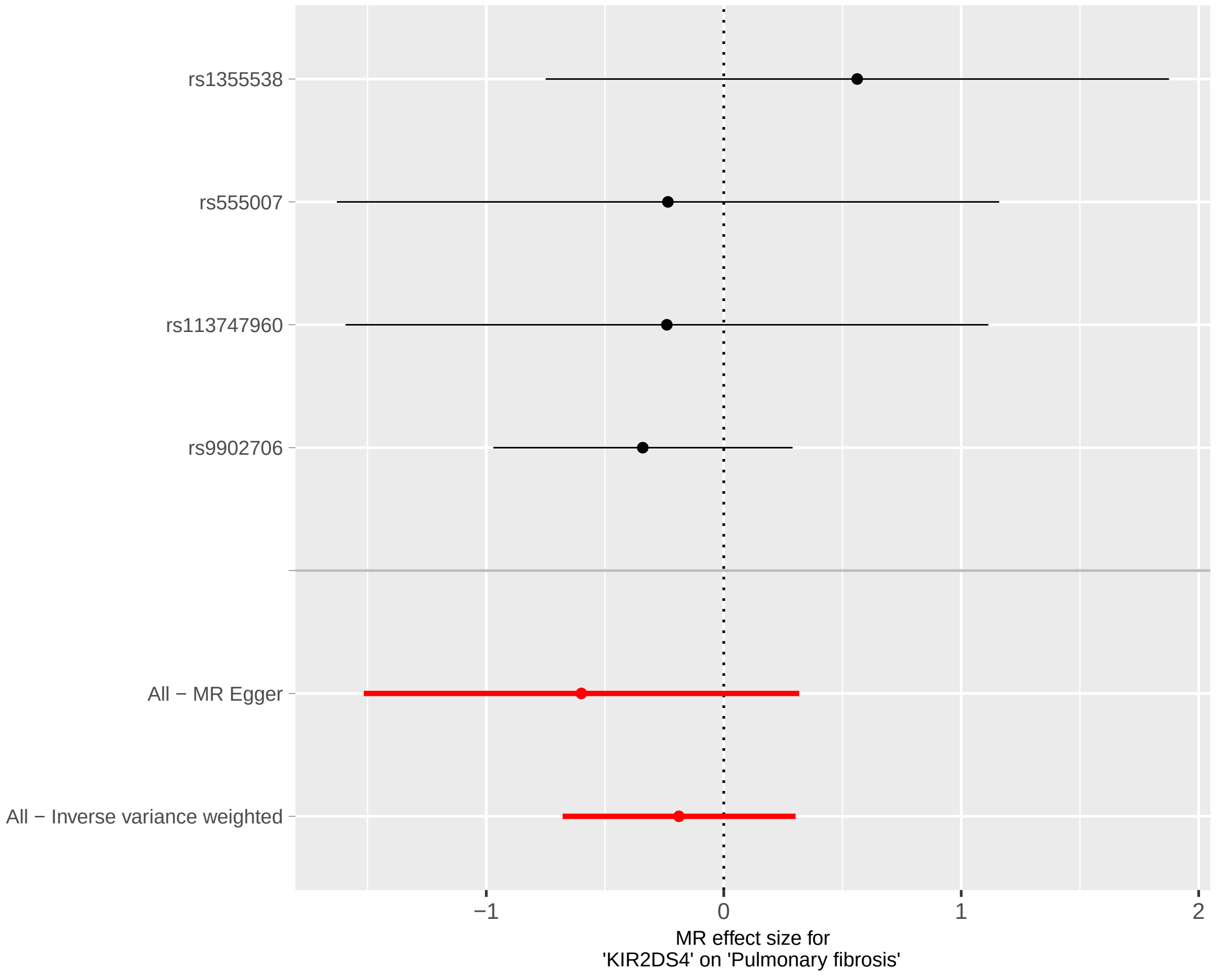
rs1355538
rs555007
rs113747960
rs9902706
All − MR Egger
All − Inverse variance weighted
−1
0
1
2
MR effect size for
'KIR2DS4' on 'Pulmonary fibrosis'

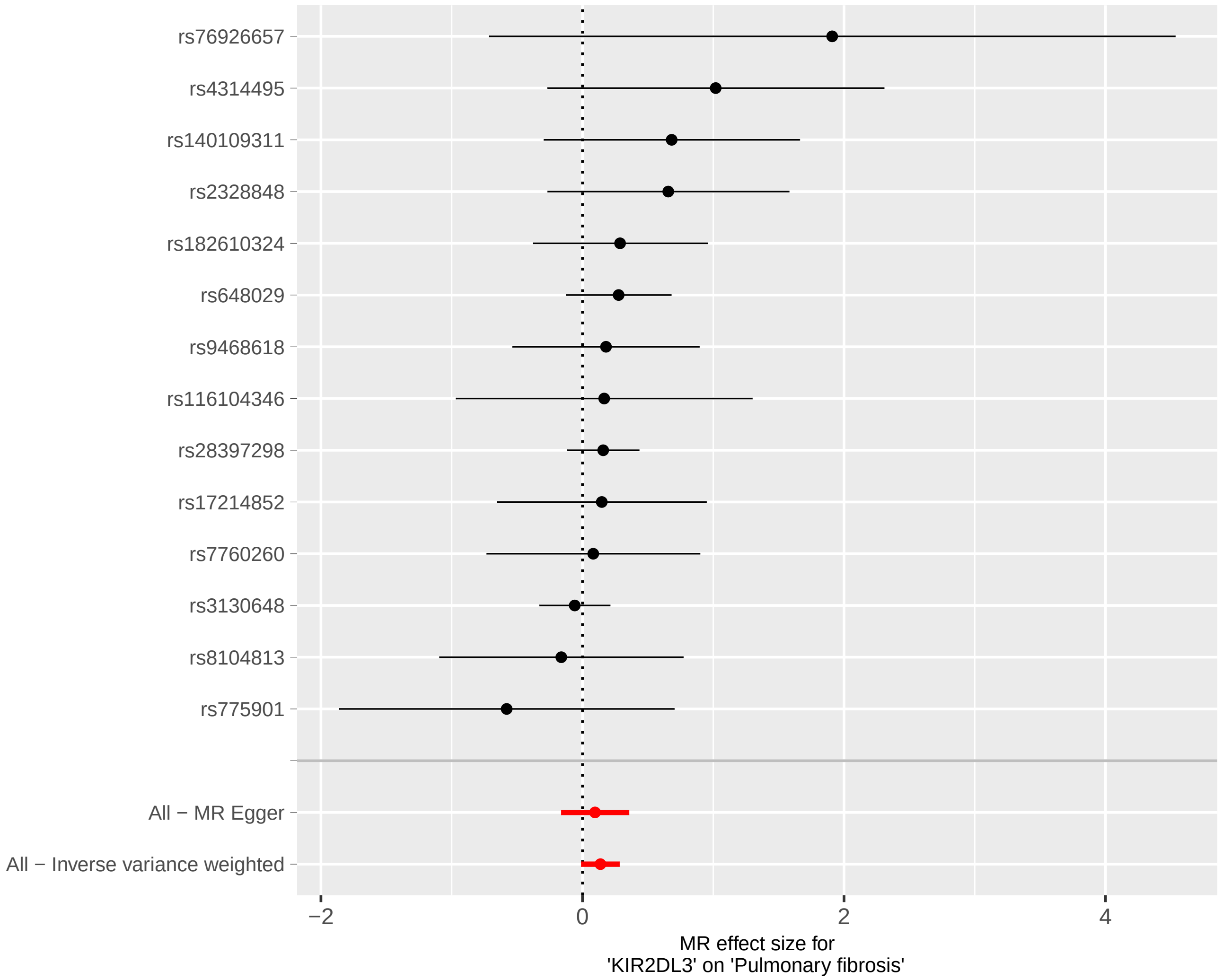

rs76926657
rs4314495
rs140109311
rs2328848
rs182610324
rs648029
rs9468618
rs116104346
rs28397298
rs17214852
rs7760260
rs3130648
rs8104813
rs775901
All − MR Egger
All − Inverse variance weighted
−2
0
2
4
MR effect size for
'KIR2DL3' on 'Pulmonary fibrosis'

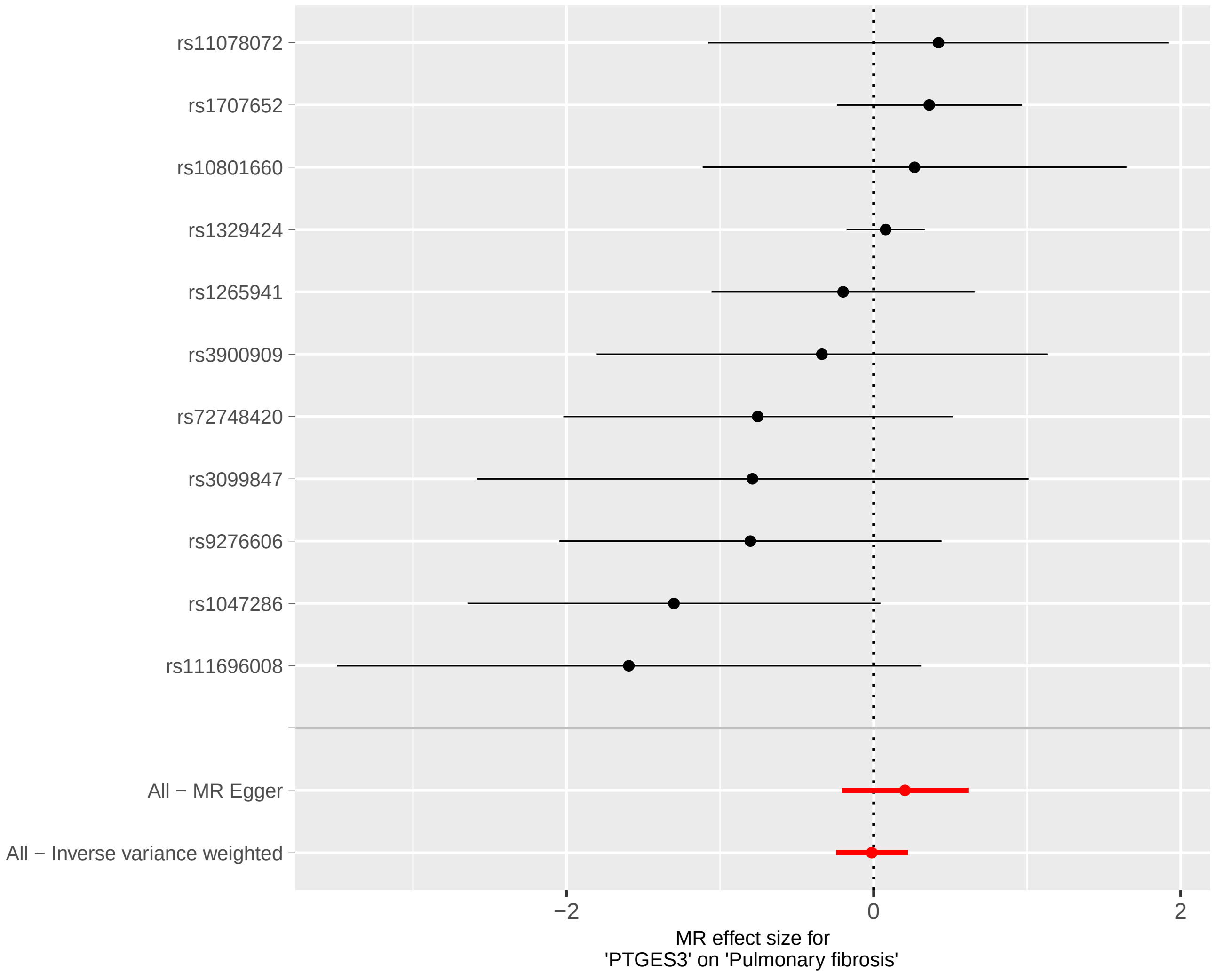
rs11078072
rs1707652
rs10801660
rs1329424
rs1265941
rs3900909
rs72748420
rs3099847
rs9276606
rs1047286
rs111696008
All − MR Egger
All − Inverse variance weighted
−2
0
2
MR effect size for
'PTGES3' on 'Pulmonary fibrosis'

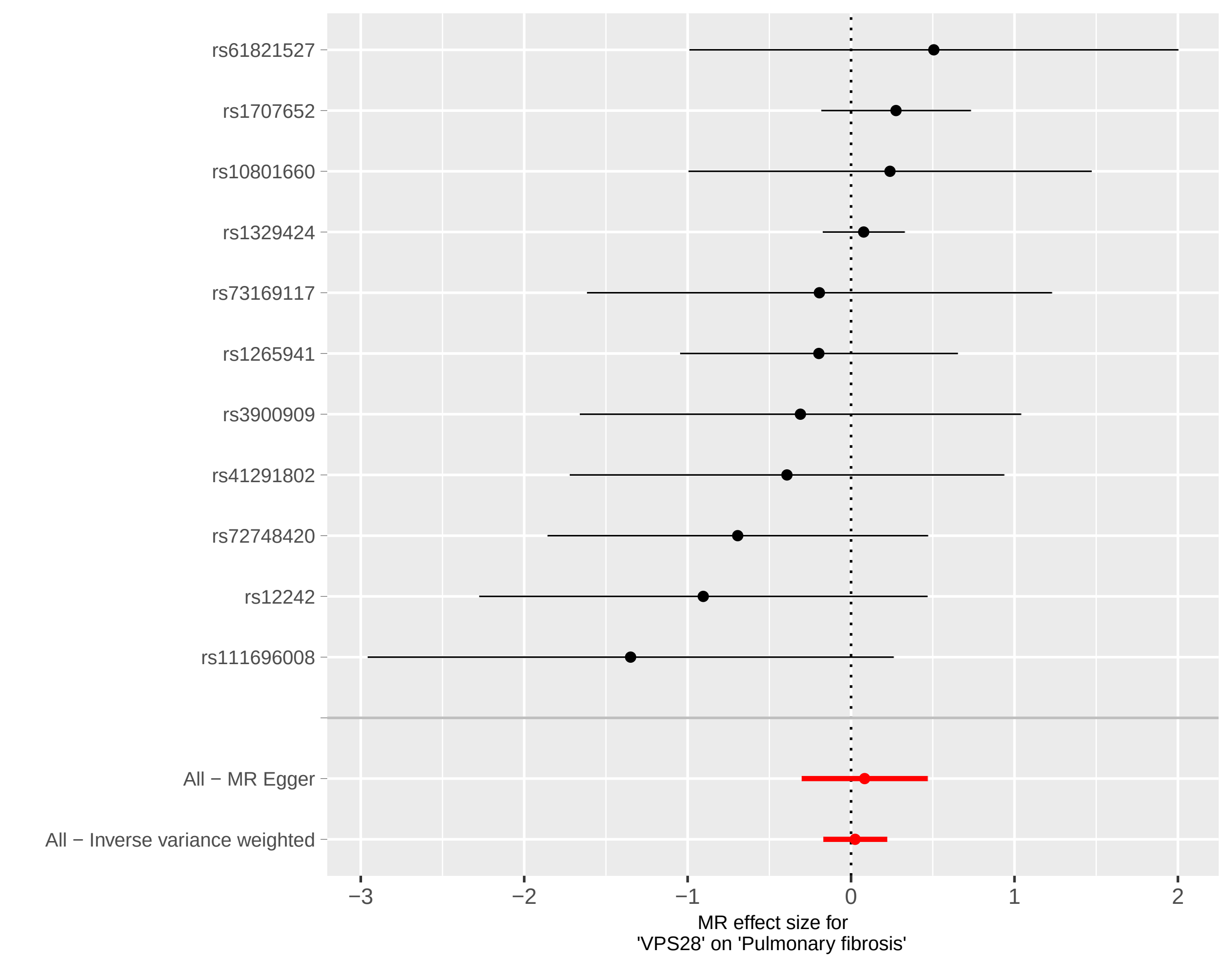

rs61821527
rs1707652
rs10801660
rs1329424
rs73169117
rs1265941
rs3900909
rs41291802
rs72748420
rs12242
rs111696008
All − MR Egger
All − Inverse variance weighted
−3
−2
−1
0
1
2
MR effect size for
'VPS28' on 'Pulmonary fibrosis'

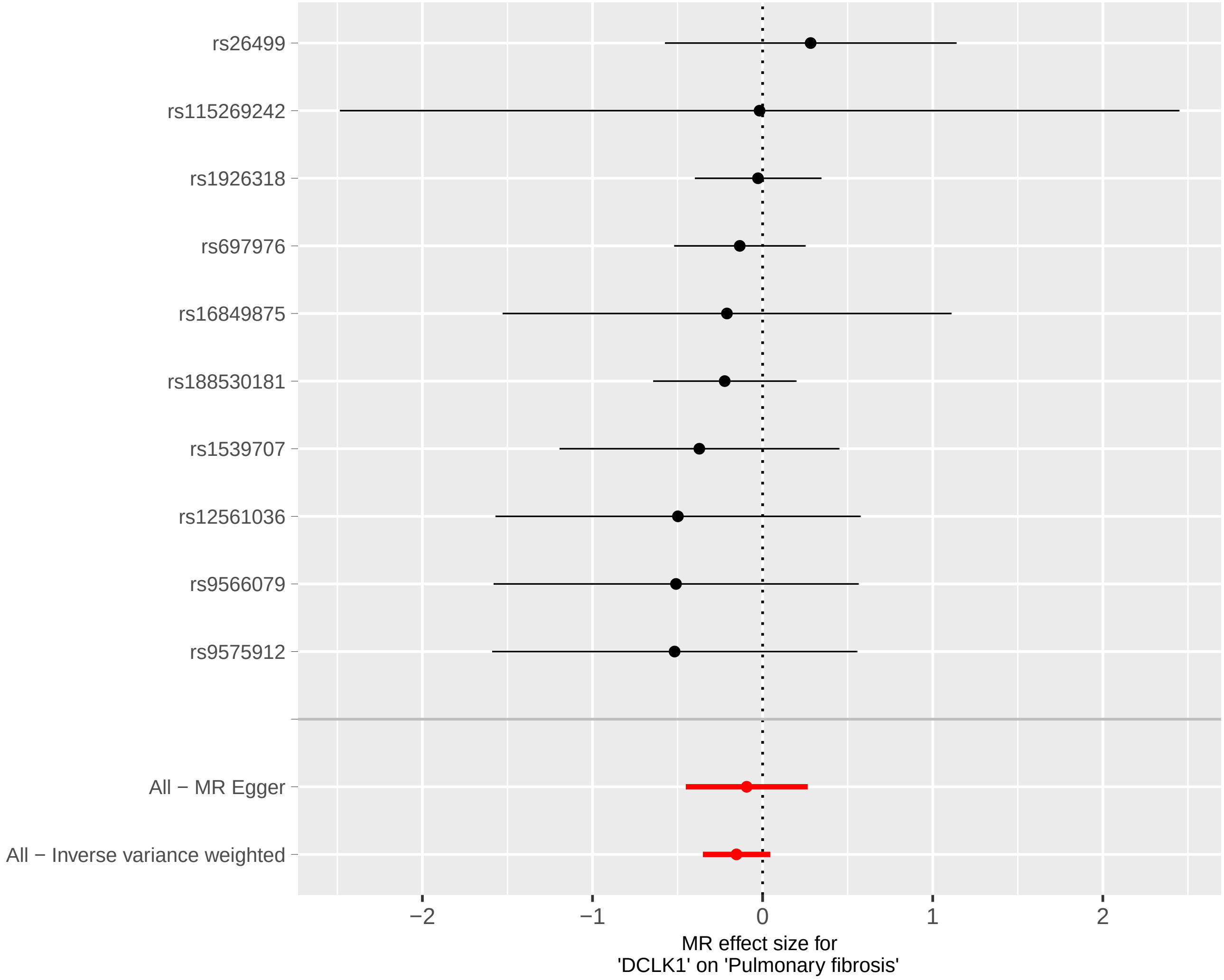

rs26499
rs115269242
rs1926318
rs697976
rs16849875
rs188530181
rs1539707
rs12561036
rs9566079
rs9575912
All − MR Egger
All − Inverse variance weighted
−2
−1
0
1
2
MR effect size for
'DCLK1' on 'Pulmonary fibrosis'

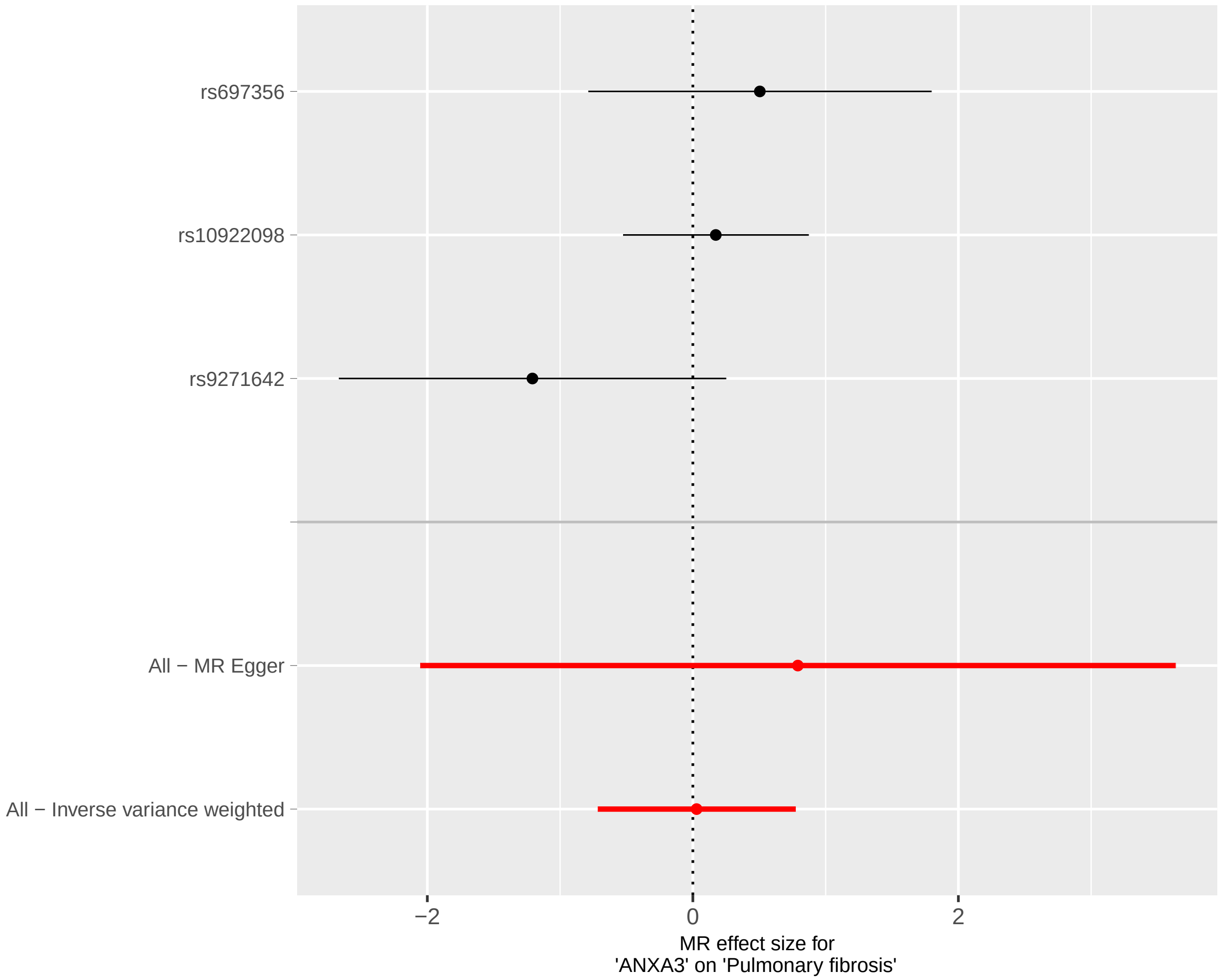
rs697356
rs10922098
rs9271642
All − MR Egger
All − Inverse variance weighted
−2
0
2
MR effect size for
'ANXA3' on 'Pulmonary fibrosis'

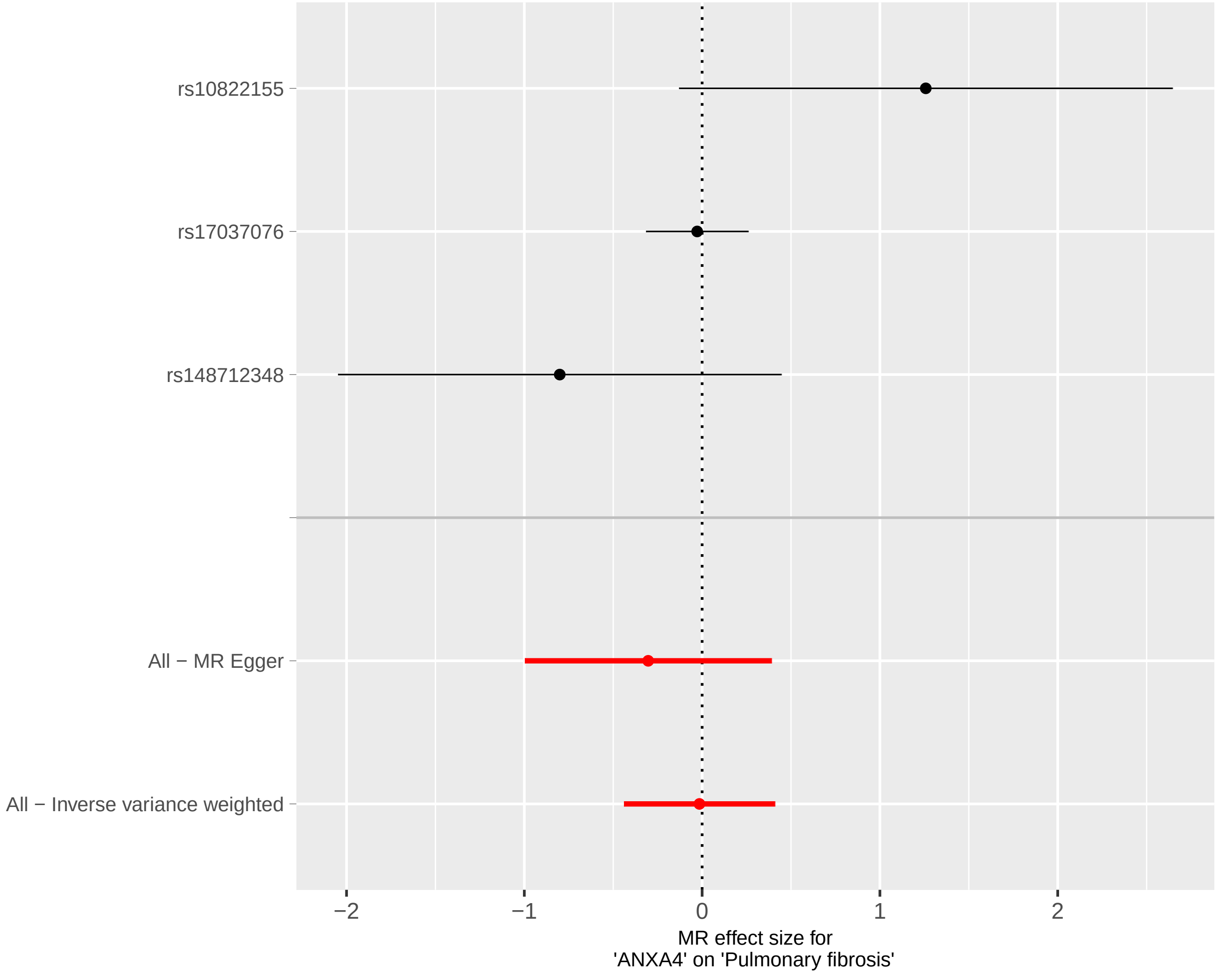

rs10822155
rs17037076
rs148712348
All − MR Egger
All − Inverse variance weighted
−2
−1
0
1
2
MR effect size for
'ANXA4' on 'Pulmonary fibrosis'

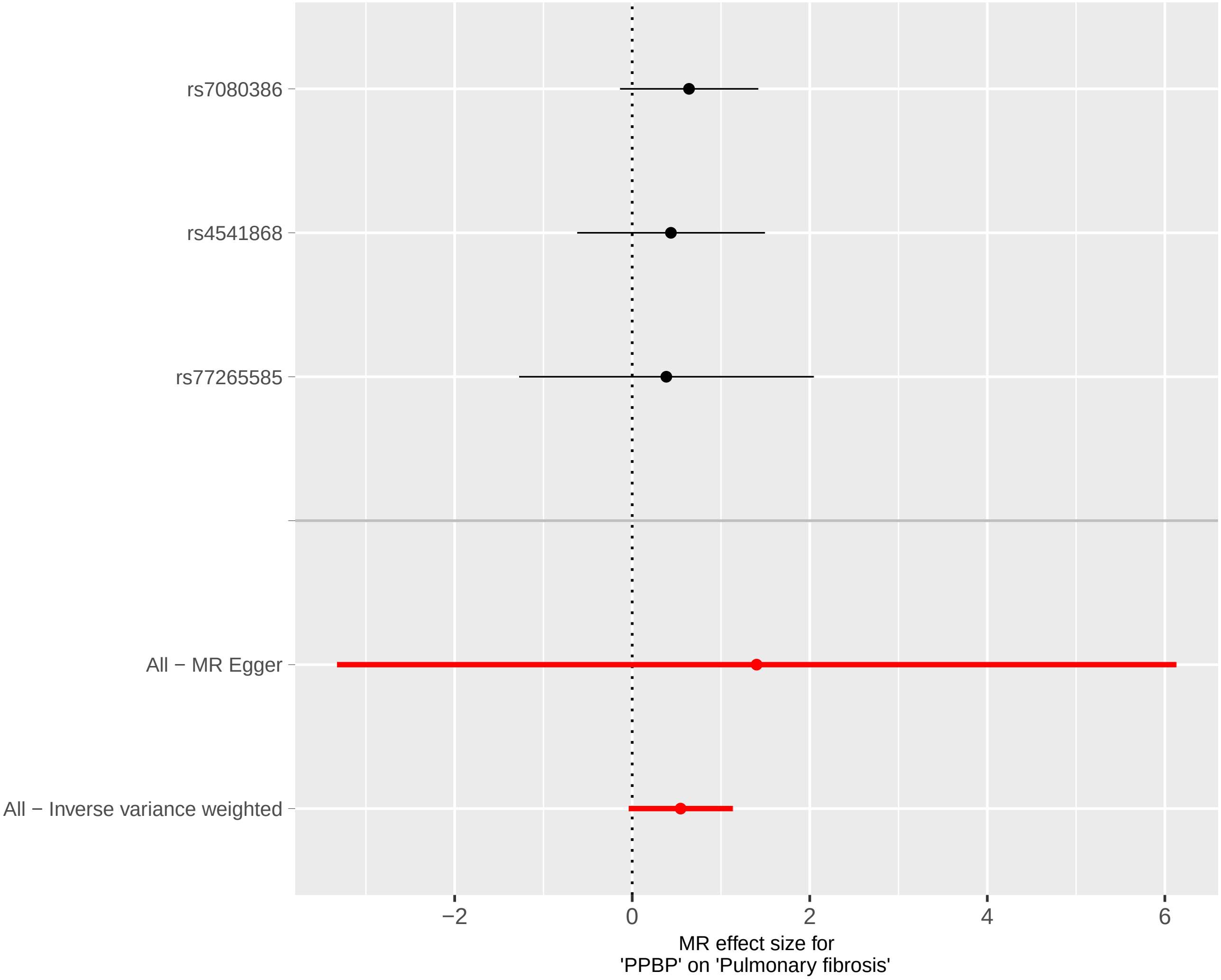
rs7080386
rs4541868
rs77265585
All − MR Egger
All − Inverse variance weighted
−2
0
2
4
6
MR effect size for
'PPBP' on 'Pulmonary fibrosis'

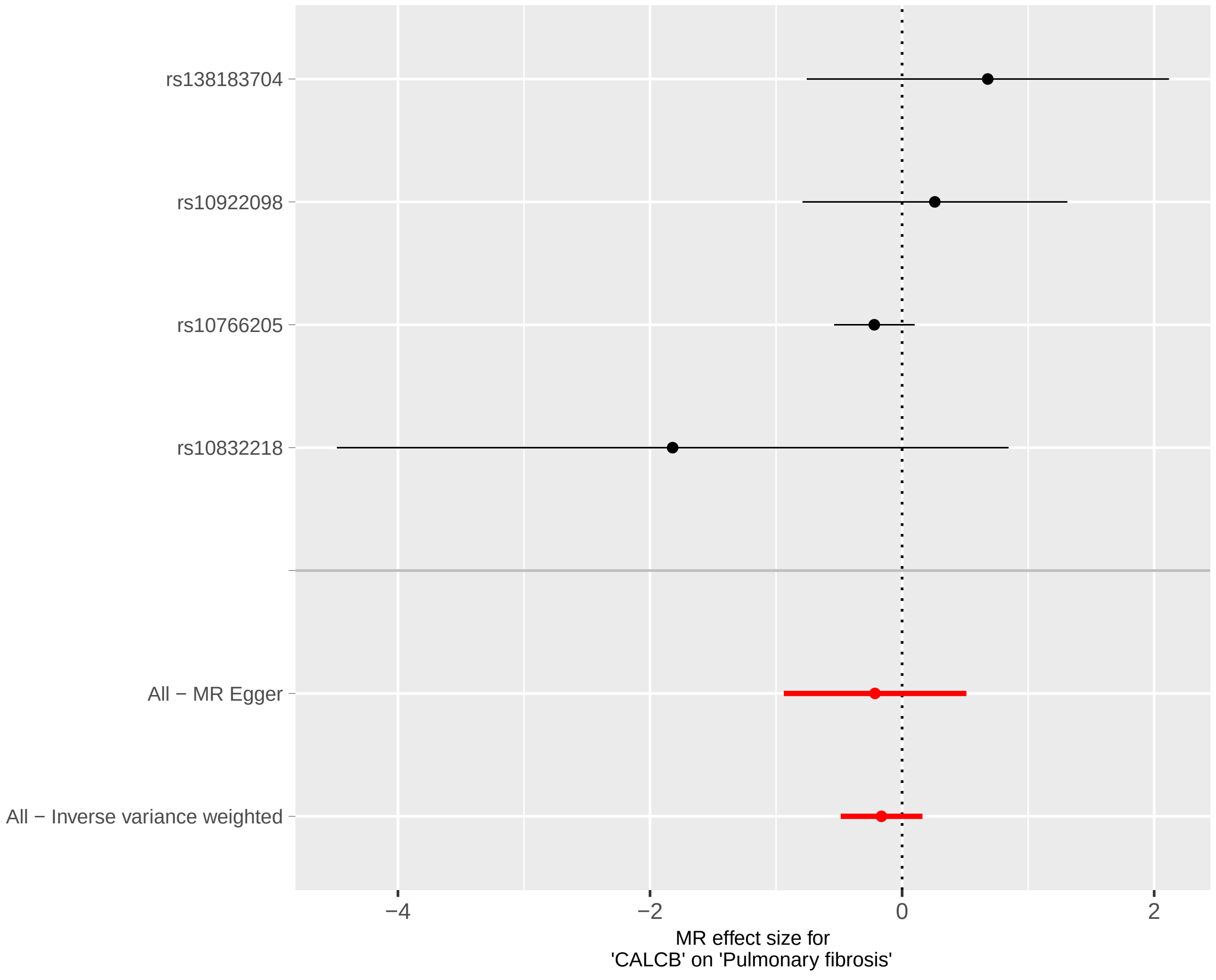

rs138183704
rs10922098
rs10766205
rs10832218
All − MR Egger
All − Inverse variance weighted
−4
−2
0
2
MR effect size for
'CALCB' on 'Pulmonary fibrosis'

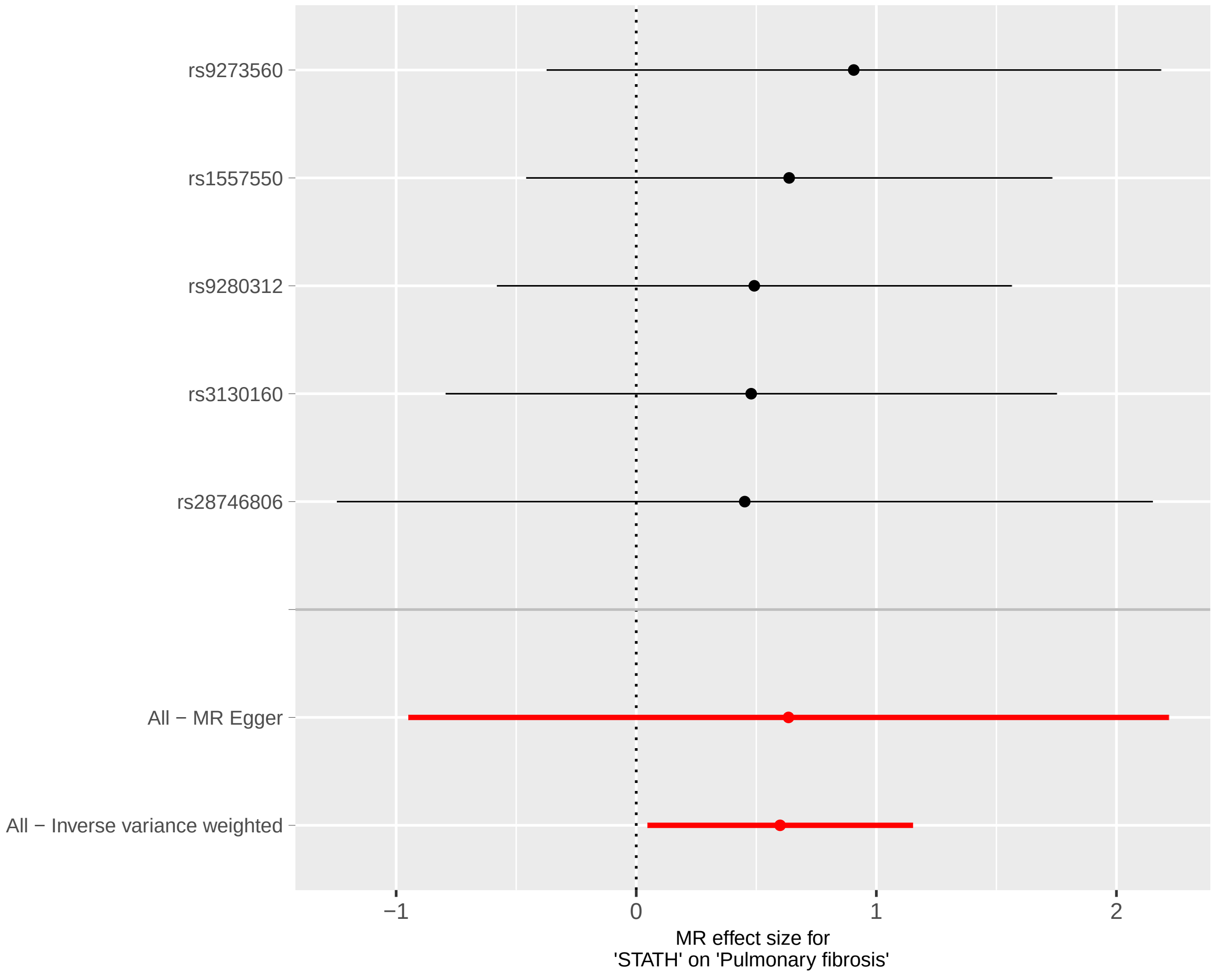

rs9273560
rs1557550
rs9280312
rs3130160
rs28746806
All − MR Egger
All − Inverse variance weighted
−1
0
1
2
MR effect size for
'STATH' on 'Pulmonary fibrosis'

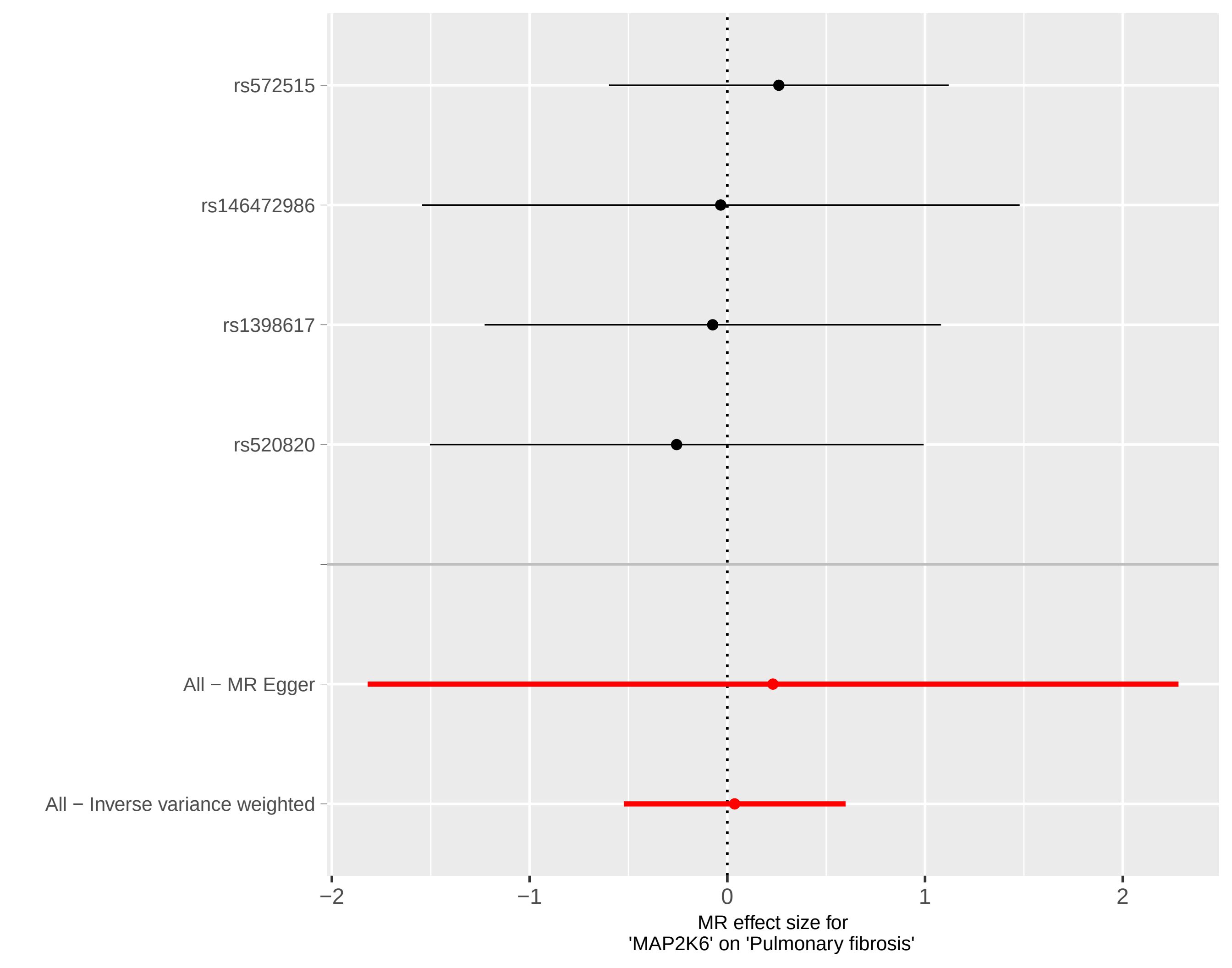
rs572515
rs146472986
rs1398617
rs520820
All − MR Egger
All − Inverse variance weighted
−2
−1
0
1
2
MR effect size for
'MAP2K6' on 'Pulmonary fibrosis'

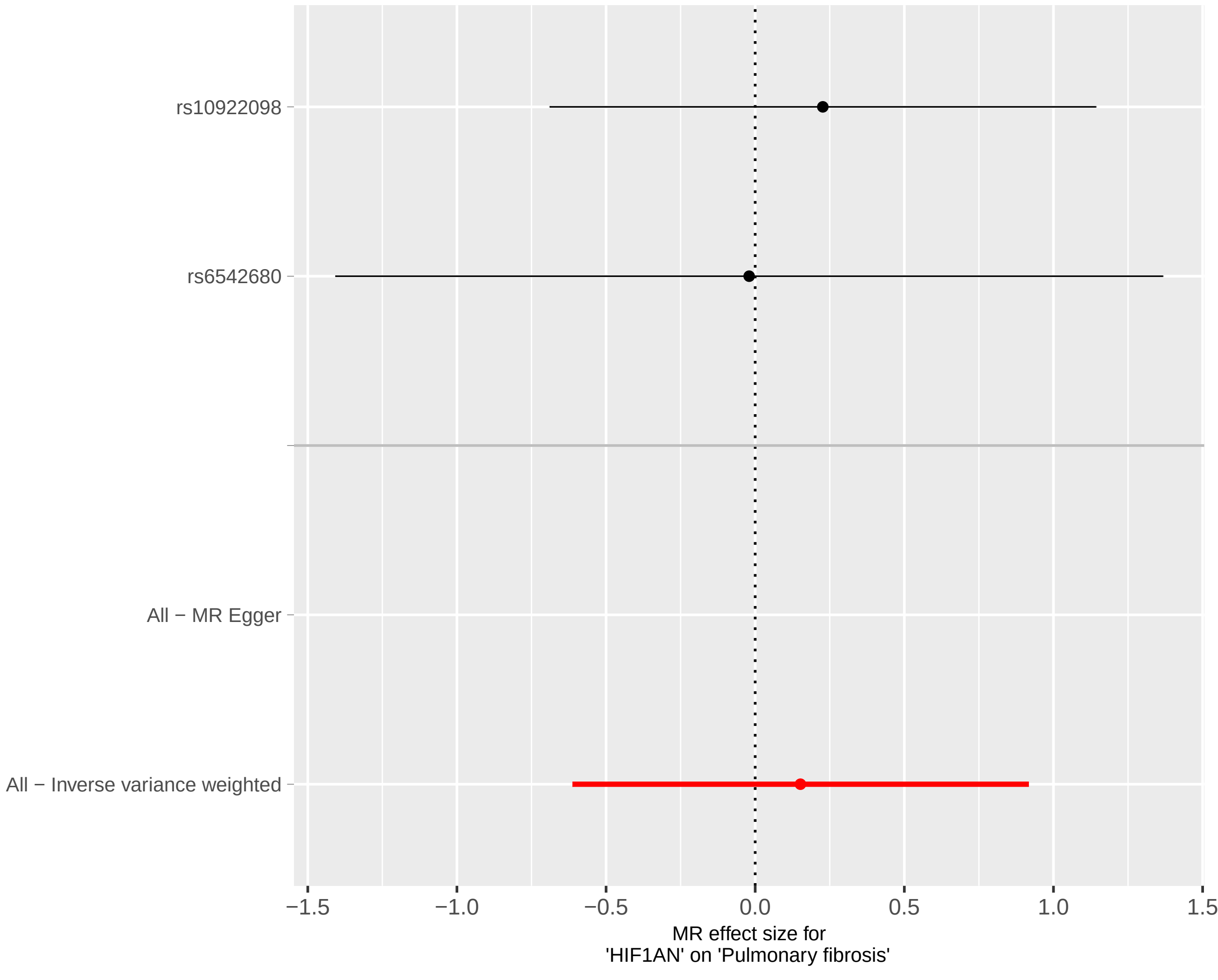
rs10922098
rs6542680
All − MR Egger
All − Inverse variance weighted
−1.5
−1.0
−0.5
0.0
0.5
1.0
1.5
MR effect size for
'HIF1AN' on 'Pulmonary fibrosis'

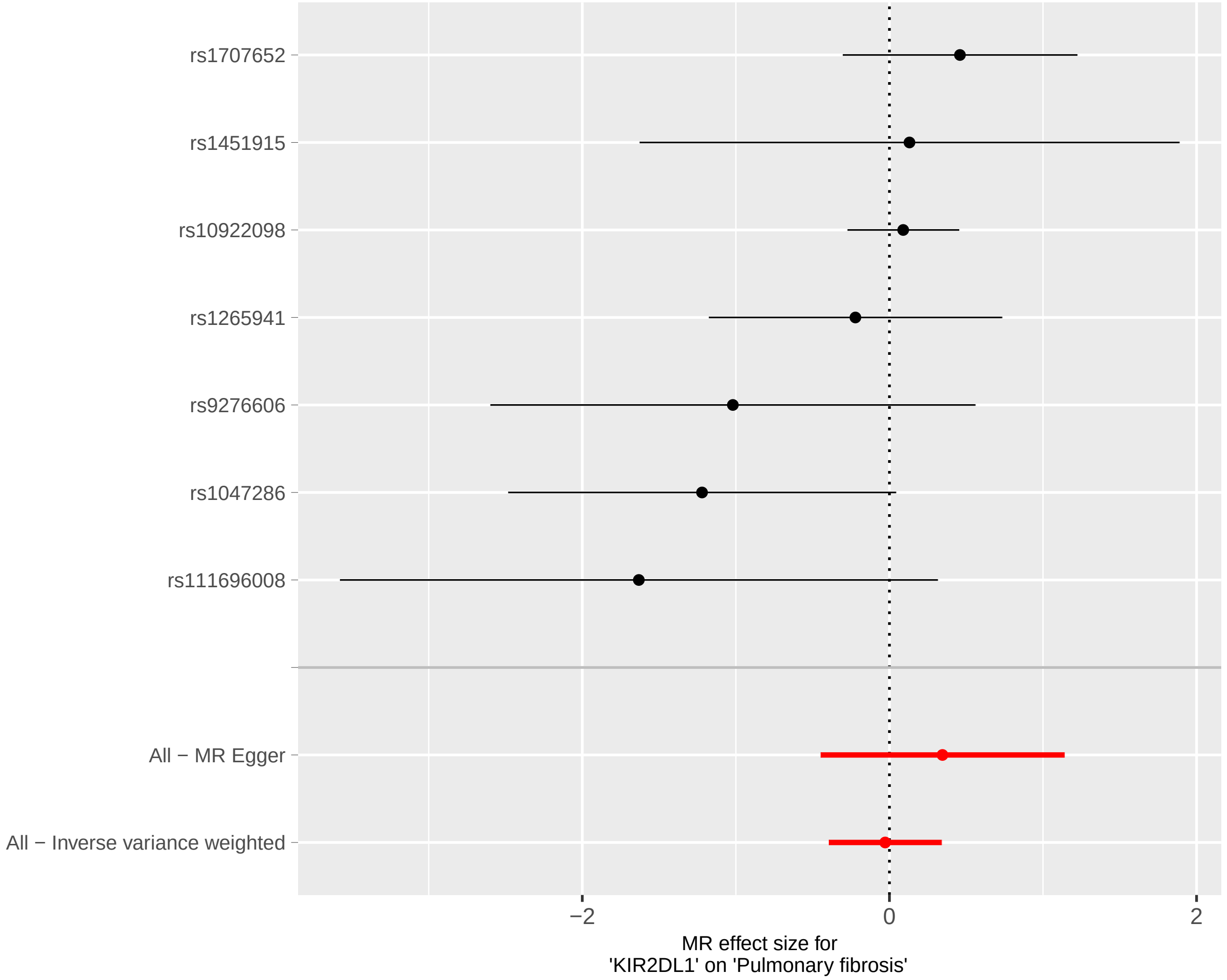

rs1707652
rs1451915
rs10922098
rs1265941
rs9276606
rs1047286
rs11696008
All − MR Egger
All − Inverse variance weighted
−2
0
2
MR effect size for
'KIR2DL1' on 'Pulmonary fibrosis'

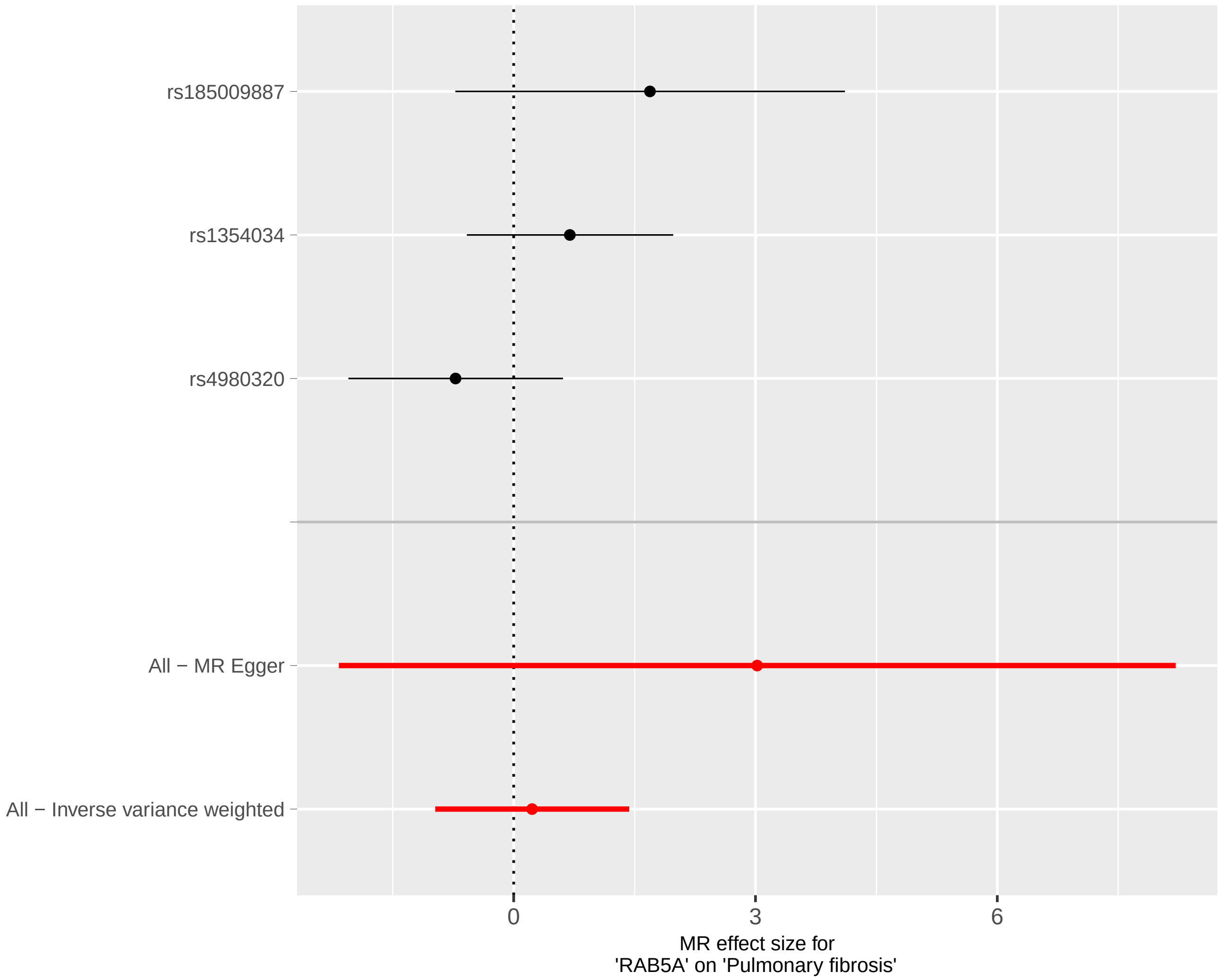
rs185009887
rs1354034
rs4980320
All − MR Egger
All − Inverse variance weighted
0
3
6
MR effect size for
'RAB5A' on 'Pulmonary fibrosis'

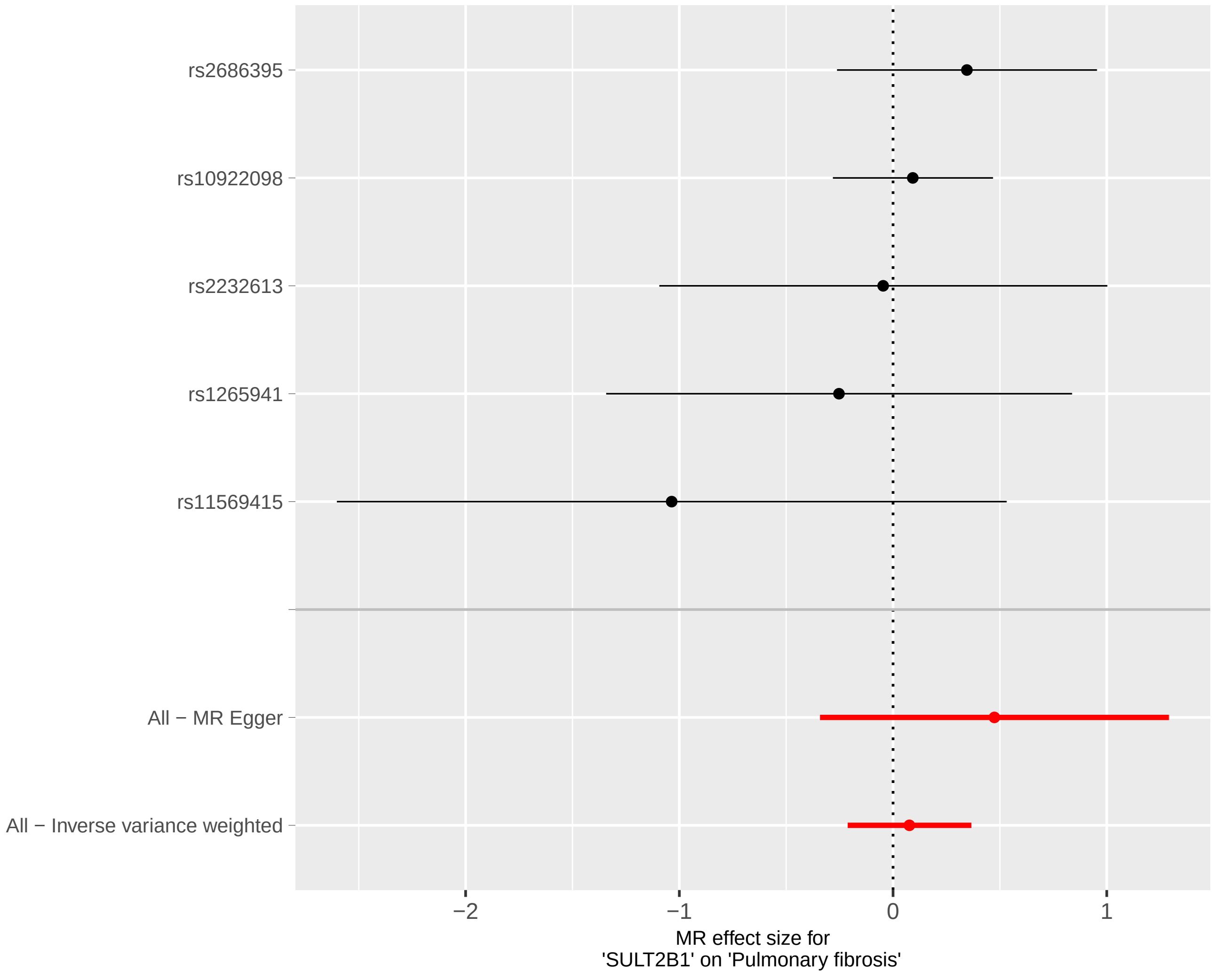

rs2686395
rs10922098
rs2232613
rs1265941
rs11569415
All − MR Egger
All − Inverse variance weighted
−2
−1
0
1
MR effect size for
'SULT2B1' on 'Pulmonary fibrosis'

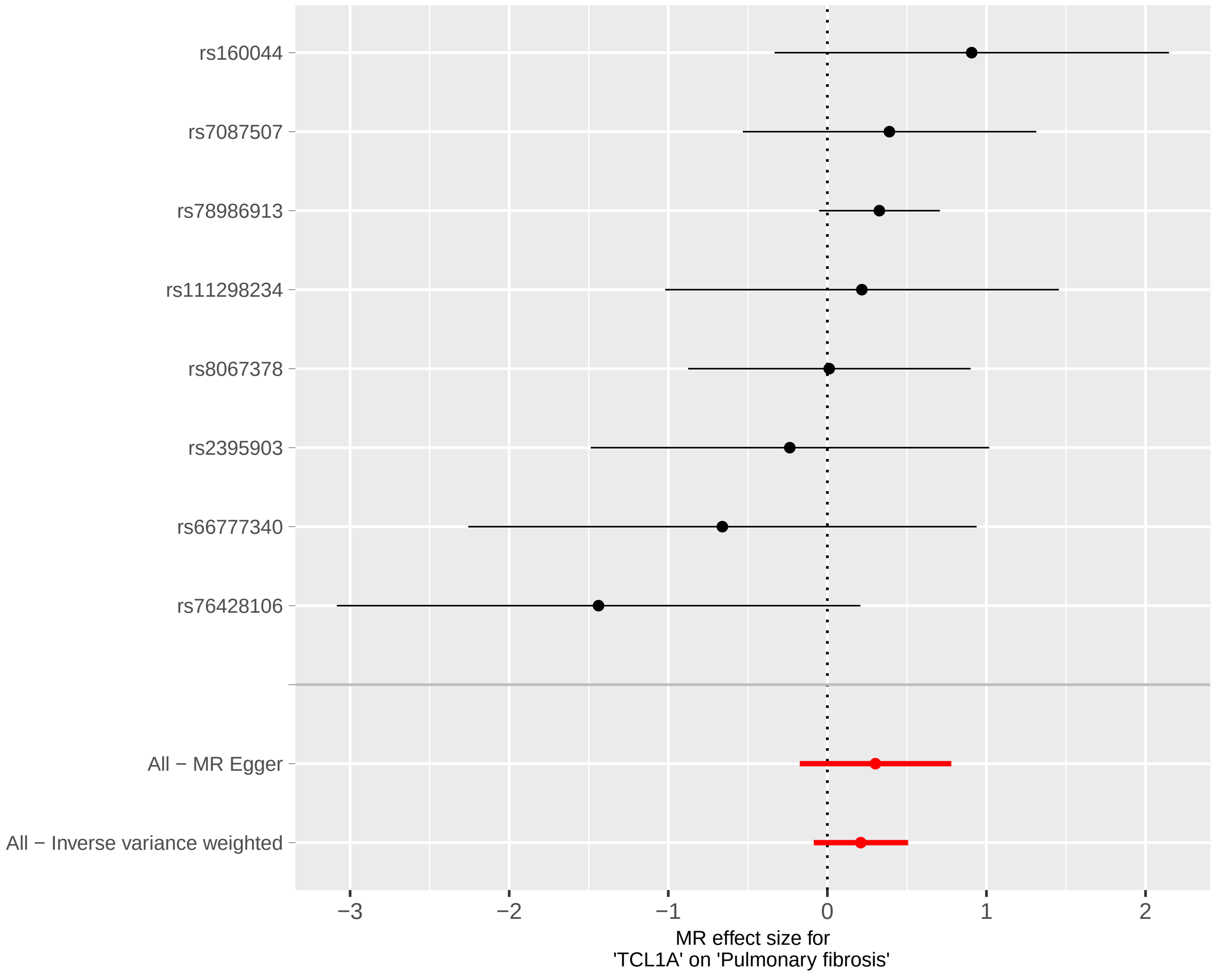
rs160044
rs7087507
rs78986913
rs111298234
rs8067378
rs2395903
rs66777340
rs76428106
All − MR Egger
All − Inverse variance weighted
−3
−2
−1
0
1
2
MR effect size for
'TCL1A' on 'Pulmonary fibrosis'

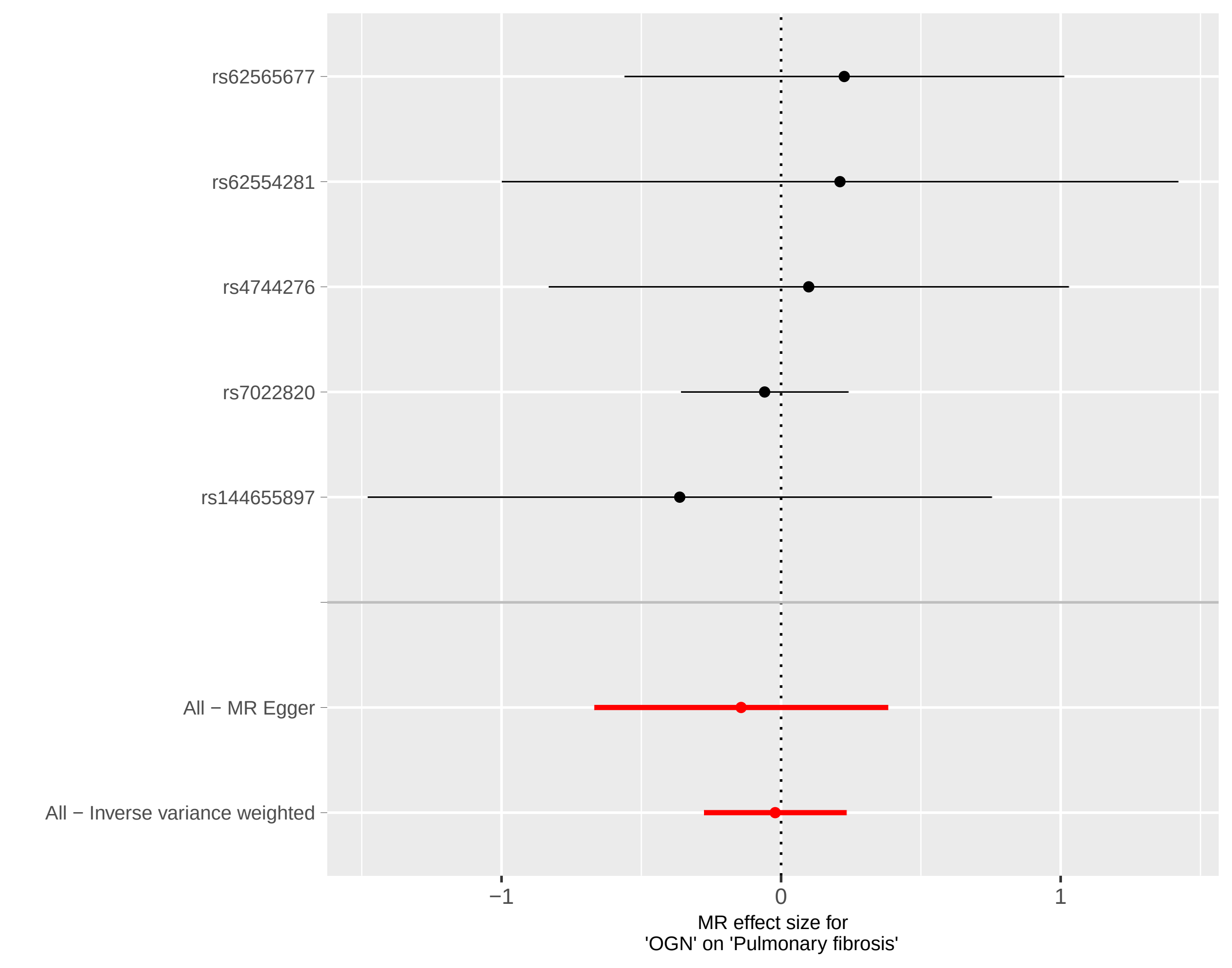
rs62565677
rs62554281
rs4744276
rs7022820
rs144655897
All − MR Egger
All − Inverse variance weighted
−1
0
1
MR effect size for
'OGN' on 'Pulmonary fibrosis'

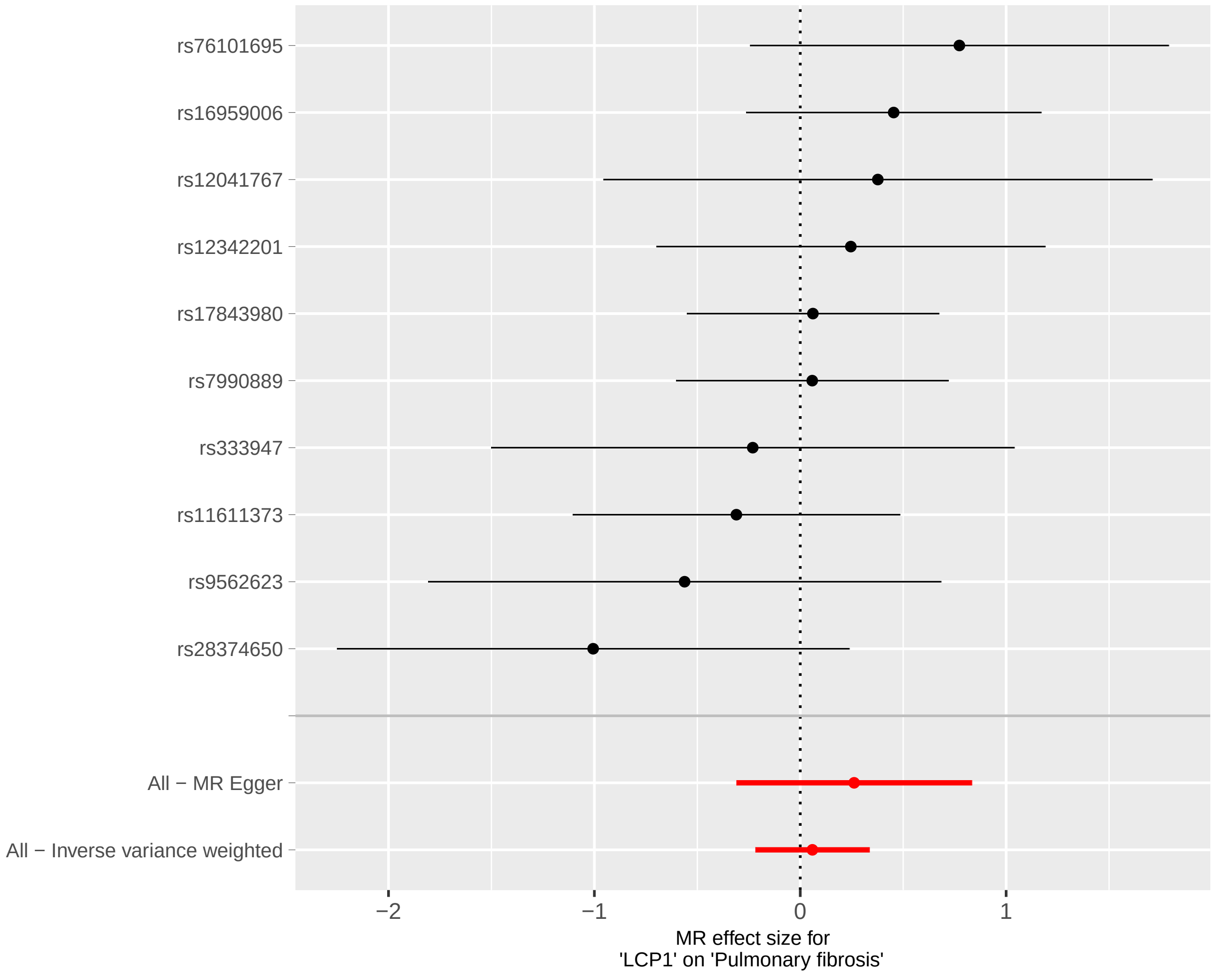
rs76101695
rs16959006
rs12041767
rs12342201
rs17843980
rs7990889
rs333947
rs11611373
rs9562623
rs28374650
All − MR Egger
All − Inverse variance weighted
−2
−1
0
1
MR effect size for
'LCP1' on 'Pulmonary fibrosis'

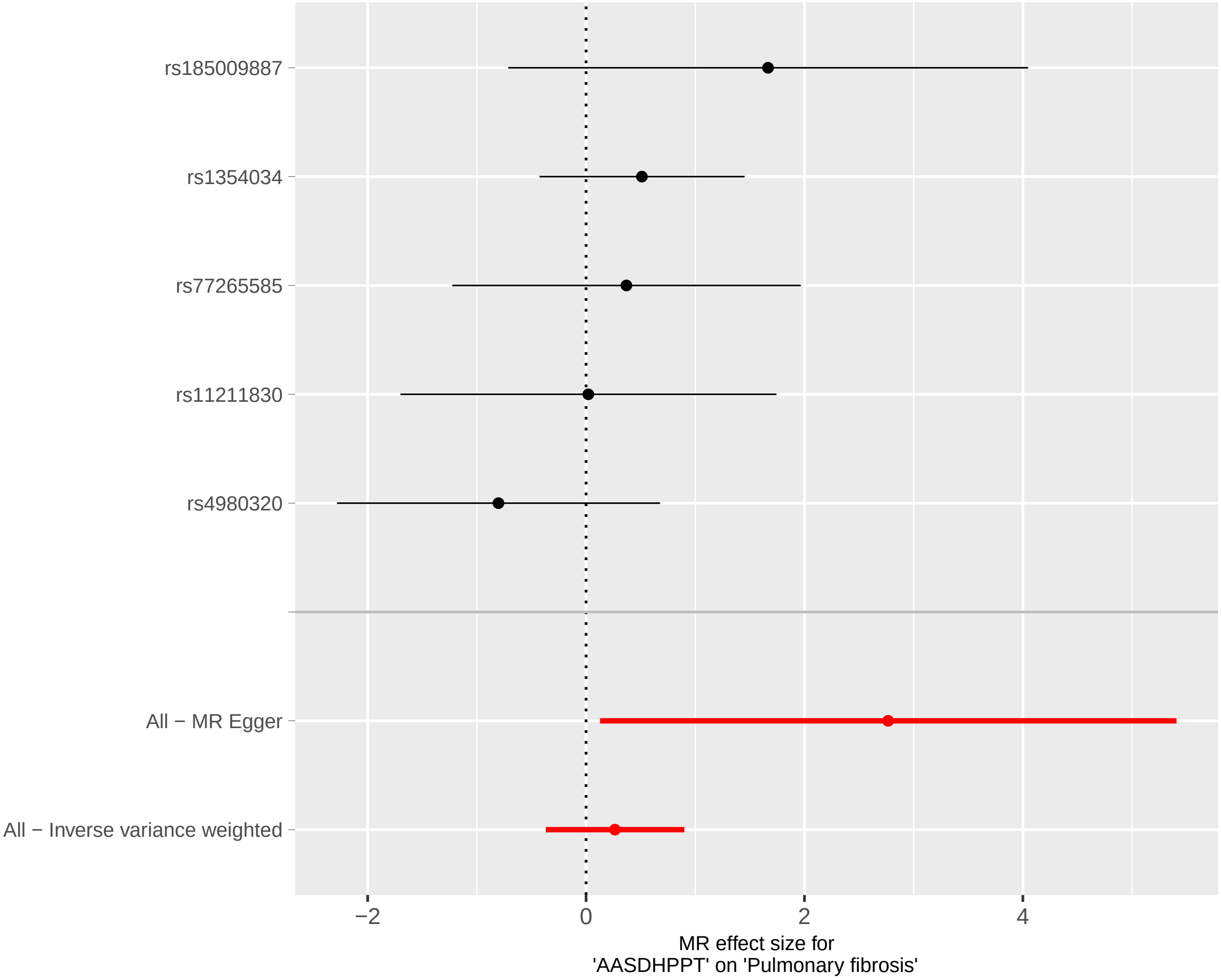
rs185009887
rs1354034
rs77265585
rs11211830
rs4980320
All − MR Egger
All − Inverse variance weighted
−2
0
2
4
MR effect size for
'AASDHPPT' on 'Pulmonary fibrosis'

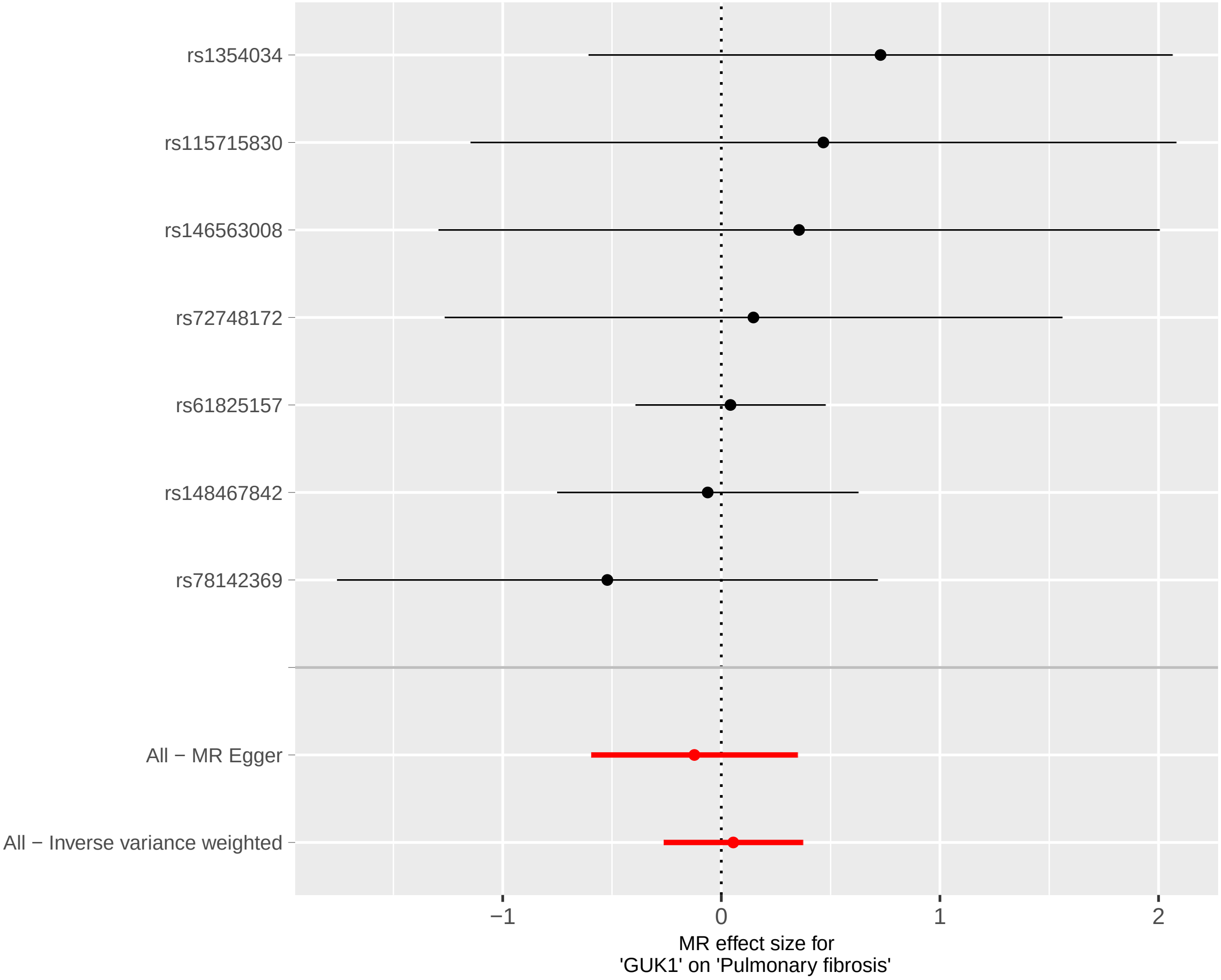
rs1354034
rs115715830
rs146563008
rs72748172
rs61825157
rs148467842
rs78142369
All − MR Egger
All − Inverse variance weighted
−1
0
1
2
MR effect size for
'GUK1' on 'Pulmonary fibrosis'

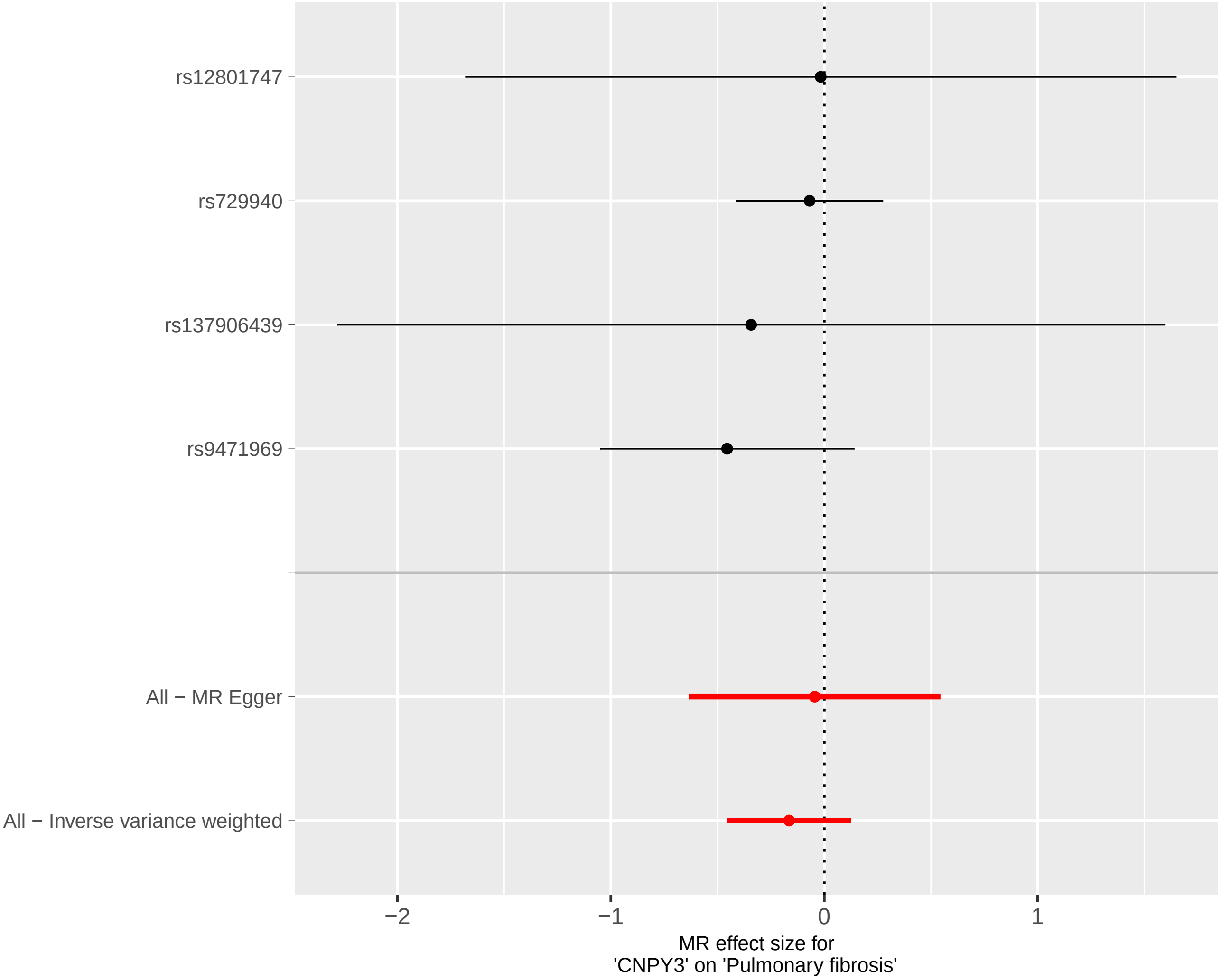
rs12801747
rs729940
rs137906439
rs9471969
All − MR Egger
All − Inverse variance weighted
−2
−1
0
1
MR effect size for
'CNPY3' on 'Pulmonary fibrosis'

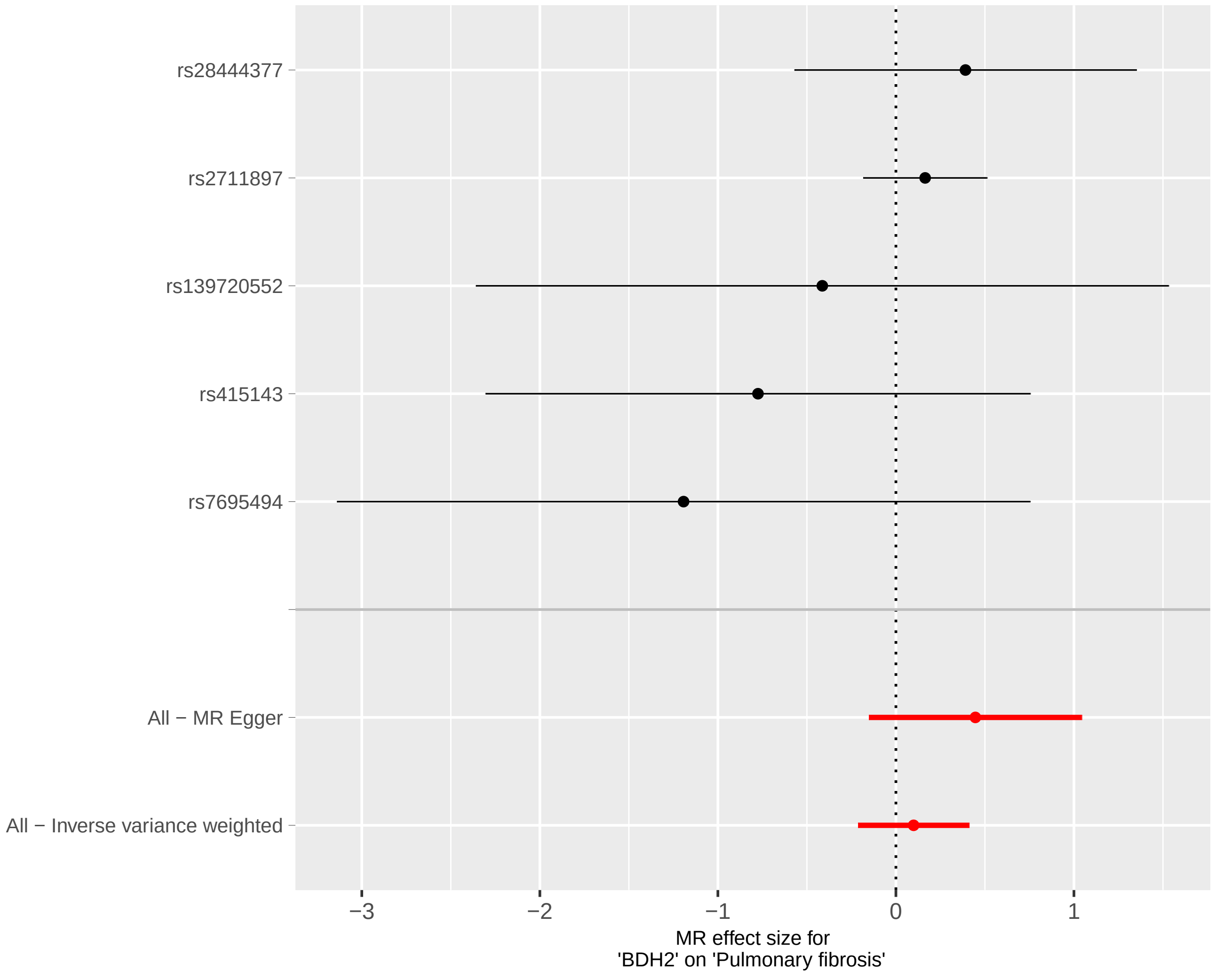
rs28444377
rs2711897
rs139720552
rs415143
rs7695494
All − MR Egger
All − Inverse variance weighted
−3
−2
−1
0
1
MR effect size for
'BDH2' on 'Pulmonary fibrosis'

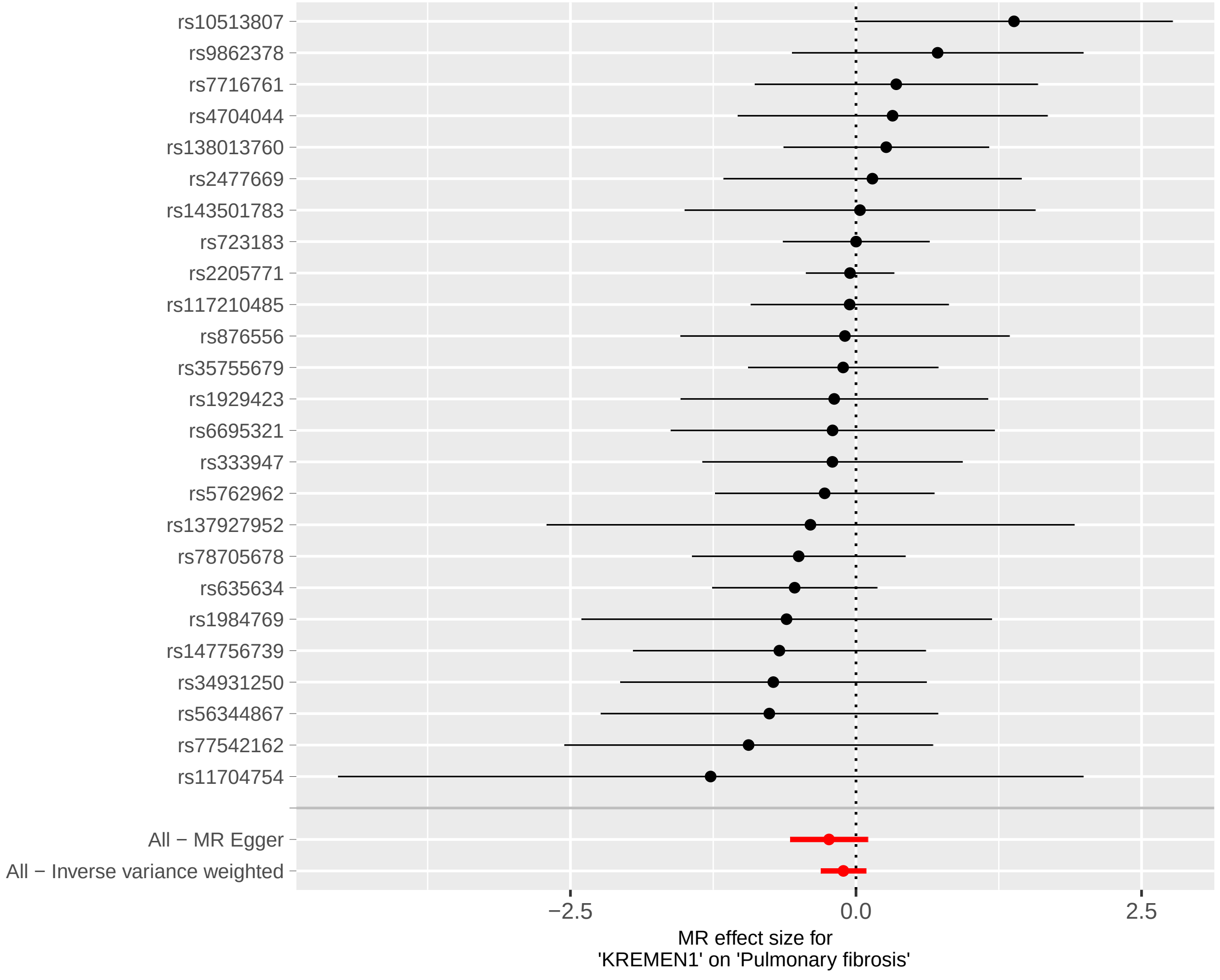
rs10513807
rs9862378
rs7716761
rs4704044
rs138013760
rs2477669
rs143501783
rs723183
rs2205771
rs117210485
rs876556
rs35755679
rs1929423
rs6695321
rs333947
rs5762962
rs137927952
rs78705678
rs635634
rs1984769
rs147756739
rs34931250
rs56344867
rs77542162
rs11704754
All − MR Egger
All − Inverse variance weighted
−2.5
0.0
2.5
MR effect size for
'KREMEN1' on 'Pulmonary fibrosis'

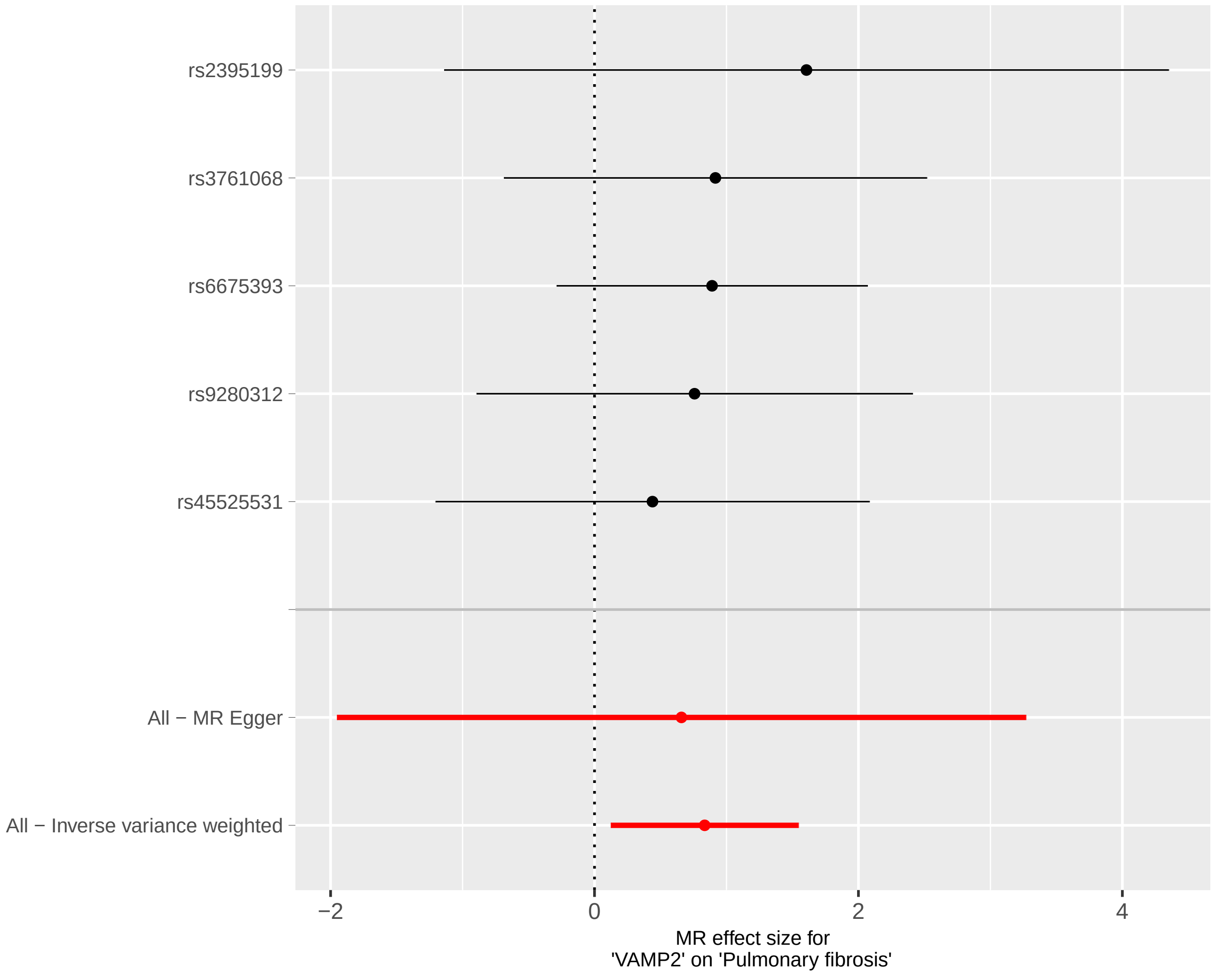

rs2395199
rs3761068
rs6675393
rs9280312
rs45525531
All − MR Egger
All − Inverse variance weighted
−2
0
2
4
MR effect size for
'VAMP2' on 'Pulmonary fibrosis'

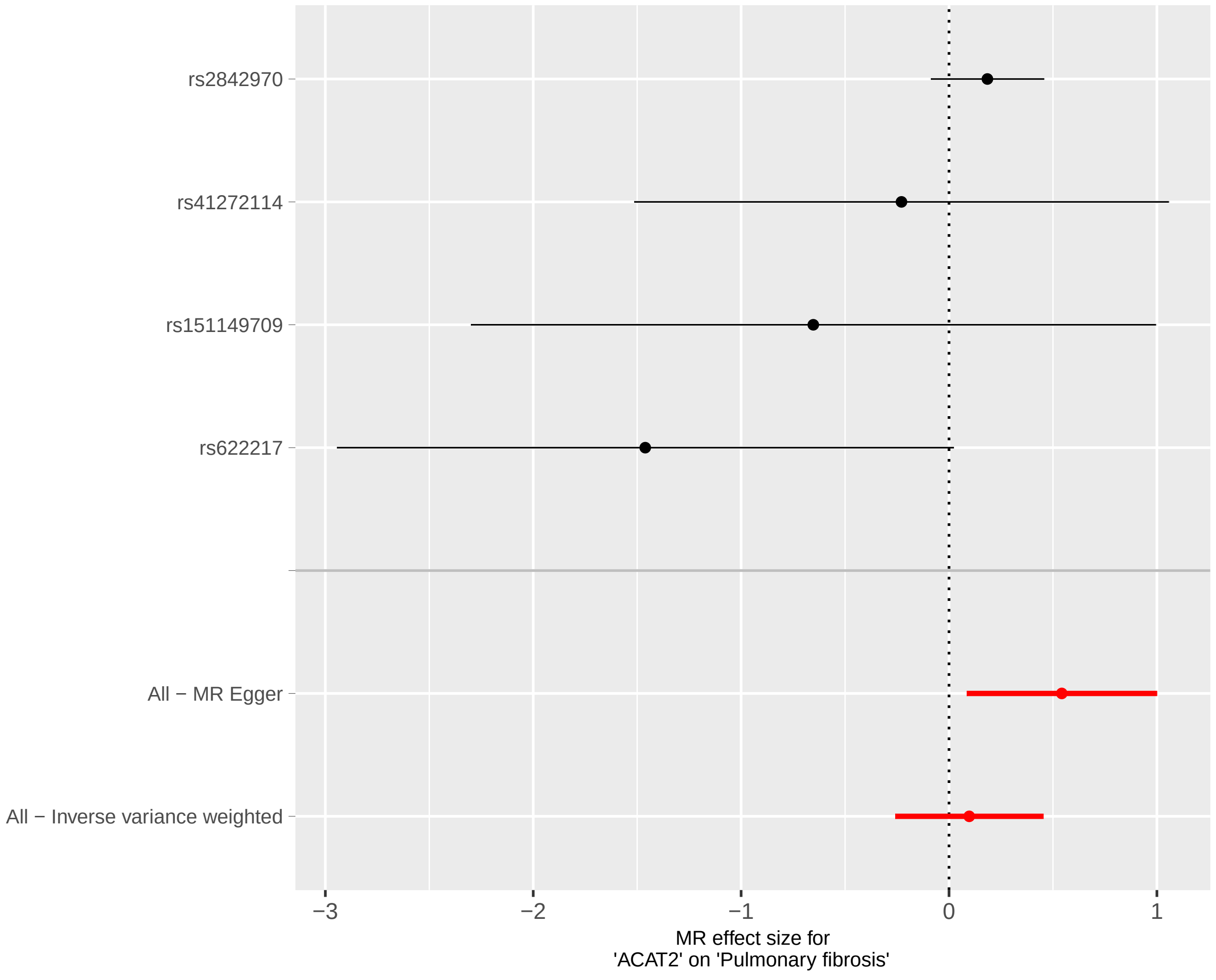

rs2842970
rs41272114
rs151149709
rs622217
All − MR Egger
All − Inverse variance weighted
−3
−2
−1
0
1
MR effect size for
'ACAT2' on 'Pulmonary fibrosis'

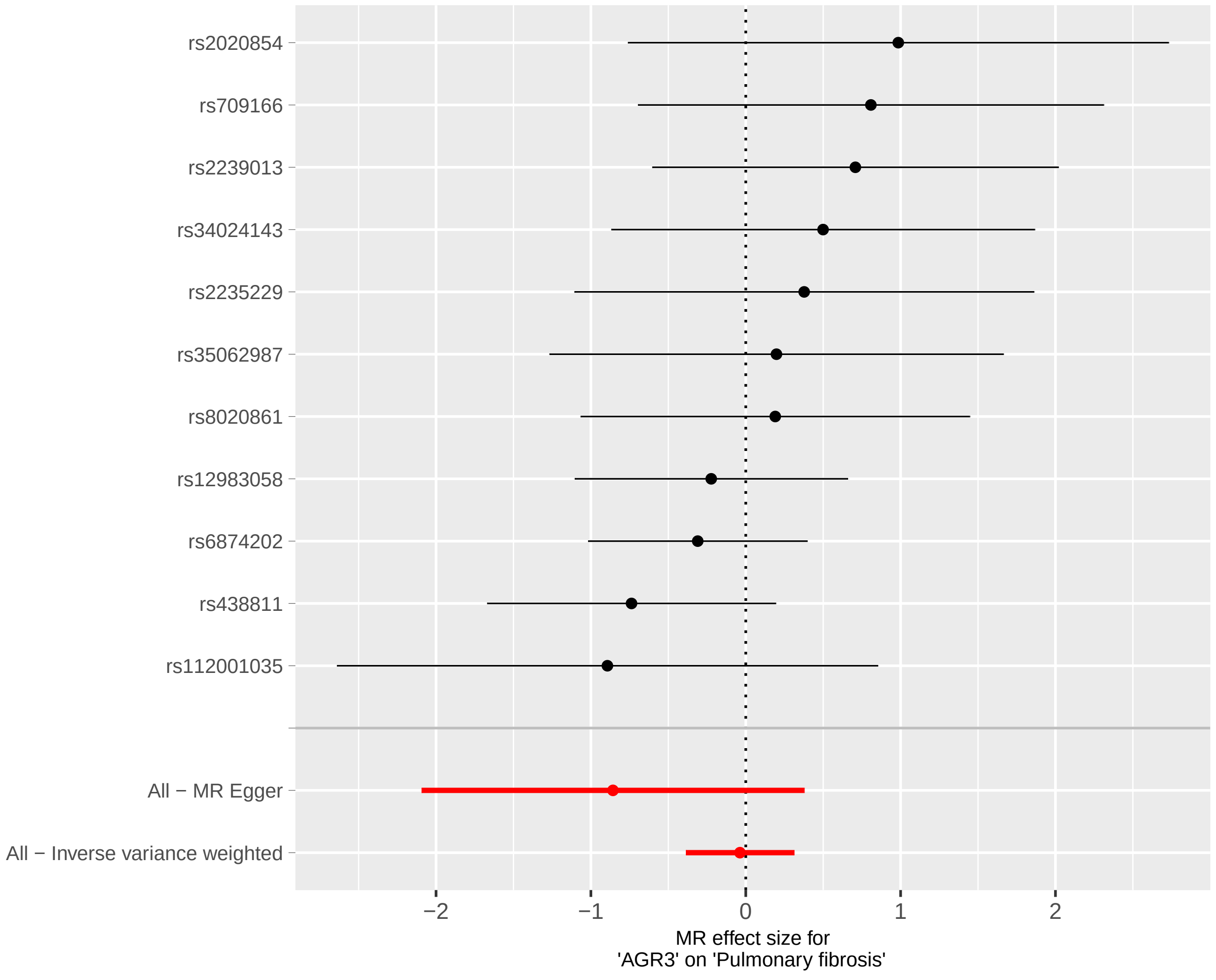
rs2020854
rs709166
rs2239013
rs34024143
rs2235229
rs35062987
rs8020861
rs12983058
rs6874202
rs438811
rs112001035
All − MR Egger
All − Inverse variance weighted
−2
−1
0
1
2
MR effect size for
'AGR3' on 'Pulmonary fibrosis'

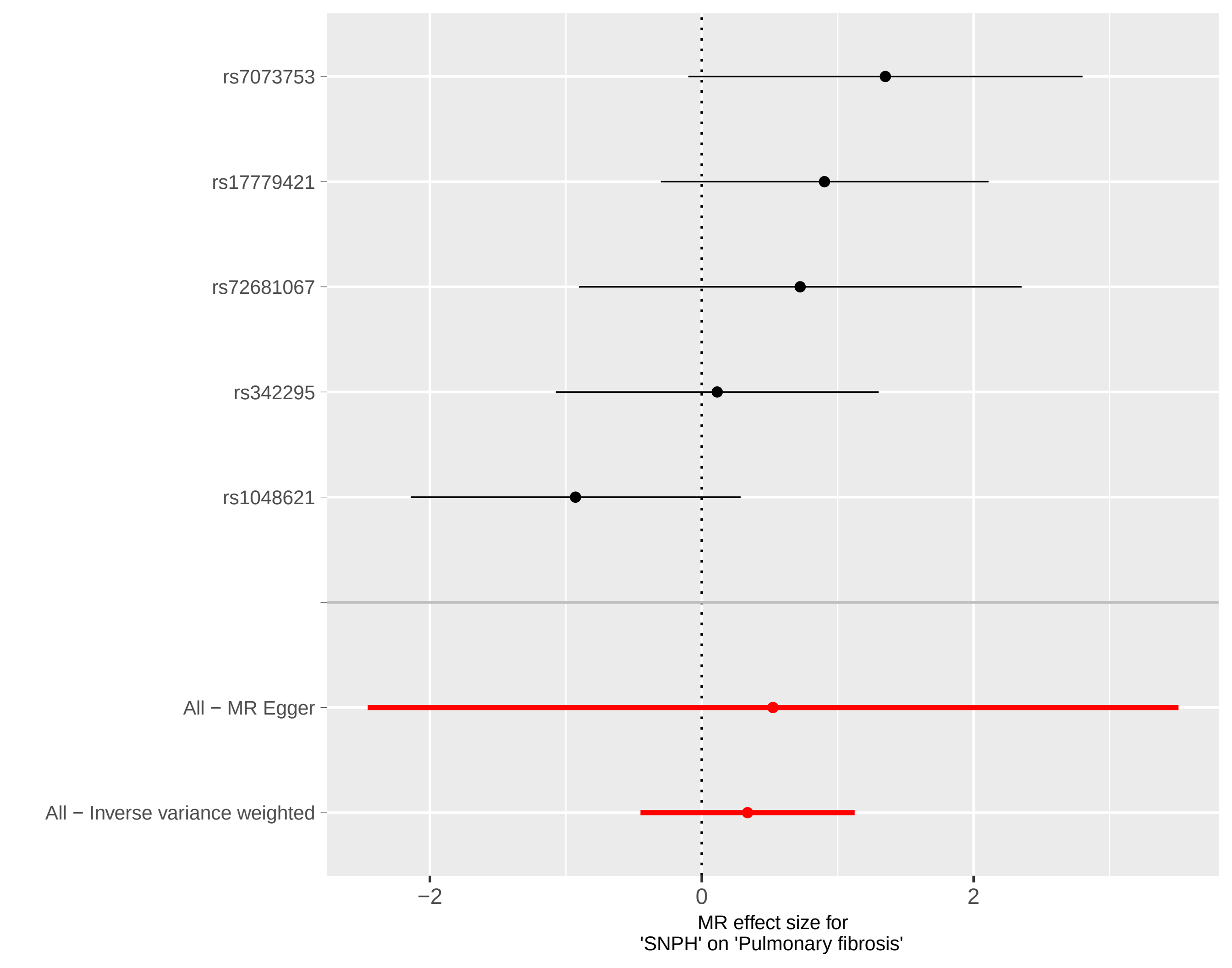
rs7073753
rs17779421
rs72681067
rs342295
rs1048621
All − MR Egger
All − Inverse variance weighted
−2
0
2
MR effect size for
'SNPH' on 'Pulmonary fibrosis'

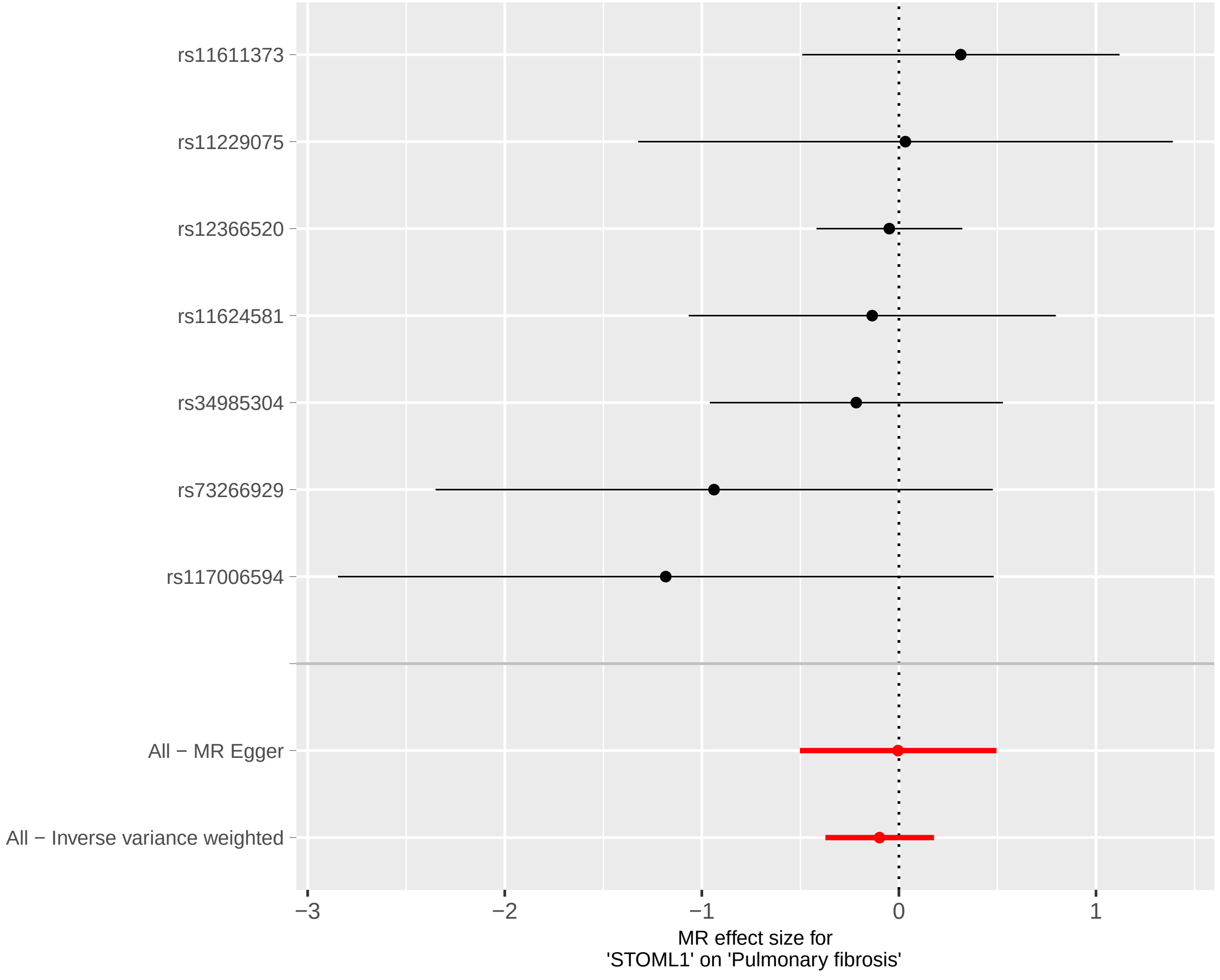
rs11611373
rs11229075
rs12366520
rs11624581
rs34985304
rs73266929
rs117006594
All − MR Egger
All − Inverse variance weighted
−3
−2
−1
0
1
MR effect size for
'STOML1' on 'Pulmonary fibrosis'

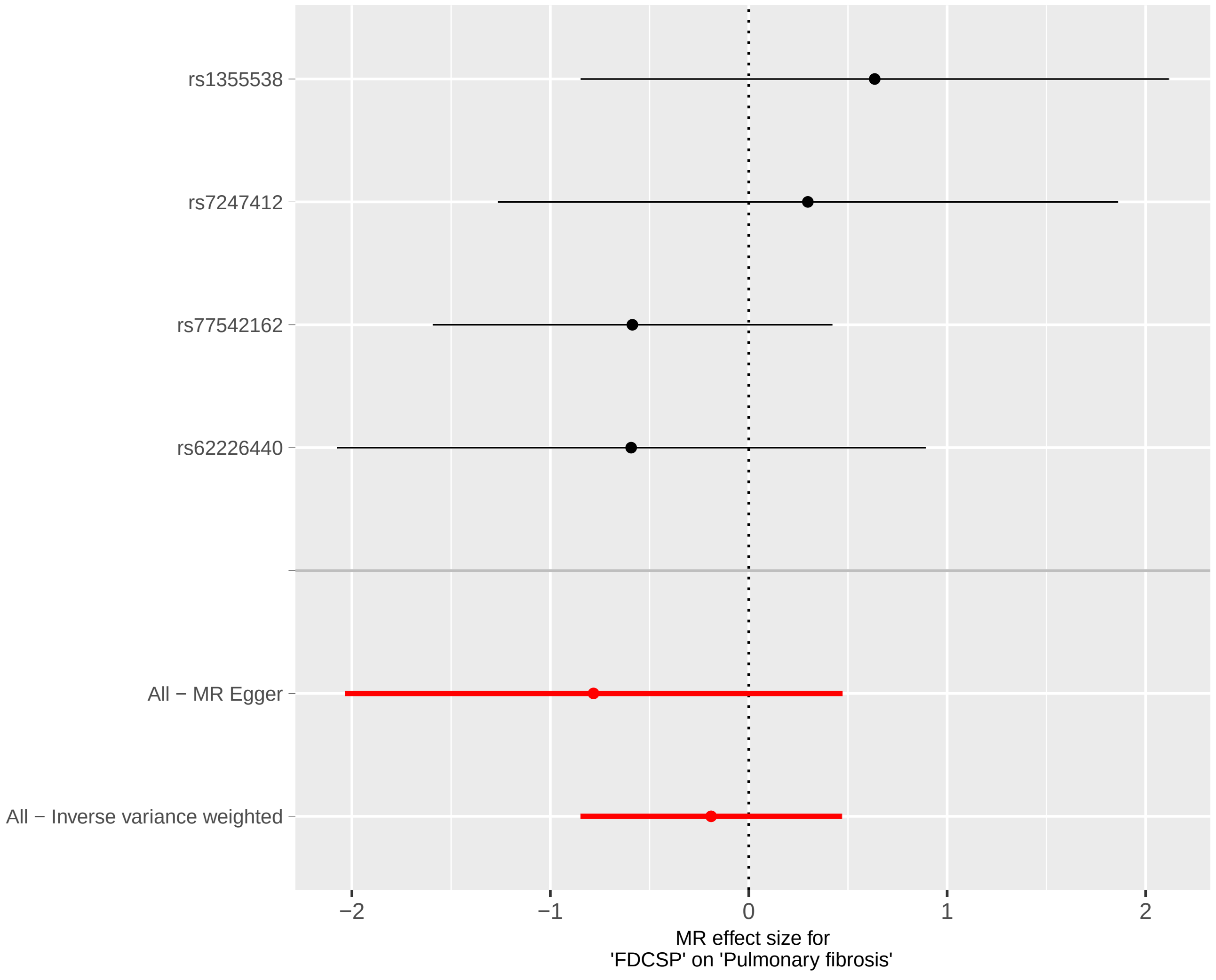
rs1355538
rs7247412
rs77542162
rs62226440
All − MR Egger
All − Inverse variance weighted
−2
−1
0
1
2
MR effect size for
'FDCSP' on 'Pulmonary fibrosis'

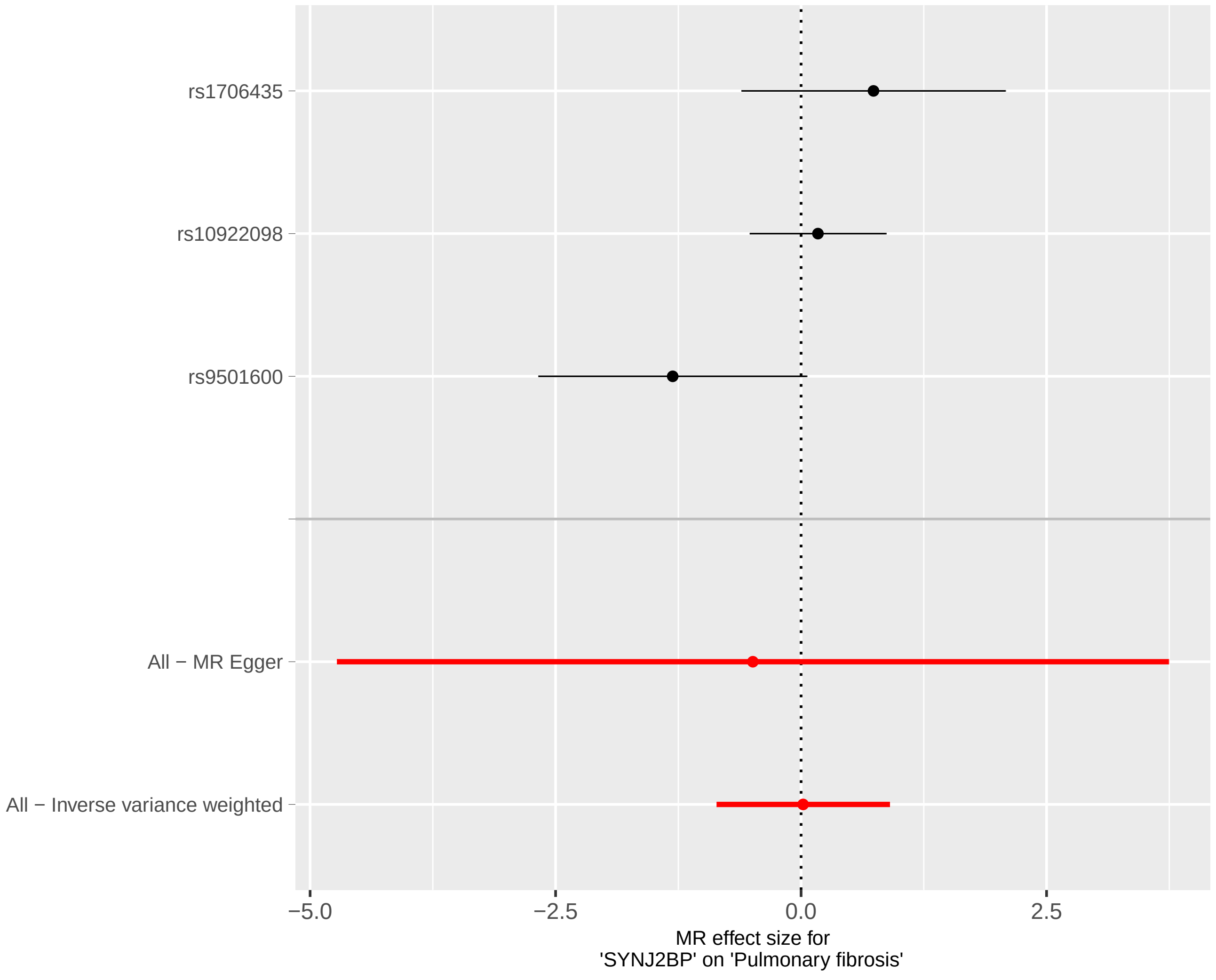

rs1706435
rs10922098
rs9501600
All − MR Egger
All − Inverse variance weighted
−5.0
−2.5
0.0
2.5
MR effect size for
'SYNJ2BP' on 'Pulmonary fibrosis'

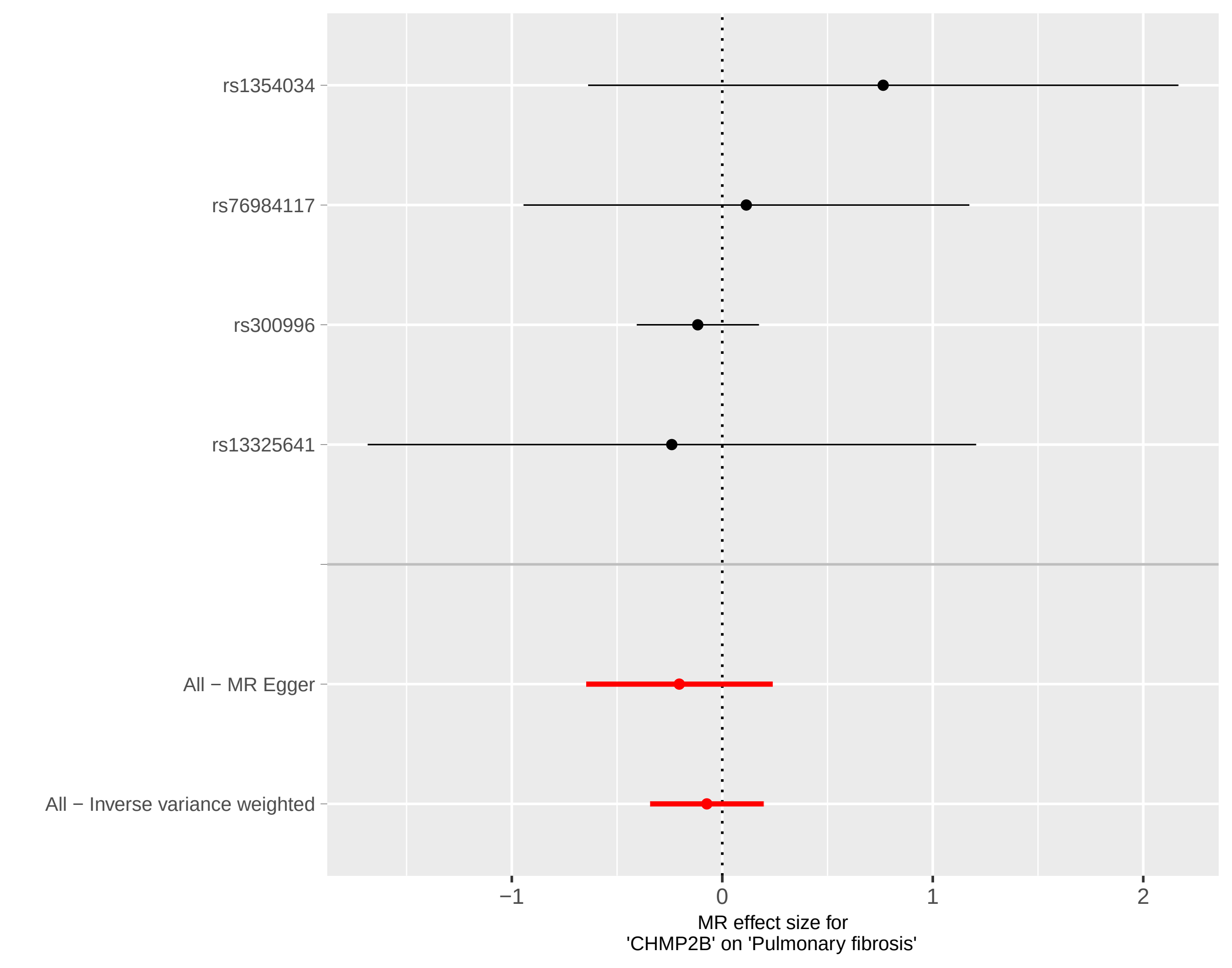
rs1354034
rs76984117
rs300996
rs13325641
All − MR Egger
All − Inverse variance weighted
−1
0
1
2
MR effect size for
'CHMP2B' on 'Pulmonary fibrosis'

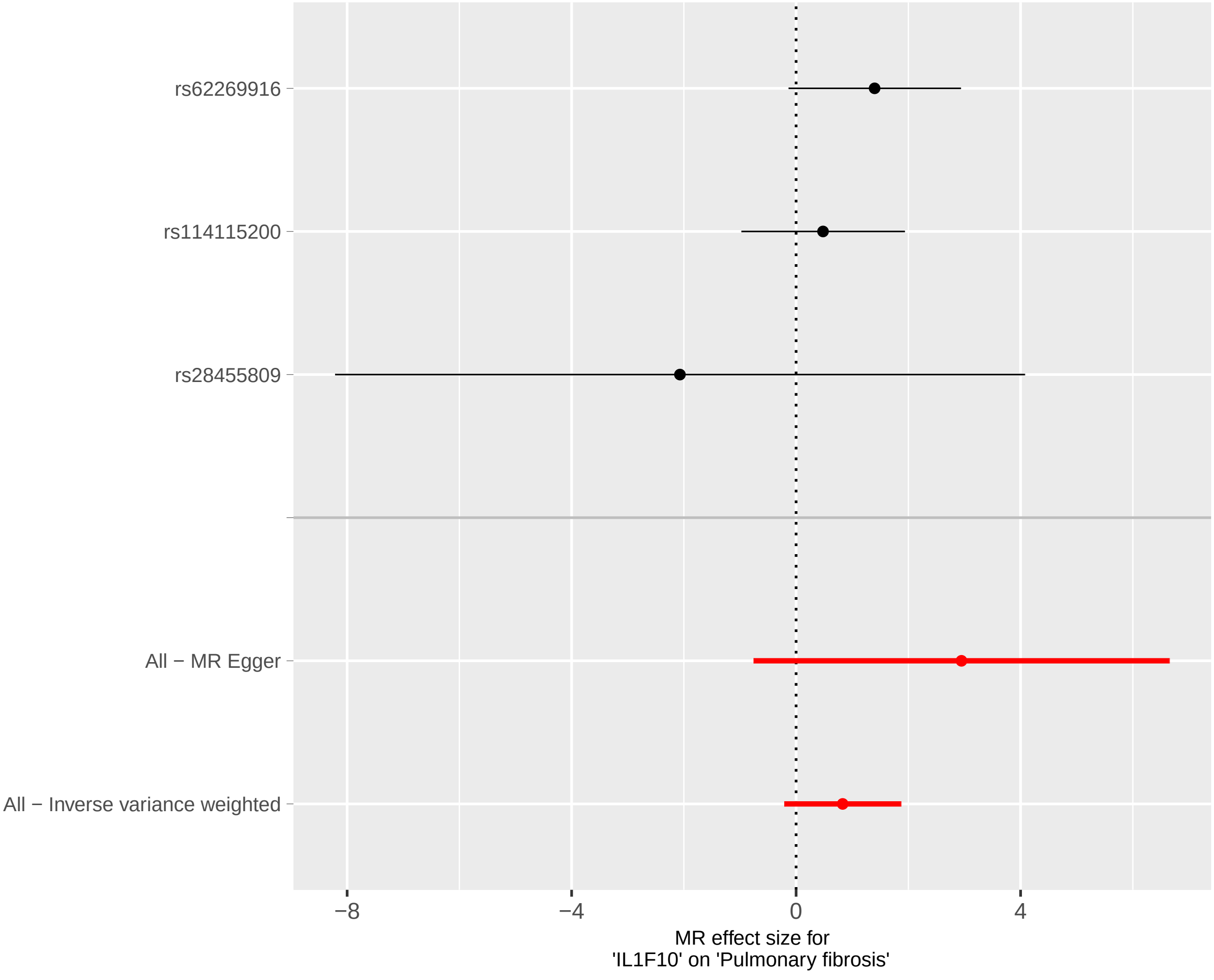
rs62269916
rs114115200
rs28455809
All − MR Egger
All − Inverse variance weighted
−8
−4
0
4
MR effect size for
'IL1F10' on 'Pulmonary fibrosis'

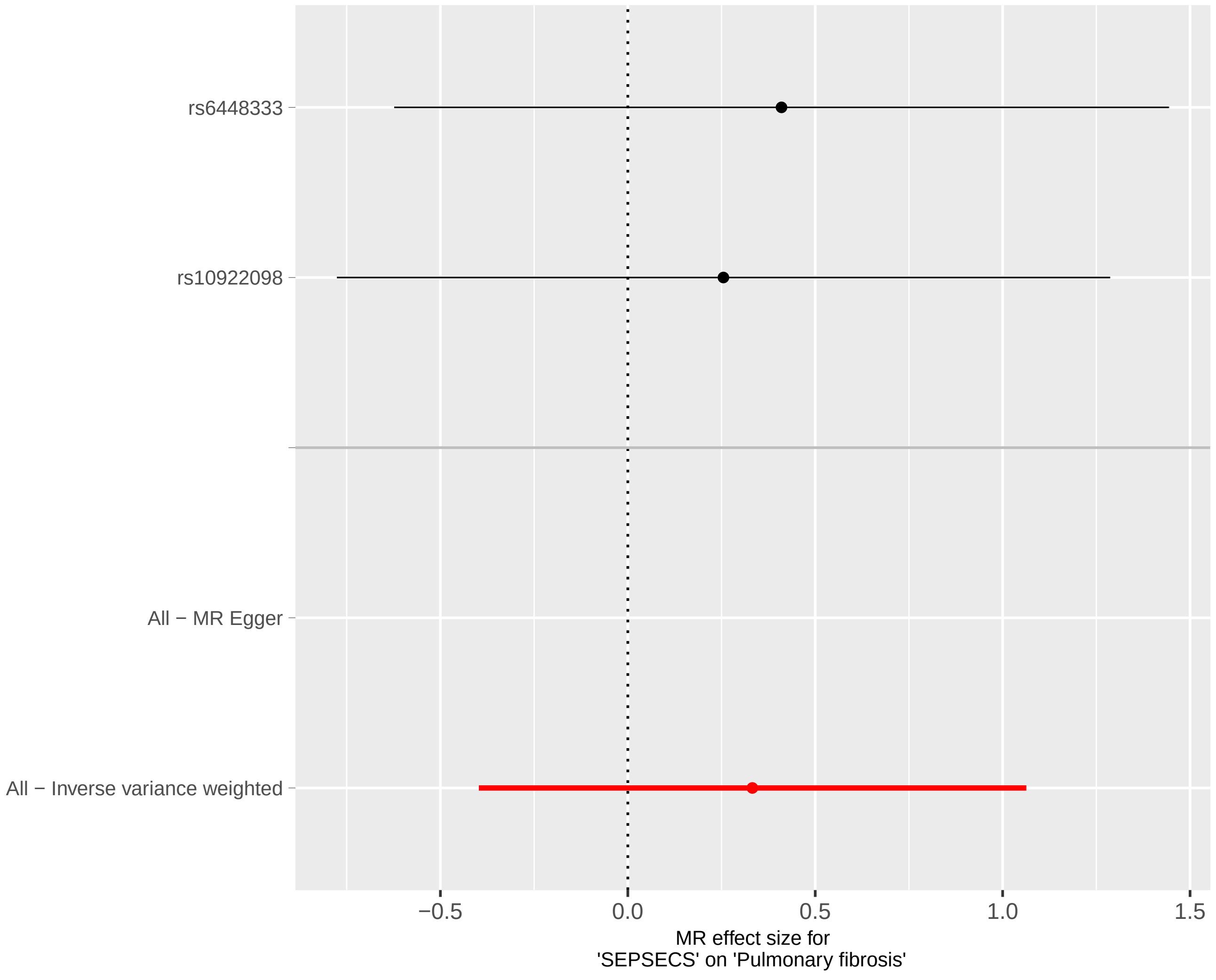
rs6448333
rs10922098
All − MR Egger
All − Inverse variance weighted
−0.5
0.0
0.5
1.0
1.5
MR effect size for
'SEPSECS' on 'Pulmonary fibrosis'

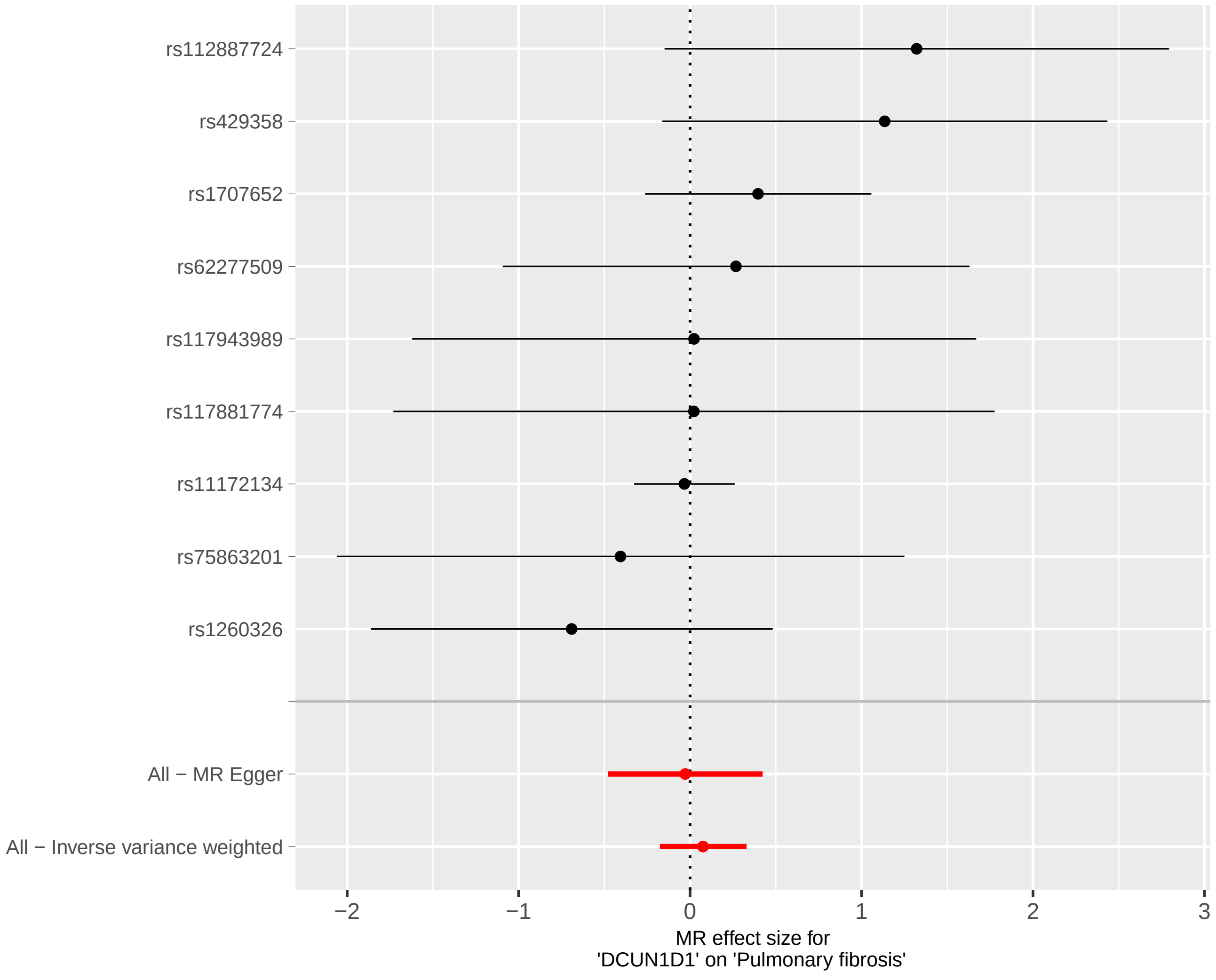

rs112887724
rs429358
rs1707652
rs62277509
rs117943989
rs117881774
rs11172134
rs75863201
rs1260326
All − MR Egger
All − Inverse variance weighted
−2
−1
0
1
2
3
MR effect size for
'DCUN1D1' on 'Pulmonary fibrosis'

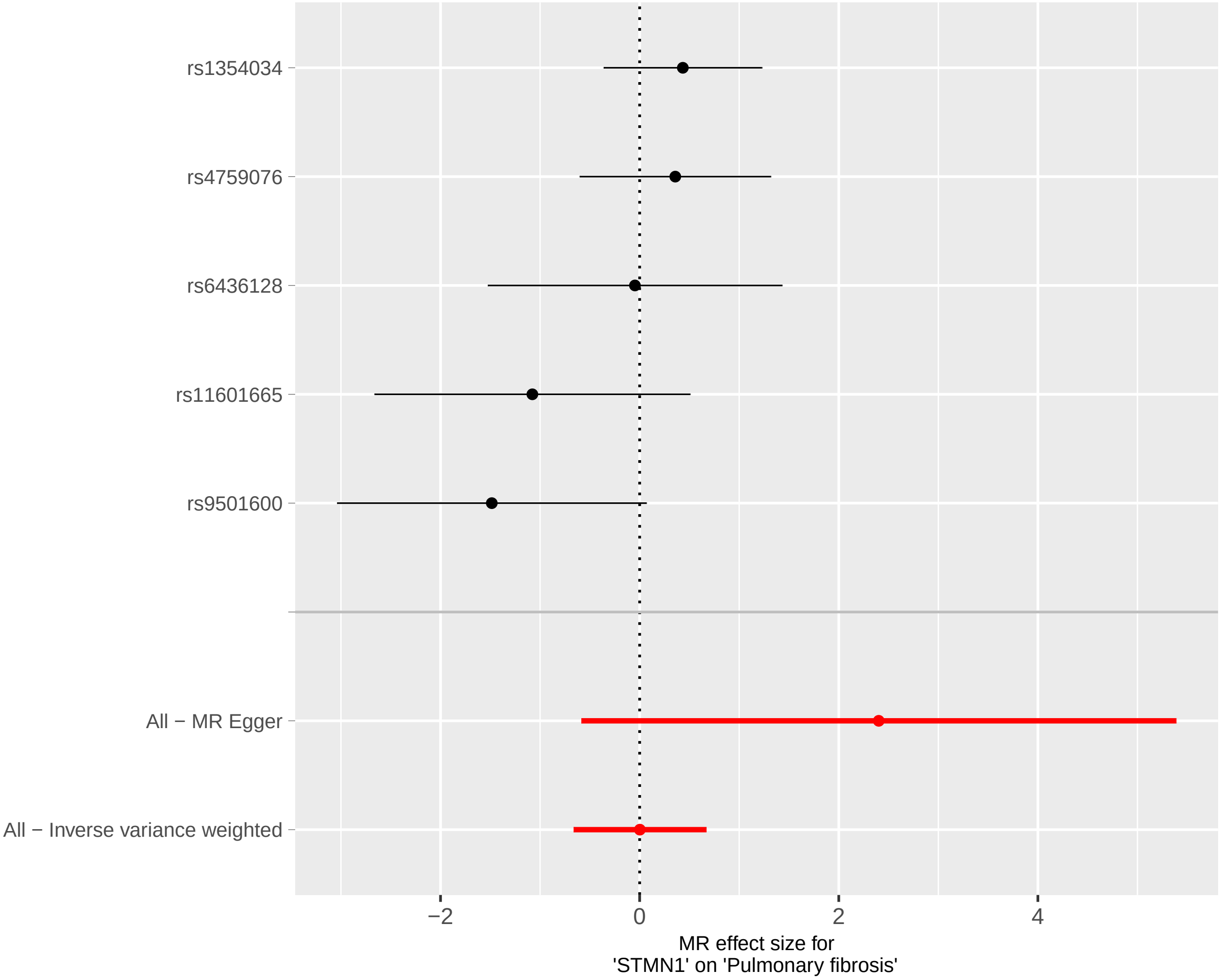
rs1354034
rs4759076
rs6436128
rs11601665
rs9501600
All − MR Egger
All − Inverse variance weighted
−2
0
2
4
MR effect size for
'STMN1' on 'Pulmonary fibrosis'

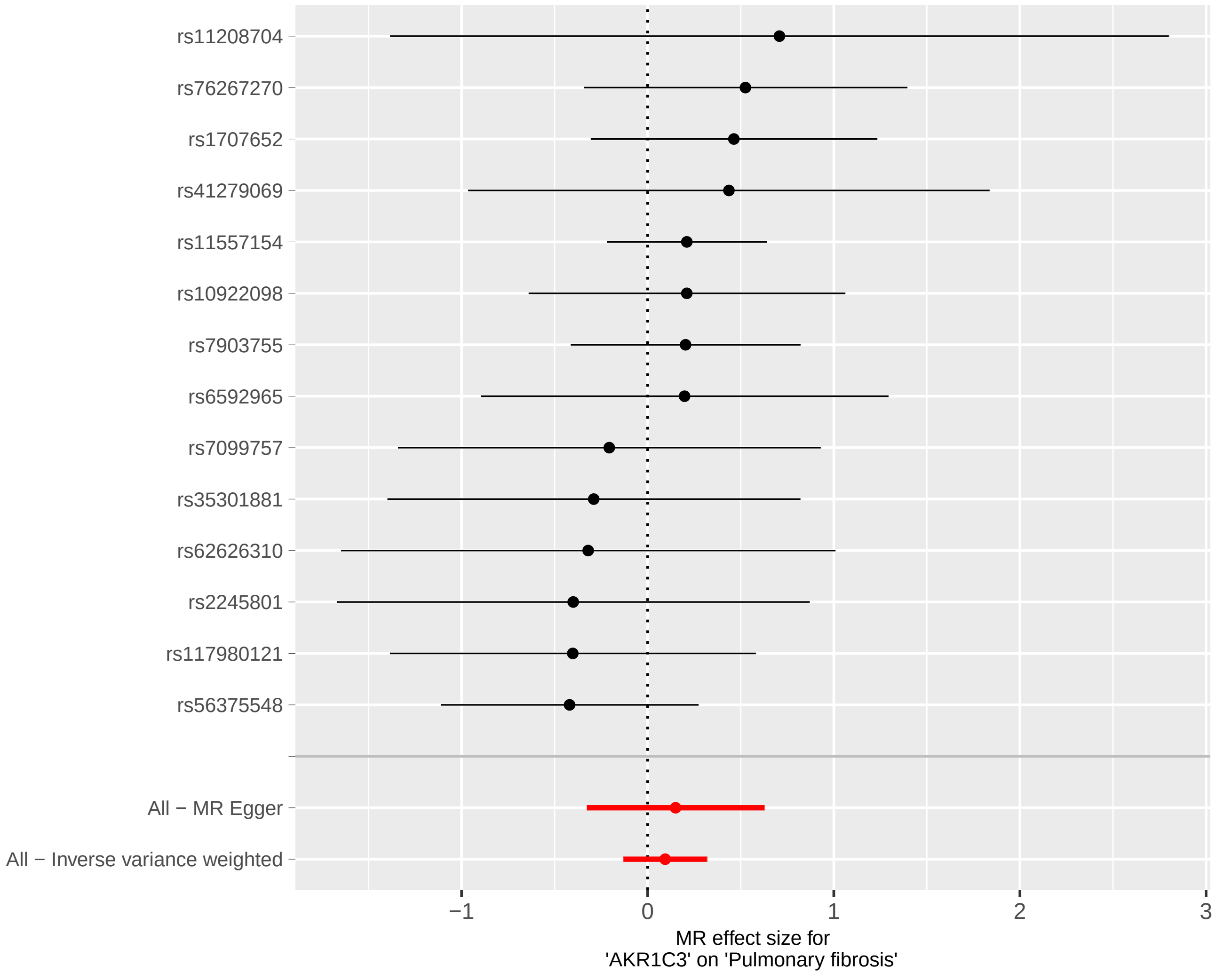

rs11208704
rs76267270
rs1707652
rs41279069
rs11557154
rs10922098
rs7903755
rs6592965
rs7099757
rs35301881
rs62626310
rs2245801
rs117980121
rs56375548
All − MR Egger
All − Inverse variance weighted
−1
0
1
2
3
MR effect size for
'AKR1C3' on 'Pulmonary fibrosis'

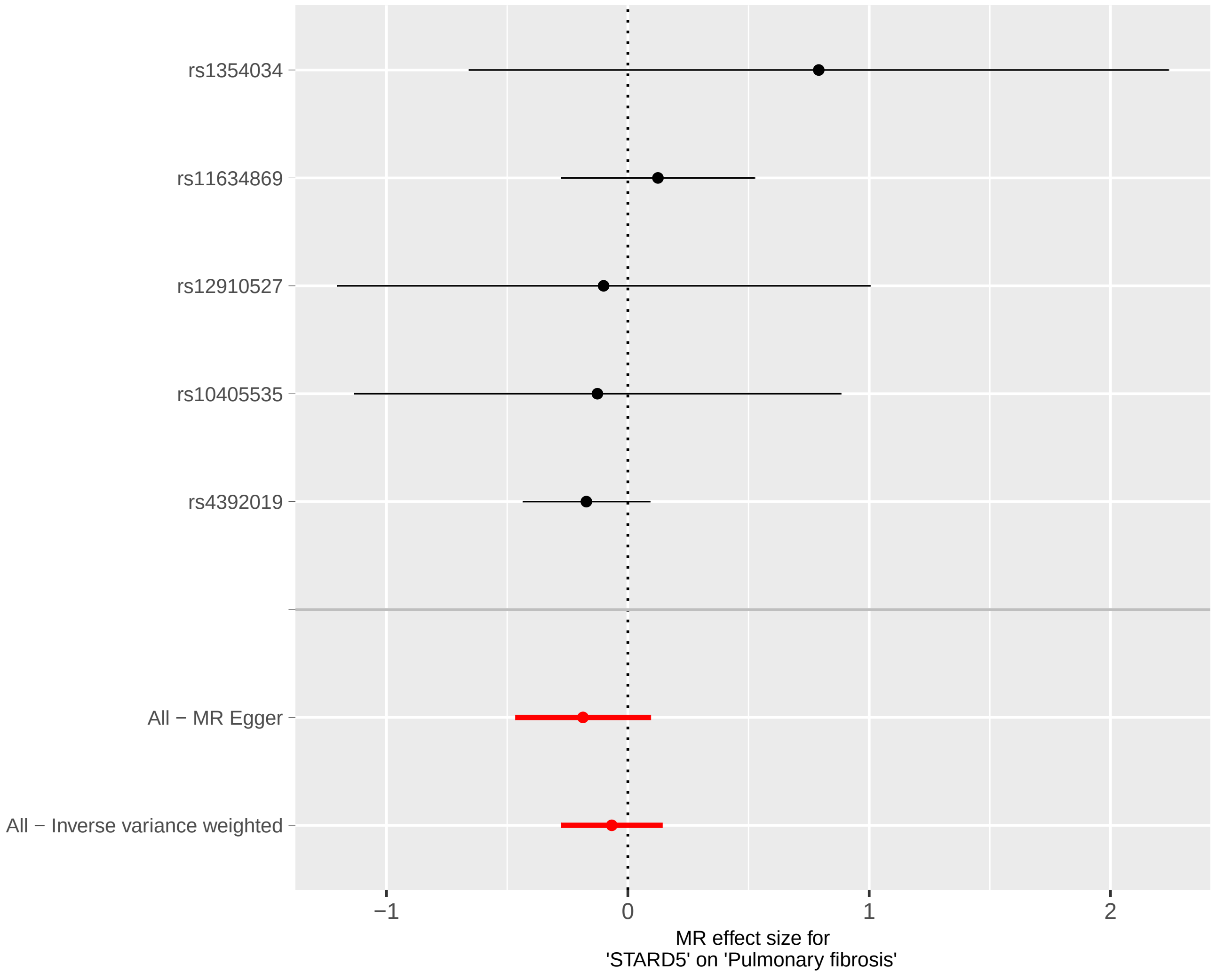

rs1354034
rs11634869
rs12910527
rs10405535
rs4392019
All − MR Egger
All − Inverse variance weighted
−1
0
1
2
MR effect size for
'STARD5' on 'Pulmonary fibrosis'

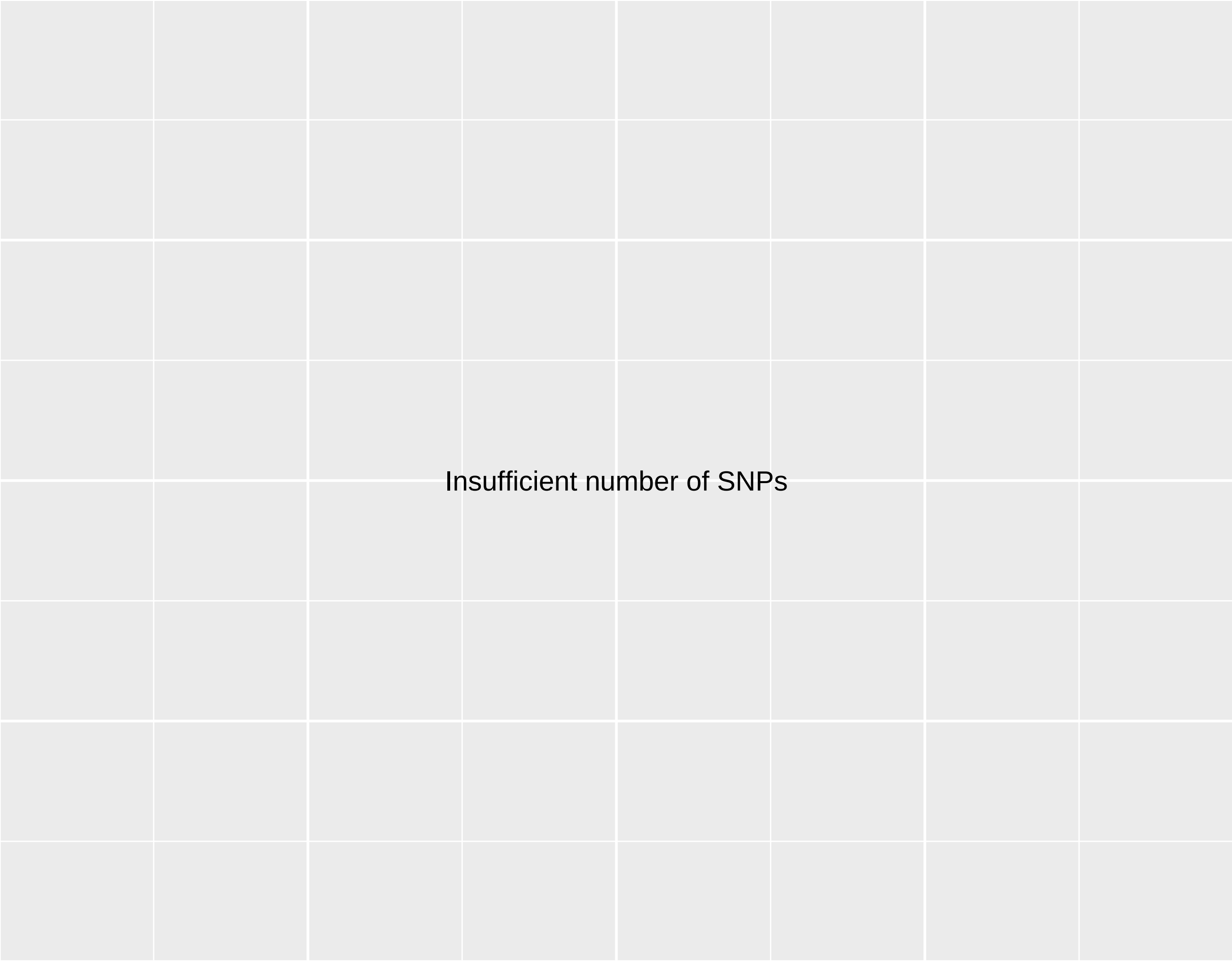
Insufficient number of SNPs

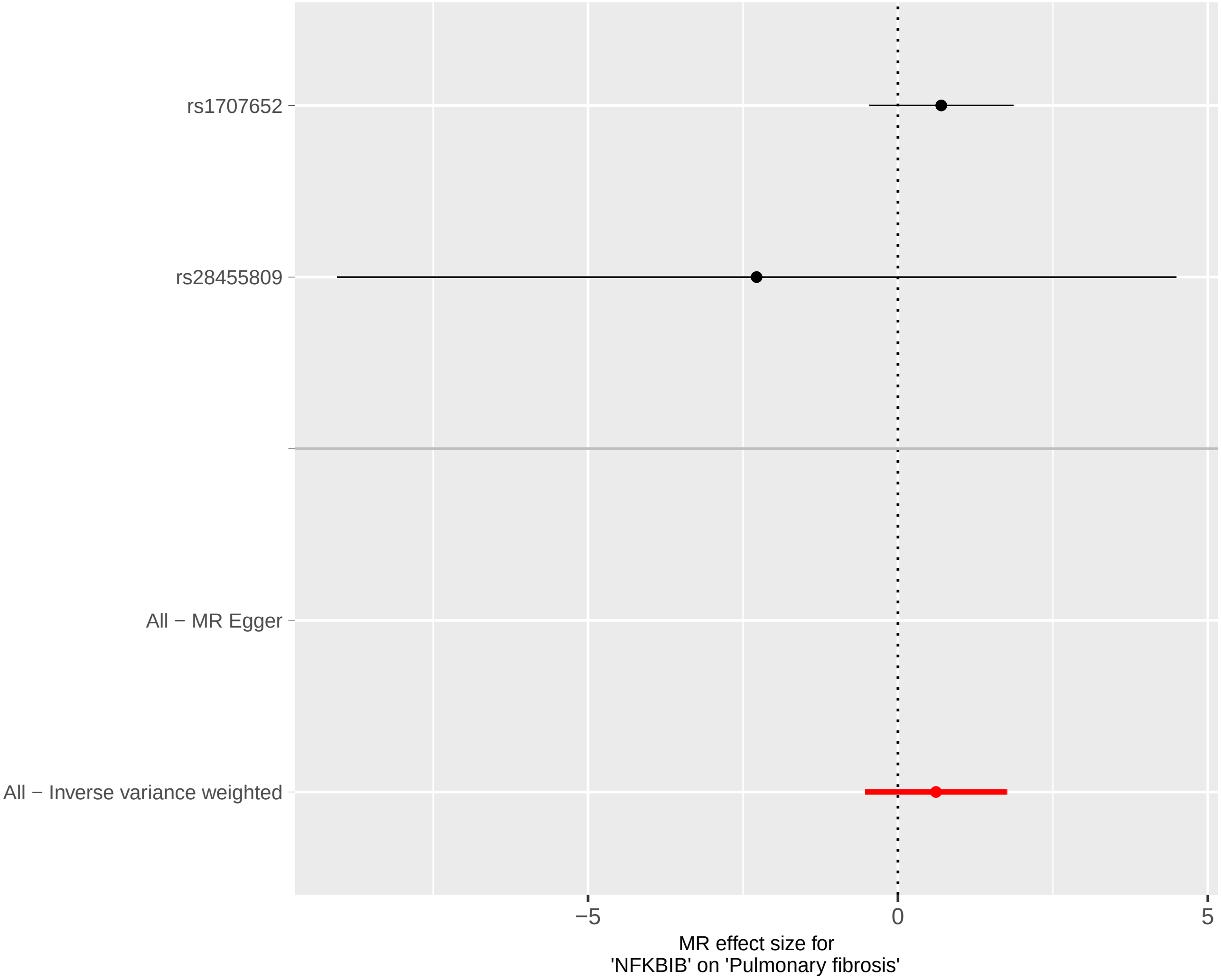

rs1707652
rs28455809
All − MR Egger
All − Inverse variance weighted
−5
0
5
MR effect size for
'NFKBIB' on 'Pulmonary fibrosis'

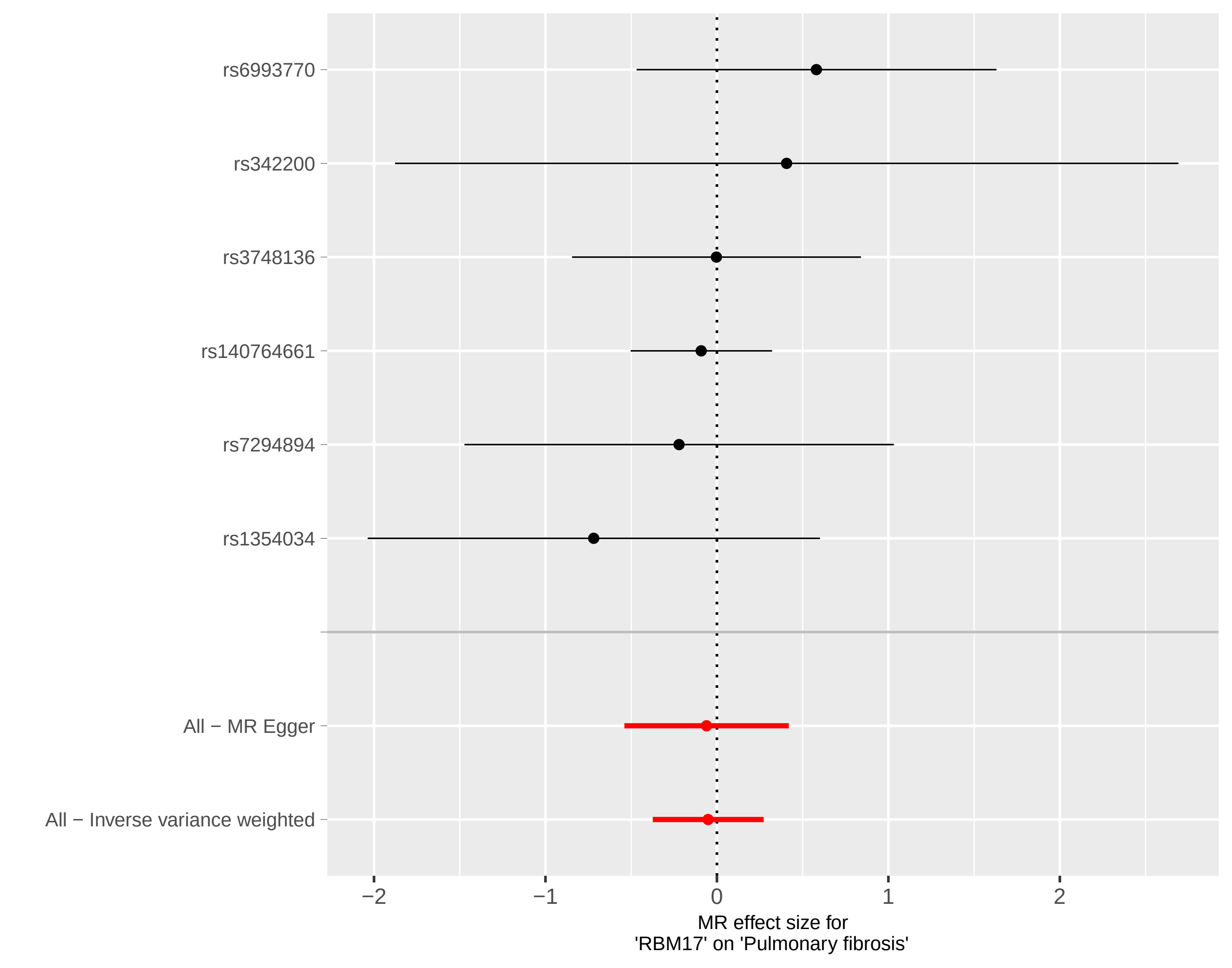
rs6993770
rs342200
rs3748136
rs140764661
rs7294894
rs1354034
All − MR Egger
All − Inverse variance weighted
−2
−1
0
1
2
MR effect size for
'RBM17' on 'Pulmonary fibrosis'

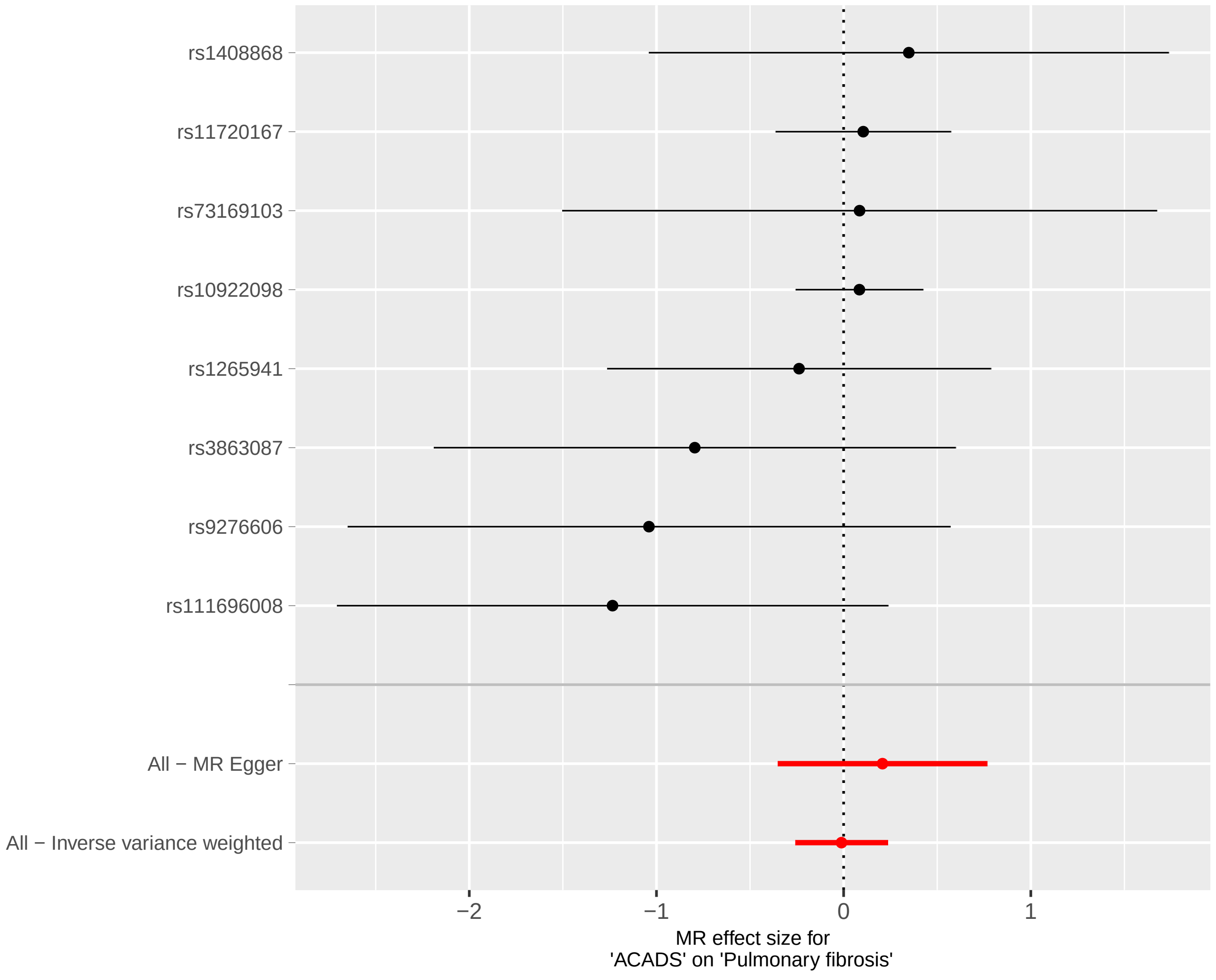

rs1408868
rs11720167
rs73169103
rs10922098
rs1265941
rs3863087
rs9276606
rs111696008
All − MR Egger
All − Inverse variance weighted
−2
−1
0
1
MR effect size for
'ACADS' on 'Pulmonary fibrosis'

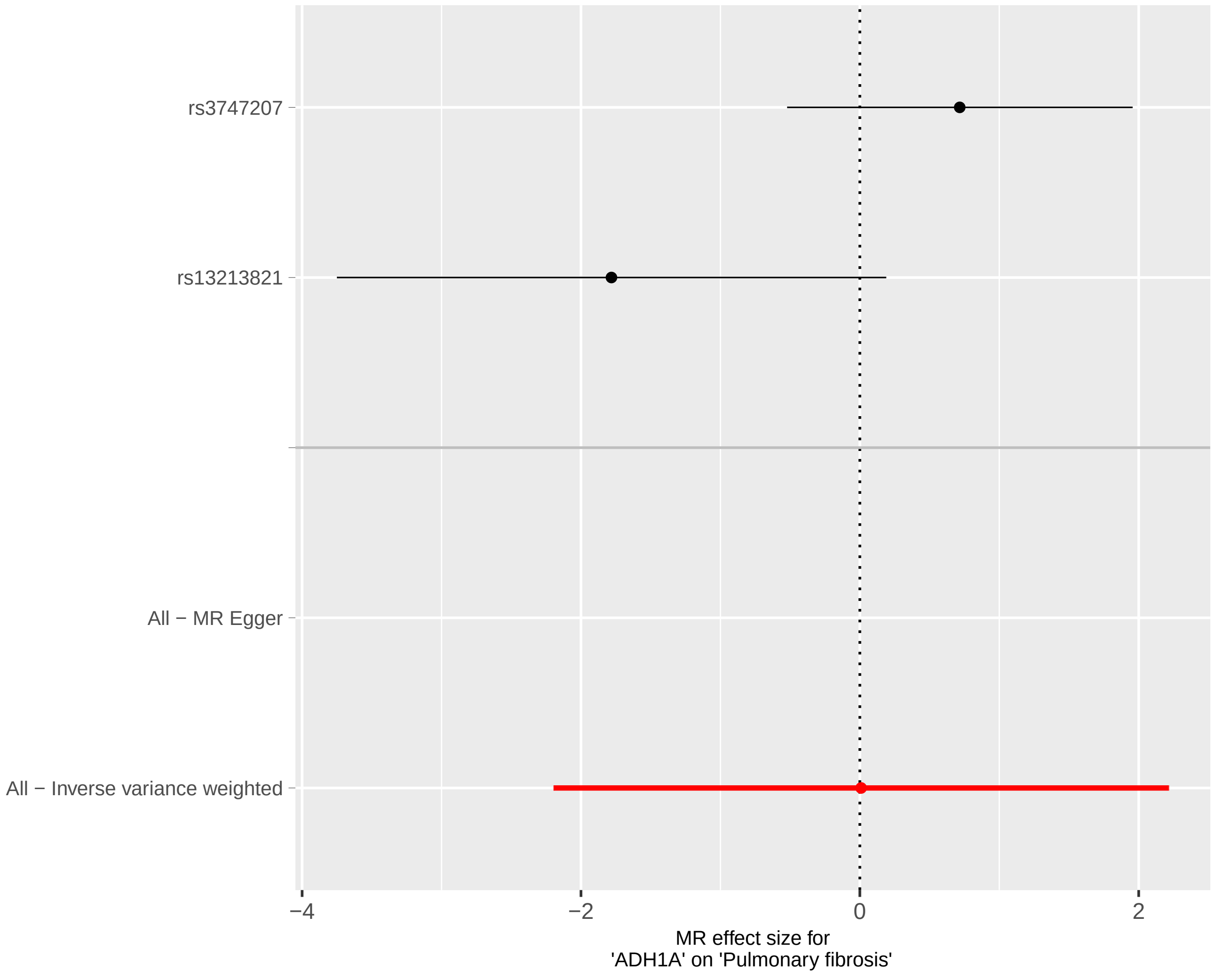
rs3747207
rs13213821
All − MR Egger
All − Inverse variance weighted
−4
−2
0
2
MR effect size for
'ADH1A' on 'Pulmonary fibrosis'

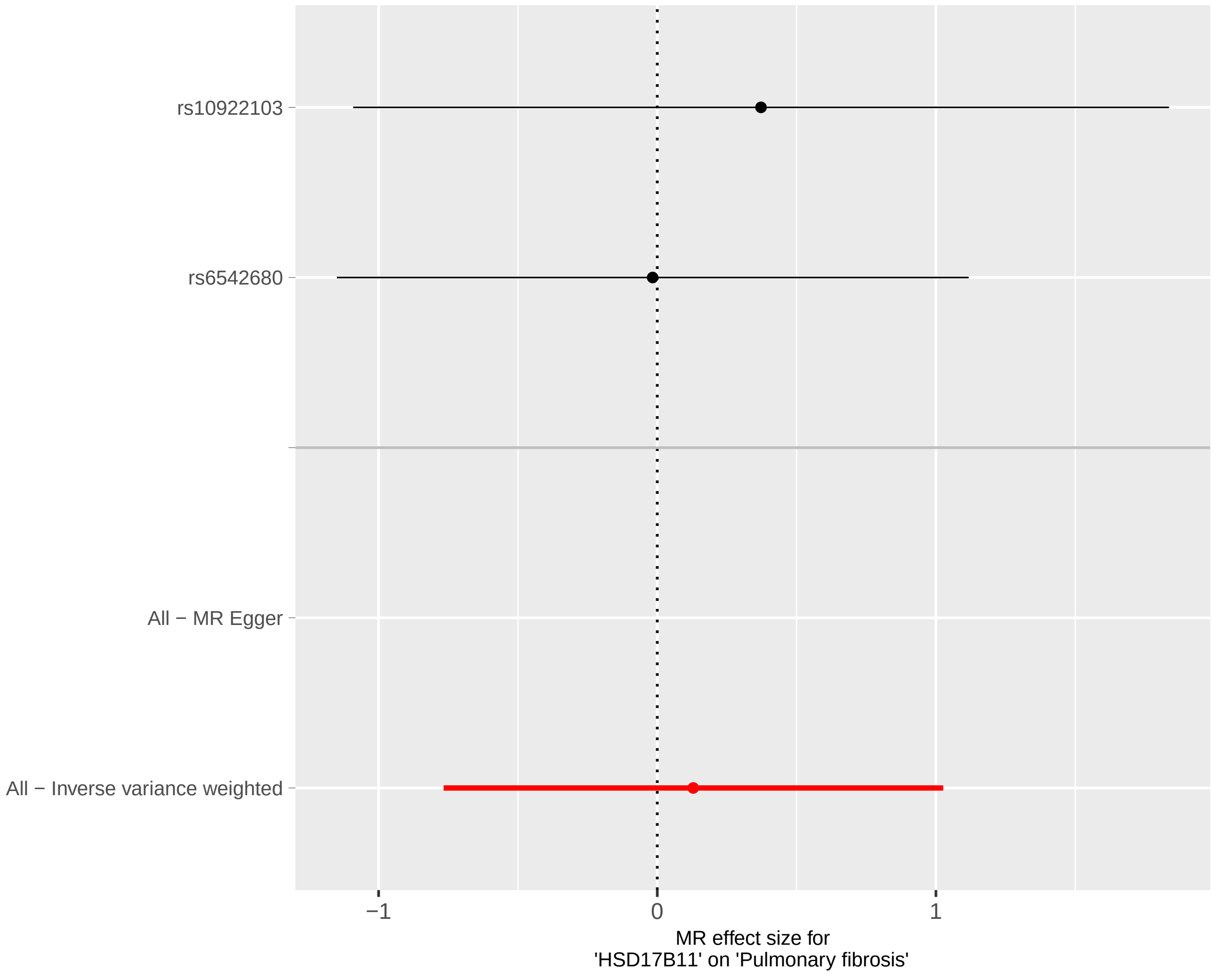

rs10922103
rs6542680
All − MR Egger
All − Inverse variance weighted
−1
0
1
MR effect size for
'HSD17B11' on 'Pulmonary fibrosis'

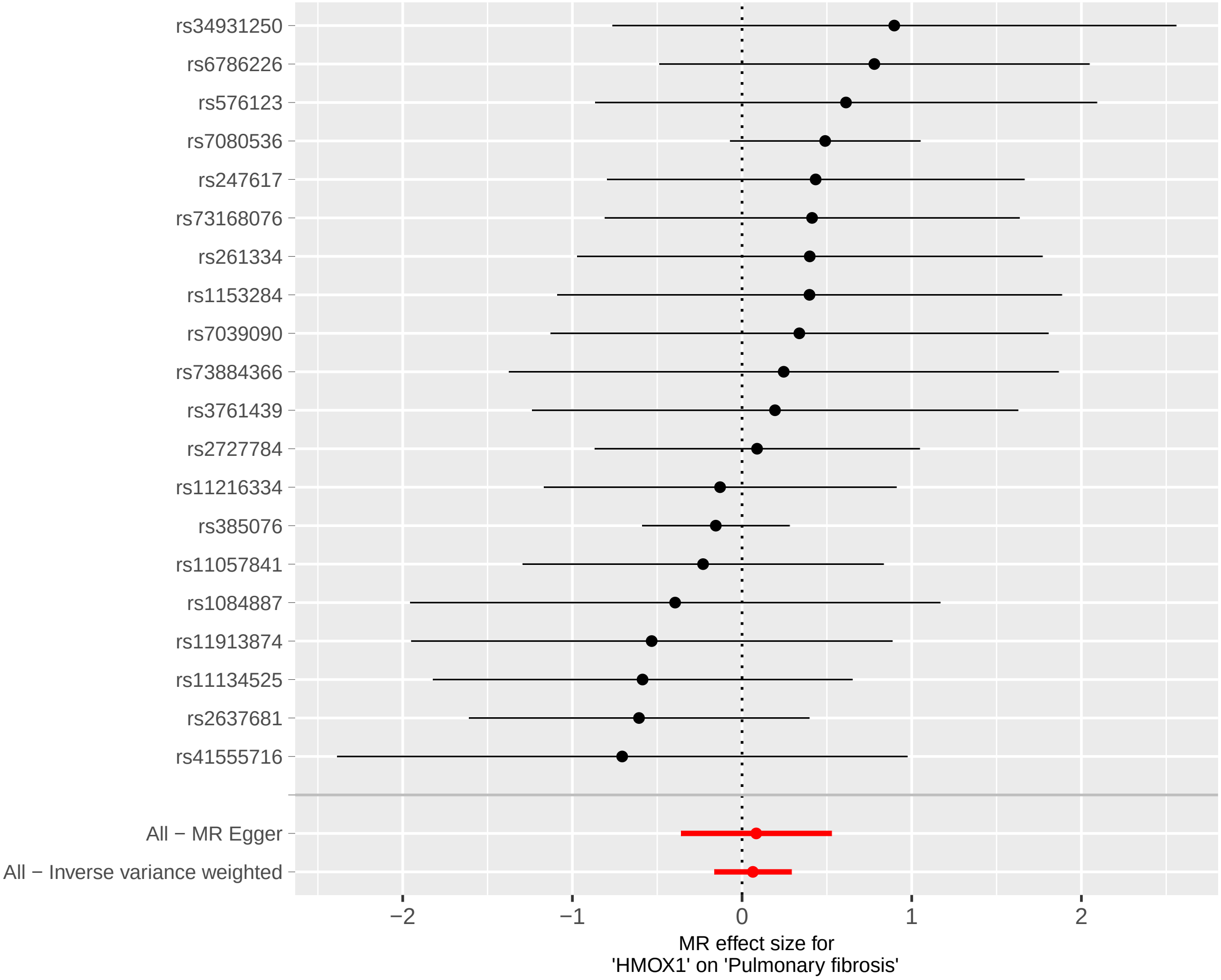
rs34931250
rs6786226
rs576123
rs7080536
rs247617
rs73168076
rs261334
rs1153284
rs7039090
rs73884366
rs3761439
rs2727784
rs11216334
rs385076
rs11057841
rs1084887
rs11913874
rs11134525
rs2637681
rs41555716
All − MR Egger
All − Inverse variance weighted
−2
−1
0
1
2
MR effect size for
'HMOX1' on 'Pulmonary fibrosis'

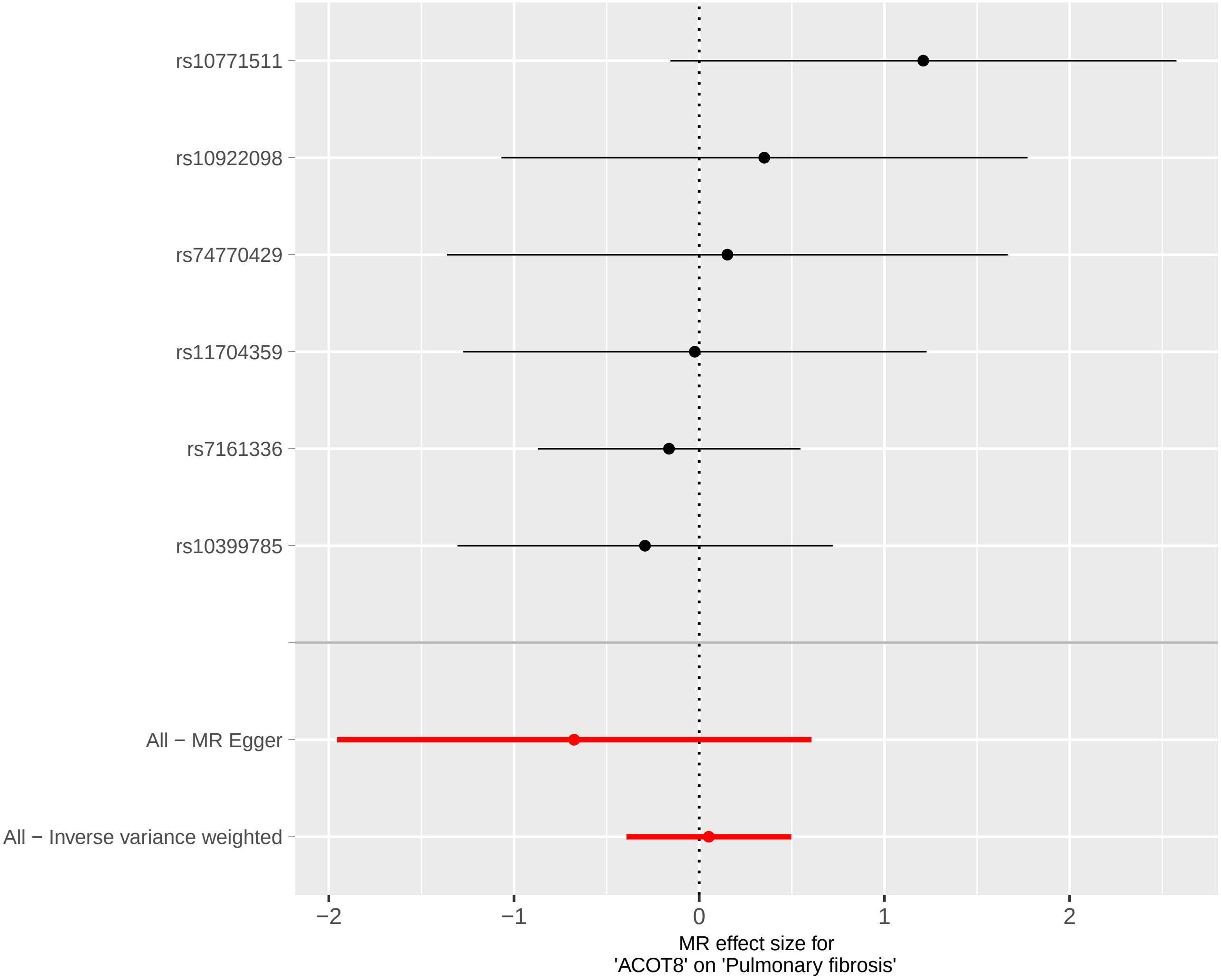

rs10771511
rs10922098
rs74770429
rs11704359
rs7161336
rs10399785
All − MR Egger
All − Inverse variance weighted
−2
−1
0
1
2
MR effect size for
'ACOT8' on 'Pulmonary fibrosis'

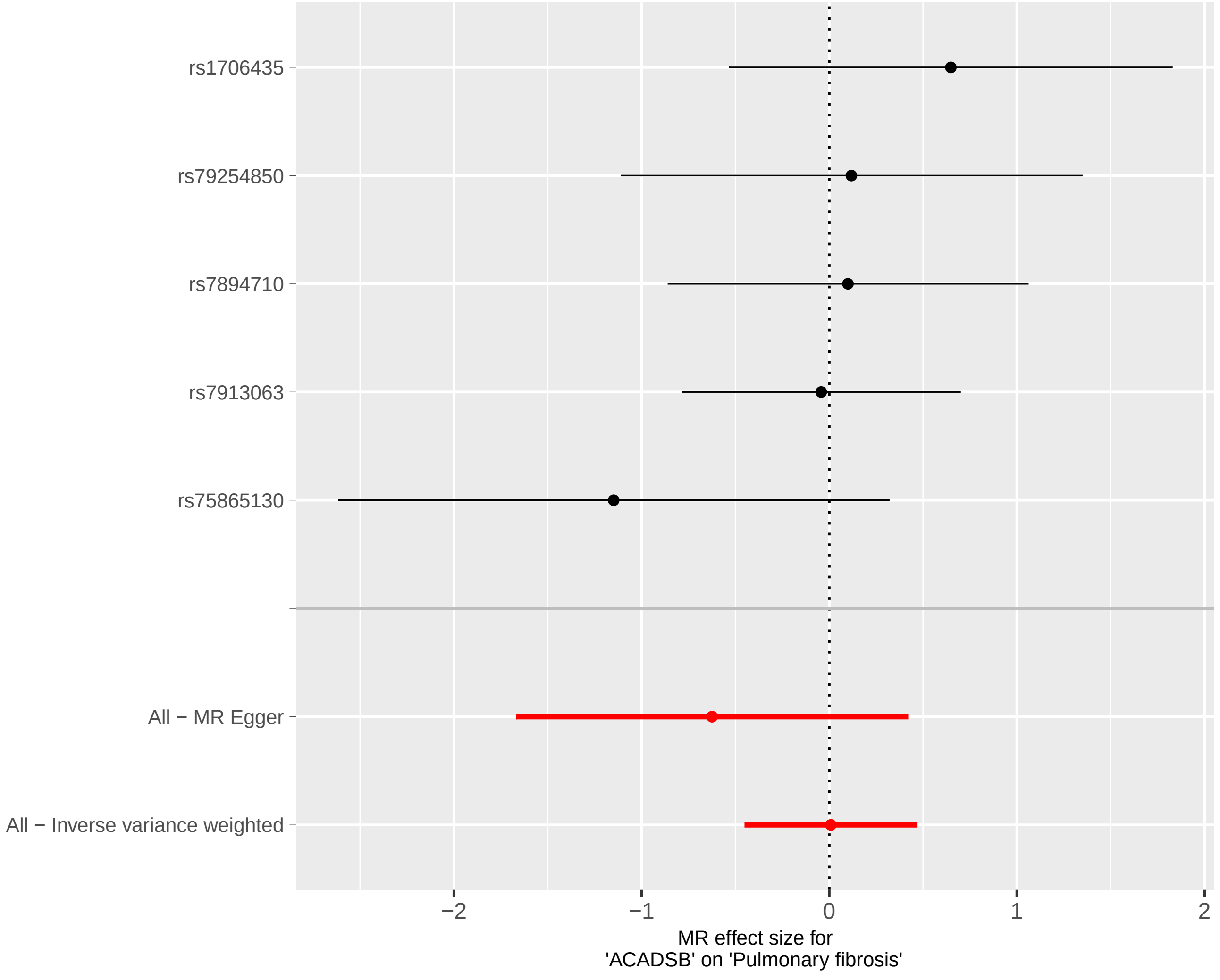
rs1706435
rs79254850
rs7894710
rs7913063
rs75865130
All − MR Egger
All − Inverse variance weighted
−2
−1
0
1
2
MR effect size for
'ACADSB' on 'Pulmonary fibrosis'

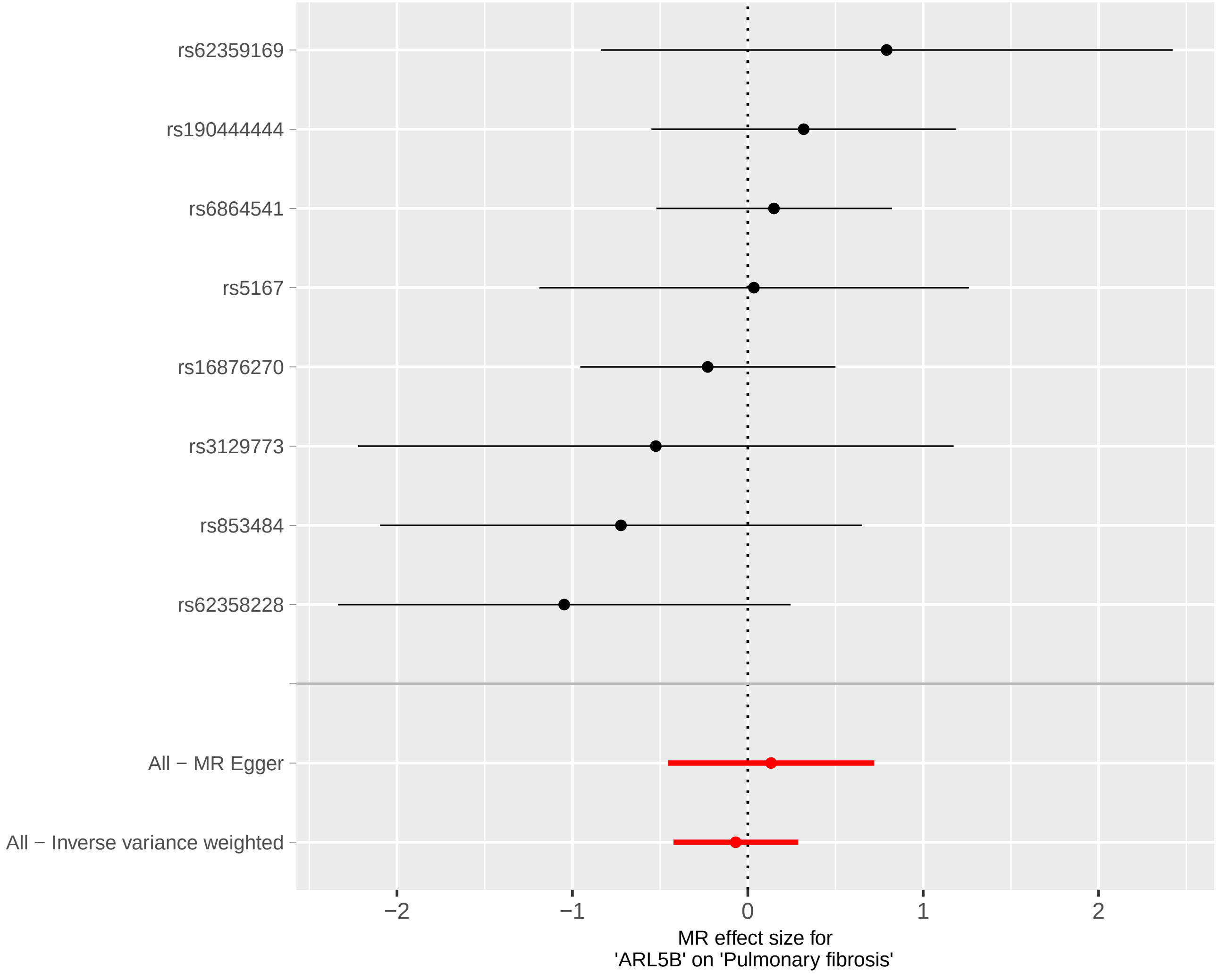

rs62359169
rs190444444
rs6864541
rs5167
rs16876270
rs3129773
rs853484
rs62358228
All − MR Egger
All − Inverse variance weighted
−2
−1
0
1
2
MR effect size for
'ARL5B' on 'Pulmonary fibrosis'

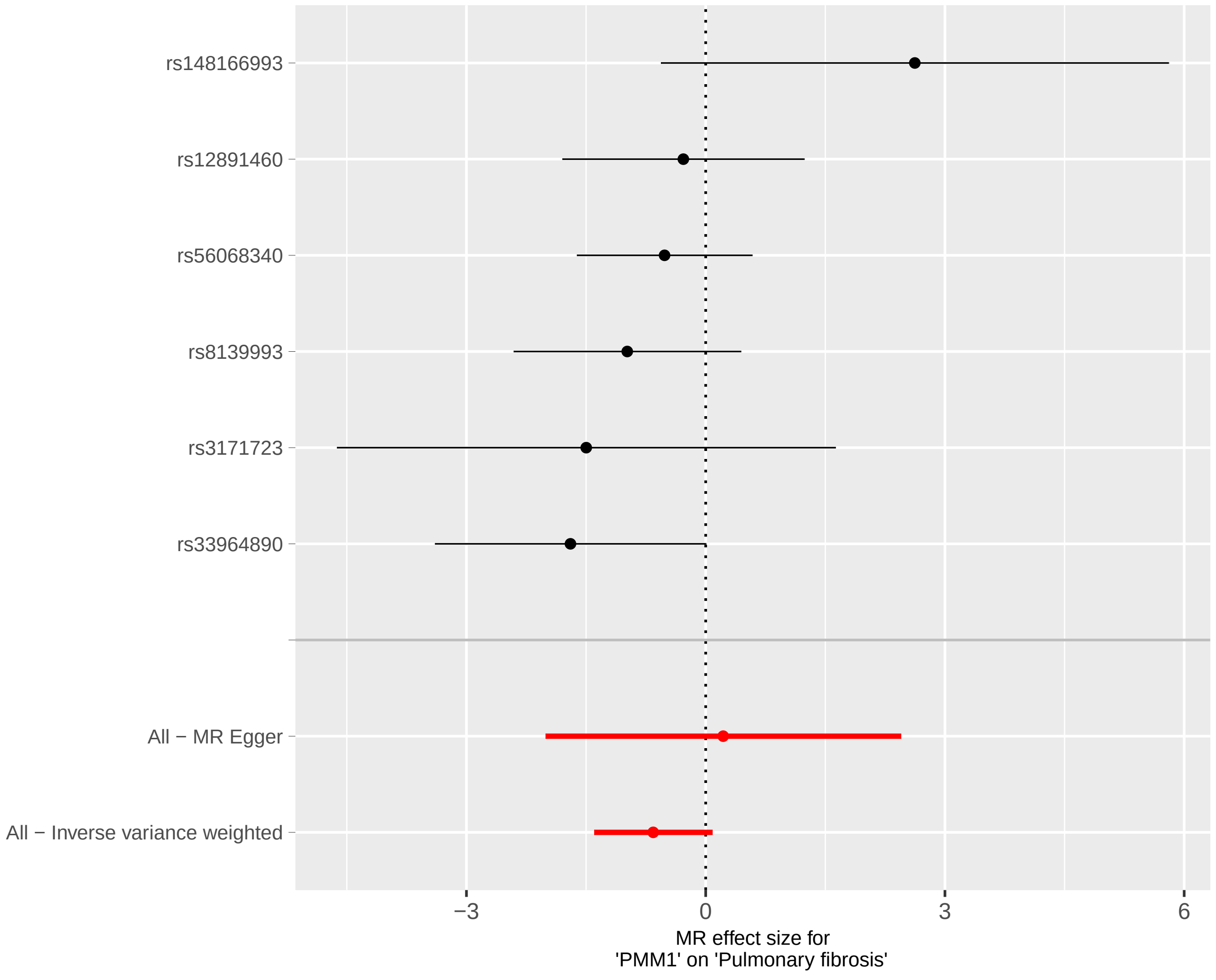
rs148166993
rs12891460
rs56068340
rs8139993
rs3171723
rs33964890
All − MR Egger
All − Inverse variance weighted
−3
0
3
6
MR effect size for
'PMM1' on 'Pulmonary fibrosis'

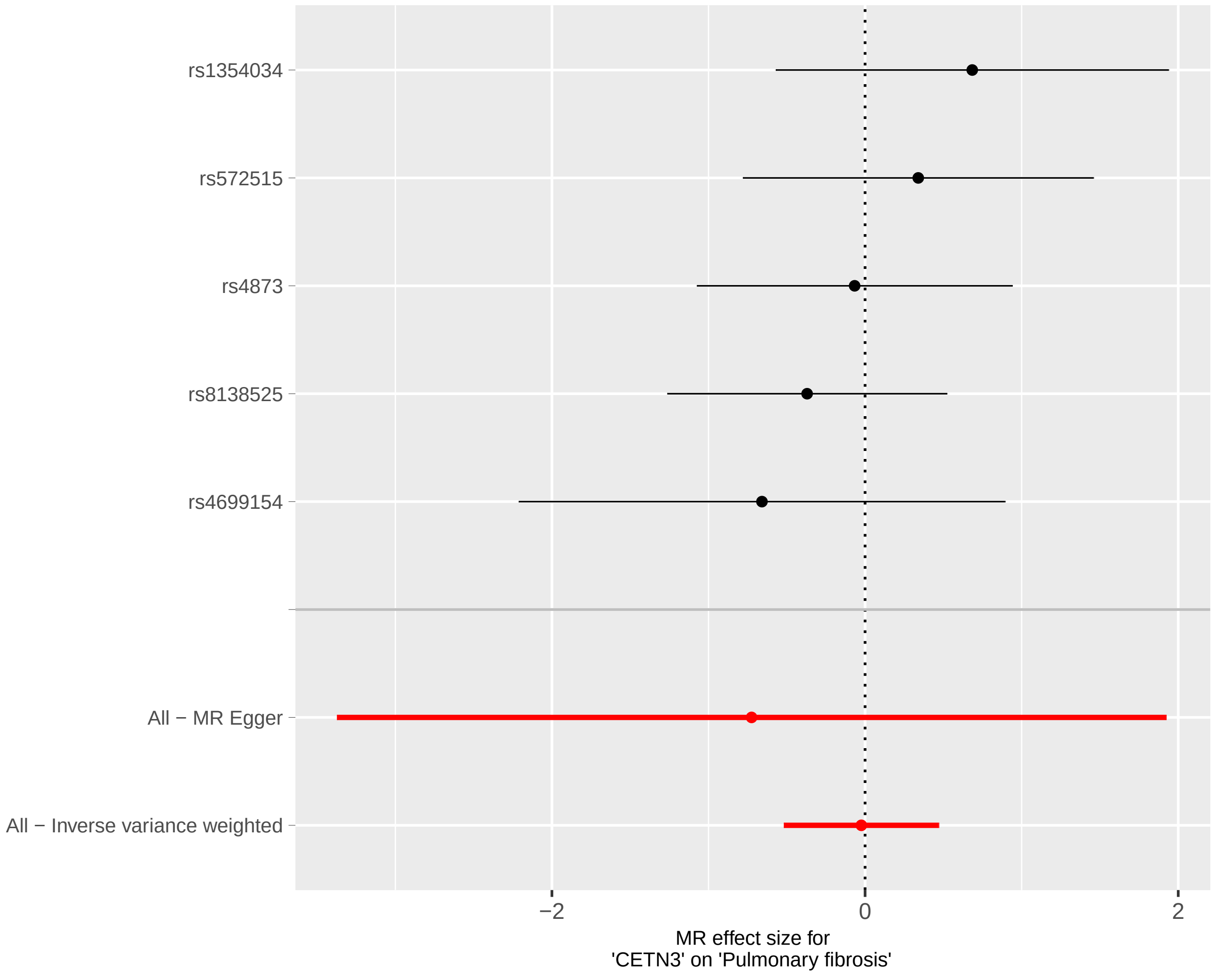

rs1354034
rs572515
rs4873
rs8138525
rs4699154
All − MR Egger
All − Inverse variance weighted
−2
0
2
MR effect size for
'CETN3' on 'Pulmonary fibrosis'

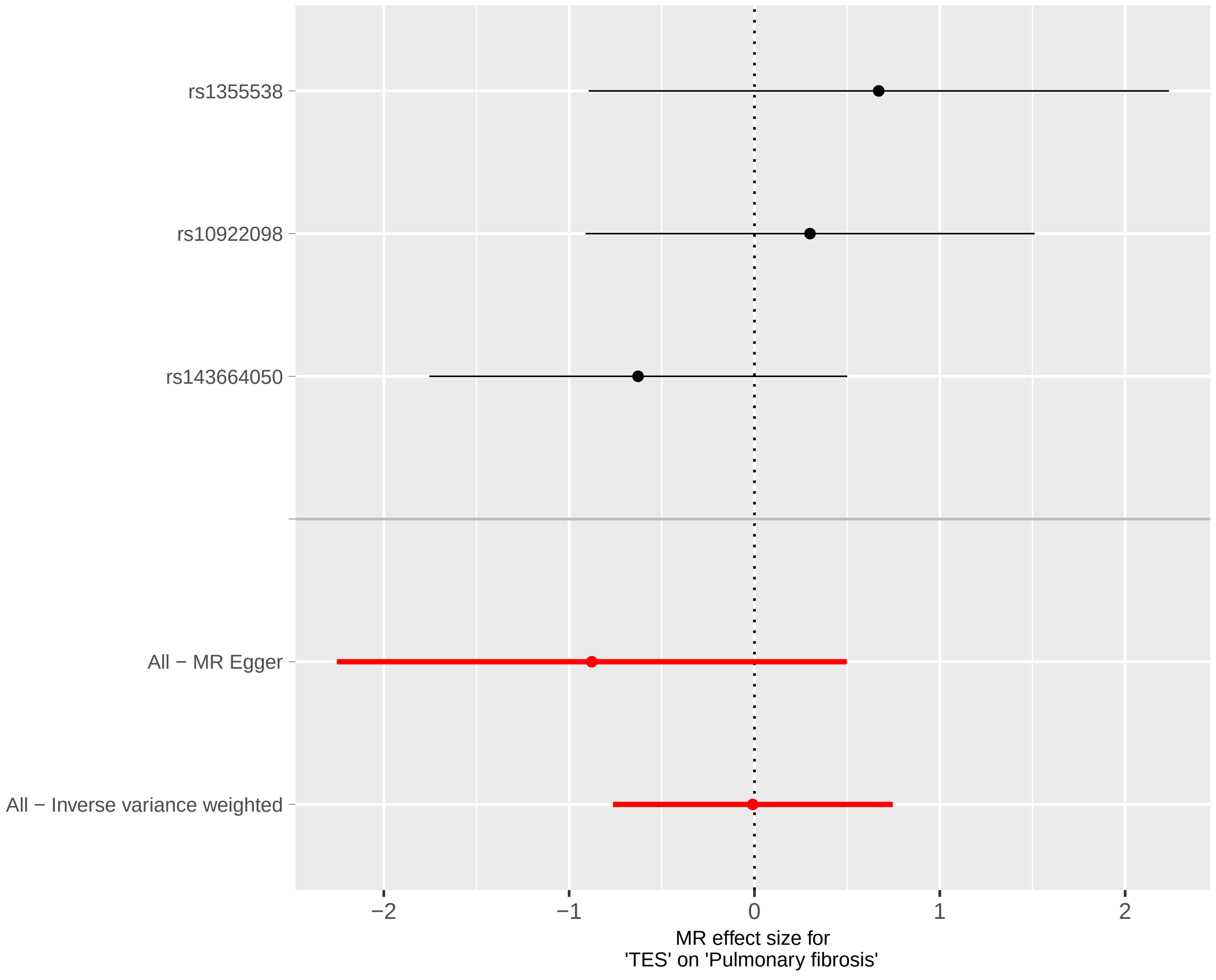

rs1355538
rs10922098
rs143664050
All − MR Egger
All − Inverse variance weighted
−2
−1
0
1
2
MR effect size for
'TES' on 'Pulmonary fibrosis'

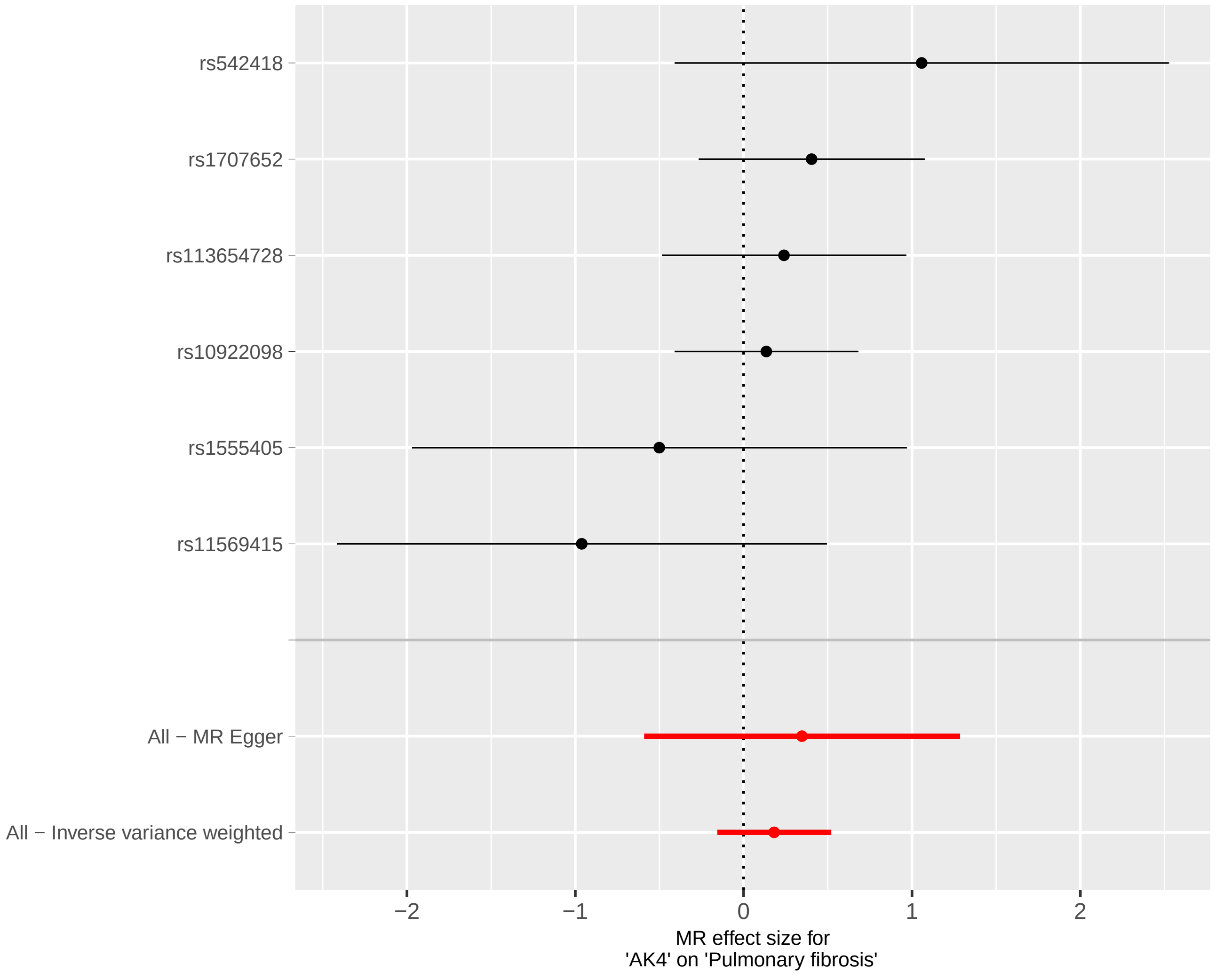

rs542418
rs1707652
rs113654728
rs10922098
rs1555405
rs11569415
All − MR Egger
All − Inverse variance weighted
−2
−1
0
1
2
MR effect size for
'AK4' on 'Pulmonary fibrosis'

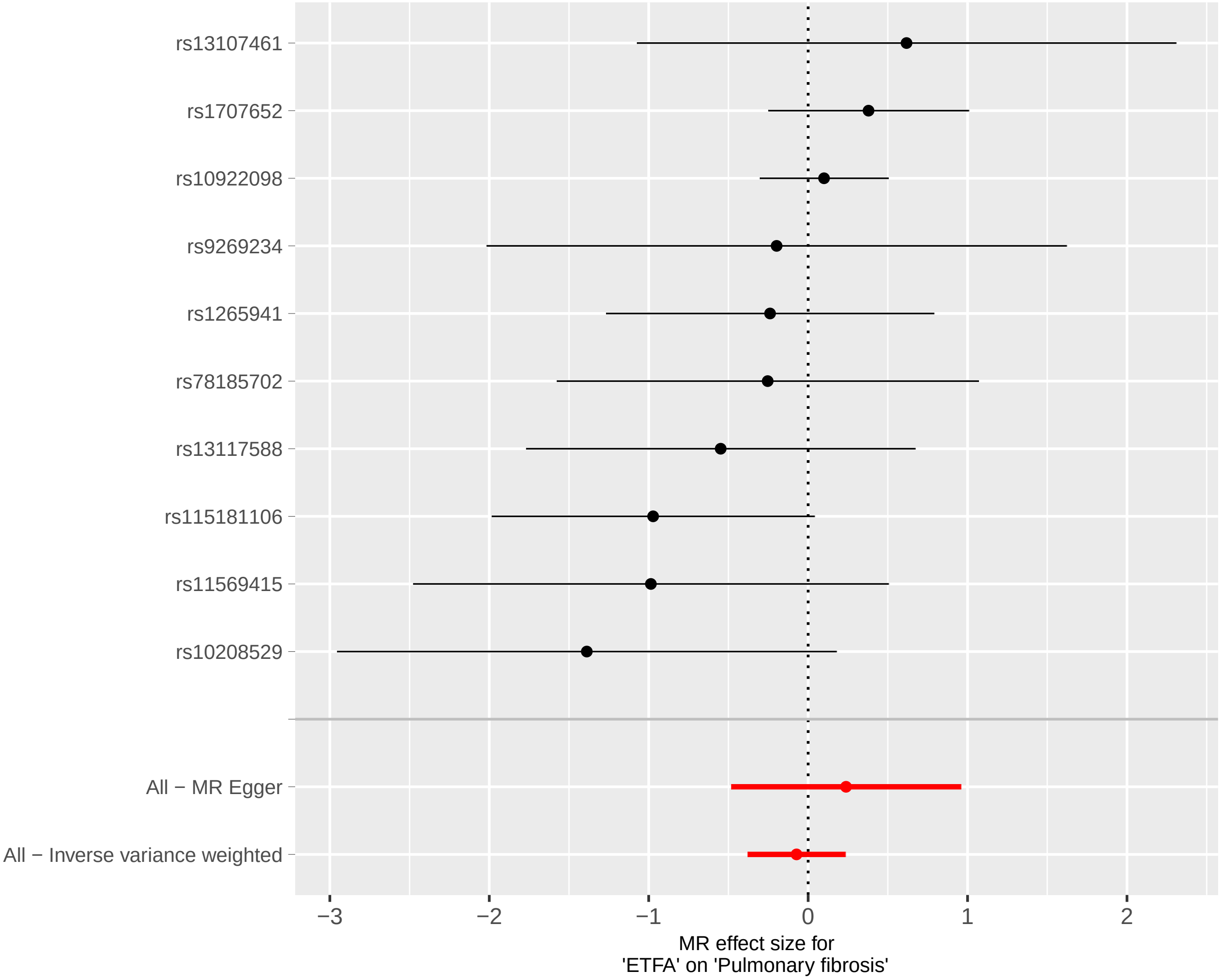

rs13107461
rs1707652
rs10922098
rs9269234
rs1265941
rs78185702
rs13117588
rs115181106
rs11569415
rs10208529
All − MR Egger
All − Inverse variance weighted
−3
−2
−1
0
1
2
MR effect size for
'ETFA' on 'Pulmonary fibrosis'

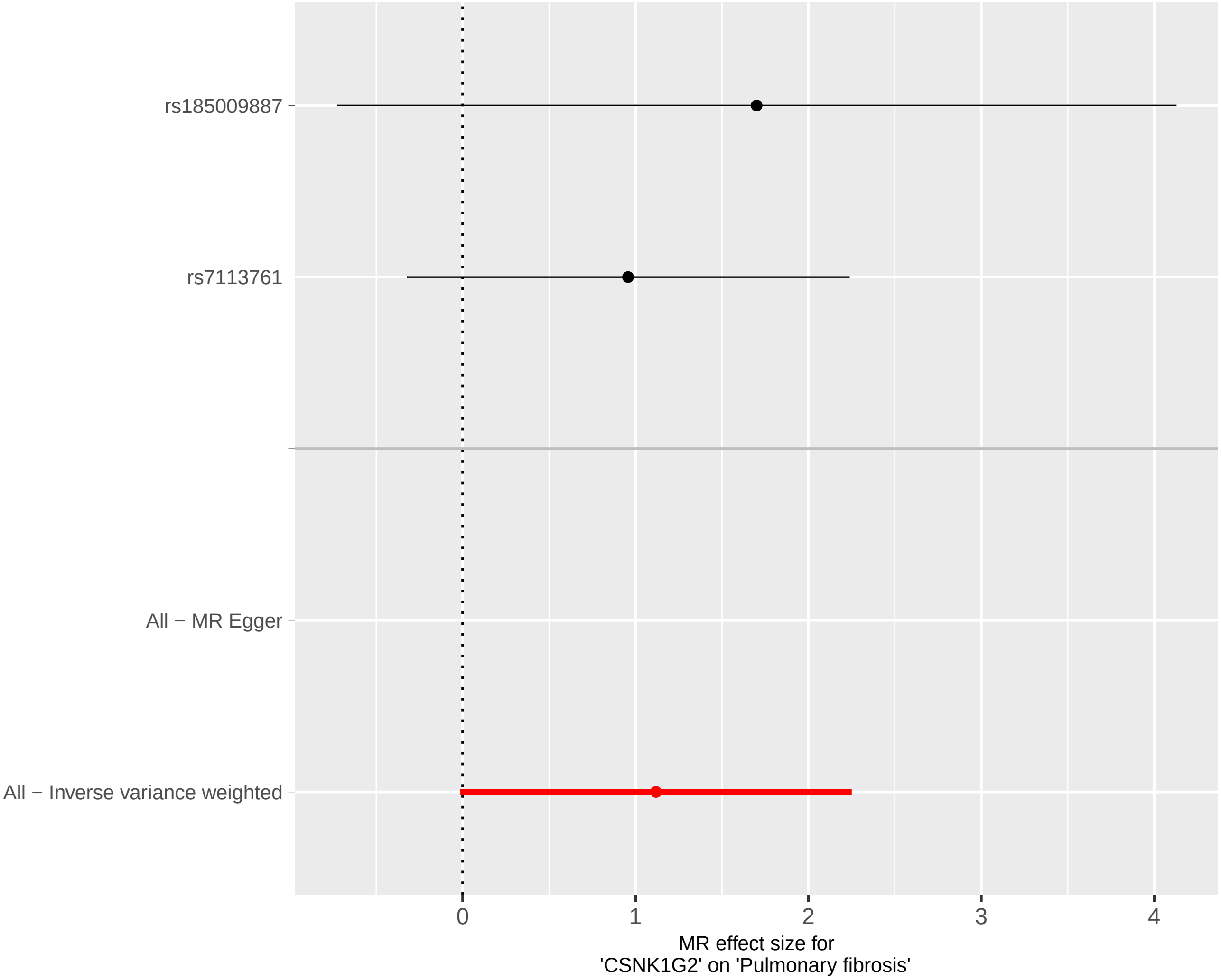
rs185009887
rs7113761
All − MR Egger
All − Inverse variance weighted
0
1
2
3
4
MR effect size for
'CSNK1G2' on 'Pulmonary fibrosis'

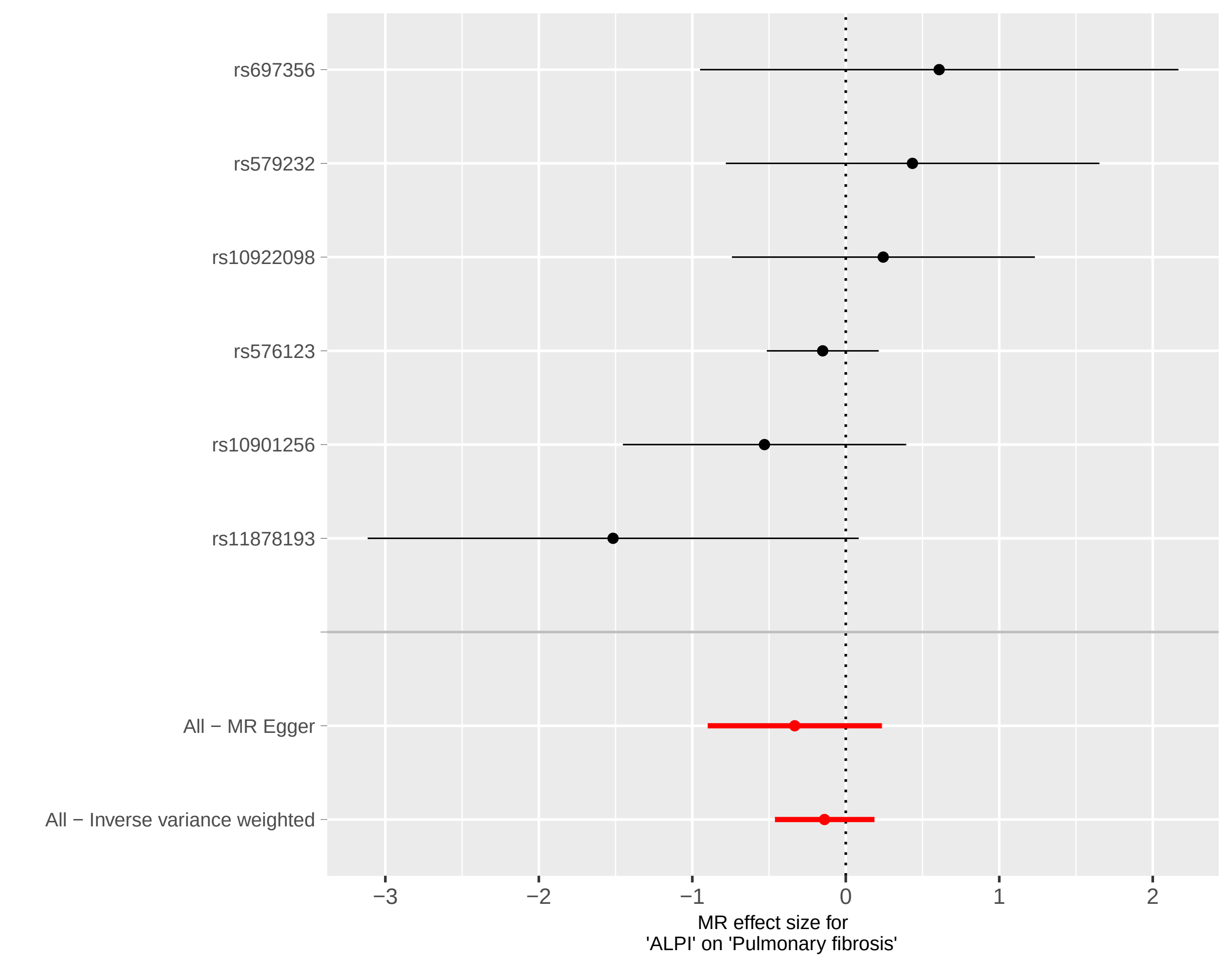

rs697356
rs579232
rs10922098
rs576123
rs10901256
rs11878193
All − MR Egger
All − Inverse variance weighted
−3
−2
−1
0
1
2
MR effect size for
'ALPI' on 'Pulmonary fibrosis'

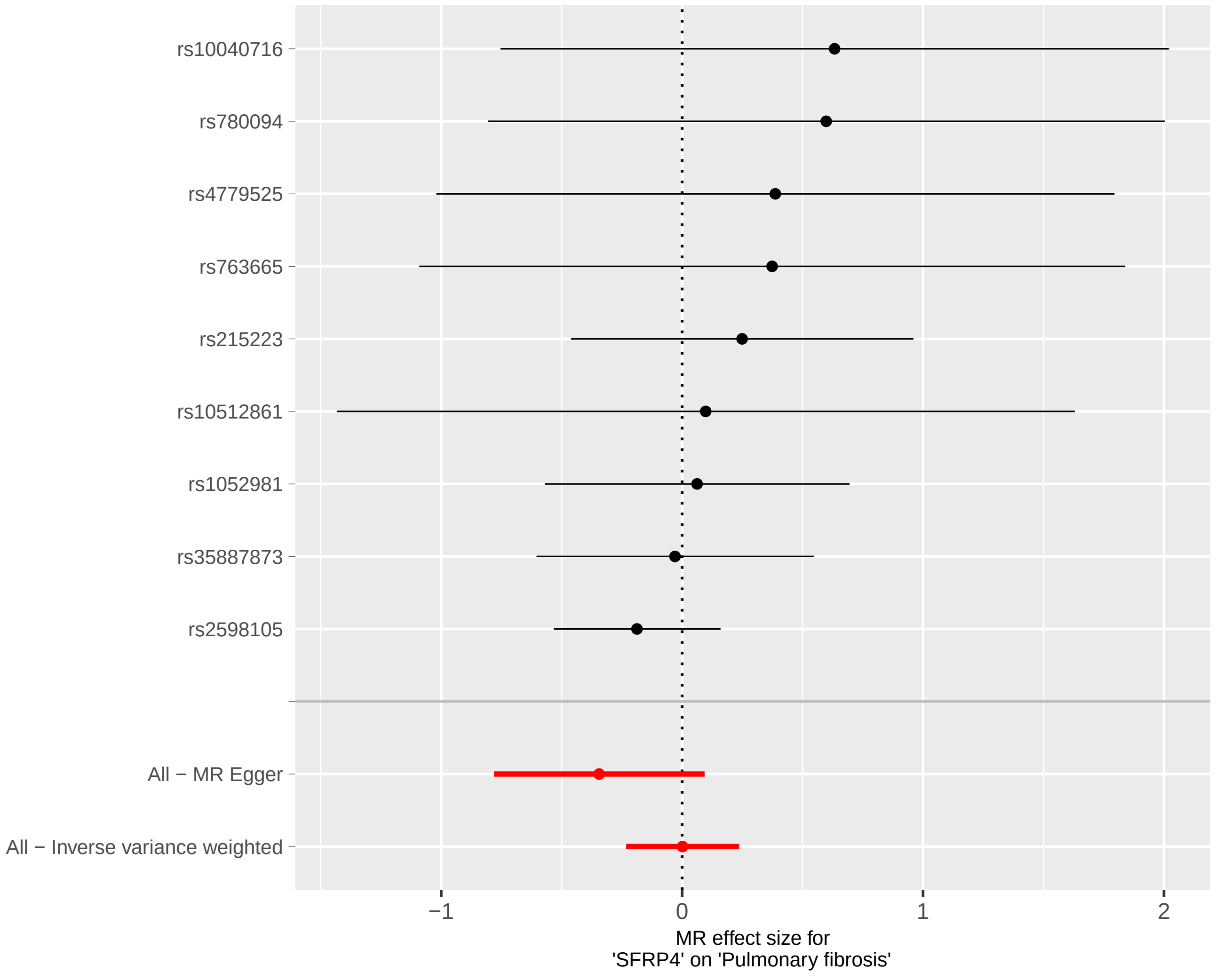

rs10040716
rs780094
rs4779525
rs763665
rs215223
rs10512861
rs1052981
rs35887873
rs2598105
All − MR Egger
All − Inverse variance weighted
−1
0
1
2
MR effect size for
'SFRP4' on 'Pulmonary fibrosis'

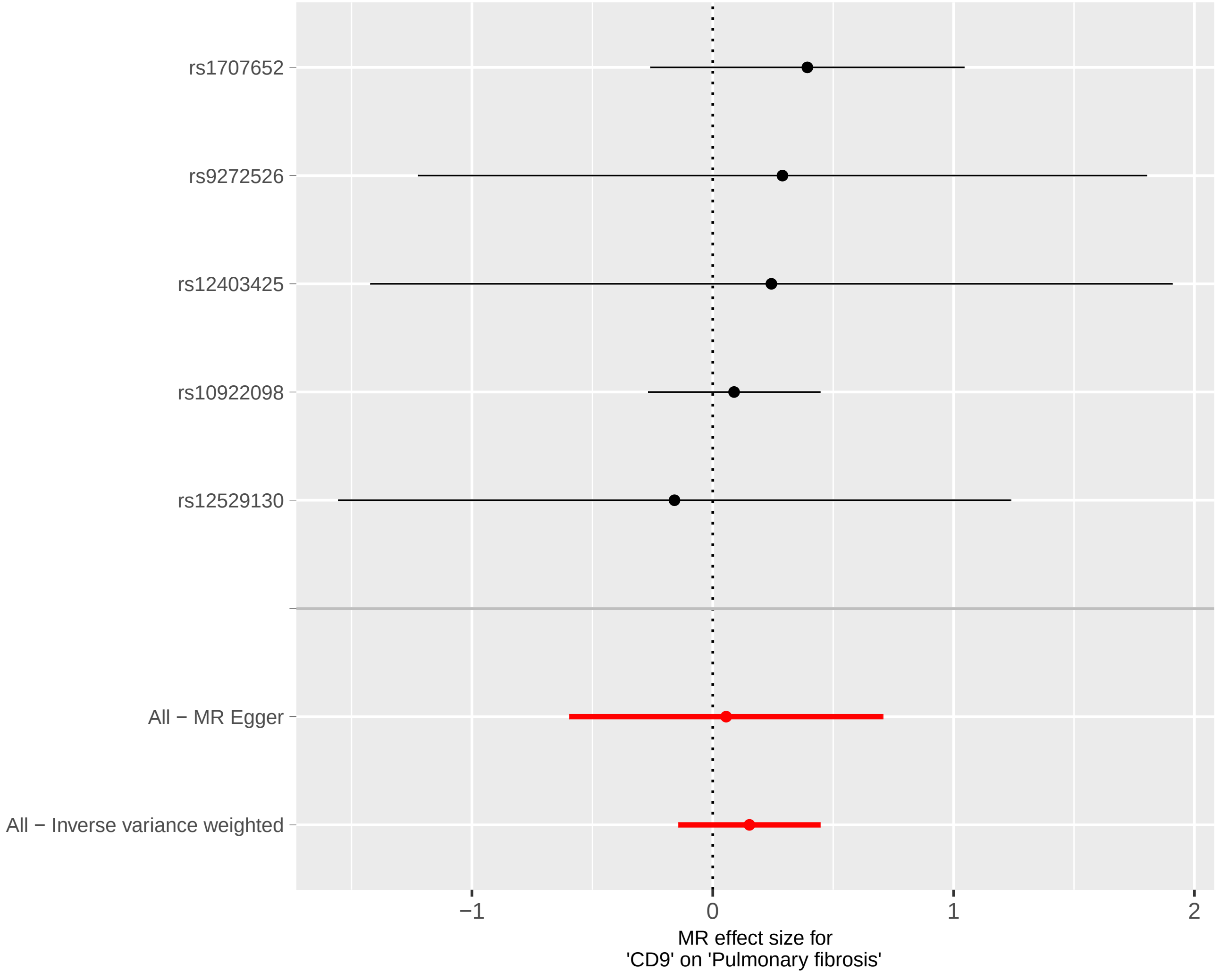
rs1707652
rs9272526
rs12403425
rs10922098
rs12529130
All − MR Egger
All − Inverse variance weighted
−1
0
1
2
MR effect size for
'CD9' on 'Pulmonary fibrosis'

Insufficient number of SNPs

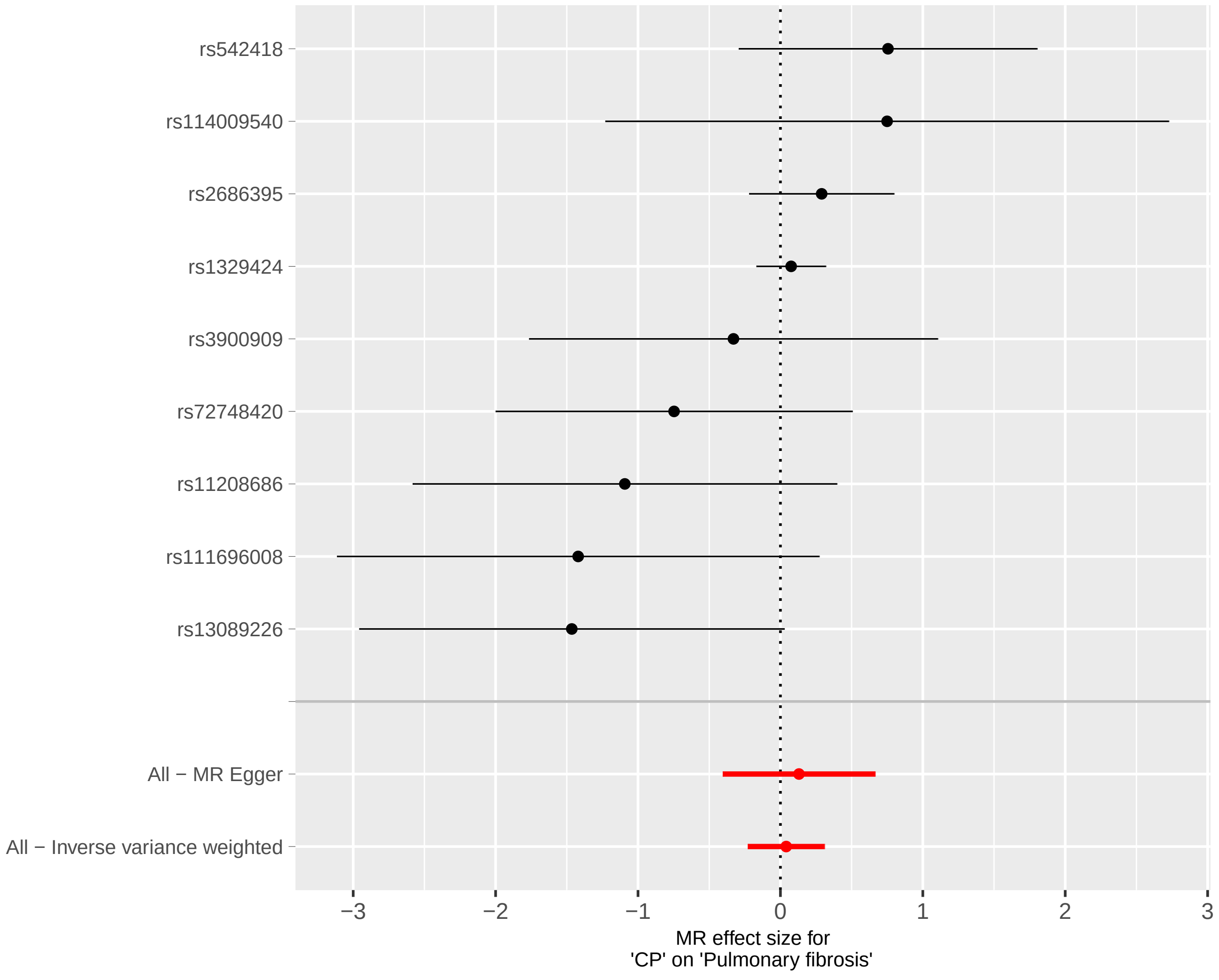

rs542418
rs114009540
rs2686395
rs1329424
rs3900909
rs72748420
rs11208686
rs111696008
rs13089226
All − MR Egger
All − Inverse variance weighted
−3
−2
−1
0
1
2
3
MR effect size for
'CP' on 'Pulmonary fibrosis'

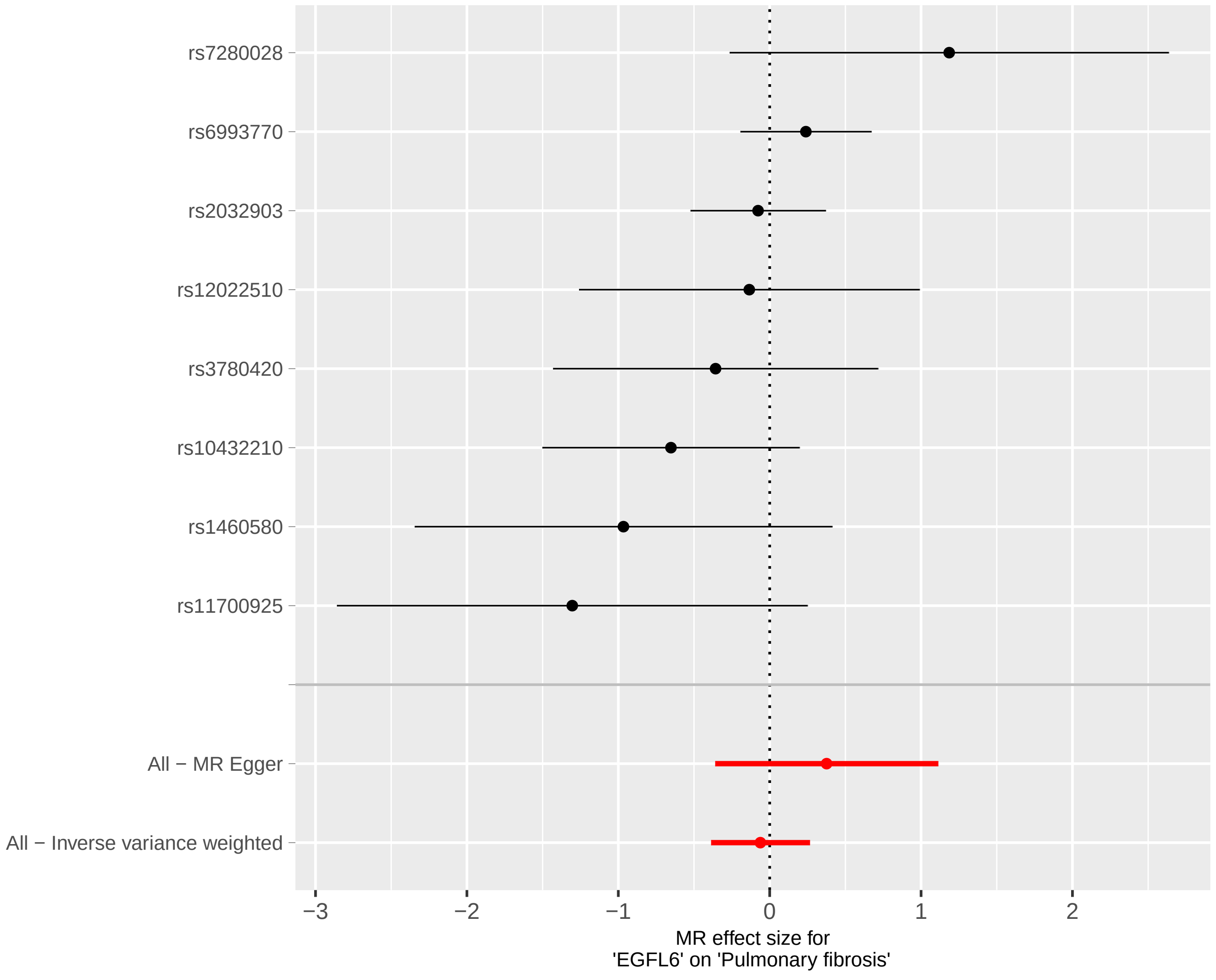
rs7280028
rs6993770
rs2032903
rs12022510
rs3780420
rs10432210
rs1460580
rs11700925
All − MR Egger
All − Inverse variance weighted
−3
−2
−1
0
1
2
MR effect size for
'EGFL6' on 'Pulmonary fibrosis'

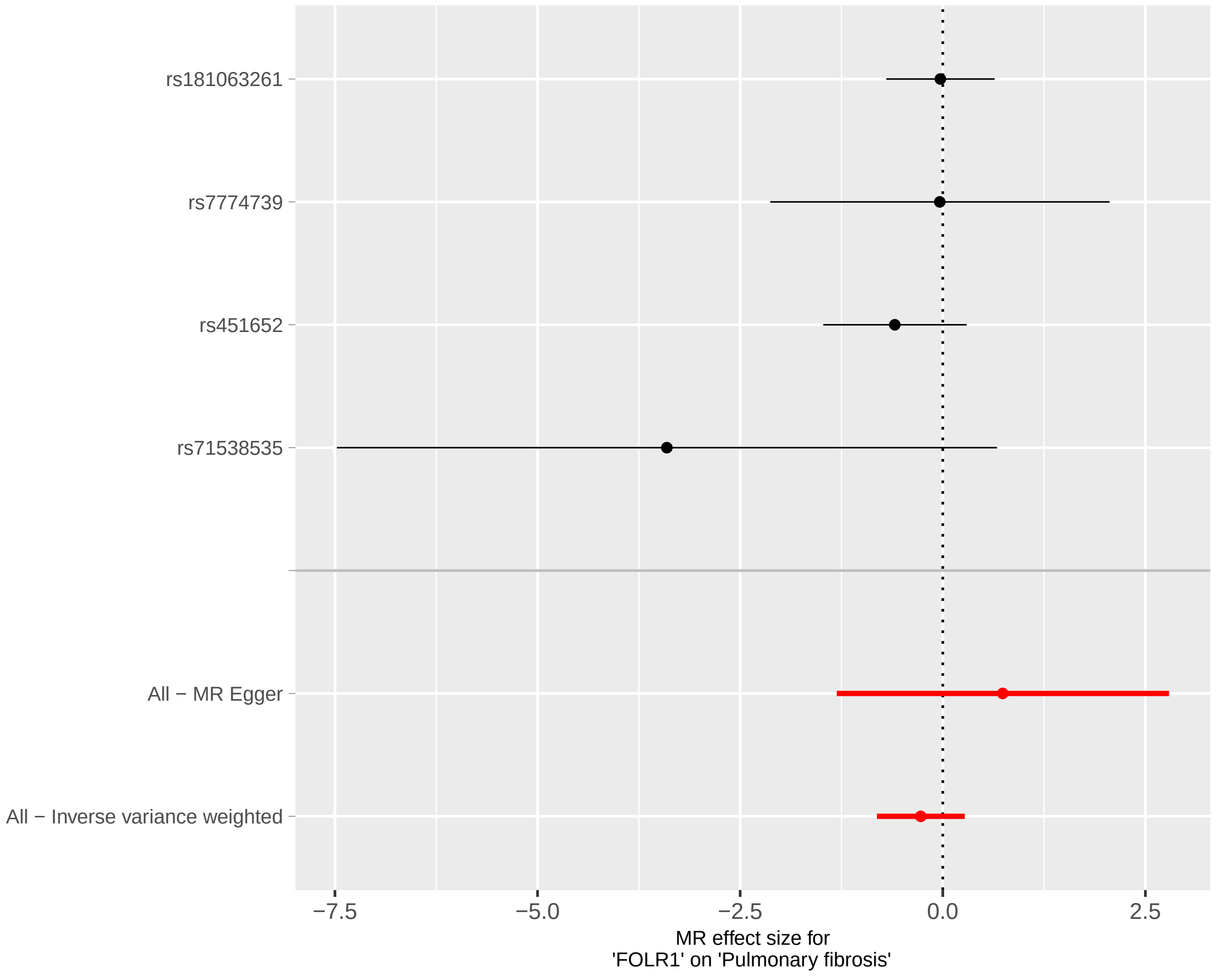
rs181063261
rs7774739
rs451652
rs71538535
All − MR Egger
All − Inverse variance weighted
−7.5
−5.0
−2.5
0.0
2.5
MR effect size for
'FOLR1' on 'Pulmonary fibrosis'

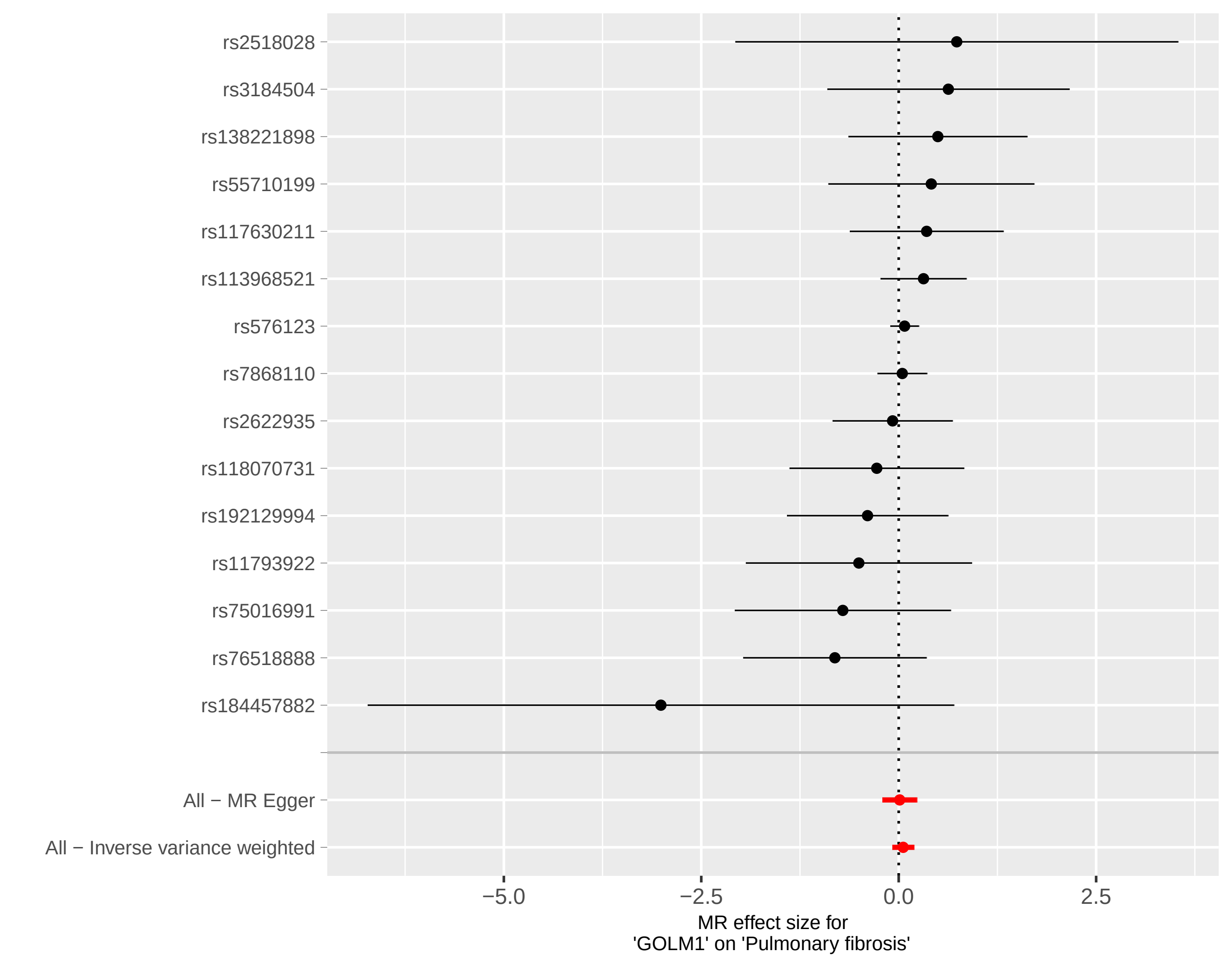
rs2518028
rs3184504
rs138221898
rs55710199
rs117630211
rs113968521
rs576123
rs7868110
rs2622935
rs118070731
rs192129994
rs11793922
rs75016991
rs76518888
rs184457882
All − MR Egger
All − Inverse variance weighted
−5.0
−2.5
0.0
2.5
MR effect size for
'GOLM1' on 'Pulmonary fibrosis'

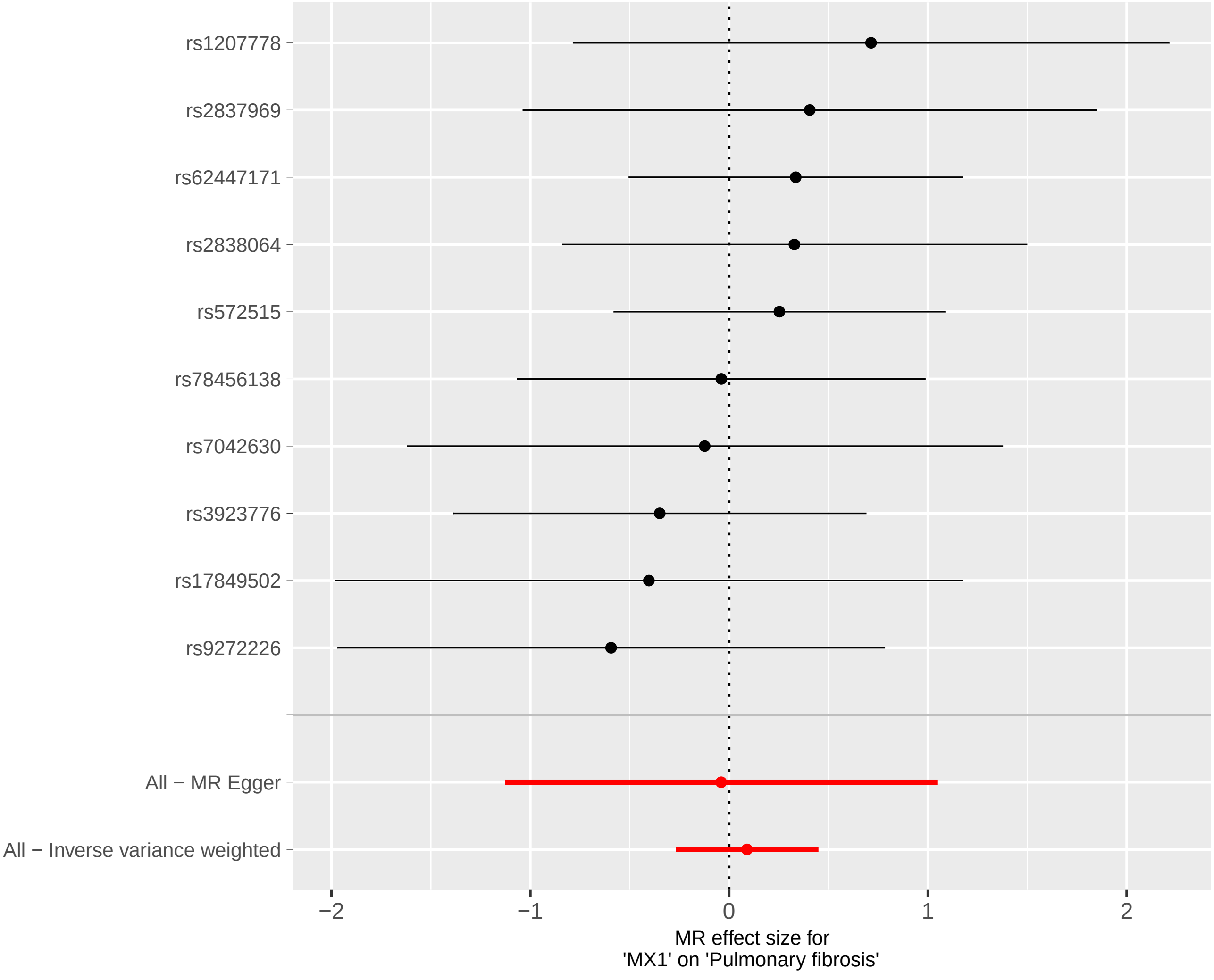
rs1207778
rs2837969
rs62447171
rs2838064
rs572515
rs78456138
rs7042630
rs3923776
rs17849502
rs9272226
All − MR Egger
All − Inverse variance weighted
−2
−1
0
1
2
MR effect size for
'MX1' on 'Pulmonary fibrosis'

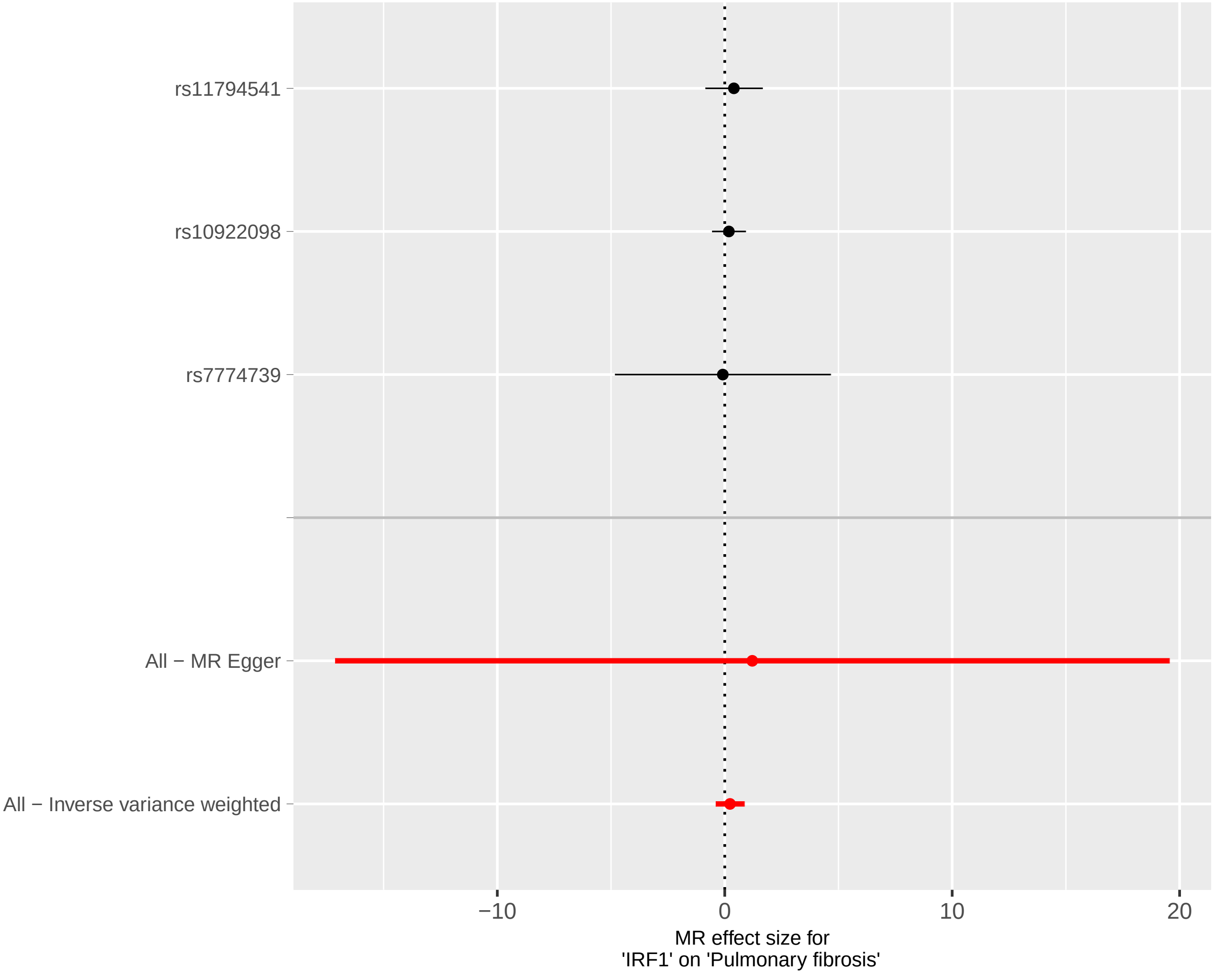

rs11794541
rs10922098
rs7774739
All − MR Egger
All − Inverse variance weighted
−10
0
10
20
MR effect size for
'IRF1' on 'Pulmonary fibrosis'

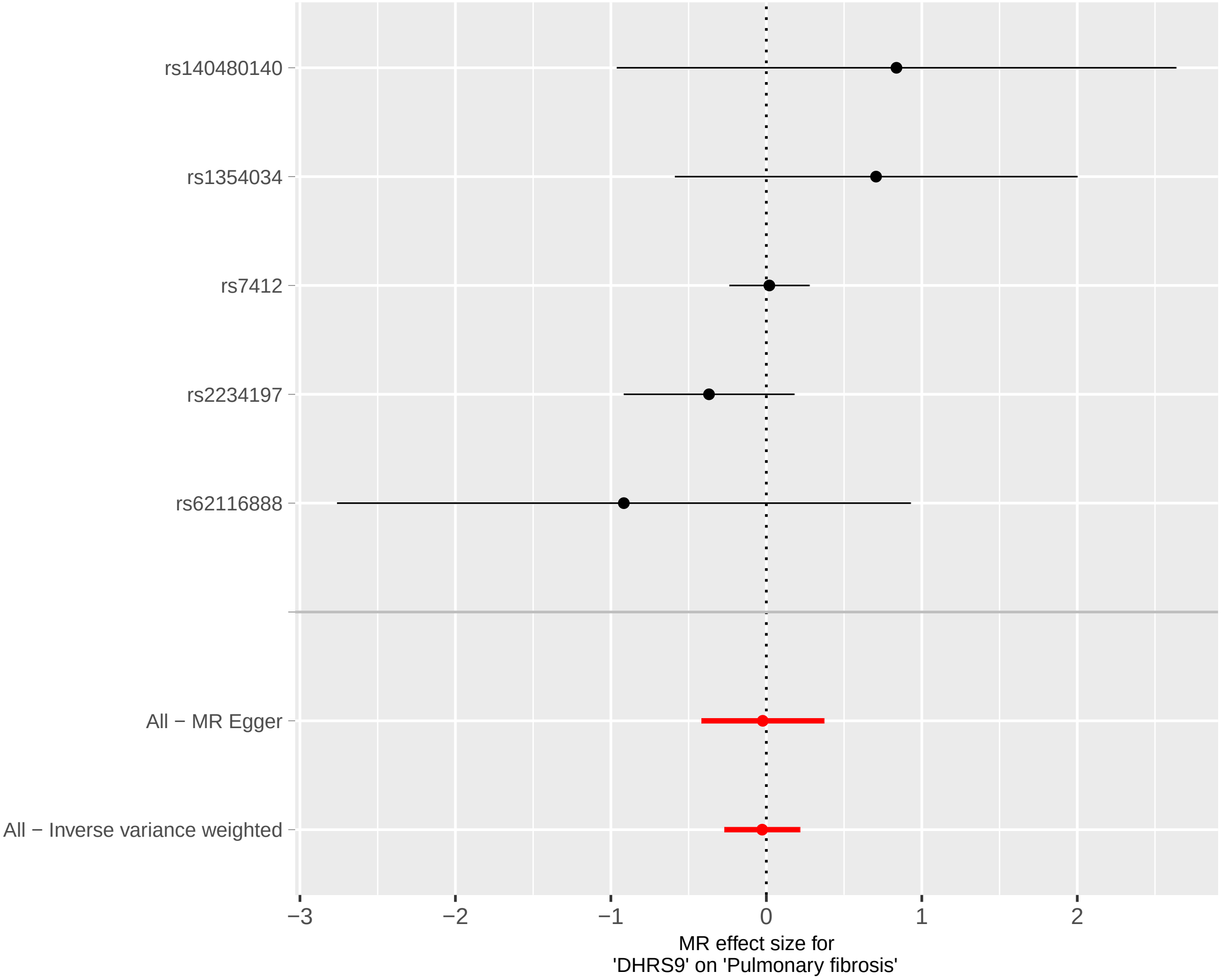

rs140480140
rs1354034
rs7412
rs2234197
rs62116888
All − MR Egger
All − Inverse variance weighted
−3
−2
−1
0
1
2
MR effect size for
'DHRS9' on 'Pulmonary fibrosis'

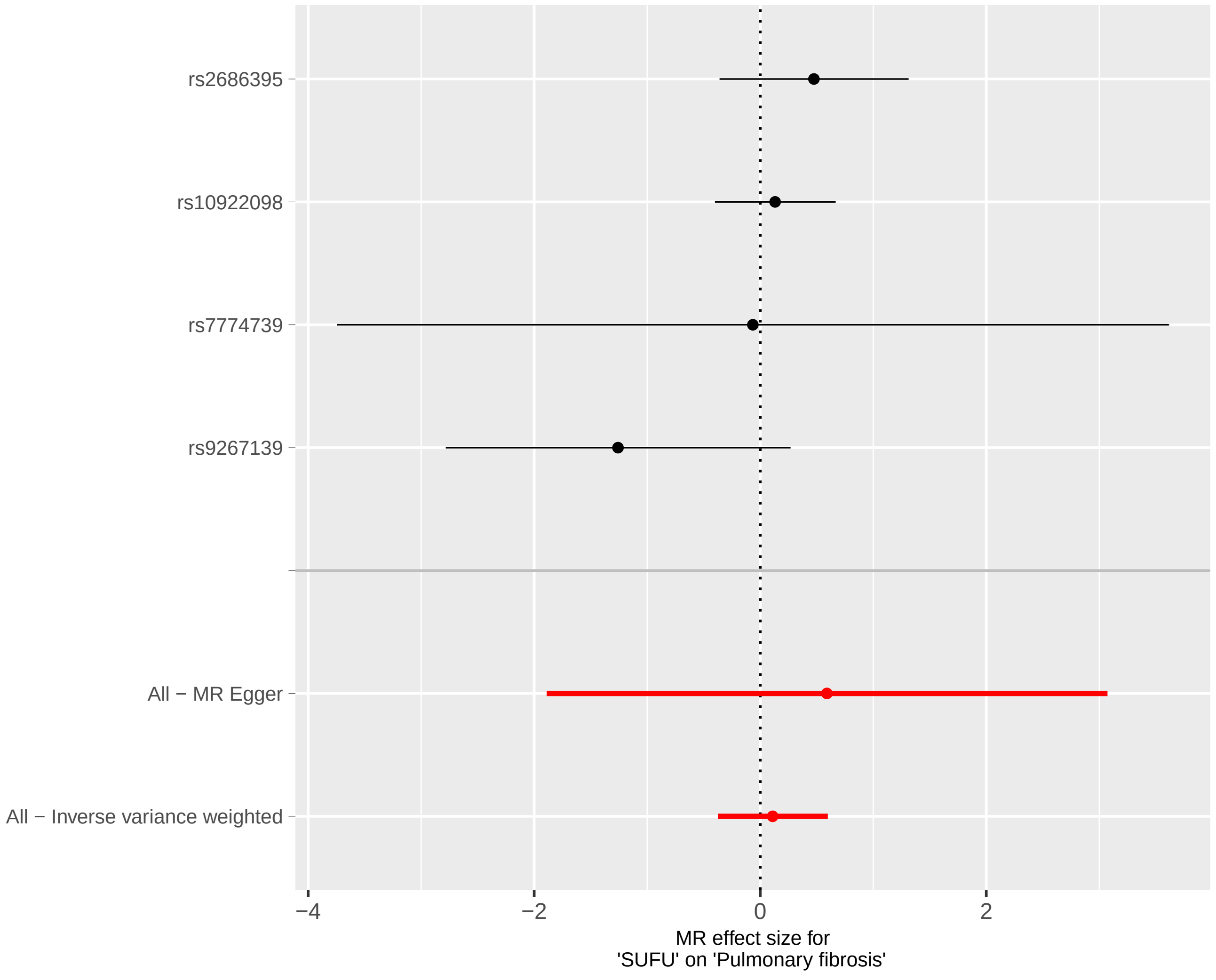

rs2686395
rs10922098
rs7774739
rs9267139
All − MR Egger
All − Inverse variance weighted
−4
−2
0
2
MR effect size for
'SUFU' on 'Pulmonary fibrosis'

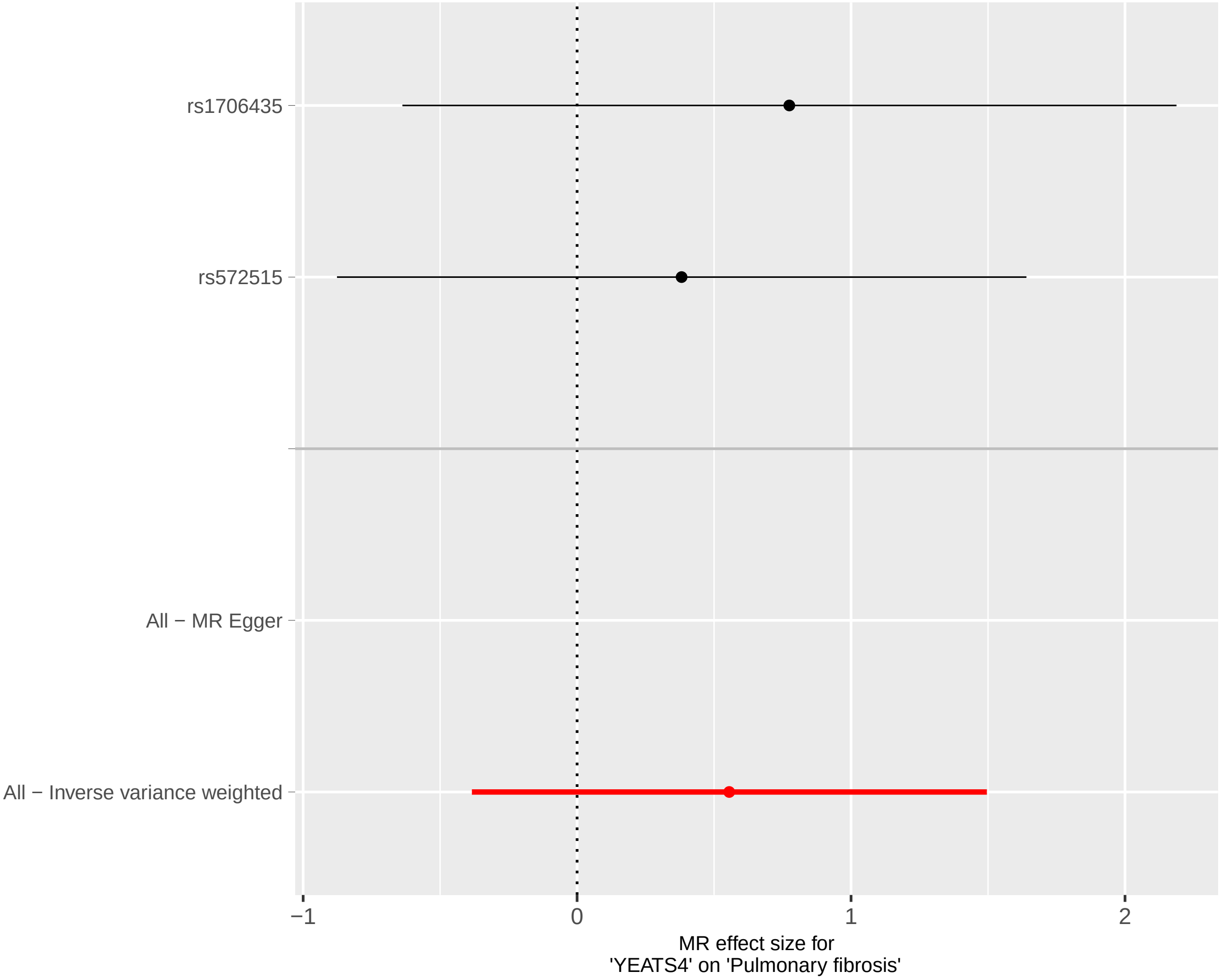

rs1706435
rs572515
All − MR Egger
All − Inverse variance weighted
−1
0
1
2
MR effect size for
'YEATS4' on 'Pulmonary fibrosis'

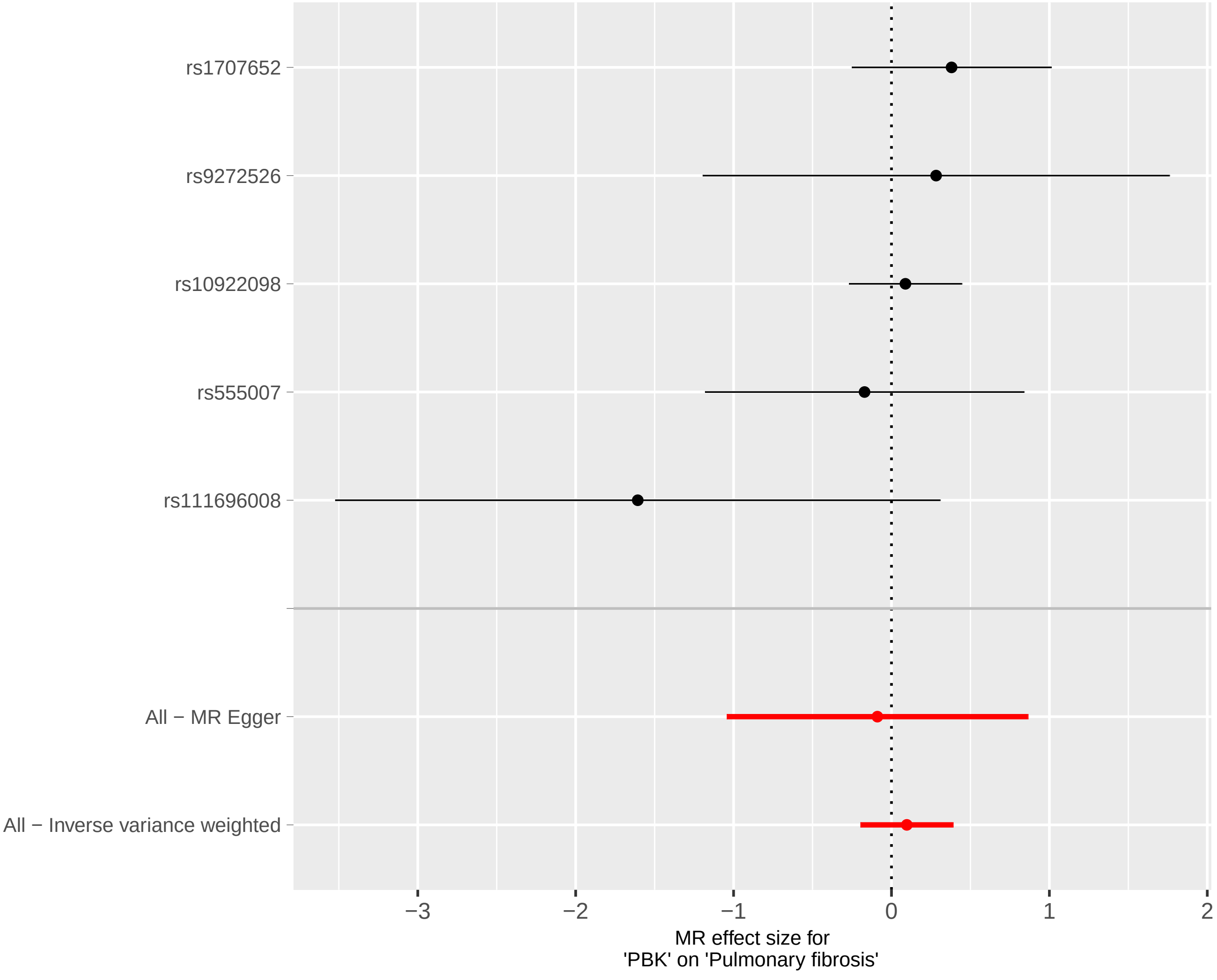

rs1707652
rs9272526
rs10922098
rs555007
rs111696008
All − MR Egger
All − Inverse variance weighted
−3
−2
−1
0
1
2
MR effect size for
'PBK' on 'Pulmonary fibrosis'

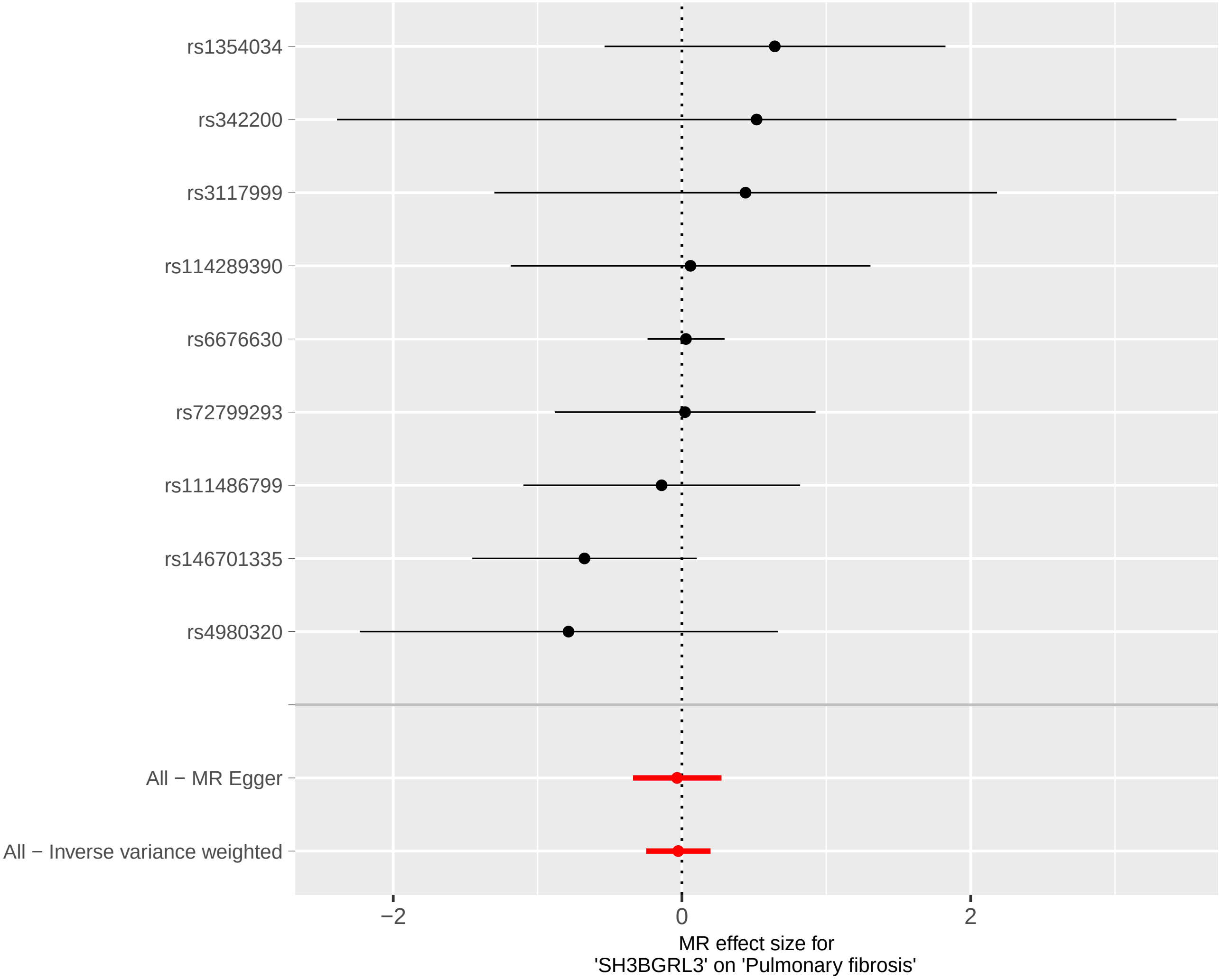

rs1354034
rs342200
rs3117999
rs114289390
rs6676630
rs72799293
rs111486799
rs146701335
rs4980320
All − MR Egger
All − Inverse variance weighted
−2
0
2
MR effect size for
'SH3BGRL3' on 'Pulmonary fibrosis'

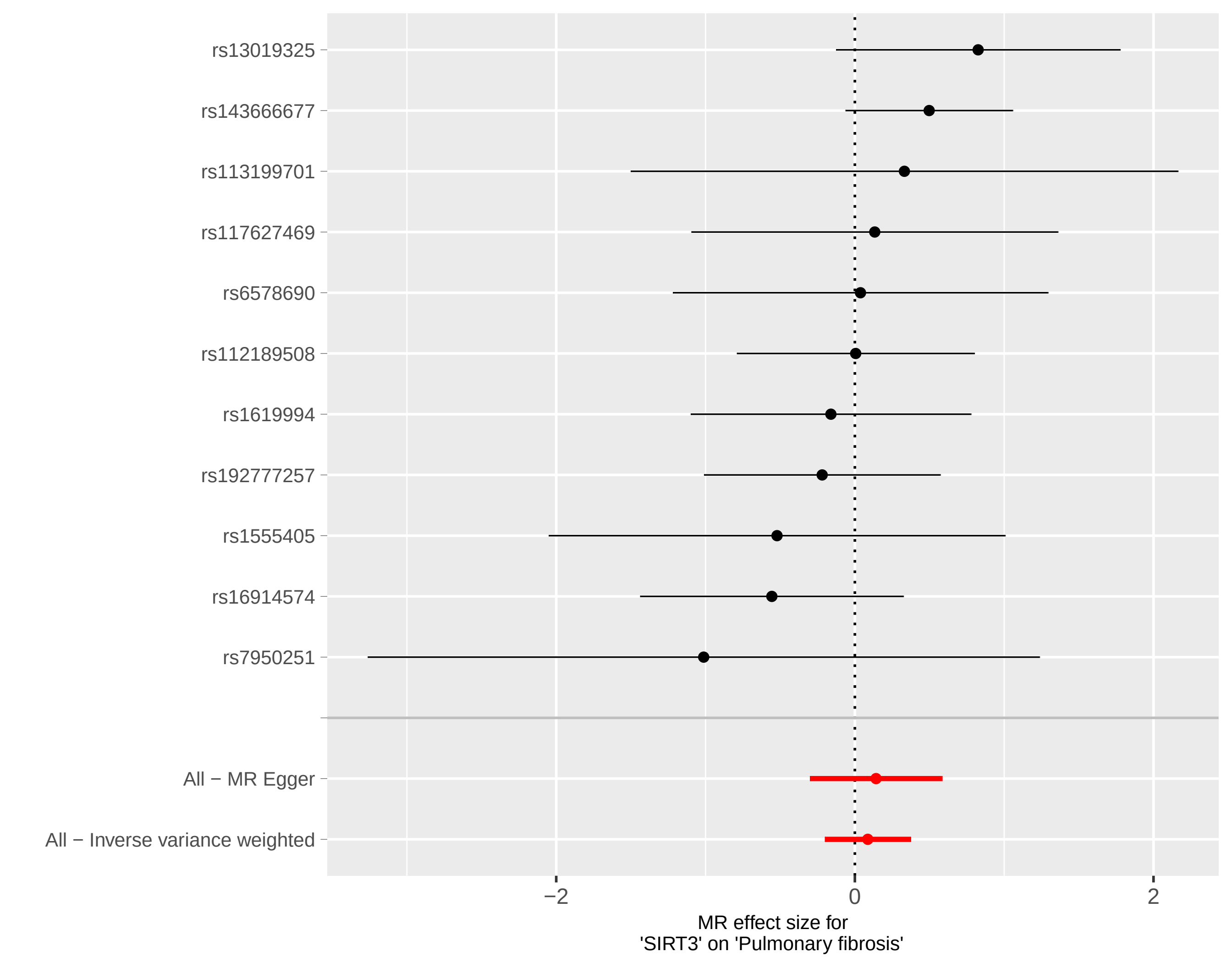

rs13019325
rs143666677
rs113199701
rs117627469
rs6578690
rs112189508
rs1619994
rs192777257
rs1555405
rs16914574
rs7950251
All − MR Egger
All − Inverse variance weighted
−2
0
2
MR effect size for
'SIRT3' on 'Pulmonary fibrosis'

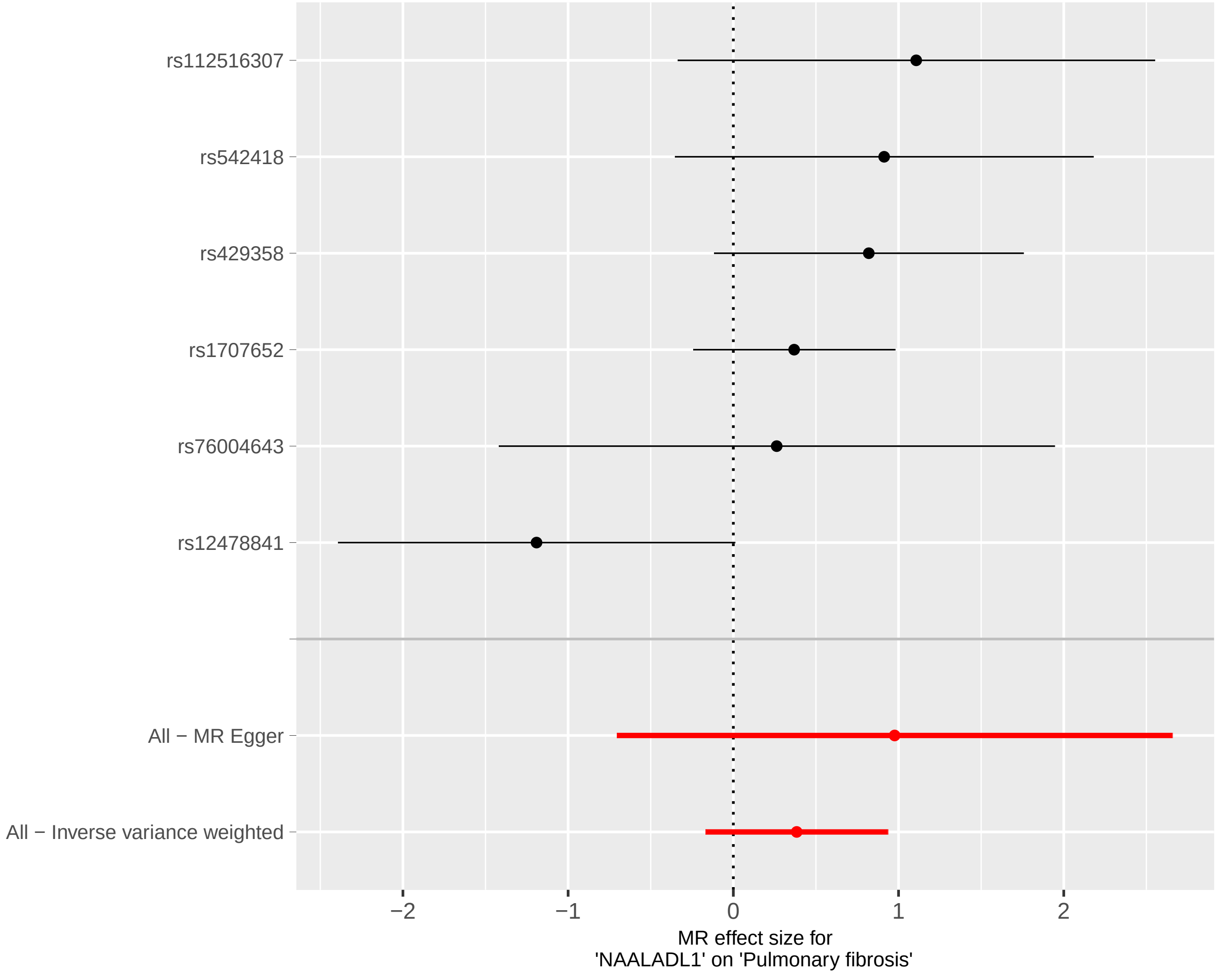

rs112516307
rs542418
rs429358
rs1707652
rs76004643
rs12478841
All − MR Egger
All − Inverse variance weighted
−2
−1
0
1
2
MR effect size for
'NAALADL1' on 'Pulmonary fibrosis'

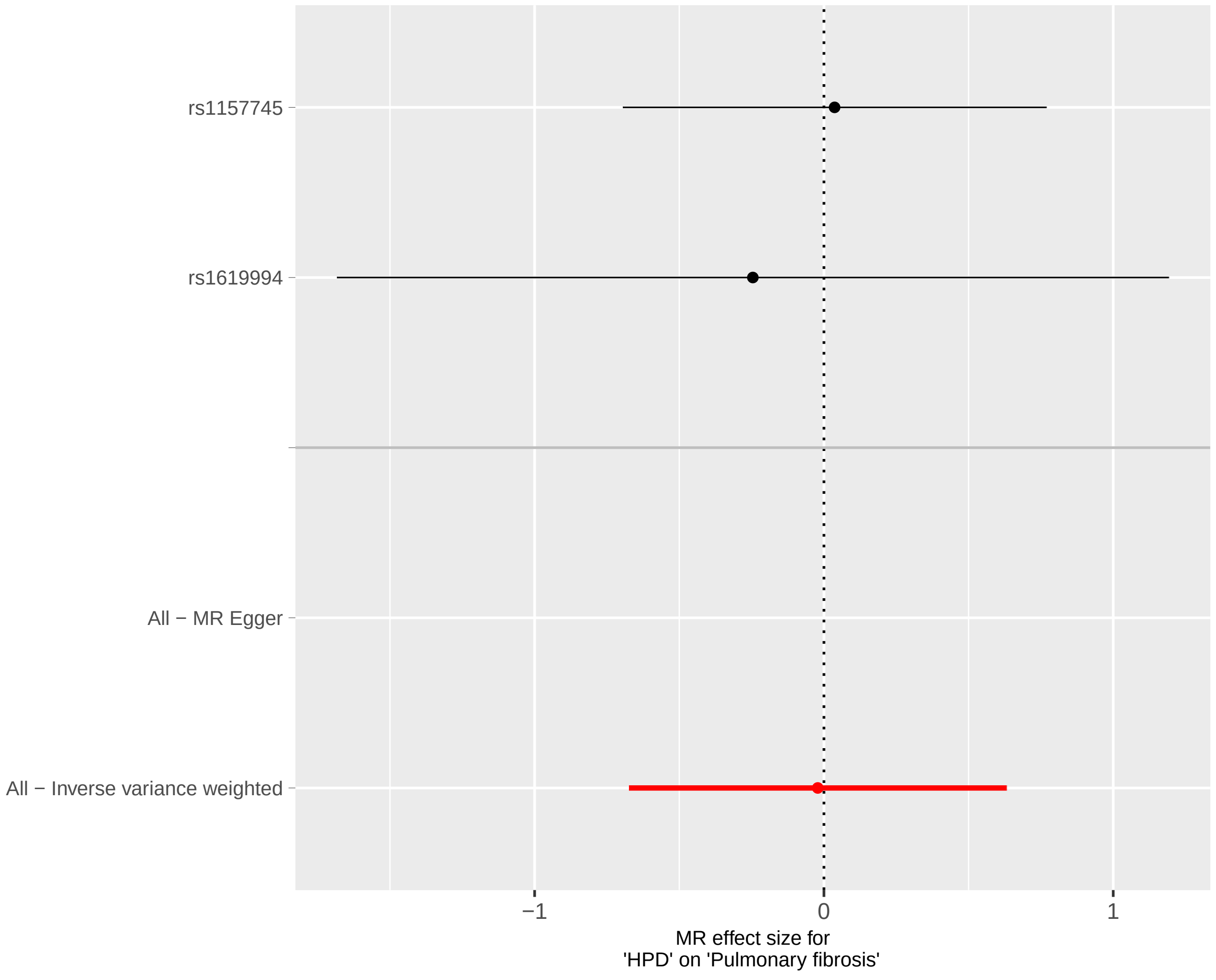
rs1157745
rs1619994
All − MR Egger
All − Inverse variance weighted
−1
0
1
MR effect size for
'HPD' on 'Pulmonary fibrosis'

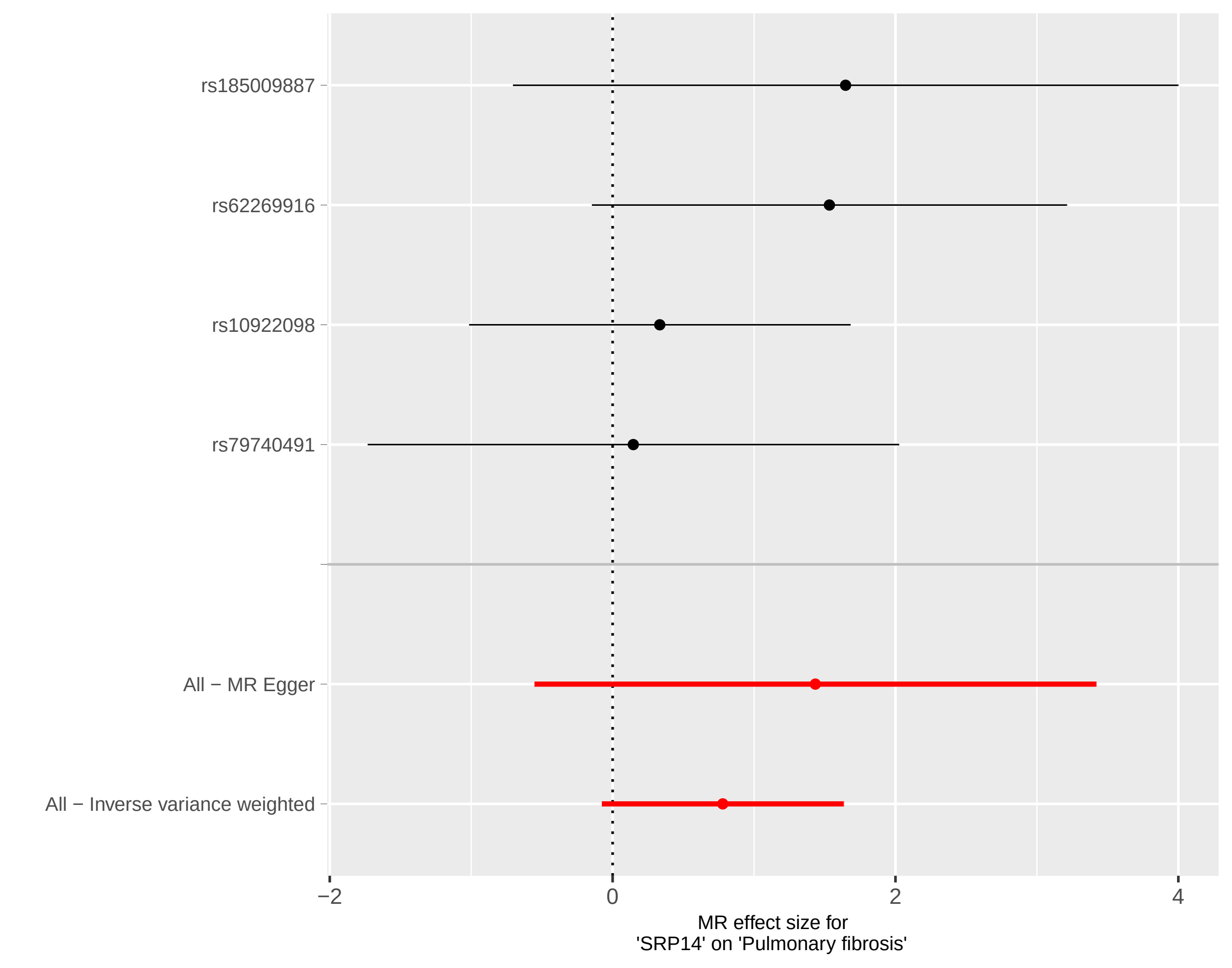

rs185009887
rs62269916
rs10922098
rs79740491
All − MR Egger
All − Inverse variance weighted
−2
0
2
4
MR effect size for
'SRP14' on 'Pulmonary fibrosis'

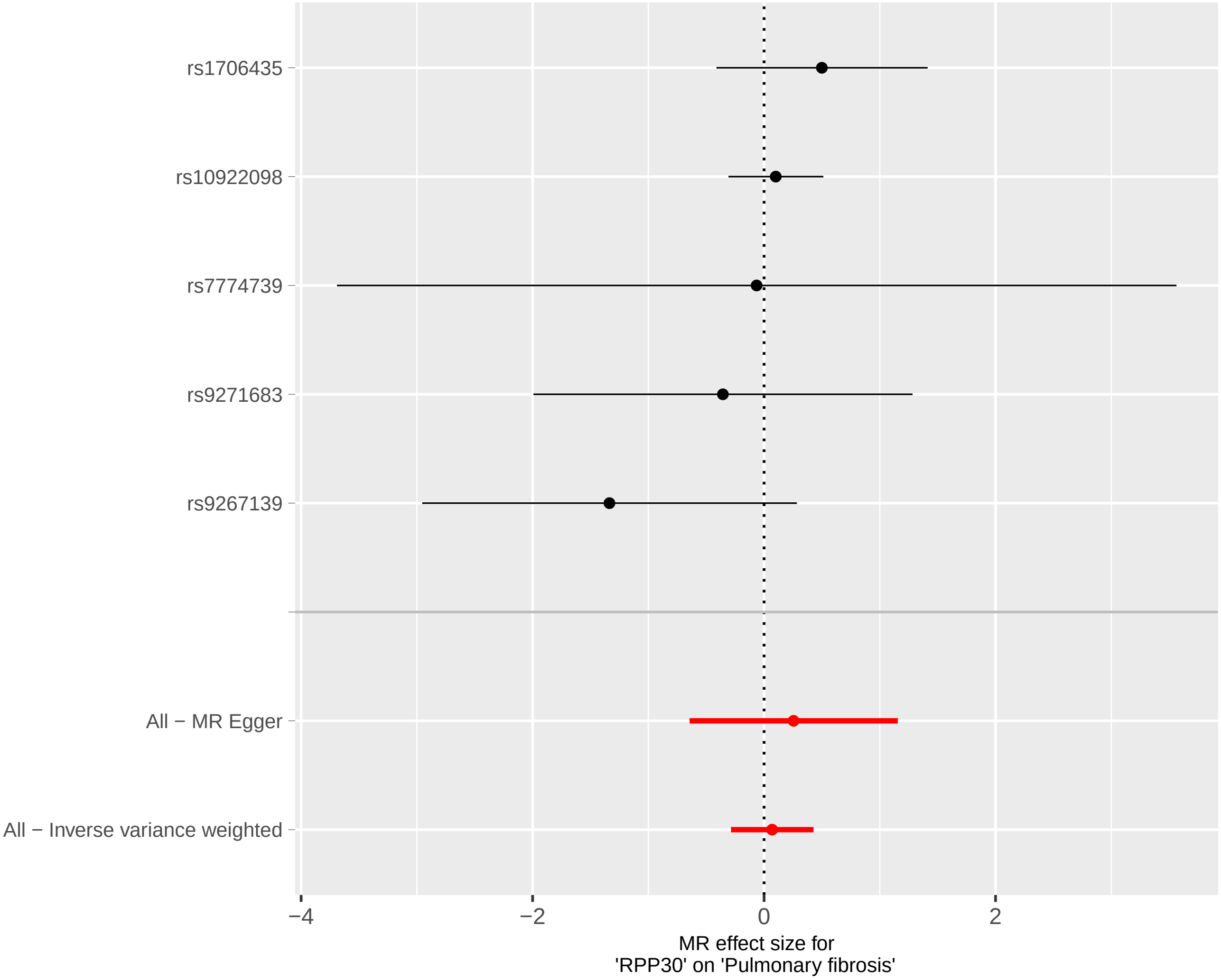

rs1706435
rs10922098
rs7774739
rs9271683
rs9267139
All − MR Egger
All − Inverse variance weighted
−4
−2
0
2
MR effect size for
'RPP30' on 'Pulmonary fibrosis'

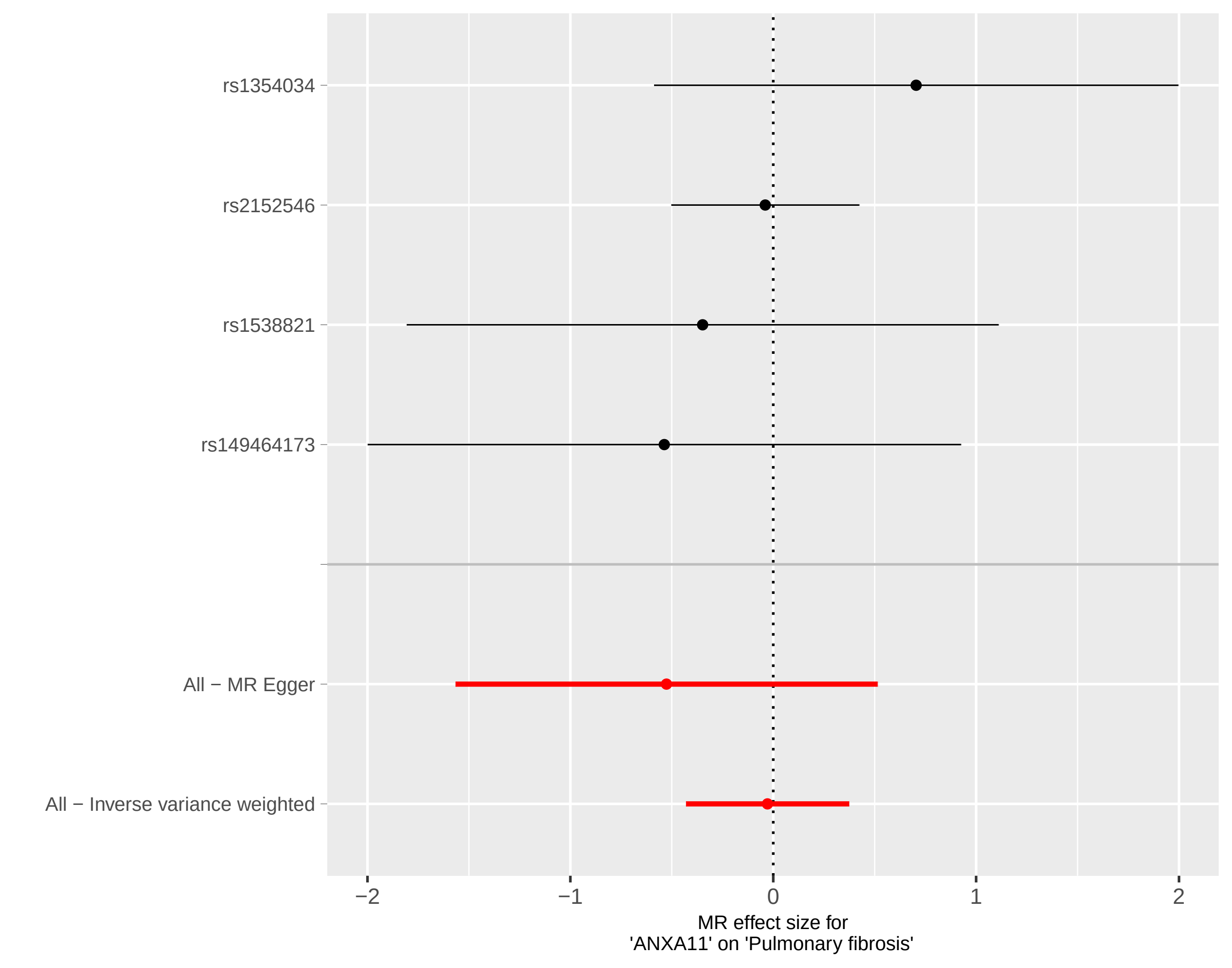
rs1354034
rs2152546
rs1538821
rs149464173
All − MR Egger
All − Inverse variance weighted
−2
−1
0
1
2
MR effect size for
'ANXA11' on 'Pulmonary fibrosis'

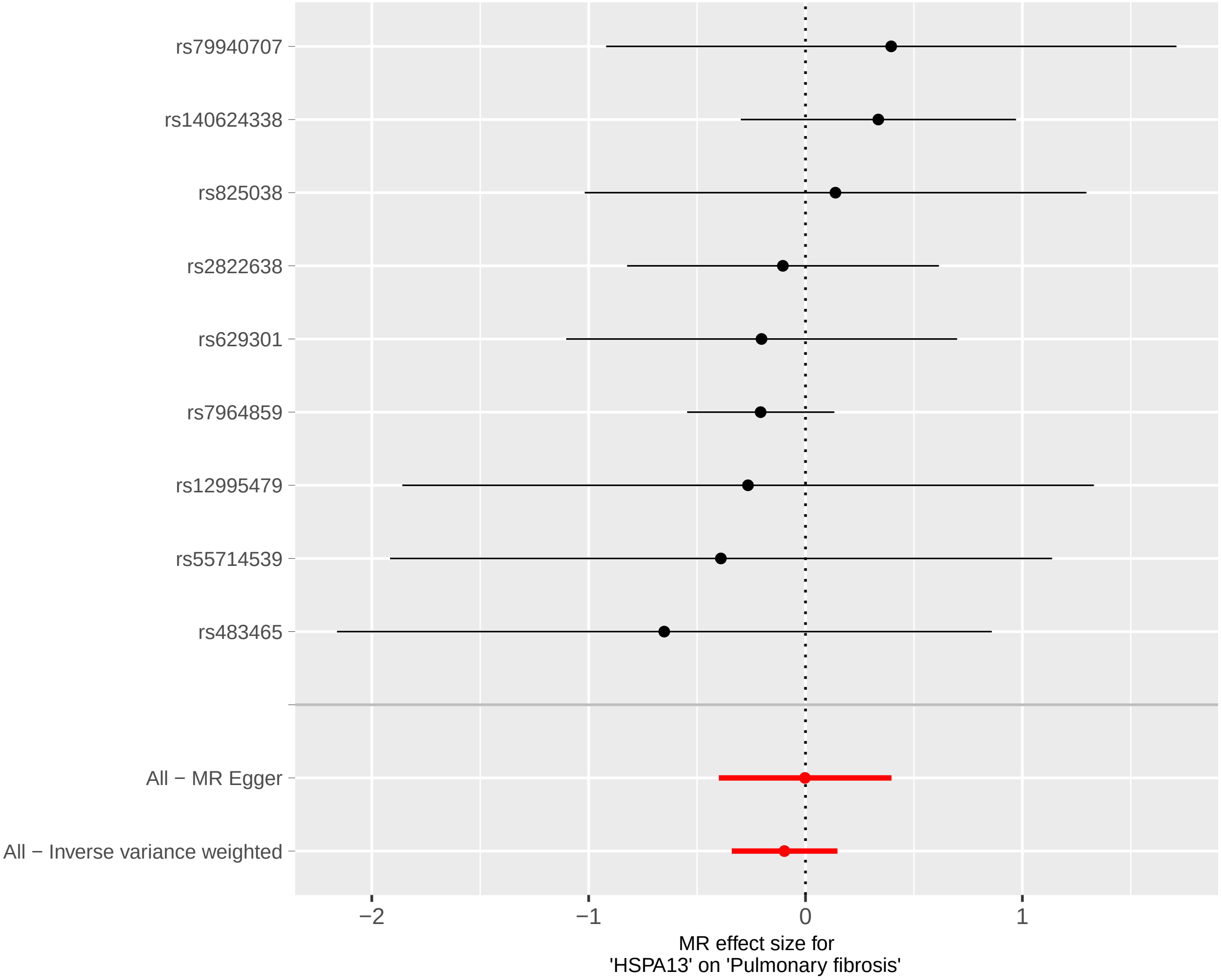

rs79940707
rs140624338
rs825038
rs2822638
rs629301
rs7964859
rs12995479
rs55714539
rs483465
All − MR Egger
All − Inverse variance weighted
−2
−1
0
1
MR effect size for
'HSPA13' on 'Pulmonary fibrosis'

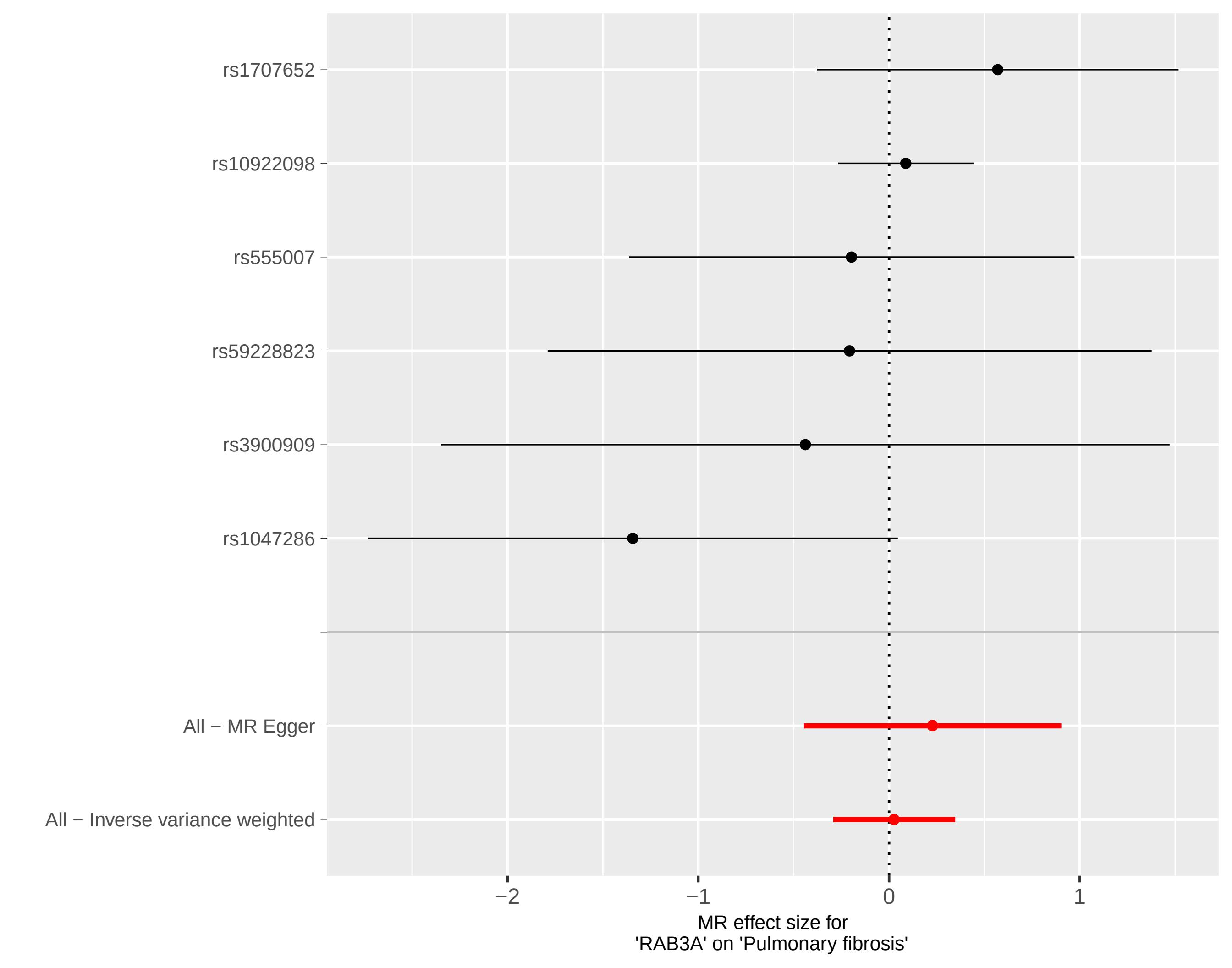

rs1707652
rs10922098
rs555007
rs59228823
rs3900909
rs1047286
All − MR Egger
All − Inverse variance weighted
−2
−1
0
1
MR effect size for
'RAB3A' on 'Pulmonary fibrosis'

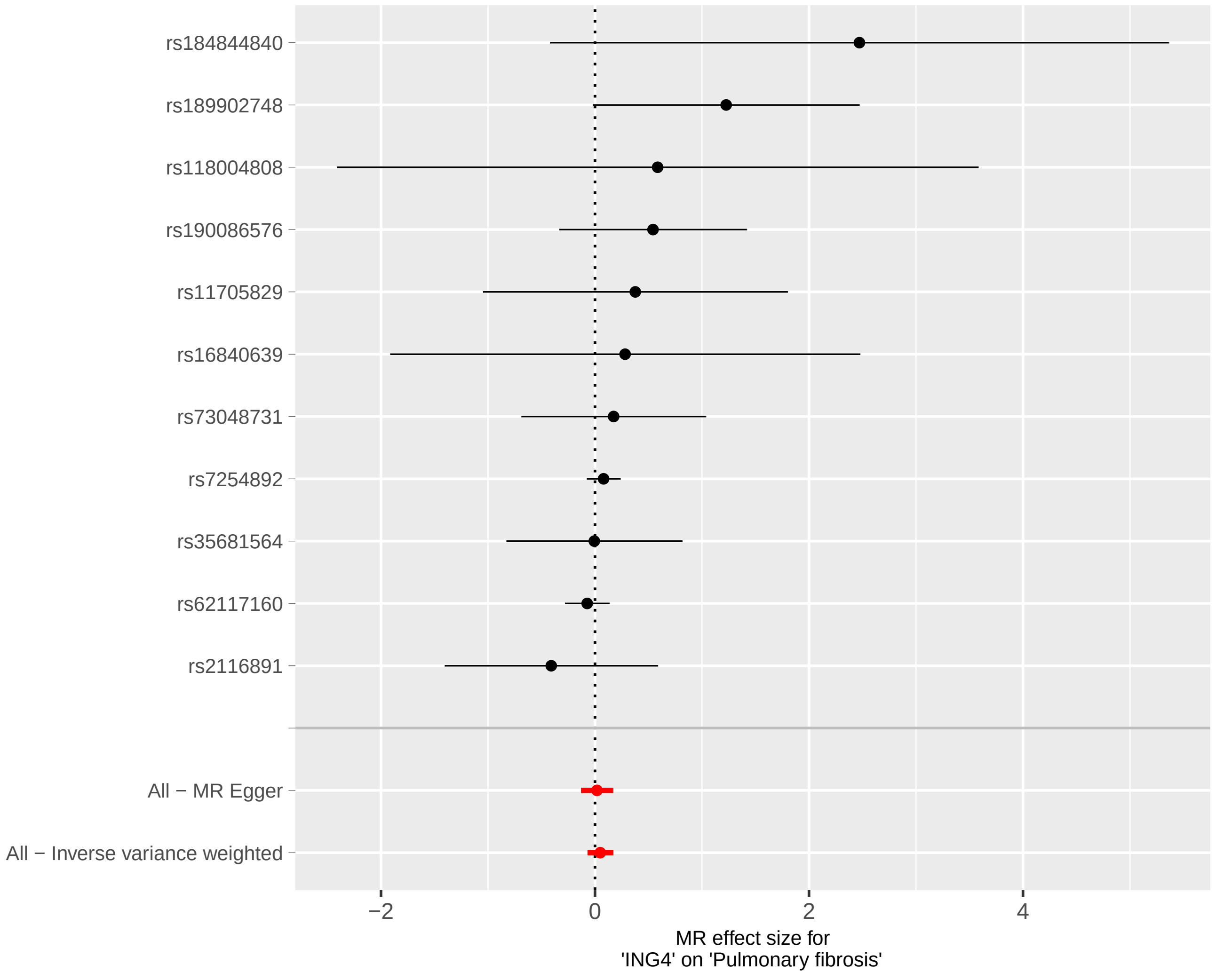

rs184844840
rs189902748
rs118004808
rs190086576
rs11705829
rs16840639
rs73048731
rs7254892
rs35681564
rs62117160
rs2116891
All − MR Egger
All − Inverse variance weighted
−2
0
2
4
MR effect size for
'ING4' on 'Pulmonary fibrosis'

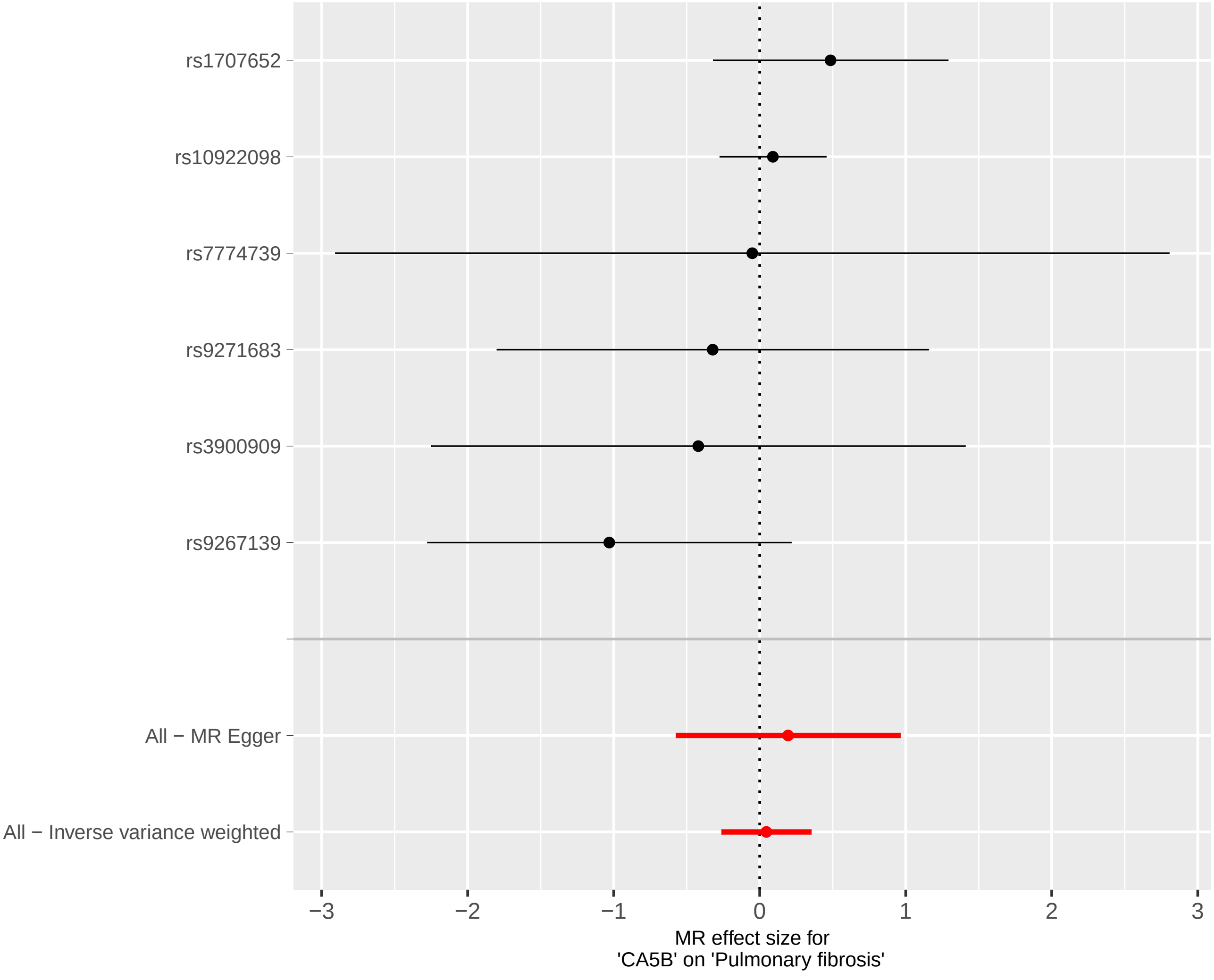

rs1707652
rs10922098
rs7774739
rs9271683
rs3900909
rs9267139
All − MR Egger
All − Inverse variance weighted
−3
−2
−1
0
1
2
3
MR effect size for
'CA5B' on 'Pulmonary fibrosis'

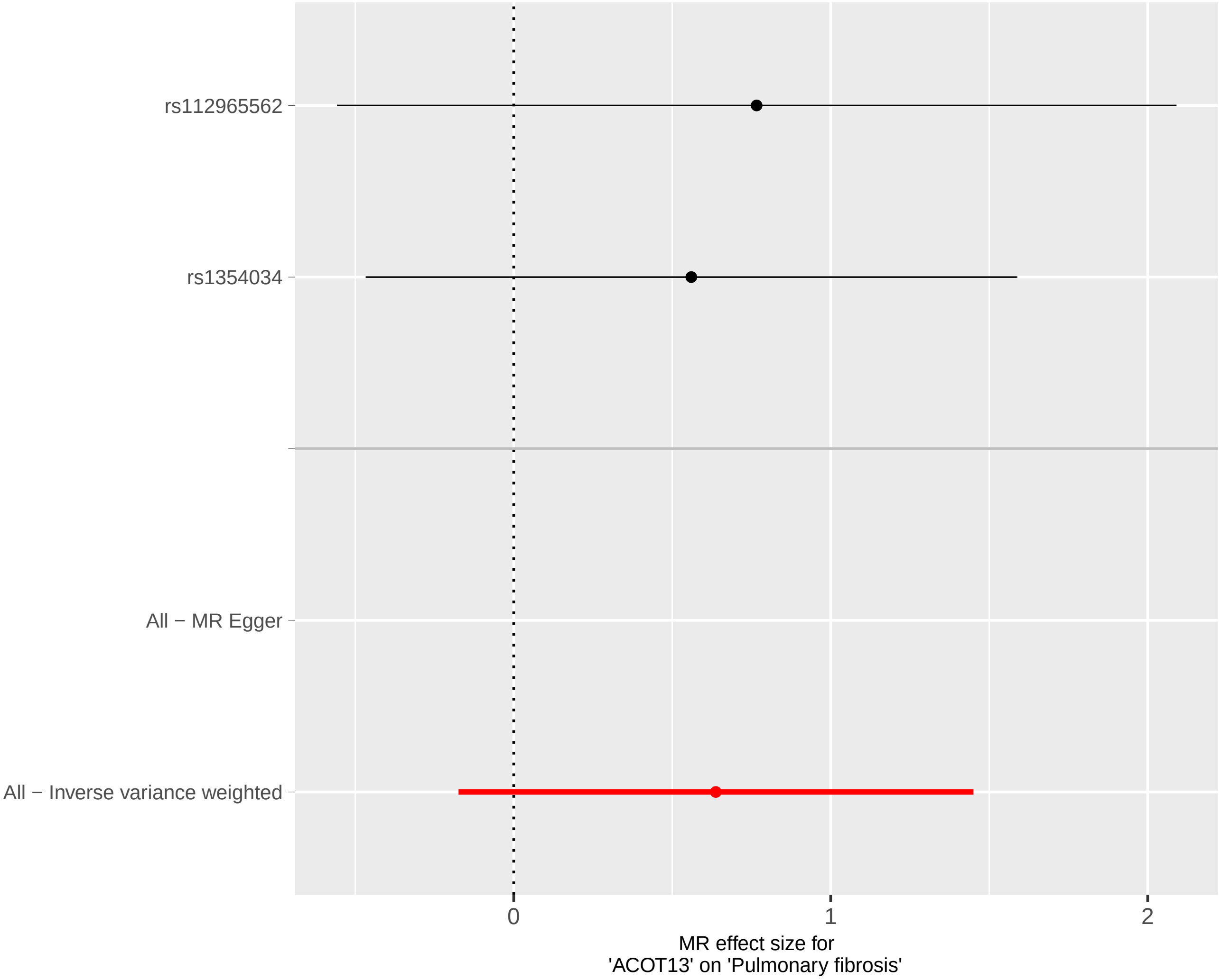
rs112965562
rs1354034
All − MR Egger
All − Inverse variance weighted
0
1
2
MR effect size for
'ACOT13' on 'Pulmonary fibrosis'

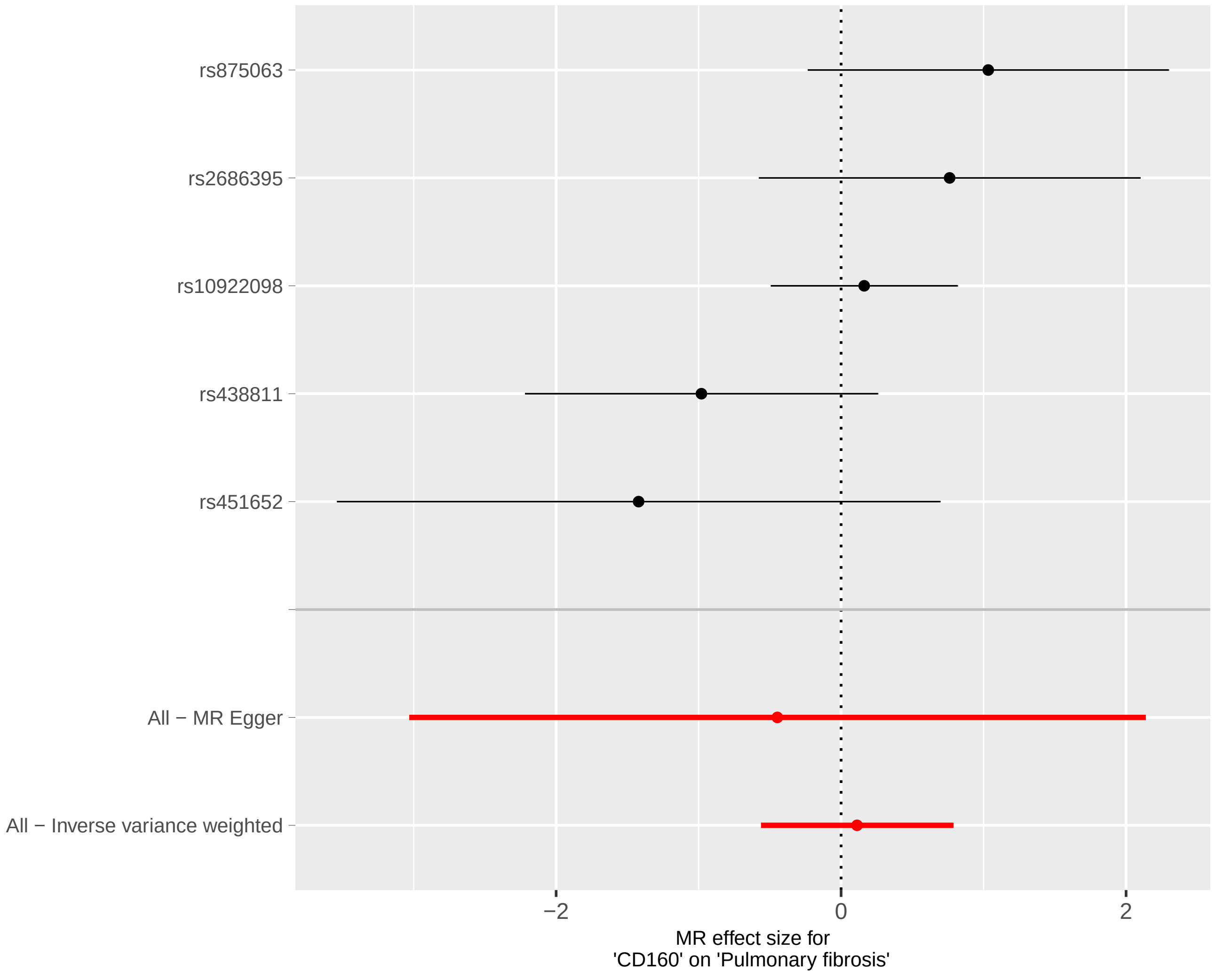

rs875063
rs2686395
rs10922098
rs438811
rs451652
All − MR Egger
All − Inverse variance weighted
−2
0
2
MR effect size for
'CD160' on 'Pulmonary fibrosis'

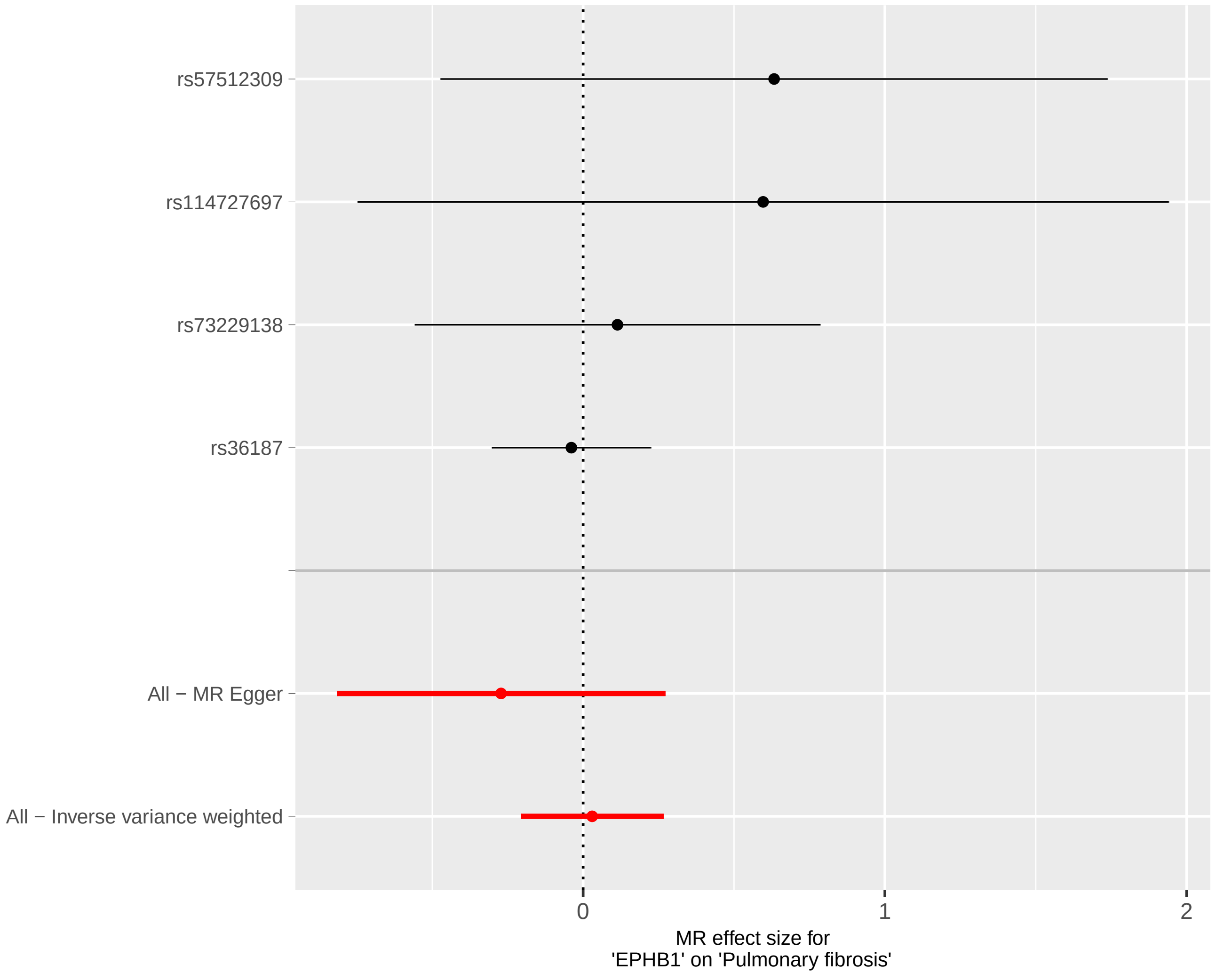

rs57512309
rs114727697
rs73229138
rs36187
All − MR Egger
All − Inverse variance weighted
0
1
2
MR effect size for
'EPHB1' on 'Pulmonary fibrosis'

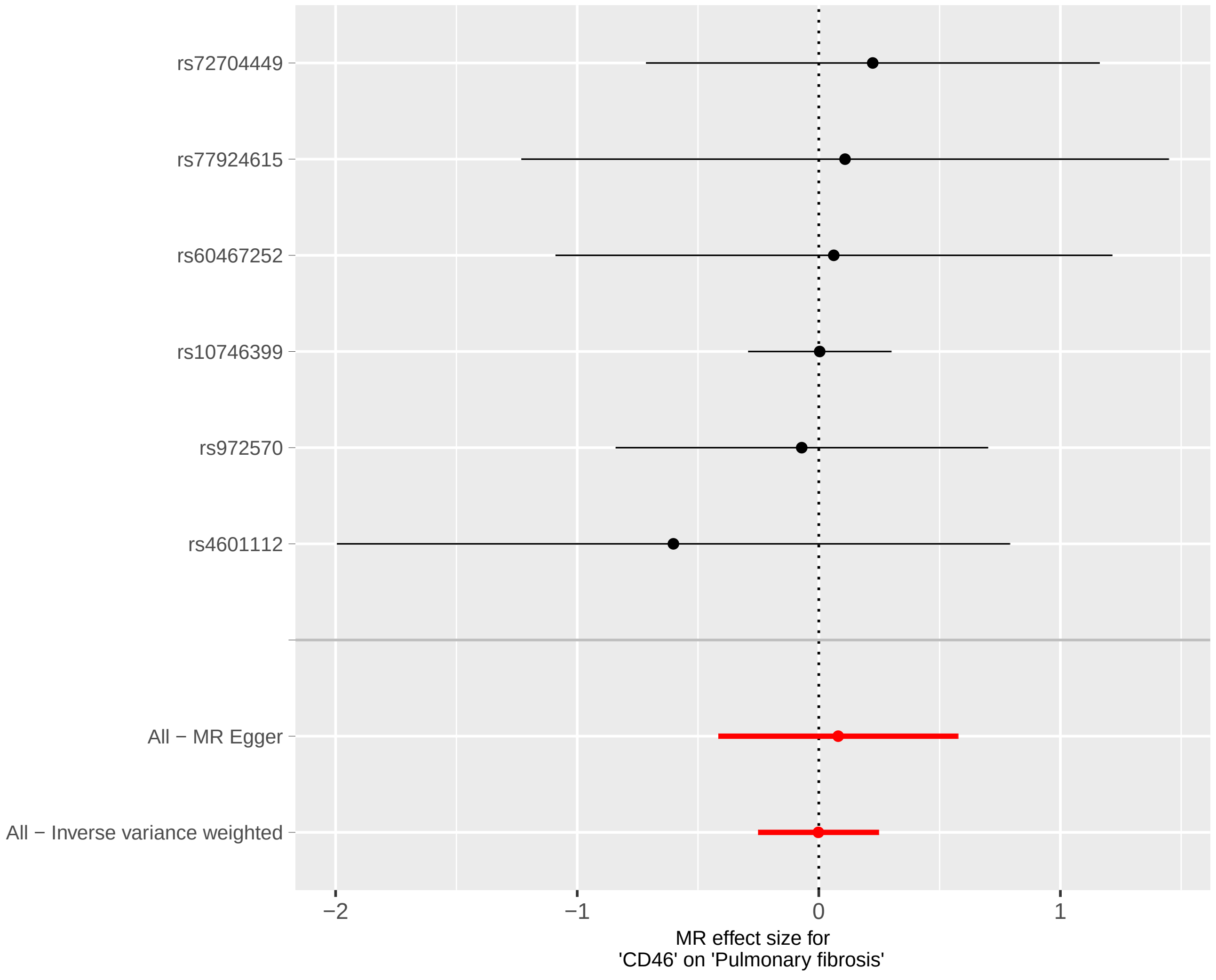
rs72704449
rs77924615
rs60467252
rs10746399
rs972570
rs4601112
All − MR Egger
All − Inverse variance weighted
−2
−1
0
1
MR effect size for
'CD46' on 'Pulmonary fibrosis'

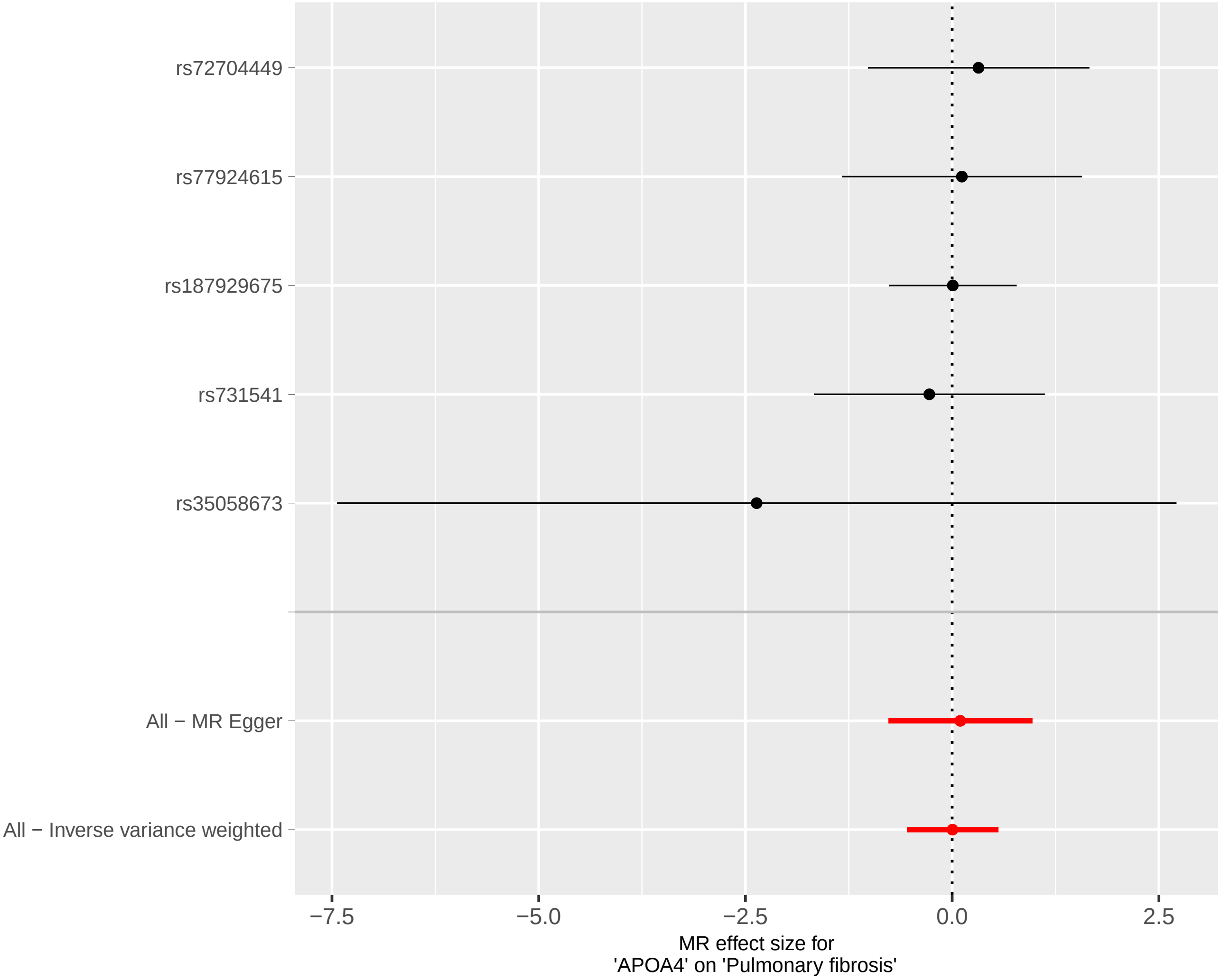
rs72704449
rs77924615
rs187929675
rs731541
rs35058673
All − MR Egger
All − Inverse variance weighted
−7.5
−5.0
−2.5
0.0
2.5
MR effect size for
'APOA4' on 'Pulmonary fibrosis'

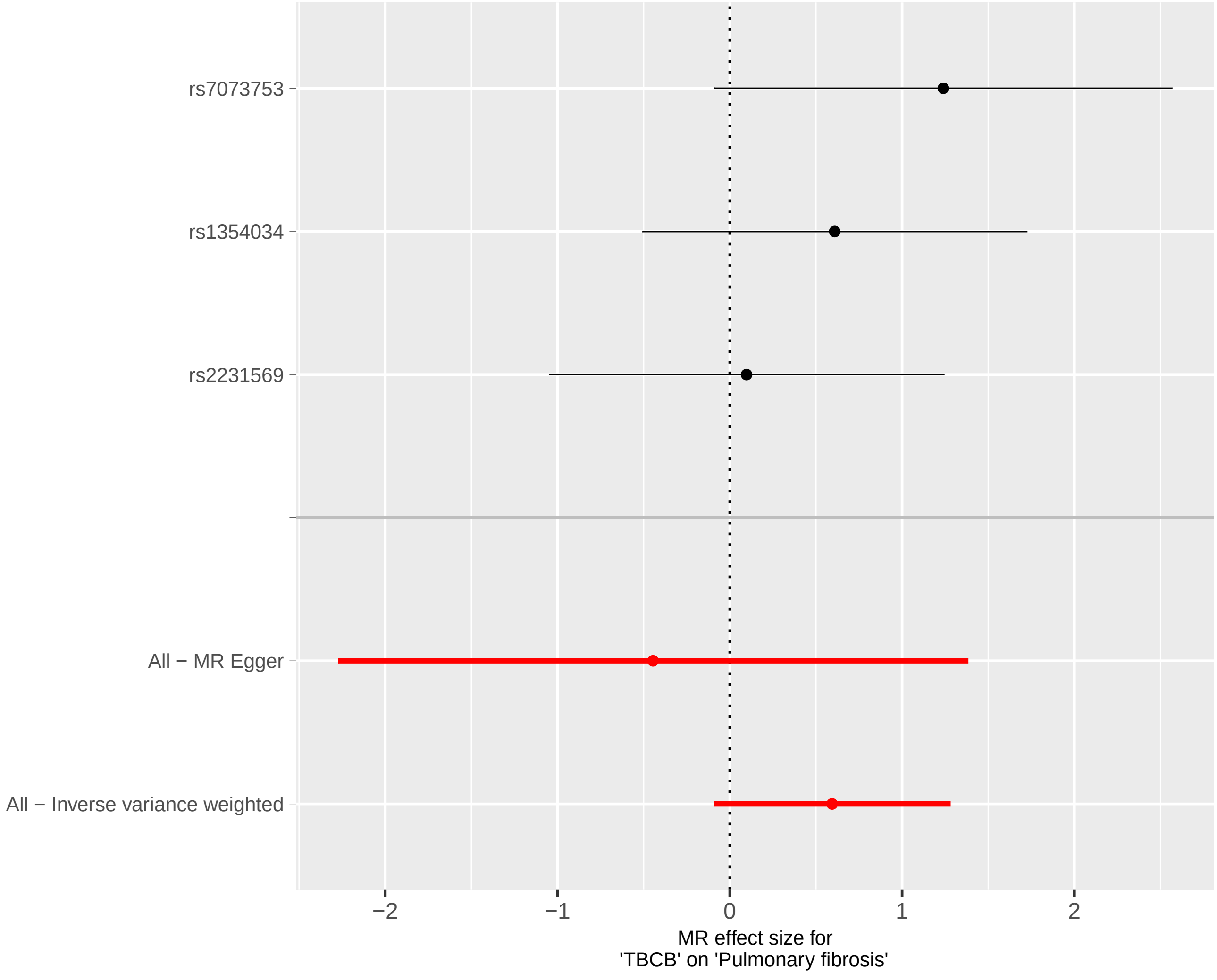
rs7073753
rs1354034
rs2231569
All − MR Egger
All − Inverse variance weighted
−2
−1
0
1
2
MR effect size for
'TBCB' on 'Pulmonary fibrosis'

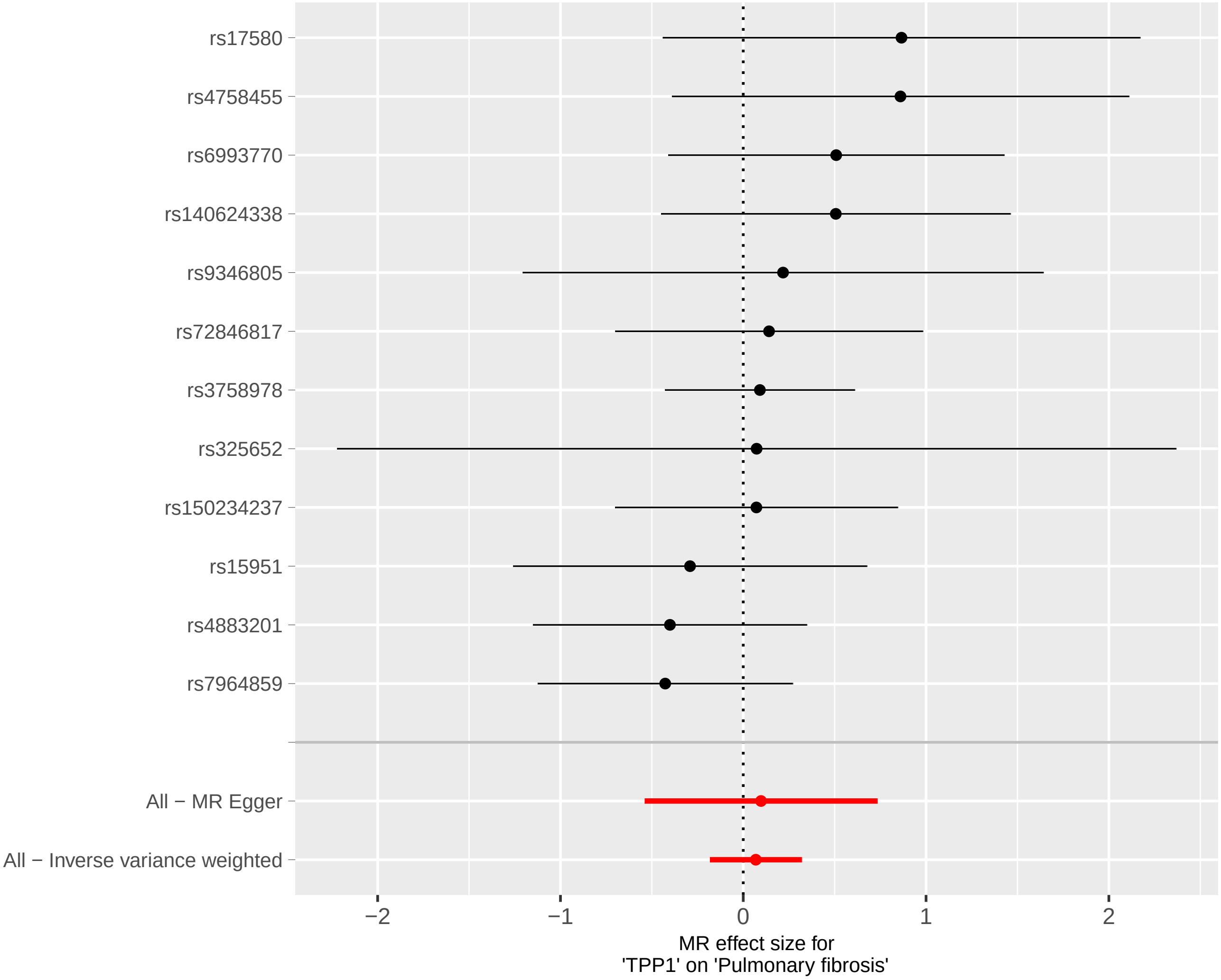
rs17580
rs4758455
rs6993770
rs140624338
rs9346805
rs72846817
rs3758978
rs325652
rs150234237
rs15951
rs4883201
rs7964859
All − MR Egger
All − Inverse variance weighted
−2
−1
0
1
2
MR effect size for
'TPP1' on 'Pulmonary fibrosis'

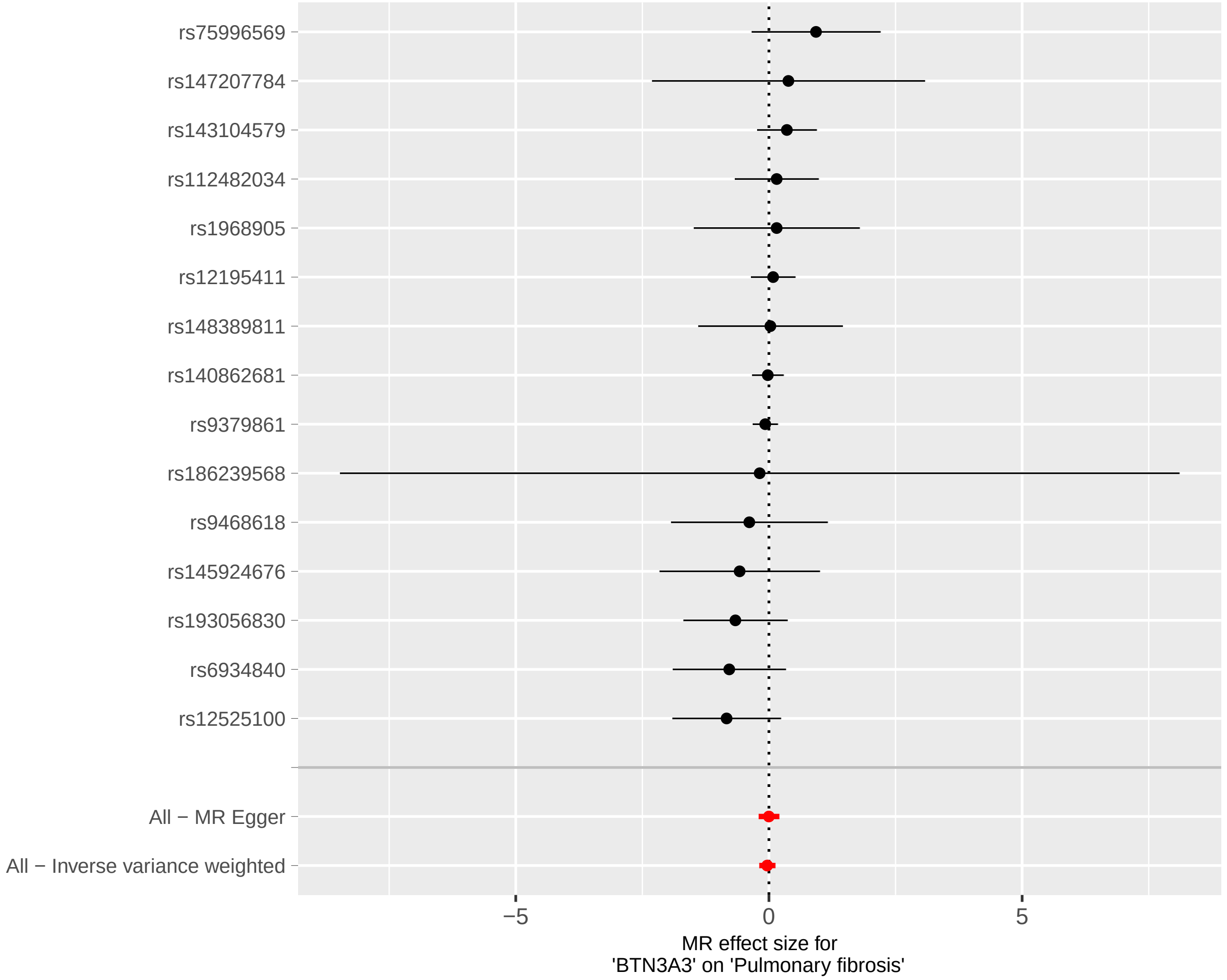

rs75996569
rs147207784
rs143104579
rs112482034
rs1968905
rs12195411
rs148389811
rs140862681
rs9379861
rs186239568
rs9468618
rs145924676
rs193056830
rs6934840
rs12525100
All − MR Egger
All − Inverse variance weighted
−5
0
5
MR effect size for
'BTN3A3' on 'Pulmonary fibrosis'

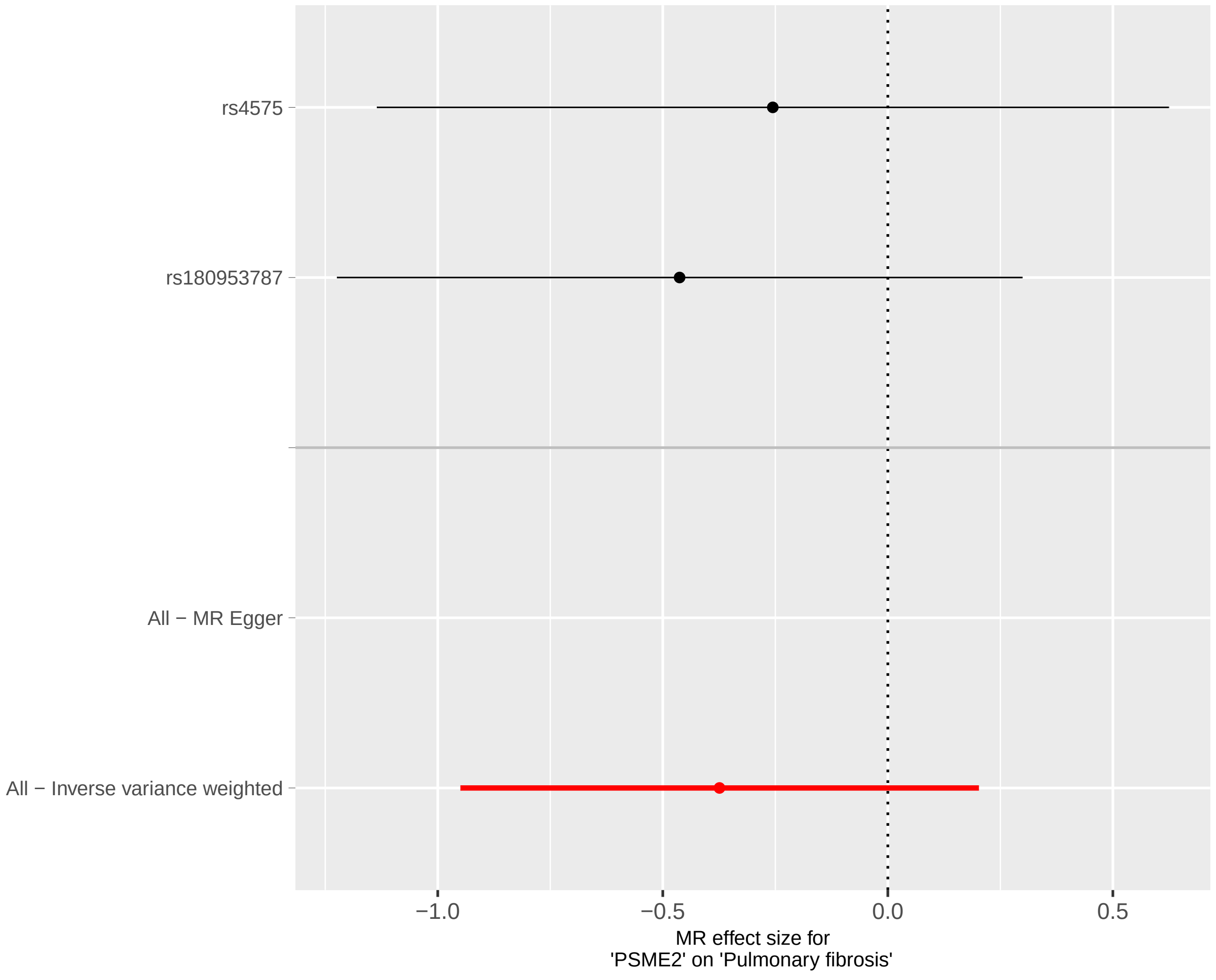

rs4575
rs180953787
All − MR Egger
All − Inverse variance weighted
−1.0
−0.5
0.0
0.5
MR effect size for
'PSME2' on 'Pulmonary fibrosis'

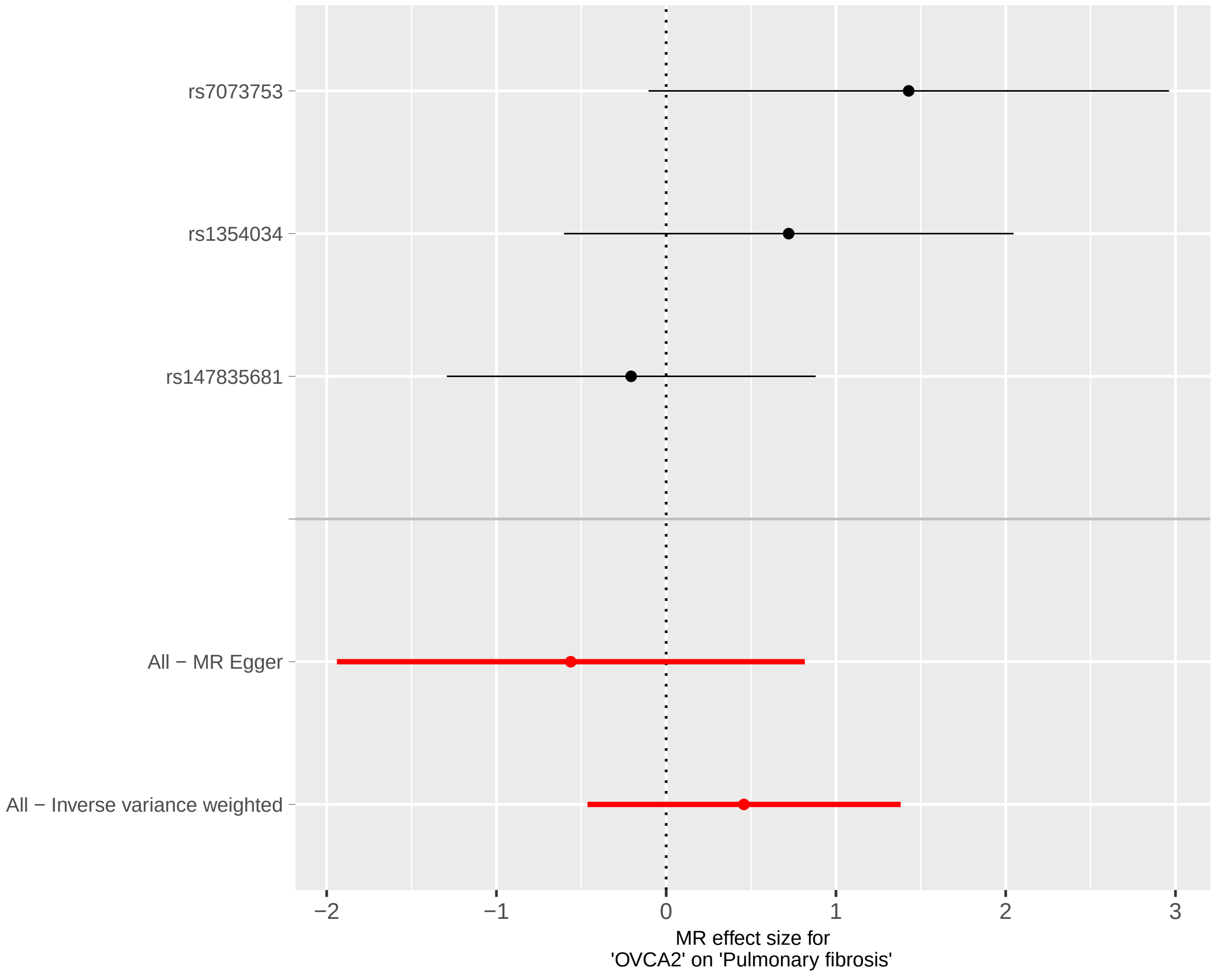

rs7073753
rs1354034
rs147835681
All − MR Egger
All − Inverse variance weighted
−2
−1
0
1
2
3
MR effect size for
'OVCA2' on 'Pulmonary fibrosis'

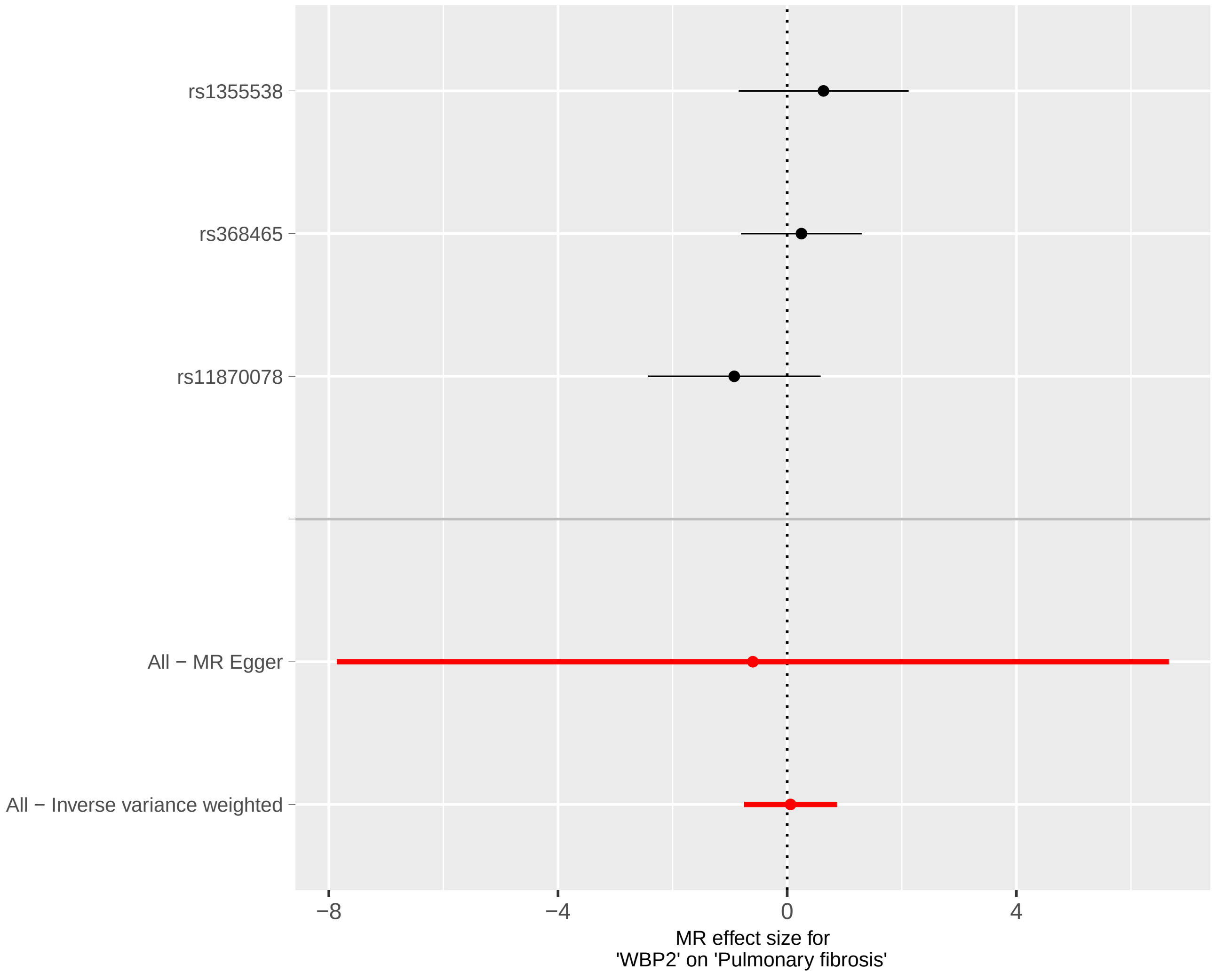

rs1355538
rs368465
rs11870078
All − MR Egger
All − Inverse variance weighted
−8
−4
0
4
MR effect size for
'WBP2' on 'Pulmonary fibrosis'

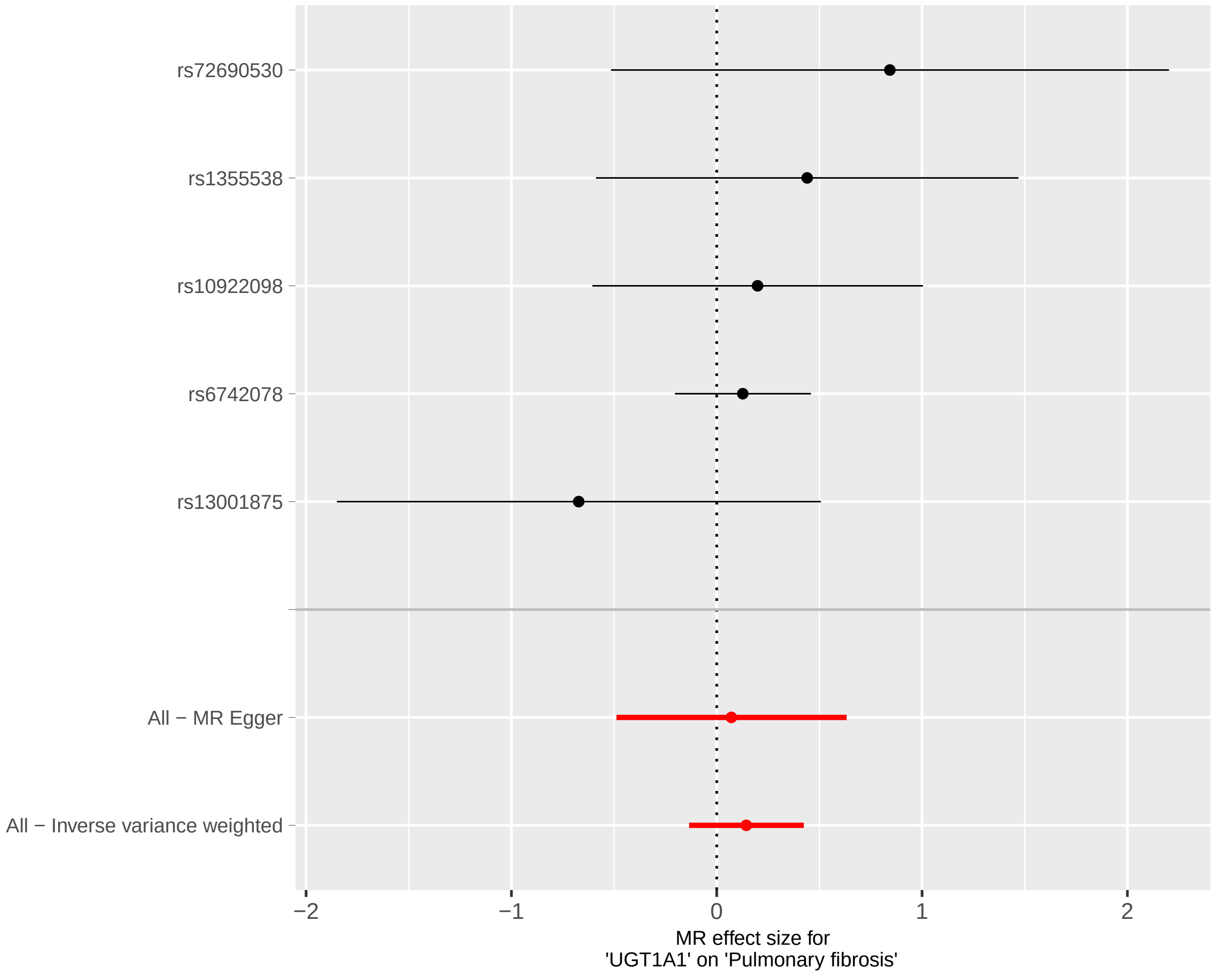
rs72690530
rs1355538
rs10922098
rs6742078
rs13001875
All − MR Egger
All − Inverse variance weighted
−2
−1
0
1
2
MR effect size for
'UGT1A1' on 'Pulmonary fibrosis'

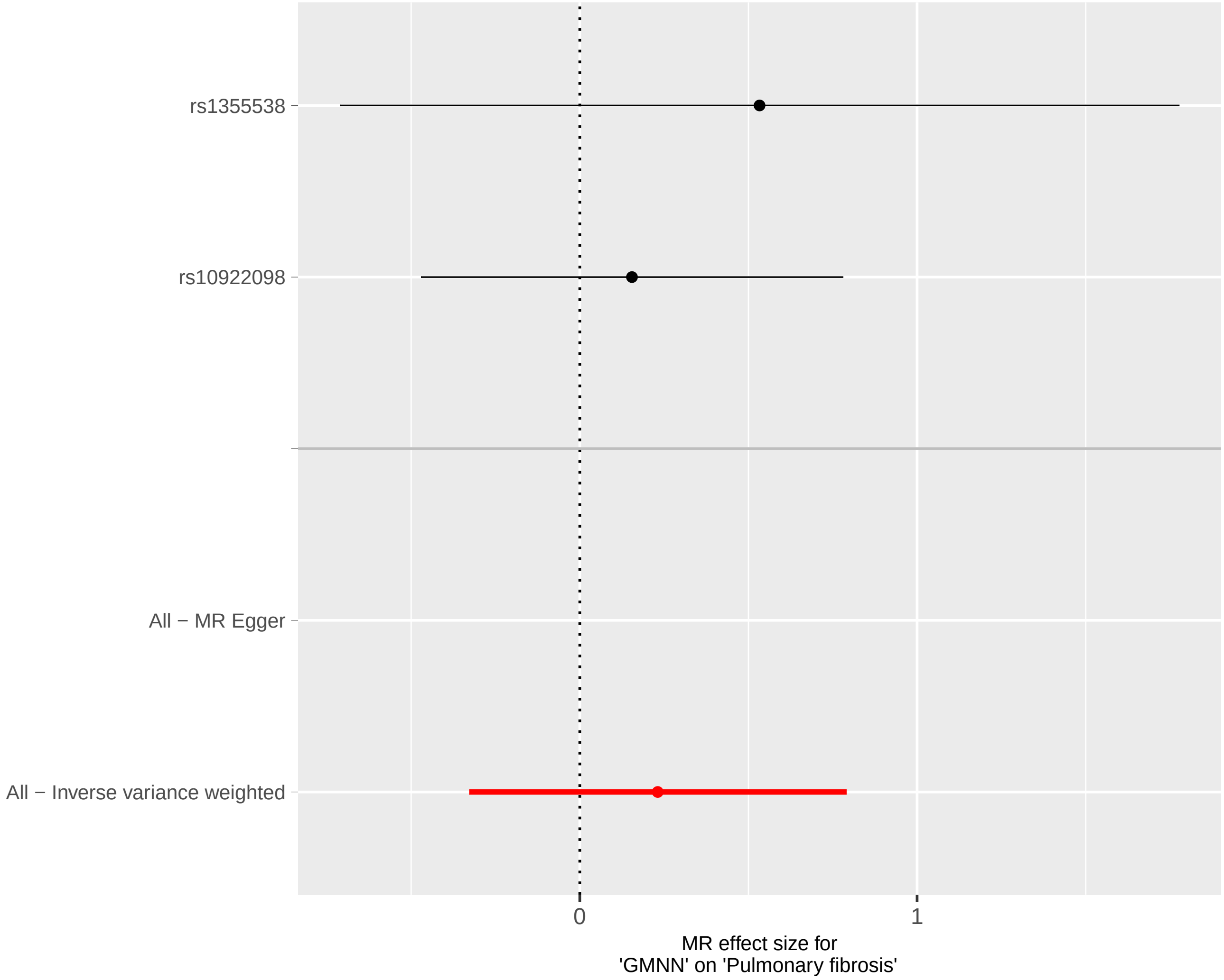
rs1355538
rs10922098
All − MR Egger
All − Inverse variance weighted
0
1
MR effect size for
'GMNN' on 'Pulmonary fibrosis'

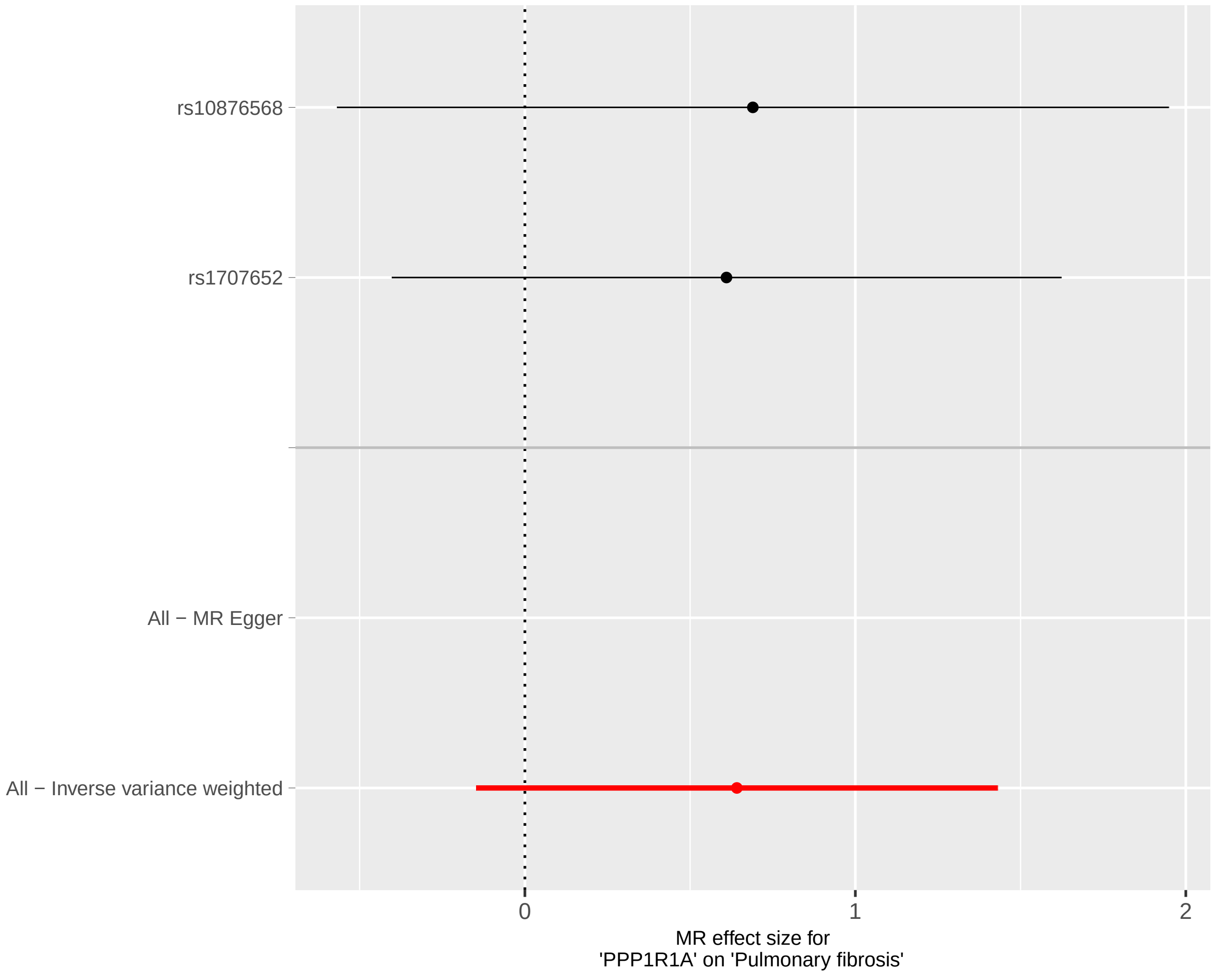
rs10876568
rs1707652
All − MR Egger
All − Inverse variance weighted
0
1
2
MR effect size for
'PPP1R1A' on 'Pulmonary fibrosis'

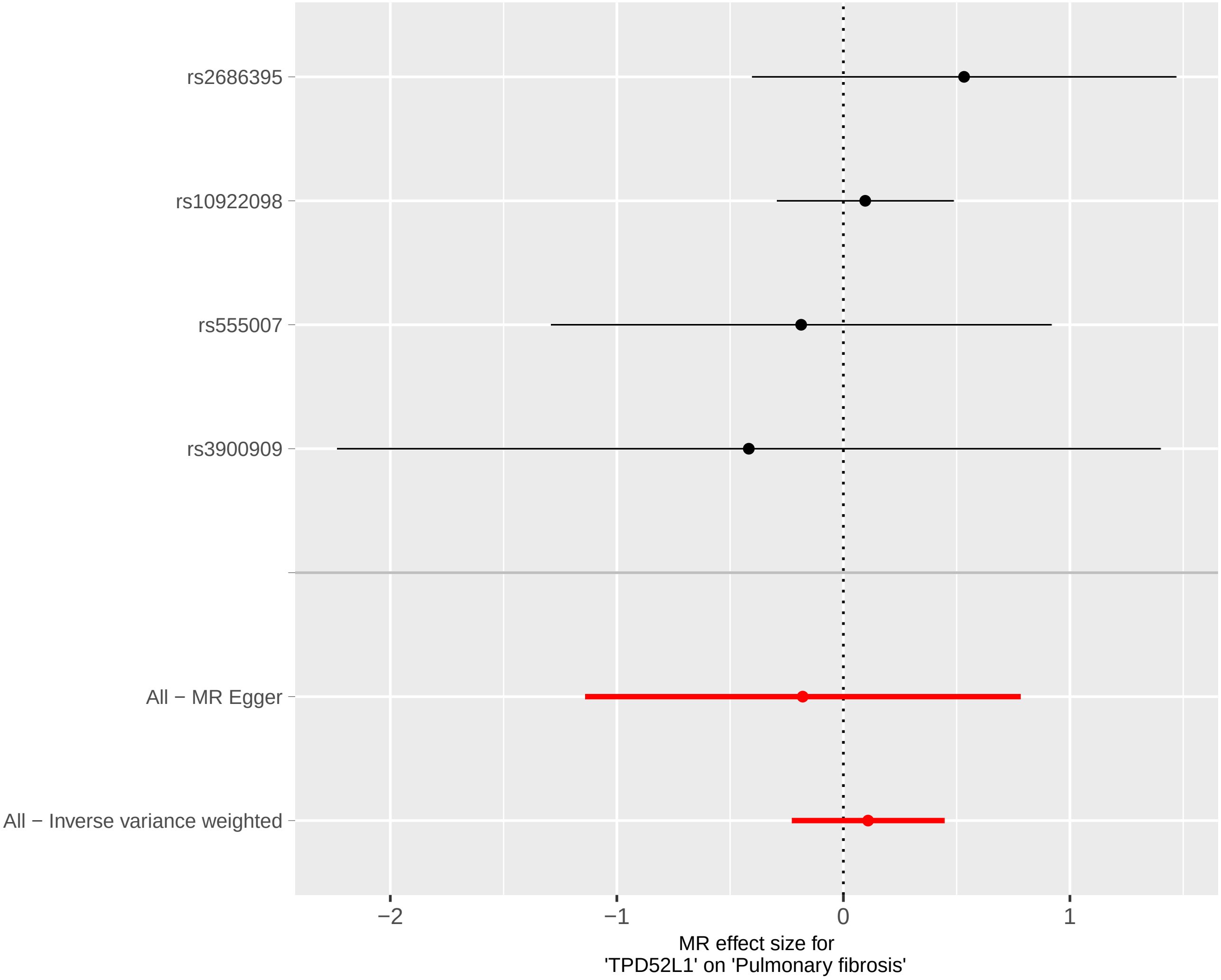

rs2686395
rs10922098
rs555007
rs3900909
All − MR Egger
All − Inverse variance weighted
−2
−1
0
1
MR effect size for
'TPD52L1' on 'Pulmonary fibrosis'

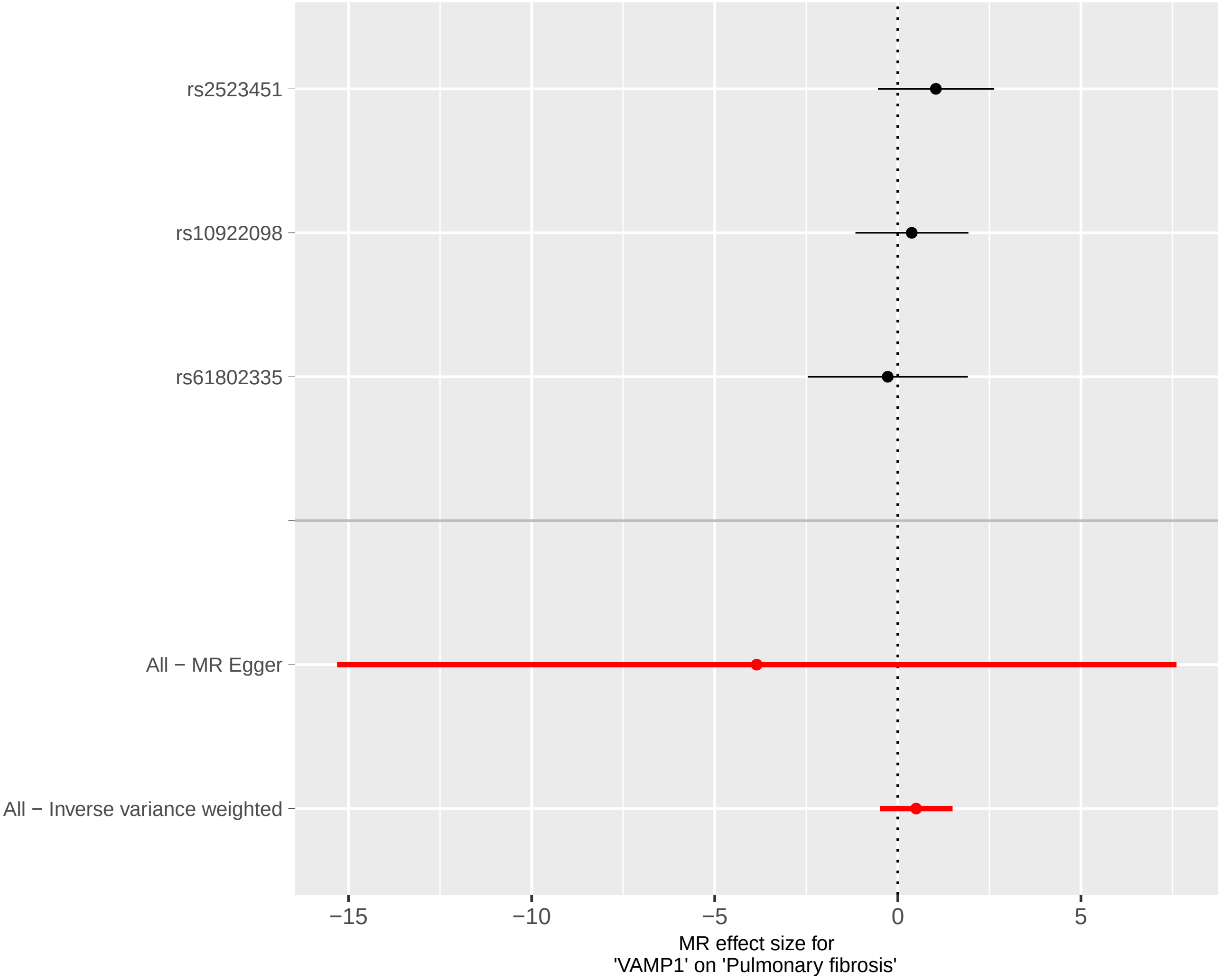

rs2523451
rs10922098
rs61802335
All − MR Egger
All − Inverse variance weighted
−15
−10
−5
0
5
MR effect size for
'VAMP1' on 'Pulmonary fibrosis'

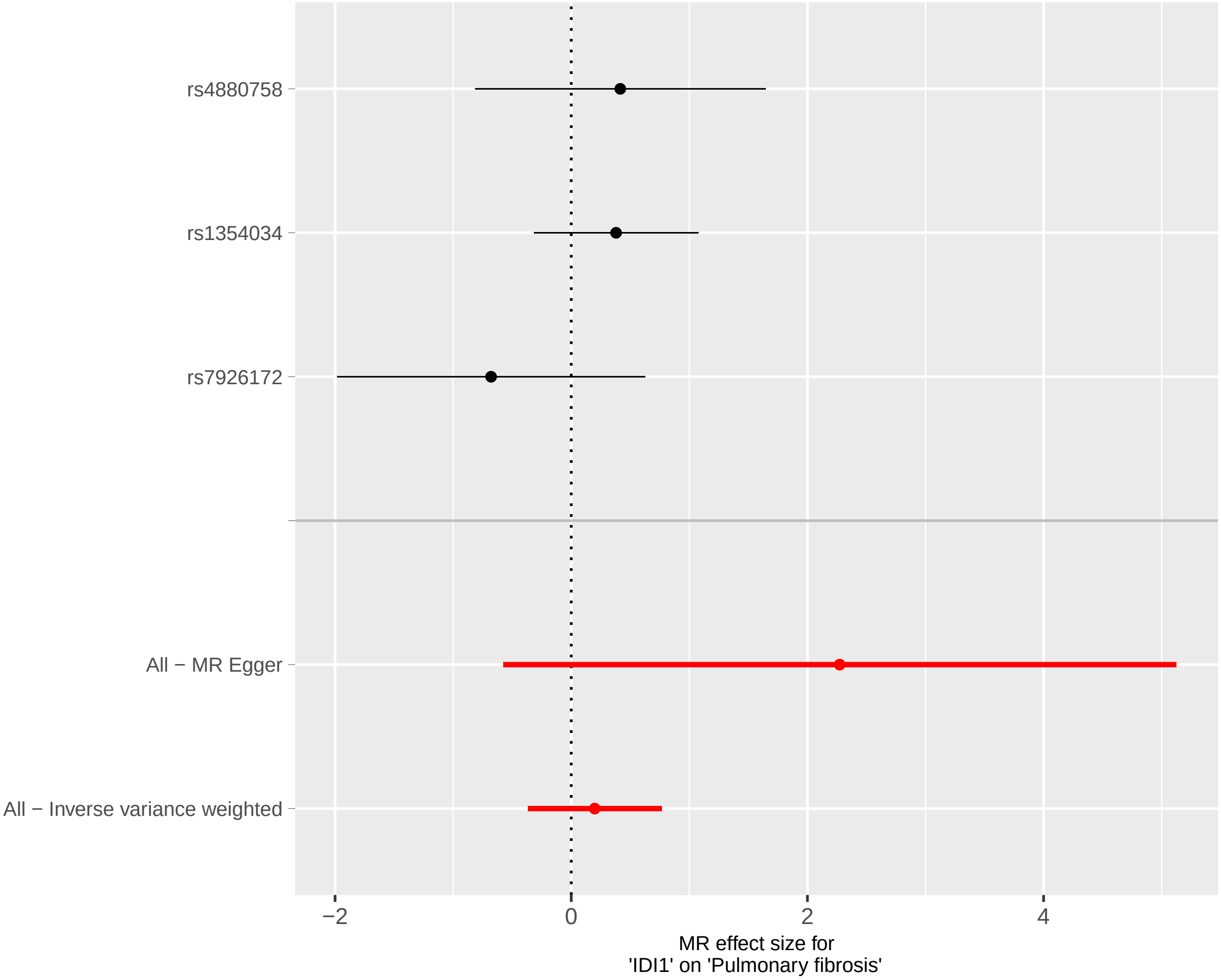

rs4880758
rs1354034
rs7926172
All − MR Egger
All − Inverse variance weighted
−2
0
2
4
MR effect size for
'IDI1' on 'Pulmonary fibrosis'

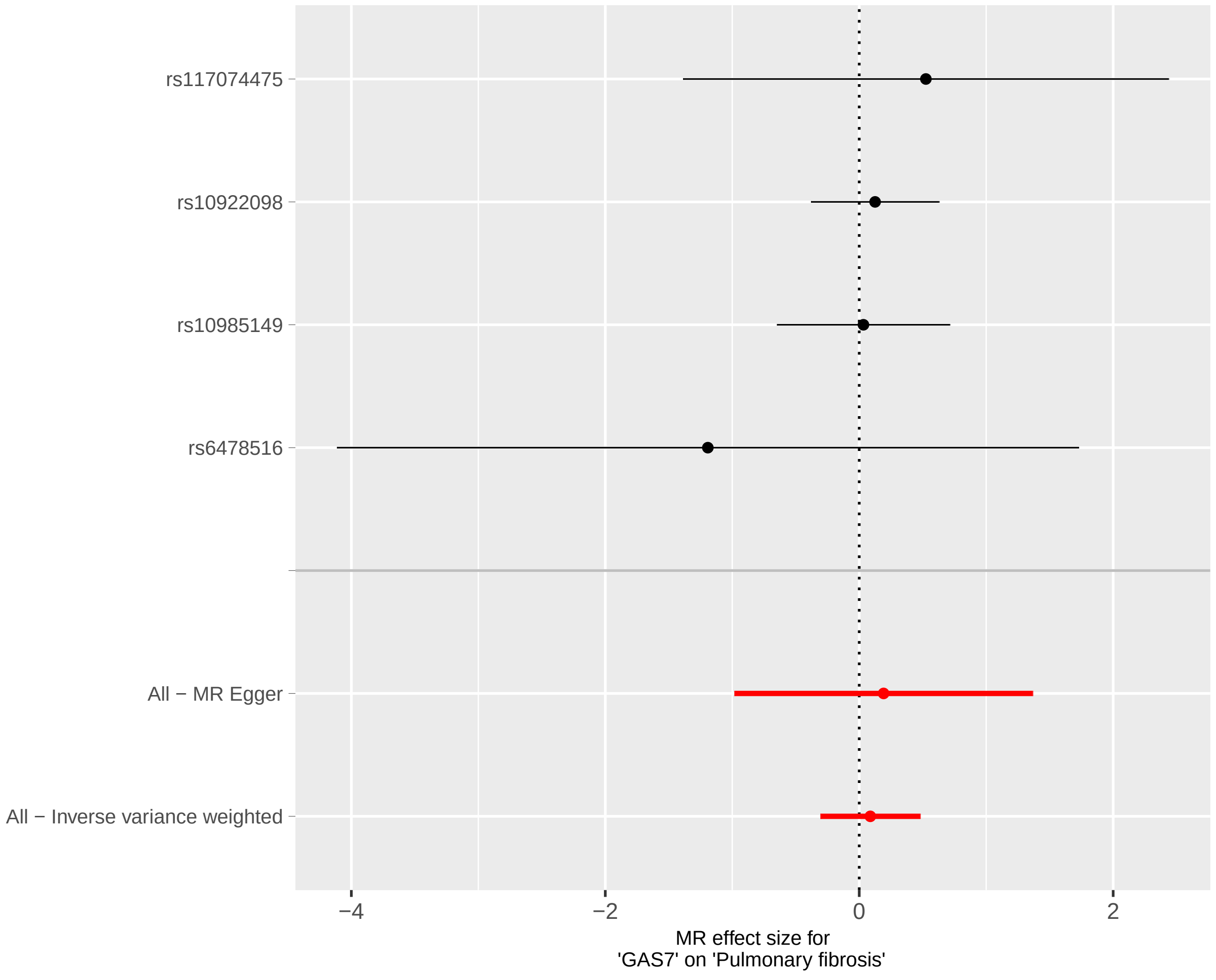
rs117074475
rs10922098
rs10985149
rs6478516
All − MR Egger
All − Inverse variance weighted
−4
−2
0
2
MR effect size for
'GAS7' on 'Pulmonary fibrosis'

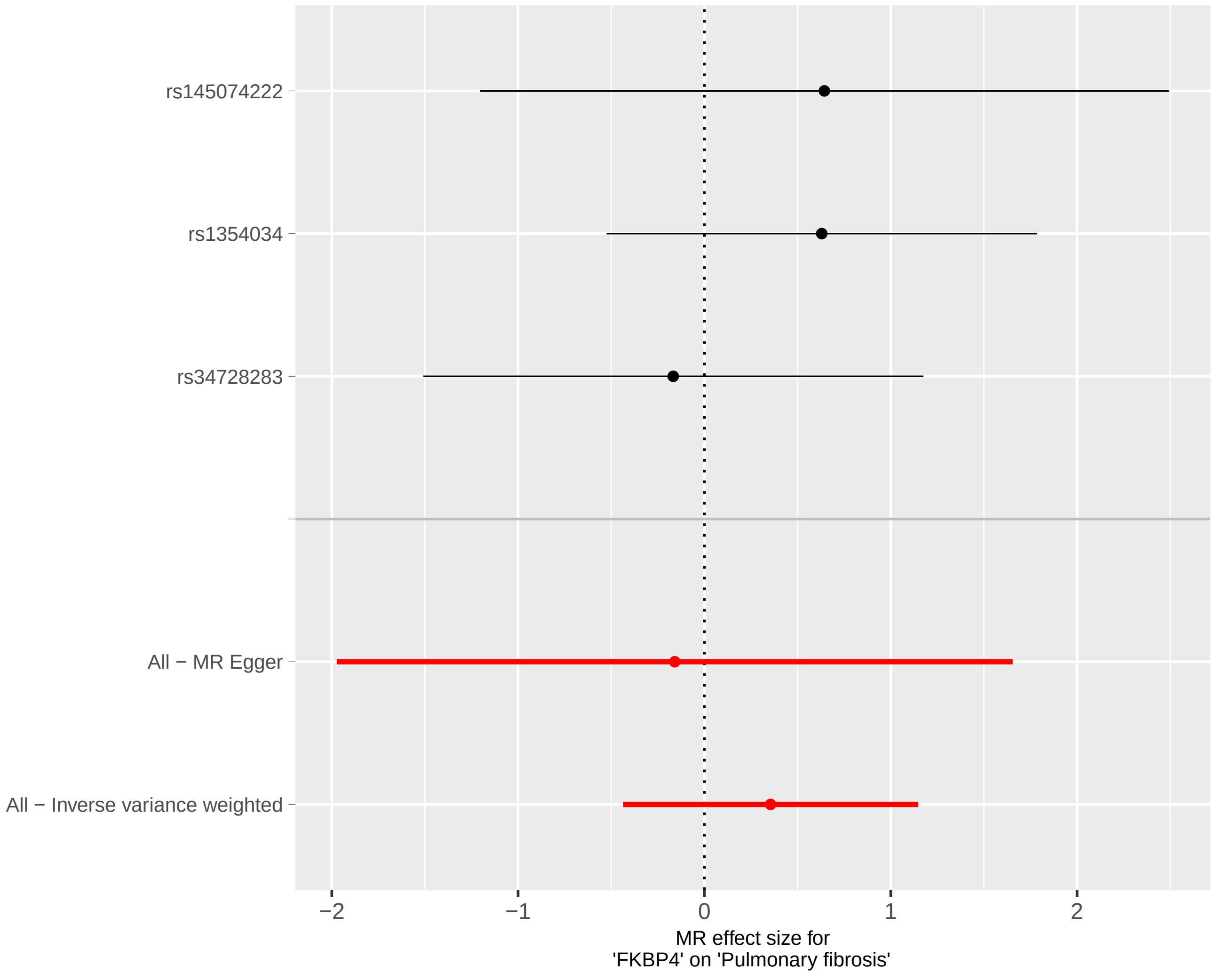

rs145074222
rs1354034
rs34728283
All − MR Egger
All − Inverse variance weighted
−2
−1
0
1
2
MR effect size for
'FKBP4' on 'Pulmonary fibrosis'

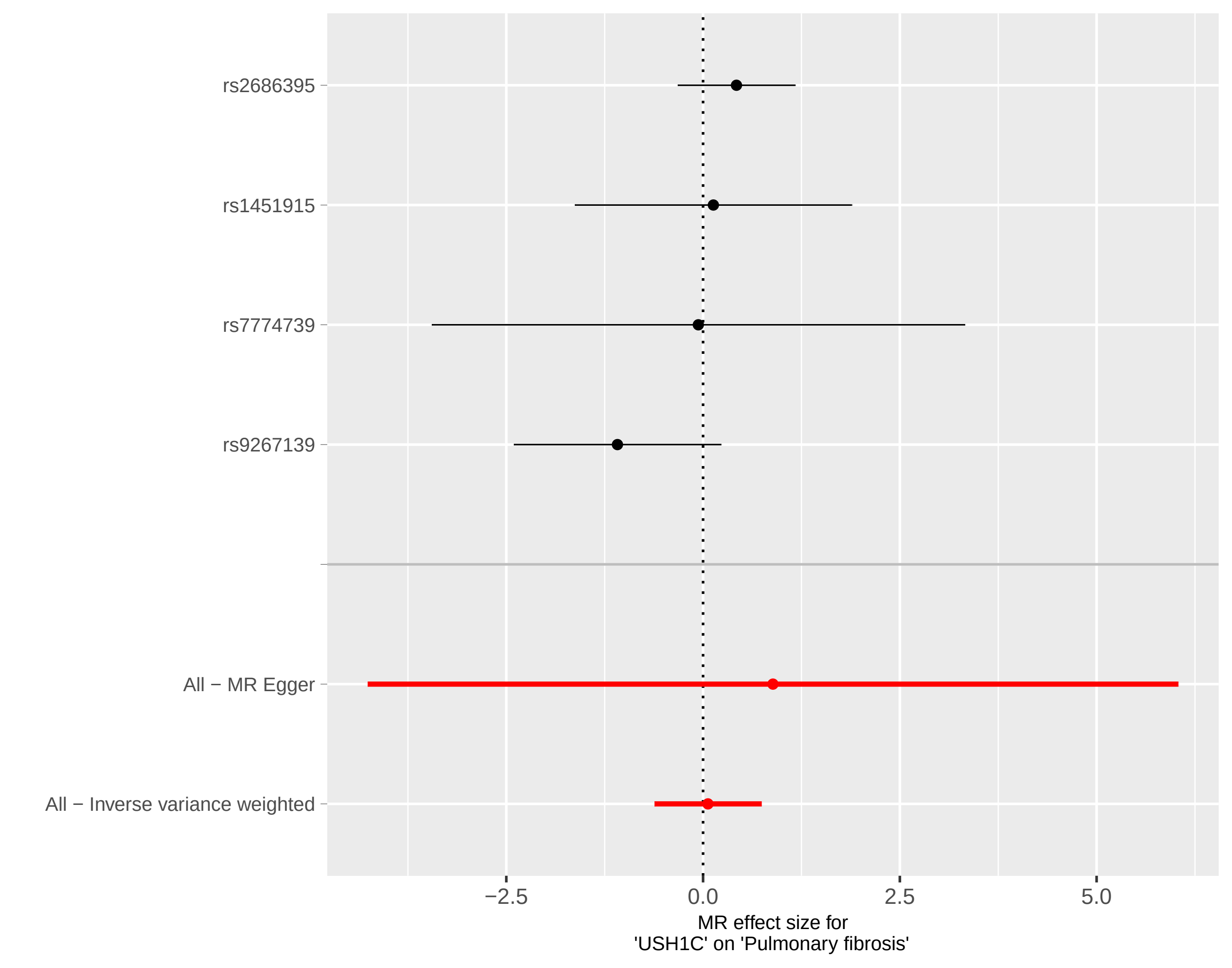

rs2686395
rs1451915
rs7774739
rs9267139
All − MR Egger
All − Inverse variance weighted
−2.5
0.0
2.5
5.0
MR effect size for
'USH1C' on 'Pulmonary fibrosis'

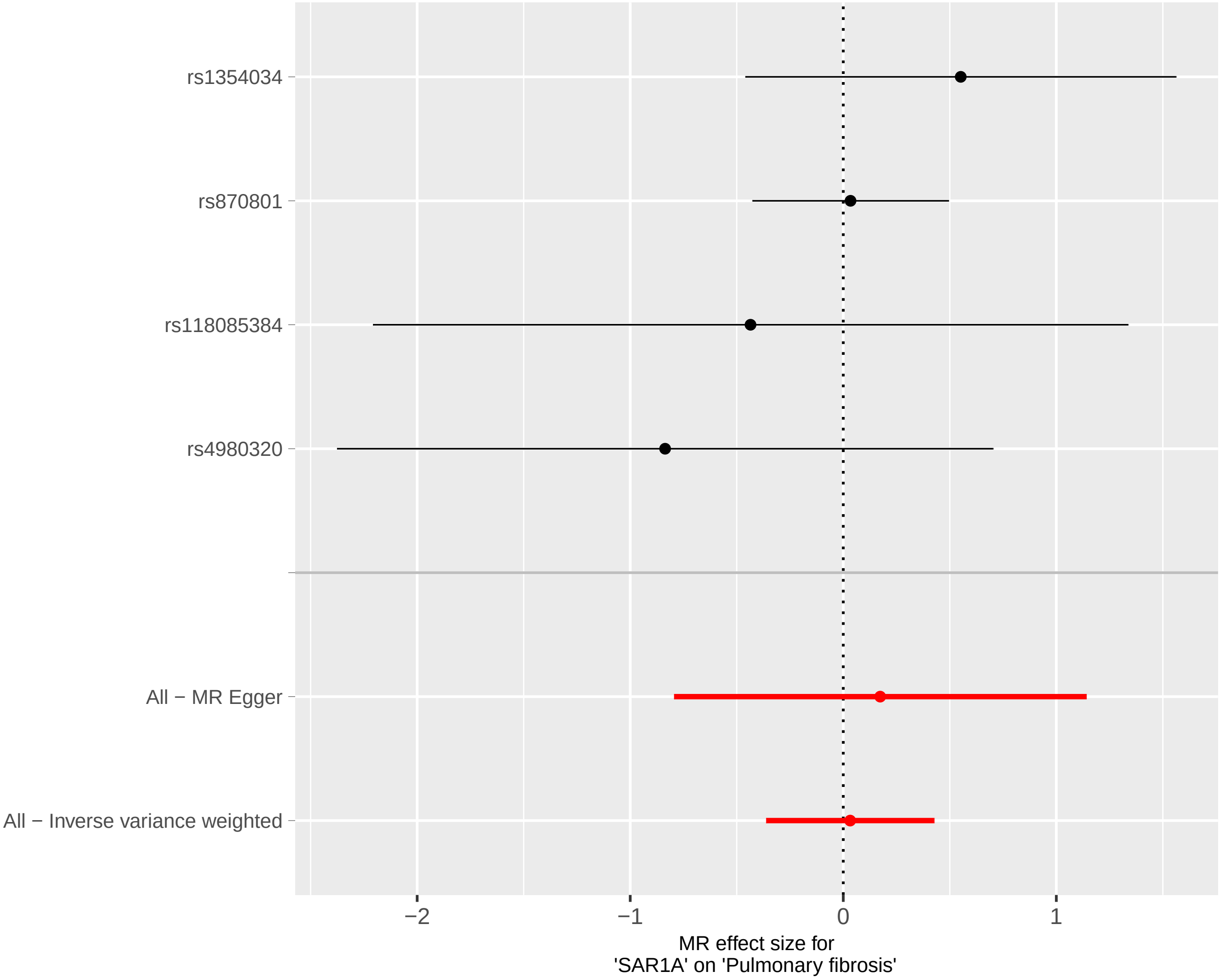
rs1354034
rs870801
rs118085384
rs4980320
All − MR Egger
All − Inverse variance weighted
−2
−1
0
1
MR effect size for
'SAR1A' on 'Pulmonary fibrosis'

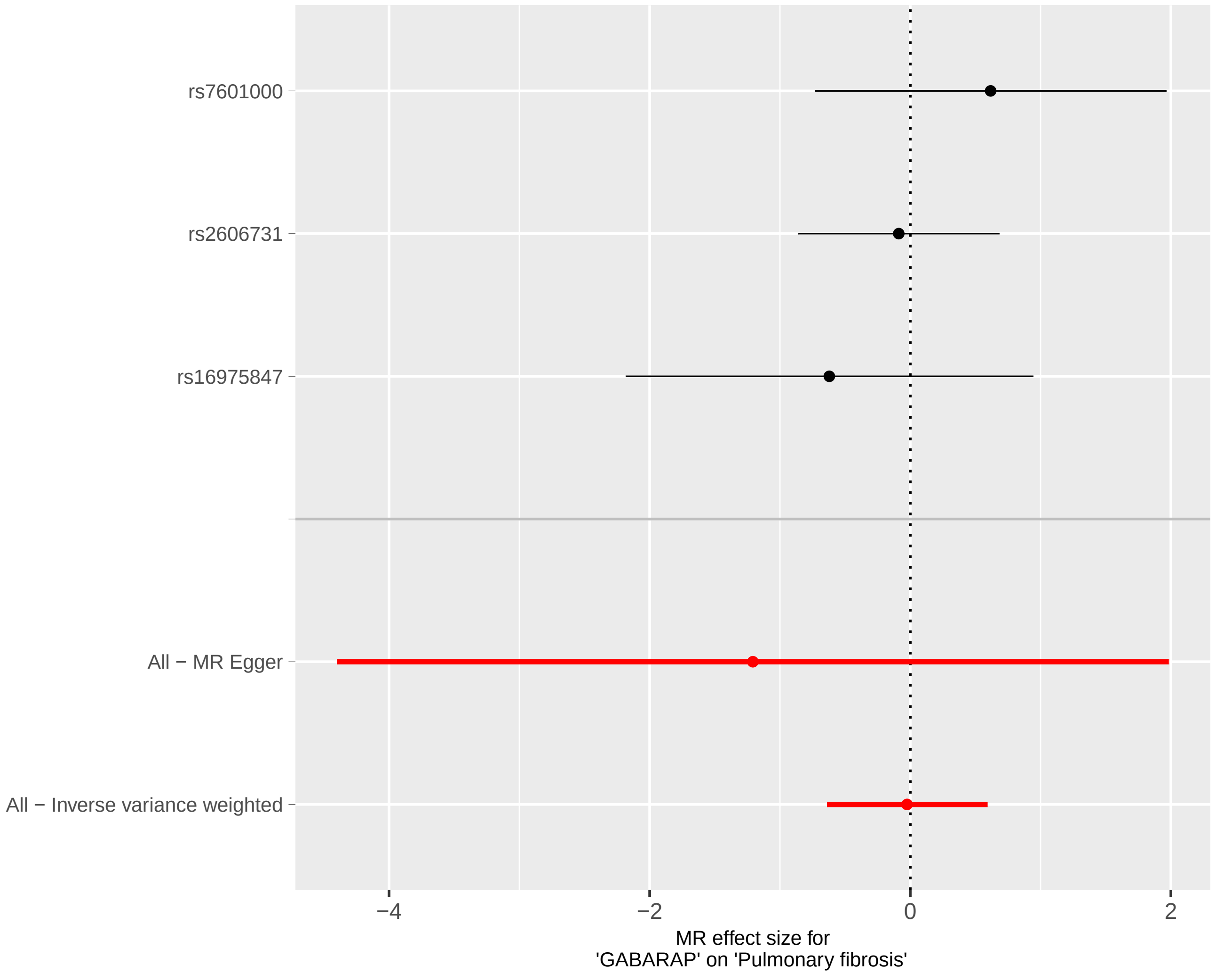
rs7601000
rs2606731
rs16975847
All − MR Egger
All − Inverse variance weighted
−4
−2
0
2
MR effect size for
'GABARAP' on 'Pulmonary fibrosis'

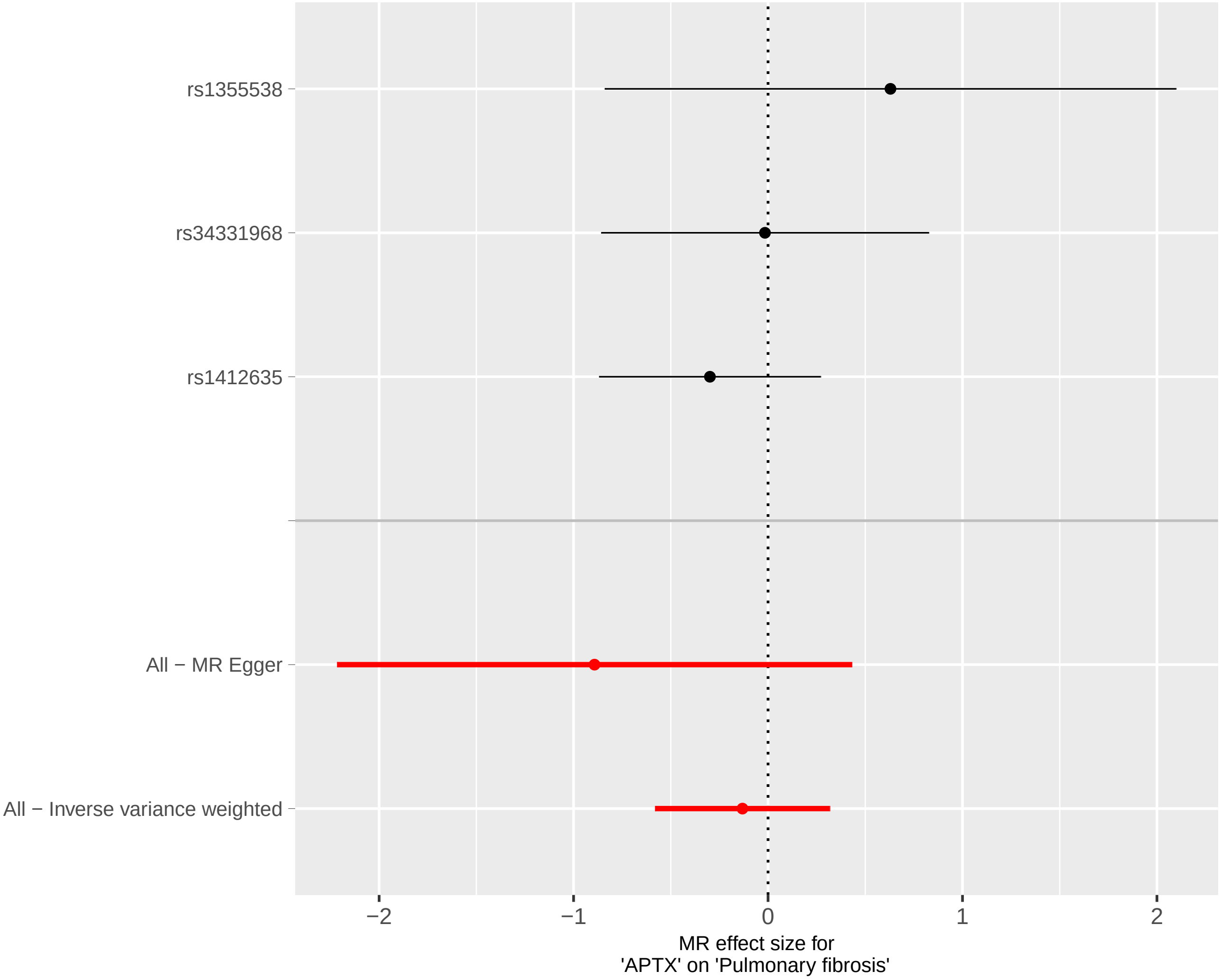

rs1355538
rs34331968
rs1412635
All − MR Egger
All − Inverse variance weighted
−2
−1
0
1
2
MR effect size for
'APTX' on 'Pulmonary fibrosis'

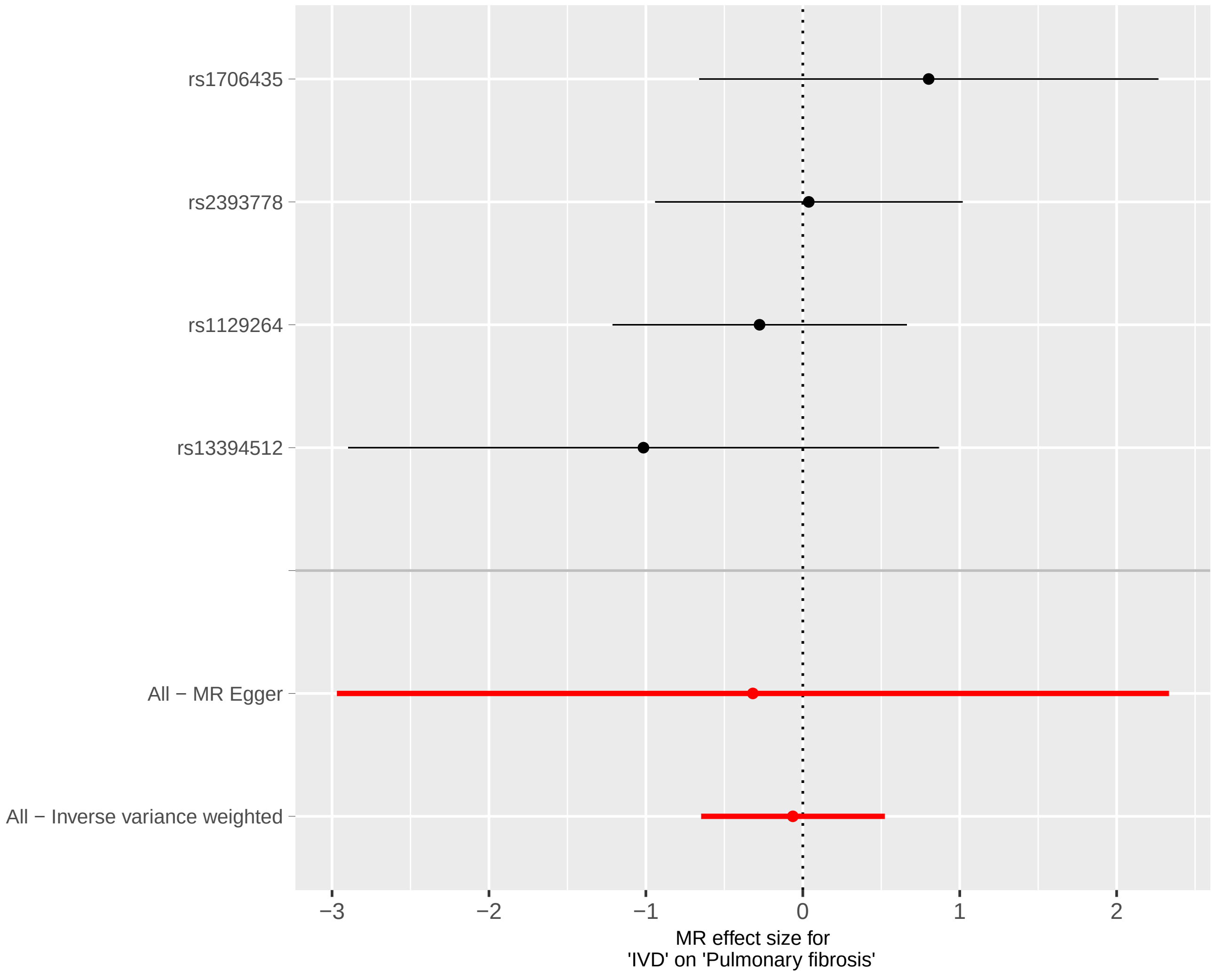
rs1706435
rs2393778
rs1129264
rs13394512
All − MR Egger
All − Inverse variance weighted
−3
−2
−1
0
1
2
MR effect size for
'IVD' on 'Pulmonary fibrosis'

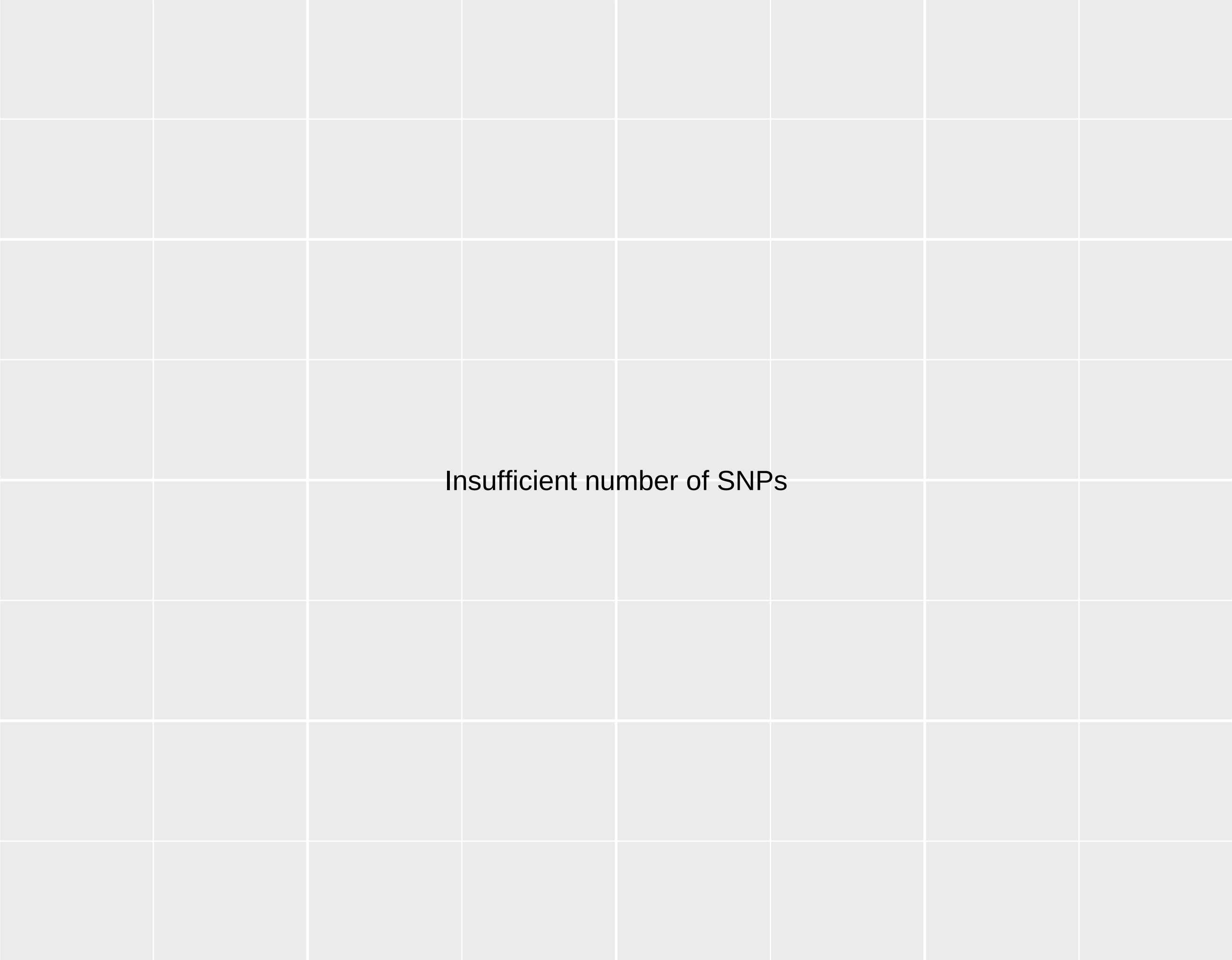
Insufficient number of SNPs

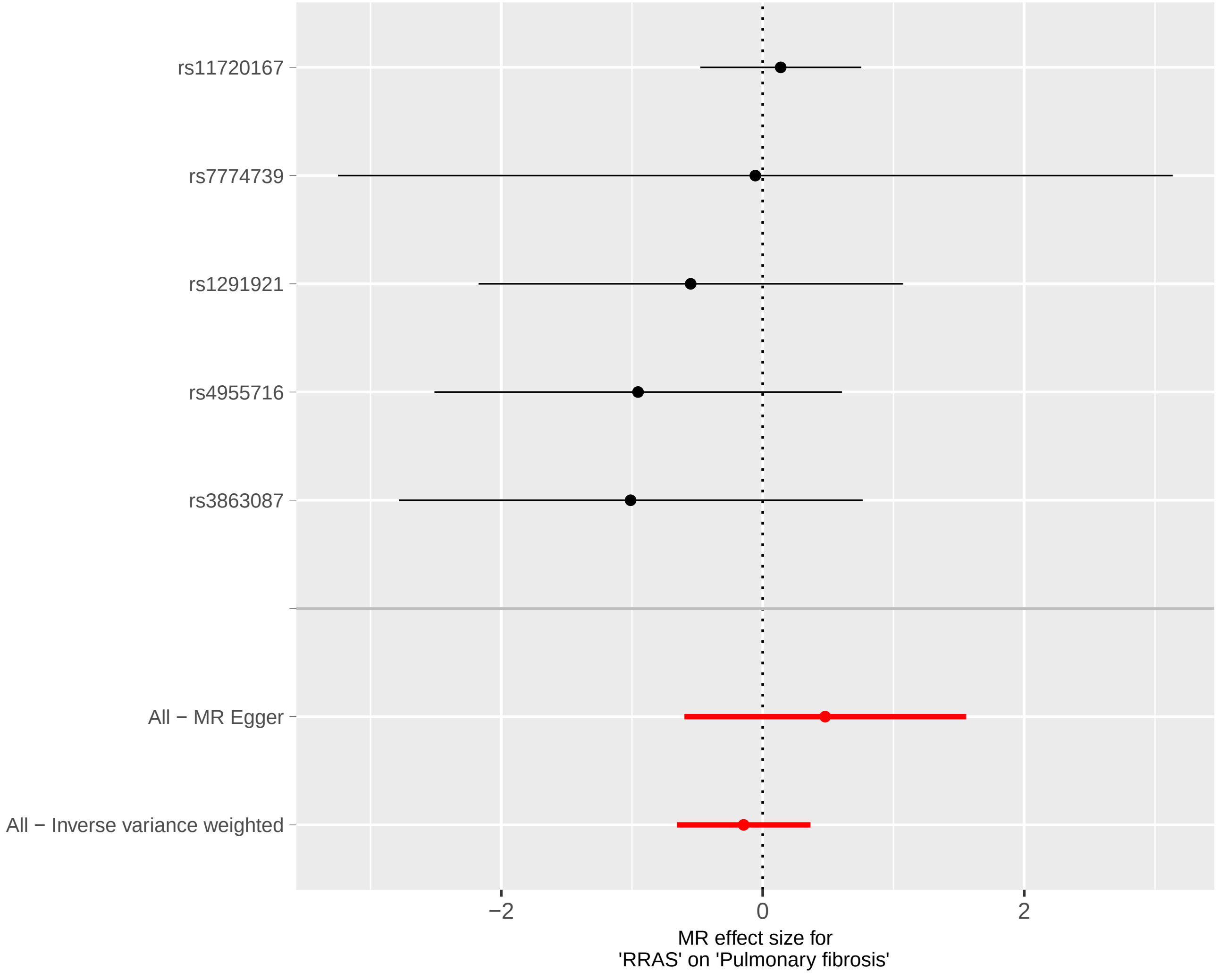
rs11720167
rs7774739
rs1291921
rs4955716
rs3863087
All − MR Egger
All − Inverse variance weighted
−2
0
2
MR effect size for
'RRAS' on 'Pulmonary fibrosis'

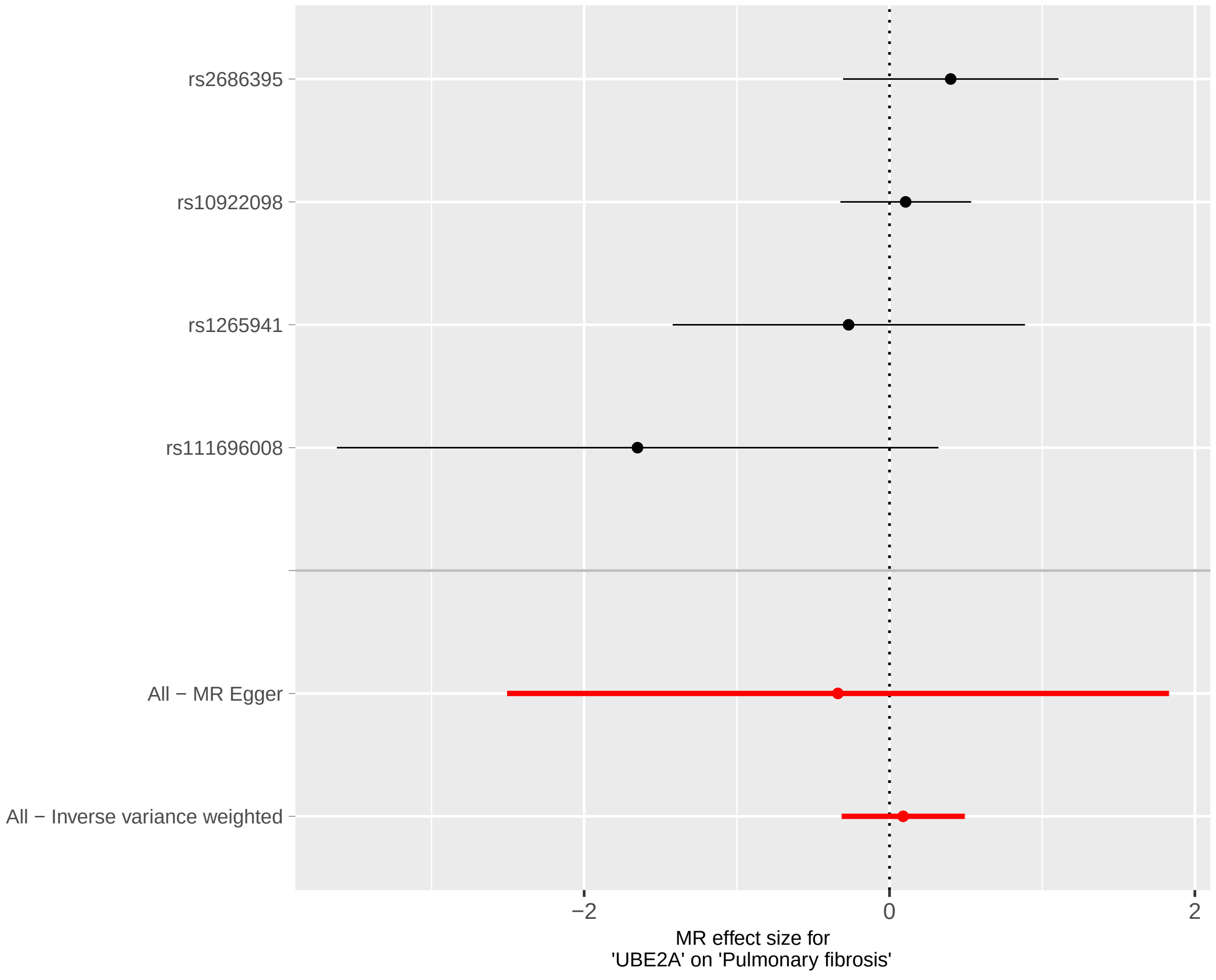

rs2686395
rs10922098
rs1265941
rs11696008
All − MR Egger
All − Inverse variance weighted
−2
0
2
MR effect size for
'UBE2A' on 'Pulmonary fibrosis'

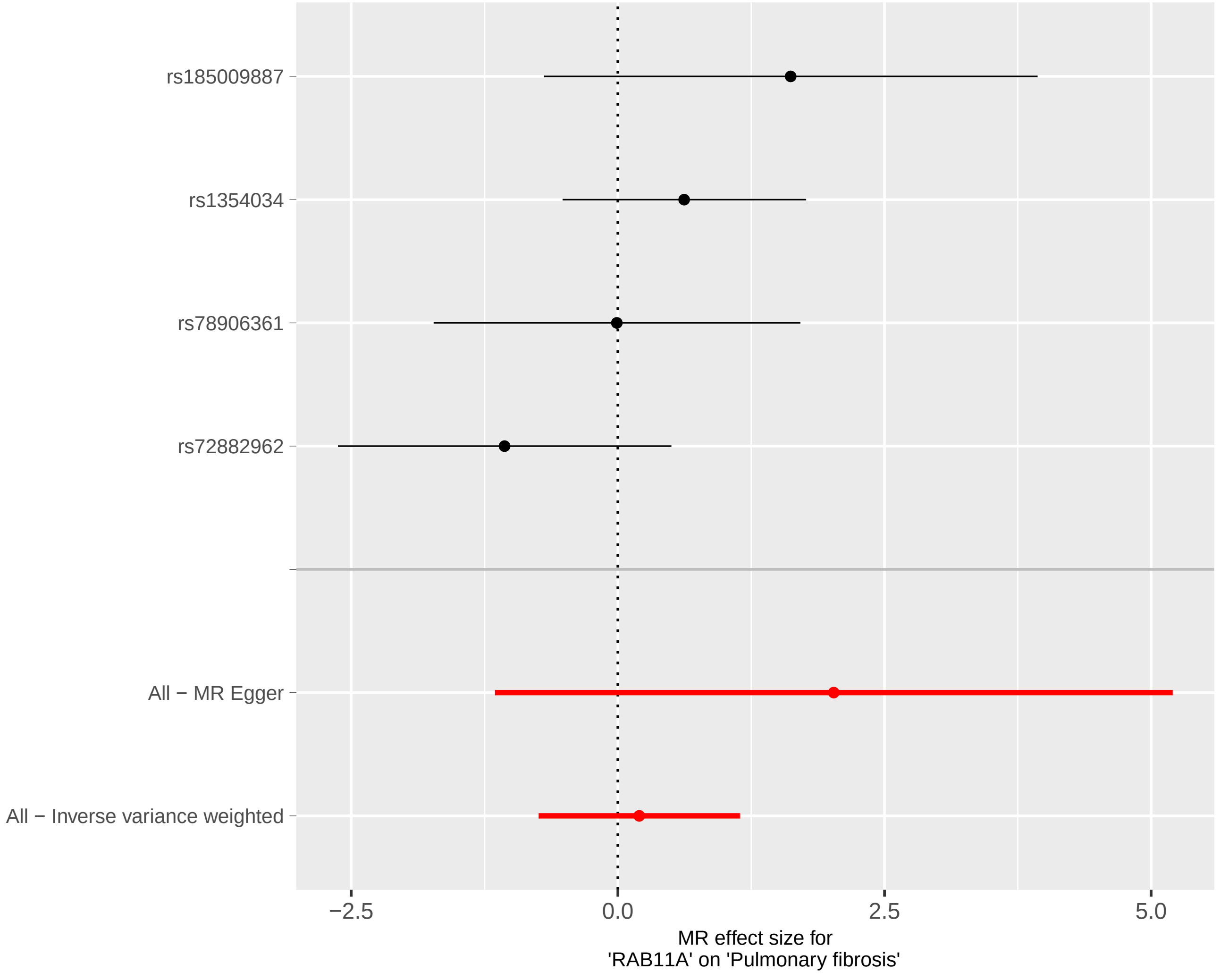

rs185009887
rs1354034
rs78906361
rs72882962
All − MR Egger
All − Inverse variance weighted
−2.5
0.0
2.5
5.0
MR effect size for
'RAB11A' on 'Pulmonary fibrosis'

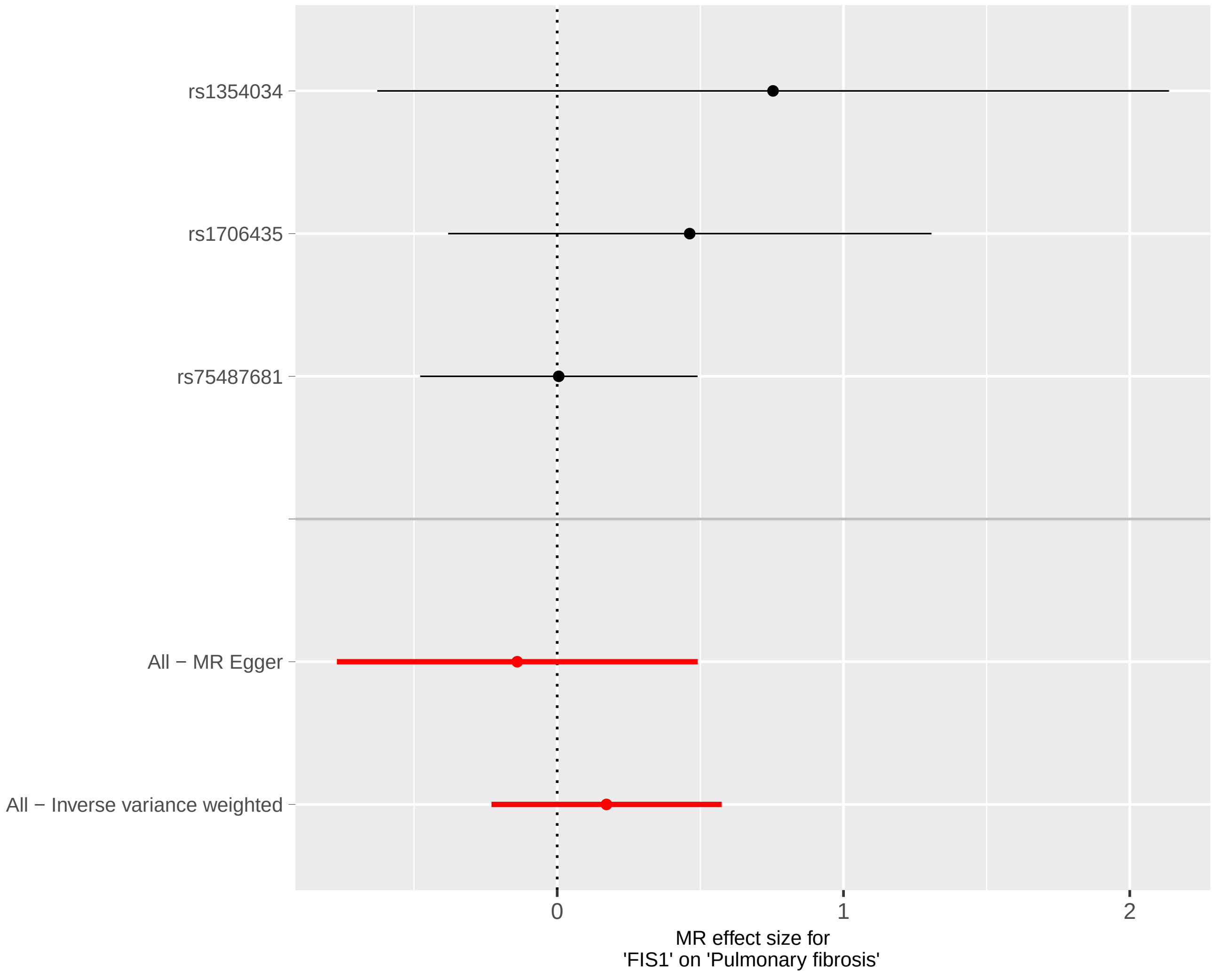
rs1354034
rs1706435
rs75487681
All − MR Egger
All − Inverse variance weighted
0
1
2
MR effect size for
'FIS1' on 'Pulmonary fibrosis'

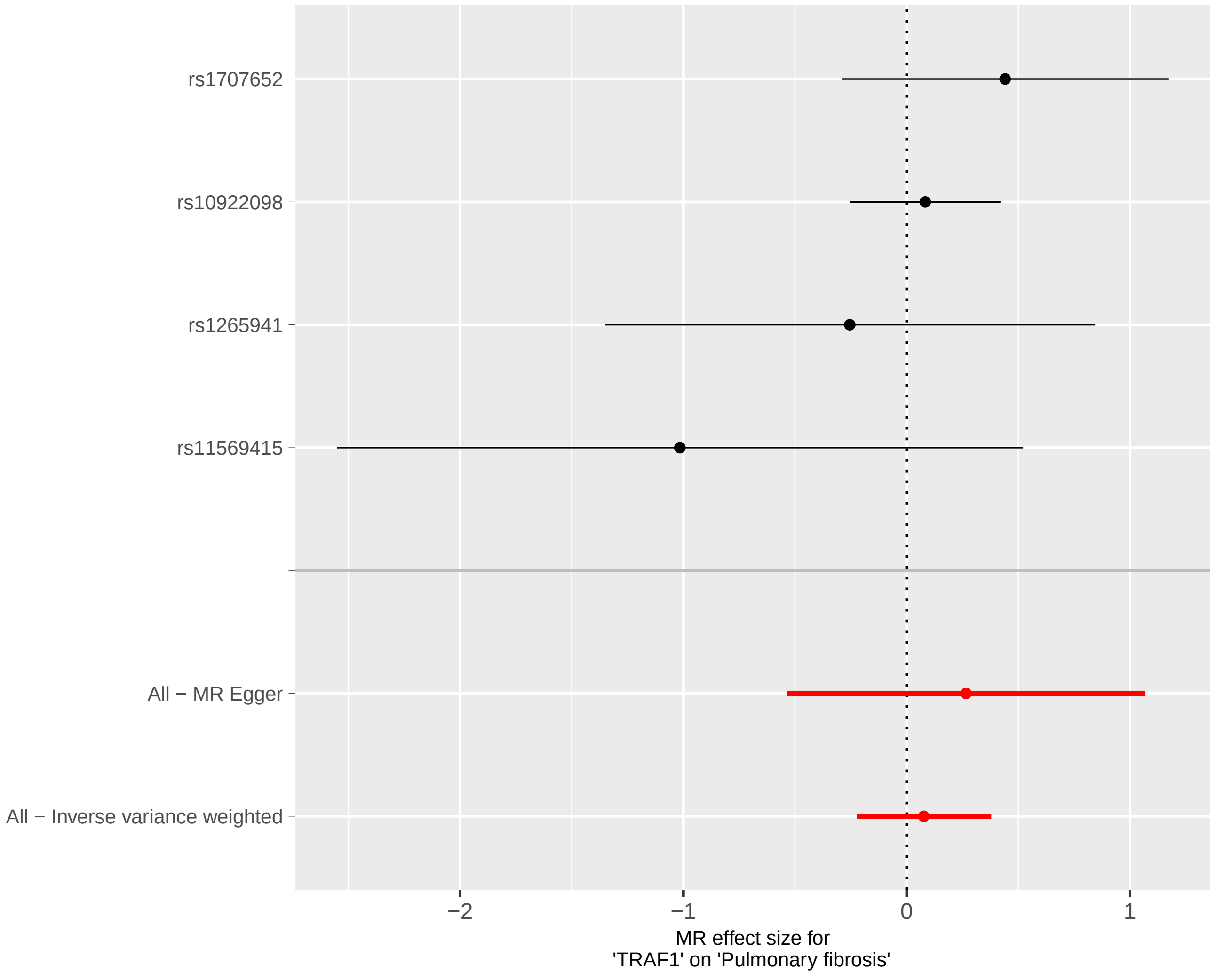
rs1707652
rs10922098
rs1265941
rs11569415
All − MR Egger
All − Inverse variance weighted
−2
−1
0
1
MR effect size for
'TRAF1' on 'Pulmonary fibrosis'

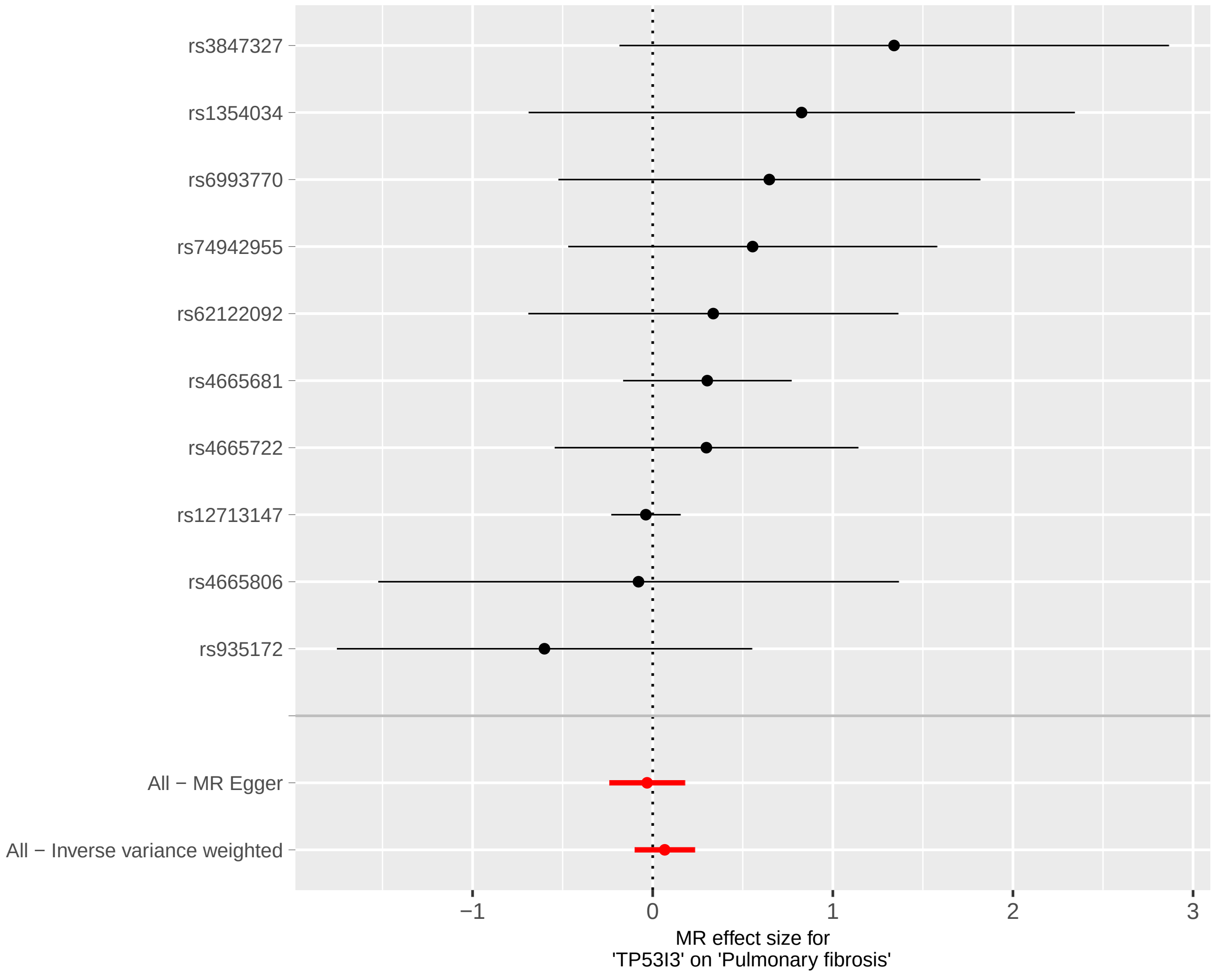

rs3847327
rs1354034
rs6993770
rs74942955
rs62122092
rs4665681
rs4665722
rs12713147
rs4665806
rs935172
All − MR Egger
All − Inverse variance weighted
−1
0
1
2
3
MR effect size for
'TP53I3' on 'Pulmonary fibrosis'

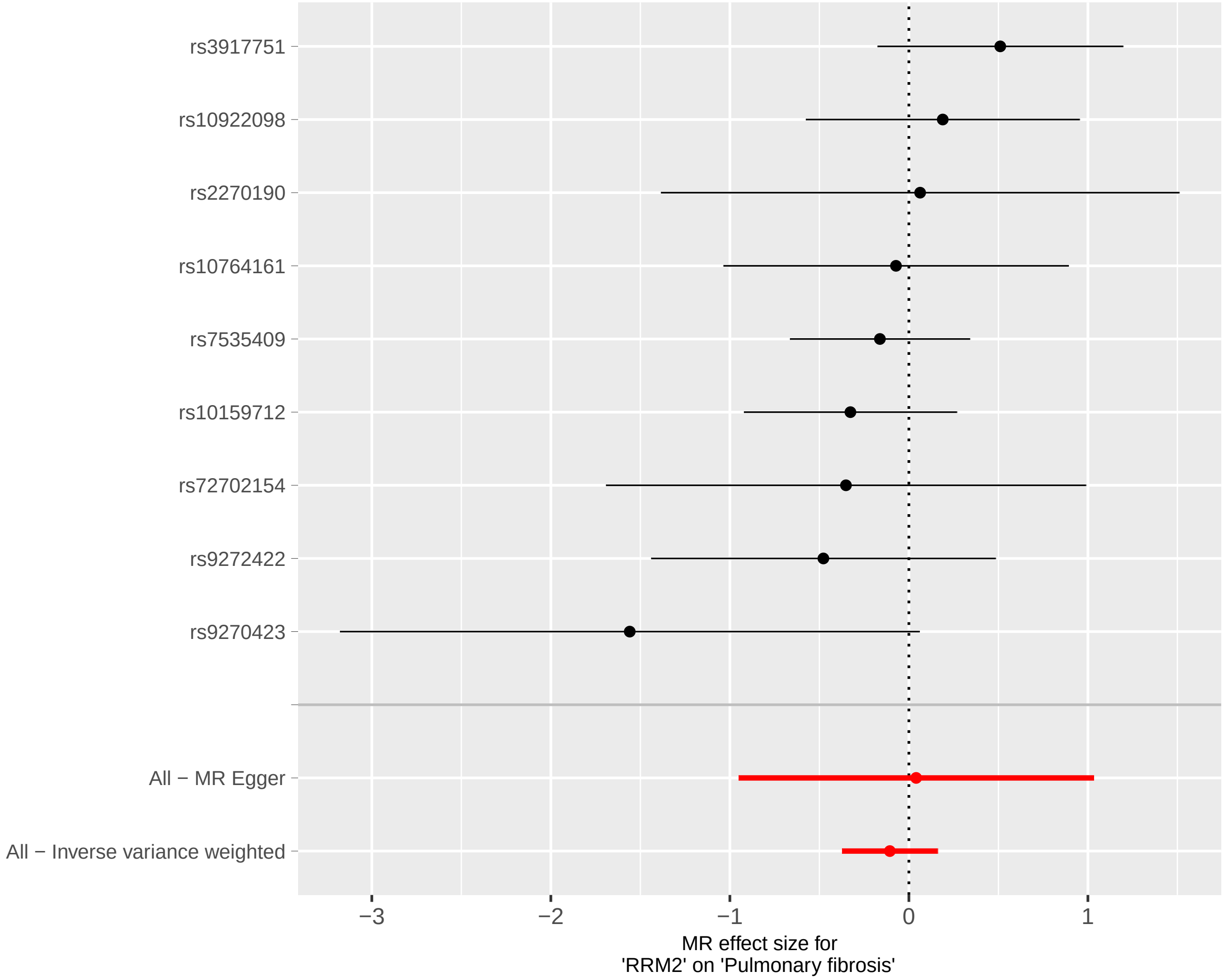

rs3917751
rs10922098
rs2270190
rs10764161
rs7535409
rs10159712
rs72702154
rs9272422
rs9270423
All − MR Egger
All − Inverse variance weighted
−3
−2
−1
0
1
MR effect size for
'RRM2' on 'Pulmonary fibrosis'

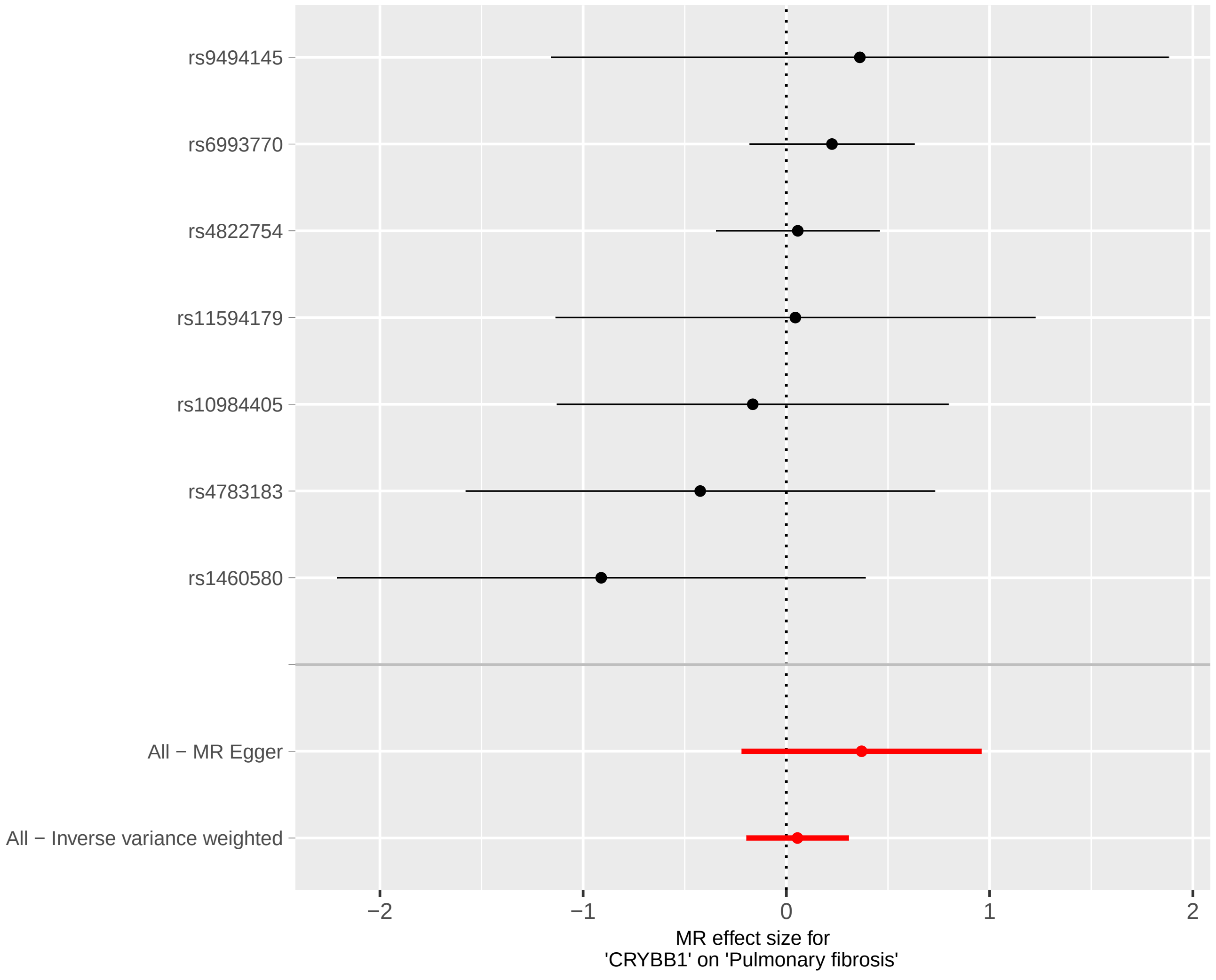
rs9494145
rs6993770
rs4822754
rs11594179
rs10984405
rs4783183
rs1460580
All − MR Egger
All − Inverse variance weighted
−2
−1
0
1
2
MR effect size for
'CRYBB1' on 'Pulmonary fibrosis'

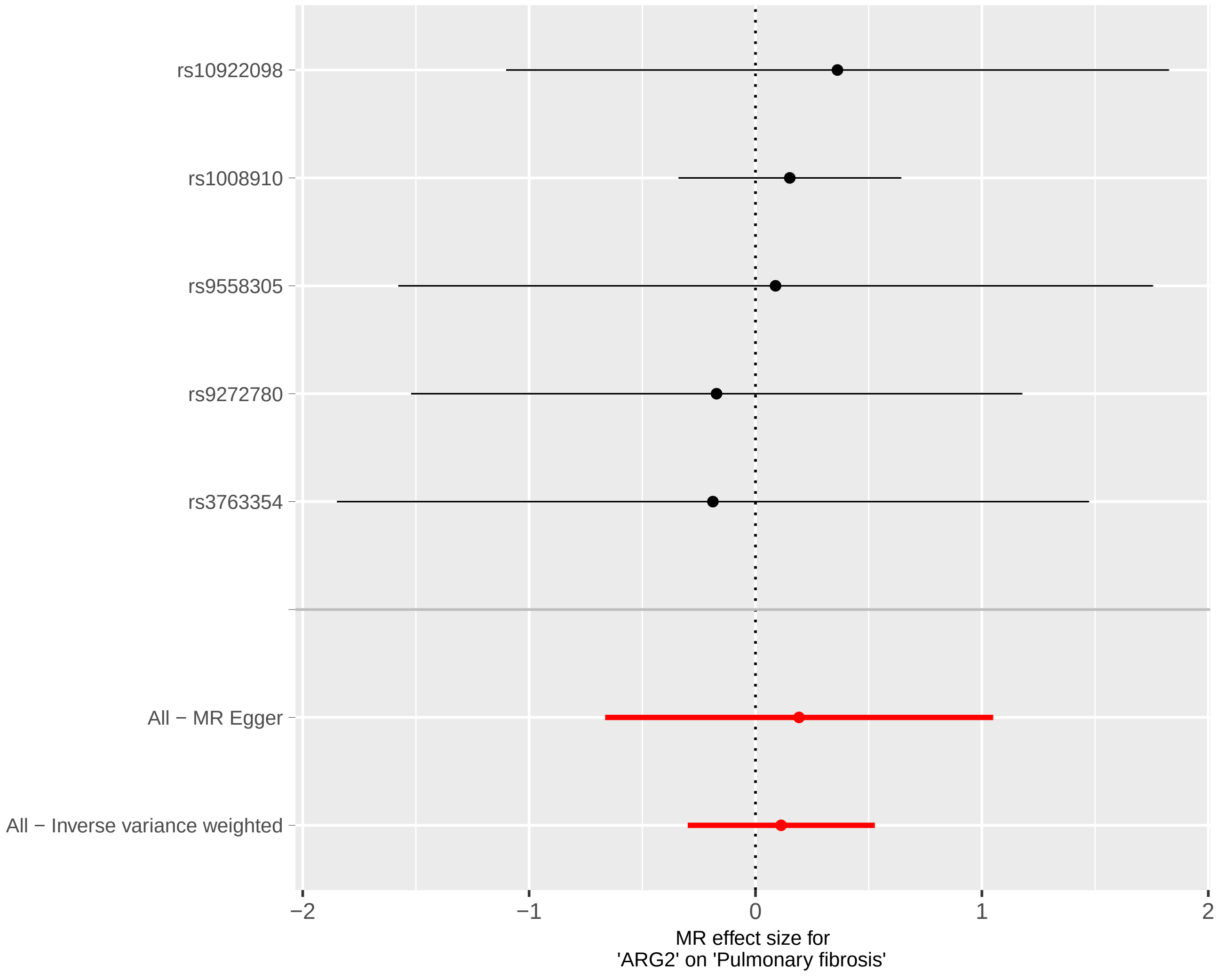
rs10922098
rs1008910
rs9558305
rs9272780
rs3763354
All − MR Egger
All − Inverse variance weighted
−2
−1
0
1
2
MR effect size for
'ARG2' on 'Pulmonary fibrosis'

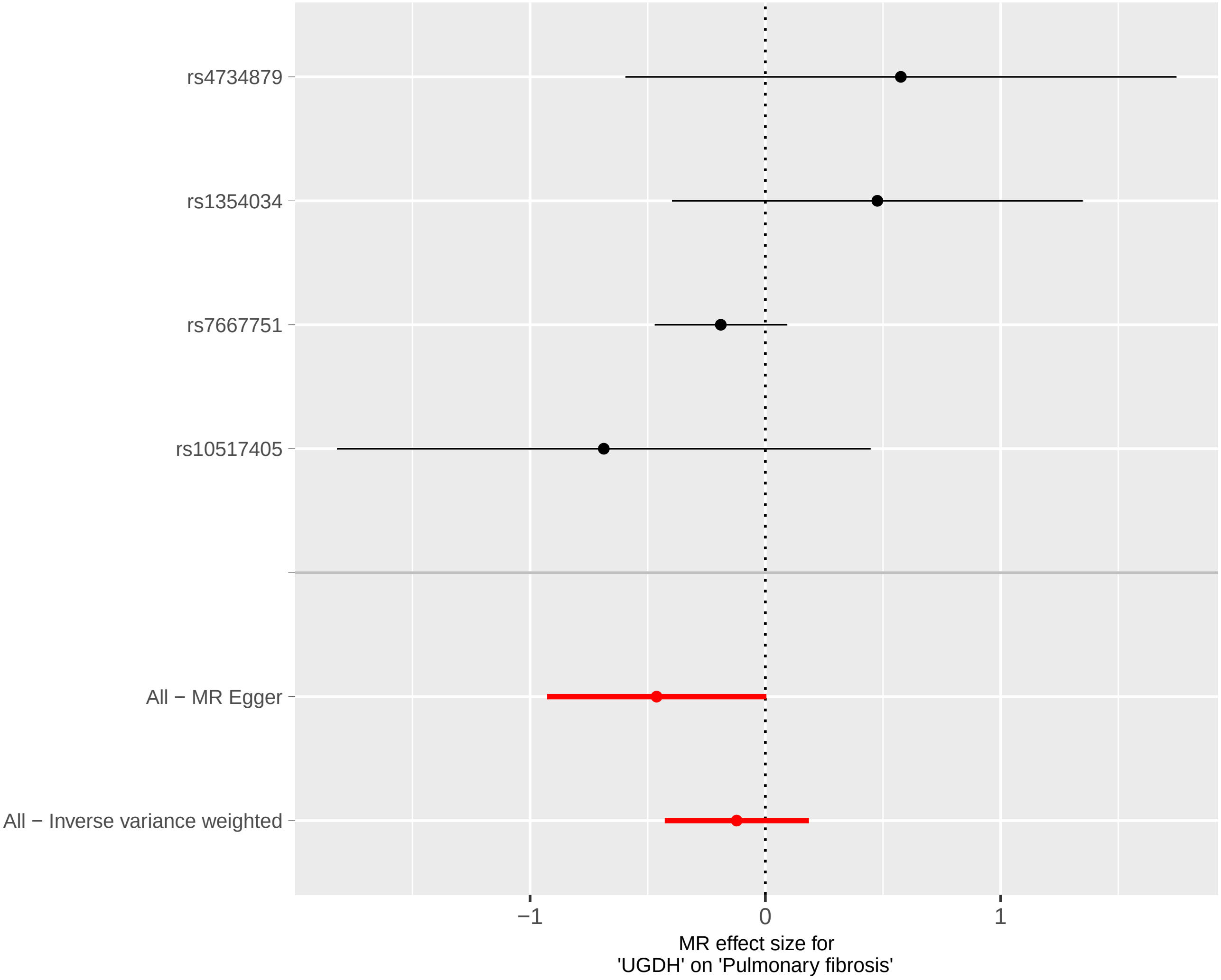
rs4734879
rs1354034
rs7667751
rs10517405
All − MR Egger
All − Inverse variance weighted
−1
0
1
MR effect size for
'UGDH' on 'Pulmonary fibrosis'

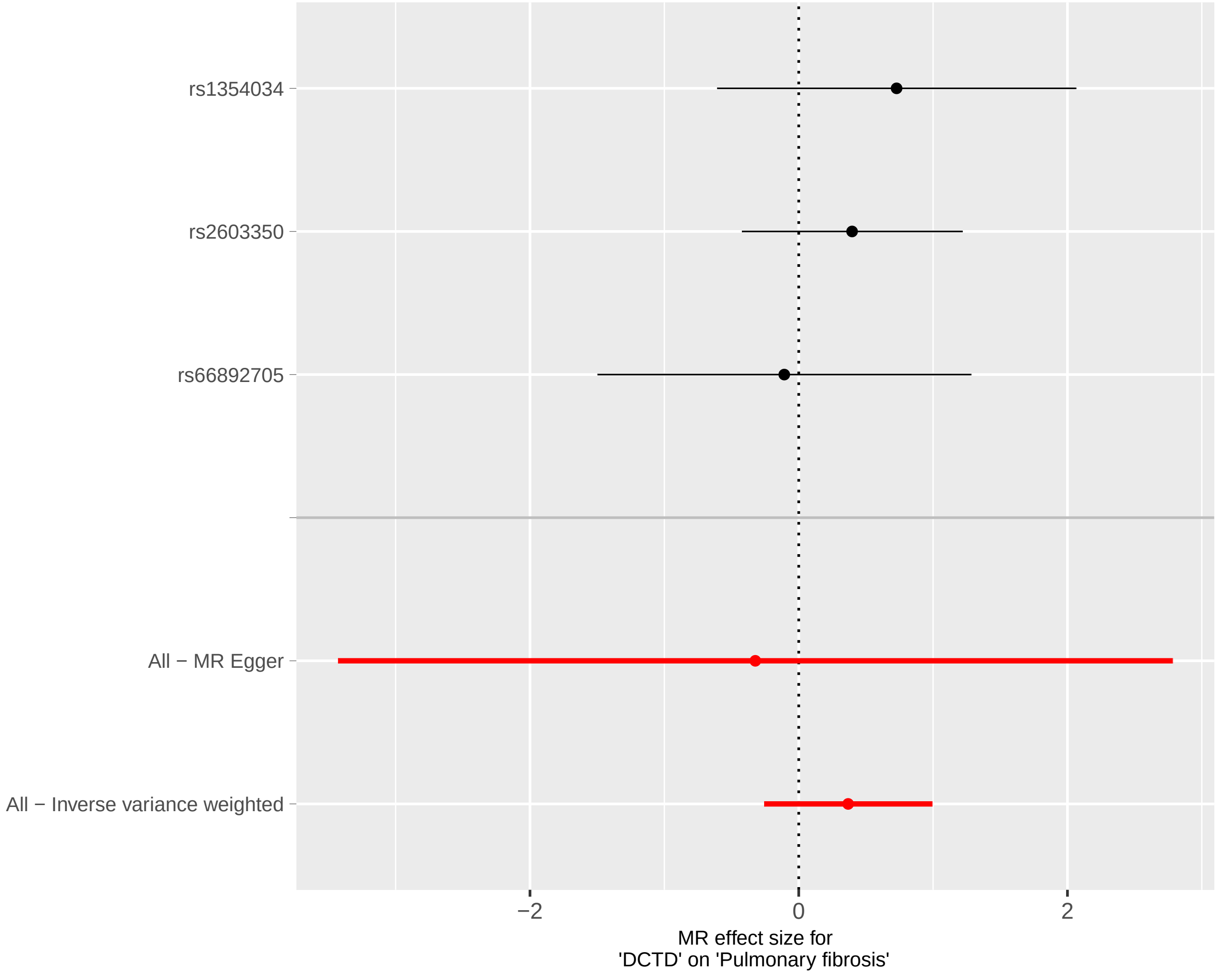

rs1354034
rs2603350
rs66892705
All − MR Egger
All − Inverse variance weighted
−2
0
2
MR effect size for
'DCTD' on 'Pulmonary fibrosis'

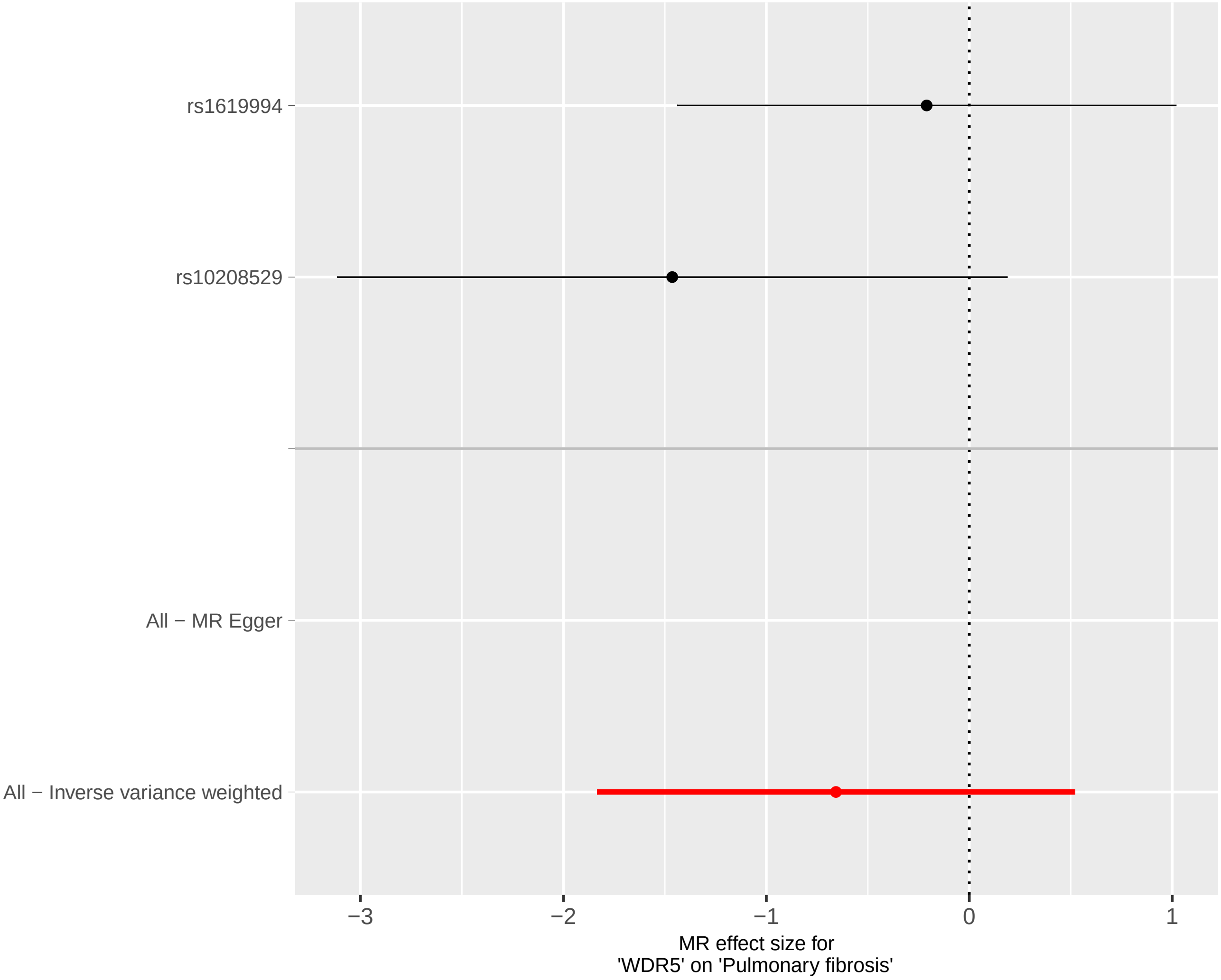

rs1619994
rs10208529
All − MR Egger
All − Inverse variance weighted
−3
−2
−1
0
1
MR effect size for
'WDR5' on 'Pulmonary fibrosis'

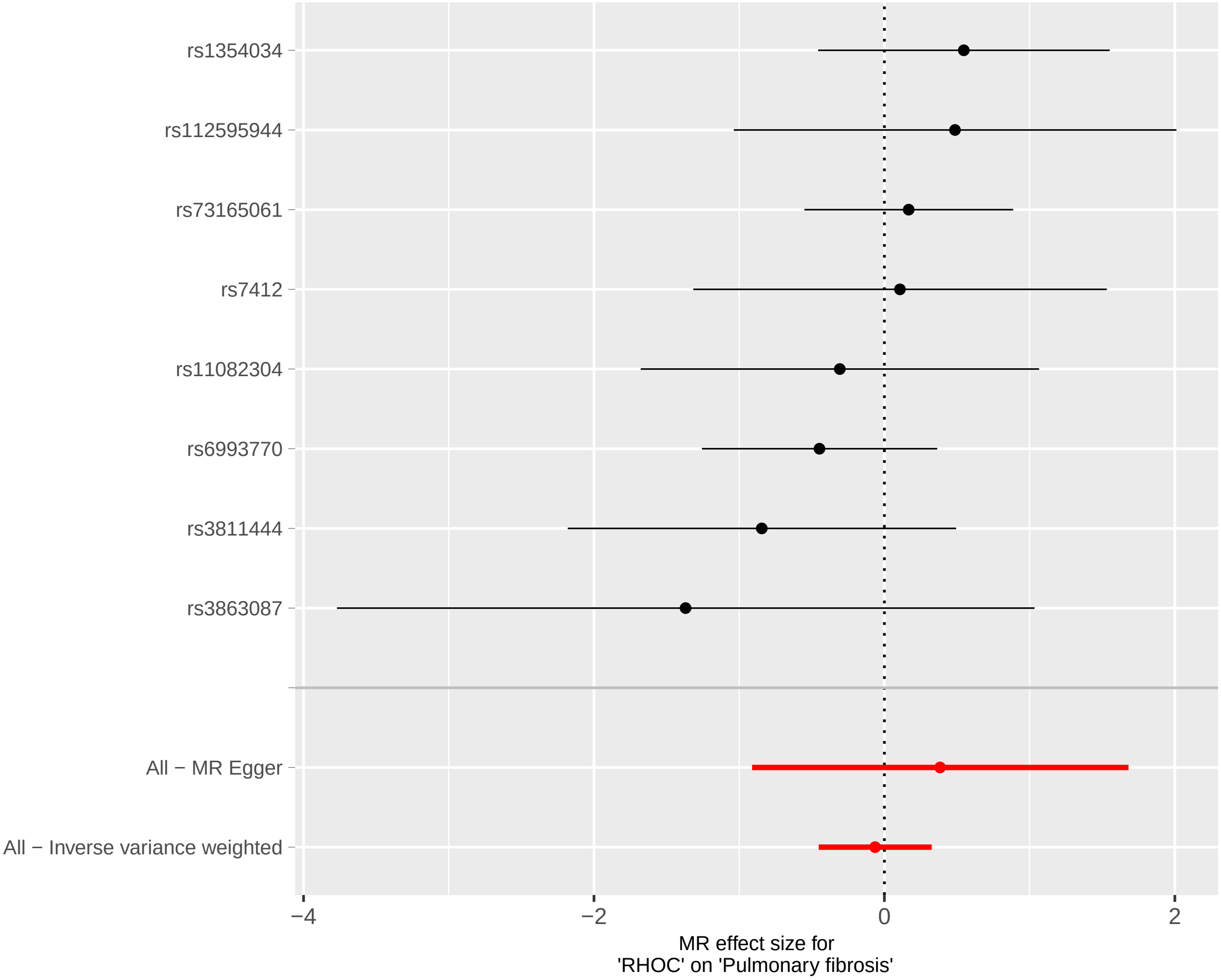
rs1354034
rs112595944
rs73165061
rs7412
rs11082304
rs6993770
rs3811444
rs3863087
All − MR Egger
All − Inverse variance weighted
−4
−2
0
2
MR effect size for
'RHOC' on 'Pulmonary fibrosis'

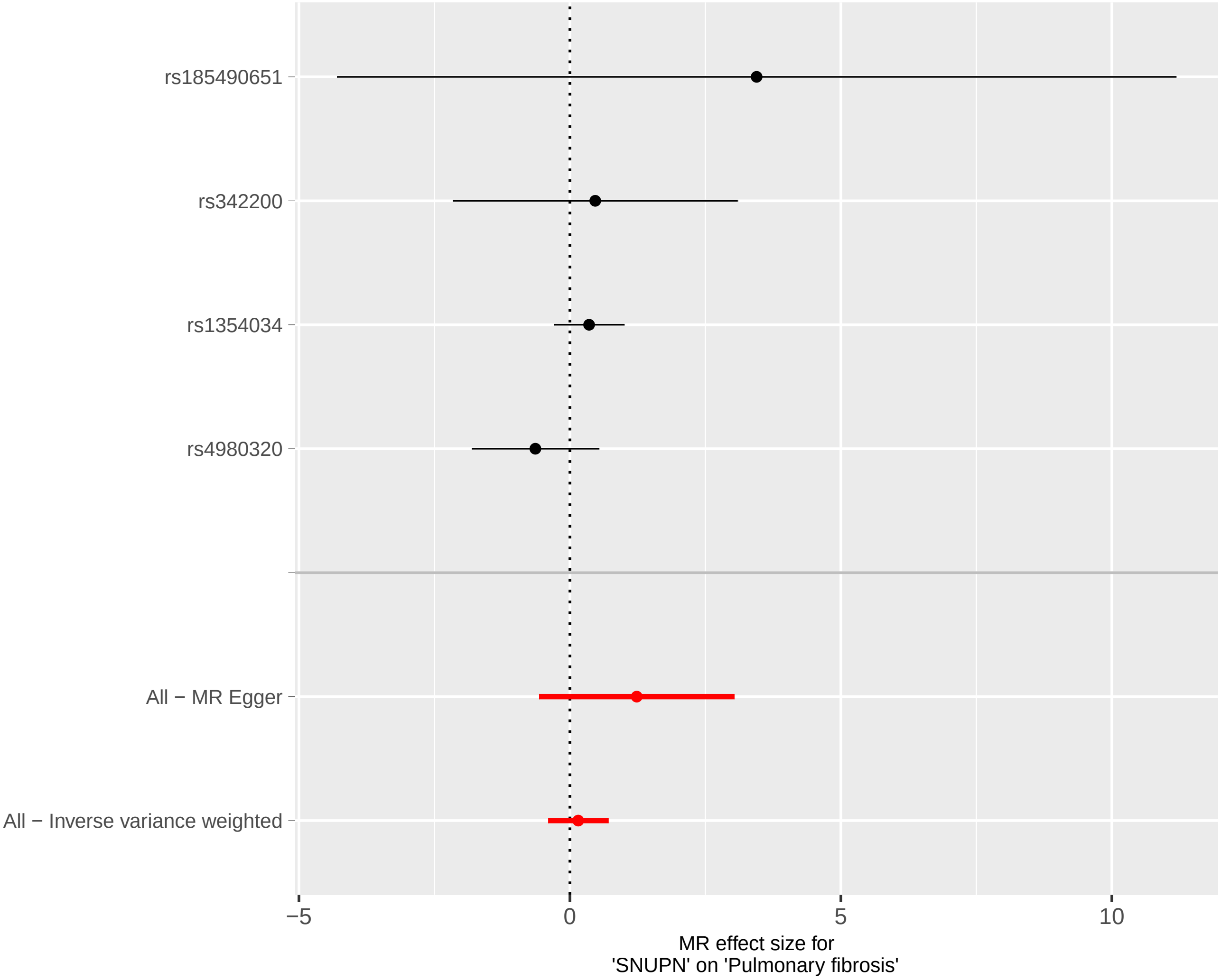
rs185490651
rs342200
rs1354034
rs4980320
All − MR Egger
All − Inverse variance weighted
−5
0
5
10
MR effect size for
'SNUPN' on 'Pulmonary fibrosis'

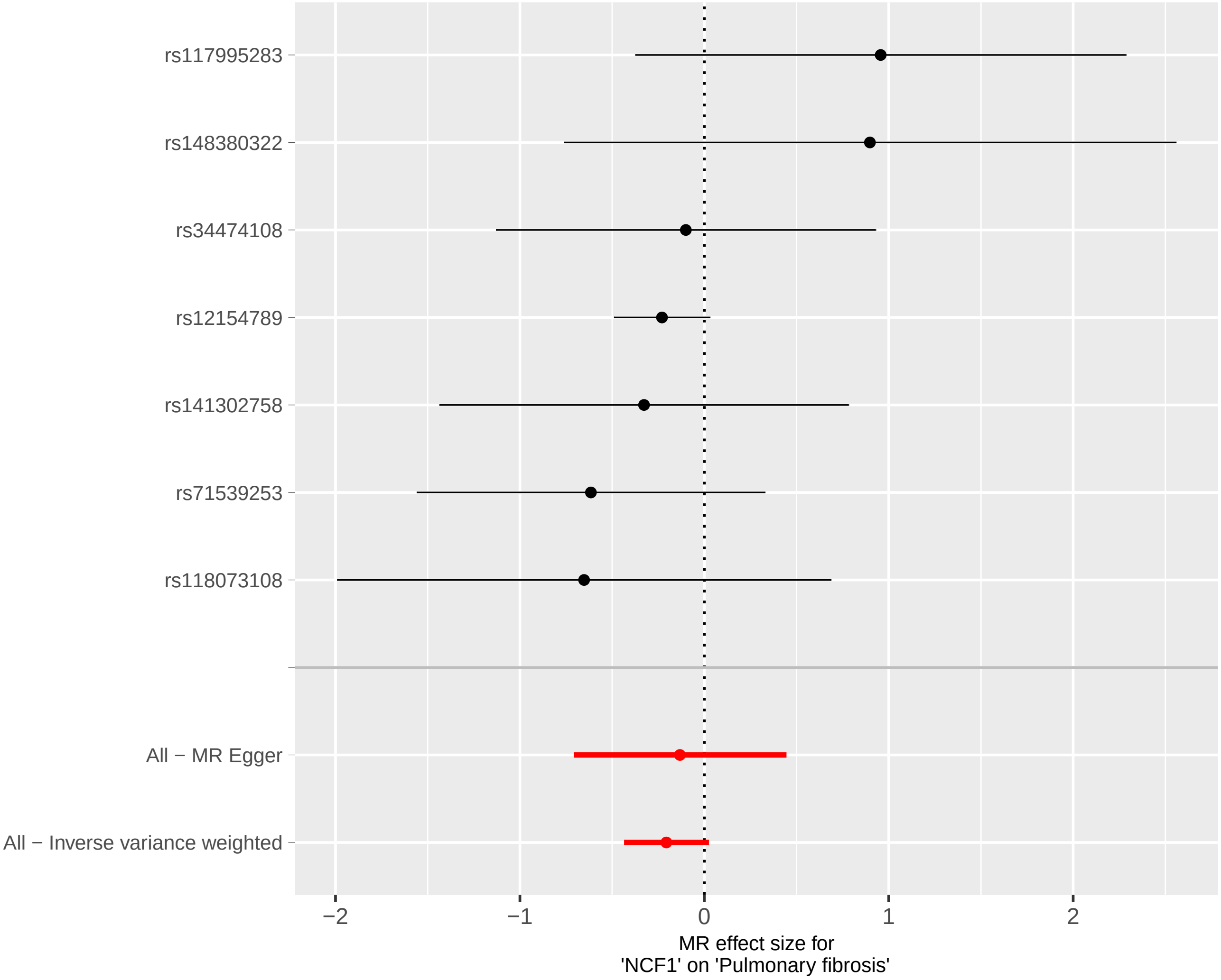
rs117995283
rs148380322
rs34474108
rs12154789
rs141302758
rs71539253
rs118073108
All − MR Egger
All − Inverse variance weighted
−2
−1
0
1
2
MR effect size for
'NCF1' on 'Pulmonary fibrosis'

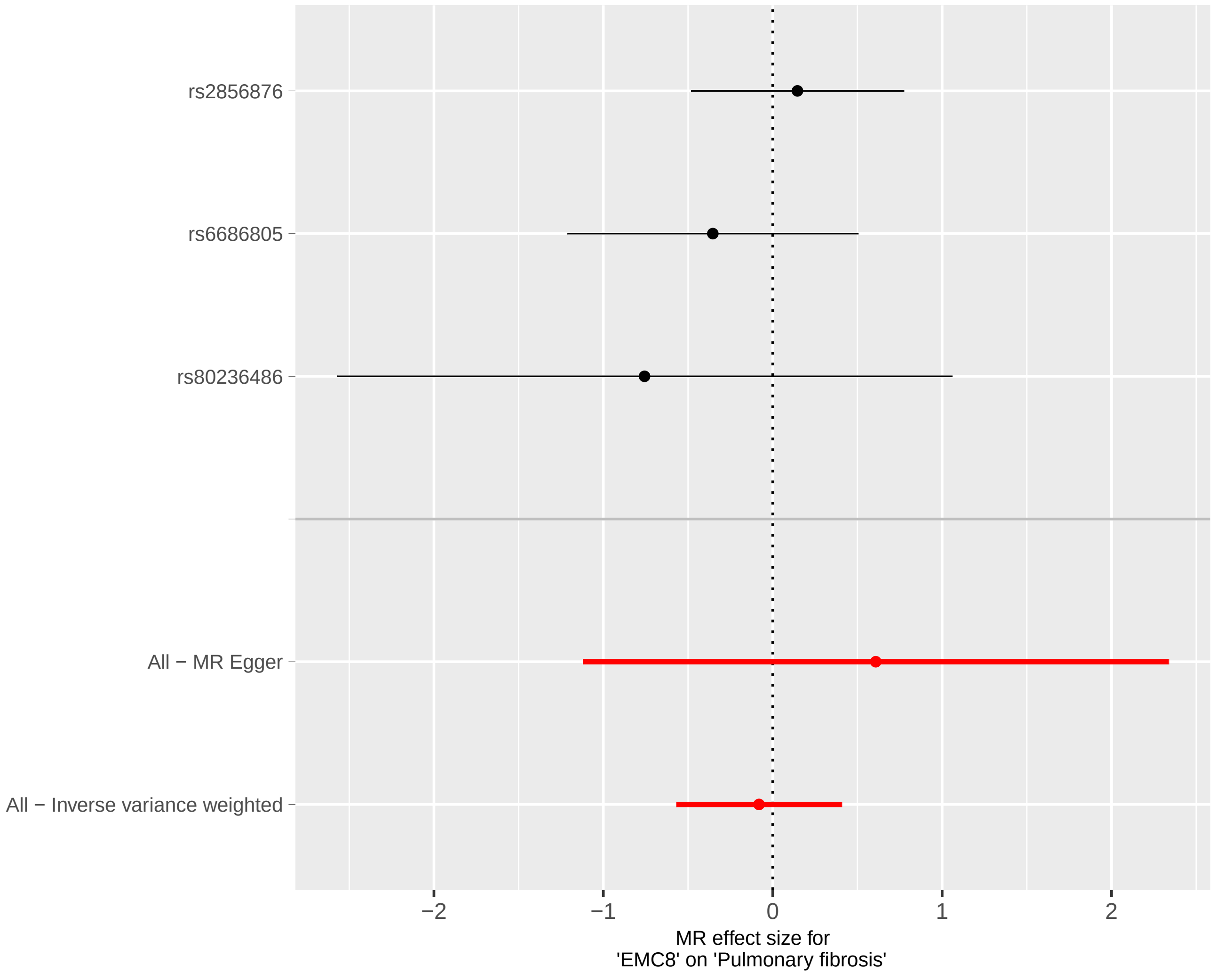
rs2856876
rs6686805
rs80236486
All − MR Egger
All − Inverse variance weighted
−2
−1
0
1
2
MR effect size for
'EMC8' on 'Pulmonary fibrosis'

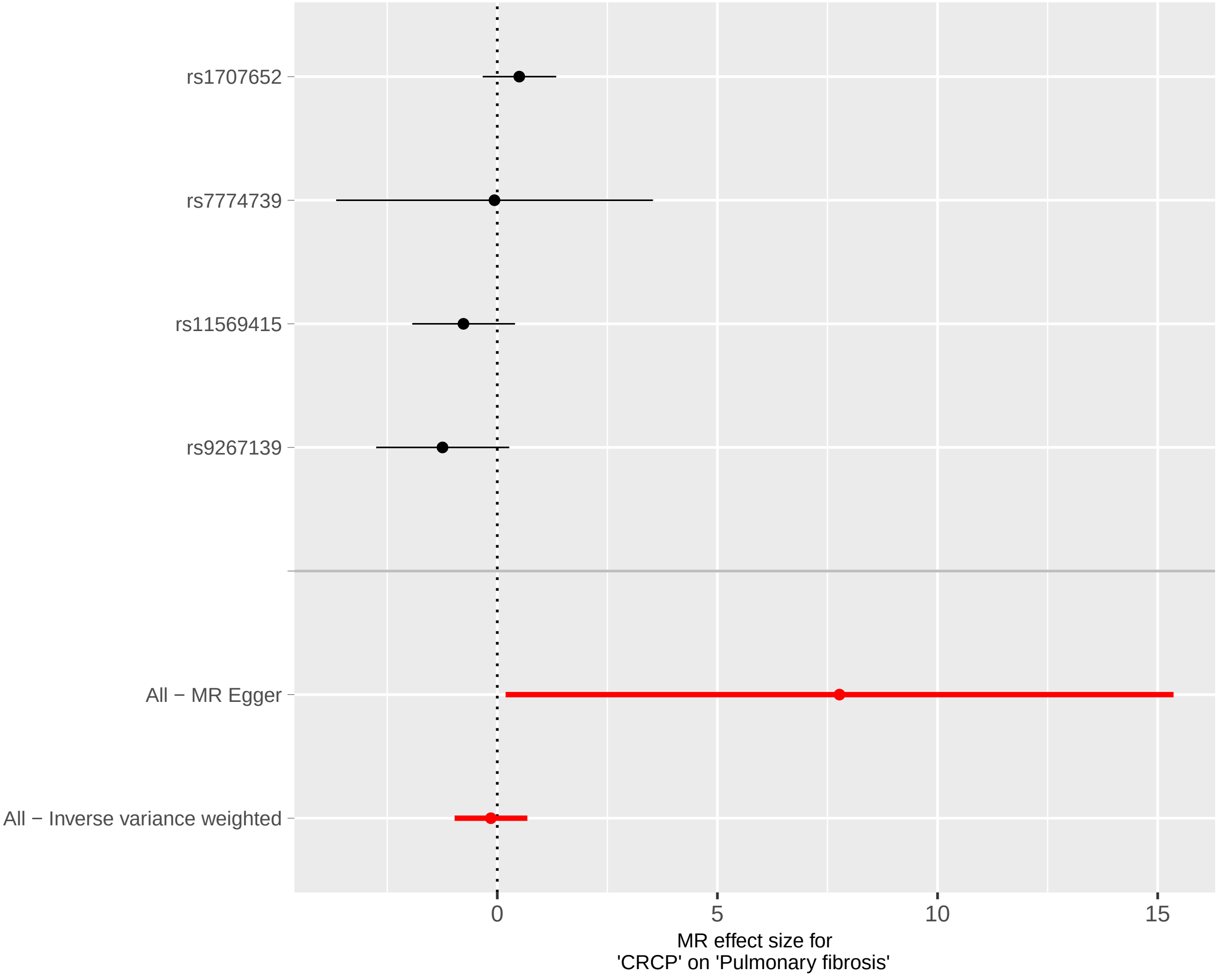

rs1707652
rs7774739
rs11569415
rs9267139
All − MR Egger
All − Inverse variance weighted
0
5
10
15
MR effect size for
'CRCP' on 'Pulmonary fibrosis'

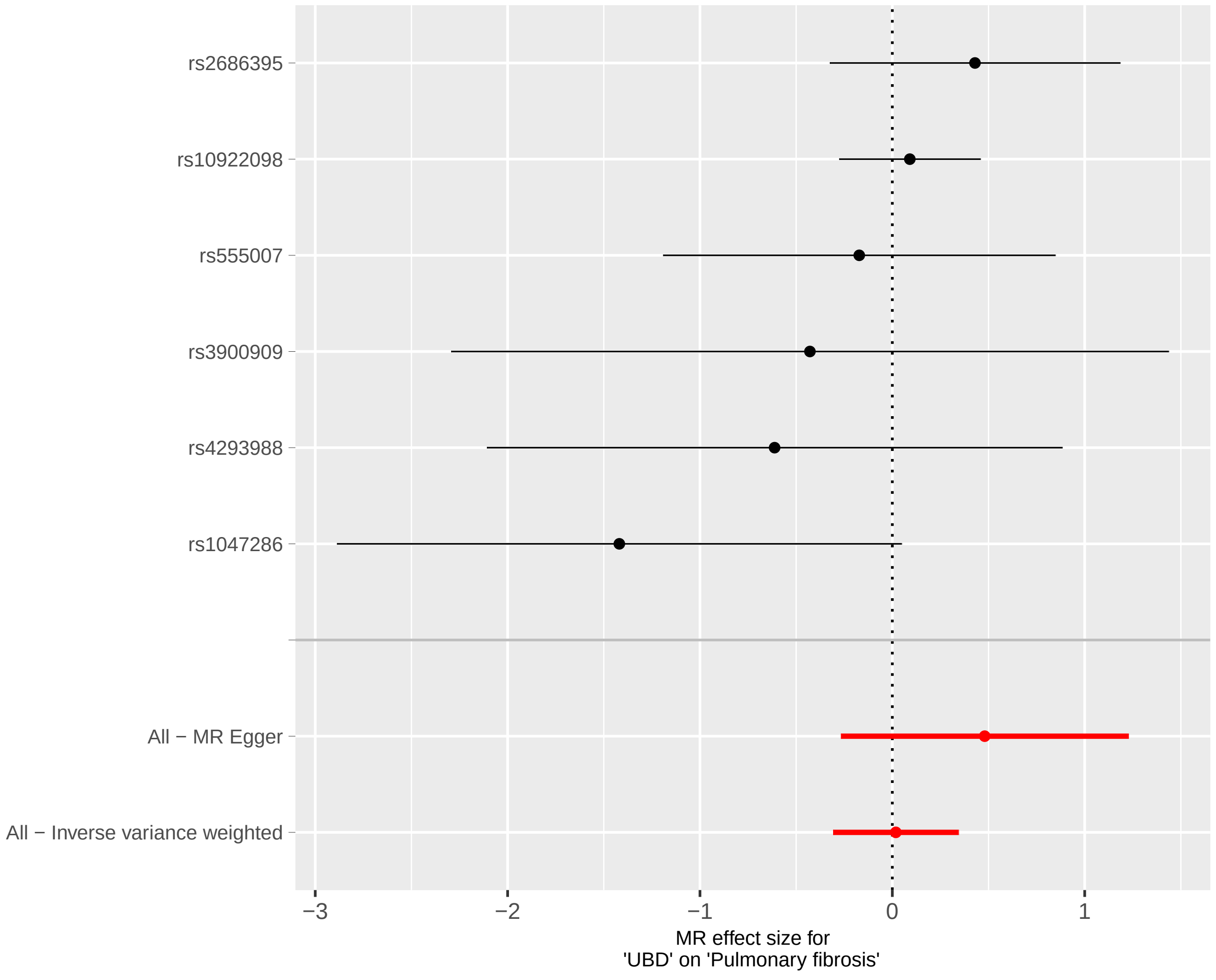
rs2686395
rs10922098
rs555007
rs3900909
rs4293988
rs1047286
All − MR Egger
All − Inverse variance weighted
−3
−2
−1
0
1
MR effect size for
'UBD' on 'Pulmonary fibrosis'

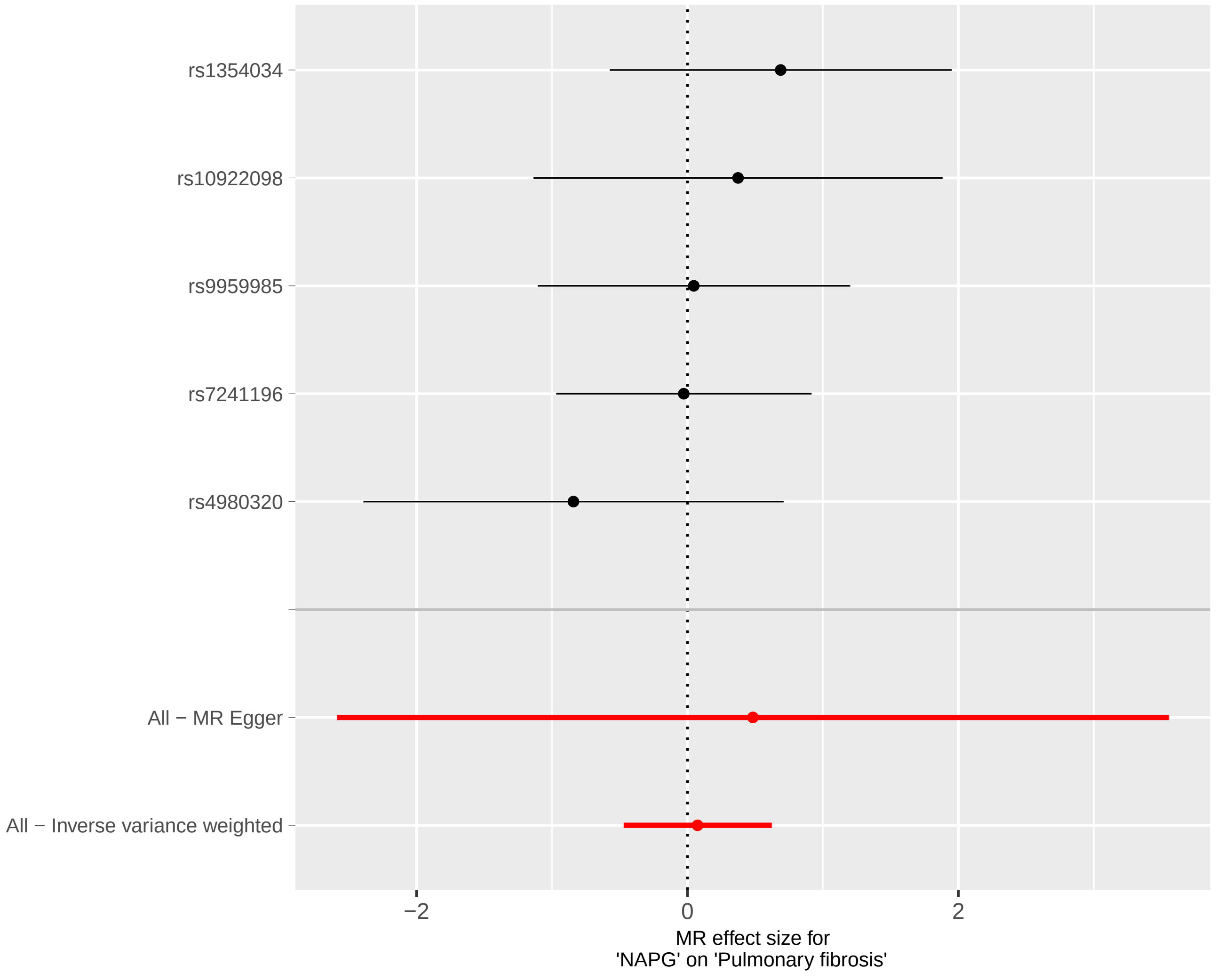

rs1354034
rs10922098
rs9959985
rs7241196
rs4980320
All − MR Egger
All − Inverse variance weighted
−2
0
2
MR effect size for
'NAPG' on 'Pulmonary fibrosis'

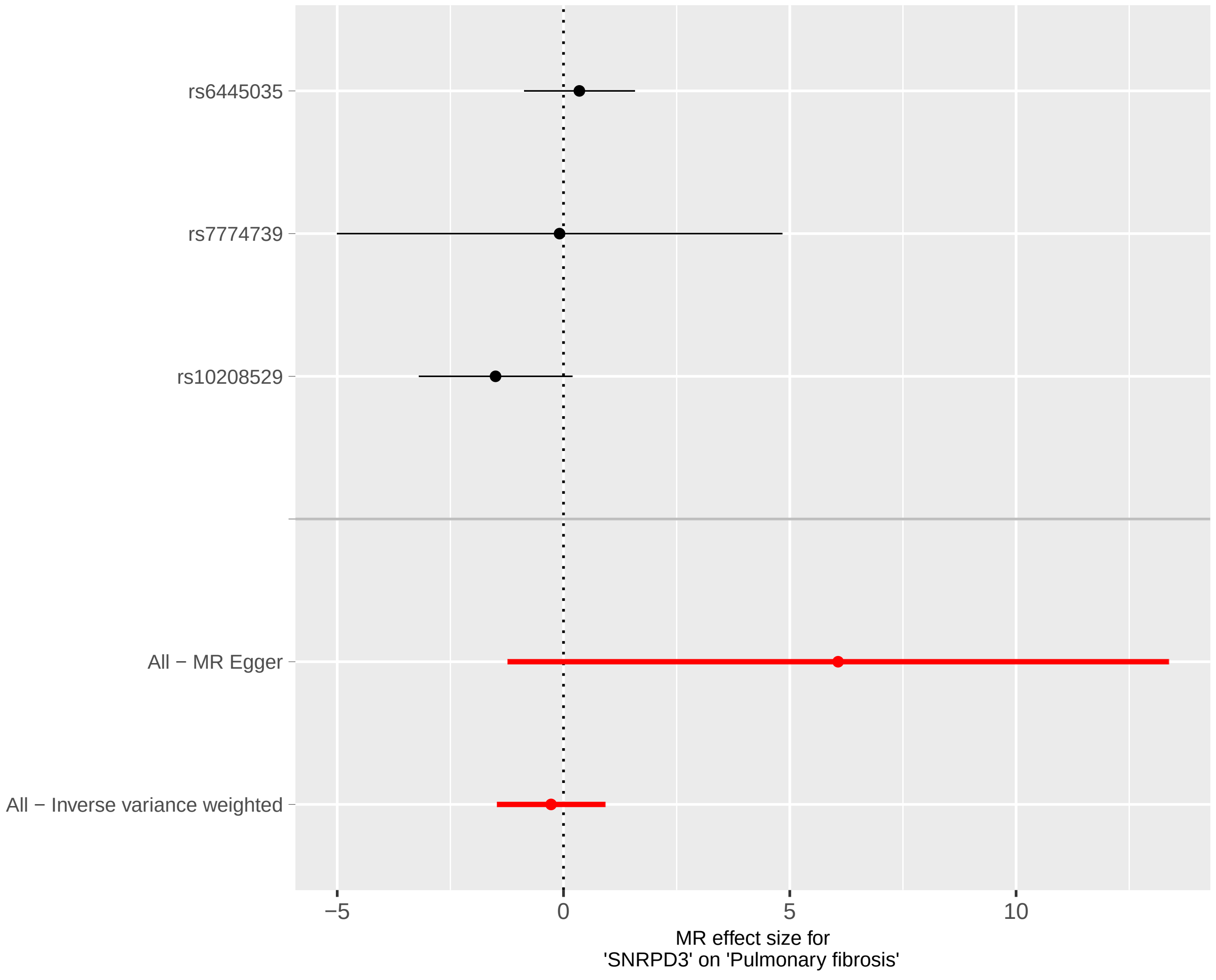
rs6445035
rs7774739
rs10208529
All − MR Egger
All − Inverse variance weighted
−5
0
5
10
MR effect size for
'SNRPD3' on 'Pulmonary fibrosis'

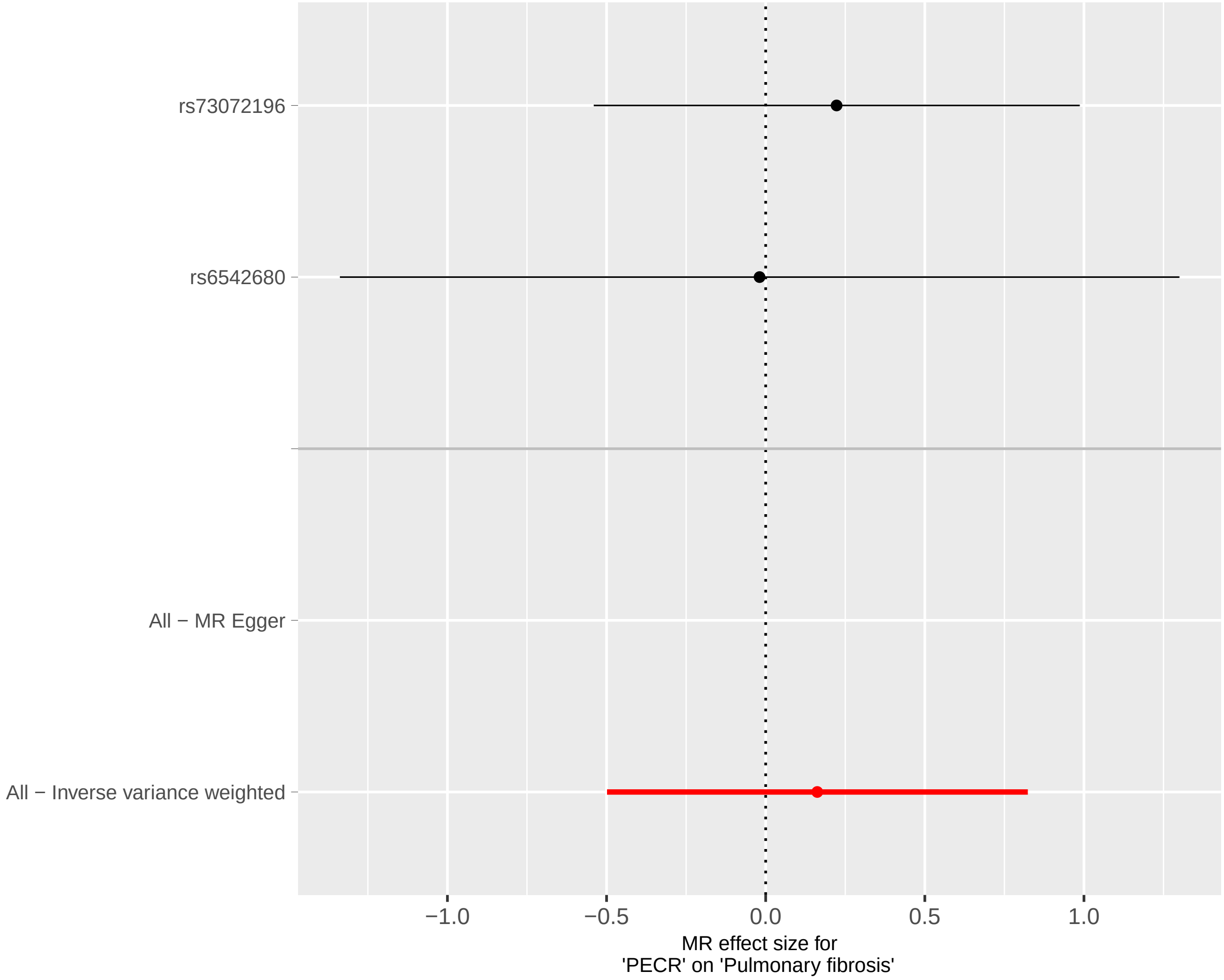
rs73072196
rs6542680
All − MR Egger
All − Inverse variance weighted
−1.0
−0.5
0.0
0.5
1.0
MR effect size for
'PECR' on 'Pulmonary fibrosis'

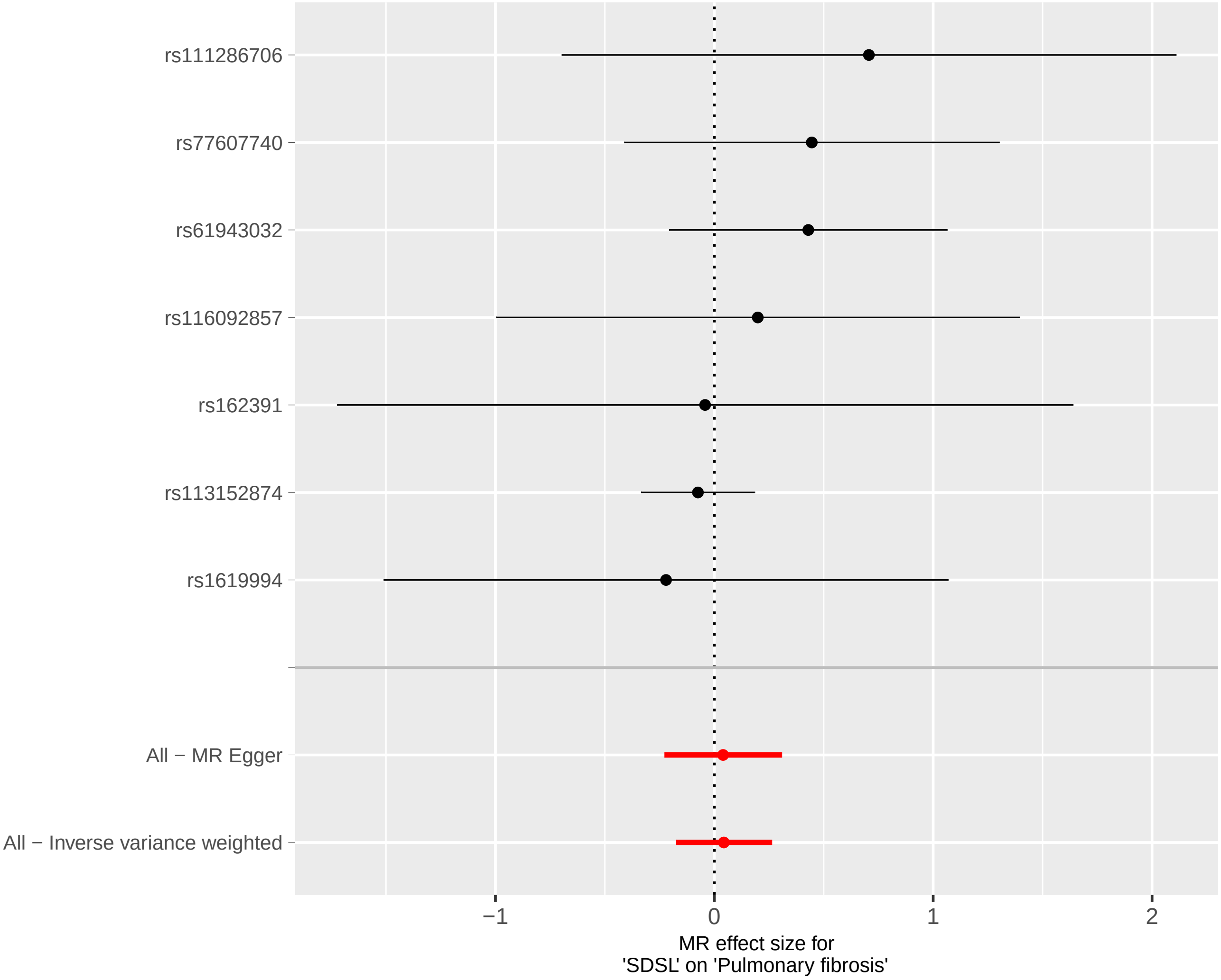

rs111286706
rs77607740
rs61943032
rs116092857
rs162391
rs113152874
rs1619994
All − MR Egger
All − Inverse variance weighted
−1
0
1
2
MR effect size for
'SDSL' on 'Pulmonary fibrosis'

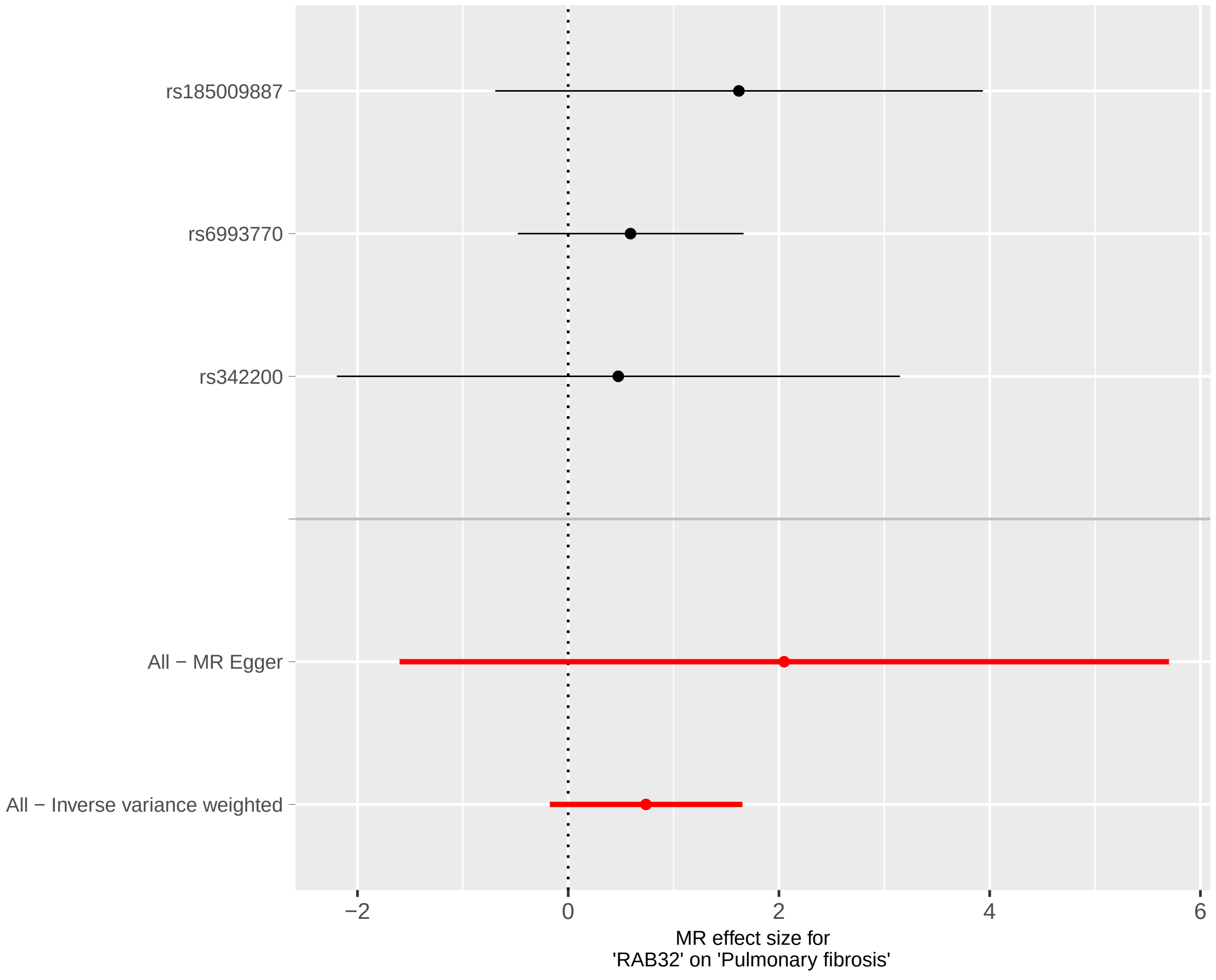

rs185009887
rs6993770
rs342200
All − MR Egger
All − Inverse variance weighted
−2
0
2
4
6
MR effect size for
'RAB32' on 'Pulmonary fibrosis'

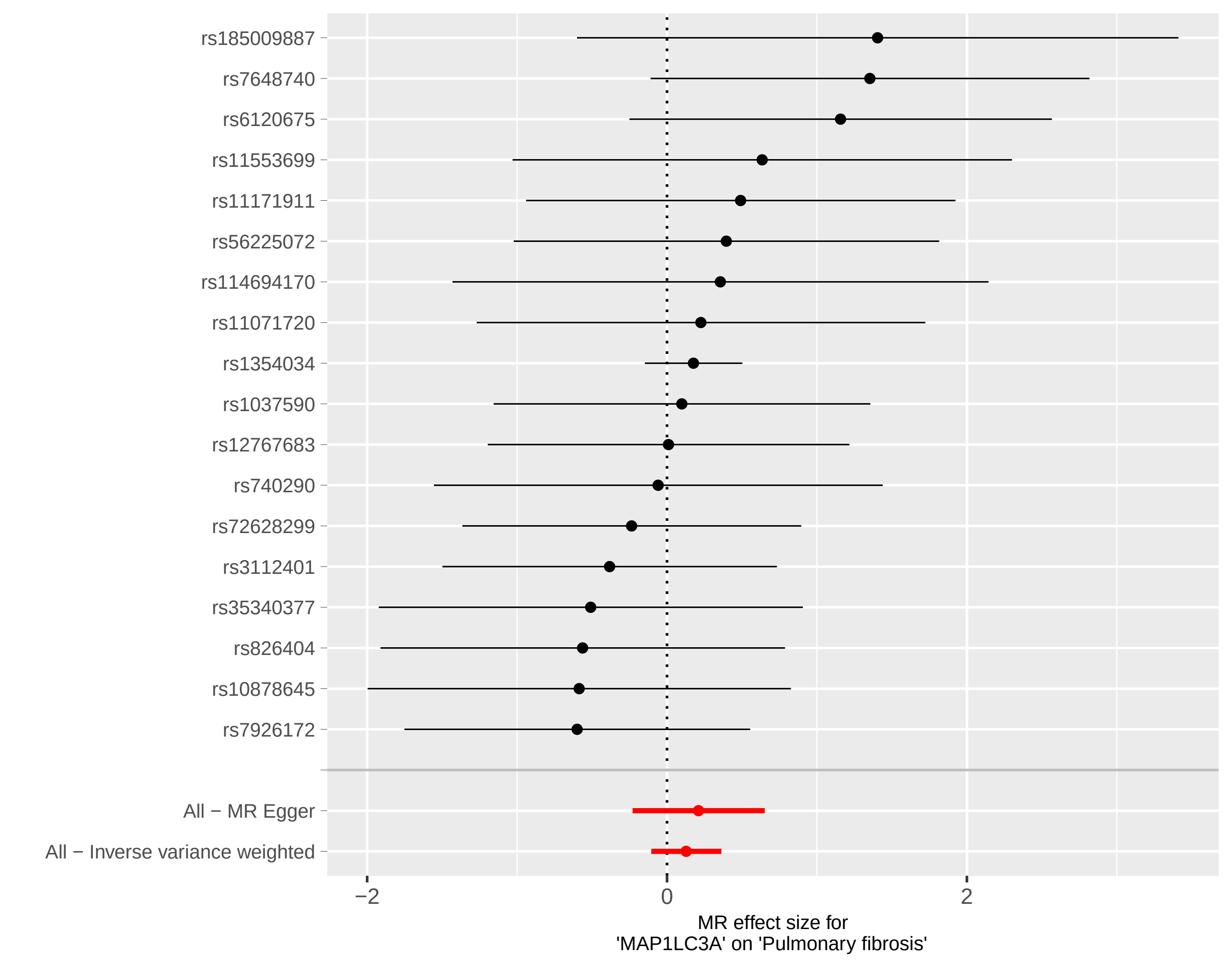
rs185009887
rs7648740
rs6120675
rs11553699
rs11171911
rs56225072
rs114694170
rs11071720
rs1354034
rs1037590
rs12767683
rs740290
rs72628299
rs3112401
rs35340377
rs826404
rs10878645
rs7926172
All − MR Egger
All − Inverse variance weighted
−2
0
2
MR effect size for
'MAP1LC3A' on 'Pulmonary fibrosis'

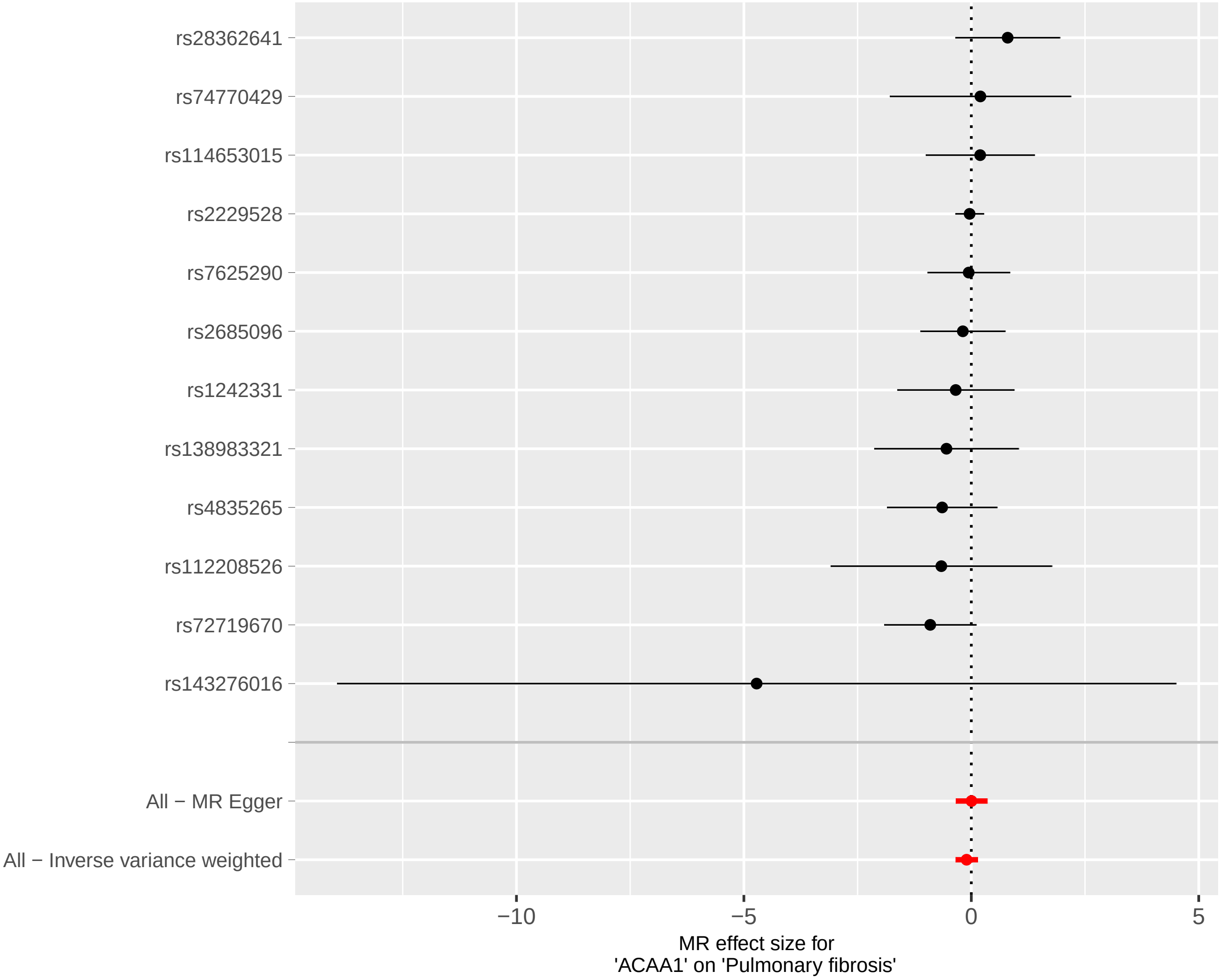
rs28362641
rs74770429
rs114653015
rs2229528
rs7625290
rs2685096
rs1242331
rs138983321
rs4835265
rs112208526
rs72719670
rs143276016
All − MR Egger
All − Inverse variance weighted
−10
−5
0
5
MR effect size for
'ACAA1' on 'Pulmonary fibrosis'

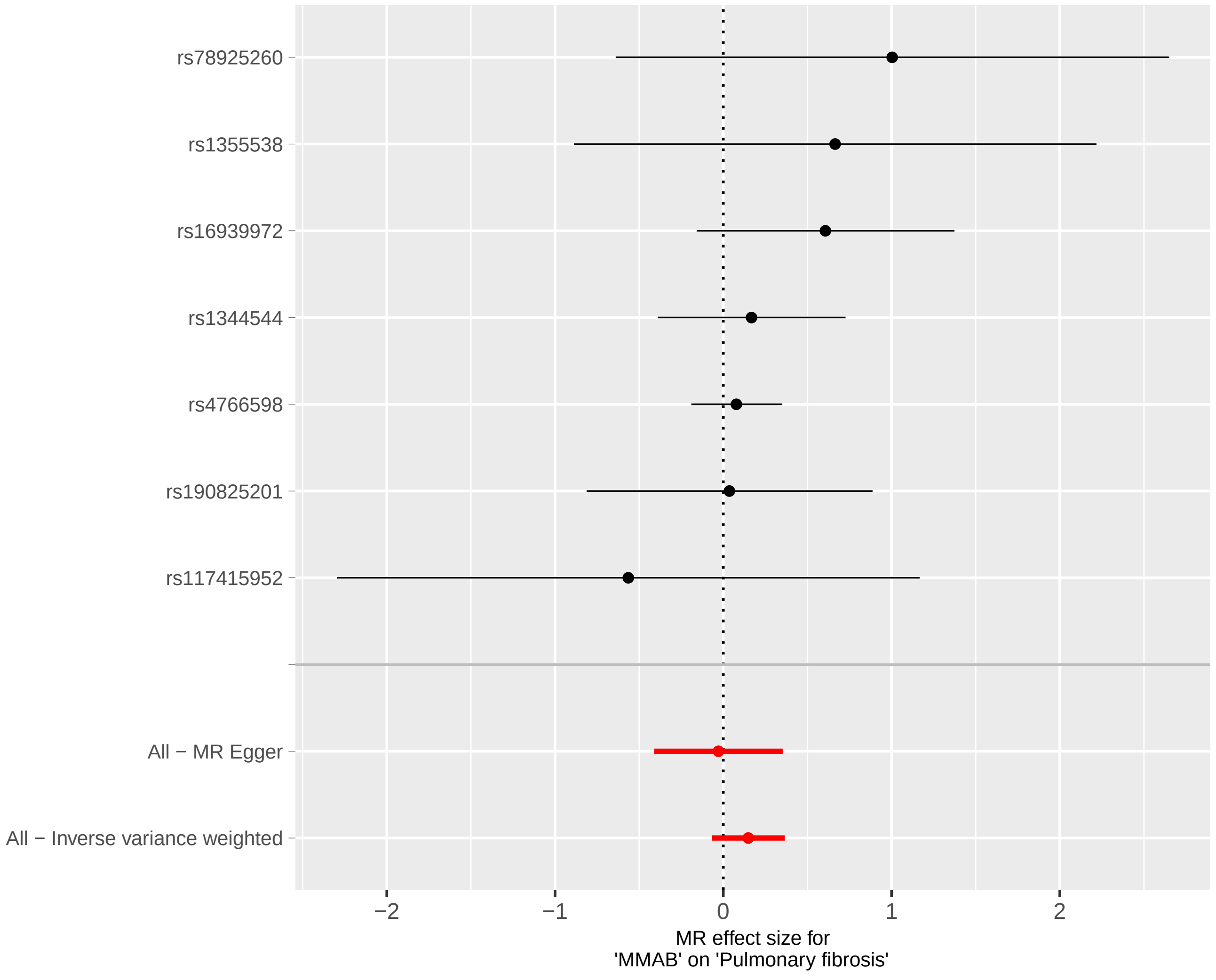
rs78925260
rs1355538
rs16939972
rs1344544
rs4766598
rs190825201
rs117415952
All − MR Egger
All − Inverse variance weighted
−2
−1
0
1
2
MR effect size for
'MMAB' on 'Pulmonary fibrosis'

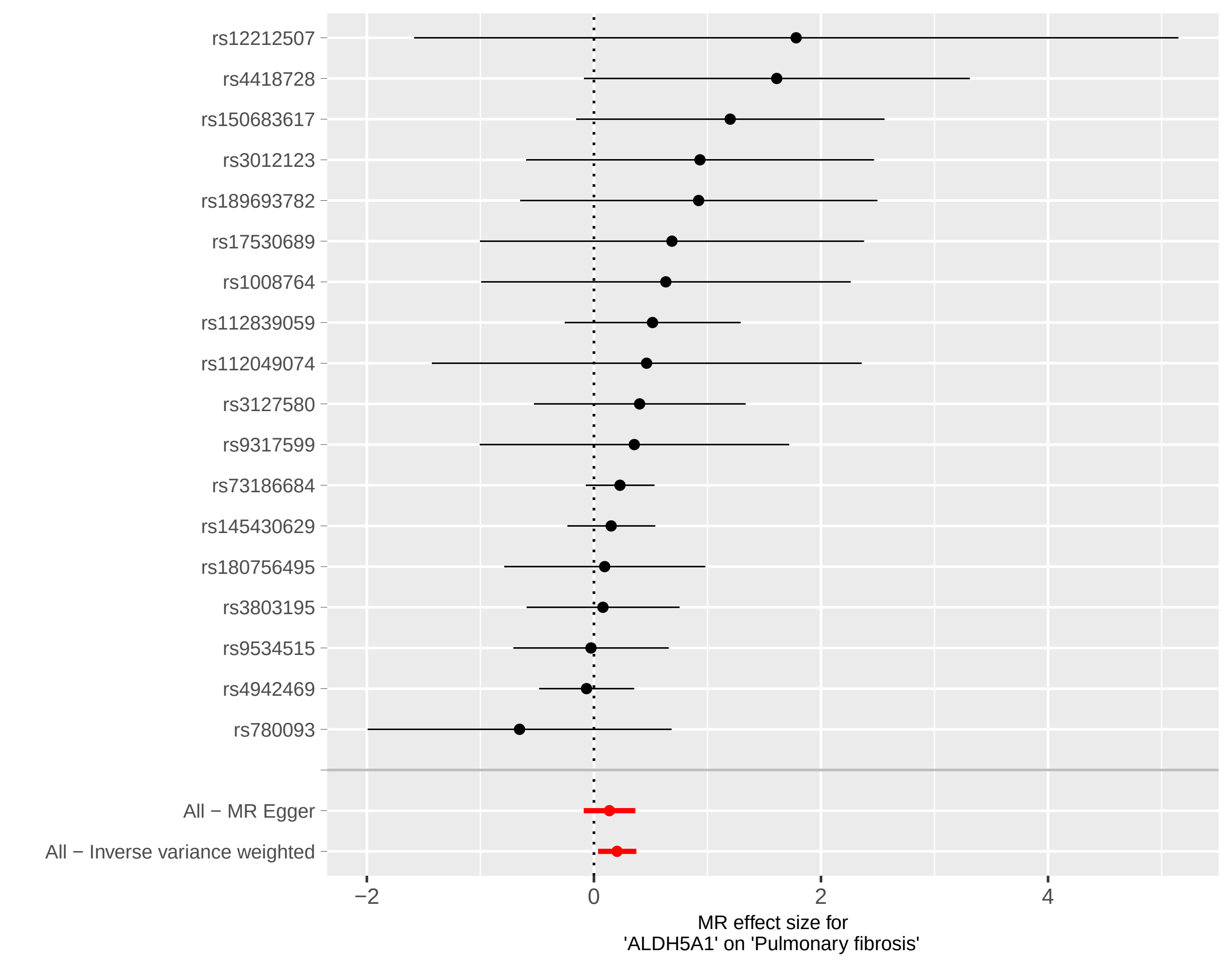

rs12212507
rs4418728
rs150683617
rs3012123
rs189693782
rs17530689
rs1008764
rs112839059
rs112049074
rs3127580
rs9317599
rs73186684
rs145430629
rs180756495
rs3803195
rs9534515
rs4942469
rs780093
All − MR Egger
All − Inverse variance weighted
−2
0
2
4
MR effect size for
'ALDH5A1' on 'Pulmonary fibrosis'

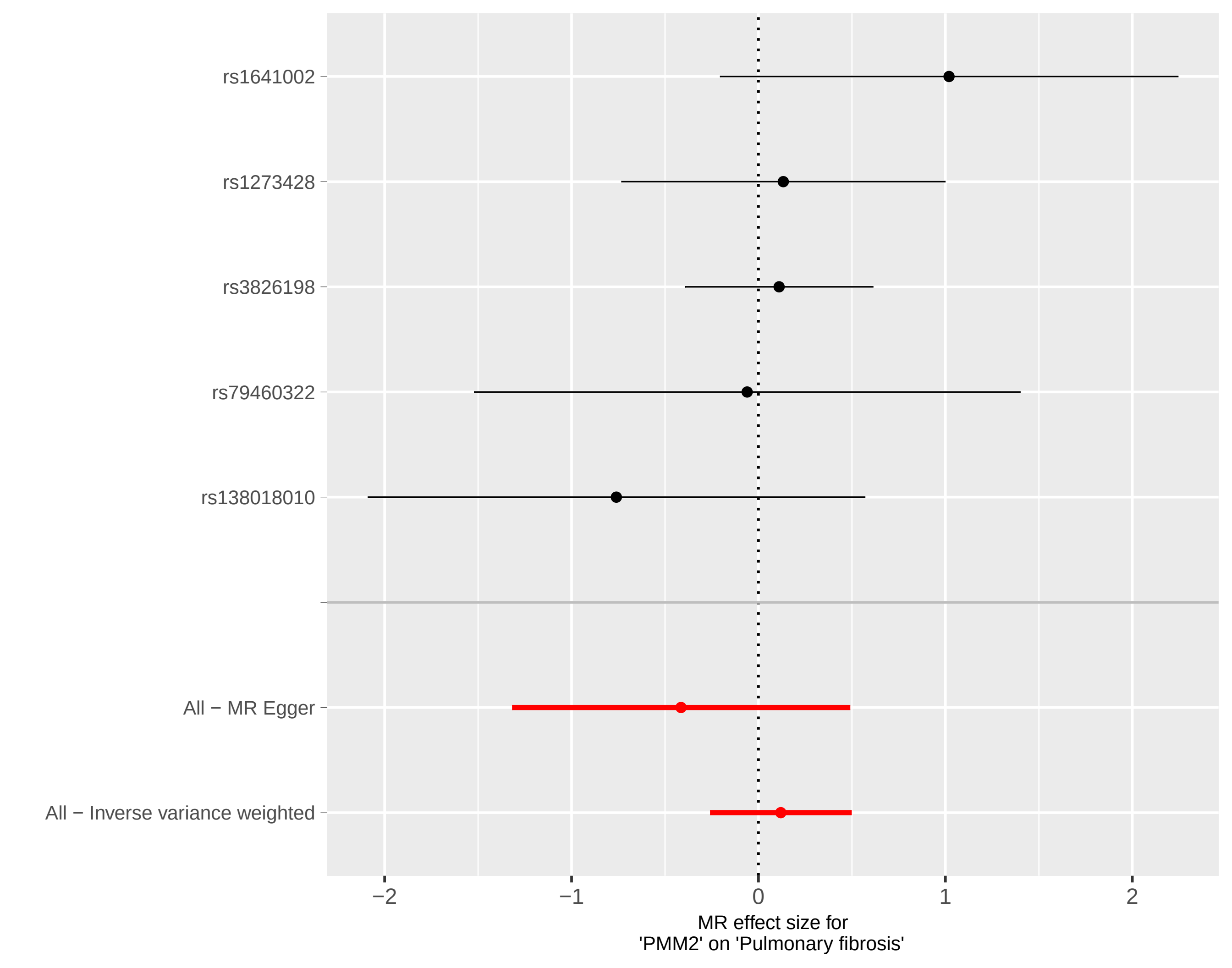

rs1641002
rs1273428
rs3826198
rs79460322
rs138018010
All − MR Egger
All − Inverse variance weighted
−2
−1
0
1
2
MR effect size for
'PMM2' on 'Pulmonary fibrosis'

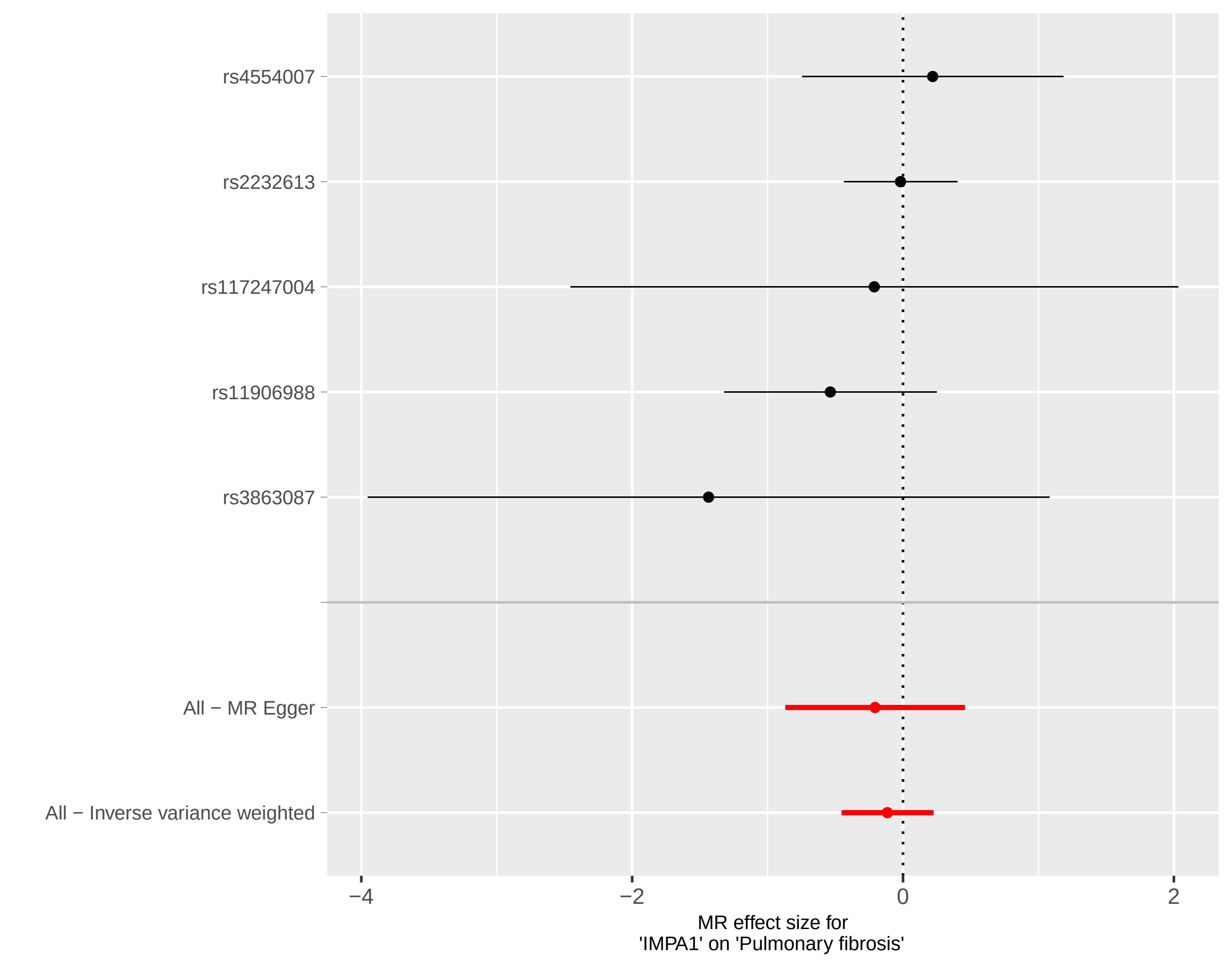

rs4554007
rs2232613
rs117247004
rs11906988
rs3863087
All − MR Egger
All − Inverse variance weighted
−4
−2
0
2
MR effect size for
'IMPA1' on 'Pulmonary fibrosis'

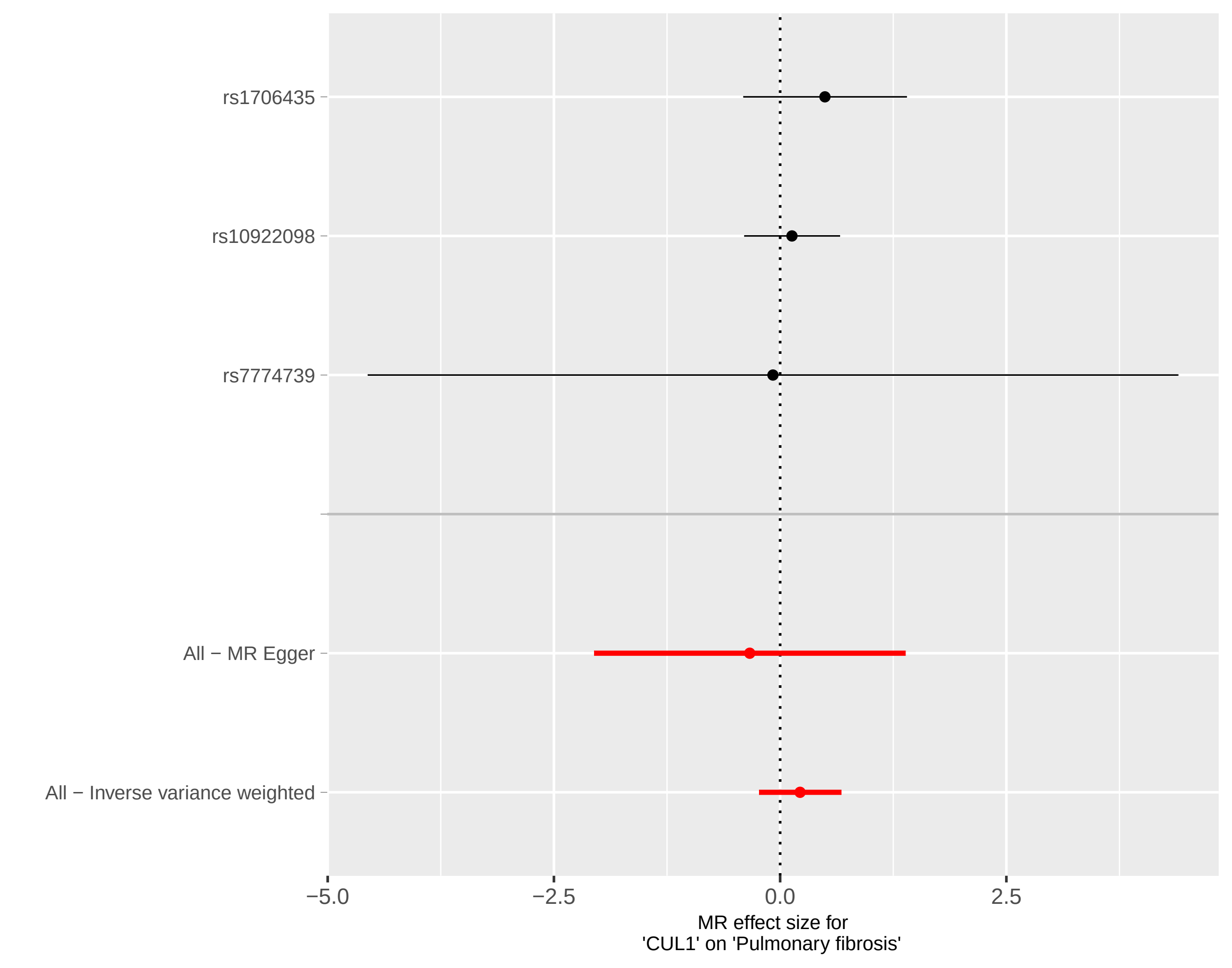
rs1706435
rs10922098
rs7774739
All − MR Egger
All − Inverse variance weighted
−5.0
−2.5
0.0
2.5
MR effect size for
'CUL1' on 'Pulmonary fibrosis'

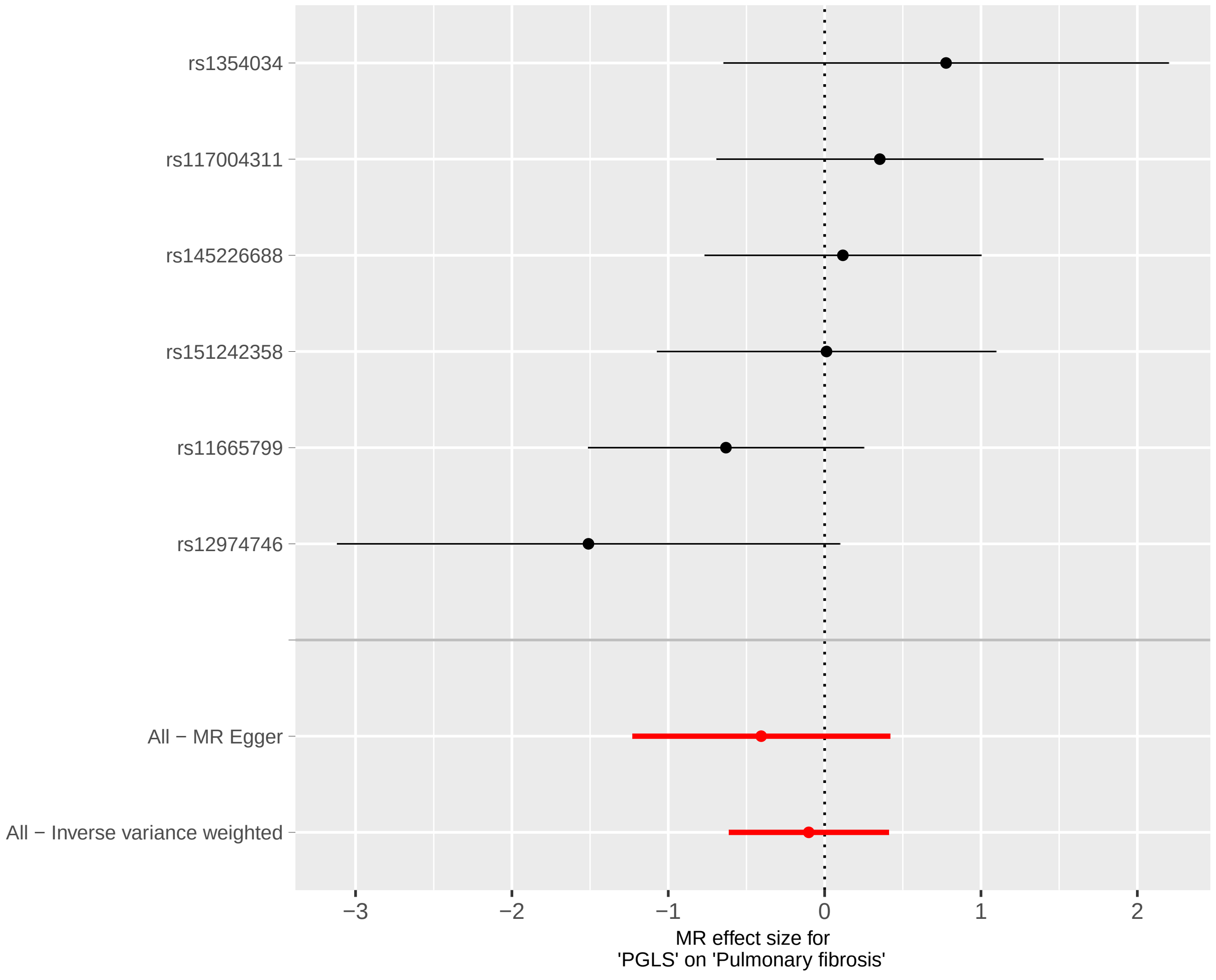

rs1354034
rs117004311
rs145226688
rs151242358
rs11665799
rs12974746
All − MR Egger
All − Inverse variance weighted
−3
−2
−1
0
1
2
MR effect size for
'PGLS' on 'Pulmonary fibrosis'

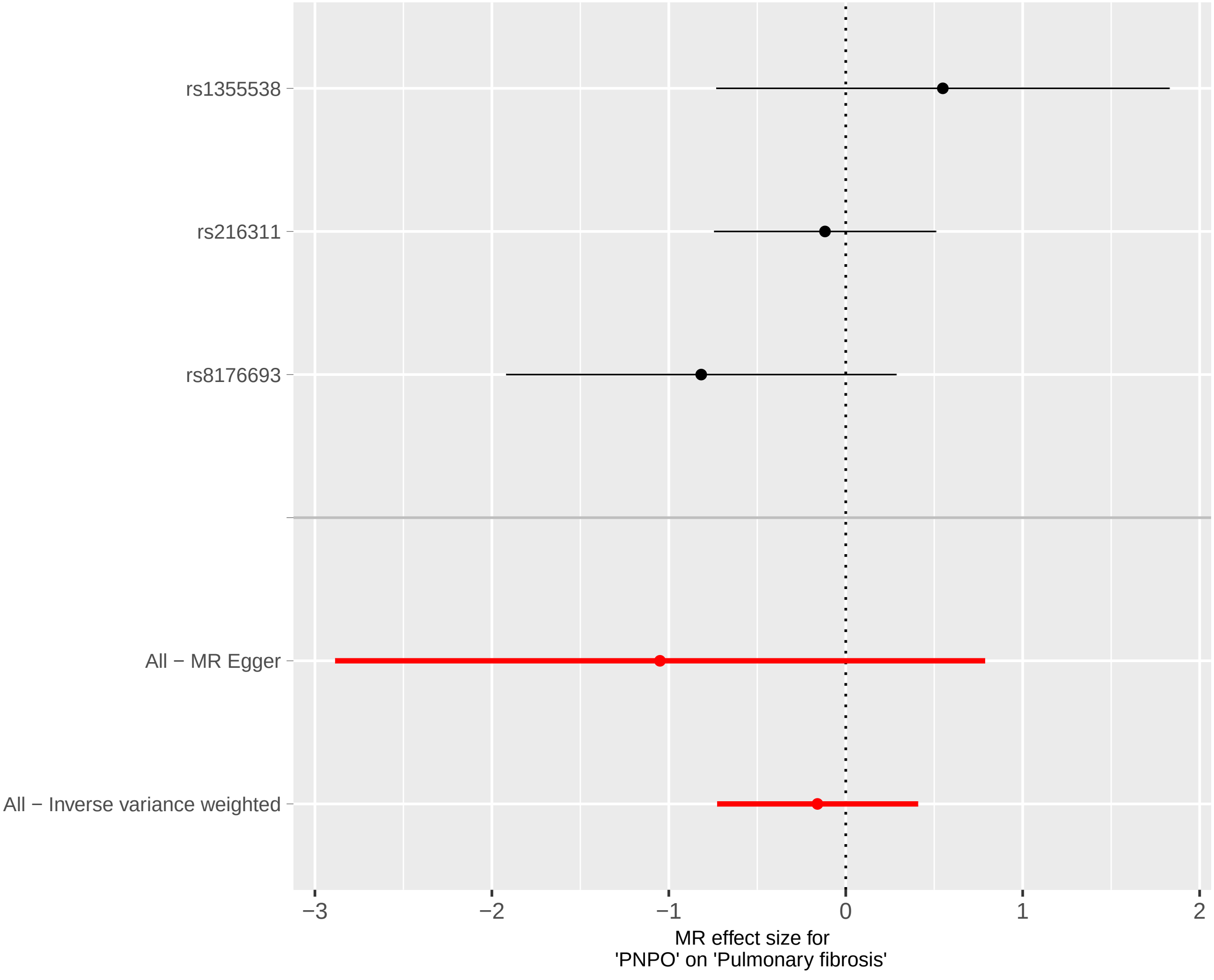
rs1355538
rs216311
rs8176693
All − MR Egger
All − Inverse variance weighted
−3
−2
−1
0
1
2
MR effect size for
'PNPO' on 'Pulmonary fibrosis'

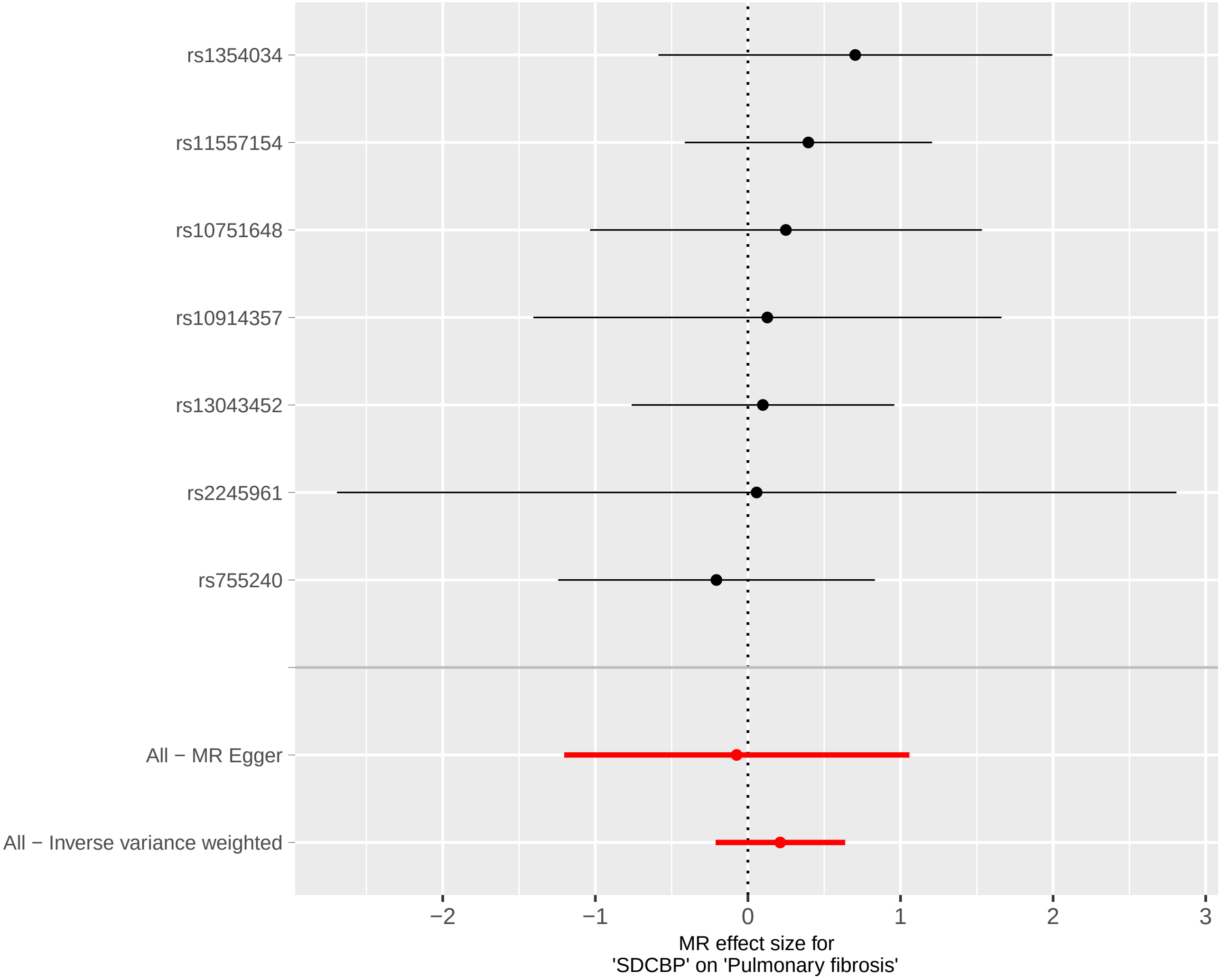

rs1354034
rs11557154
rs10751648
rs10914357
rs13043452
rs2245961
rs755240
All − MR Egger
All − Inverse variance weighted
−2
−1
0
1
2
3
MR effect size for
'SDCBP' on 'Pulmonary fibrosis'

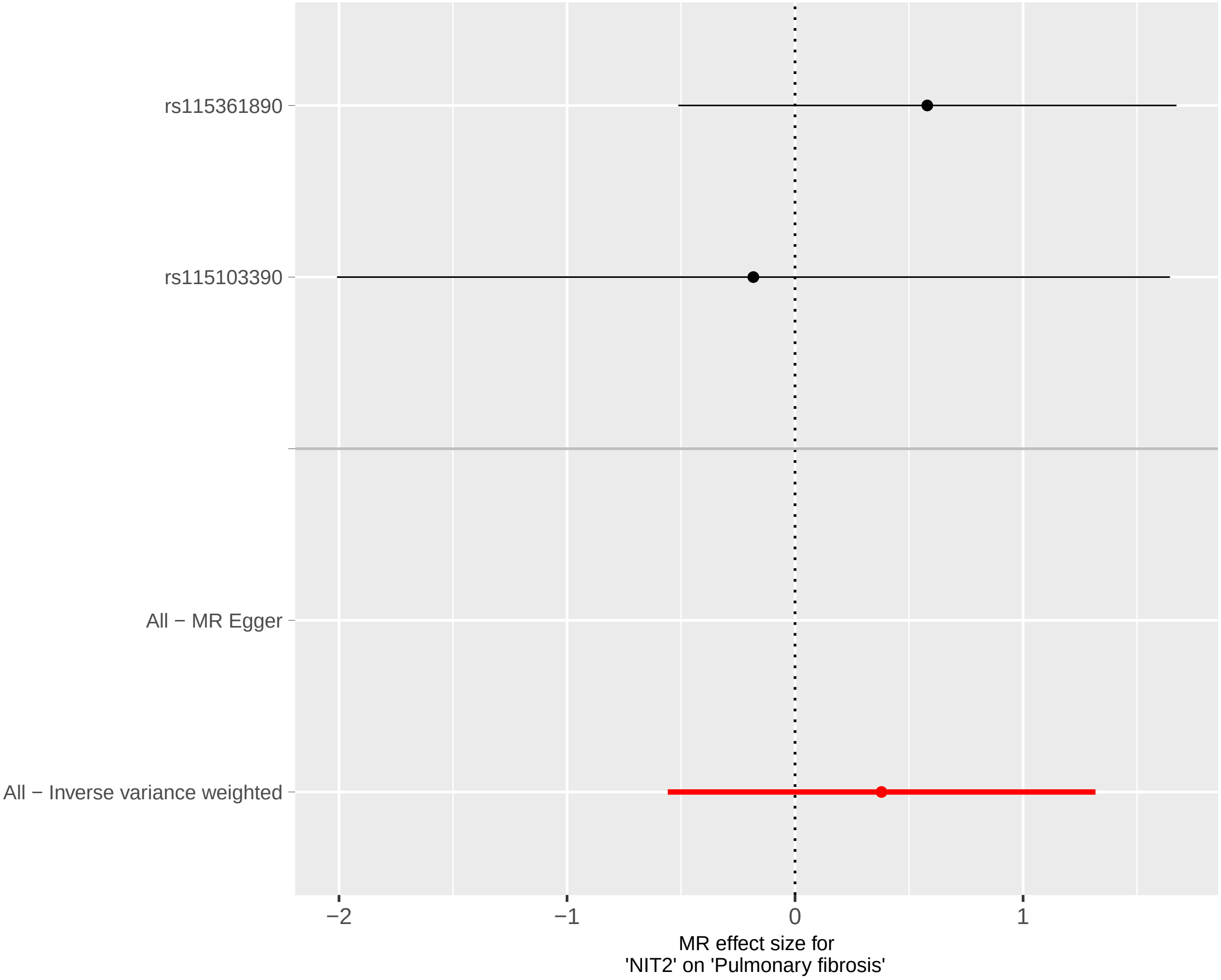

rs115361890
rs115103390
All − MR Egger
All − Inverse variance weighted
−2
−1
0
1
MR effect size for
'NIT2' on 'Pulmonary fibrosis'

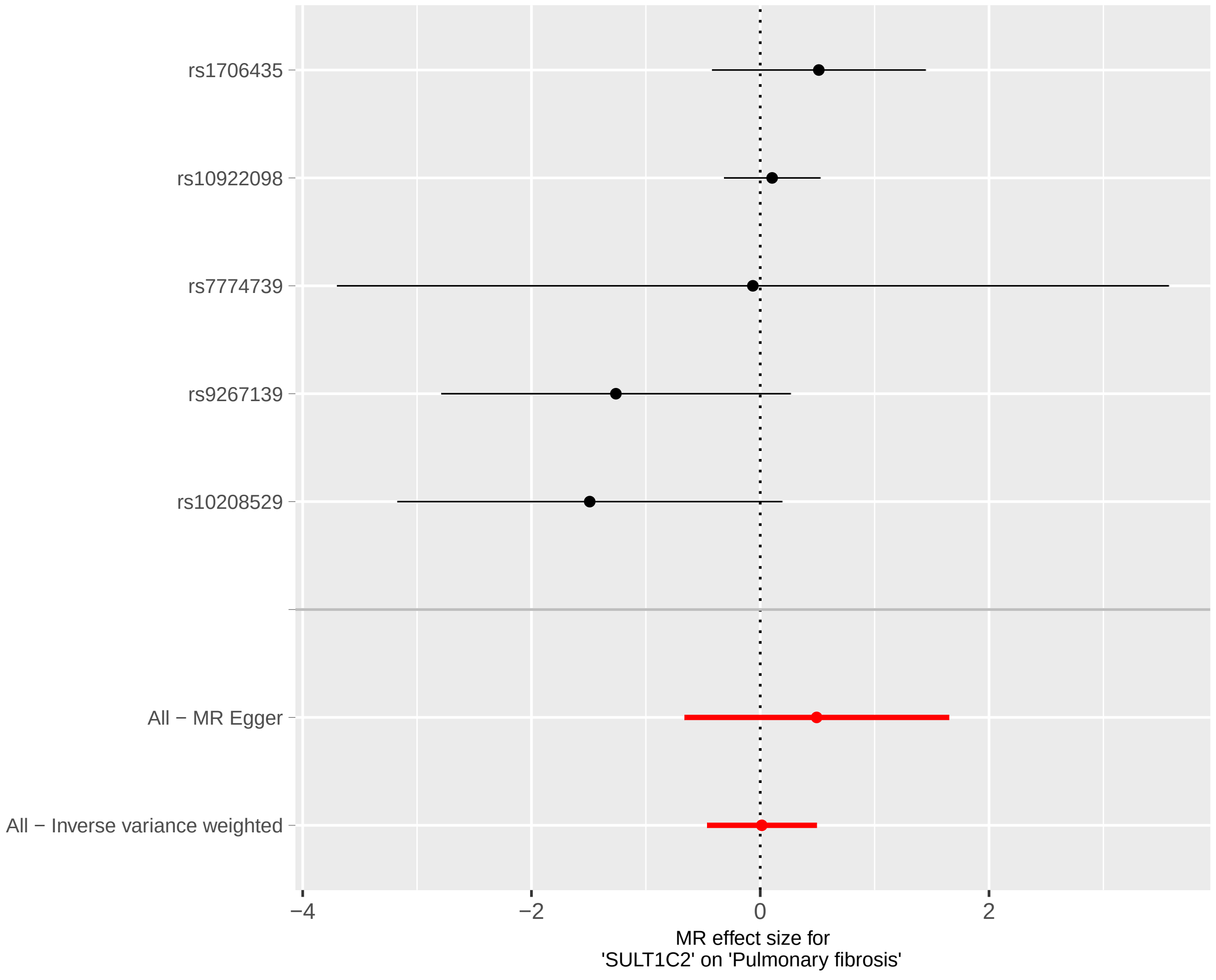
rs1706435
rs10922098
rs7774739
rs9267139
rs10208529
All − MR Egger
All − Inverse variance weighted
−4
−2
0
2
MR effect size for
'SULT1C2' on 'Pulmonary fibrosis'

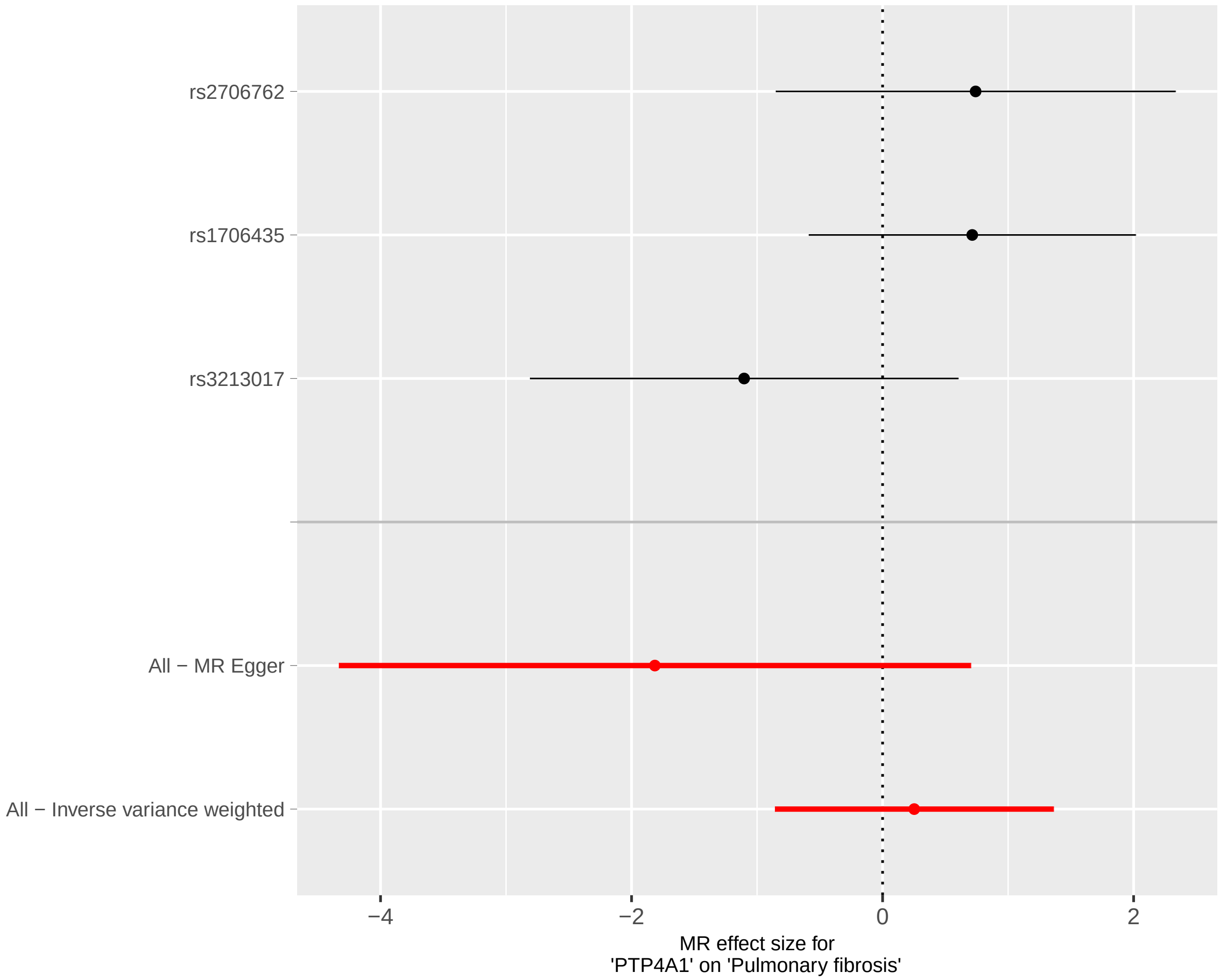

rs2706762
rs1706435
rs3213017
All − MR Egger
All − Inverse variance weighted
−4
−2
0
2
MR effect size for
'PTP4A1' on 'Pulmonary fibrosis'

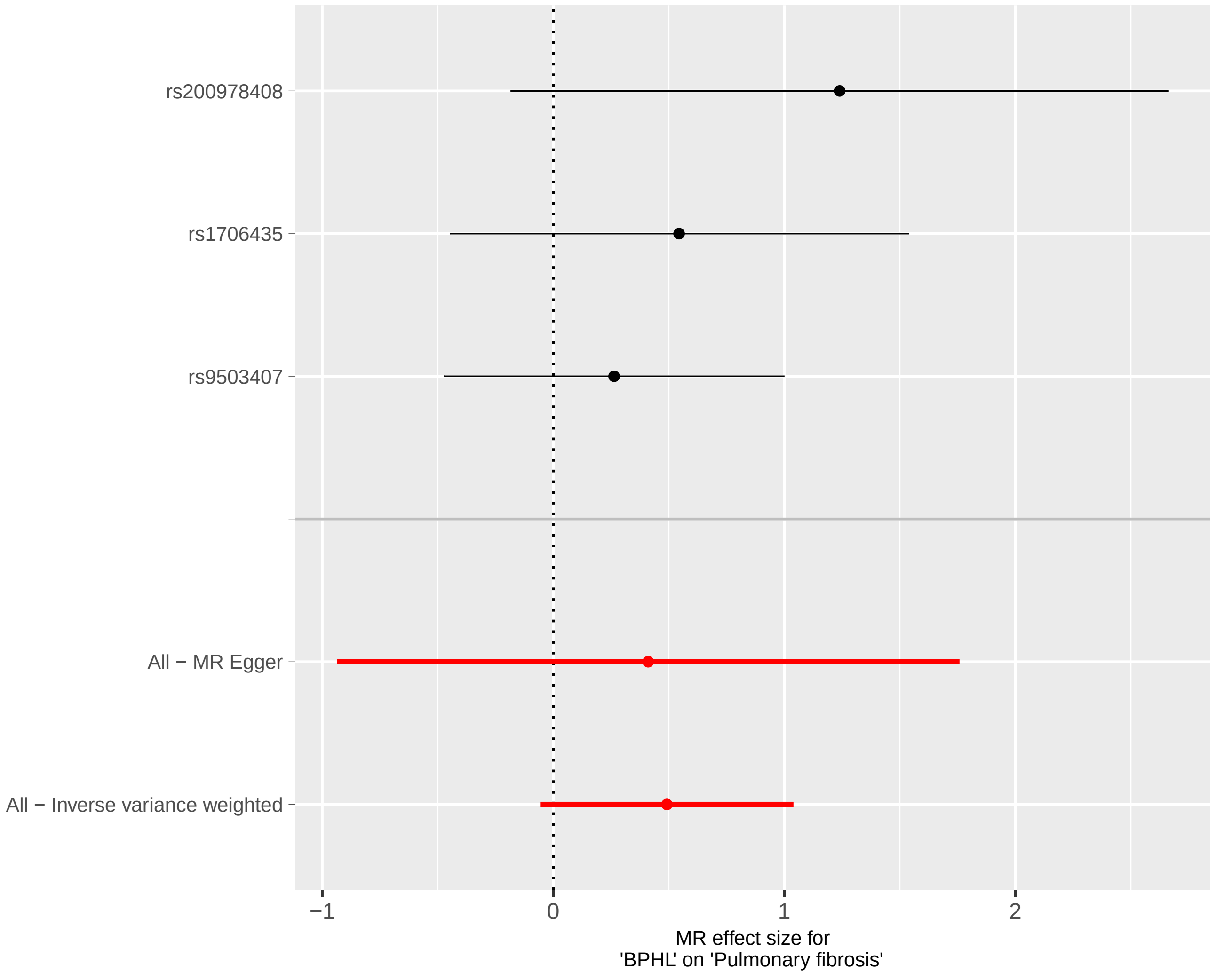

rs200978408
rs1706435
rs9503407
All − MR Egger
All − Inverse variance weighted
−1
0
1
2
MR effect size for
'BPHL' on 'Pulmonary fibrosis'

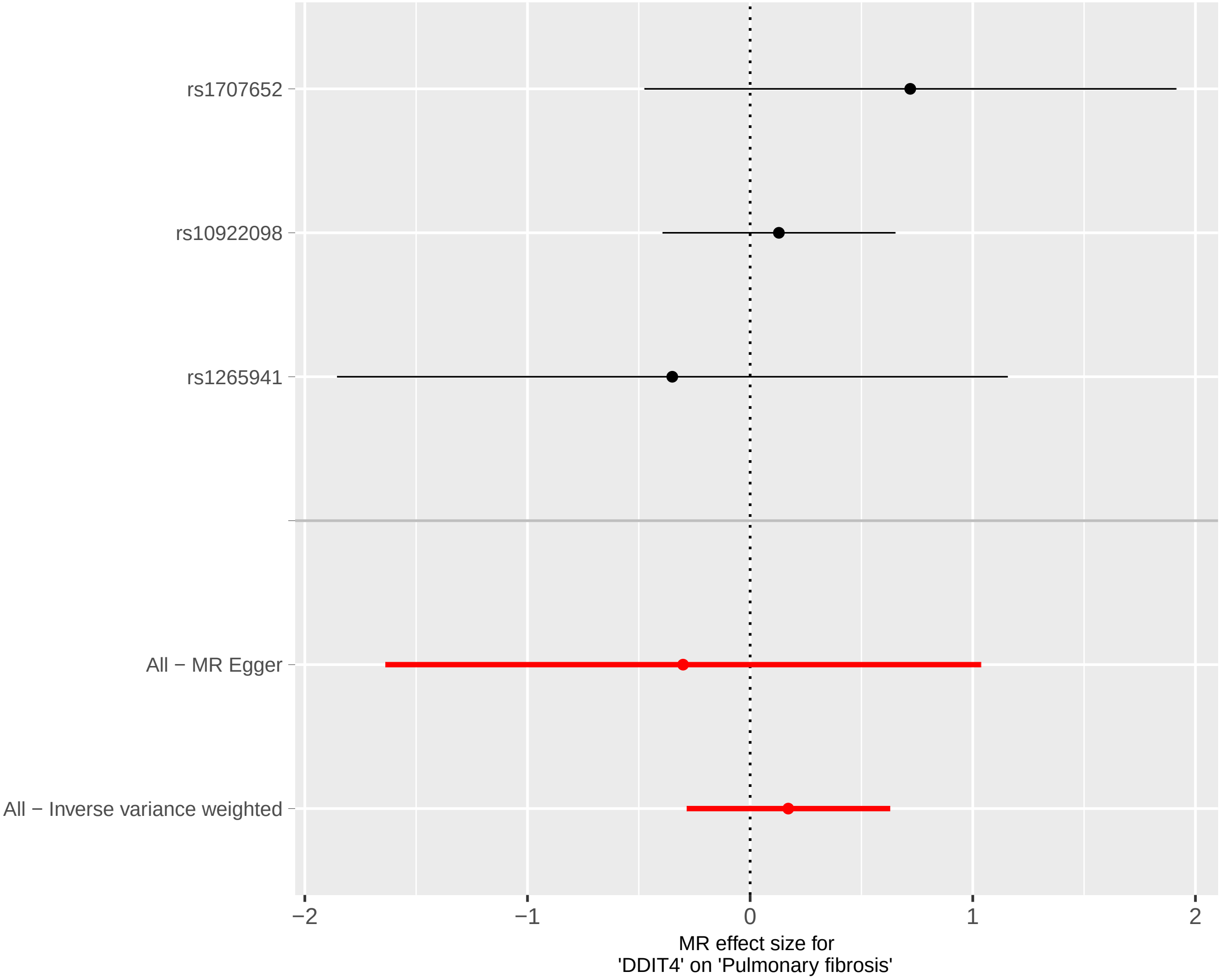

rs1707652
rs10922098
rs1265941
All − MR Egger
All − Inverse variance weighted
−2
−1
0
1
2
MR effect size for
'DDIT4' on 'Pulmonary fibrosis'

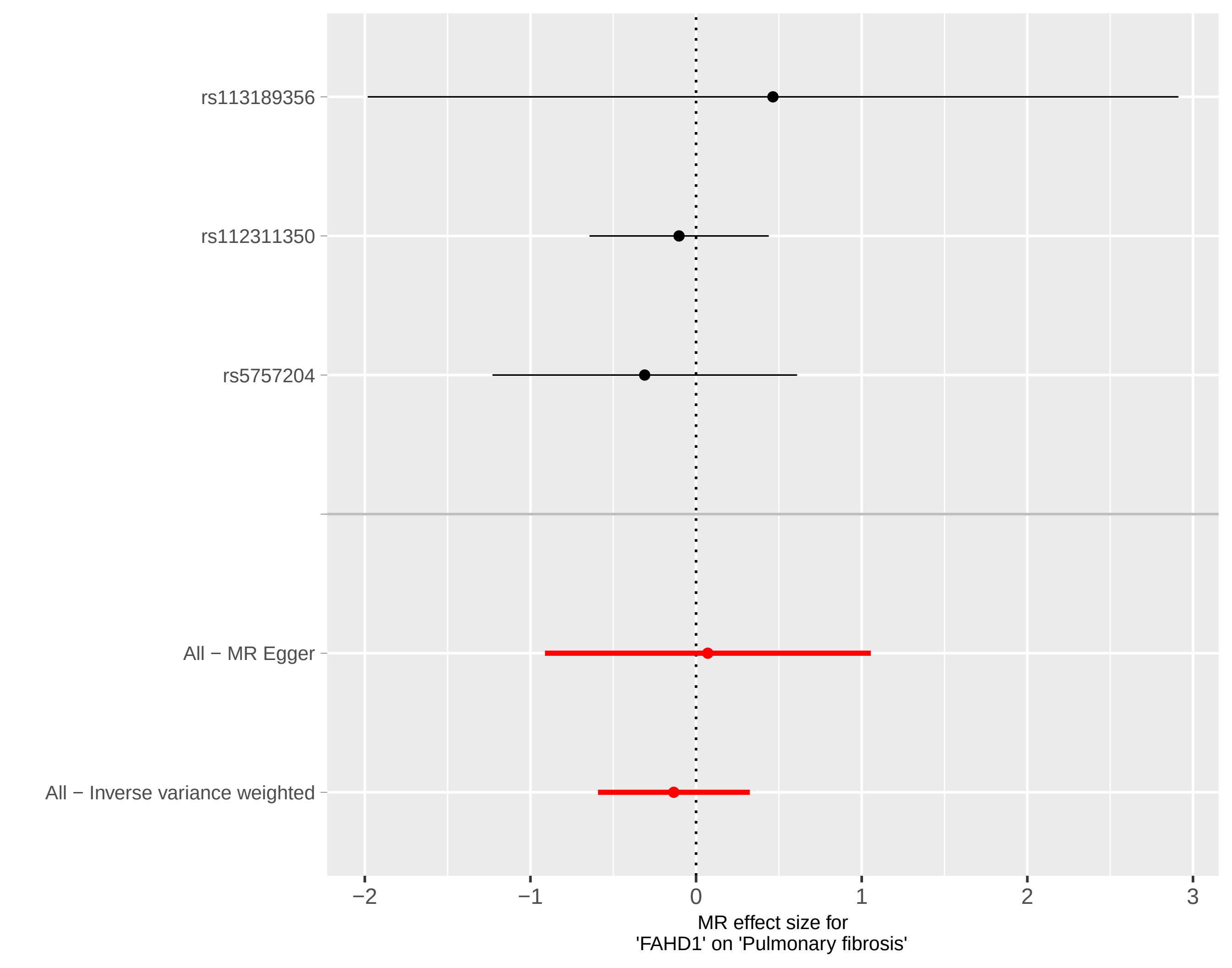
rs113189356
rs112311350
rs5757204
All − MR Egger
All − Inverse variance weighted
−2
−1
0
1
2
3
MR effect size for
'FAHD1' on 'Pulmonary fibrosis'

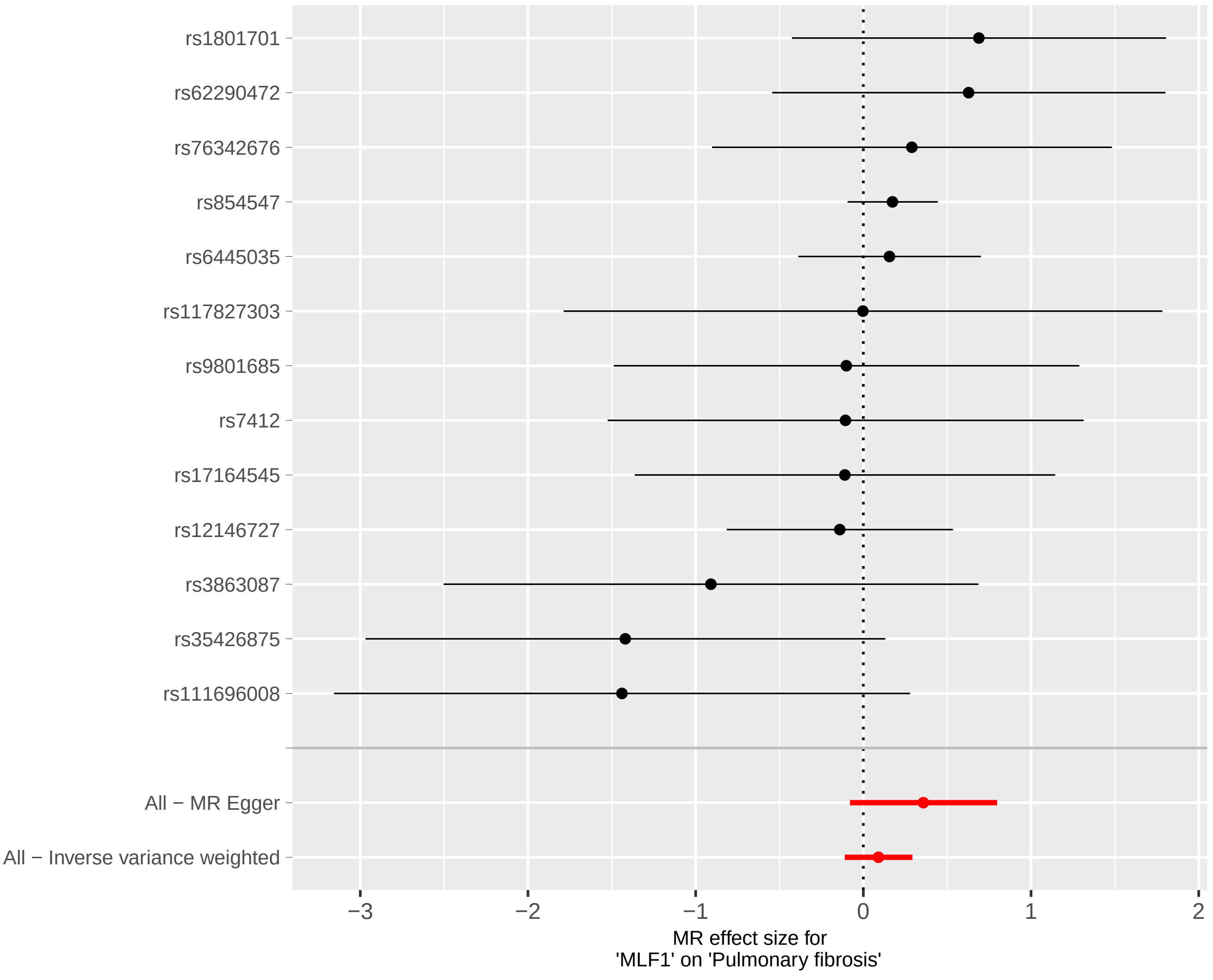

rs1801701
rs62290472
rs76342676
rs854547
rs6445035
rs117827303
rs9801685
rs7412
rs17164545
rs12146727
rs3863087
rs35426875
rs111696008
All − MR Egger
All − Inverse variance weighted
−3
−2
−1
0
1
2
MR effect size for
'MLF1' on 'Pulmonary fibrosis'

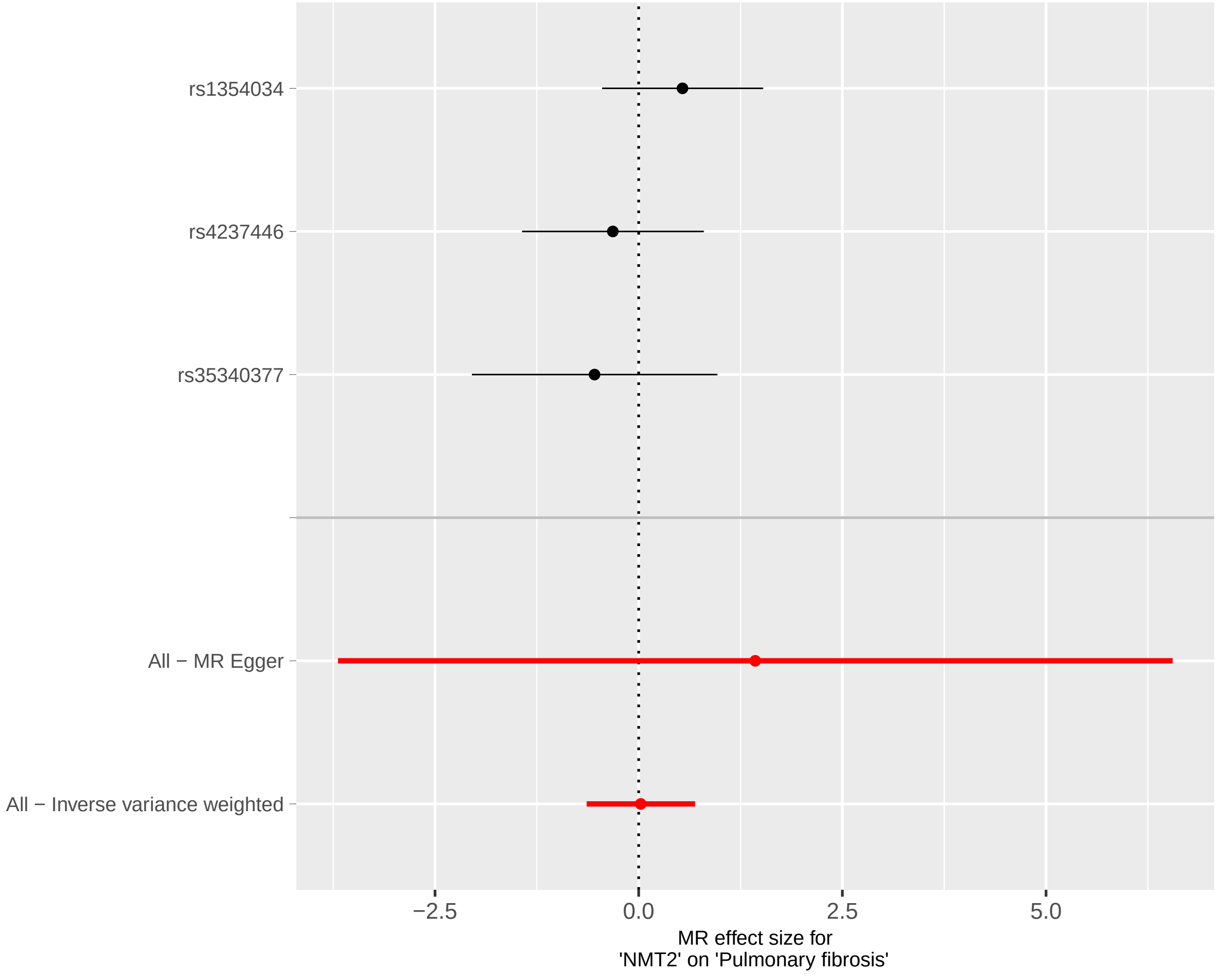

rs1354034
rs4237446
rs35340377
All − MR Egger
All − Inverse variance weighted
−2.5
0.0
2.5
5.0
MR effect size for
'NMT2' on 'Pulmonary fibrosis'

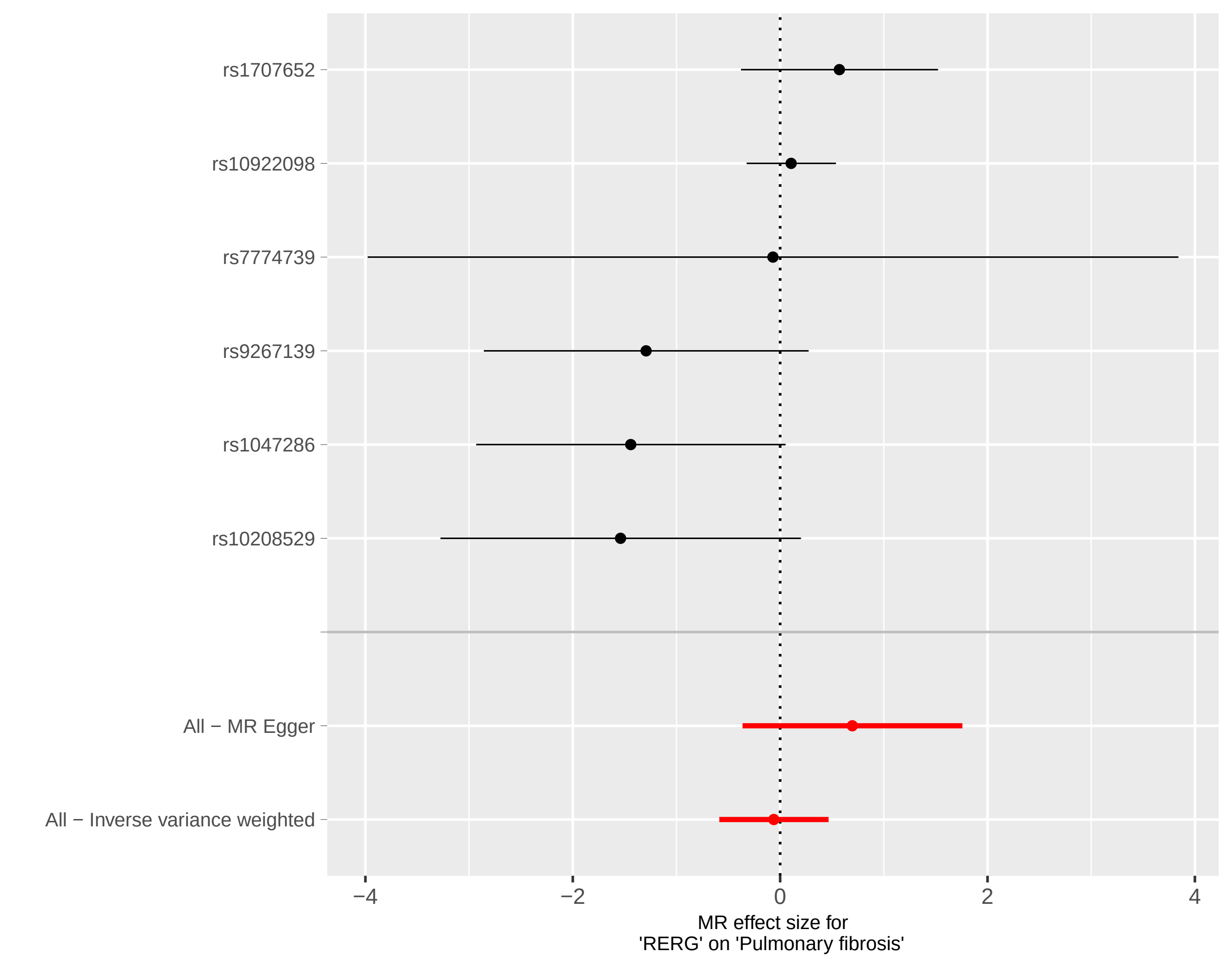

rs1707652
rs10922098
rs7774739
rs9267139
rs1047286
rs10208529
All − MR Egger
All − Inverse variance weighted
−4
−2
0
2
4
MR effect size for
'RERG' on 'Pulmonary fibrosis'

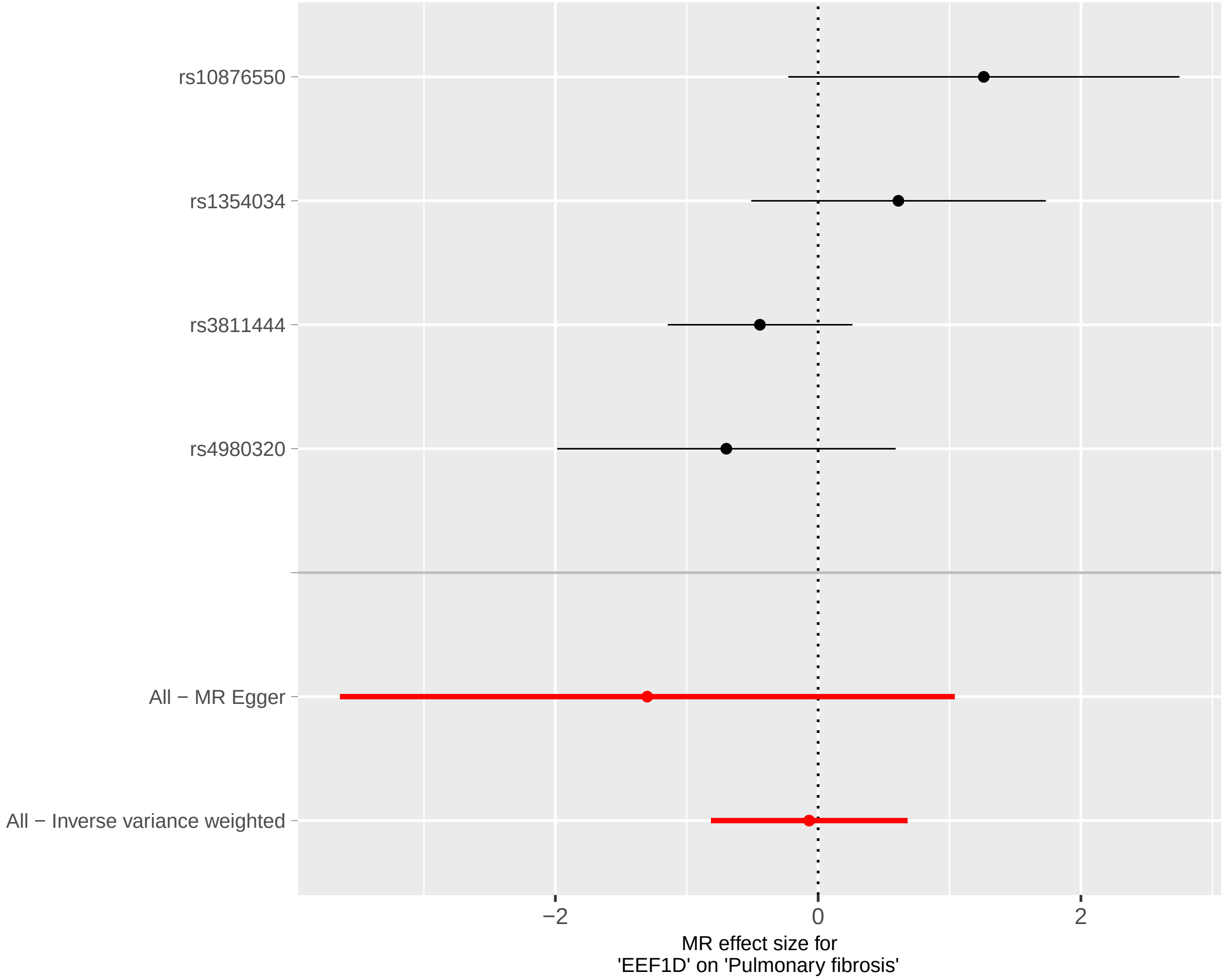
rs10876550
rs1354034
rs3811444
rs4980320
All − MR Egger
All − Inverse variance weighted
−2
0
2
MR effect size for
'EEF1D' on 'Pulmonary fibrosis'

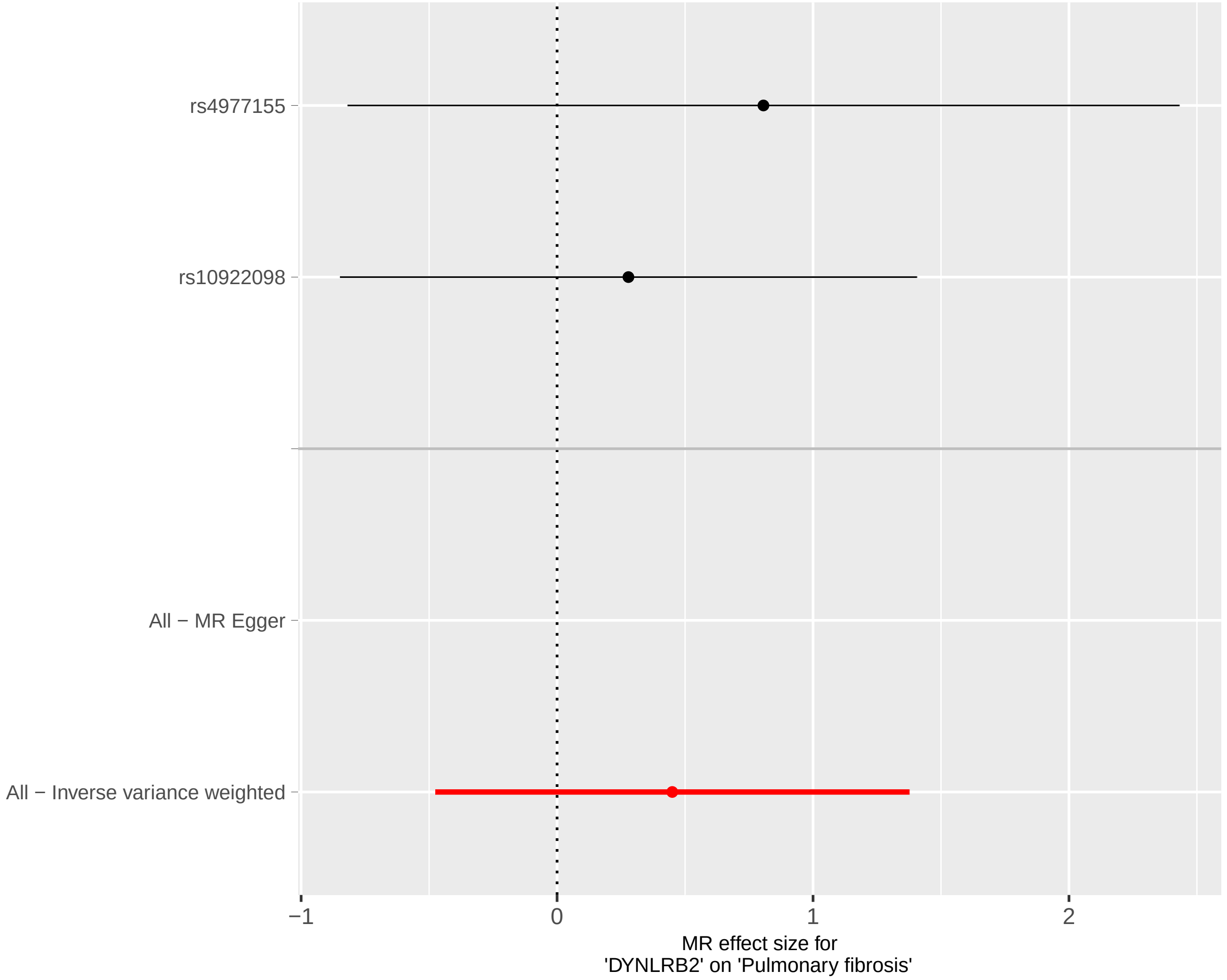
rs4977155
rs10922098
All − MR Egger
All − Inverse variance weighted
−1
0
1
2
MR effect size for
'DYNLRB2' on 'Pulmonary fibrosis'

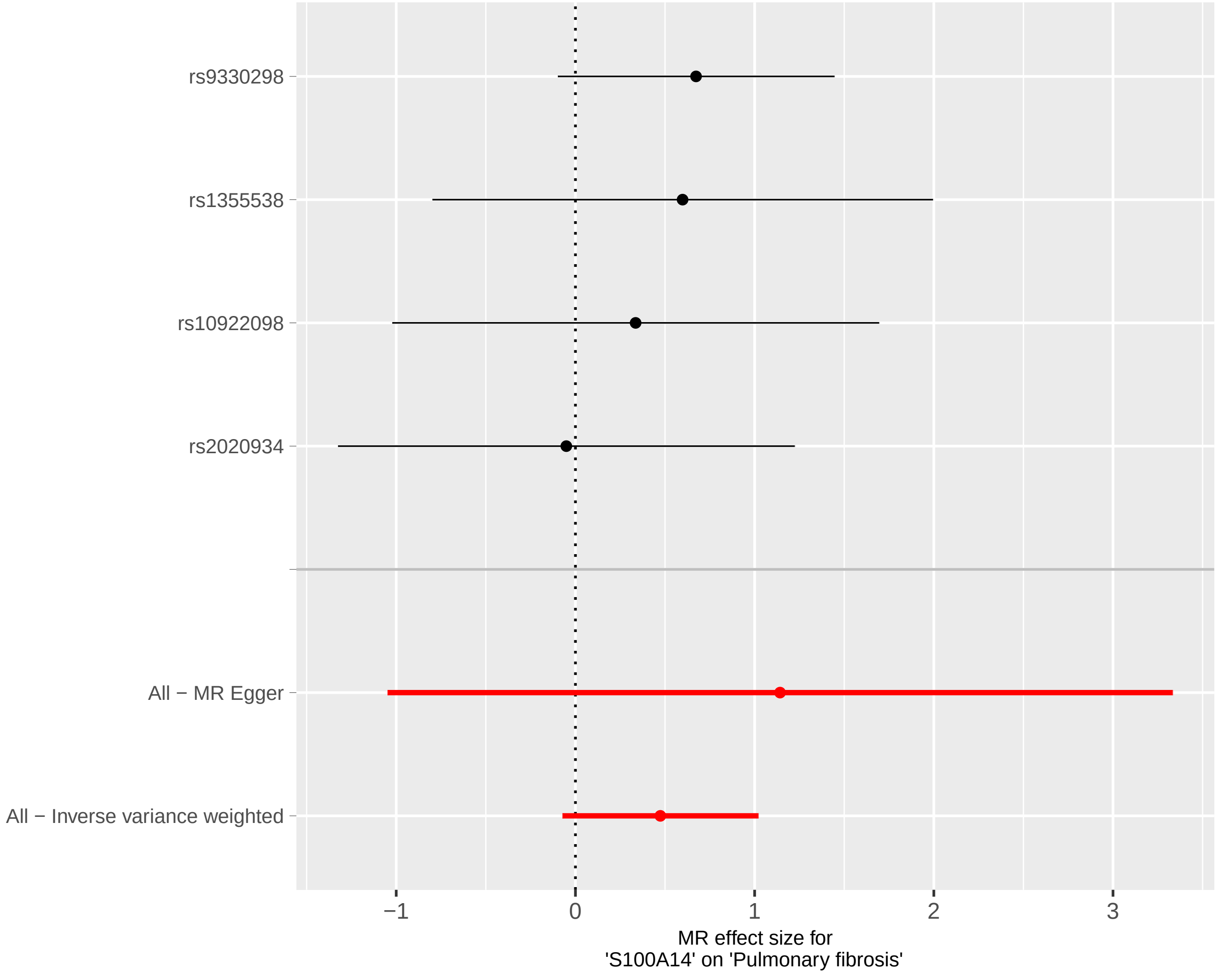
rs9330298
rs1355538
rs10922098
rs2020934
All − MR Egger
All − Inverse variance weighted
−1
0
1
2
3
MR effect size for
'S100A14' on 'Pulmonary fibrosis'

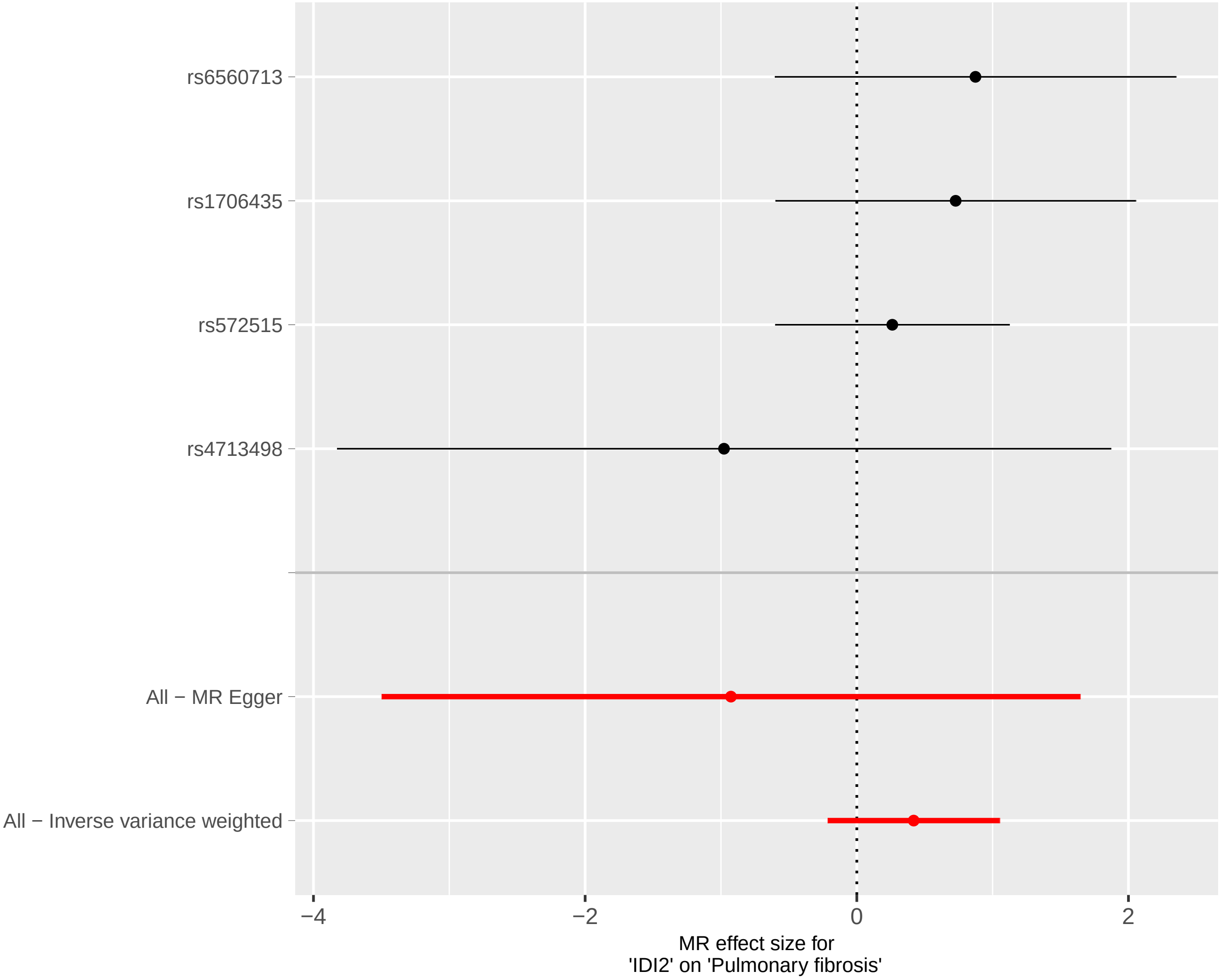
rs6560713
rs1706435
rs572515
rs4713498
All − MR Egger
All − Inverse variance weighted
−4
−2
0
2
MR effect size for
'IDI2' on 'Pulmonary fibrosis'

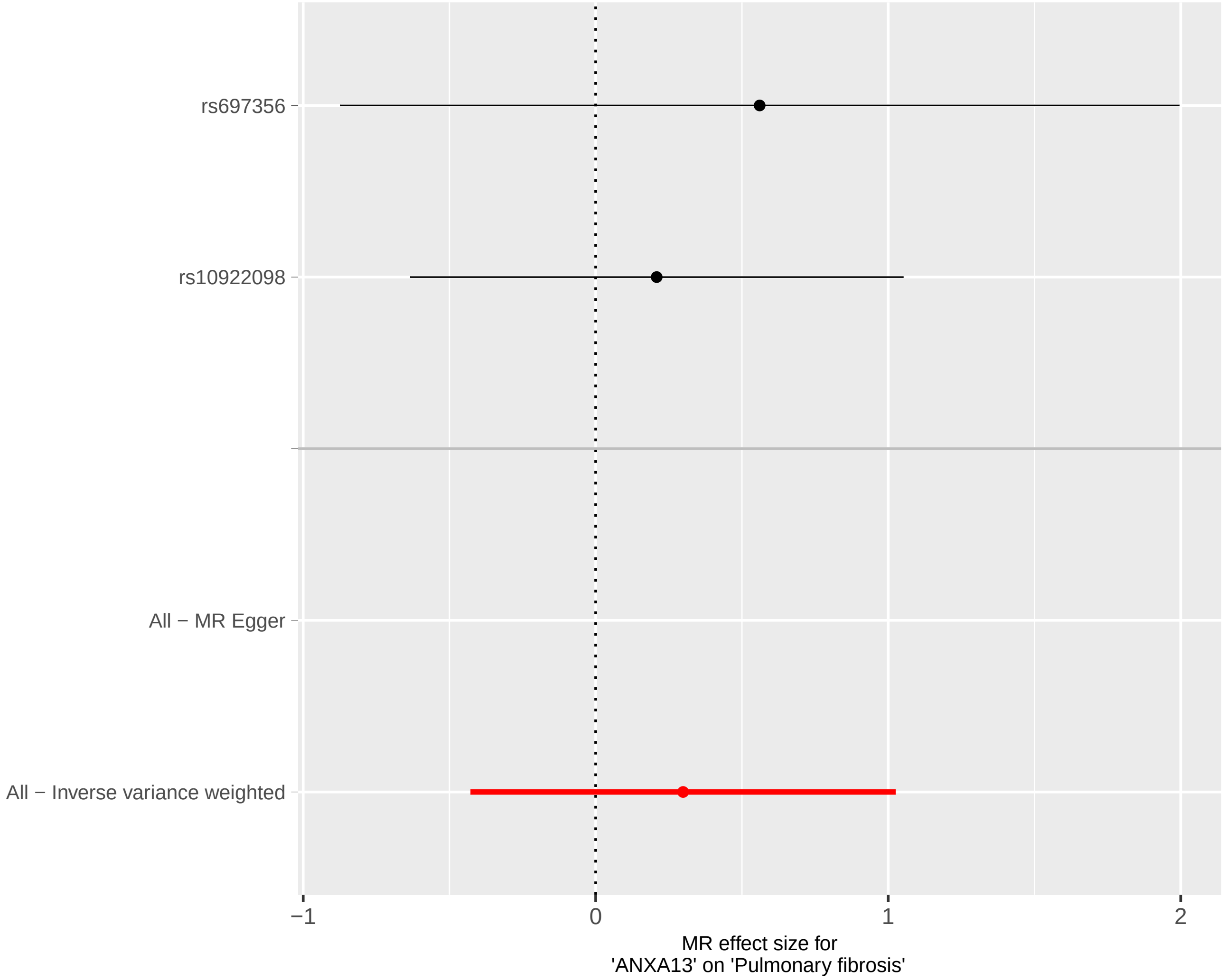

rs697356
rs10922098
All − MR Egger
All − Inverse variance weighted
−1
0
1
2
MR effect size for
'ANXA13' on 'Pulmonary fibrosis'

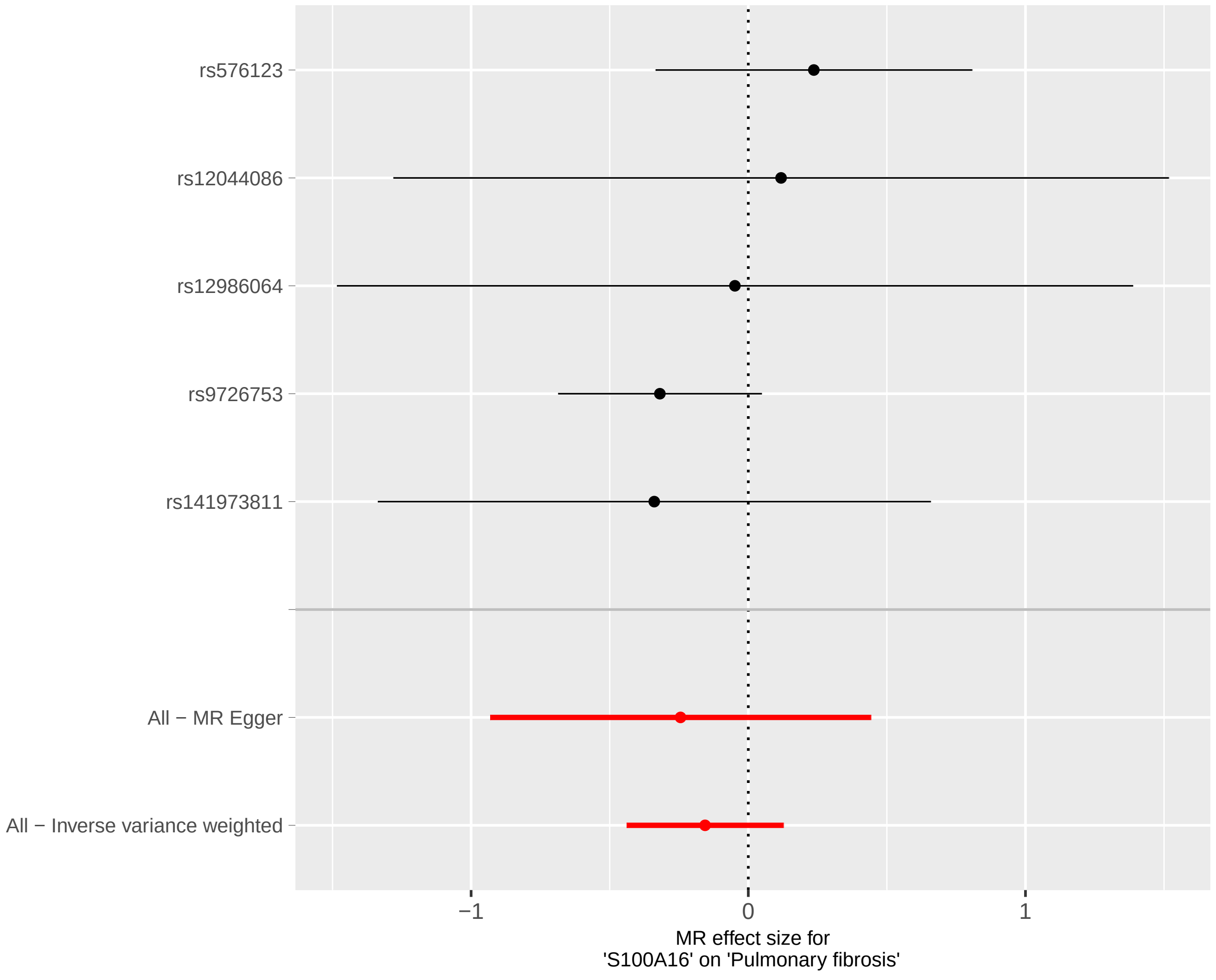
rs576123
rs12044086
rs12986064
rs9726753
rs141973811
All − MR Egger
All − Inverse variance weighted
−1
0
1
MR effect size for
'S100A16' on 'Pulmonary fibrosis'

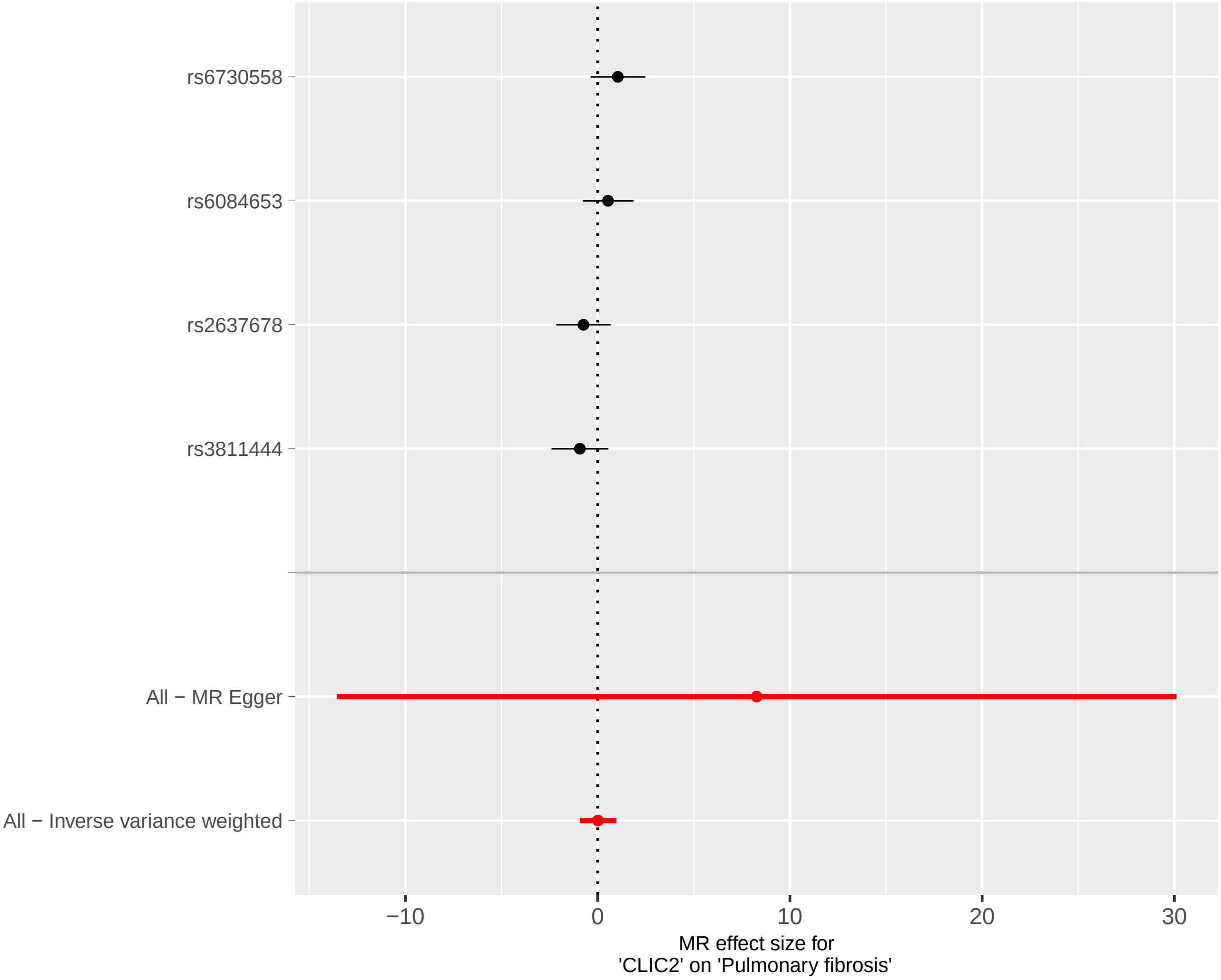
rs6730558
rs6084653
rs2637678
rs3811444
All − MR Egger
All − Inverse variance weighted
−10
0
10
20
30
MR effect size for
'CLIC2' on 'Pulmonary fibrosis'

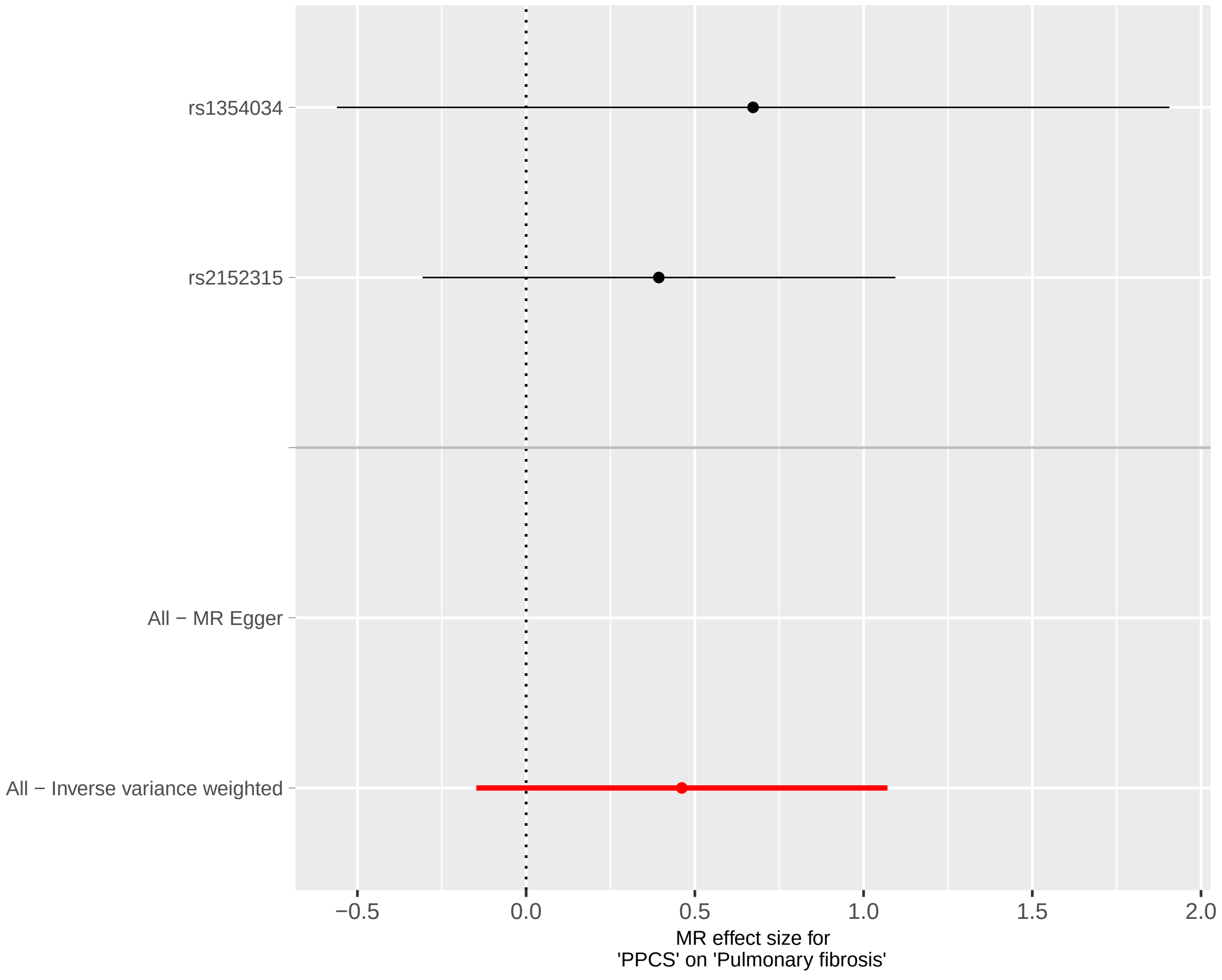

rs1354034
rs2152315
All − MR Egger
All − Inverse variance weighted
−0.5
0.0
0.5
1.0
1.5
2.0
MR effect size for
'PPCS' on 'Pulmonary fibrosis'

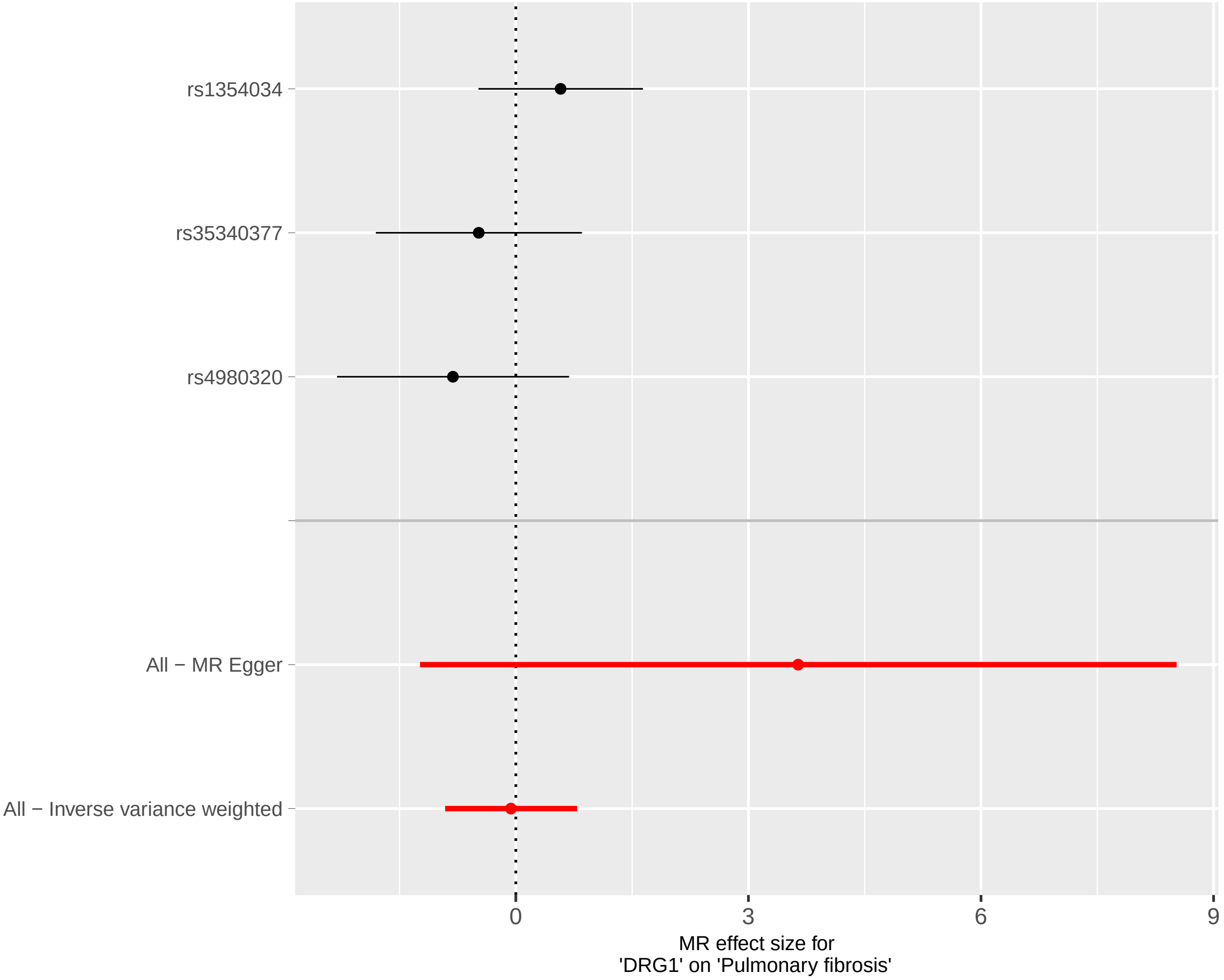

rs1354034
rs35340377
rs4980320
All − MR Egger
All − Inverse variance weighted
0
3
6
9
MR effect size for
'DRG1' on 'Pulmonary fibrosis'

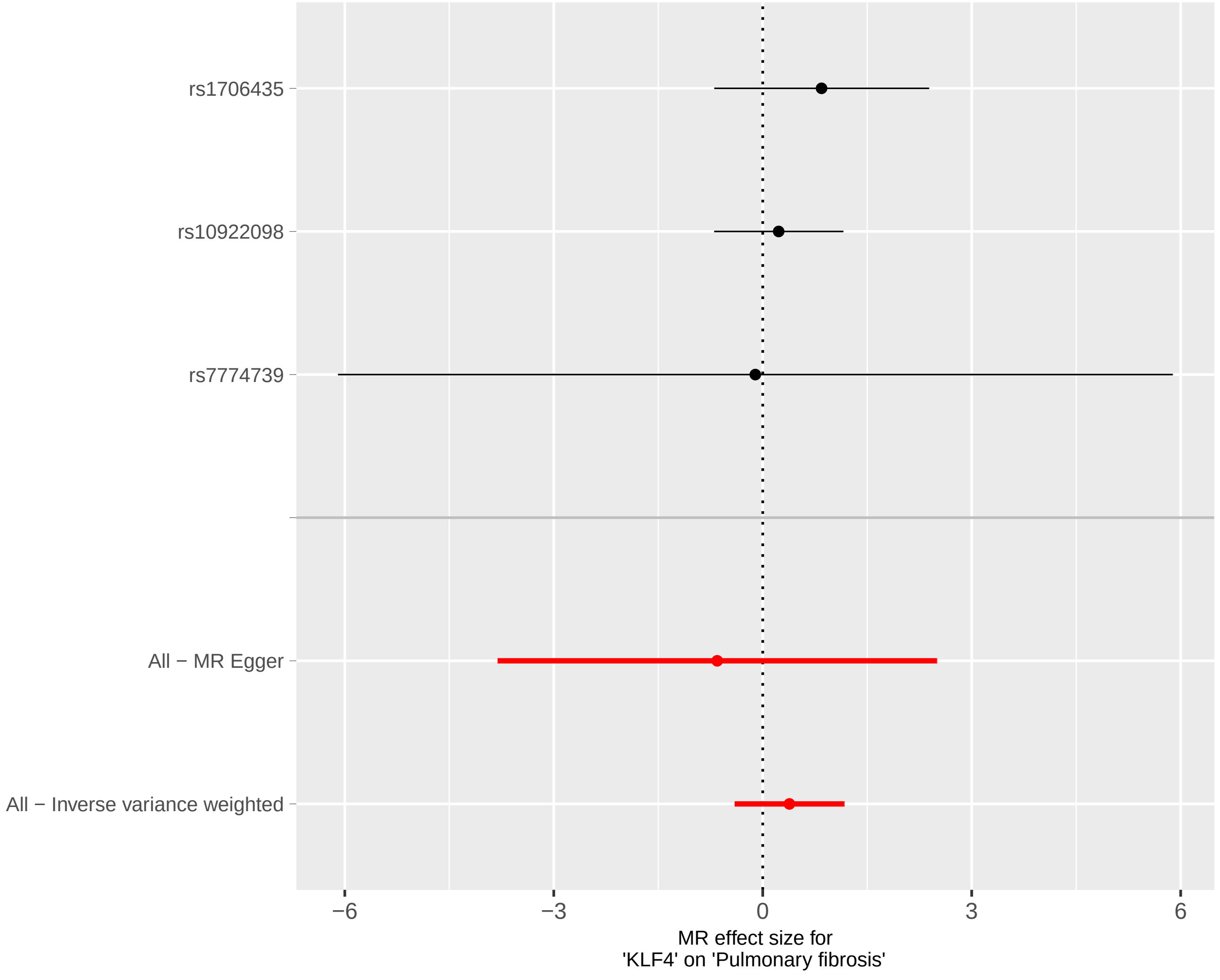
rs1706435
rs10922098
rs7774739
All − MR Egger
All − Inverse variance weighted
−6
−3
0
3
6
MR effect size for
'KLF4' on 'Pulmonary fibrosis'

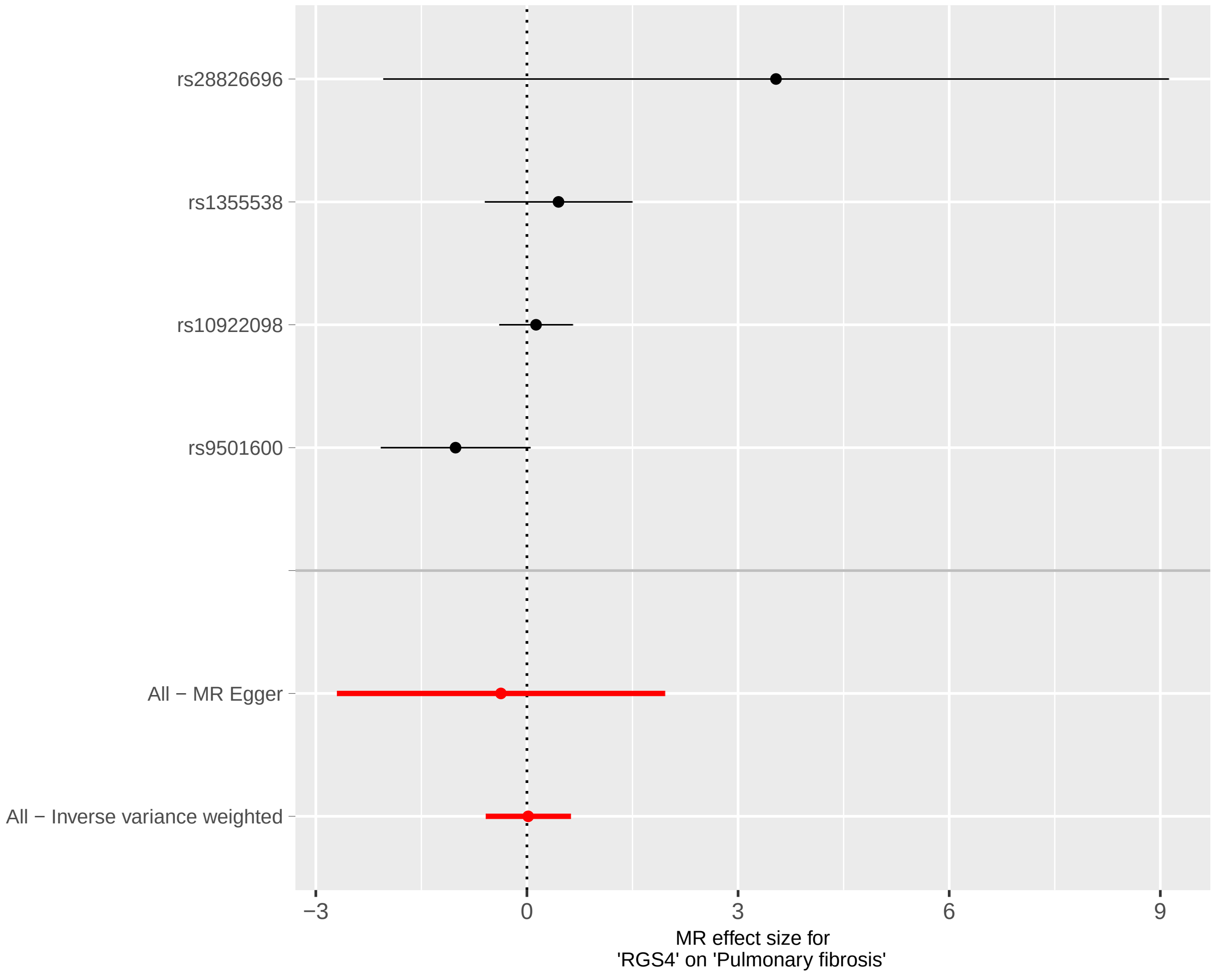
rs28826696
rs1355538
rs10922098
rs9501600
All − MR Egger
All − Inverse variance weighted
−3
0
3
6
9
MR effect size for
'RGS4' on 'Pulmonary fibrosis'

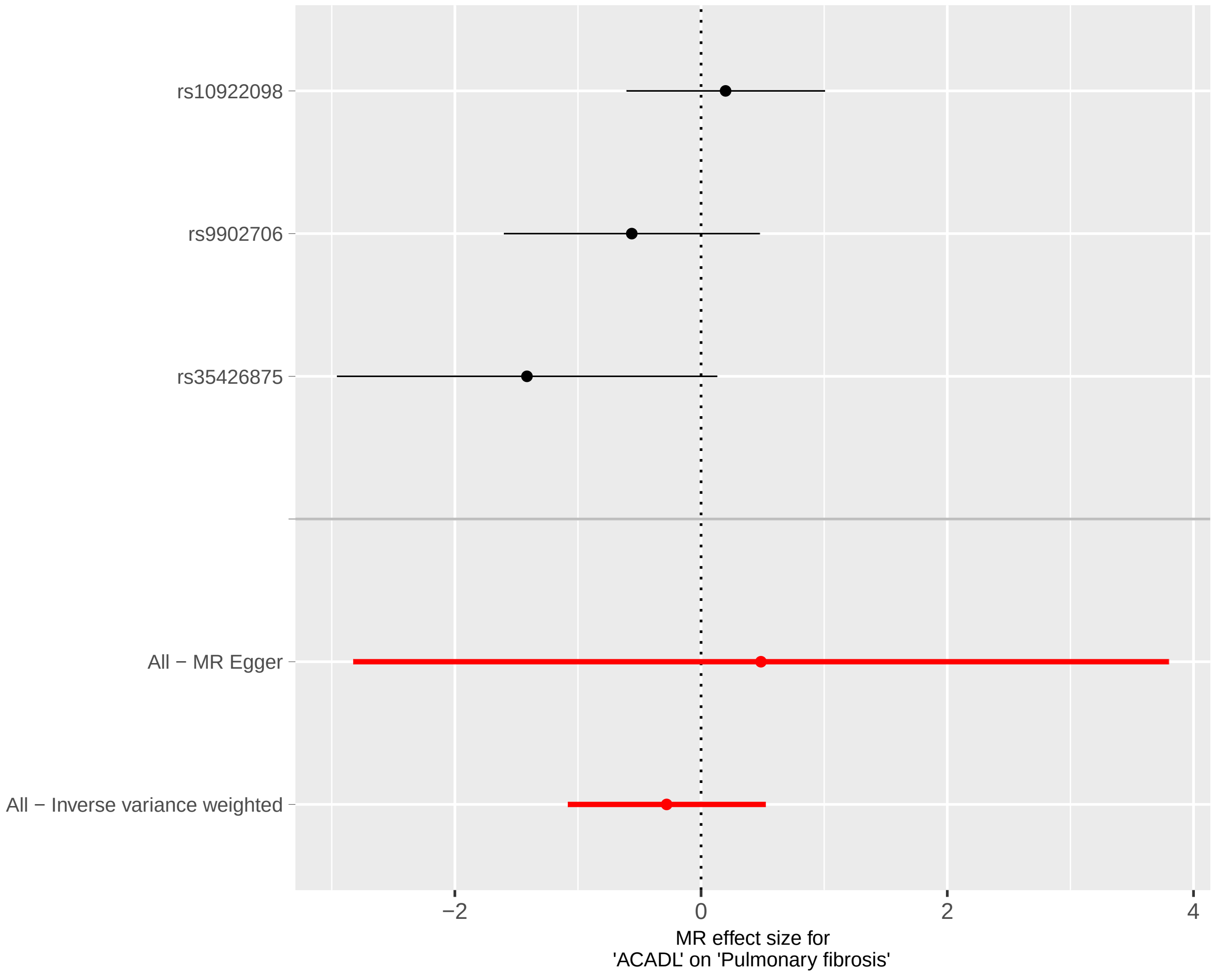

rs10922098
rs9902706
rs35426875
All − MR Egger
All − Inverse variance weighted
−2
0
2
4
MR effect size for
'ACADL' on 'Pulmonary fibrosis'

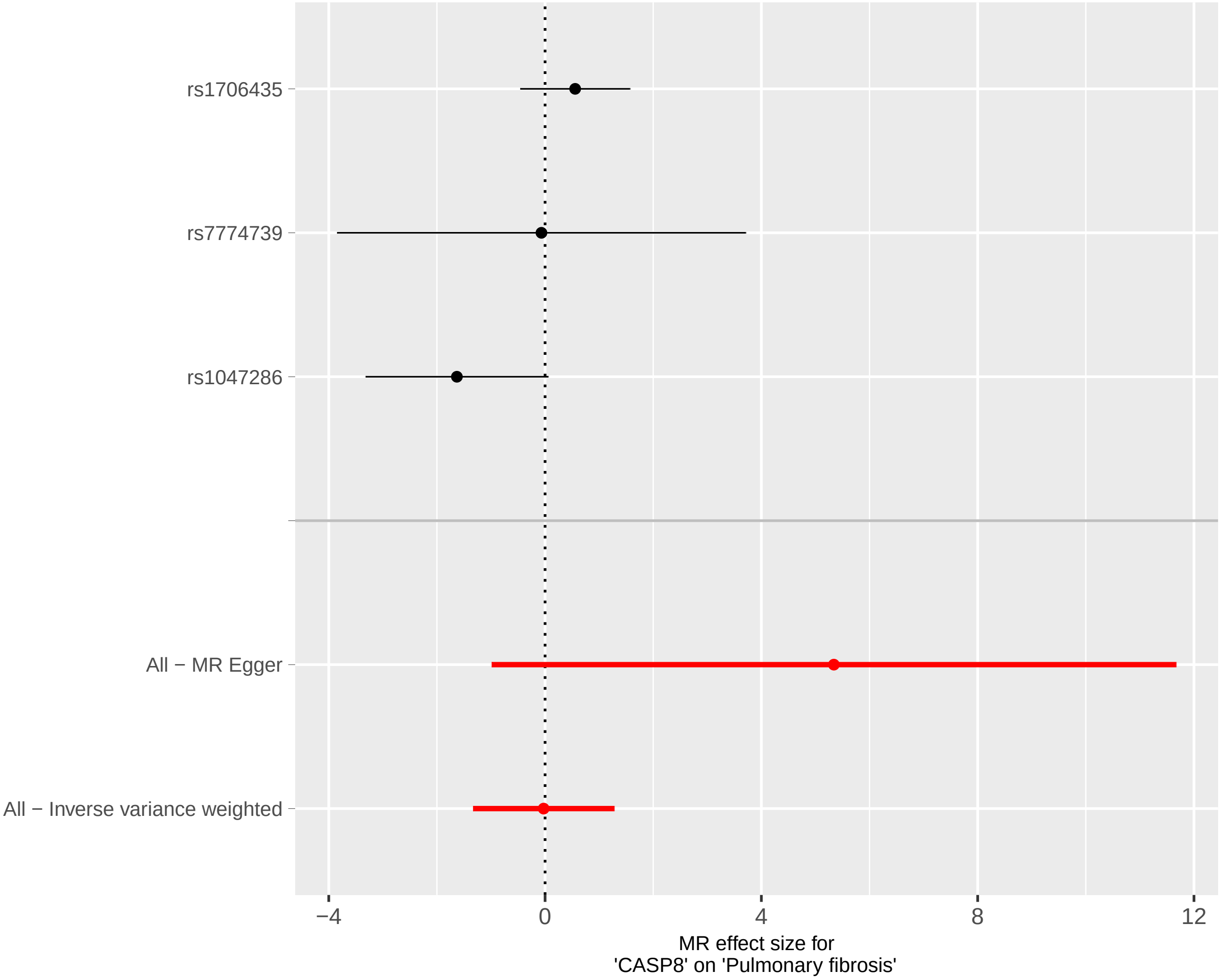

rs1706435
rs7774739
rs1047286
All − MR Egger
All − Inverse variance weighted
−4
0
4
8
12
MR effect size for
'CASP8' on 'Pulmonary fibrosis'

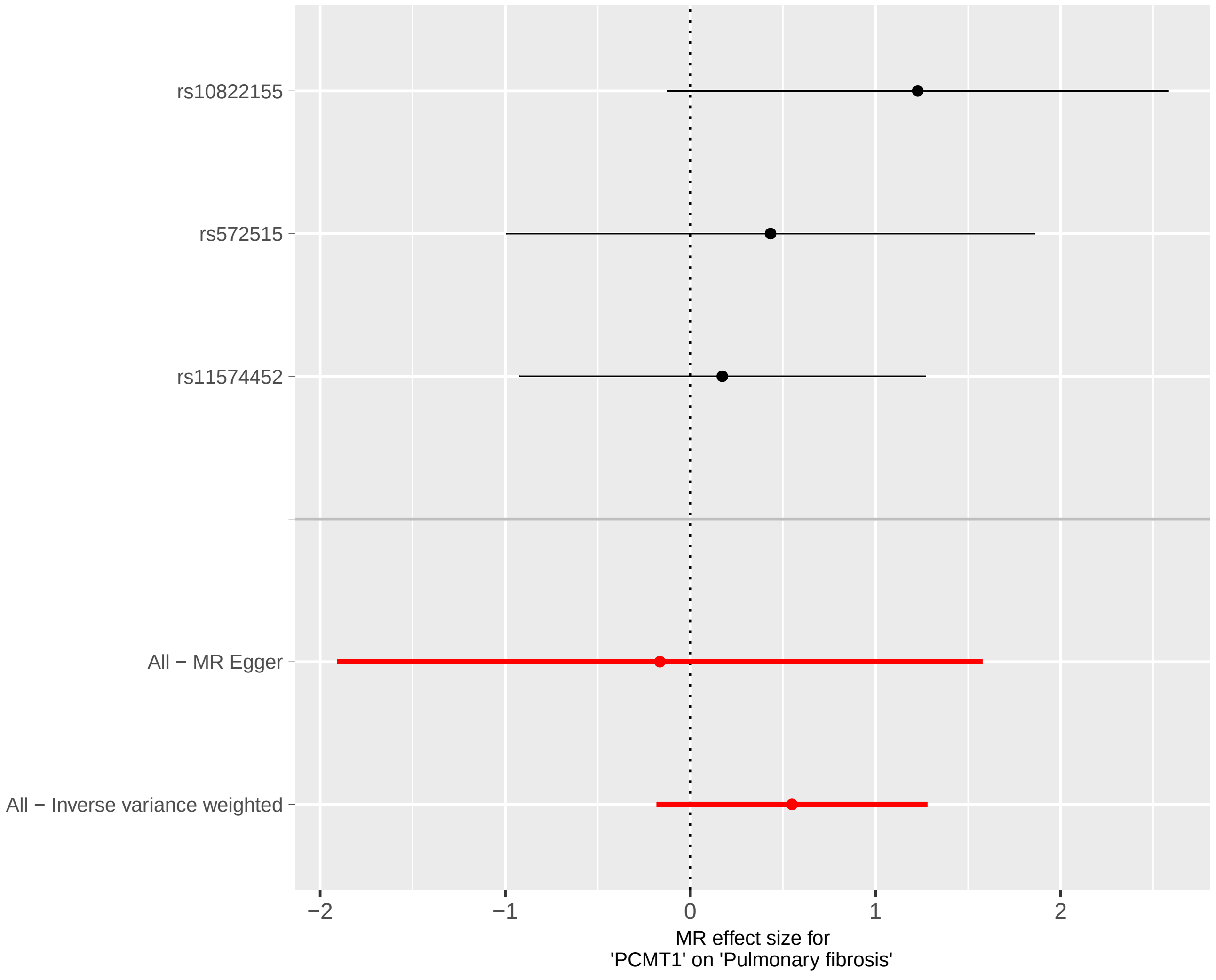
rs10822155
rs572515
rs11574452
All − MR Egger
All − Inverse variance weighted
−2
−1
0
1
2
MR effect size for
'PCMT1' on 'Pulmonary fibrosis'

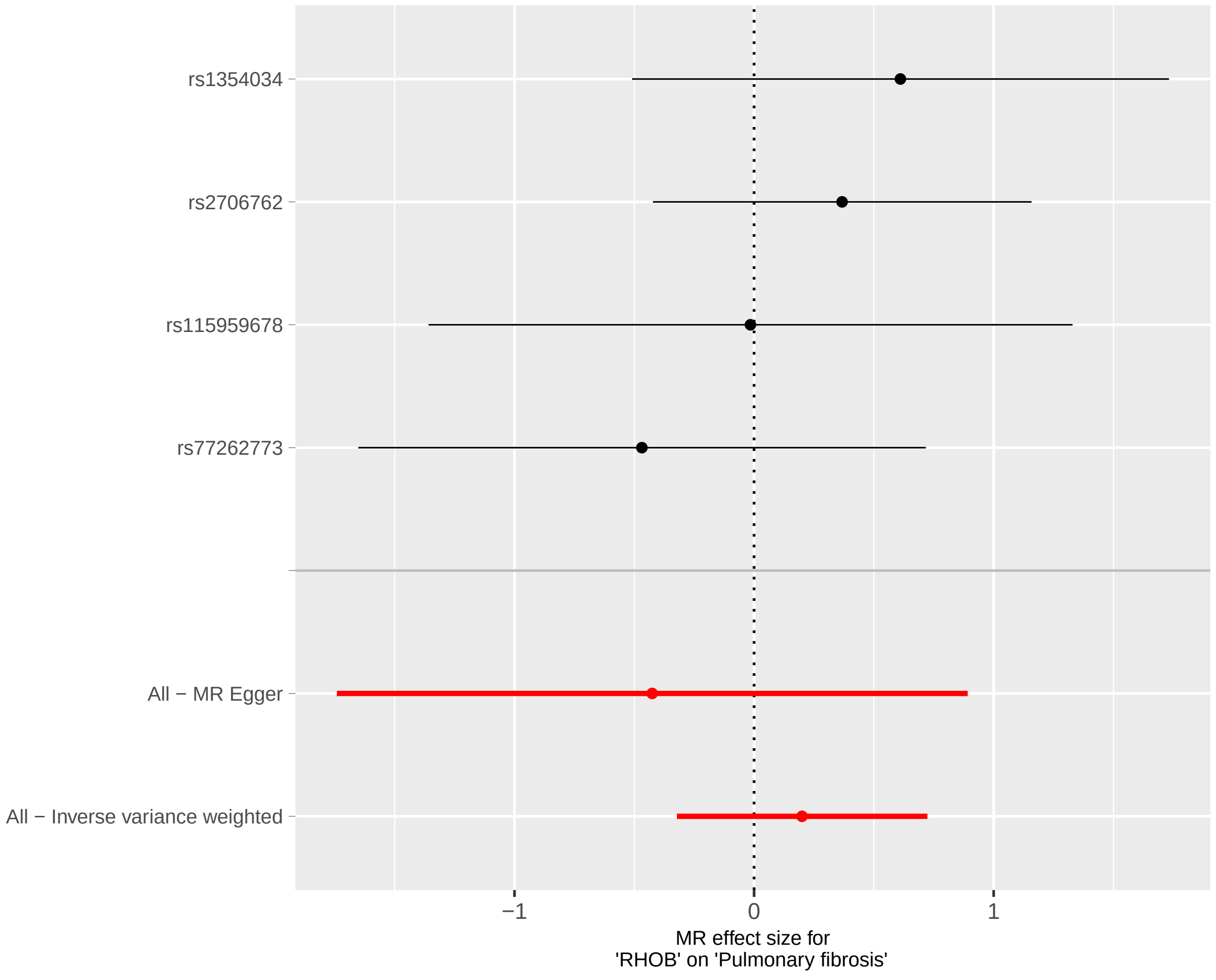
rs1354034
rs2706762
rs115959678
rs77262773
All − MR Egger
All − Inverse variance weighted
−1
0
1
MR effect size for
'RHOB' on 'Pulmonary fibrosis'

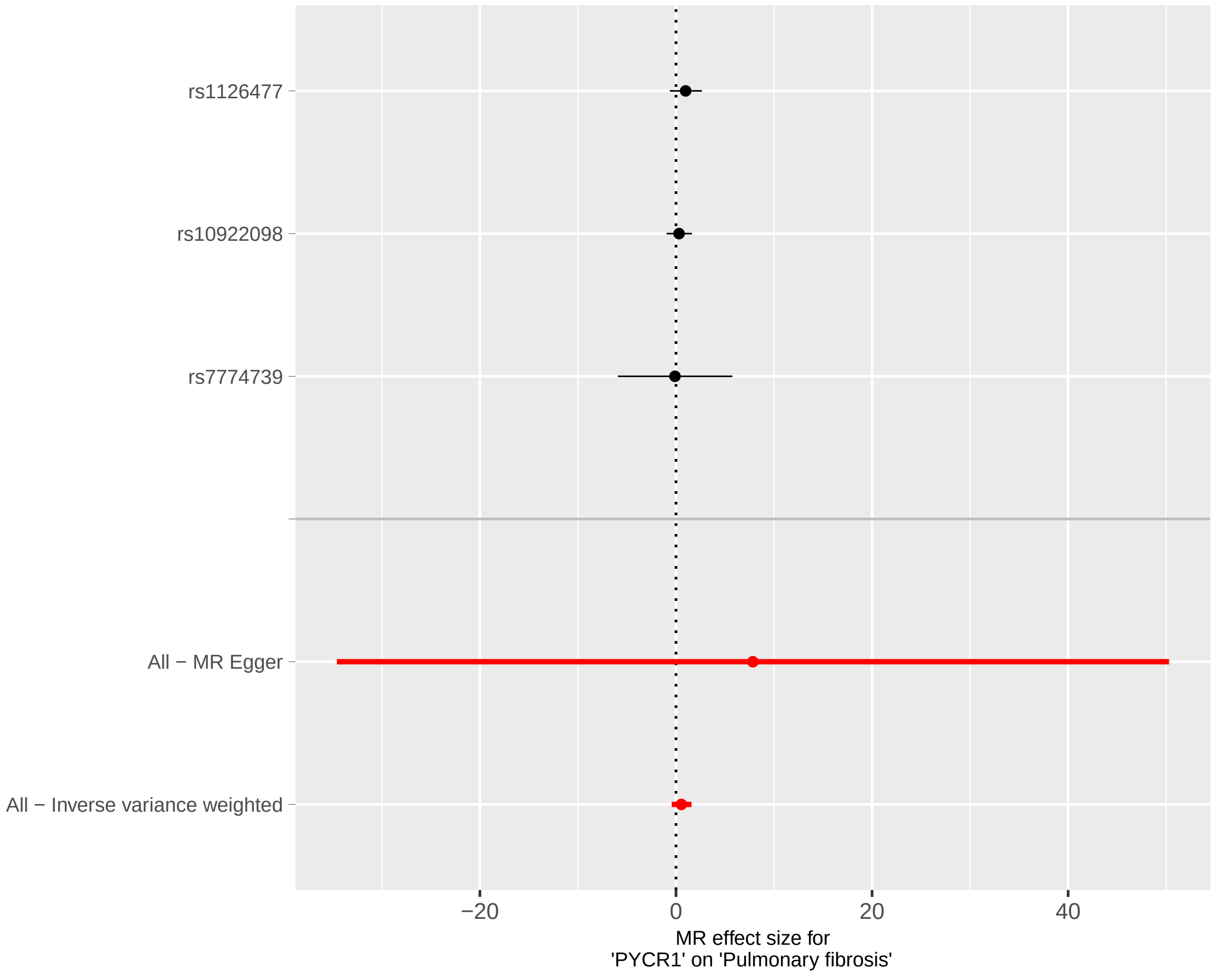

rs1126477
rs10922098
rs7774739
All − MR Egger
All − Inverse variance weighted
−20
0
20
40
MR effect size for
'PYCR1' on 'Pulmonary fibrosis'

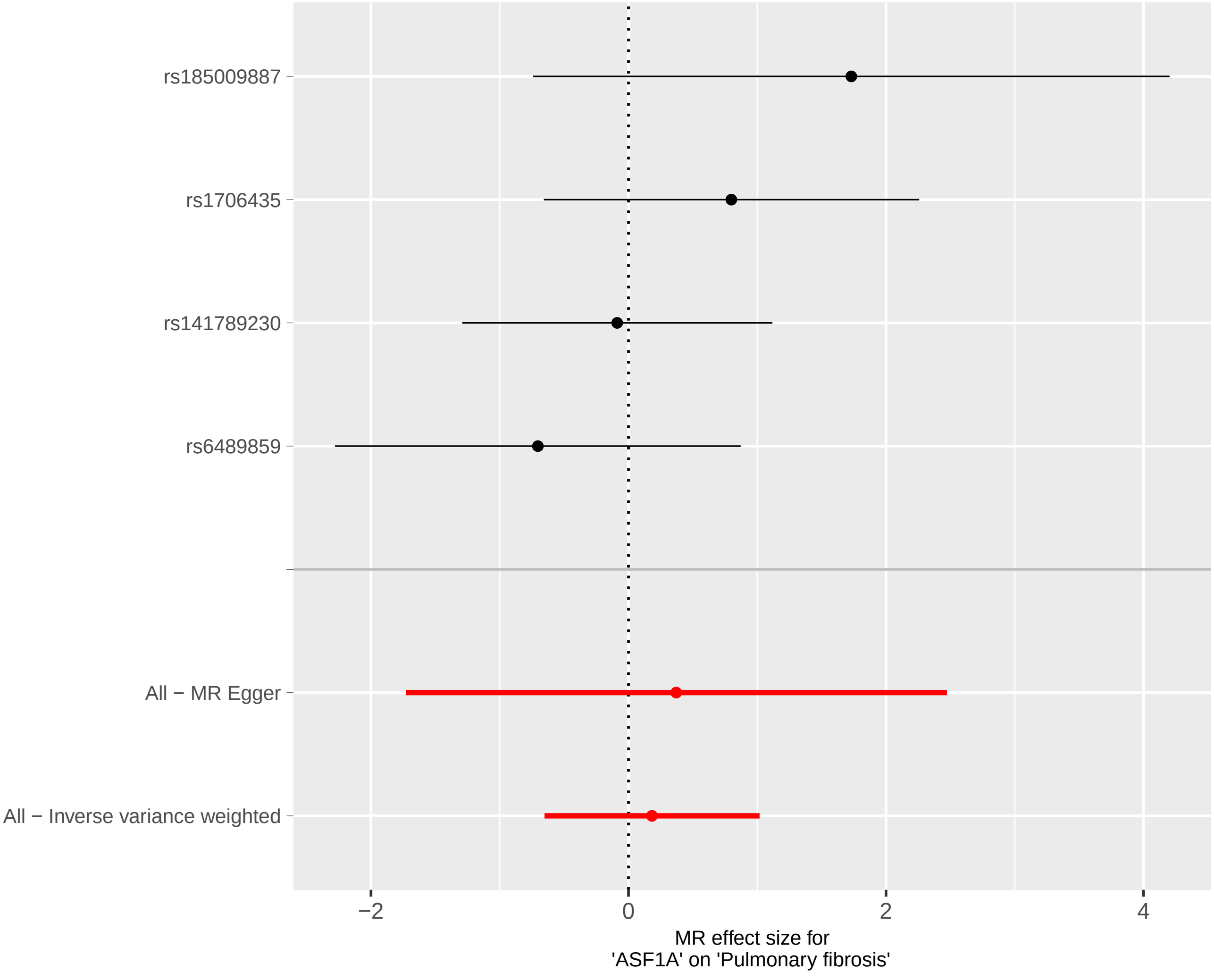
rs185009887
rs1706435
rs141789230
rs6489859
All − MR Egger
All − Inverse variance weighted
−2
0
2
4
MR effect size for
'ASF1A' on 'Pulmonary fibrosis'

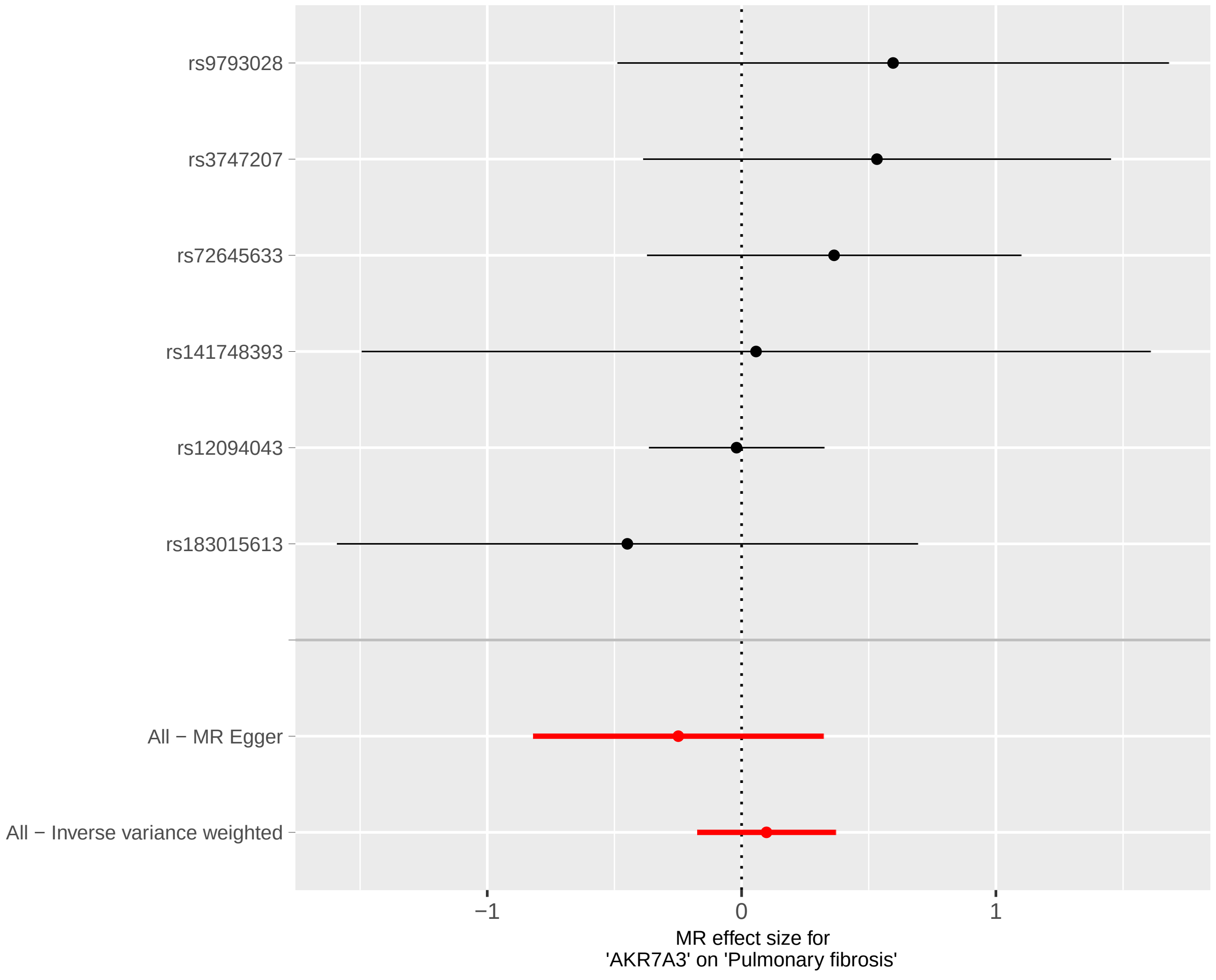

rs9793028
rs3747207
rs72645633
rs141748393
rs12094043
rs183015613
All − MR Egger
All − Inverse variance weighted
−1
0
1
MR effect size for
'AKR7A3' on 'Pulmonary fibrosis'

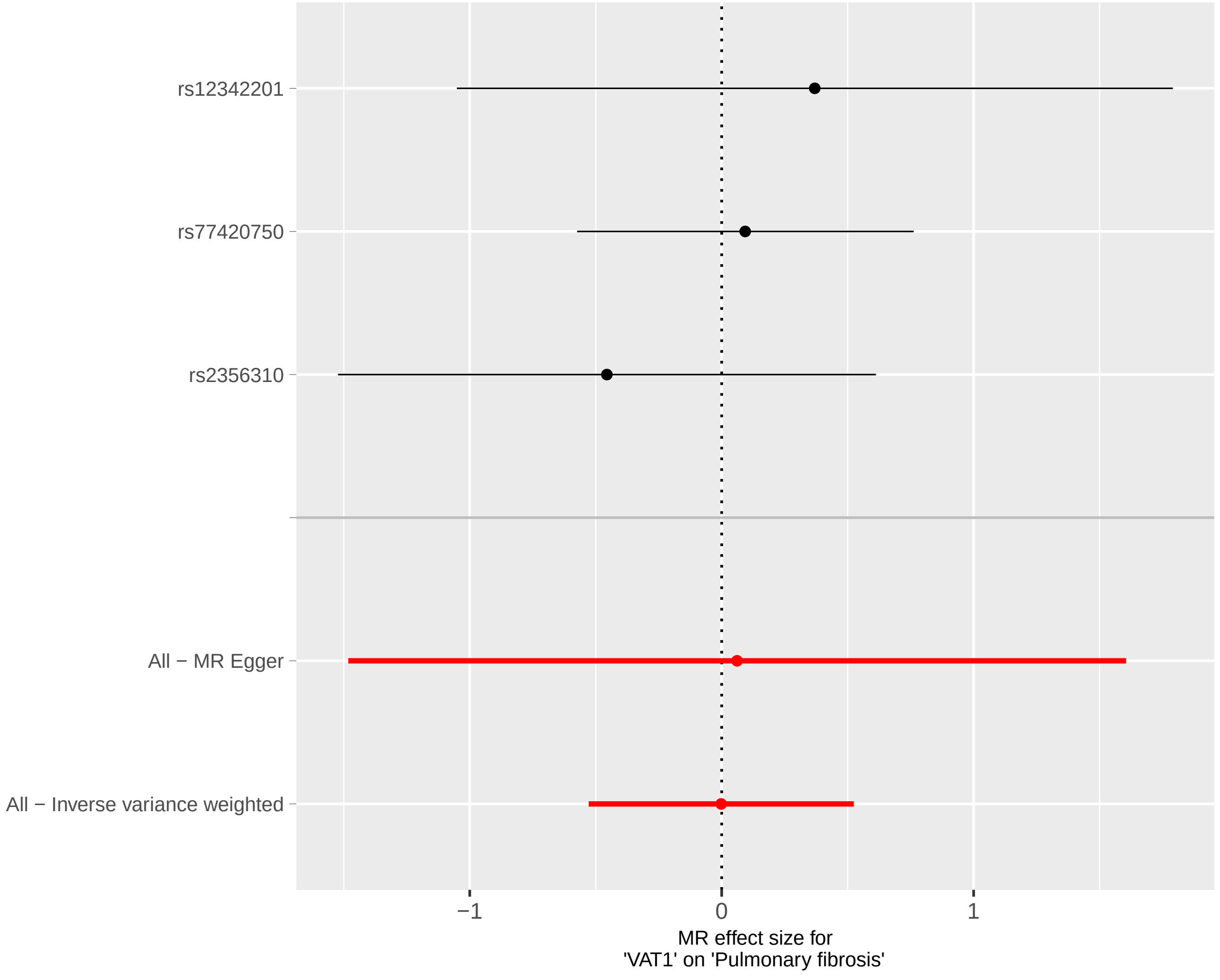

rs12342201
rs77420750
rs2356310
All − MR Egger
All − Inverse variance weighted
−1
0
1
MR effect size for
'VAT1' on 'Pulmonary fibrosis'

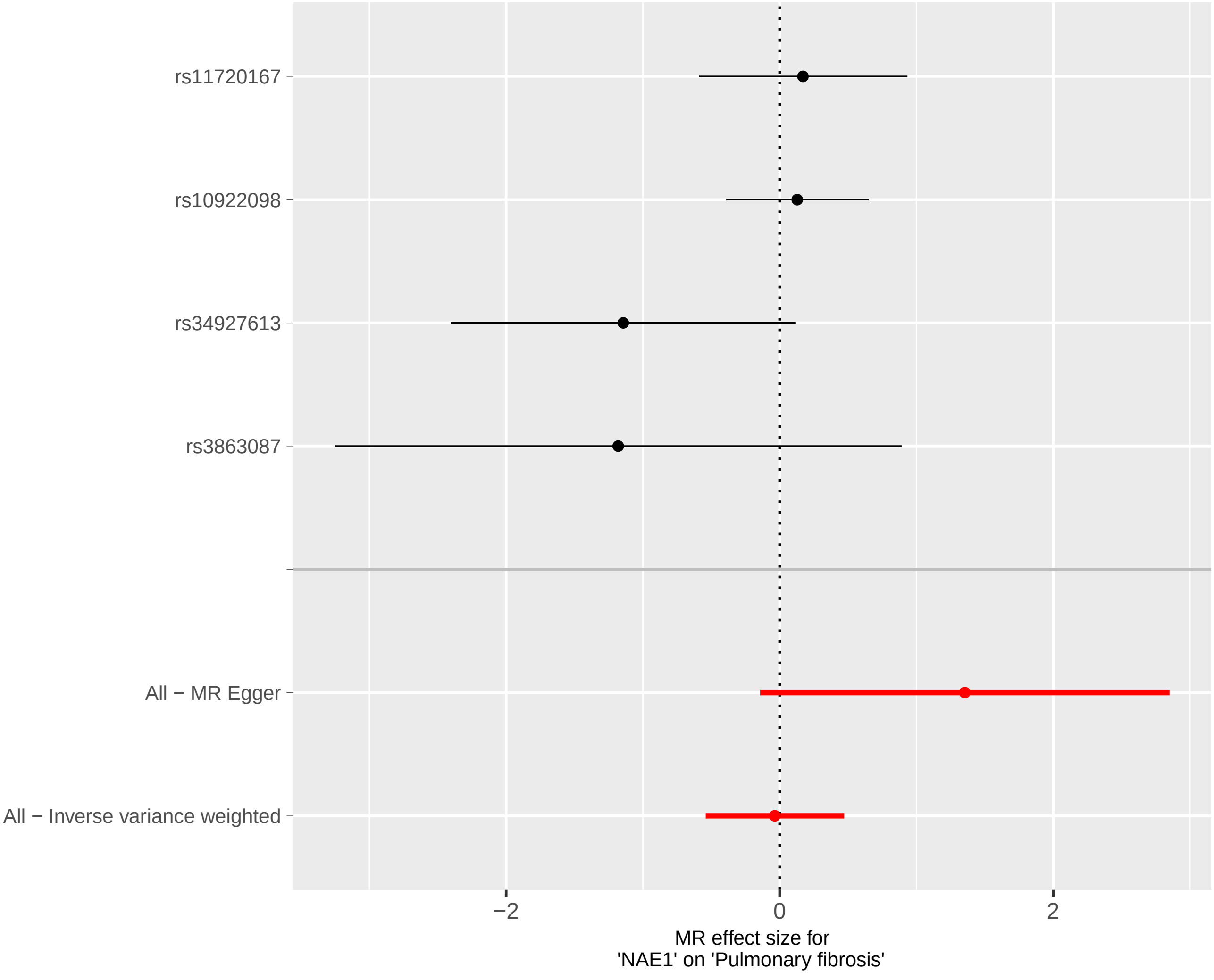

rs11720167
rs10922098
rs34927613
rs3863087
All − MR Egger
All − Inverse variance weighted
−2
0
2
MR effect size for
'NAE1' on 'Pulmonary fibrosis'

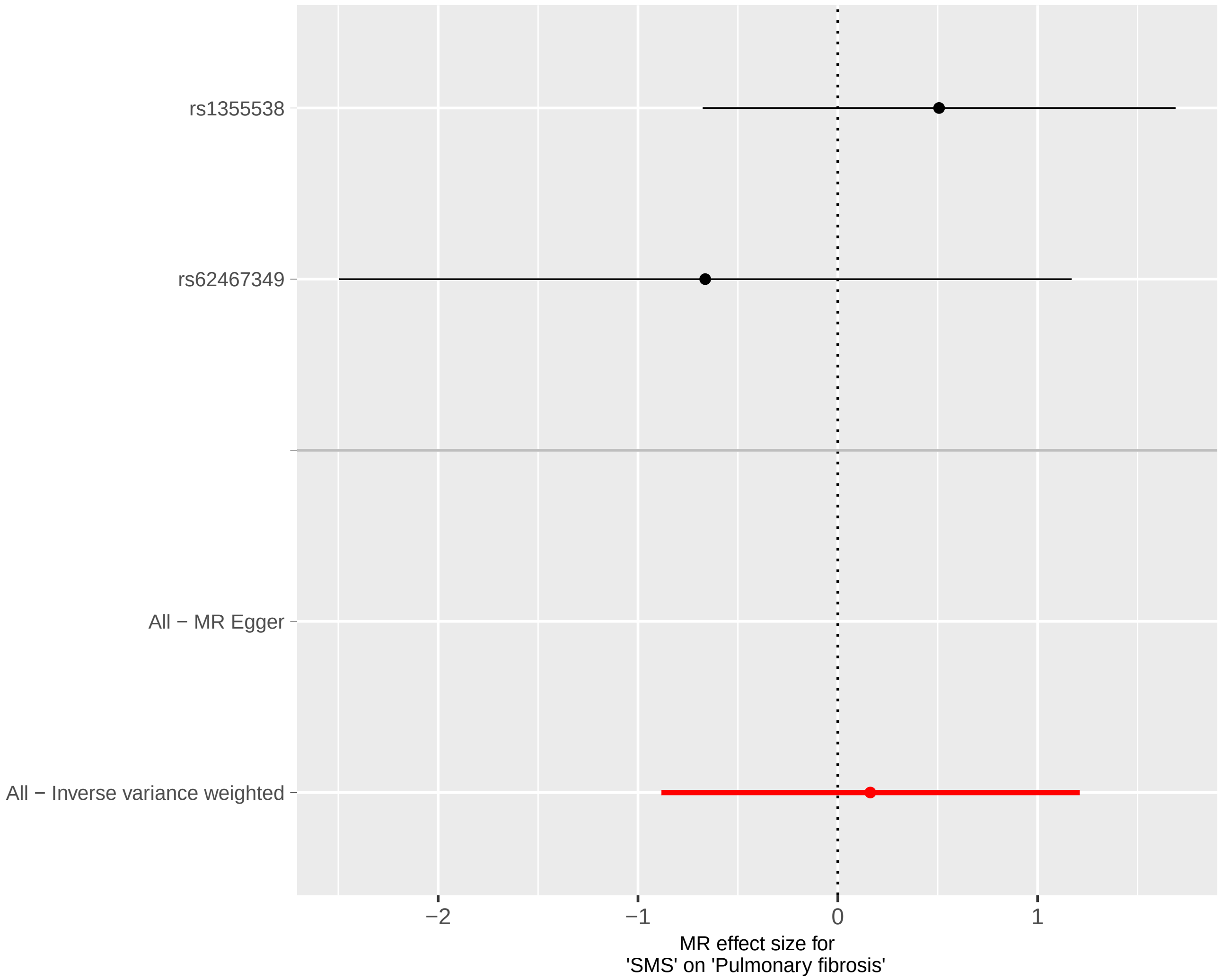

rs1355538
rs62467349
All − MR Egger
All − Inverse variance weighted
−2
−1
0
1
MR effect size for
'SMS' on 'Pulmonary fibrosis'

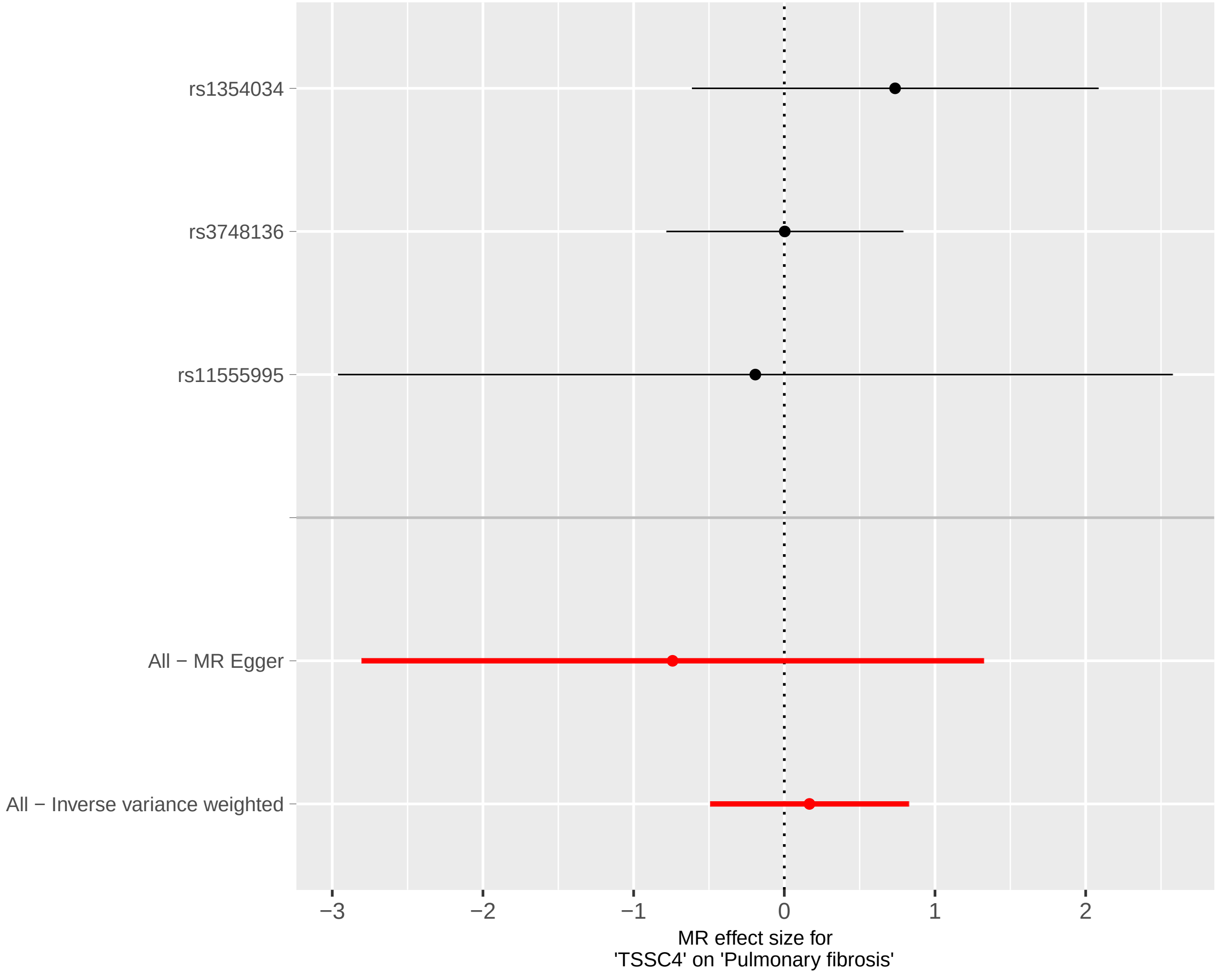
rs1354034
rs3748136
rs11555995
All − MR Egger
All − Inverse variance weighted
−3
−2
−1
0
1
2
MR effect size for
'TSSC4' on 'Pulmonary fibrosis'

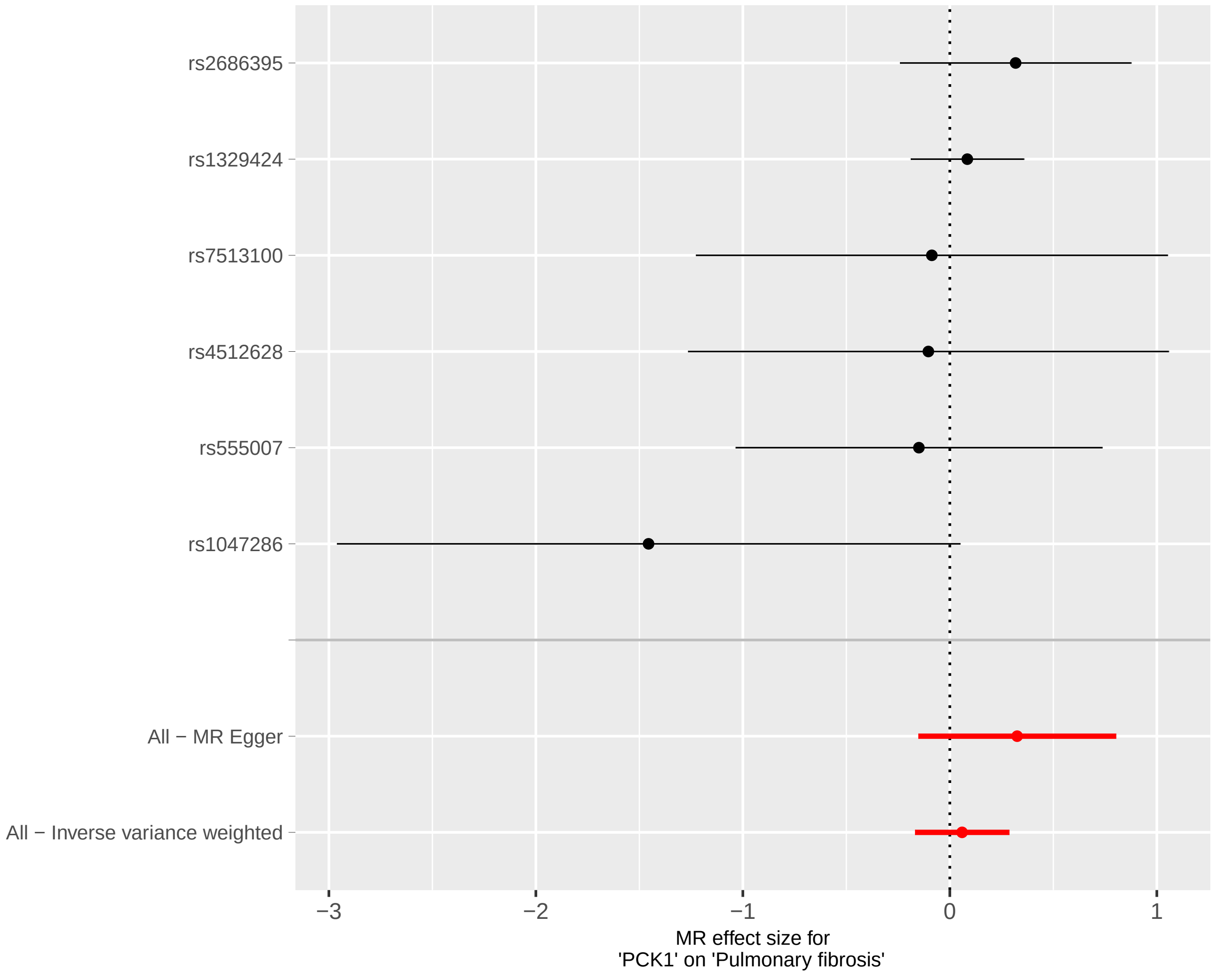

rs2686395
rs1329424
rs7513100
rs4512628
rs555007
rs1047286
All − MR Egger
All − Inverse variance weighted
−3
−2
−1
0
1
MR effect size for
'PCK1' on 'Pulmonary fibrosis'

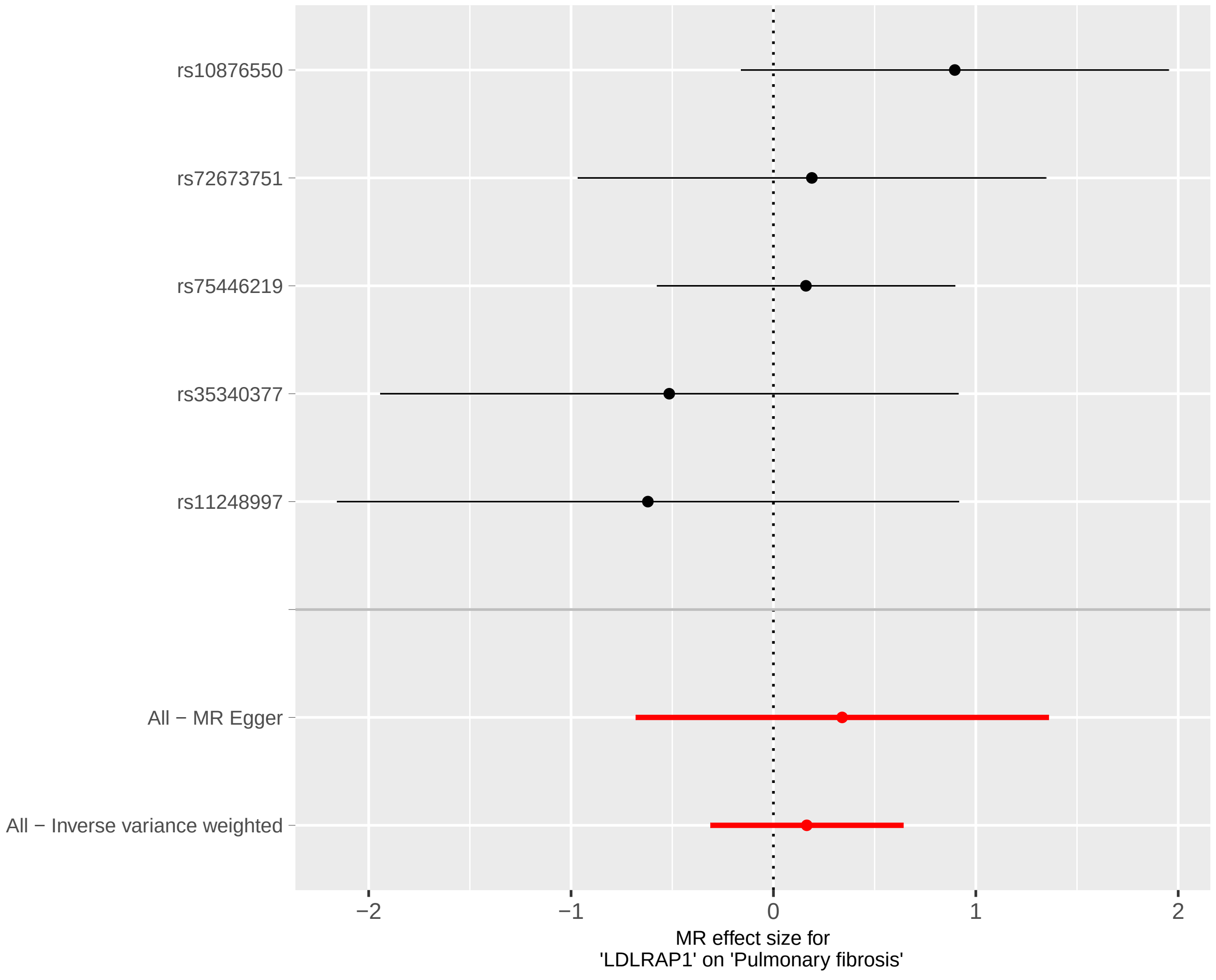
rs10876550
rs72673751
rs75446219
rs35340377
rs11248997
All − MR Egger
All − Inverse variance weighted
−2
−1
0
1
2
MR effect size for
'LDLRAP1' on 'Pulmonary fibrosis'

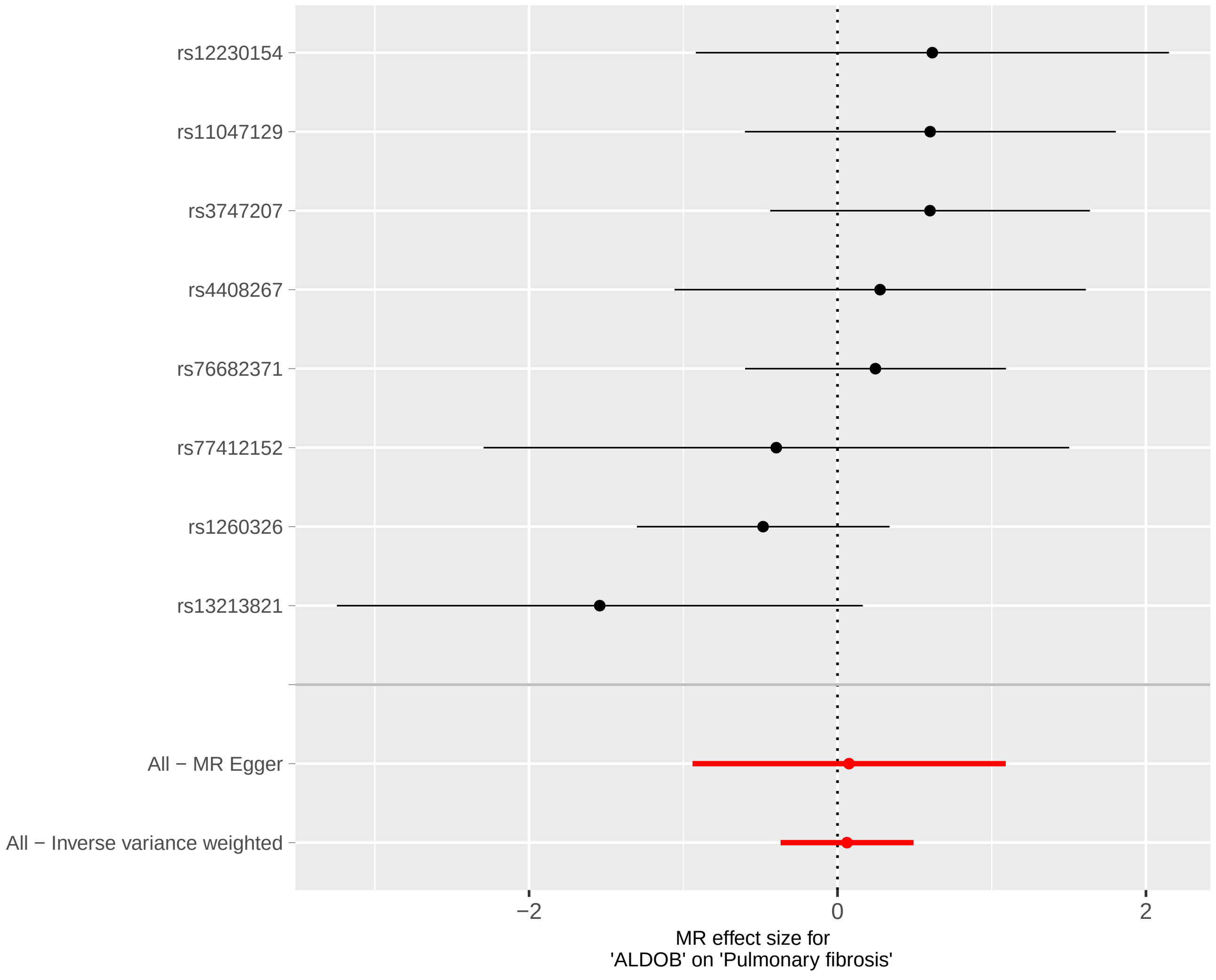
rs12230154
rs11047129
rs3747207
rs4408267
rs76682371
rs77412152
rs1260326
rs13213821
All − MR Egger
All − Inverse variance weighted
−2
0
2
MR effect size for
'ALDOB' on 'Pulmonary fibrosis'

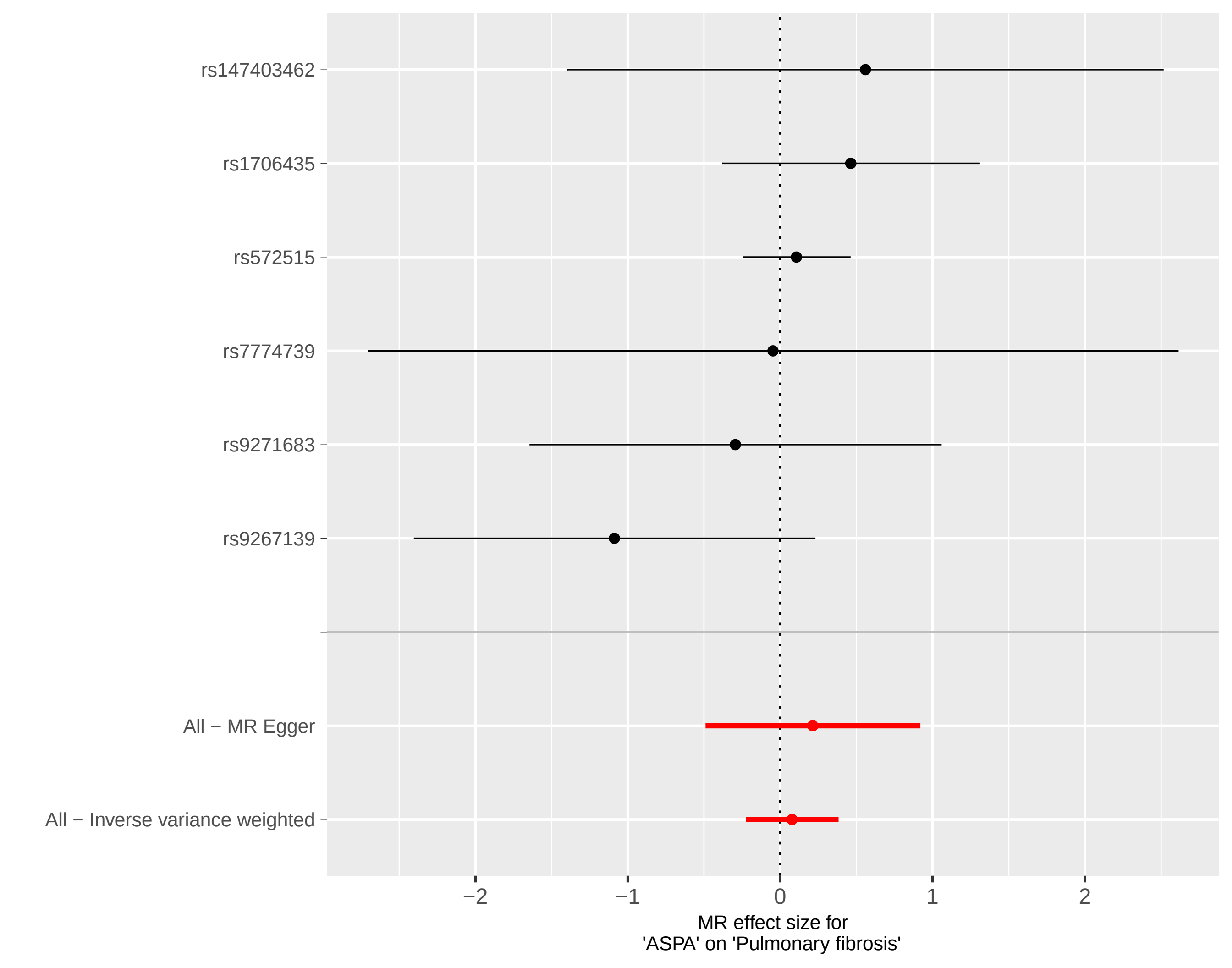
rs147403462
rs1706435
rs572515
rs7774739
rs9271683
rs9267139
All − MR Egger
All − Inverse variance weighted
−2
−1
0
1
2
MR effect size for
'ASPA' on 'Pulmonary fibrosis'

Insufficient number of SNPs

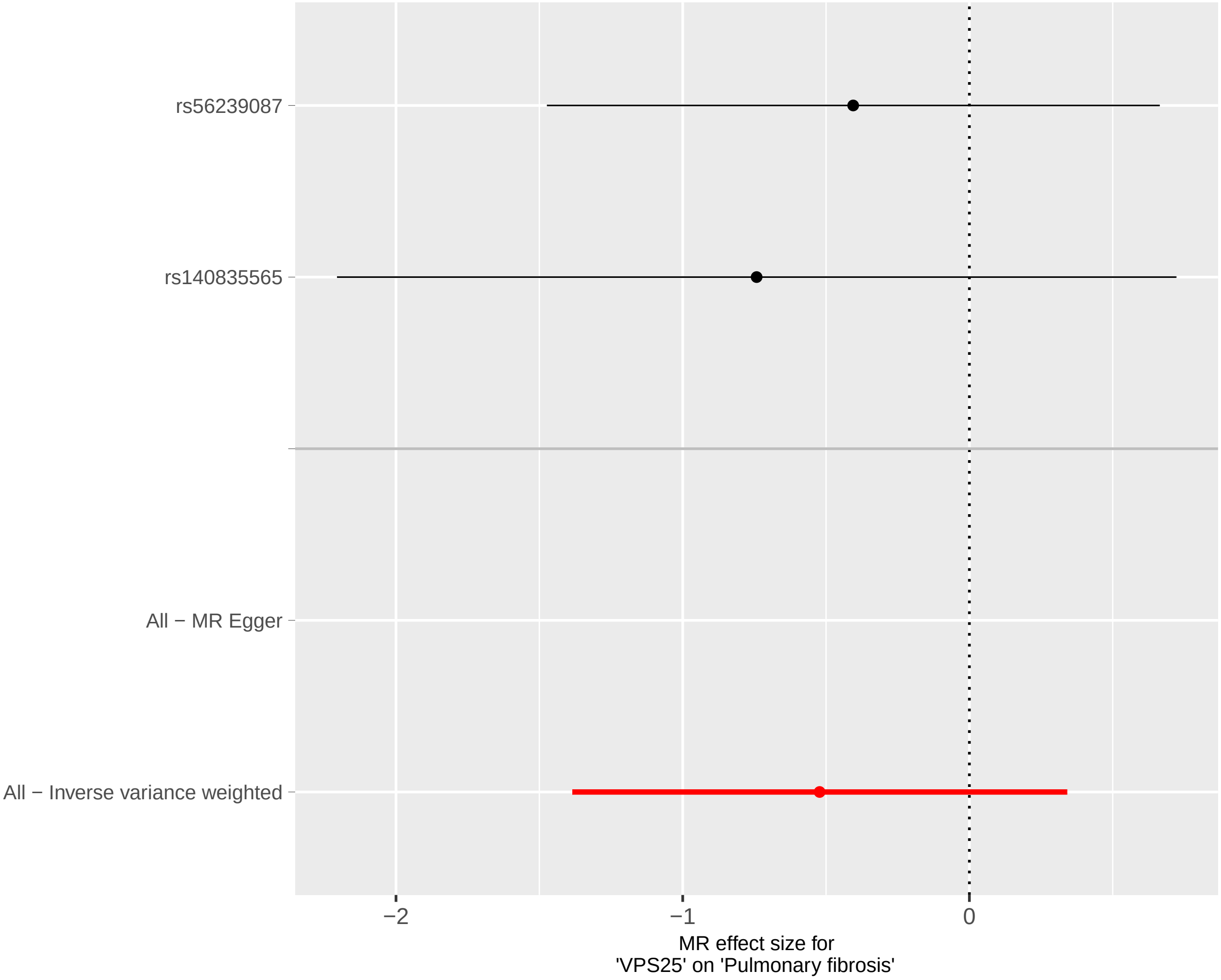
rs56239087
rs140835565
All − MR Egger
All − Inverse variance weighted
−2
−1
0
MR effect size for
'VPS25' on 'Pulmonary fibrosis'

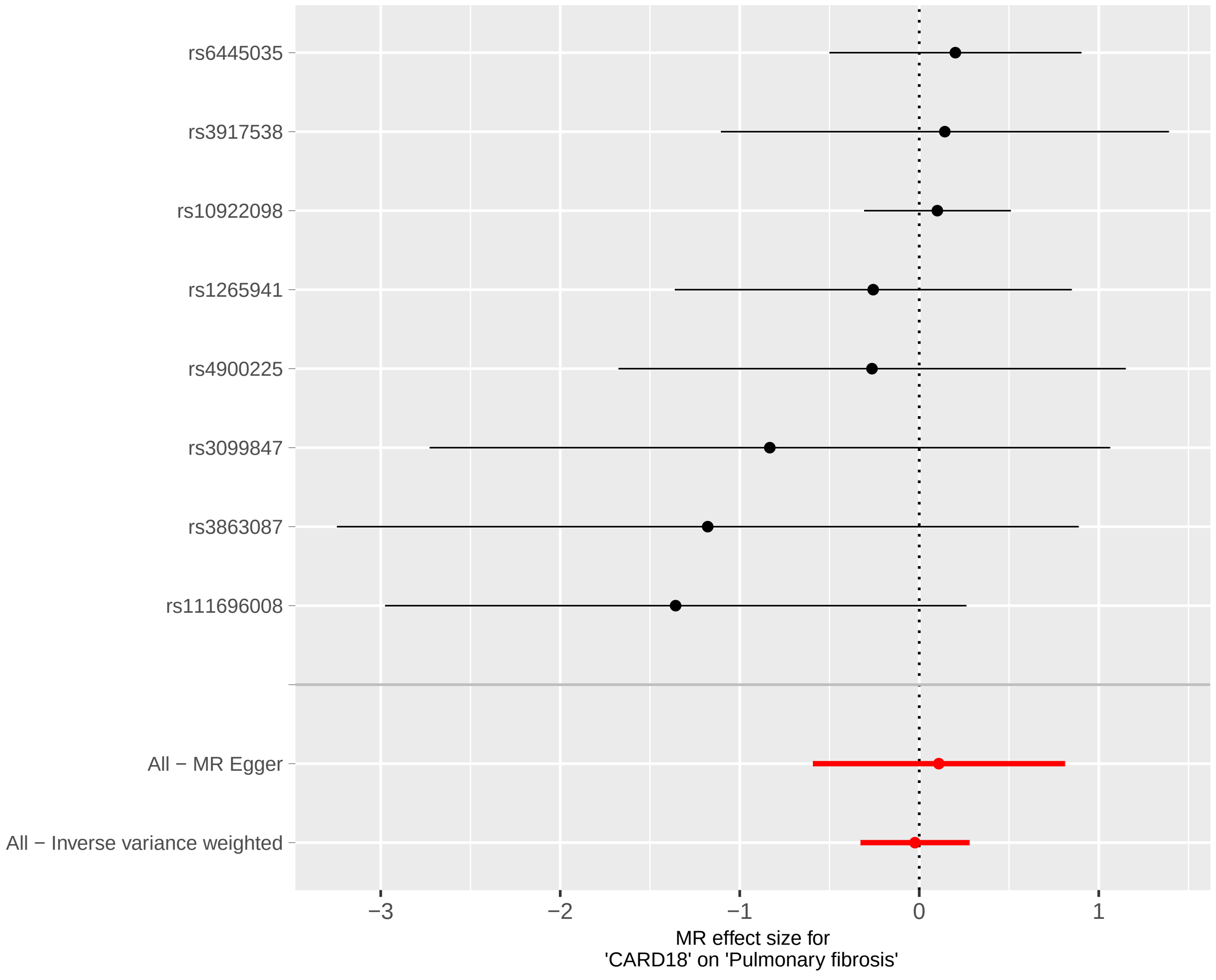

rs6445035
rs3917538
rs10922098
rs1265941
rs4900225
rs3099847
rs3863087
rs111696008
All − MR Egger
All − Inverse variance weighted
−3
−2
−1
0
1
MR effect size for
'CARD18' on 'Pulmonary fibrosis'

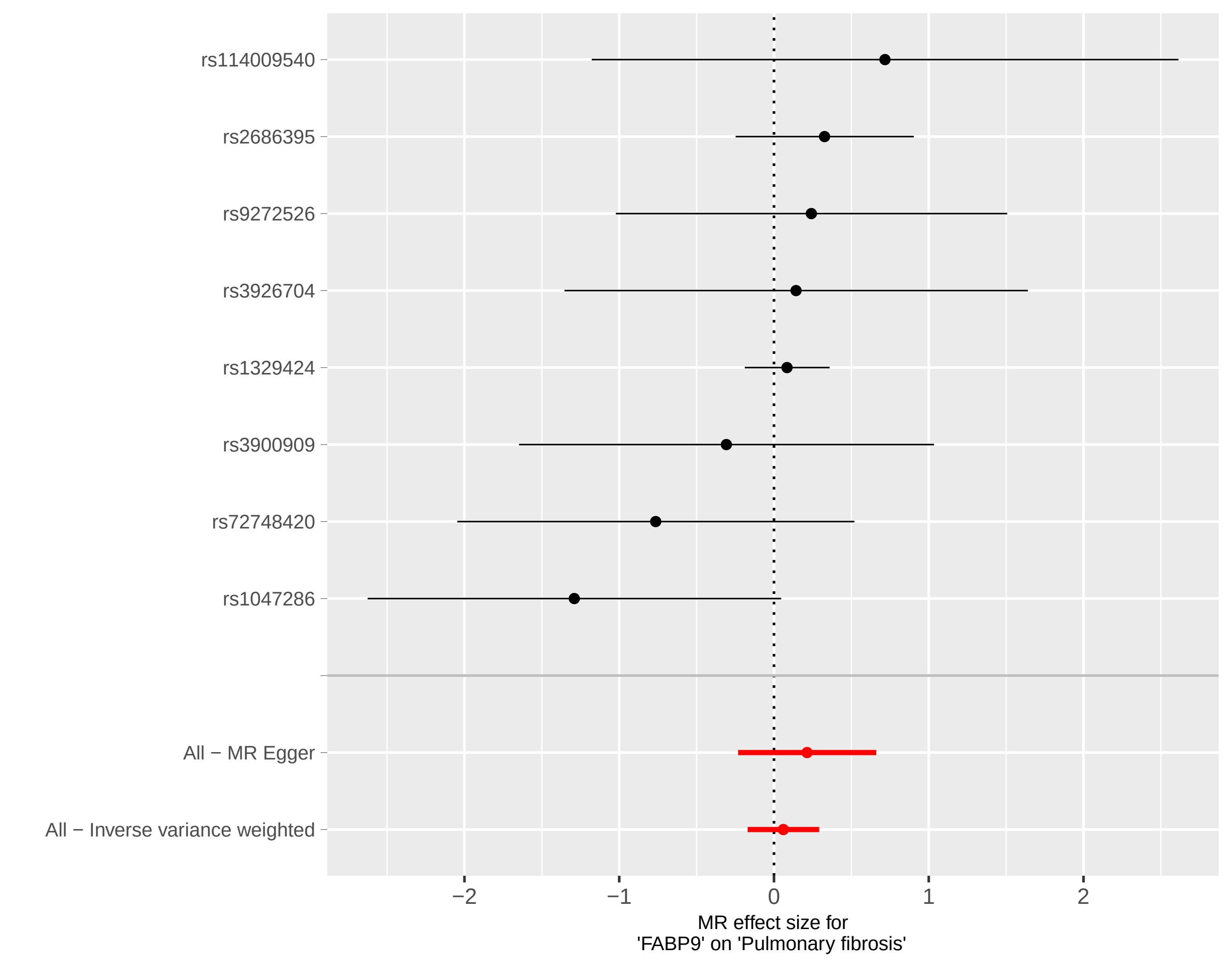

rs114009540
rs2686395
rs9272526
rs3926704
rs1329424
rs3900909
rs72748420
rs1047286
All − MR Egger
All − Inverse variance weighted
−2
−1
0
1
2
MR effect size for
'FABP9' on 'Pulmonary fibrosis'

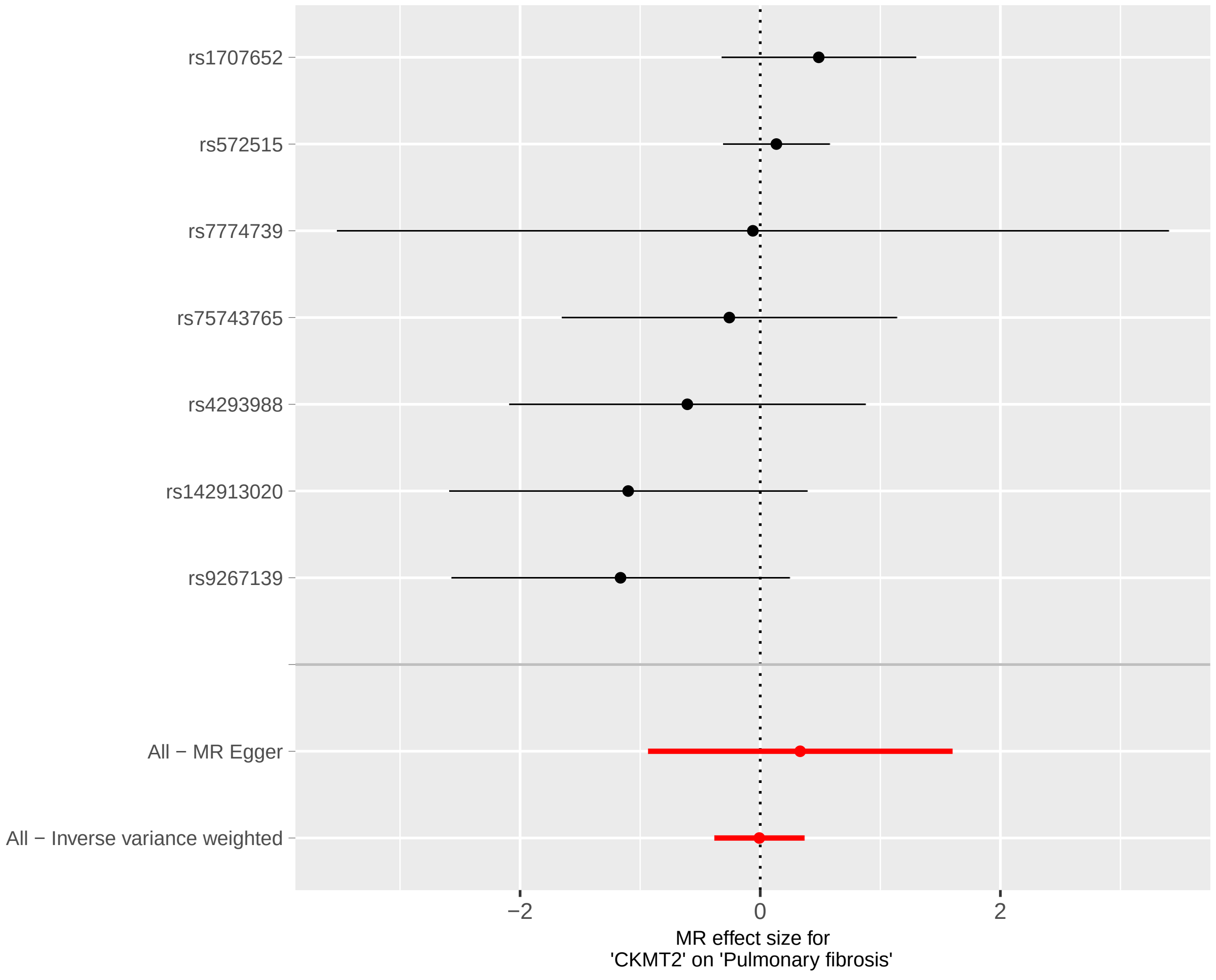
rs1707652
rs572515
rs7774739
rs75743765
rs4293988
rs142913020
rs9267139
All − MR Egger
All − Inverse variance weighted
−2
0
2
MR effect size for
'CKMT2' on 'Pulmonary fibrosis'

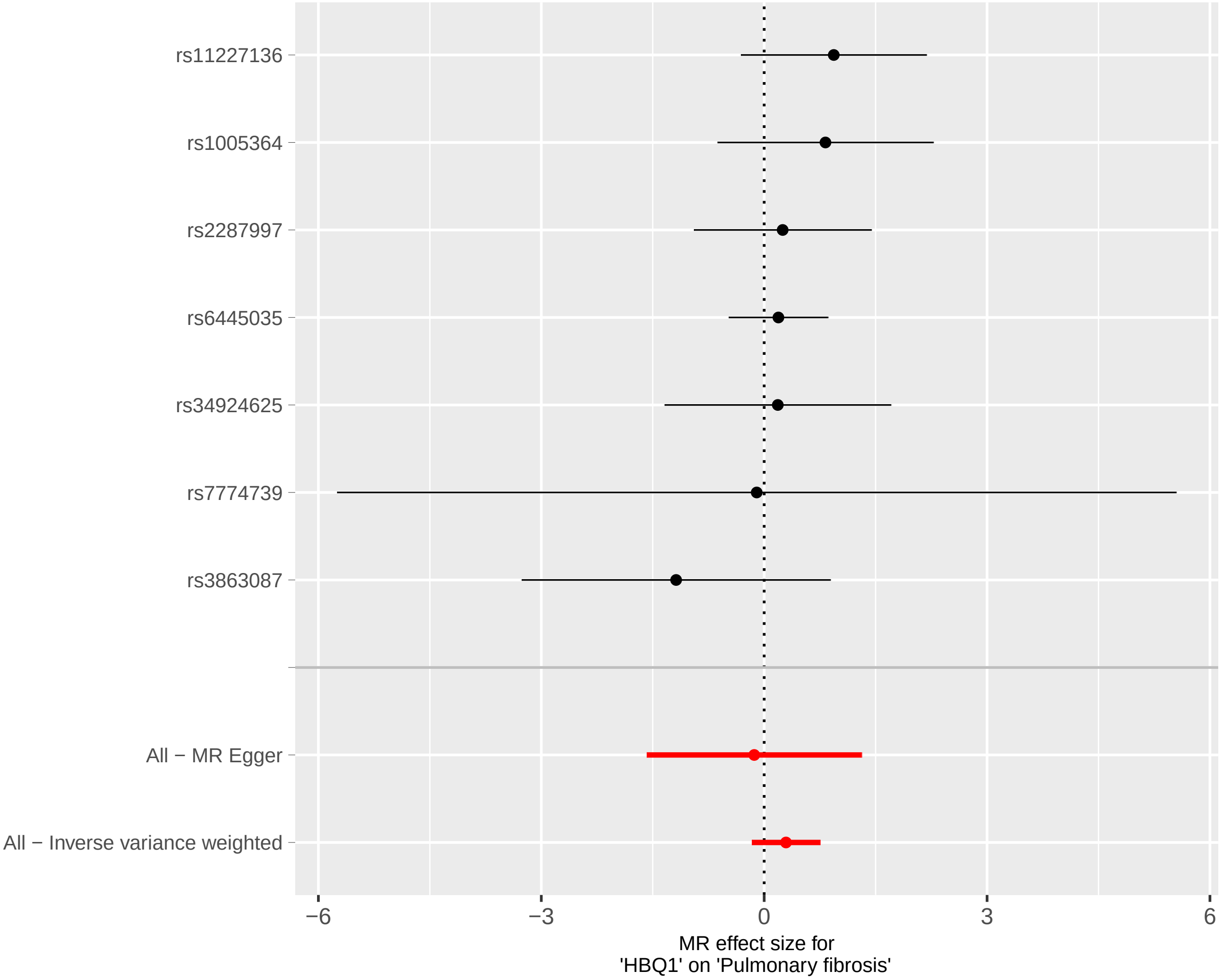
rs11227136
rs1005364
rs2287997
rs6445035
rs34924625
rs7774739
rs3863087
All − MR Egger
All − Inverse variance weighted
−6
−3
0
3
6
MR effect size for
'HBQ1' on 'Pulmonary fibrosis'

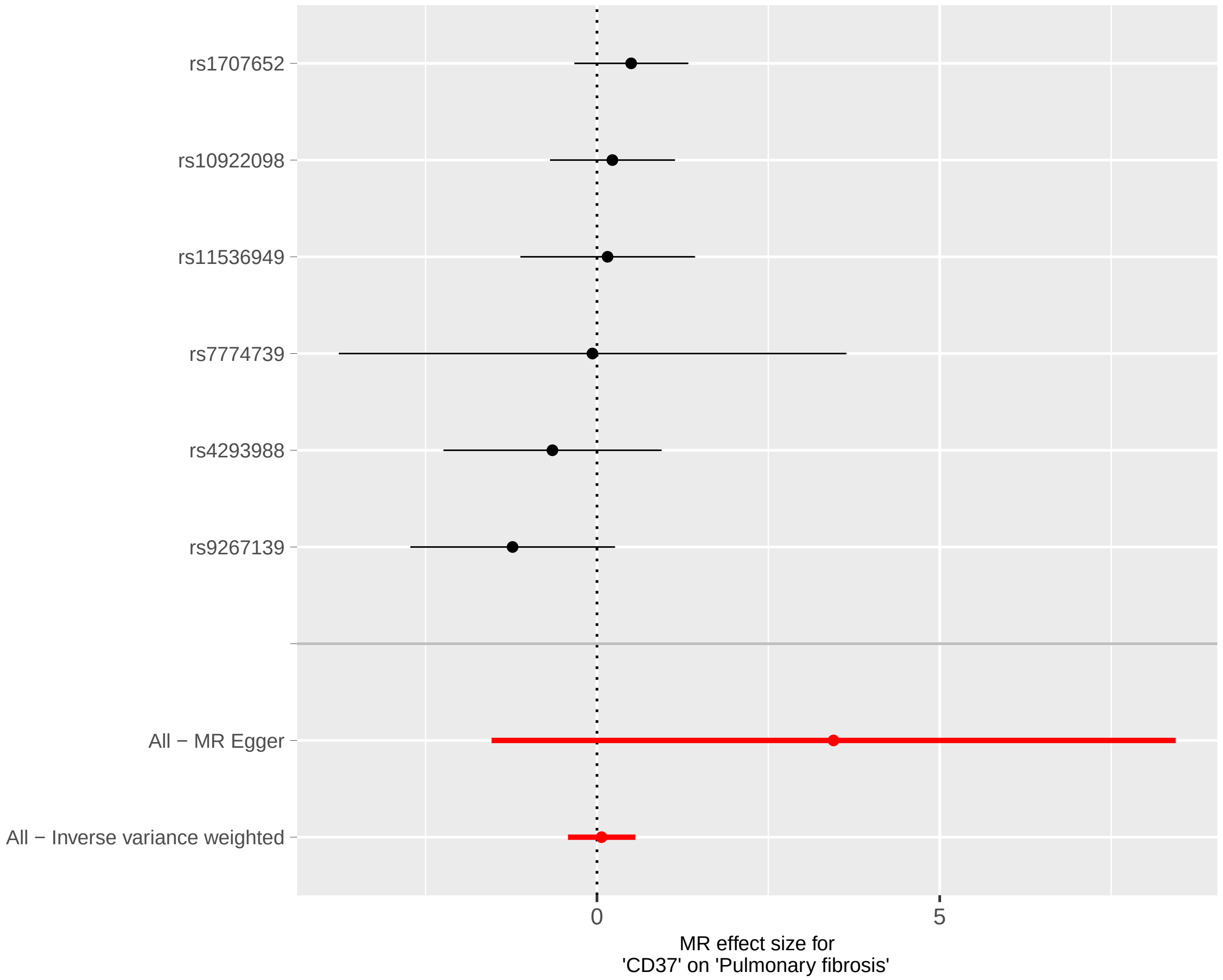

rs1707652
rs10922098
rs11536949
rs7774739
rs4293988
rs9267139
All − MR Egger
All − Inverse variance weighted
0
5
MR effect size for
'CD37' on 'Pulmonary fibrosis'

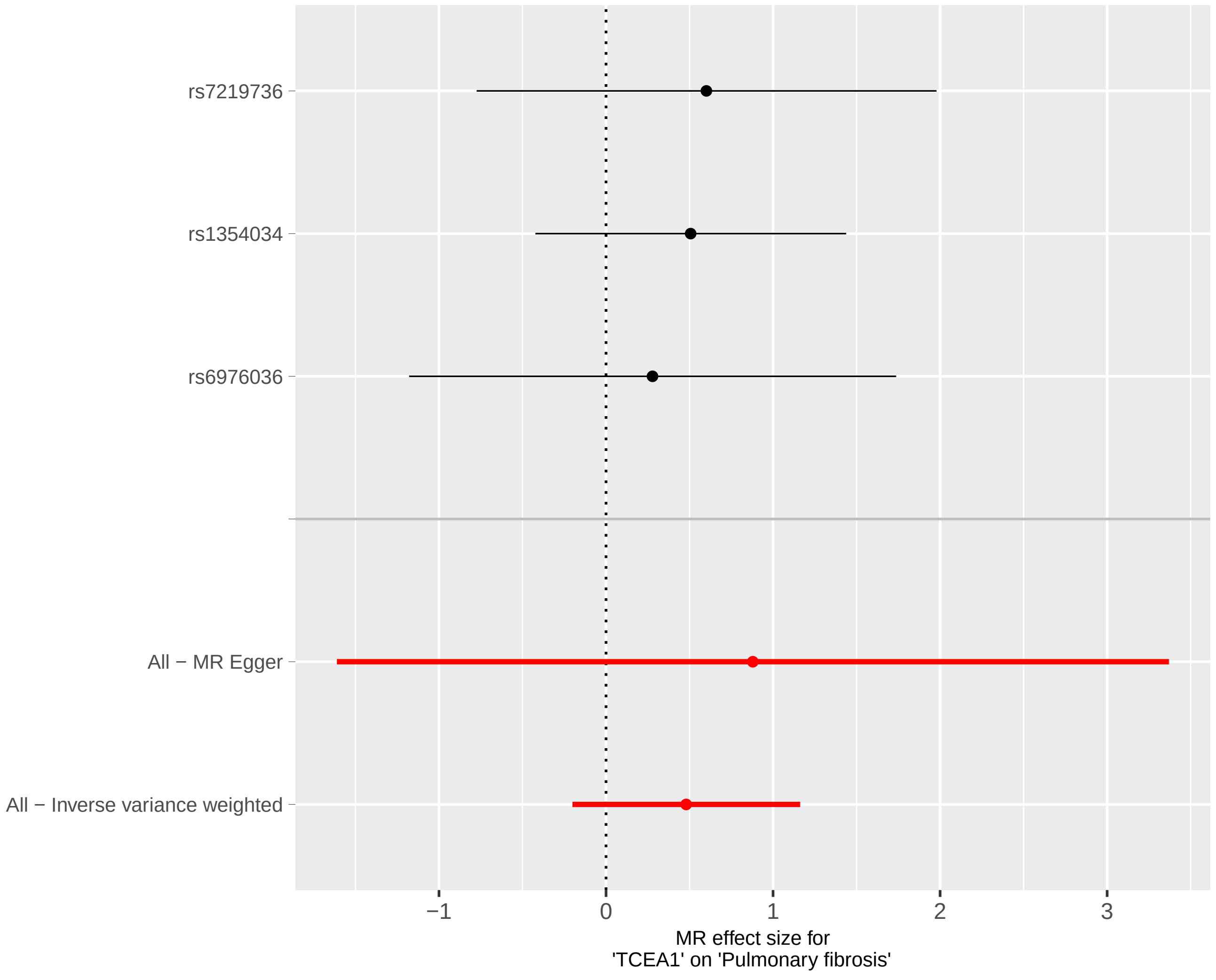
rs7219736
rs1354034
rs6976036
All − MR Egger
All − Inverse variance weighted
−1
0
1
2
3
MR effect size for
'TCEA1' on 'Pulmonary fibrosis'

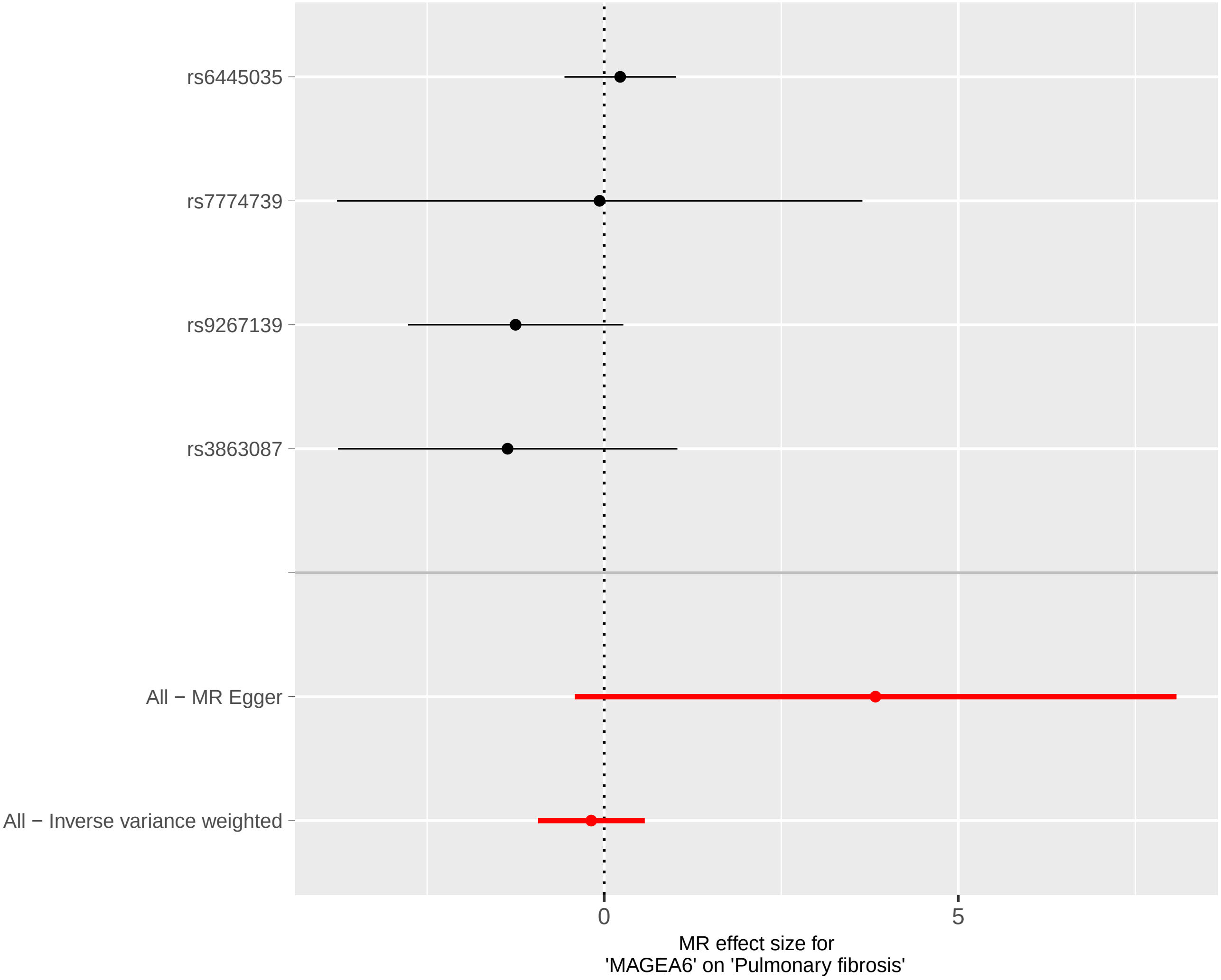

rs6445035
rs7774739
rs9267139
rs3863087
All − MR Egger
All − Inverse variance weighted
0
5
MR effect size for
'MAGEA6' on 'Pulmonary fibrosis'

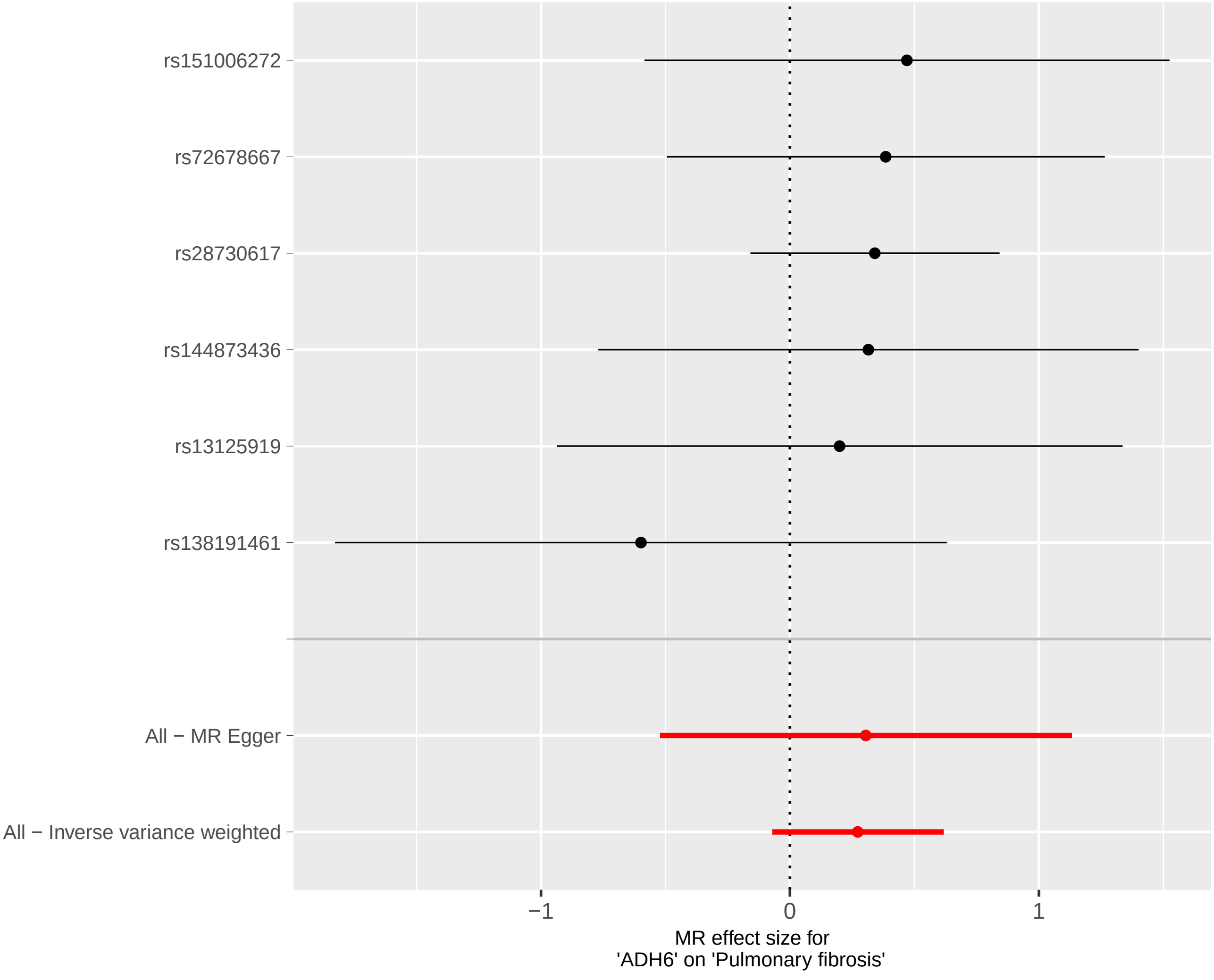

rs151006272
rs72678667
rs28730617
rs144873436
rs13125919
rs138191461
All − MR Egger
All − Inverse variance weighted
−1
0
1
MR effect size for
'ADH6' on 'Pulmonary fibrosis'

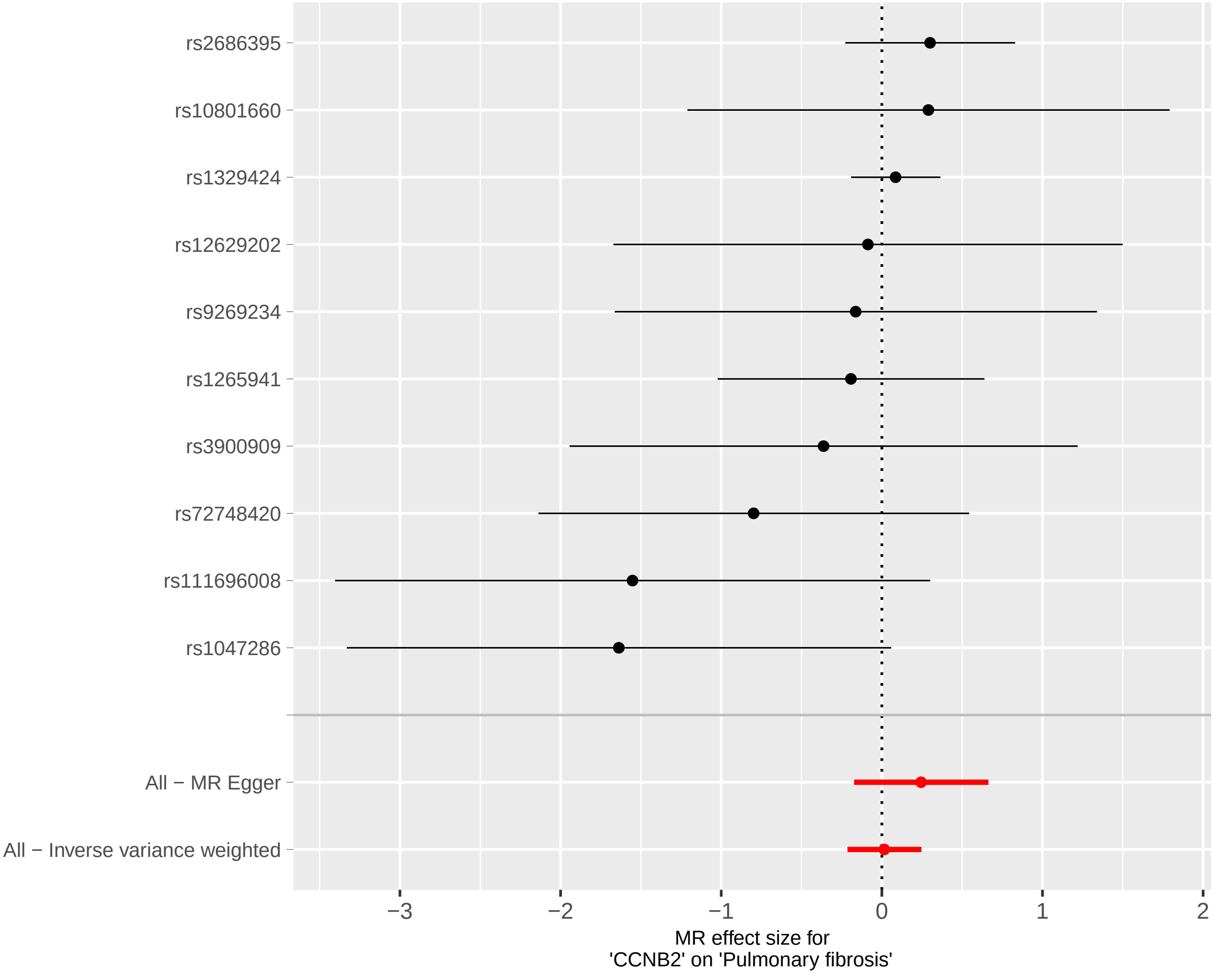

rs2686395
rs10801660
rs1329424
rs12629202
rs9269234
rs1265941
rs3900909
rs72748420
rs111696008
rs1047286
All − MR Egger
All − Inverse variance weighted
−3
−2
−1
0
1
2
MR effect size for
'CCNB2' on 'Pulmonary fibrosis'

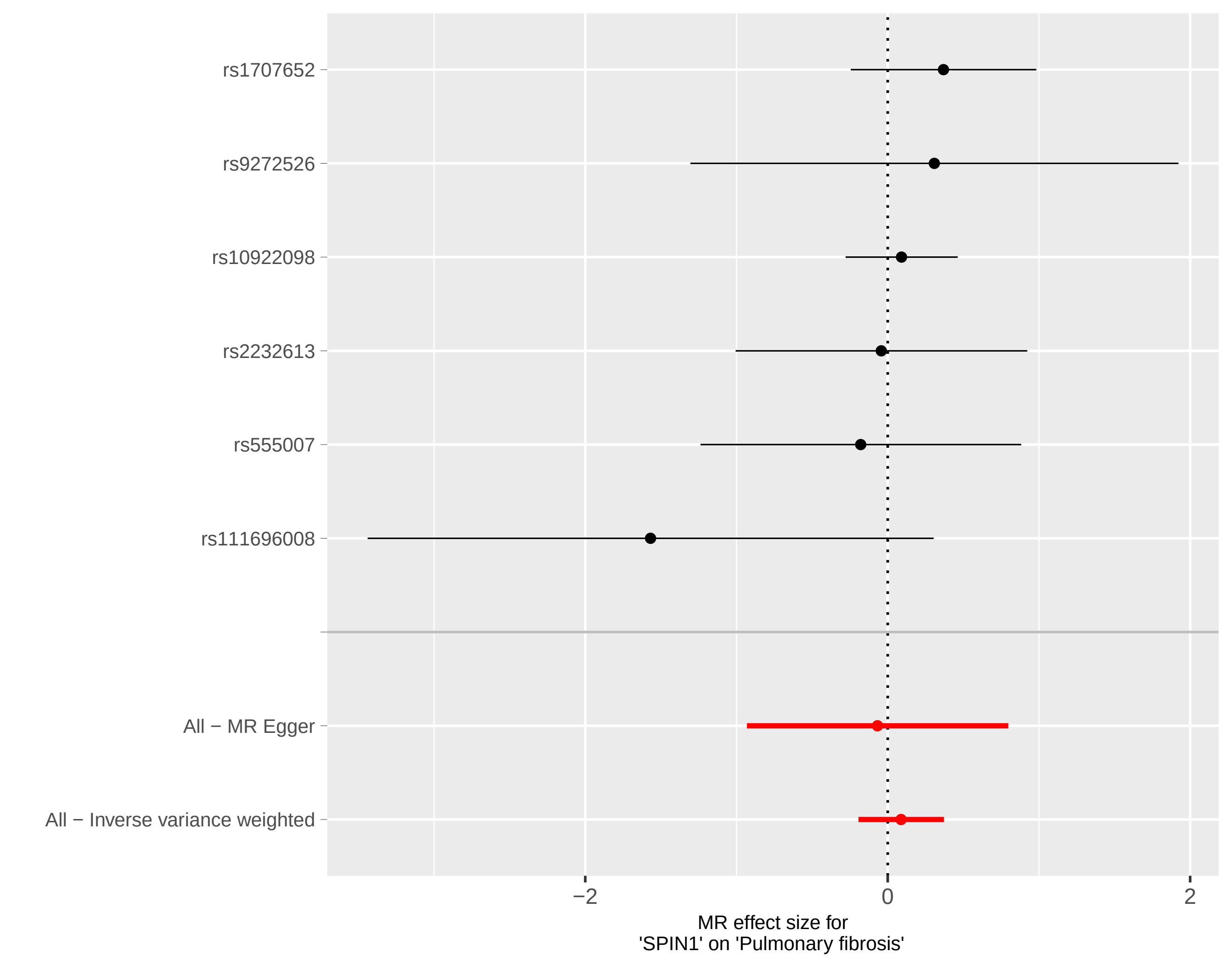

rs1707652
rs9272526
rs10922098
rs2232613
rs555007
rs111696008
All − MR Egger
All − Inverse variance weighted
−2
0
2
MR effect size for
'SPIN1' on 'Pulmonary fibrosis'

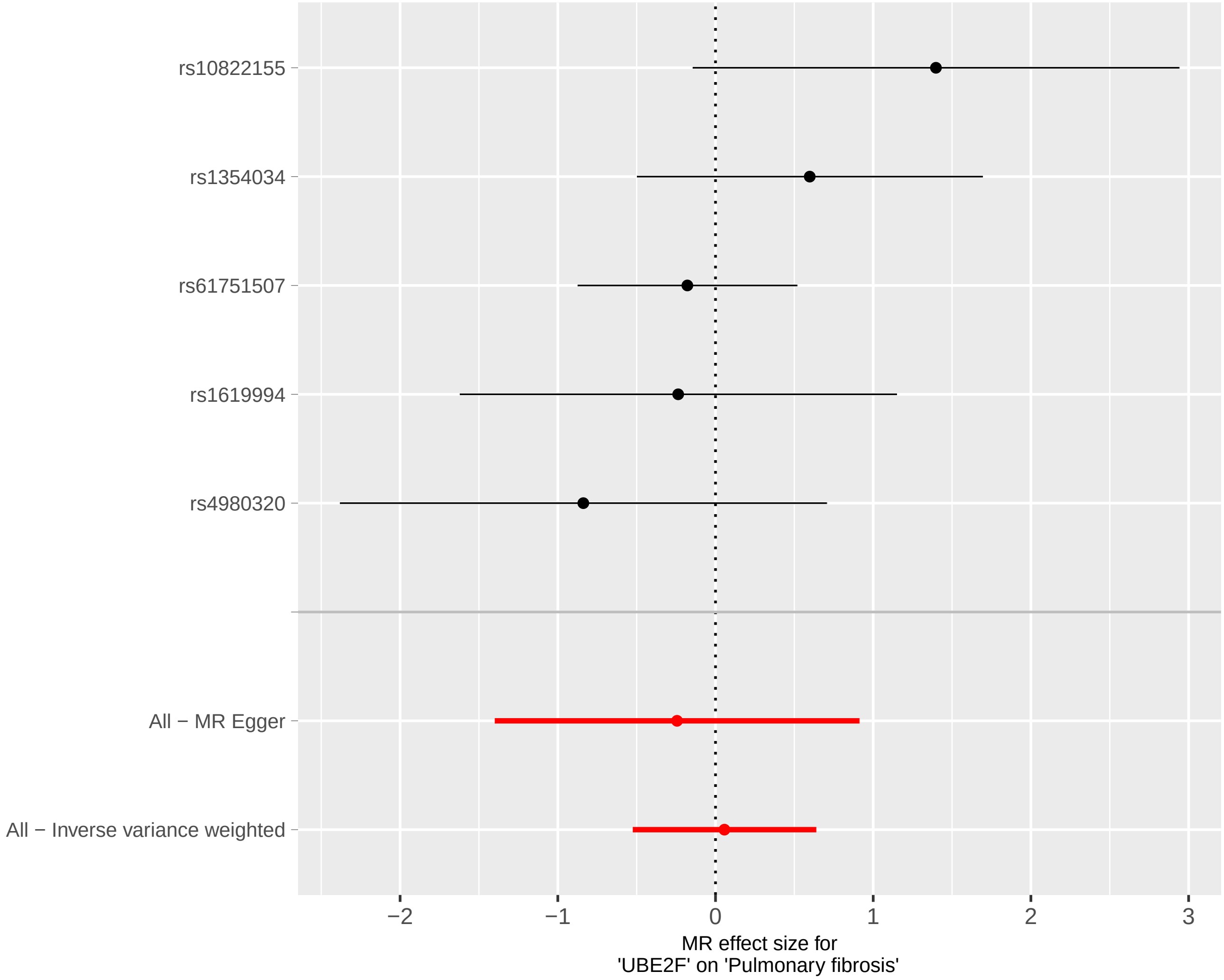
rs10822155
rs1354034
rs61751507
rs1619994
rs4980320
All − MR Egger
All − Inverse variance weighted
−2
−1
0
1
2
3
MR effect size for
'UBE2F' on 'Pulmonary fibrosis'

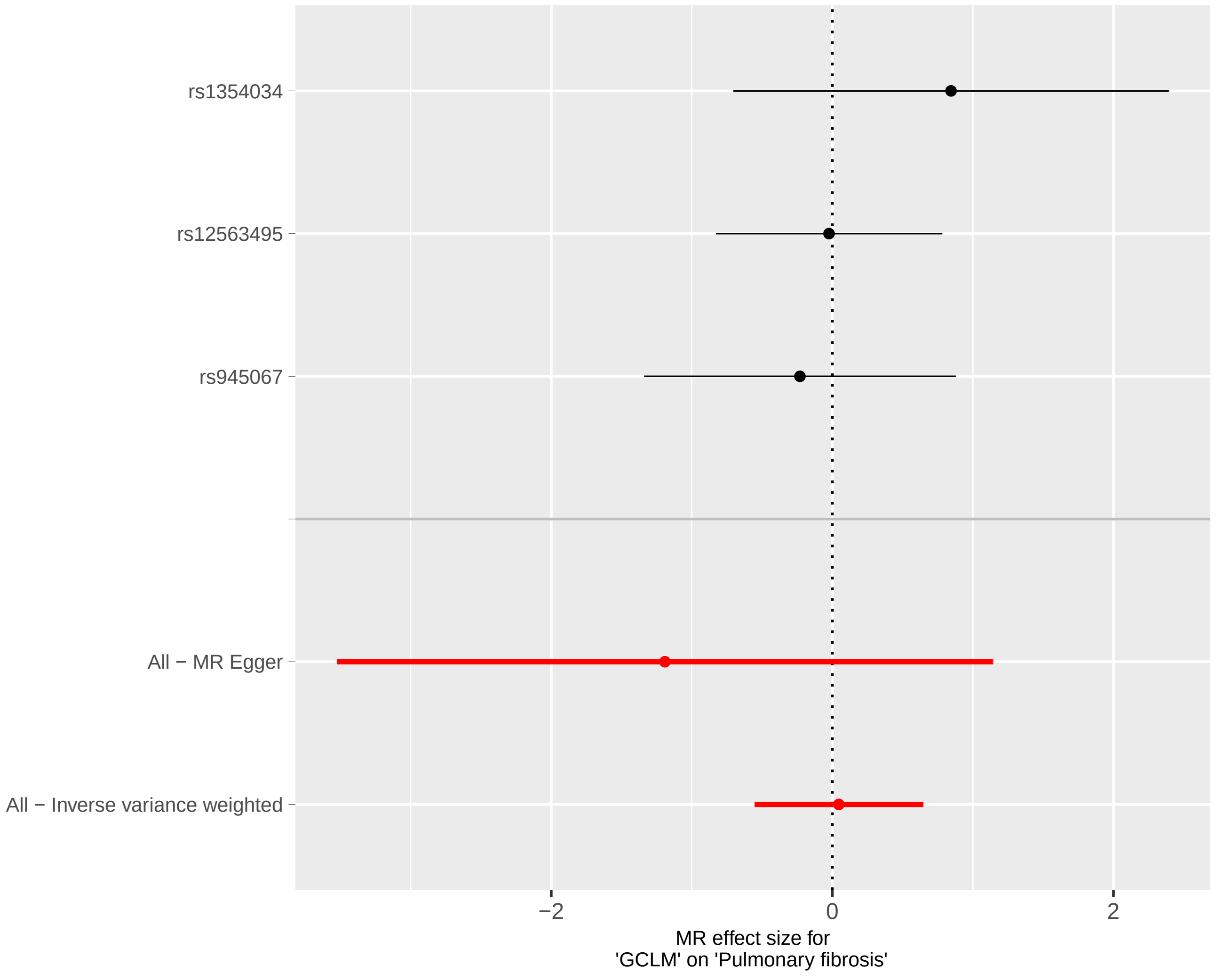

rs1354034
rs12563495
rs945067
All − MR Egger
All − Inverse variance weighted
−2
0
2
MR effect size for
'GCLM' on 'Pulmonary fibrosis'

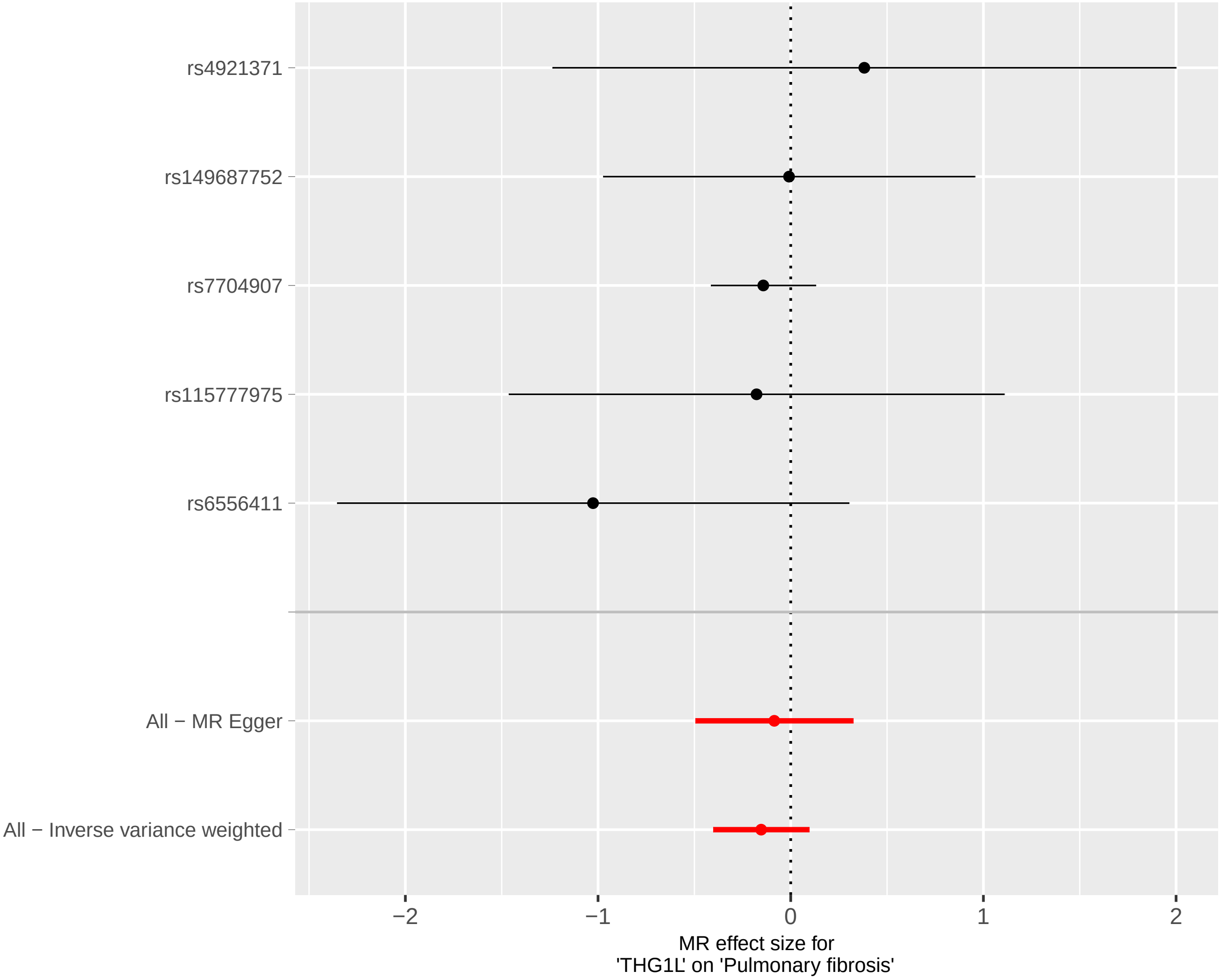

rs4921371
rs149687752
rs7704907
rs115777975
rs6556411
All − MR Egger
All − Inverse variance weighted
−2
−1
0
1
2
MR effect size for
'THG1L' on 'Pulmonary fibrosis'

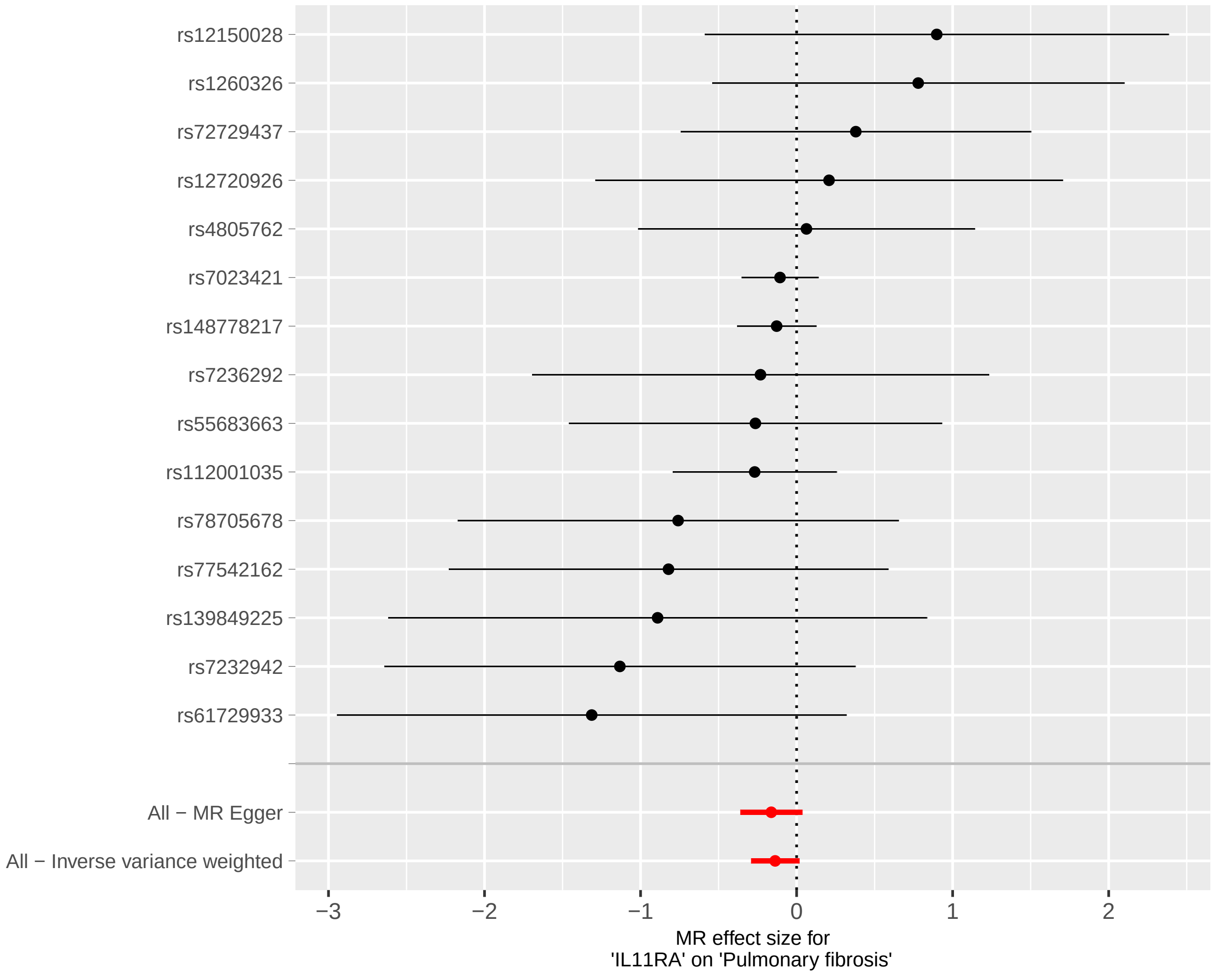
rs12150028
rs1260326
rs72729437
rs12720926
rs4805762
rs7023421
rs148778217
rs7236292
rs55683663
rs112001035
rs78705678
rs77542162
rs139849225
rs7232942
rs61729933
All − MR Egger
All − Inverse variance weighted
−3
−2
−1
0
1
2
MR effect size for
'IL11RA' on 'Pulmonary fibrosis'

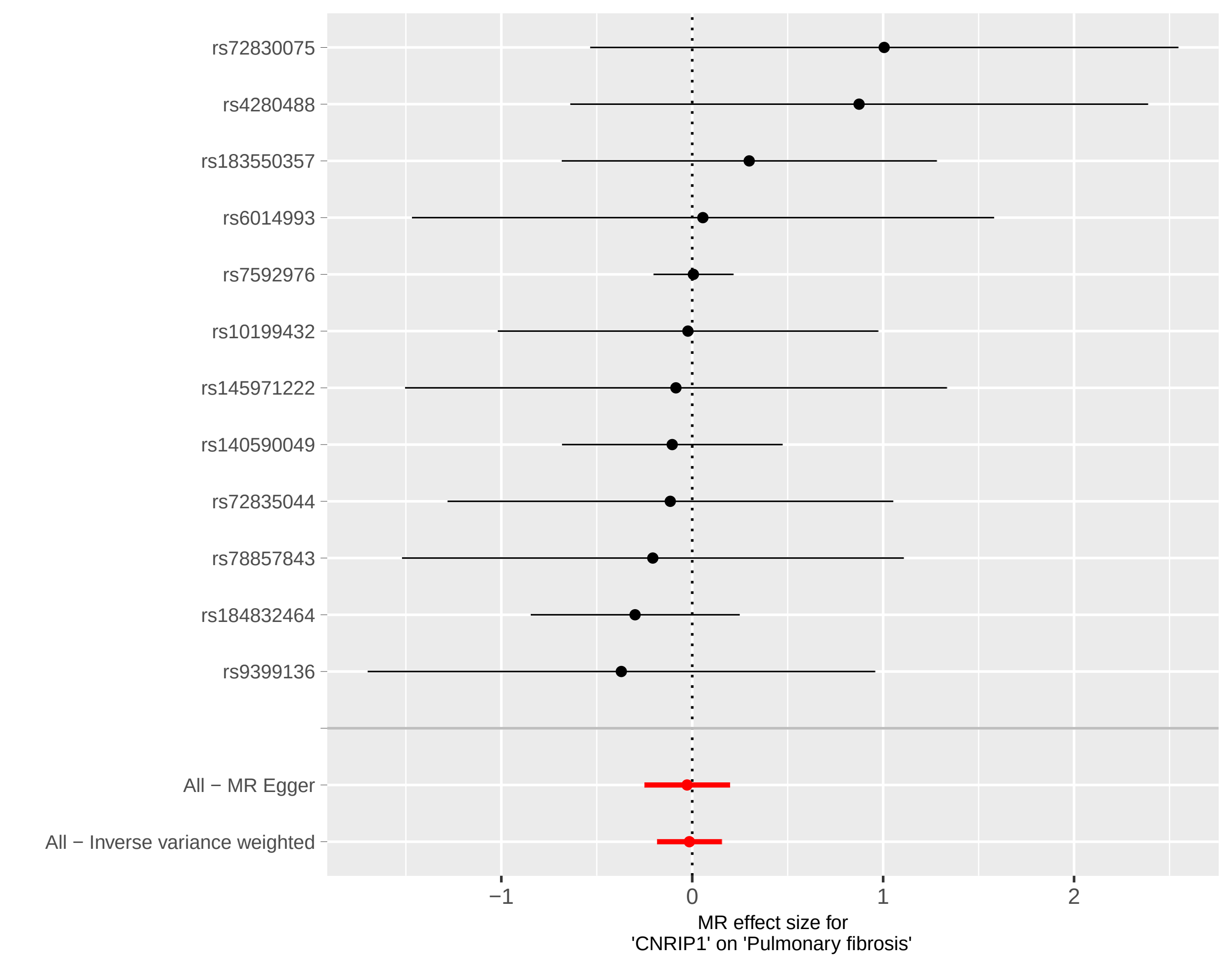
rs72830075
rs4280488
rs183550357
rs6014993
rs7592976
rs10199432
rs145971222
rs140590049
rs72835044
rs78857843
rs184832464
rs9399136
All − MR Egger
All − Inverse variance weighted
−1
0
1
2
MR effect size for
'CNRIP1' on 'Pulmonary fibrosis'

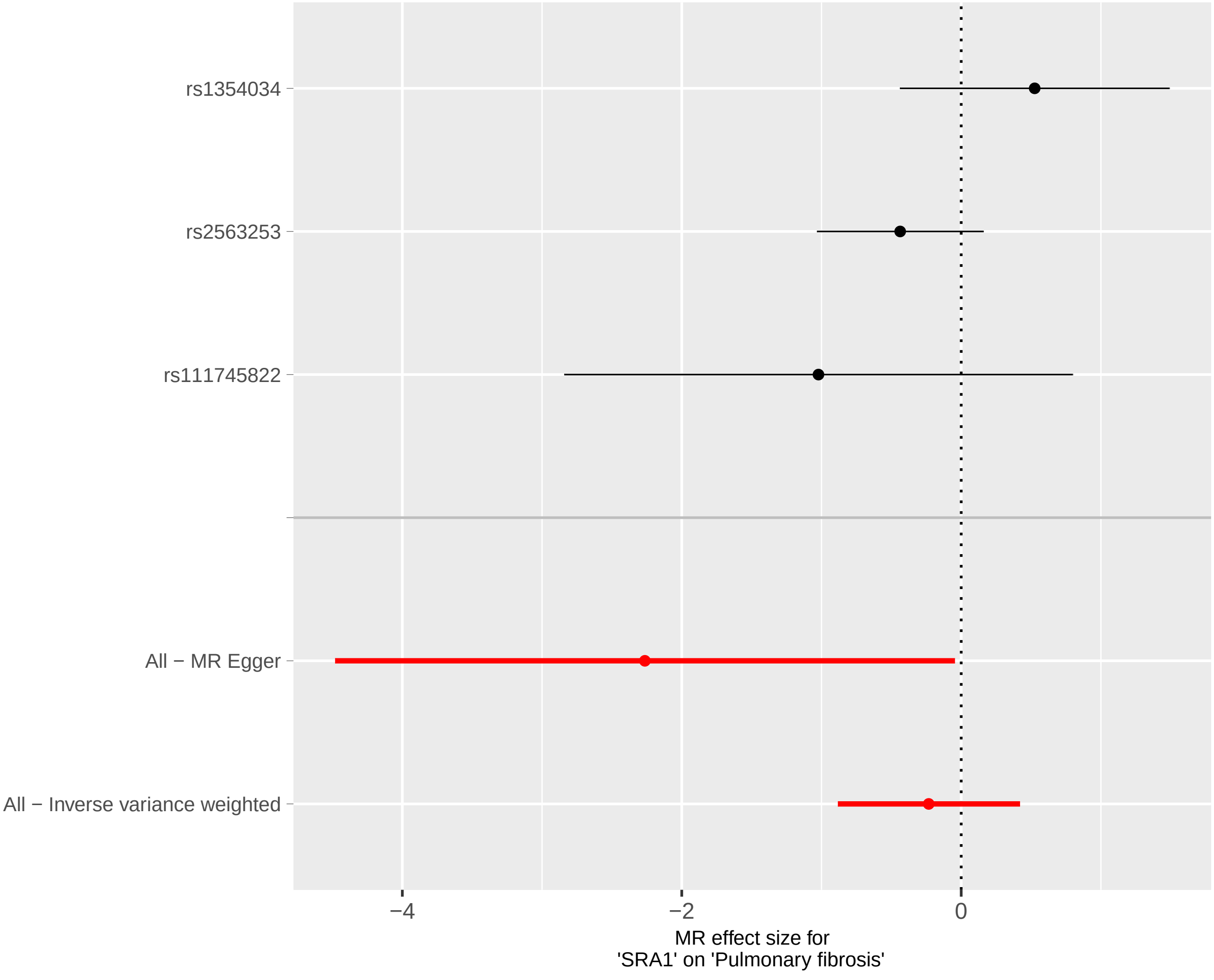
rs1354034
rs2563253
rs111745822
All − MR Egger
All − Inverse variance weighted
−4
−2
0
MR effect size for
'SRA1' on 'Pulmonary fibrosis'

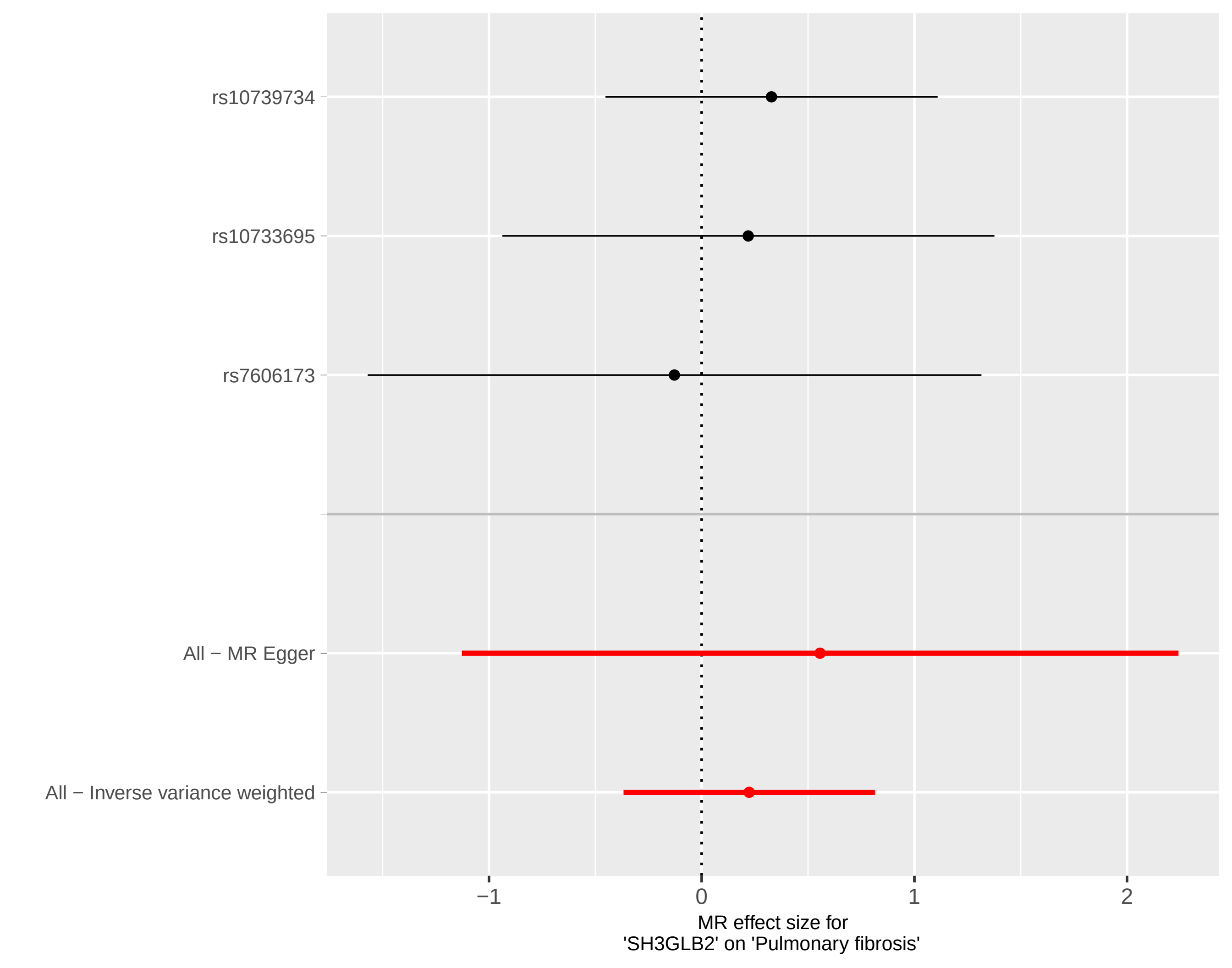

rs10739734
rs10733695
rs7606173
All − MR Egger
All − Inverse variance weighted
−1
0
1
2
MR effect size for
'SH3GLB2' on 'Pulmonary fibrosis'

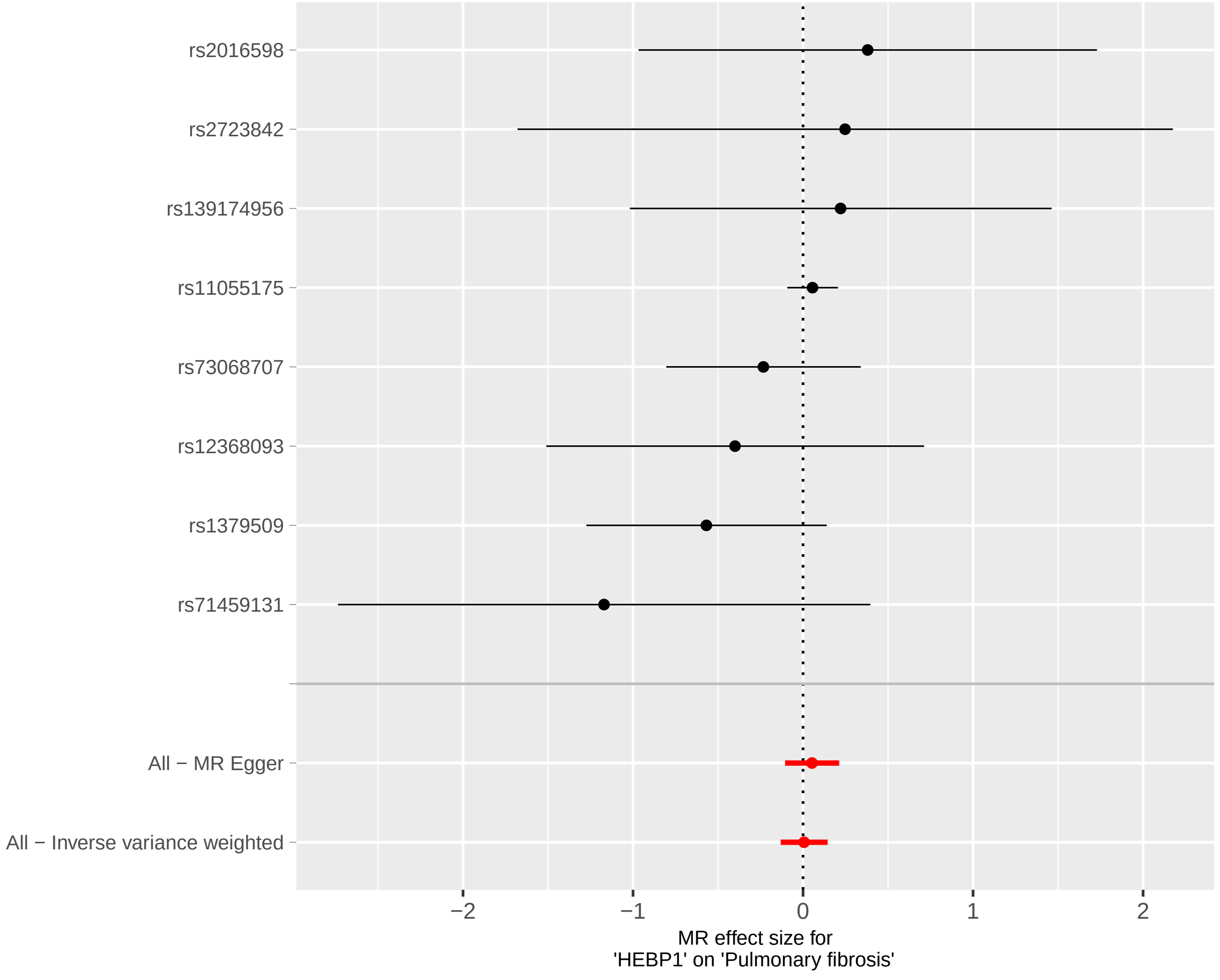
rs2016598
rs2723842
rs139174956
rs11055175
rs73068707
rs12368093
rs1379509
rs71459131
All − MR Egger
All − Inverse variance weighted
−2
−1
0
1
2
MR effect size for
'HEBP1' on 'Pulmonary fibrosis'

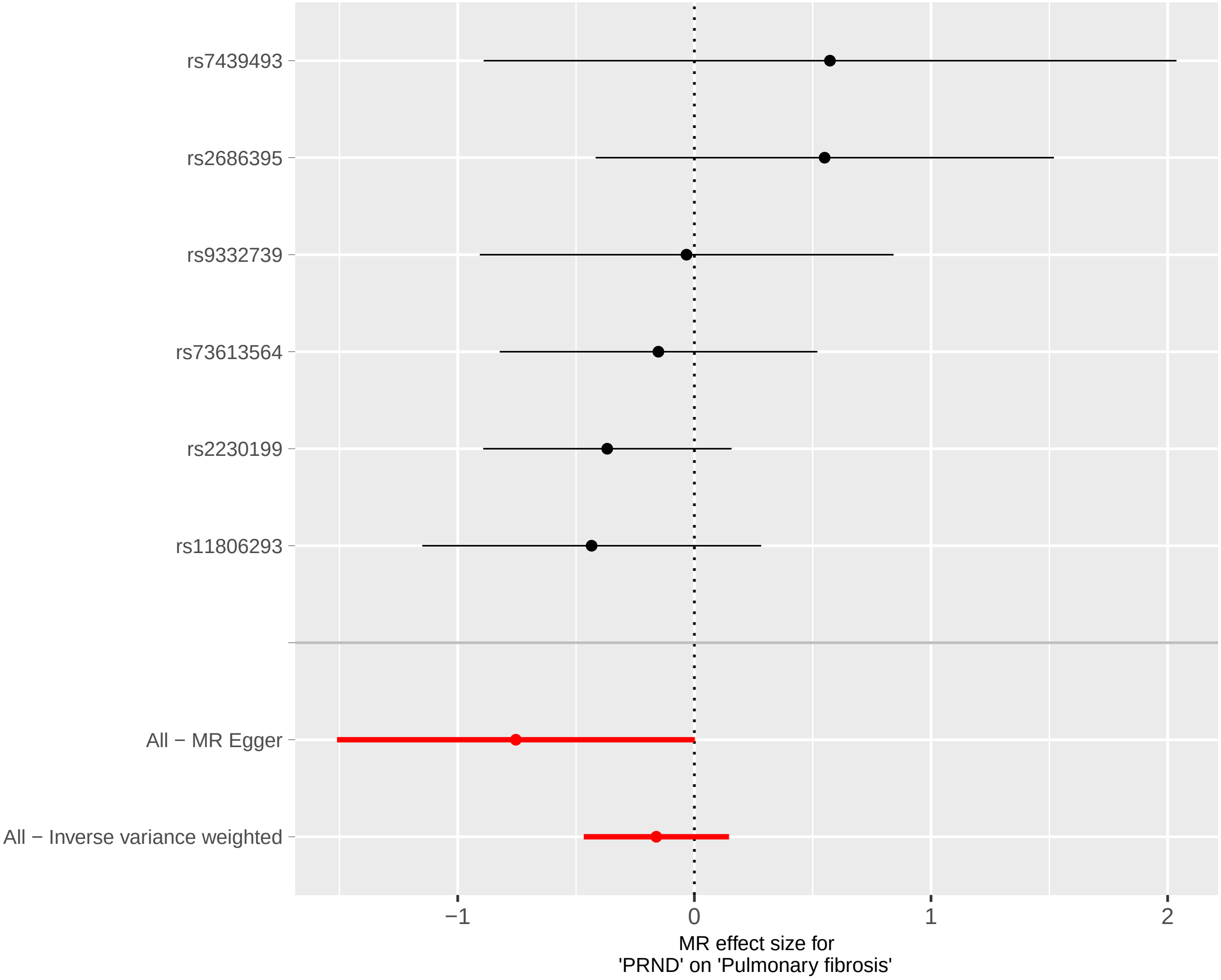

rs7439493
rs2686395
rs9332739
rs73613564
rs2230199
rs11806293
All − MR Egger
All − Inverse variance weighted
−1
0
1
2
MR effect size for
'PRND' on 'Pulmonary fibrosis'

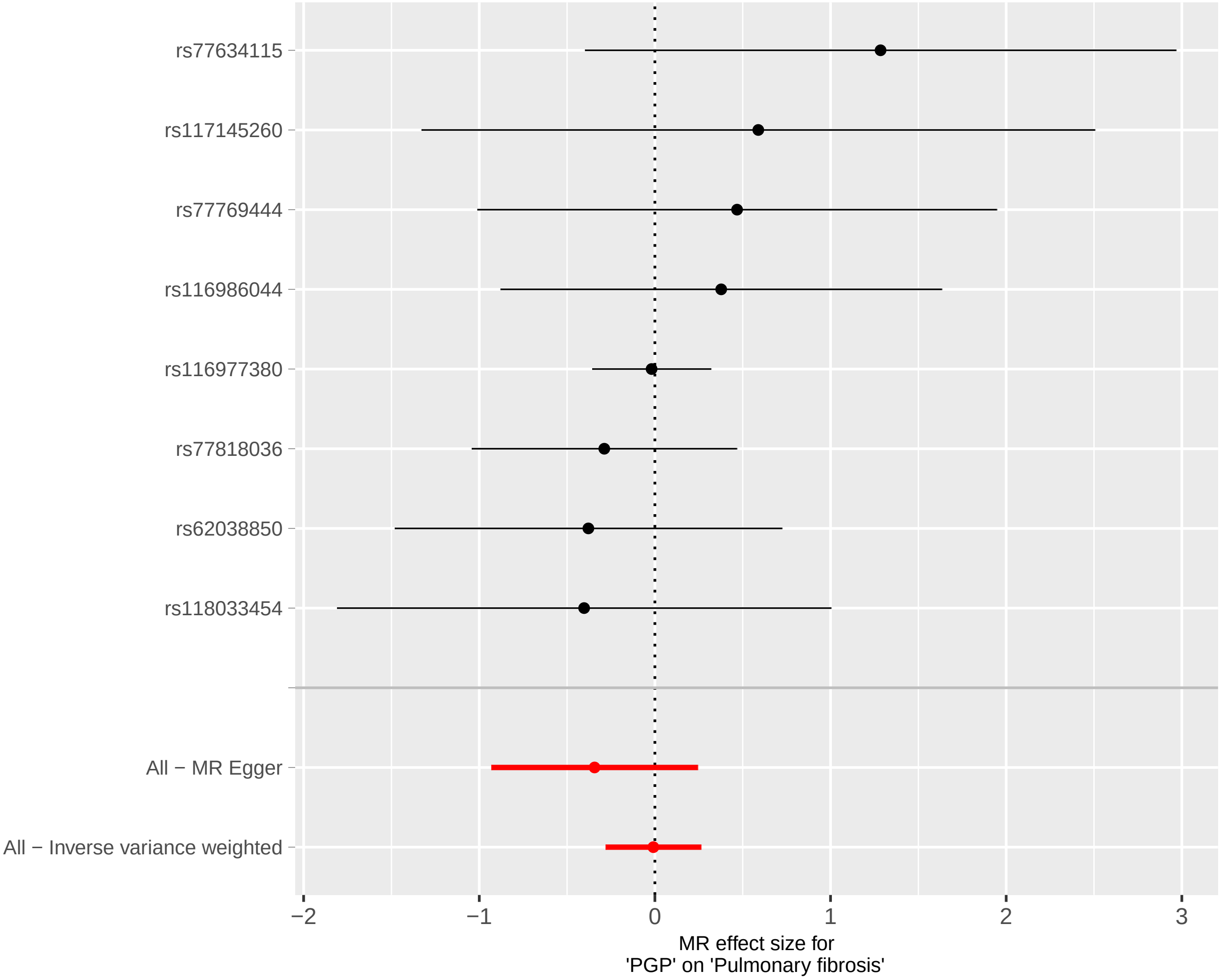
rs77634115
rs117145260
rs77769444
rs116986044
rs116977380
rs77818036
rs62038850
rs118033454
All − MR Egger
All − Inverse variance weighted
−2
−1
0
1
2
3
MR effect size for
'PGP' on 'Pulmonary fibrosis'

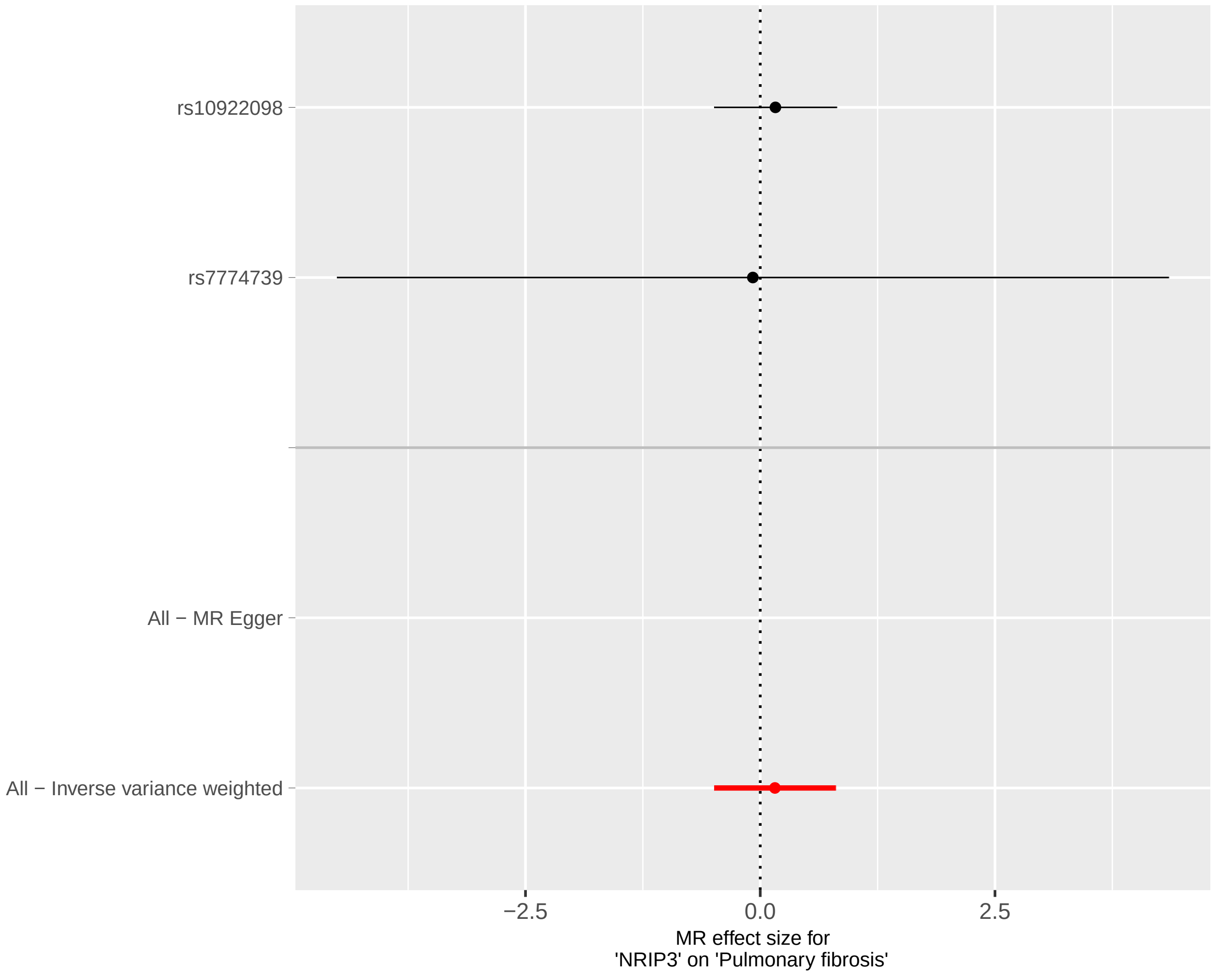
rs10922098
rs7774739
All − MR Egger
All − Inverse variance weighted
−2.5
0.0
2.5
MR effect size for
'NRIP3' on 'Pulmonary fibrosis'

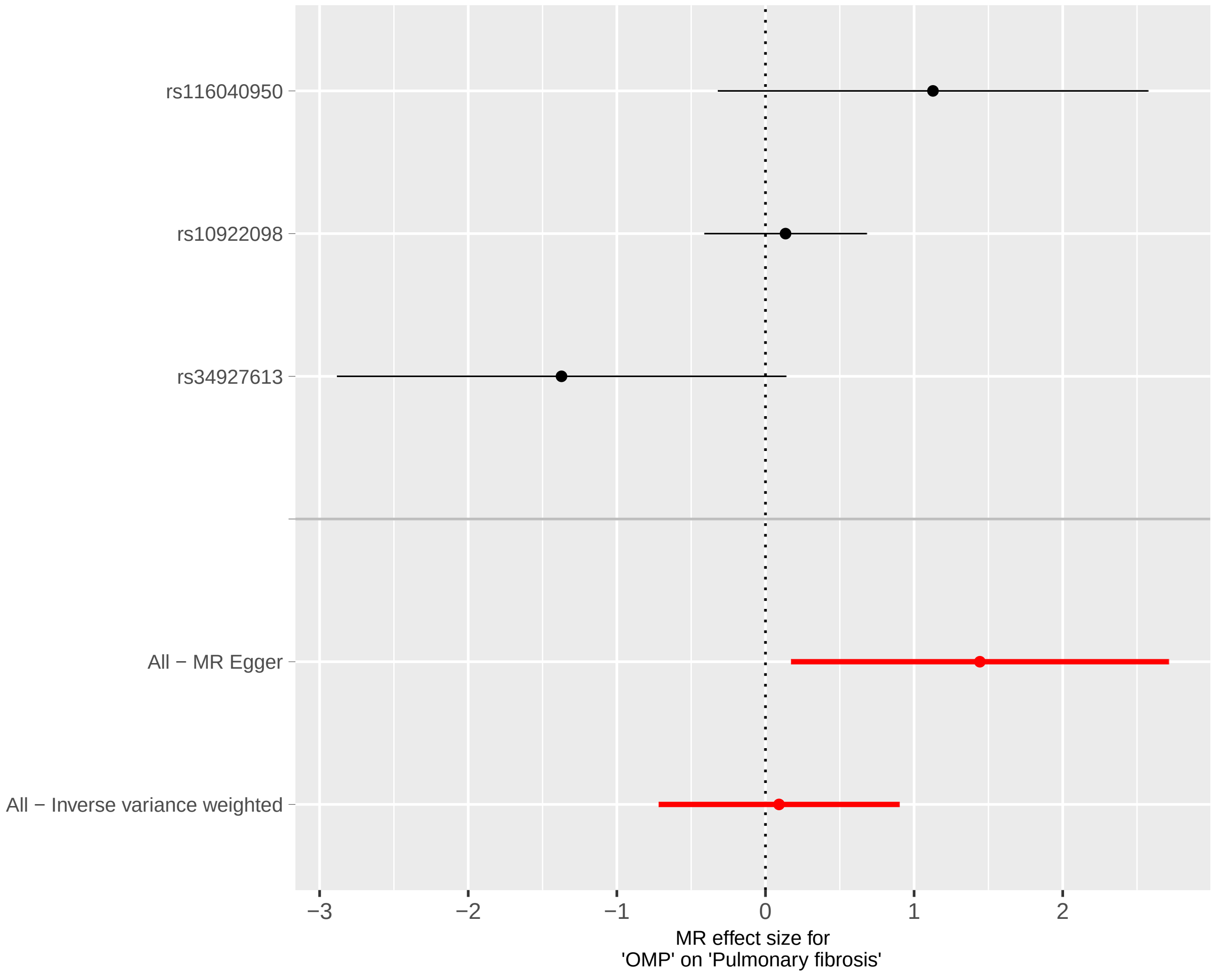

rs116040950
rs10922098
rs34927613
All − MR Egger
All − Inverse variance weighted
−3
−2
−1
0
1
2
MR effect size for
'OMP' on 'Pulmonary fibrosis'

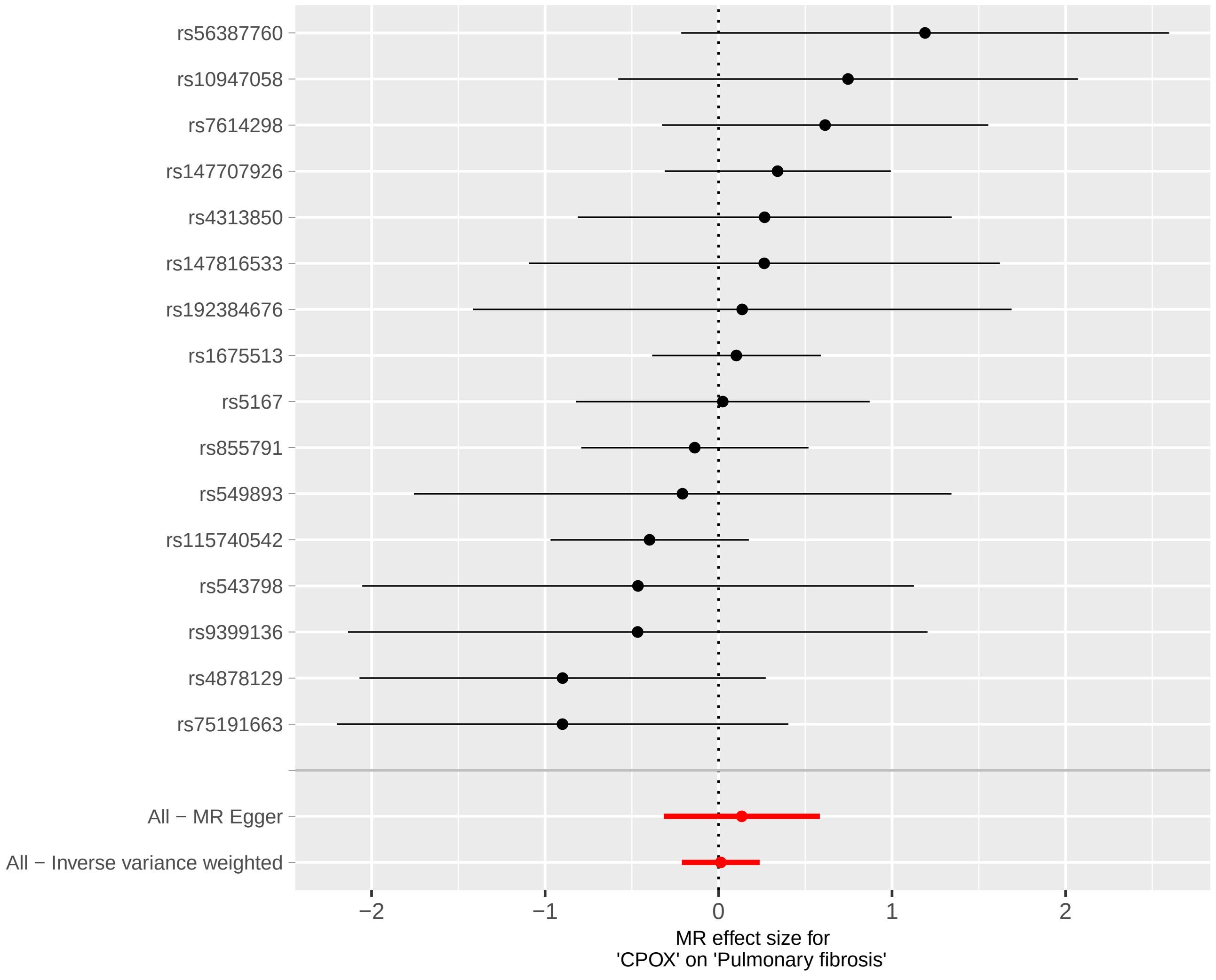

rs56387760
rs10947058
rs7614298
rs147707926
rs4313850
rs147816533
rs192384676
rs1675513
rs5167
rs855791
rs549893
rs115740542
rs543798
rs9399136
rs4878129
rs75191663
All − MR Egger
All − Inverse variance weighted
−2
−1
0
1
2
MR effect size for
'CPOX' on 'Pulmonary fibrosis'

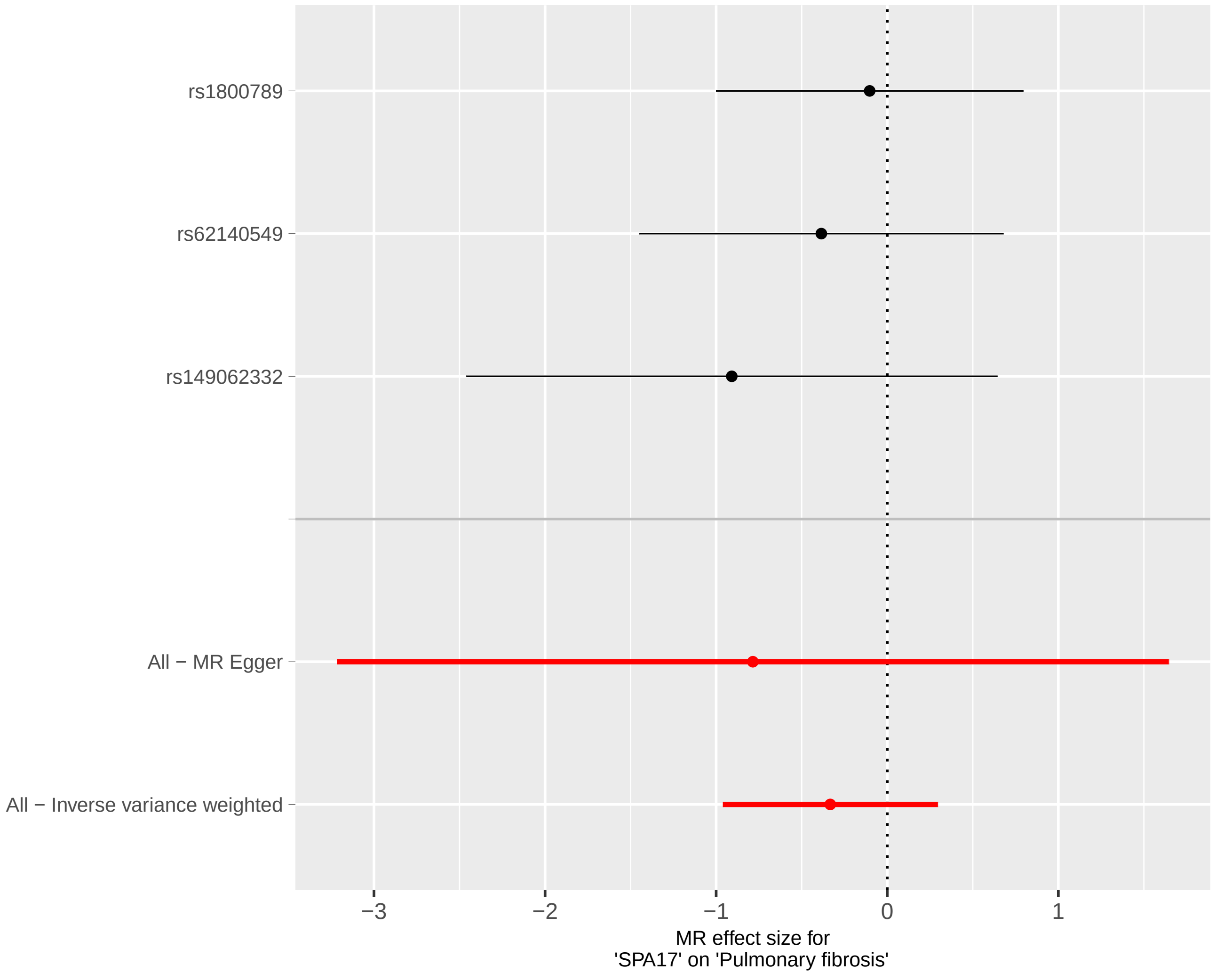

rs1800789
rs62140549
rs149062332
All − MR Egger
All − Inverse variance weighted
−3
−2
−1
0
1
MR effect size for
'SPA17' on 'Pulmonary fibrosis'

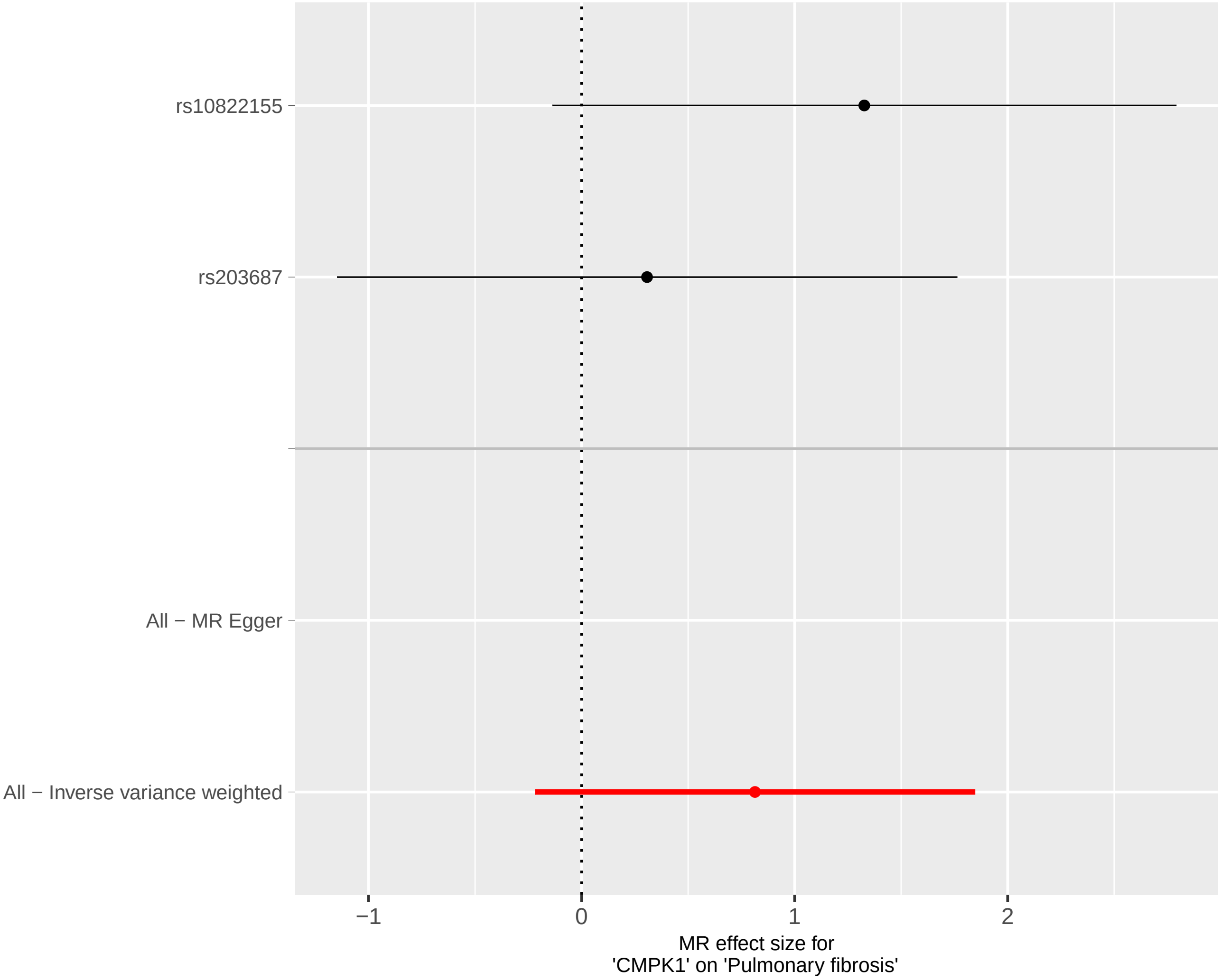
rs10822155
rs203687
All − MR Egger
All − Inverse variance weighted
−1
0
1
2
MR effect size for
'CMPK1' on 'Pulmonary fibrosis'

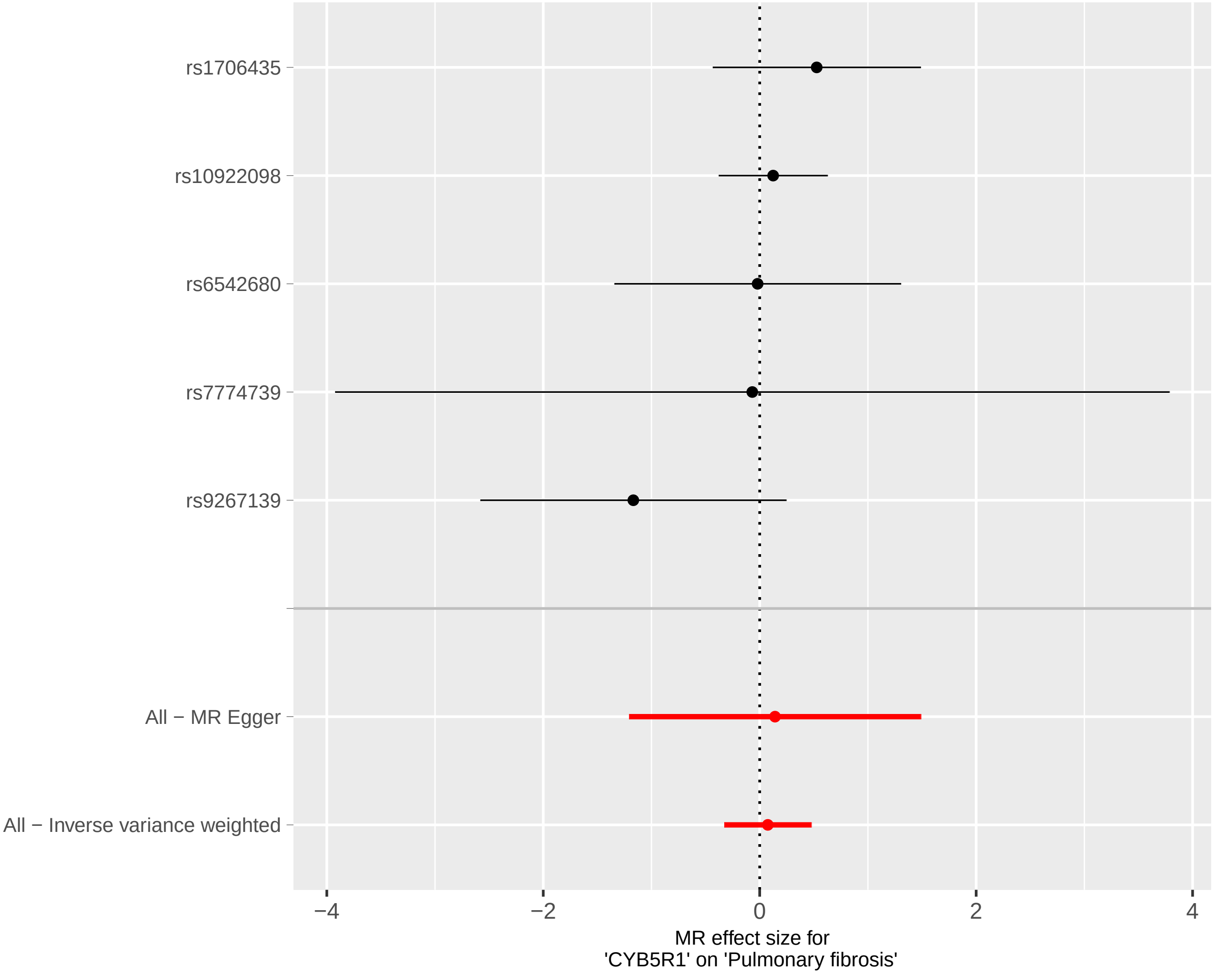
rs1706435
rs10922098
rs6542680
rs7774739
rs9267139
All − MR Egger
All − Inverse variance weighted
−4
−2
0
2
4
MR effect size for
'CYB5R1' on 'Pulmonary fibrosis'

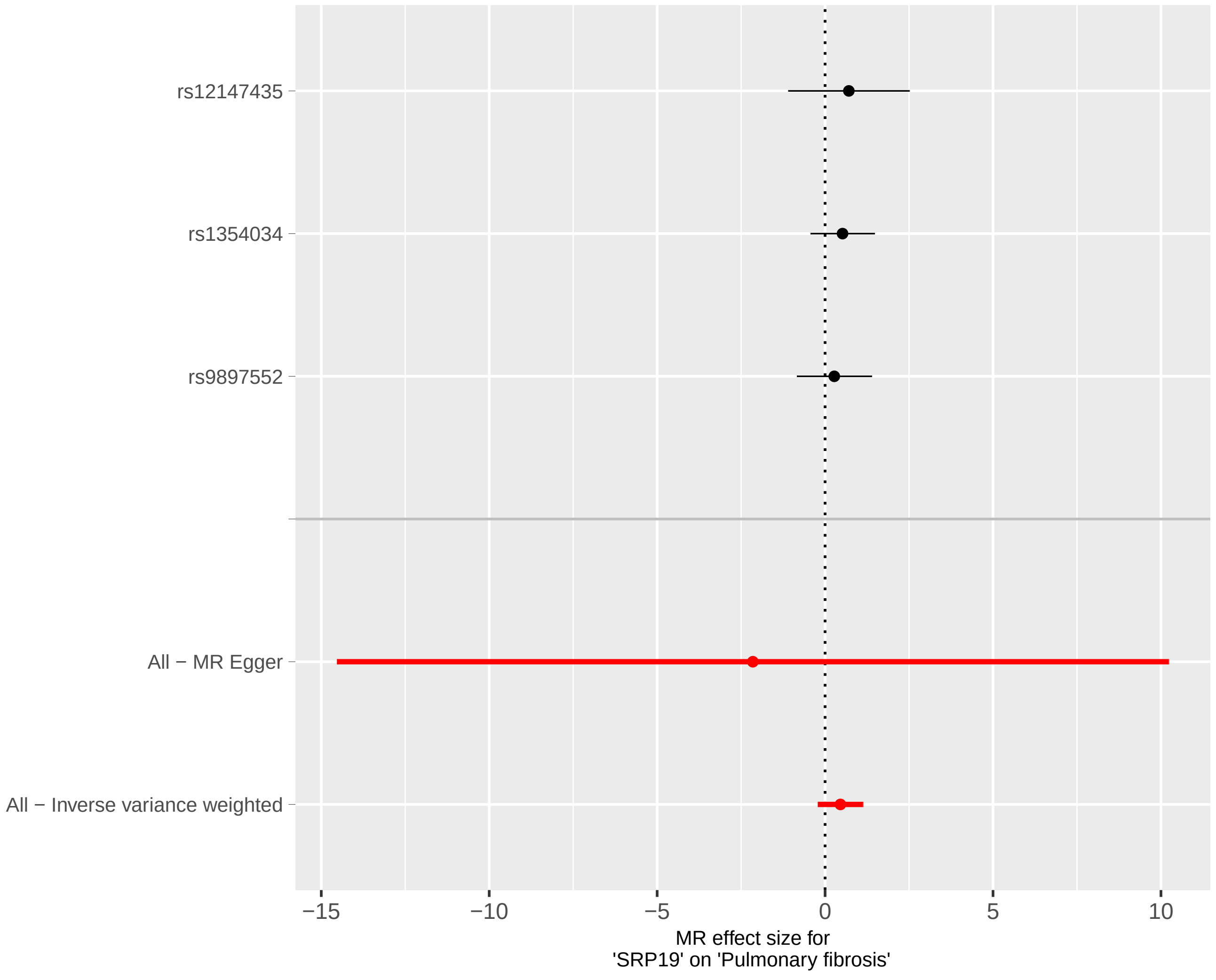

rs12147435
rs1354034
rs9897552
All − MR Egger
All − Inverse variance weighted
−15
−10
−5
0
5
10
MR effect size for
'SRP19' on 'Pulmonary fibrosis'

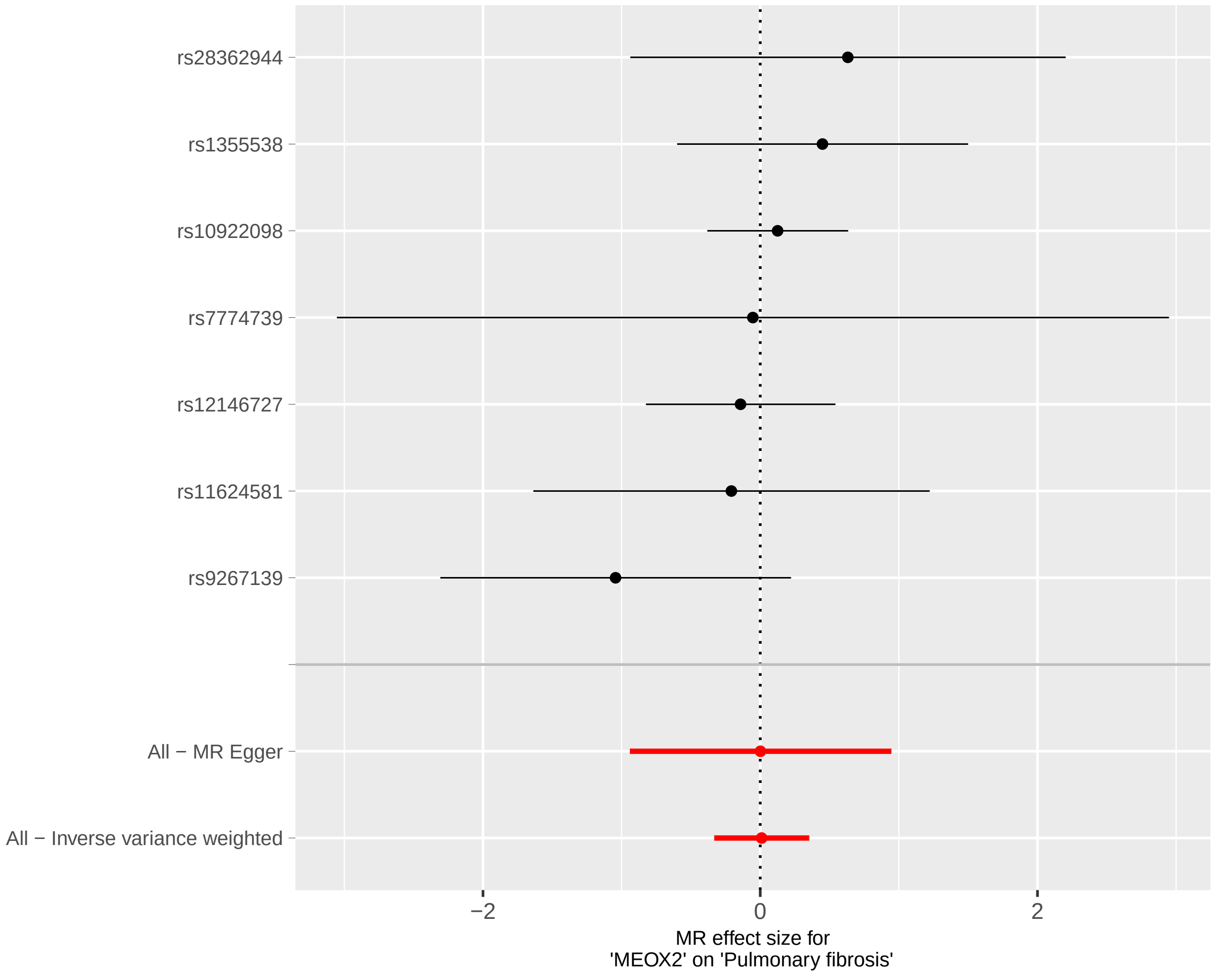

rs28362944
rs1355538
rs10922098
rs7774739
rs12146727
rs11624581
rs9267139
All − MR Egger
All − Inverse variance weighted
−2
0
2
MR effect size for
'MEOX2' on 'Pulmonary fibrosis'

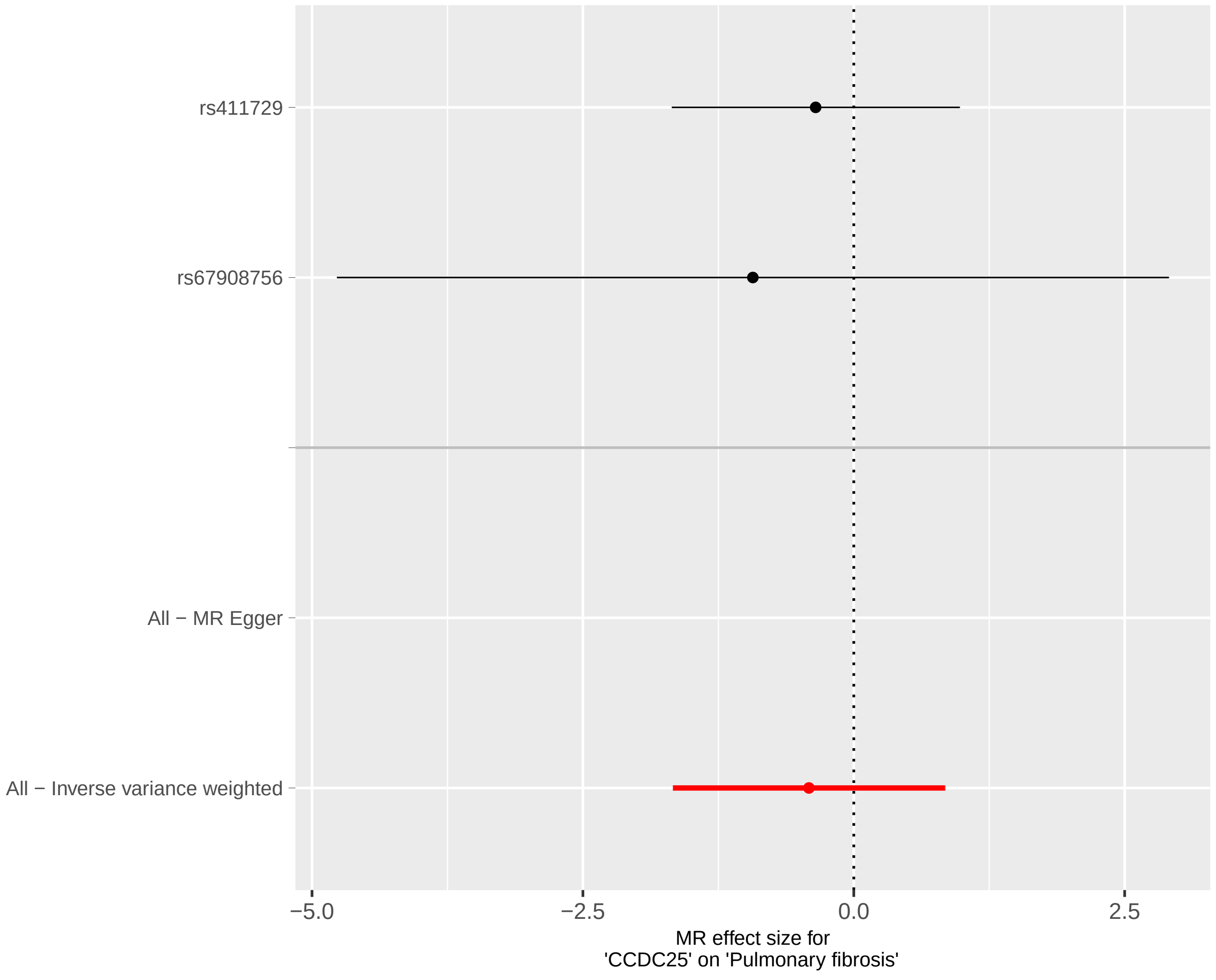

rs411729
rs67908756
All − MR Egger
All − Inverse variance weighted
−5.0
−2.5
0.0
2.5
MR effect size for
'CCDC25' on 'Pulmonary fibrosis'

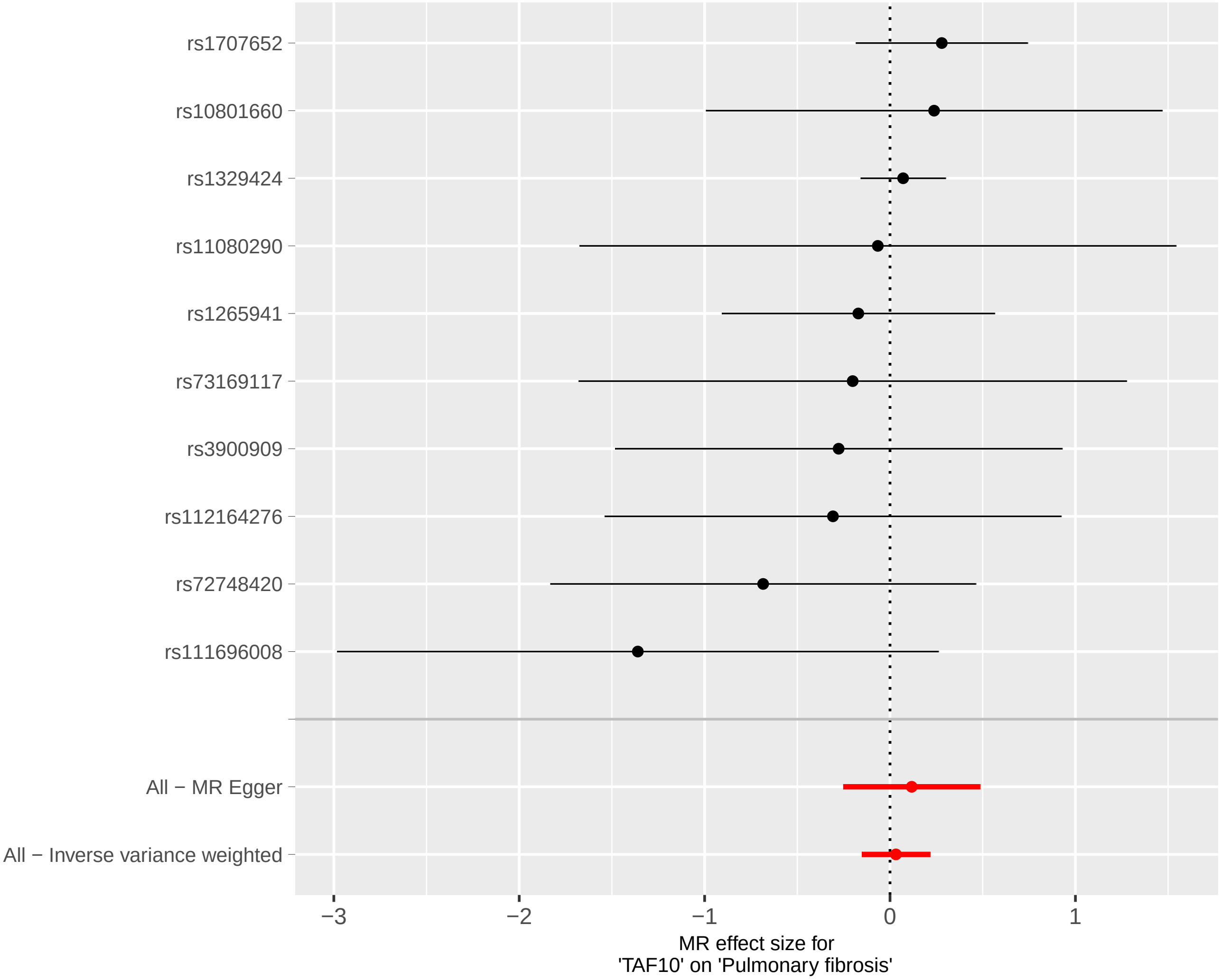
rs1707652
rs10801660
rs1329424
rs11080290
rs1265941
rs73169117
rs3900909
rs112164276
rs72748420
rs111696008
All − MR Egger
All − Inverse variance weighted
−3
−2
−1
0
1
MR effect size for
'TAF10' on 'Pulmonary fibrosis'

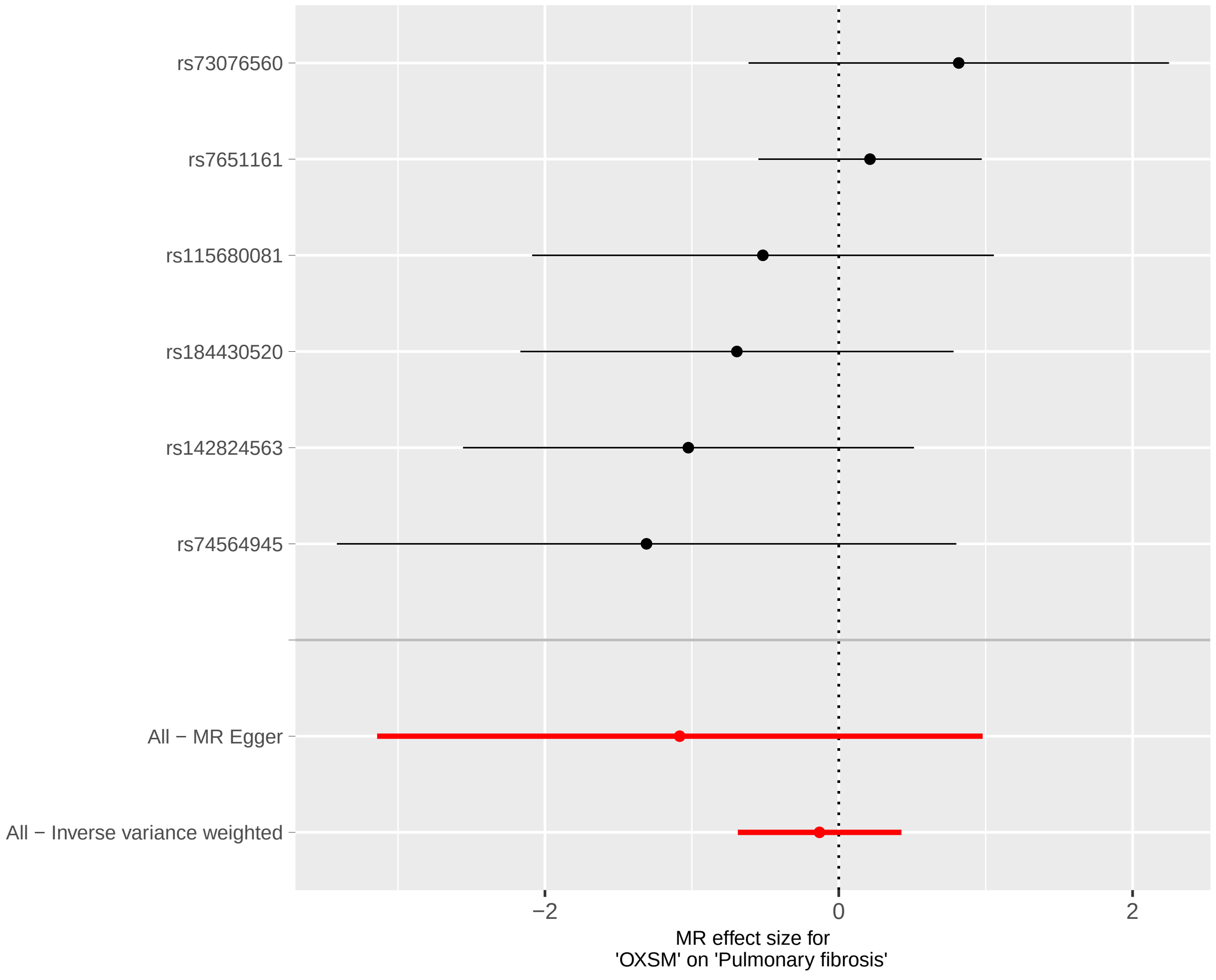

rs73076560
rs7651161
rs115680081
rs184430520
rs142824563
rs74564945
All − MR Egger
All − Inverse variance weighted
−2
0
2
MR effect size for
'OXSM' on 'Pulmonary fibrosis'

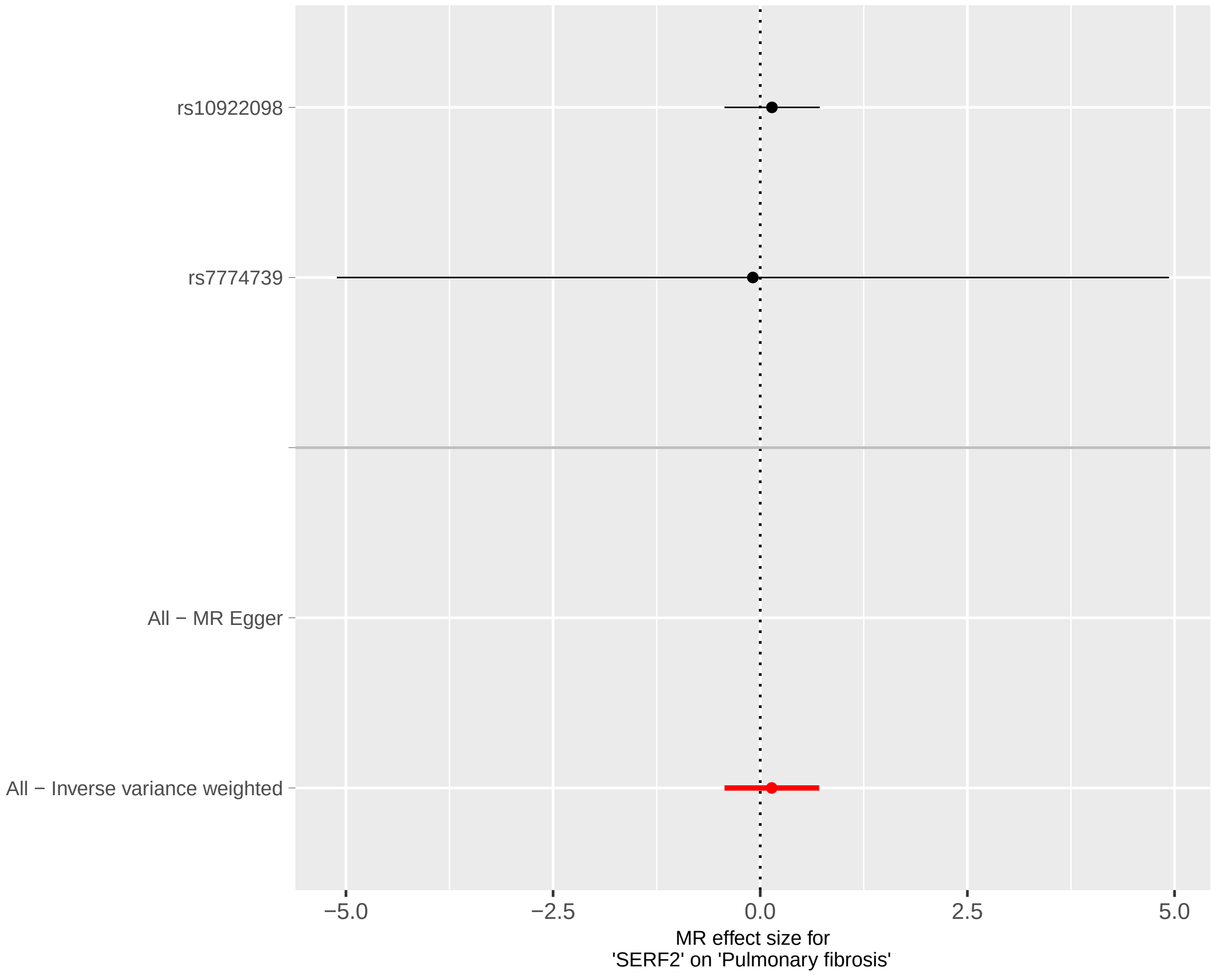

rs10922098
rs7774739
All − MR Egger
All − Inverse variance weighted
−5.0
−2.5
0.0
2.5
5.0
MR effect size for
'SERF2' on 'Pulmonary fibrosis'

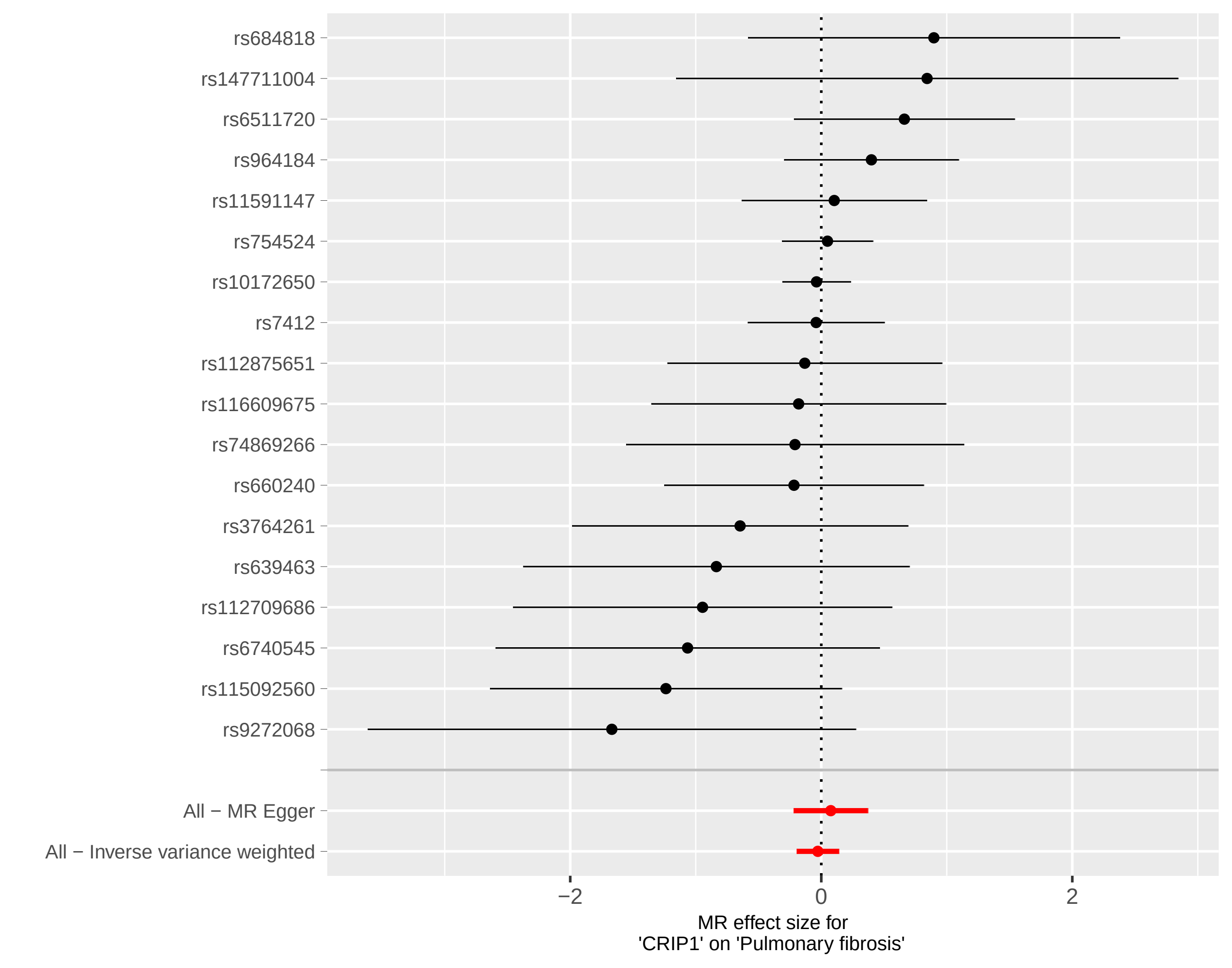

rs684818
rs147711004
rs6511720
rs964184
rs11591147
rs754524
rs10172650
rs7412
rs112875651
rs116609675
rs74869266
rs660240
rs3764261
rs639463
rs112709686
rs6740545
rs115092560
rs9272068
All − MR Egger
All − Inverse variance weighted
−2
0
2
MR effect size for
'CRIP1' on 'Pulmonary fibrosis'

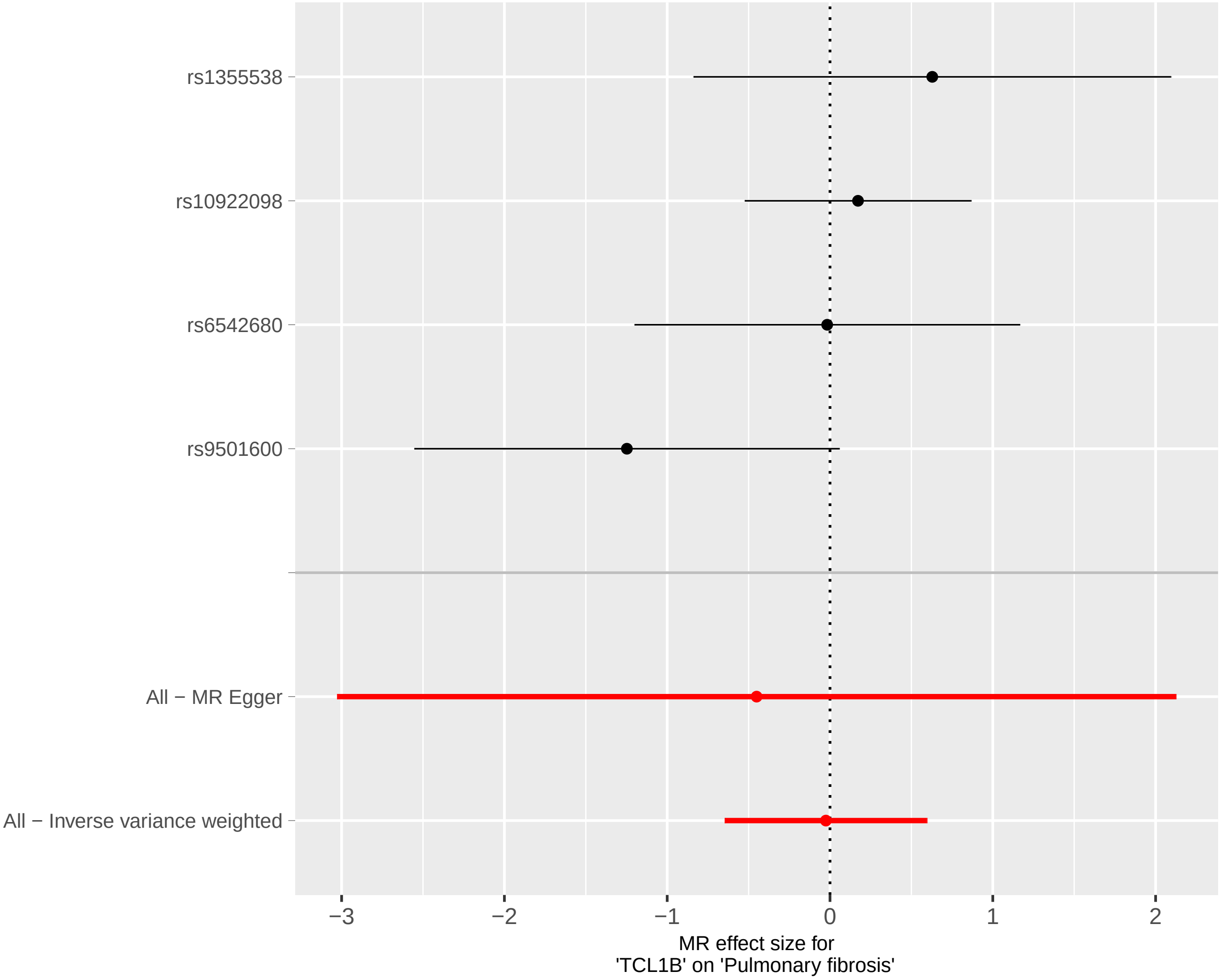

rs1355538
rs10922098
rs6542680
rs9501600
All − MR Egger
All − Inverse variance weighted
−3
−2
−1
0
1
2
MR effect size for
'TCL1B' on 'Pulmonary fibrosis'

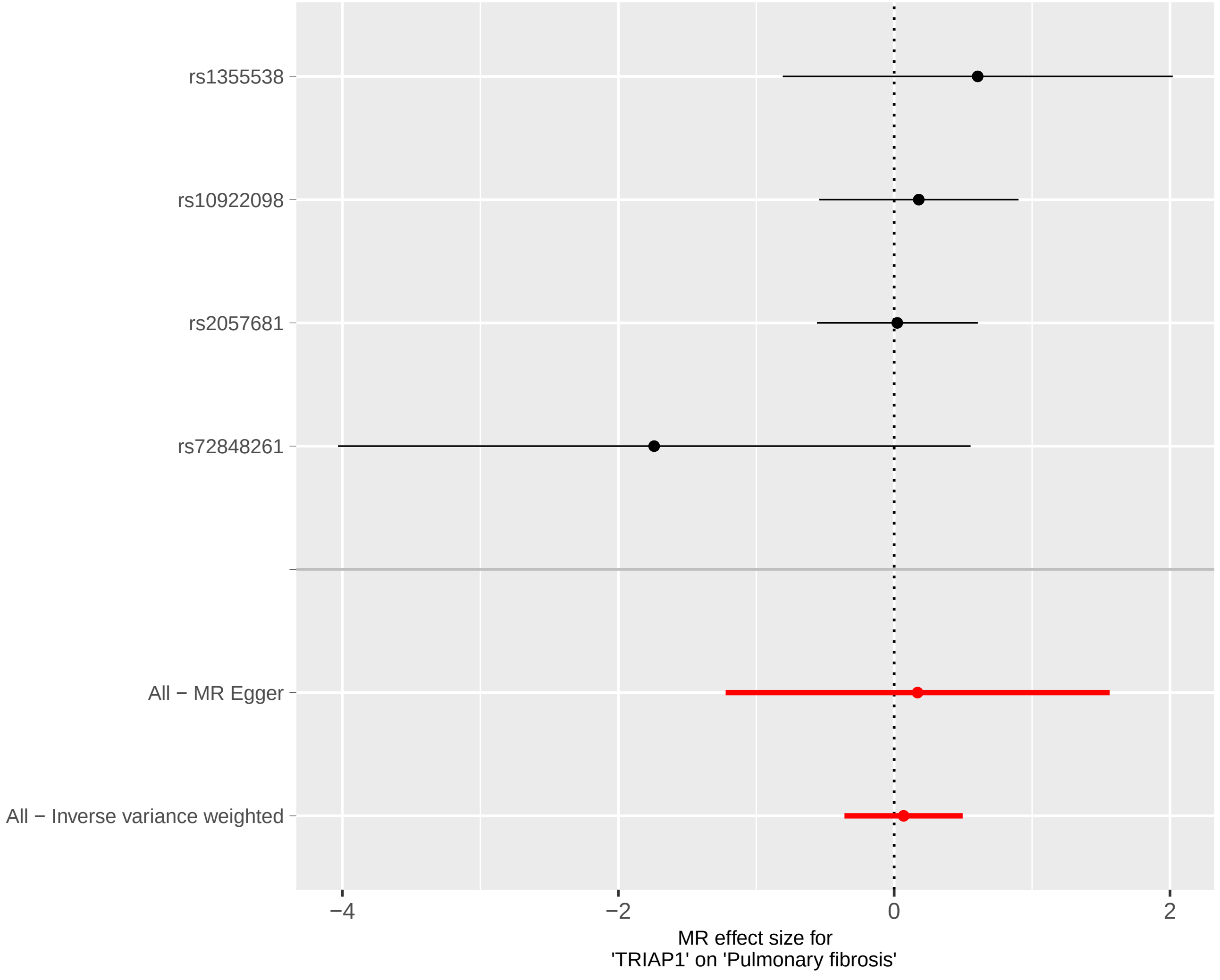

rs1355538
rs10922098
rs2057681
rs72848261
All − MR Egger
All − Inverse variance weighted
−4
−2
0
2
MR effect size for
'TRIAP1' on 'Pulmonary fibrosis'

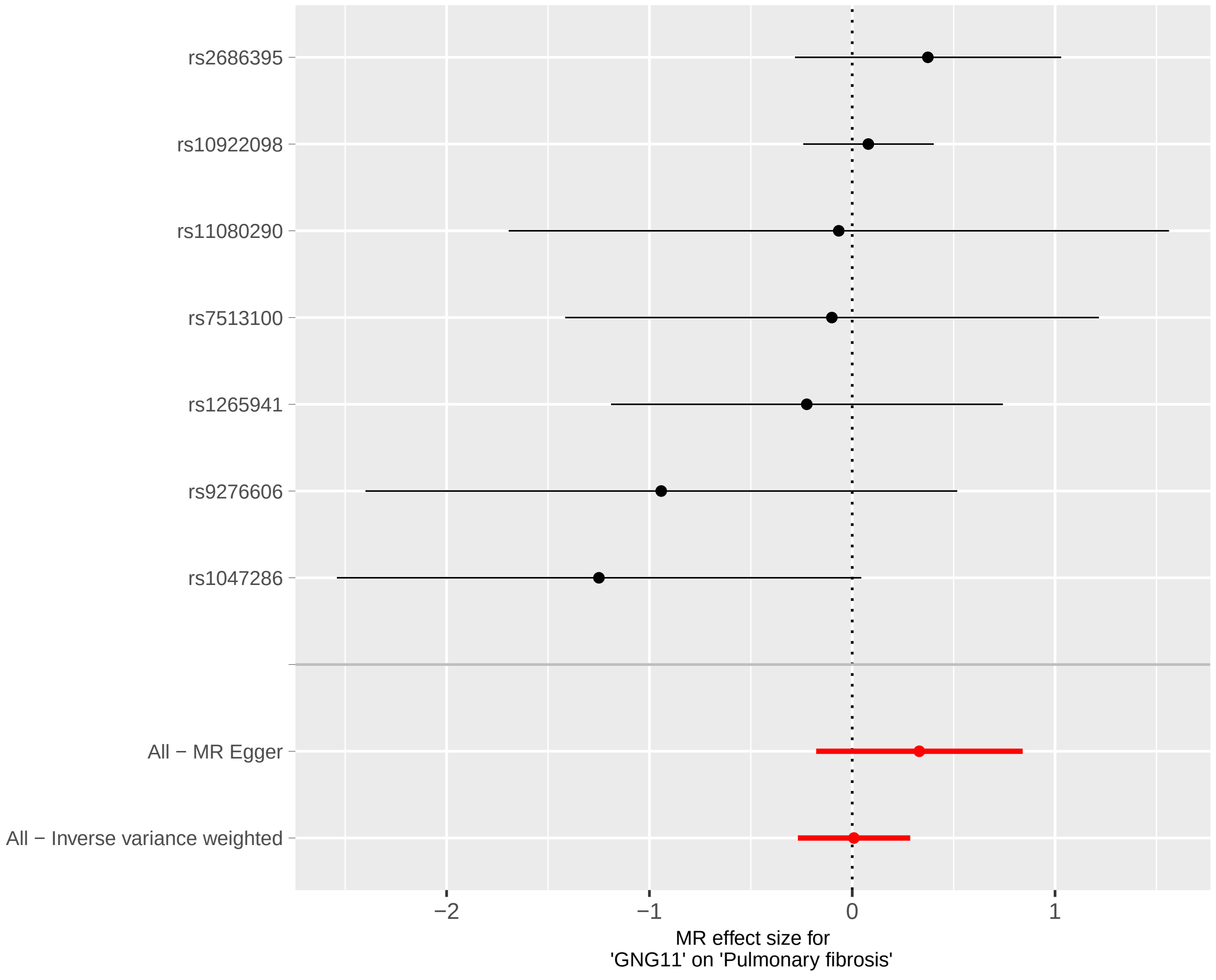

rs2686395
rs10922098
rs11080290
rs7513100
rs1265941
rs9276606
rs1047286
All − MR Egger
All − Inverse variance weighted
−2
−1
0
1
MR effect size for
'GNG11' on 'Pulmonary fibrosis'

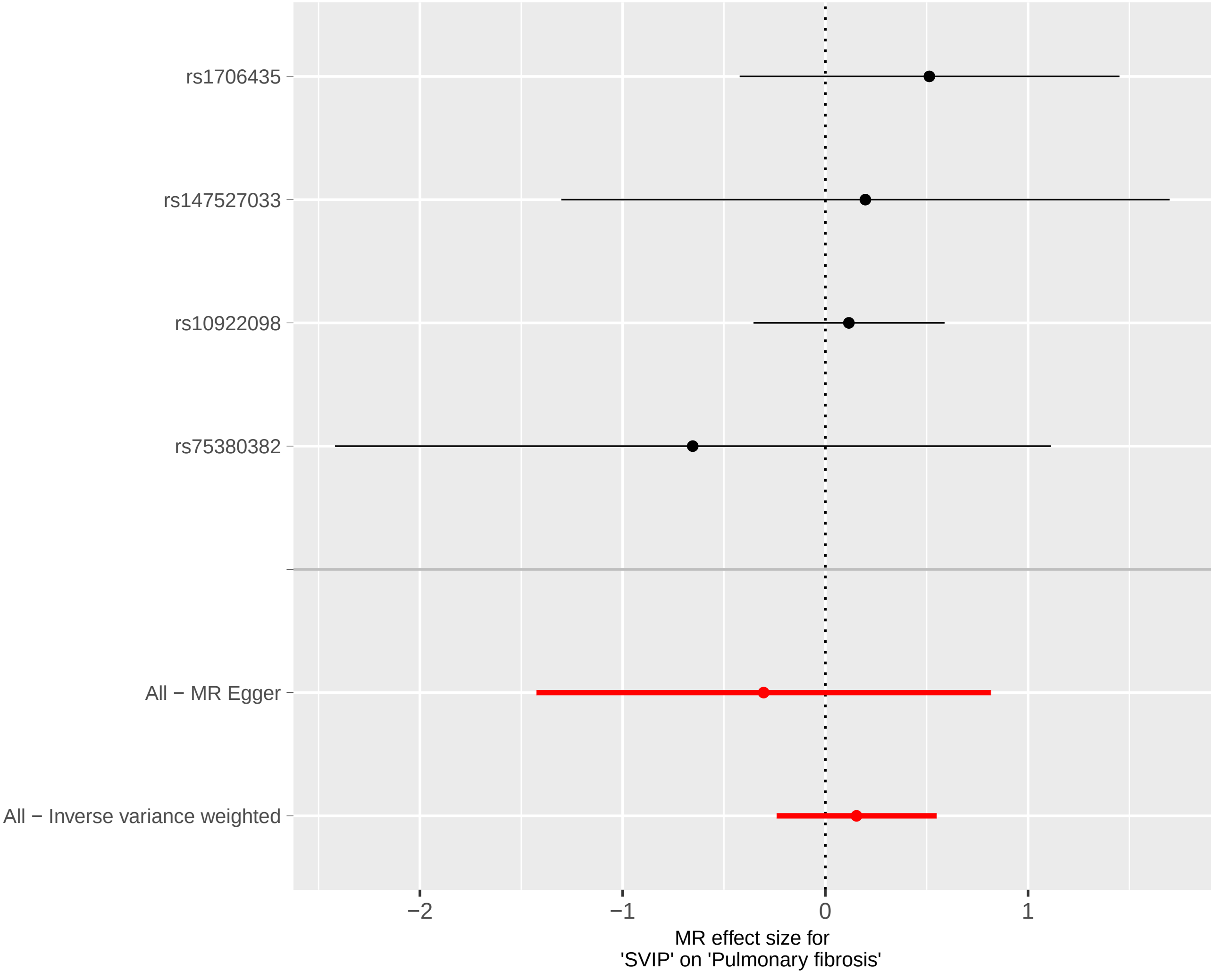

rs1706435
rs147527033
rs10922098
rs75380382
All − MR Egger
All − Inverse variance weighted
−2
−1
0
1
MR effect size for
'SVIP' on 'Pulmonary fibrosis'

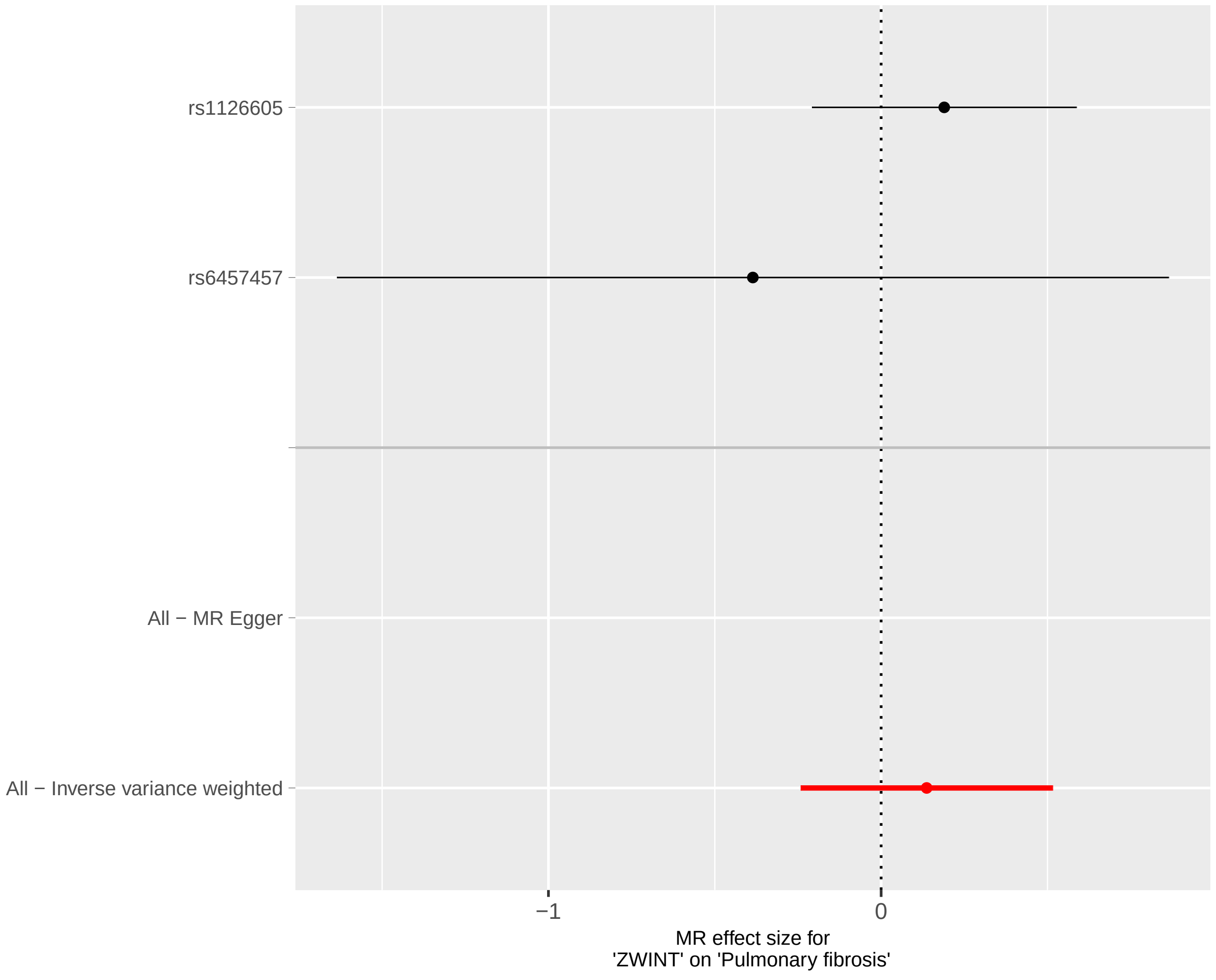
rs1126605
rs6457457
All − MR Egger
All − Inverse variance weighted
−1
0
MR effect size for
'ZWINT' on 'Pulmonary fibrosis'

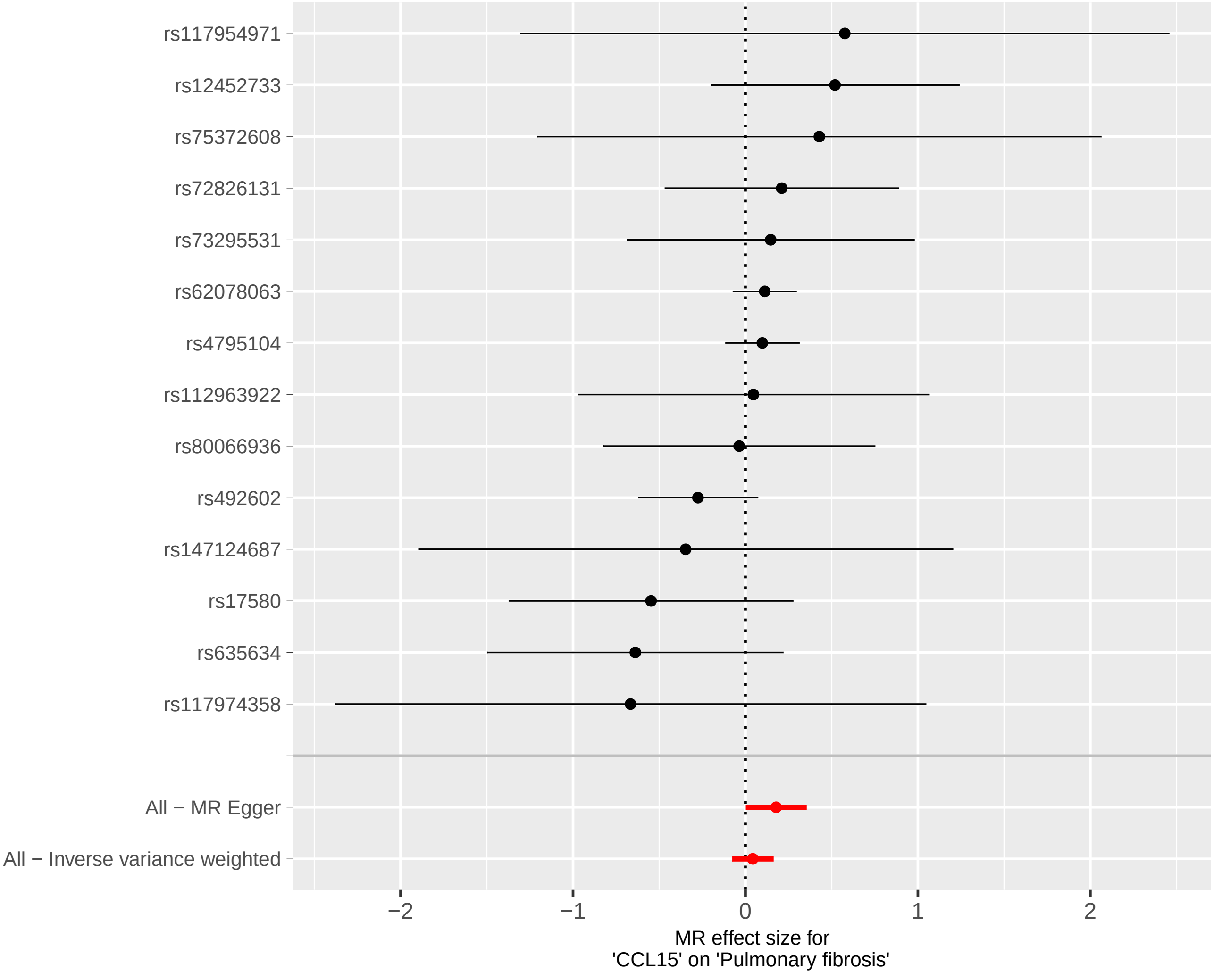
rs117954971
rs12452733
rs75372608
rs72826131
rs73295531
rs62078063
rs4795104
rs112963922
rs80066936
rs492602
rs147124687
rs17580
rs635634
rs117974358
All − MR Egger
All − Inverse variance weighted
−2
−1
0
1
2
MR effect size for
'CCL15' on 'Pulmonary fibrosis'

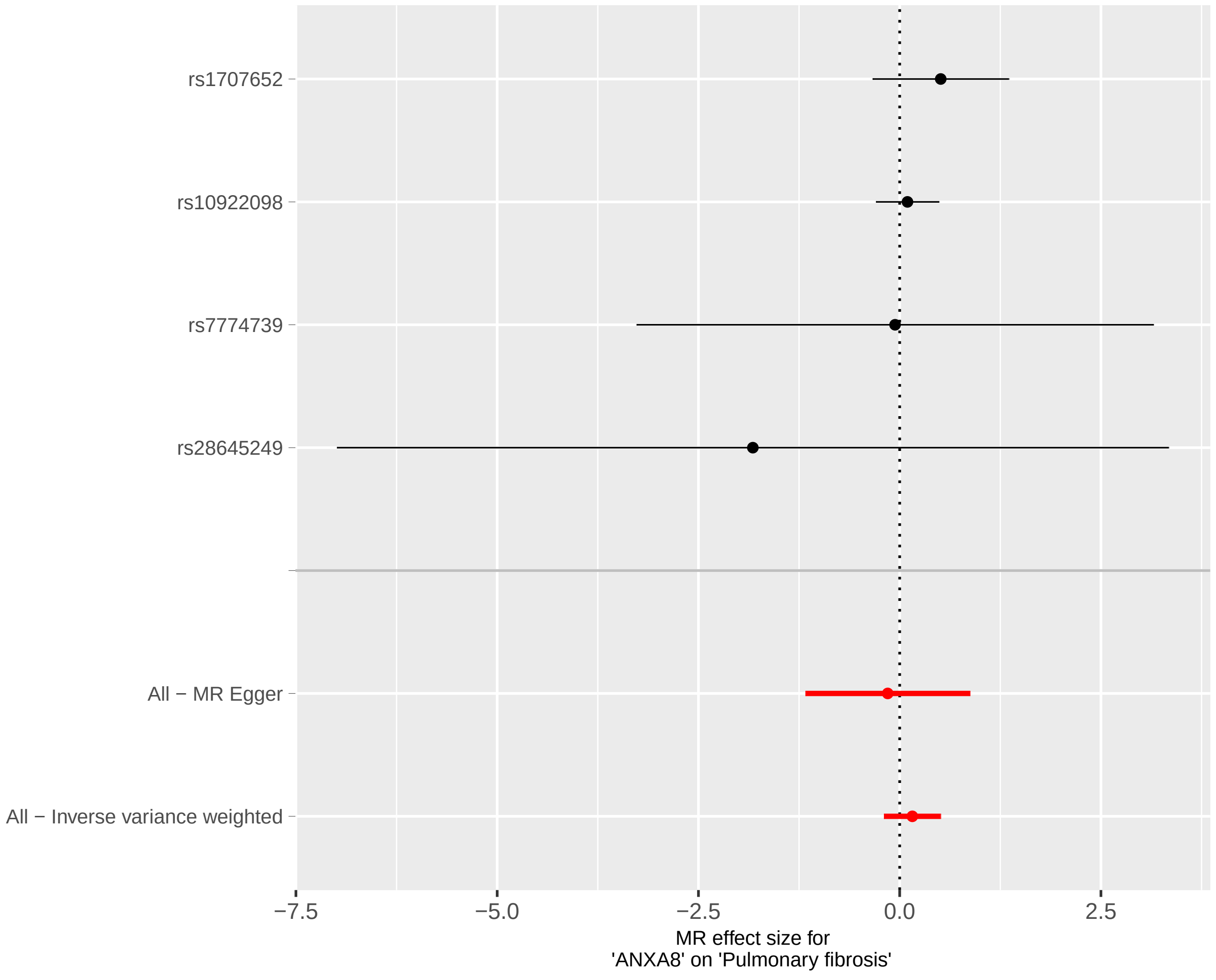

rs1707652
rs10922098
rs7774739
rs28645249
All − MR Egger
All − Inverse variance weighted
−7.5
−5.0
−2.5
0.0
2.5
MR effect size for
'ANXA8' on 'Pulmonary fibrosis'

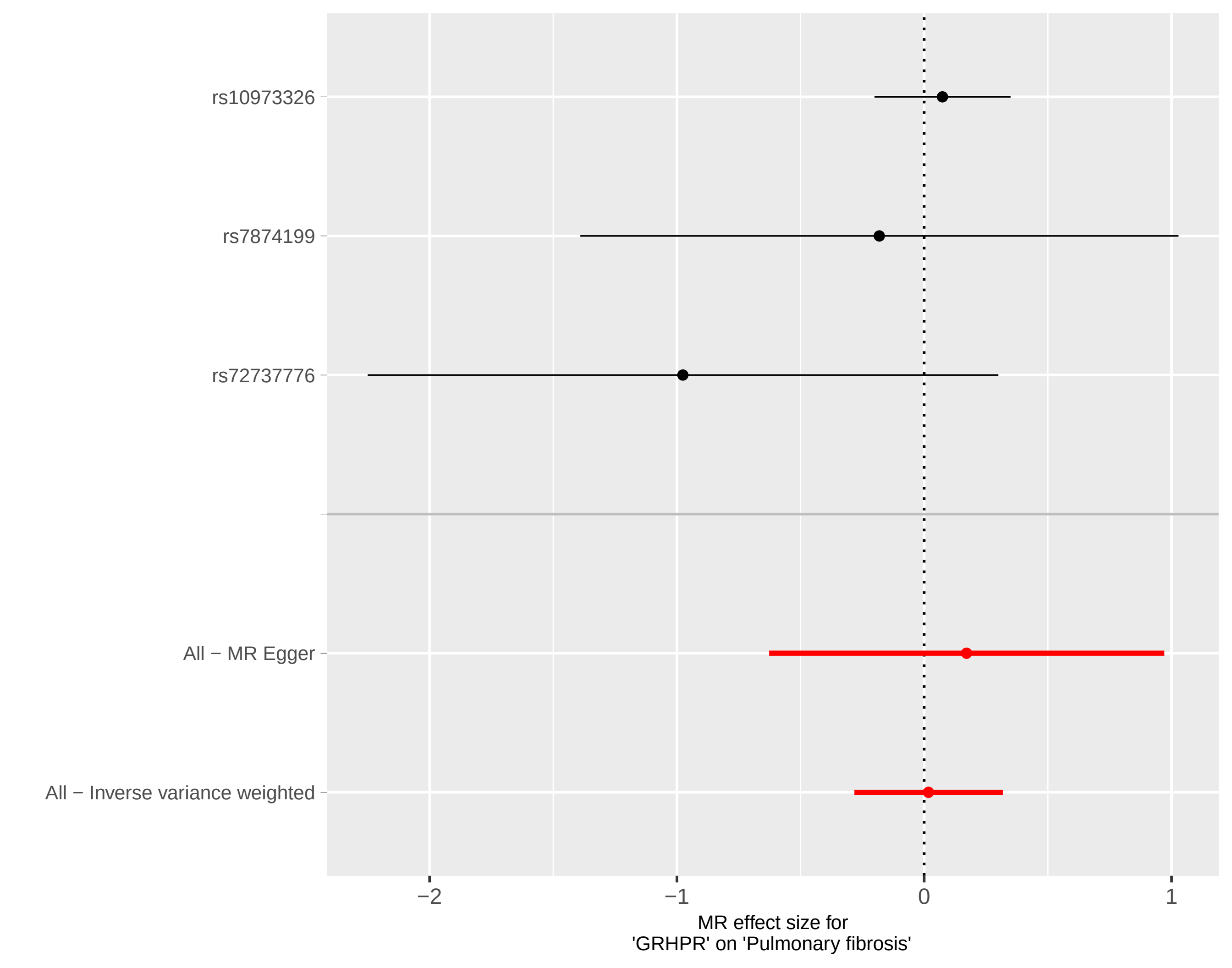

rs10973326
rs7874199
rs72737776
All − MR Egger
All − Inverse variance weighted
−2
−1
0
1
MR effect size for
'GRHPR' on 'Pulmonary fibrosis'

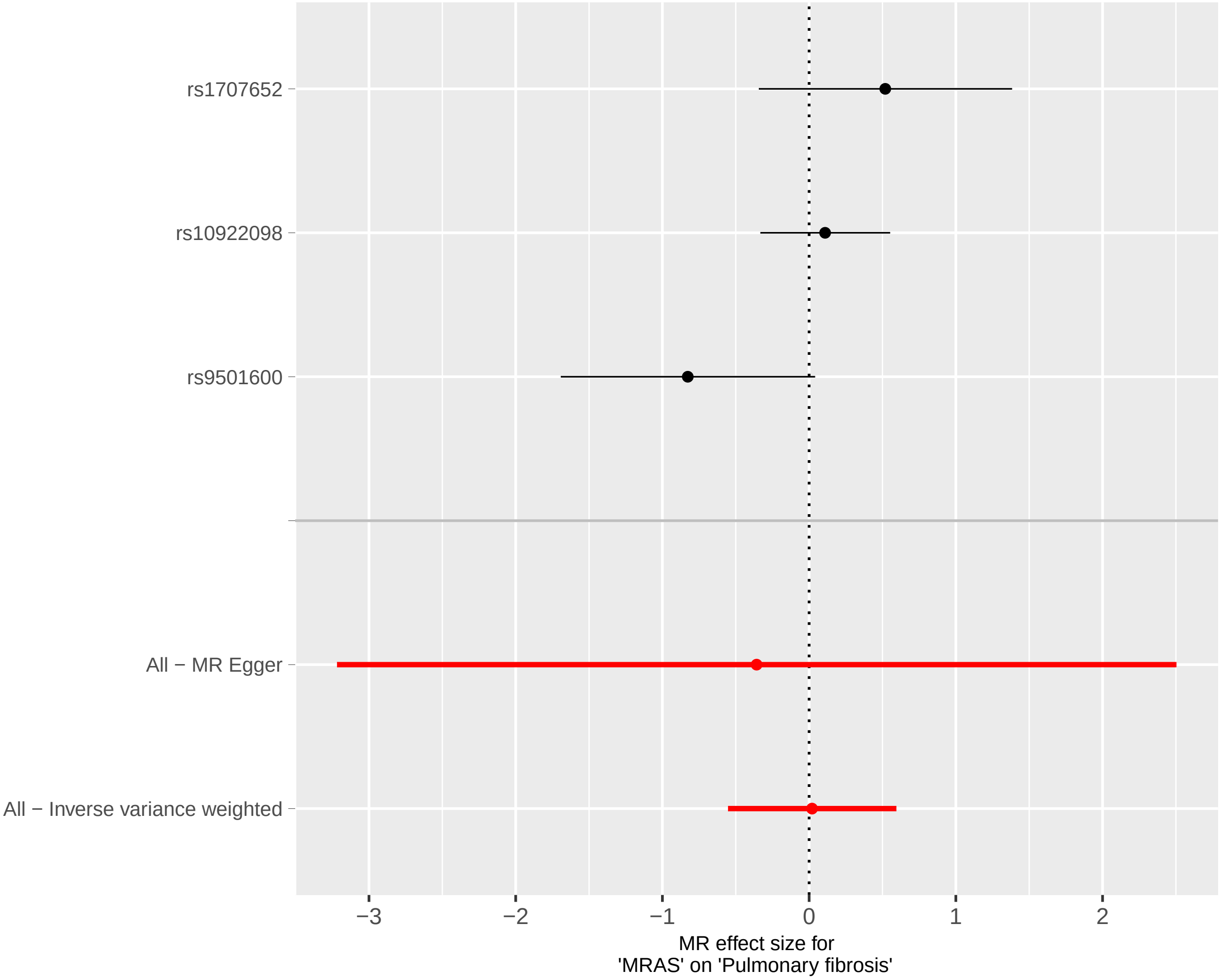

rs1707652
rs10922098
rs9501600
All − MR Egger
All − Inverse variance weighted
−3
−2
−1
0
1
2
MR effect size for
'MRAS' on 'Pulmonary fibrosis'

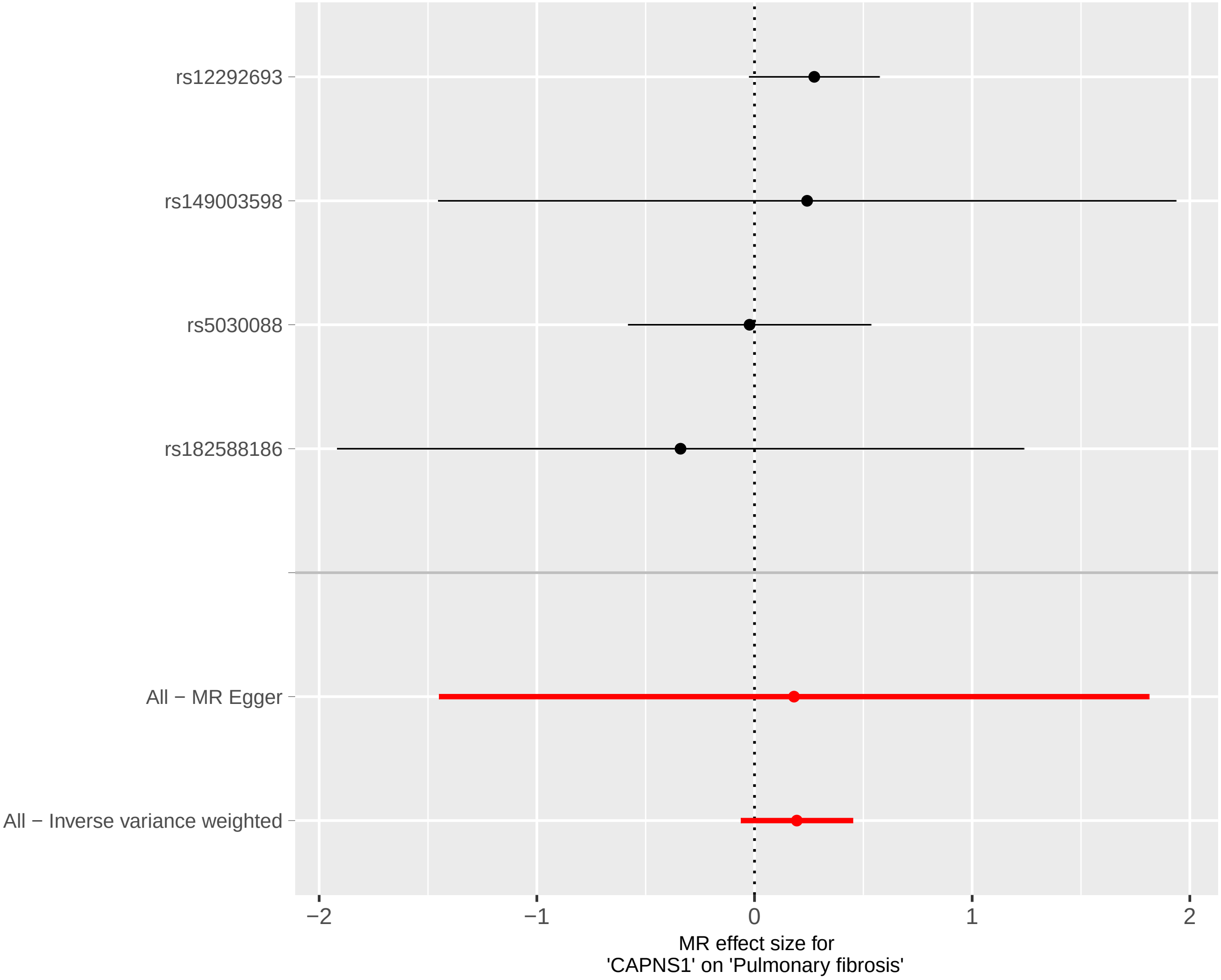
rs12292693
rs149003598
rs5030088
rs182588186
All − MR Egger
All − Inverse variance weighted
−2
−1
0
1
2
MR effect size for
'CAPNS1' on 'Pulmonary fibrosis'

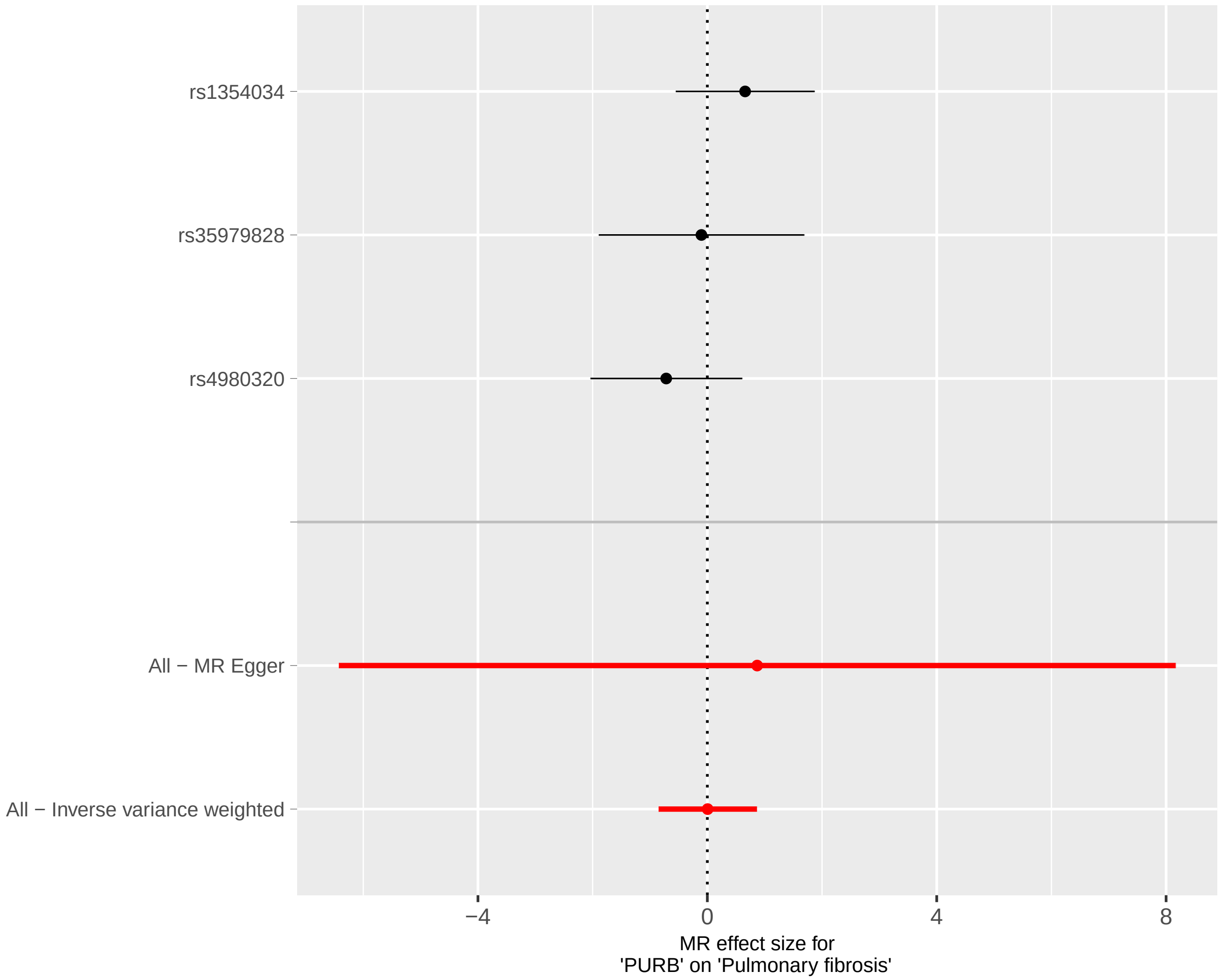

rs1354034
rs35979828
rs4980320
All − MR Egger
All − Inverse variance weighted
−4
0
4
8
MR effect size for
'PURB' on 'Pulmonary fibrosis'

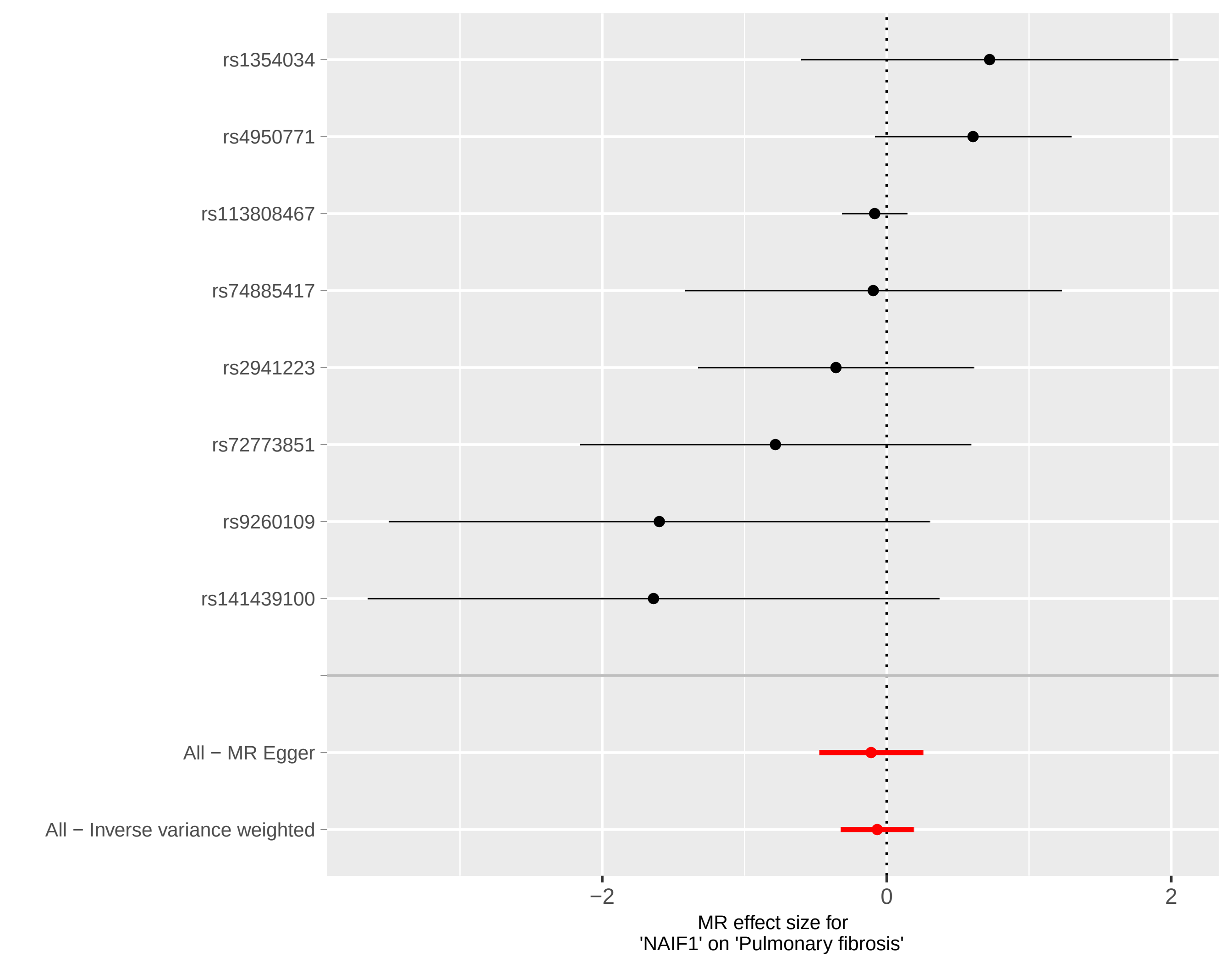

rs1354034
rs4950771
rs113808467
rs74885417
rs2941223
rs72773851
rs9260109
rs141439100
All − MR Egger
All − Inverse variance weighted
−2
0
2
MR effect size for
'NAIF1' on 'Pulmonary fibrosis'

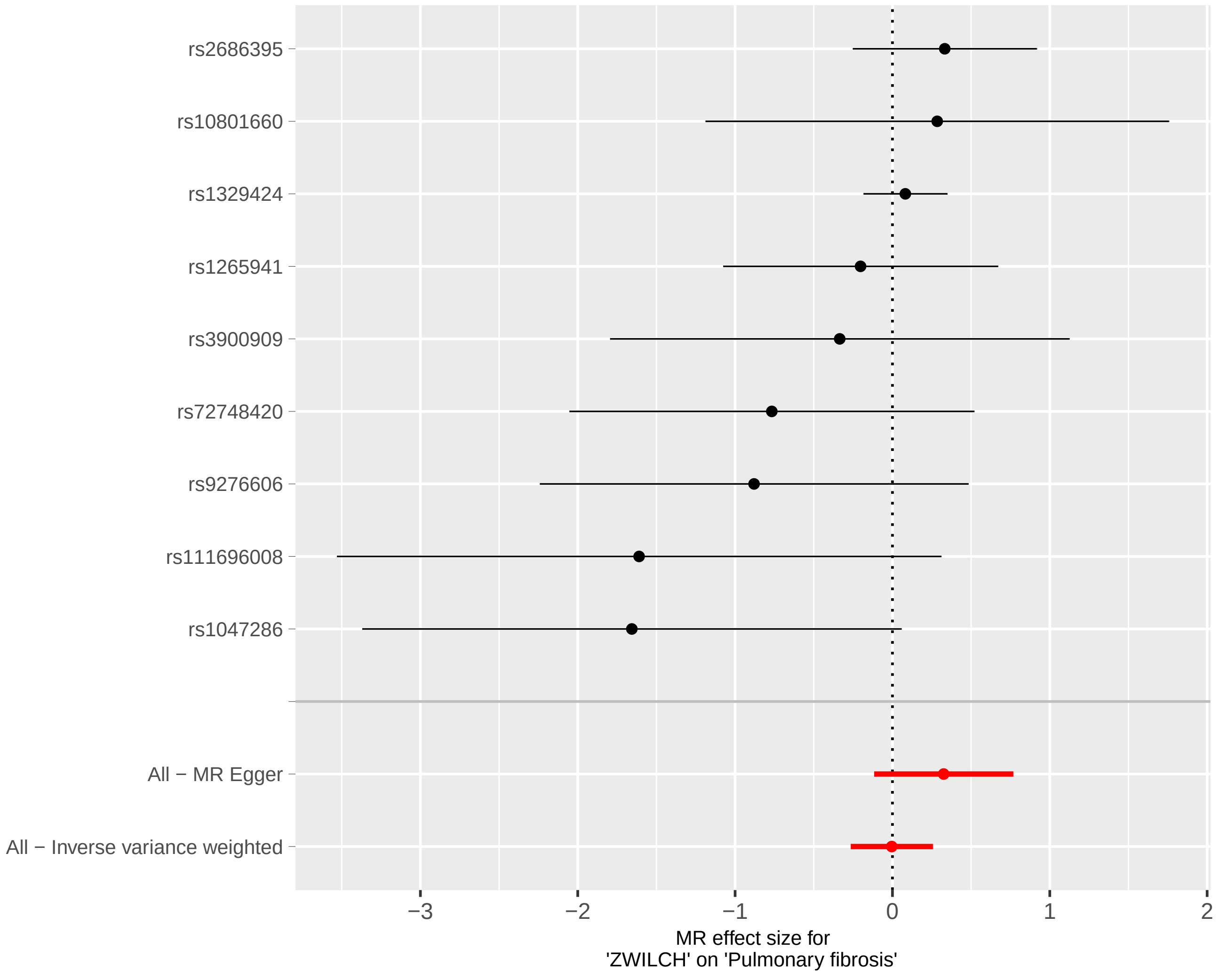

rs2686395
rs10801660
rs1329424
rs1265941
rs3900909
rs72748420
rs9276606
rs111696008
rs1047286
All − MR Egger
All − Inverse variance weighted
−3
−2
−1
0
1
2
MR effect size for
'ZWILCH' on 'Pulmonary fibrosis'

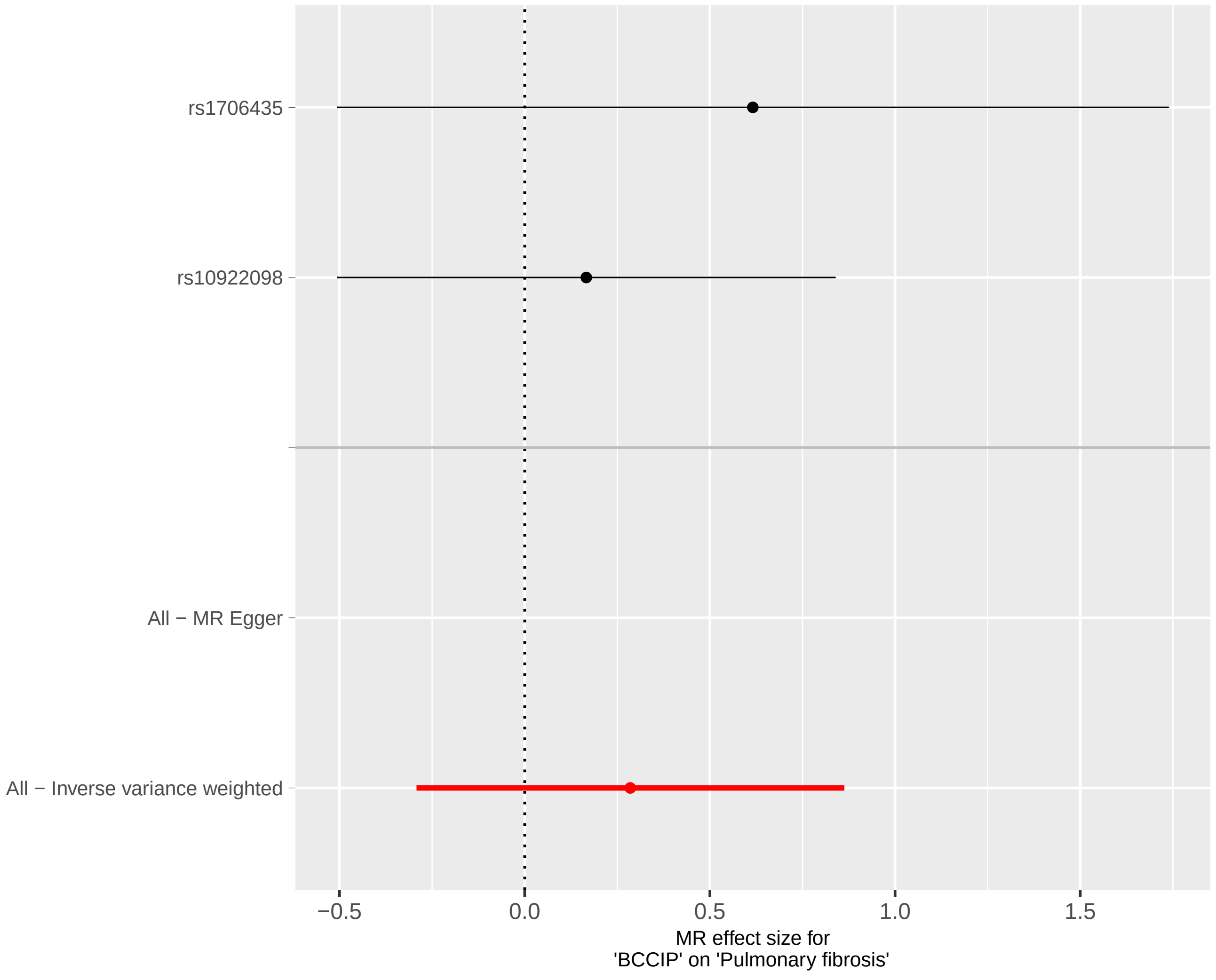
rs1706435
rs10922098
All − MR Egger
All − Inverse variance weighted
−0.5
0.0
0.5
1.0
1.5
MR effect size for
'BCCIP' on 'Pulmonary fibrosis'

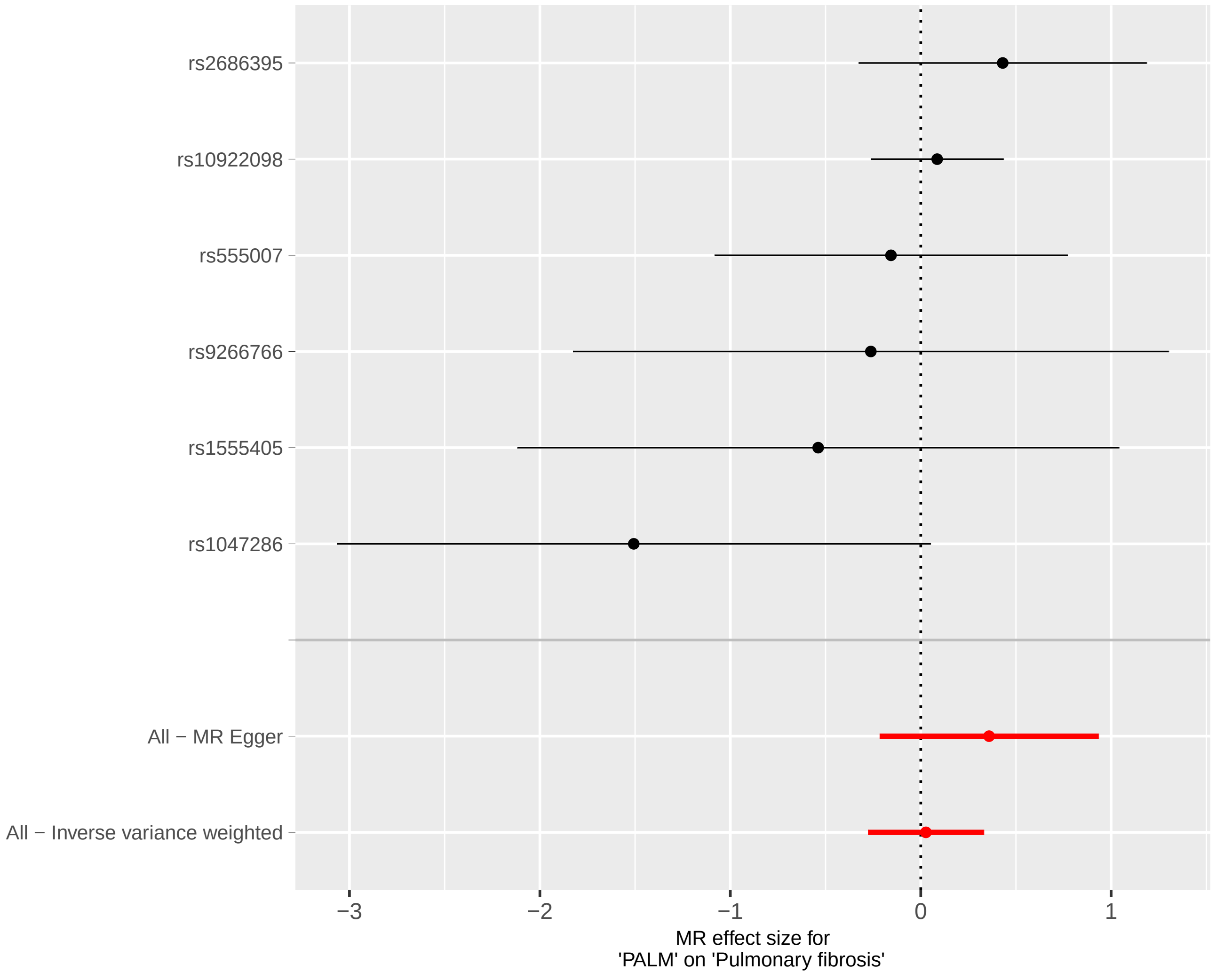

rs2686395
rs10922098
rs555007
rs9266766
rs1555405
rs1047286
All − MR Egger
All − Inverse variance weighted
−3
−2
−1
0
1
MR effect size for
'PALM' on 'Pulmonary fibrosis'

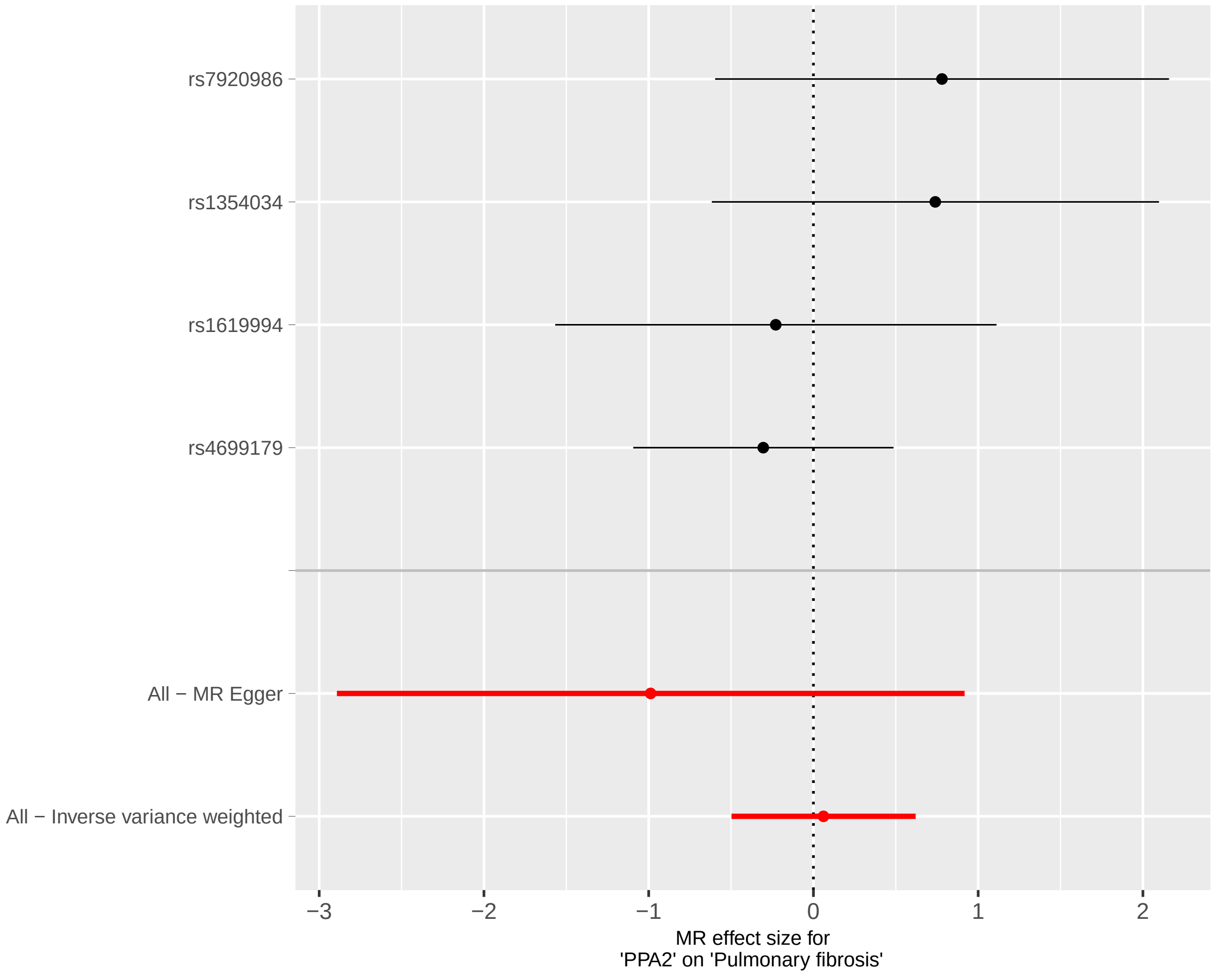

rs7920986
rs1354034
rs1619994
rs4699179
All − MR Egger
All − Inverse variance weighted
−3
−2
−1
0
1
2
MR effect size for
'PPA2' on 'Pulmonary fibrosis'

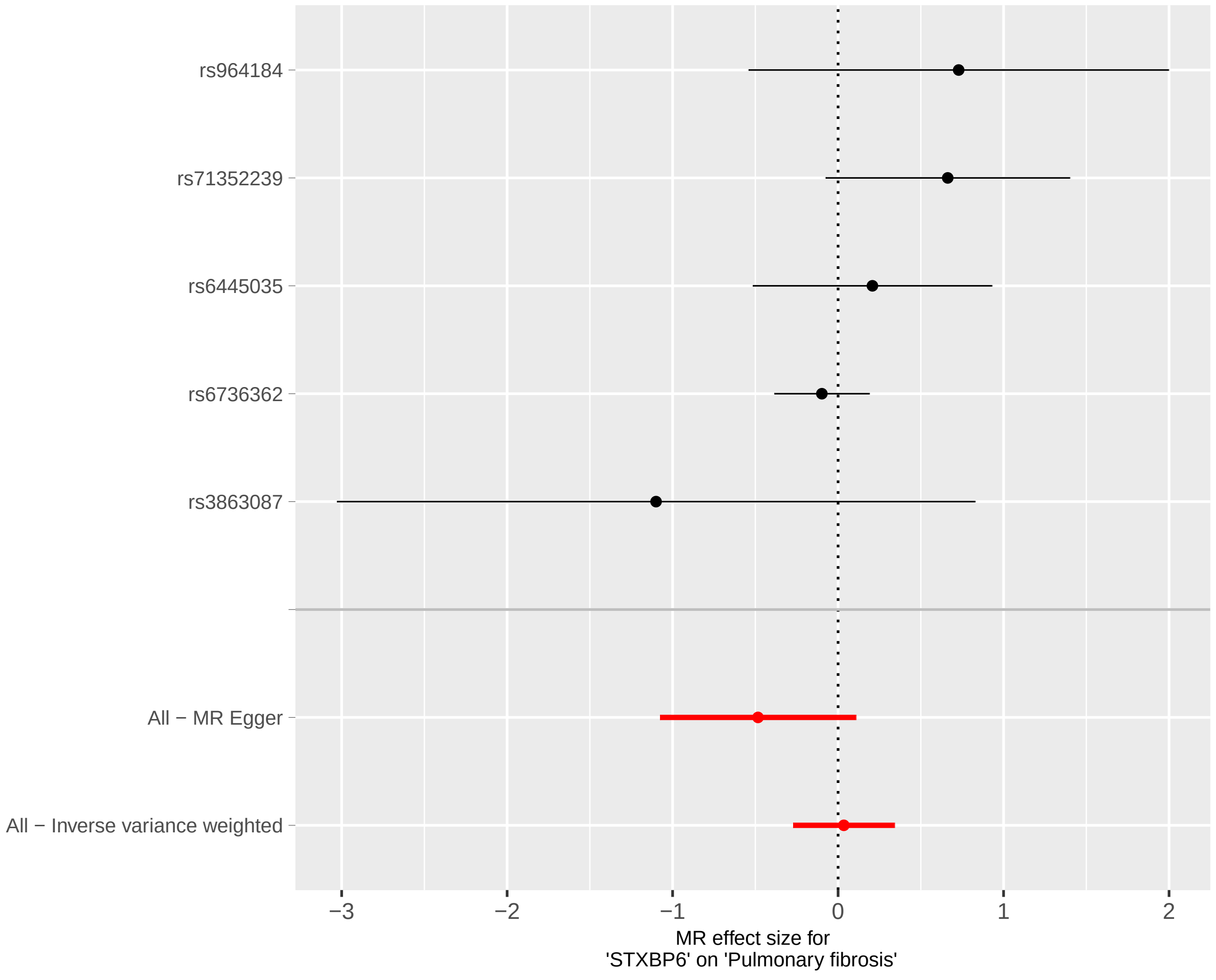
rs964184
rs71352239
rs6445035
rs6736362
rs3863087
All − MR Egger
All − Inverse variance weighted
−3
−2
−1
0
1
2
MR effect size for
'STXBP6' on 'Pulmonary fibrosis'

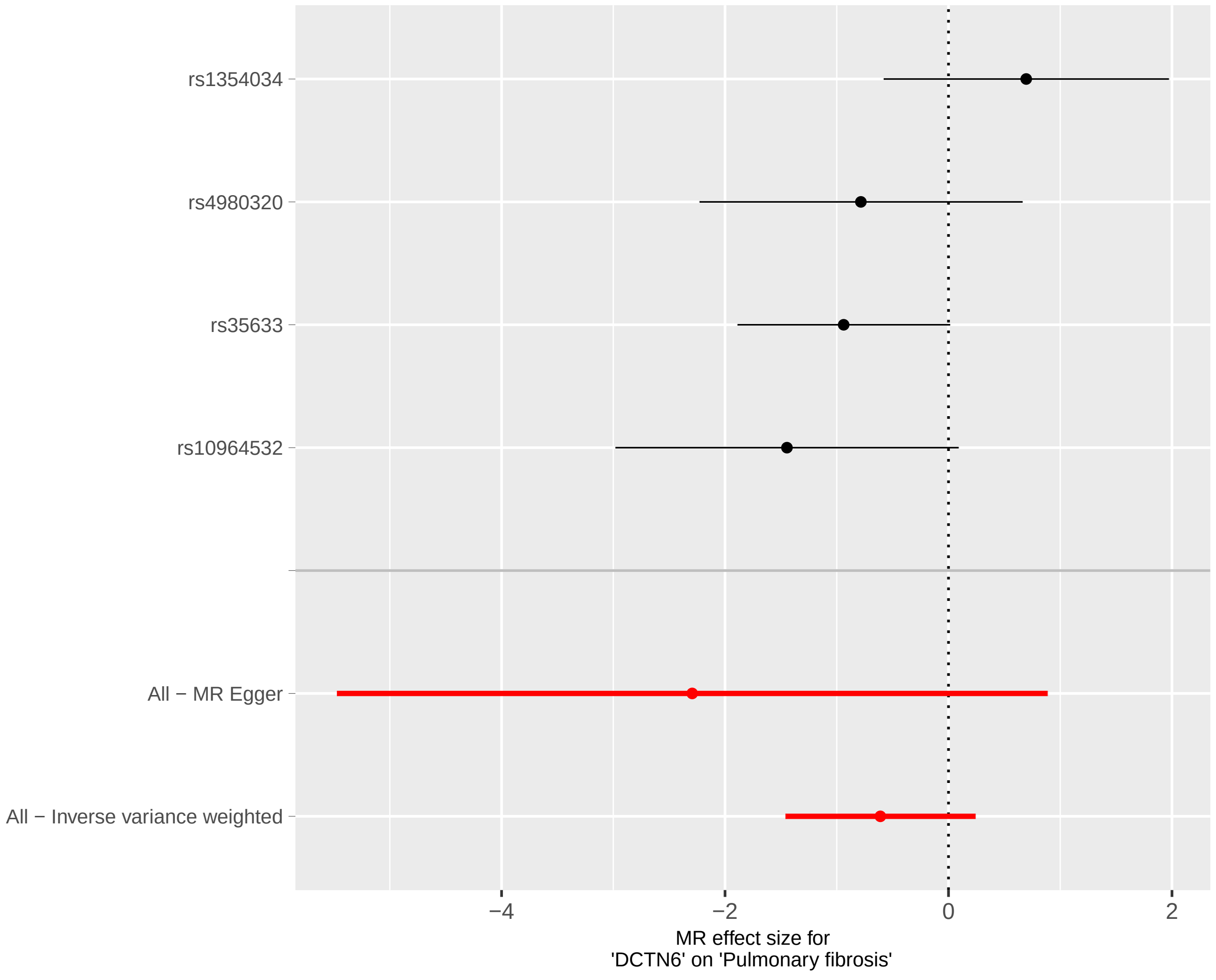

rs1354034
rs4980320
rs35633
rs10964532
All − MR Egger
All − Inverse variance weighted
−4
−2
0
2
MR effect size for
'DCTN6' on 'Pulmonary fibrosis'

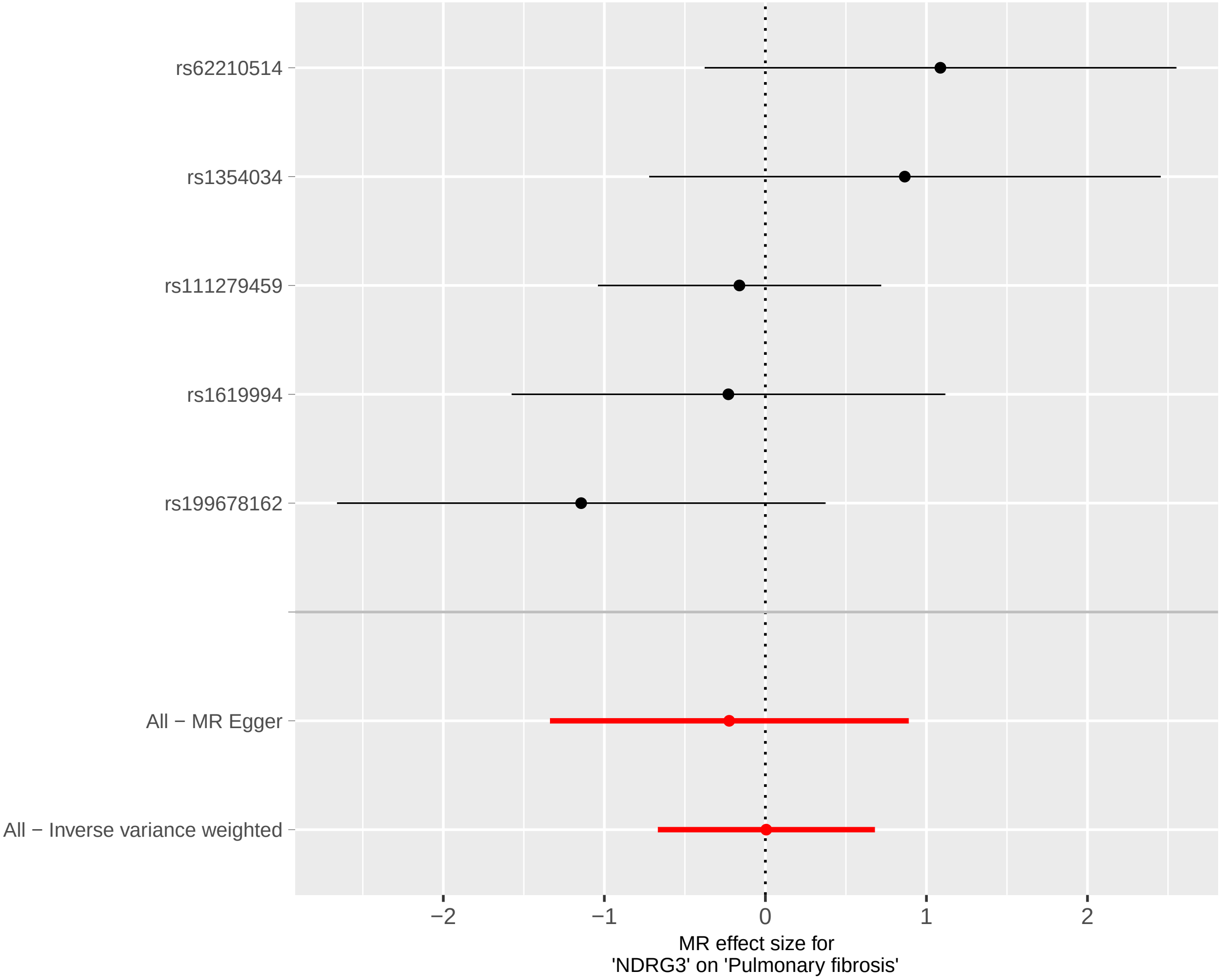

rs62210514
rs1354034
rs111279459
rs1619994
rs199678162
All − MR Egger
All − Inverse variance weighted
−2
−1
0
1
2
MR effect size for
'NDRG3' on 'Pulmonary fibrosis'

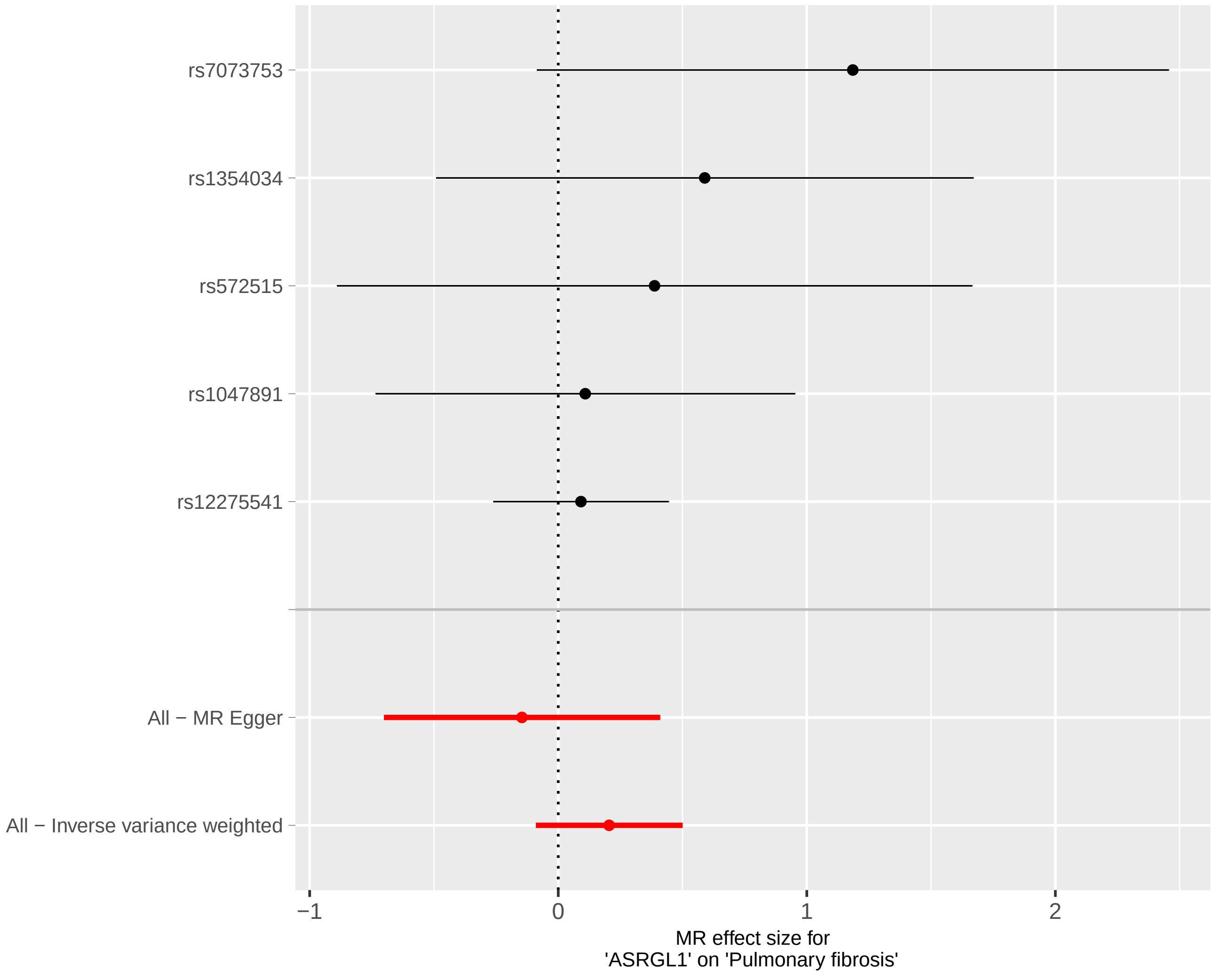

rs7073753
rs1354034
rs572515
rs1047891
rs12275541
All − MR Egger
All − Inverse variance weighted
−1
0
1
2
MR effect size for
'ASRGL1' on 'Pulmonary fibrosis'

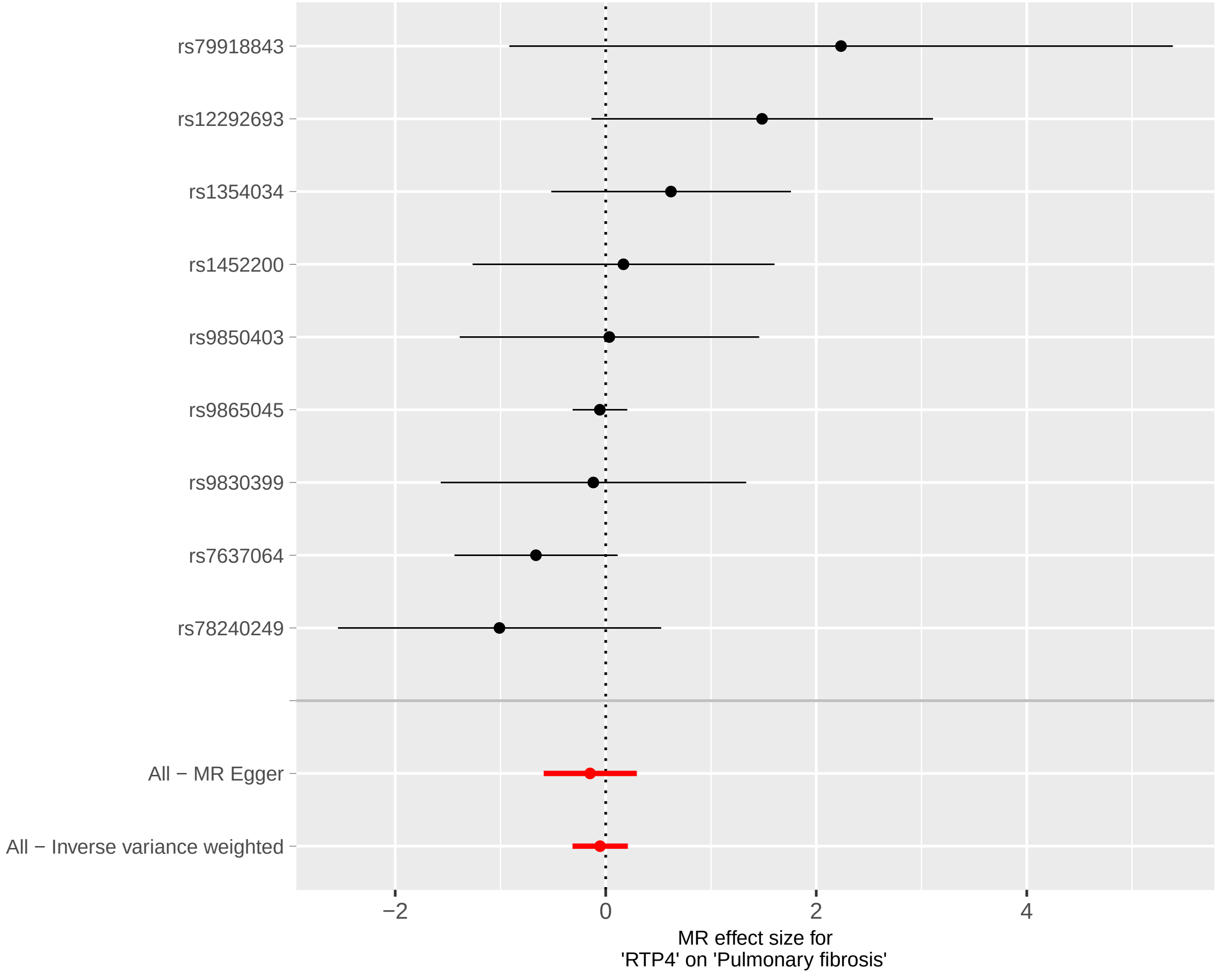

rs79918843
rs12292693
rs1354034
rs1452200
rs9850403
rs9865045
rs9830399
rs7637064
rs78240249
All − MR Egger
All − Inverse variance weighted
−2
0
2
4
MR effect size for
'RTP4' on 'Pulmonary fibrosis'

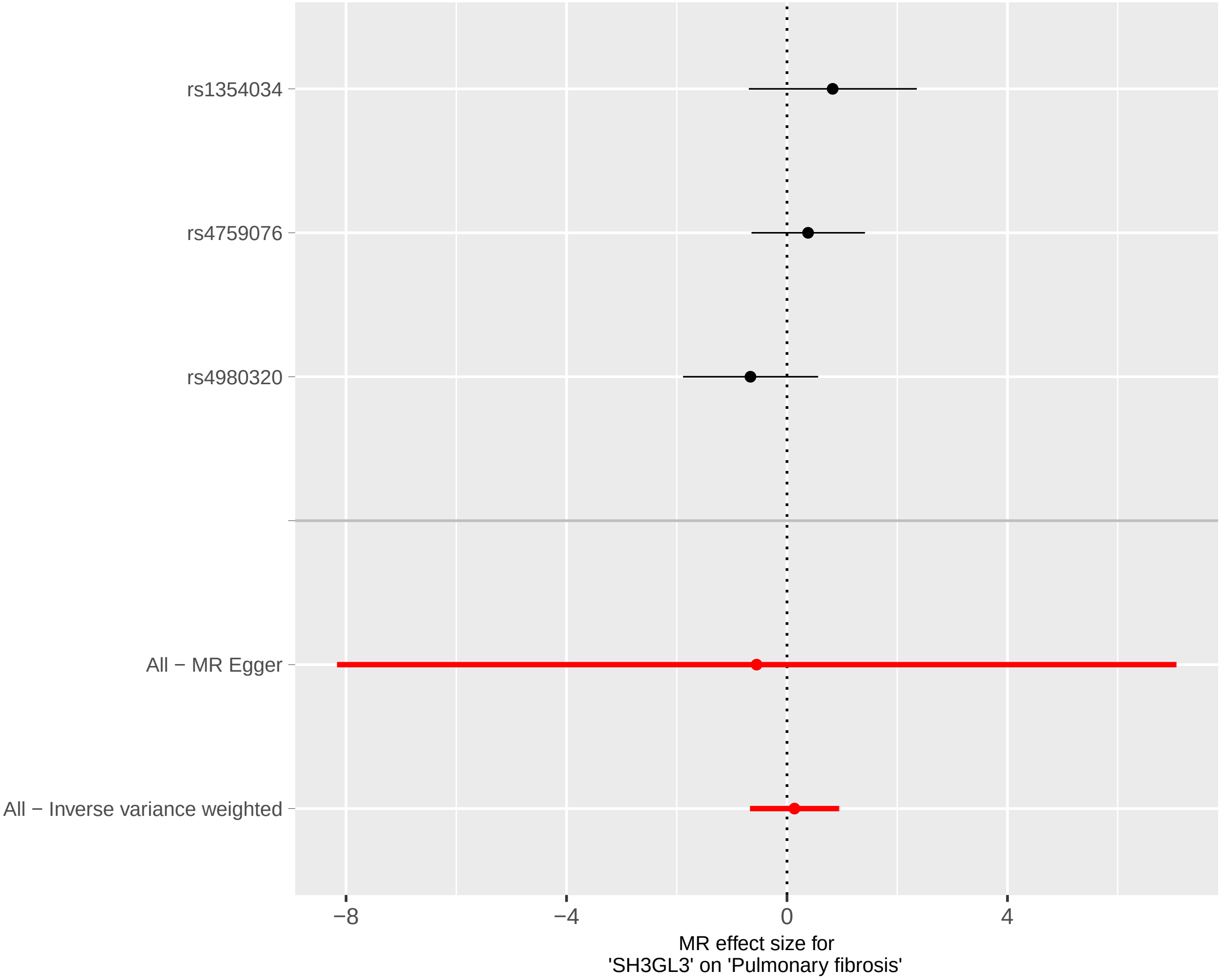
rs1354034
rs4759076
rs4980320
All − MR Egger
All − Inverse variance weighted
−8
−4
0
4
MR effect size for
'SH3GL3' on 'Pulmonary fibrosis'

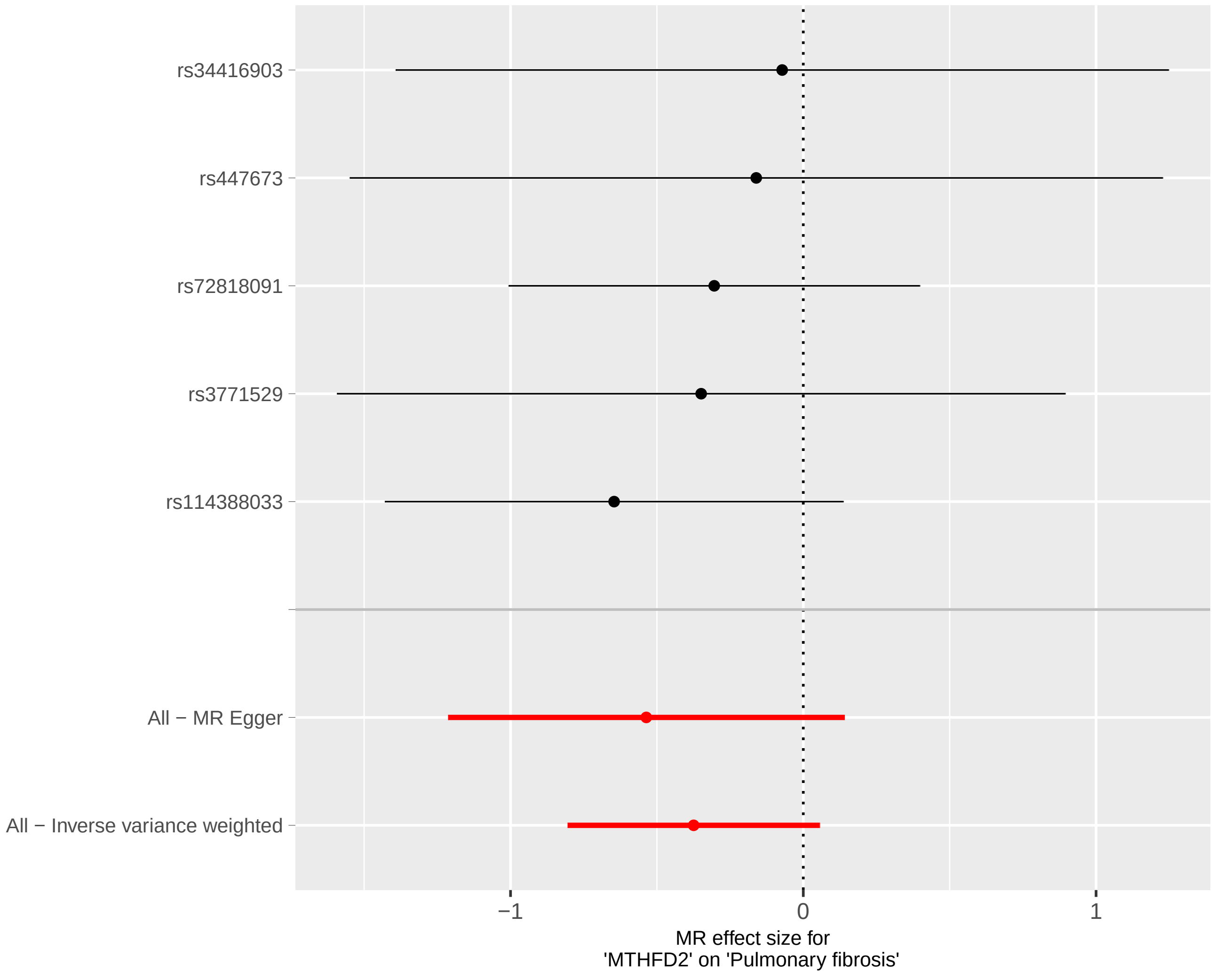
rs34416903
rs447673
rs72818091
rs3771529
rs114388033
All − MR Egger
All − Inverse variance weighted
−1
0
1
MR effect size for
'MTHFD2' on 'Pulmonary fibrosis'

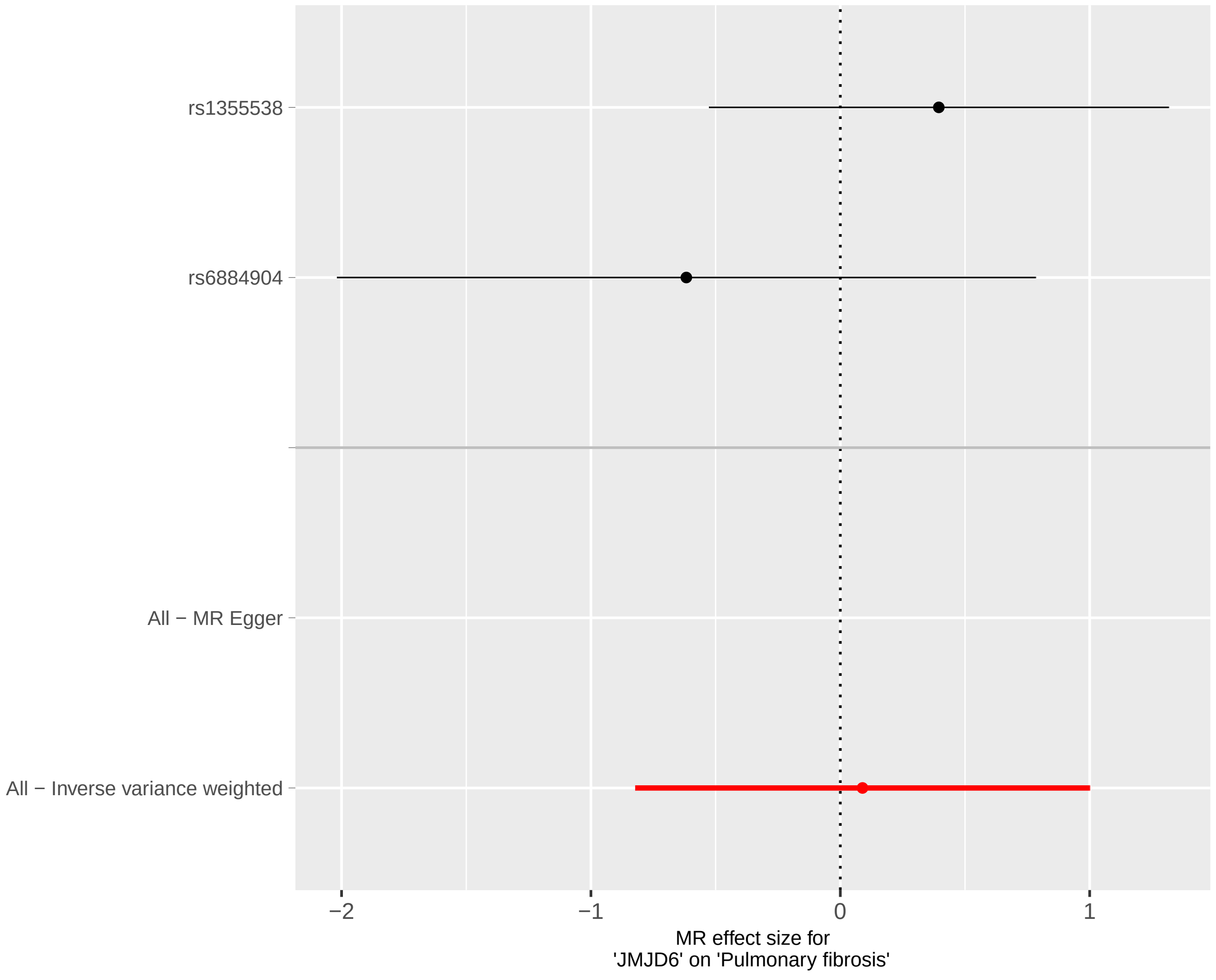

rs1355538
rs6884904
All − MR Egger
All − Inverse variance weighted
−2
−1
0
1
MR effect size for
'JMJD6' on 'Pulmonary fibrosis'

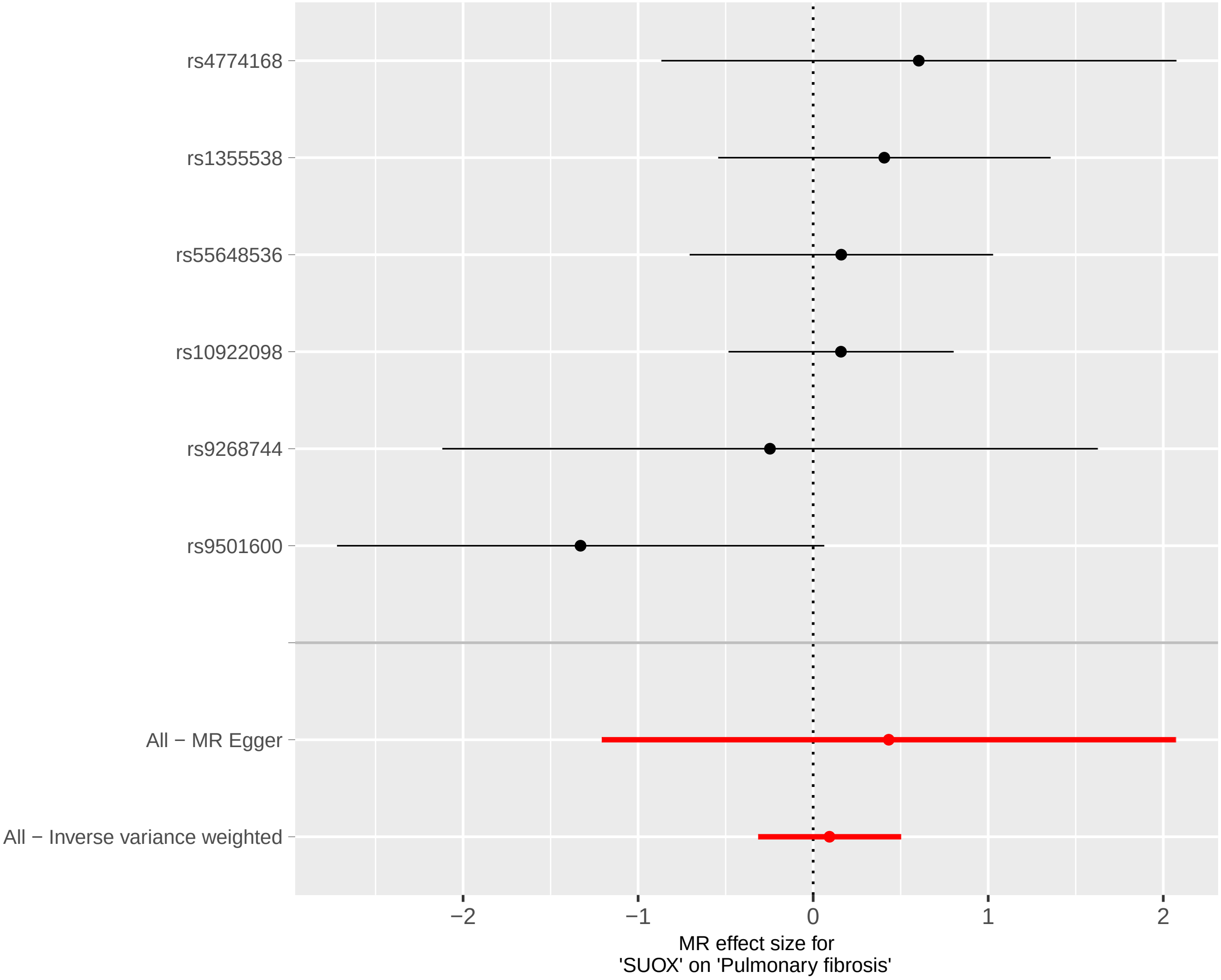

rs4774168
rs1355538
rs55648536
rs10922098
rs9268744
rs9501600
All − MR Egger
All − Inverse variance weighted
−2
−1
0
1
2
MR effect size for
'SUOX' on 'Pulmonary fibrosis'

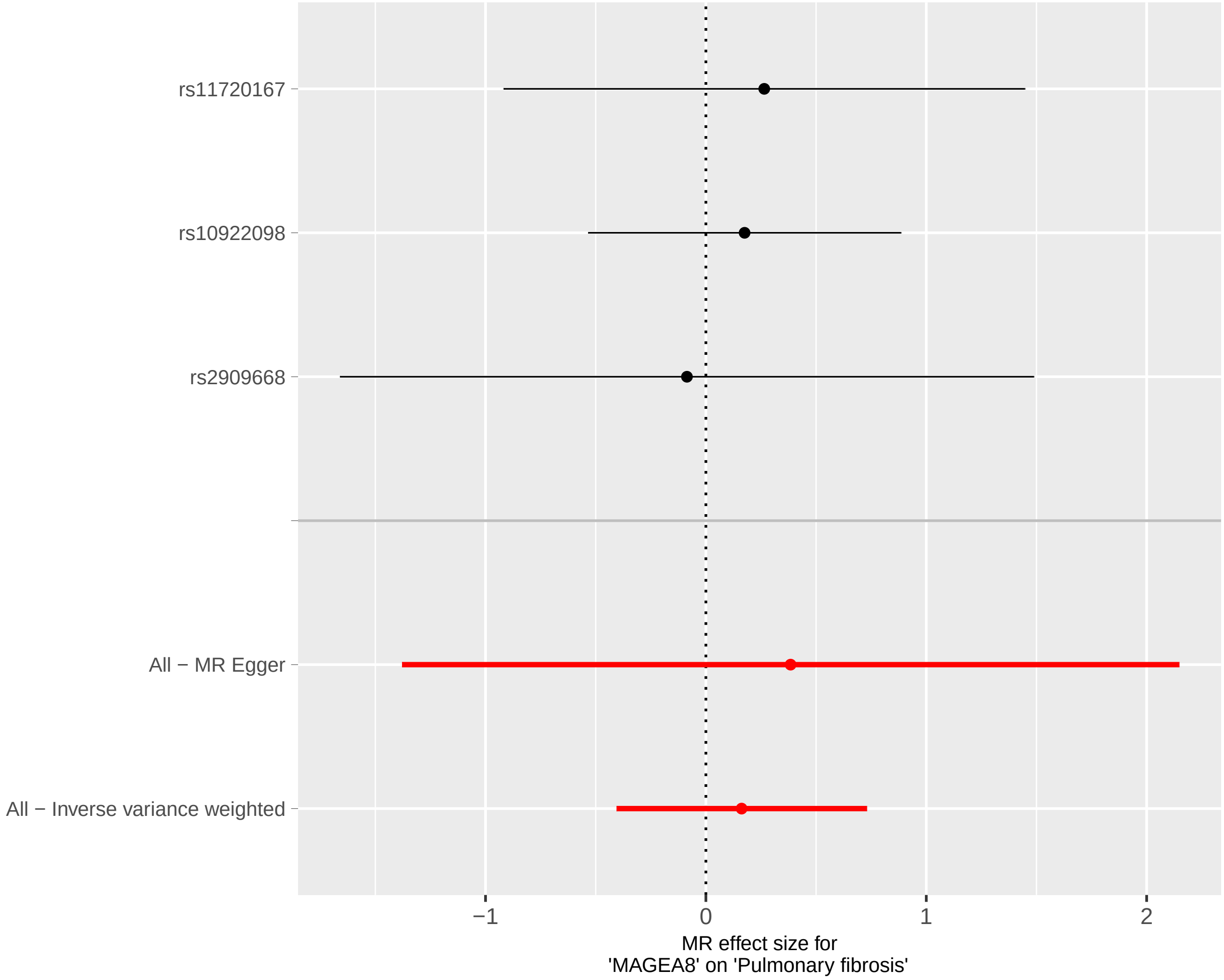

rs11720167
rs10922098
rs2909668
All − MR Egger
All − Inverse variance weighted
−1
0
1
2
MR effect size for
'MAGEA8' on 'Pulmonary fibrosis'

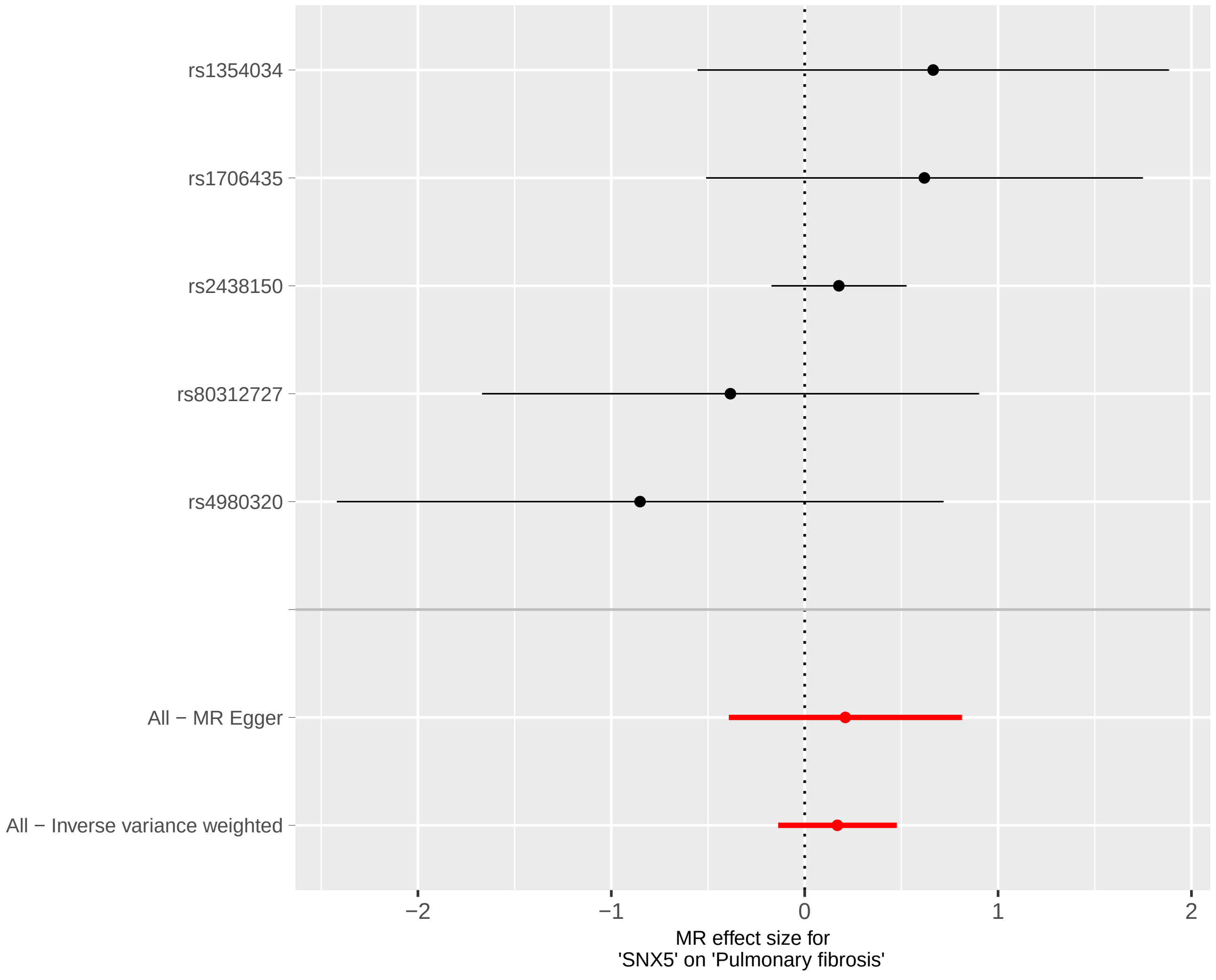

rs1354034
rs1706435
rs2438150
rs80312727
rs4980320
All − MR Egger
All − Inverse variance weighted
−2
−1
0
1
2
MR effect size for
'SNX5' on 'Pulmonary fibrosis'

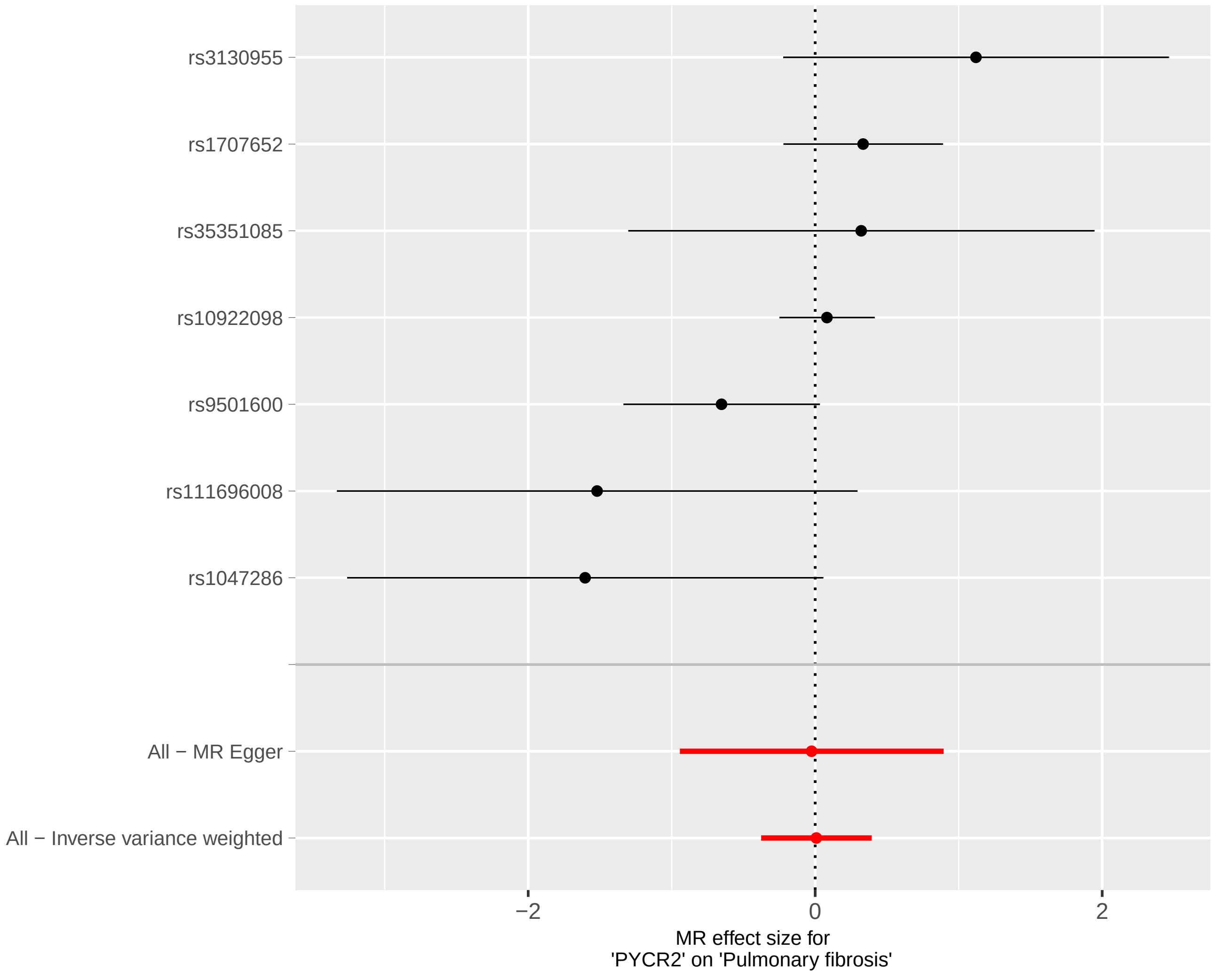

rs3130955
rs1707652
rs35351085
rs10922098
rs9501600
rs111696008
rs1047286
All − MR Egger
All − Inverse variance weighted
−2
0
2
MR effect size for
'PYCR2' on 'Pulmonary fibrosis'

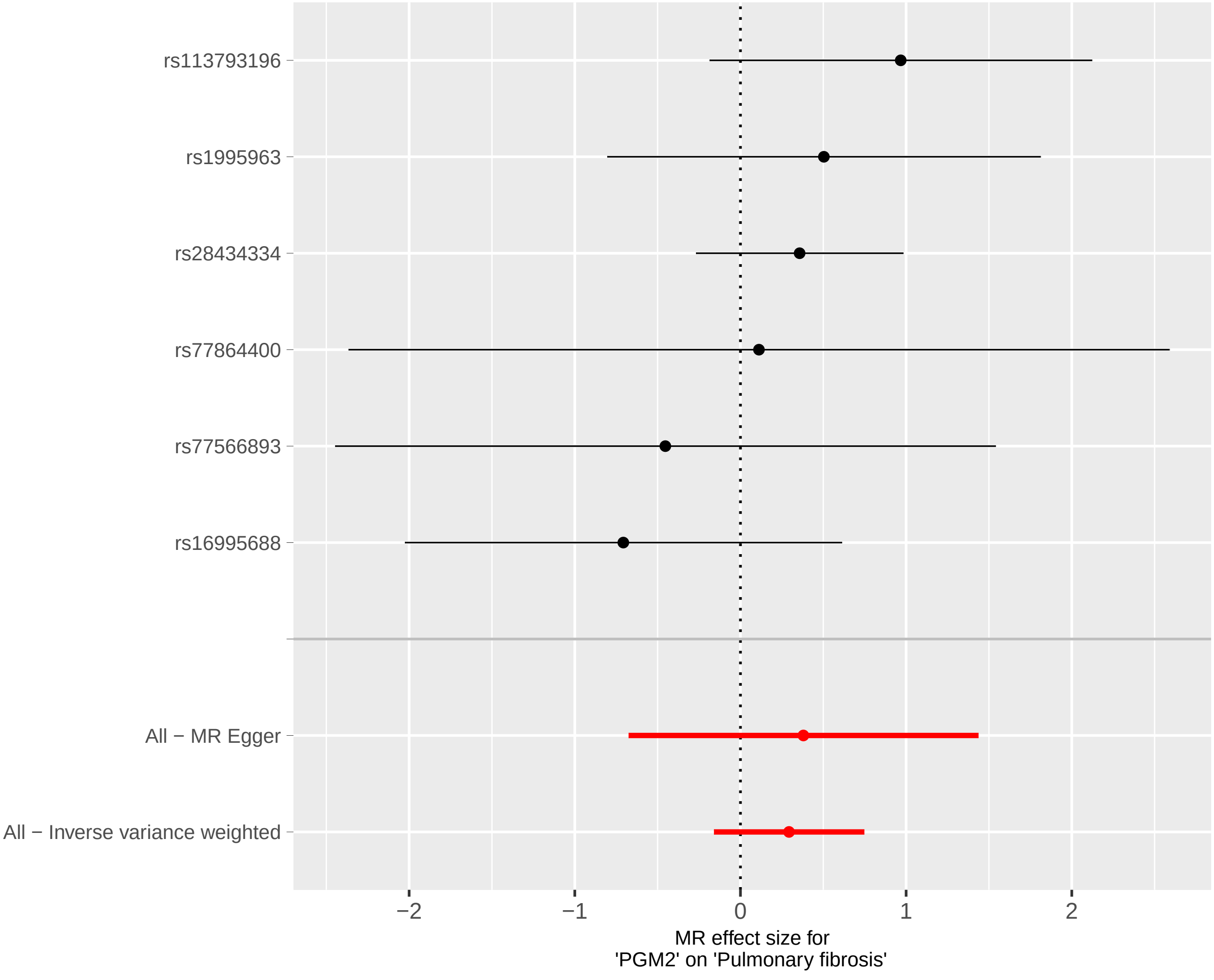

rs113793196
rs1995963
rs28434334
rs77864400
rs77566893
rs16995688
All − MR Egger
All − Inverse variance weighted
−2
−1
0
1
2
MR effect size for
'PGM2' on 'Pulmonary fibrosis'

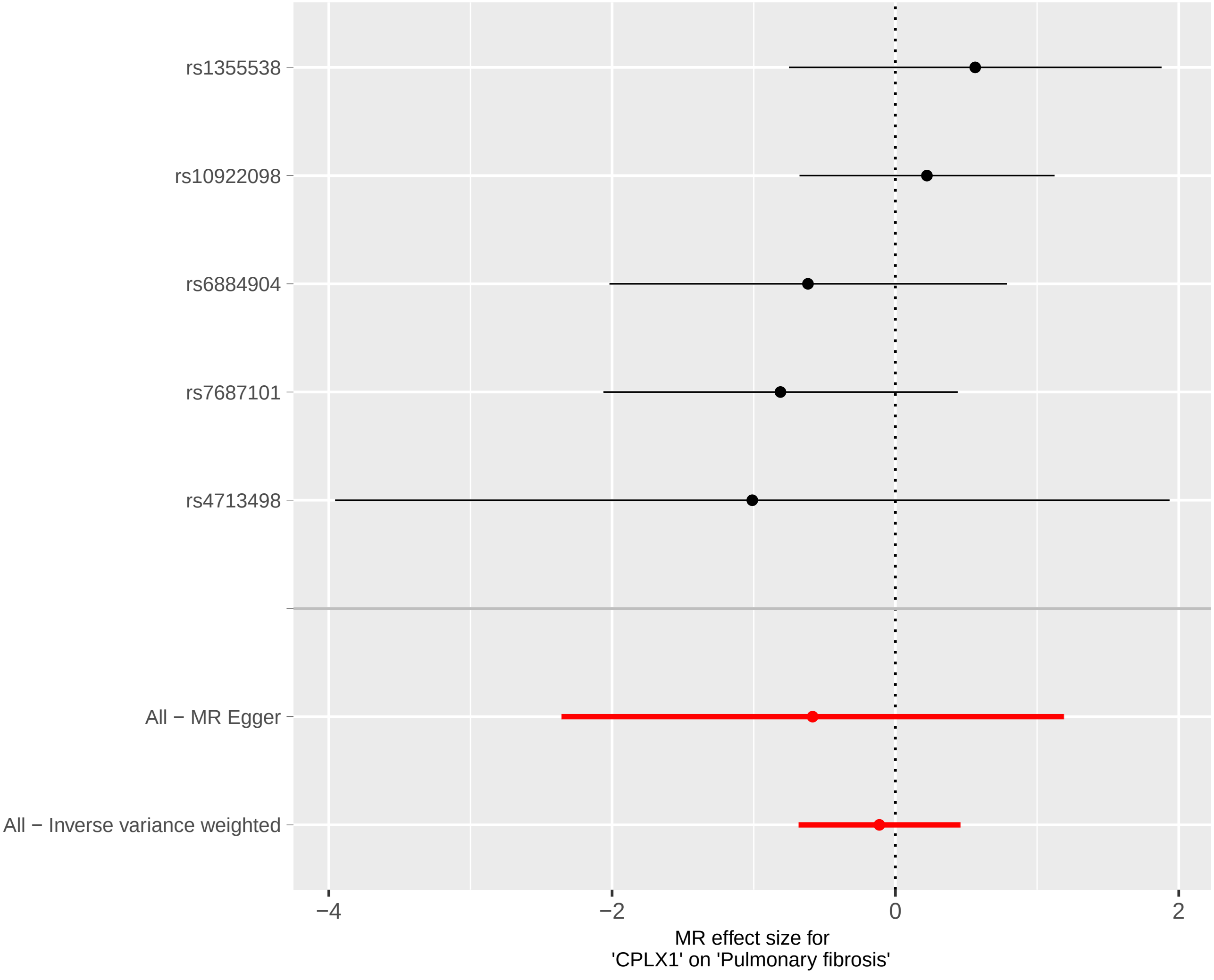

rs1355538
rs10922098
rs6884904
rs7687101
rs4713498
All − MR Egger
All − Inverse variance weighted
−4
−2
0
2
MR effect size for
'CPLX1' on 'Pulmonary fibrosis'

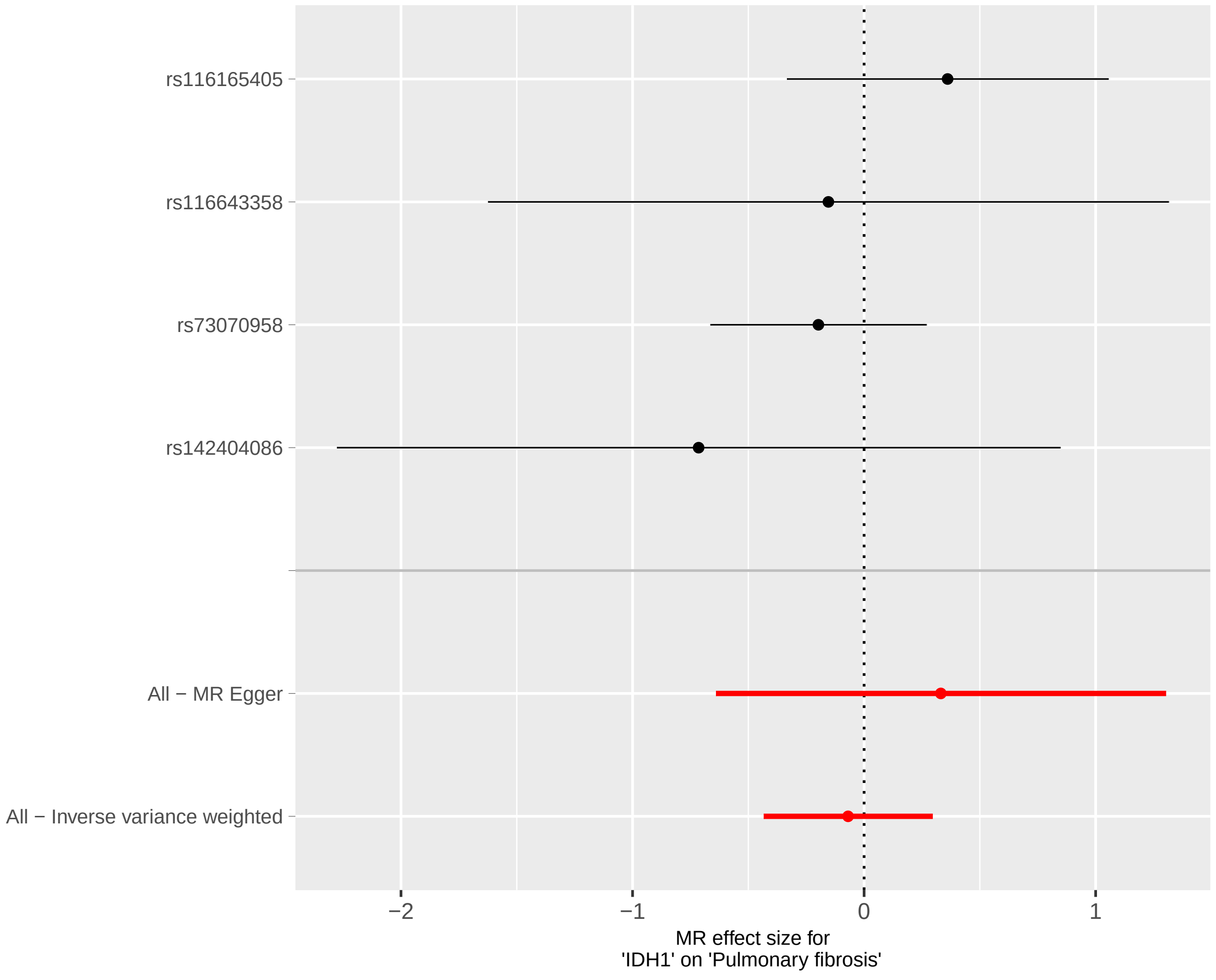
rs116165405
rs116643358
rs73070958
rs142404086
All − MR Egger
All − Inverse variance weighted
−2
−1
0
1
MR effect size for
'IDH1' on 'Pulmonary fibrosis'

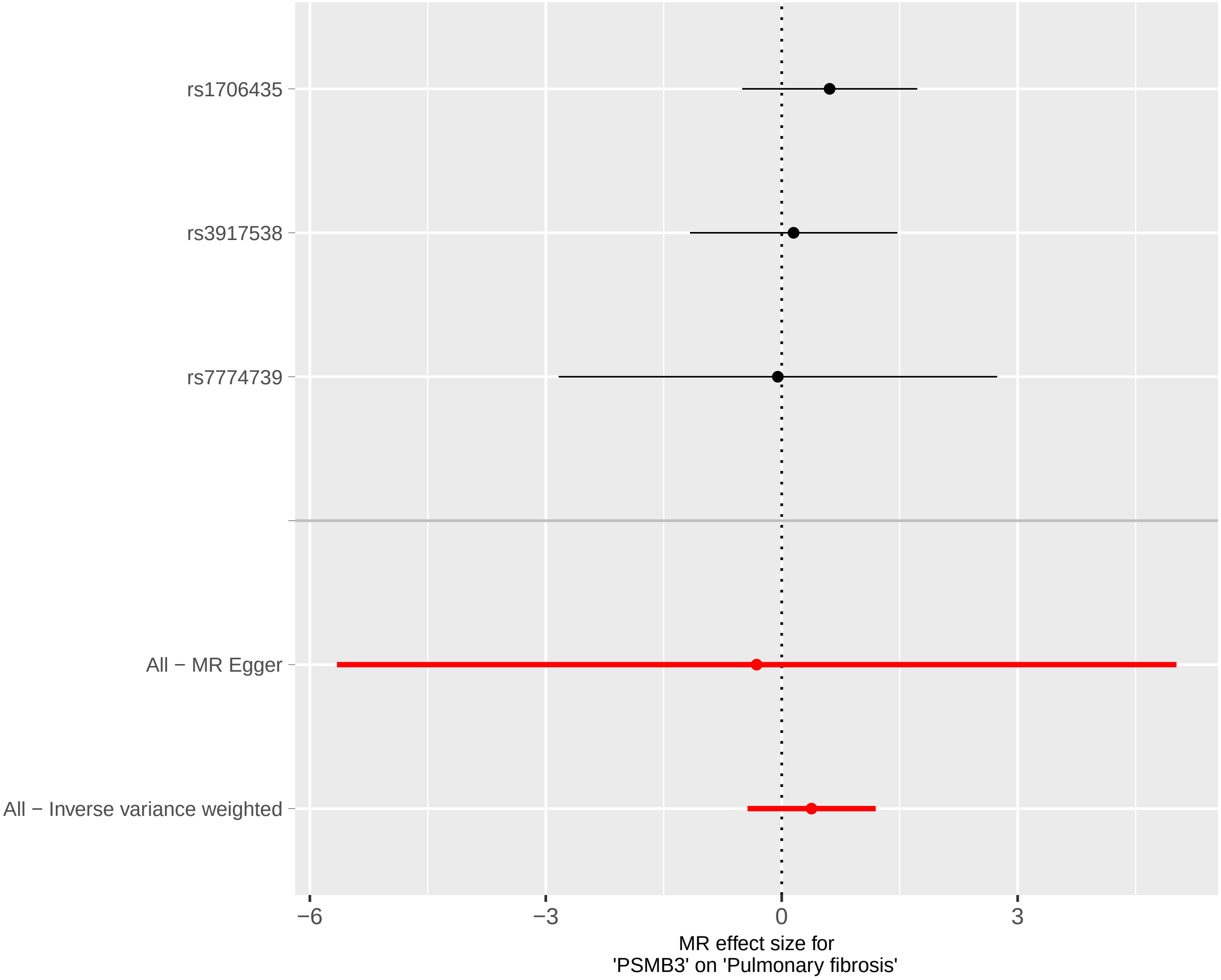

rs1706435
rs3917538
rs7774739
All − MR Egger
All − Inverse variance weighted
−6
−3
0
3
MR effect size for
'PSMB3' on 'Pulmonary fibrosis'

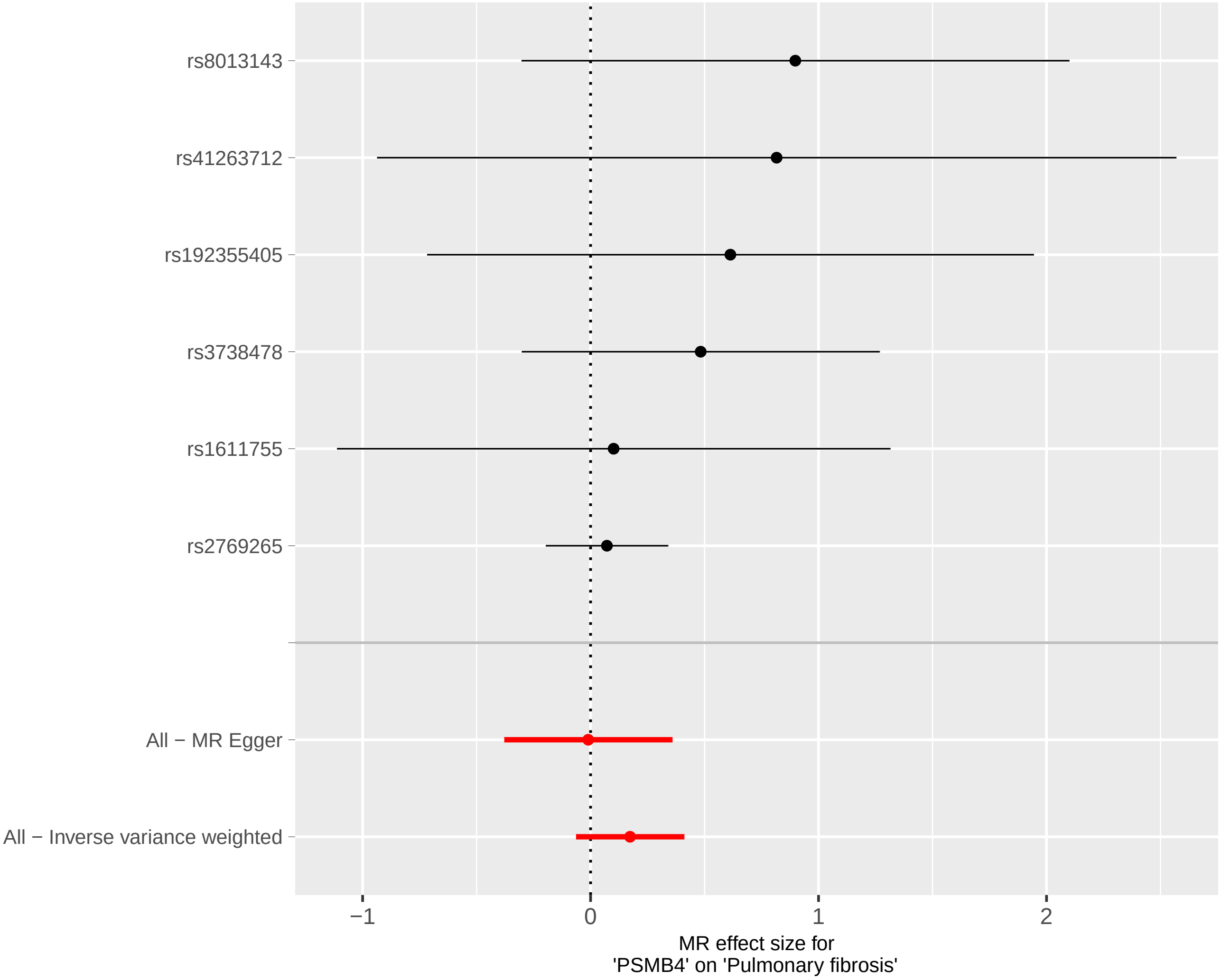

rs8013143
rs41263712
rs192355405
rs3738478
rs1611755
rs2769265
All − MR Egger
All − Inverse variance weighted
−1
0
1
2
MR effect size for
'PSMB4' on 'Pulmonary fibrosis'

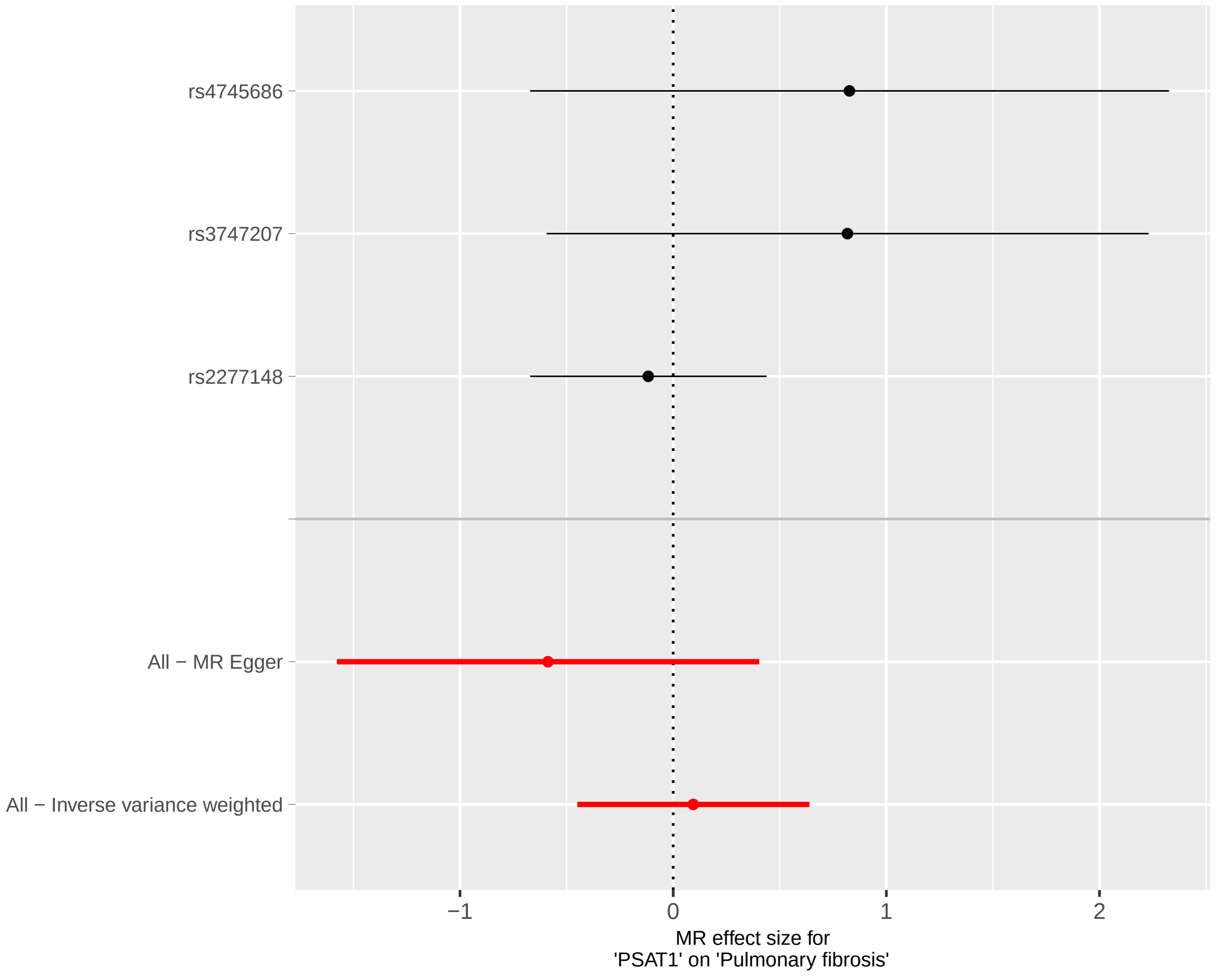
rs4745686
rs3747207
rs2277148
All − MR Egger
All − Inverse variance weighted
−1
0
1
2
MR effect size for
'PSAT1' on 'Pulmonary fibrosis'

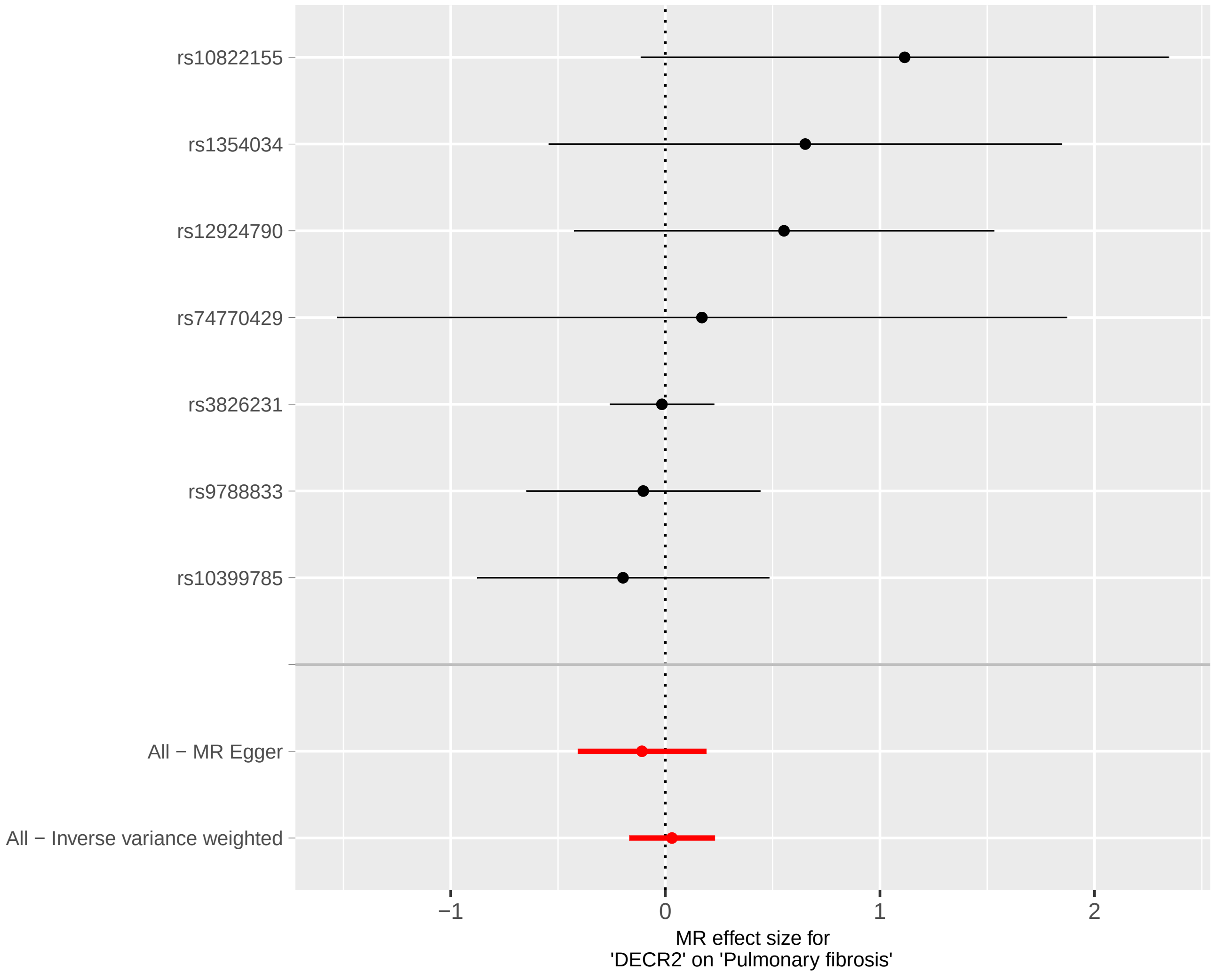
rs10822155
rs1354034
rs12924790
rs74770429
rs3826231
rs9788833
rs10399785
All − MR Egger
All − Inverse variance weighted
−1
0
1
2
MR effect size for
'DECR2' on 'Pulmonary fibrosis'

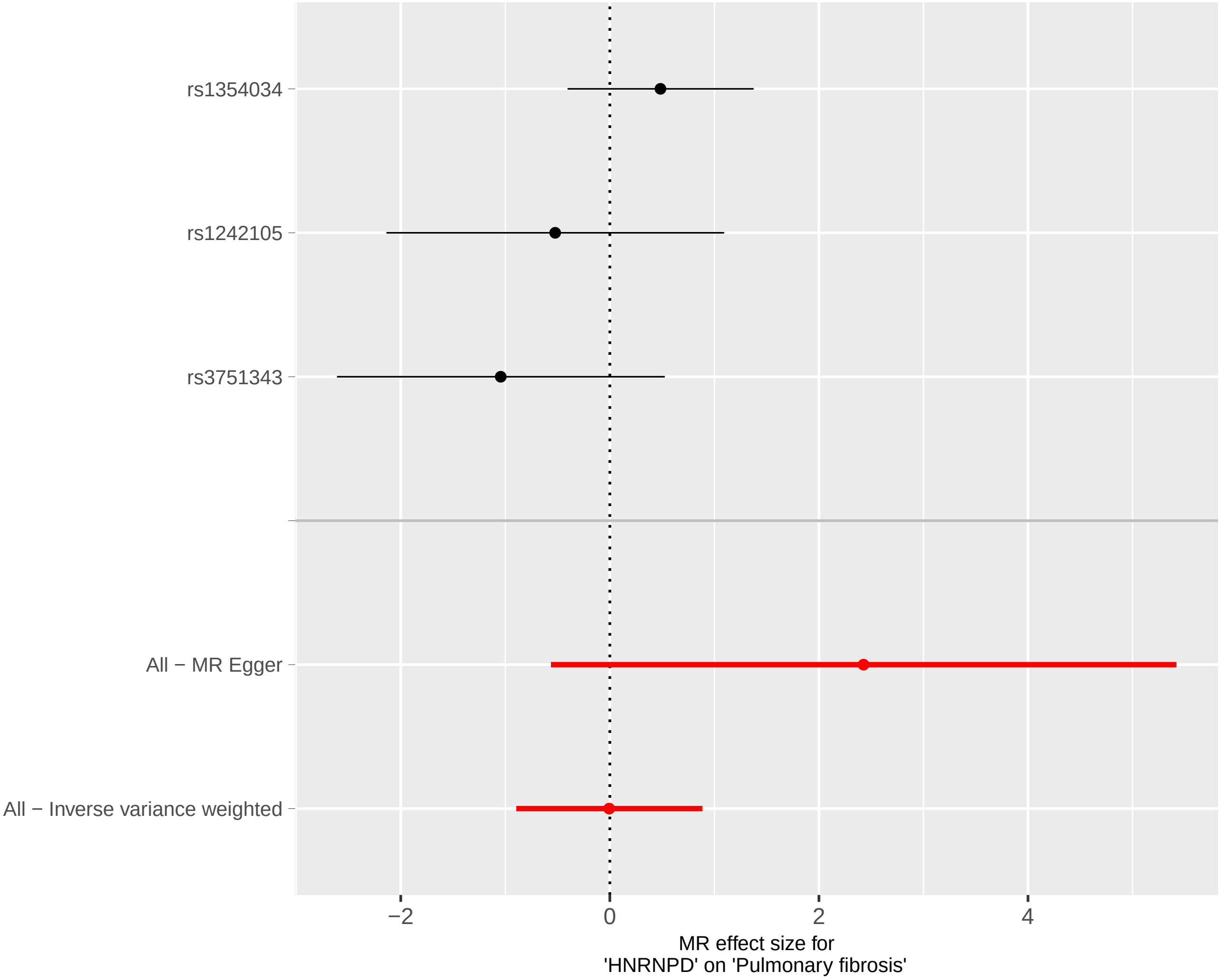
rs1354034
rs1242105
rs3751343
All − MR Egger
All − Inverse variance weighted
−2
0
2
4
MR effect size for
'HNRNPD' on 'Pulmonary fibrosis'

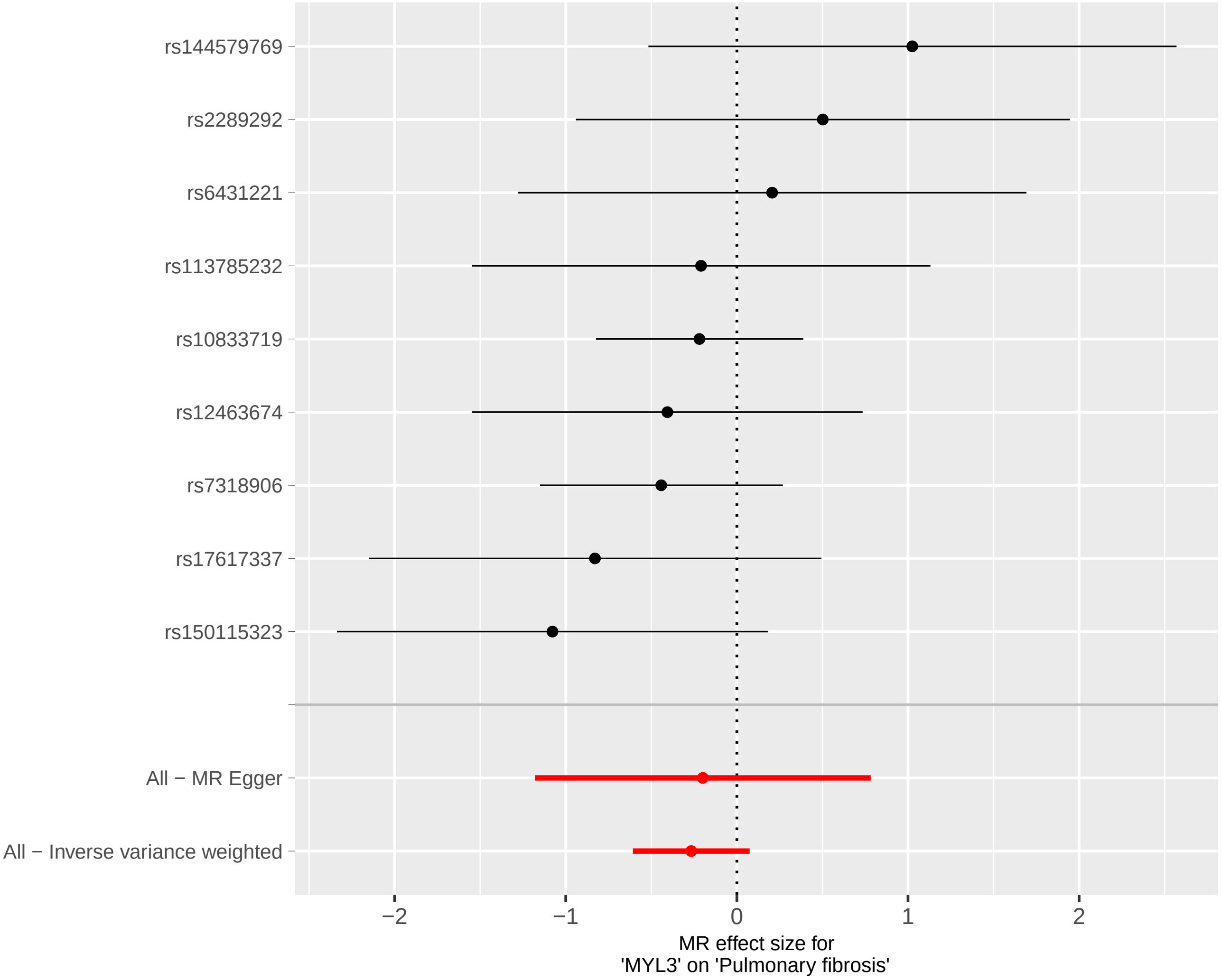
rs144579769
rs2289292
rs6431221
rs113785232
rs10833719
rs12463674
rs7318906
rs17617337
rs150115323
All − MR Egger
All − Inverse variance weighted
−2
−1
0
1
2
MR effect size for
'MYL3' on 'Pulmonary fibrosis'

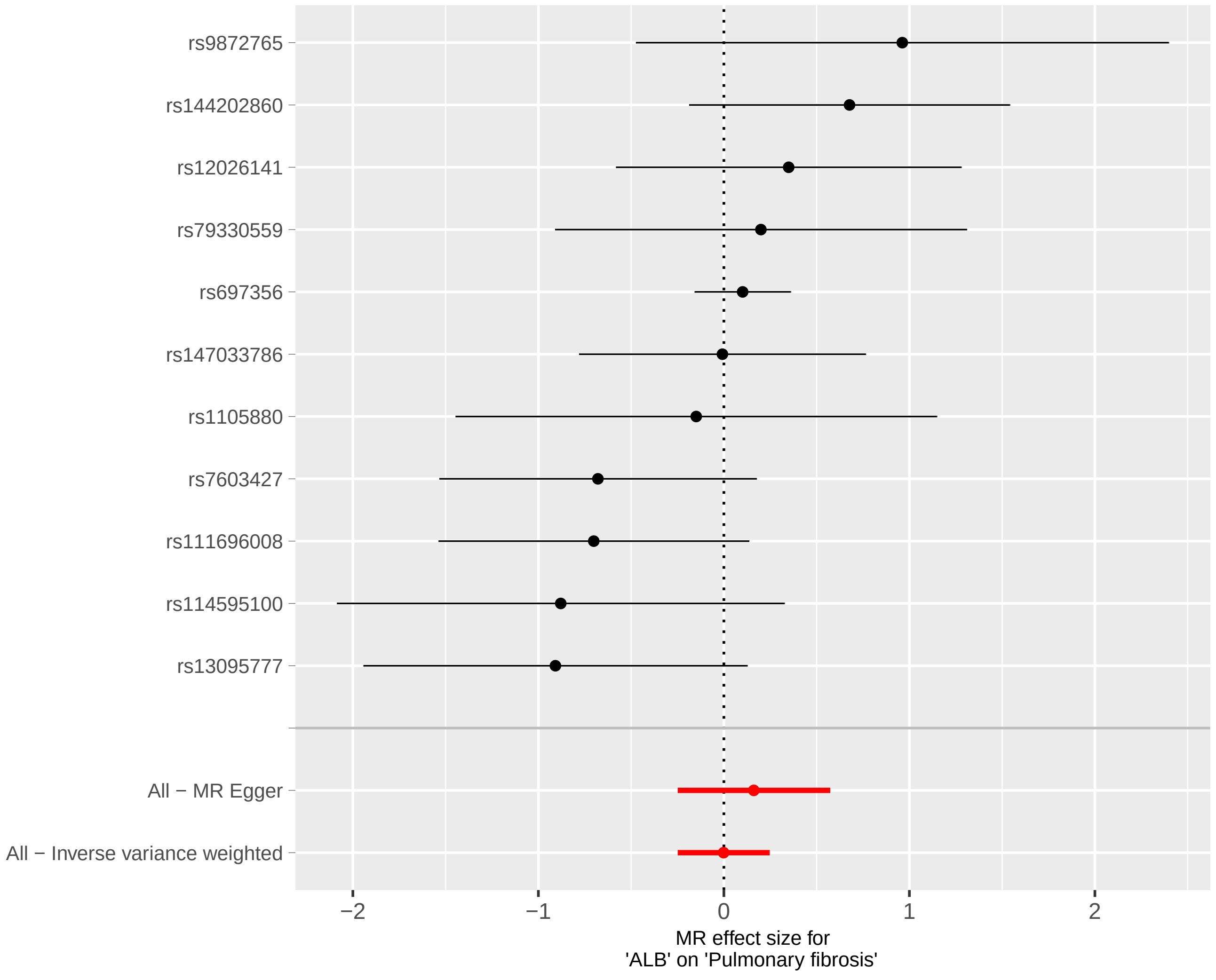
rs9872765
rs144202860
rs12026141
rs79330559
rs697356
rs147033786
rs1105880
rs7603427
rs111696008
rs114595100
rs13095777
All − MR Egger
All − Inverse variance weighted
−2
−1
0
1
2
MR effect size for
'ALB' on 'Pulmonary fibrosis'

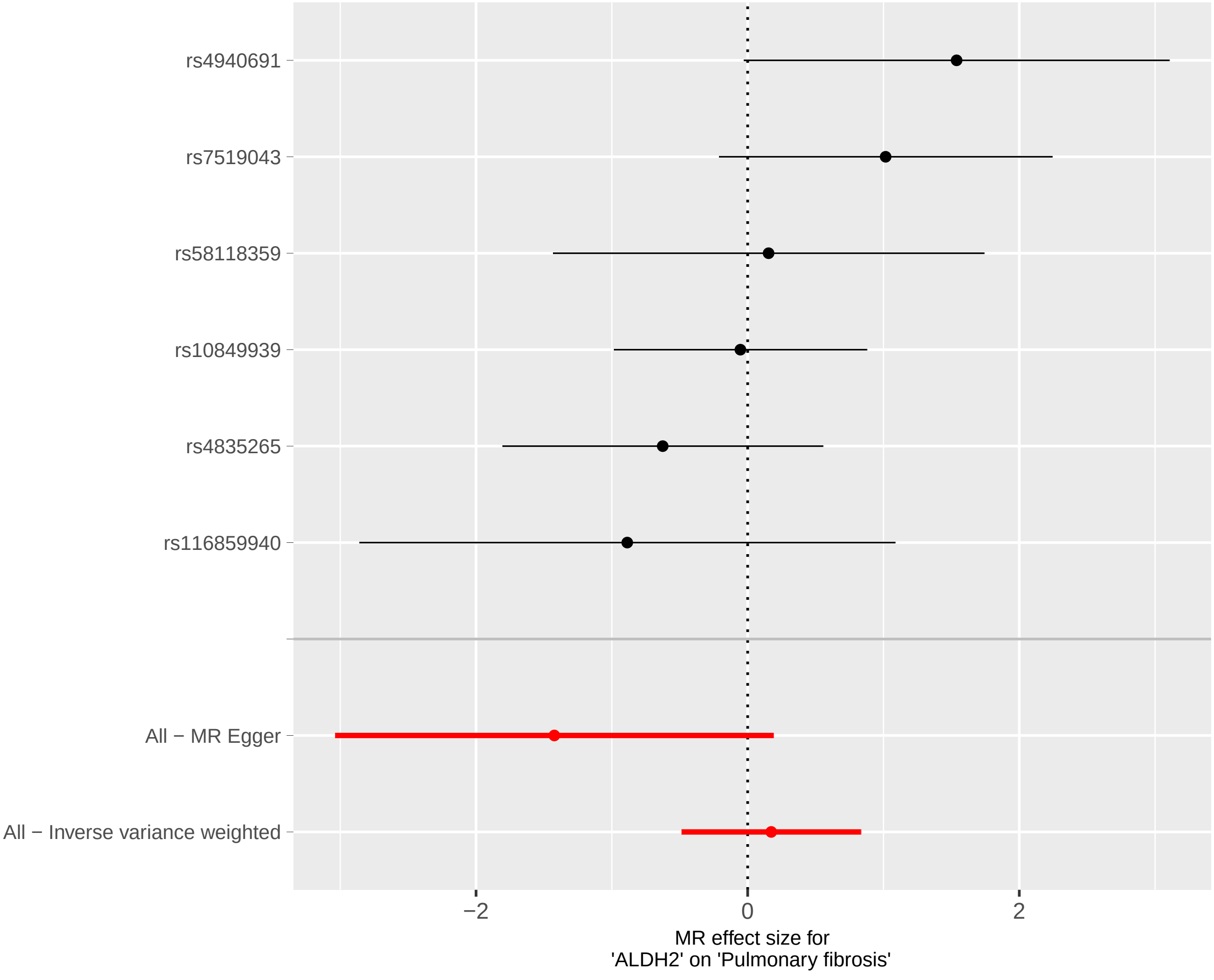

rs4940691
rs7519043
rs58118359
rs10849939
rs4835265
rs116859940
All − MR Egger
All − Inverse variance weighted
−2
0
2
MR effect size for
'ALDH2' on 'Pulmonary fibrosis'

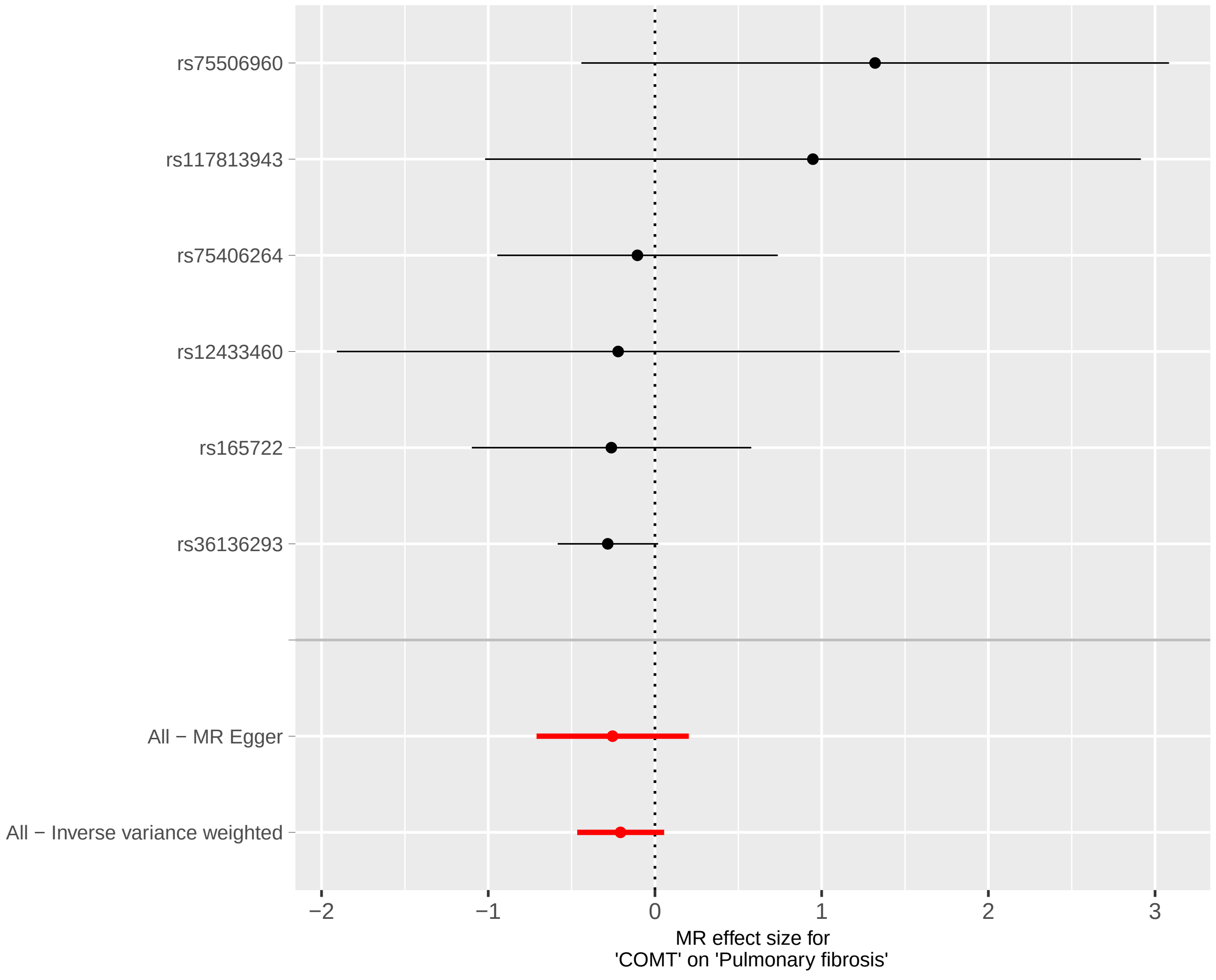
rs75506960
rs117813943
rs75406264
rs12433460
rs165722
rs36136293
All − MR Egger
All − Inverse variance weighted
−2
−1
0
1
2
3
MR effect size for
'COMT' on 'Pulmonary fibrosis'

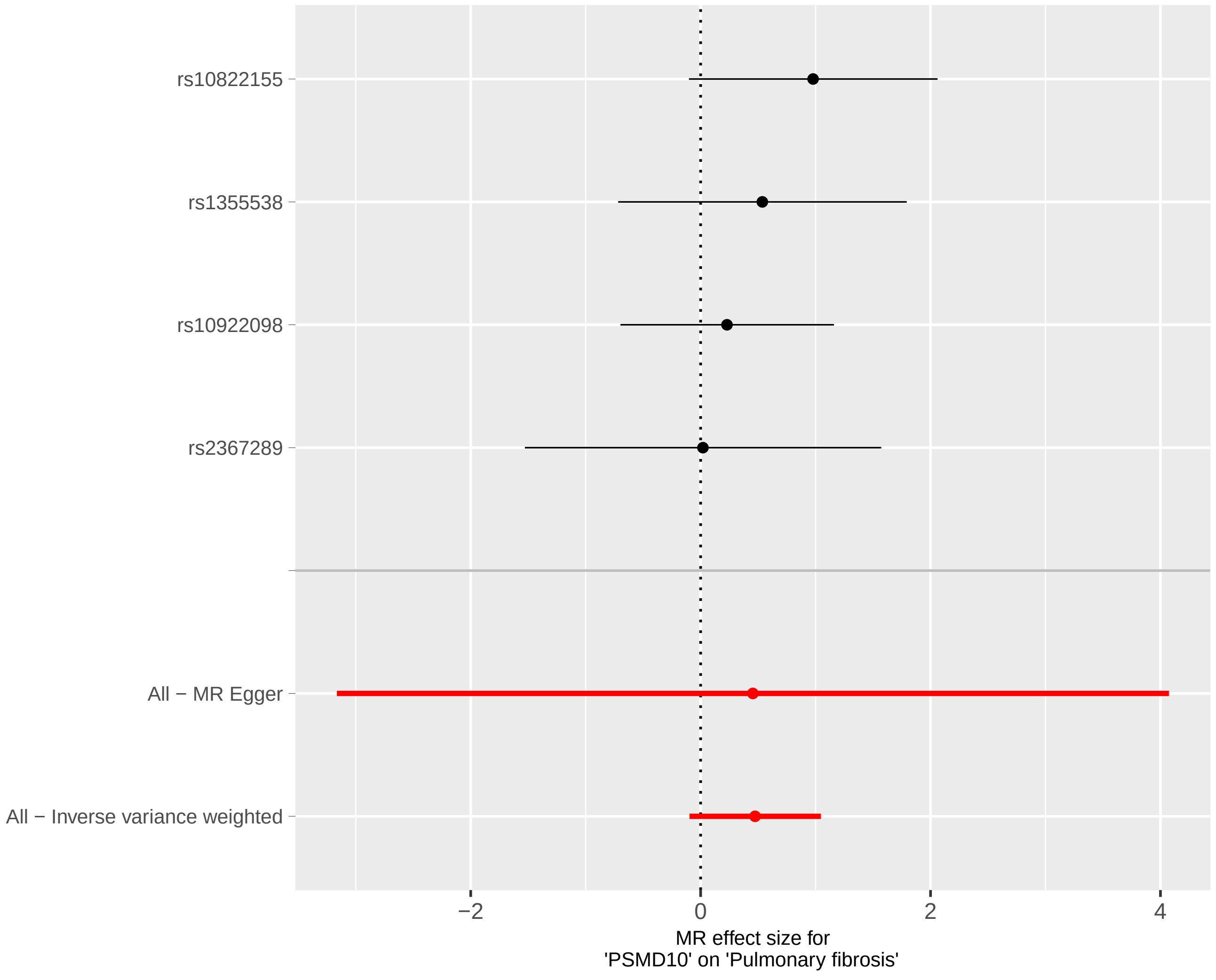
rs10822155
rs1355538
rs10922098
rs2367289
All − MR Egger
All − Inverse variance weighted
−2
0
2
4
MR effect size for
'PSMD10' on 'Pulmonary fibrosis'

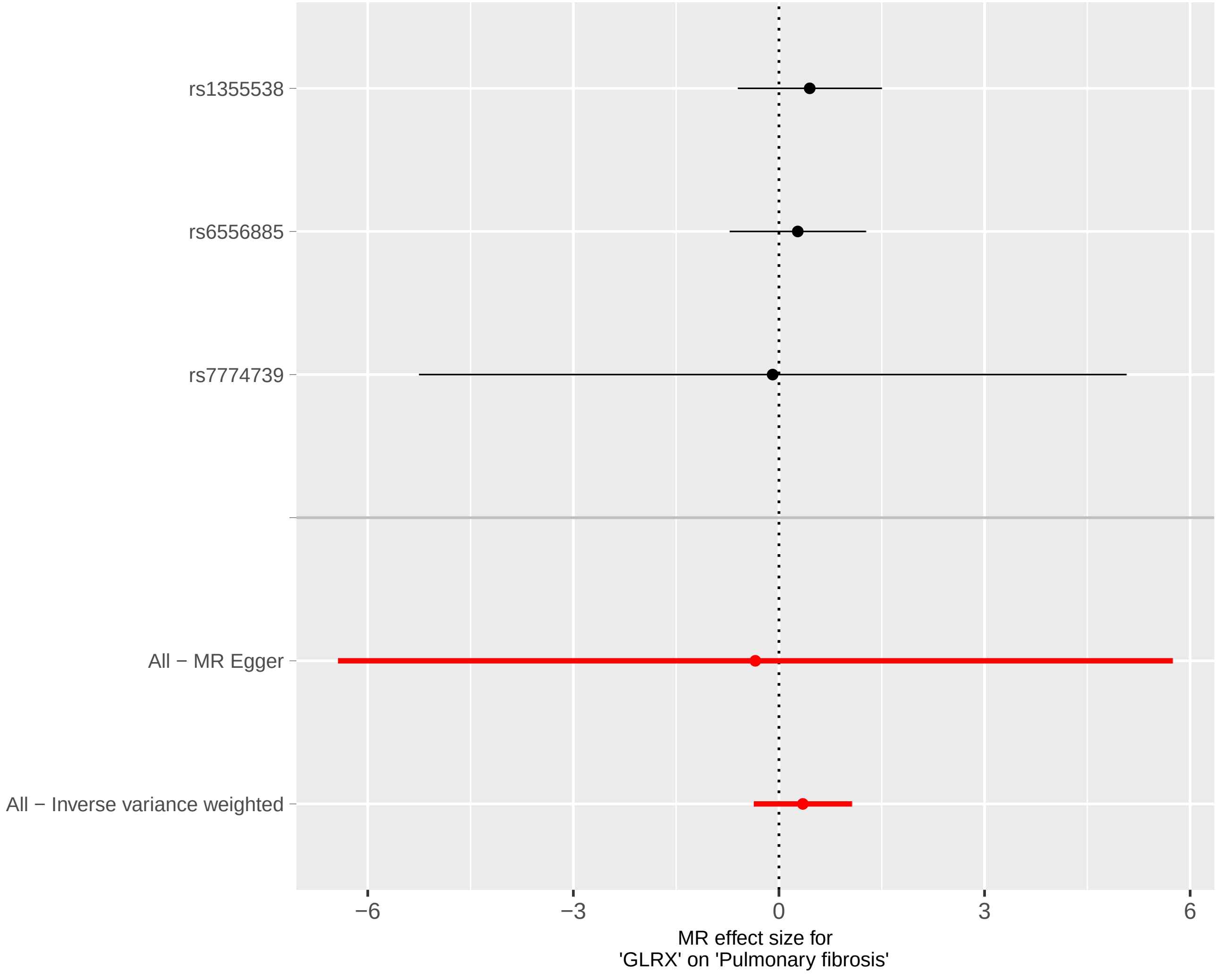
rs1355538
rs6556885
rs7774739
All − MR Egger
All − Inverse variance weighted
−6
−3
0
3
6
MR effect size for
'GLRX' on 'Pulmonary fibrosis'

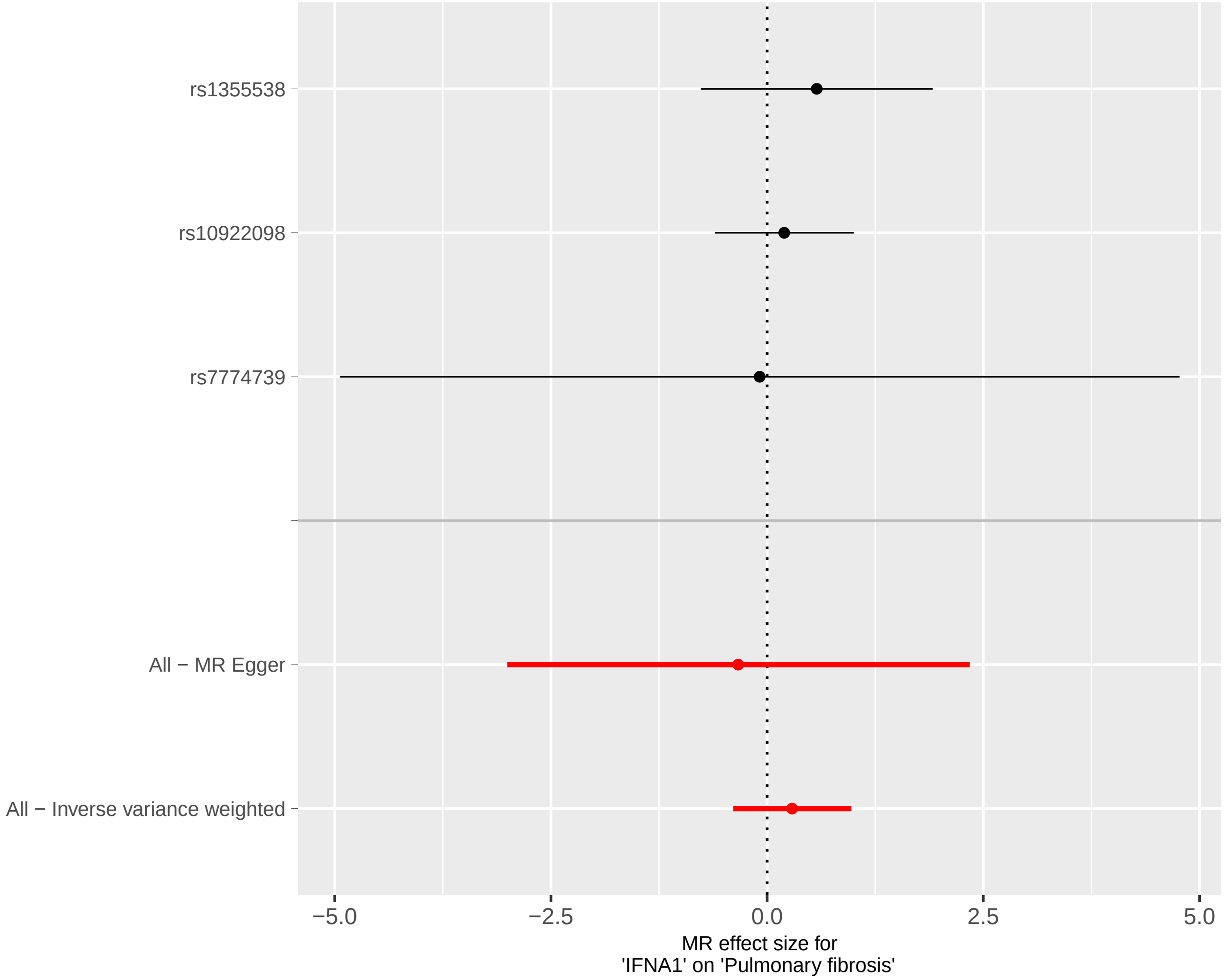
rs1355538
rs10922098
rs7774739
All − MR Egger
All − Inverse variance weighted
−5.0
−2.5
0.0
2.5
5.0
MR effect size for
'IFNA1' on 'Pulmonary fibrosis'

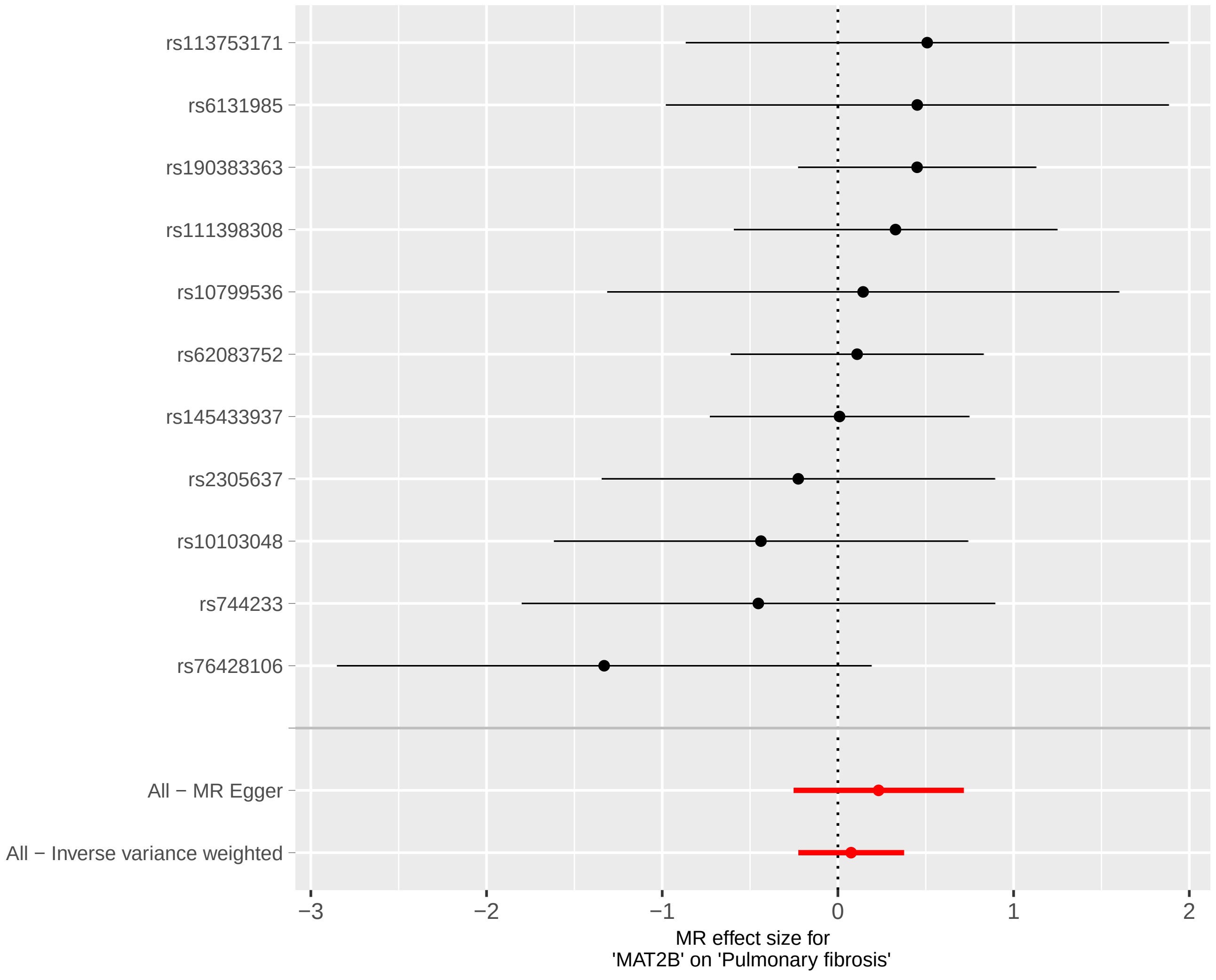

rs113753171
rs6131985
rs190383363
rs111398308
rs10799536
rs62083752
rs145433937
rs2305637
rs10103048
rs744233
rs76428106
All − MR Egger
All − Inverse variance weighted
−3
−2
−1
0
1
2
MR effect size for
'MAT2B' on 'Pulmonary fibrosis'

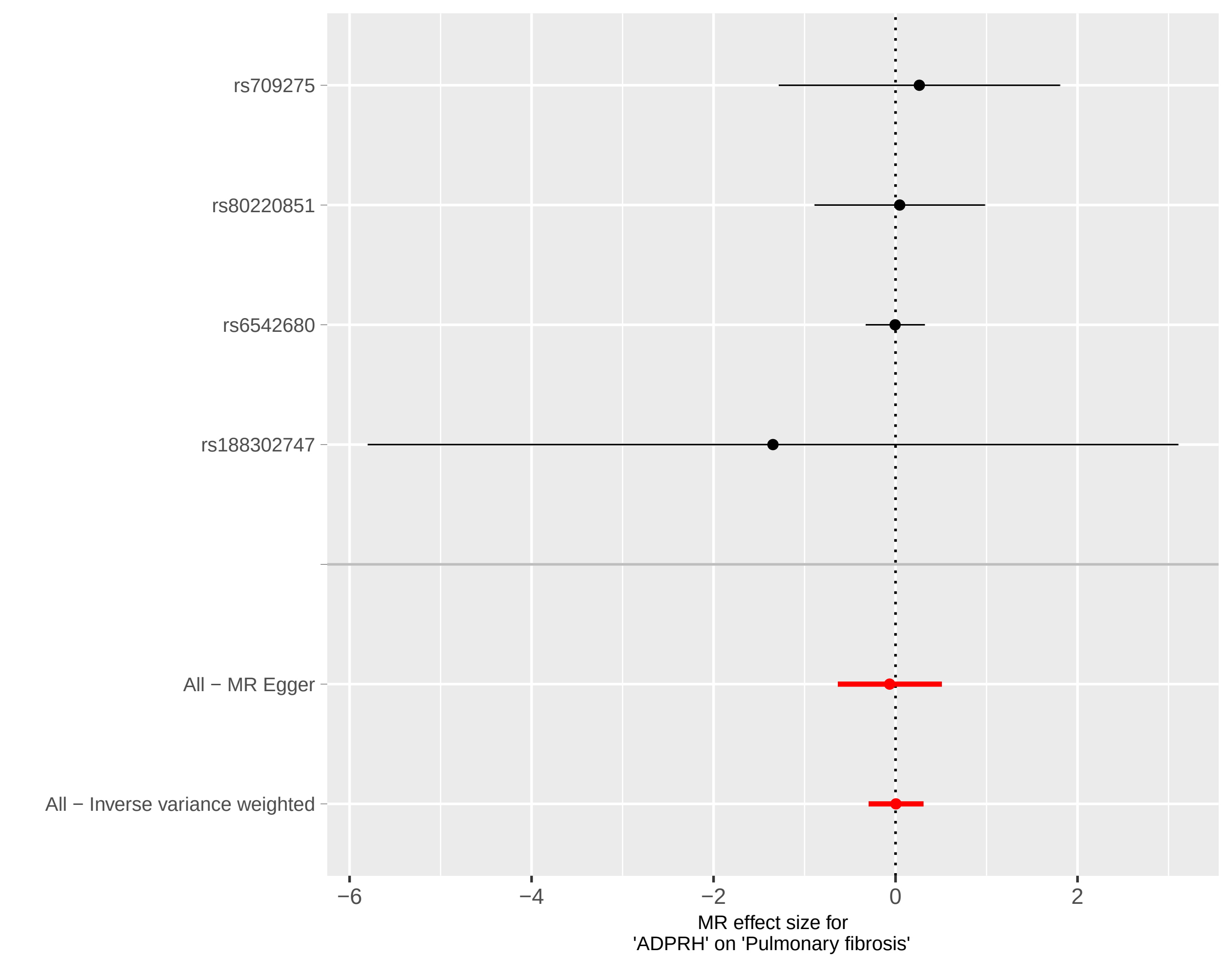
rs709275
rs80220851
rs6542680
rs188302747
All − MR Egger
All − Inverse variance weighted
−6
−4
−2
0
2
MR effect size for
'ADPRH' on 'Pulmonary fibrosis'

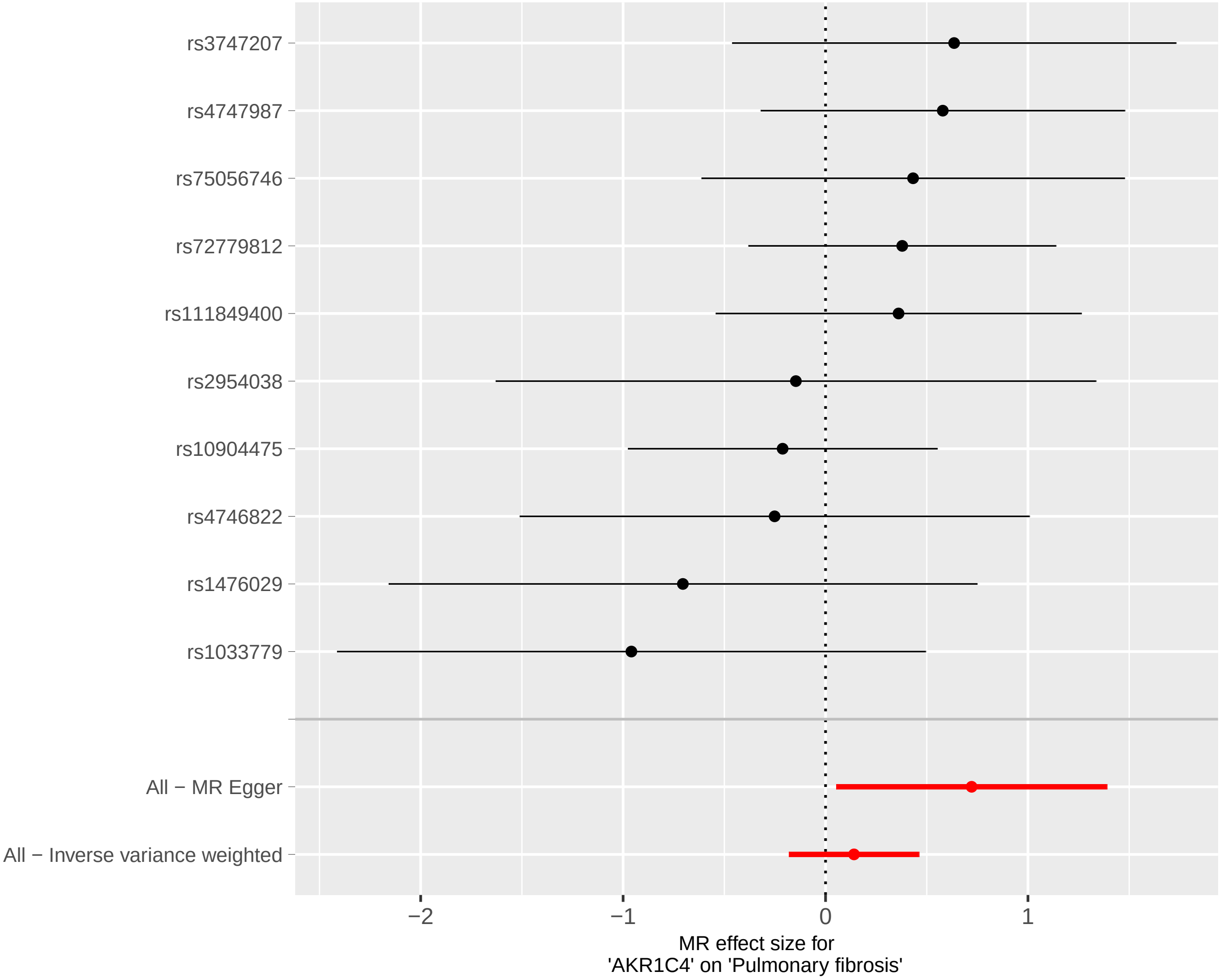
rs3747207
rs4747987
rs75056746
rs72779812
rs111849400
rs2954038
rs10904475
rs4746822
rs1476029
rs1033779
All − MR Egger
All − Inverse variance weighted
−2
−1
0
1
MR effect size for
'AKR1C4' on 'Pulmonary fibrosis'

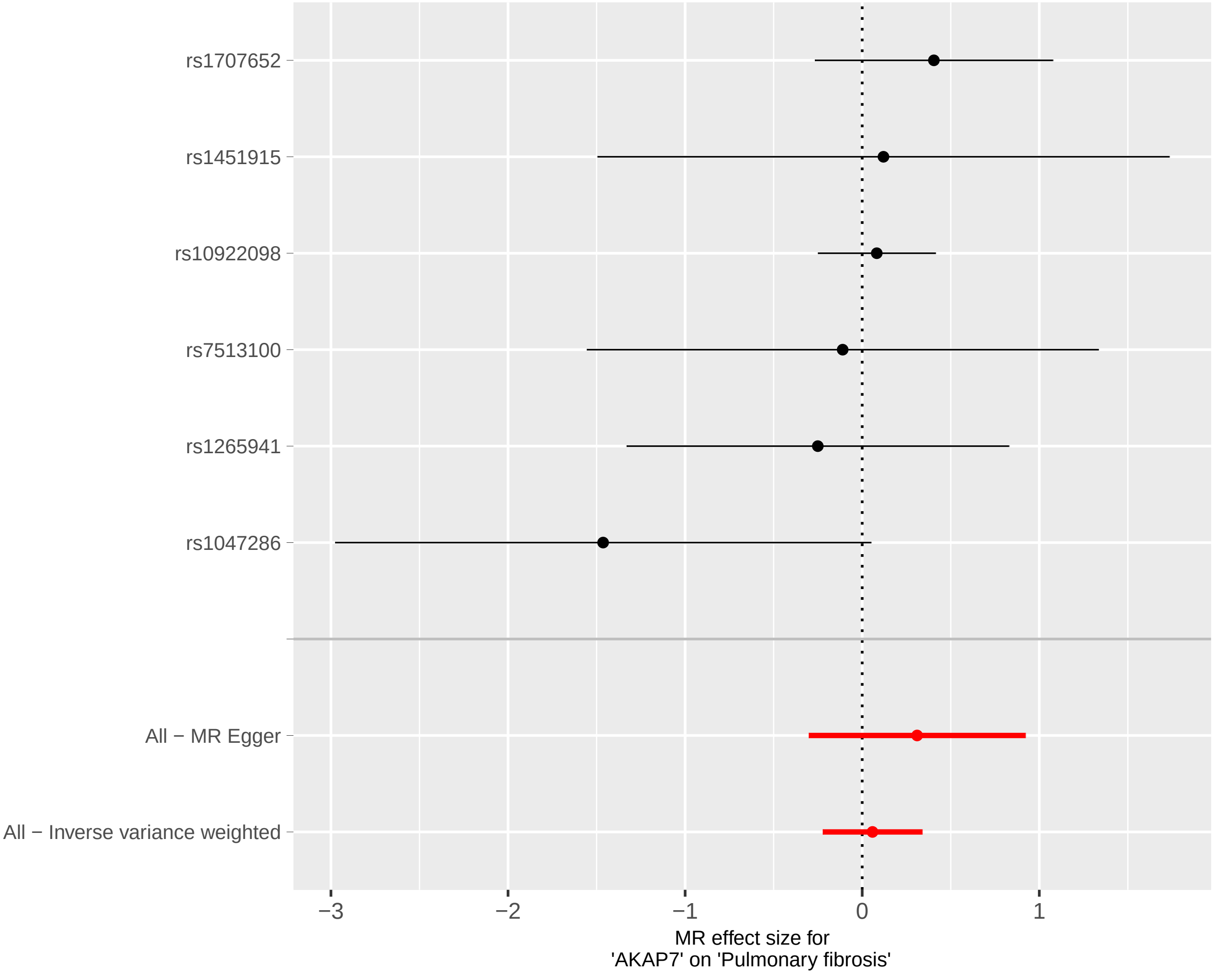

rs1707652
rs1451915
rs10922098
rs7513100
rs1265941
rs1047286
All − MR Egger
All − Inverse variance weighted
−3
−2
−1
0
1
MR effect size for
'AKAP7' on 'Pulmonary fibrosis'

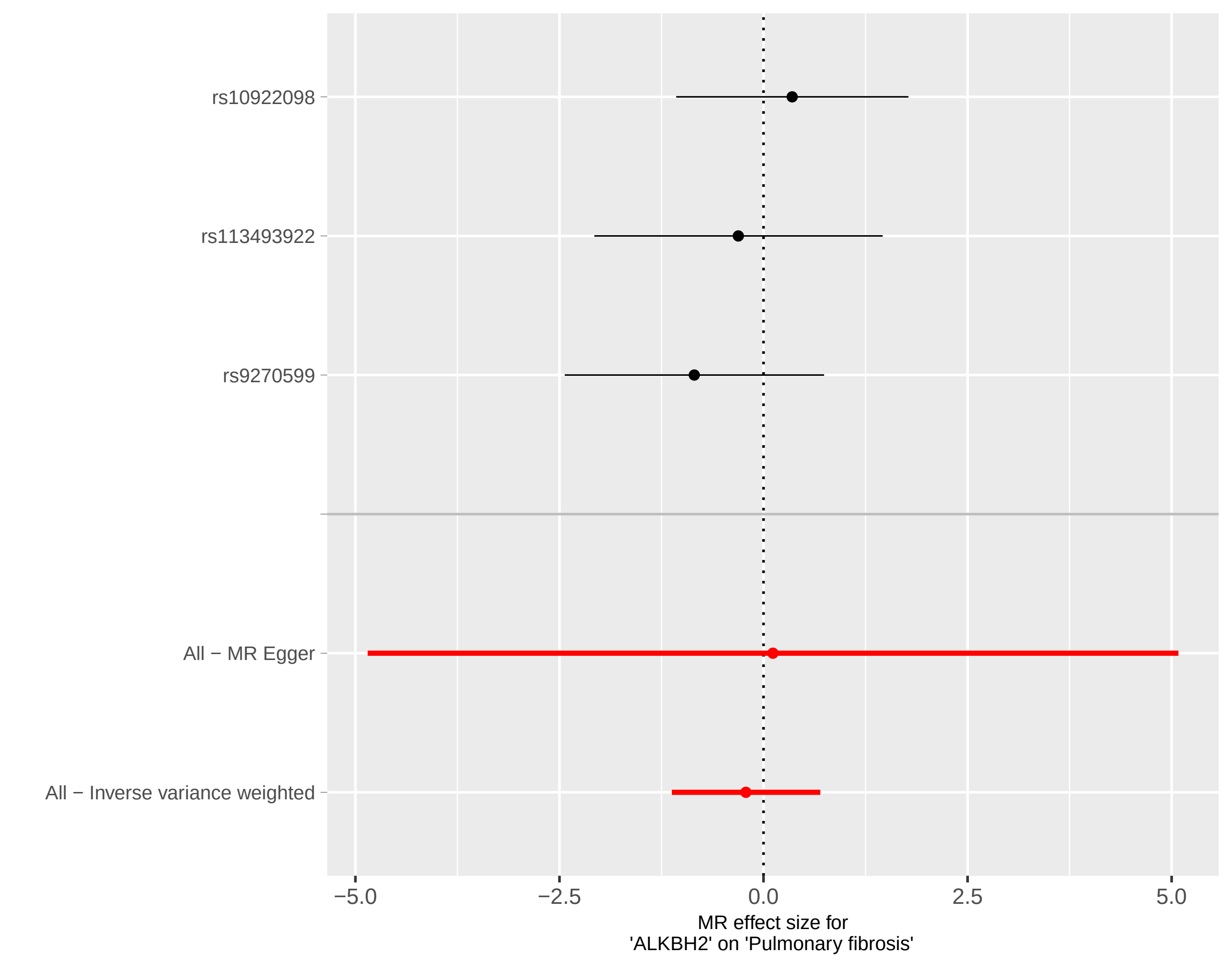

rs10922098
rs113493922
rs9270599
All − MR Egger
All − Inverse variance weighted
−5.0
−2.5
0.0
2.5
5.0
MR effect size for
'ALKBH2' on 'Pulmonary fibrosis'

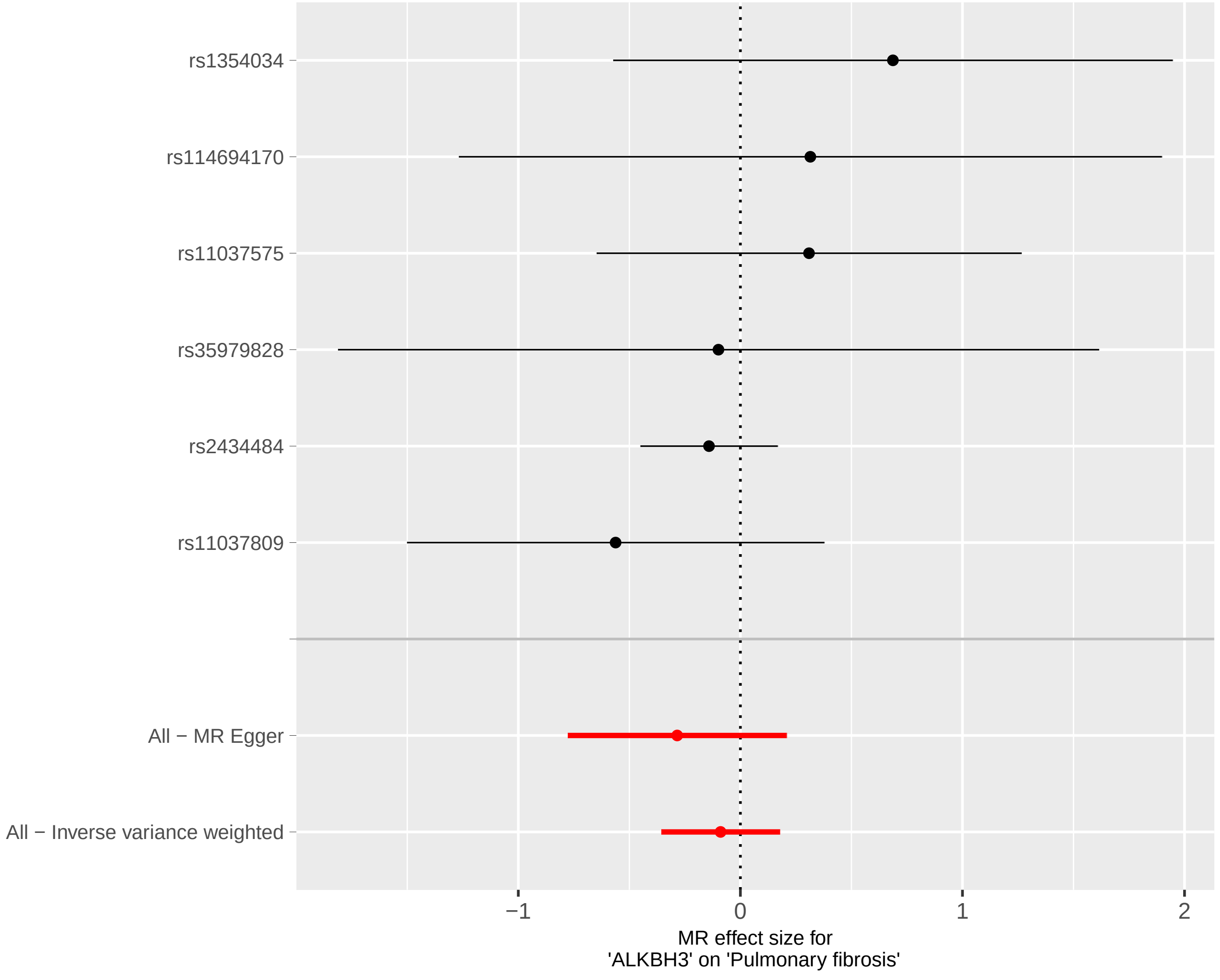

rs1354034
rs114694170
rs11037575
rs35979828
rs2434484
rs11037809
All − MR Egger
All − Inverse variance weighted
−1
0
1
2
MR effect size for
'ALKBH3' on 'Pulmonary fibrosis'

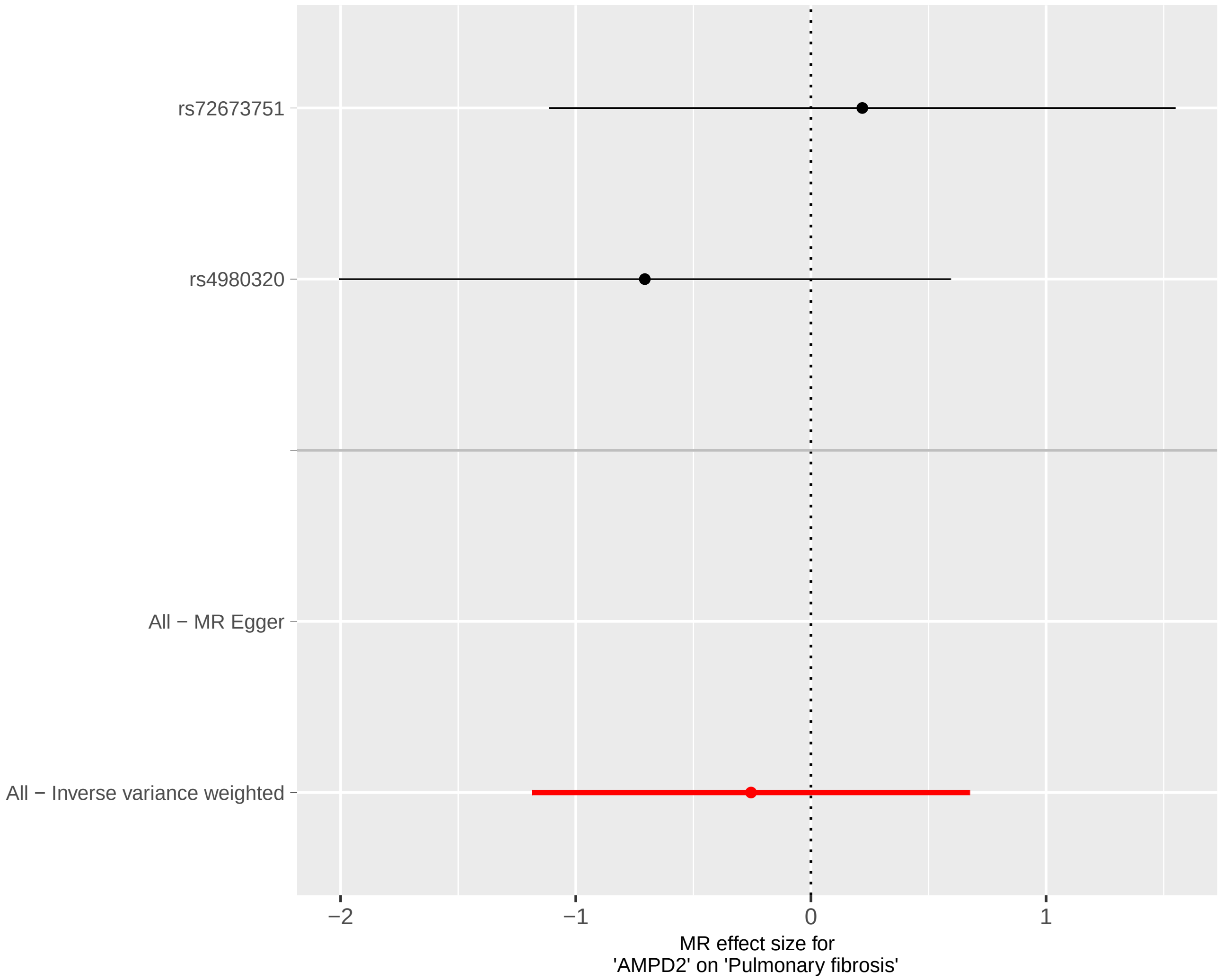

rs72673751
rs4980320
All − MR Egger
All − Inverse variance weighted
−2
−1
0
1
MR effect size for
'AMPD2' on 'Pulmonary fibrosis'

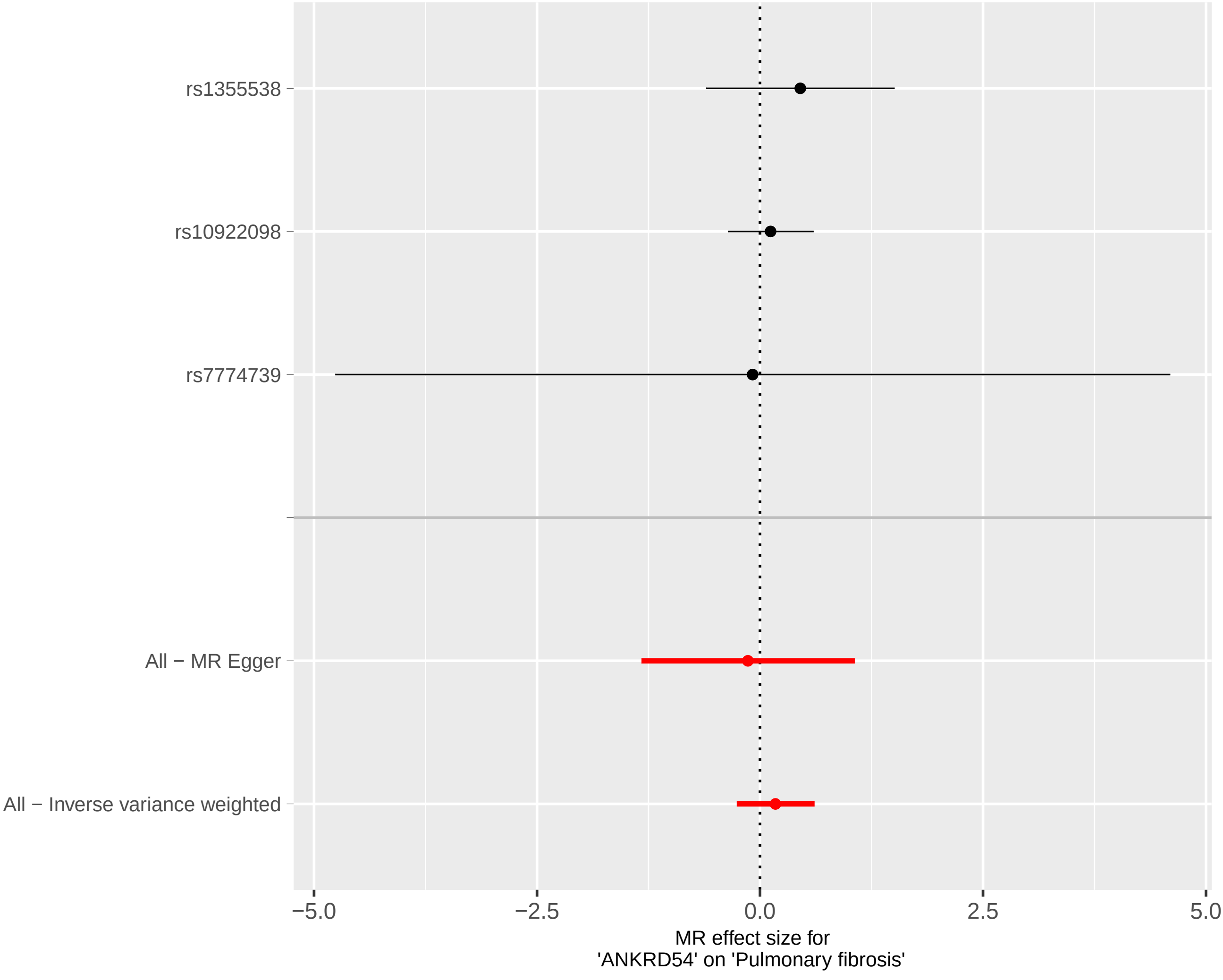
rs1355538
rs10922098
rs7774739
All − MR Egger
All − Inverse variance weighted
−5.0
−2.5
0.0
2.5
5.0
MR effect size for
'ANKRD54' on 'Pulmonary fibrosis'

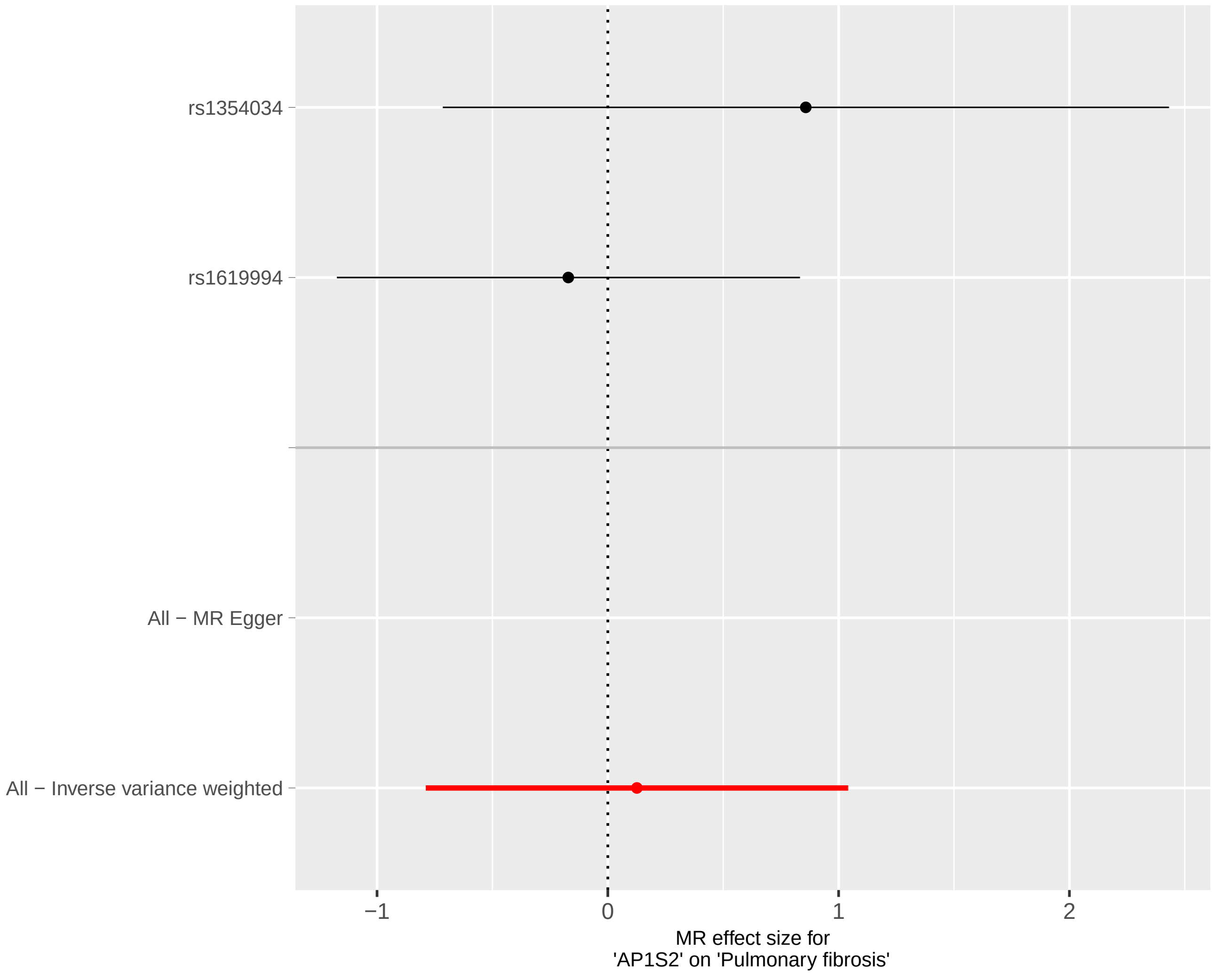
rs1354034
rs1619994
All − MR Egger
All − Inverse variance weighted
−1
0
1
2
MR effect size for
'AP1S2' on 'Pulmonary fibrosis'

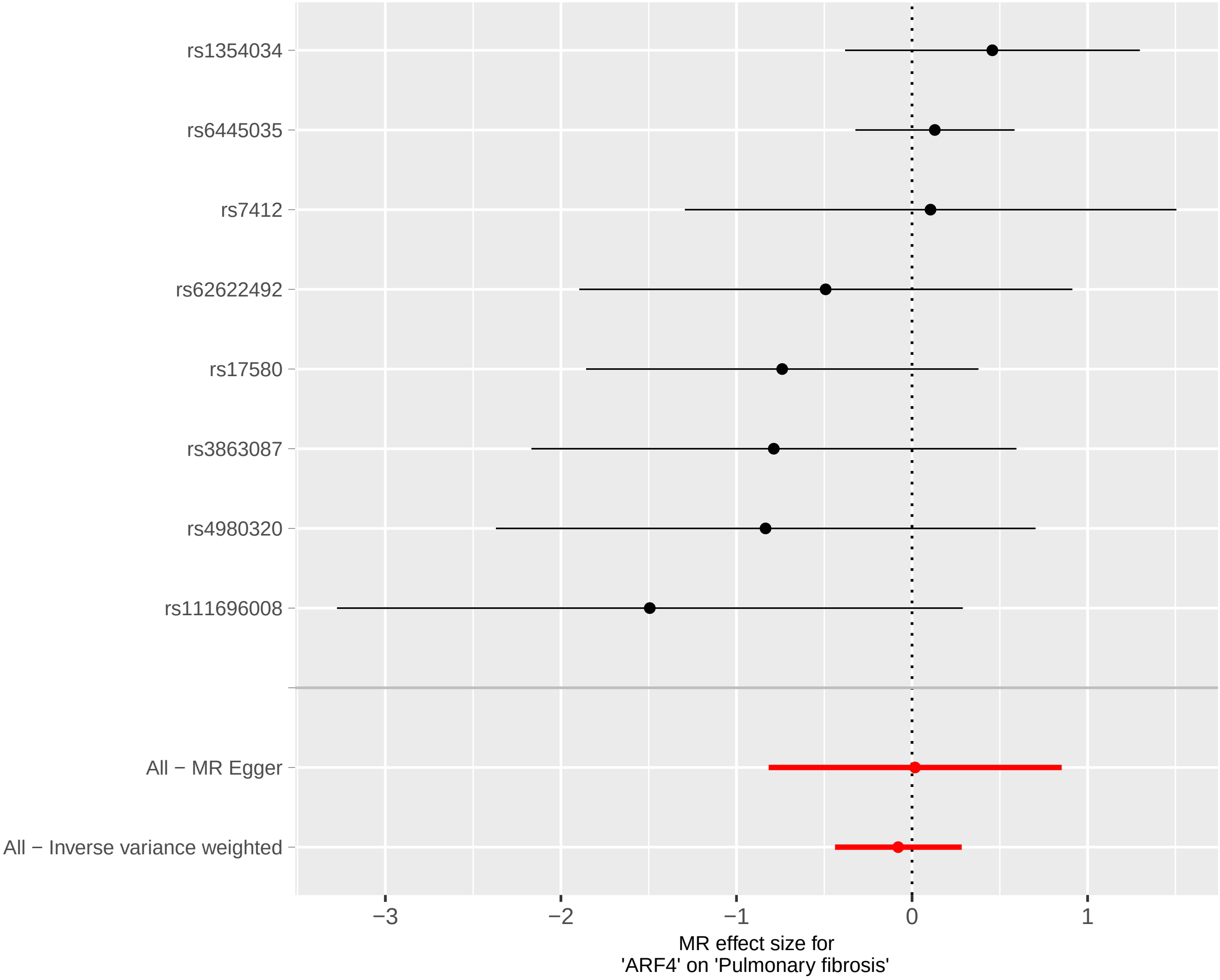

rs1354034
rs6445035
rs7412
rs62622492
rs17580
rs3863087
rs4980320
rs111696008
All − MR Egger
All − Inverse variance weighted
−3
−2
−1
0
1
MR effect size for
'ARF4' on 'Pulmonary fibrosis'

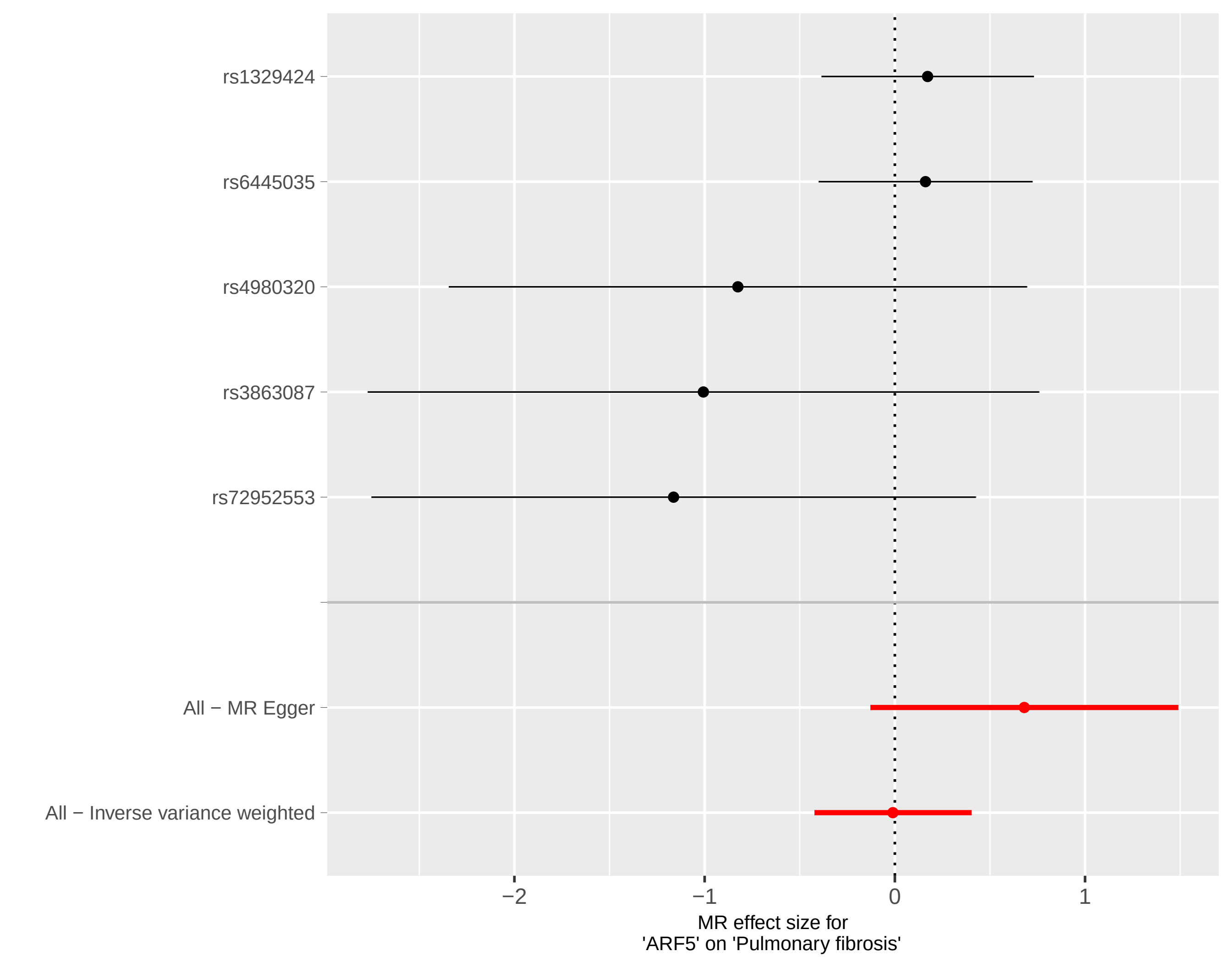

rs1329424
rs6445035
rs4980320
rs3863087
rs72952553
All − MR Egger
All − Inverse variance weighted
−2
−1
0
1
MR effect size for
'ARF5' on 'Pulmonary fibrosis'

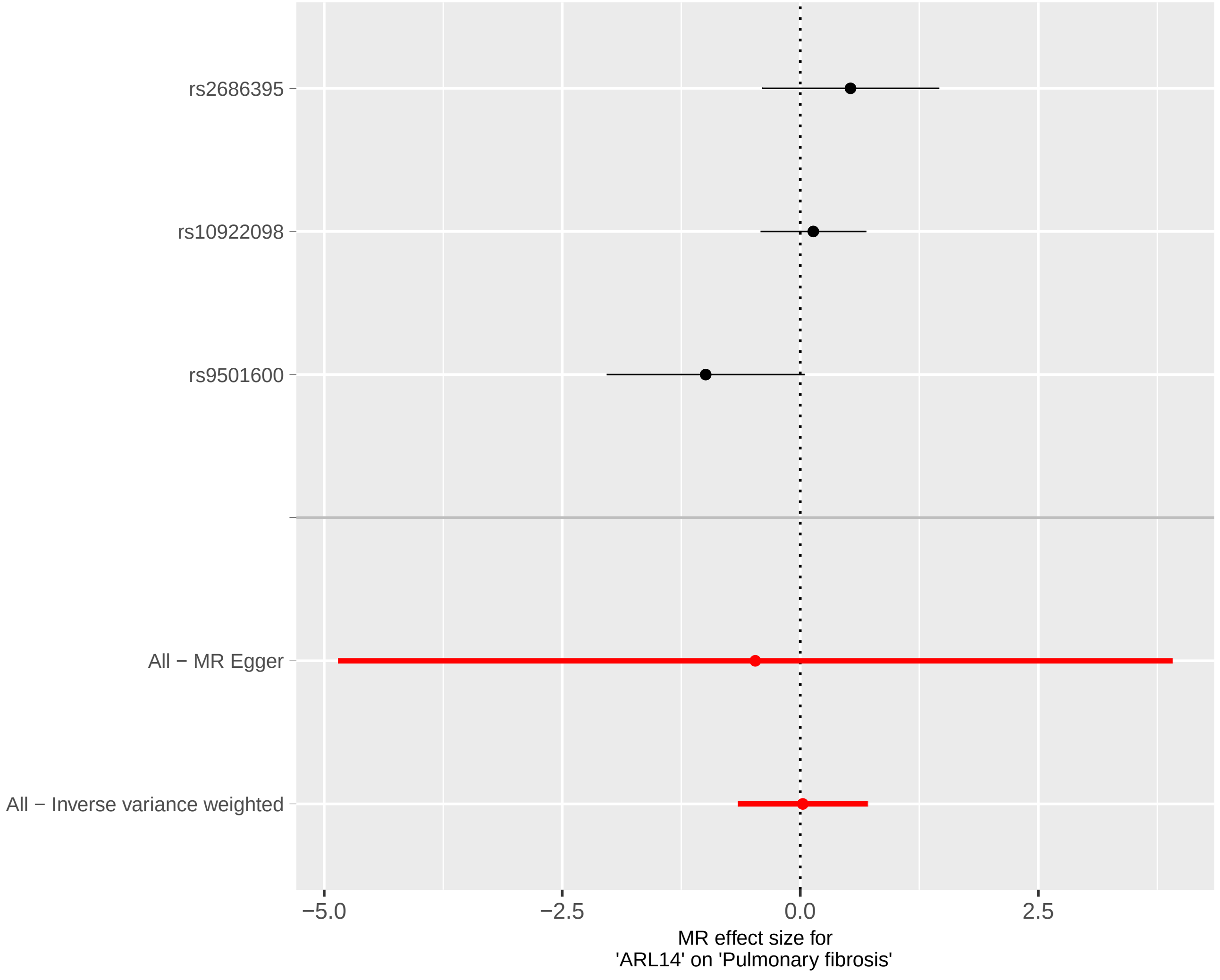

rs2686395
rs10922098
rs9501600
All − MR Egger
All − Inverse variance weighted
−5.0
−2.5
0.0
2.5
MR effect size for
'ARL14' on 'Pulmonary fibrosis'

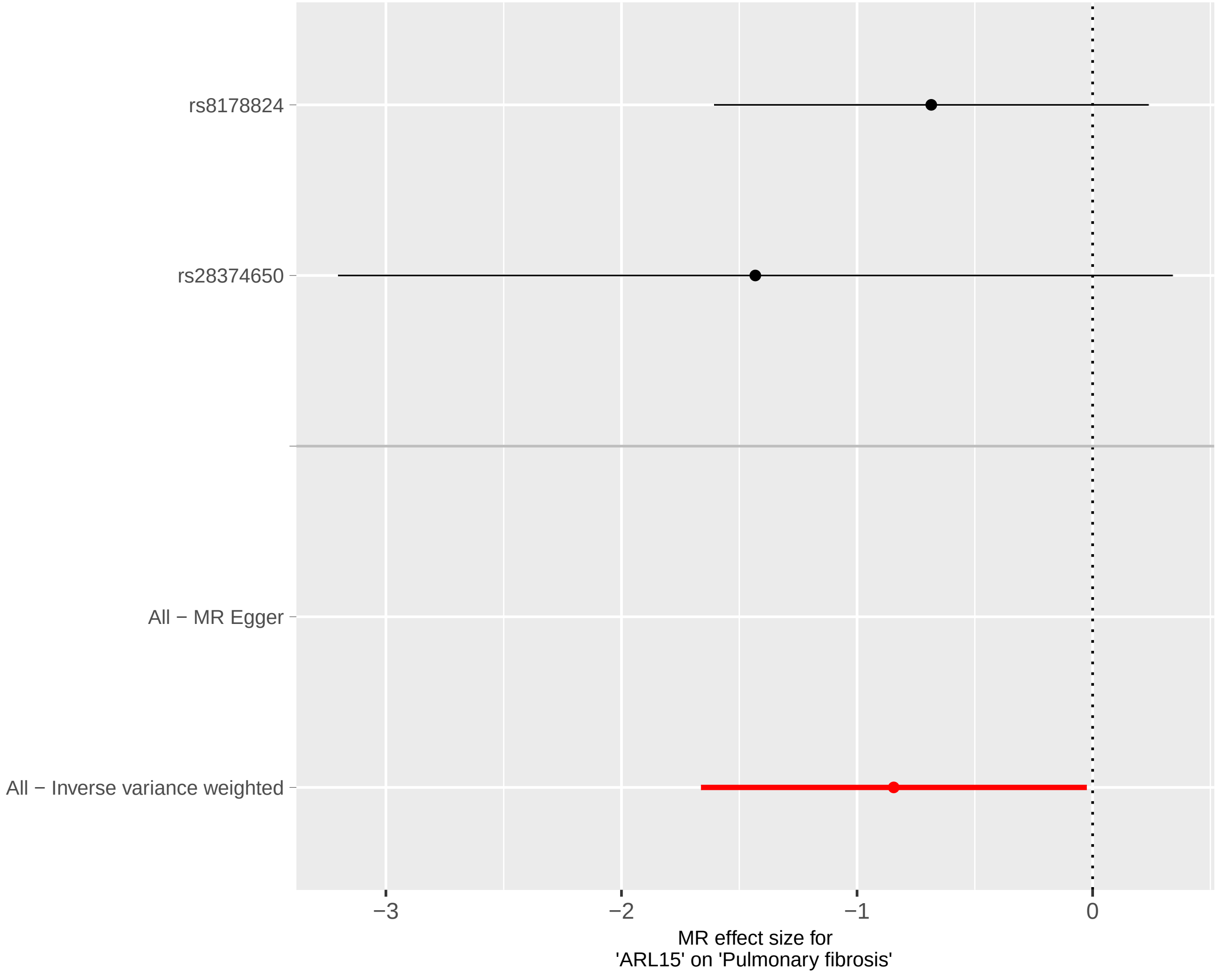

rs8178824
rs28374650
All − MR Egger
All − Inverse variance weighted
−3
−2
−1
0
MR effect size for
'ARL15' on 'Pulmonary fibrosis'

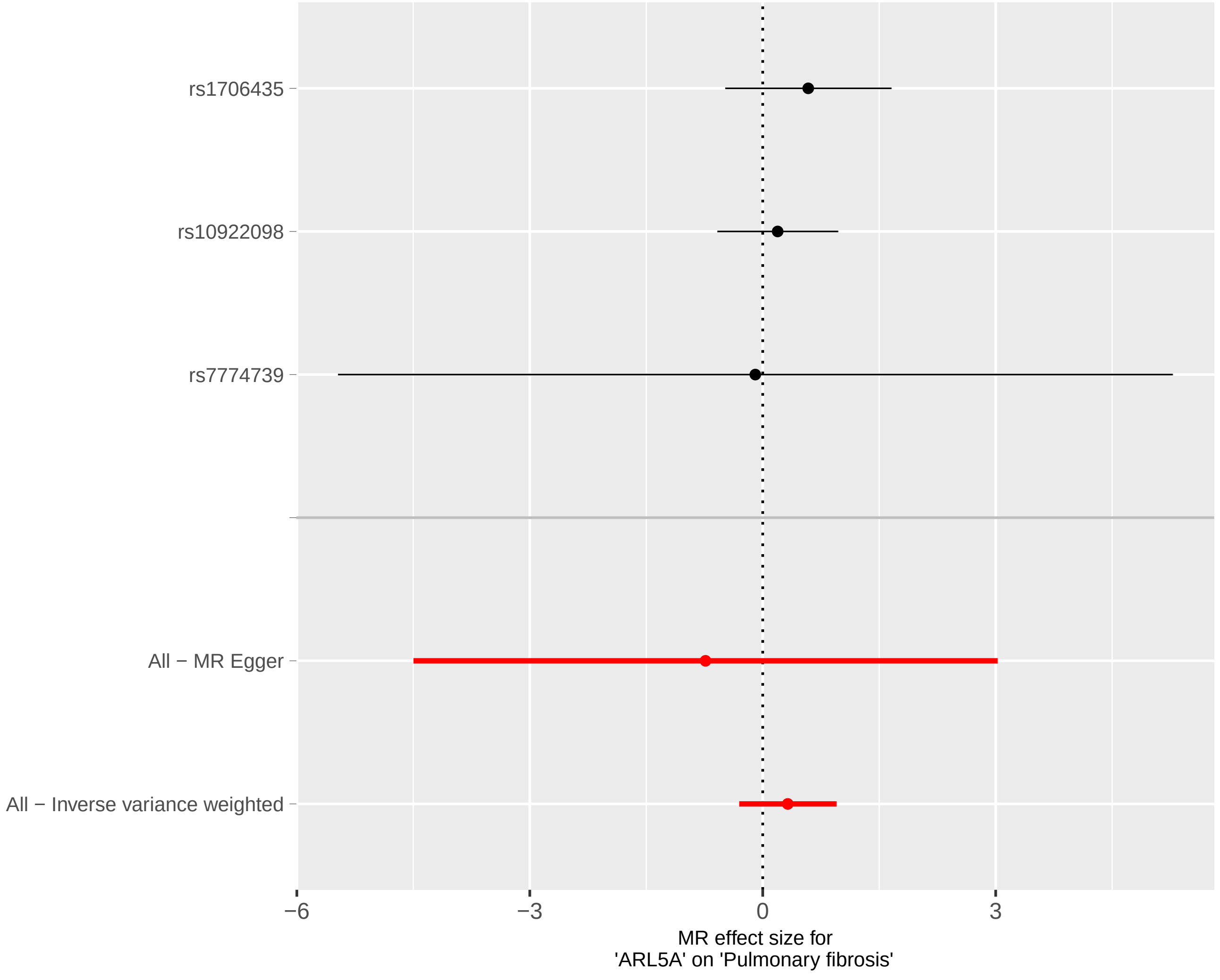

rs1706435
rs10922098
rs7774739
All − MR Egger
All − Inverse variance weighted
−6
−3
0
3
MR effect size for
'ARL5A' on 'Pulmonary fibrosis'

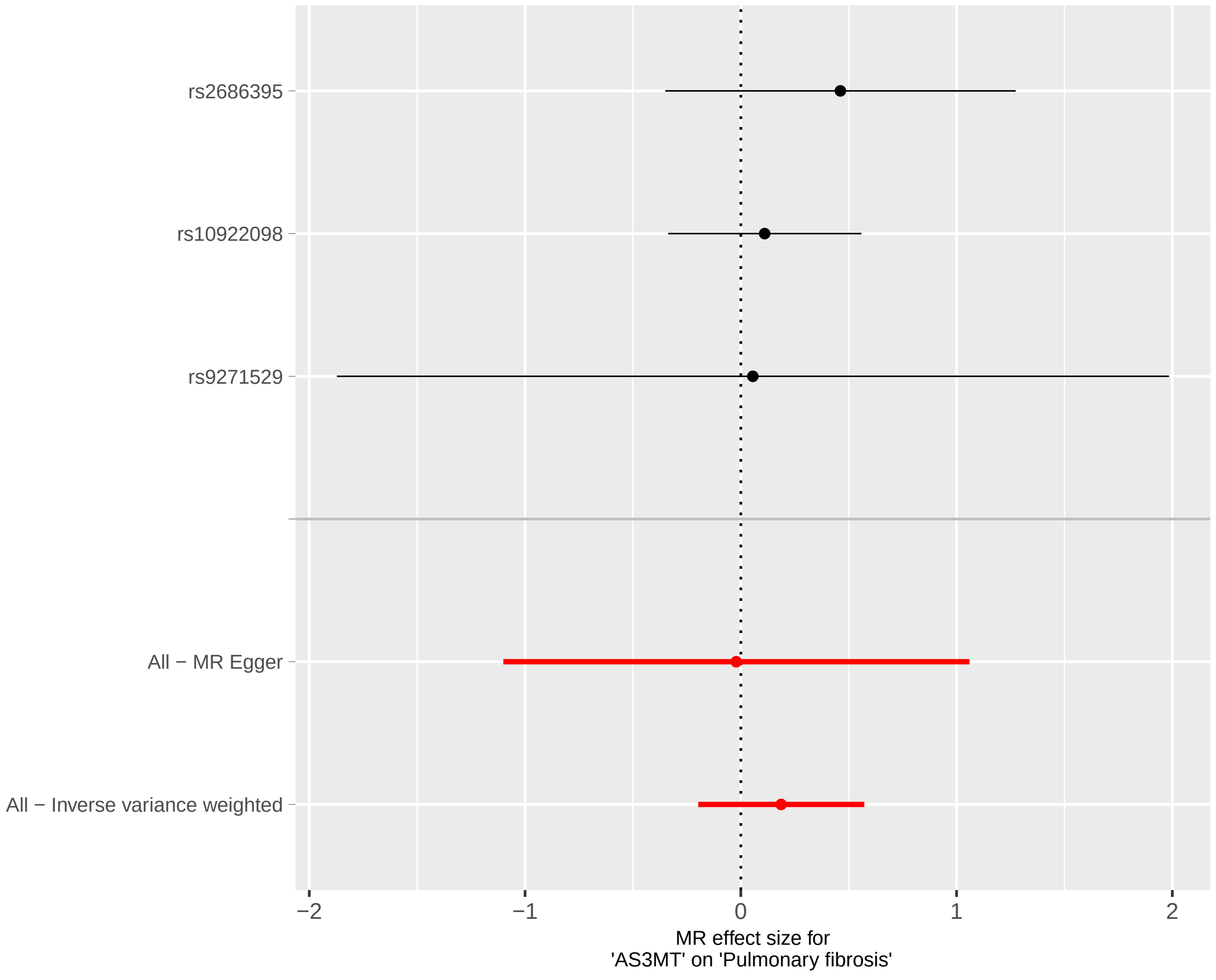

rs2686395
rs10922098
rs9271529
All − MR Egger
All − Inverse variance weighted
−2
−1
0
1
2
MR effect size for
'AS3MT' on 'Pulmonary fibrosis'

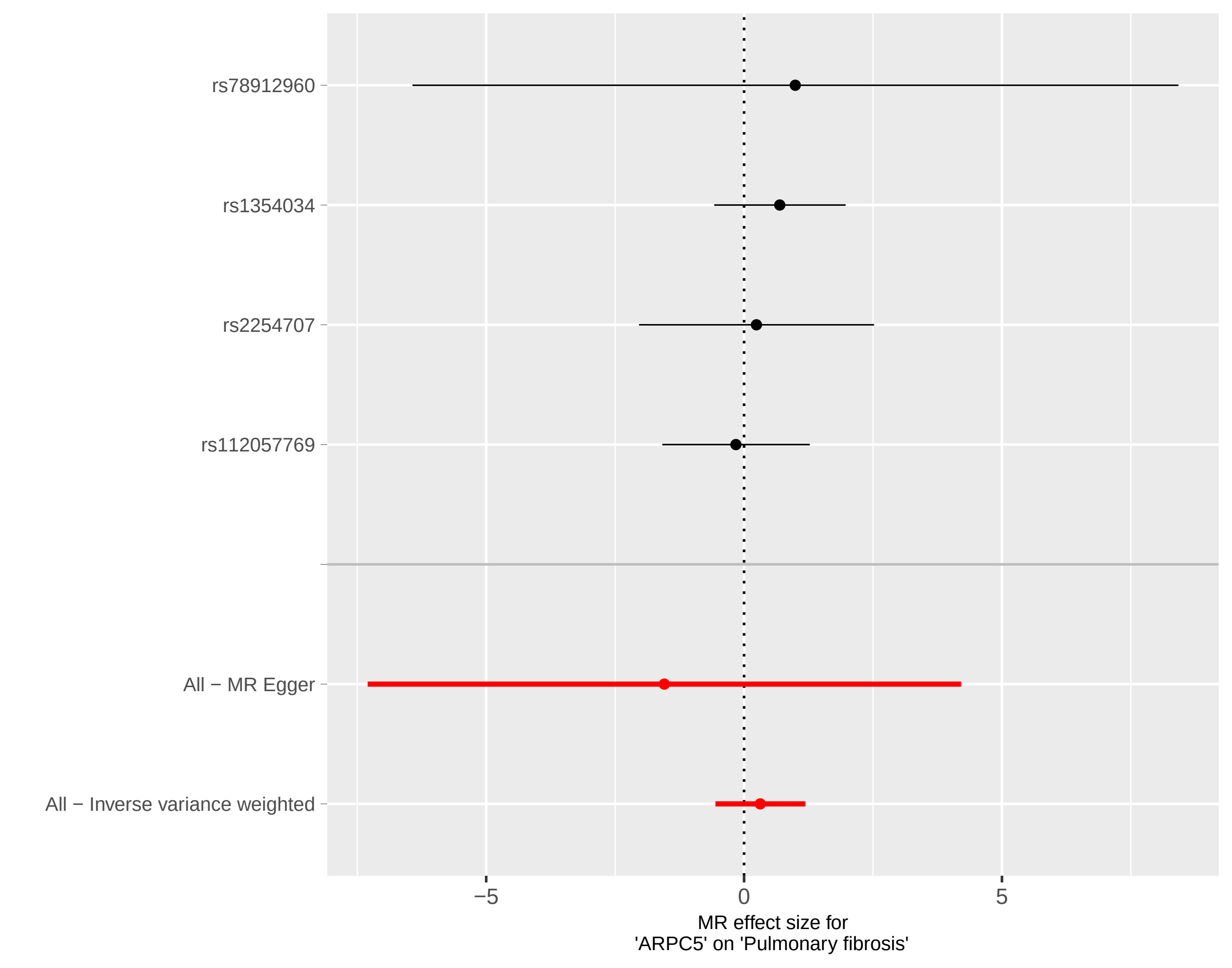

rs78912960
rs1354034
rs2254707
rs112057769
All − MR Egger
All − Inverse variance weighted
−5
0
5
MR effect size for
'ARPC5' on 'Pulmonary fibrosis'

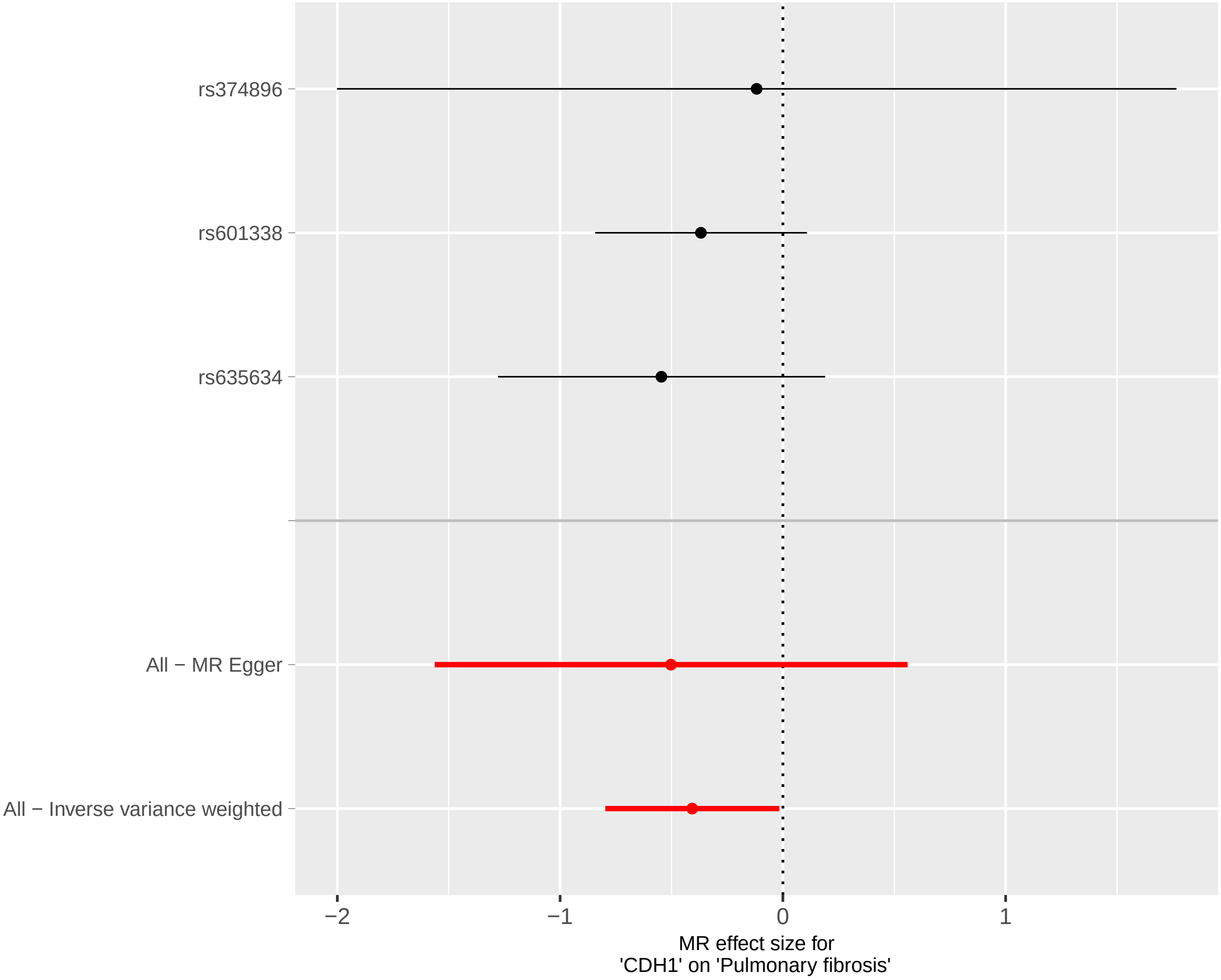

rs374896
rs601338
rs635634
All − MR Egger
All − Inverse variance weighted
−2
−1
0
1
MR effect size for
'CDH1' on 'Pulmonary fibrosis'

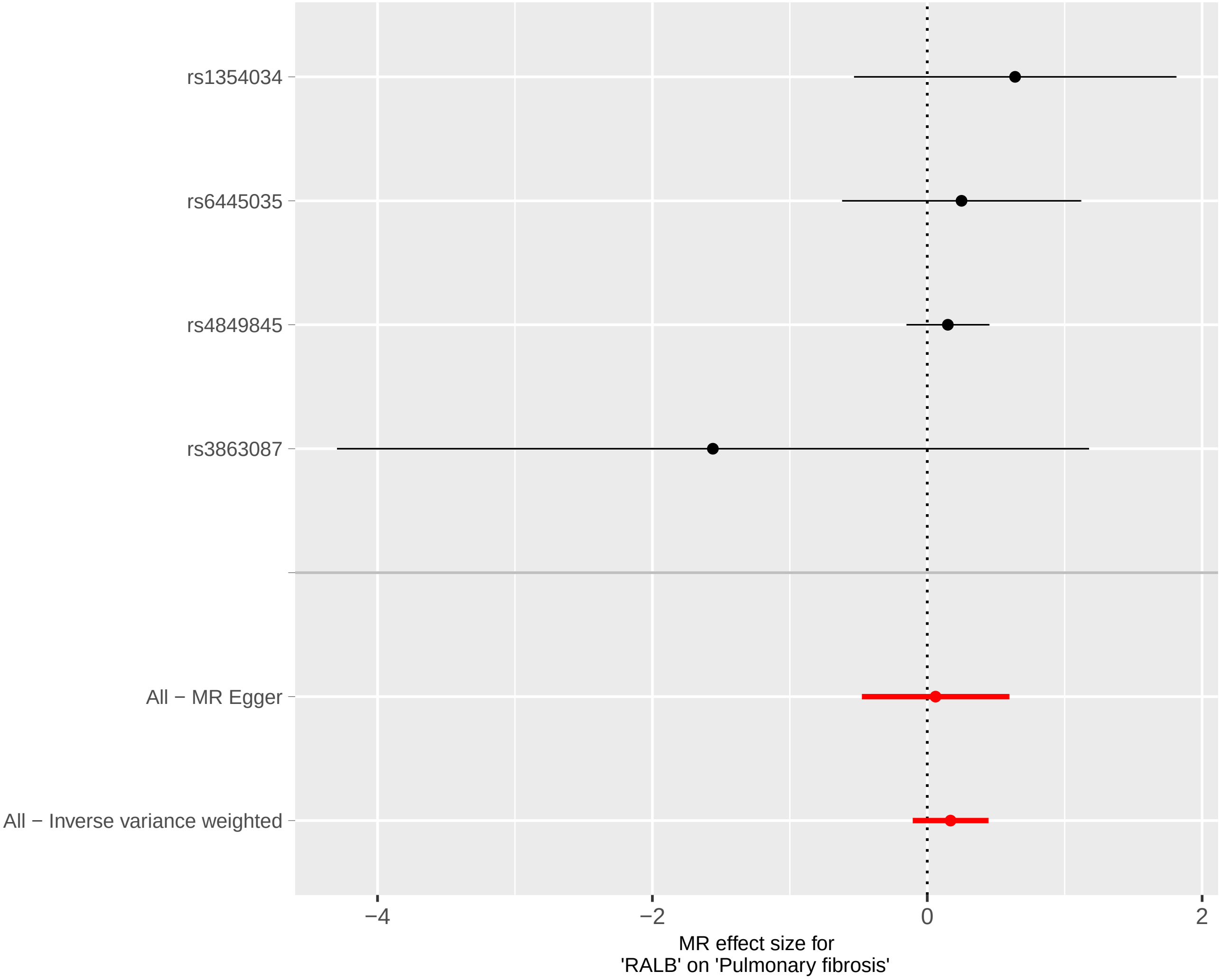

rs1354034
rs6445035
rs4849845
rs3863087
All − MR Egger
All − Inverse variance weighted
−4
−2
0
2
MR effect size for
'RALB' on 'Pulmonary fibrosis'

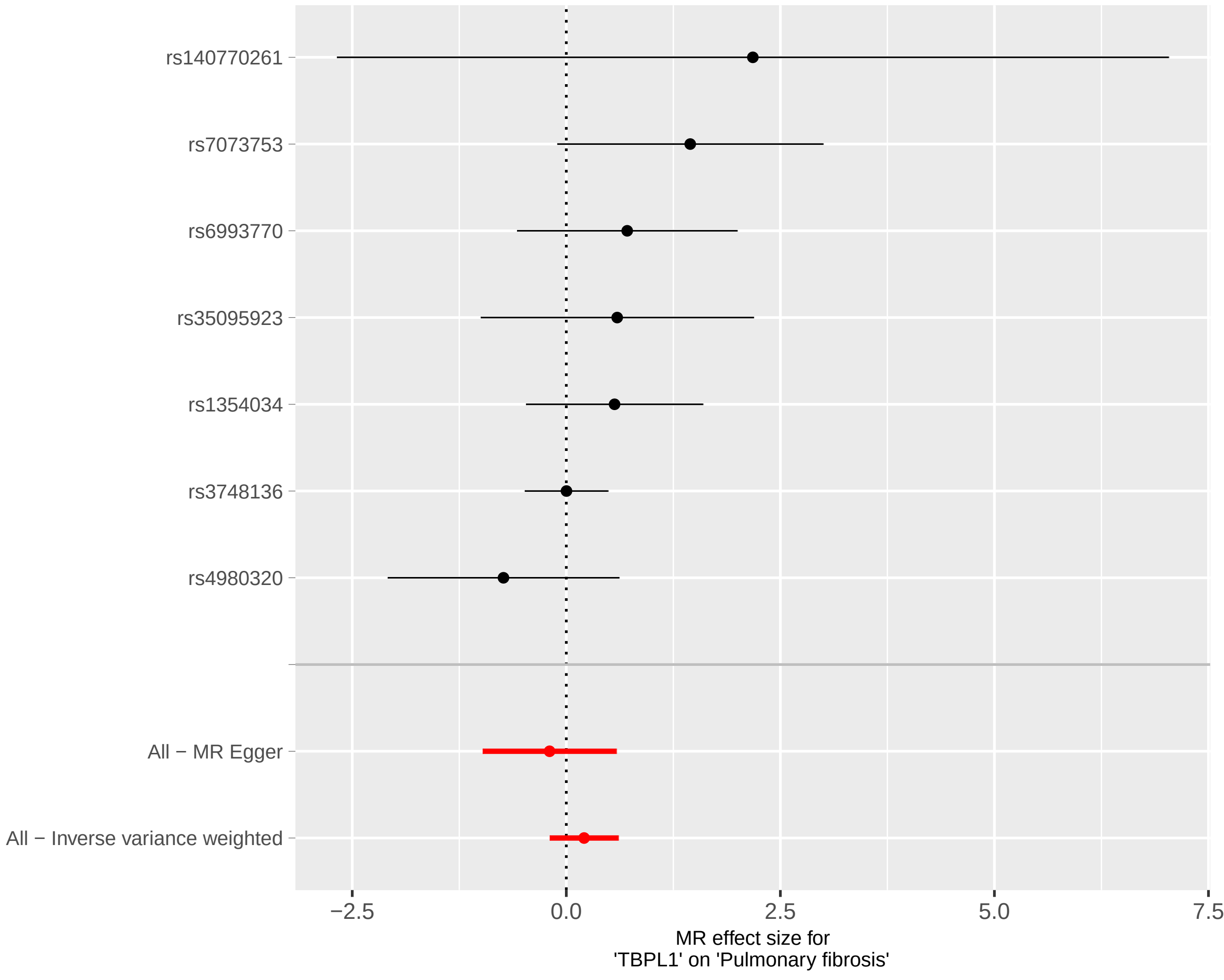

rs140770261
rs7073753
rs6993770
rs35095923
rs1354034
rs3748136
rs4980320
All − MR Egger
All − Inverse variance weighted
−2.5
0.0
2.5
5.0
7.5
MR effect size for
'TBPL1' on 'Pulmonary fibrosis'

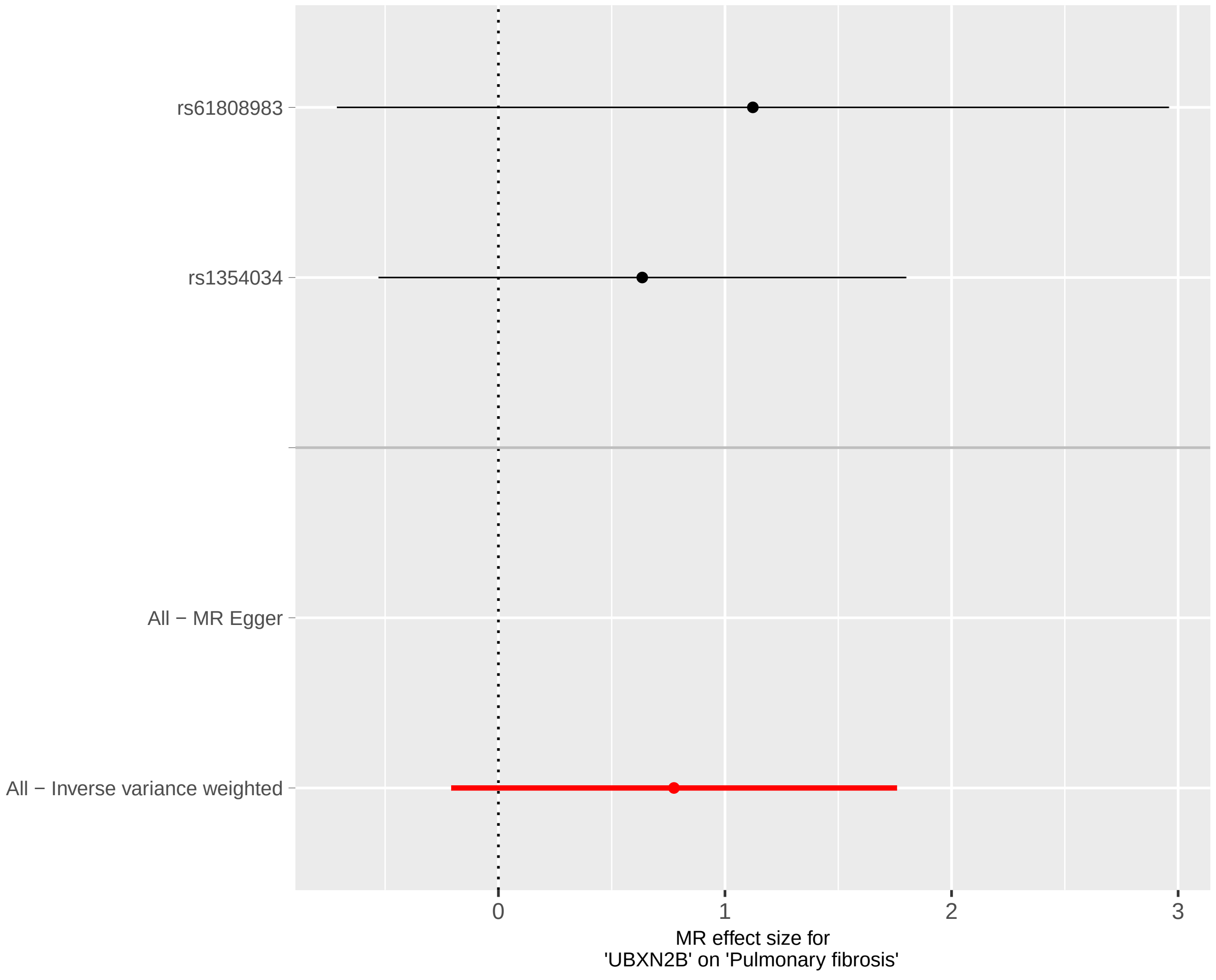
rs61808983
rs1354034
All − MR Egger
All − Inverse variance weighted
0
1
2
3
MR effect size for
'UBXN2B' on 'Pulmonary fibrosis'

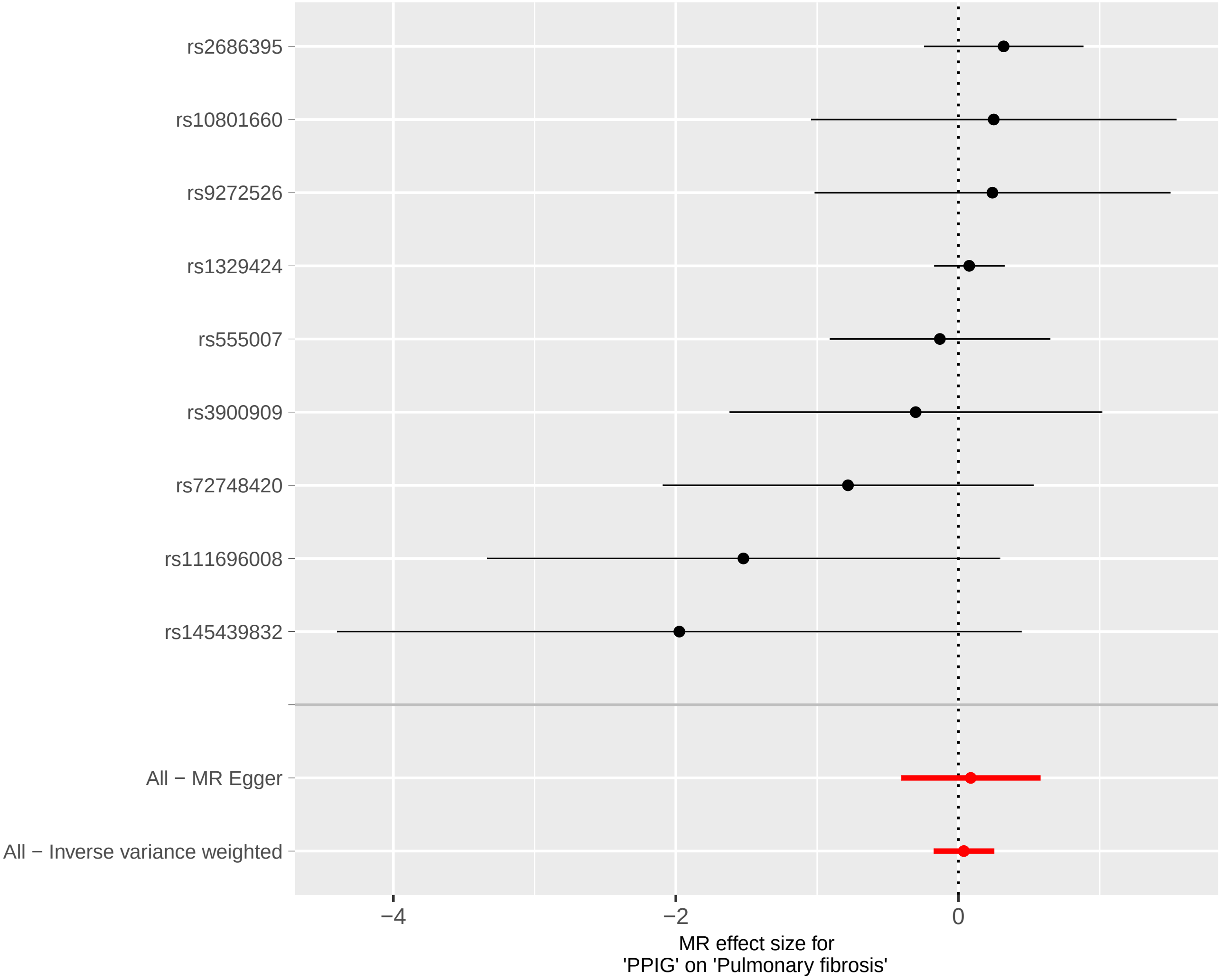

rs2686395
rs10801660
rs9272526
rs1329424
rs555007
rs3900909
rs72748420
rs111696008
rs145439832
All − MR Egger
All − Inverse variance weighted
−4
−2
0
MR effect size for
'PPIG' on 'Pulmonary fibrosis'

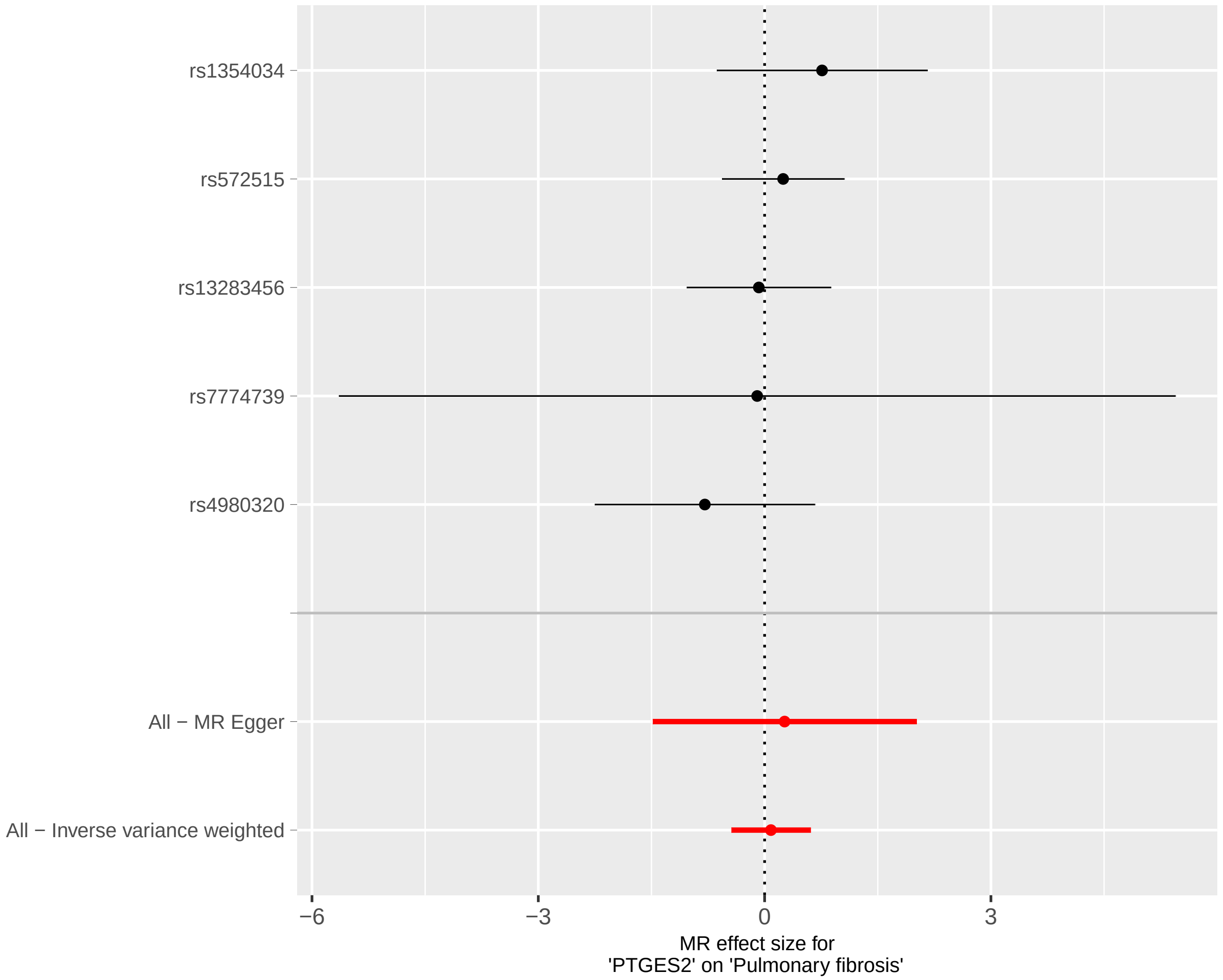
rs1354034
rs572515
rs13283456
rs7774739
rs4980320
All − MR Egger
All − Inverse variance weighted
−6
−3
0
3
MR effect size for
'PTGES2' on 'Pulmonary fibrosis'

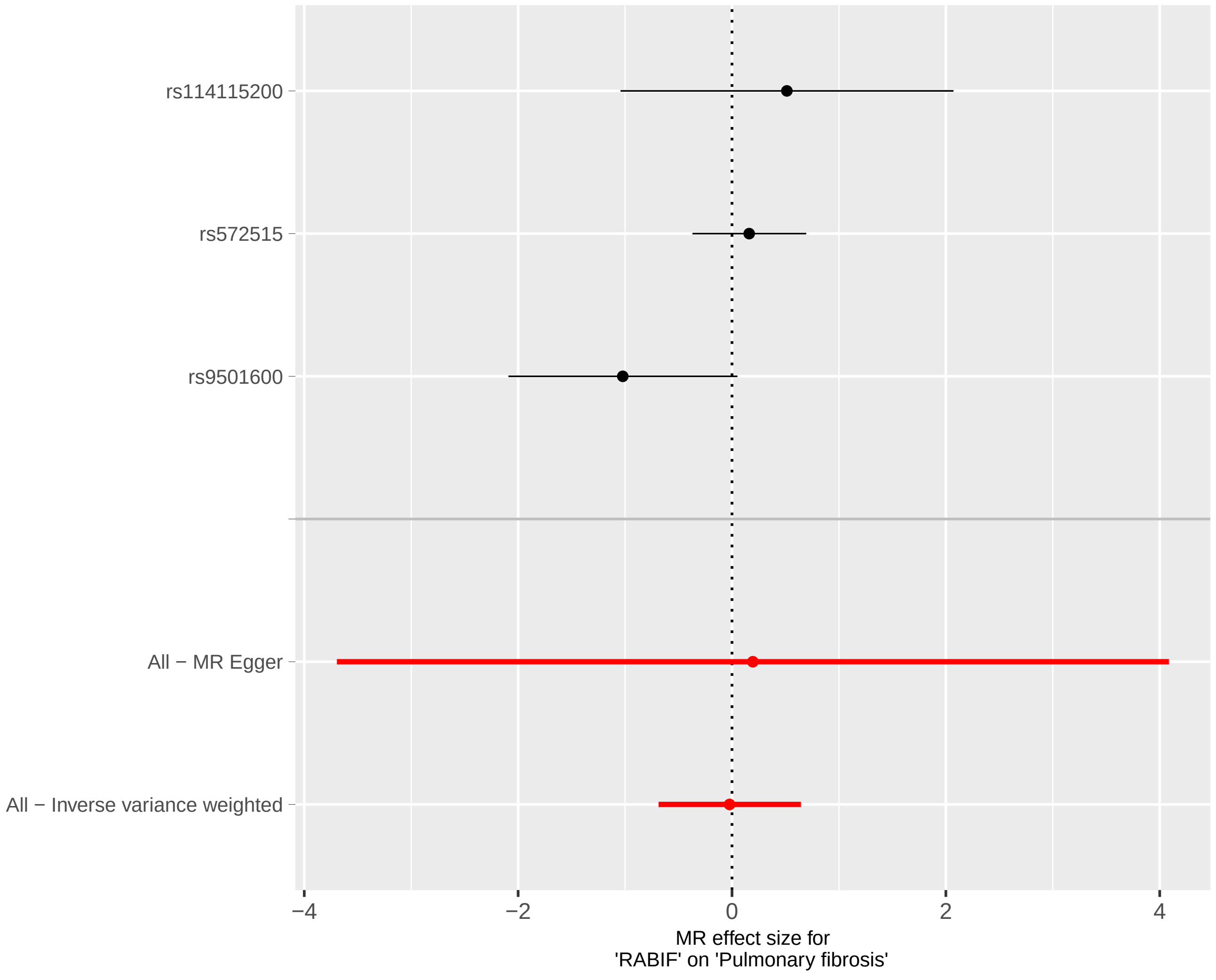

rs114115200
rs572515
rs9501600
All − MR Egger
All − Inverse variance weighted
−4
−2
0
2
4
MR effect size for
'RABIF' on 'Pulmonary fibrosis'

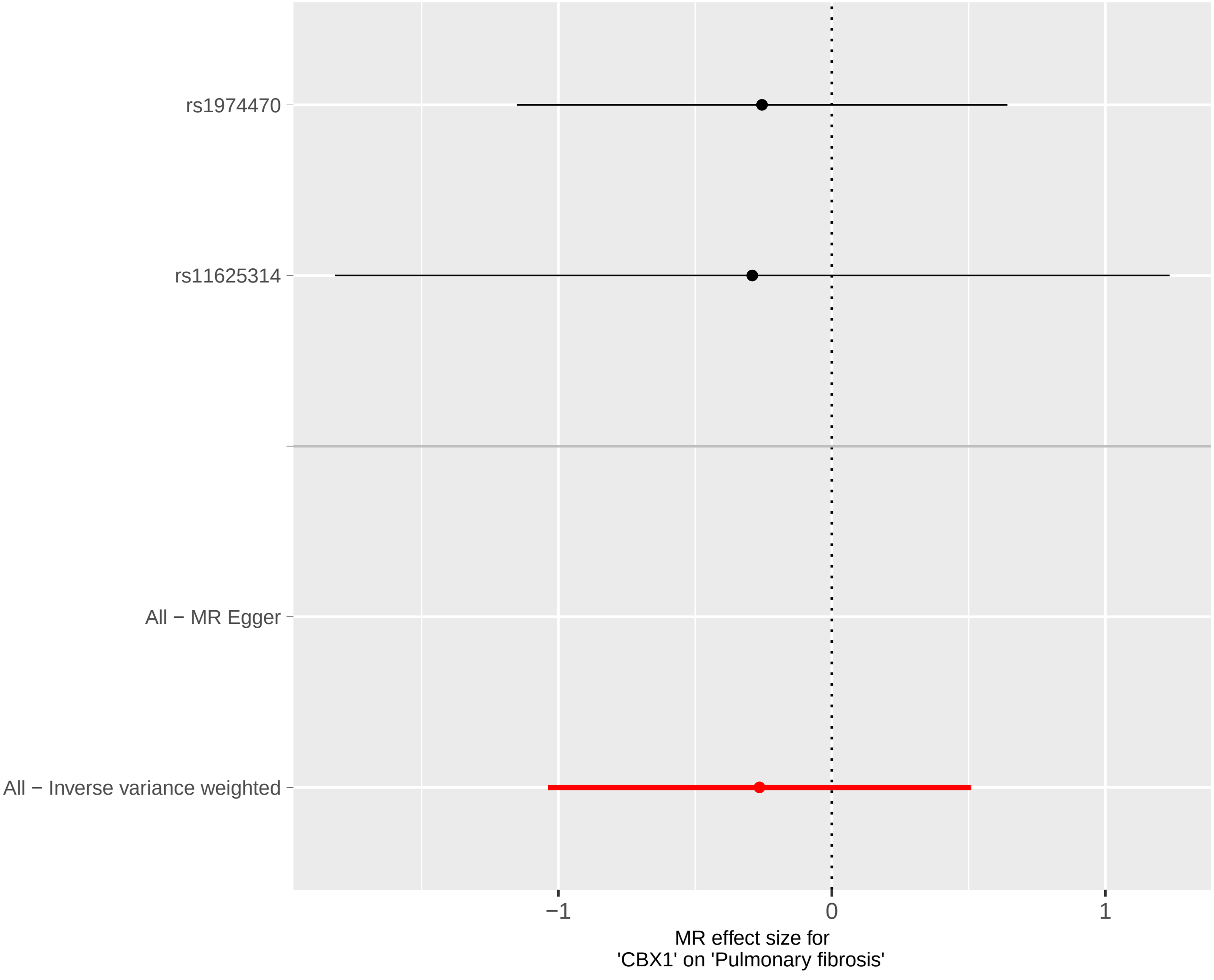

rs1974470
rs11625314
All − MR Egger
All − Inverse variance weighted
−1
0
1
MR effect size for
'CBX1' on 'Pulmonary fibrosis'

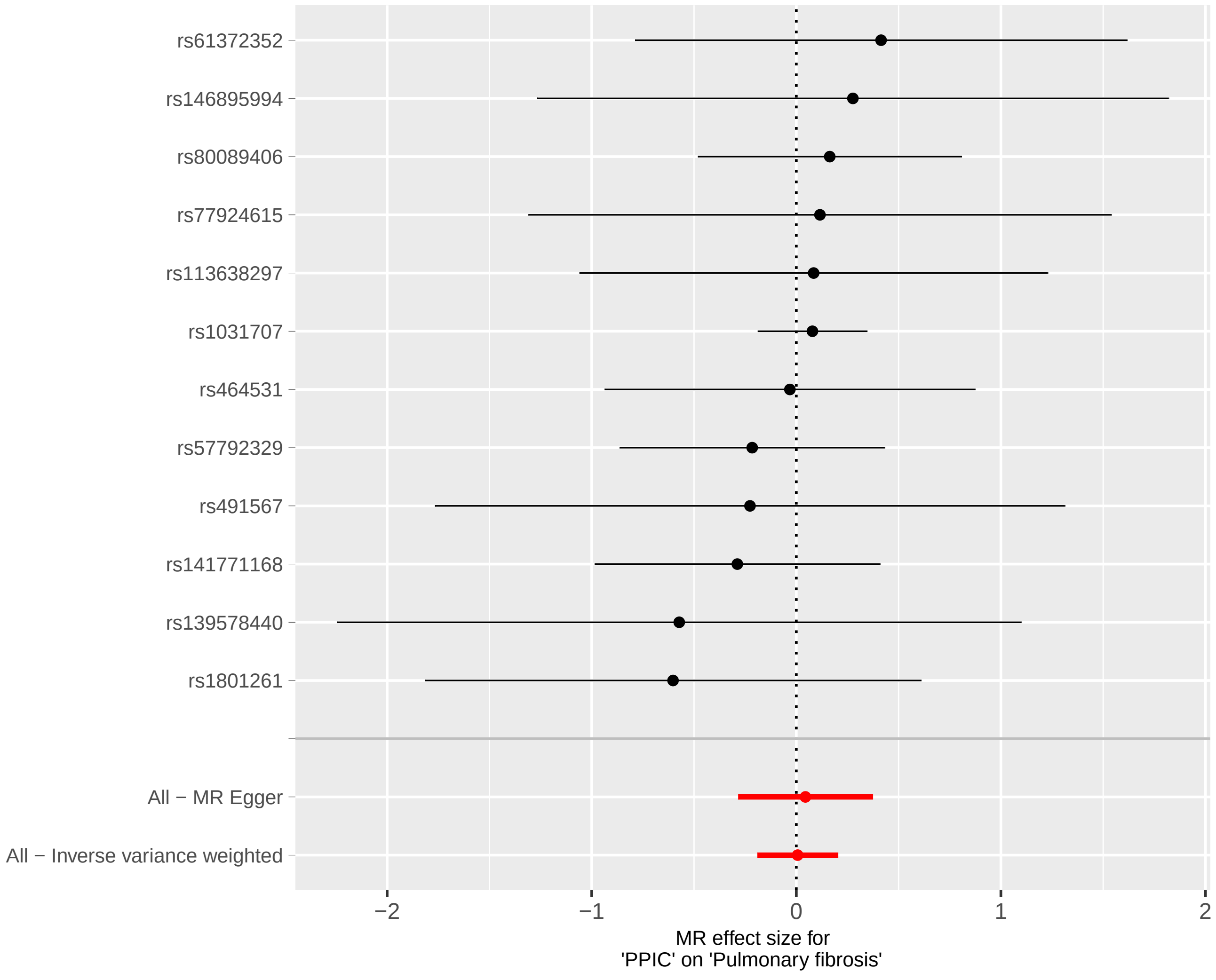

rs61372352
rs146895994
rs80089406
rs77924615
rs113638297
rs1031707
rs464531
rs57792329
rs491567
rs141771168
rs139578440
rs1801261
All − MR Egger
All − Inverse variance weighted
−2
−1
0
1
2
MR effect size for
'PPIC' on 'Pulmonary fibrosis'

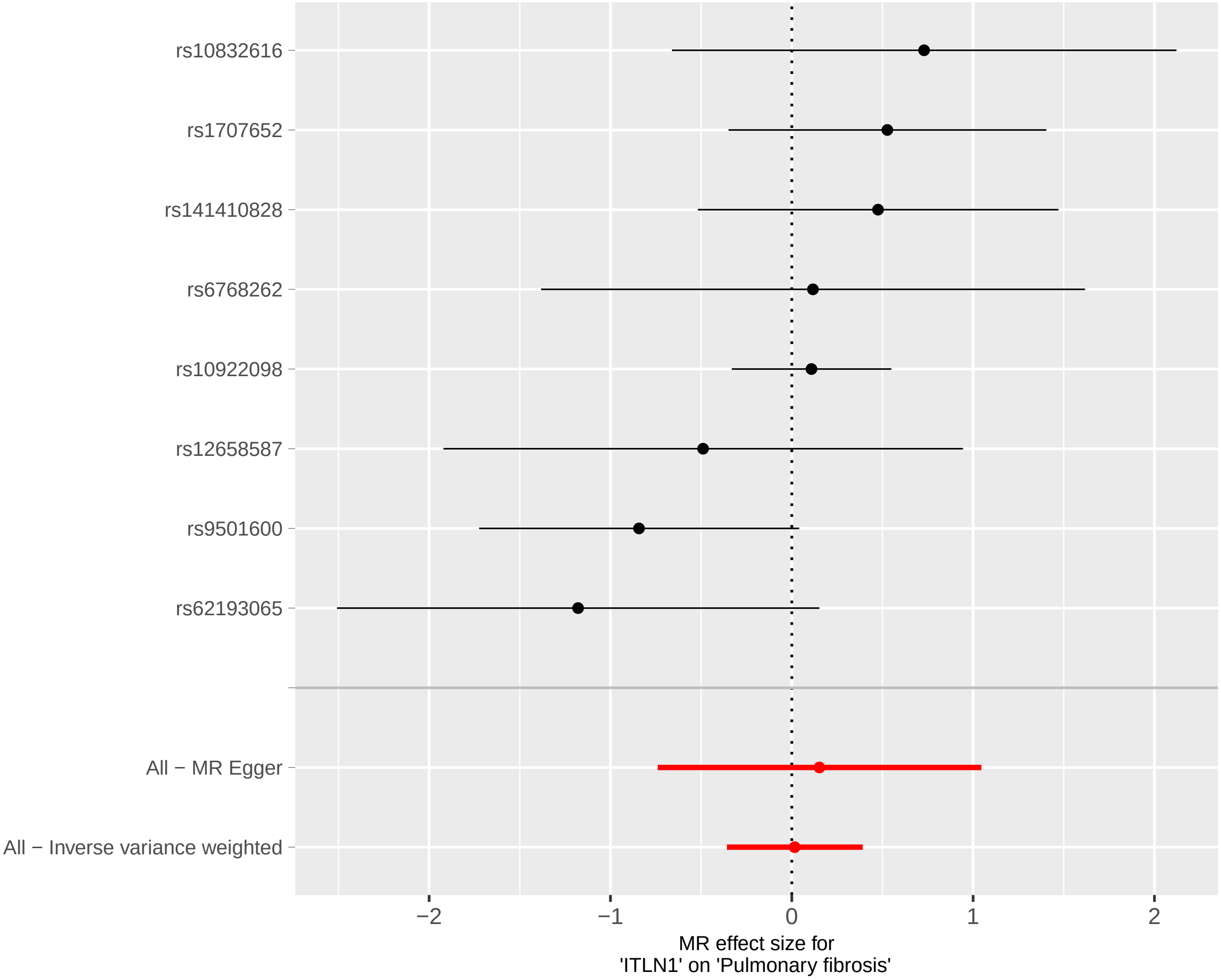

rs10832616
rs1707652
rs141410828
rs6768262
rs10922098
rs12658587
rs9501600
rs62193065
All − MR Egger
All − Inverse variance weighted
−2
−1
0
1
2
MR effect size for
'ITLN1' on 'Pulmonary fibrosis'

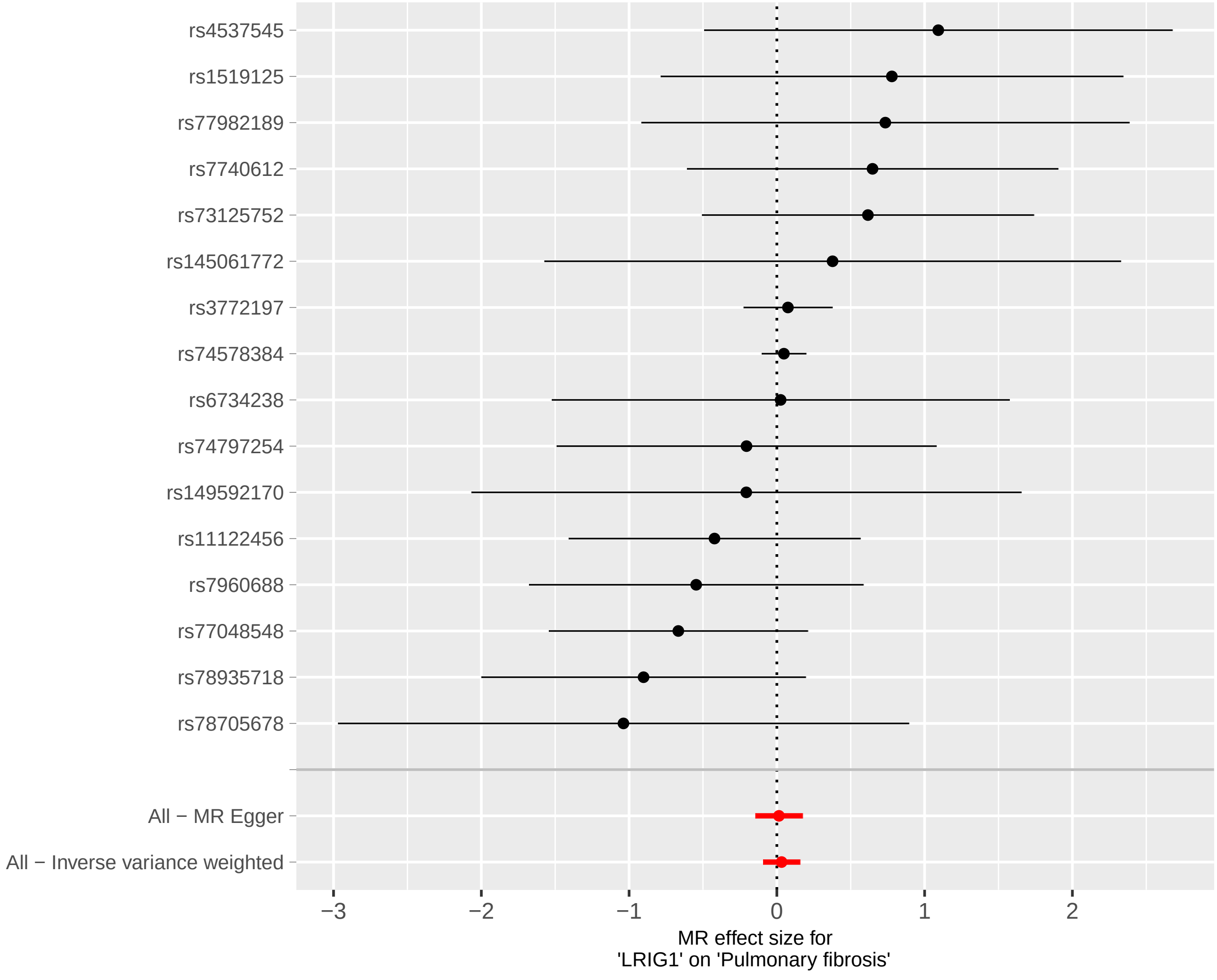

rs4537545
rs1519125
rs77982189
rs7740612
rs73125752
rs145061772
rs3772197
rs74578384
rs6734238
rs74797254
rs149592170
rs11122456
rs7960688
rs77048548
rs78935718
rs78705678
All − MR Egger
All − Inverse variance weighted
−3
−2
−1
0
1
2
MR effect size for
'LRIG1' on 'Pulmonary fibrosis'

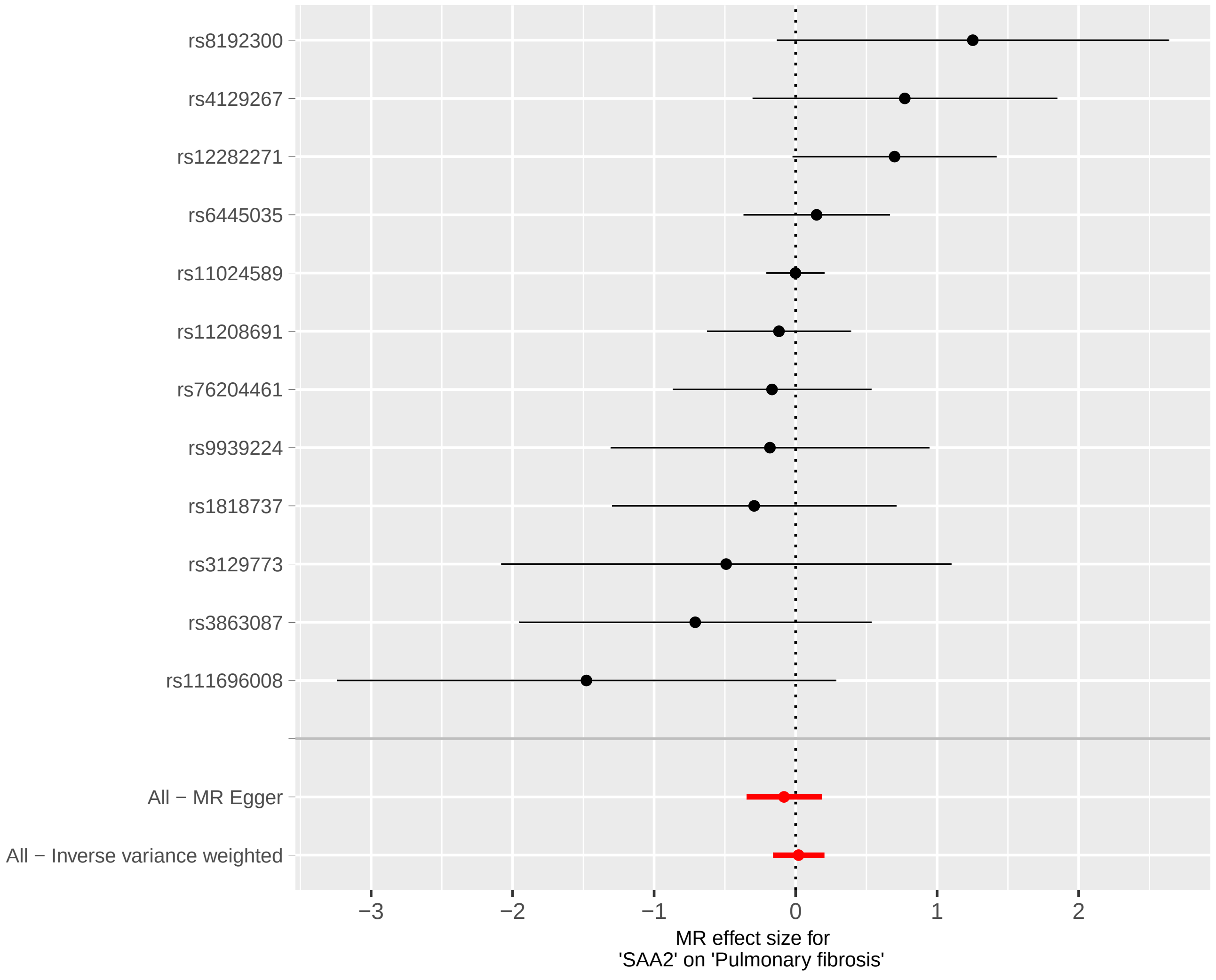

rs8192300
rs4129267
rs12282271
rs6445035
rs11024589
rs11208691
rs76204461
rs9939224
rs1818737
rs3129773
rs3863087
rs111696008
All − MR Egger
All − Inverse variance weighted
−3
−2
−1
0
1
2
MR effect size for
'SAA2' on 'Pulmonary fibrosis'

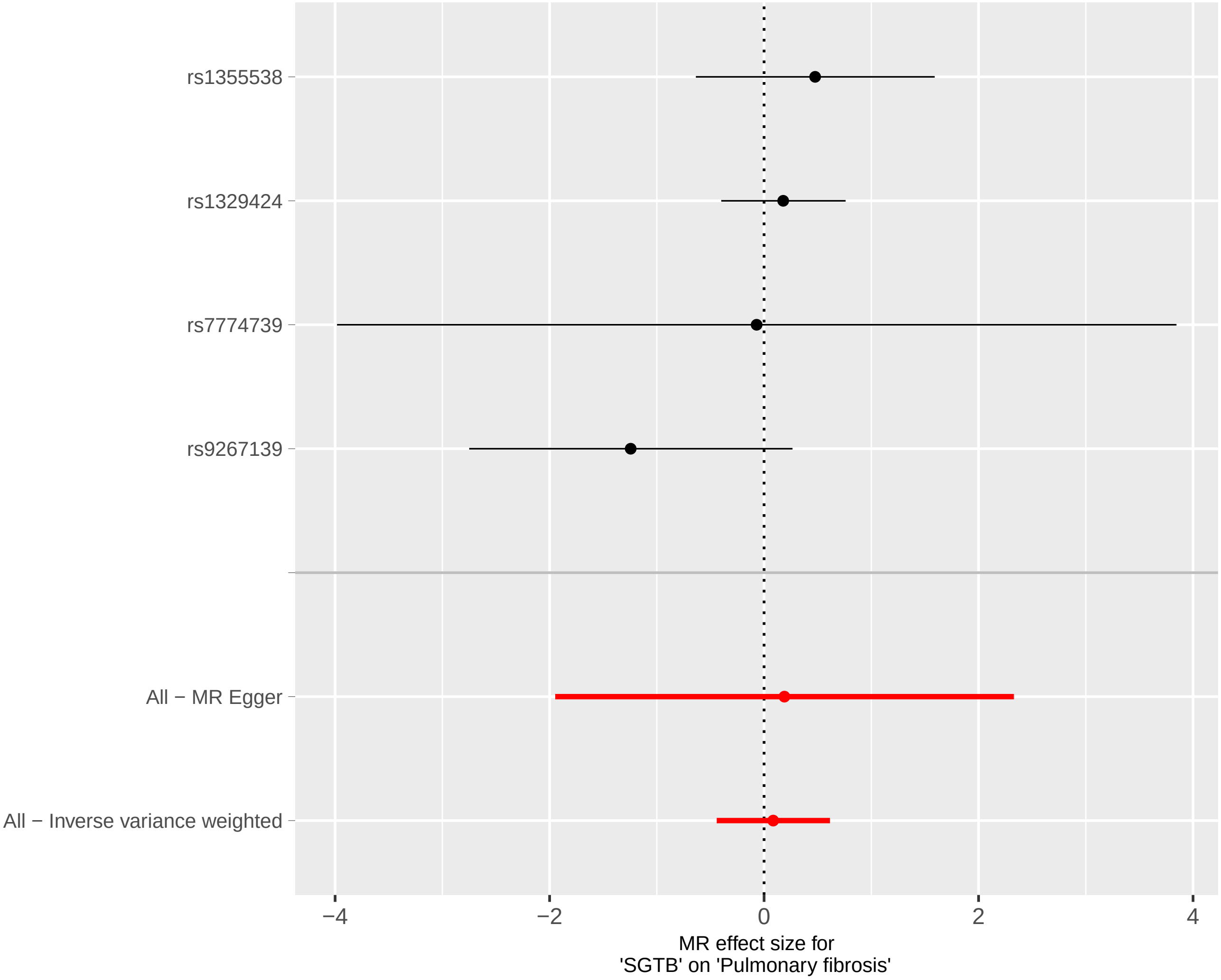

rs1355538
rs1329424
rs7774739
rs9267139
All − MR Egger
All − Inverse variance weighted
−4
−2
0
2
4
MR effect size for
'SGTB' on 'Pulmonary fibrosis'

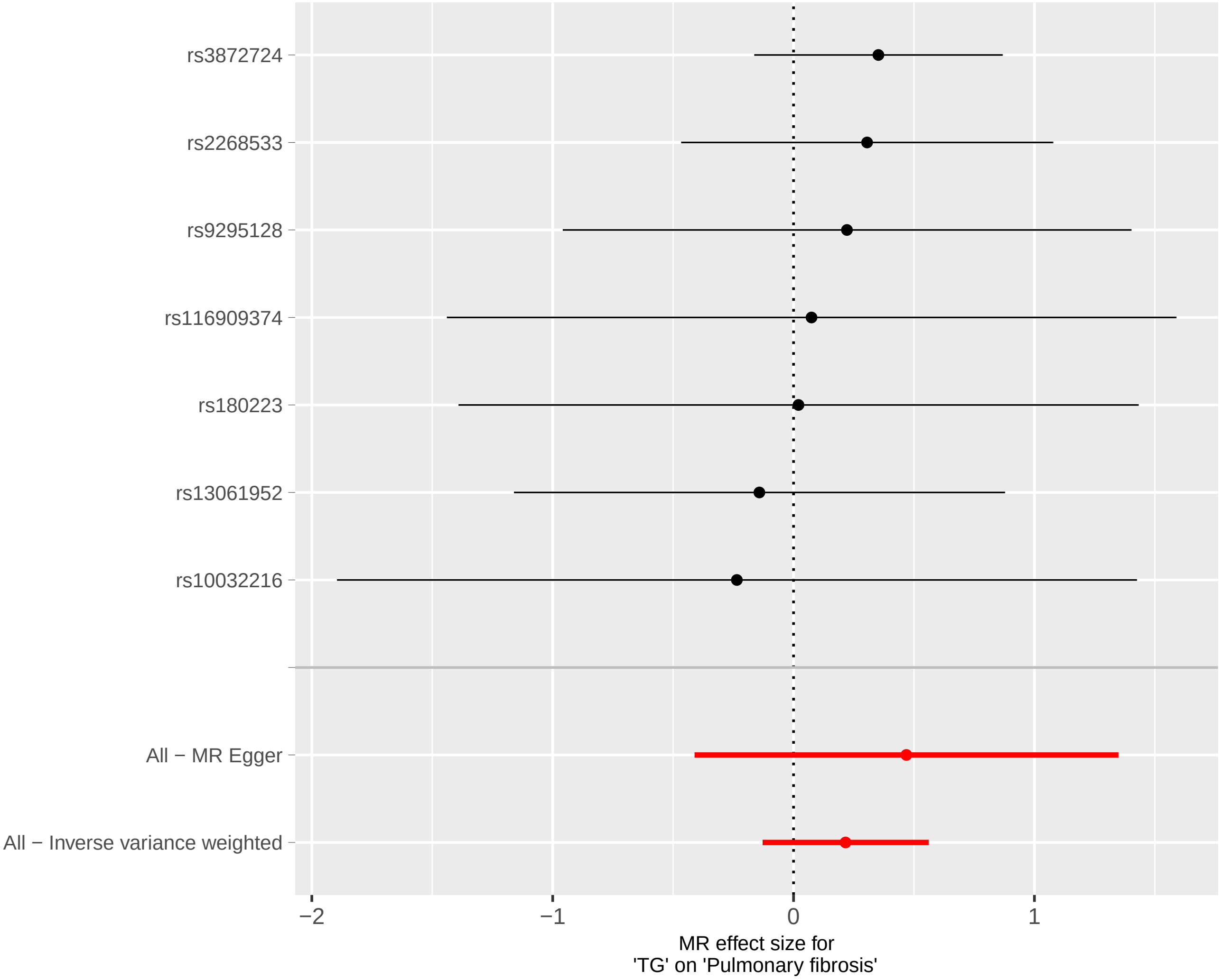
rs3872724
rs2268533
rs9295128
rs116909374
rs180223
rs13061952
rs10032216
All − MR Egger
All − Inverse variance weighted
−2
−1
0
1
MR effect size for
'TG' on 'Pulmonary fibrosis'

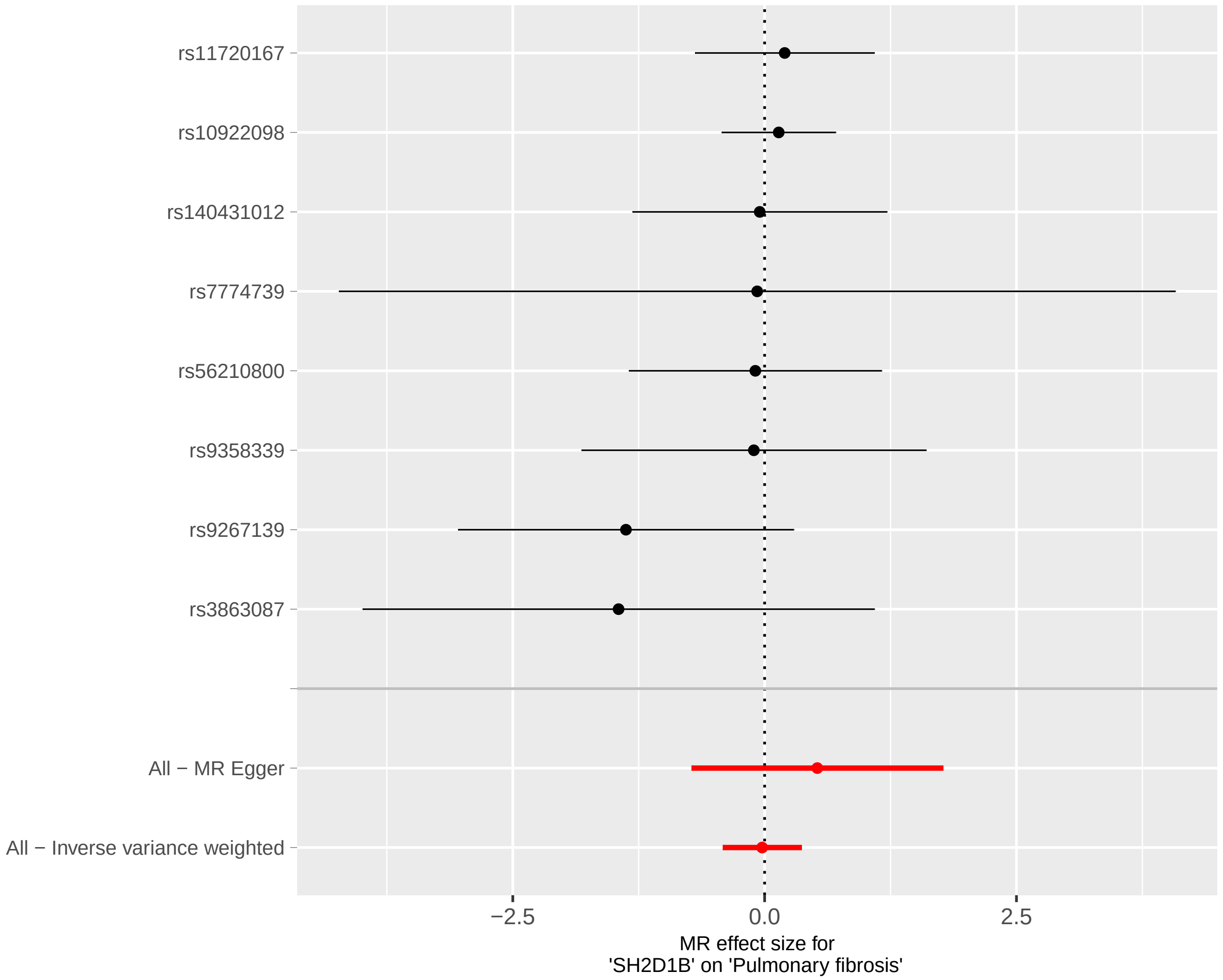

rs11720167
rs10922098
rs140431012
rs7774739
rs56210800
rs9358339
rs9267139
rs3863087
All − MR Egger
All − Inverse variance weighted
−2.5
0.0
2.5
MR effect size for
'SH2D1B' on 'Pulmonary fibrosis'

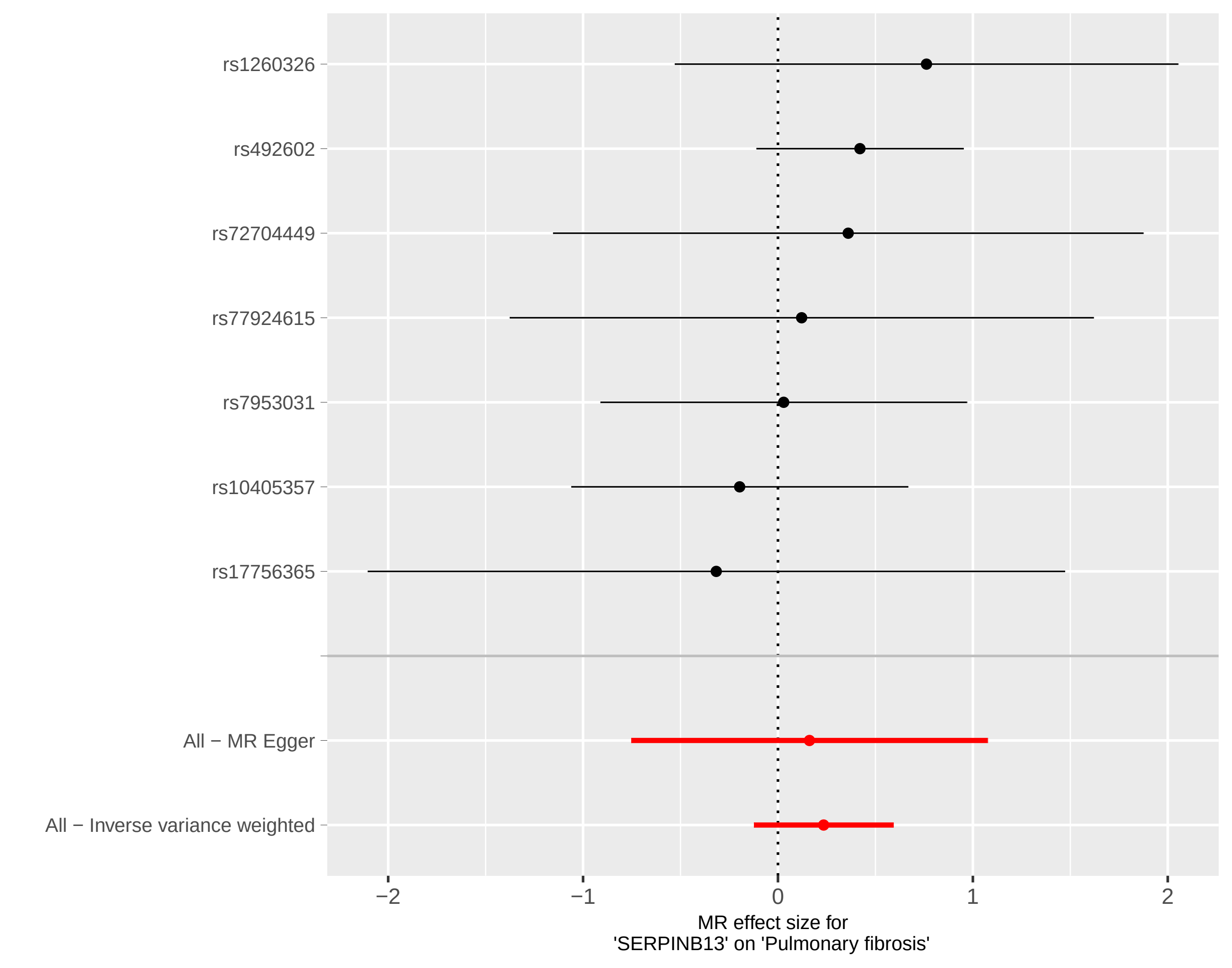
rs1260326
rs492602
rs72704449
rs77924615
rs7953031
rs10405357
rs17756365
All − MR Egger
All − Inverse variance weighted
−2
−1
0
1
2
MR effect size for
'SERPINB13' on 'Pulmonary fibrosis'

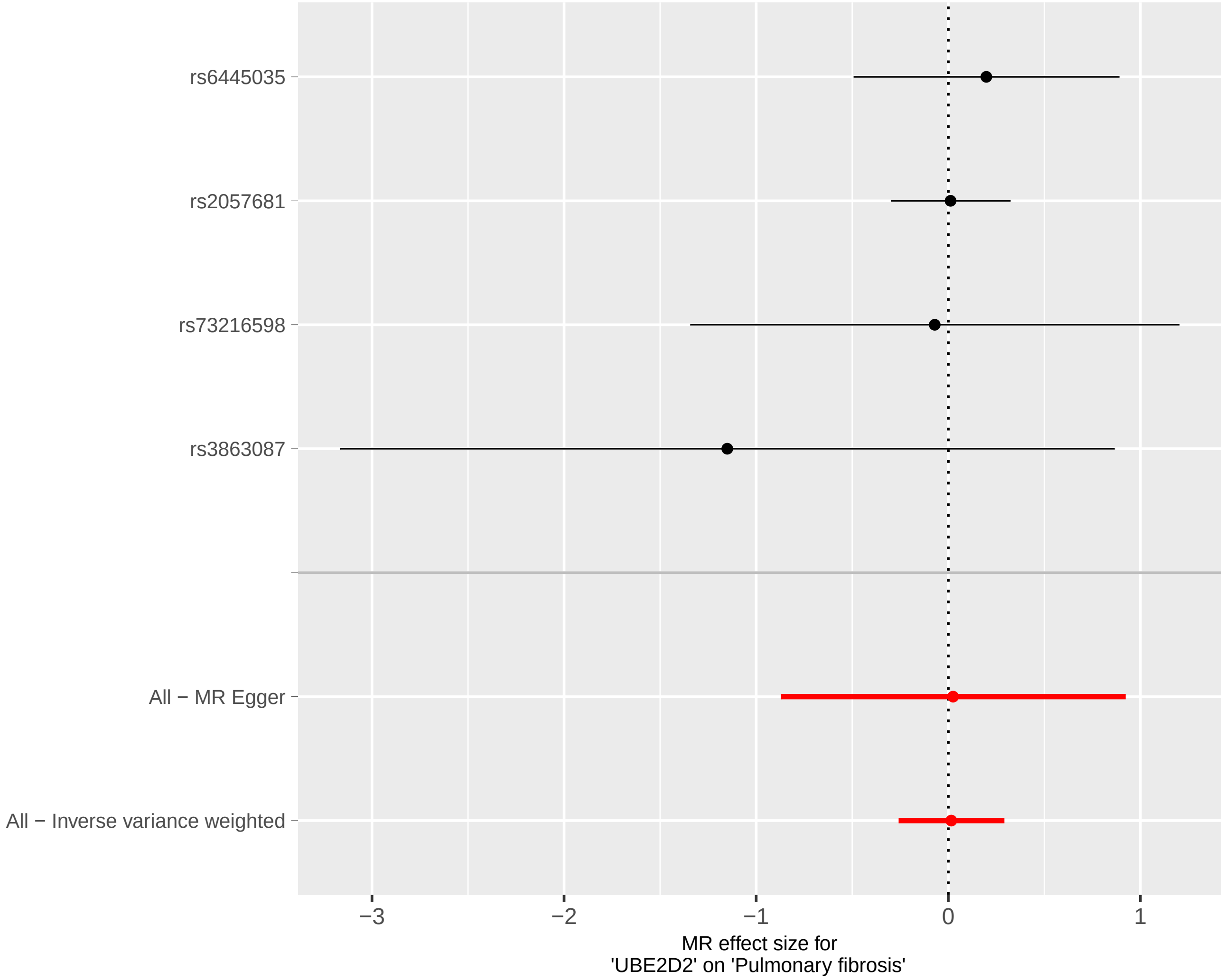

rs6445035
rs2057681
rs73216598
rs3863087
All − MR Egger
All − Inverse variance weighted
−3
−2
−1
0
1
MR effect size for
'UBE2D2' on 'Pulmonary fibrosis'

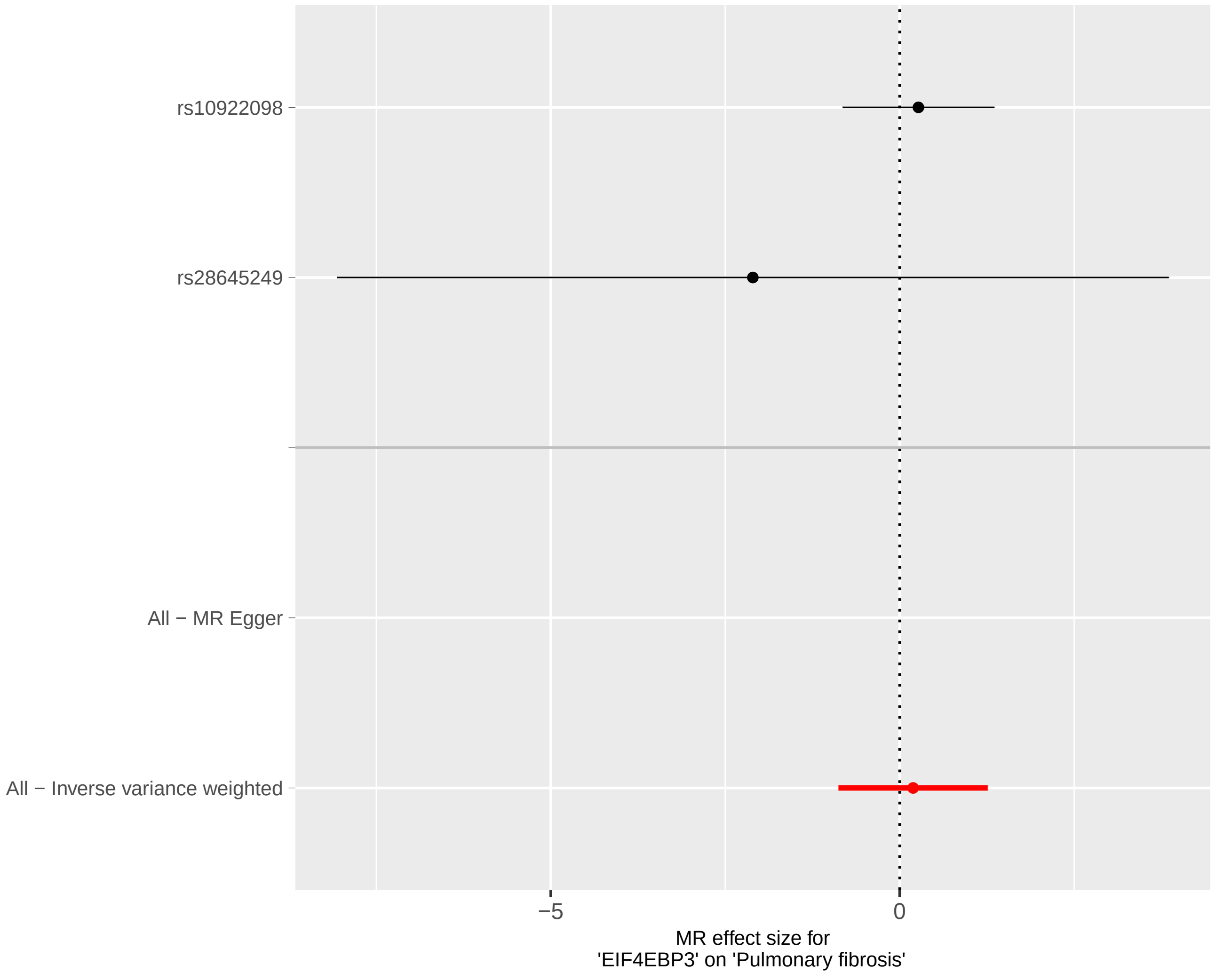
rs10922098
rs28645249
All − MR Egger
All − Inverse variance weighted
−5
0
MR effect size for
'EIF4EBP3' on 'Pulmonary fibrosis'

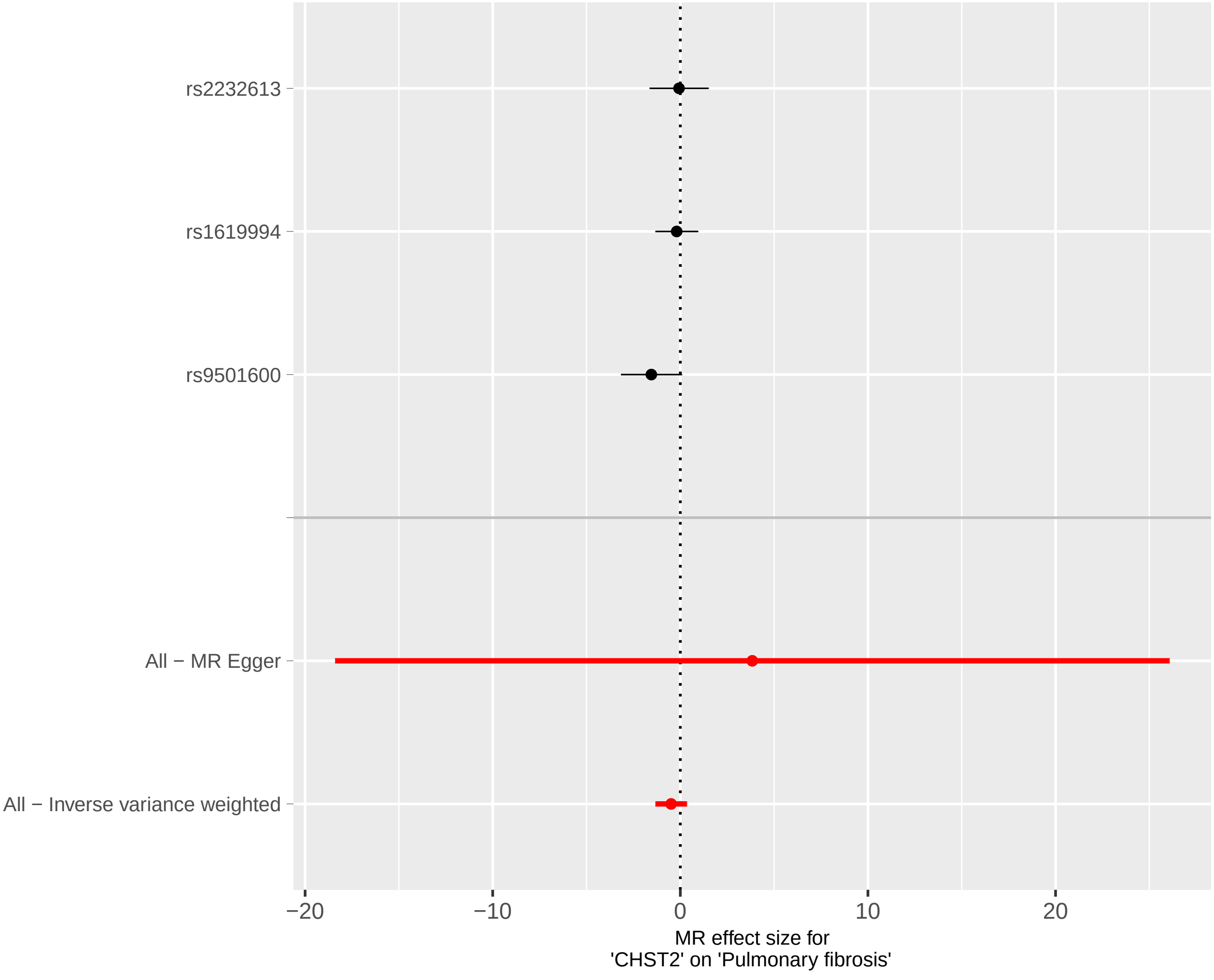

rs2232613
rs1619994
rs9501600
All − MR Egger
All − Inverse variance weighted
−20
−10
0
10
20
MR effect size for
'CHST2' on 'Pulmonary fibrosis'

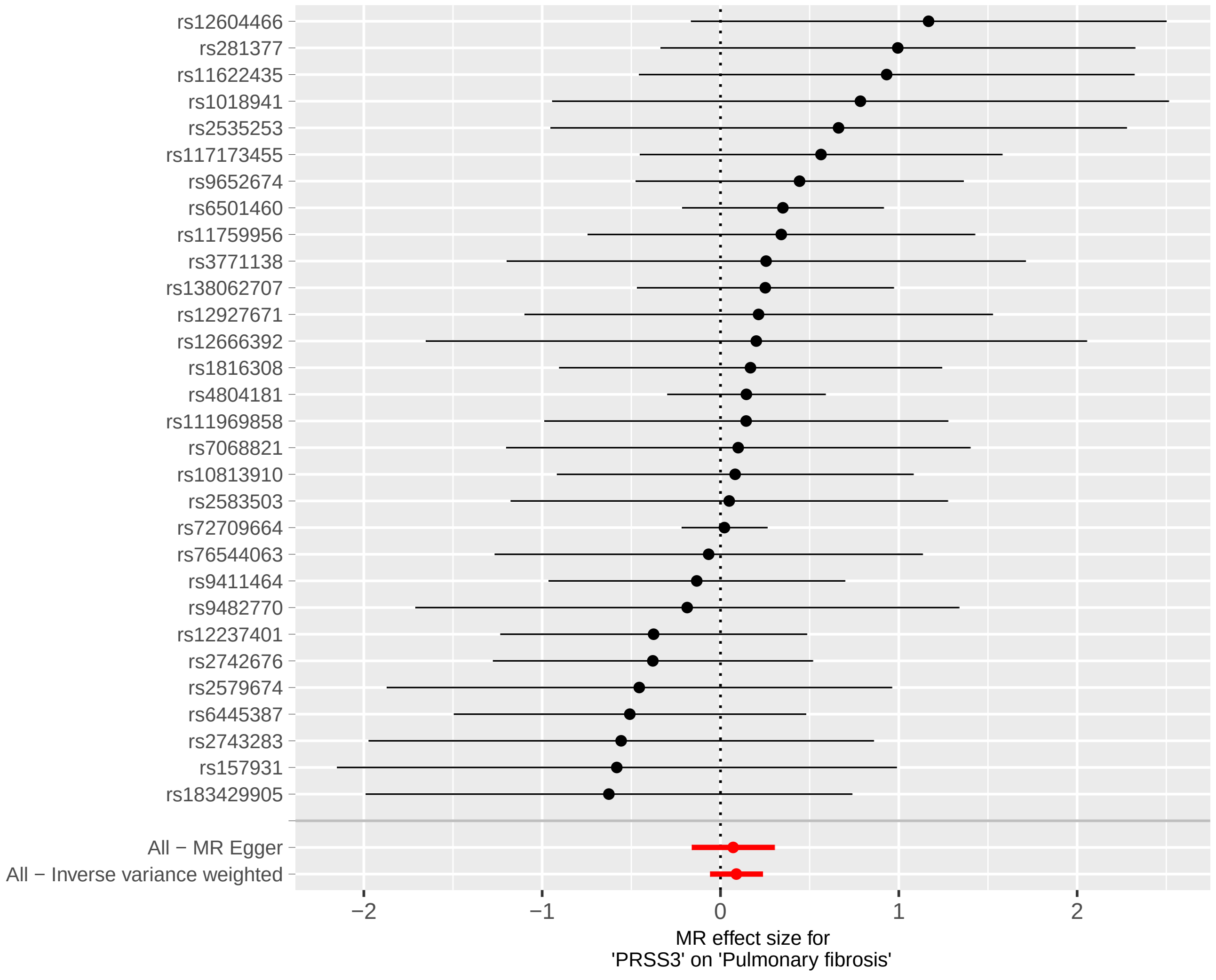

rs12604466
rs281377
rs11622435
rs1018941
rs2535253
rs117173455
rs9652674
rs6501460
rs11759956
rs3771138
rs138062707
rs12927671
rs12666392
rs1816308
rs4804181
rs111969858
rs7068821
rs10813910
rs2583503
rs72709664
rs76544063
rs9411464
rs9482770
rs12237401
rs2742676
rs2579674
rs6445387
rs2743283
rs157931
rs183429905
All − MR Egger
All − Inverse variance weighted
−2
−1
0
1
2
MR effect size for
'PRSS3' on 'Pulmonary fibrosis'

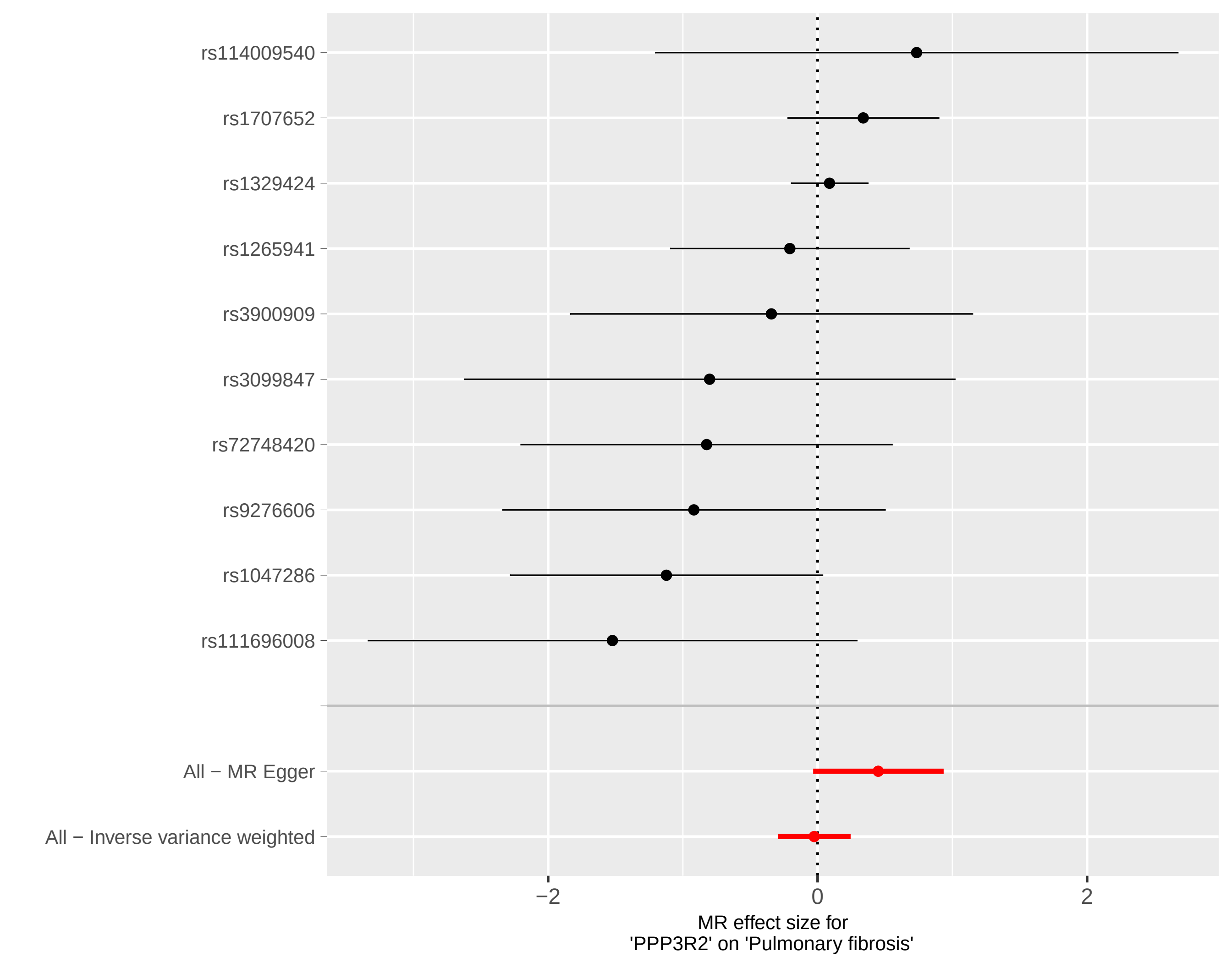

rs114009540
rs1707652
rs1329424
rs1265941
rs3900909
rs3099847
rs72748420
rs9276606
rs1047286
rs111696008
All − MR Egger
All − Inverse variance weighted
−2
0
2
MR effect size for
'PPP3R2' on 'Pulmonary fibrosis'

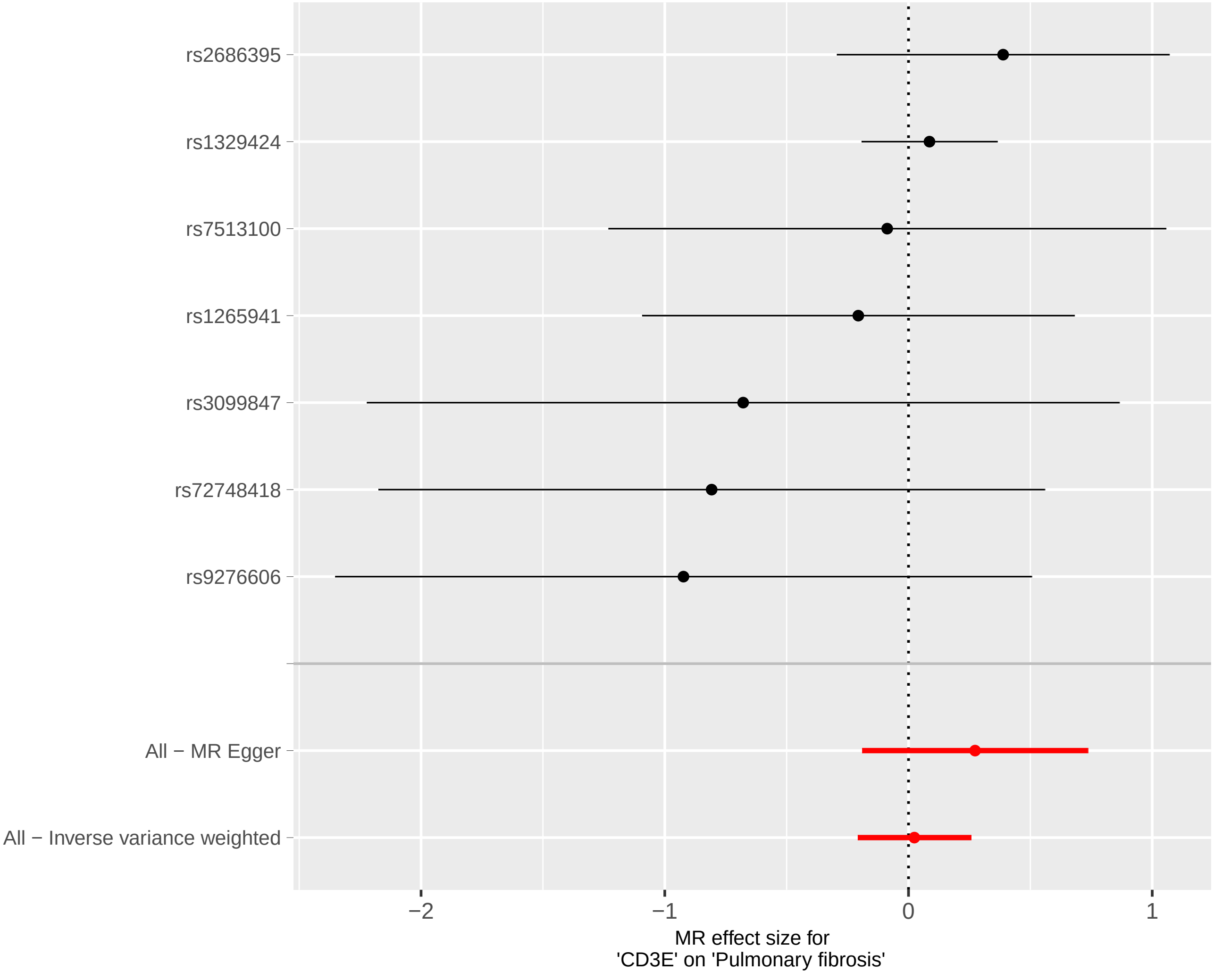

rs2686395
rs1329424
rs7513100
rs1265941
rs3099847
rs72748418
rs9276606
All − MR Egger
All − Inverse variance weighted
−2
−1
0
1
MR effect size for
'CD3E' on 'Pulmonary fibrosis'

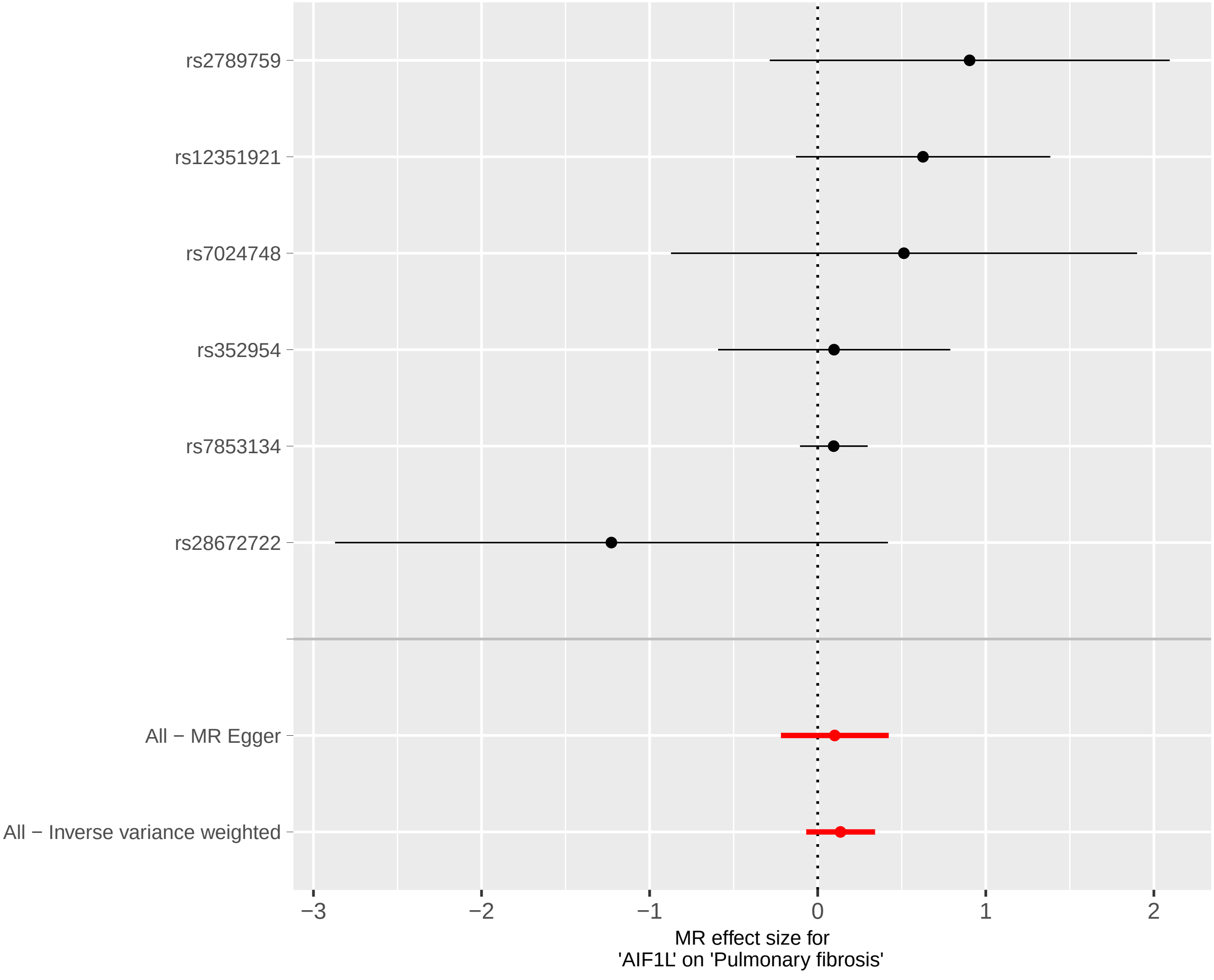

rs2789759
rs12351921
rs7024748
rs352954
rs7853134
rs28672722
All − MR Egger
All − Inverse variance weighted
−3
−2
−1
0
1
2
MR effect size for
'AIF1L' on 'Pulmonary fibrosis'

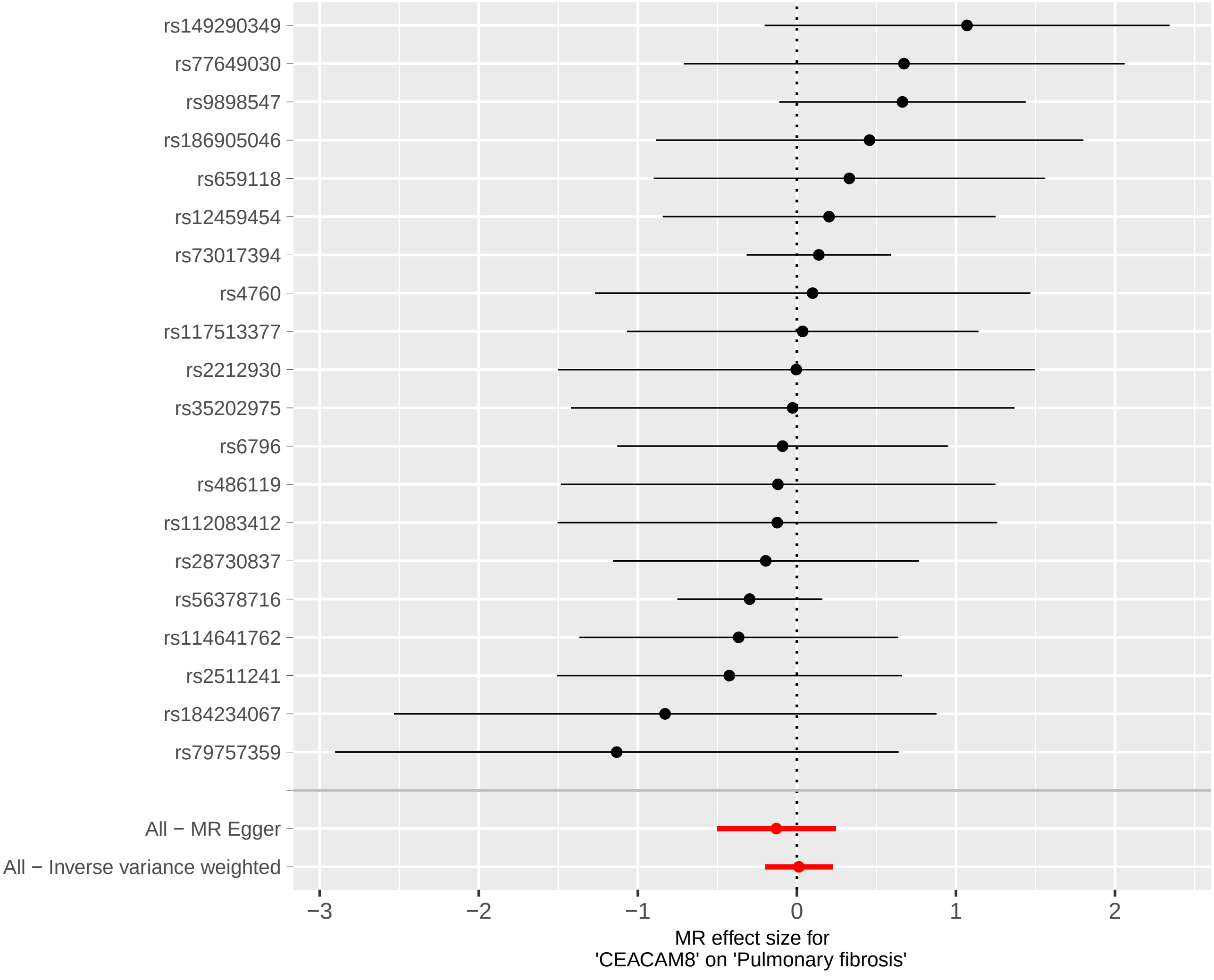
rs149290349
rs77649030
rs9898547
rs186905046
rs659118
rs12459454
rs73017394
rs4760
rs117513377
rs2212930
rs35202975
rs6796
rs486119
rs112083412
rs28730837
rs56378716
rs114641762
rs2511241
rs184234067
rs79757359
All − MR Egger
All − Inverse variance weighted
−3
−2
−1
0
1
2
MR effect size for
'CEACAM8' on 'Pulmonary fibrosis'

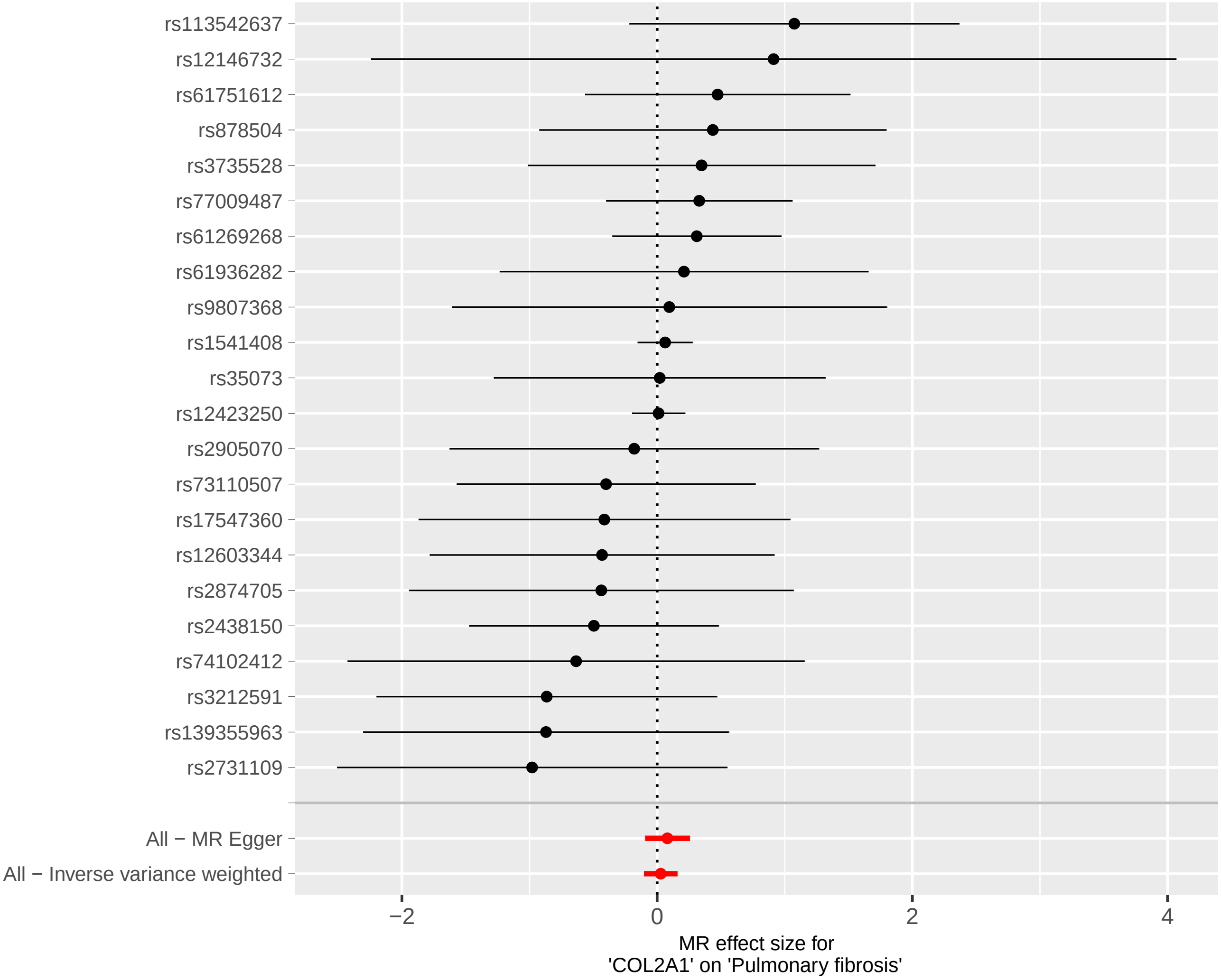

rs113542637
rs12146732
rs61751612
rs878504
rs3735528
rs77009487
rs61269268
rs61936282
rs9807368
rs1541408
rs35073
rs12423250
rs2905070
rs73110507
rs17547360
rs12603344
rs2874705
rs2438150
rs74102412
rs3212591
rs139355963
rs2731109
All − MR Egger
All − Inverse variance weighted
−2
0
2
4
MR effect size for
'COL2A1' on 'Pulmonary fibrosis'

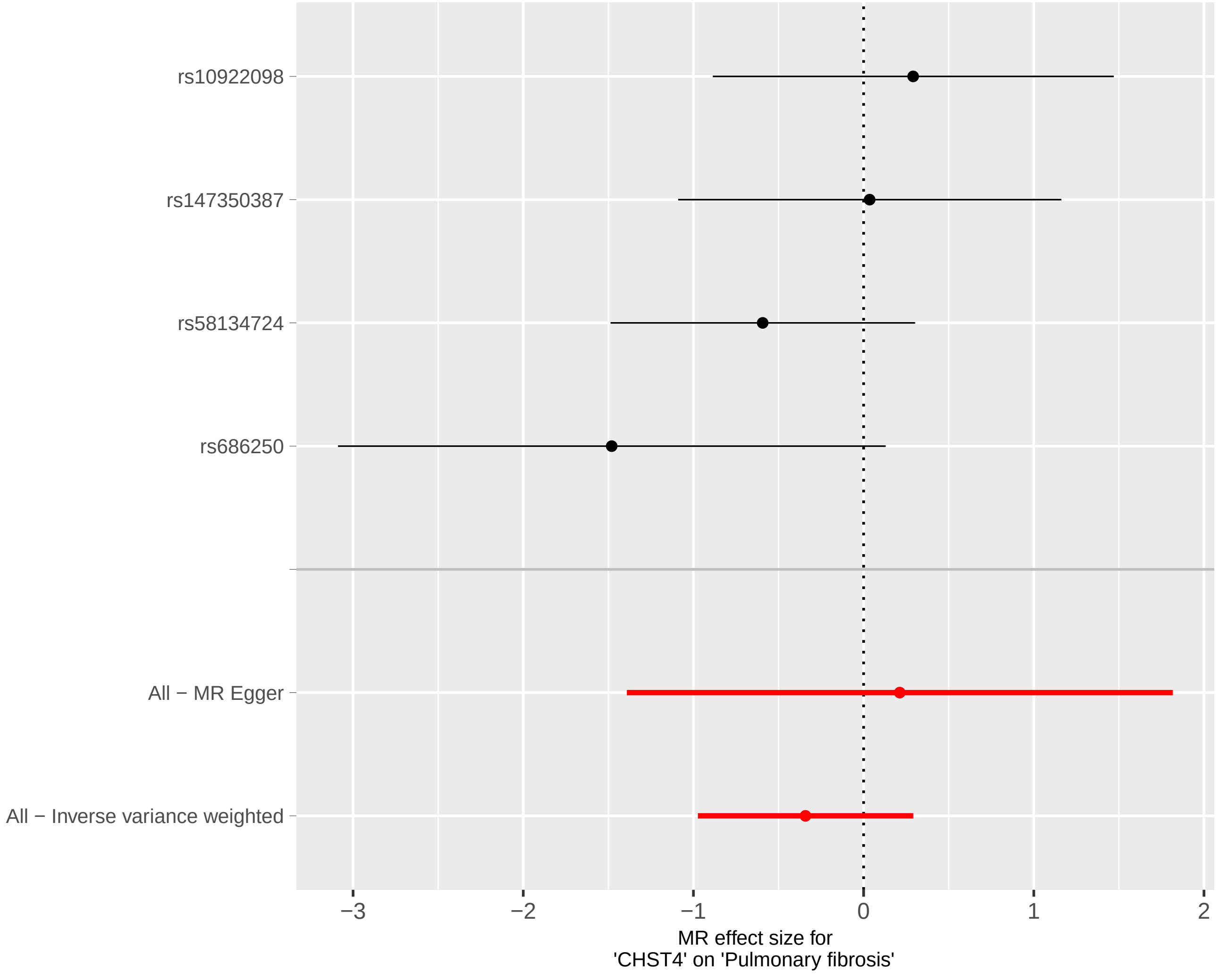
rs10922098
rs147350387
rs58134724
rs686250
All − MR Egger
All − Inverse variance weighted
−3
−2
−1
0
1
2
MR effect size for
'CHST4' on 'Pulmonary fibrosis'

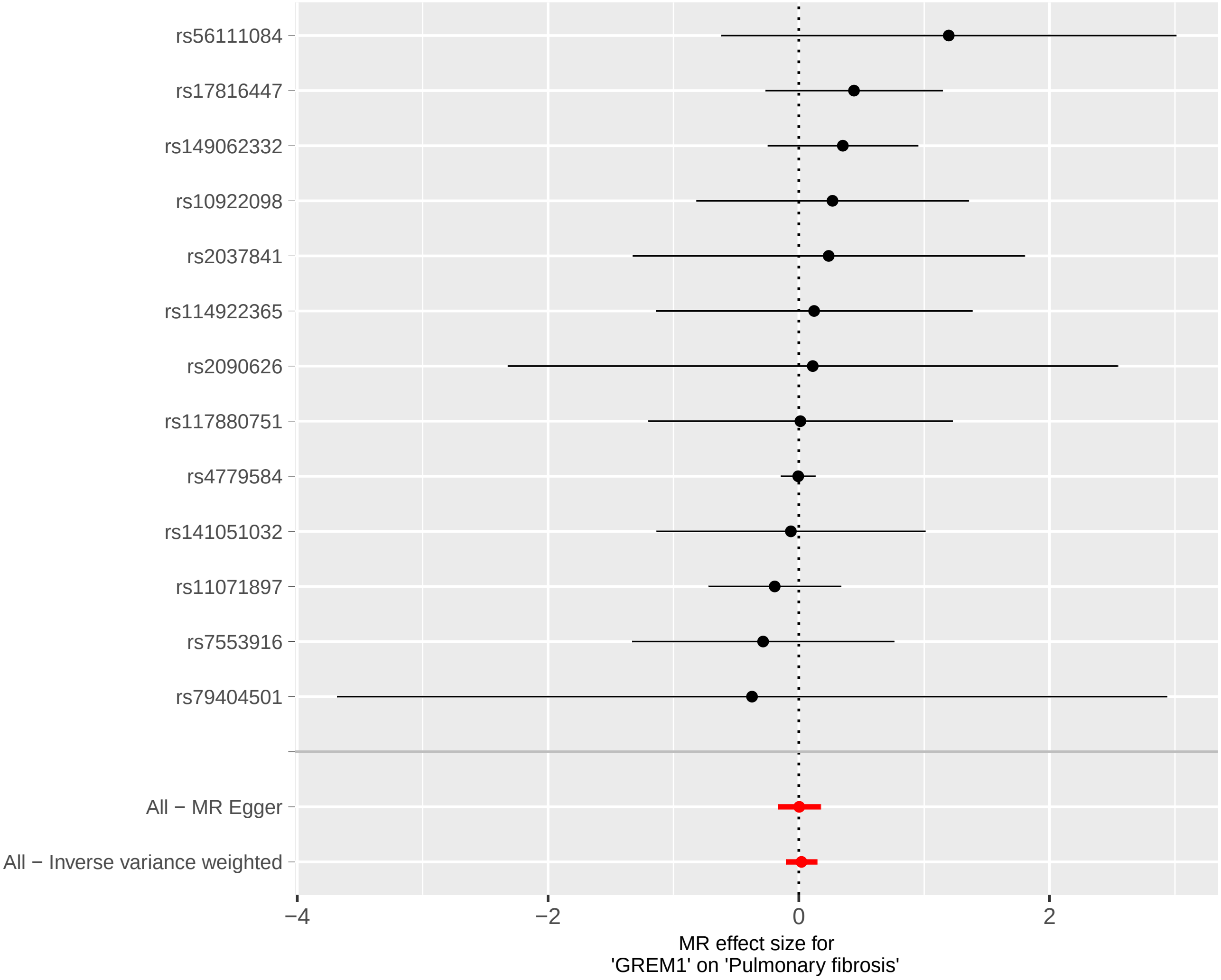

rs56111084
rs17816447
rs149062332
rs10922098
rs2037841
rs114922365
rs2090626
rs117880751
rs4779584
rs141051032
rs11071897
rs7553916
rs79404501
All − MR Egger
All − Inverse variance weighted
−4
−2
0
2
MR effect size for
'GREM1' on 'Pulmonary fibrosis'

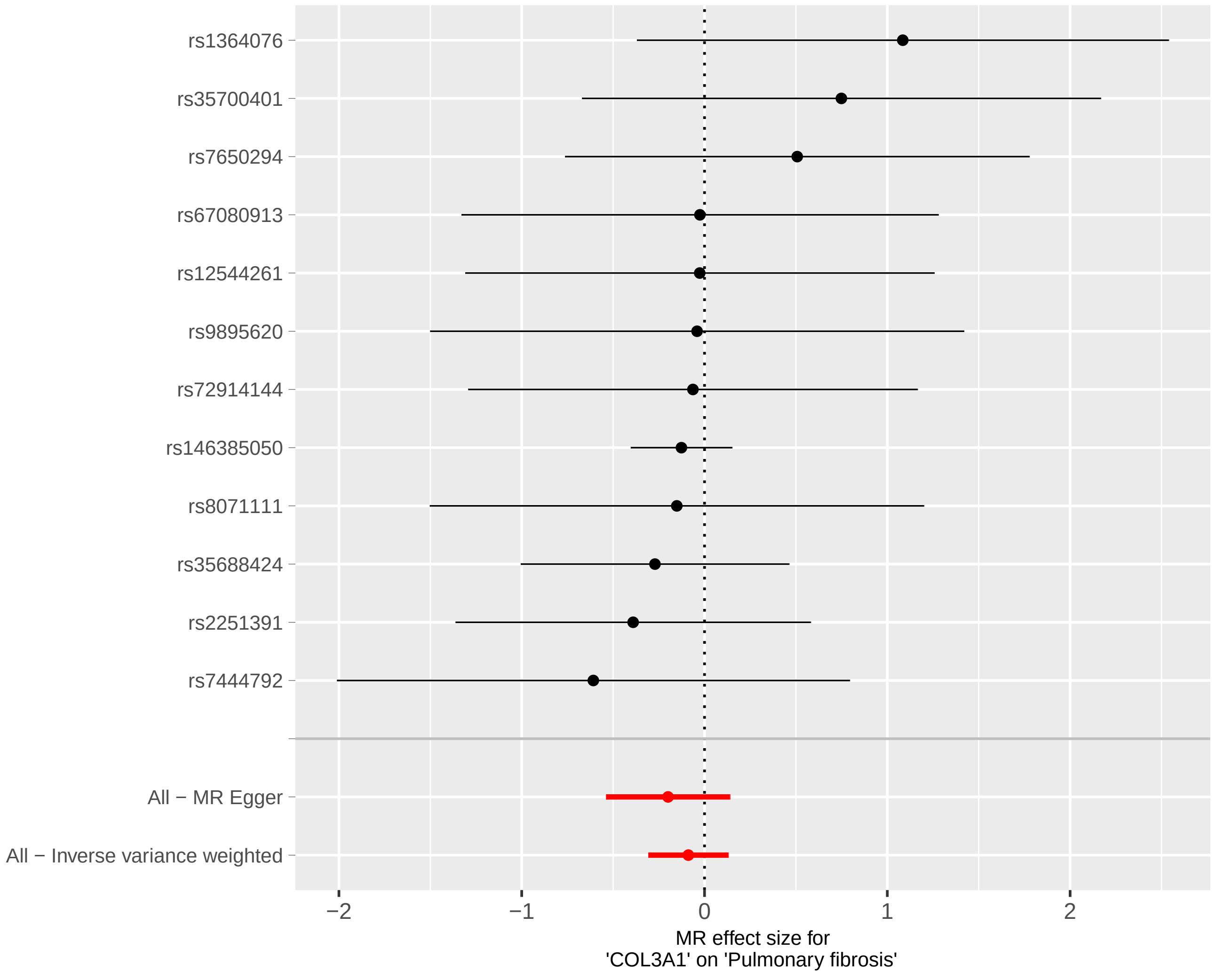

rs1364076
rs35700401
rs7650294
rs67080913
rs12544261
rs9895620
rs72914144
rs146385050
rs8071111
rs35688424
rs2251391
rs7444792
All − MR Egger
All − Inverse variance weighted
−2
−1
0
1
2
MR effect size for
'COL3A1' on 'Pulmonary fibrosis'

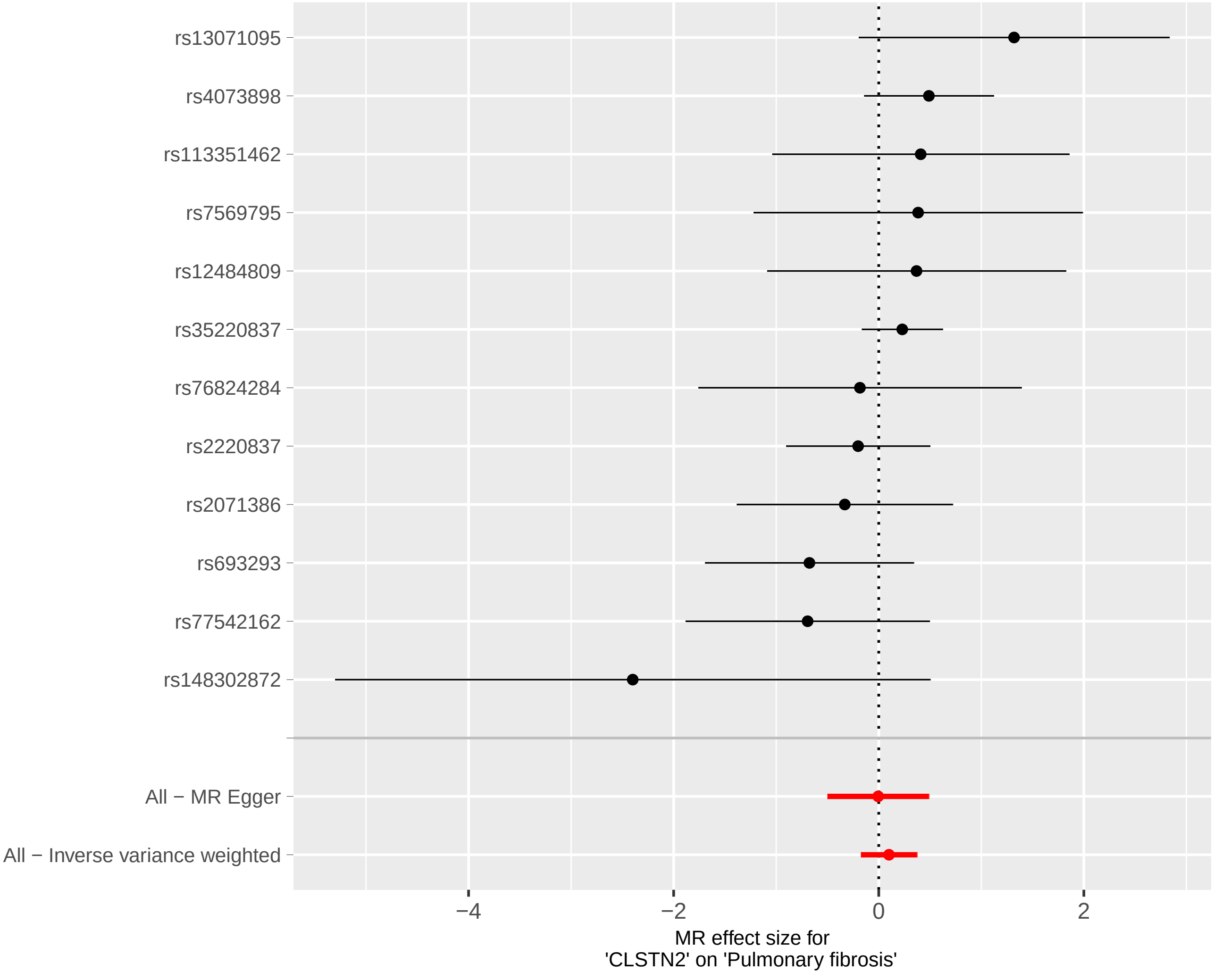

rs13071095
rs4073898
rs113351462
rs7569795
rs12484809
rs35220837
rs76824284
rs2220837
rs2071386
rs693293
rs77542162
rs148302872
All − MR Egger
All − Inverse variance weighted
−4
−2
0
2
MR effect size for
'CLSTN2' on 'Pulmonary fibrosis'

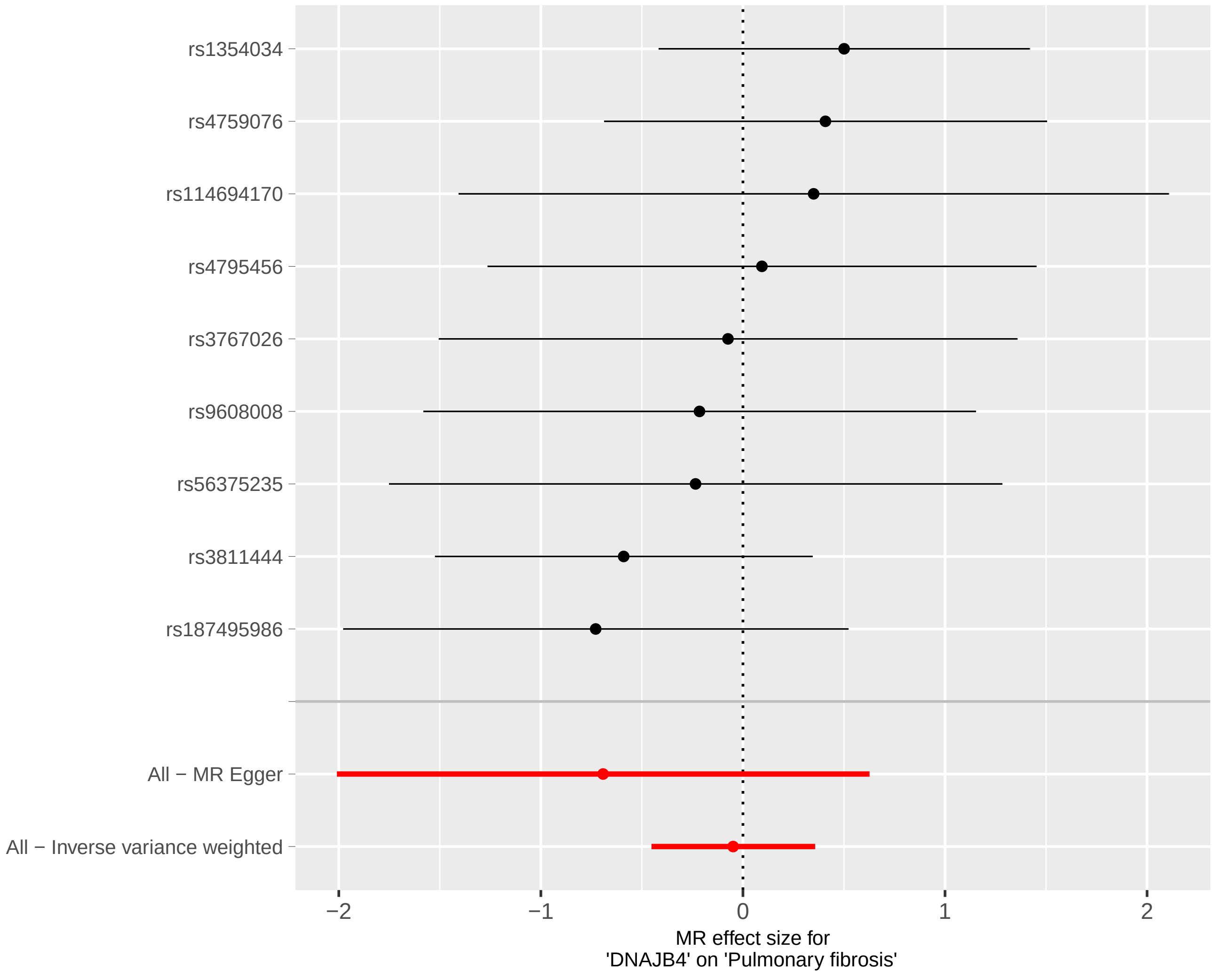
rs1354034
rs4759076
rs114694170
rs4795456
rs3767026
rs9608008
rs56375235
rs3811444
rs187495986
All − MR Egger
All − Inverse variance weighted
−2
−1
0
1
2
MR effect size for
'DNAJB4' on 'Pulmonary fibrosis'

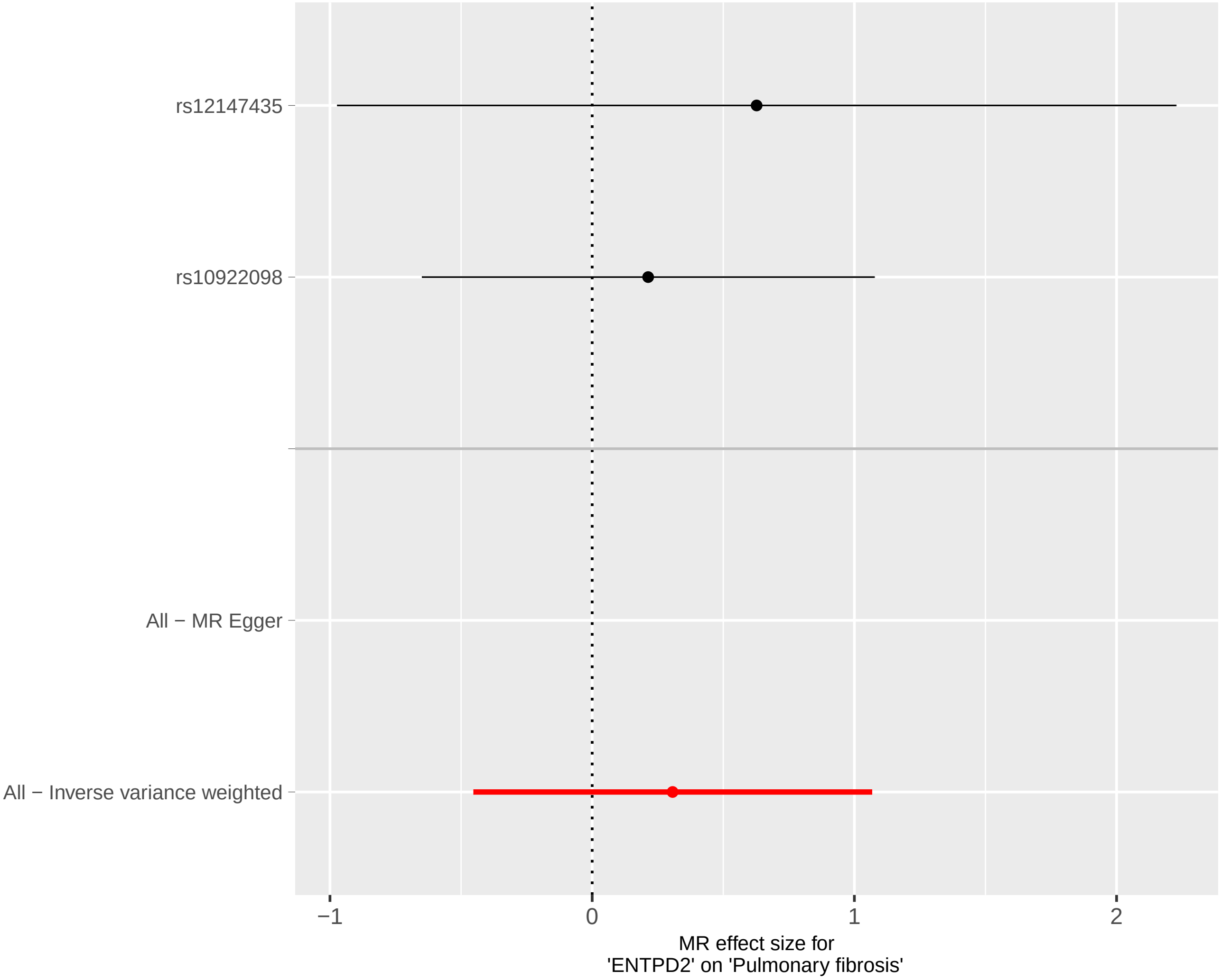

rs12147435
rs10922098
All − MR Egger
All − Inverse variance weighted
−1
0
1
2
MR effect size for
'ENTPD2' on 'Pulmonary fibrosis'

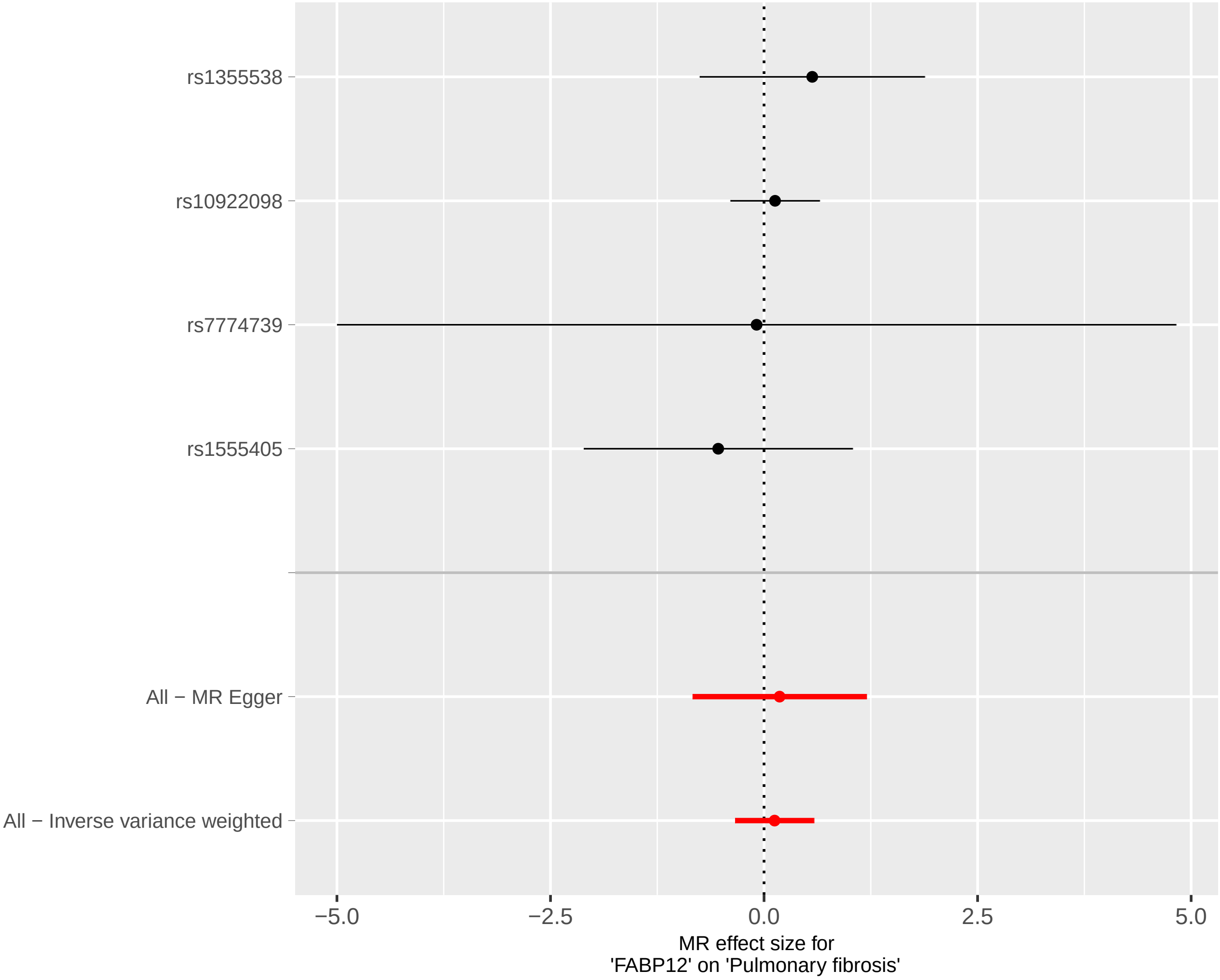
rs1355538
rs10922098
rs7774739
rs1555405
All − MR Egger
All − Inverse variance weighted
−5.0
−2.5
0.0
2.5
5.0
MR effect size for
'FABP12' on 'Pulmonary fibrosis'

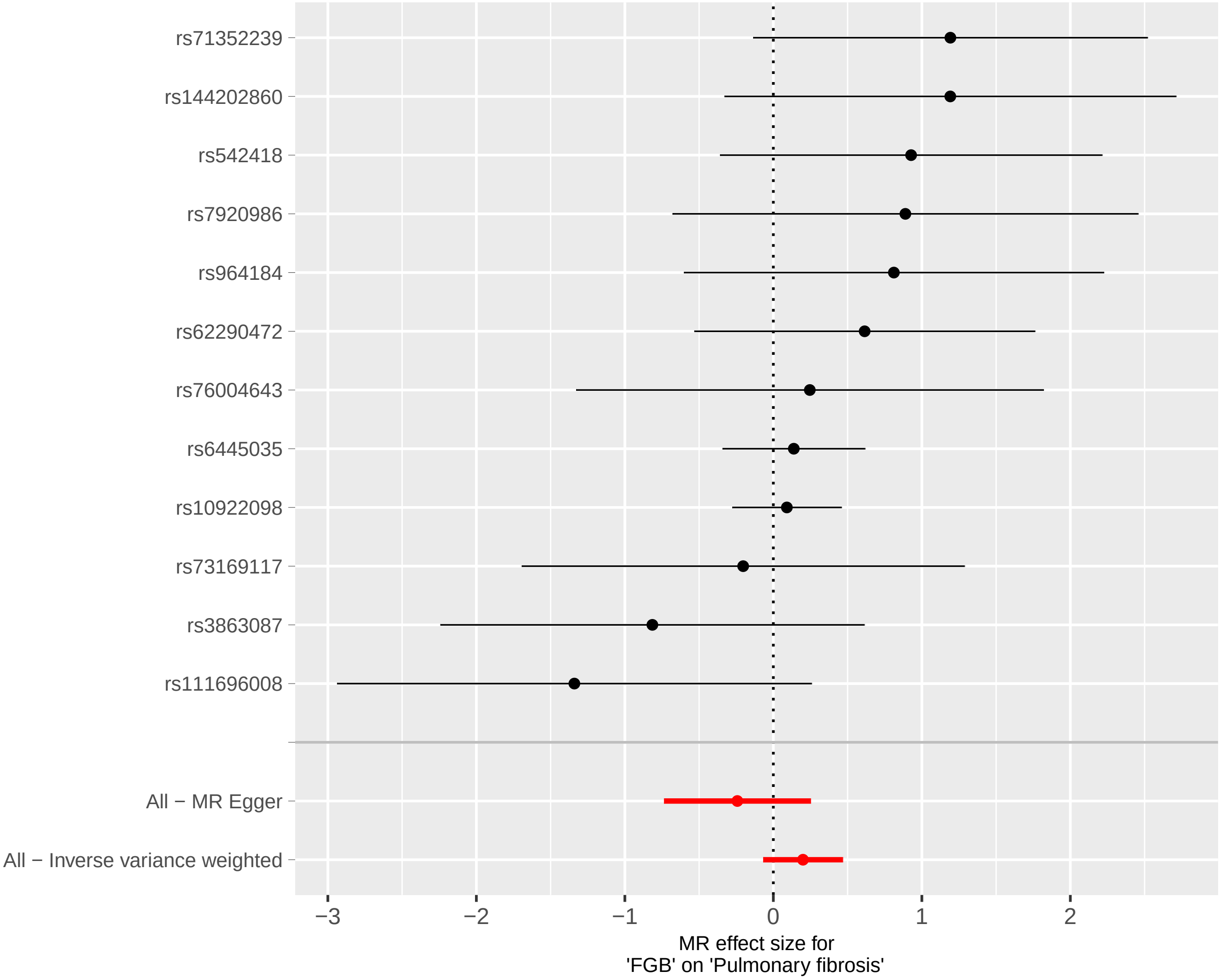
rs71352239
rs144202860
rs542418
rs7920986
rs964184
rs62290472
rs76004643
rs6445035
rs10922098
rs73169117
rs3863087
rs111696008
All − MR Egger
All − Inverse variance weighted
−3
−2
−1
0
1
2
MR effect size for
'FGB' on 'Pulmonary fibrosis'

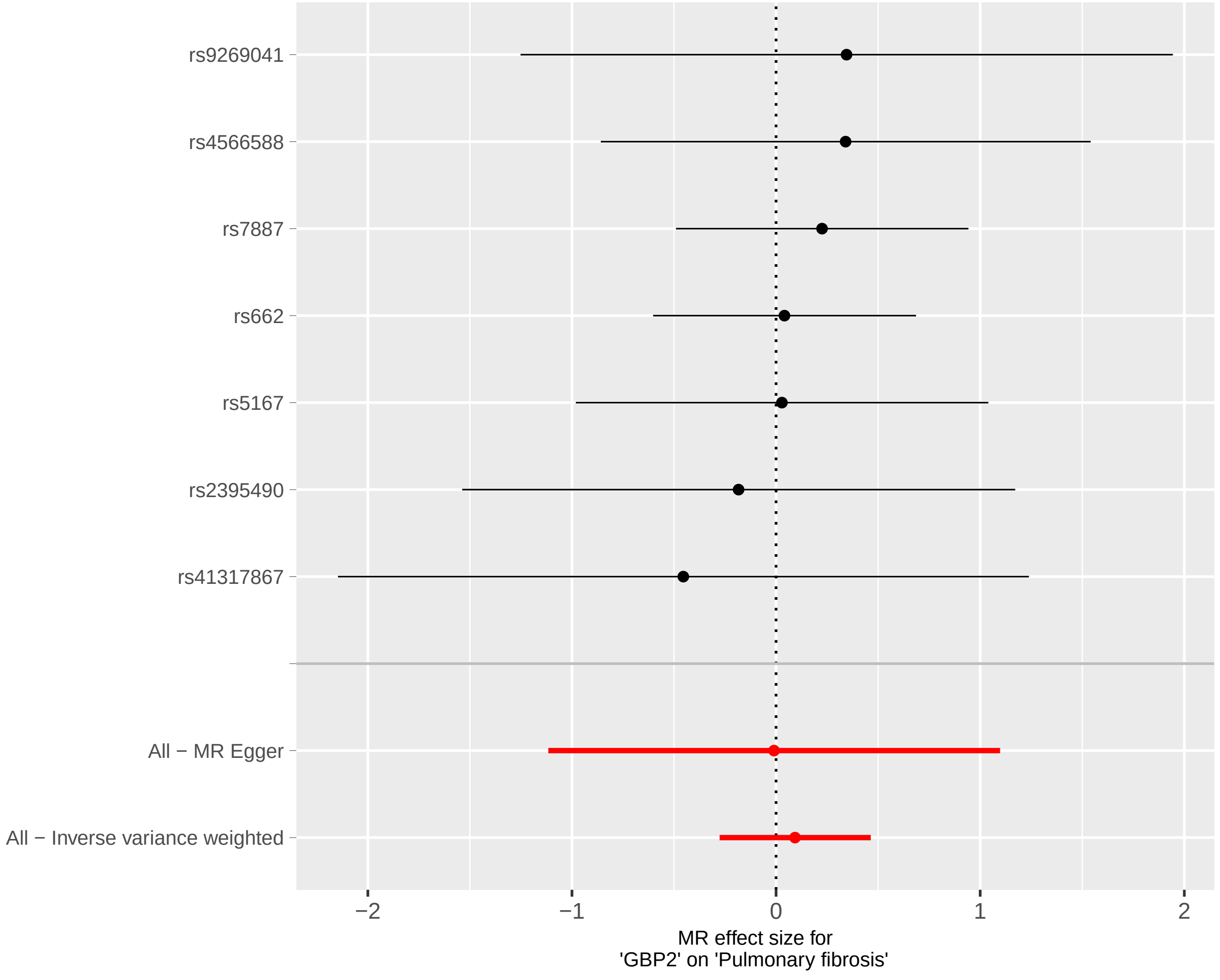

rs9269041
rs4566588
rs7887
rs662
rs5167
rs2395490
rs41317867
All − MR Egger
All − Inverse variance weighted
−2
−1
0
1
2
MR effect size for
'GBP2' on 'Pulmonary fibrosis'

Insufficient number of SNPs

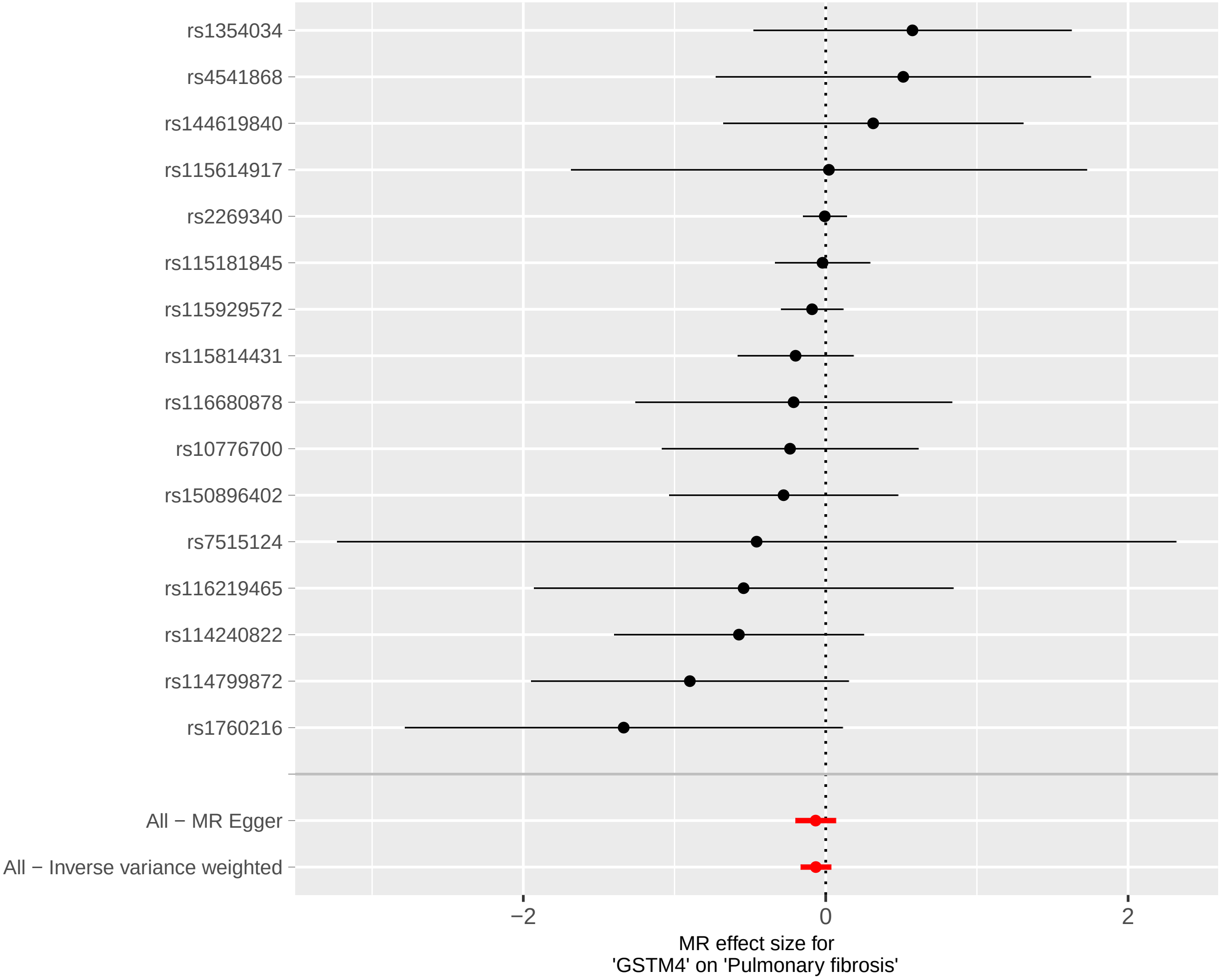
rs1354034
rs4541868
rs144619840
rs115614917
rs2269340
rs115181845
rs115929572
rs115814431
rs116680878
rs10776700
rs150896402
rs7515124
rs116219465
rs114240822
rs114799872
rs1760216
All − MR Egger
All − Inverse variance weighted
−2
0
2
MR effect size for
'GSTM4' on 'Pulmonary fibrosis'

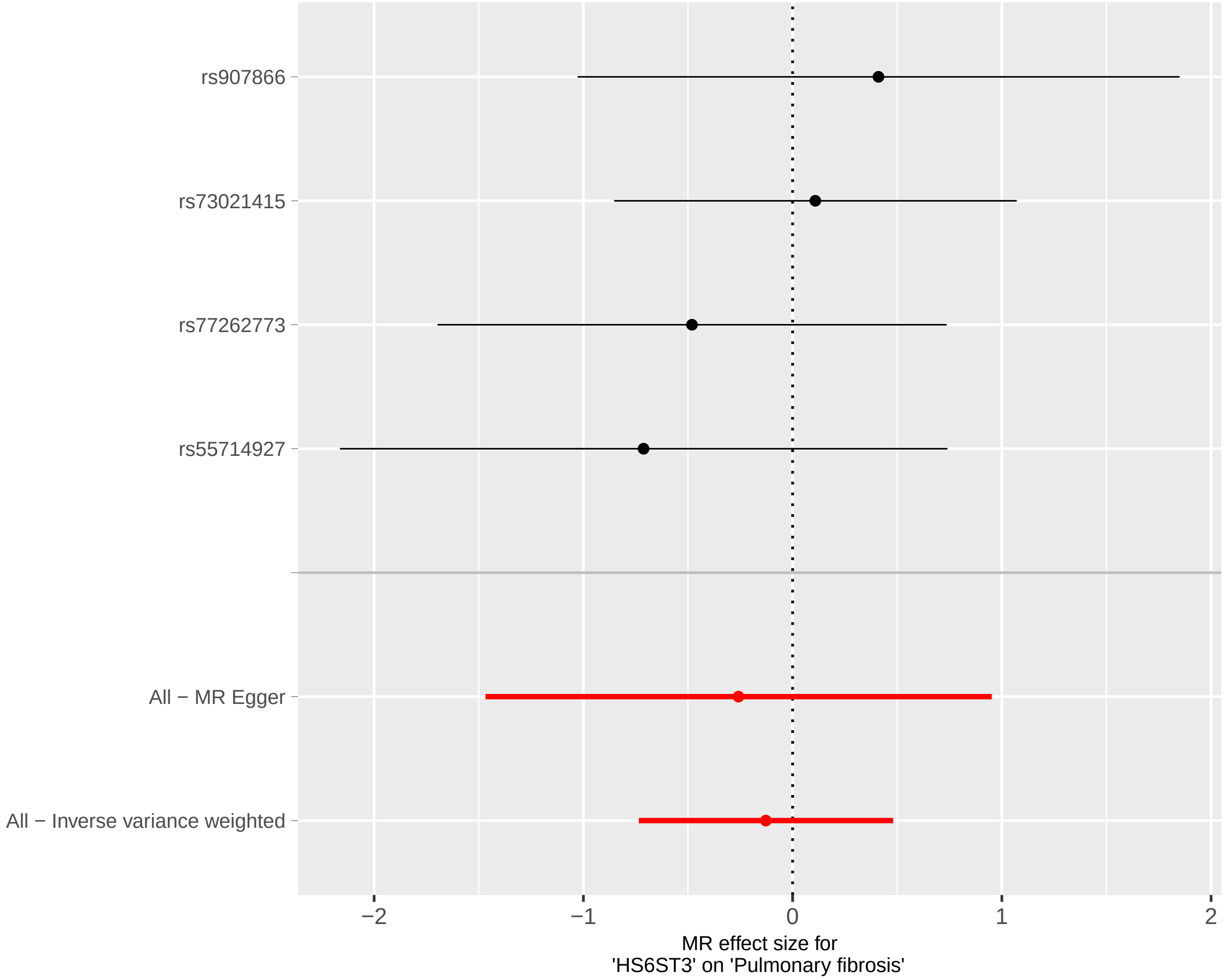
rs907866
rs73021415
rs77262773
rs55714927
All − MR Egger
All − Inverse variance weighted
−2
−1
0
1
2
MR effect size for
'HS6ST3' on 'Pulmonary fibrosis'

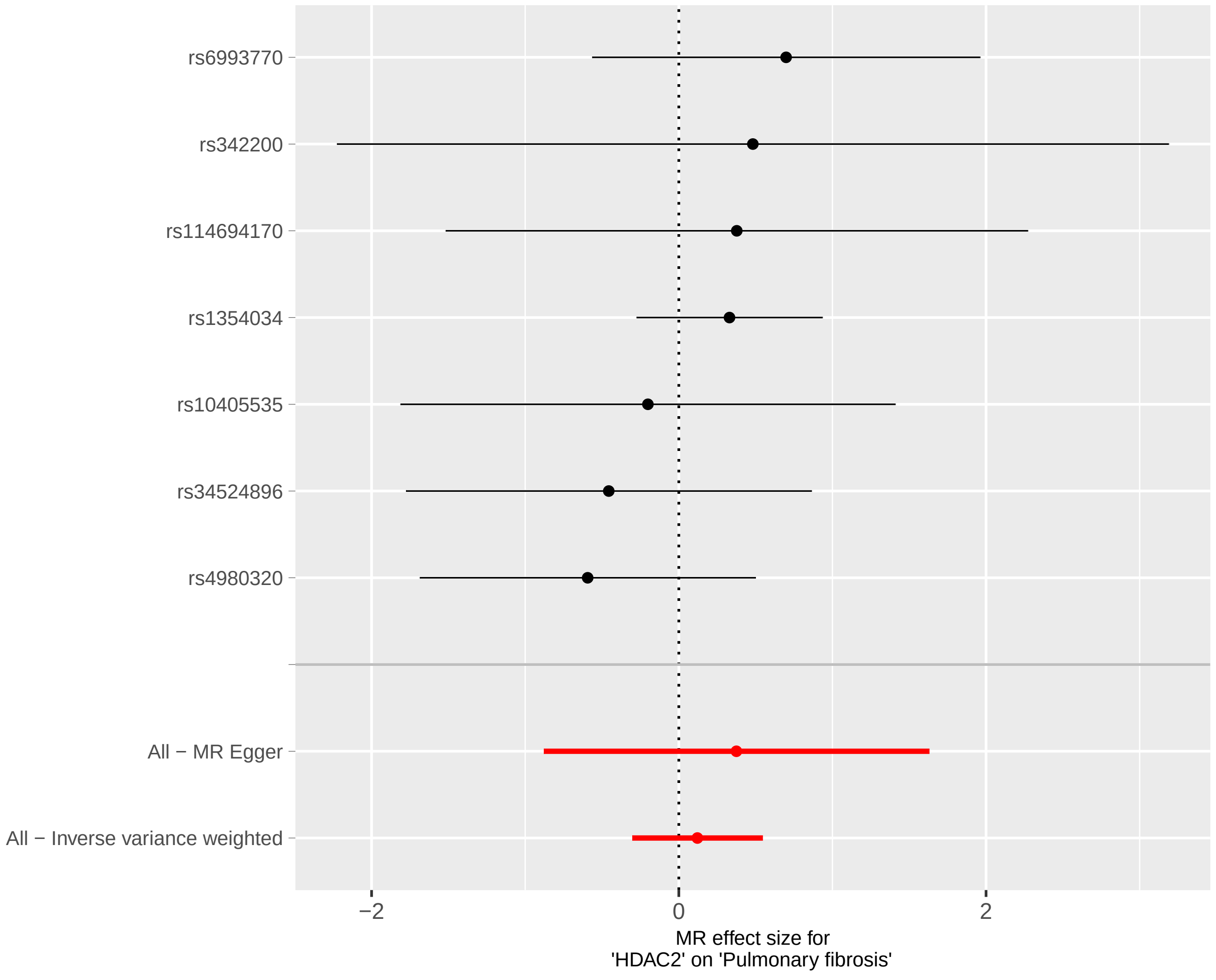
rs6993770
rs342200
rs114694170
rs1354034
rs10405535
rs34524896
rs4980320
All − MR Egger
All − Inverse variance weighted
−2
0
2
MR effect size for
'HDAC2' on 'Pulmonary fibrosis'

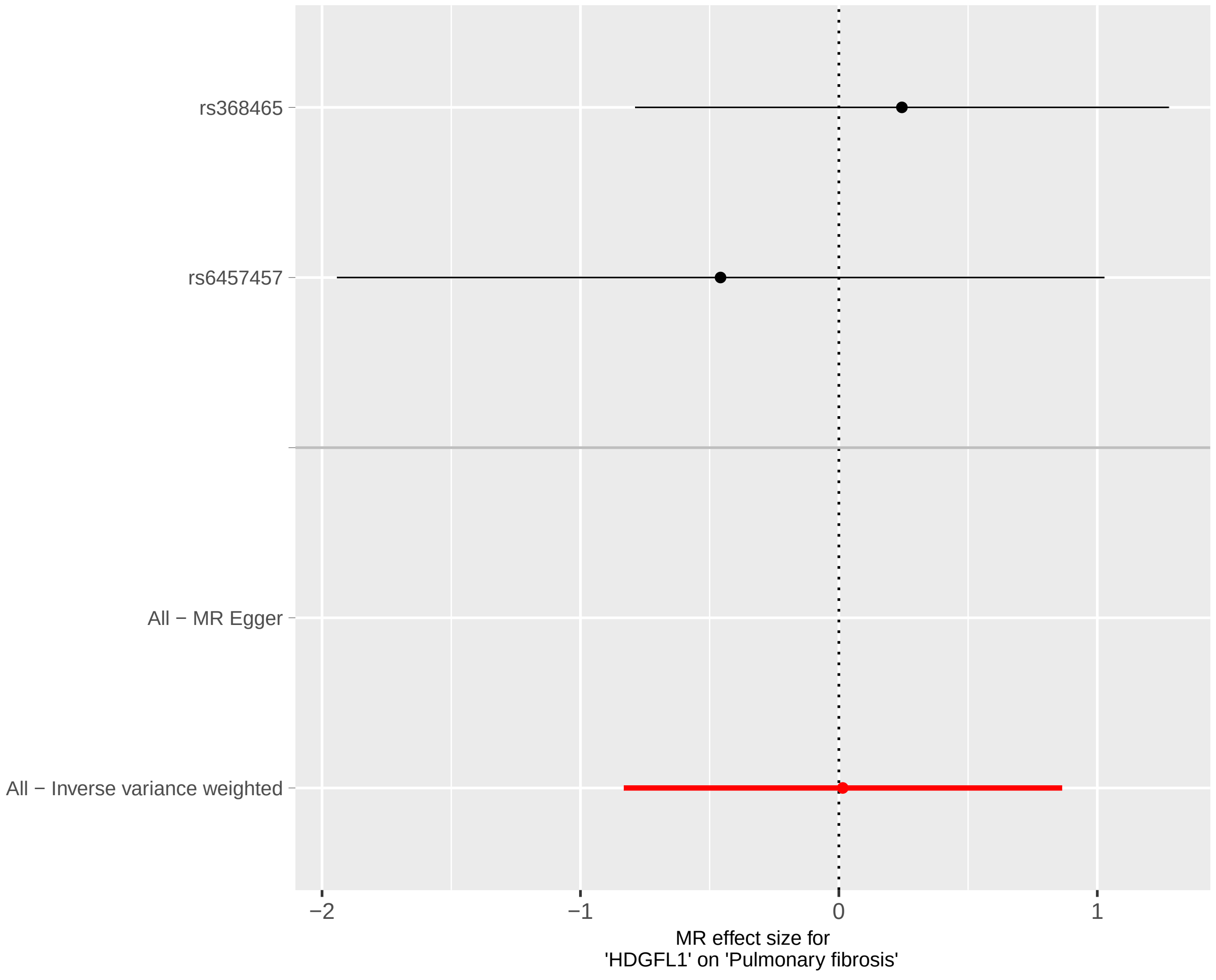
rs368465
rs6457457
All − MR Egger
All − Inverse variance weighted
−2
−1
0
1
MR effect size for
'HDGFL1' on 'Pulmonary fibrosis'

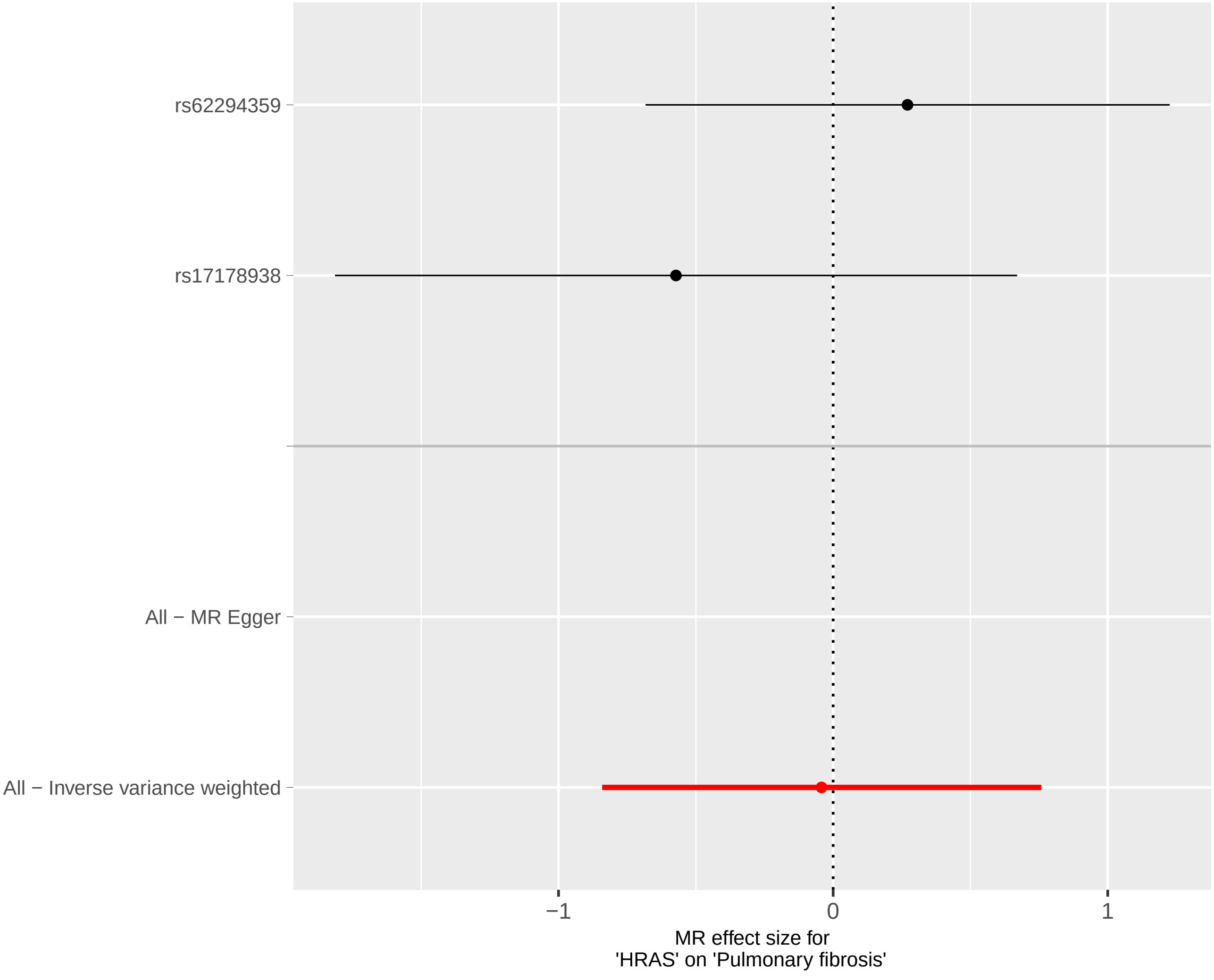
rs62294359
rs17178938
All − MR Egger
All − Inverse variance weighted
−1
0
1
MR effect size for
'HRAS' on 'Pulmonary fibrosis'

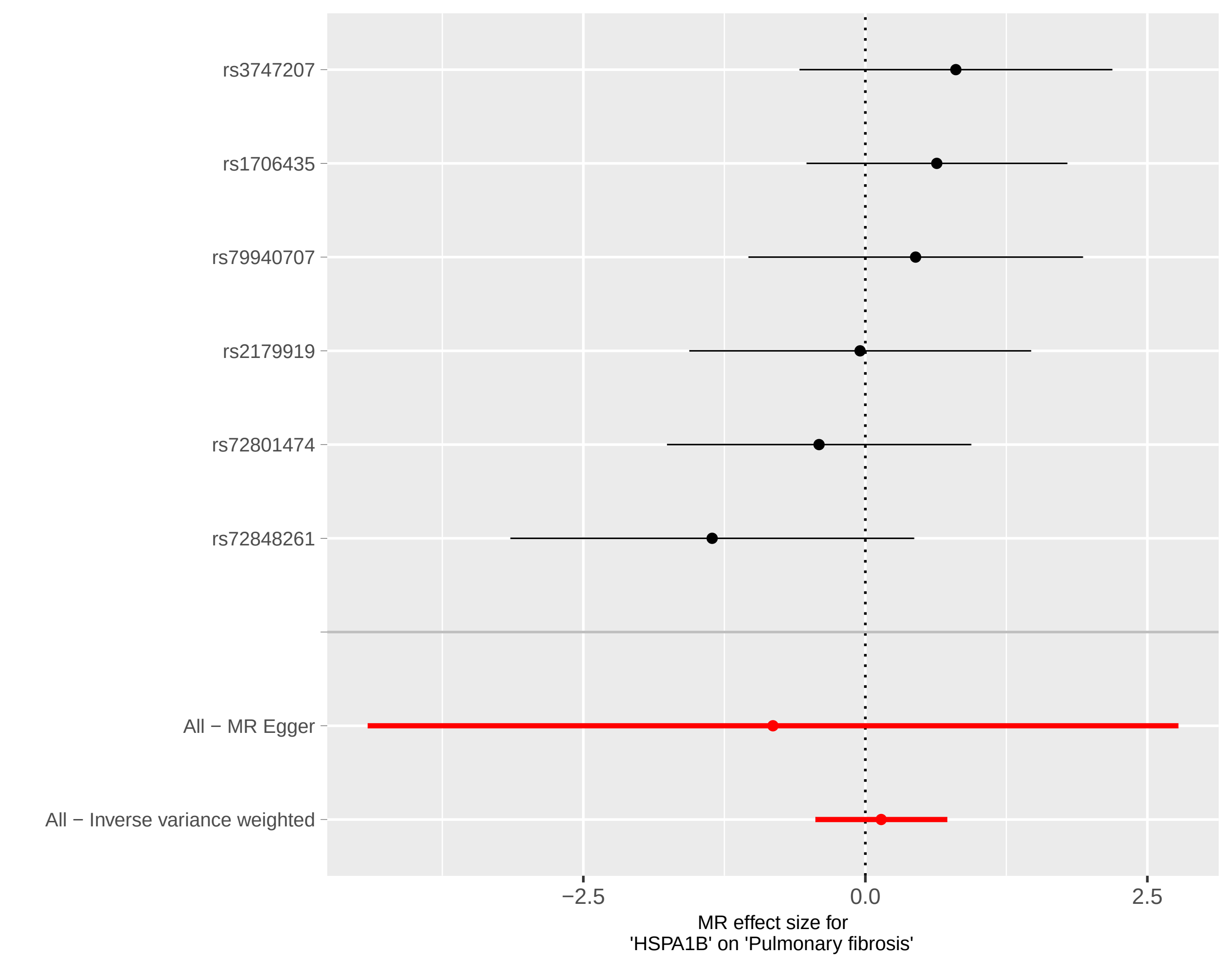

rs3747207
rs1706435
rs79940707
rs2179919
rs72801474
rs72848261
All − MR Egger
All − Inverse variance weighted
−2.5
0.0
2.5
MR effect size for
'HSPA1B' on 'Pulmonary fibrosis'

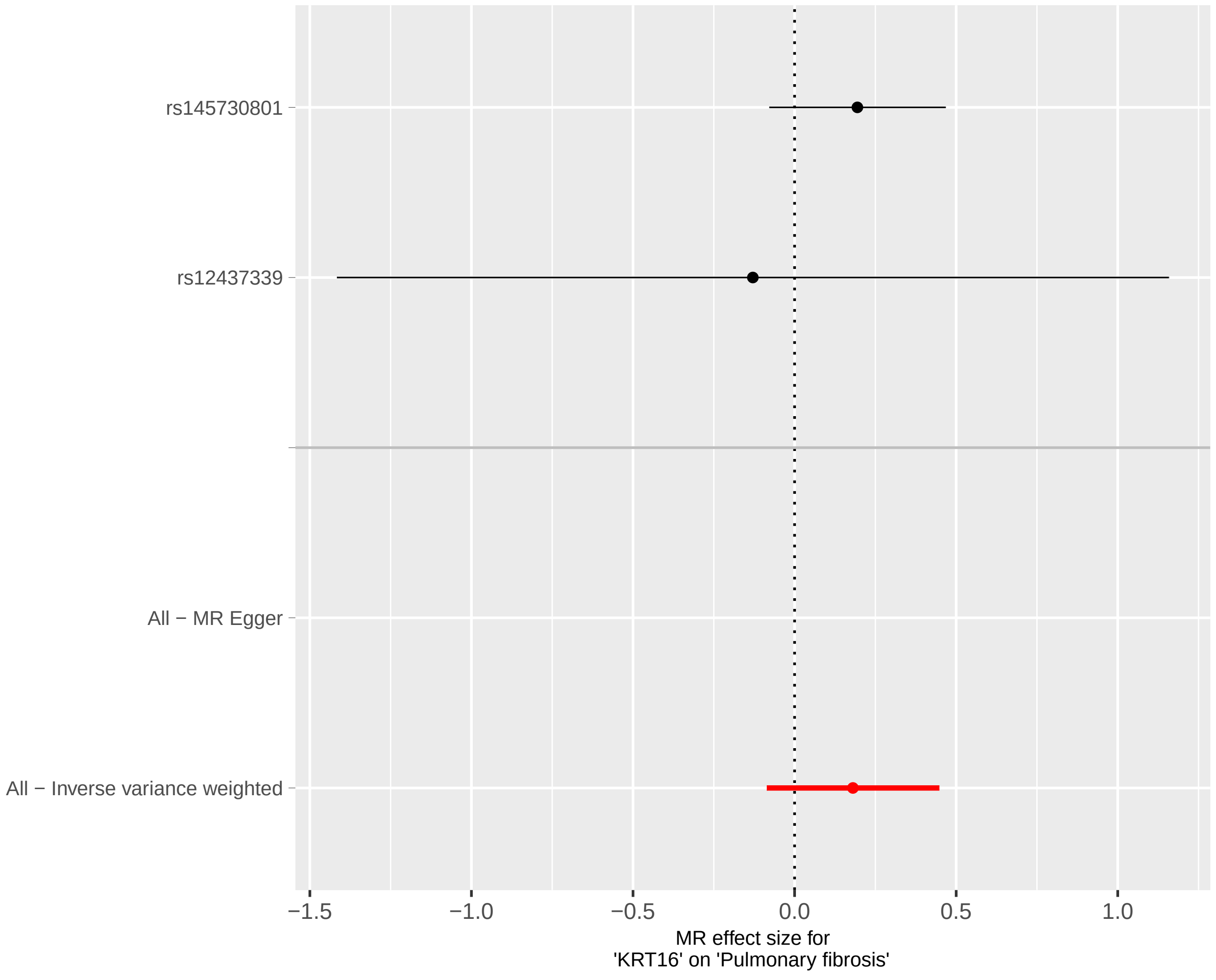

rs145730801
rs12437339
All − MR Egger
All − Inverse variance weighted
−1.5
−1.0
−0.5
0.0
0.5
1.0
MR effect size for
'KRT16' on 'Pulmonary fibrosis'

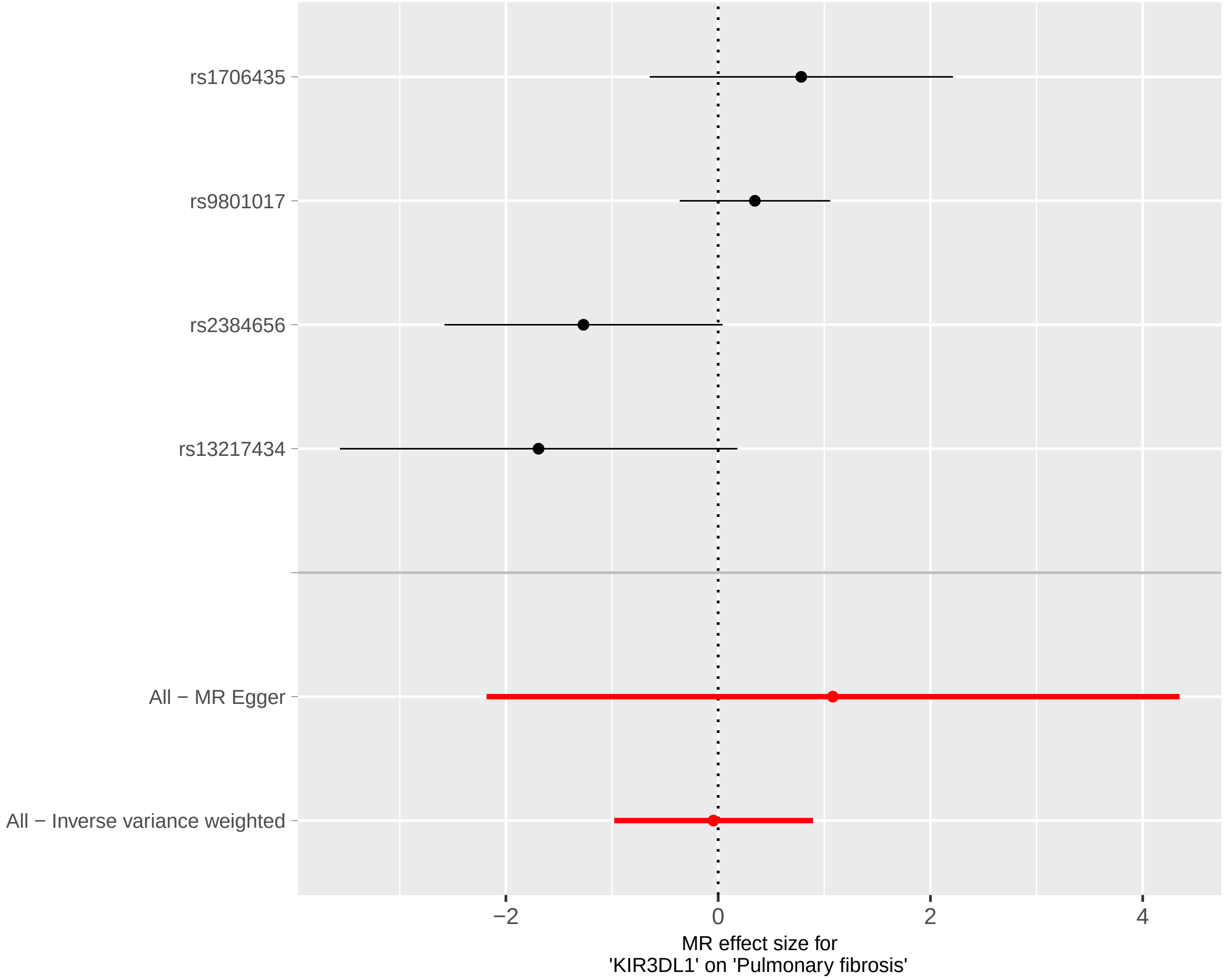

rs1706435
rs9801017
rs2384656
rs13217434
All − MR Egger
All − Inverse variance weighted
−2
0
2
4
MR effect size for
'KIR3DL1' on 'Pulmonary fibrosis'

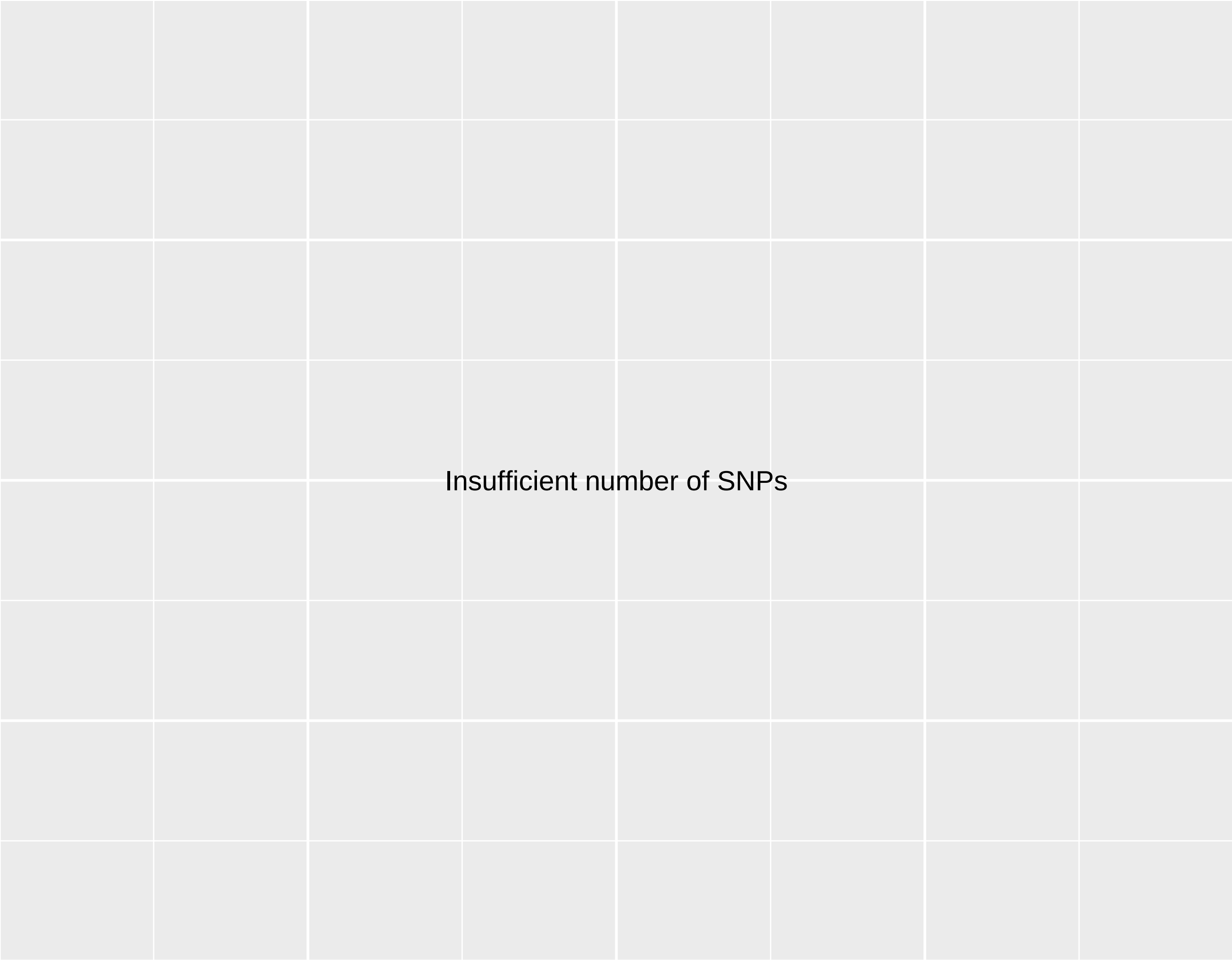
Insufficient number of SNPs

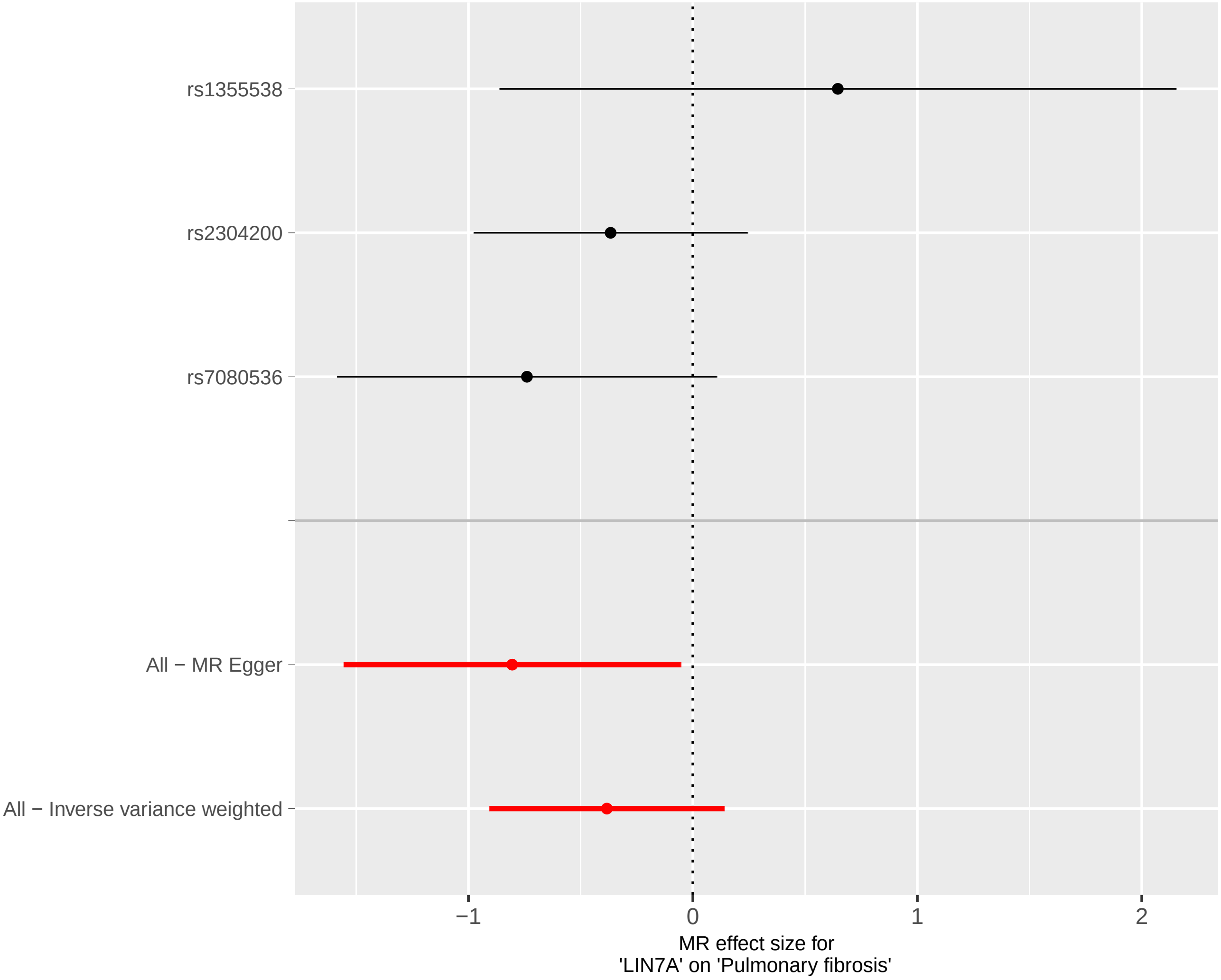

rs1355538
rs2304200
rs7080536
All − MR Egger
All − Inverse variance weighted
−1
0
1
2
MR effect size for
'LIN7A' on 'Pulmonary fibrosis'

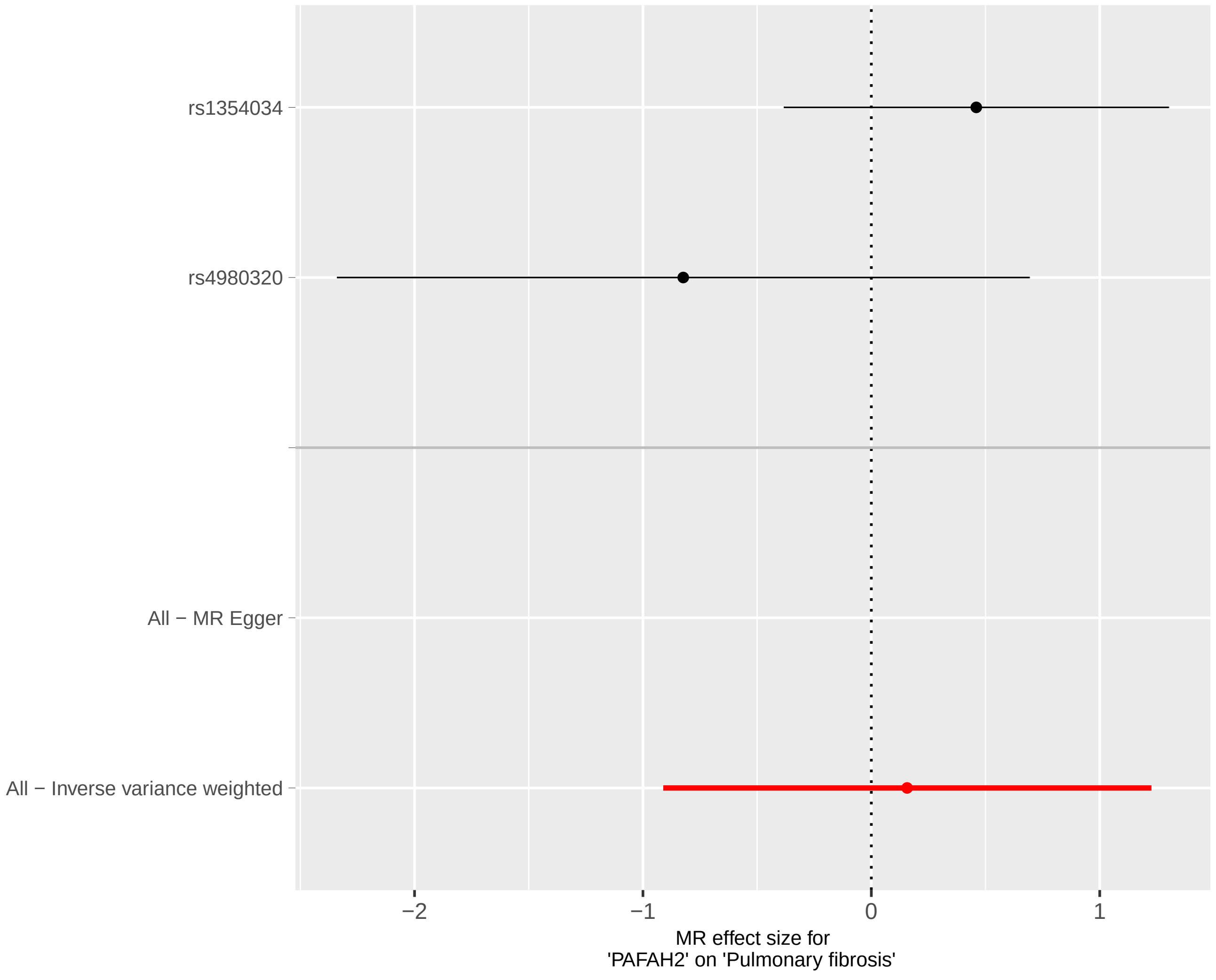
rs1354034
rs4980320
All − MR Egger
All − Inverse variance weighted
−2
−1
0
1
MR effect size for
'PAFAH2' on 'Pulmonary fibrosis'

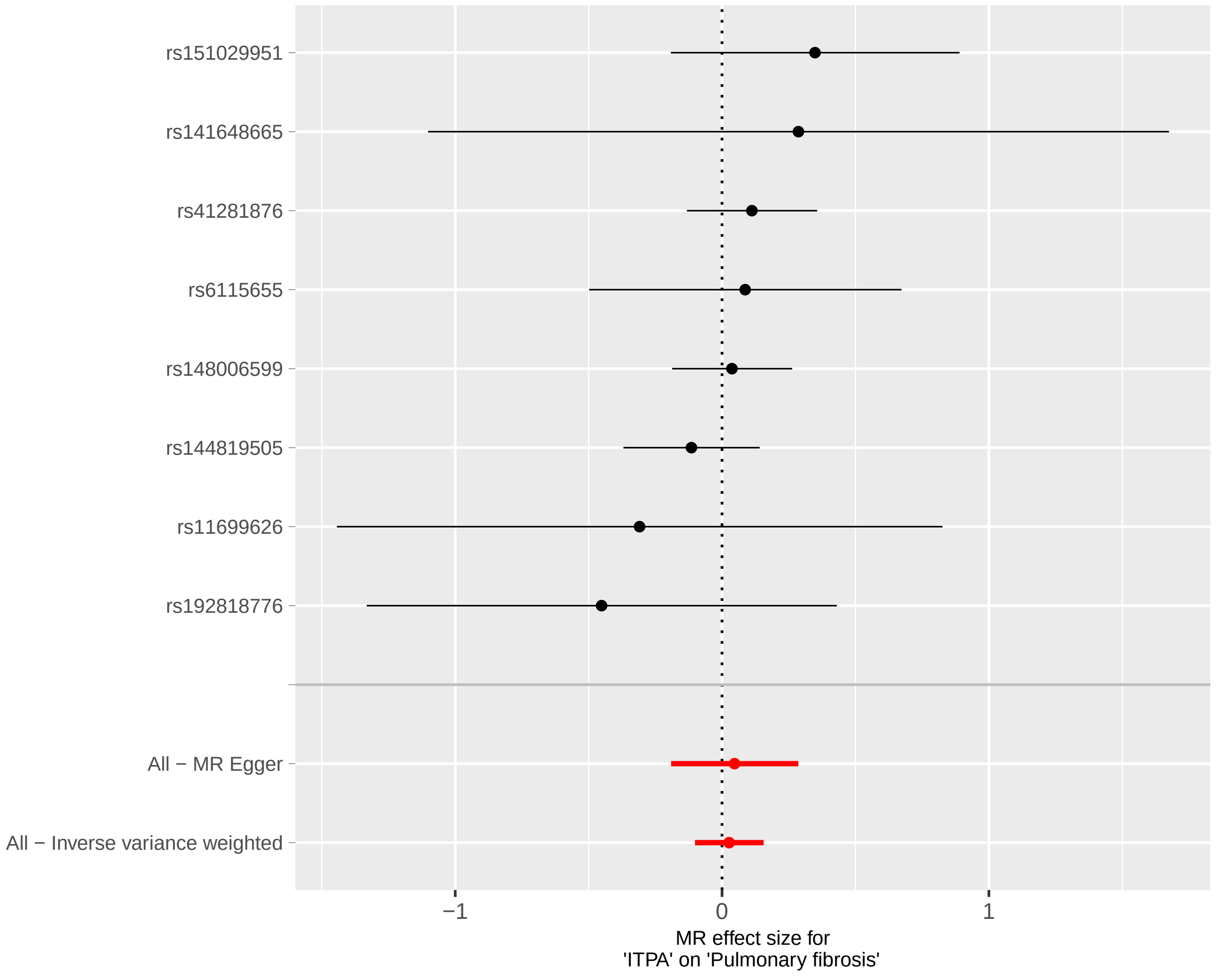
rs151029951
rs141648665
rs41281876
rs6115655
rs148006599
rs144819505
rs11699626
rs192818776
All − MR Egger
All − Inverse variance weighted
−1
0
1
MR effect size for
'ITPA' on 'Pulmonary fibrosis'

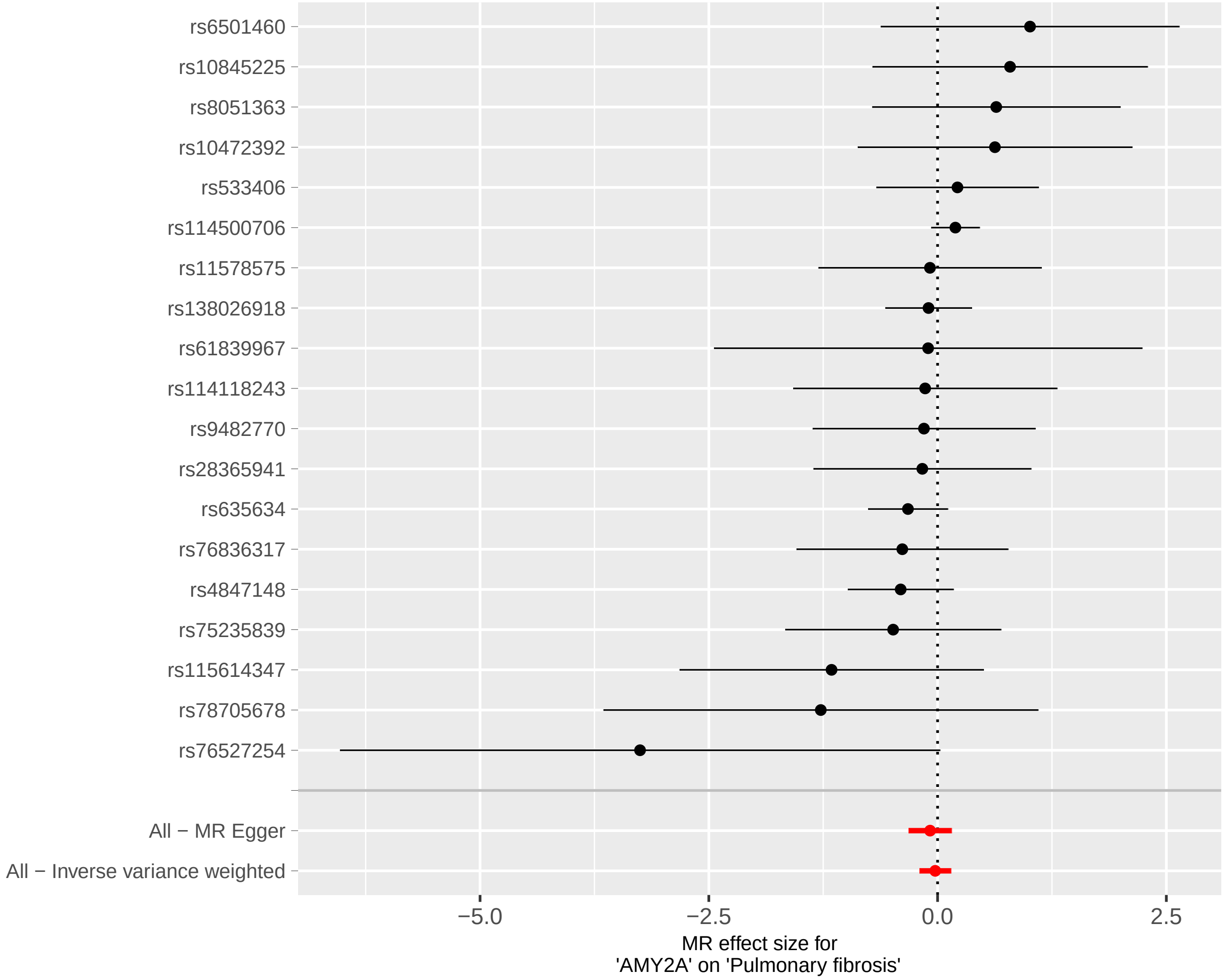

rs6501460
rs10845225
rs8051363
rs10472392
rs533406
rs114500706
rs11578575
rs138026918
rs61839967
rs114118243
rs9482770
rs28365941
rs635634
rs76836317
rs4847148
rs75235839
rs115614347
rs78705678
rs76527254
All − MR Egger
All − Inverse variance weighted
−5.0
−2.5
0.0
2.5
MR effect size for
'AMY2A' on 'Pulmonary fibrosis'

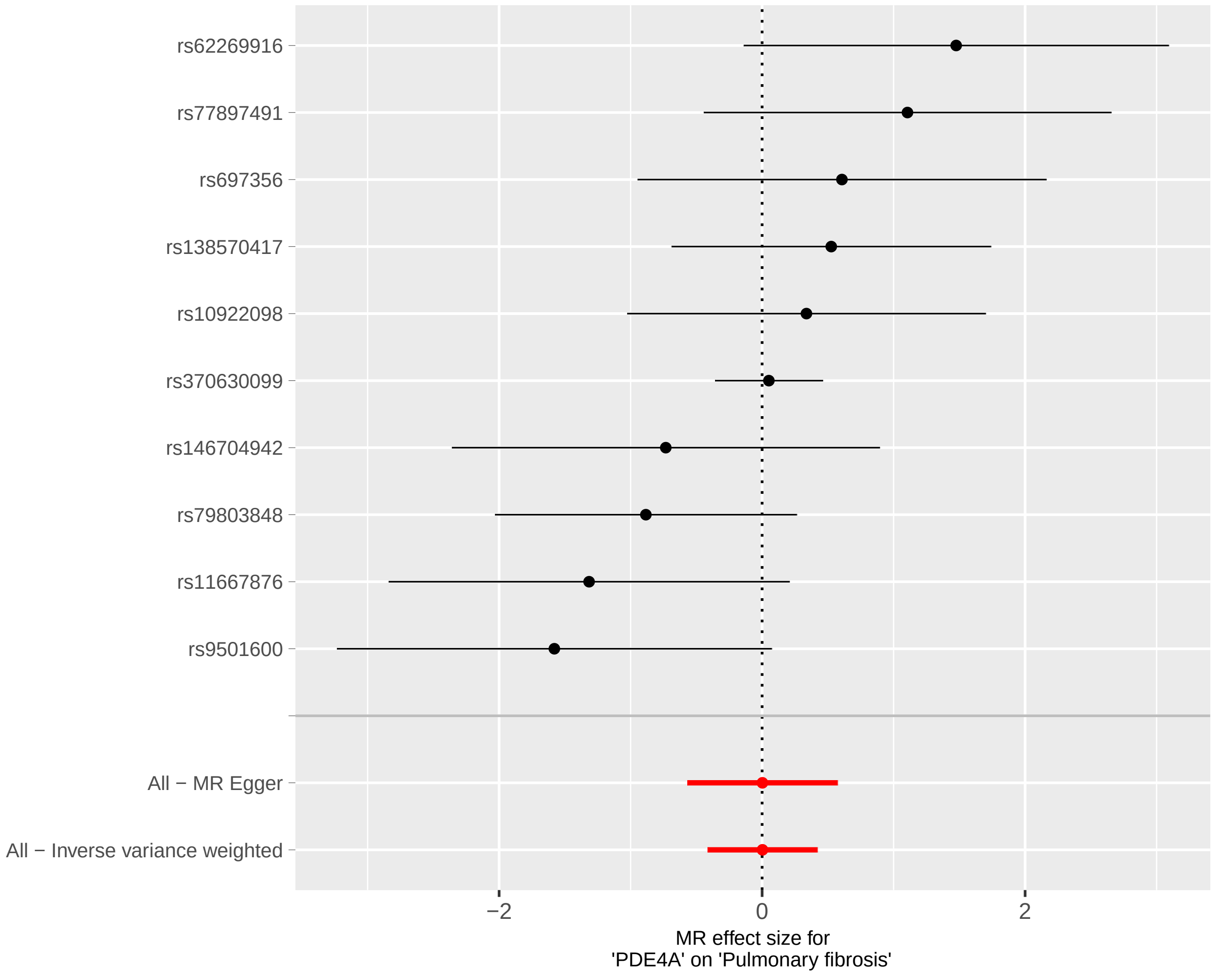

rs62269916
rs77897491
rs697356
rs138570417
rs10922098
rs370630099
rs146704942
rs79803848
rs11667876
rs9501600
All − MR Egger
All − Inverse variance weighted
−2
0
2
MR effect size for
'PDE4A' on 'Pulmonary fibrosis'

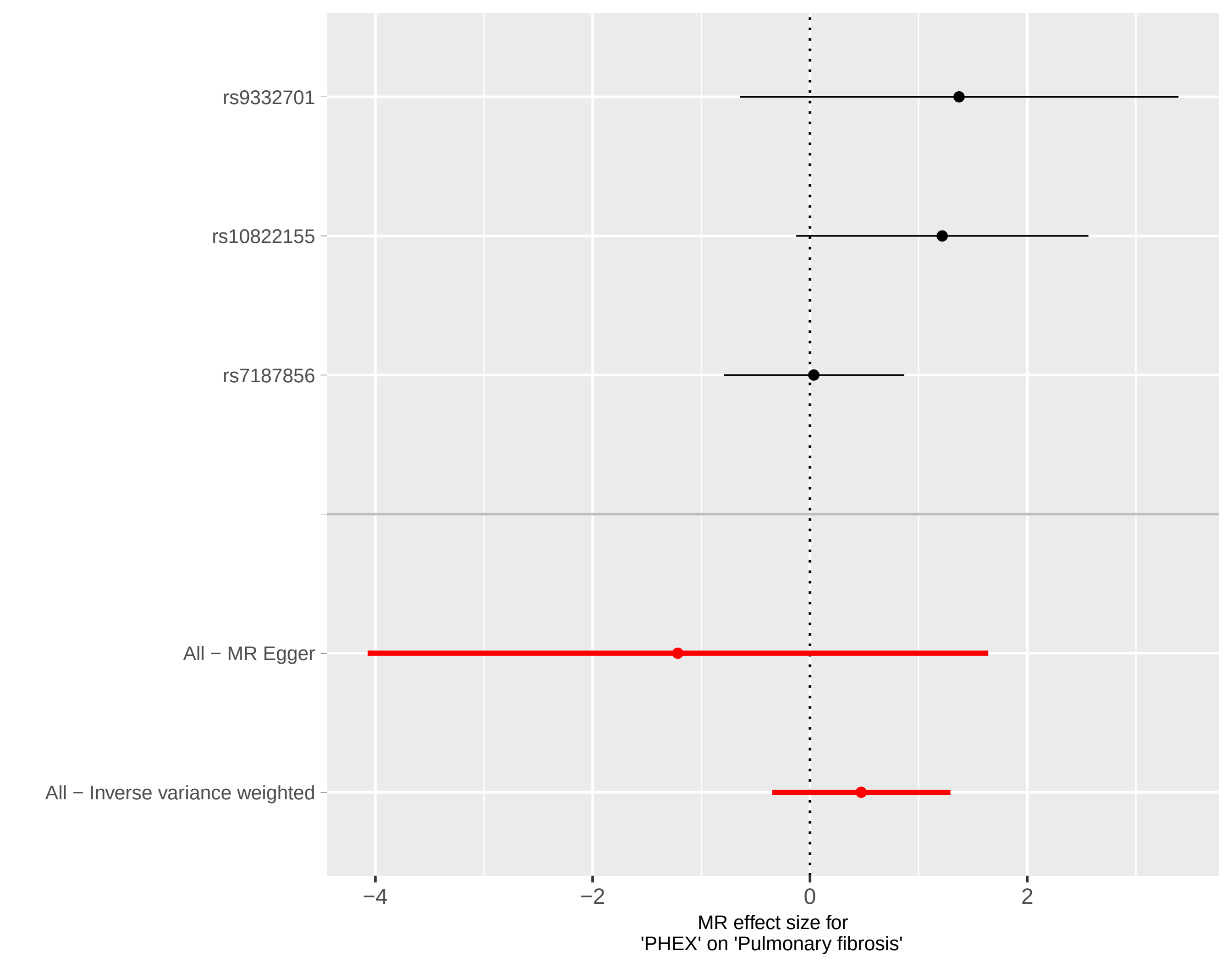
rs9332701
rs10822155
rs7187856
All − MR Egger
All − Inverse variance weighted
−4
−2
0
2
MR effect size for
'PHEX' on 'Pulmonary fibrosis'

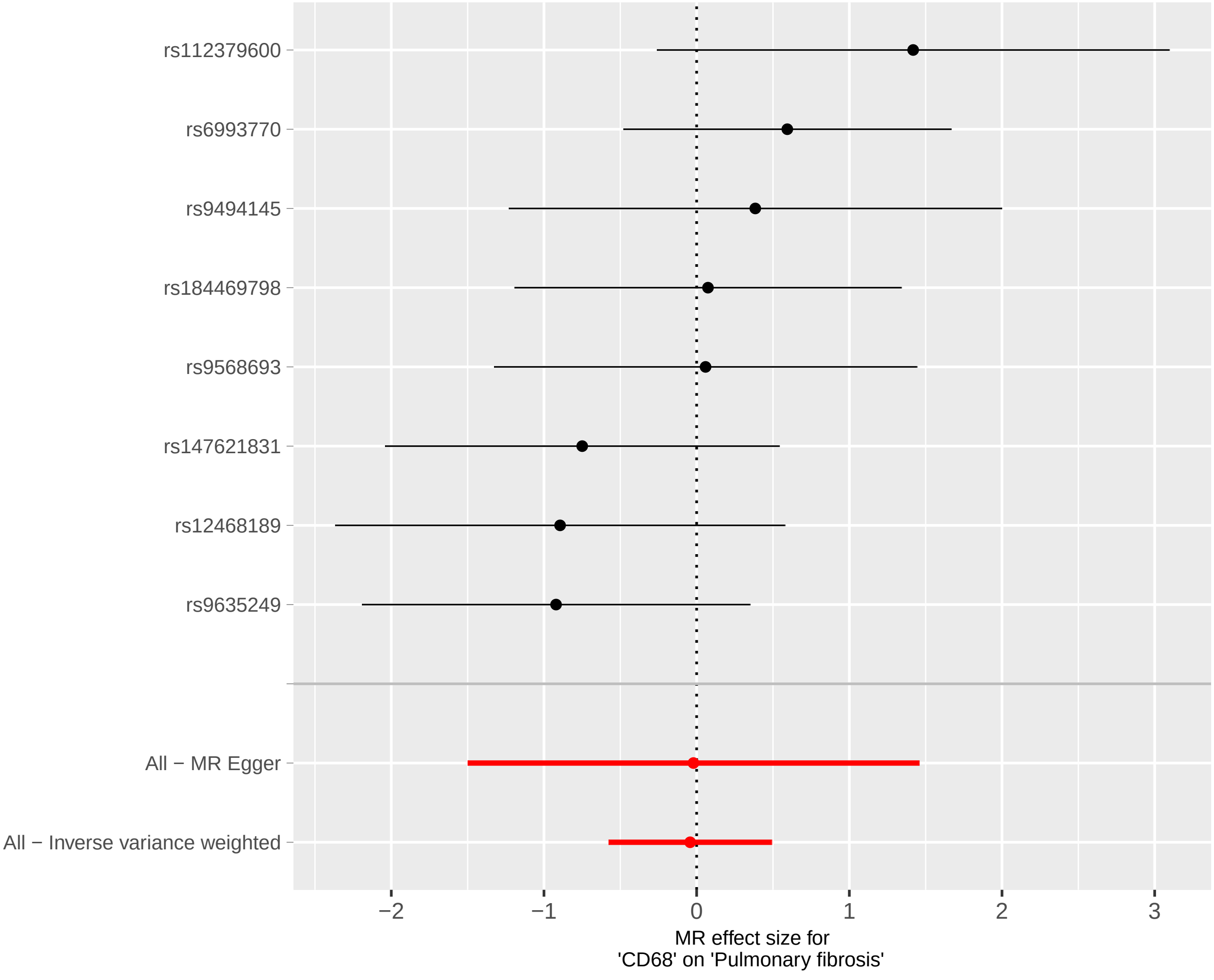
rs112379600
rs6993770
rs9494145
rs184469798
rs9568693
rs147621831
rs12468189
rs9635249
All − MR Egger
All − Inverse variance weighted
−2
−1
0
1
2
3
MR effect size for
'CD68' on 'Pulmonary fibrosis'

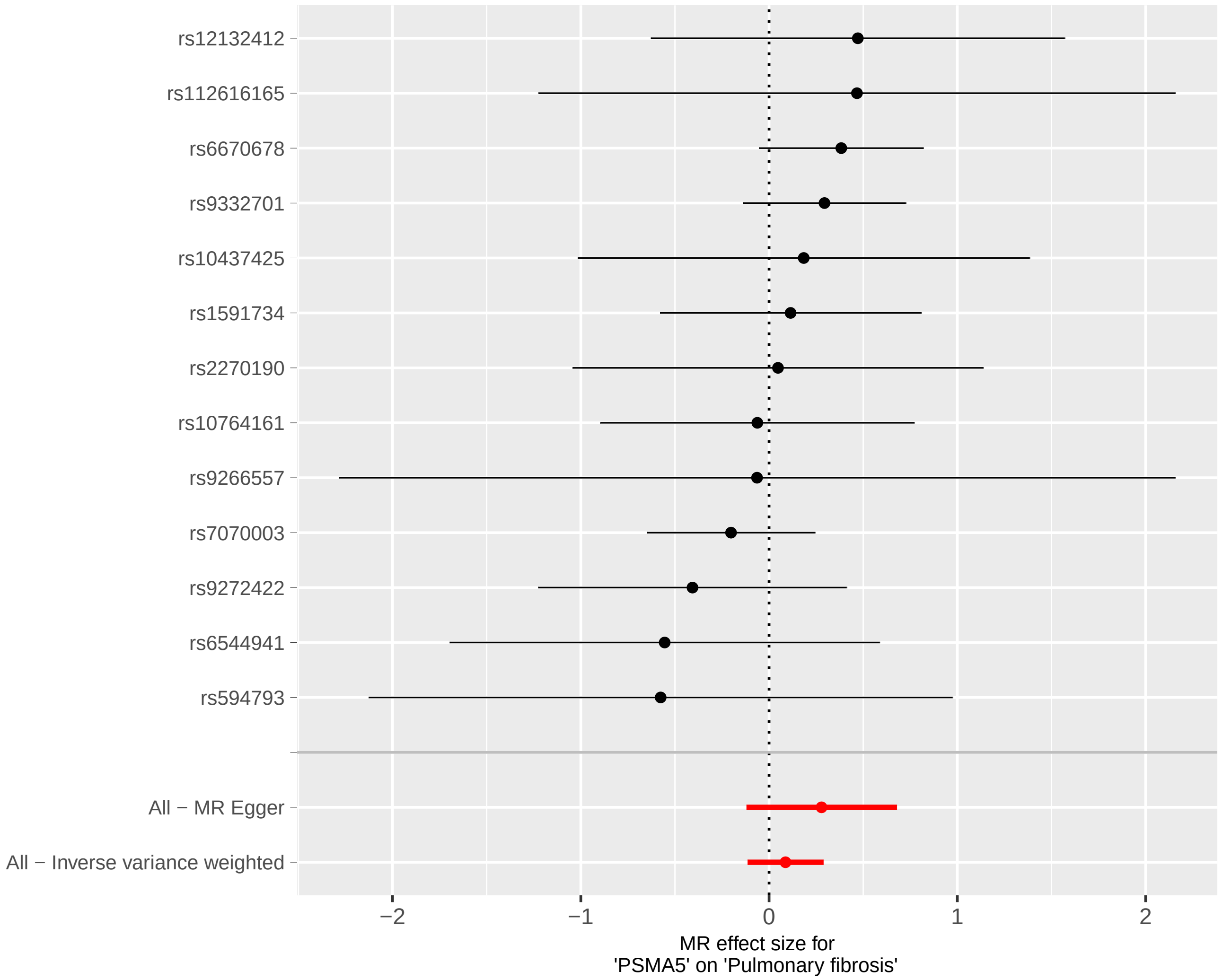

rs12132412
rs112616165
rs6670678
rs9332701
rs10437425
rs1591734
rs2270190
rs10764161
rs9266557
rs7070003
rs9272422
rs6544941
rs594793
All − MR Egger
All − Inverse variance weighted
−2
−1
0
1
2
MR effect size for
'PSMA5' on 'Pulmonary fibrosis'

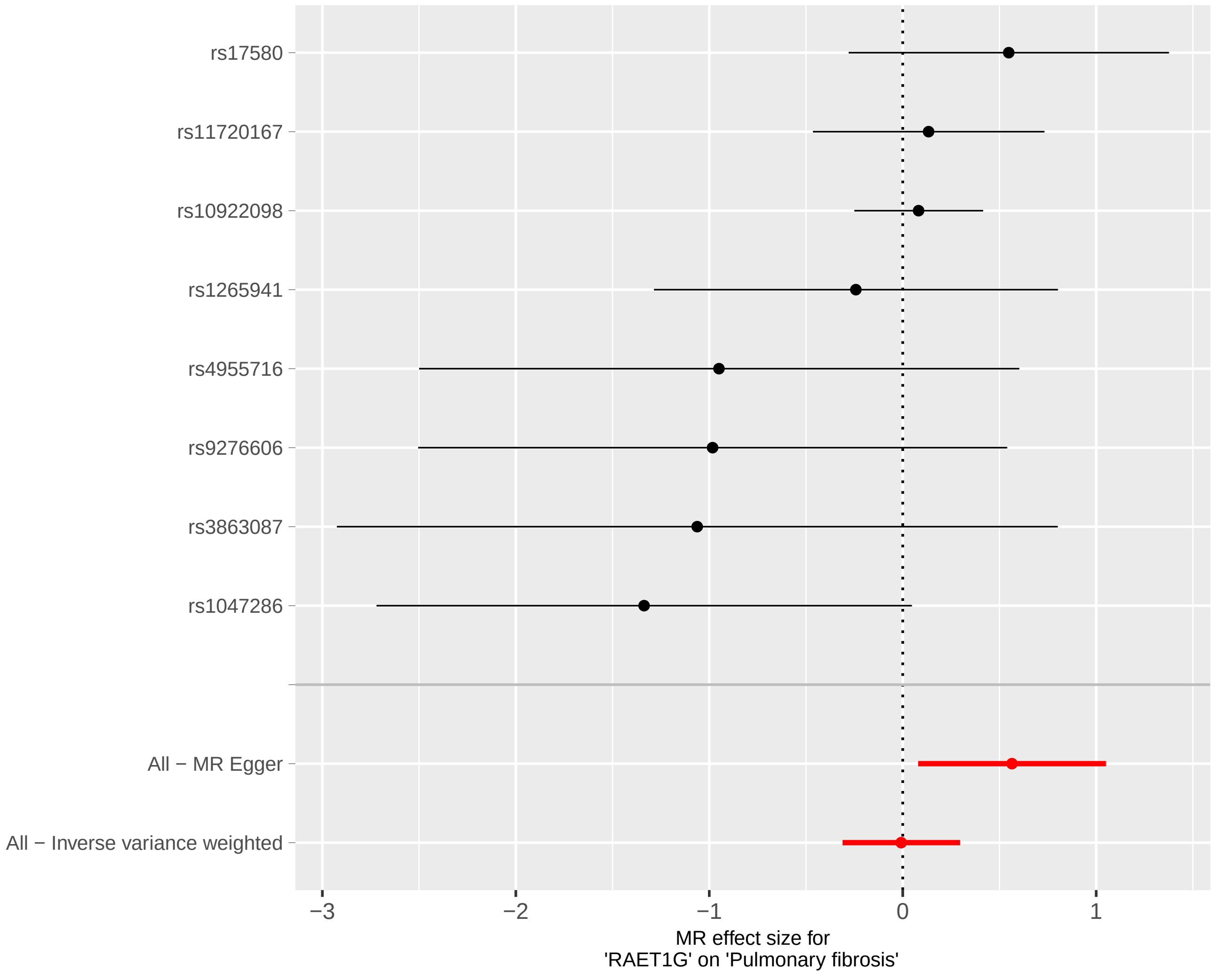

rs17580
rs11720167
rs10922098
rs1265941
rs4955716
rs9276606
rs3863087
rs1047286
All − MR Egger
All − Inverse variance weighted
−3
−2
−1
0
1
MR effect size for
'RAET1G' on 'Pulmonary fibrosis'

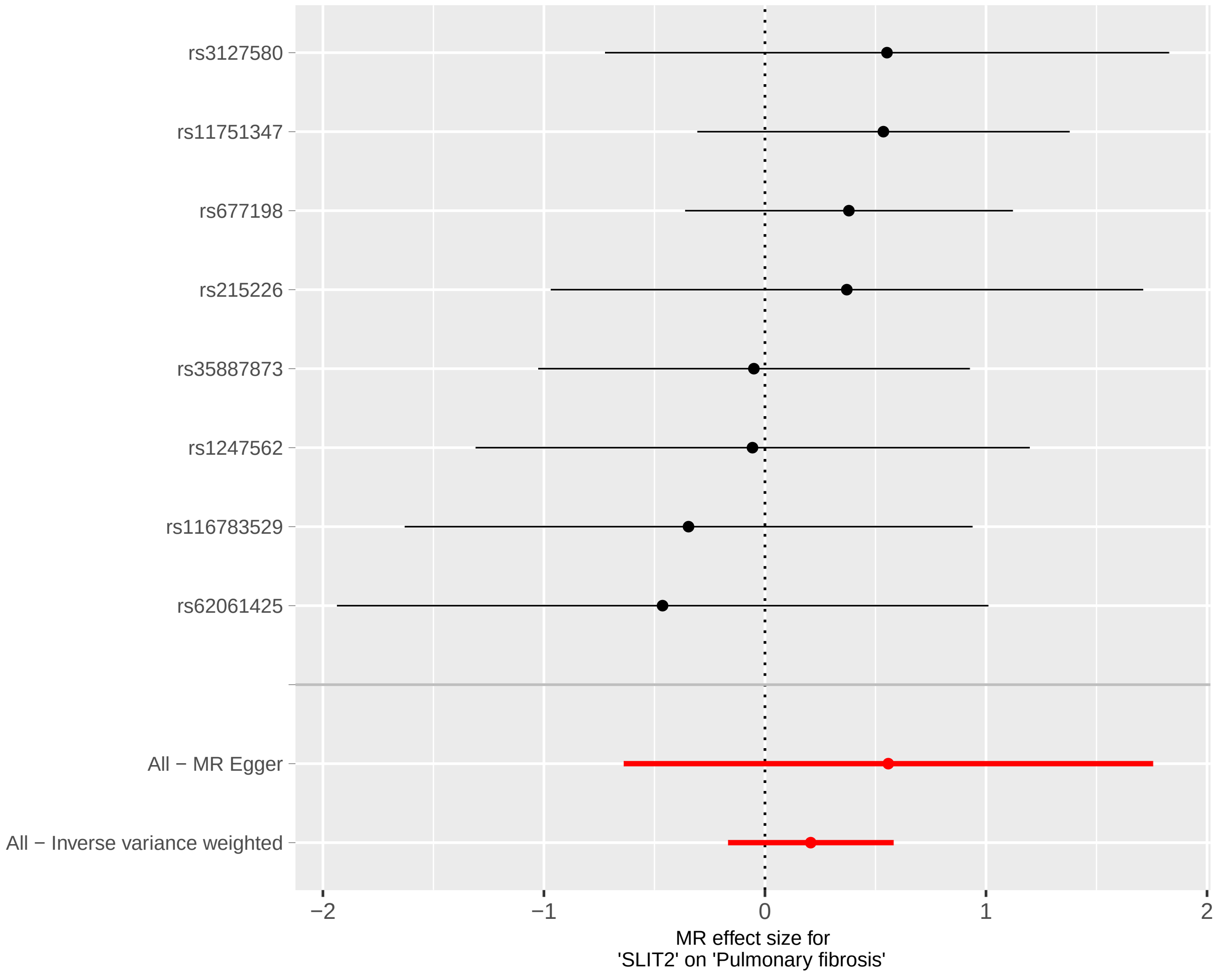

rs3127580
rs11751347
rs677198
rs215226
rs35887873
rs1247562
rs116783529
rs62061425
All − MR Egger
All − Inverse variance weighted
−2
−1
0
1
2
MR effect size for
'SLIT2' on 'Pulmonary fibrosis'

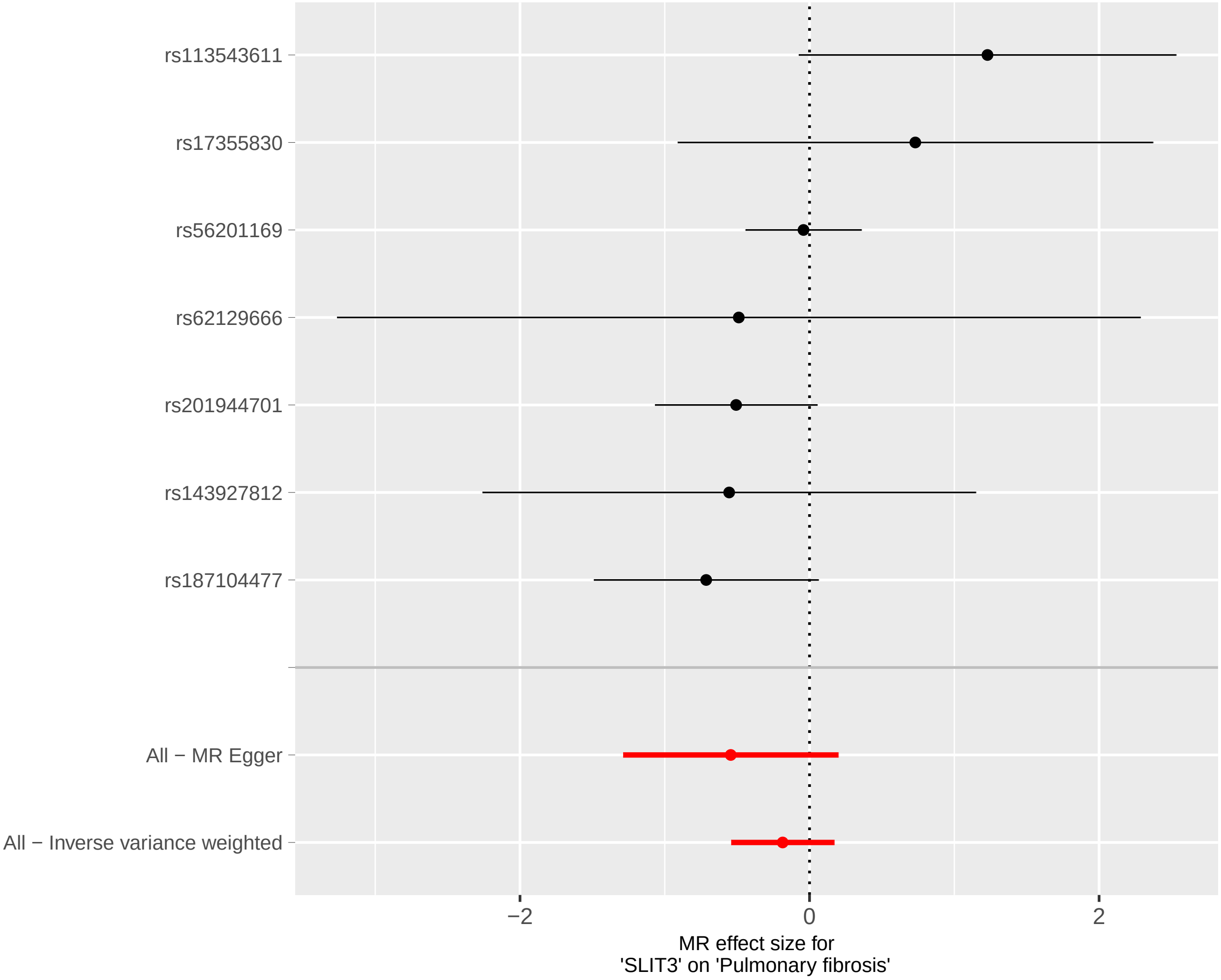

rs113543611
rs17355830
rs56201169
rs62129666
rs201944701
rs143927812
rs187104477
All − MR Egger
All − Inverse variance weighted
−2
0
2
MR effect size for
'SLIT3' on 'Pulmonary fibrosis'

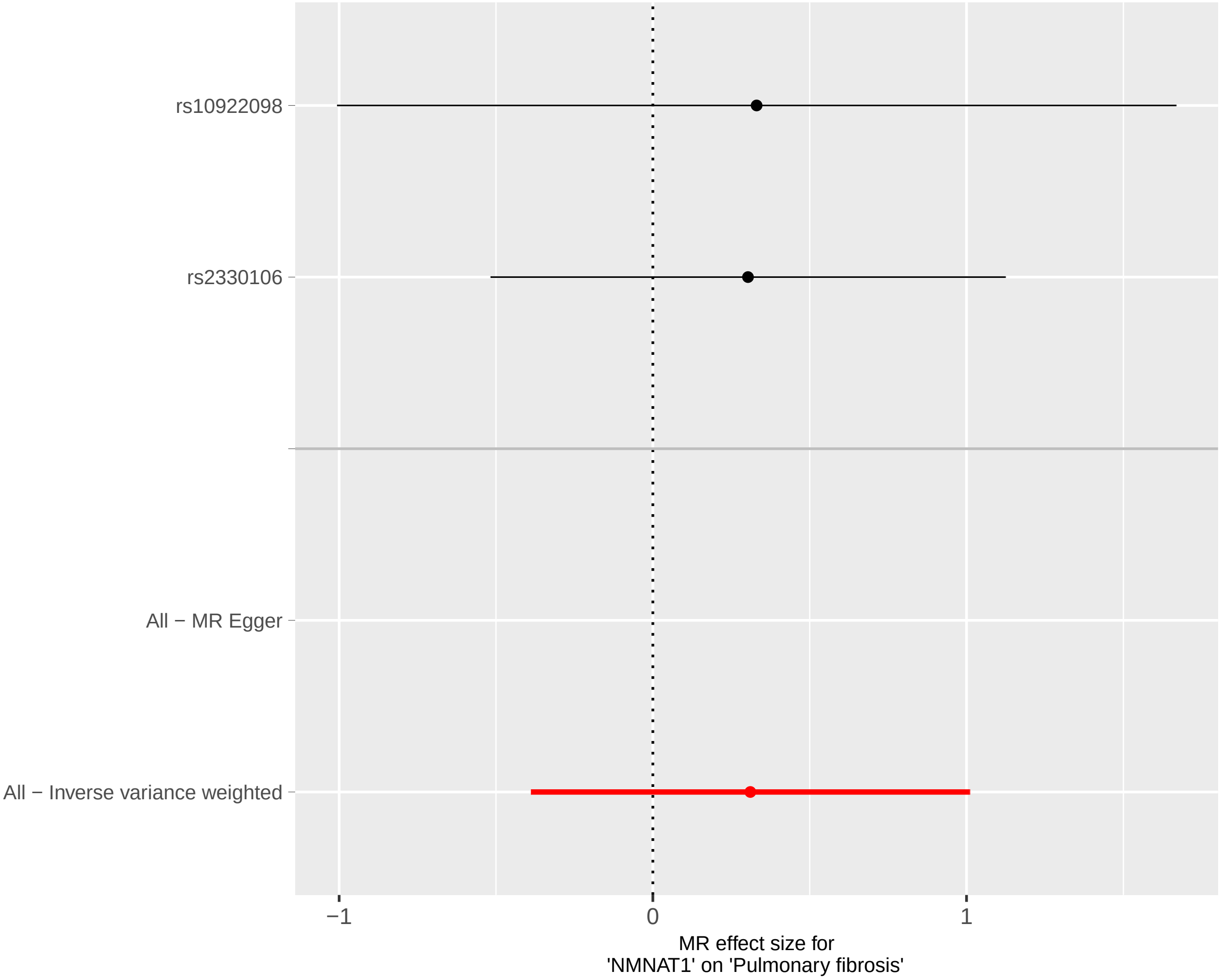
rs10922098
rs2330106
All − MR Egger
All − Inverse variance weighted
−1
0
1
MR effect size for
'NMNAT1' on 'Pulmonary fibrosis'

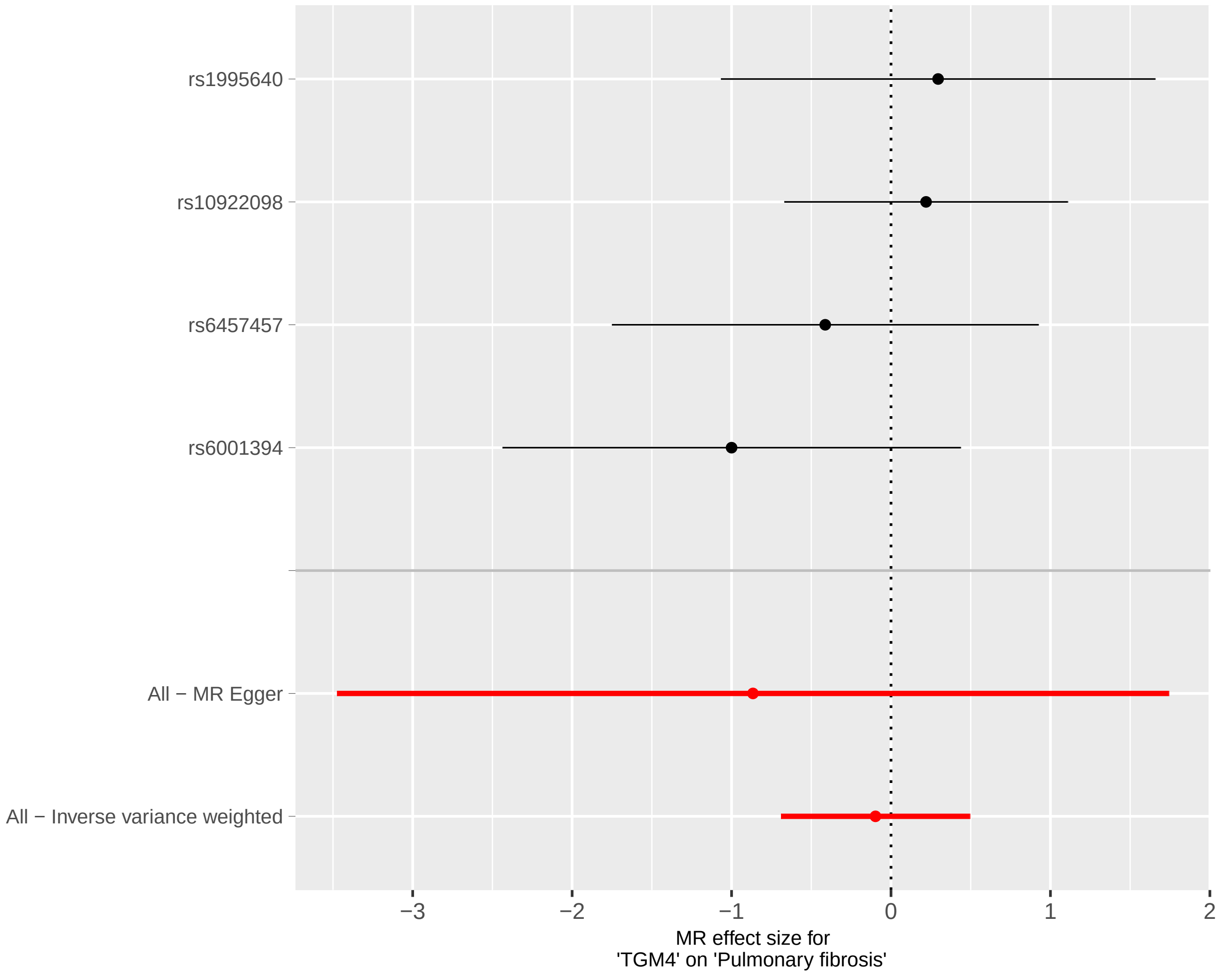

rs1995640
rs10922098
rs6457457
rs6001394
All − MR Egger
All − Inverse variance weighted
−3
−2
−1
0
1
2
MR effect size for
'TGM4' on 'Pulmonary fibrosis'

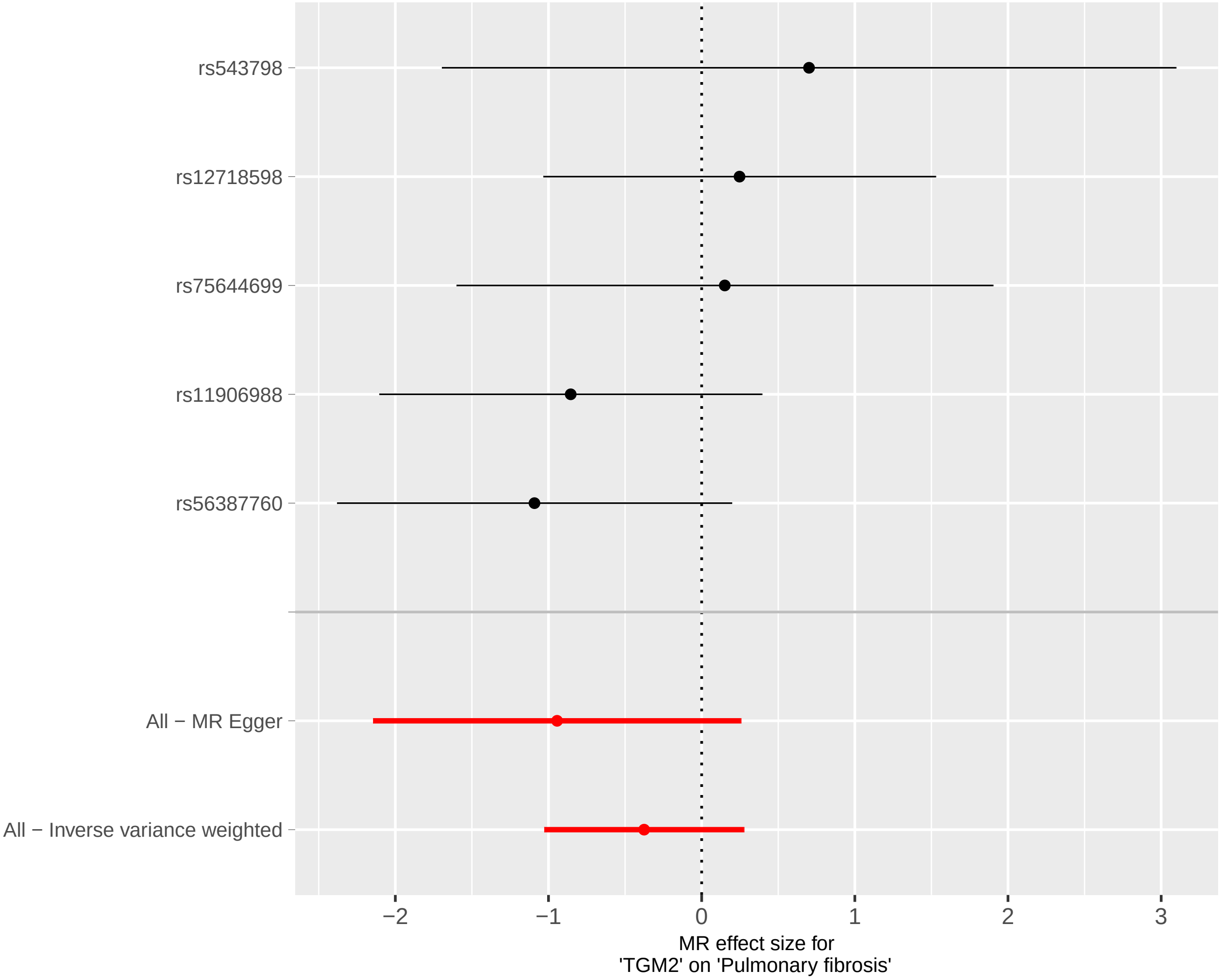

rs543798
rs12718598
rs75644699
rs11906988
rs56387760
All − MR Egger
All − Inverse variance weighted
−2
−1
0
1
2
3
MR effect size for
'TGM2' on 'Pulmonary fibrosis'

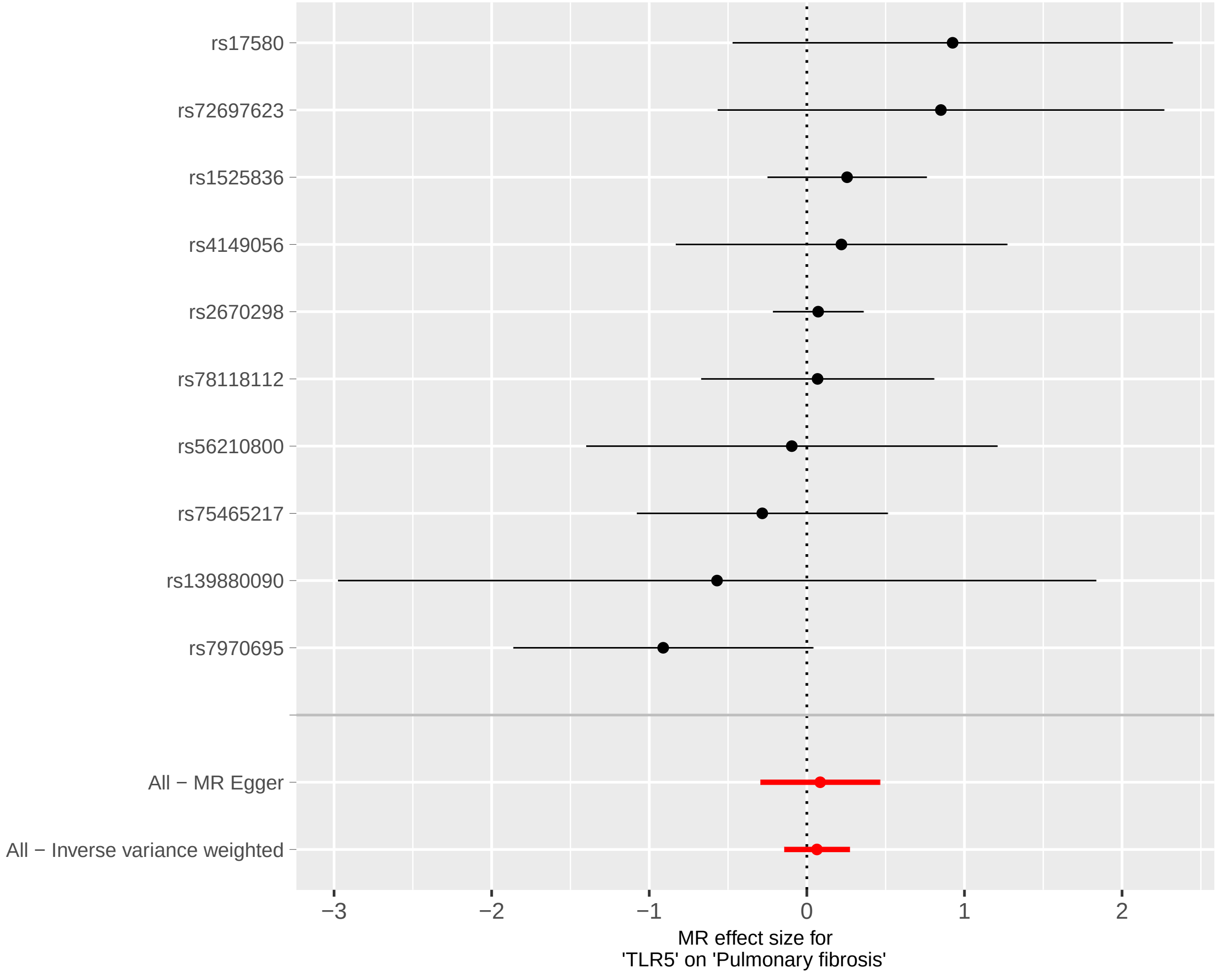
rs17580
rs72697623
rs1525836
rs4149056
rs2670298
rs78118112
rs56210800
rs75465217
rs139880090
rs7970695
All − MR Egger
All − Inverse variance weighted
−3
−2
−1
0
1
2
MR effect size for
'TLR5' on 'Pulmonary fibrosis'

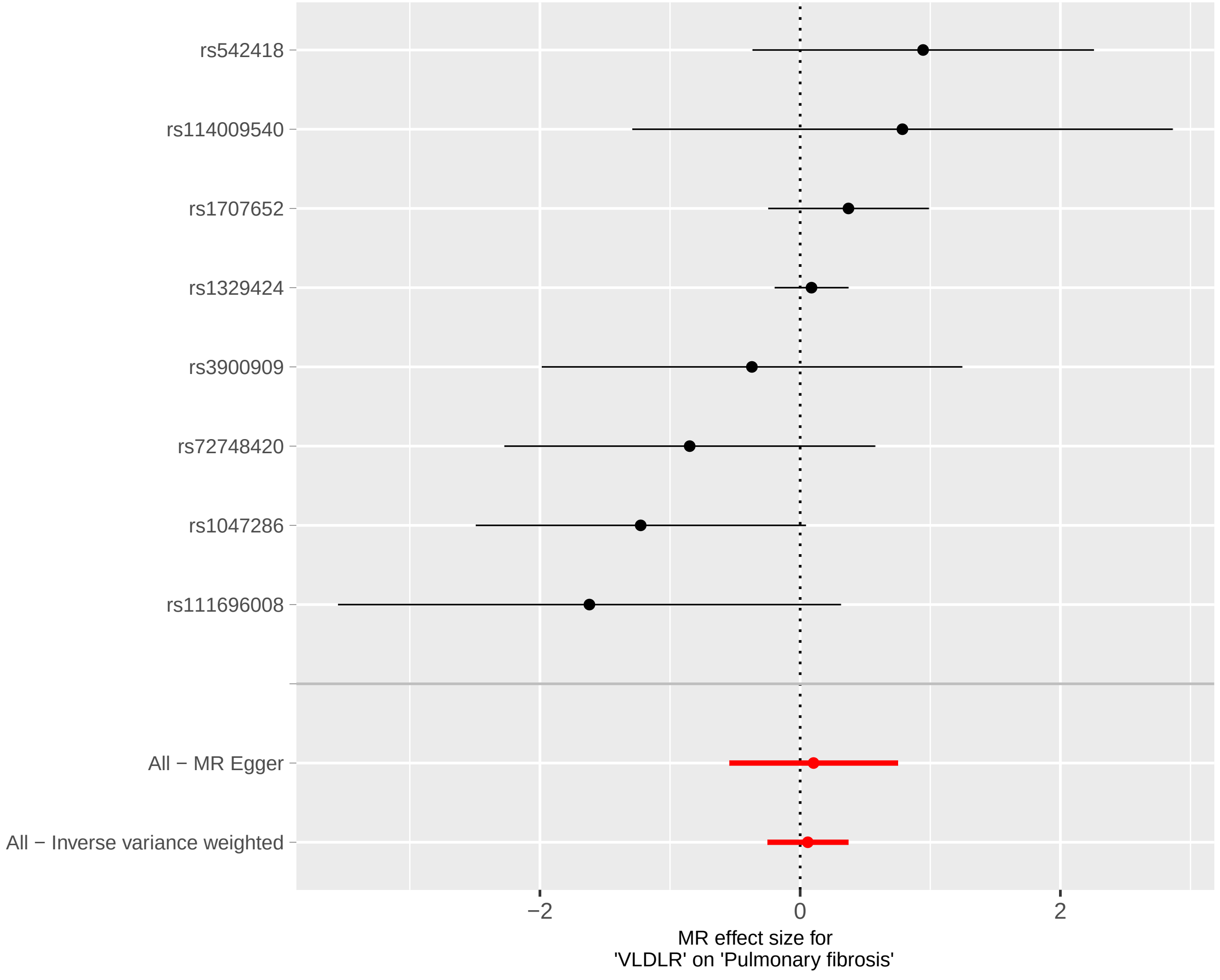
rs542418
rs114009540
rs1707652
rs1329424
rs3900909
rs72748420
rs1047286
rs111696008
All − MR Egger
All − Inverse variance weighted
−2
0
2
MR effect size for
'VLDLR' on 'Pulmonary fibrosis'

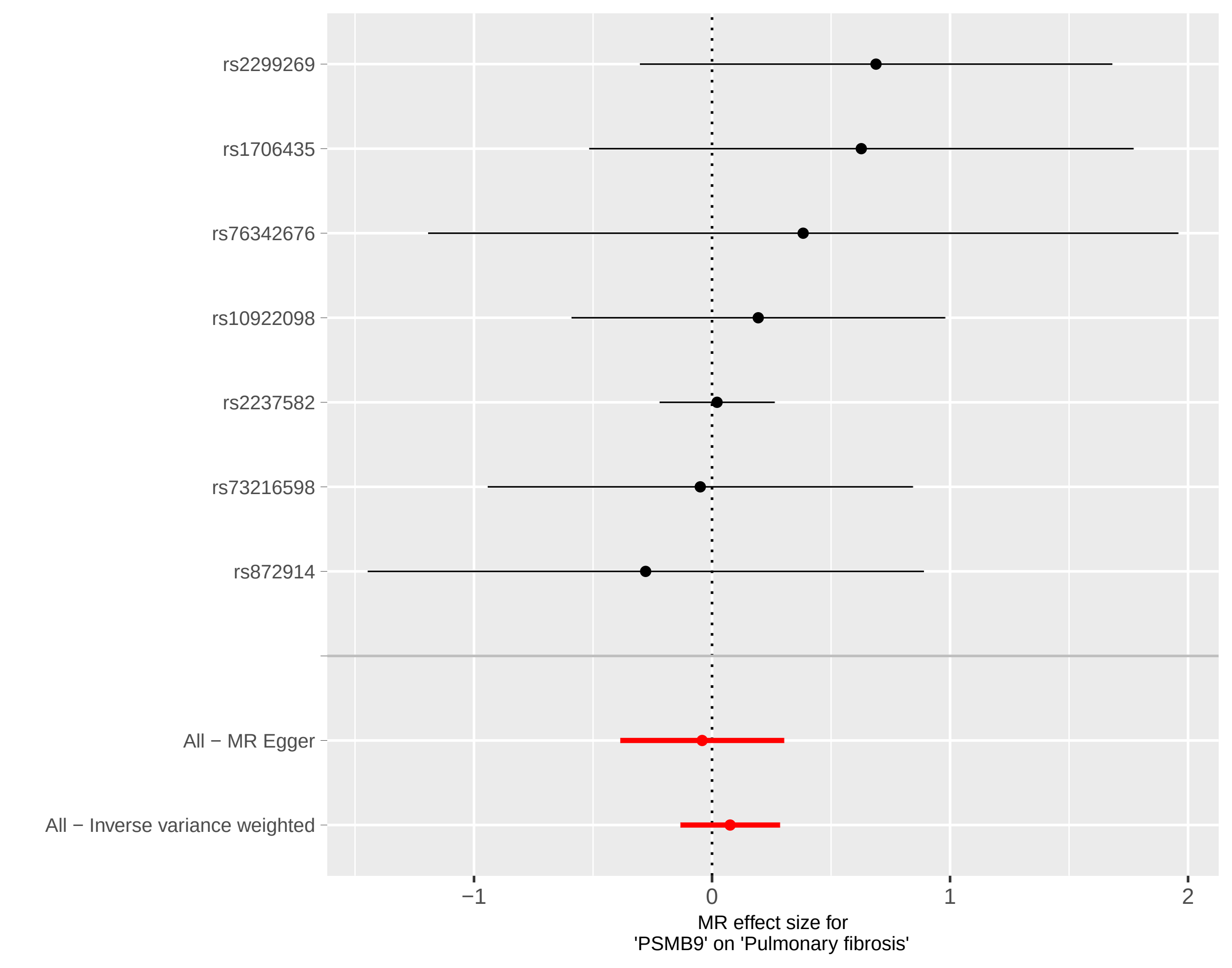
rs2299269
rs1706435
rs76342676
rs10922098
rs2237582
rs73216598
rs872914
All − MR Egger
All − Inverse variance weighted
−1
0
1
2
MR effect size for
'PSMB9' on 'Pulmonary fibrosis'

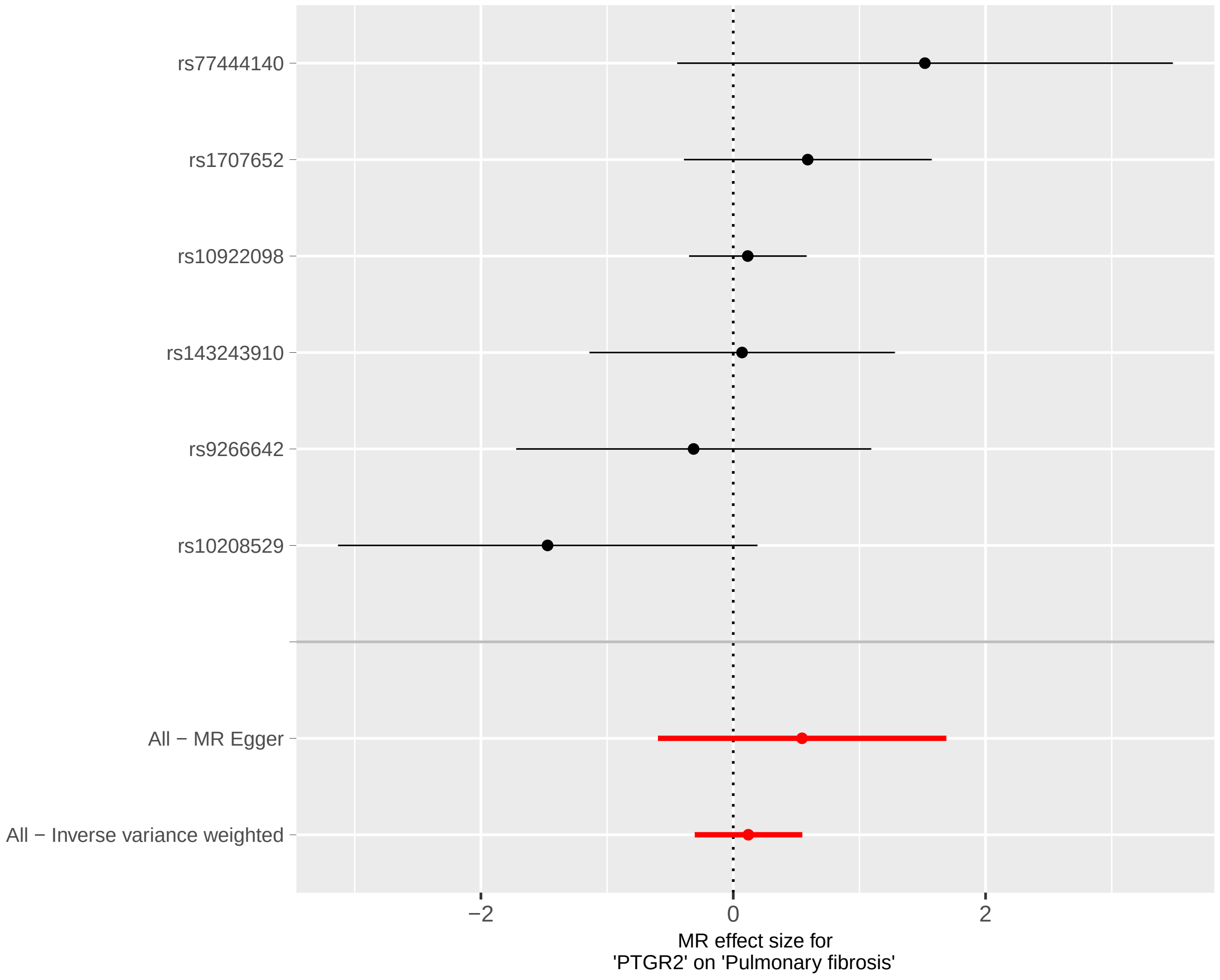

rs77444140
rs1707652
rs10922098
rs143243910
rs9266642
rs10208529
All − MR Egger
All − Inverse variance weighted
−2
0
2
MR effect size for
'PTGR2' on 'Pulmonary fibrosis'

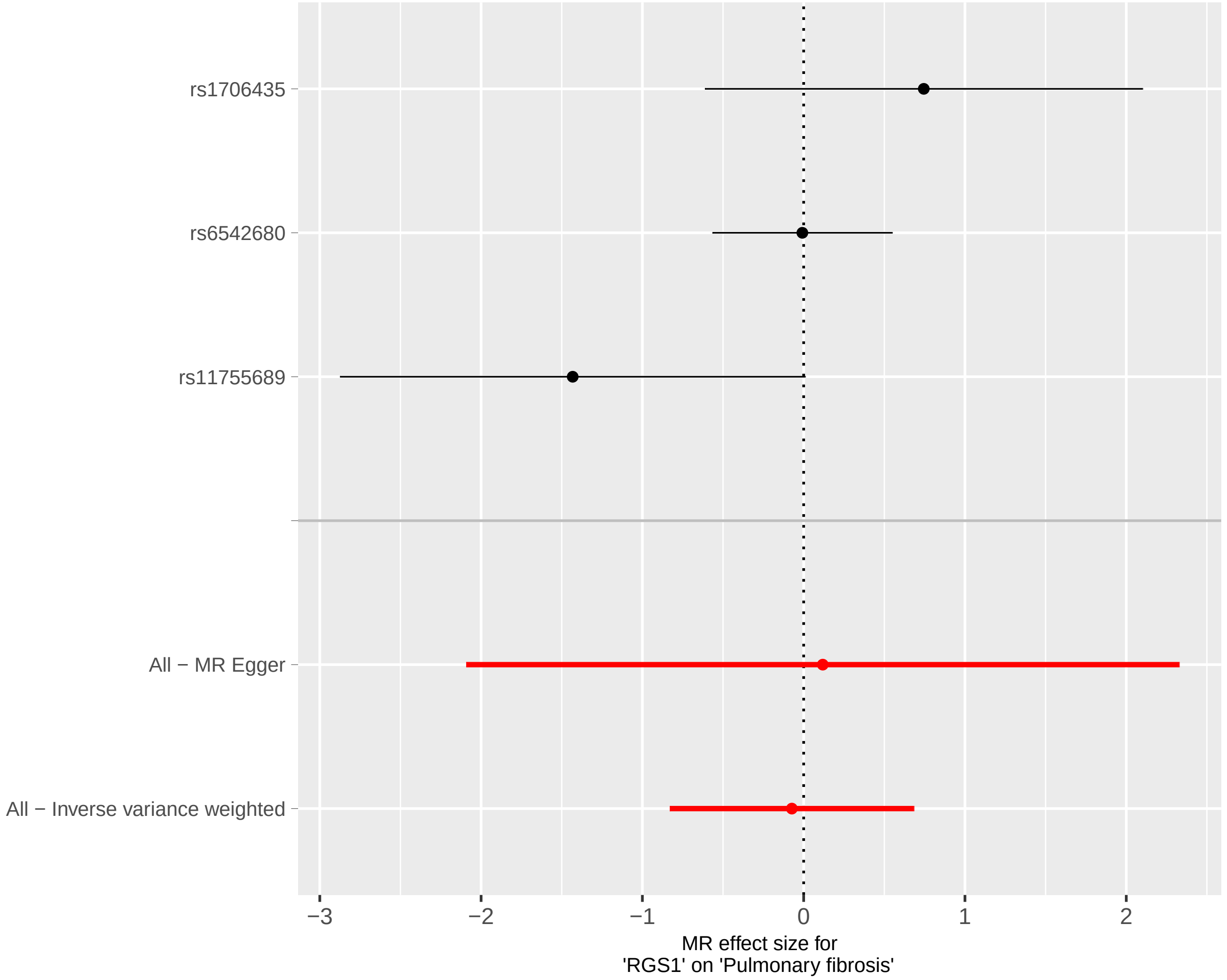
rs1706435
rs6542680
rs11755689
All − MR Egger
All − Inverse variance weighted
−3
−2
−1
0
1
2
MR effect size for
'RGS1' on 'Pulmonary fibrosis'

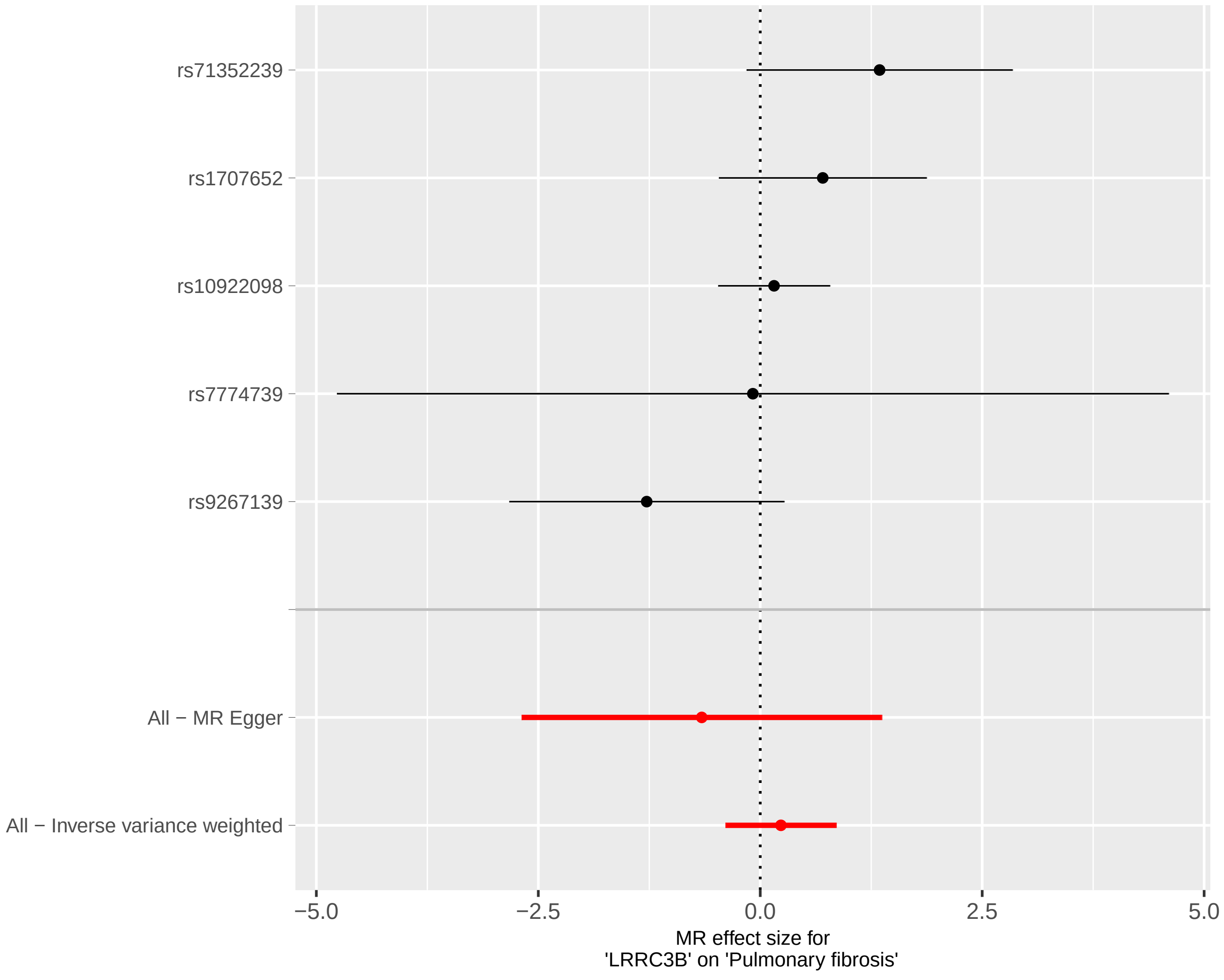
rs71352239
rs1707652
rs10922098
rs7774739
rs9267139
All − MR Egger
All − Inverse variance weighted
−5.0
−2.5
0.0
2.5
5.0
MR effect size for
'LRRC3B' on 'Pulmonary fibrosis'

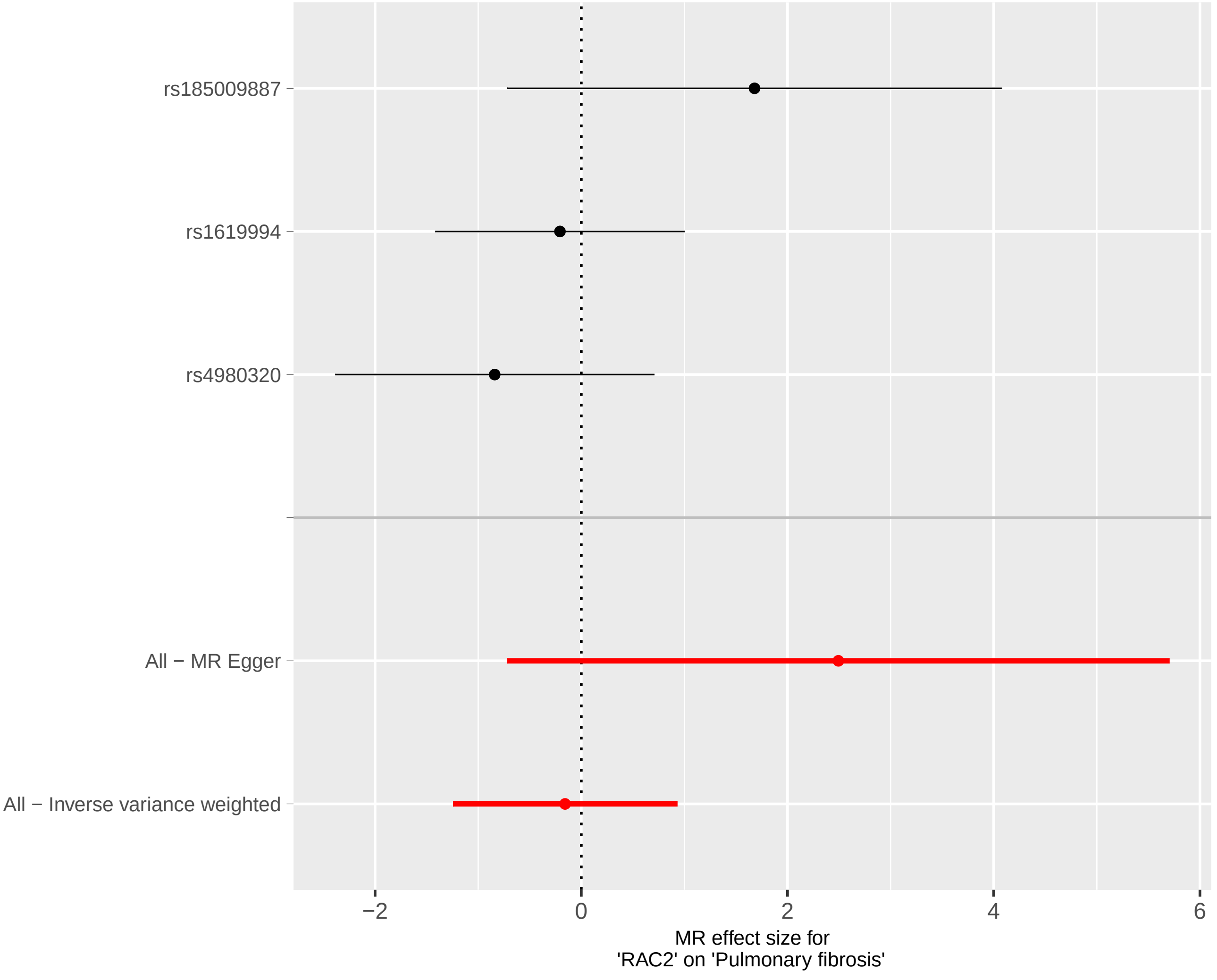
rs185009887
rs1619994
rs4980320
All − MR Egger
All − Inverse variance weighted
−2
0
2
4
6
MR effect size for
'RAC2' on 'Pulmonary fibrosis'

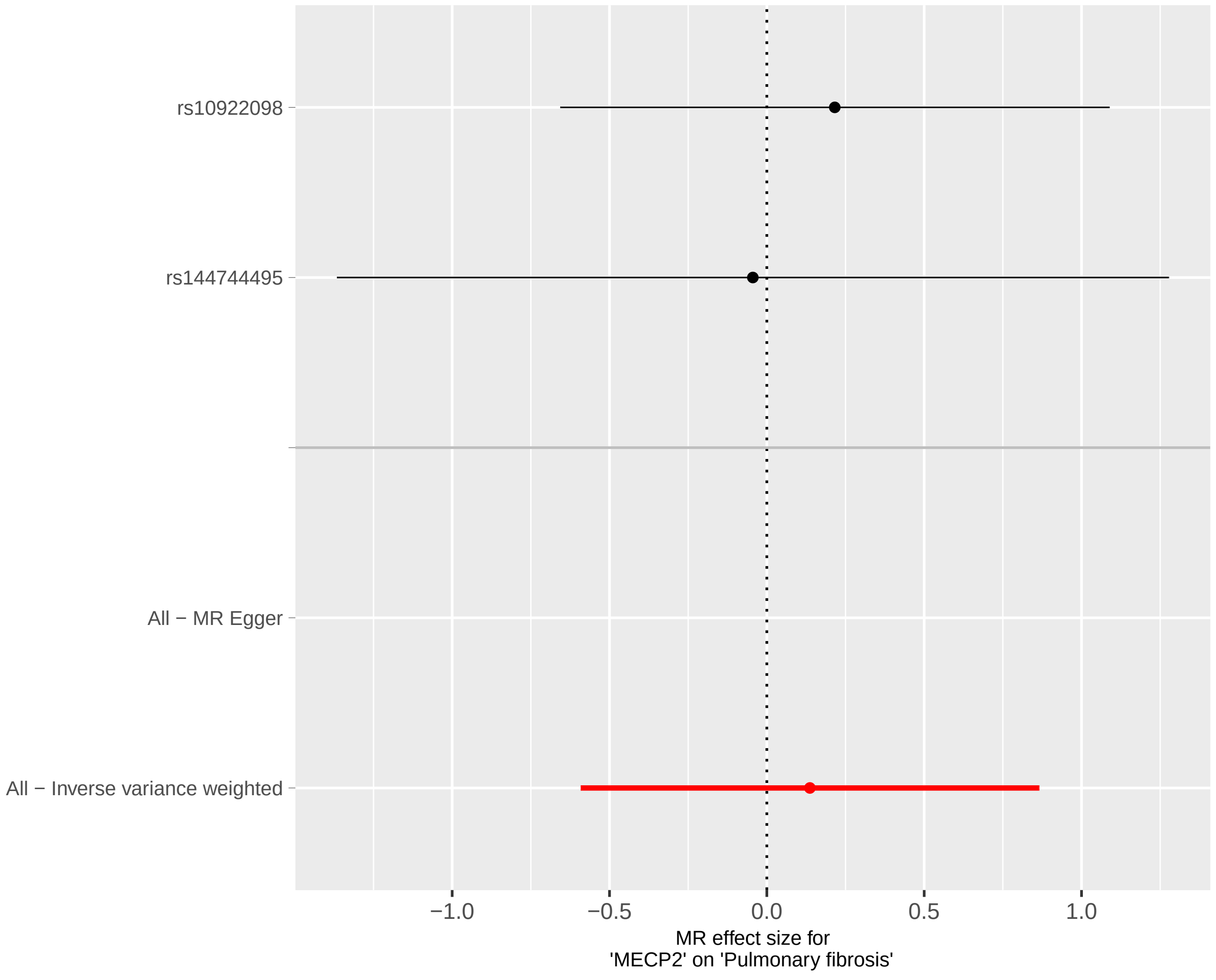

rs10922098
rs144744495
All − MR Egger
All − Inverse variance weighted
−1.0
−0.5
0.0
0.5
1.0
MR effect size for
'MECP2' on 'Pulmonary fibrosis'

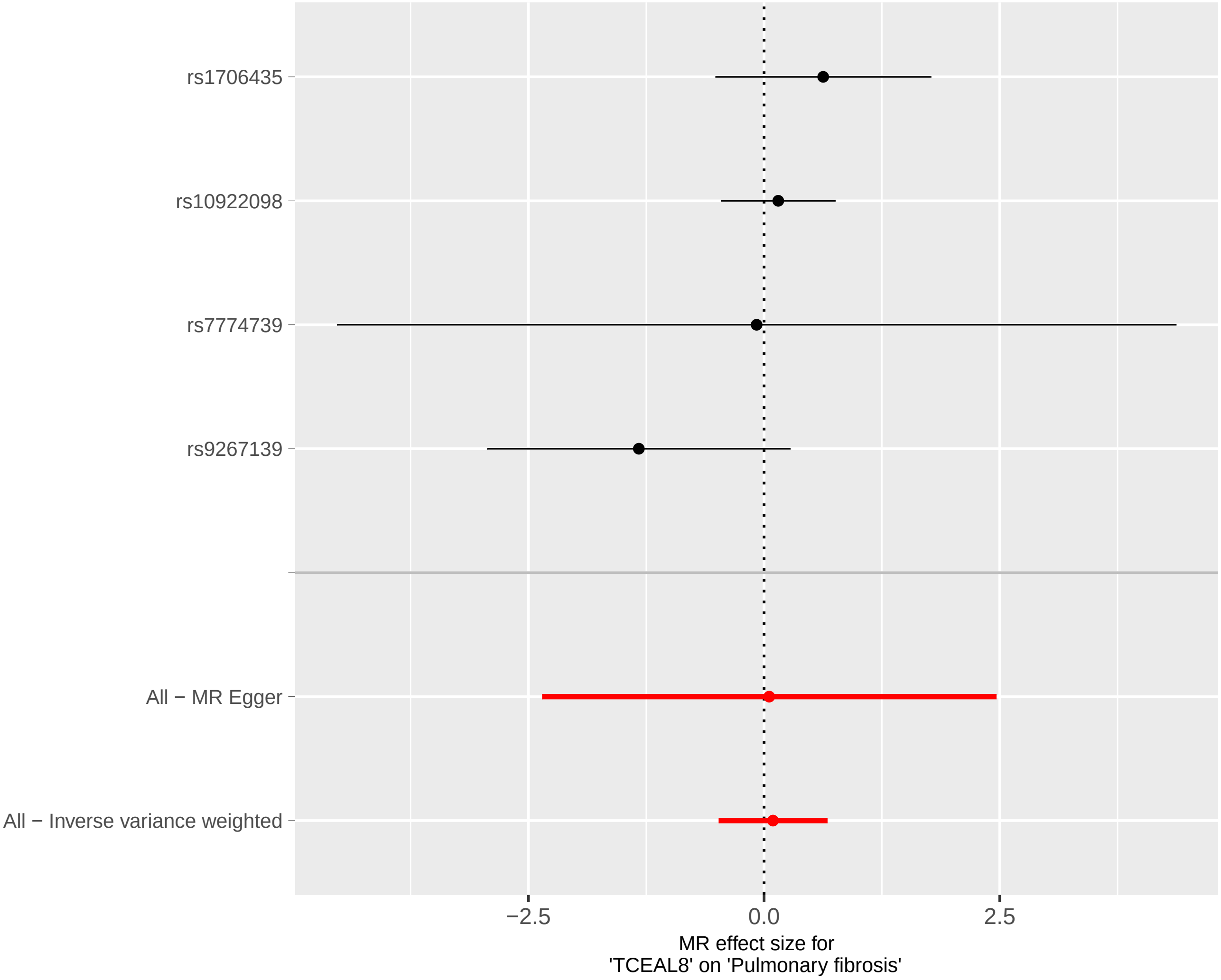
rs1706435
rs10922098
rs7774739
rs9267139
All − MR Egger
All − Inverse variance weighted
−2.5
0.0
2.5
MR effect size for
'TCEAL8' on 'Pulmonary fibrosis'

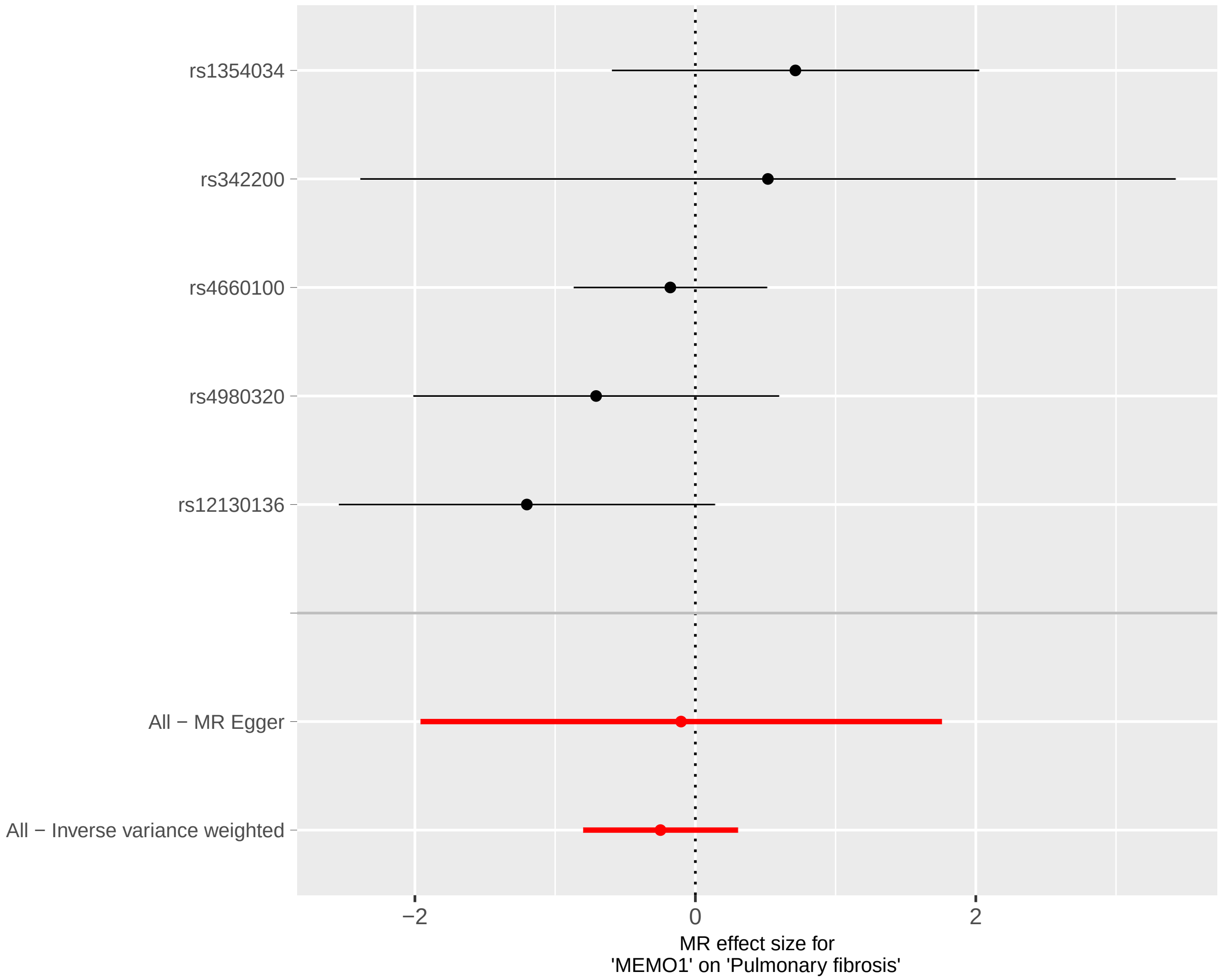
rs1354034
rs342200
rs4660100
rs4980320
rs12130136
All − MR Egger
All − Inverse variance weighted
−2
0
2
MR effect size for
'MEMO1' on 'Pulmonary fibrosis'

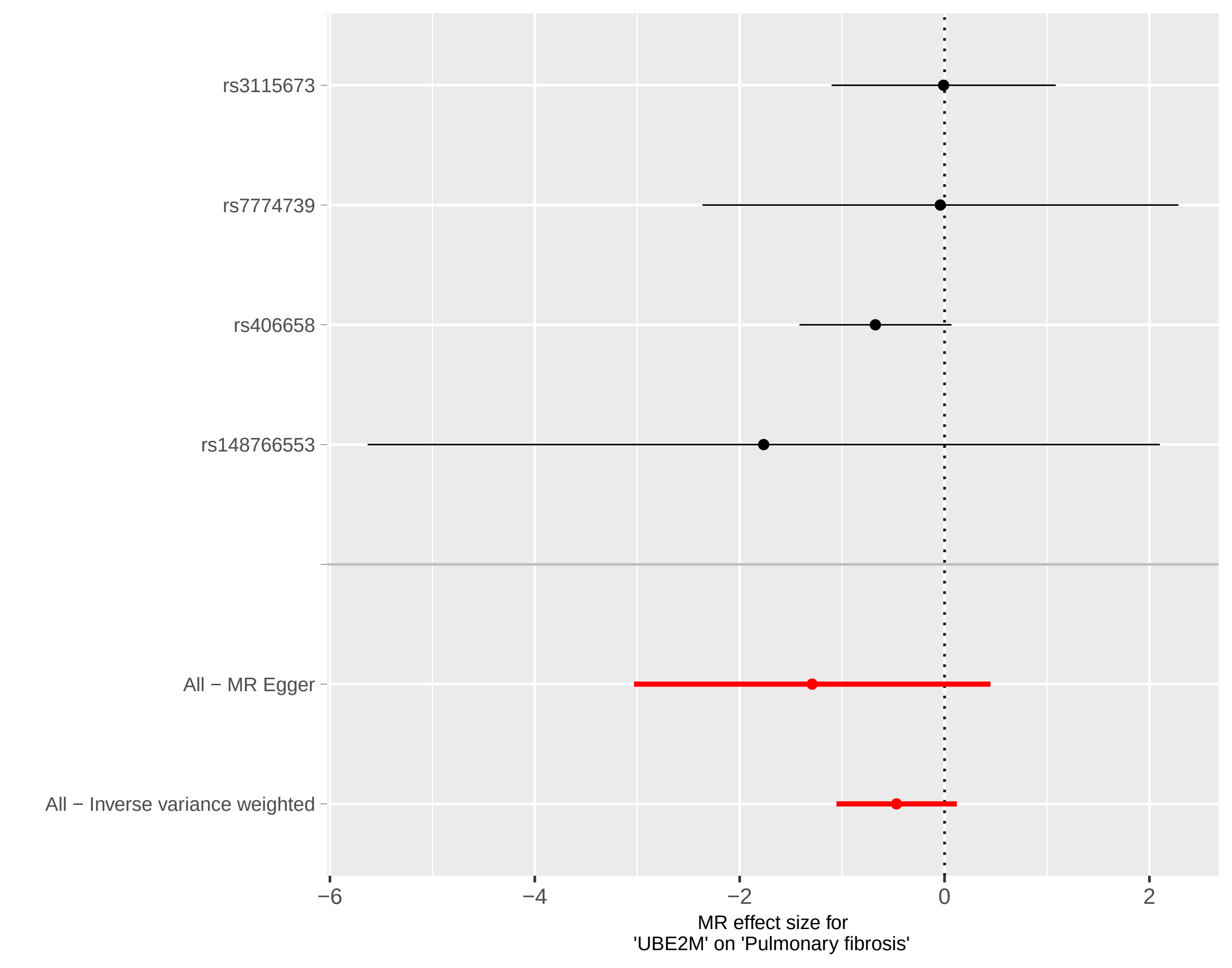

rs3115673
rs7774739
rs406658
rs148766553
All − MR Egger
All − Inverse variance weighted
−6
−4
−2
0
2
MR effect size for
'UBE2M' on 'Pulmonary fibrosis'

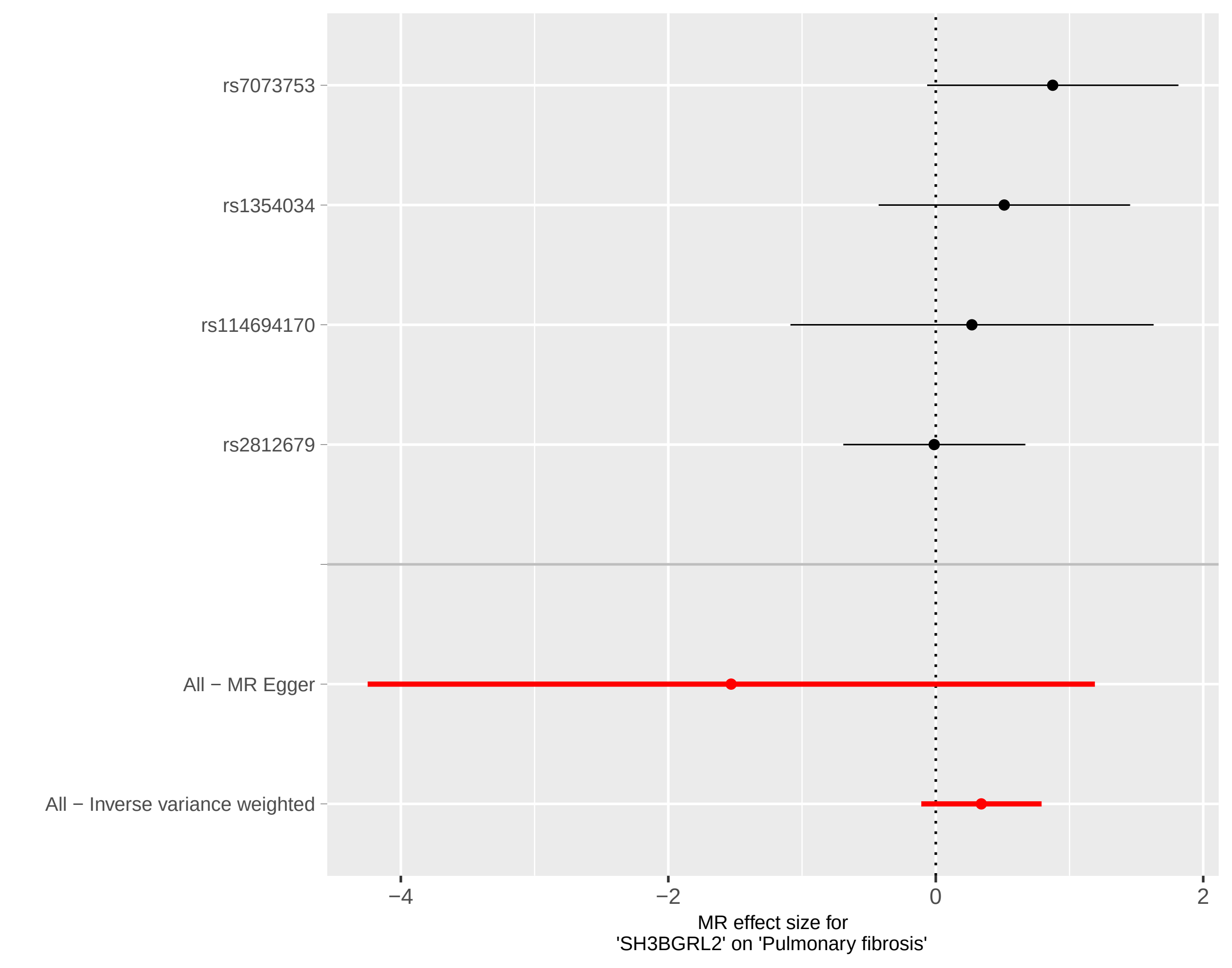
rs7073753
rs1354034
rs114694170
rs2812679
All − MR Egger
All − Inverse variance weighted
−4
−2
0
2
MR effect size for
'SH3BGRL2' on 'Pulmonary fibrosis'

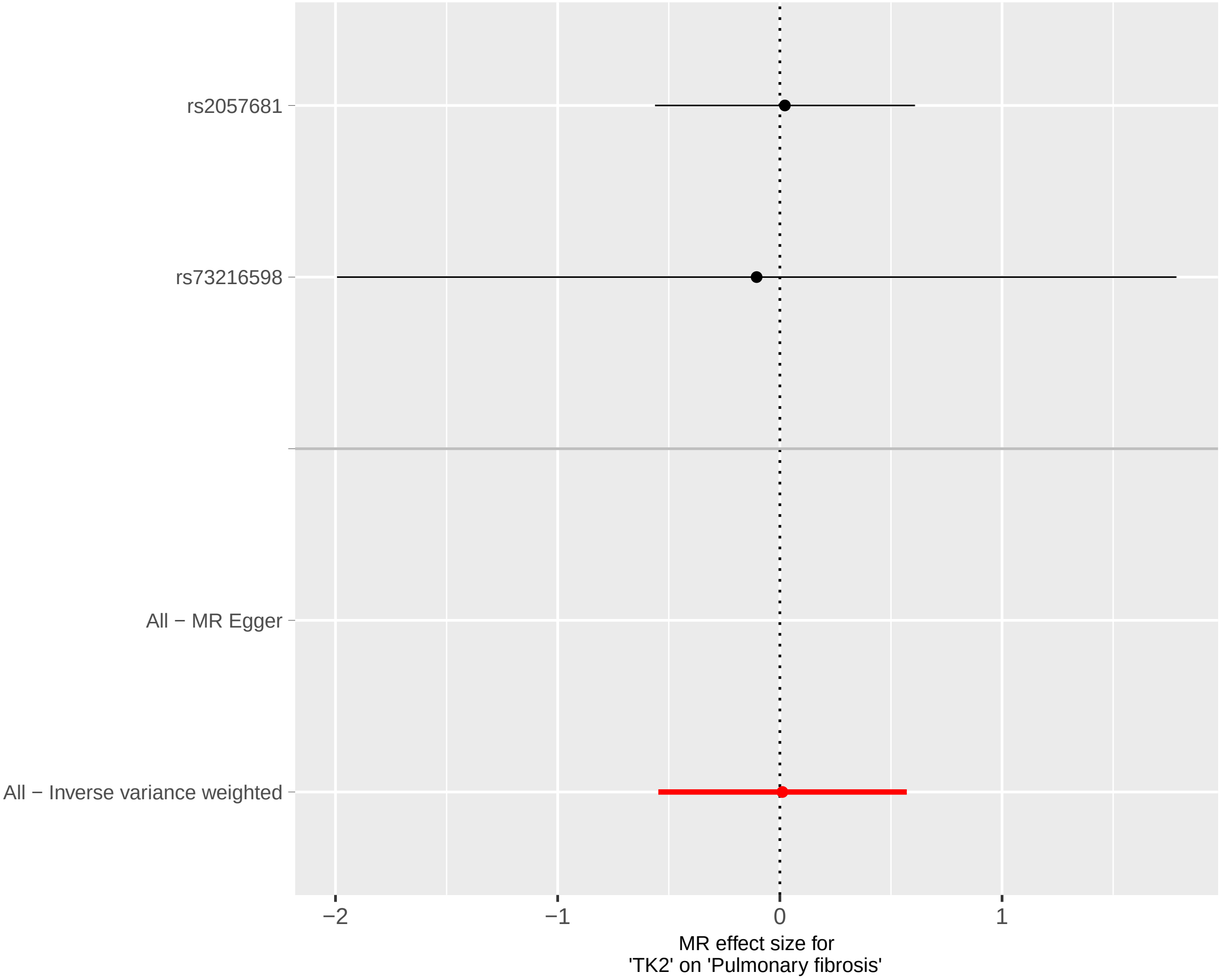
rs2057681
rs73216598
All − MR Egger
All − Inverse variance weighted
−2
−1
0
1
MR effect size for
'TK2' on 'Pulmonary fibrosis'

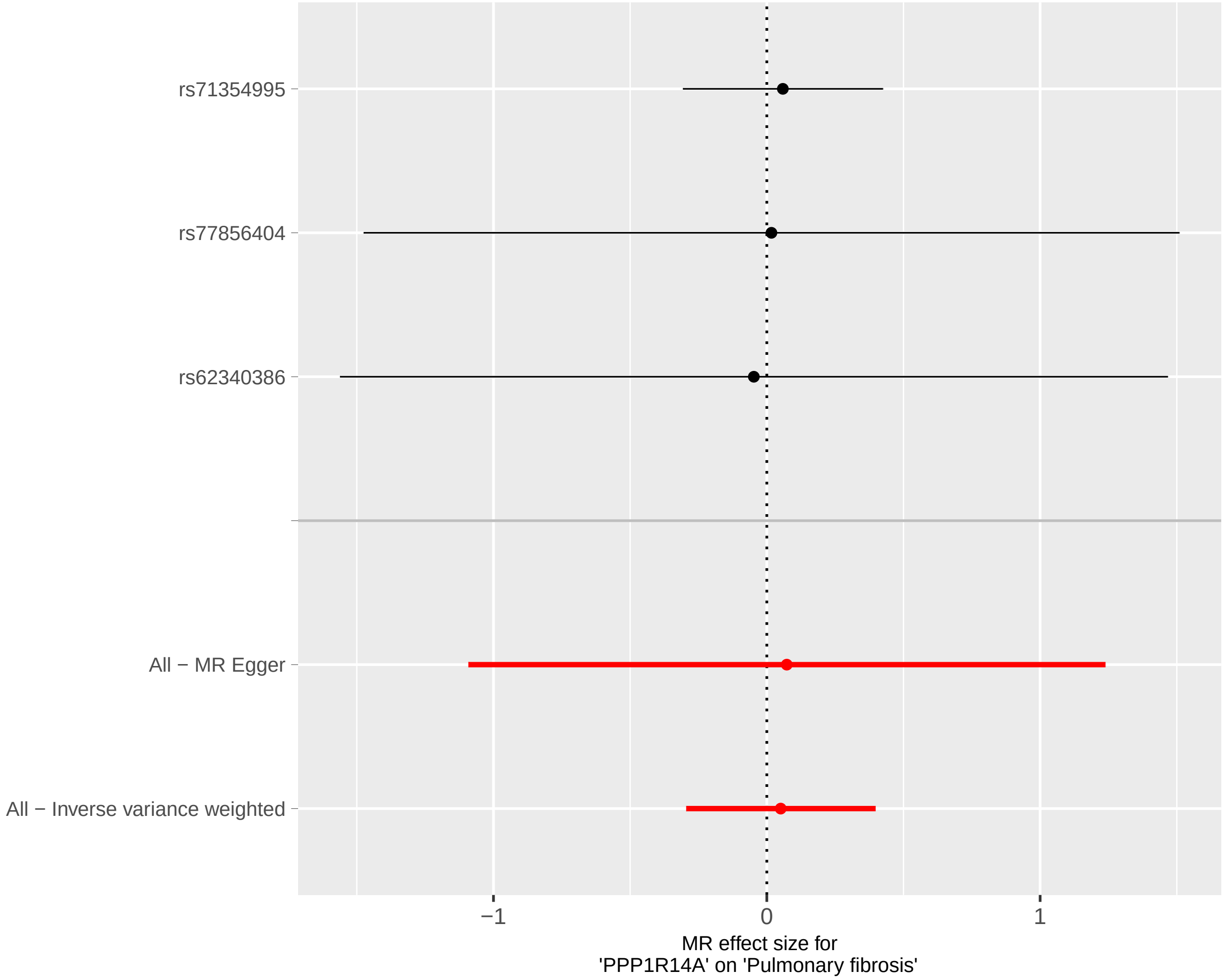

rs71354995
rs77856404
rs62340386
All − MR Egger
All − Inverse variance weighted
−1
0
1
MR effect size for
'PPP1R14A' on 'Pulmonary fibrosis'

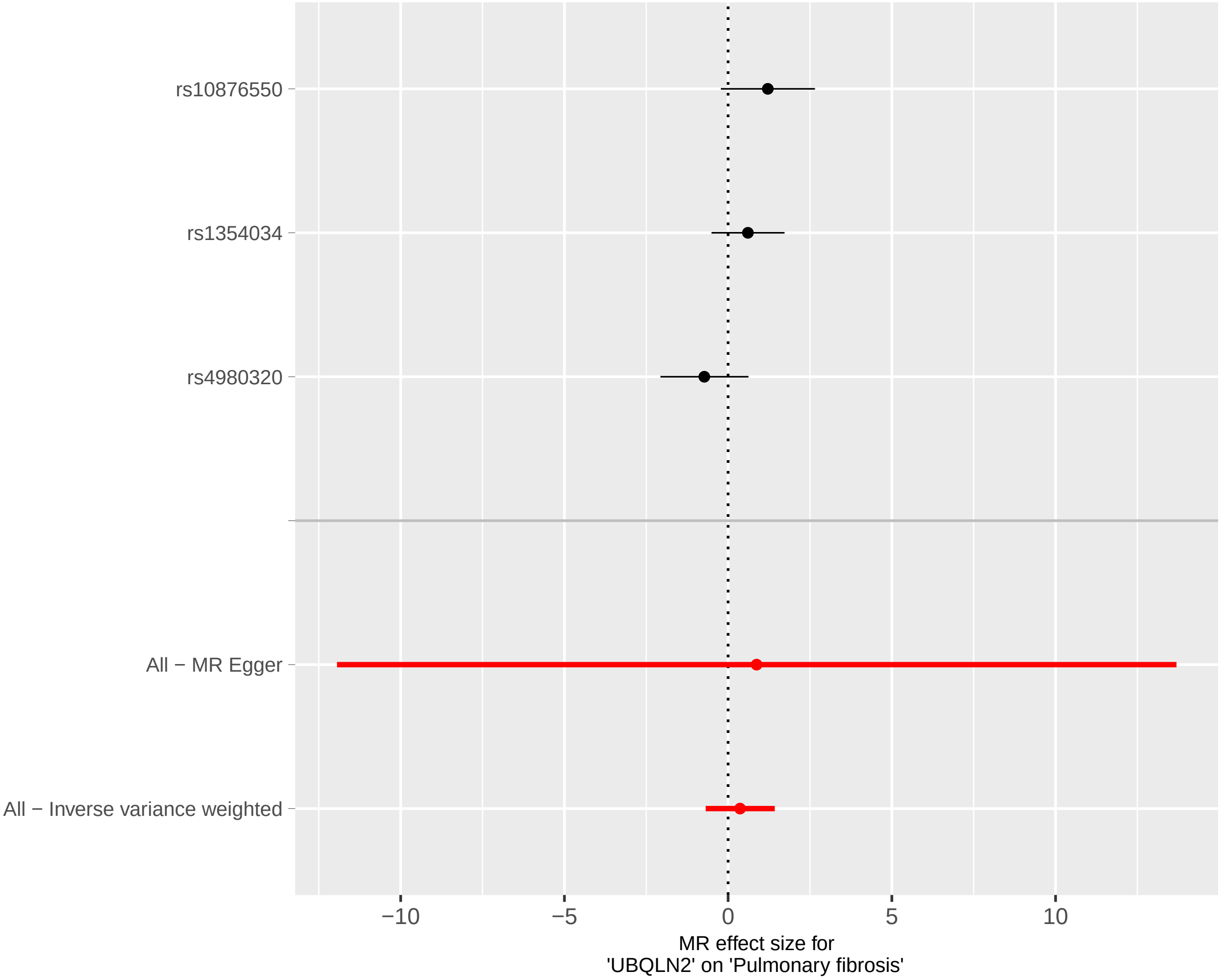
rs10876550
rs1354034
rs4980320
All − MR Egger
All − Inverse variance weighted
−10
−5
0
5
10
MR effect size for
'UBQLN2' on 'Pulmonary fibrosis'

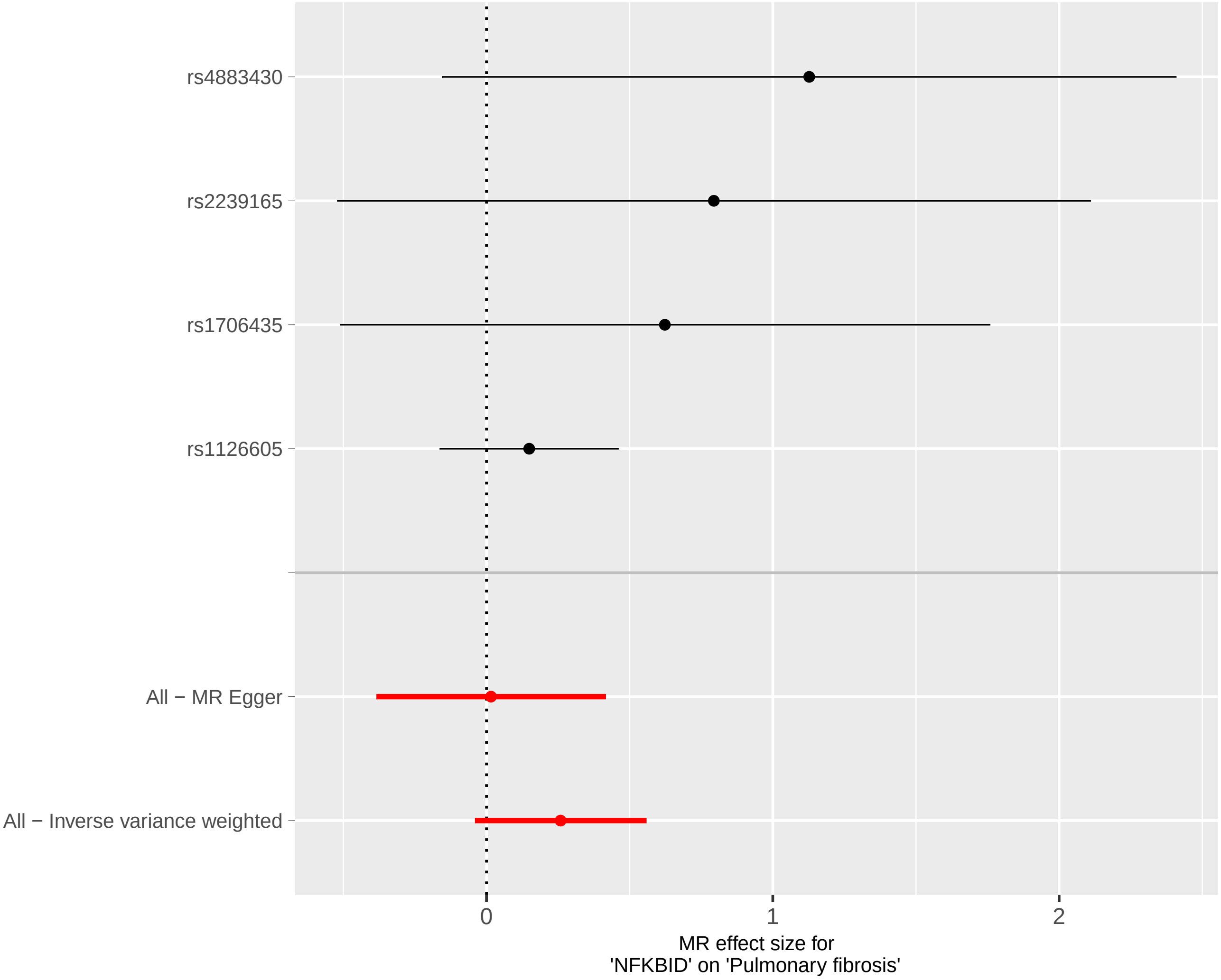

rs4883430
rs2239165
rs1706435
rs1126605
All − MR Egger
All − Inverse variance weighted
0
1
2
MR effect size for
'NFKBID' on 'Pulmonary fibrosis'

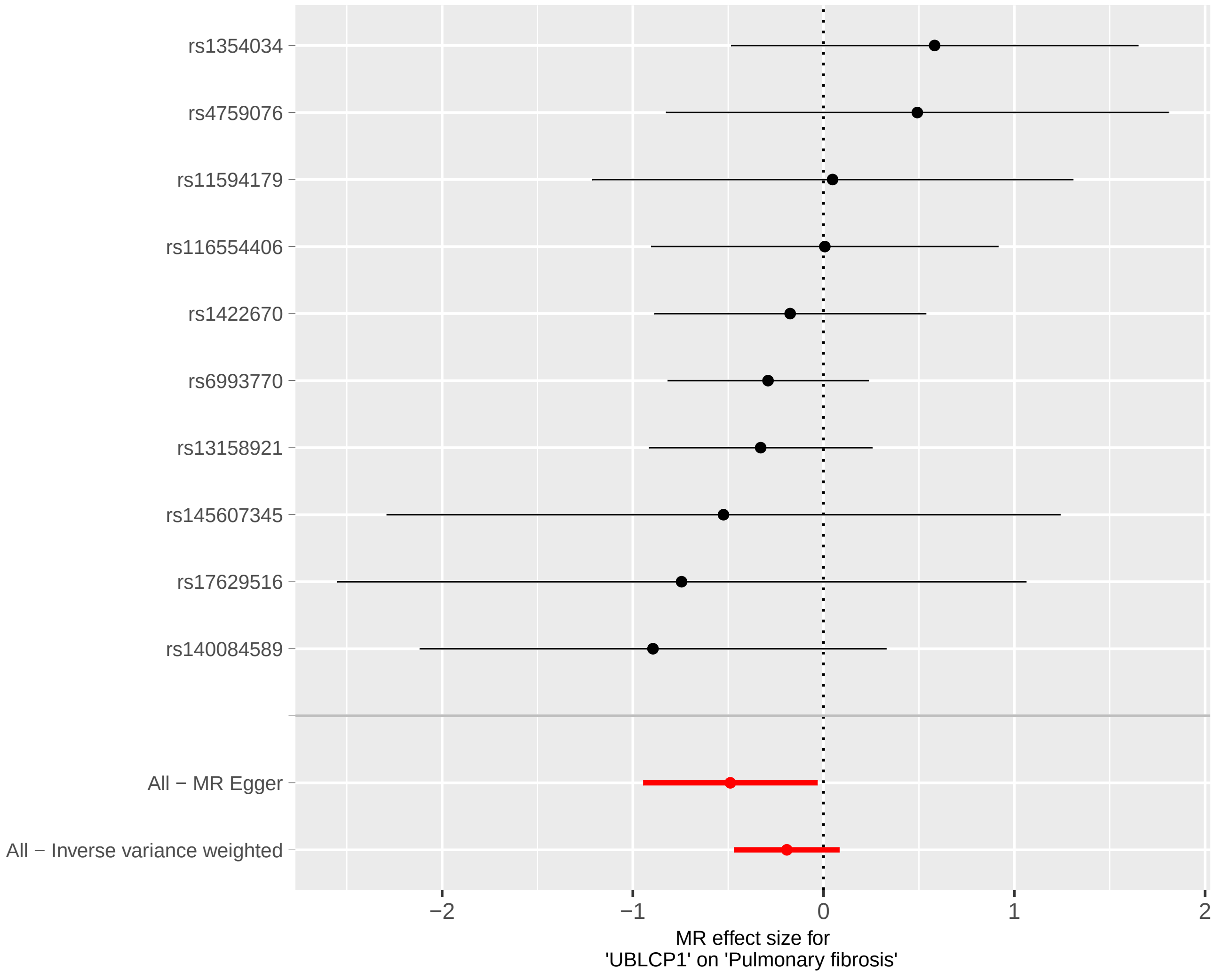

rs1354034
rs4759076
rs11594179
rs116554406
rs1422670
rs6993770
rs13158921
rs145607345
rs17629516
rs140084589
All − MR Egger
All − Inverse variance weighted
−2
−1
0
1
2
MR effect size for
'UBLCP1' on 'Pulmonary fibrosis'

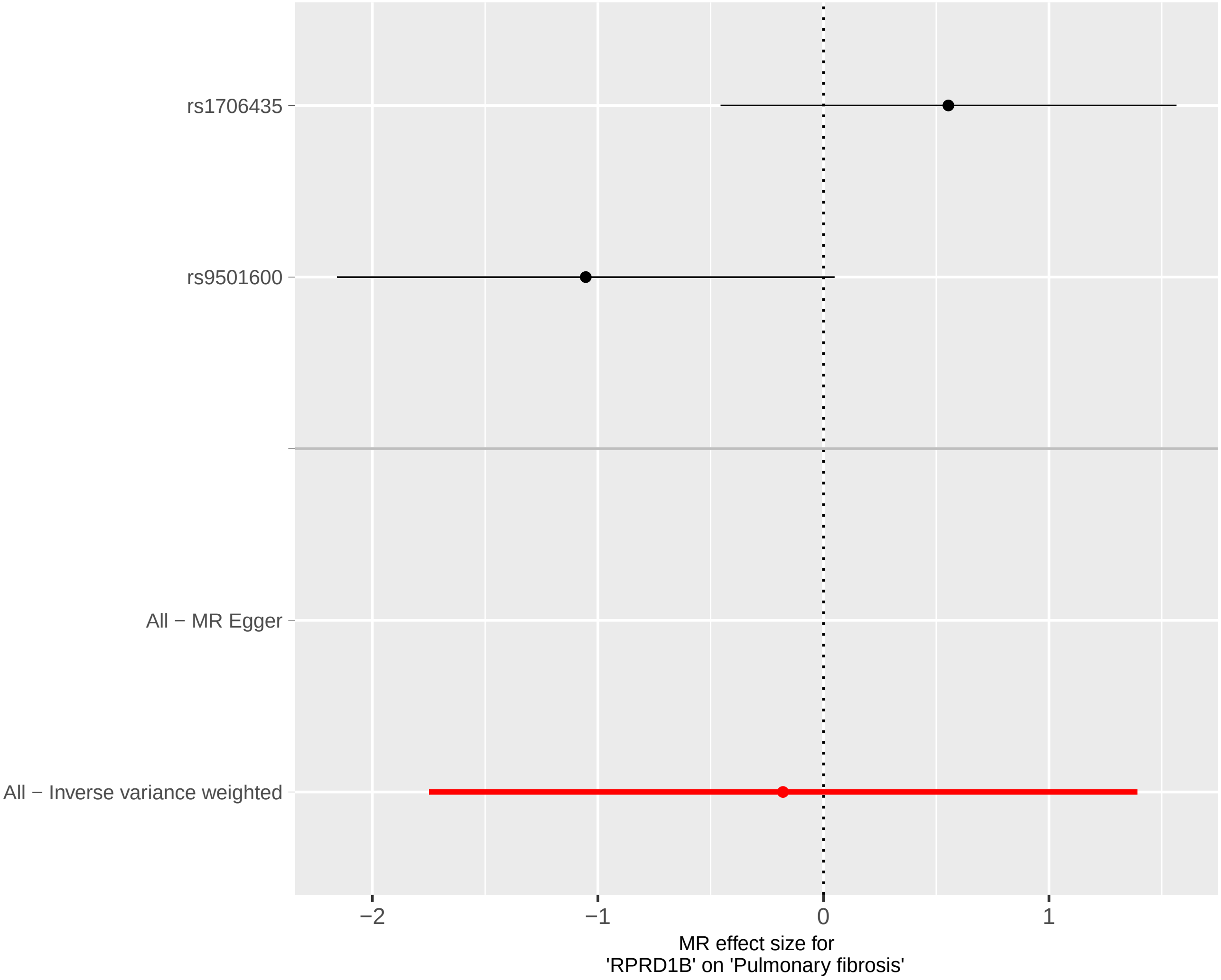

rs1706435
rs9501600
All − MR Egger
All − Inverse variance weighted
−2
−1
0
1
MR effect size for
'RPRD1B' on 'Pulmonary fibrosis'

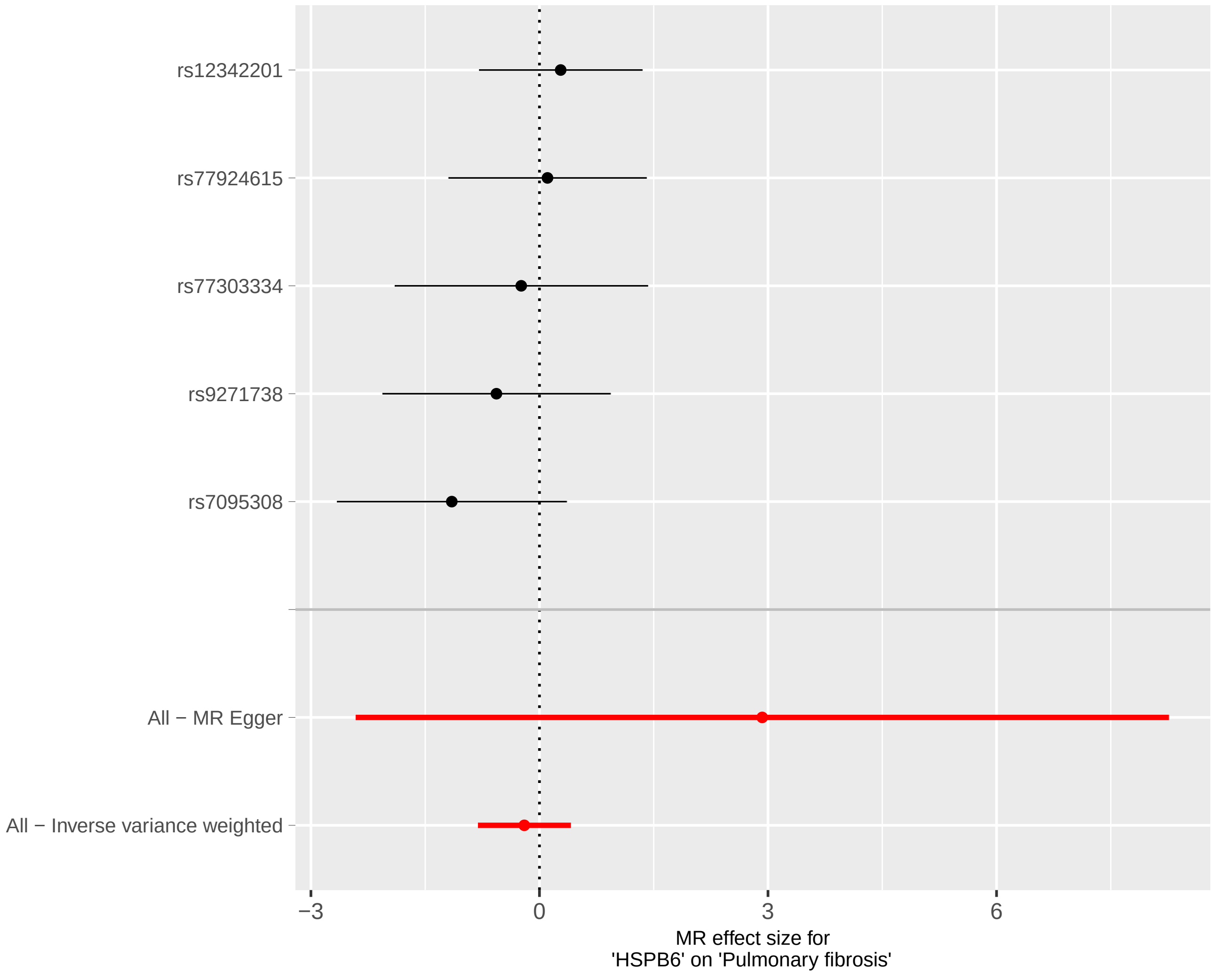
rs12342201
rs77924615
rs77303334
rs9271738
rs7095308
All − MR Egger
All − Inverse variance weighted
−3
0
3
6
MR effect size for
'HSPB6' on 'Pulmonary fibrosis'

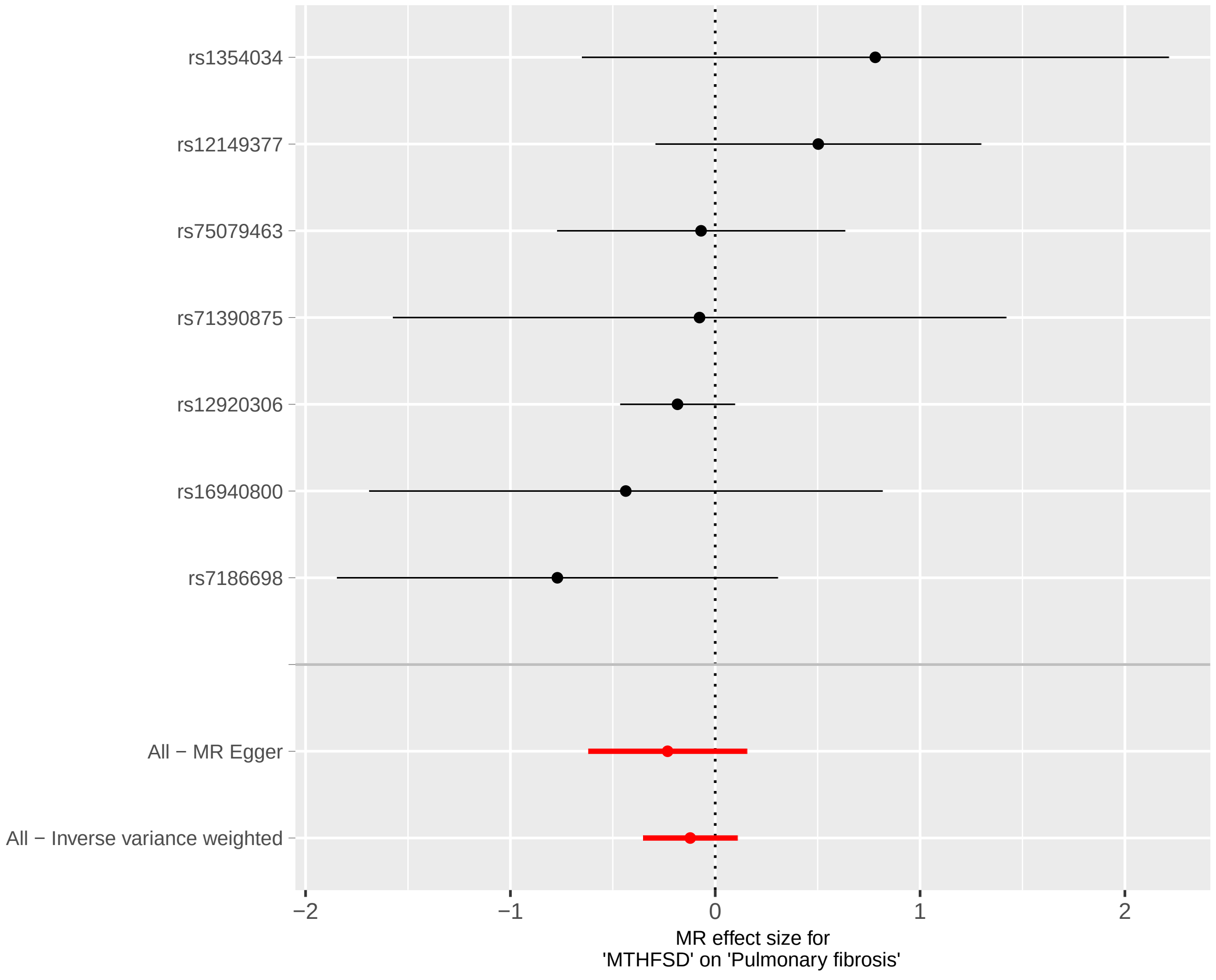

rs1354034
rs12149377
rs75079463
rs71390875
rs12920306
rs16940800
rs7186698
All − MR Egger
All − Inverse variance weighted
−2
−1
0
1
2
MR effect size for
'MTHFSD' on 'Pulmonary fibrosis'

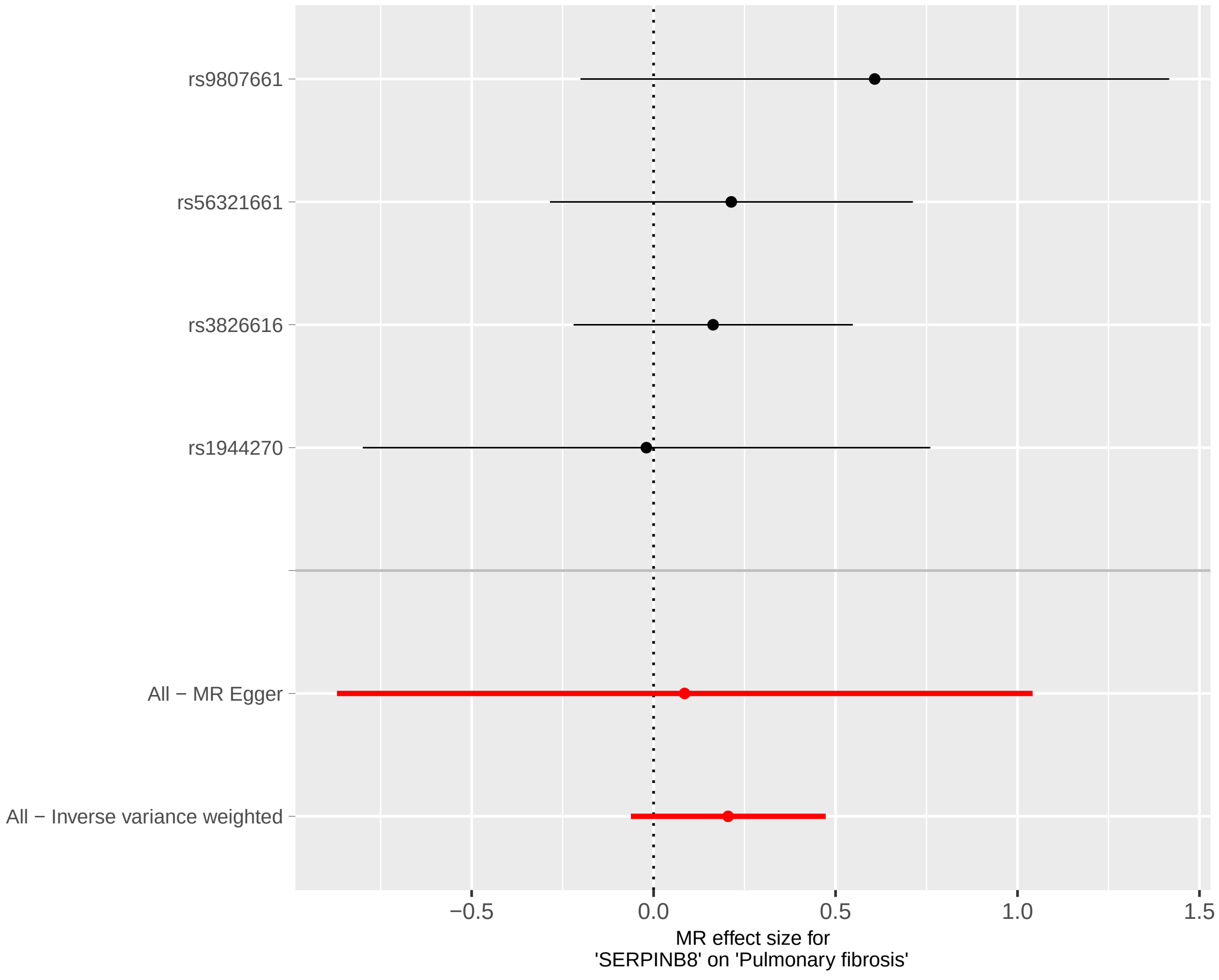
rs9807661
rs56321661
rs3826616
rs1944270
All − MR Egger
All − Inverse variance weighted
−0.5
0.0
0.5
1.0
1.5
MR effect size for
'SERPINB8' on 'Pulmonary fibrosis'

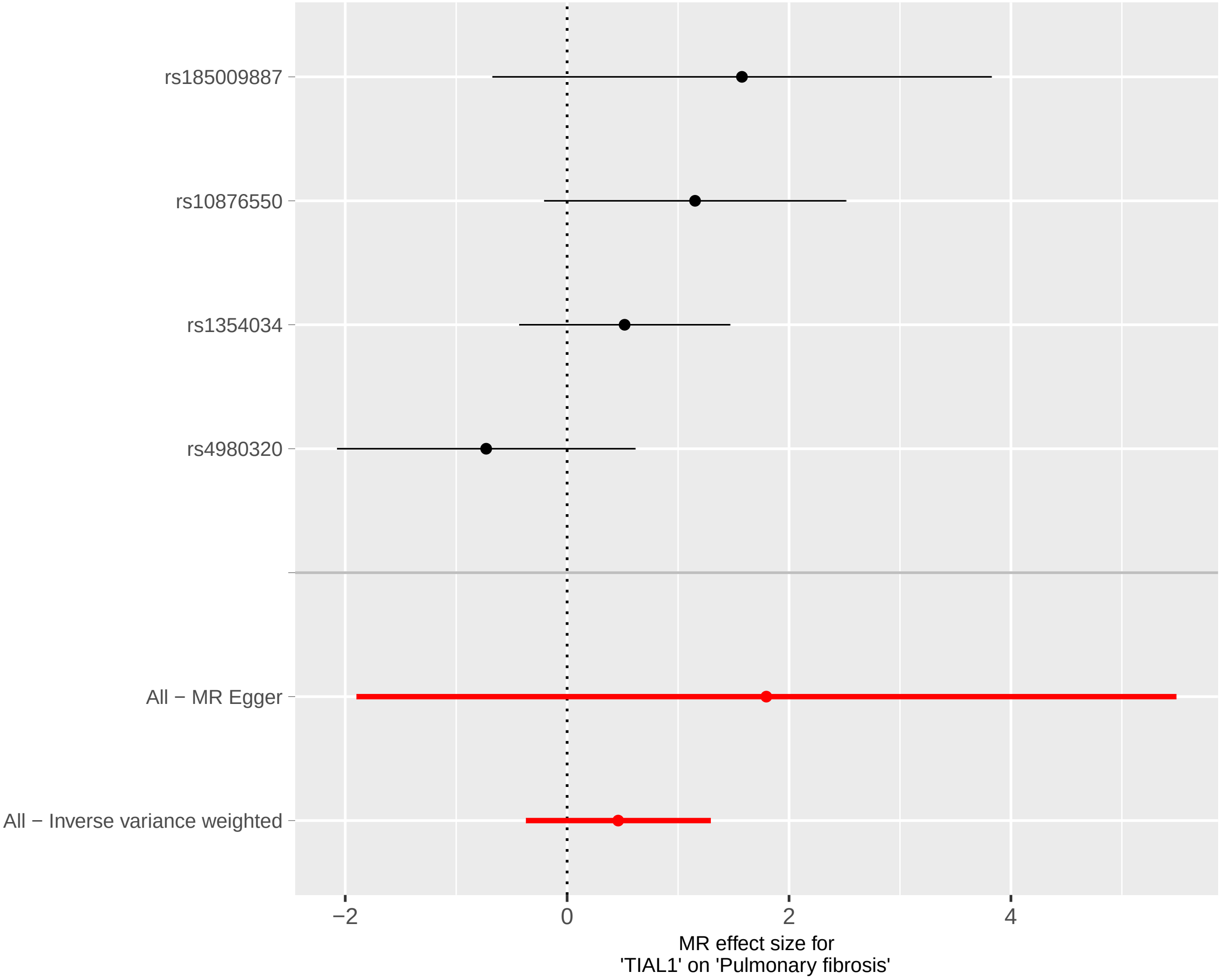

rs185009887
rs10876550
rs1354034
rs4980320
All − MR Egger
All − Inverse variance weighted
−2
0
2
4
MR effect size for
'TIAL1' on 'Pulmonary fibrosis'

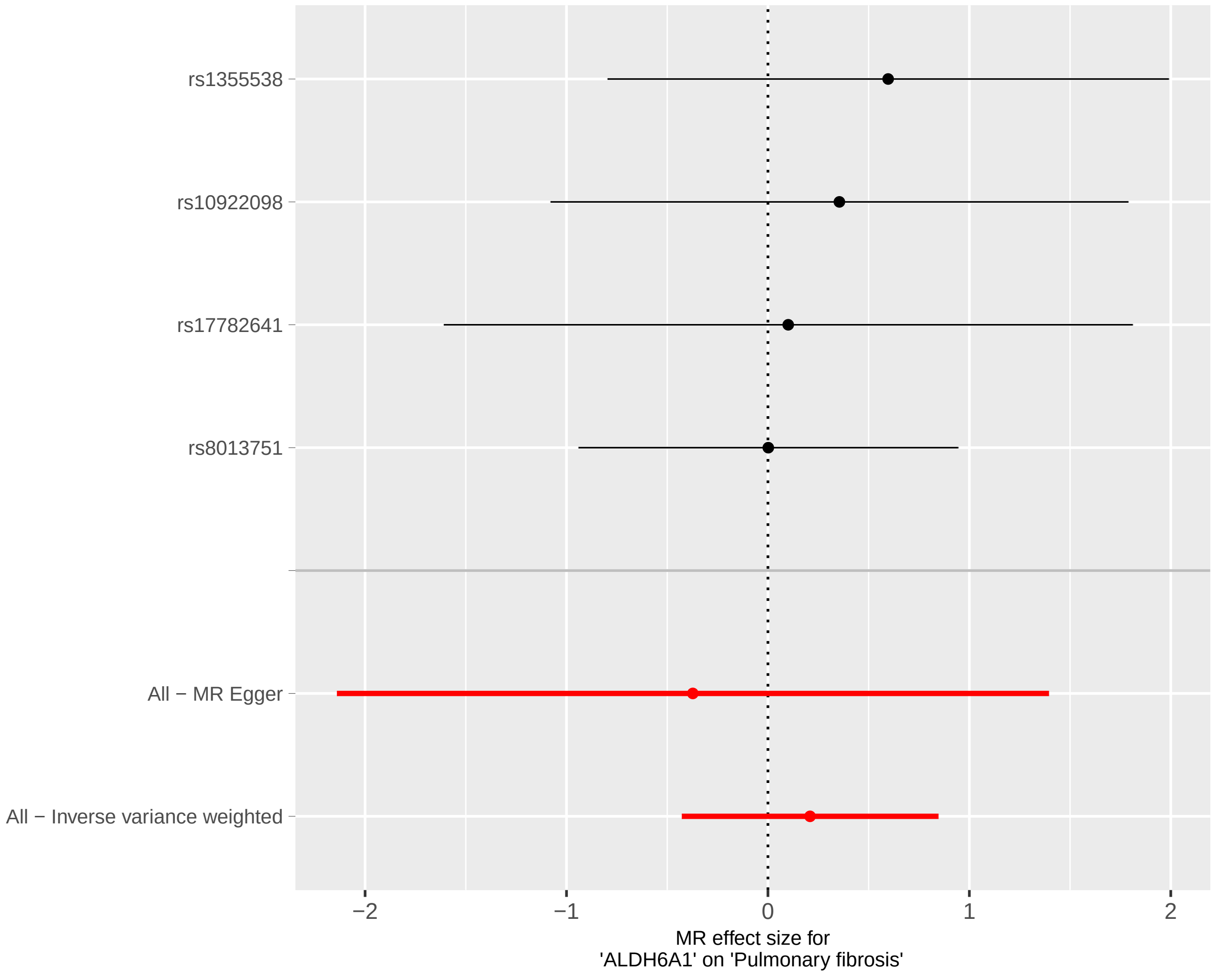
rs1355538
rs10922098
rs17782641
rs8013751
All − MR Egger
All − Inverse variance weighted
−2
−1
0
1
2
MR effect size for
'ALDH6A1' on 'Pulmonary fibrosis'

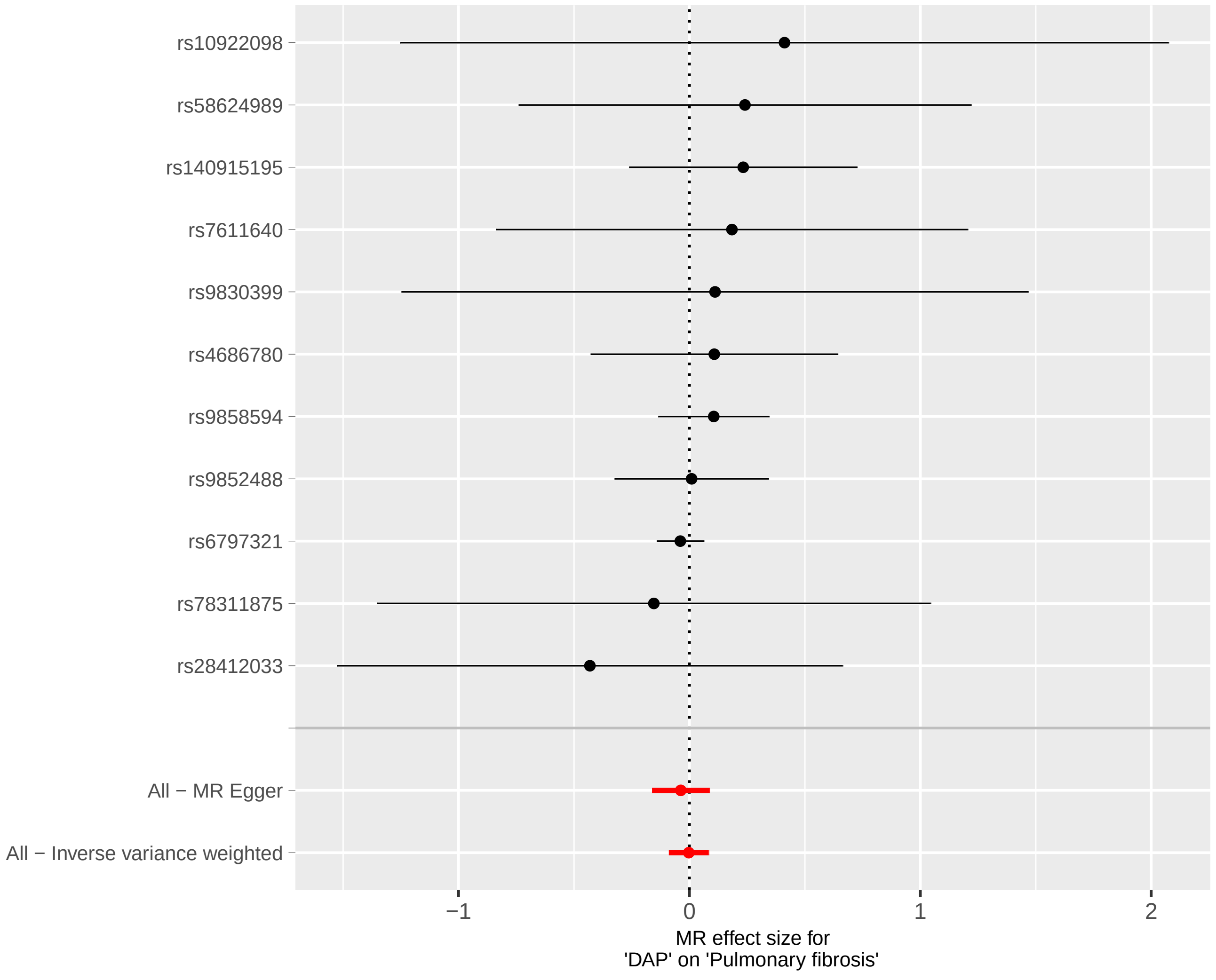
rs10922098
rs58624989
rs140915195
rs7611640
rs9830399
rs4686780
rs9858594
rs9852488
rs6797321
rs78311875
rs28412033
All − MR Egger
All − Inverse variance weighted
−1
0
1
2
MR effect size for
'DAP' on 'Pulmonary fibrosis'

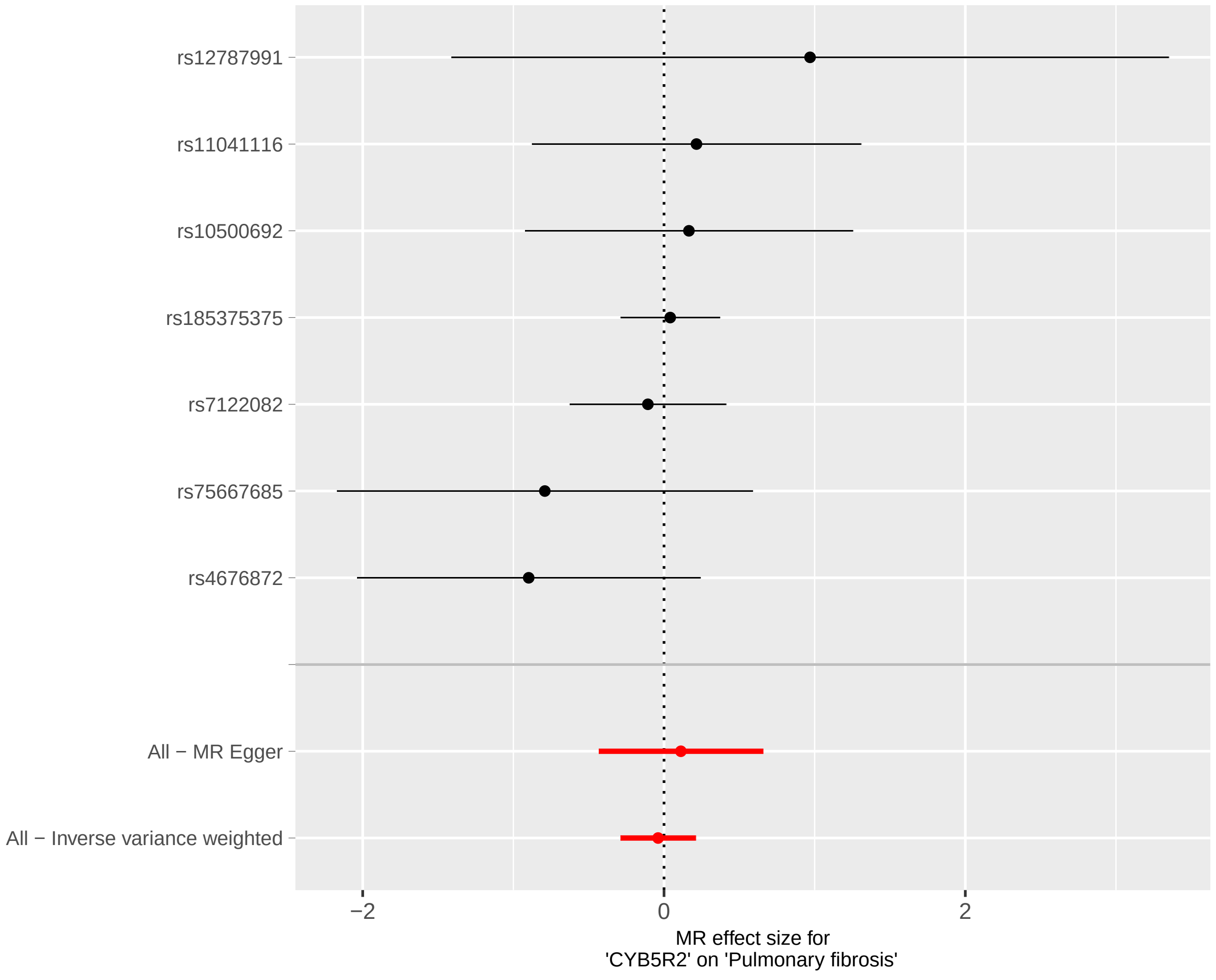

rs12787991
rs11041116
rs10500692
rs185375375
rs7122082
rs75667685
rs4676872
All − MR Egger
All − Inverse variance weighted
−2
0
2
MR effect size for
'CYB5R2' on 'Pulmonary fibrosis'

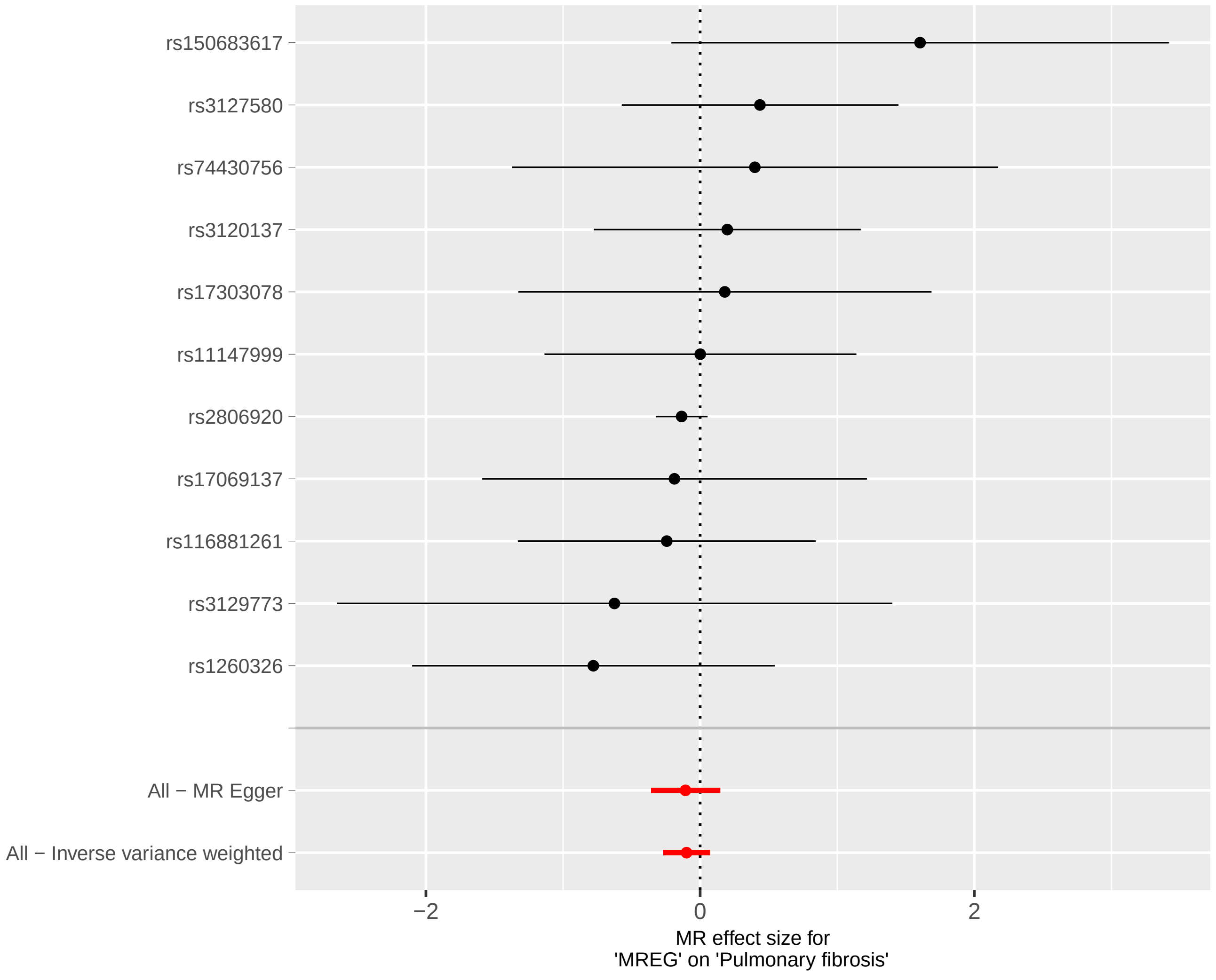

rs150683617
rs3127580
rs74430756
rs3120137
rs17303078
rs11147999
rs2806920
rs17069137
rs116881261
rs3129773
rs1260326
All − MR Egger
All − Inverse variance weighted
−2
0
2
MR effect size for
'MREG' on 'Pulmonary fibrosis'

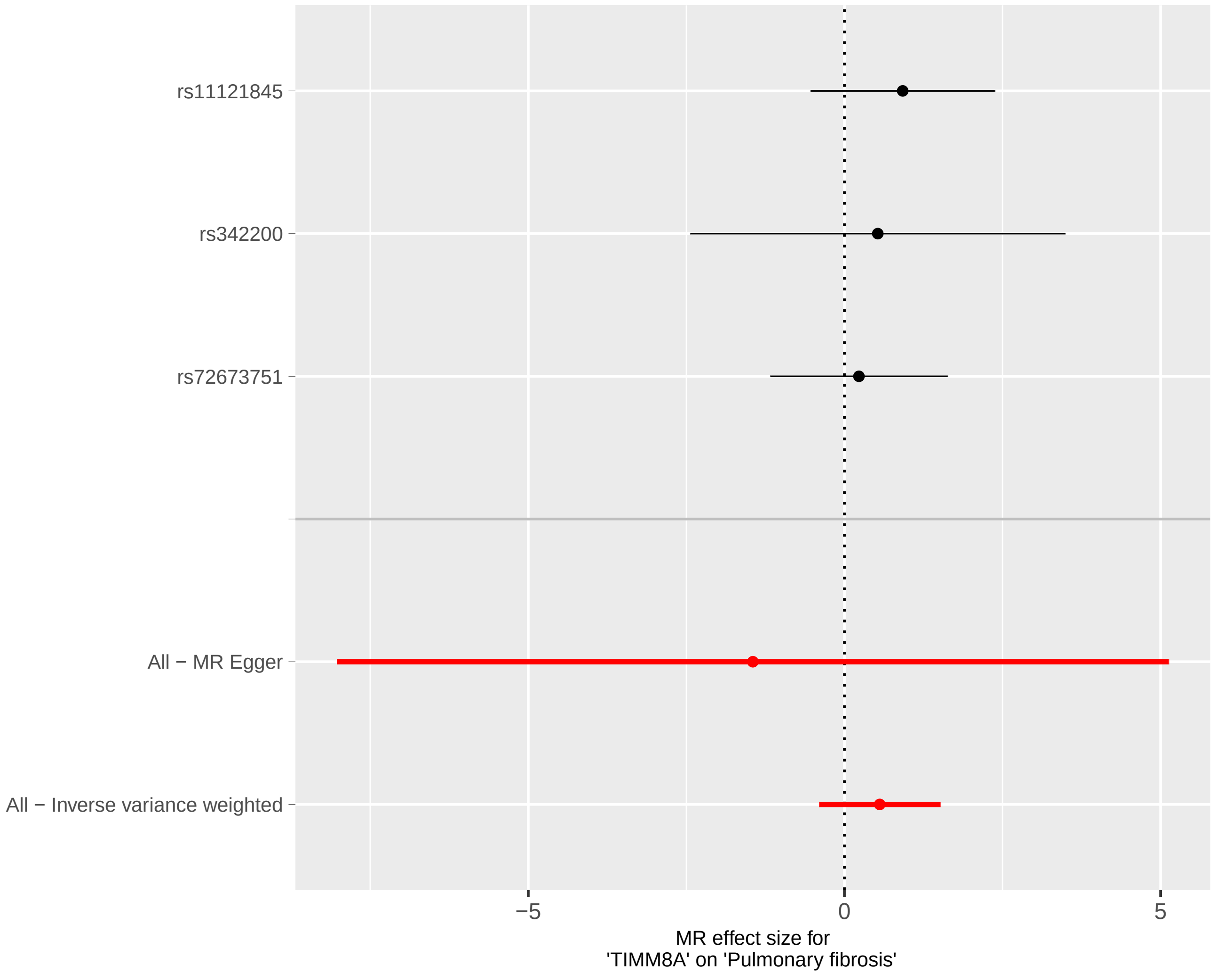

rs11121845
rs342200
rs72673751
All − MR Egger
All − Inverse variance weighted
−5
0
5
MR effect size for
'TIMM8A' on 'Pulmonary fibrosis'

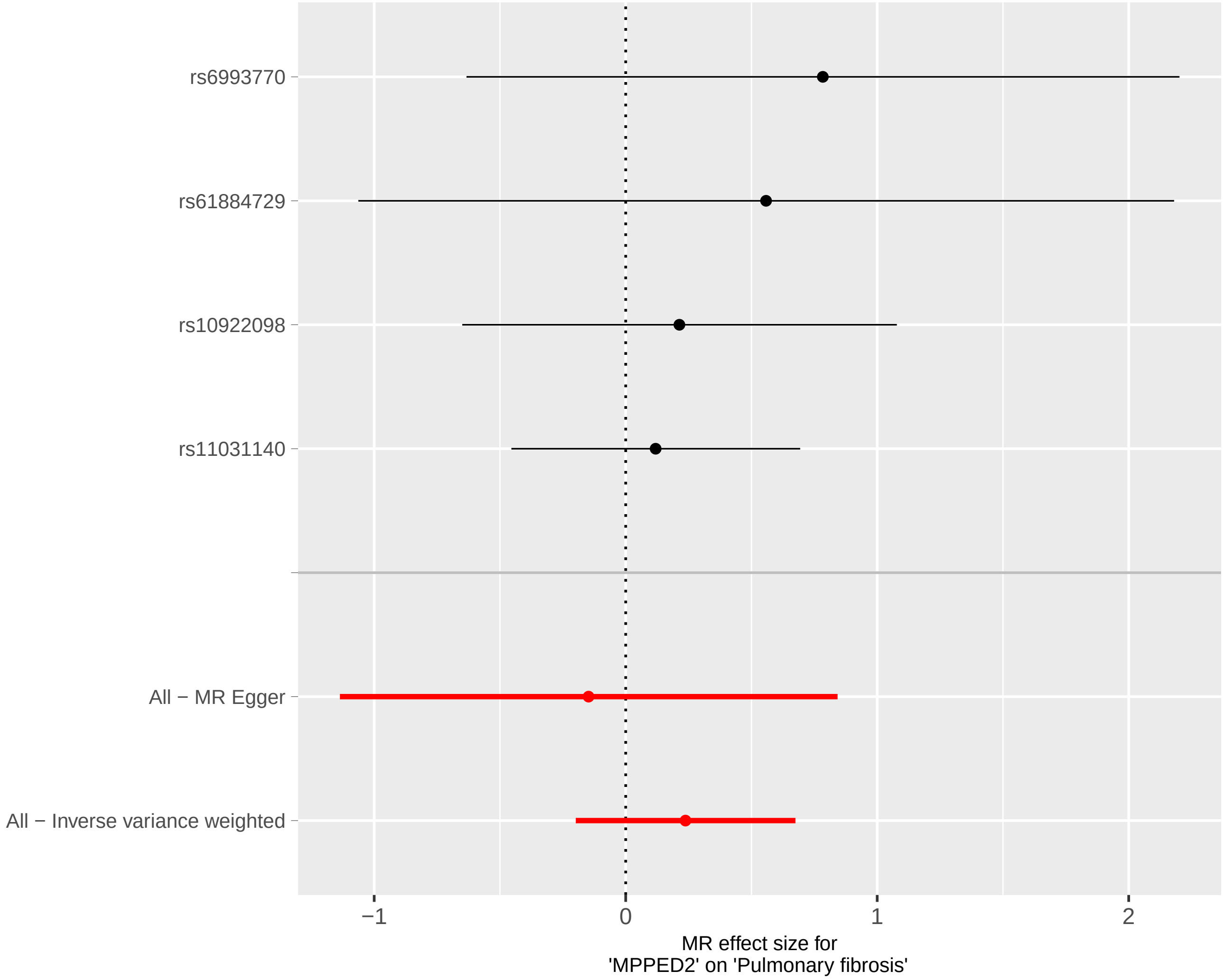

rs6993770
rs61884729
rs10922098
rs11031140
All − MR Egger
All − Inverse variance weighted
−1
0
1
2
MR effect size for
'MPPED2' on 'Pulmonary fibrosis'

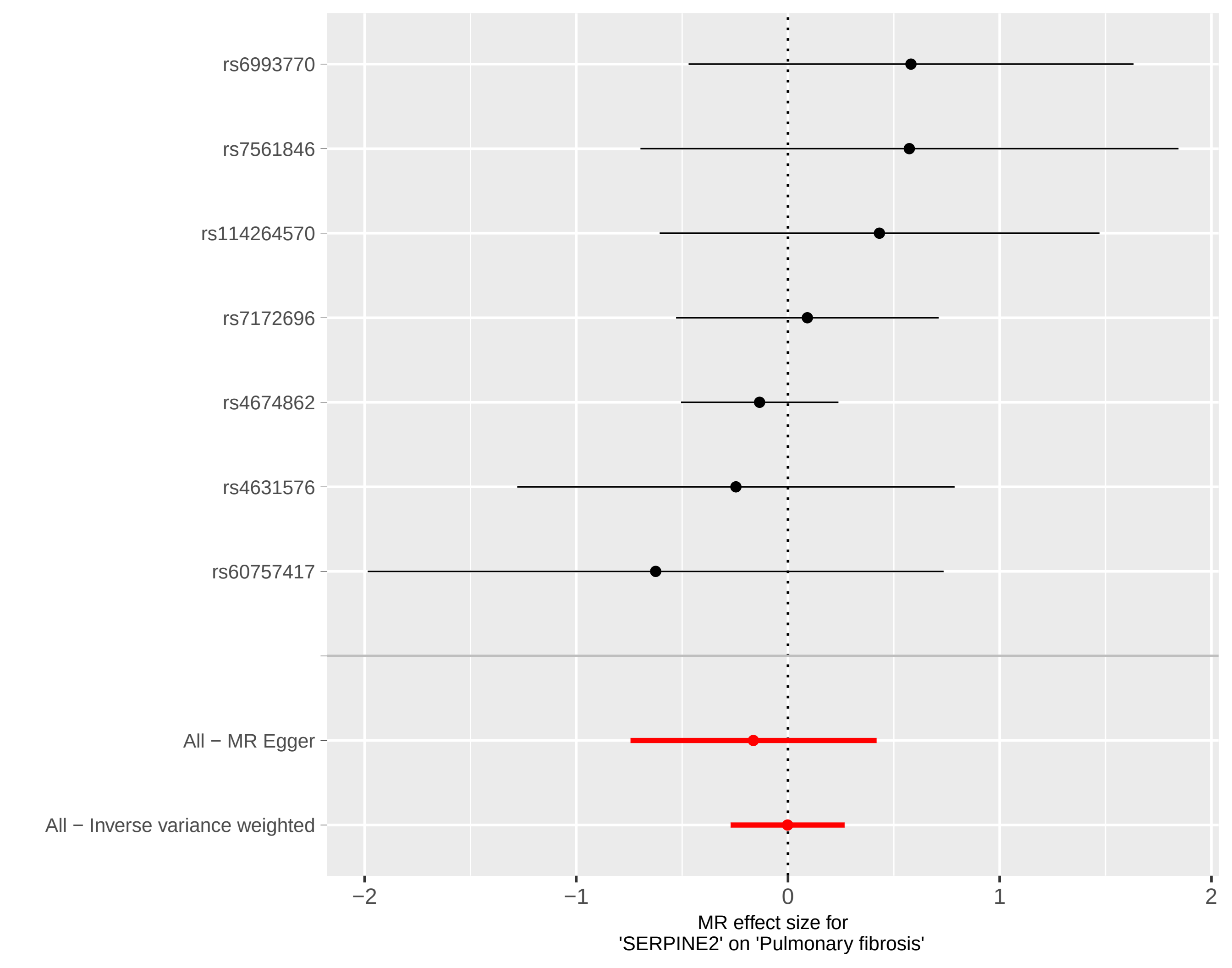

rs6993770
rs7561846
rs114264570
rs7172696
rs4674862
rs4631576
rs60757417
All − MR Egger
All − Inverse variance weighted
−2
−1
0
1
2
MR effect size for
'SERPINE2' on 'Pulmonary fibrosis'

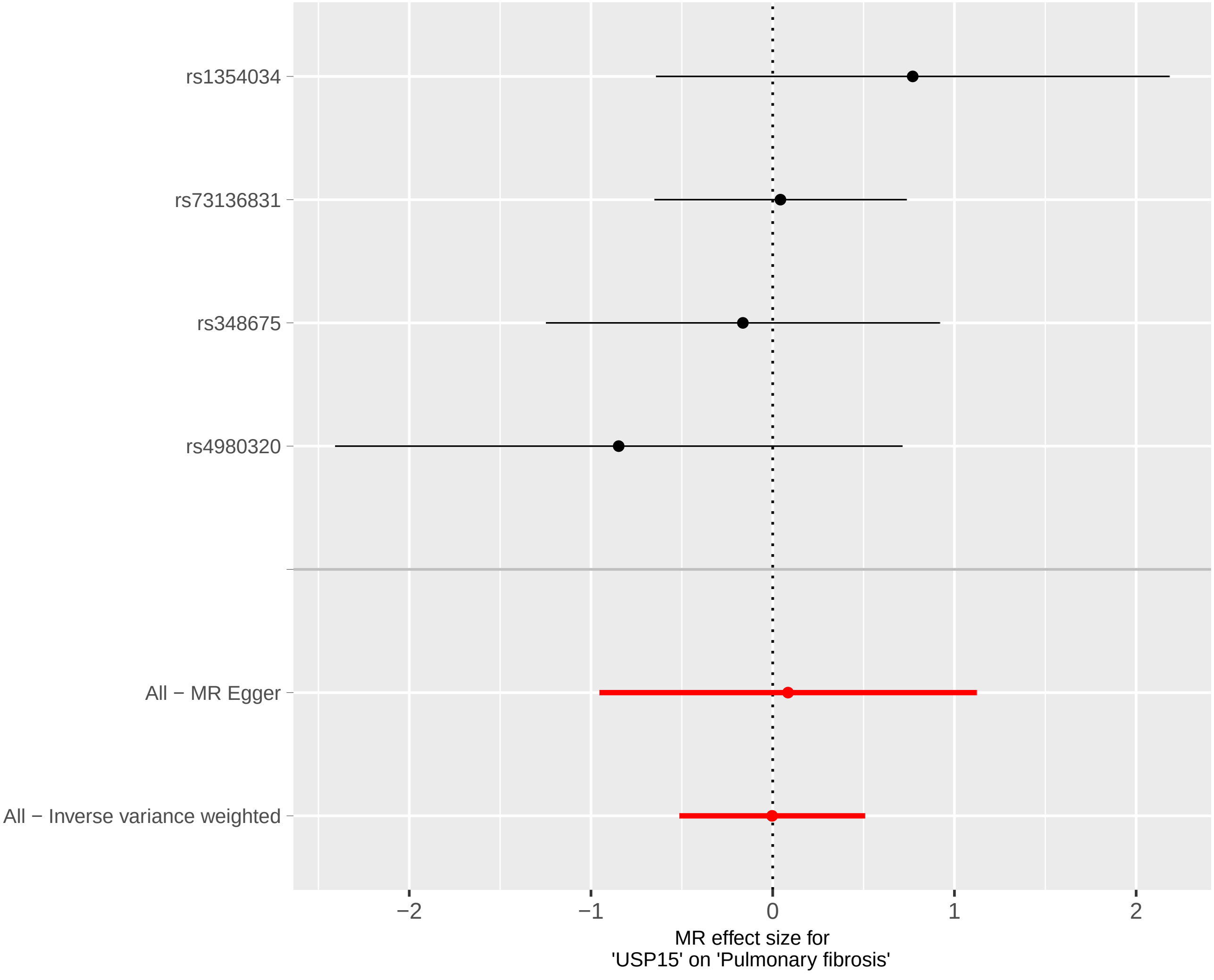
rs1354034
rs73136831
rs348675
rs4980320
All − MR Egger
All − Inverse variance weighted
−2
−1
0
1
2
MR effect size for
'USP15' on 'Pulmonary fibrosis'

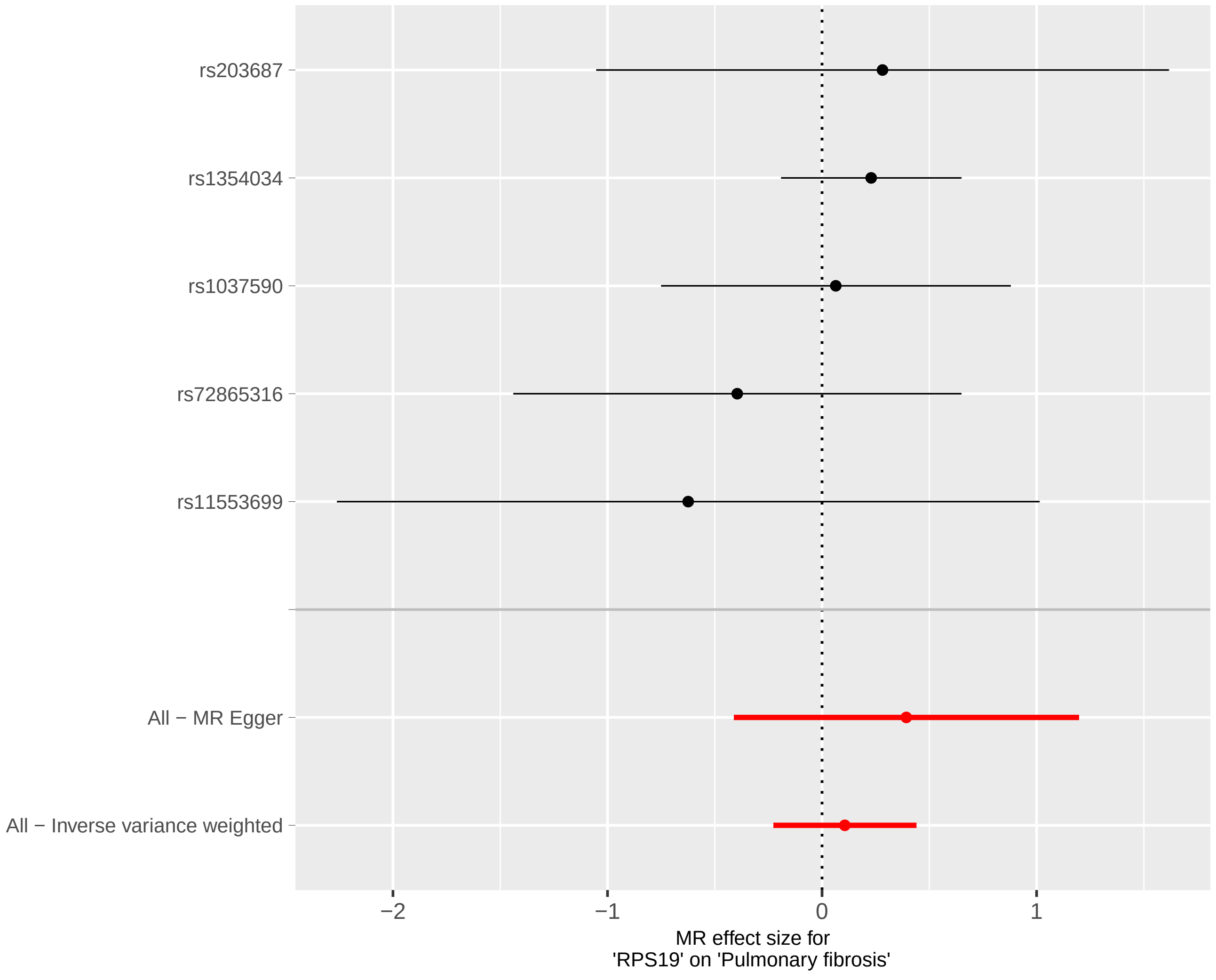
rs203687
rs1354034
rs1037590
rs72865316
rs11553699
All − MR Egger
All − Inverse variance weighted
−2
−1
0
1
MR effect size for
'RPS19' on 'Pulmonary fibrosis'

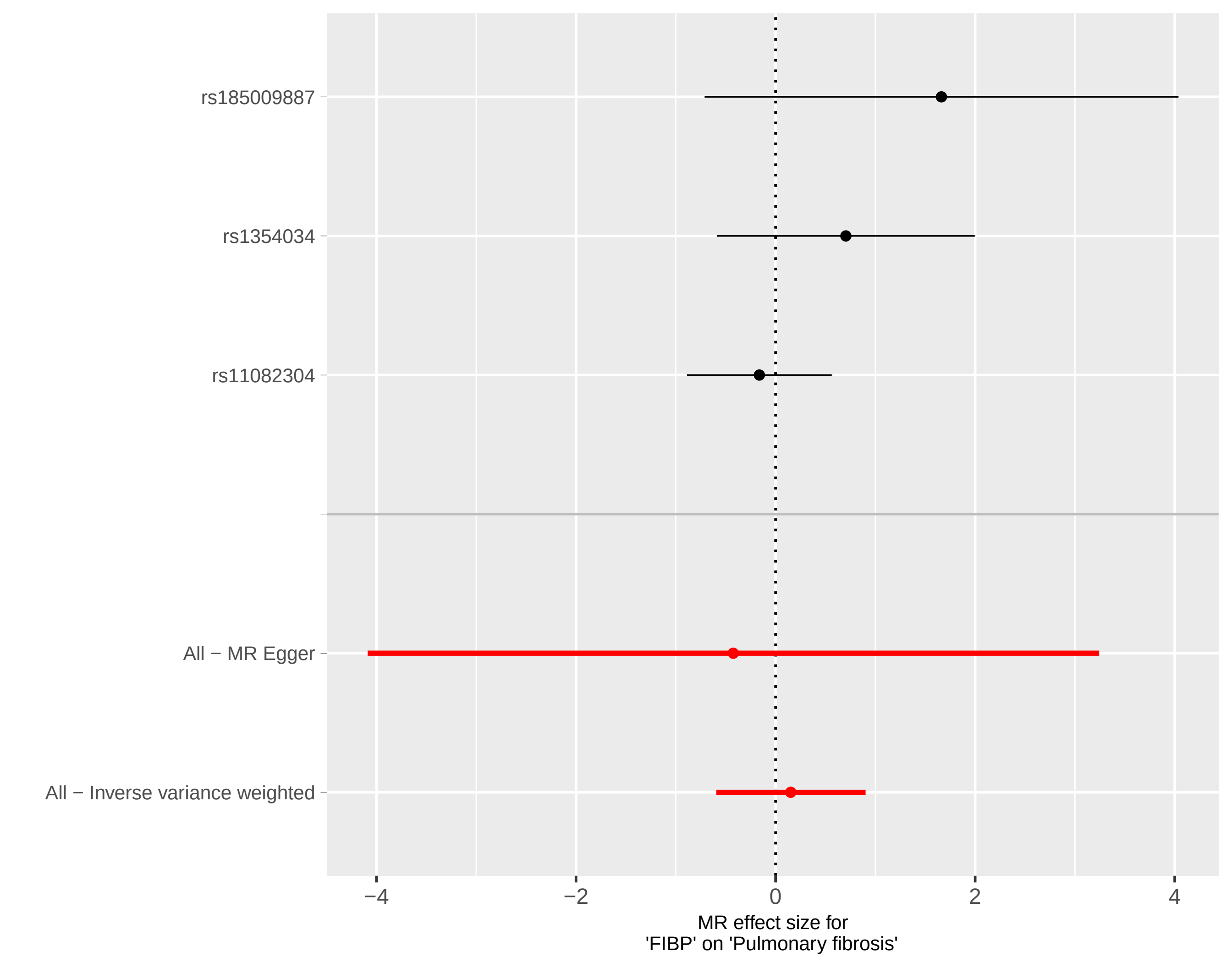

rs185009887
rs1354034
rs11082304
All − MR Egger
All − Inverse variance weighted
−4
−2
0
2
4
MR effect size for
'FIBP' on 'Pulmonary fibrosis'

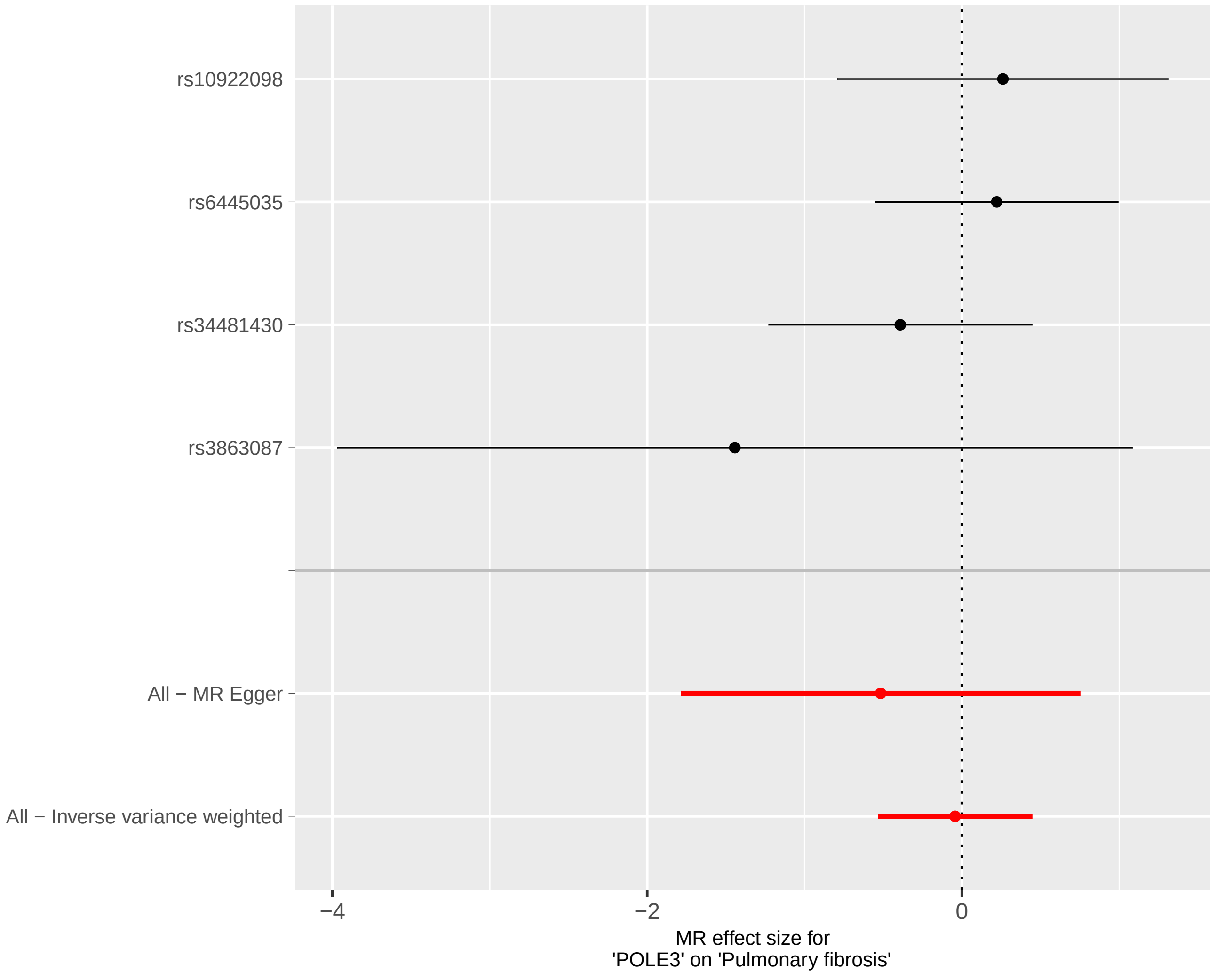

rs10922098
rs6445035
rs34481430
rs3863087
All − MR Egger
All − Inverse variance weighted
−4
−2
0
MR effect size for
'POLE3' on 'Pulmonary fibrosis'

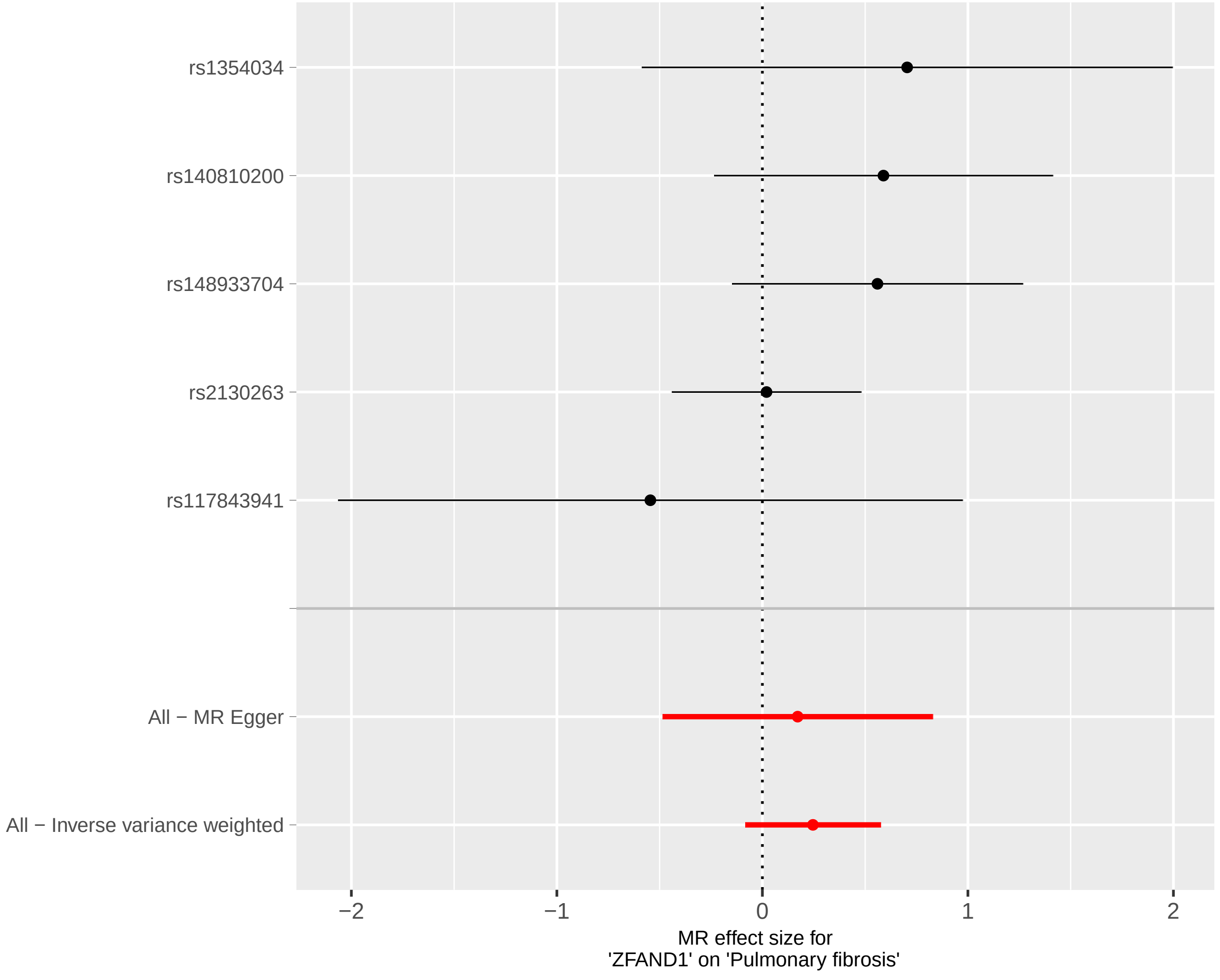

rs1354034
rs140810200
rs148933704
rs2130263
rs117843941
All − MR Egger
All − Inverse variance weighted
−2
−1
0
1
2
MR effect size for
'ZFAND1' on 'Pulmonary fibrosis'

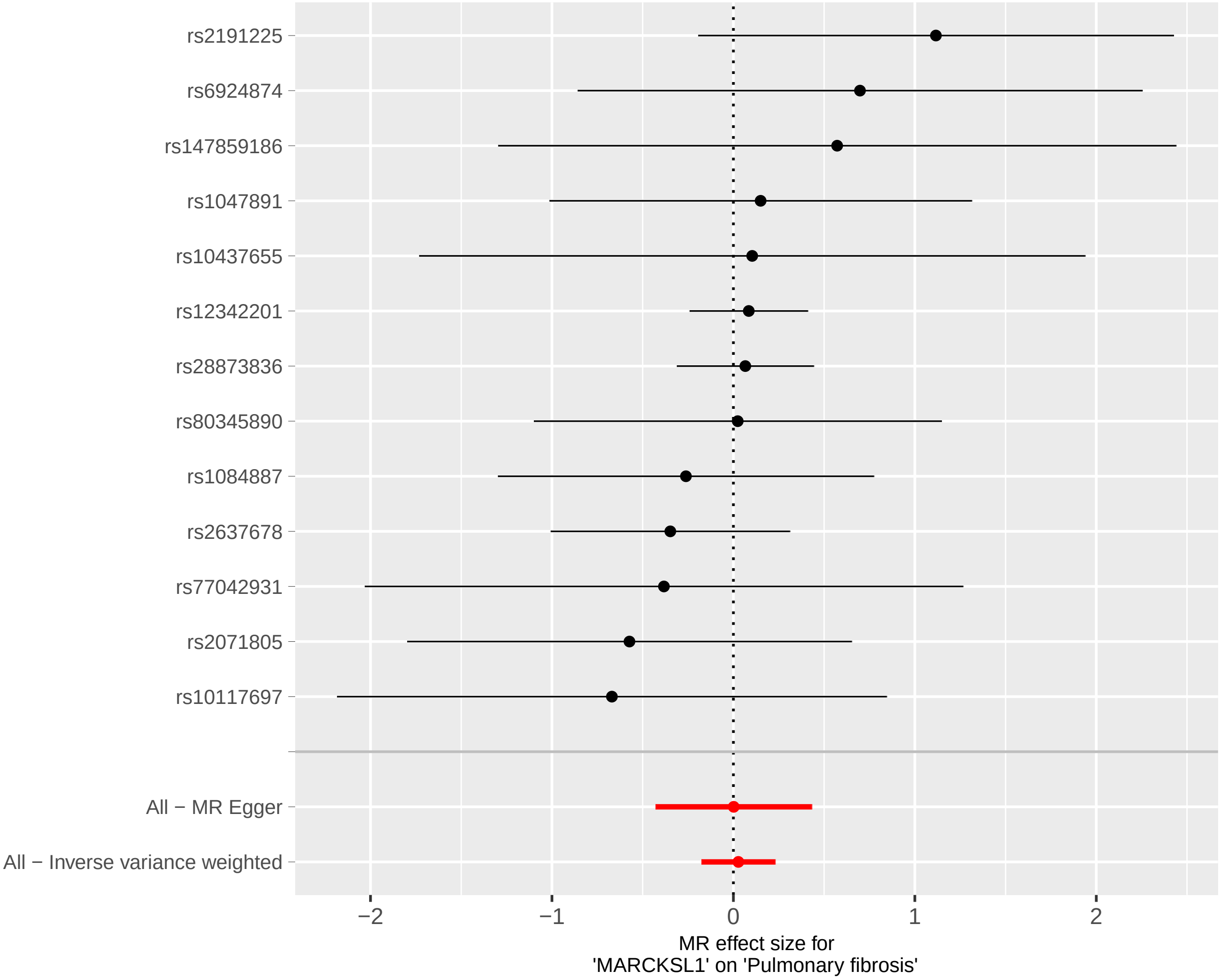
rs2191225
rs6924874
rs147859186
rs1047891
rs10437655
rs12342201
rs28873836
rs80345890
rs1084887
rs2637678
rs77042931
rs2071805
rs10117697
All − MR Egger
All − Inverse variance weighted
−2
−1
0
1
2
MR effect size for
'MARCKSL1' on 'Pulmonary fibrosis'

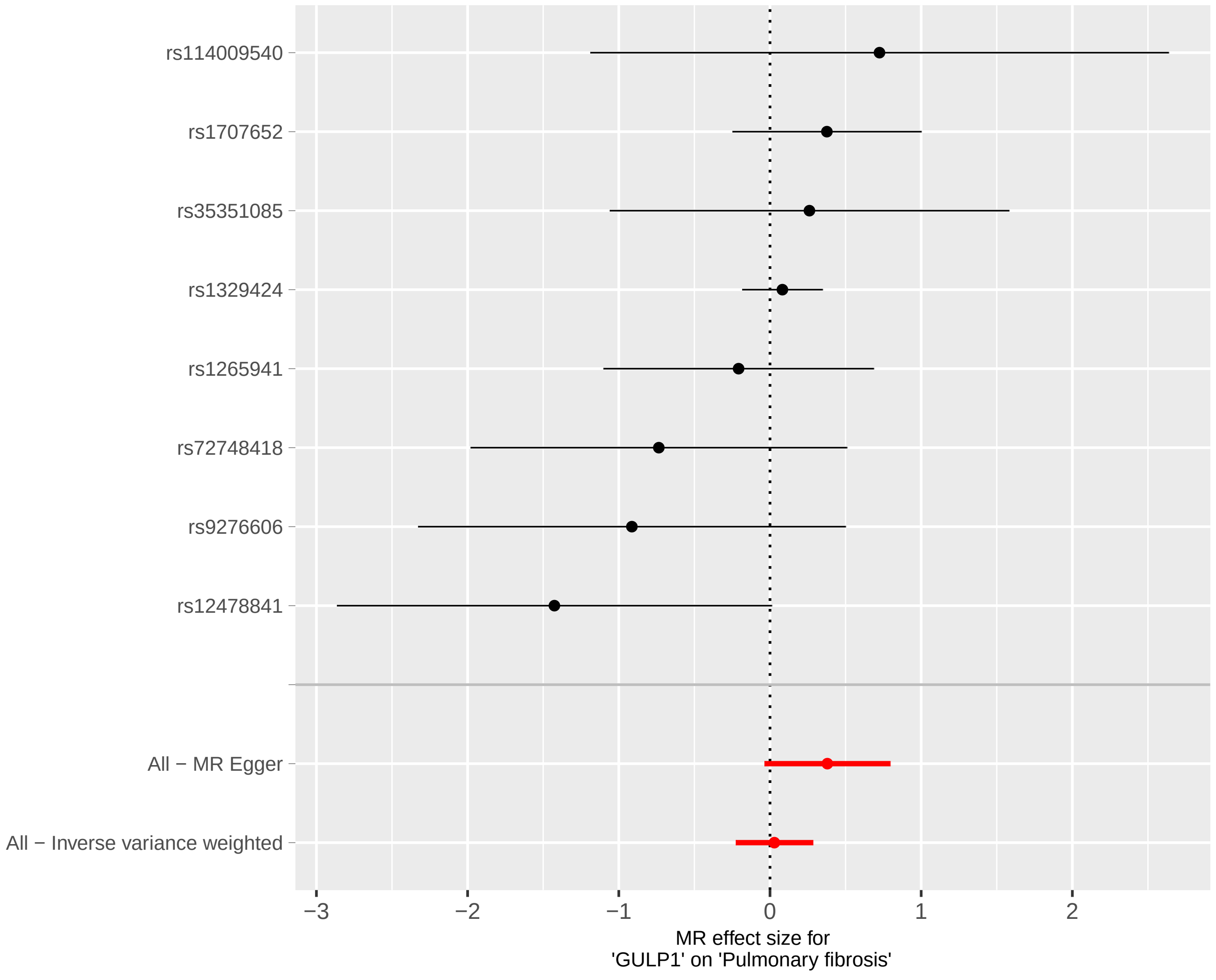
rs114009540
rs1707652
rs35351085
rs1329424
rs1265941
rs72748418
rs9276606
rs12478841
All − MR Egger
All − Inverse variance weighted
−3
−2
−1
0
1
2
MR effect size for
'GULP1' on 'Pulmonary fibrosis'

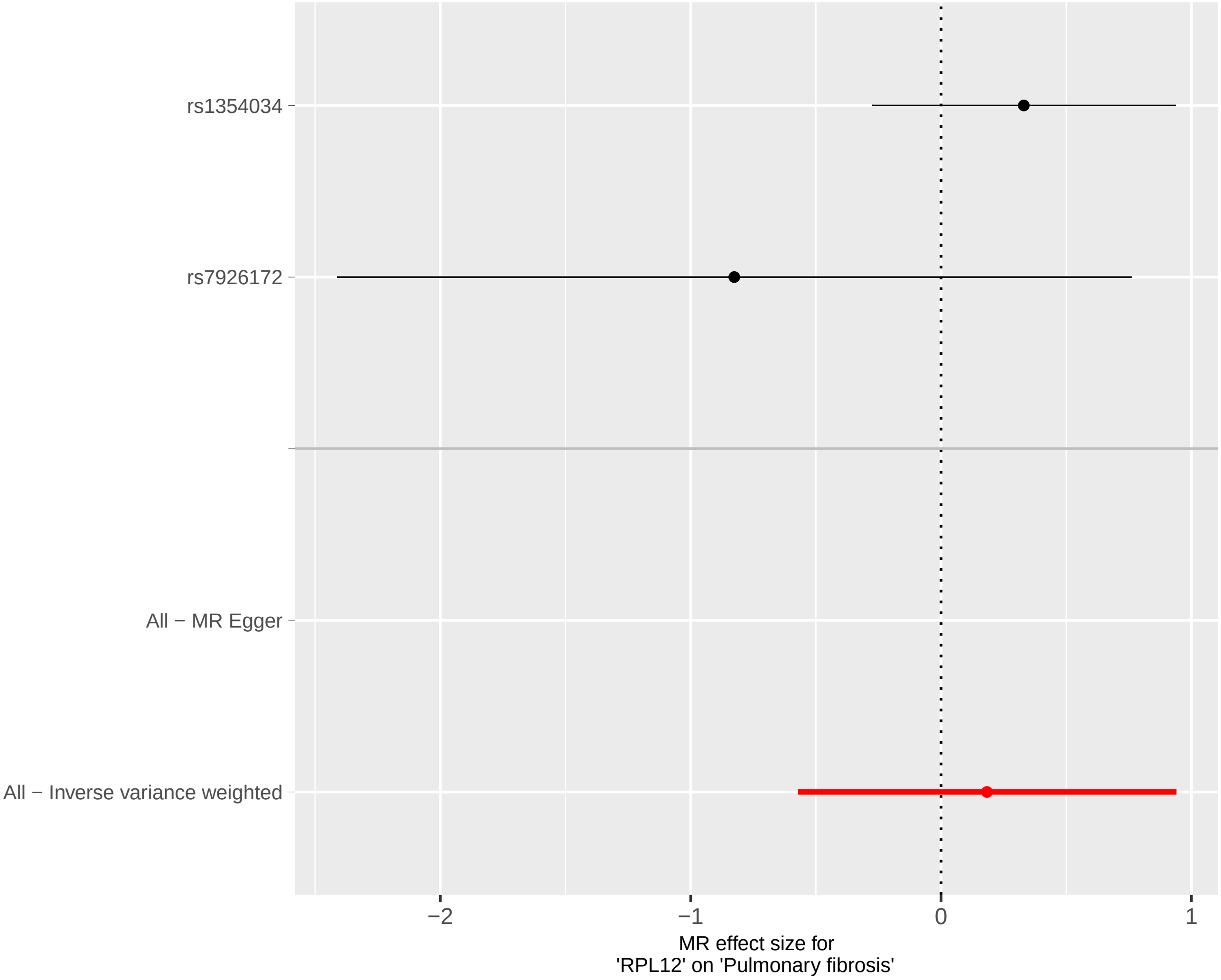
rs1354034
rs7926172
All − MR Egger
All − Inverse variance weighted
−2
−1
0
1
MR effect size for
'RPL12' on 'Pulmonary fibrosis'

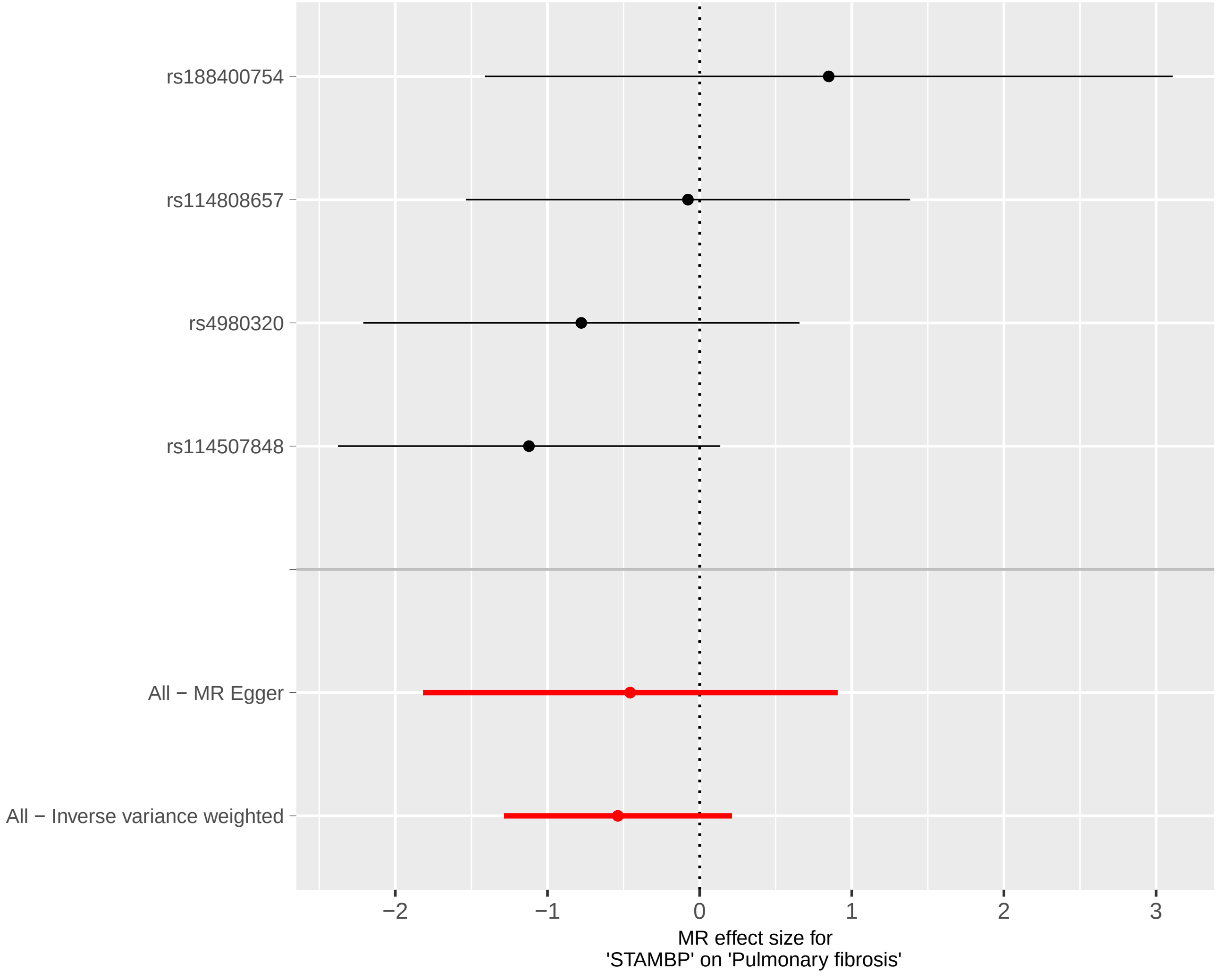
rs188400754
rs114808657
rs4980320
rs114507848
All − MR Egger
All − Inverse variance weighted
−2
−1
0
1
2
3
MR effect size for
'STAMBP' on 'Pulmonary fibrosis'

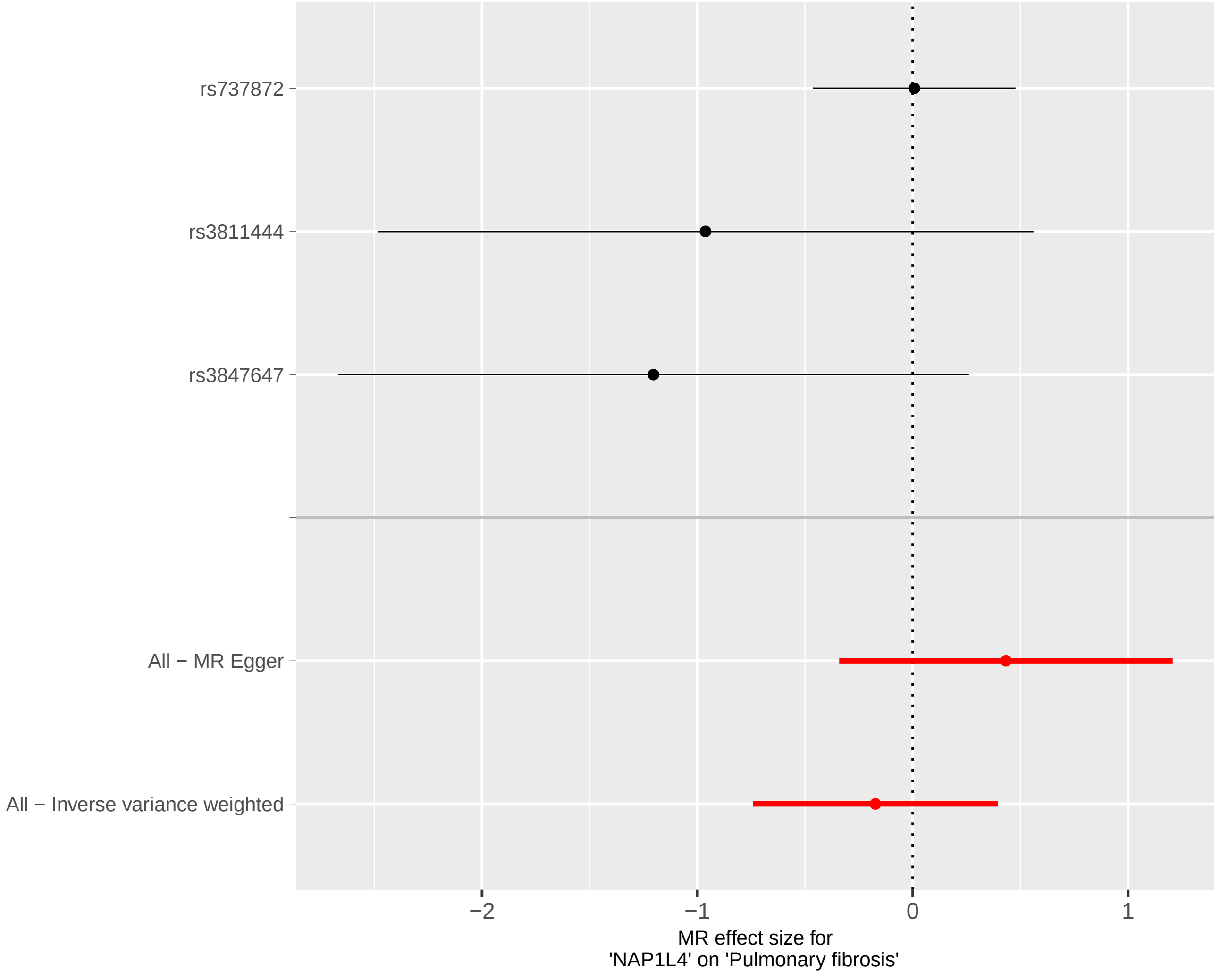

rs737872
rs3811444
rs3847647
All − MR Egger
All − Inverse variance weighted
−2
−1
0
1
MR effect size for
'NAP1L4' on 'Pulmonary fibrosis'

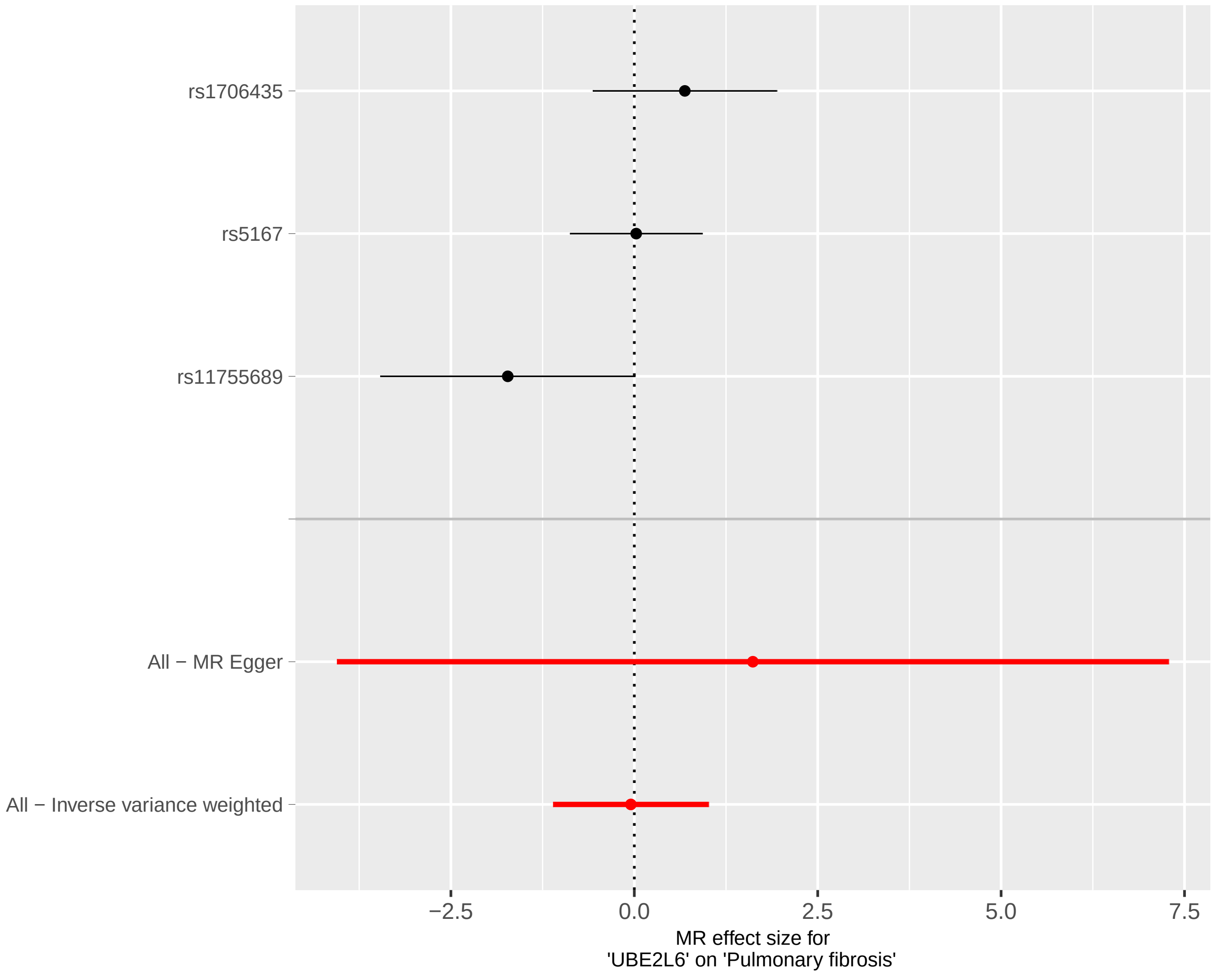

rs1706435
rs5167
rs11755689
All − MR Egger
All − Inverse variance weighted
−2.5
0.0
2.5
5.0
7.5
MR effect size for
'UBE2L6' on 'Pulmonary fibrosis'

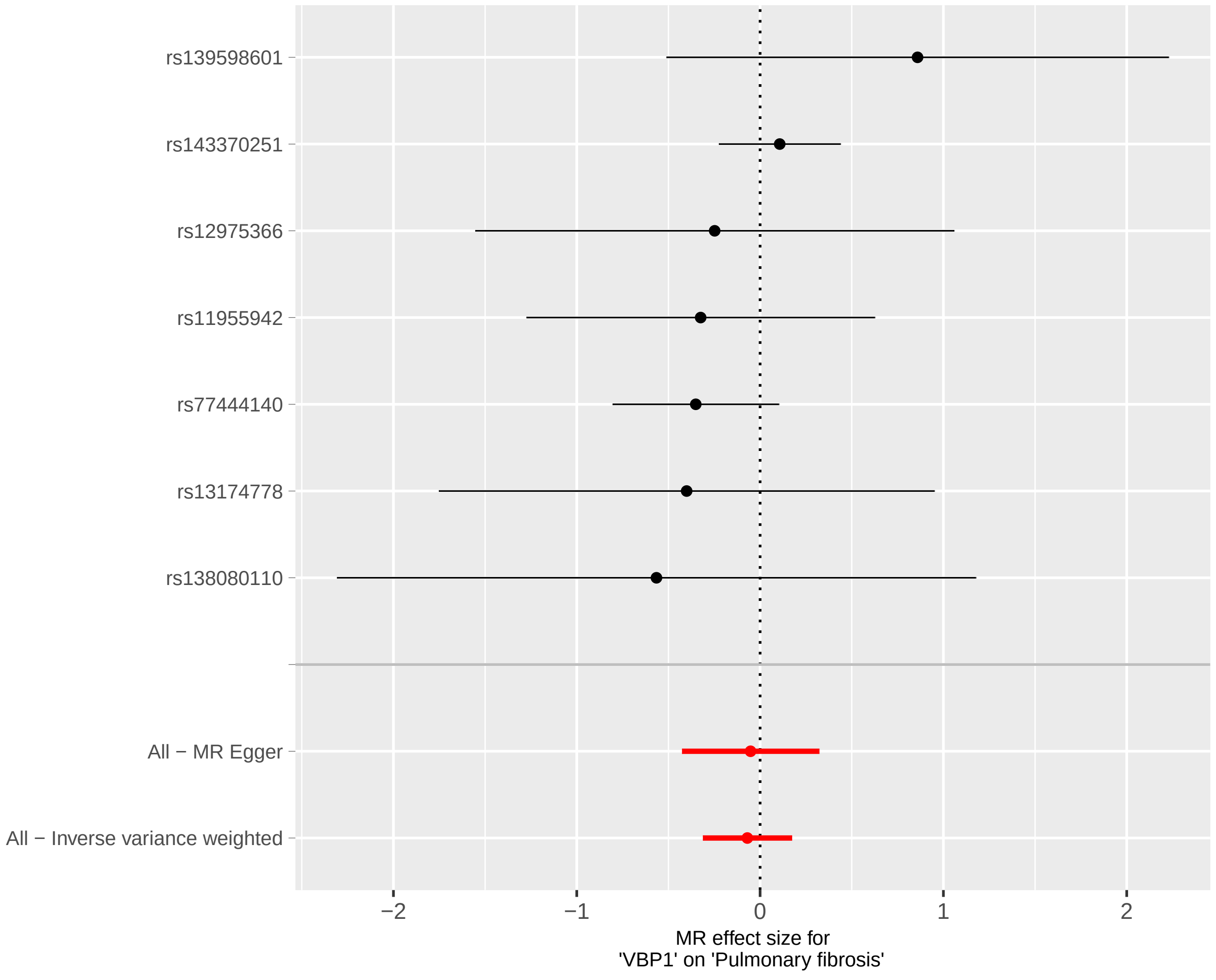
rs139598601
rs143370251
rs12975366
rs11955942
rs77444140
rs13174778
rs138080110
All − MR Egger
All − Inverse variance weighted
−2
−1
0
1
2
MR effect size for
'VBP1' on 'Pulmonary fibrosis'

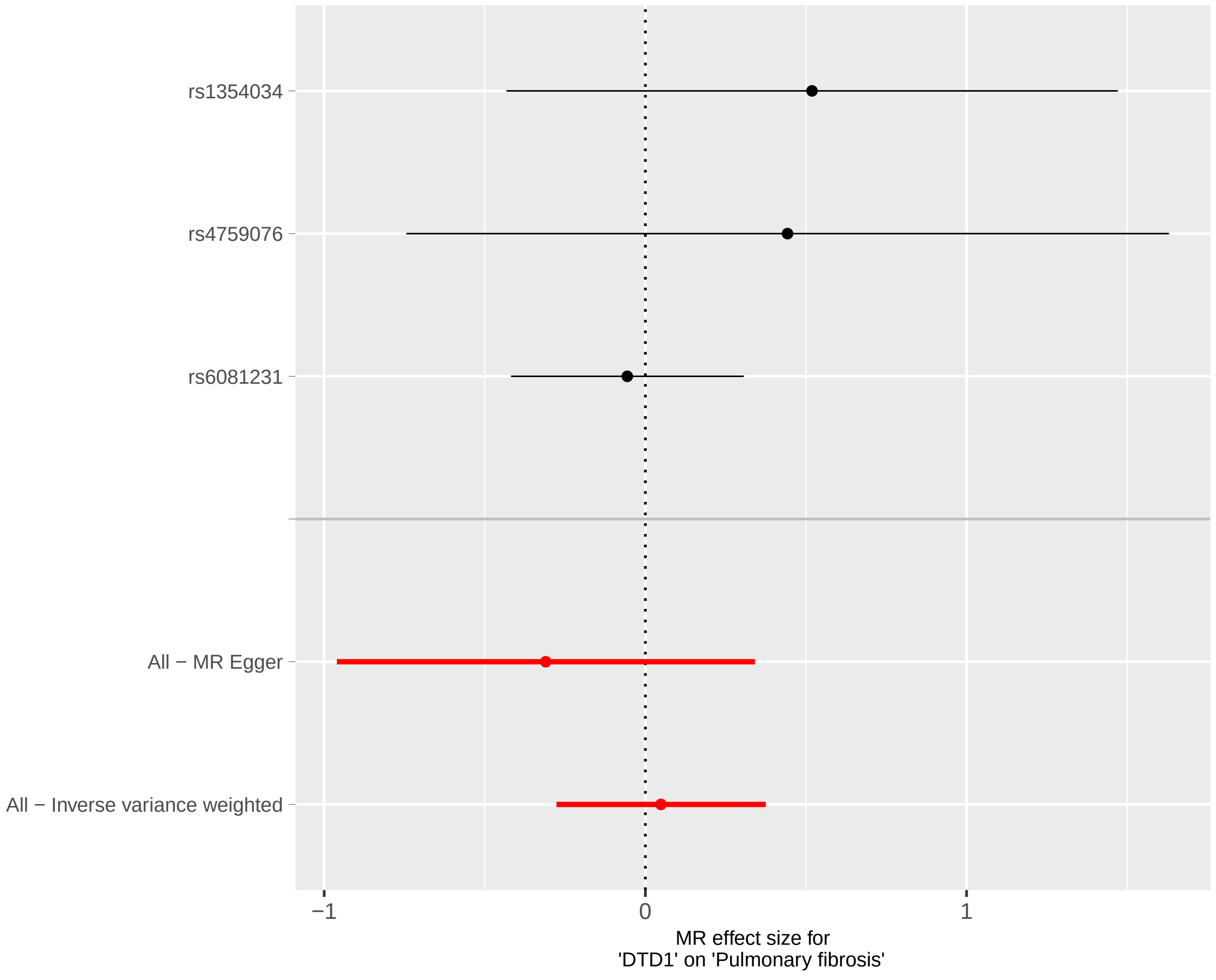

rs1354034
rs4759076
rs6081231
All − MR Egger
All − Inverse variance weighted
−1
0
1
MR effect size for
'DTD1' on 'Pulmonary fibrosis'

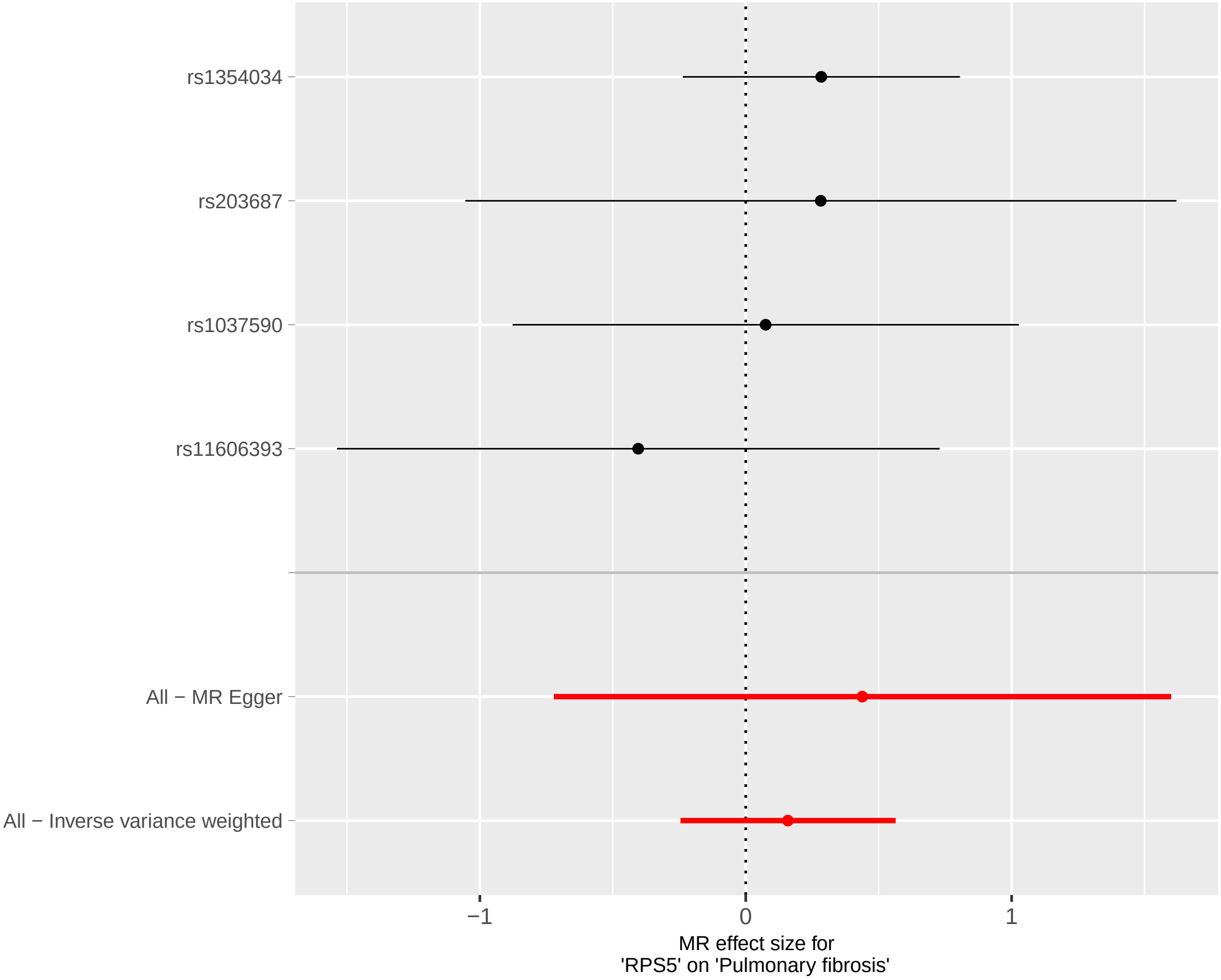

rs1354034
rs203687
rs1037590
rs11606393
All − MR Egger
All − Inverse variance weighted
−1
0
1
MR effect size for
'RPS5' on 'Pulmonary fibrosis'

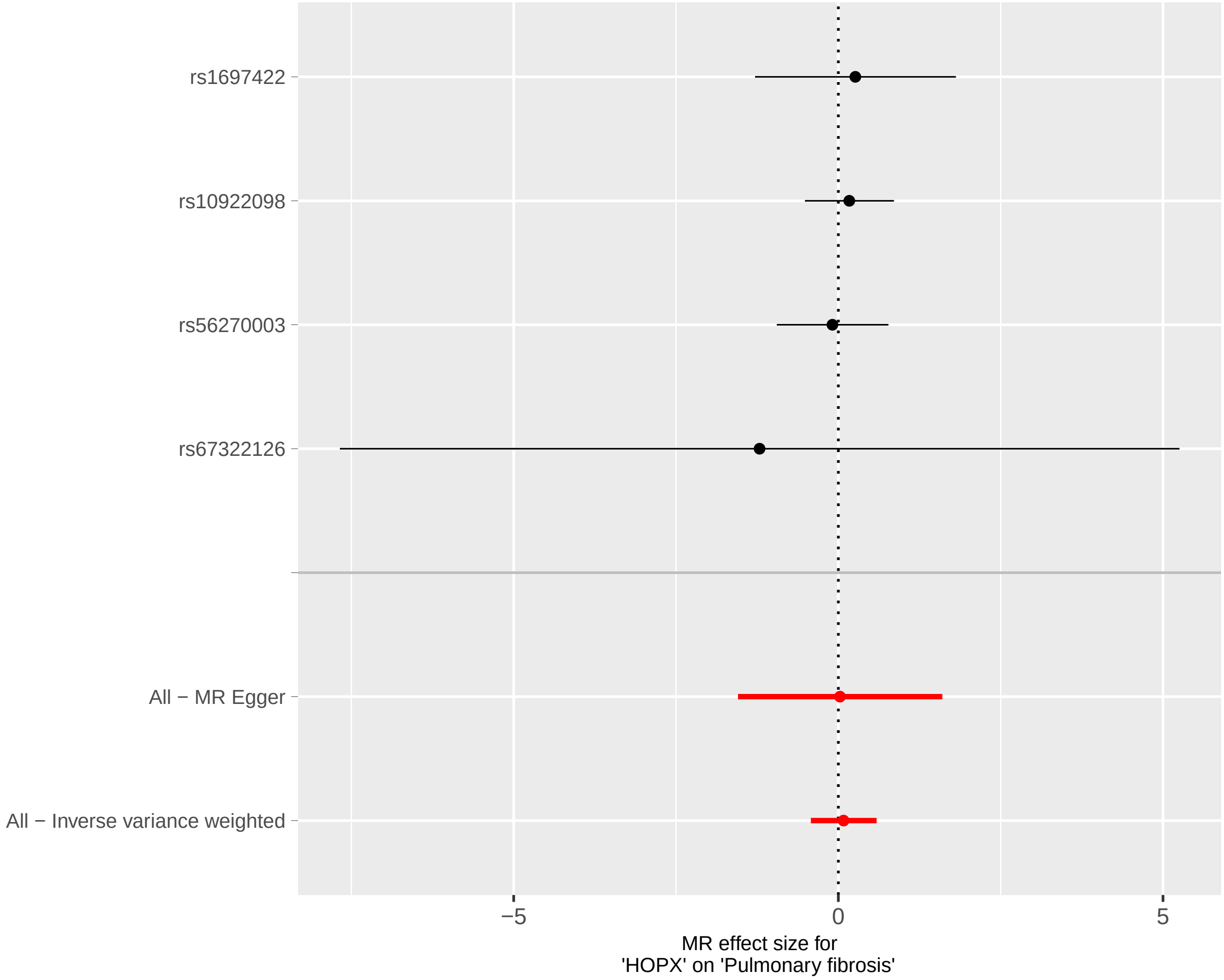

rs1697422
rs10922098
rs56270003
rs67322126
All − MR Egger
All − Inverse variance weighted
−5
0
5
MR effect size for
'HOPX' on 'Pulmonary fibrosis'

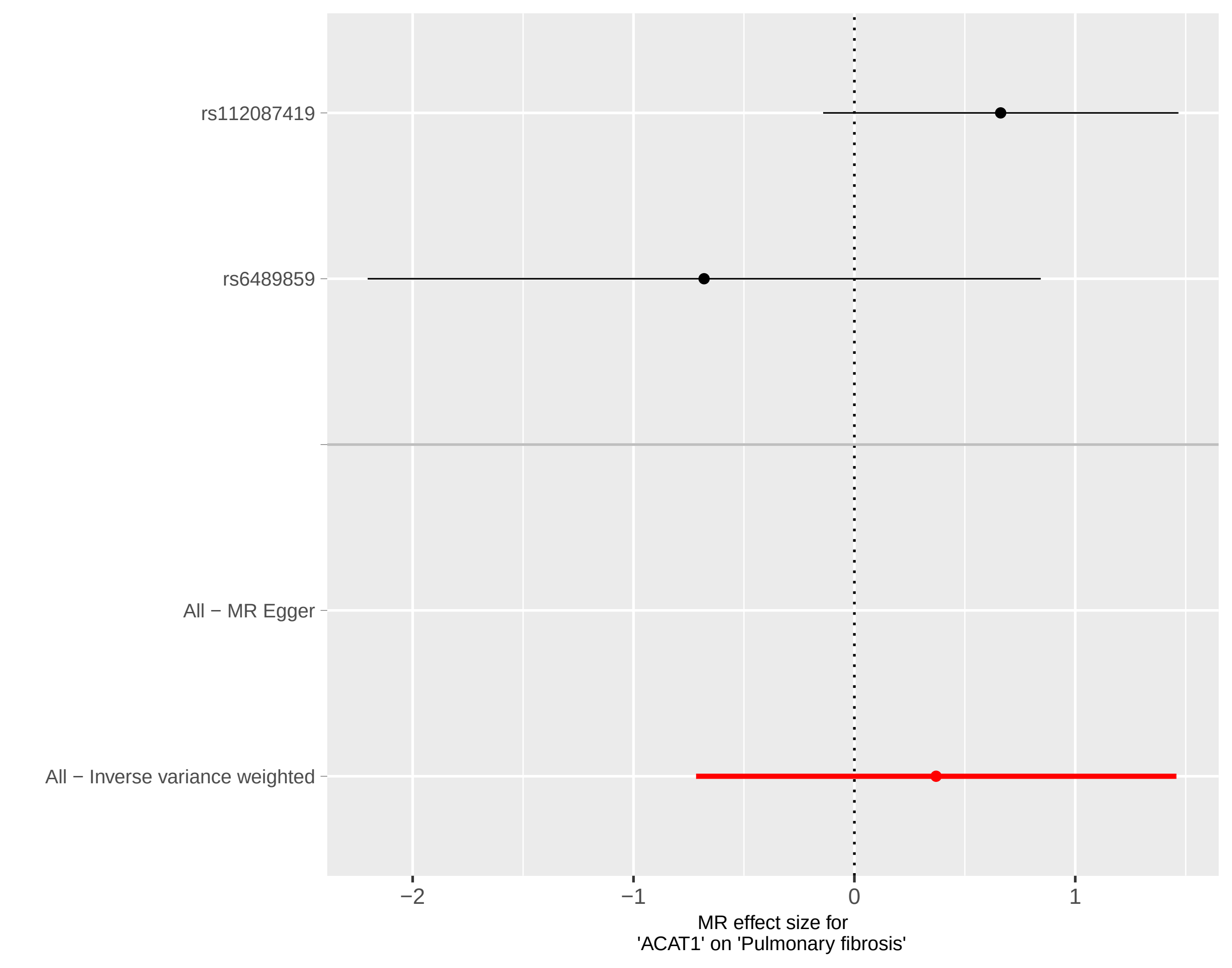

rs112087419
rs6489859
All − MR Egger
All − Inverse variance weighted
−2
−1
0
1
MR effect size for
'ACAT1' on 'Pulmonary fibrosis'

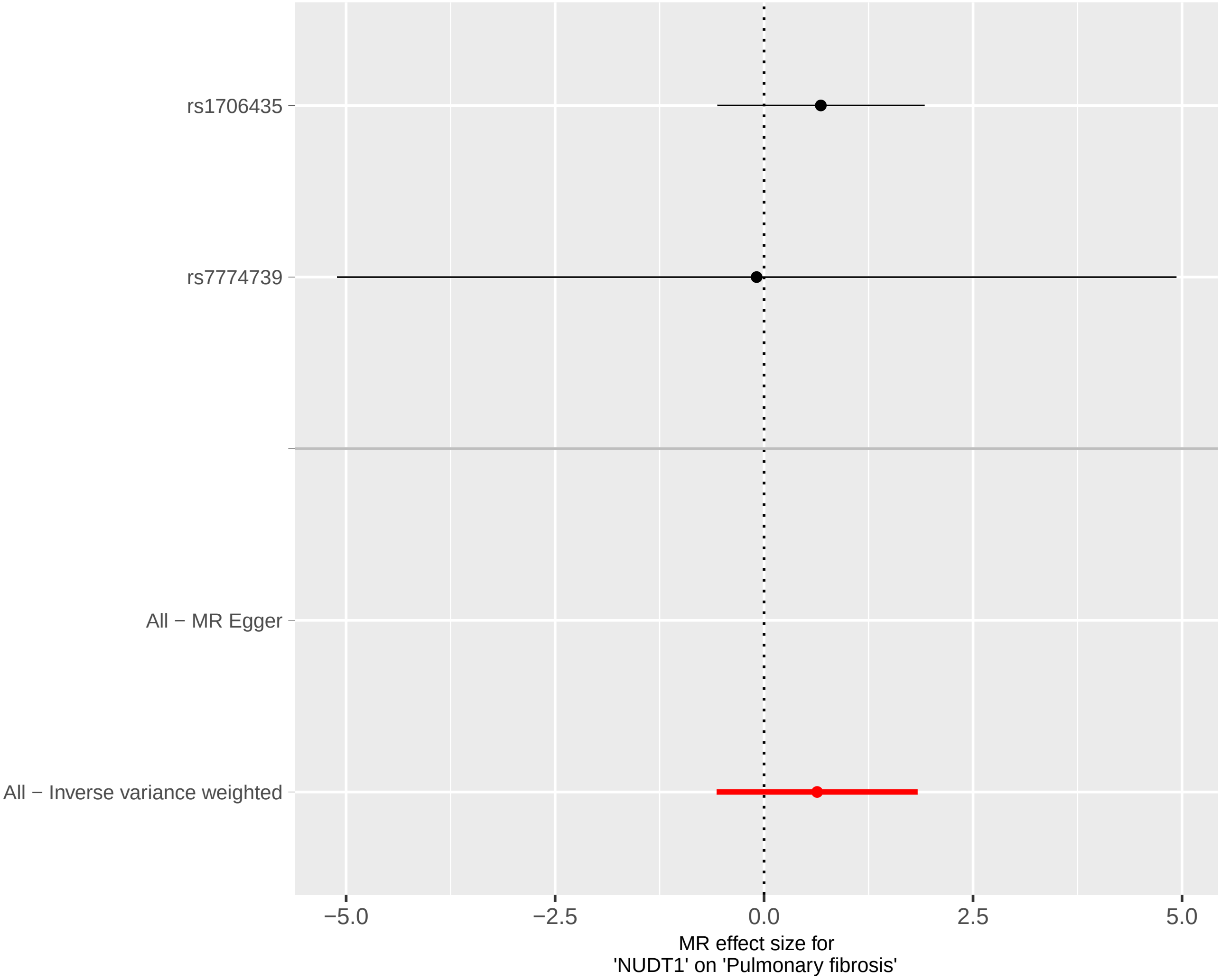
rs1706435
rs7774739
All − MR Egger
All − Inverse variance weighted
−5.0
−2.5
0.0
2.5
5.0
MR effect size for
'NUDT1' on 'Pulmonary fibrosis'

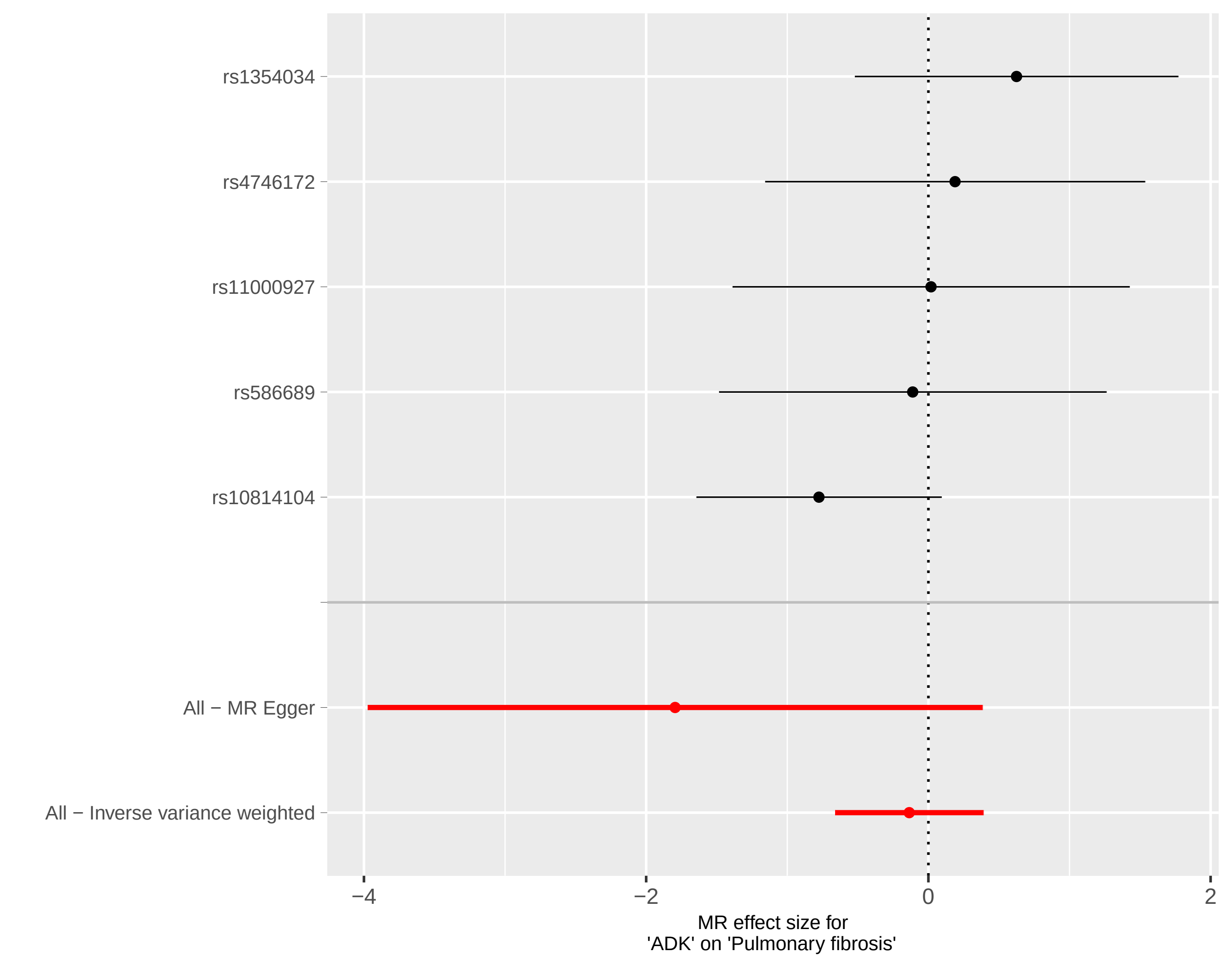

rs1354034
rs4746172
rs11000927
rs586689
rs10814104
All − MR Egger
All − Inverse variance weighted
−4
−2
0
2
MR effect size for
'ADK' on 'Pulmonary fibrosis'

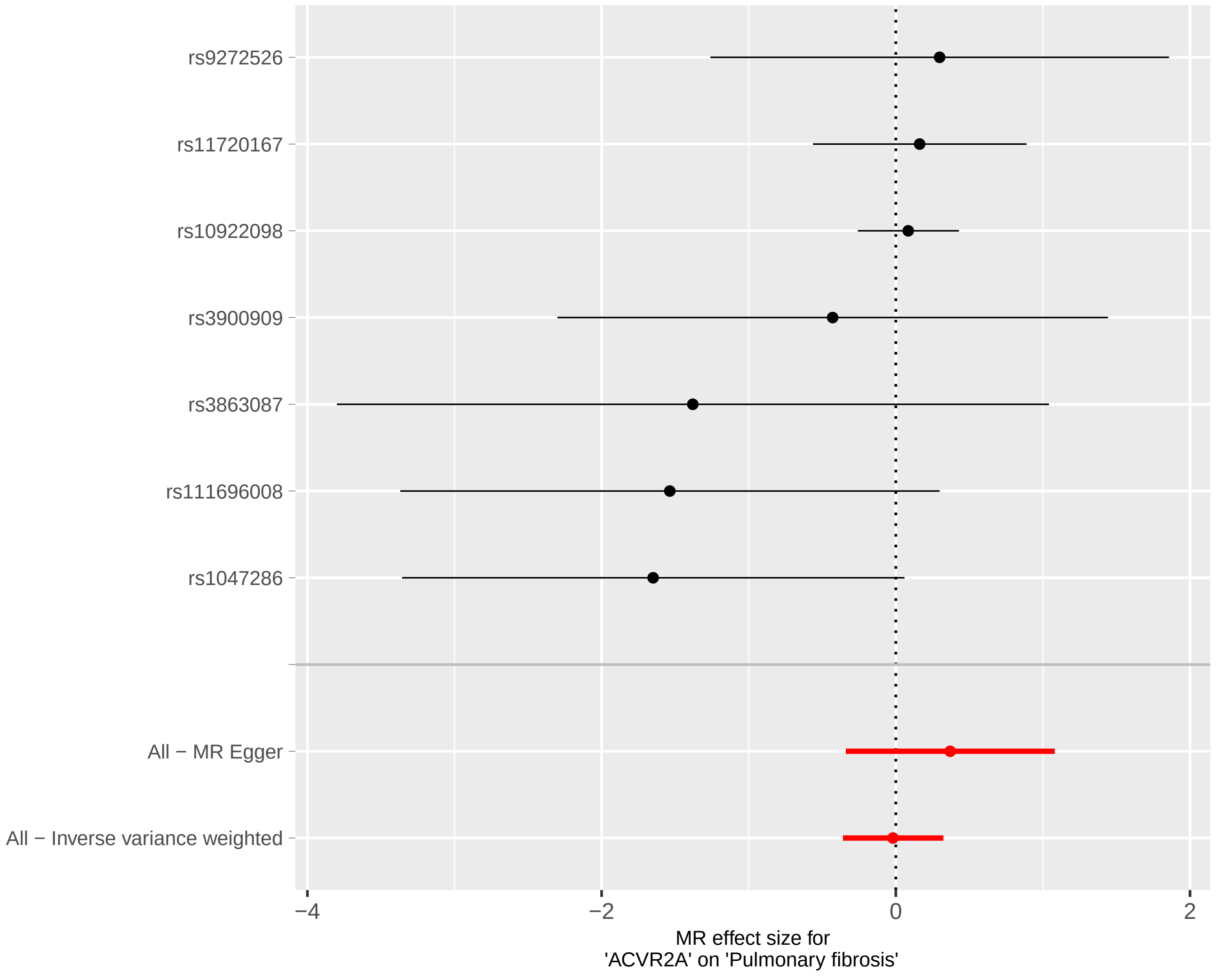
rs9272526
rs11720167
rs10922098
rs3900909
rs3863087
rs111696008
rs1047286
All − MR Egger
All − Inverse variance weighted
−4
−2
0
2
MR effect size for
'ACVR2A' on 'Pulmonary fibrosis'

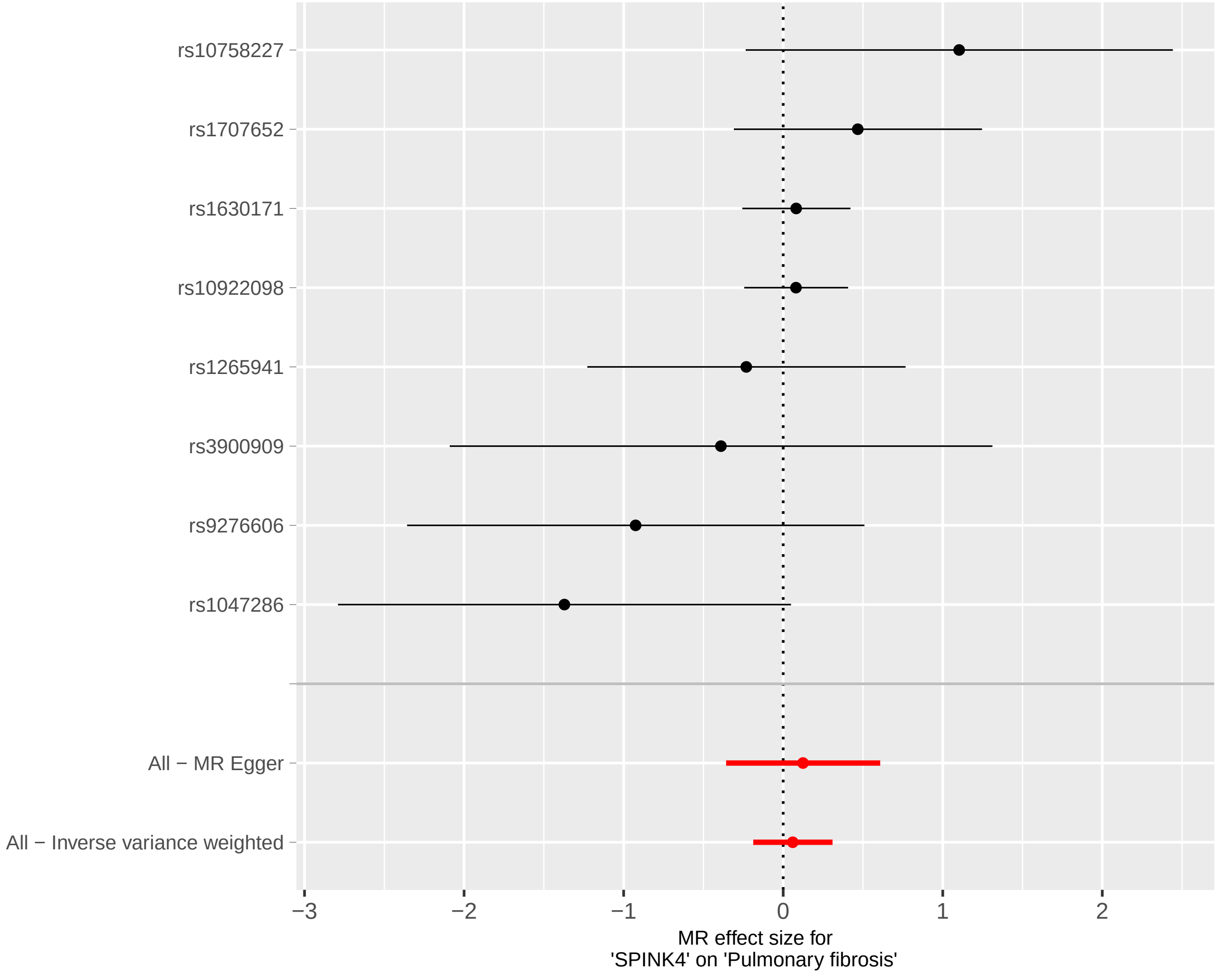
rs10758227
rs1707652
rs1630171
rs10922098
rs1265941
rs3900909
rs9276606
rs1047286
All − MR Egger
All − Inverse variance weighted
−3
−2
−1
0
1
2
MR effect size for
'SPINK4' on 'Pulmonary fibrosis'

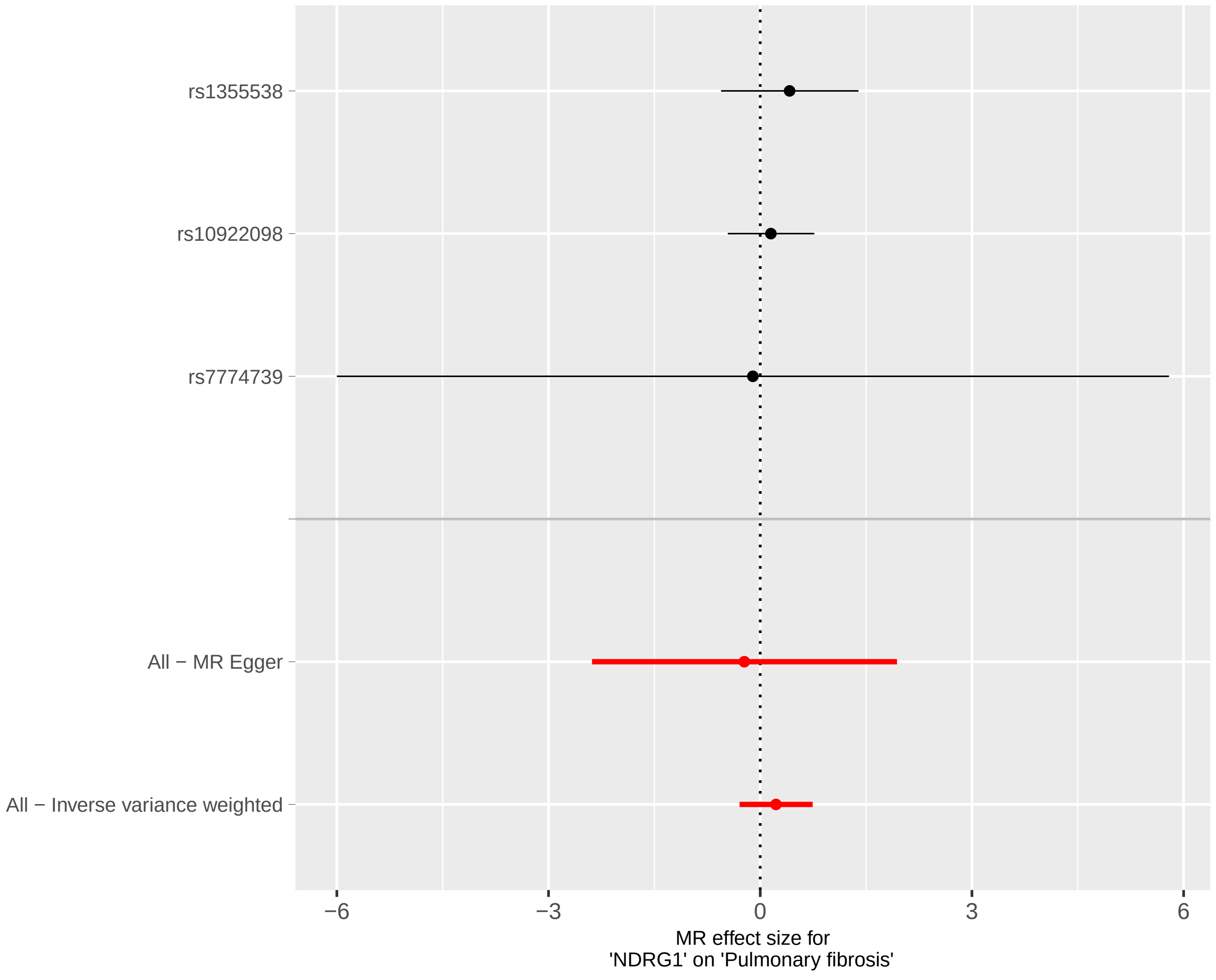

rs1355538
rs10922098
rs7774739
All − MR Egger
All − Inverse variance weighted
−6
−3
0
3
6
MR effect size for
'NDRG1' on 'Pulmonary fibrosis'

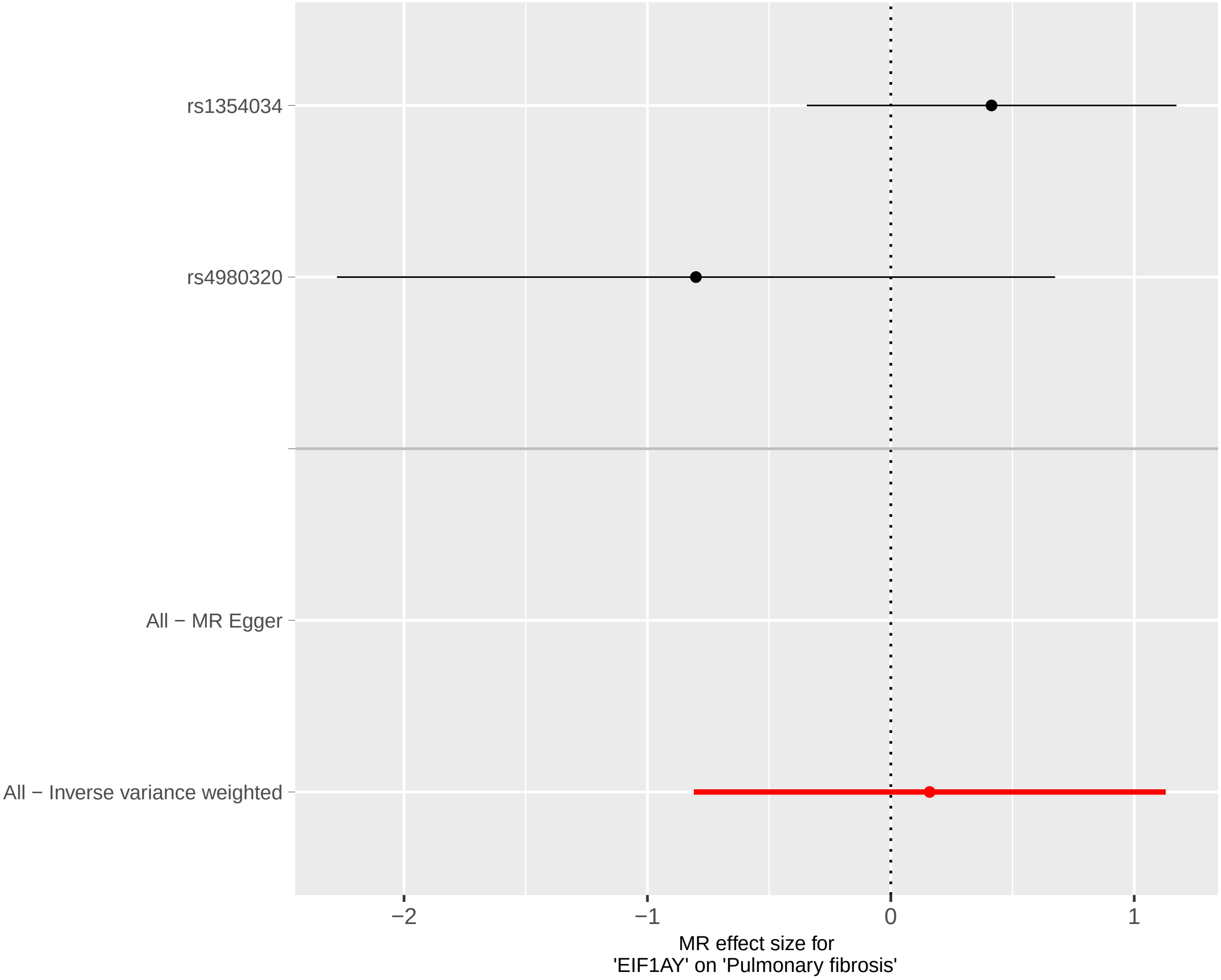
rs1354034
rs4980320
All − MR Egger
All − Inverse variance weighted
−2
−1
0
1
MR effect size for
'EIF1AY' on 'Pulmonary fibrosis'

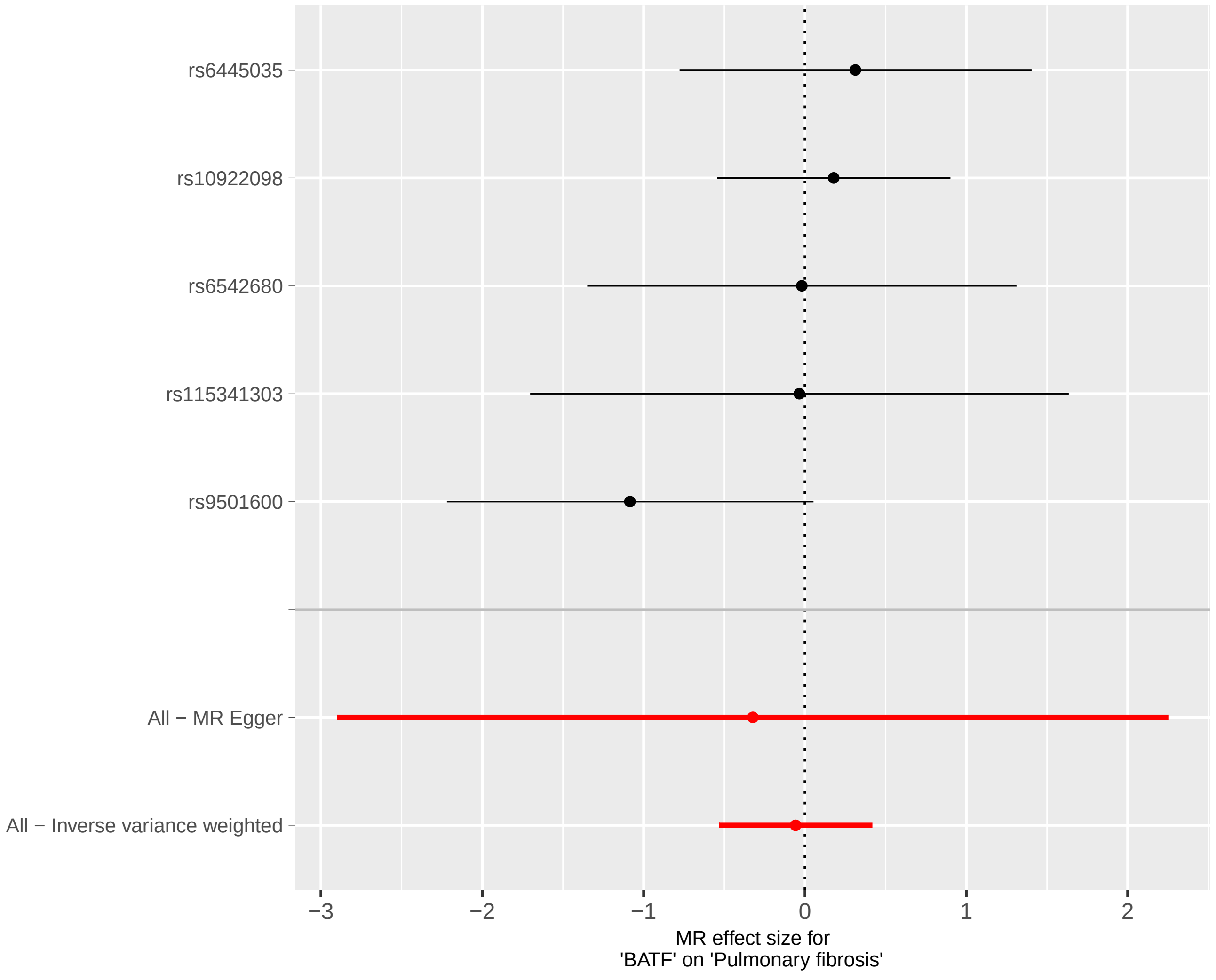
rs6445035
rs10922098
rs6542680
rs115341303
rs9501600
All − MR Egger
All − Inverse variance weighted
−3
−2
−1
0
1
2
MR effect size for
'BATF' on 'Pulmonary fibrosis'

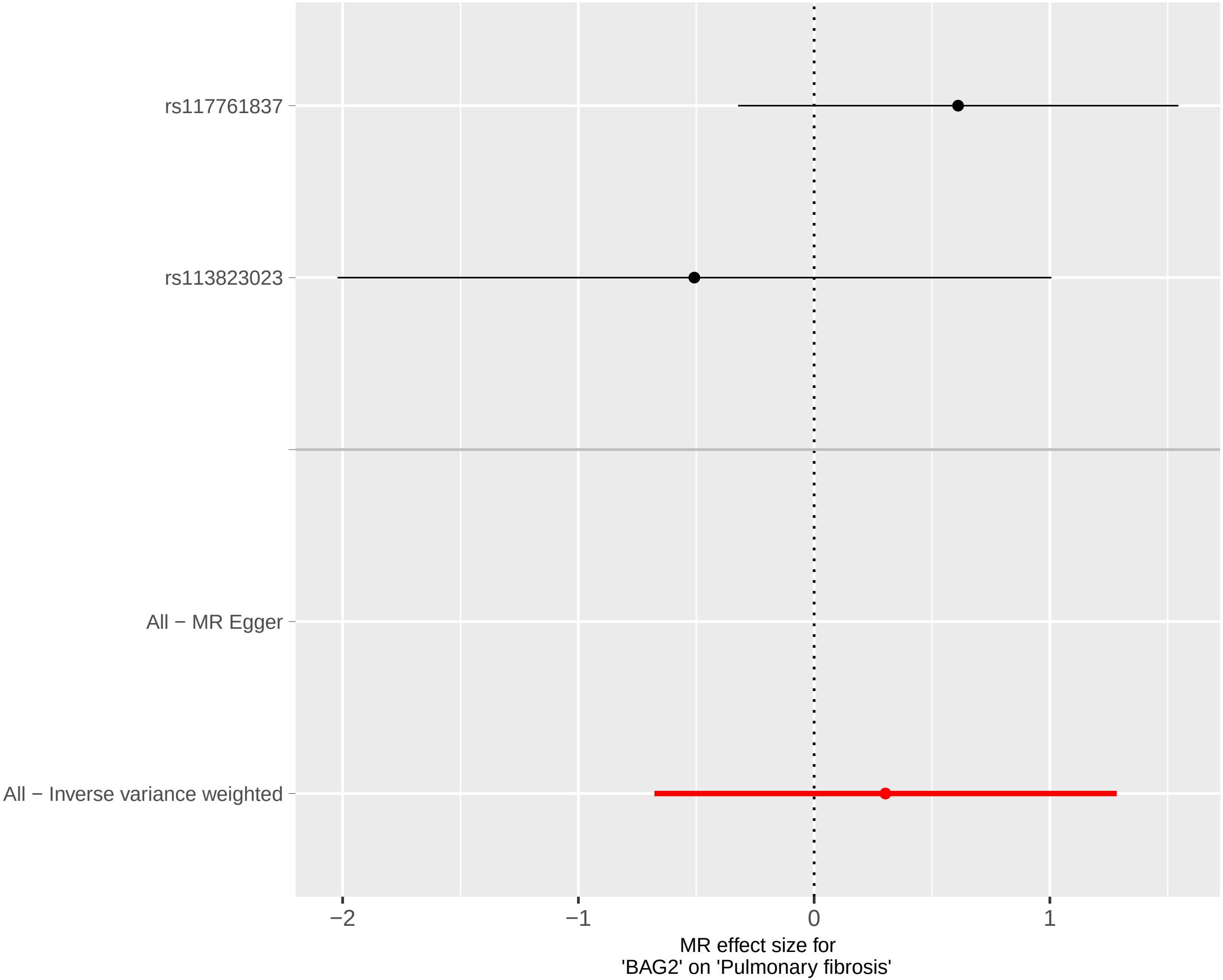

rs117761837
rs113823023
All − MR Egger
All − Inverse variance weighted
−2
−1
0
1
MR effect size for
'BAG2' on 'Pulmonary fibrosis'

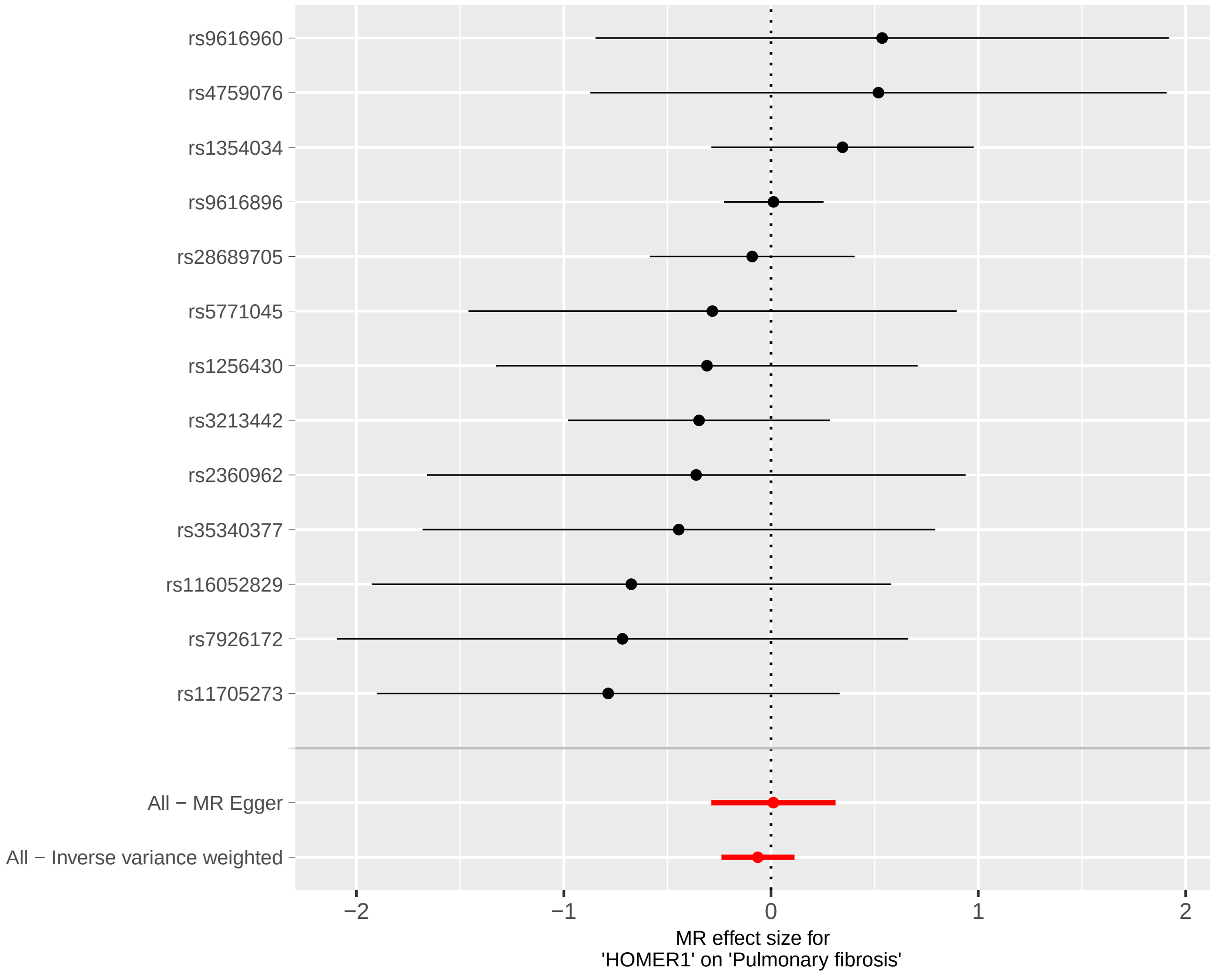

rs9616960
rs4759076
rs1354034
rs9616896
rs28689705
rs5771045
rs1256430
rs3213442
rs2360962
rs35340377
rs116052829
rs7926172
rs11705273
All − MR Egger
All − Inverse variance weighted
−2
−1
0
1
2
MR effect size for
'HOMER1' on 'Pulmonary fibrosis'

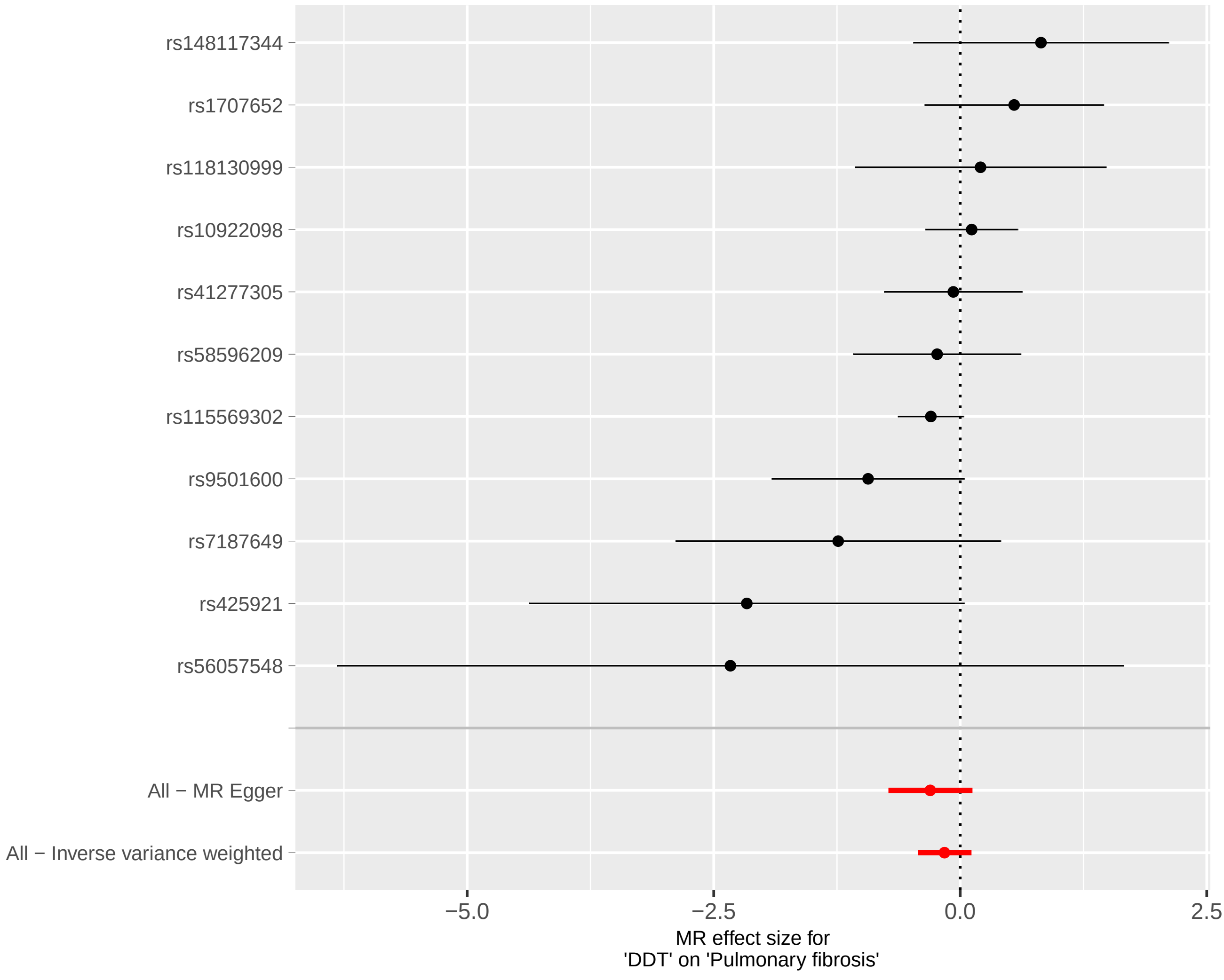
rs148117344
rs1707652
rs118130999
rs10922098
rs41277305
rs58596209
rs115569302
rs9501600
rs7187649
rs425921
rs56057548
All − MR Egger
All − Inverse variance weighted
−5.0
−2.5
0.0
2.5
MR effect size for
'DDT' on 'Pulmonary fibrosis'

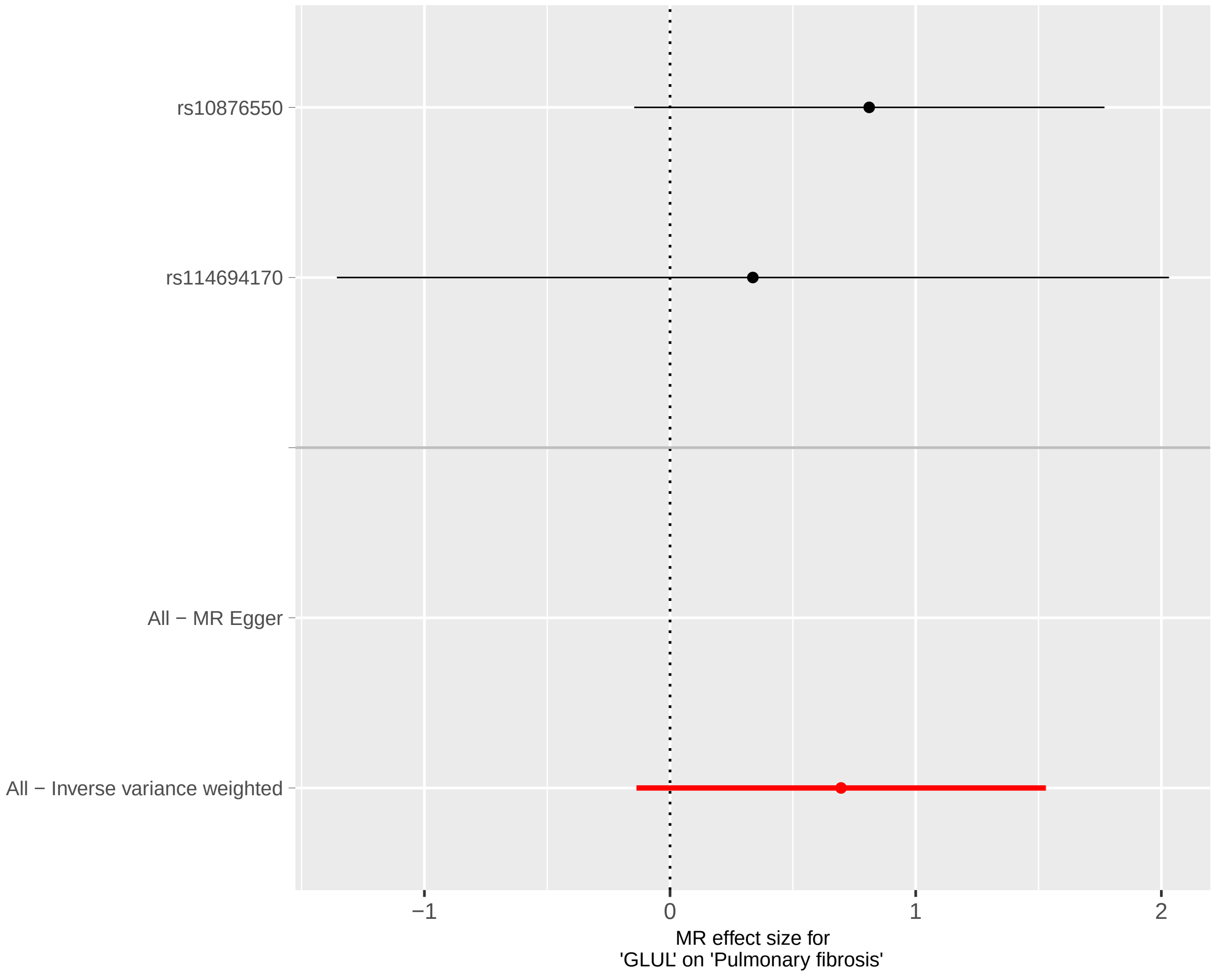

rs10876550
rs114694170
All − MR Egger
All − Inverse variance weighted
−1
0
1
2
MR effect size for
'GLUL' on 'Pulmonary fibrosis'

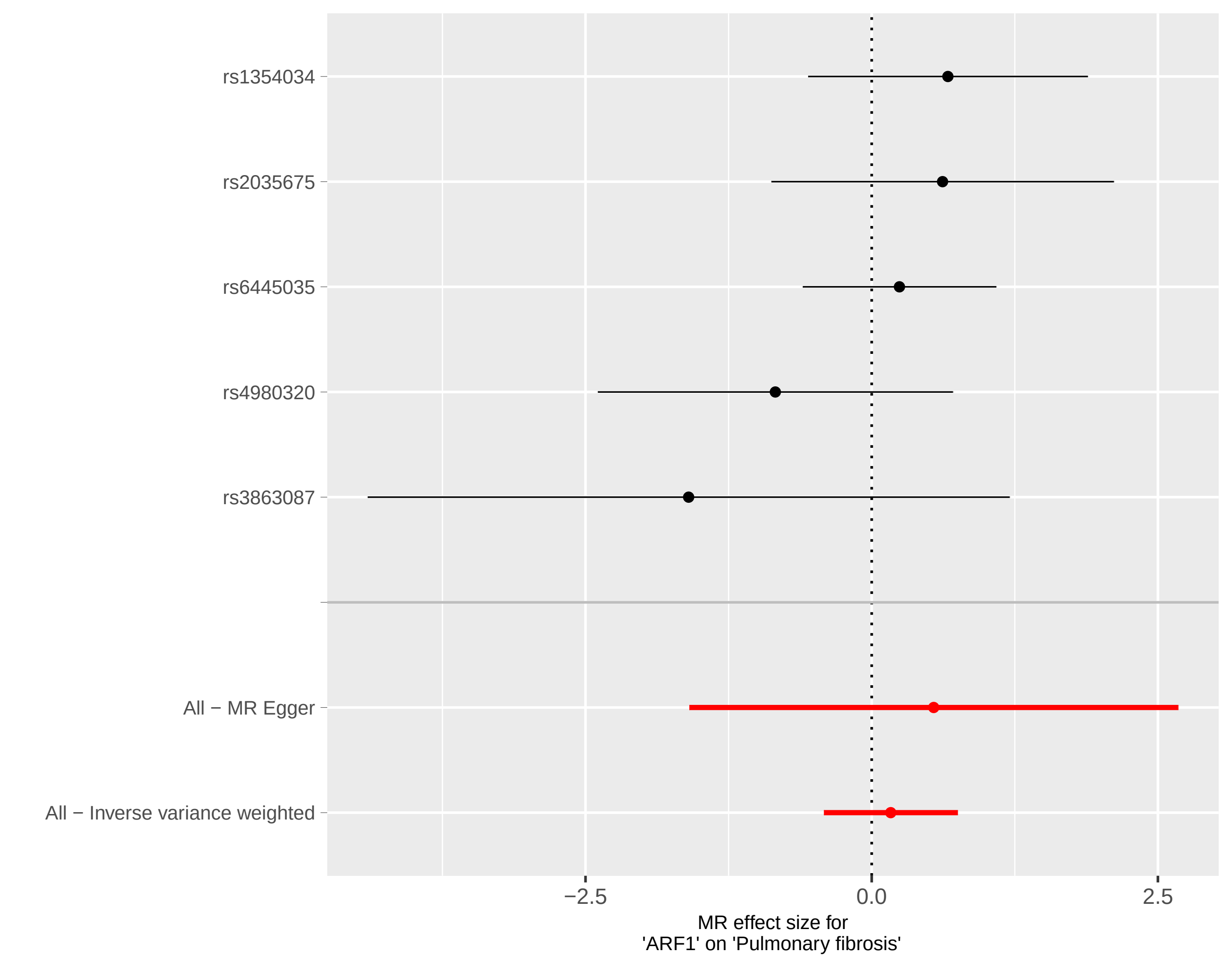
rs1354034
rs2035675
rs6445035
rs4980320
rs3863087
All − MR Egger
All − Inverse variance weighted
−2.5
0.0
2.5
MR effect size for
'ARF1' on 'Pulmonary fibrosis'

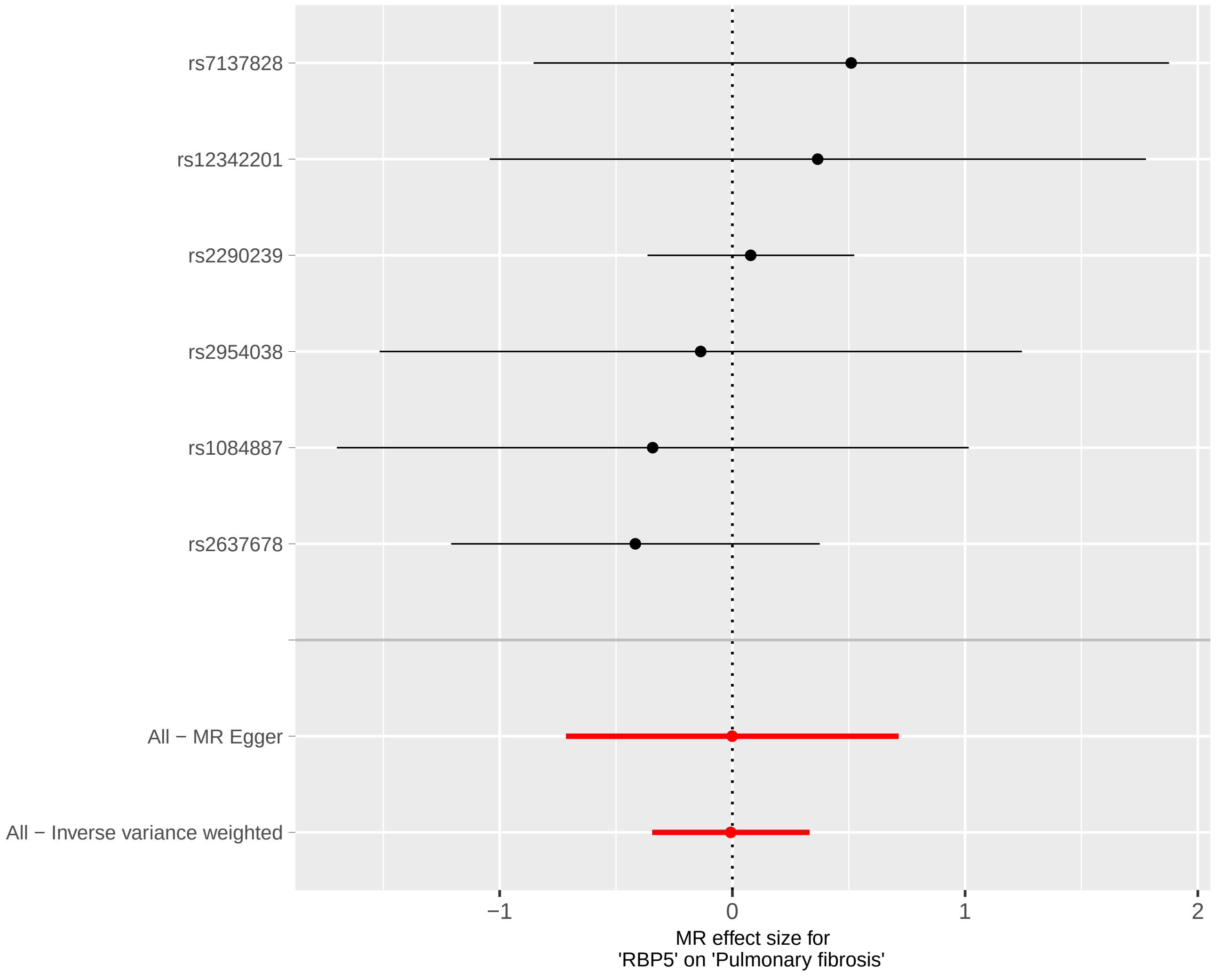
rs7137828
rs12342201
rs2290239
rs2954038
rs1084887
rs2637678
All − MR Egger
All − Inverse variance weighted
−1
0
1
2
MR effect size for
'RBP5' on 'Pulmonary fibrosis'

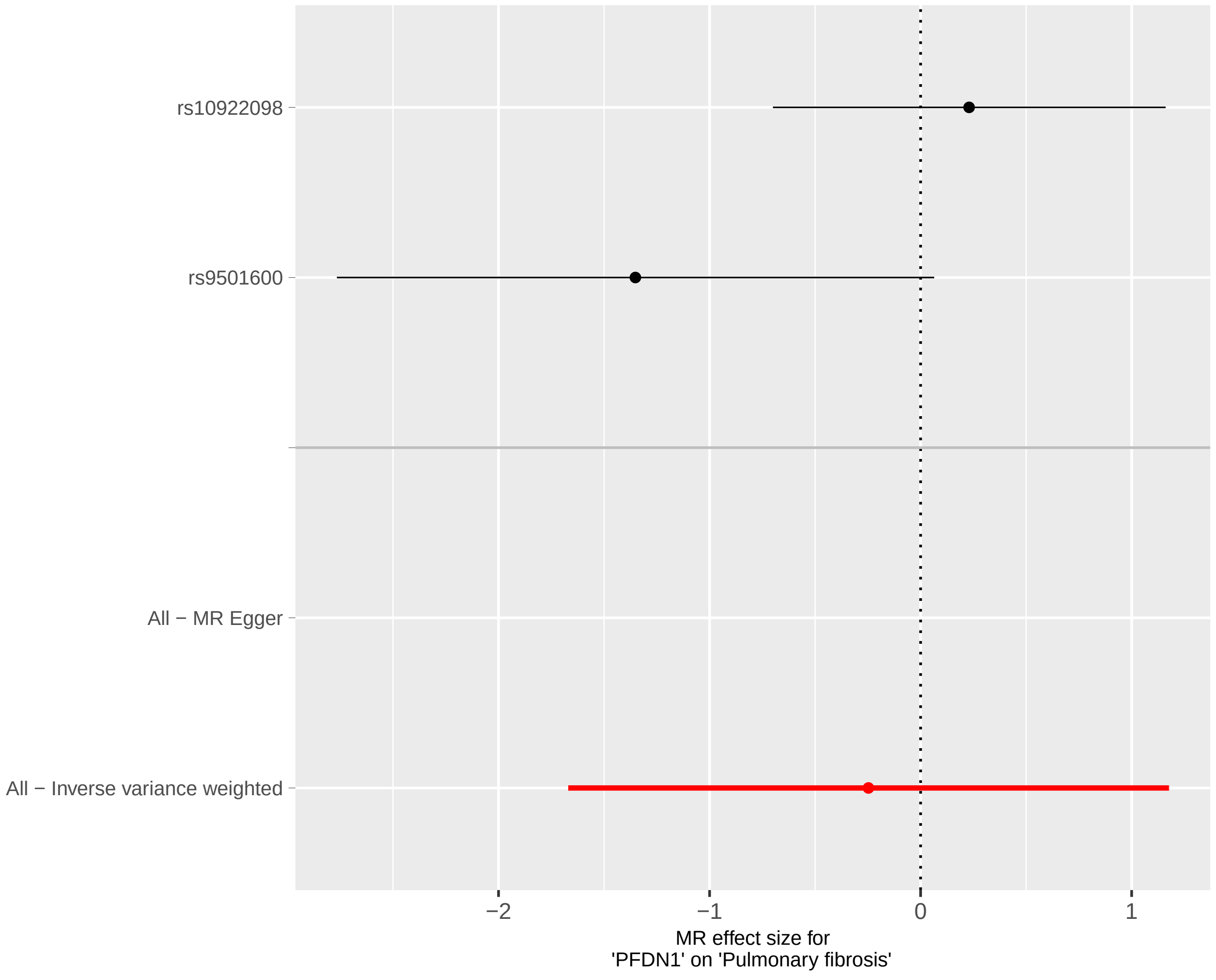

rs10922098
rs9501600
All − MR Egger
All − Inverse variance weighted
−2
−1
0
1
MR effect size for
'PFDN1' on 'Pulmonary fibrosis'

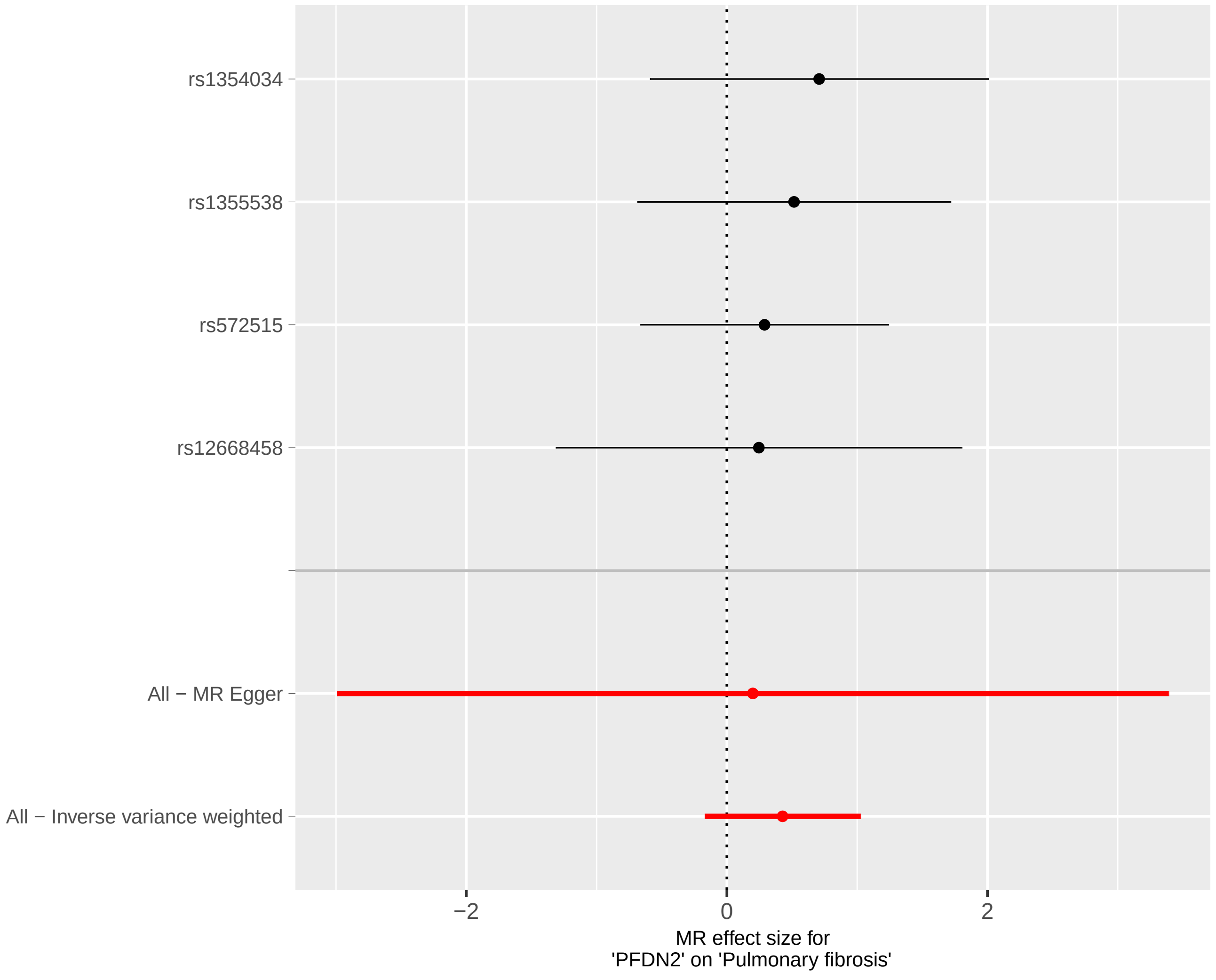
rs1354034
rs1355538
rs572515
rs12668458
All − MR Egger
All − Inverse variance weighted
−2
0
2
MR effect size for
'PFDN2' on 'Pulmonary fibrosis'

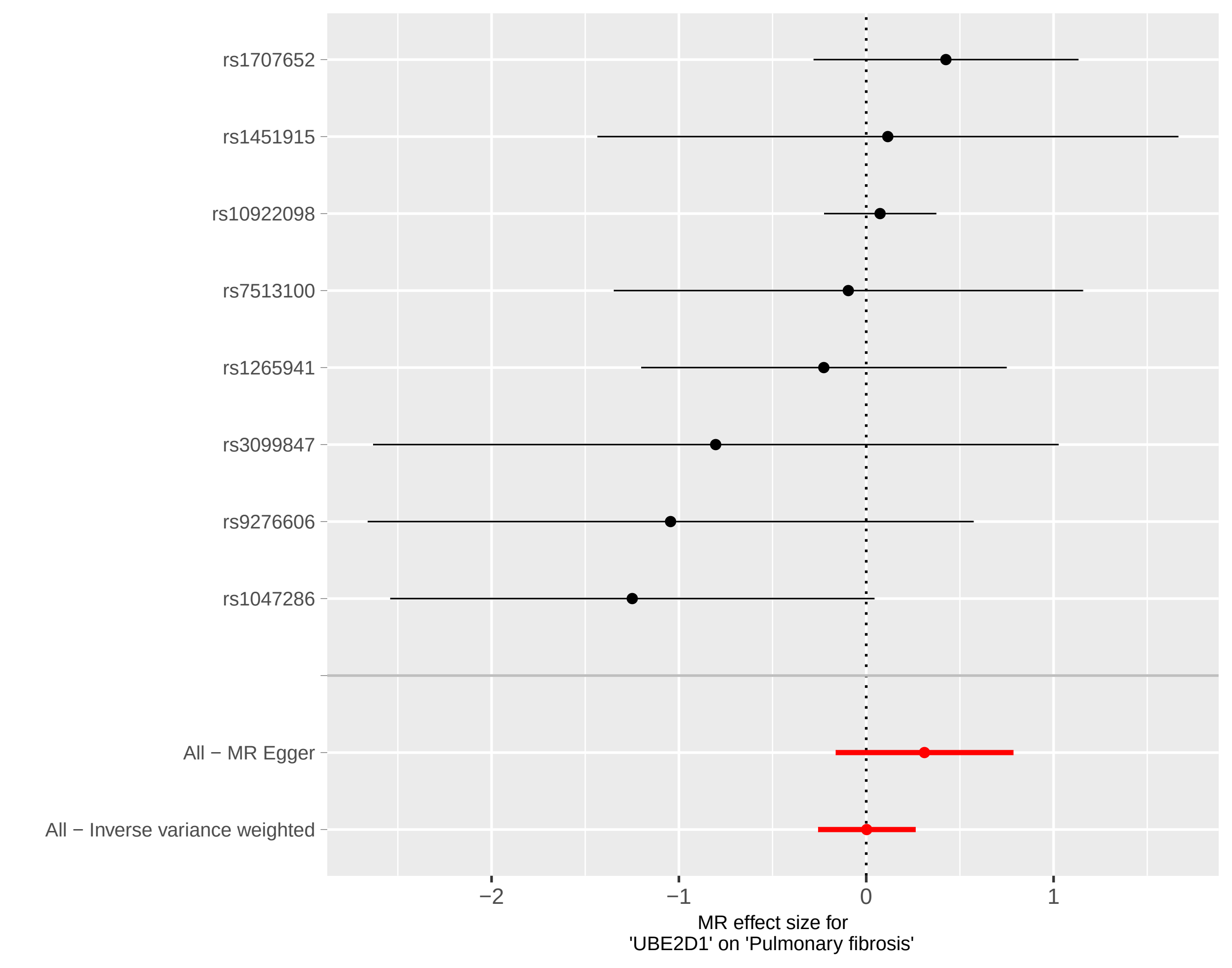
rs1707652
rs1451915
rs10922098
rs7513100
rs1265941
rs3099847
rs9276606
rs1047286
All − MR Egger
All − Inverse variance weighted
−2
−1
0
1
MR effect size for
'UBE2D1' on 'Pulmonary fibrosis'

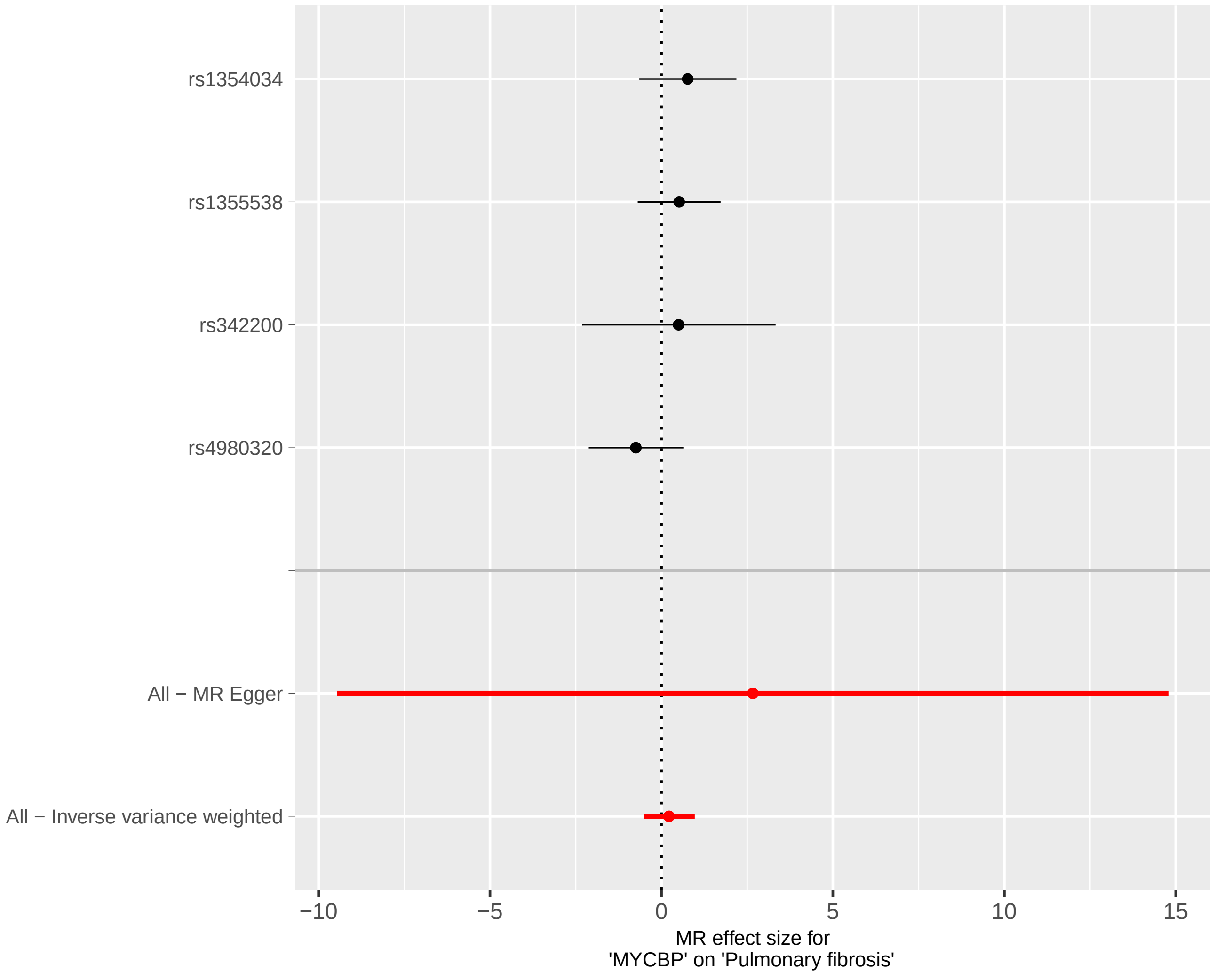

rs1354034
rs1355538
rs342200
rs4980320
All − MR Egger
All − Inverse variance weighted
−10
−5
0
5
10
15
MR effect size for
'MYCBP' on 'Pulmonary fibrosis'

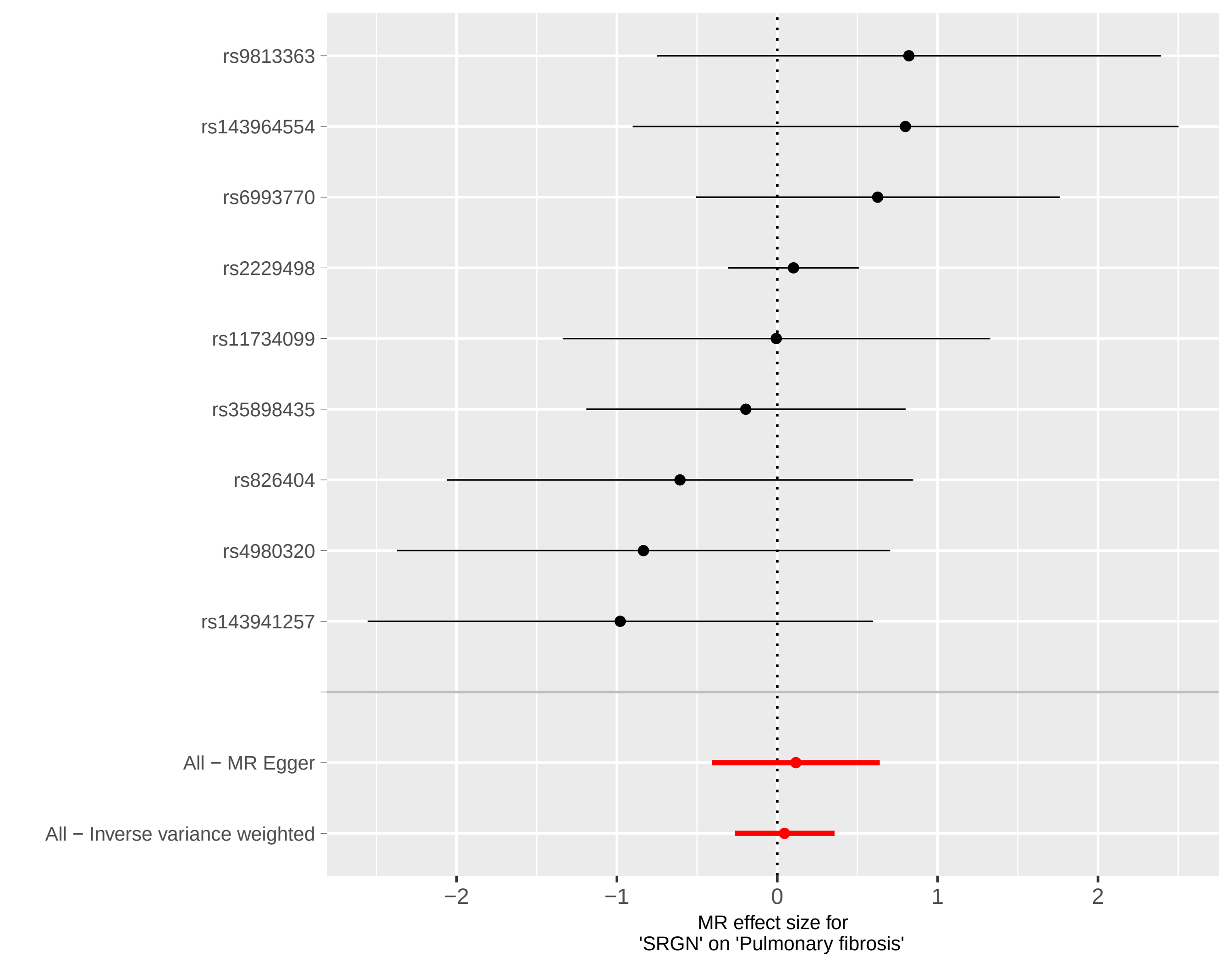

rs9813363
rs143964554
rs6993770
rs2229498
rs11734099
rs35898435
rs826404
rs4980320
rs143941257
All − MR Egger
All − Inverse variance weighted
−2
−1
0
1
2
MR effect size for
'SRGN' on 'Pulmonary fibrosis'

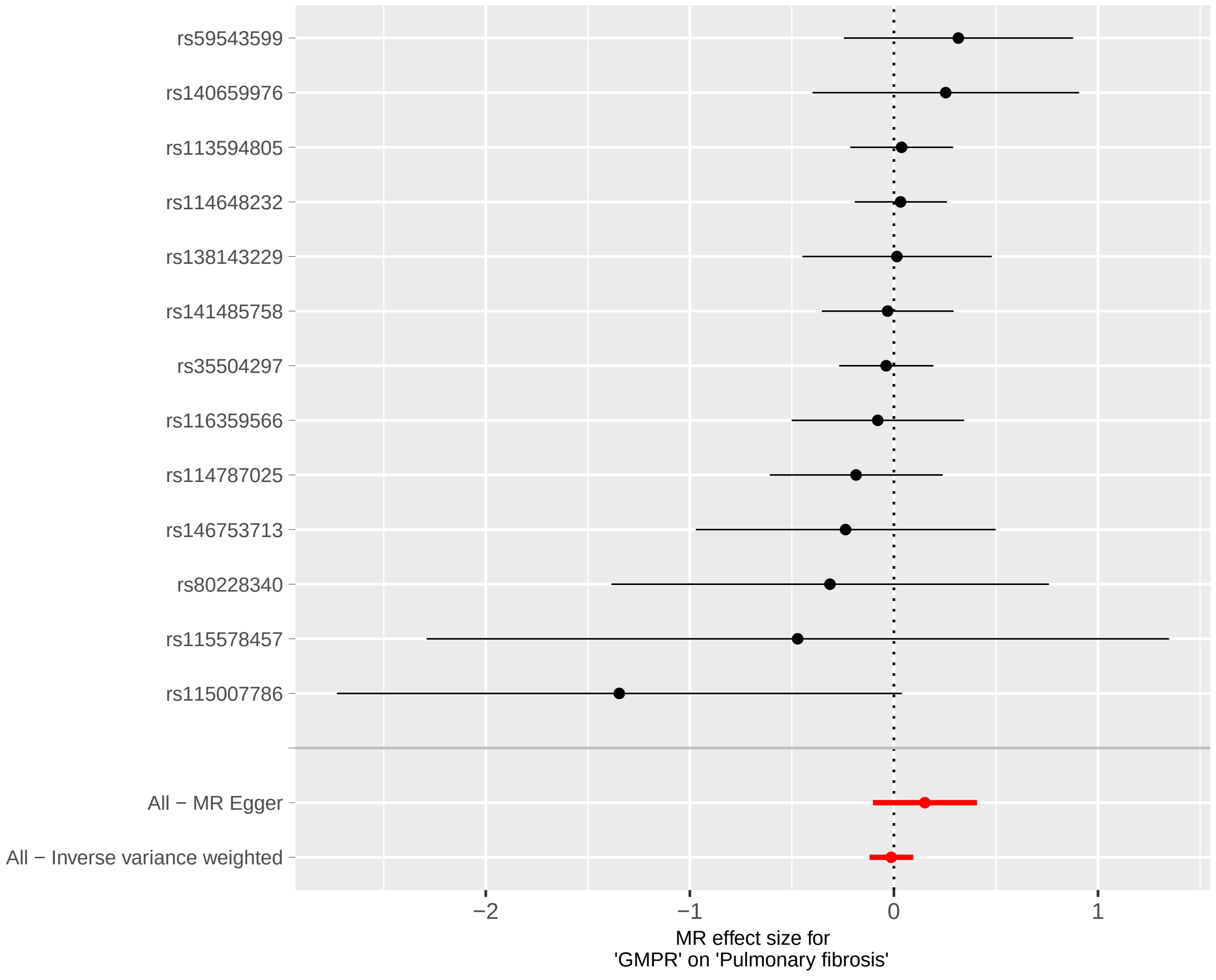
rs59543599
rs140659976
rs113594805
rs114648232
rs138143229
rs141485758
rs35504297
rs116359566
rs114787025
rs146753713
rs80228340
rs115578457
rs115007786
All − MR Egger
All − Inverse variance weighted
−2
−1
0
1
MR effect size for
'GMPR' on 'Pulmonary fibrosis'

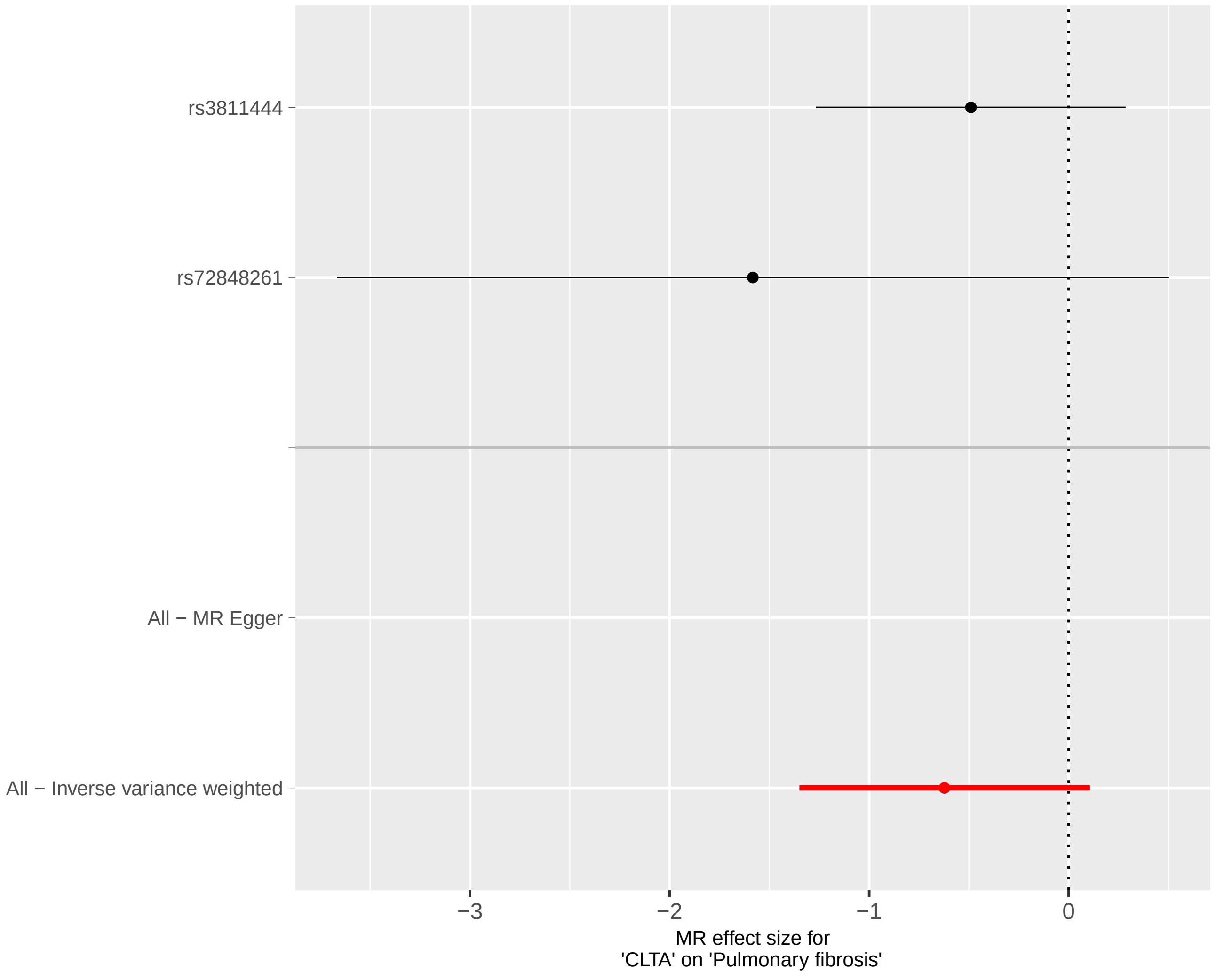

rs3811444
rs72848261
All − MR Egger
All − Inverse variance weighted
−3
−2
−1
0
MR effect size for
'CLTA' on 'Pulmonary fibrosis'

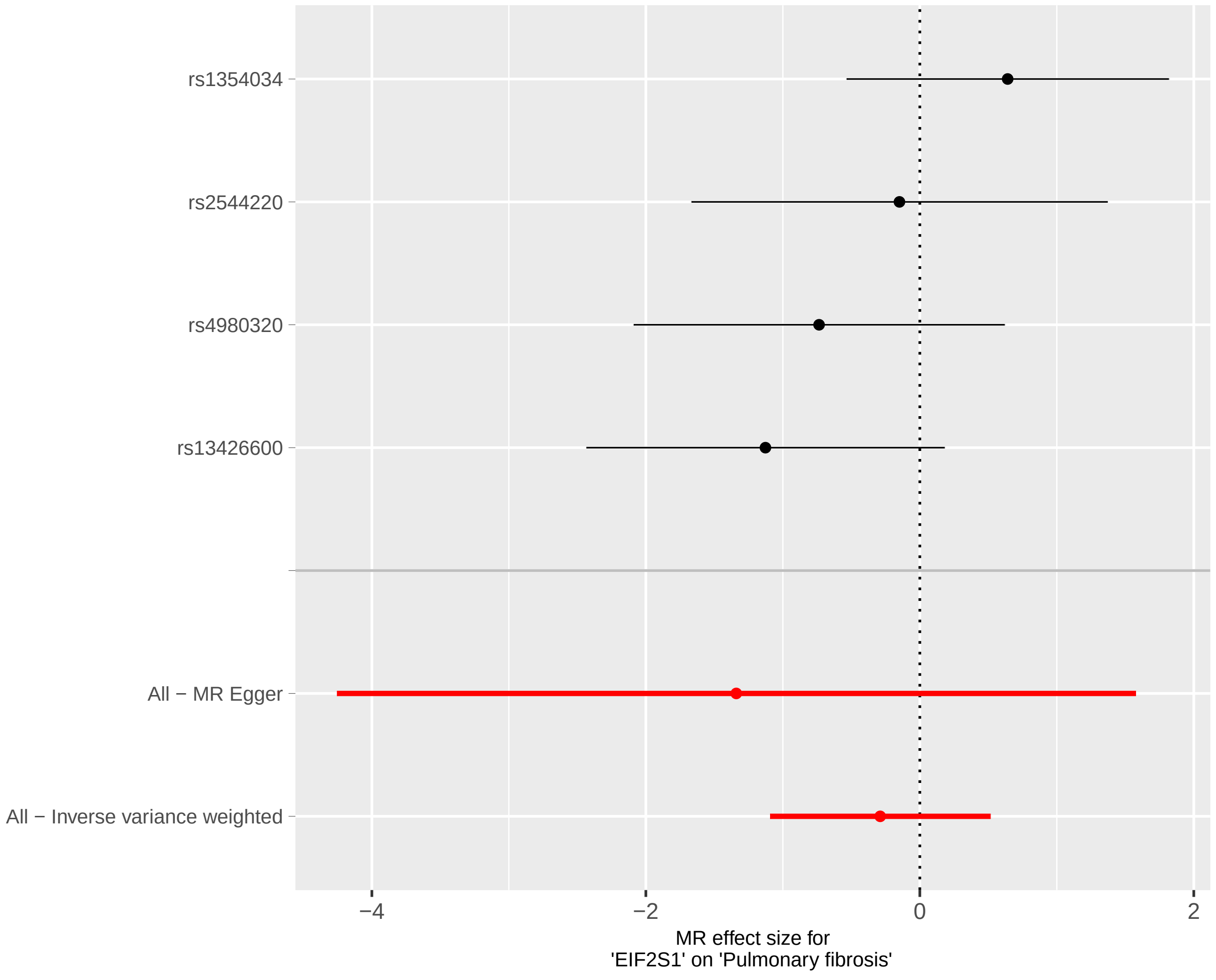
rs1354034
rs2544220
rs4980320
rs13426600
All − MR Egger
All − Inverse variance weighted
−4
−2
0
2
MR effect size for
'EIF2S1' on 'Pulmonary fibrosis'

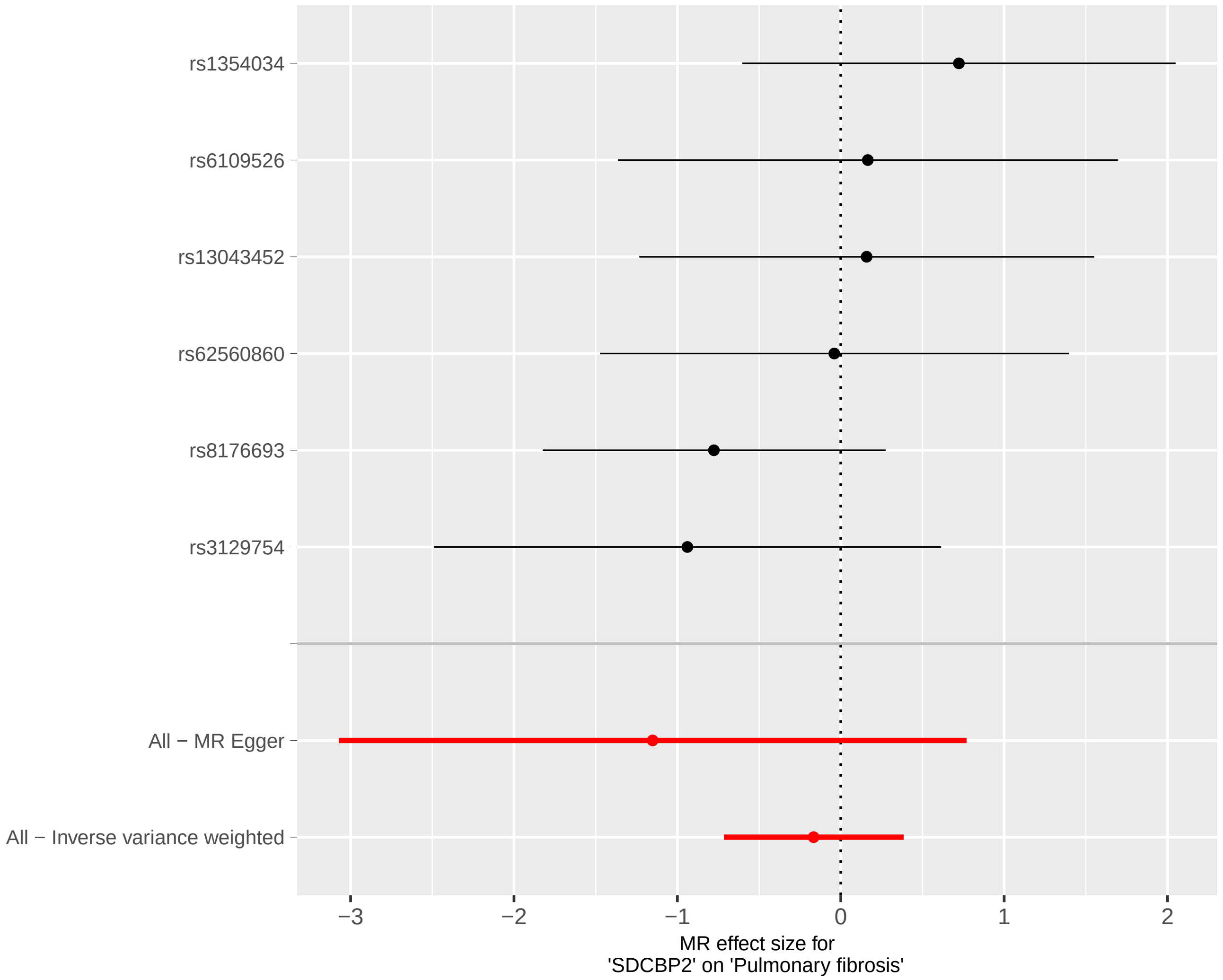

rs1354034
rs6109526
rs13043452
rs62560860
rs8176693
rs3129754
All − MR Egger
All − Inverse variance weighted
−3
−2
−1
0
1
2
MR effect size for
'SDCBP2' on 'Pulmonary fibrosis'

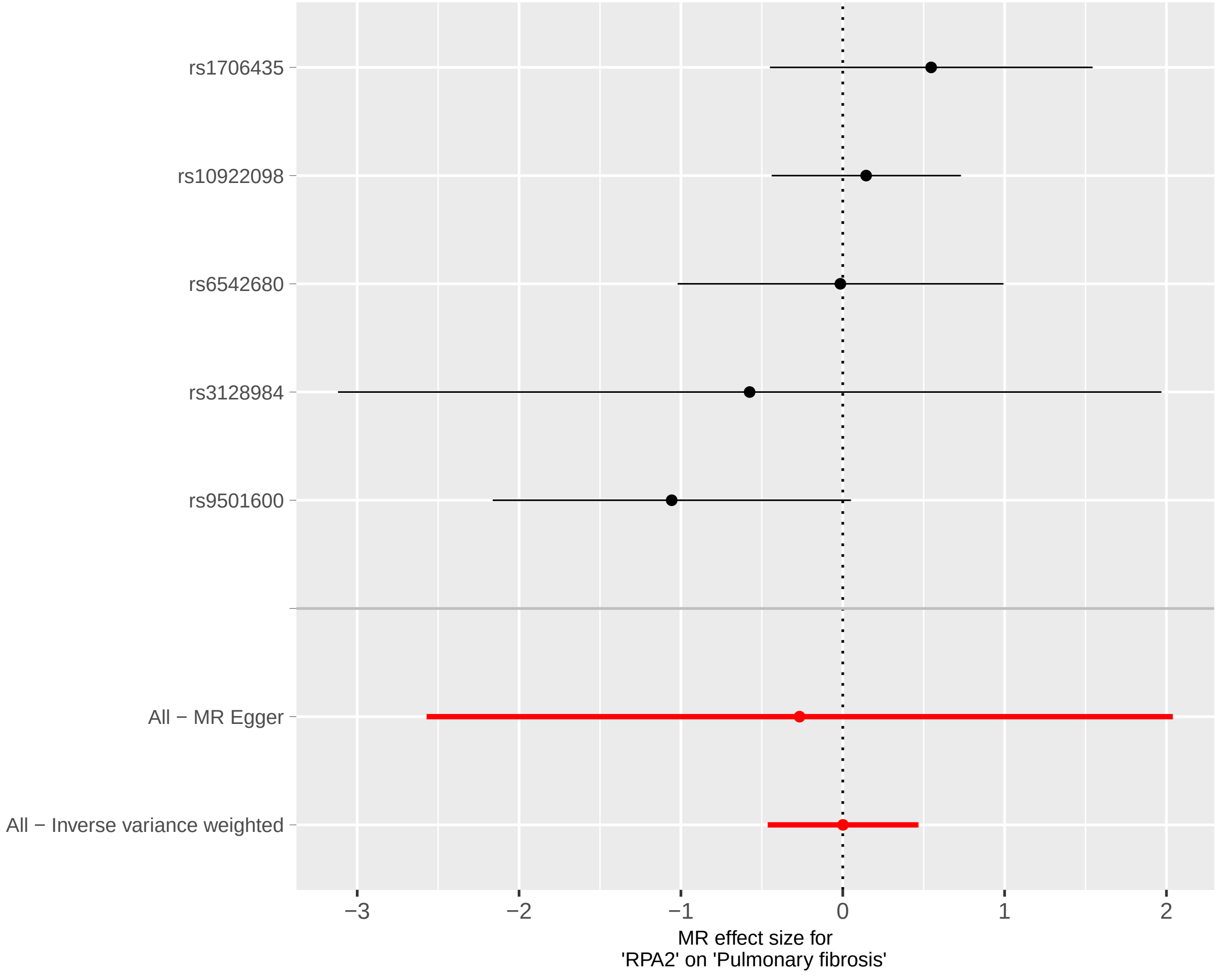
rs1706435
rs10922098
rs6542680
rs3128984
rs9501600
All − MR Egger
All − Inverse variance weighted
−3
−2
−1
0
1
2
MR effect size for
'RPA2' on 'Pulmonary fibrosis'

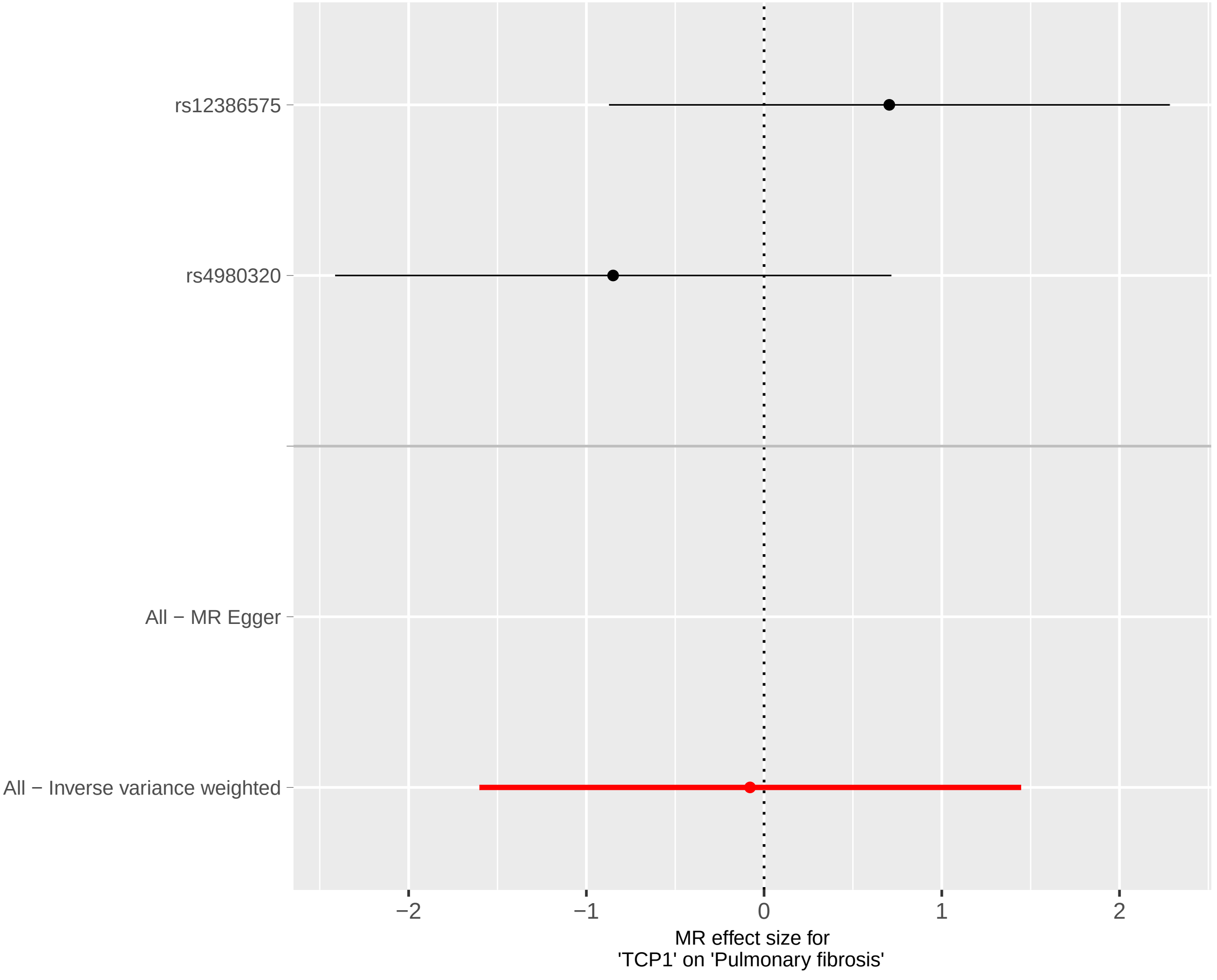
rs12386575
rs4980320
All − MR Egger
All − Inverse variance weighted
−2
−1
0
1
2
MR effect size for
'TCP1' on 'Pulmonary fibrosis'

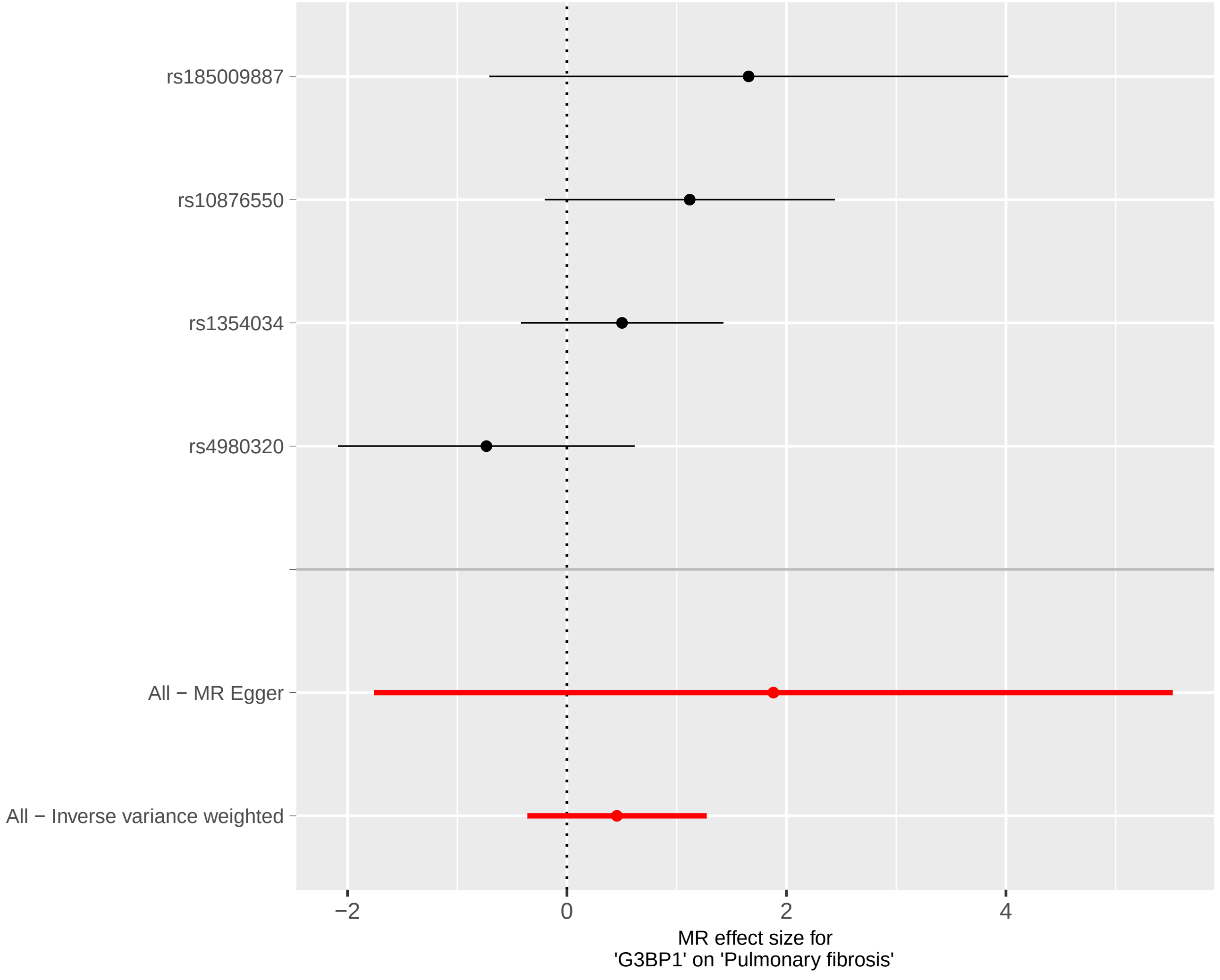
rs185009887
rs10876550
rs1354034
rs4980320
All − MR Egger
All − Inverse variance weighted
−2
0
2
4
MR effect size for
'G3BP1' on 'Pulmonary fibrosis'

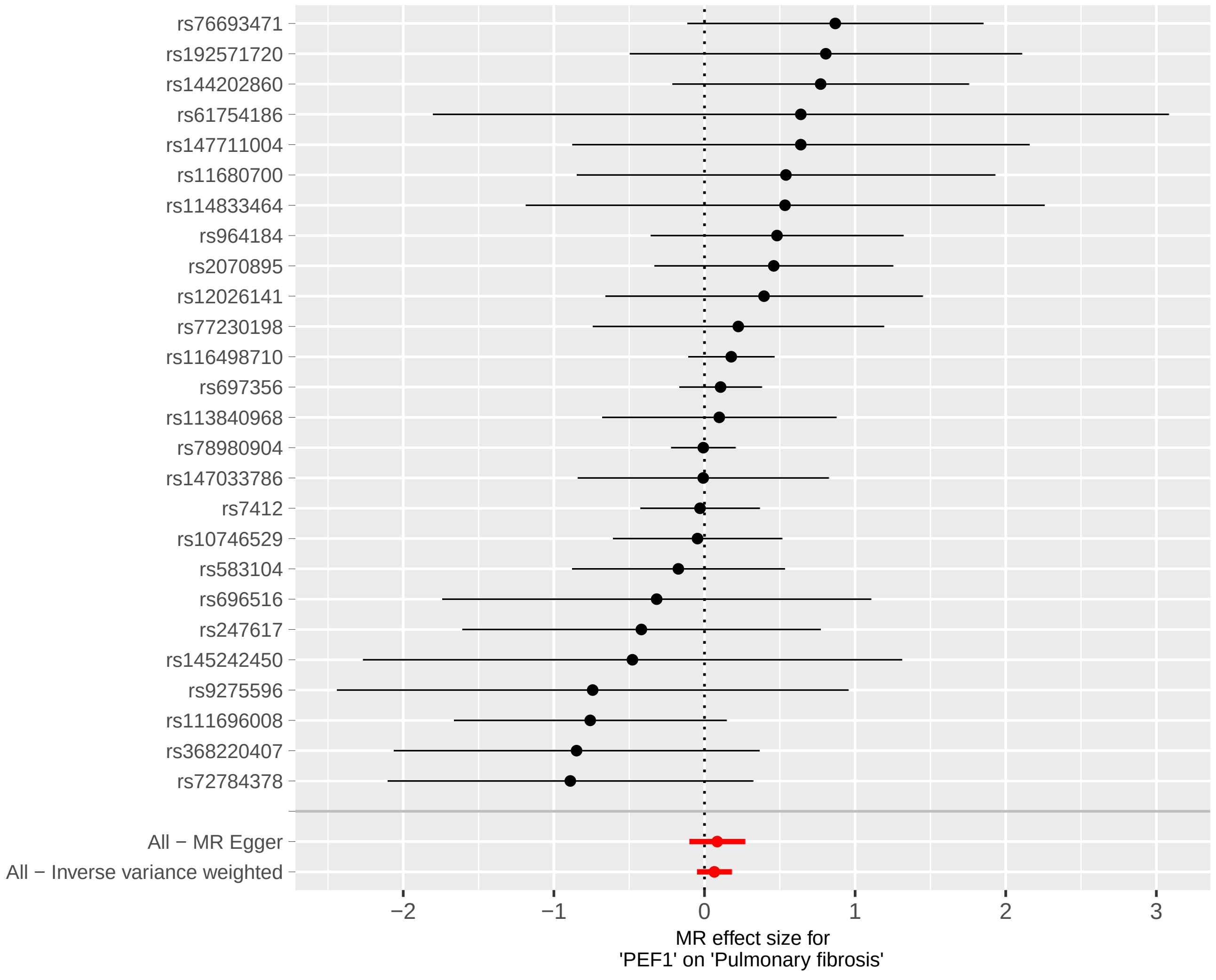

rs76693471
rs192571720
rs144202860
rs61754186
rs147711004
rs11680700
rs114833464
rs964184
rs2070895
rs12026141
rs77230198
rs116498710
rs697356
rs113840968
rs78980904
rs147033786
rs7412
rs10746529
rs583104
rs696516
rs247617
rs145242450
rs9275596
rs111696008
rs368220407
rs72784378
All − MR Egger
All − Inverse variance weighted
−2
−1
0
1
2
3
MR effect size for
'PEF1' on 'Pulmonary fibrosis'

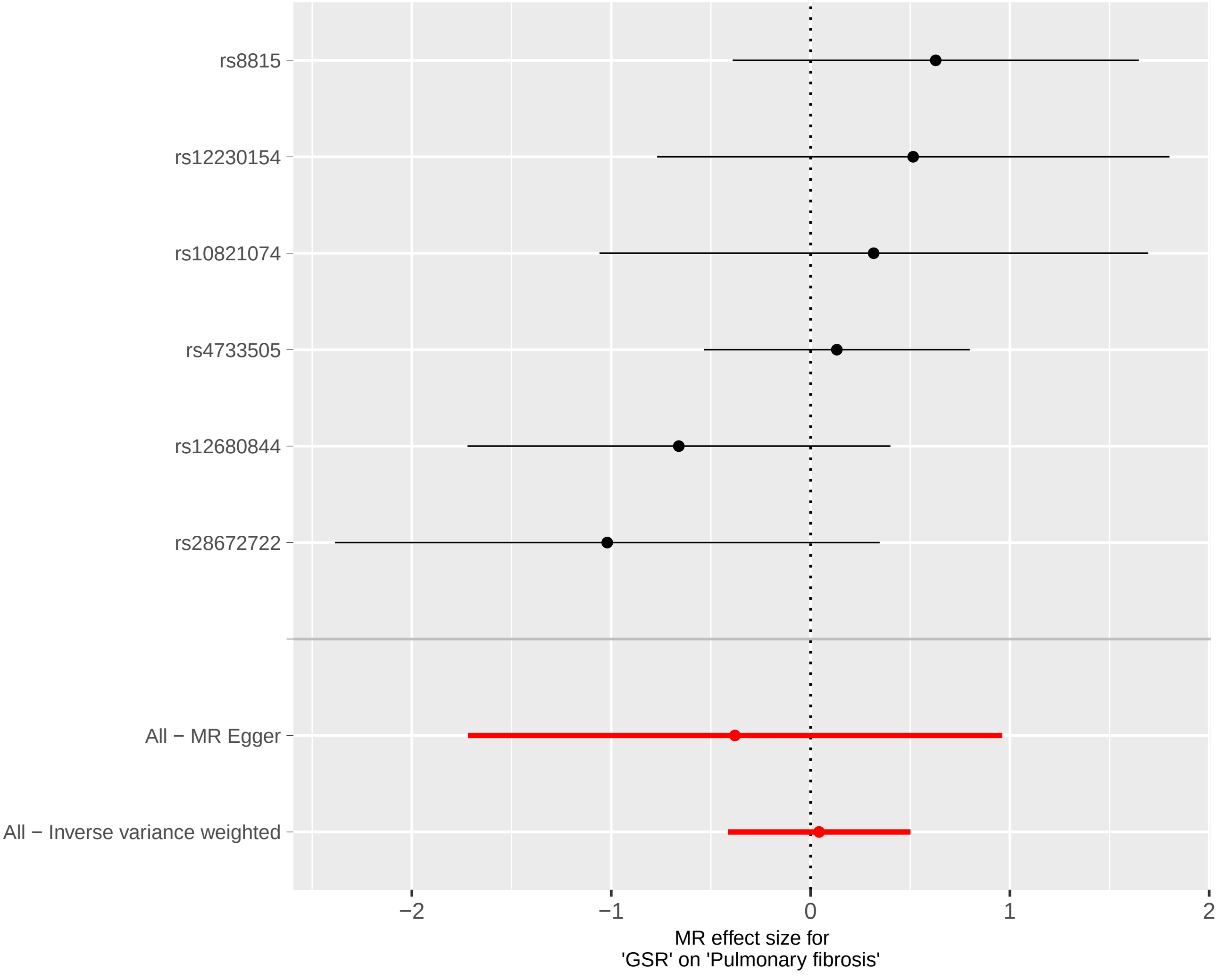

rs8815
rs12230154
rs10821074
rs4733505
rs12680844
rs28672722
All − MR Egger
All − Inverse variance weighted
−2
−1
0
1
2
MR effect size for
'GSR' on 'Pulmonary fibrosis'

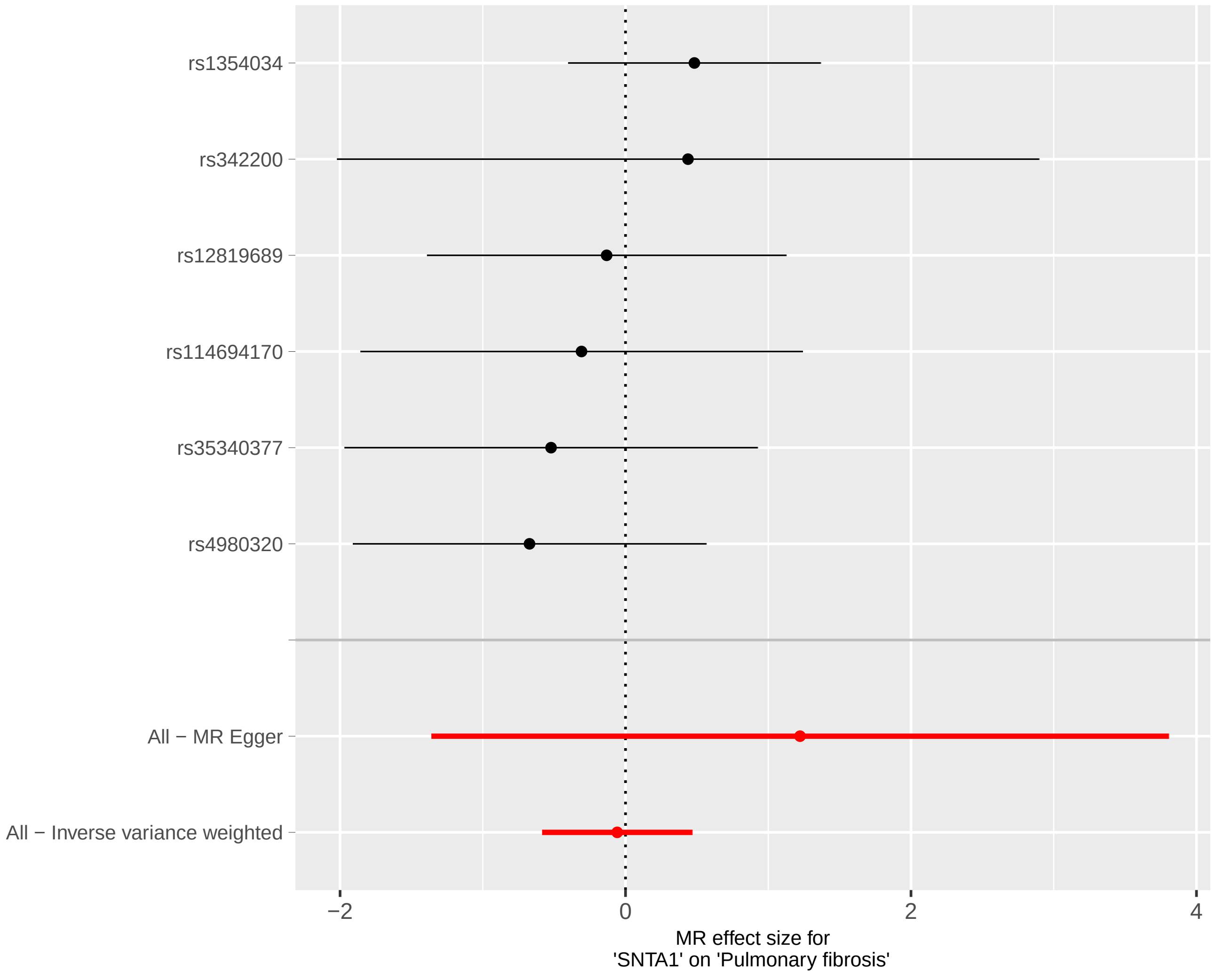
rs1354034
rs342200
rs12819689
rs114694170
rs35340377
rs4980320
All − MR Egger
All − Inverse variance weighted
−2
0
2
4
MR effect size for
'SNTA1' on 'Pulmonary fibrosis'

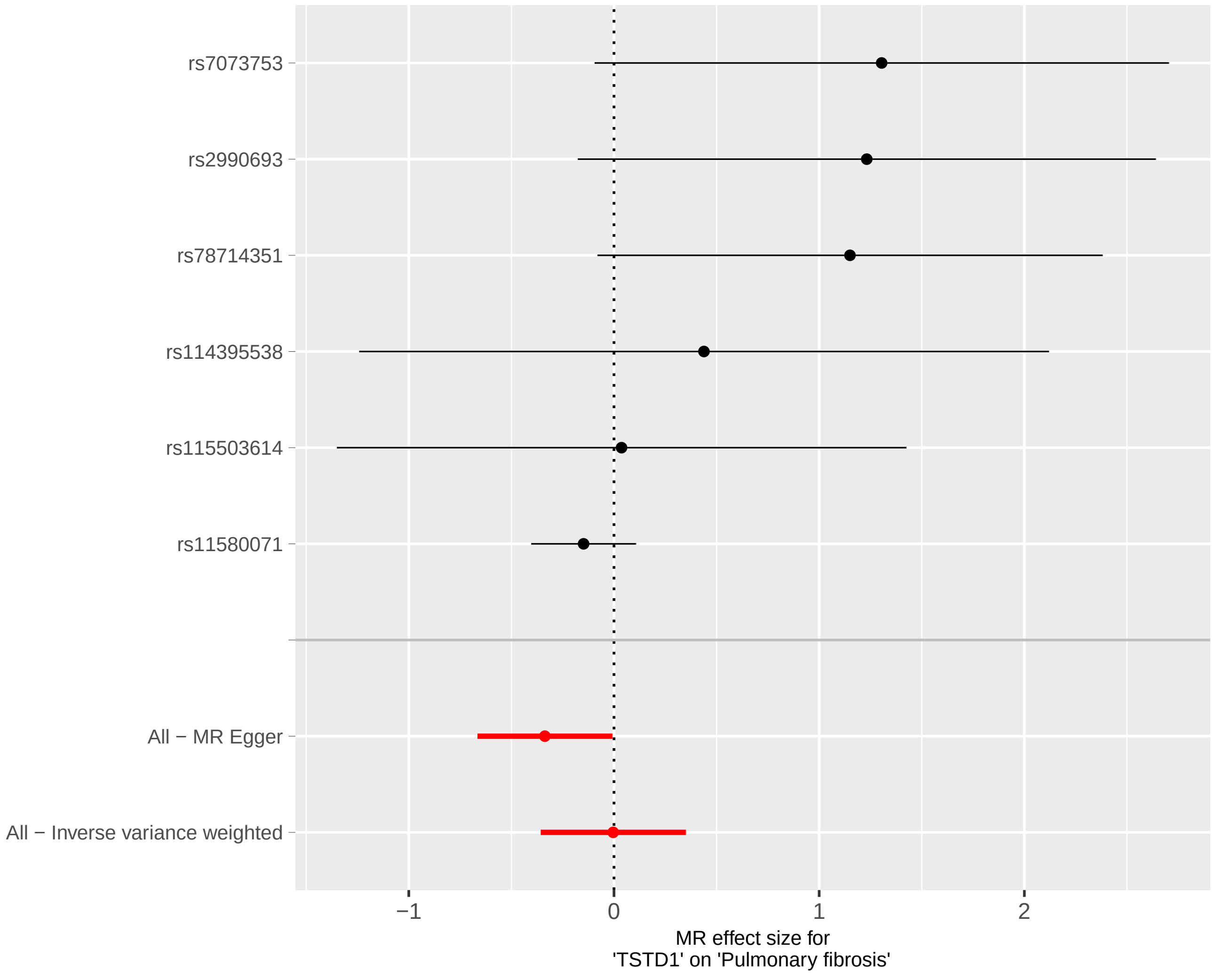
rs7073753
rs2990693
rs78714351
rs114395538
rs115503614
rs11580071
All − MR Egger
All − Inverse variance weighted
−1
0
1
2
MR effect size for
'TSTD1' on 'Pulmonary fibrosis'

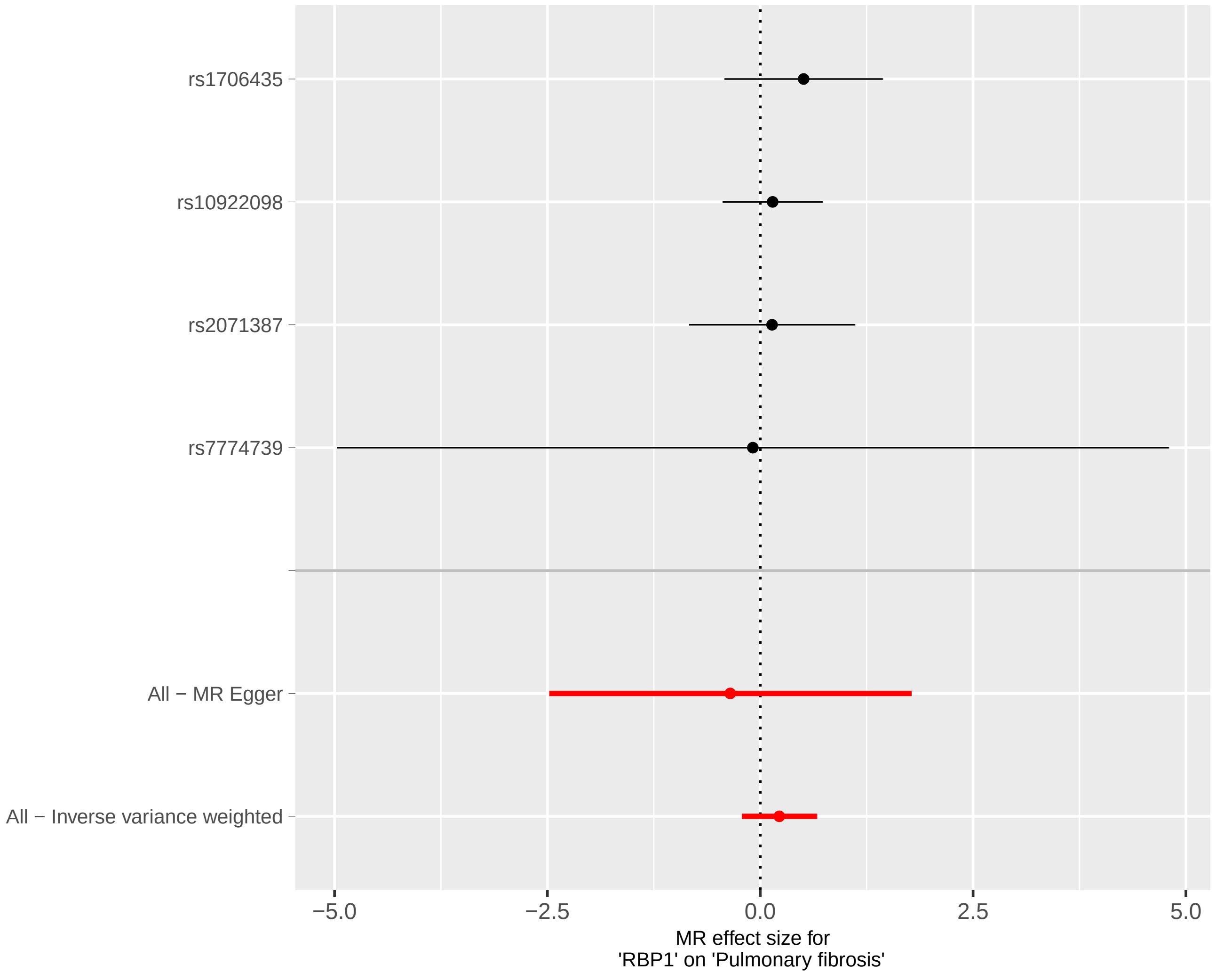

rs1706435
rs10922098
rs2071387
rs7774739
All − MR Egger
All − Inverse variance weighted
−5.0
−2.5
0.0
2.5
5.0
MR effect size for
'RBP1' on 'Pulmonary fibrosis'

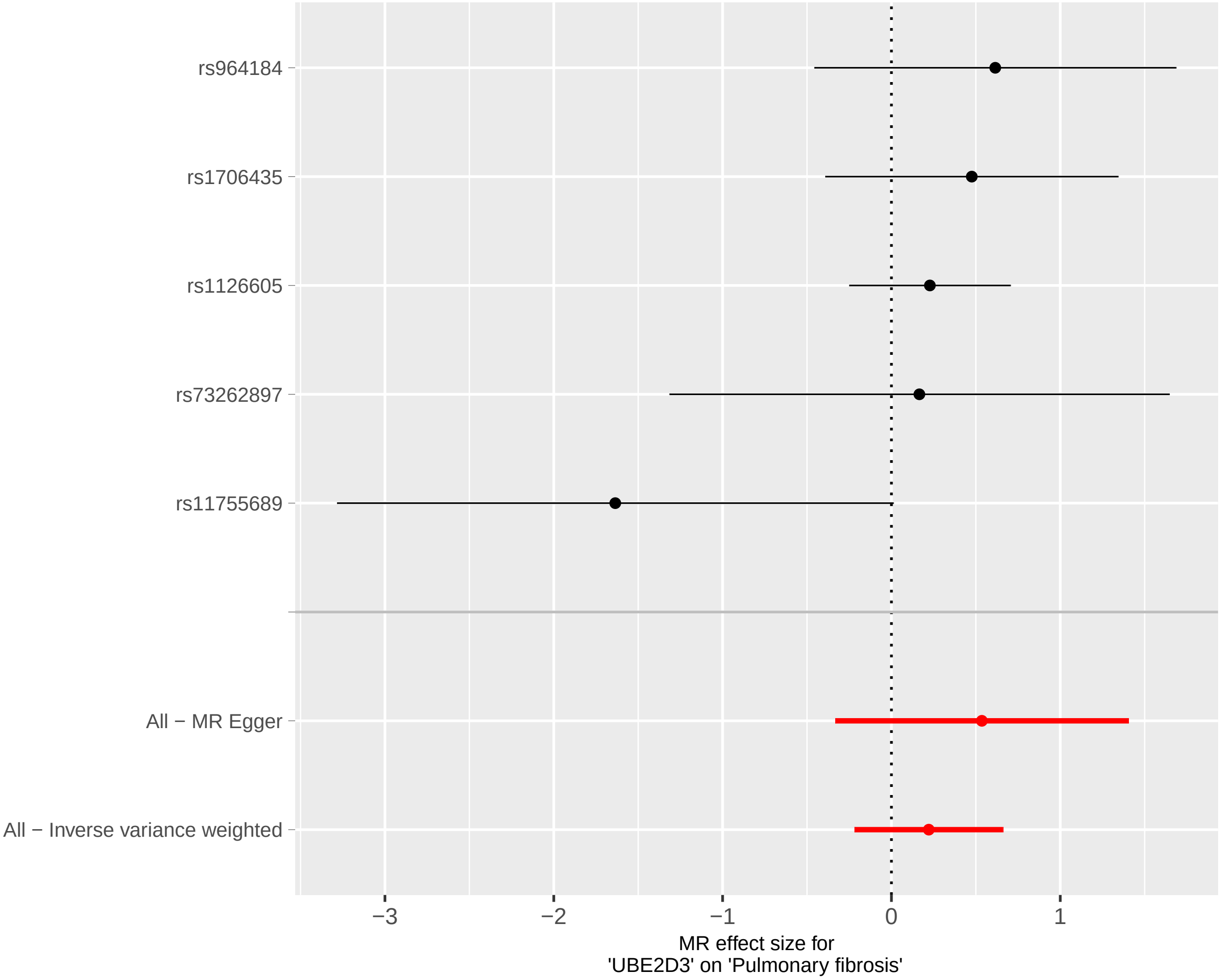

rs964184
rs1706435
rs1126605
rs73262897
rs11755689
All − MR Egger
All − Inverse variance weighted
−3
−2
−1
0
1
MR effect size for
'UBE2D3' on 'Pulmonary fibrosis'

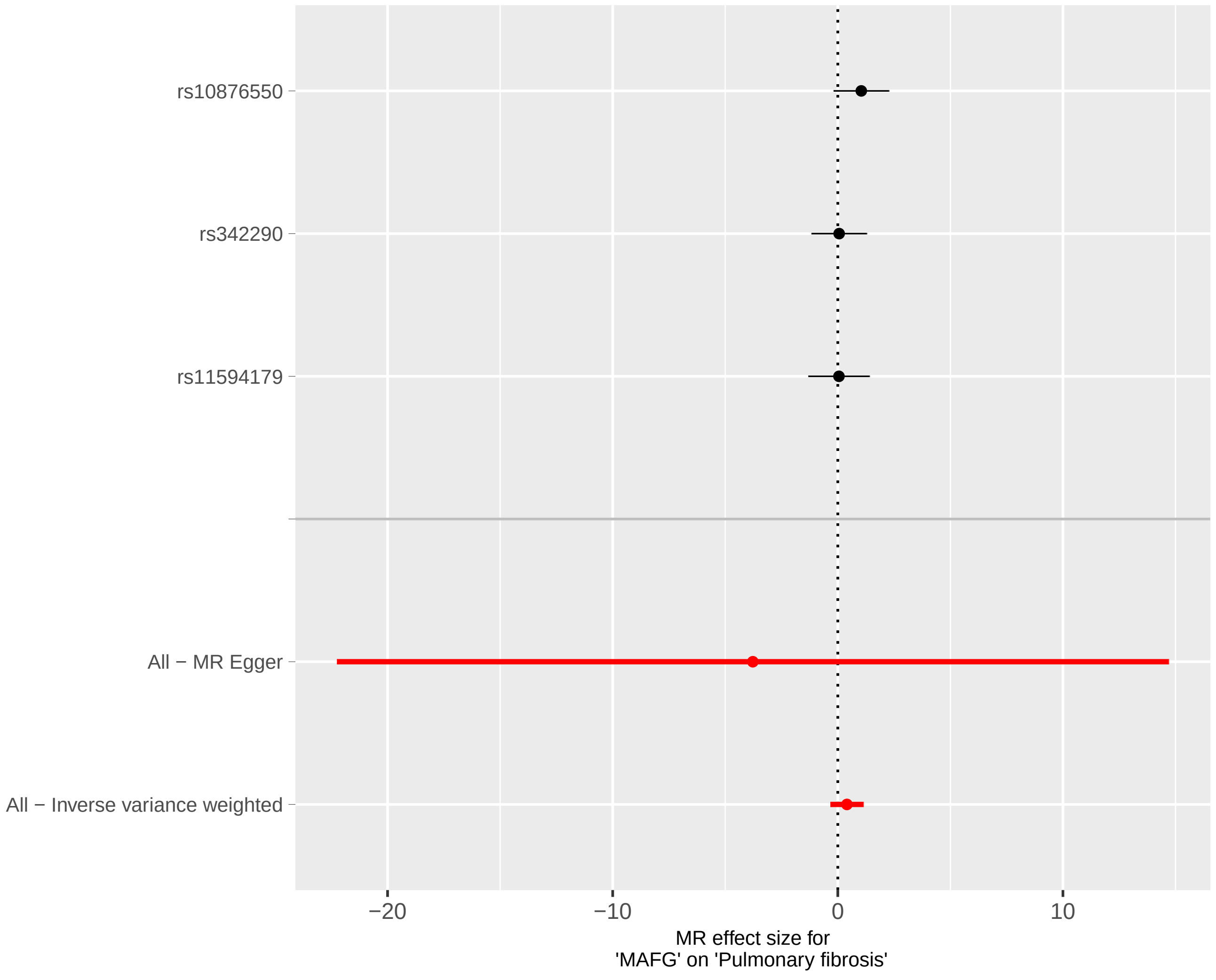

rs10876550
rs342290
rs11594179
All − MR Egger
All − Inverse variance weighted
−20
−10
0
10
MR effect size for
'MAFG' on 'Pulmonary fibrosis'

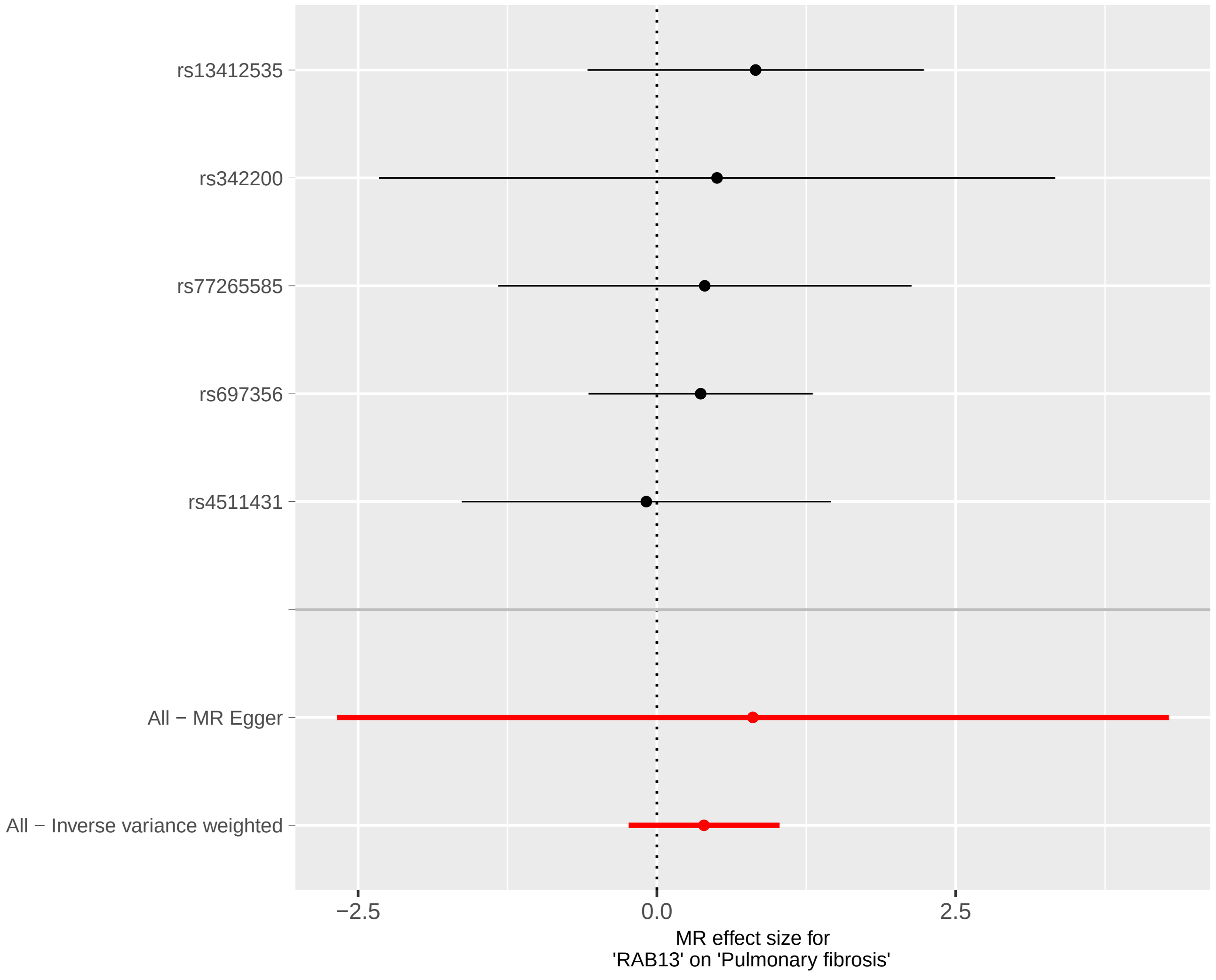
rs13412535
rs342200
rs77265585
rs697356
rs4511431
All − MR Egger
All − Inverse variance weighted
−2.5
0.0
2.5
MR effect size for
'RAB13' on 'Pulmonary fibrosis'

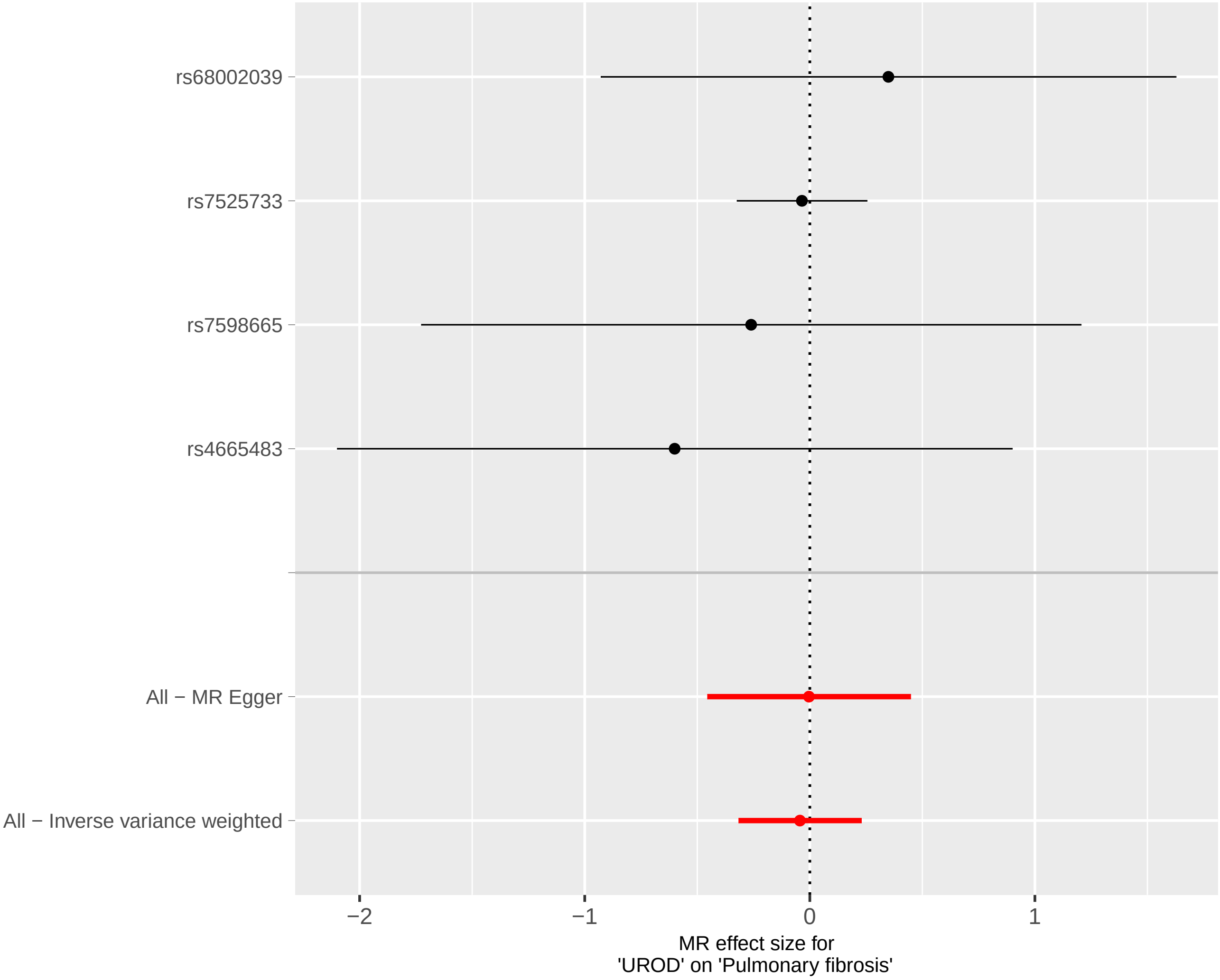

rs68002039
rs7525733
rs7598665
rs4665483
All − MR Egger
All − Inverse variance weighted
−2
−1
0
1
MR effect size for
'UROD' on 'Pulmonary fibrosis'

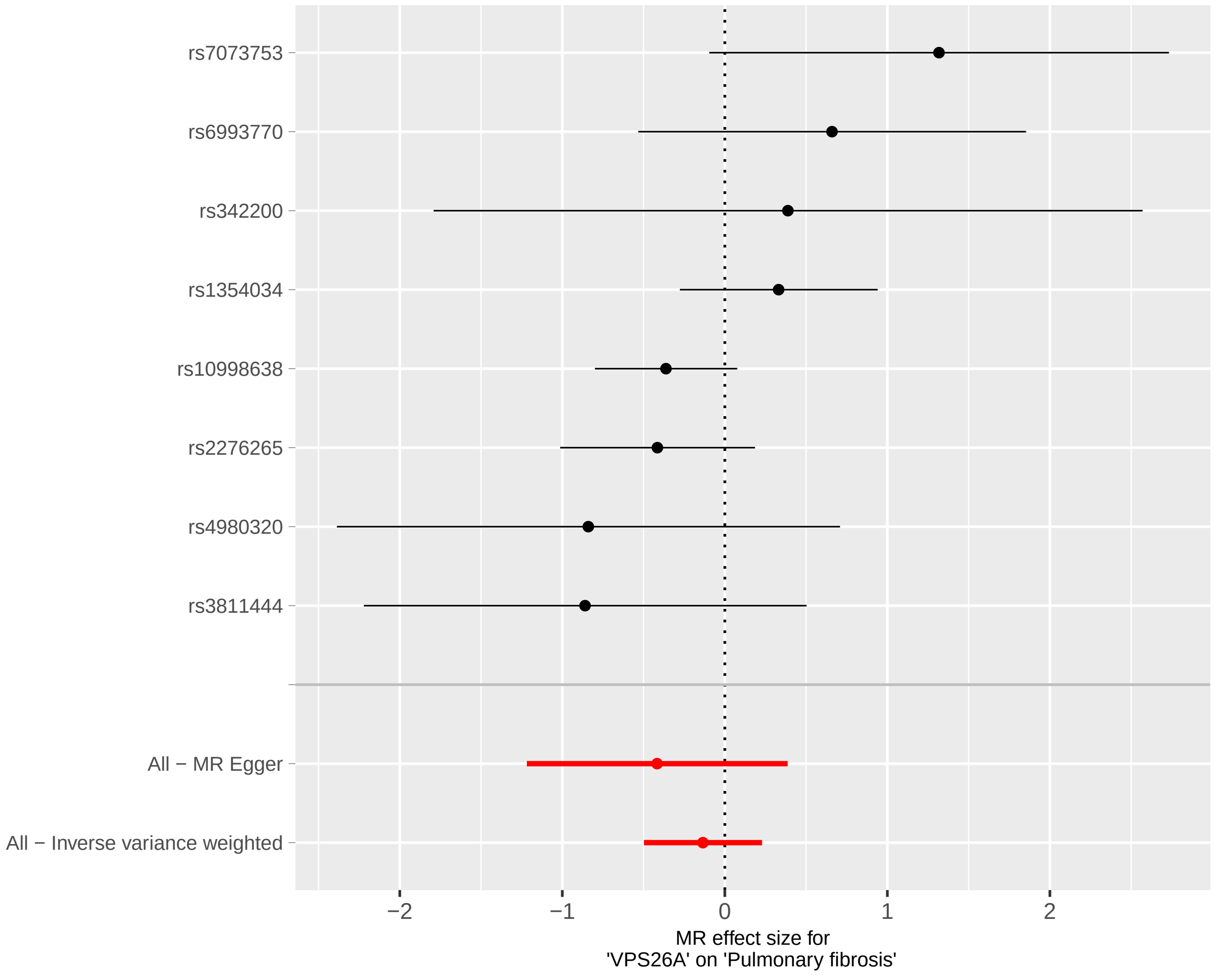

rs7073753
rs6993770
rs342200
rs1354034
rs10998638
rs2276265
rs4980320
rs3811444
All − MR Egger
All − Inverse variance weighted
−2
−1
0
1
2
MR effect size for
'VPS26A' on 'Pulmonary fibrosis'

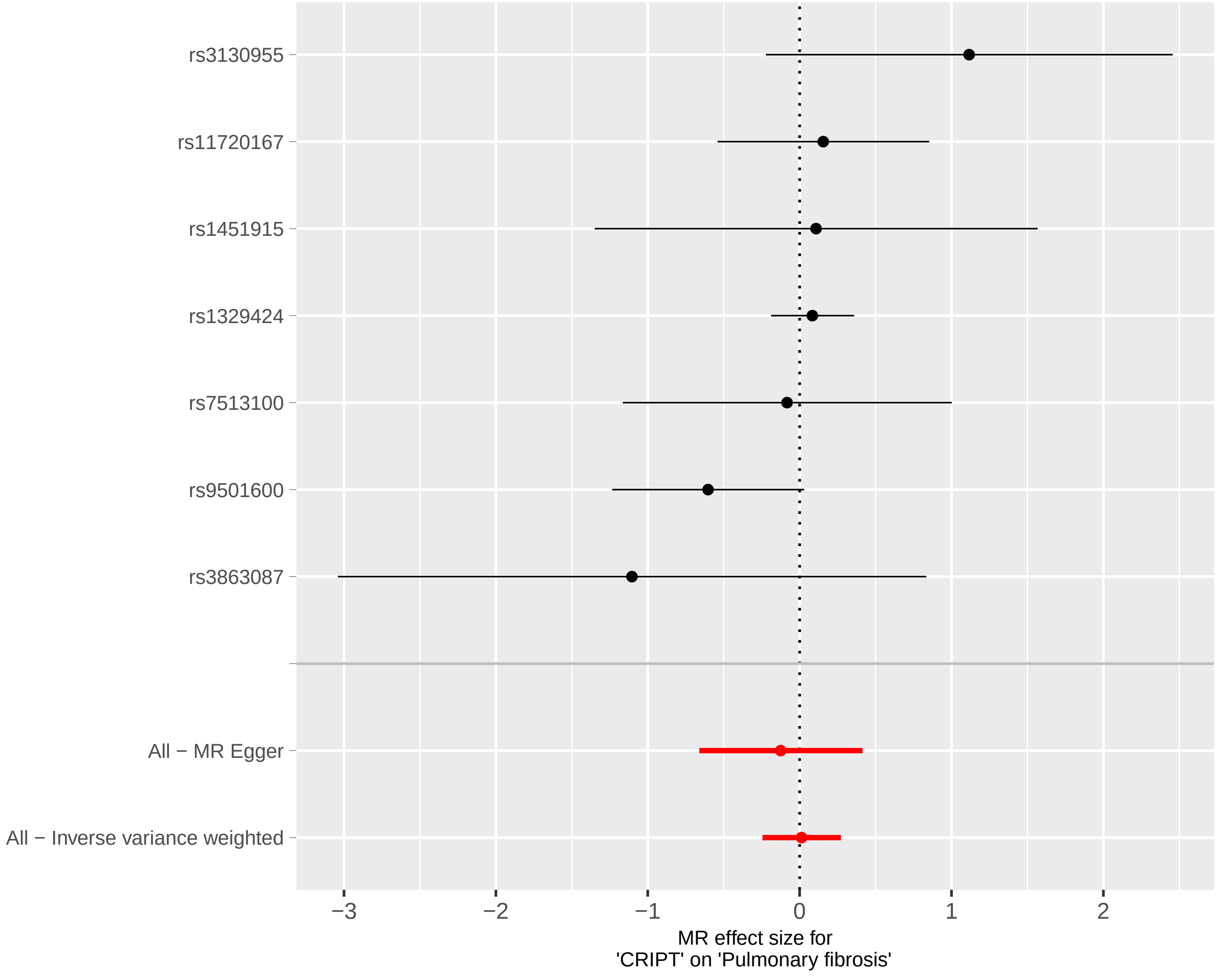
rs3130955
rs11720167
rs1451915
rs1329424
rs7513100
rs9501600
rs3863087
All − MR Egger
All − Inverse variance weighted
−3
−2
−1
0
1
2
MR effect size for
'CRIPT' on 'Pulmonary fibrosis'

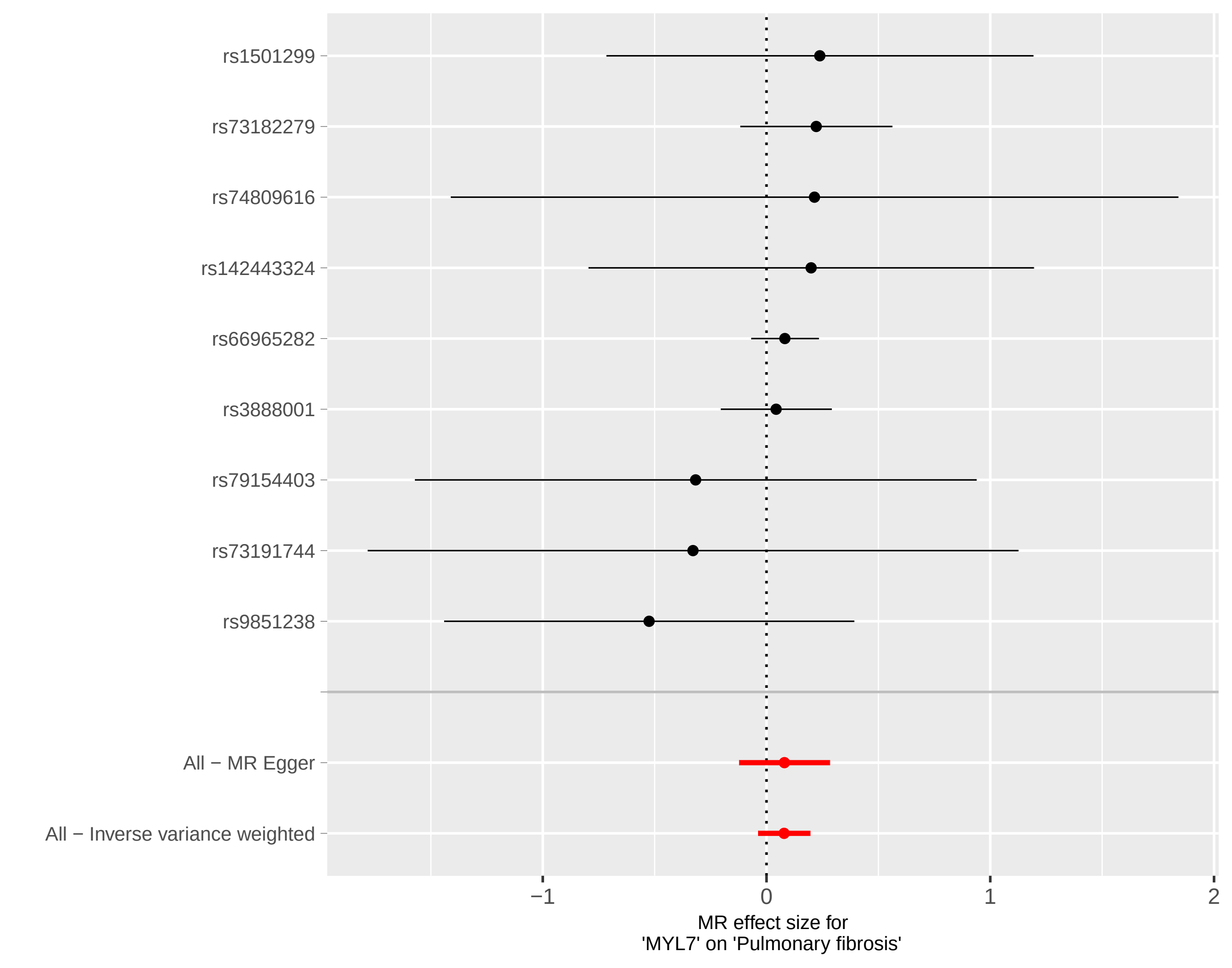
rs1501299
rs73182279
rs74809616
rs142443324
rs66965282
rs3888001
rs79154403
rs73191744
rs9851238
All − MR Egger
All − Inverse variance weighted
−1
0
1
2
MR effect size for
'MYL7' on 'Pulmonary fibrosis'

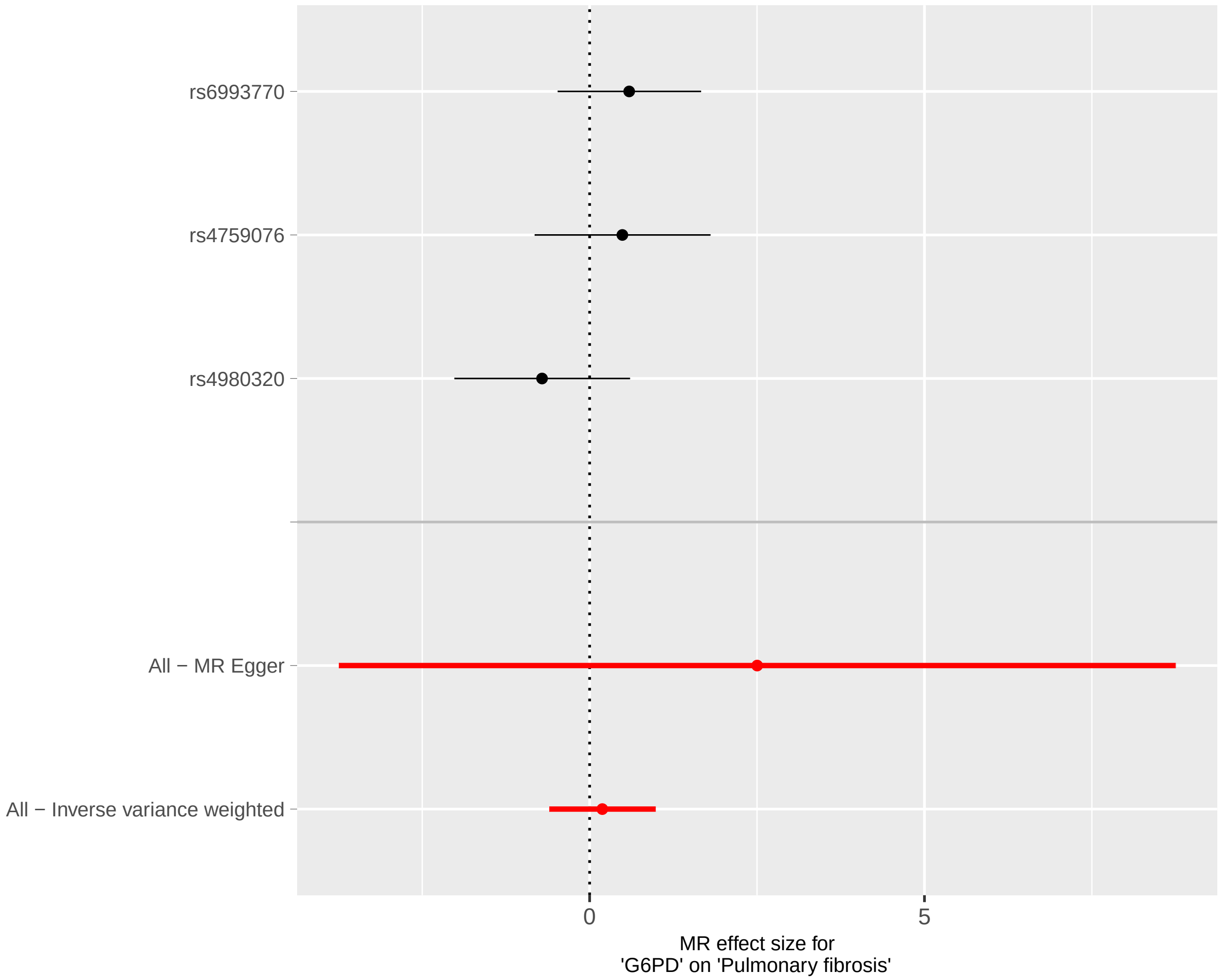
rs6993770
rs4759076
rs4980320
All − MR Egger
All − Inverse variance weighted
0
5
MR effect size for
'G6PD' on 'Pulmonary fibrosis'

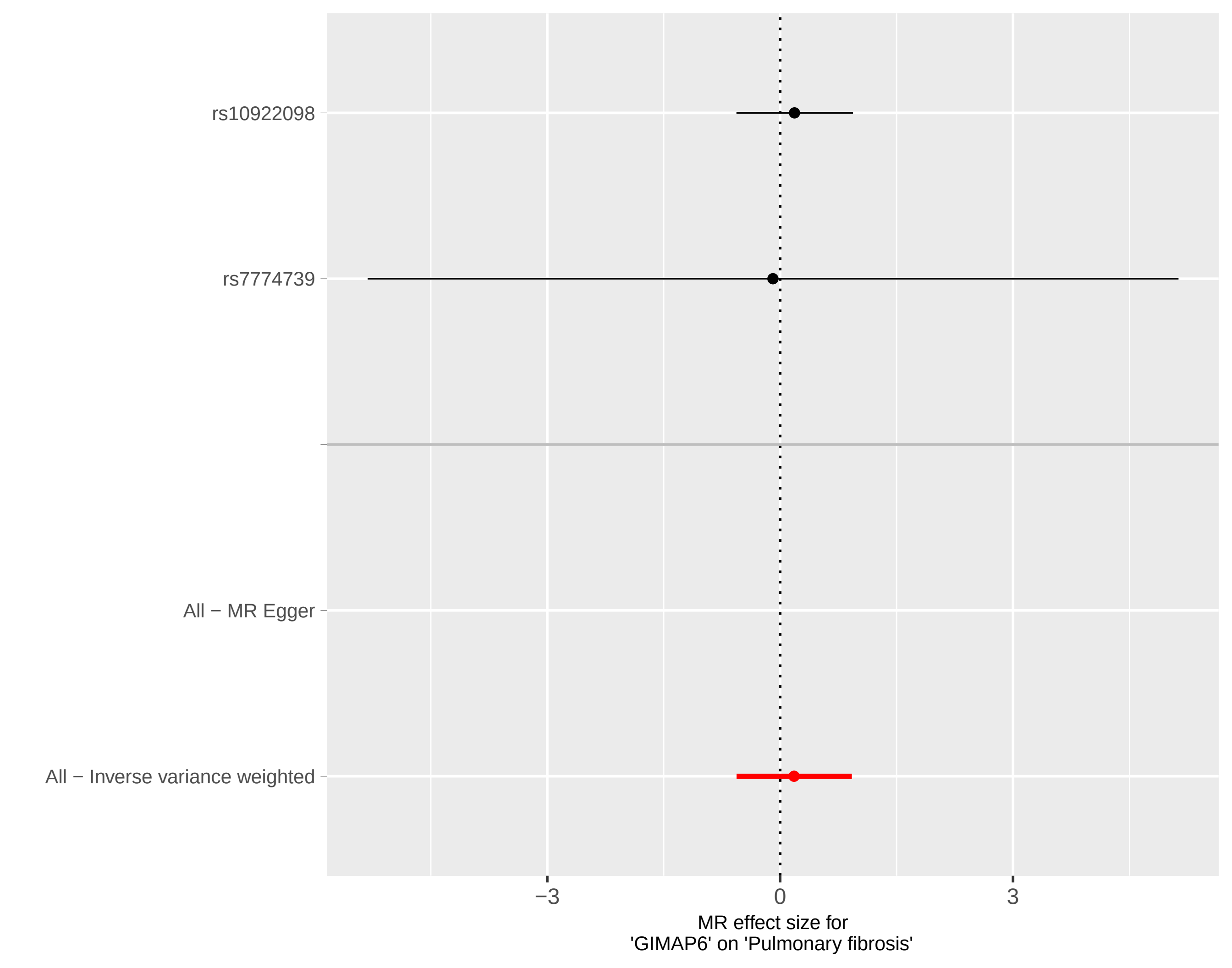
rs10922098
rs7774739
All − MR Egger
All − Inverse variance weighted
−3
0
3
MR effect size for
'GIMAP6' on 'Pulmonary fibrosis'

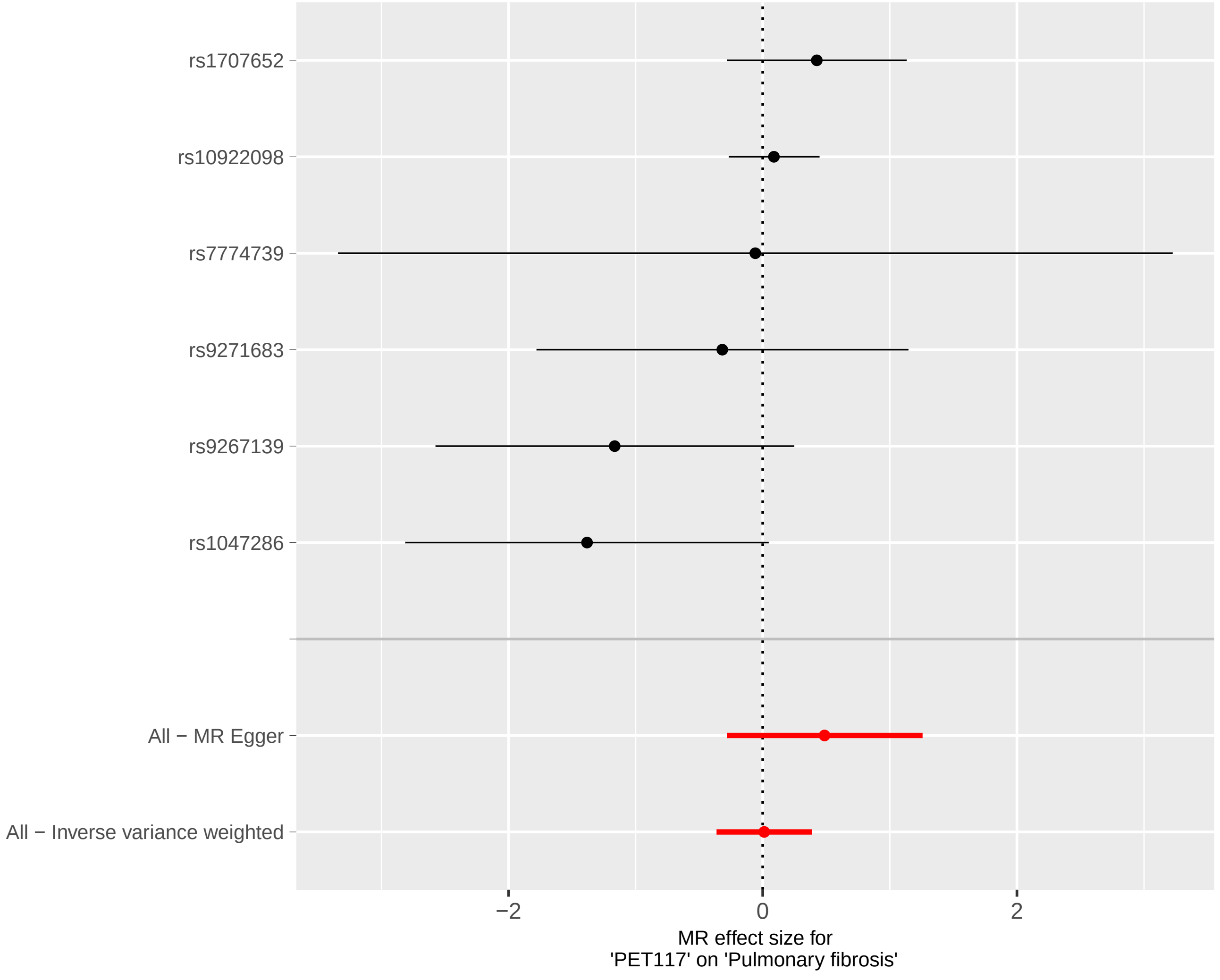
rs1707652
rs10922098
rs7774739
rs9271683
rs9267139
rs1047286
All − MR Egger
All − Inverse variance weighted
−2
0
2
MR effect size for
'PET117' on 'Pulmonary fibrosis'

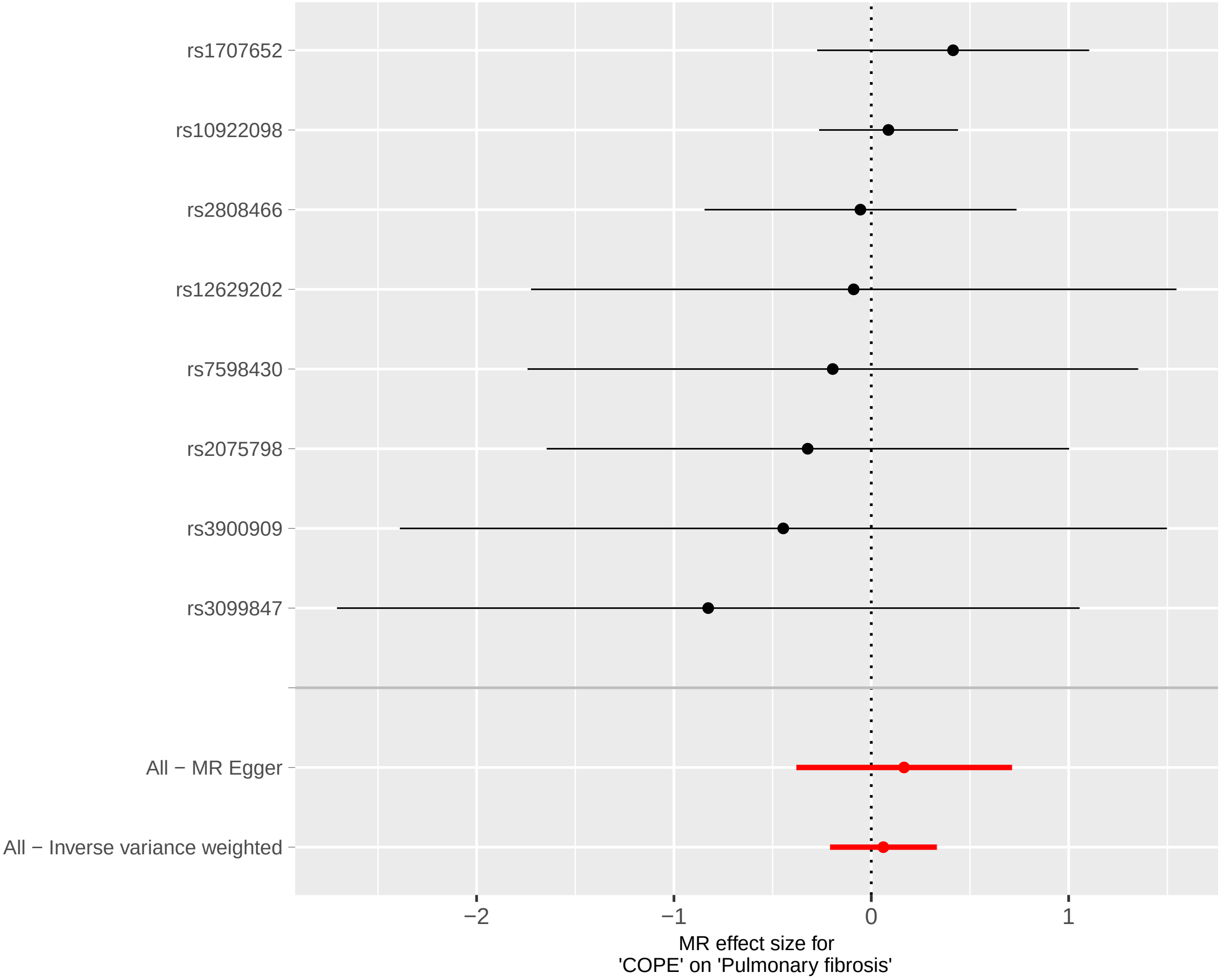
rs1707652
rs10922098
rs2808466
rs12629202
rs7598430
rs2075798
rs3900909
rs3099847
All − MR Egger
All − Inverse variance weighted
−2
−1
0
1
MR effect size for
'COPE' on 'Pulmonary fibrosis'

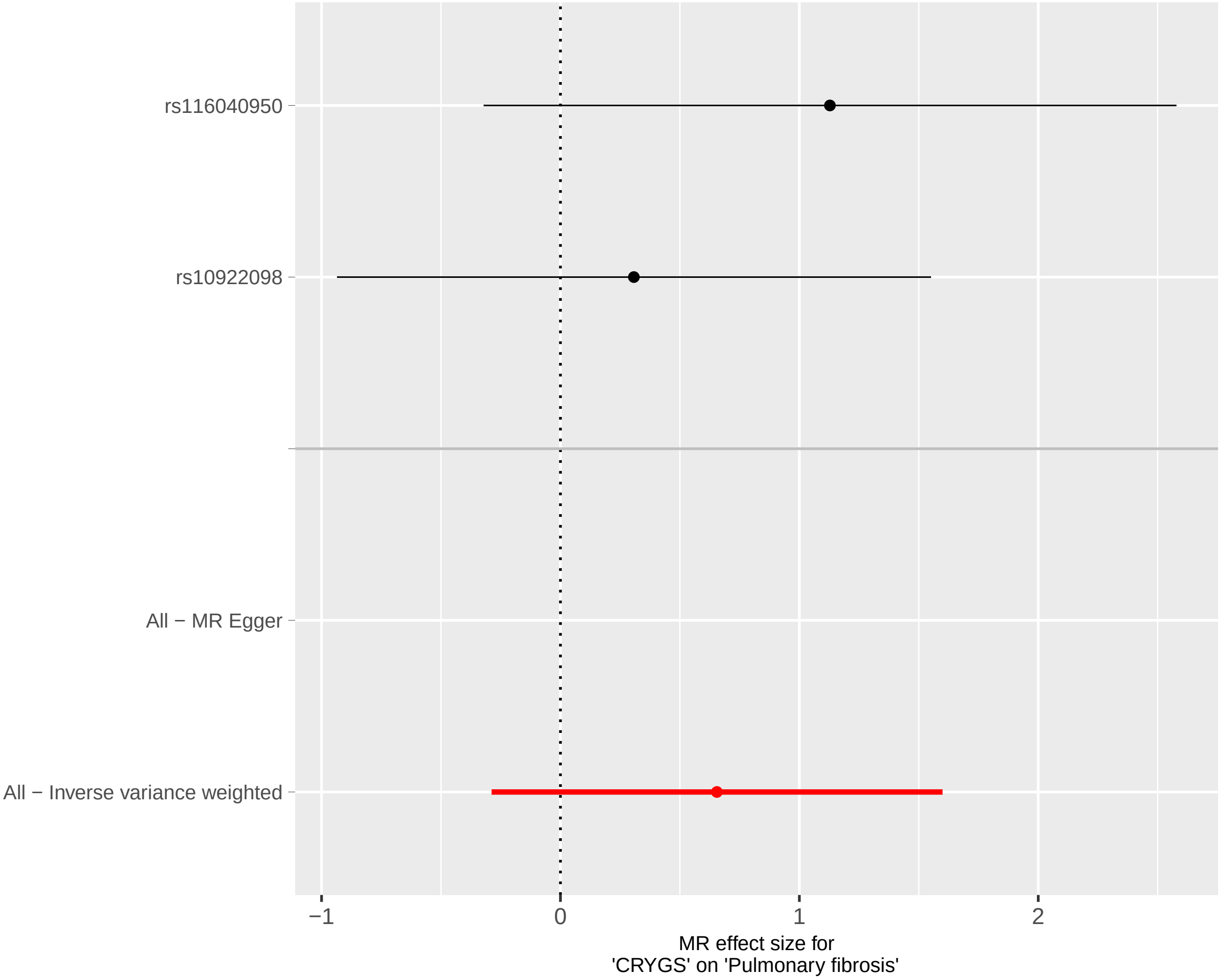

rs116040950
rs10922098
All − MR Egger
All − Inverse variance weighted
−1
0
1
2
MR effect size for
'CRYGS' on 'Pulmonary fibrosis'

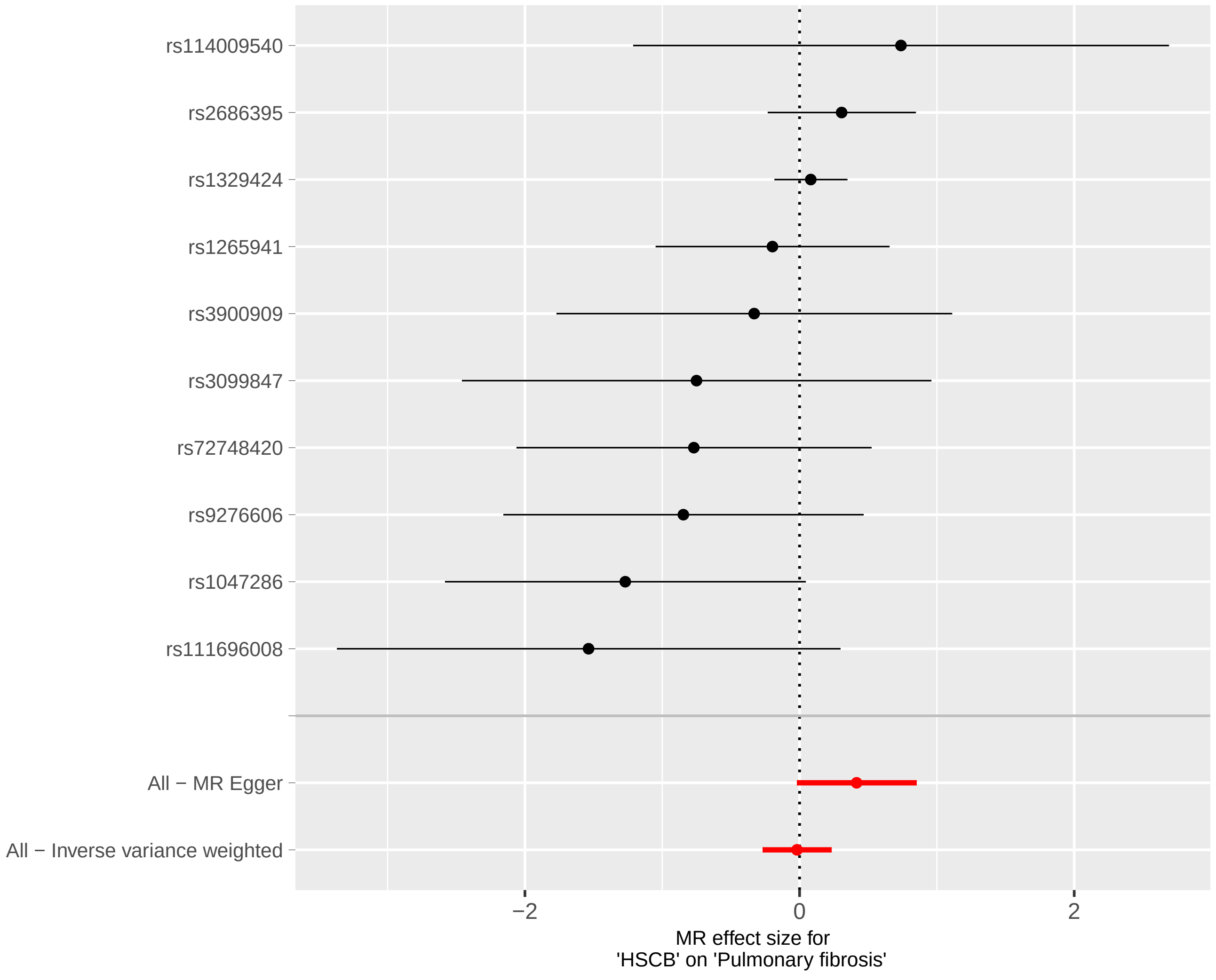
rs114009540
rs2686395
rs1329424
rs1265941
rs3900909
rs3099847
rs72748420
rs9276606
rs1047286
rs111696008
All − MR Egger
All − Inverse variance weighted
−2
0
2
MR effect size for
'HSCB' on 'Pulmonary fibrosis'

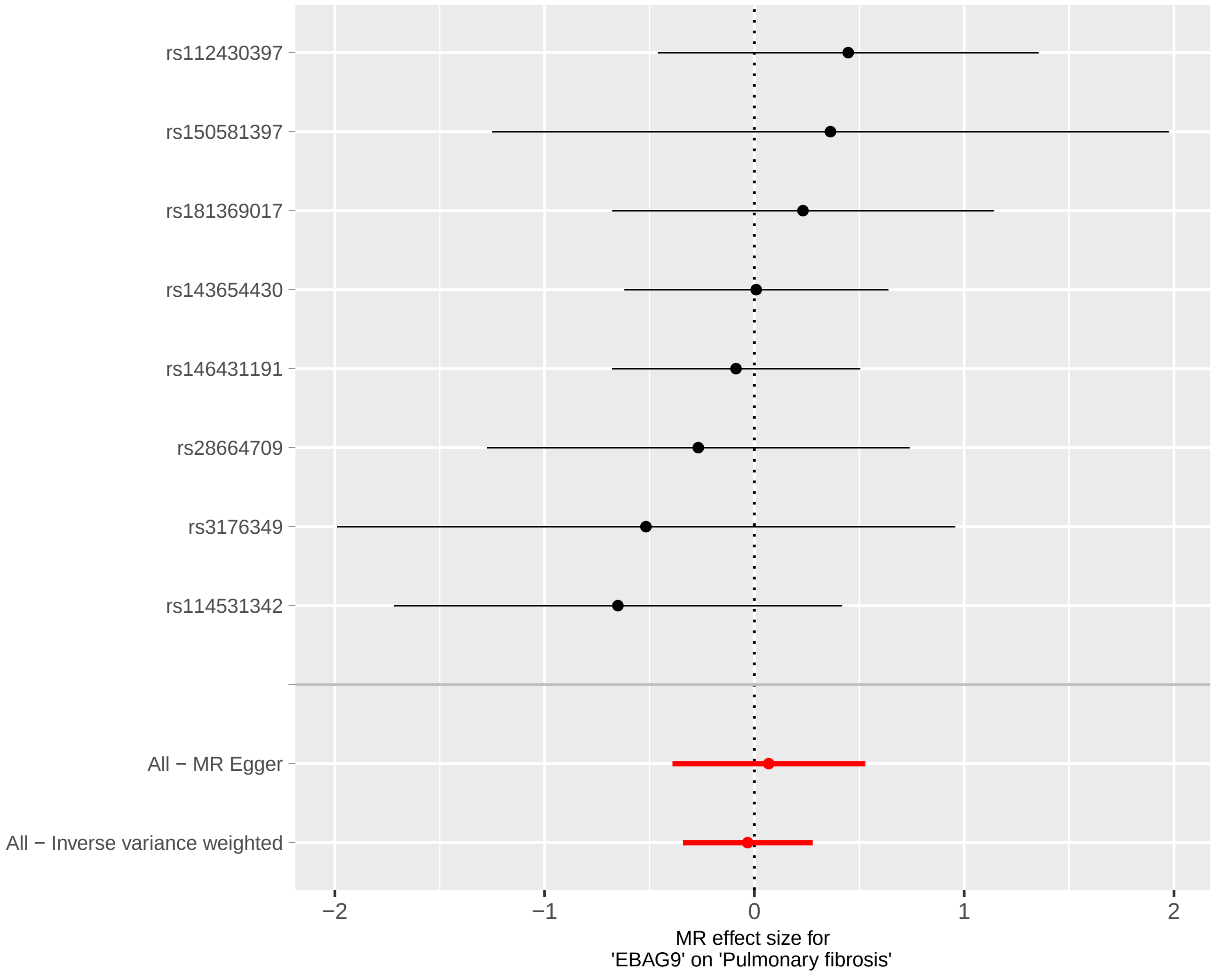

rs112430397
rs150581397
rs181369017
rs143654430
rs146431191
rs28664709
rs3176349
rs114531342
All − MR Egger
All − Inverse variance weighted
−2
−1
0
1
2
MR effect size for
'EBAG9' on 'Pulmonary fibrosis'

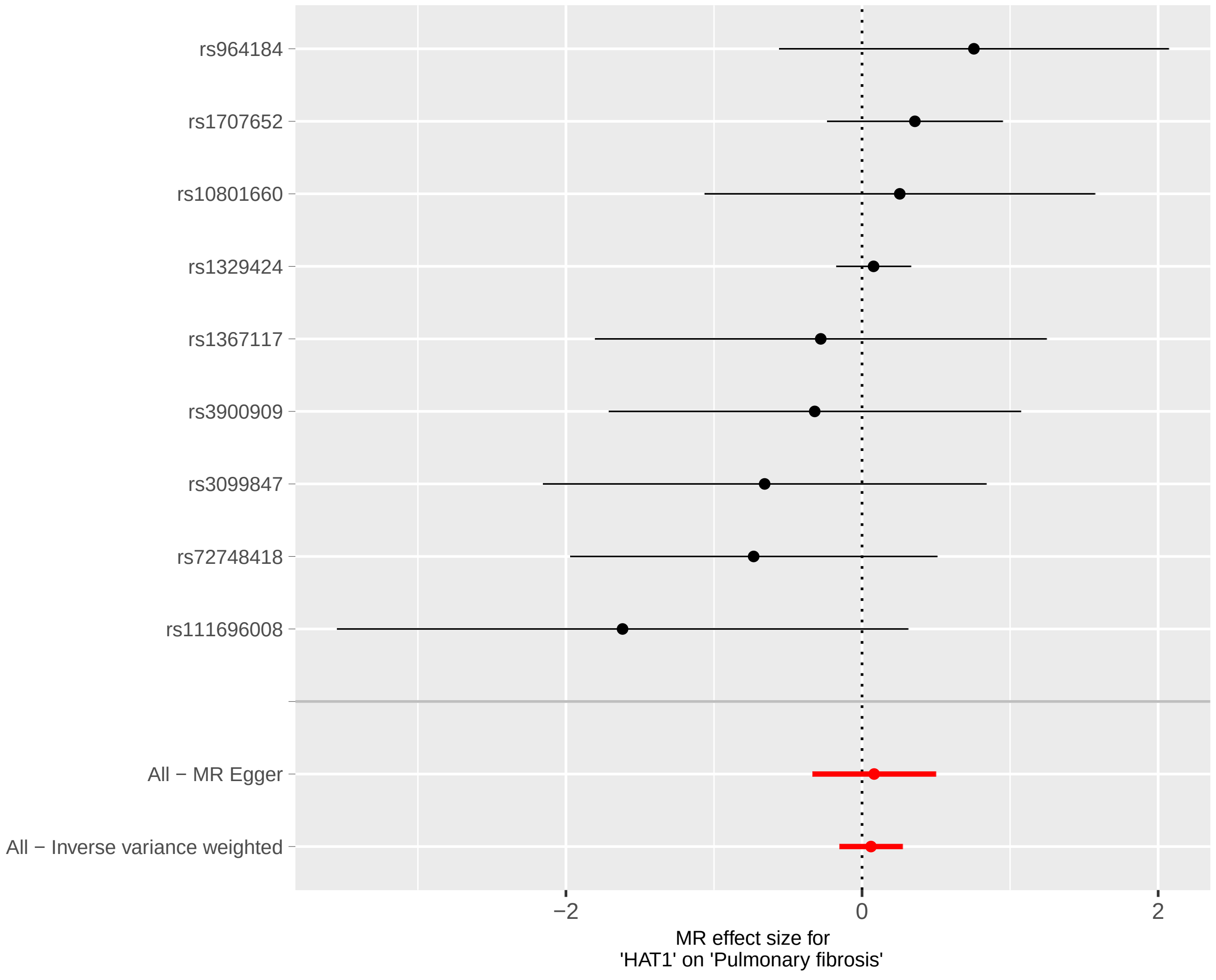
rs964184
rs1707652
rs10801660
rs1329424
rs1367117
rs3900909
rs3099847
rs72748418
rs111696008
All − MR Egger
All − Inverse variance weighted
−2
0
2
MR effect size for
'HAT1' on 'Pulmonary fibrosis'

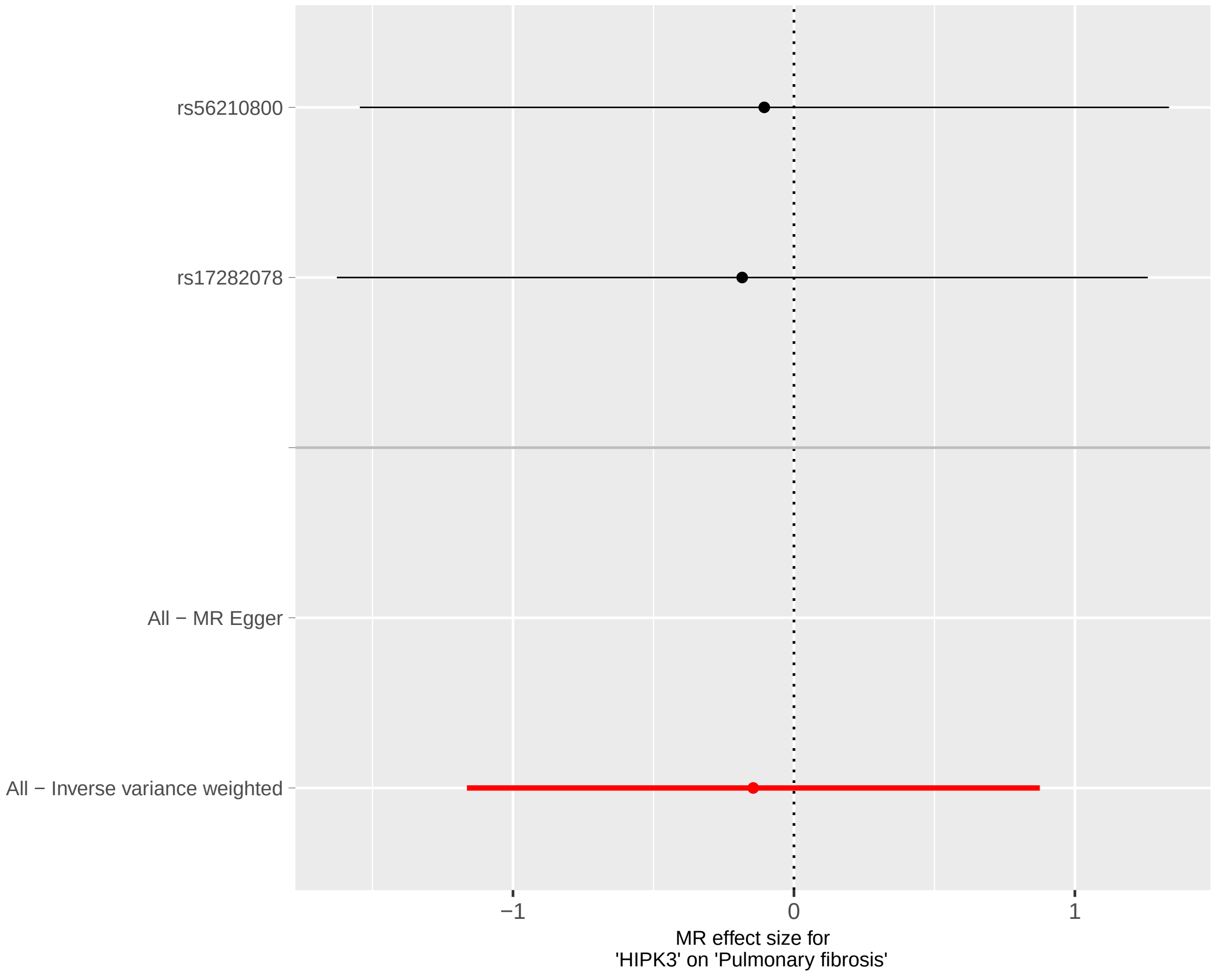
rs56210800
rs17282078
All − MR Egger
All − Inverse variance weighted
−1
0
1
MR effect size for
'HIPK3' on 'Pulmonary fibrosis'

Insufficient number of SNPs

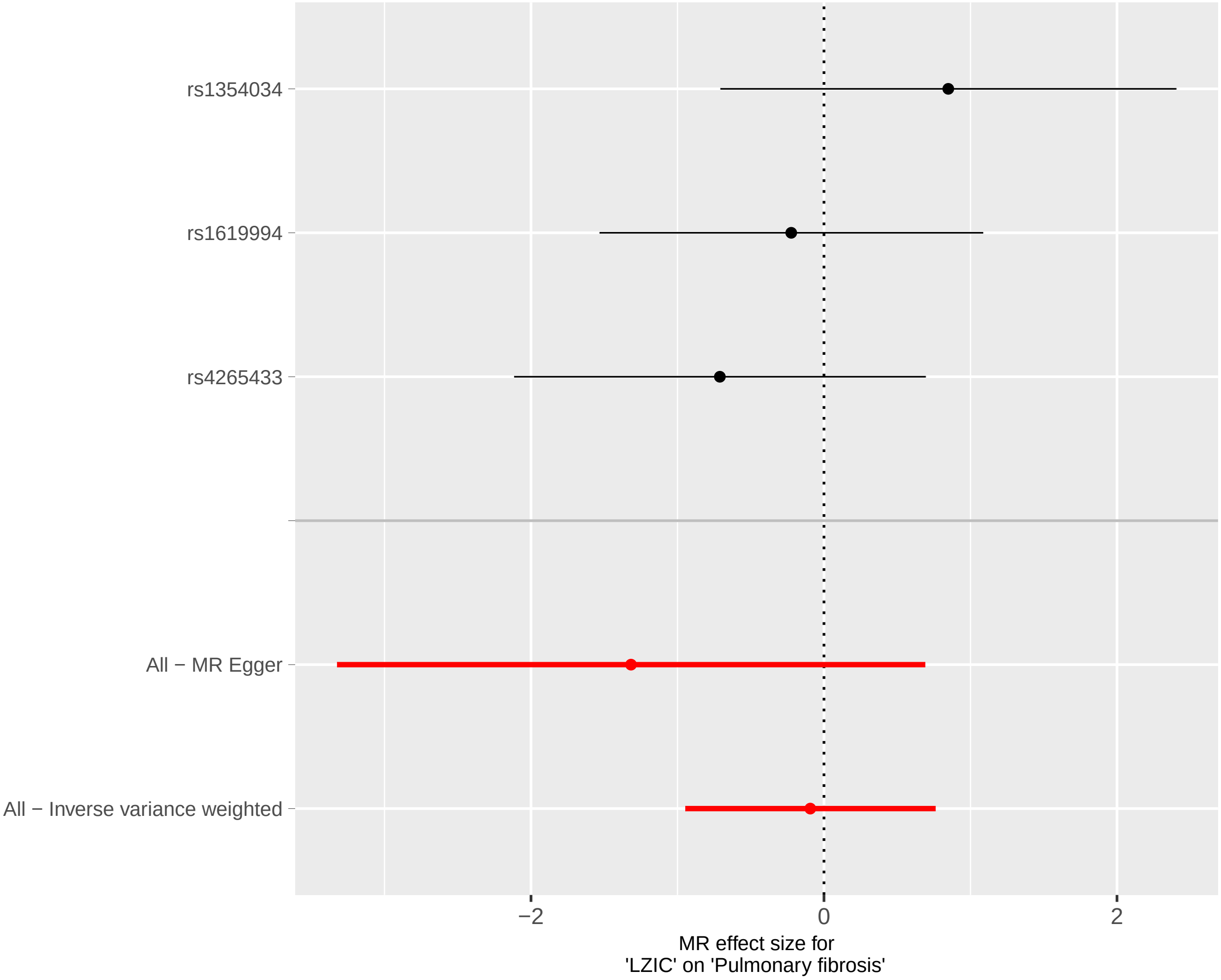

rs1354034
rs1619994
rs4265433
All − MR Egger
All − Inverse variance weighted
−2
0
2
MR effect size for
'LZIC' on 'Pulmonary fibrosis'

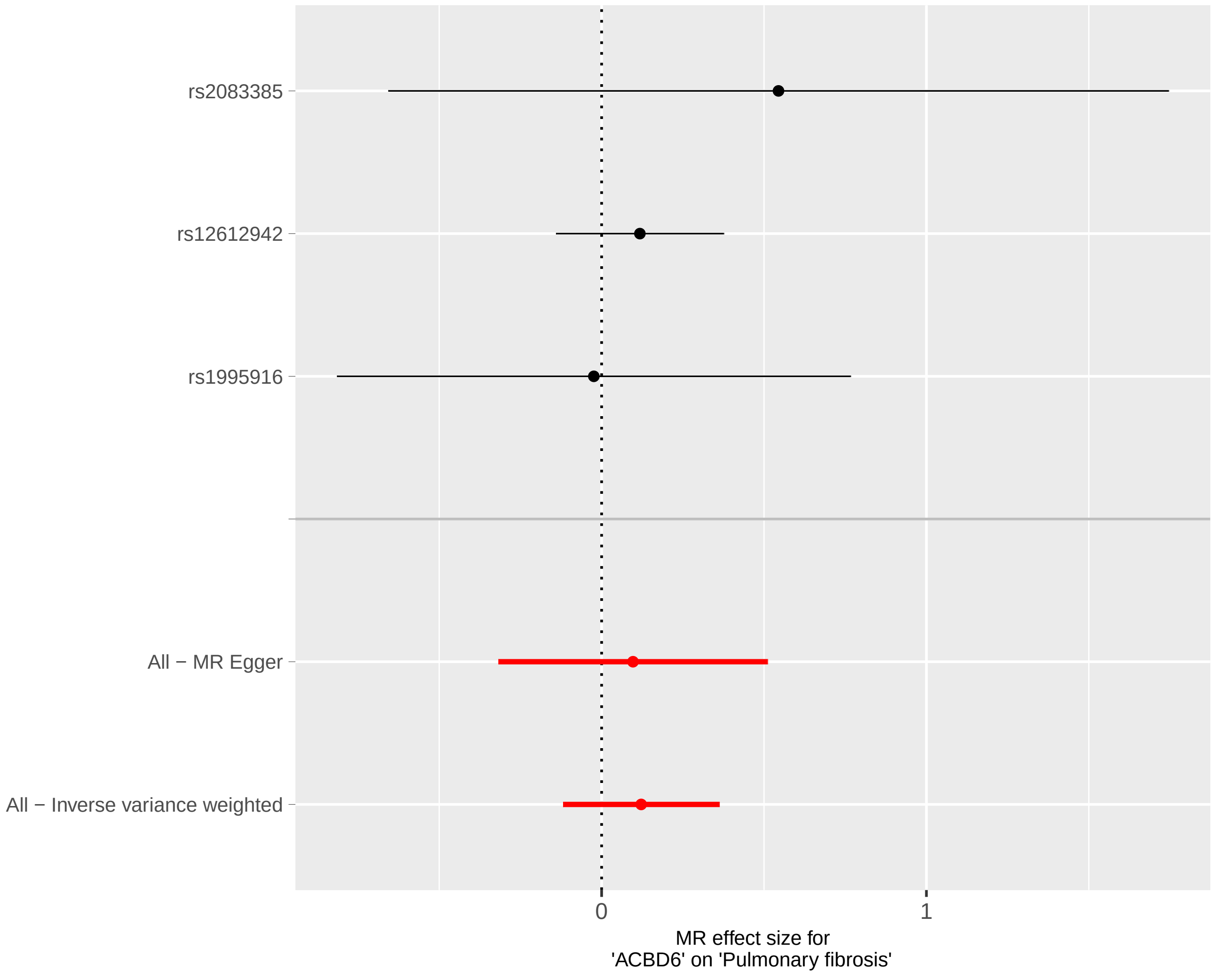
rs2083385
rs12612942
rs1995916
All − MR Egger
All − Inverse variance weighted
0
1
MR effect size for
'ACBD6' on 'Pulmonary fibrosis'

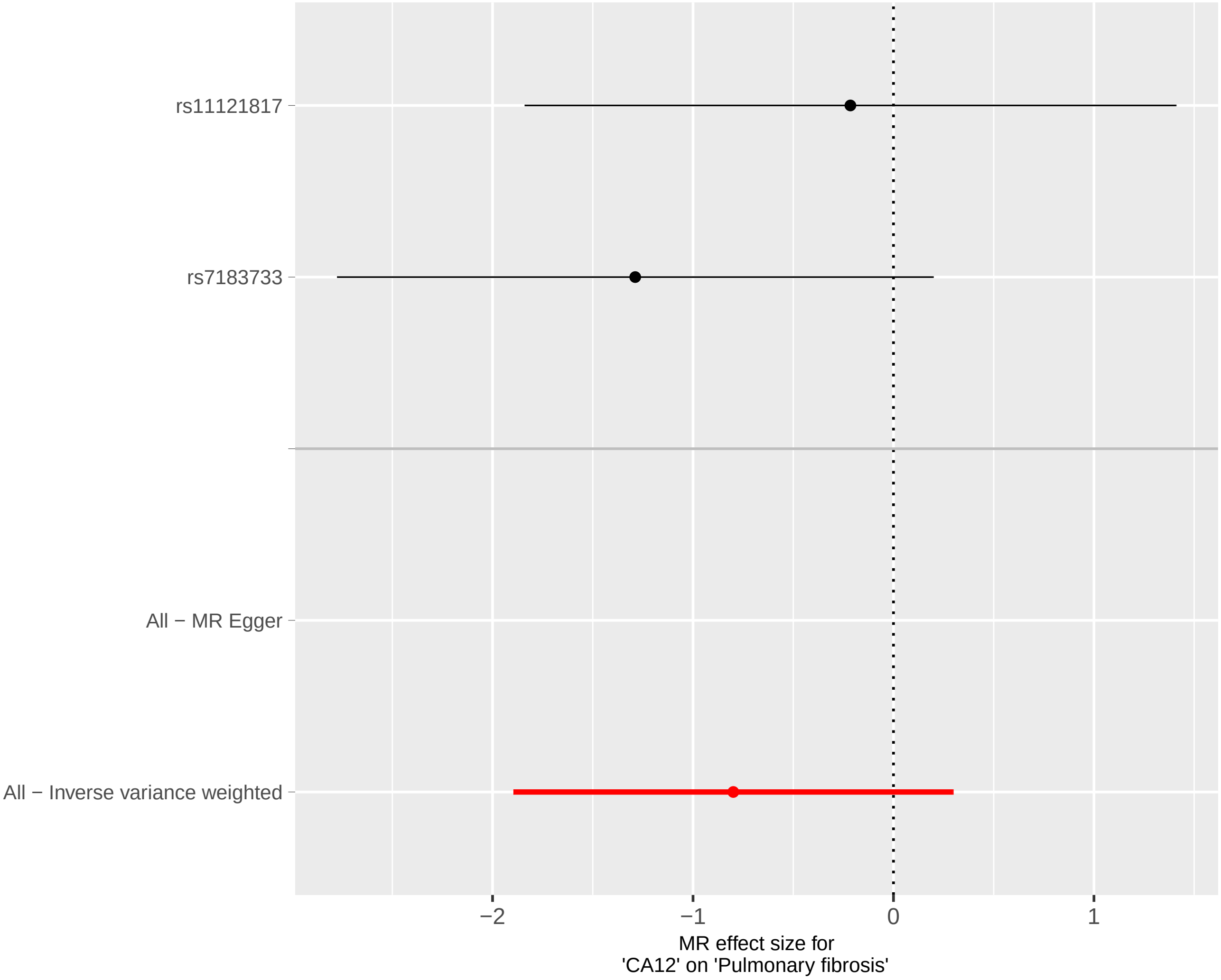

rs11121817
rs7183733
All − MR Egger
All − Inverse variance weighted
−2
−1
0
1
MR effect size for
'CA12' on 'Pulmonary fibrosis'

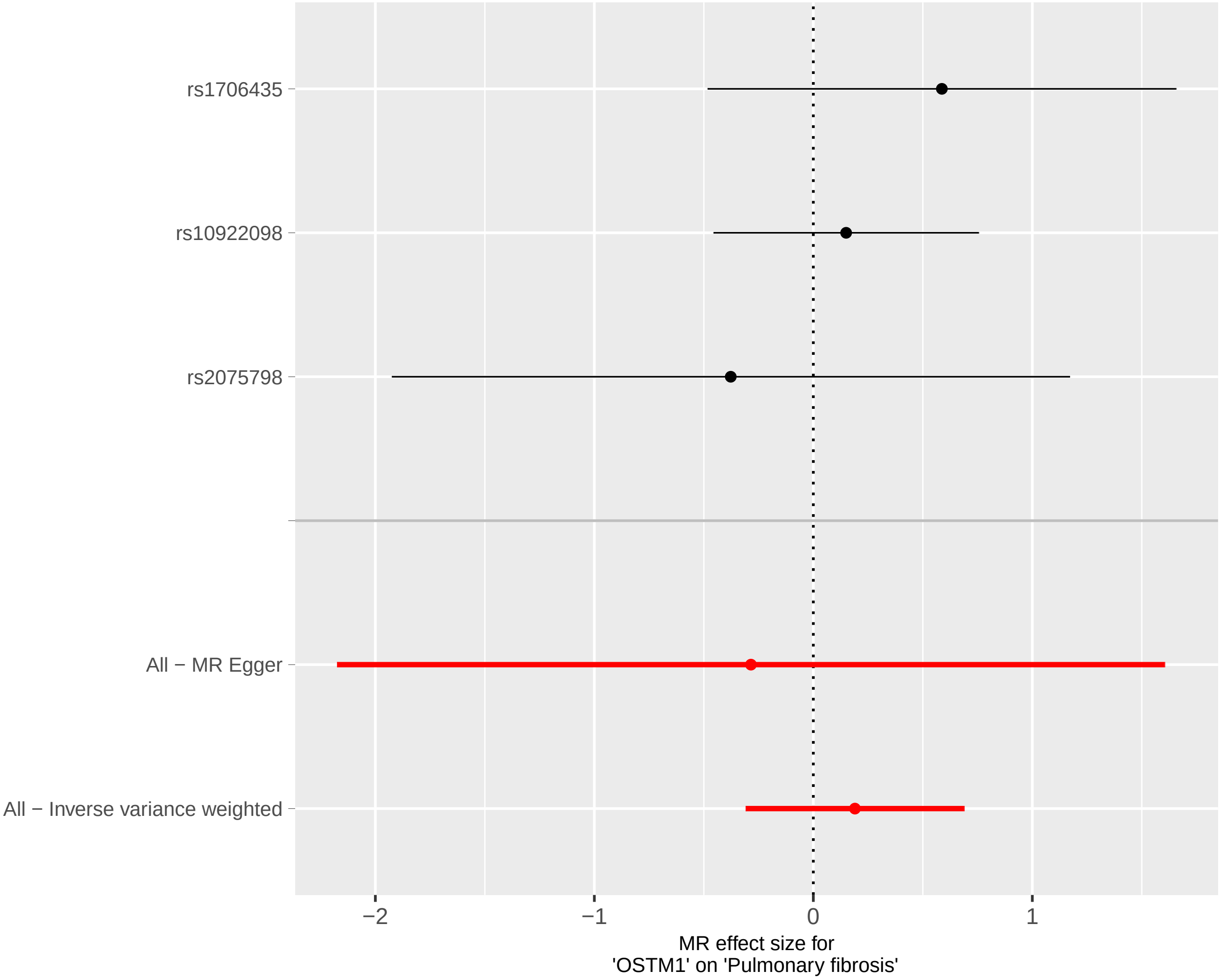

rs1706435
rs10922098
rs2075798
All − MR Egger
All − Inverse variance weighted
−2
−1
0
1
MR effect size for
'OSTM1' on 'Pulmonary fibrosis'

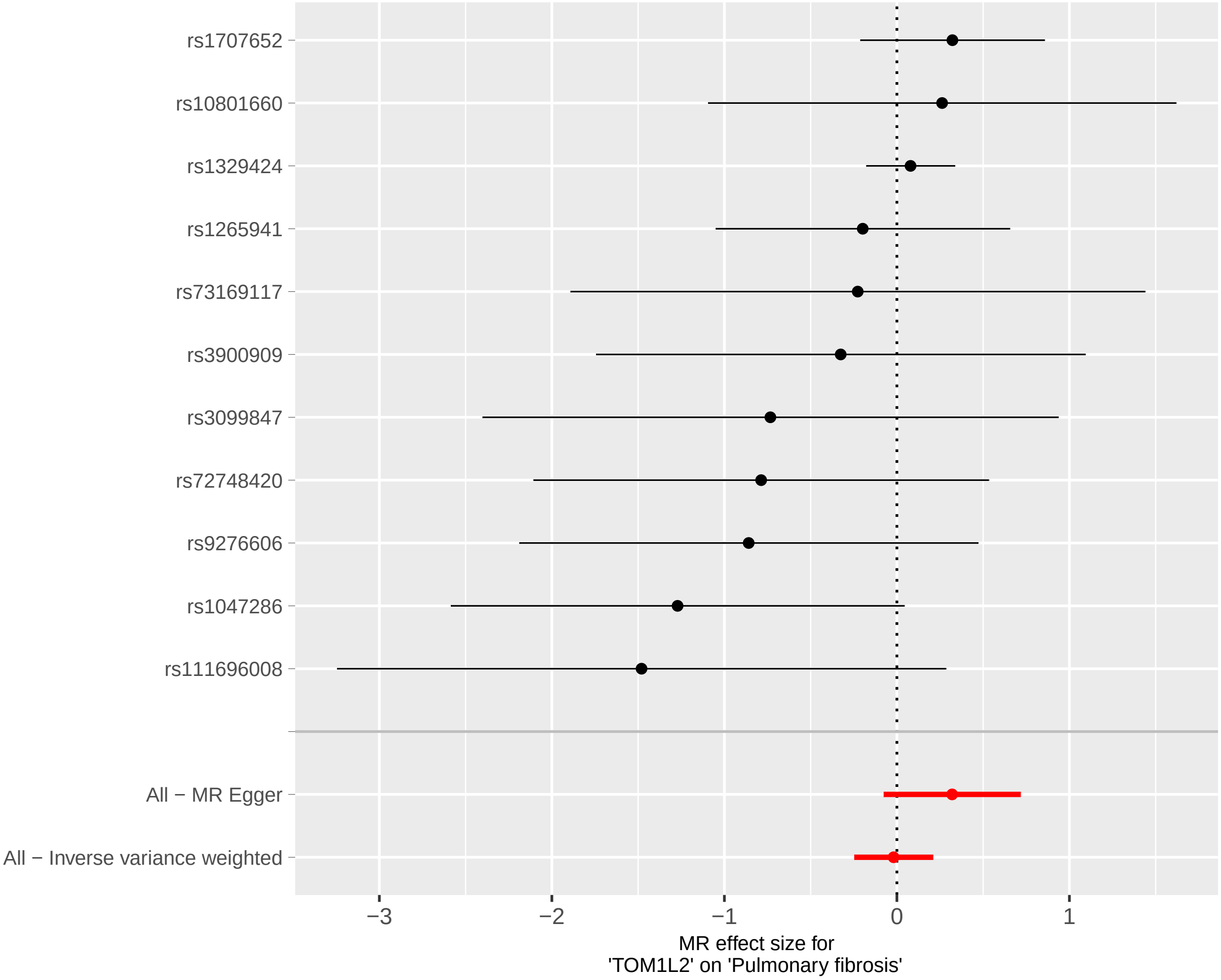

rs1707652
rs10801660
rs1329424
rs1265941
rs73169117
rs3900909
rs3099847
rs72748420
rs9276606
rs1047286
rs111696008
All − MR Egger
All − Inverse variance weighted
−3
−2
−1
0
1
MR effect size for
'TOM1L2' on 'Pulmonary fibrosis'

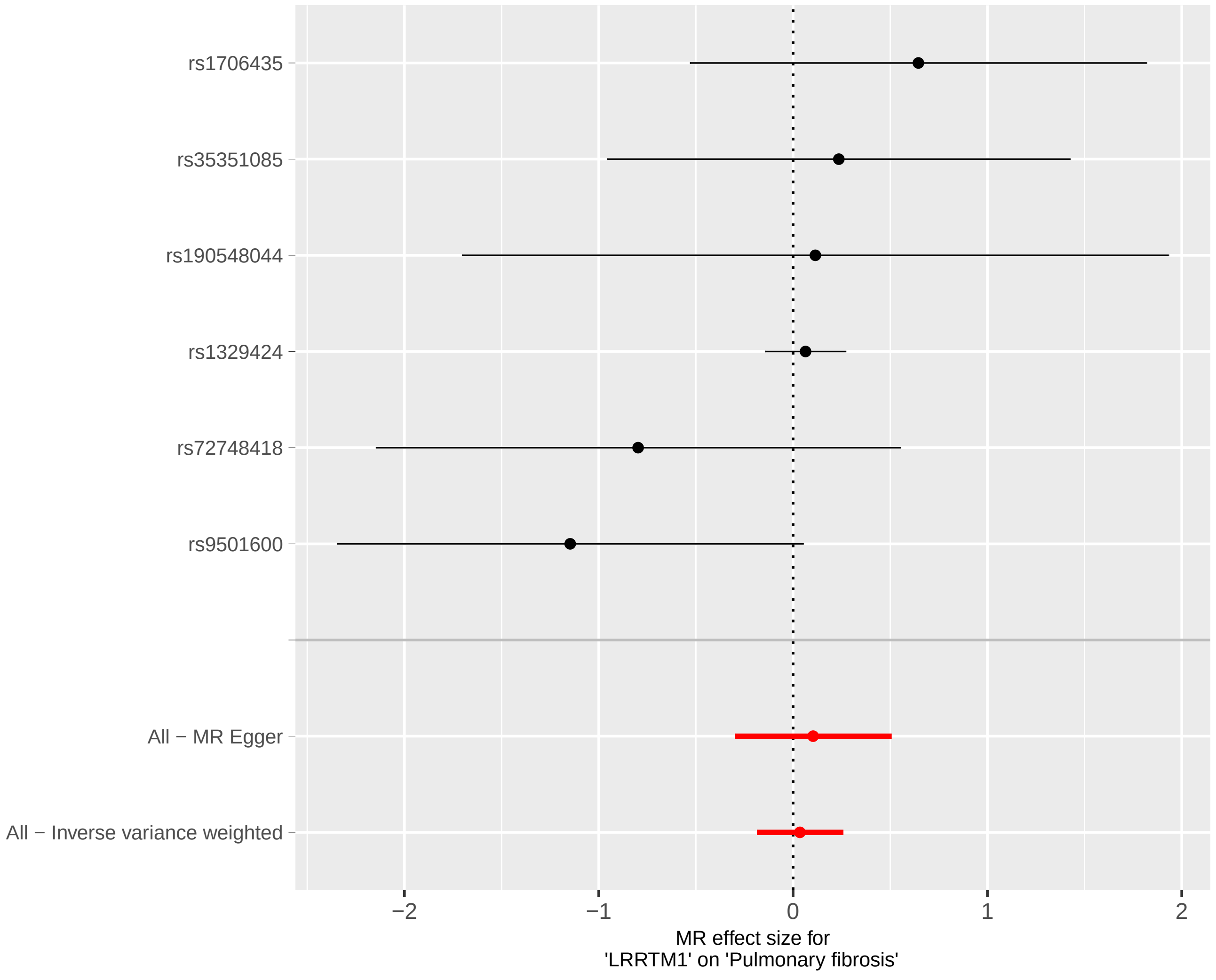

rs1706435
rs35351085
rs190548044
rs1329424
rs72748418
rs9501600
All − MR Egger
All − Inverse variance weighted
−2
−1
0
1
2
MR effect size for
'LRRTM1' on 'Pulmonary fibrosis'

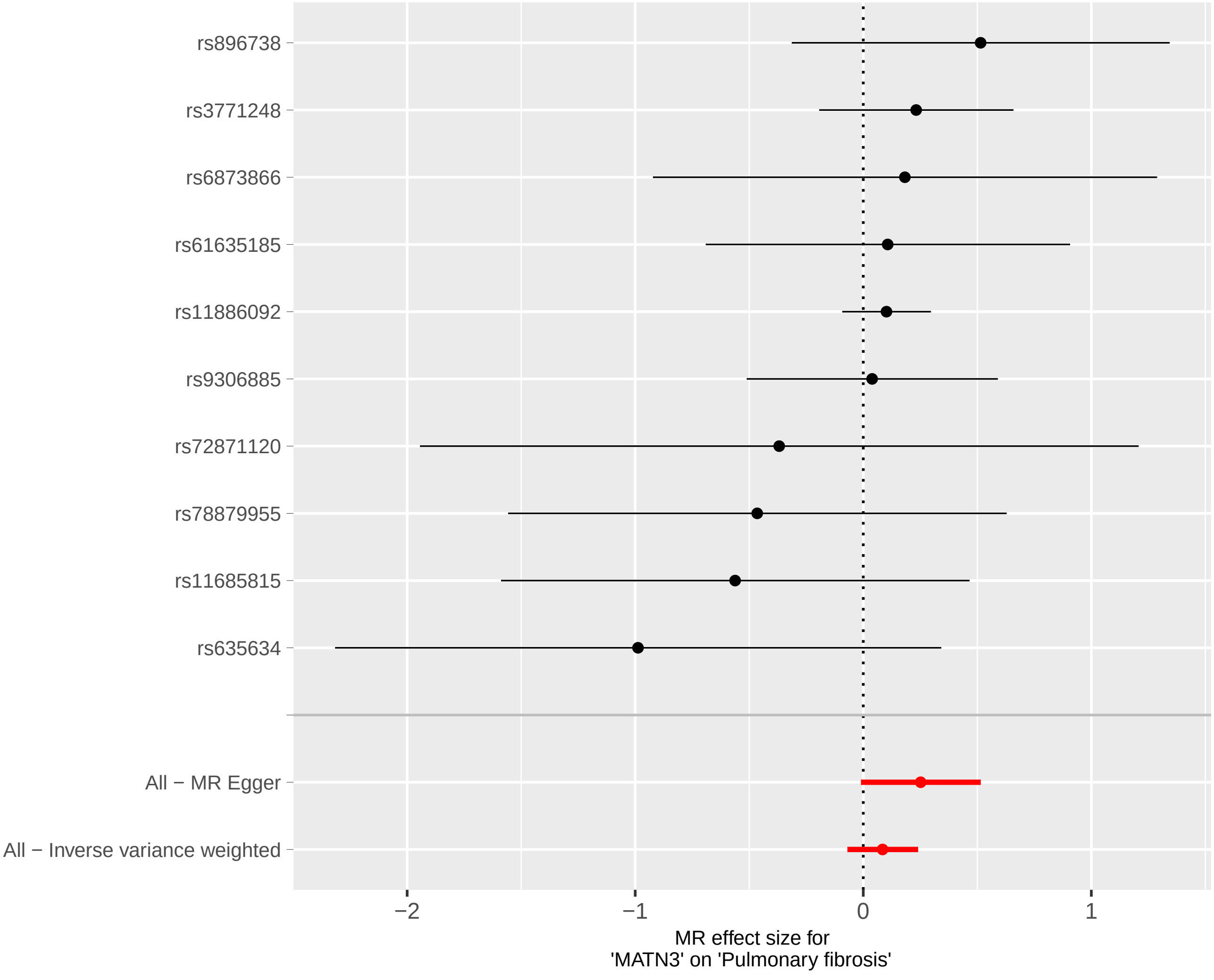

rs896738
rs3771248
rs6873866
rs61635185
rs11886092
rs9306885
rs72871120
rs78879955
rs11685815
rs635634
All − MR Egger
All − Inverse variance weighted
−2
−1
0
1
MR effect size for
'MATN3' on 'Pulmonary fibrosis'

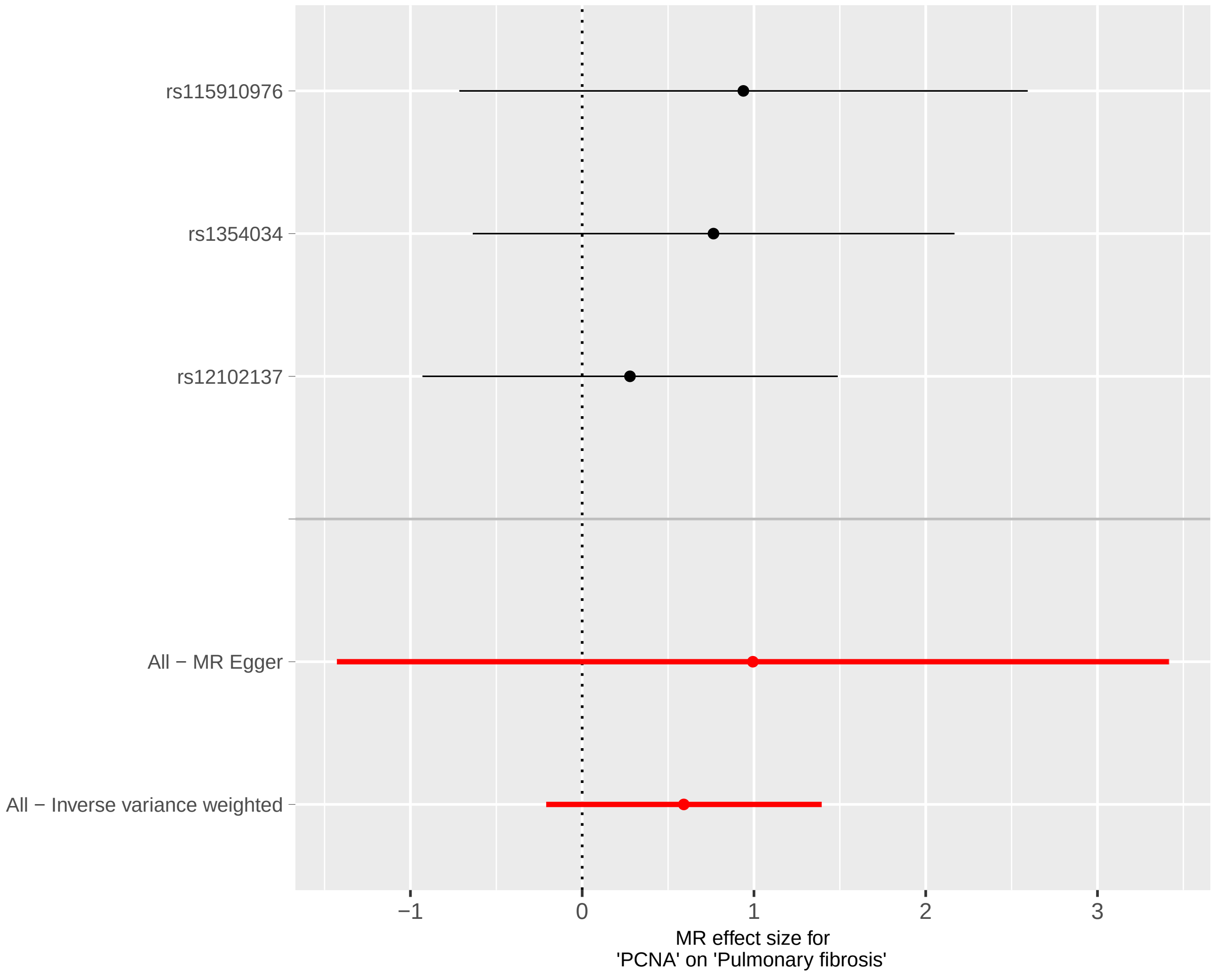
rs115910976
rs1354034
rs12102137
All − MR Egger
All − Inverse variance weighted
−1
0
1
2
3
MR effect size for
'PCNA' on 'Pulmonary fibrosis'

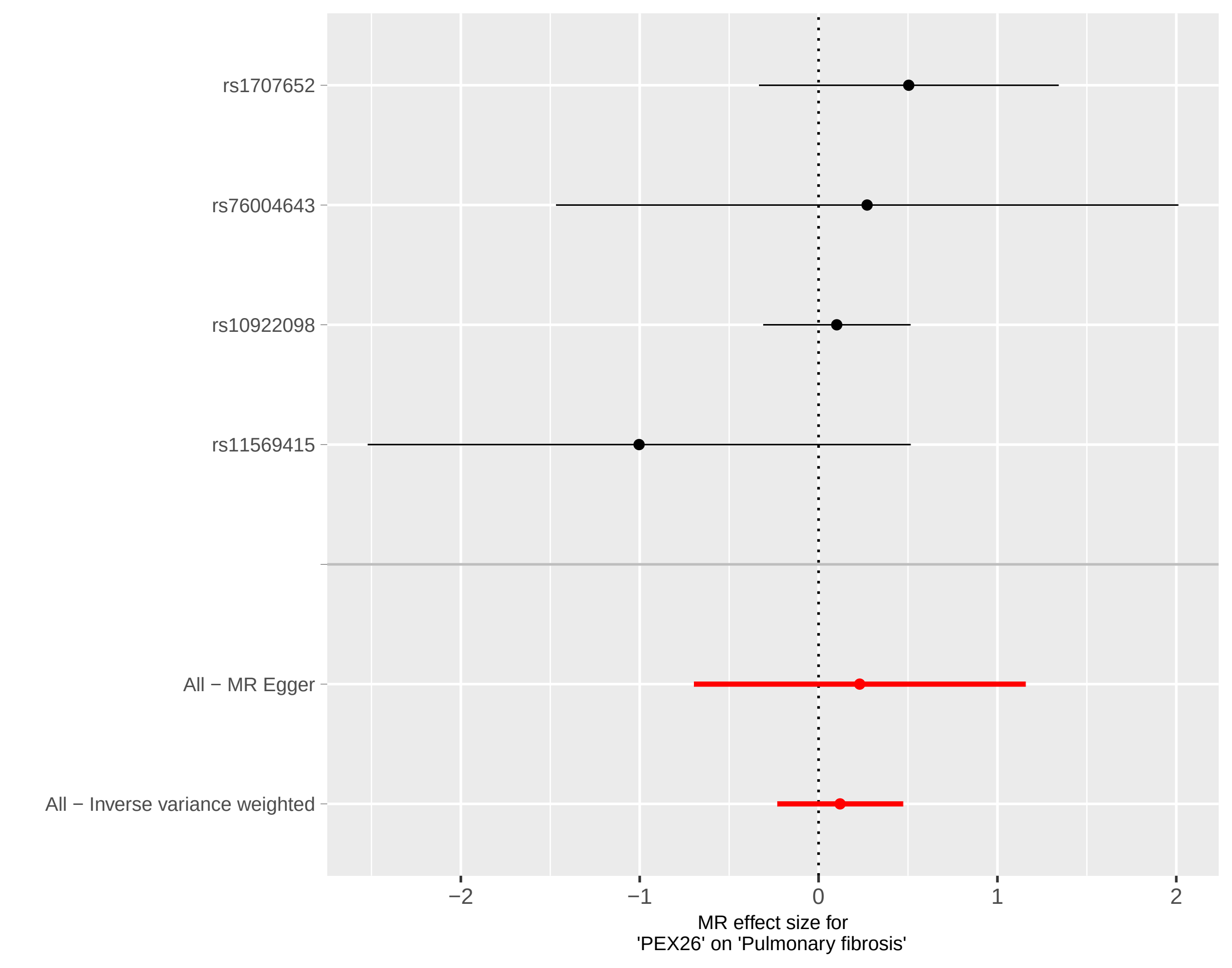

rs1707652
rs76004643
rs10922098
rs11569415
All − MR Egger
All − Inverse variance weighted
−2
−1
0
1
2
MR effect size for
'PEX26' on 'Pulmonary fibrosis'

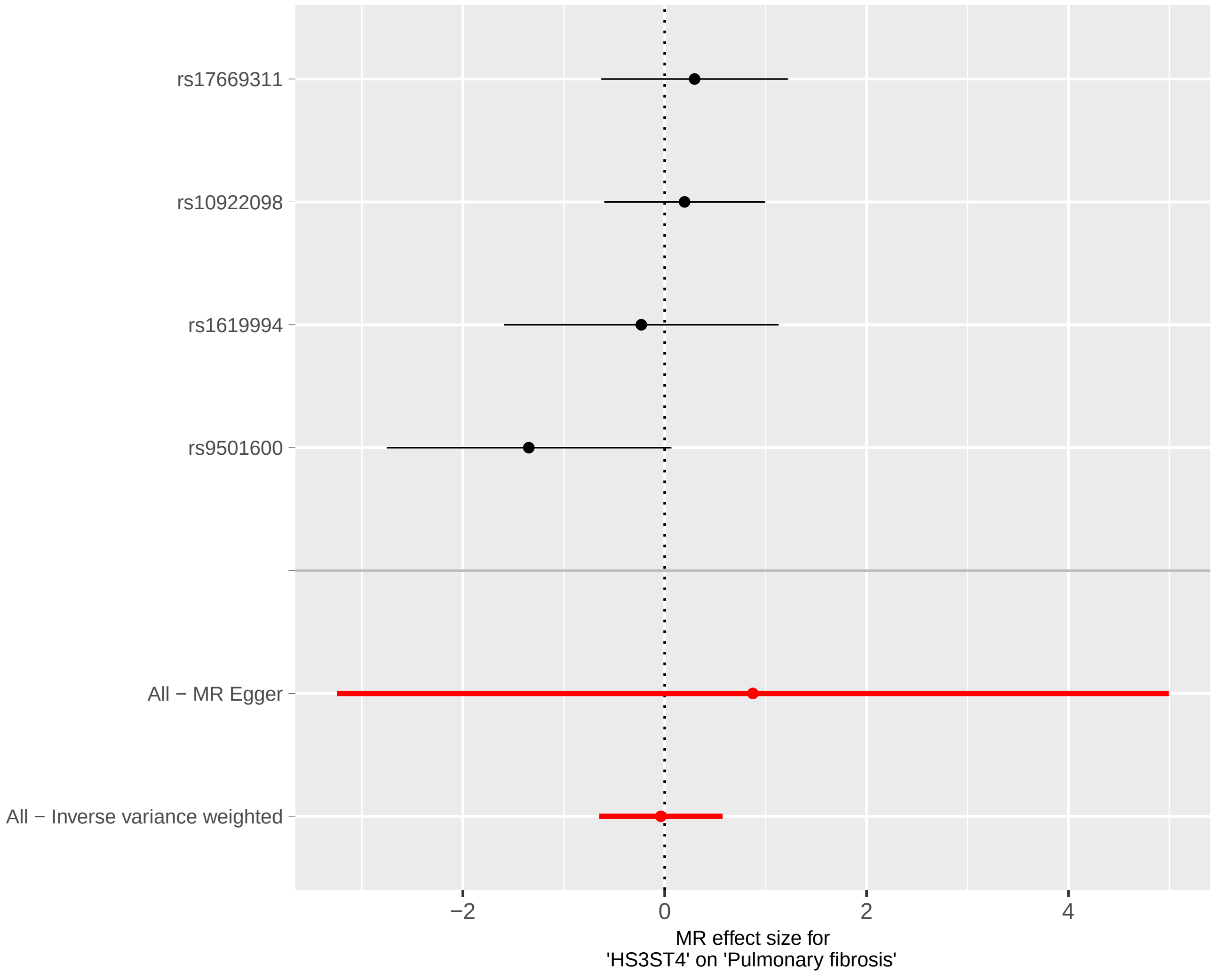
rs17669311
rs10922098
rs1619994
rs9501600
All − MR Egger
All − Inverse variance weighted
−2
0
2
4
MR effect size for
'HS3ST4' on 'Pulmonary fibrosis'

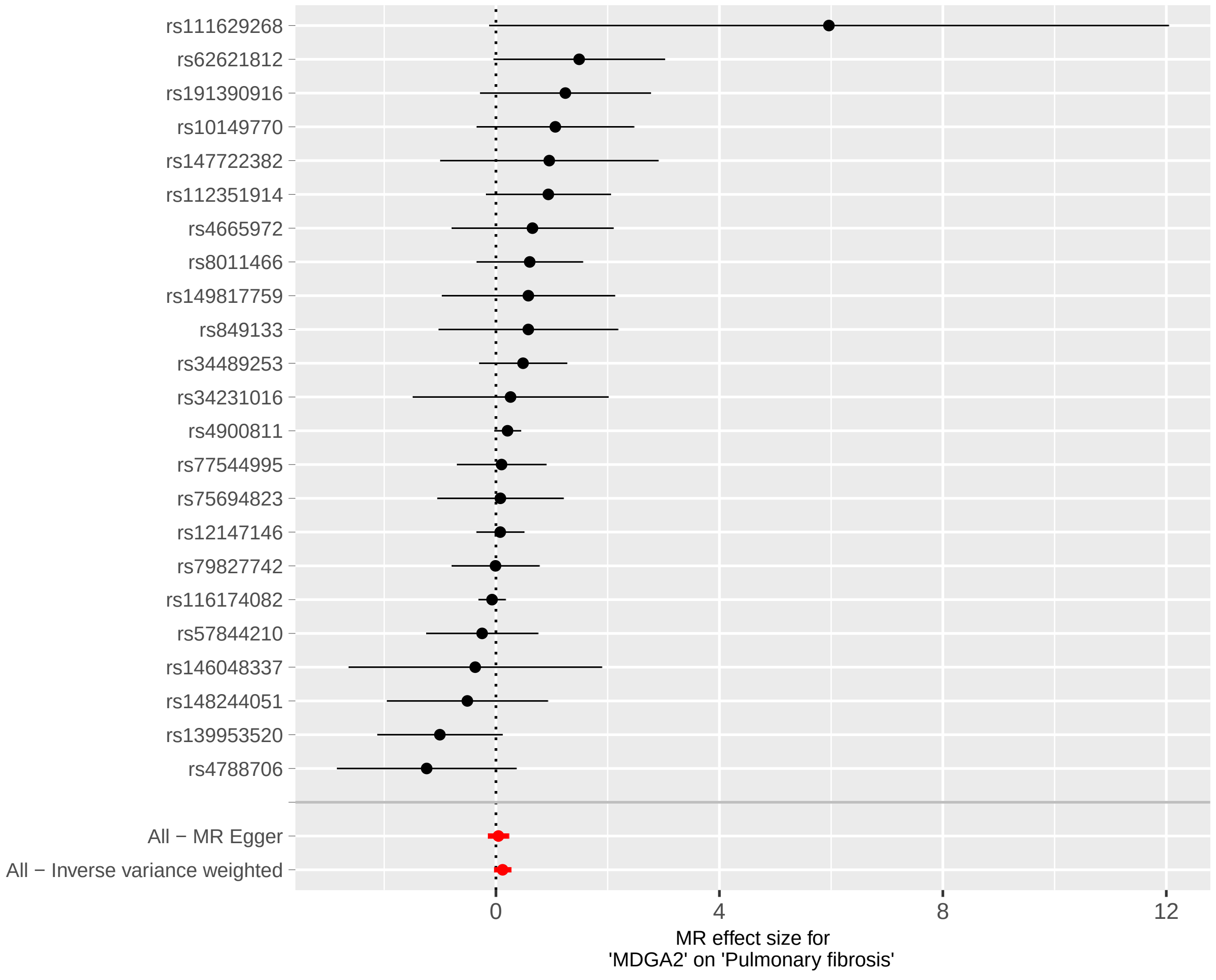
rs111629268
rs62621812
rs191390916
rs10149770
rs147722382
rs112351914
rs4665972
rs8011466
rs149817759
rs849133
rs34489253
rs34231016
rs4900811
rs77544995
rs75694823
rs12147146
rs79827742
rs116174082
rs57844210
rs146048337
rs148244051
rs139953520
rs4788706
All − MR Egger
All − Inverse variance weighted
0
4
8
12
MR effect size for
'MDGA2' on 'Pulmonary fibrosis'

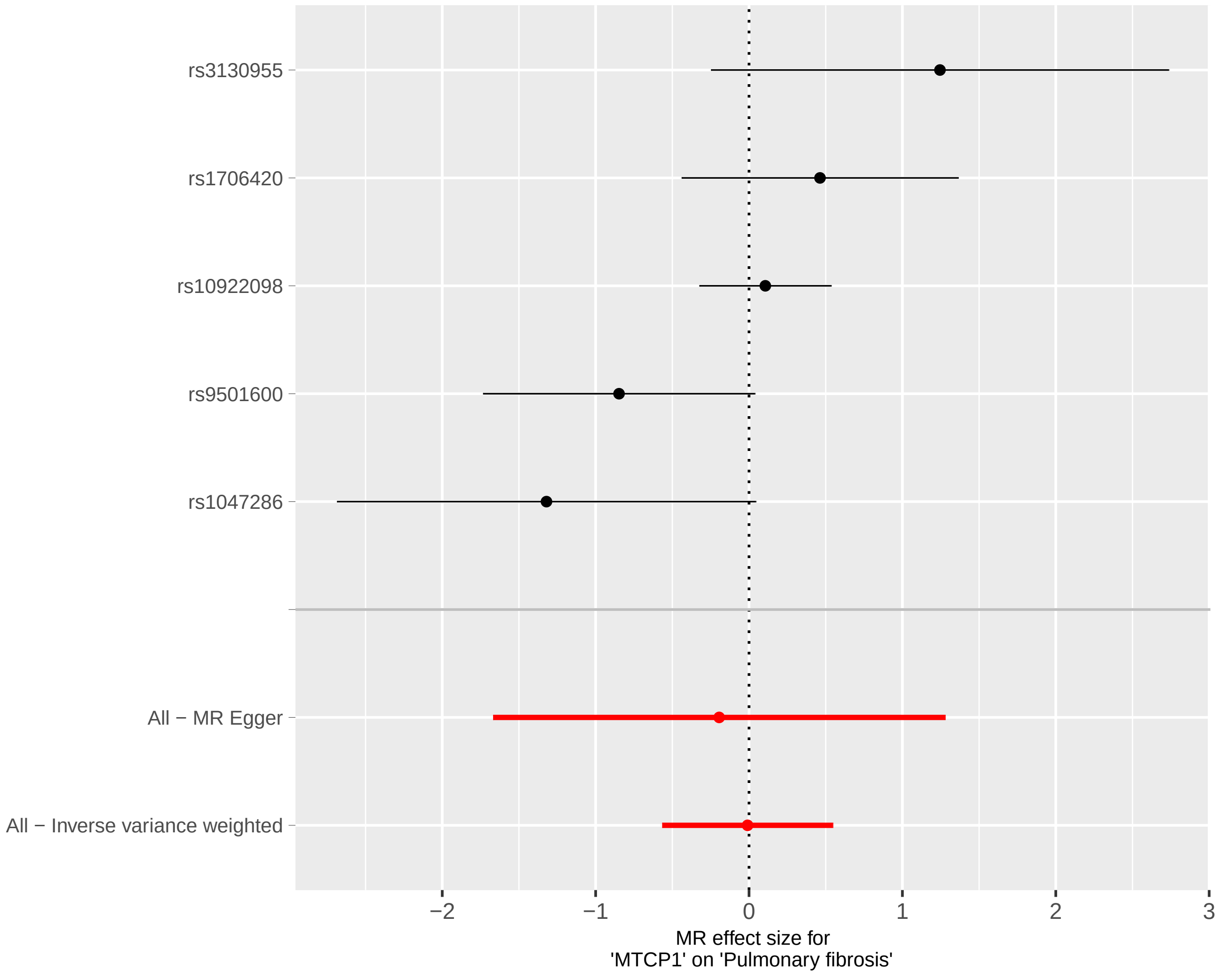
rs3130955
rs1706420
rs10922098
rs9501600
rs1047286
All − MR Egger
All − Inverse variance weighted
−2
−1
0
1
2
3
MR effect size for
'MTCP1' on 'Pulmonary fibrosis'

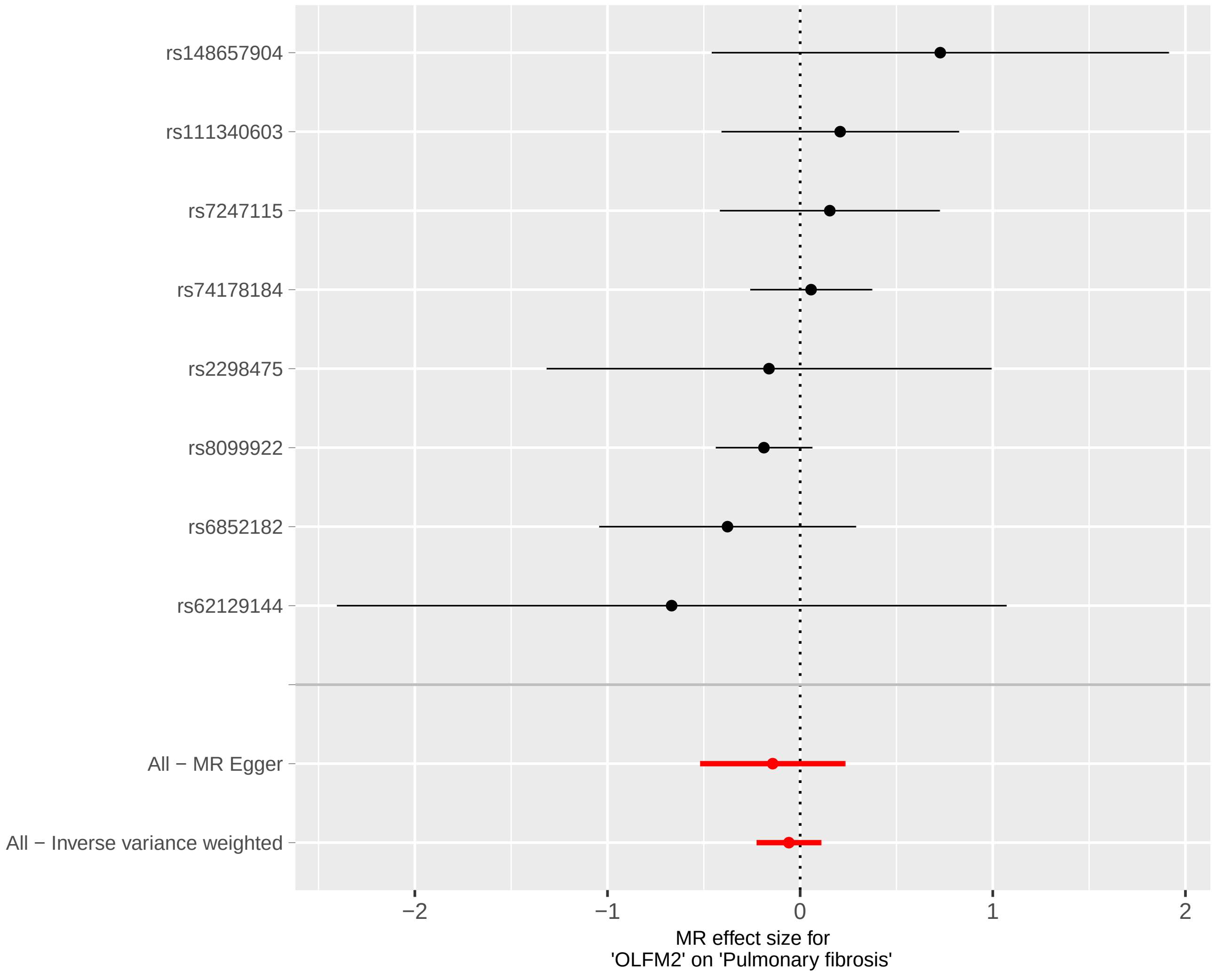

rs148657904
rs111340603
rs7247115
rs74178184
rs2298475
rs8099922
rs6852182
rs62129144
All − MR Egger
All − Inverse variance weighted
−2
−1
0
1
2
MR effect size for
'OLFM2' on 'Pulmonary fibrosis'

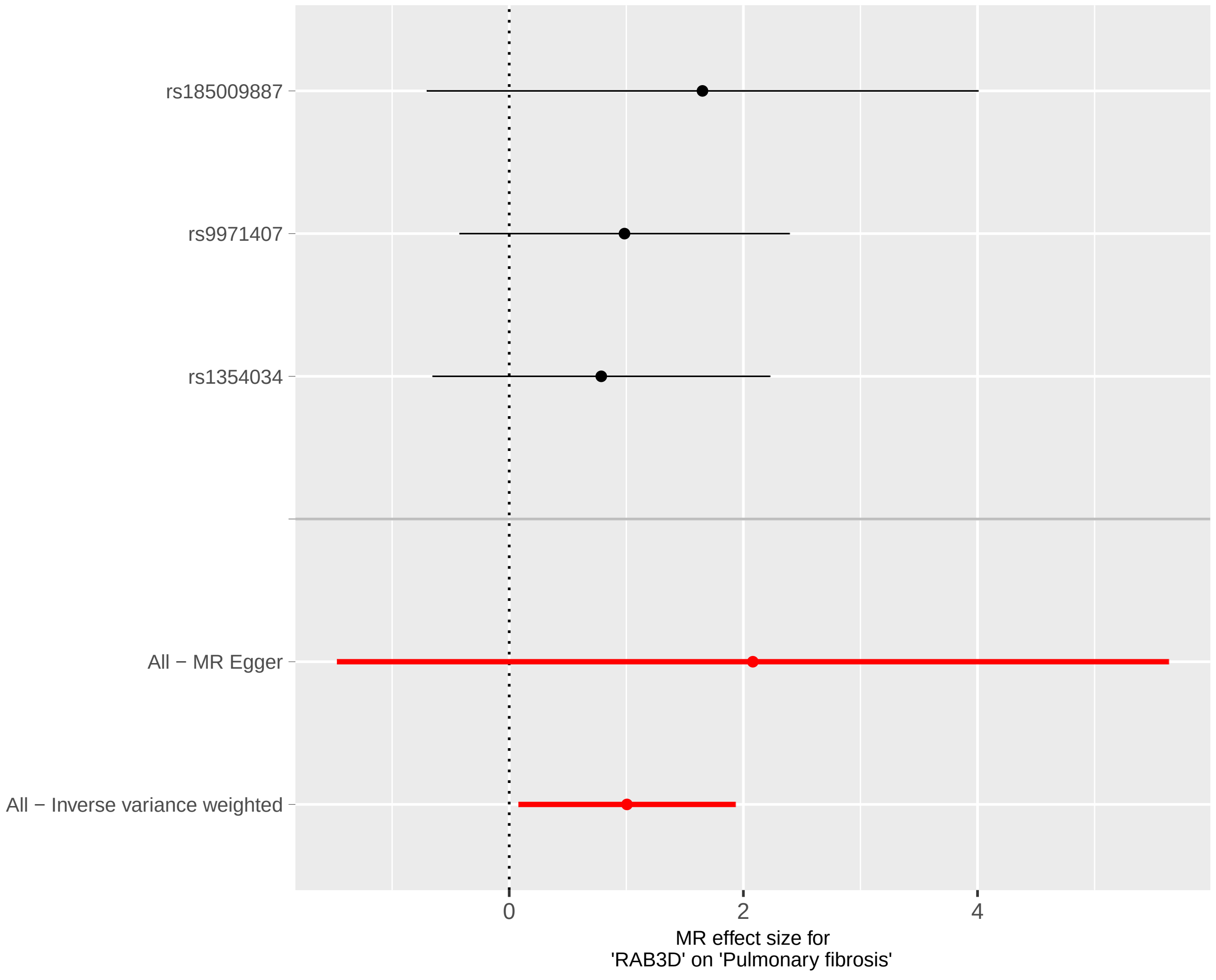
rs185009887
rs9971407
rs1354034
All − MR Egger
All − Inverse variance weighted
0
2
4
MR effect size for
'RAB3D' on 'Pulmonary fibrosis'

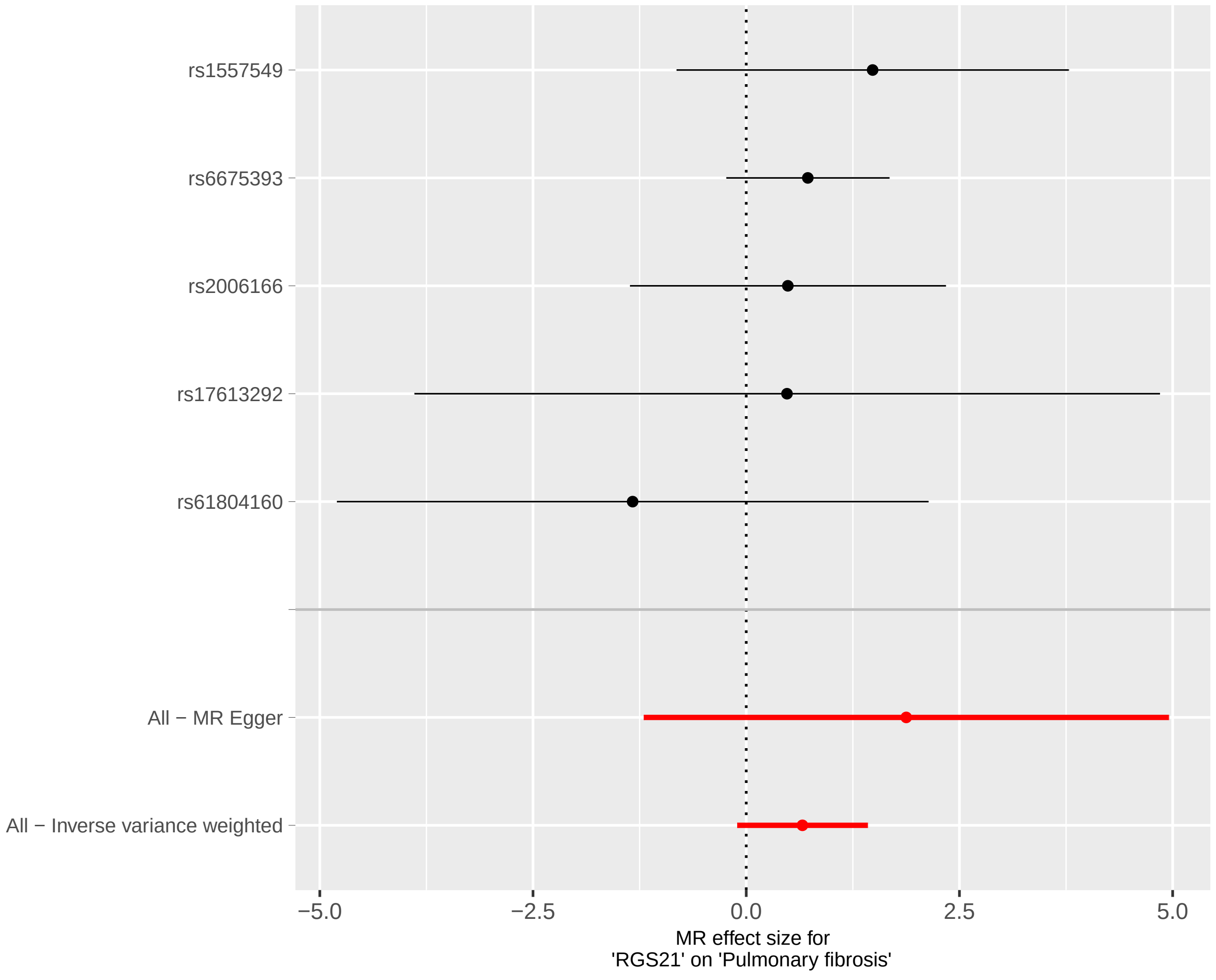
rs1557549
rs6675393
rs2006166
rs17613292
rs61804160
All − MR Egger
All − Inverse variance weighted
−5.0
−2.5
0.0
2.5
5.0
MR effect size for
'RGS21' on 'Pulmonary fibrosis'

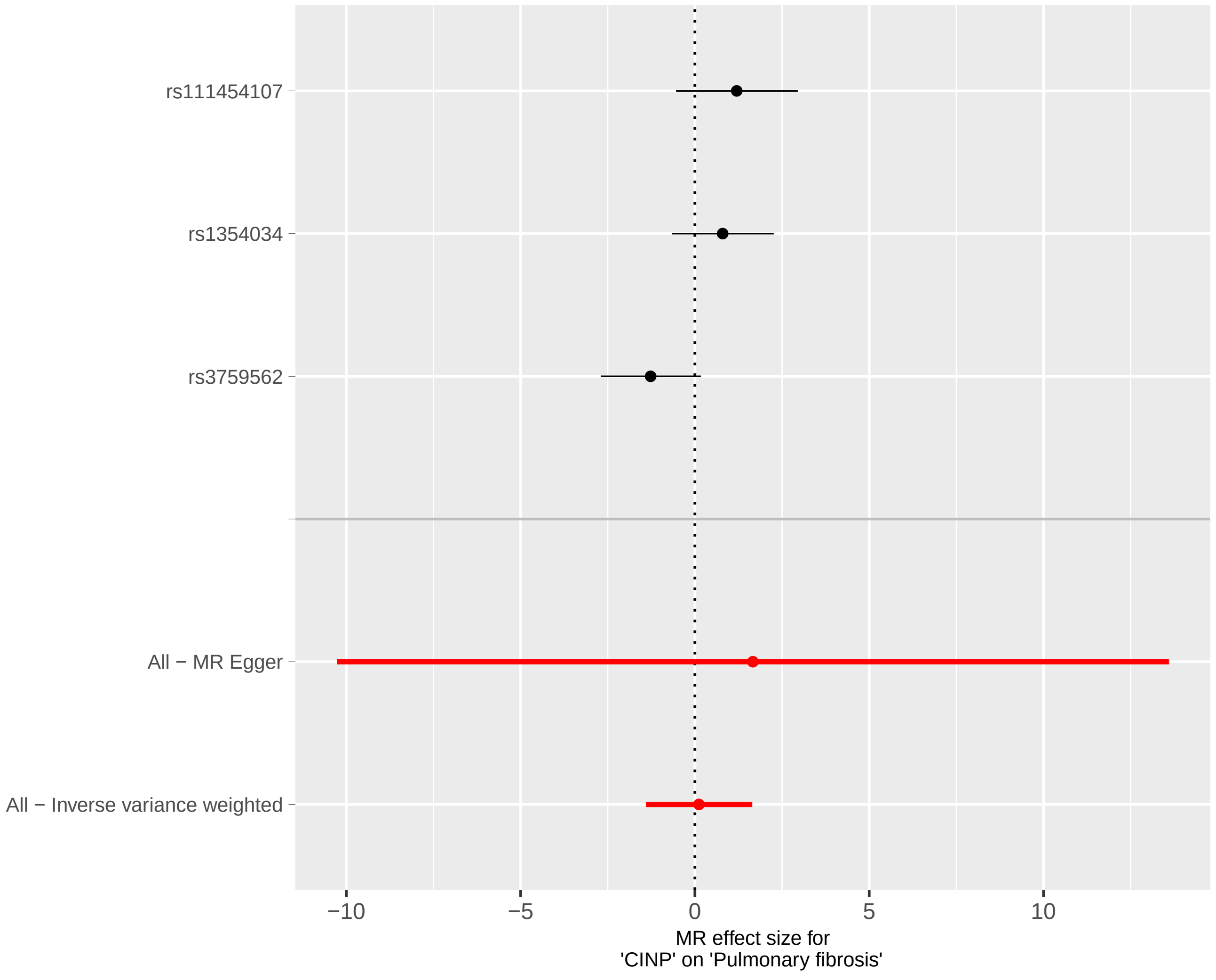

rs111454107
rs1354034
rs3759562
All − MR Egger
All − Inverse variance weighted
−10
−5
0
5
10
MR effect size for
'CINP' on 'Pulmonary fibrosis'

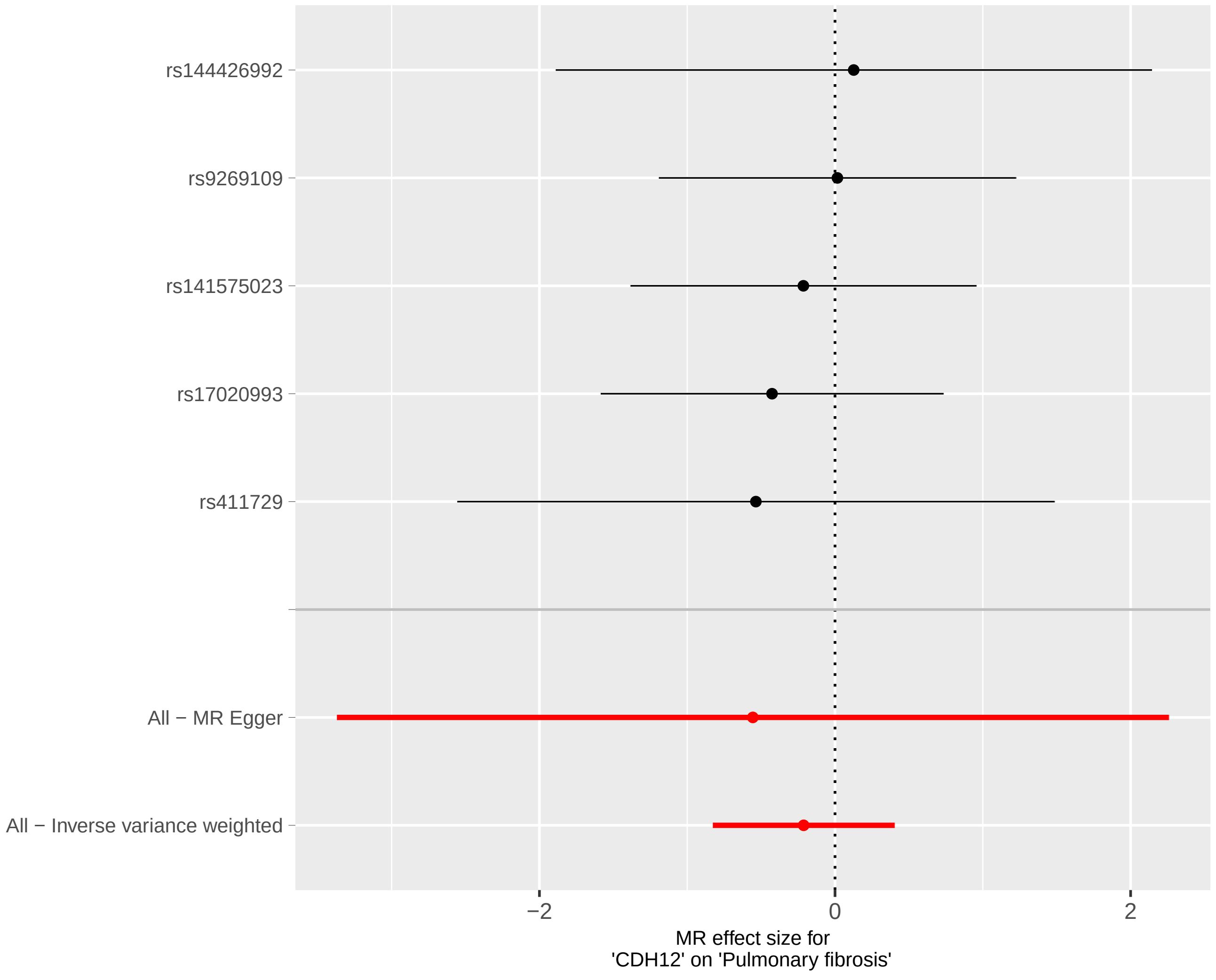

rs144426992
rs9269109
rs141575023
rs17020993
rs411729
All − MR Egger
All − Inverse variance weighted
−2
0
2
MR effect size for
'CDH12' on 'Pulmonary fibrosis'

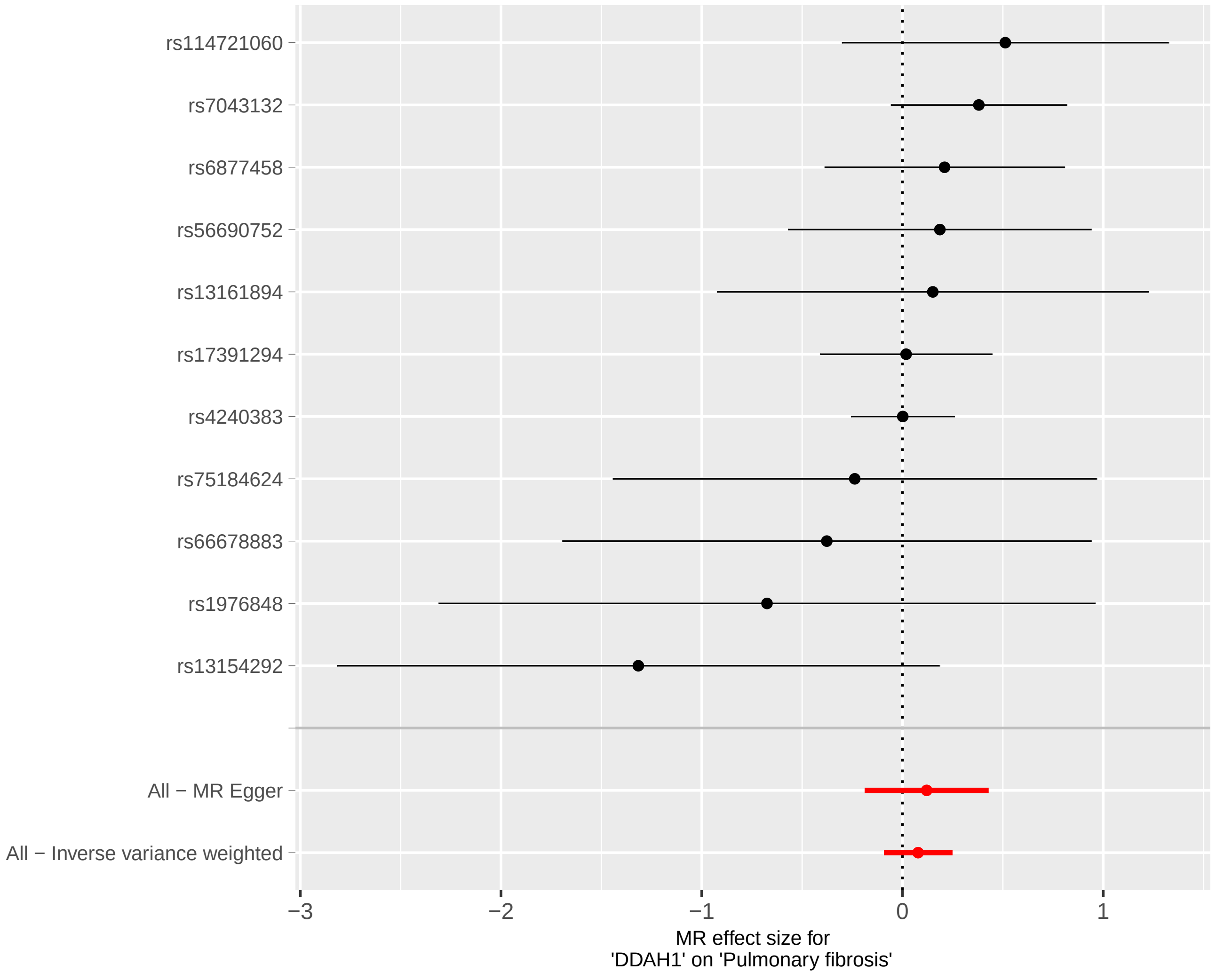

rs114721060
rs7043132
rs6877458
rs56690752
rs13161894
rs17391294
rs4240383
rs75184624
rs66678883
rs1976848
rs13154292
All − MR Egger
All − Inverse variance weighted
−3
−2
−1
0
1
MR effect size for
'DDAH1' on 'Pulmonary fibrosis'

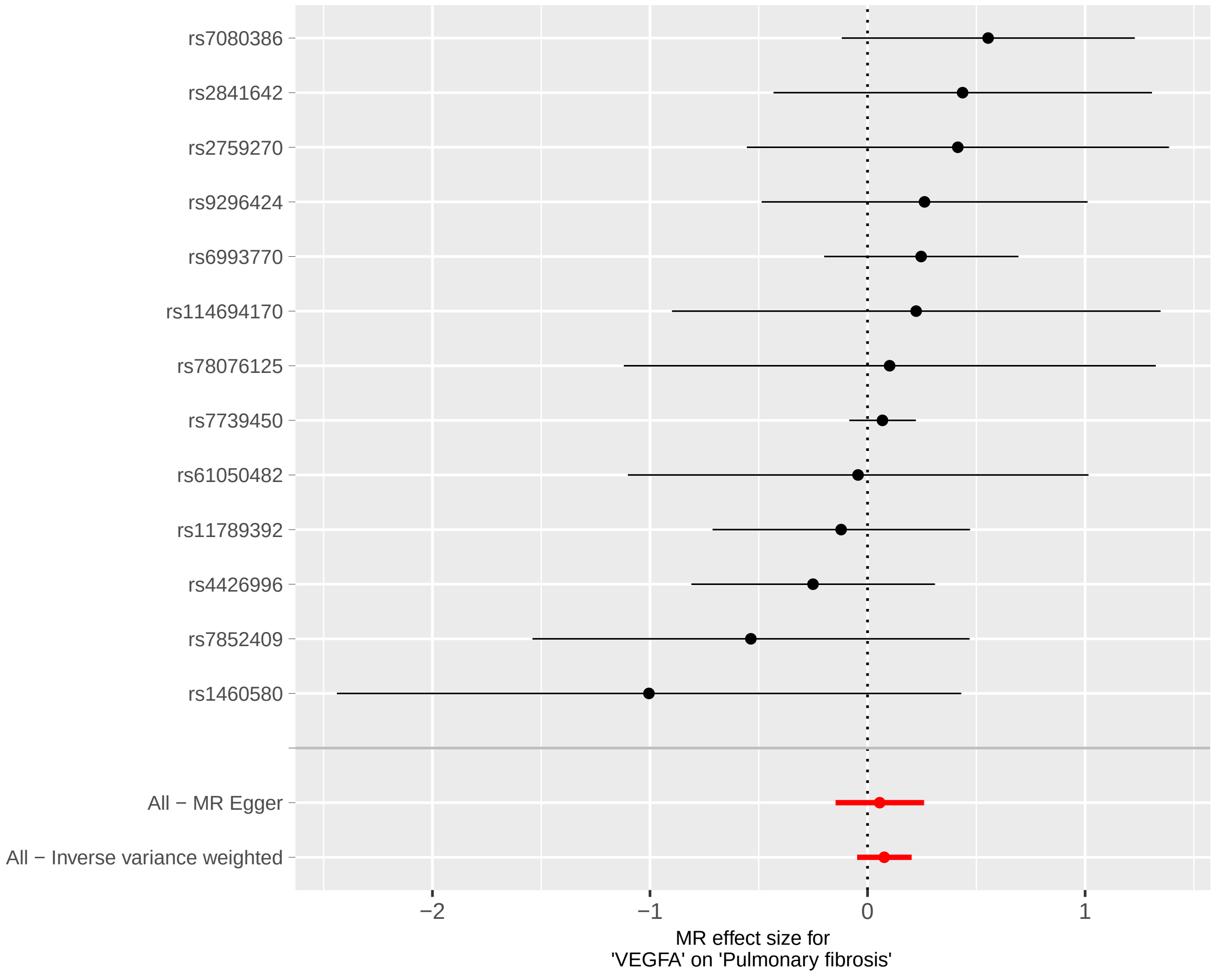

rs7080386
rs2841642
rs2759270
rs9296424
rs6993770
rs114694170
rs78076125
rs7739450
rs61050482
rs11789392
rs4426996
rs7852409
rs1460580
All − MR Egger
All − Inverse variance weighted
−2
−1
0
1
MR effect size for
'VEGFA' on 'Pulmonary fibrosis'

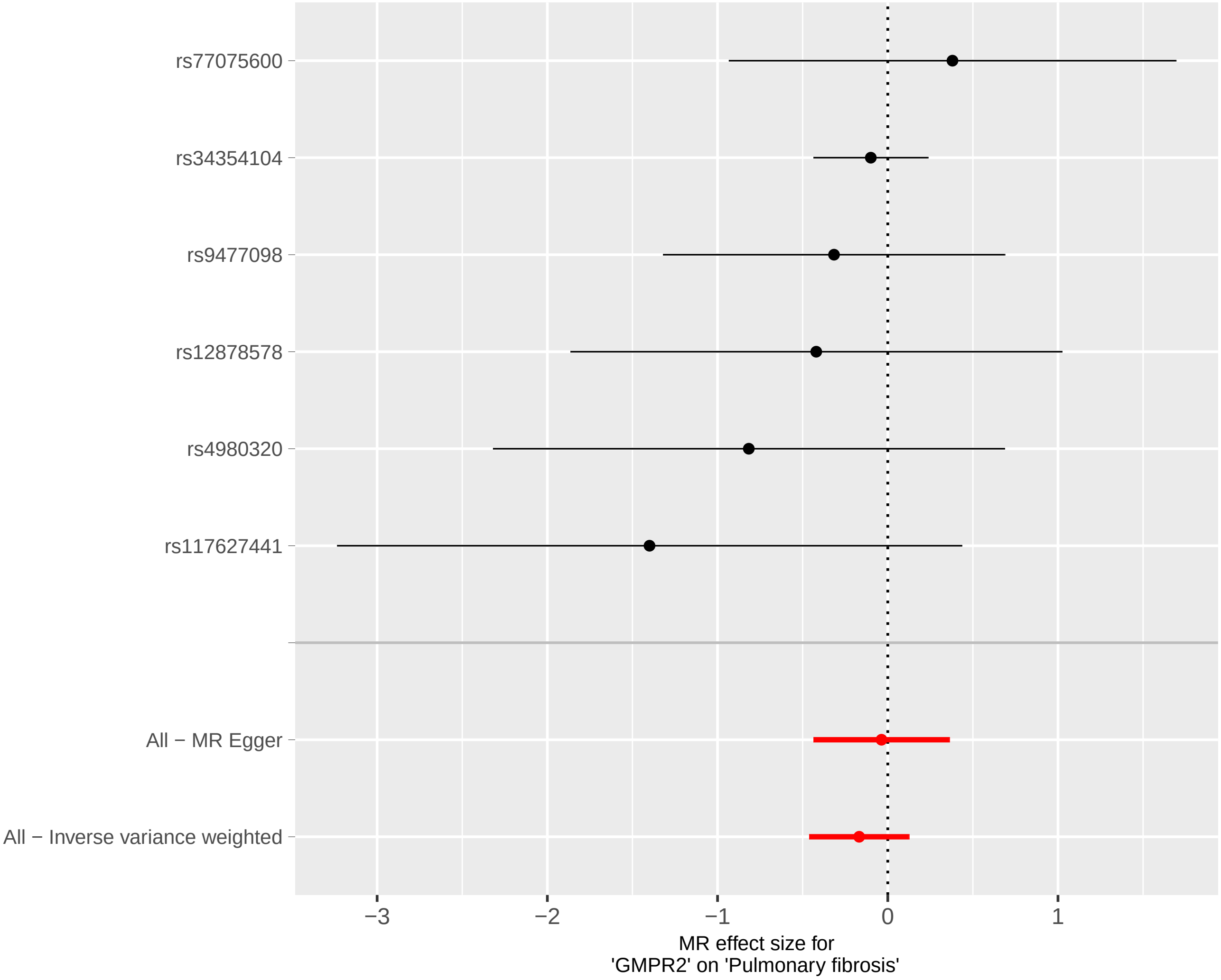
rs77075600
rs34354104
rs9477098
rs12878578
rs4980320
rs117627441
All − MR Egger
All − Inverse variance weighted
−3
−2
−1
0
1
MR effect size for
'GMPR2' on 'Pulmonary fibrosis'

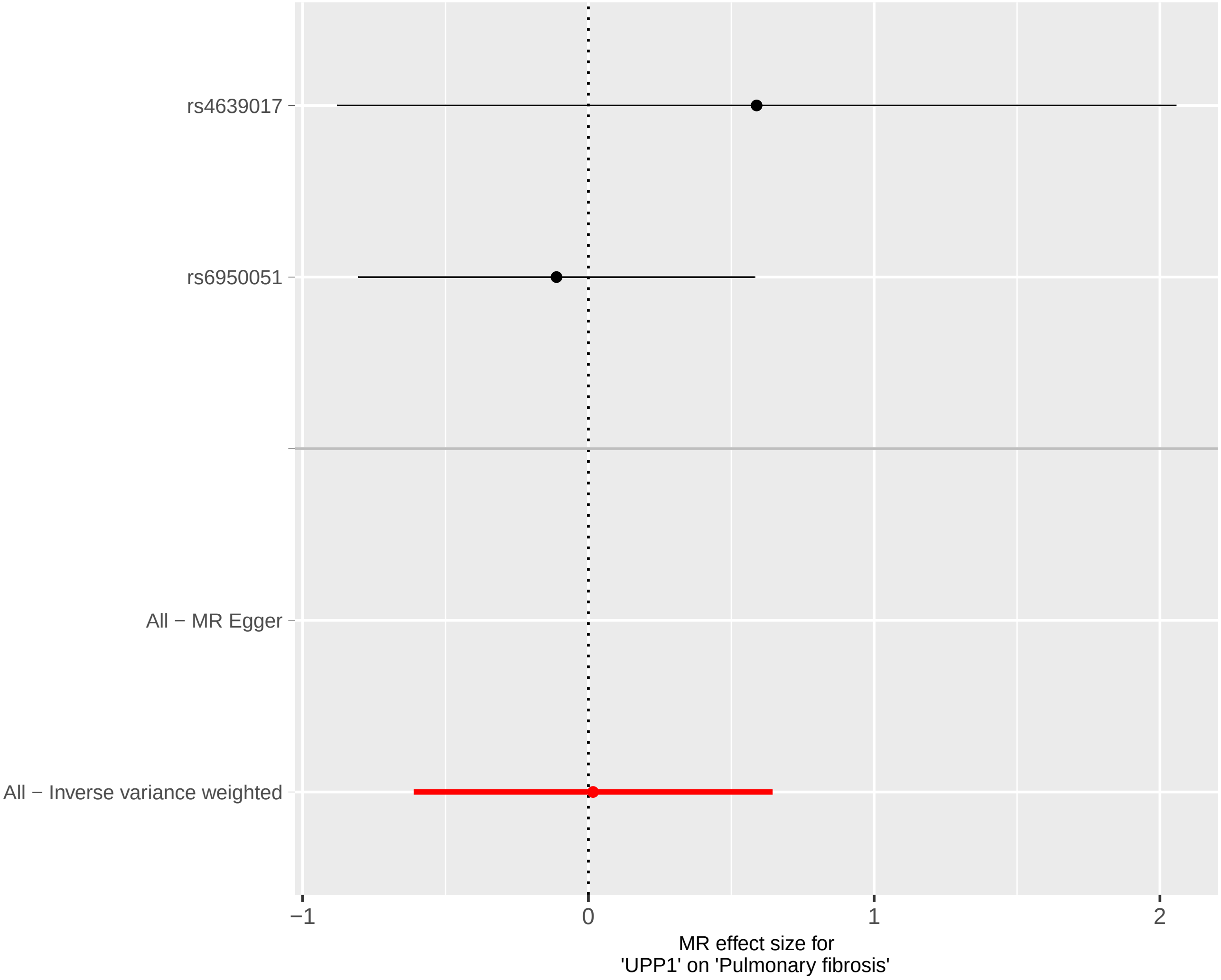

rs4639017
rs6950051
All − MR Egger
All − Inverse variance weighted
−1
0
1
2
MR effect size for
'UPP1' on 'Pulmonary fibrosis'

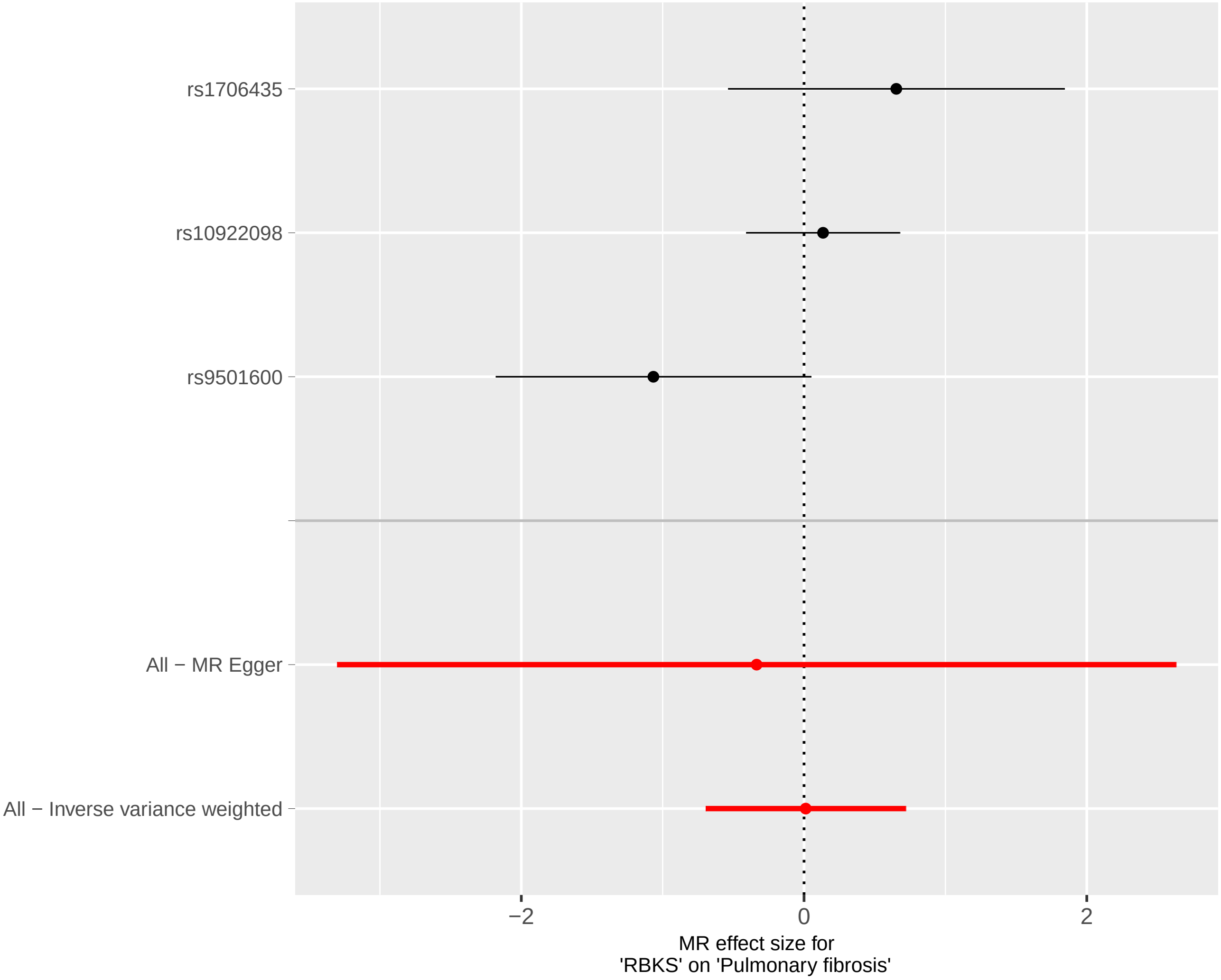
rs1706435
rs10922098
rs9501600
All − MR Egger
All − Inverse variance weighted
−2
0
2
MR effect size for
'RBKS' on 'Pulmonary fibrosis'

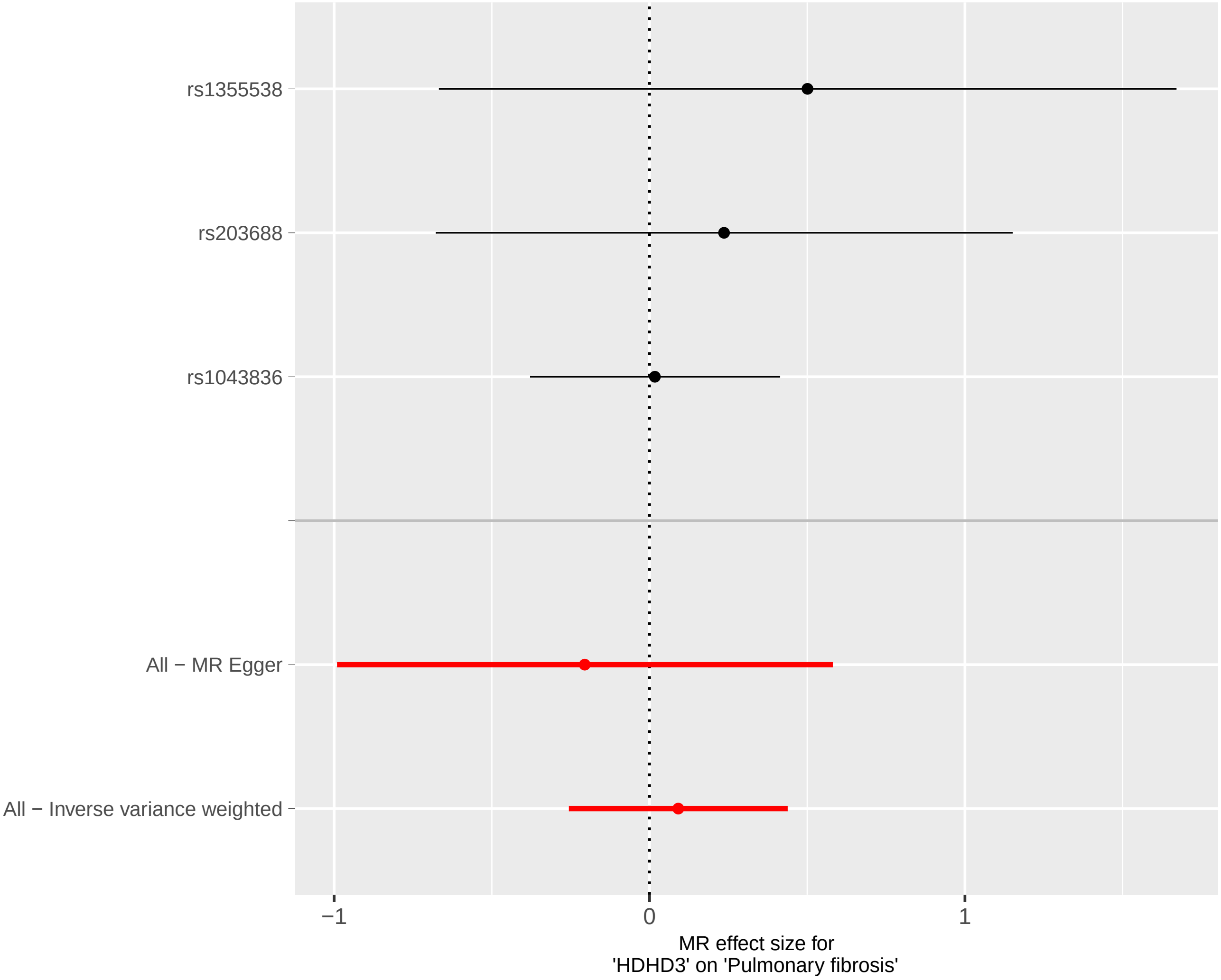

rs1355538
rs203688
rs1043836
All − MR Egger
All − Inverse variance weighted
−1
0
1
MR effect size for
'HDHD3' on 'Pulmonary fibrosis'

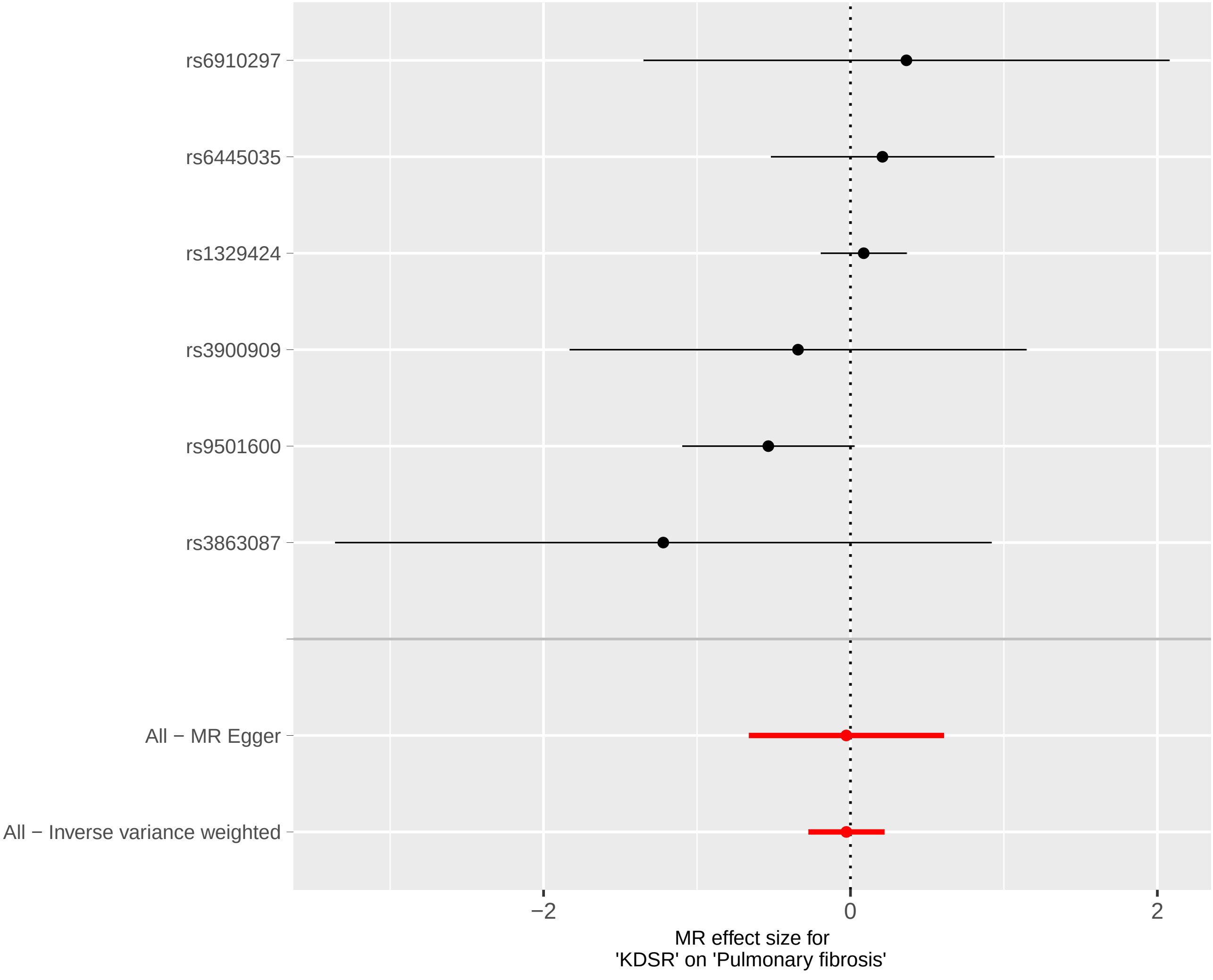

rs6910297
rs6445035
rs1329424
rs3900909
rs9501600
rs3863087
All − MR Egger
All − Inverse variance weighted
−2
0
2
MR effect size for
'KDSR' on 'Pulmonary fibrosis'

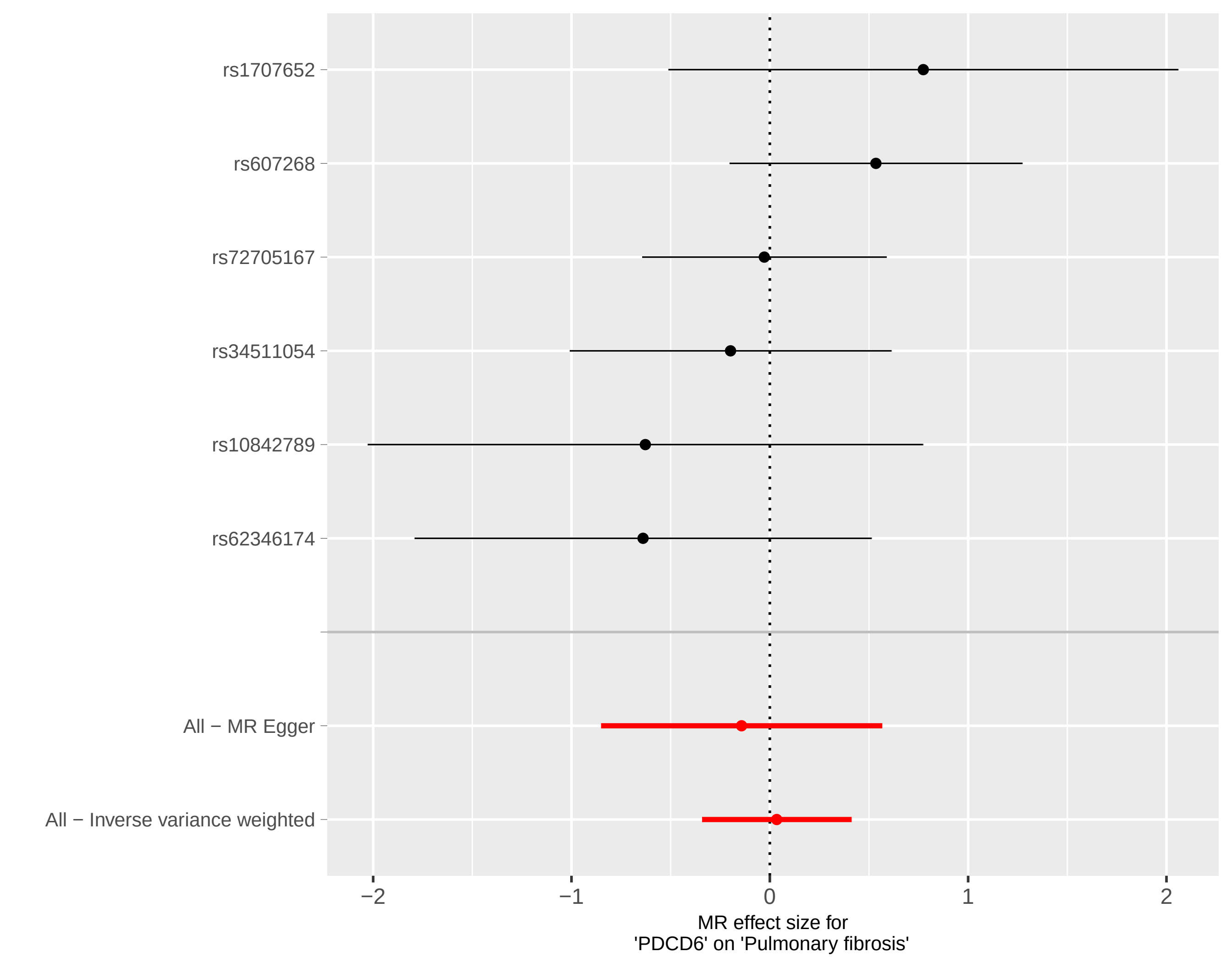

rs1707652
rs607268
rs72705167
rs34511054
rs10842789
rs62346174
All − MR Egger
All − Inverse variance weighted
−2
−1
0
1
2
MR effect size for
'PDCD6' on 'Pulmonary fibrosis'

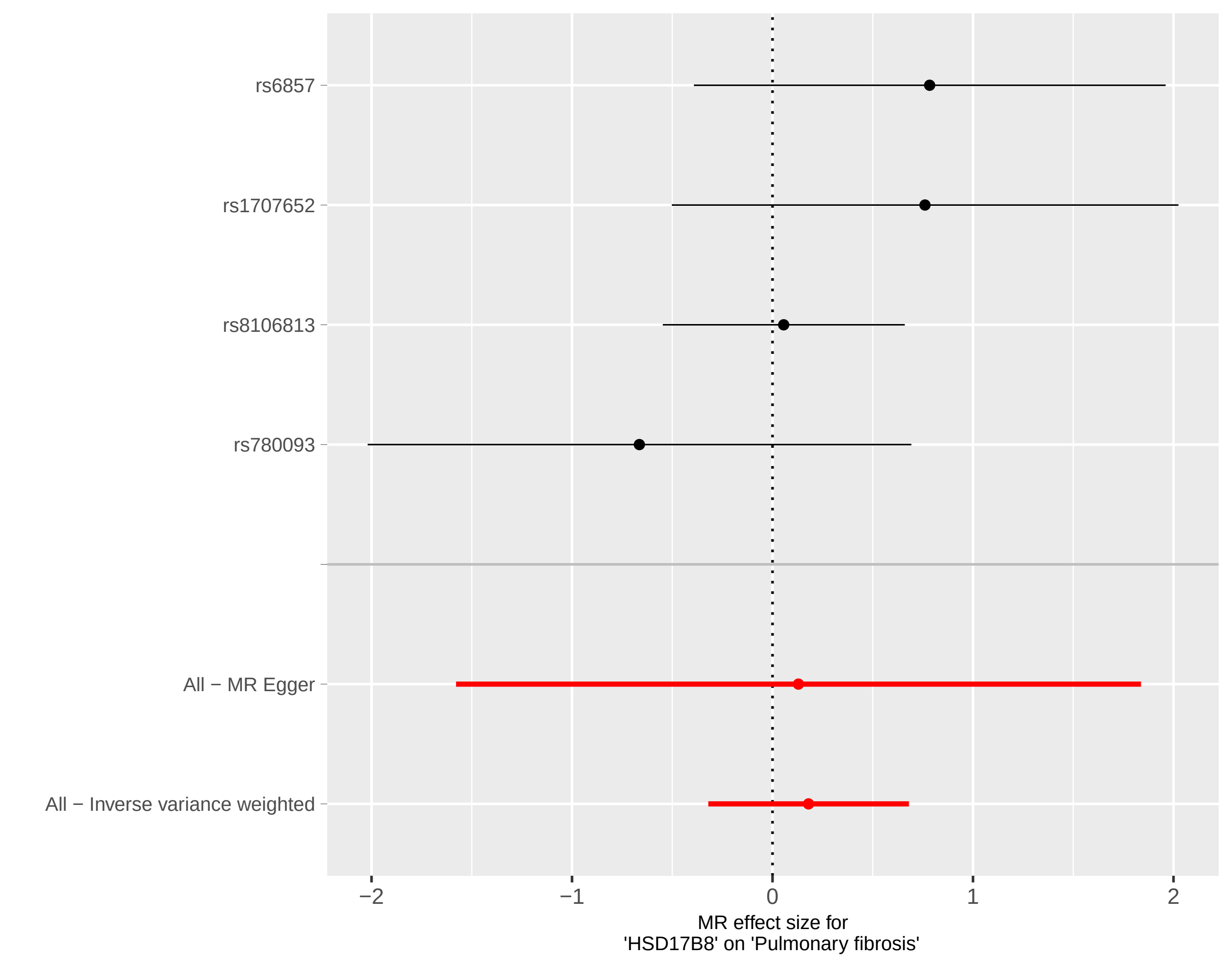
rs6857
rs1707652
rs8106813
rs780093
All − MR Egger
All − Inverse variance weighted
−2
−1
0
1
2
MR effect size for
'HSD17B8' on 'Pulmonary fibrosis'

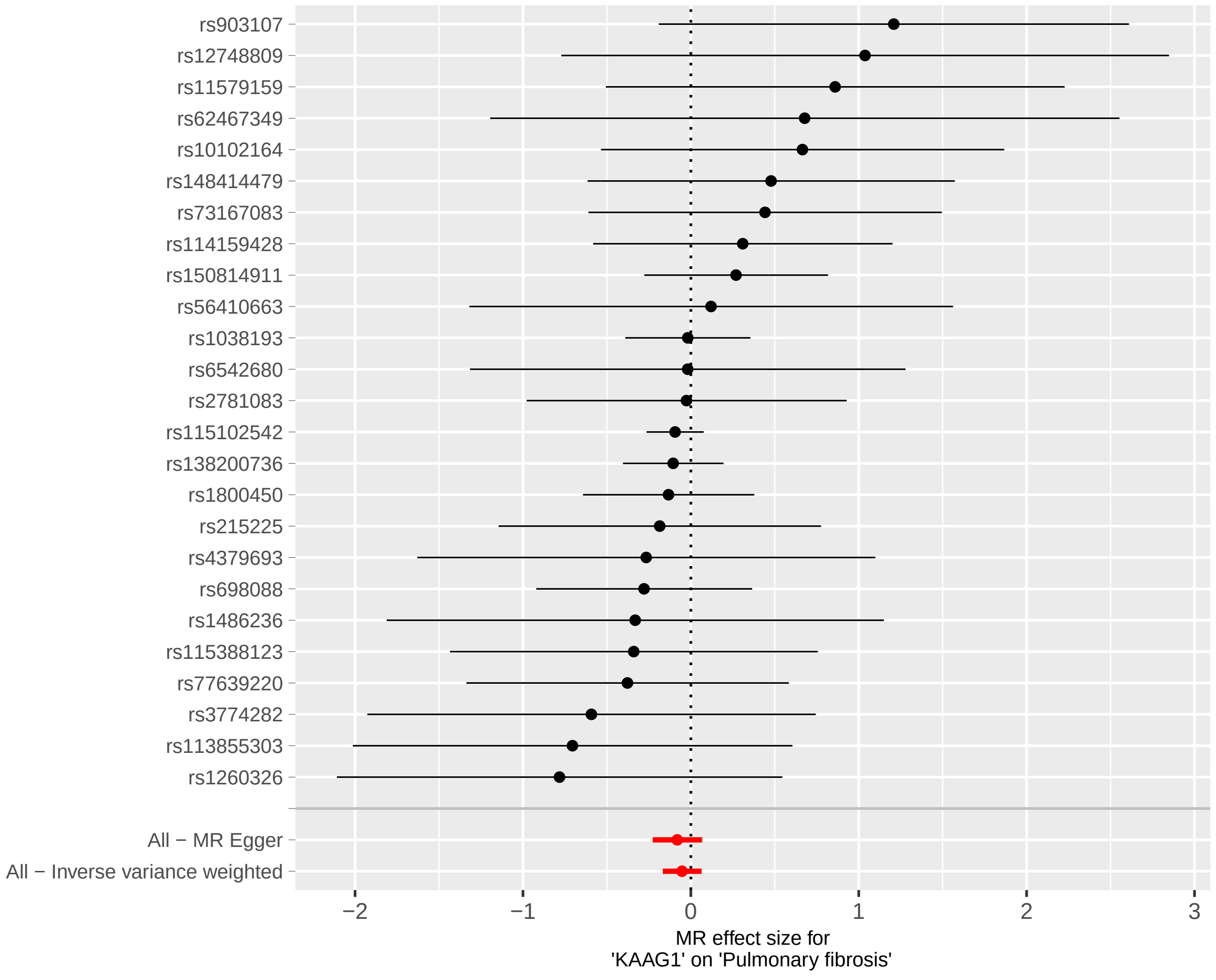
rs903107
rs12748809
rs11579159
rs62467349
rs10102164
rs148414479
rs73167083
rs114159428
rs150814911
rs56410663
rs1038193
rs6542680
rs2781083
rs115102542
rs138200736
rs1800450
rs215225
rs4379693
rs698088
rs1486236
rs115388123
rs77639220
rs3774282
rs113855303
rs1260326
All − MR Egger
All − Inverse variance weighted
−2
−1
0
1
2
3
MR effect size for
'KAAG1' on 'Pulmonary fibrosis'

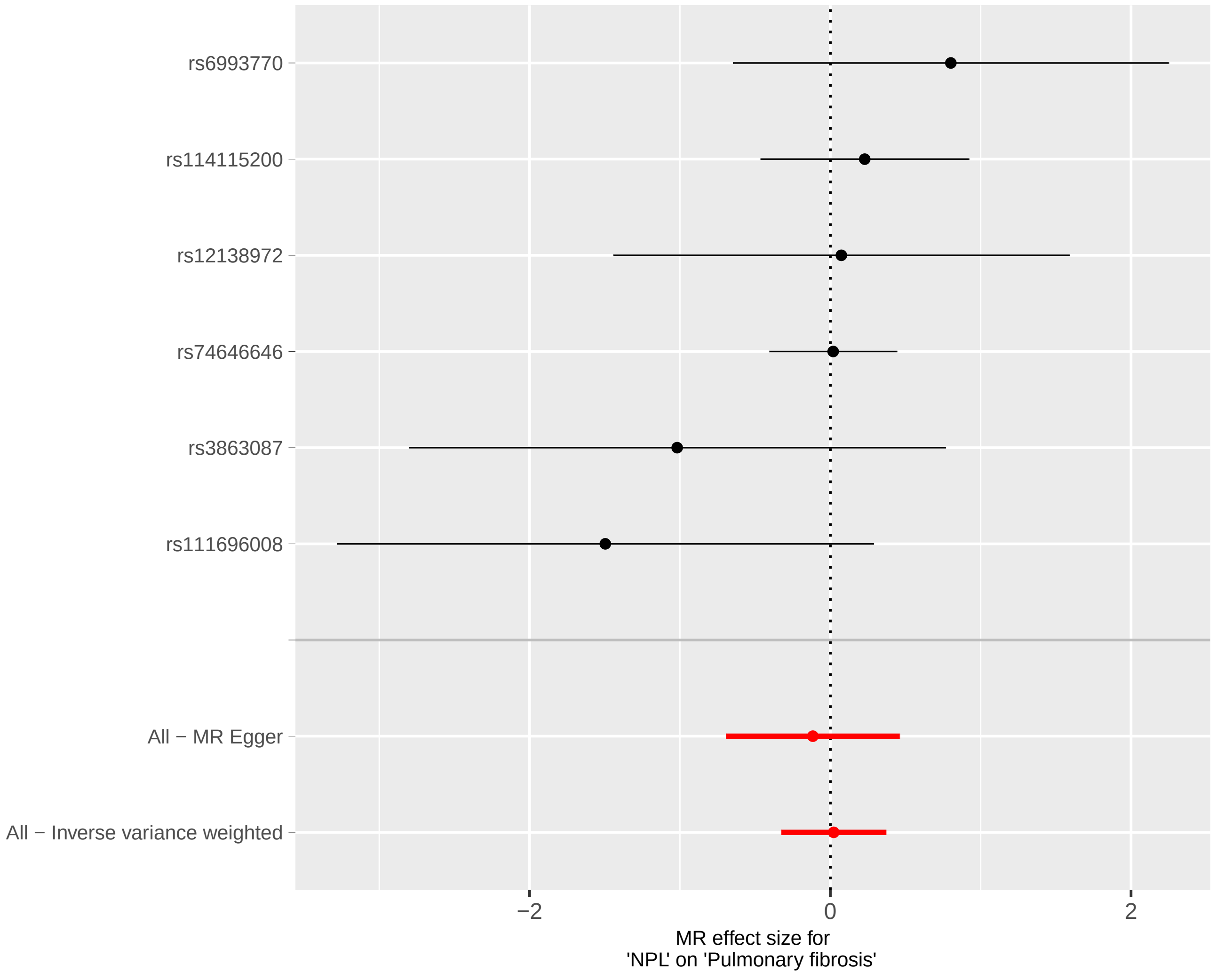
rs6993770
rs114115200
rs12138972
rs74646646
rs3863087
rs111696008
All − MR Egger
All − Inverse variance weighted
−2
0
2
MR effect size for
'NPL' on 'Pulmonary fibrosis'

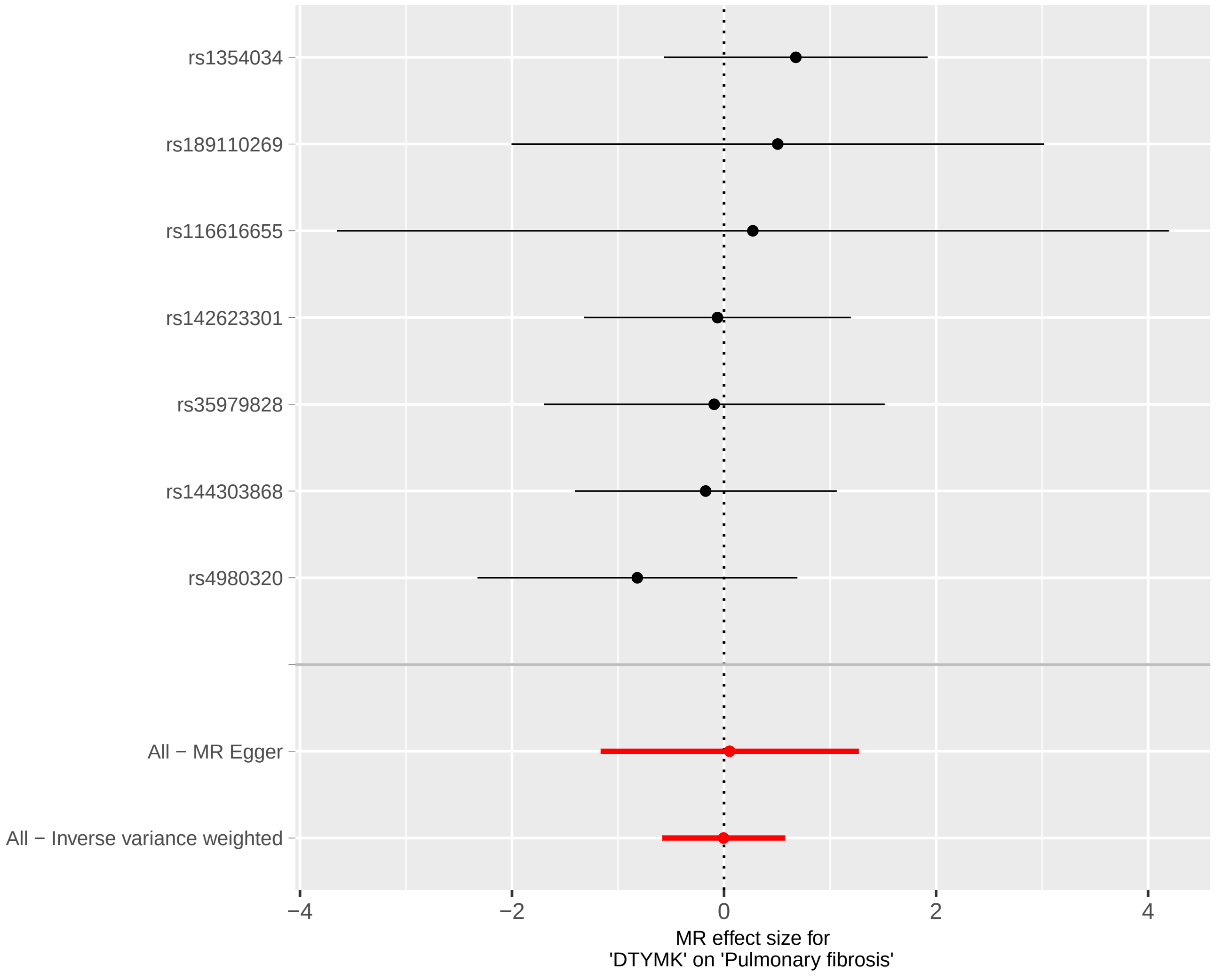

rs1354034
rs189110269
rs116616655
rs142623301
rs35979828
rs144303868
rs4980320
All − MR Egger
All − Inverse variance weighted
−4
−2
0
2
4
MR effect size for
'DTYMK' on 'Pulmonary fibrosis'

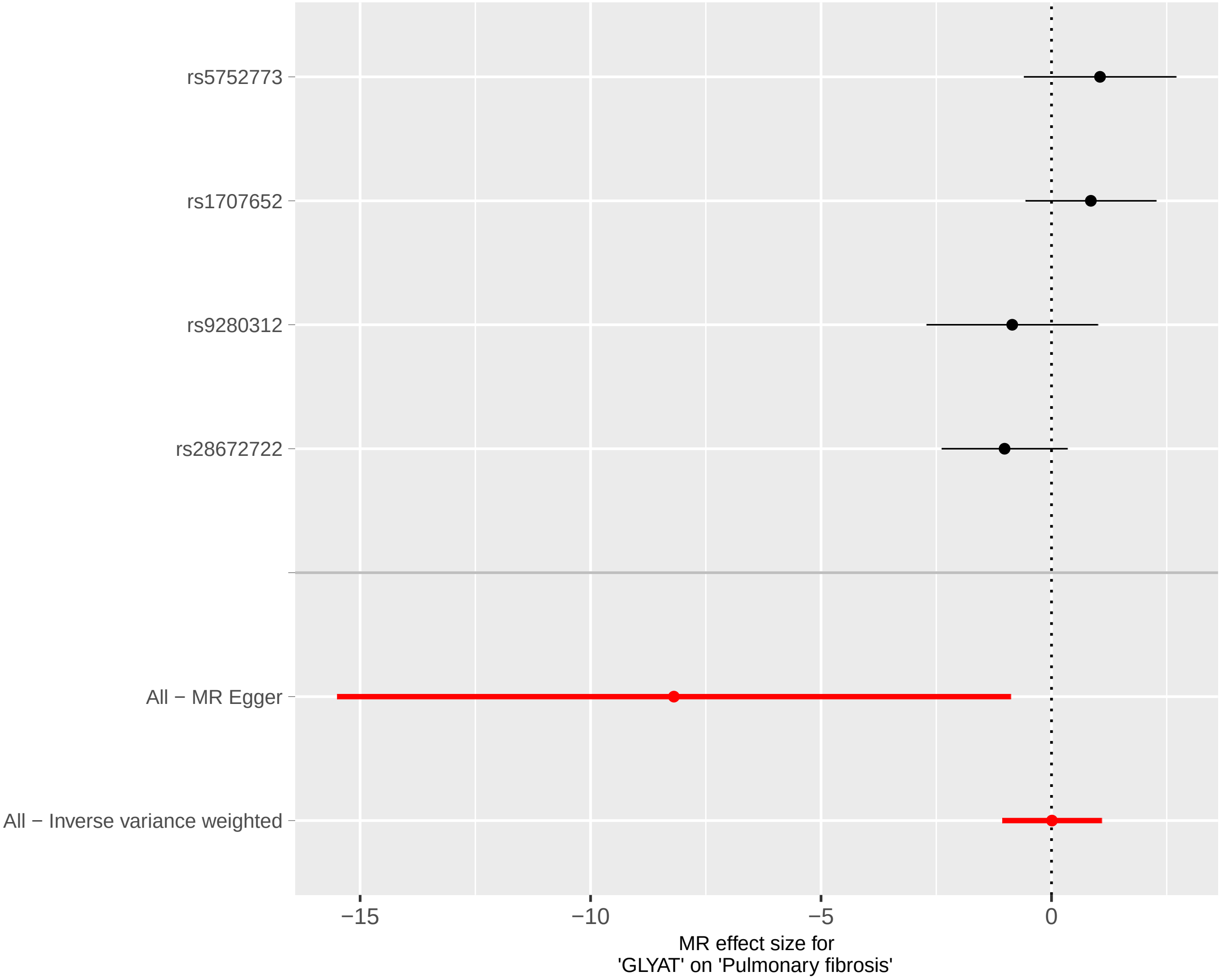
rs5752773
rs1707652
rs9280312
rs28672722
All − MR Egger
All − Inverse variance weighted
−15
−10
−5
0
MR effect size for
'GLYAT' on 'Pulmonary fibrosis'

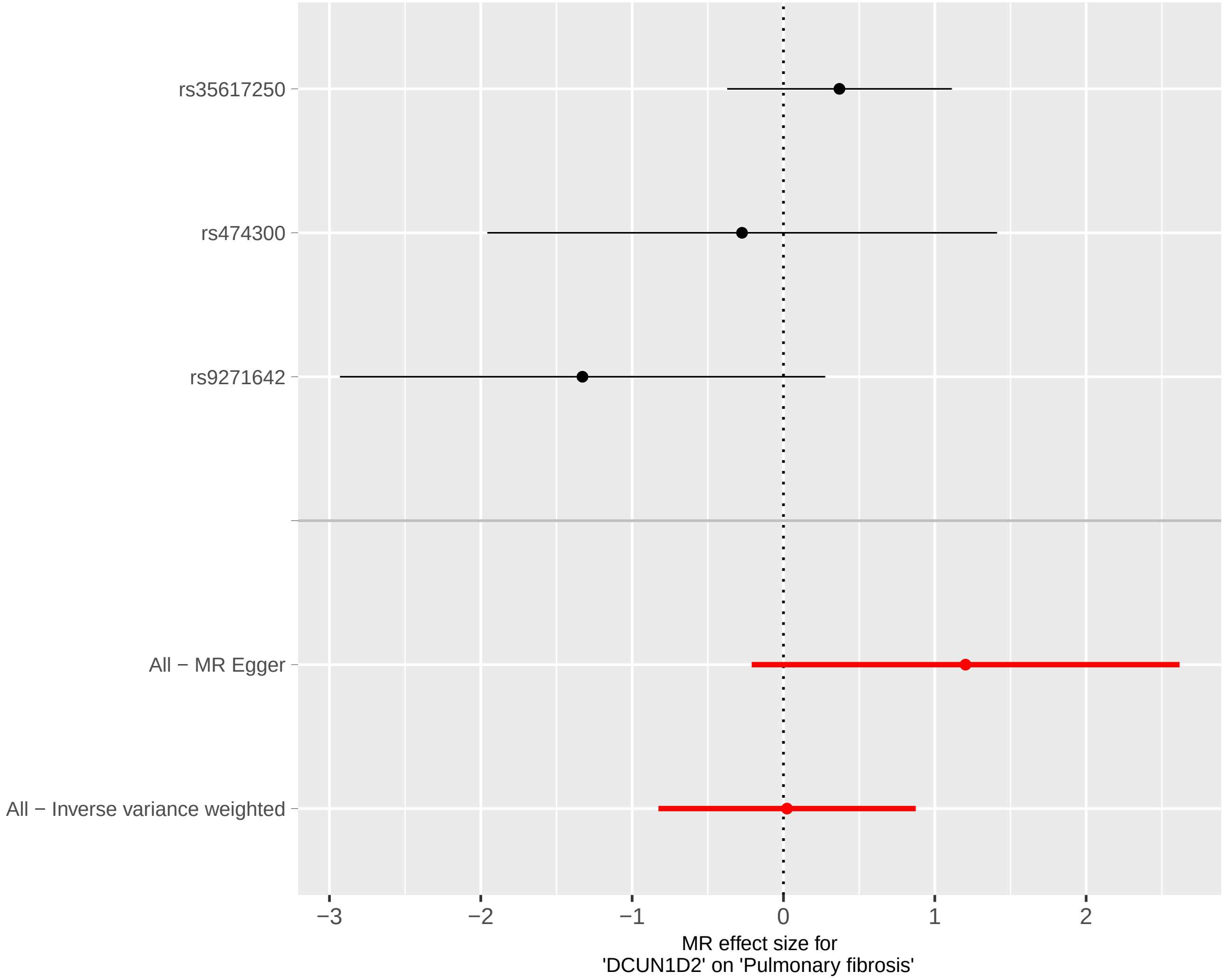
rs35617250
rs474300
rs9271642
All − MR Egger
All − Inverse variance weighted
−3
−2
−1
0
1
2
MR effect size for
'DCUN1D2' on 'Pulmonary fibrosis'

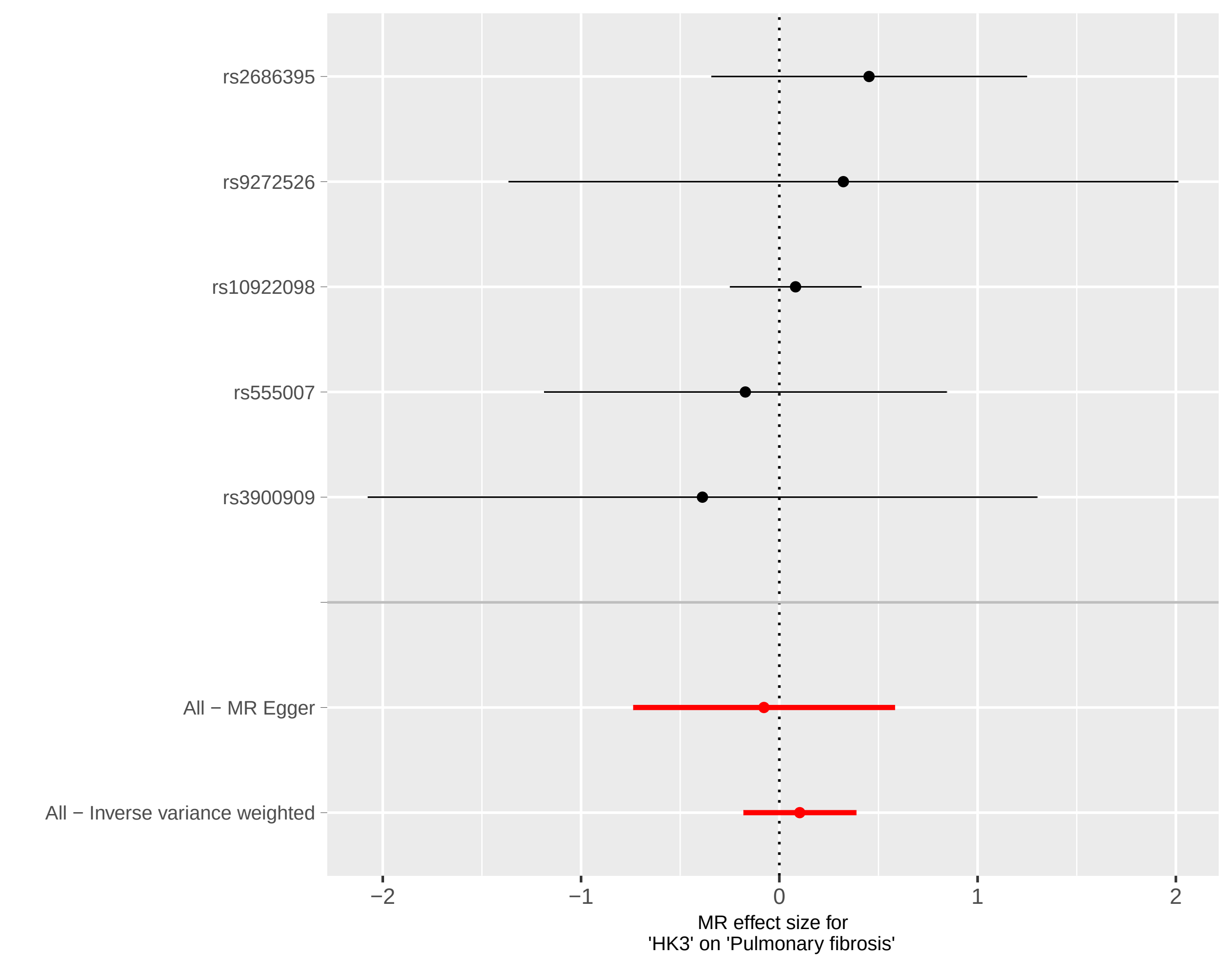
rs2686395
rs9272526
rs10922098
rs555007
rs3900909
All − MR Egger
All − Inverse variance weighted
−2
−1
0
1
2
MR effect size for
'HK3' on 'Pulmonary fibrosis'

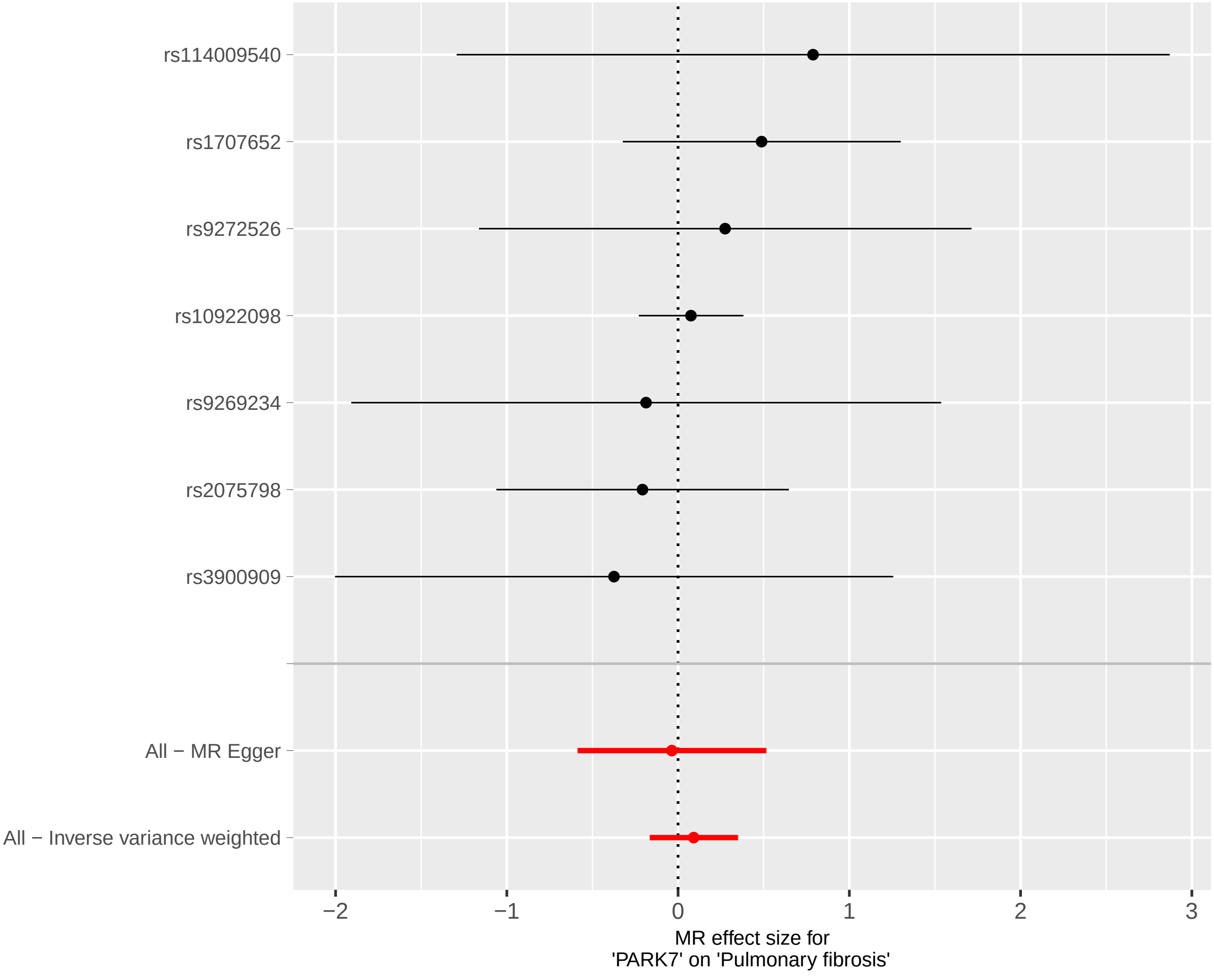

rs114009540
rs1707652
rs9272526
rs10922098
rs9269234
rs2075798
rs3900909
All − MR Egger
All − Inverse variance weighted
−2
−1
0
1
2
3
MR effect size for
'PARK7' on 'Pulmonary fibrosis'

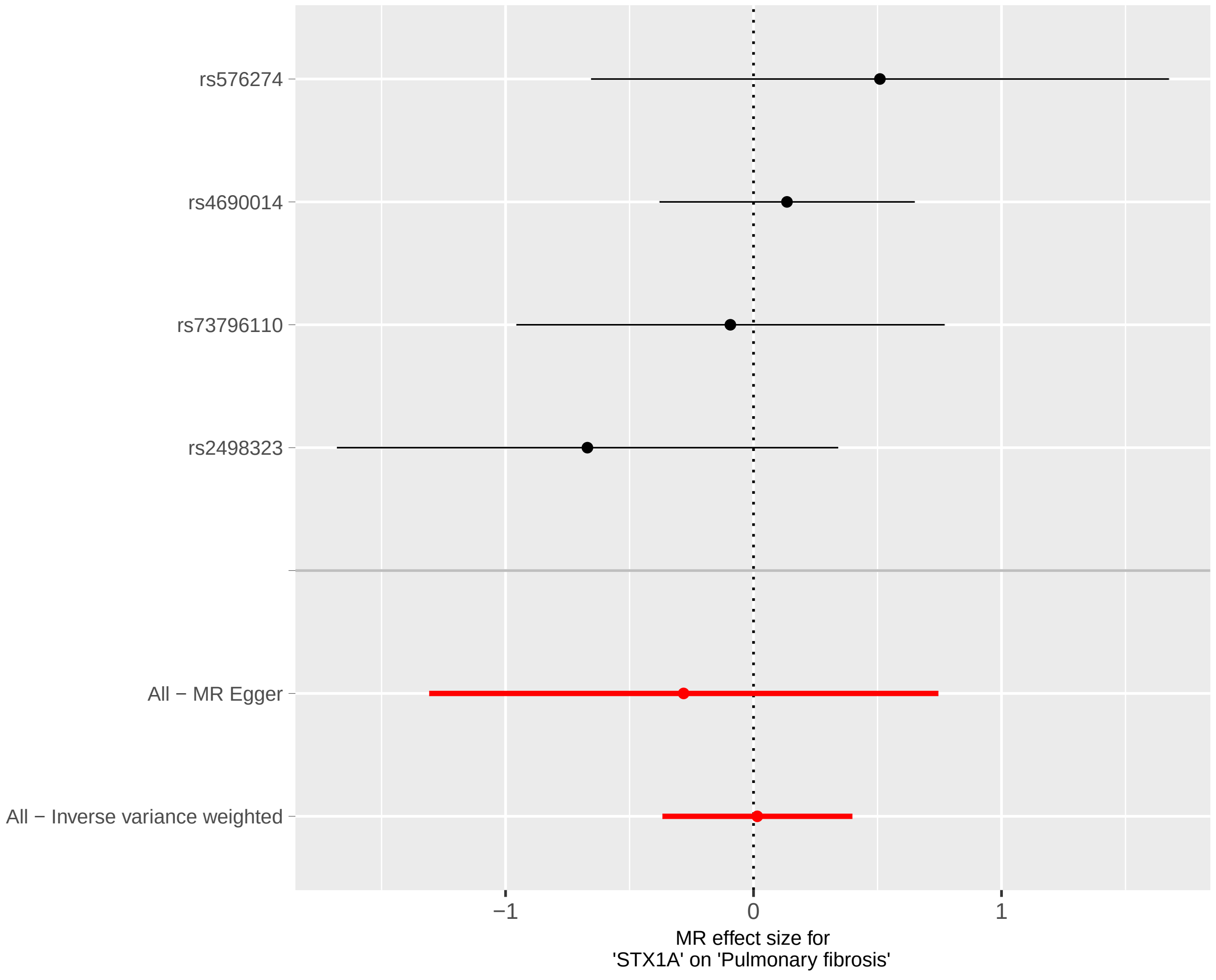
rs576274
rs4690014
rs73796110
rs2498323
All − MR Egger
All − Inverse variance weighted
−1
0
1
MR effect size for
'STX1A' on 'Pulmonary fibrosis'

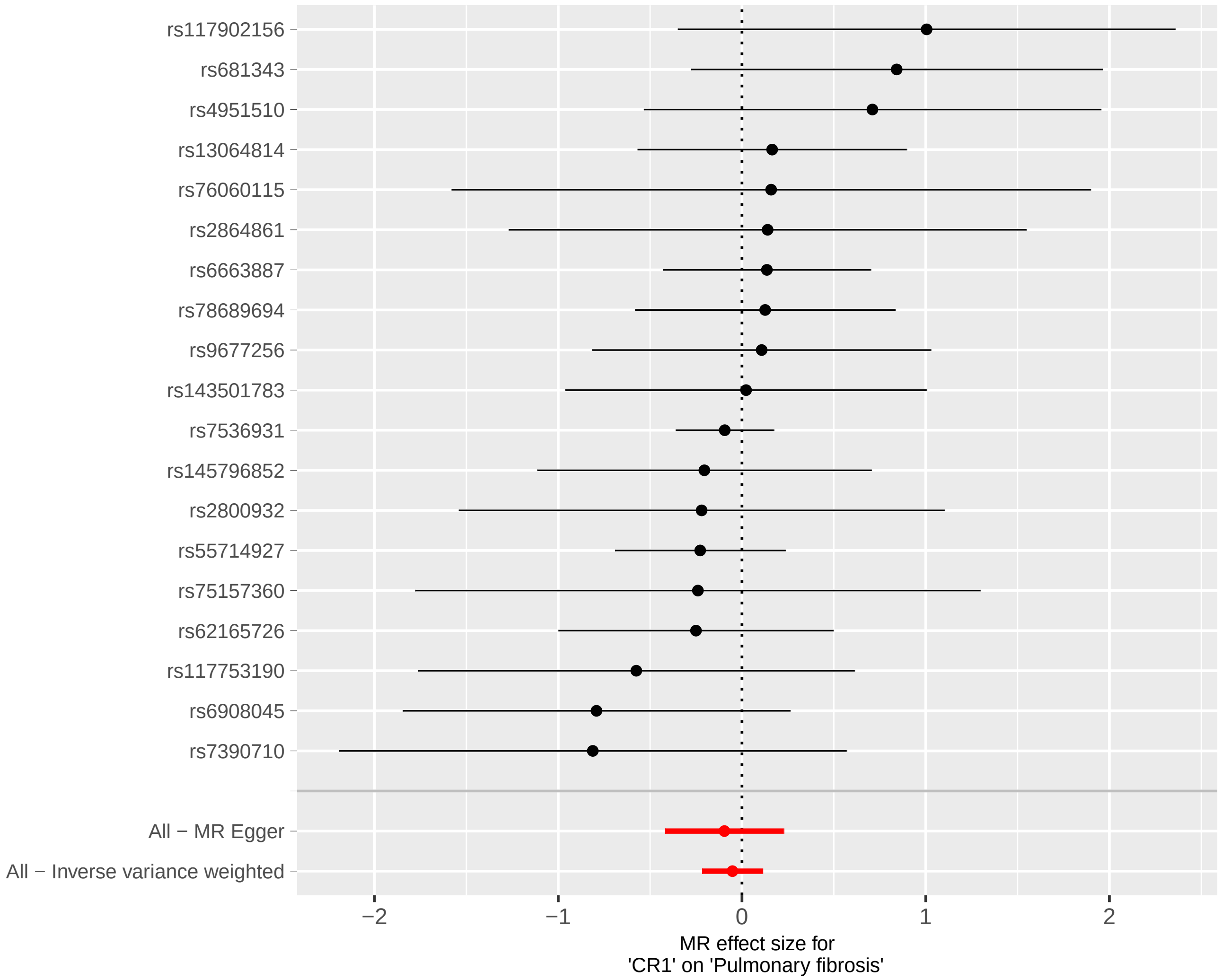

rs117902156
rs681343
rs4951510
rs13064814
rs76060115
rs2864861
rs6663887
rs78689694
rs9677256
rs143501783
rs7536931
rs145796852
rs2800932
rs55714927
rs75157360
rs62165726
rs117753190
rs6908045
rs7390710
All − MR Egger
All − Inverse variance weighted
−2
−1
0
1
2
MR effect size for
'CR1' on 'Pulmonary fibrosis'

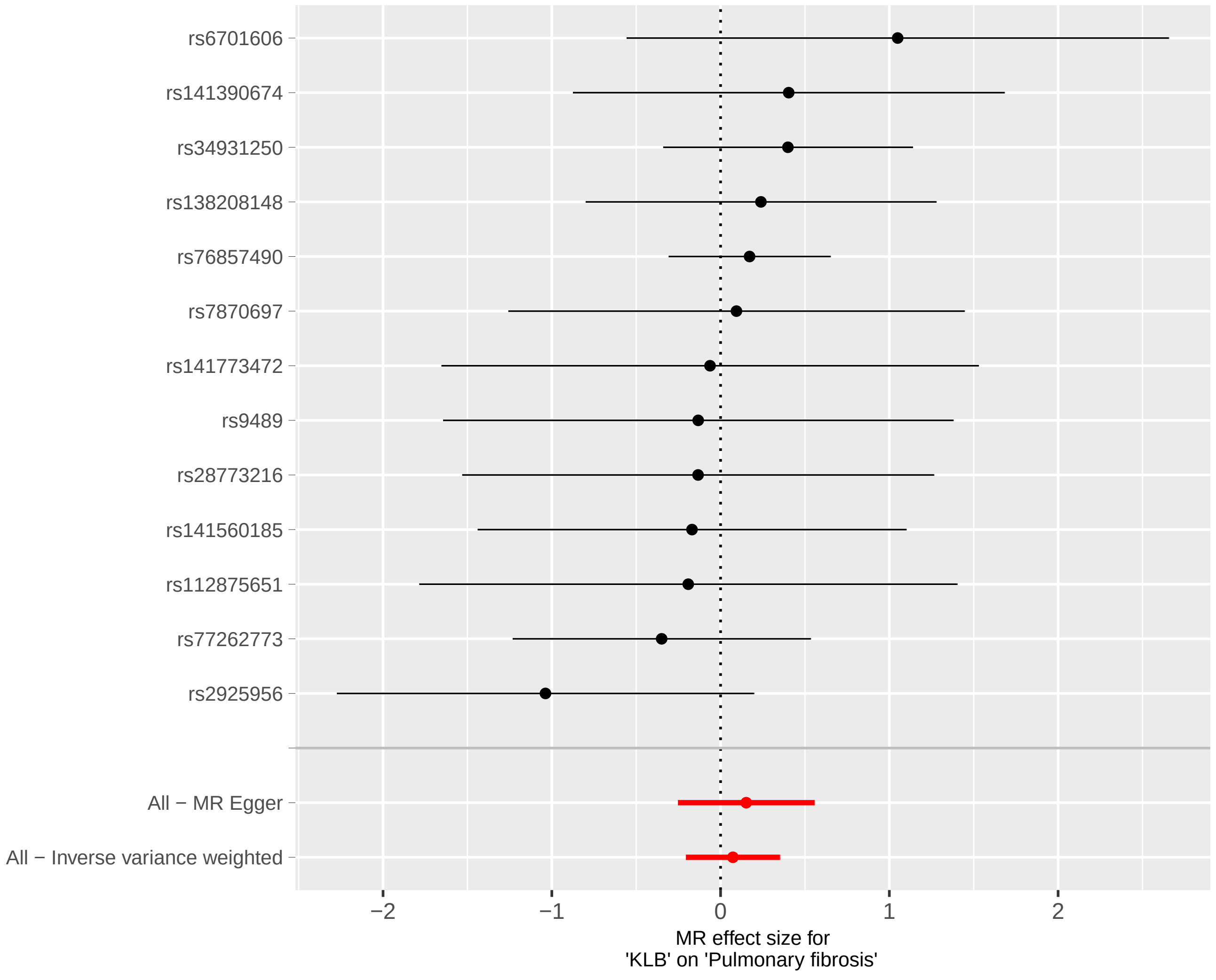
rs6701606
rs141390674
rs34931250
rs138208148
rs76857490
rs7870697
rs141773472
rs9489
rs28773216
rs141560185
rs112875651
rs77262773
rs2925956
All − MR Egger
All − Inverse variance weighted
−2
−1
0
1
2
MR effect size for
'KLB' on 'Pulmonary fibrosis'

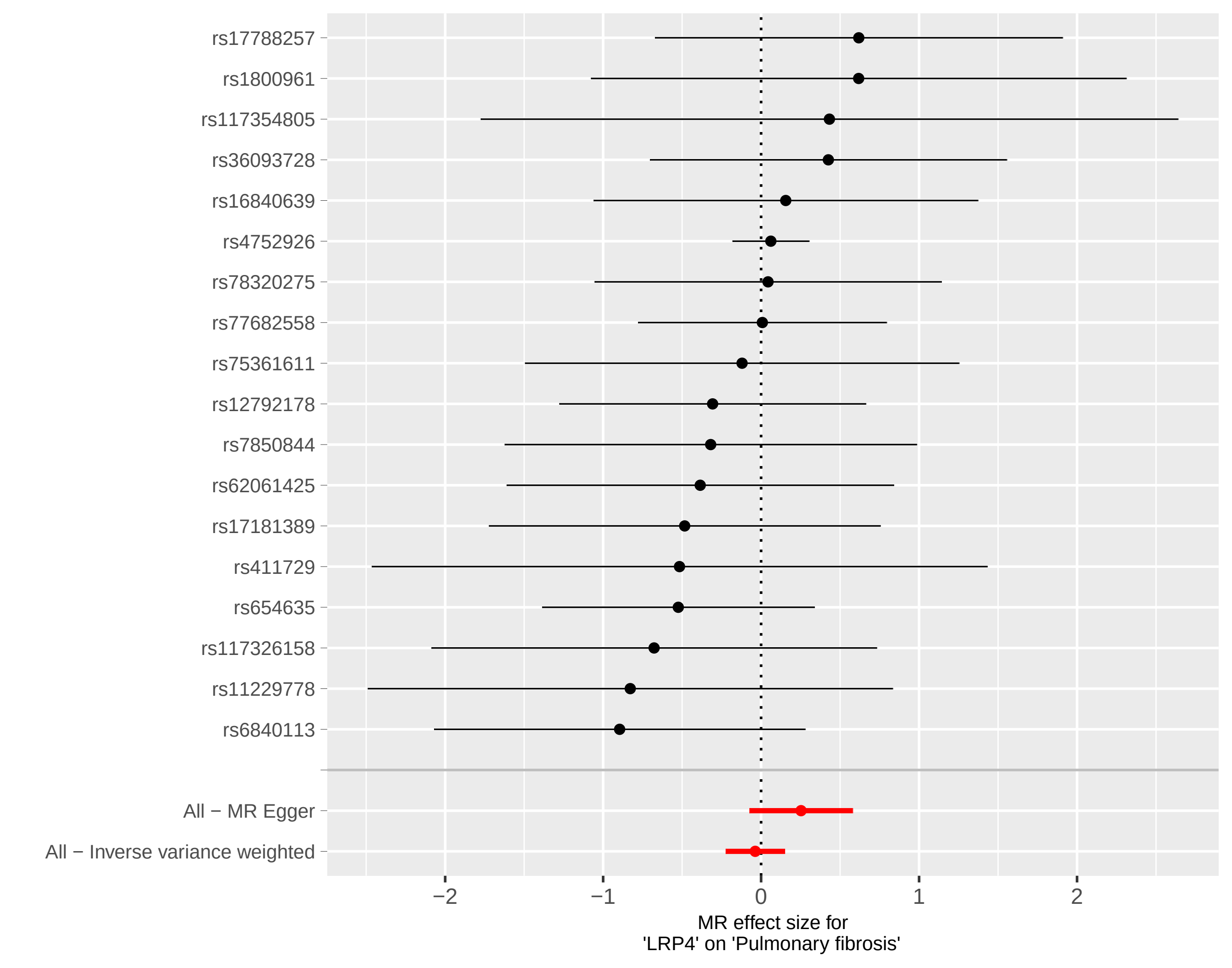

rs17788257
rs1800961
rs117354805
rs36093728
rs16840639
rs4752926
rs78320275
rs77682558
rs75361611
rs12792178
rs7850844
rs62061425
rs17181389
rs411729
rs654635
rs117326158
rs11229778
rs6840113
All − MR Egger
All − Inverse variance weighted
−2
−1
0
1
2
MR effect size for
'LRP4' on 'Pulmonary fibrosis'

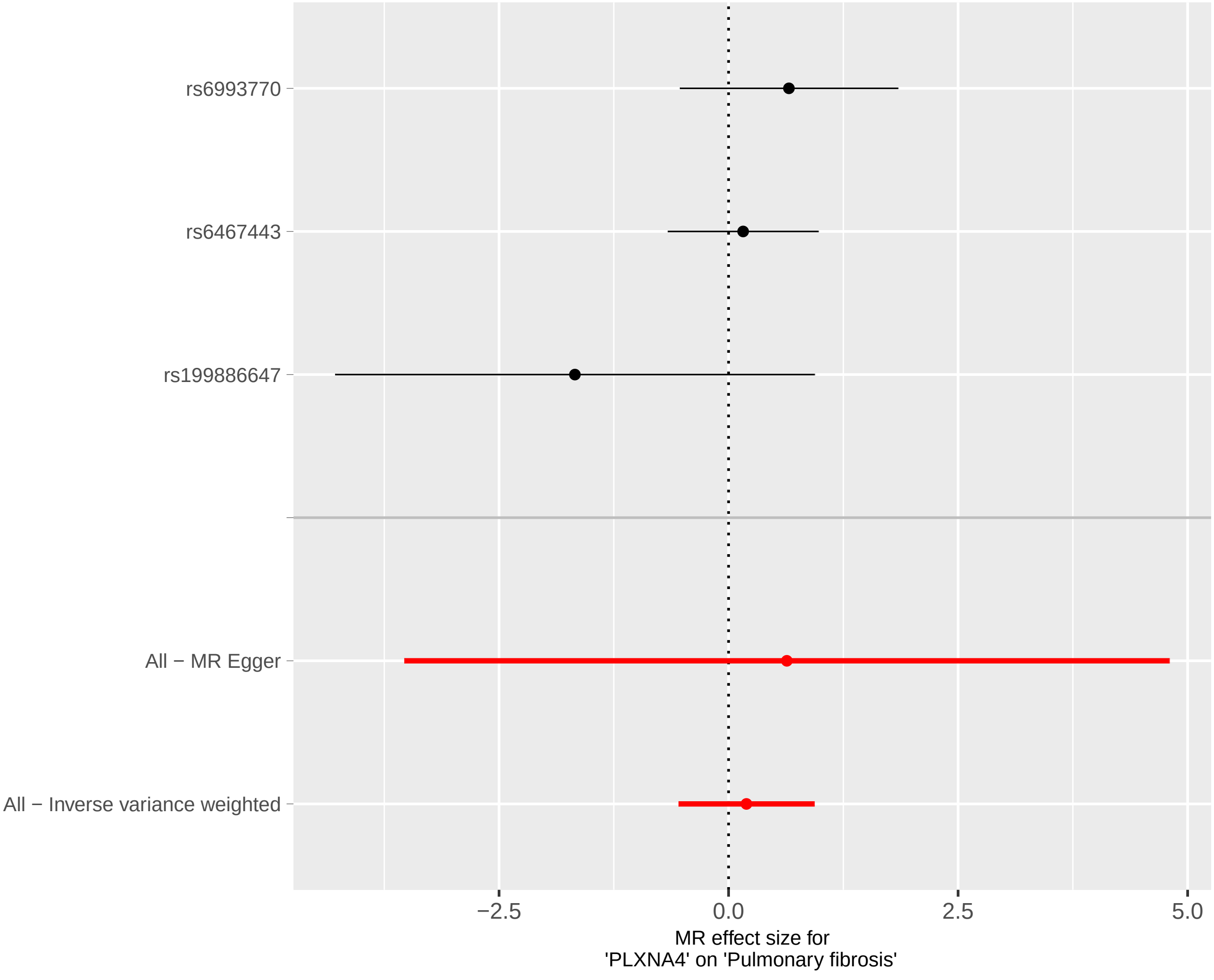

rs6993770
rs6467443
rs199886647
All − MR Egger
All − Inverse variance weighted
−2.5
0.0
2.5
5.0
MR effect size for
'PLXNA4' on 'Pulmonary fibrosis'

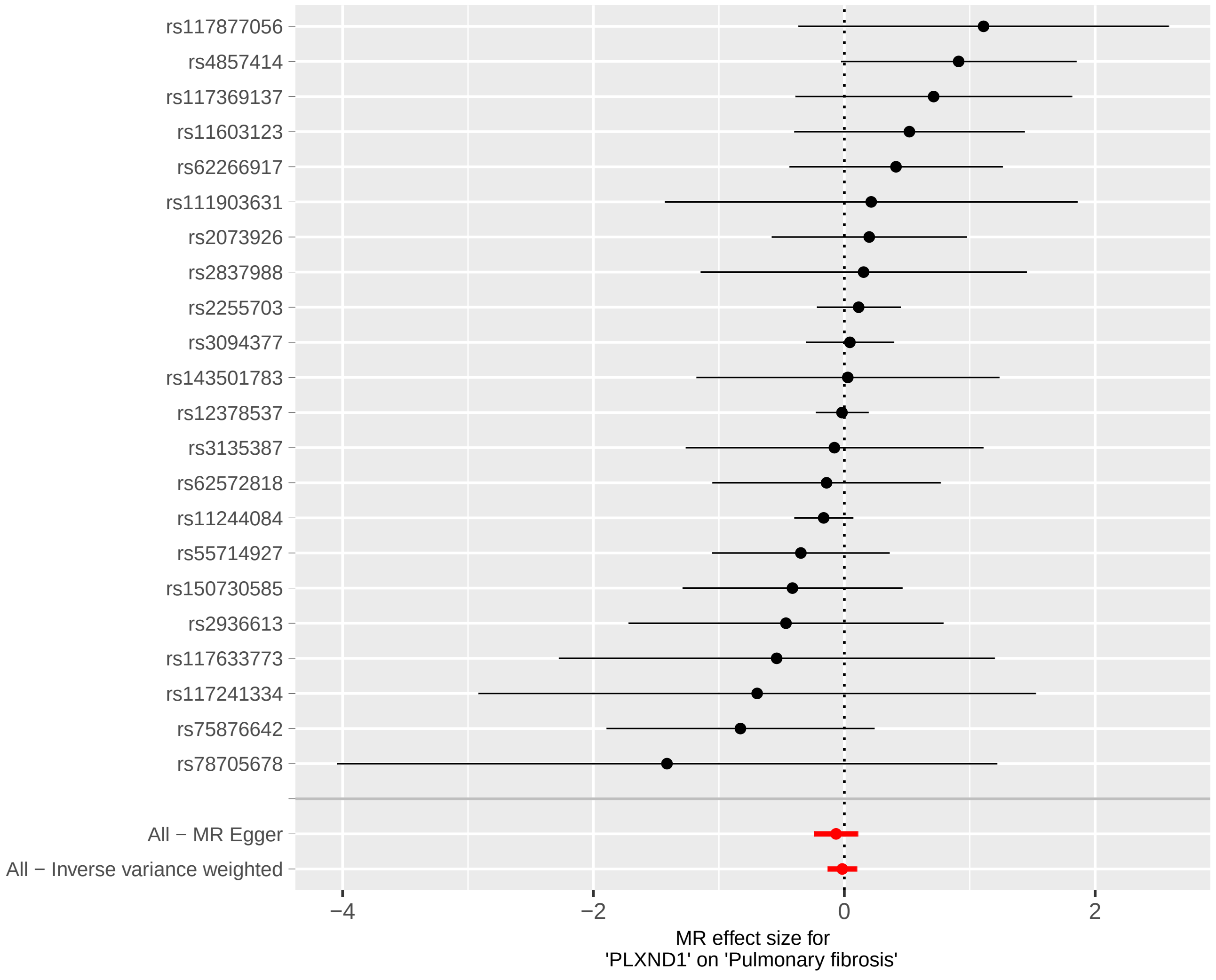
rs117877056
rs4857414
rs117369137
rs11603123
rs62266917
rs111903631
rs2073926
rs2837988
rs2255703
rs3094377
rs143501783
rs12378537
rs3135387
rs62572818
rs11244084
rs55714927
rs150730585
rs2936613
rs117633773
rs117241334
rs75876642
rs78705678
All − MR Egger
All − Inverse variance weighted
−4
−2
0
2
MR effect size for
'PLXND1' on 'Pulmonary fibrosis'

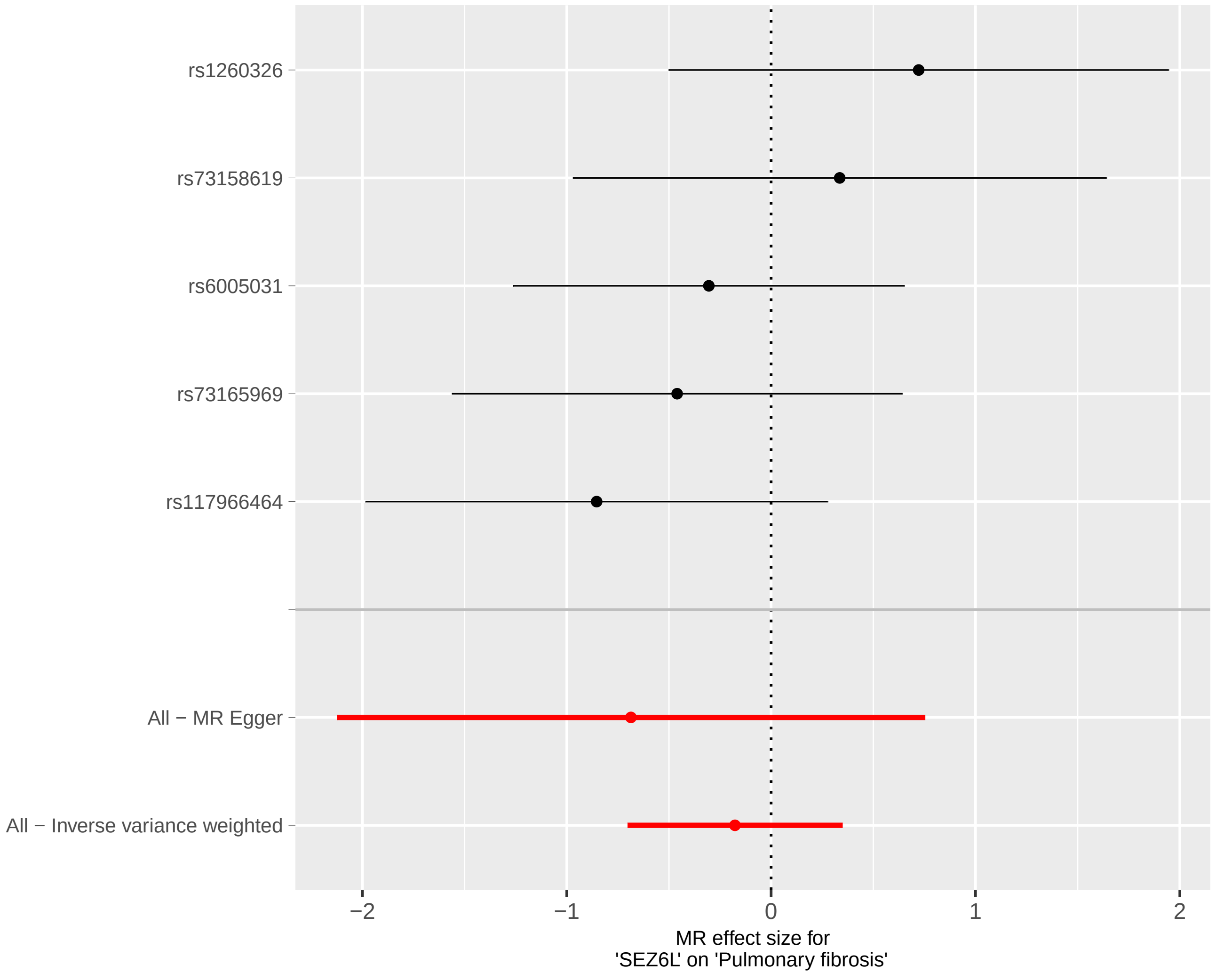
rs1260326
rs73158619
rs6005031
rs73165969
rs117966464
All − MR Egger
All − Inverse variance weighted
−2
−1
0
1
2
MR effect size for
'SEZ6L' on 'Pulmonary fibrosis'

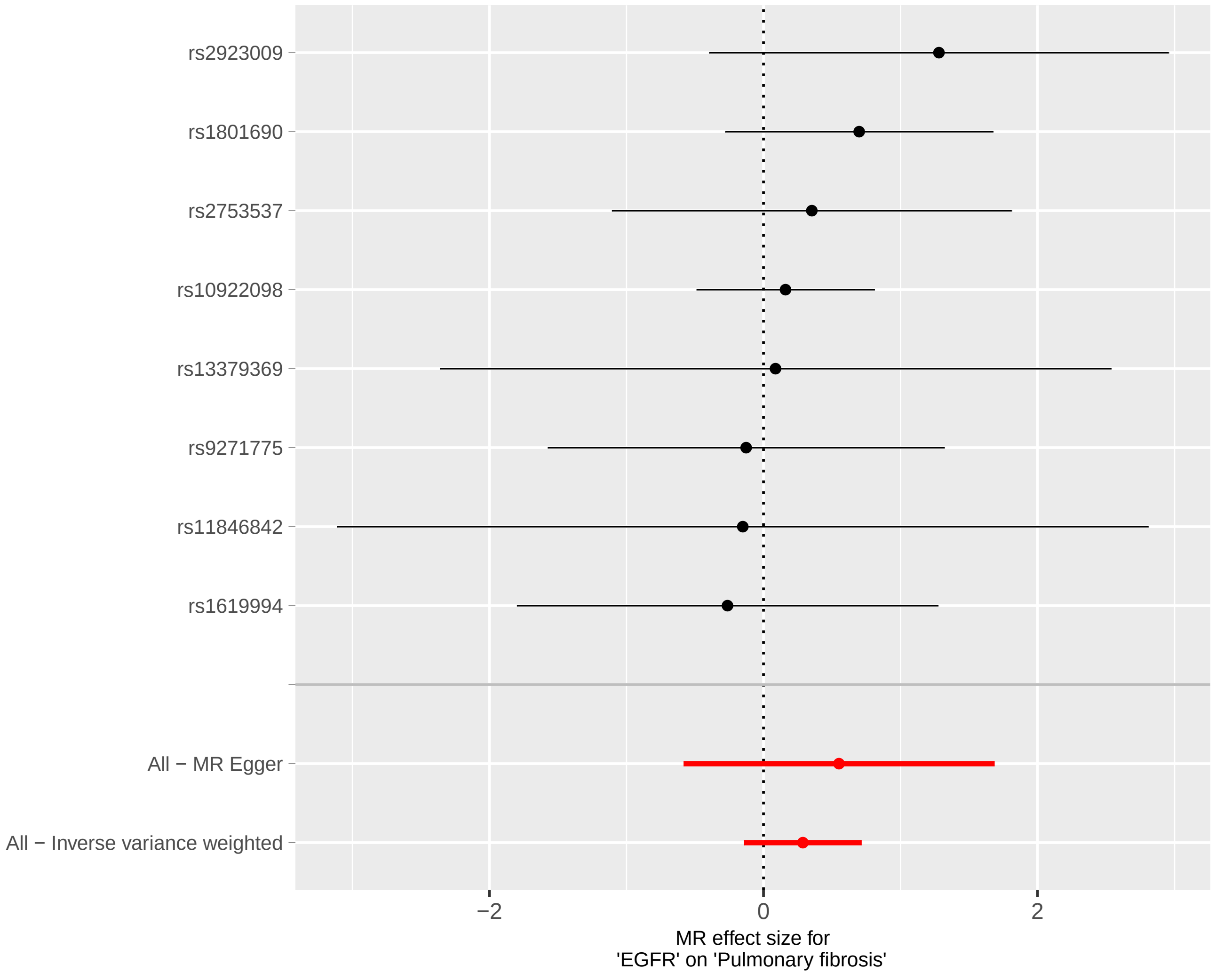
rs2923009
rs1801690
rs2753537
rs10922098
rs13379369
rs9271775
rs11846842
rs1619994
All − MR Egger
All − Inverse variance weighted
−2
0
2
MR effect size for
'EGFR' on 'Pulmonary fibrosis'

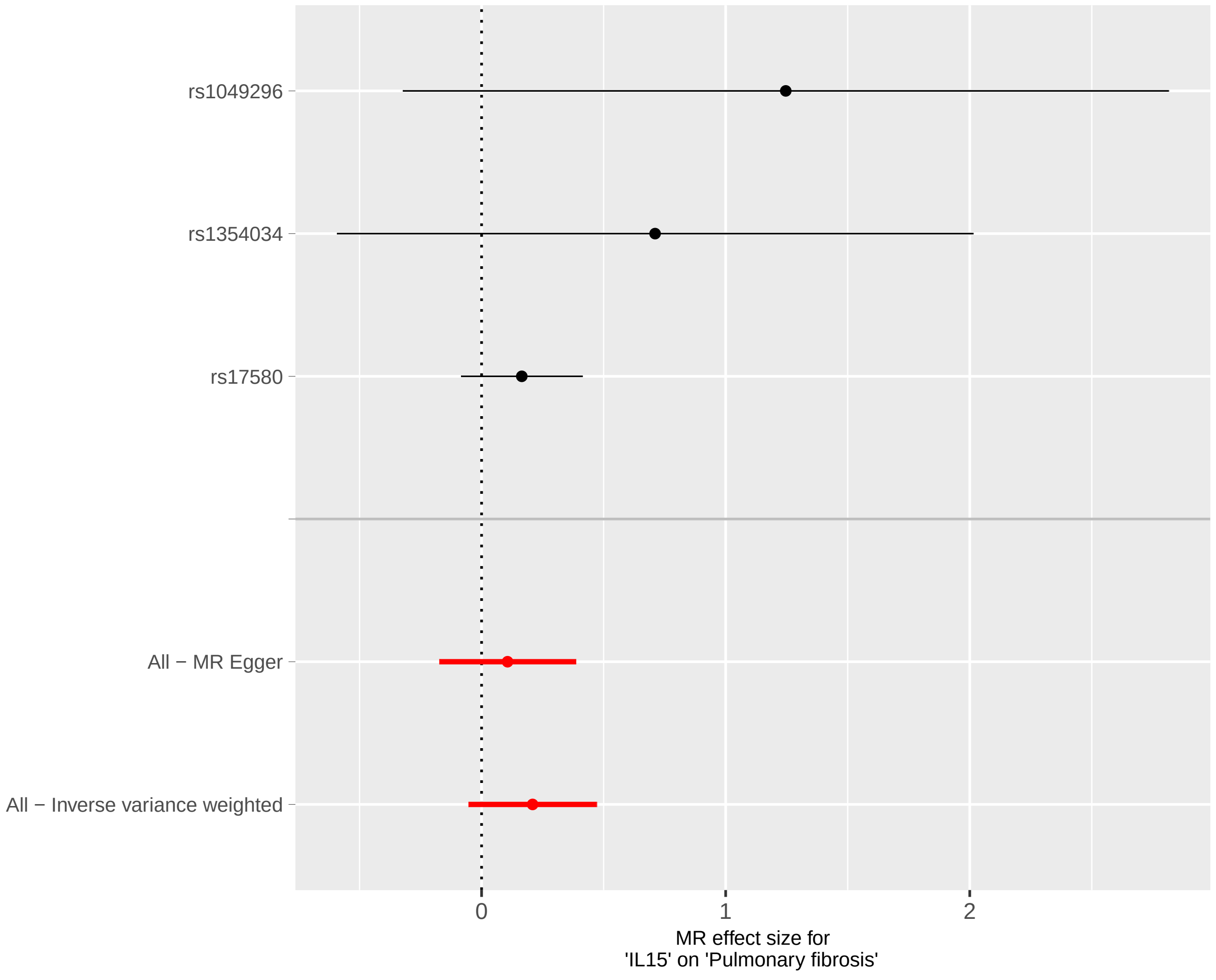
rs1049296
rs1354034
rs17580
All − MR Egger
All − Inverse variance weighted
0
1
2
MR effect size for
'IL15' on 'Pulmonary fibrosis'

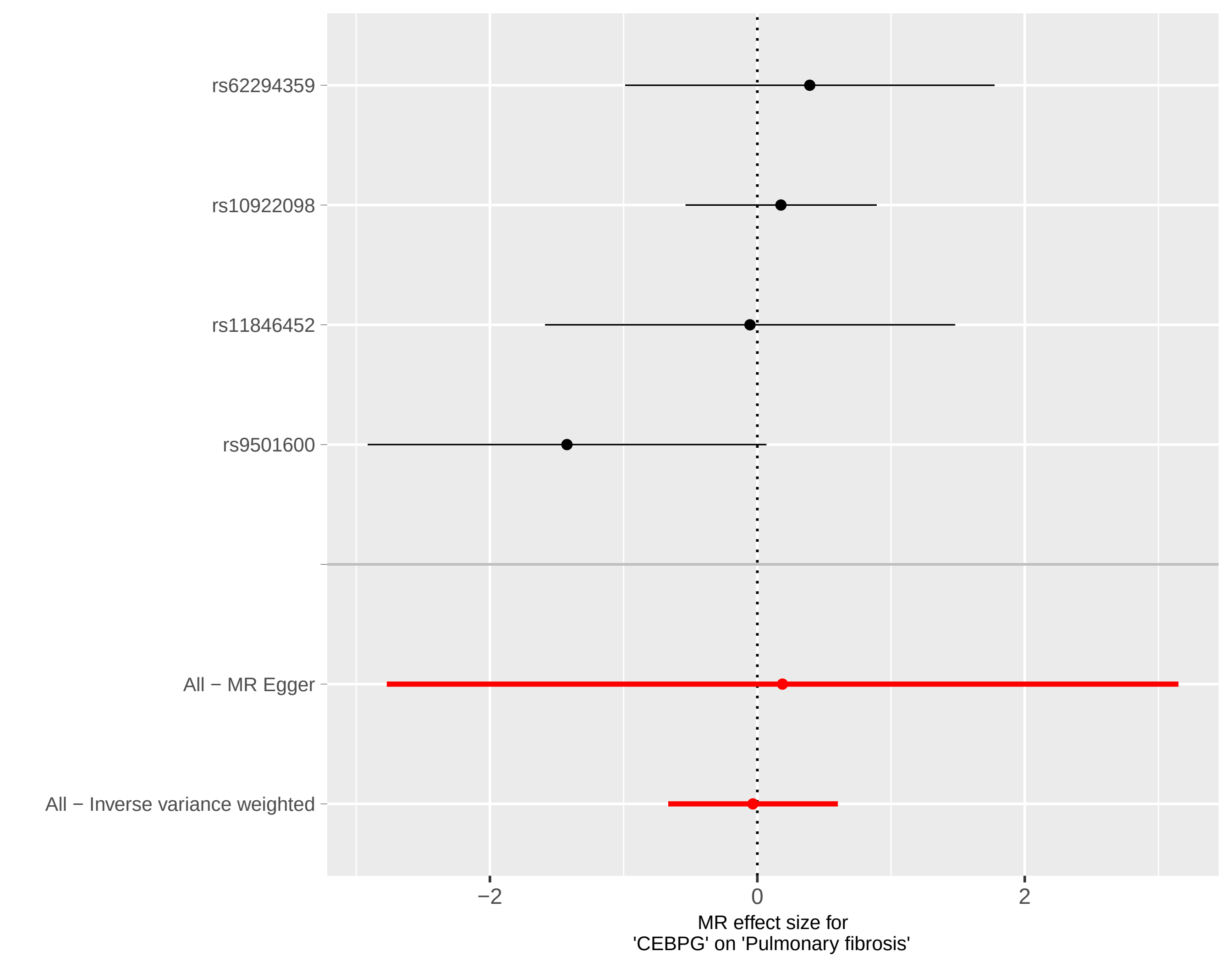
rs62294359
rs10922098
rs11846452
rs9501600
All − MR Egger
All − Inverse variance weighted
−2
0
2
MR effect size for
'CEBPG' on 'Pulmonary fibrosis'

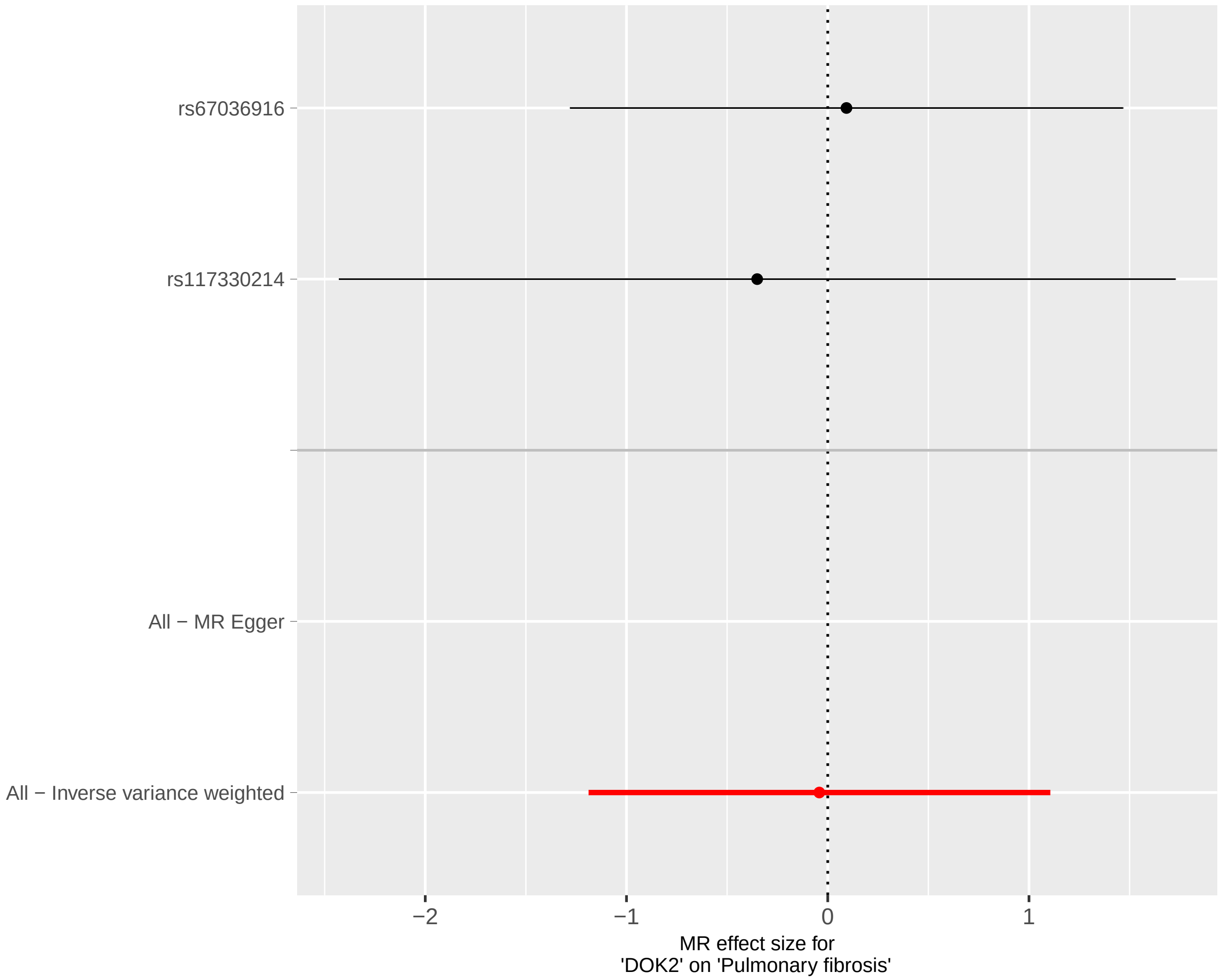
rs67036916
rs117330214
All − MR Egger
All − Inverse variance weighted
−2
−1
0
1
MR effect size for
'DOK2' on 'Pulmonary fibrosis'

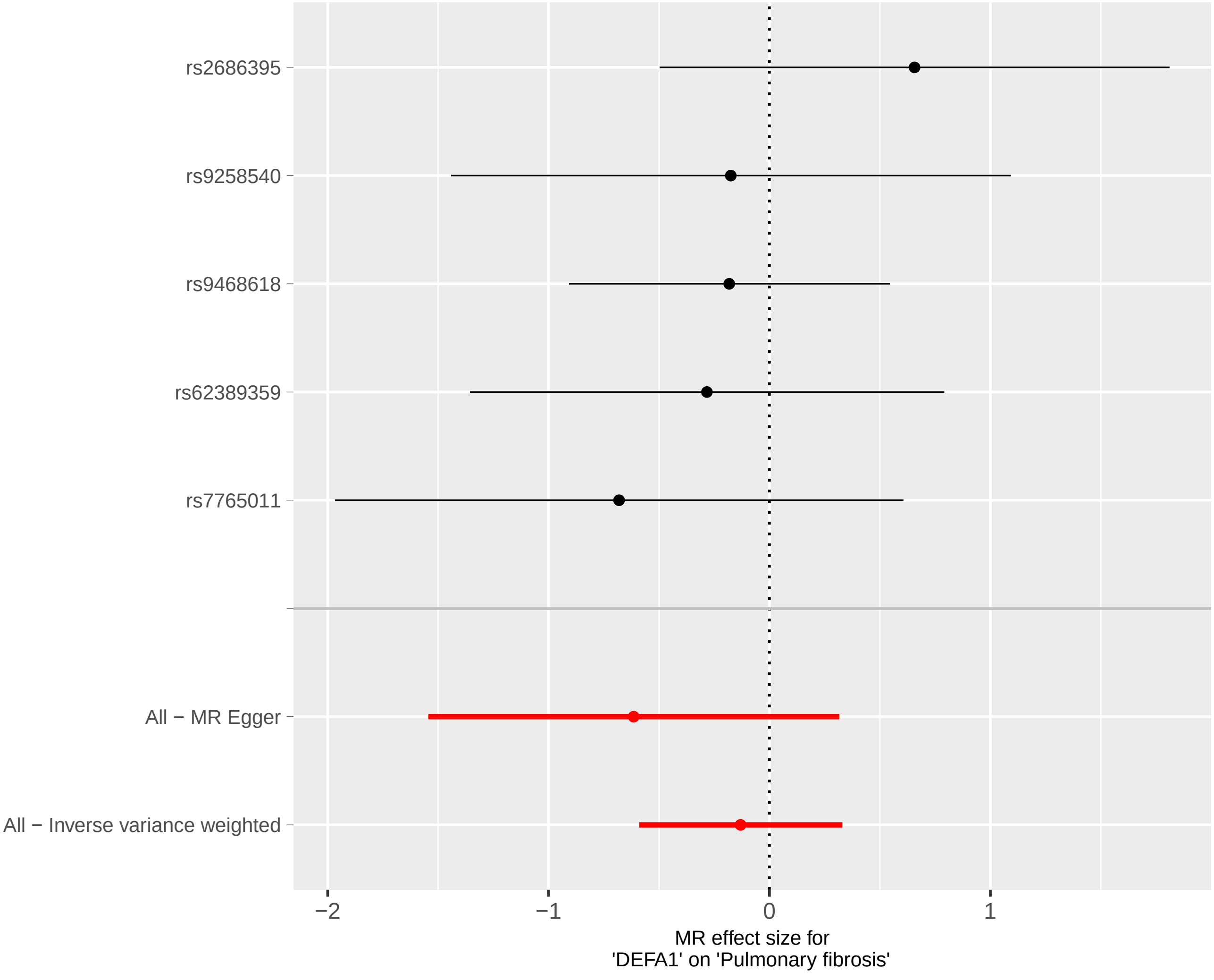
rs2686395
rs9258540
rs9468618
rs62389359
rs7765011
All − MR Egger
All − Inverse variance weighted
−2
−1
0
1
MR effect size for
'DEFA1' on 'Pulmonary fibrosis'

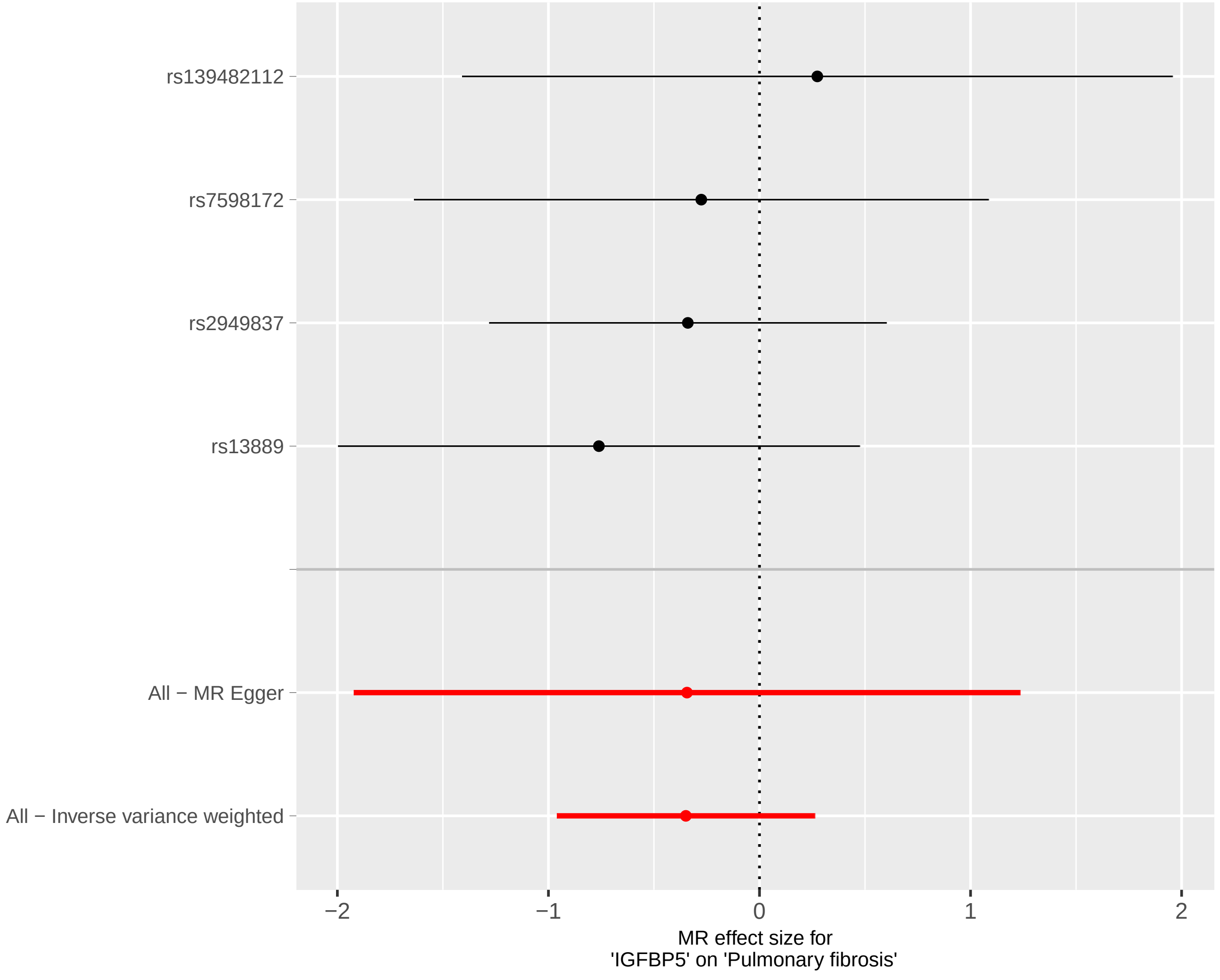
rs139482112
rs7598172
rs2949837
rs13889
All − MR Egger
All − Inverse variance weighted
−2
−1
0
1
2
MR effect size for
'IGFBP5' on 'Pulmonary fibrosis'

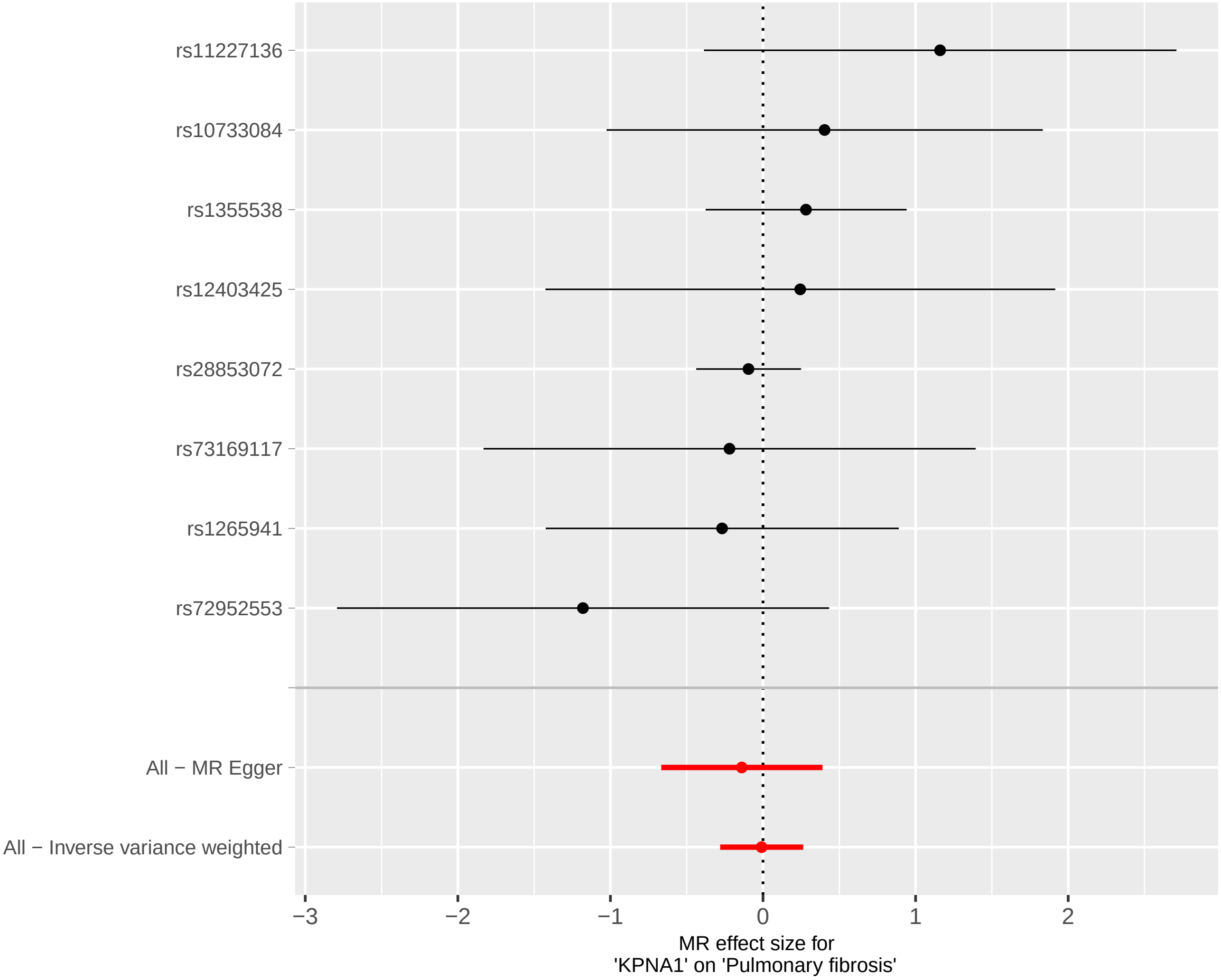

rs11227136
rs10733084
rs1355538
rs12403425
rs28853072
rs73169117
rs1265941
rs72952553
All − MR Egger
All − Inverse variance weighted
−3
−2
−1
0
1
2
MR effect size for
'KPNA1' on 'Pulmonary fibrosis'

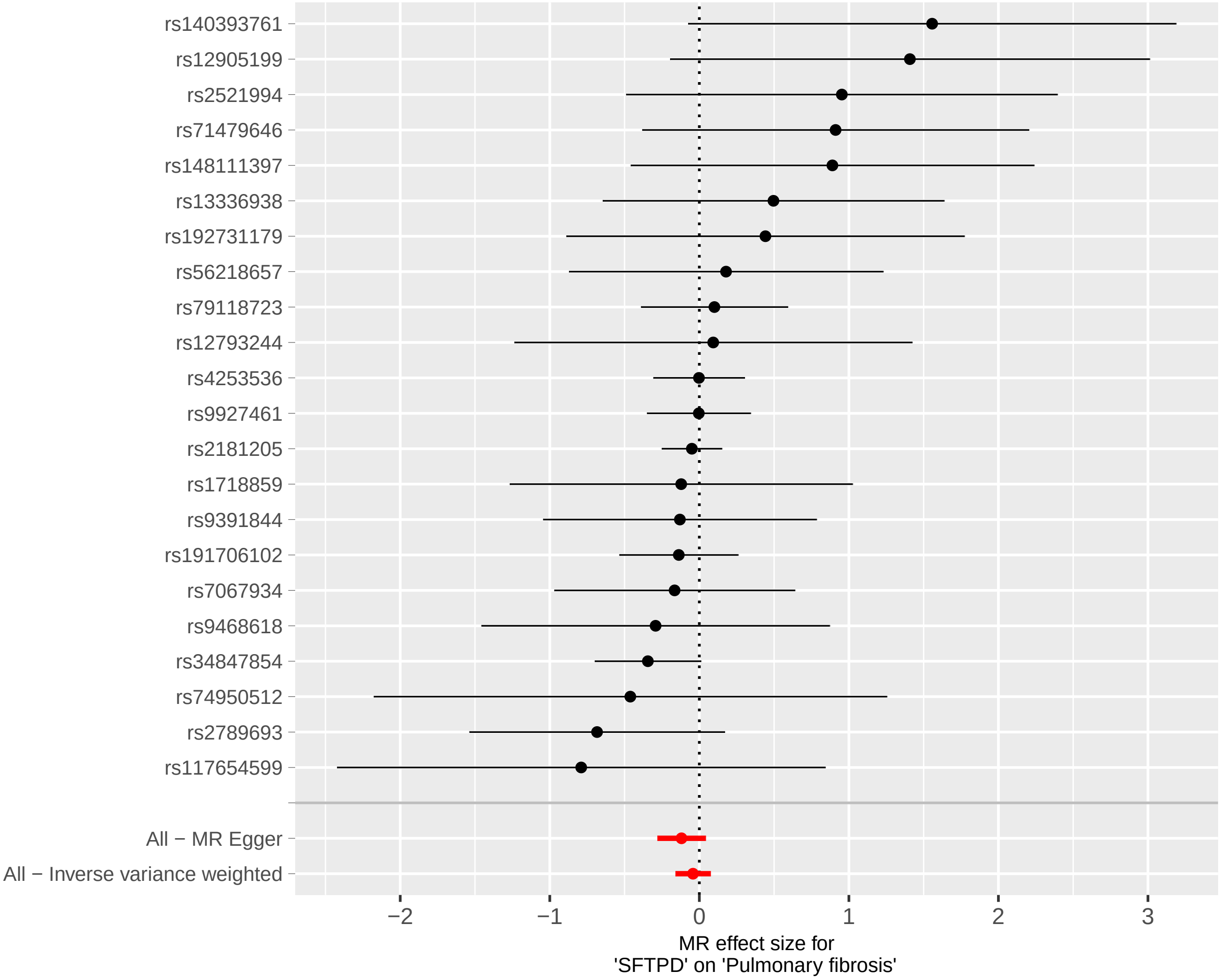
rs140393761
rs12905199
rs2521994
rs71479646
rs148111397
rs13336938
rs192731179
rs56218657
rs79118723
rs12793244
rs4253536
rs9927461
rs2181205
rs1718859
rs9391844
rs191706102
rs7067934
rs9468618
rs34847854
rs74950512
rs2789693
rs117654599
All − MR Egger
All − Inverse variance weighted
−2
−1
0
1
2
3
MR effect size for
'SFTPD' on 'Pulmonary fibrosis'

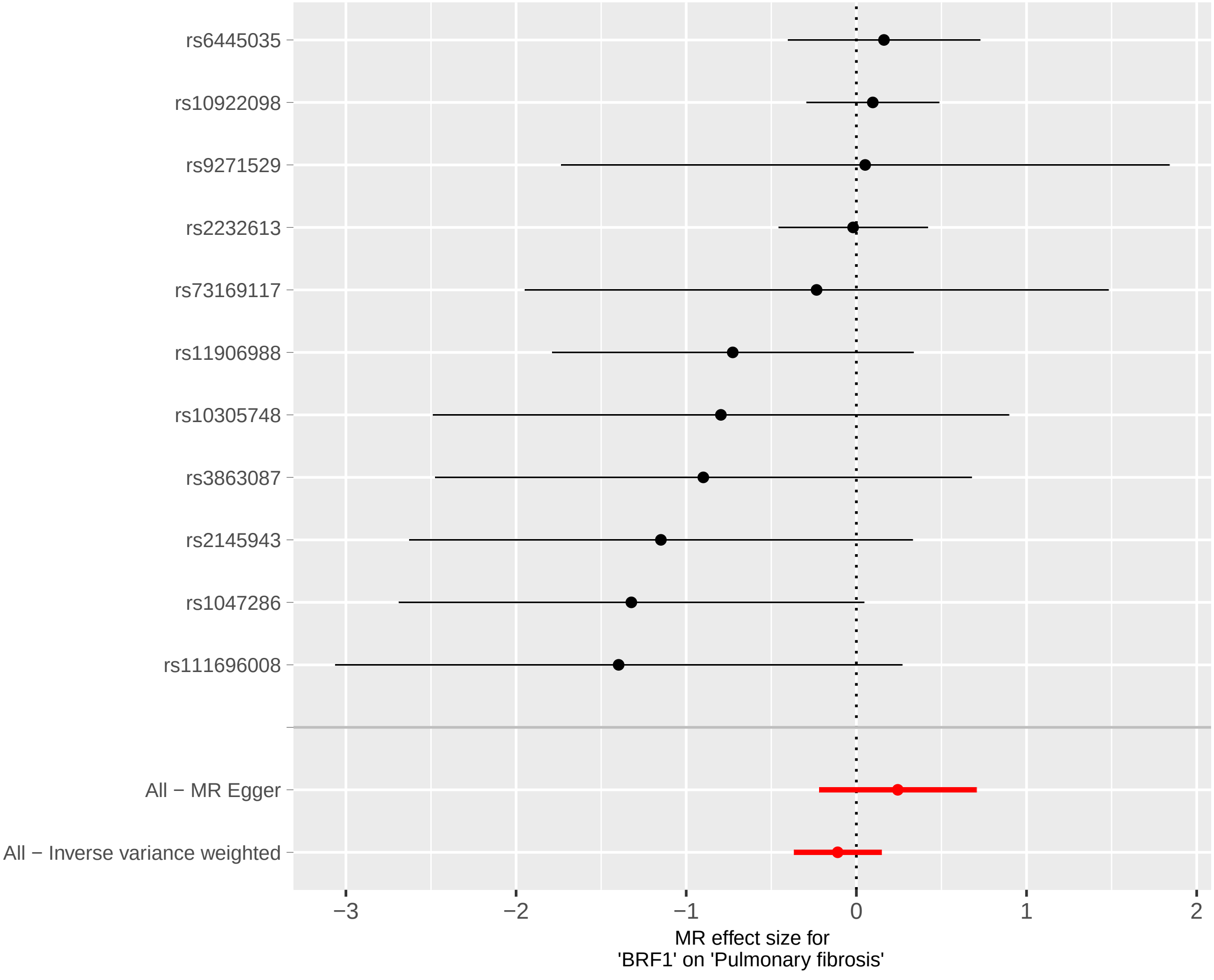

rs6445035
rs10922098
rs9271529
rs2232613
rs73169117
rs11906988
rs10305748
rs3863087
rs2145943
rs1047286
rs111696008
All − MR Egger
All − Inverse variance weighted
−3
−2
−1
0
1
2
MR effect size for
'BRF1' on 'Pulmonary fibrosis'

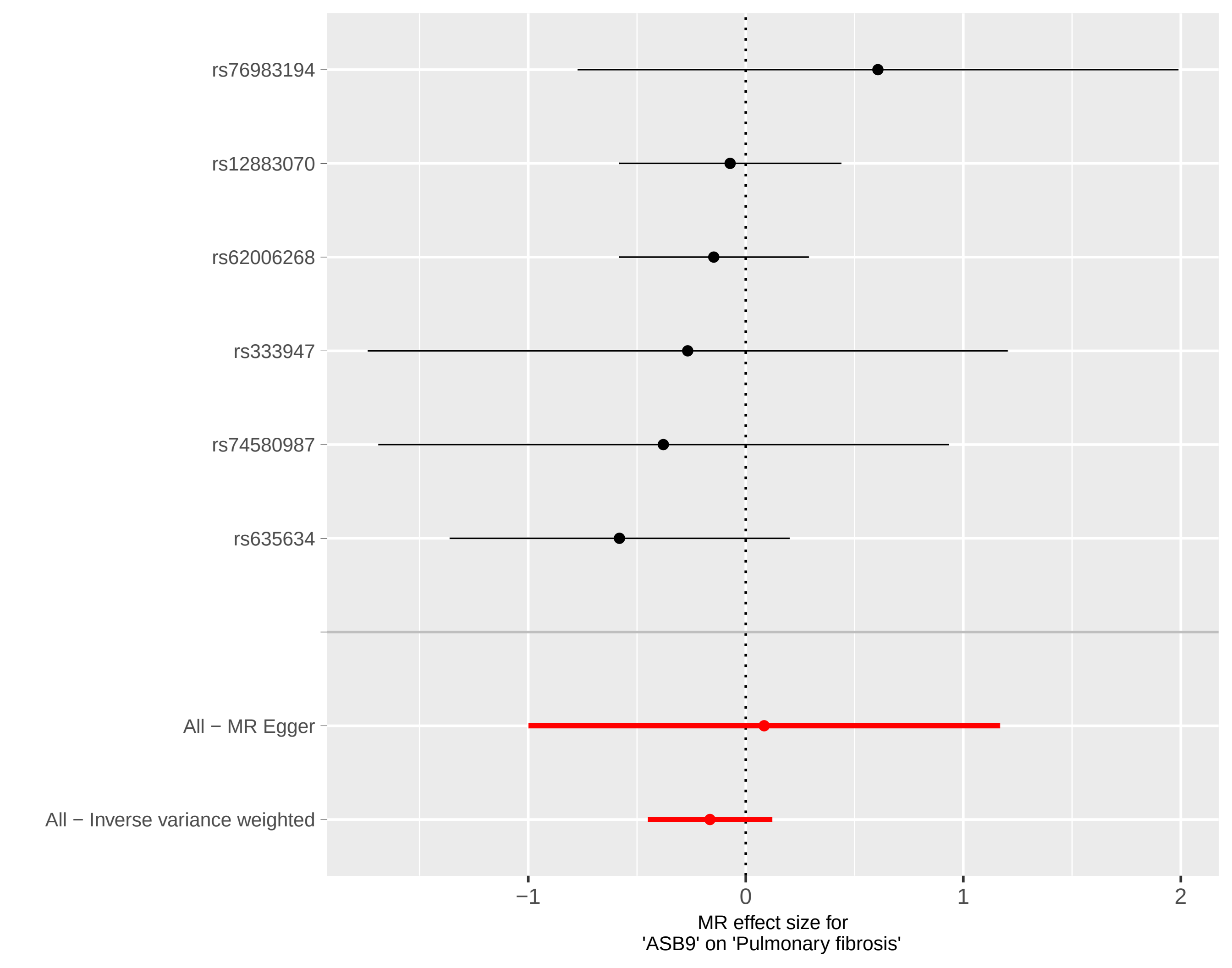
rs76983194
rs12883070
rs62006268
rs333947
rs74580987
rs635634
All − MR Egger
All − Inverse variance weighted
−1
0
1
2
MR effect size for
'ASB9' on 'Pulmonary fibrosis'

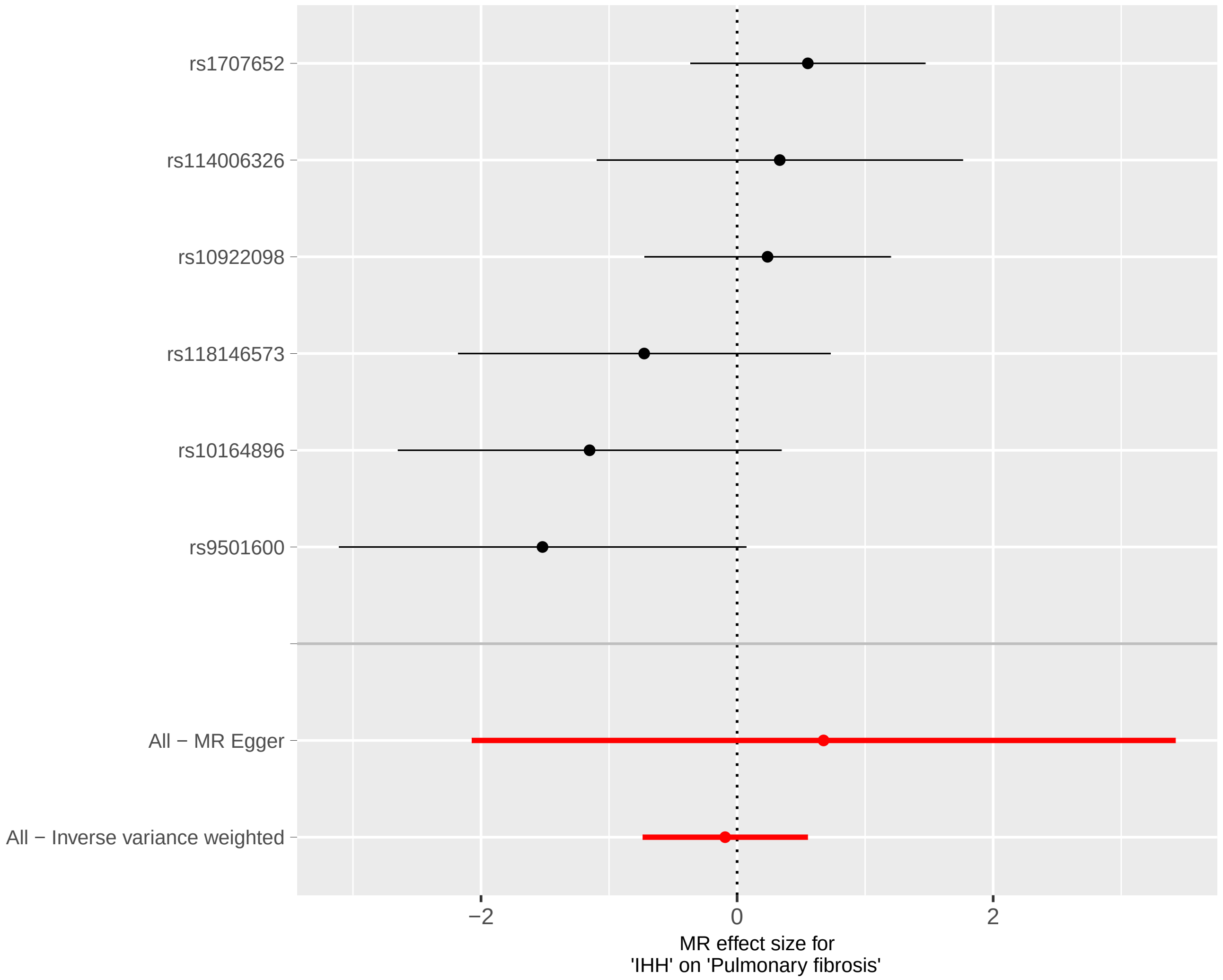
rs1707652
rs114006326
rs10922098
rs118146573
rs10164896
rs9501600
All − MR Egger
All − Inverse variance weighted
−2
0
2
MR effect size for
'IHH' on 'Pulmonary fibrosis'

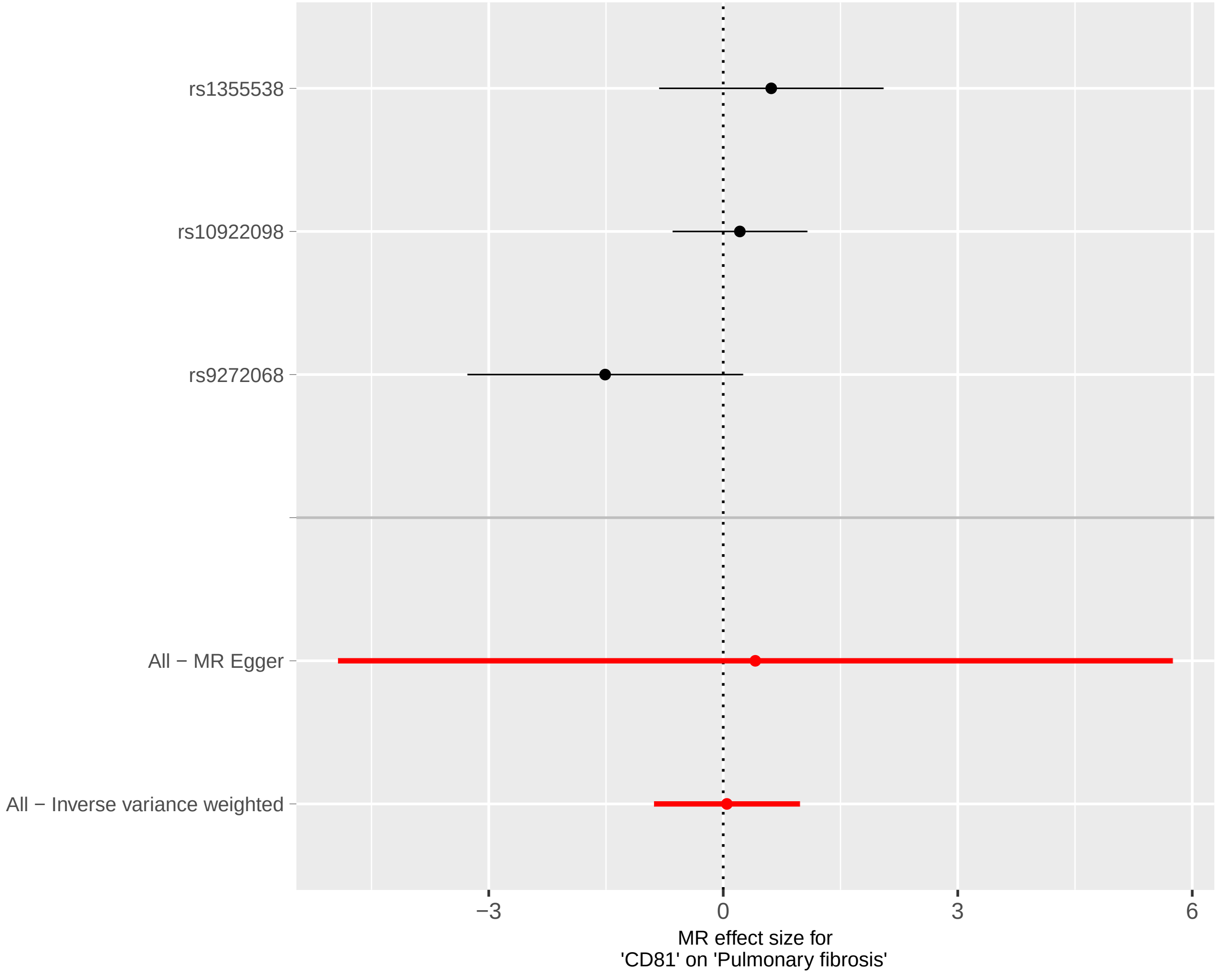
rs1355538
rs10922098
rs9272068
All − MR Egger
All − Inverse variance weighted
−3
0
3
6
MR effect size for
'CD81' on 'Pulmonary fibrosis'

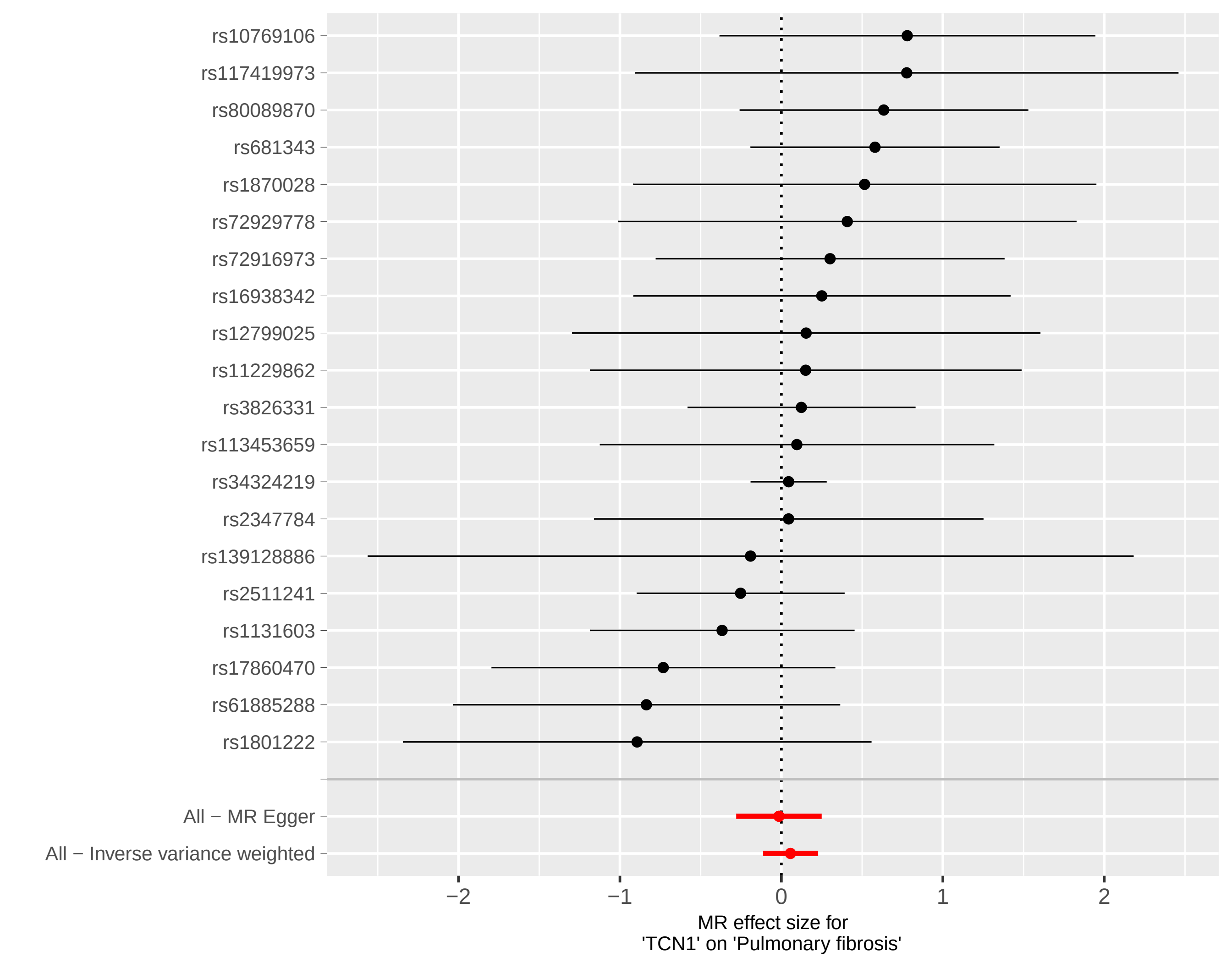
rs10769106
rs117419973
rs80089870
rs681343
rs1870028
rs72929778
rs72916973
rs16938342
rs12799025
rs11229862
rs3826331
rs113453659
rs34324219
rs2347784
rs139128886
rs2511241
rs1131603
rs17860470
rs61885288
rs1801222
All − MR Egger
All − Inverse variance weighted
−2
−1
0
1
2
MR effect size for
'TCN1' on 'Pulmonary fibrosis'

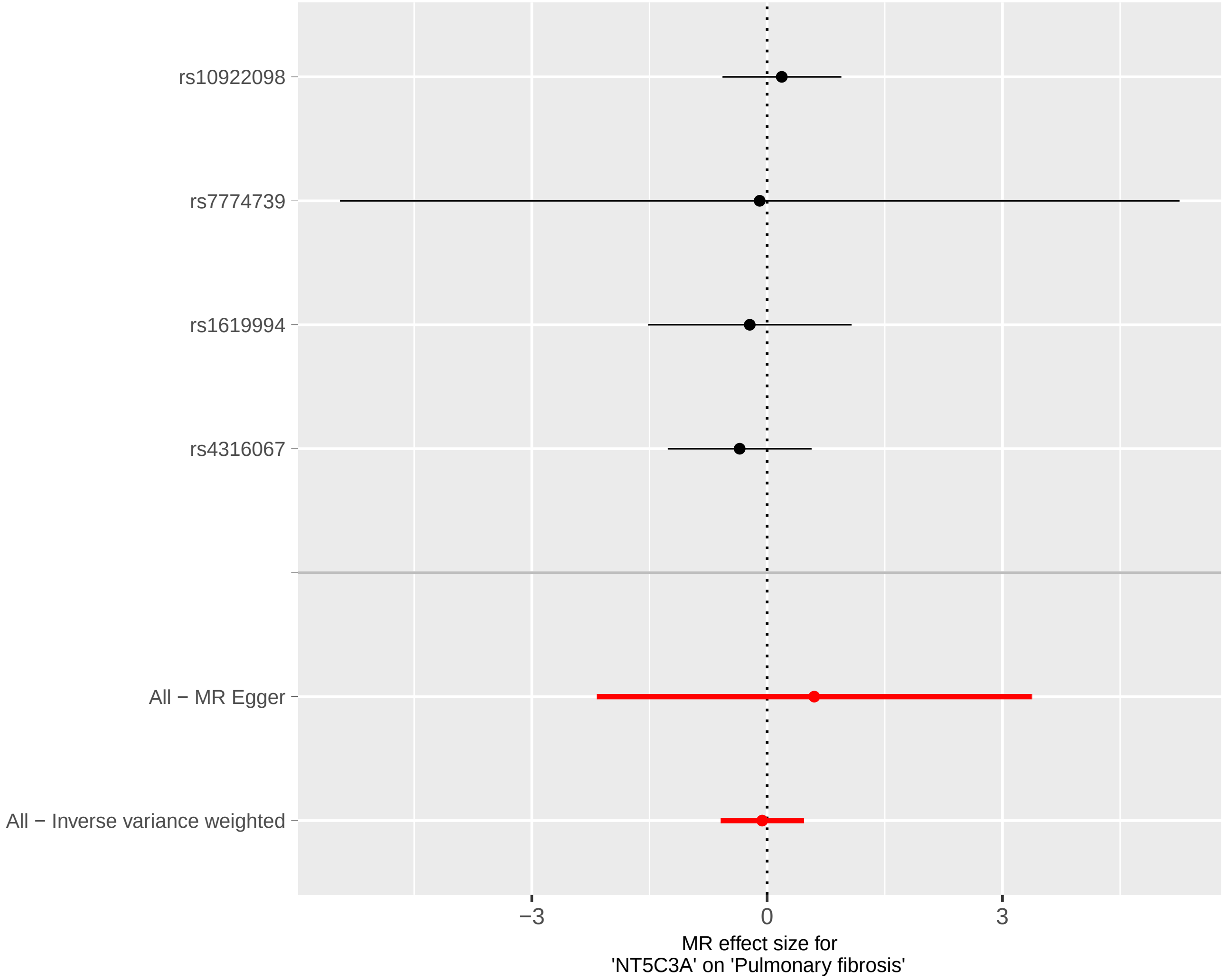

rs10922098
rs7774739
rs1619994
rs4316067
All − MR Egger
All − Inverse variance weighted
−3
0
3
MR effect size for
'NT5C3A' on 'Pulmonary fibrosis'

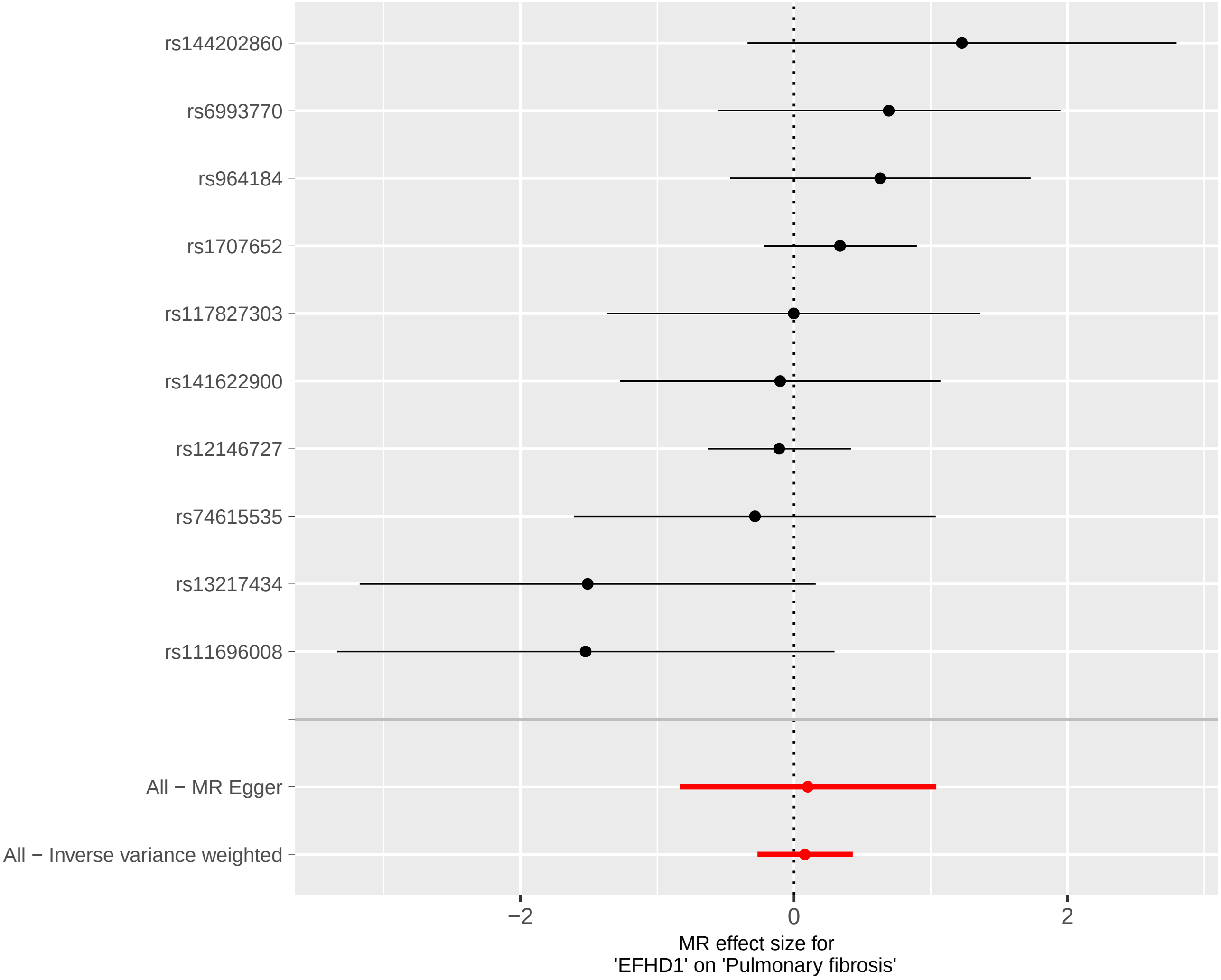
rs144202860
rs6993770
rs964184
rs1707652
rs117827303
rs141622900
rs12146727
rs74615535
rs13217434
rs111696008
All − MR Egger
All − Inverse variance weighted
−2
0
2
MR effect size for
'EFHD1' on 'Pulmonary fibrosis'

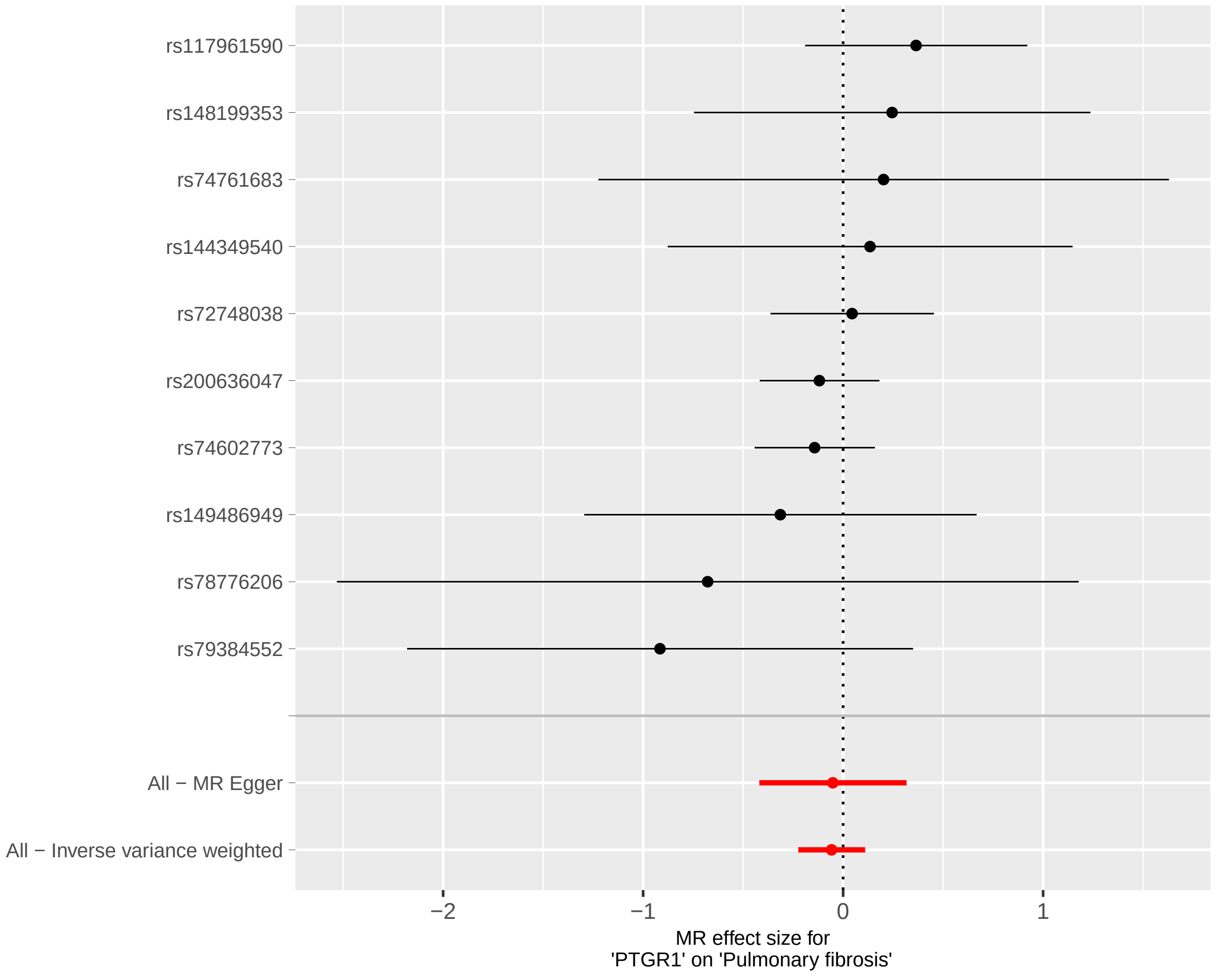

rs117961590
rs148199353
rs74761683
rs144349540
rs72748038
rs200636047
rs74602773
rs149486949
rs78776206
rs79384552
All − MR Egger
All − Inverse variance weighted
−2
−1
0
1
MR effect size for
'PTGR1' on 'Pulmonary fibrosis'

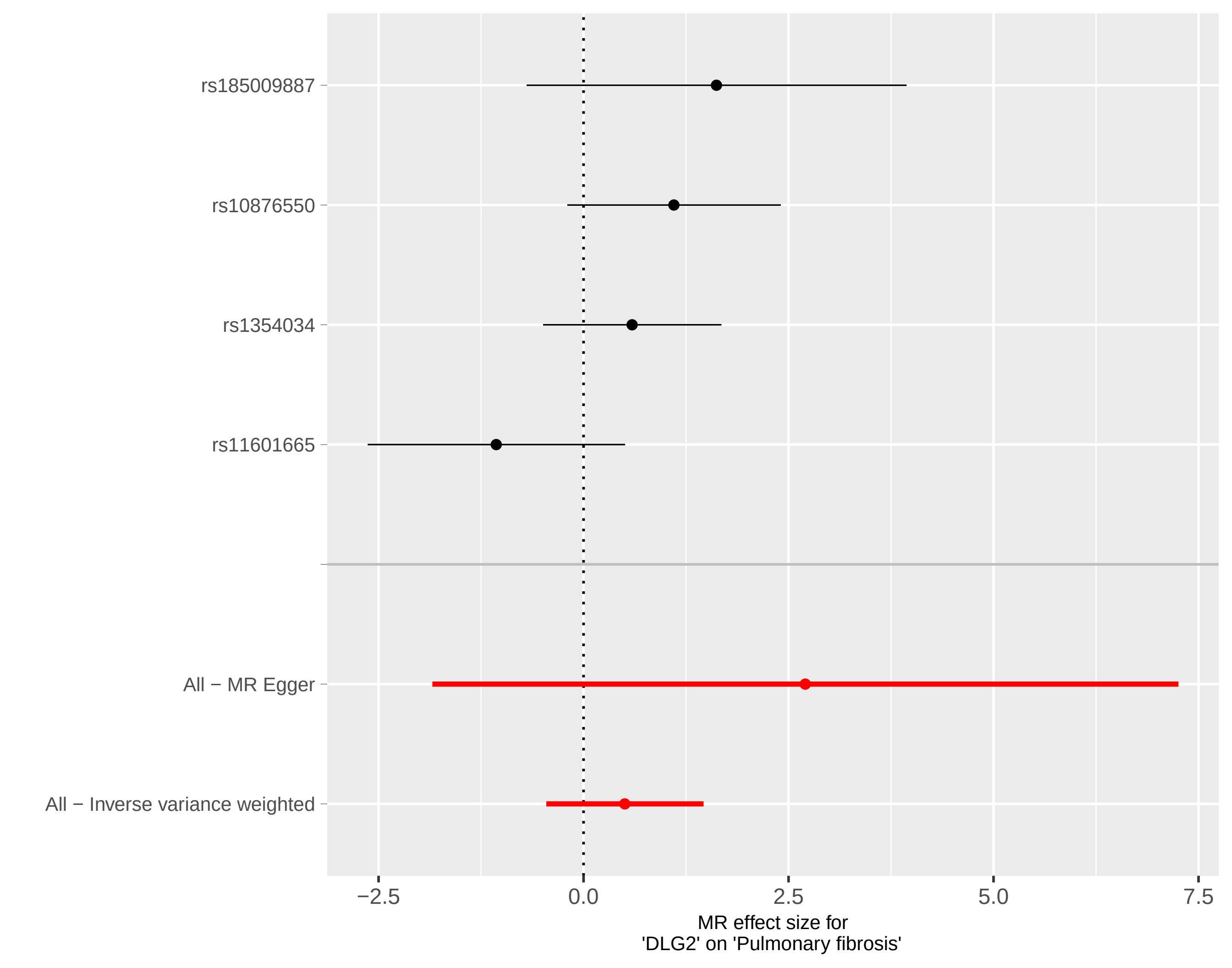

rs185009887
rs10876550
rs1354034
rs11601665
All − MR Egger
All − Inverse variance weighted
−2.5
0.0
2.5
5.0
7.5
MR effect size for
'DLG2' on 'Pulmonary fibrosis'

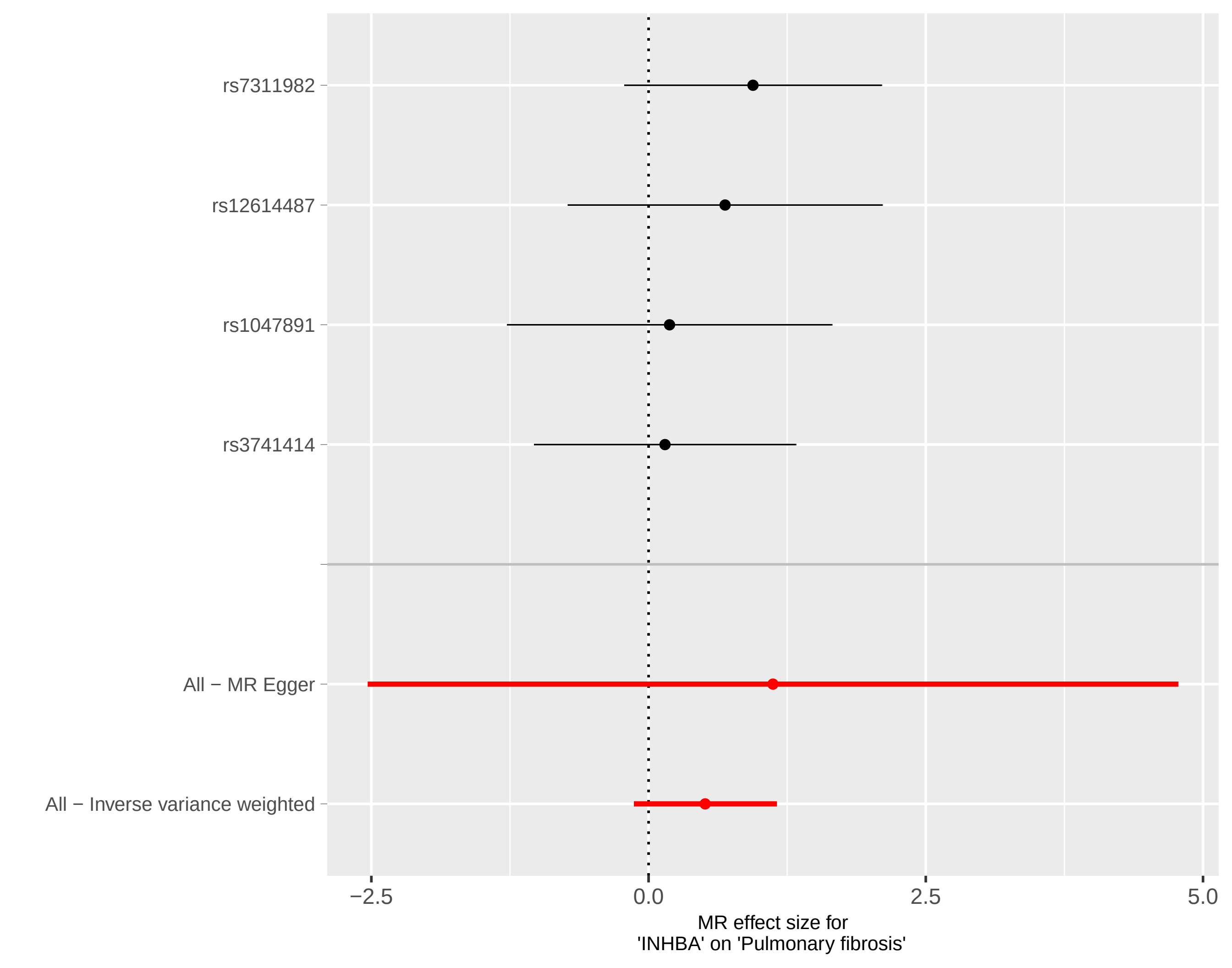
rs7311982
rs12614487
rs1047891
rs3741414
All − MR Egger
All − Inverse variance weighted
−2.5
0.0
2.5
5.0
MR effect size for
'INHBA' on 'Pulmonary fibrosis'

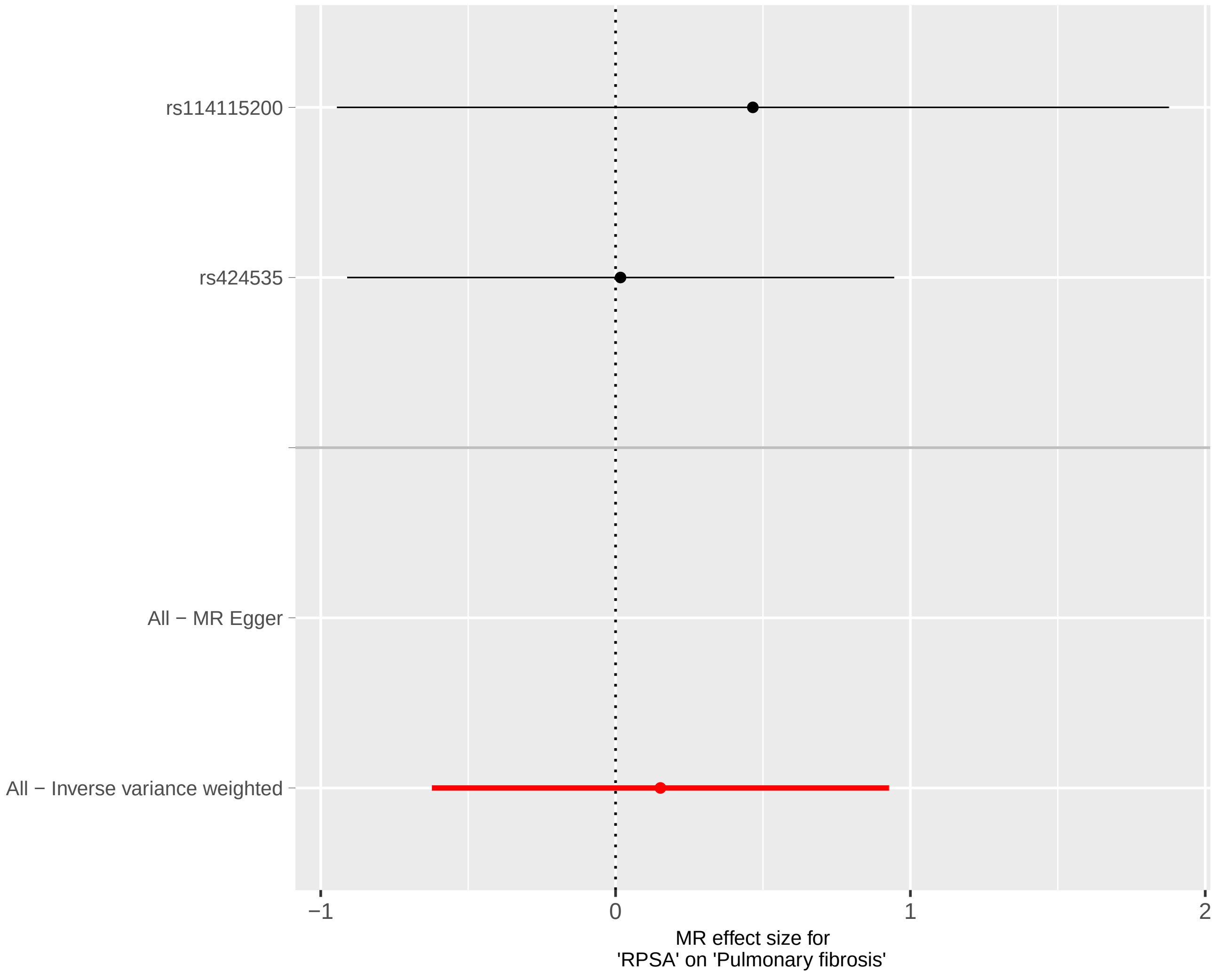
rs114115200
rs424535
All − MR Egger
All − Inverse variance weighted
−1
0
1
2
MR effect size for
'RPSA' on 'Pulmonary fibrosis'

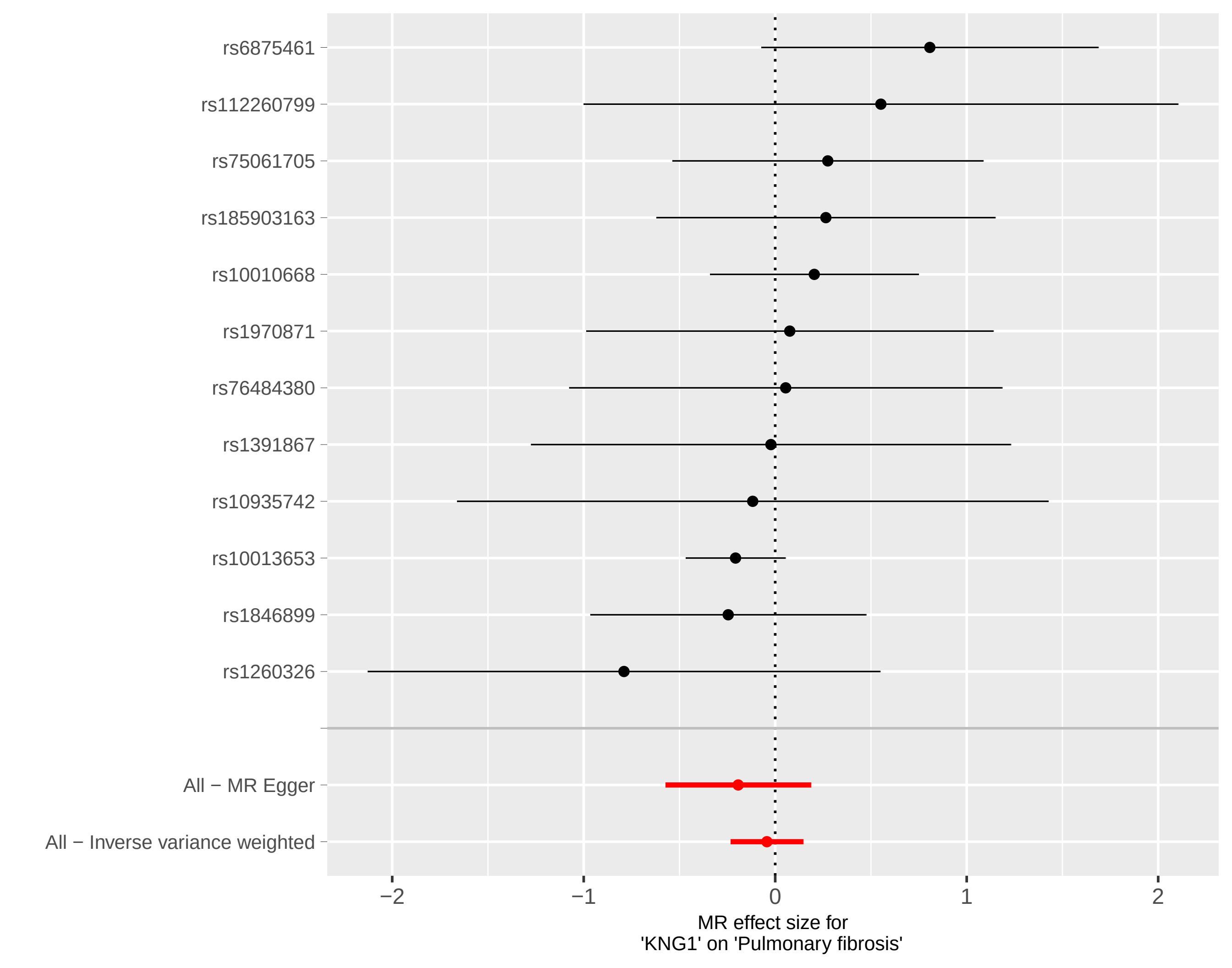

rs6875461
rs112260799
rs75061705
rs185903163
rs10010668
rs1970871
rs76484380
rs1391867
rs10935742
rs10013653
rs1846899
rs1260326
All − MR Egger
All − Inverse variance weighted
−2
−1
0
1
2
MR effect size for
'KNG1' on 'Pulmonary fibrosis'

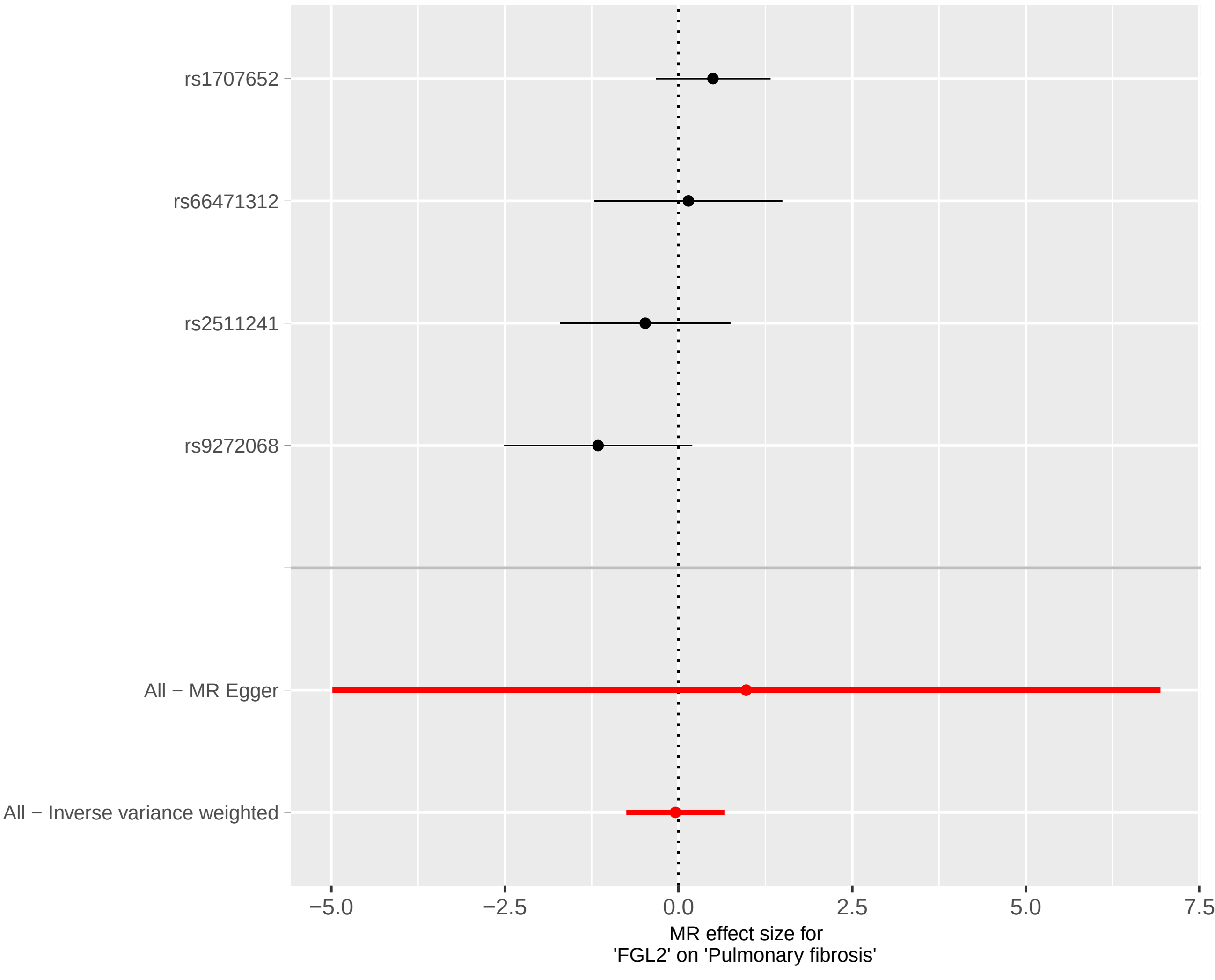
rs1707652
rs66471312
rs2511241
rs9272068
All − MR Egger
All − Inverse variance weighted
−5.0
−2.5
0.0
2.5
5.0
7.5
MR effect size for
'FGL2' on 'Pulmonary fibrosis'

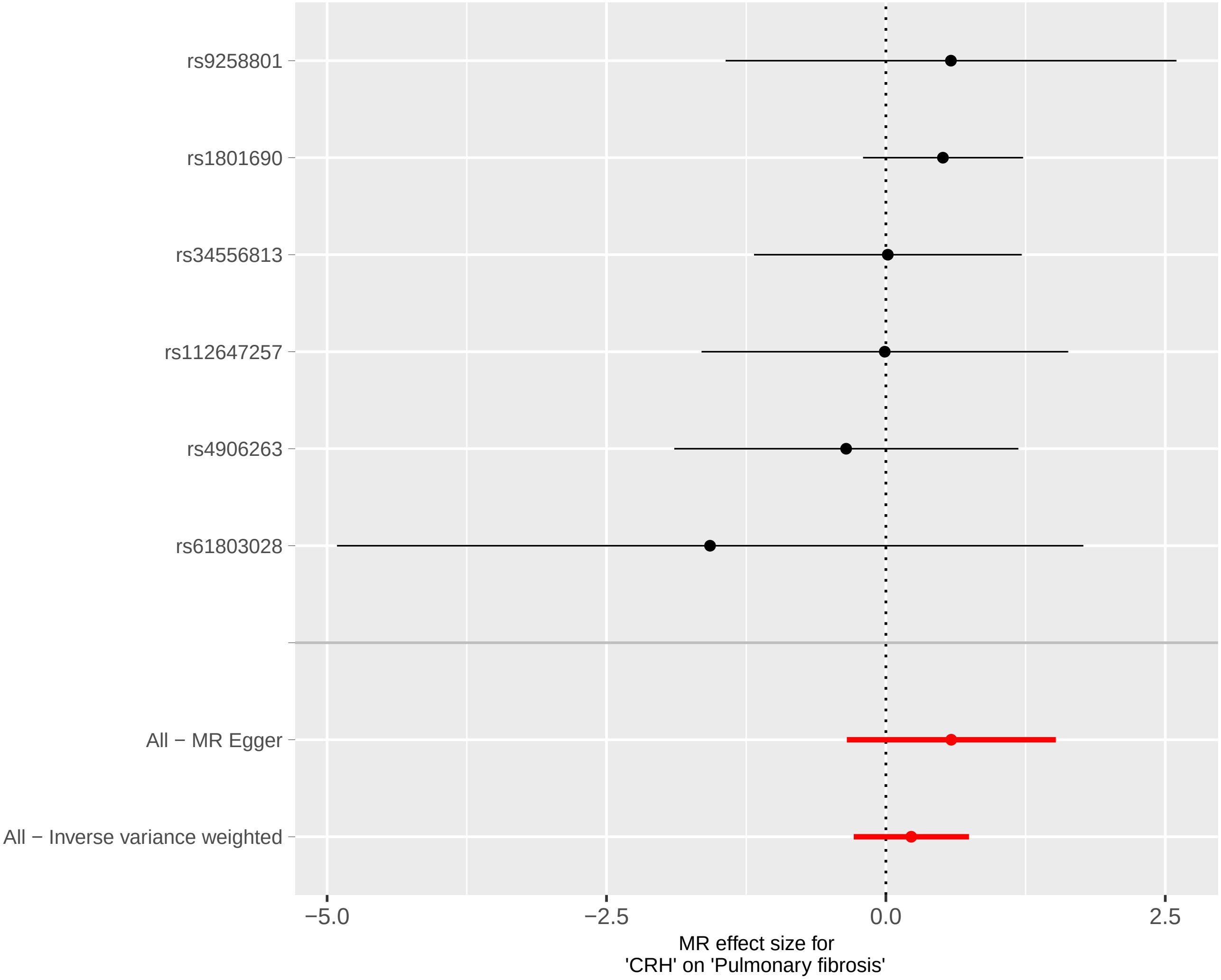

rs9258801
rs1801690
rs34556813
rs112647257
rs4906263
rs61803028
All − MR Egger
All − Inverse variance weighted
−5.0
−2.5
0.0
2.5
MR effect size for
'CRH' on 'Pulmonary fibrosis'

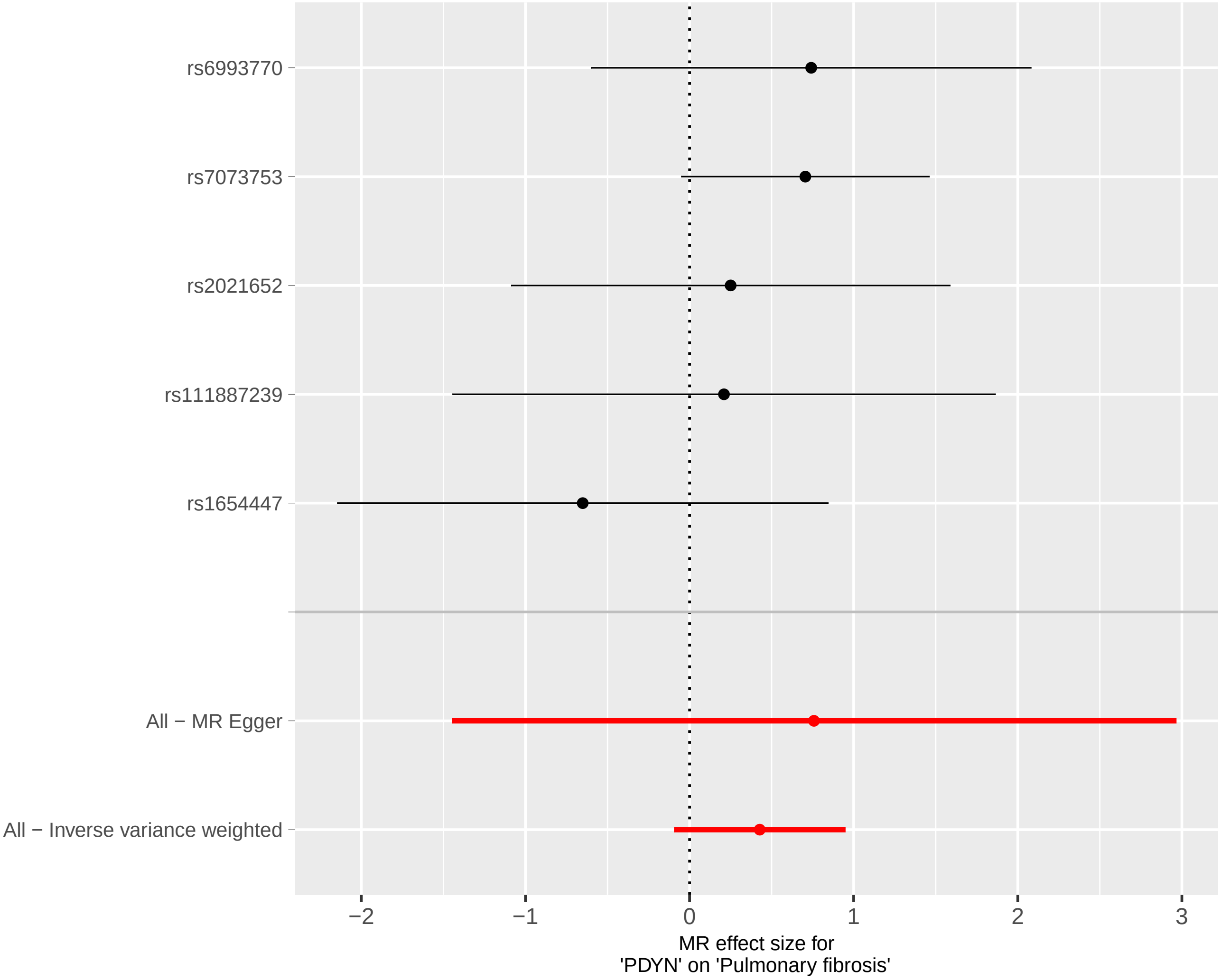

rs6993770
rs7073753
rs2021652
rs111887239
rs1654447
All − MR Egger
All − Inverse variance weighted
−2
−1
0
1
2
3
MR effect size for
'PDYN' on 'Pulmonary fibrosis'

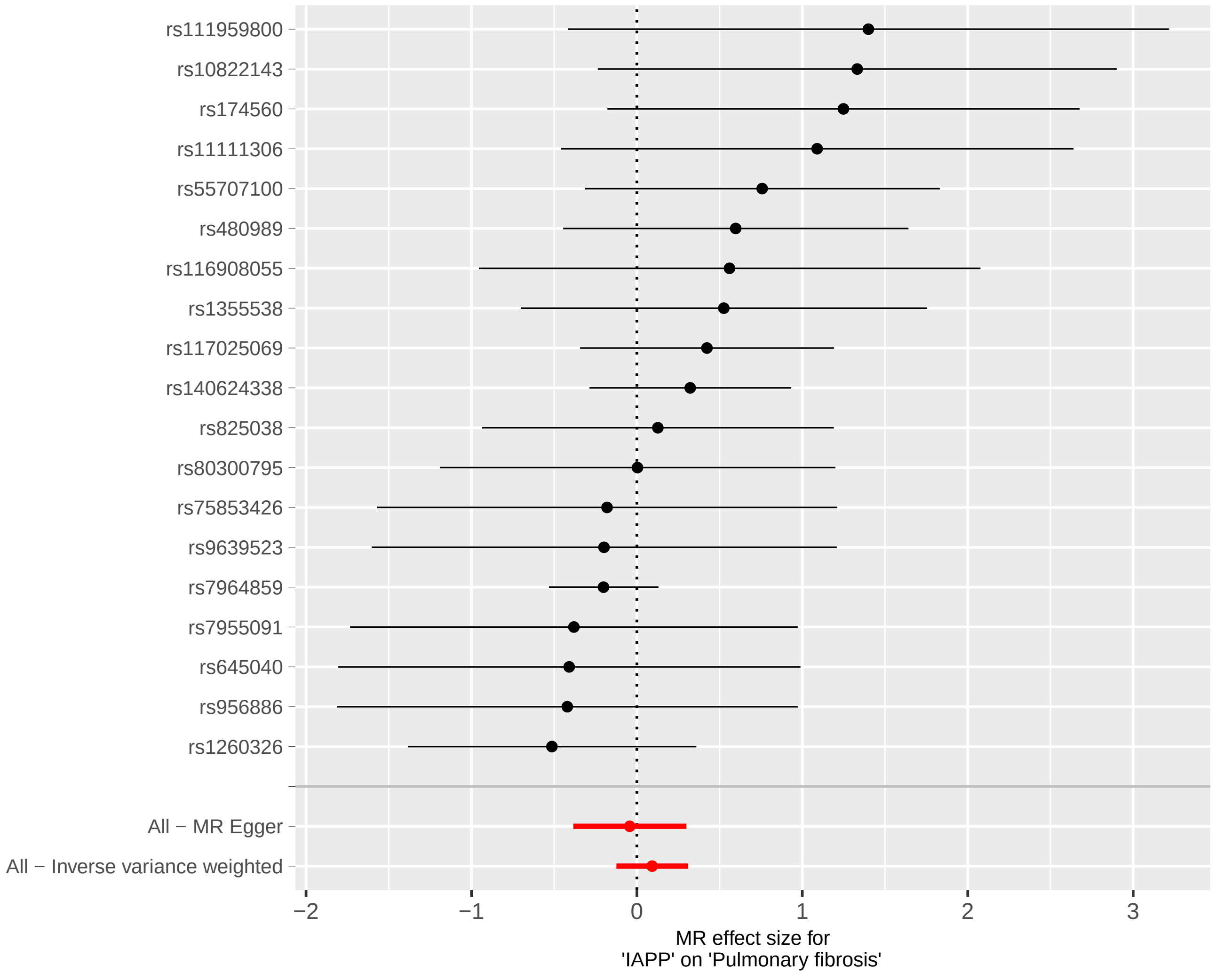
rs111959800
rs10822143
rs174560
rs11111306
rs55707100
rs480989
rs116908055
rs1355538
rs117025069
rs140624338
rs825038
rs80300795
rs75853426
rs9639523
rs7964859
rs7955091
rs645040
rs956886
rs1260326
All − MR Egger
All − Inverse variance weighted
−2
−1
0
1
2
3
MR effect size for
'IAPP' on 'Pulmonary fibrosis'

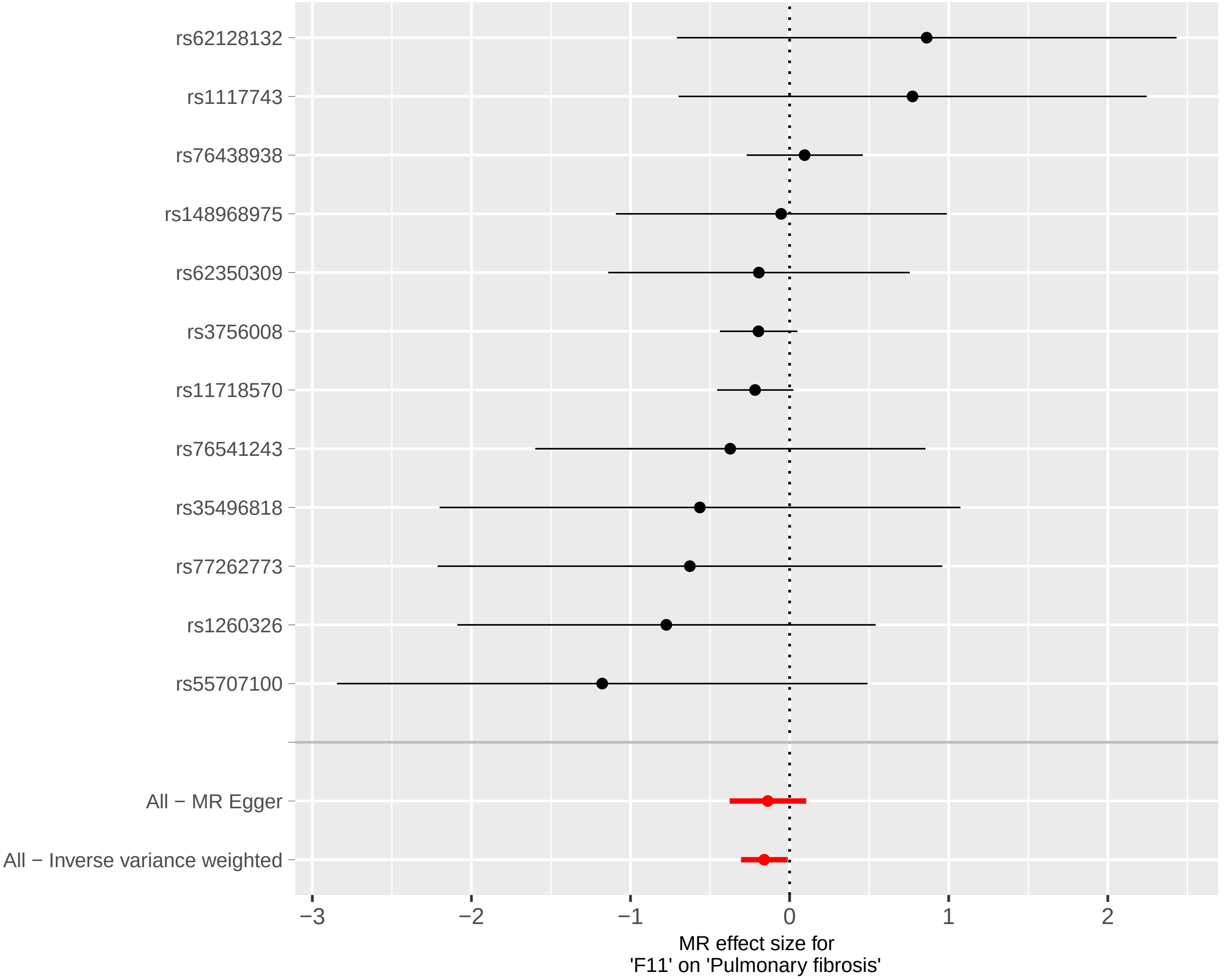

rs62128132
rs1117743
rs76438938
rs148968975
rs62350309
rs3756008
rs11718570
rs76541243
rs35496818
rs77262773
rs1260326
rs55707100
All − MR Egger
All − Inverse variance weighted
−3
−2
−1
0
1
2
MR effect size for
'F11' on 'Pulmonary fibrosis'

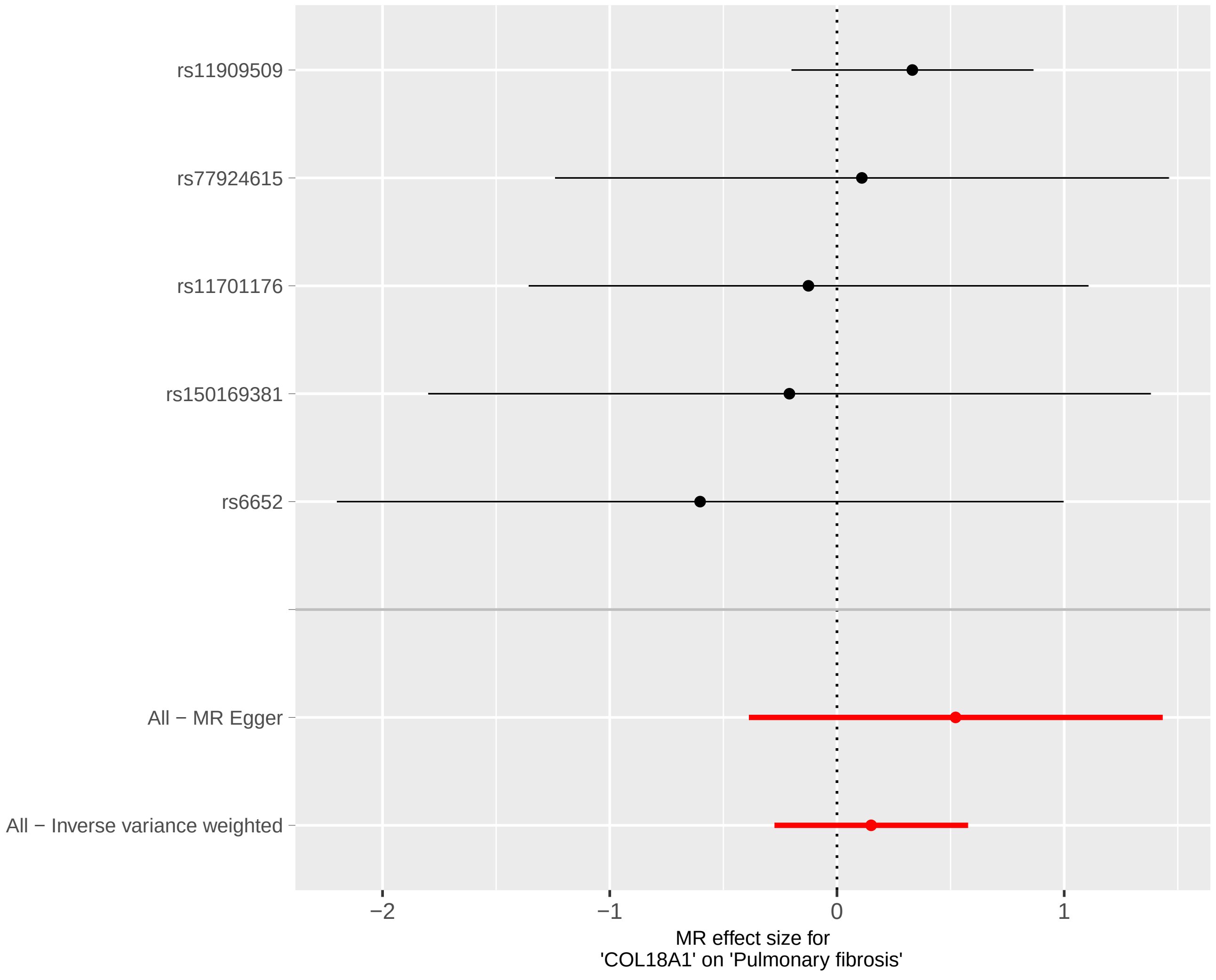

rs11909509
rs77924615
rs11701176
rs150169381
rs6652
All − MR Egger
All − Inverse variance weighted
−2
−1
0
1
MR effect size for
'COL18A1' on 'Pulmonary fibrosis'

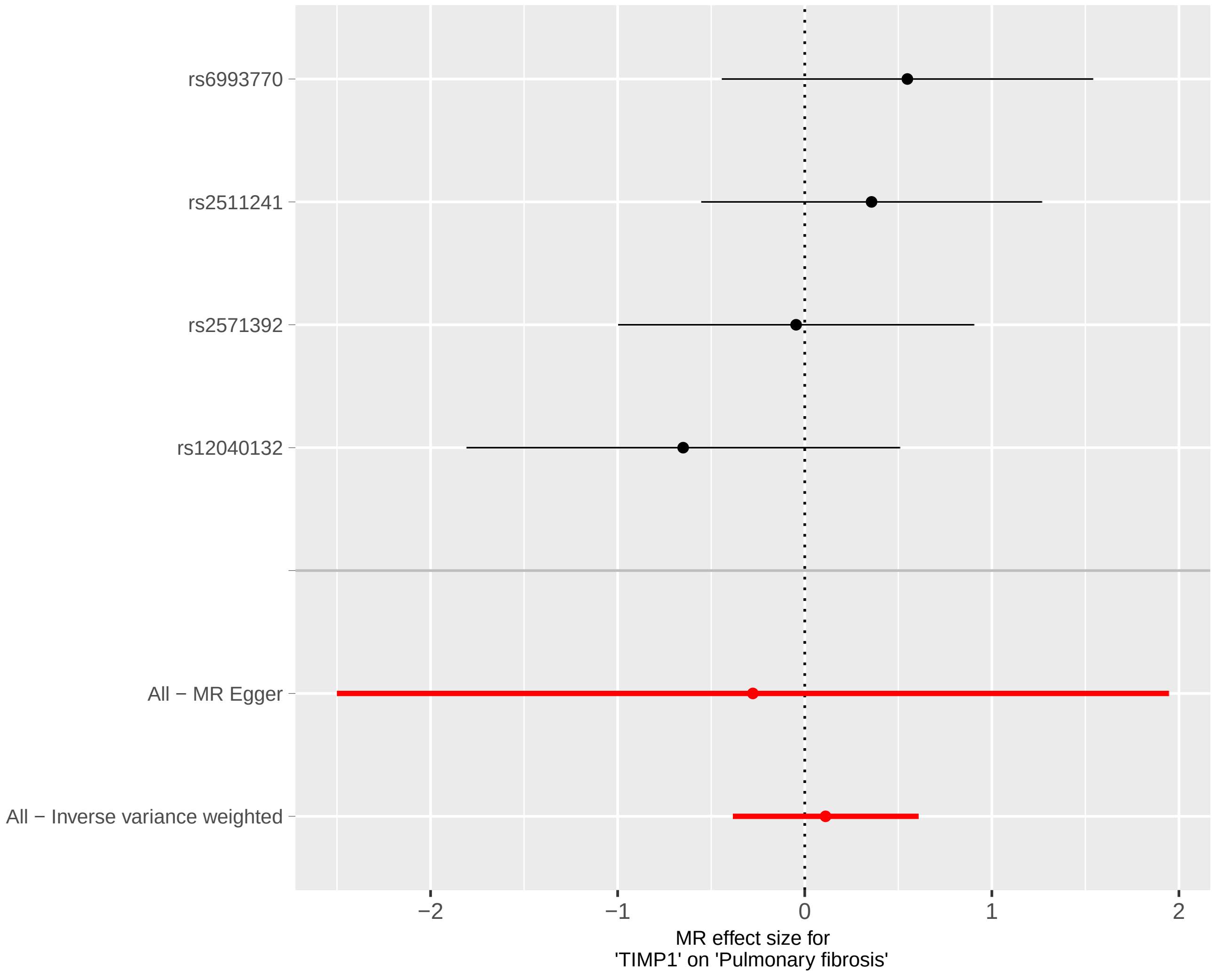

rs6993770
rs2511241
rs2571392
rs12040132
All − MR Egger
All − Inverse variance weighted
−2
−1
0
1
2
MR effect size for
'TIMP1' on 'Pulmonary fibrosis'

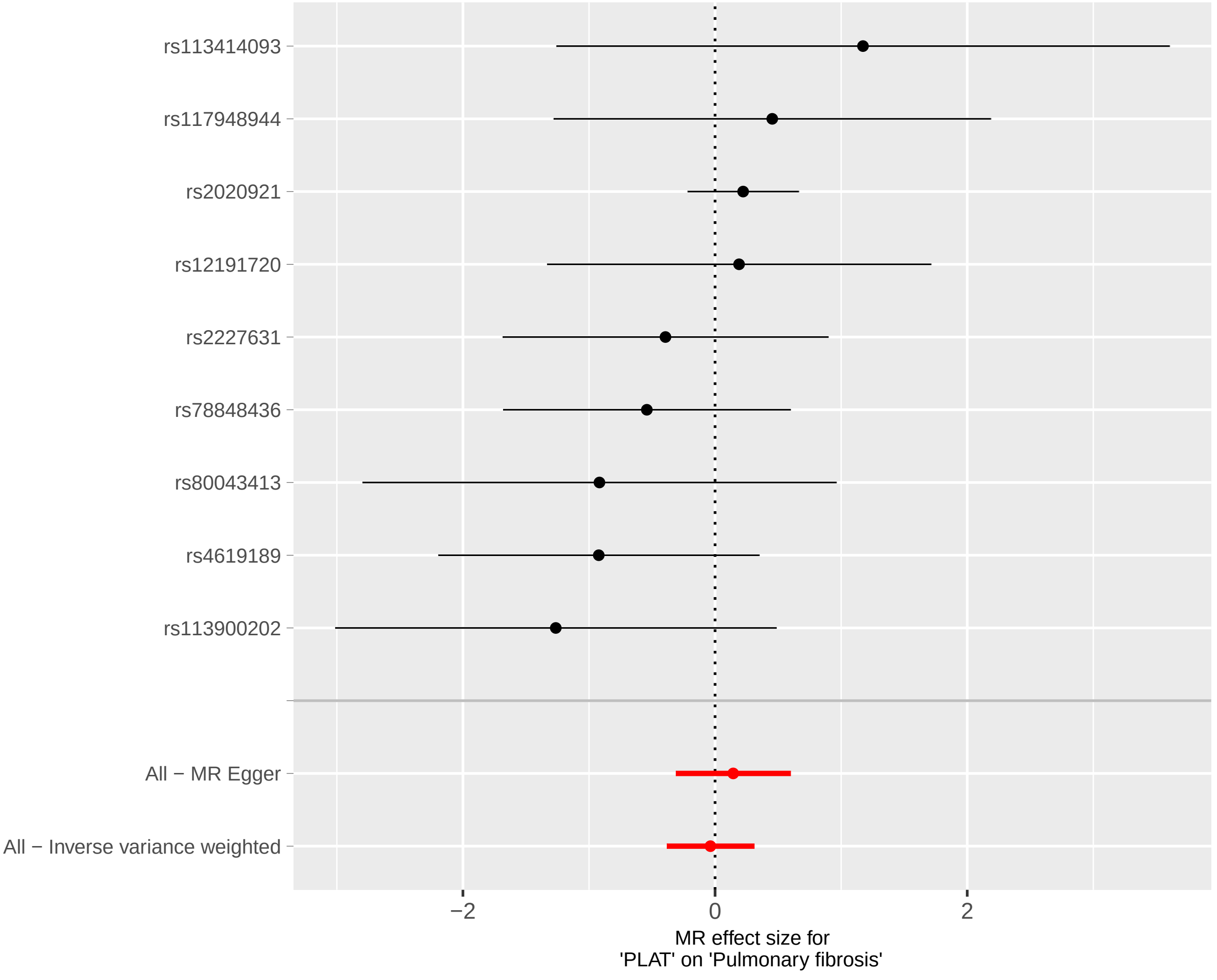

rs113414093
rs117948944
rs2020921
rs12191720
rs2227631
rs78848436
rs80043413
rs4619189
rs113900202
All − MR Egger
All − Inverse variance weighted
−2
0
2
MR effect size for
'PLAT' on 'Pulmonary fibrosis'

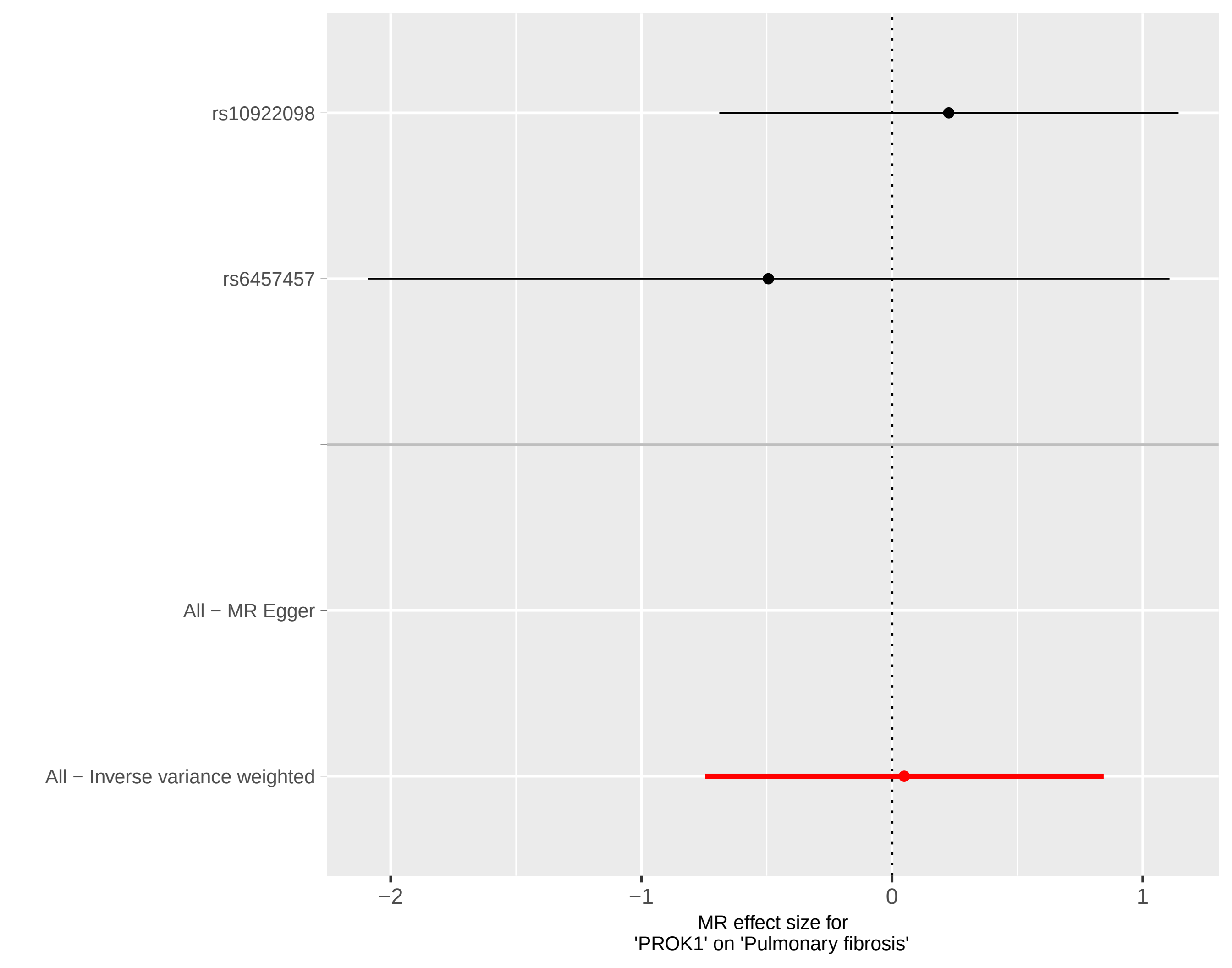

rs10922098
rs6457457
All − MR Egger
All − Inverse variance weighted
−2
−1
0
1
MR effect size for
'PROK1' on 'Pulmonary fibrosis'

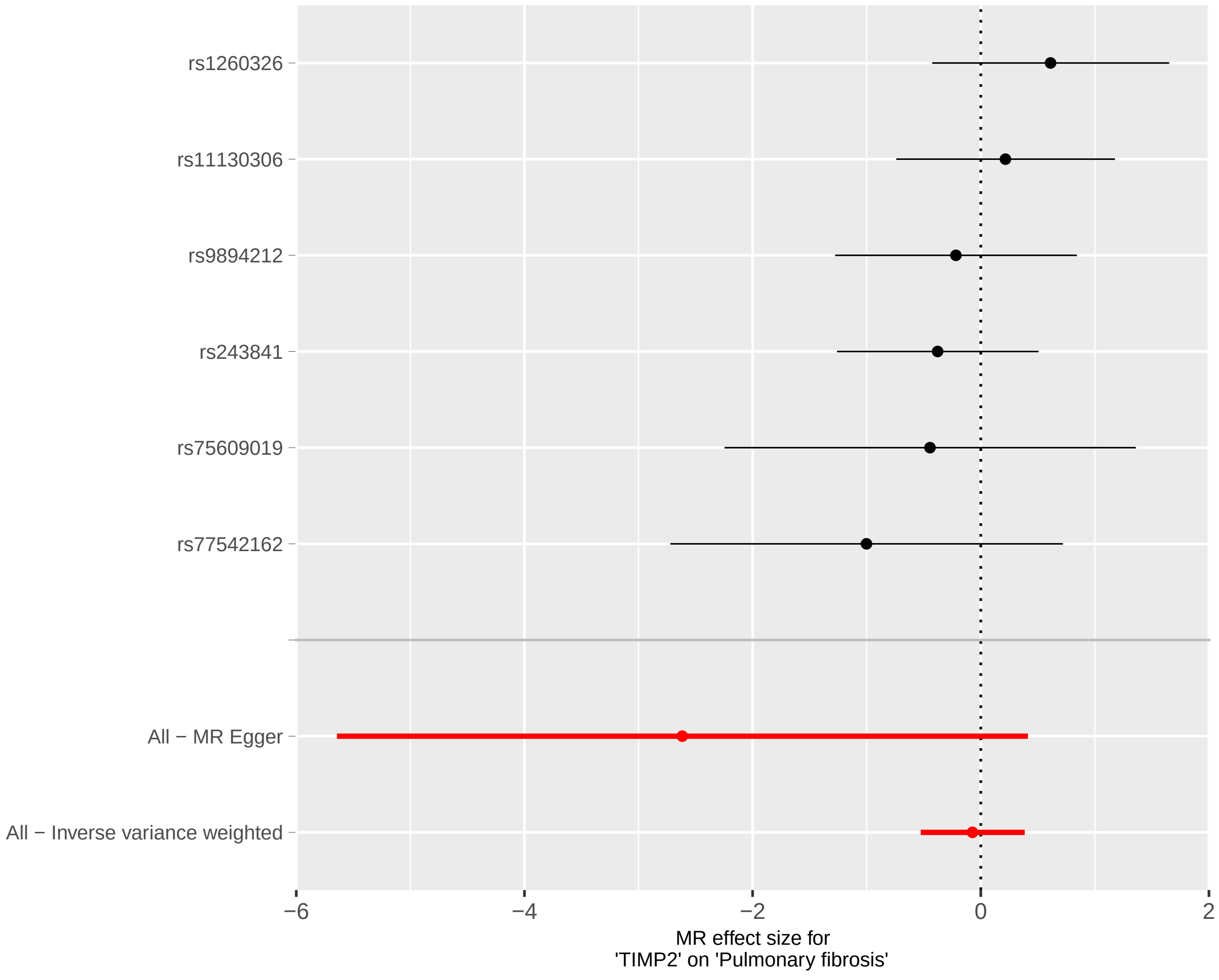
rs1260326
rs11130306
rs9894212
rs243841
rs75609019
rs77542162
All − MR Egger
All − Inverse variance weighted
−6
−4
−2
0
2
MR effect size for
'TIMP2' on 'Pulmonary fibrosis'

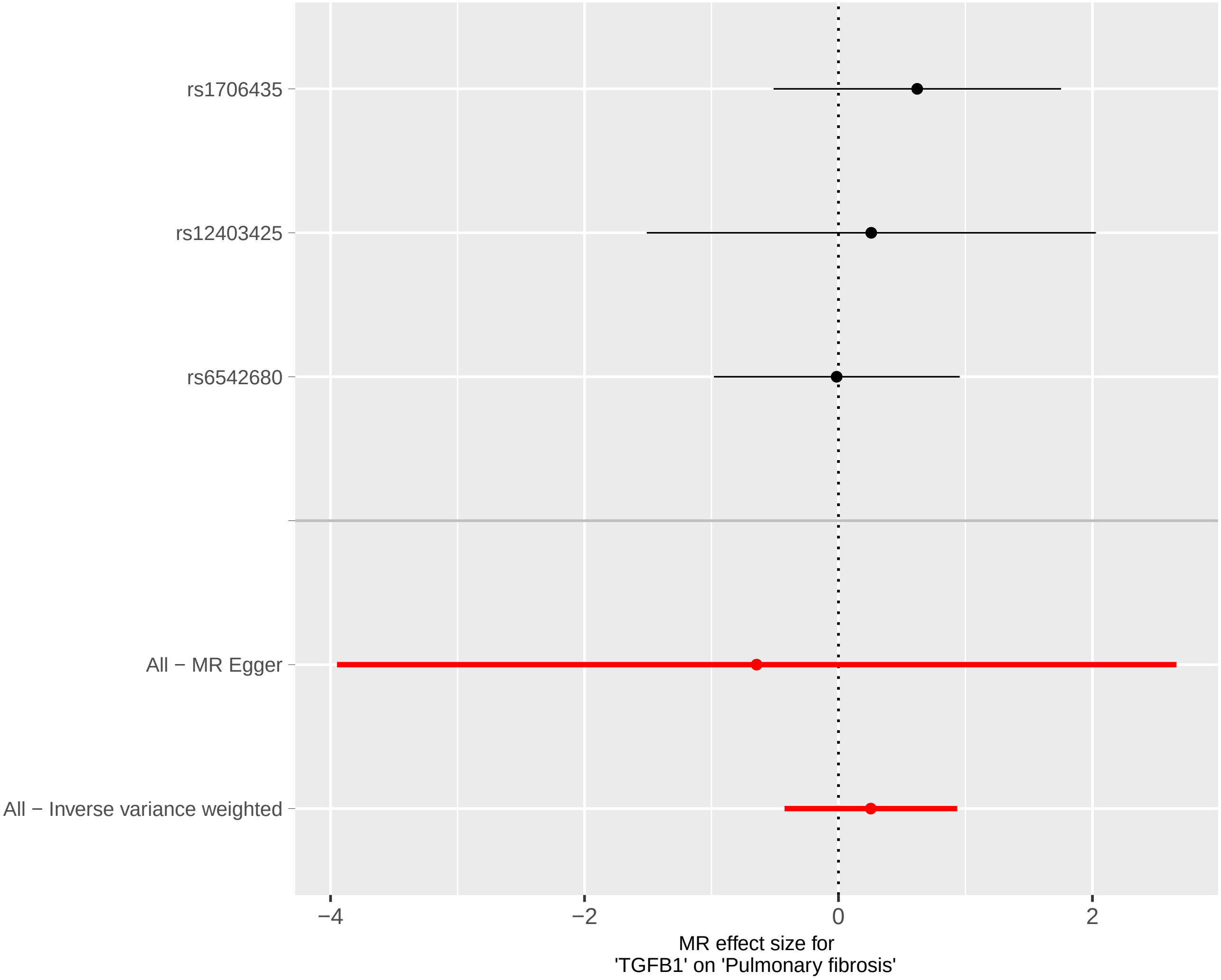

rs1706435
rs12403425
rs6542680
All − MR Egger
All − Inverse variance weighted
−4
−2
0
2
MR effect size for
'TGFB1' on 'Pulmonary fibrosis'

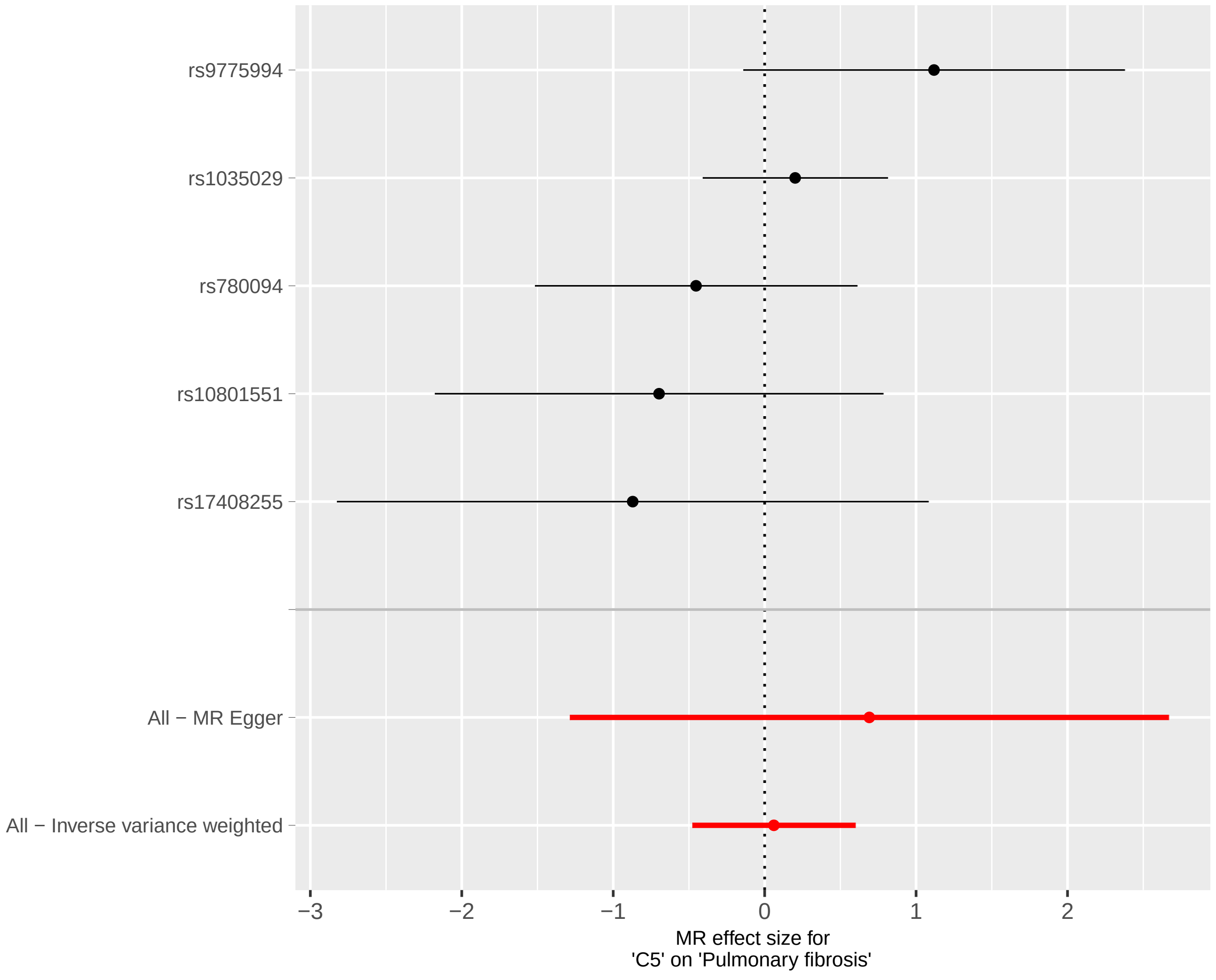

rs9775994
rs1035029
rs780094
rs10801551
rs17408255
All − MR Egger
All − Inverse variance weighted
−3
−2
−1
0
1
2
MR effect size for
'C5' on 'Pulmonary fibrosis'

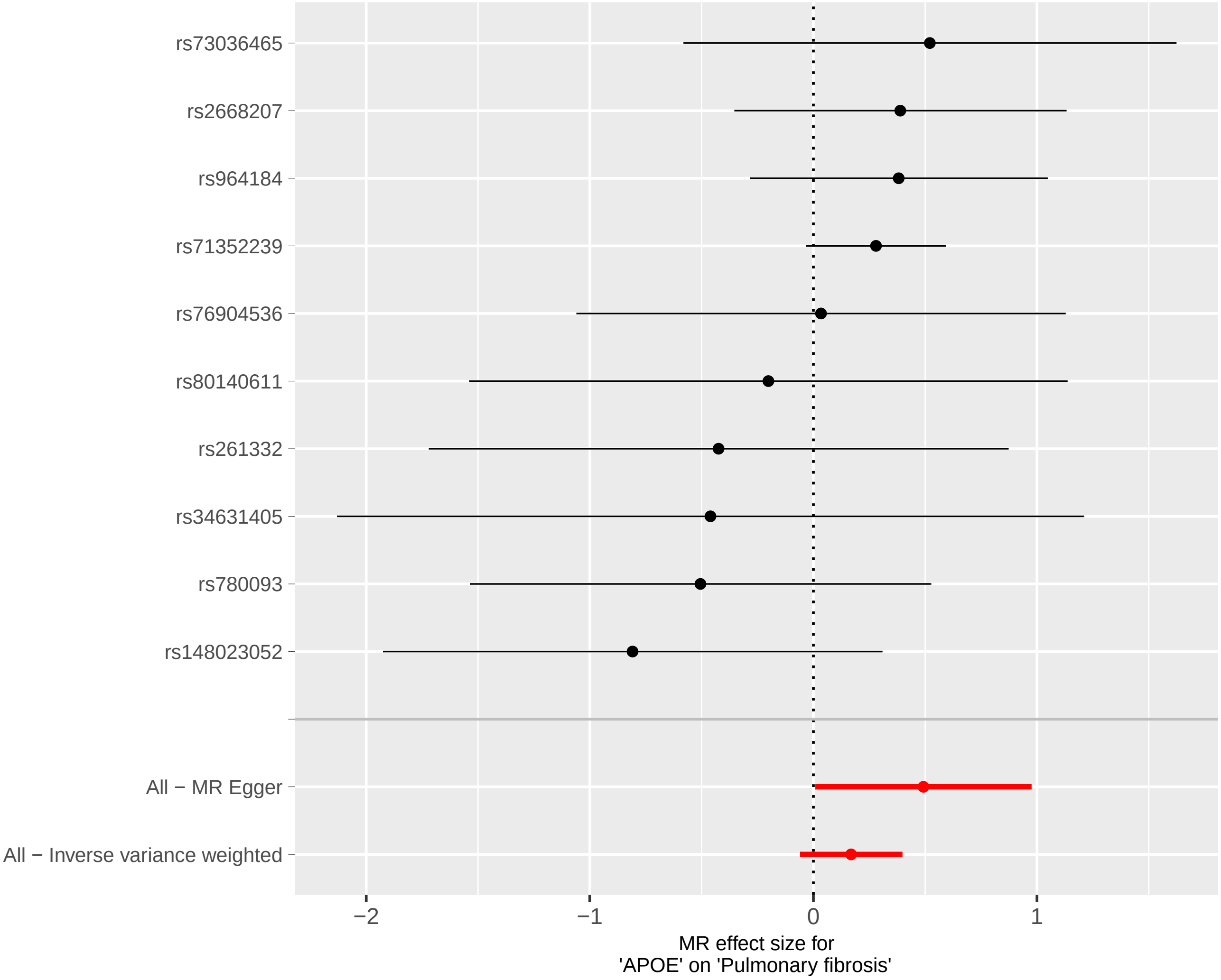
rs73036465
rs2668207
rs964184
rs71352239
rs76904536
rs80140611
rs261332
rs34631405
rs780093
rs148023052
All − MR Egger
All − Inverse variance weighted
−2
−1
0
1
MR effect size for
'APOE' on 'Pulmonary fibrosis'

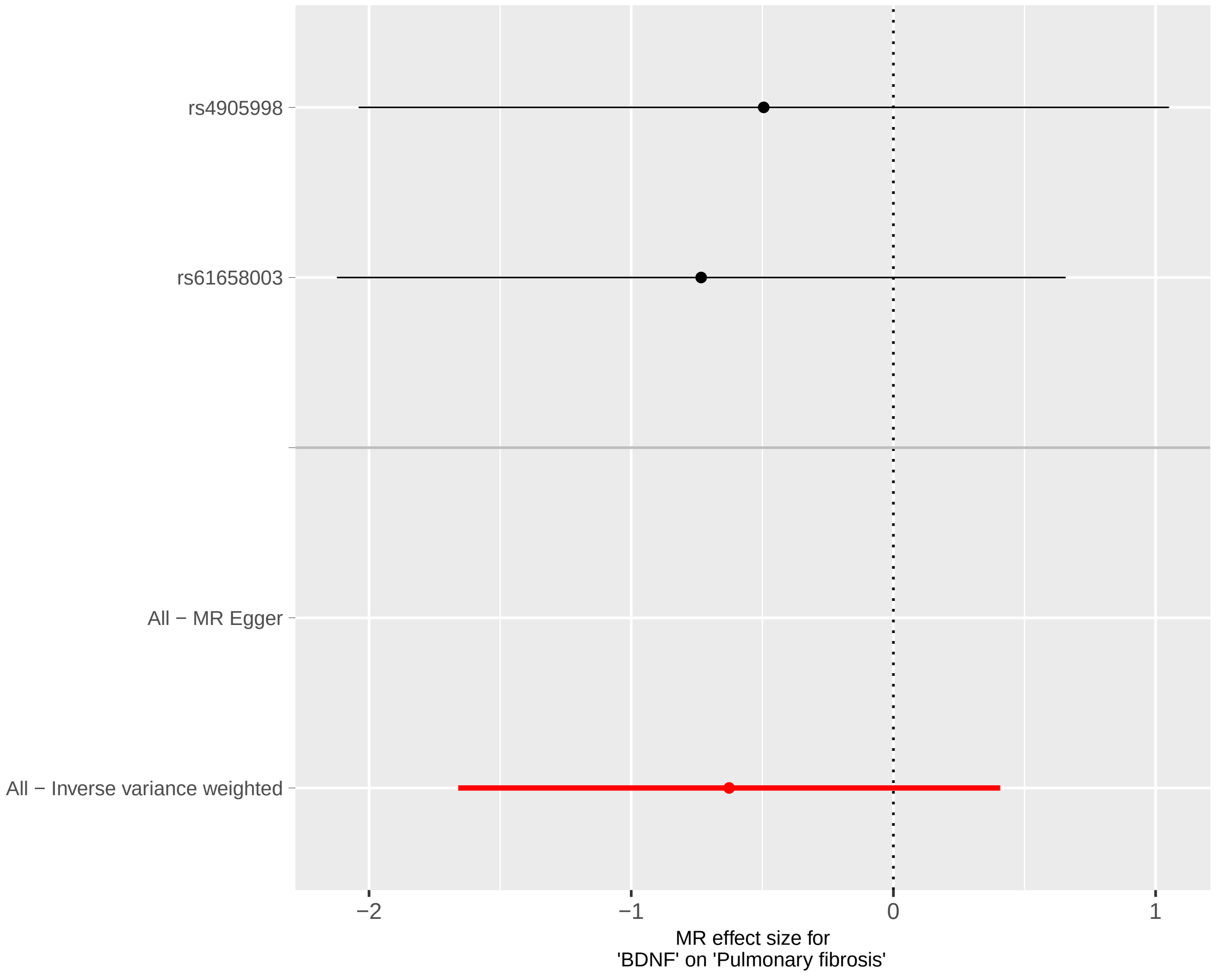

rs4905998
rs61658003
All − MR Egger
All − Inverse variance weighted
−2
−1
0
1
MR effect size for
'BDNF' on 'Pulmonary fibrosis'

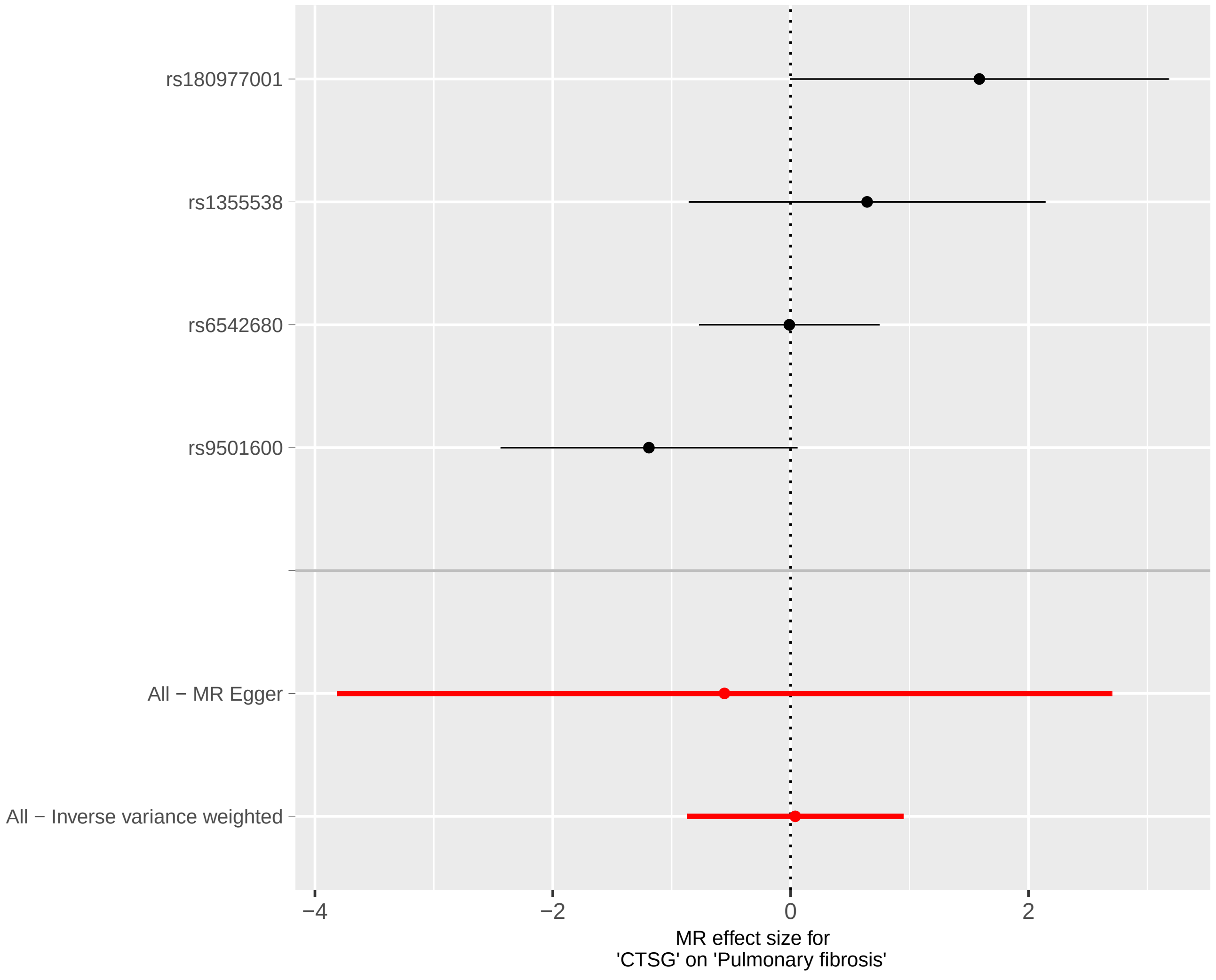

rs180977001
rs1355538
rs6542680
rs9501600
All − MR Egger
All − Inverse variance weighted
−4
−2
0
2
MR effect size for
'CTSG' on 'Pulmonary fibrosis'

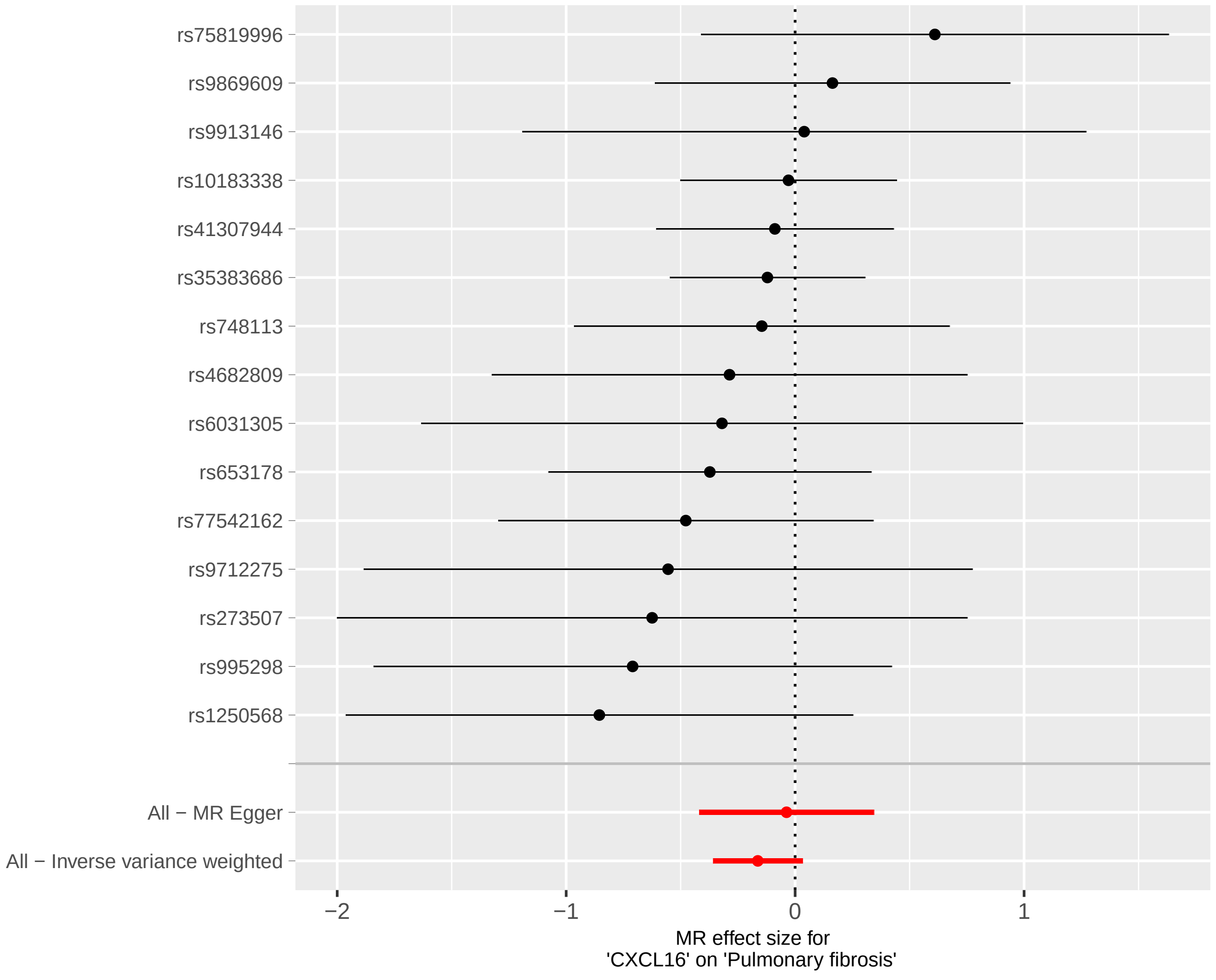

rs75819996
rs9869609
rs9913146
rs10183338
rs41307944
rs35383686
rs748113
rs4682809
rs6031305
rs653178
rs77542162
rs9712275
rs273507
rs995298
rs1250568
All − MR Egger
All − Inverse variance weighted
−2
−1
0
1
MR effect size for
'CXCL16' on 'Pulmonary fibrosis'

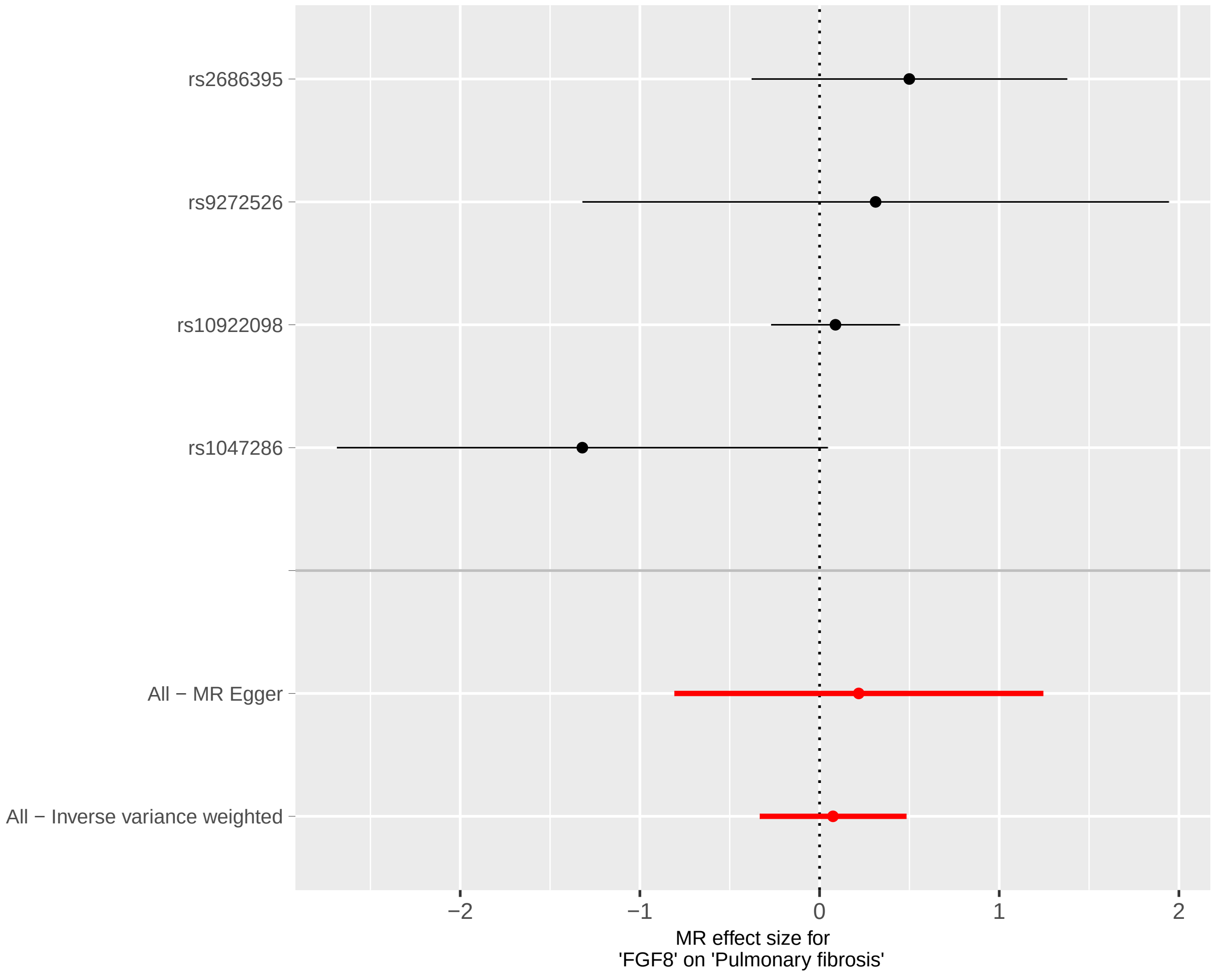
rs2686395
rs9272526
rs10922098
rs1047286
All − MR Egger
All − Inverse variance weighted
−2
−1
0
1
2
MR effect size for
'FGF8' on 'Pulmonary fibrosis'

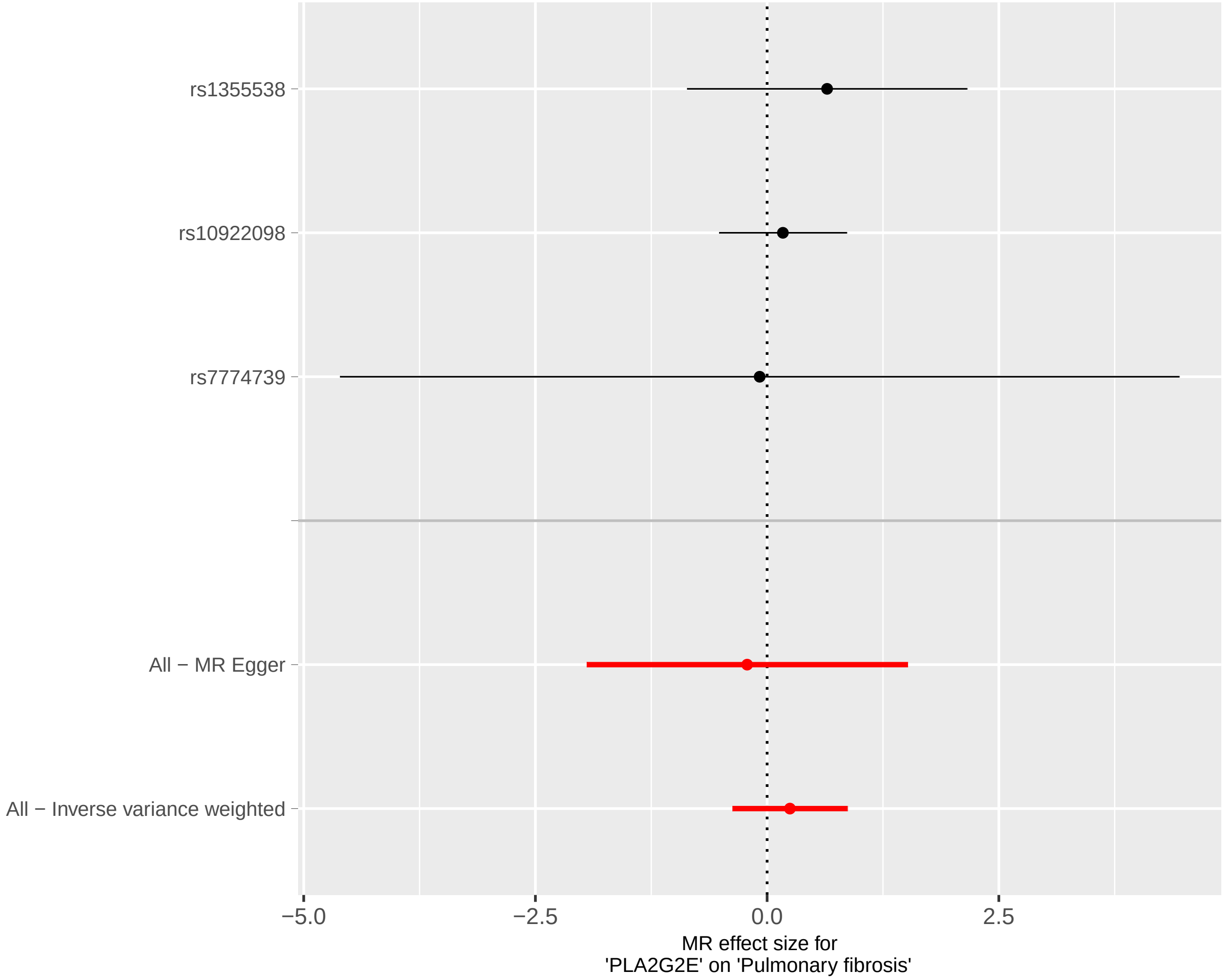
rs1355538
rs10922098
rs7774739
All − MR Egger
All − Inverse variance weighted
−5.0
−2.5
0.0
2.5
MR effect size for
'PLA2G2E' on 'Pulmonary fibrosis'

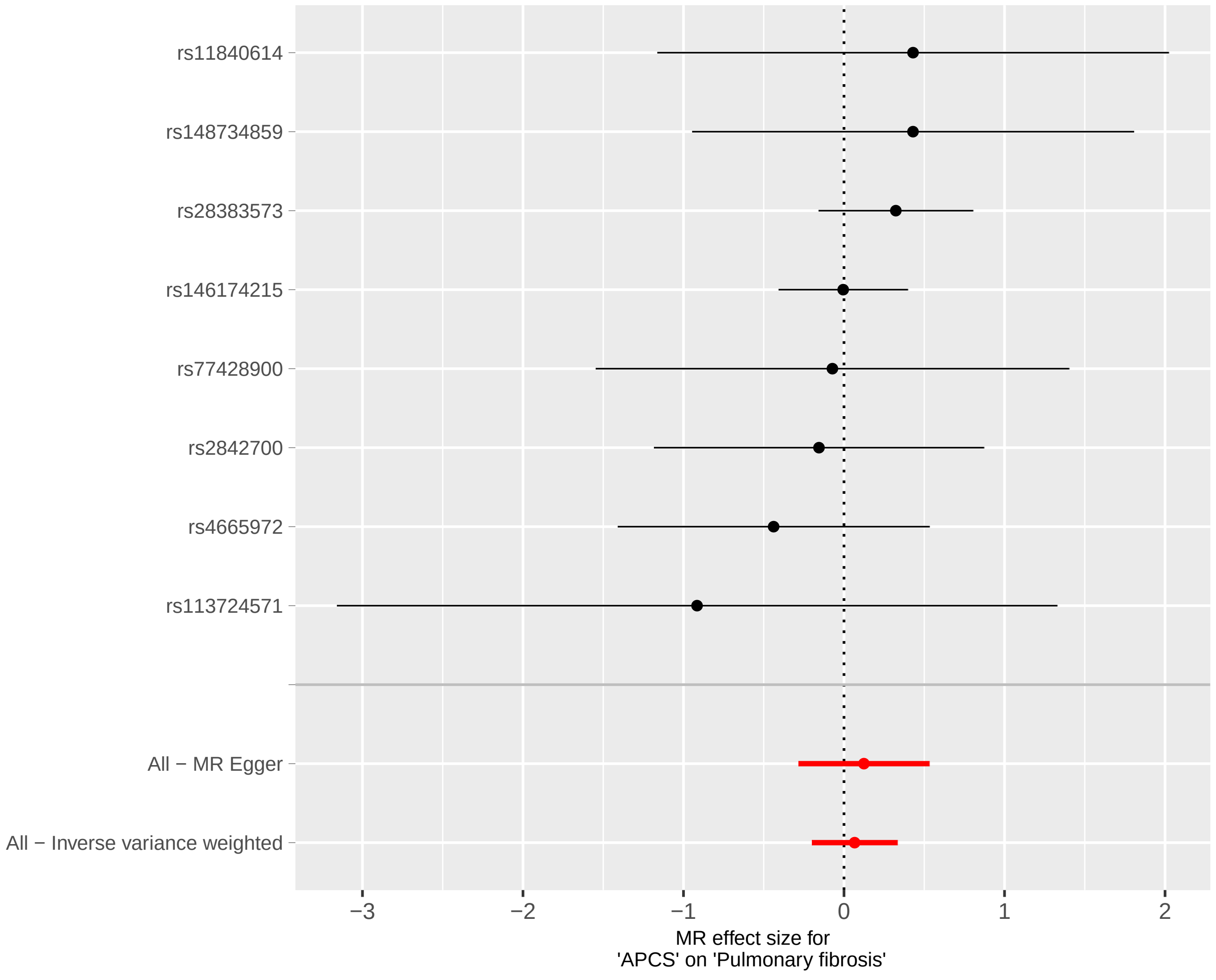
rs11840614
rs148734859
rs28383573
rs146174215
rs77428900
rs2842700
rs4665972
rs113724571
All − MR Egger
All − Inverse variance weighted
−3
−2
−1
0
1
2
MR effect size for
'APCS' on 'Pulmonary fibrosis'

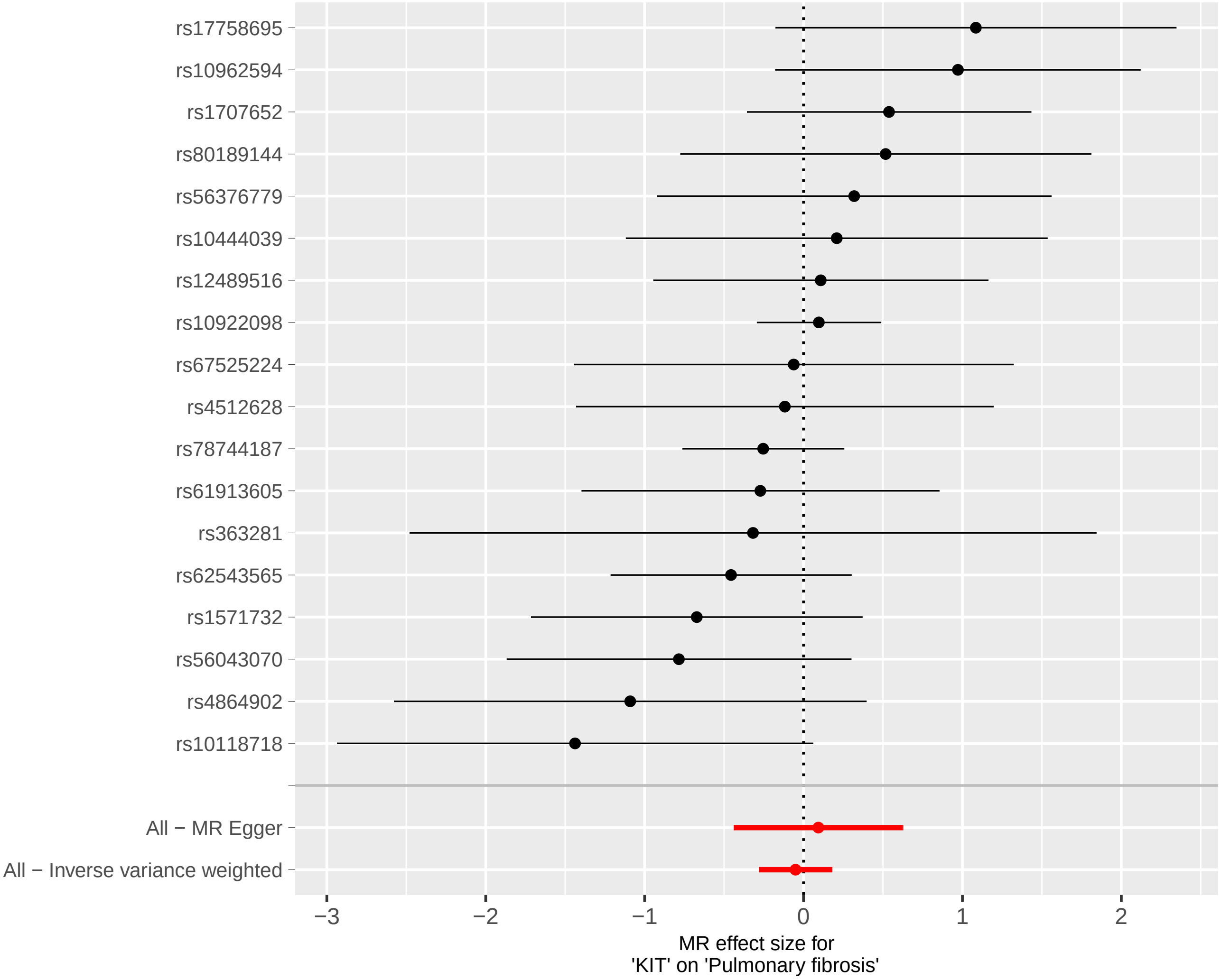

rs17758695
rs10962594
rs1707652
rs80189144
rs56376779
rs10444039
rs12489516
rs10922098
rs67525224
rs4512628
rs78744187
rs61913605
rs363281
rs62543565
rs1571732
rs56043070
rs4864902
rs10118718
All − MR Egger
All − Inverse variance weighted
−3
−2
−1
0
1
2
MR effect size for
'KIT' on 'Pulmonary fibrosis'

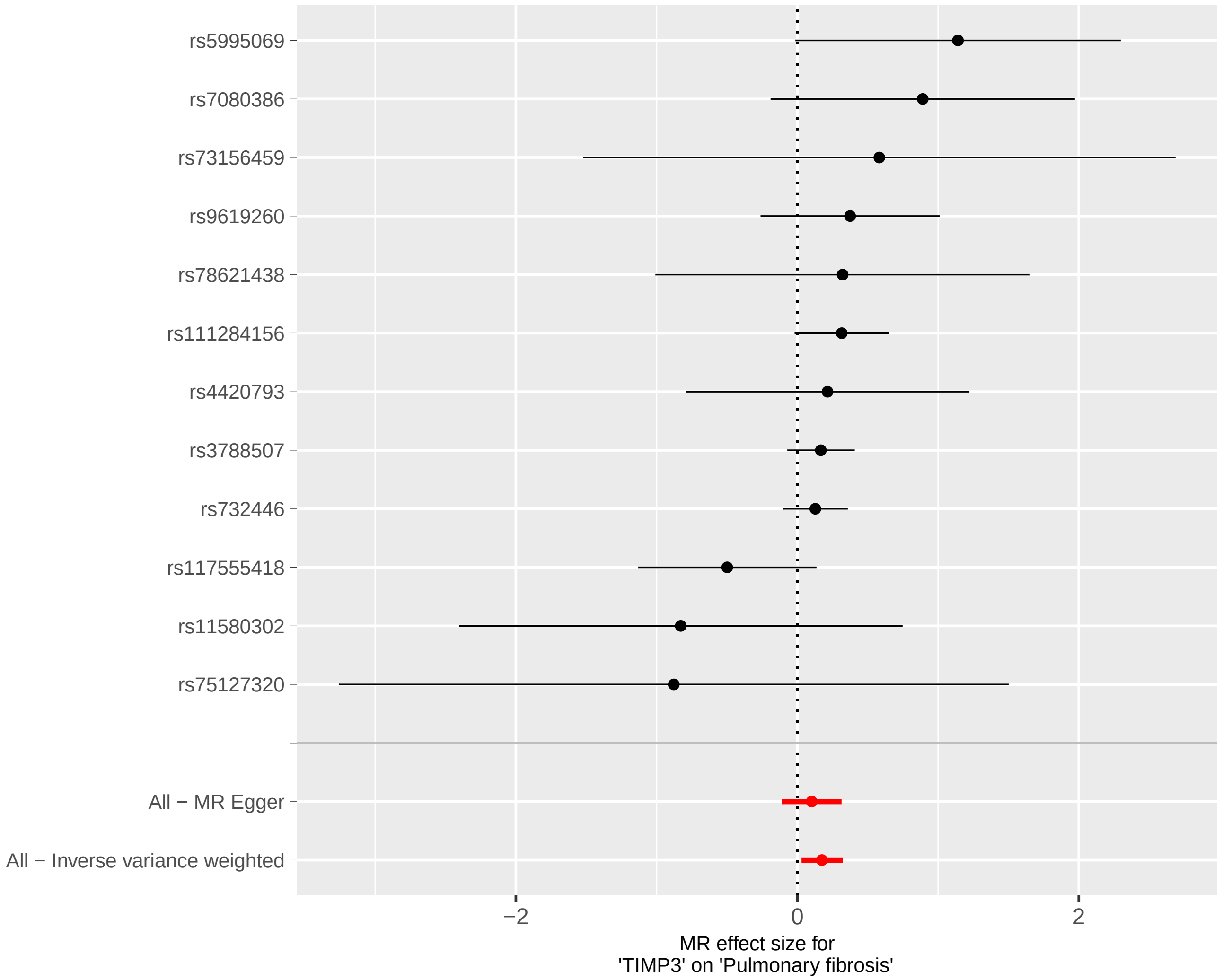

rs5995069
rs7080386
rs73156459
rs9619260
rs78621438
rs111284156
rs4420793
rs3788507
rs732446
rs117555418
rs11580302
rs75127320
All − MR Egger
All − Inverse variance weighted
−2
0
2
MR effect size for
'TIMP3' on 'Pulmonary fibrosis'

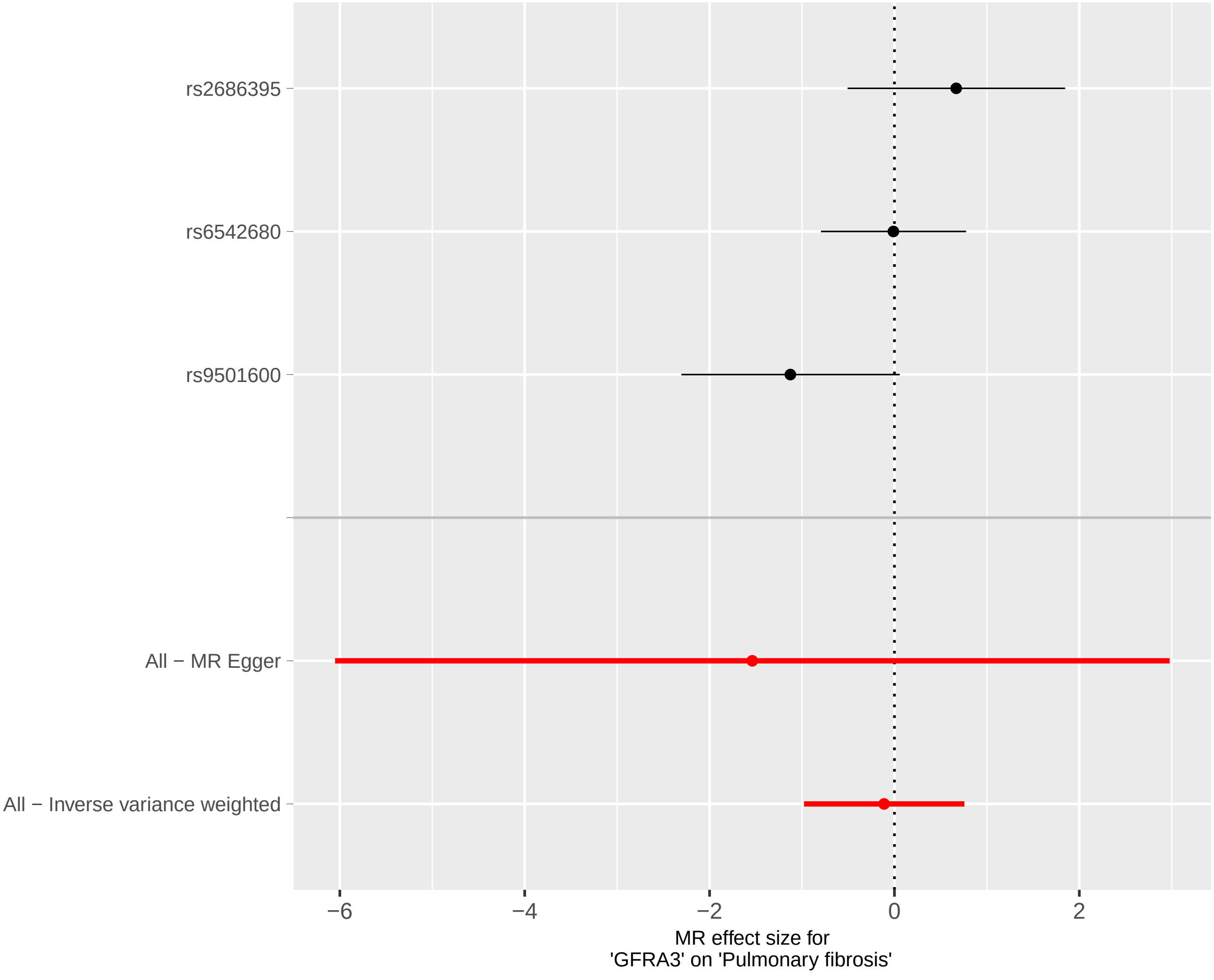
rs2686395
rs6542680
rs9501600
All − MR Egger
All − Inverse variance weighted
−6
−4
−2
0
2
MR effect size for
'GFRA3' on 'Pulmonary fibrosis'

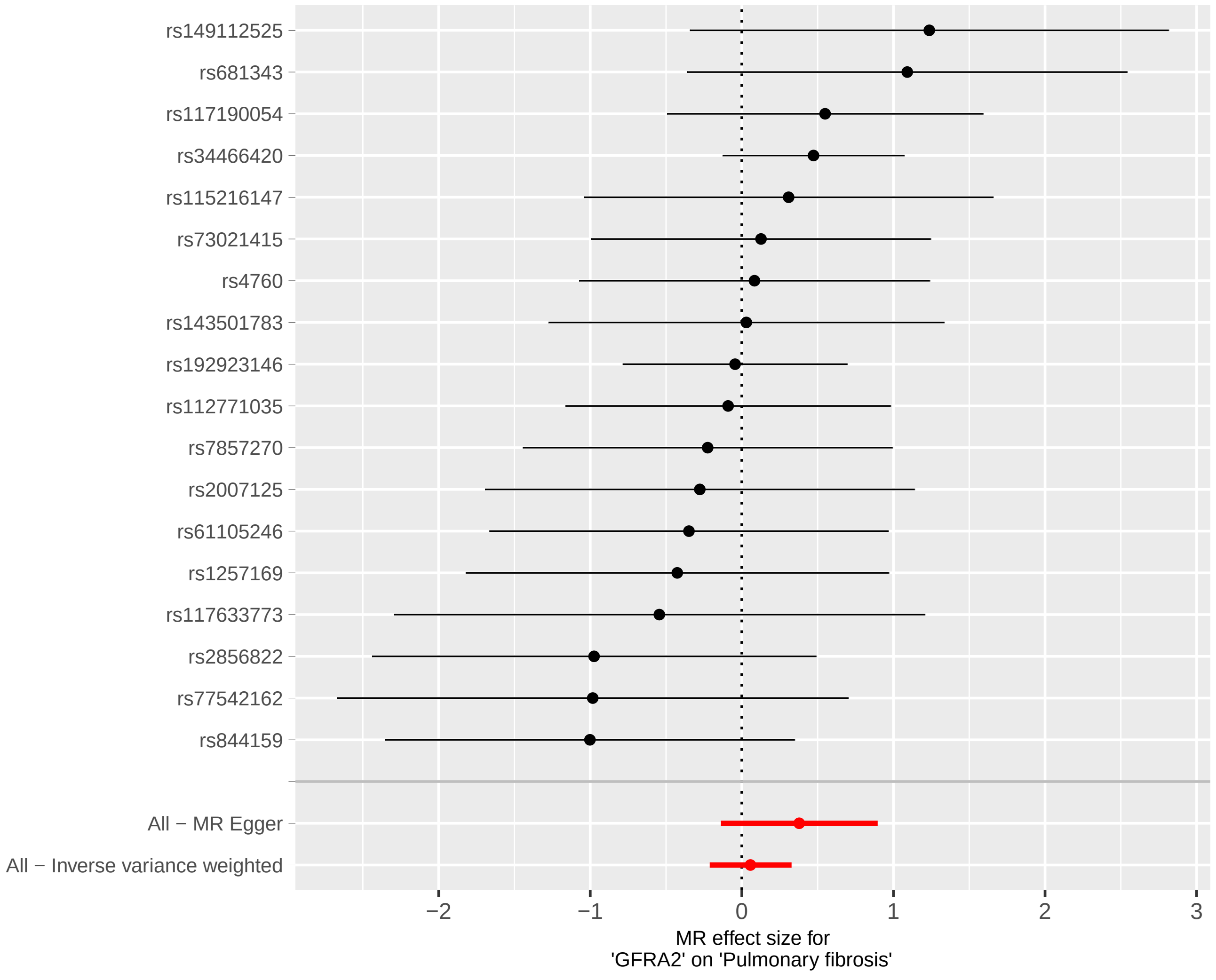

rs149112525
rs681343
rs117190054
rs34466420
rs115216147
rs73021415
rs4760
rs143501783
rs192923146
rs112771035
rs7857270
rs2007125
rs61105246
rs1257169
rs117633773
rs2856822
rs77542162
rs844159
All − MR Egger
All − Inverse variance weighted
−2
−1
0
1
2
3
MR effect size for
'GFRA2' on 'Pulmonary fibrosis'

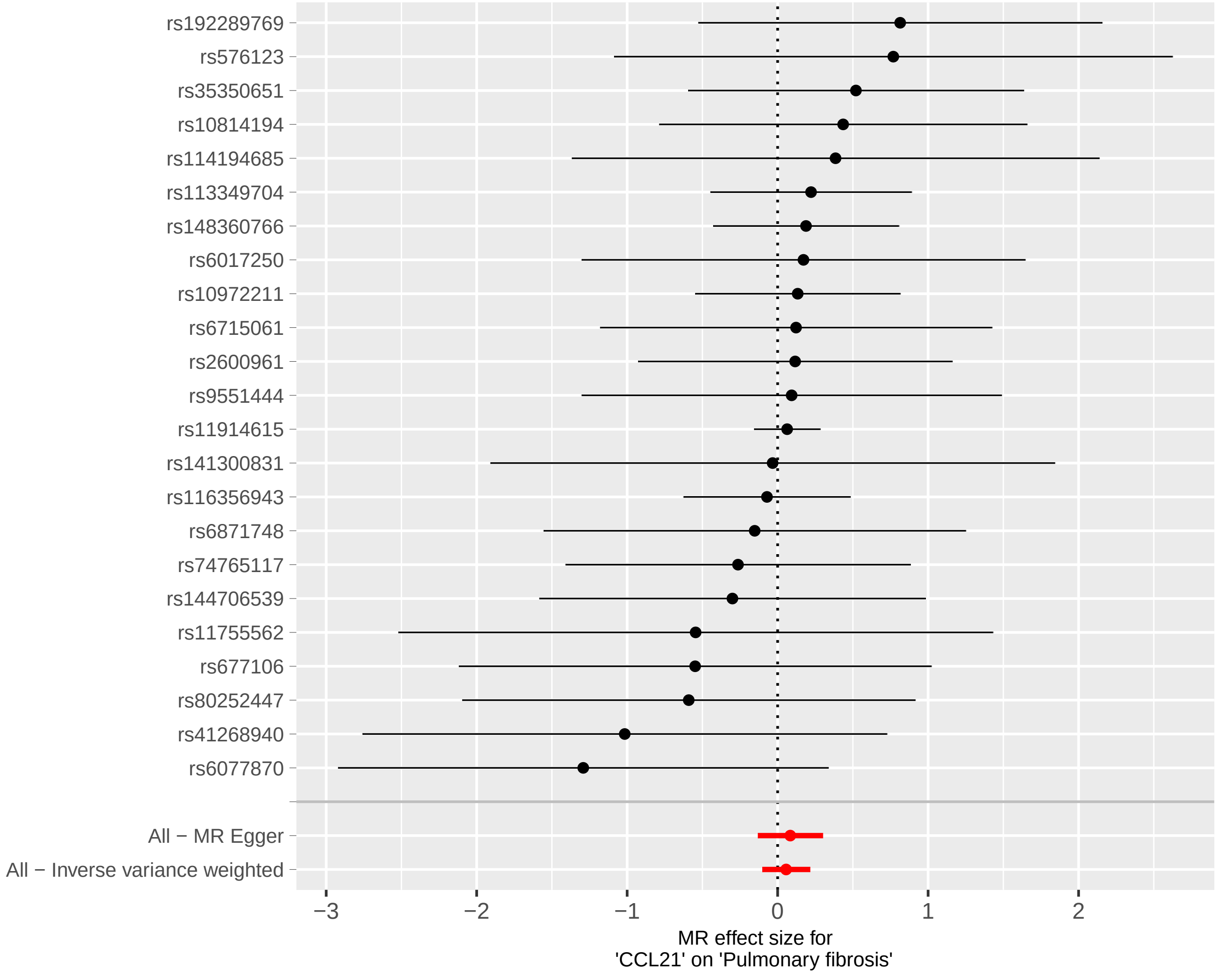

rs192289769
rs576123
rs35350651
rs10814194
rs114194685
rs113349704
rs148360766
rs6017250
rs10972211
rs6715061
rs2600961
rs9551444
rs11914615
rs141300831
rs116356943
rs6871748
rs74765117
rs144706539
rs11755562
rs677106
rs80252447
rs41268940
rs6077870
All − MR Egger
All − Inverse variance weighted
−3
−2
−1
0
1
2
MR effect size for
'CCL21' on 'Pulmonary fibrosis'

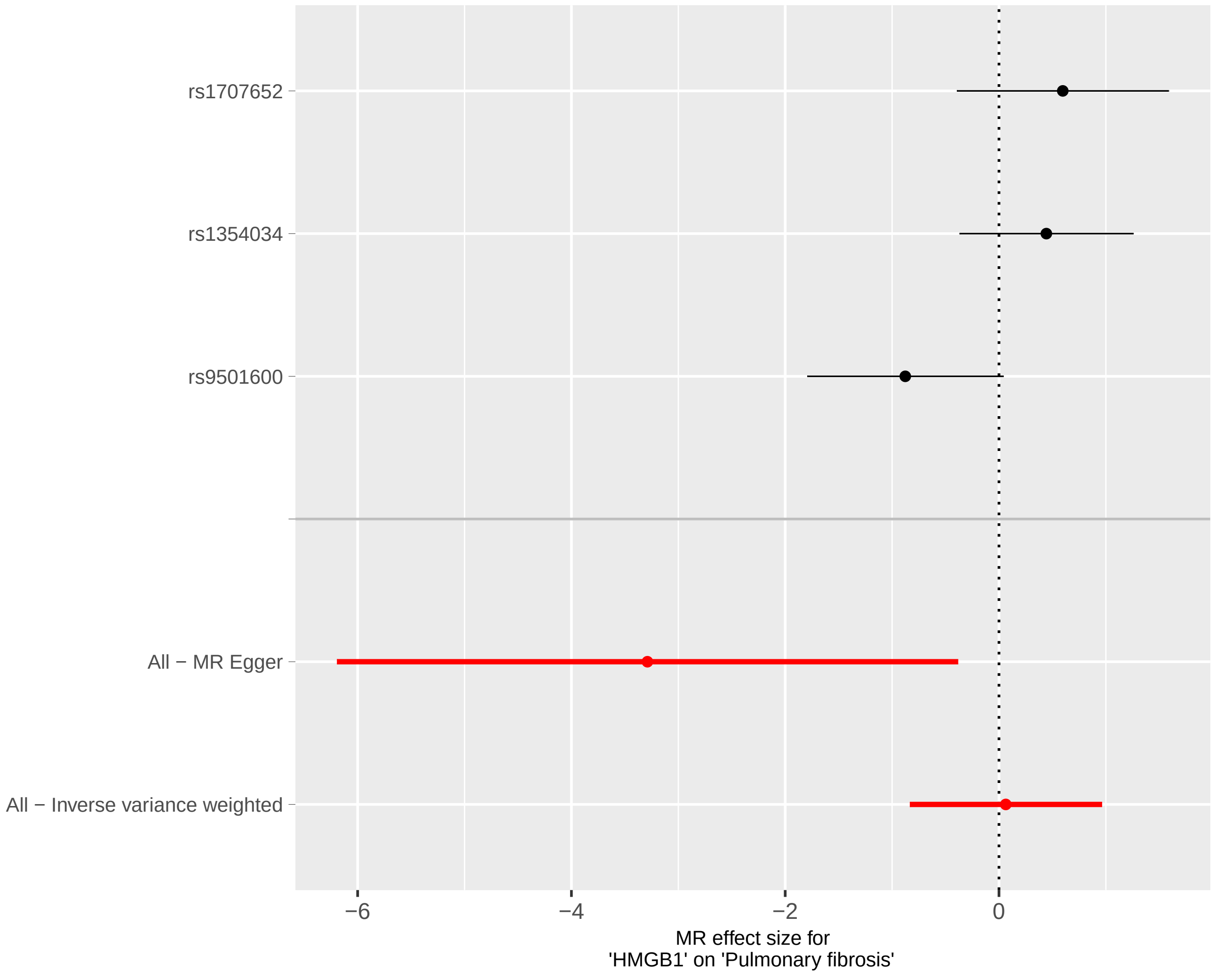
rs1707652
rs1354034
rs9501600
All − MR Egger
All − Inverse variance weighted
−6
−4
−2
0
MR effect size for
'HMGB1' on 'Pulmonary fibrosis'

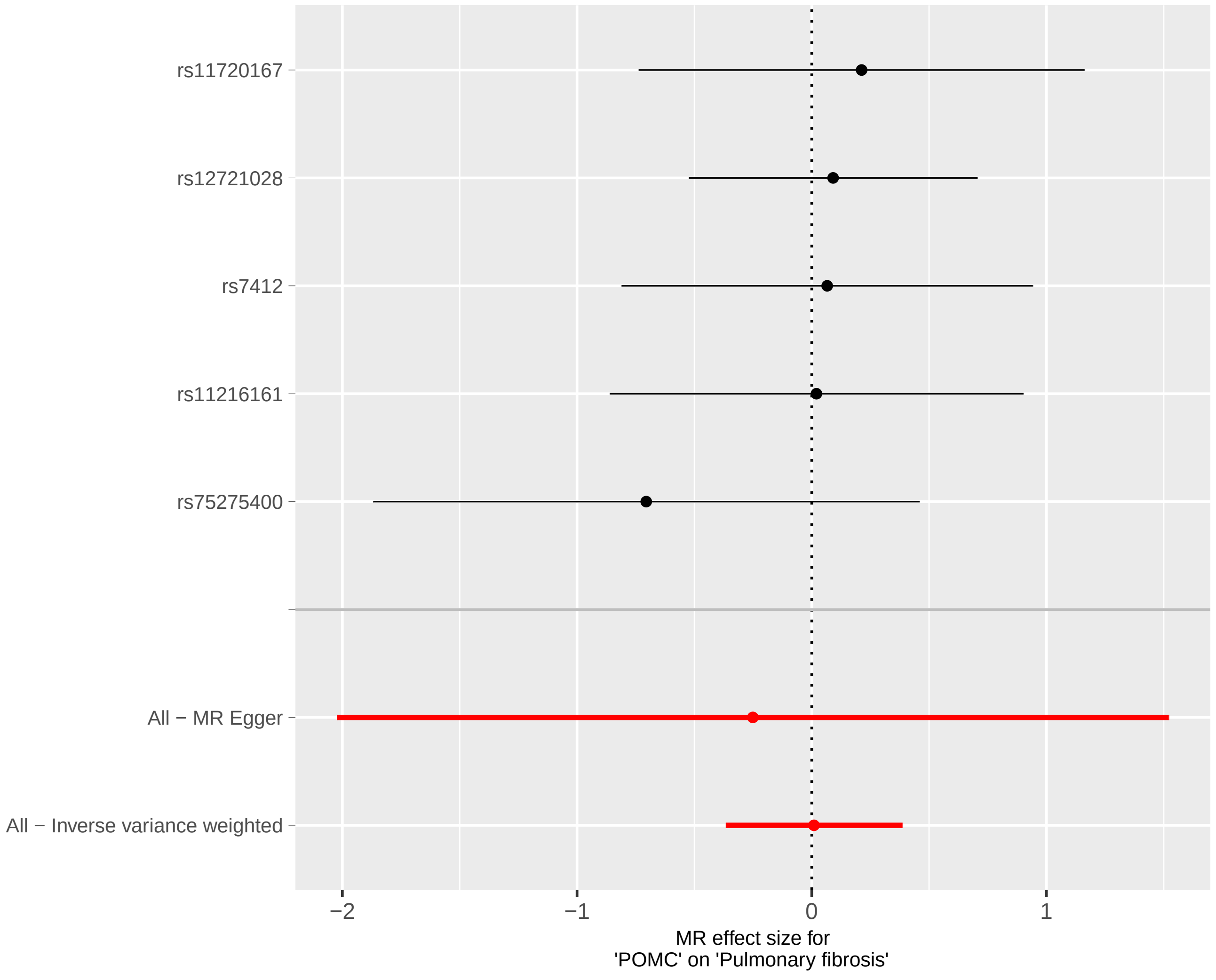
rs11720167
rs12721028
rs7412
rs11216161
rs75275400
All − MR Egger
All − Inverse variance weighted
−2
−1
0
1
MR effect size for
'POMC' on 'Pulmonary fibrosis'

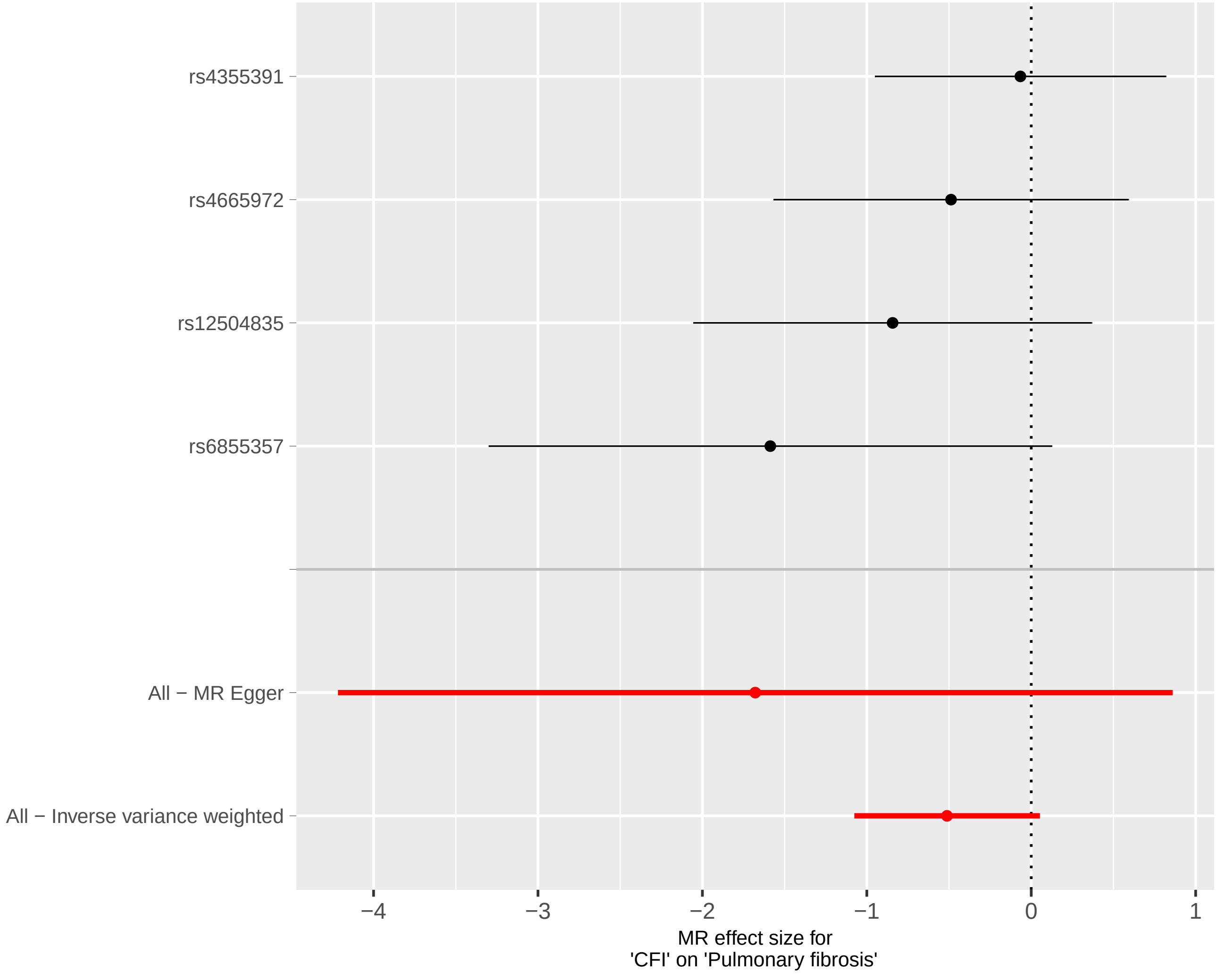
rs4355391
rs4665972
rs12504835
rs6855357
All − MR Egger
All − Inverse variance weighted
−4
−3
−2
−1
0
1
MR effect size for
'CFI' on 'Pulmonary fibrosis'

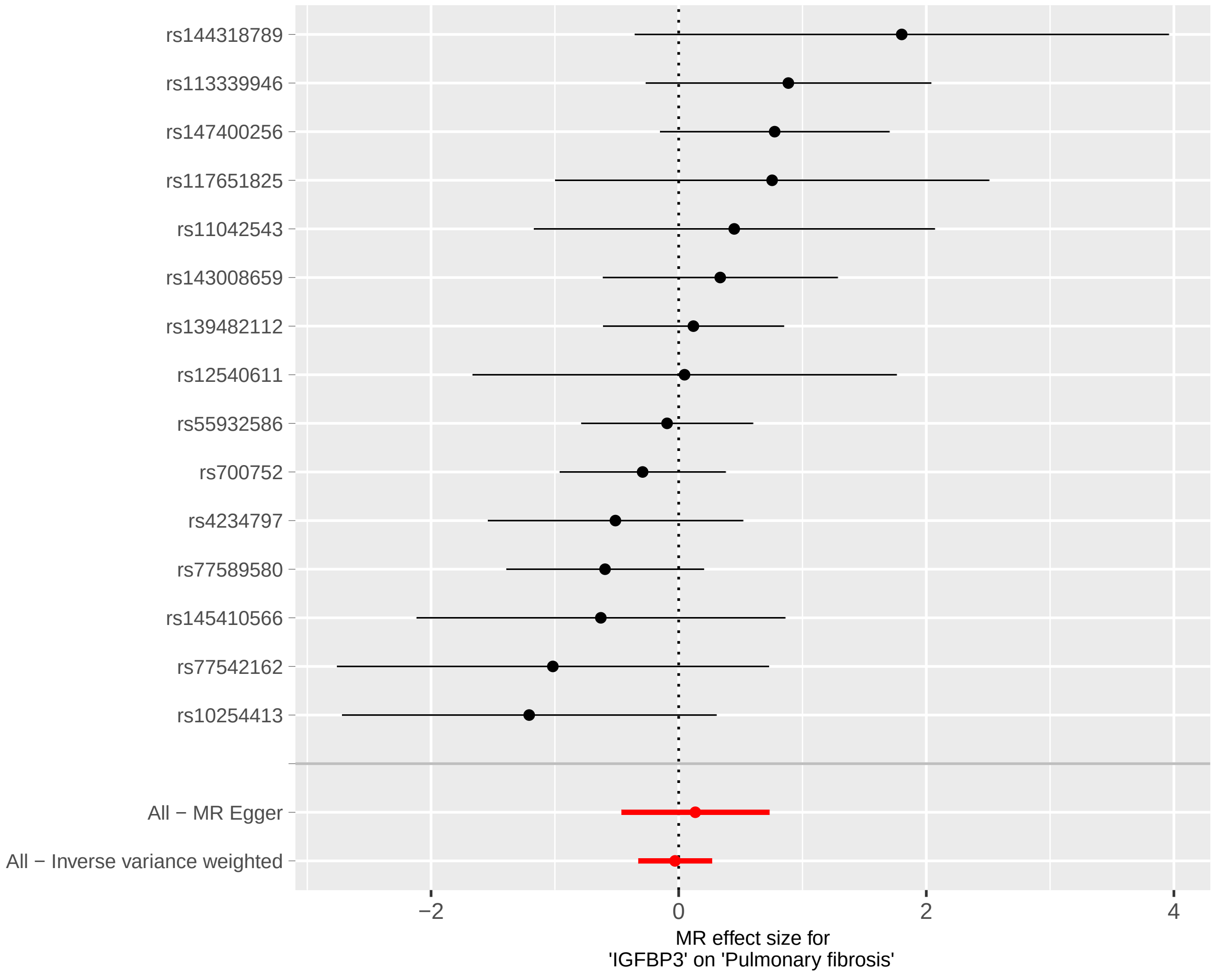
rs144318789
rs113339946
rs147400256
rs117651825
rs11042543
rs143008659
rs139482112
rs12540611
rs55932586
rs700752
rs4234797
rs77589580
rs145410566
rs77542162
rs10254413
All − MR Egger
All − Inverse variance weighted
−2
0
2
4
MR effect size for
'IGFBP3' on 'Pulmonary fibrosis'

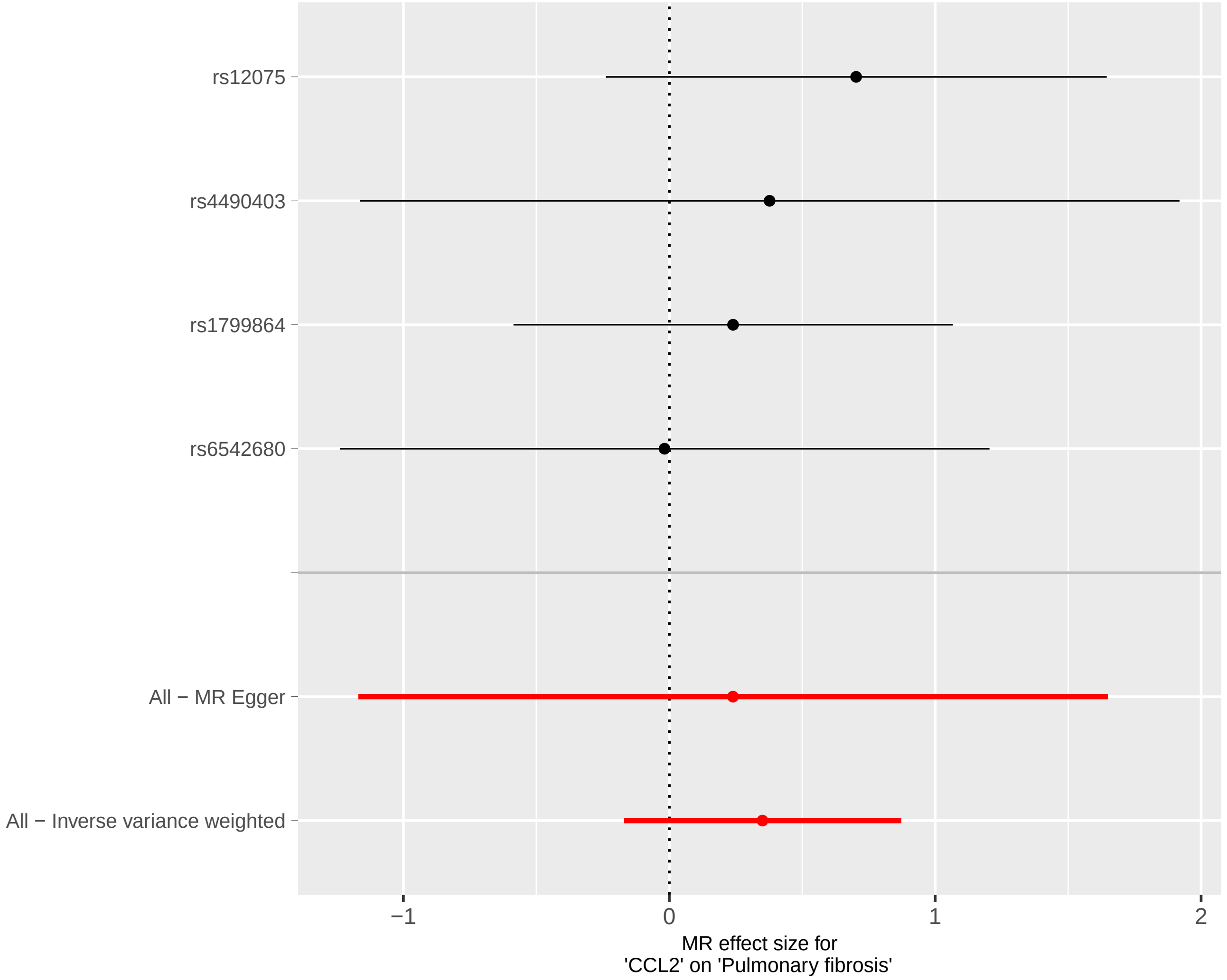

rs12075
rs4490403
rs1799864
rs6542680
All − MR Egger
All − Inverse variance weighted
−1
0
1
2
MR effect size for
'CCL2' on 'Pulmonary fibrosis'

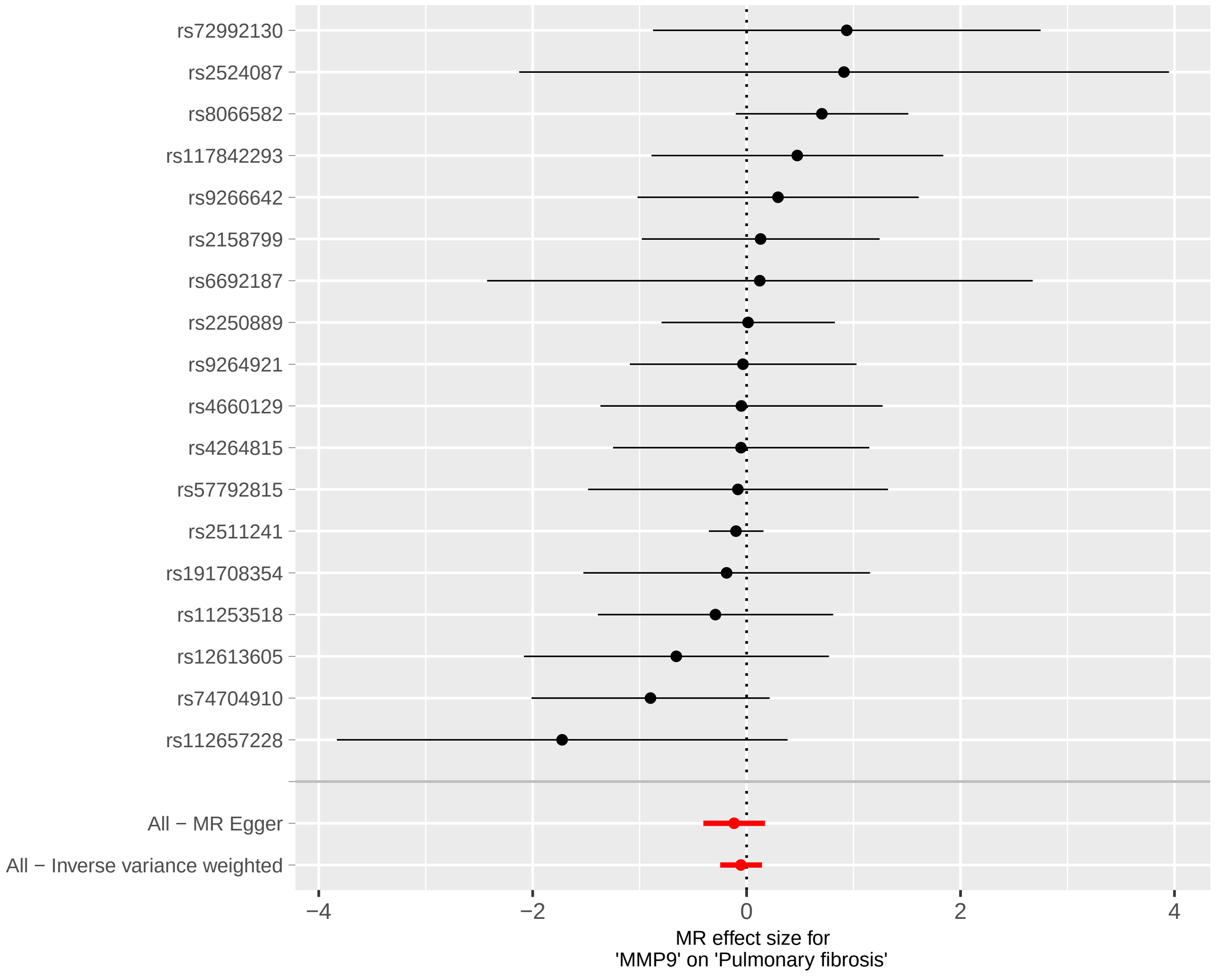
rs72992130
rs2524087
rs8066582
rs117842293
rs9266642
rs2158799
rs6692187
rs2250889
rs9264921
rs4660129
rs4264815
rs57792815
rs2511241
rs191708354
rs11253518
rs12613605
rs74704910
rs112657228
All − MR Egger
All − Inverse variance weighted
−4
−2
0
2
4
MR effect size for
'MMP9' on 'Pulmonary fibrosis'

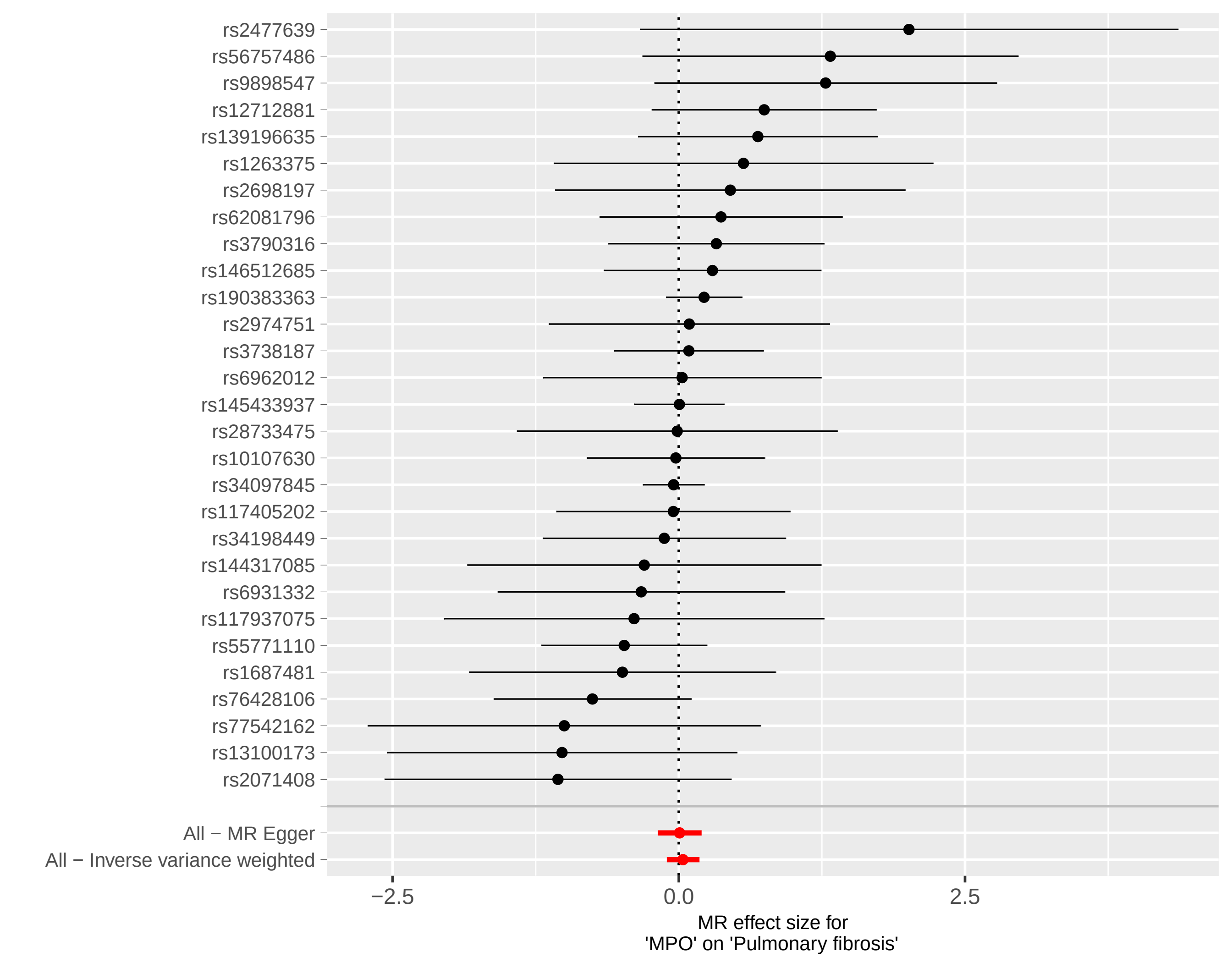

rs2477639
rs56757486
rs9898547
rs12712881
rs139196635
rs1263375
rs2698197
rs62081796
rs3790316
rs146512685
rs190383363
rs2974751
rs3738187
rs6962012
rs145433937
rs28733475
rs10107630
rs34097845
rs117405202
rs34198449
rs144317085
rs6931332
rs117937075
rs55771110
rs1687481
rs76428106
rs77542162
rs13100173
rs2071408
All − MR Egger
All − Inverse variance weighted
−2.5
0.0
2.5
MR effect size for
'MPO' on 'Pulmonary fibrosis'

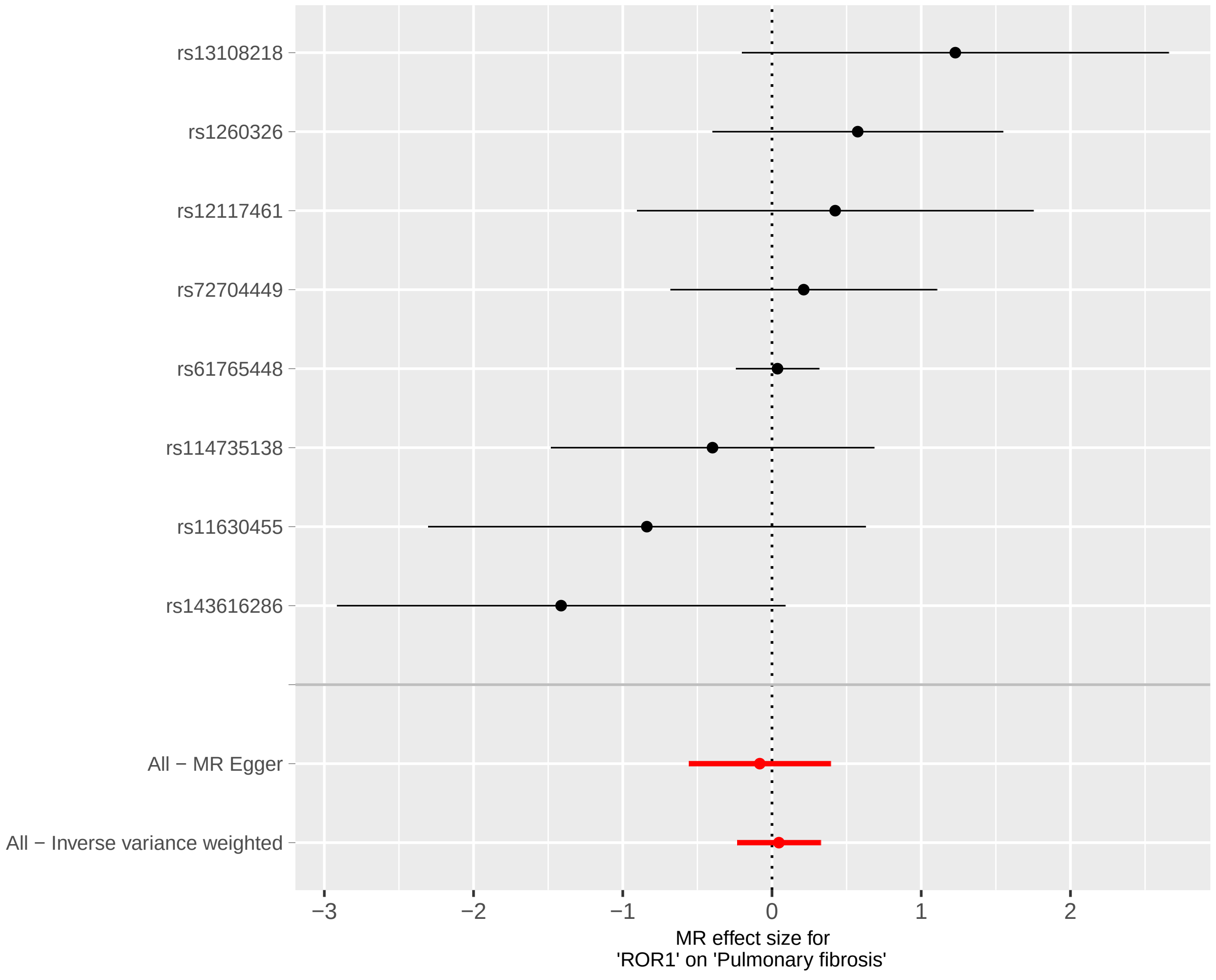

rs13108218
rs1260326
rs12117461
rs72704449
rs61765448
rs114735138
rs11630455
rs143616286
All − MR Egger
All − Inverse variance weighted
−3
−2
−1
0
1
2
MR effect size for
'ROR1' on 'Pulmonary fibrosis'

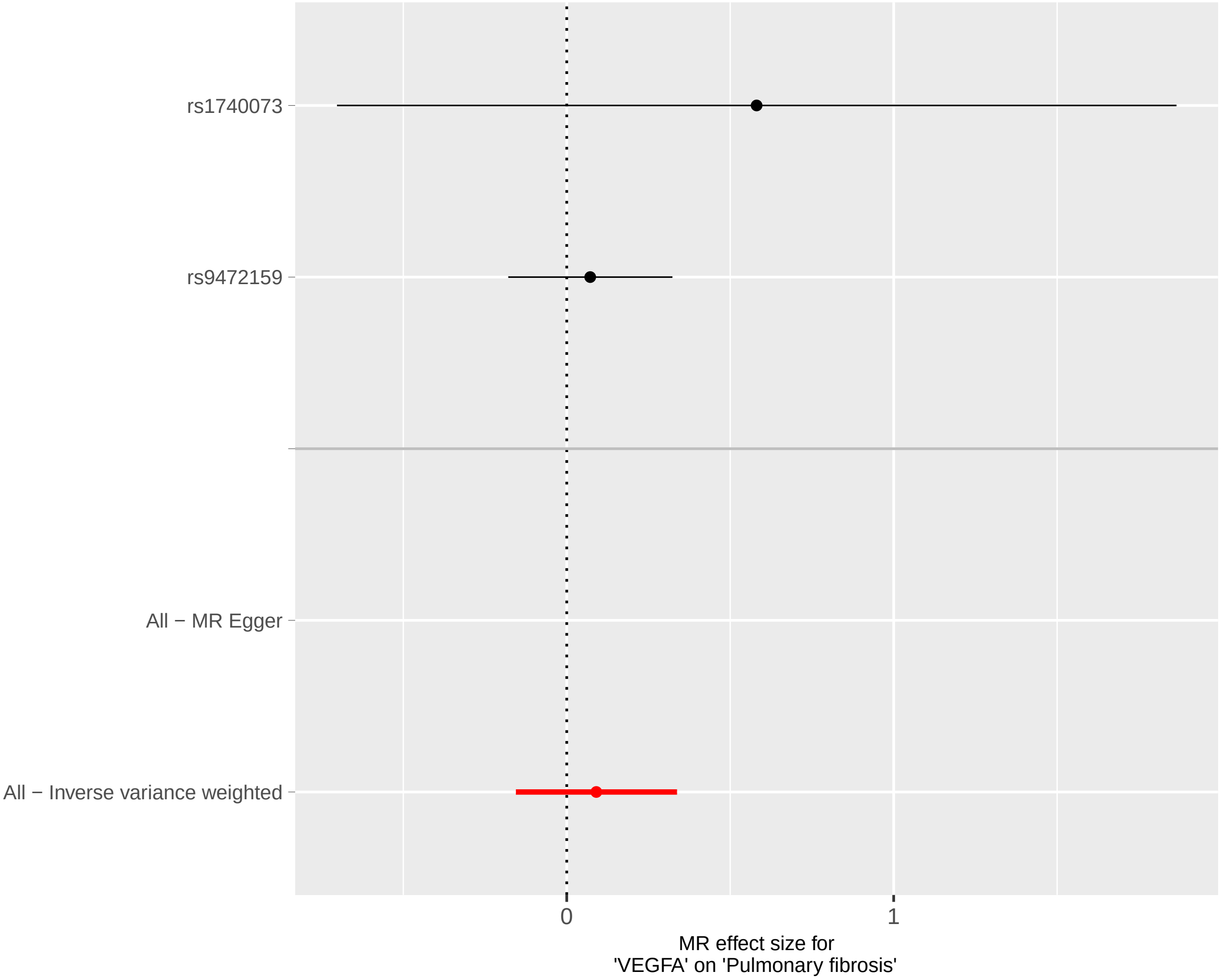
rs1740073
rs9472159
All − MR Egger
All − Inverse variance weighted
0
1
MR effect size for
'VEGFA' on 'Pulmonary fibrosis'

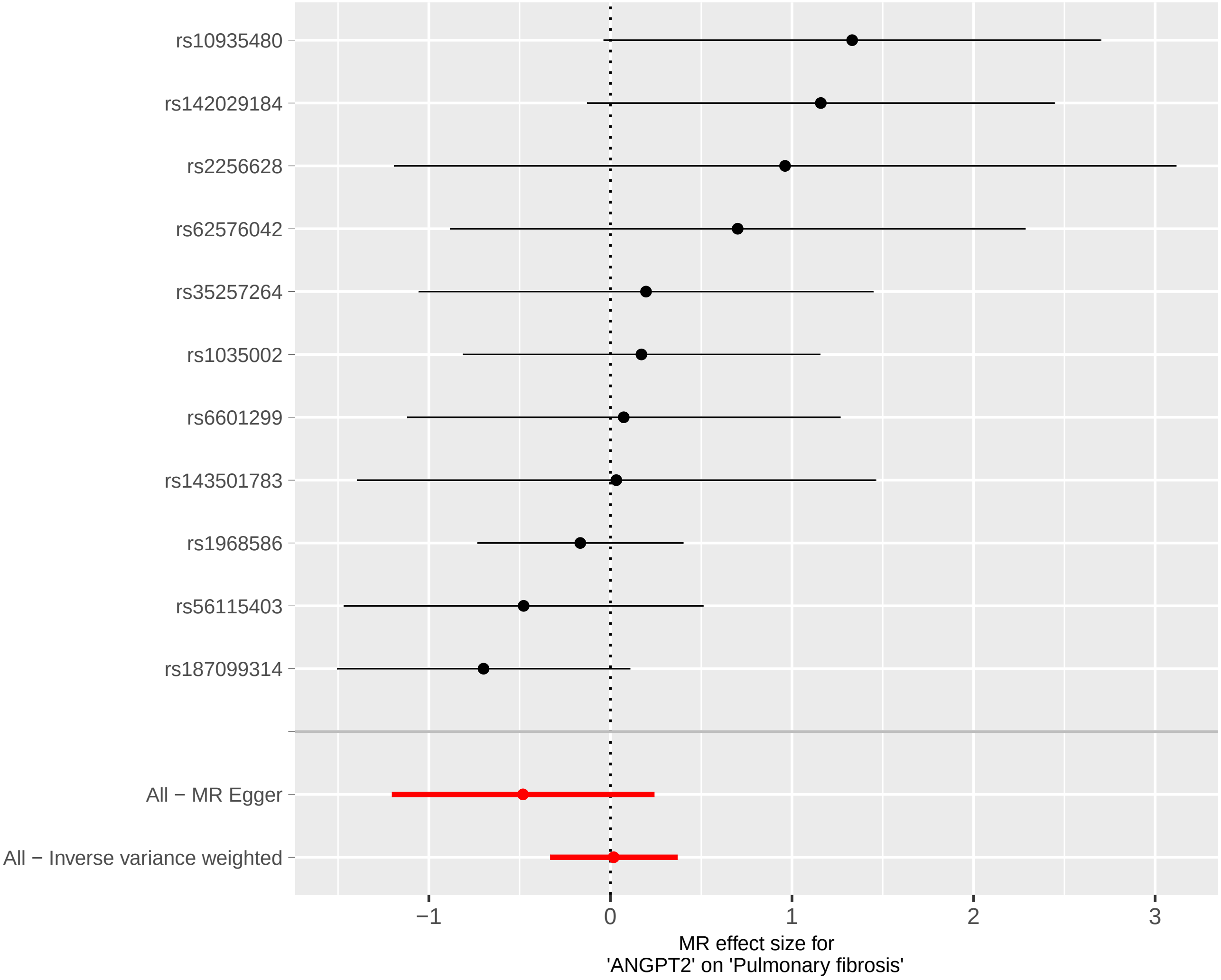
rs10935480
rs142029184
rs2256628
rs62576042
rs35257264
rs1035002
rs6601299
rs143501783
rs1968586
rs56115403
rs187099314
All − MR Egger
All − Inverse variance weighted
−1
0
1
2
3
MR effect size for
'ANGPT2' on 'Pulmonary fibrosis'

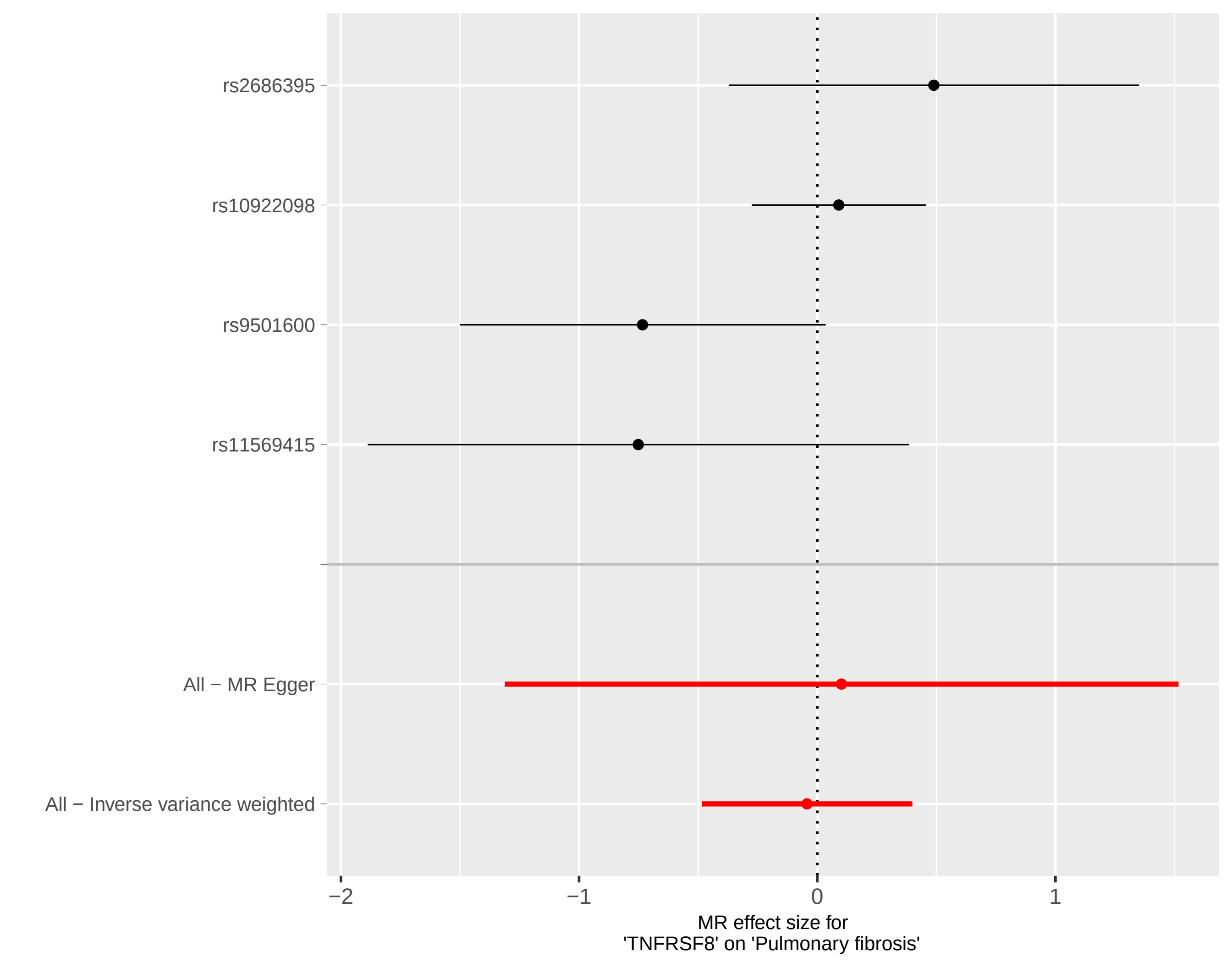

rs2686395
rs10922098
rs9501600
rs11569415
All − MR Egger
All − Inverse variance weighted
−2
−1
0
1
MR effect size for
'TNFRSF8' on 'Pulmonary fibrosis'

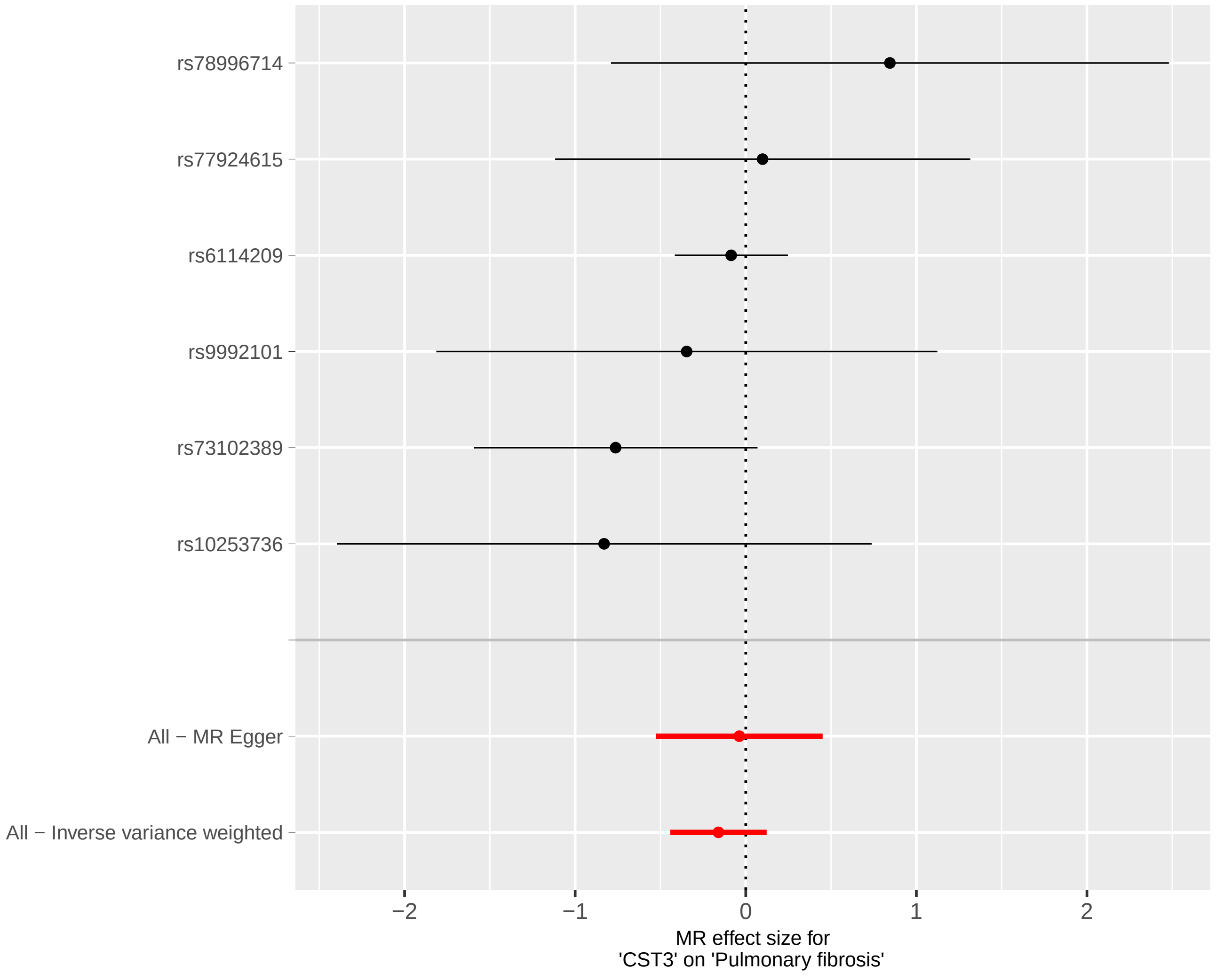
rs78996714
rs77924615
rs6114209
rs9992101
rs73102389
rs10253736
All − MR Egger
All − Inverse variance weighted
−2
−1
0
1
2
MR effect size for
'CST3' on 'Pulmonary fibrosis'

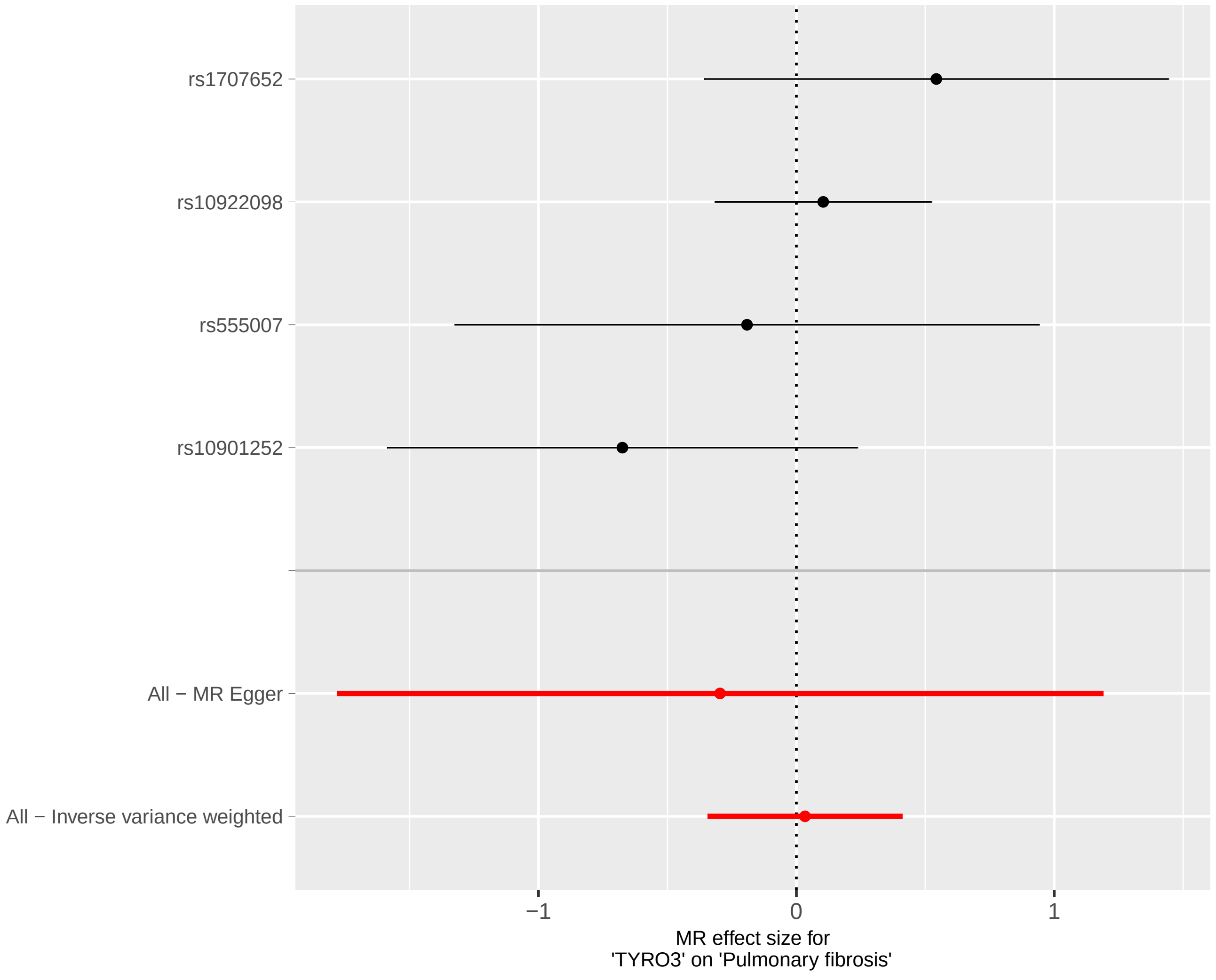
rs1707652
rs10922098
rs555007
rs10901252
All − MR Egger
All − Inverse variance weighted
−1
0
1
MR effect size for
'TYRO3' on 'Pulmonary fibrosis'

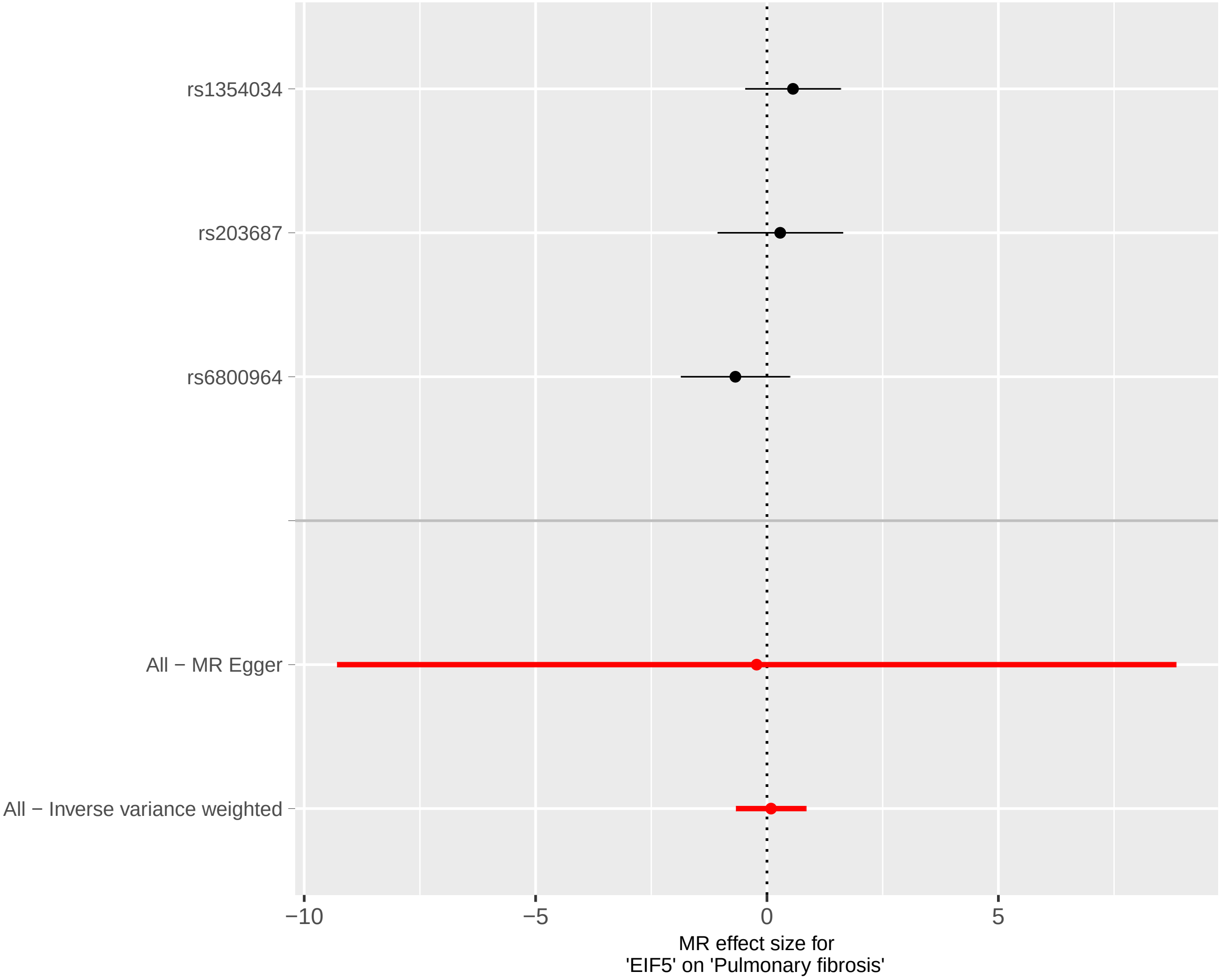
rs1354034
rs203687
rs6800964
All − MR Egger
All − Inverse variance weighted
−10
−5
0
5
MR effect size for
'EIF5' on 'Pulmonary fibrosis'

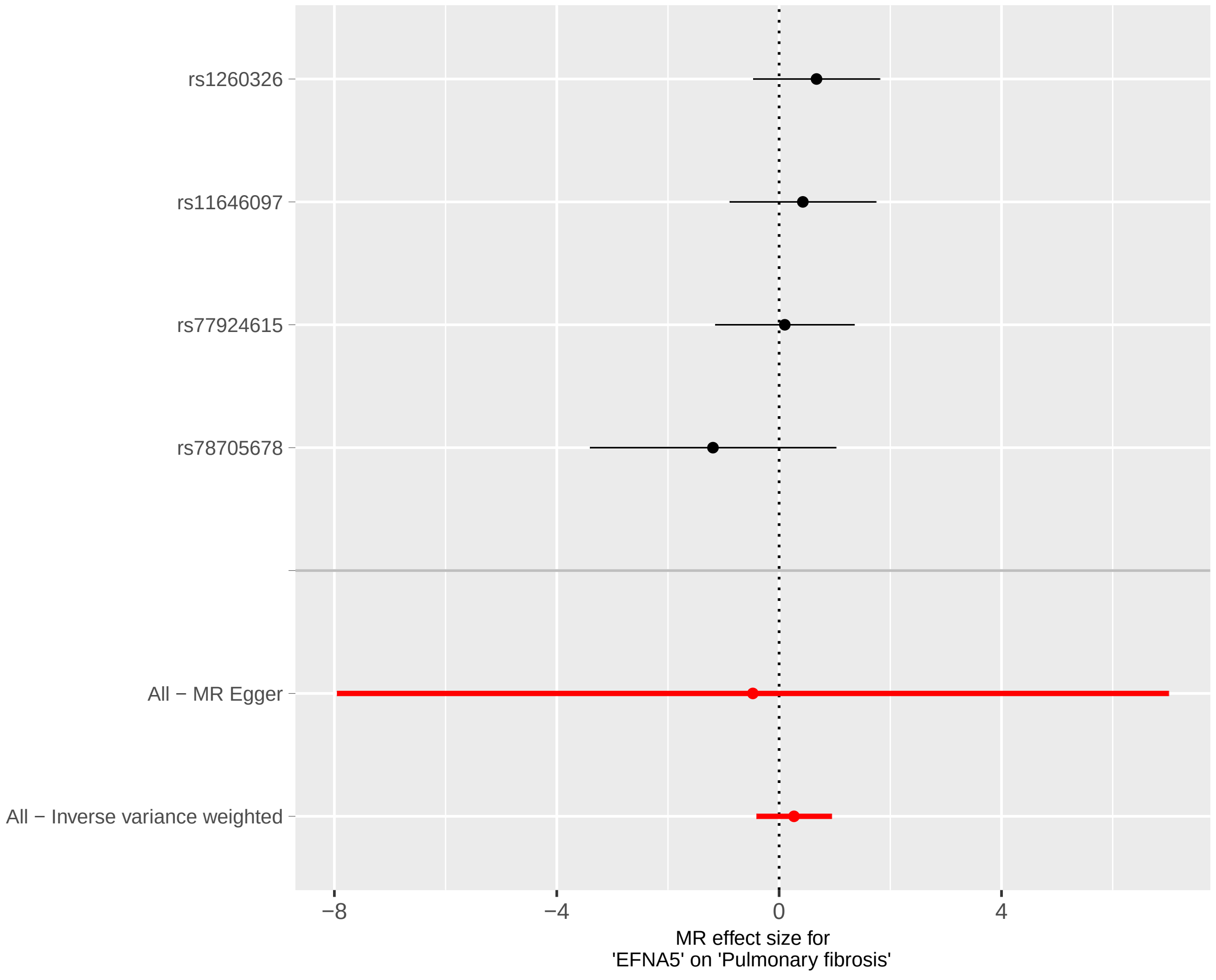
rs1260326
rs11646097
rs77924615
rs78705678
All − MR Egger
All − Inverse variance weighted
−8
−4
0
4
MR effect size for
'EFNA5' on 'Pulmonary fibrosis'

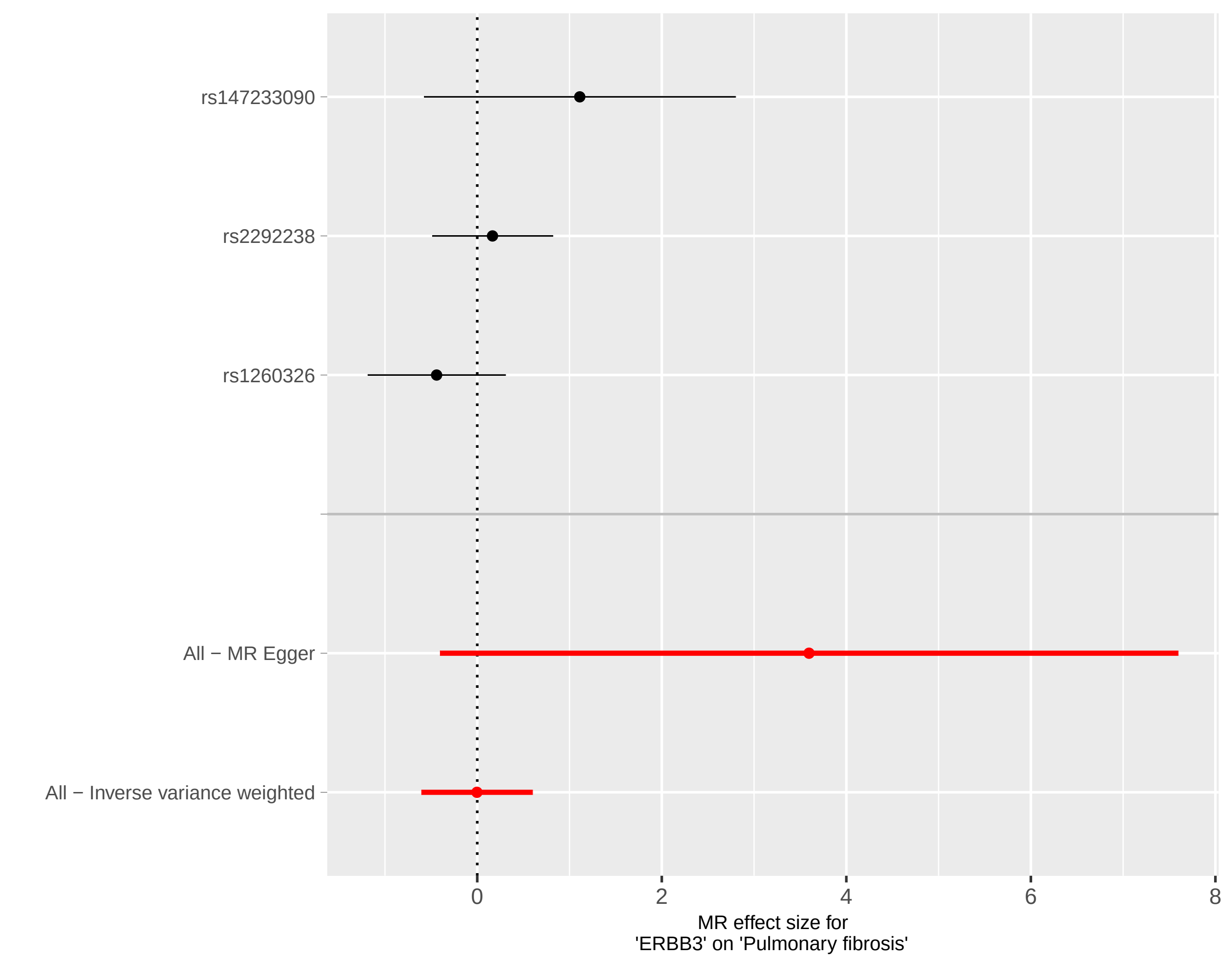
rs147233090
rs2292238
rs1260326
All − MR Egger
All − Inverse variance weighted
0
2
4
6
8
MR effect size for
'ERBB3' on 'Pulmonary fibrosis'

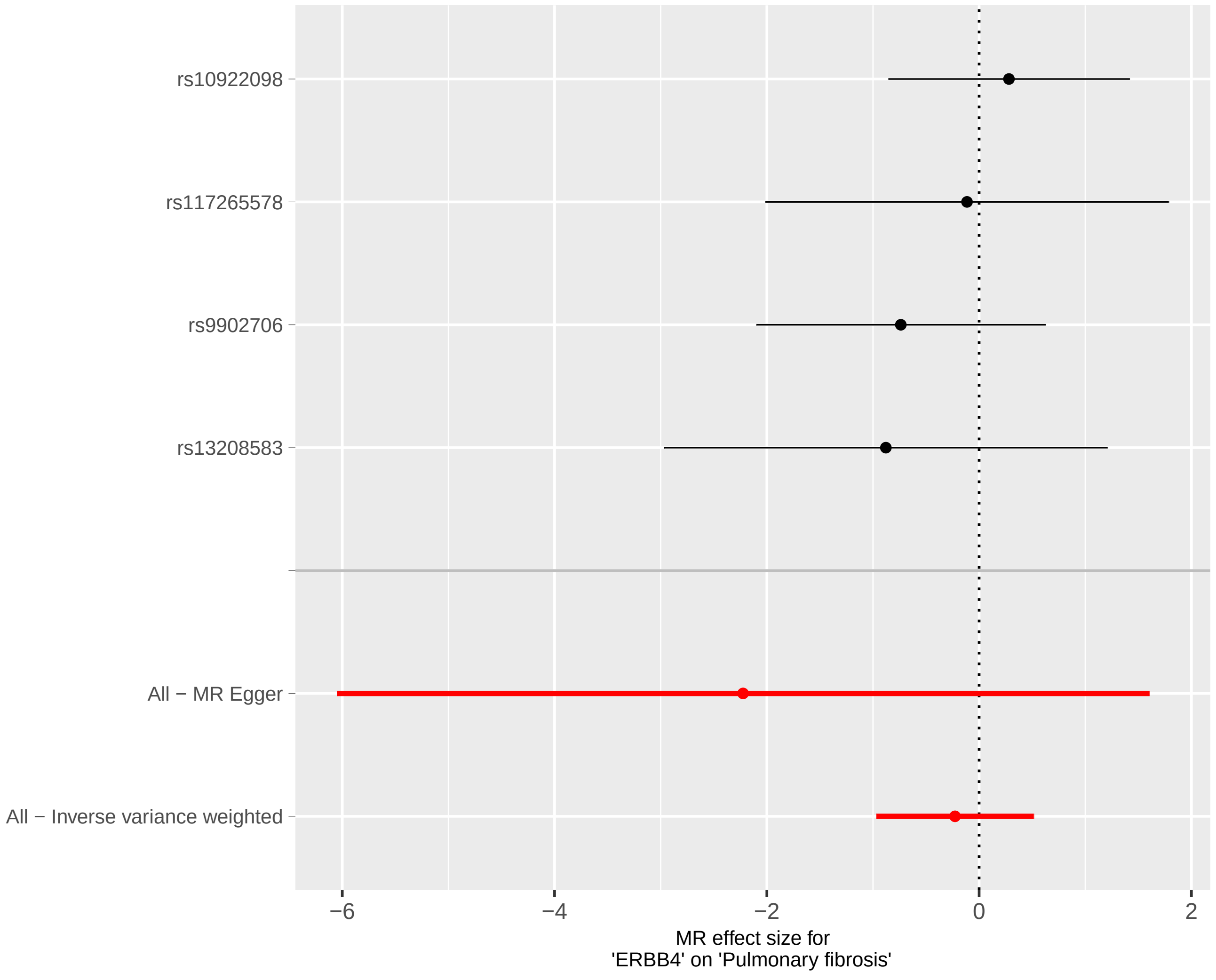

rs10922098
rs117265578
rs9902706
rs13208583
All − MR Egger
All − Inverse variance weighted
−6
−4
−2
0
2
MR effect size for
'ERBB4' on 'Pulmonary fibrosis'

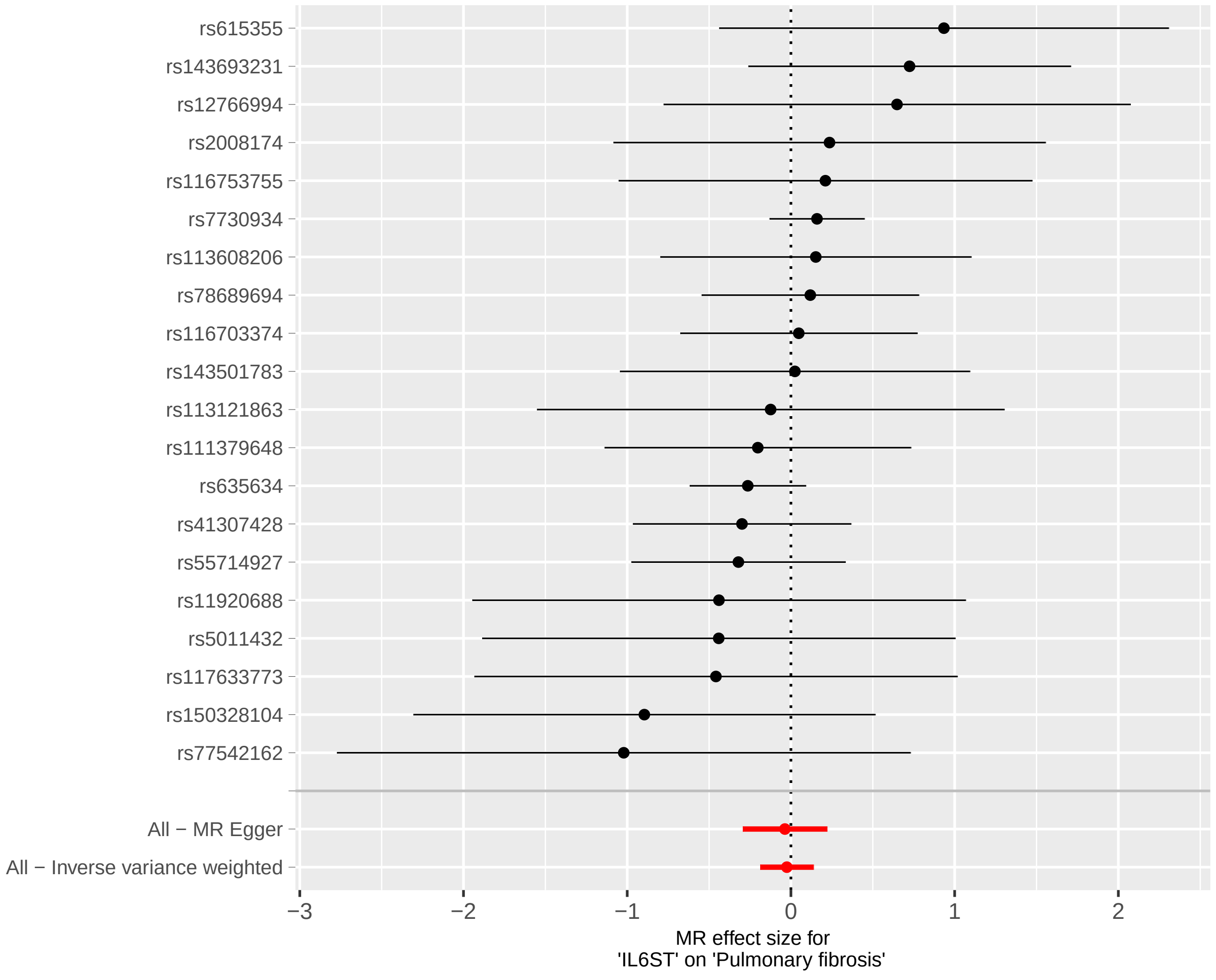
rs615355
rs143693231
rs12766994
rs2008174
rs116753755
rs7730934
rs113608206
rs78689694
rs116703374
rs143501783
rs113121863
rs111379648
rs635634
rs41307428
rs55714927
rs11920688
rs5011432
rs117633773
rs150328104
rs77542162
All − MR Egger
All − Inverse variance weighted
−3
−2
−1
0
1
2
MR effect size for
'IL6ST' on 'Pulmonary fibrosis'

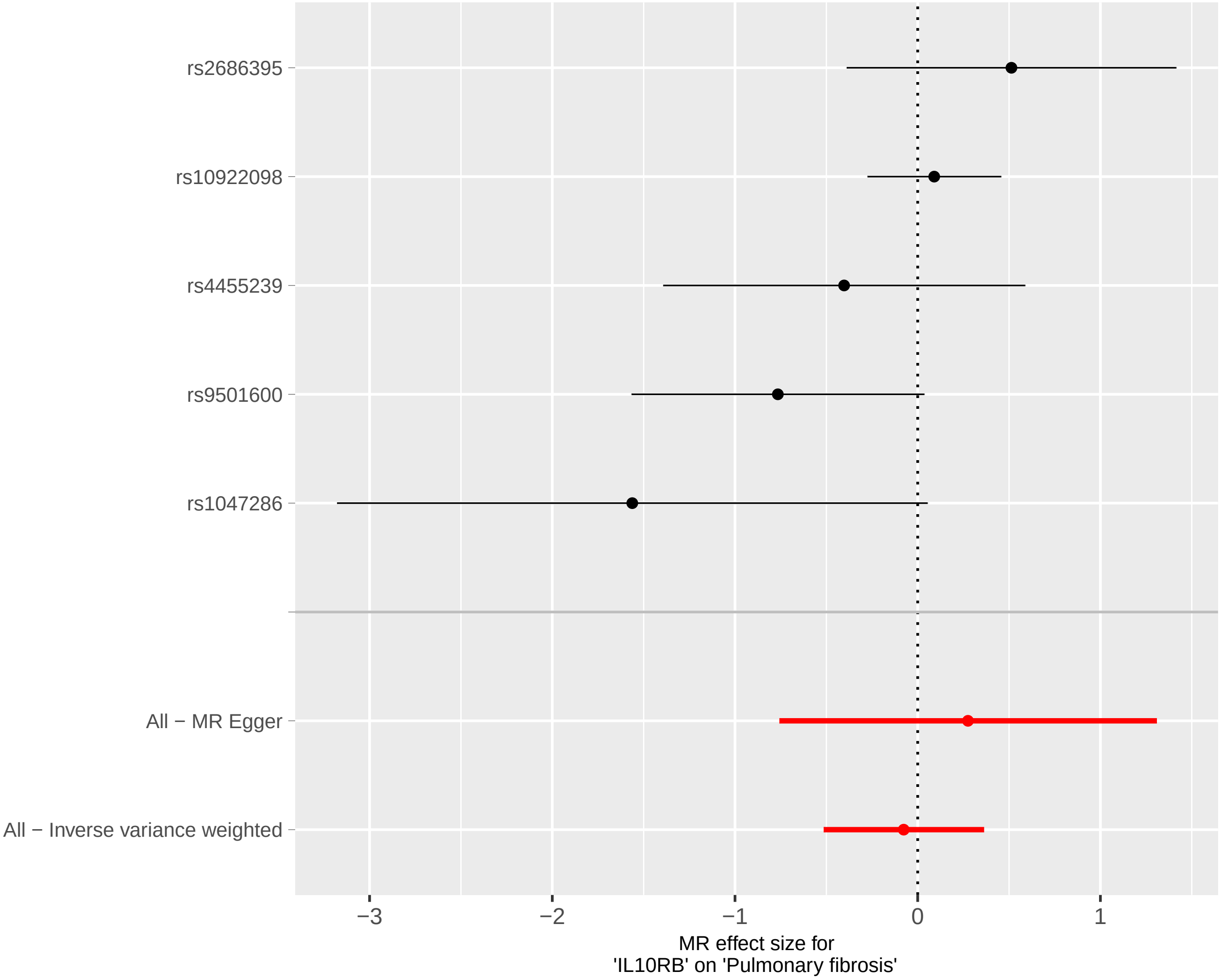

rs2686395
rs10922098
rs4455239
rs9501600
rs1047286
All − MR Egger
All − Inverse variance weighted
−3
−2
−1
0
1
MR effect size for
'IL10RB' on 'Pulmonary fibrosis'

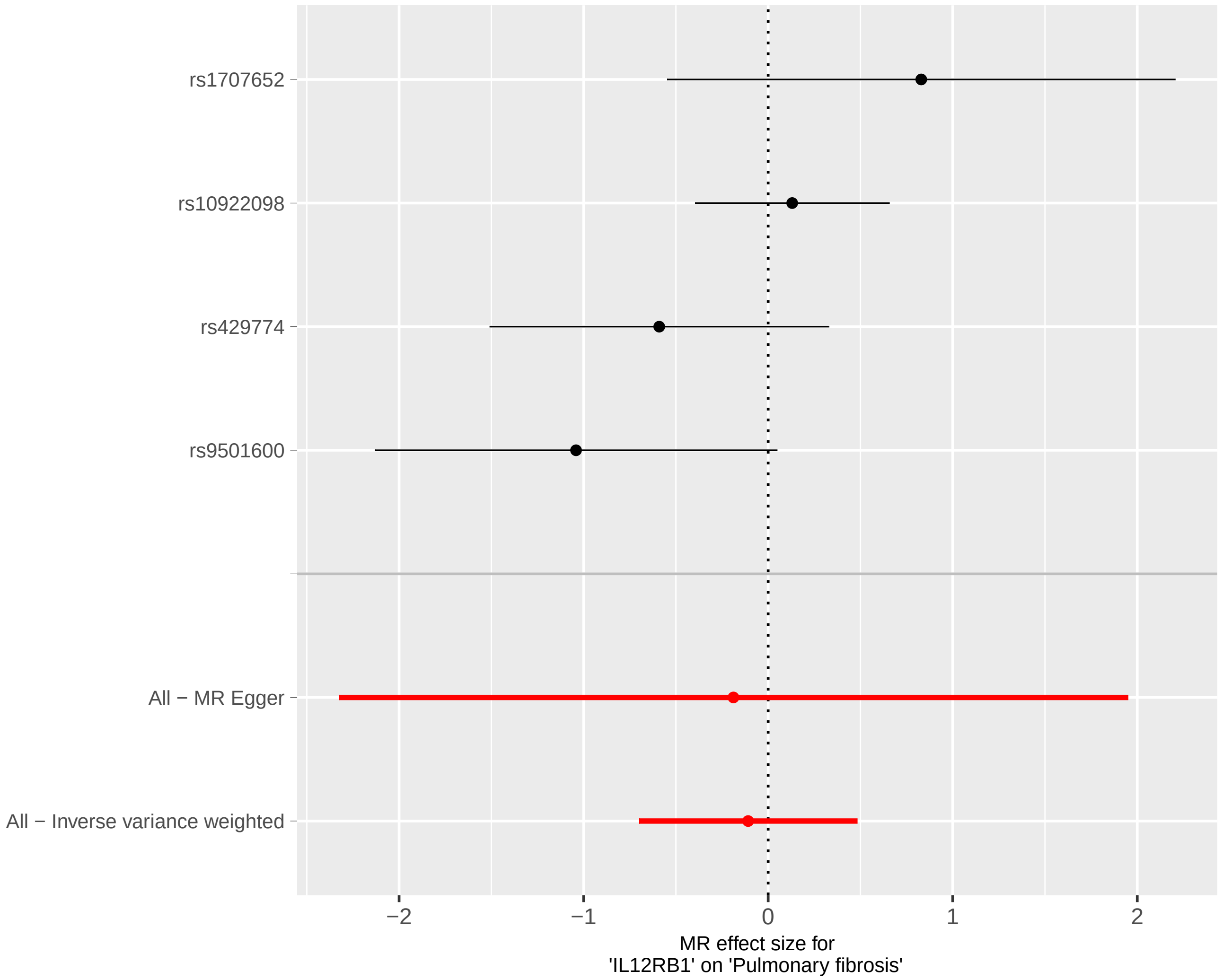

rs1707652
rs10922098
rs429774
rs9501600
All − MR Egger
All − Inverse variance weighted
−2
−1
0
1
2
MR effect size for
'IL12RB1' on 'Pulmonary fibrosis'

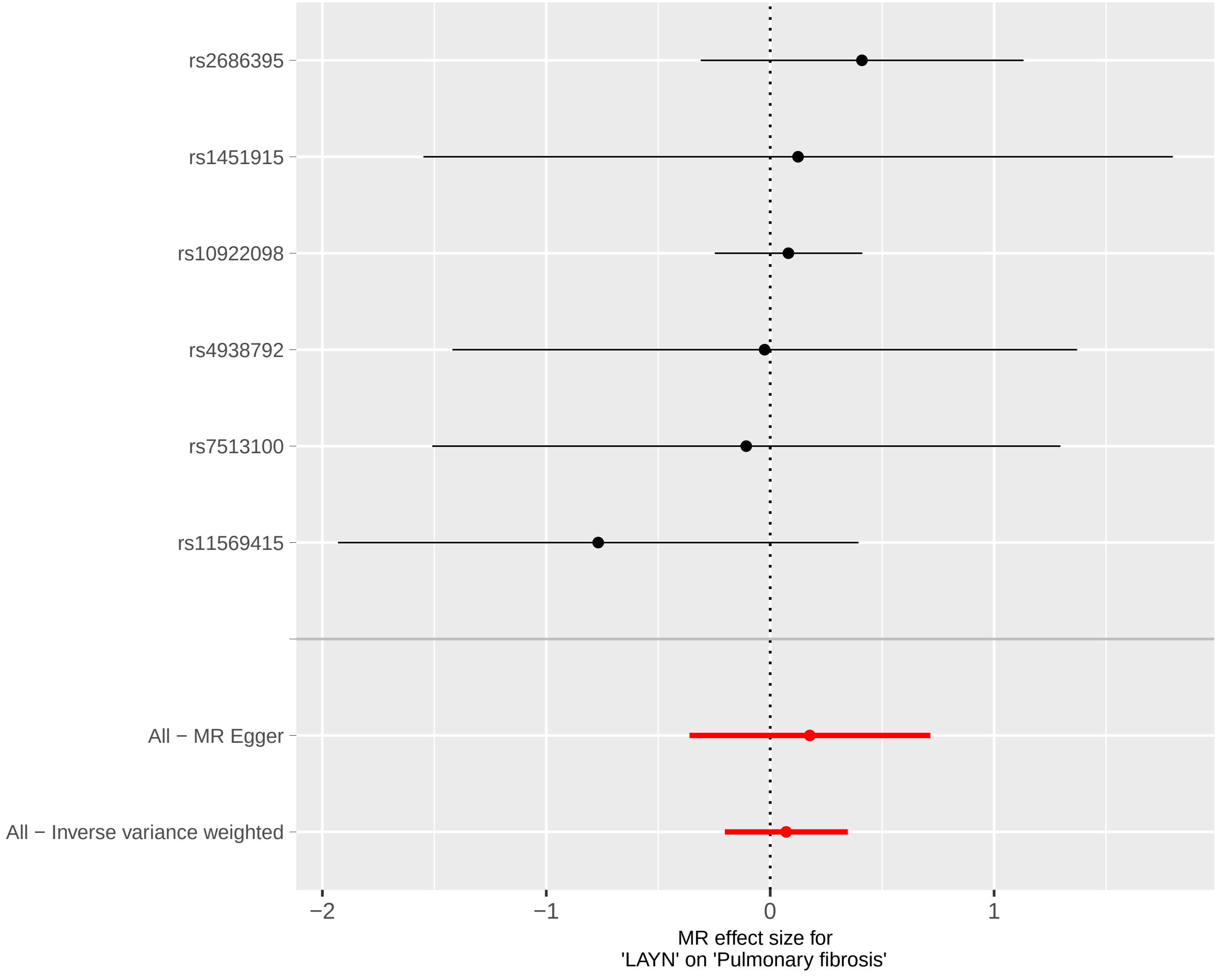
rs2686395
rs1451915
rs10922098
rs4938792
rs7513100
rs11569415
All − MR Egger
All − Inverse variance weighted
−2
−1
0
1
MR effect size for
'LAYN' on 'Pulmonary fibrosis'

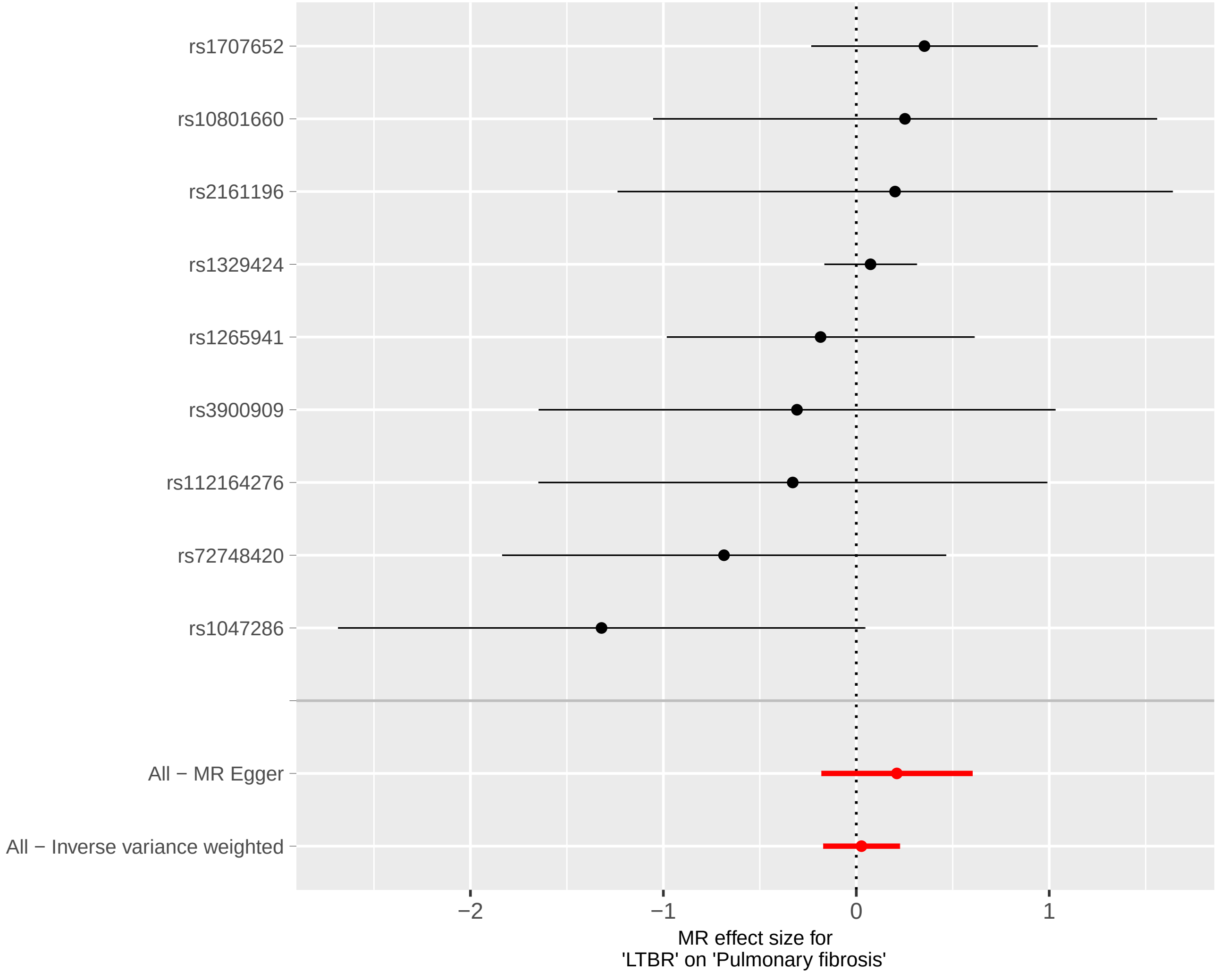
rs1707652
rs10801660
rs2161196
rs1329424
rs1265941
rs3900909
rs112164276
rs72748420
rs1047286
All − MR Egger
All − Inverse variance weighted
−2
−1
0
1
MR effect size for
'LTBR' on 'Pulmonary fibrosis'

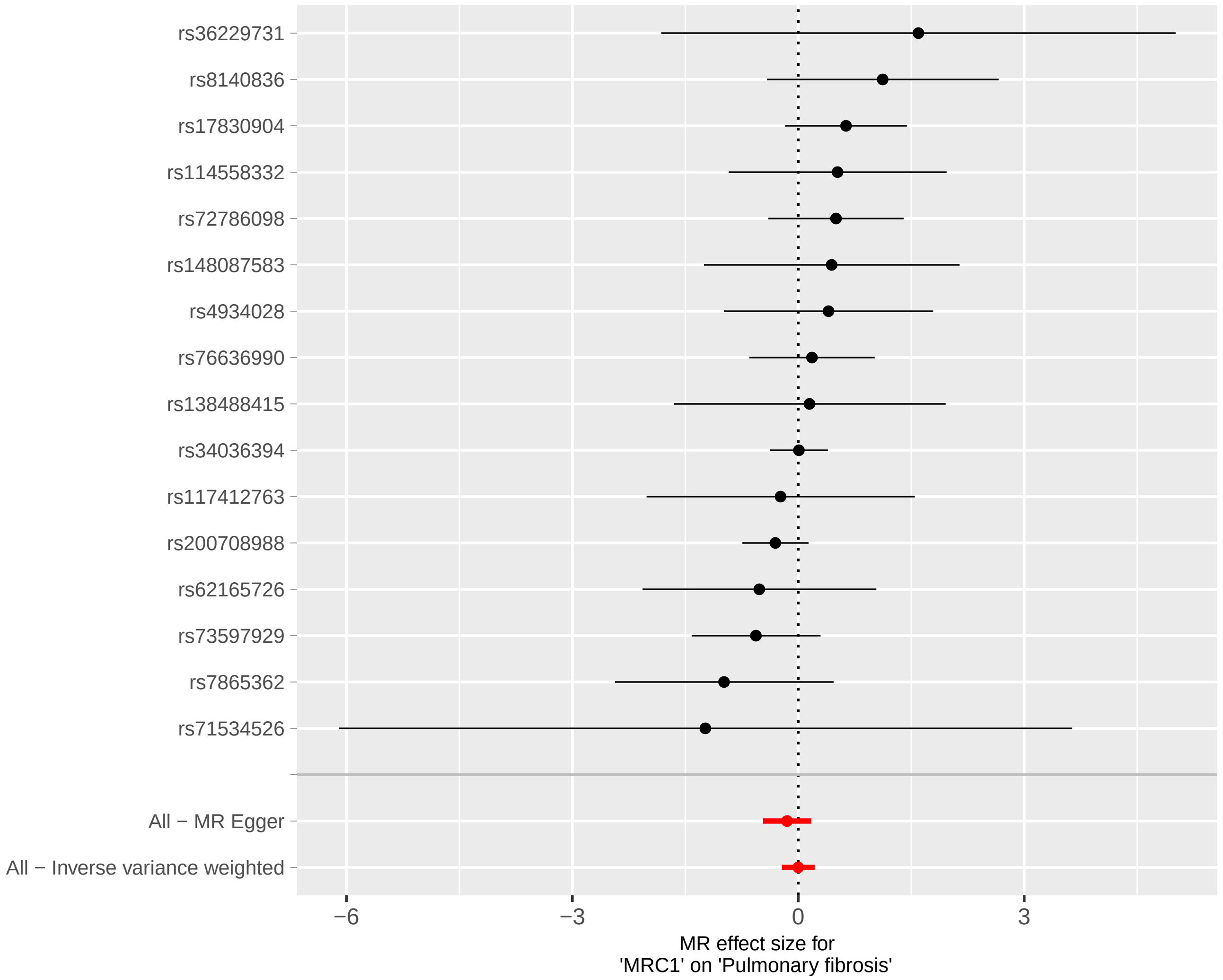
rs36229731
rs8140836
rs17830904
rs114558332
rs72786098
rs148087583
rs4934028
rs76636990
rs138488415
rs34036394
rs117412763
rs200708988
rs62165726
rs73597929
rs7865362
rs71534526
All − MR Egger
All − Inverse variance weighted
−6
−3
0
3
MR effect size for
'MRC1' on 'Pulmonary fibrosis'

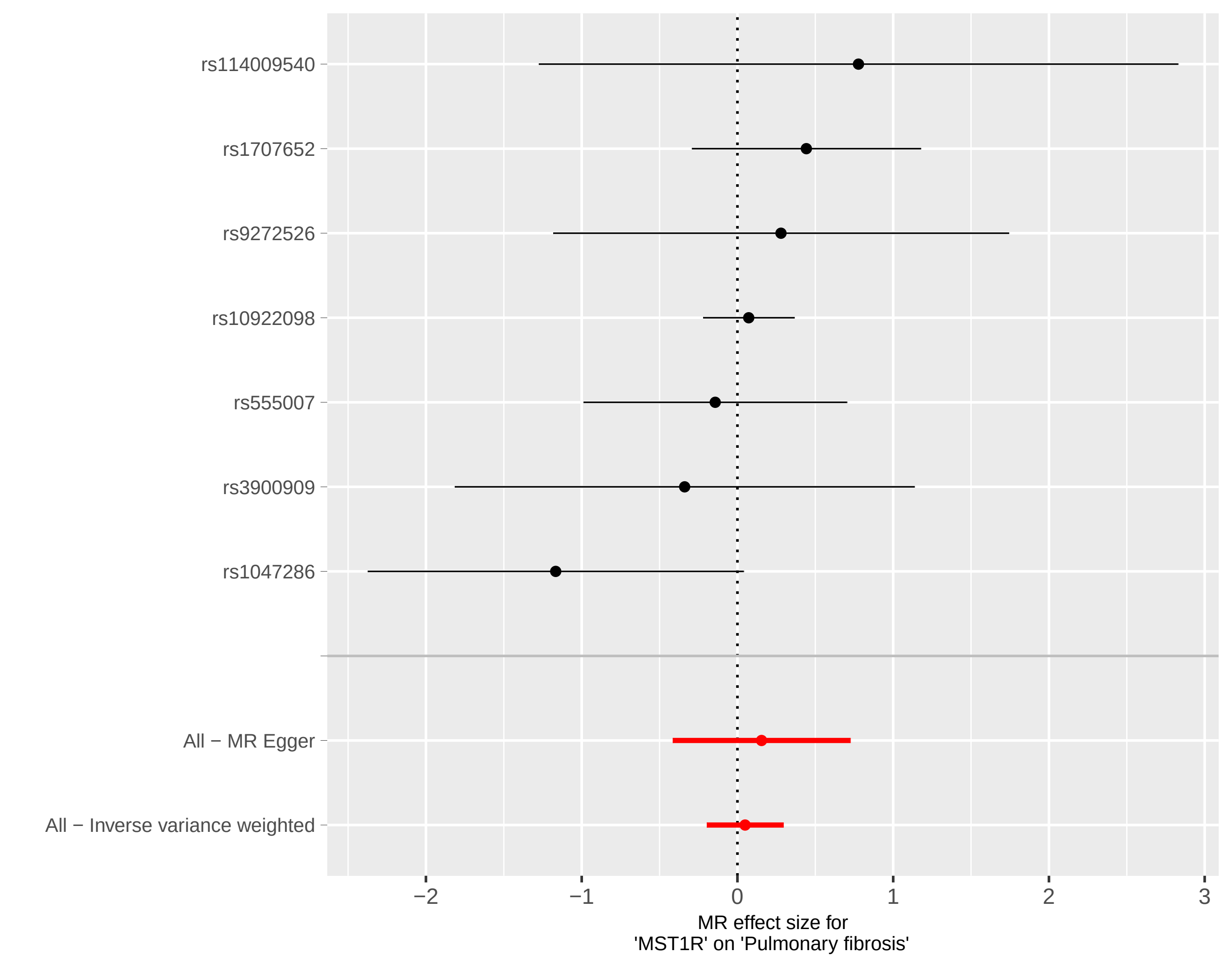
rs114009540
rs1707652
rs9272526
rs10922098
rs555007
rs3900909
rs1047286
All − MR Egger
All − Inverse variance weighted
−2
−1
0
1
2
3
MR effect size for
'MST1R' on 'Pulmonary fibrosis'

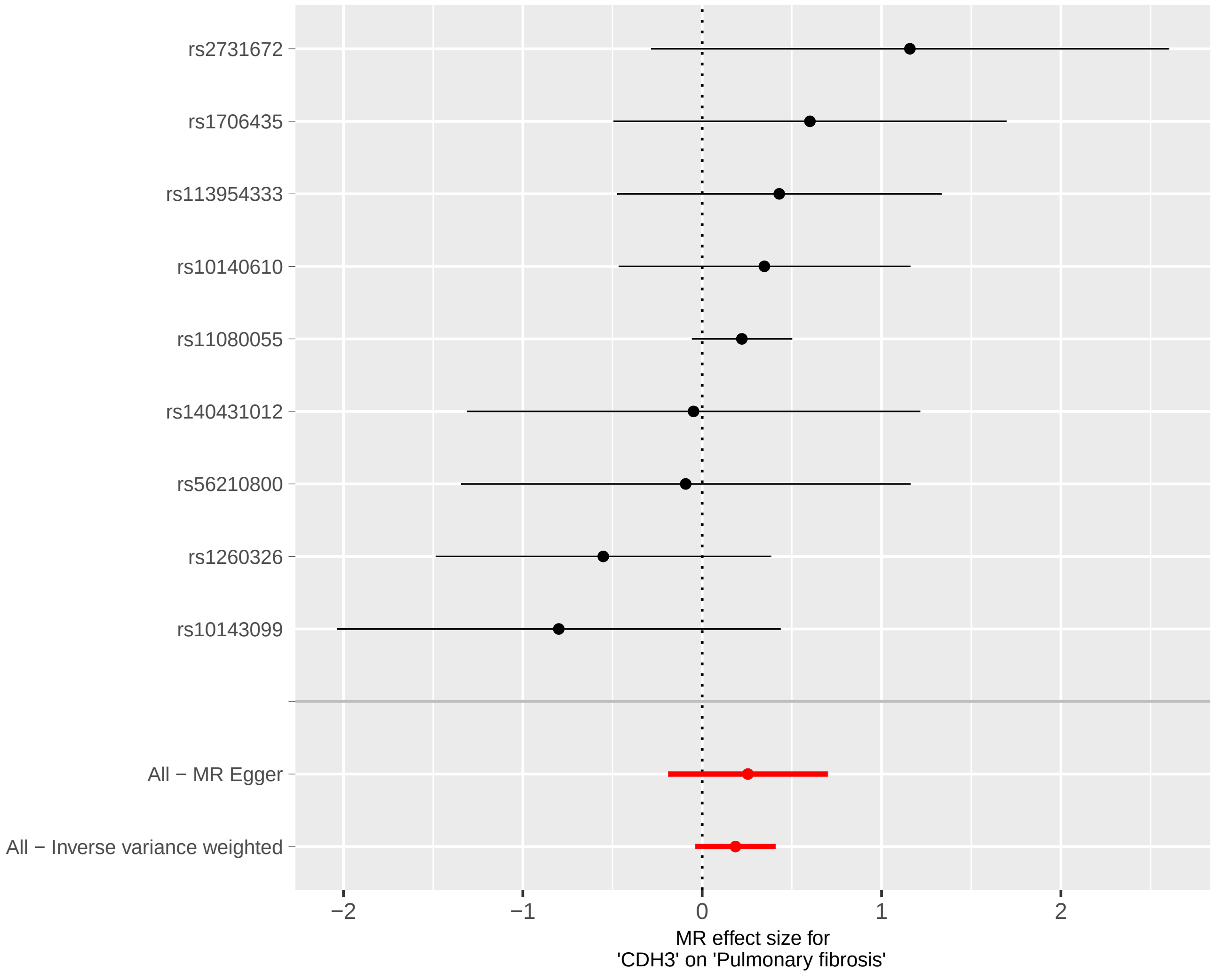
rs2731672
rs1706435
rs113954333
rs10140610
rs11080055
rs140431012
rs56210800
rs1260326
rs10143099
All − MR Egger
All − Inverse variance weighted
−2
−1
0
1
2
MR effect size for
'CDH3' on 'Pulmonary fibrosis'

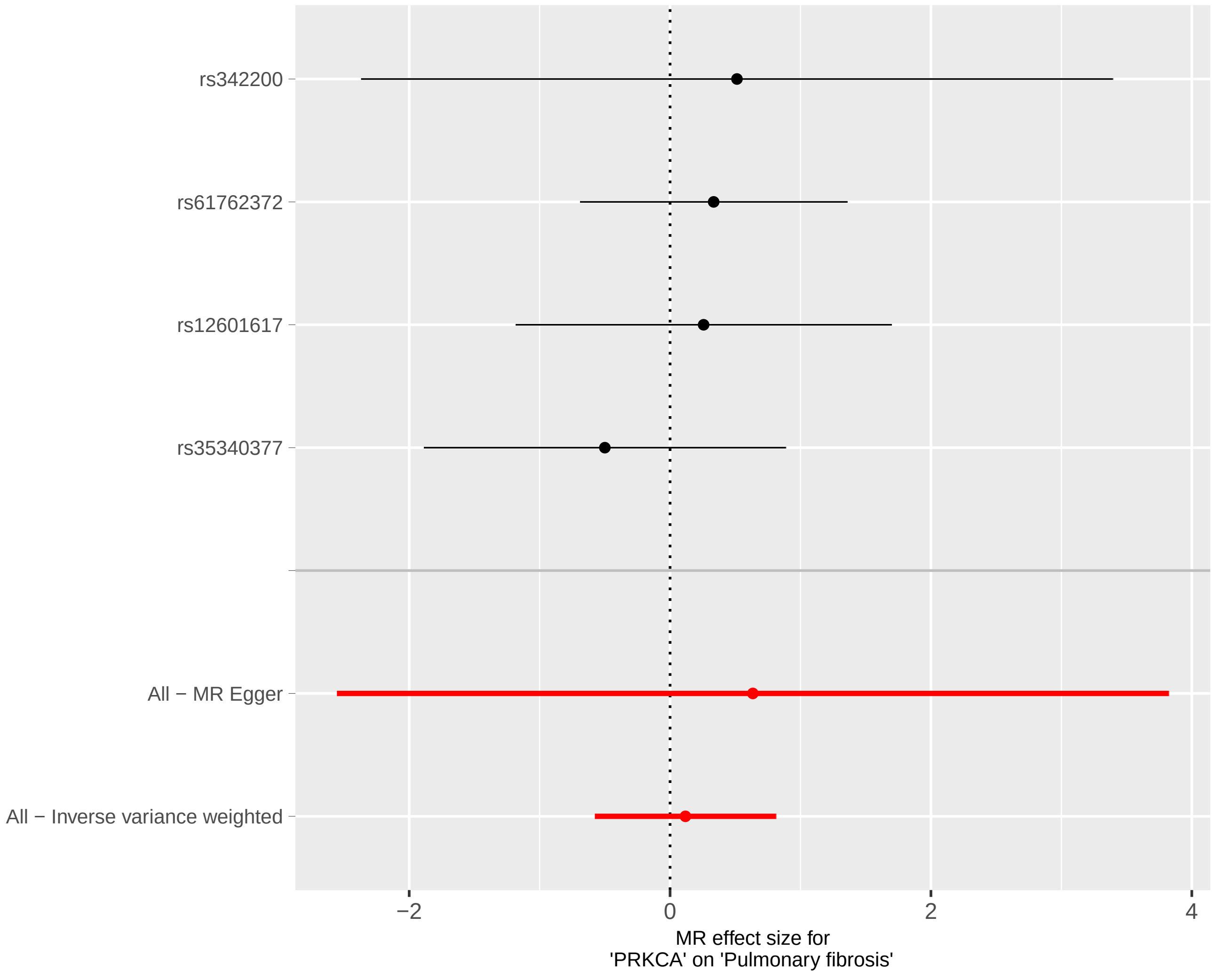

rs342200
rs61762372
rs12601617
rs35340377
All − MR Egger
All − Inverse variance weighted
−2
0
2
4
MR effect size for
'PRKCA' on 'Pulmonary fibrosis'

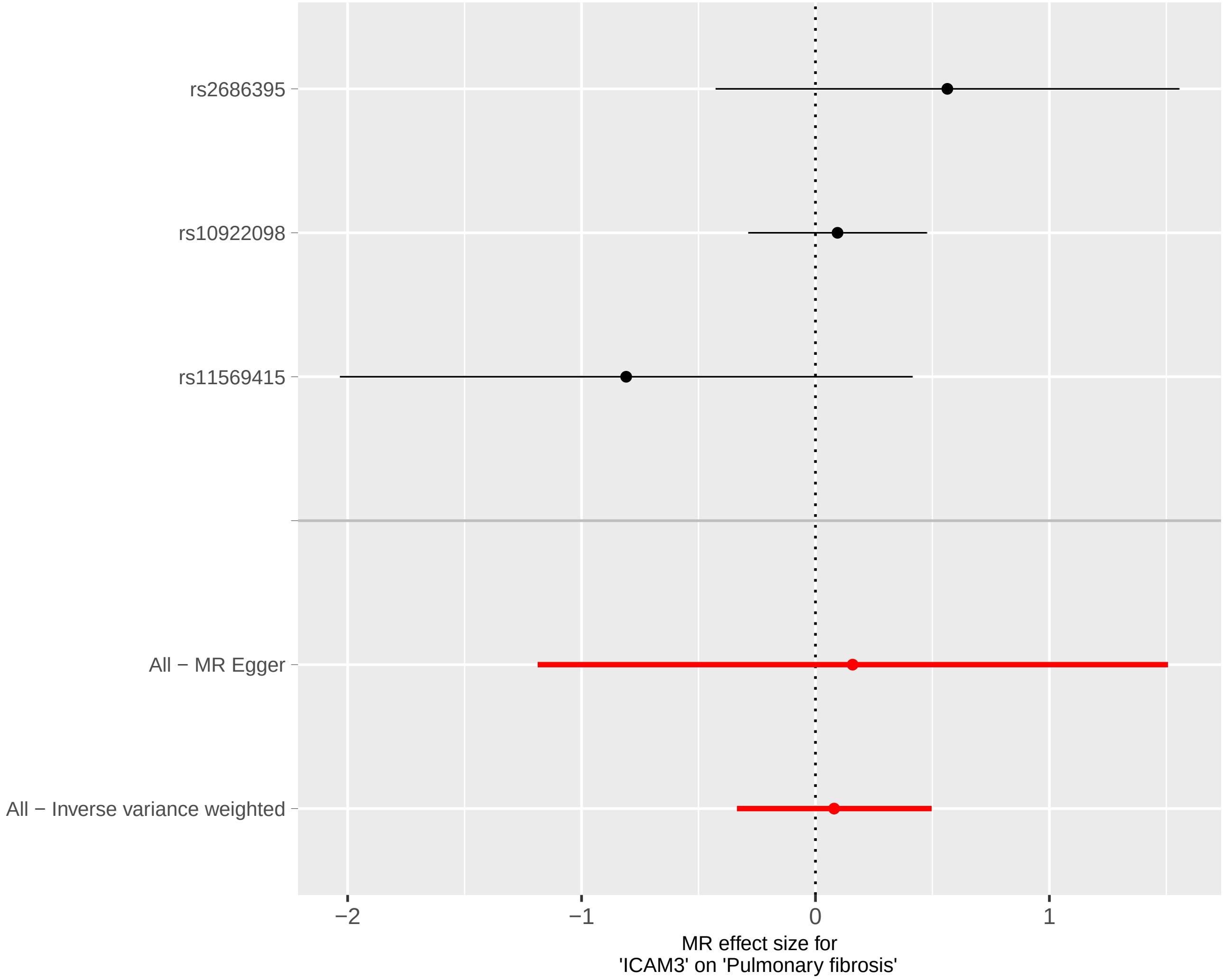

rs2686395
rs10922098
rs11569415
All − MR Egger
All − Inverse variance weighted
−2
−1
0
1
MR effect size for
'ICAM3' on 'Pulmonary fibrosis'

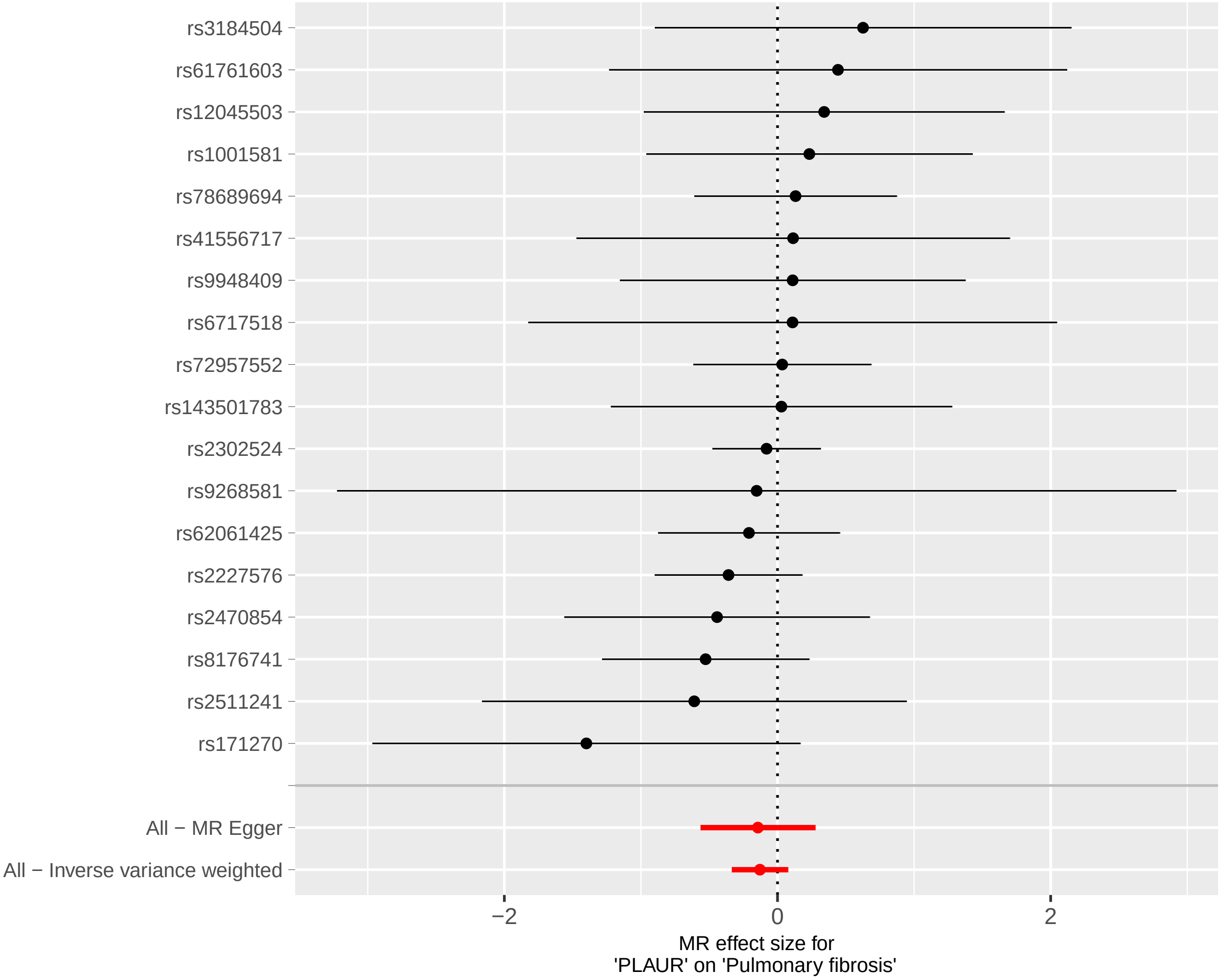

rs3184504
rs61761603
rs12045503
rs1001581
rs78689694
rs41556717
rs9948409
rs6717518
rs72957552
rs143501783
rs2302524
rs9268581
rs62061425
rs2227576
rs2470854
rs8176741
rs2511241
rs171270
All − MR Egger
All − Inverse variance weighted
−2
0
2
MR effect size for
'PLAUR' on 'Pulmonary fibrosis'

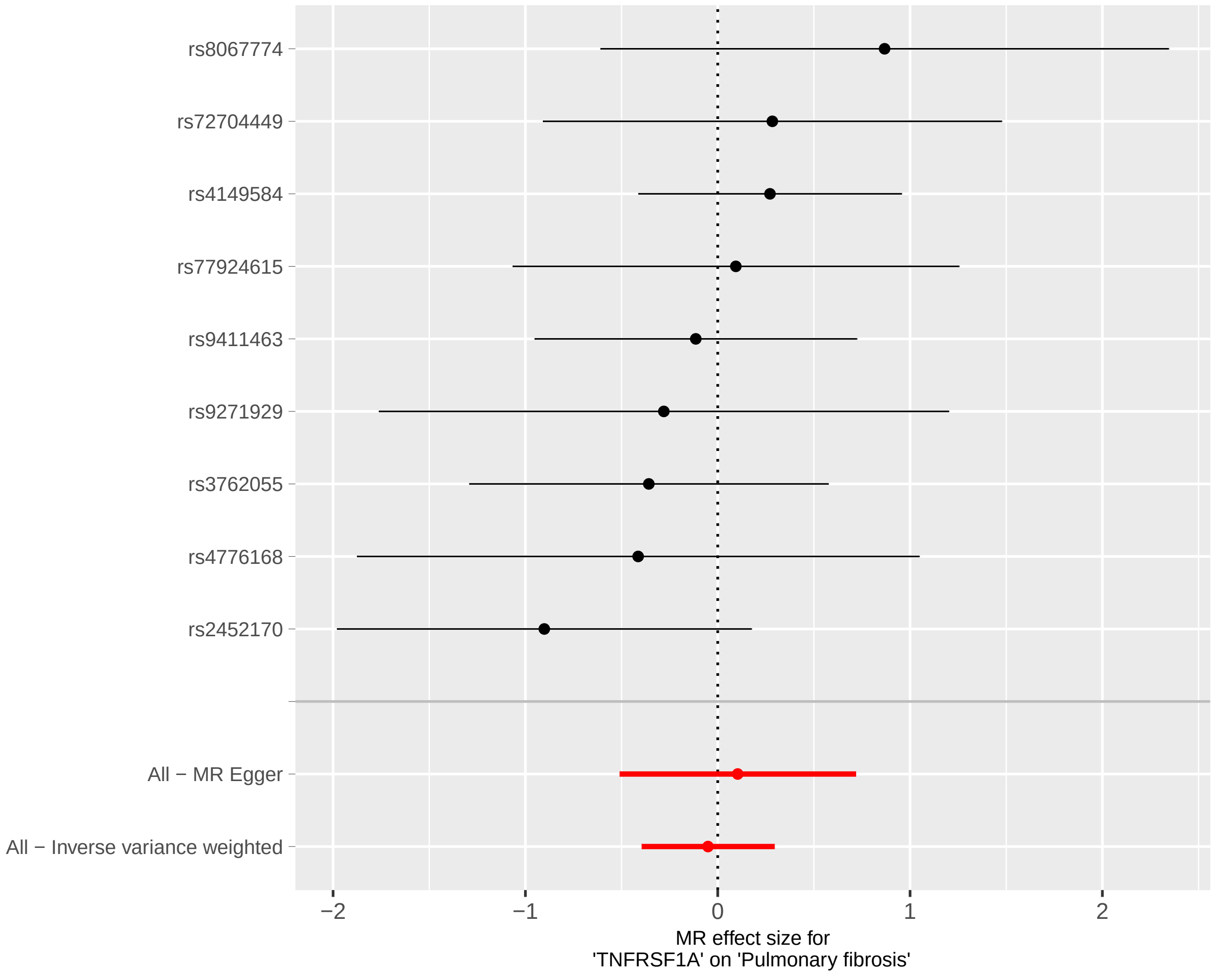

rs8067774
rs72704449
rs4149584
rs77924615
rs9411463
rs9271929
rs3762055
rs4776168
rs2452170
All − MR Egger
All − Inverse variance weighted
−2
−1
0
1
2
MR effect size for
'TNFRSF1A' on 'Pulmonary fibrosis'

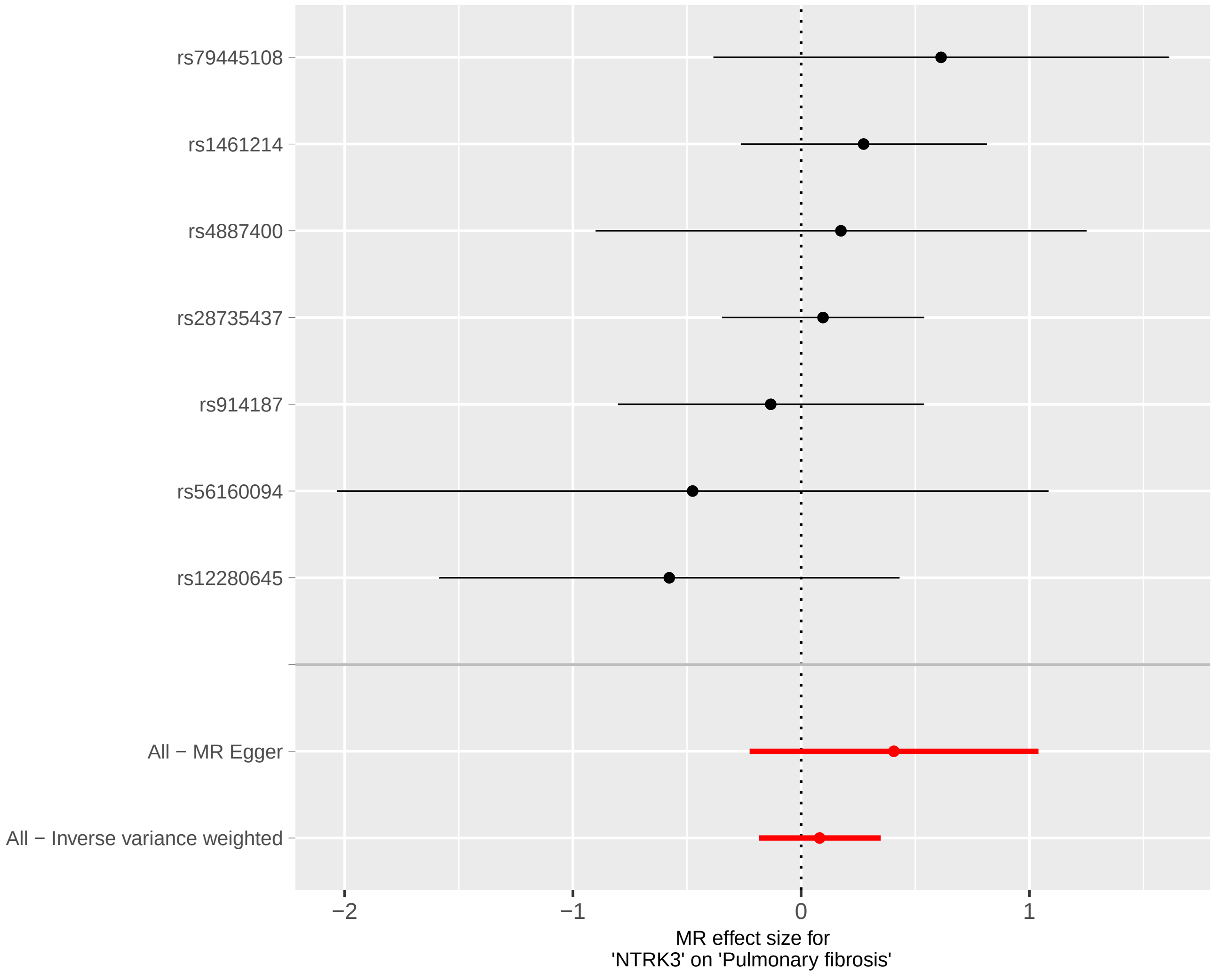
rs79445108
rs1461214
rs4887400
rs28735437
rs914187
rs56160094
rs12280645
All − MR Egger
All − Inverse variance weighted
−2
−1
0
1
MR effect size for
'NTRK3' on 'Pulmonary fibrosis'

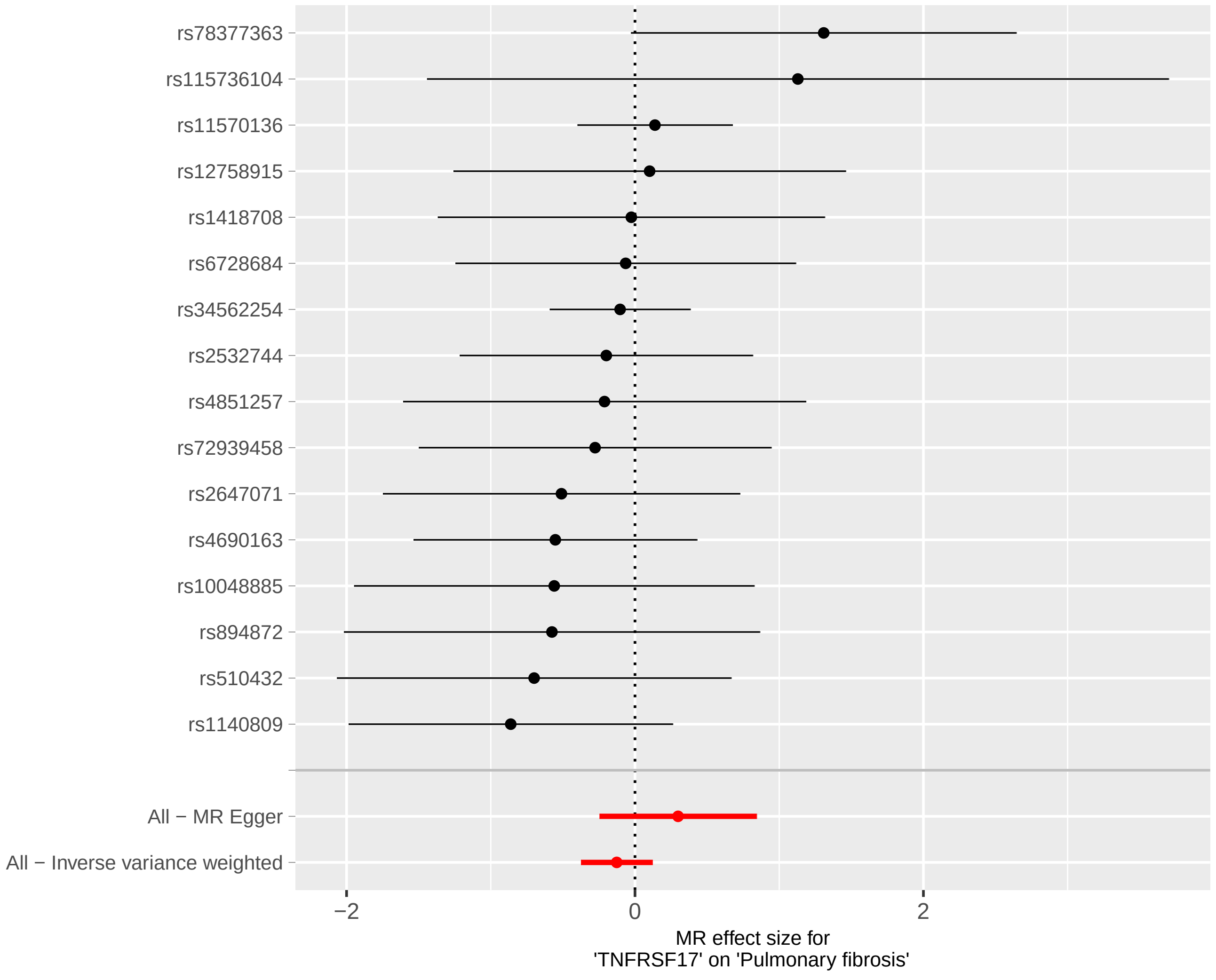
rs78377363
rs115736104
rs11570136
rs12758915
rs1418708
rs6728684
rs34562254
rs2532744
rs4851257
rs72939458
rs2647071
rs4690163
rs10048885
rs894872
rs510432
rs1140809
All − MR Egger
All − Inverse variance weighted
−2
0
2
MR effect size for
'TNFRSF17' on 'Pulmonary fibrosis'

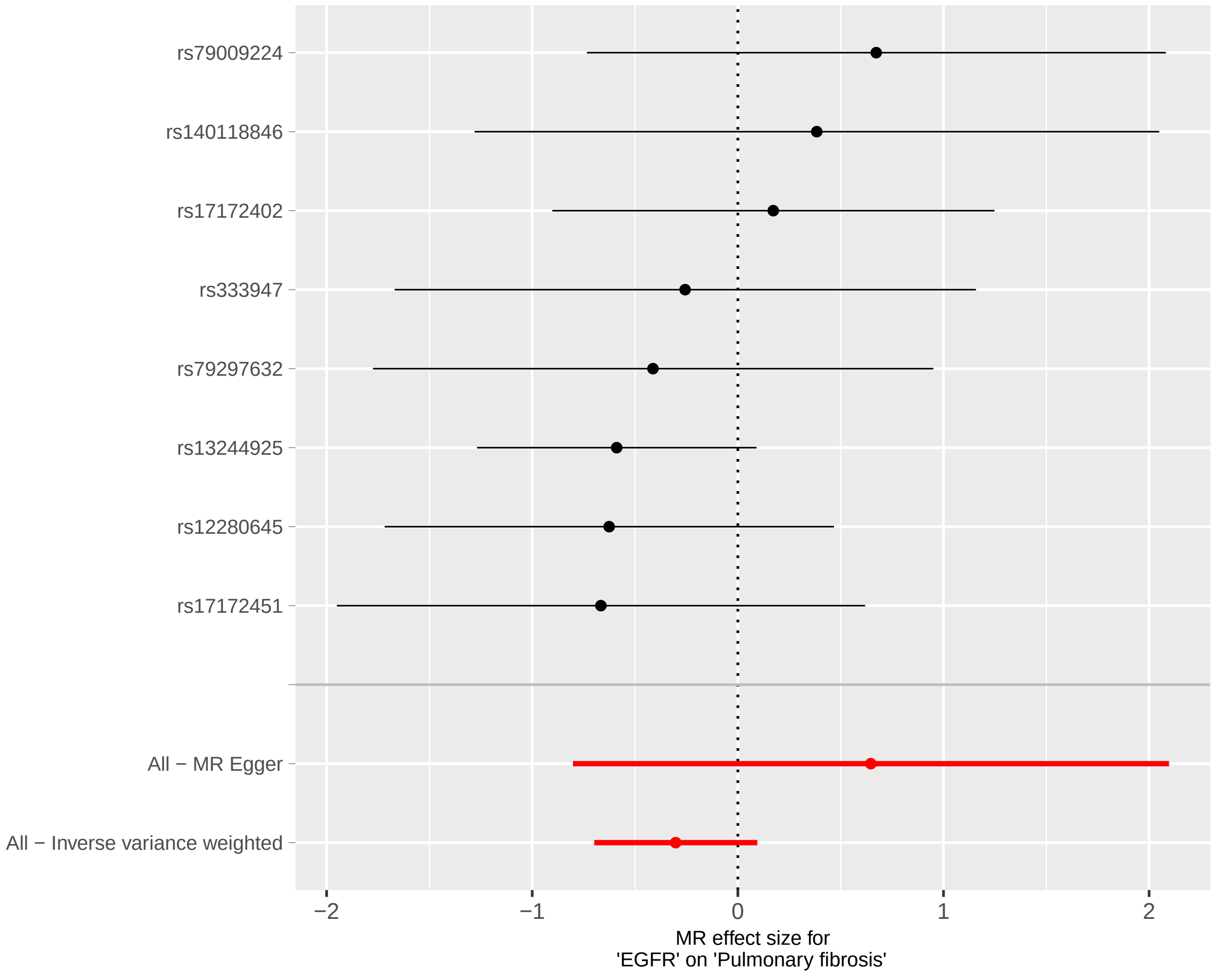
rs79009224
rs140118846
rs17172402
rs333947
rs79297632
rs13244925
rs12280645
rs17172451
All − MR Egger
All − Inverse variance weighted
−2
−1
0
1
2
MR effect size for
'EGFR' on 'Pulmonary fibrosis'

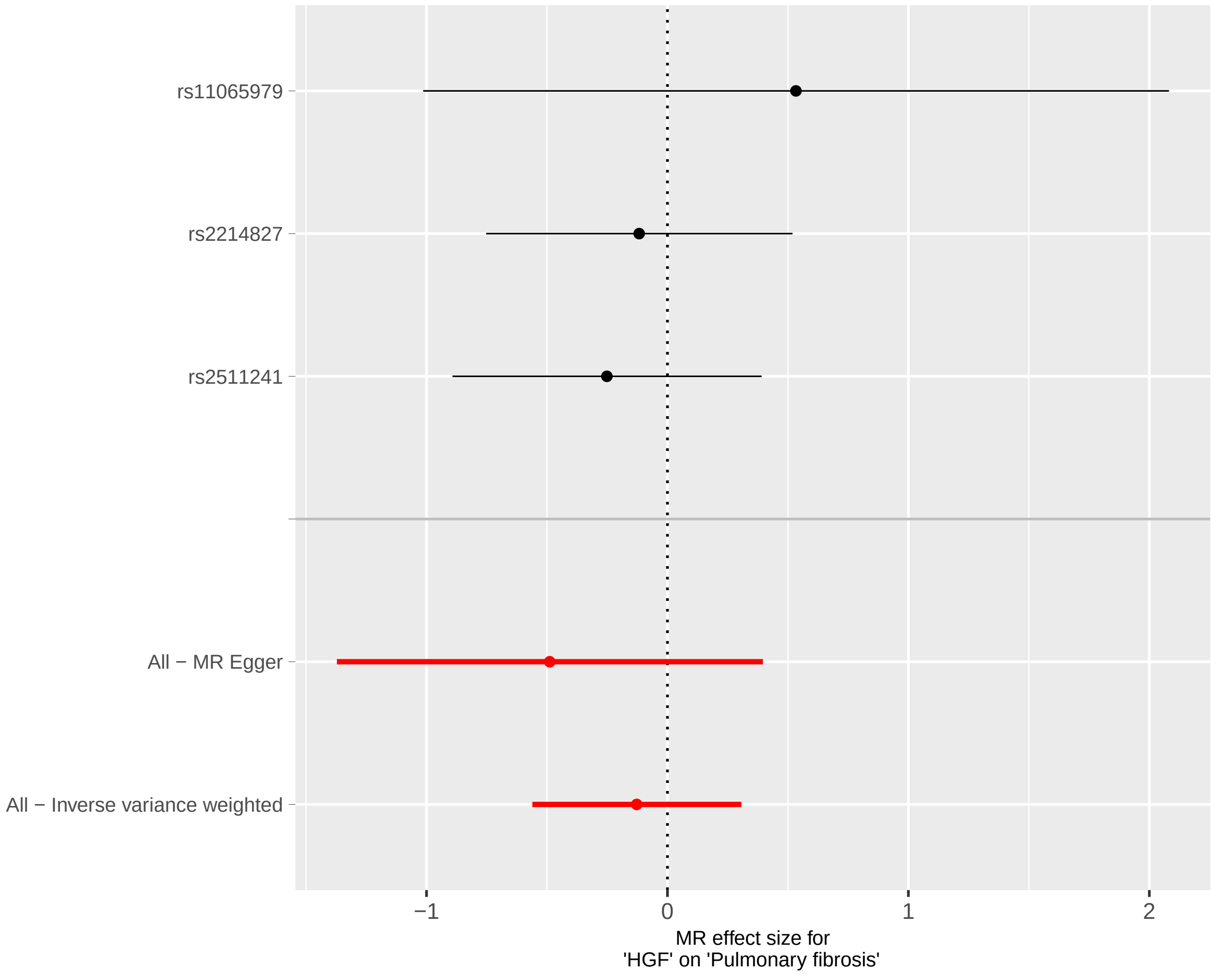

rs11065979
rs2214827
rs2511241
All − MR Egger
All − Inverse variance weighted
−1
0
1
2
MR effect size for
'HGF' on 'Pulmonary fibrosis'

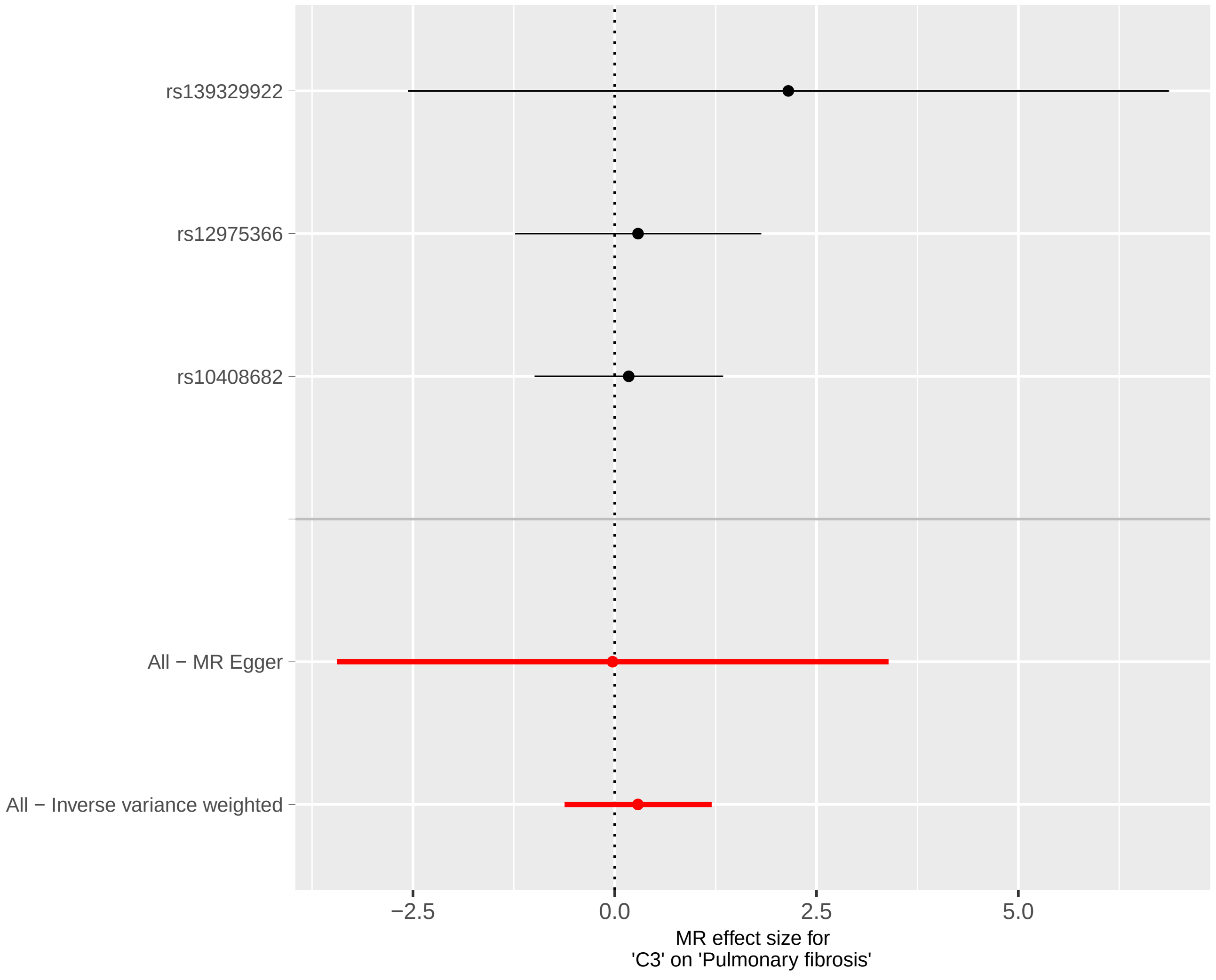
rs139329922
rs12975366
rs10408682
All − MR Egger
All − Inverse variance weighted
−2.5
0.0
2.5
5.0
MR effect size for
'C3' on 'Pulmonary fibrosis'

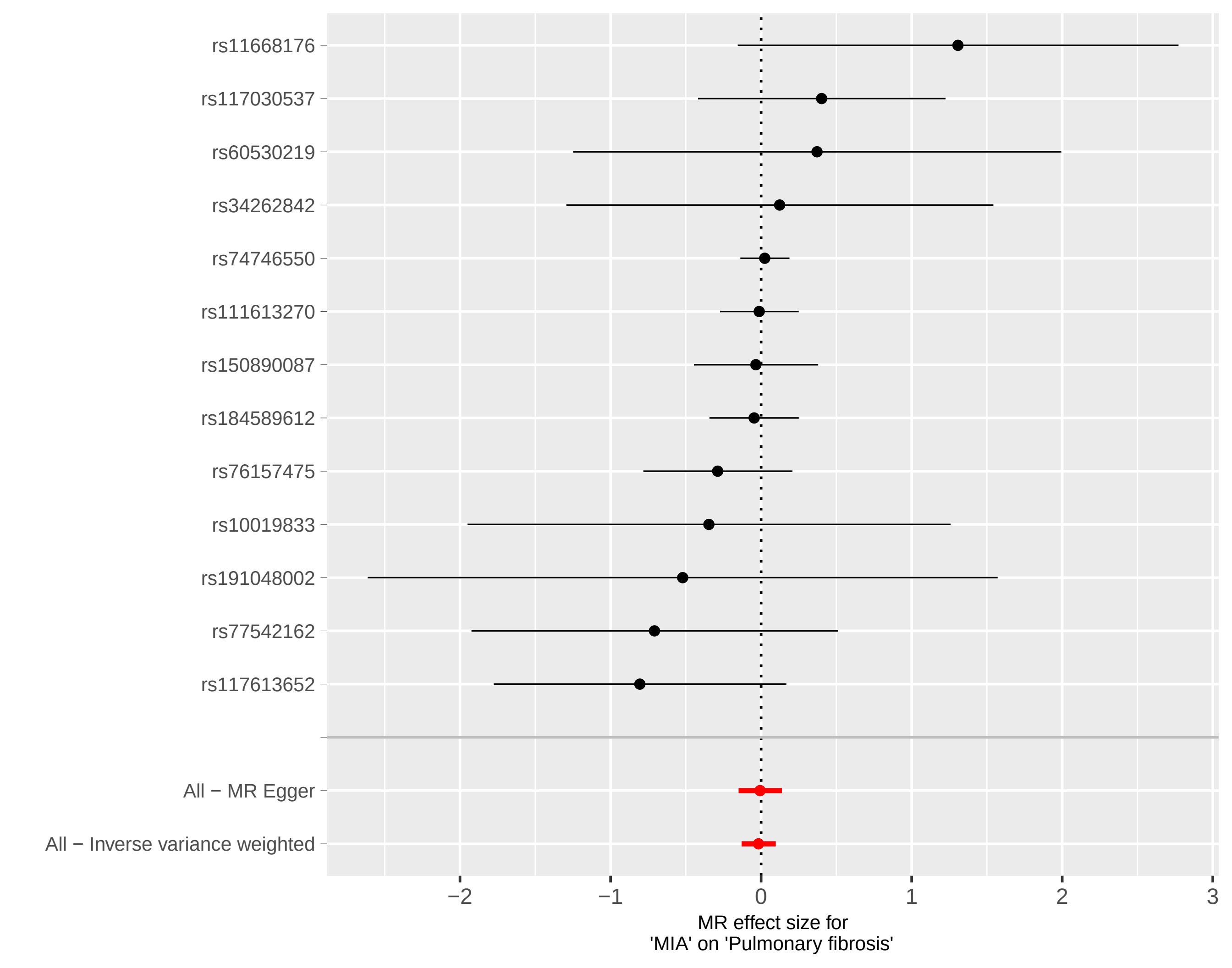
rs11668176
rs117030537
rs60530219
rs34262842
rs74746550
rs111613270
rs150890087
rs184589612
rs76157475
rs10019833
rs191048002
rs77542162
rs117613652
All − MR Egger
All − Inverse variance weighted
−2
−1
0
1
2
3
MR effect size for
'MIA' on 'Pulmonary fibrosis'

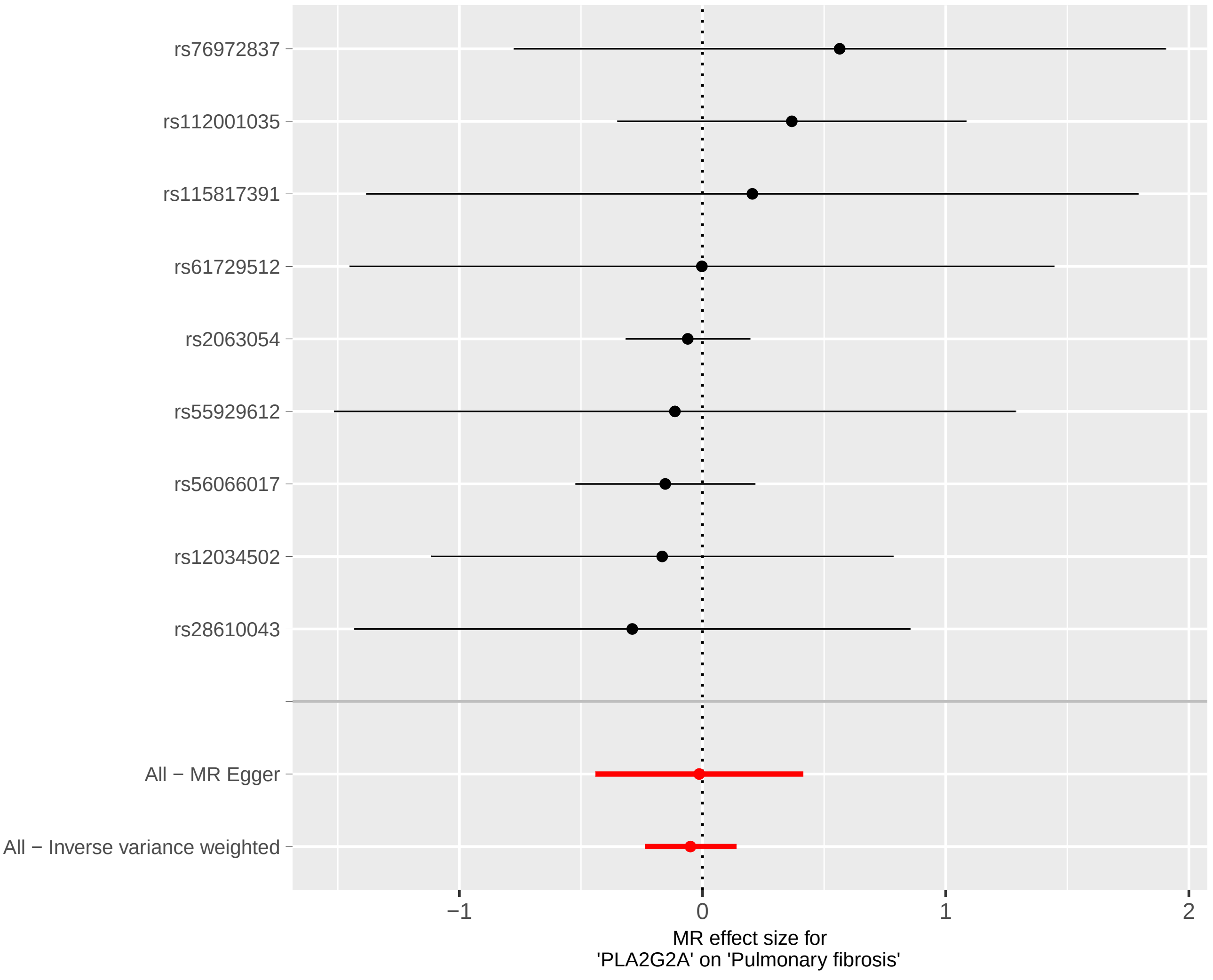

rs76972837
rs112001035
rs115817391
rs61729512
rs2063054
rs55929612
rs56066017
rs12034502
rs28610043
All − MR Egger
All − Inverse variance weighted
−1
0
1
2
MR effect size for
'PLA2G2A' on 'Pulmonary fibrosis'

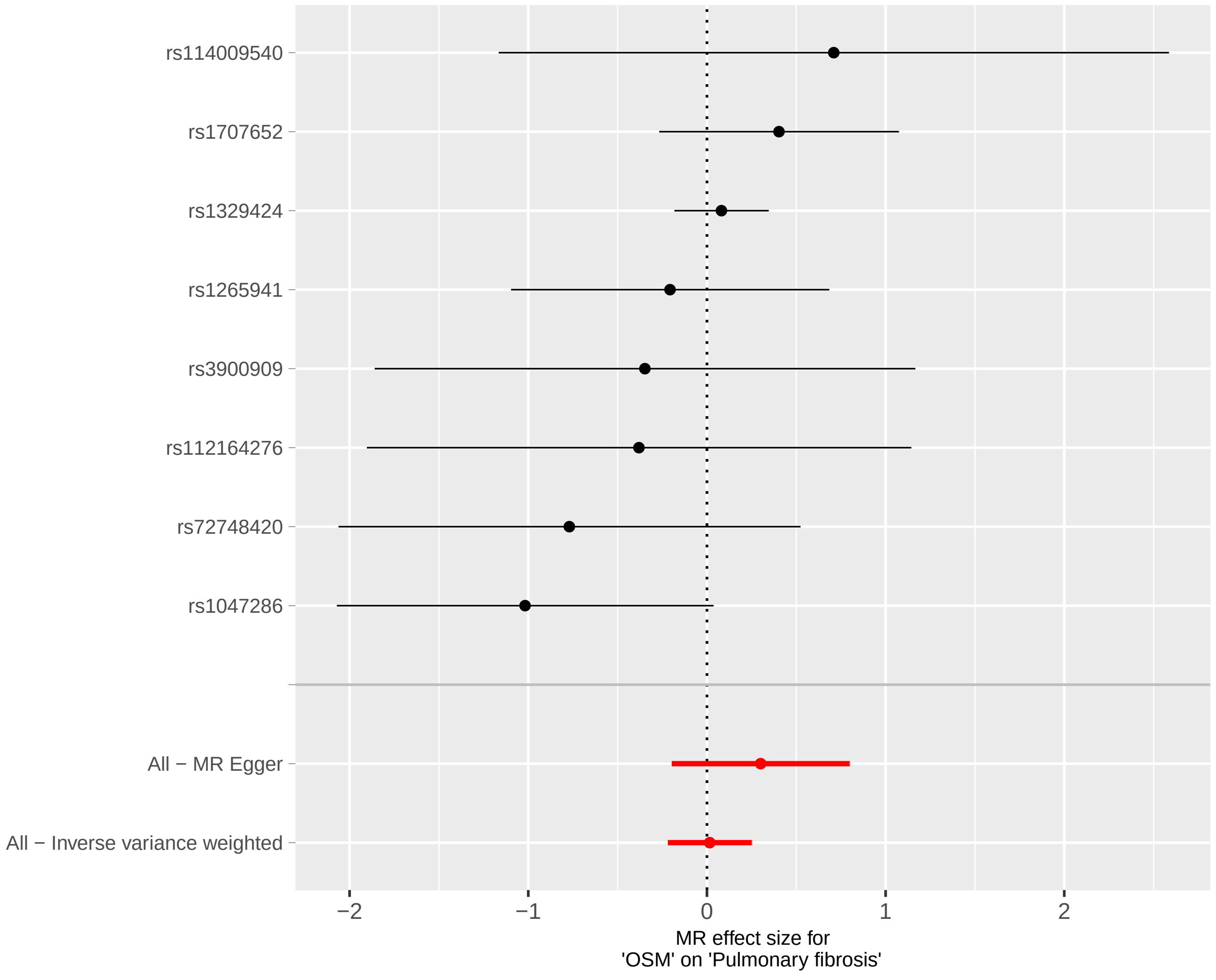
rs114009540
rs1707652
rs1329424
rs1265941
rs3900909
rs112164276
rs72748420
rs1047286
All − MR Egger
All − Inverse variance weighted
−2
−1
0
1
2
MR effect size for
'OSM' on 'Pulmonary fibrosis'

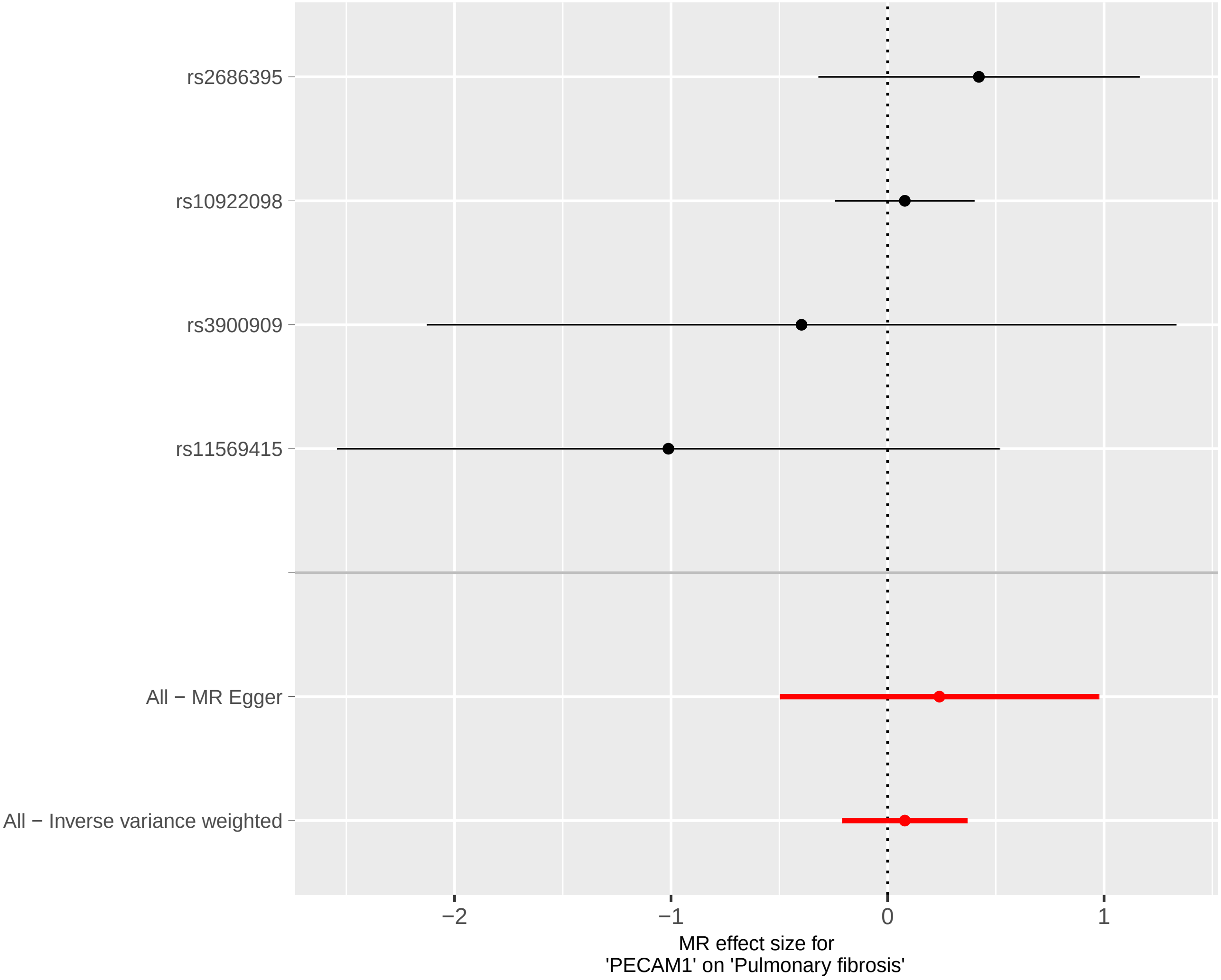
rs2686395
rs10922098
rs3900909
rs11569415
All − MR Egger
All − Inverse variance weighted
−2
−1
0
1
MR effect size for
'PECAM1' on 'Pulmonary fibrosis'

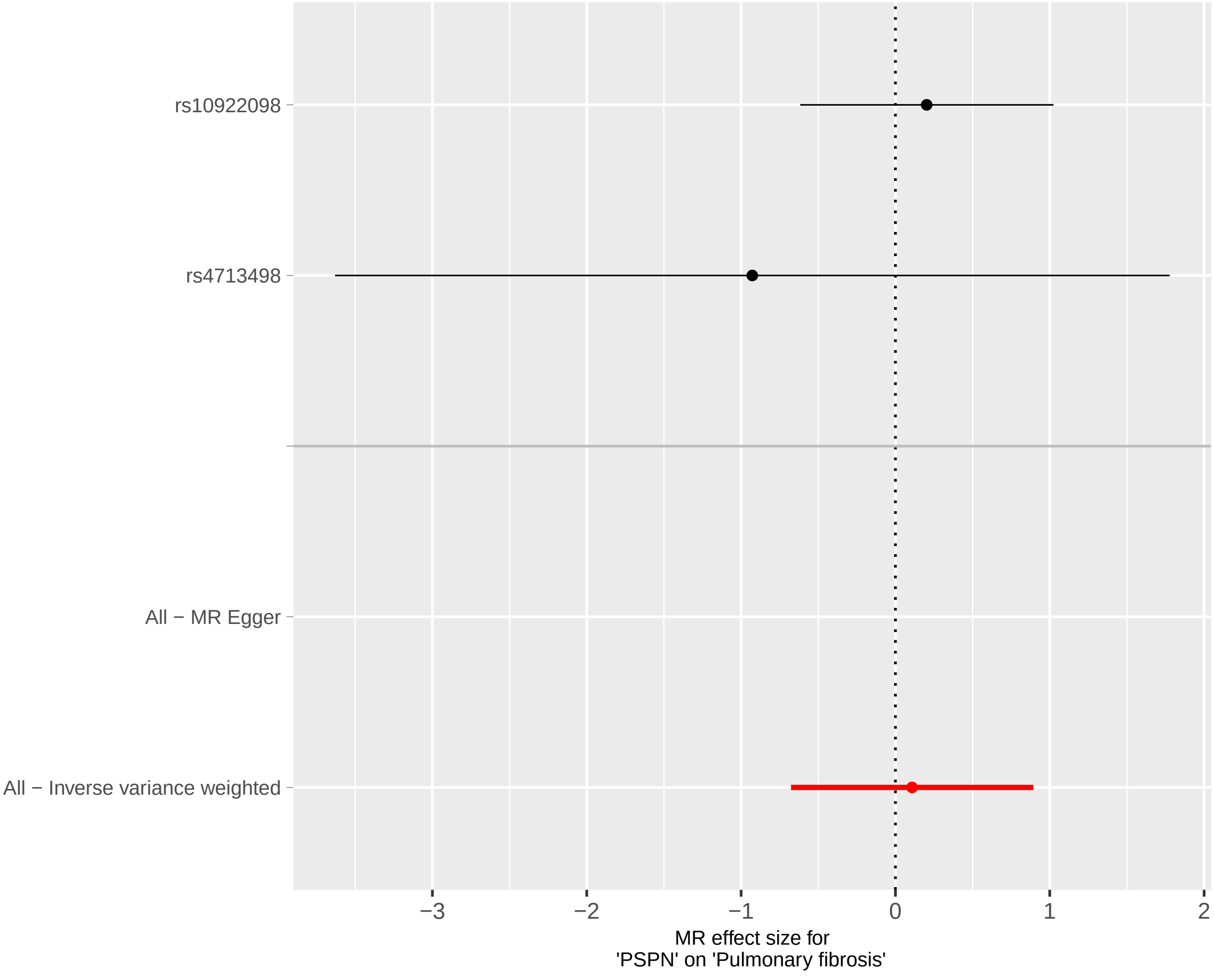

rs10922098
rs4713498
All − MR Egger
All − Inverse variance weighted
−3
−2
−1
0
1
2
MR effect size for
'PSPN' on 'Pulmonary fibrosis'

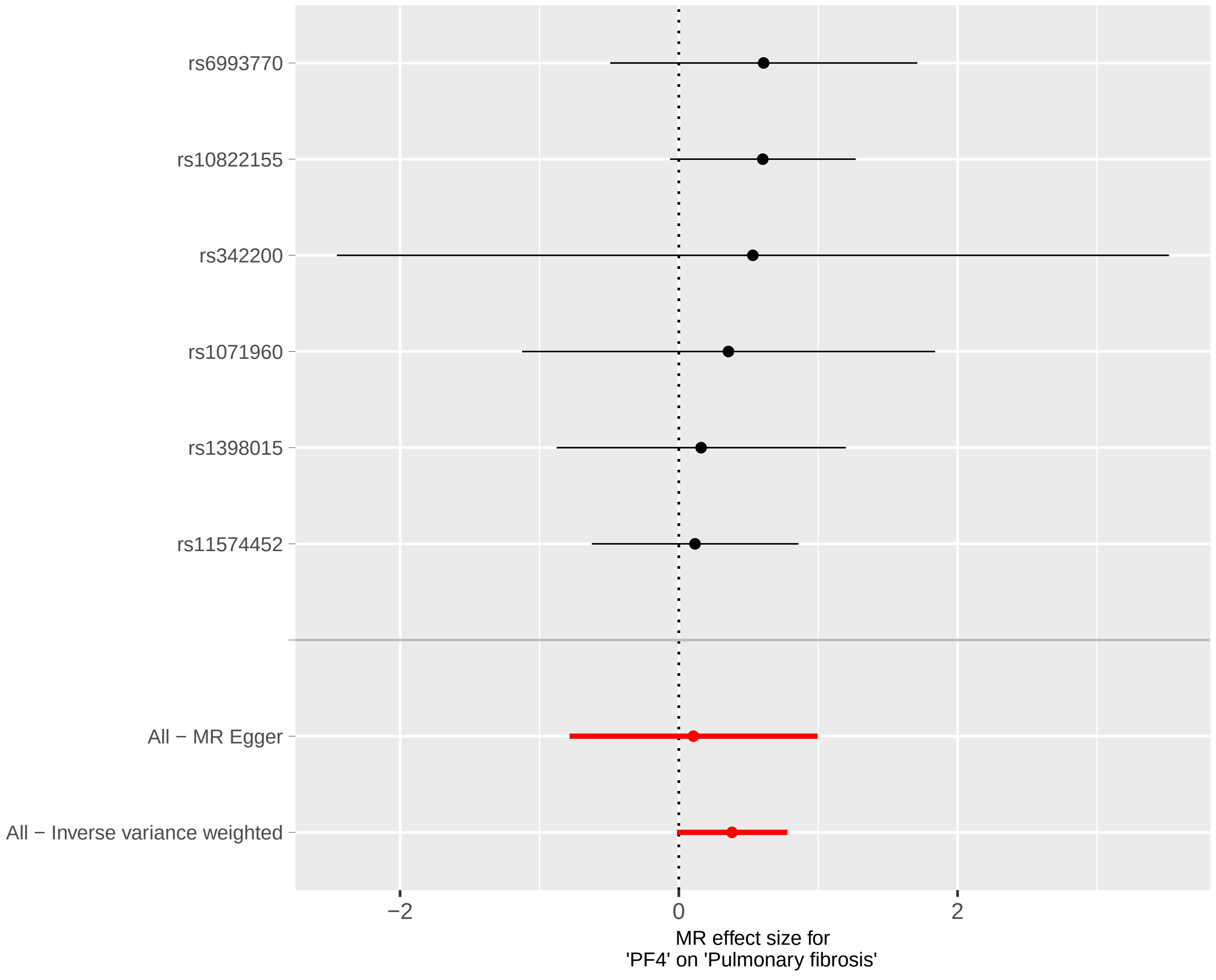

rs6993770
rs10822155
rs342200
rs1071960
rs1398015
rs11574452
All − MR Egger
All − Inverse variance weighted
−2
0
2
MR effect size for
'PF4' on 'Pulmonary fibrosis'

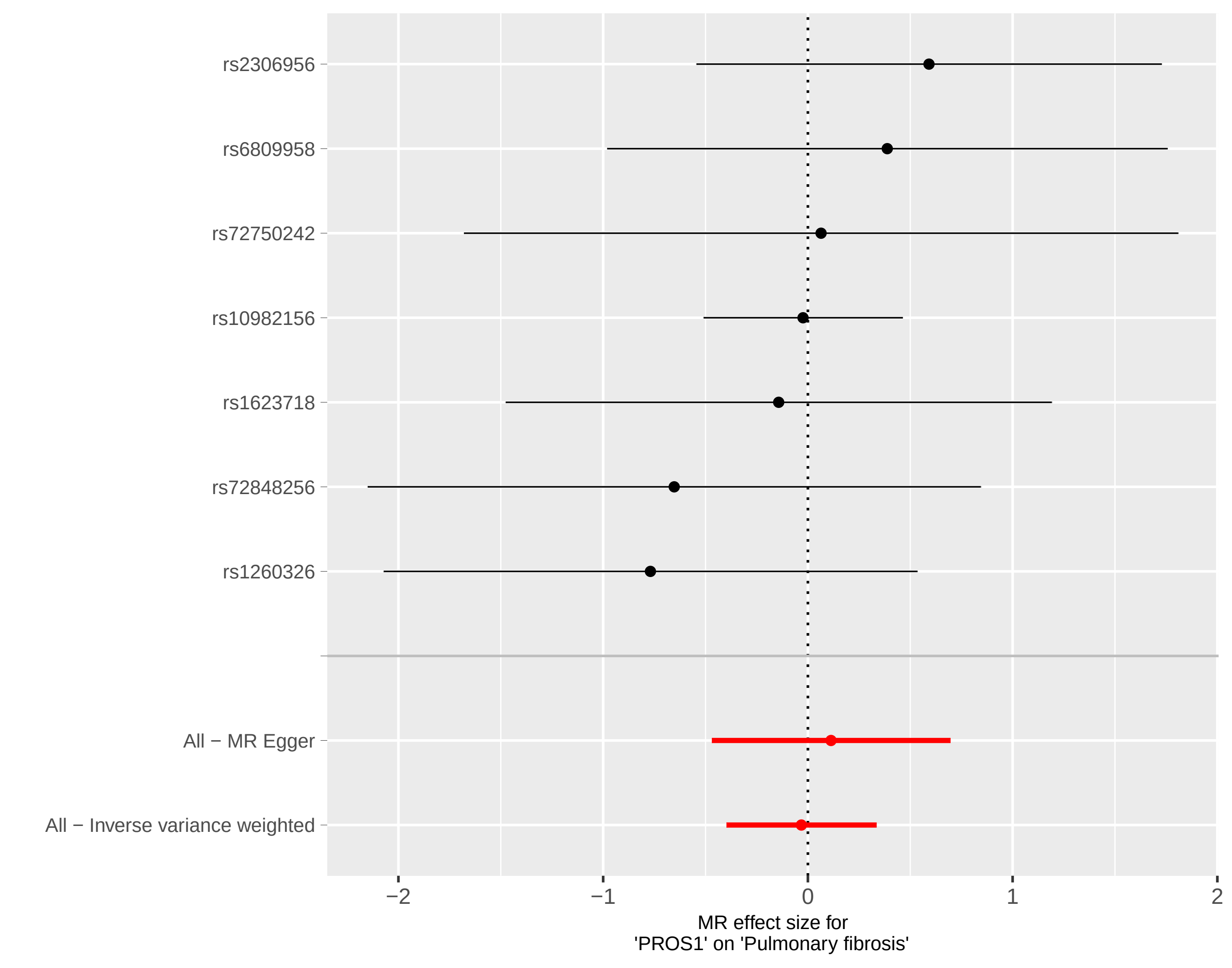

rs2306956
rs6809958
rs72750242
rs10982156
rs1623718
rs72848256
rs1260326
All − MR Egger
All − Inverse variance weighted
−2
−1
0
1
2
MR effect size for
'PROS1' on 'Pulmonary fibrosis'

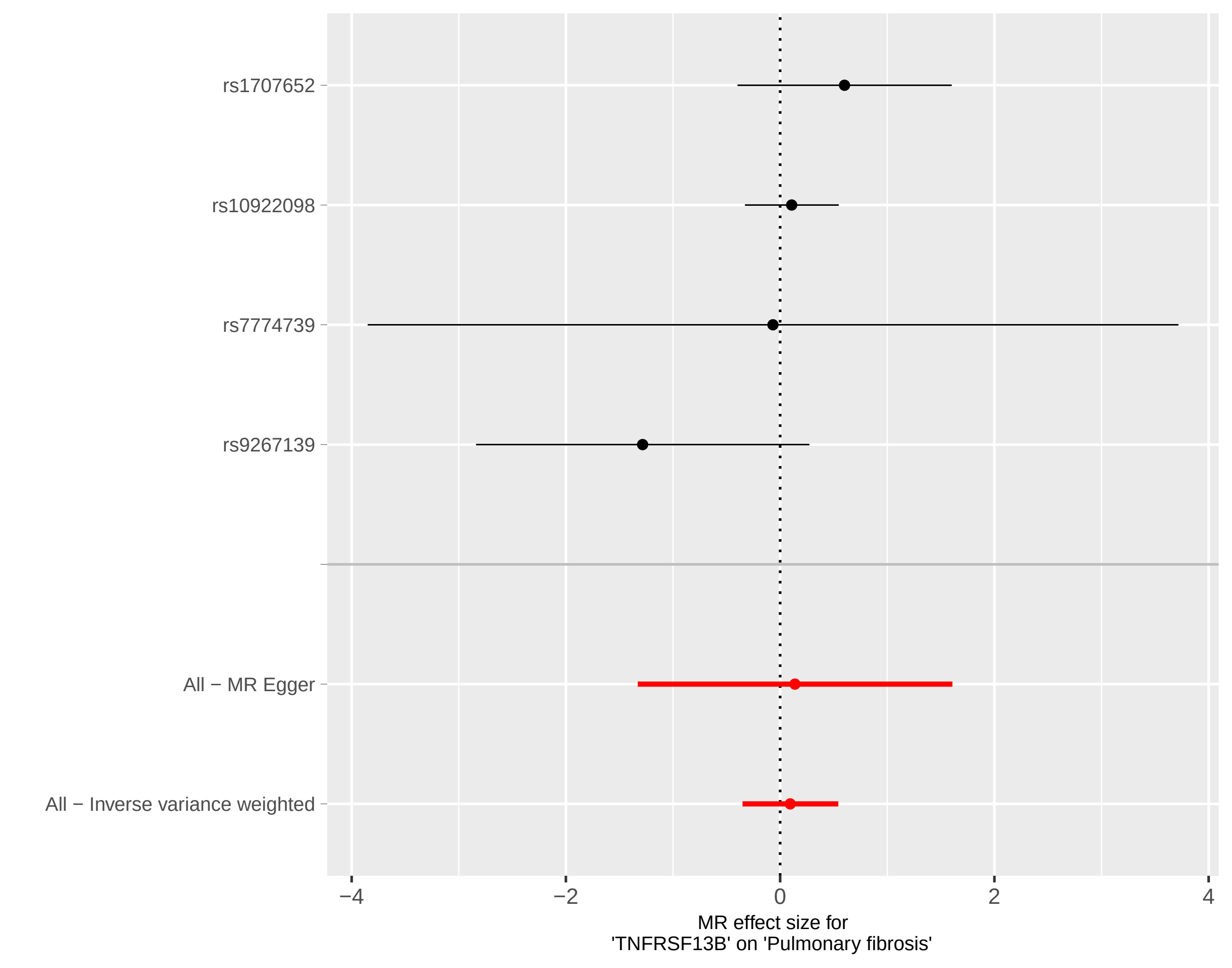
rs1707652
rs10922098
rs7774739
rs9267139
All − MR Egger
All − Inverse variance weighted
−4
−2
0
2
4
MR effect size for
'TNFRSF13B' on 'Pulmonary fibrosis'

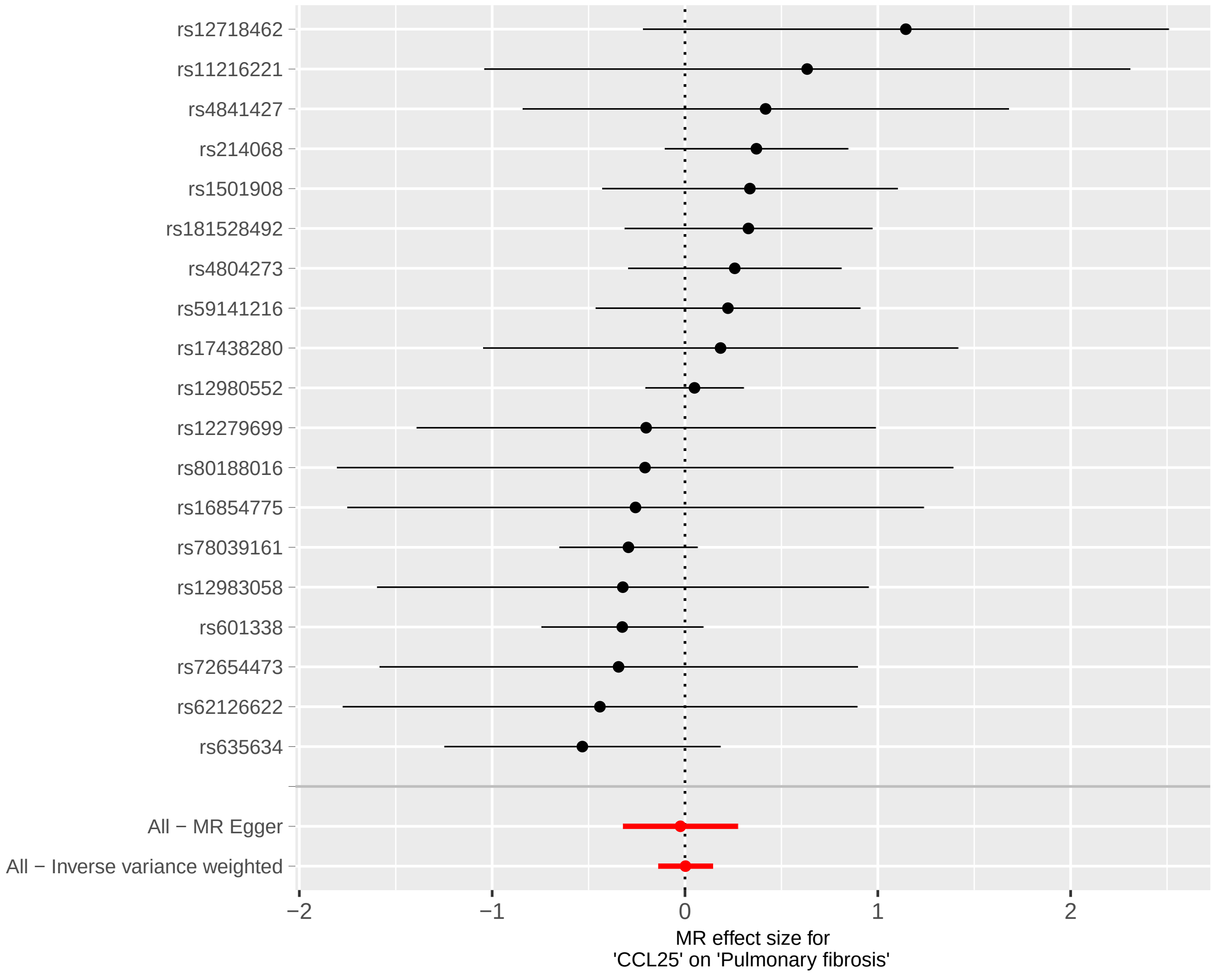

rs12718462
rs11216221
rs4841427
rs214068
rs1501908
rs181528492
rs4804273
rs59141216
rs17438280
rs12980552
rs12279699
rs80188016
rs16854775
rs78039161
rs12983058
rs601338
rs72654473
rs62126622
rs635634
All − MR Egger
All − Inverse variance weighted
−2
−1
0
1
2
MR effect size for
'CCL25' on 'Pulmonary fibrosis'

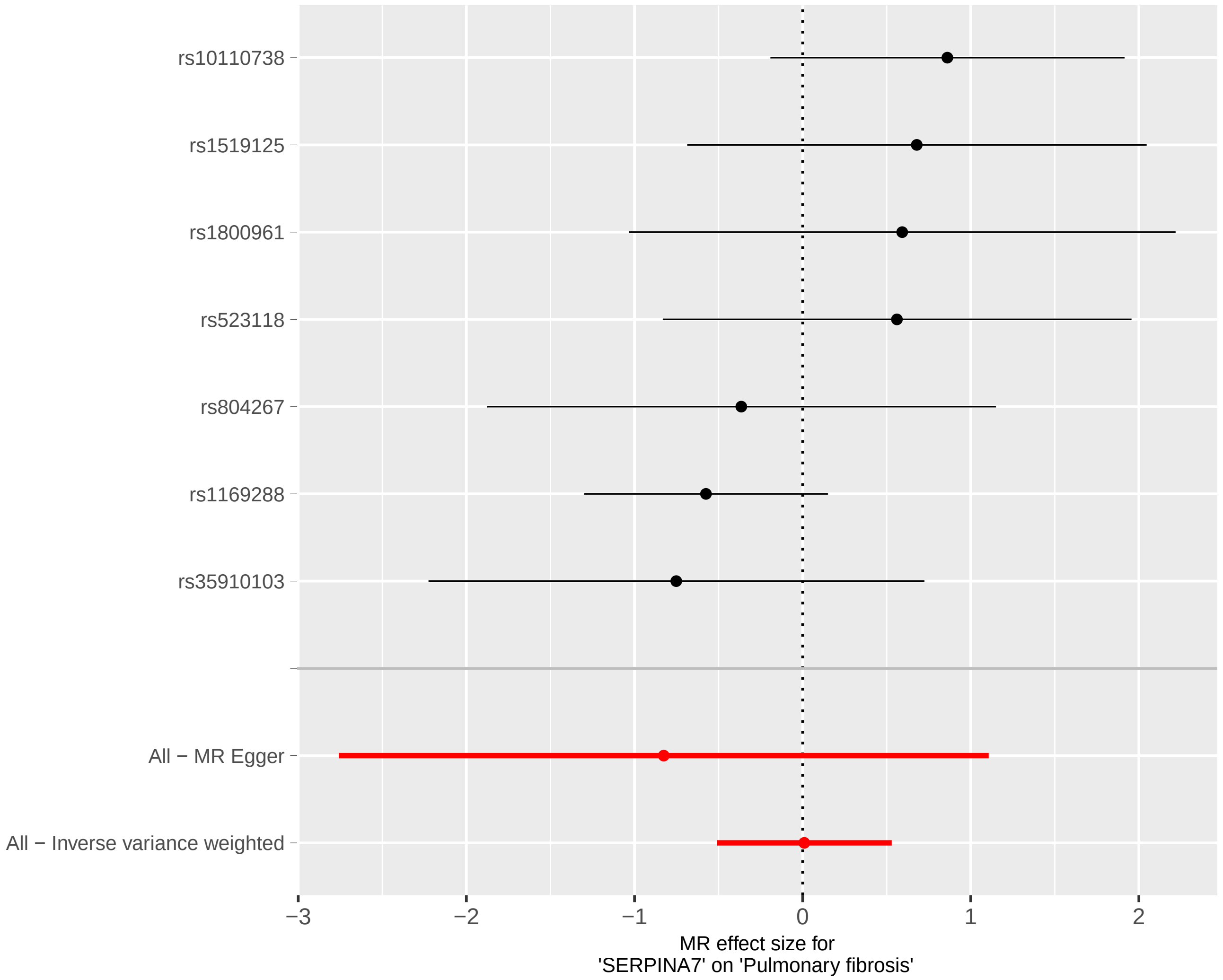

rs10110738
rs1519125
rs1800961
rs523118
rs804267
rs1169288
rs35910103
All − MR Egger
All − Inverse variance weighted
−3
−2
−1
0
1
2
MR effect size for
'SERPINA7' on 'Pulmonary fibrosis'

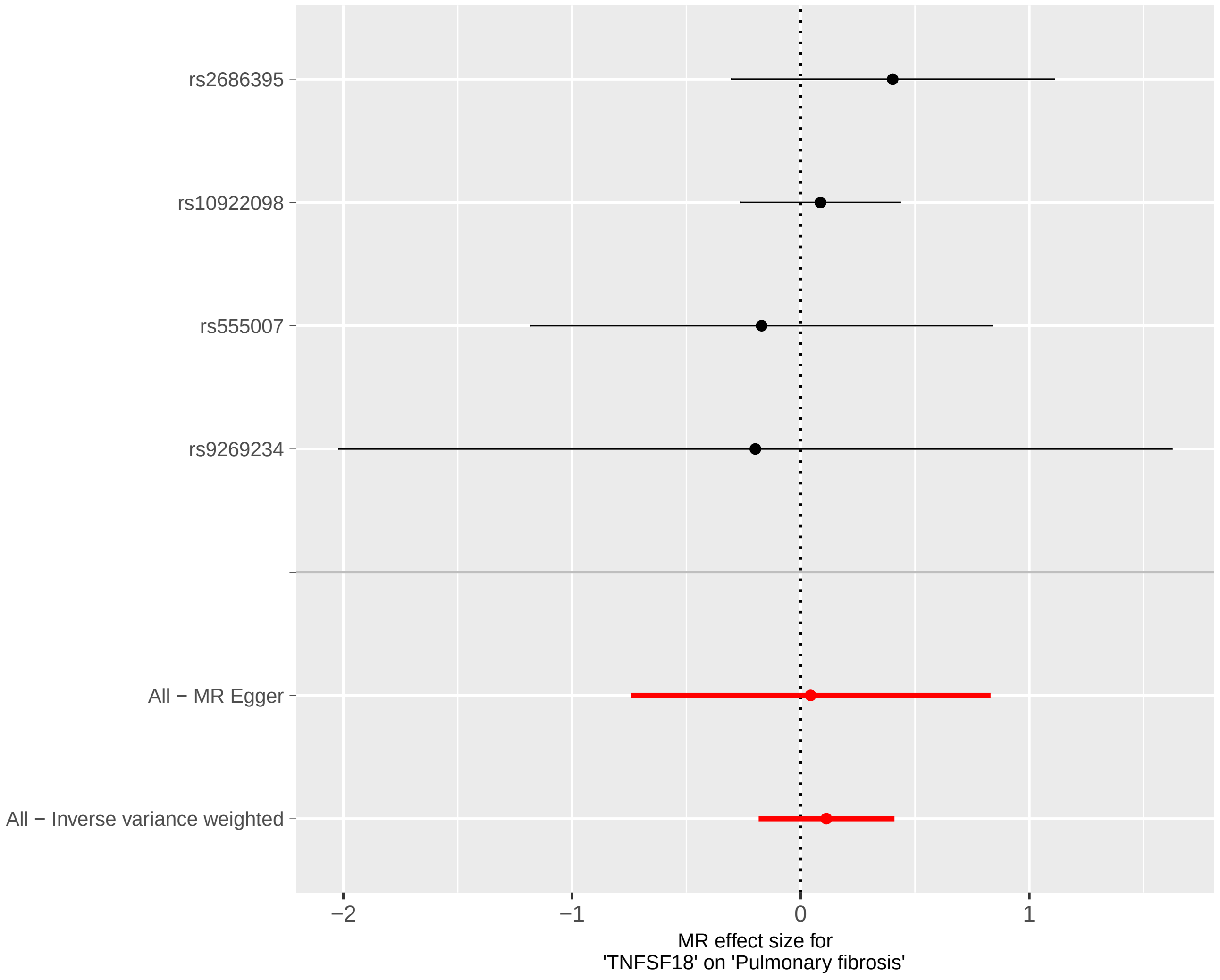

rs2686395
rs10922098
rs555007
rs9269234
All − MR Egger
All − Inverse variance weighted
−2
−1
0
1
MR effect size for
'TNFSF18' on 'Pulmonary fibrosis'

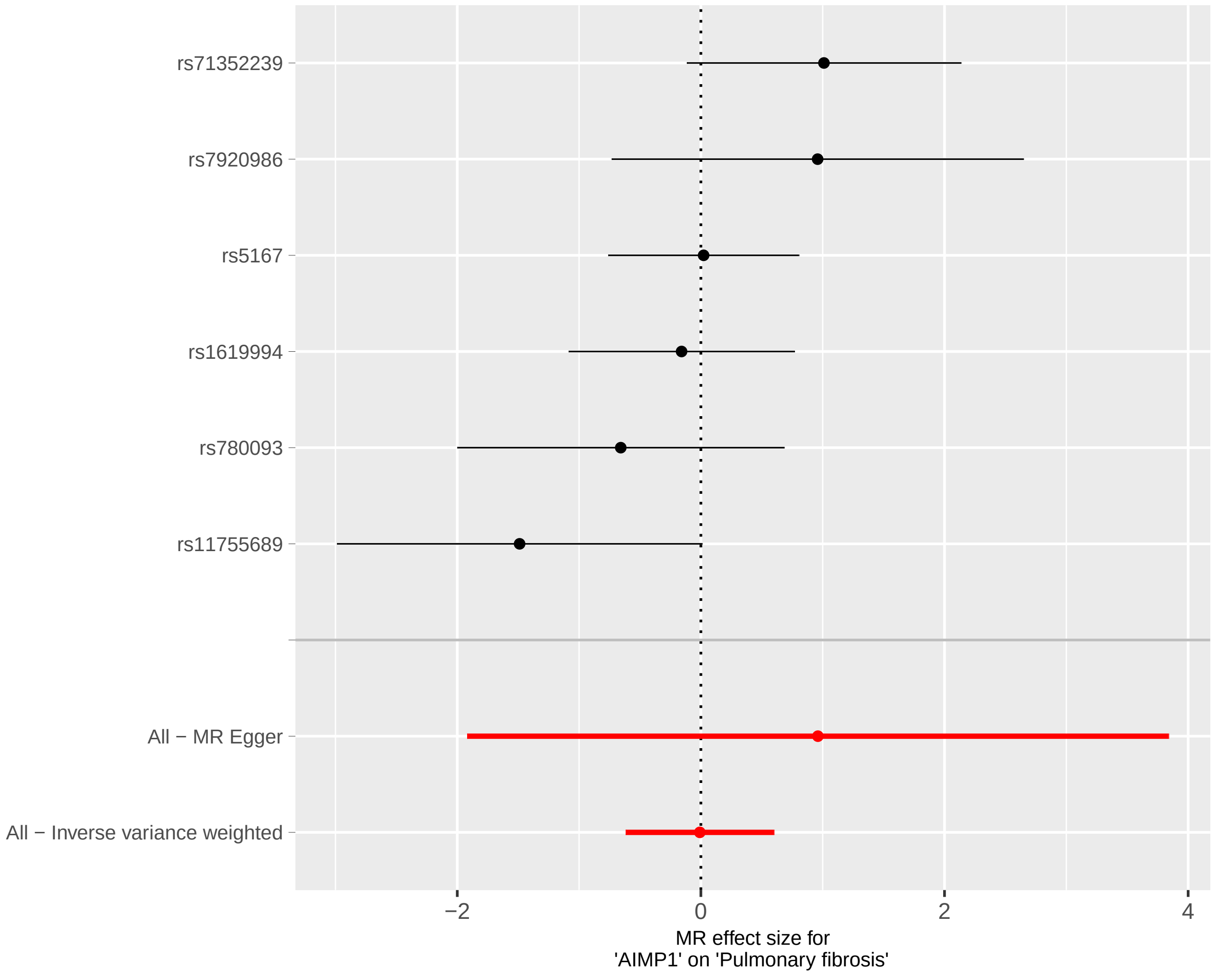
rs71352239
rs7920986
rs5167
rs1619994
rs780093
rs11755689
All − MR Egger
All − Inverse variance weighted
−2
0
2
4
MR effect size for
'AIMP1' on 'Pulmonary fibrosis'

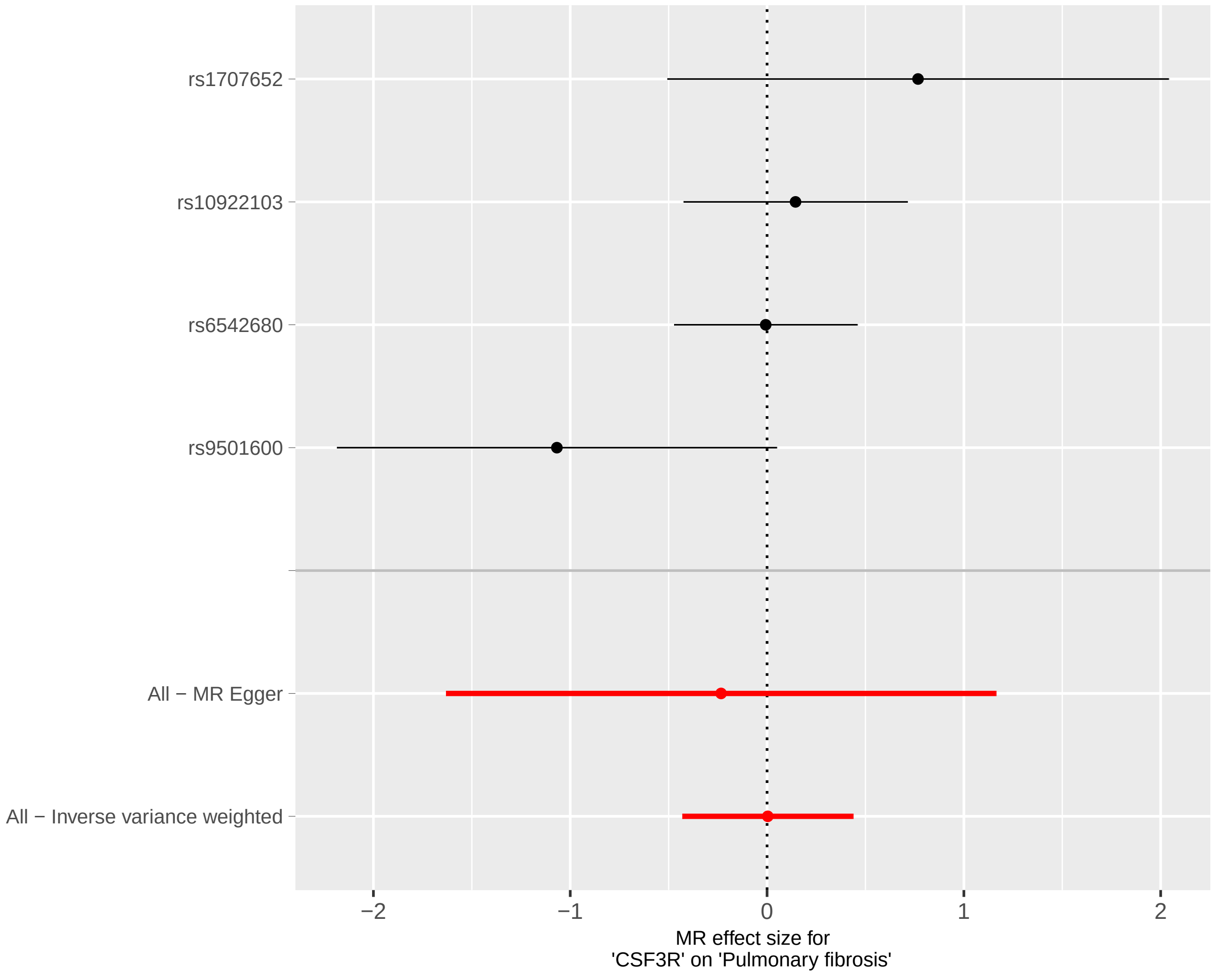

rs1707652
rs10922103
rs6542680
rs9501600
All − MR Egger
All − Inverse variance weighted
−2
−1
0
1
2
MR effect size for
'CSF3R' on 'Pulmonary fibrosis'

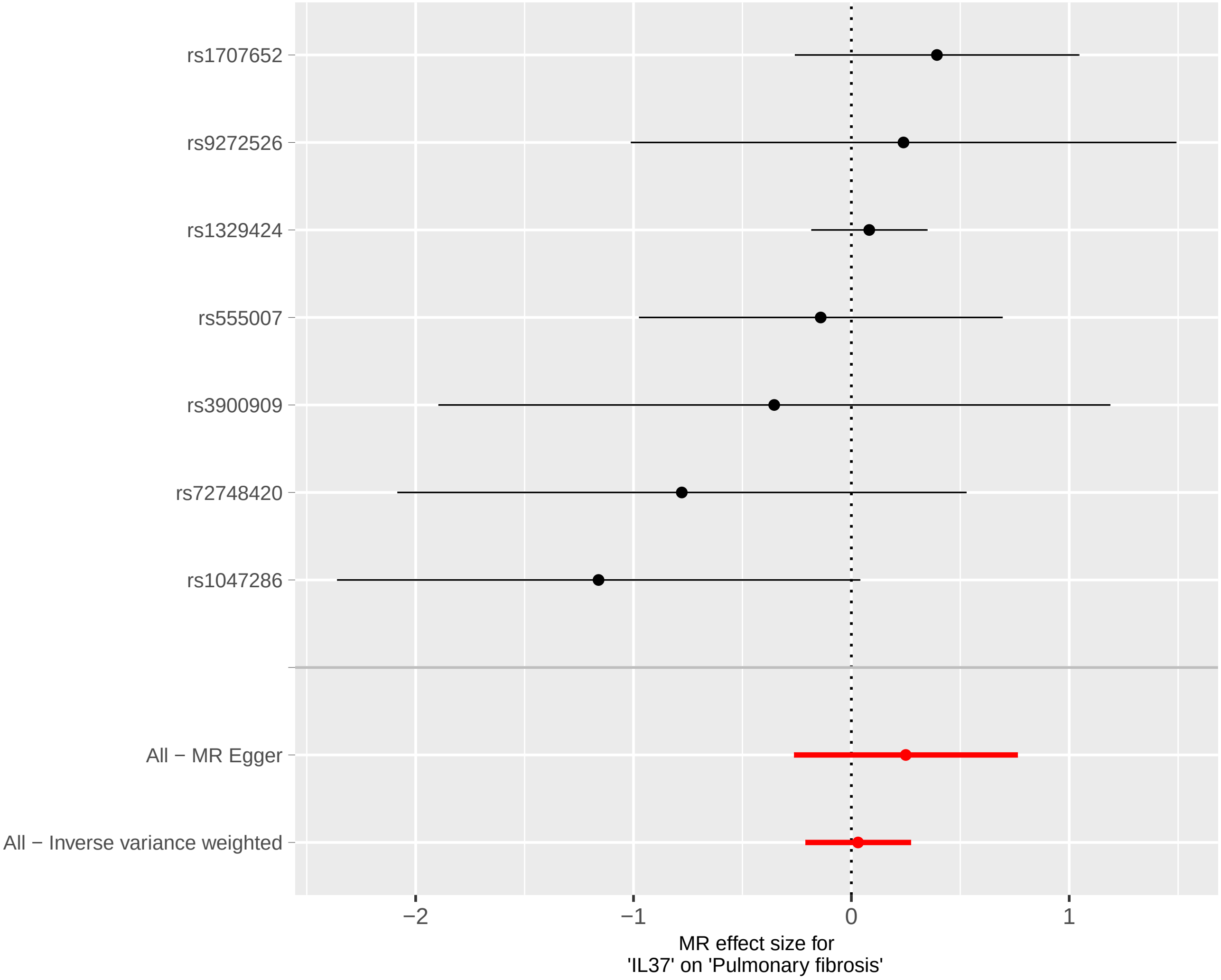

rs1707652
rs9272526
rs1329424
rs555007
rs3900909
rs72748420
rs1047286
All − MR Egger
All − Inverse variance weighted
−2
−1
0
1
MR effect size for
'IL37' on 'Pulmonary fibrosis'

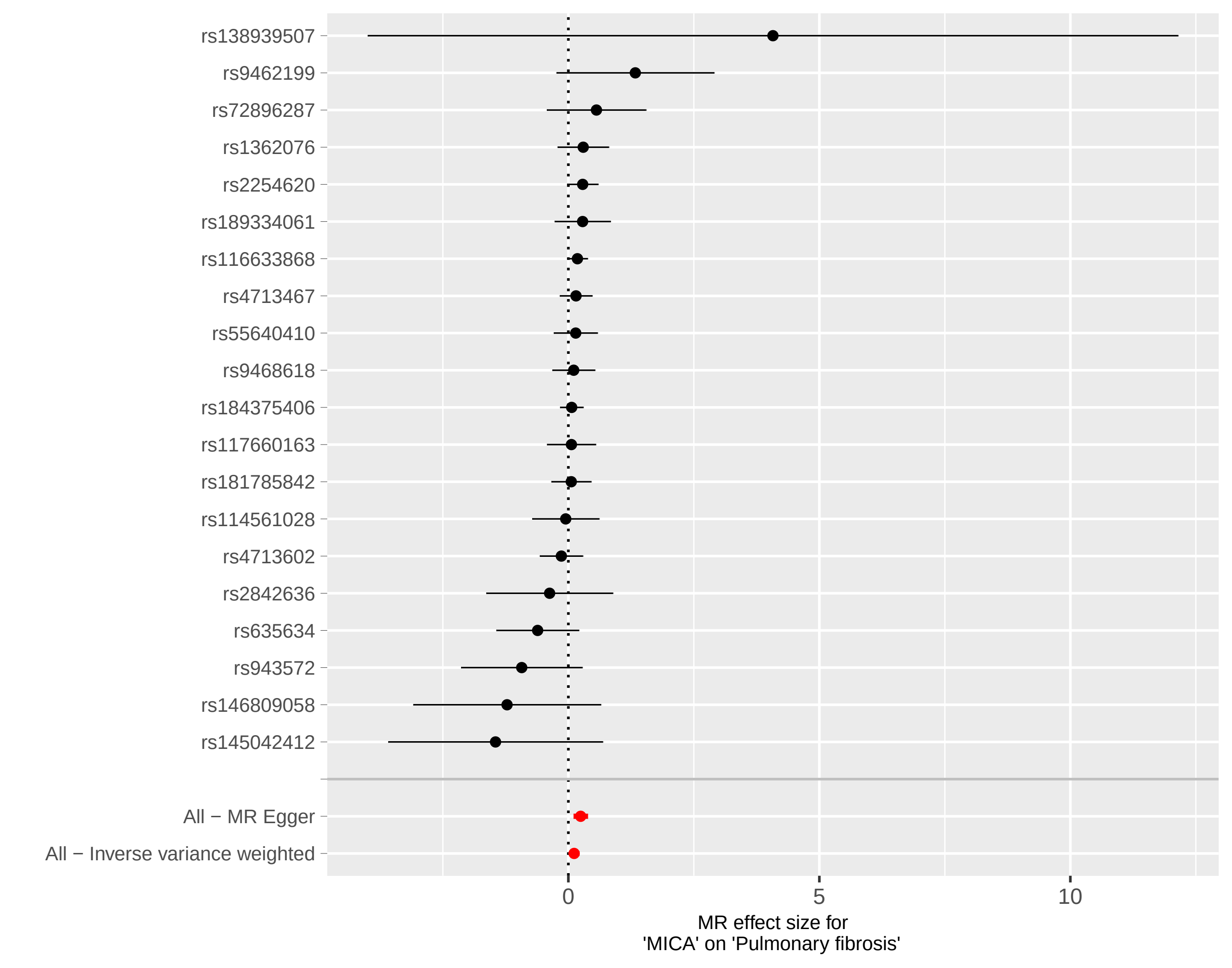
rs138939507
rs9462199
rs72896287
rs1362076
rs2254620
rs189334061
rs116633868
rs4713467
rs55640410
rs9468618
rs184375406
rs117660163
rs181785842
rs114561028
rs4713602
rs2842636
rs635634
rs943572
rs146809058
rs145042412
All − MR Egger
All − Inverse variance weighted
0
5
10
MR effect size for
'MICA' on 'Pulmonary fibrosis'

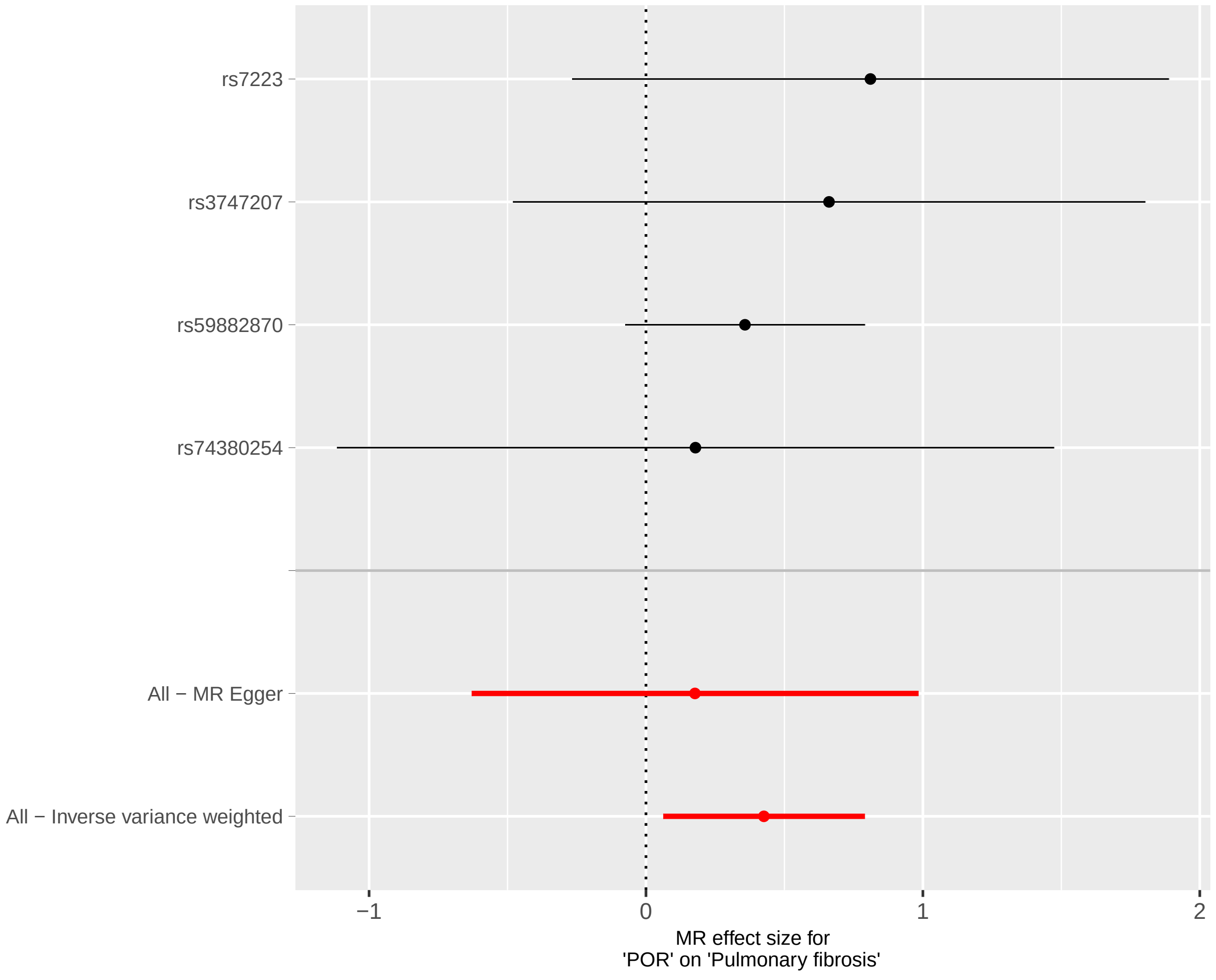
rs7223
rs3747207
rs59882870
rs74380254
All − MR Egger
All − Inverse variance weighted
−1
0
1
2
MR effect size for
'POR' on 'Pulmonary fibrosis'

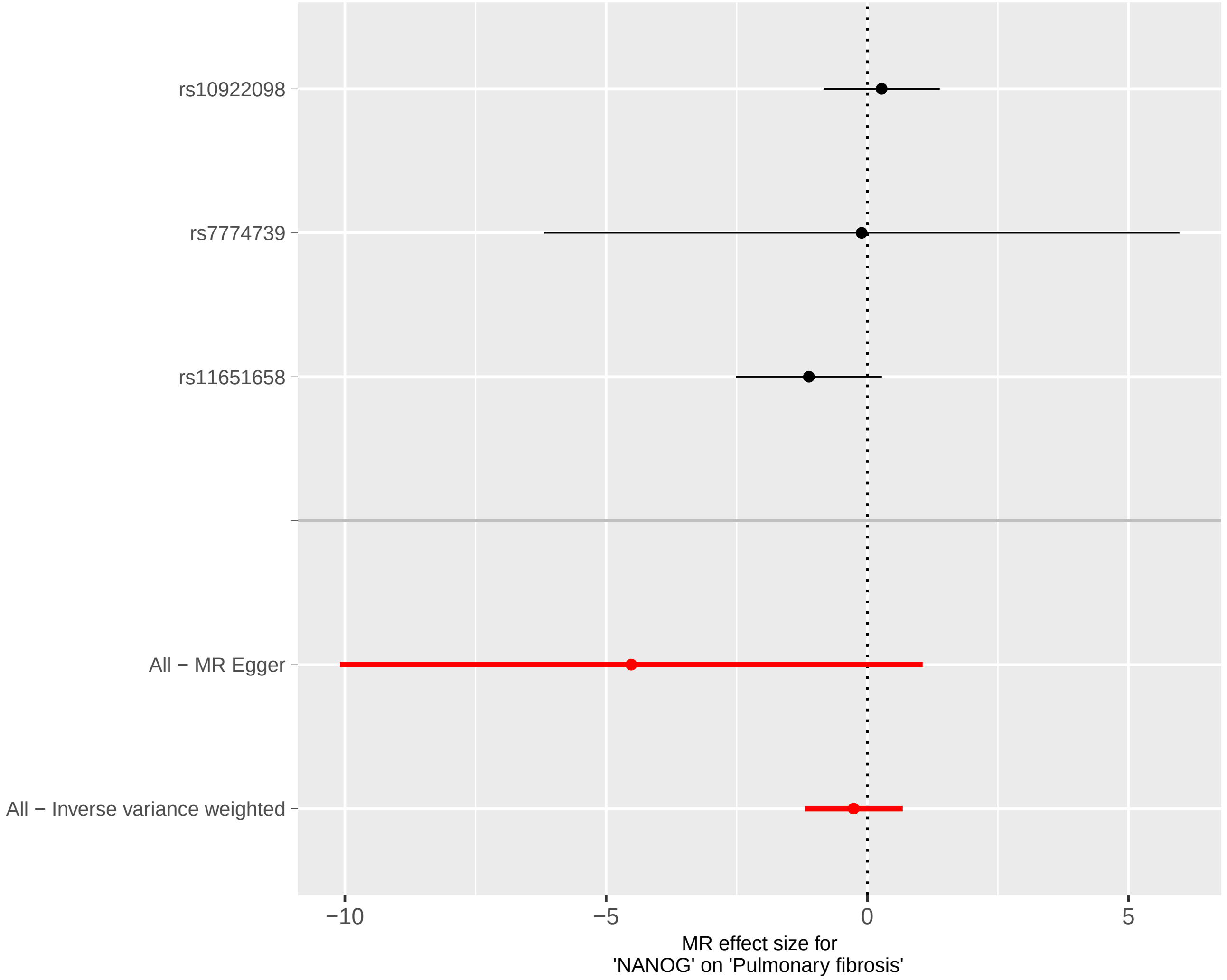
rs10922098
rs7774739
rs11651658
All − MR Egger
All − Inverse variance weighted
−10
−5
0
5
MR effect size for
'NANOG' on 'Pulmonary fibrosis'

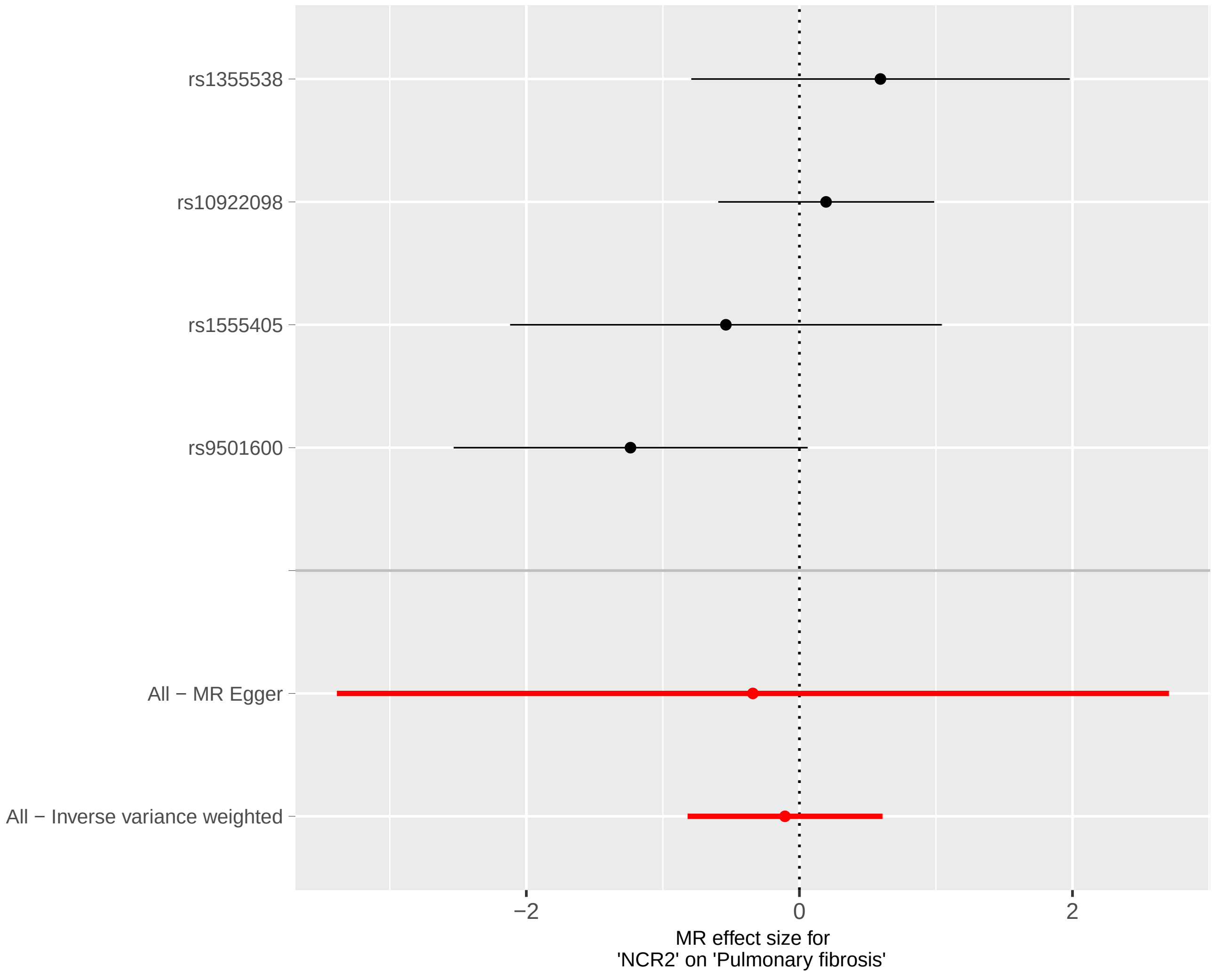
rs1355538
rs10922098
rs1555405
rs9501600
All − MR Egger
All − Inverse variance weighted
−2
0
2
MR effect size for
'NCR2' on 'Pulmonary fibrosis'

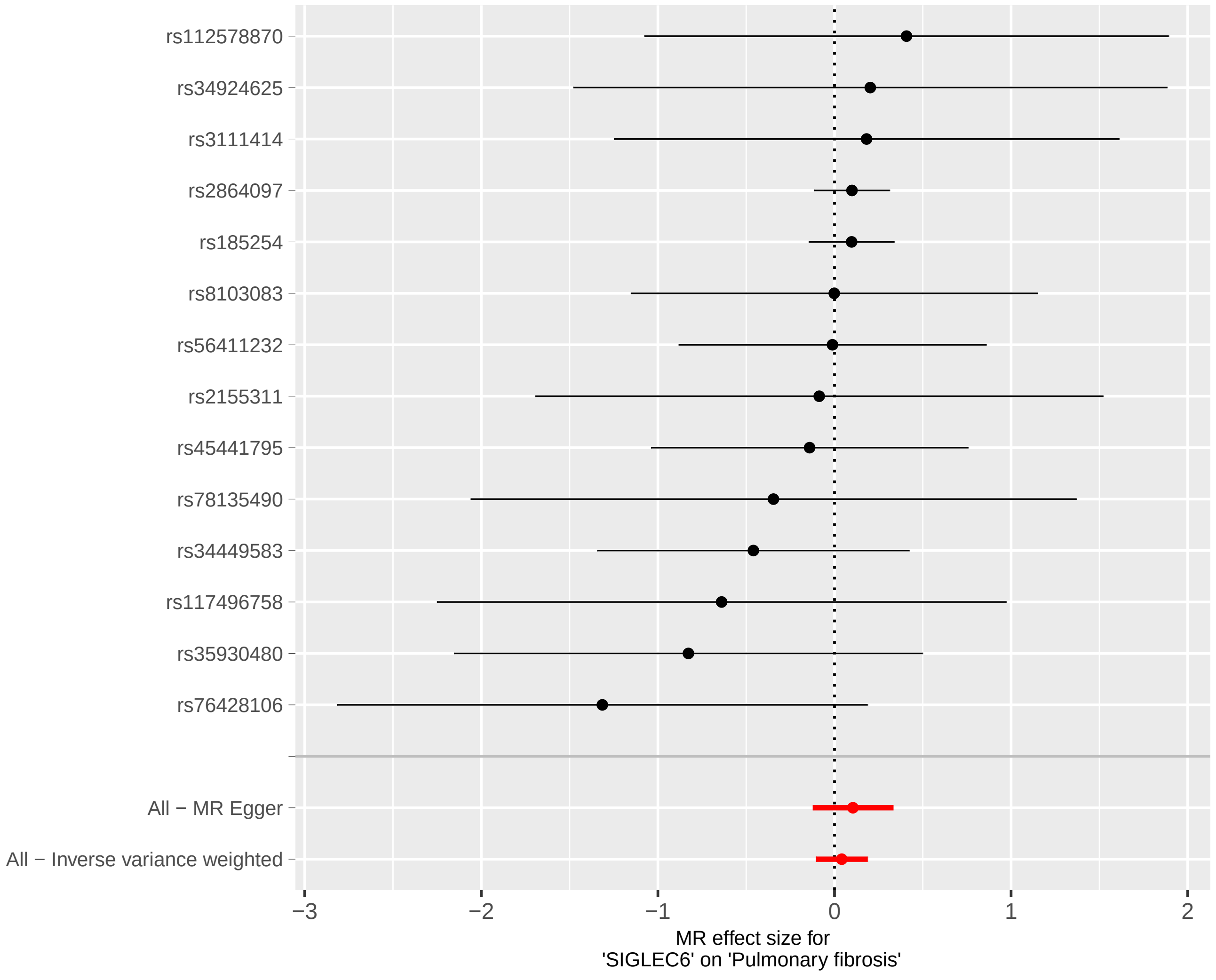
rs112578870
rs34924625
rs3111414
rs2864097
rs185254
rs8103083
rs56411232
rs2155311
rs45441795
rs78135490
rs34449583
rs117496758
rs35930480
rs76428106
All − MR Egger
All − Inverse variance weighted
−3
−2
−1
0
1
2
MR effect size for
'SIGLEC6' on 'Pulmonary fibrosis'

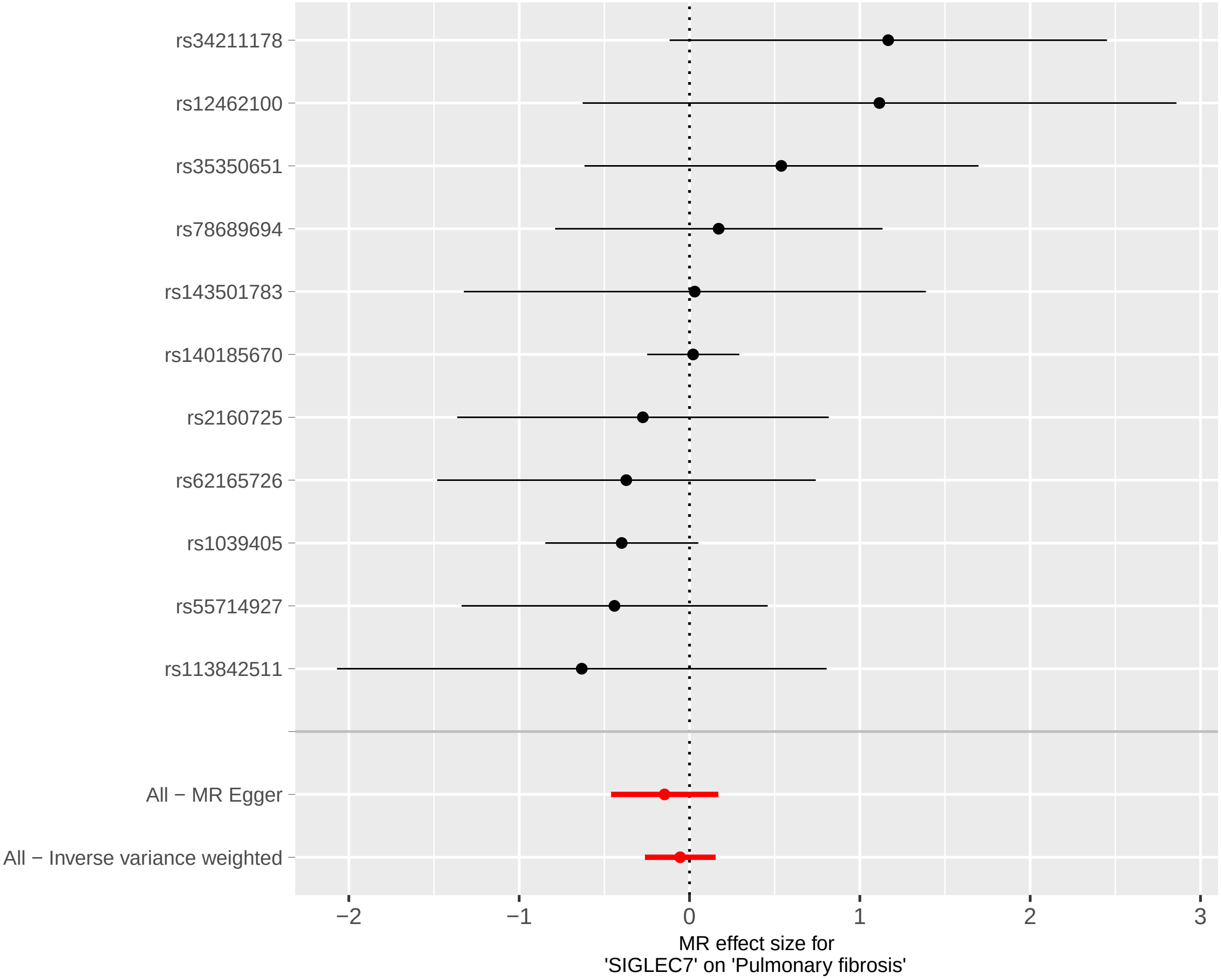
rs34211178
rs12462100
rs35350651
rs78689694
rs143501783
rs140185670
rs2160725
rs62165726
rs1039405
rs55714927
rs113842511
All − MR Egger
All − Inverse variance weighted
−2
−1
0
1
2
3
MR effect size for
'SIGLEC7' on 'Pulmonary fibrosis'

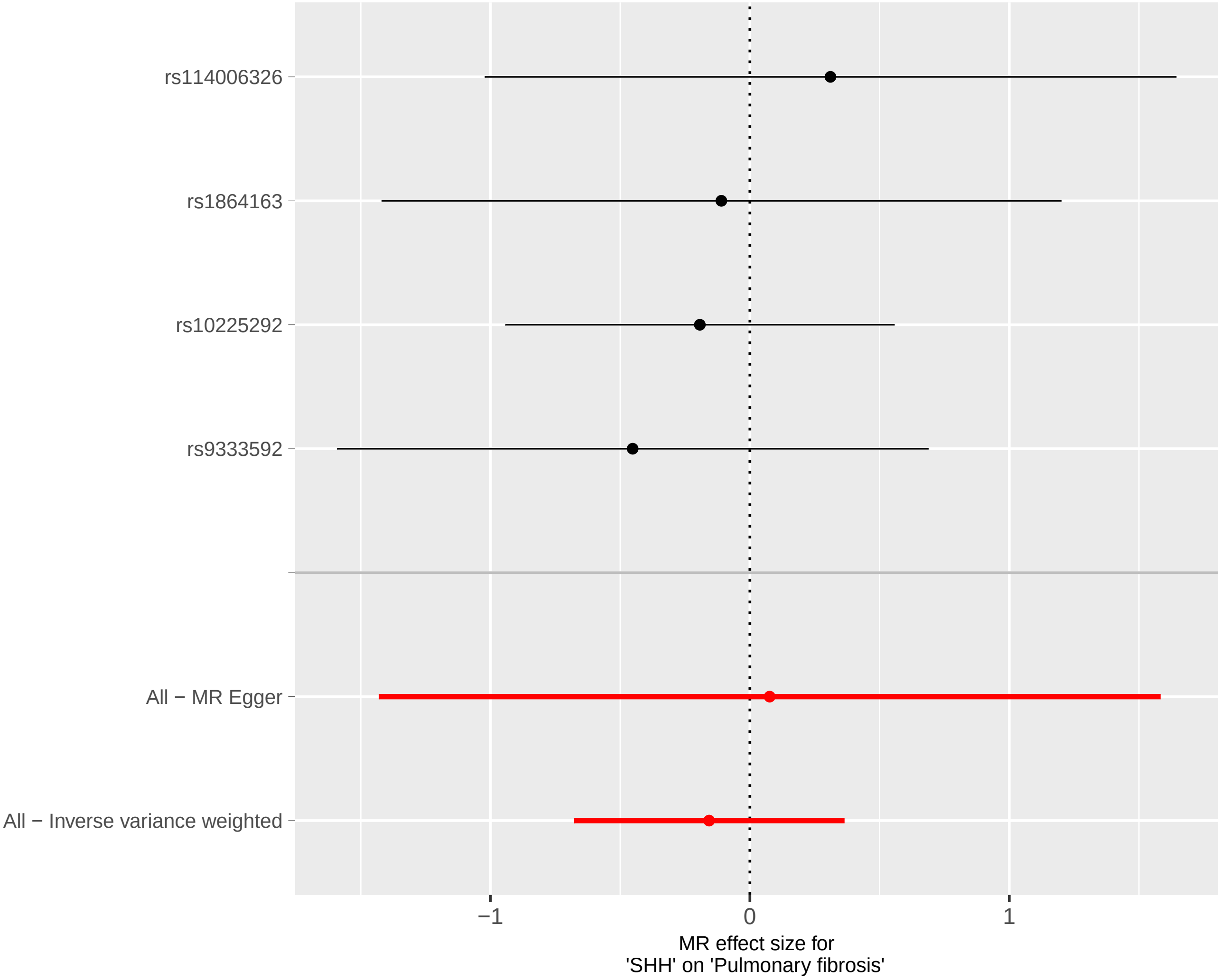

rs114006326
rs1864163
rs10225292
rs9333592
All − MR Egger
All − Inverse variance weighted
−1
0
1
MR effect size for
'SHH' on 'Pulmonary fibrosis'

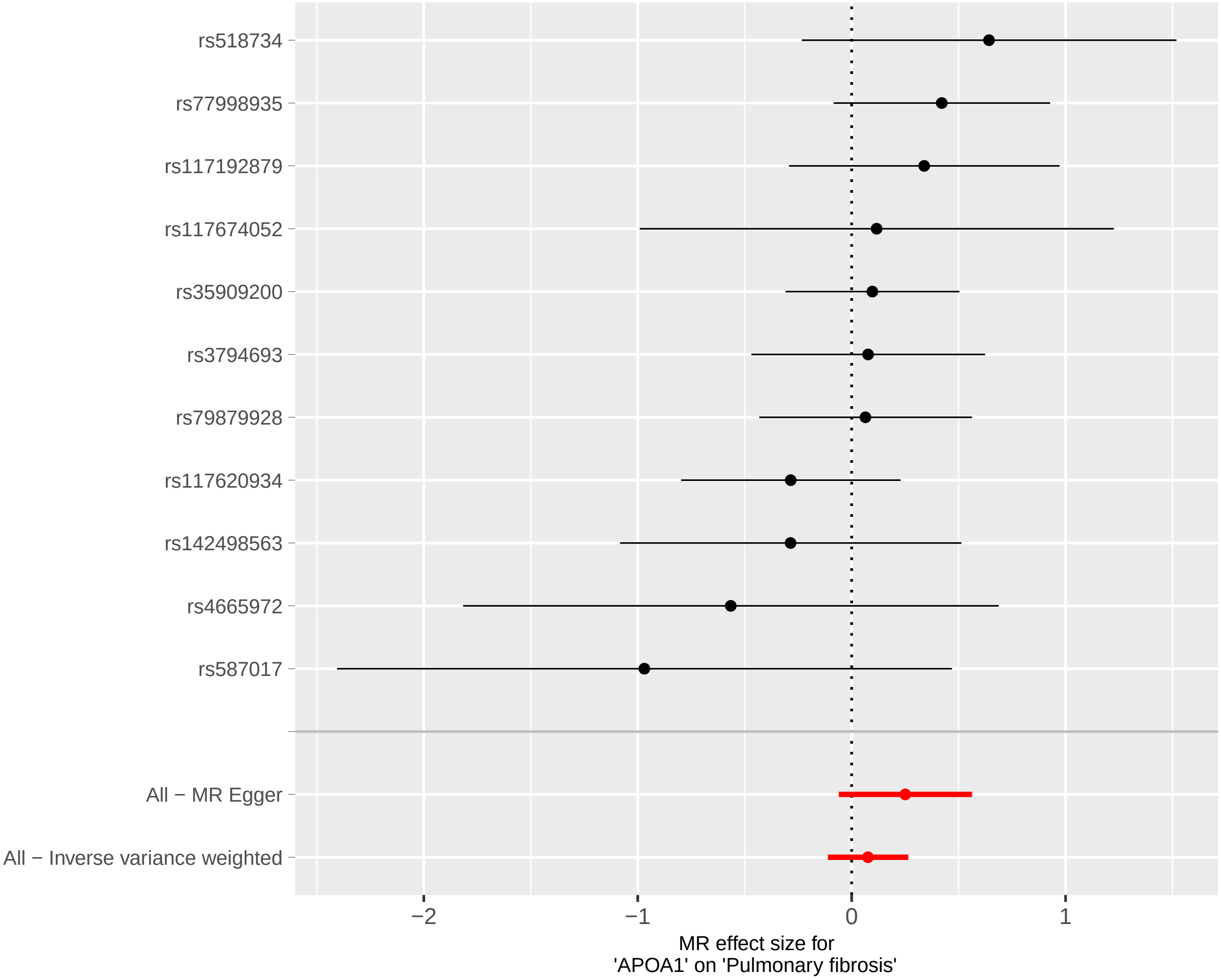

rs518734
rs77998935
rs117192879
rs117674052
rs35909200
rs3794693
rs79879928
rs117620934
rs142498563
rs4665972
rs587017
All − MR Egger
All − Inverse variance weighted
−2
−1
0
1
MR effect size for
'APOA1' on 'Pulmonary fibrosis'

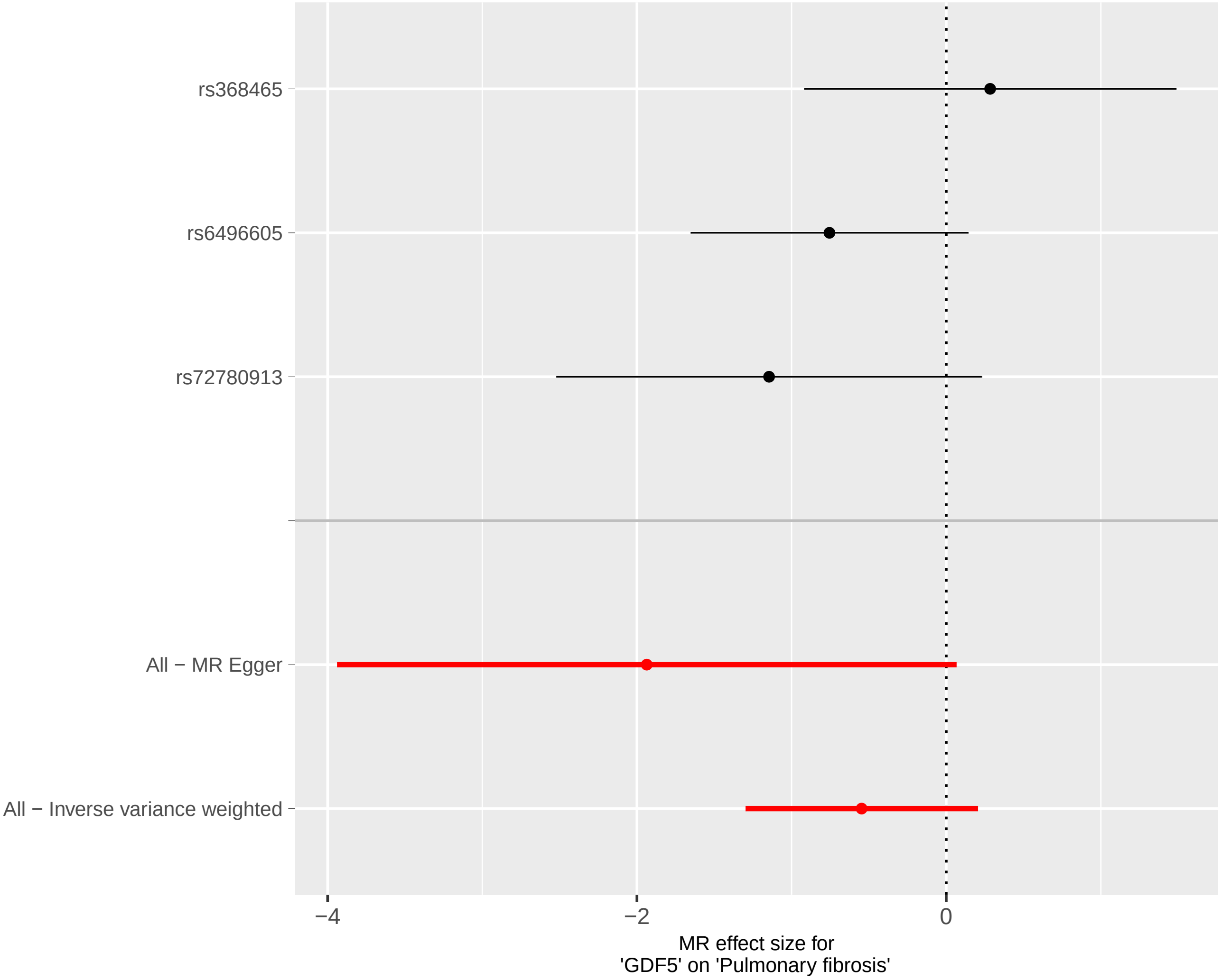
rs368465
rs6496605
rs72780913
All − MR Egger
All − Inverse variance weighted
−4
−2
0
MR effect size for
'GDF5' on 'Pulmonary fibrosis'

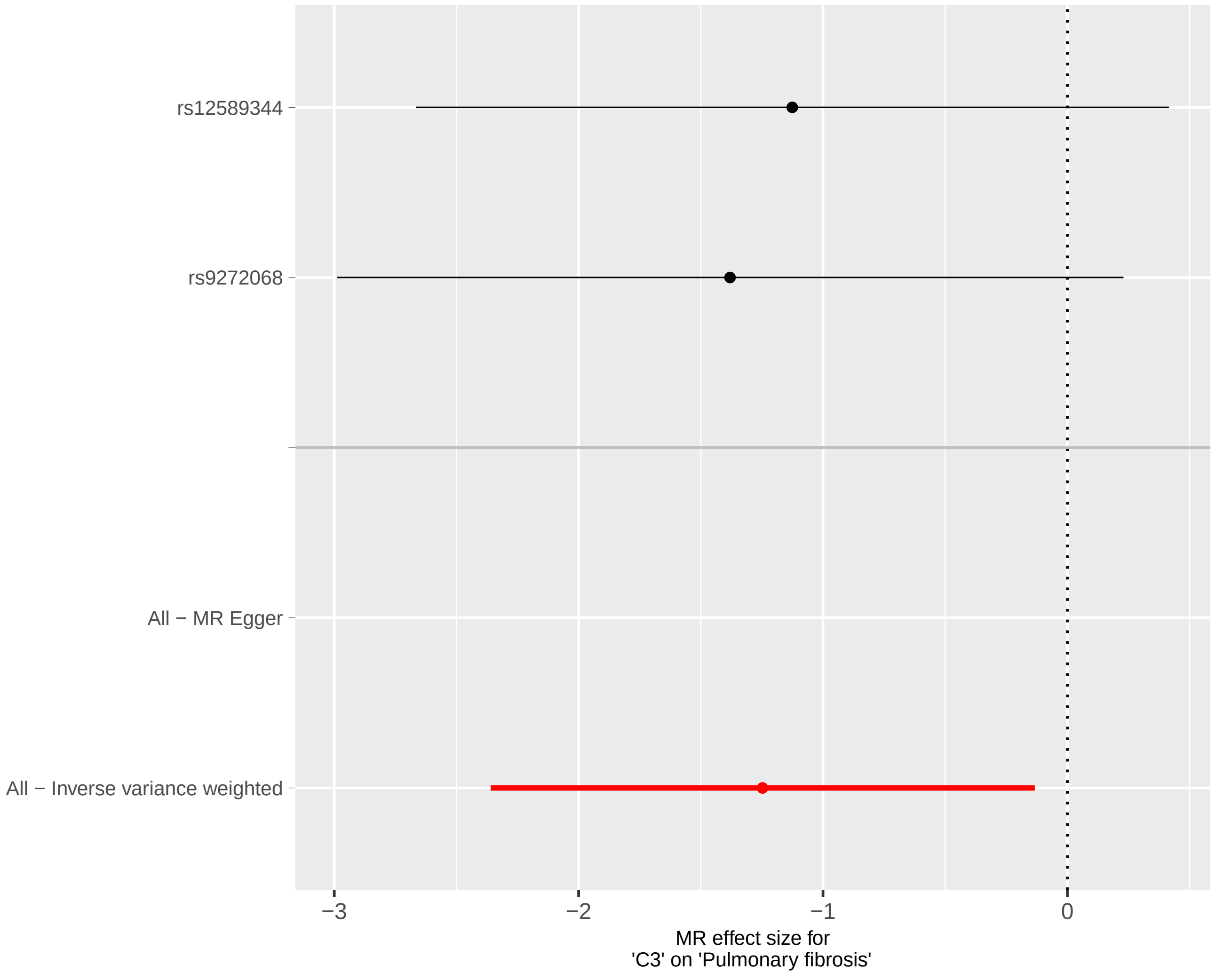

rs12589344
rs9272068
All − MR Egger
All − Inverse variance weighted
−3
−2
−1
0
MR effect size for
'C3' on 'Pulmonary fibrosis'

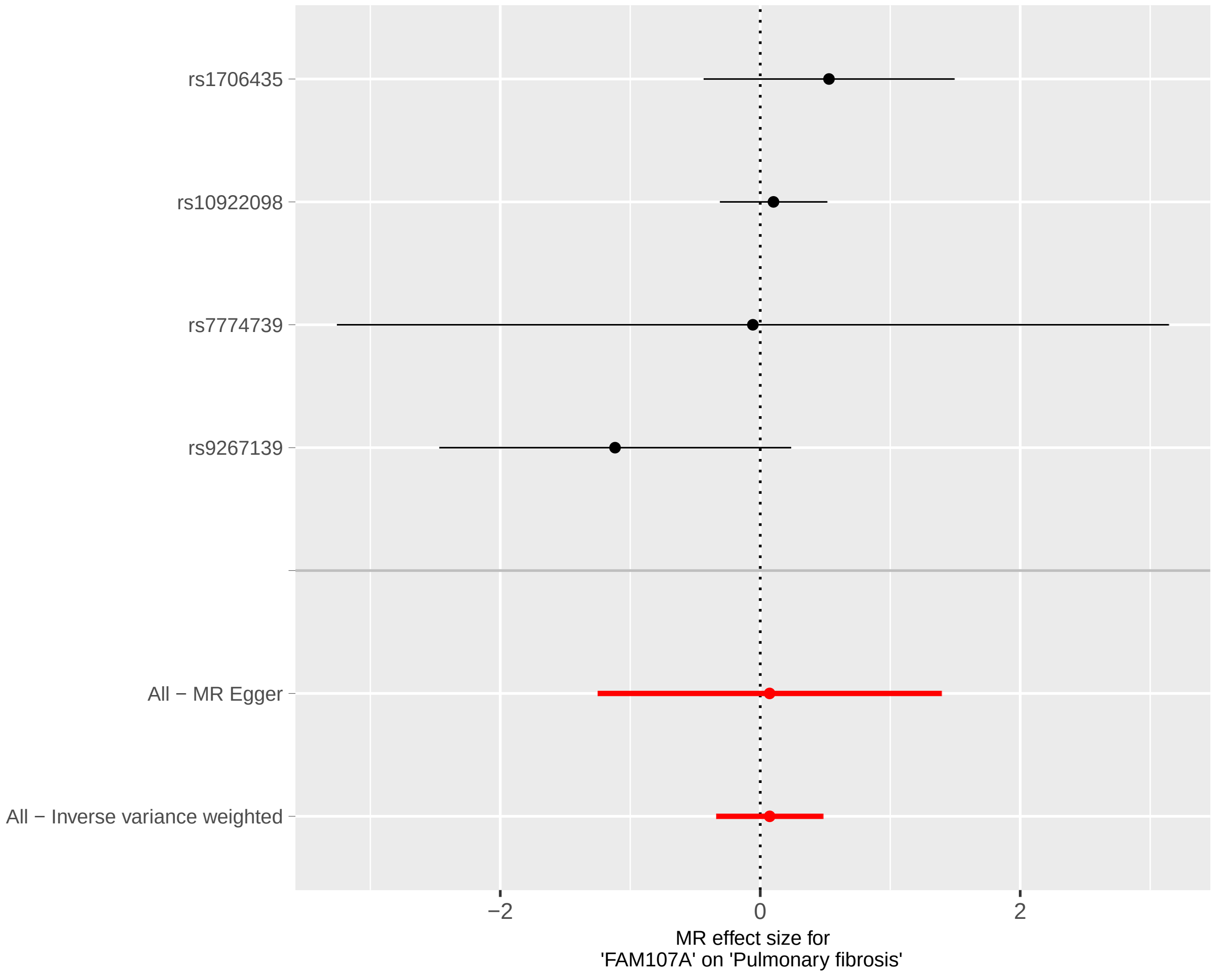
rs1706435
rs10922098
rs7774739
rs9267139
All − MR Egger
All − Inverse variance weighted
−2
0
2
MR effect size for
'FAM107A' on 'Pulmonary fibrosis'

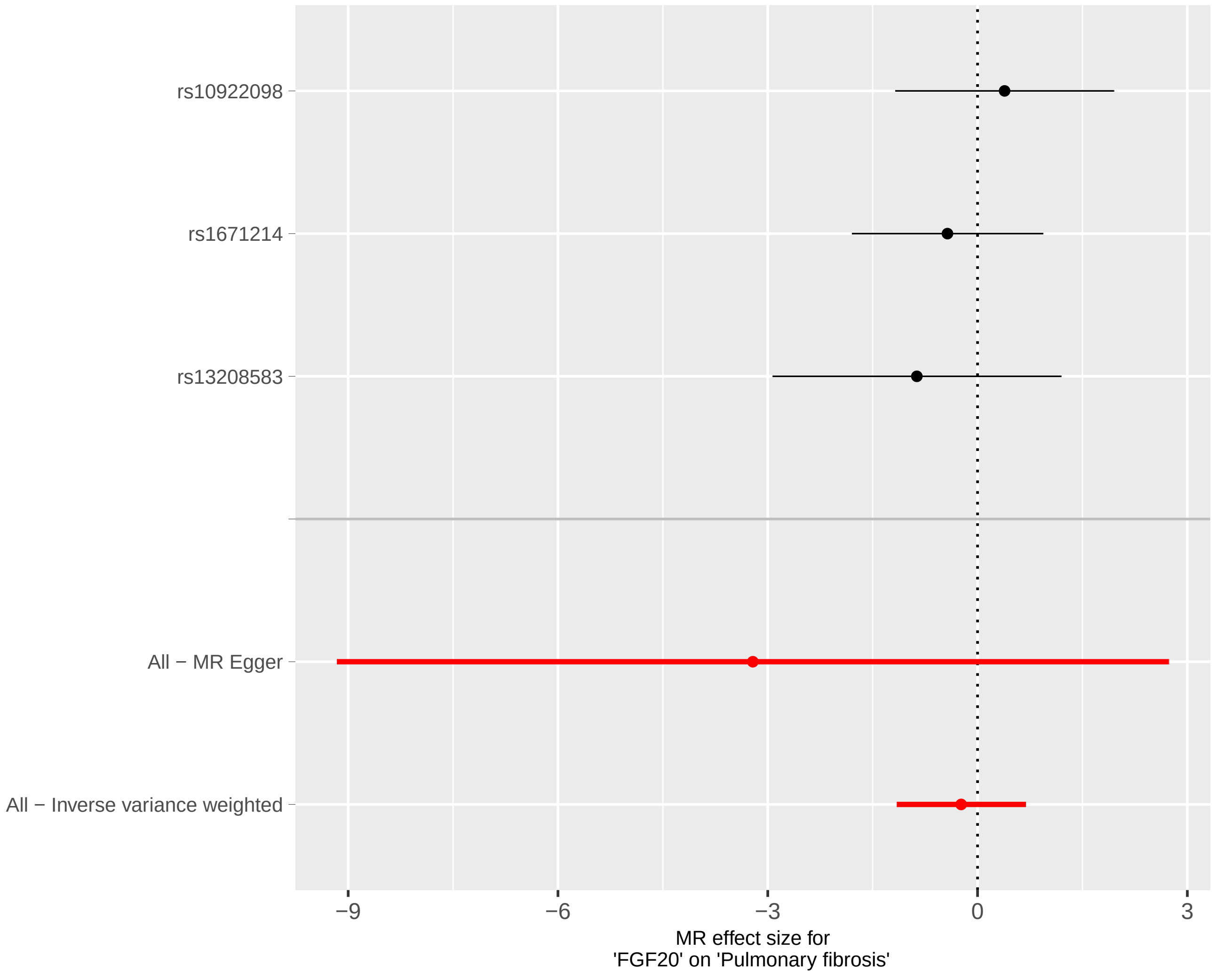
rs10922098
rs1671214
rs13208583
All − MR Egger
All − Inverse variance weighted
−9
−6
−3
0
3
MR effect size for
'FGF20' on 'Pulmonary fibrosis'

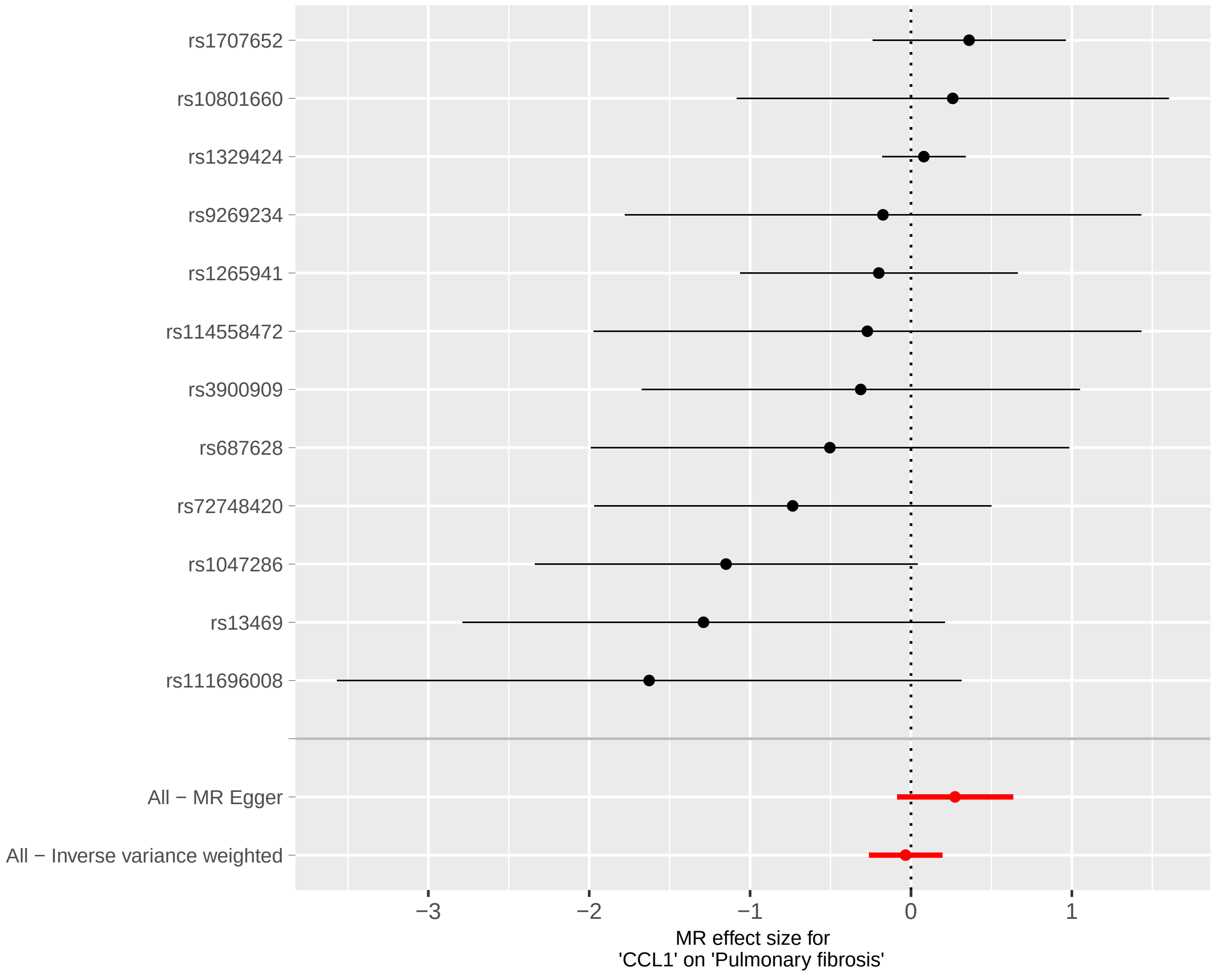
rs1707652
rs10801660
rs1329424
rs9269234
rs1265941
rs114558472
rs3900909
rs687628
rs72748420
rs1047286
rs13469
rs111696008
All − MR Egger
All − Inverse variance weighted
−3
−2
−1
0
1
MR effect size for
'CCL1' on 'Pulmonary fibrosis'

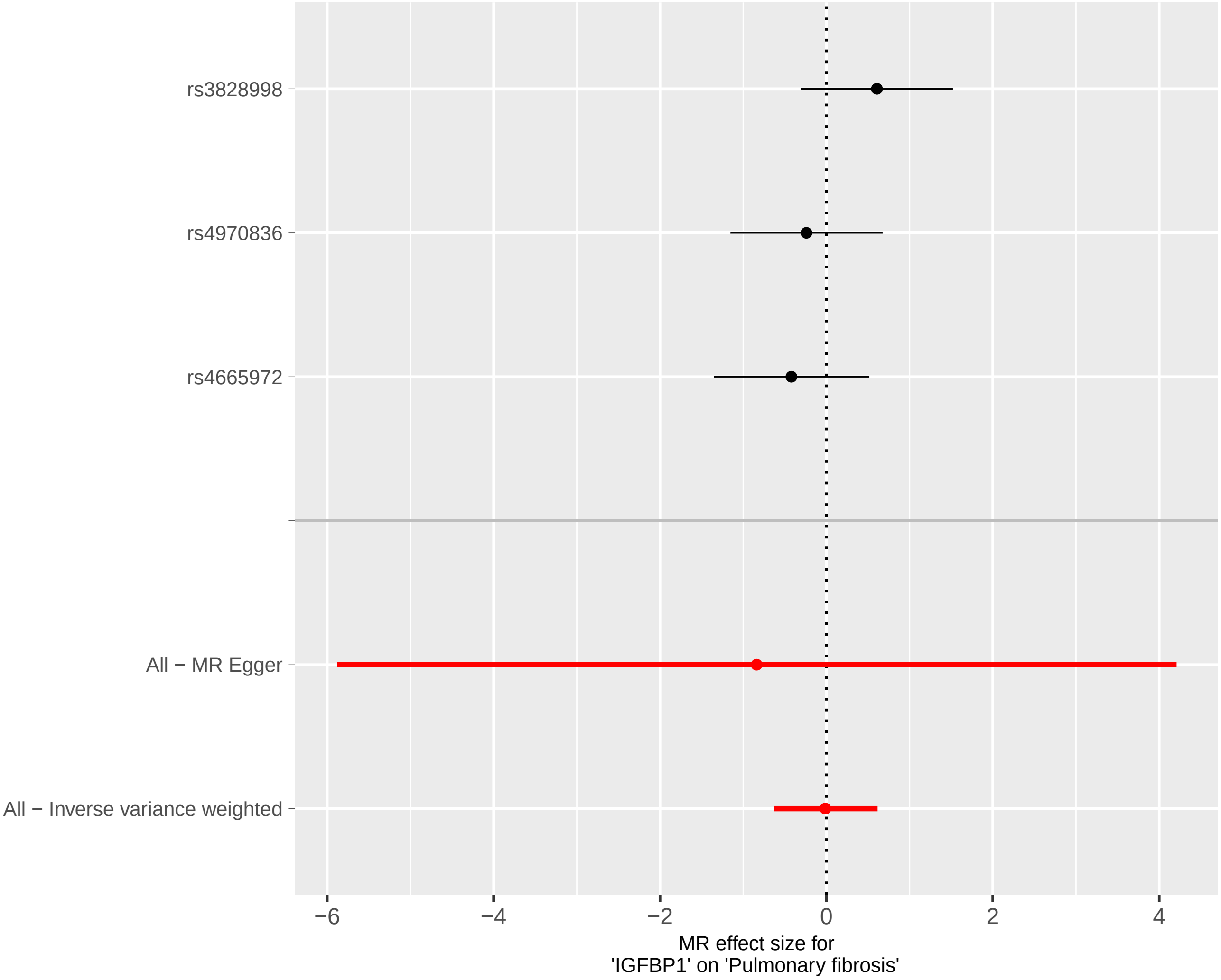
rs3828998
rs4970836
rs4665972
All − MR Egger
All − Inverse variance weighted
−6
−4
−2
0
2
4
MR effect size for
'IGFBP1' on 'Pulmonary fibrosis'

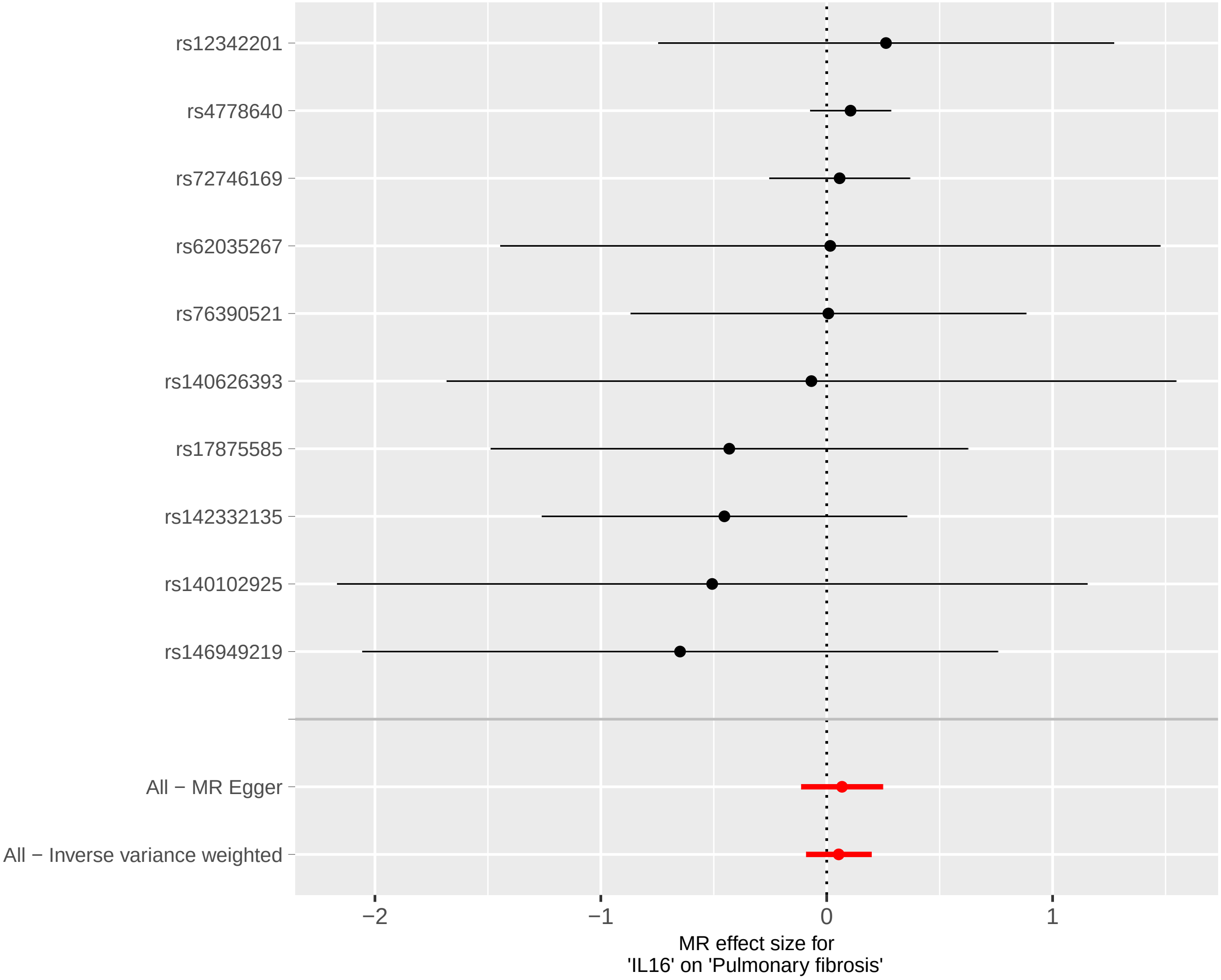

rs12342201
rs4778640
rs72746169
rs62035267
rs76390521
rs140626393
rs17875585
rs142332135
rs140102925
rs146949219
All − MR Egger
All − Inverse variance weighted
−2
−1
0
1
MR effect size for
'IL16' on 'Pulmonary fibrosis'

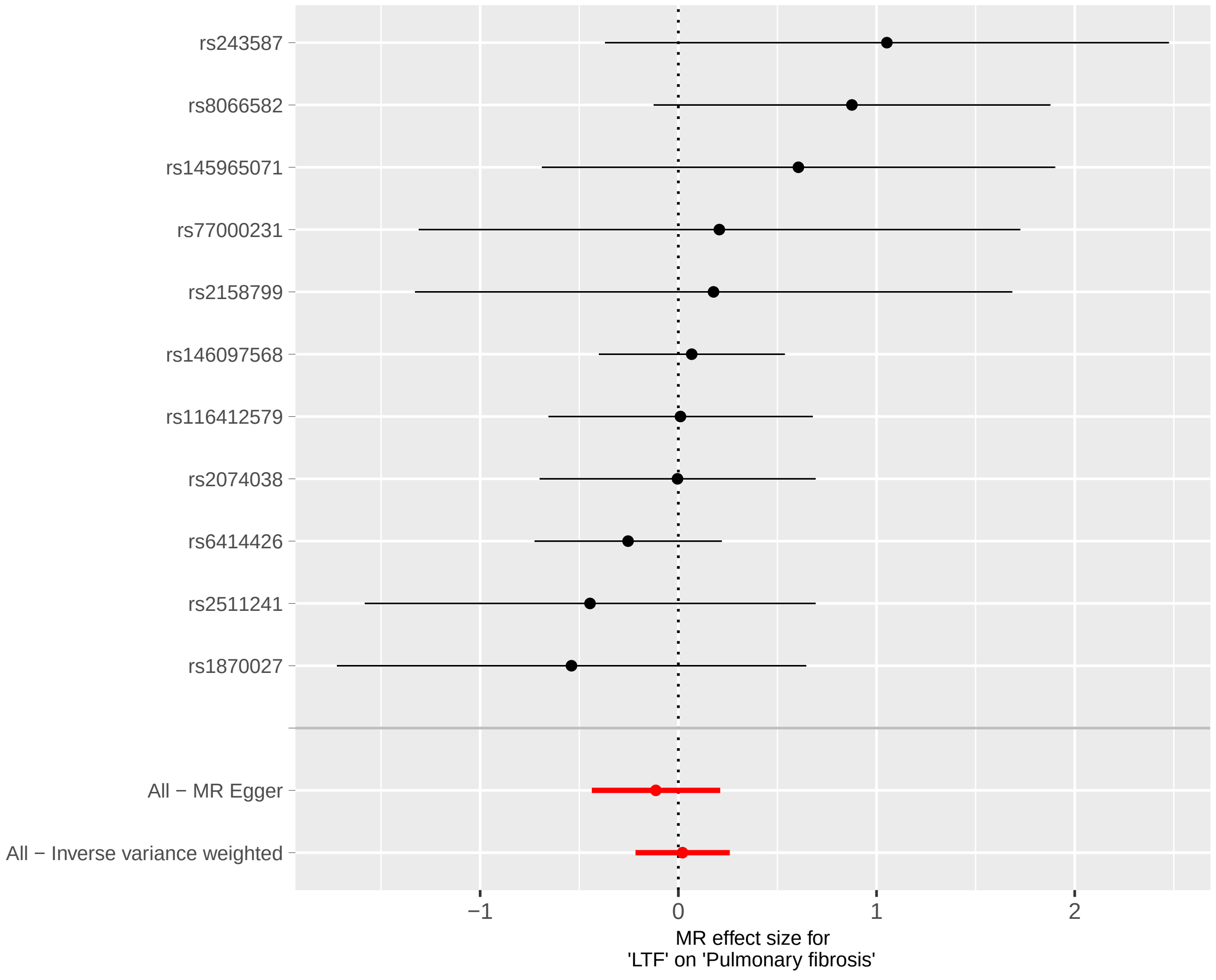

rs243587
rs8066582
rs145965071
rs77000231
rs2158799
rs146097568
rs116412579
rs2074038
rs6414426
rs2511241
rs1870027
All − MR Egger
All − Inverse variance weighted
−1
0
1
2
MR effect size for
'LTF' on 'Pulmonary fibrosis'

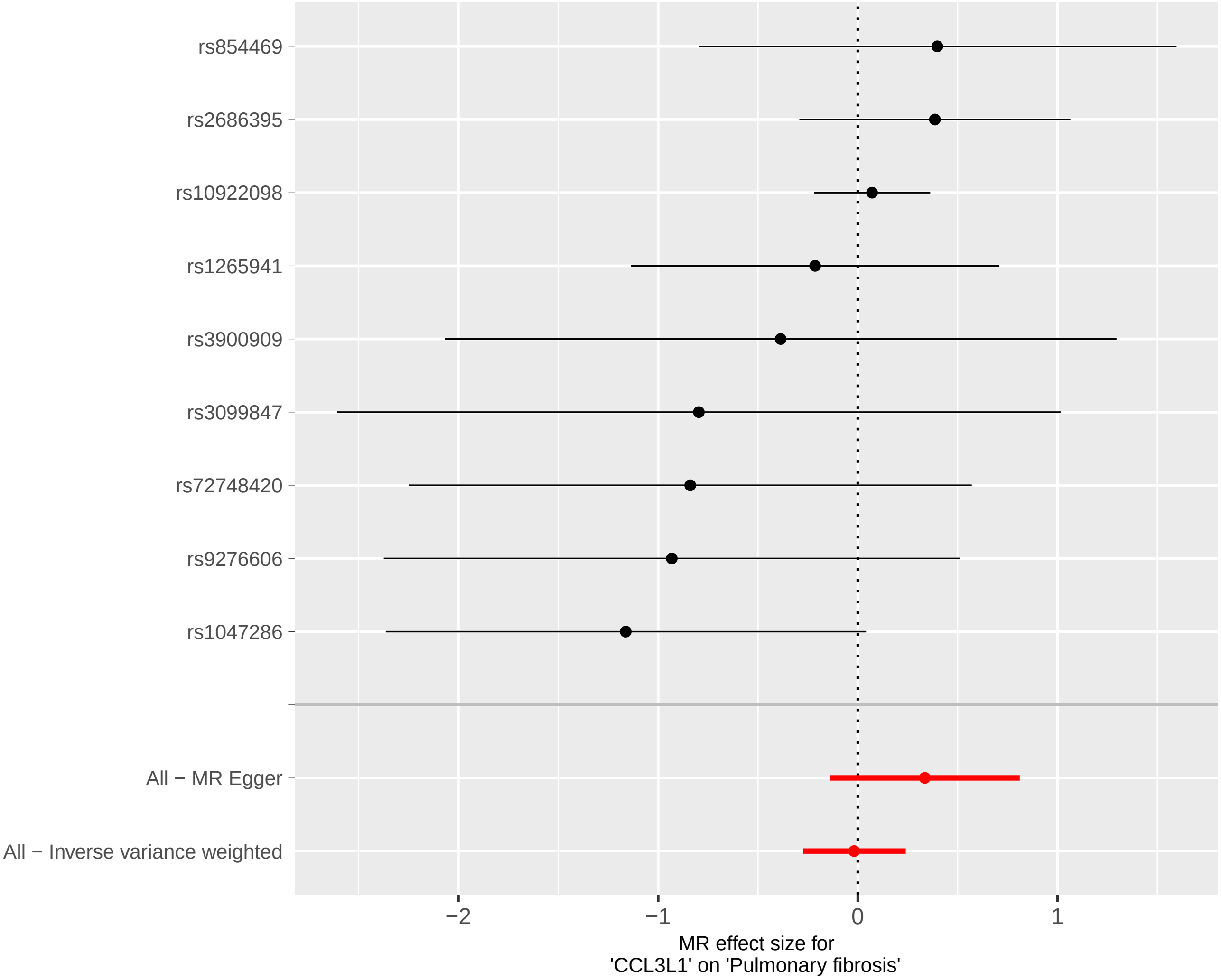

rs854469
rs2686395
rs10922098
rs1265941
rs3900909
rs3099847
rs72748420
rs9276606
rs1047286
All − MR Egger
All − Inverse variance weighted
−2
−1
0
1
MR effect size for
'CCL3L1' on 'Pulmonary fibrosis'

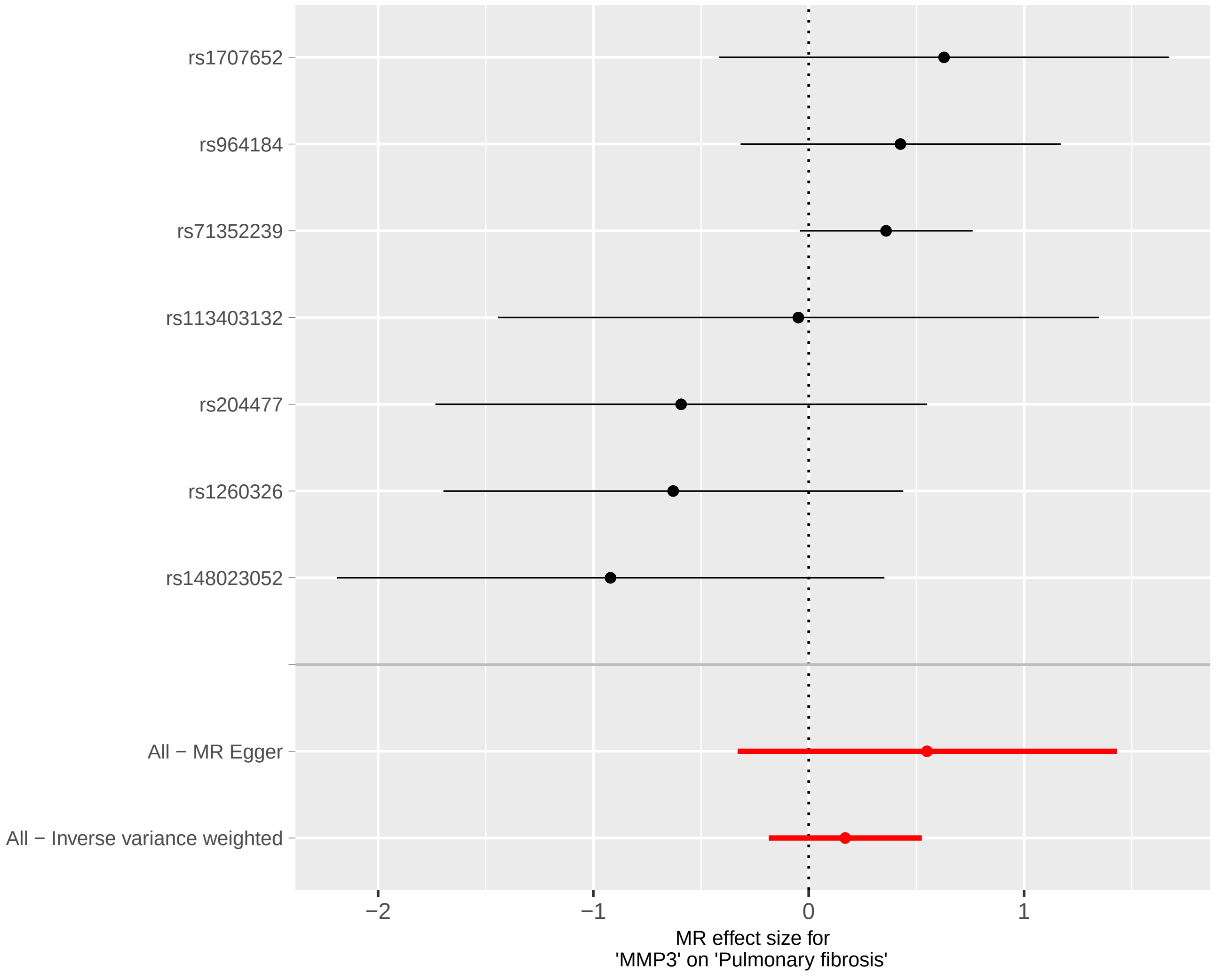
rs1707652
rs964184
rs71352239
rs113403132
rs204477
rs1260326
rs148023052
All − MR Egger
All − Inverse variance weighted
−2
−1
0
1
MR effect size for
'MMP3' on 'Pulmonary fibrosis'

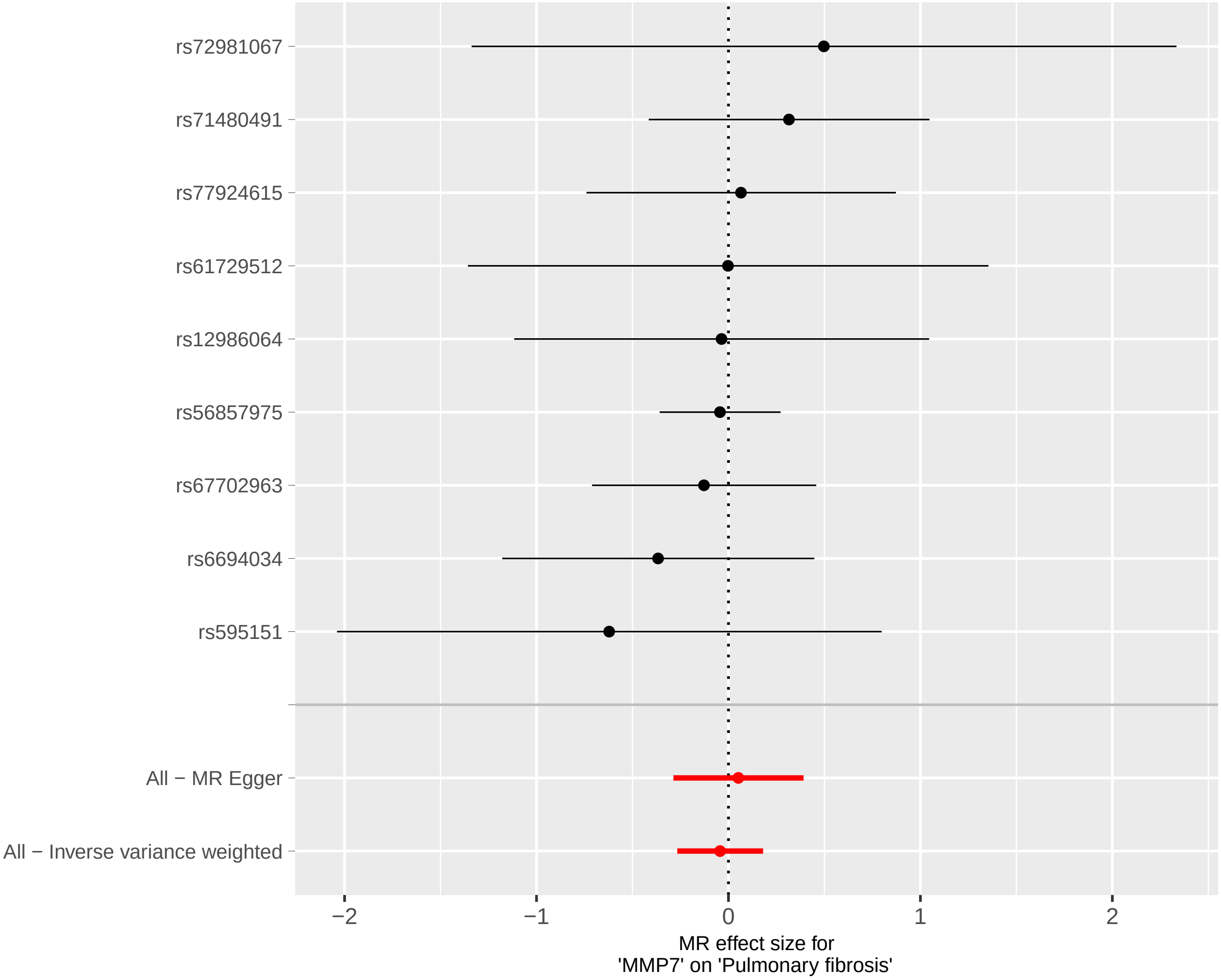

rs72981067
rs71480491
rs77924615
rs61729512
rs12986064
rs56857975
rs67702963
rs6694034
rs595151
All − MR Egger
All − Inverse variance weighted
−2
−1
0
1
2
MR effect size for
'MMP7' on 'Pulmonary fibrosis'

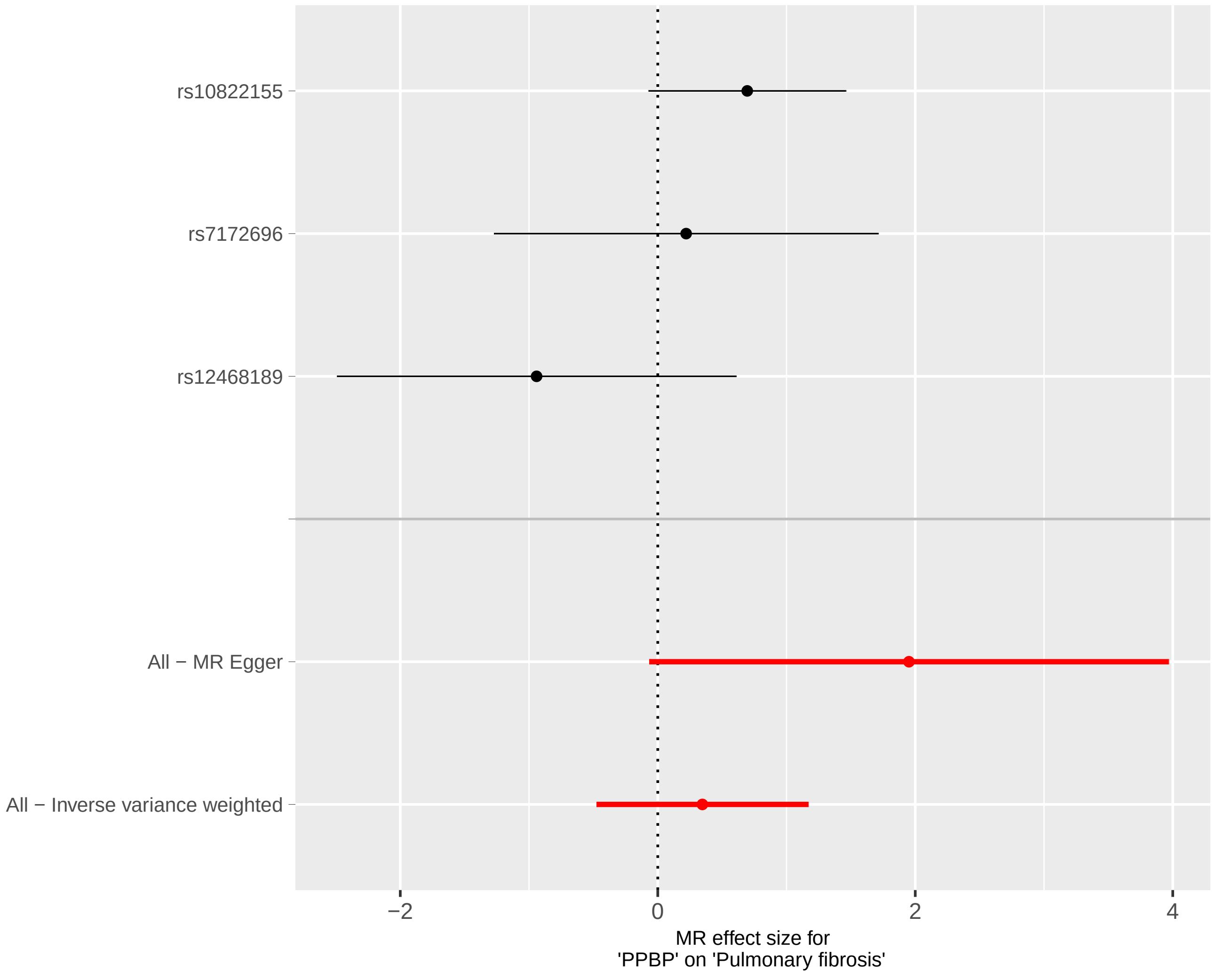
rs10822155
rs7172696
rs12468189
All − MR Egger
All − Inverse variance weighted
−2
0
2
4
MR effect size for
'PPBP' on 'Pulmonary fibrosis'

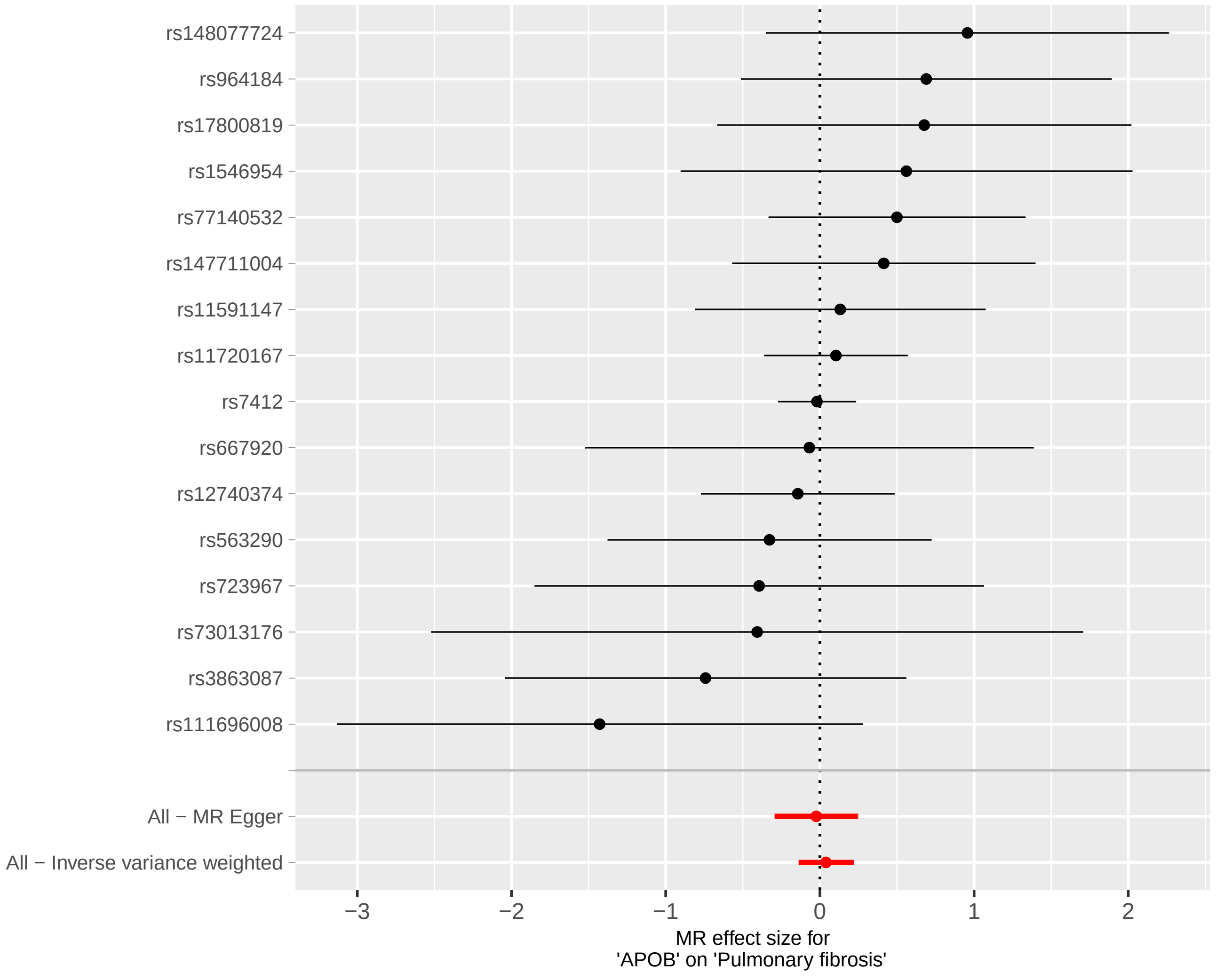

rs148077724
rs964184
rs17800819
rs1546954
rs77140532
rs147711004
rs11591147
rs11720167
rs7412
rs667920
rs12740374
rs563290
rs723967
rs73013176
rs3863087
rs111696008
All − MR Egger
All − Inverse variance weighted
−3
−2
−1
0
1
2
MR effect size for
'APOB' on 'Pulmonary fibrosis'

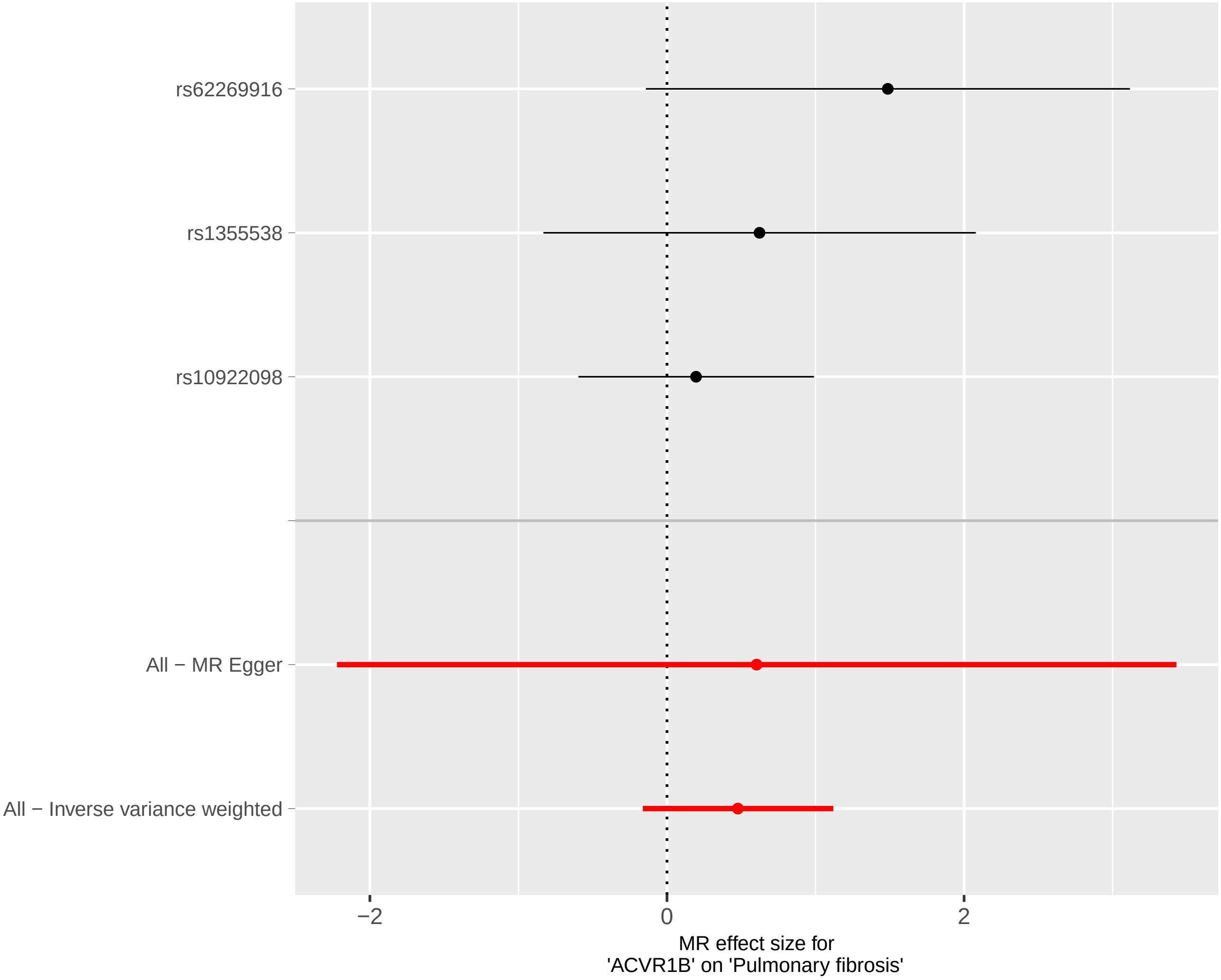

rs62269916
rs1355538
rs10922098
All − MR Egger
All − Inverse variance weighted
−2
0
2
MR effect size for
'ACVR1B' on 'Pulmonary fibrosis'

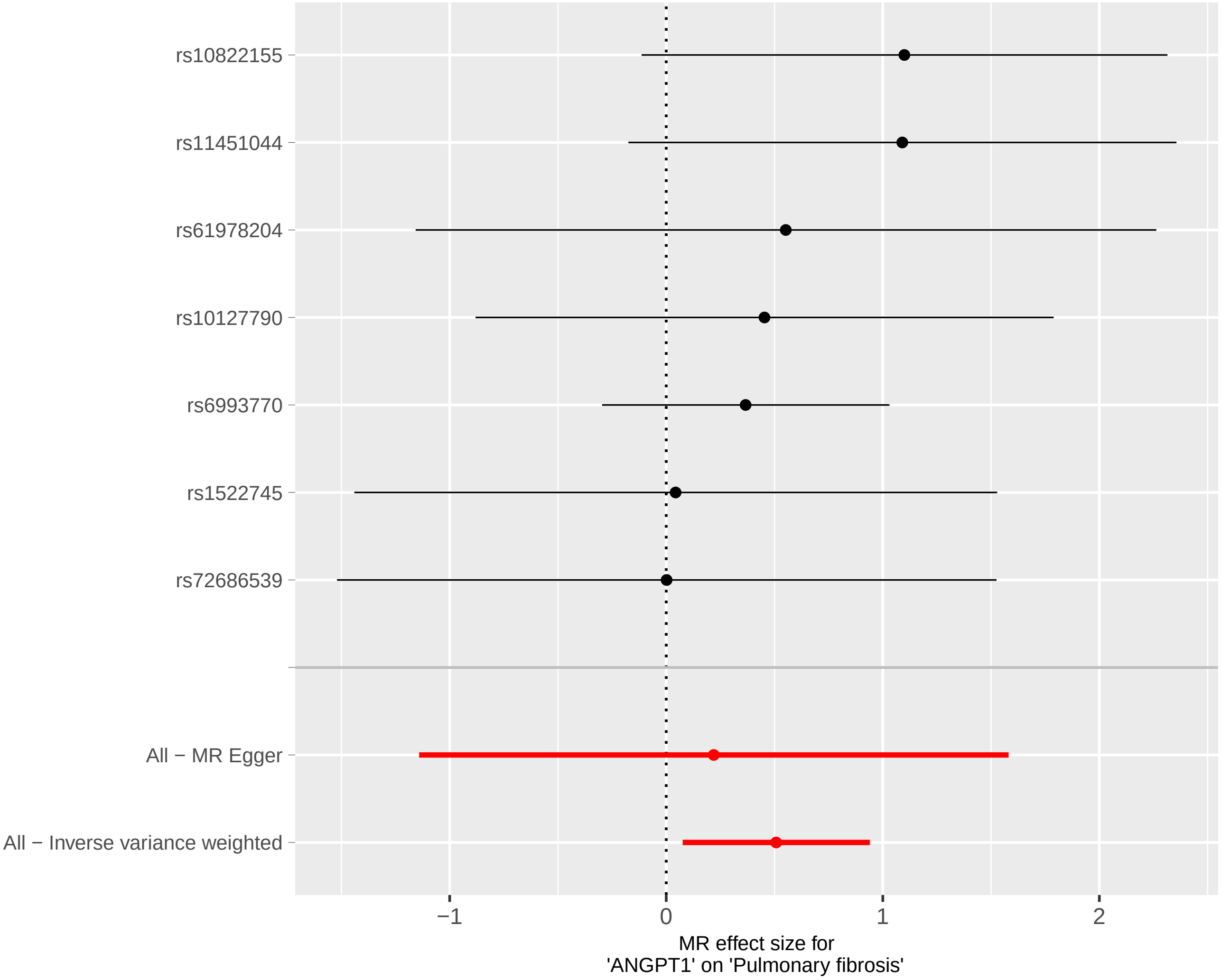

rs10822155
rs11451044
rs61978204
rs10127790
rs6993770
rs1522745
rs72686539
All − MR Egger
All − Inverse variance weighted
−1
0
1
2
MR effect size for
'ANGPT1' on 'Pulmonary fibrosis'

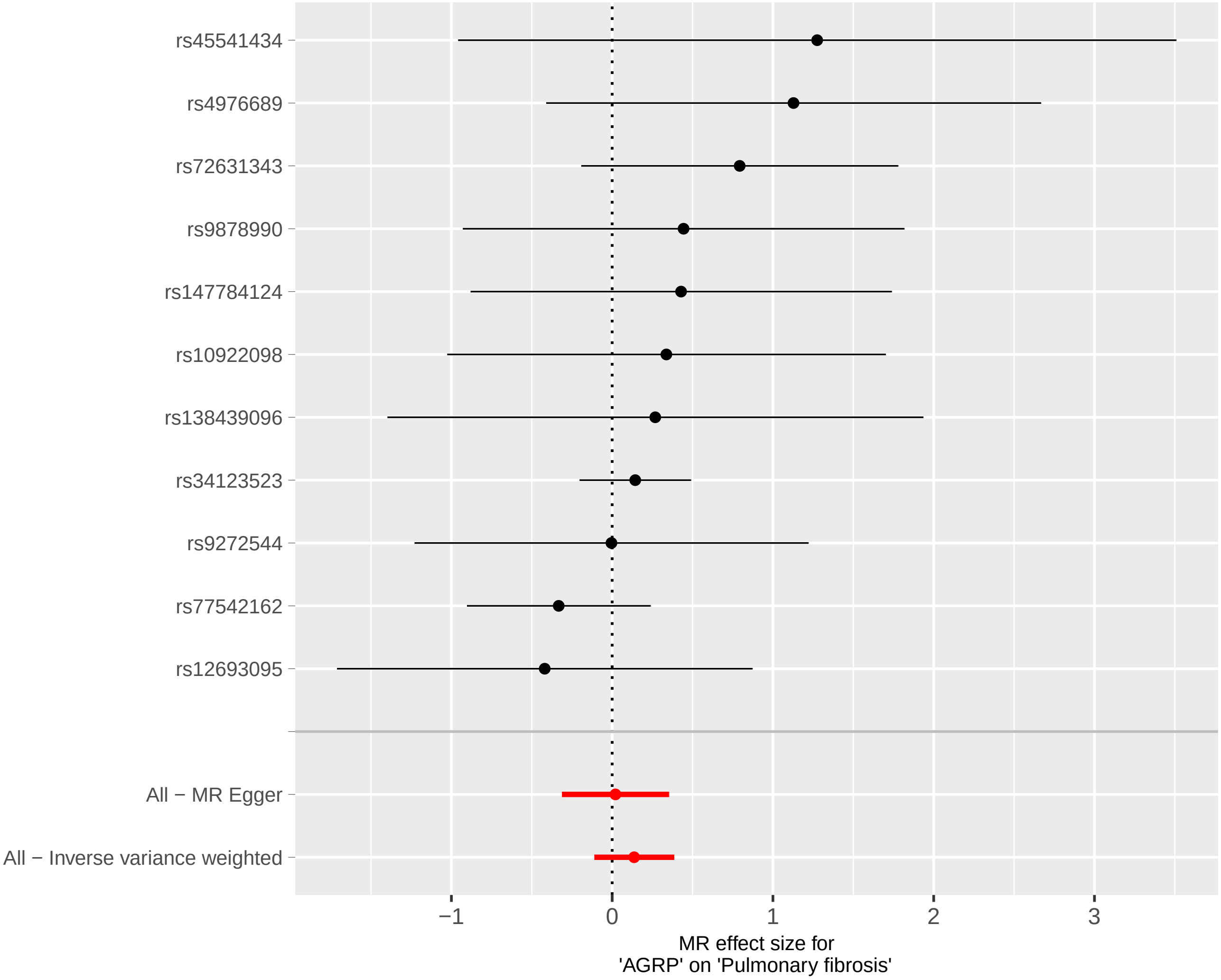
rs45541434
rs4976689
rs72631343
rs9878990
rs147784124
rs10922098
rs138439096
rs34123523
rs9272544
rs77542162
rs12693095
All − MR Egger
All − Inverse variance weighted
−1
0
1
2
3
MR effect size for
'AGRP' on 'Pulmonary fibrosis'

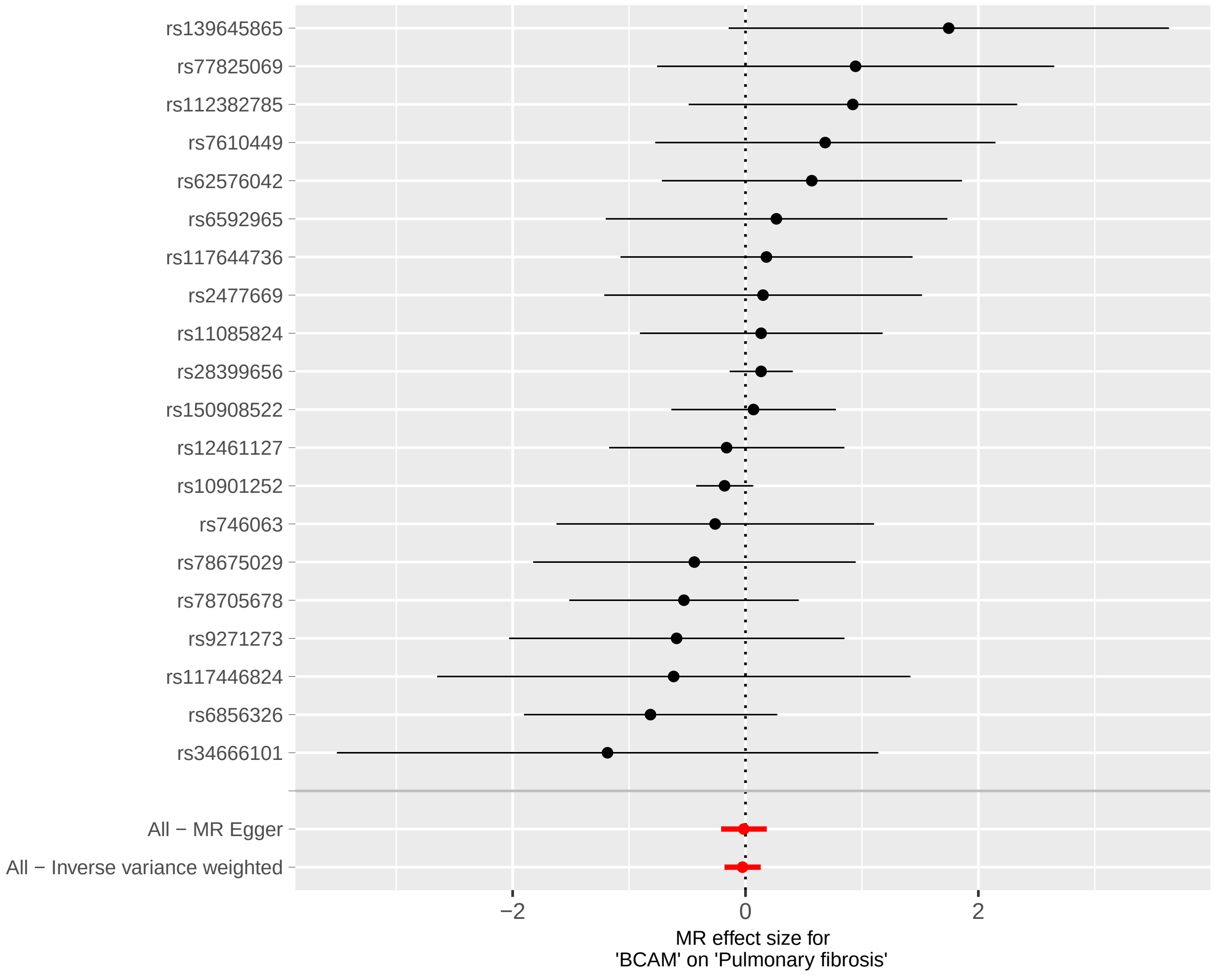

rs139645865
rs77825069
rs112382785
rs7610449
rs62576042
rs6592965
rs117644736
rs2477669
rs11085824
rs28399656
rs150908522
rs12461127
rs10901252
rs746063
rs78675029
rs78705678
rs9271273
rs117446824
rs6856326
rs34666101
All − MR Egger
All − Inverse variance weighted
−2
0
2
MR effect size for
'BCAM' on 'Pulmonary fibrosis'

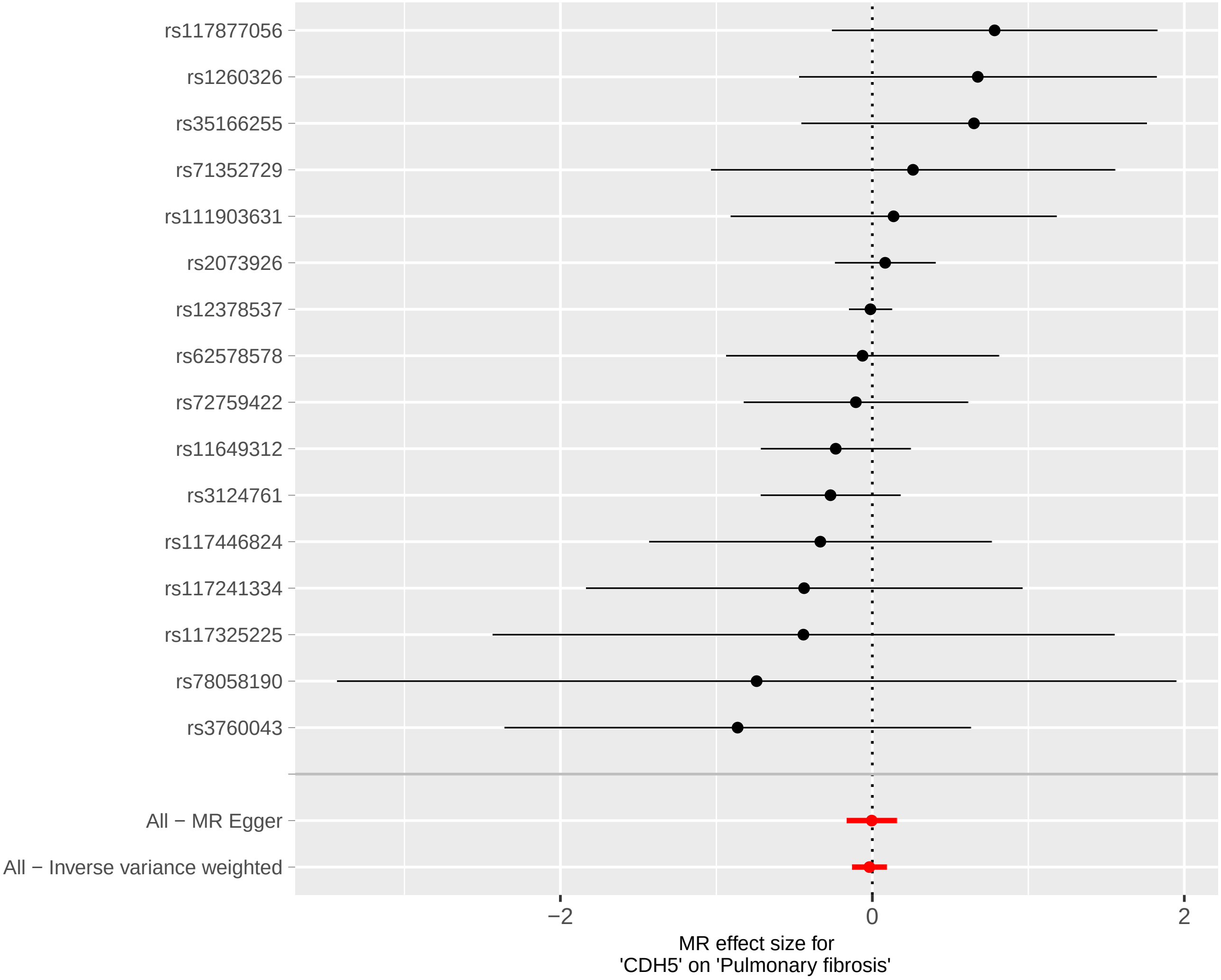
rs117877056
rs1260326
rs35166255
rs71352729
rs111903631
rs2073926
rs12378537
rs62578578
rs72759422
rs11649312
rs3124761
rs117446824
rs117241334
rs117325225
rs78058190
rs3760043
All − MR Egger
All − Inverse variance weighted
−2
0
2
MR effect size for
'CDH5' on 'Pulmonary fibrosis'

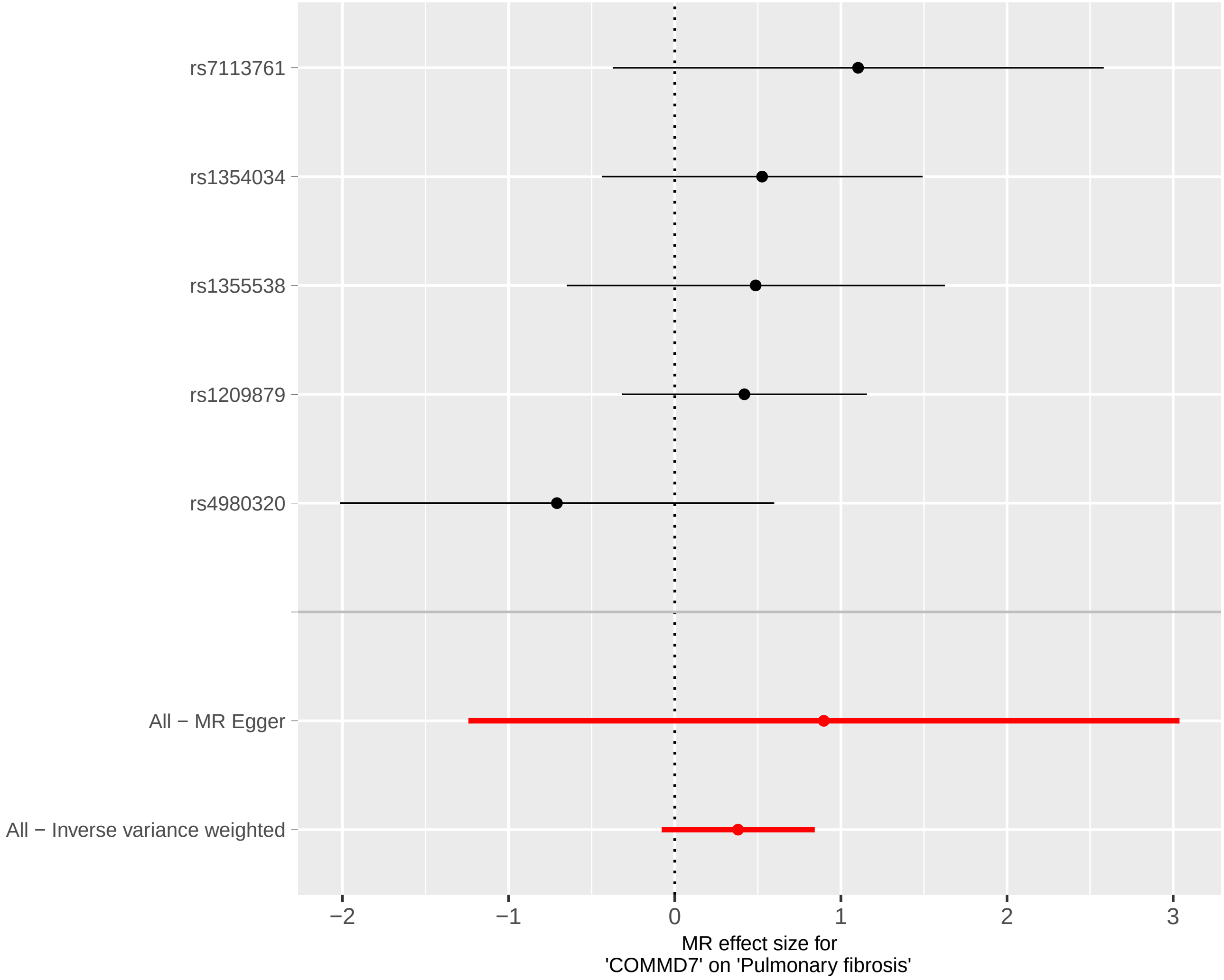
rs7113761
rs1354034
rs1355538
rs1209879
rs4980320
All − MR Egger
All − Inverse variance weighted
−2
−1
0
1
2
3
MR effect size for
'COMMD7' on 'Pulmonary fibrosis'

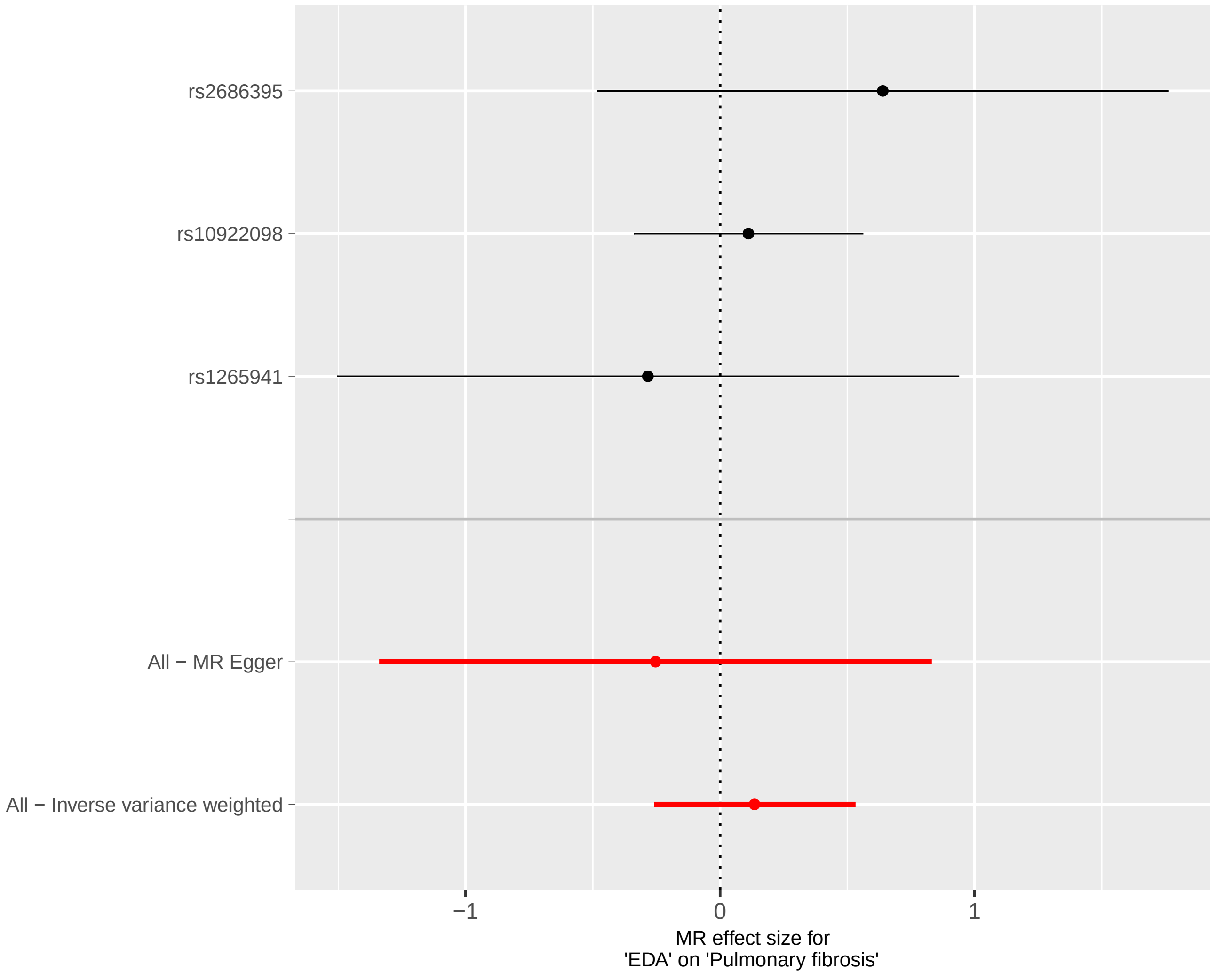

rs2686395
rs10922098
rs1265941
All − MR Egger
All − Inverse variance weighted
−1
0
1
MR effect size for
'EDA' on 'Pulmonary fibrosis'

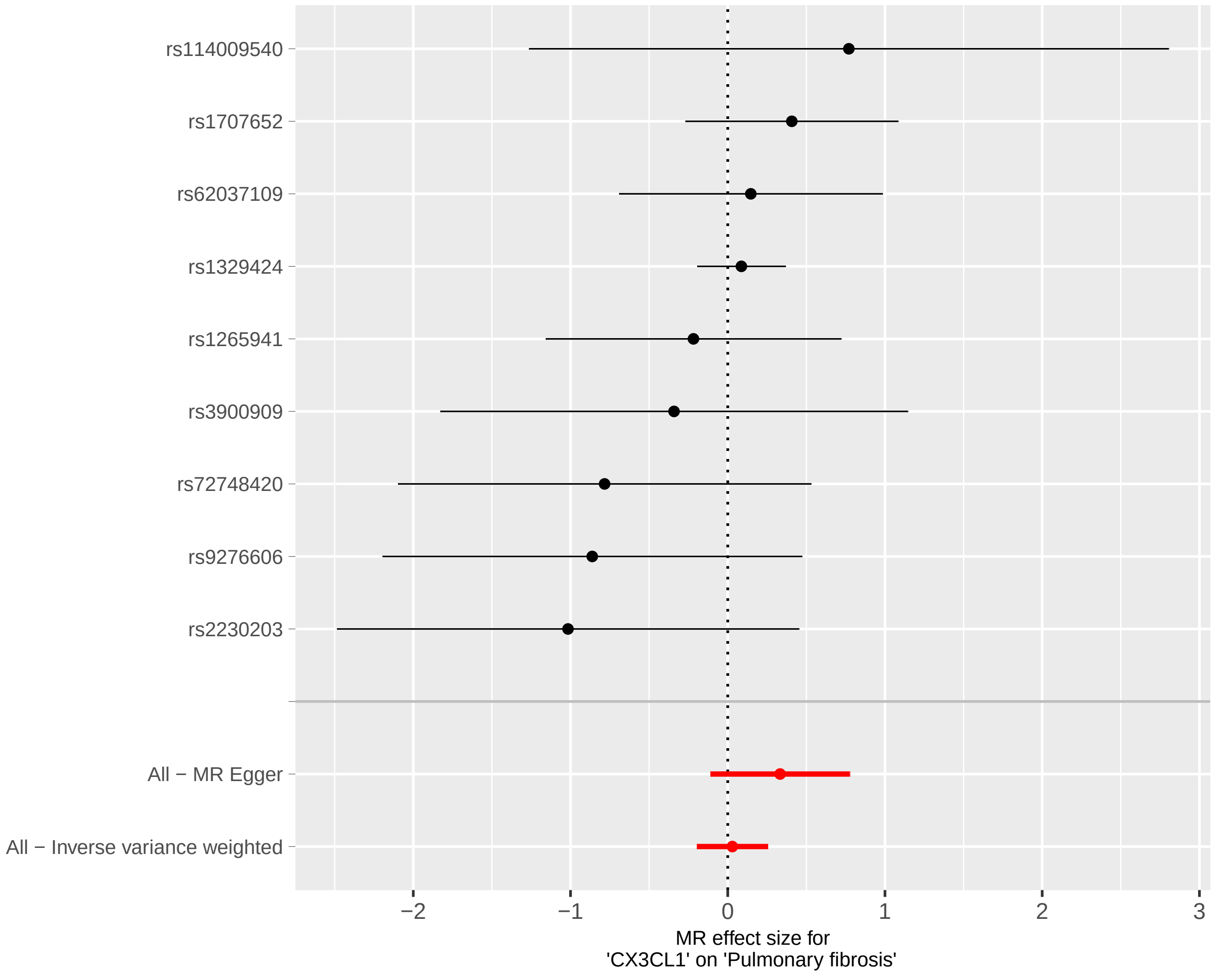

rs114009540
rs1707652
rs62037109
rs1329424
rs1265941
rs3900909
rs72748420
rs9276606
rs2230203
All − MR Egger
All − Inverse variance weighted
−2
−1
0
1
2
3
MR effect size for
'CX3CL1' on 'Pulmonary fibrosis'

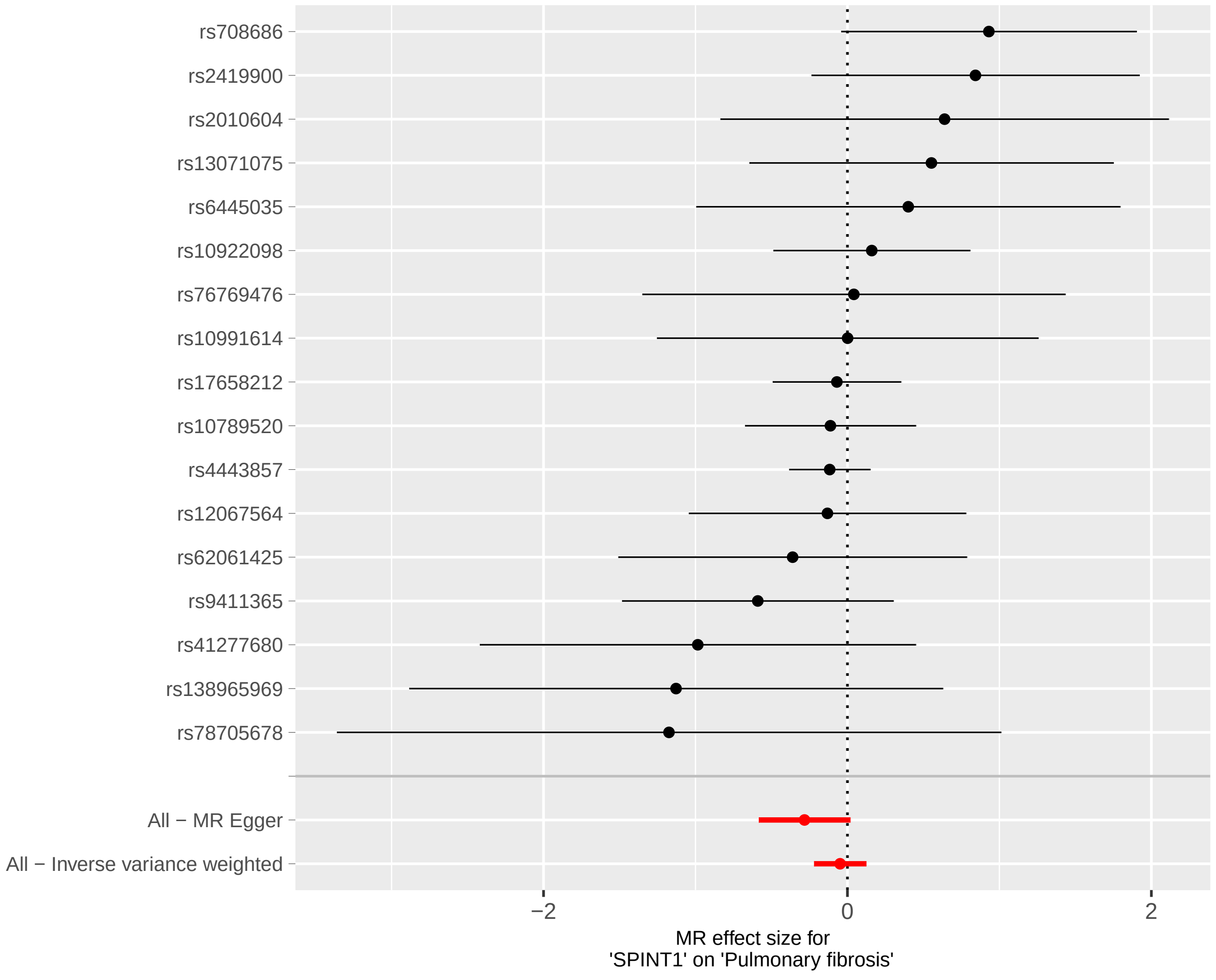
rs708686
rs2419900
rs2010604
rs13071075
rs6445035
rs10922098
rs76769476
rs10991614
rs17658212
rs10789520
rs4443857
rs12067564
rs62061425
rs9411365
rs41277680
rs138965969
rs78705678
All − MR Egger
All − Inverse variance weighted
−2
0
2
MR effect size for
'SPINT1' on 'Pulmonary fibrosis'

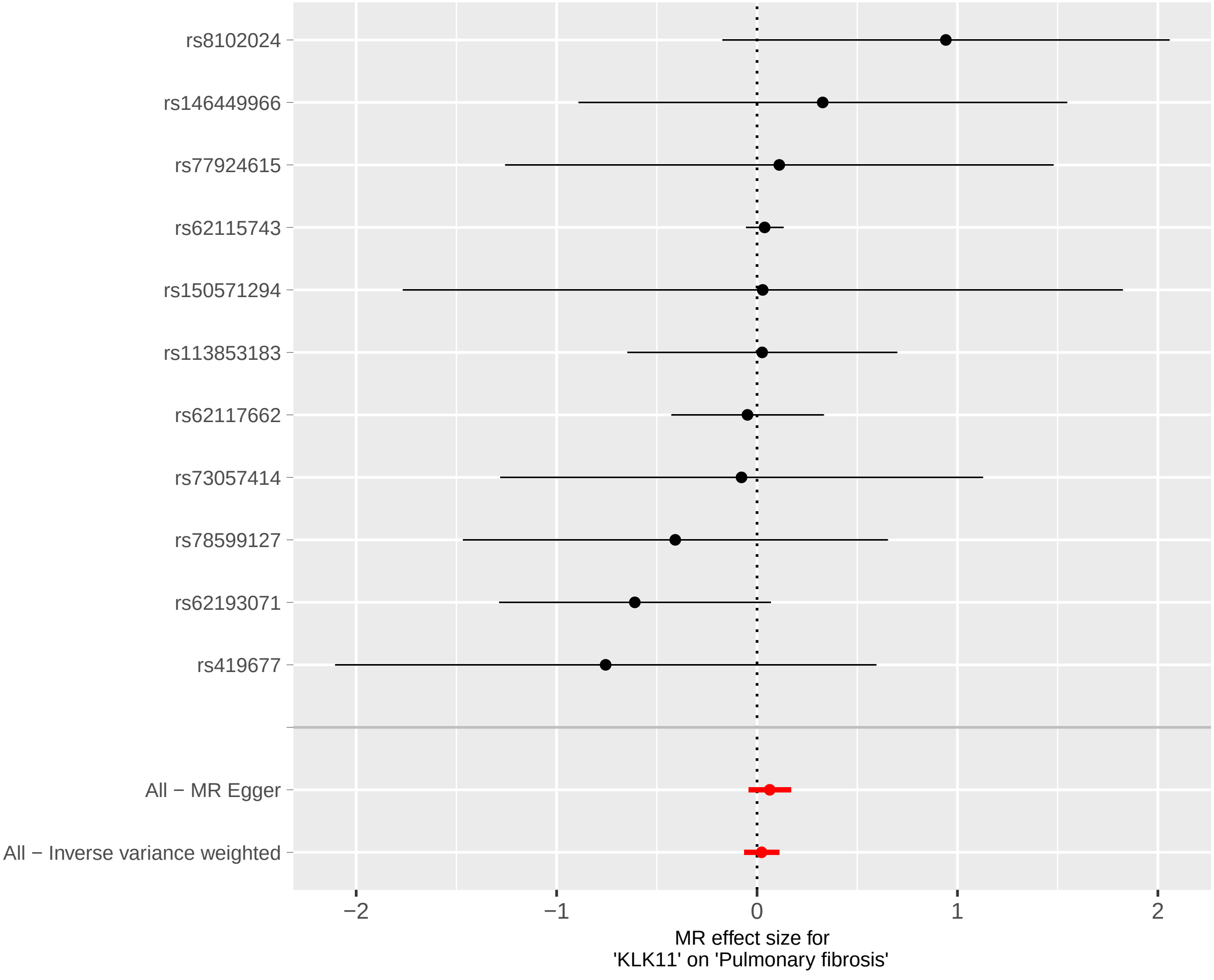
rs8102024
rs146449966
rs77924615
rs62115743
rs150571294
rs113853183
rs62117662
rs73057414
rs78599127
rs62193071
rs419677
All − MR Egger
All − Inverse variance weighted
−2
−1
0
1
2
MR effect size for
'KLK11' on 'Pulmonary fibrosis'

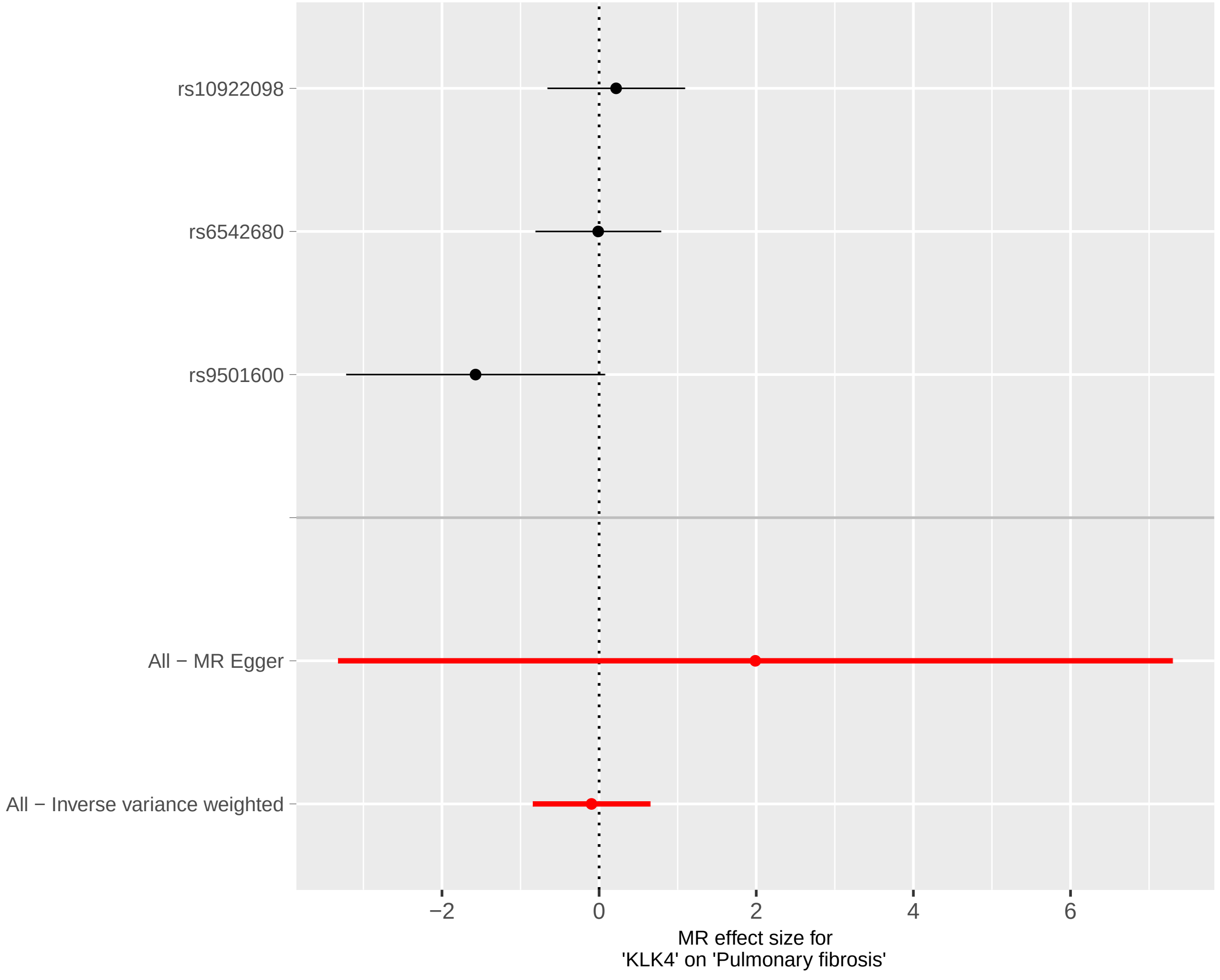
rs10922098
rs6542680
rs9501600
All − MR Egger
All − Inverse variance weighted
−2
0
2
4
6
MR effect size for
'KLK4' on 'Pulmonary fibrosis'

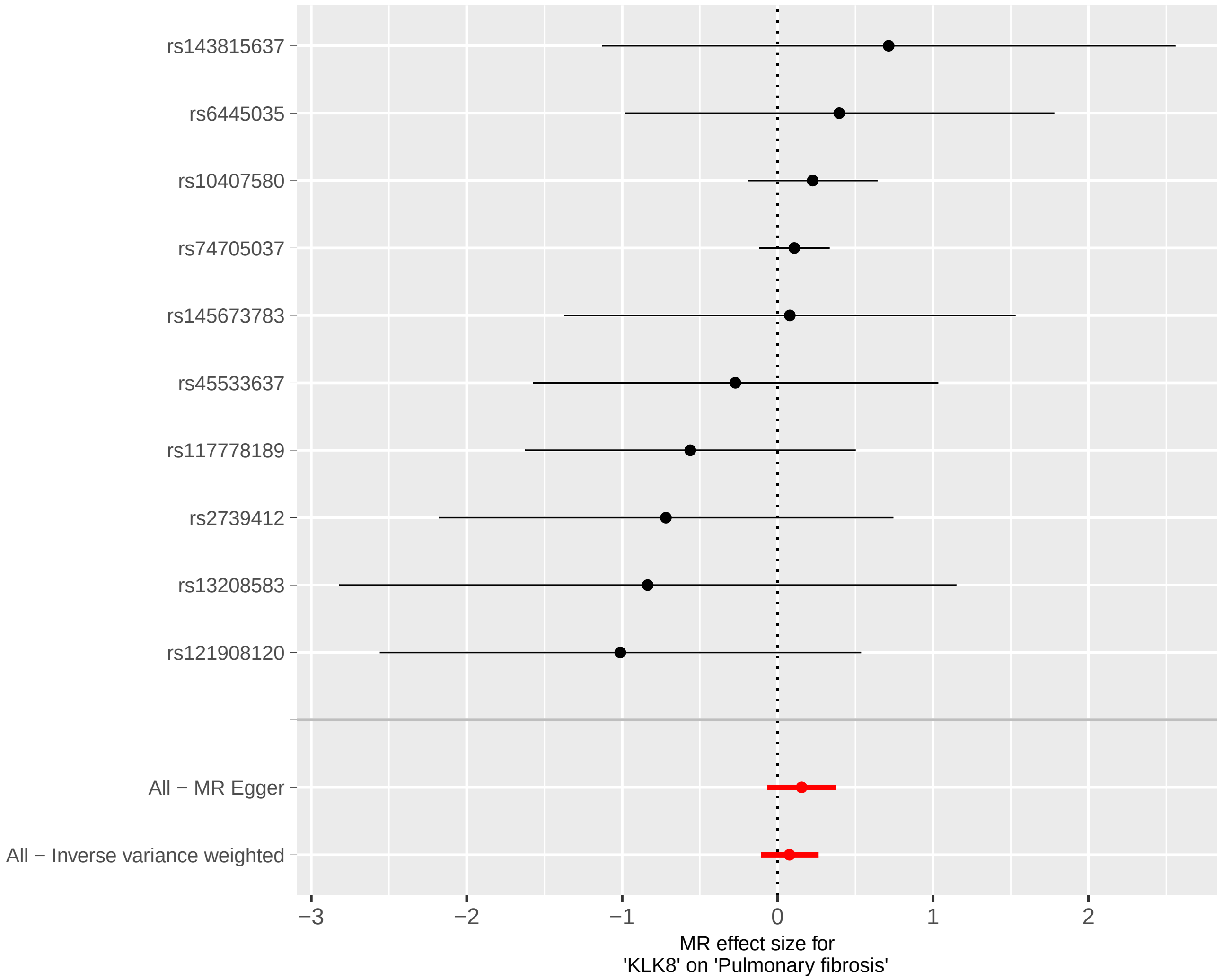

rs143815637
rs6445035
rs10407580
rs74705037
rs145673783
rs45533637
rs117778189
rs2739412
rs13208583
rs121908120
All − MR Egger
All − Inverse variance weighted
−3
−2
−1
0
1
2
MR effect size for
'KLK8' on 'Pulmonary fibrosis'

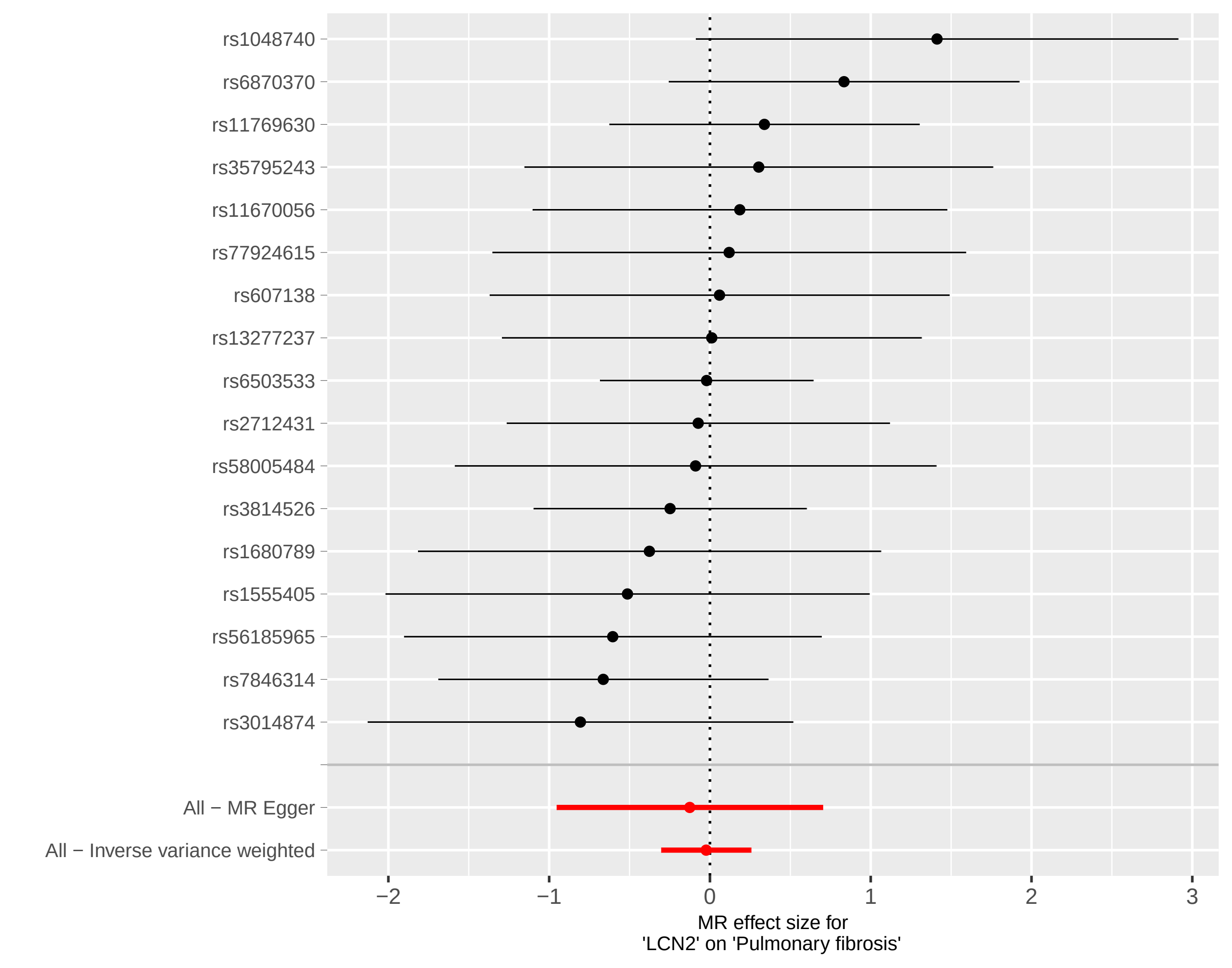
rs1048740
rs6870370
rs11769630
rs35795243
rs11670056
rs77924615
rs607138
rs13277237
rs6503533
rs2712431
rs58005484
rs3814526
rs1680789
rs1555405
rs56185965
rs7846314
rs3014874
All − MR Egger
All − Inverse variance weighted
−2
−1
0
1
2
3
MR effect size for
'LCN2' on 'Pulmonary fibrosis'

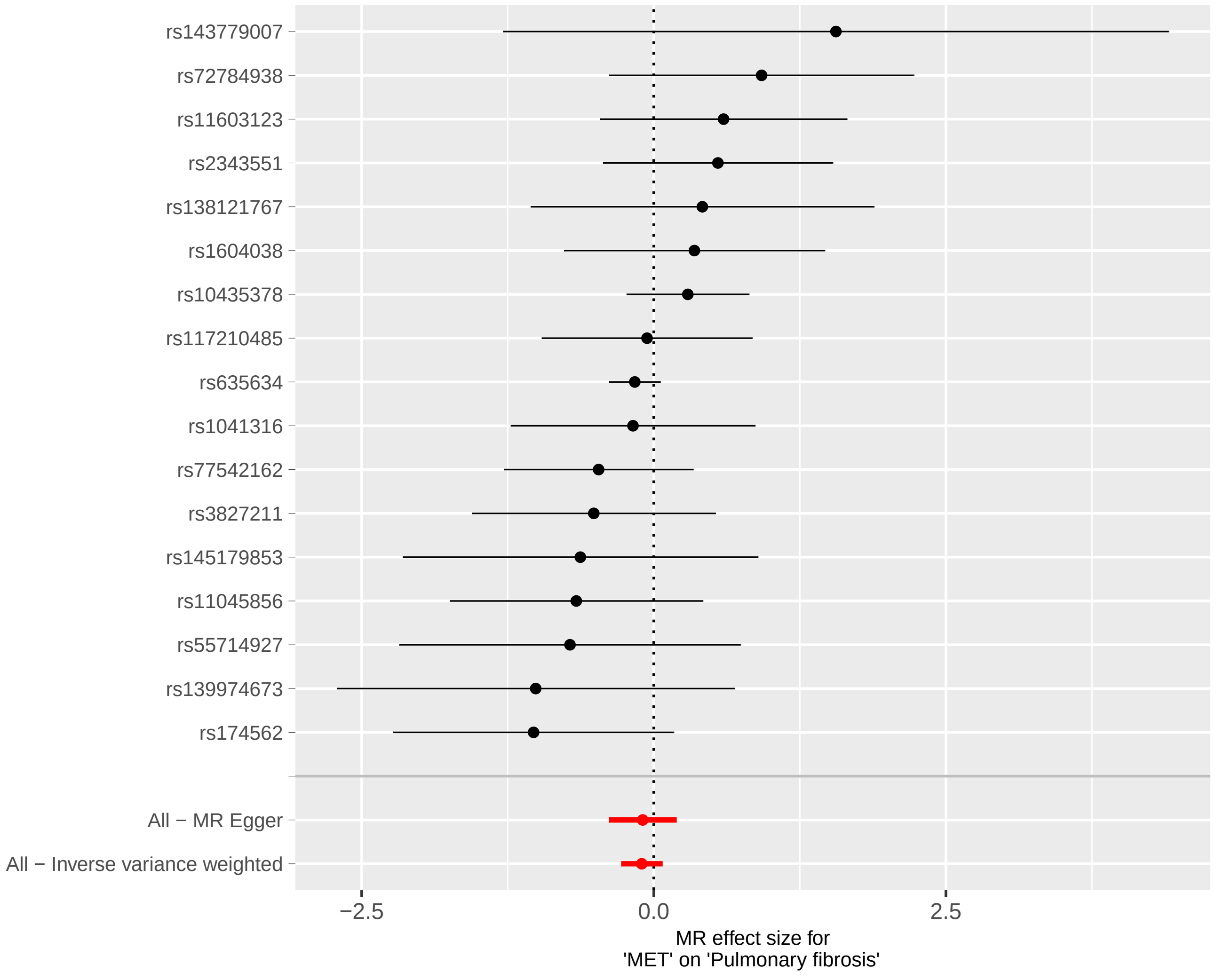
rs143779007
rs72784938
rs11603123
rs2343551
rs138121767
rs1604038
rs10435378
rs117210485
rs635634
rs1041316
rs77542162
rs3827211
rs145179853
rs11045856
rs55714927
rs139974673
rs174562
All − MR Egger
All − Inverse variance weighted
−2.5
0.0
2.5
MR effect size for
'MET' on 'Pulmonary fibrosis'

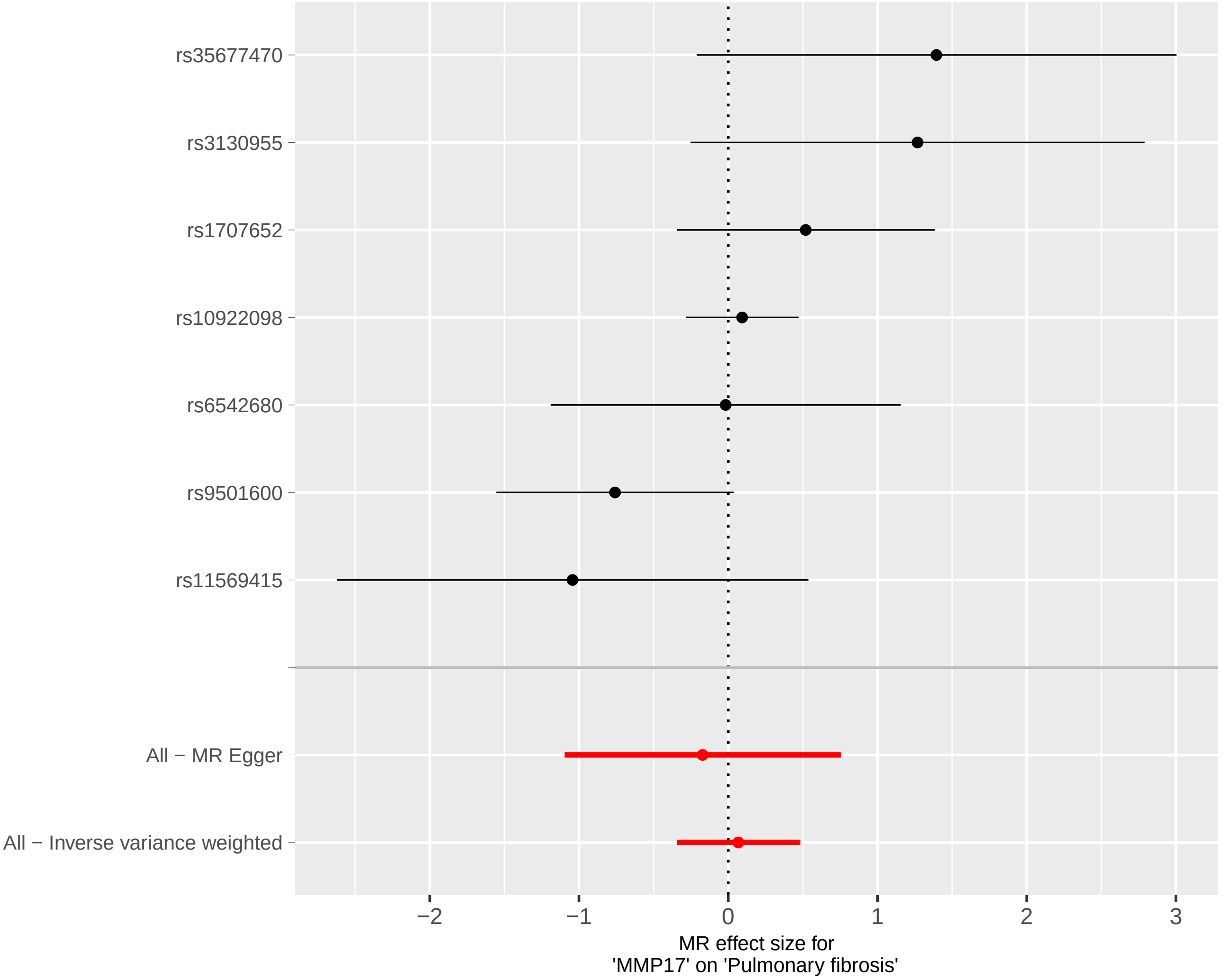

rs35677470
rs3130955
rs1707652
rs10922098
rs6542680
rs9501600
rs11569415
All − MR Egger
All − Inverse variance weighted
−2
−1
0
1
2
3
MR effect size for
'MMP17' on 'Pulmonary fibrosis'

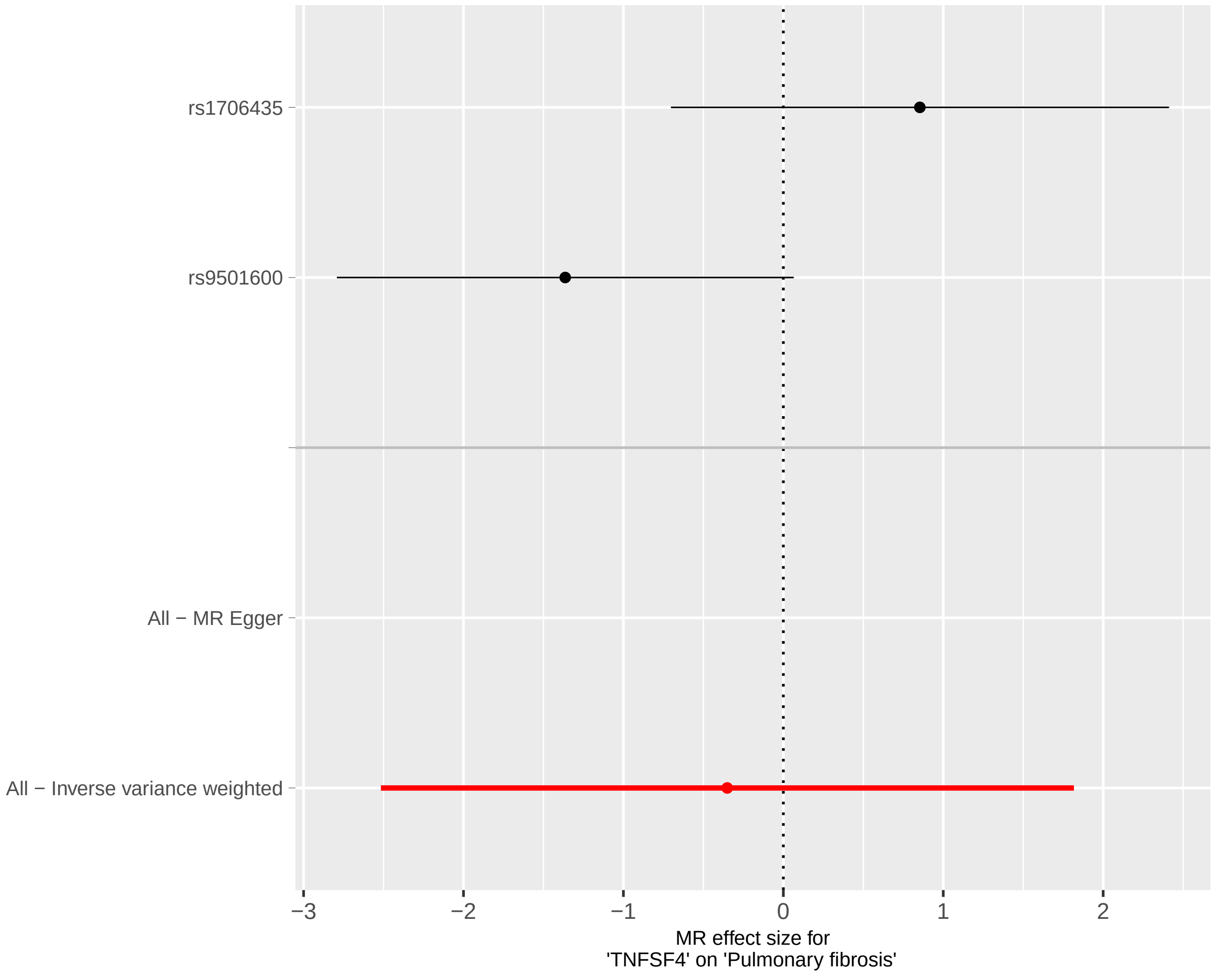
rs1706435
rs9501600
All − MR Egger
All − Inverse variance weighted
−3
−2
−1
0
1
2
MR effect size for
'TNFSF4' on 'Pulmonary fibrosis'

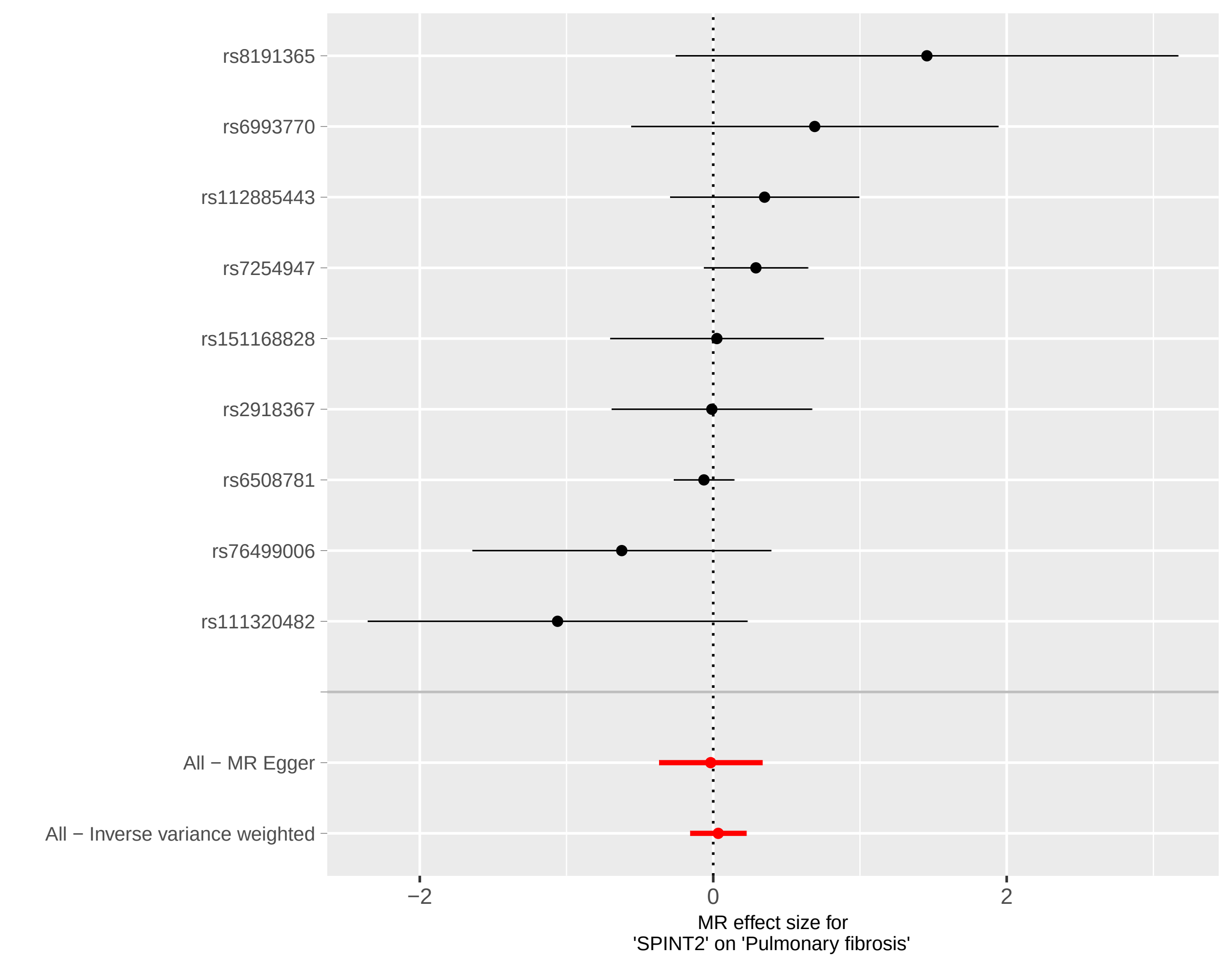

rs8191365
rs6993770
rs112885443
rs7254947
rs151168828
rs2918367
rs6508781
rs76499006
rs111320482
All − MR Egger
All − Inverse variance weighted
−2
0
2
MR effect size for
'SPINT2' on 'Pulmonary fibrosis'

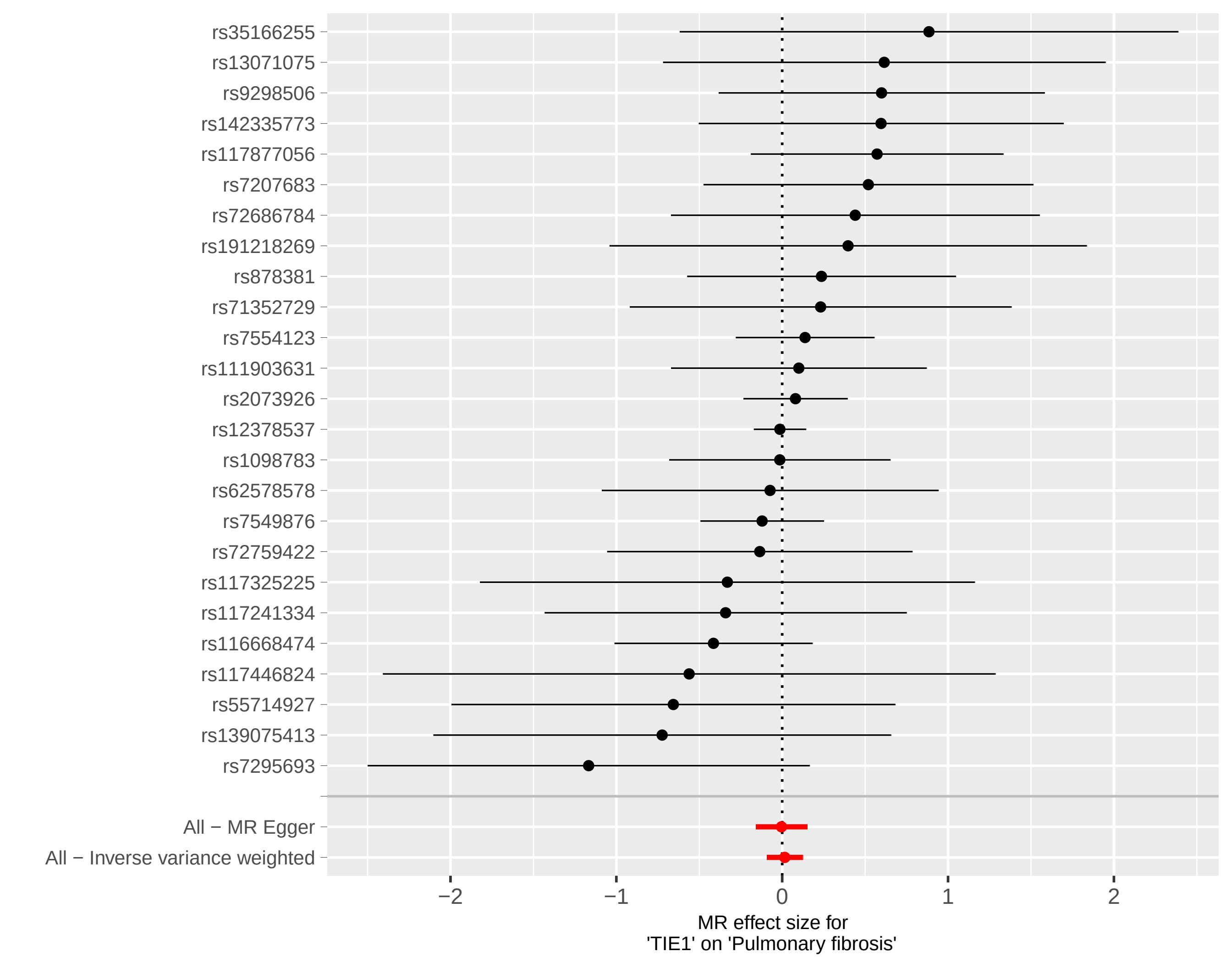
rs35166255
rs13071075
rs9298506
rs142335773
rs117877056
rs7207683
rs72686784
rs191218269
rs878381
rs71352729
rs7554123
rs111903631
rs2073926
rs12378537
rs1098783
rs62578578
rs7549876
rs72759422
rs117325225
rs117241334
rs116668474
rs117446824
rs55714927
rs139075413
rs7295693
All − MR Egger
All − Inverse variance weighted
−2
−1
0
1
2
MR effect size for
'TIE1' on 'Pulmonary fibrosis'

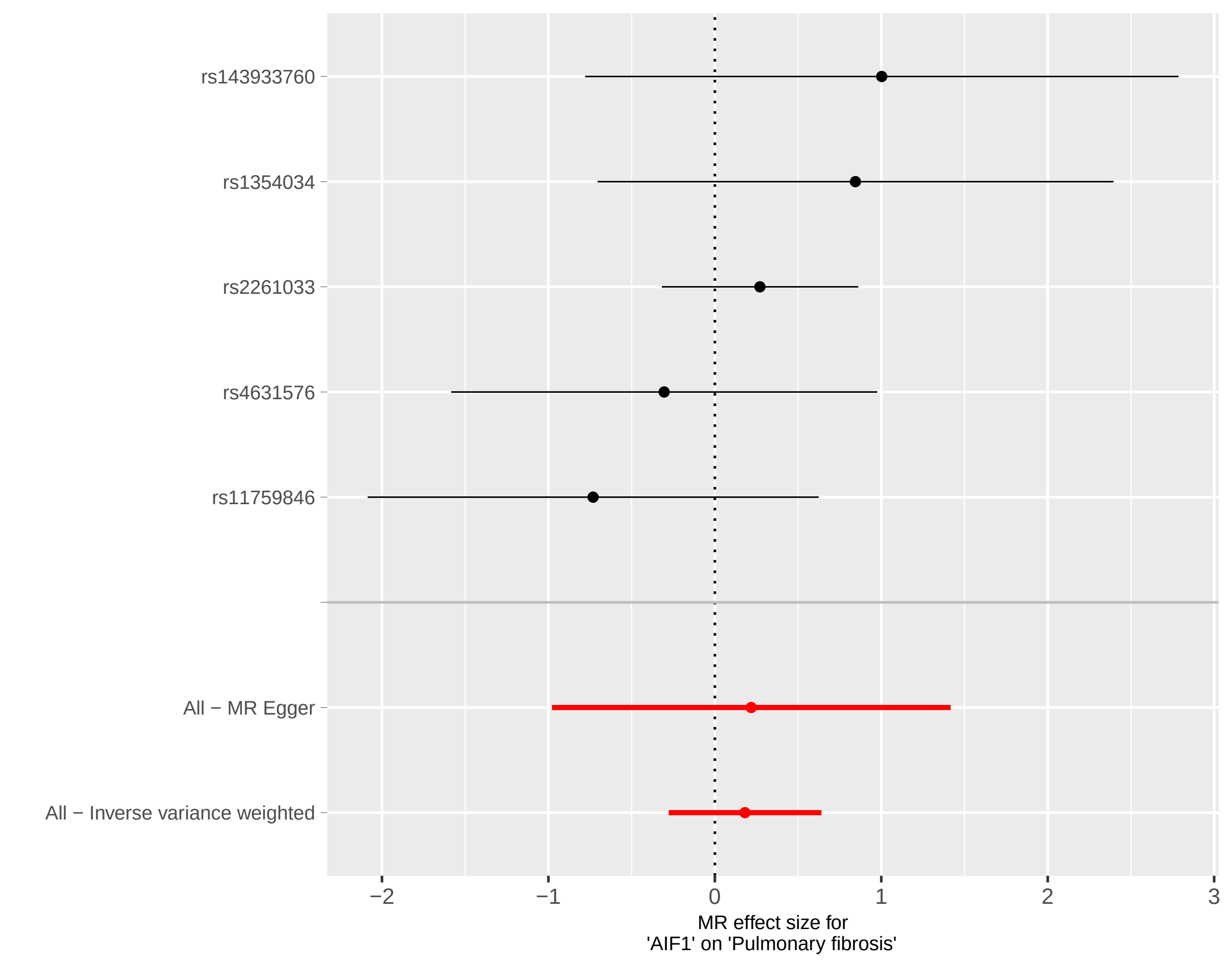

rs143933760
rs1354034
rs2261033
rs4631576
rs11759846
All − MR Egger
All − Inverse variance weighted
−2
−1
0
1
2
3
MR effect size for
'AIF1' on 'Pulmonary fibrosis'

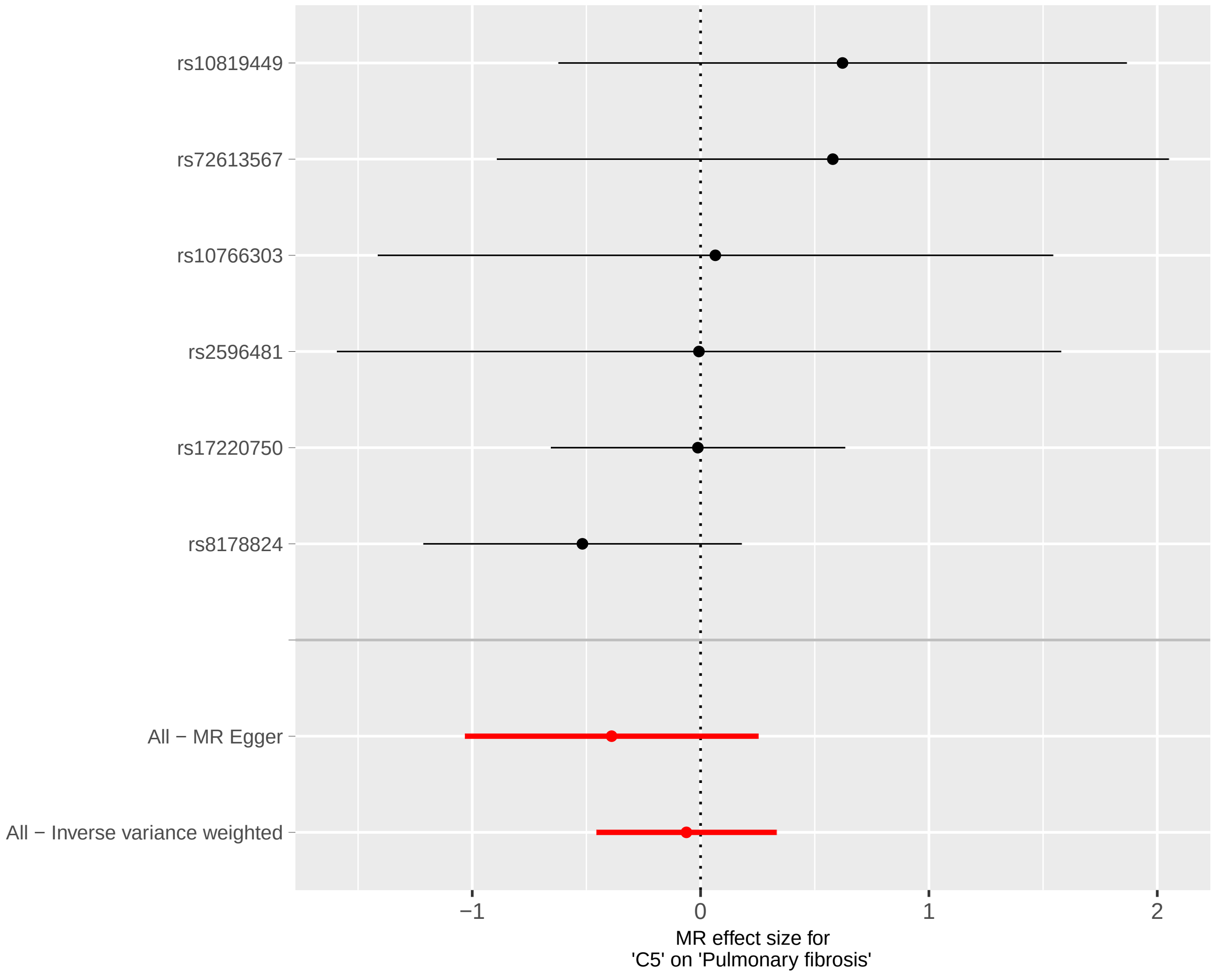
rs10819449
rs72613567
rs10766303
rs2596481
rs17220750
rs8178824
All − MR Egger
All − Inverse variance weighted
−1
0
1
2
MR effect size for
'C5' on 'Pulmonary fibrosis'

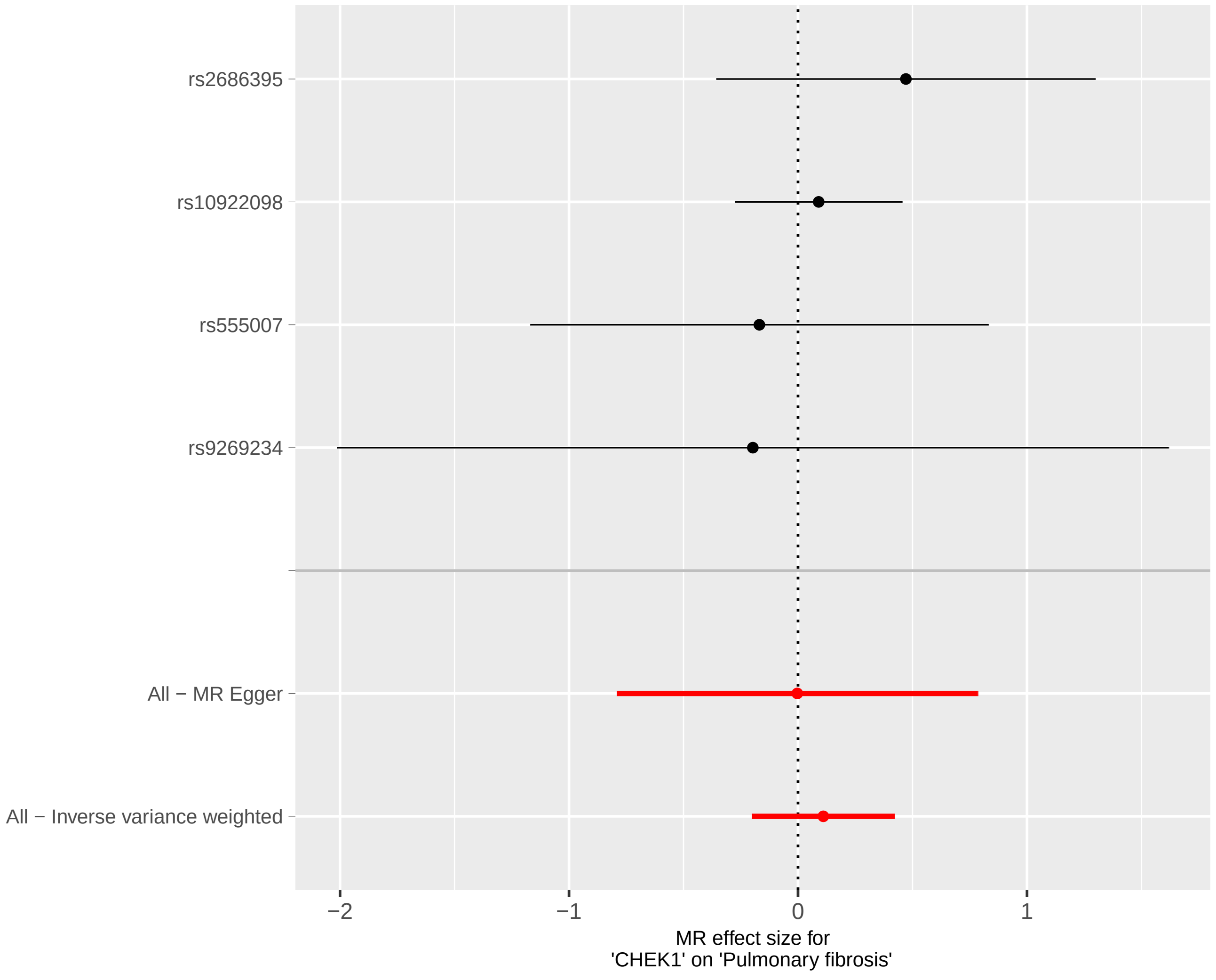

rs2686395
rs10922098
rs555007
rs9269234
All − MR Egger
All − Inverse variance weighted
−2
−1
0
1
MR effect size for
'CHEK1' on 'Pulmonary fibrosis'

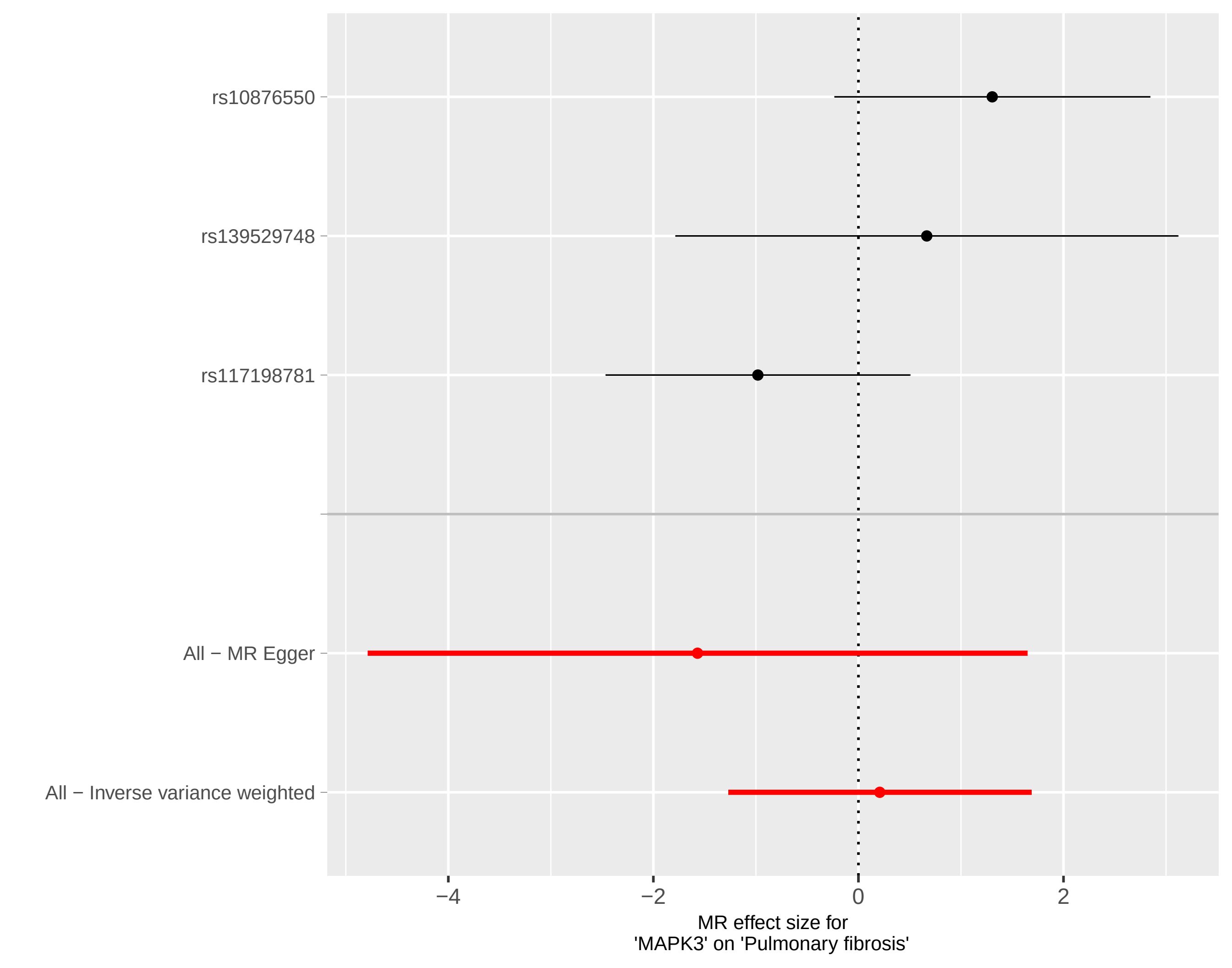

rs10876550
rs139529748
rs117198781
All − MR Egger
All − Inverse variance weighted
−4
−2
0
2
MR effect size for
'MAPK3' on 'Pulmonary fibrosis'

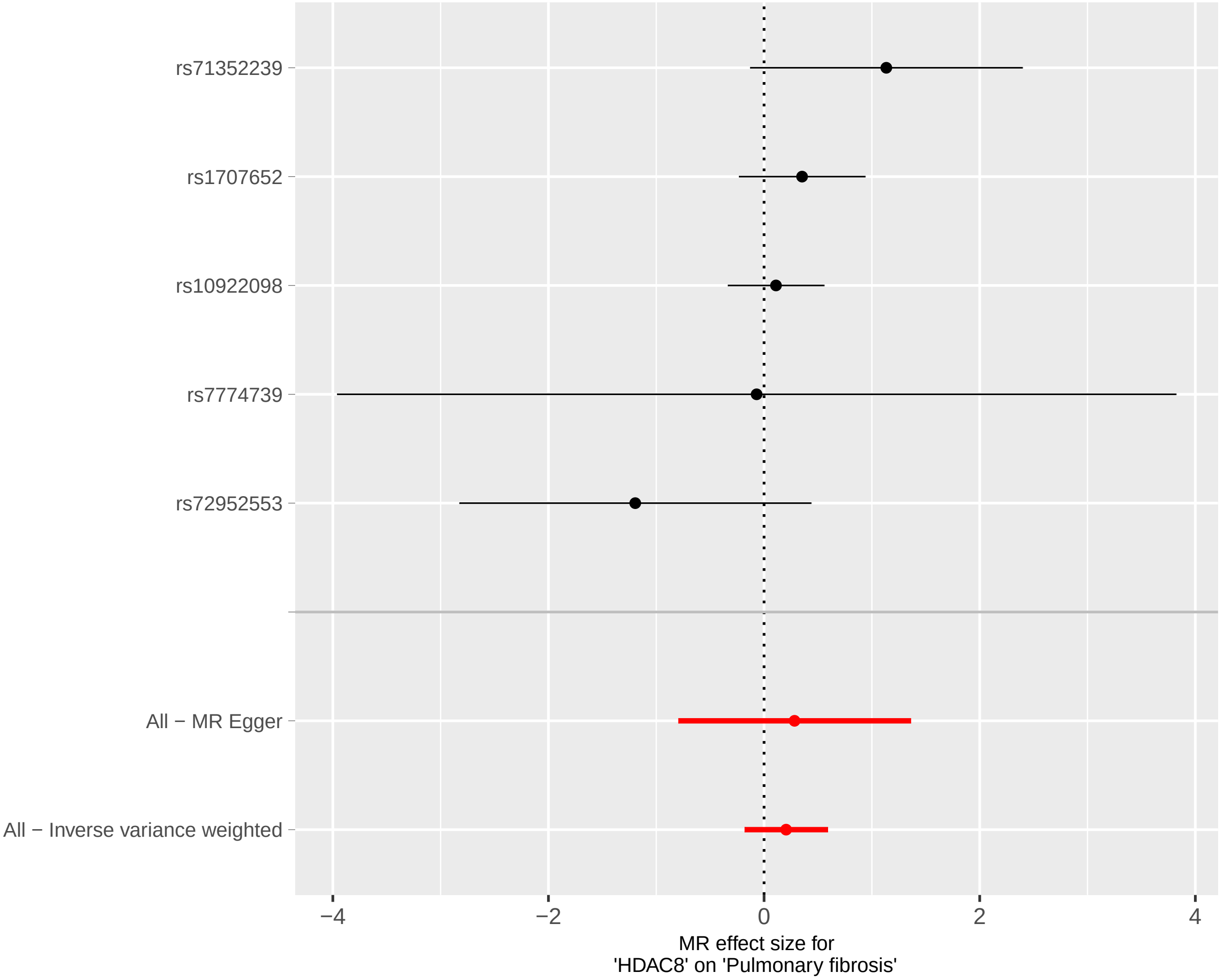
rs71352239
rs1707652
rs10922098
rs7774739
rs72952553
All − MR Egger
All − Inverse variance weighted
−4
−2
0
2
4
MR effect size for
'HDAC8' on 'Pulmonary fibrosis'

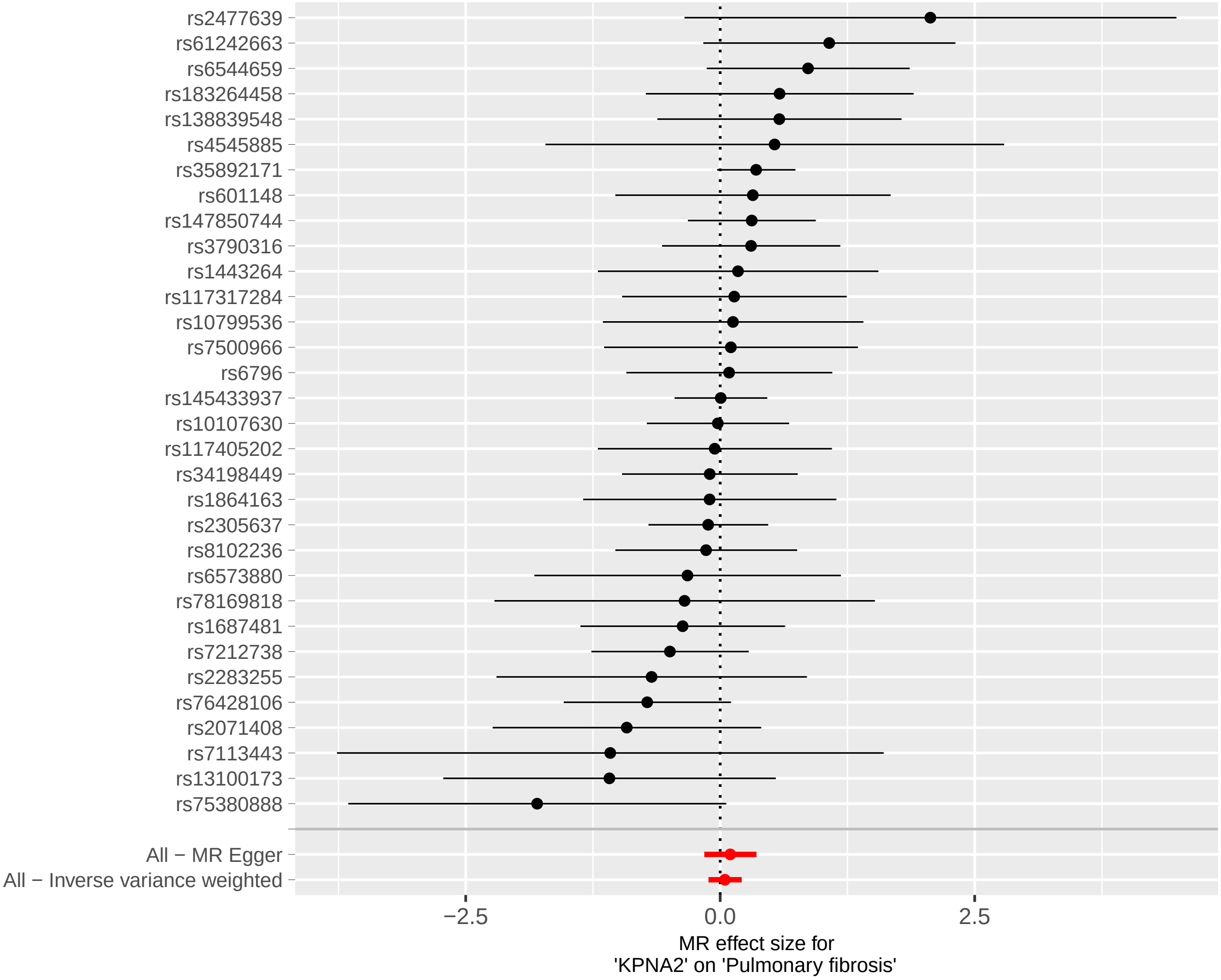
rs2477639
rs61242663
rs6544659
rs183264458
rs138839548
rs4545885
rs35892171
rs601148
rs147850744
rs3790316
rs1443264
rs117317284
rs10799536
rs7500966
rs6796
rs145433937
rs10107630
rs117405202
rs34198449
rs1864163
rs2305637
rs8102236
rs6573880
rs78169818
rs1687481
rs7212738
rs2283255
rs76428106
rs2071408
rs7113443
rs13100173
rs75380888
All − MR Egger
All − Inverse variance weighted
−2.5
0.0
2.5
MR effect size for
'KPNA2' on 'Pulmonary fibrosis'

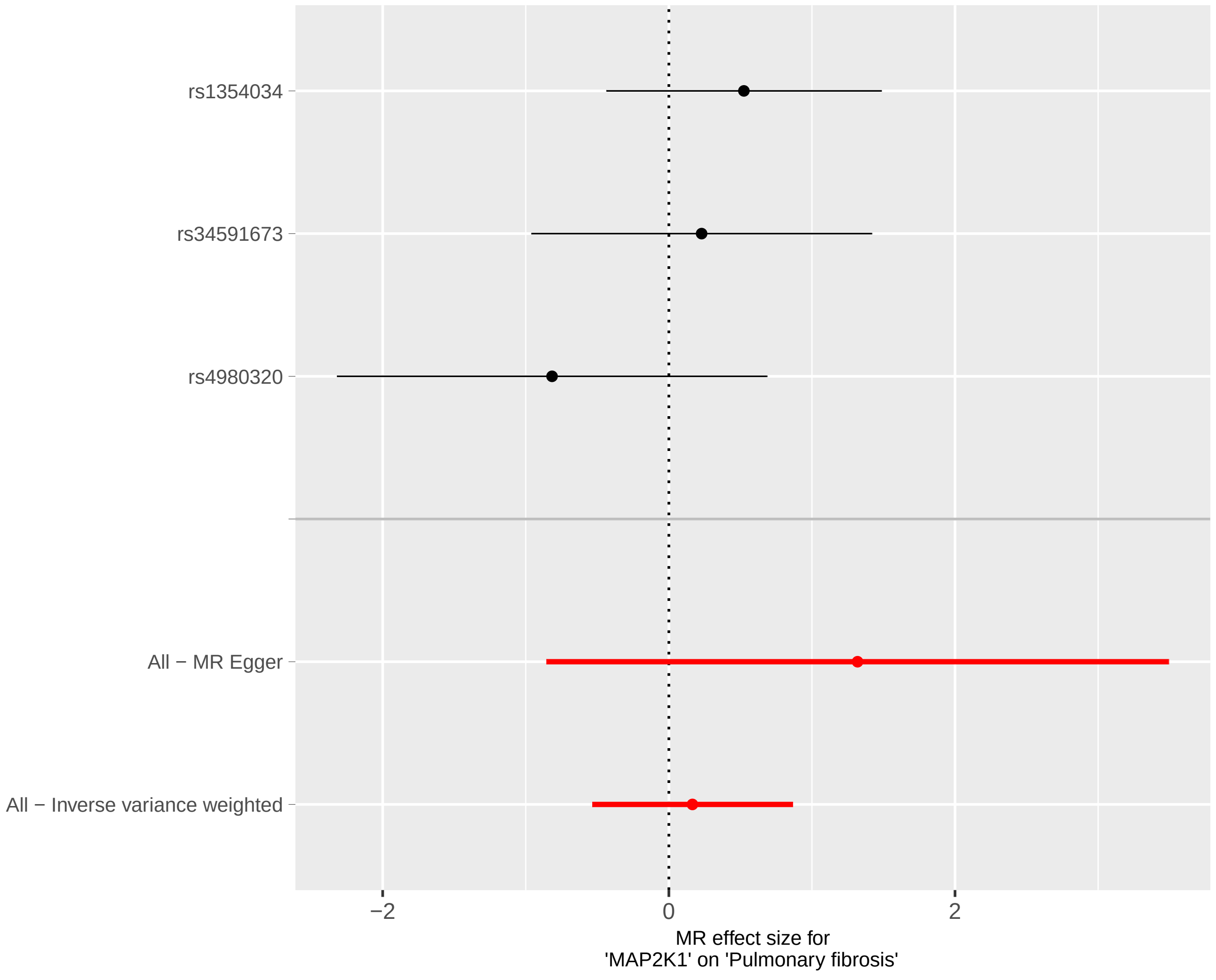

rs1354034
rs34591673
rs4980320
All − MR Egger
All − Inverse variance weighted
−2
0
2
MR effect size for
'MAP2K1' on 'Pulmonary fibrosis'

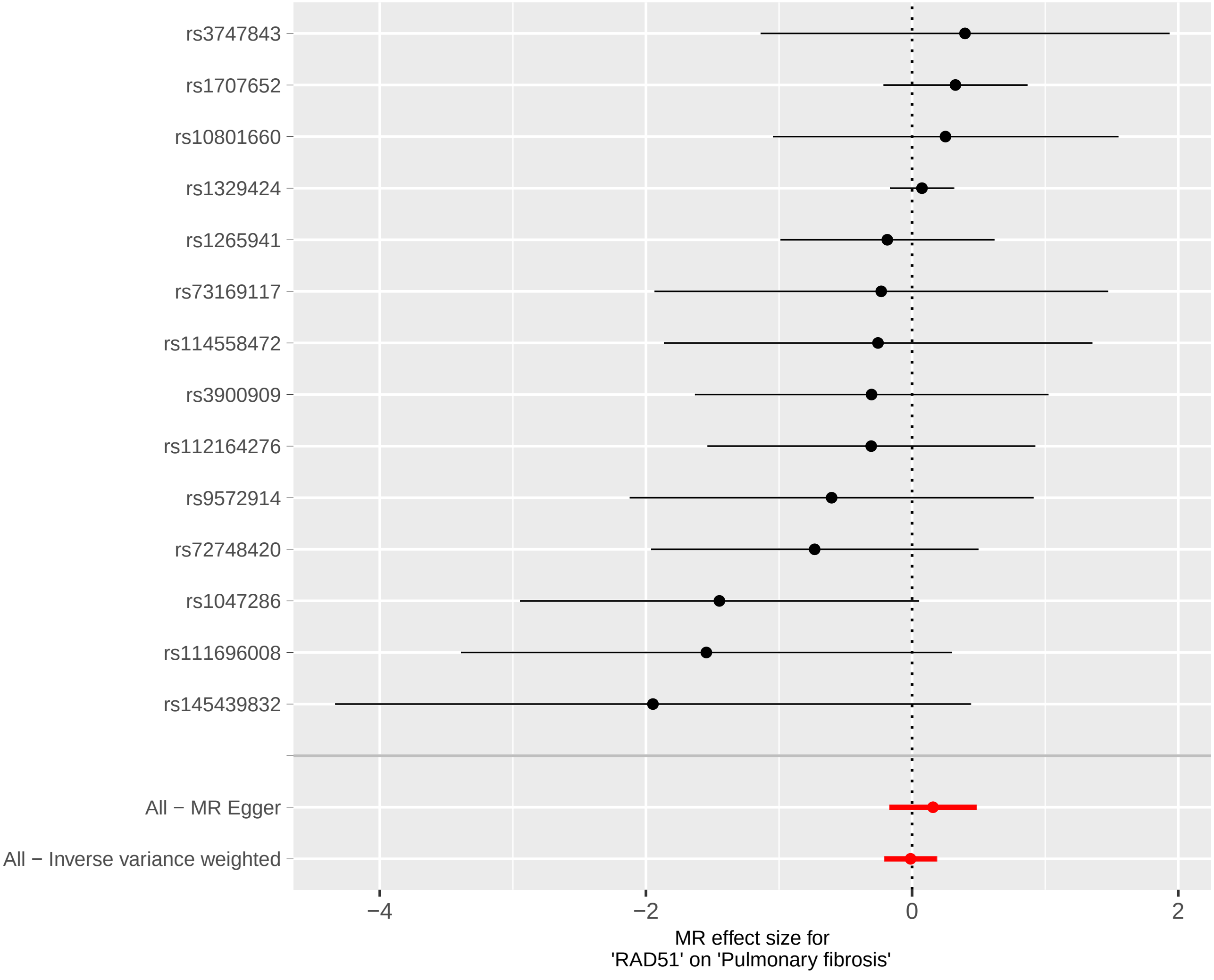

rs3747843
rs1707652
rs10801660
rs1329424
rs1265941
rs73169117
rs114558472
rs3900909
rs112164276
rs9572914
rs72748420
rs1047286
rs111696008
rs145439832
All − MR Egger
All − Inverse variance weighted
−4
−2
0
2
MR effect size for
'RAD51' on 'Pulmonary fibrosis'

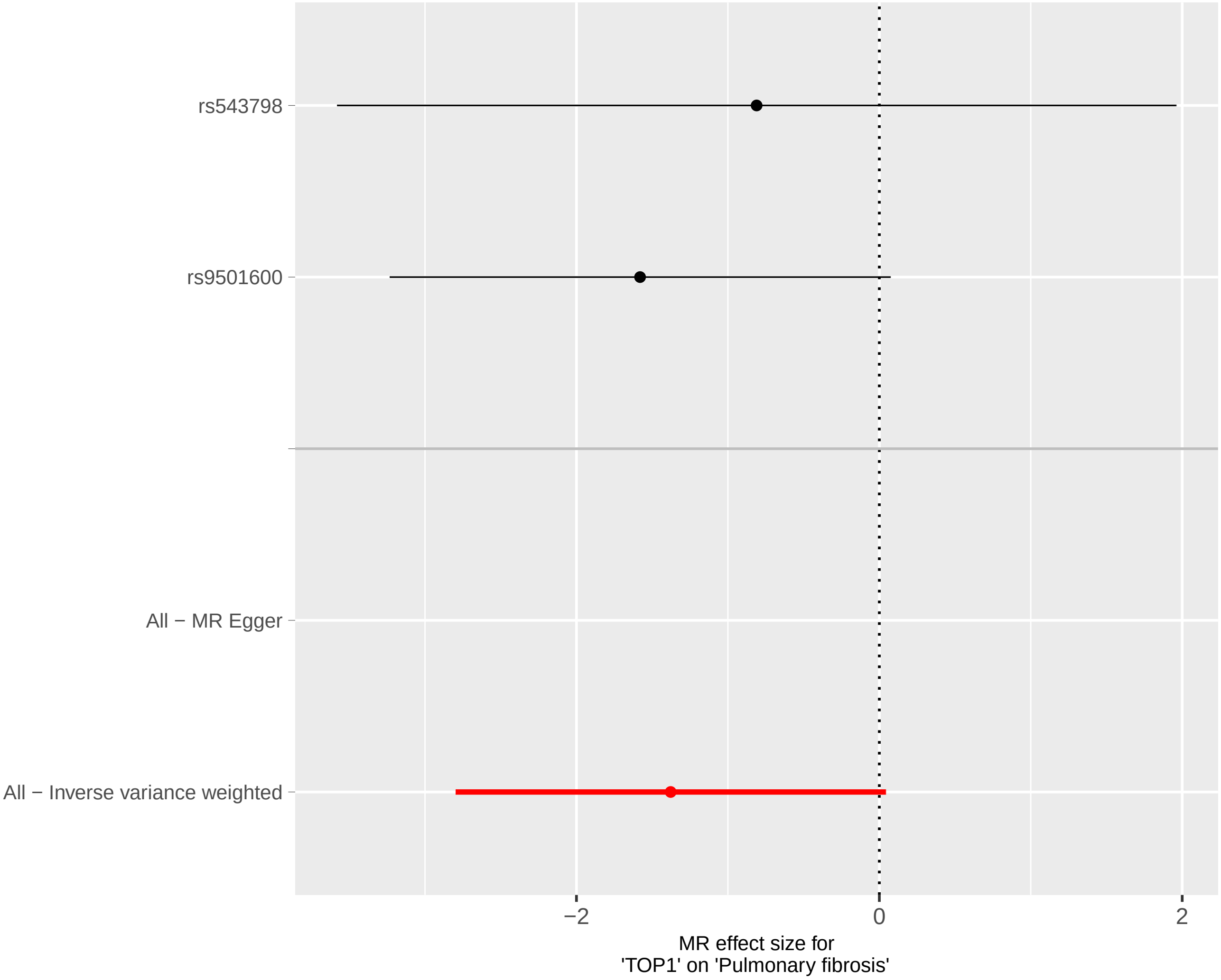

rs543798
rs9501600
All − MR Egger
All − Inverse variance weighted
−2
0
2
MR effect size for
'TOP1' on 'Pulmonary fibrosis'

Insufficient number of SNPs

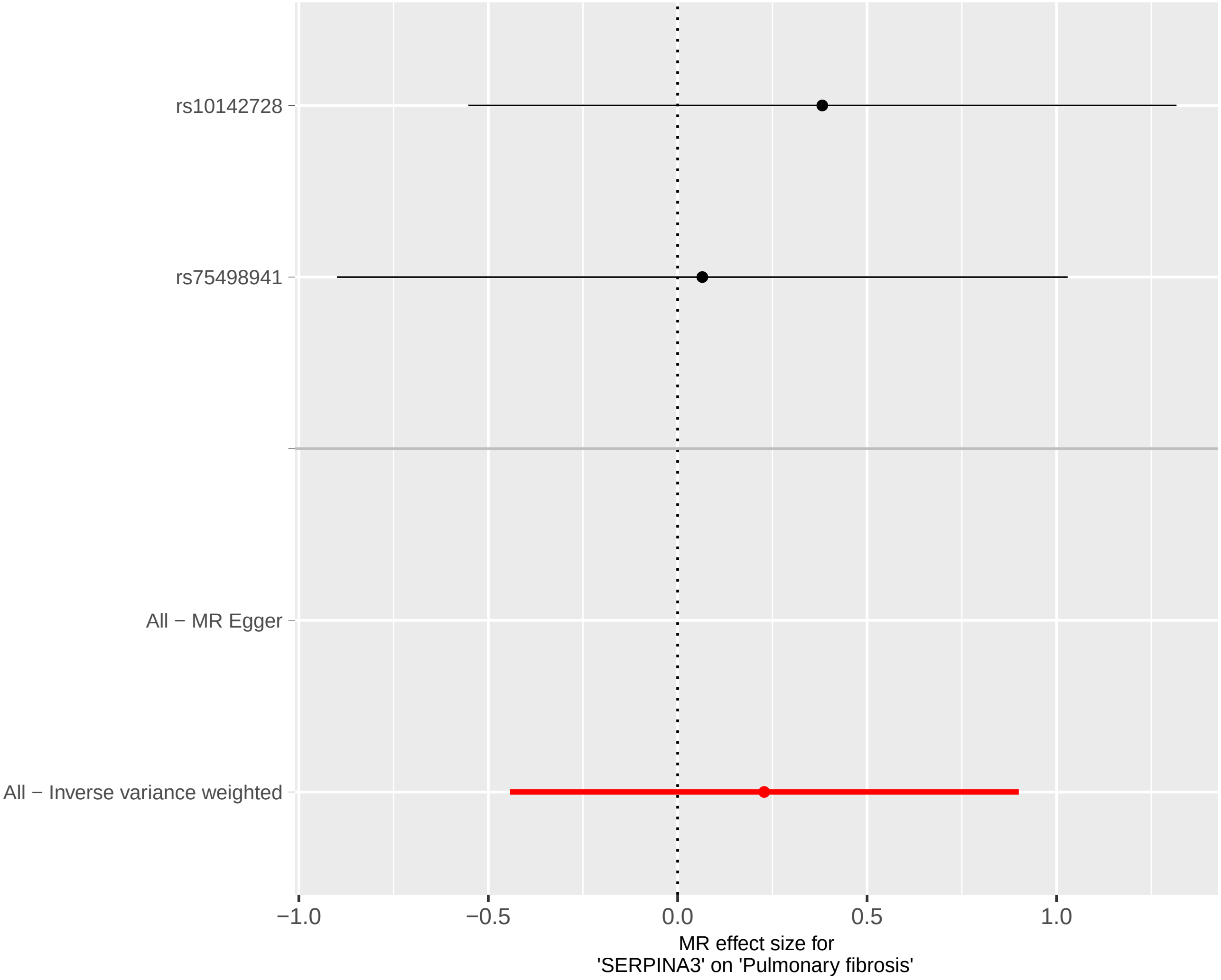
rs10142728
rs75498941
All − MR Egger
All − Inverse variance weighted
−1.0
−0.5
0.0
0.5
1.0
MR effect size for
'SERPINA3' on 'Pulmonary fibrosis'

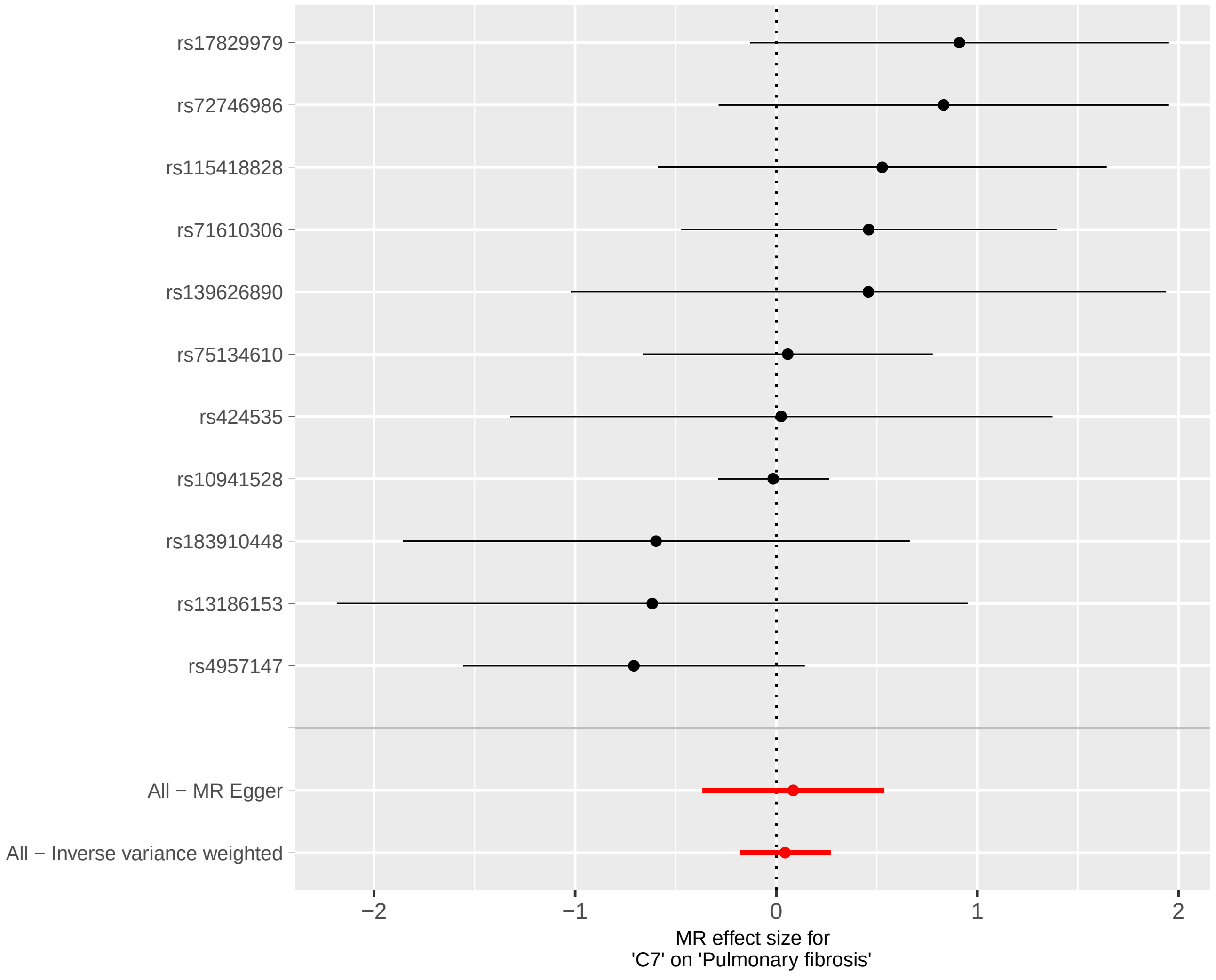
rs17829979
rs72746986
rs115418828
rs71610306
rs139626890
rs75134610
rs424535
rs10941528
rs183910448
rs13186153
rs4957147
All − MR Egger
All − Inverse variance weighted
−2
−1
0
1
2
MR effect size for
'C7' on 'Pulmonary fibrosis'

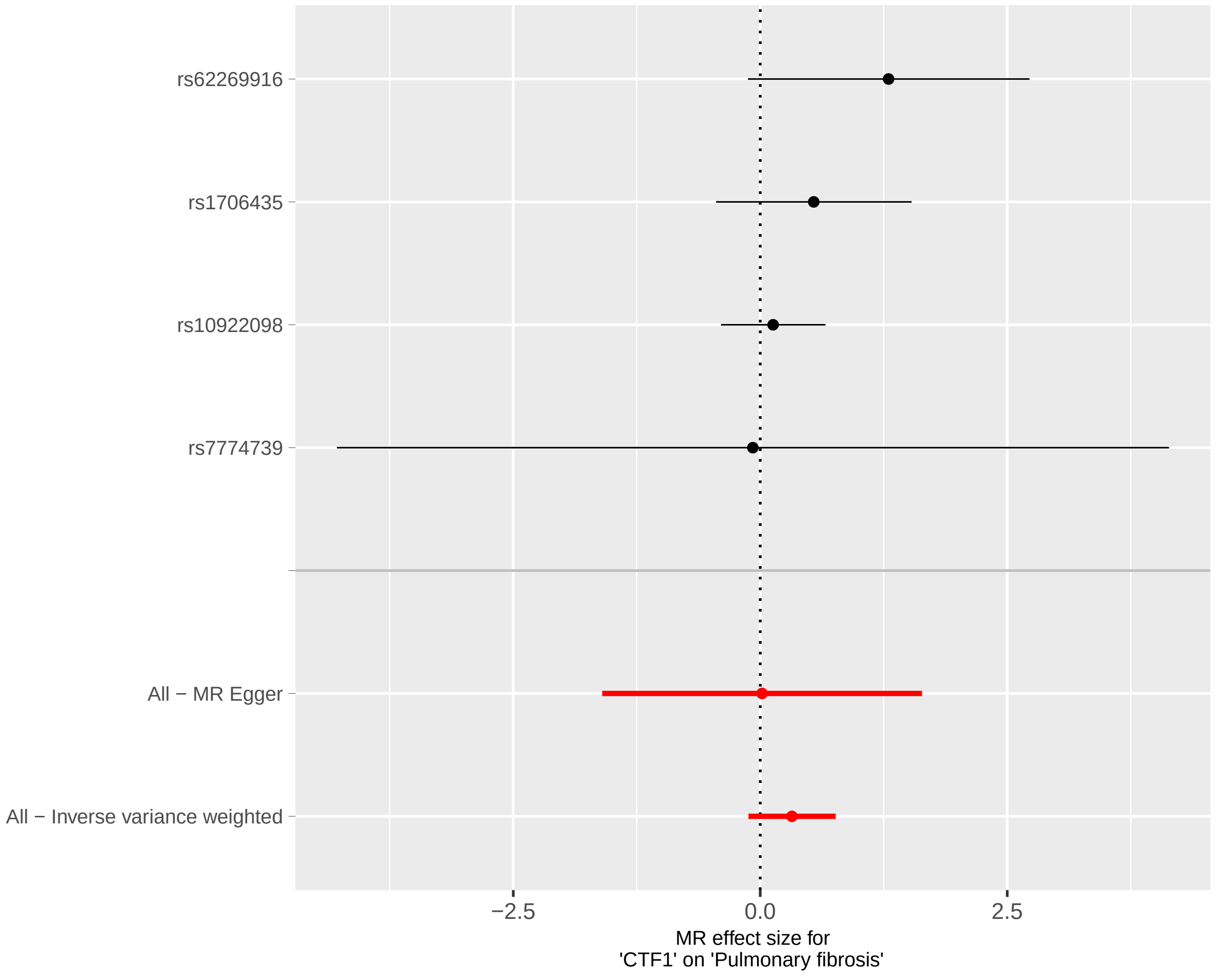
rs62269916
rs1706435
rs10922098
rs7774739
All − MR Egger
All − Inverse variance weighted
−2.5
0.0
2.5
MR effect size for
'CTF1' on 'Pulmonary fibrosis'

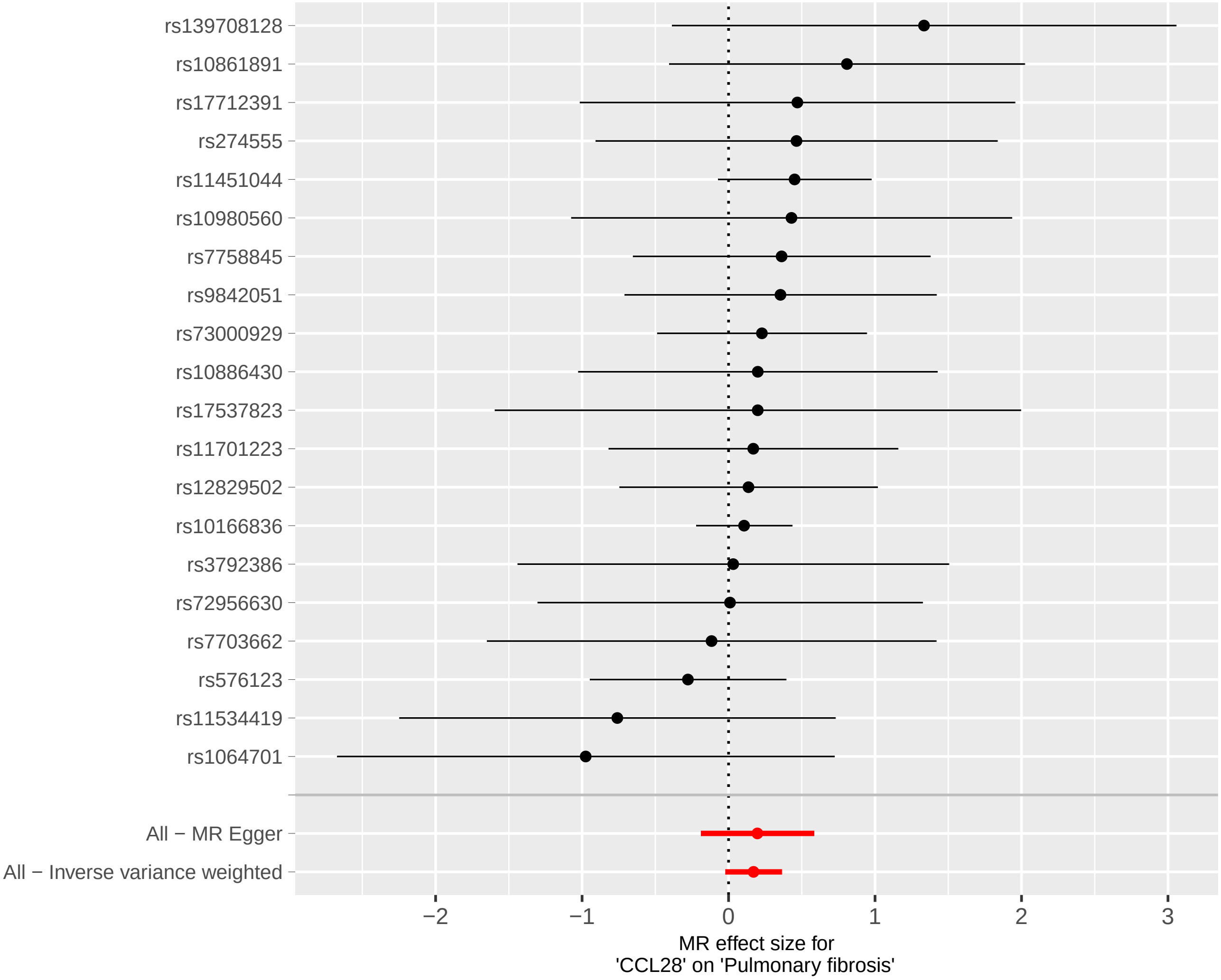

rs139708128
rs10861891
rs17712391
rs274555
rs11451044
rs10980560
rs7758845
rs9842051
rs73000929
rs10886430
rs17537823
rs11701223
rs12829502
rs10166836
rs3792386
rs72956630
rs7703662
rs576123
rs11534419
rs1064701
All − MR Egger
All − Inverse variance weighted
−2
−1
0
1
2
3
MR effect size for
'CCL28' on 'Pulmonary fibrosis'

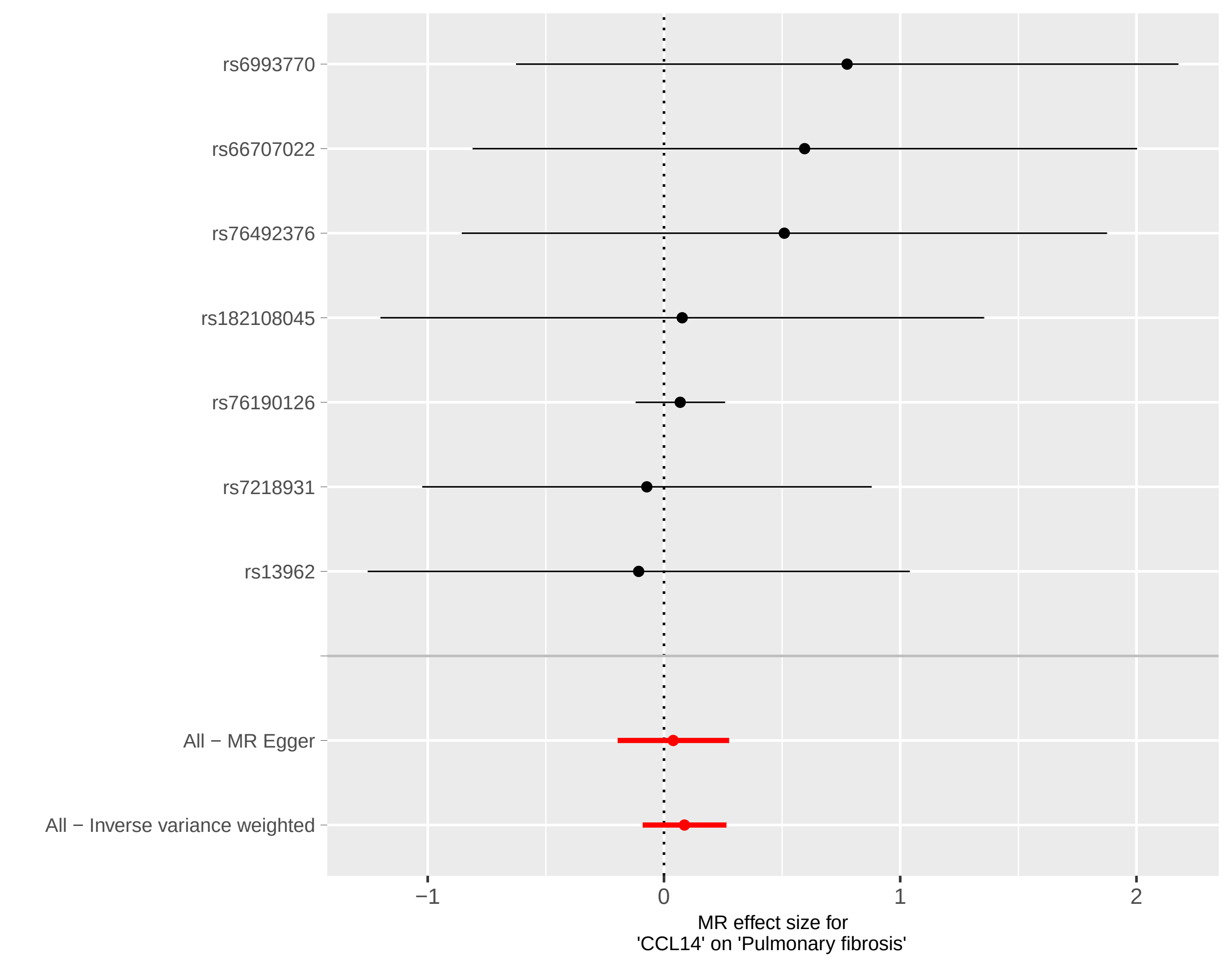

rs6993770
rs66707022
rs76492376
rs182108045
rs76190126
rs7218931
rs13962
All − MR Egger
All − Inverse variance weighted
−1
0
1
2
MR effect size for
'CCL14' on 'Pulmonary fibrosis'

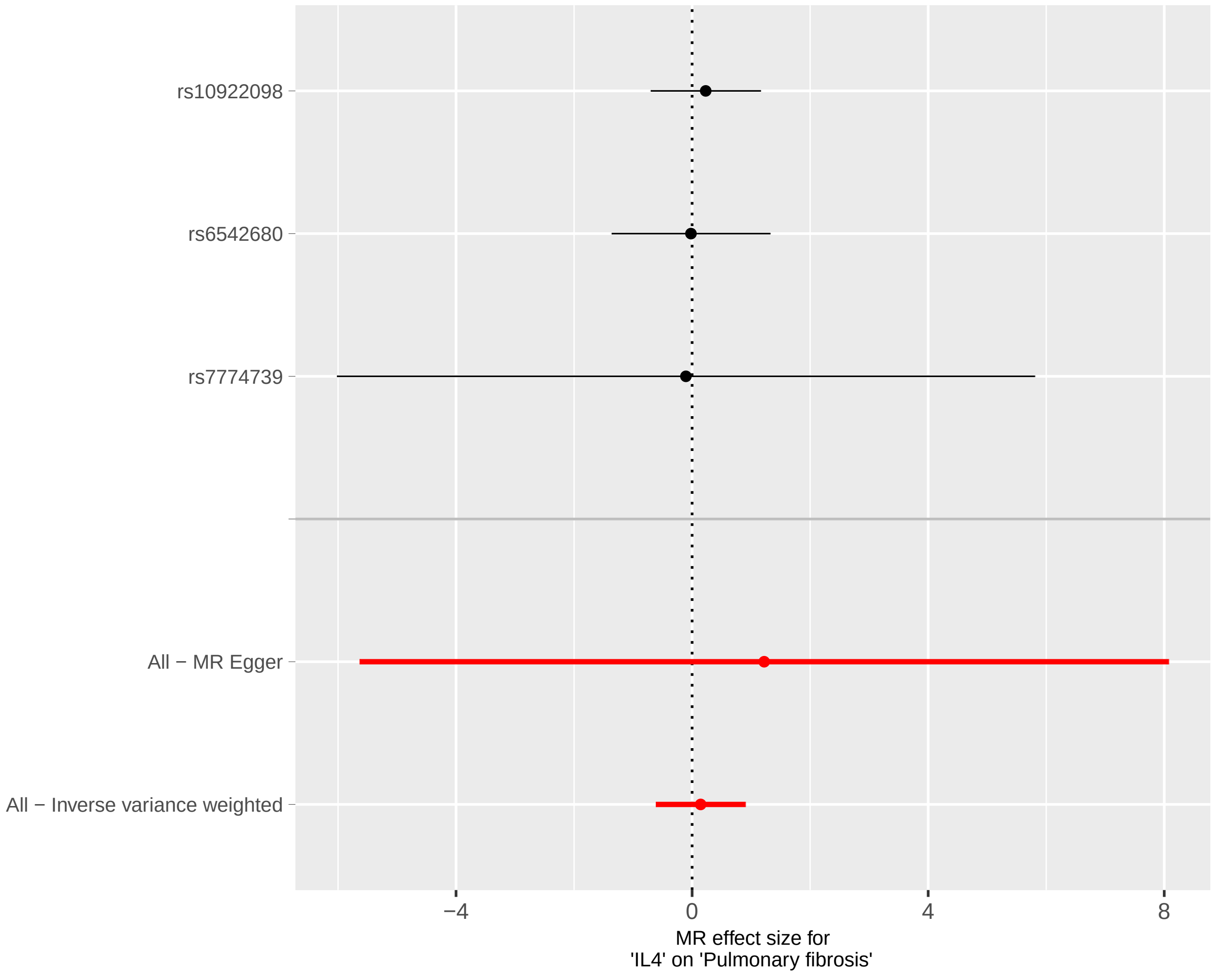

rs10922098
rs6542680
rs7774739
All − MR Egger
All − Inverse variance weighted
−4
0
4
8
MR effect size for
'IL4' on 'Pulmonary fibrosis'

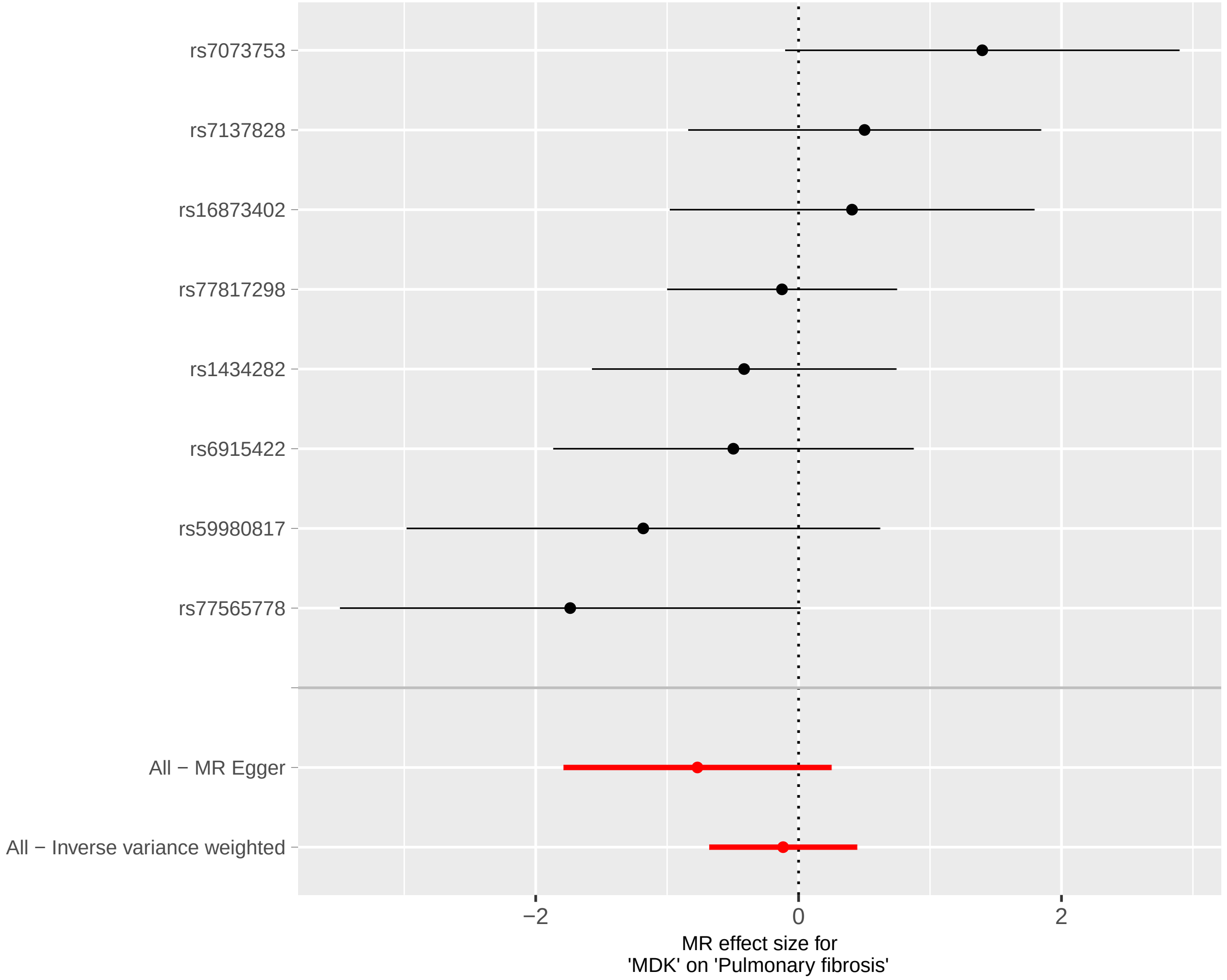

rs7073753
rs7137828
rs16873402
rs77817298
rs1434282
rs6915422
rs59980817
rs77565778
All − MR Egger
All − Inverse variance weighted
−2
0
2
MR effect size for
'MDK' on 'Pulmonary fibrosis'

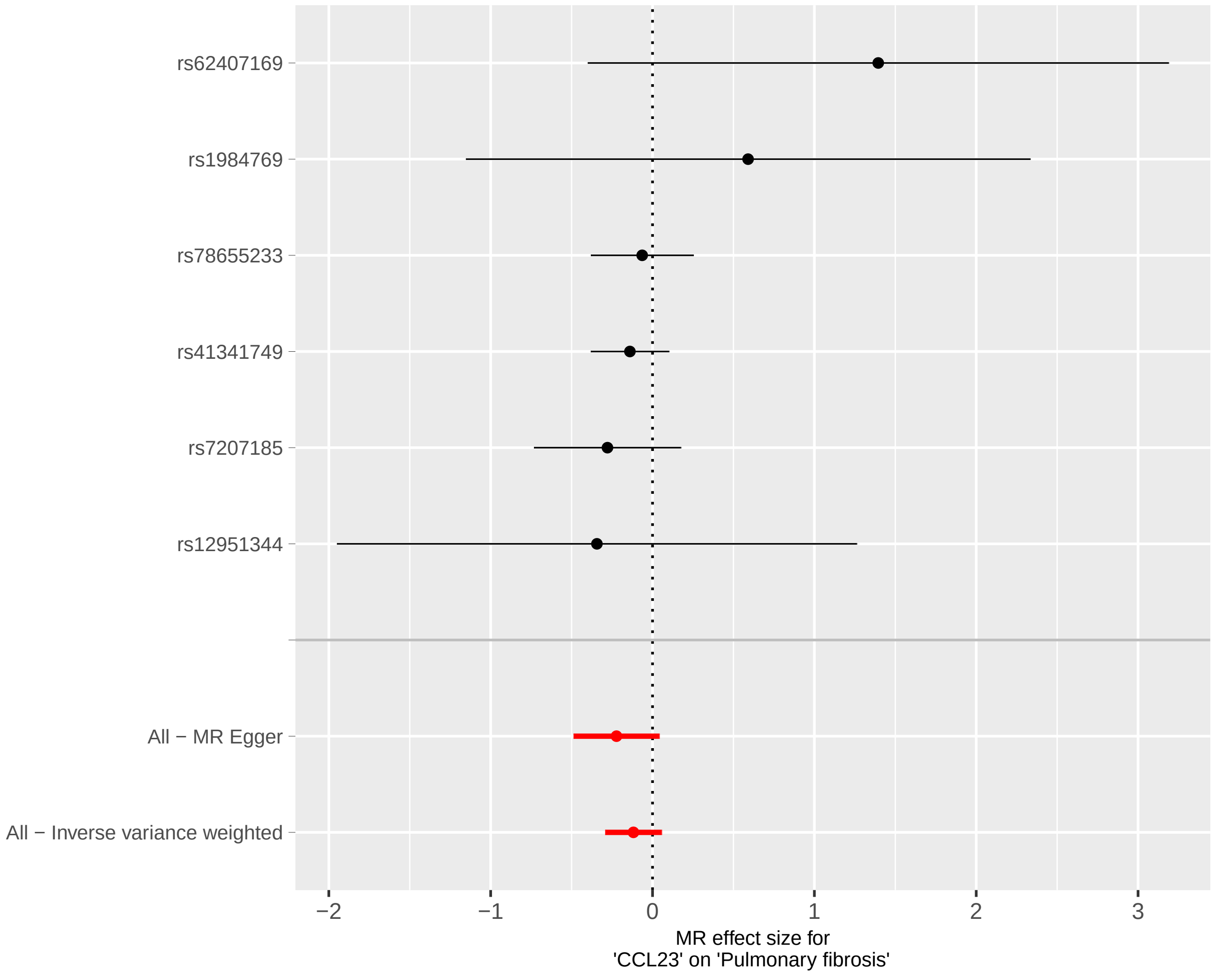

rs62407169
rs1984769
rs78655233
rs41341749
rs7207185
rs12951344
All − MR Egger
All − Inverse variance weighted
−2
−1
0
1
2
3
MR effect size for
'CCL23' on 'Pulmonary fibrosis'

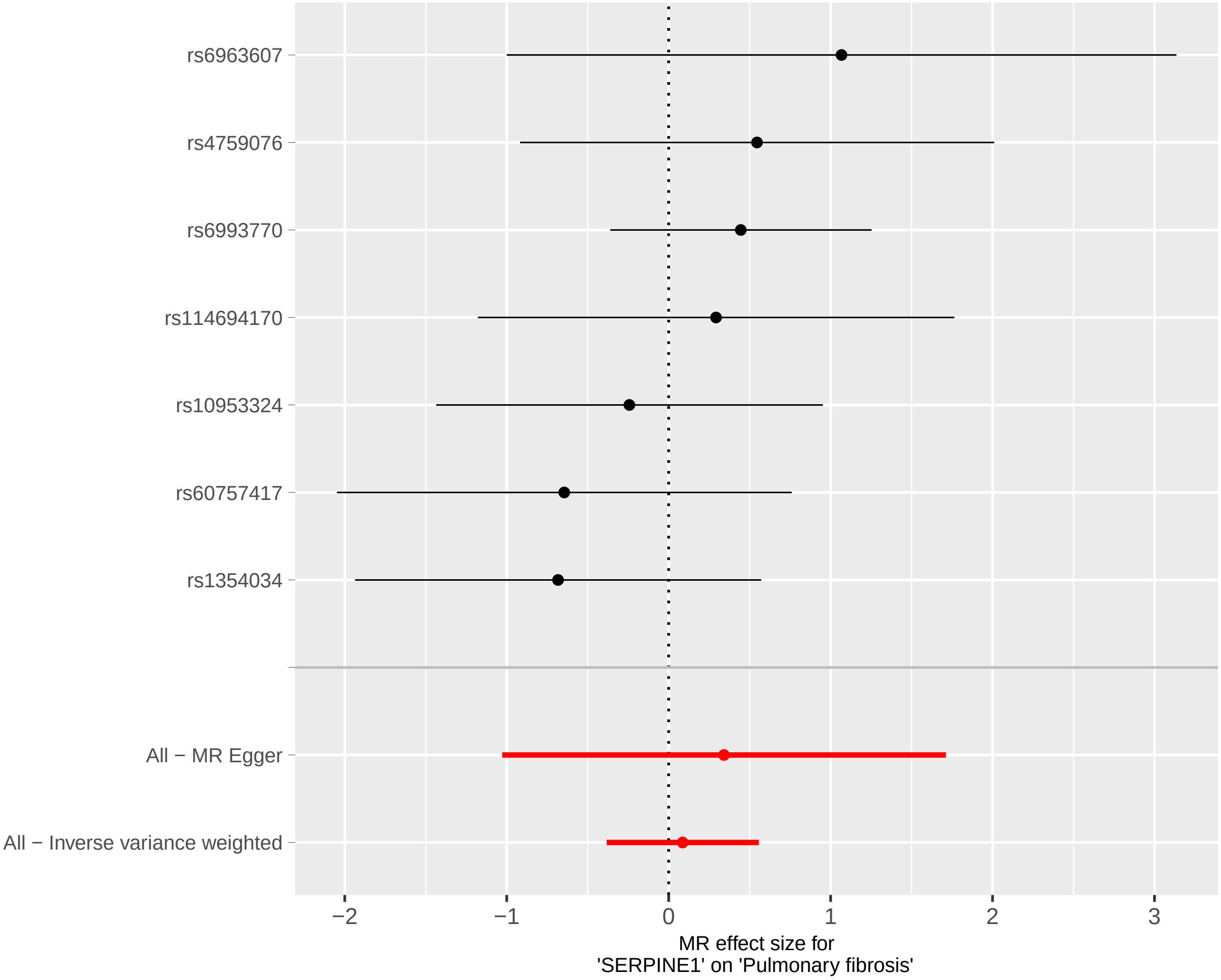
rs6963607
rs4759076
rs6993770
rs114694170
rs10953324
rs60757417
rs1354034
All − MR Egger
All − Inverse variance weighted
−2
−1
0
1
2
3
MR effect size for
'SERPINE1' on 'Pulmonary fibrosis'

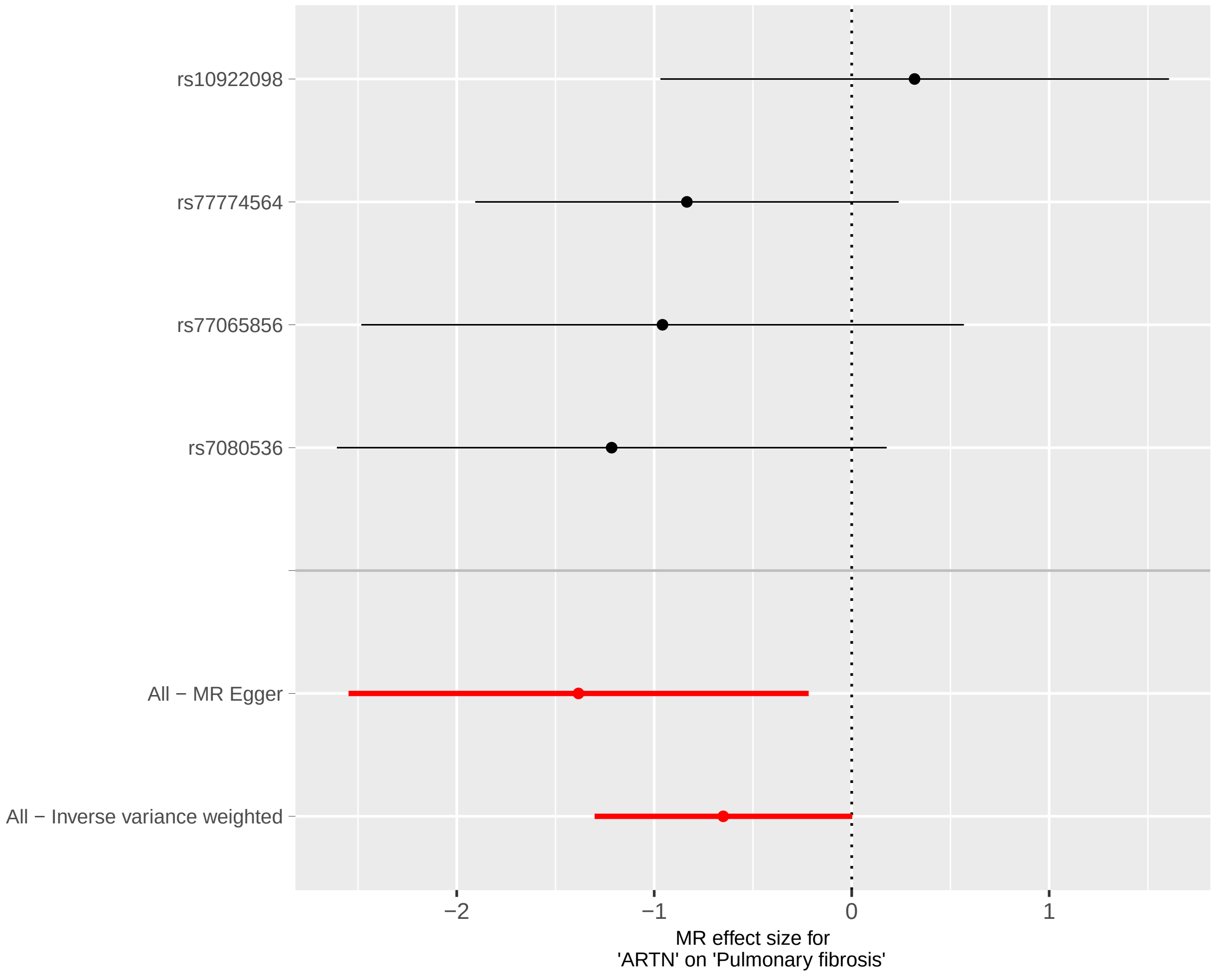
rs10922098
rs77774564
rs77065856
rs7080536
All − MR Egger
All − Inverse variance weighted
−2
−1
0
1
MR effect size for
'ARTN' on 'Pulmonary fibrosis'

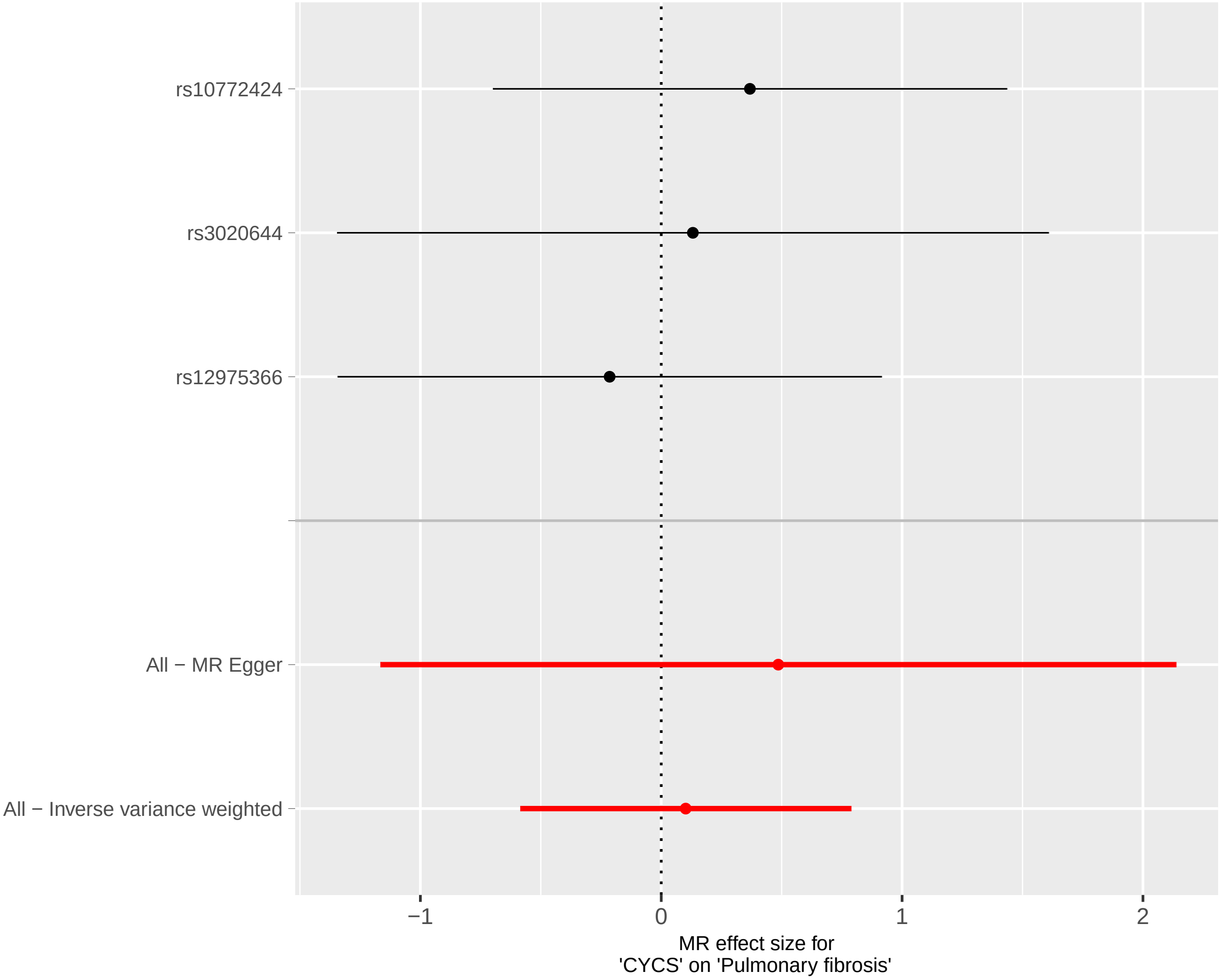
rs10772424
rs3020644
rs12975366
All − MR Egger
All − Inverse variance weighted
−1
0
1
2
MR effect size for
'CYCS' on 'Pulmonary fibrosis'

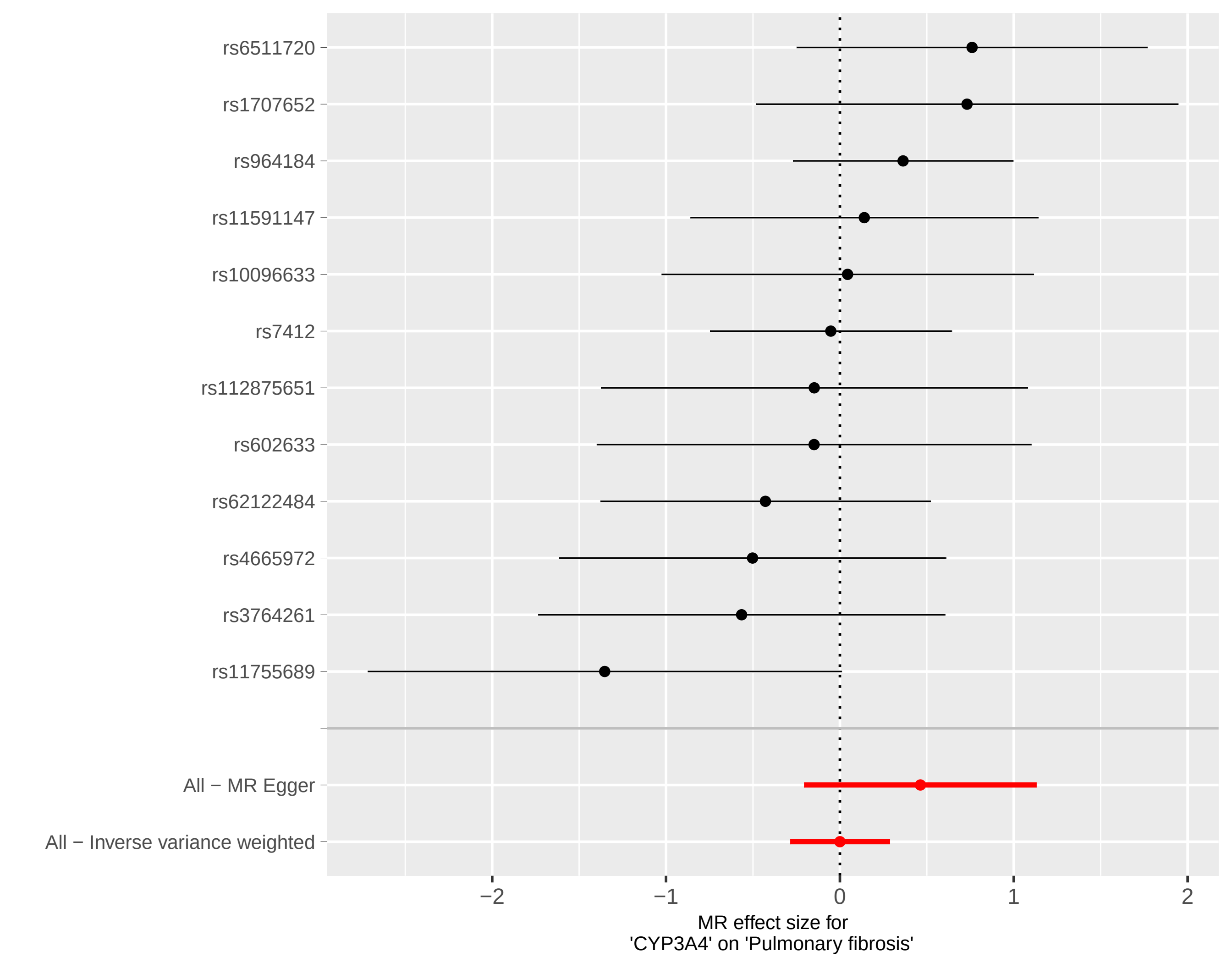

rs6511720
rs1707652
rs964184
rs11591147
rs10096633
rs7412
rs112875651
rs602633
rs62122484
rs4665972
rs3764261
rs11755689
All − MR Egger
All − Inverse variance weighted
−2
−1
0
1
2
MR effect size for
'CYP3A4' on 'Pulmonary fibrosis'

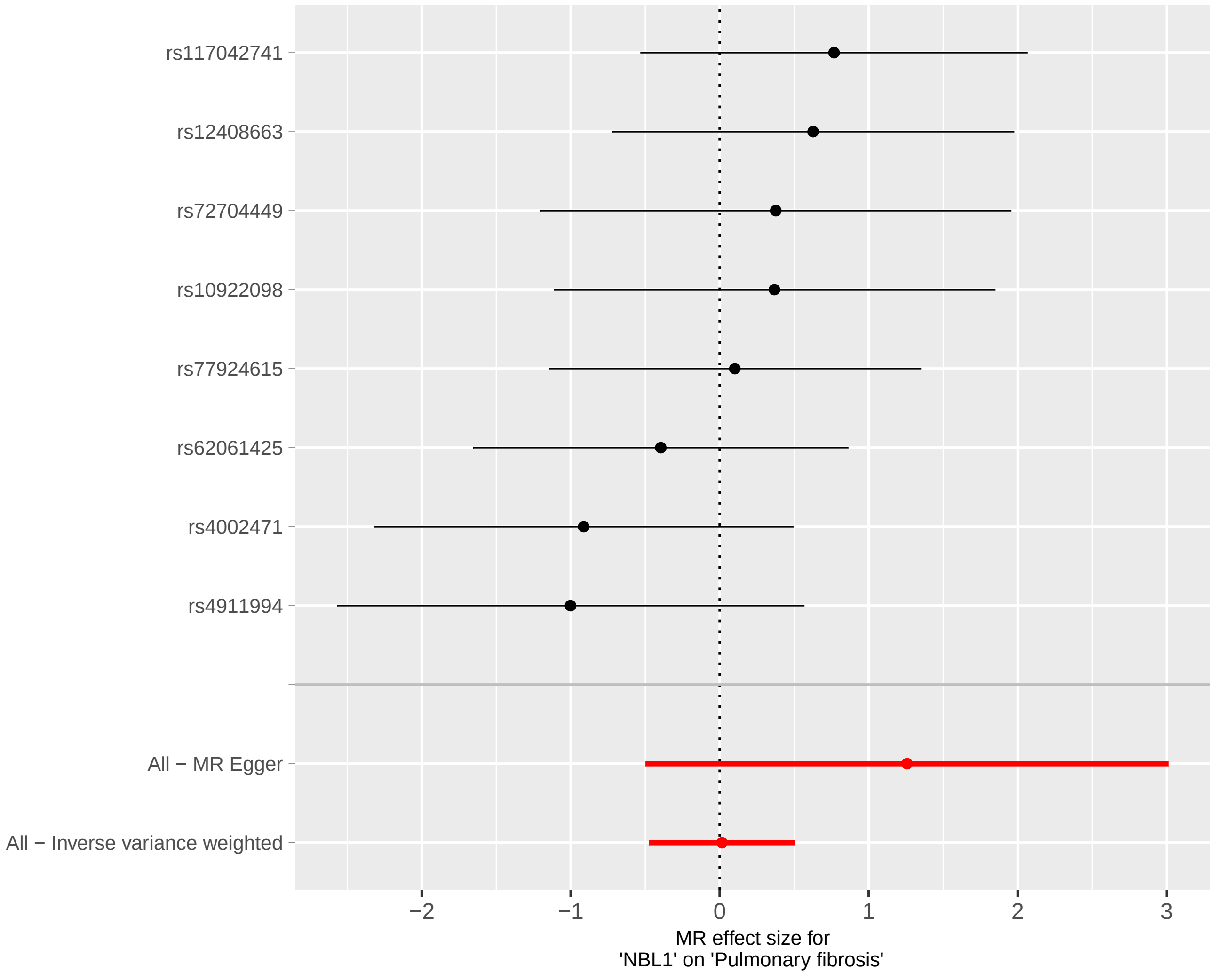
rs117042741
rs12408663
rs72704449
rs10922098
rs77924615
rs62061425
rs4002471
rs4911994
All − MR Egger
All − Inverse variance weighted
−2
−1
0
1
2
3
MR effect size for
'NBL1' on 'Pulmonary fibrosis'

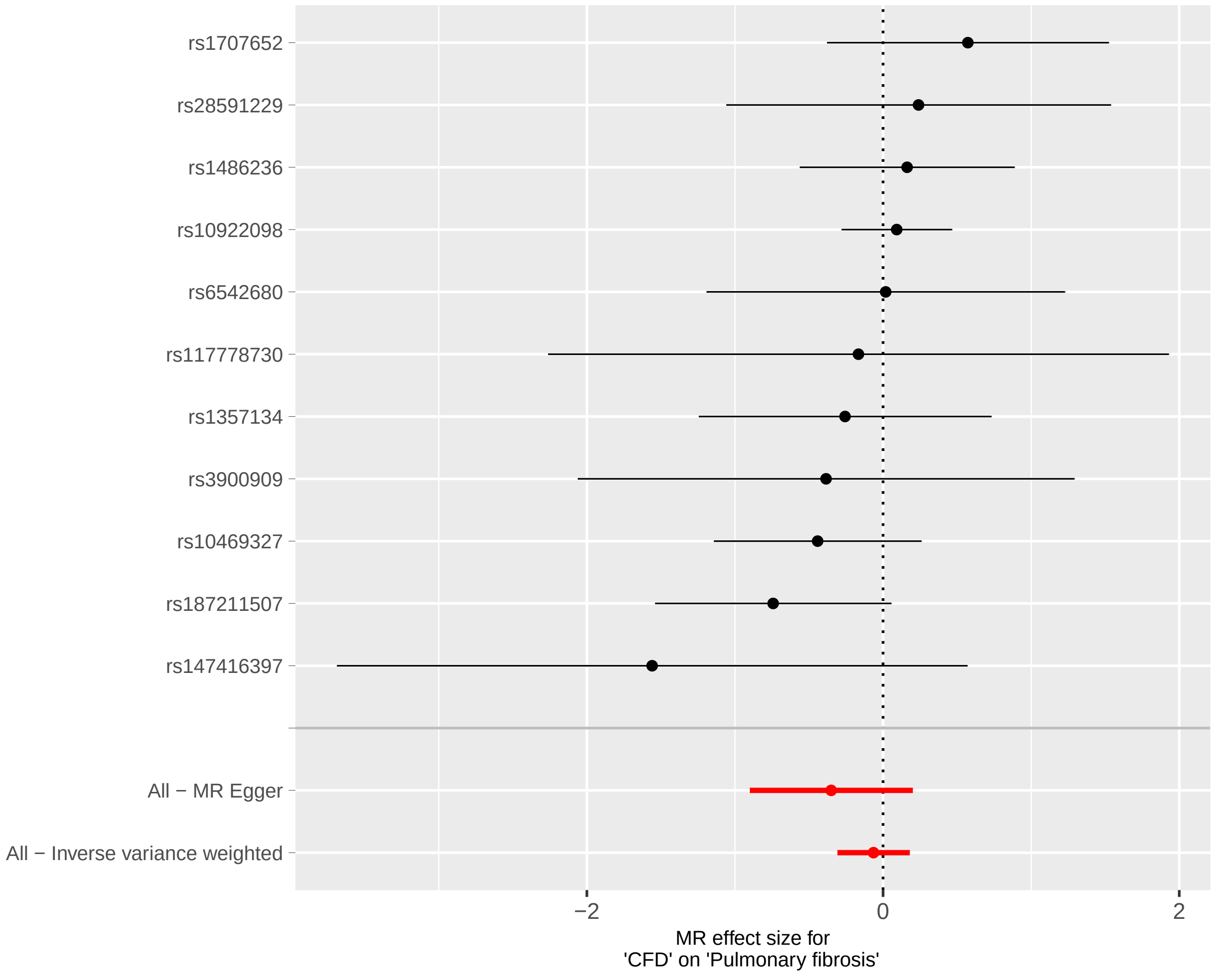
rs1707652
rs28591229
rs1486236
rs10922098
rs6542680
rs117778730
rs1357134
rs3900909
rs10469327
rs187211507
rs147416397
All − MR Egger
All − Inverse variance weighted
−2
0
2
MR effect size for
'CFD' on 'Pulmonary fibrosis'

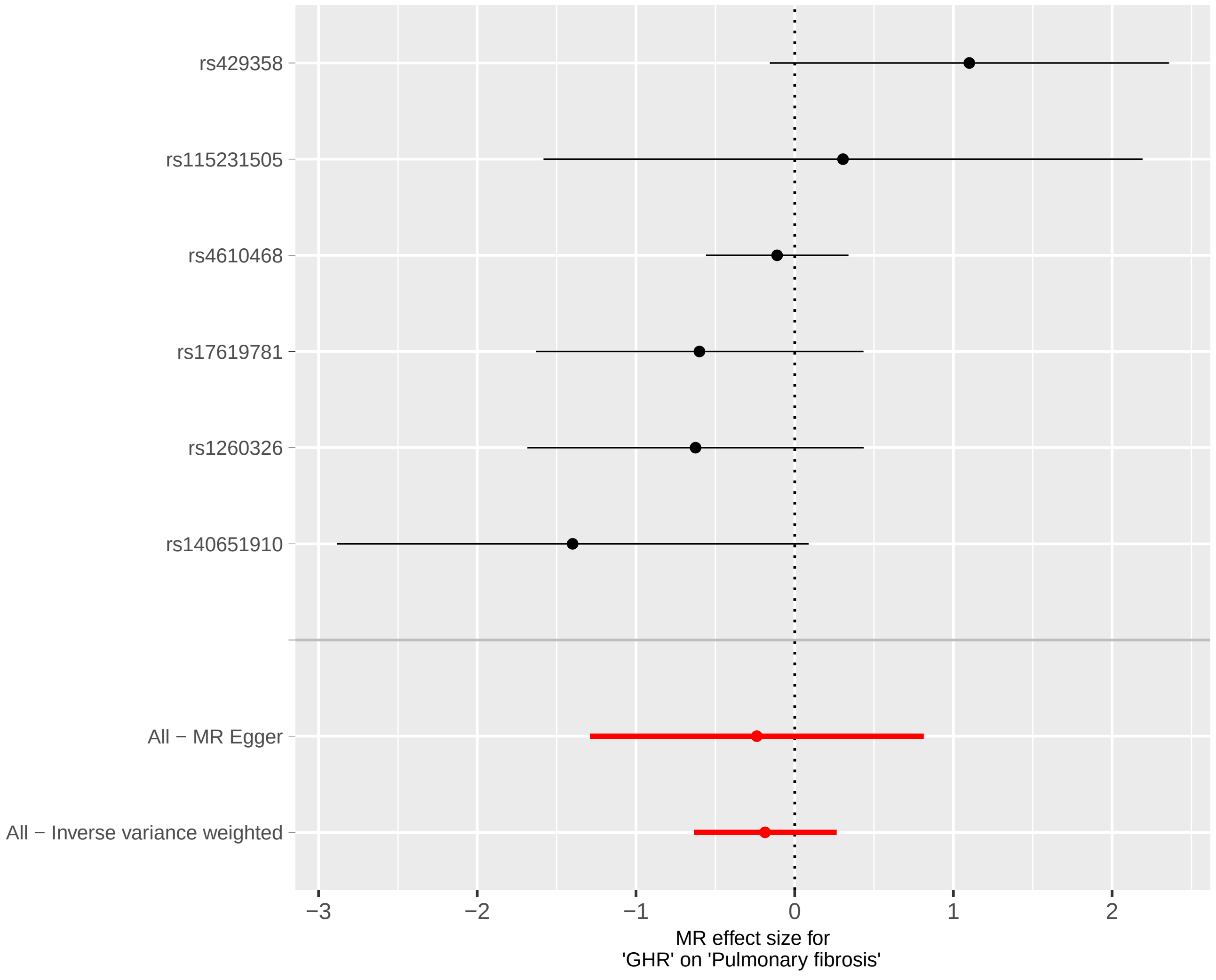

rs429358
rs115231505
rs4610468
rs17619781
rs1260326
rs140651910
All − MR Egger
All − Inverse variance weighted
−3
−2
−1
0
1
2
MR effect size for
'GHR' on 'Pulmonary fibrosis'

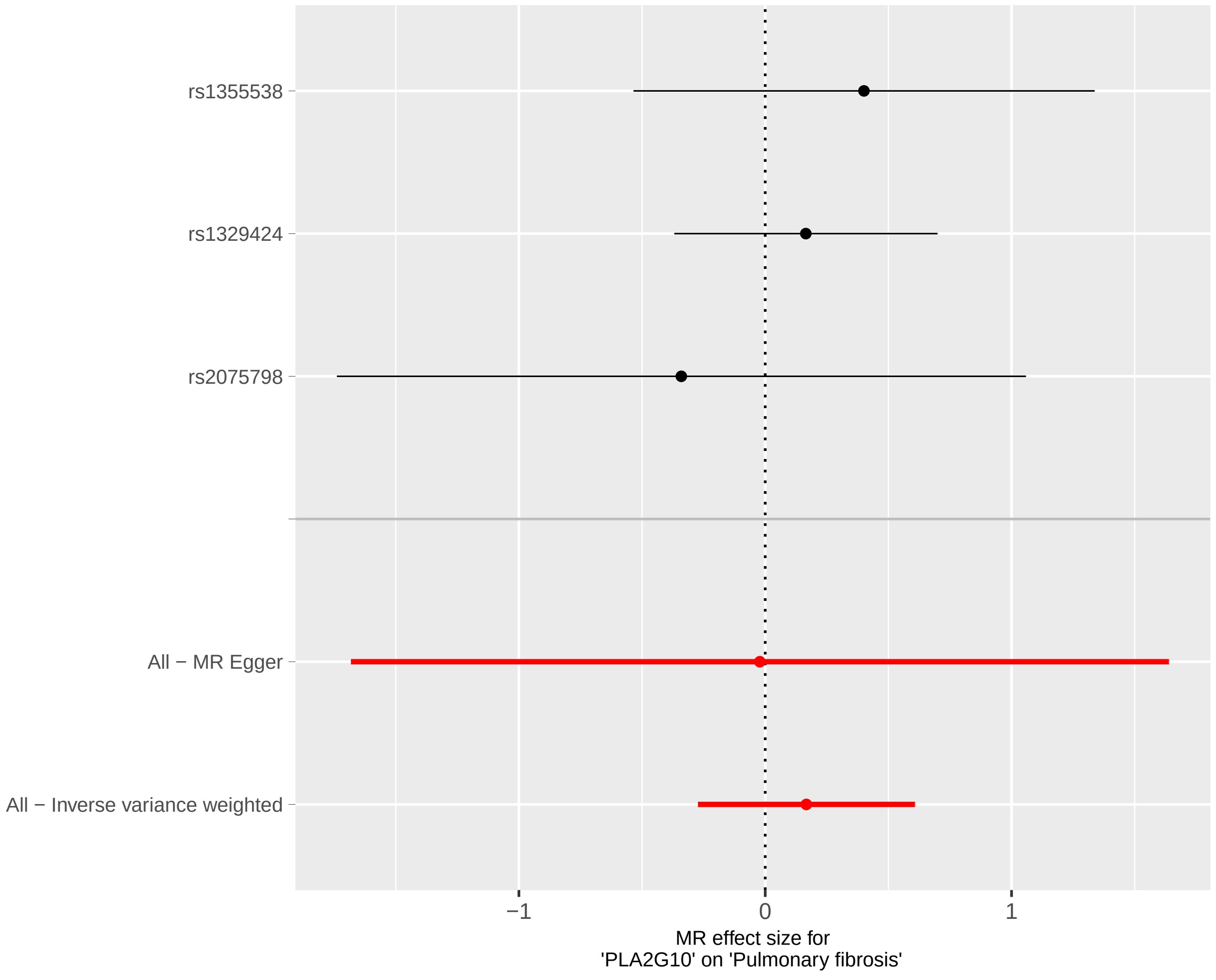

rs1355538
rs1329424
rs2075798
All − MR Egger
All − Inverse variance weighted
−1
0
1
MR effect size for
'PLA2G10' on 'Pulmonary fibrosis'

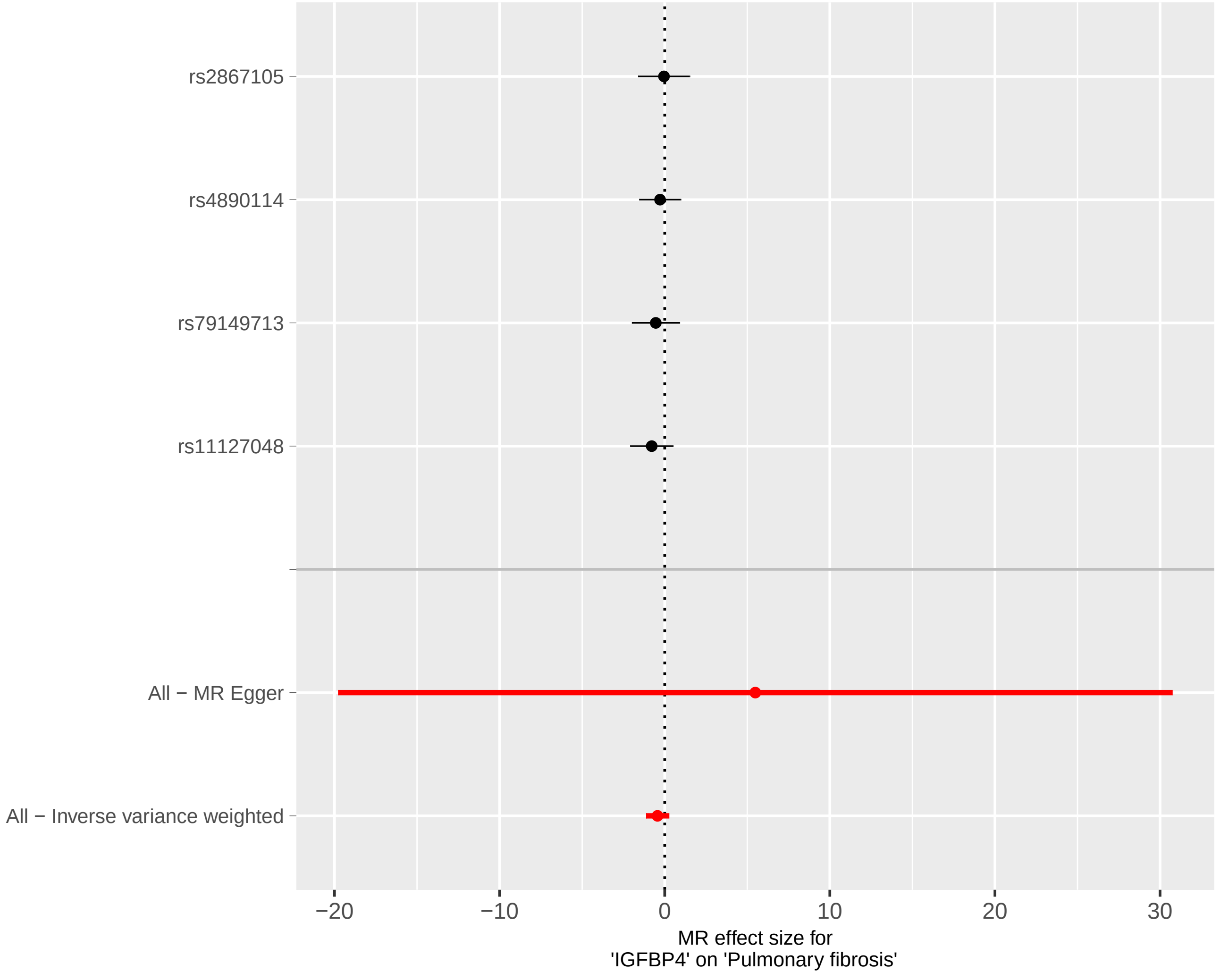

rs2867105
rs4890114
rs79149713
rs11127048
All − MR Egger
All − Inverse variance weighted
−20
−10
0
10
20
30
MR effect size for
'IGFBP4' on 'Pulmonary fibrosis'

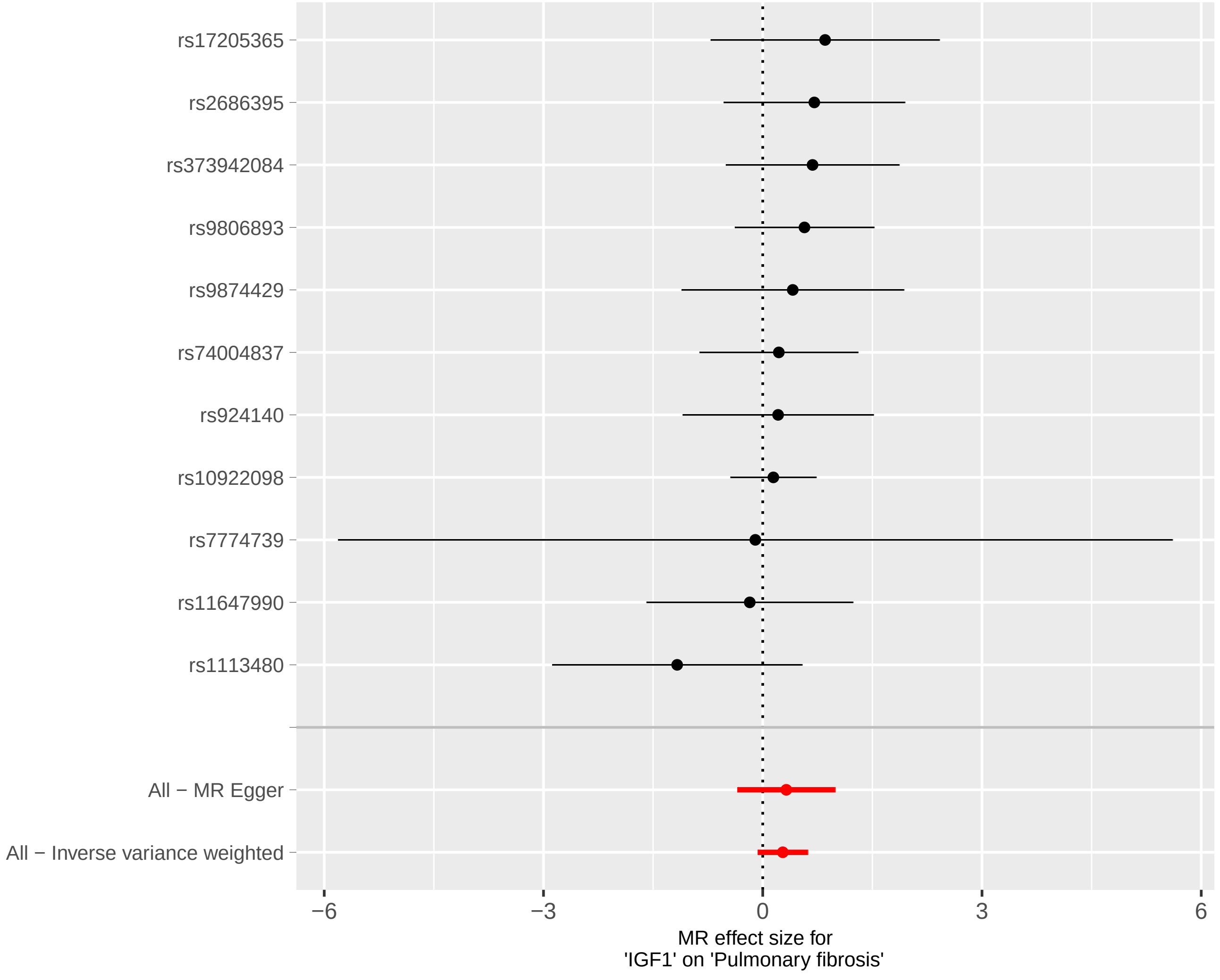

rs17205365
rs2686395
rs373942084
rs9806893
rs9874429
rs74004837
rs924140
rs10922098
rs7774739
rs11647990
rs1113480
All − MR Egger
All − Inverse variance weighted
−6
−3
0
3
6
MR effect size for
'IGF1' on 'Pulmonary fibrosis'

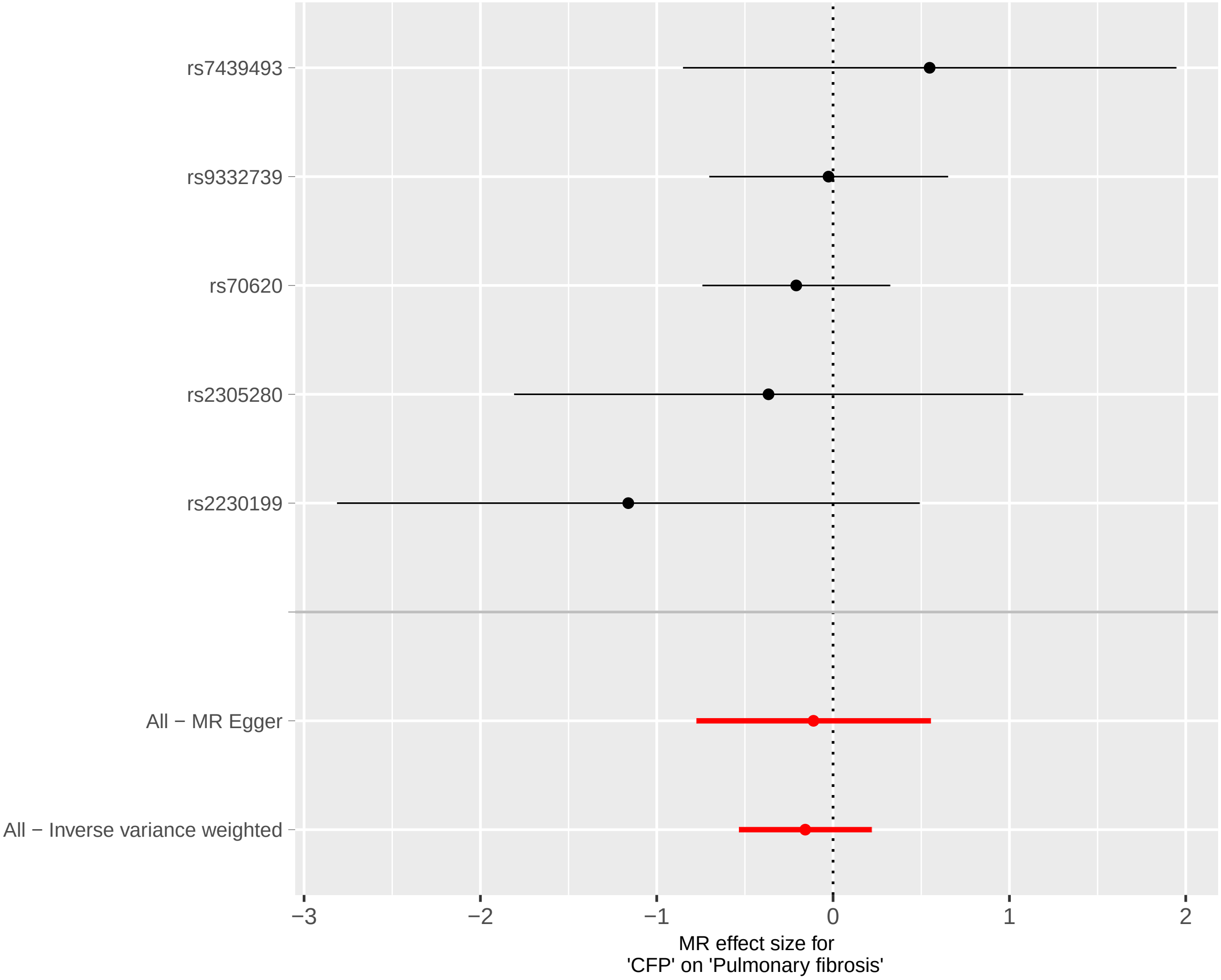
rs7439493
rs9332739
rs70620
rs2305280
rs2230199
All − MR Egger
All − Inverse variance weighted
−3
−2
−1
0
1
2
MR effect size for
'CFP' on 'Pulmonary fibrosis'

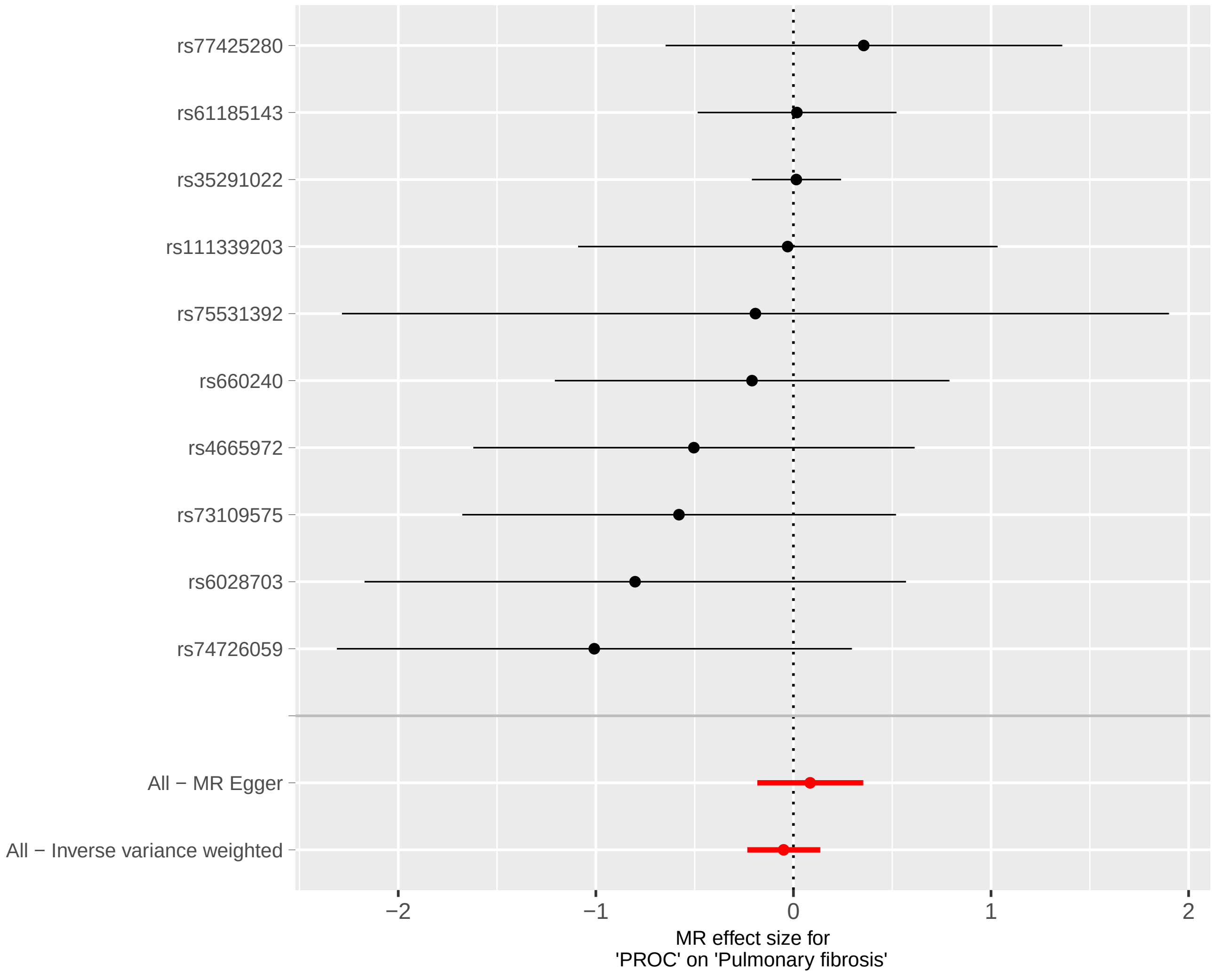

rs77425280
rs61185143
rs35291022
rs111339203
rs75531392
rs660240
rs4665972
rs73109575
rs6028703
rs74726059
All − MR Egger
All − Inverse variance weighted
−2
−1
0
1
2
MR effect size for
'PROC' on 'Pulmonary fibrosis'

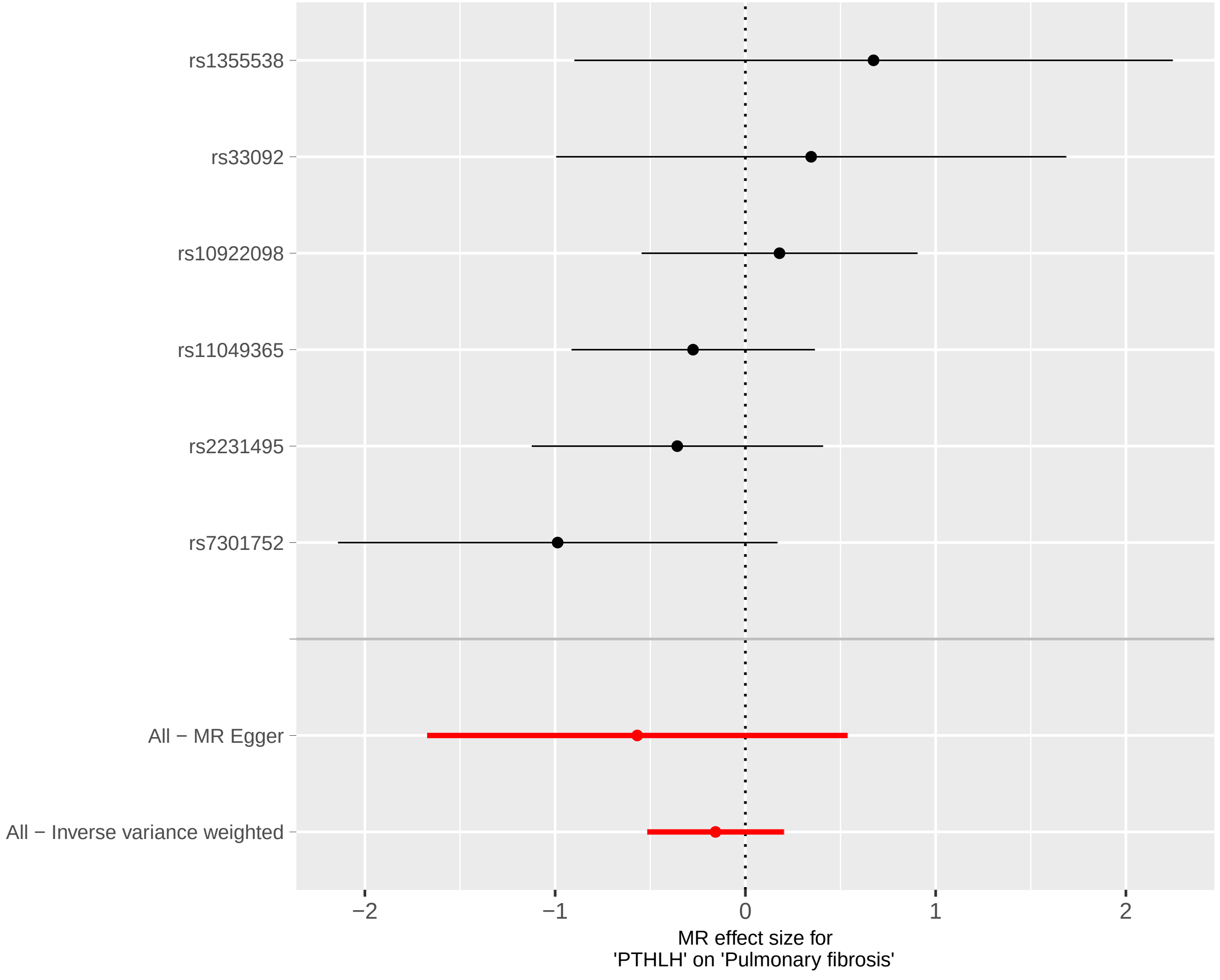
rs1355538
rs33092
rs10922098
rs11049365
rs2231495
rs7301752
All − MR Egger
All − Inverse variance weighted
−2
−1
0
1
2
MR effect size for
'PTHLH' on 'Pulmonary fibrosis'

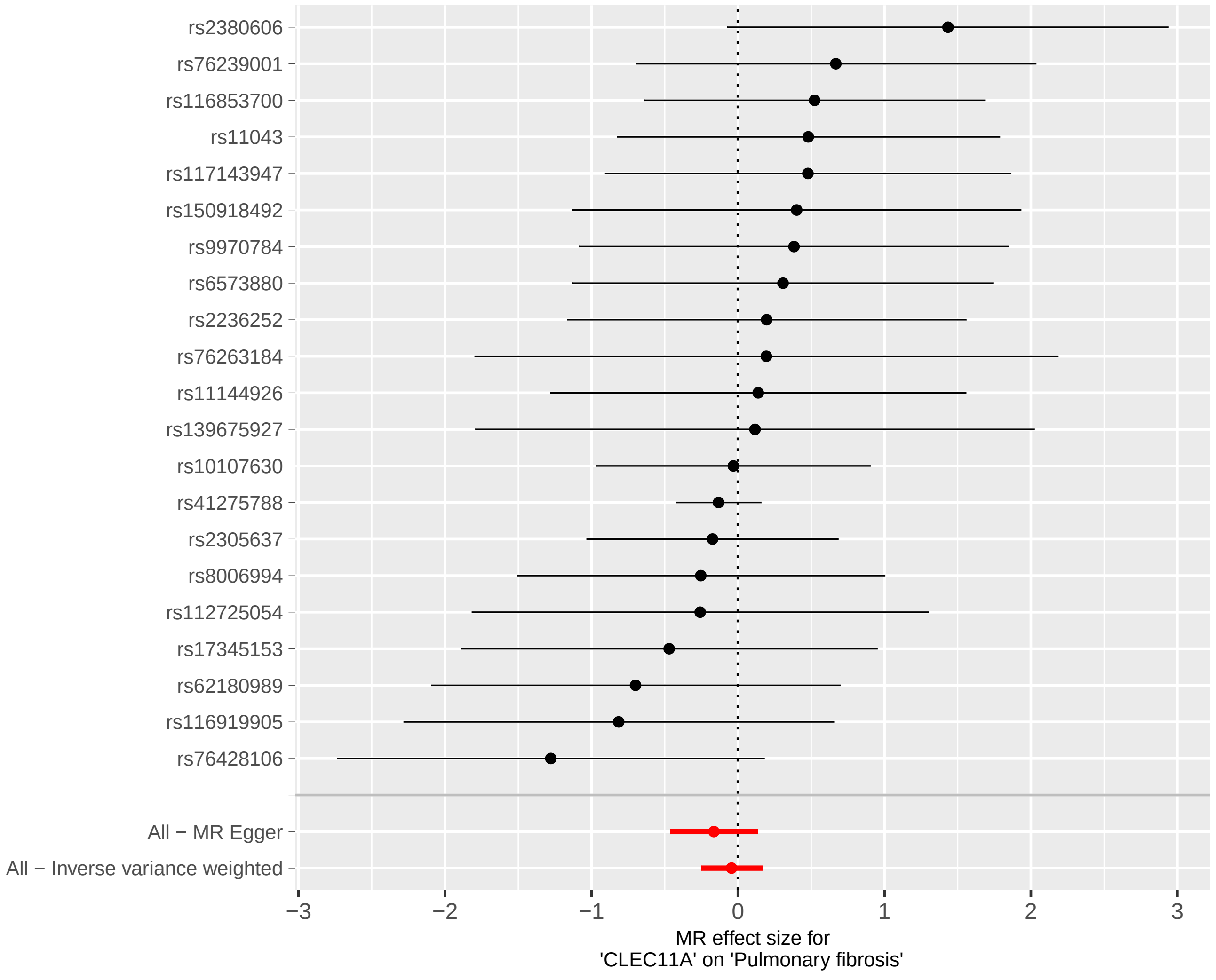

rs2380606
rs76239001
rs116853700
rs11043
rs117143947
rs150918492
rs9970784
rs6573880
rs2236252
rs76263184
rs11144926
rs139675927
rs10107630
rs41275788
rs2305637
rs8006994
rs112725054
rs17345153
rs62180989
rs116919905
rs76428106
All − MR Egger
All − Inverse variance weighted
−3
−2
−1
0
1
2
3
MR effect size for
'CLEC11A' on 'Pulmonary fibrosis'

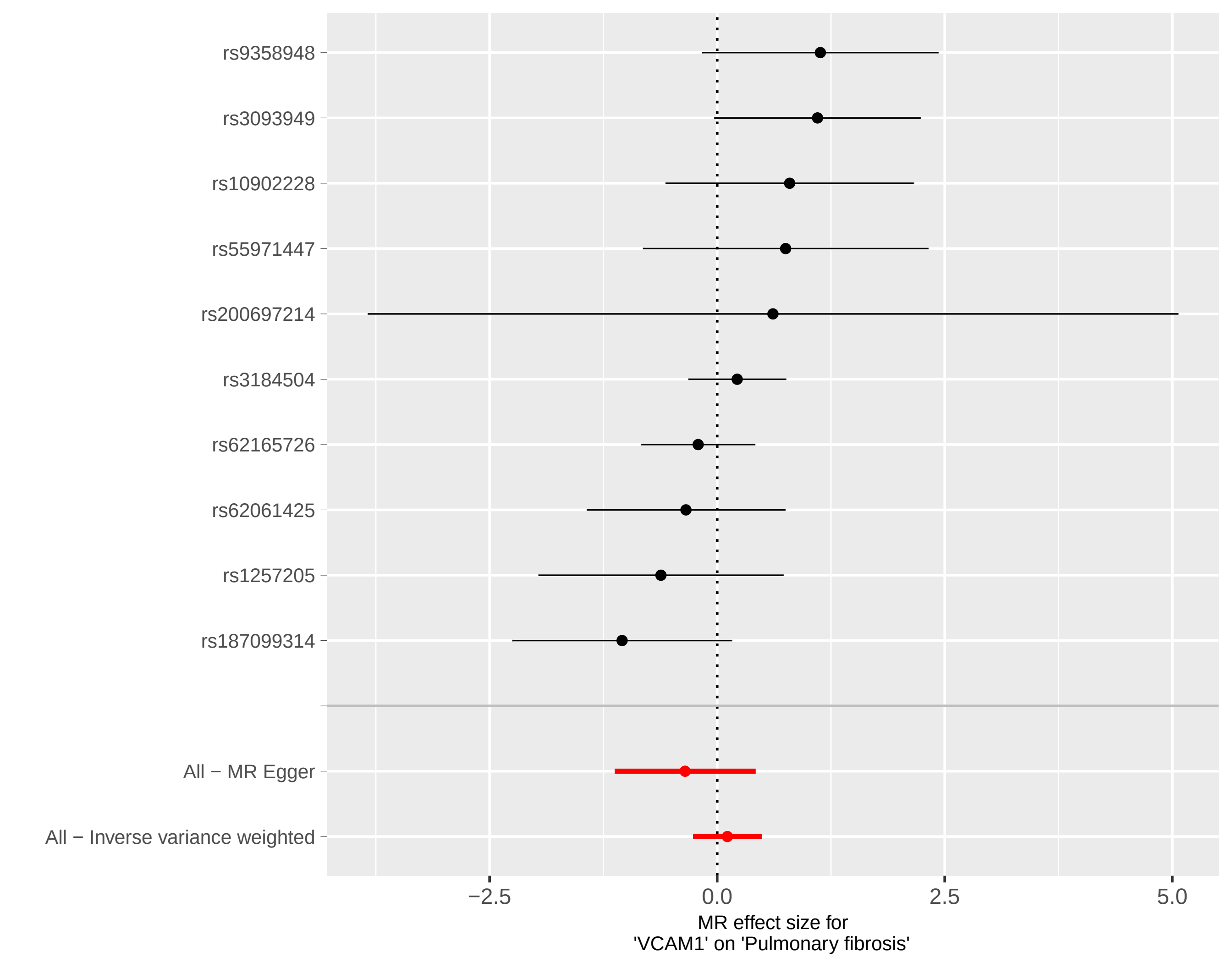
rs9358948
rs3093949
rs10902228
rs55971447
rs200697214
rs3184504
rs62165726
rs62061425
rs1257205
rs187099314
All − MR Egger
All − Inverse variance weighted
−2.5
0.0
2.5
5.0
MR effect size for
'VCAM1' on 'Pulmonary fibrosis'

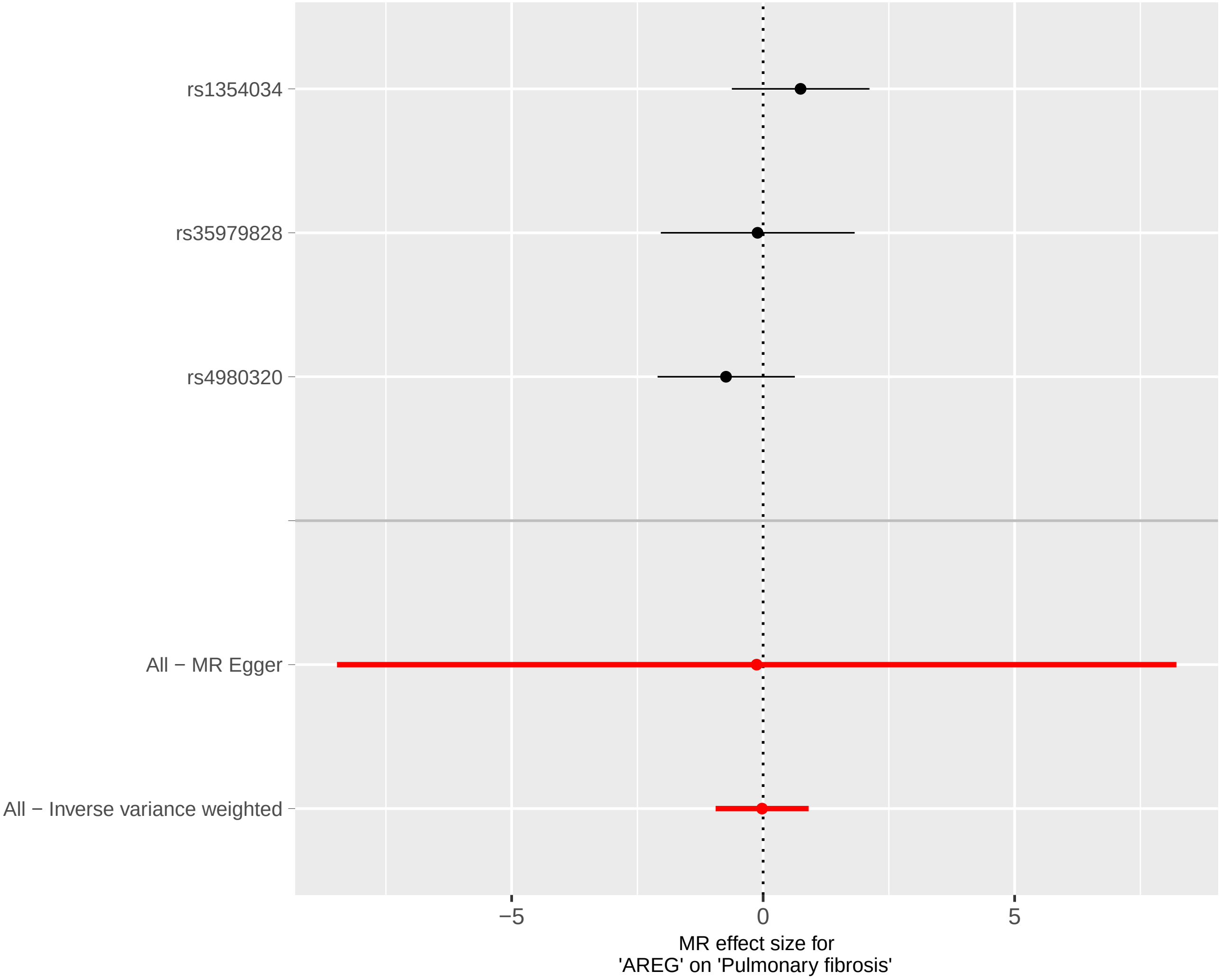
rs1354034
rs35979828
rs4980320
All − MR Egger
All − Inverse variance weighted
−5
0
5
MR effect size for
'AREG' on 'Pulmonary fibrosis'

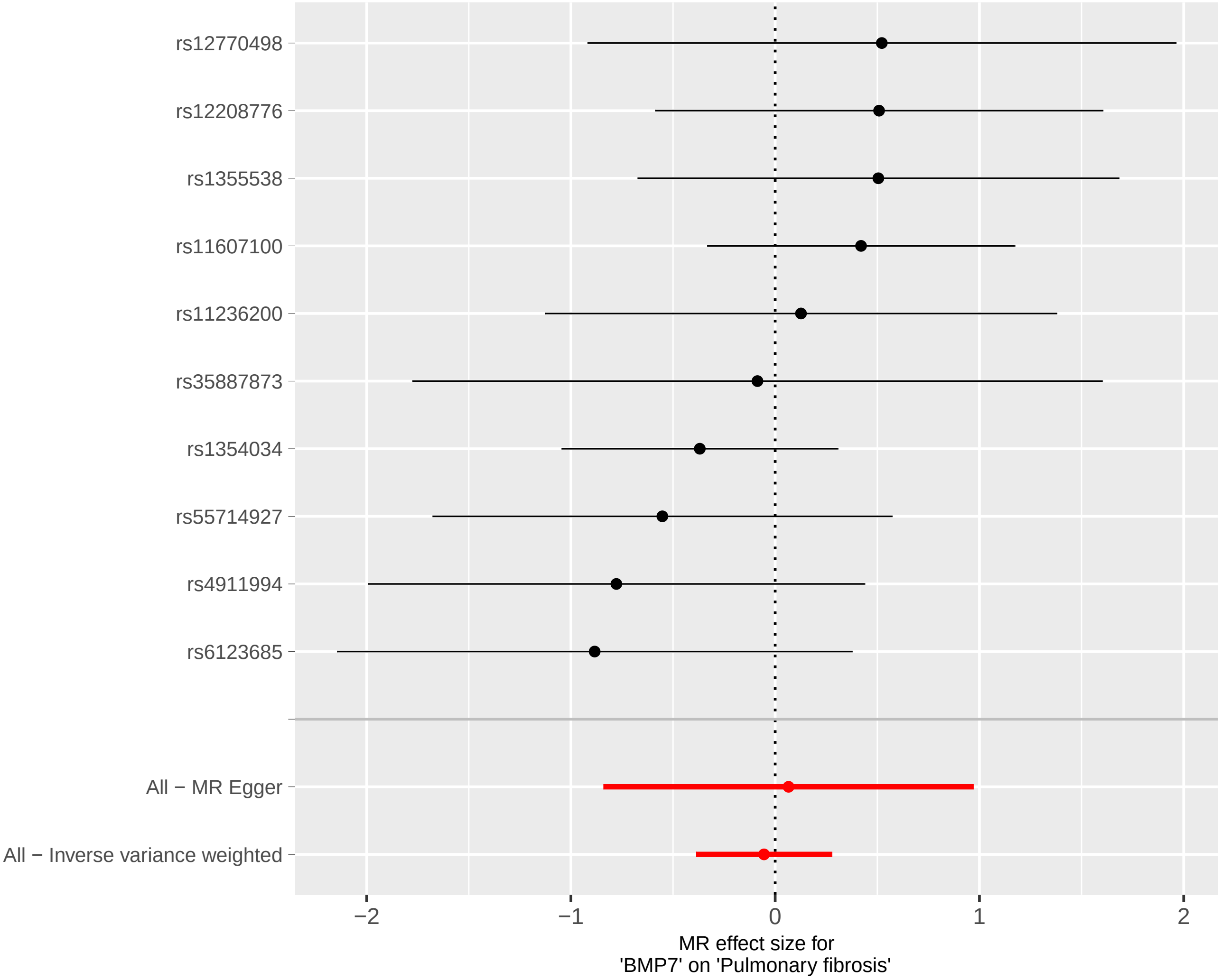

rs12770498
rs12208776
rs1355538
rs11607100
rs11236200
rs35887873
rs1354034
rs55714927
rs4911994
rs6123685
All − MR Egger
All − Inverse variance weighted
−2
−1
0
1
2
MR effect size for
'BMP7' on 'Pulmonary fibrosis'

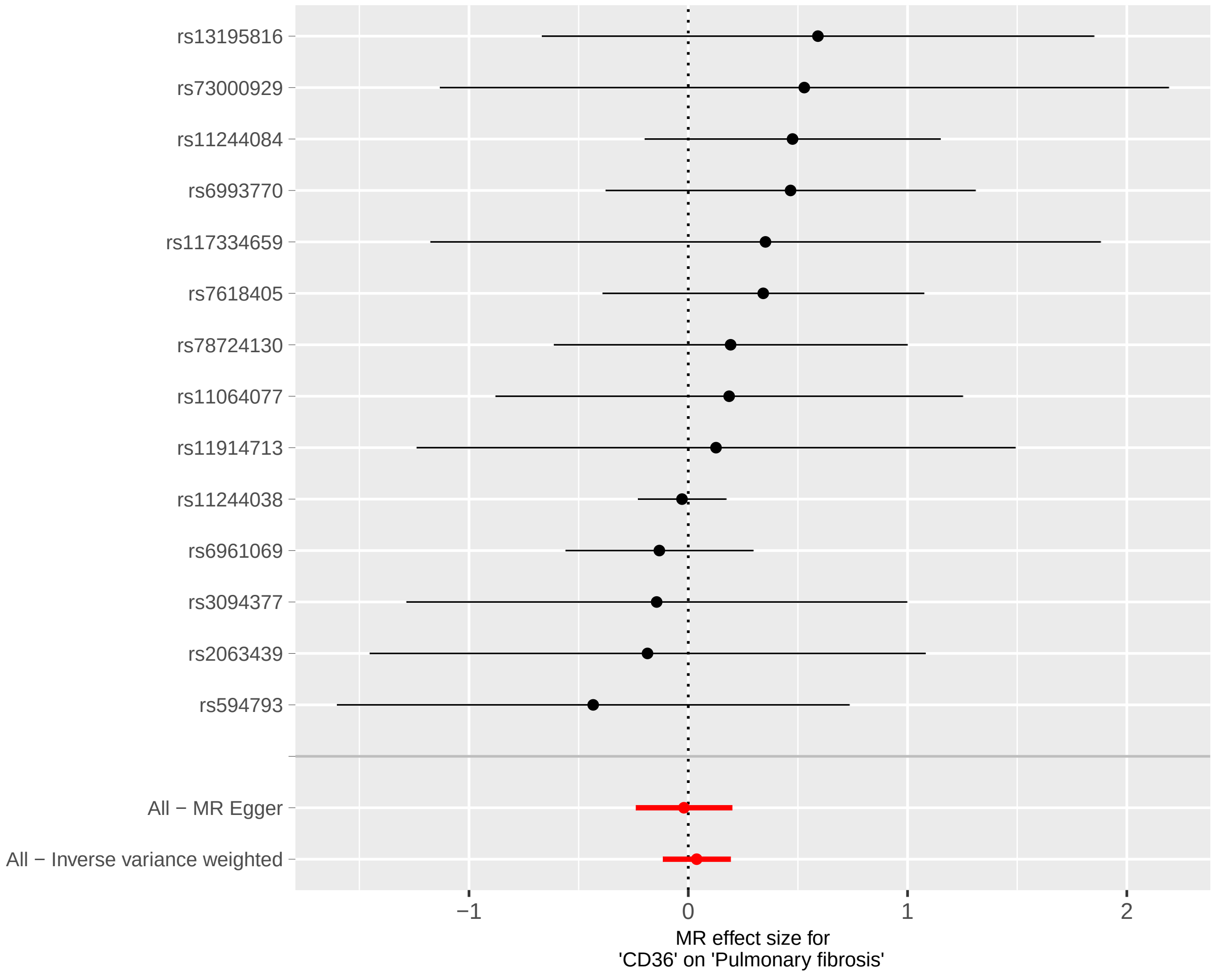
rs13195816
rs73000929
rs11244084
rs6993770
rs117334659
rs7618405
rs78724130
rs11064077
rs11914713
rs11244038
rs6961069
rs3094377
rs2063439
rs594793
All − MR Egger
All − Inverse variance weighted
−1
0
1
2
MR effect size for
'CD36' on 'Pulmonary fibrosis'

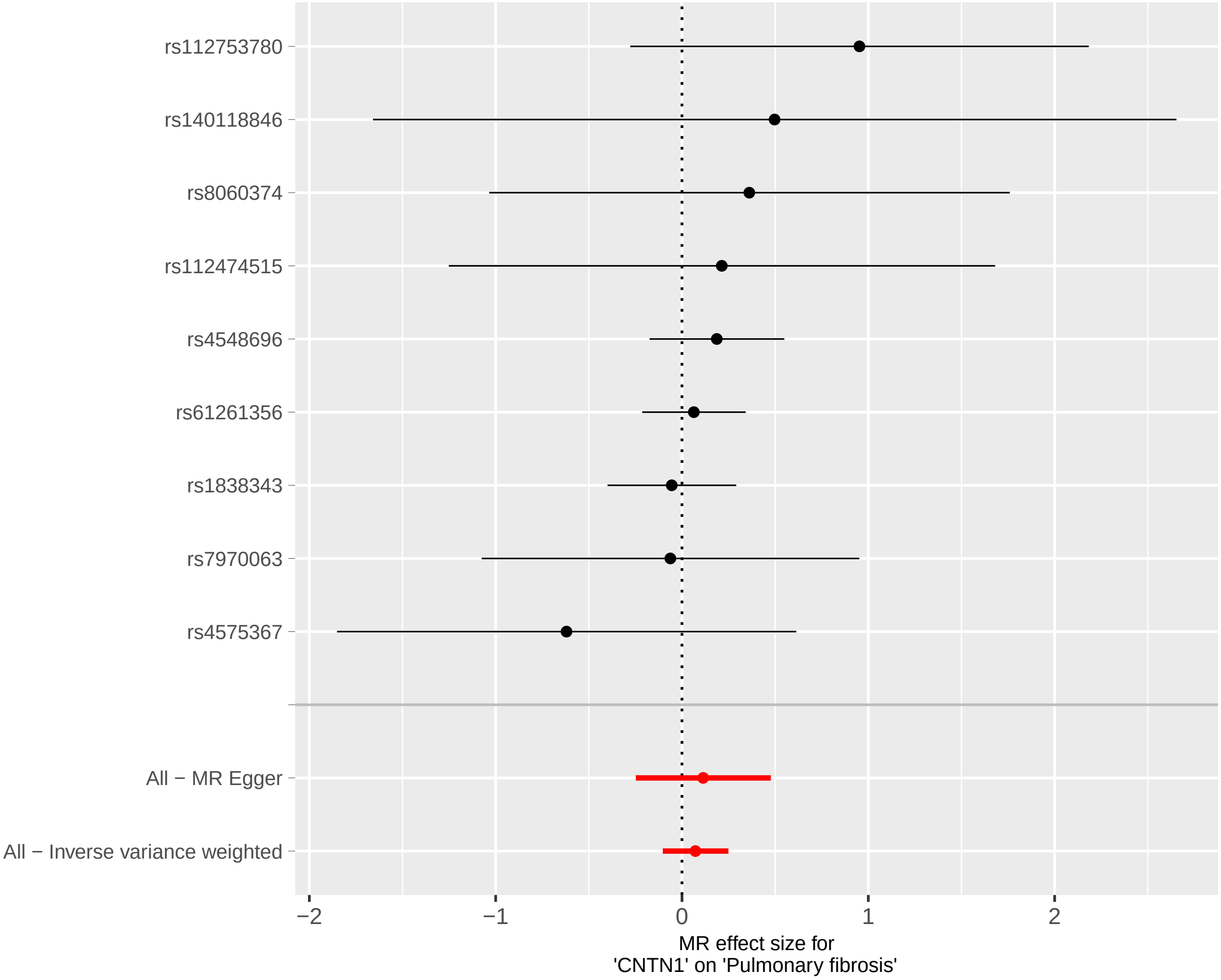
rs112753780
rs140118846
rs8060374
rs112474515
rs4548696
rs61261356
rs1838343
rs7970063
rs4575367
All − MR Egger
All − Inverse variance weighted
−2
−1
0
1
2
MR effect size for
'CNTN1' on 'Pulmonary fibrosis'

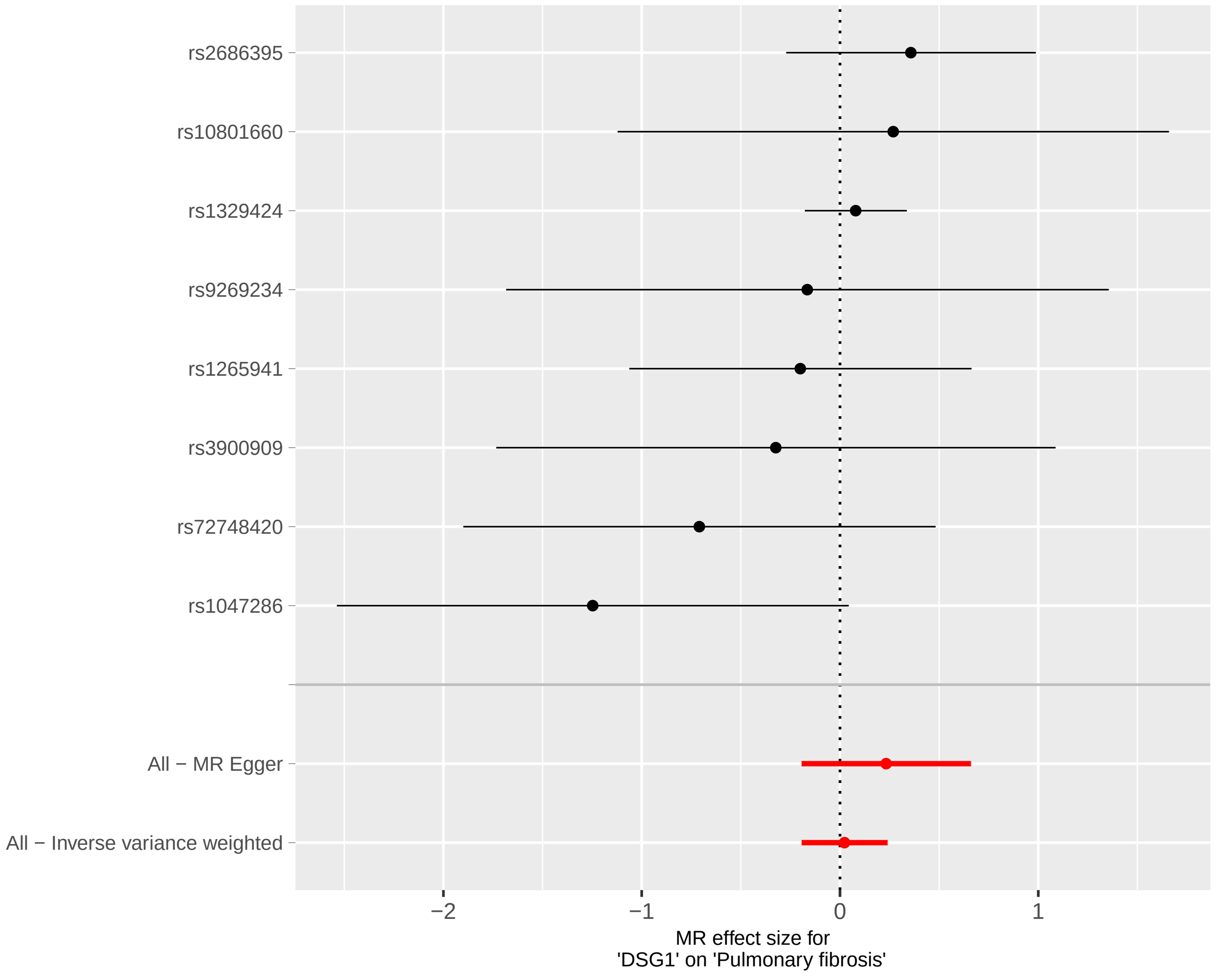

rs2686395
rs10801660
rs1329424
rs9269234
rs1265941
rs3900909
rs72748420
rs1047286
All − MR Egger
All − Inverse variance weighted
−2
−1
0
1
MR effect size for
'DSG1' on 'Pulmonary fibrosis'

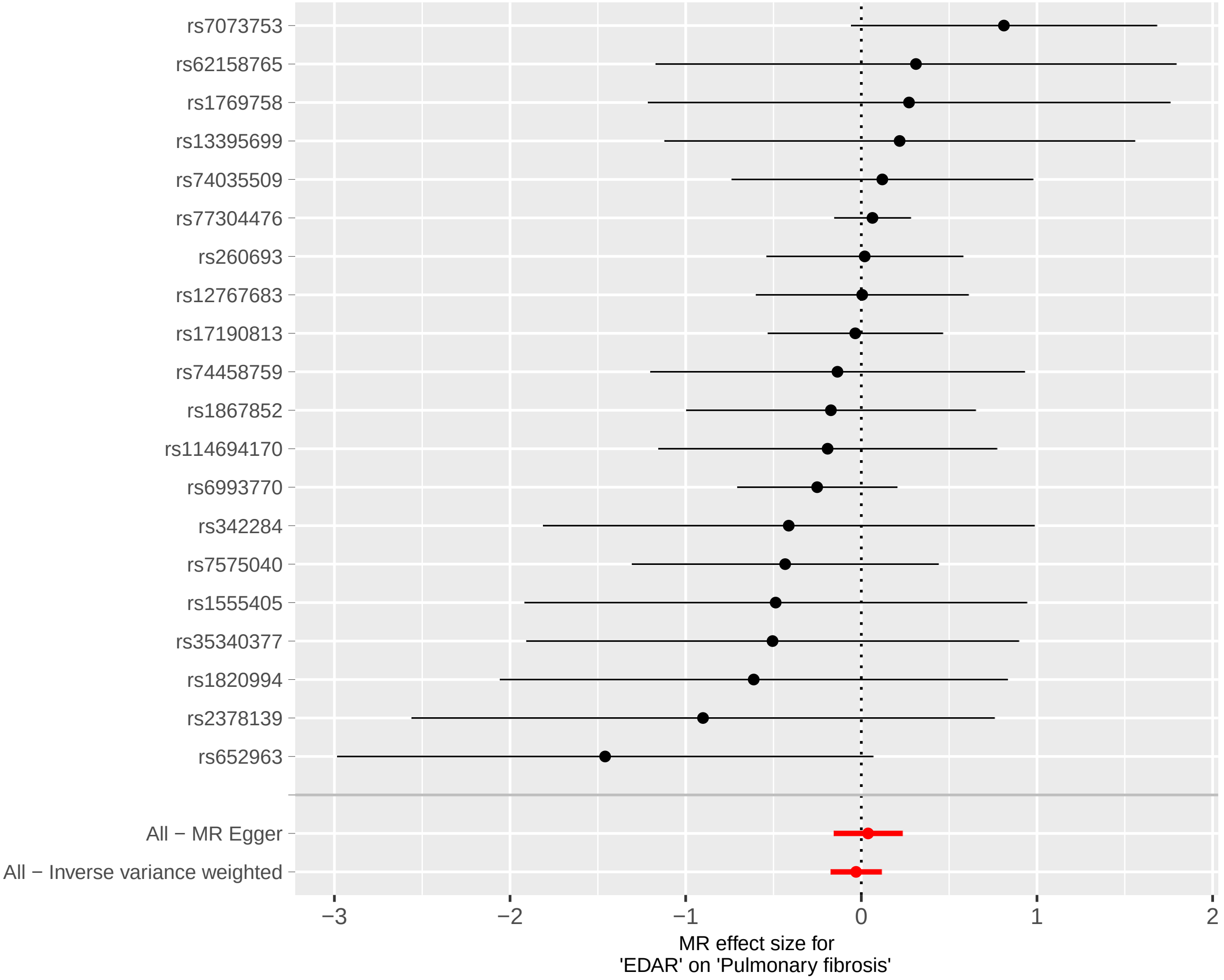
rs7073753
rs62158765
rs1769758
rs13395699
rs74035509
rs77304476
rs260693
rs12767683
rs17190813
rs74458759
rs1867852
rs114694170
rs6993770
rs342284
rs7575040
rs1555405
rs35340377
rs1820994
rs2378139
rs652963
All − MR Egger
All − Inverse variance weighted
−3
−2
−1
0
1
2
MR effect size for
'EDAR' on 'Pulmonary fibrosis'

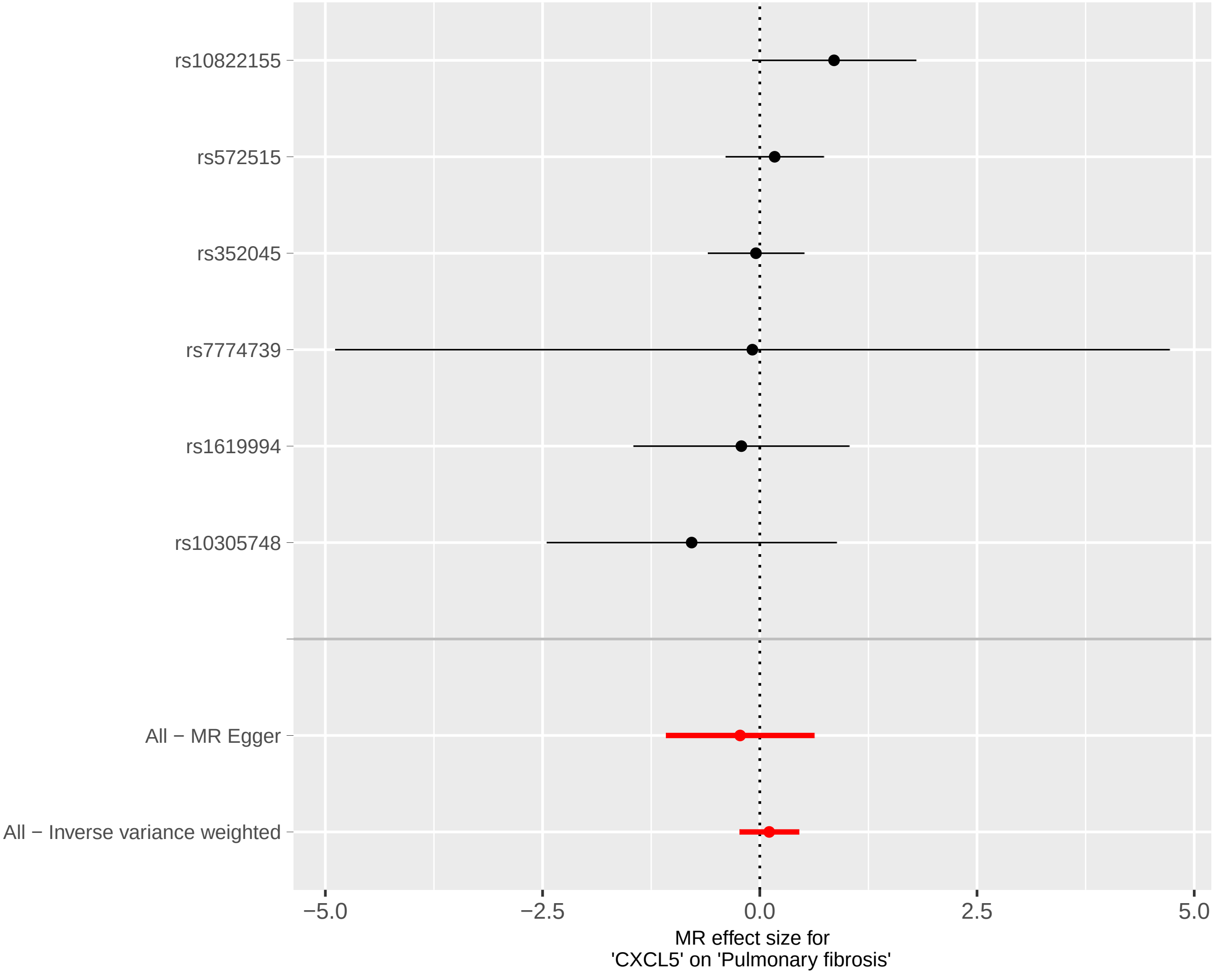
rs10822155
rs572515
rs352045
rs7774739
rs1619994
rs10305748
All − MR Egger
All − Inverse variance weighted
−5.0
−2.5
0.0
2.5
5.0
MR effect size for
'CXCL5' on 'Pulmonary fibrosis'

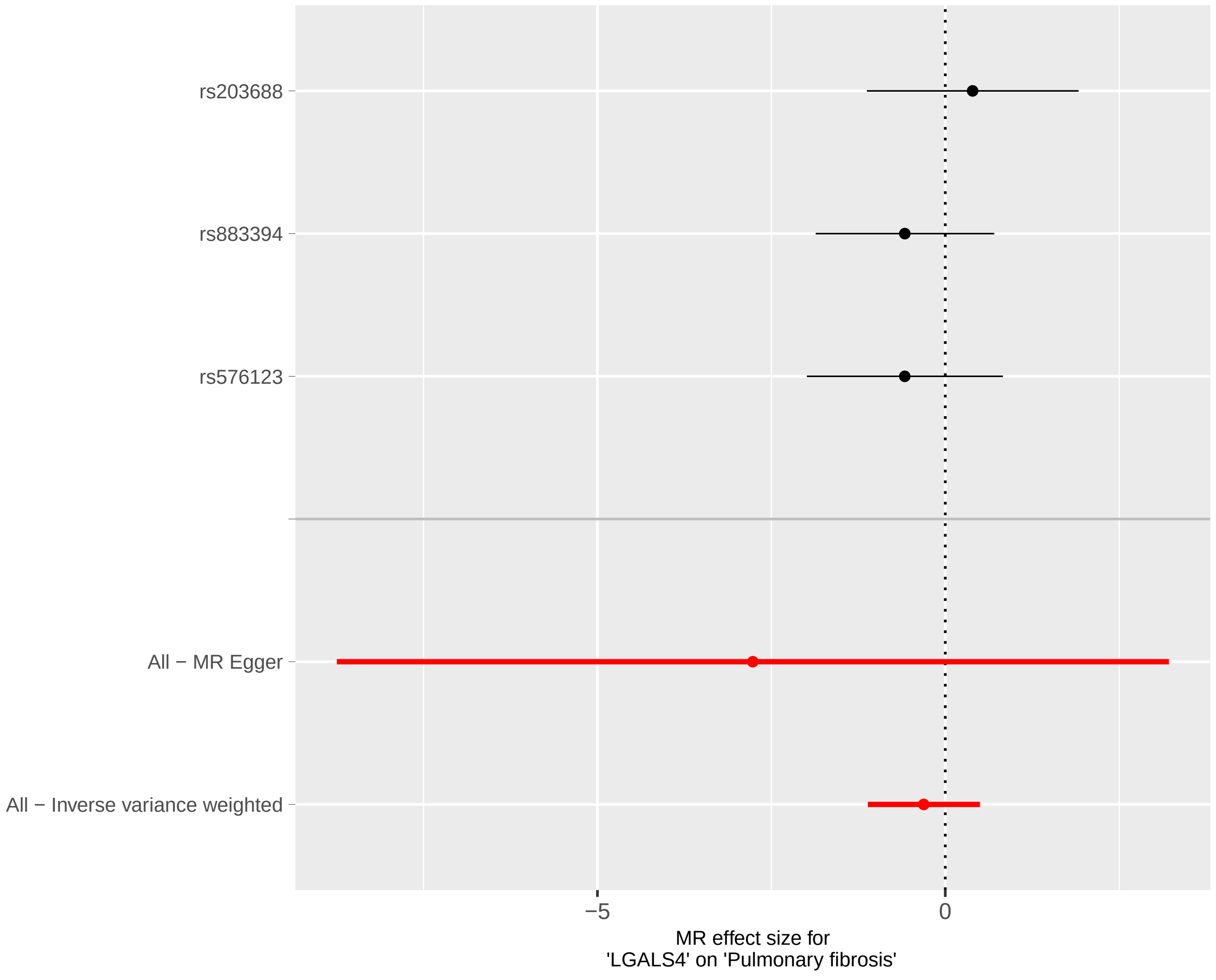

rs203688
rs883394
rs576123
All − MR Egger
All − Inverse variance weighted
−5
0
MR effect size for
'LGALS4' on 'Pulmonary fibrosis'

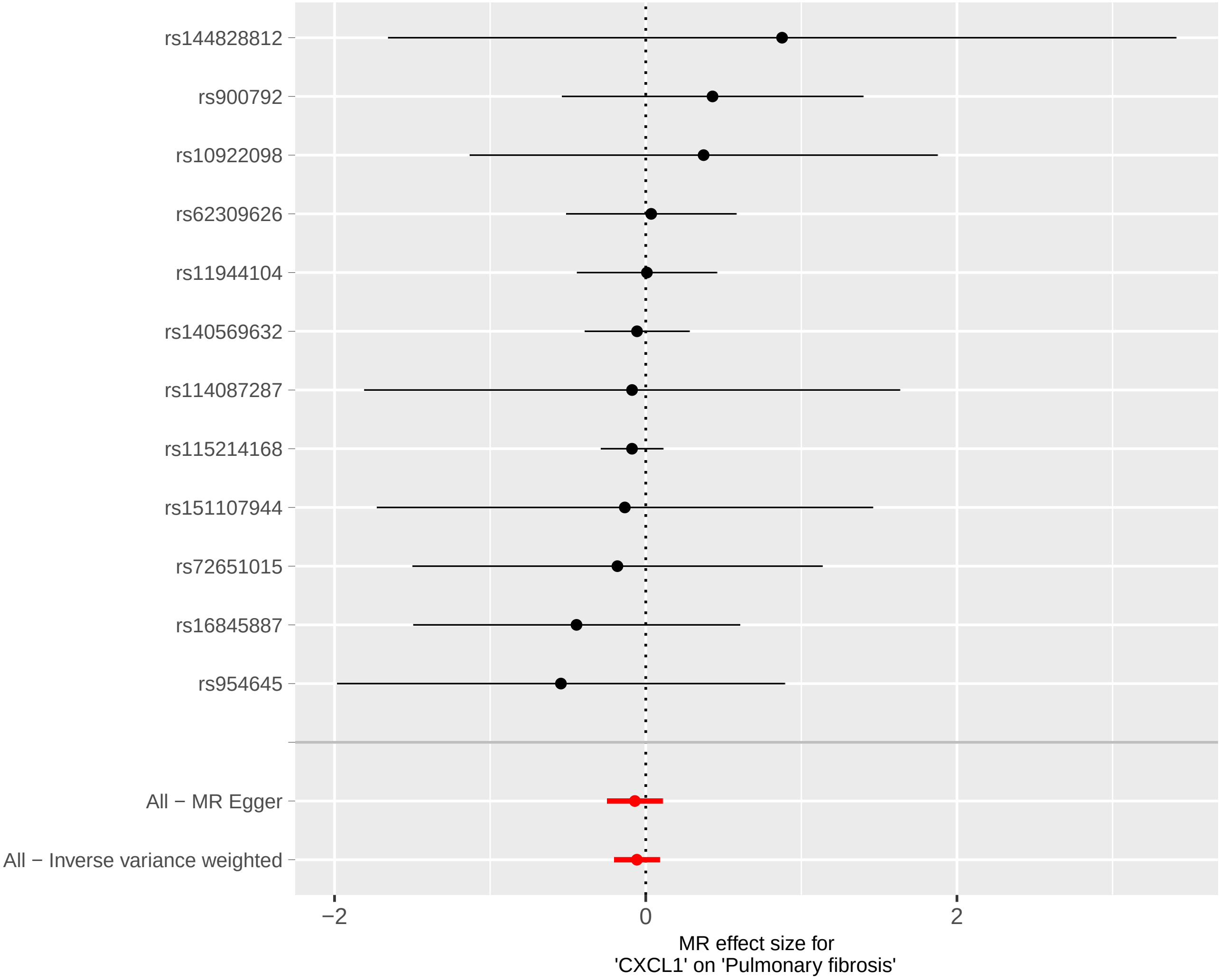

rs144828812
rs900792
rs10922098
rs62309626
rs11944104
rs140569632
rs114087287
rs115214168
rs151107944
rs72651015
rs16845887
rs954645
All − MR Egger
All − Inverse variance weighted
−2
0
2
MR effect size for
'CXCL1' on 'Pulmonary fibrosis'

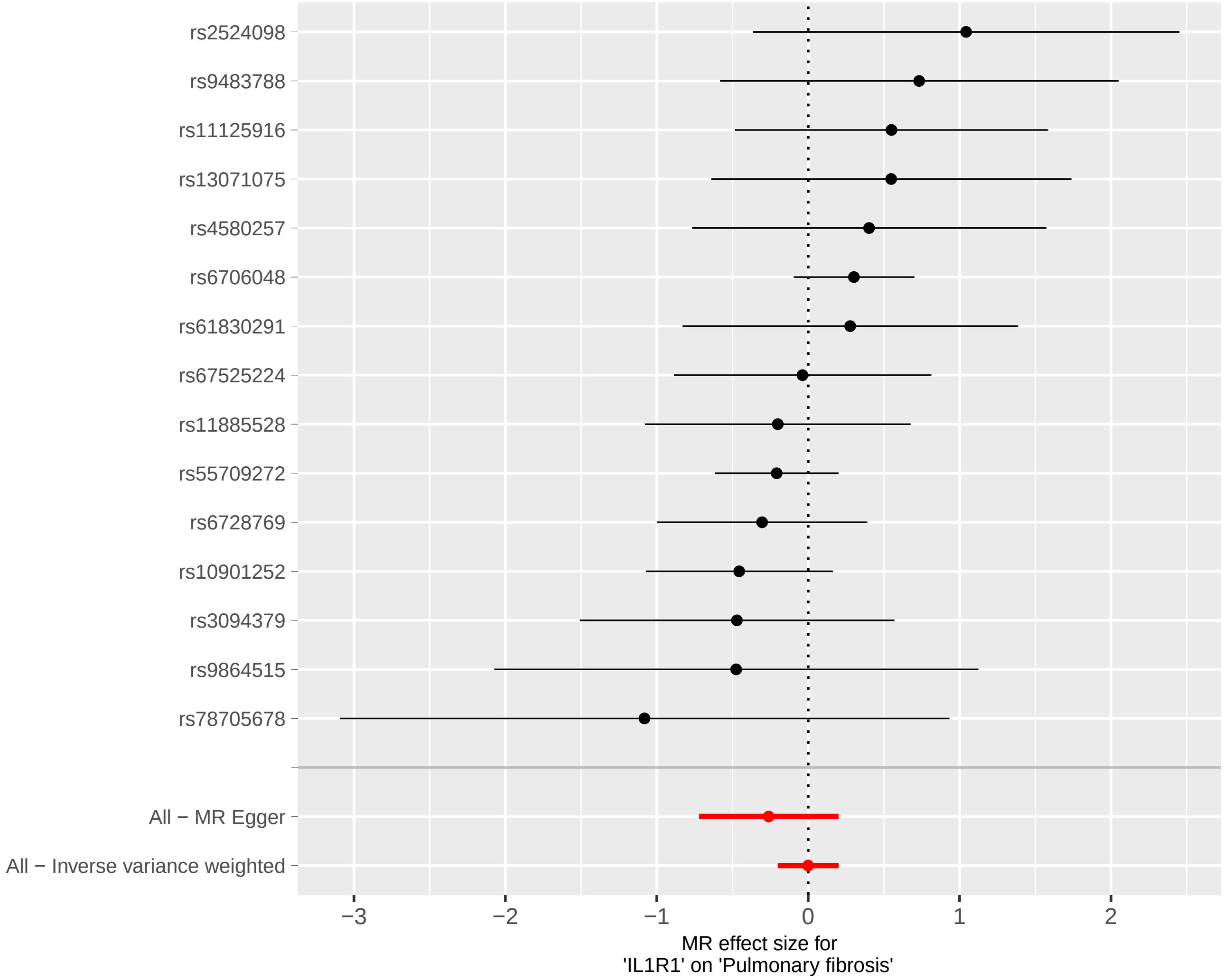

rs2524098
rs9483788
rs11125916
rs13071075
rs4580257
rs6706048
rs61830291
rs67525224
rs11885528
rs55709272
rs6728769
rs10901252
rs3094379
rs9864515
rs78705678
All − MR Egger
All − Inverse variance weighted
−3
−2
−1
0
1
2
MR effect size for
'IL1R1' on 'Pulmonary fibrosis'

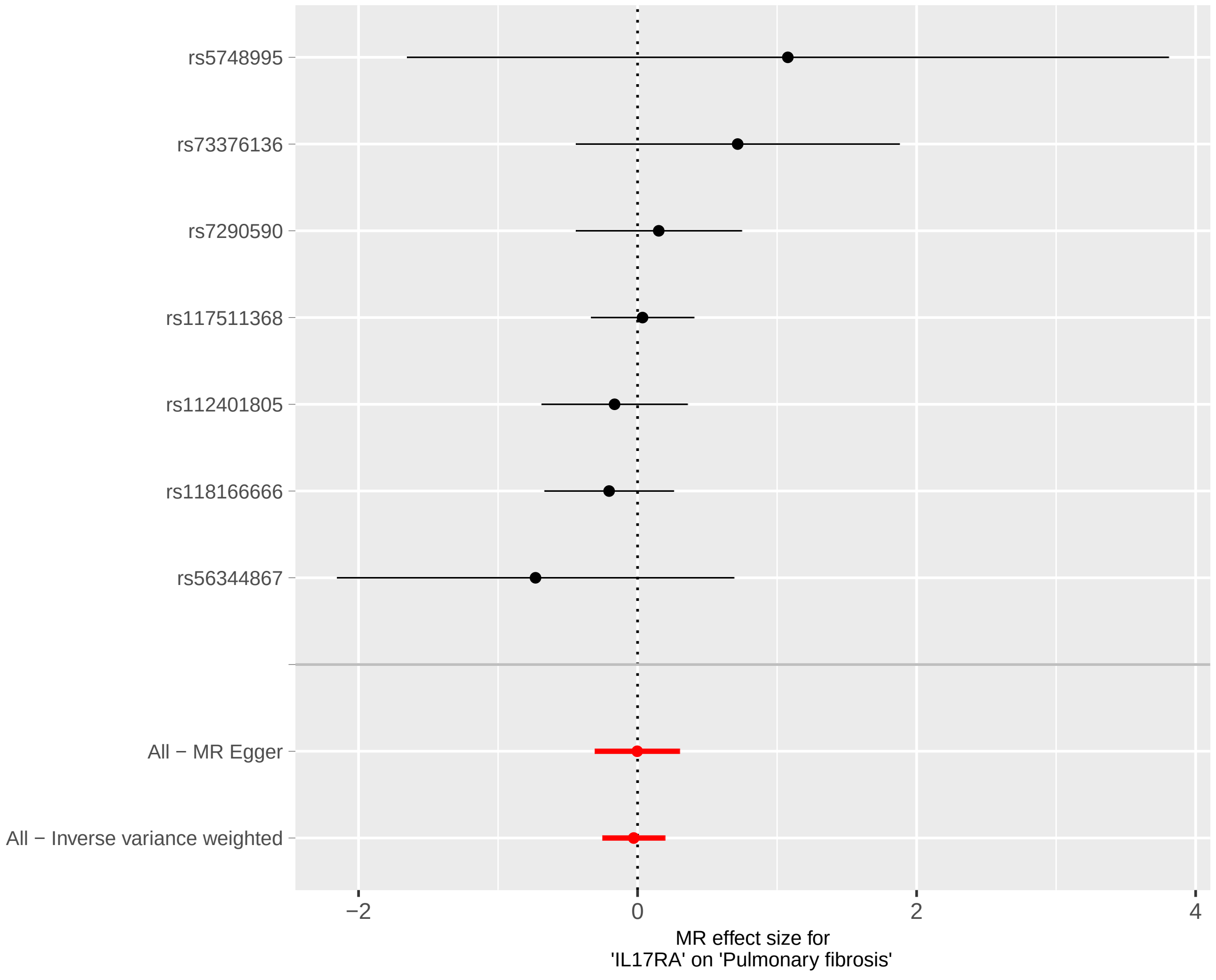

rs5748995
rs73376136
rs7290590
rs117511368
rs112401805
rs118166666
rs56344867
All − MR Egger
All − Inverse variance weighted
−2
0
2
4
MR effect size for
'IL17RA' on 'Pulmonary fibrosis'

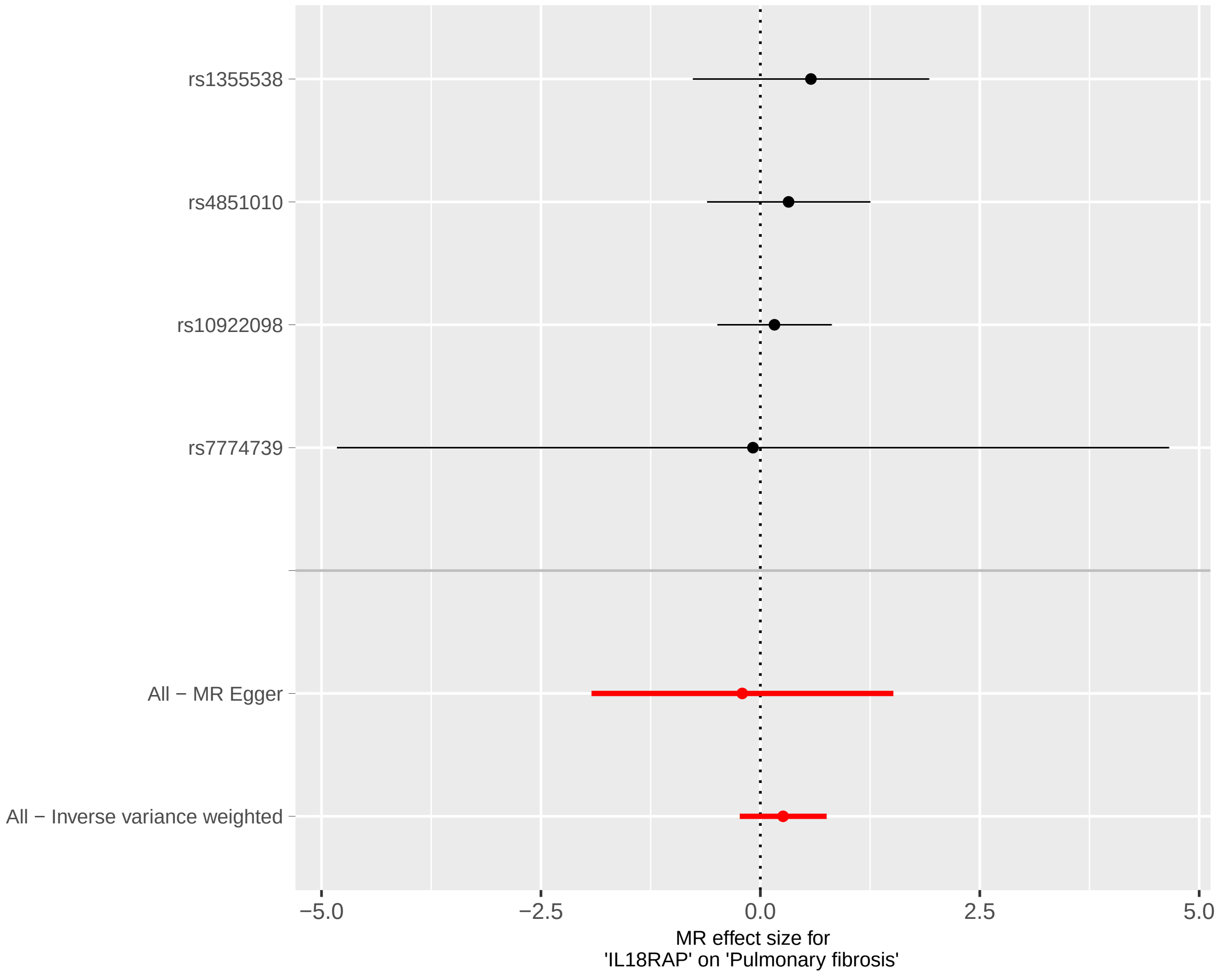

rs1355538
rs4851010
rs10922098
rs7774739
All − MR Egger
All − Inverse variance weighted
−5.0
−2.5
0.0
2.5
5.0
MR effect size for
'IL18RAP' on 'Pulmonary fibrosis'

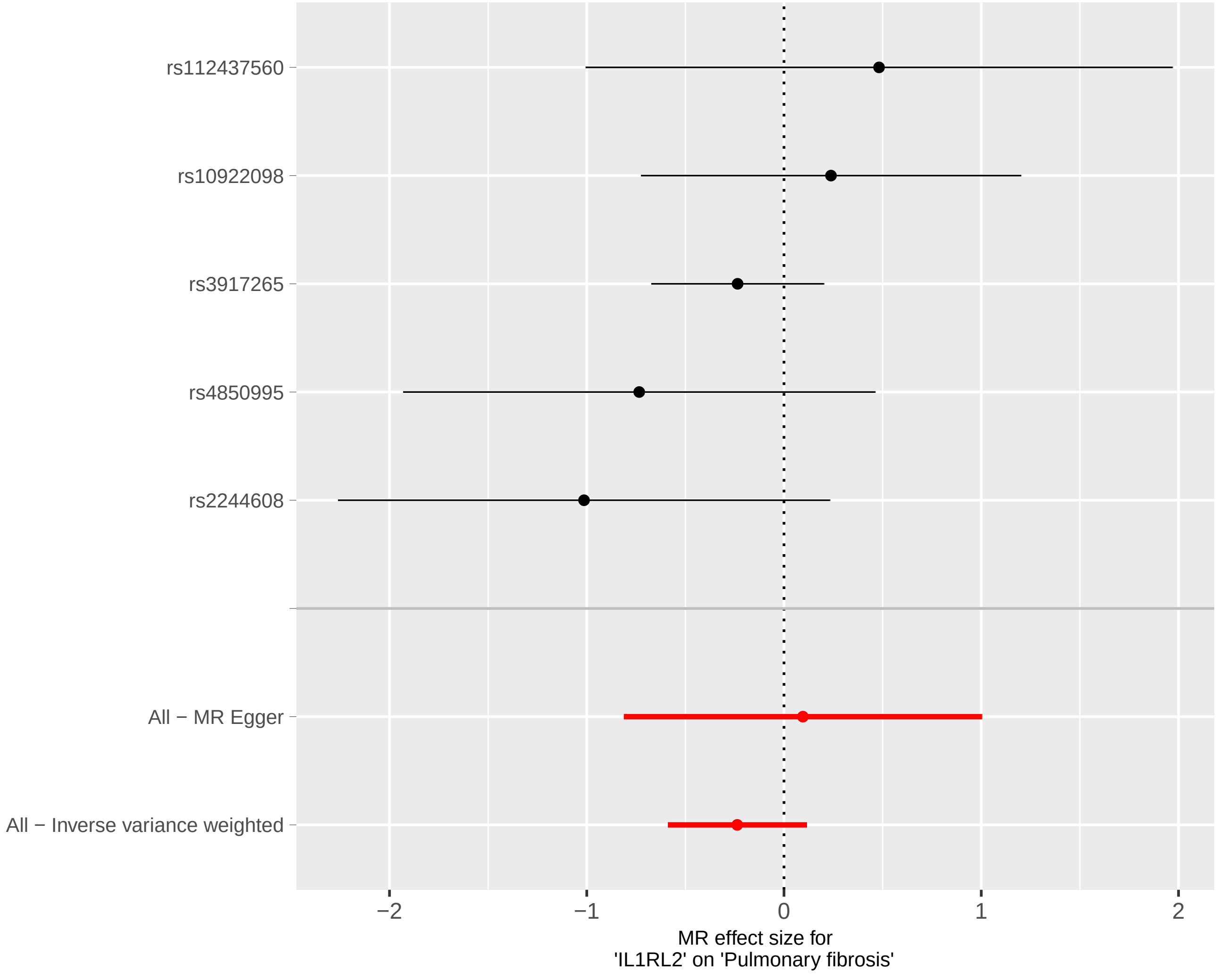
rs112437560
rs10922098
rs3917265
rs4850995
rs2244608
All − MR Egger
All − Inverse variance weighted
−2
−1
0
1
2
MR effect size for
'IL1RL2' on 'Pulmonary fibrosis'

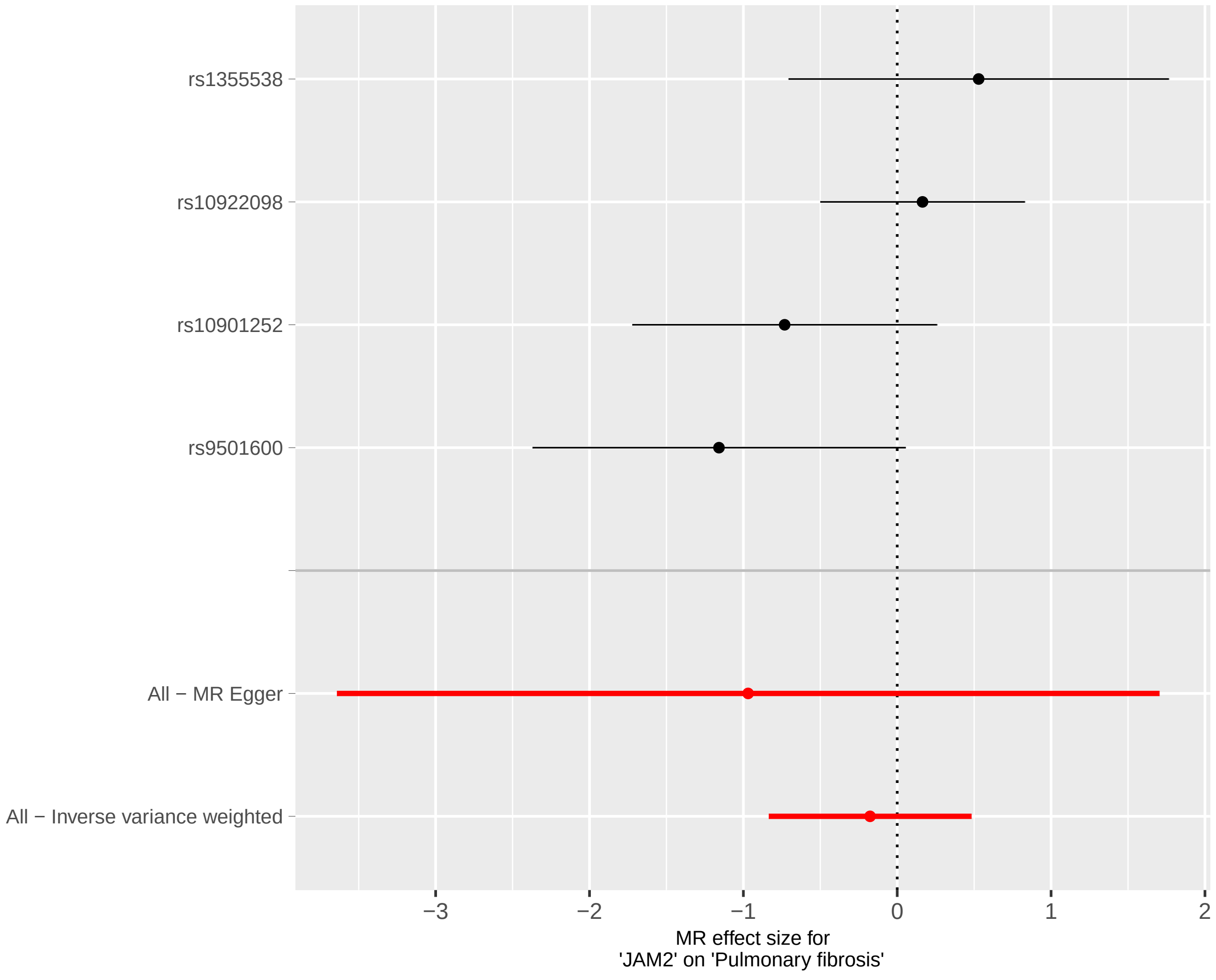
rs1355538
rs10922098
rs10901252
rs9501600
All − MR Egger
All − Inverse variance weighted
−3
−2
−1
0
1
2
MR effect size for
'JAM2' on 'Pulmonary fibrosis'

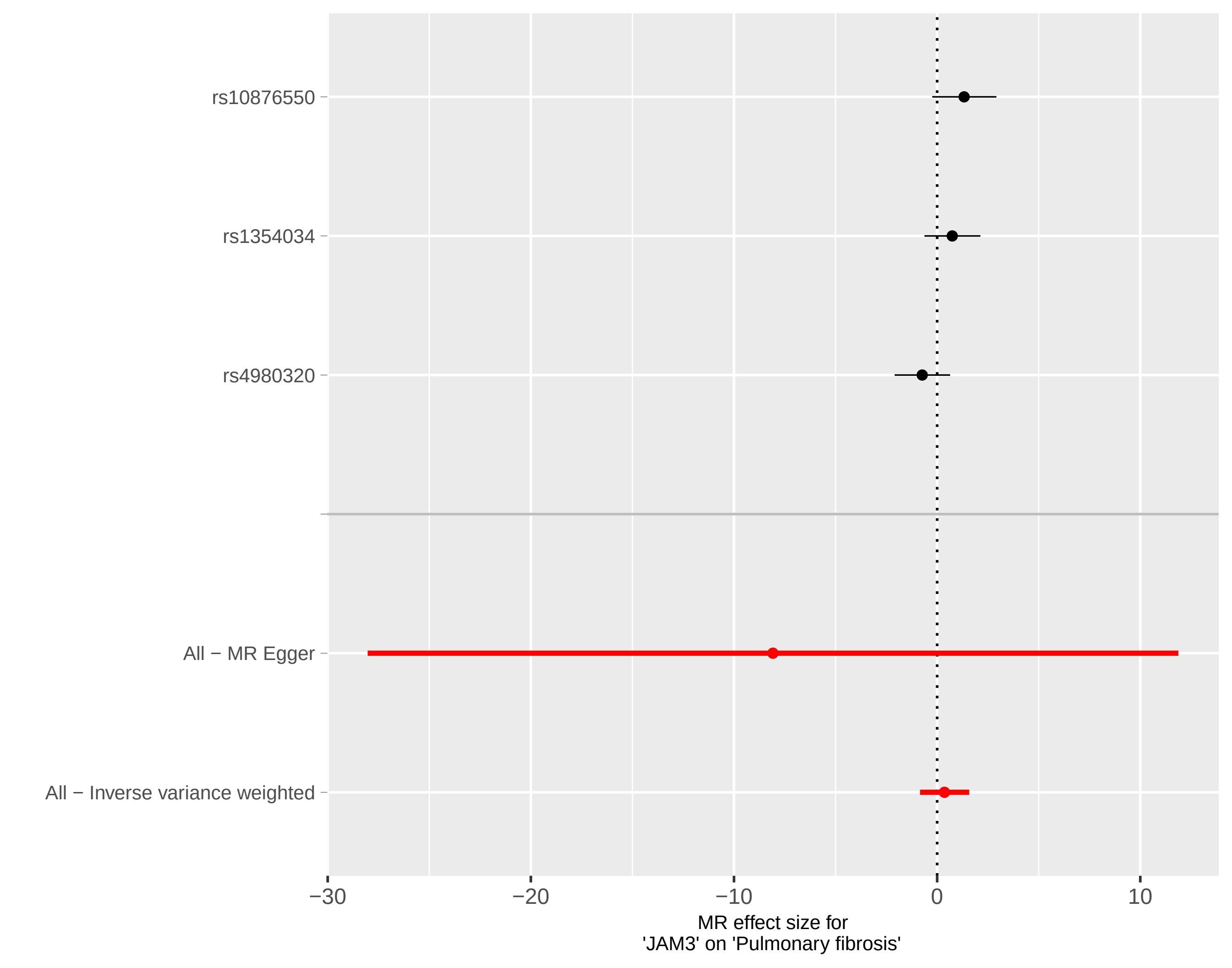
rs10876550
rs1354034
rs4980320
All − MR Egger
All − Inverse variance weighted
−30
−20
−10
0
10
MR effect size for
'JAM3' on 'Pulmonary fibrosis'

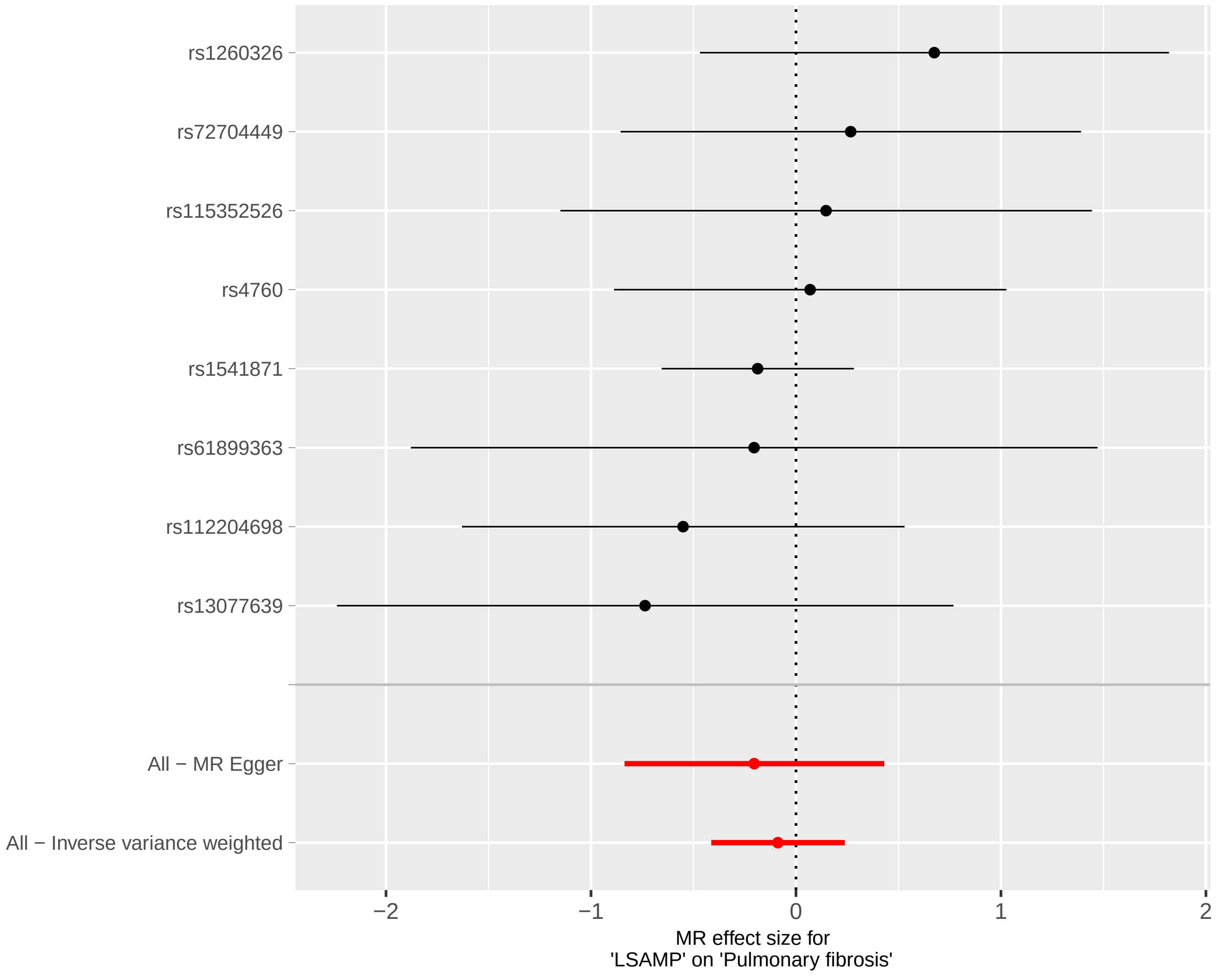

rs1260326
rs72704449
rs115352526
rs4760
rs1541871
rs61899363
rs112204698
rs13077639
All − MR Egger
All − Inverse variance weighted
−2
−1
0
1
2
MR effect size for
'LSAMP' on 'Pulmonary fibrosis'

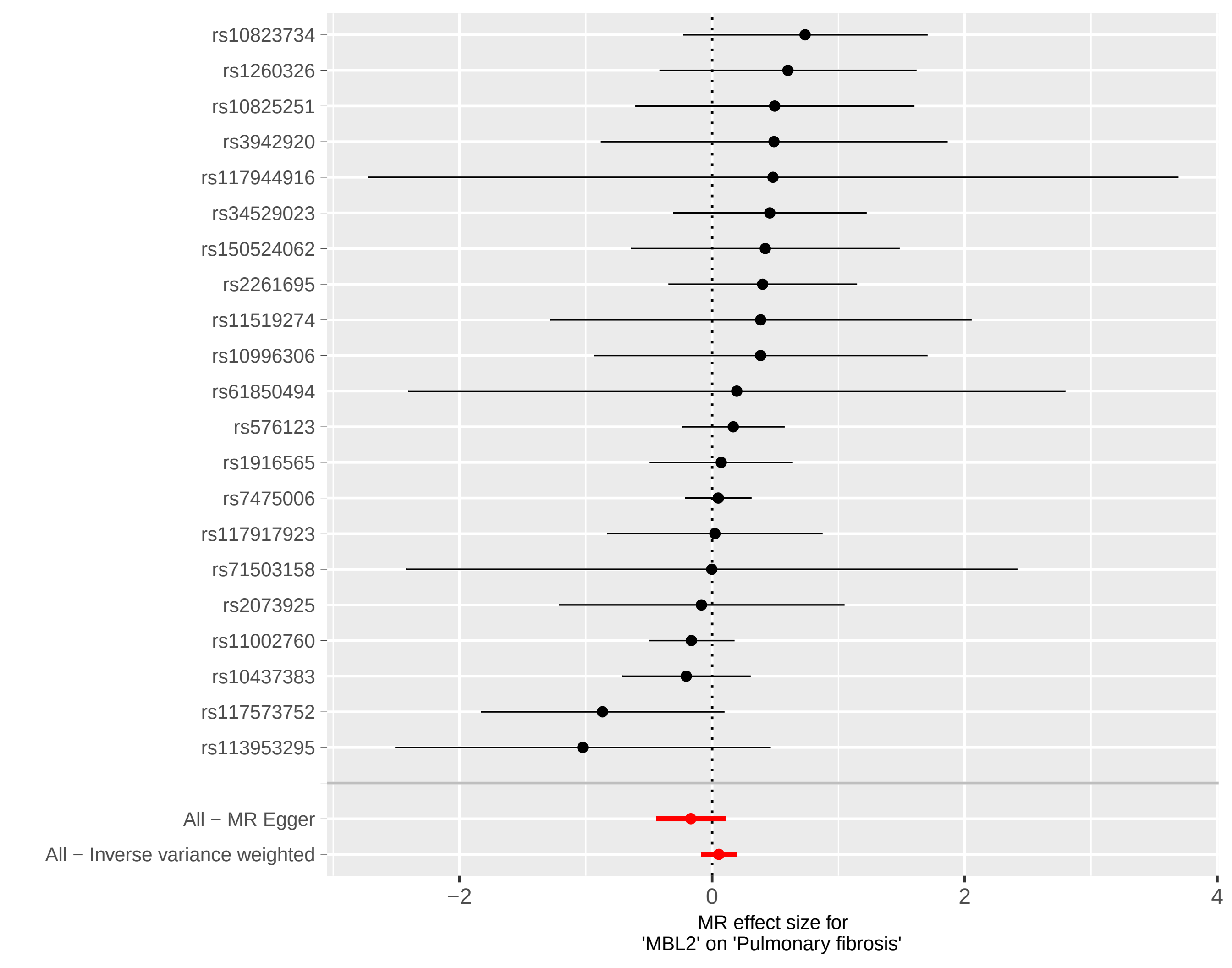

rs10823734
rs1260326
rs10825251
rs3942920
rs117944916
rs34529023
rs150524062
rs2261695
rs11519274
rs10996306
rs61850494
rs576123
rs1916565
rs7475006
rs117917923
rs71503158
rs2073925
rs11002760
rs10437383
rs117573752
rs113953295
All − MR Egger
All − Inverse variance weighted
−2
0
2
4
MR effect size for
'MBL2' on 'Pulmonary fibrosis'

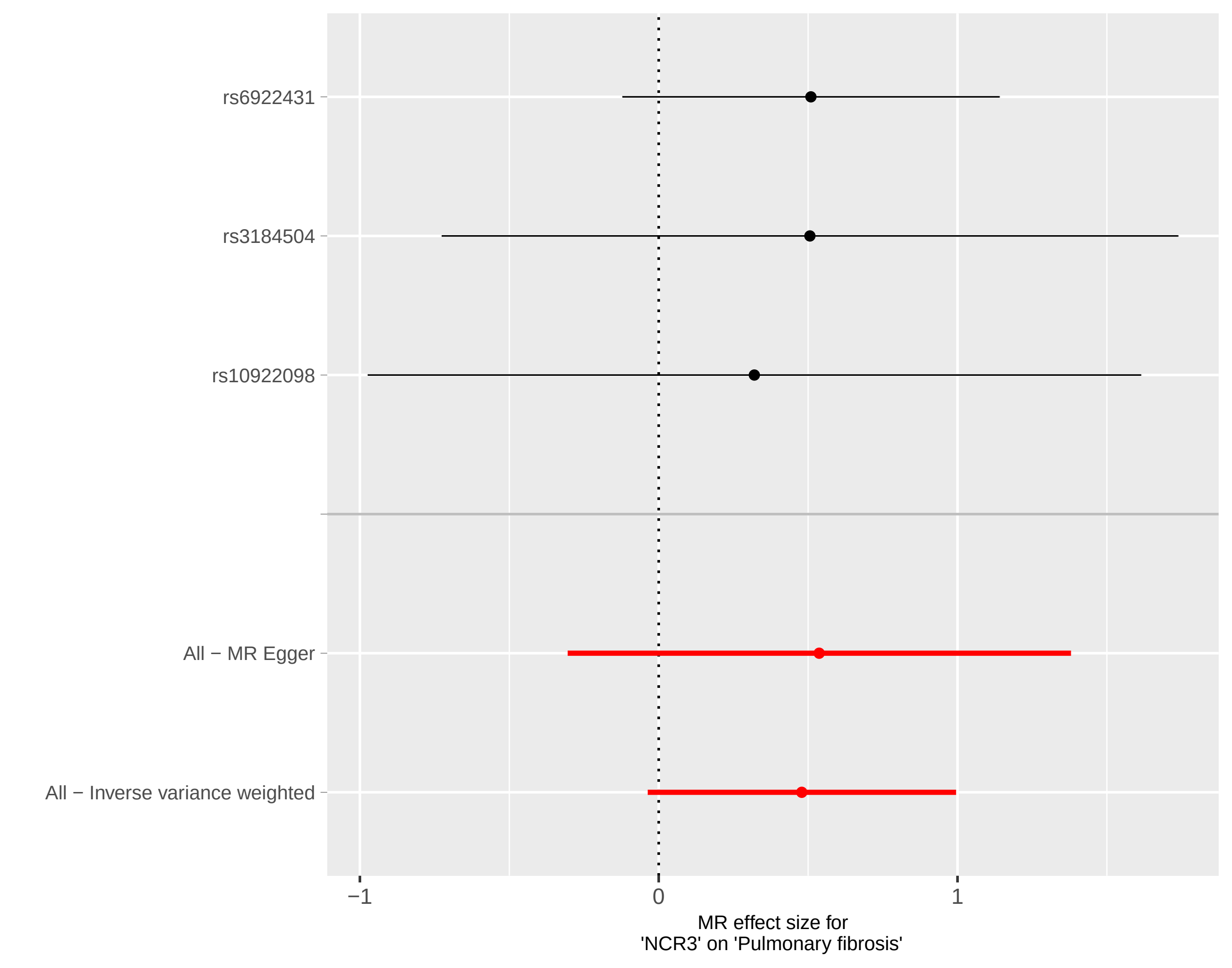

rs6922431
rs3184504
rs10922098
All − MR Egger
All − Inverse variance weighted
−1
0
1
MR effect size for
'NCR3' on 'Pulmonary fibrosis'

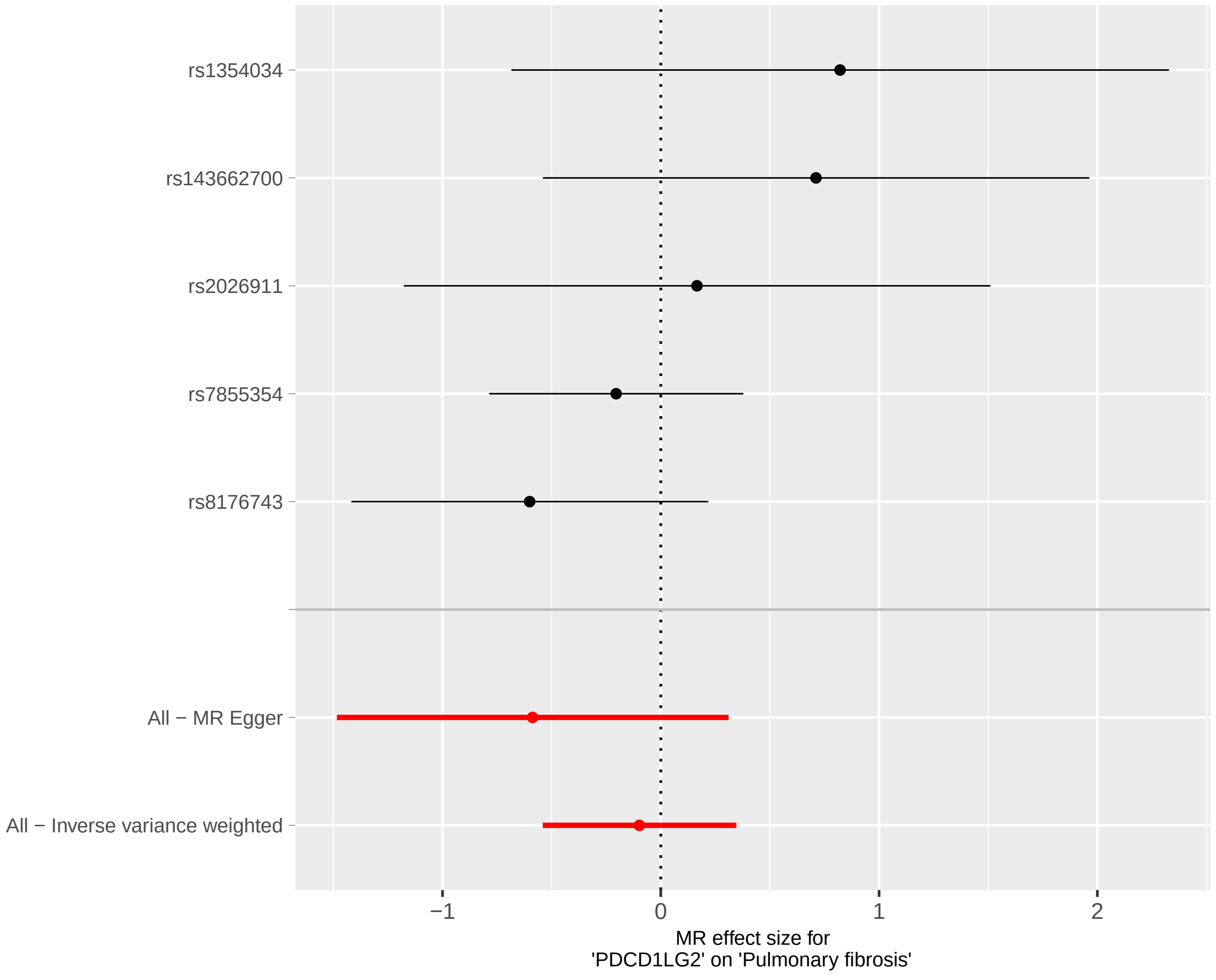
rs1354034
rs143662700
rs2026911
rs7855354
rs8176743
All − MR Egger
All − Inverse variance weighted
−1
0
1
2
MR effect size for
'PDCD1LG2' on 'Pulmonary fibrosis'

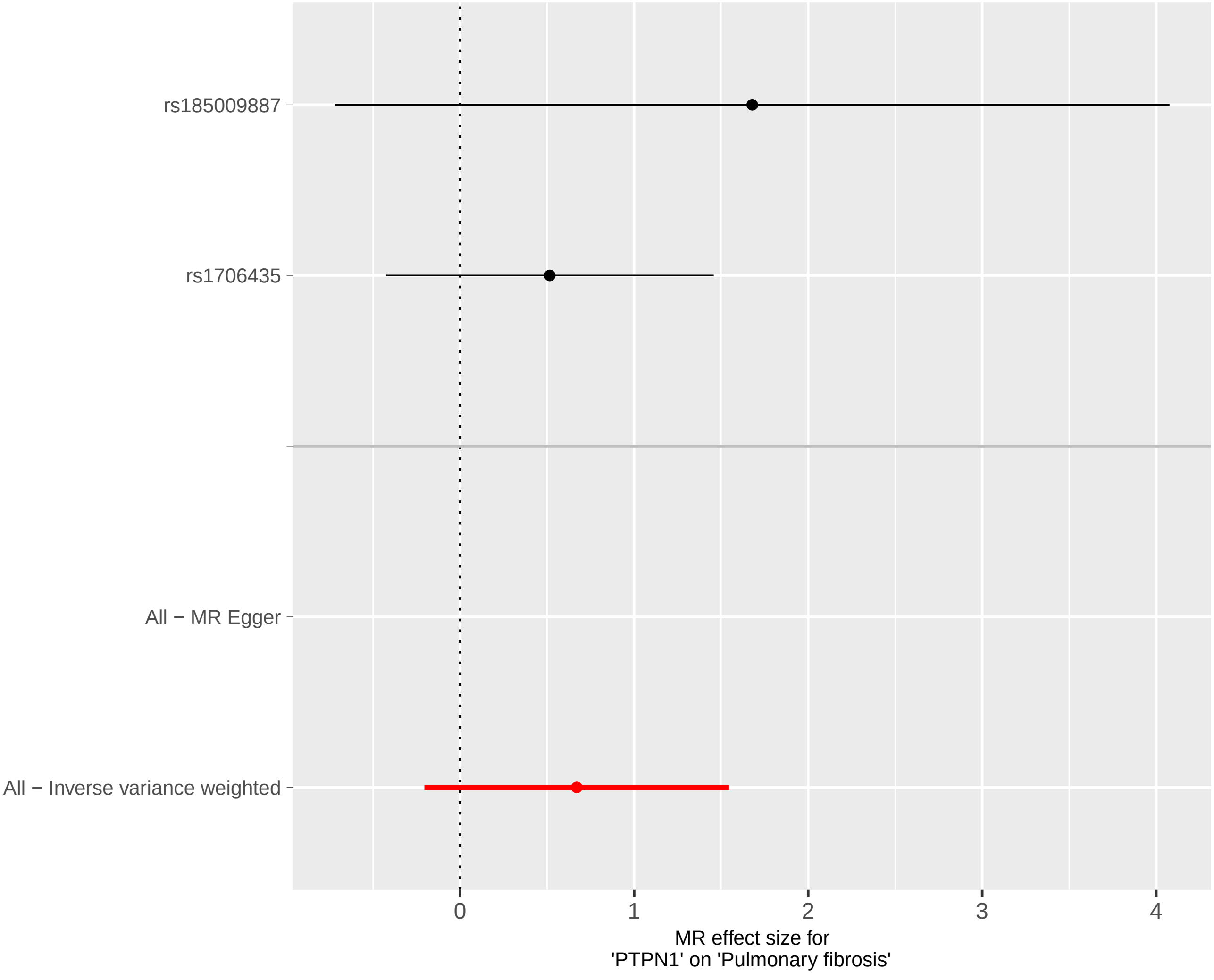

rs185009887
rs1706435
All − MR Egger
All − Inverse variance weighted
0
1
2
3
4
MR effect size for
'PTPN1' on 'Pulmonary fibrosis'

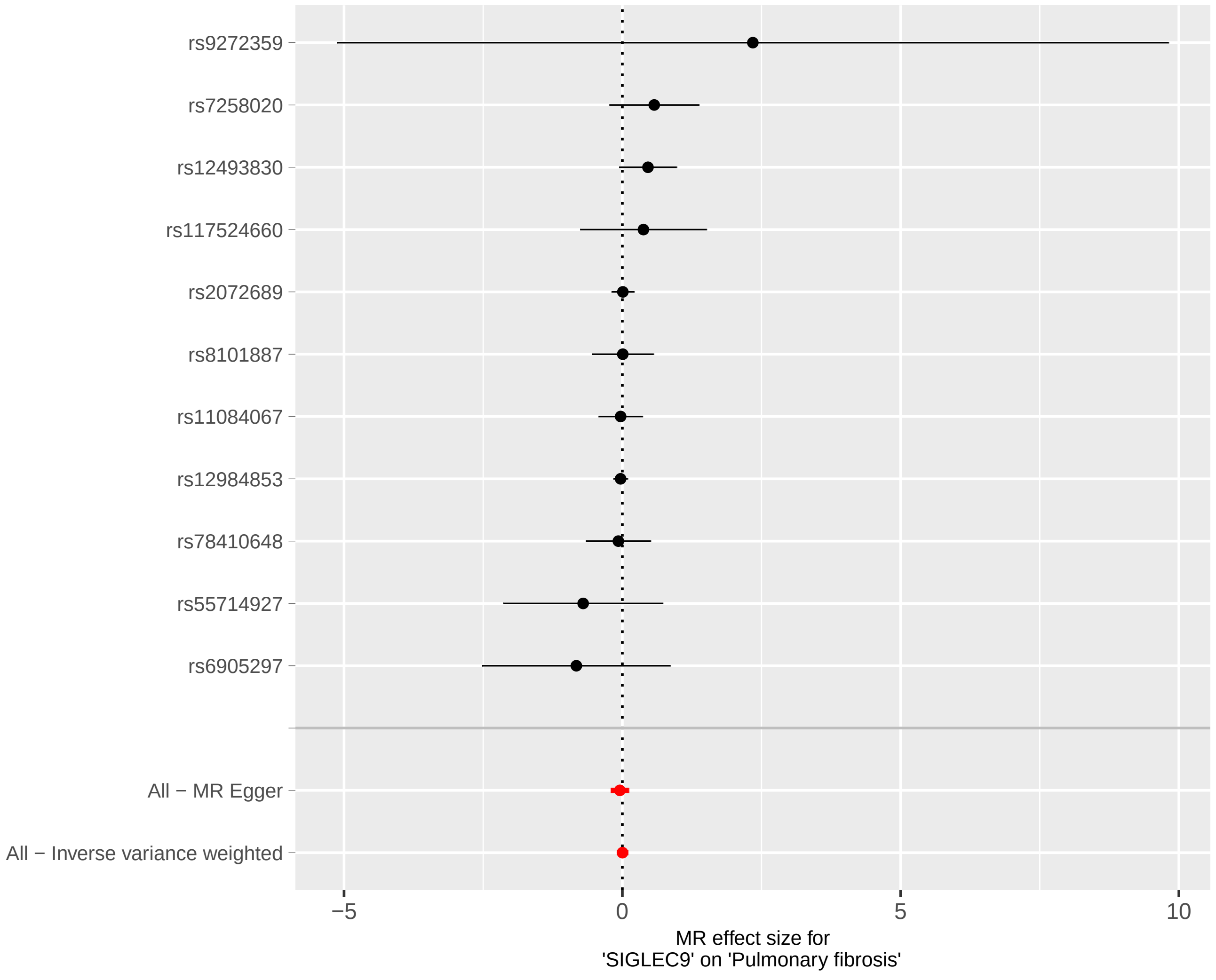
rs9272359
rs7258020
rs12493830
rs117524660
rs2072689
rs8101887
rs11084067
rs12984853
rs78410648
rs55714927
rs6905297
All − MR Egger
All − Inverse variance weighted
−5
0
5
10
MR effect size for
'SIGLEC9' on 'Pulmonary fibrosis'

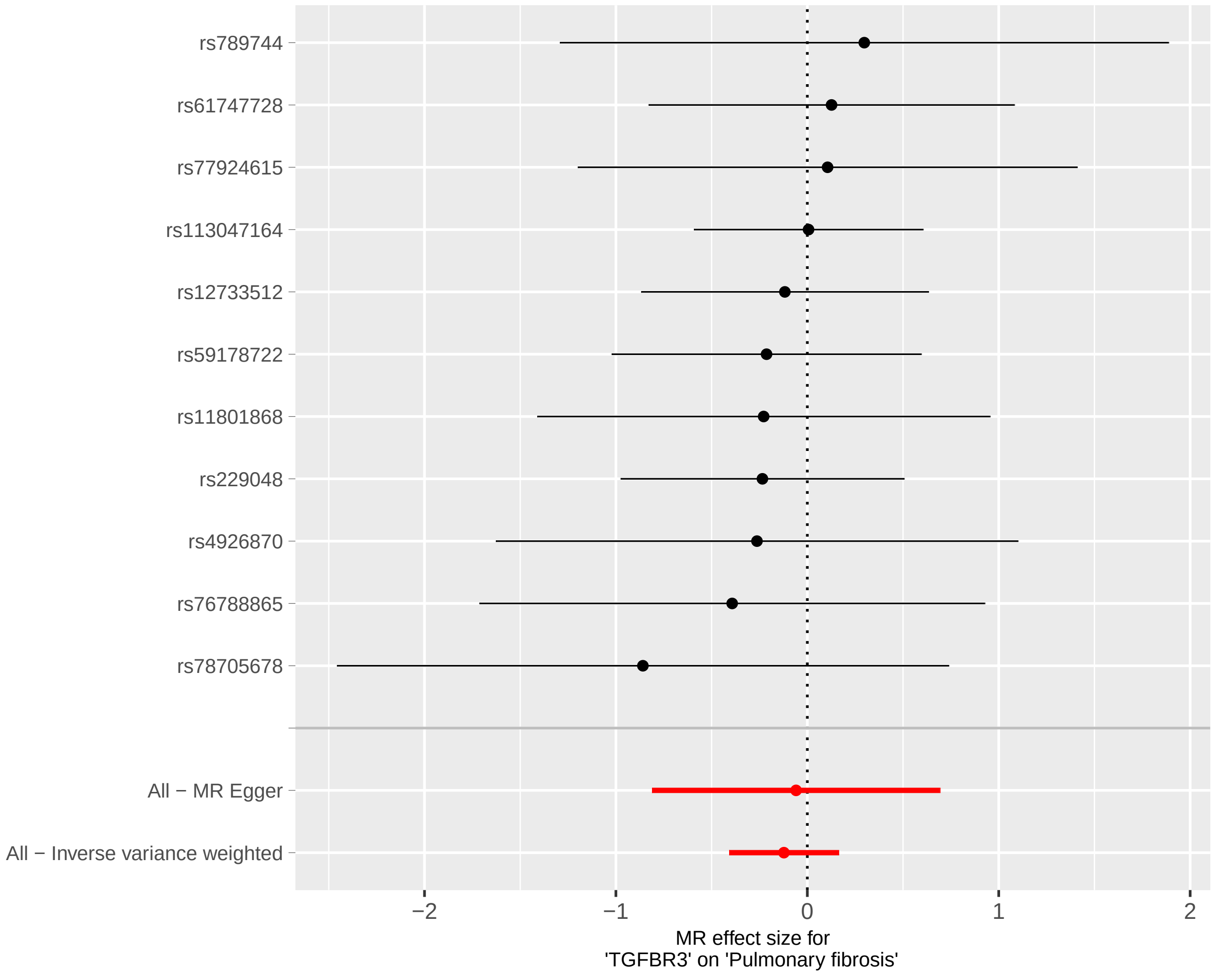
rs789744
rs61747728
rs77924615
rs113047164
rs12733512
rs59178722
rs11801868
rs229048
rs4926870
rs76788865
rs78705678
All − MR Egger
All − Inverse variance weighted
−2
−1
0
1
2
MR effect size for
'TGFBR3' on 'Pulmonary fibrosis'

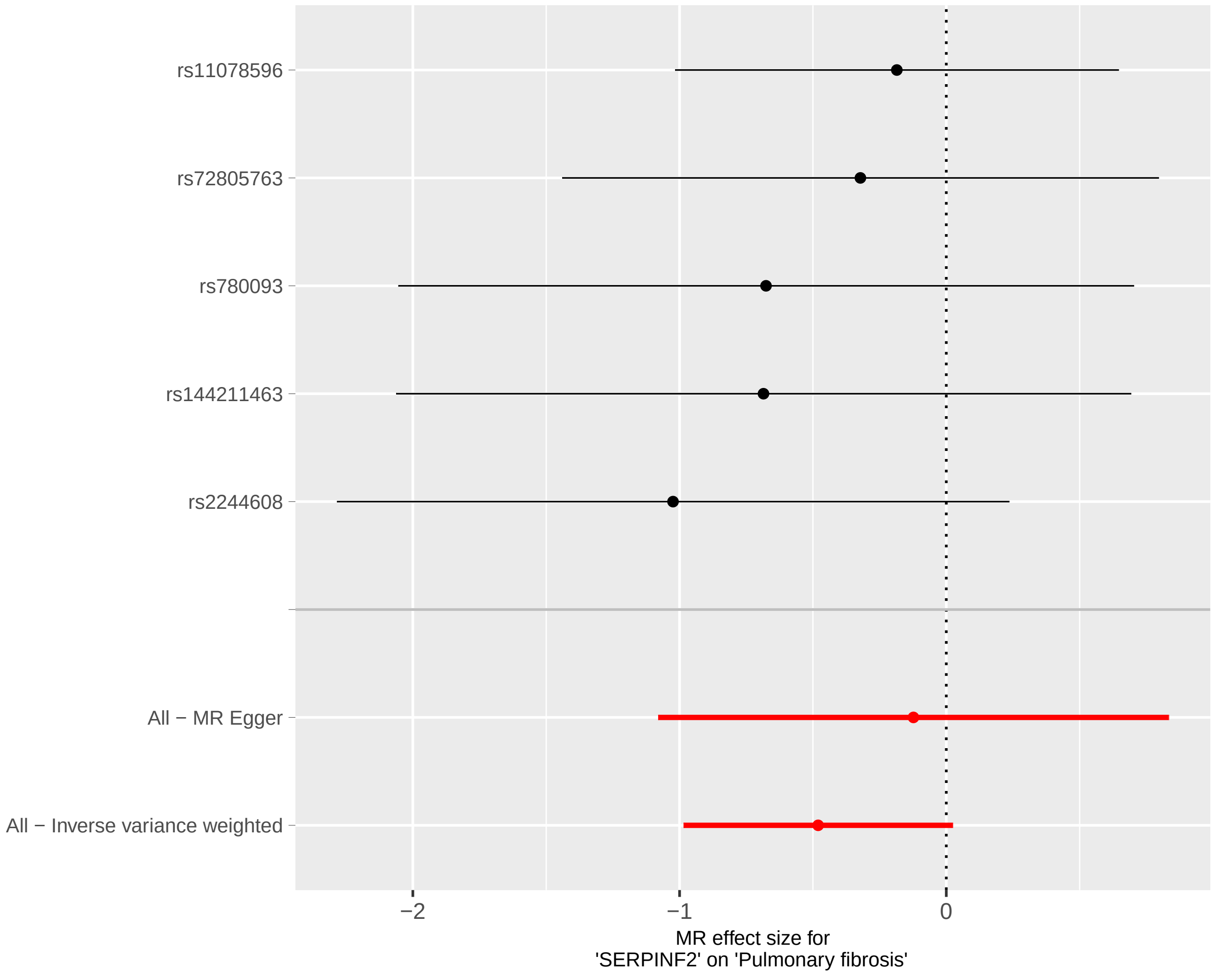

rs11078596
rs72805763
rs780093
rs144211463
rs2244608
All − MR Egger
All − Inverse variance weighted
−2
−1
0
MR effect size for
'SERPINF2' on 'Pulmonary fibrosis'

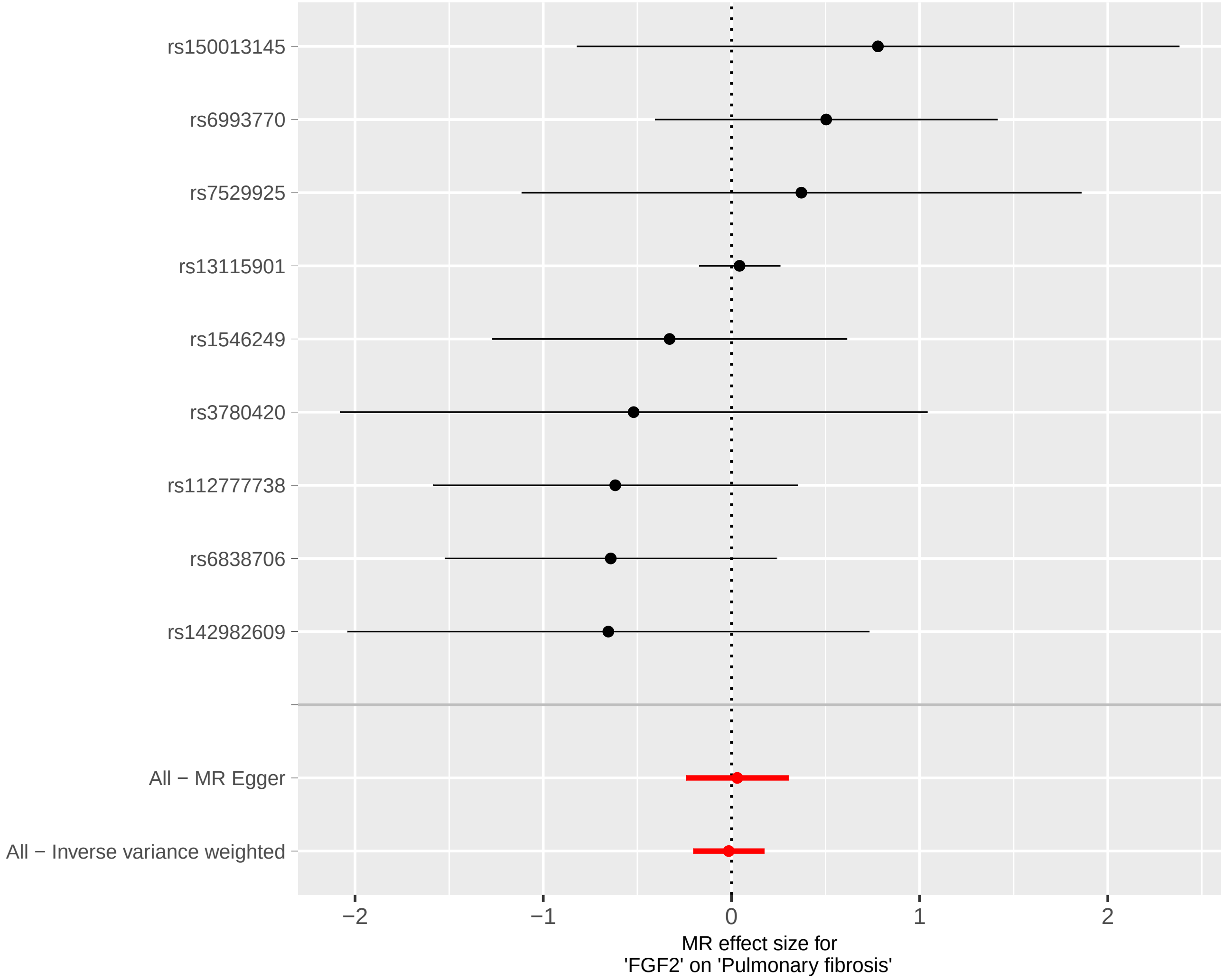

rs150013145
rs6993770
rs7529925
rs13115901
rs1546249
rs3780420
rs112777738
rs6838706
rs142982609
All − MR Egger
All − Inverse variance weighted
−2
−1
0
1
2
MR effect size for
'FGF2' on 'Pulmonary fibrosis'

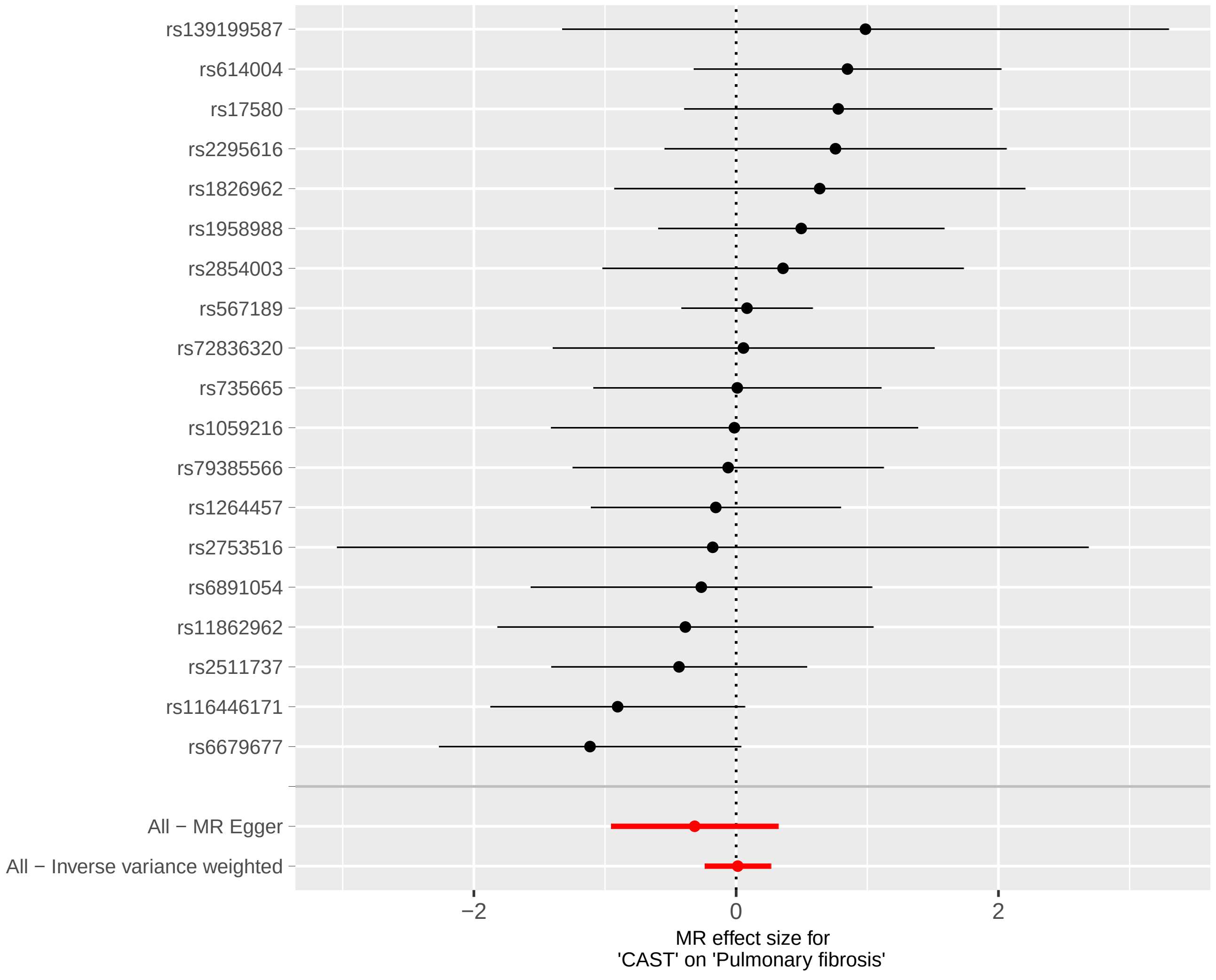

rs139199587
rs614004
rs17580
rs2295616
rs1826962
rs1958988
rs2854003
rs567189
rs72836320
rs735665
rs1059216
rs79385566
rs1264457
rs2753516
rs6891054
rs11862962
rs2511737
rs116446171
rs6679677
All − MR Egger
All − Inverse variance weighted
−2
0
2
MR effect size for
'CAST' on 'Pulmonary fibrosis'

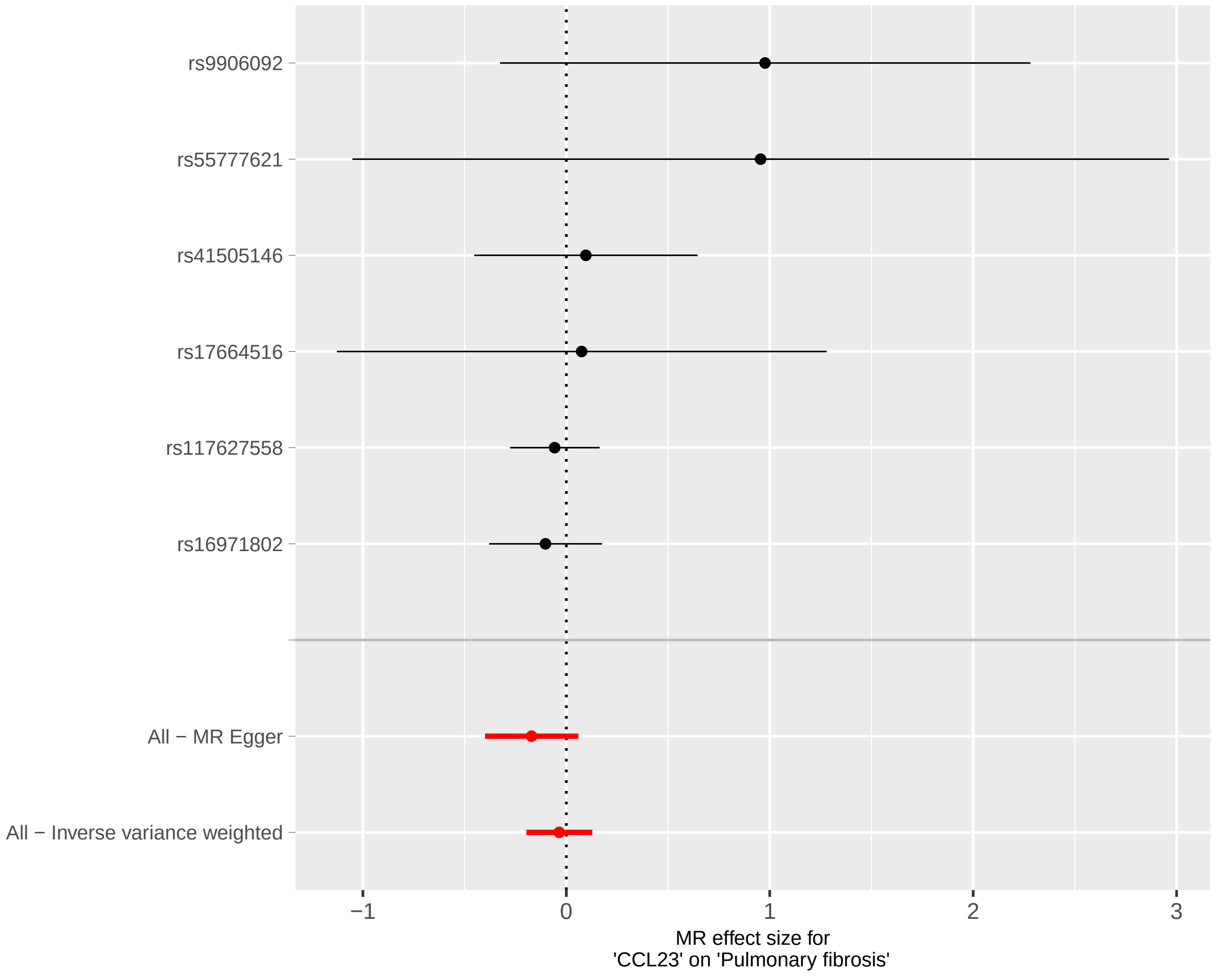

rs9906092
rs55777621
rs41505146
rs17664516
rs117627558
rs16971802
All − MR Egger
All − Inverse variance weighted
−1
0
1
2
3
MR effect size for
'CCL23' on 'Pulmonary fibrosis'

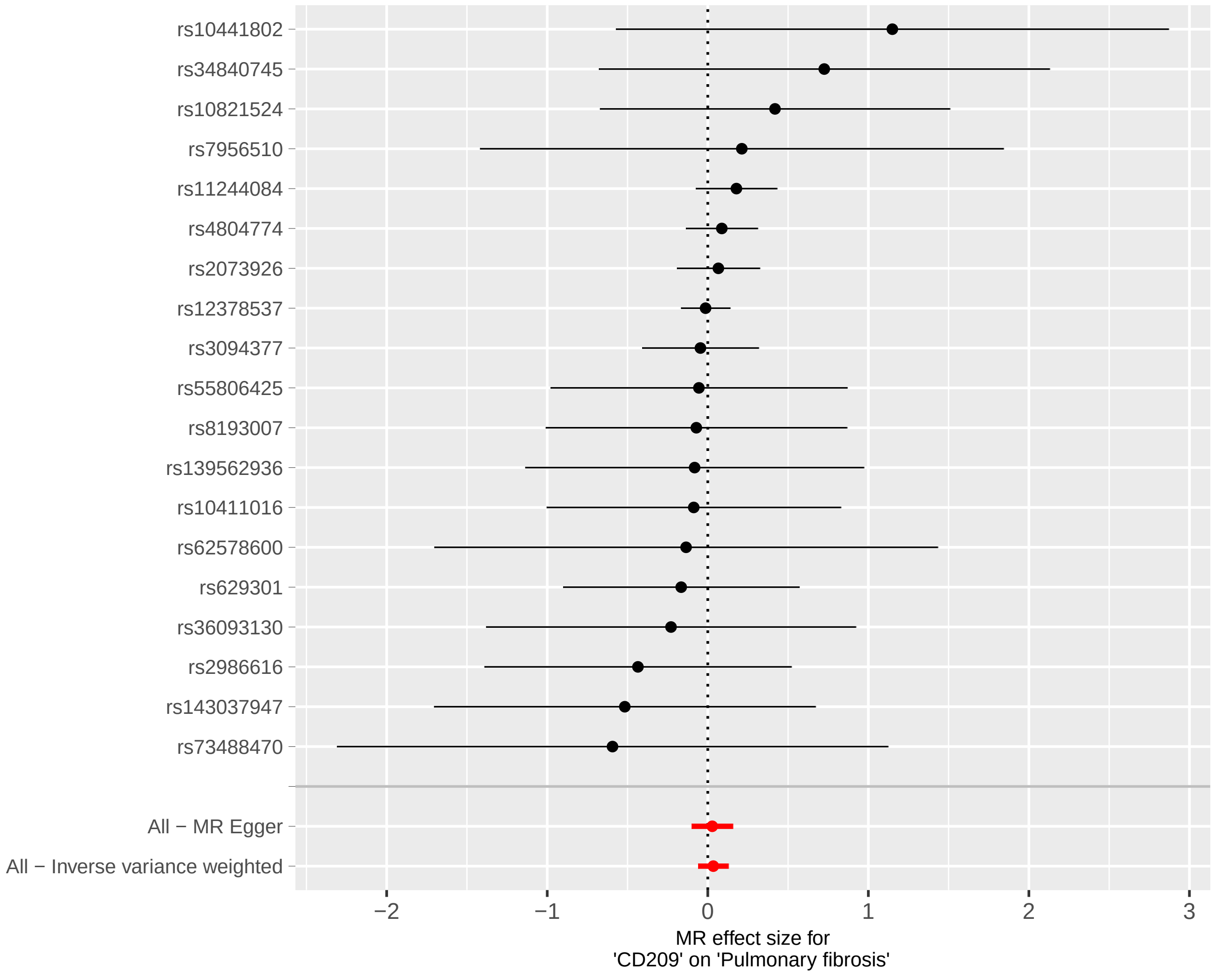

rs10441802
rs34840745
rs10821524
rs7956510
rs11244084
rs4804774
rs2073926
rs12378537
rs3094377
rs55806425
rs8193007
rs139562936
rs10411016
rs62578600
rs629301
rs36093130
rs2986616
rs143037947
rs73488470
All − MR Egger
All − Inverse variance weighted
−2
−1
0
1
2
3
MR effect size for
'CD209' on 'Pulmonary fibrosis'

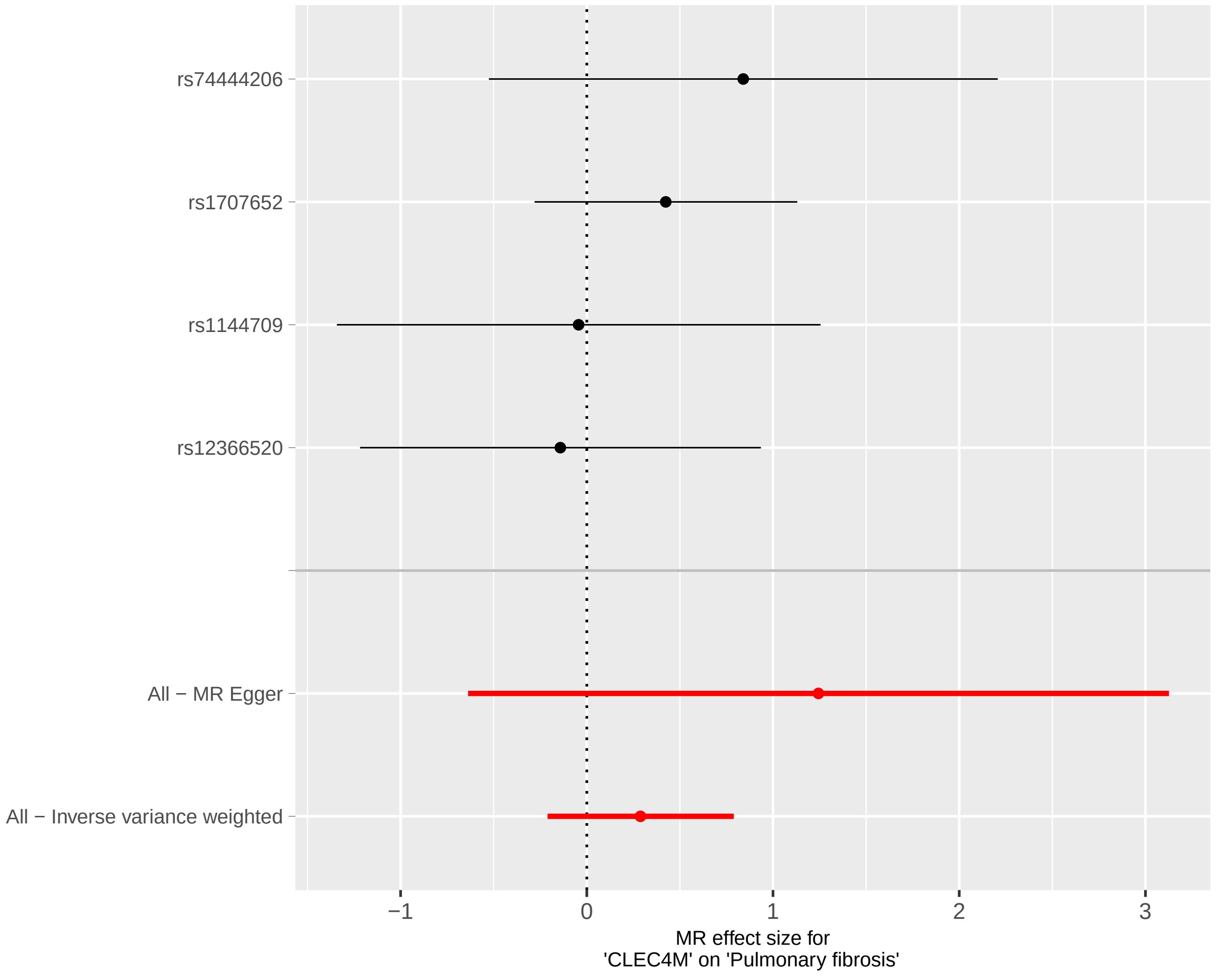

rs74444206
rs1707652
rs1144709
rs12366520
All − MR Egger
All − Inverse variance weighted
−1
0
1
2
3
MR effect size for
'CLEC4M' on 'Pulmonary fibrosis'

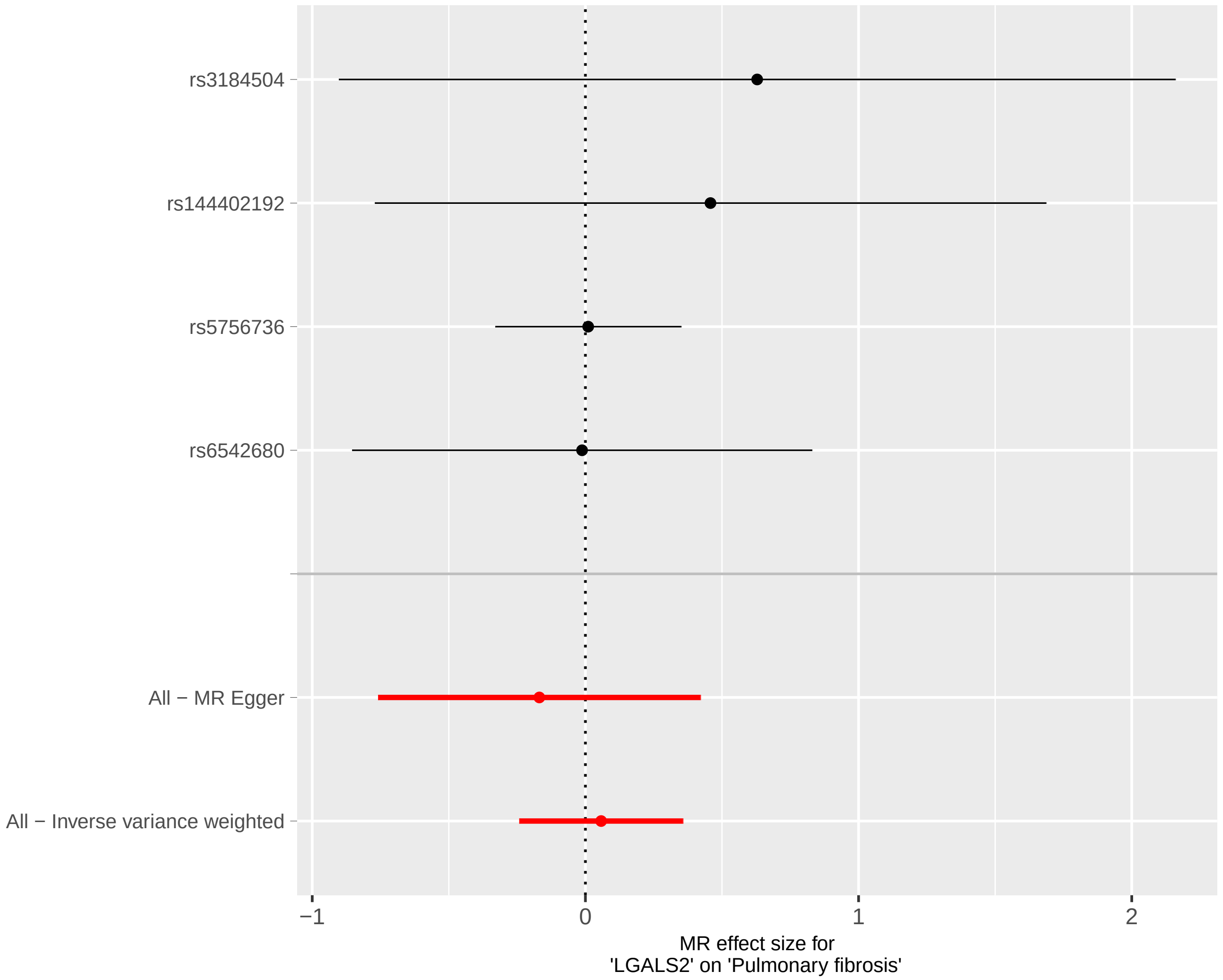

rs3184504
rs144402192
rs5756736
rs6542680
All − MR Egger
All − Inverse variance weighted
−1
0
1
2
MR effect size for
'LGALS2' on 'Pulmonary fibrosis'

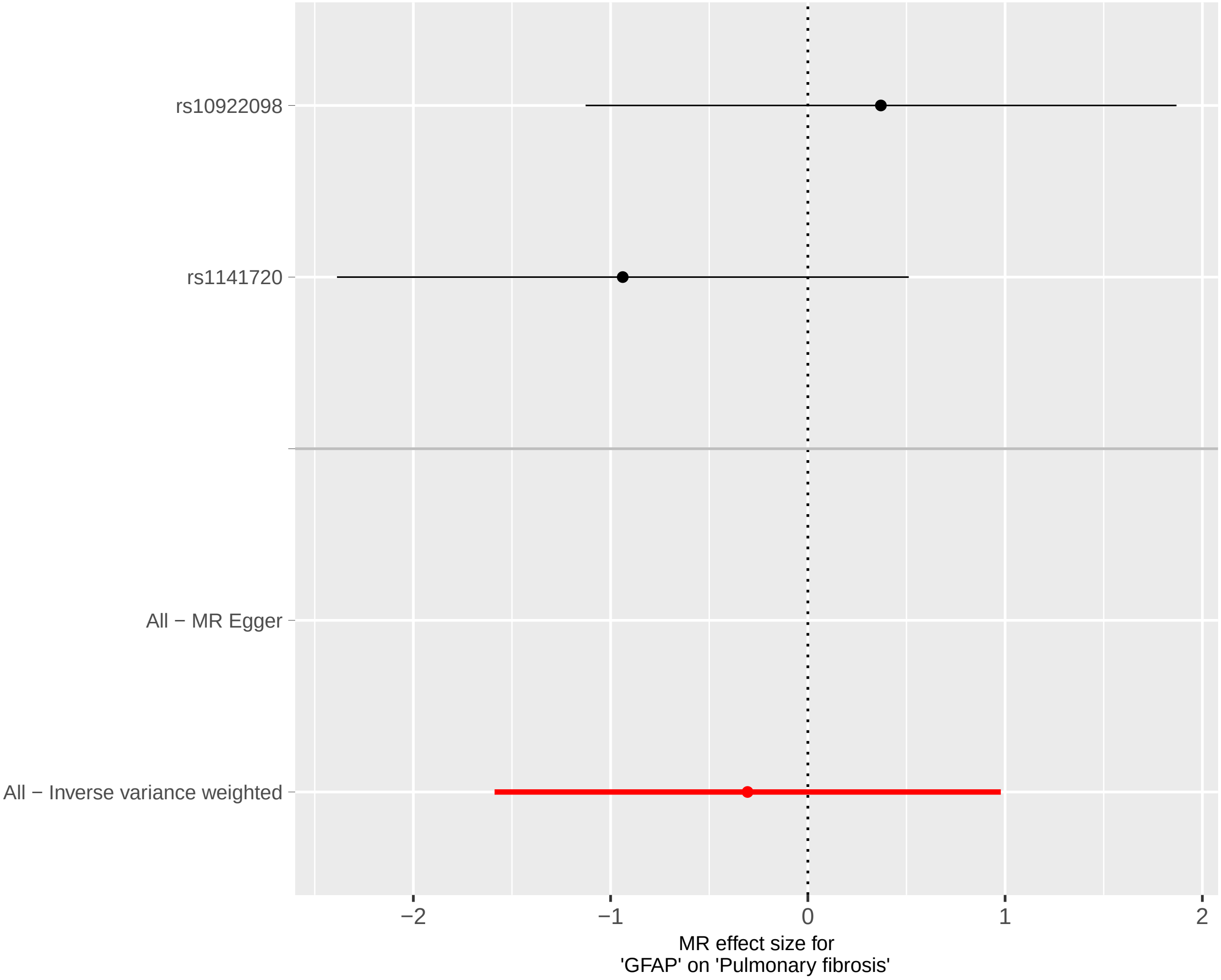
rs10922098
rs1141720
All − MR Egger
All − Inverse variance weighted
−2
−1
0
1
2
MR effect size for
'GFAP' on 'Pulmonary fibrosis'

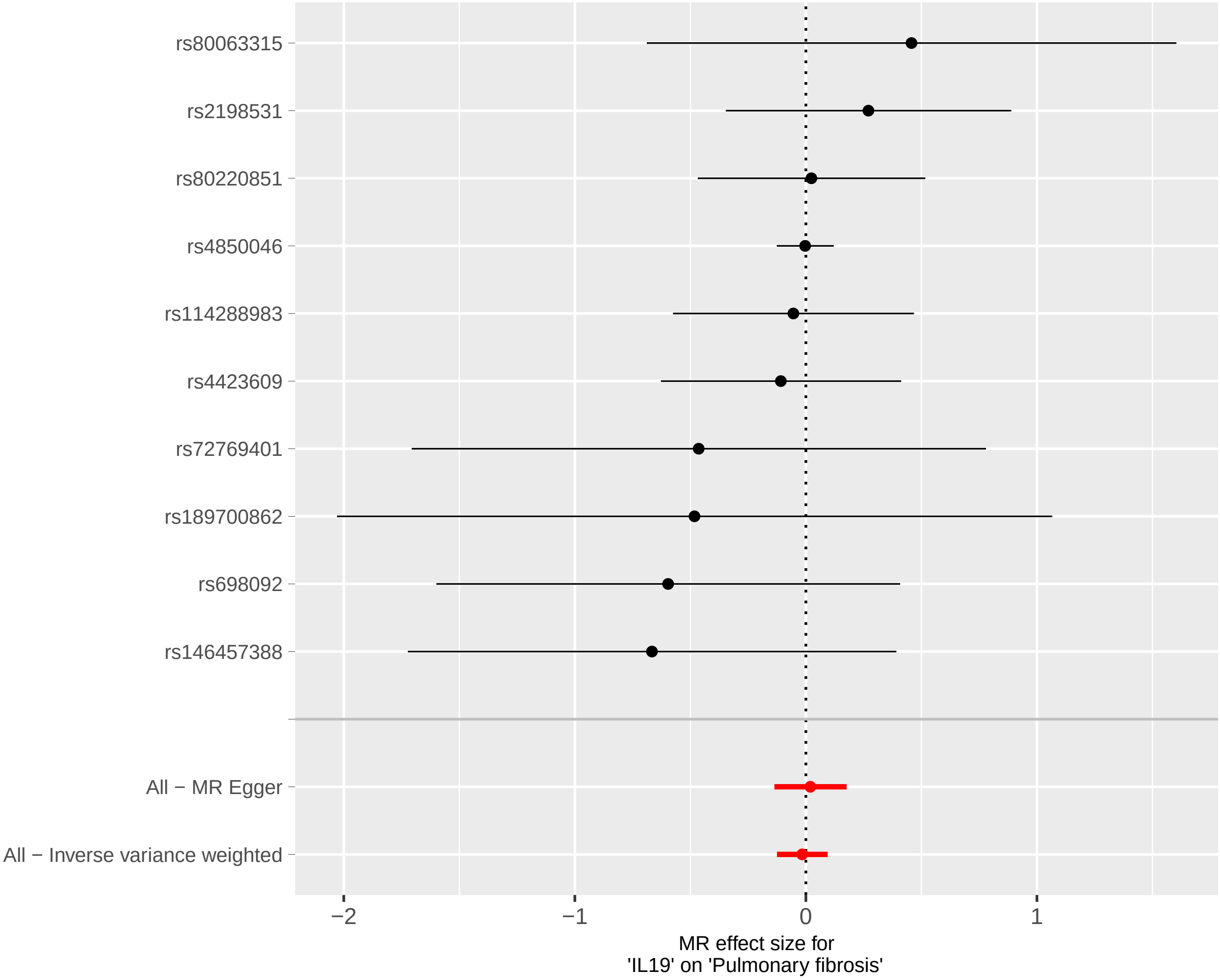
rs80063315
rs2198531
rs80220851
rs4850046
rs114288983
rs4423609
rs72769401
rs189700862
rs698092
rs146457388
All − MR Egger
All − Inverse variance weighted
−2
−1
0
1
MR effect size for
'IL19' on 'Pulmonary fibrosis'

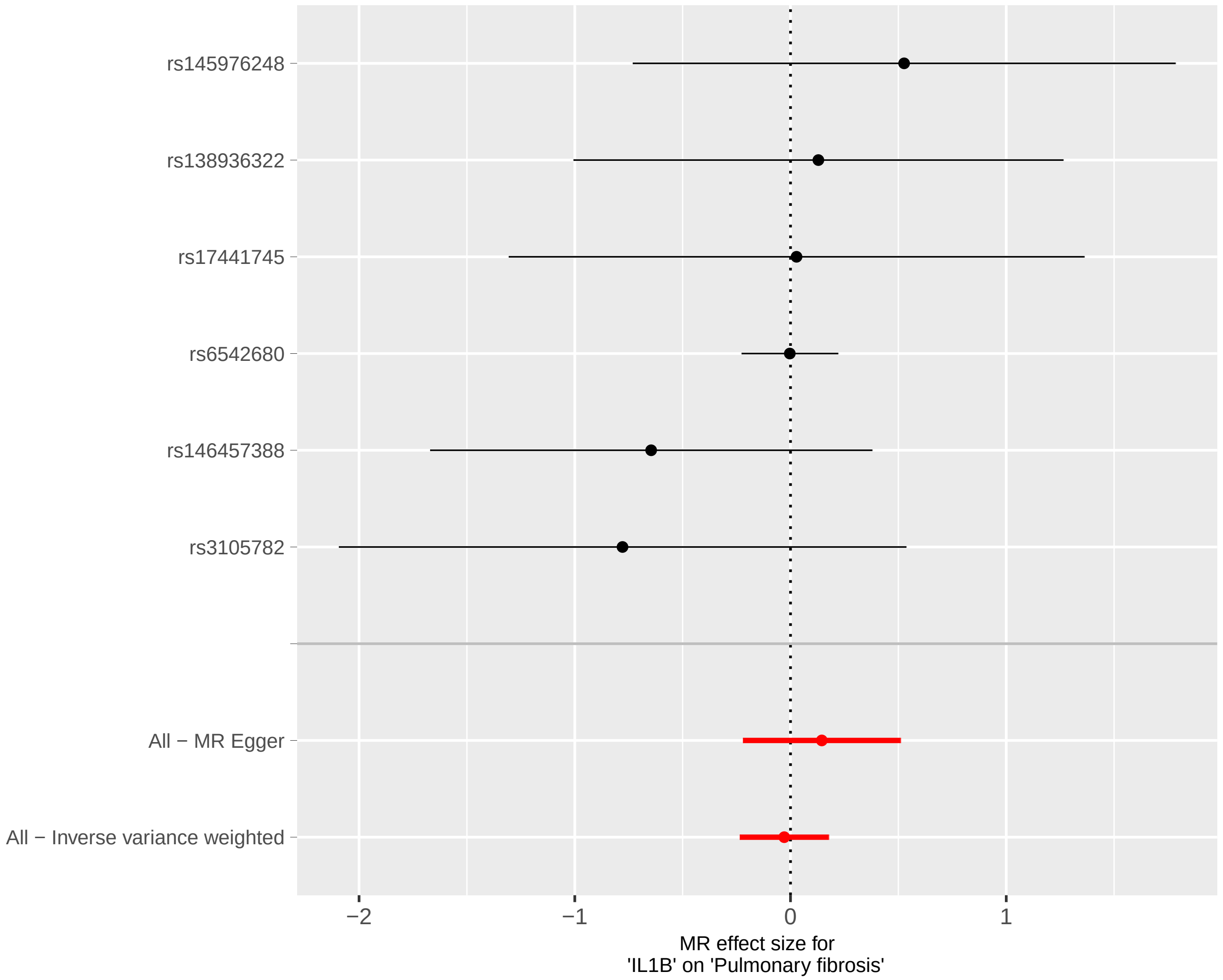
rs145976248
rs138936322
rs17441745
rs6542680
rs146457388
rs3105782
All − MR Egger
All − Inverse variance weighted
−2
−1
0
1
MR effect size for
'IL1B' on 'Pulmonary fibrosis'

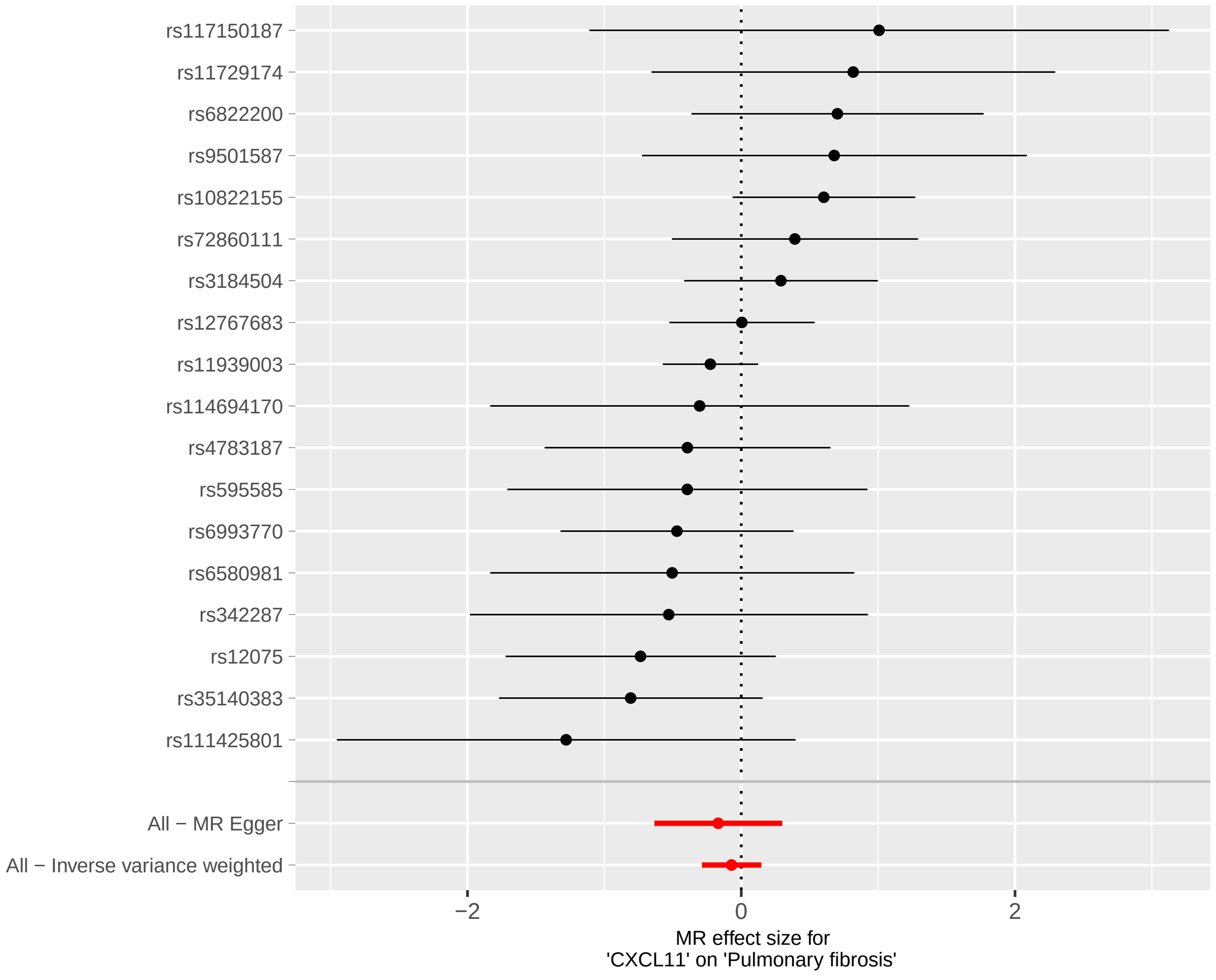
rs117150187
rs11729174
rs6822200
rs9501587
rs10822155
rs72860111
rs3184504
rs12767683
rs11939003
rs114694170
rs4783187
rs595585
rs6993770
rs6580981
rs342287
rs12075
rs35140383
rs111425801
All − MR Egger
All − Inverse variance weighted
−2
0
2
MR effect size for
'CXCL11' on 'Pulmonary fibrosis'

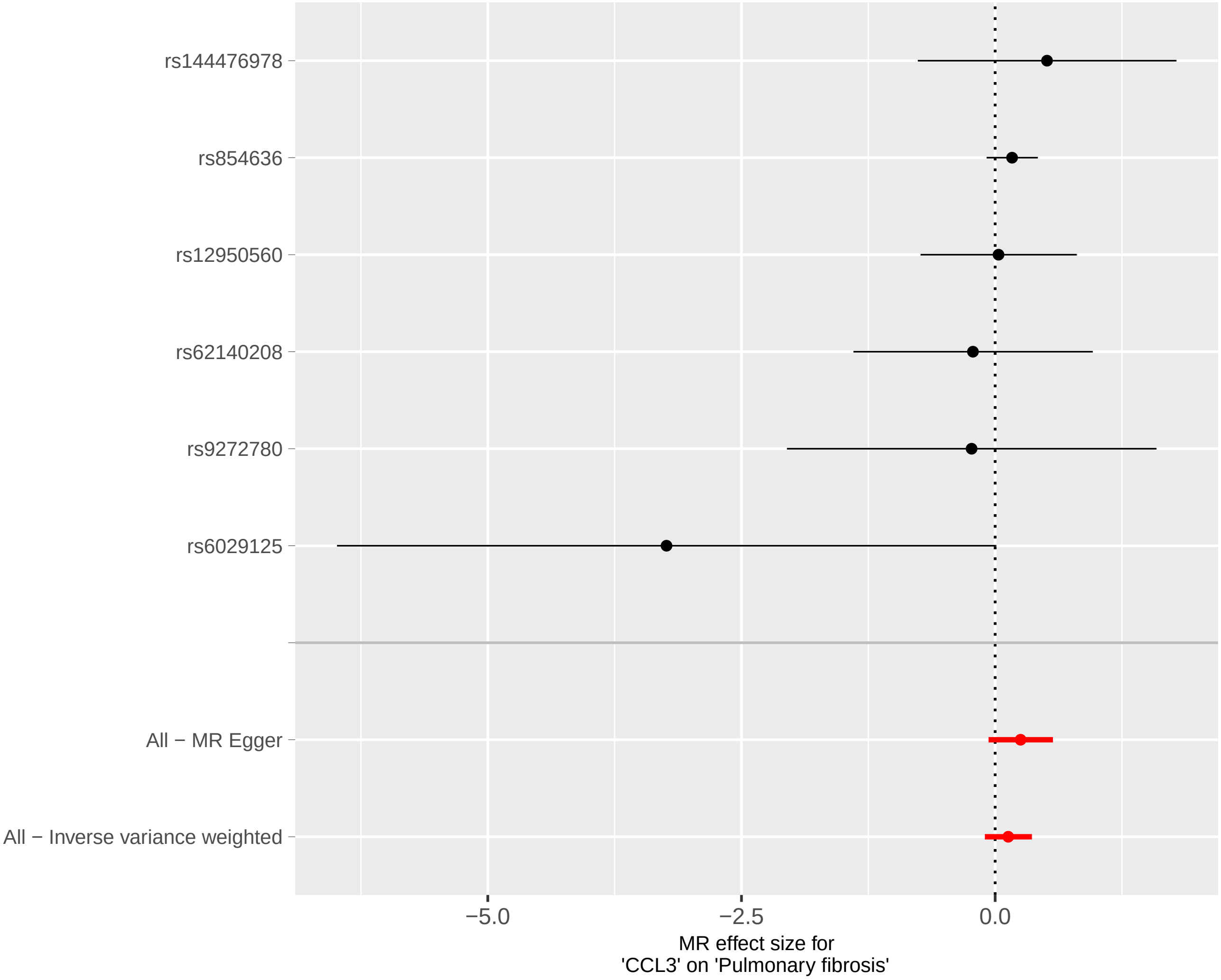
rs144476978
rs854636
rs12950560
rs62140208
rs9272780
rs6029125
All − MR Egger
All − Inverse variance weighted
−5.0
−2.5
0.0
MR effect size for
'CCL3' on 'Pulmonary fibrosis'

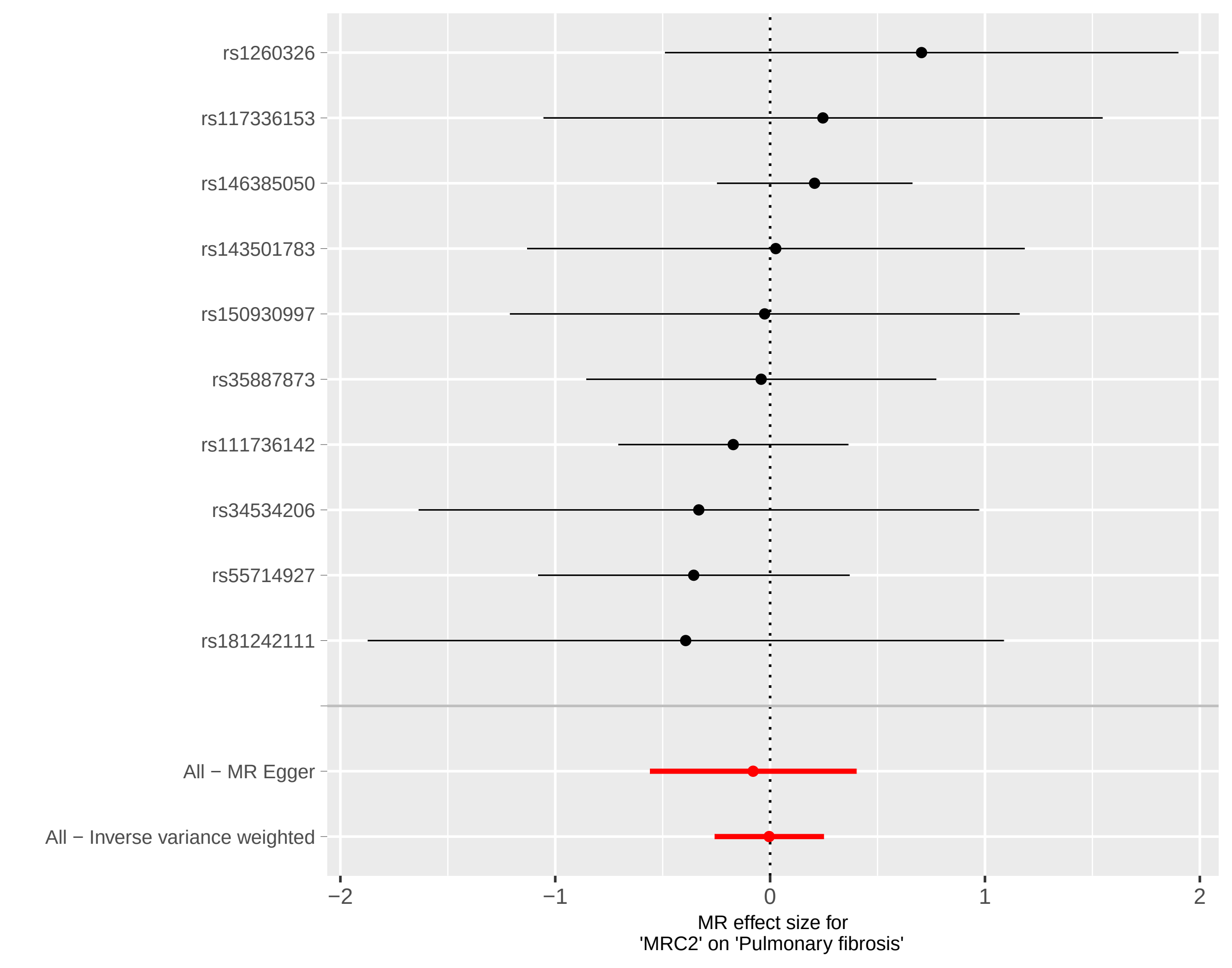
rs1260326
rs117336153
rs146385050
rs143501783
rs150930997
rs35887873
rs111736142
rs34534206
rs55714927
rs181242111
All − MR Egger
All − Inverse variance weighted
−2
−1
0
1
2
MR effect size for
'MRC2' on 'Pulmonary fibrosis'

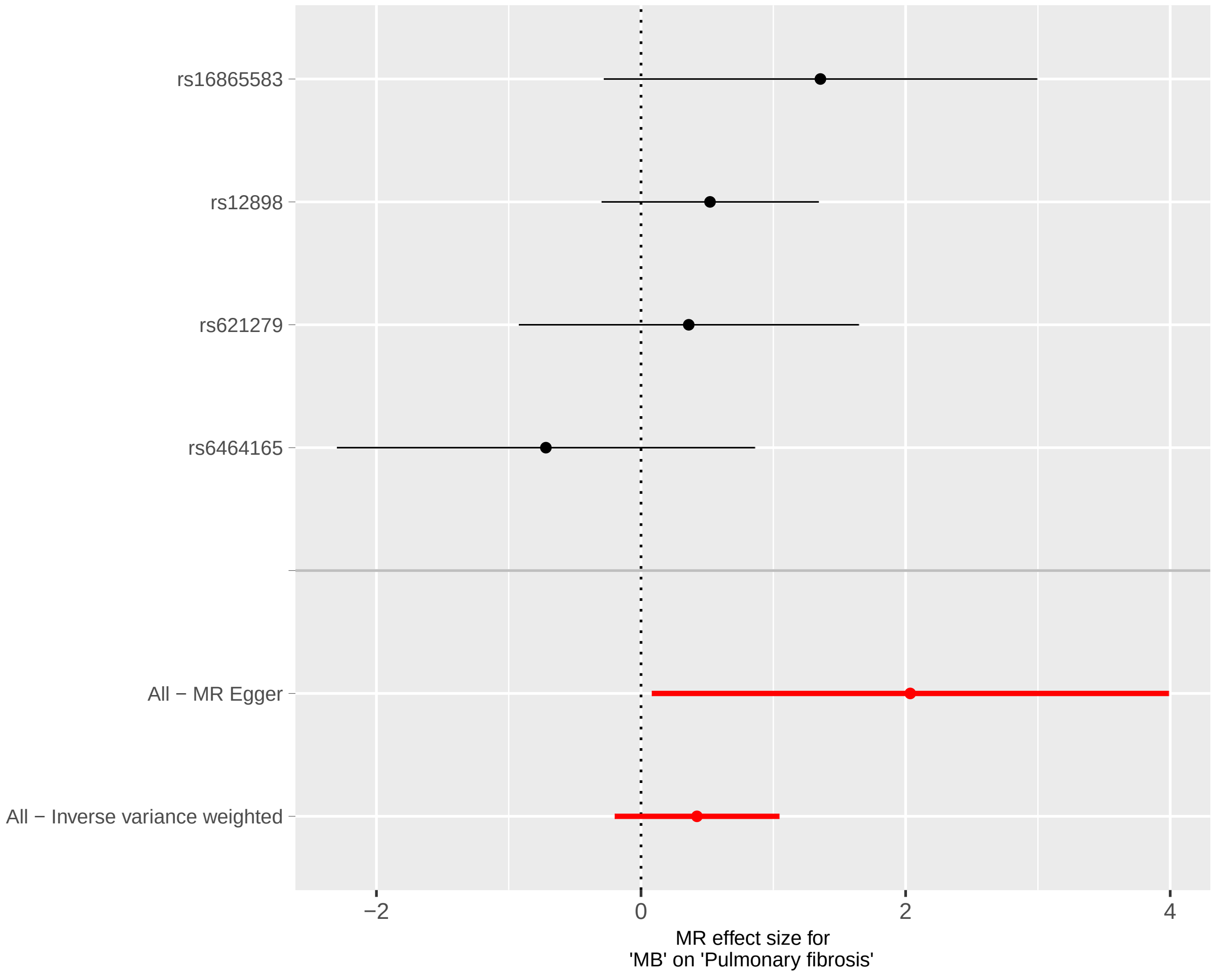

rs16865583
rs12898
rs621279
rs6464165
All − MR Egger
All − Inverse variance weighted
−2
0
2
4
MR effect size for
'MB' on 'Pulmonary fibrosis'

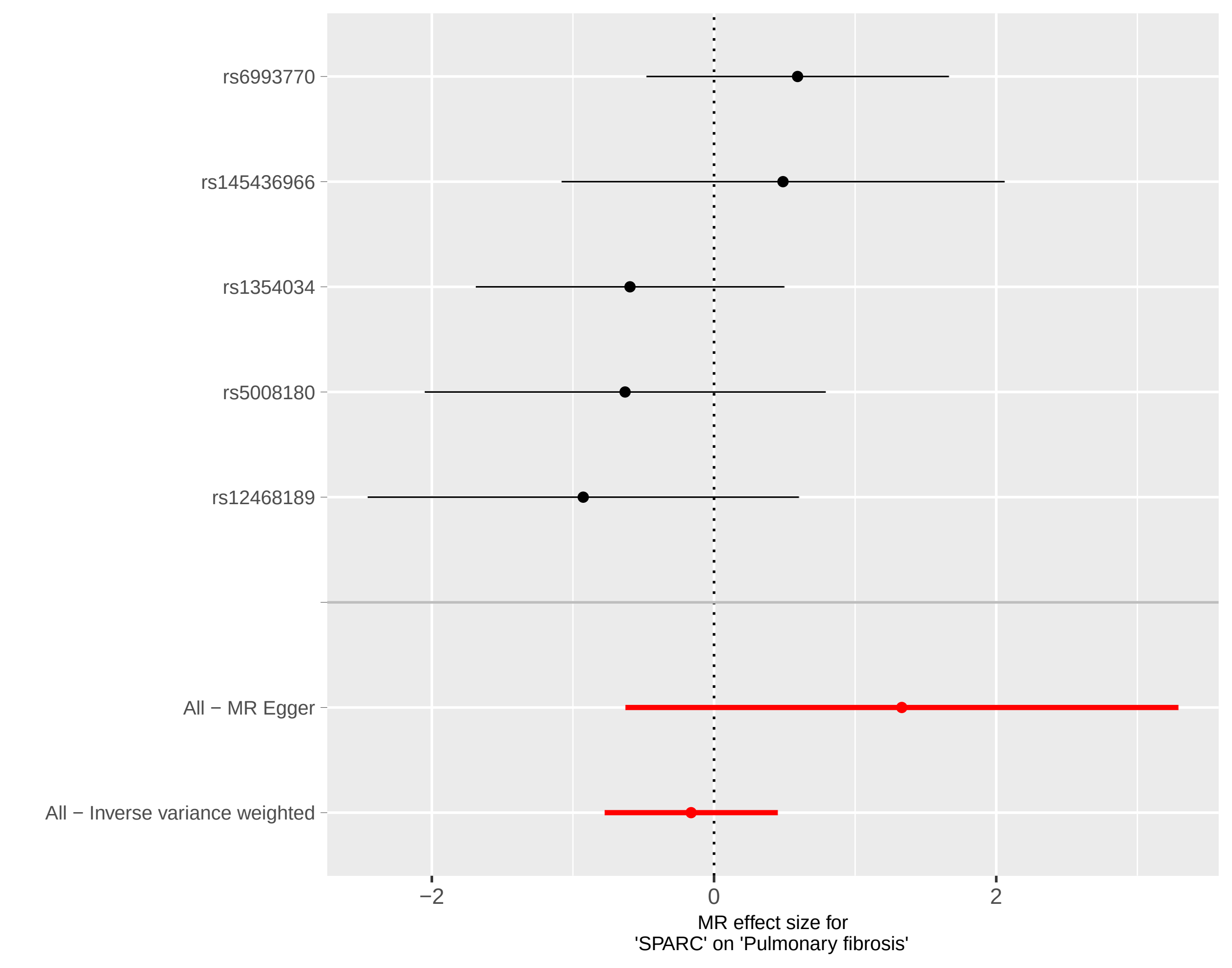

rs6993770
rs145436966
rs1354034
rs5008180
rs12468189
All − MR Egger
All − Inverse variance weighted
−2
0
2
MR effect size for
'SPARC' on 'Pulmonary fibrosis'

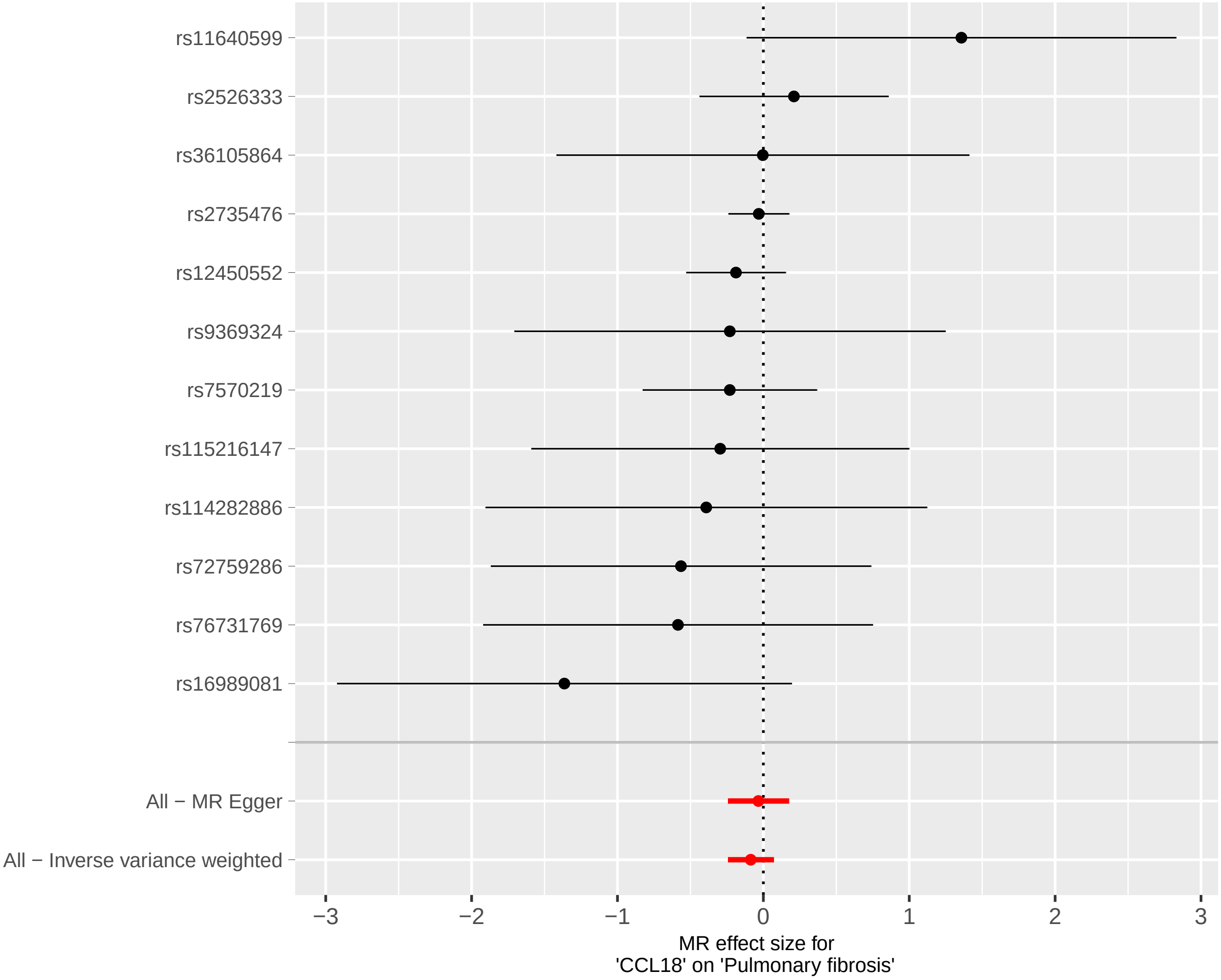
rs11640599
rs2526333
rs36105864
rs2735476
rs12450552
rs9369324
rs7570219
rs115216147
rs114282886
rs72759286
rs76731769
rs16989081
All − MR Egger
All − Inverse variance weighted
−3
−2
−1
0
1
2
3
MR effect size for
'CCL18' on 'Pulmonary fibrosis'

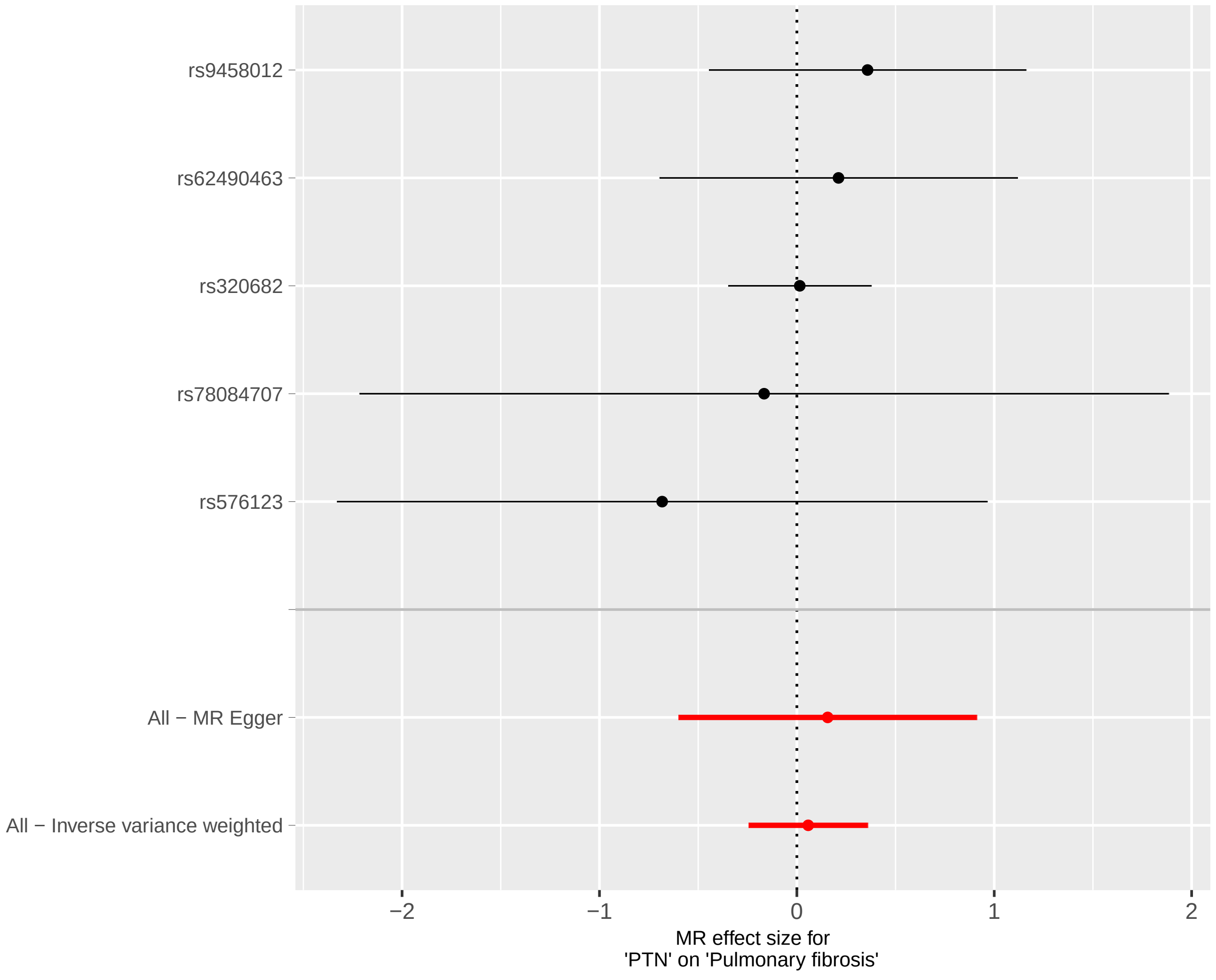

rs9458012
rs62490463
rs320682
rs78084707
rs576123
All − MR Egger
All − Inverse variance weighted
−2
−1
0
1
2
MR effect size for
'PTN' on 'Pulmonary fibrosis'

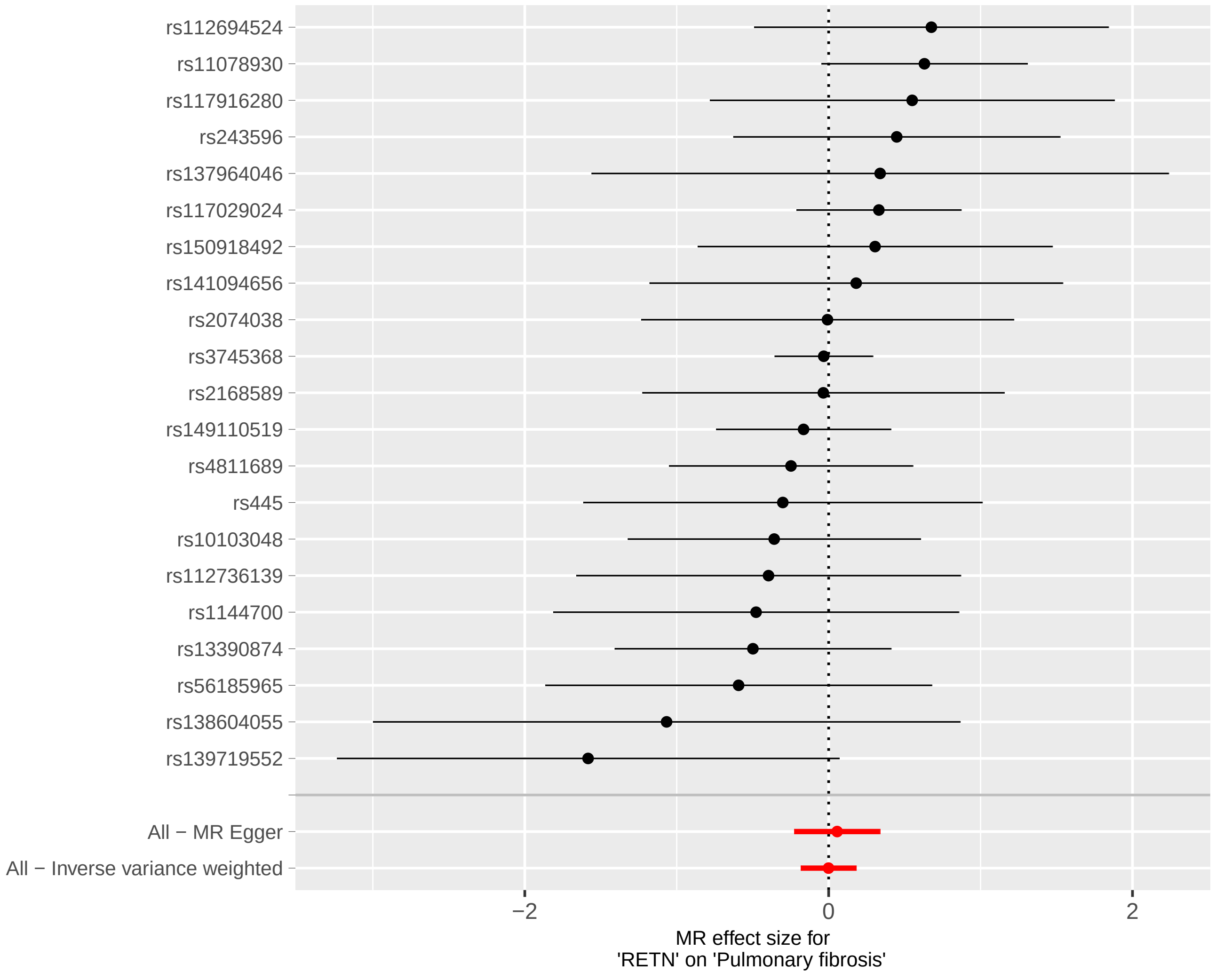
rs112694524
rs11078930
rs117916280
rs243596
rs137964046
rs117029024
rs150918492
rs141094656
rs2074038
rs3745368
rs2168589
rs149110519
rs4811689
rs445
rs10103048
rs112736139
rs1144700
rs13390874
rs56185965
rs138604055
rs139719552
All − MR Egger
All − Inverse variance weighted
−2
0
2
MR effect size for
'RETN' on 'Pulmonary fibrosis'

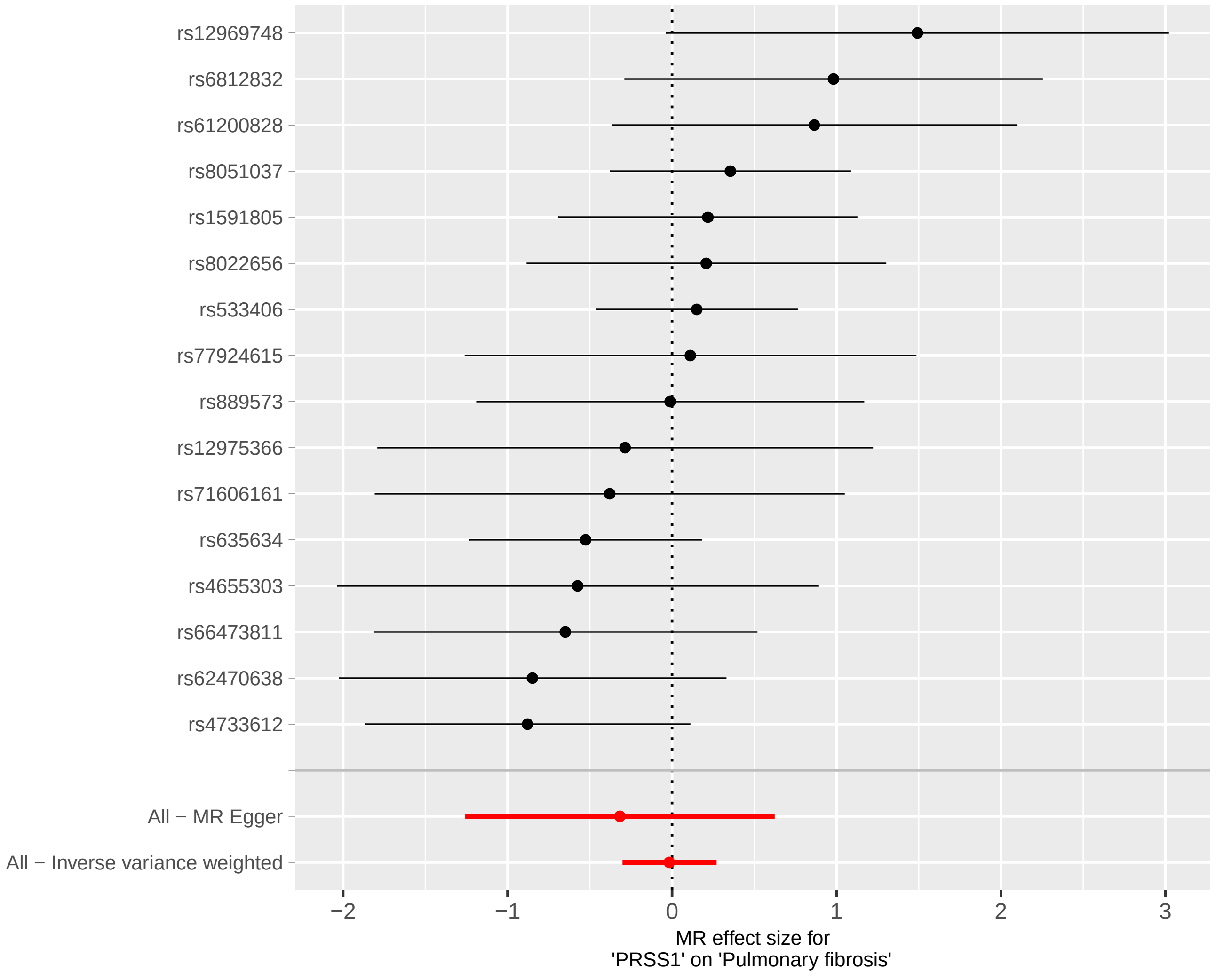
rs12969748
rs6812832
rs61200828
rs8051037
rs1591805
rs8022656
rs533406
rs77924615
rs889573
rs12975366
rs71606161
rs635634
rs4655303
rs66473811
rs62470638
rs4733612
All − MR Egger
All − Inverse variance weighted
−2
−1
0
1
2
3
MR effect size for
'PRSS1' on 'Pulmonary fibrosis'

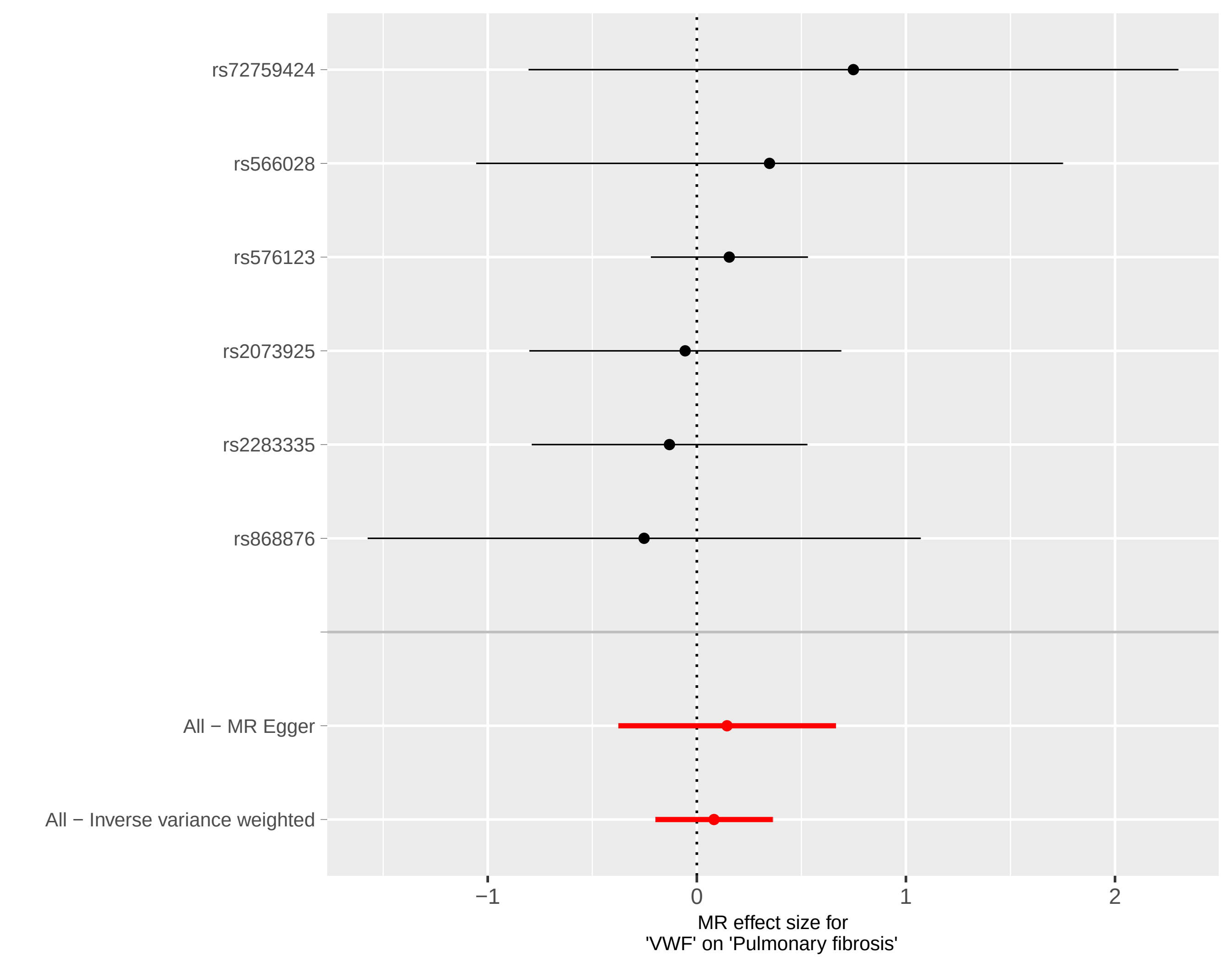

rs72759424
rs566028
rs576123
rs2073925
rs2283335
rs868876
All − MR Egger
All − Inverse variance weighted
−1
0
1
2
MR effect size for
'VWF' on 'Pulmonary fibrosis'

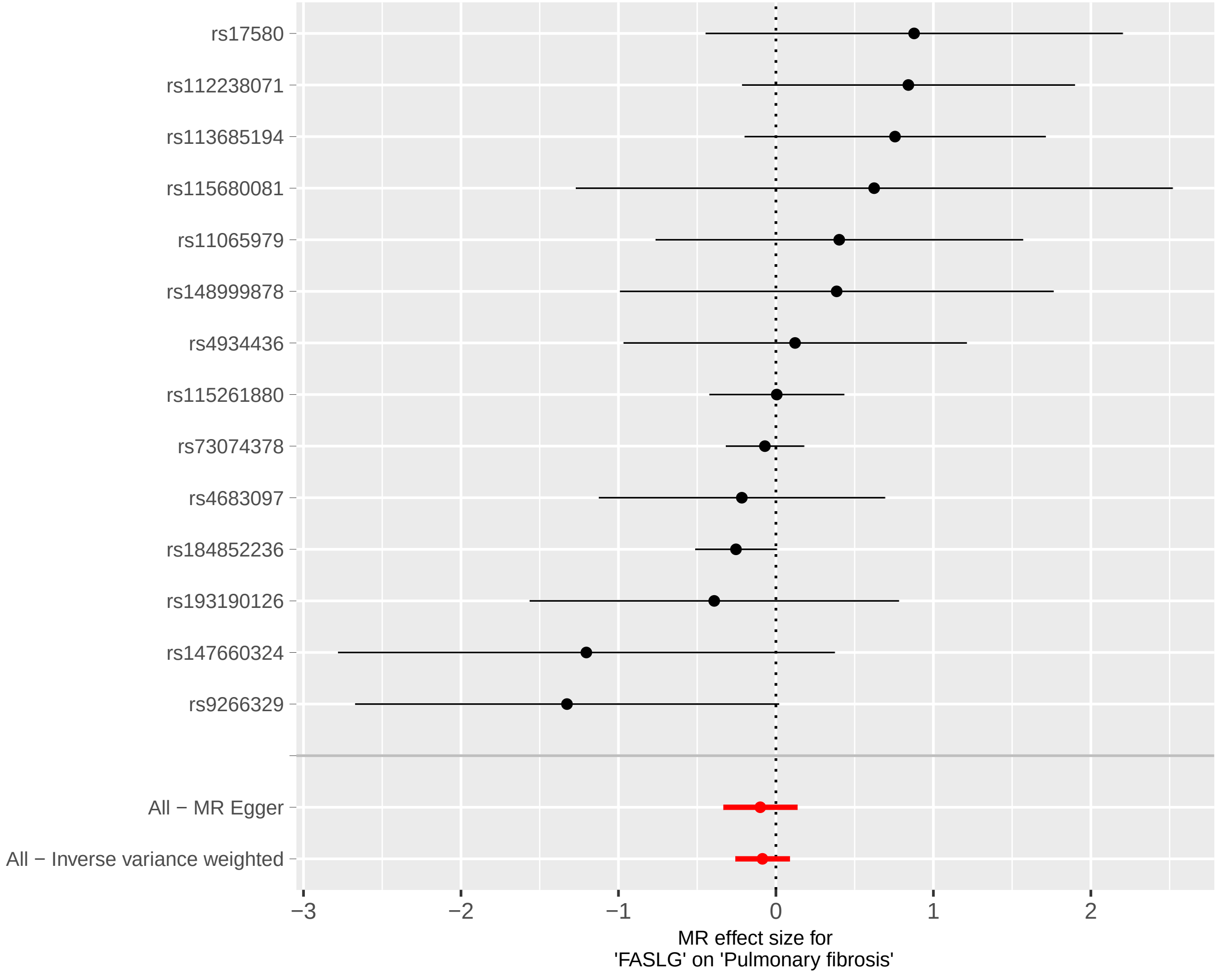
rs17580
rs112238071
rs113685194
rs115680081
rs11065979
rs148999878
rs4934436
rs115261880
rs73074378
rs4683097
rs184852236
rs193190126
rs147660324
rs9266329
All − MR Egger
All − Inverse variance weighted
−3
−2
−1
0
1
2
MR effect size for
'FASLG' on 'Pulmonary fibrosis'

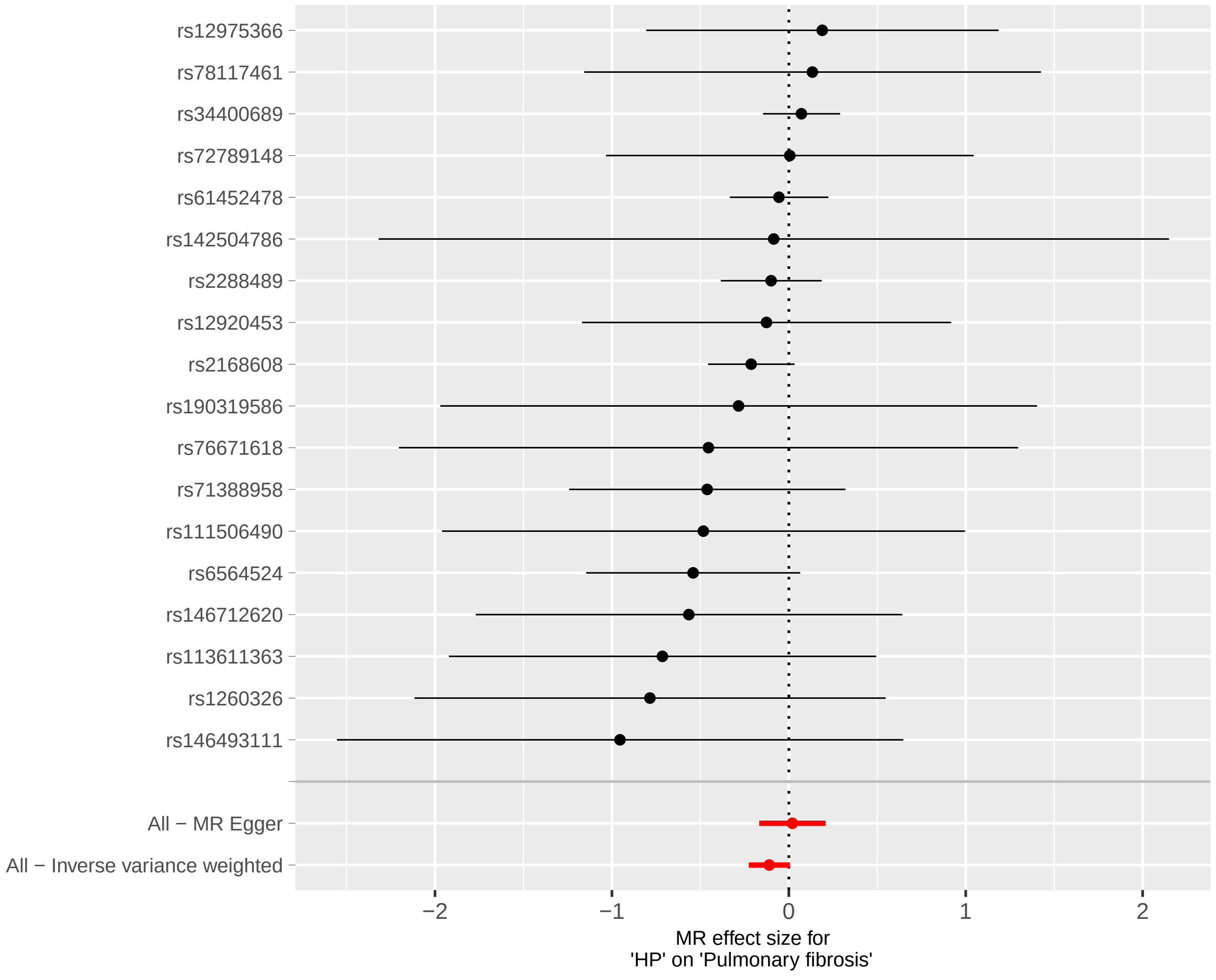
rs12975366
rs78117461
rs34400689
rs72789148
rs61452478
rs142504786
rs2288489
rs12920453
rs2168608
rs190319586
rs76671618
rs71388958
rs111506490
rs6564524
rs146712620
rs113611363
rs1260326
rs146493111
All − MR Egger
All − Inverse variance weighted
−2
−1
0
1
2
MR effect size for
'HP' on 'Pulmonary fibrosis'

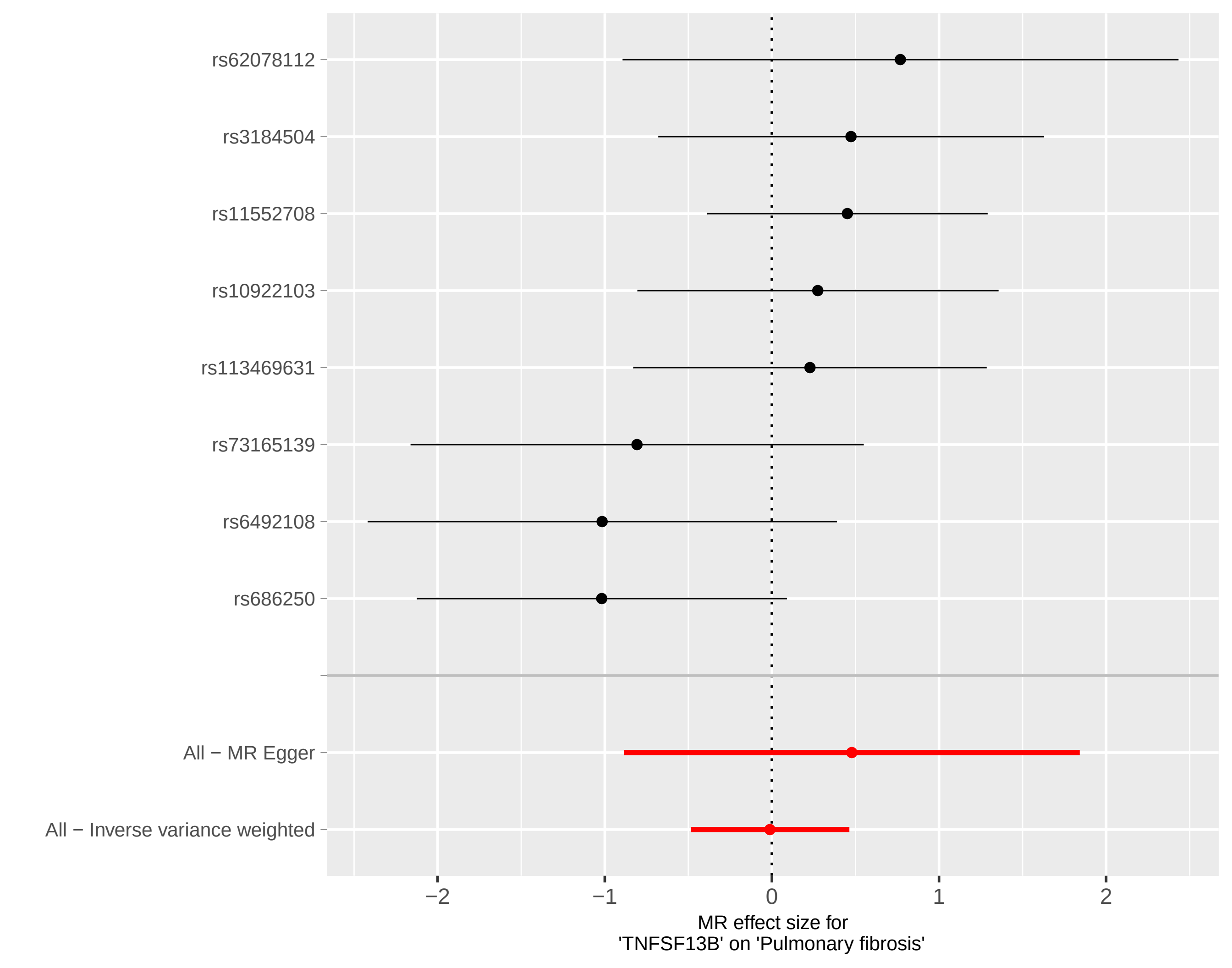
rs62078112
rs3184504
rs11552708
rs10922103
rs113469631
rs73165139
rs6492108
rs686250
All − MR Egger
All − Inverse variance weighted
−2
−1
0
1
2
MR effect size for
'TNFSF13B' on 'Pulmonary fibrosis'

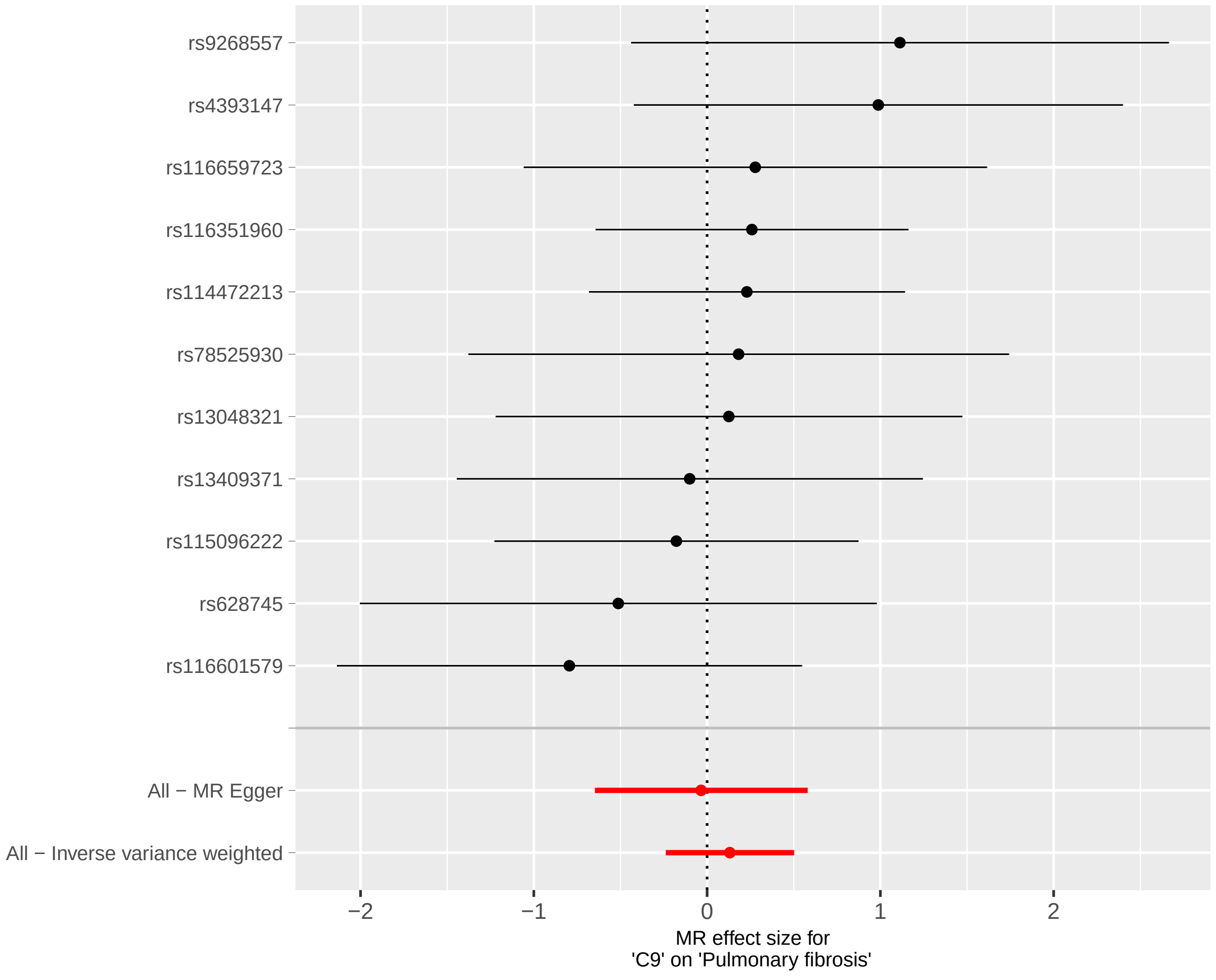
rs9268557
rs4393147
rs116659723
rs116351960
rs114472213
rs78525930
rs13048321
rs13409371
rs115096222
rs628745
rs116601579
All − MR Egger
All − Inverse variance weighted
−2
−1
0
1
2
MR effect size for
'C9' on 'Pulmonary fibrosis'

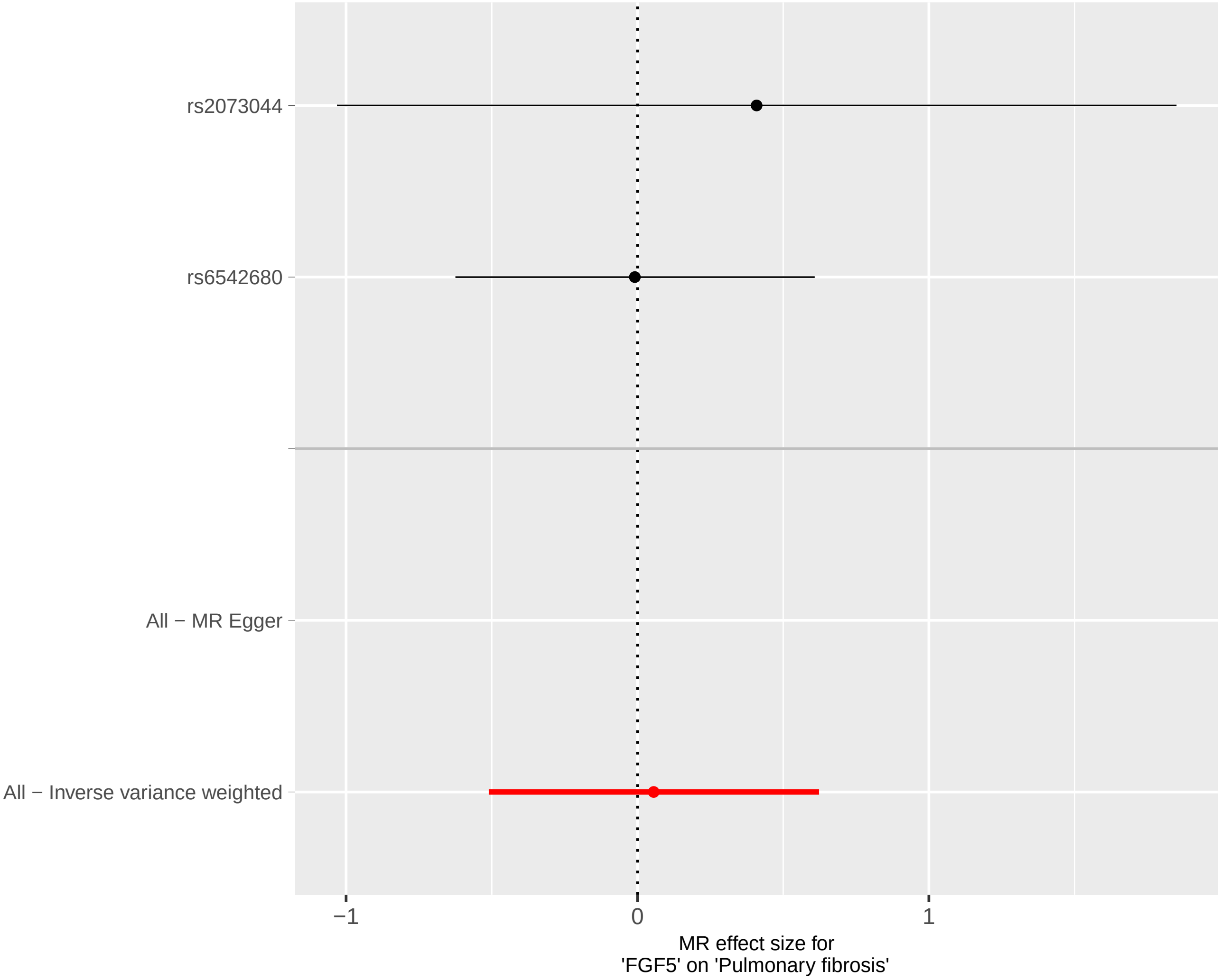
rs2073044
rs6542680
All − MR Egger
All − Inverse variance weighted
−1
0
1
MR effect size for
'FGF5' on 'Pulmonary fibrosis'

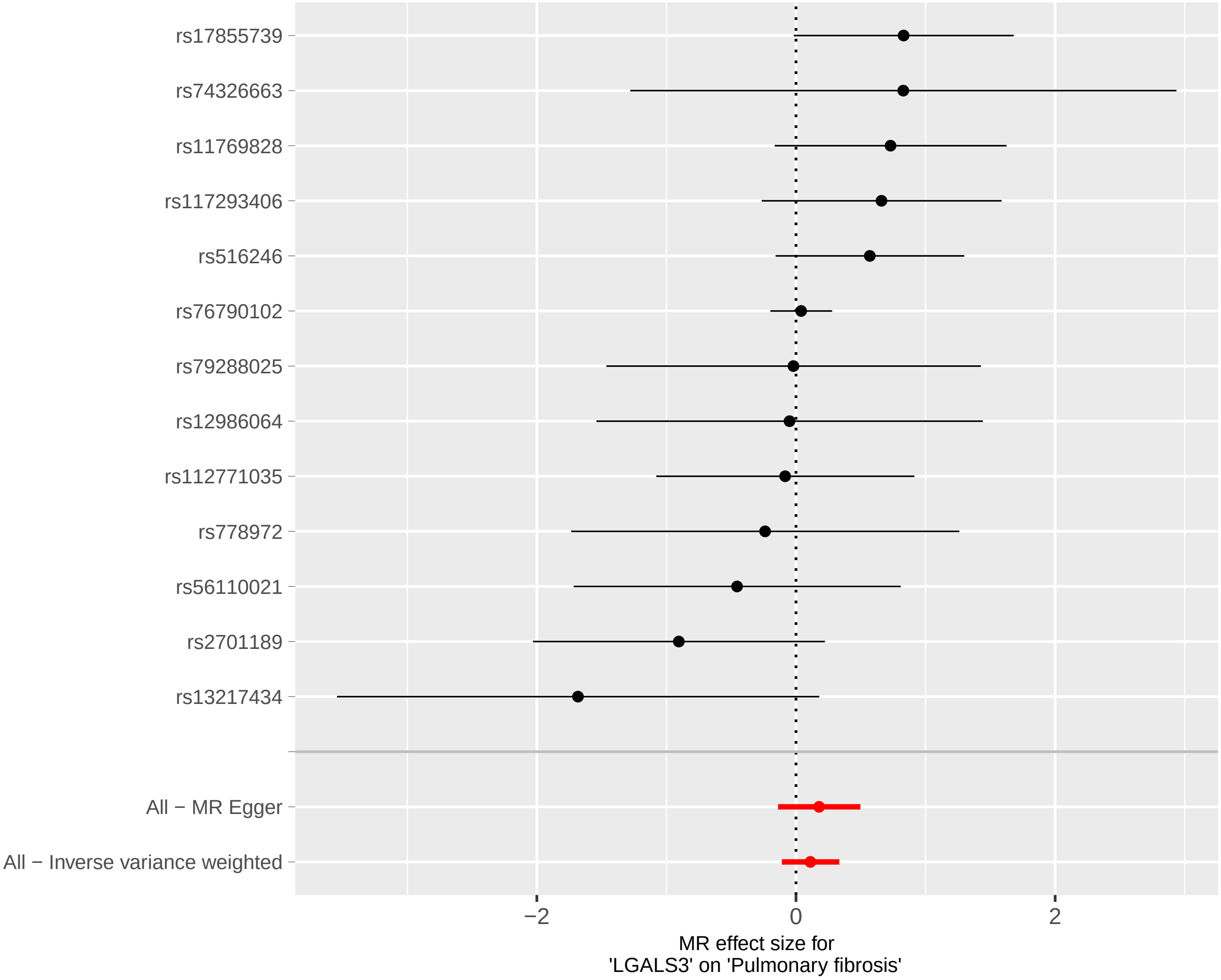

rs17855739
rs74326663
rs11769828
rs117293406
rs516246
rs76790102
rs79288025
rs12986064
rs112771035
rs778972
rs56110021
rs2701189
rs13217434
All − MR Egger
All − Inverse variance weighted
−2
0
2
MR effect size for
'LGALS3' on 'Pulmonary fibrosis'

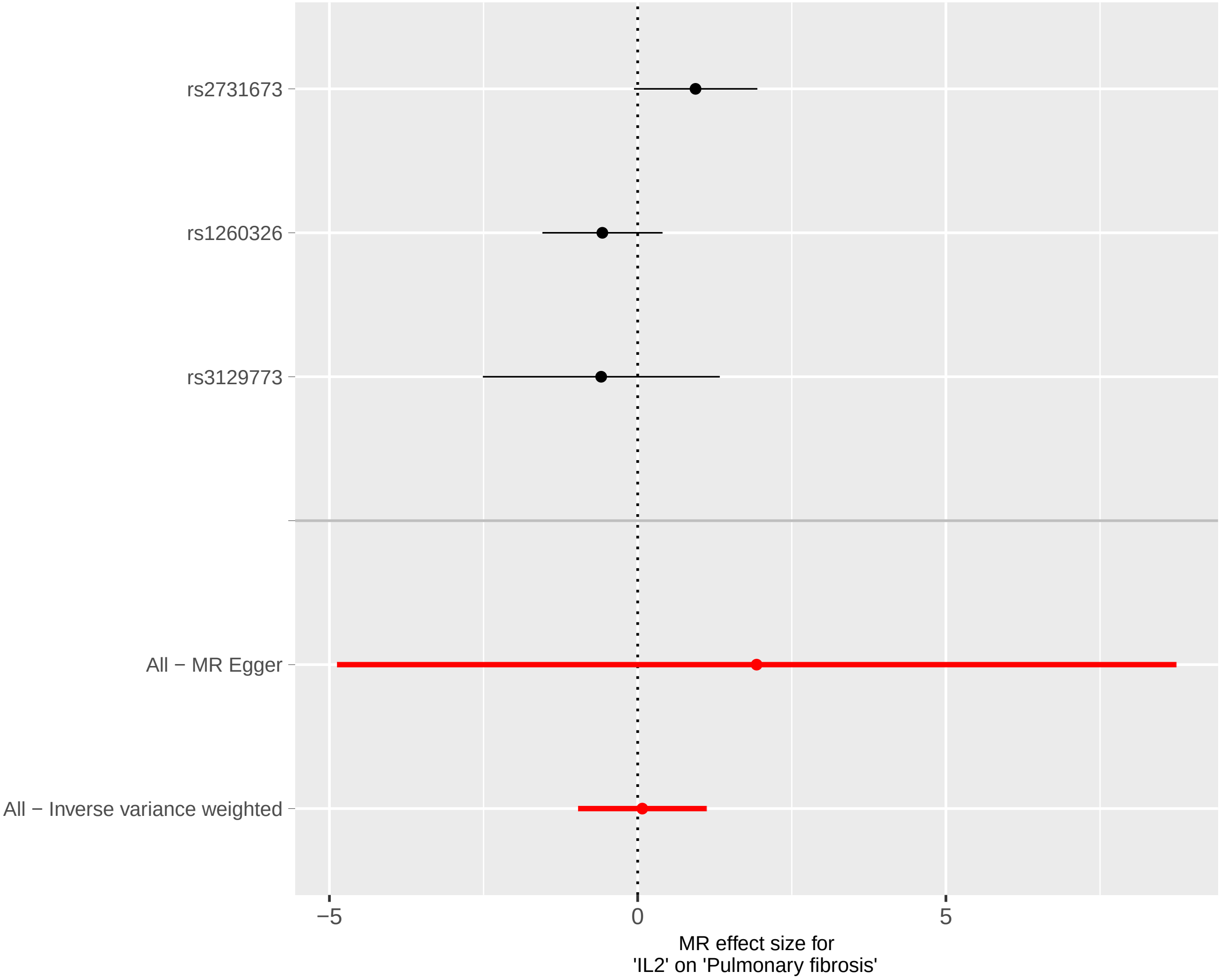

rs2731673
rs1260326
rs3129773
All − MR Egger
All − Inverse variance weighted
−5
0
5
MR effect size for
'IL2' on 'Pulmonary fibrosis'

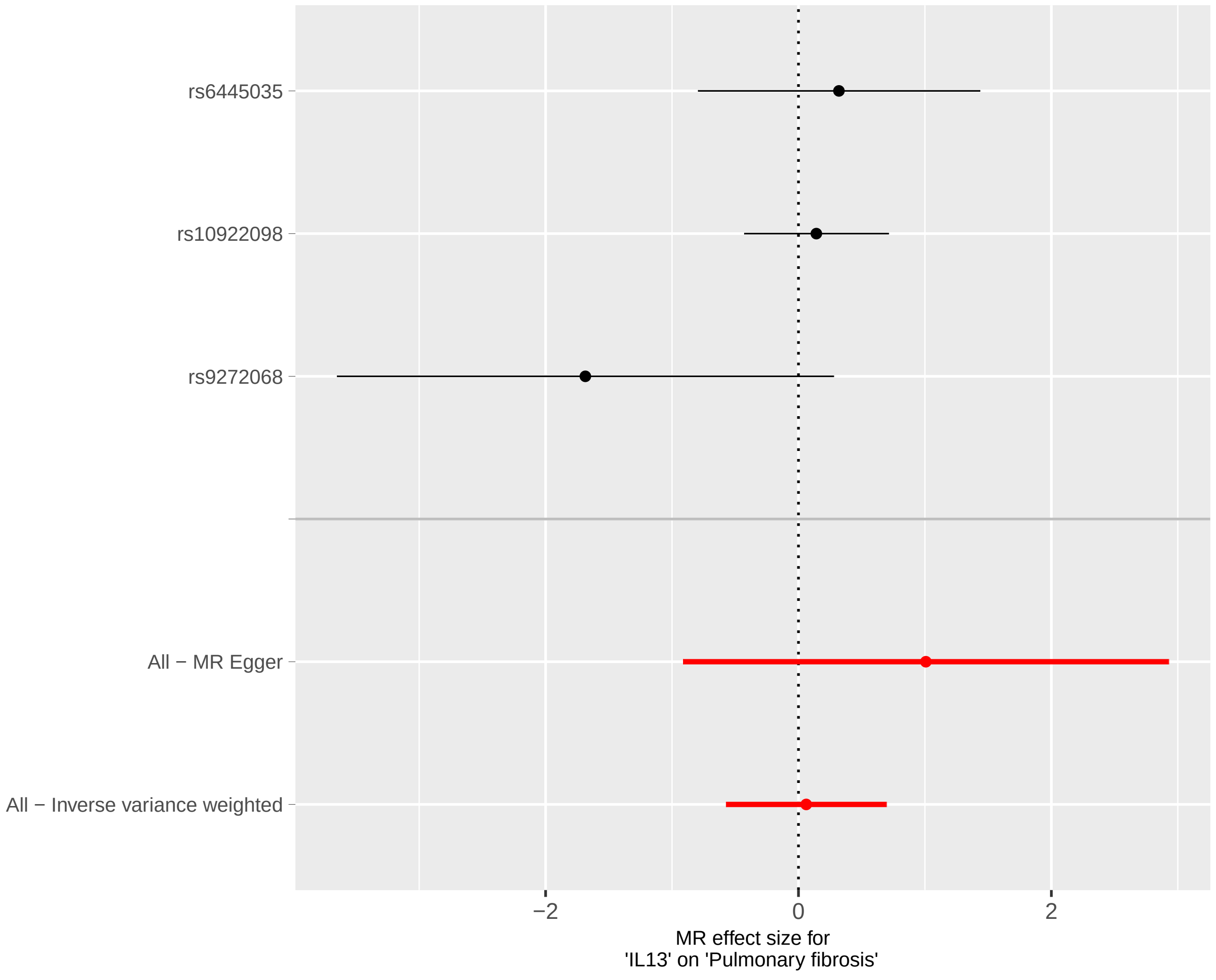

rs6445035
rs10922098
rs9272068
All − MR Egger
All − Inverse variance weighted
−2
0
2
MR effect size for
'IL13' on 'Pulmonary fibrosis'

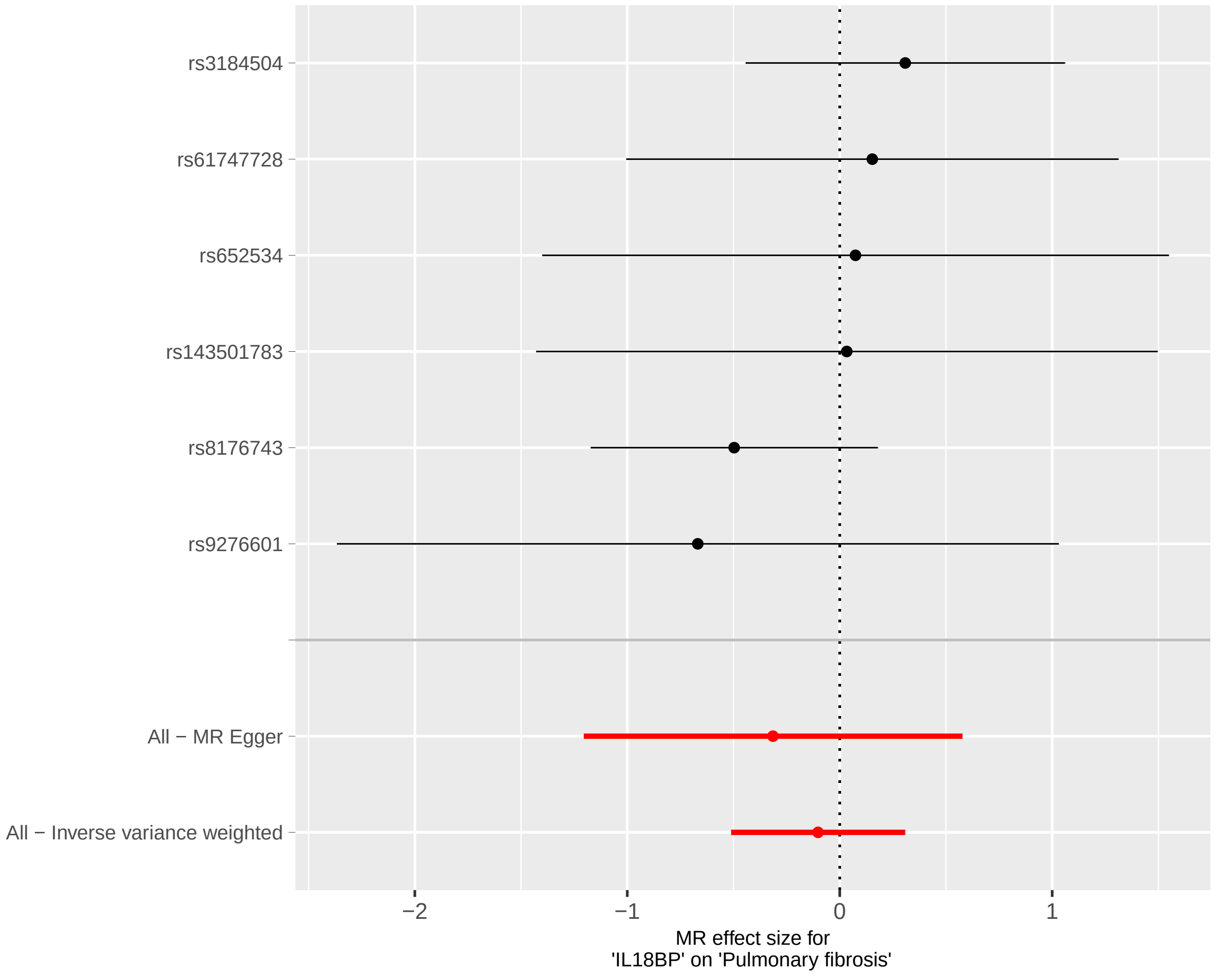

rs3184504
rs61747728
rs652534
rs143501783
rs8176743
rs9276601
All − MR Egger
All − Inverse variance weighted
−2
−1
0
1
MR effect size for
'IL18BP' on 'Pulmonary fibrosis'

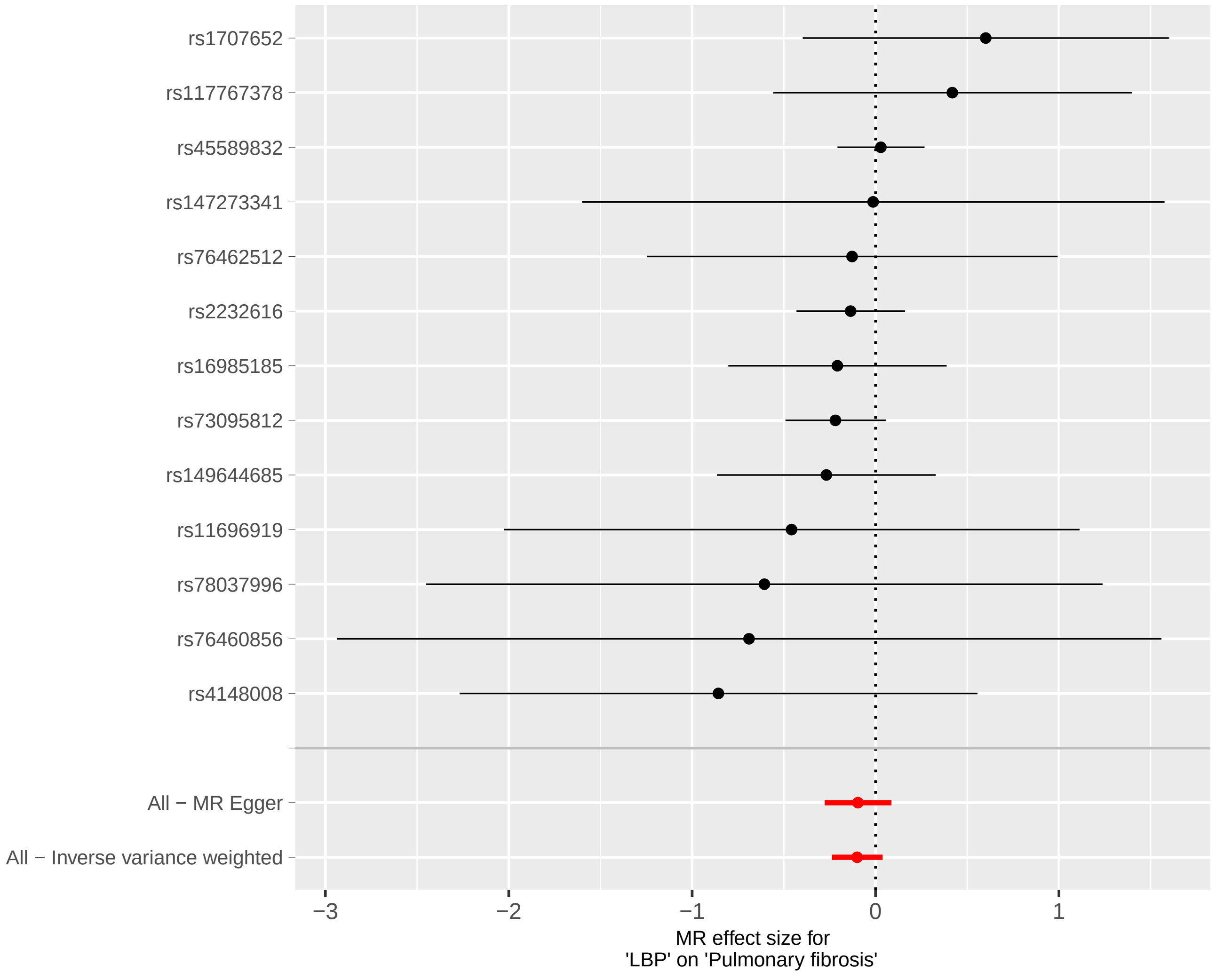
rs1707652
rs117767378
rs45589832
rs147273341
rs76462512
rs2232616
rs16985185
rs73095812
rs149644685
rs11696919
rs78037996
rs76460856
rs4148008
All − MR Egger
All − Inverse variance weighted
−3
−2
−1
0
1
MR effect size for
'LBP' on 'Pulmonary fibrosis'

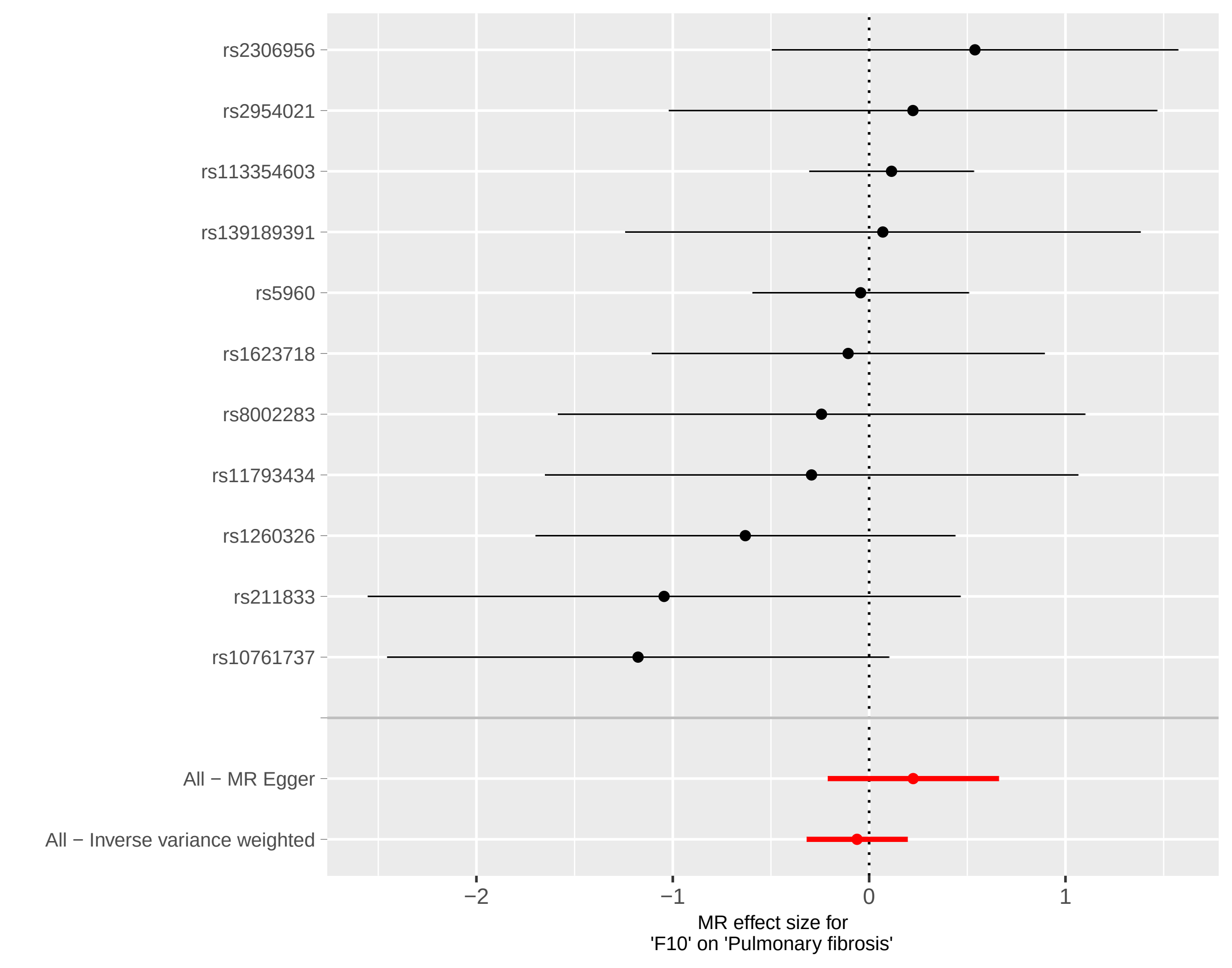
rs2306956
rs2954021
rs113354603
rs139189391
rs5960
rs1623718
rs8002283
rs11793434
rs1260326
rs211833
rs10761737
All − MR Egger
All − Inverse variance weighted
−2
−1
0
1
MR effect size for
'F10' on 'Pulmonary fibrosis'

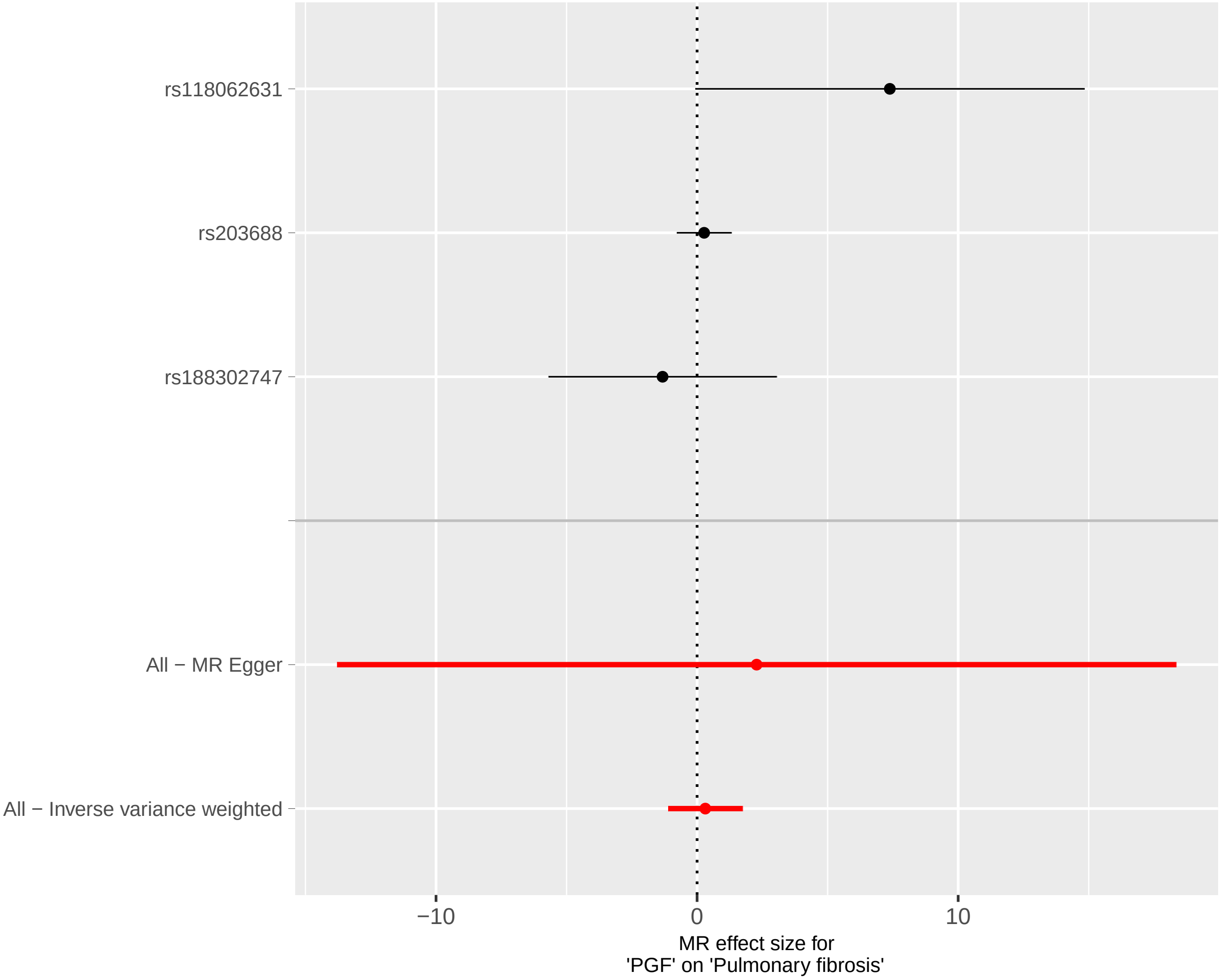
rs118062631
rs203688
rs188302747
All − MR Egger
All − Inverse variance weighted
−10
0
10
MR effect size for
'PGF' on 'Pulmonary fibrosis'

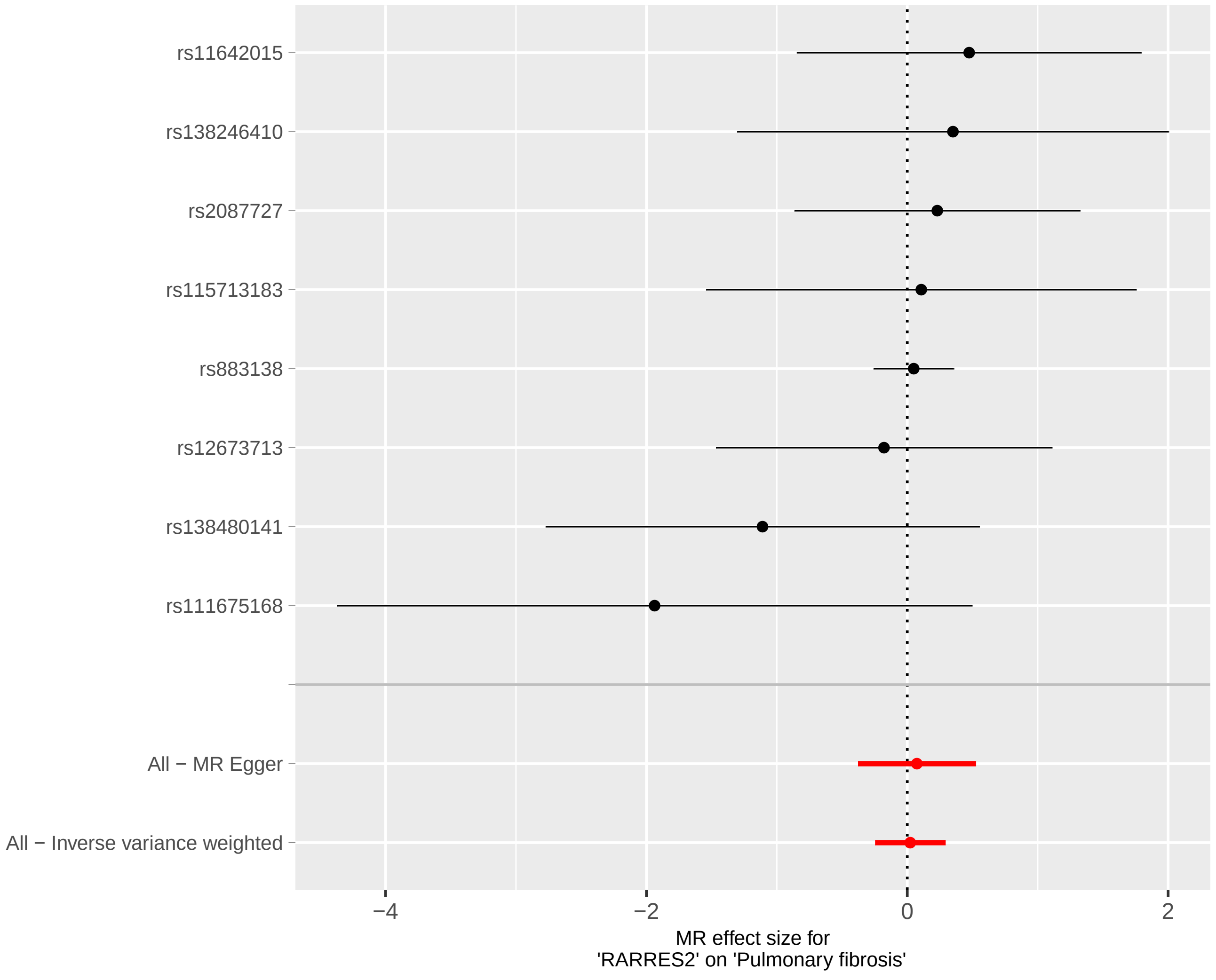
rs11642015
rs138246410
rs2087727
rs115713183
rs883138
rs12673713
rs138480141
rs111675168
All − MR Egger
All − Inverse variance weighted
−4
−2
0
2
MR effect size for
'RARRES2' on 'Pulmonary fibrosis'

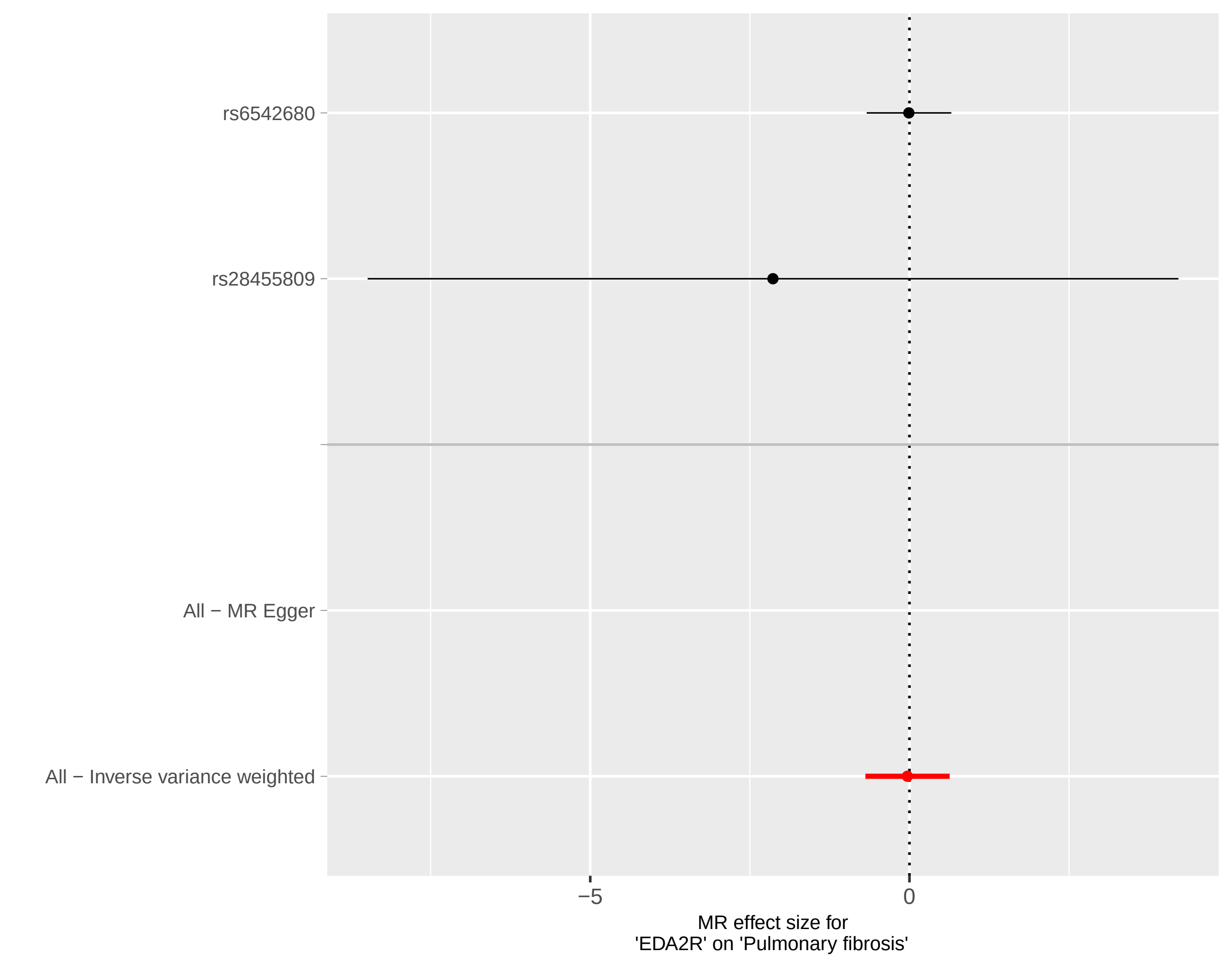
rs6542680
rs28455809
All − MR Egger
All − Inverse variance weighted
−5
0
MR effect size for
'EDA2R' on 'Pulmonary fibrosis'

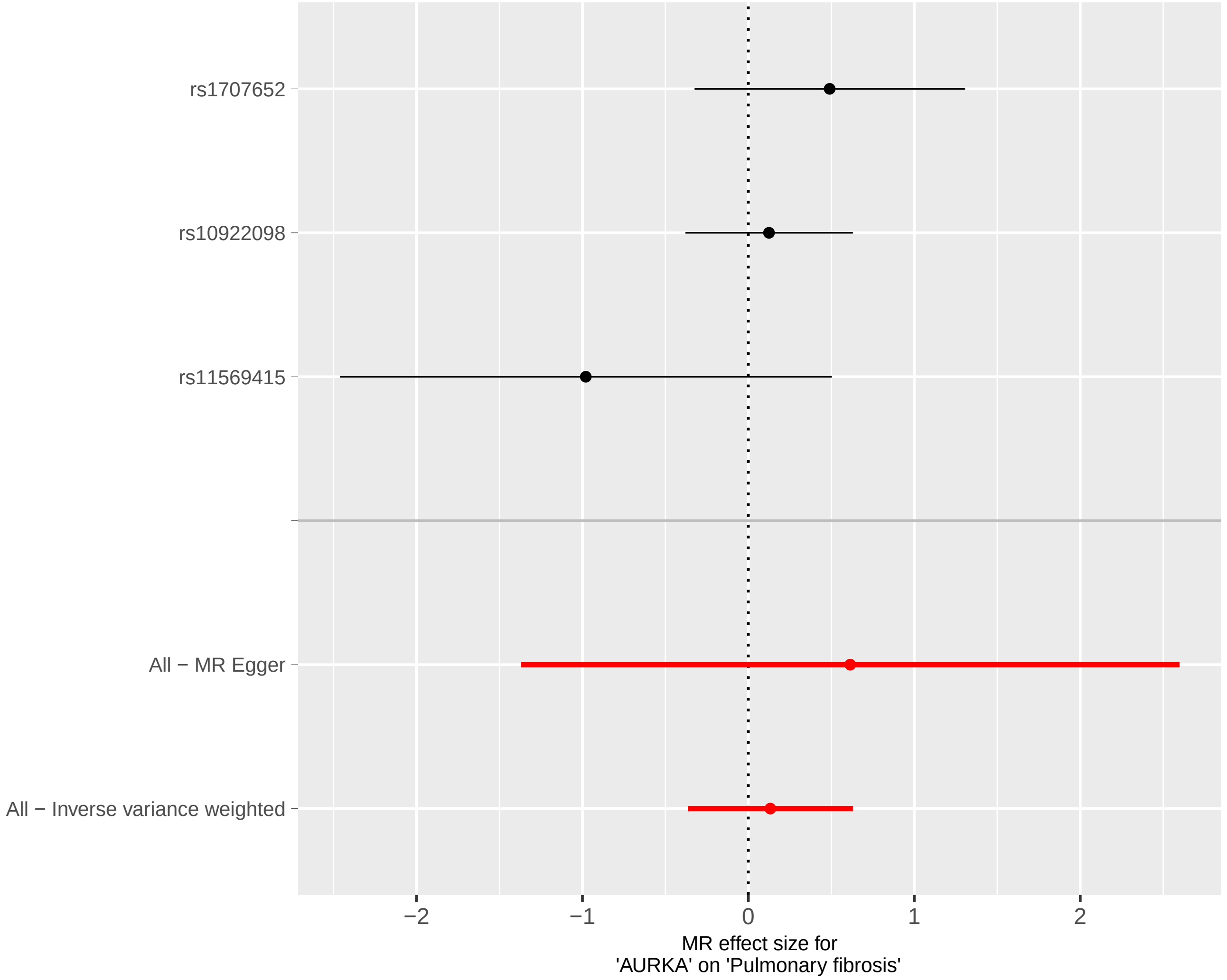
rs1707652
rs10922098
rs11569415
All − MR Egger
All − Inverse variance weighted
−2
−1
0
1
2
MR effect size for
'AURKA' on 'Pulmonary fibrosis'

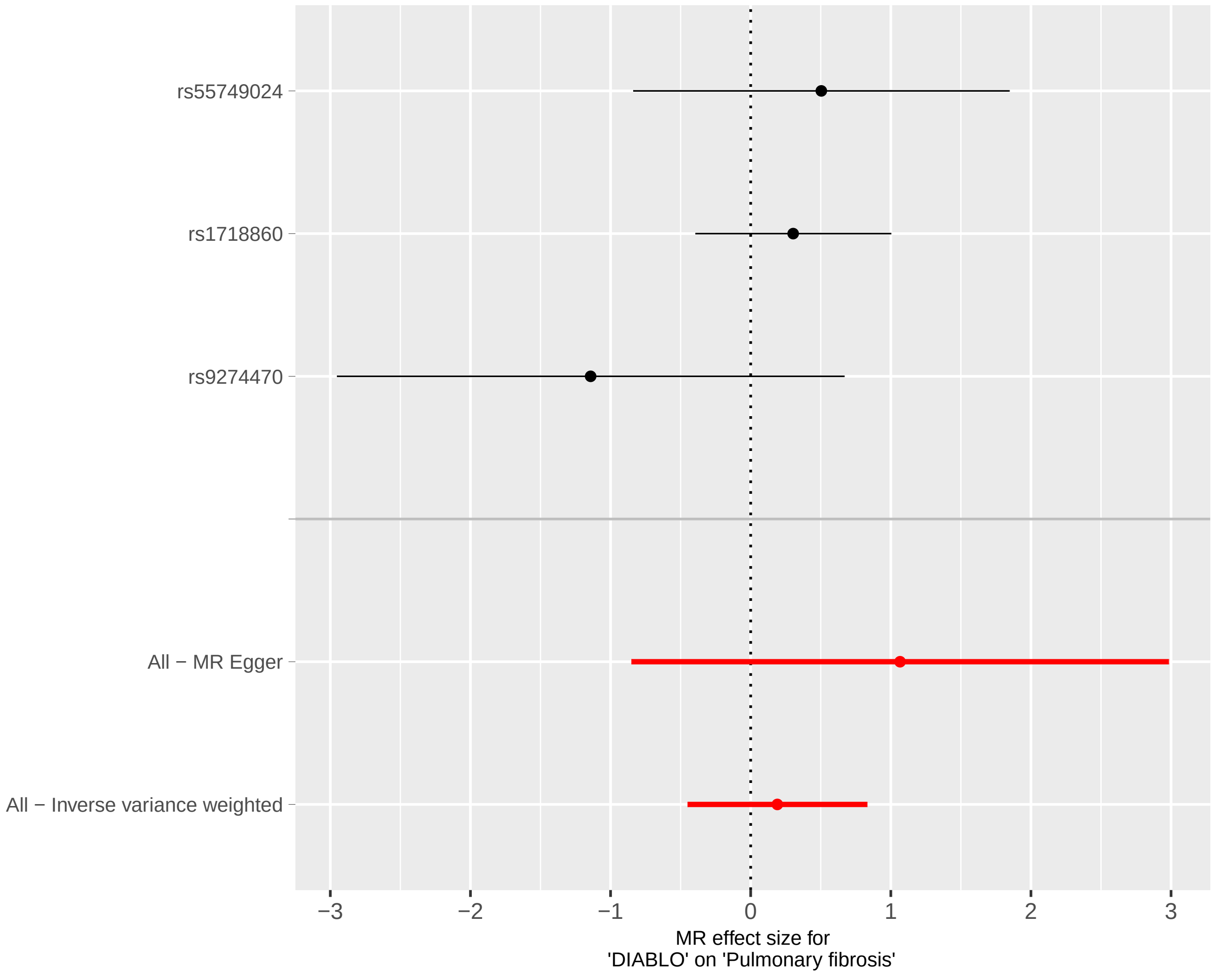

rs55749024
rs1718860
rs9274470
All − MR Egger
All − Inverse variance weighted
−3
−2
−1
0
1
2
3
MR effect size for
'DIABLO' on 'Pulmonary fibrosis'

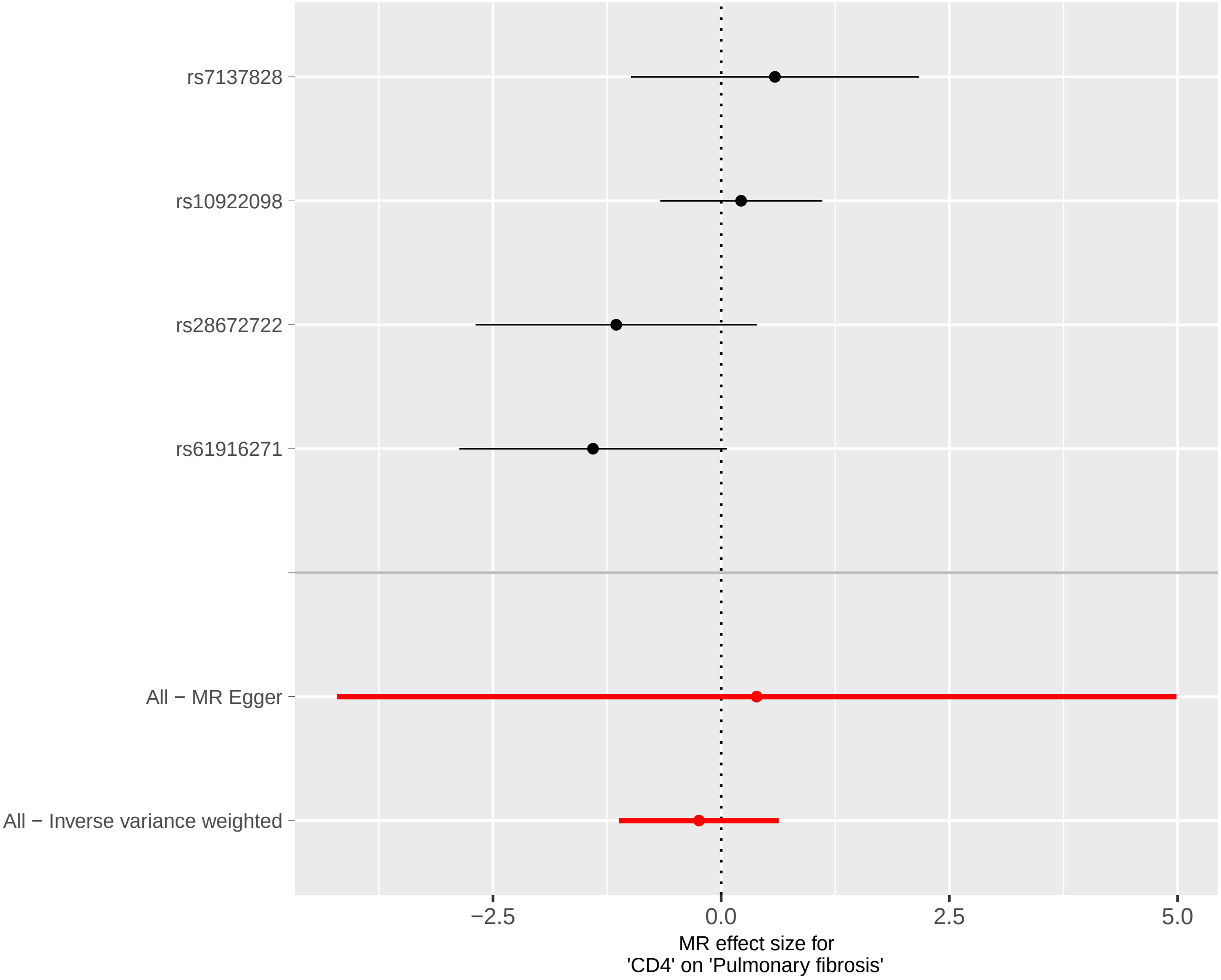
rs7137828
rs10922098
rs28672722
rs61916271
All − MR Egger
All − Inverse variance weighted
−2.5
0.0
2.5
5.0
MR effect size for
'CD4' on 'Pulmonary fibrosis'

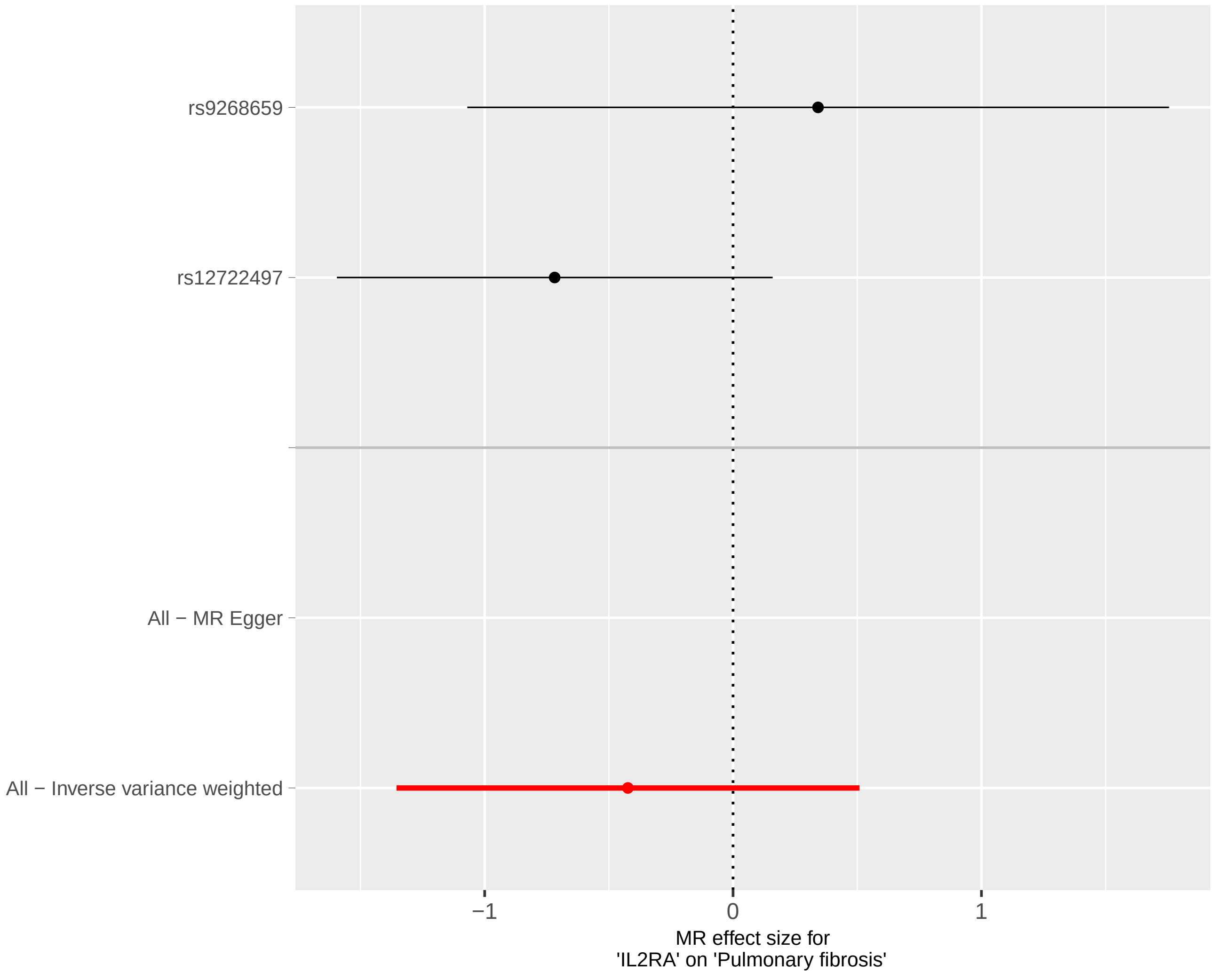
rs9268659
rs12722497
All − MR Egger
All − Inverse variance weighted
−1
0
1
MR effect size for
'IL2RA' on 'Pulmonary fibrosis'

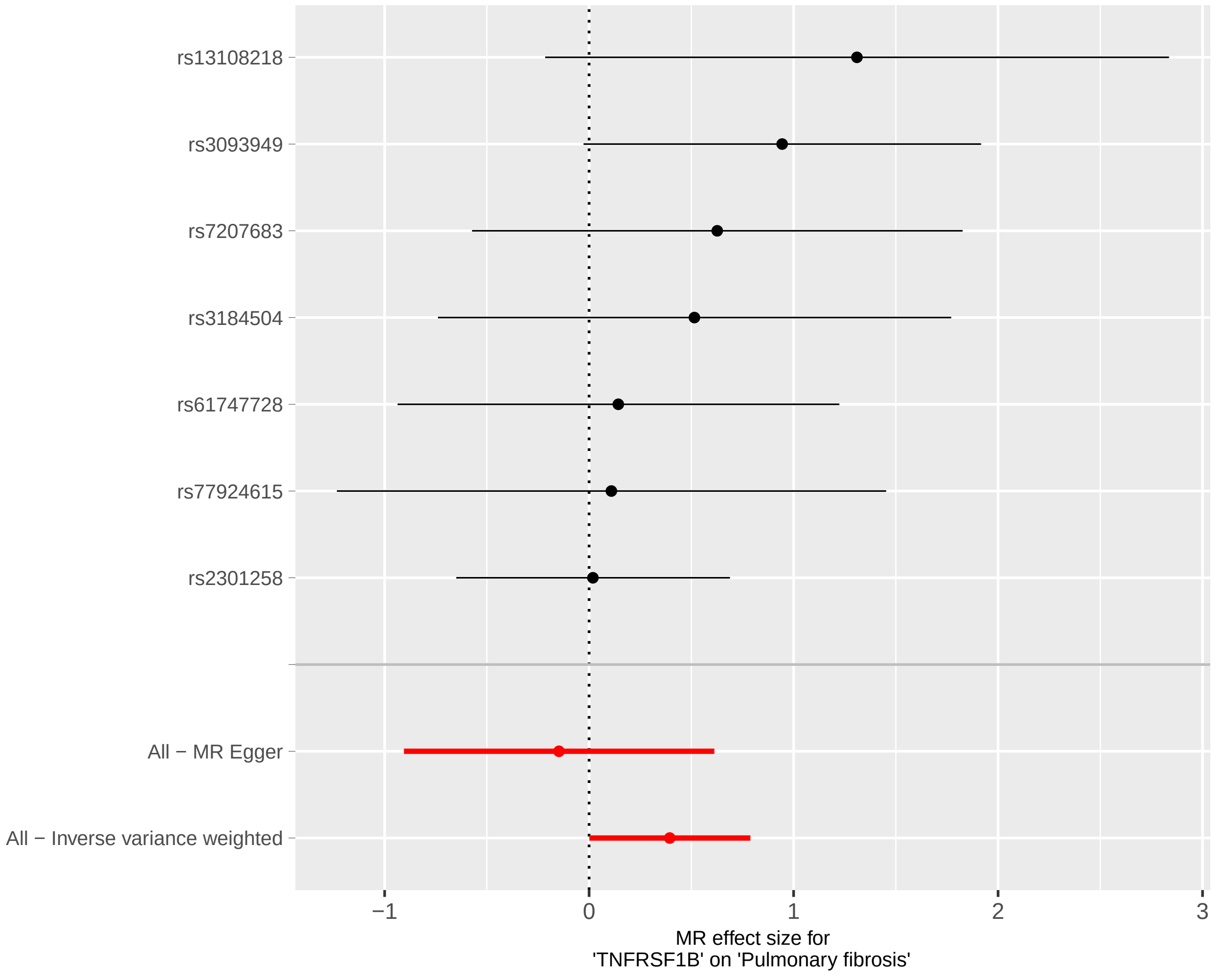

rs13108218
rs3093949
rs7207683
rs3184504
rs61747728
rs77924615
rs2301258
All − MR Egger
All − Inverse variance weighted
−1
0
1
2
3
MR effect size for
'TNFRSF1B' on 'Pulmonary fibrosis'

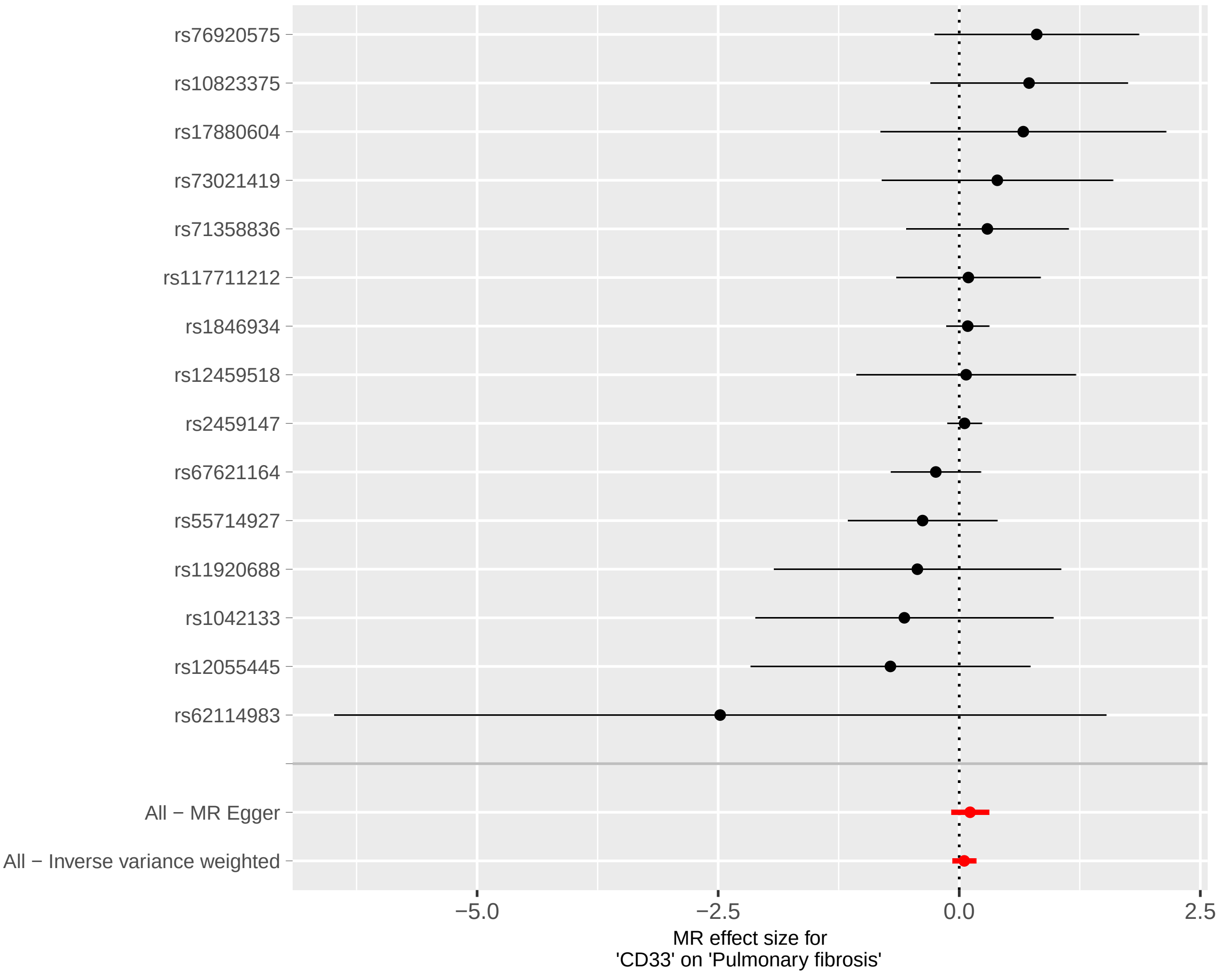
rs76920575
rs10823375
rs17880604
rs73021419
rs71358836
rs117711212
rs1846934
rs12459518
rs2459147
rs67621164
rs55714927
rs11920688
rs1042133
rs12055445
rs62114983
All − MR Egger
All − Inverse variance weighted
−5.0
−2.5
0.0
2.5
MR effect size for
'CD33' on 'Pulmonary fibrosis'

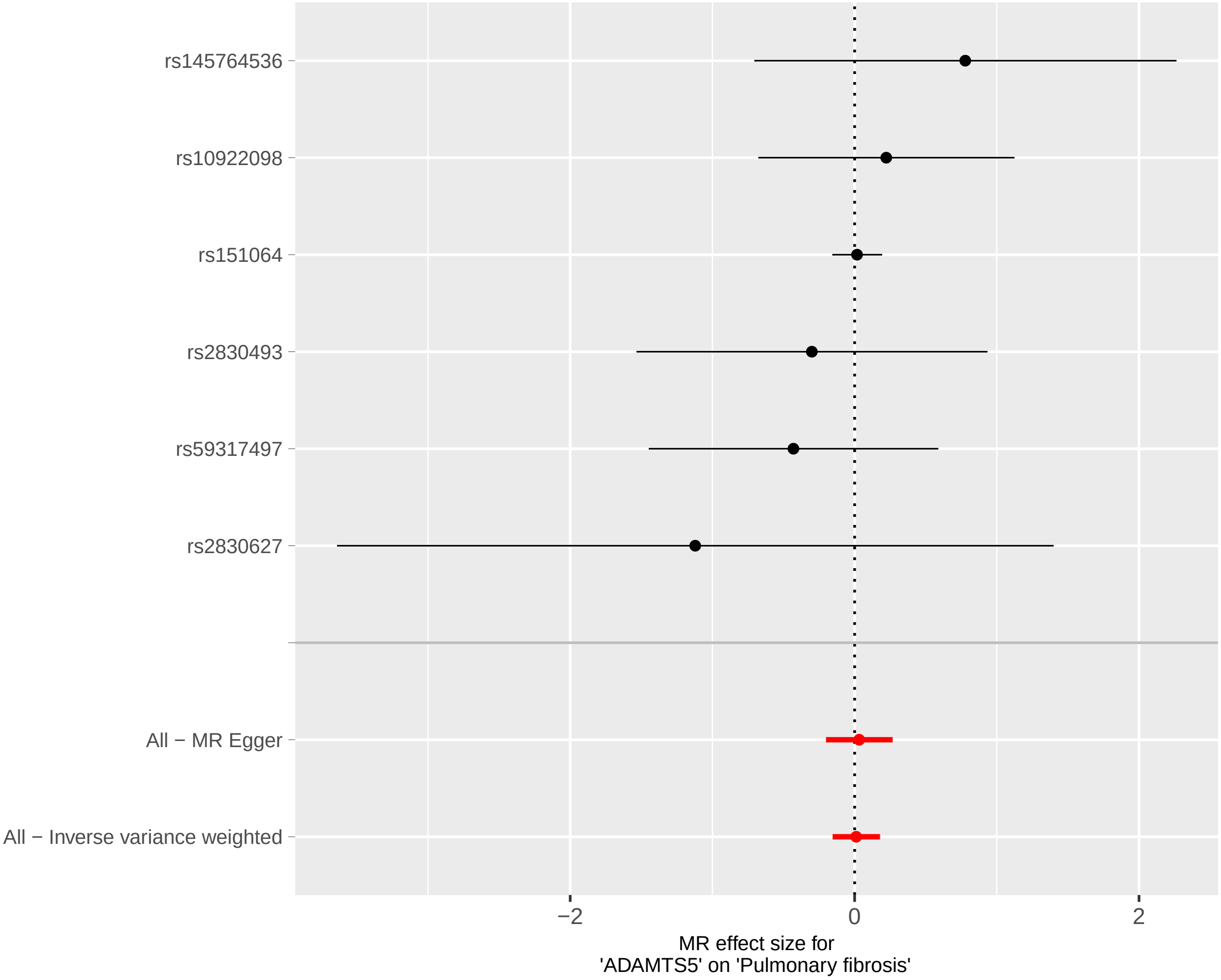
rs145764536
rs10922098
rs151064
rs2830493
rs59317497
rs2830627
All − MR Egger
All − Inverse variance weighted
−2
0
2
MR effect size for
'ADAMTS5' on 'Pulmonary fibrosis'

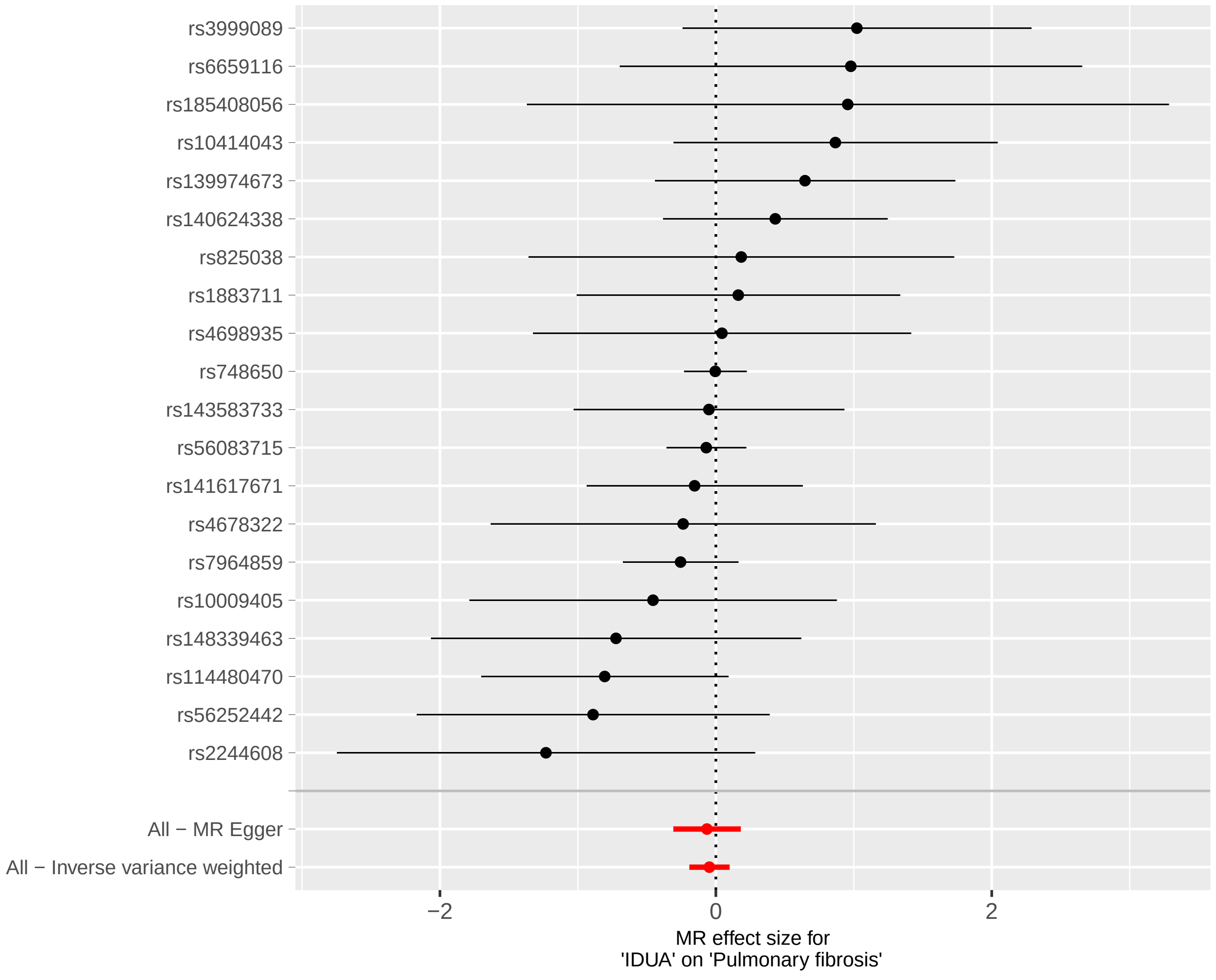

rs3999089
rs6659116
rs185408056
rs10414043
rs139974673
rs140624338
rs825038
rs1883711
rs4698935
rs748650
rs143583733
rs56083715
rs141617671
rs4678322
rs7964859
rs10009405
rs148339463
rs114480470
rs56252442
rs2244608
All − MR Egger
All − Inverse variance weighted
−2
0
2
MR effect size for
'IDUA' on 'Pulmonary fibrosis'

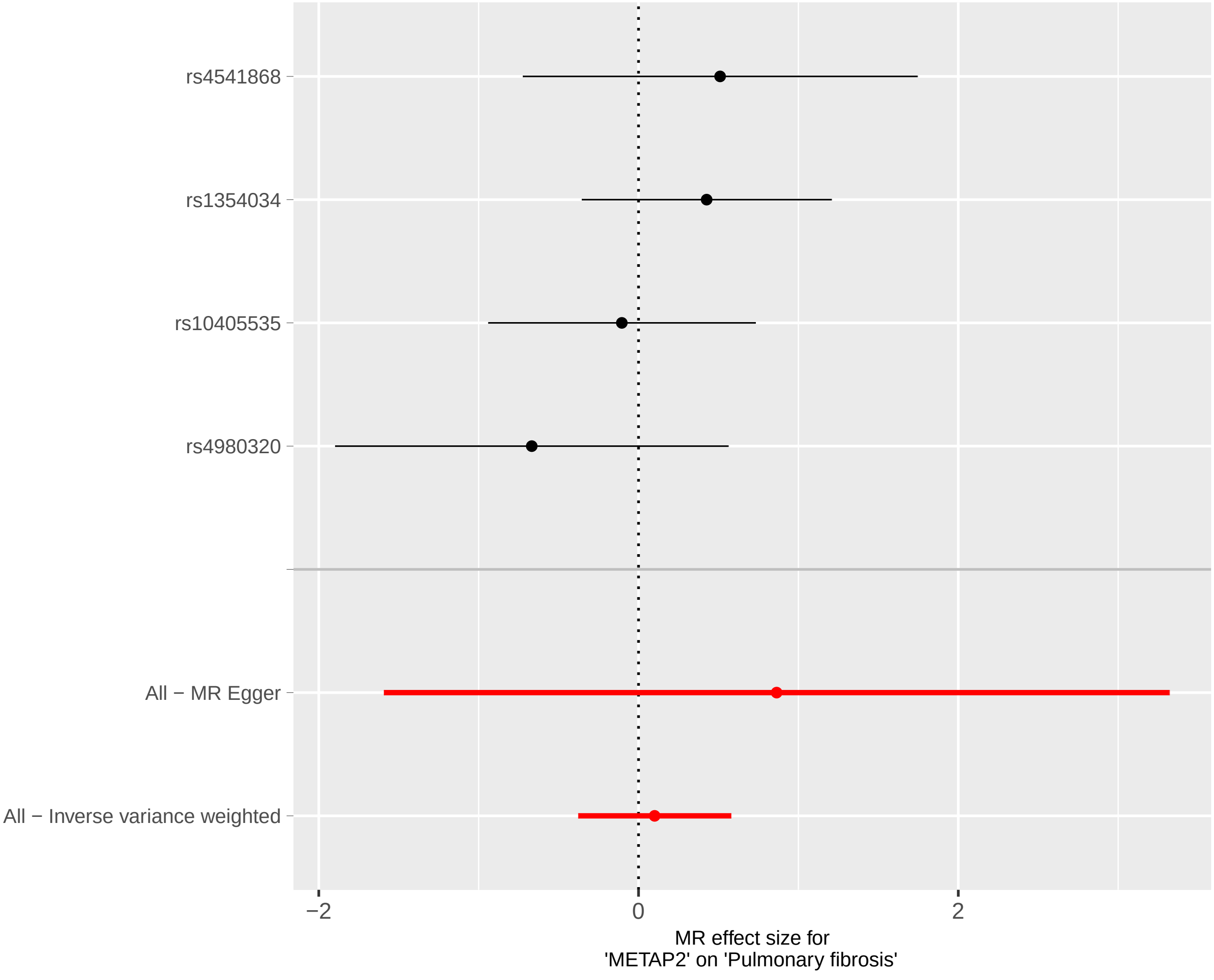
rs4541868
rs1354034
rs10405535
rs4980320
All − MR Egger
All − Inverse variance weighted
−2
0
2
MR effect size for
'METAP2' on 'Pulmonary fibrosis'

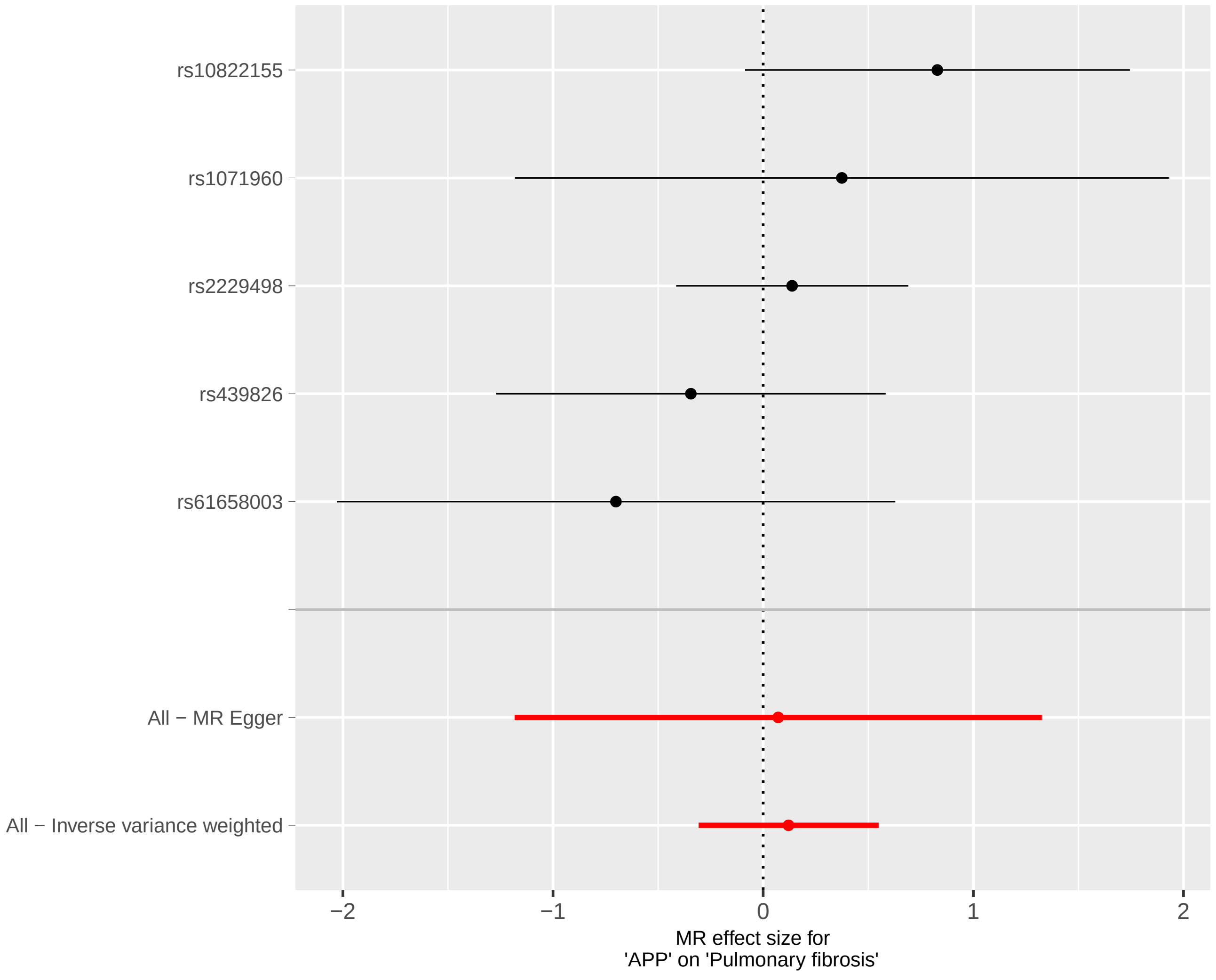

rs10822155
rs1071960
rs2229498
rs439826
rs61658003
All − MR Egger
All − Inverse variance weighted
−2
−1
0
1
2
MR effect size for
'APP' on 'Pulmonary fibrosis'

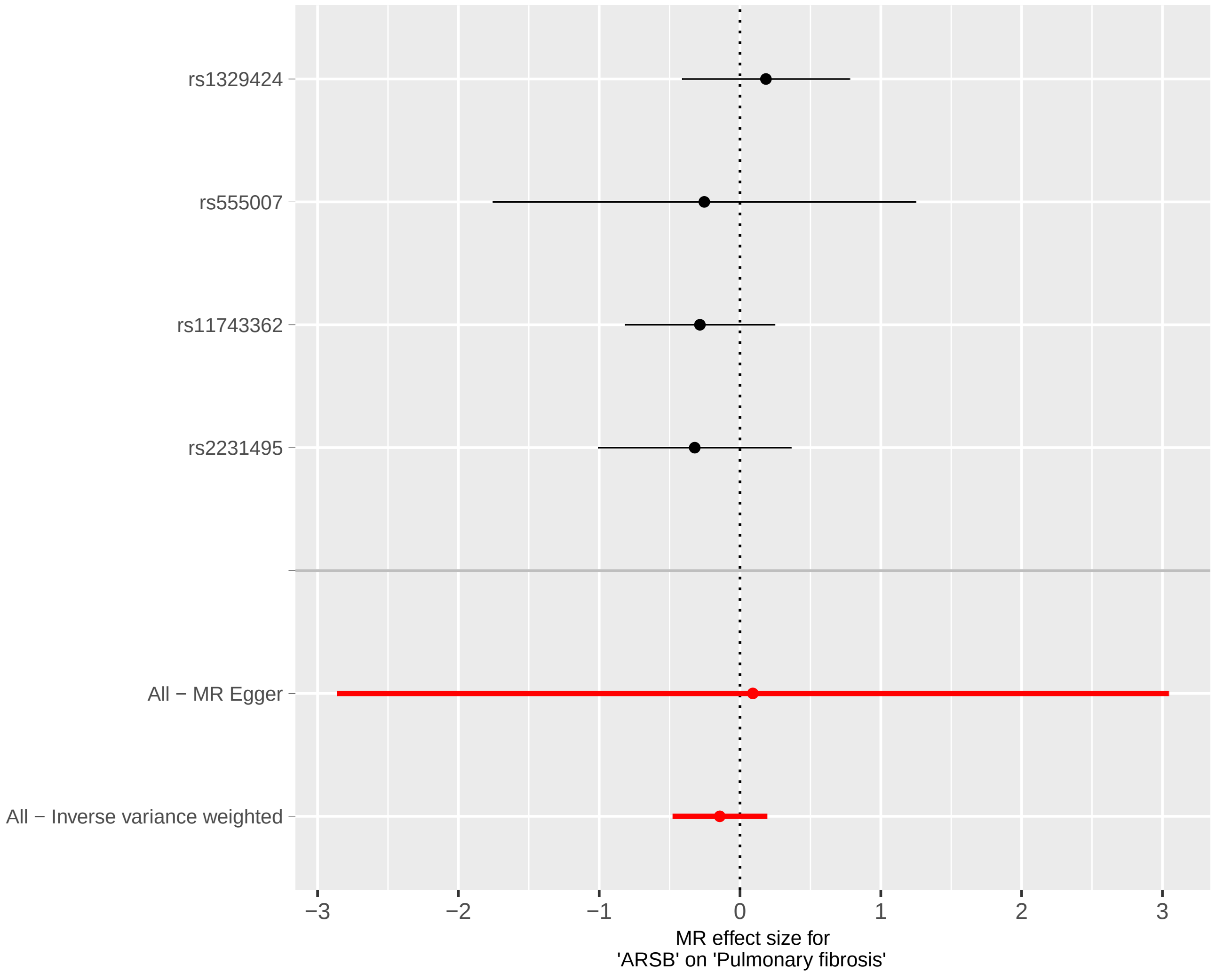
rs1329424
rs555007
rs11743362
rs2231495
All − MR Egger
All − Inverse variance weighted
−3
−2
−1
0
1
2
3
MR effect size for
'ARSB' on 'Pulmonary fibrosis'

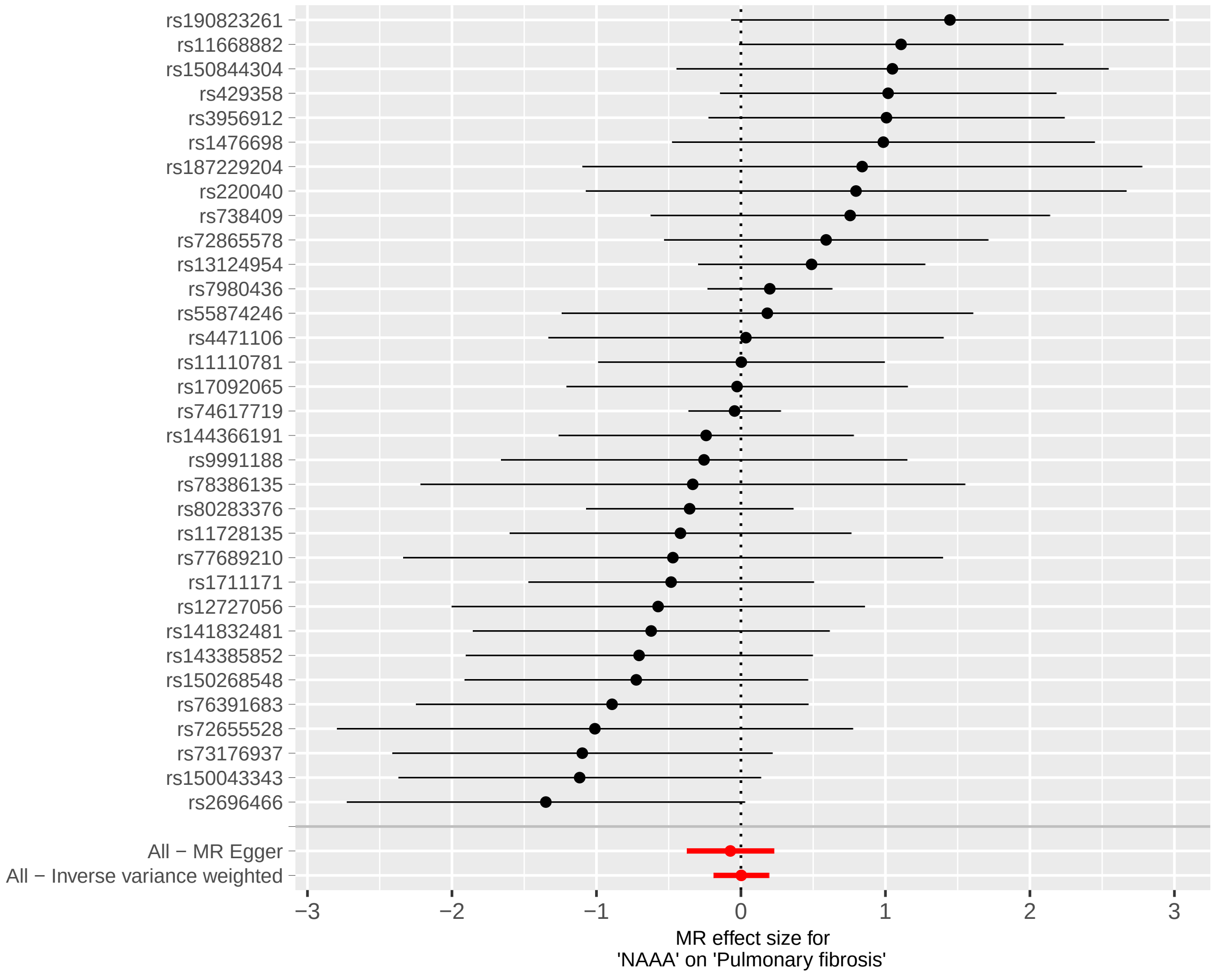

rs190823261
rs11668882
rs150844304
rs429358
rs3956912
rs1476698
rs187229204
rs220040
rs738409
rs72865578
rs13124954
rs7980436
rs55874246
rs4471106
rs11110781
rs17092065
rs74617719
rs144366191
rs9991188
rs78386135
rs80283376
rs11728135
rs77689210
rs1711171
rs12727056
rs141832481
rs143385852
rs150268548
rs76391683
rs72655528
rs73176937
rs150043343
rs2696466
All − MR Egger
All − Inverse variance weighted
−3
−2
−1
0
1
2
3
MR effect size for
'NAAA' on 'Pulmonary fibrosis'

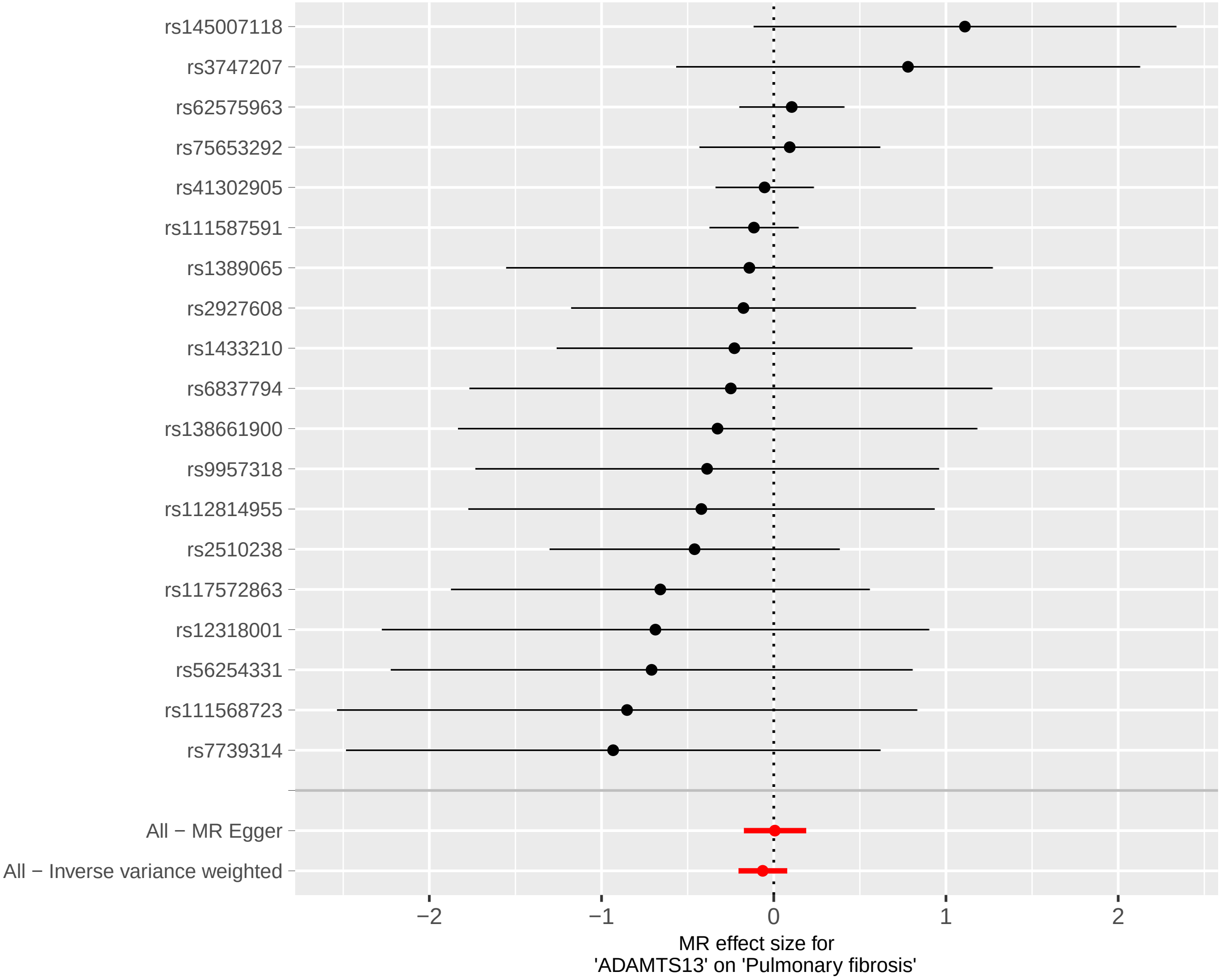
rs145007118
rs3747207
rs62575963
rs75653292
rs41302905
rs111587591
rs1389065
rs2927608
rs1433210
rs6837794
rs138661900
rs9957318
rs112814955
rs2510238
rs117572863
rs12318001
rs56254331
rs111568723
rs7739314
All − MR Egger
All − Inverse variance weighted
−2
−1
0
1
2
MR effect size for
'ADAMTS13' on 'Pulmonary fibrosis'

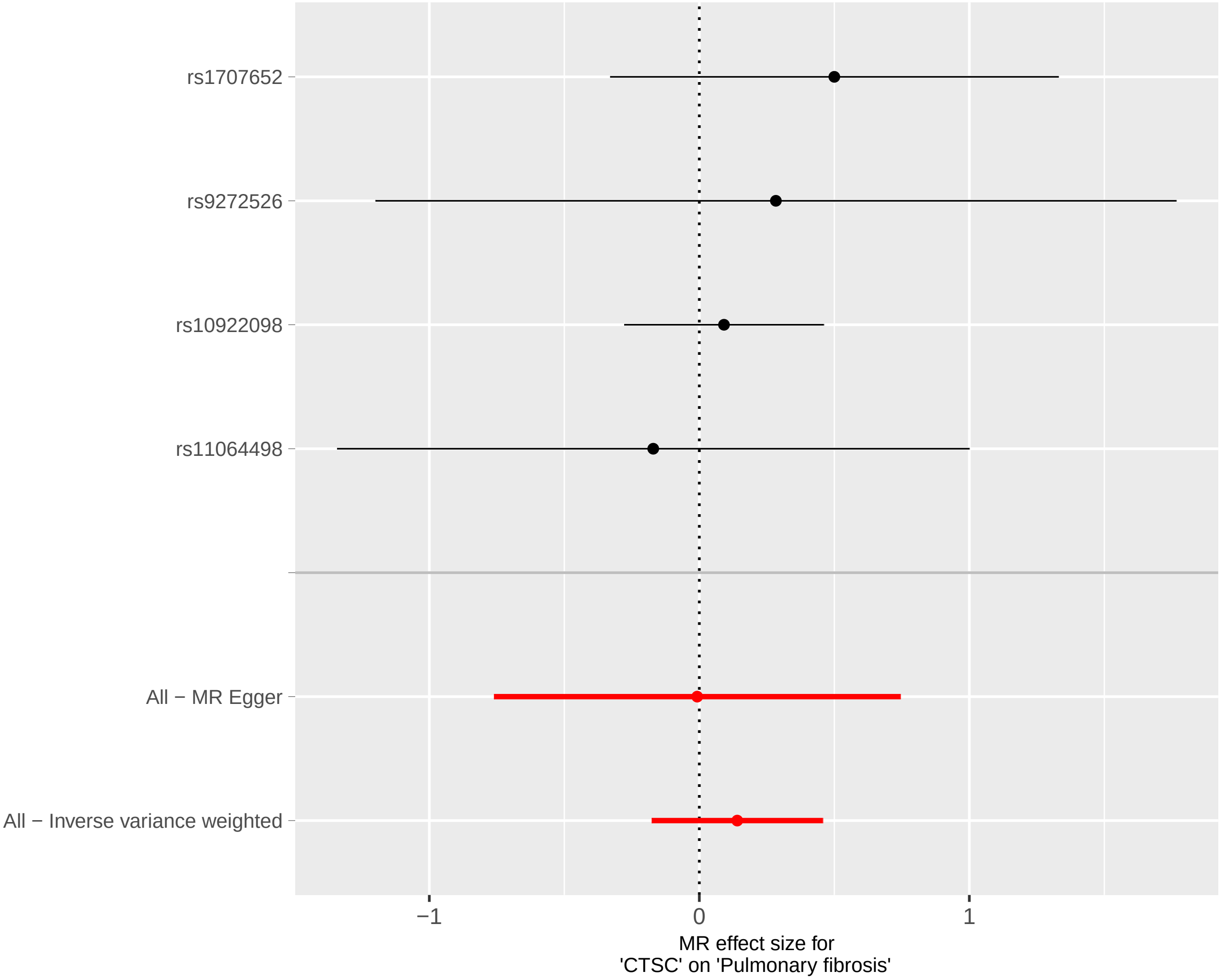

rs1707652
rs9272526
rs10922098
rs11064498
All − MR Egger
All − Inverse variance weighted
−1
0
1
MR effect size for
'CTSC' on 'Pulmonary fibrosis'

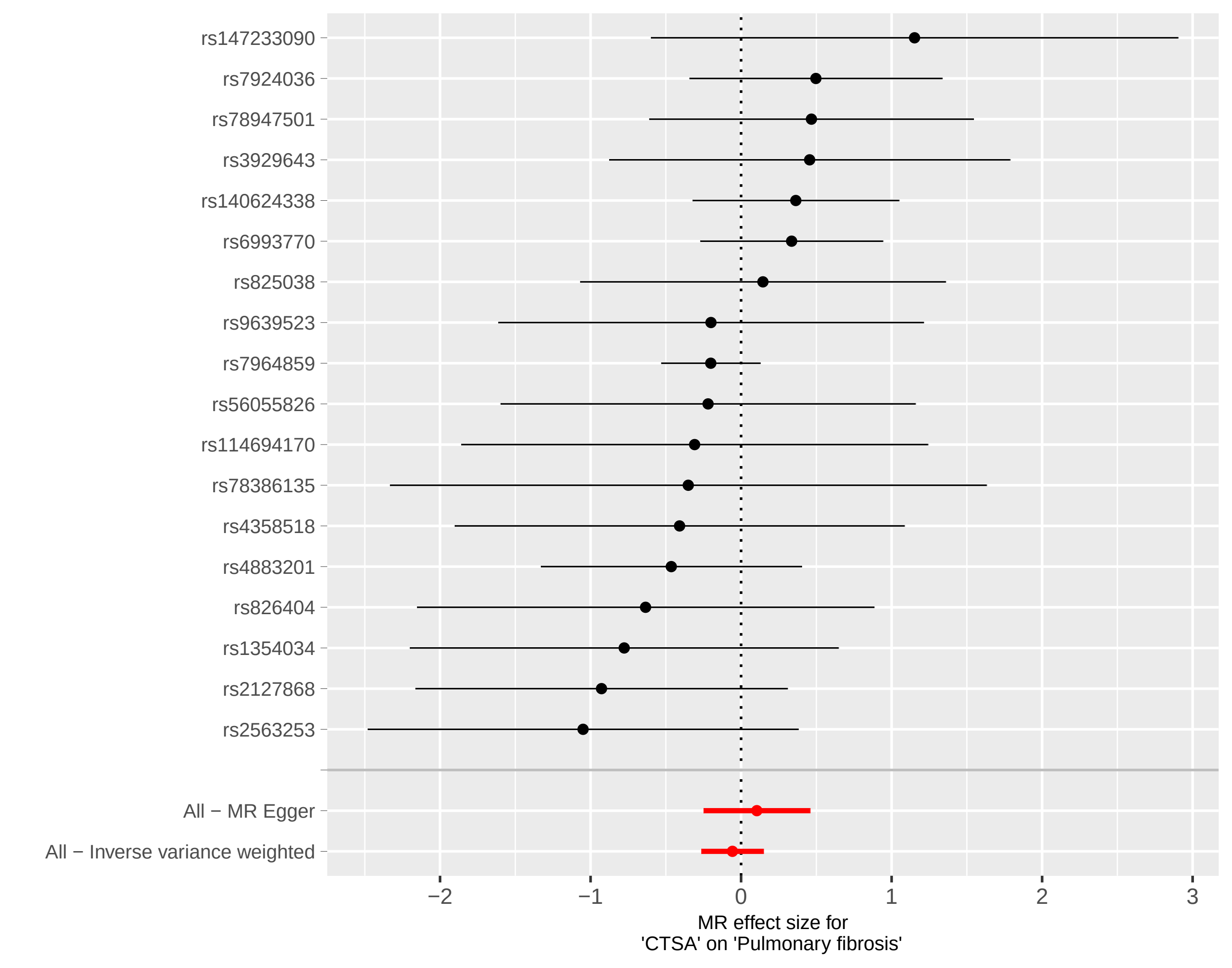

rs147233090
rs7924036
rs78947501
rs3929643
rs140624338
rs6993770
rs825038
rs9639523
rs7964859
rs56055826
rs114694170
rs78386135
rs4358518
rs4883201
rs826404
rs1354034
rs2127868
rs2563253
All − MR Egger
All − Inverse variance weighted
−2
−1
0
1
2
3
MR effect size for
'CTSA' on 'Pulmonary fibrosis'

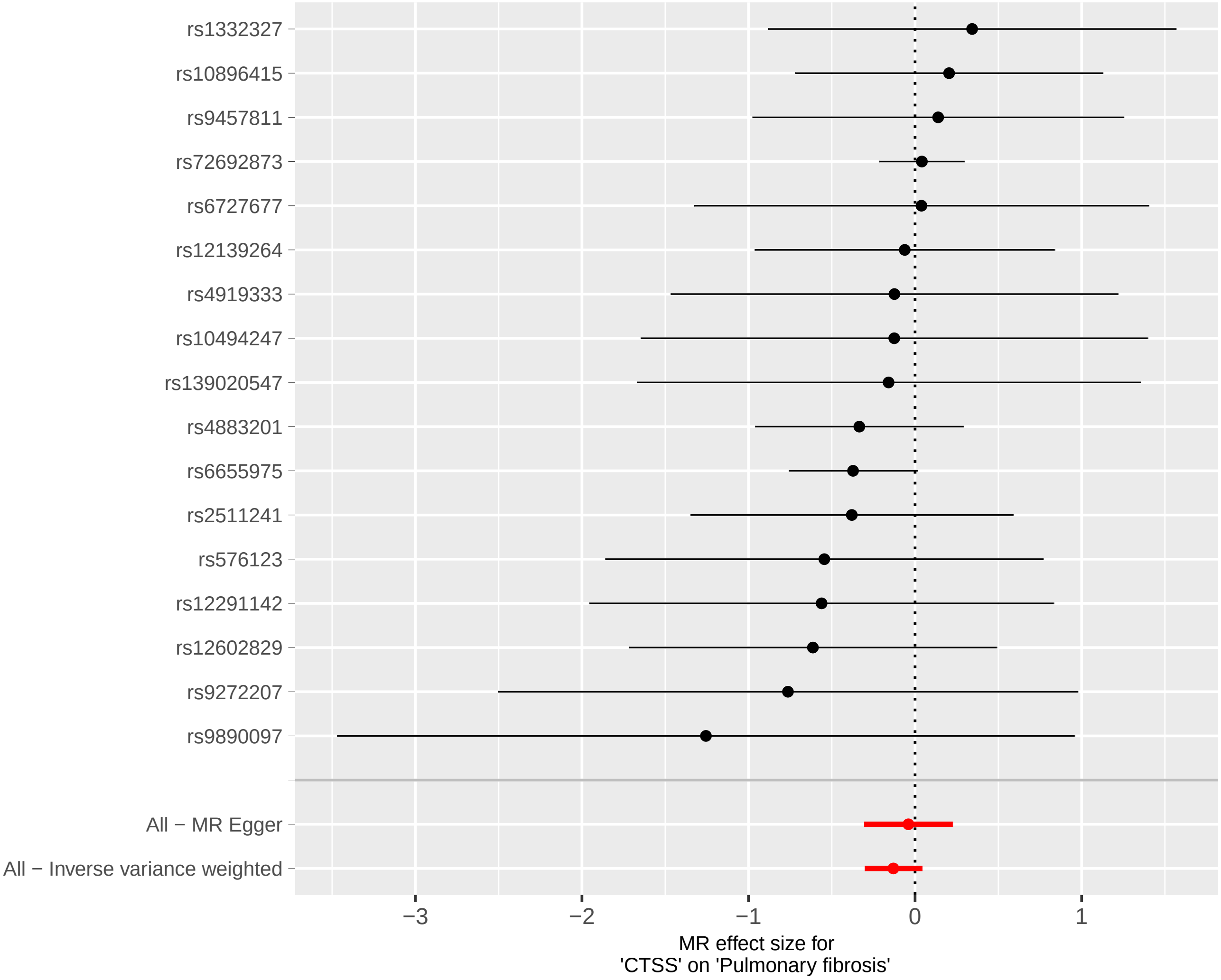

rs1332327
rs10896415
rs9457811
rs72692873
rs6727677
rs12139264
rs4919333
rs10494247
rs139020547
rs4883201
rs6655975
rs2511241
rs576123
rs12291142
rs12602829
rs9272207
rs9890097
All − MR Egger
All − Inverse variance weighted
−3
−2
−1
0
1
MR effect size for
'CTSS' on 'Pulmonary fibrosis'

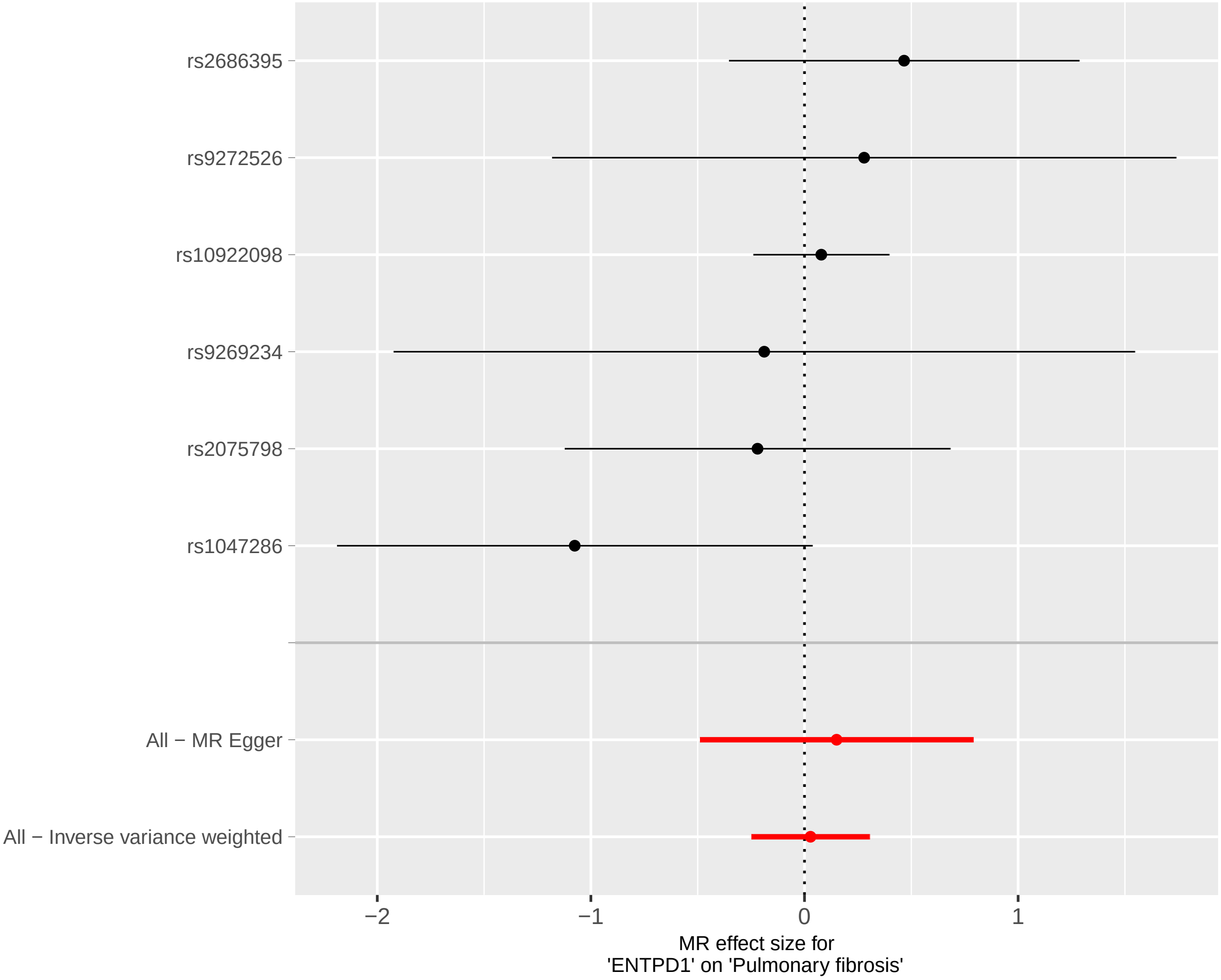
rs2686395
rs9272526
rs10922098
rs9269234
rs2075798
rs1047286
All − MR Egger
All − Inverse variance weighted
−2
−1
0
1
MR effect size for
'ENTPD1' on 'Pulmonary fibrosis'

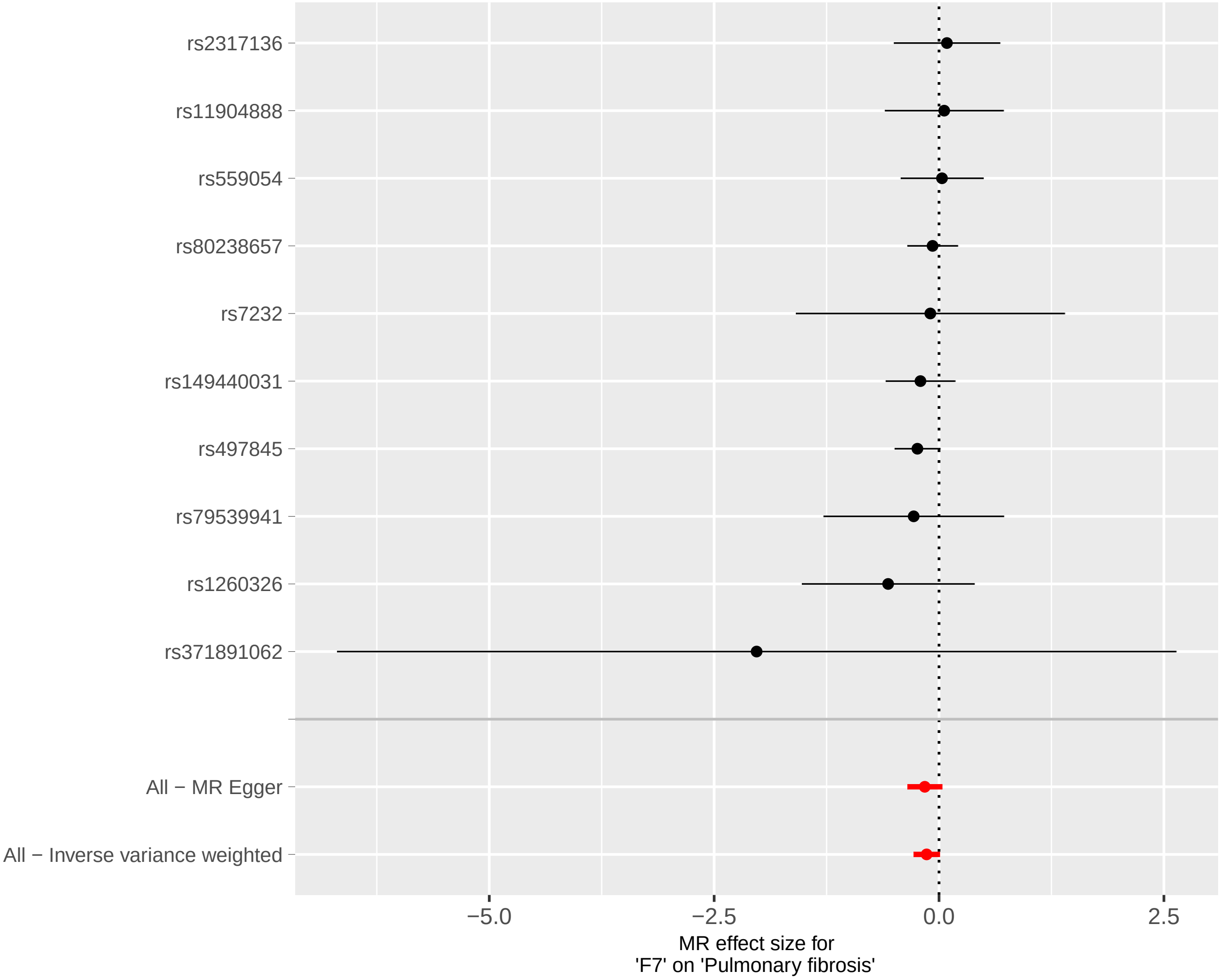
rs2317136
rs11904888
rs559054
rs80238657
rs7232
rs149440031
rs497845
rs79539941
rs1260326
rs371891062
All − MR Egger
All − Inverse variance weighted
−5.0
−2.5
0.0
2.5
MR effect size for
'F7' on 'Pulmonary fibrosis'

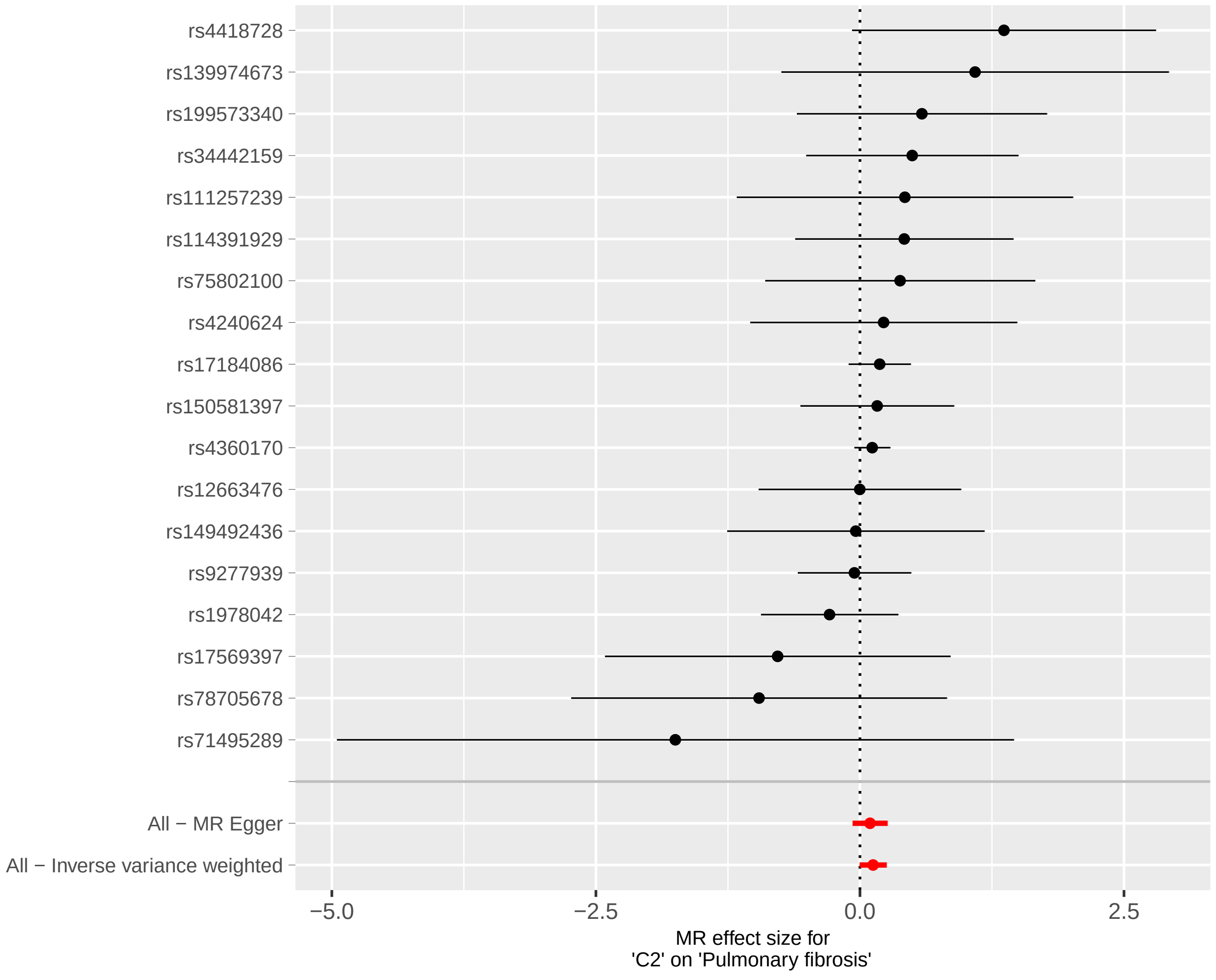

rs4418728
rs139974673
rs199573340
rs34442159
rs111257239
rs114391929
rs75802100
rs4240624
rs17184086
rs150581397
rs4360170
rs12663476
rs149492436
rs9277939
rs1978042
rs17569397
rs78705678
rs71495289
All − MR Egger
All − Inverse variance weighted
−5.0
−2.5
0.0
2.5
MR effect size for
'C2' on 'Pulmonary fibrosis'

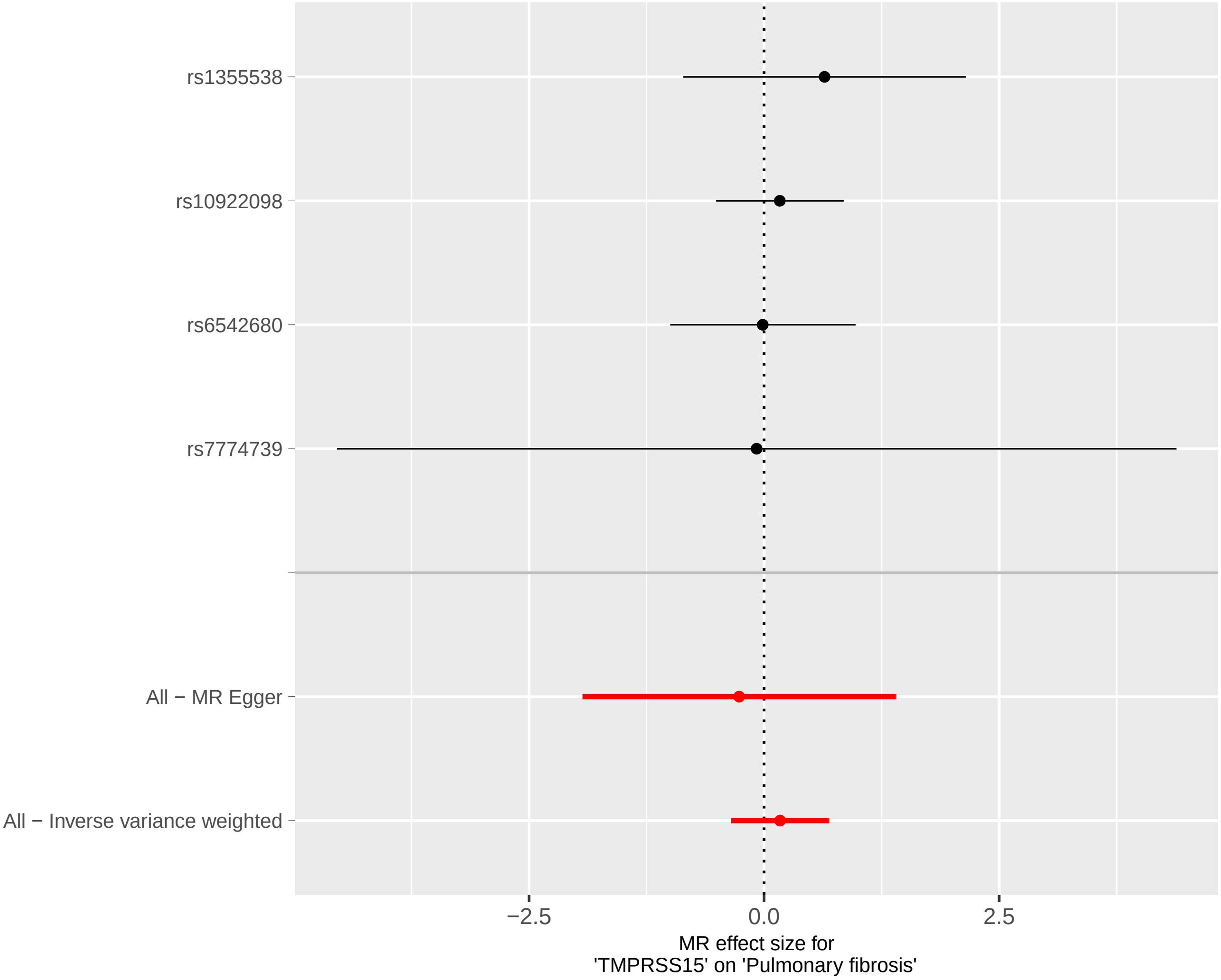
rs1355538
rs10922098
rs6542680
rs7774739
All − MR Egger
All − Inverse variance weighted
−2.5
0.0
2.5
MR effect size for
'TMPRSS15' on 'Pulmonary fibrosis'

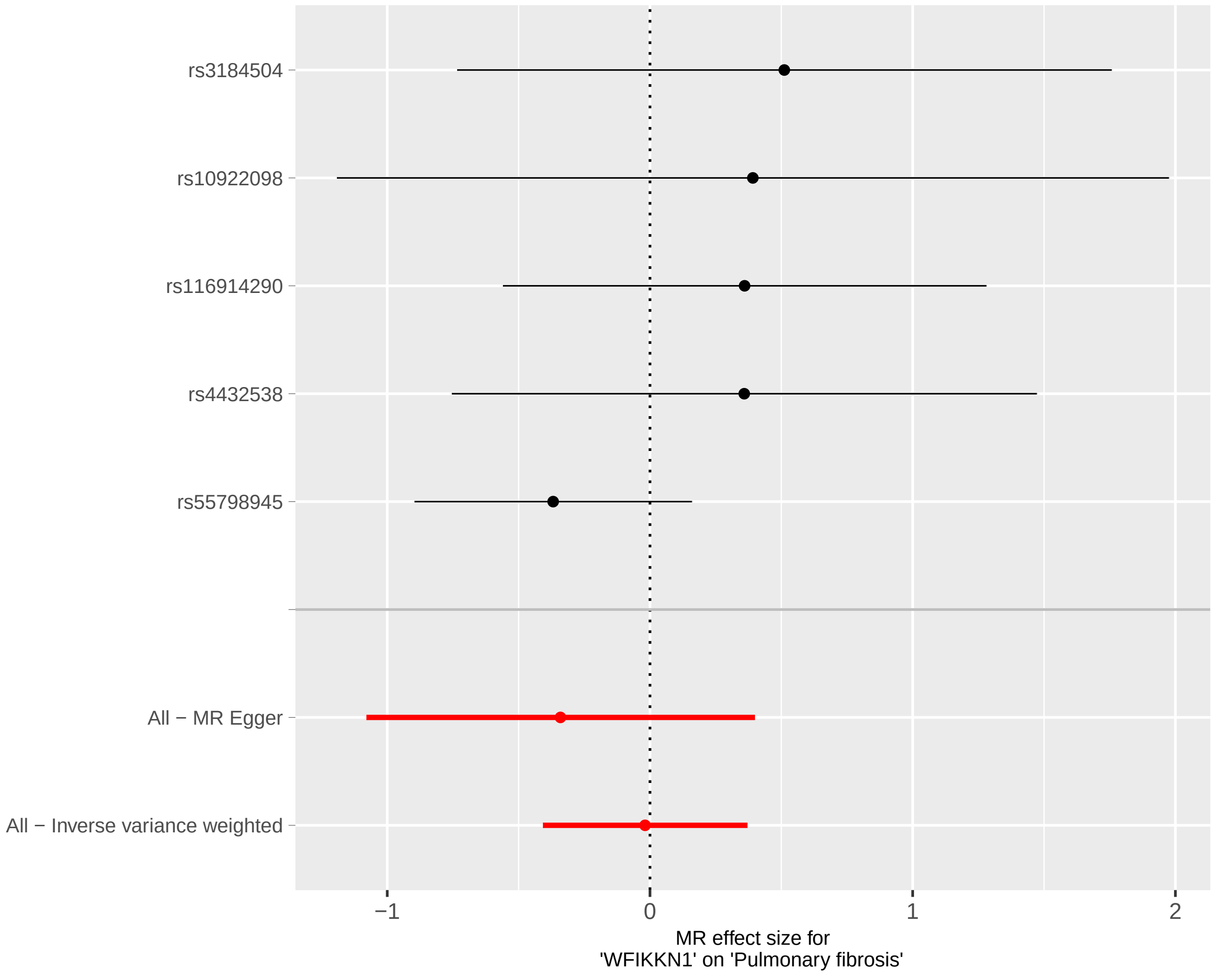
rs3184504
rs10922098
rs116914290
rs4432538
rs55798945
All − MR Egger
All − Inverse variance weighted
−1
0
1
2
MR effect size for
'WFIKKN1' on 'Pulmonary fibrosis'

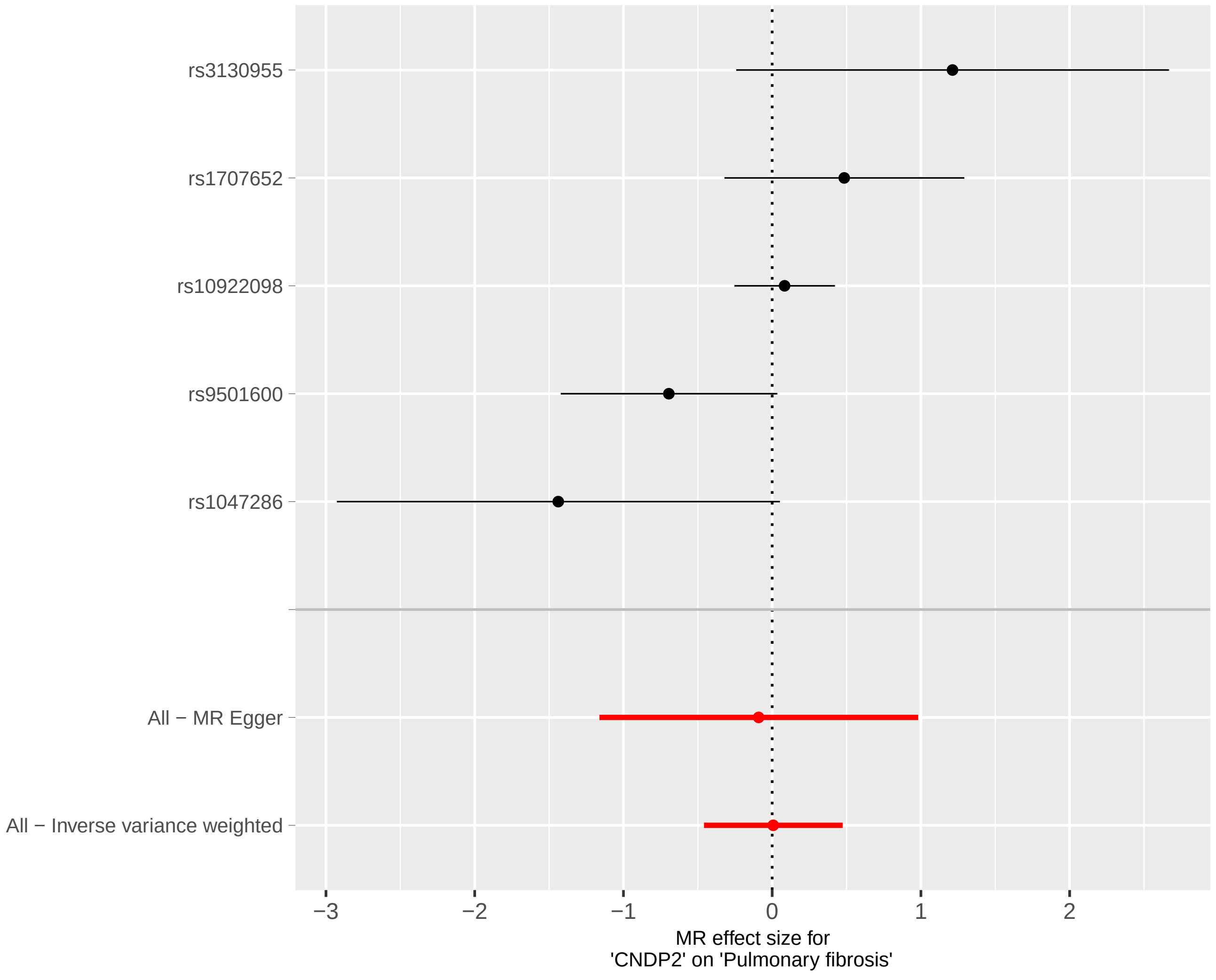
rs3130955
rs1707652
rs10922098
rs9501600
rs1047286
All − MR Egger
All − Inverse variance weighted
−3
−2
−1
0
1
2
MR effect size for
'CNDP2' on 'Pulmonary fibrosis'

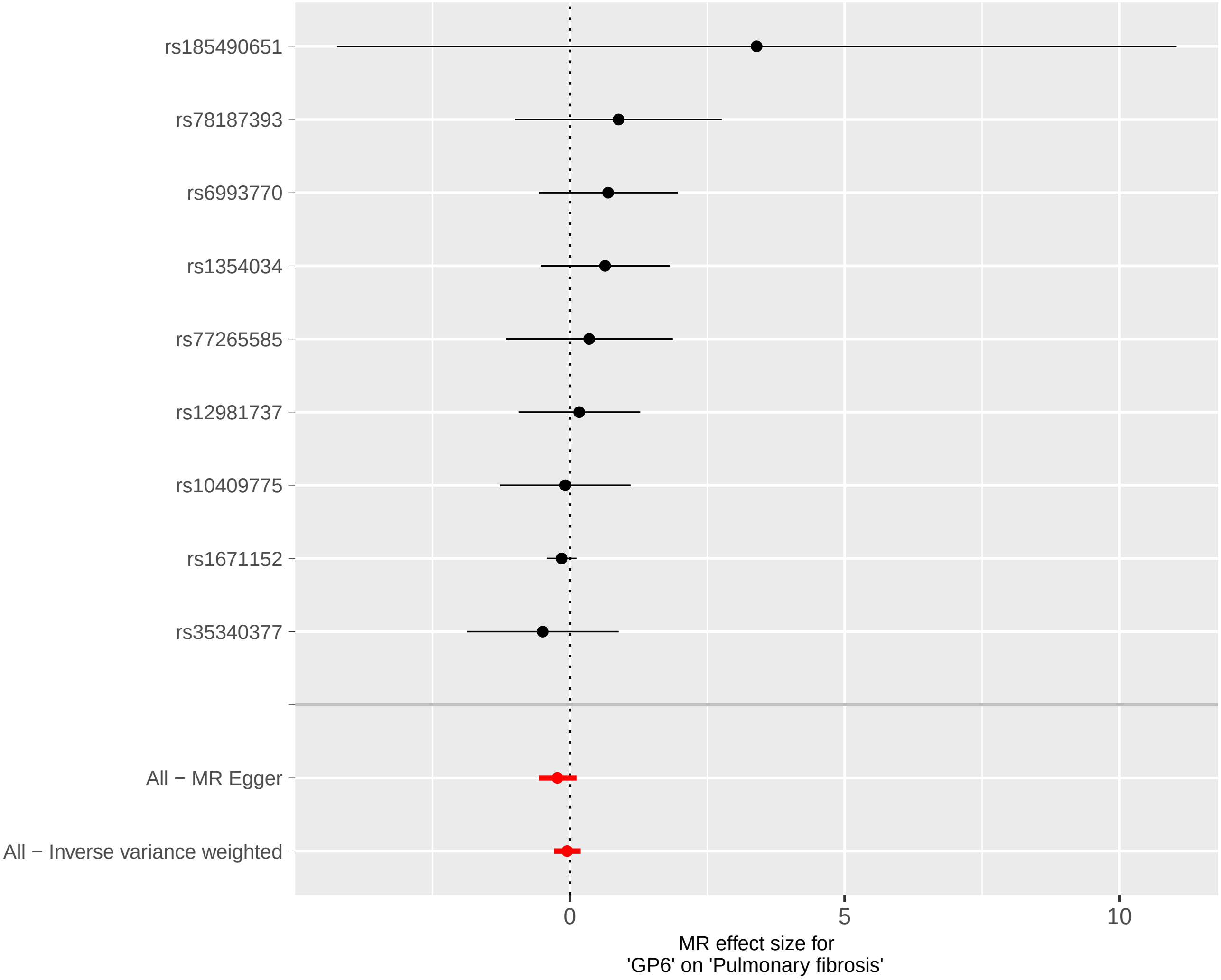
rs185490651
rs78187393
rs6993770
rs1354034
rs77265585
rs12981737
rs10409775
rs1671152
rs35340377
All − MR Egger
All − Inverse variance weighted
0
5
10
MR effect size for
'GP6' on 'Pulmonary fibrosis'

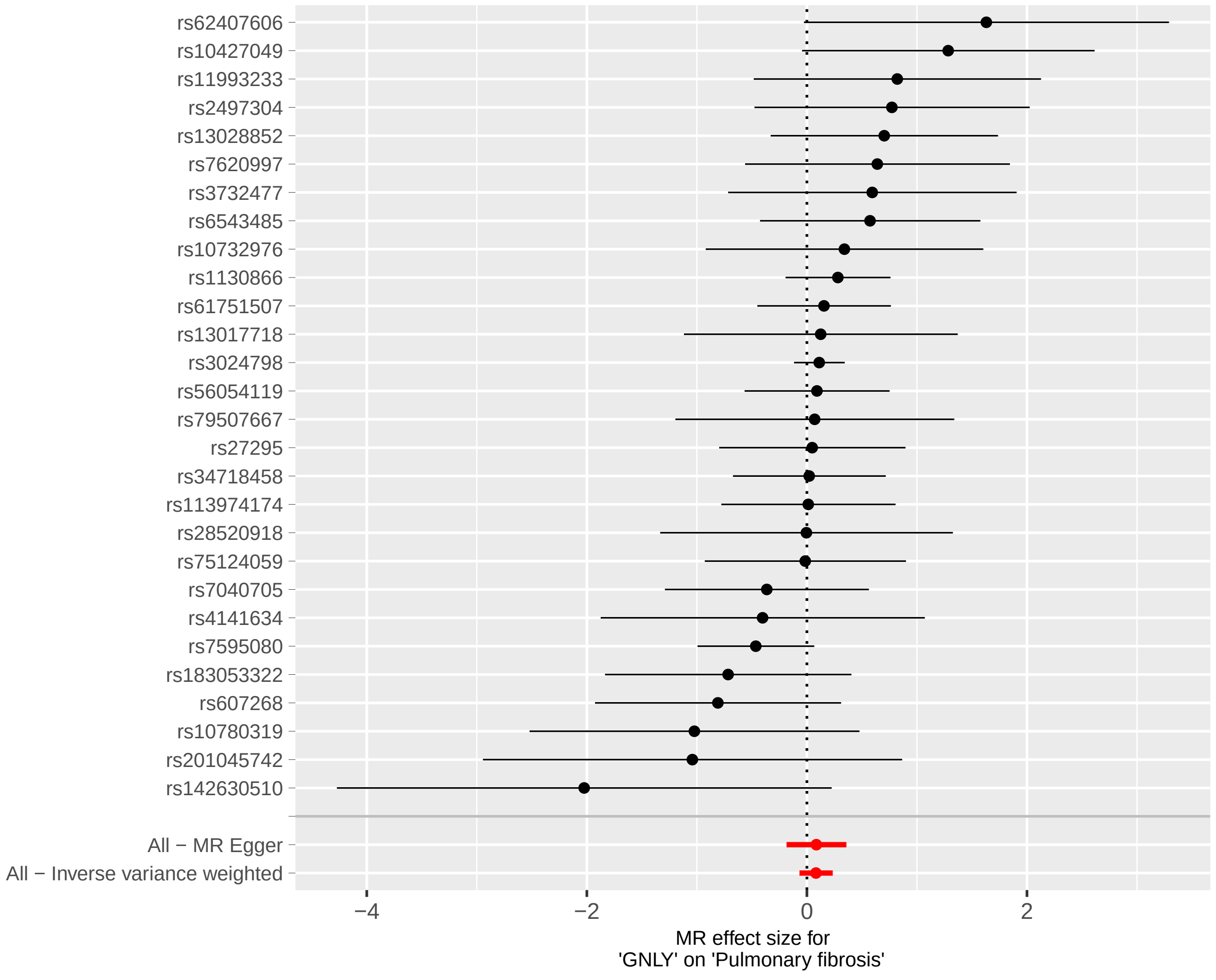
rs62407606
rs10427049
rs11993233
rs2497304
rs13028852
rs7620997
rs3732477
rs6543485
rs10732976
rs1130866
rs61751507
rs13017718
rs3024798
rs56054119
rs79507667
rs27295
rs34718458
rs113974174
rs28520918
rs75124059
rs7040705
rs4141634
rs7595080
rs183053322
rs607268
rs10780319
rs201045742
rs142630510
All − MR Egger
All − Inverse variance weighted
−4
−2
0
2
MR effect size for
'GNLY' on 'Pulmonary fibrosis'

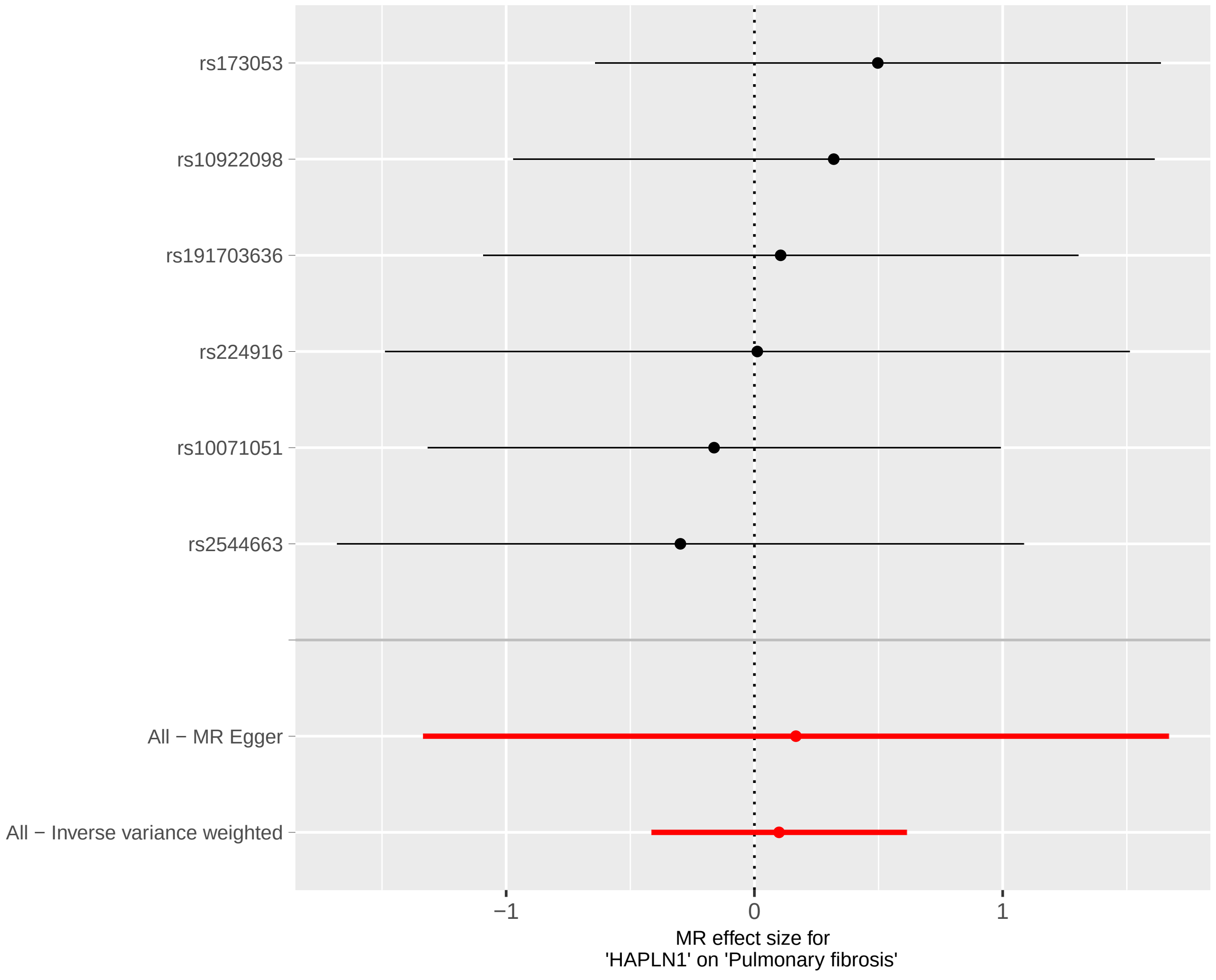
rs173053
rs10922098
rs191703636
rs224916
rs10071051
rs2544663
All − MR Egger
All − Inverse variance weighted
−1
0
1
MR effect size for
'HAPLN1' on 'Pulmonary fibrosis'

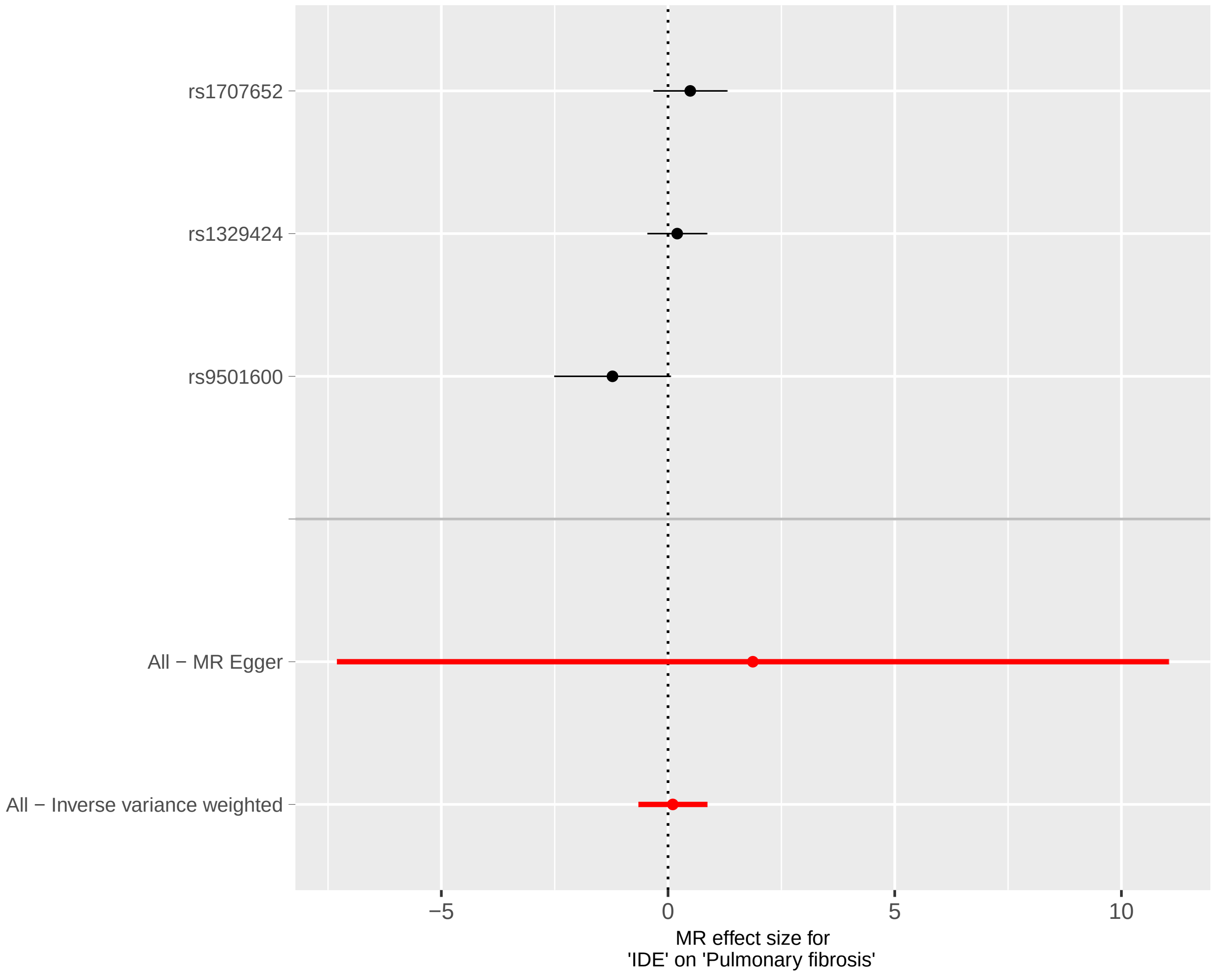

rs1707652
rs1329424
rs9501600
All − MR Egger
All − Inverse variance weighted
−5
0
5
10
MR effect size for
'IDE' on 'Pulmonary fibrosis'

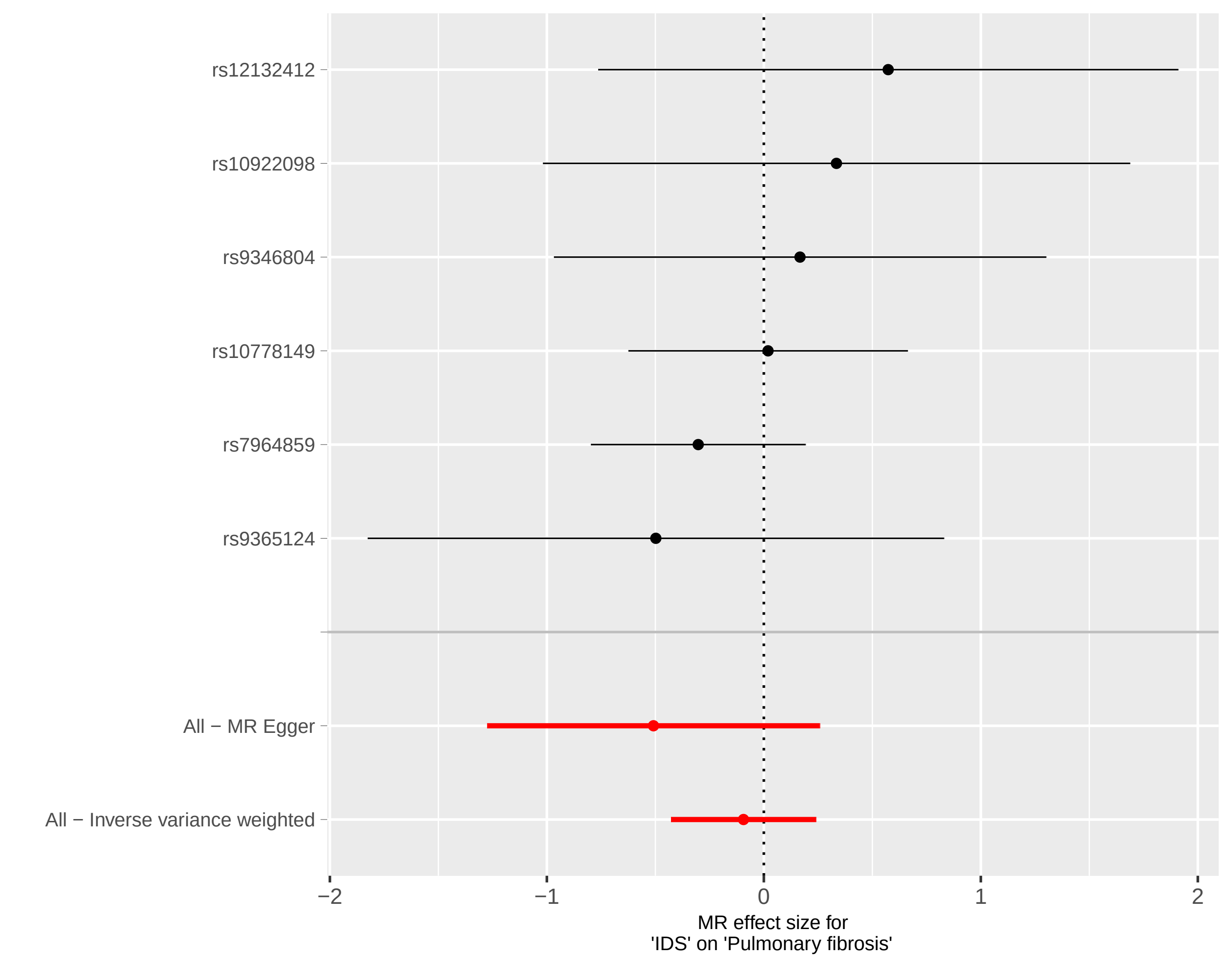

rs12132412
rs10922098
rs9346804
rs10778149
rs7964859
rs9365124
All − MR Egger
All − Inverse variance weighted
−2
−1
0
1
2
MR effect size for
'IDS' on 'Pulmonary fibrosis'

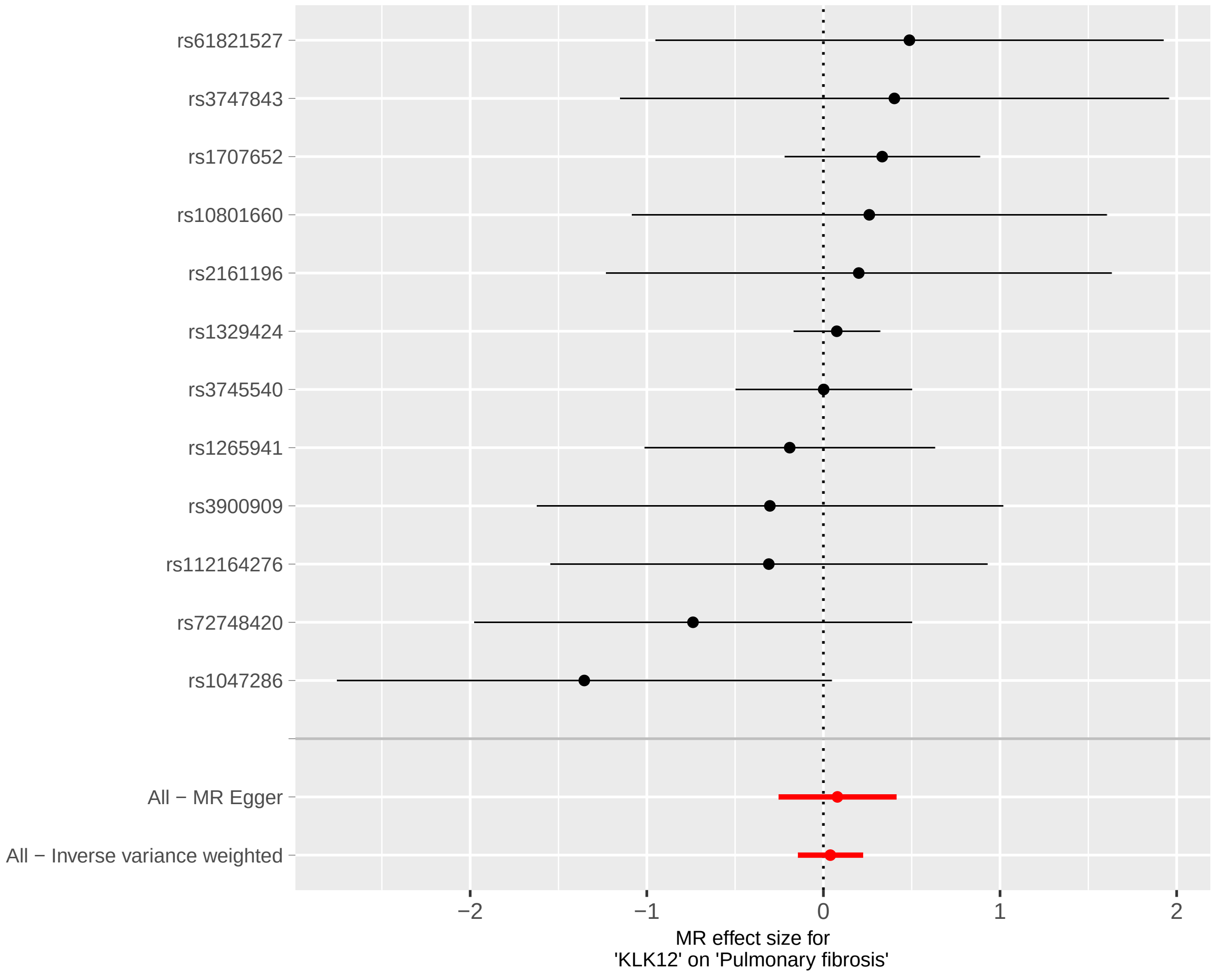

rs61821527
rs3747843
rs1707652
rs10801660
rs2161196
rs1329424
rs3745540
rs1265941
rs3900909
rs112164276
rs72748420
rs1047286
All − MR Egger
All − Inverse variance weighted
−2
−1
0
1
2
MR effect size for
'KLK12' on 'Pulmonary fibrosis'

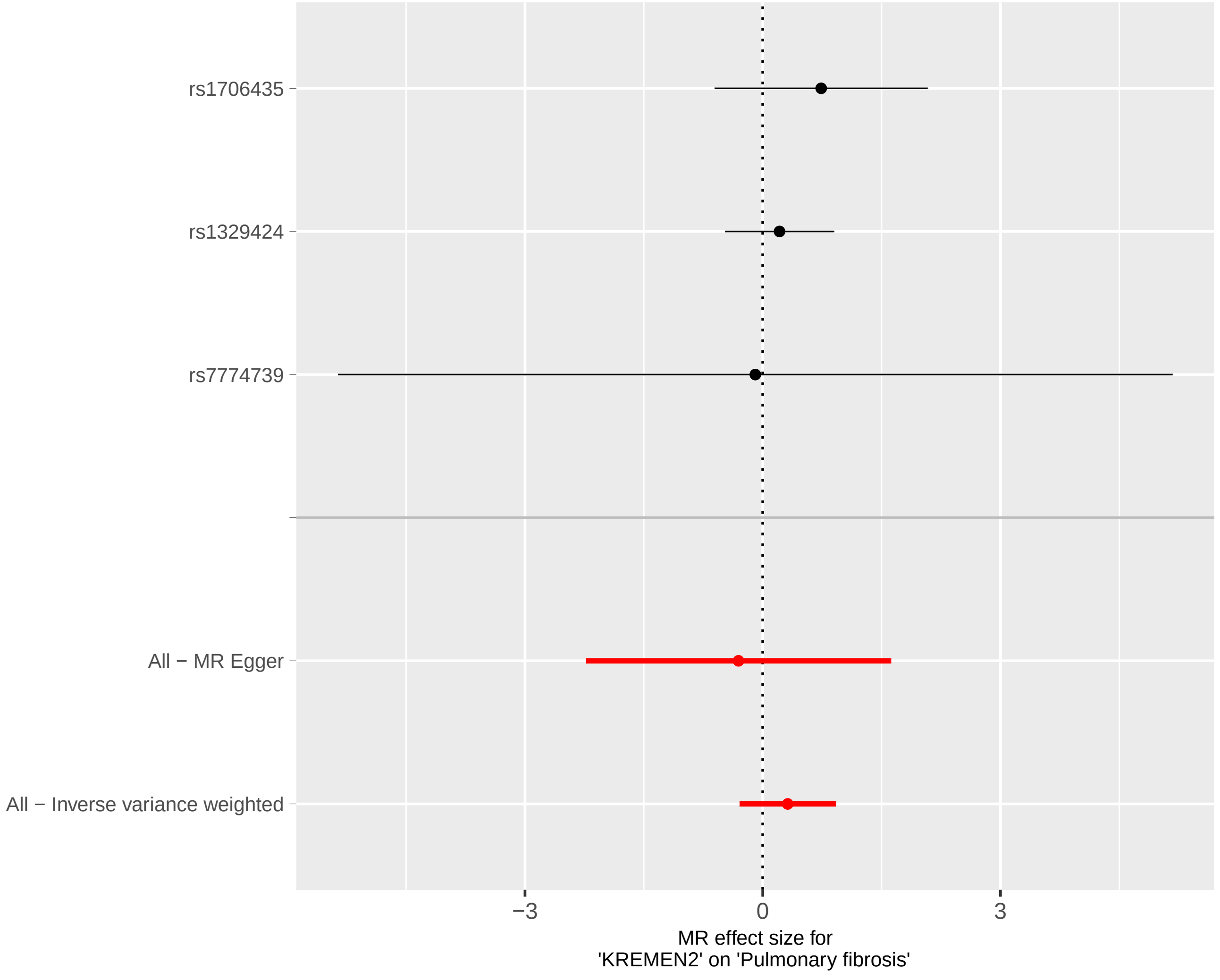

rs1706435
rs1329424
rs7774739
All − MR Egger
All − Inverse variance weighted
−3
0
3
MR effect size for
'KREMEN2' on 'Pulmonary fibrosis'

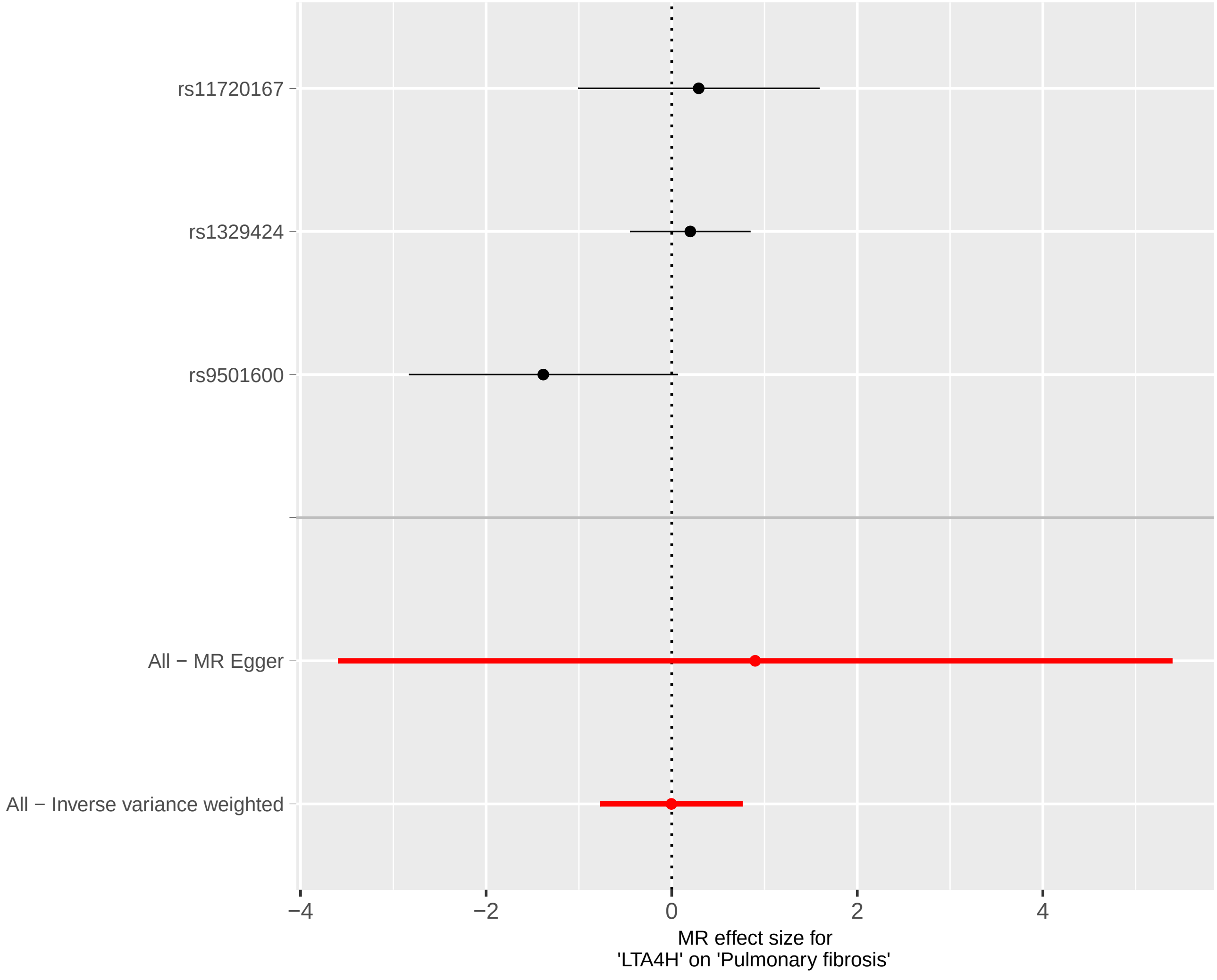
rs11720167
rs1329424
rs9501600
All − MR Egger
All − Inverse variance weighted
−4
−2
0
2
4
MR effect size for
'LTA4H' on 'Pulmonary fibrosis'

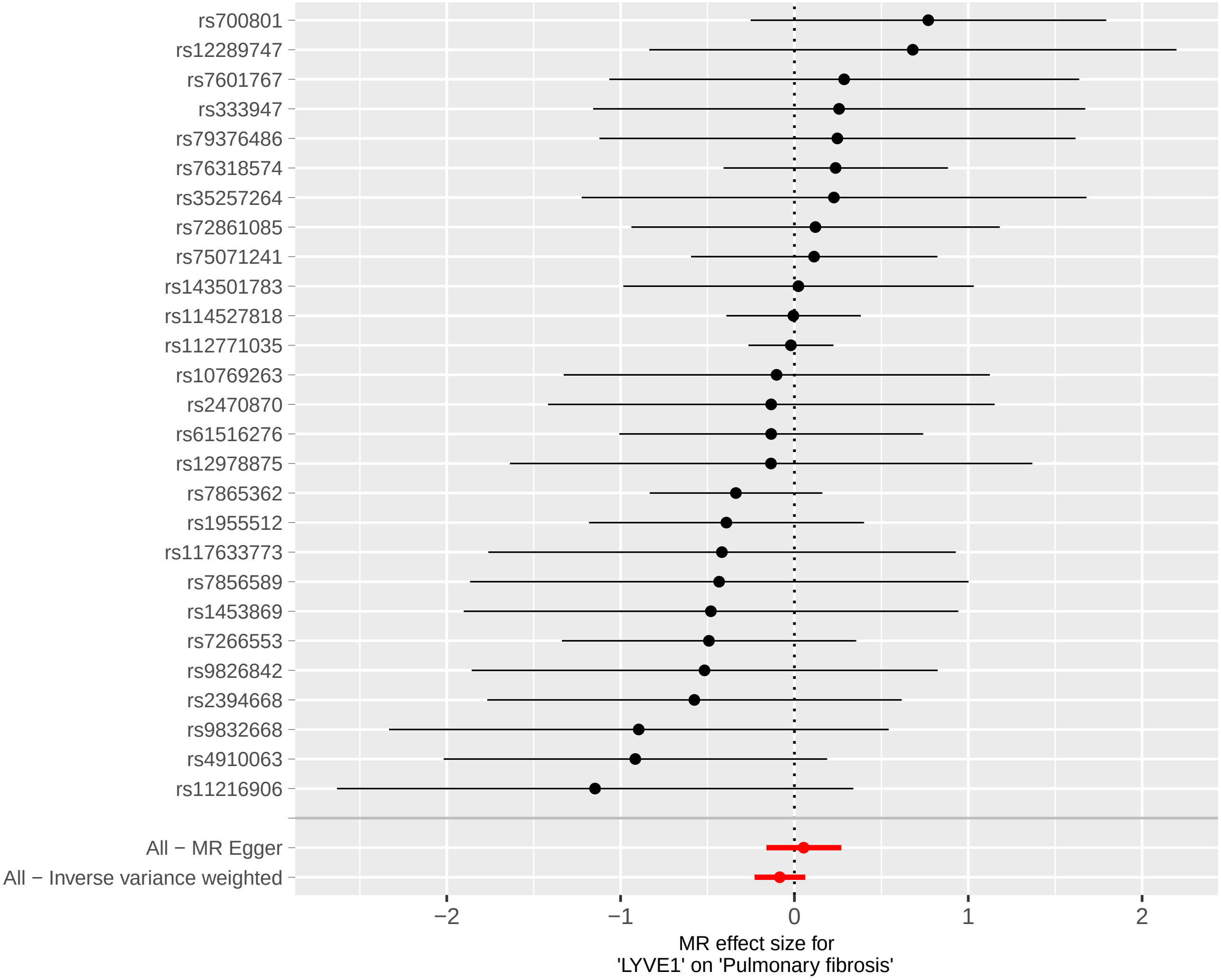

rs700801
rs12289747
rs7601767
rs333947
rs79376486
rs76318574
rs35257264
rs72861085
rs75071241
rs143501783
rs114527818
rs112771035
rs10769263
rs2470870
rs61516276
rs12978875
rs7865362
rs1955512
rs117633773
rs7856589
rs1453869
rs7266553
rs9826842
rs2394668
rs9832668
rs4910063
rs11216906
All − MR Egger
All − Inverse variance weighted
−2
−1
0
1
2
MR effect size for
'LYVE1' on 'Pulmonary fibrosis'

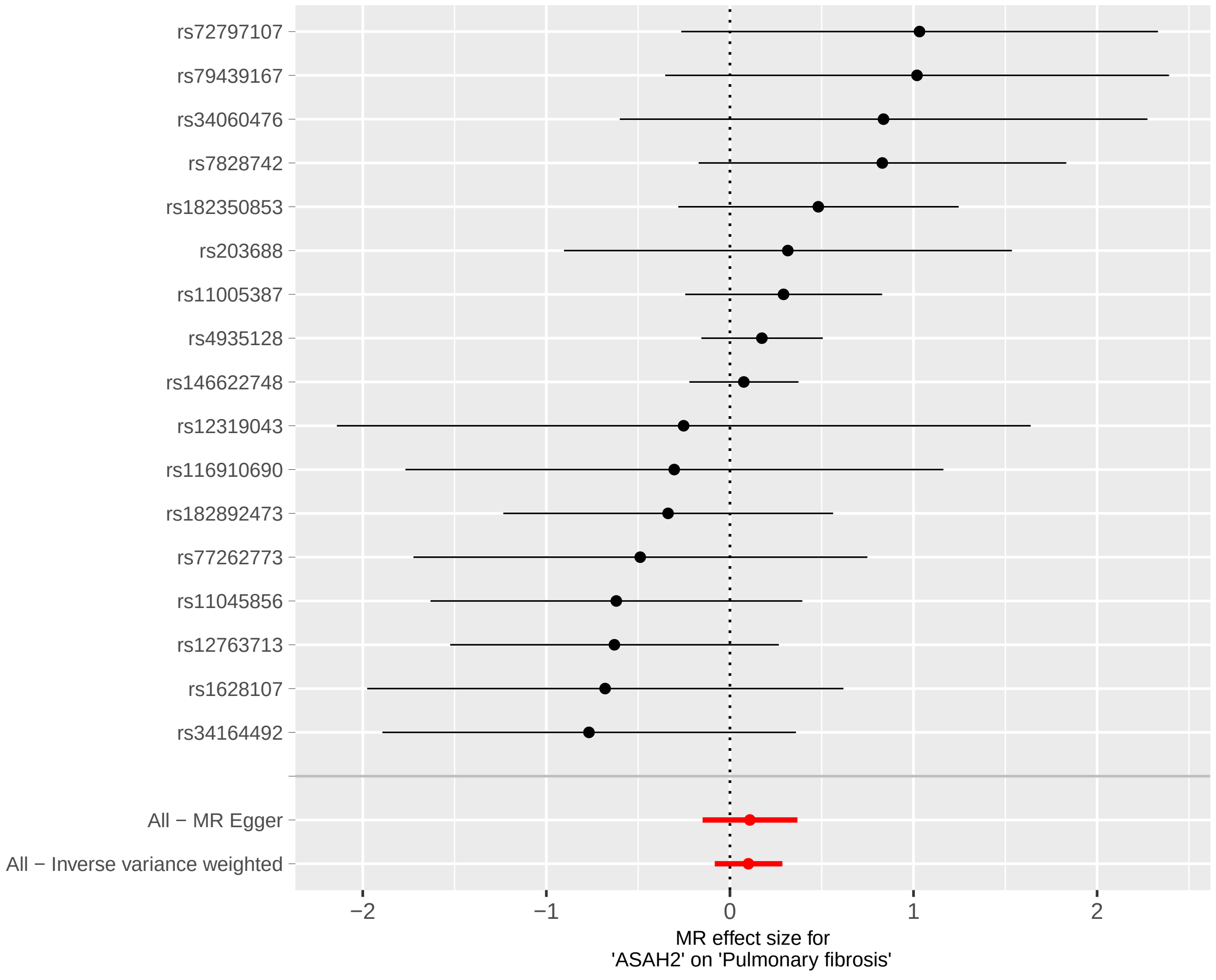
rs72797107
rs79439167
rs34060476
rs7828742
rs182350853
rs203688
rs11005387
rs4935128
rs146622748
rs12319043
rs116910690
rs182892473
rs77262773
rs11045856
rs12763713
rs1628107
rs34164492
All − MR Egger
All − Inverse variance weighted
−2
−1
0
1
2
MR effect size for
'ASAH2' on 'Pulmonary fibrosis'

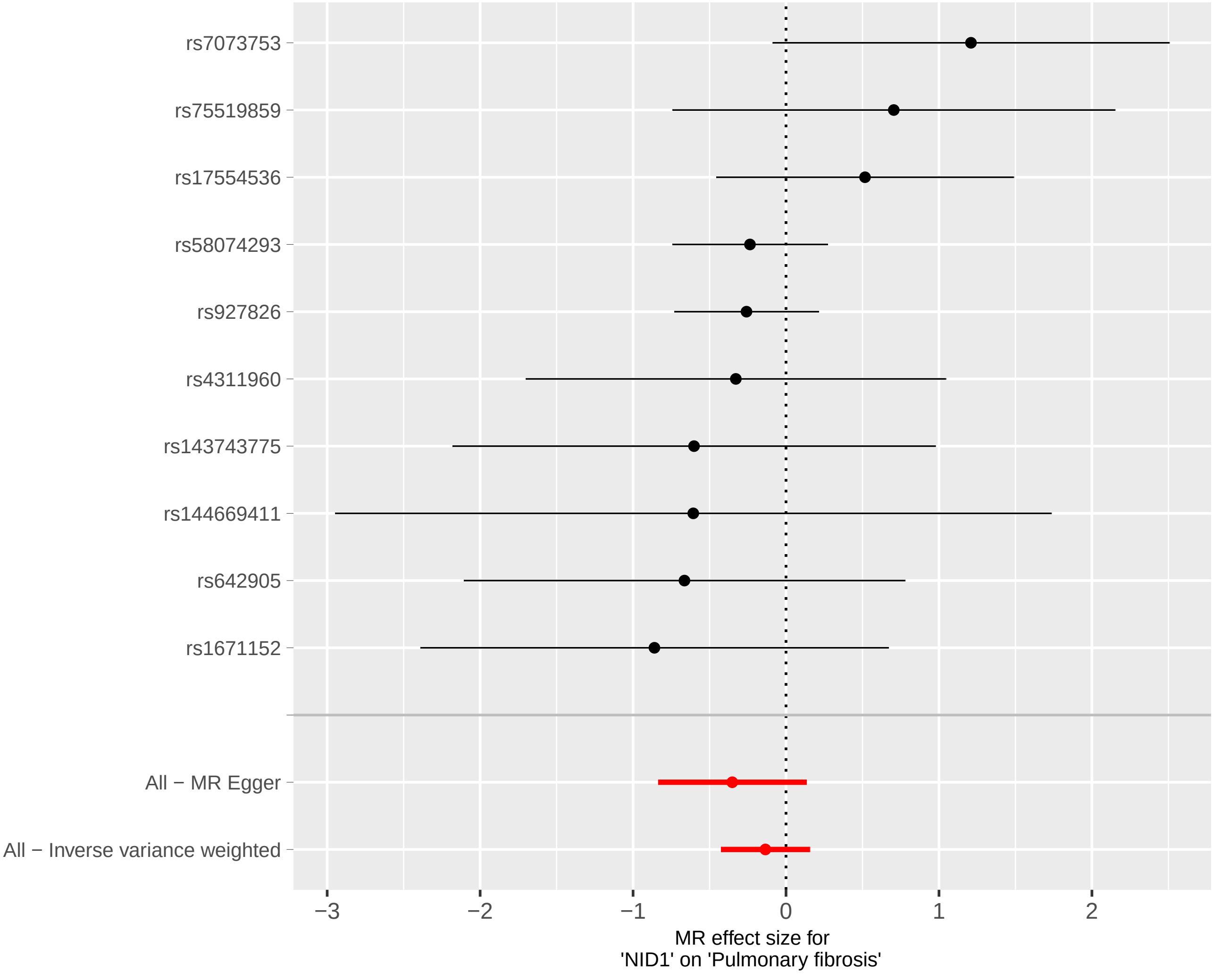

rs7073753
rs75519859
rs17554536
rs58074293
rs927826
rs4311960
rs143743775
rs144669411
rs642905
rs1671152
All − MR Egger
All − Inverse variance weighted
−3
−2
−1
0
1
2
MR effect size for
'NID1' on 'Pulmonary fibrosis'

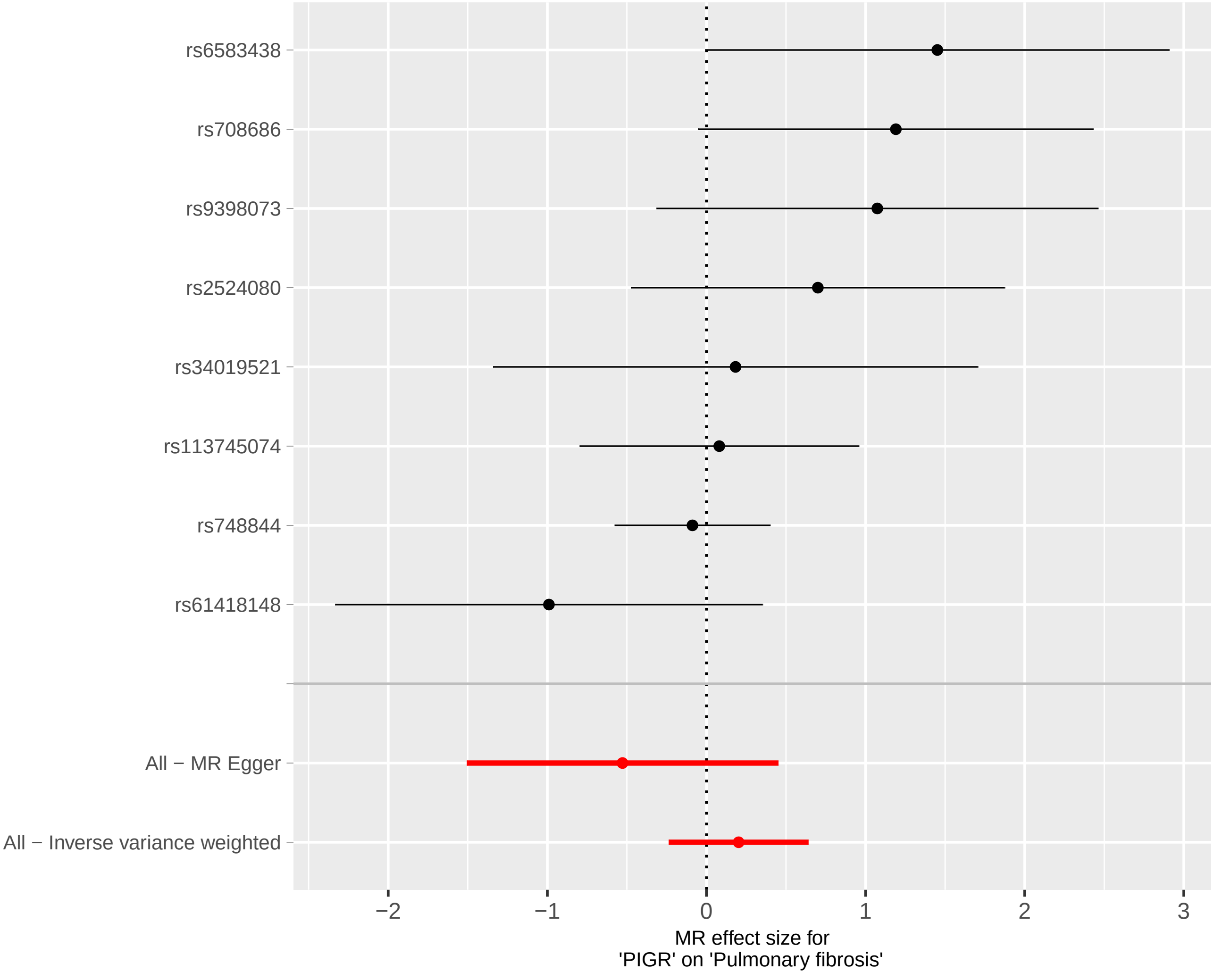

rs6583438
rs708686
rs9398073
rs2524080
rs34019521
rs113745074
rs748844
rs61418148
All − MR Egger
All − Inverse variance weighted
−2
−1
0
1
2
3
MR effect size for
'PIGR' on 'Pulmonary fibrosis'

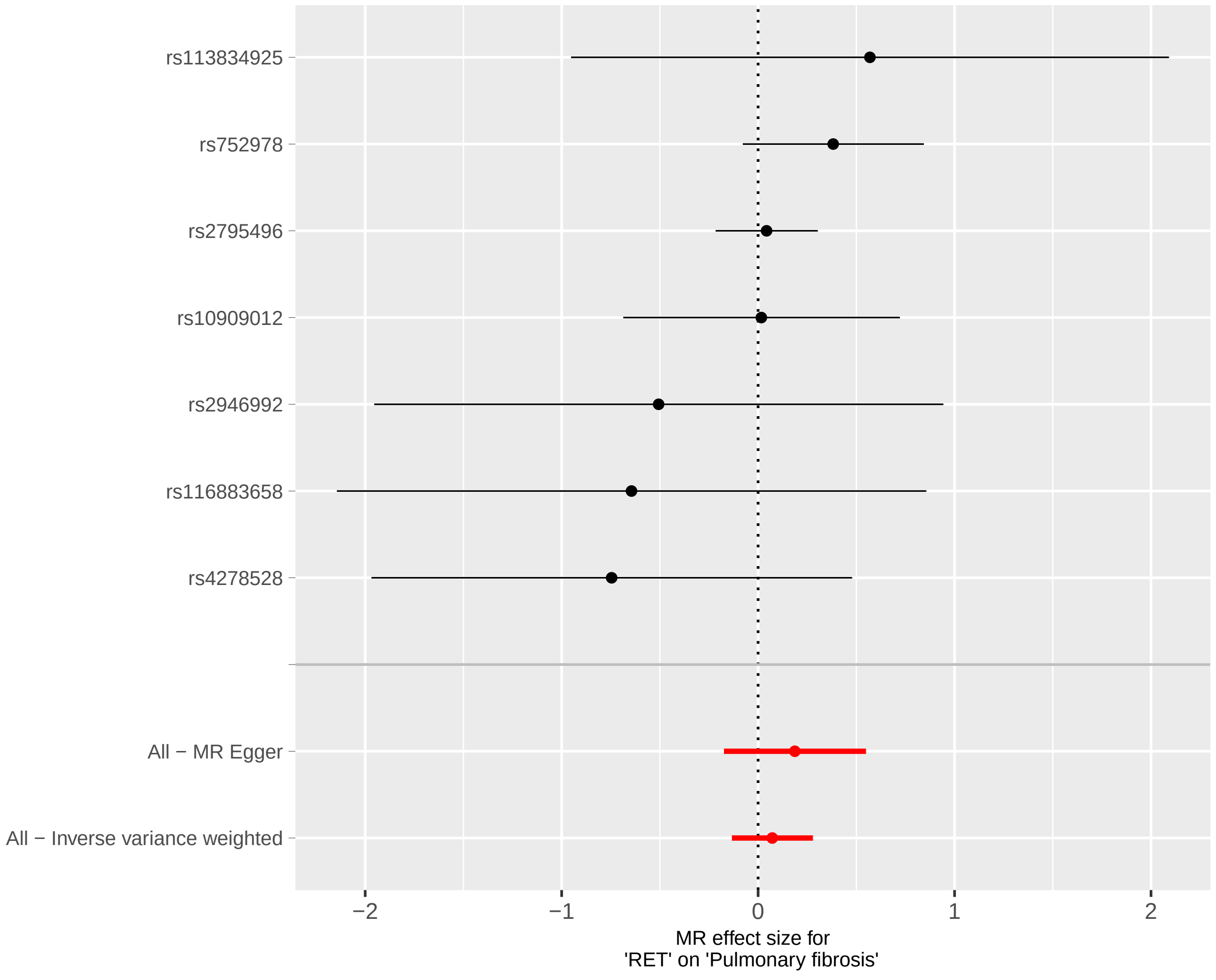

rs113834925
rs752978
rs2795496
rs10909012
rs2946992
rs116883658
rs4278528
All − MR Egger
All − Inverse variance weighted
−2
−1
0
1
2
MR effect size for
'RET' on 'Pulmonary fibrosis'

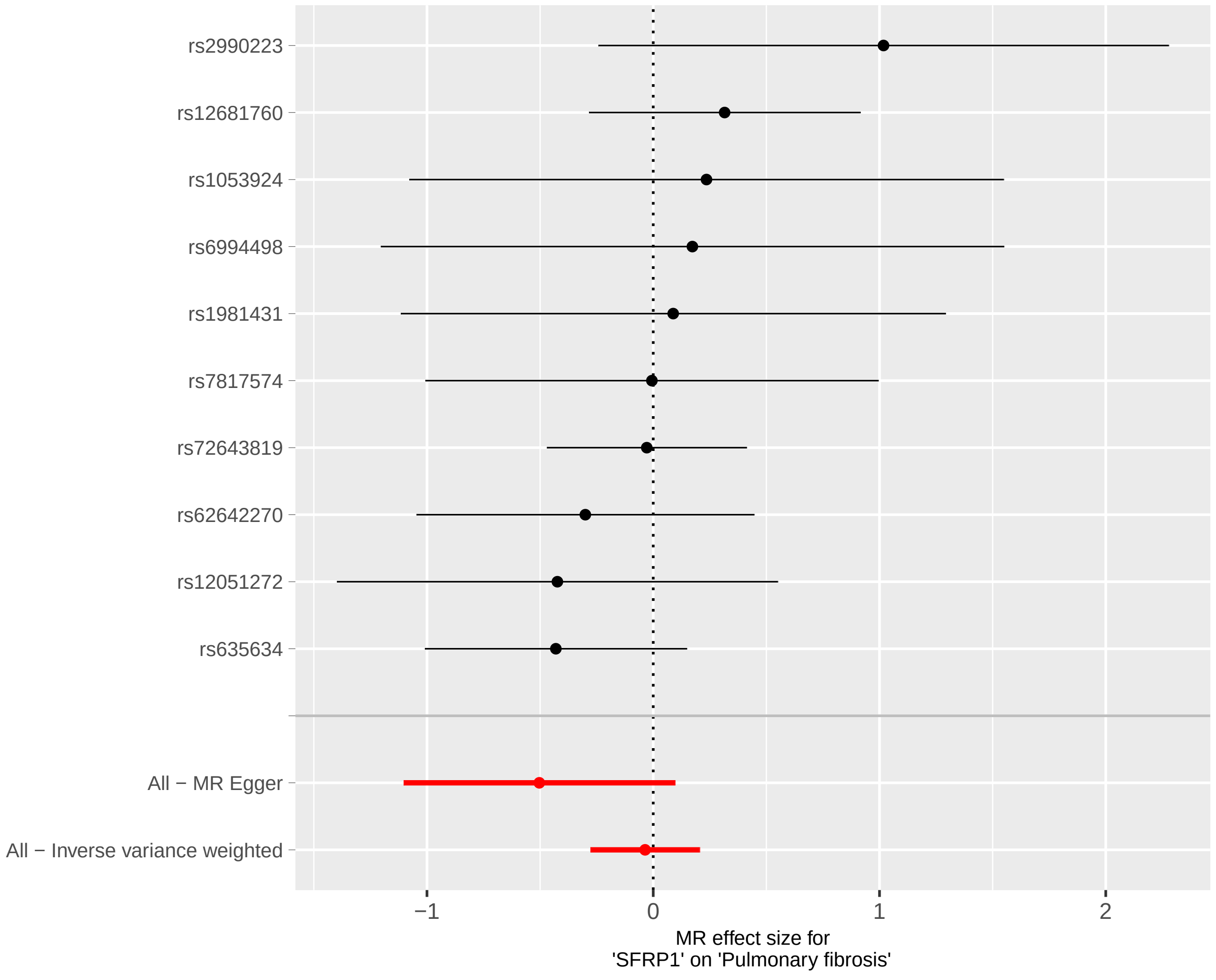

rs2990223
rs12681760
rs1053924
rs6994498
rs1981431
rs7817574
rs72643819
rs62642270
rs12051272
rs635634
All − MR Egger
All − Inverse variance weighted
−1
0
1
2
MR effect size for
'SFRP1' on 'Pulmonary fibrosis'

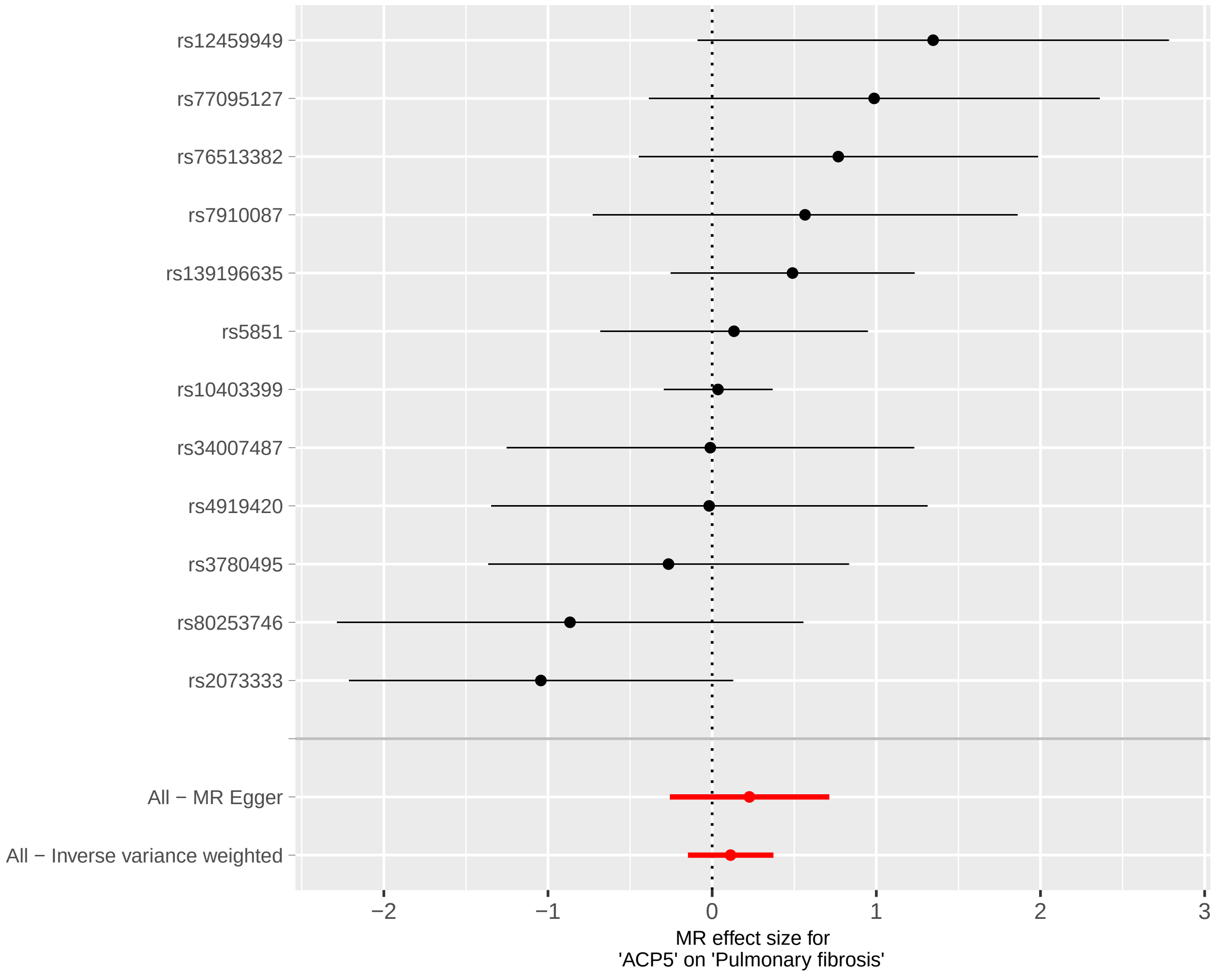

rs12459949
rs77095127
rs76513382
rs7910087
rs139196635
rs5851
rs10403399
rs34007487
rs4919420
rs3780495
rs80253746
rs2073333
All − MR Egger
All − Inverse variance weighted
−2
−1
0
1
2
3
MR effect size for
'ACP5' on 'Pulmonary fibrosis'

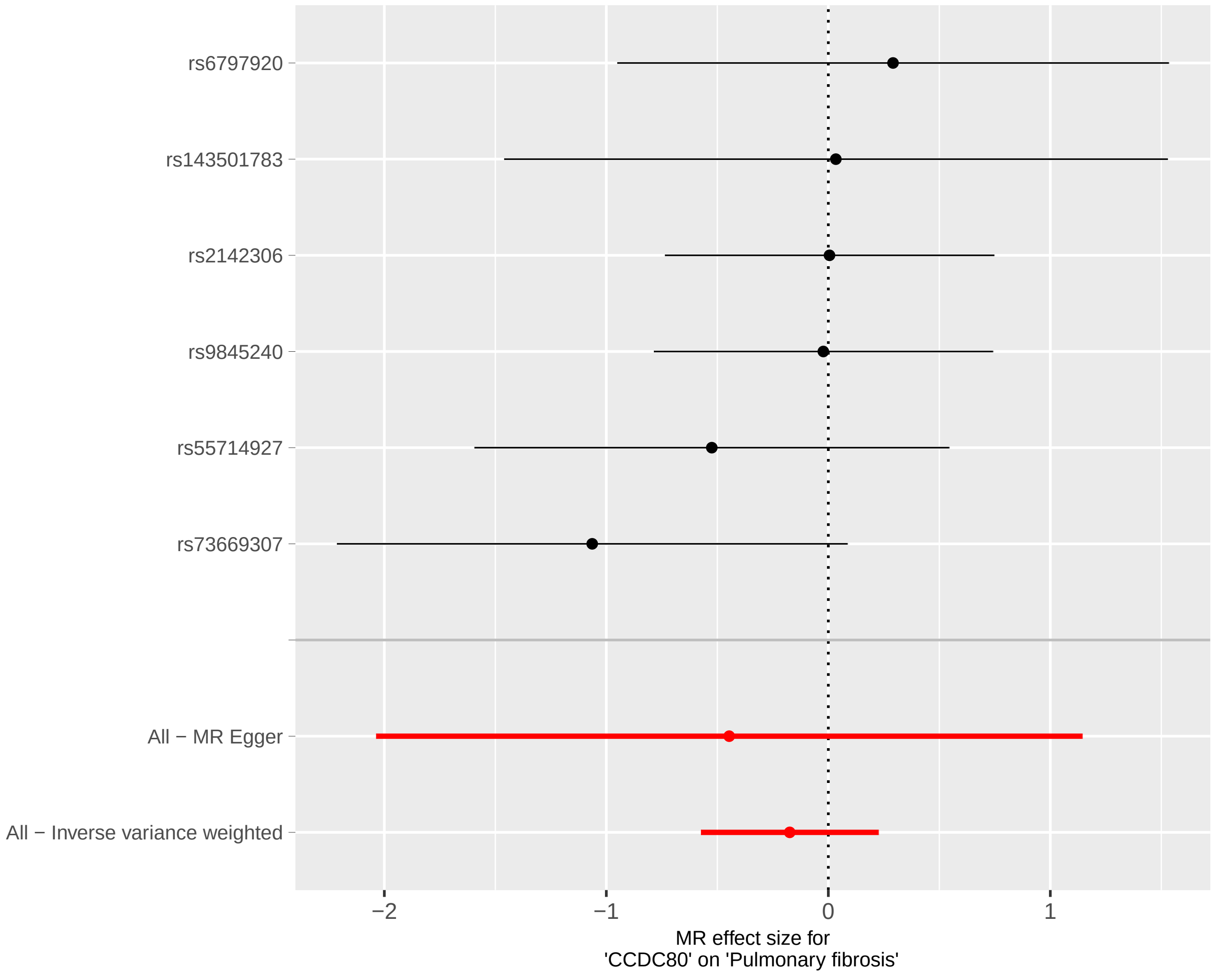

rs6797920
rs143501783
rs2142306
rs9845240
rs55714927
rs73669307
All − MR Egger
All − Inverse variance weighted
−2
−1
0
1
MR effect size for
'CCDC80' on 'Pulmonary fibrosis'

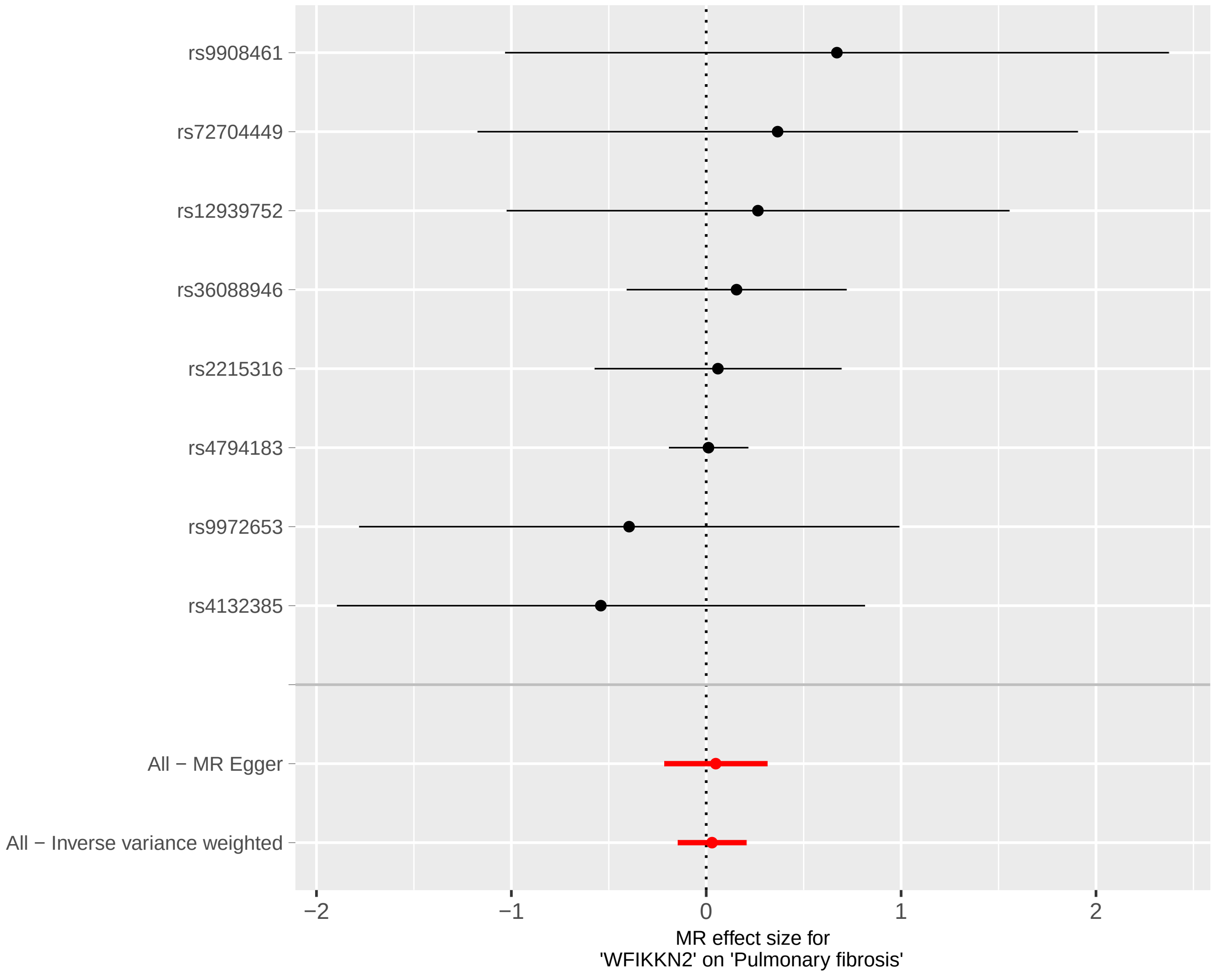

rs9908461
rs72704449
rs12939752
rs36088946
rs2215316
rs4794183
rs9972653
rs4132385
All − MR Egger
All − Inverse variance weighted
−2
−1
0
1
2
MR effect size for
'WFIKKN2' on 'Pulmonary fibrosis'

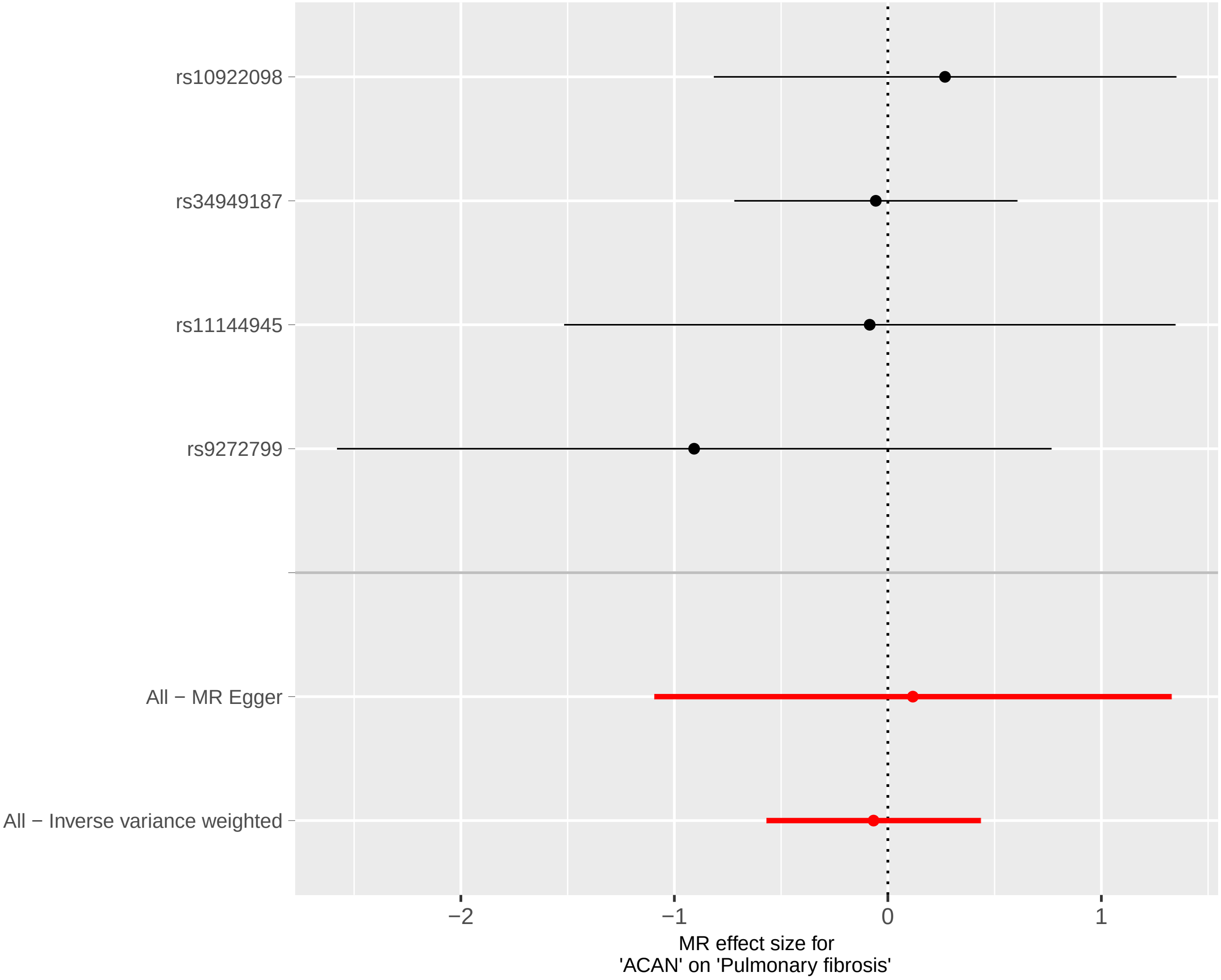

rs10922098
rs34949187
rs11144945
rs9272799
All − MR Egger
All − Inverse variance weighted
−2
−1
0
1
MR effect size for
'ACAN' on 'Pulmonary fibrosis'

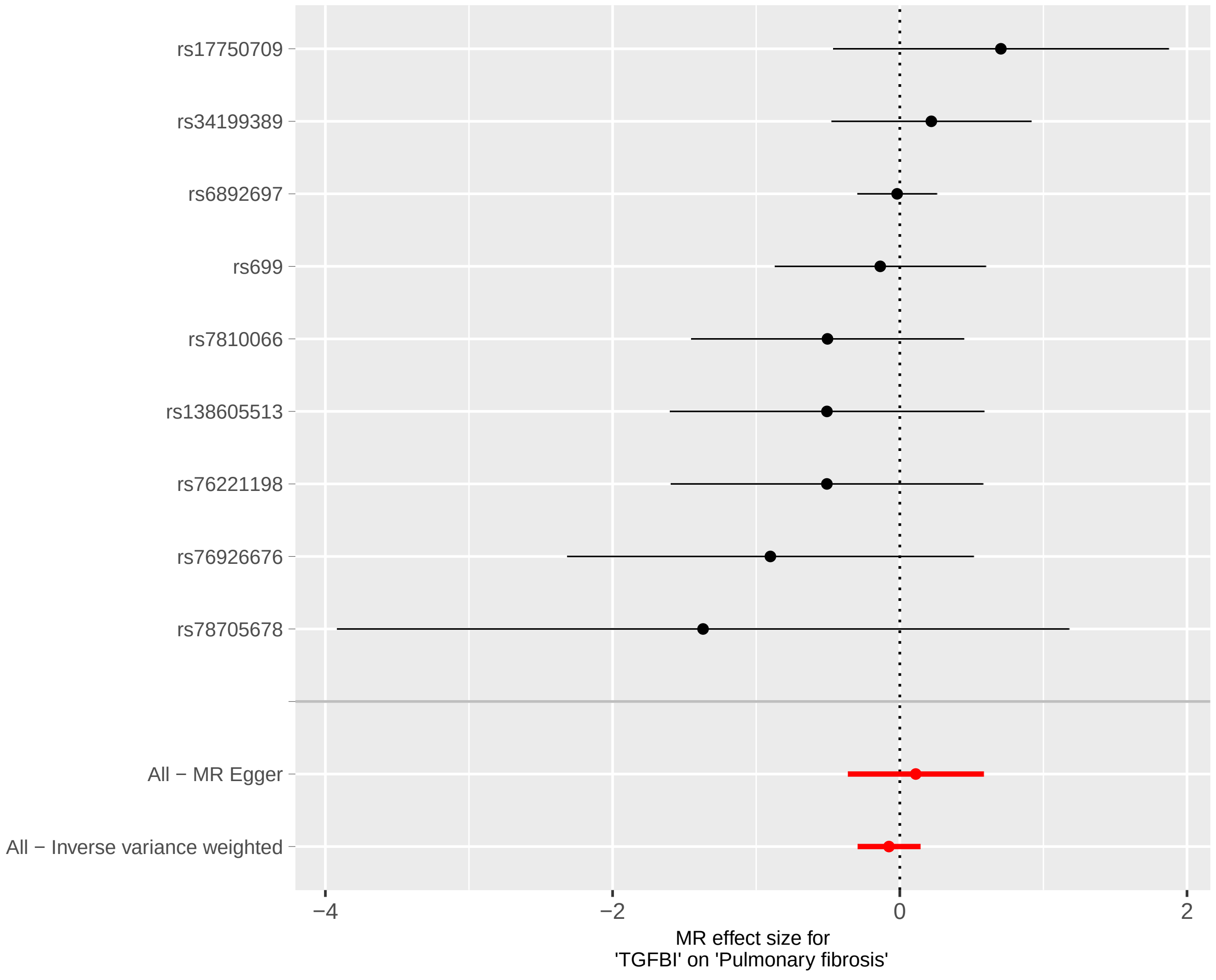

rs17750709
rs34199389
rs6892697
rs699
rs7810066
rs138605513
rs76221198
rs76926676
rs78705678
All − MR Egger
All − Inverse variance weighted
−4
−2
0
2
MR effect size for
'TGFBI' on 'Pulmonary fibrosis'

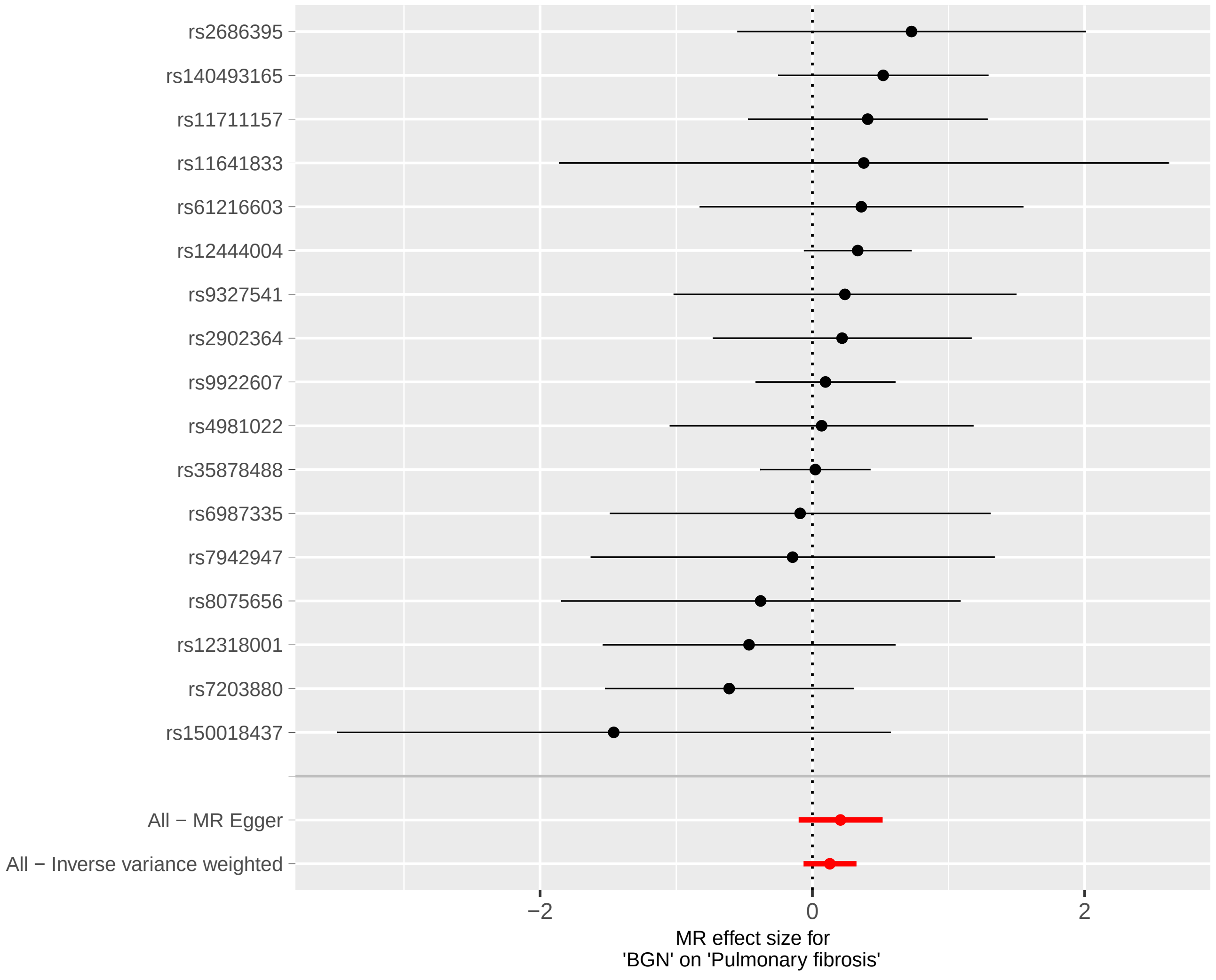

rs2686395
rs140493165
rs11711157
rs11641833
rs61216603
rs12444004
rs9327541
rs2902364
rs9922607
rs4981022
rs35878488
rs6987335
rs7942947
rs8075656
rs12318001
rs7203880
rs150018437
All − MR Egger
All − Inverse variance weighted
−2
0
2
MR effect size for
'BGN' on 'Pulmonary fibrosis'

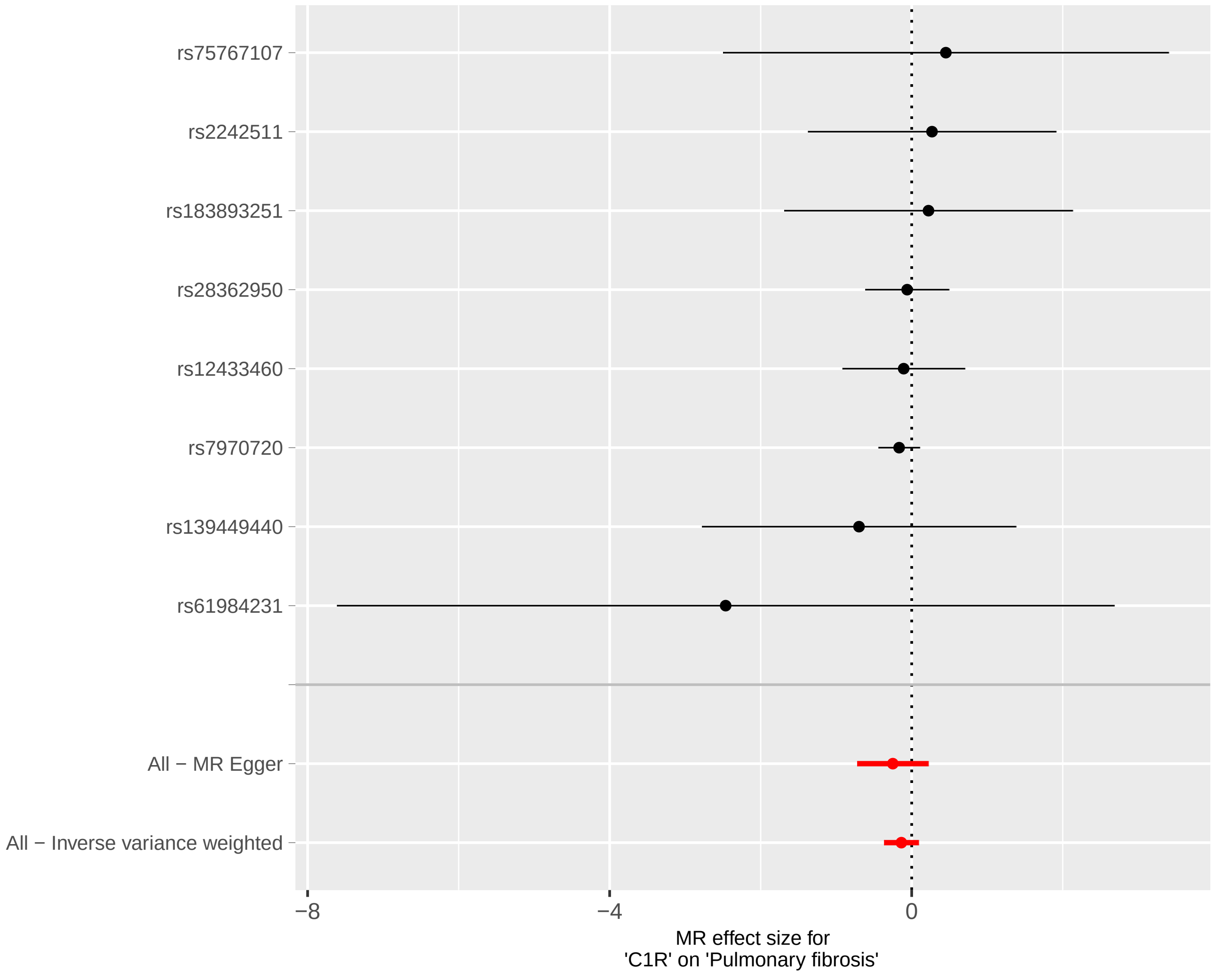

rs75767107
rs2242511
rs183893251
rs28362950
rs12433460
rs7970720
rs139449440
rs61984231
All − MR Egger
All − Inverse variance weighted
−8
−4
0
MR effect size for
'C1R' on 'Pulmonary fibrosis'

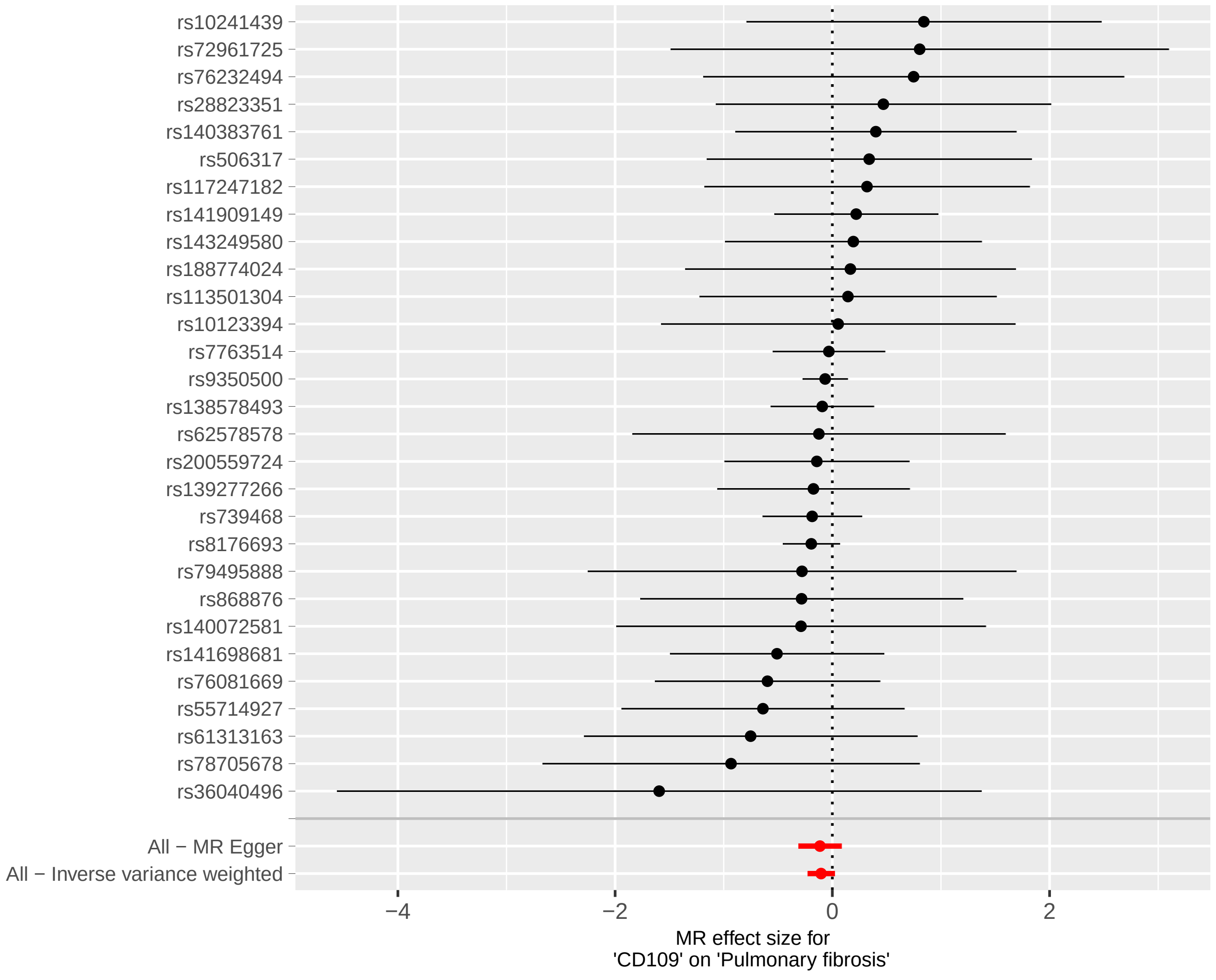
rs10241439
rs72961725
rs76232494
rs28823351
rs140383761
rs506317
rs117247182
rs141909149
rs143249580
rs188774024
rs113501304
rs10123394
rs7763514
rs9350500
rs138578493
rs62578578
rs200559724
rs139277266
rs739468
rs8176693
rs79495888
rs868876
rs140072581
rs141698681
rs76081669
rs55714927
rs61313163
rs78705678
rs36040496
All − MR Egger
All − Inverse variance weighted
−4
−2
0
2
MR effect size for
'CD109' on 'Pulmonary fibrosis'

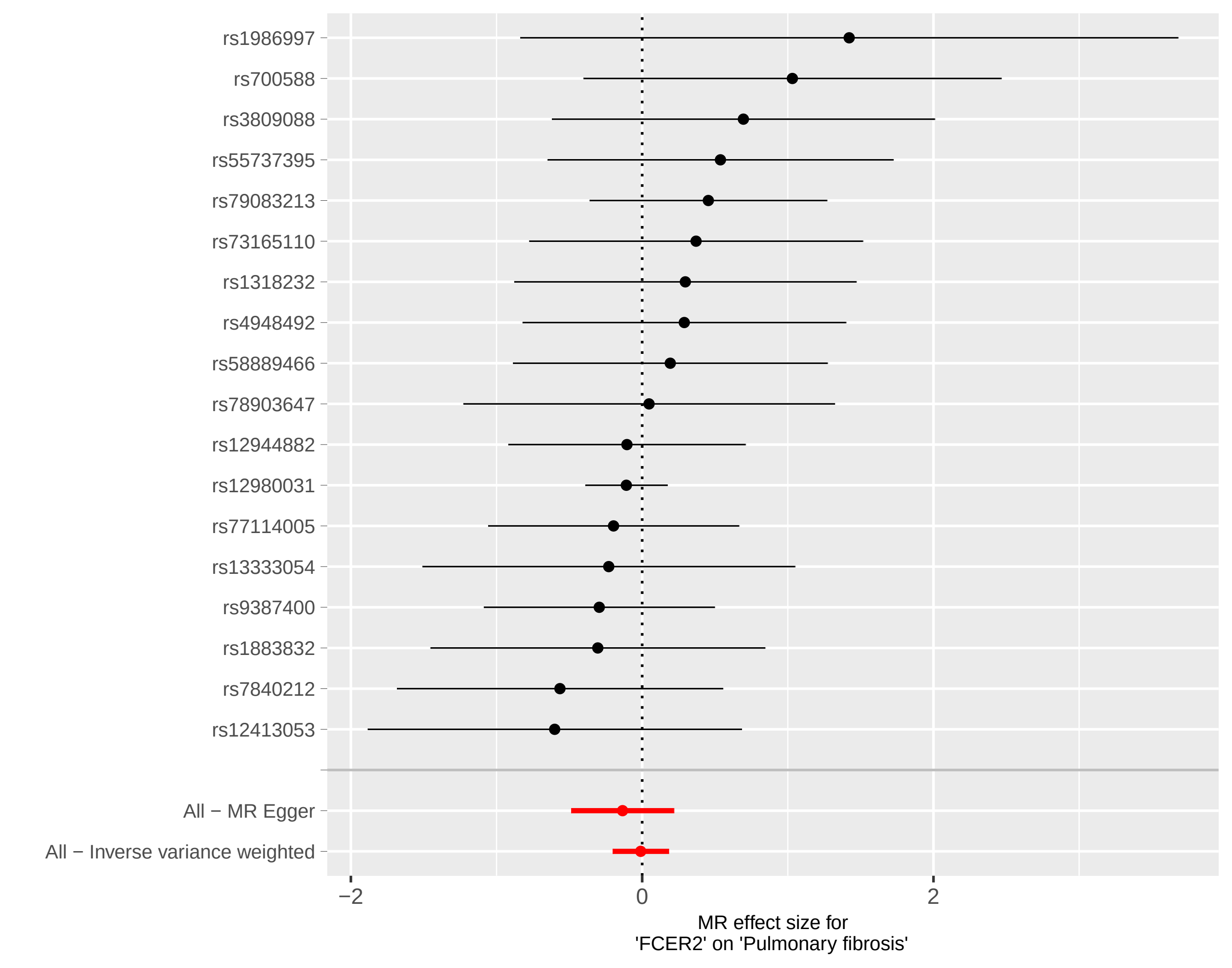
rs1986997
rs700588
rs3809088
rs55737395
rs79083213
rs73165110
rs1318232
rs4948492
rs58889466
rs78903647
rs12944882
rs12980031
rs77114005
rs13333054
rs9387400
rs1883832
rs7840212
rs12413053
All − MR Egger
All − Inverse variance weighted
−2
0
2
MR effect size for
'FCER2' on 'Pulmonary fibrosis'

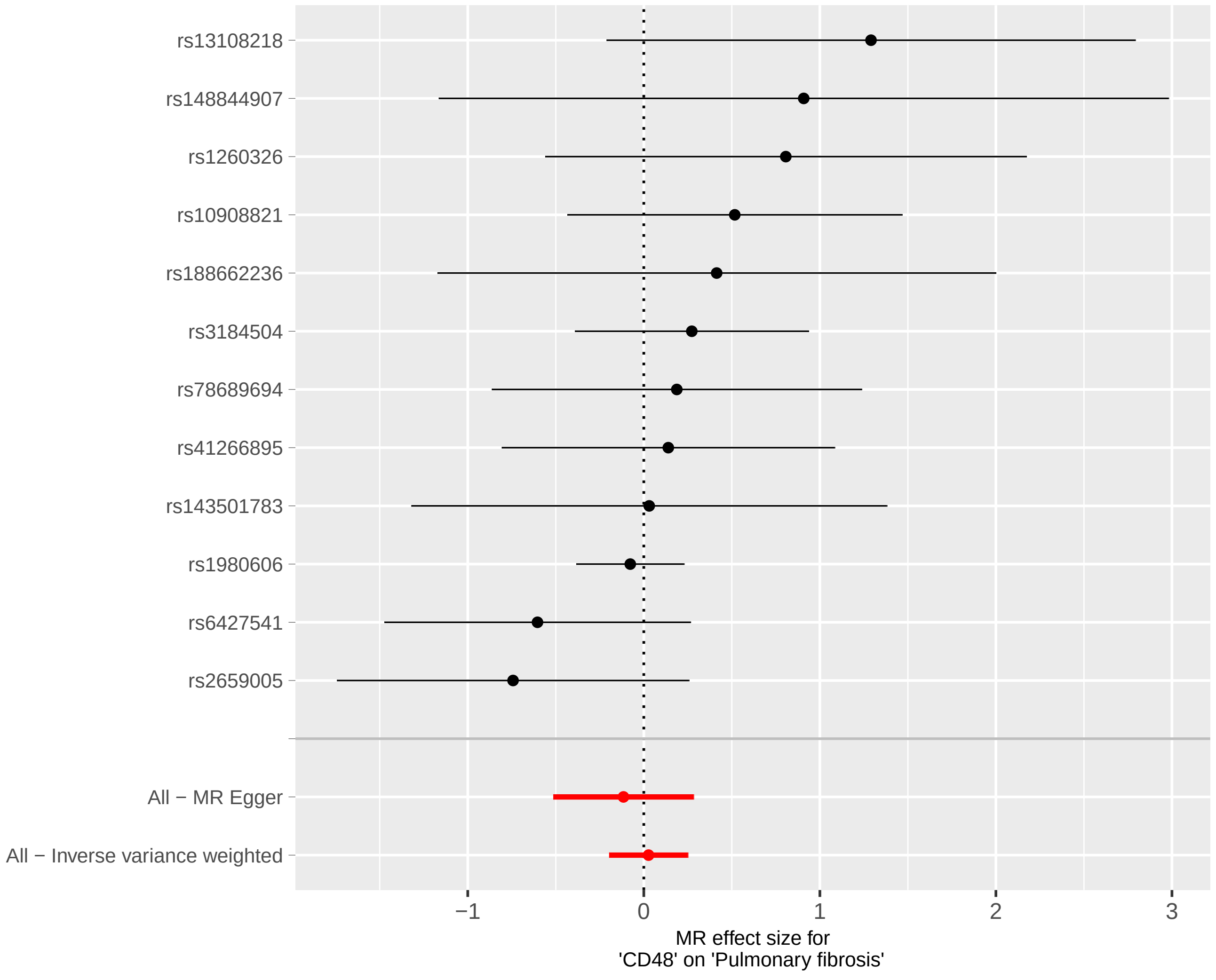

rs13108218
rs148844907
rs1260326
rs10908821
rs188662236
rs3184504
rs78689694
rs41266895
rs143501783
rs1980606
rs6427541
rs2659005
All − MR Egger
All − Inverse variance weighted
−1
0
1
2
3
MR effect size for
'CD48' on 'Pulmonary fibrosis'

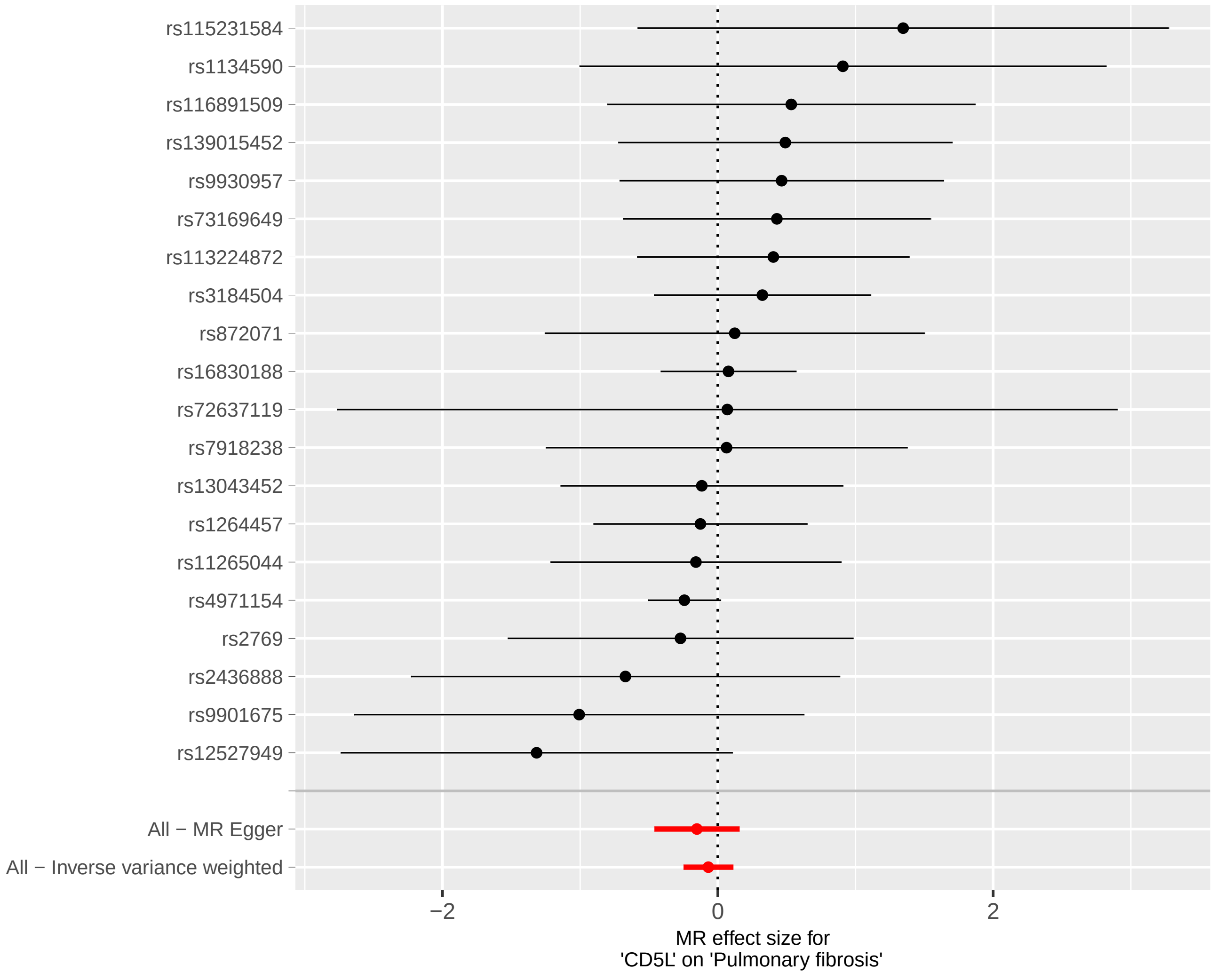

rs115231584
rs1134590
rs116891509
rs139015452
rs9930957
rs73169649
rs113224872
rs3184504
rs872071
rs16830188
rs72637119
rs7918238
rs13043452
rs1264457
rs11265044
rs4971154
rs2769
rs2436888
rs9901675
rs12527949
All − MR Egger
All − Inverse variance weighted
−2
0
2
MR effect size for
'CD5L' on 'Pulmonary fibrosis'

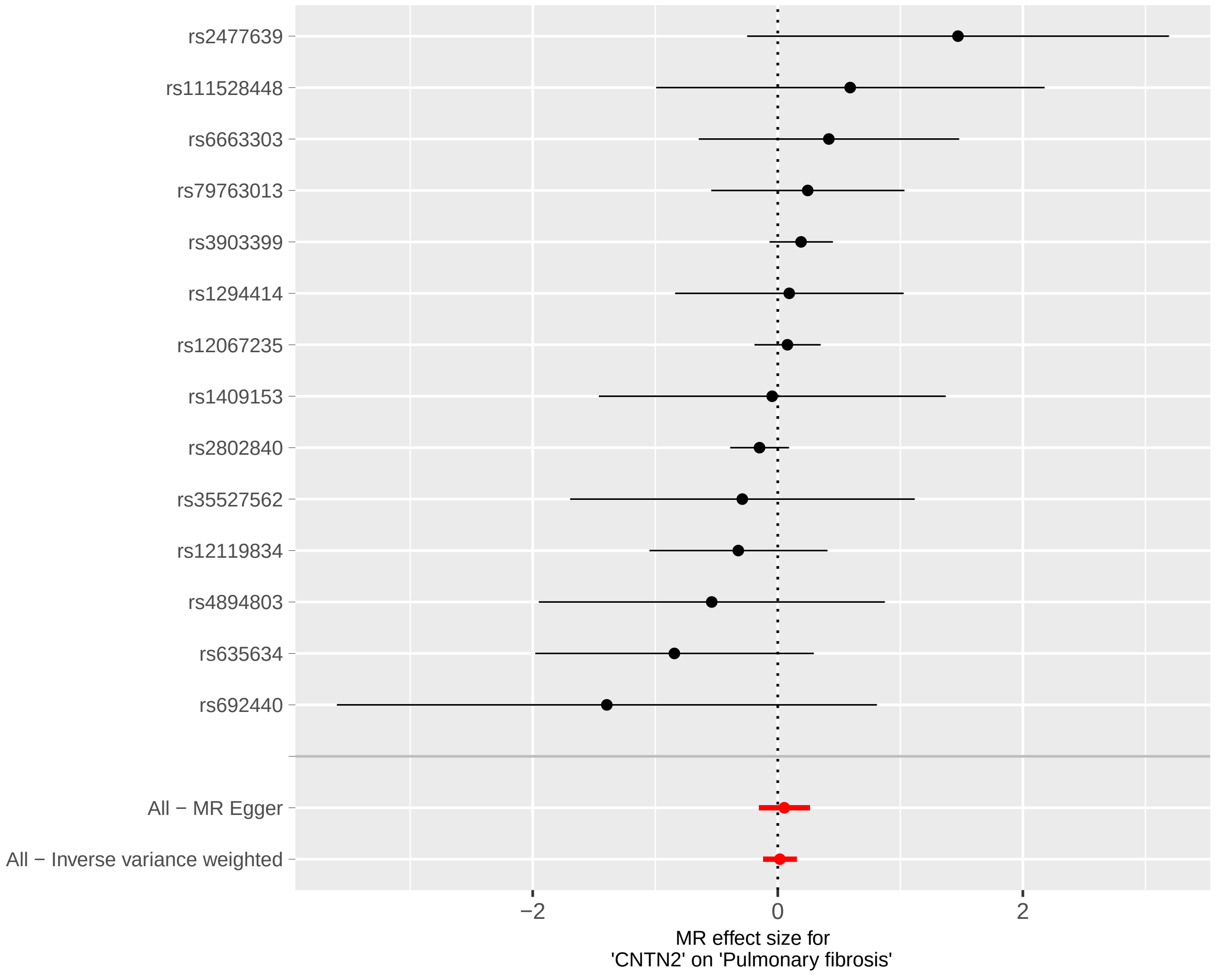

rs2477639
rs111528448
rs6663303
rs79763013
rs3903399
rs1294414
rs12067235
rs1409153
rs2802840
rs35527562
rs12119834
rs4894803
rs635634
rs692440
All − MR Egger
All − Inverse variance weighted
−2
0
2
MR effect size for
'CNTN2' on 'Pulmonary fibrosis'

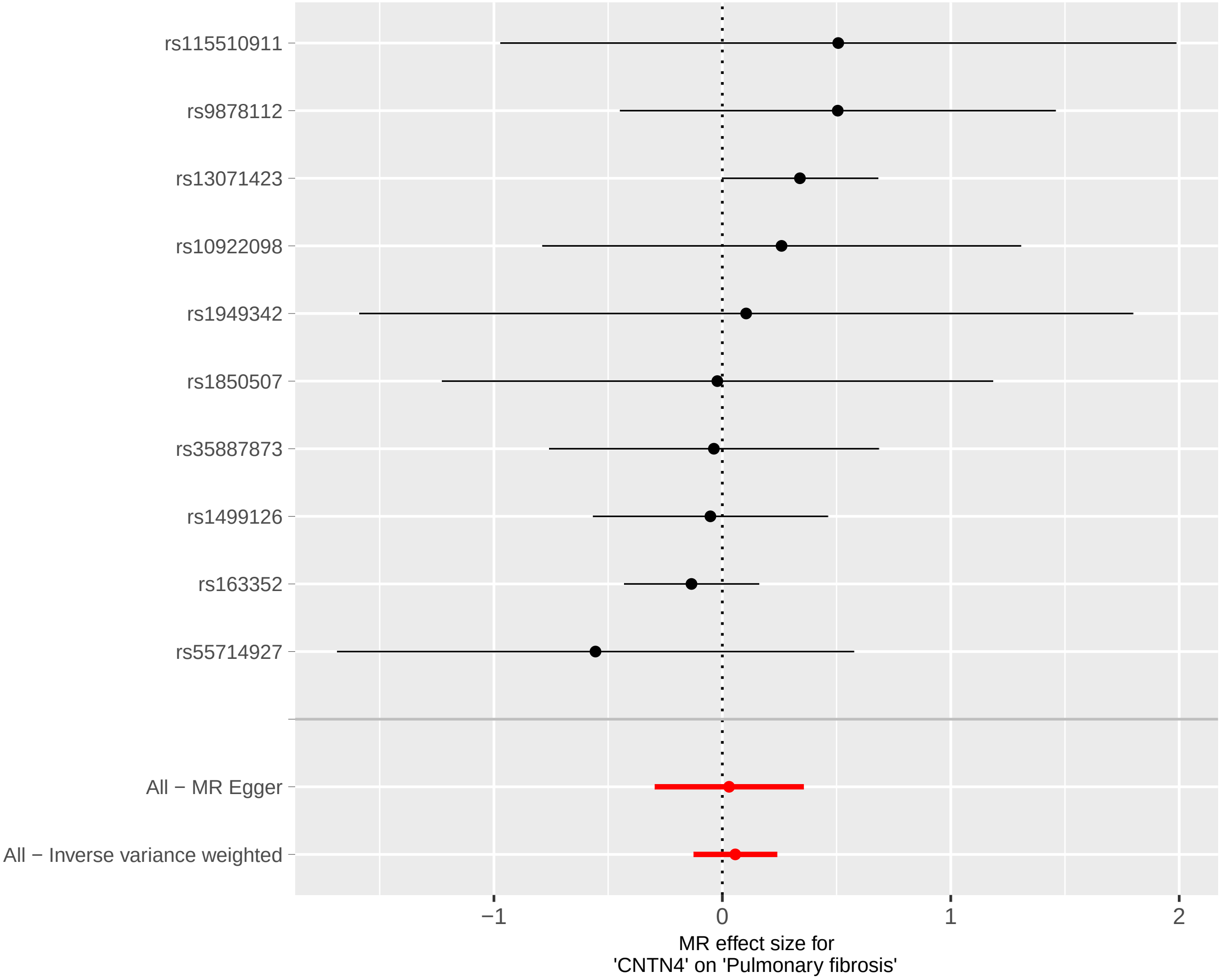
rs115510911
rs9878112
rs13071423
rs10922098
rs1949342
rs1850507
rs35887873
rs1499126
rs163352
rs55714927
All − MR Egger
All − Inverse variance weighted
−1
0
1
2
MR effect size for
'CNTN4' on 'Pulmonary fibrosis'

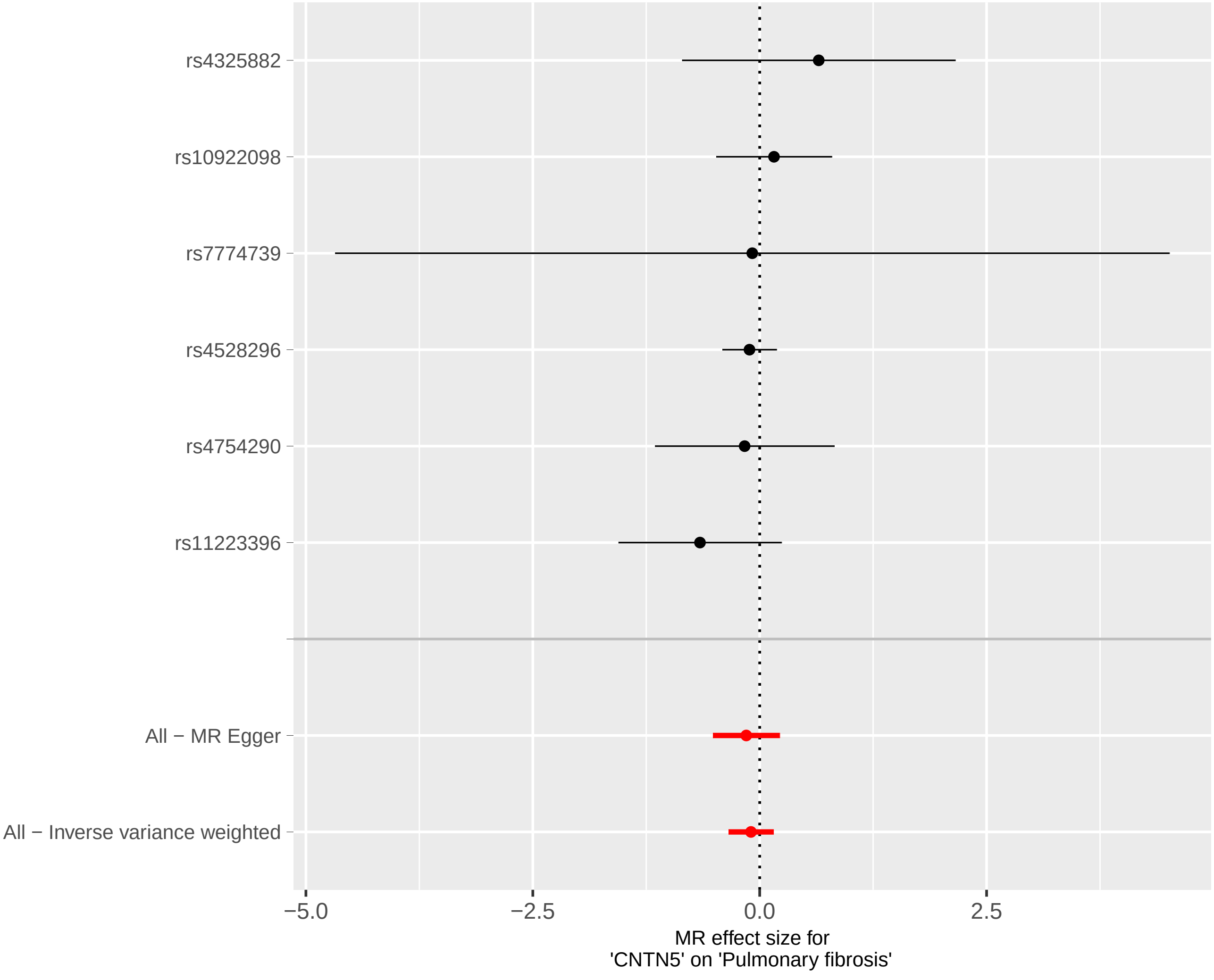
rs4325882
rs10922098
rs7774739
rs4528296
rs4754290
rs11223396
All − MR Egger
All − Inverse variance weighted
−5.0
−2.5
0.0
2.5
MR effect size for
'CNTN5' on 'Pulmonary fibrosis'

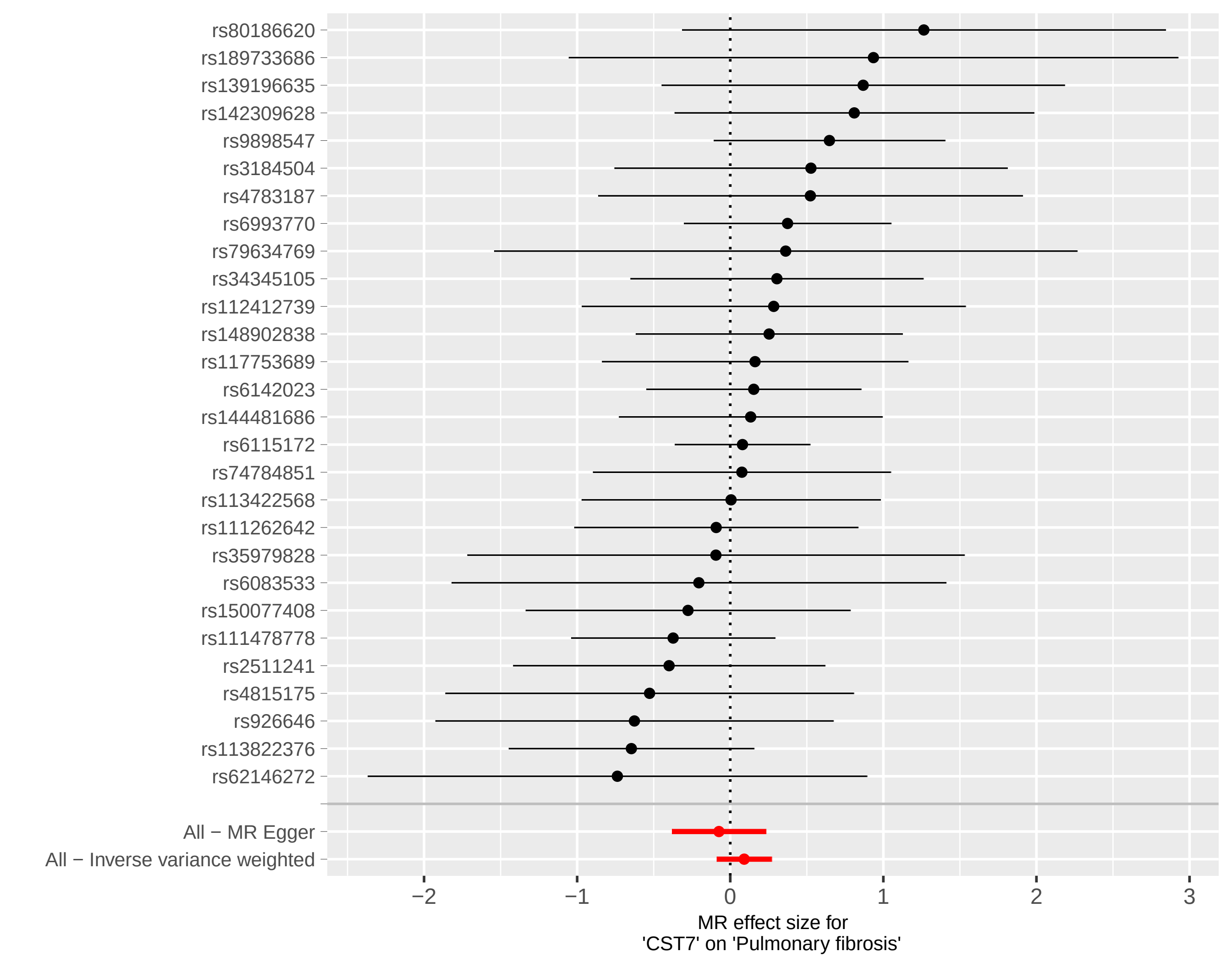
rs80186620
rs189733686
rs139196635
rs142309628
rs9898547
rs3184504
rs4783187
rs6993770
rs79634769
rs34345105
rs112412739
rs148902838
rs117753689
rs6142023
rs144481686
rs6115172
rs74784851
rs113422568
rs111262642
rs35979828
rs6083533
rs150077408
rs111478778
rs2511241
rs4815175
rs926646
rs113822376
rs62146272
All − MR Egger
All − Inverse variance weighted
−2
−1
0
1
2
3
MR effect size for
'CST7' on 'Pulmonary fibrosis'

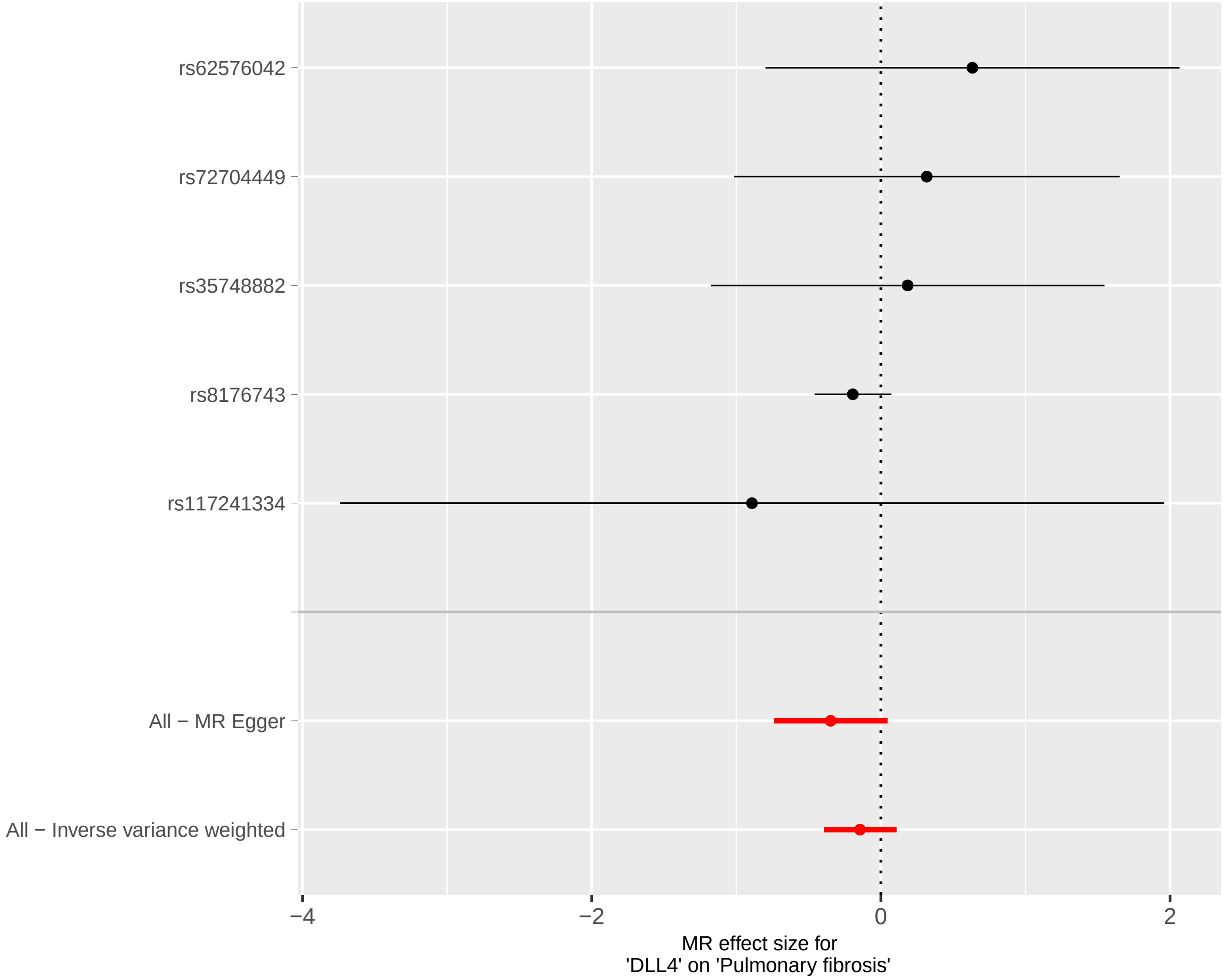

rs62576042
rs72704449
rs35748882
rs8176743
rs117241334
All − MR Egger
All − Inverse variance weighted
−4
−2
0
2
MR effect size for
'DLL4' on 'Pulmonary fibrosis'

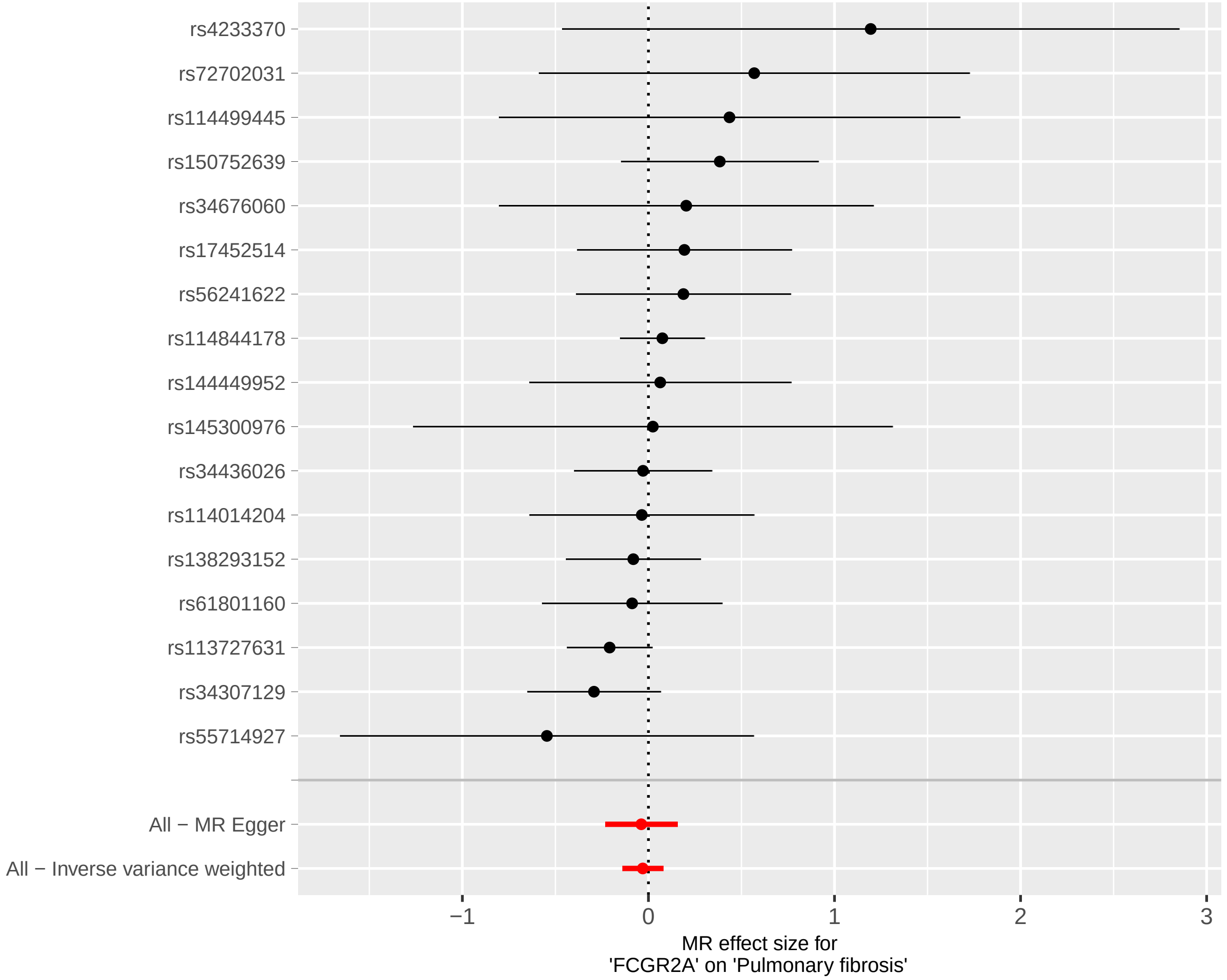

rs4233370
rs72702031
rs114499445
rs150752639
rs34676060
rs17452514
rs56241622
rs114844178
rs144449952
rs145300976
rs34436026
rs114014204
rs138293152
rs61801160
rs113727631
rs34307129
rs55714927
All − MR Egger
All − Inverse variance weighted
−1
0
1
2
3
MR effect size for
'FCGR2A' on 'Pulmonary fibrosis'

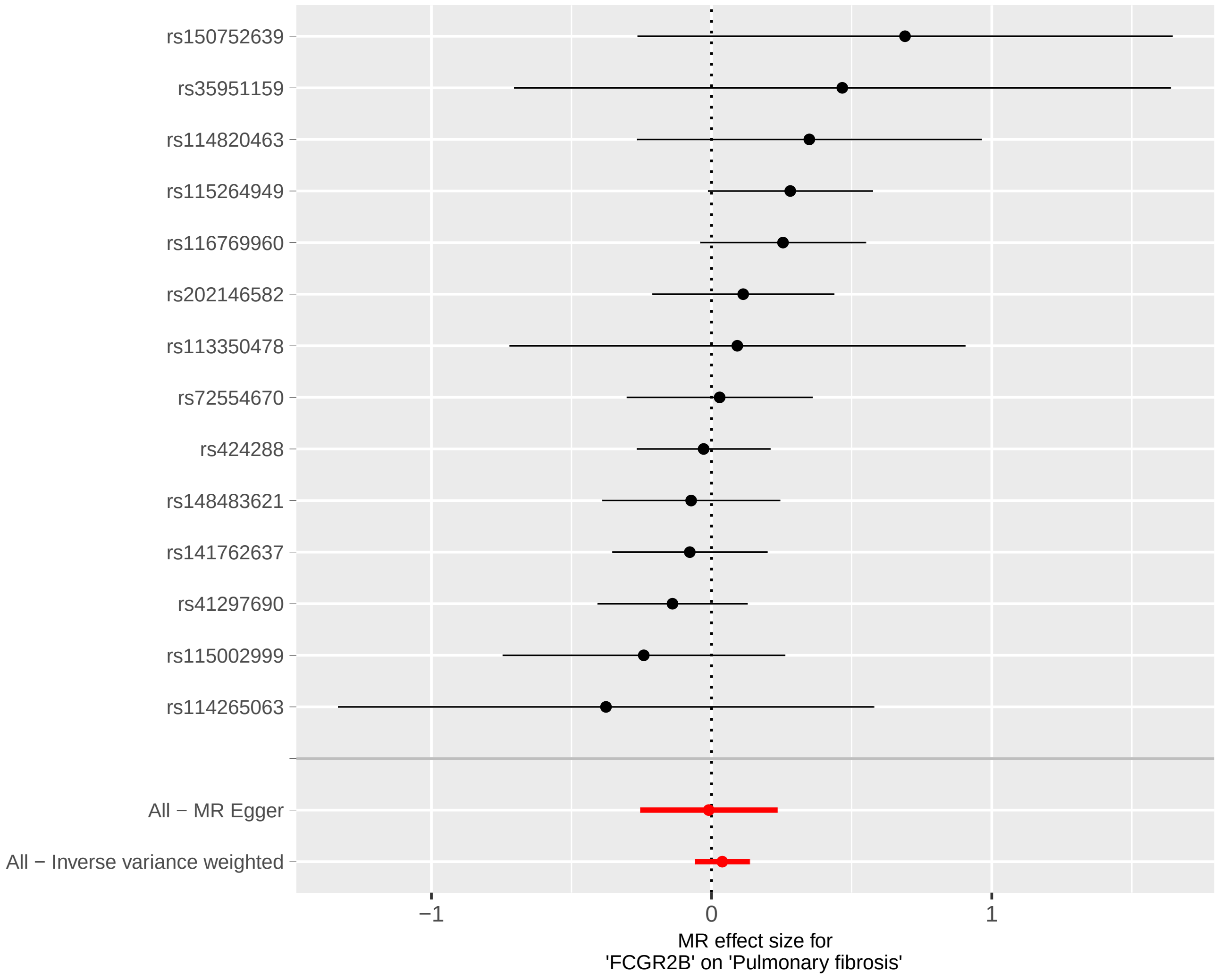

rs150752639
rs35951159
rs114820463
rs115264949
rs116769960
rs202146582
rs113350478
rs72554670
rs424288
rs148483621
rs141762637
rs41297690
rs115002999
rs114265063
All − MR Egger
All − Inverse variance weighted
−1
0
1
MR effect size for
'FCGR2B' on 'Pulmonary fibrosis'

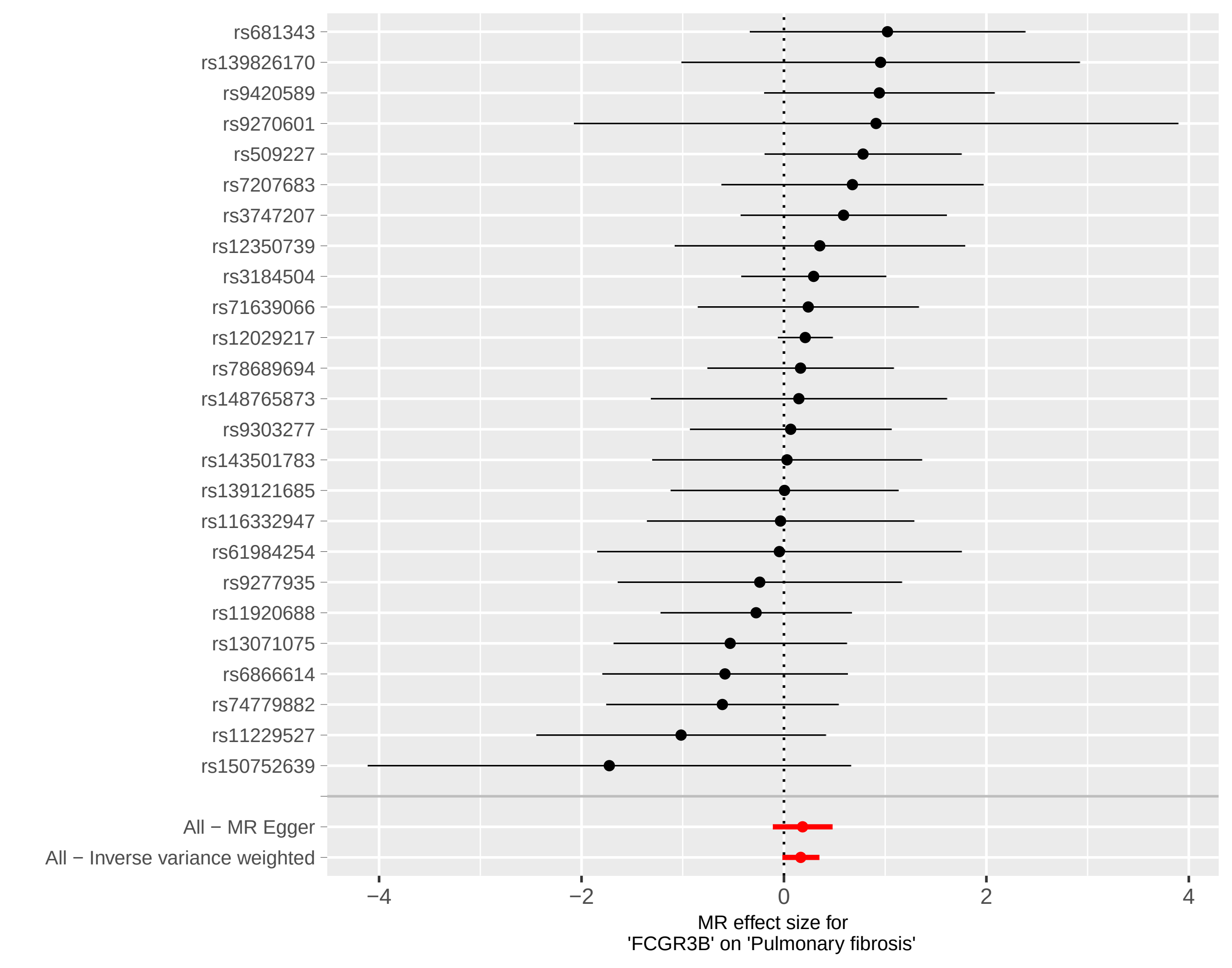
rs681343
rs139826170
rs9420589
rs9270601
rs509227
rs7207683
rs3747207
rs12350739
rs3184504
rs71639066
rs12029217
rs78689694
rs148765873
rs9303277
rs143501783
rs139121685
rs116332947
rs61984254
rs9277935
rs11920688
rs13071075
rs6866614
rs74779882
rs11229527
rs150752639
All − MR Egger
All − Inverse variance weighted
−4
−2
0
2
4
MR effect size for
'FCGR3B' on 'Pulmonary fibrosis'

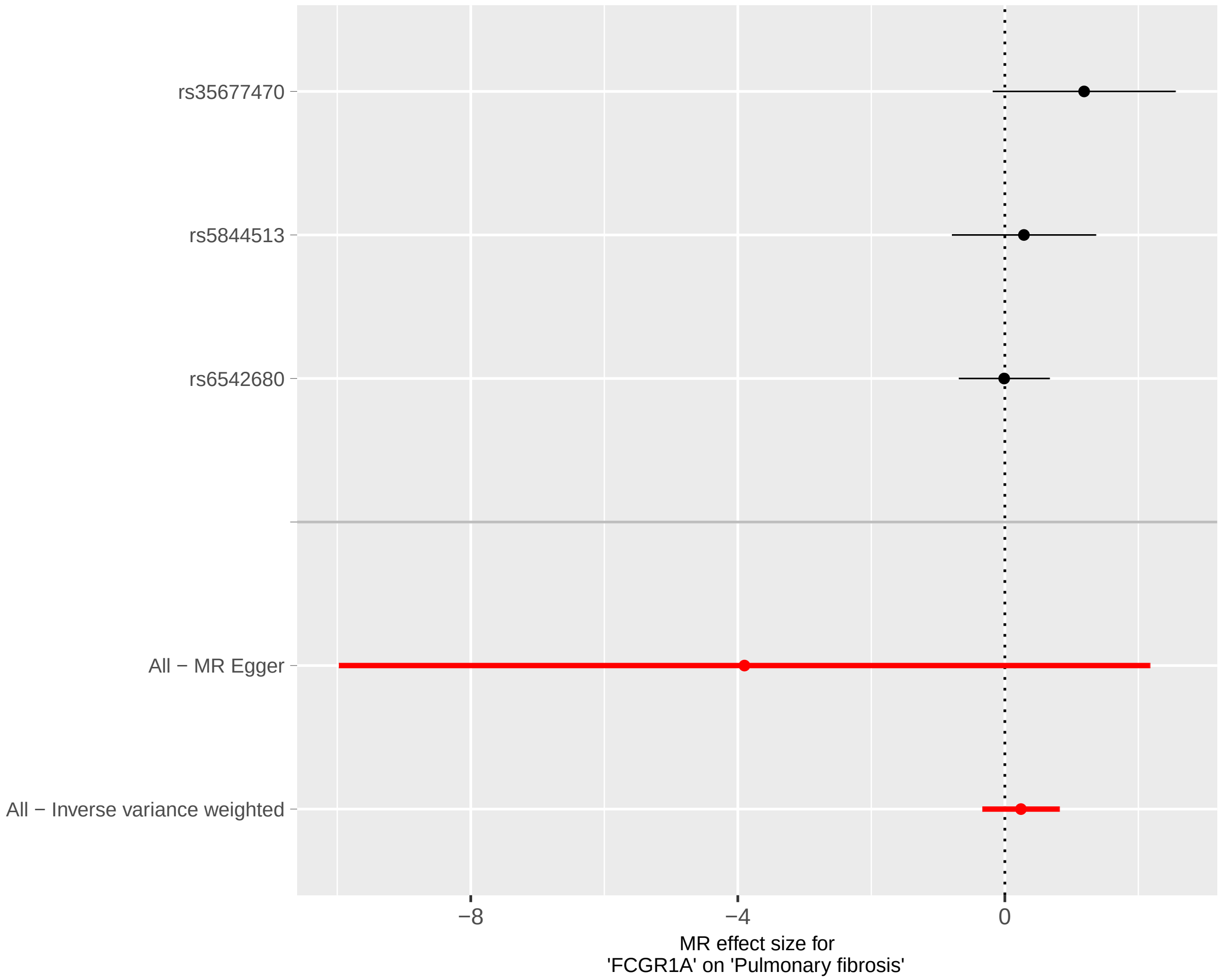

rs35677470
rs5844513
rs6542680
All − MR Egger
All − Inverse variance weighted
−8
−4
0
MR effect size for
'FCGR1A' on 'Pulmonary fibrosis'

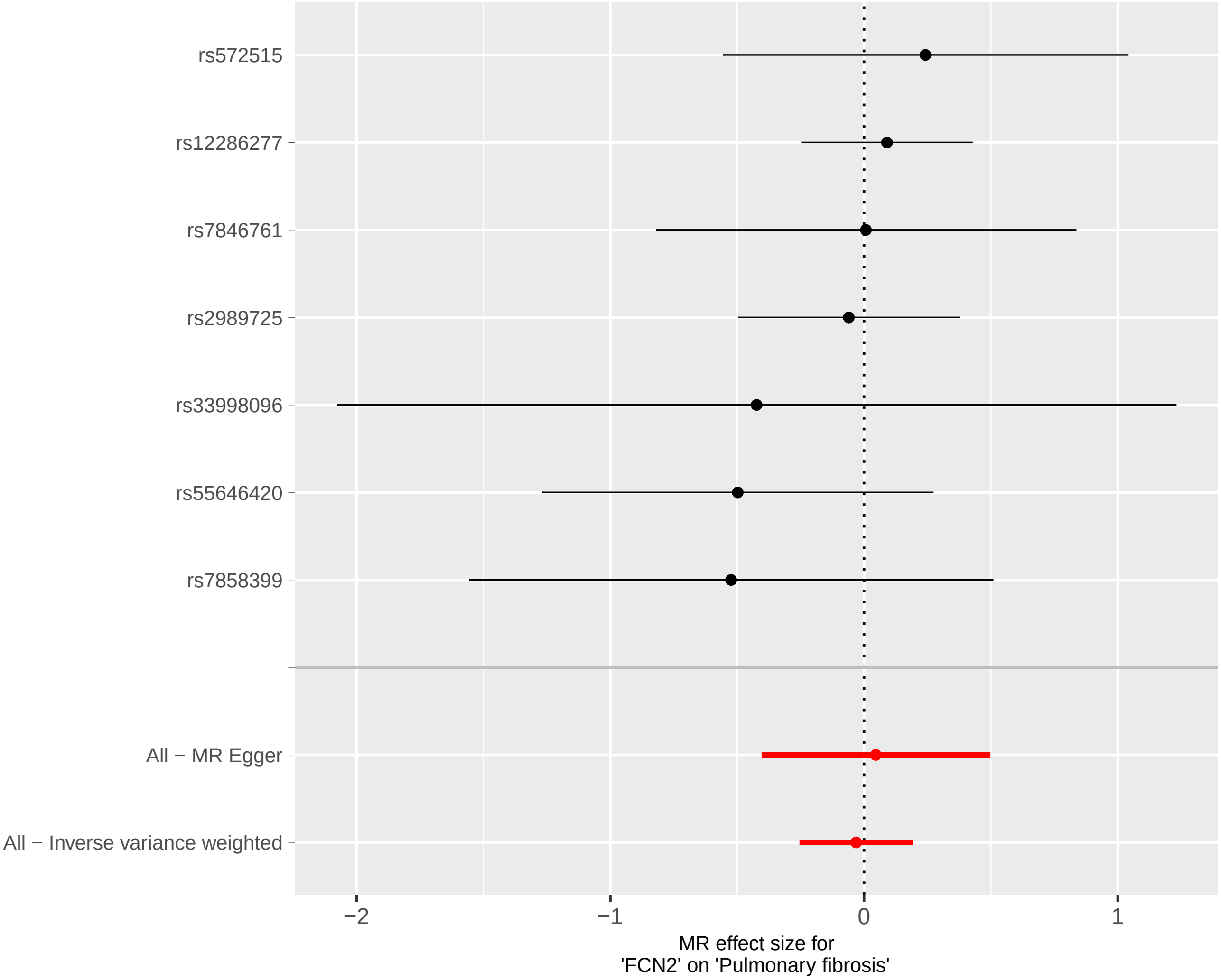

rs572515
rs12286277
rs7846761
rs2989725
rs33998096
rs55646420
rs7858399
All − MR Egger
All − Inverse variance weighted
−2
−1
0
1
MR effect size for
'FCN2' on 'Pulmonary fibrosis'

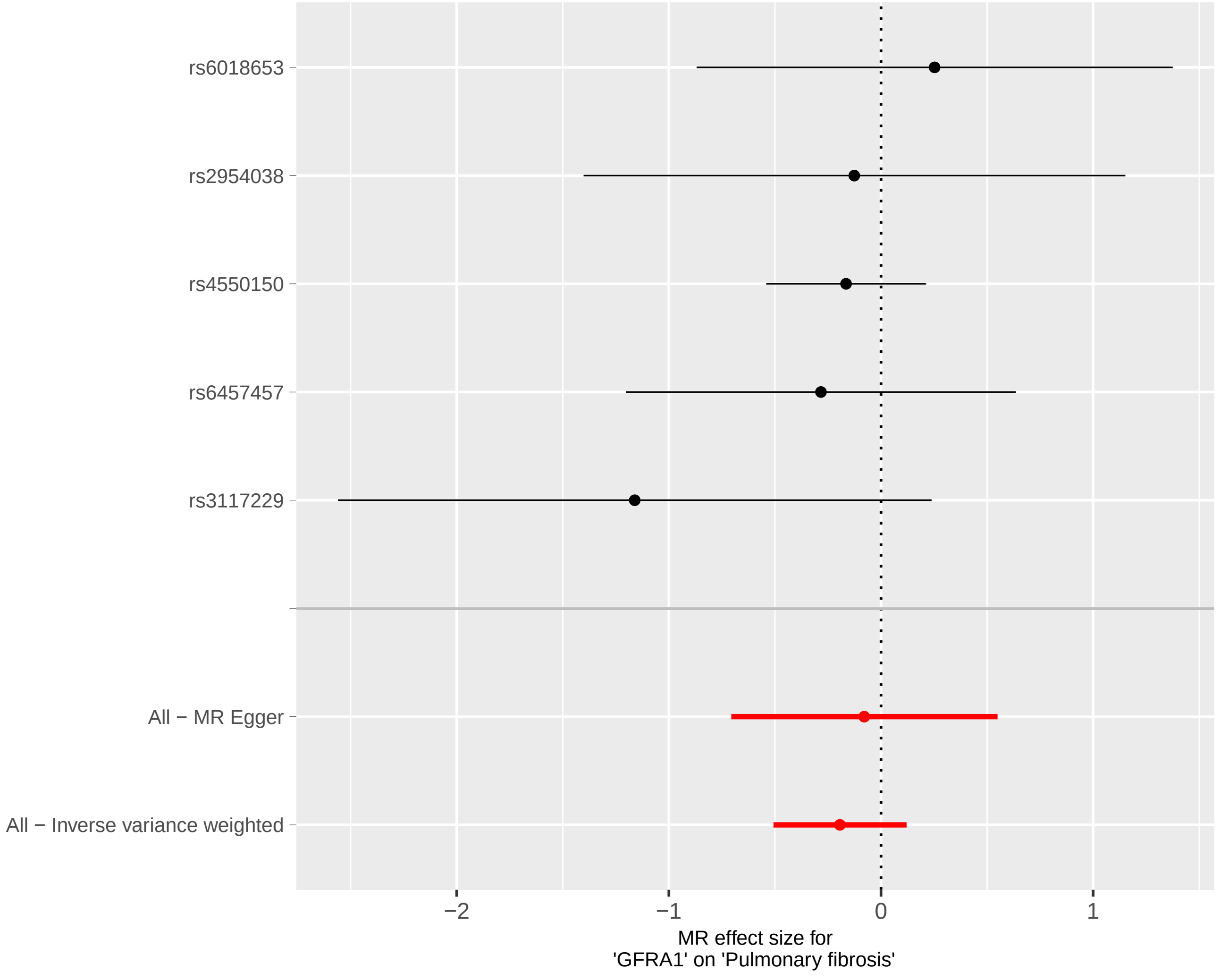

rs6018653
rs2954038
rs4550150
rs6457457
rs3117229
All − MR Egger
All − Inverse variance weighted
−2
−1
0
1
MR effect size for
'GFRA1' on 'Pulmonary fibrosis'

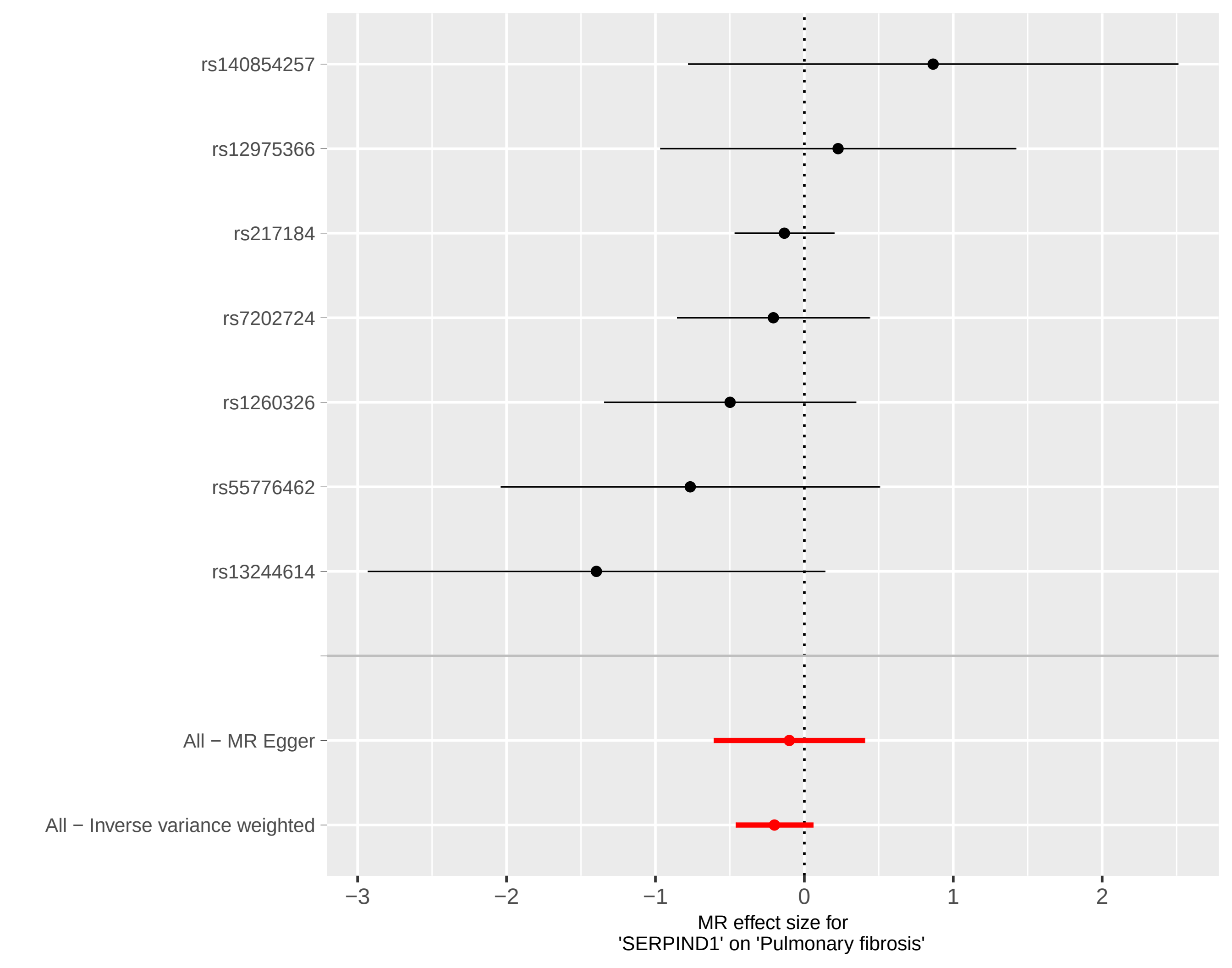

rs140854257
rs12975366
rs217184
rs7202724
rs1260326
rs55776462
rs13244614
All − MR Egger
All − Inverse variance weighted
−3
−2
−1
0
1
2
MR effect size for
'SERPIND1' on 'Pulmonary fibrosis'

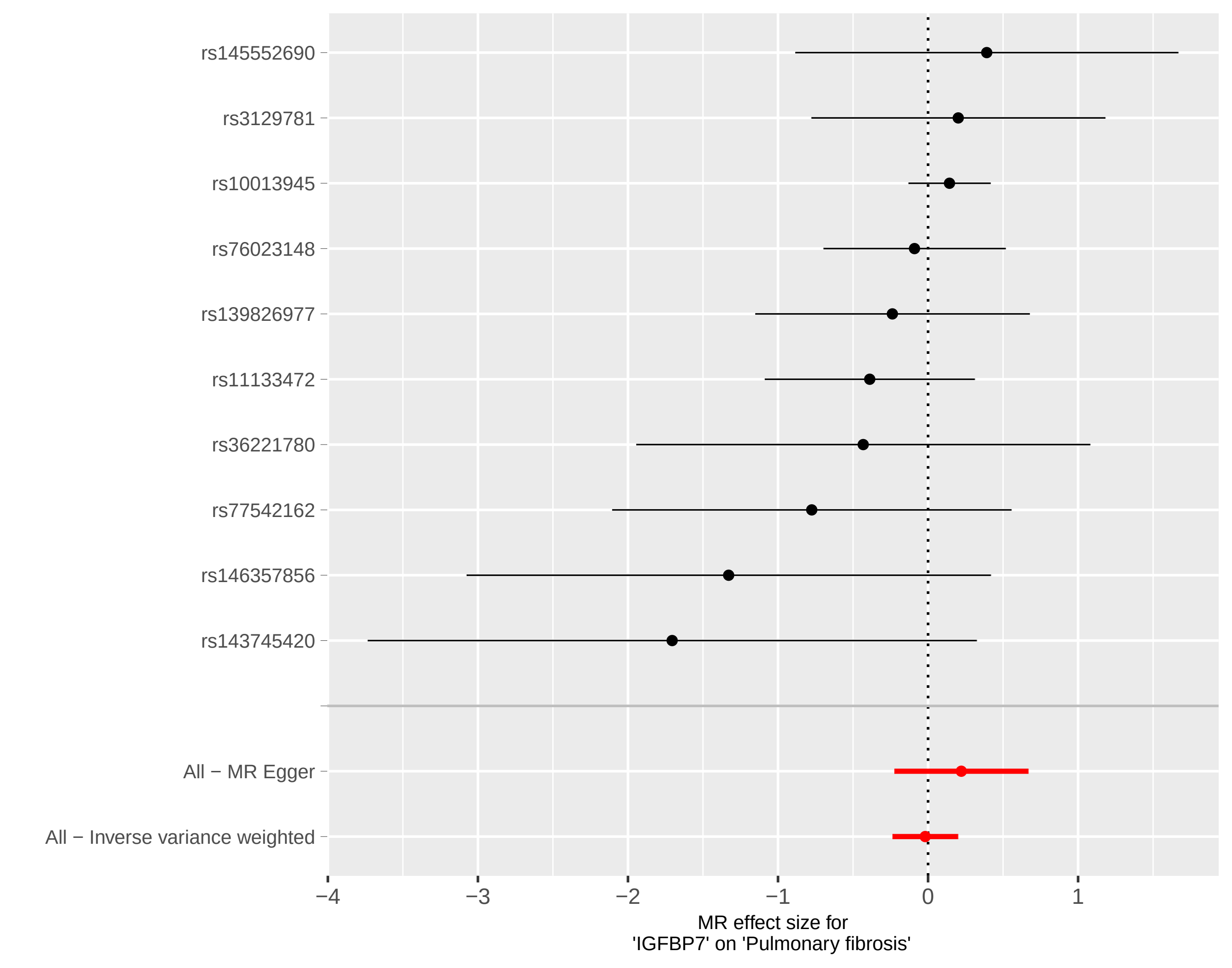
rs145552690
rs3129781
rs10013945
rs76023148
rs139826977
rs11133472
rs36221780
rs77542162
rs146357856
rs143745420
All − MR Egger
All − Inverse variance weighted
−4
−3
−2
−1
0
1
MR effect size for
'IGFBP7' on 'Pulmonary fibrosis'

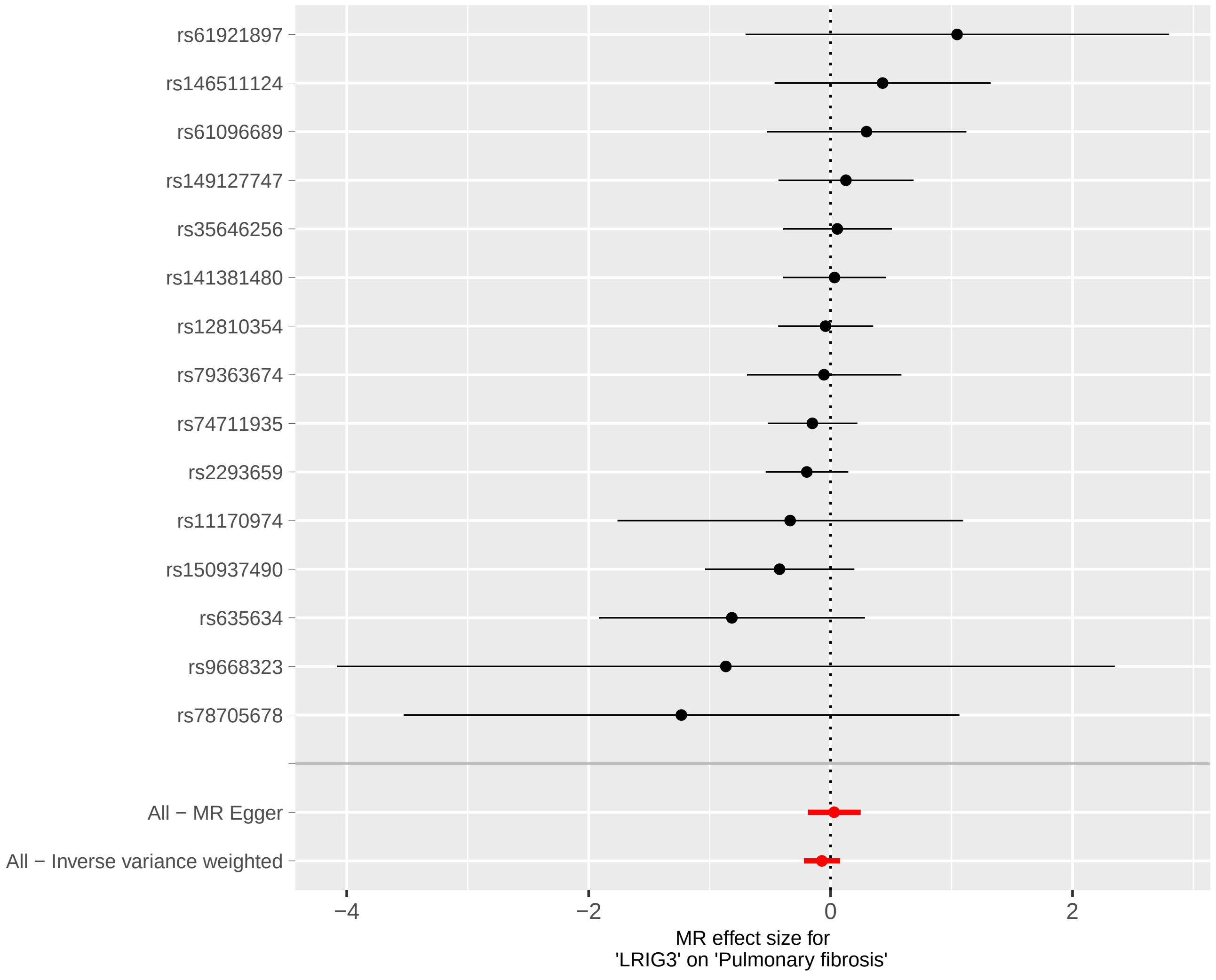

rs61921897
rs146511124
rs61096689
rs149127747
rs35646256
rs141381480
rs12810354
rs79363674
rs74711935
rs2293659
rs11170974
rs150937490
rs635634
rs9668323
rs78705678
All − MR Egger
All − Inverse variance weighted
−4
−2
0
2
MR effect size for
'LRIG3' on 'Pulmonary fibrosis'

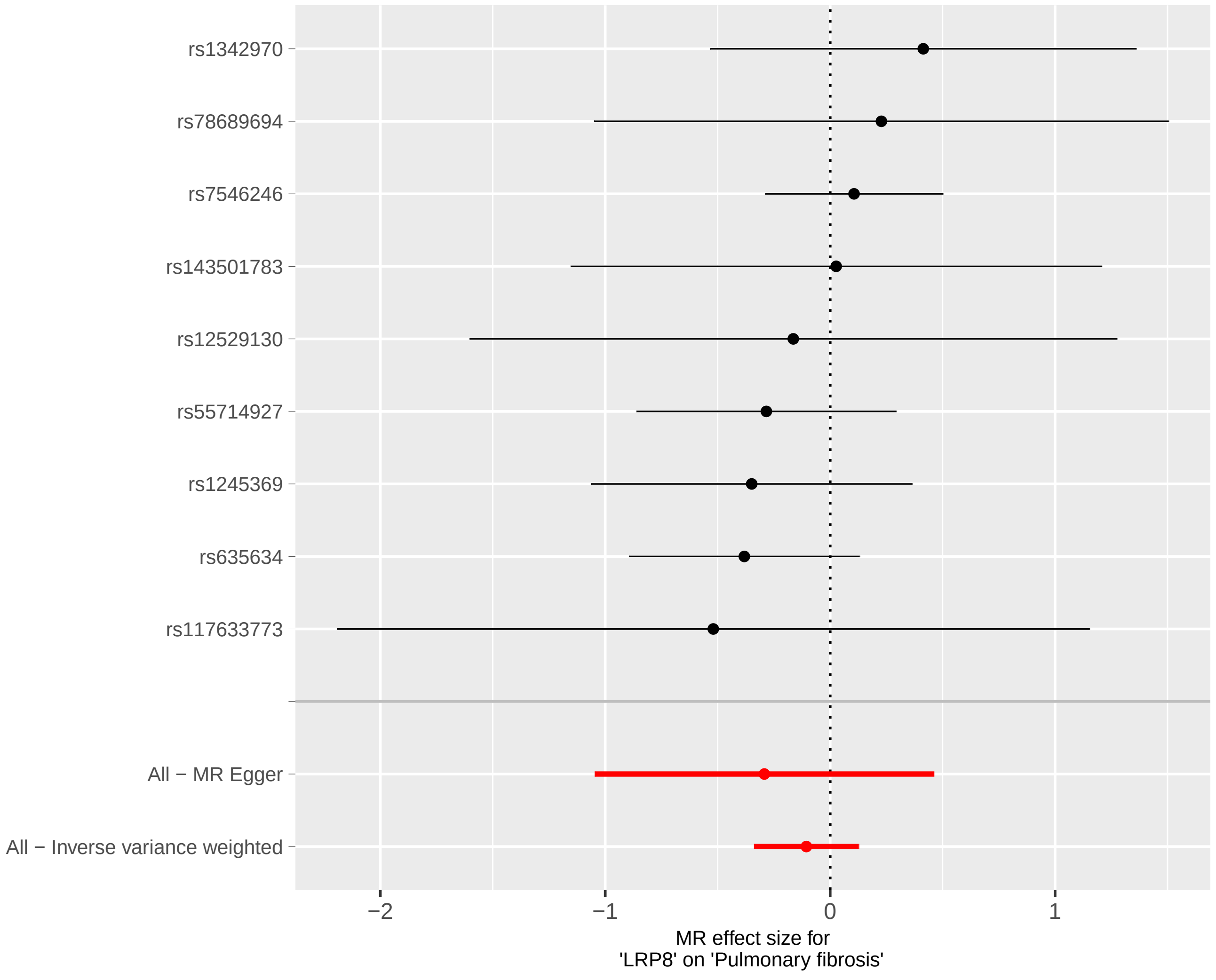

rs1342970
rs78689694
rs7546246
rs143501783
rs12529130
rs55714927
rs1245369
rs635634
rs117633773
All − MR Egger
All − Inverse variance weighted
−2
−1
0
1
MR effect size for
'LRP8' on 'Pulmonary fibrosis'

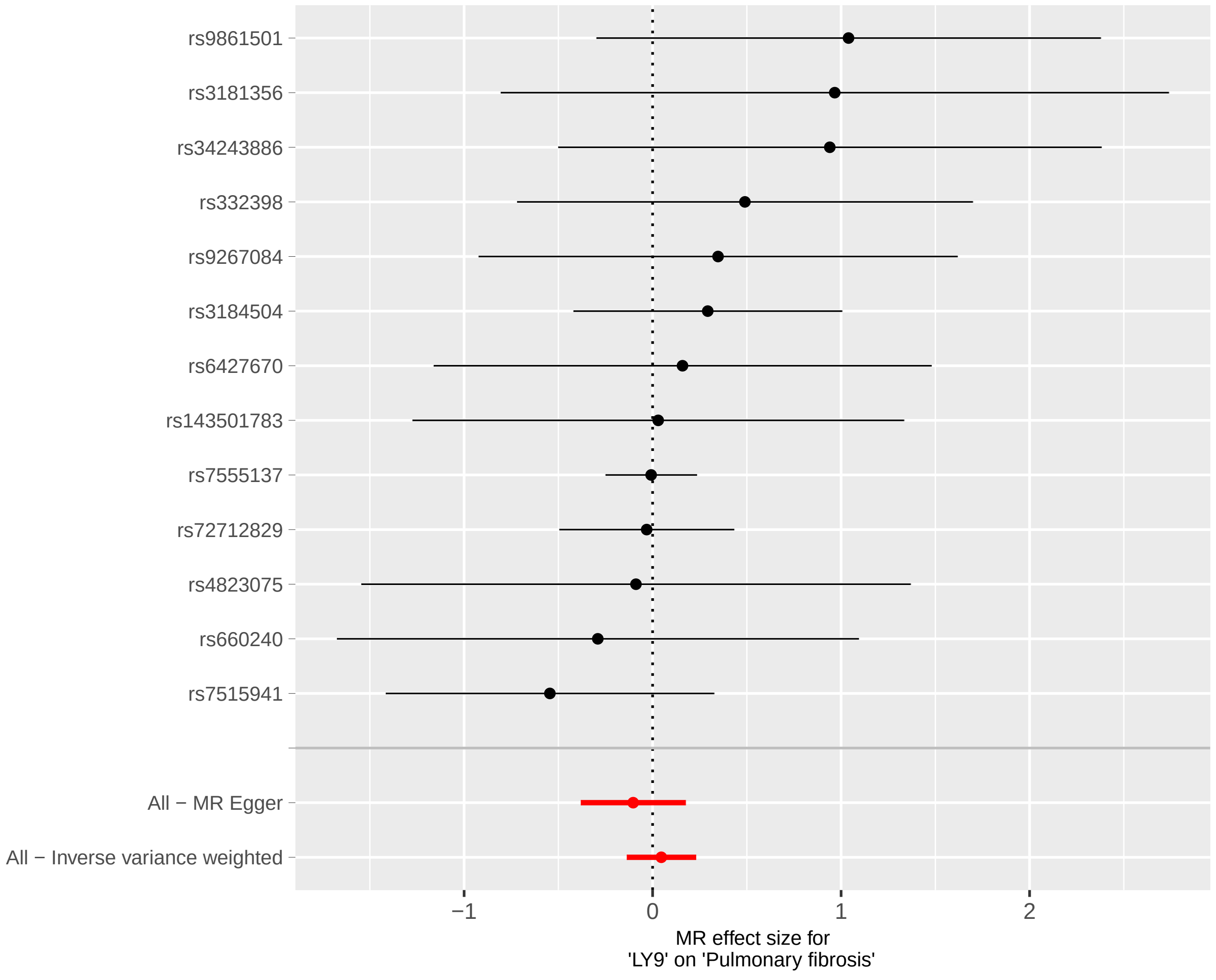

rs9861501
rs3181356
rs34243886
rs332398
rs9267084
rs3184504
rs6427670
rs143501783
rs7555137
rs72712829
rs4823075
rs660240
rs7515941
All − MR Egger
All − Inverse variance weighted
−1
0
1
2
MR effect size for
'LY9' on 'Pulmonary fibrosis'

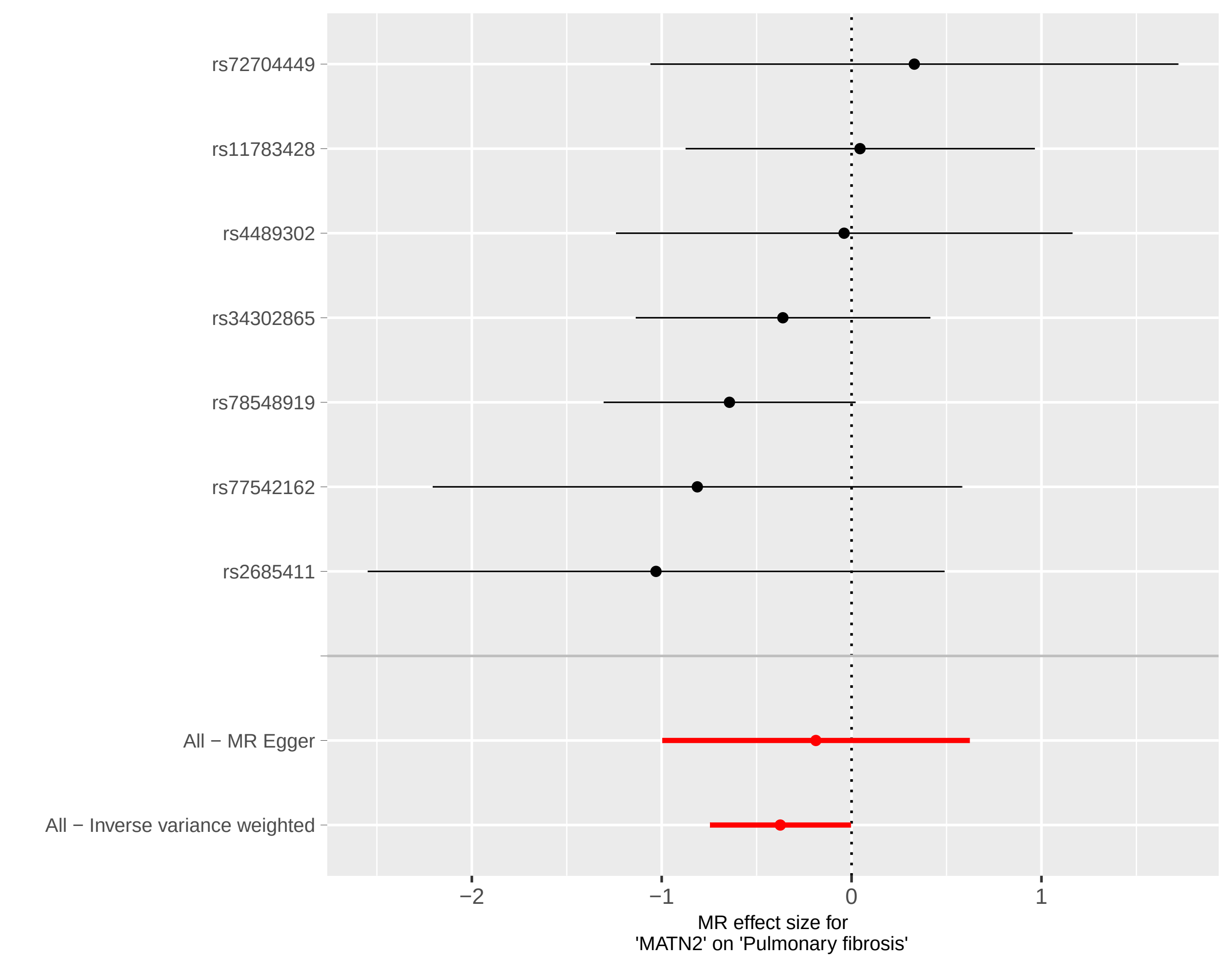

rs72704449
rs11783428
rs4489302
rs34302865
rs78548919
rs77542162
rs2685411
All − MR Egger
All − Inverse variance weighted
−2
−1
0
1
MR effect size for
'MATN2' on 'Pulmonary fibrosis'

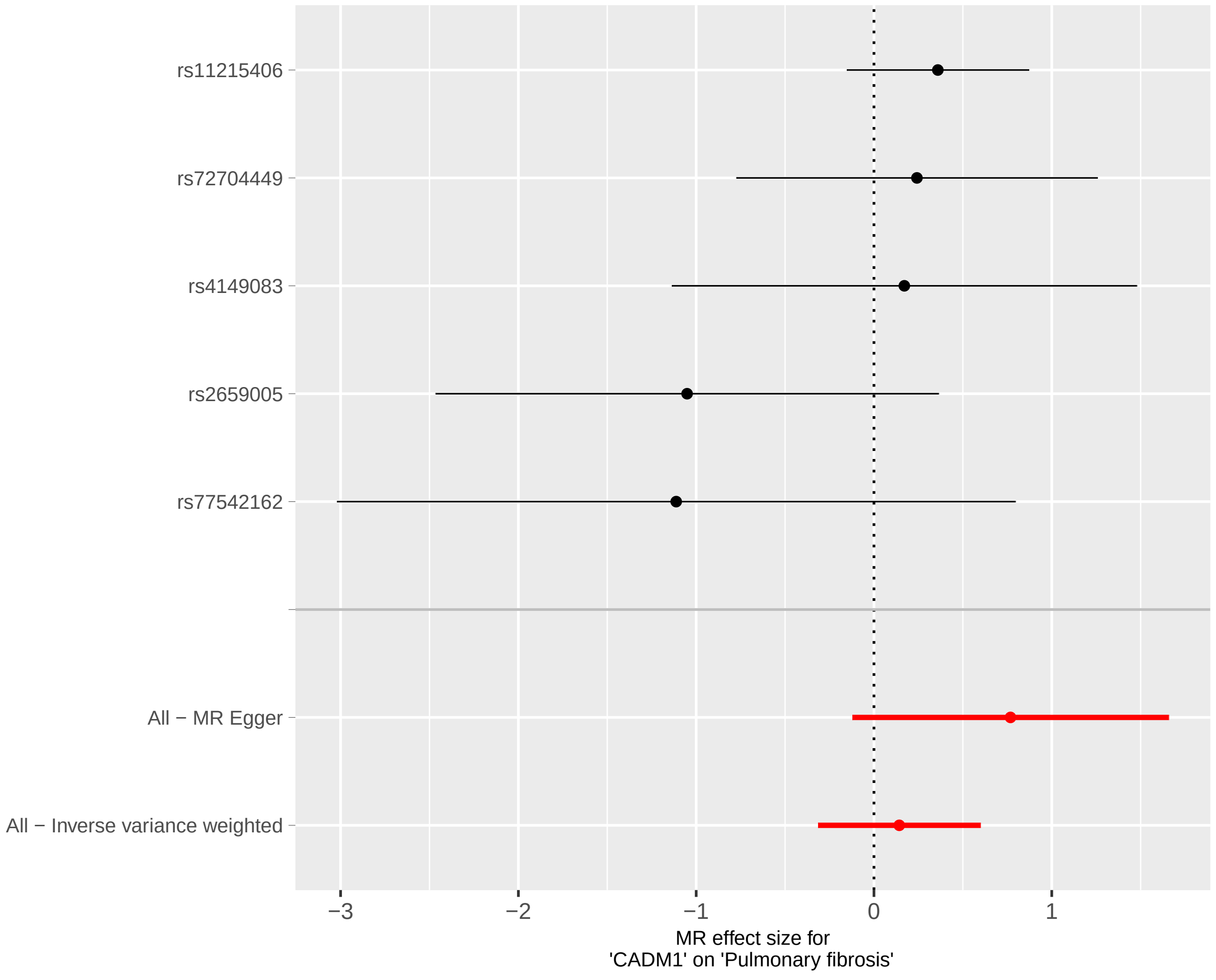
rs11215406
rs72704449
rs4149083
rs2659005
rs77542162
All − MR Egger
All − Inverse variance weighted
−3
−2
−1
0
1
MR effect size for
'CADM1' on 'Pulmonary fibrosis'

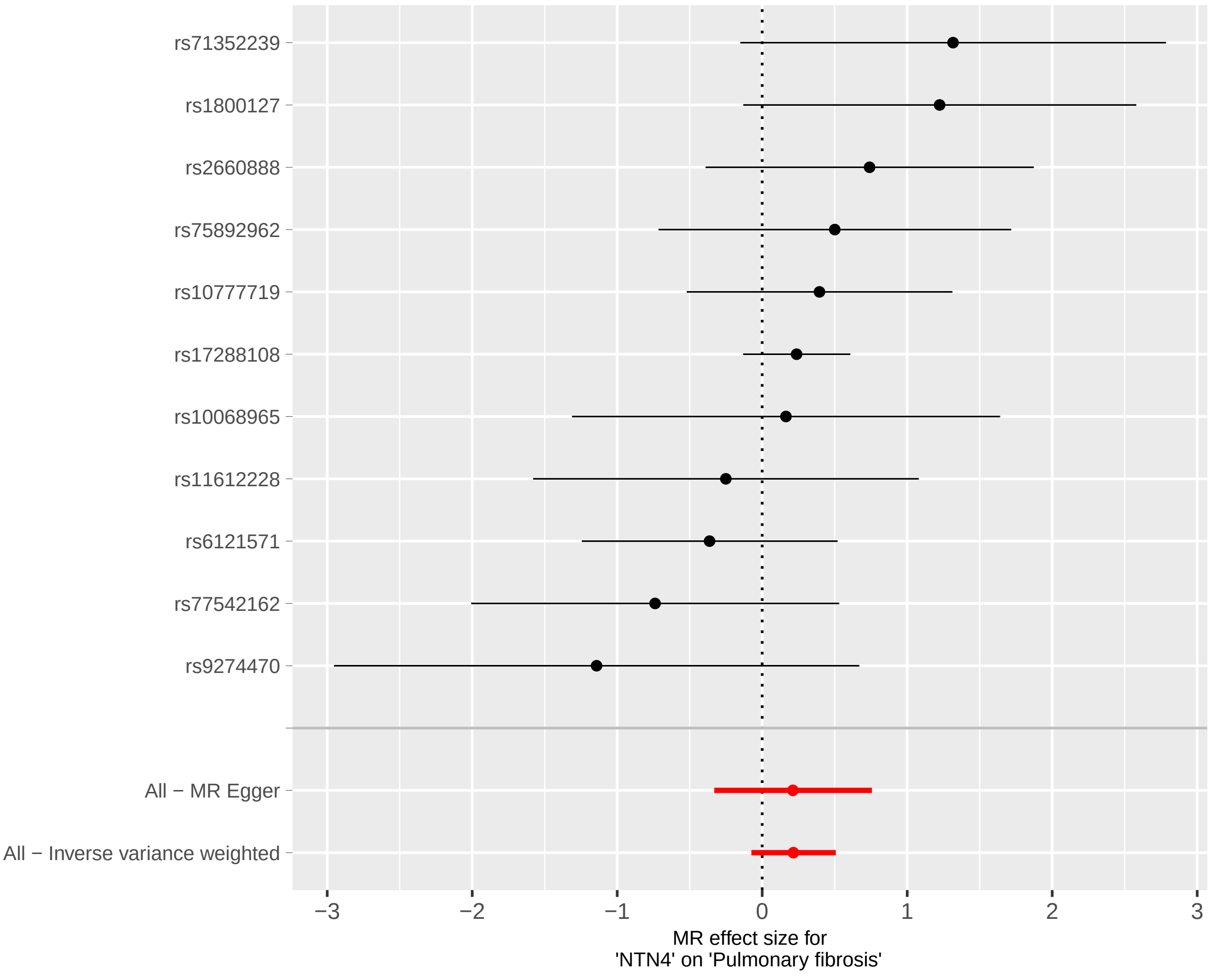
rs71352239
rs1800127
rs2660888
rs75892962
rs10777719
rs17288108
rs10068965
rs11612228
rs6121571
rs77542162
rs9274470
All − MR Egger
All − Inverse variance weighted
−3
−2
−1
0
1
2
3
MR effect size for
'NTN4' on 'Pulmonary fibrosis'

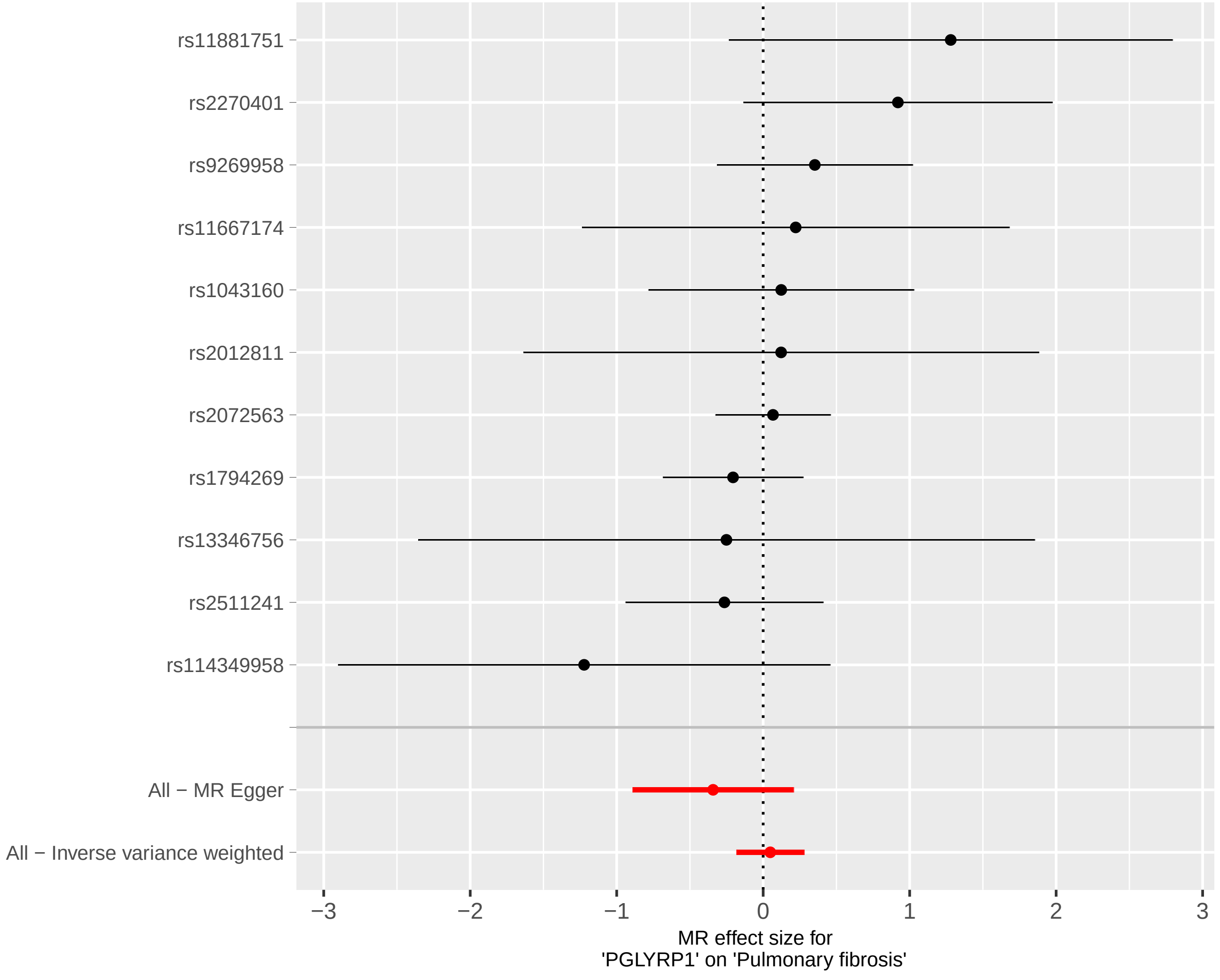
rs11881751
rs2270401
rs9269958
rs11667174
rs1043160
rs2012811
rs2072563
rs1794269
rs13346756
rs2511241
rs114349958
All − MR Egger
All − Inverse variance weighted
−3
−2
−1
0
1
2
3
MR effect size for
'PGLYRP1' on 'Pulmonary fibrosis'

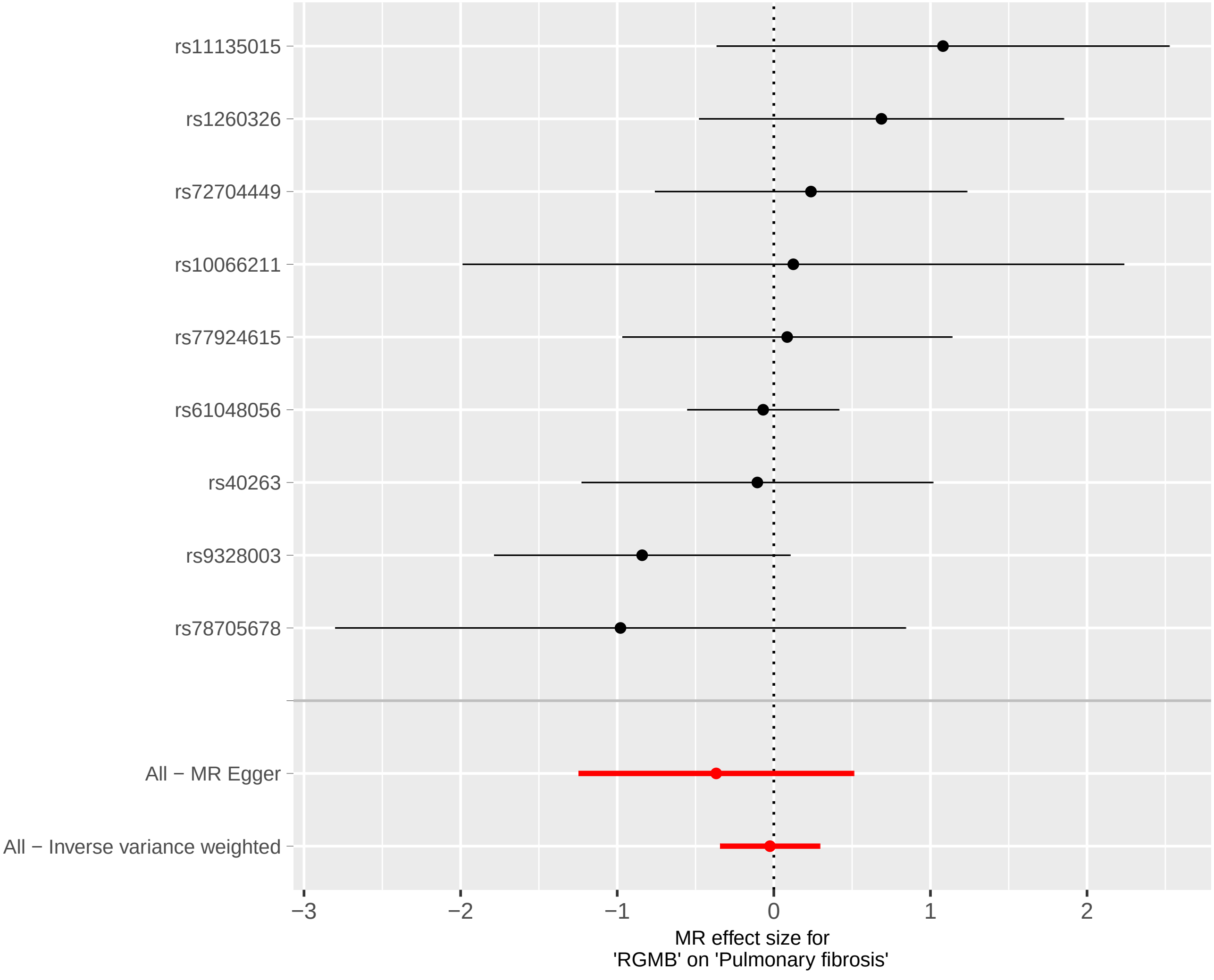
rs11135015
rs1260326
rs72704449
rs10066211
rs77924615
rs61048056
rs40263
rs9328003
rs78705678
All − MR Egger
All − Inverse variance weighted
−3
−2
−1
0
1
2
MR effect size for
'RGMB' on 'Pulmonary fibrosis'

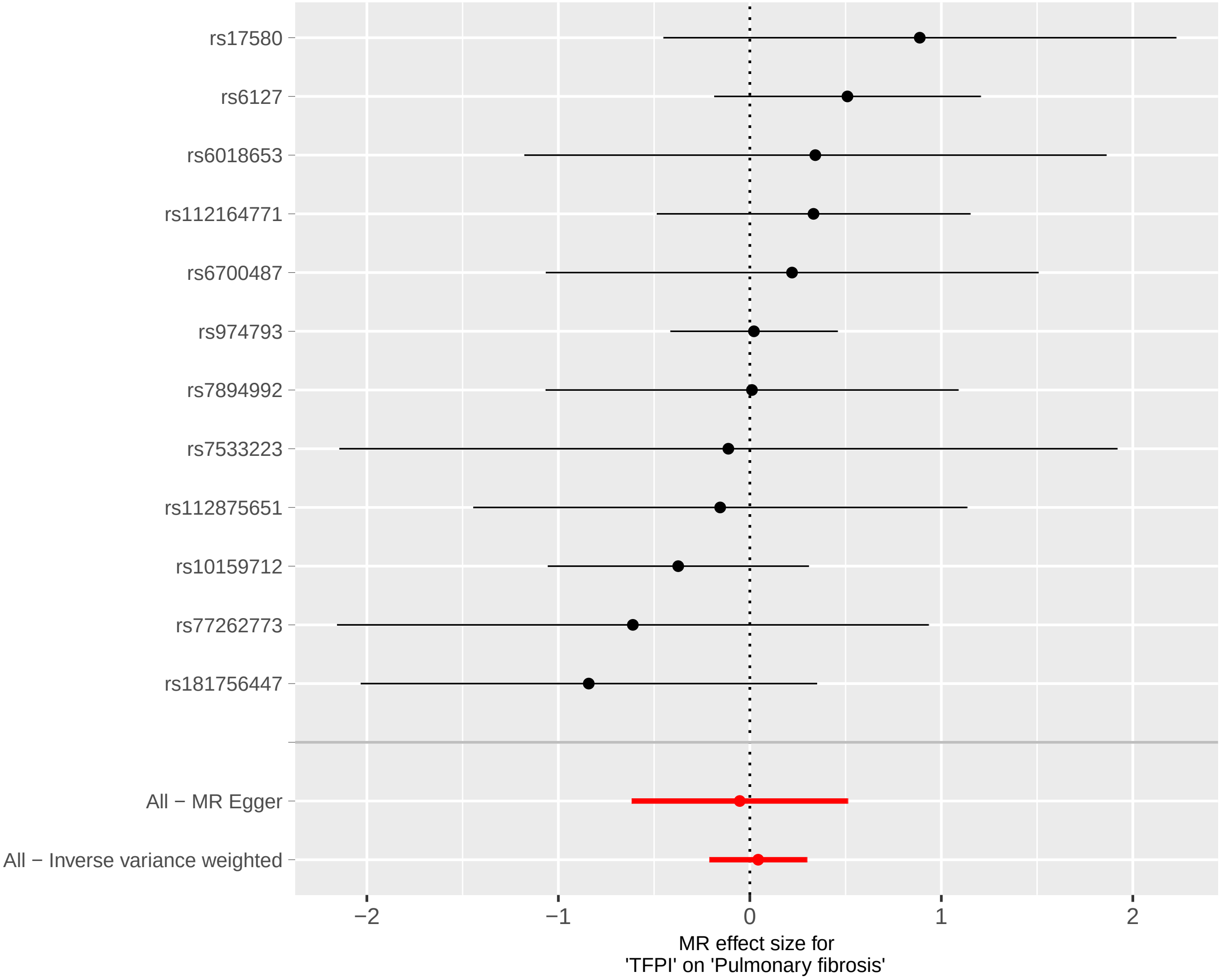

rs17580
rs6127
rs6018653
rs112164771
rs6700487
rs974793
rs7894992
rs7533223
rs112875651
rs10159712
rs77262773
rs181756447
All − MR Egger
All − Inverse variance weighted
−2
−1
0
1
2
MR effect size for
'TFPI' on 'Pulmonary fibrosis'

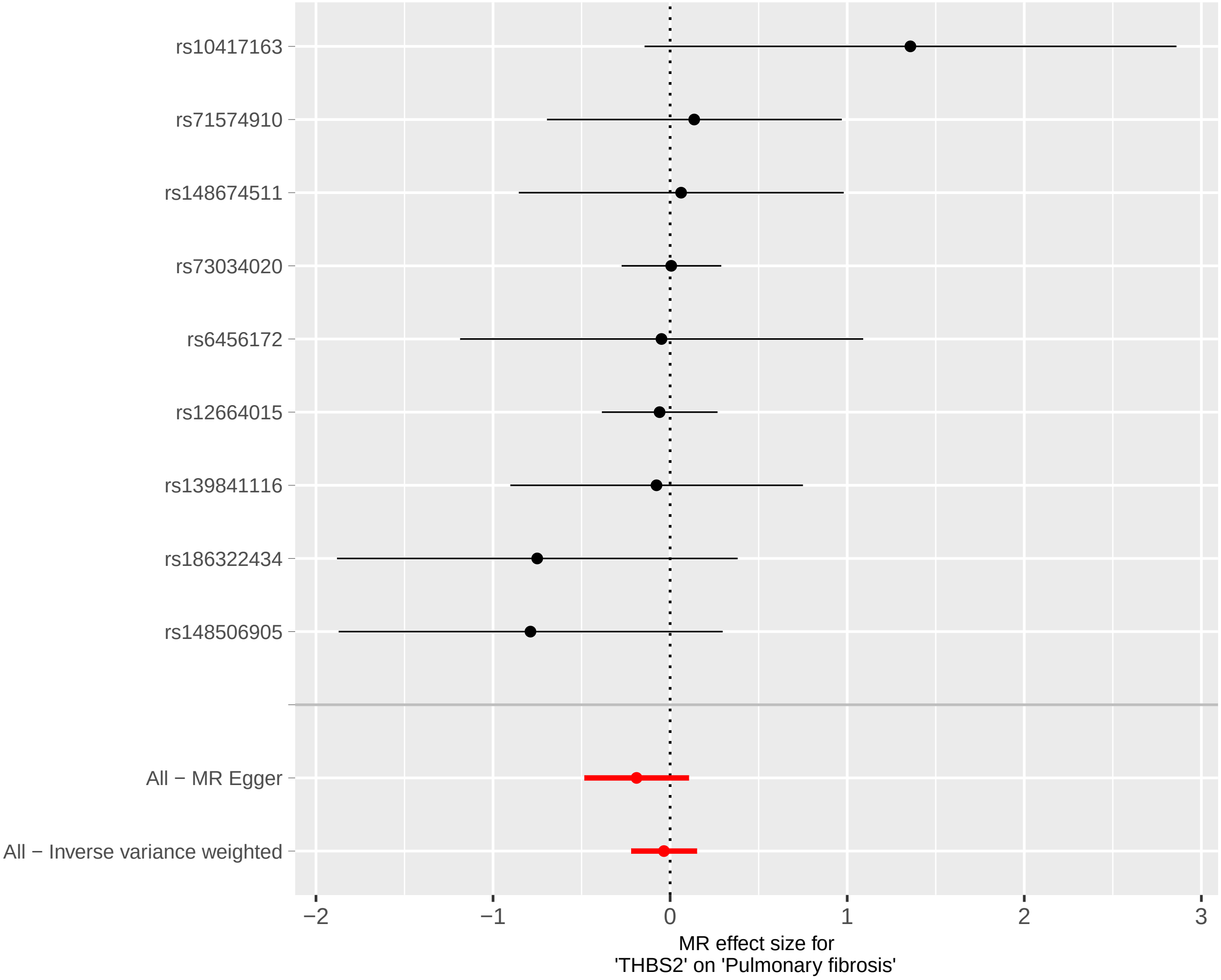

rs10417163
rs71574910
rs148674511
rs73034020
rs6456172
rs12664015
rs139841116
rs186322434
rs148506905
All − MR Egger
All − Inverse variance weighted
−2
−1
0
1
2
3
MR effect size for
'THBS2' on 'Pulmonary fibrosis'

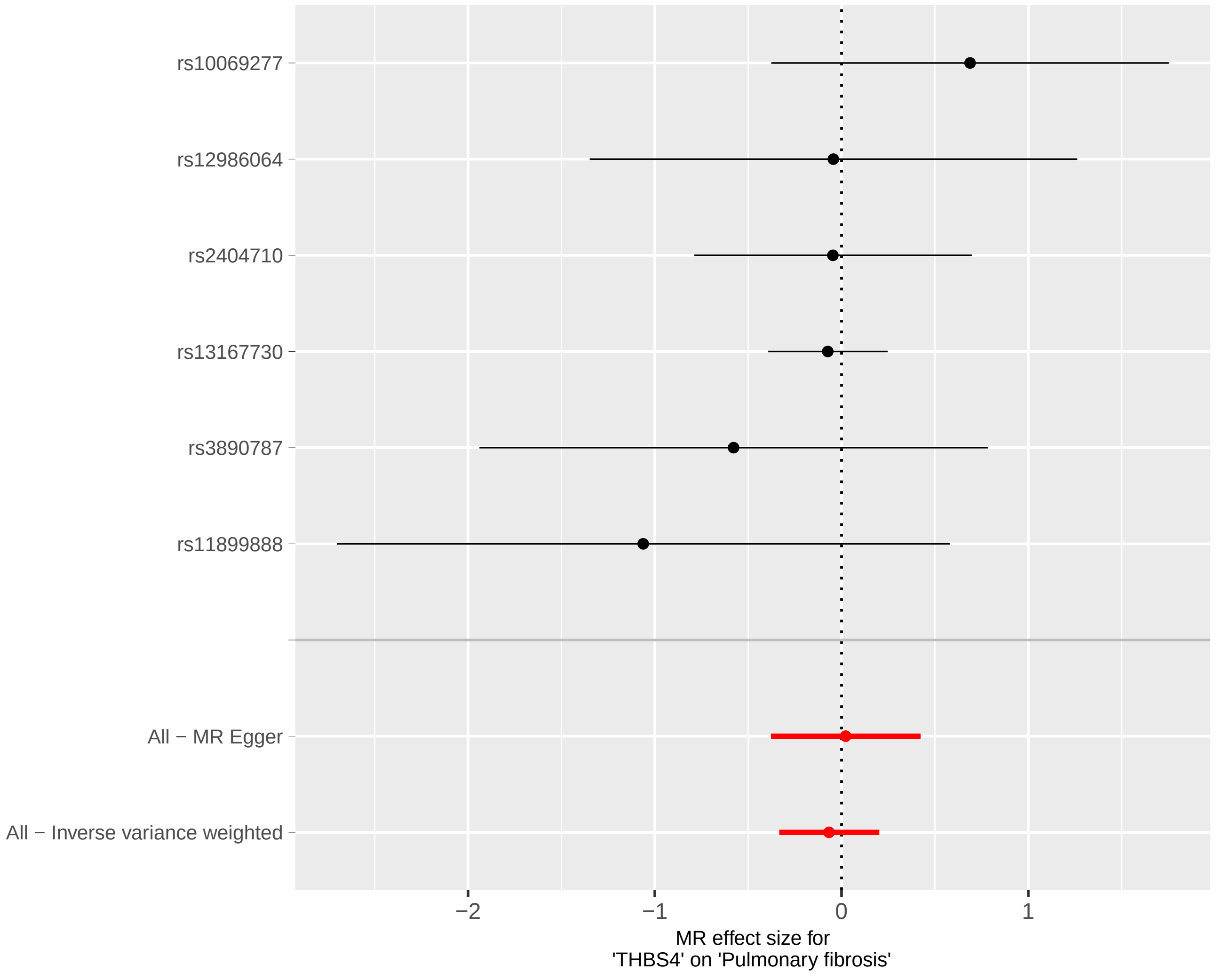

rs10069277
rs12986064
rs2404710
rs13167730
rs3890787
rs11899888
All − MR Egger
All − Inverse variance weighted
−2
−1
0
1
MR effect size for
'THBS4' on 'Pulmonary fibrosis'

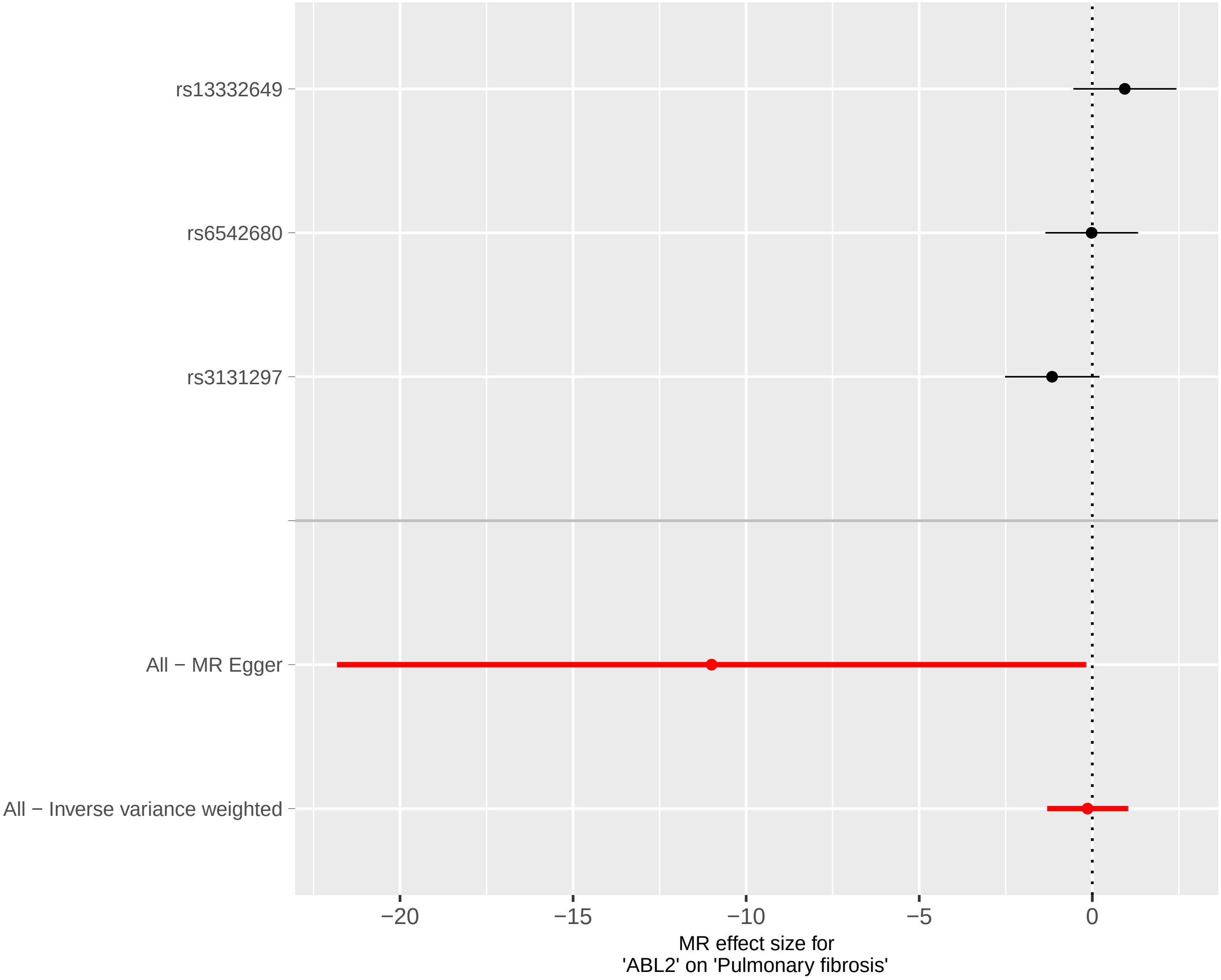
rs13332649
rs6542680
rs3131297
All − MR Egger
All − Inverse variance weighted
−20
−15
−10
−5
0
MR effect size for
'ABL2' on 'Pulmonary fibrosis'

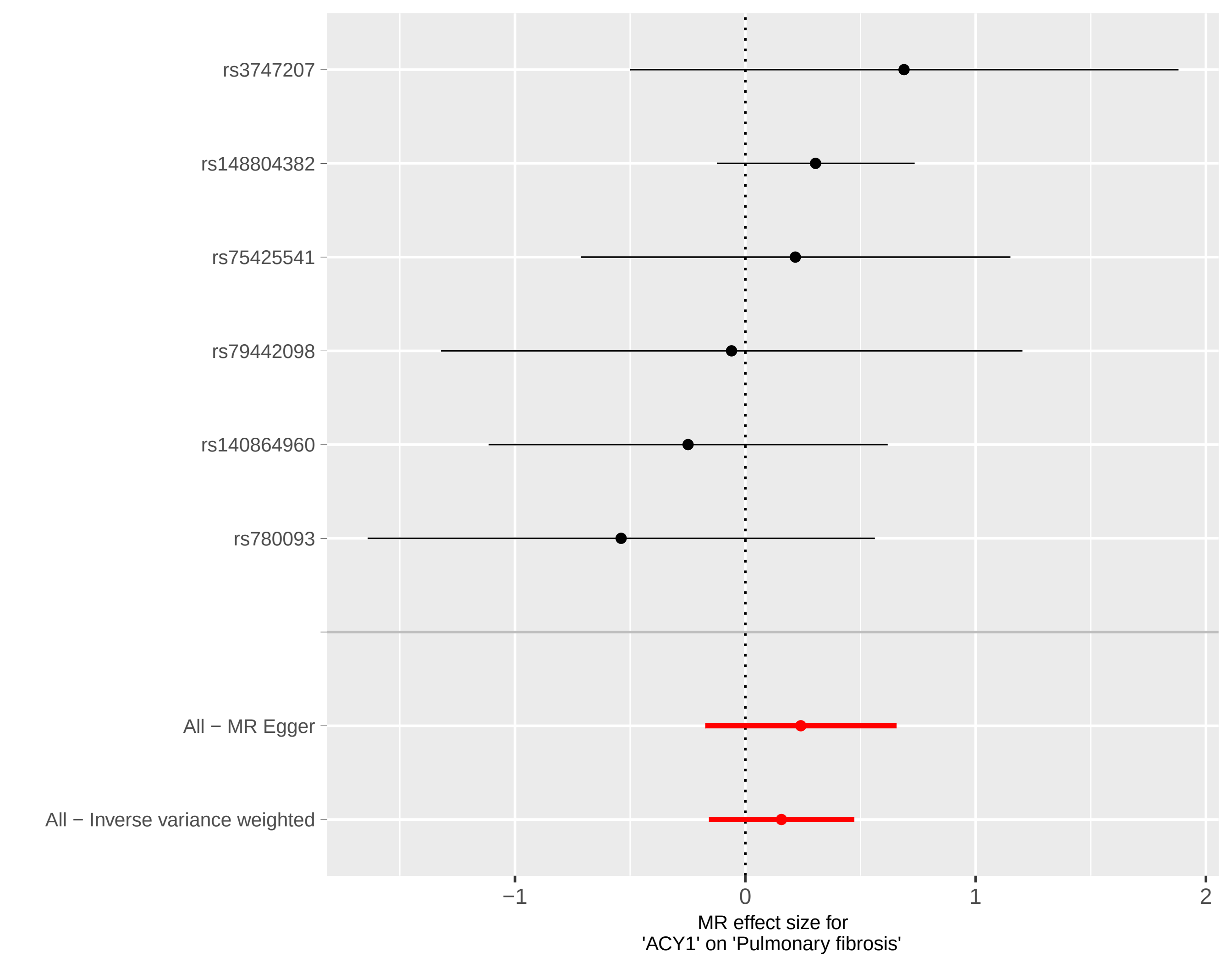

rs3747207
rs148804382
rs75425541
rs79442098
rs140864960
rs780093
All − MR Egger
All − Inverse variance weighted
−1
0
1
2
MR effect size for
'ACY1' on 'Pulmonary fibrosis'

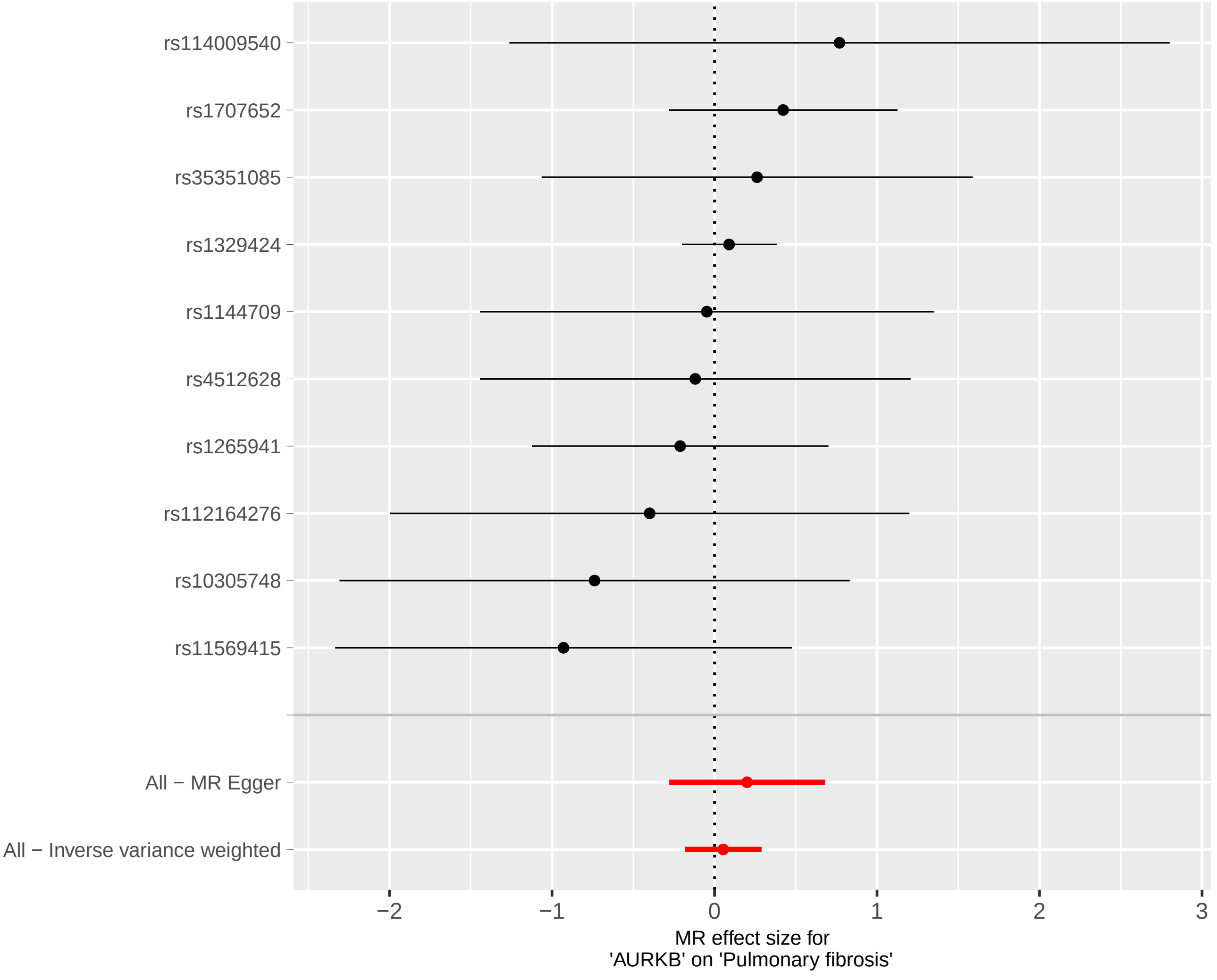
rs114009540
rs1707652
rs35351085
rs1329424
rs1144709
rs4512628
rs1265941
rs112164276
rs10305748
rs11569415
All − MR Egger
All − Inverse variance weighted
−2
−1
0
1
2
3
MR effect size for
'AURKB' on 'Pulmonary fibrosis'

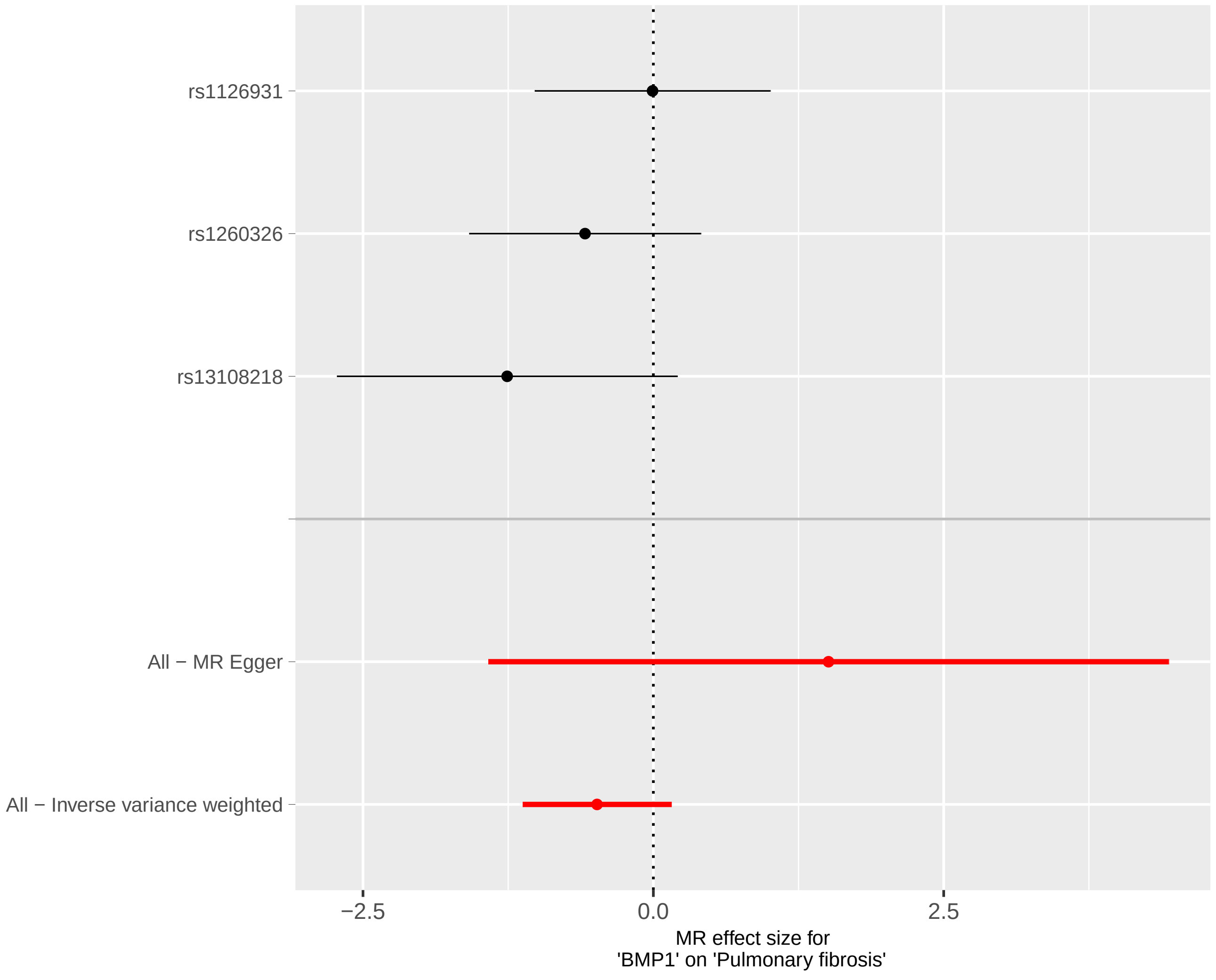

rs1126931
rs1260326
rs13108218
All − MR Egger
All − Inverse variance weighted
−2.5
0.0
2.5
MR effect size for
'BMP1' on 'Pulmonary fibrosis'

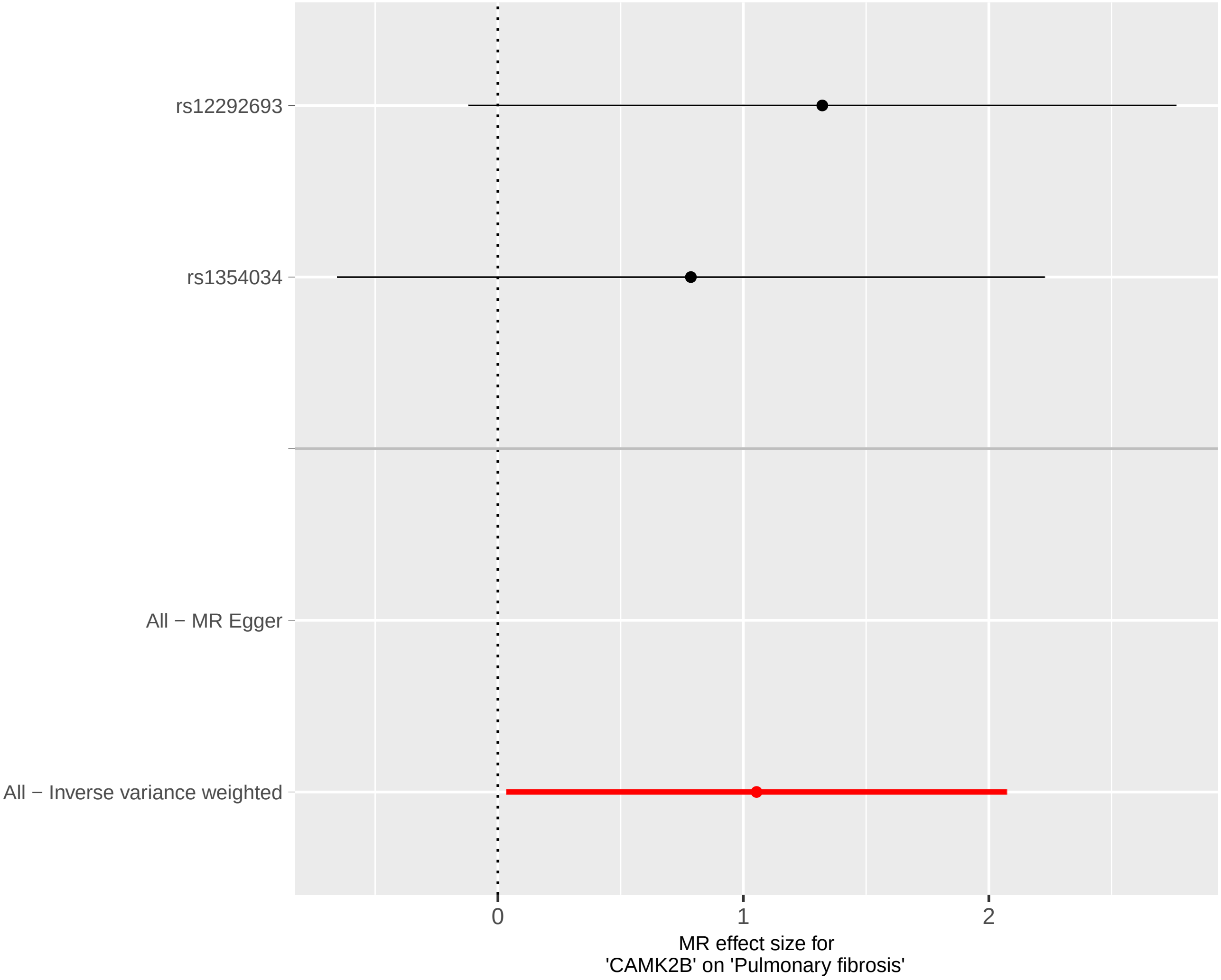

rs12292693
rs1354034
All − MR Egger
All − Inverse variance weighted
0
1
2
MR effect size for
'CAMK2B' on 'Pulmonary fibrosis'

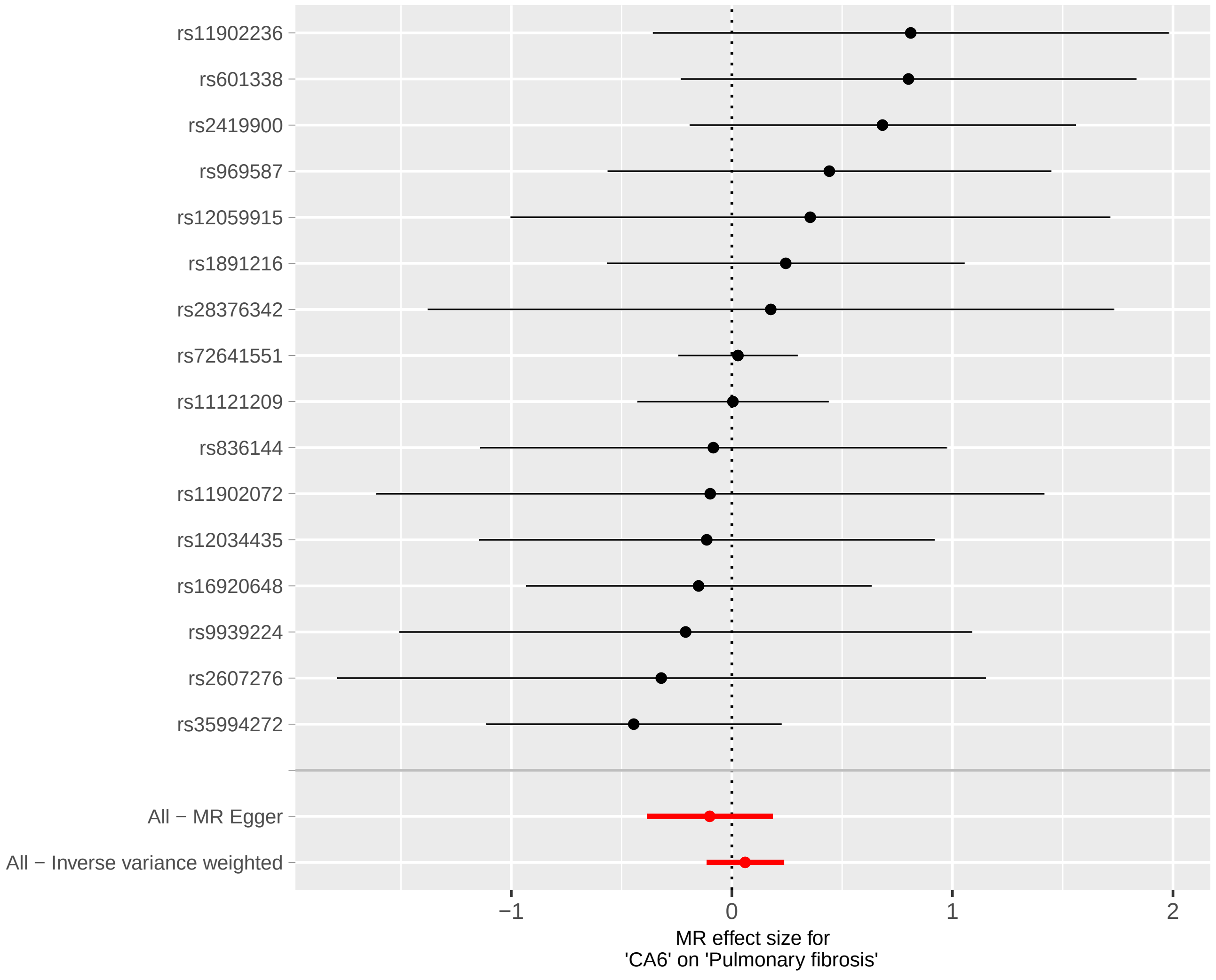
rs11902236
rs601338
rs2419900
rs969587
rs12059915
rs1891216
rs28376342
rs72641551
rs11121209
rs836144
rs11902072
rs12034435
rs16920648
rs9939224
rs2607276
rs35994272
All − MR Egger
All − Inverse variance weighted
−1
0
1
2
MR effect size for
'CA6' on 'Pulmonary fibrosis'

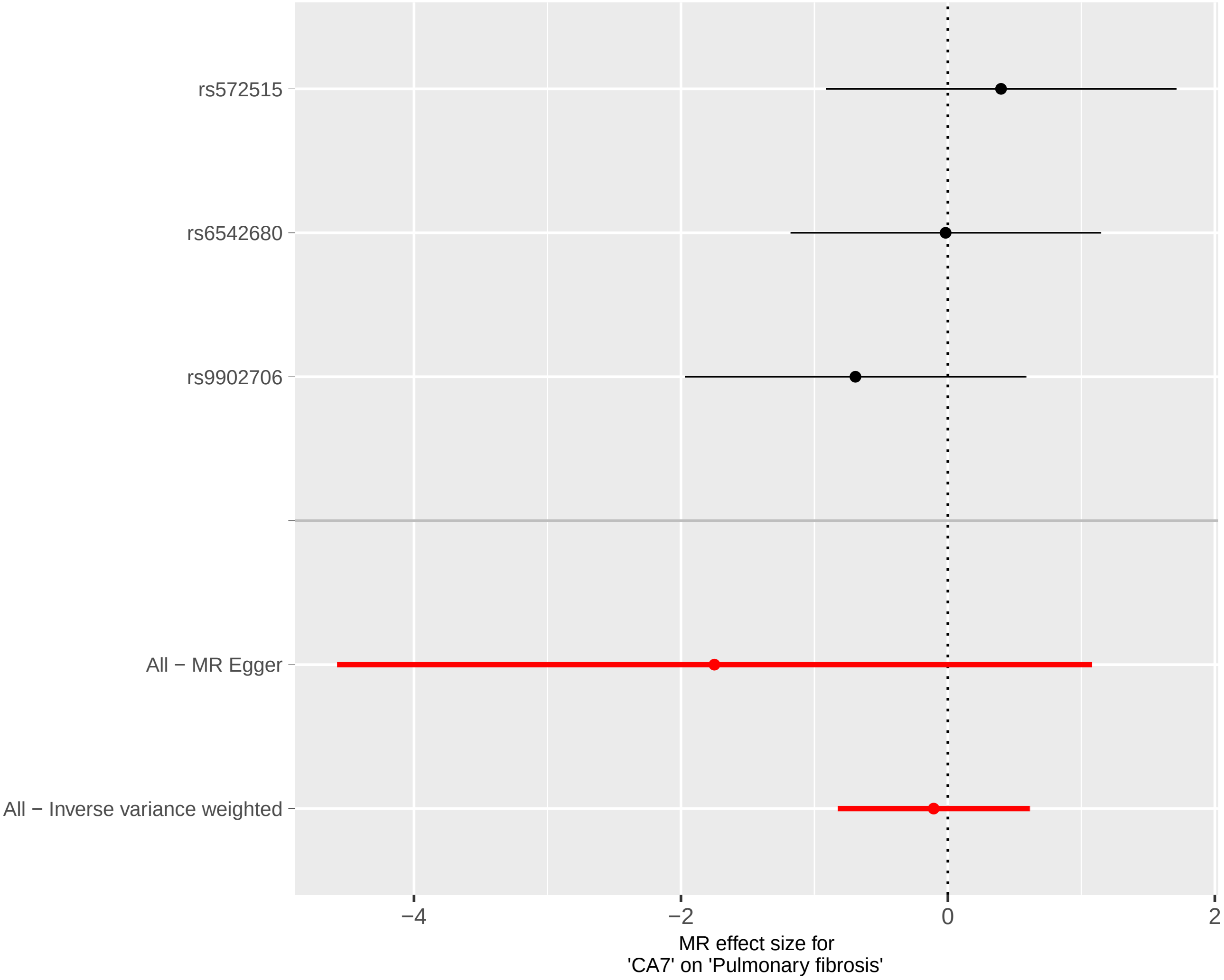
rs572515
rs6542680
rs9902706
All − MR Egger
All − Inverse variance weighted
−4
−2
0
2
MR effect size for
'CA7' on 'Pulmonary fibrosis'

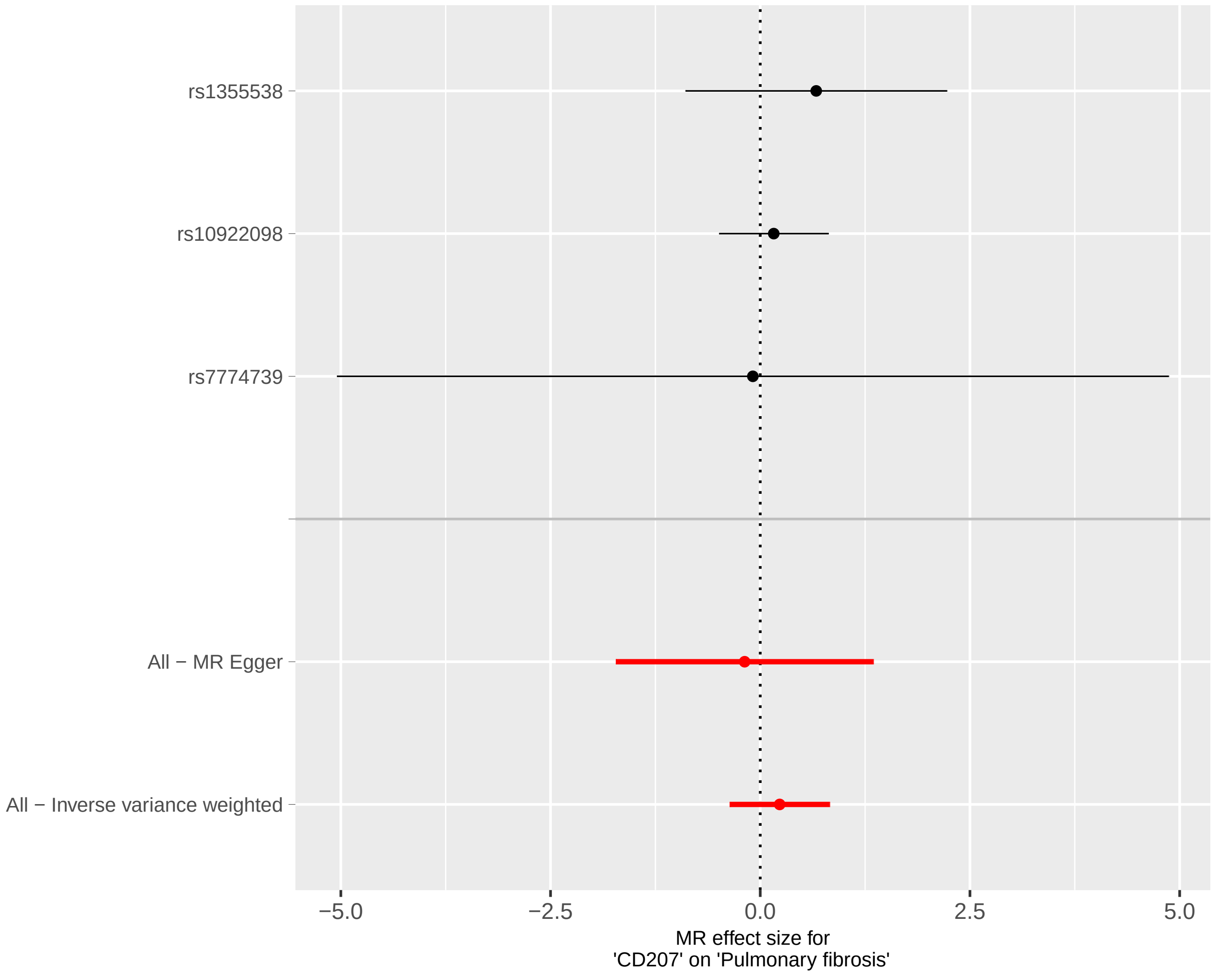
rs1355538
rs10922098
rs7774739
All − MR Egger
All − Inverse variance weighted
−5.0
−2.5
0.0
2.5
5.0
MR effect size for
'CD207' on 'Pulmonary fibrosis'

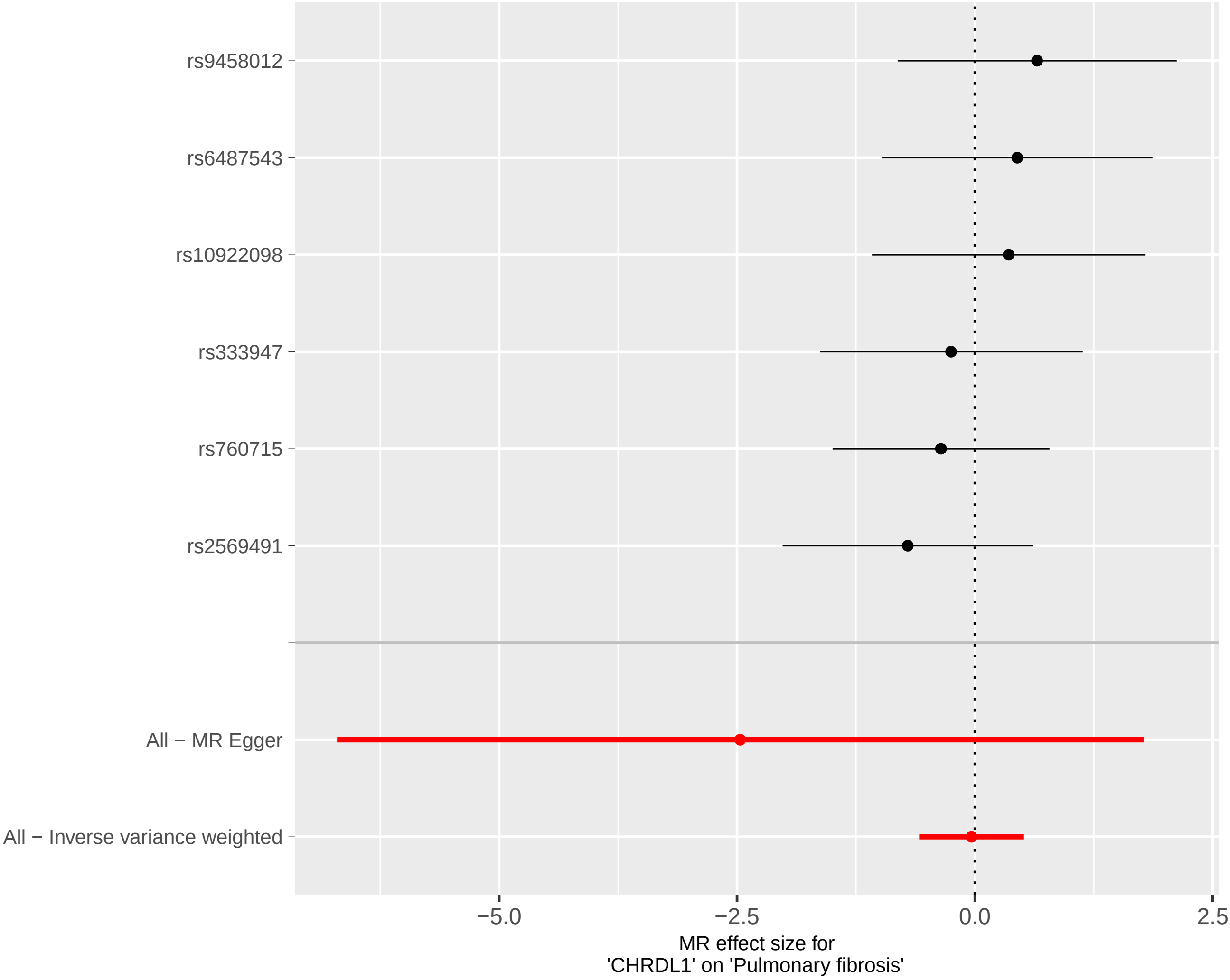

rs9458012
rs6487543
rs10922098
rs333947
rs760715
rs2569491
All − MR Egger
All − Inverse variance weighted
−5.0
−2.5
0.0
2.5
MR effect size for
'CHRDL1' on 'Pulmonary fibrosis'

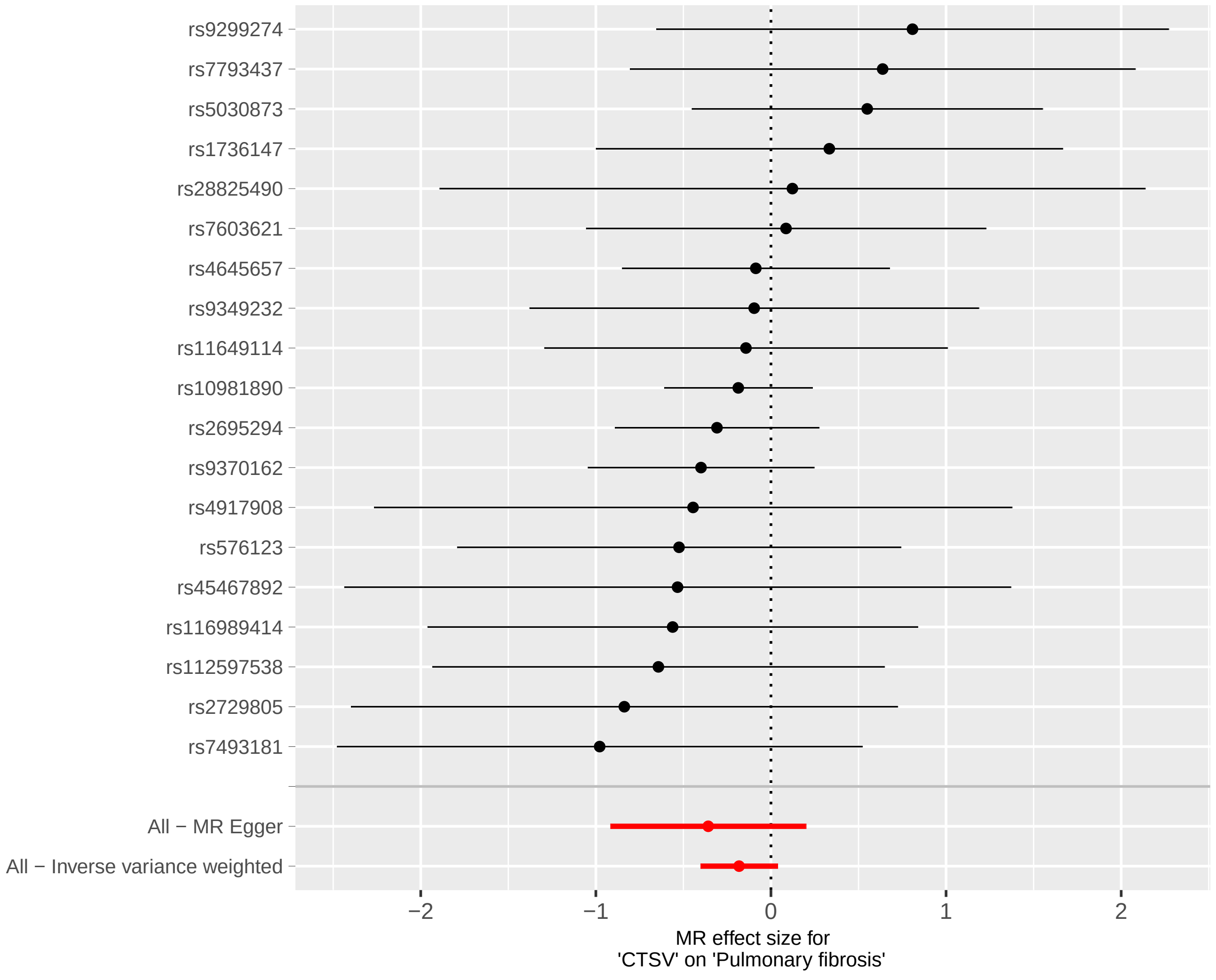
rs9299274
rs7793437
rs5030873
rs1736147
rs28825490
rs7603621
rs4645657
rs9349232
rs11649114
rs10981890
rs2695294
rs9370162
rs4917908
rs576123
rs45467892
rs116989414
rs112597538
rs2729805
rs7493181
All − MR Egger
All − Inverse variance weighted
−2
−1
0
1
2
MR effect size for
'CTSV' on 'Pulmonary fibrosis'

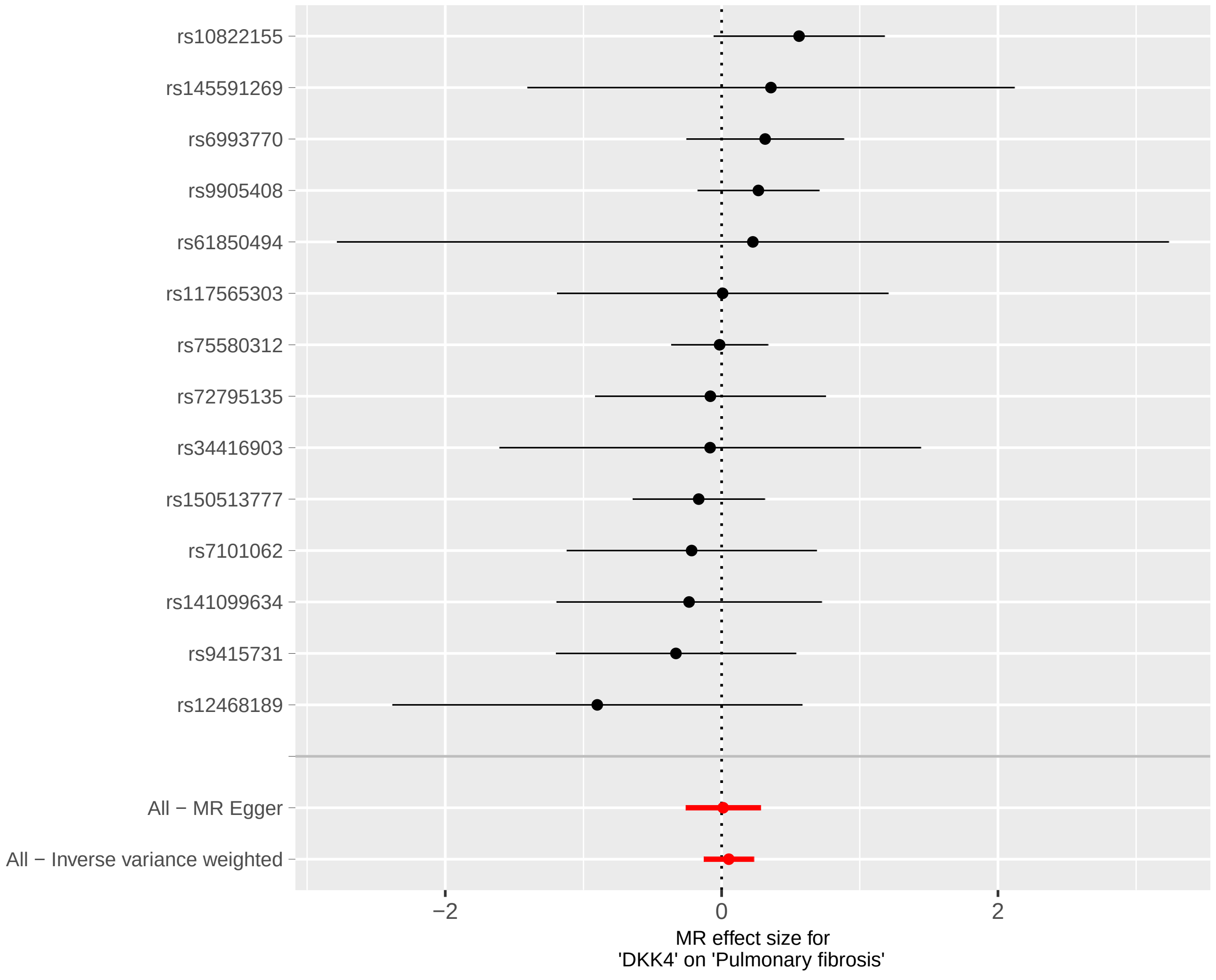

rs10822155
rs145591269
rs6993770
rs9905408
rs61850494
rs117565303
rs75580312
rs72795135
rs34416903
rs150513777
rs7101062
rs141099634
rs9415731
rs12468189
All − MR Egger
All − Inverse variance weighted
−2
0
2
MR effect size for
'DKK4' on 'Pulmonary fibrosis'

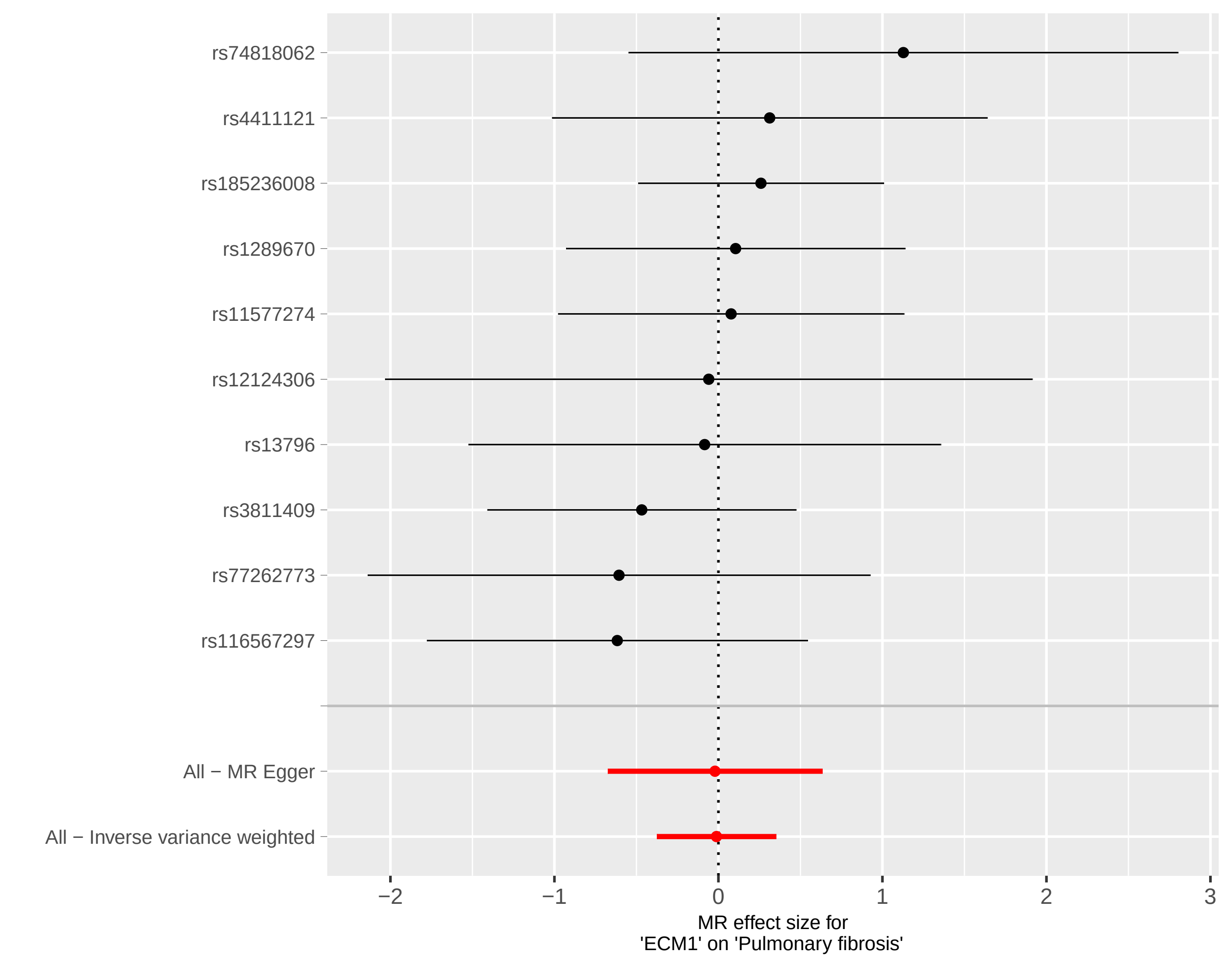

rs74818062
rs4411121
rs185236008
rs1289670
rs11577274
rs12124306
rs13796
rs3811409
rs77262773
rs116567297
All − MR Egger
All − Inverse variance weighted
−2
−1
0
1
2
3
MR effect size for
'ECM1' on 'Pulmonary fibrosis'

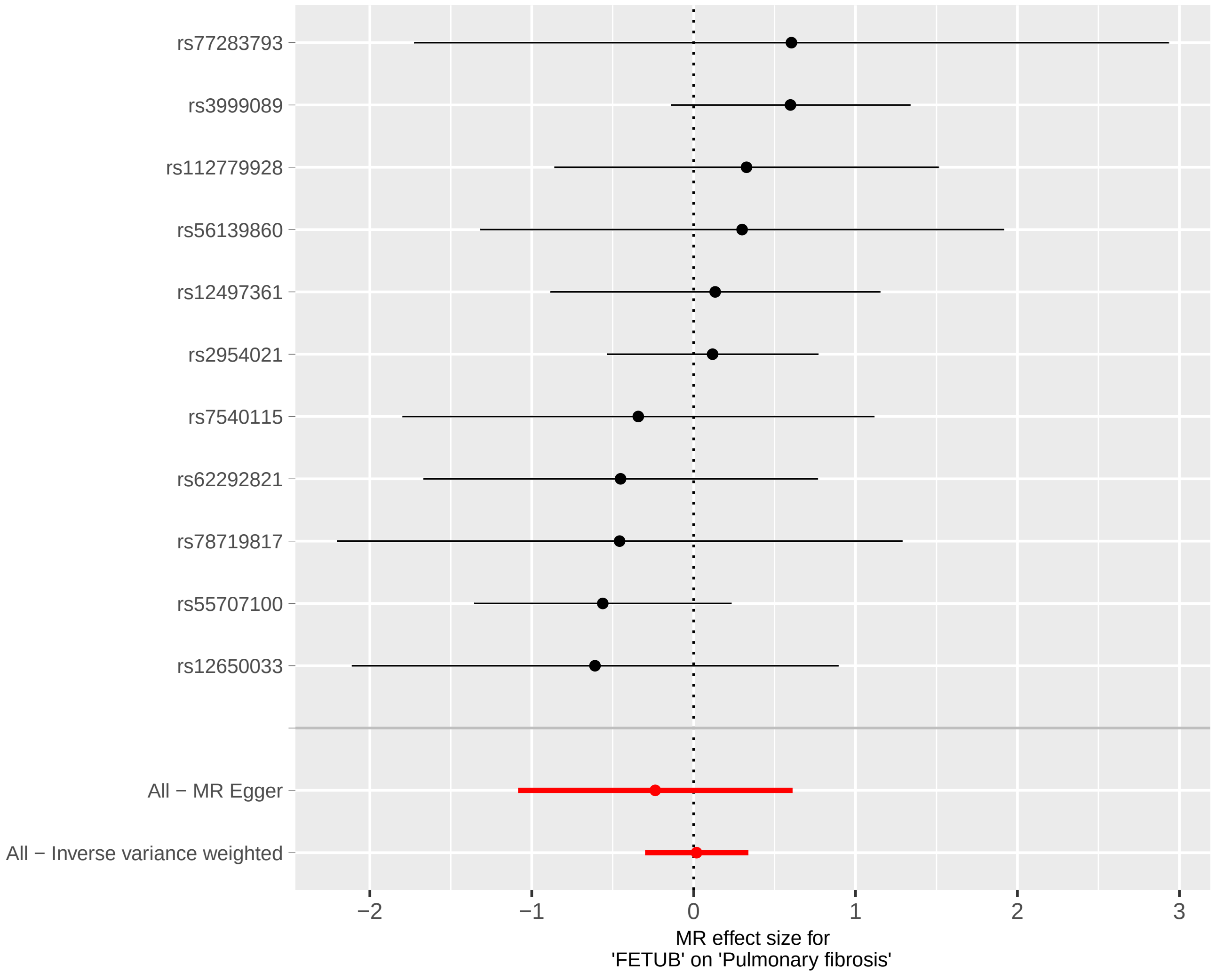

rs77283793
rs3999089
rs112779928
rs56139860
rs12497361
rs2954021
rs7540115
rs62292821
rs78719817
rs55707100
rs12650033
All − MR Egger
All − Inverse variance weighted
−2
−1
0
1
2
3
MR effect size for
'FETUB' on 'Pulmonary fibrosis'

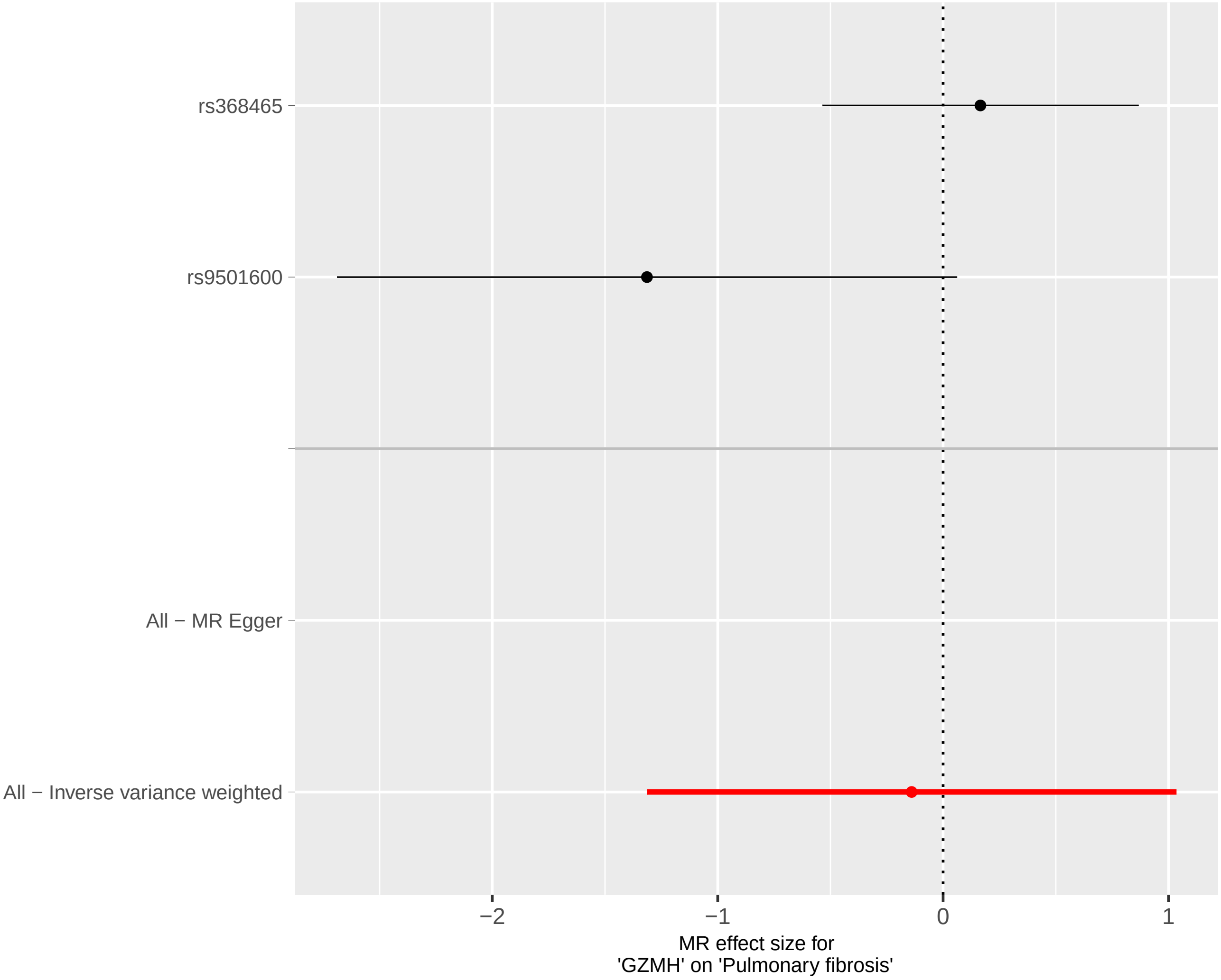

rs368465
rs9501600
All − MR Egger
All − Inverse variance weighted
−2
−1
0
1
MR effect size for
'GZMH' on 'Pulmonary fibrosis'

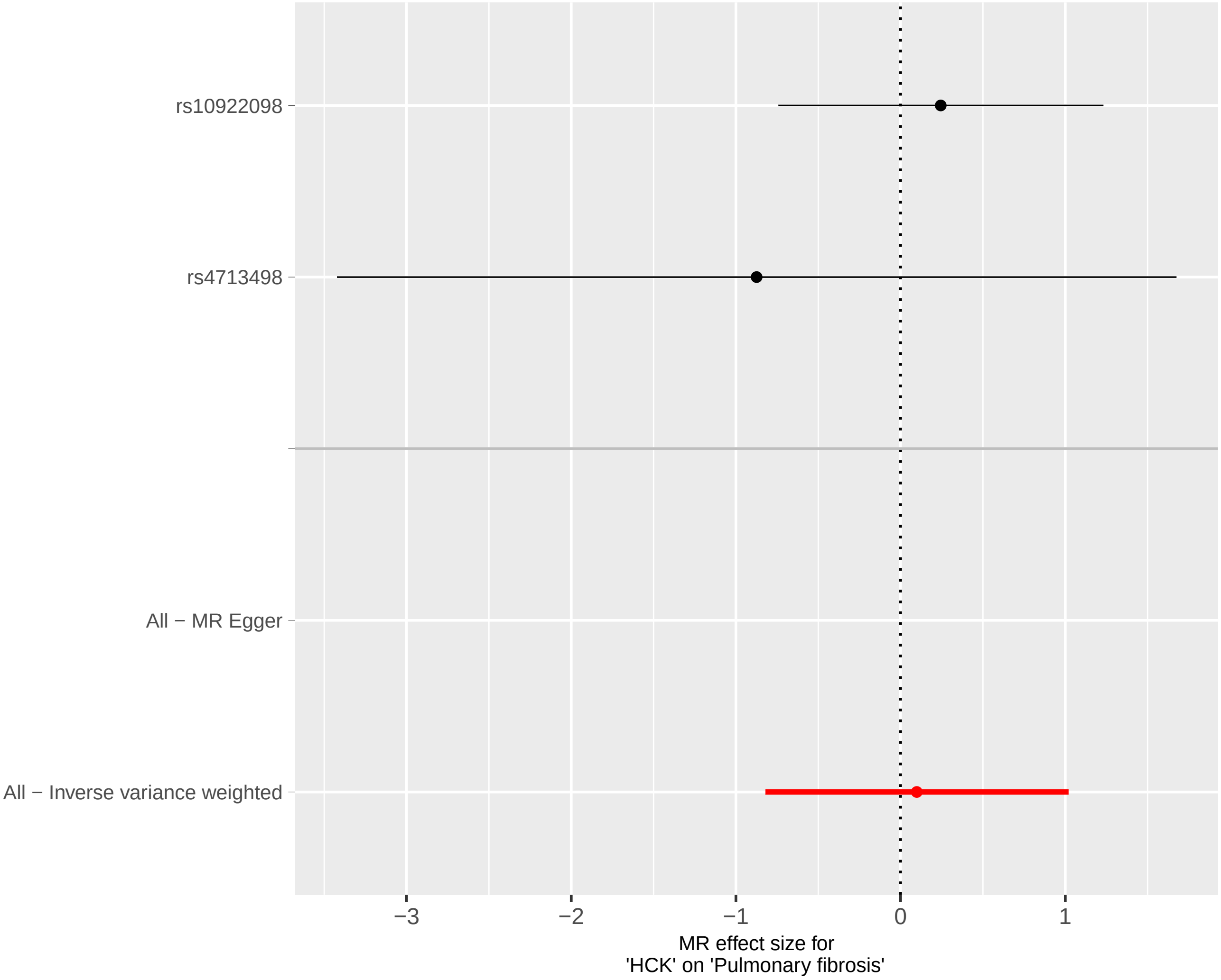
rs10922098
rs4713498
All − MR Egger
All − Inverse variance weighted
−3
−2
−1
0
1
MR effect size for
'HCK' on 'Pulmonary fibrosis'

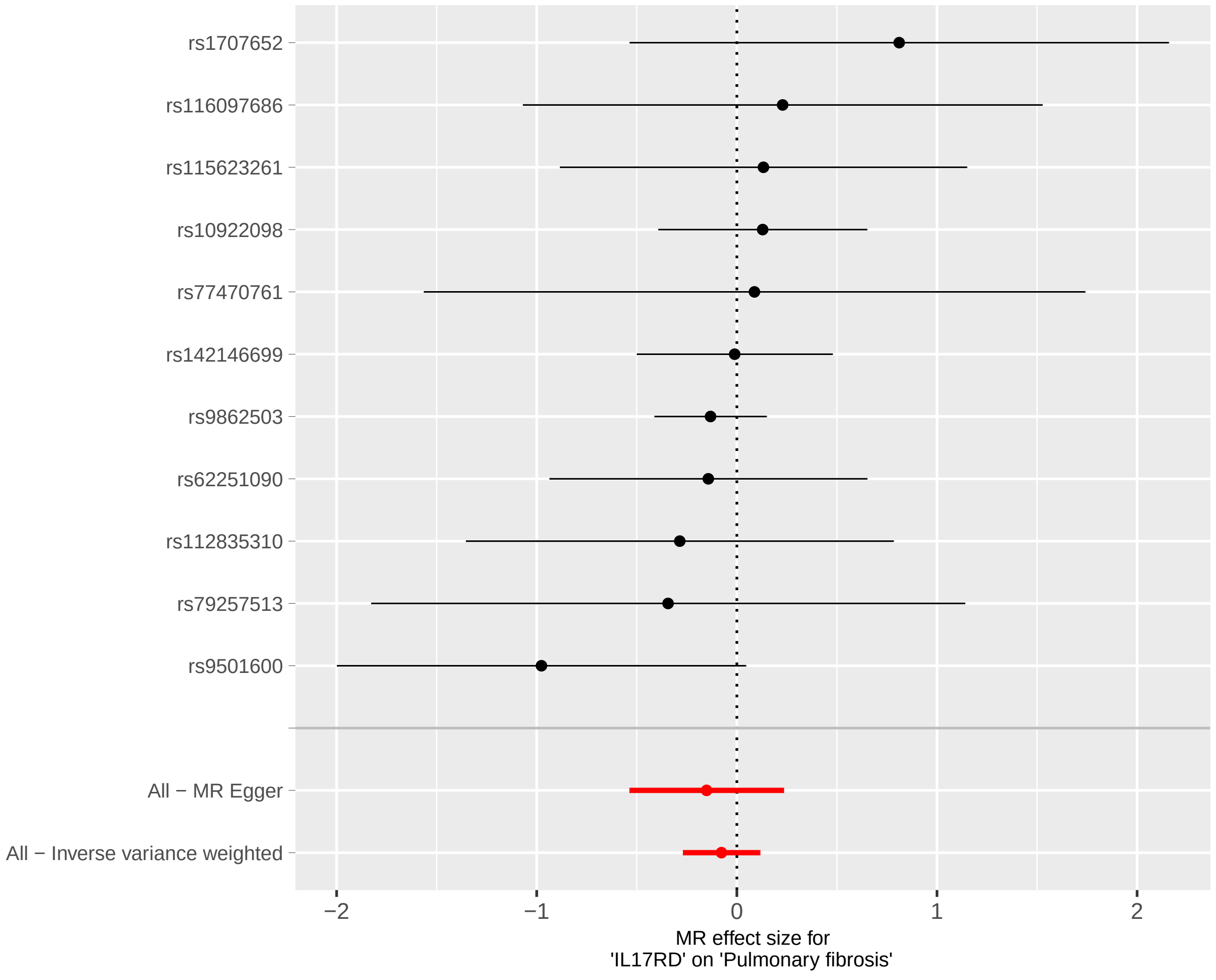
rs1707652
rs116097686
rs115623261
rs10922098
rs77470761
rs142146699
rs9862503
rs62251090
rs112835310
rs79257513
rs9501600
All − MR Egger
All − Inverse variance weighted
−2
−1
0
1
2
MR effect size for
'IL17RD' on 'Pulmonary fibrosis'

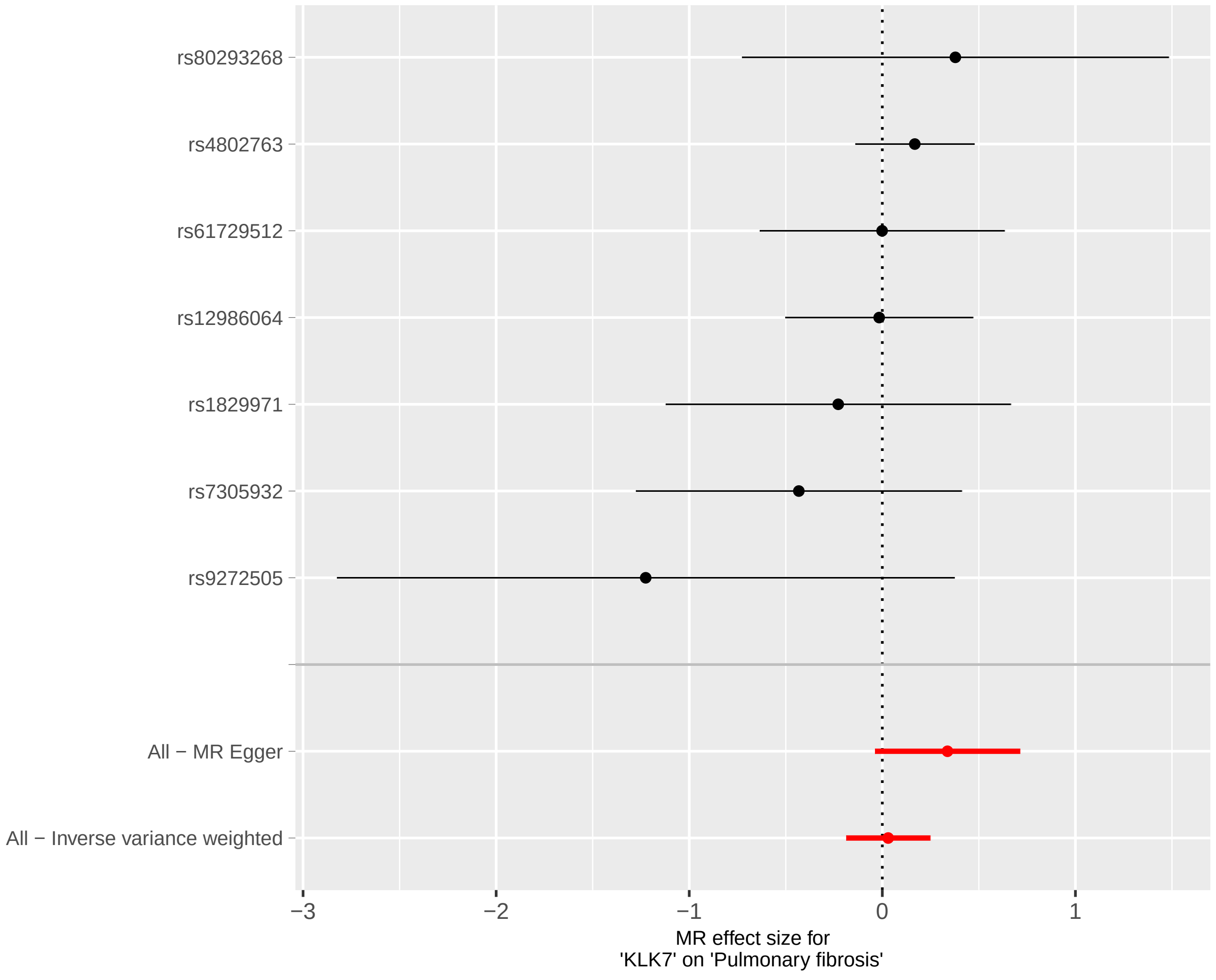

rs80293268
rs4802763
rs61729512
rs12986064
rs1829971
rs7305932
rs9272505
All − MR Egger
All − Inverse variance weighted
−3
−2
−1
0
1
MR effect size for
'KLK7' on 'Pulmonary fibrosis'

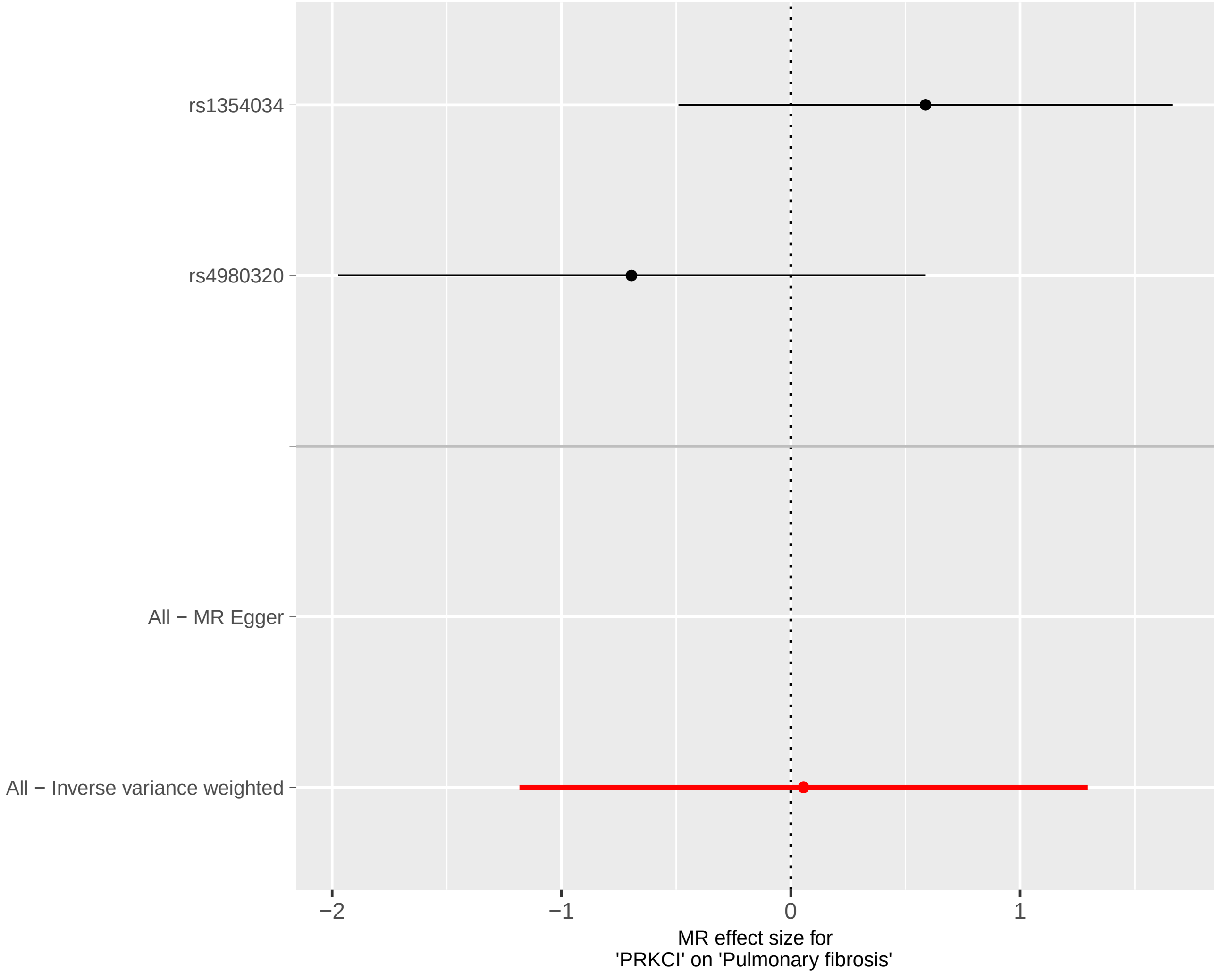
rs1354034
rs4980320
All − MR Egger
All − Inverse variance weighted
−2
−1
0
1
MR effect size for
'PRKCI' on 'Pulmonary fibrosis'

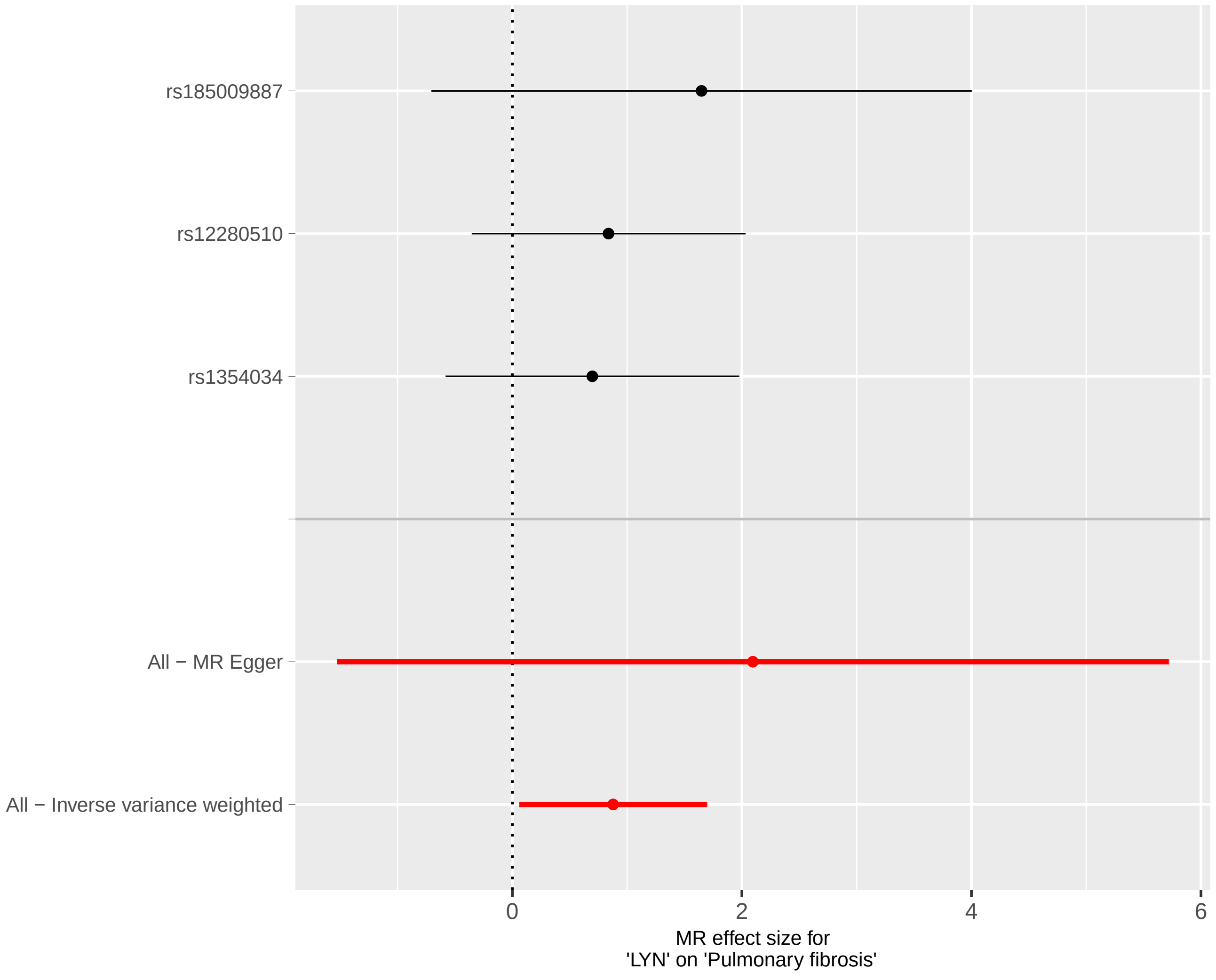

rs185009887
rs12280510
rs1354034
All − MR Egger
All − Inverse variance weighted
0
2
4
6
MR effect size for
'LYN' on 'Pulmonary fibrosis'

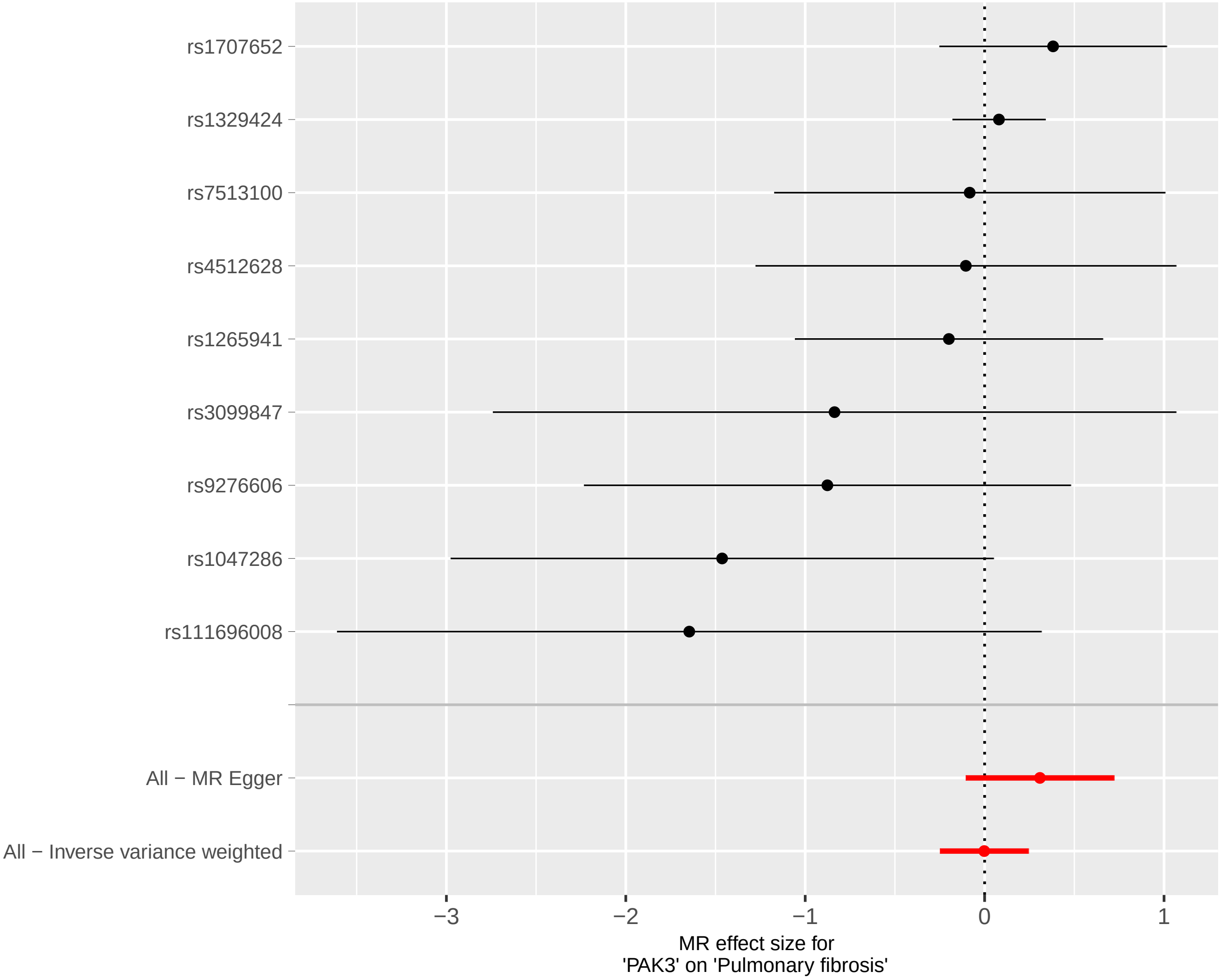
rs1707652
rs1329424
rs7513100
rs4512628
rs1265941
rs3099847
rs9276606
rs1047286
rs111696008
All − MR Egger
All − Inverse variance weighted
−3
−2
−1
0
1
MR effect size for
'PAK3' on 'Pulmonary fibrosis'

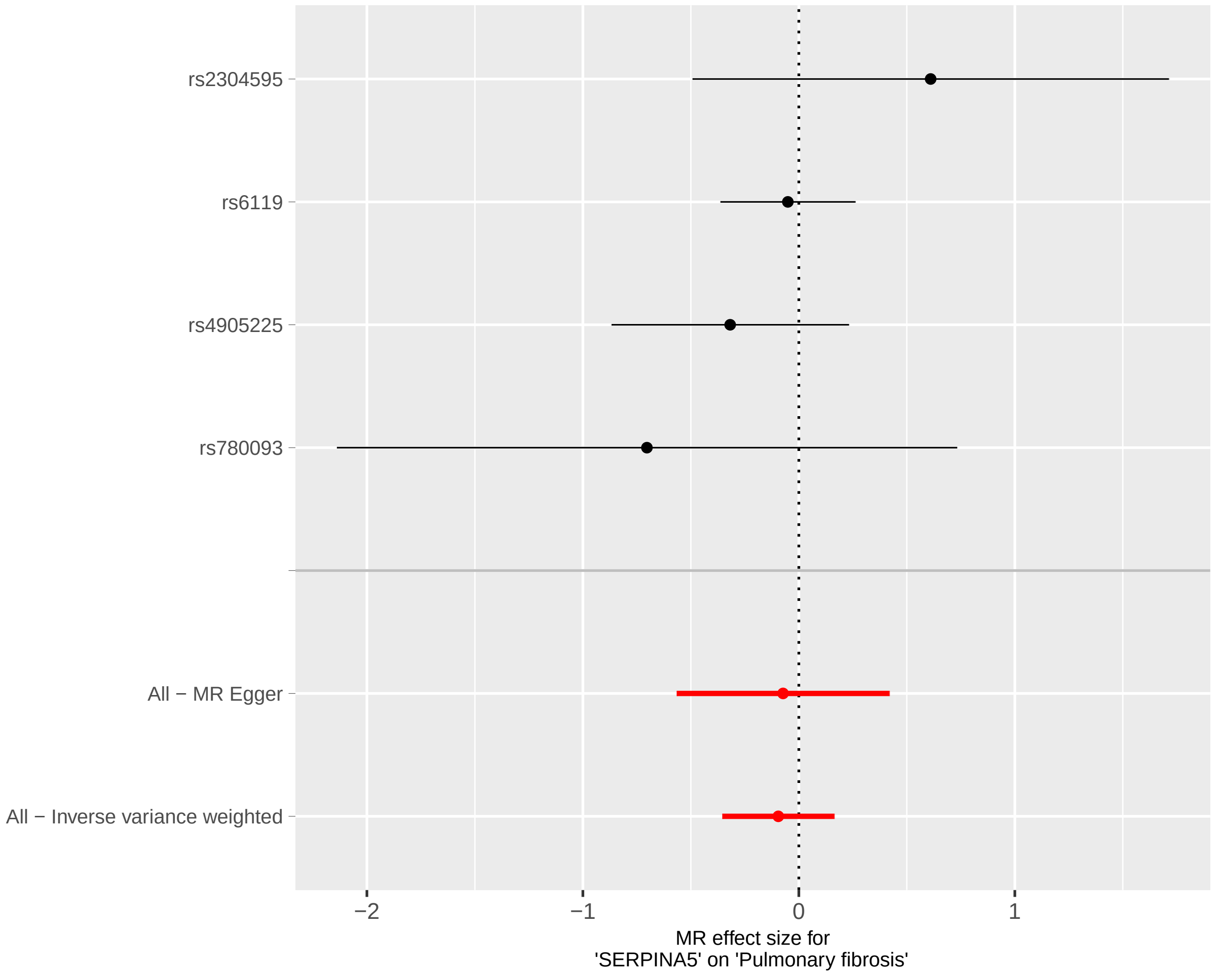
rs2304595
rs6119
rs4905225
rs780093
All − MR Egger
All − Inverse variance weighted
−2
−1
0
1
MR effect size for
'SERPINA5' on 'Pulmonary fibrosis'

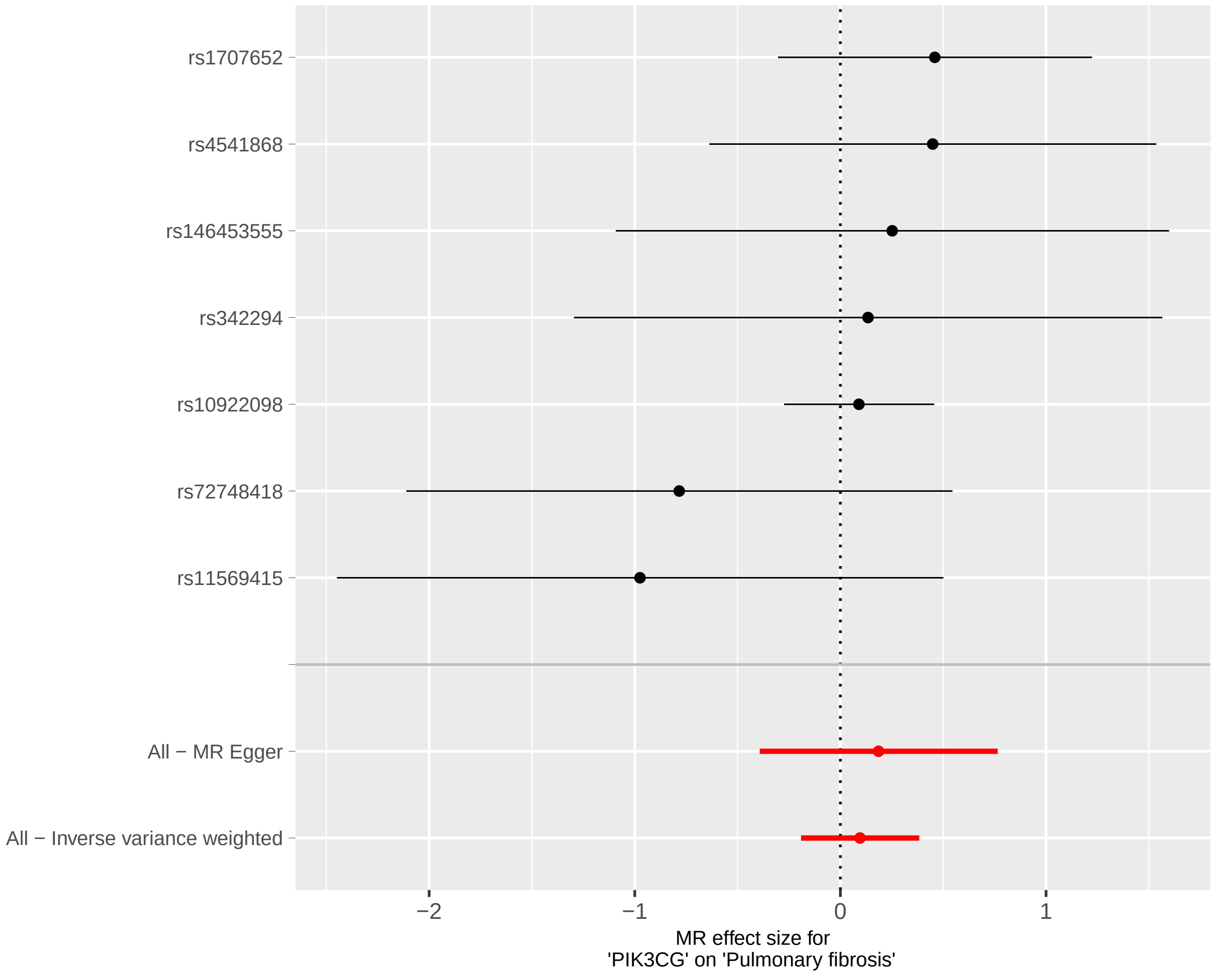
rs1707652
rs4541868
rs146453555
rs342294
rs10922098
rs72748418
rs11569415
All − MR Egger
All − Inverse variance weighted
−2
−1
0
1
MR effect size for
'PIK3CG' on 'Pulmonary fibrosis'

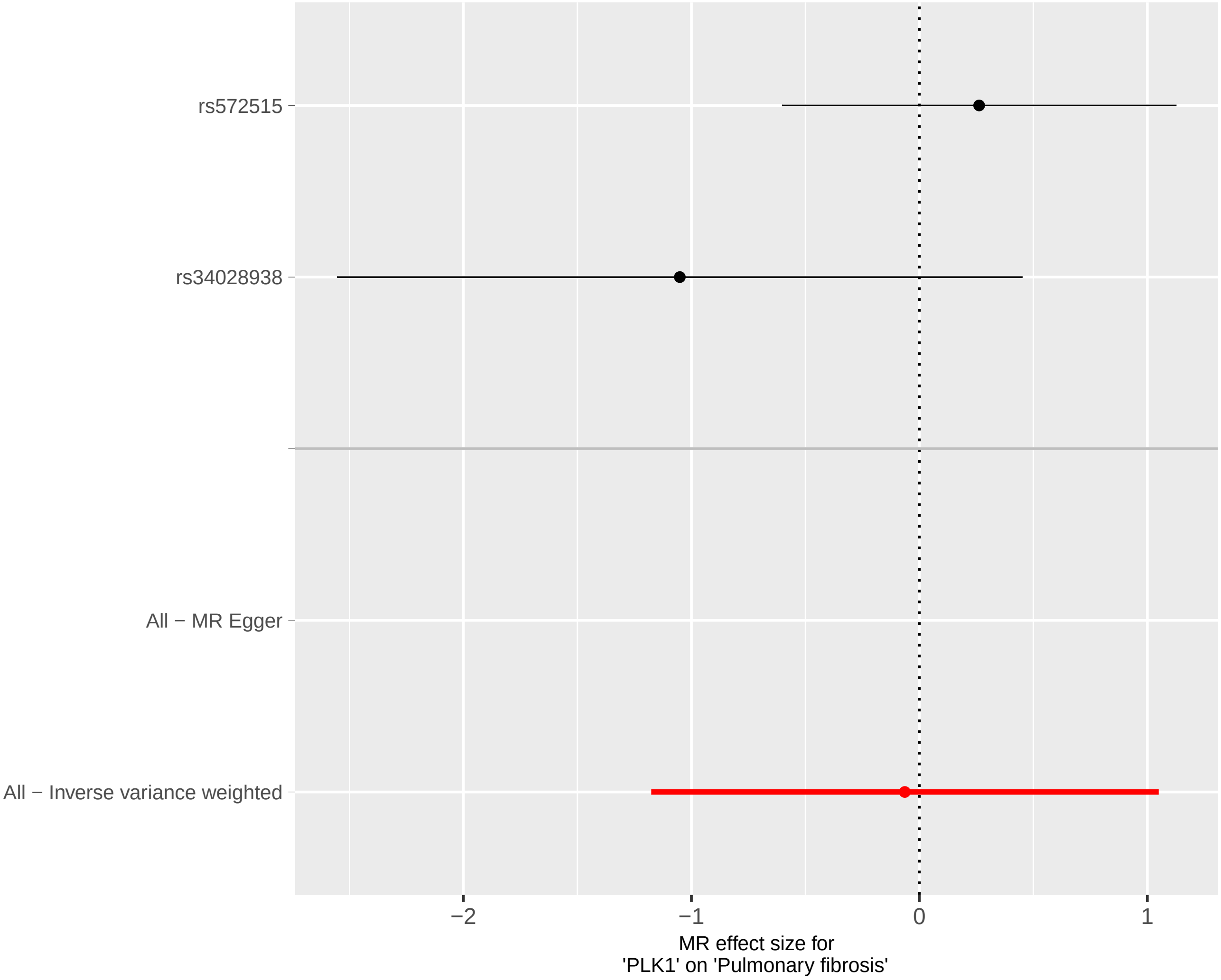

rs572515
rs34028938
All − MR Egger
All − Inverse variance weighted
−2
−1
0
1
MR effect size for
'PLK1' on 'Pulmonary fibrosis'

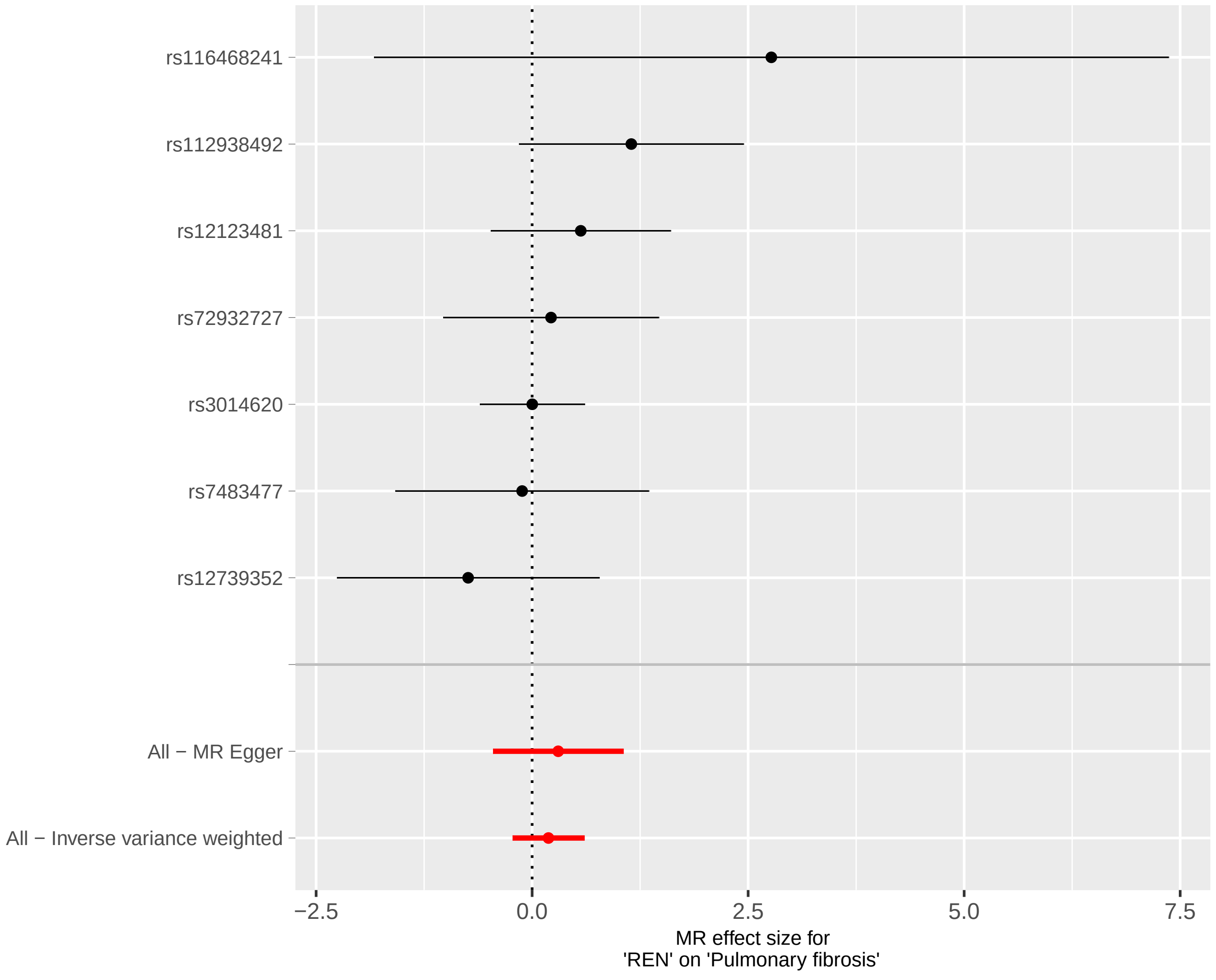
rs116468241
rs112938492
rs12123481
rs72932727
rs3014620
rs7483477
rs12739352
All − MR Egger
All − Inverse variance weighted
−2.5
0.0
2.5
5.0
7.5
MR effect size for
'REN' on 'Pulmonary fibrosis'

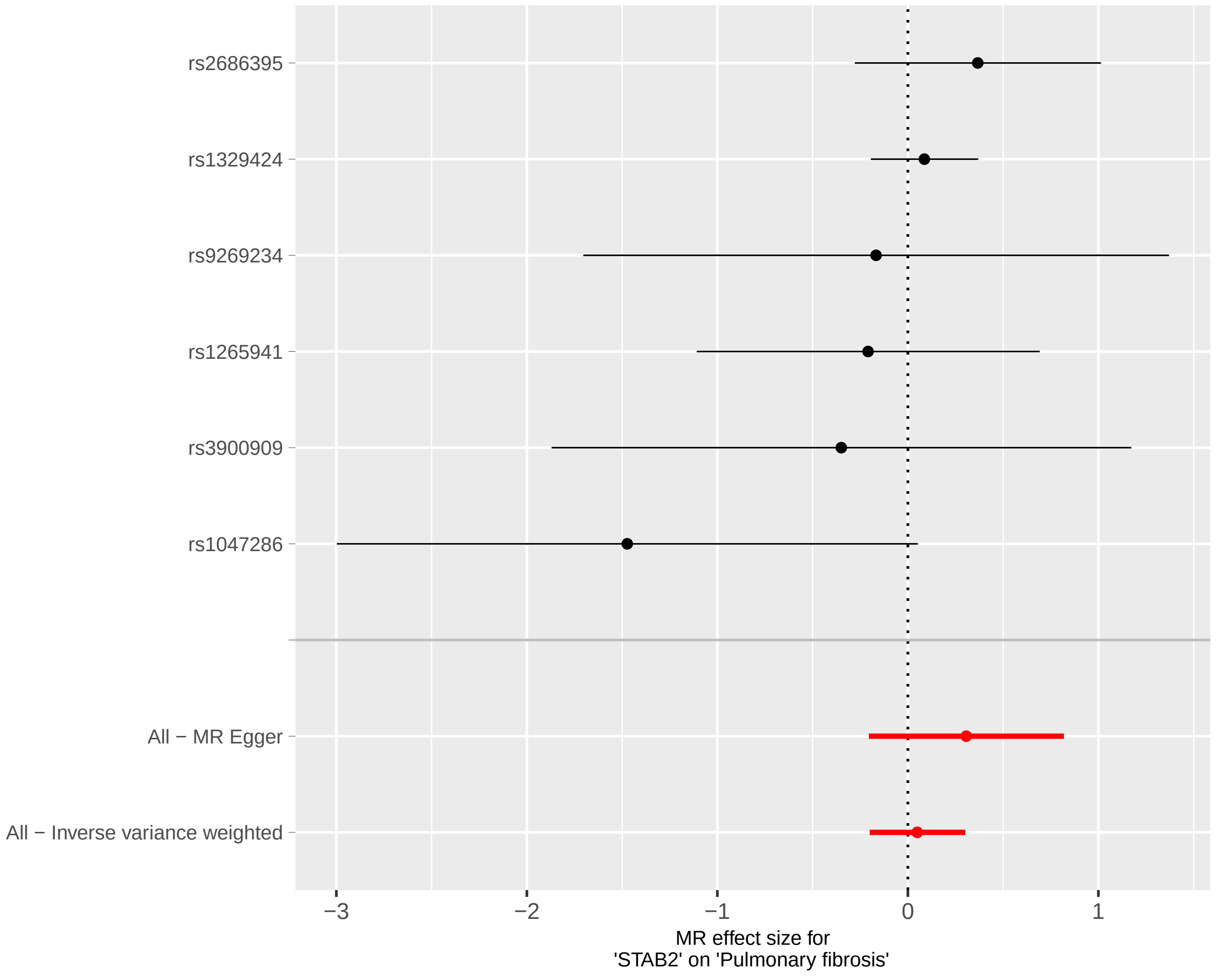
rs2686395
rs1329424
rs9269234
rs1265941
rs3900909
rs1047286
All − MR Egger
All − Inverse variance weighted
−3
−2
−1
0
1
MR effect size for
'STAB2' on 'Pulmonary fibrosis'

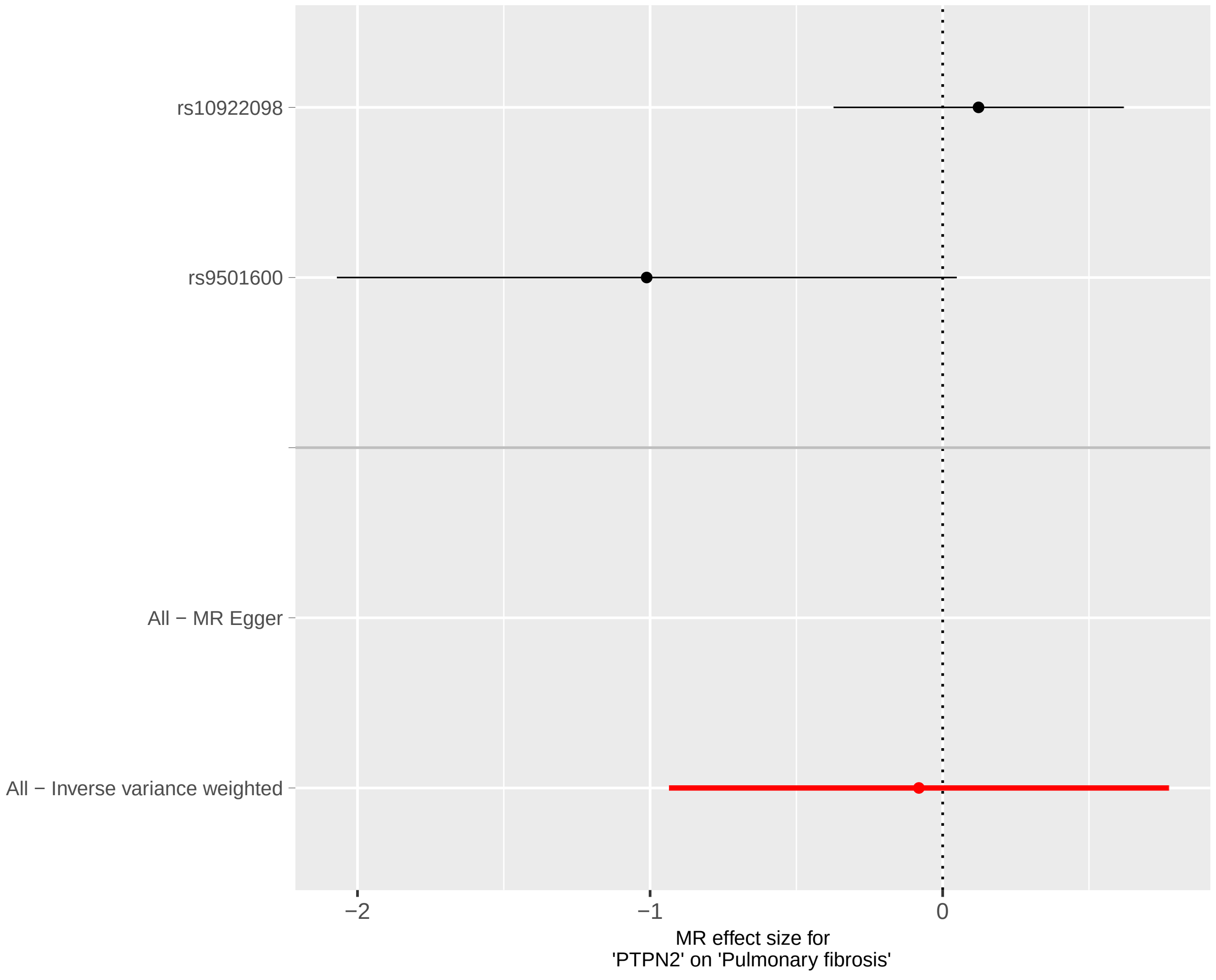
rs10922098
rs9501600
All − MR Egger
All − Inverse variance weighted
−2
−1
0
MR effect size for
'PTPN2' on 'Pulmonary fibrosis'

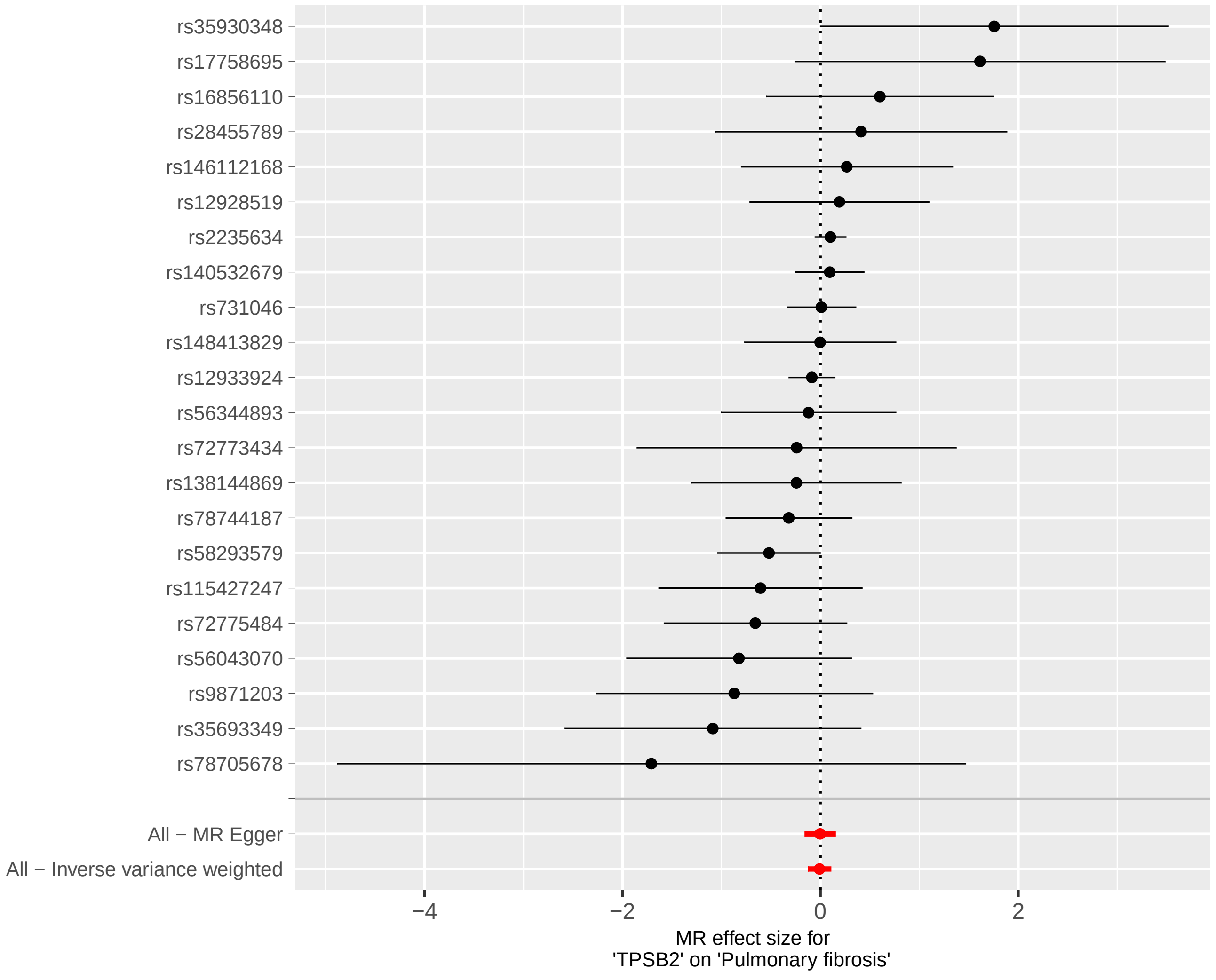
rs35930348
rs17758695
rs16856110
rs28455789
rs146112168
rs12928519
rs2235634
rs140532679
rs731046
rs148413829
rs12933924
rs56344893
rs72773434
rs138144869
rs78744187
rs58293579
rs115427247
rs72775484
rs56043070
rs9871203
rs35693349
rs78705678
All − MR Egger
All − Inverse variance weighted
−4
−2
0
2
MR effect size for
'TPSB2' on 'Pulmonary fibrosis'

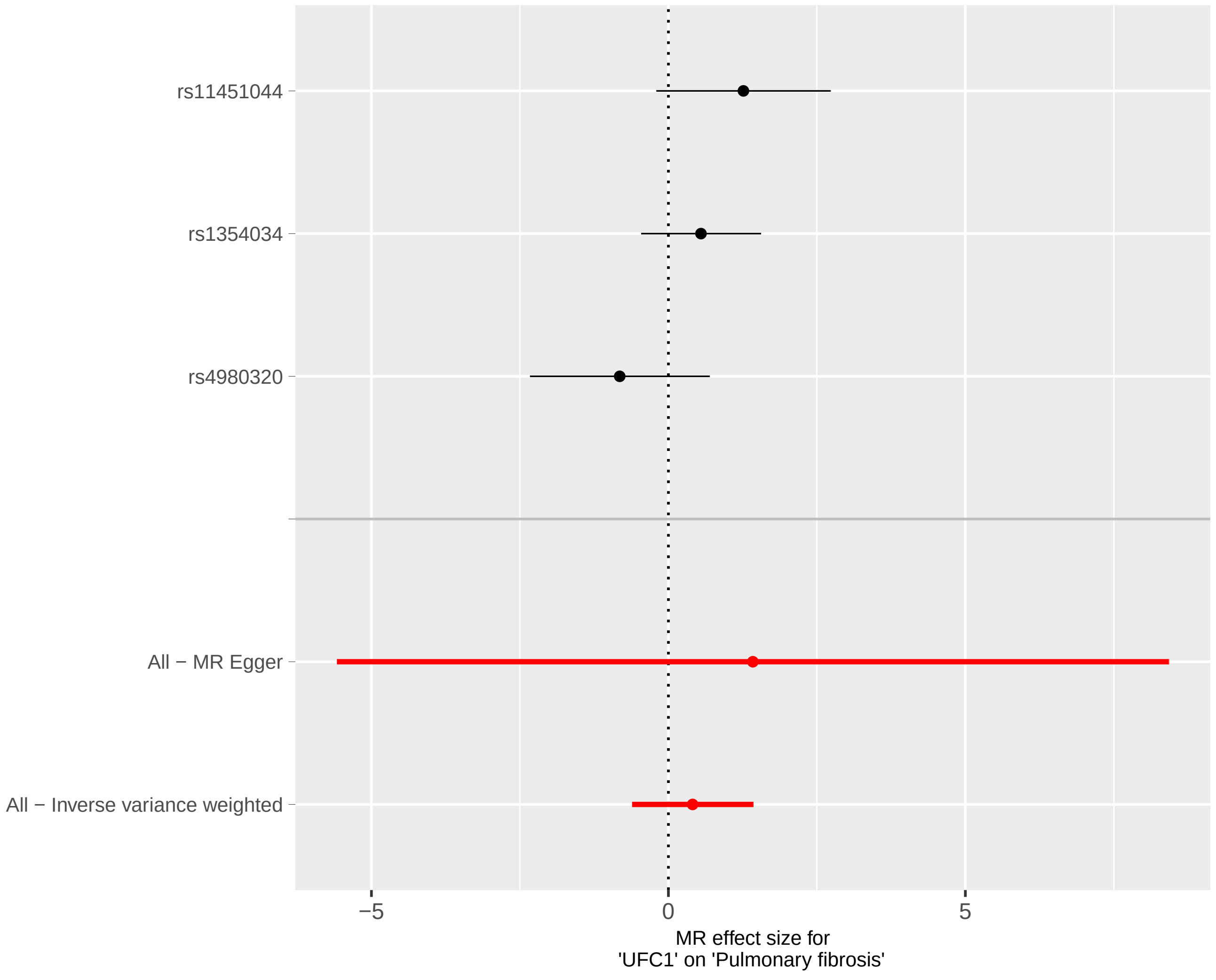
rs11451044
rs1354034
rs4980320
All − MR Egger
All − Inverse variance weighted
−5
0
5
MR effect size for
'UFC1' on 'Pulmonary fibrosis'

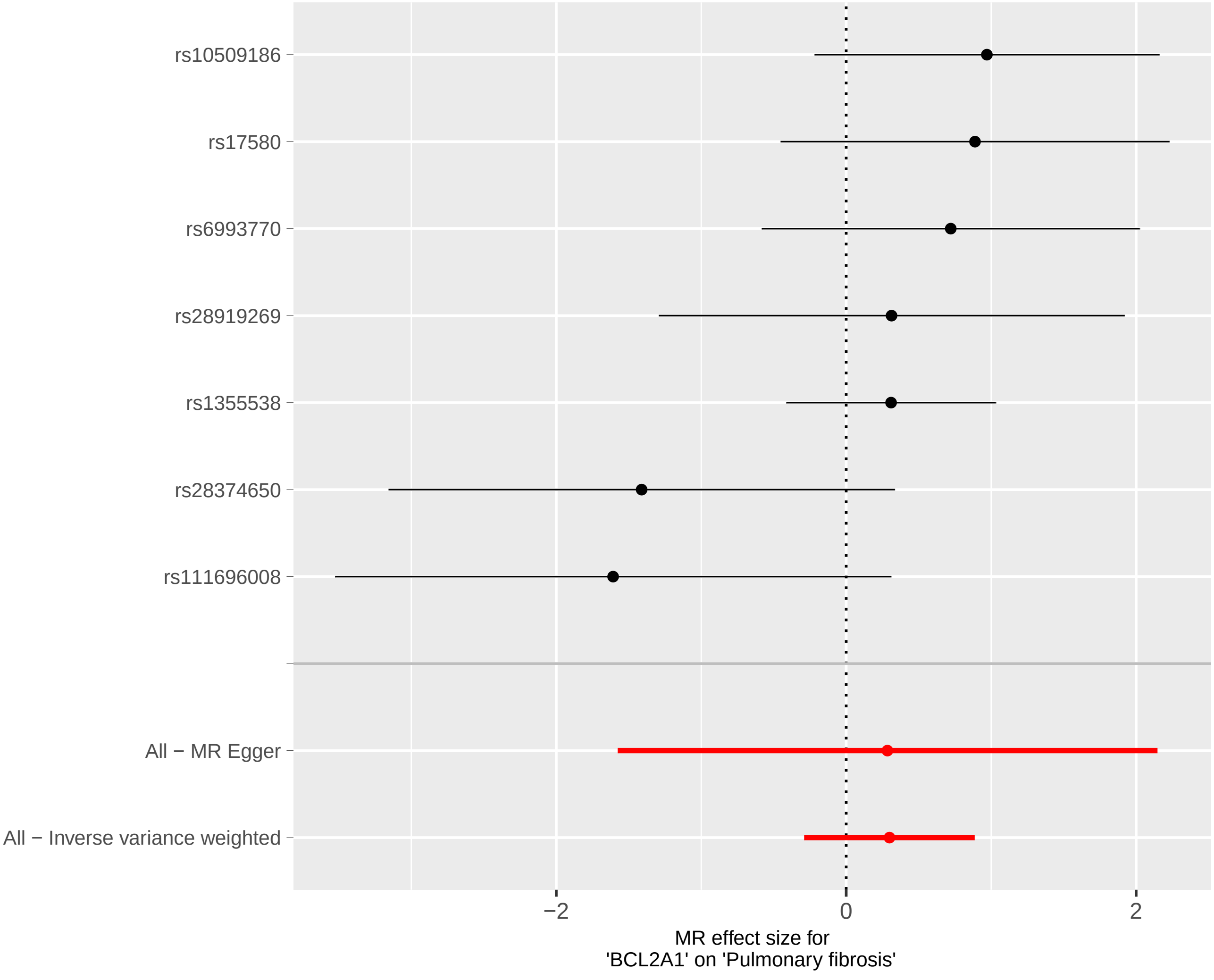
rs10509186
rs17580
rs6993770
rs28919269
rs1355538
rs28374650
rs111696008
All − MR Egger
All − Inverse variance weighted
−2
0
2
MR effect size for
'BCL2A1' on 'Pulmonary fibrosis'

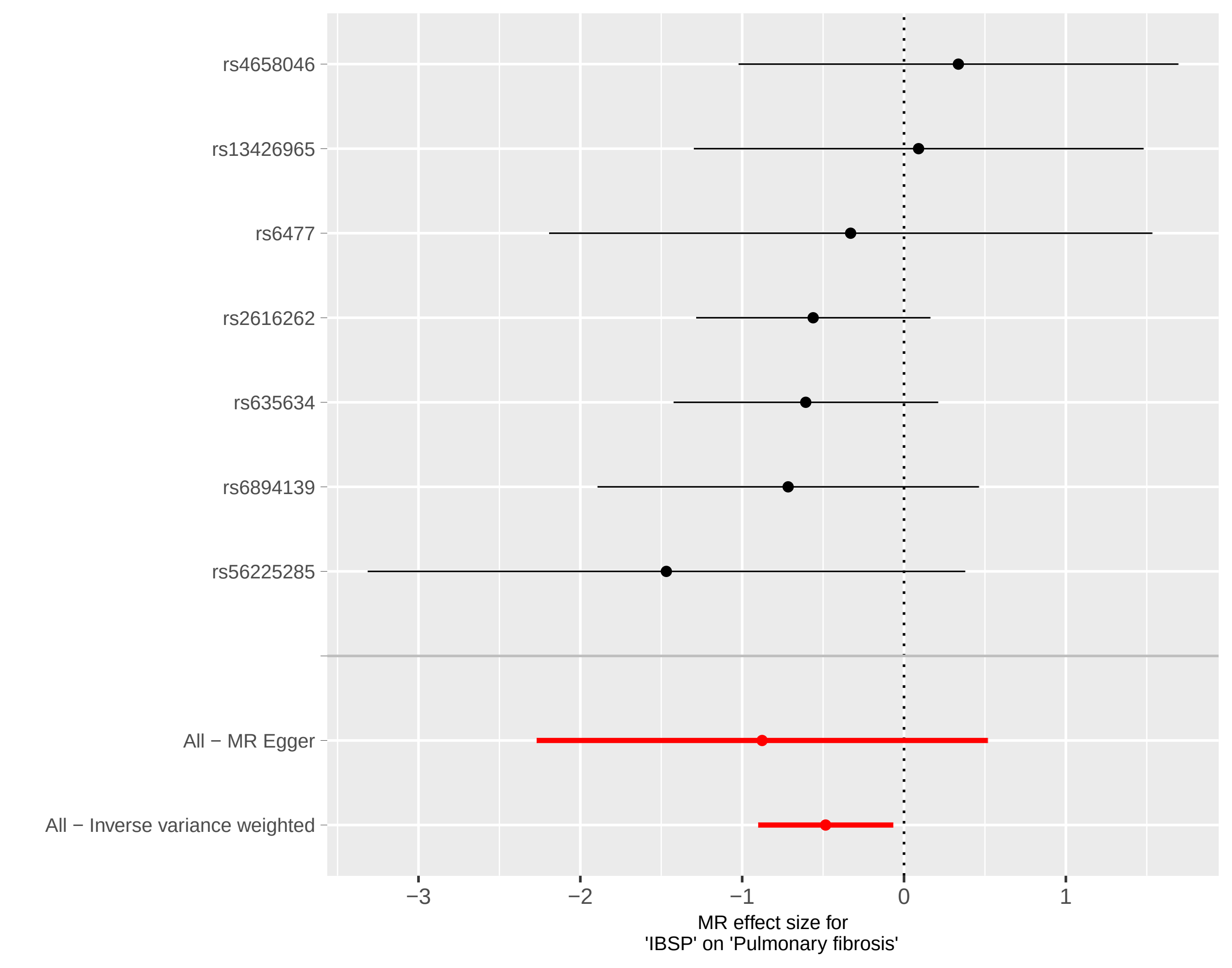
rs4658046
rs13426965
rs6477
rs2616262
rs635634
rs6894139
rs56225285
All − MR Egger
All − Inverse variance weighted
−3
−2
−1
0
1
MR effect size for
'IBSP' on 'Pulmonary fibrosis'

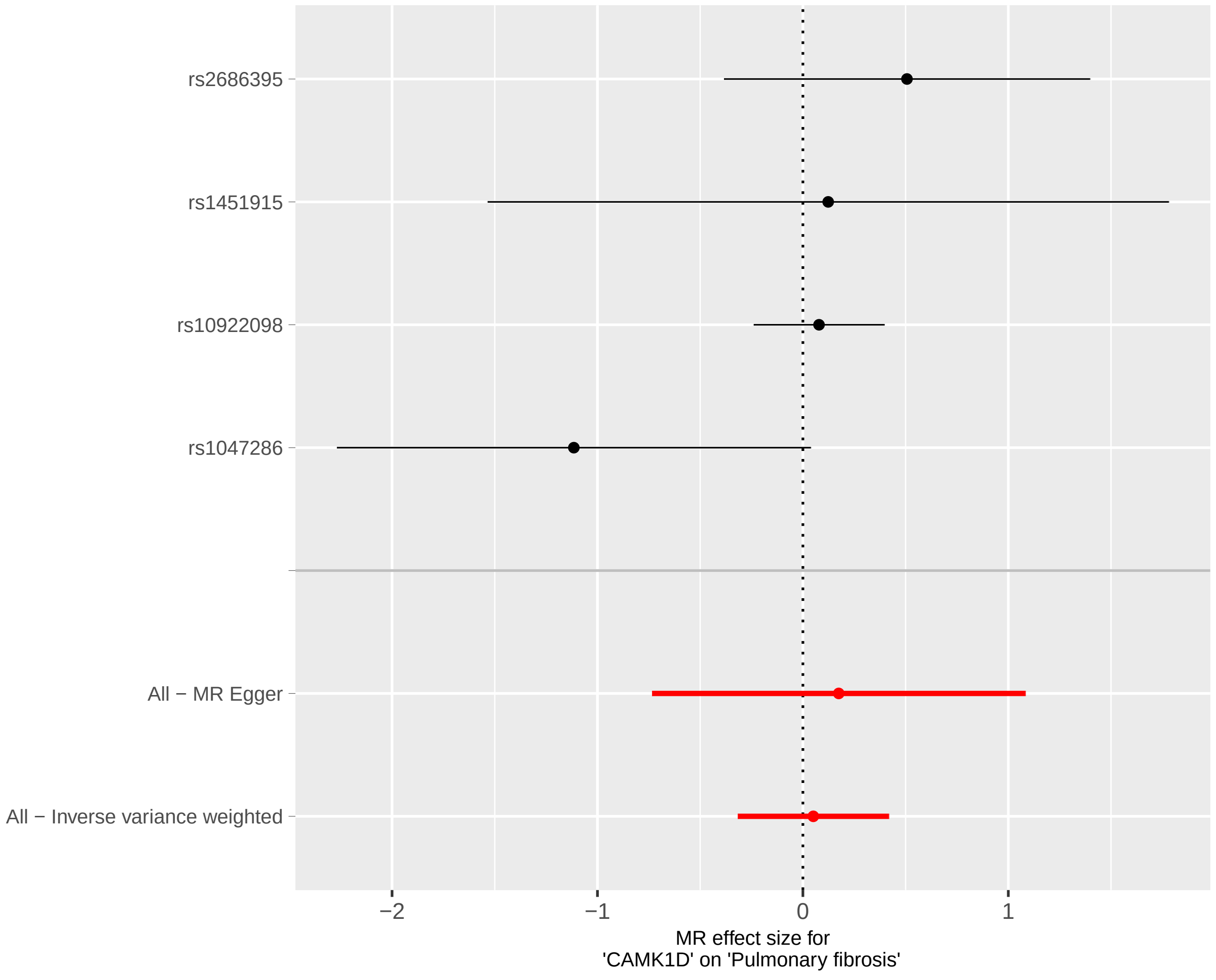
rs2686395
rs1451915
rs10922098
rs1047286
All − MR Egger
All − Inverse variance weighted
−2
−1
0
1
MR effect size for
'CAMK1D' on 'Pulmonary fibrosis'

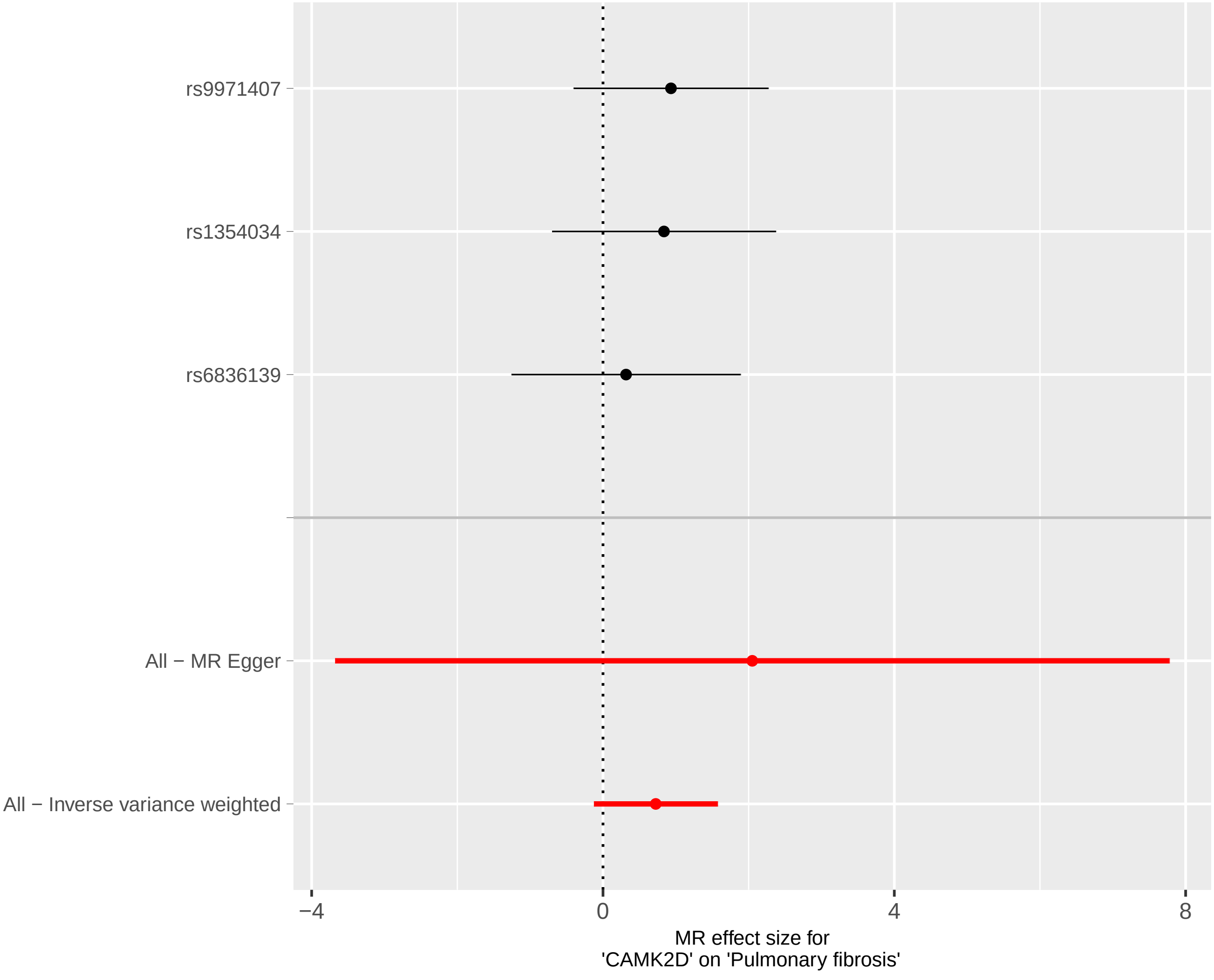
rs9971407
rs1354034
rs6836139
All − MR Egger
All − Inverse variance weighted
−4
0
4
8
MR effect size for
'CAMK2D' on 'Pulmonary fibrosis'

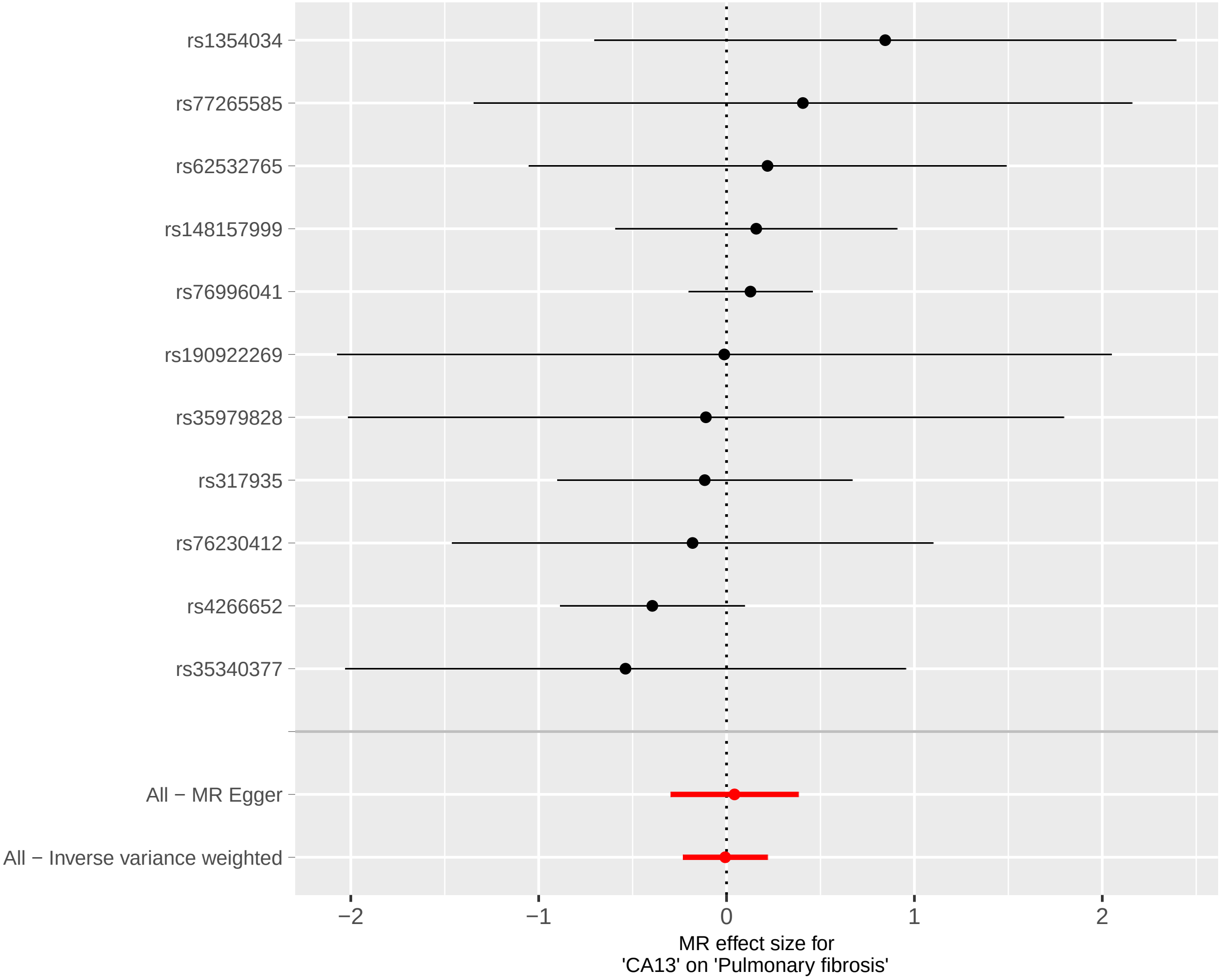

rs1354034
rs77265585
rs62532765
rs148157999
rs76996041
rs190922269
rs35979828
rs317935
rs76230412
rs4266652
rs35340377
All − MR Egger
All − Inverse variance weighted
−2
−1
0
1
2
MR effect size for
'CA13' on 'Pulmonary fibrosis'

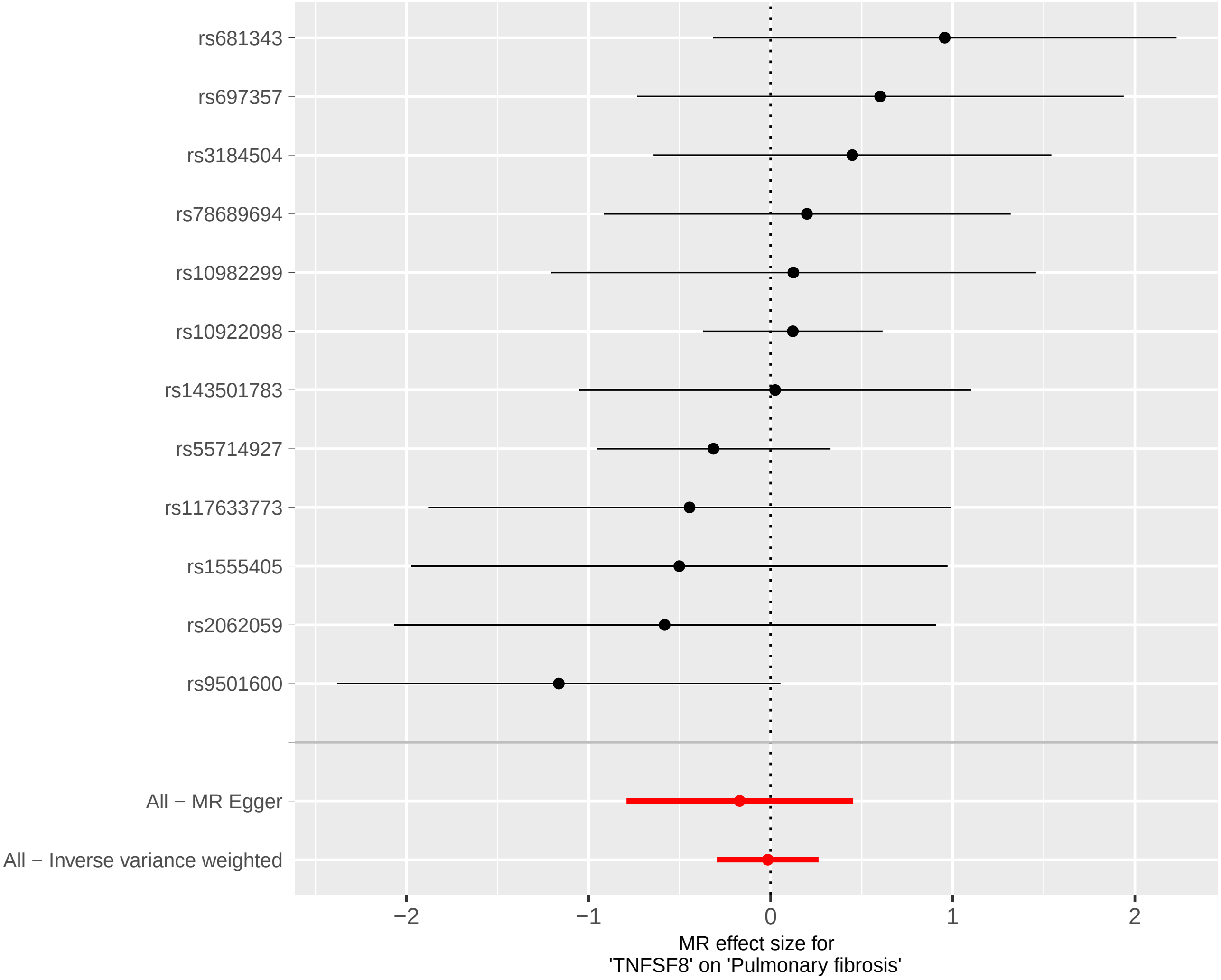

rs681343
rs697357
rs3184504
rs78689694
rs10982299
rs10922098
rs143501783
rs55714927
rs117633773
rs1555405
rs2062059
rs9501600
All − MR Egger
All − Inverse variance weighted
−2
−1
0
1
2
MR effect size for
'TNFSF8' on 'Pulmonary fibrosis'

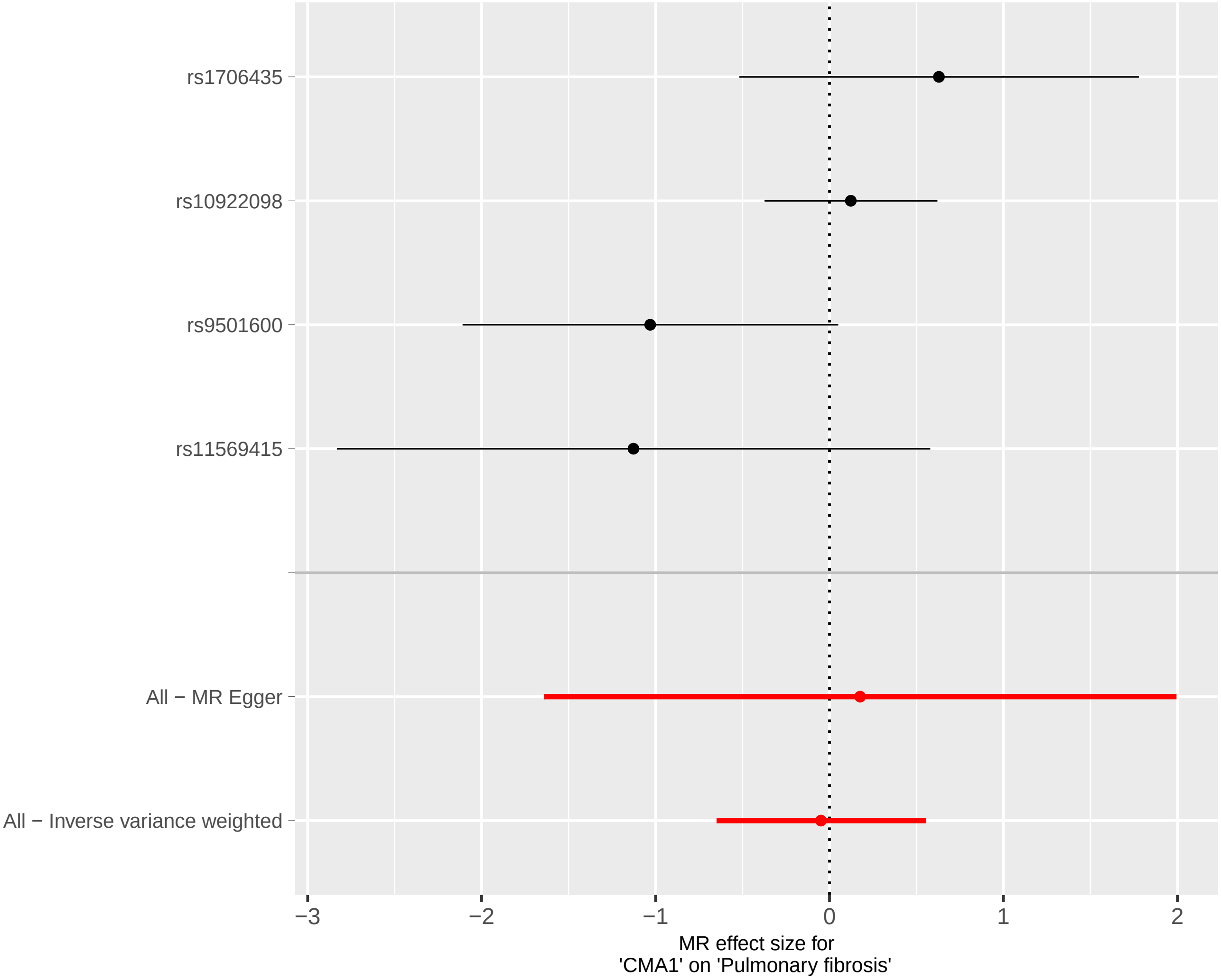

rs1706435
rs10922098
rs9501600
rs11569415
All − MR Egger
All − Inverse variance weighted
−3
−2
−1
0
1
2
MR effect size for
'CMA1' on 'Pulmonary fibrosis'

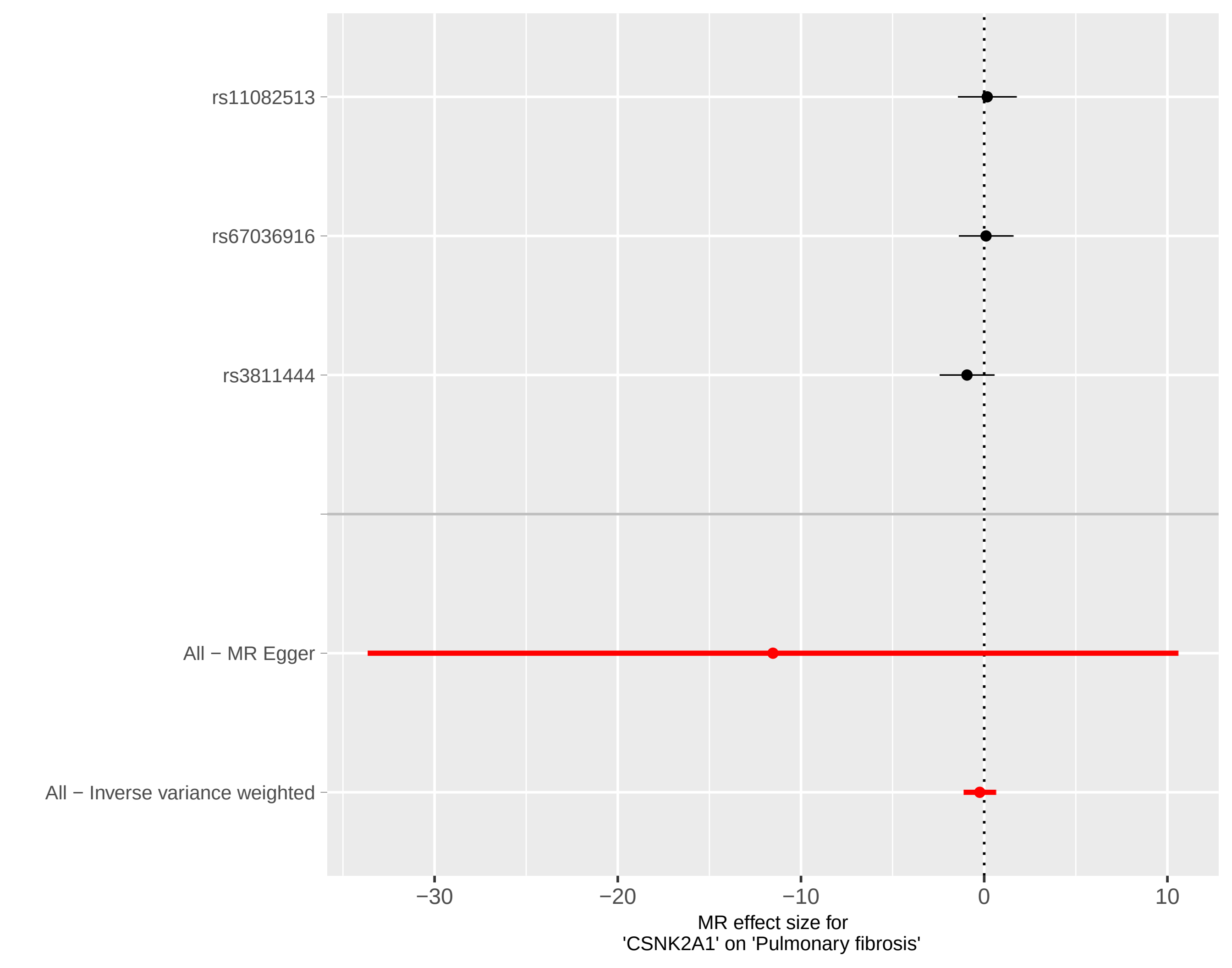
rs11082513
rs67036916
rs3811444
All − MR Egger
All − Inverse variance weighted
−30
−20
−10
0
10
MR effect size for
'CSNK2A1' on 'Pulmonary fibrosis'

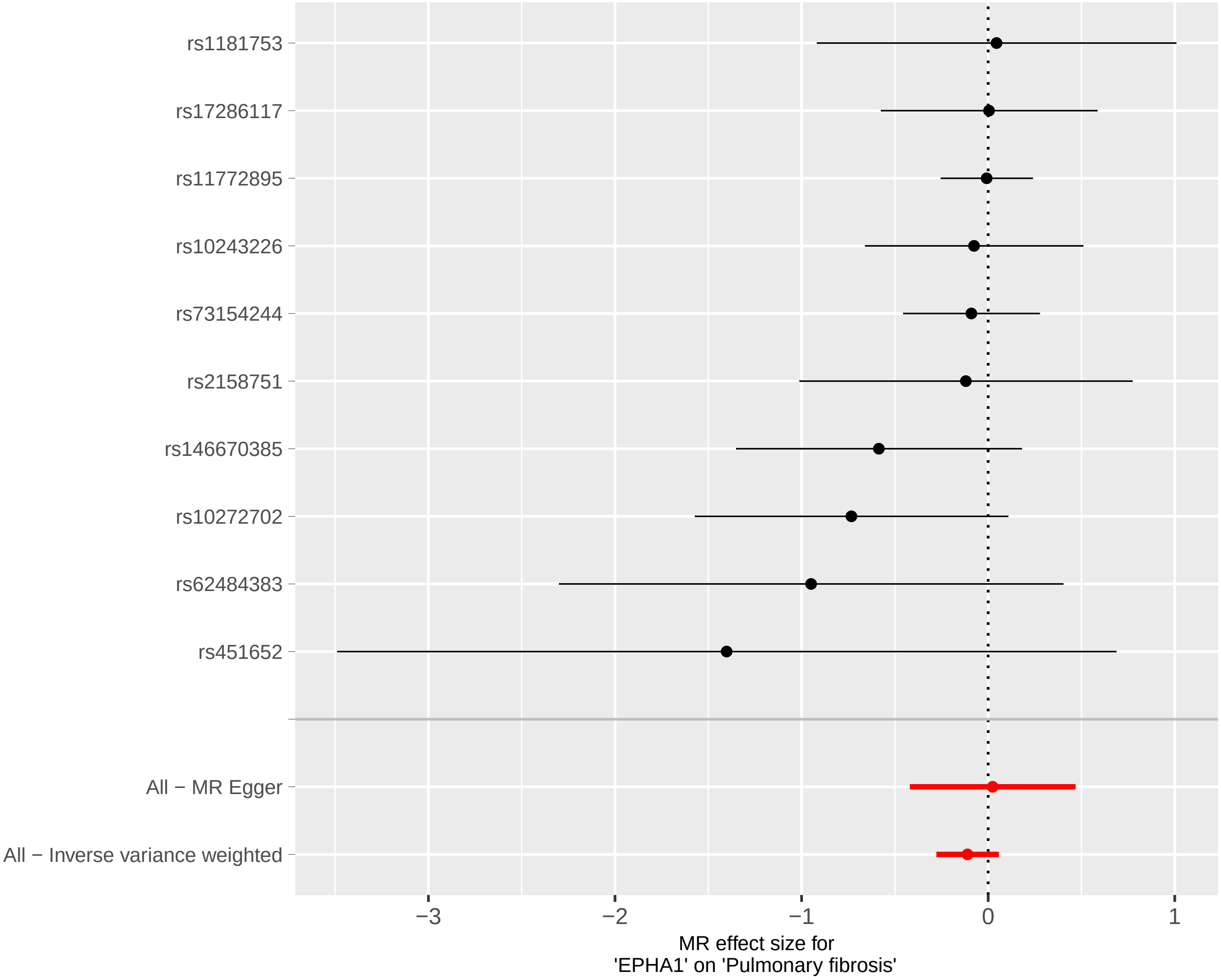
rs1181753
rs17286117
rs11772895
rs10243226
rs73154244
rs2158751
rs146670385
rs10272702
rs62484383
rs451652
All − MR Egger
All − Inverse variance weighted
−3
−2
−1
0
1
MR effect size for
'EPHA1' on 'Pulmonary fibrosis'

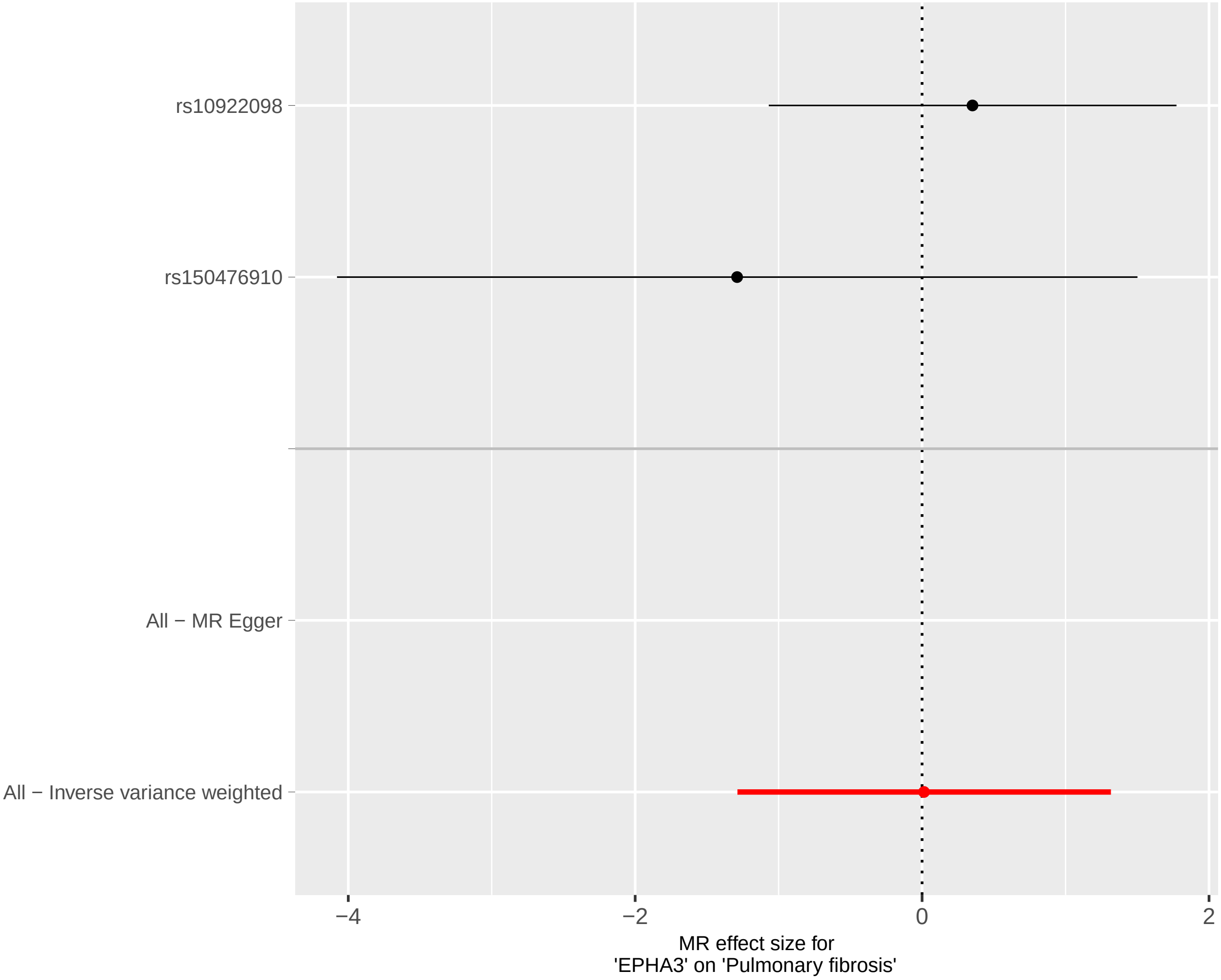

rs10922098
rs150476910
All − MR Egger
All − Inverse variance weighted
−4
−2
0
2
MR effect size for
'EPHA3' on 'Pulmonary fibrosis'

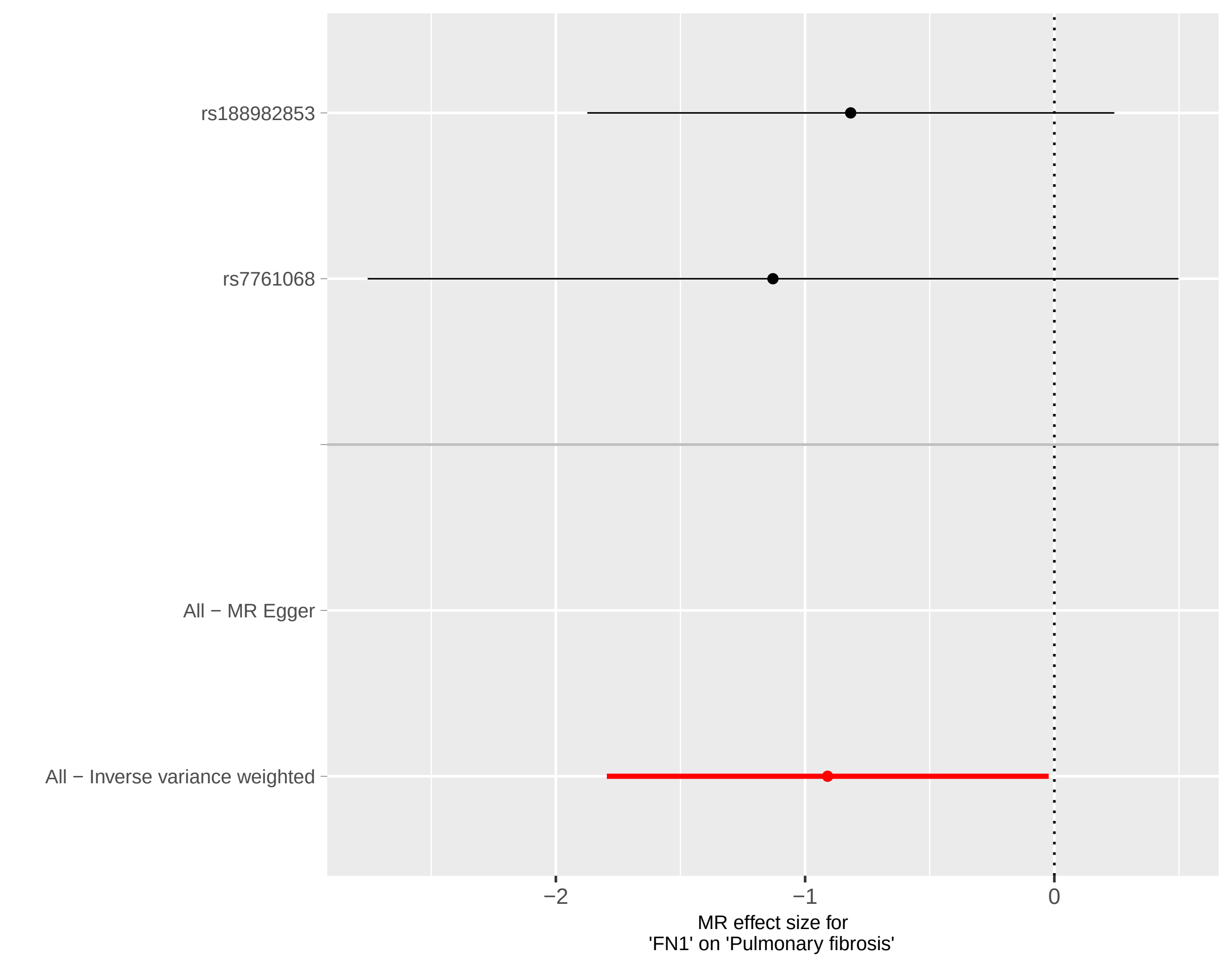

rs188982853
rs7761068
All − MR Egger
All − Inverse variance weighted
−2
−1
0
MR effect size for
'FN1' on 'Pulmonary fibrosis'

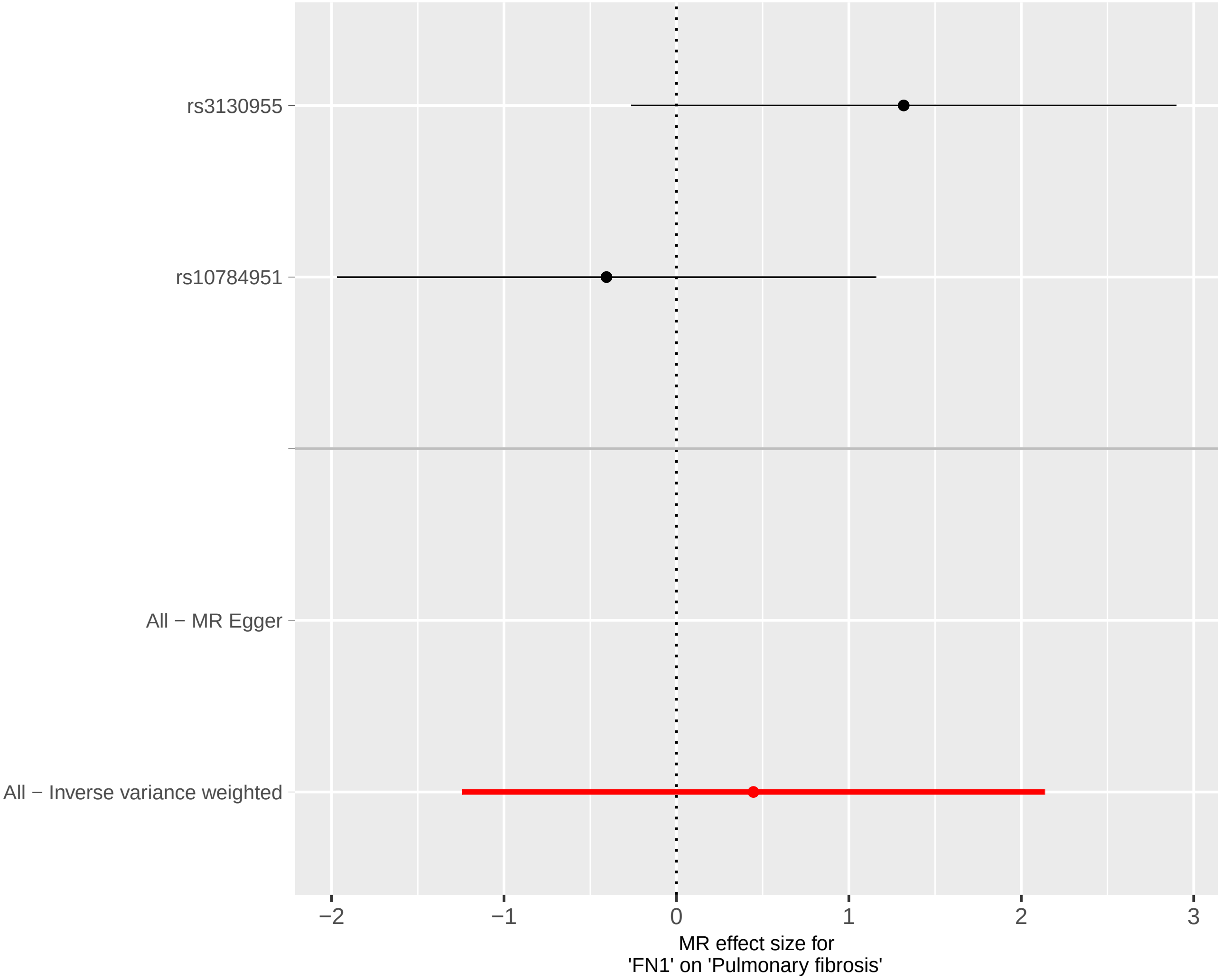
rs3130955
rs10784951
All − MR Egger
All − Inverse variance weighted
−2
−1
0
1
2
3
MR effect size for
'FN1' on 'Pulmonary fibrosis'

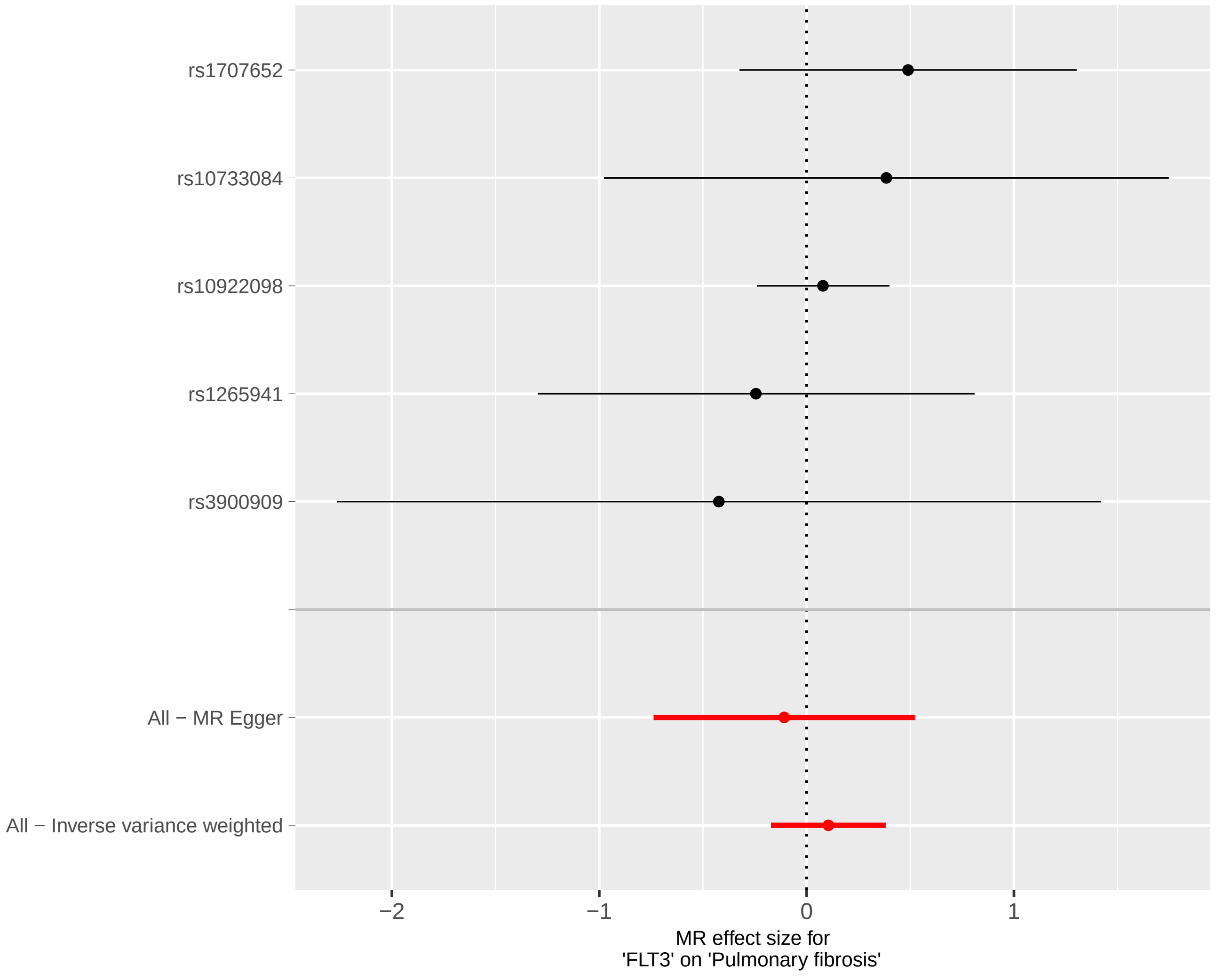
rs1707652
rs10733084
rs10922098
rs1265941
rs3900909
All − MR Egger
All − Inverse variance weighted
−2
−1
0
1
MR effect size for
'FLT3' on 'Pulmonary fibrosis'

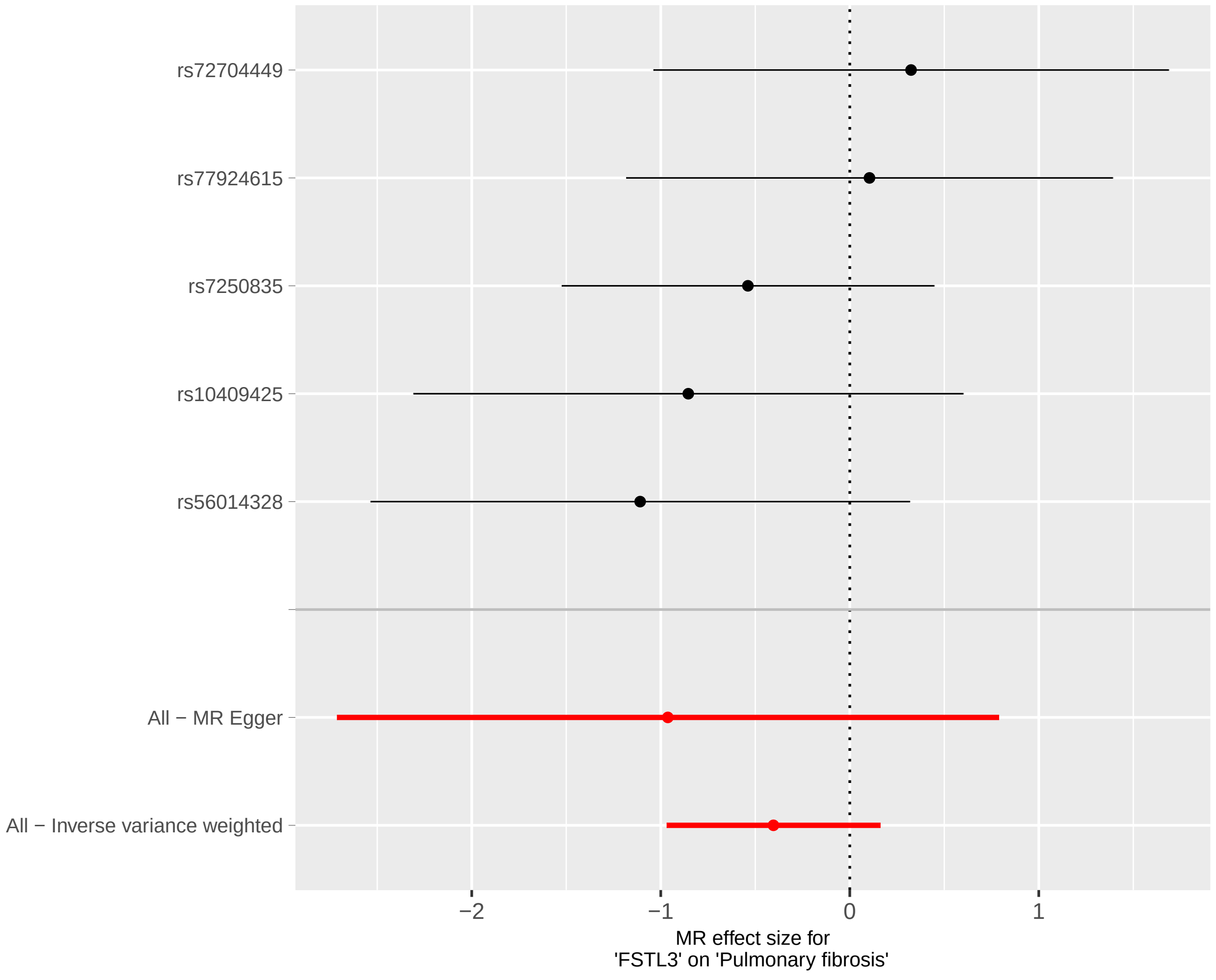

rs72704449
rs77924615
rs7250835
rs10409425
rs56014328
All − MR Egger
All − Inverse variance weighted
−2
−1
0
1
MR effect size for
'FSTL3' on 'Pulmonary fibrosis'

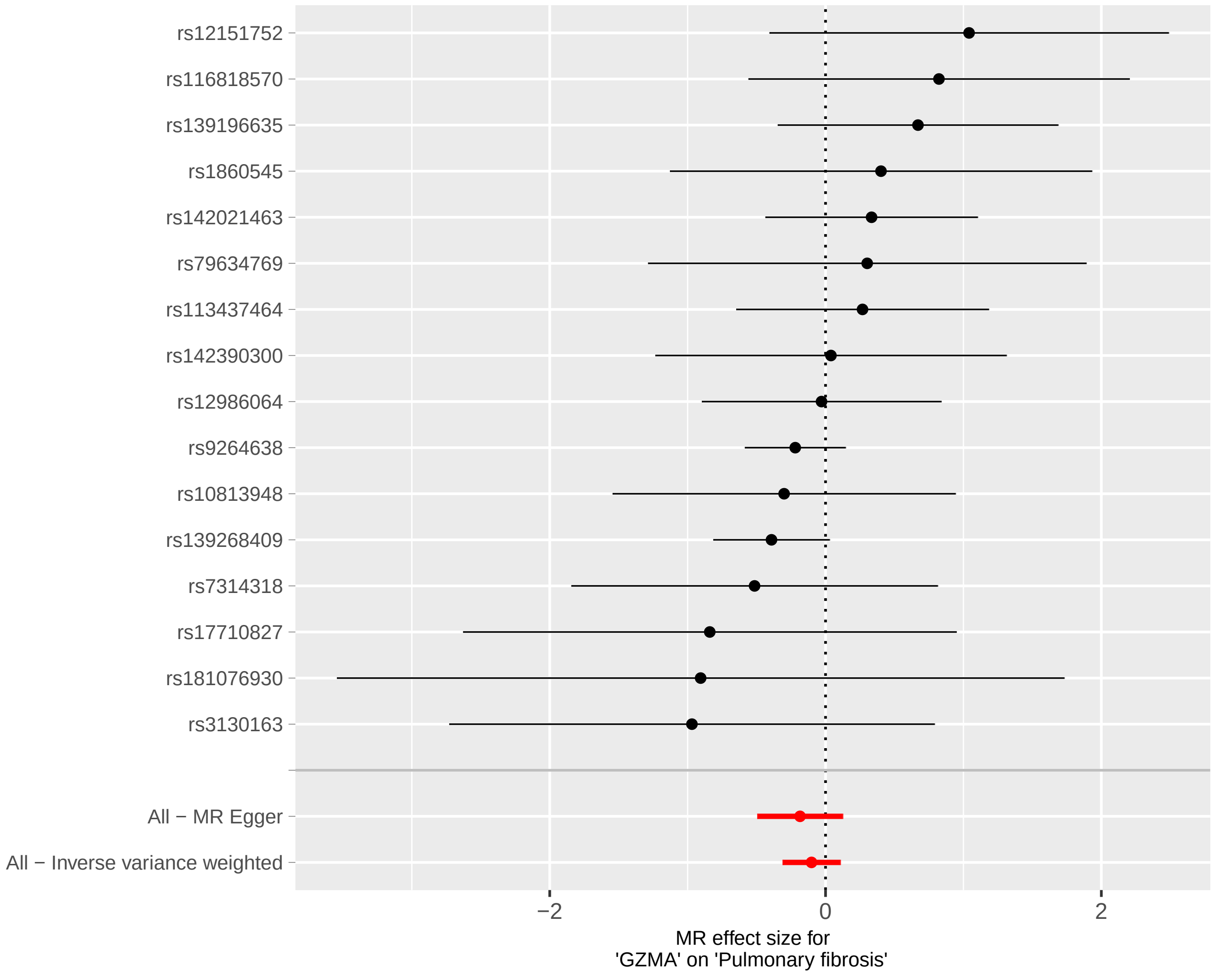

rs12151752
rs116818570
rs139196635
rs1860545
rs142021463
rs79634769
rs113437464
rs142390300
rs12986064
rs9264638
rs10813948
rs139268409
rs7314318
rs17710827
rs181076930
rs3130163
All − MR Egger
All − Inverse variance weighted
−2
0
2
MR effect size for
'GZMA' on 'Pulmonary fibrosis'

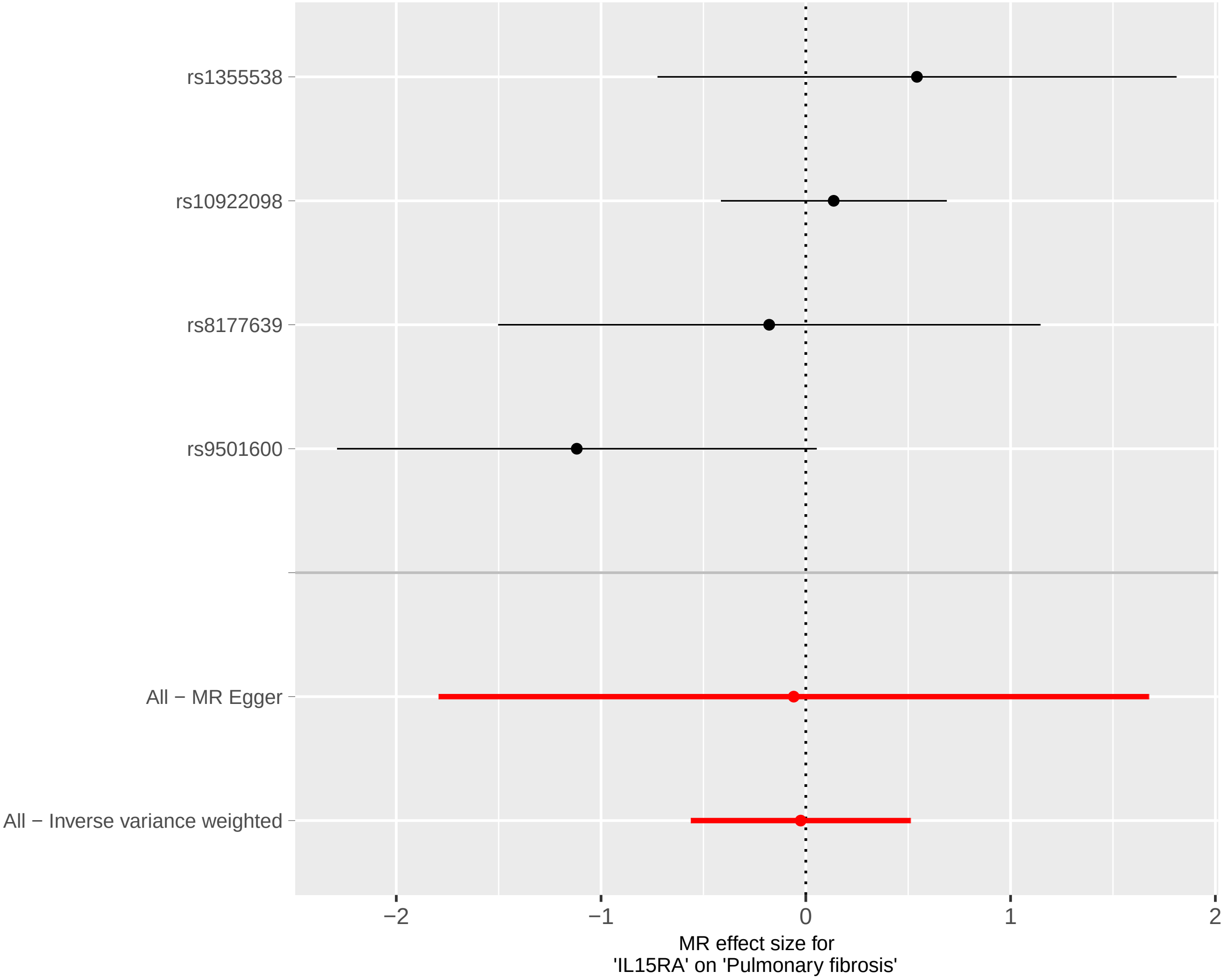

rs1355538
rs10922098
rs8177639
rs9501600
All − MR Egger
All − Inverse variance weighted
−2
−1
0
1
2
MR effect size for
'IL15RA' on 'Pulmonary fibrosis'

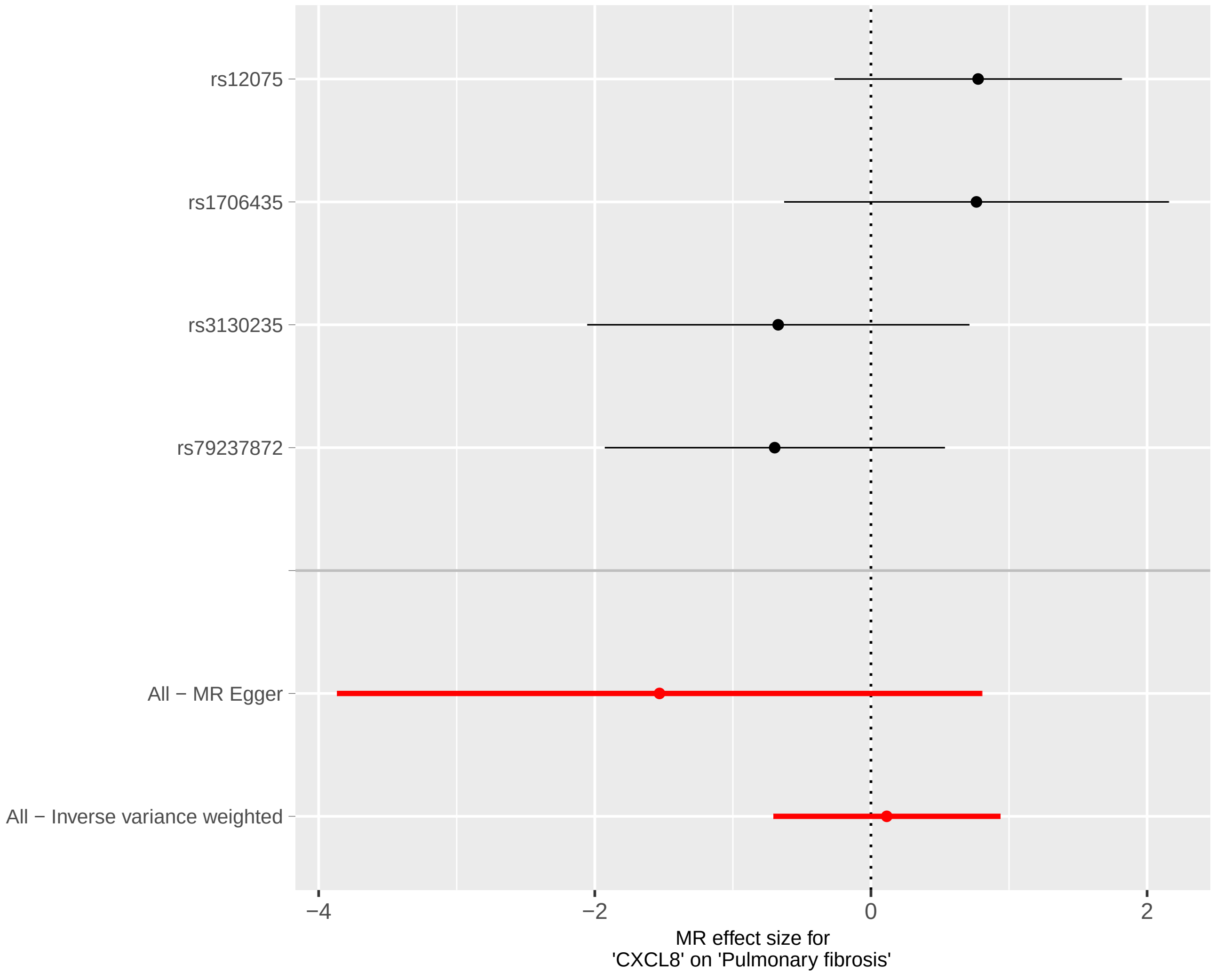
rs12075
rs1706435
rs3130235
rs79237872
All − MR Egger
All − Inverse variance weighted
−4
−2
0
2
MR effect size for
'CXCL8' on 'Pulmonary fibrosis'

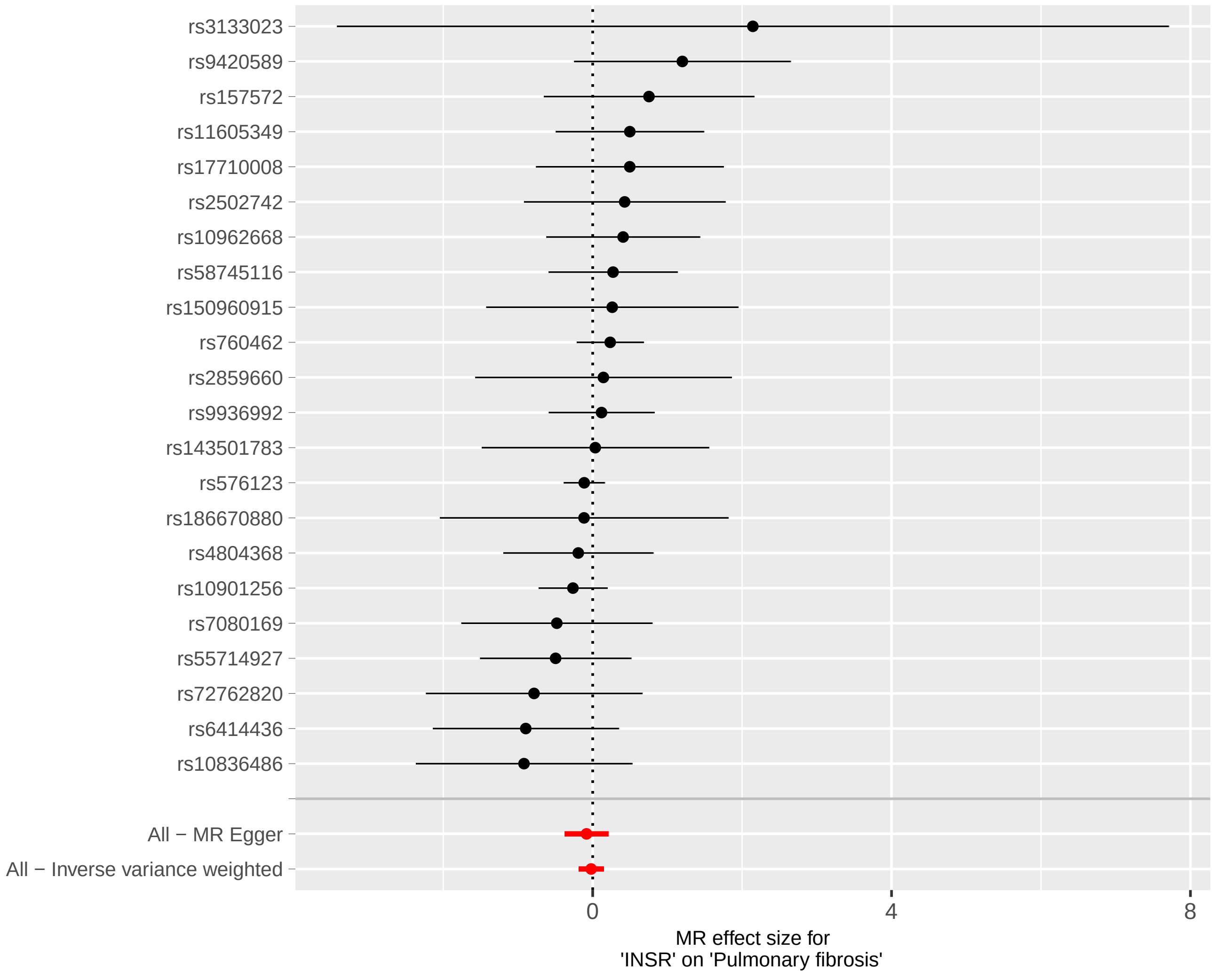

rs3133023
rs9420589
rs157572
rs11605349
rs17710008
rs2502742
rs10962668
rs58745116
rs150960915
rs760462
rs2859660
rs9936992
rs143501783
rs576123
rs186670880
rs4804368
rs10901256
rs7080169
rs55714927
rs72762820
rs6414436
rs10836486
All − MR Egger
All − Inverse variance weighted
0
4
8
MR effect size for
'INSR' on 'Pulmonary fibrosis'

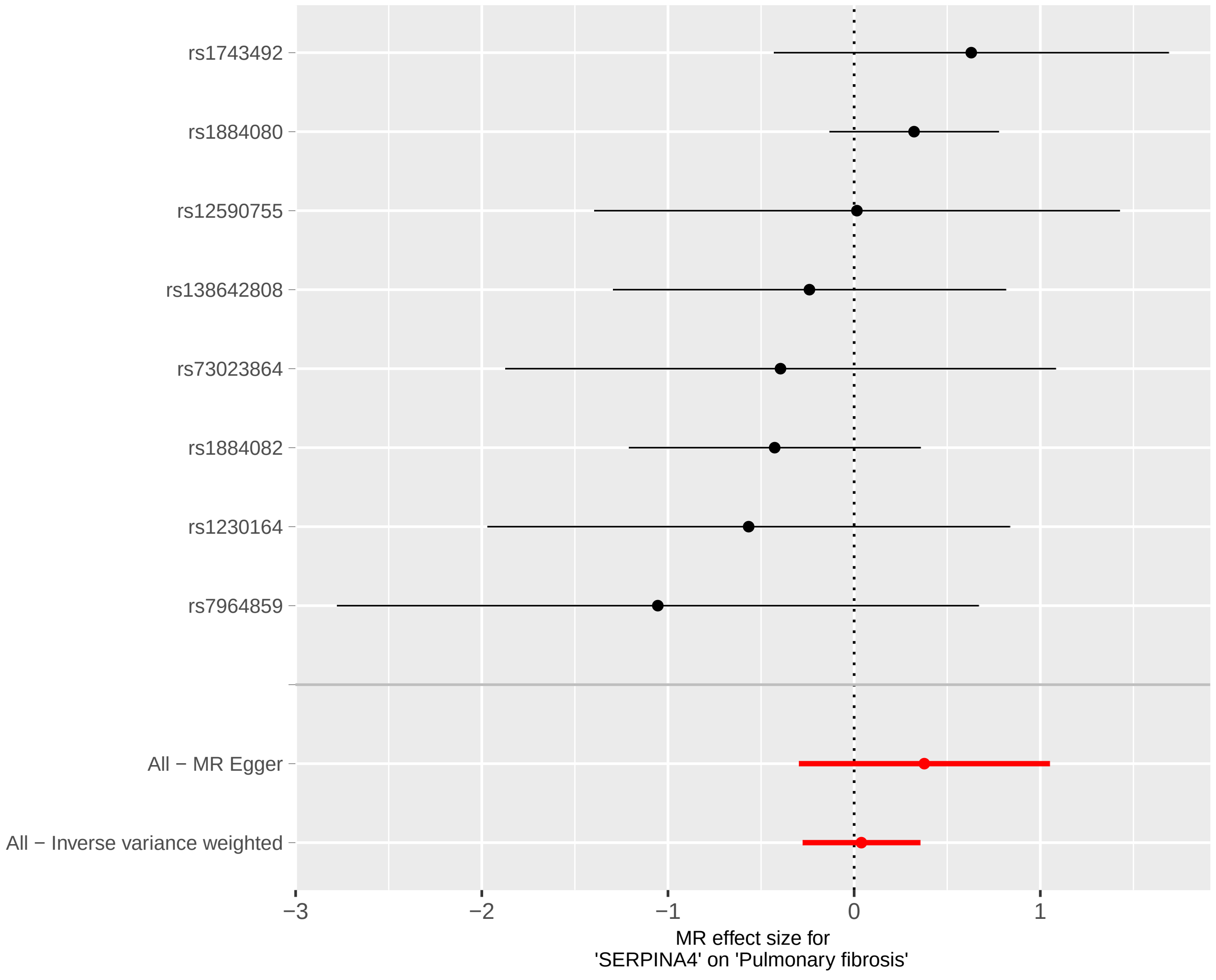
rs1743492
rs1884080
rs12590755
rs138642808
rs73023864
rs1884082
rs1230164
rs7964859
All − MR Egger
All − Inverse variance weighted
−3
−2
−1
0
1
MR effect size for
'SERPINA4' on 'Pulmonary fibrosis'

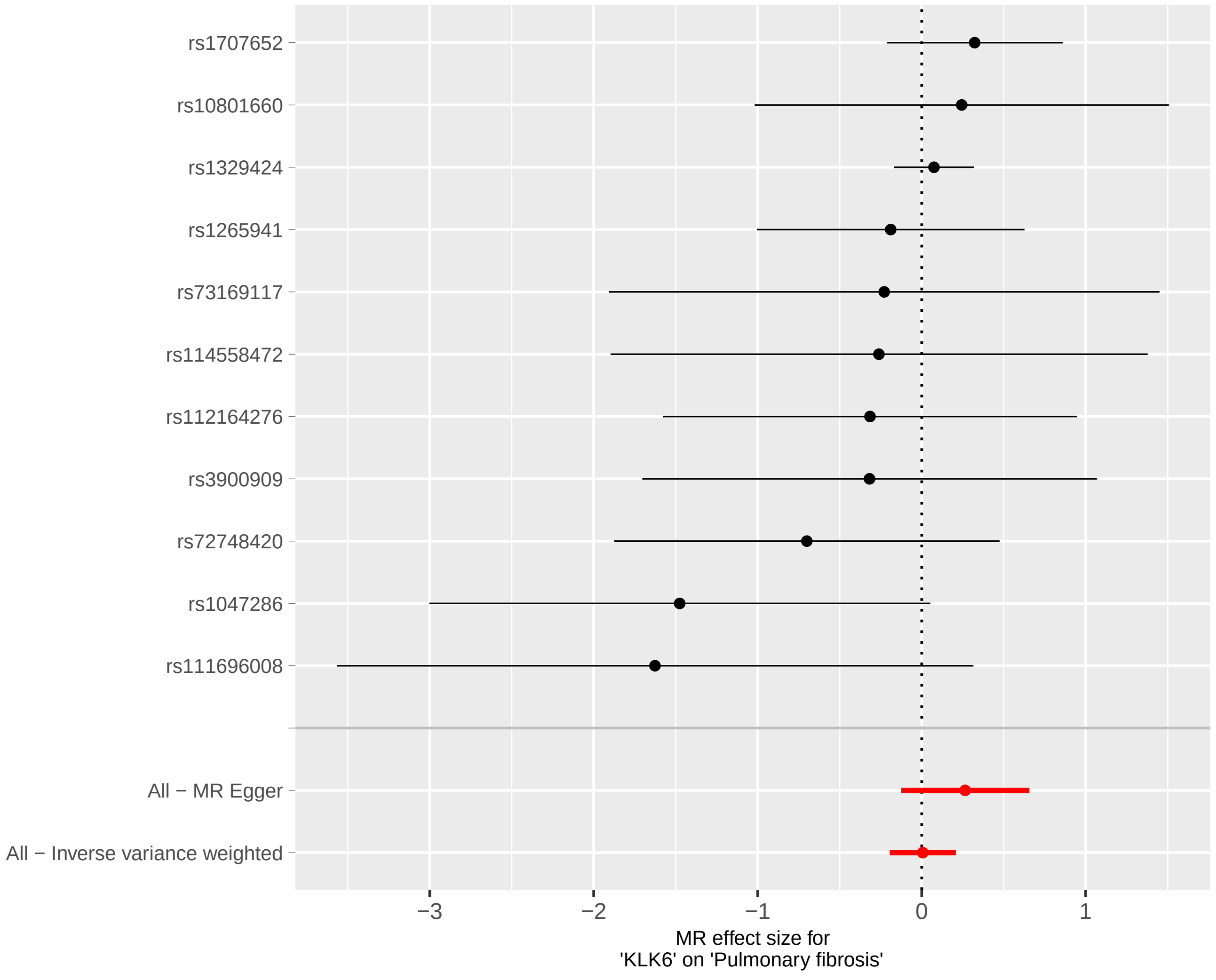

rs1707652
rs10801660
rs1329424
rs1265941
rs73169117
rs114558472
rs112164276
rs3900909
rs72748420
rs1047286
rs111696008
All − MR Egger
All − Inverse variance weighted
−3
−2
−1
0
1
MR effect size for
'KLK6' on 'Pulmonary fibrosis'

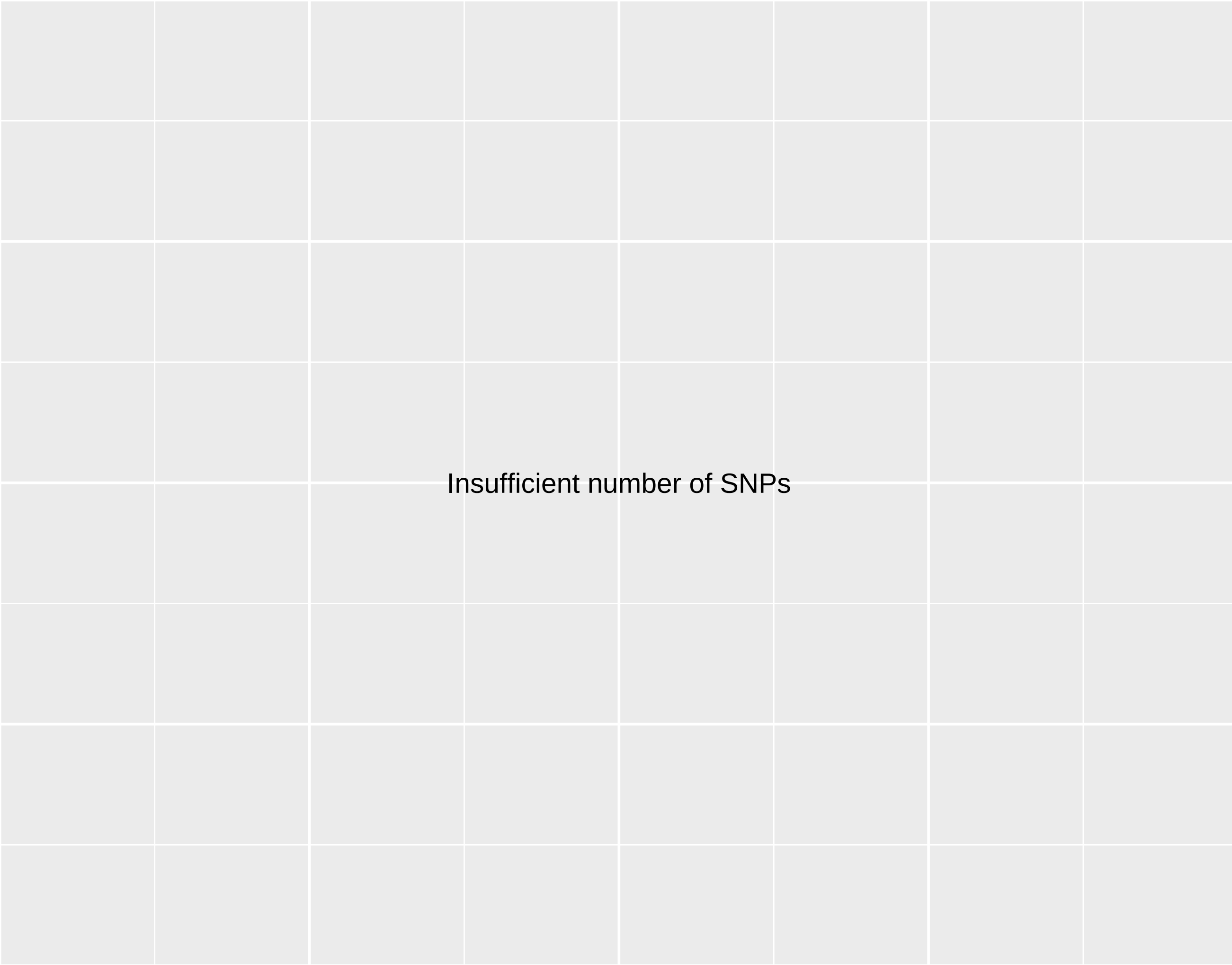
Insufficient number of SNPs

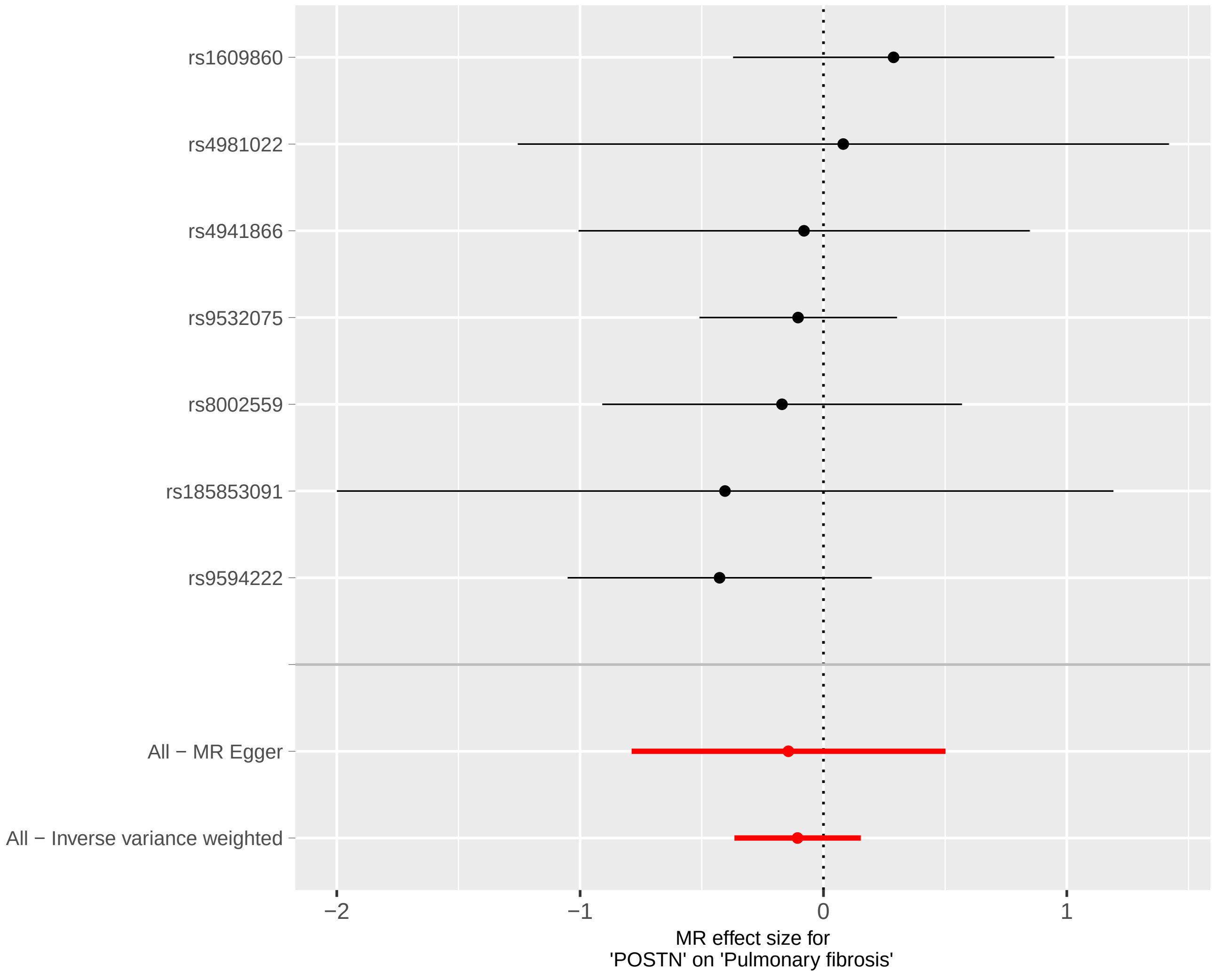

rs1609860
rs4981022
rs4941866
rs9532075
rs8002559
rs185853091
rs9594222
All − MR Egger
All − Inverse variance weighted
−2
−1
0
1
MR effect size for
'POSTN' on 'Pulmonary fibrosis'

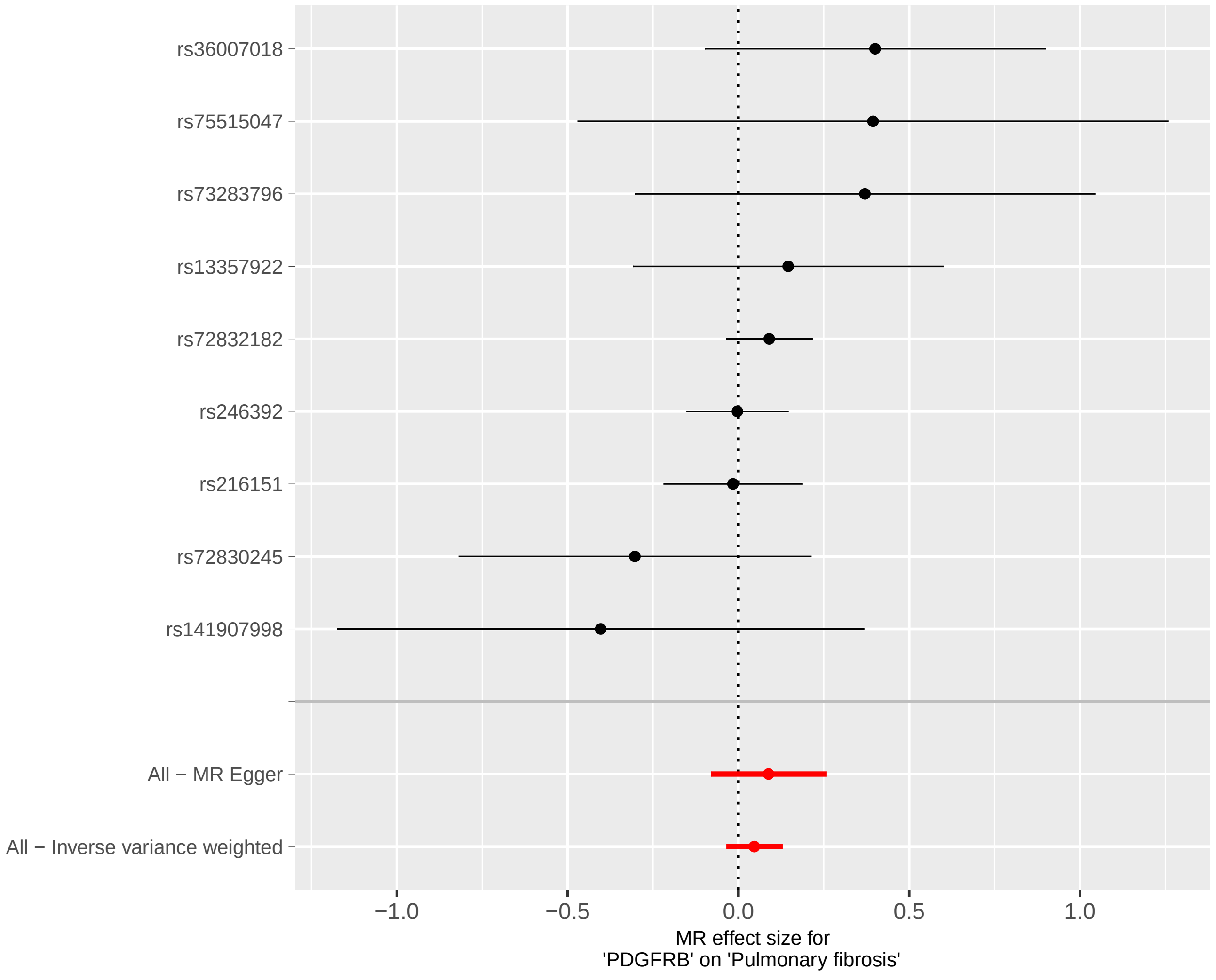

rs36007018
rs75515047
rs73283796
rs13357922
rs72832182
rs246392
rs216151
rs72830245
rs141907998
All − MR Egger
All − Inverse variance weighted
−1.0
−0.5
0.0
0.5
1.0
MR effect size for
'PDGFRB' on 'Pulmonary fibrosis'

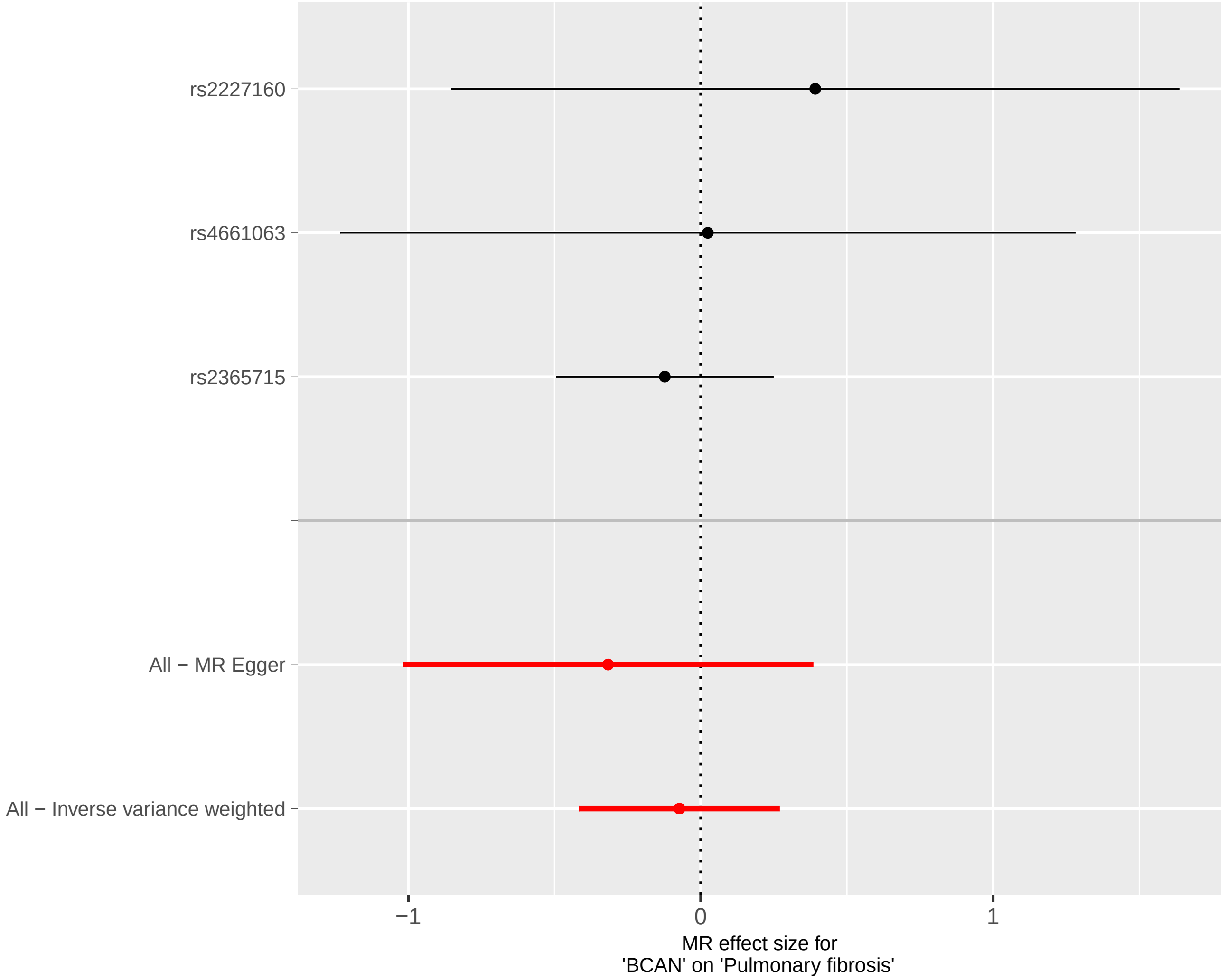
rs2227160
rs4661063
rs2365715
All − MR Egger
All − Inverse variance weighted
−1
0
1
MR effect size for
'BCAN' on 'Pulmonary fibrosis'

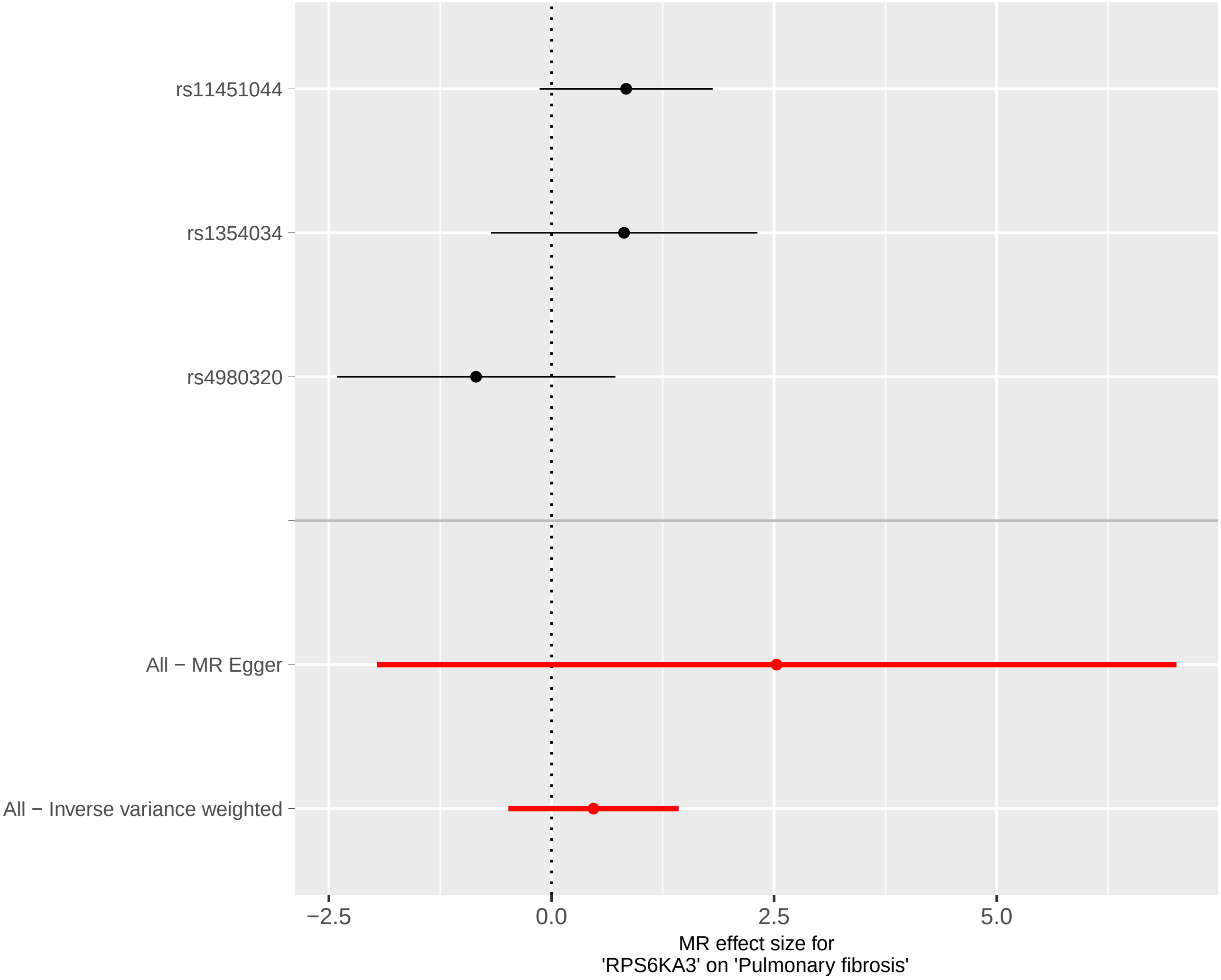

rs11451044
rs1354034
rs4980320
All − MR Egger
All − Inverse variance weighted
−2.5
0.0
2.5
5.0
MR effect size for
'RPS6KA3' on 'Pulmonary fibrosis'

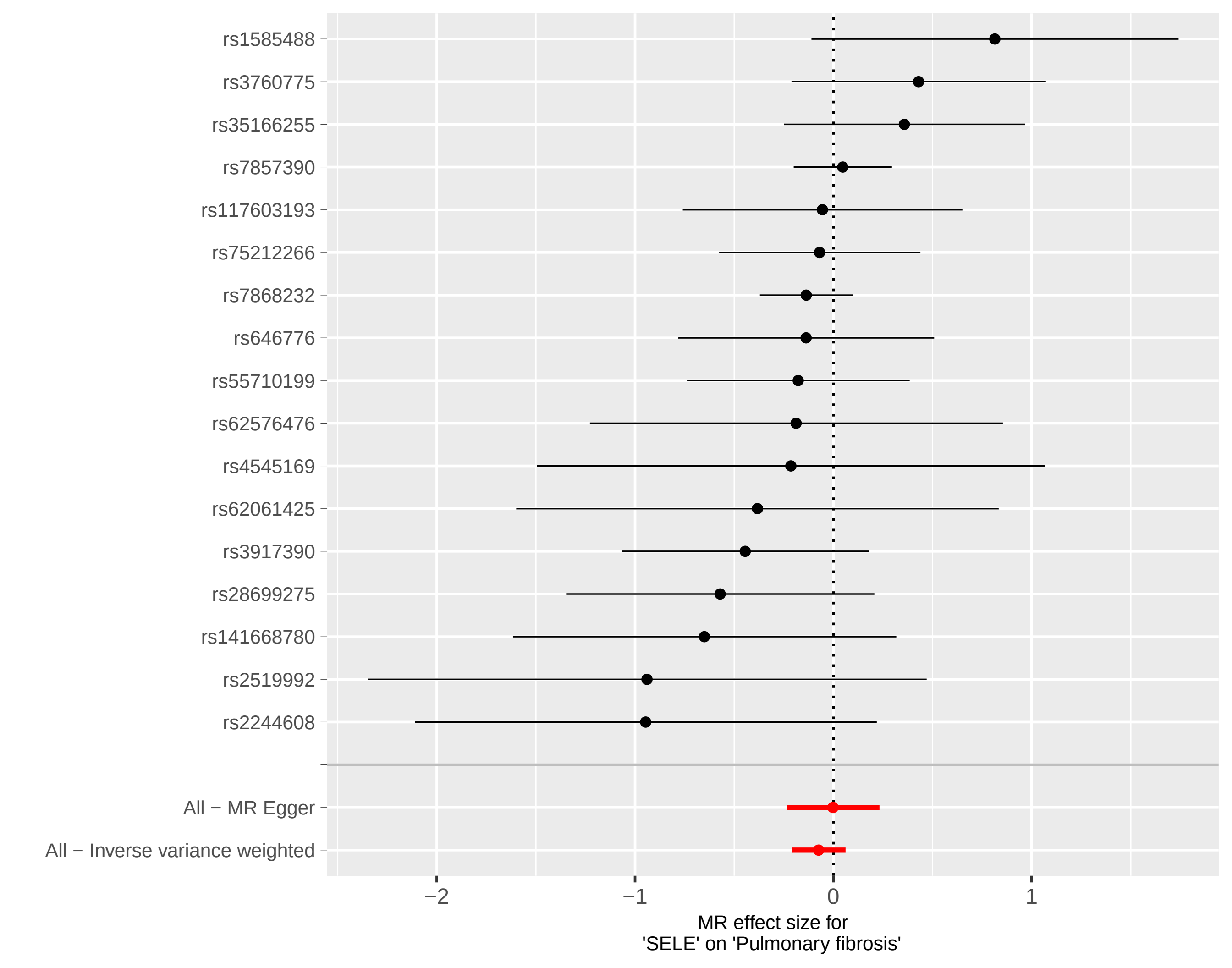
rs1585488
rs3760775
rs35166255
rs7857390
rs117603193
rs75212266
rs7868232
rs646776
rs55710199
rs62576476
rs4545169
rs62061425
rs3917390
rs28699275
rs141668780
rs2519992
rs2244608
All − MR Egger
All − Inverse variance weighted
−2
−1
0
1
MR effect size for
'SELE' on 'Pulmonary fibrosis'

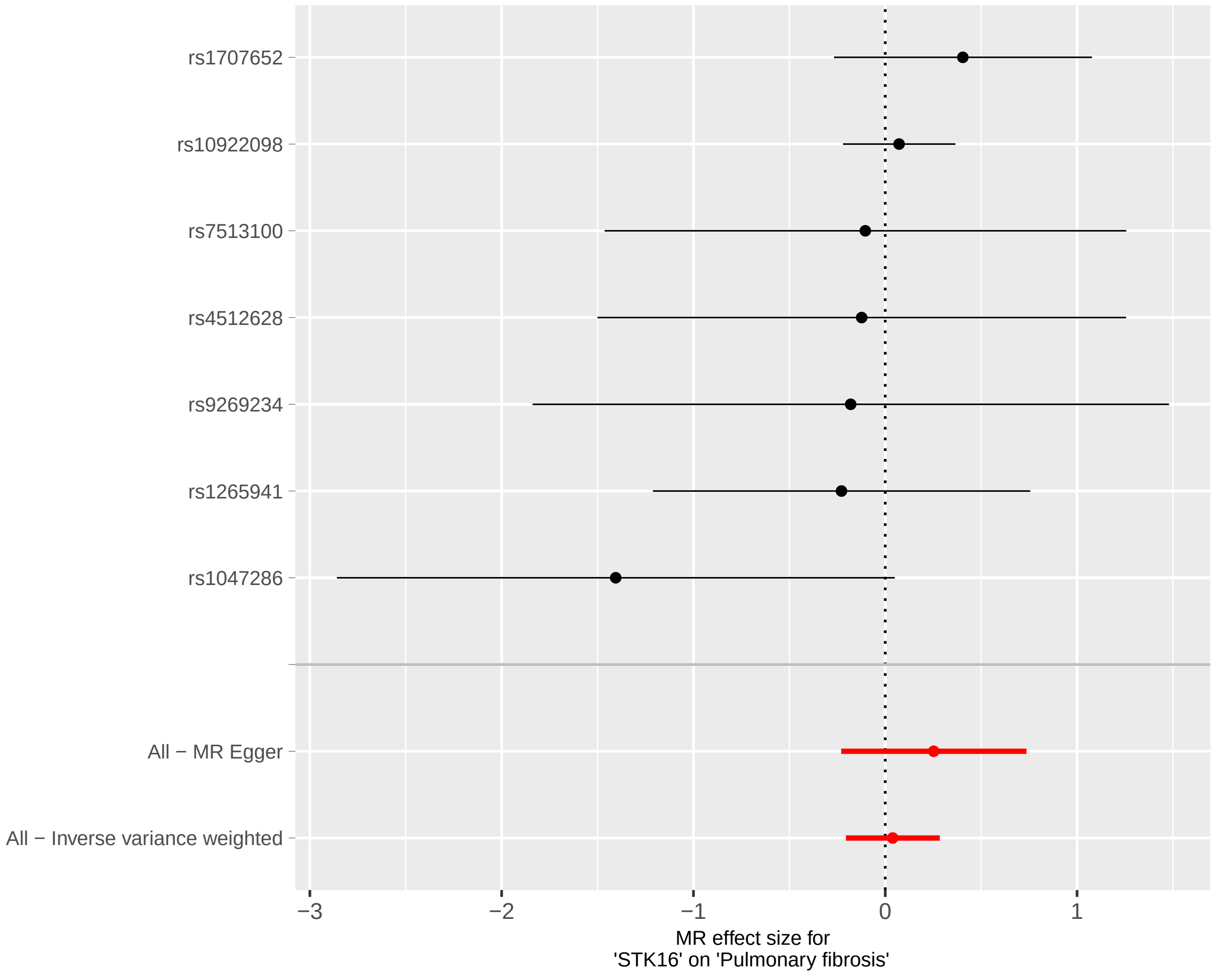
rs1707652
rs10922098
rs7513100
rs4512628
rs9269234
rs1265941
rs1047286
All − MR Egger
All − Inverse variance weighted
−3
−2
−1
0
1
MR effect size for
'STK16' on 'Pulmonary fibrosis'

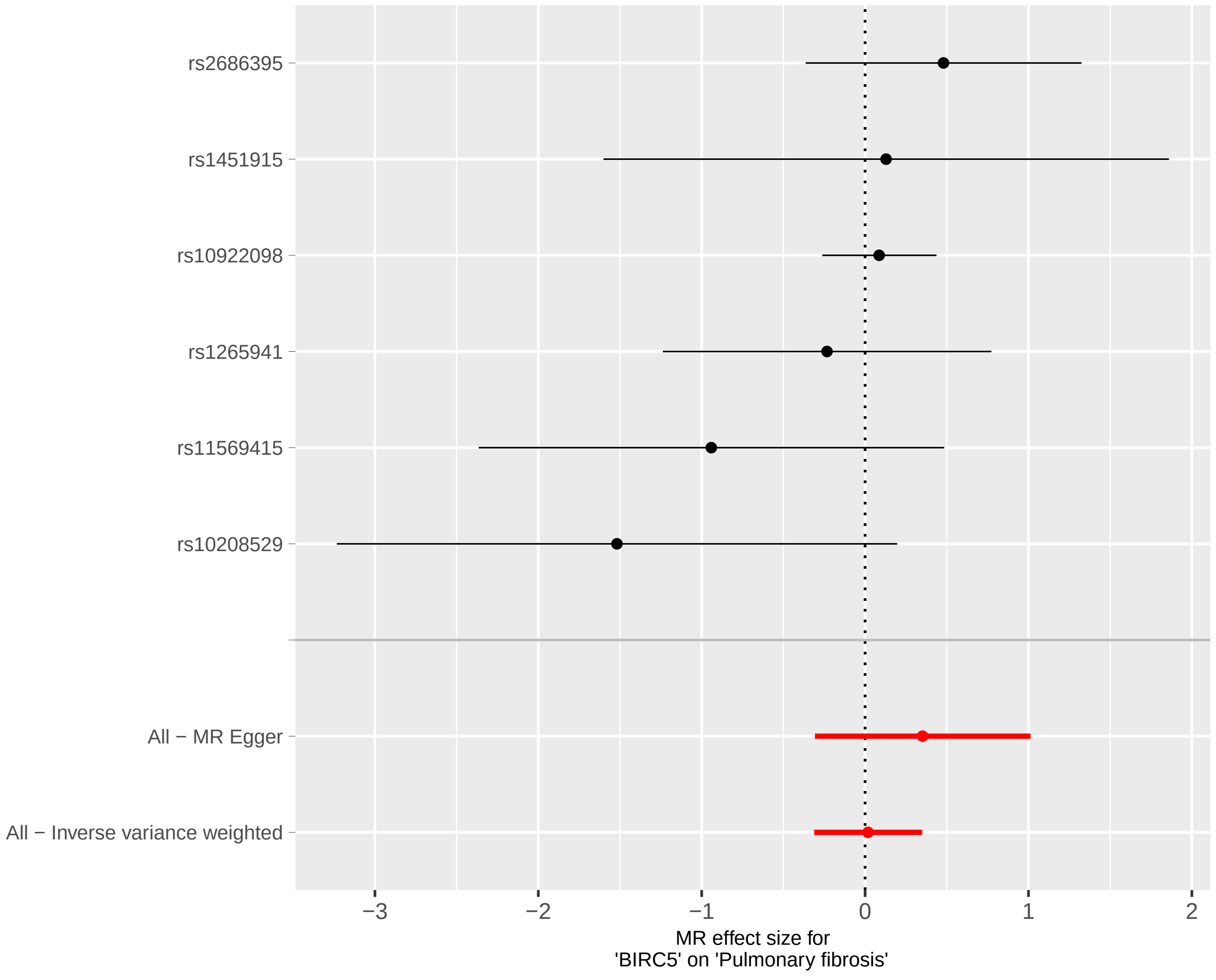
rs2686395
rs1451915
rs10922098
rs1265941
rs11569415
rs10208529
All − MR Egger
All − Inverse variance weighted
−3
−2
−1
0
1
2
MR effect size for
'BIRC5' on 'Pulmonary fibrosis'

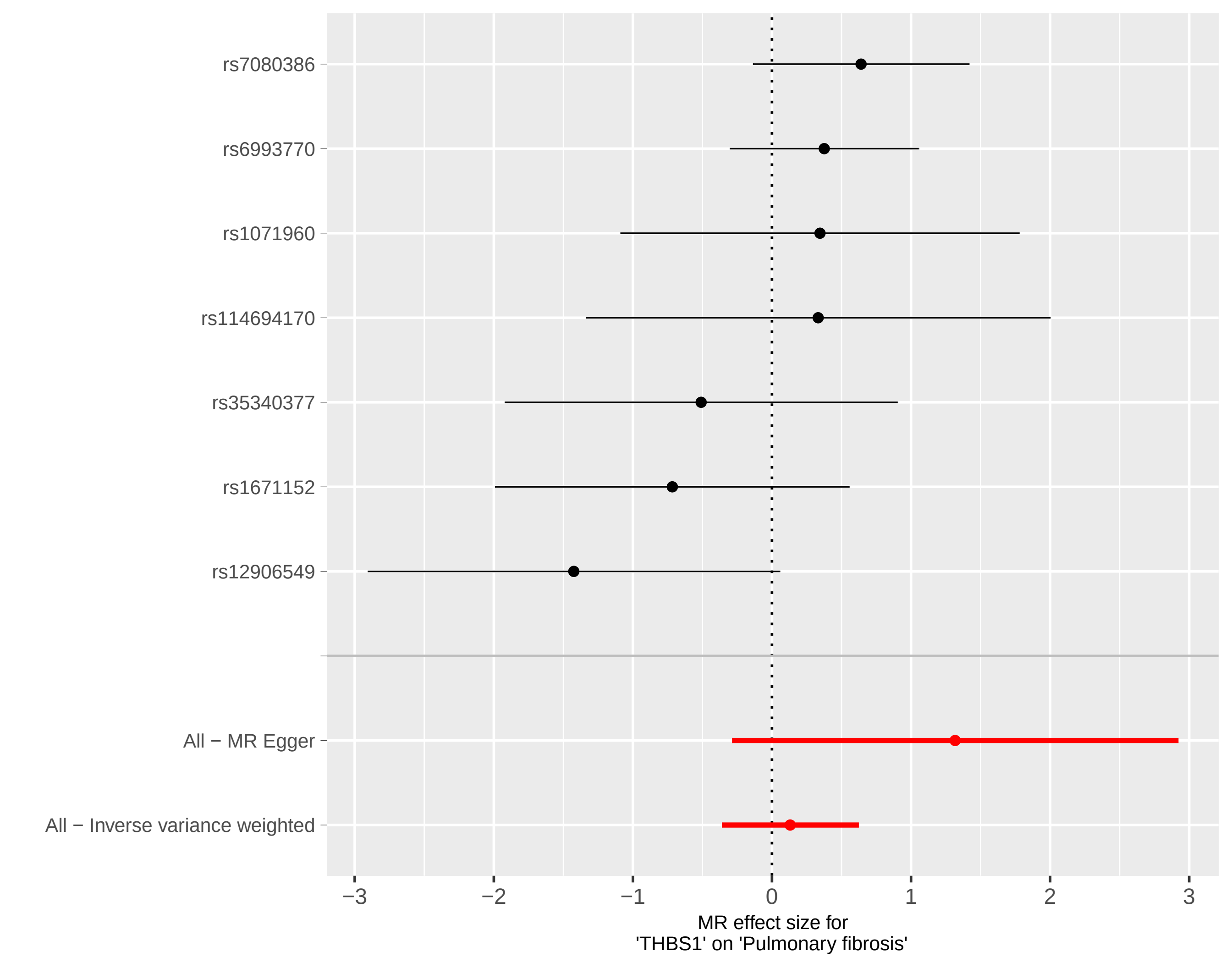

rs7080386
rs6993770
rs1071960
rs114694170
rs35340377
rs1671152
rs12906549
All − MR Egger
All − Inverse variance weighted
−3
−2
−1
0
1
2
3
MR effect size for
'THBS1' on 'Pulmonary fibrosis'

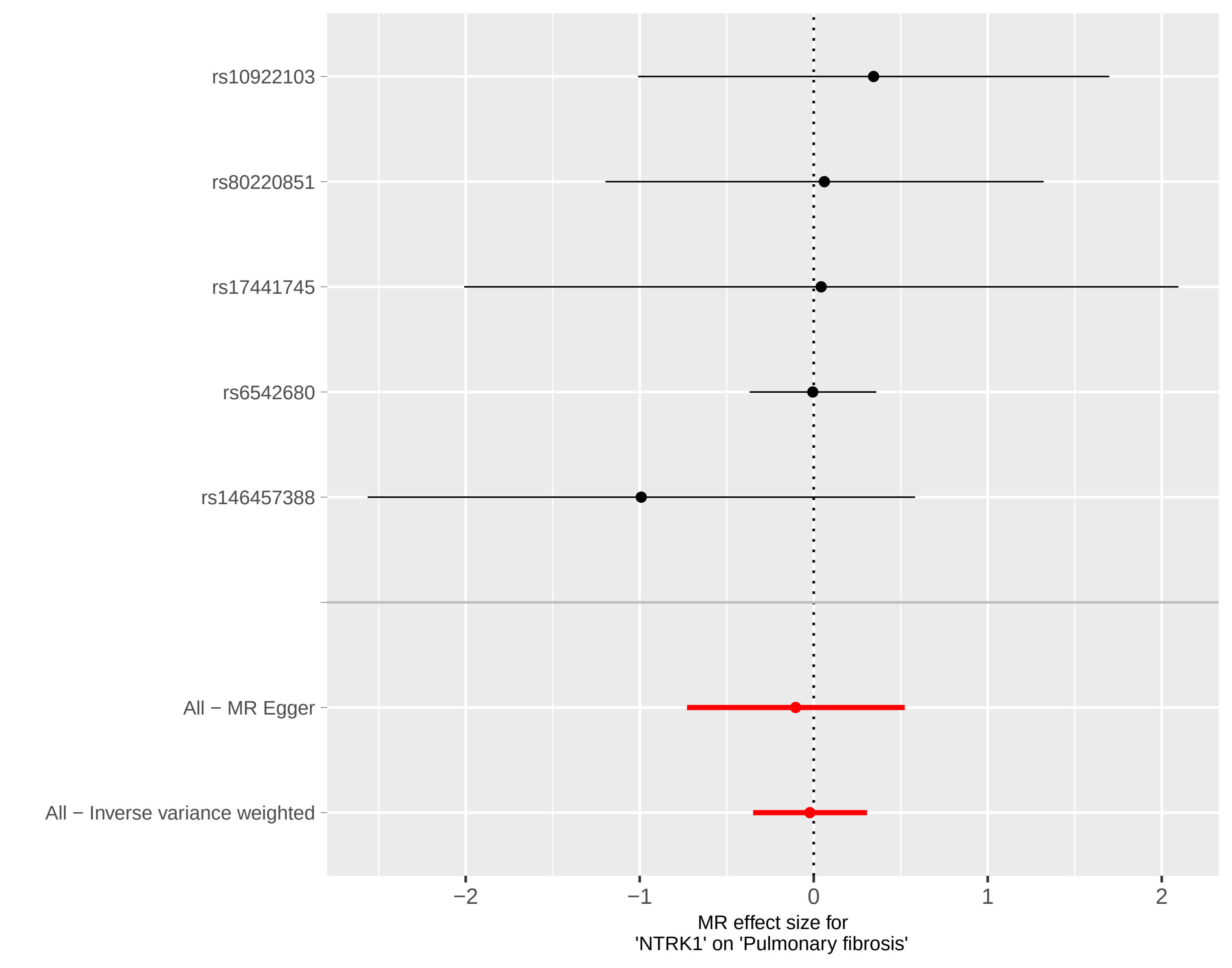

rs10922103
rs80220851
rs17441745
rs6542680
rs146457388
All − MR Egger
All − Inverse variance weighted
−2
−1
0
1
2
MR effect size for
'NTRK1' on 'Pulmonary fibrosis'

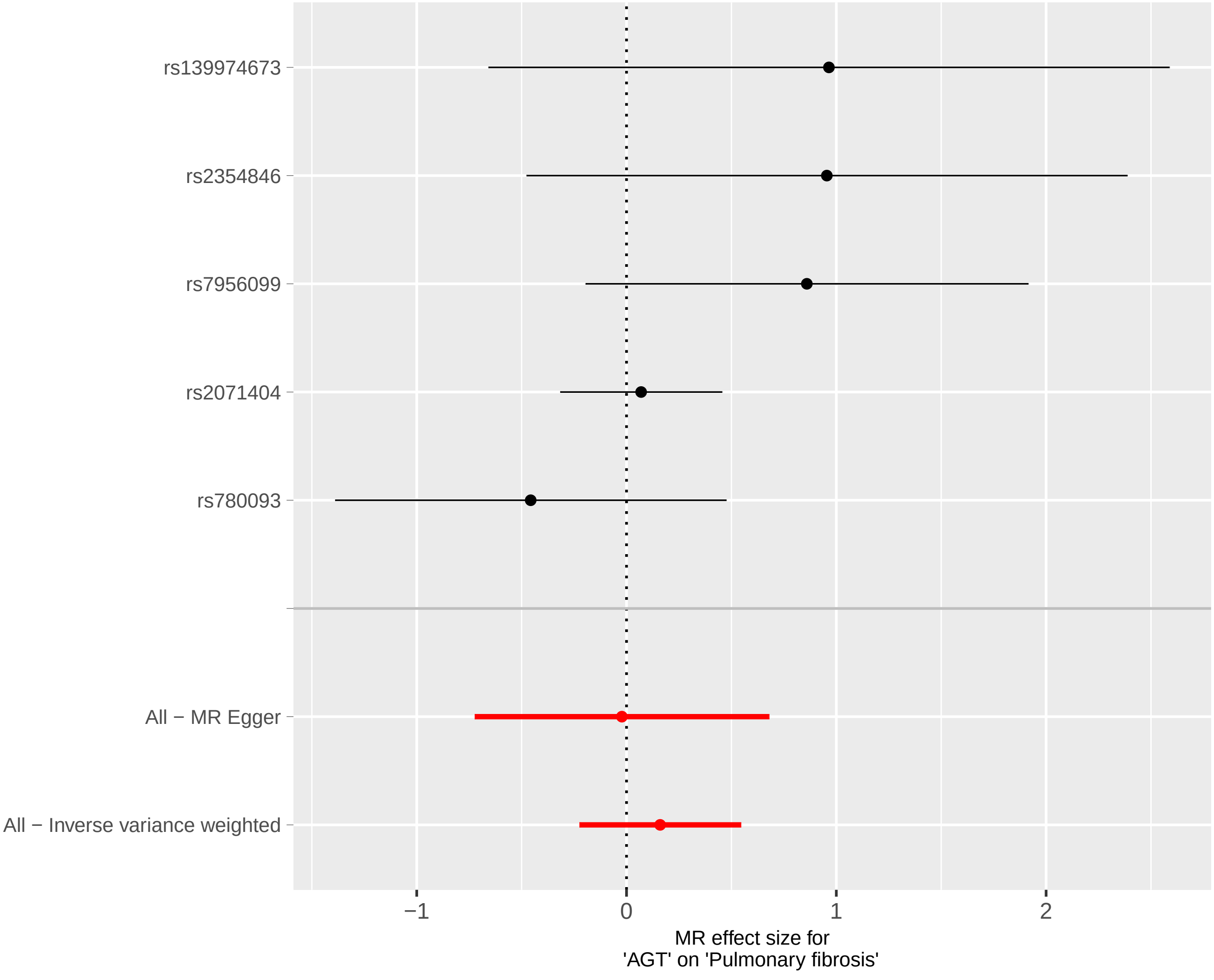

rs139974673
rs2354846
rs7956099
rs2071404
rs780093
All − MR Egger
All − Inverse variance weighted
−1
0
1
2
MR effect size for
'AGT' on 'Pulmonary fibrosis'

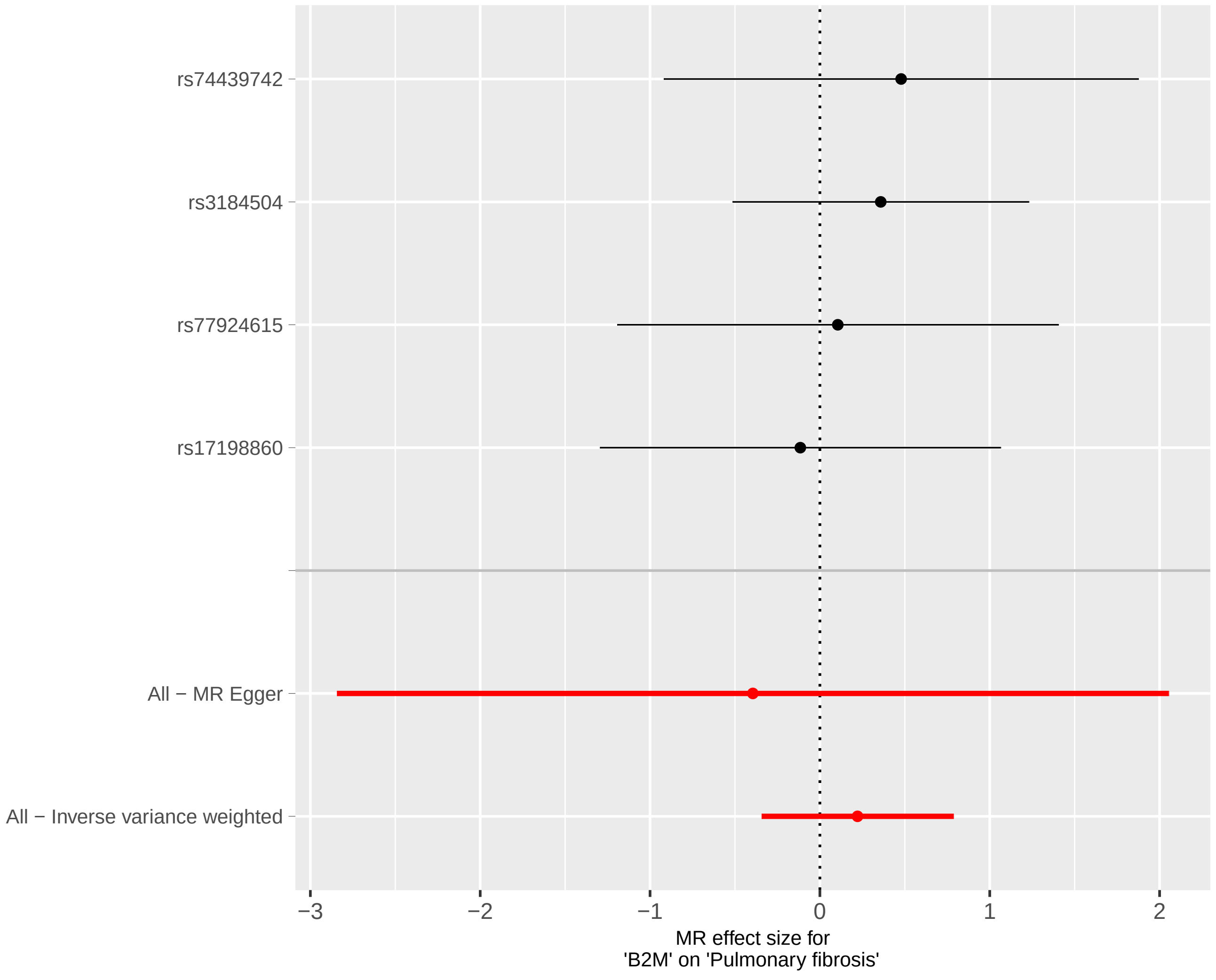
rs74439742
rs3184504
rs77924615
rs17198860
All − MR Egger
All − Inverse variance weighted
−3
−2
−1
0
1
2
MR effect size for
'B2M' on 'Pulmonary fibrosis'

Insufficient number of SNPs

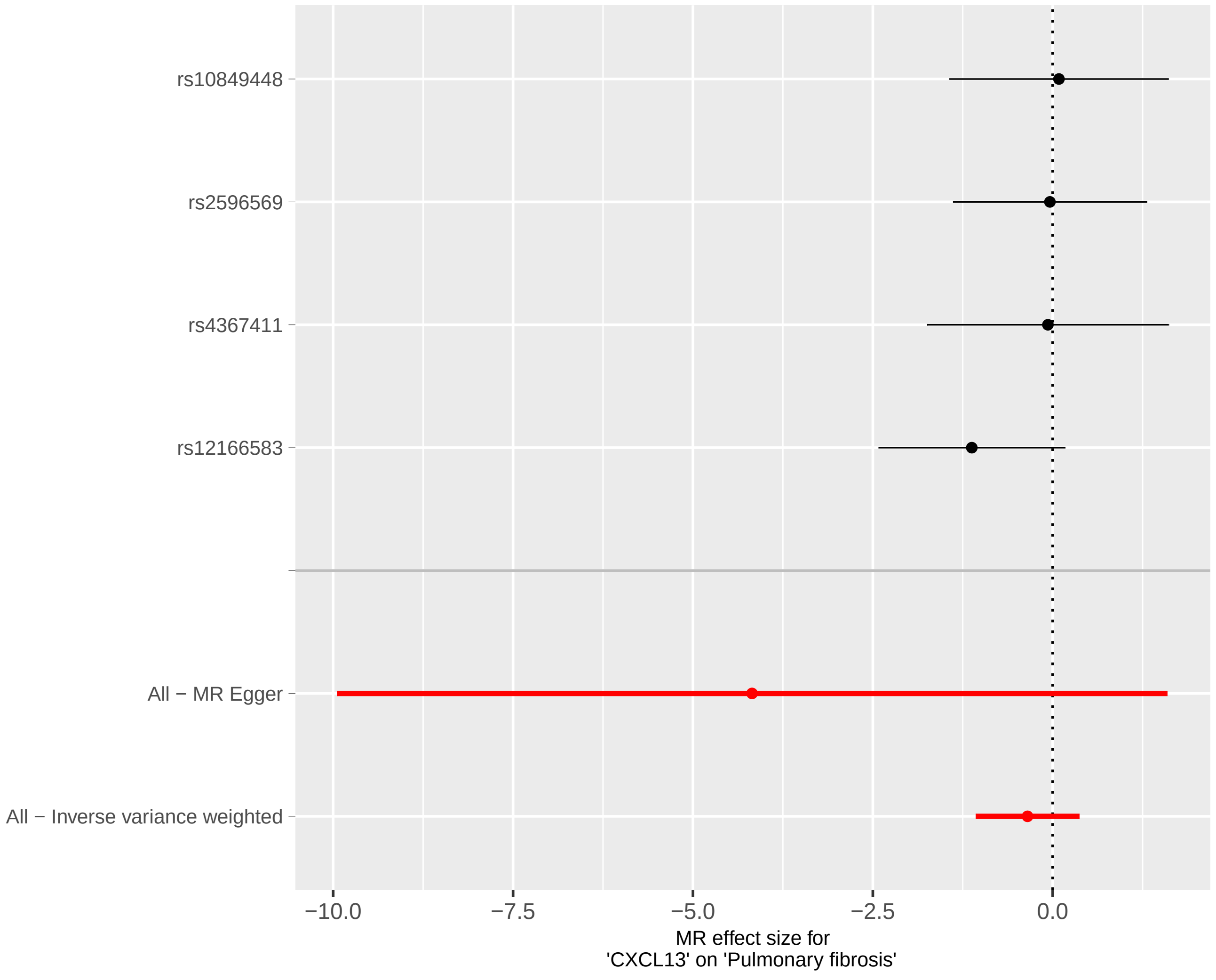

rs10849448
rs2596569
rs4367411
rs12166583
All − MR Egger
All − Inverse variance weighted
−10.0
−7.5
−5.0
−2.5
0.0
MR effect size for
'CXCL13' on 'Pulmonary fibrosis'

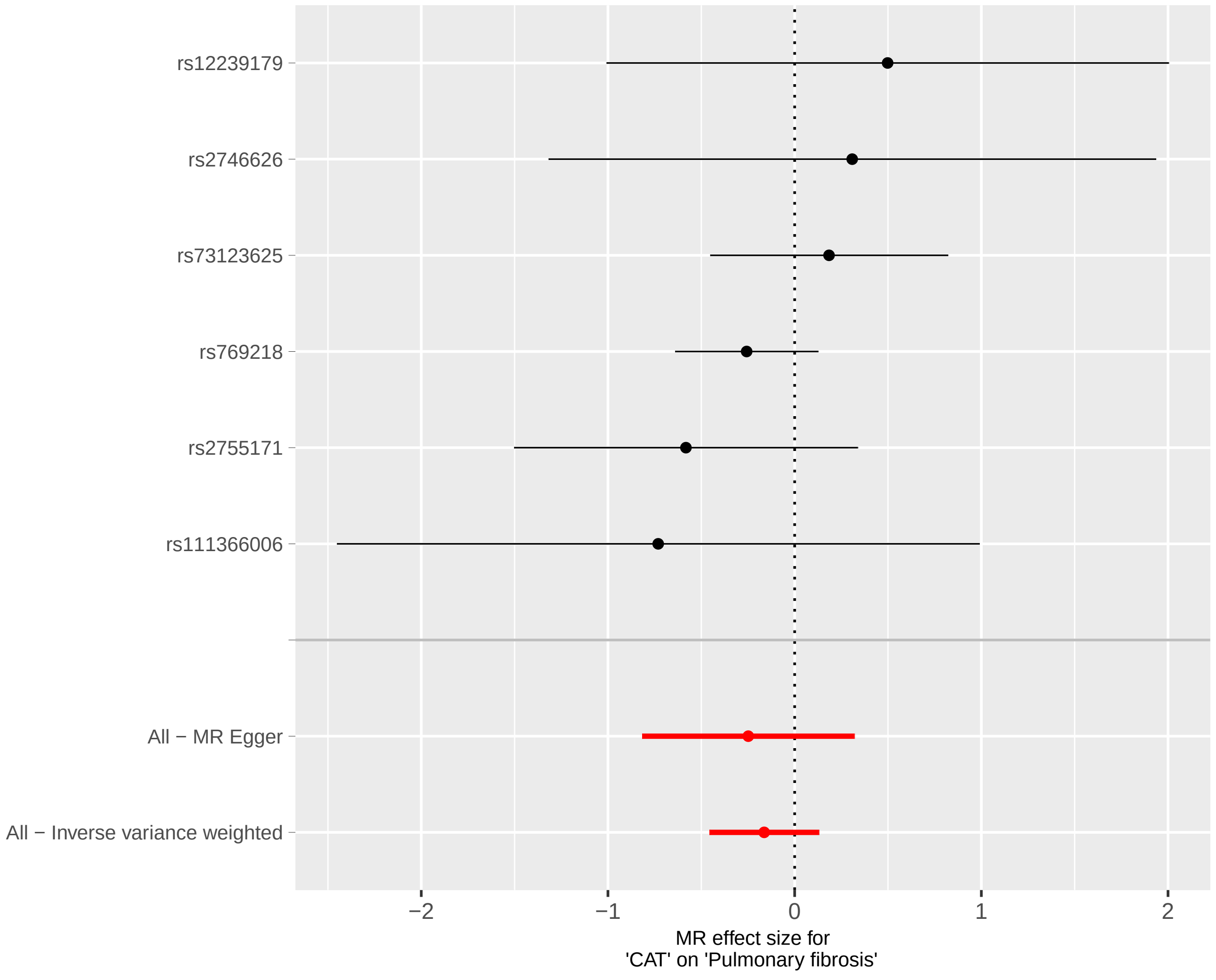
rs12239179
rs2746626
rs73123625
rs769218
rs2755171
rs111366006
All − MR Egger
All − Inverse variance weighted
−2
−1
0
1
2
MR effect size for
'CAT' on 'Pulmonary fibrosis'

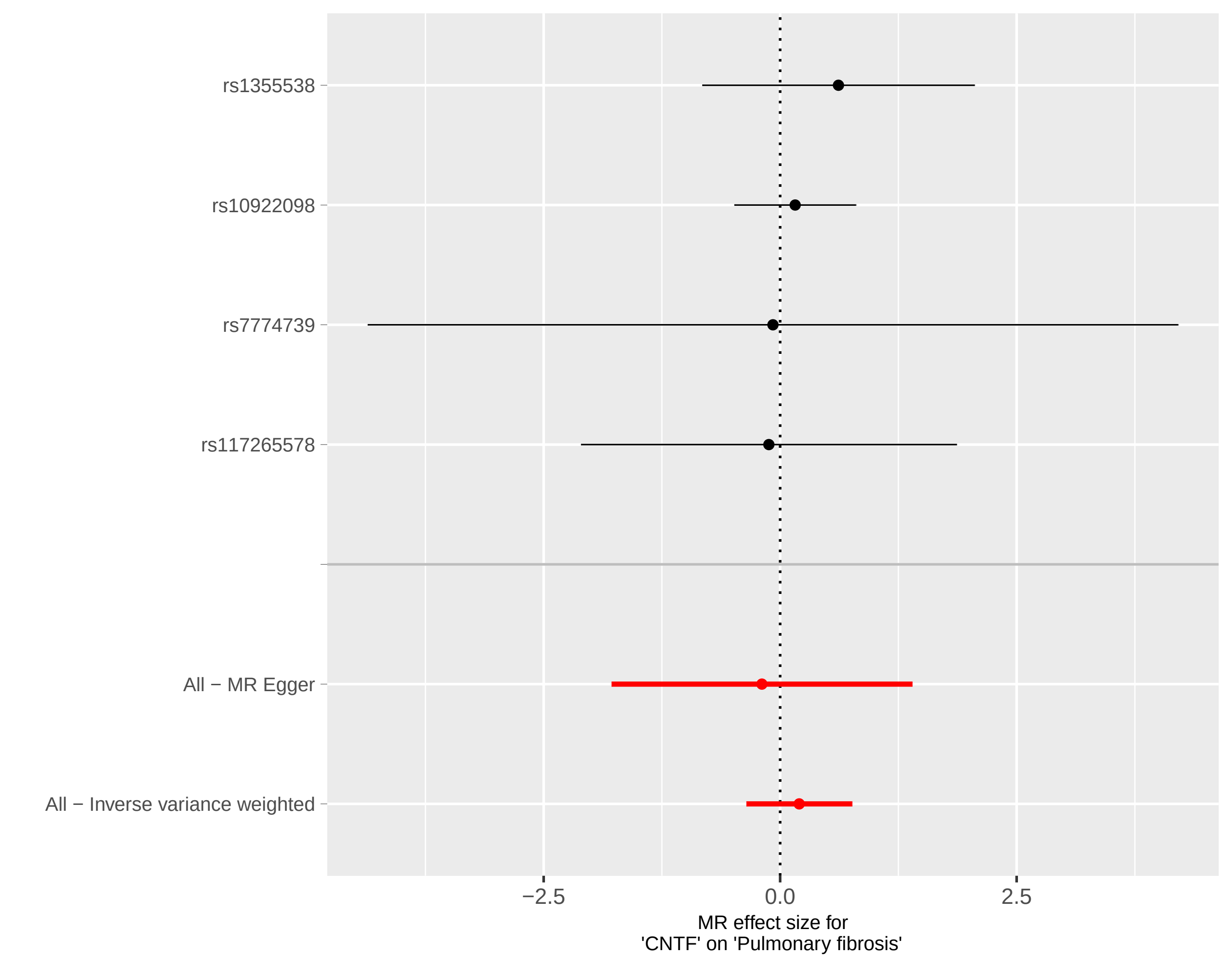
rs1355538
rs10922098
rs7774739
rs117265578
All − MR Egger
All − Inverse variance weighted
−2.5
0.0
2.5
MR effect size for
'CNTF' on 'Pulmonary fibrosis'

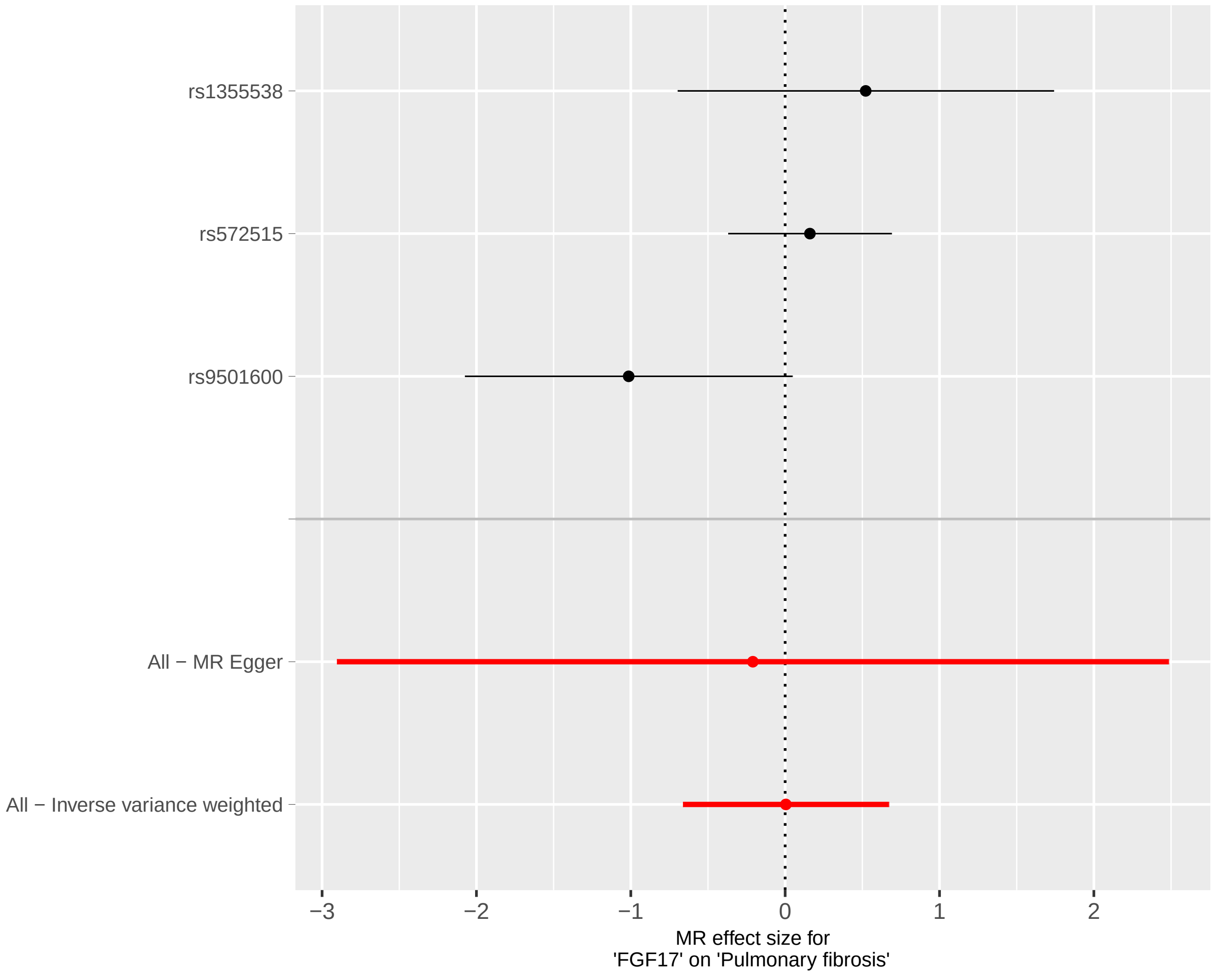

rs1355538
rs572515
rs9501600
All − MR Egger
All − Inverse variance weighted
−3
−2
−1
0
1
2
MR effect size for
'FGF17' on 'Pulmonary fibrosis'

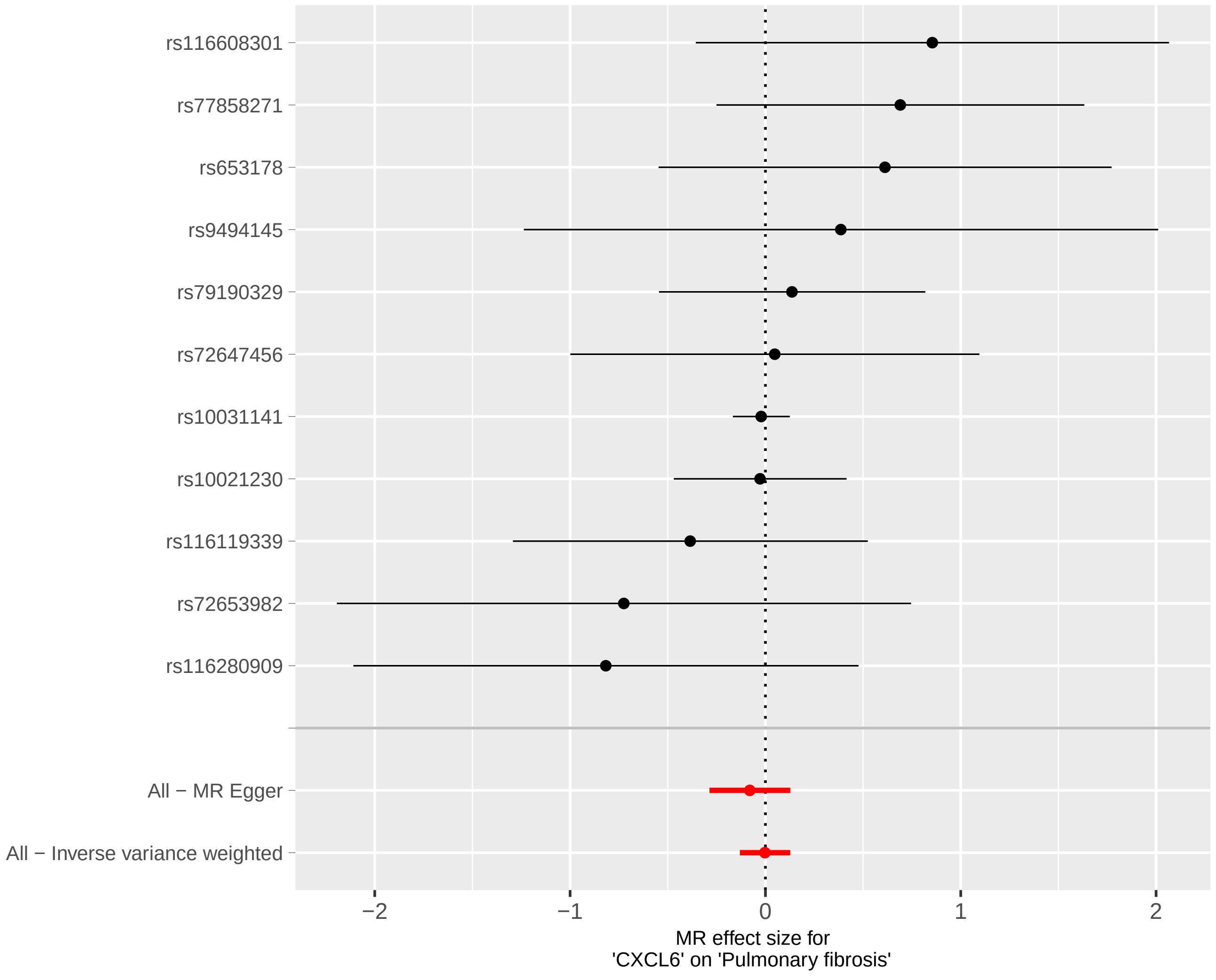
rs116608301
rs77858271
rs653178
rs9494145
rs79190329
rs72647456
rs10031141
rs10021230
rs116119339
rs72653982
rs116280909
All − MR Egger
All − Inverse variance weighted
−2
−1
0
1
2
MR effect size for
'CXCL6' on 'Pulmonary fibrosis'

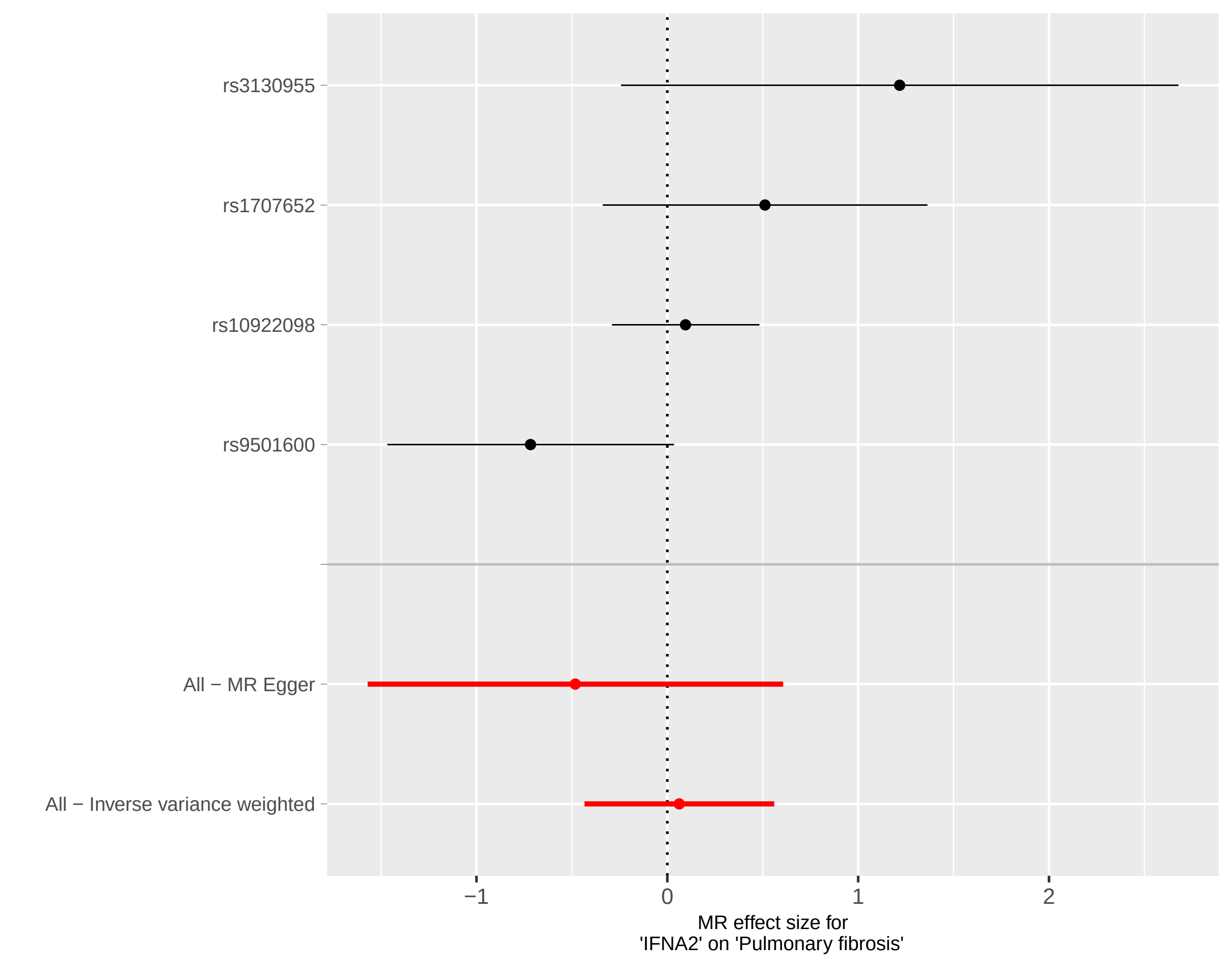
rs3130955
rs1707652
rs10922098
rs9501600
All − MR Egger
All − Inverse variance weighted
−1
0
1
2
MR effect size for
'IFNA2' on 'Pulmonary fibrosis'

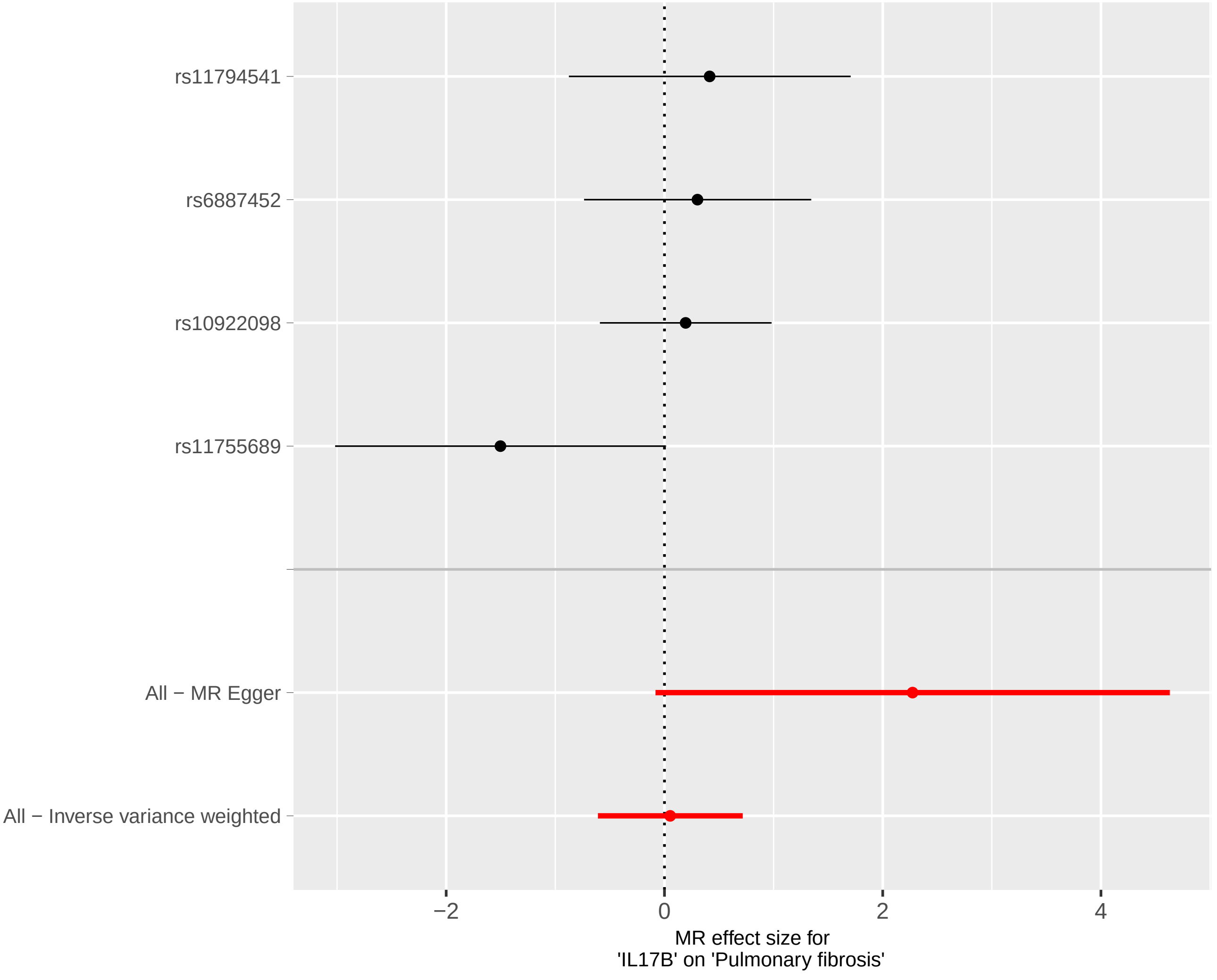

rs11794541
rs6887452
rs10922098
rs11755689
All − MR Egger
All − Inverse variance weighted
−2
0
2
4
MR effect size for
'IL17B' on 'Pulmonary fibrosis'

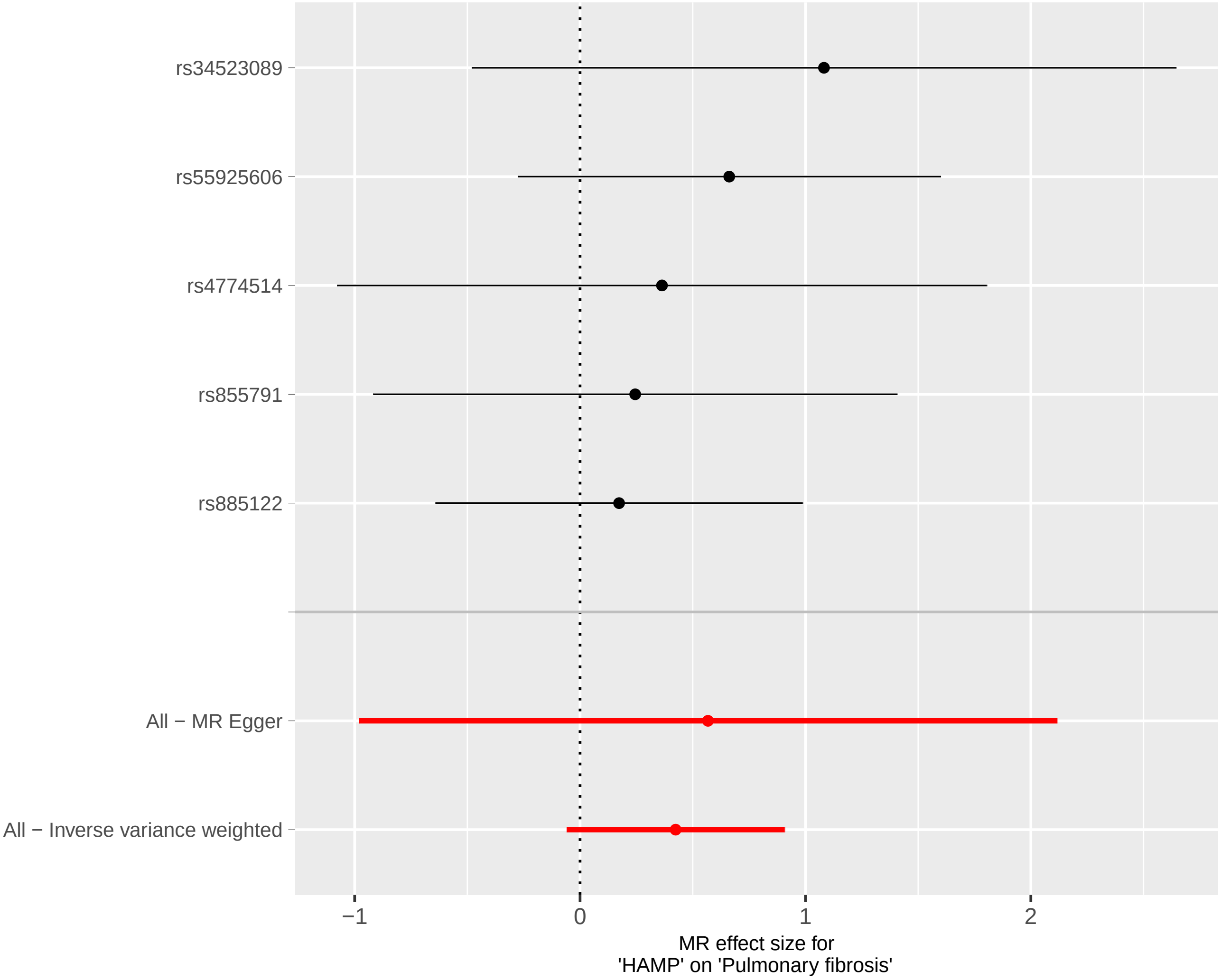
rs34523089
rs55925606
rs4774514
rs855791
rs885122
All − MR Egger
All − Inverse variance weighted
−1
0
1
2
MR effect size for
'HAMP' on 'Pulmonary fibrosis'

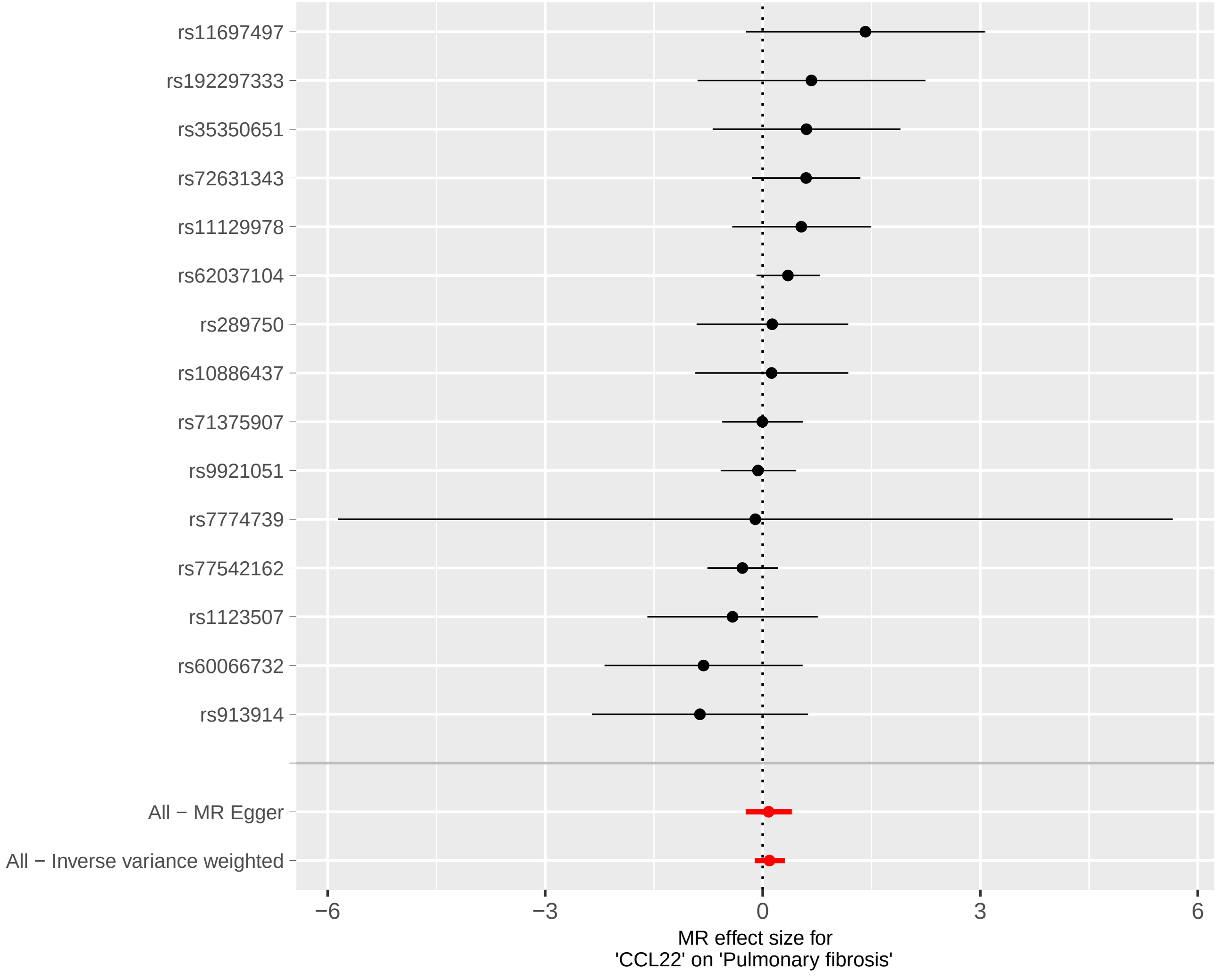

rs11697497
rs192297333
rs35350651
rs72631343
rs11129978
rs62037104
rs289750
rs10886437
rs71375907
rs9921051
rs7774739
rs77542162
rs1123507
rs60066732
rs913914
All − MR Egger
All − Inverse variance weighted
−6
−3
0
3
6
MR effect size for
'CCL22' on 'Pulmonary fibrosis'

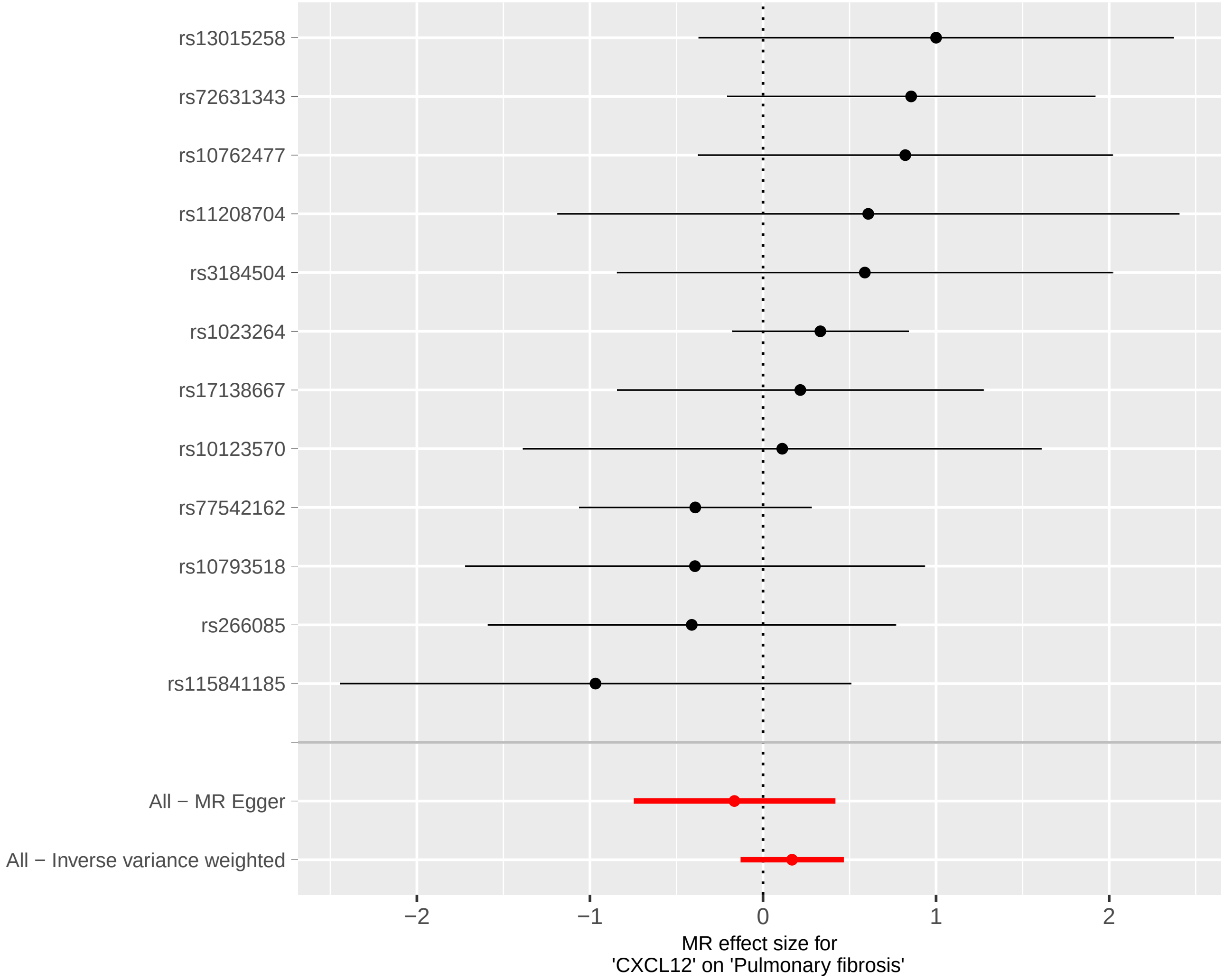

rs13015258
rs72631343
rs10762477
rs11208704
rs3184504
rs1023264
rs17138667
rs10123570
rs77542162
rs10793518
rs266085
rs115841185
All − MR Egger
All − Inverse variance weighted
−2
−1
0
1
2
MR effect size for
'CXCL12' on 'Pulmonary fibrosis'

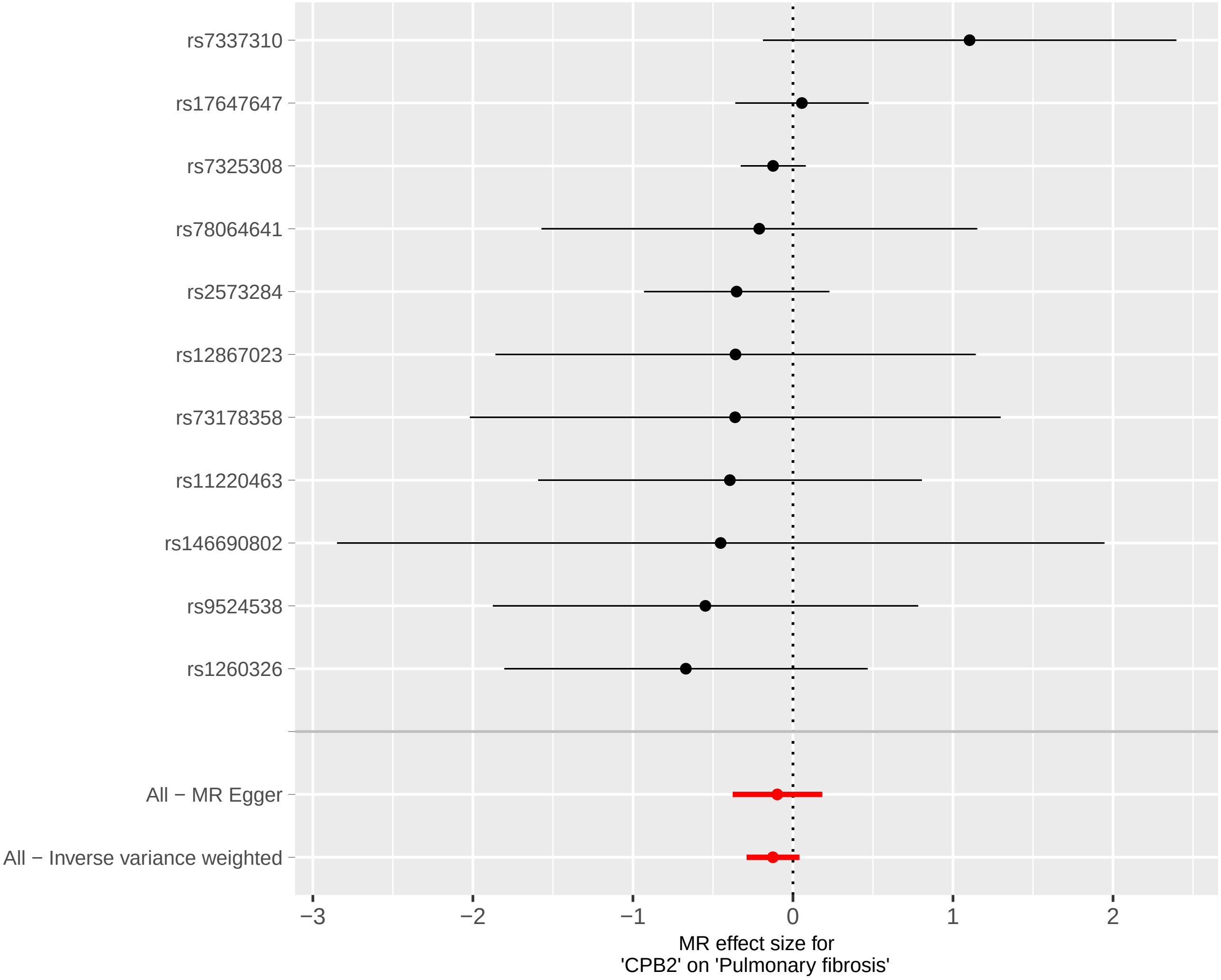

rs7337310
rs17647647
rs7325308
rs78064641
rs2573284
rs12867023
rs73178358
rs11220463
rs146690802
rs9524538
rs1260326
All − MR Egger
All − Inverse variance weighted
−3
−2
−1
0
1
2
MR effect size for
'CPB2' on 'Pulmonary fibrosis'

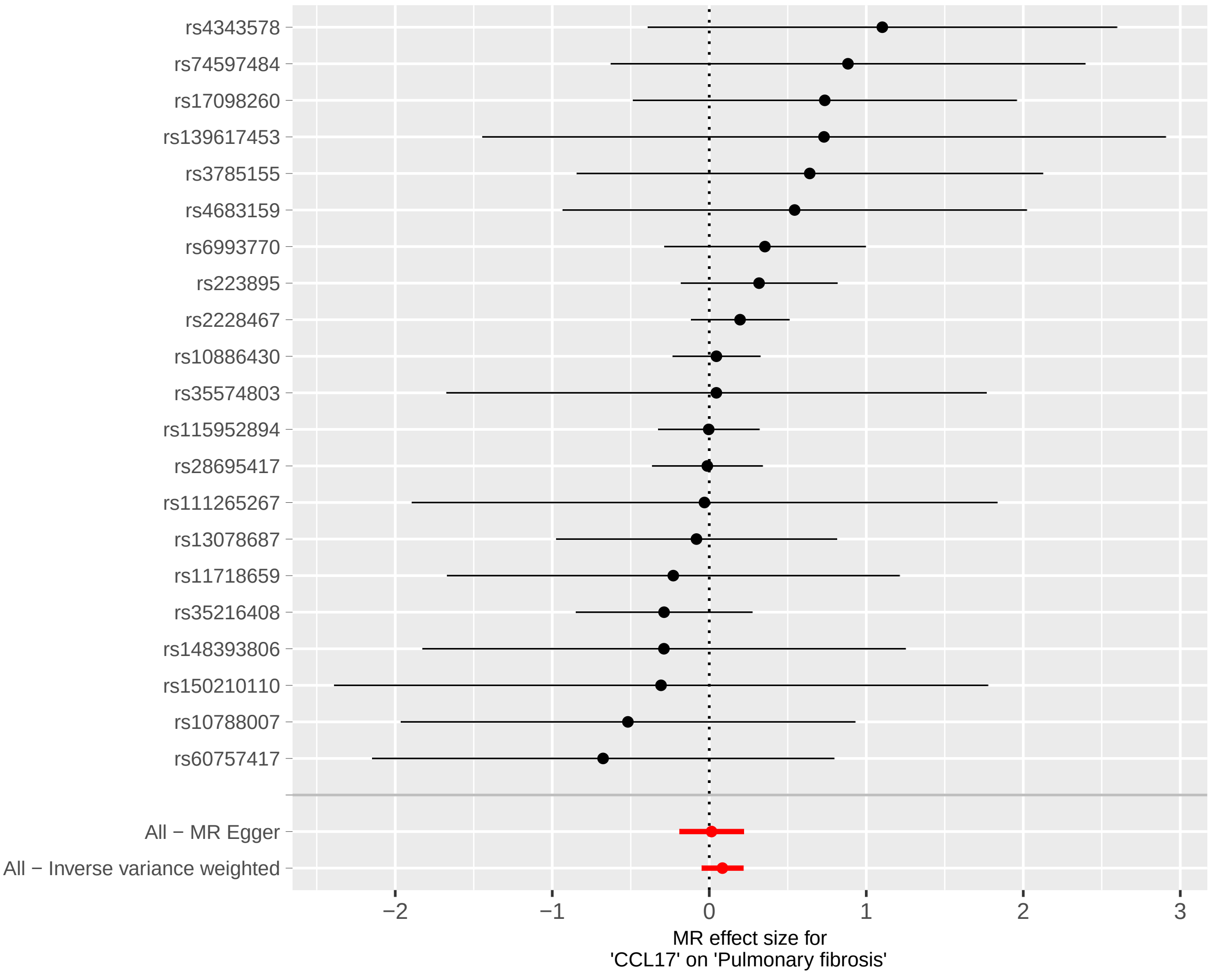

rs4343578
rs74597484
rs17098260
rs139617453
rs3785155
rs4683159
rs6993770
rs223895
rs2228467
rs10886430
rs35574803
rs115952894
rs28695417
rs111265267
rs13078687
rs11718659
rs35216408
rs148393806
rs150210110
rs10788007
rs60757417
All − MR Egger
All − Inverse variance weighted
−2
−1
0
1
2
3
MR effect size for
'CCL17' on 'Pulmonary fibrosis'

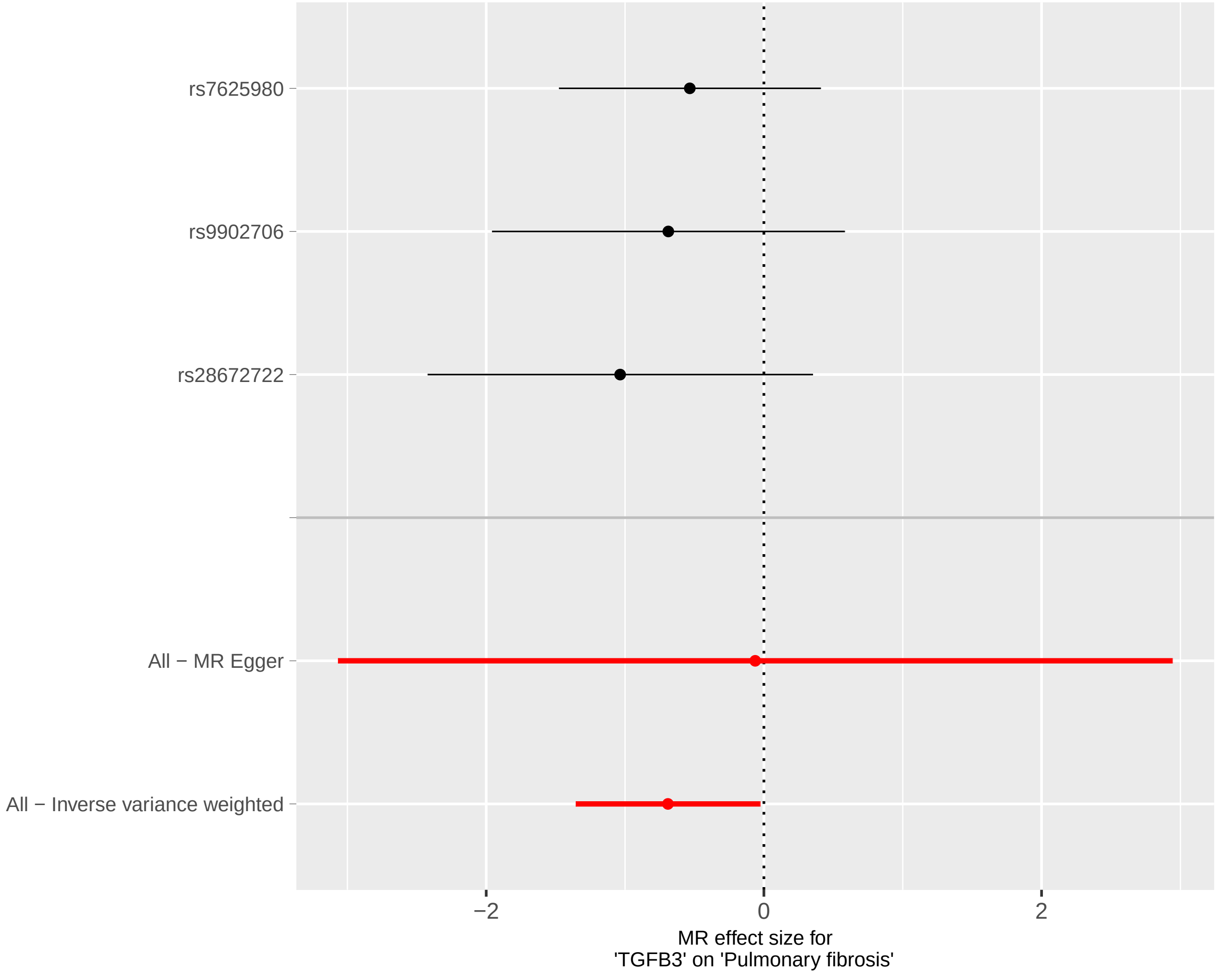

rs7625980
rs9902706
rs28672722
All − MR Egger
All − Inverse variance weighted
−2
0
2
MR effect size for
'TGFB3' on 'Pulmonary fibrosis'

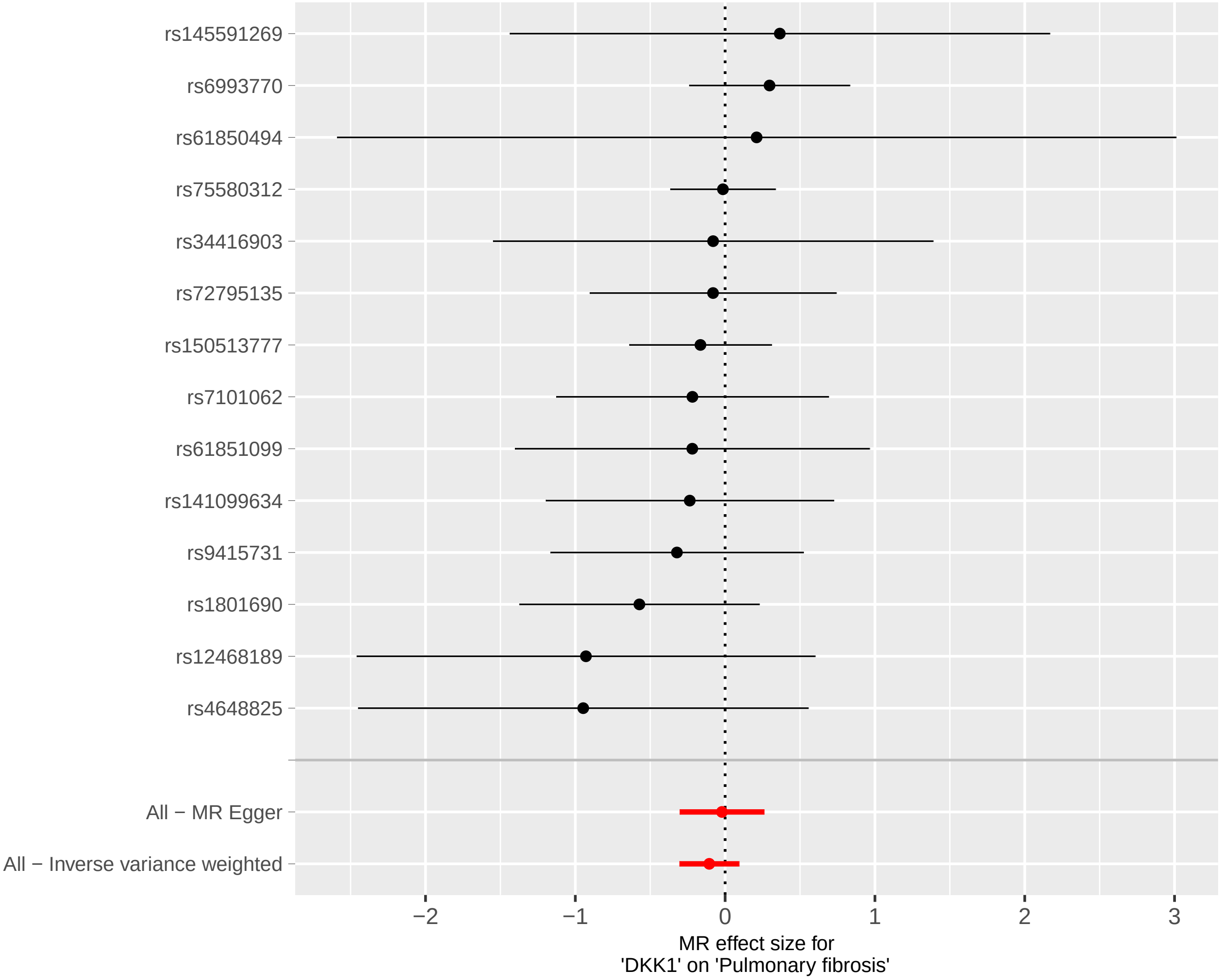

rs145591269
rs6993770
rs61850494
rs75580312
rs34416903
rs72795135
rs150513777
rs7101062
rs61851099
rs141099634
rs9415731
rs1801690
rs12468189
rs4648825
All − MR Egger
All − Inverse variance weighted
−2
−1
0
1
2
3
MR effect size for
'DKK1' on 'Pulmonary fibrosis'

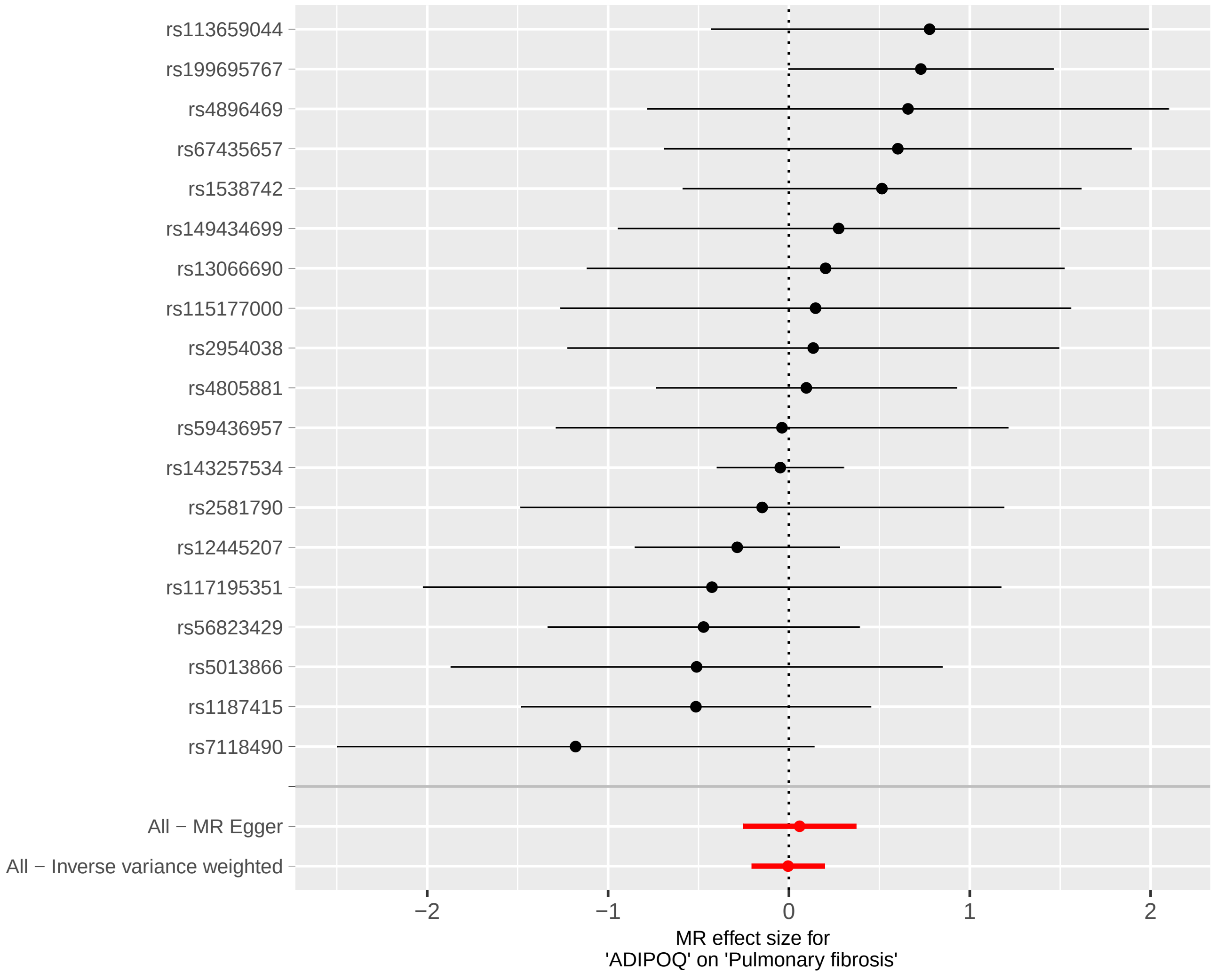

rs113659044
rs199695767
rs4896469
rs67435657
rs1538742
rs149434699
rs13066690
rs115177000
rs2954038
rs4805881
rs59436957
rs143257534
rs2581790
rs12445207
rs117195351
rs56823429
rs5013866
rs1187415
rs7118490
All − MR Egger
All − Inverse variance weighted
−2
−1
0
1
2
MR effect size for
'ADIPOQ' on 'Pulmonary fibrosis'

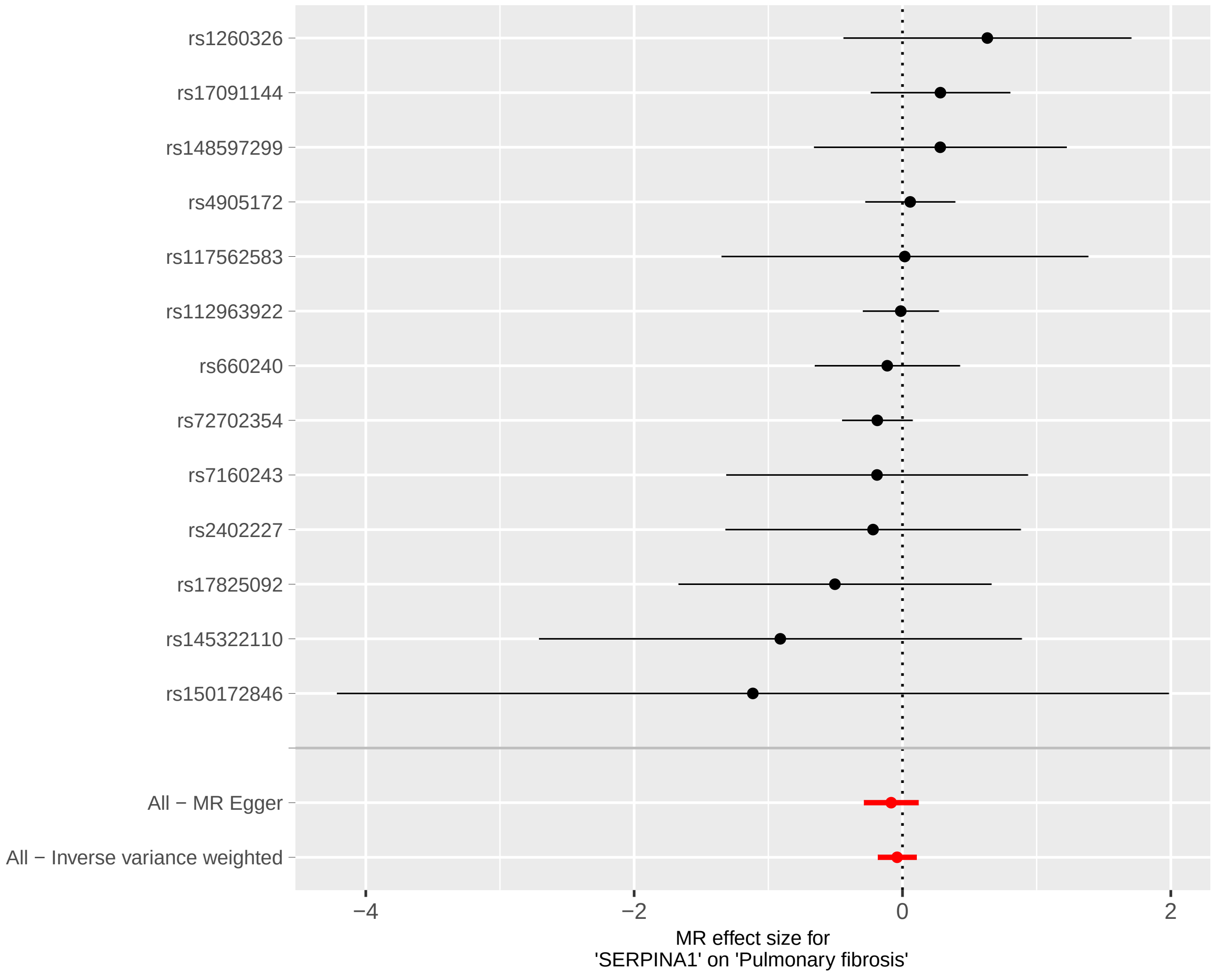

rs1260326
rs17091144
rs148597299
rs4905172
rs117562583
rs112963922
rs660240
rs72702354
rs7160243
rs2402227
rs17825092
rs145322110
rs150172846
All − MR Egger
All − Inverse variance weighted
−4
−2
0
2
MR effect size for
'SERPINA1' on 'Pulmonary fibrosis'

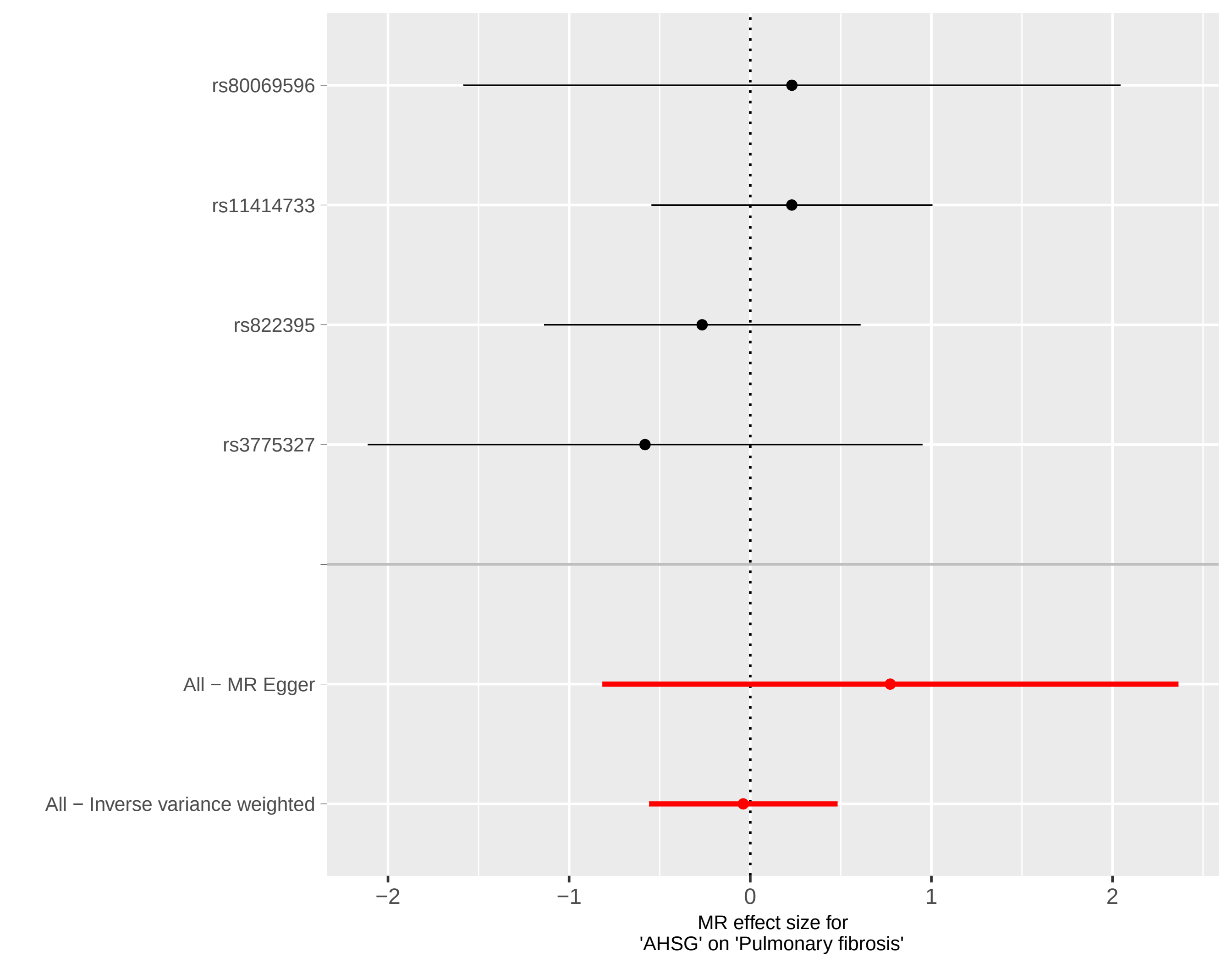

rs80069596
rs11414733
rs822395
rs3775327
All − MR Egger
All − Inverse variance weighted
−2
−1
0
1
2
MR effect size for
'AHSG' on 'Pulmonary fibrosis'

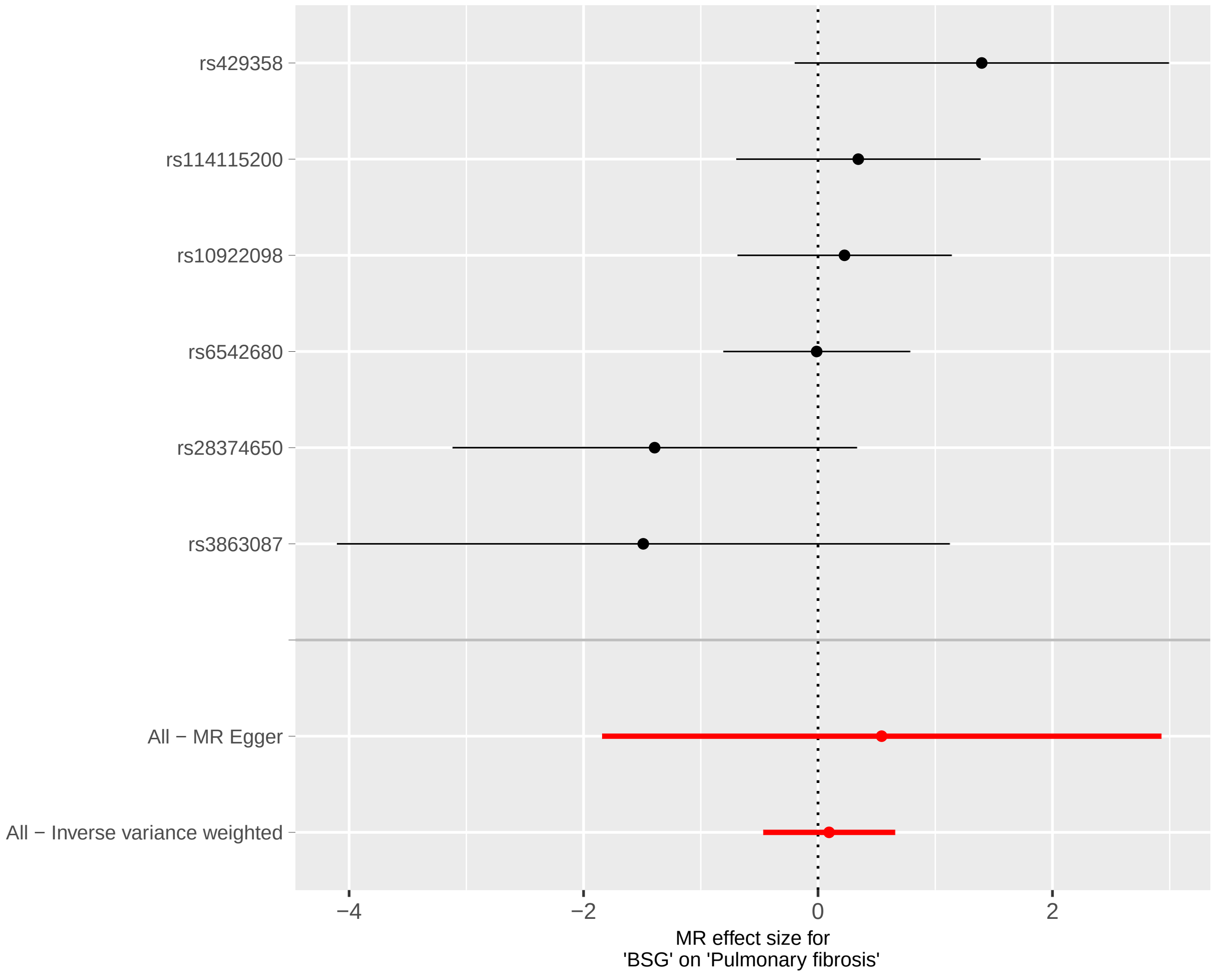
rs429358
rs114115200
rs10922098
rs6542680
rs28374650
rs3863087
All − MR Egger
All − Inverse variance weighted
−4
−2
0
2
MR effect size for
'BSG' on 'Pulmonary fibrosis'

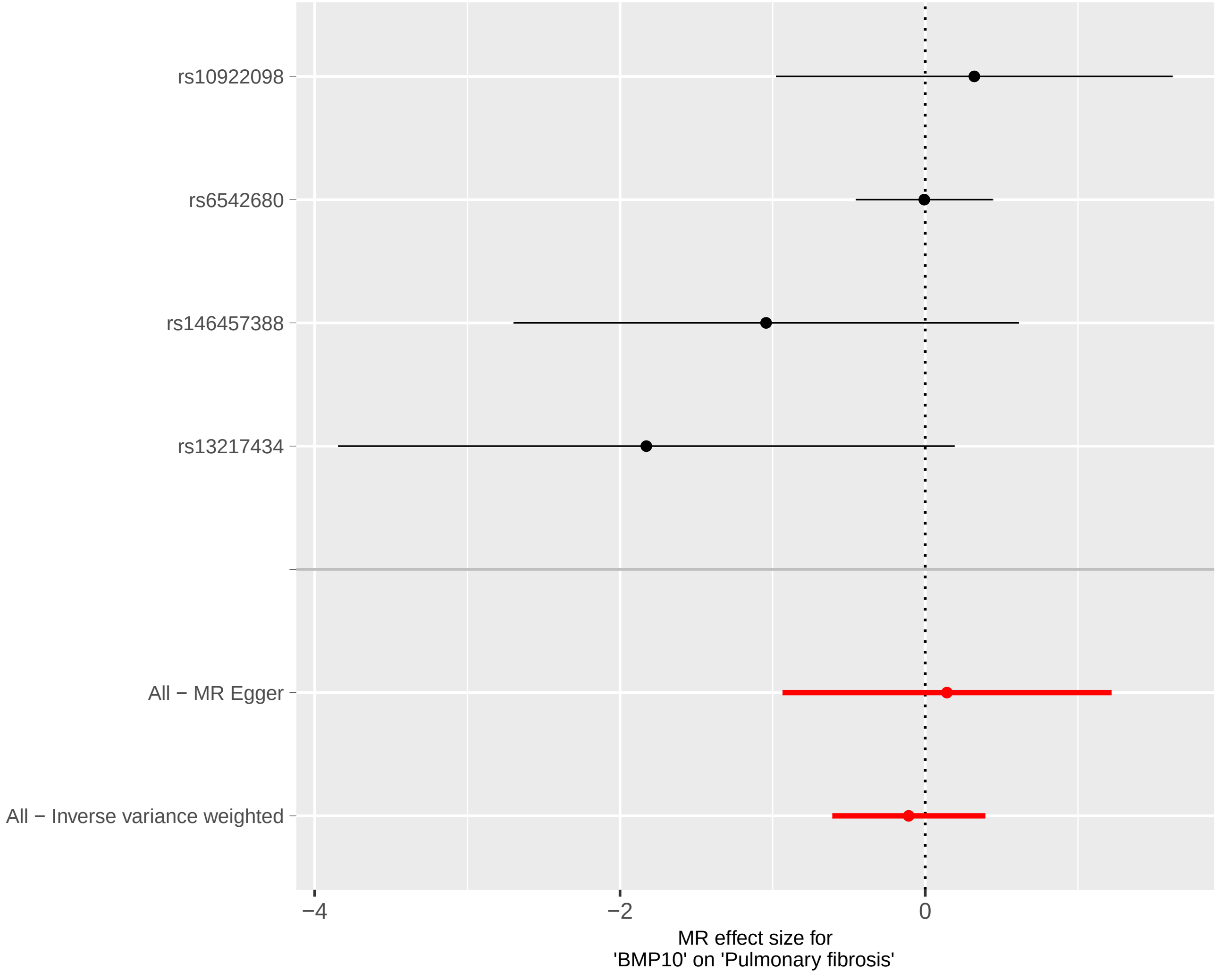
rs10922098
rs6542680
rs146457388
rs13217434
All − MR Egger
All − Inverse variance weighted
−4
−2
0
MR effect size for
'BMP10' on 'Pulmonary fibrosis'

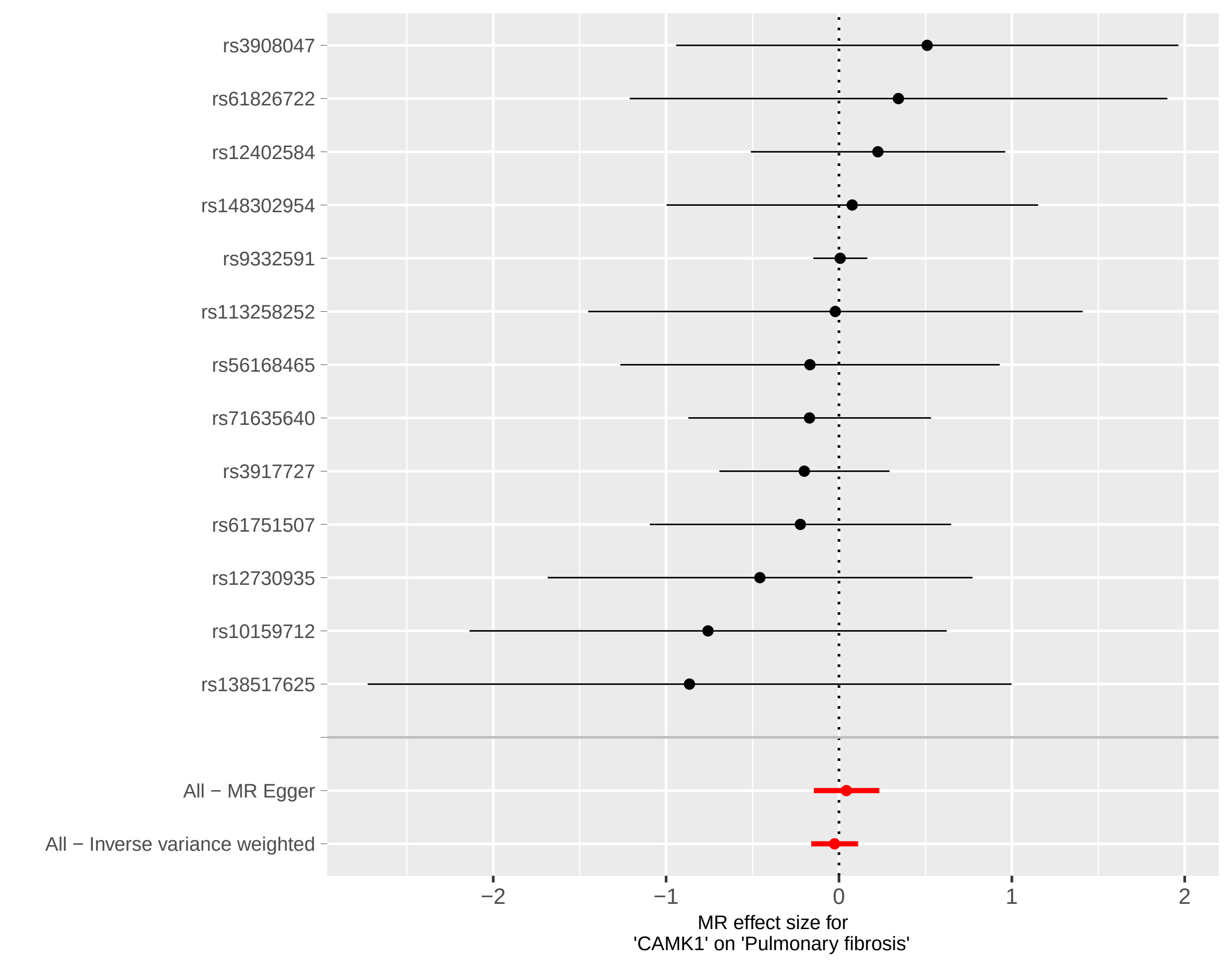
rs3908047
rs61826722
rs12402584
rs148302954
rs9332591
rs113258252
rs56168465
rs71635640
rs3917727
rs61751507
rs12730935
rs10159712
rs138517625
All − MR Egger
All − Inverse variance weighted
−2
−1
0
1
2
MR effect size for
'CAMK1' on 'Pulmonary fibrosis'

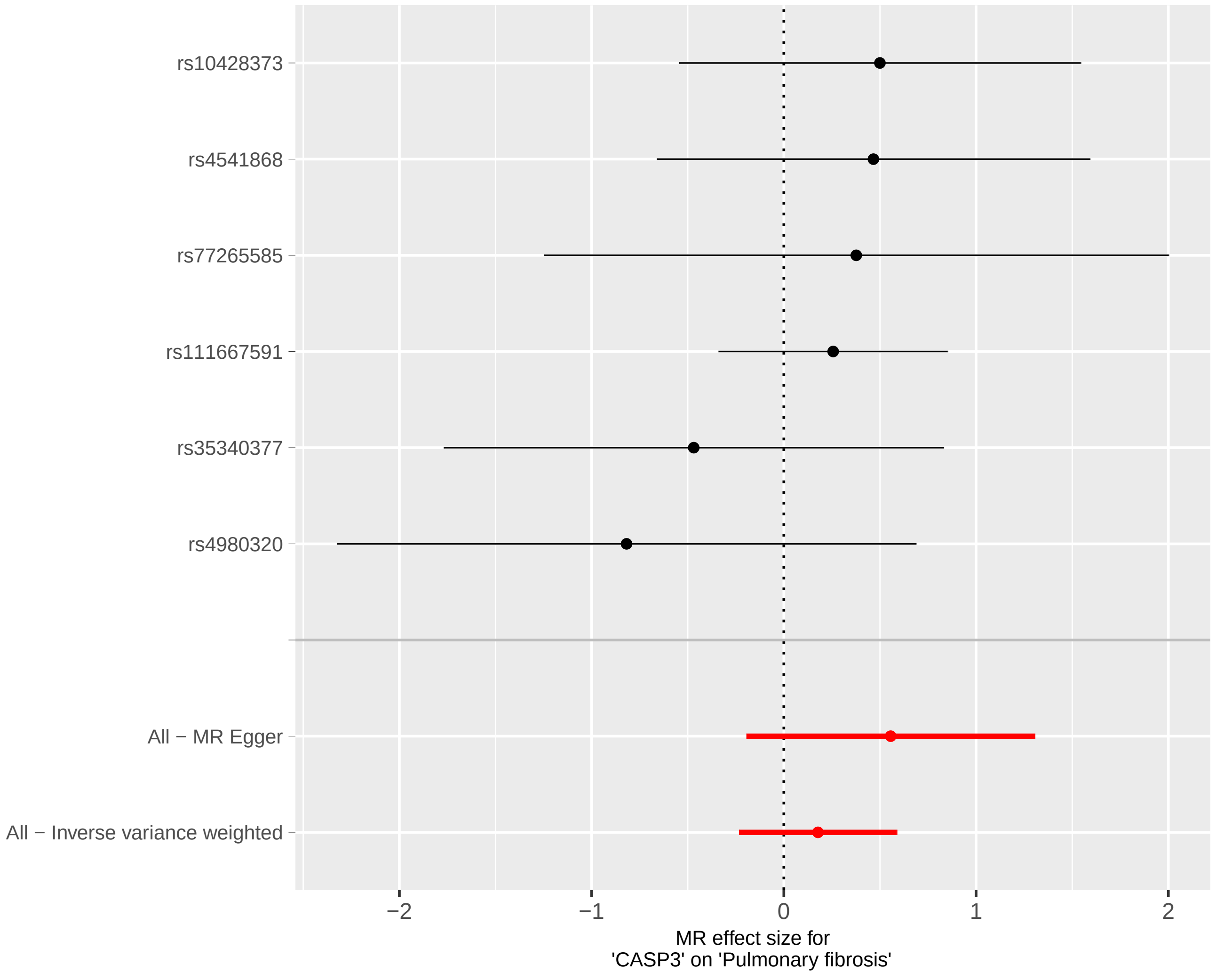
rs10428373
rs4541868
rs77265585
rs111667591
rs35340377
rs4980320
All − MR Egger
All − Inverse variance weighted
−2
−1
0
1
2
MR effect size for
'CASP3' on 'Pulmonary fibrosis'

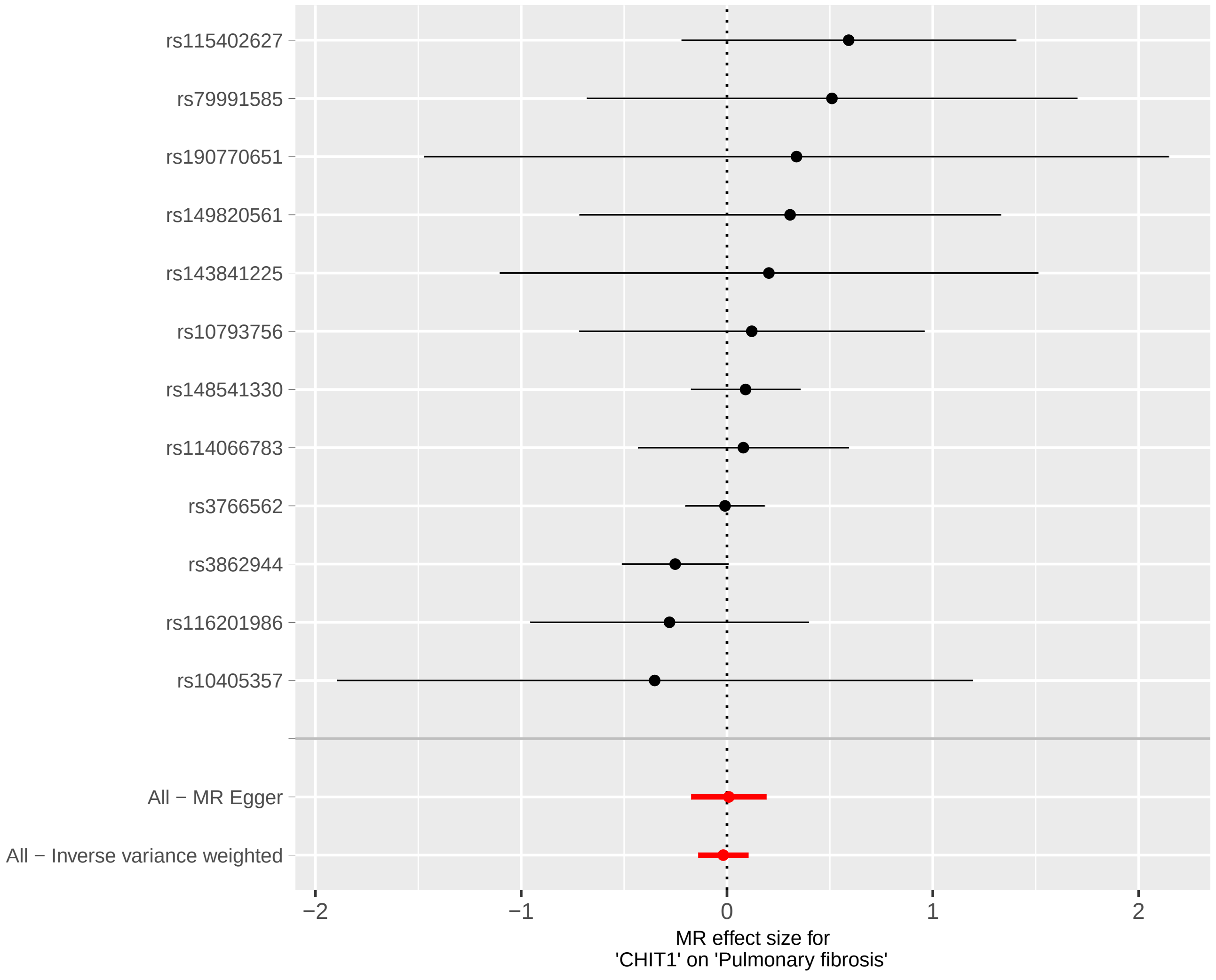
rs115402627
rs79991585
rs190770651
rs149820561
rs143841225
rs10793756
rs148541330
rs114066783
rs3766562
rs3862944
rs116201986
rs10405357
All − MR Egger
All − Inverse variance weighted
−2
−1
0
1
2
MR effect size for
'CHIT1' on 'Pulmonary fibrosis'

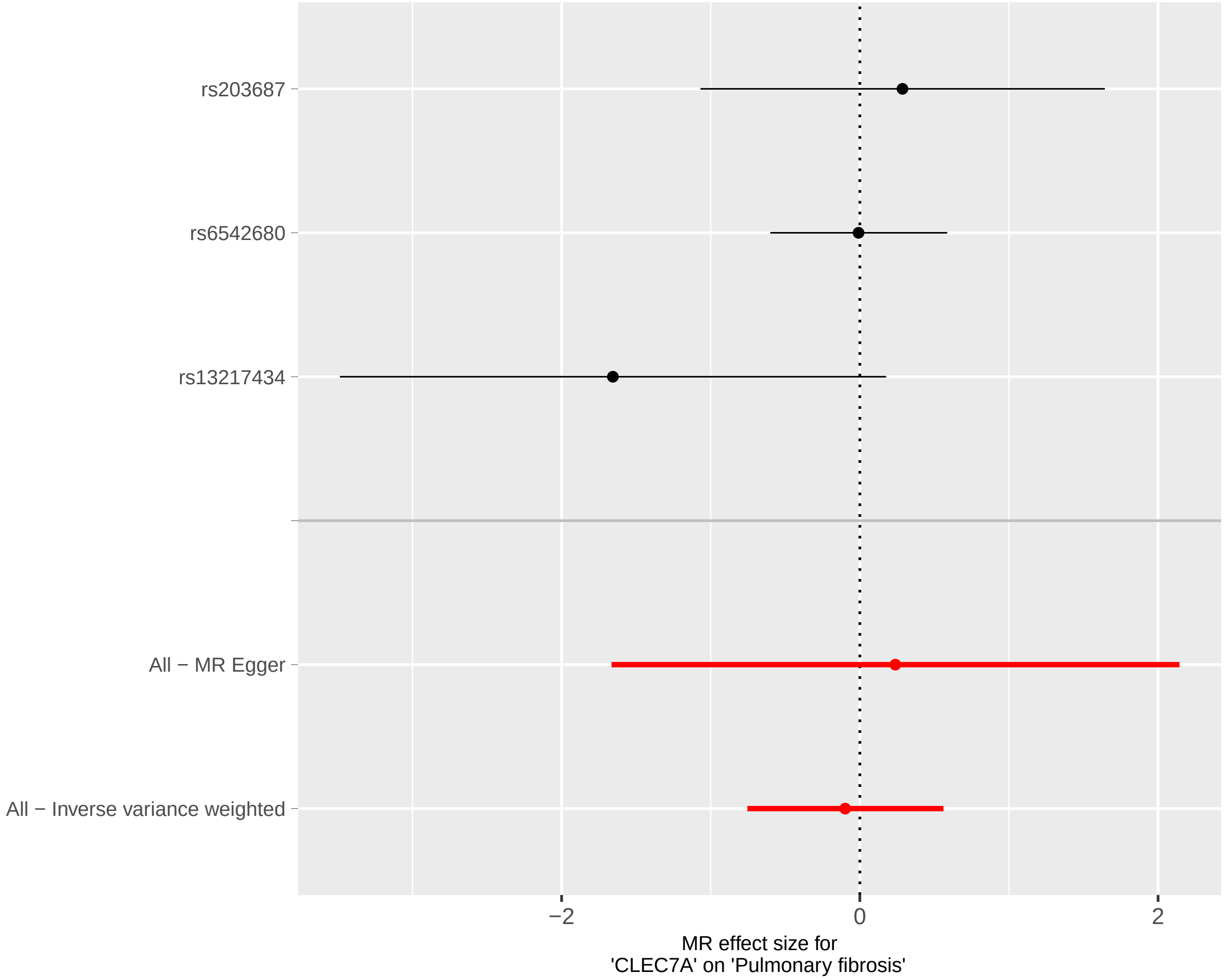

rs203687
rs6542680
rs13217434
All − MR Egger
All − Inverse variance weighted
−2
0
2
MR effect size for
'CLEC7A' on 'Pulmonary fibrosis'

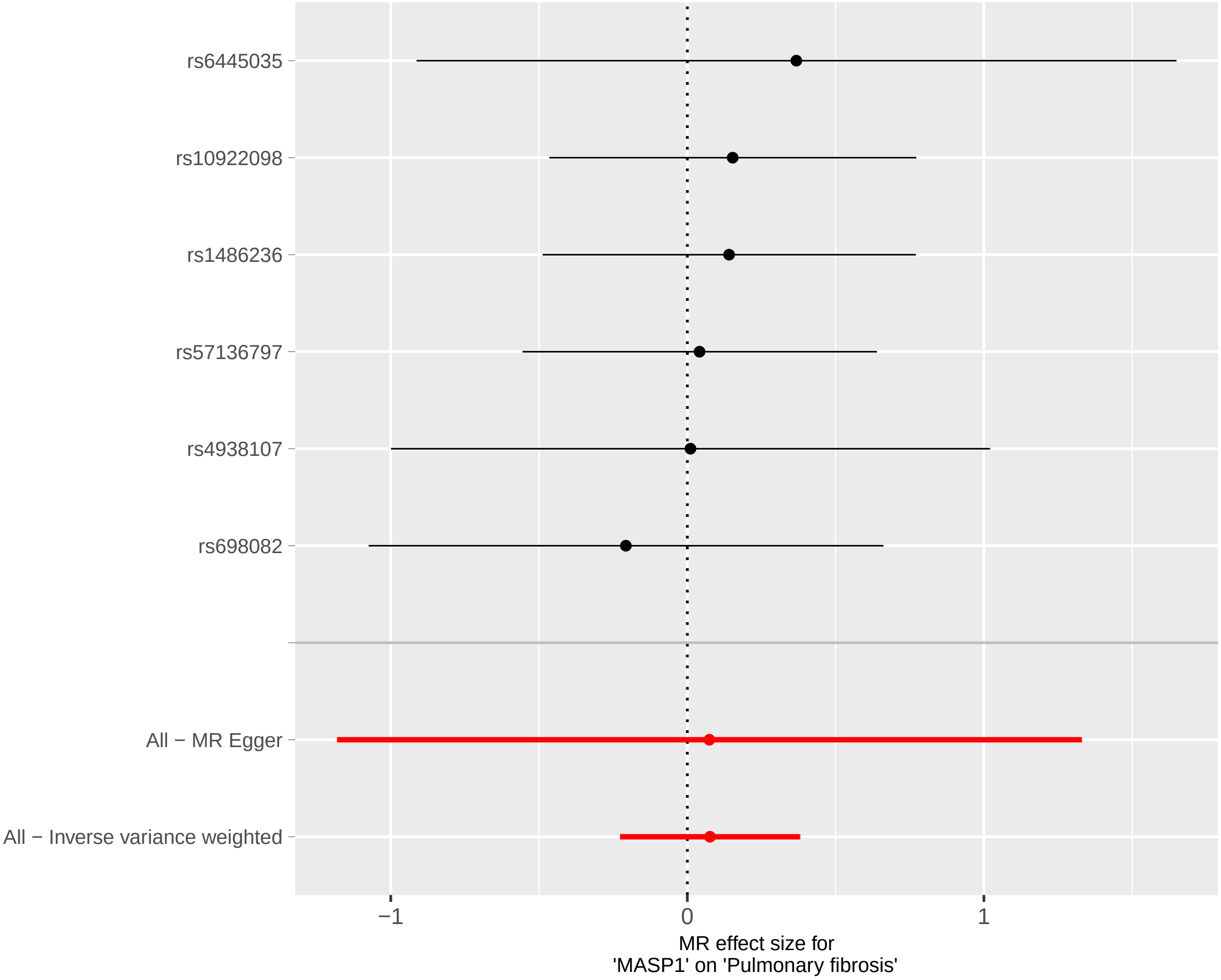

rs6445035
rs10922098
rs1486236
rs57136797
rs4938107
rs698082
All − MR Egger
All − Inverse variance weighted
−1
0
1
MR effect size for
'MASP1' on 'Pulmonary fibrosis'

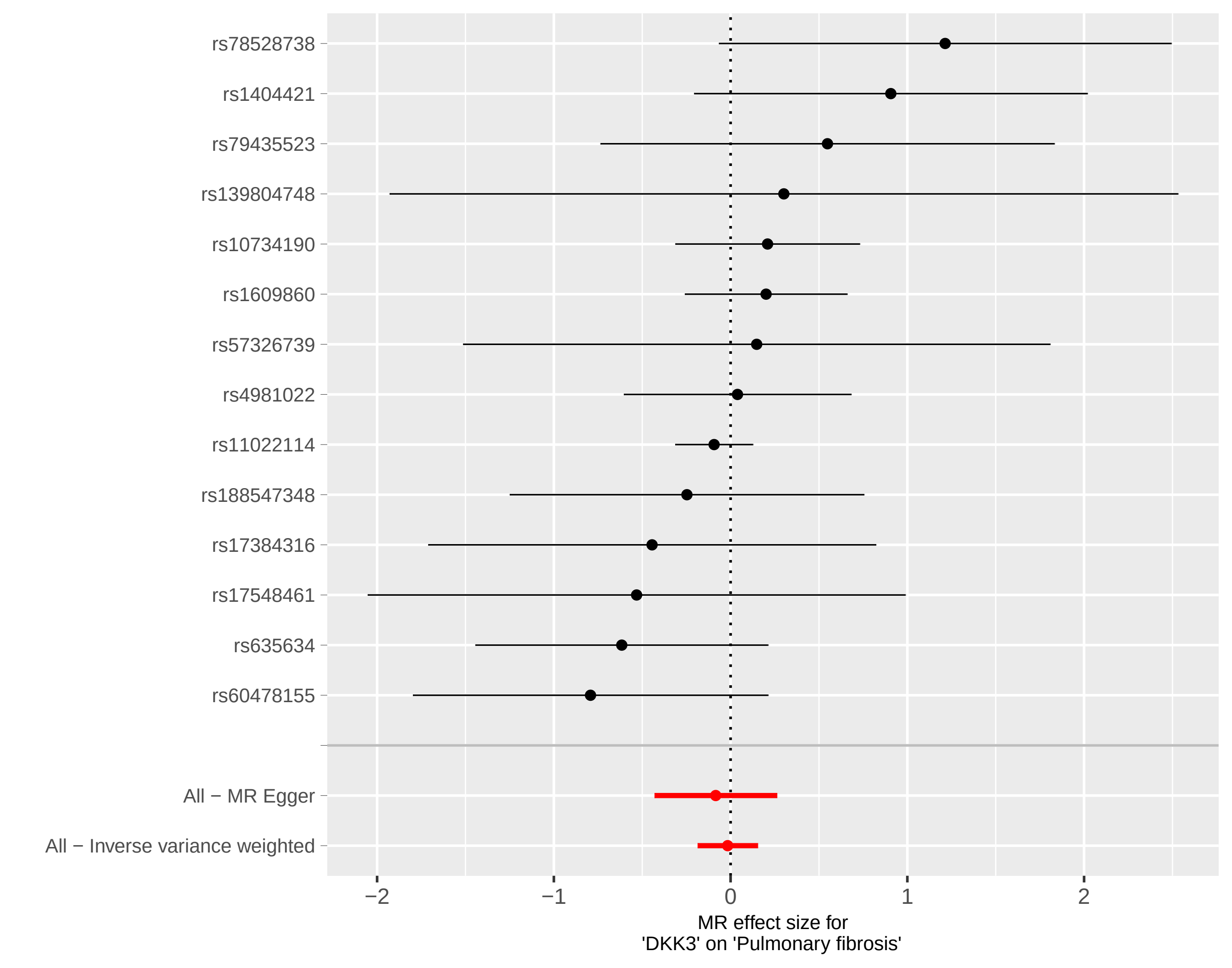
rs78528738
rs1404421
rs79435523
rs139804748
rs10734190
rs1609860
rs57326739
rs4981022
rs11022114
rs188547348
rs17384316
rs17548461
rs635634
rs60478155
All − MR Egger
All − Inverse variance weighted
−2
−1
0
1
2
MR effect size for
'DKK3' on 'Pulmonary fibrosis'

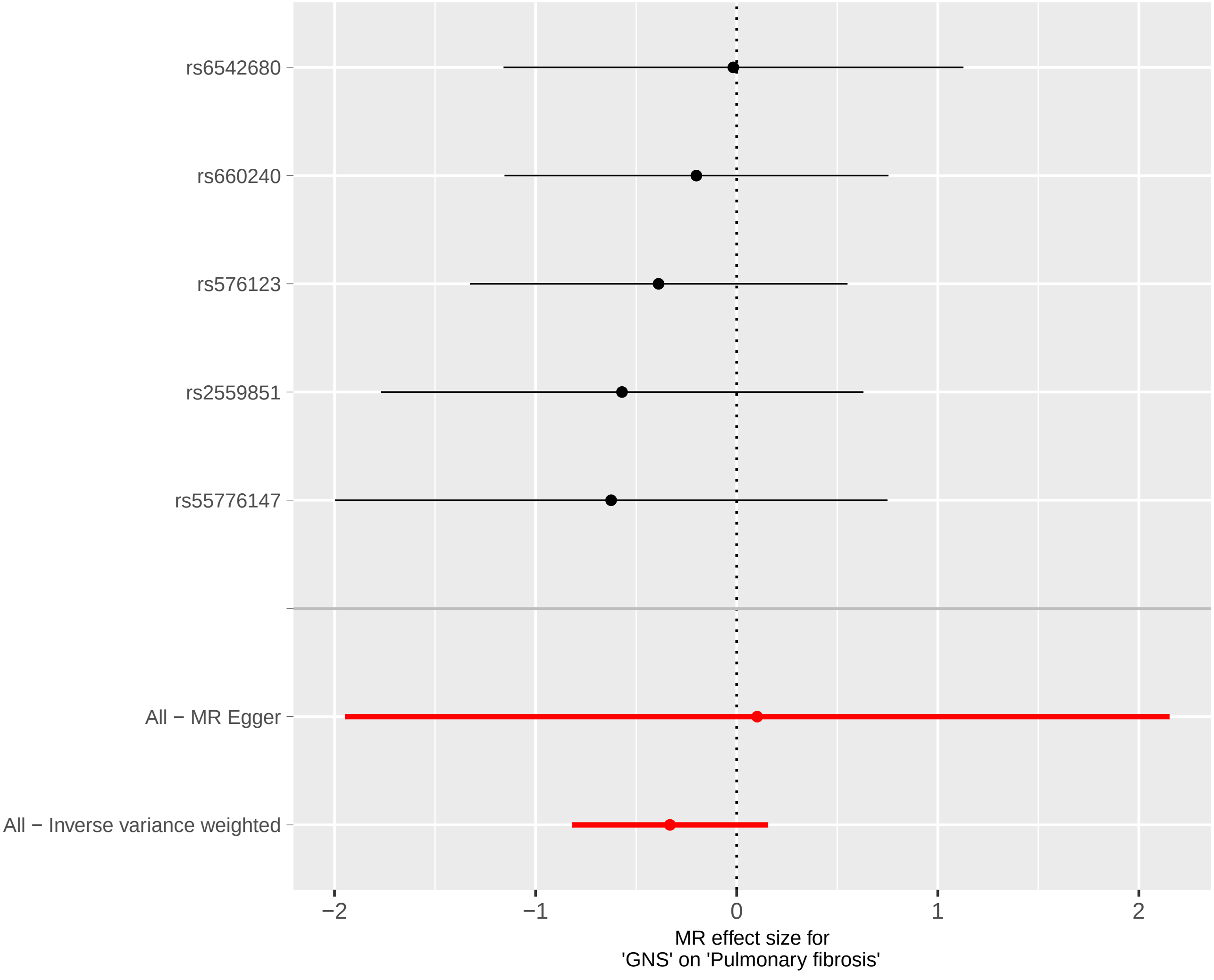

rs6542680
rs660240
rs576123
rs2559851
rs55776147
All − MR Egger
All − Inverse variance weighted
−2
−1
0
1
2
MR effect size for
'GNS' on 'Pulmonary fibrosis'

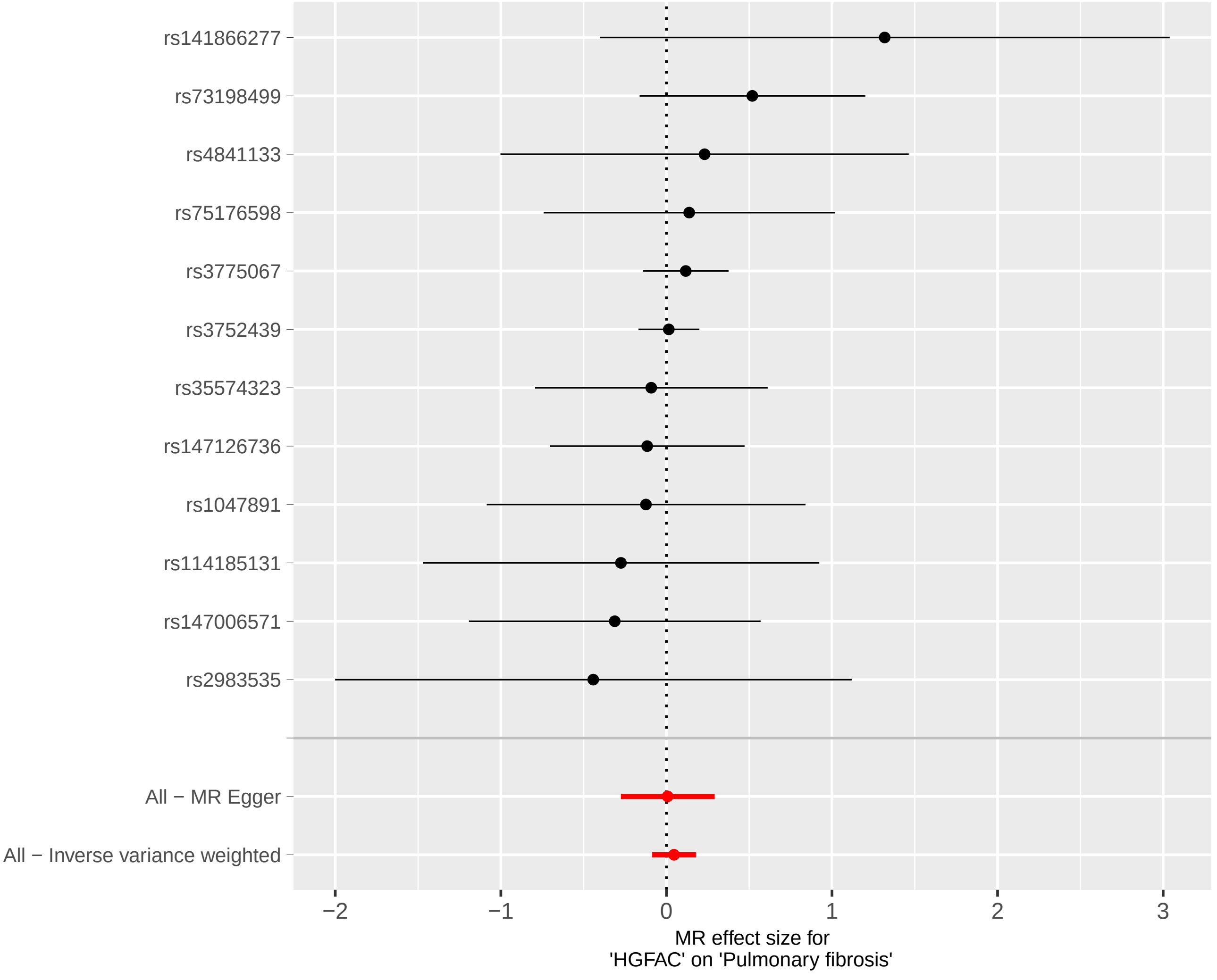

rs141866277
rs73198499
rs4841133
rs75176598
rs3775067
rs3752439
rs35574323
rs147126736
rs1047891
rs114185131
rs147006571
rs2983535
All − MR Egger
All − Inverse variance weighted
−2
−1
0
1
2
3
MR effect size for
'HGFAC' on 'Pulmonary fibrosis'

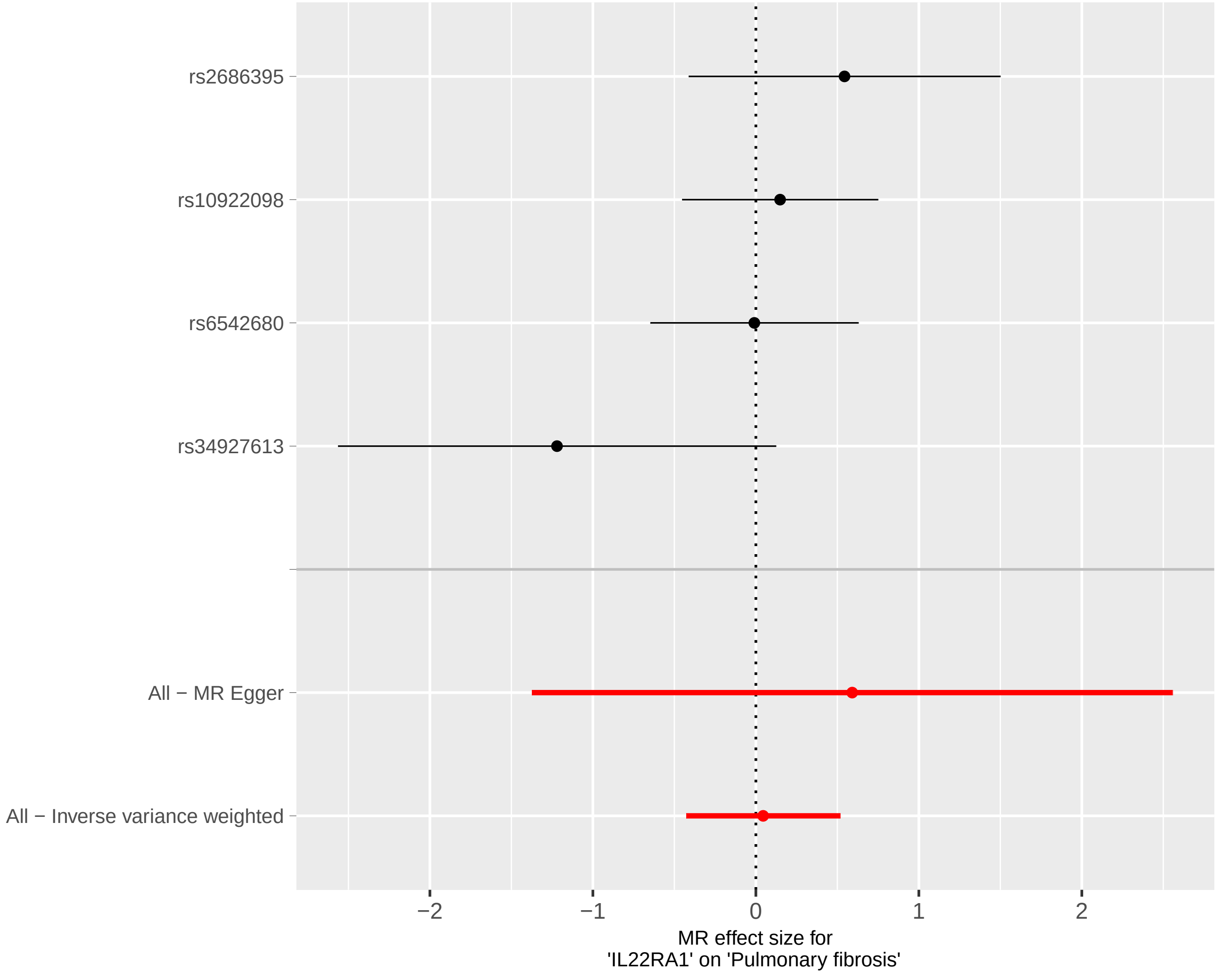

rs2686395
rs10922098
rs6542680
rs34927613
All − MR Egger
All − Inverse variance weighted
−2
−1
0
1
2
MR effect size for
'IL22RA1' on 'Pulmonary fibrosis'

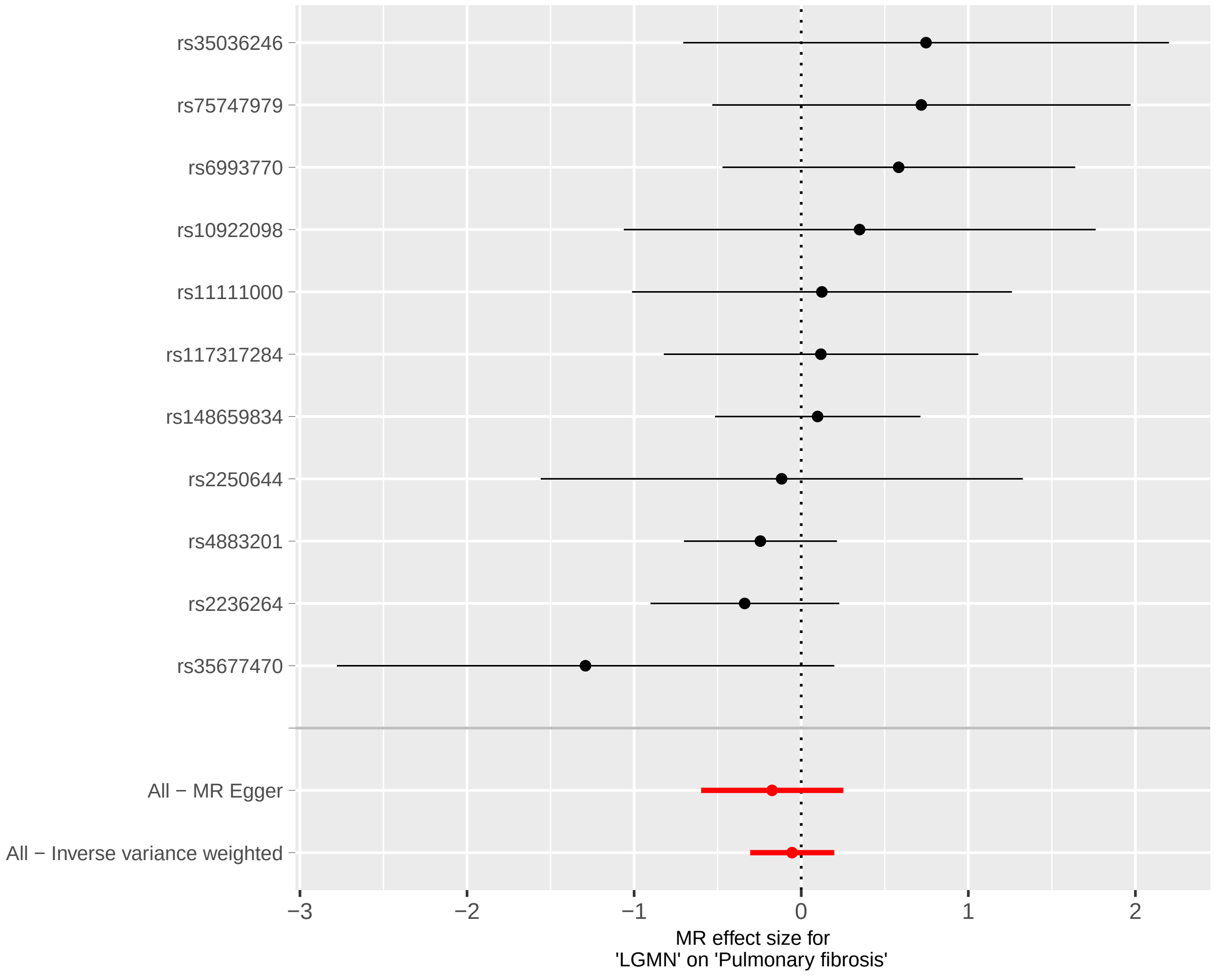
rs35036246
rs75747979
rs6993770
rs10922098
rs11111000
rs117317284
rs148659834
rs2250644
rs4883201
rs2236264
rs35677470
All − MR Egger
All − Inverse variance weighted
−3
−2
−1
0
1
2
MR effect size for
'LGMN' on 'Pulmonary fibrosis'

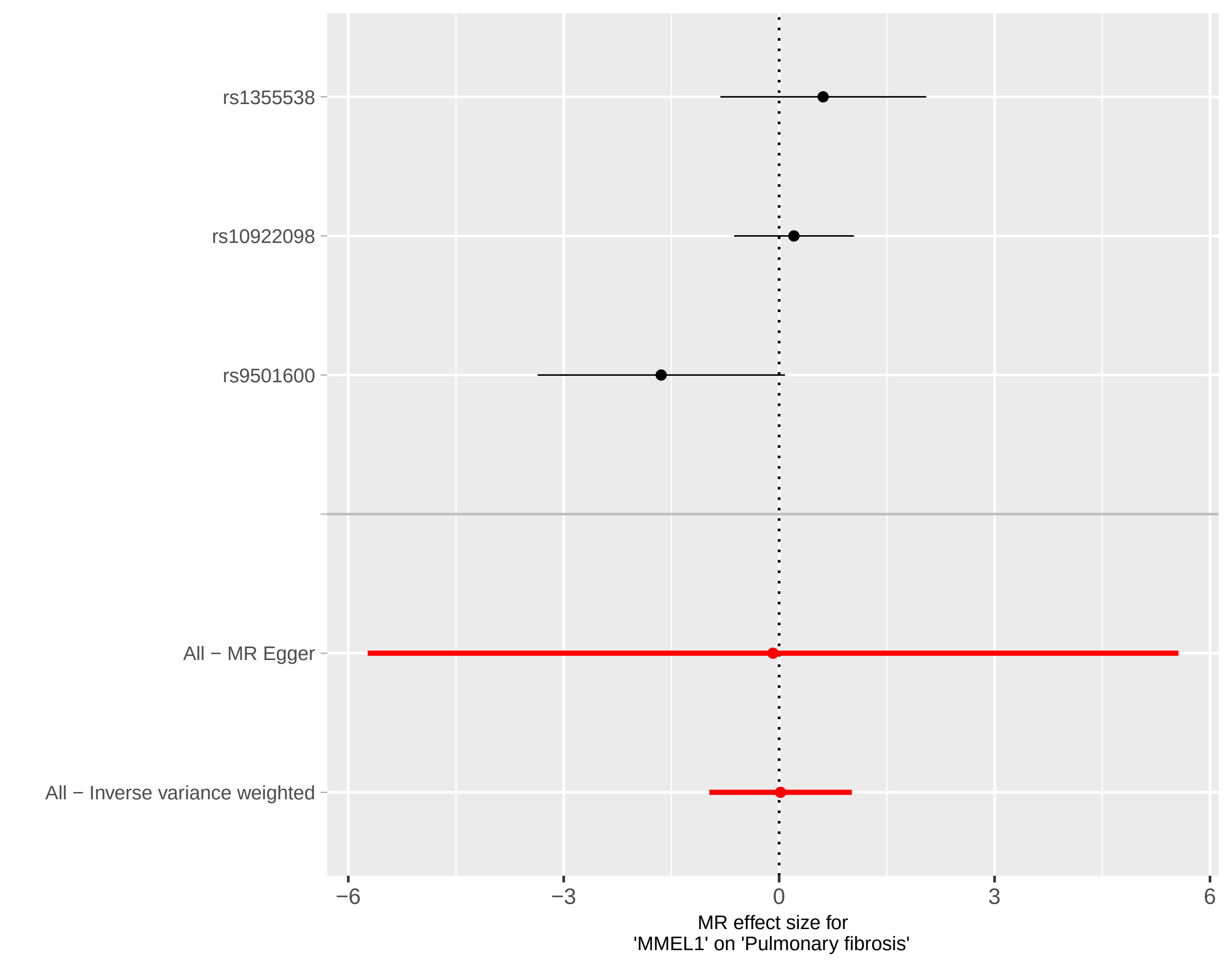
rs1355538
rs10922098
rs9501600
All − MR Egger
All − Inverse variance weighted
−6
−3
0
3
6
MR effect size for
'MMEL1' on 'Pulmonary fibrosis'

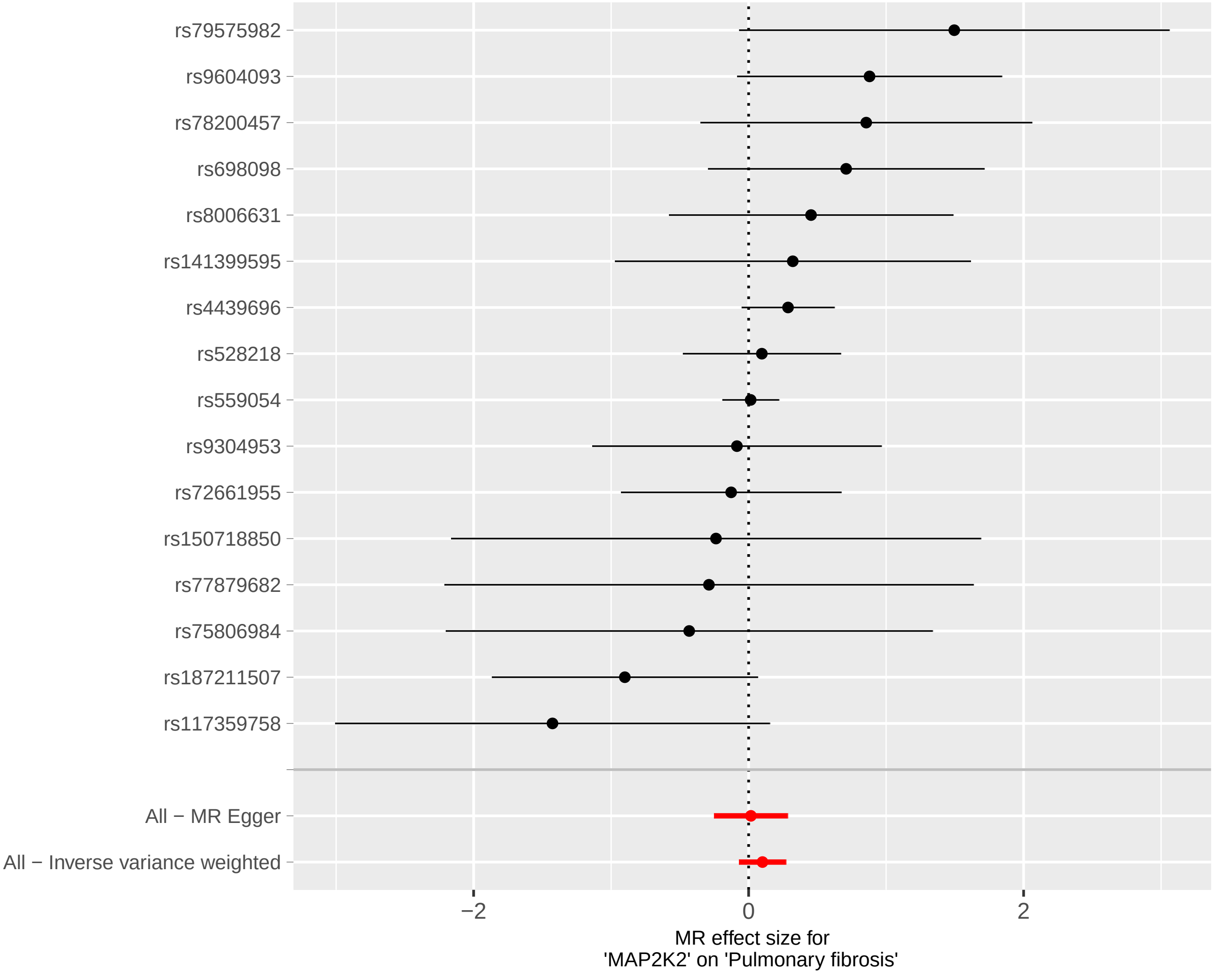
rs79575982
rs9604093
rs78200457
rs698098
rs8006631
rs141399595
rs4439696
rs528218
rs559054
rs9304953
rs72661955
rs150718850
rs77879682
rs75806984
rs187211507
rs117359758
All − MR Egger
All − Inverse variance weighted
−2
0
2
MR effect size for
'MAP2K2' on 'Pulmonary fibrosis'

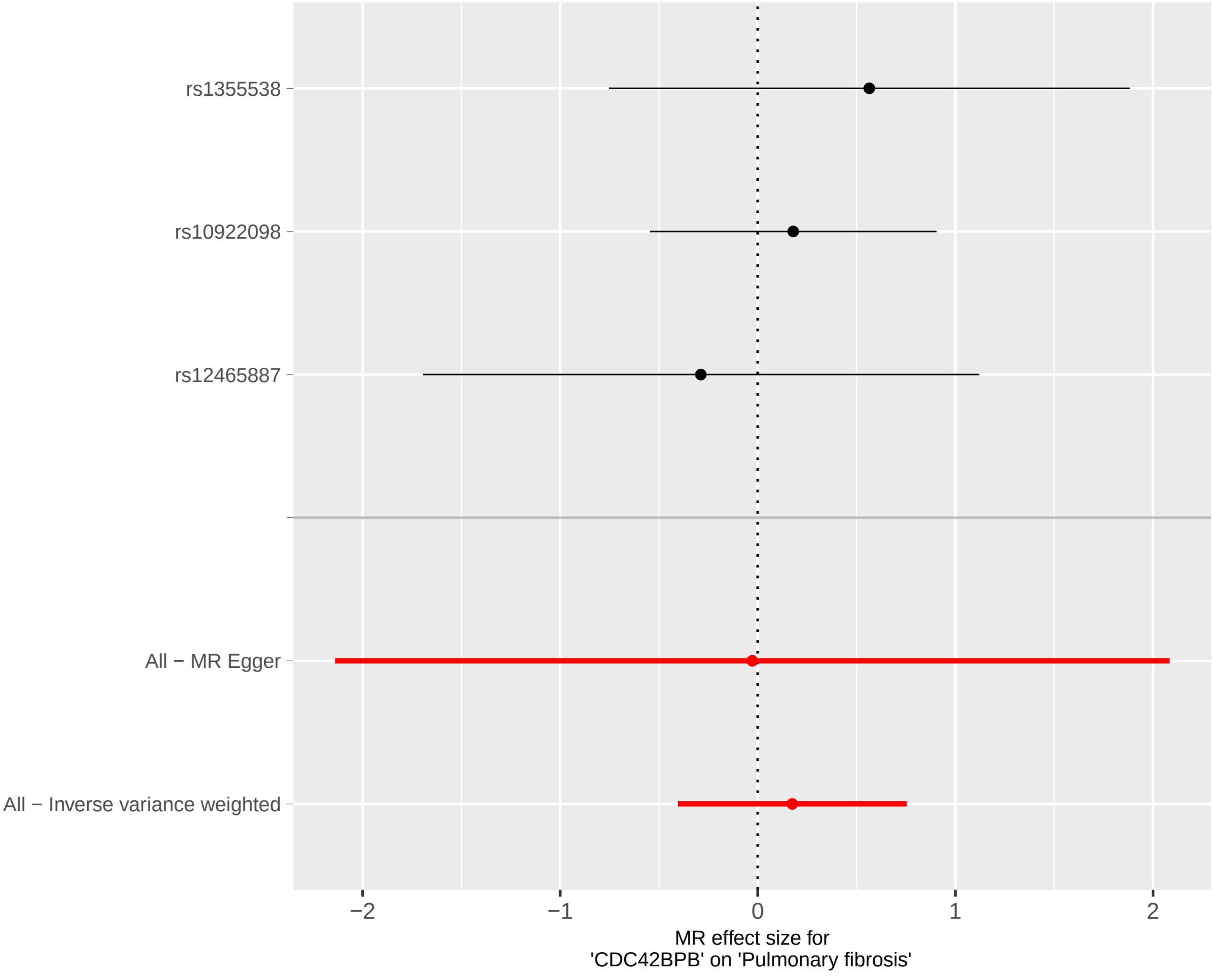

rs1355538
rs10922098
rs12465887
All − MR Egger
All − Inverse variance weighted
−2
−1
0
1
2
MR effect size for
'CDC42BPB' on 'Pulmonary fibrosis'

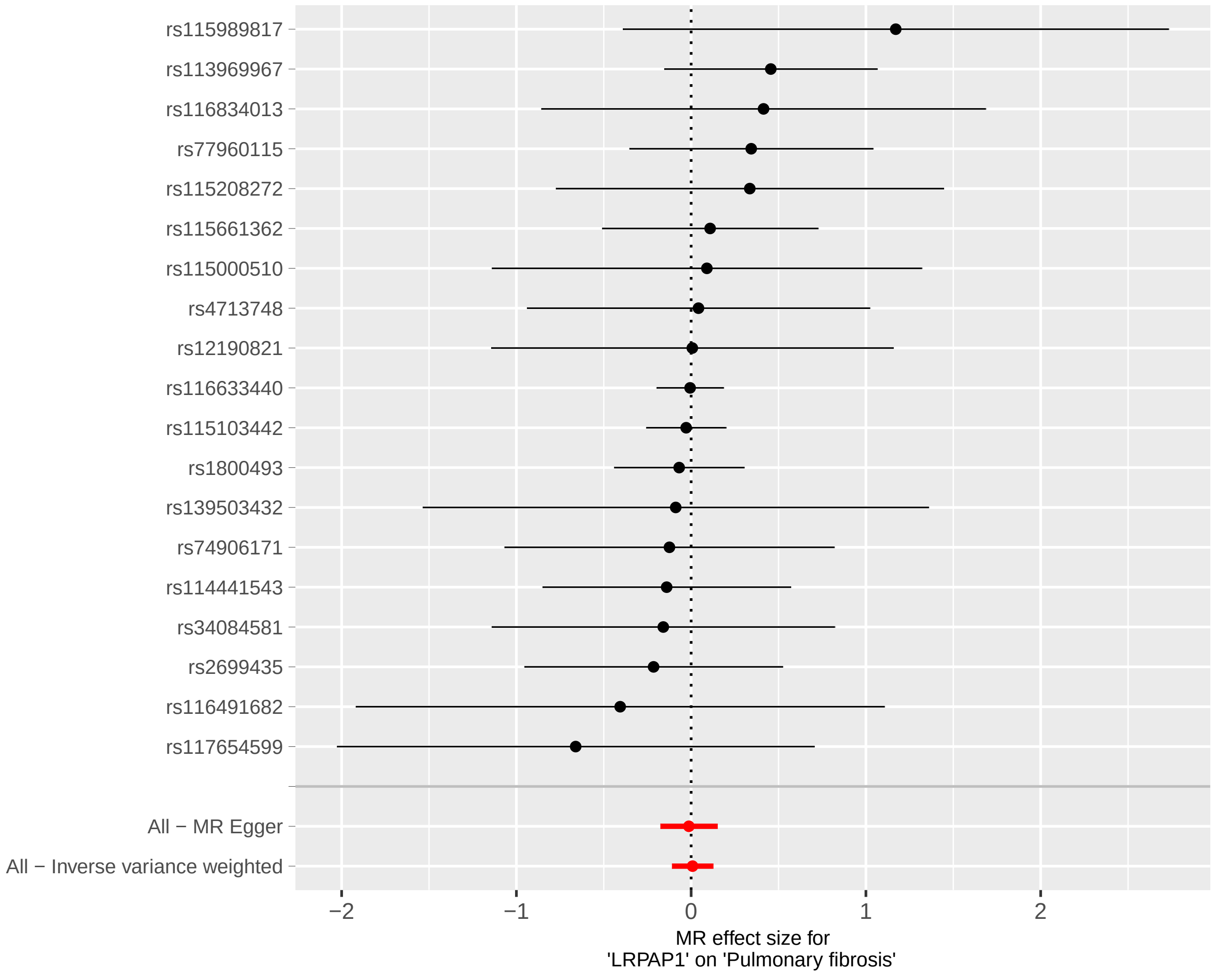

rs115989817
rs113969967
rs116834013
rs77960115
rs115208272
rs115661362
rs115000510
rs4713748
rs12190821
rs116633440
rs115103442
rs1800493
rs139503432
rs74906171
rs114441543
rs34084581
rs2699435
rs116491682
rs117654599
All − MR Egger
All − Inverse variance weighted
−2
−1
0
1
2
MR effect size for
'LRPAP1' on 'Pulmonary fibrosis'

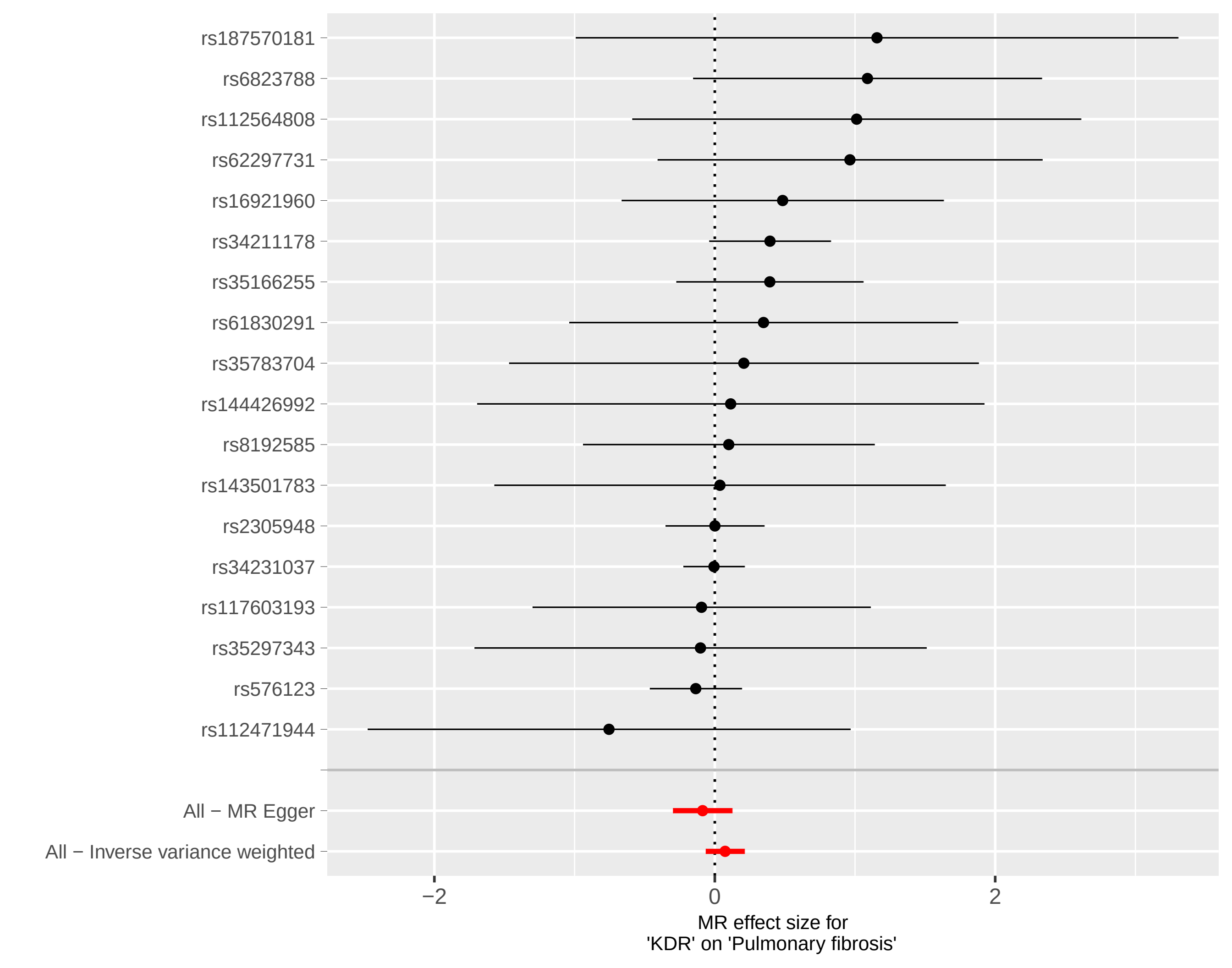
rs187570181
rs6823788
rs112564808
rs62297731
rs16921960
rs34211178
rs35166255
rs61830291
rs35783704
rs144426992
rs8192585
rs143501783
rs2305948
rs34231037
rs117603193
rs35297343
rs576123
rs112471944
All − MR Egger
All − Inverse variance weighted
−2
0
2
MR effect size for
'KDR' on 'Pulmonary fibrosis'

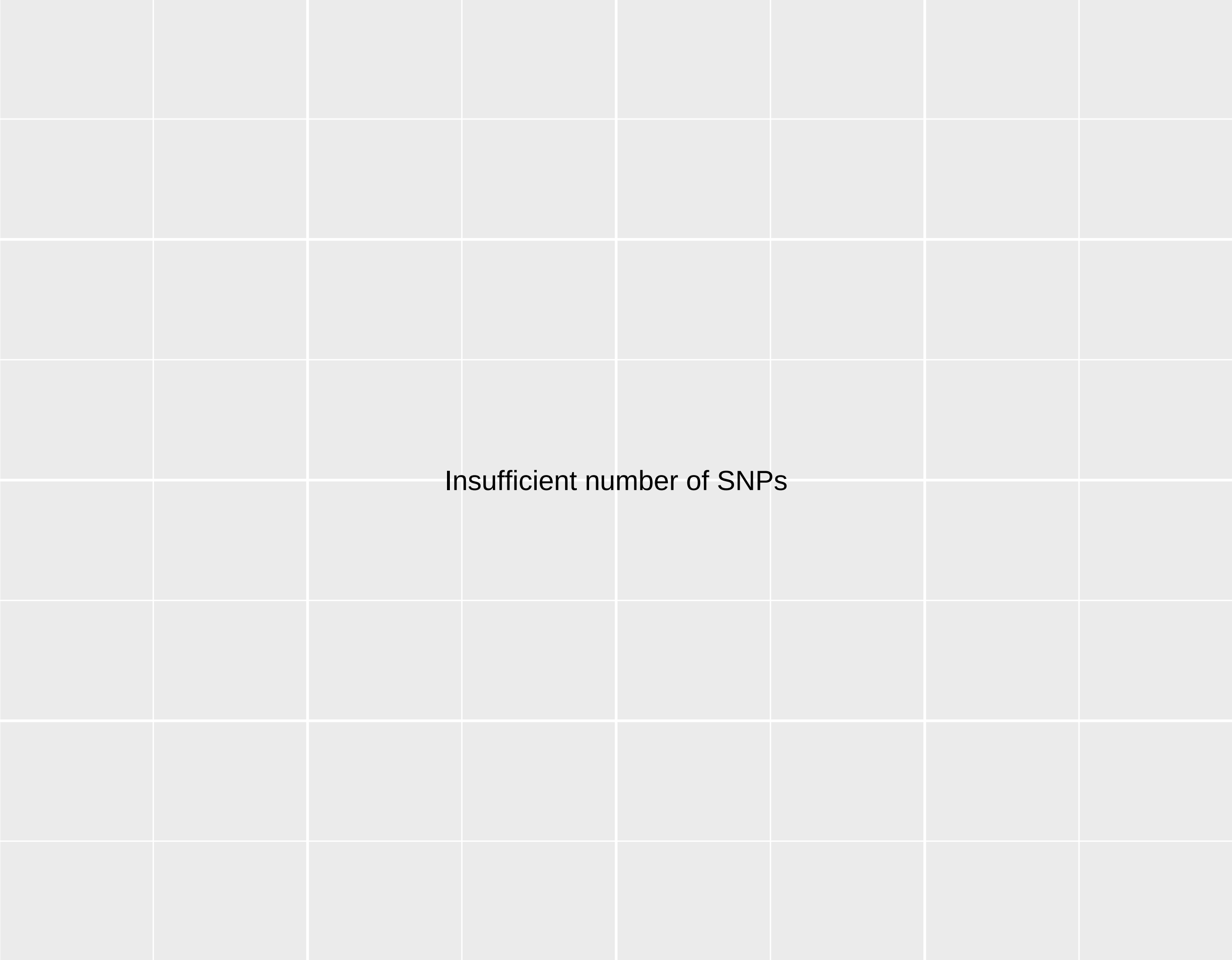
Insufficient number of SNPs

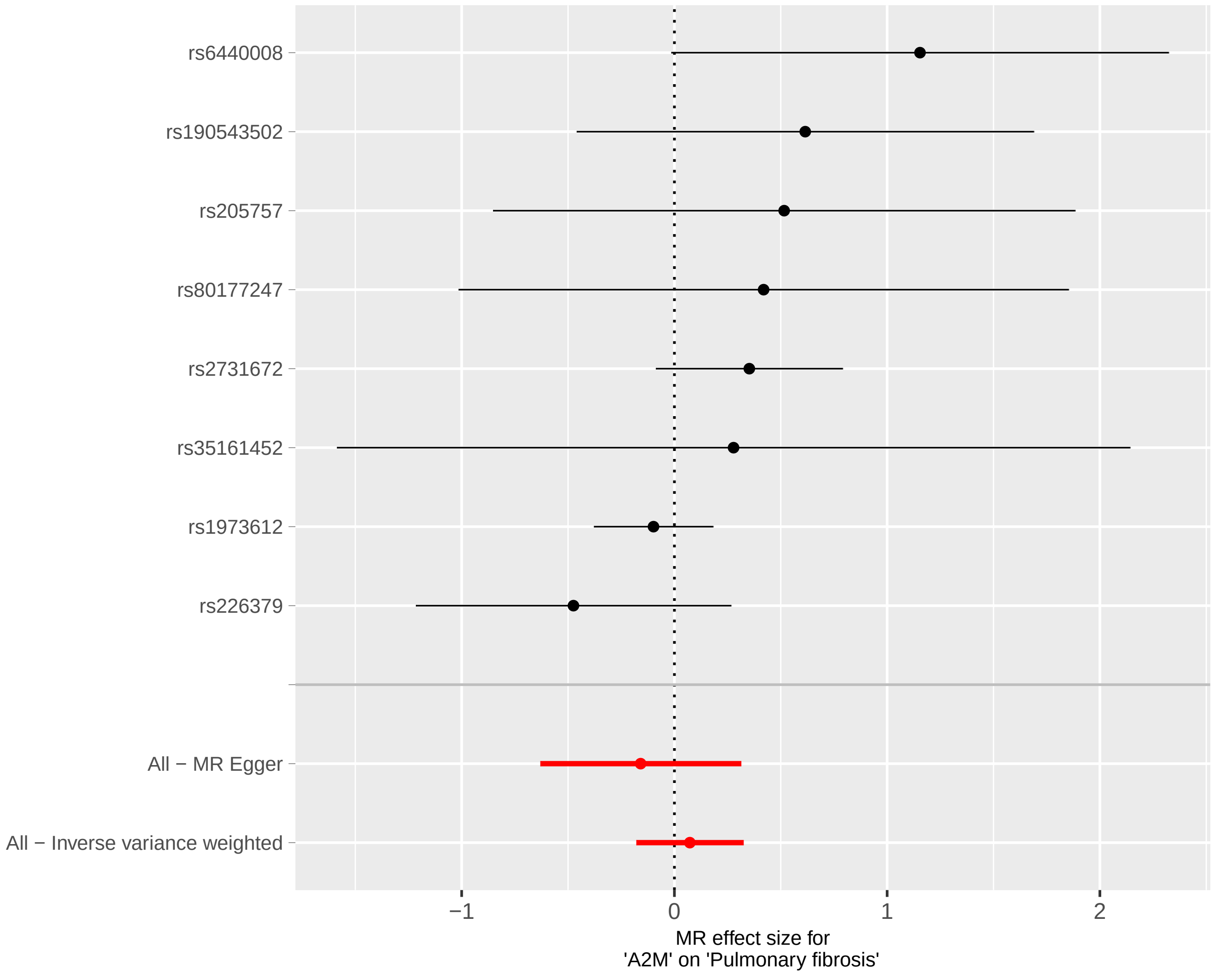
rs6440008
rs190543502
rs205757
rs80177247
rs2731672
rs35161452
rs1973612
rs226379
All − MR Egger
All − Inverse variance weighted
−1
0
1
2
MR effect size for
'A2M' on 'Pulmonary fibrosis'

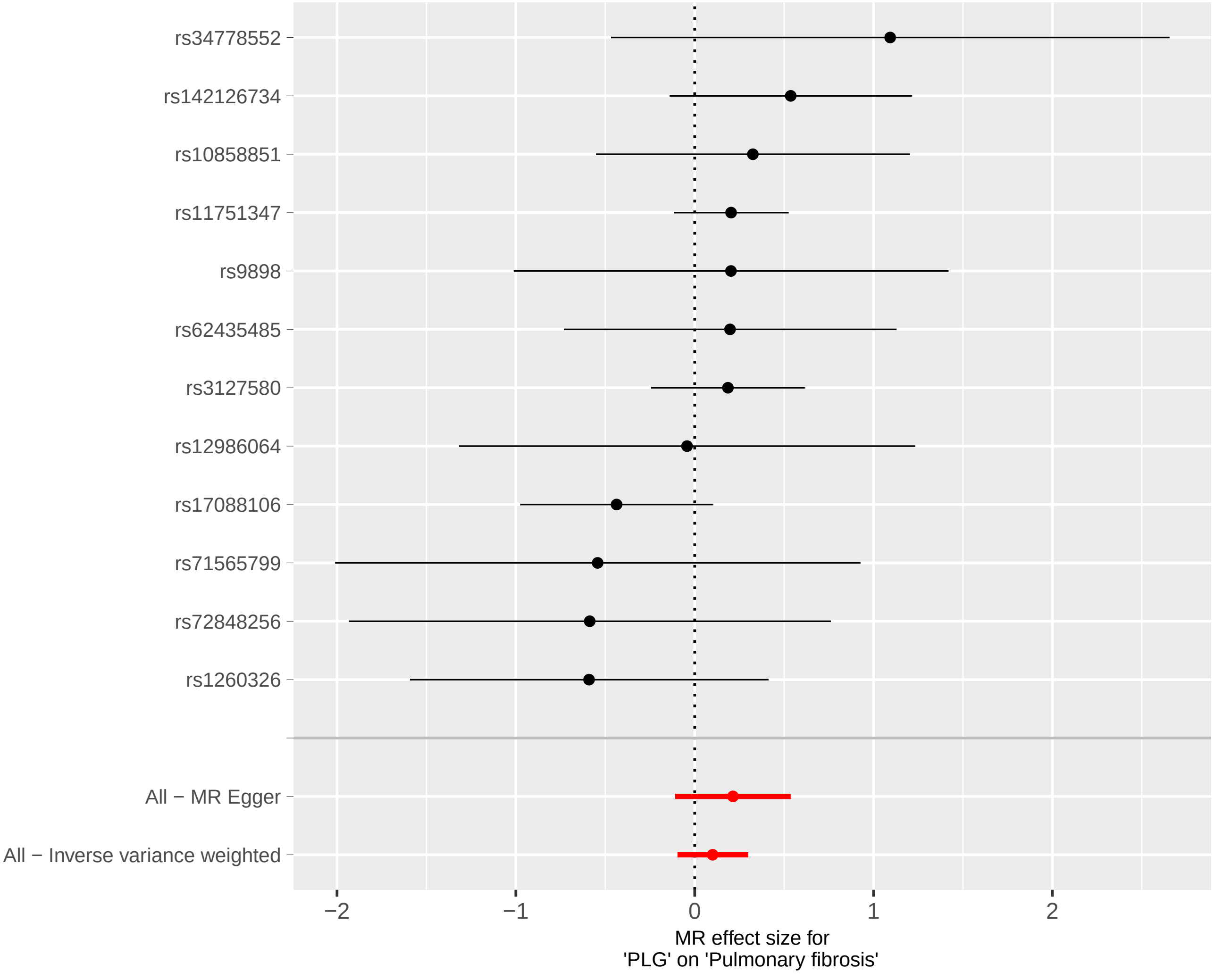

rs34778552
rs142126734
rs10858851
rs11751347
rs9898
rs62435485
rs3127580
rs12986064
rs17088106
rs71565799
rs72848256
rs1260326
All − MR Egger
All − Inverse variance weighted
−2
−1
0
1
2
MR effect size for
'PLG' on 'Pulmonary fibrosis'

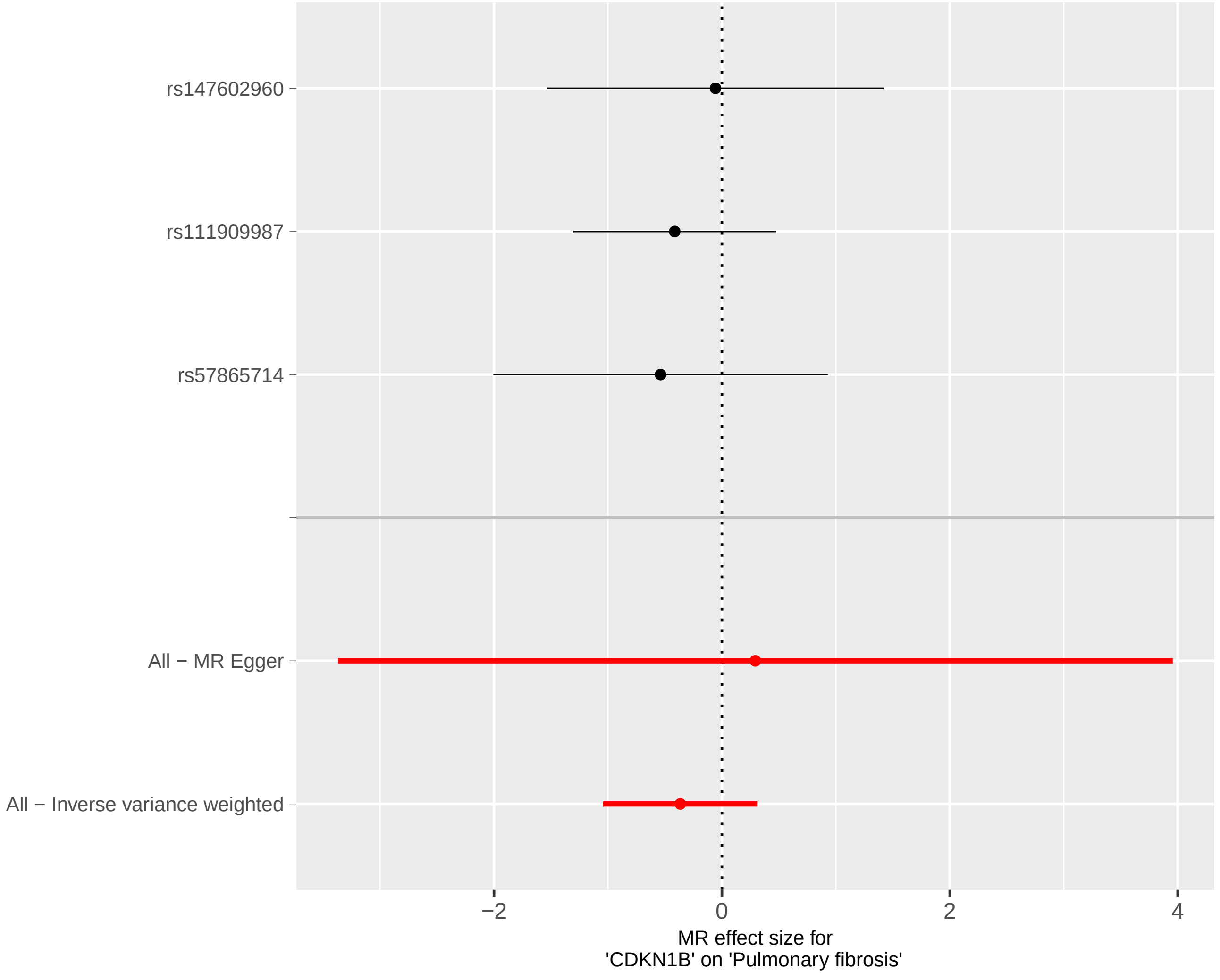
rs147602960
rs111909987
rs57865714
All − MR Egger
All − Inverse variance weighted
−2
0
2
4
MR effect size for
'CDKN1B' on 'Pulmonary fibrosis'

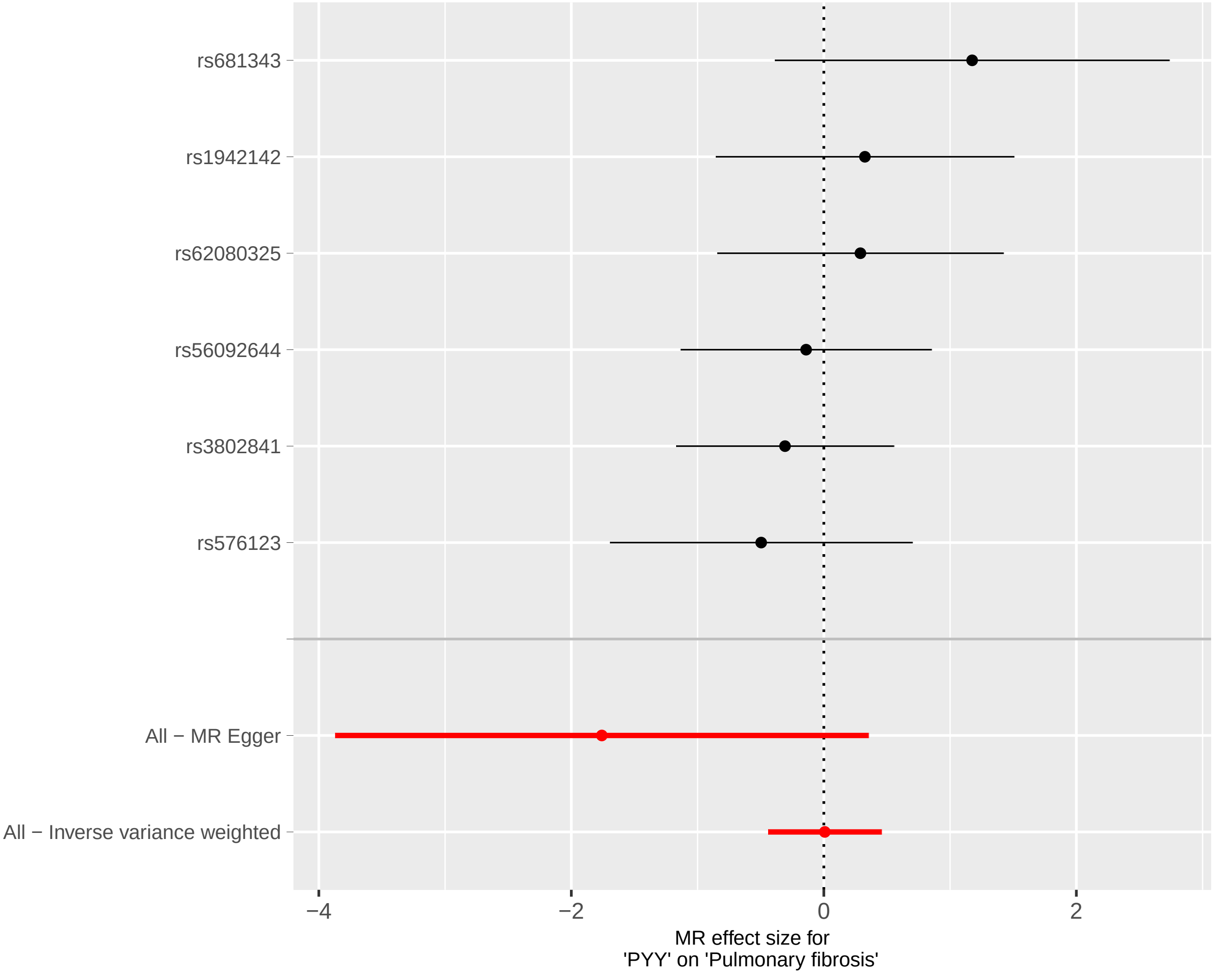

rs681343
rs1942142
rs62080325
rs56092644
rs3802841
rs576123
All − MR Egger
All − Inverse variance weighted
−4
−2
0
2
MR effect size for
'PYY' on 'Pulmonary fibrosis'

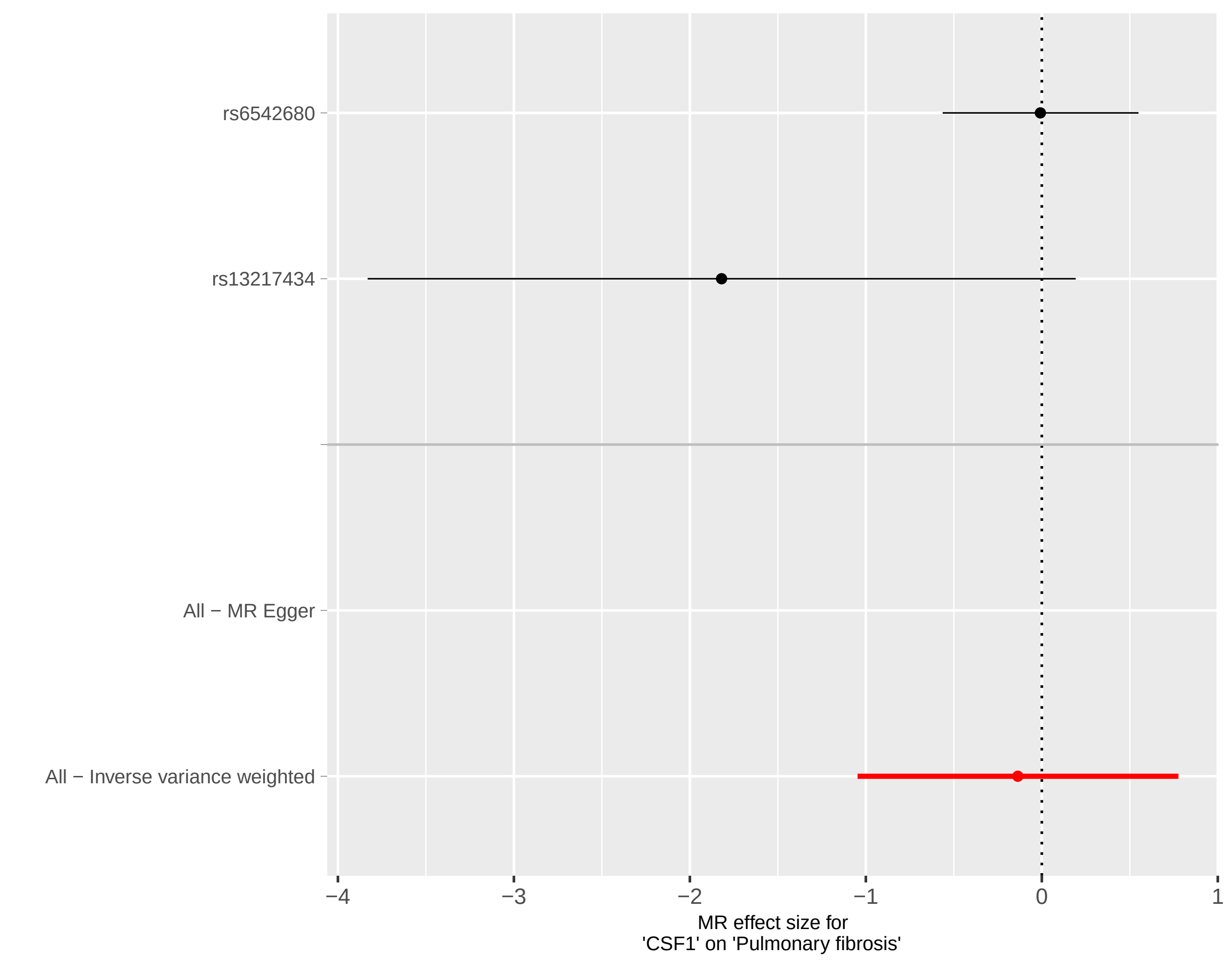

rs6542680
rs13217434
All − MR Egger
All − Inverse variance weighted
−4
−3
−2
−1
0
1
MR effect size for
'CSF1' on 'Pulmonary fibrosis'

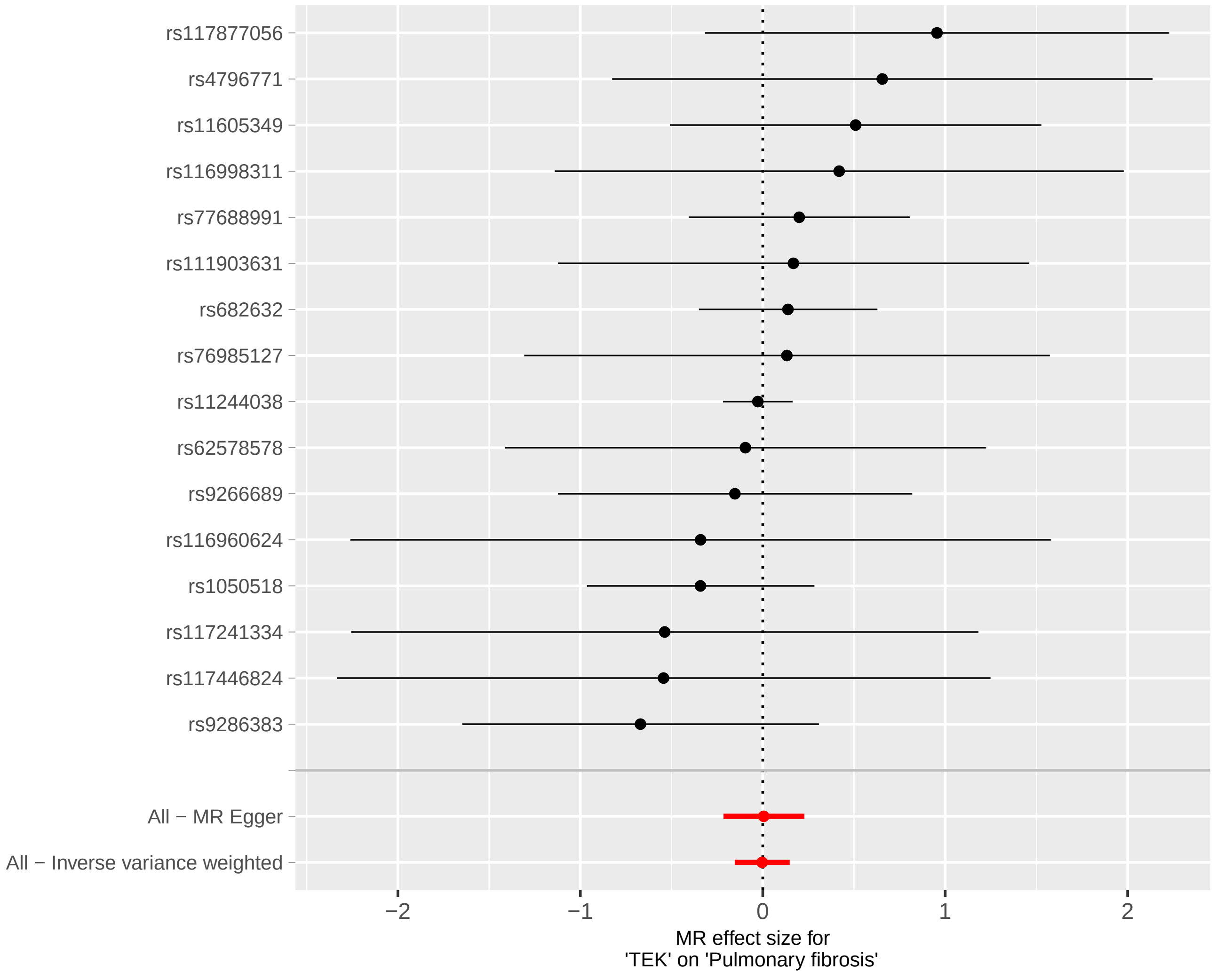

rs117877056
rs4796771
rs11605349
rs116998311
rs77688991
rs111903631
rs682632
rs76985127
rs11244038
rs62578578
rs9266689
rs116960624
rs1050518
rs117241334
rs117446824
rs9286383
All − MR Egger
All − Inverse variance weighted
−2
−1
0
1
2
MR effect size for
'TEK' on 'Pulmonary fibrosis'

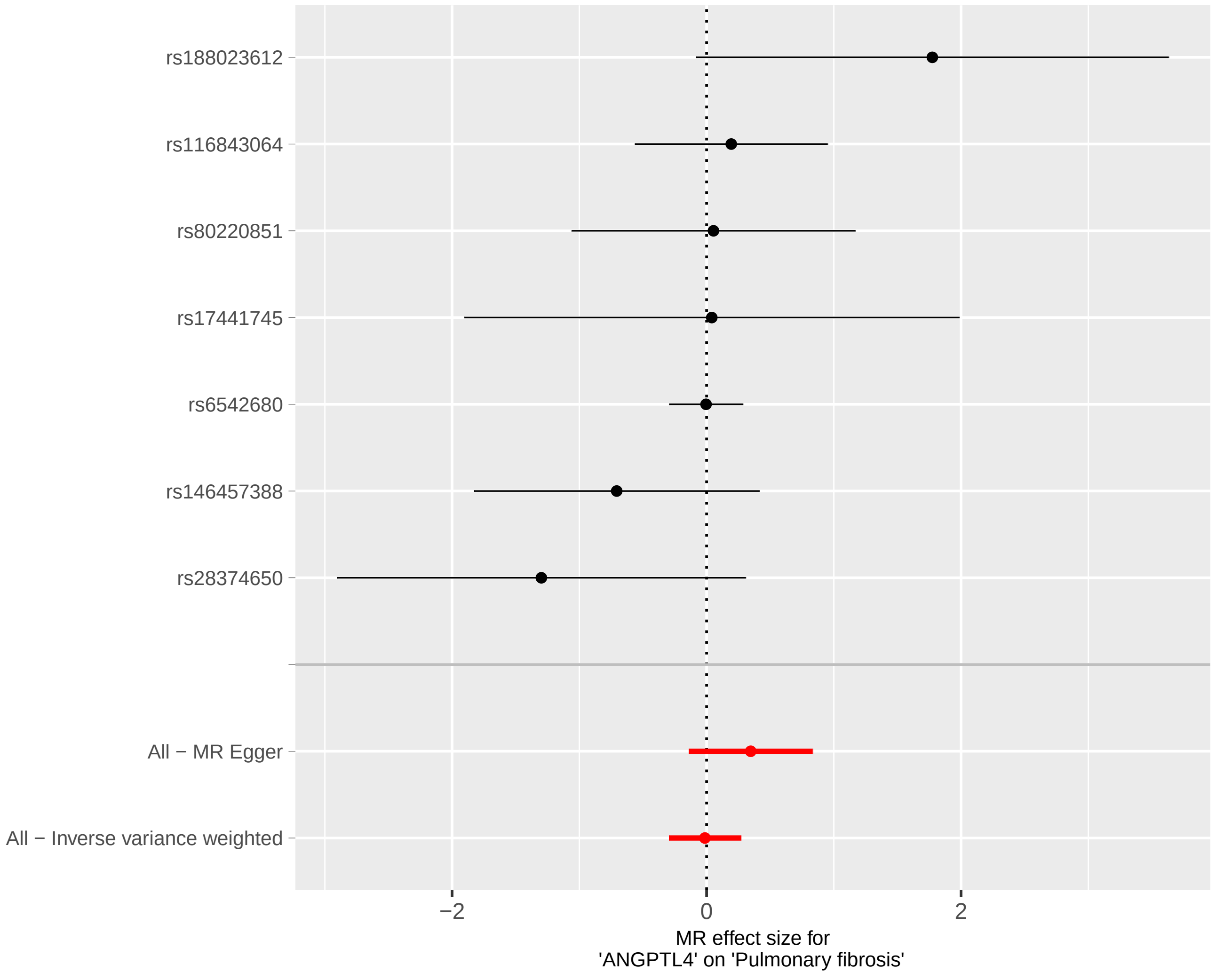
rs188023612
rs116843064
rs80220851
rs17441745
rs6542680
rs146457388
rs28374650
All − MR Egger
All − Inverse variance weighted
−2
0
2
MR effect size for
'ANGPTL4' on 'Pulmonary fibrosis'

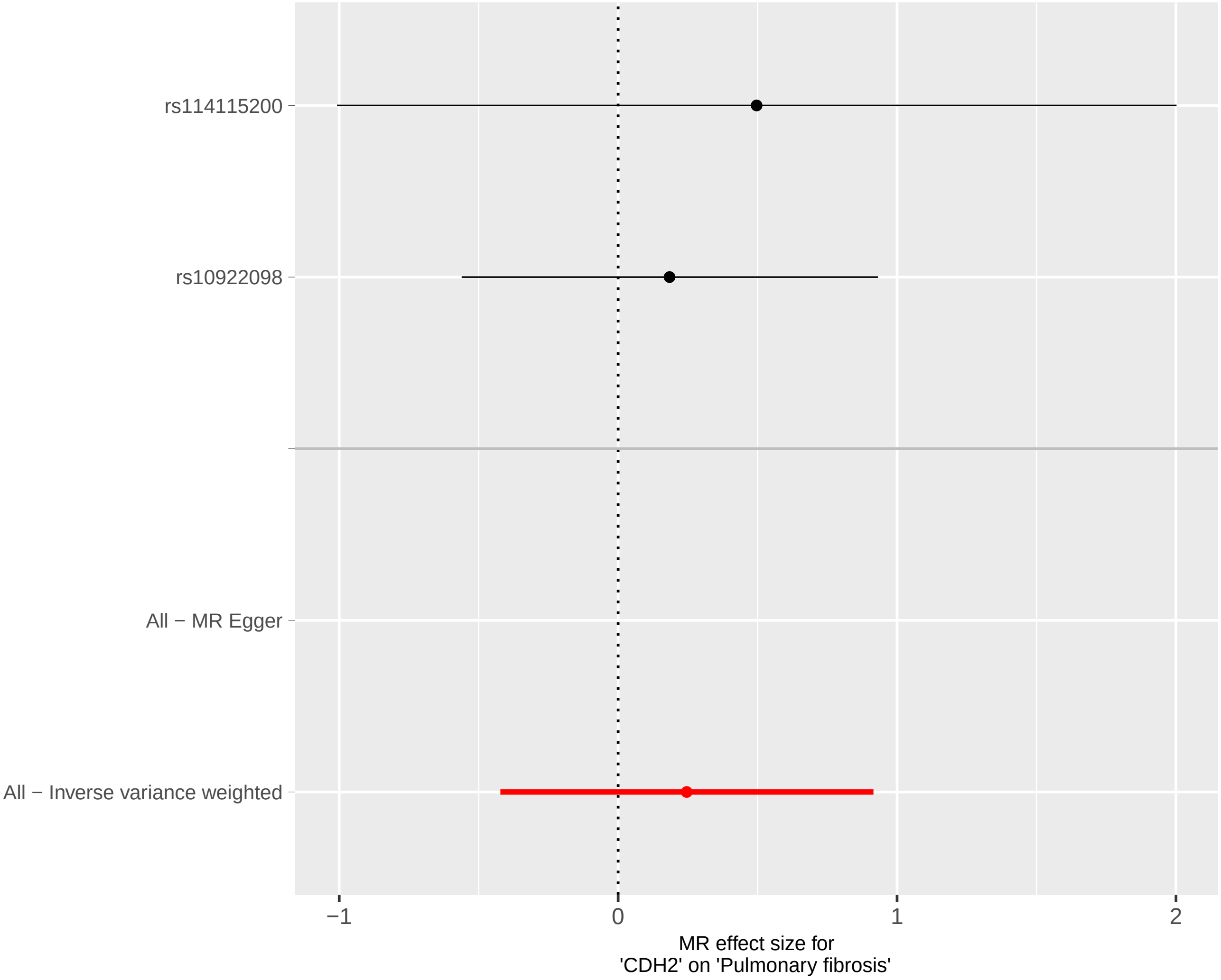
rs114115200
rs10922098
All − MR Egger
All − Inverse variance weighted
−1
0
1
2
MR effect size for
'CDH2' on 'Pulmonary fibrosis'

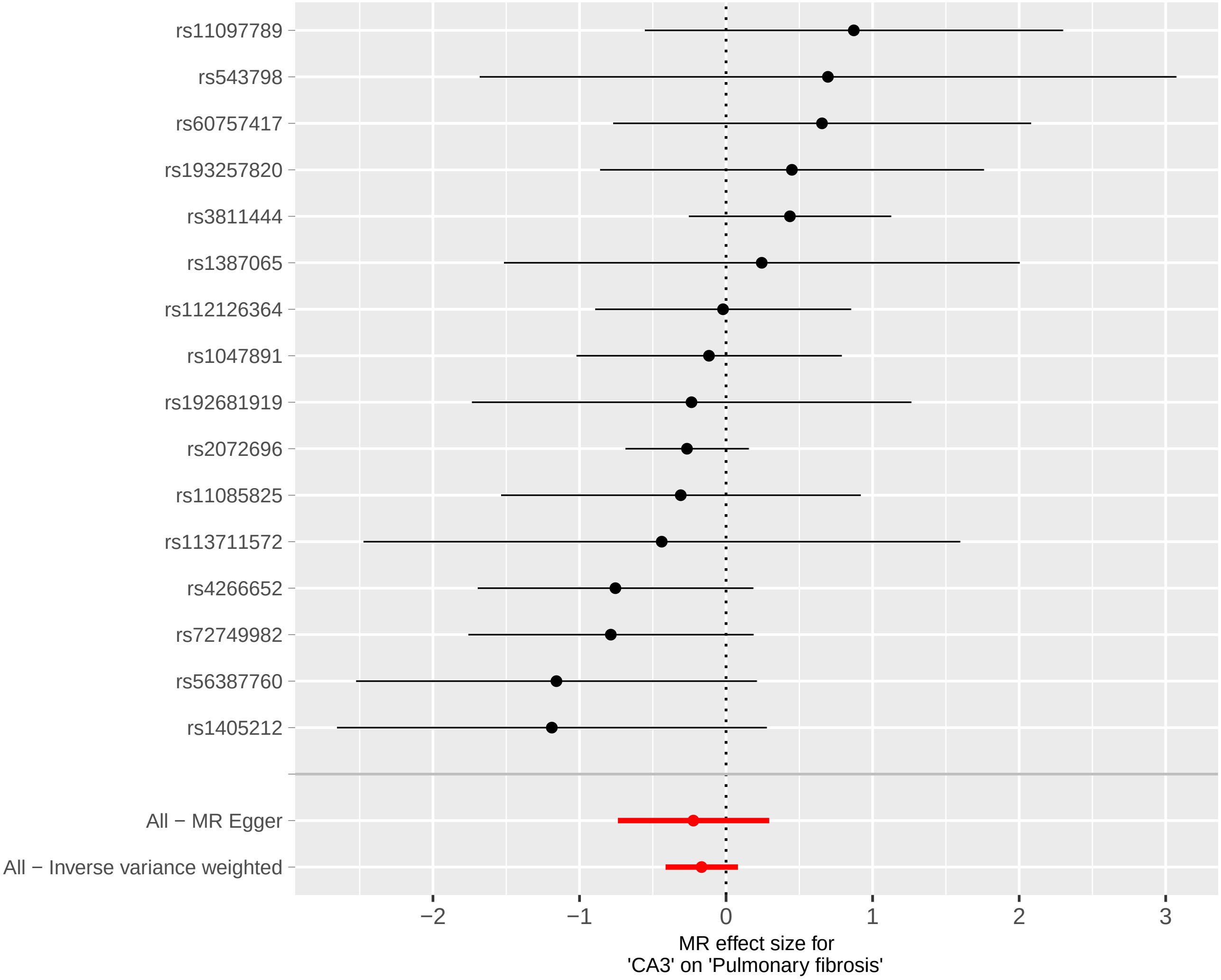

rs11097789
rs543798
rs60757417
rs193257820
rs3811444
rs1387065
rs112126364
rs1047891
rs192681919
rs2072696
rs11085825
rs113711572
rs4266652
rs72749982
rs56387760
rs1405212
All − MR Egger
All − Inverse variance weighted
−2
−1
0
1
2
3
MR effect size for
'CA3' on 'Pulmonary fibrosis'

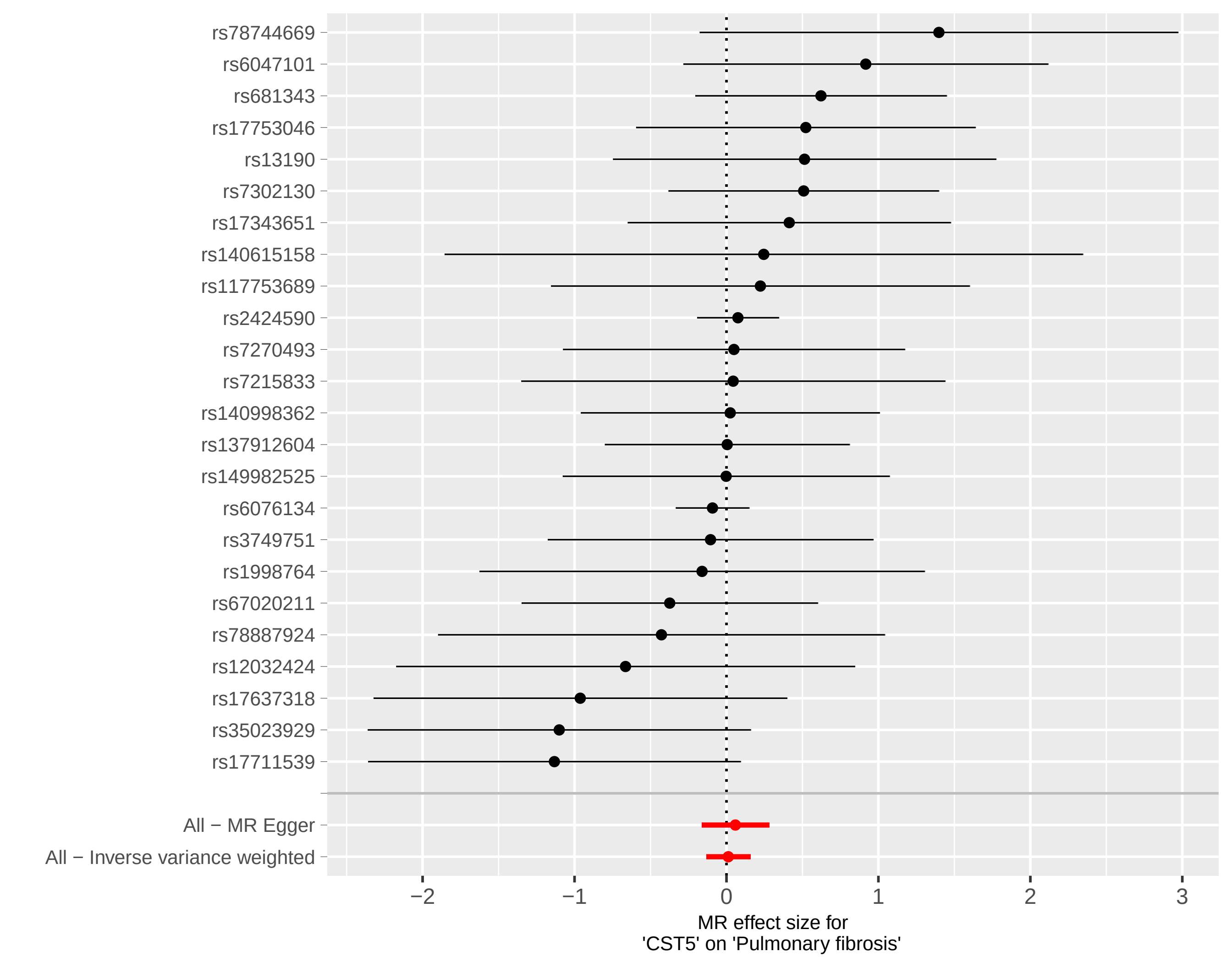
rs78744669
rs6047101
rs681343
rs17753046
rs13190
rs7302130
rs17343651
rs140615158
rs117753689
rs2424590
rs7270493
rs7215833
rs140998362
rs137912604
rs149982525
rs6076134
rs3749751
rs1998764
rs67020211
rs78887924
rs12032424
rs17637318
rs35023929
rs17711539
All − MR Egger
All − Inverse variance weighted
−2
−1
0
1
2
3
MR effect size for
'CST5' on 'Pulmonary fibrosis'

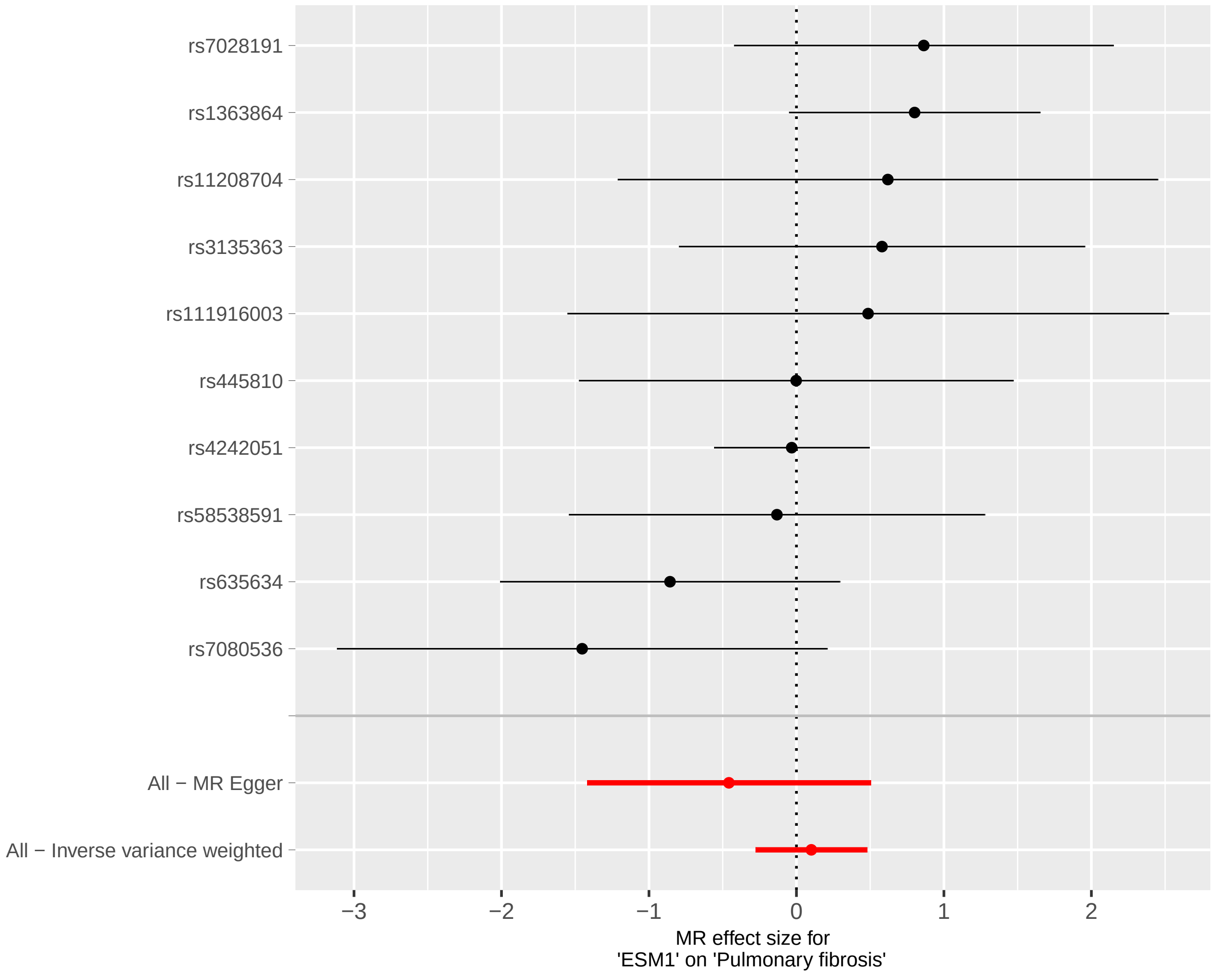

rs7028191
rs1363864
rs11208704
rs3135363
rs111916003
rs445810
rs4242051
rs58538591
rs635634
rs7080536
All − MR Egger
All − Inverse variance weighted
−3
−2
−1
0
1
2
MR effect size for
'ESM1' on 'Pulmonary fibrosis'

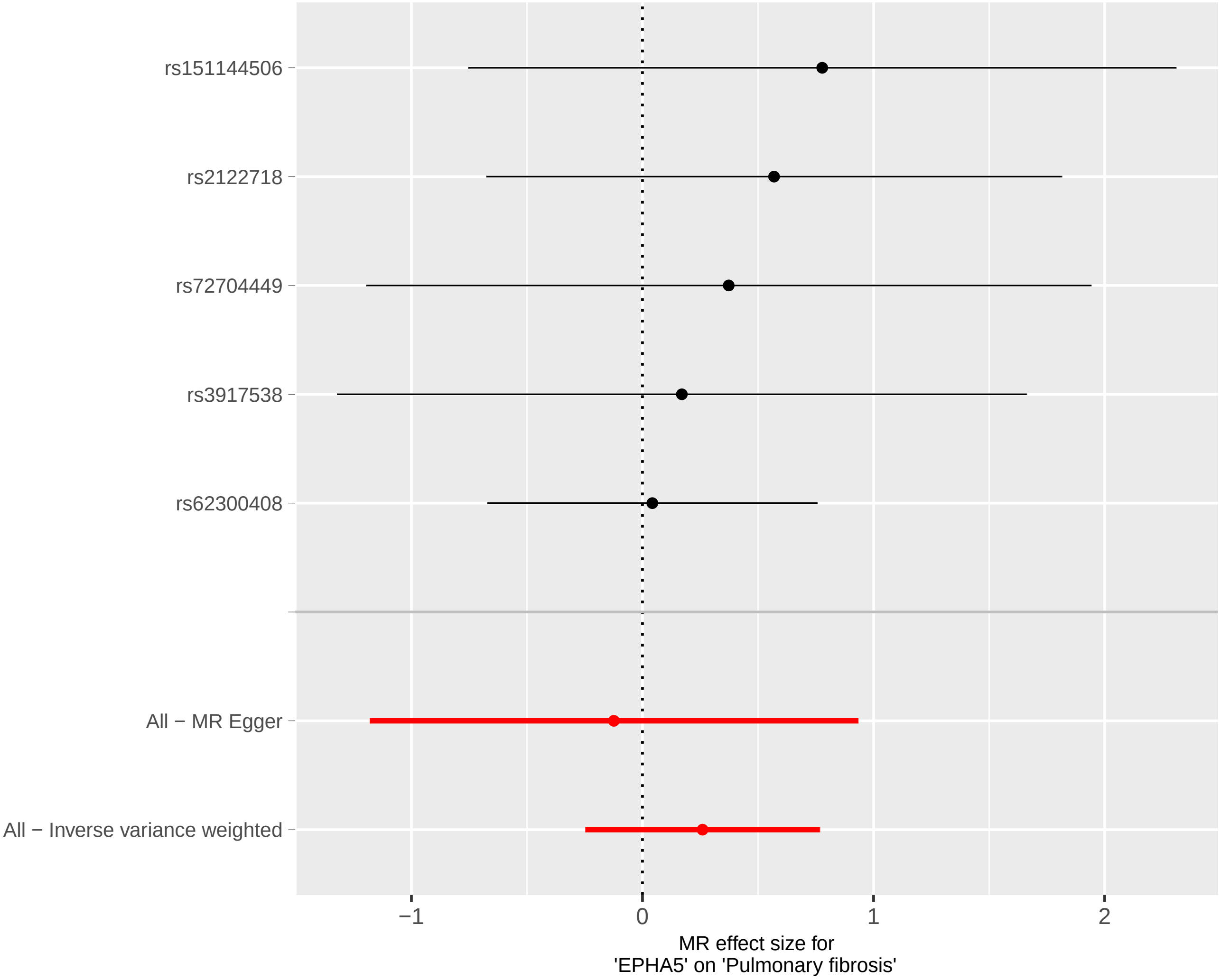

rs151144506
rs2122718
rs72704449
rs3917538
rs62300408
All − MR Egger
All − Inverse variance weighted
−1
0
1
2
MR effect size for
'EPHA5' on 'Pulmonary fibrosis'

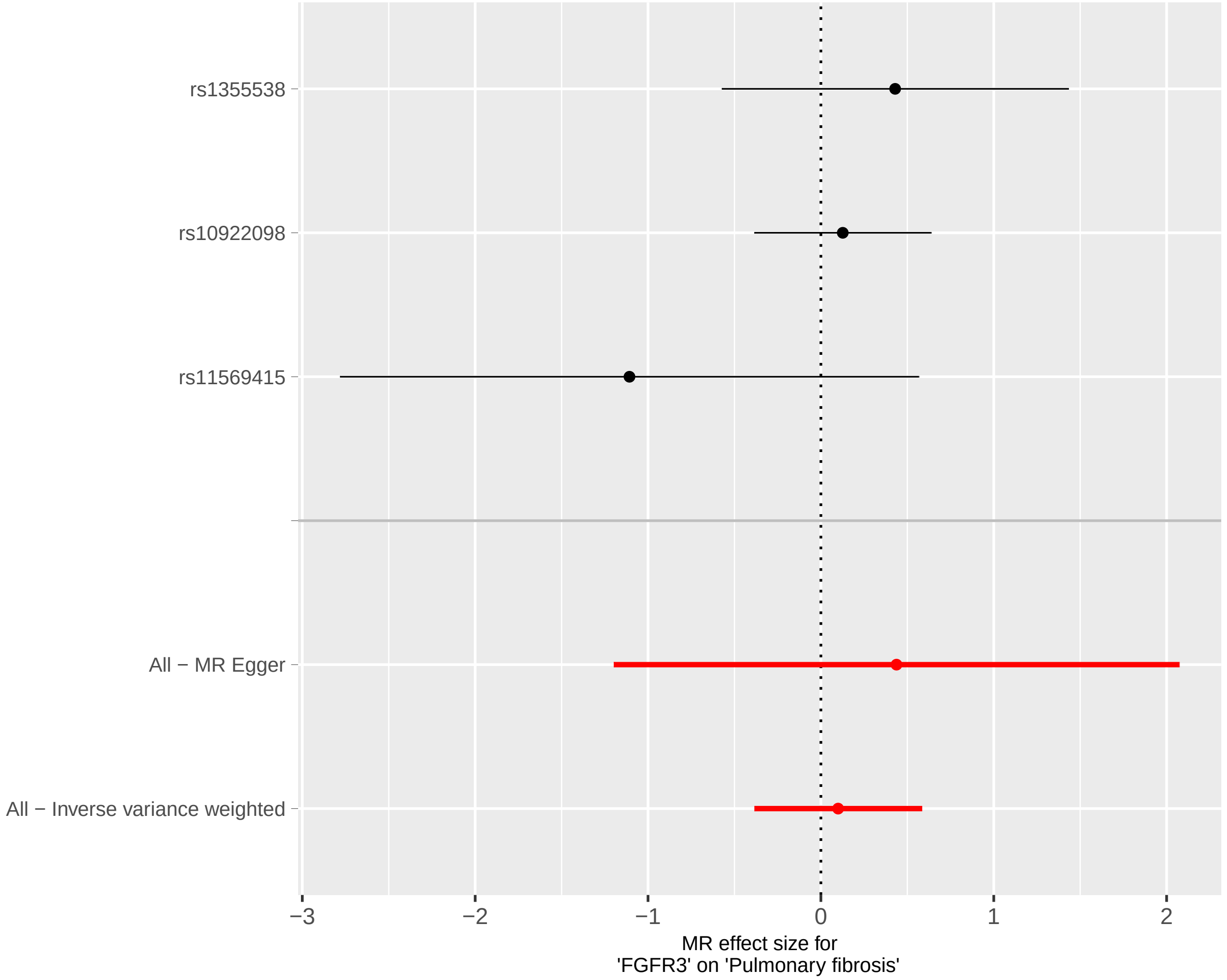

rs1355538
rs10922098
rs11569415
All − MR Egger
All − Inverse variance weighted
−3
−2
−1
0
1
2
MR effect size for
'FGFR3' on 'Pulmonary fibrosis'

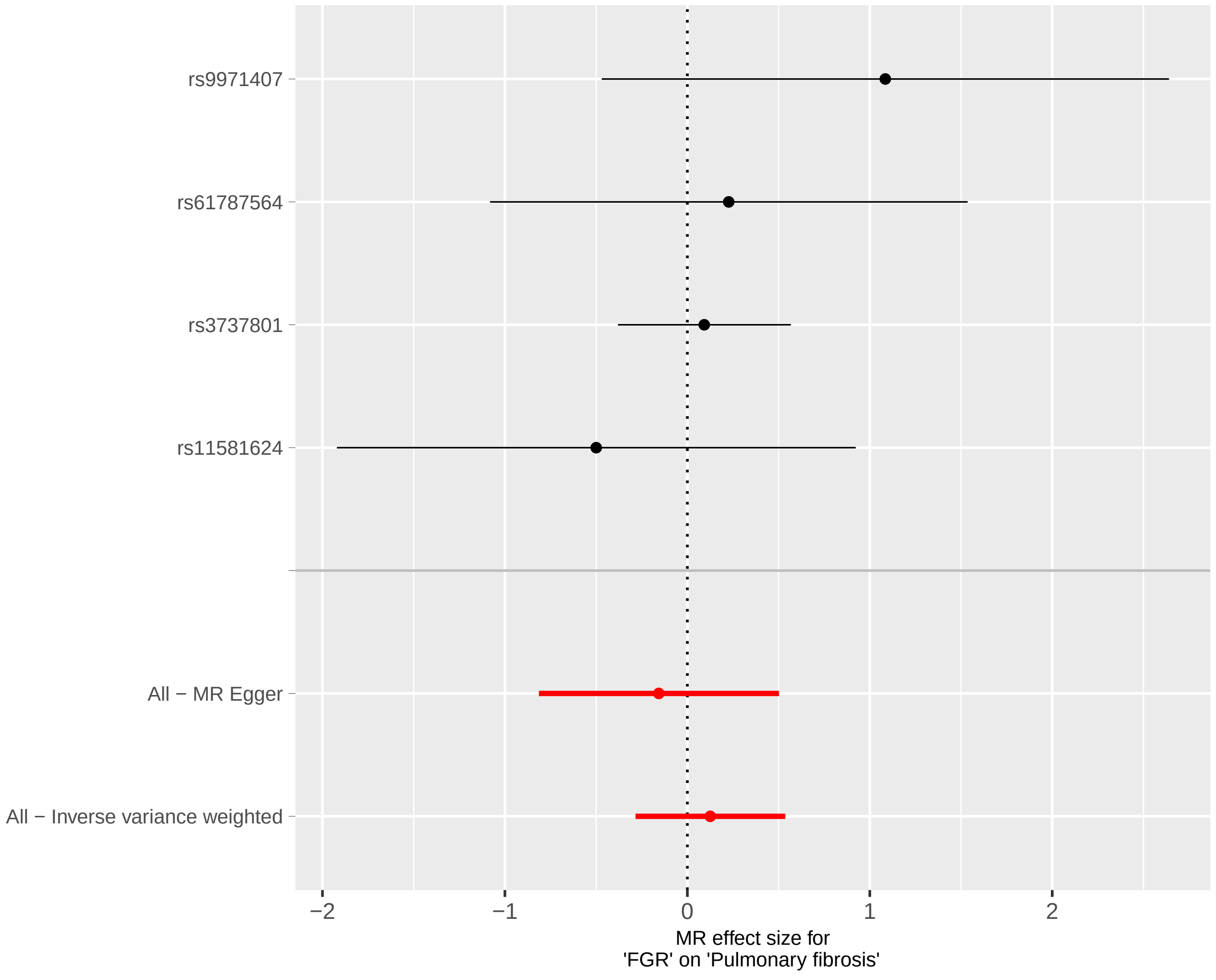
rs9971407
rs61787564
rs3737801
rs11581624
All − MR Egger
All − Inverse variance weighted
−2
−1
0
1
2
MR effect size for
'FGR' on 'Pulmonary fibrosis'

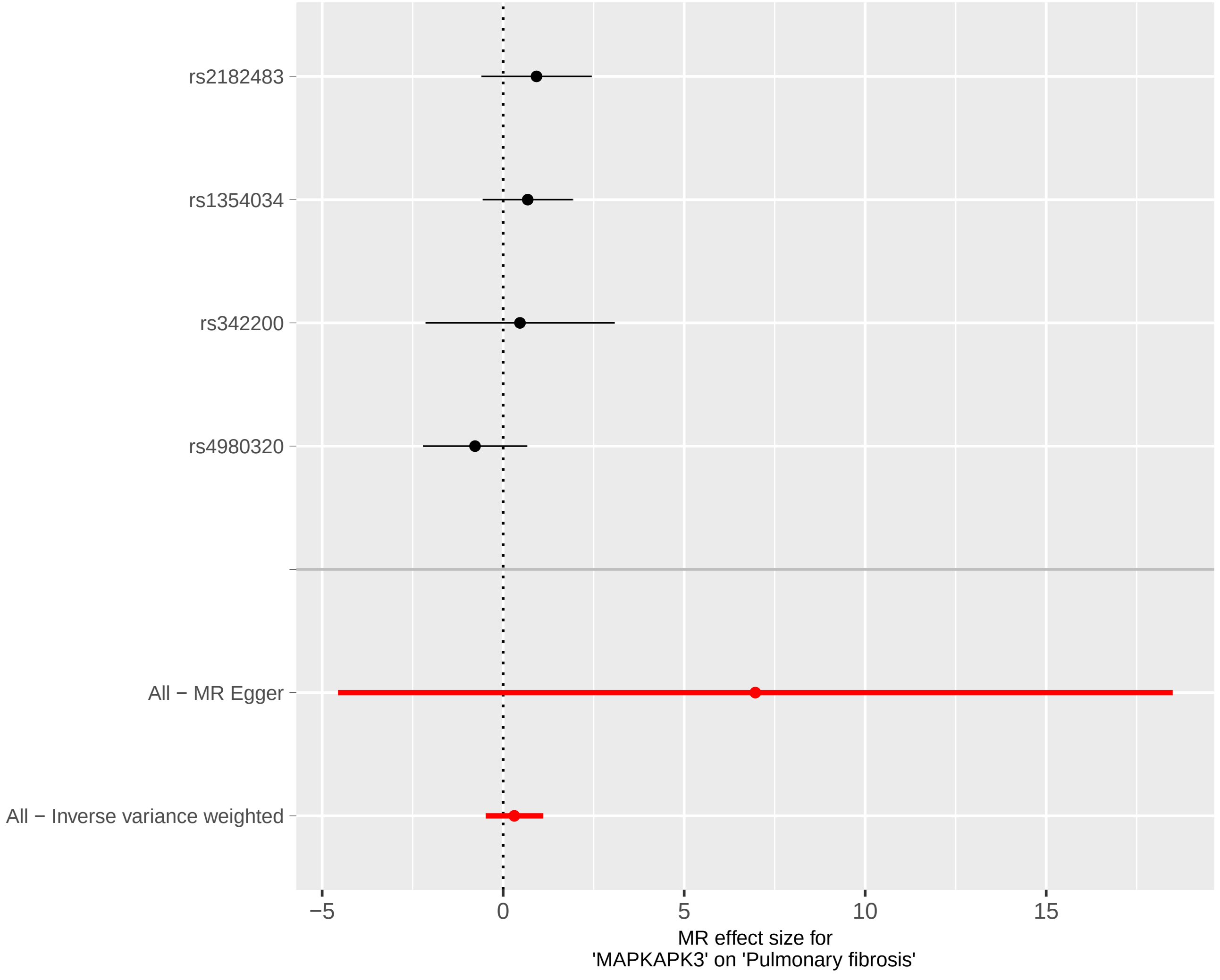
rs2182483
rs1354034
rs342200
rs4980320
All − MR Egger
All − Inverse variance weighted
−5
0
5
10
15
MR effect size for
'MAPKAPK3' on 'Pulmonary fibrosis'

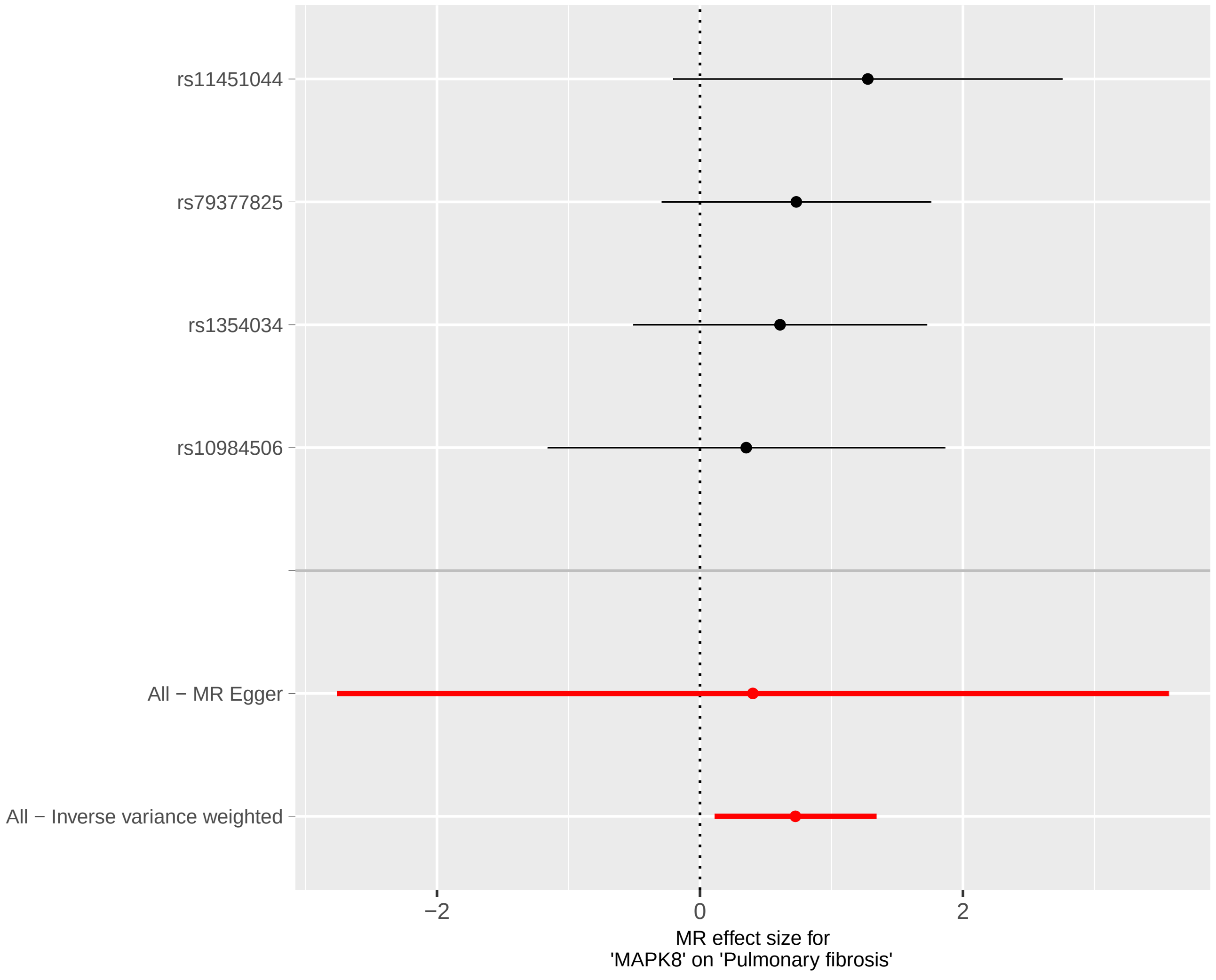

rs11451044
rs79377825
rs1354034
rs10984506
All − MR Egger
All − Inverse variance weighted
−2
0
2
MR effect size for
'MAPK8' on 'Pulmonary fibrosis'

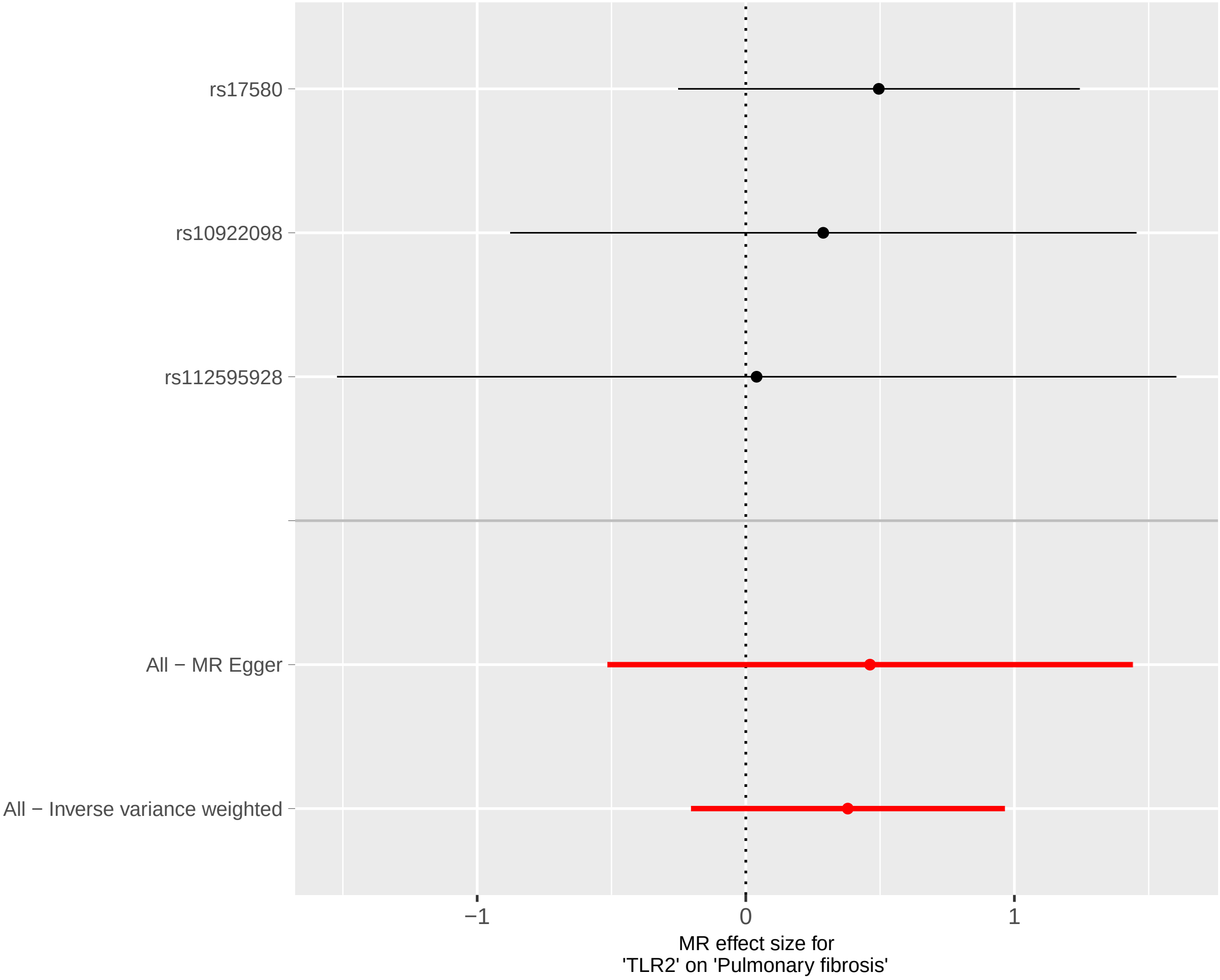

rs17580
rs10922098
rs112595928
All − MR Egger
All − Inverse variance weighted
−1
0
1
MR effect size for
'TLR2' on 'Pulmonary fibrosis'

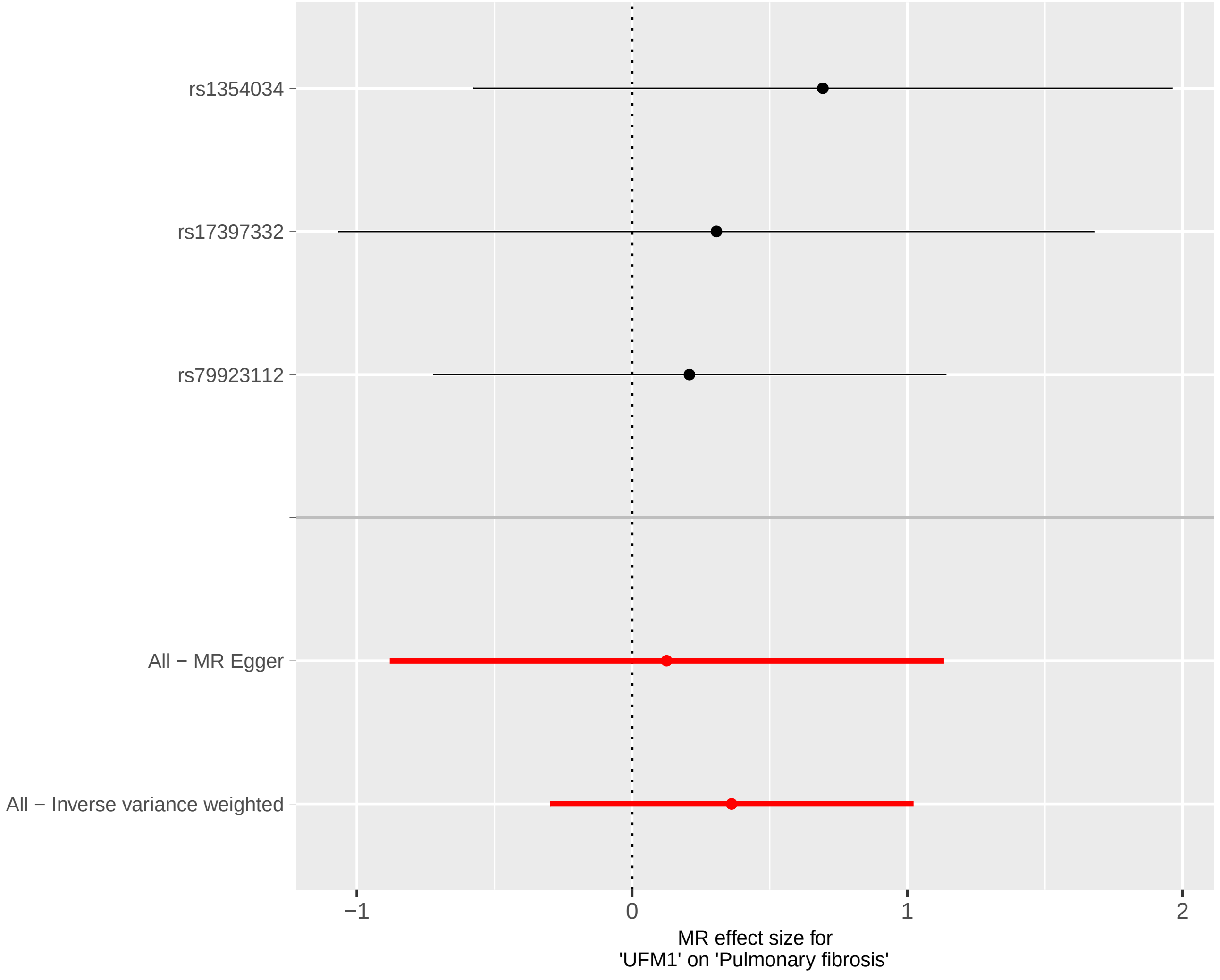

rs1354034
rs17397332
rs79923112
All − MR Egger
All − Inverse variance weighted
−1
0
1
2
MR effect size for
'UFM1' on 'Pulmonary fibrosis'

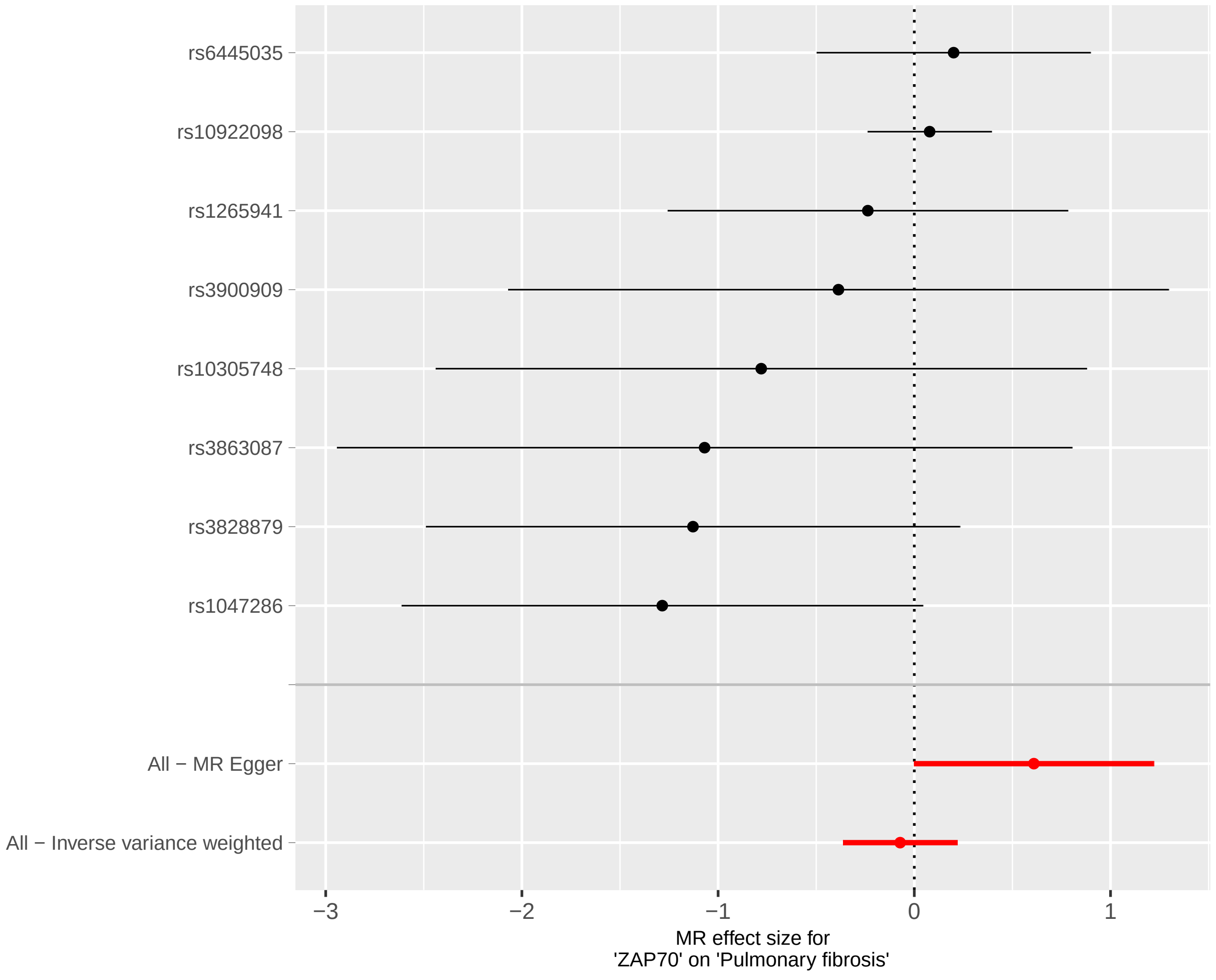

rs6445035
rs10922098
rs1265941
rs3900909
rs10305748
rs3863087
rs3828879
rs1047286
All − MR Egger
All − Inverse variance weighted
−3
−2
−1
0
1
MR effect size for
'ZAP70' on 'Pulmonary fibrosis'

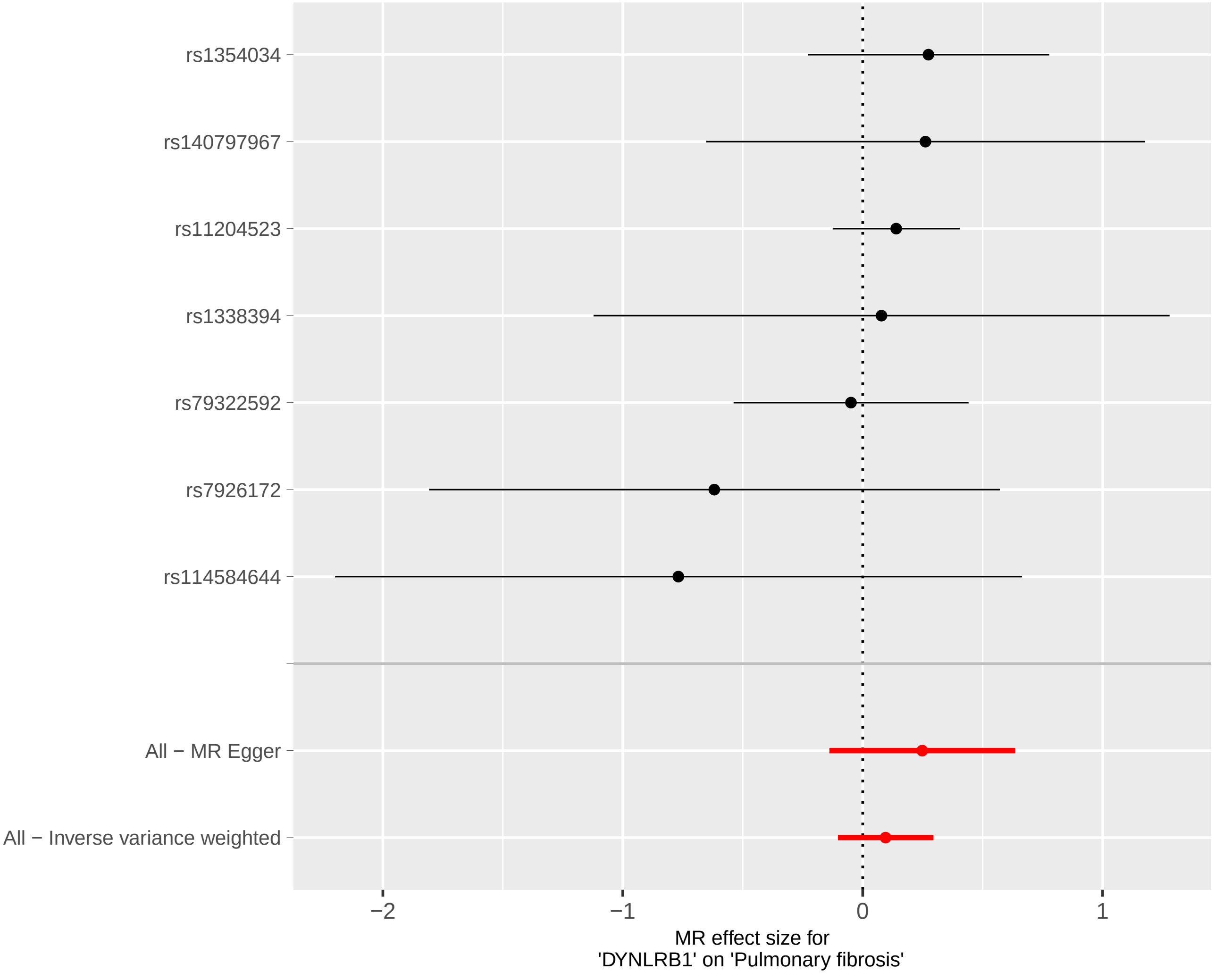
rs1354034
rs140797967
rs11204523
rs1338394
rs79322592
rs7926172
rs114584644
All − MR Egger
All − Inverse variance weighted
−2
−1
0
1
MR effect size for
'DYNLRB1' on 'Pulmonary fibrosis'

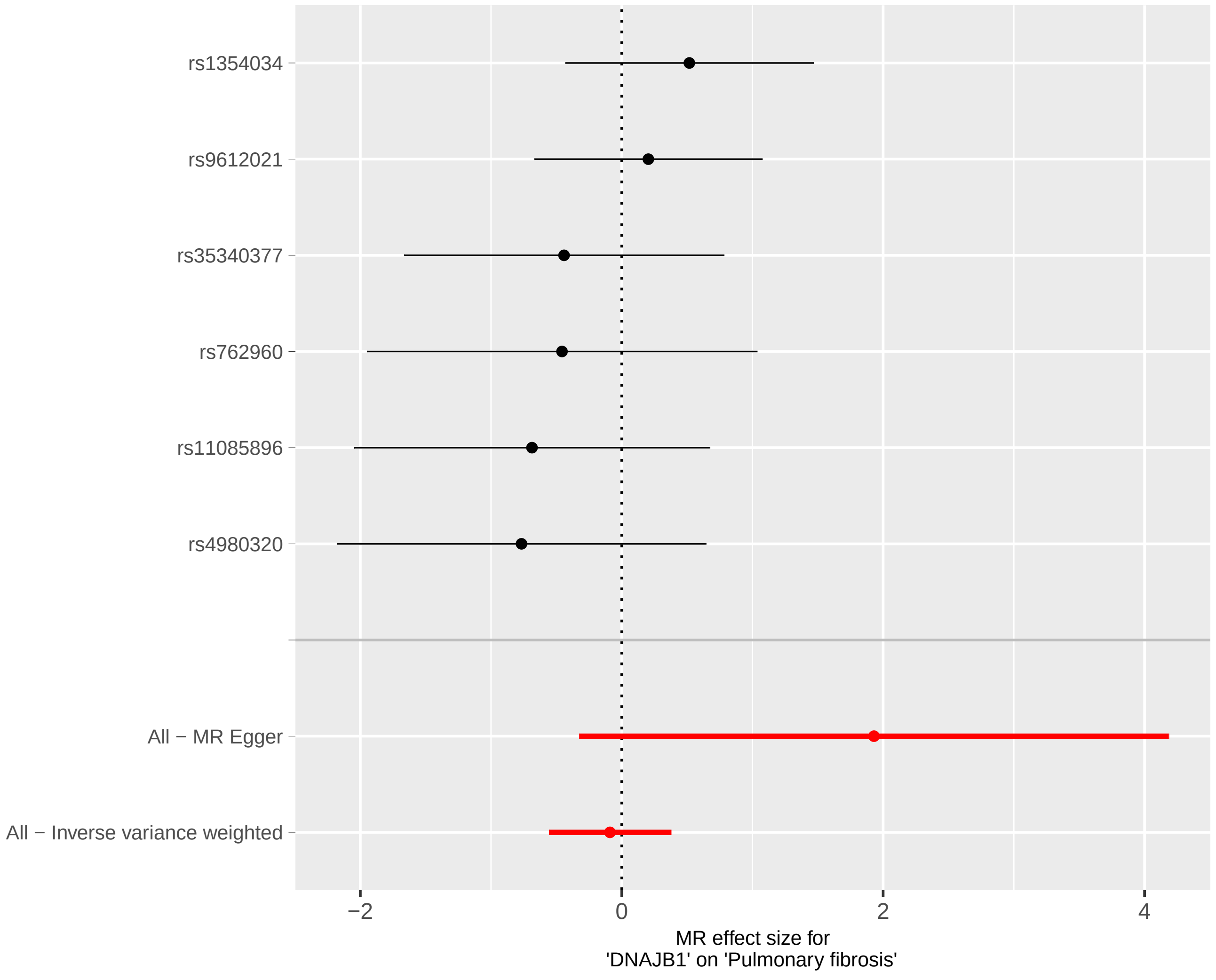

rs1354034
rs9612021
rs35340377
rs762960
rs11085896
rs4980320
All − MR Egger
All − Inverse variance weighted
−2
0
2
4
MR effect size for
'DNAJB1' on 'Pulmonary fibrosis'

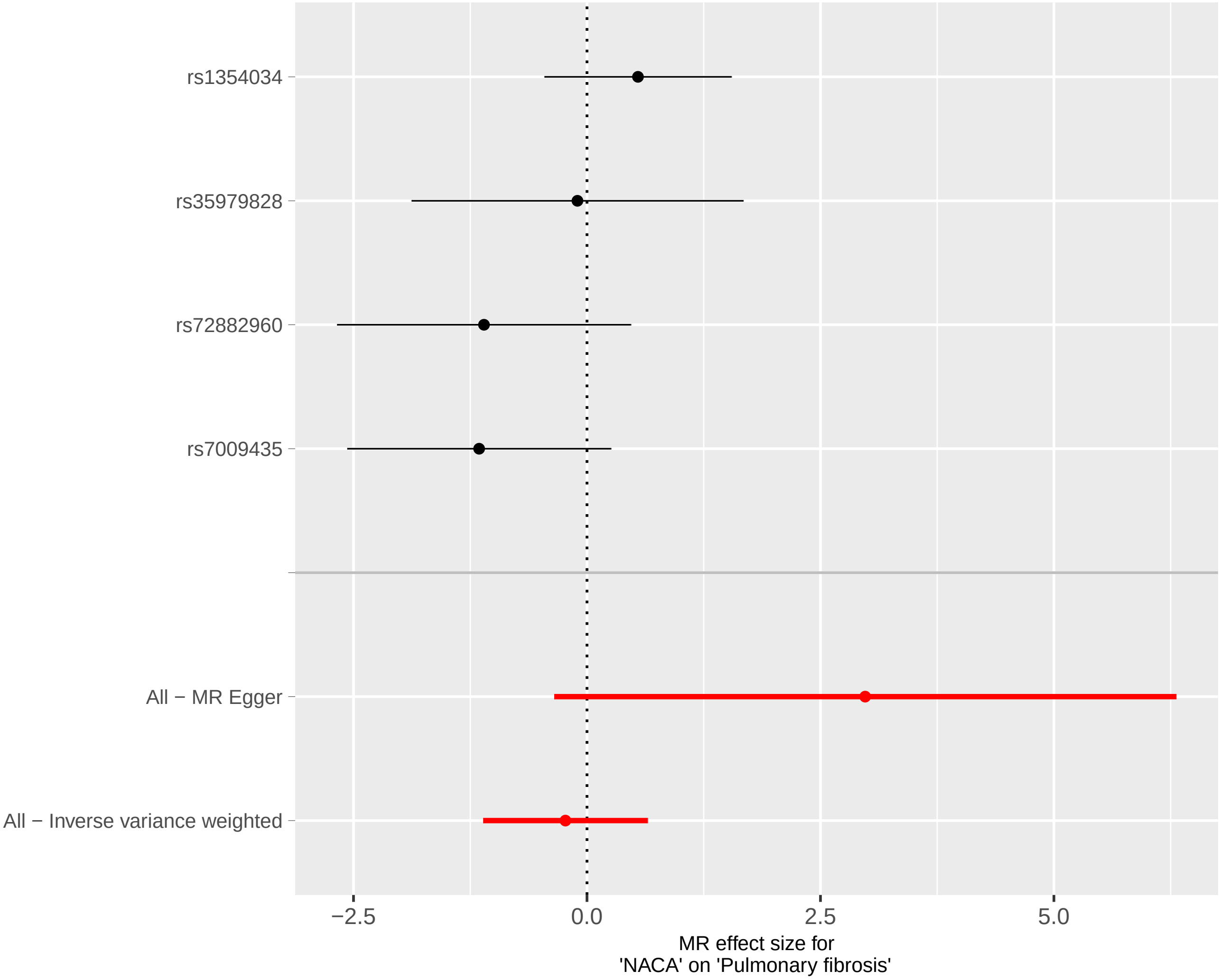

rs1354034
rs35979828
rs72882960
rs7009435
All − MR Egger
All − Inverse variance weighted
−2.5
0.0
2.5
5.0
MR effect size for
'NACA' on 'Pulmonary fibrosis'

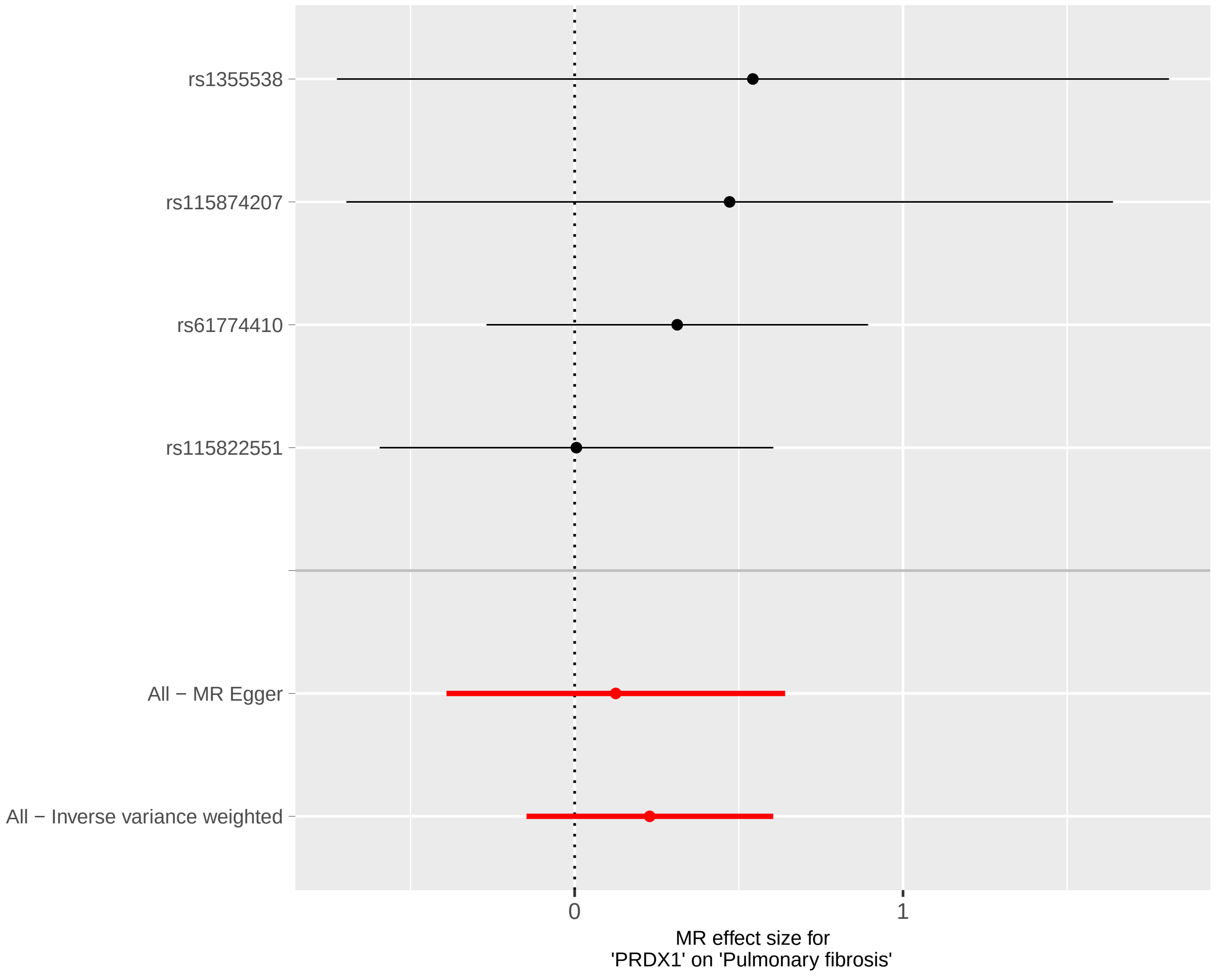
rs1355538
rs115874207
rs61774410
rs115822551
All − MR Egger
All − Inverse variance weighted
0
1
MR effect size for
'PRDX1' on 'Pulmonary fibrosis'

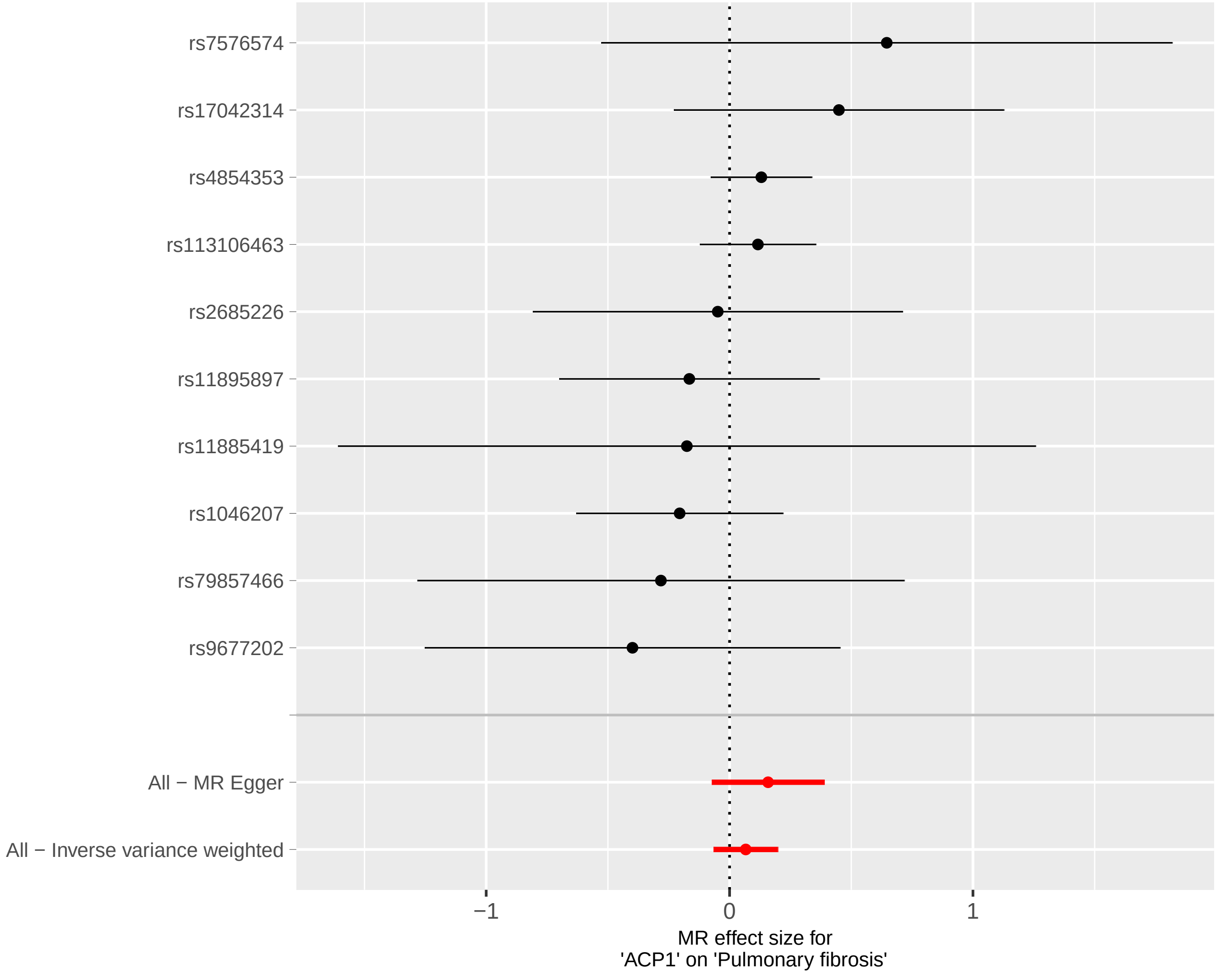

rs7576574
rs17042314
rs4854353
rs113106463
rs2685226
rs11895897
rs11885419
rs1046207
rs79857466
rs9677202
All − MR Egger
All − Inverse variance weighted
−1
0
1
MR effect size for
'ACP1' on 'Pulmonary fibrosis'

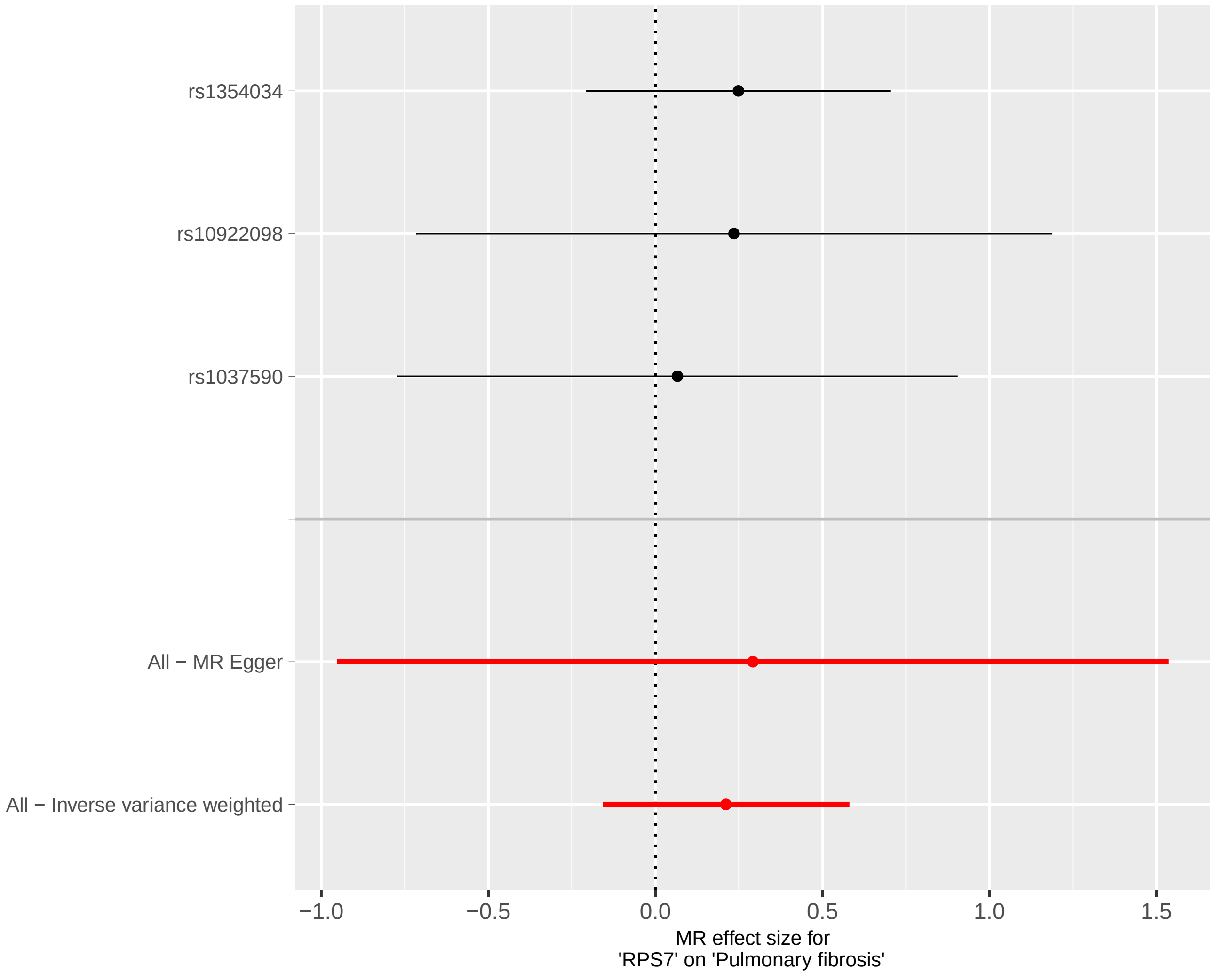
rs1354034
rs10922098
rs1037590
All − MR Egger
All − Inverse variance weighted
−1.0
−0.5
0.0
0.5
1.0
1.5
MR effect size for
'RPS7' on 'Pulmonary fibrosis'

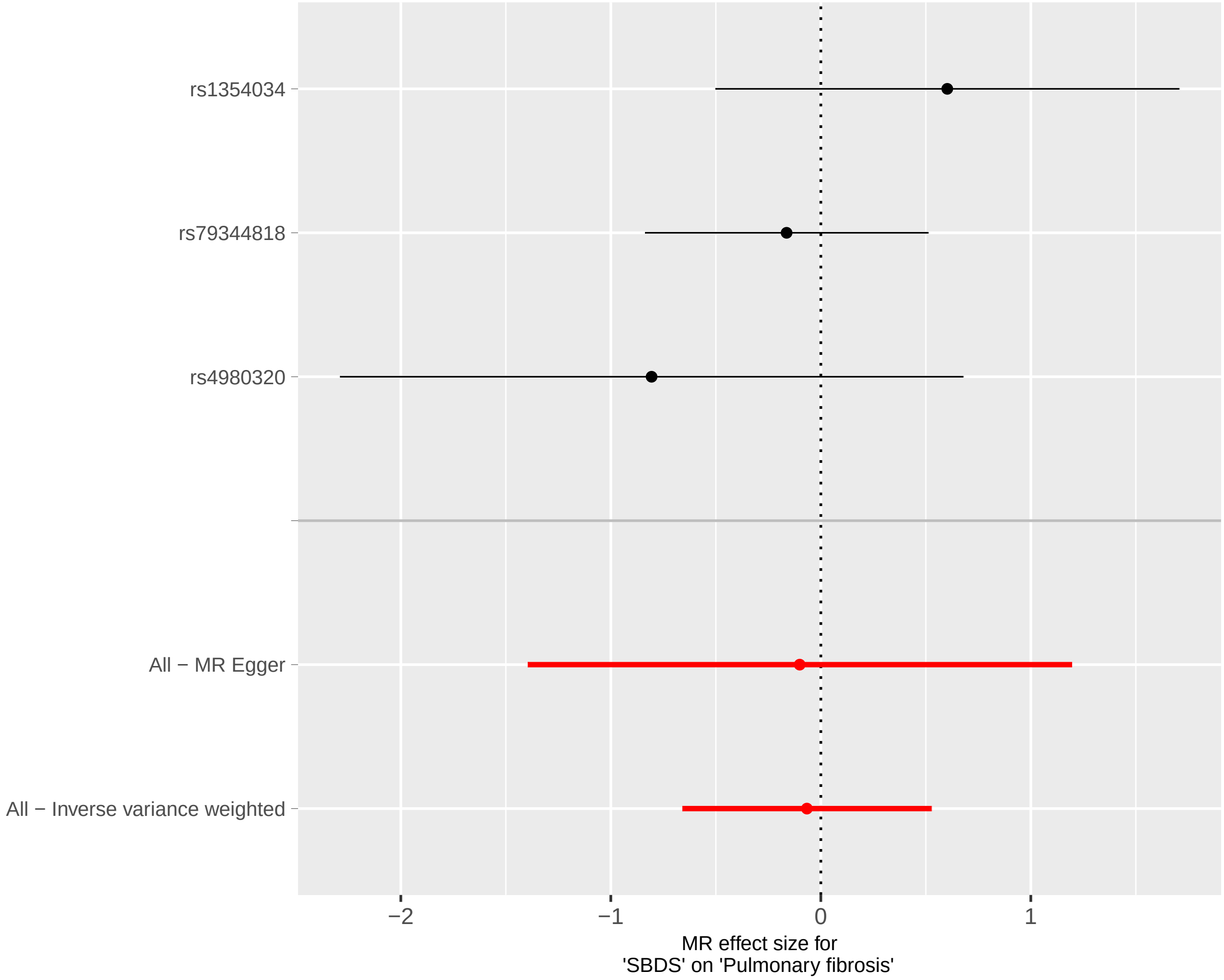
rs1354034
rs79344818
rs4980320
All − MR Egger
All − Inverse variance weighted
−2
−1
0
1
MR effect size for
'SBDS' on 'Pulmonary fibrosis'

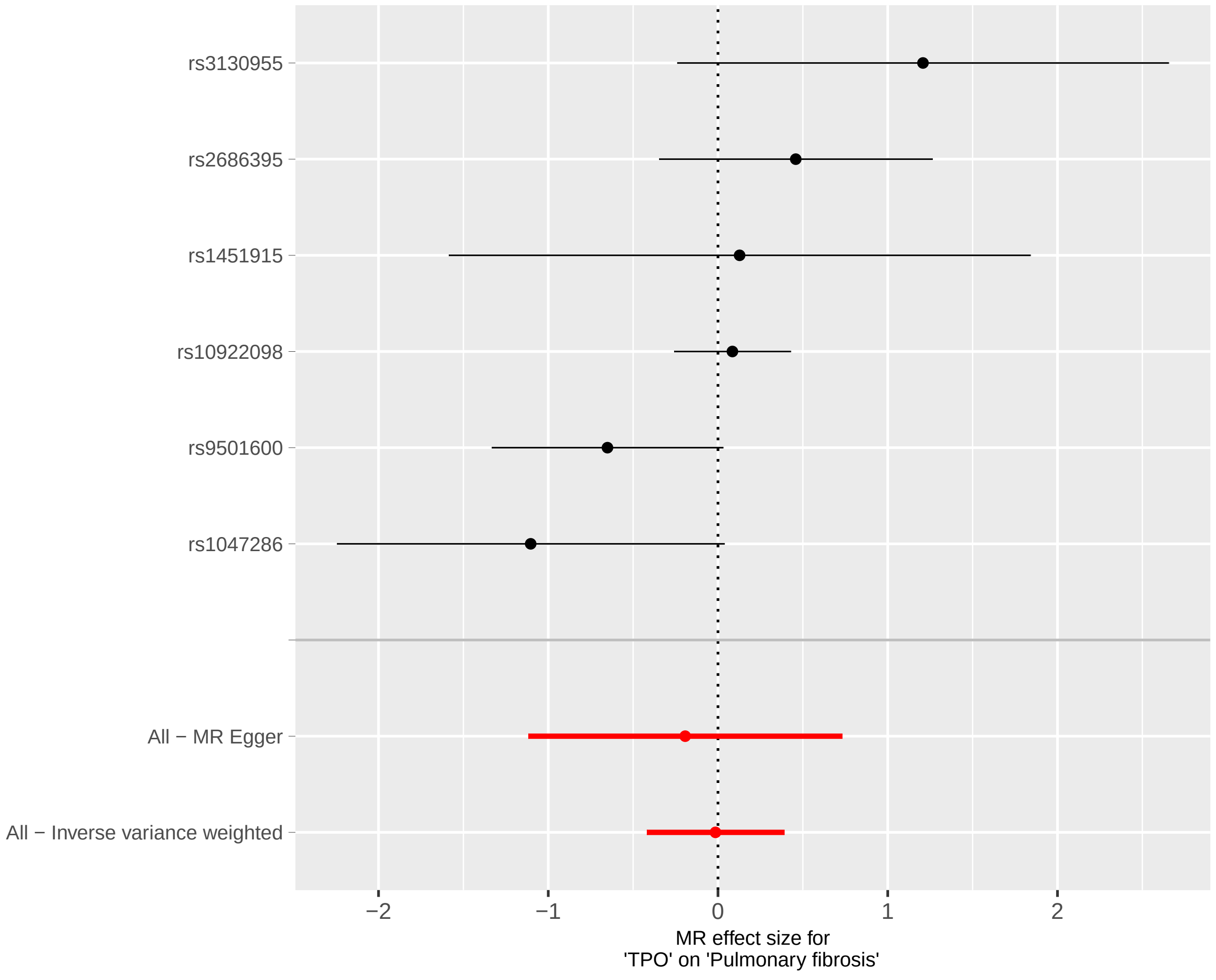

rs3130955
rs2686395
rs1451915
rs10922098
rs9501600
rs1047286
All − MR Egger
All − Inverse variance weighted
−2
−1
0
1
2
MR effect size for
'TPO' on 'Pulmonary fibrosis'

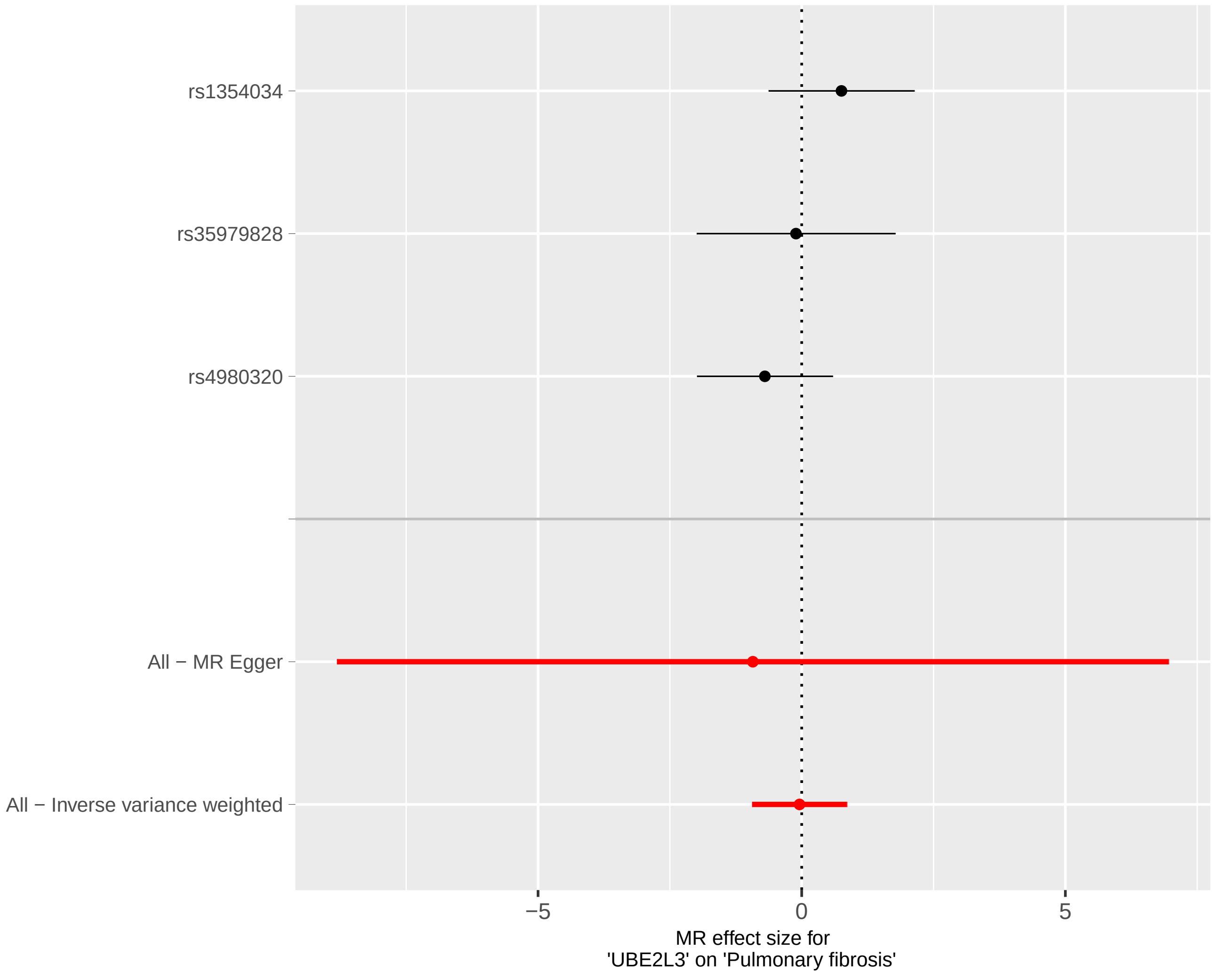
rs1354034
rs35979828
rs4980320
All − MR Egger
All − Inverse variance weighted
−5
0
5
MR effect size for
'UBE2L3' on 'Pulmonary fibrosis'

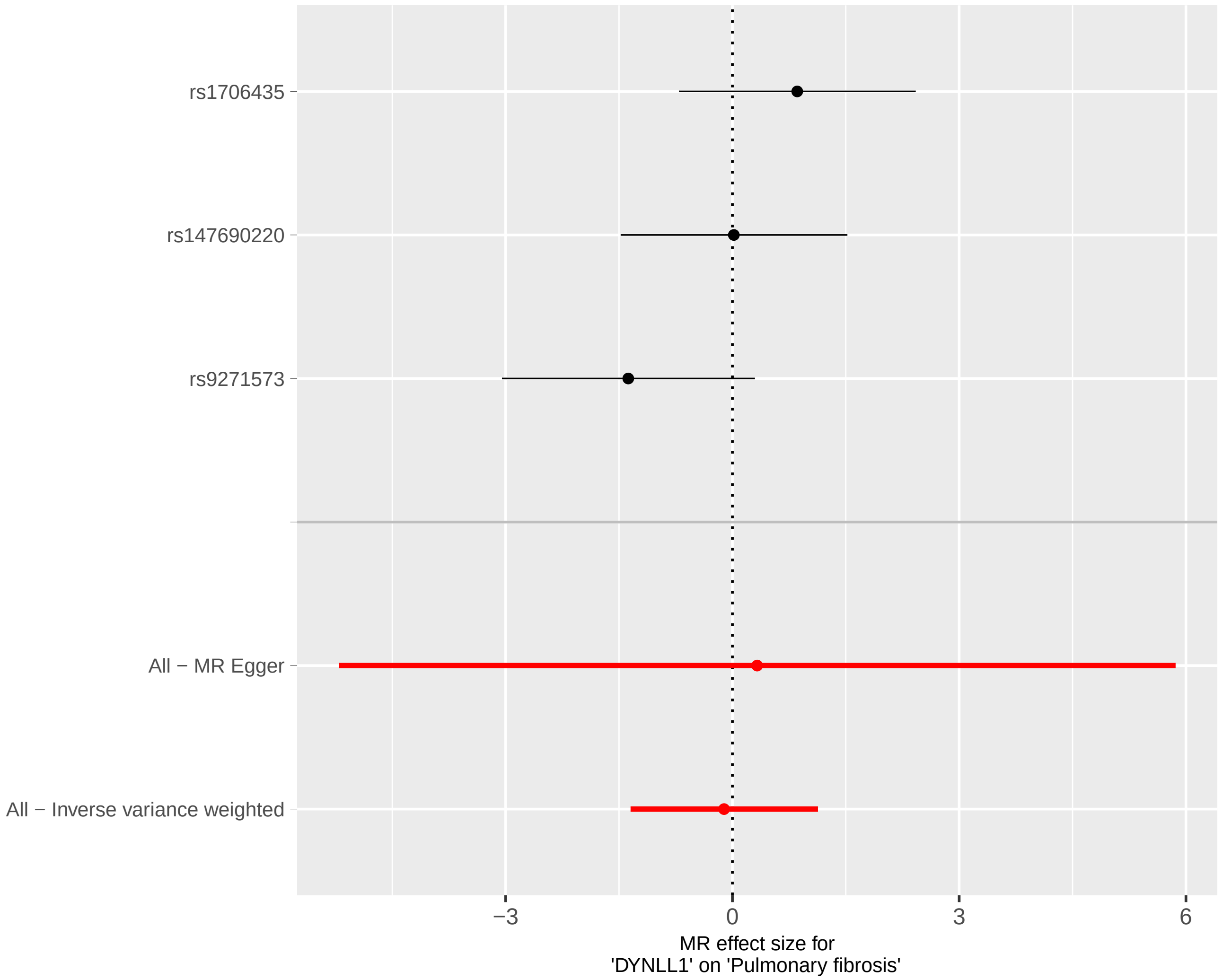

rs1706435
rs147690220
rs9271573
All − MR Egger
All − Inverse variance weighted
−3
0
3
6
MR effect size for
'DYNLL1' on 'Pulmonary fibrosis'

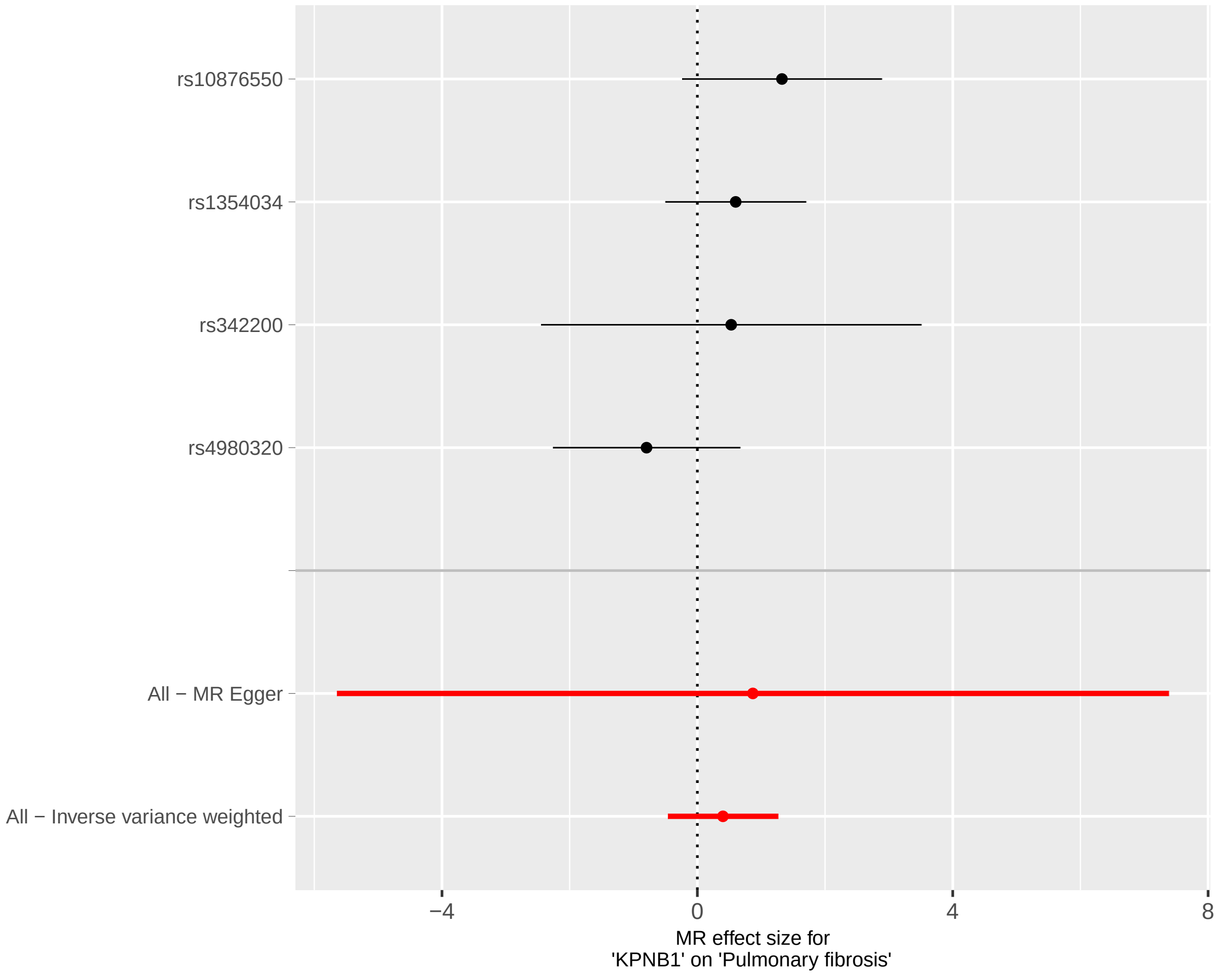

rs10876550
rs1354034
rs342200
rs4980320
All − MR Egger
All − Inverse variance weighted
−4
0
4
8
MR effect size for
'KPNB1' on 'Pulmonary fibrosis'

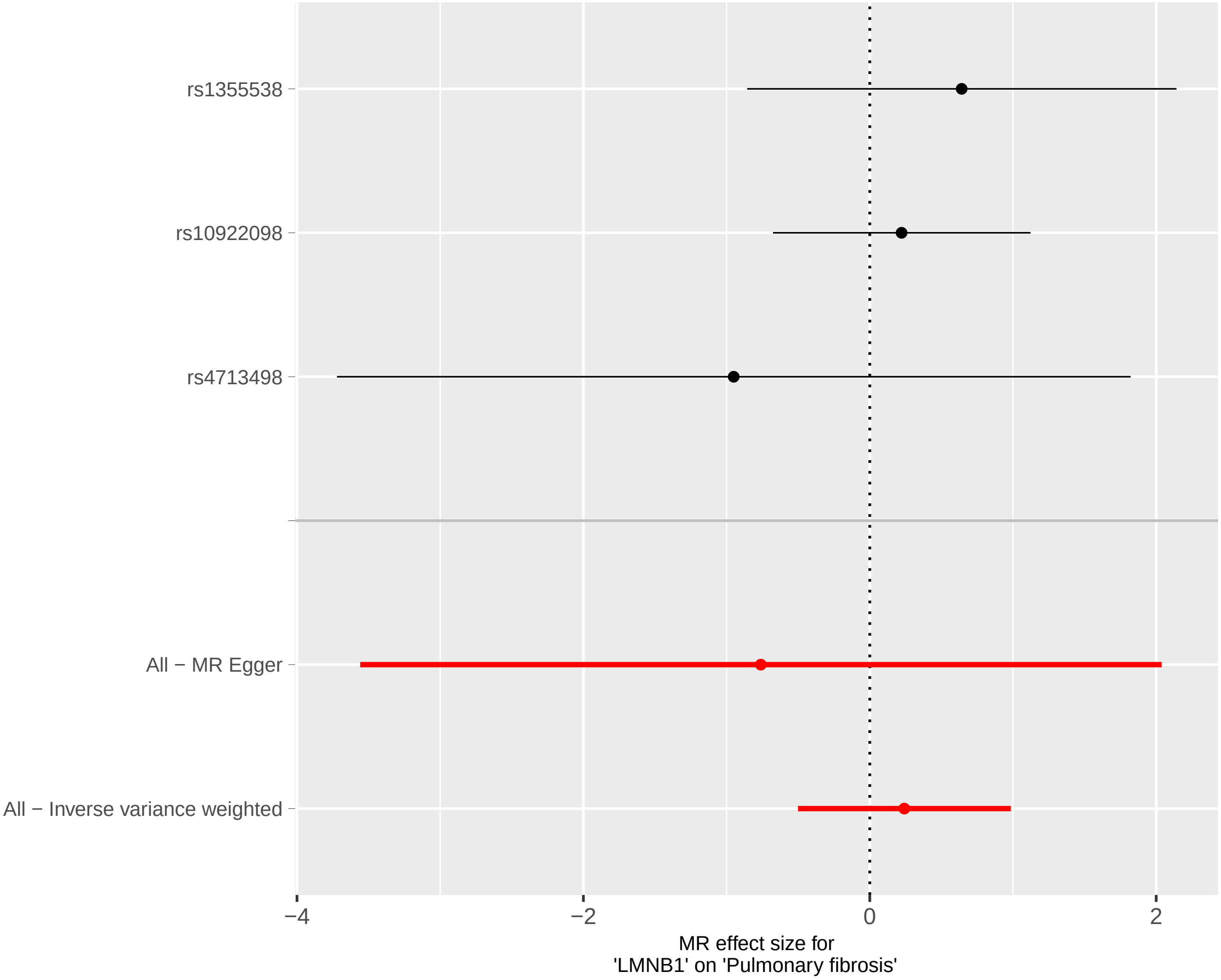

rs1355538
rs10922098
rs4713498
All − MR Egger
All − Inverse variance weighted
−4
−2
0
2
MR effect size for
'LMNB1' on 'Pulmonary fibrosis'

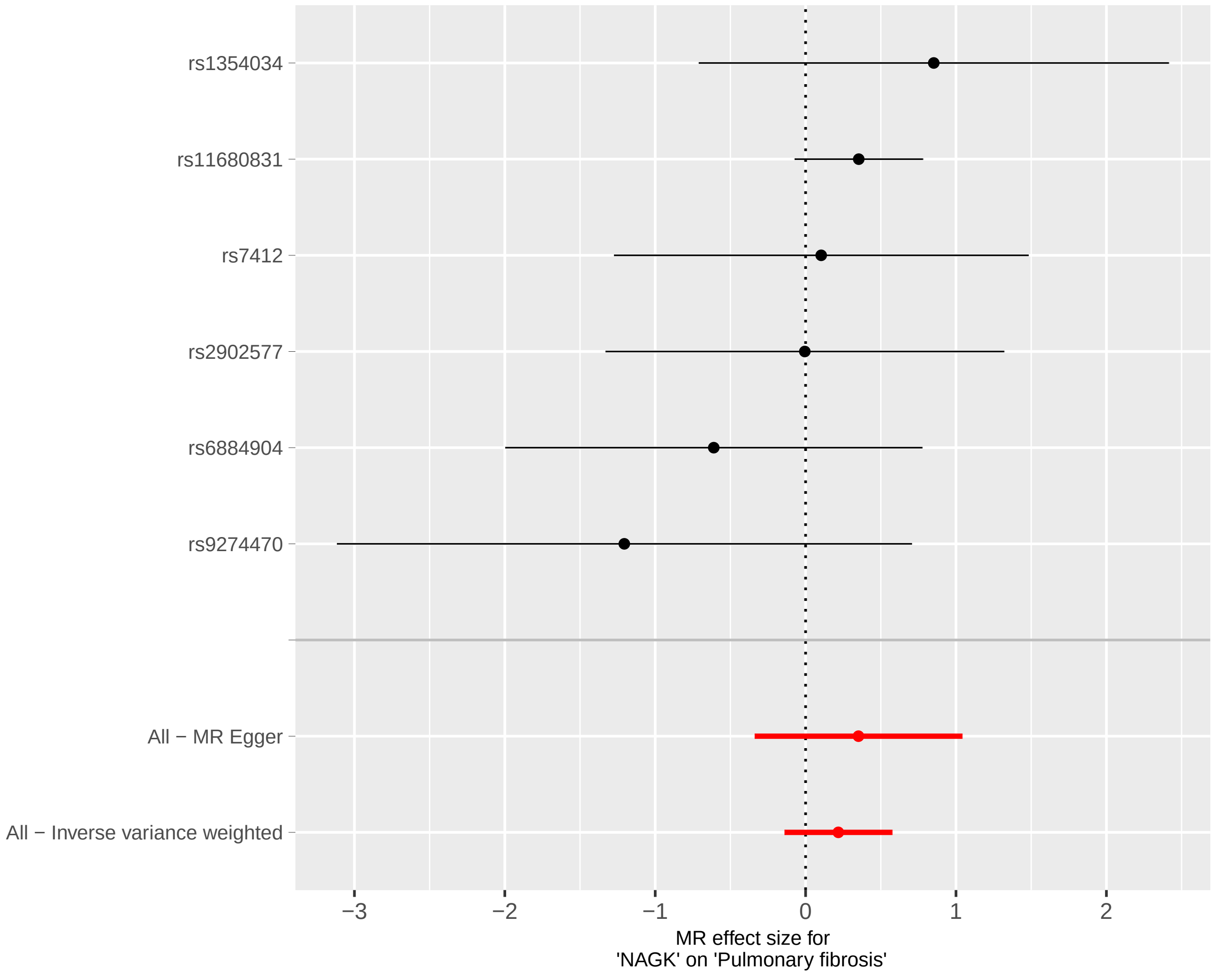
rs1354034
rs11680831
rs7412
rs2902577
rs6884904
rs9274470
All − MR Egger
All − Inverse variance weighted
−3
−2
−1
0
1
2
MR effect size for
'NAGK' on 'Pulmonary fibrosis'

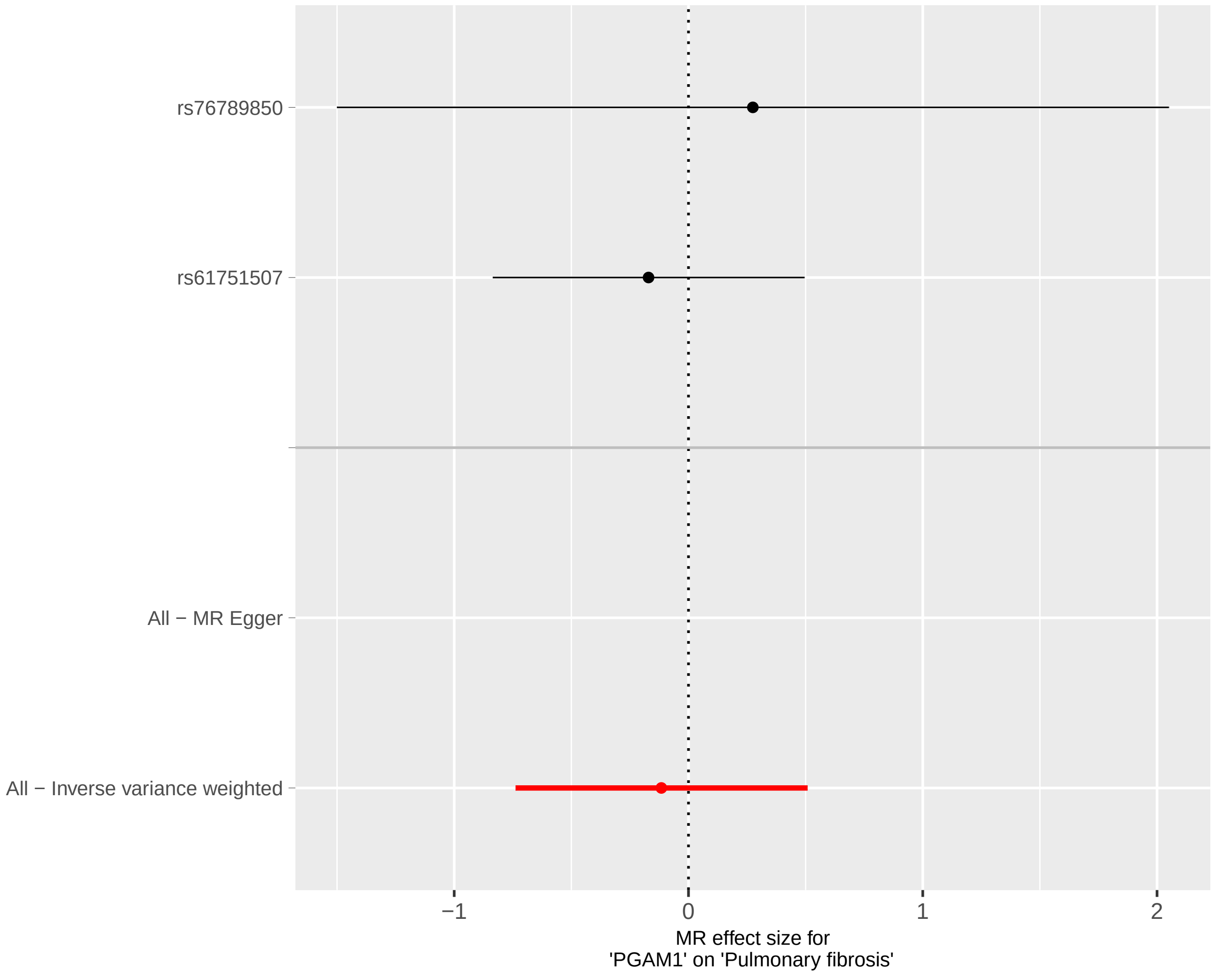

rs76789850
rs61751507
All − MR Egger
All − Inverse variance weighted
−1
0
1
2
MR effect size for
'PGAM1' on 'Pulmonary fibrosis'

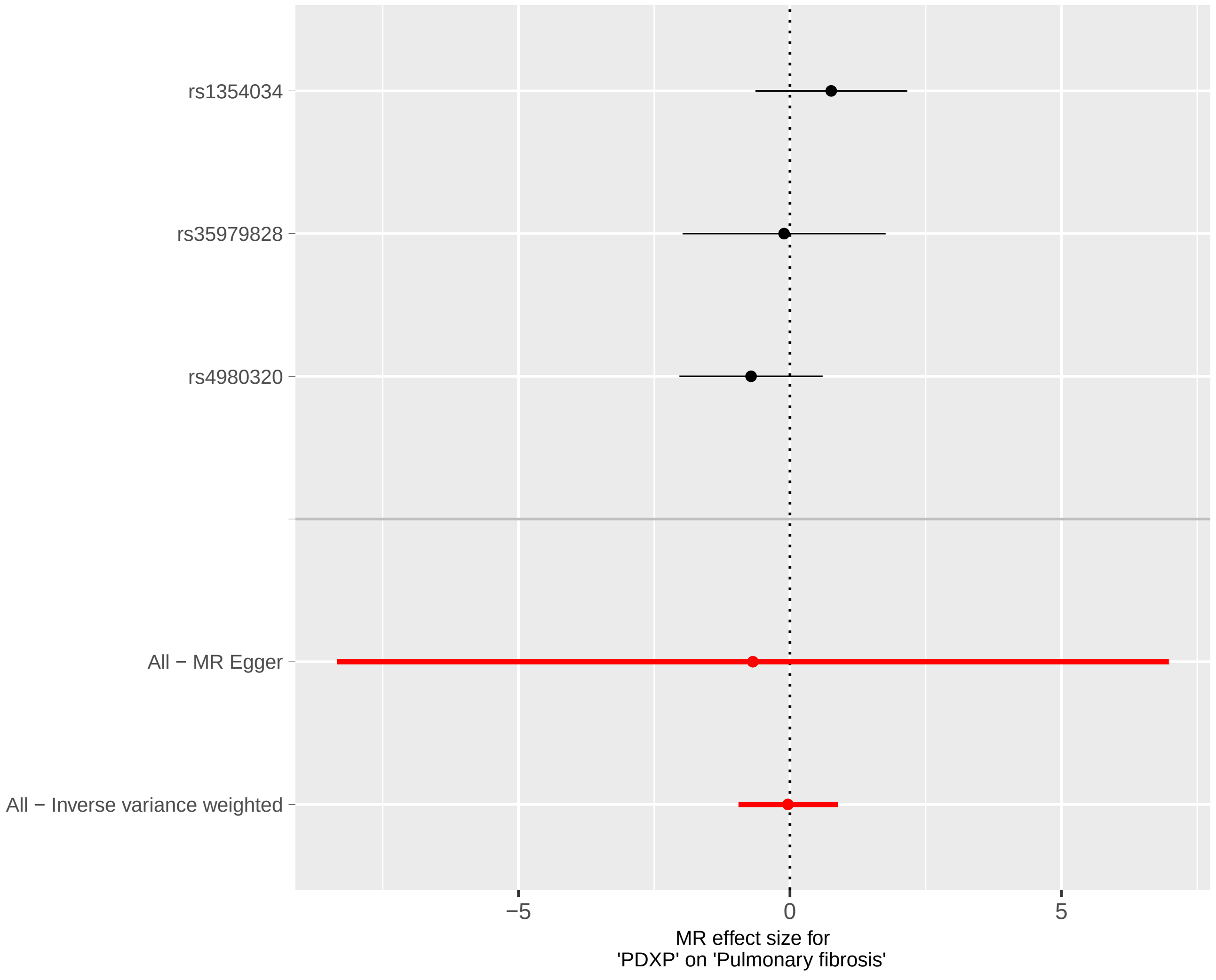

rs1354034
rs35979828
rs4980320
All − MR Egger
All − Inverse variance weighted
−5
0
5
MR effect size for
'PDXP' on 'Pulmonary fibrosis'

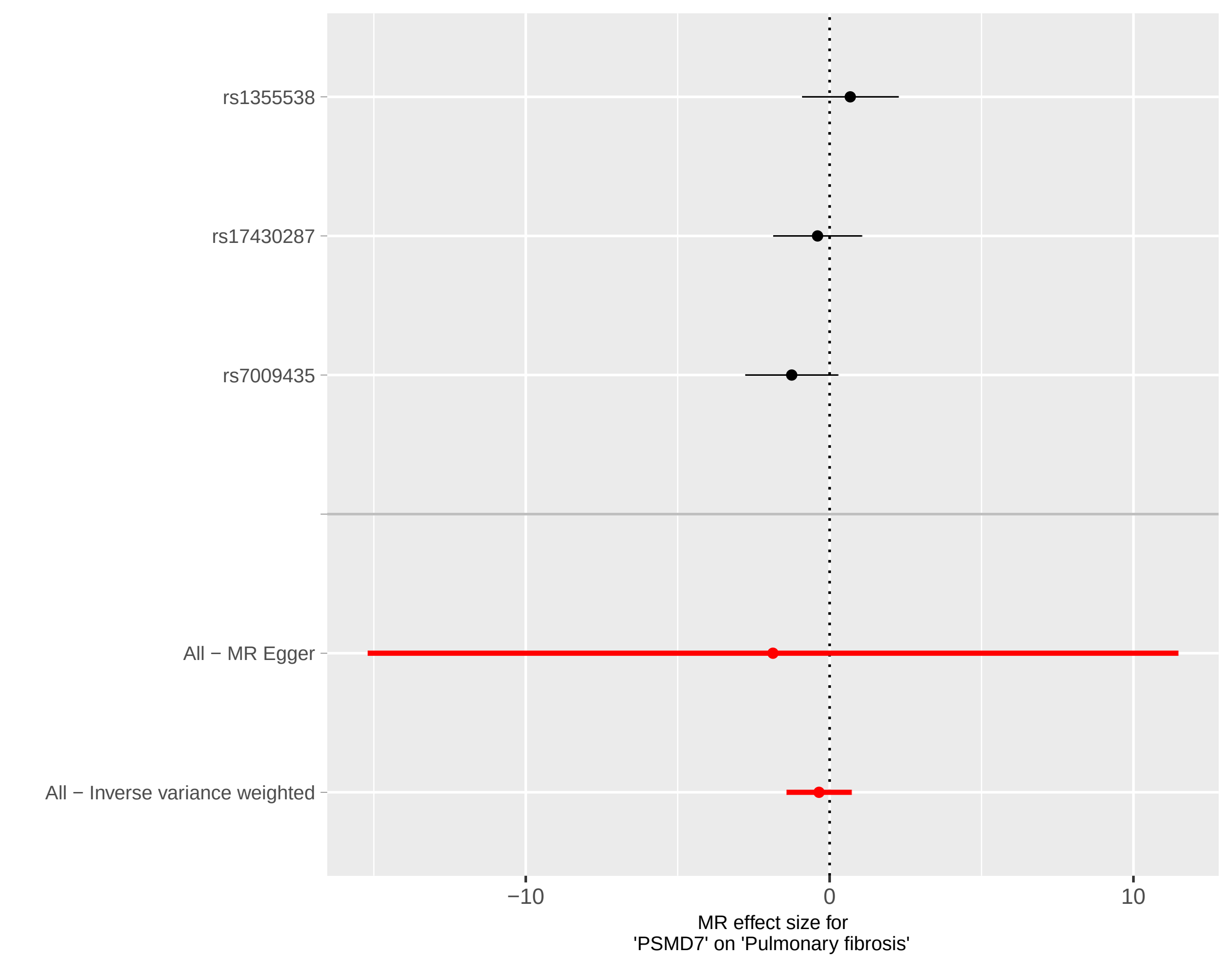

rs1355538
rs17430287
rs7009435
All − MR Egger
All − Inverse variance weighted
−10
0
10
MR effect size for
'PSMD7' on 'Pulmonary fibrosis'

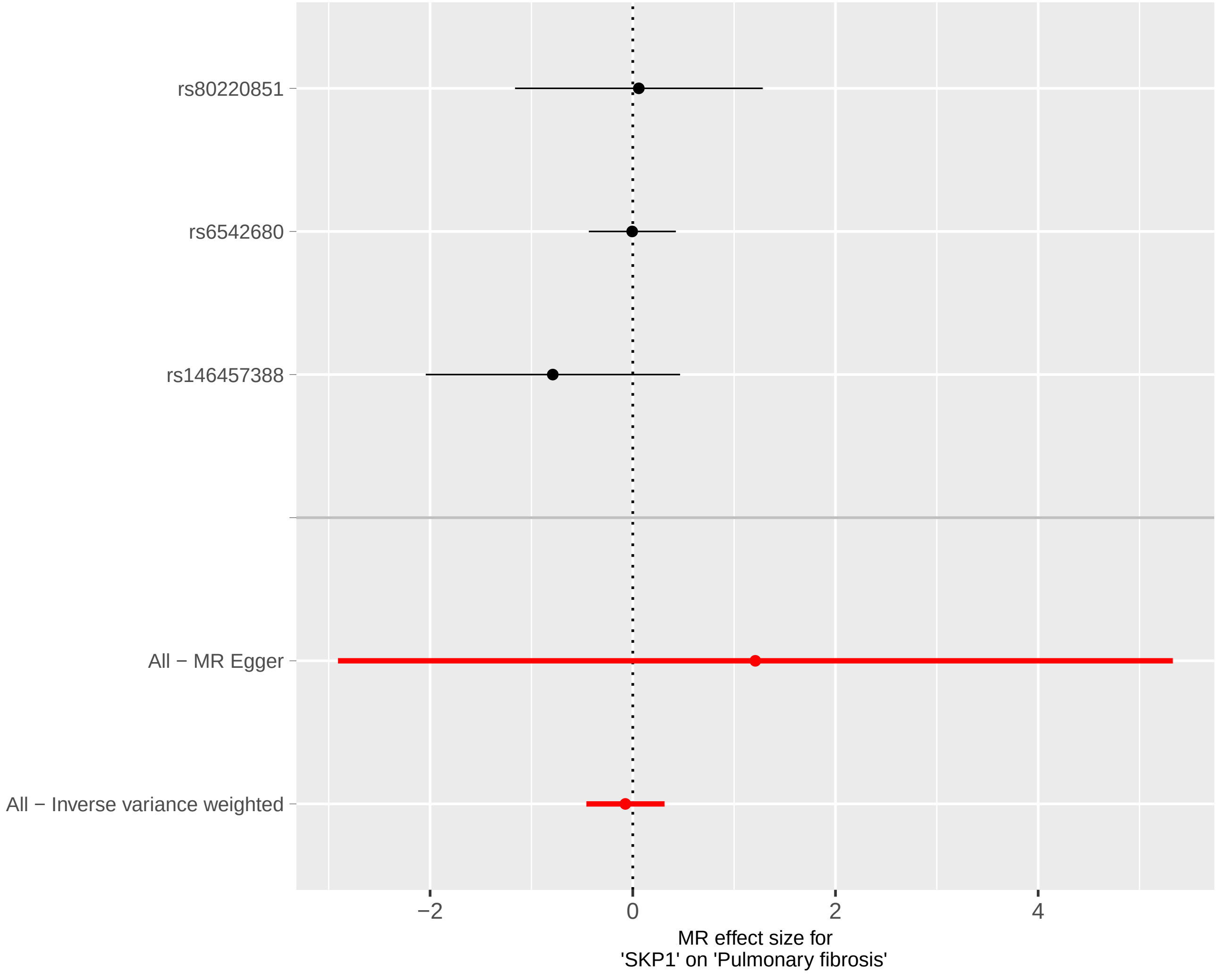
rs80220851
rs6542680
rs146457388
All − MR Egger
All − Inverse variance weighted
−2
0
2
4
MR effect size for
'SKP1' on 'Pulmonary fibrosis'

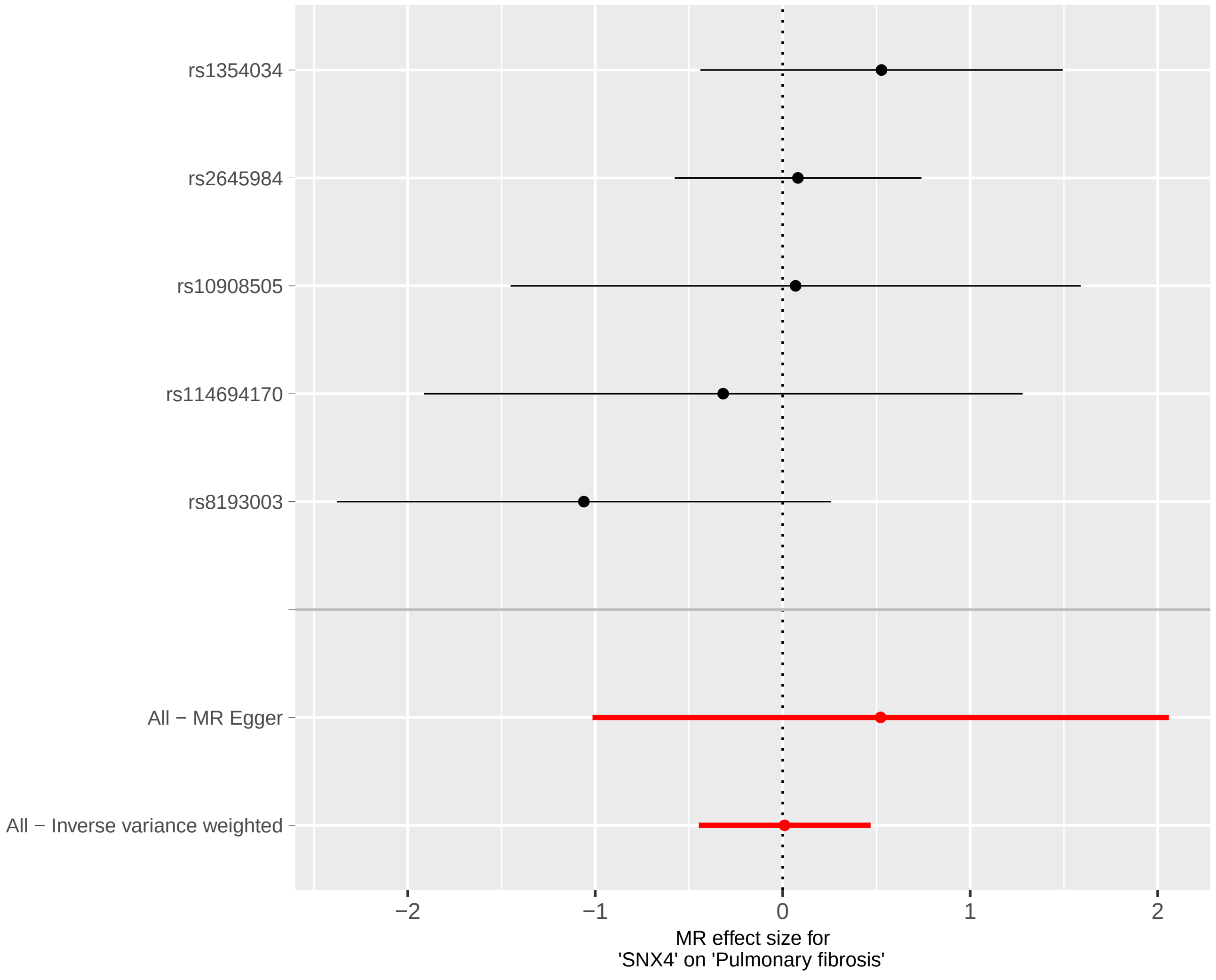
rs1354034
rs2645984
rs10908505
rs114694170
rs8193003
All − MR Egger
All − Inverse variance weighted
−2
−1
0
1
2
MR effect size for
'SNX4' on 'Pulmonary fibrosis'

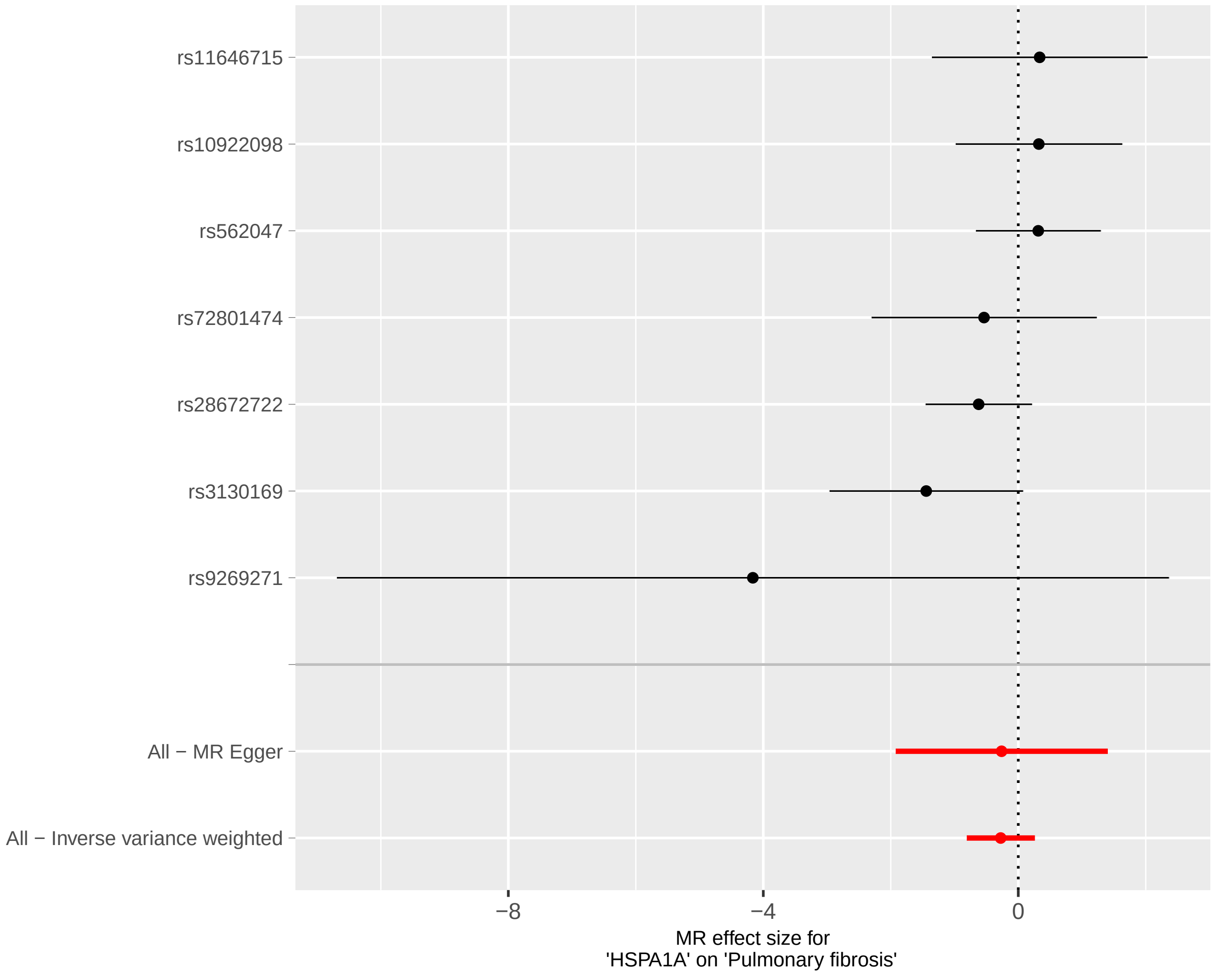
rs11646715
rs10922098
rs562047
rs72801474
rs28672722
rs3130169
rs9269271
All − MR Egger
All − Inverse variance weighted
−8
−4
0
MR effect size for
'HSPA1A' on 'Pulmonary fibrosis'

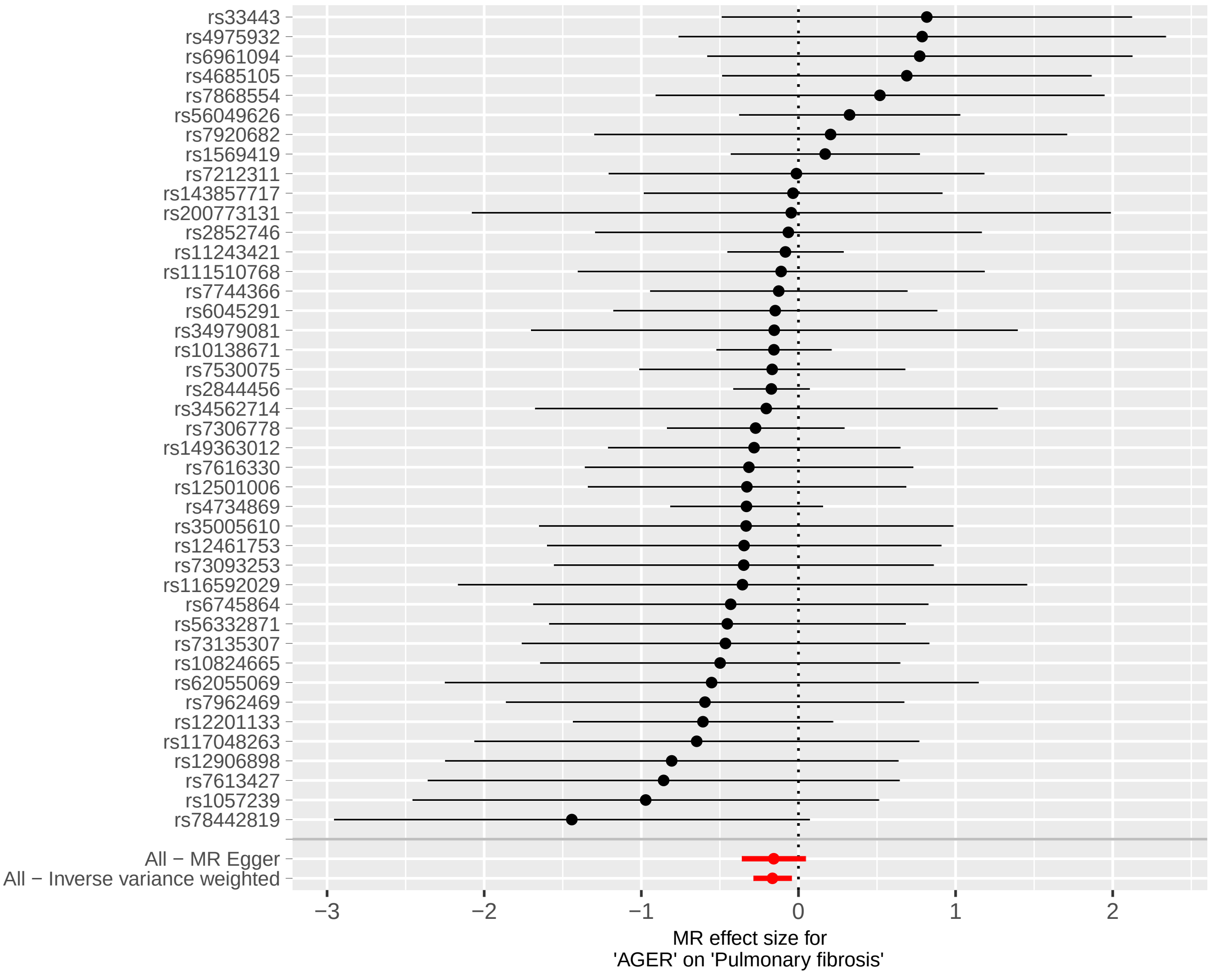

rs33443
rs4975932
rs6961094
rs4685105
rs7868554
rs56049626
rs7920682
rs1569419
rs7212311
rs143857717
rs200773131
rs2852746
rs11243421
rs111510768
rs7744366
rs6045291
rs34979081
rs10138671
rs7530075
rs2844456
rs34562714
rs7306778
rs149363012
rs7616330
rs12501006
rs4734869
rs35005610
rs12461753
rs73093253
rs116592029
rs6745864
rs56332871
rs73135307
rs10824665
rs62055069
rs7962469
rs12201133
rs117048263
rs12906898
rs7613427
rs1057239
rs78442819
All − MR Egger
All − Inverse variance weighted
−3
−2
−1
0
1
2
MR effect size for
'AGER' on 'Pulmonary fibrosis'

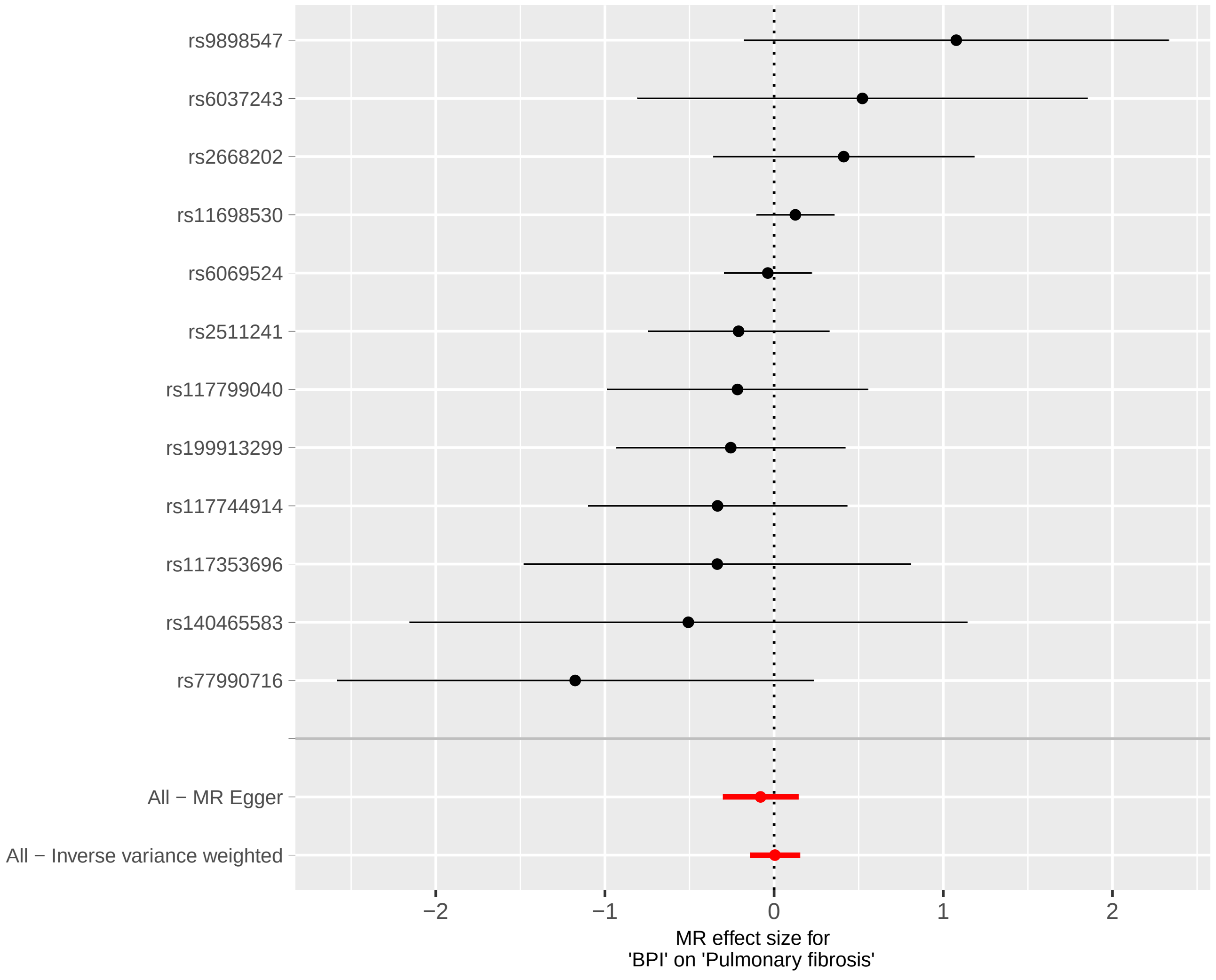

rs9898547
rs6037243
rs2668202
rs11698530
rs6069524
rs2511241
rs117799040
rs199913299
rs117744914
rs117353696
rs140465583
rs77990716
All − MR Egger
All − Inverse variance weighted
−2
−1
0
1
2
MR effect size for
'BPI' on 'Pulmonary fibrosis'

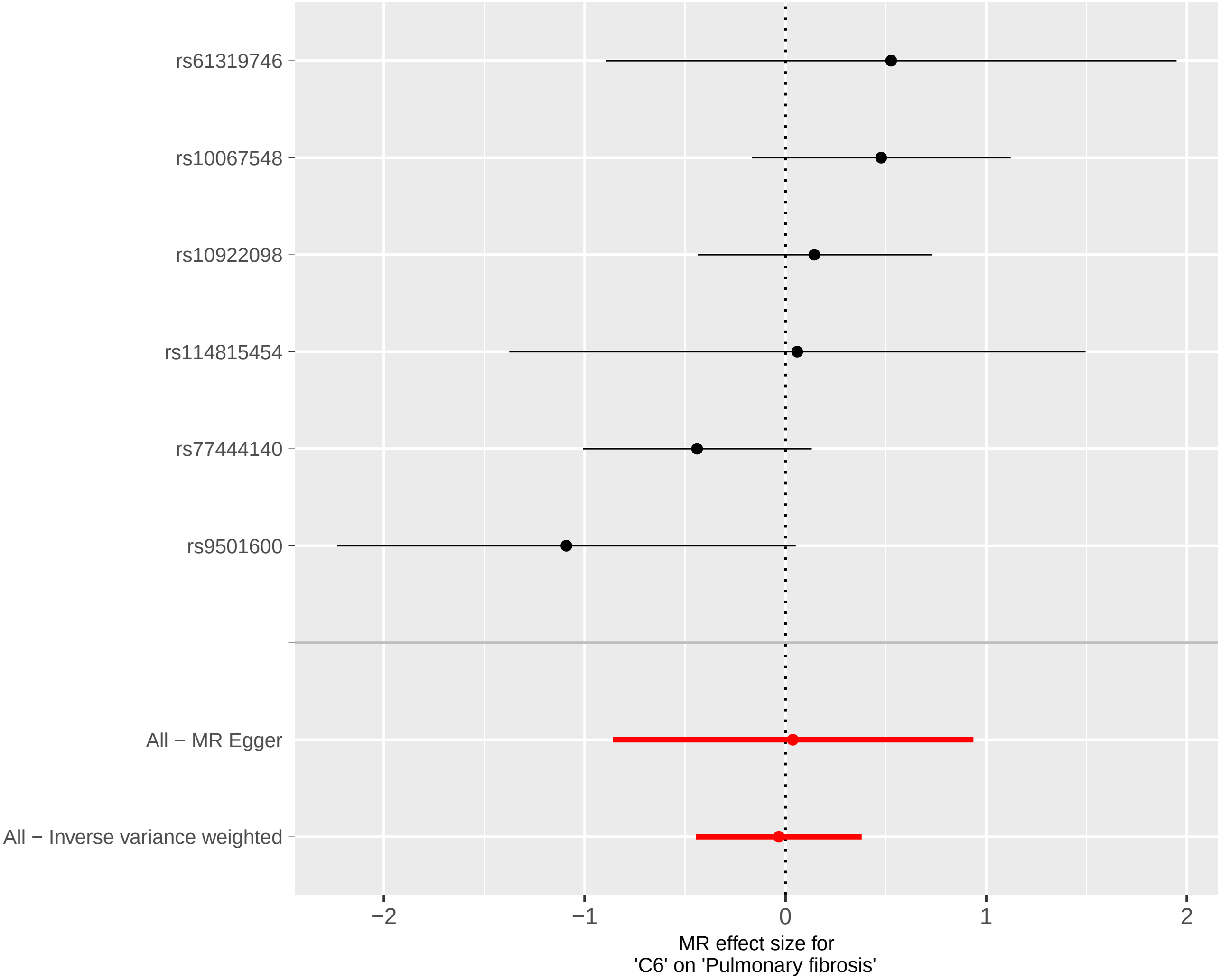

rs61319746
rs10067548
rs10922098
rs114815454
rs77444140
rs9501600
All − MR Egger
All − Inverse variance weighted
−2
−1
0
1
2
MR effect size for
'C6' on 'Pulmonary fibrosis'

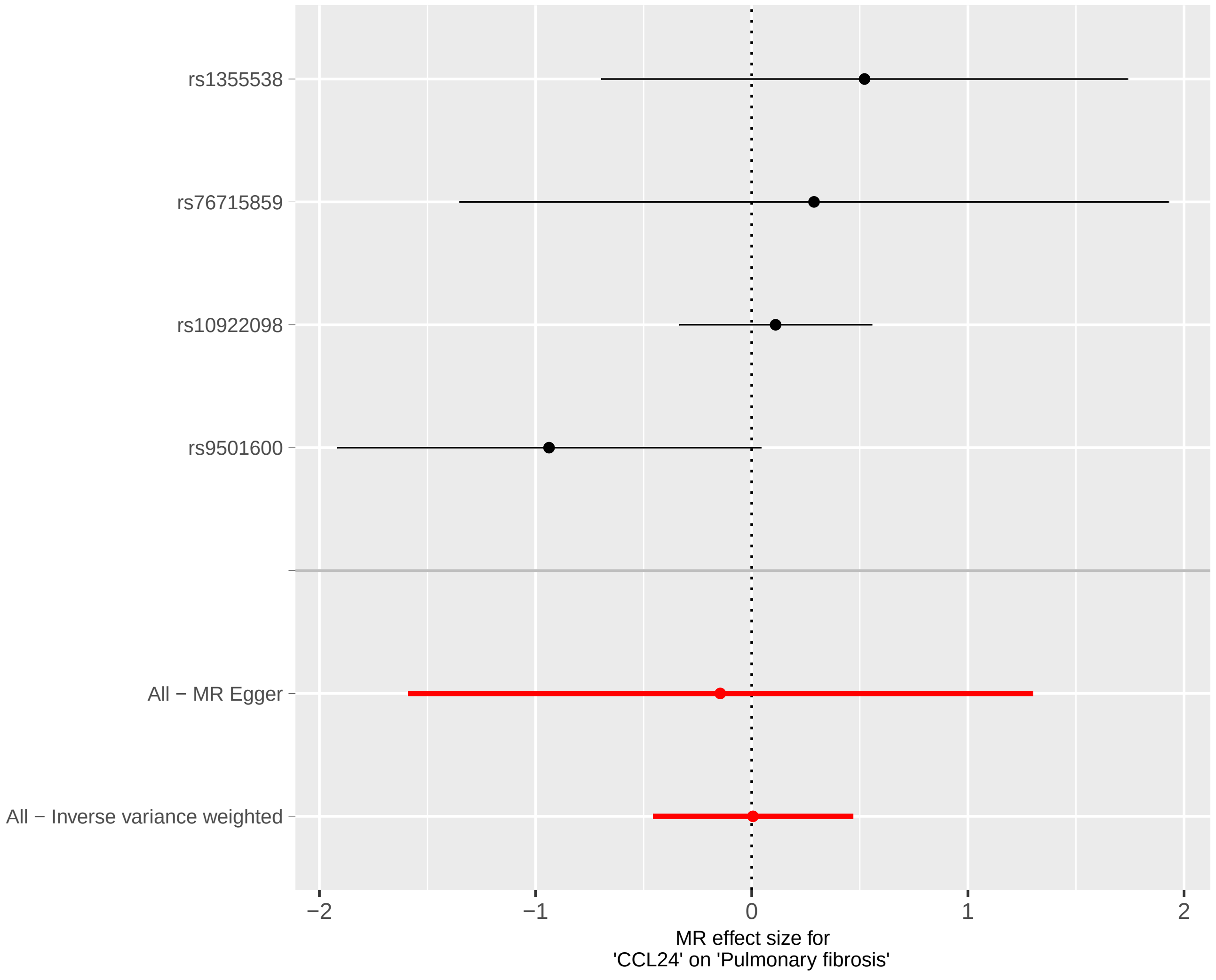

rs1355538
rs76715859
rs10922098
rs9501600
All − MR Egger
All − Inverse variance weighted
−2
−1
0
1
2
MR effect size for
'CCL24' on 'Pulmonary fibrosis'

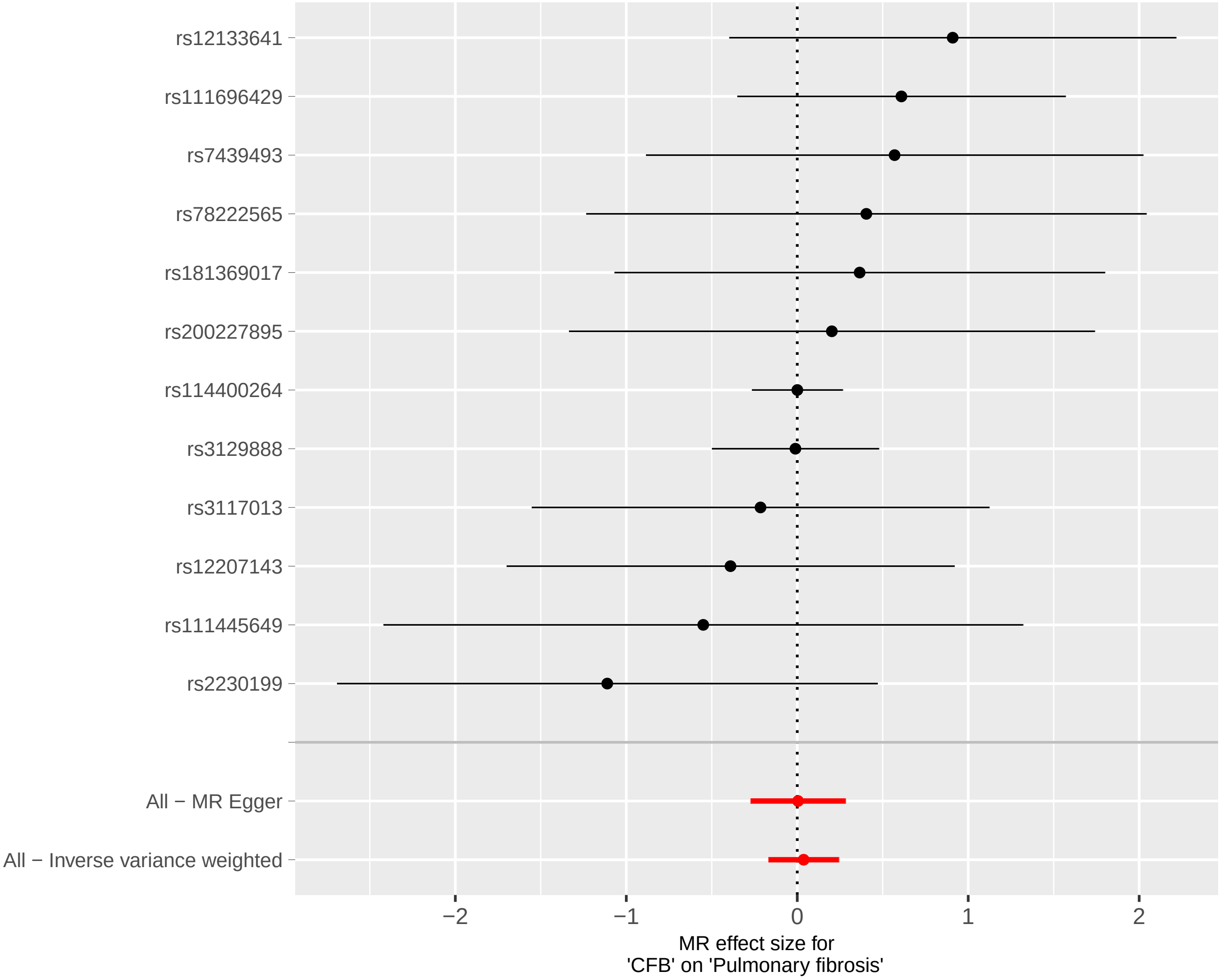
rs12133641
rs111696429
rs7439493
rs78222565
rs181369017
rs200227895
rs114400264
rs3129888
rs3117013
rs12207143
rs111445649
rs2230199
All − MR Egger
All − Inverse variance weighted
−2
−1
0
1
2
MR effect size for
'CFB' on 'Pulmonary fibrosis'

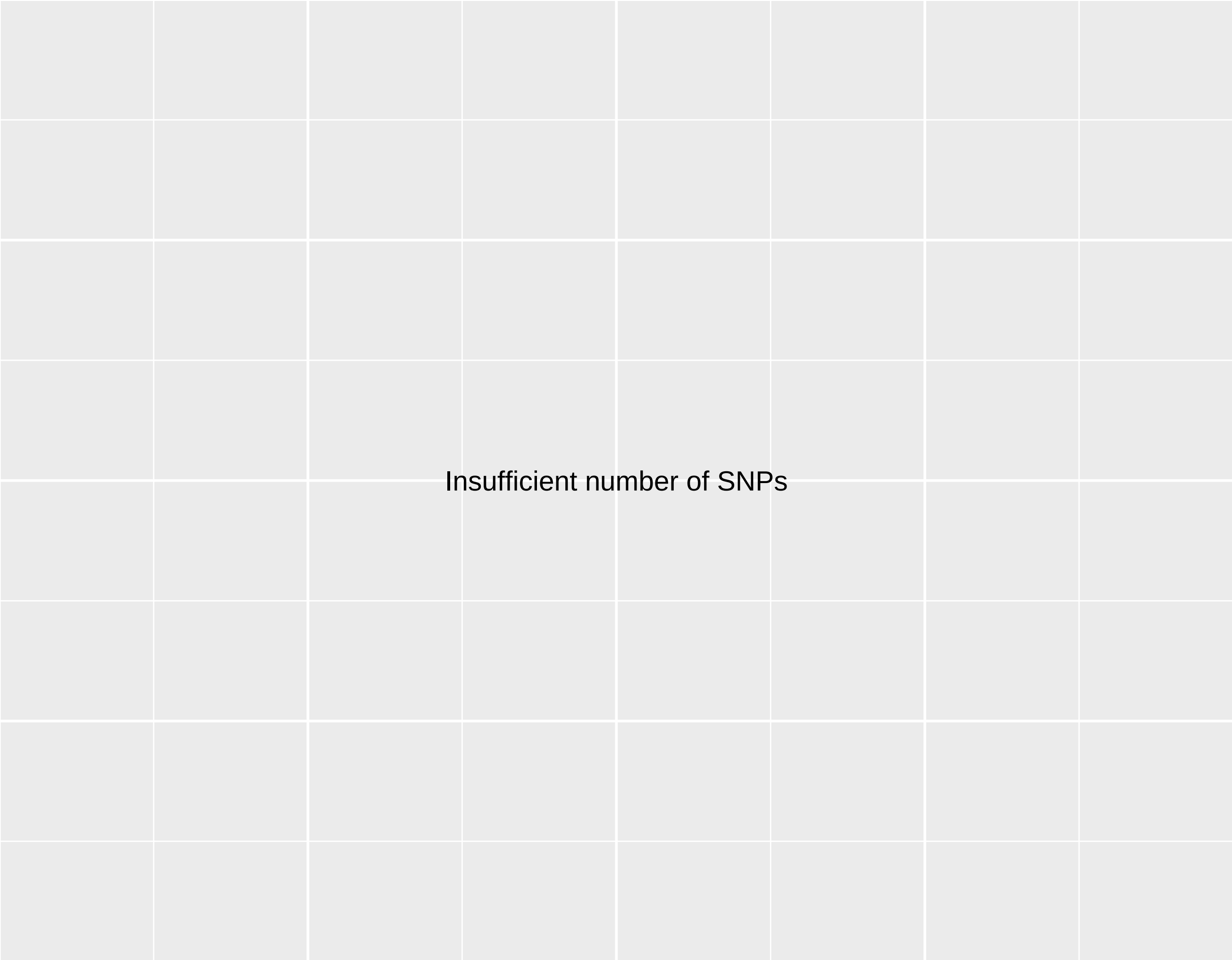
Insufficient number of SNPs

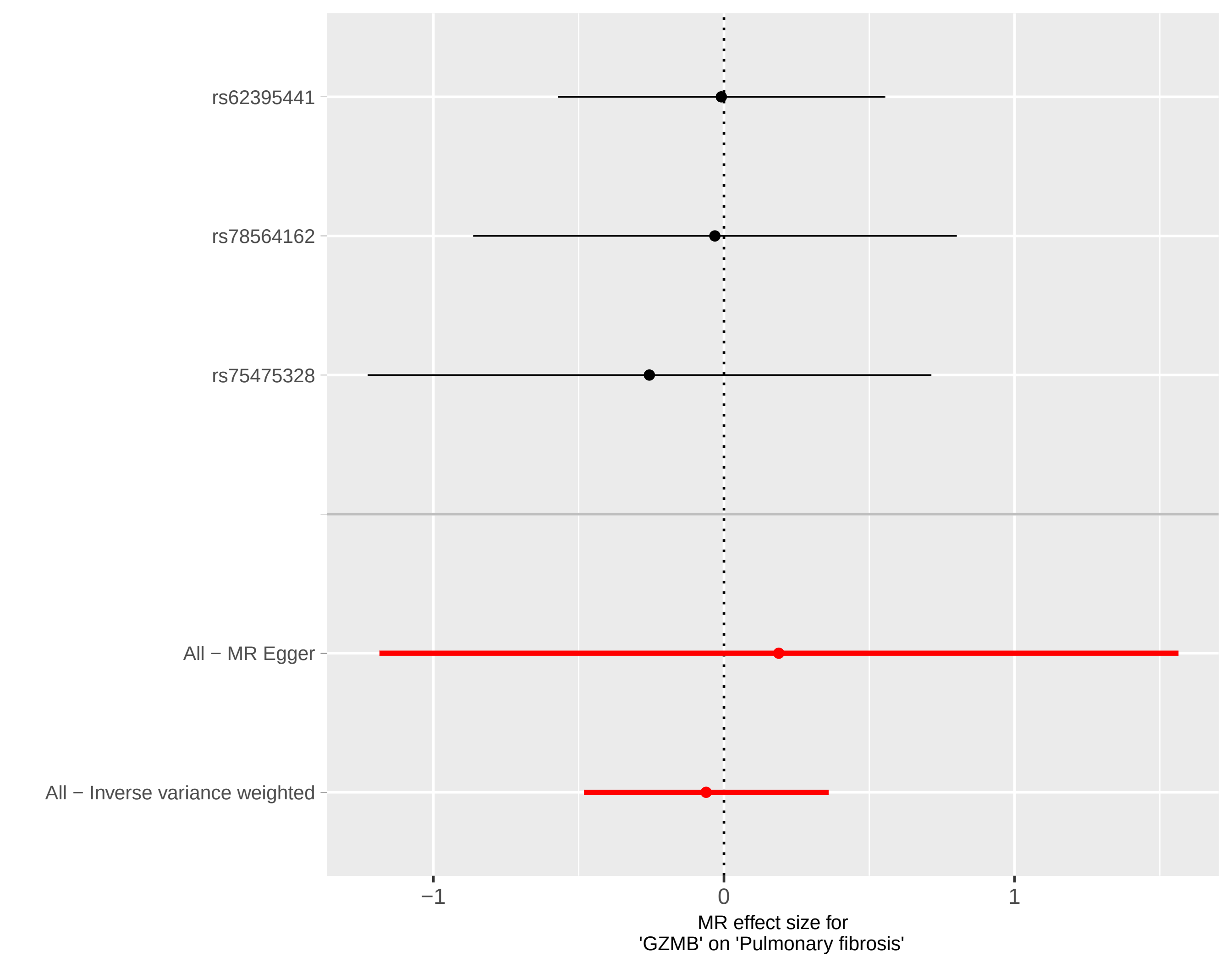

rs62395441
rs78564162
rs75475328
All − MR Egger
All − Inverse variance weighted
−1
0
1
MR effect size for
'GZMB' on 'Pulmonary fibrosis'

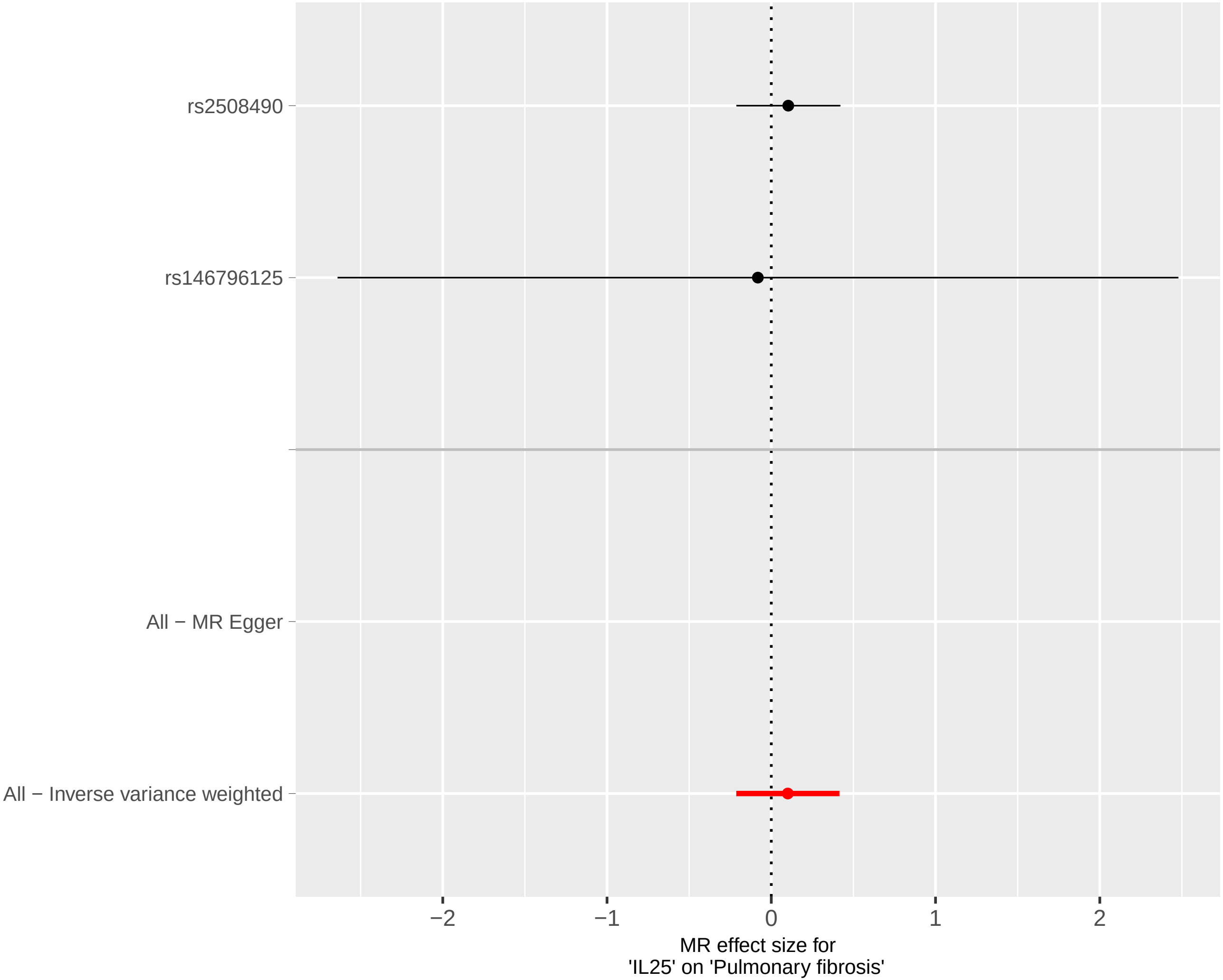
rs2508490
rs146796125
All − MR Egger
All − Inverse variance weighted
−2
−1
0
1
2
MR effect size for
'IL25' on 'Pulmonary fibrosis'

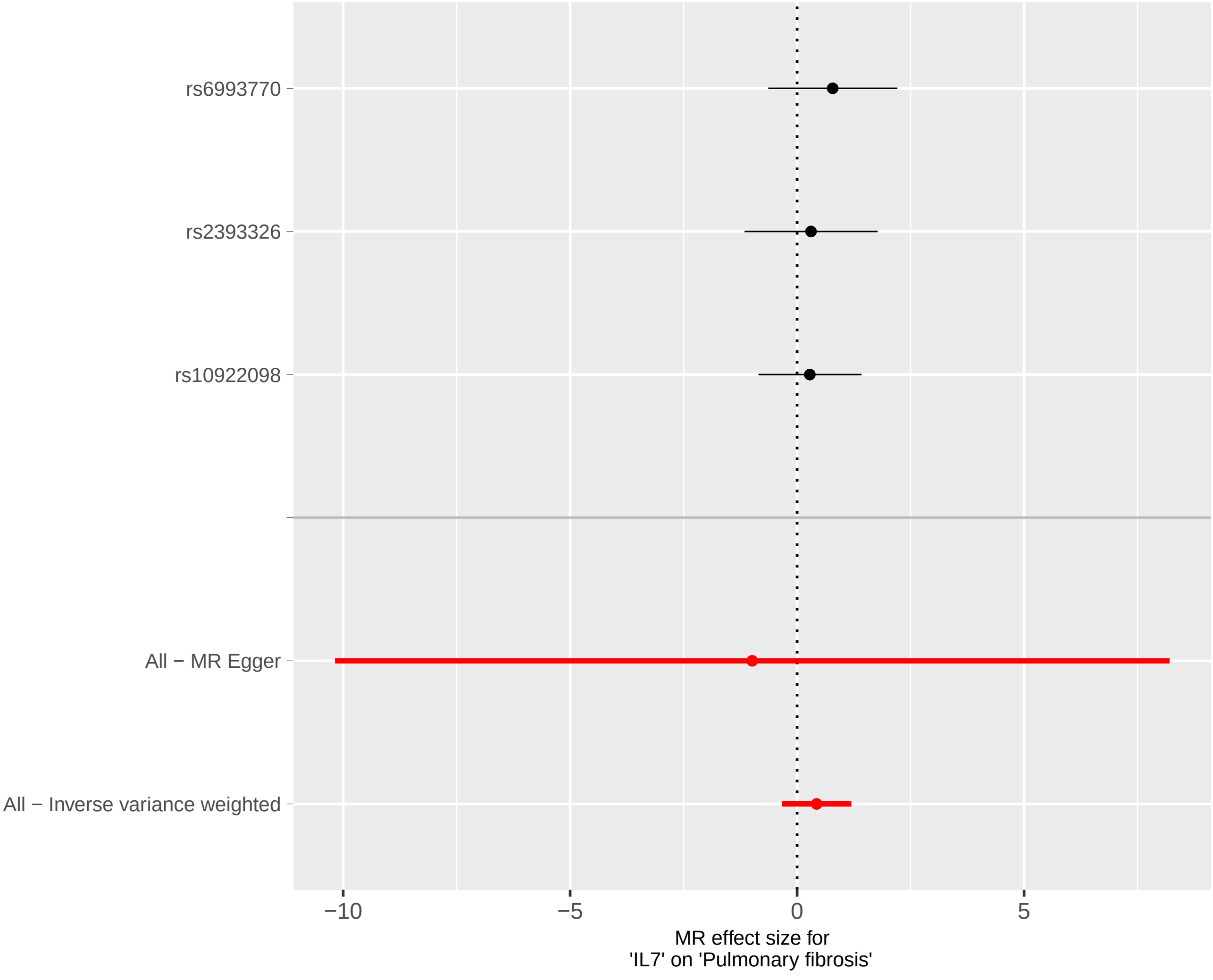
rs6993770
rs2393326
rs10922098
All − MR Egger
All − Inverse variance weighted
−10
−5
0
5
MR effect size for
'IL7' on 'Pulmonary fibrosis'

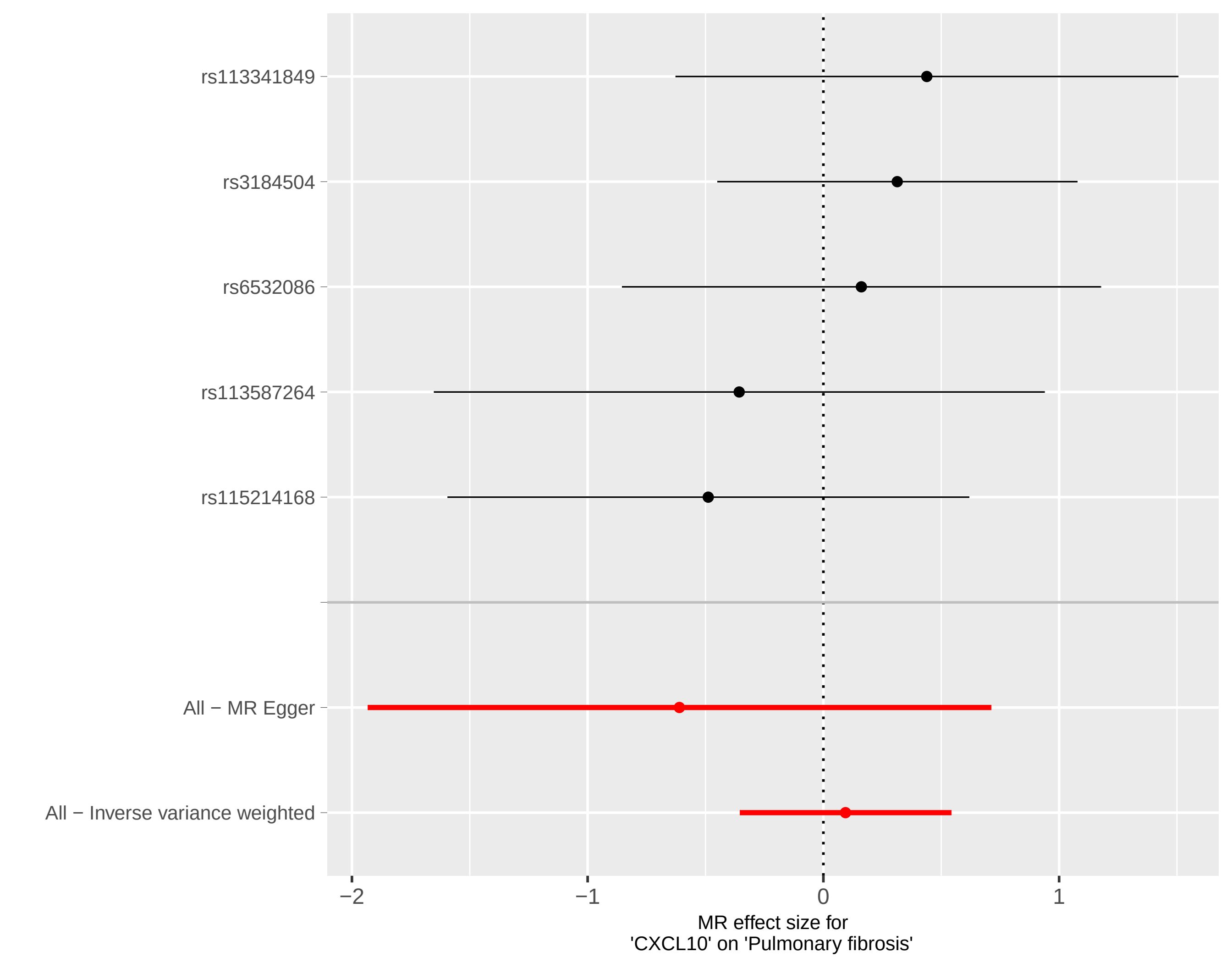
rs113341849
rs3184504
rs6532086
rs113587264
rs115214168
All − MR Egger
All − Inverse variance weighted
−2
−1
0
1
MR effect size for
'CXCL10' on 'Pulmonary fibrosis'

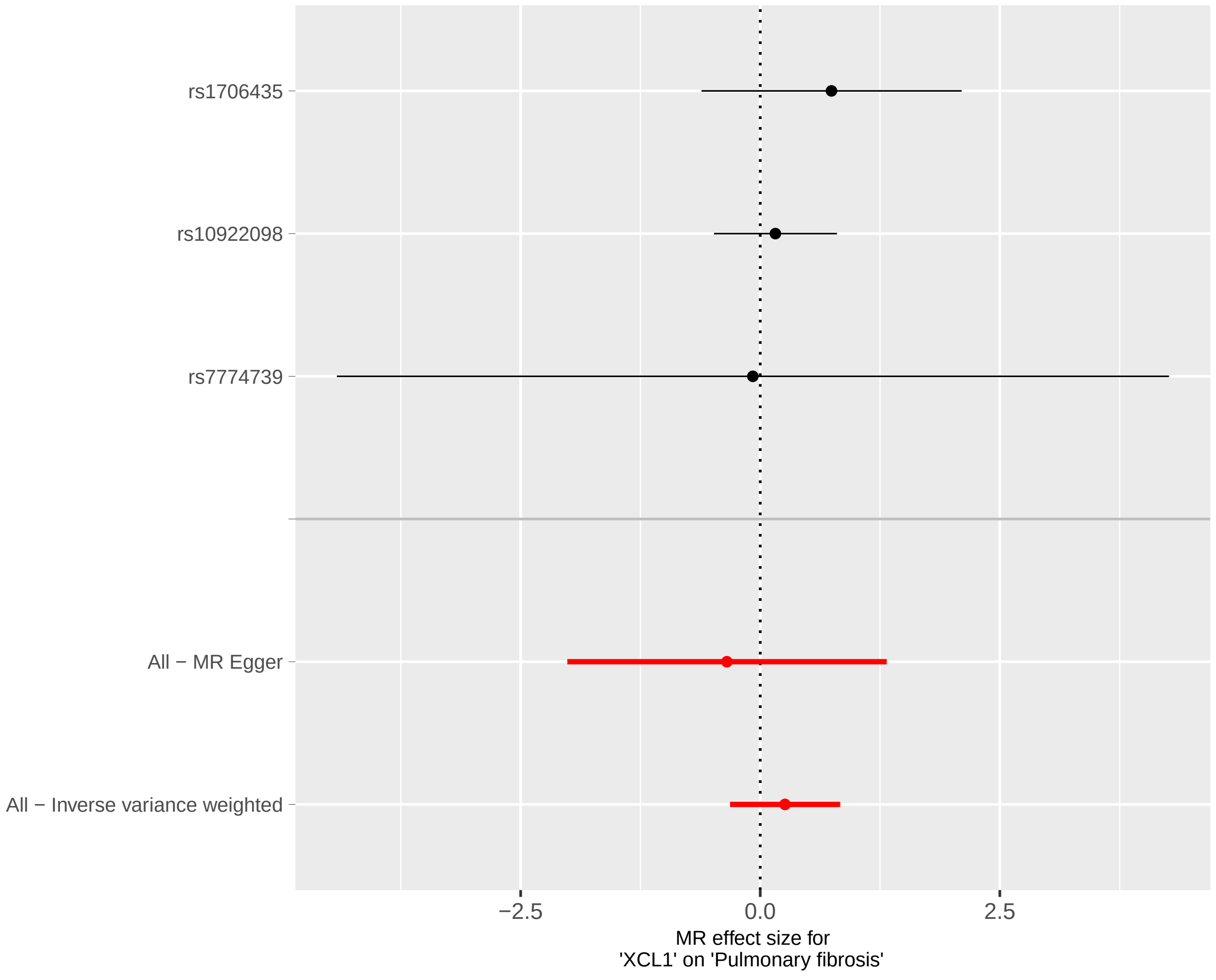

rs1706435
rs10922098
rs7774739
All − MR Egger
All − Inverse variance weighted
−2.5
0.0
2.5
MR effect size for
'XCL1' on 'Pulmonary fibrosis'

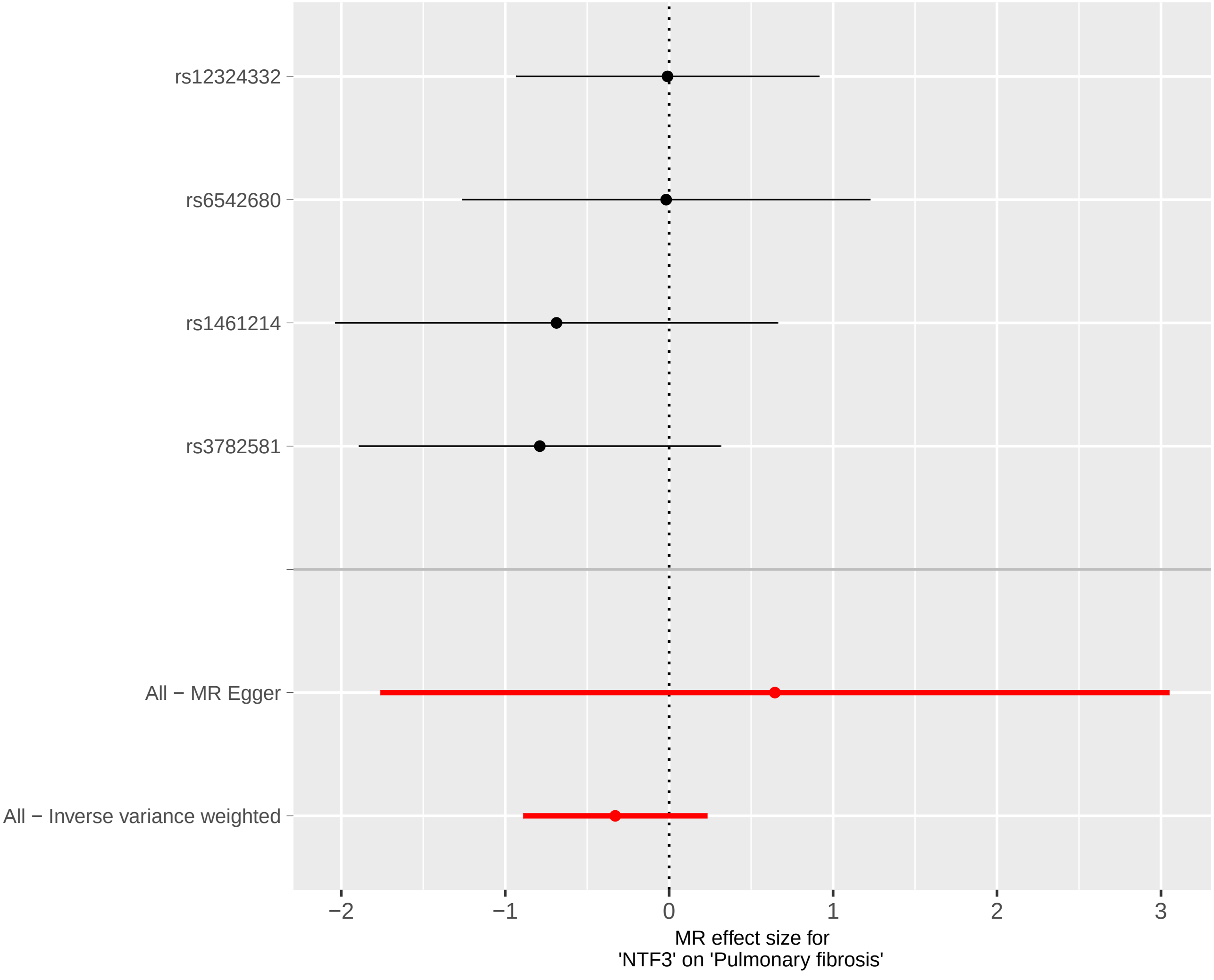
rs12324332
rs6542680
rs1461214
rs3782581
All − MR Egger
All − Inverse variance weighted
−2
−1
0
1
2
3
MR effect size for
'NTF3' on 'Pulmonary fibrosis'

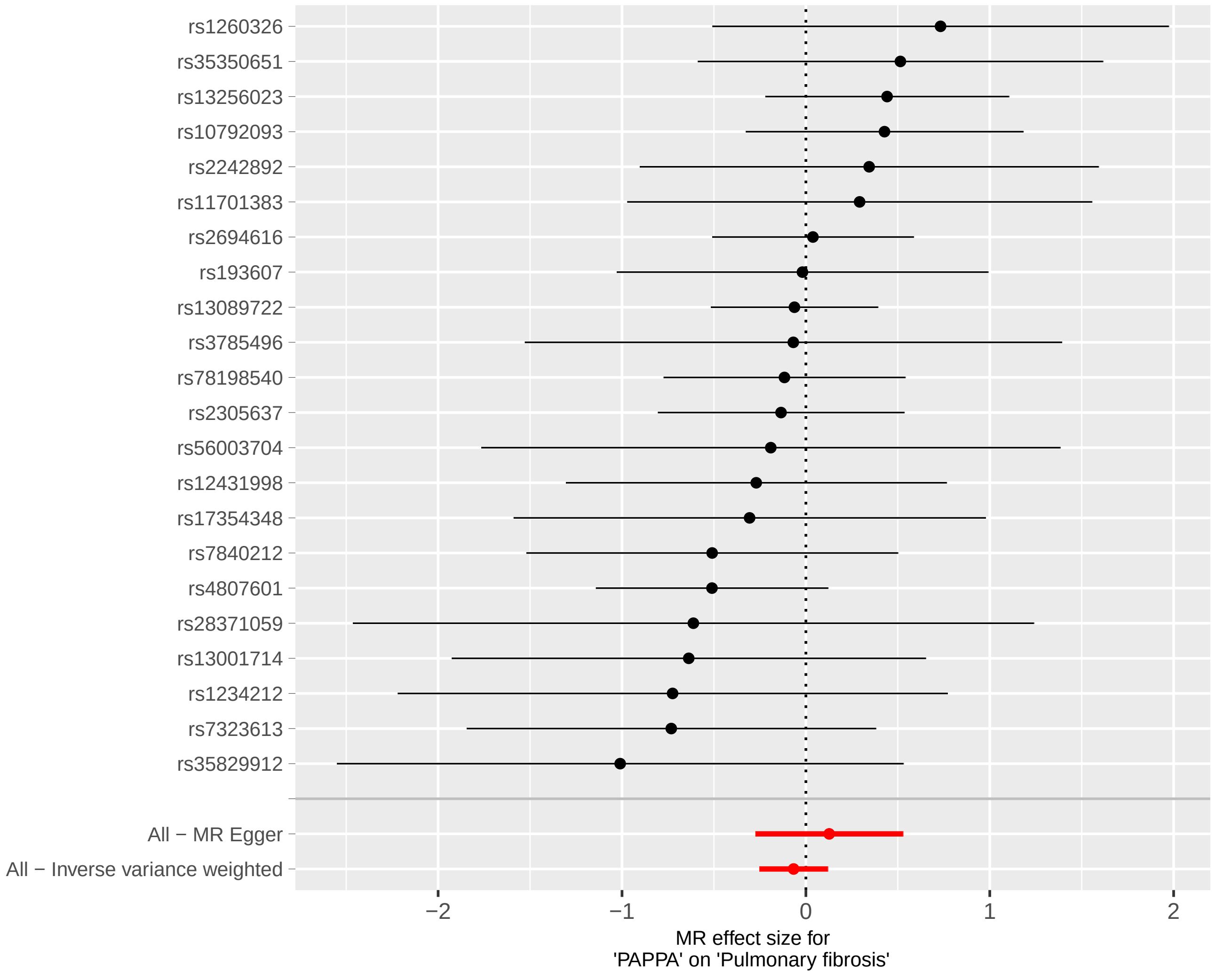

rs1260326
rs35350651
rs13256023
rs10792093
rs2242892
rs11701383
rs2694616
rs193607
rs13089722
rs3785496
rs78198540
rs2305637
rs56003704
rs12431998
rs17354348
rs7840212
rs4807601
rs28371059
rs13001714
rs1234212
rs7323613
rs35829912
All − MR Egger
All − Inverse variance weighted
−2
−1
0
1
2
MR effect size for
'PAPPA' on 'Pulmonary fibrosis'

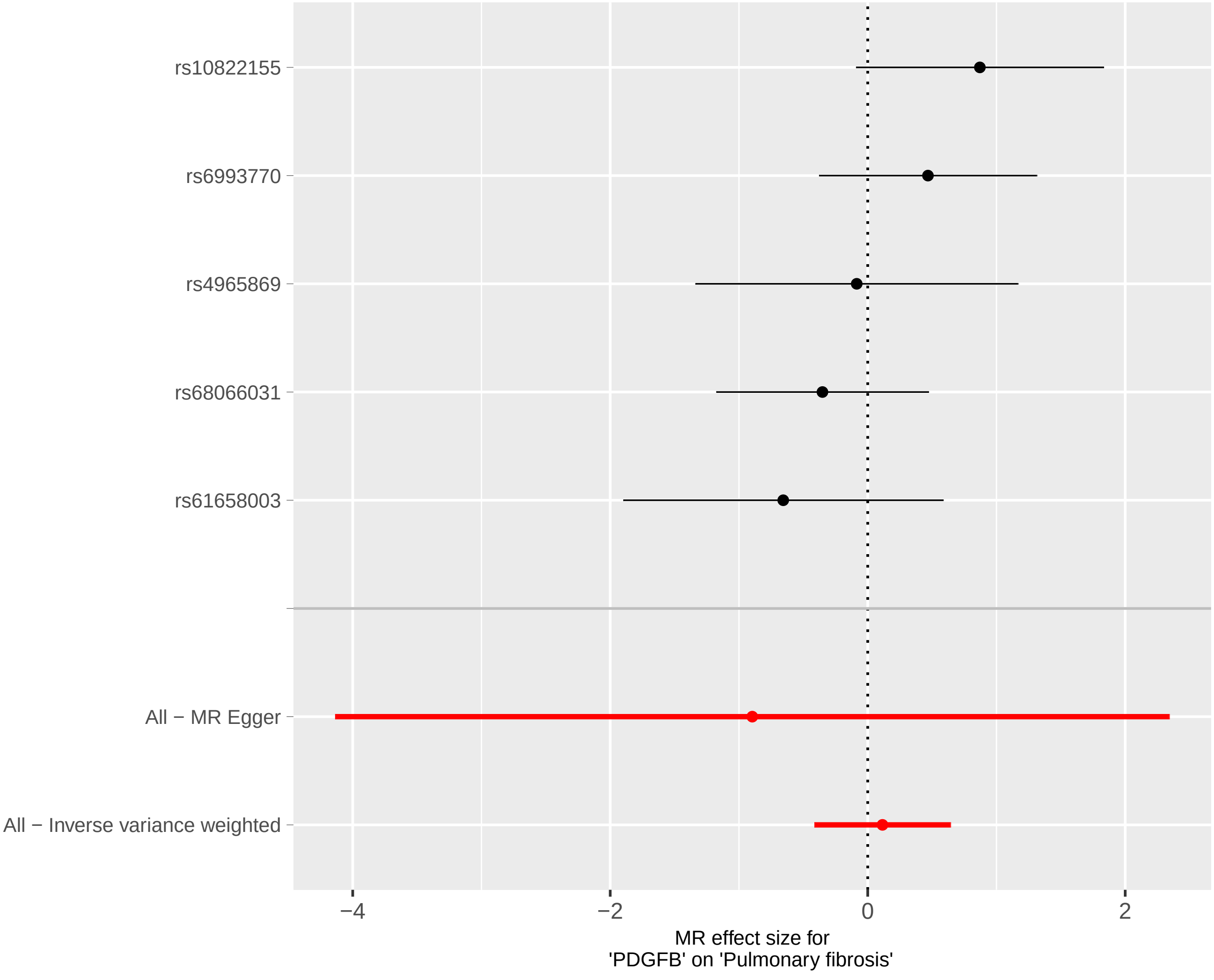
rs10822155
rs6993770
rs4965869
rs68066031
rs61658003
All − MR Egger
All − Inverse variance weighted
−4
−2
0
2
MR effect size for
'PDGFB' on 'Pulmonary fibrosis'

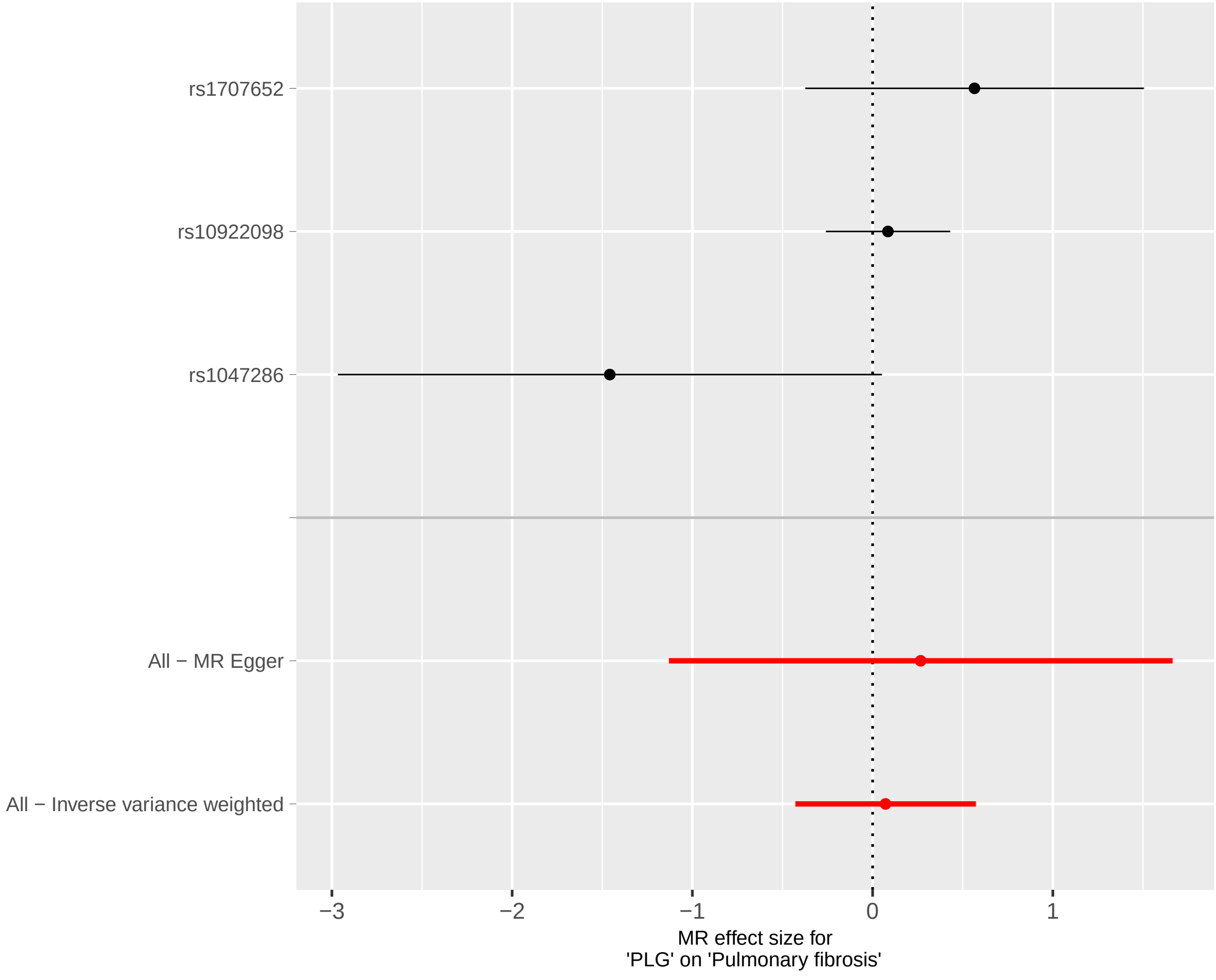

rs1707652
rs10922098
rs1047286
All − MR Egger
All − Inverse variance weighted
−3
−2
−1
0
1
MR effect size for
'PLG' on 'Pulmonary fibrosis'

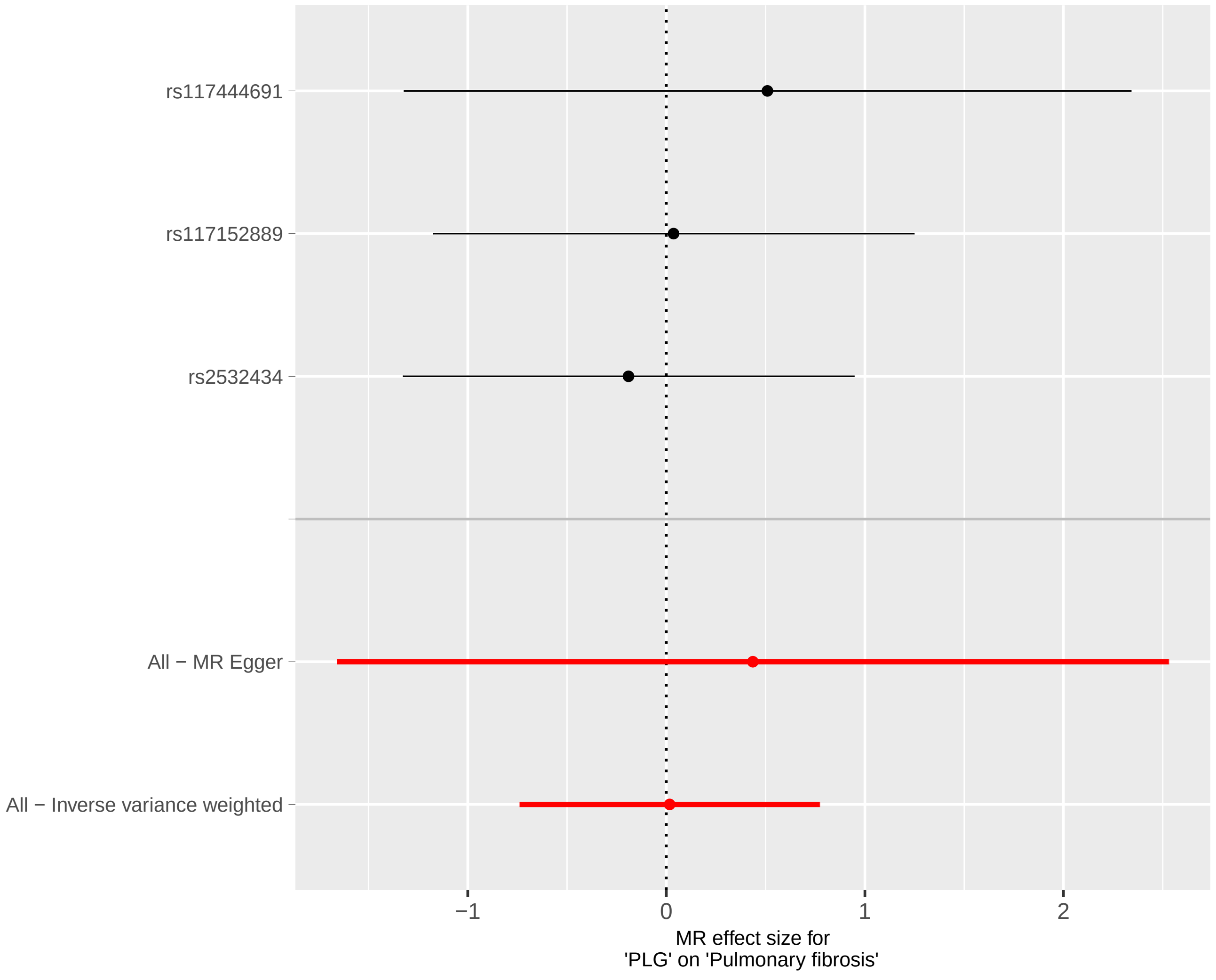

rs117444691
rs117152889
rs2532434
All − MR Egger
All − Inverse variance weighted
−1
0
1
2
MR effect size for
'PLG' on 'Pulmonary fibrosis'

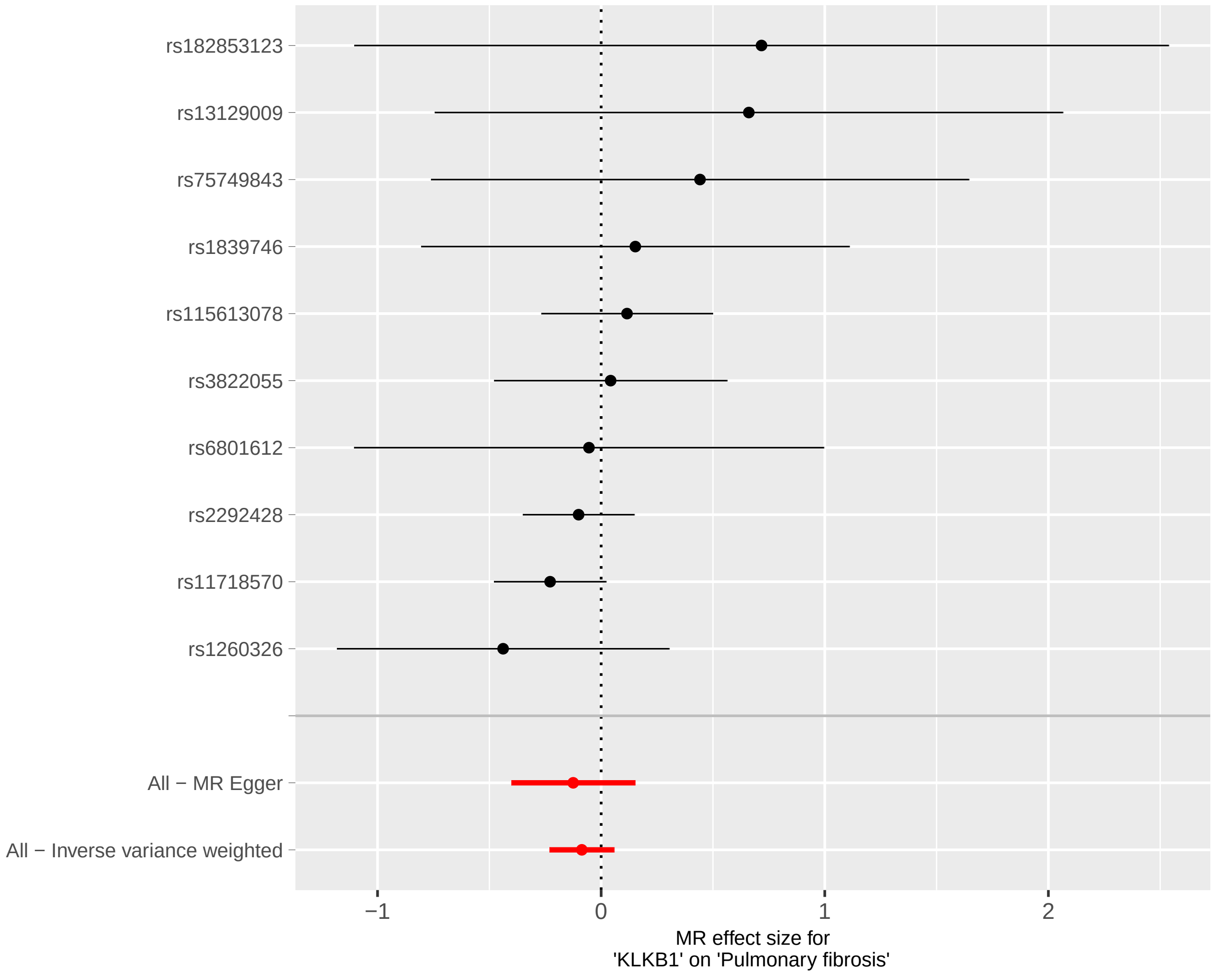

rs182853123
rs13129009
rs75749843
rs1839746
rs115613078
rs3822055
rs6801612
rs2292428
rs11718570
rs1260326
All − MR Egger
All − Inverse variance weighted
−1
0
1
2
MR effect size for
'KLKB1' on 'Pulmonary fibrosis'

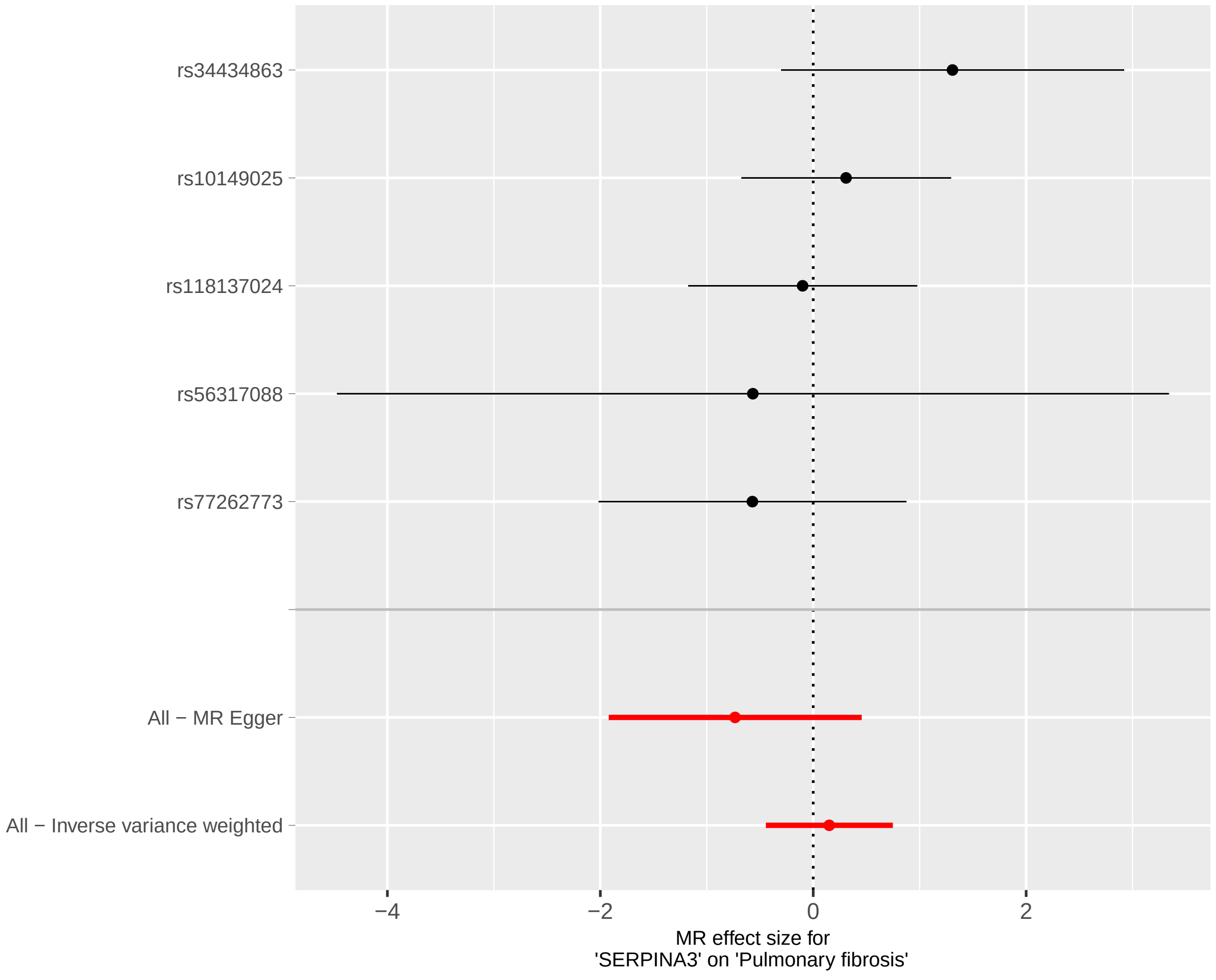

rs34434863
rs10149025
rs118137024
rs56317088
rs77262773
All − MR Egger
All − Inverse variance weighted
−4
−2
0
2
MR effect size for
'SERPINA3' on 'Pulmonary fibrosis'

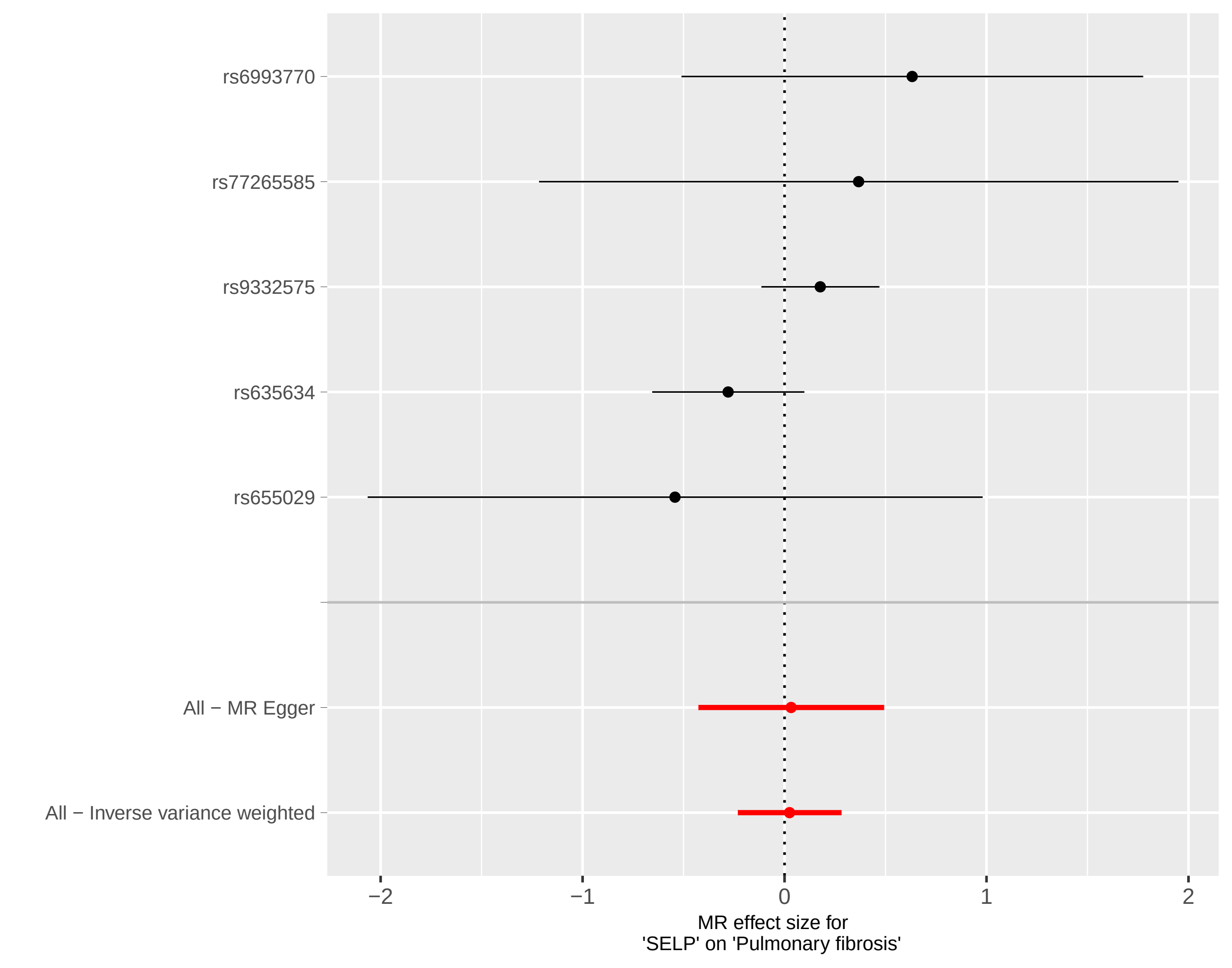

rs6993770
rs77265585
rs9332575
rs635634
rs655029
All − MR Egger
All − Inverse variance weighted
−2
−1
0
1
2
MR effect size for
'SELP' on 'Pulmonary fibrosis'

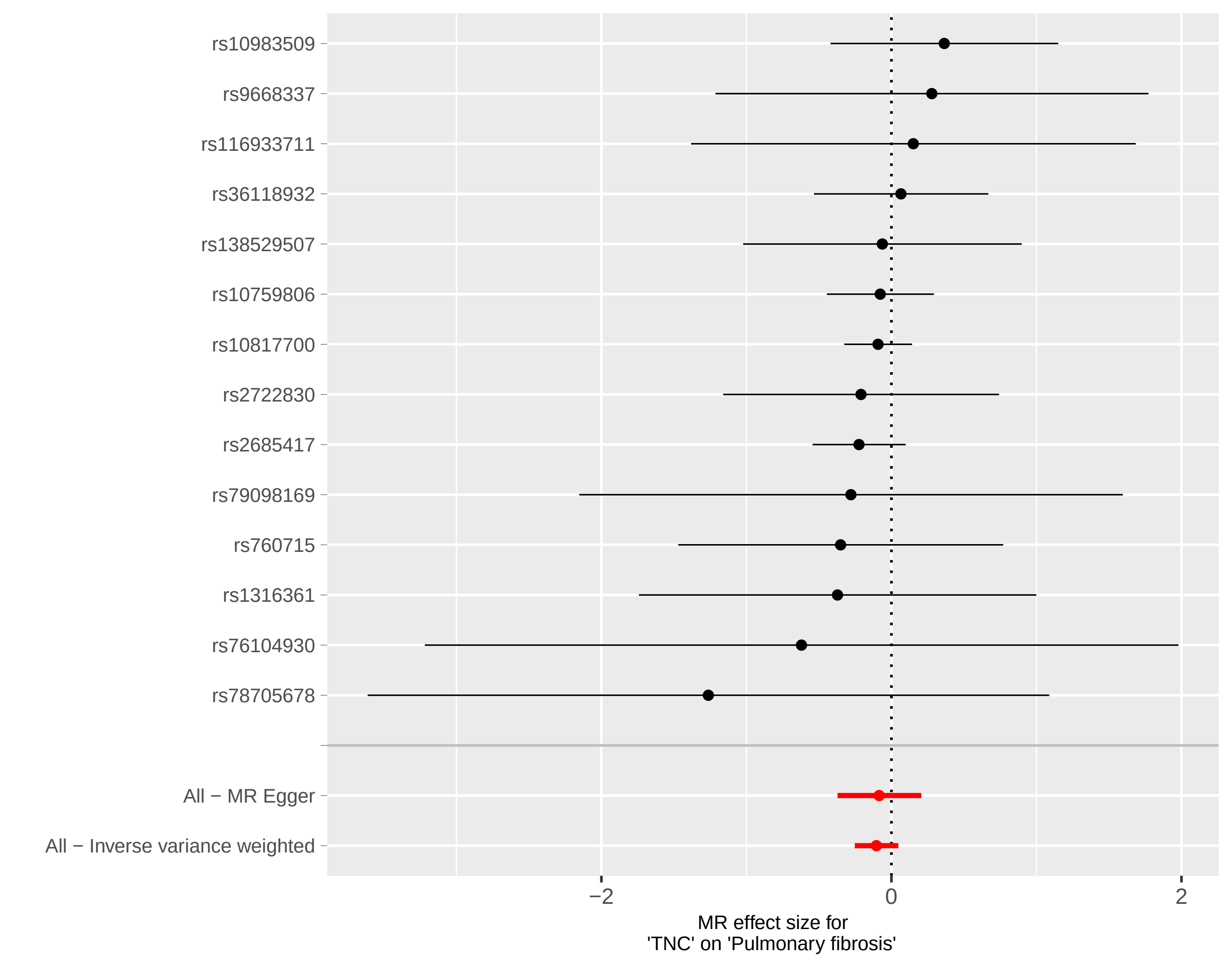

rs10983509
rs9668337
rs116933711
rs36118932
rs138529507
rs10759806
rs10817700
rs2722830
rs2685417
rs79098169
rs760715
rs1316361
rs76104930
rs78705678
All − MR Egger
All − Inverse variance weighted
−2
0
2
MR effect size for
'TNC' on 'Pulmonary fibrosis'

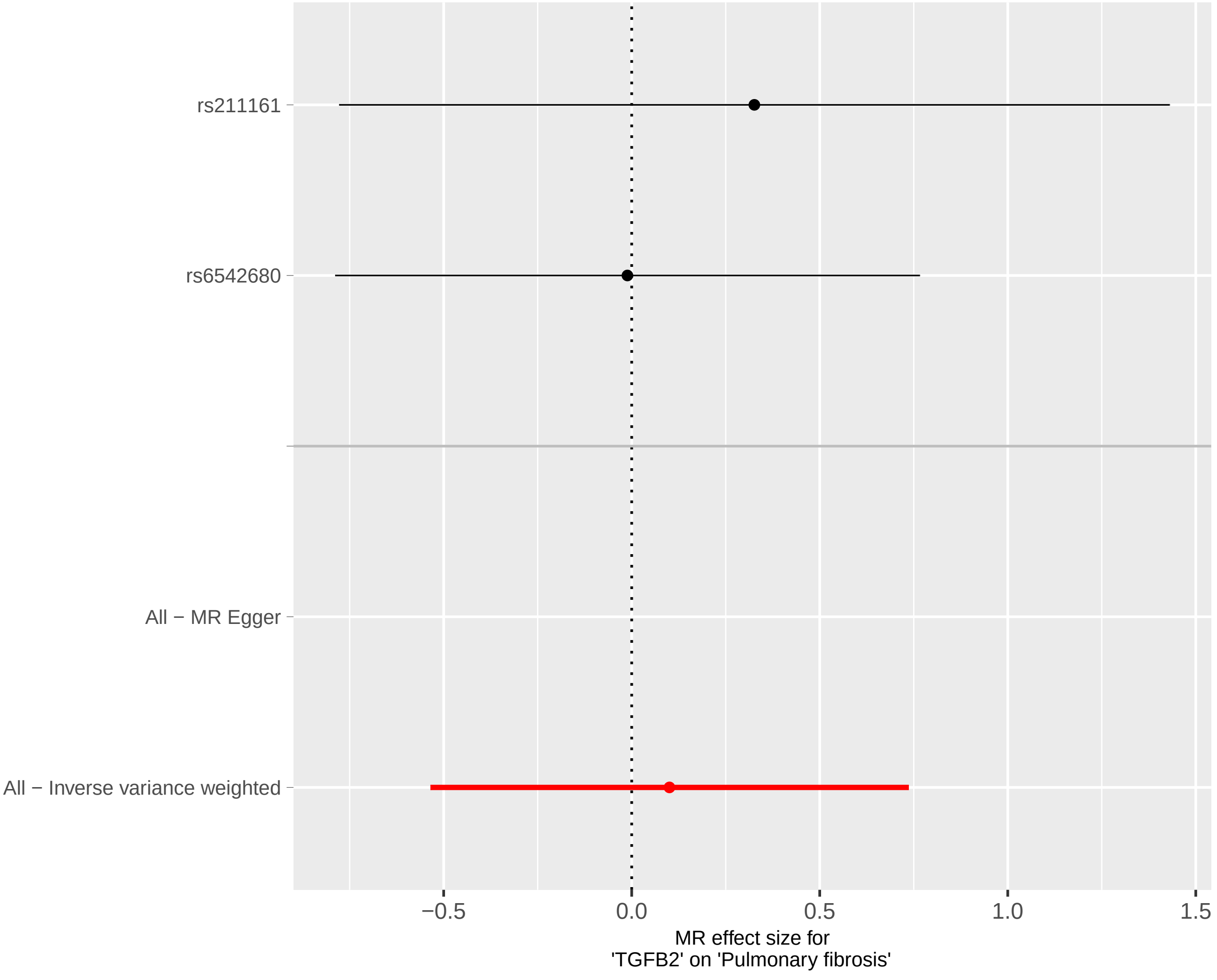
rs211161
rs6542680
All − MR Egger
All − Inverse variance weighted
−0.5
0.0
0.5
1.0
1.5
MR effect size for
'TGFB2' on 'Pulmonary fibrosis'

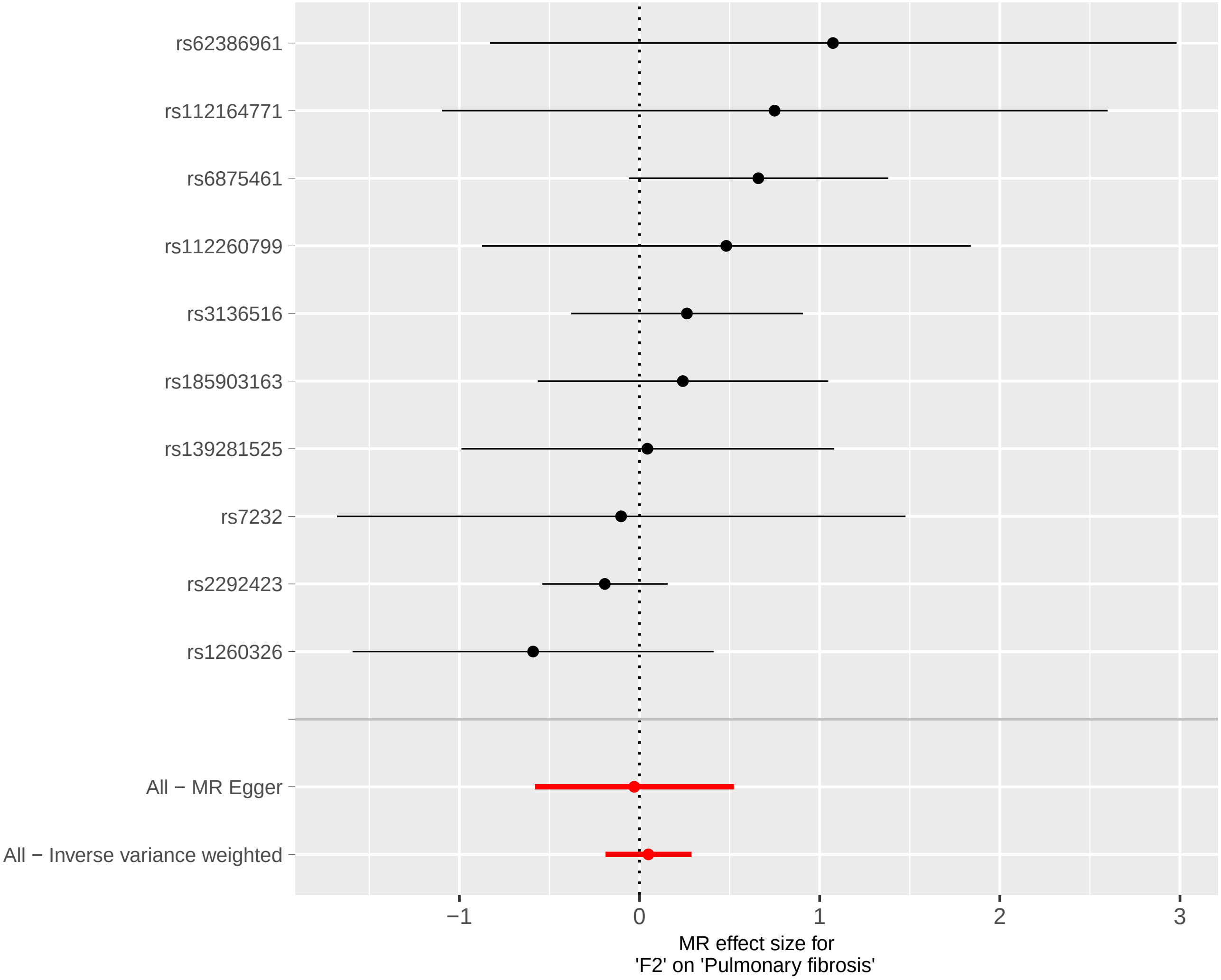
rs62386961
rs112164771
rs6875461
rs112260799
rs3136516
rs185903163
rs139281525
rs7232
rs2292423
rs1260326
All − MR Egger
All − Inverse variance weighted
−1
0
1
2
3
MR effect size for
'F2' on 'Pulmonary fibrosis'

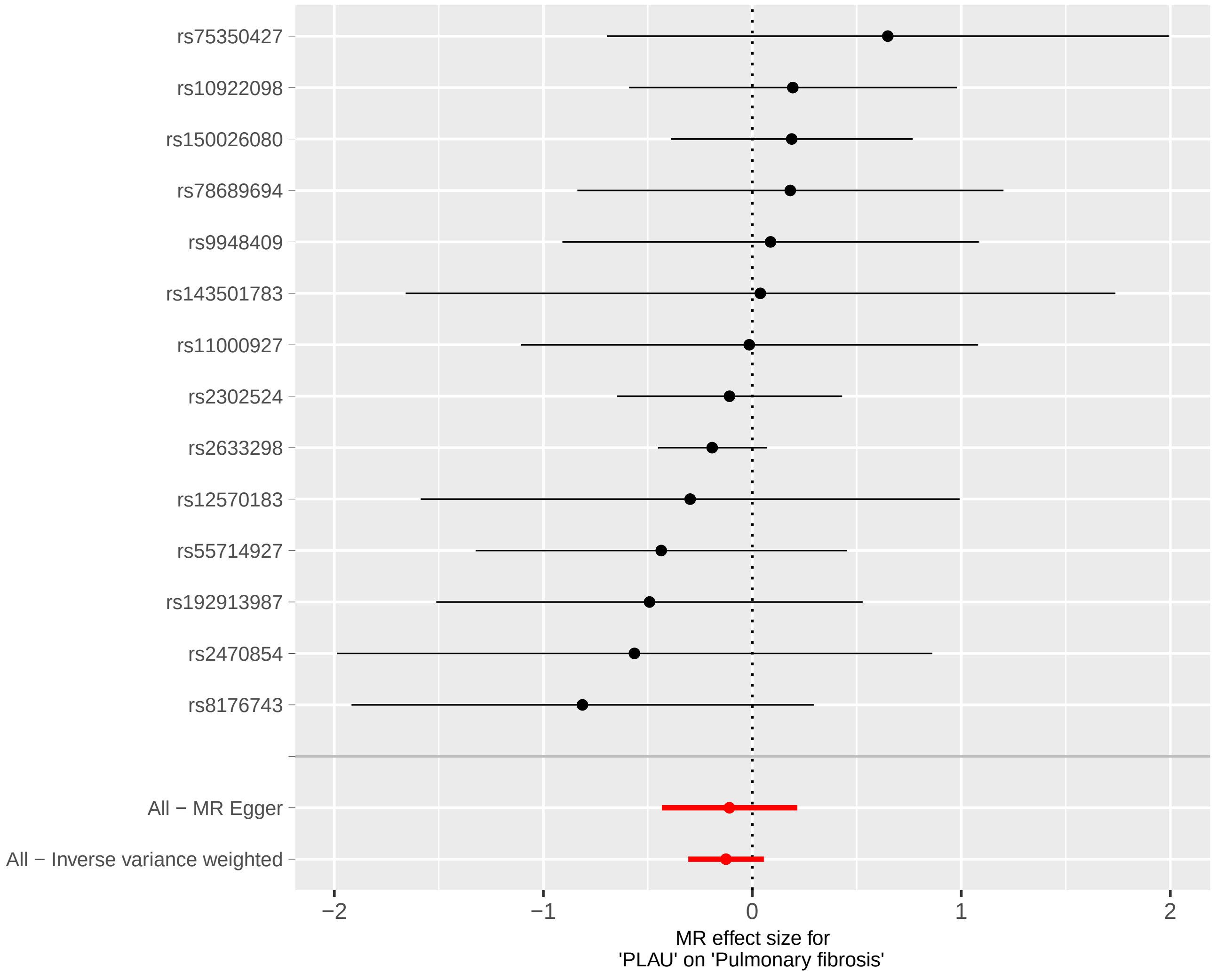
rs75350427
rs10922098
rs150026080
rs78689694
rs9948409
rs143501783
rs11000927
rs2302524
rs2633298
rs12570183
rs55714927
rs192913987
rs2470854
rs8176743
All − MR Egger
All − Inverse variance weighted
−2
−1
0
1
2
MR effect size for
'PLAU' on 'Pulmonary fibrosis'

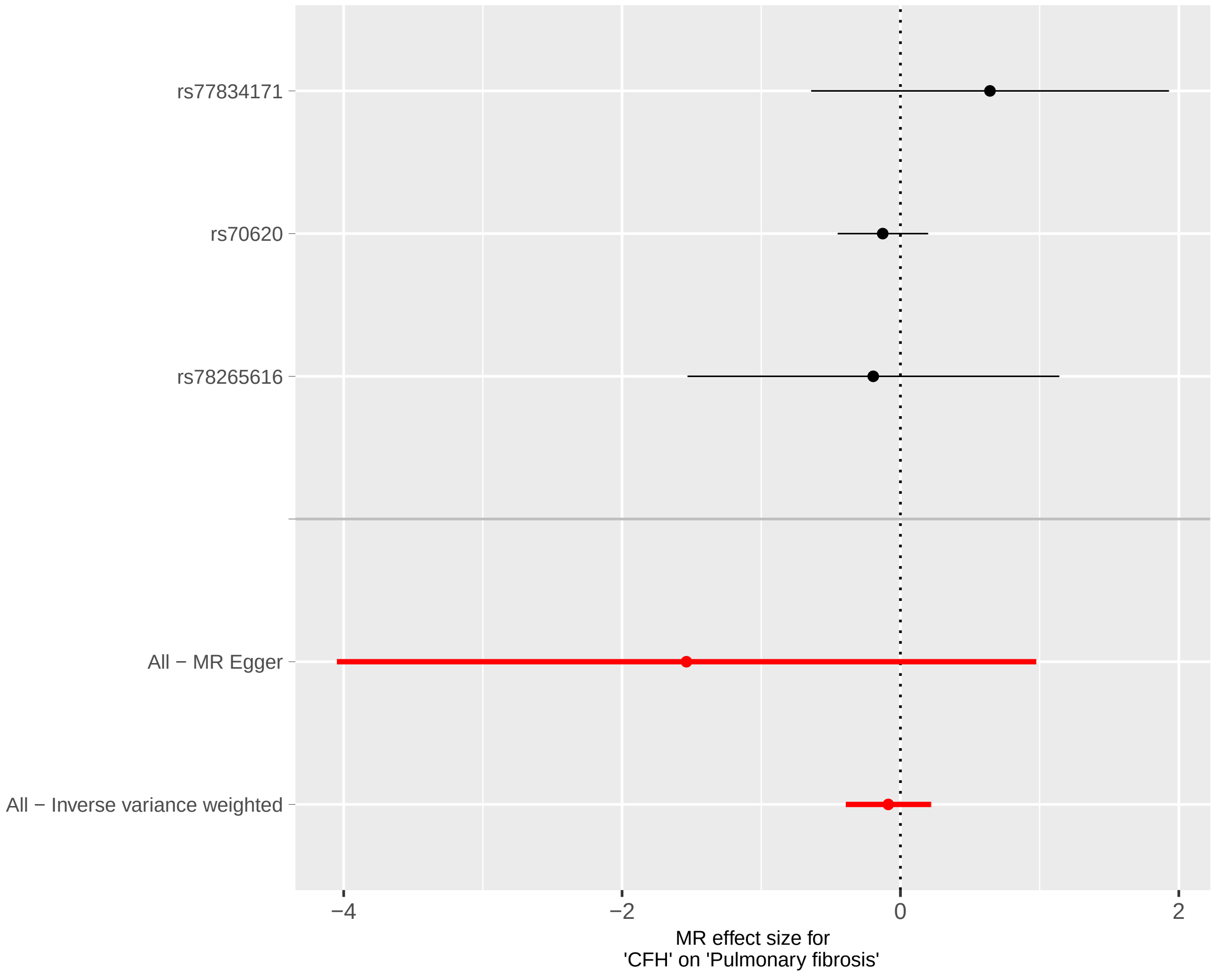

rs77834171
rs70620
rs78265616
All − MR Egger
All − Inverse variance weighted
−4
−2
0
2
MR effect size for
'CFH' on 'Pulmonary fibrosis'

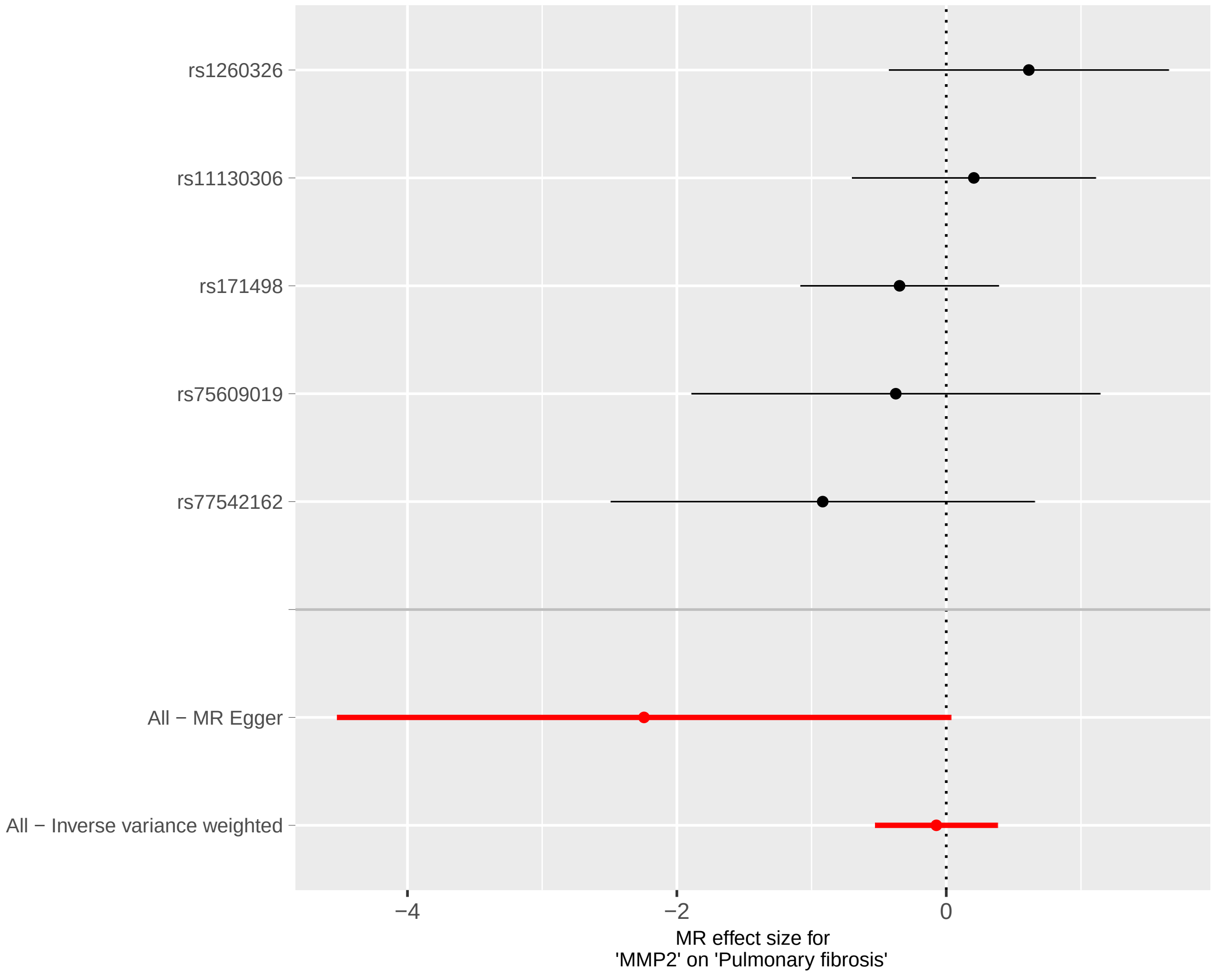

rs1260326
rs11130306
rs171498
rs75609019
rs77542162
All − MR Egger
All − Inverse variance weighted
−4
−2
0
MR effect size for
'MMP2' on 'Pulmonary fibrosis'

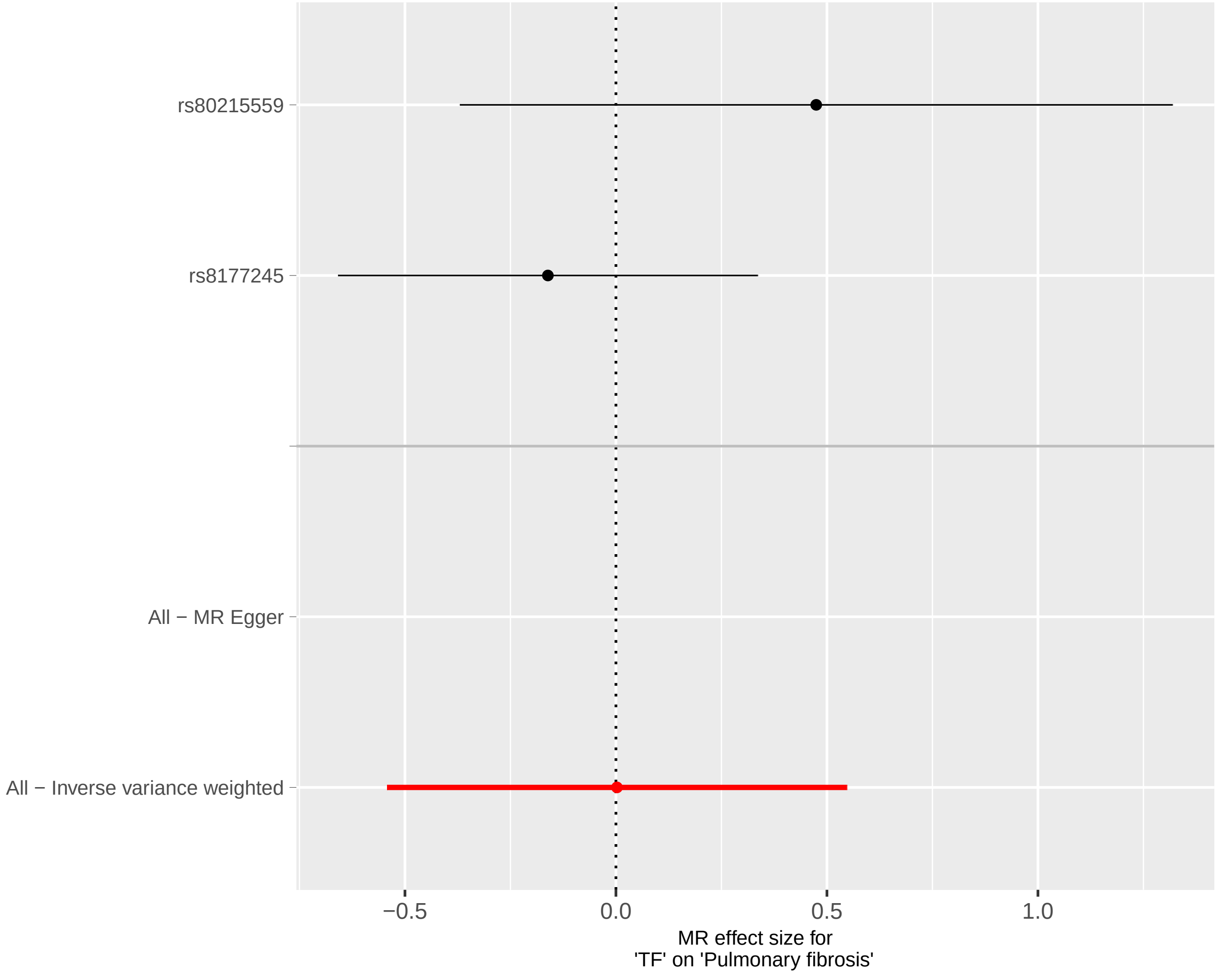
rs80215559
rs8177245
All − MR Egger
All − Inverse variance weighted
−0.5
0.0
0.5
1.0
MR effect size for
'TF' on 'Pulmonary fibrosis'

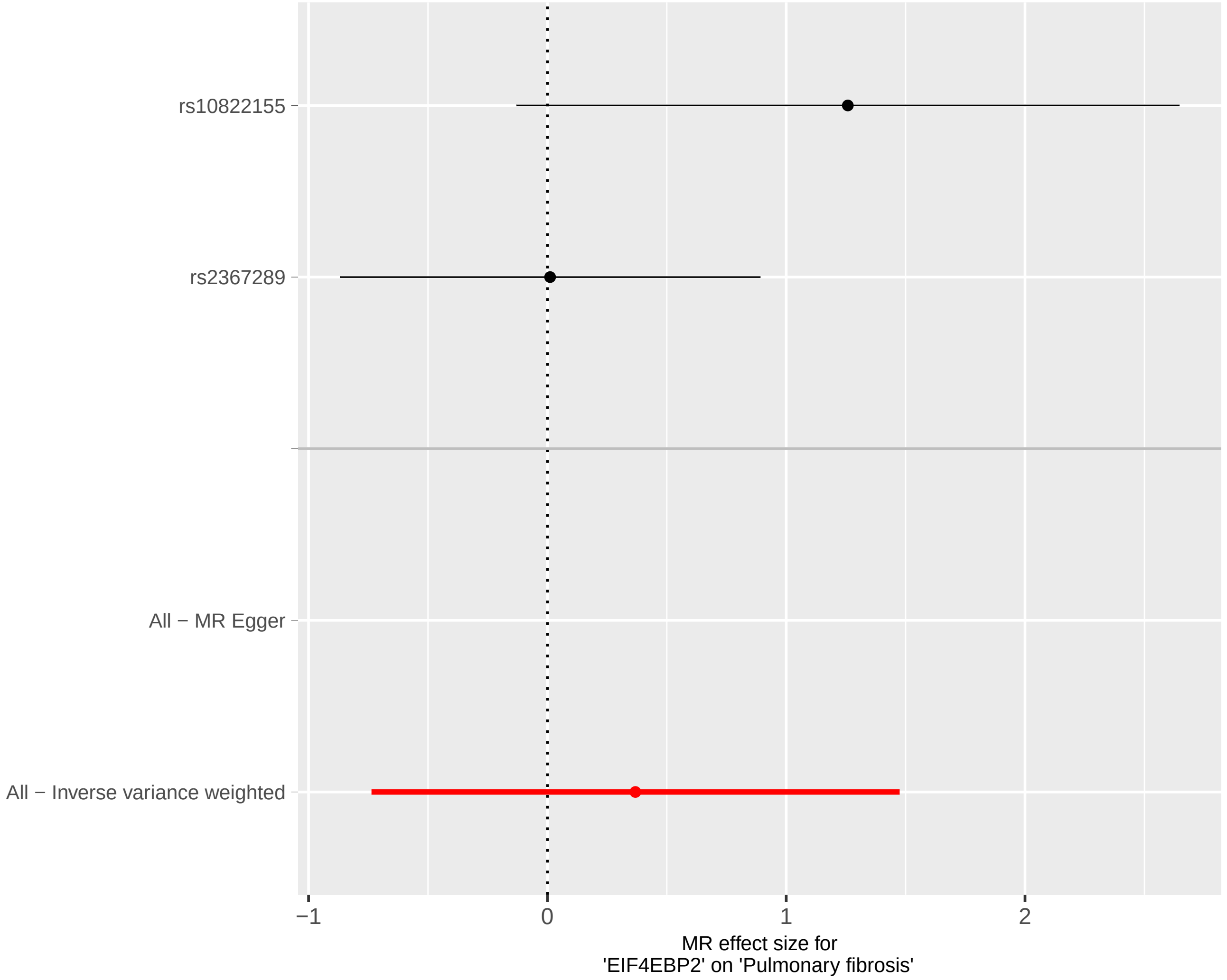

rs10822155
rs2367289
All − MR Egger
All − Inverse variance weighted
−1
0
1
2
MR effect size for
'EIF4EBP2' on 'Pulmonary fibrosis'

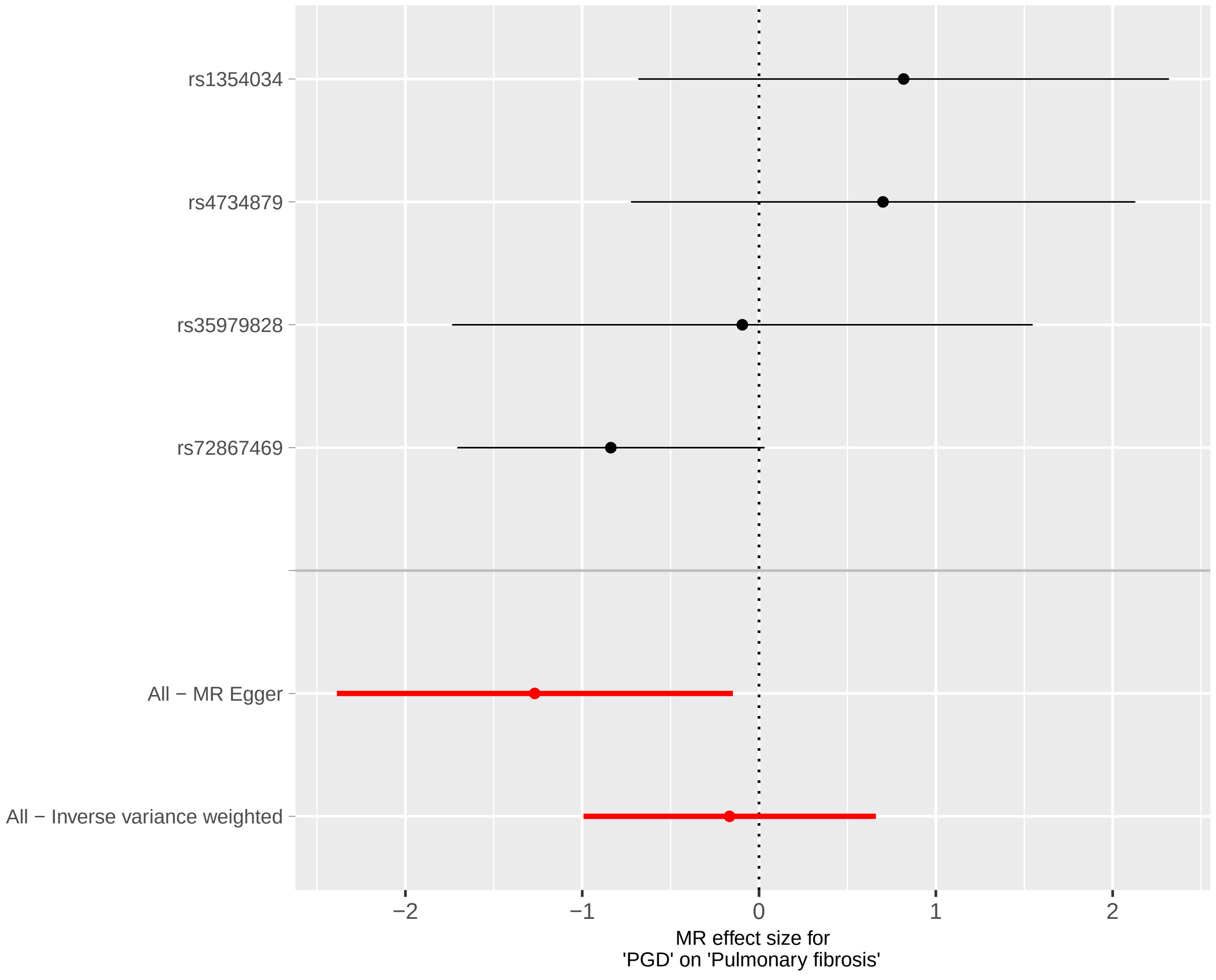
rs1354034
rs4734879
rs35979828
rs72867469
All − MR Egger
All − Inverse variance weighted
−2
−1
0
1
2
MR effect size for
'PGD' on 'Pulmonary fibrosis'

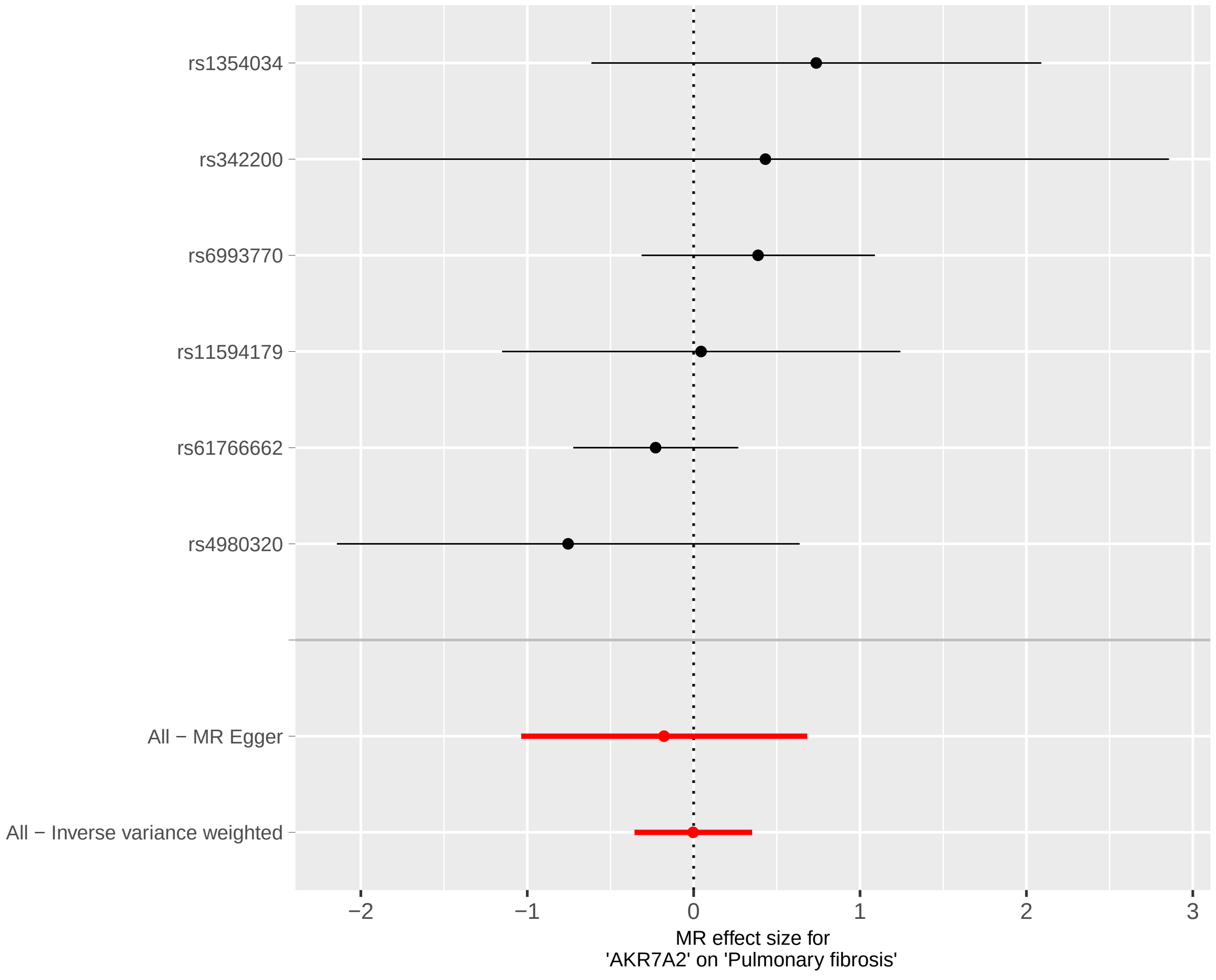

rs1354034
rs342200
rs6993770
rs11594179
rs61766662
rs4980320
All − MR Egger
All − Inverse variance weighted
−2
−1
0
1
2
3
MR effect size for
'AKR7A2' on 'Pulmonary fibrosis'

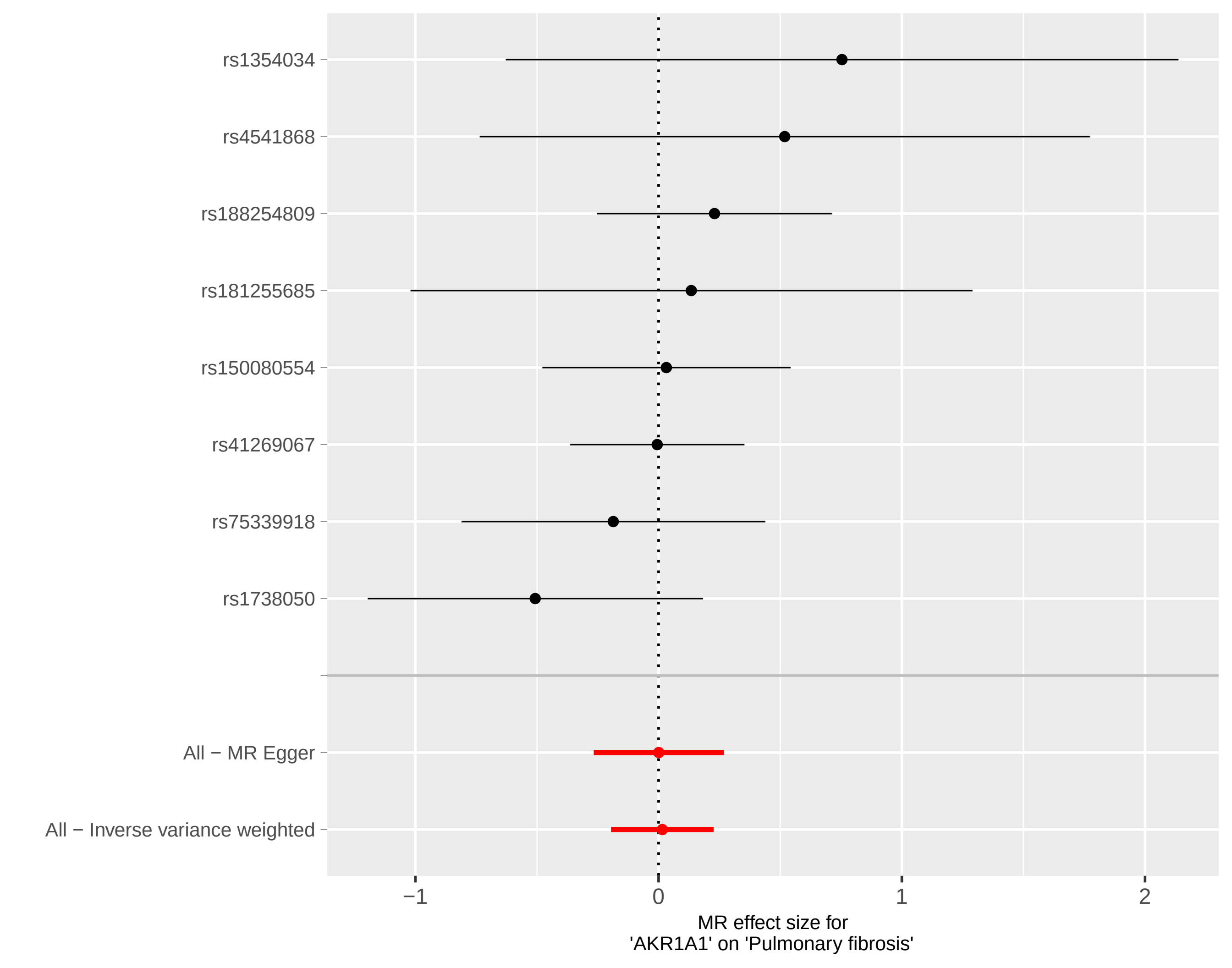
rs1354034
rs4541868
rs188254809
rs181255685
rs150080554
rs41269067
rs75339918
rs1738050
All − MR Egger
All − Inverse variance weighted
−1
0
1
2
MR effect size for
'AKR1A1' on 'Pulmonary fibrosis'

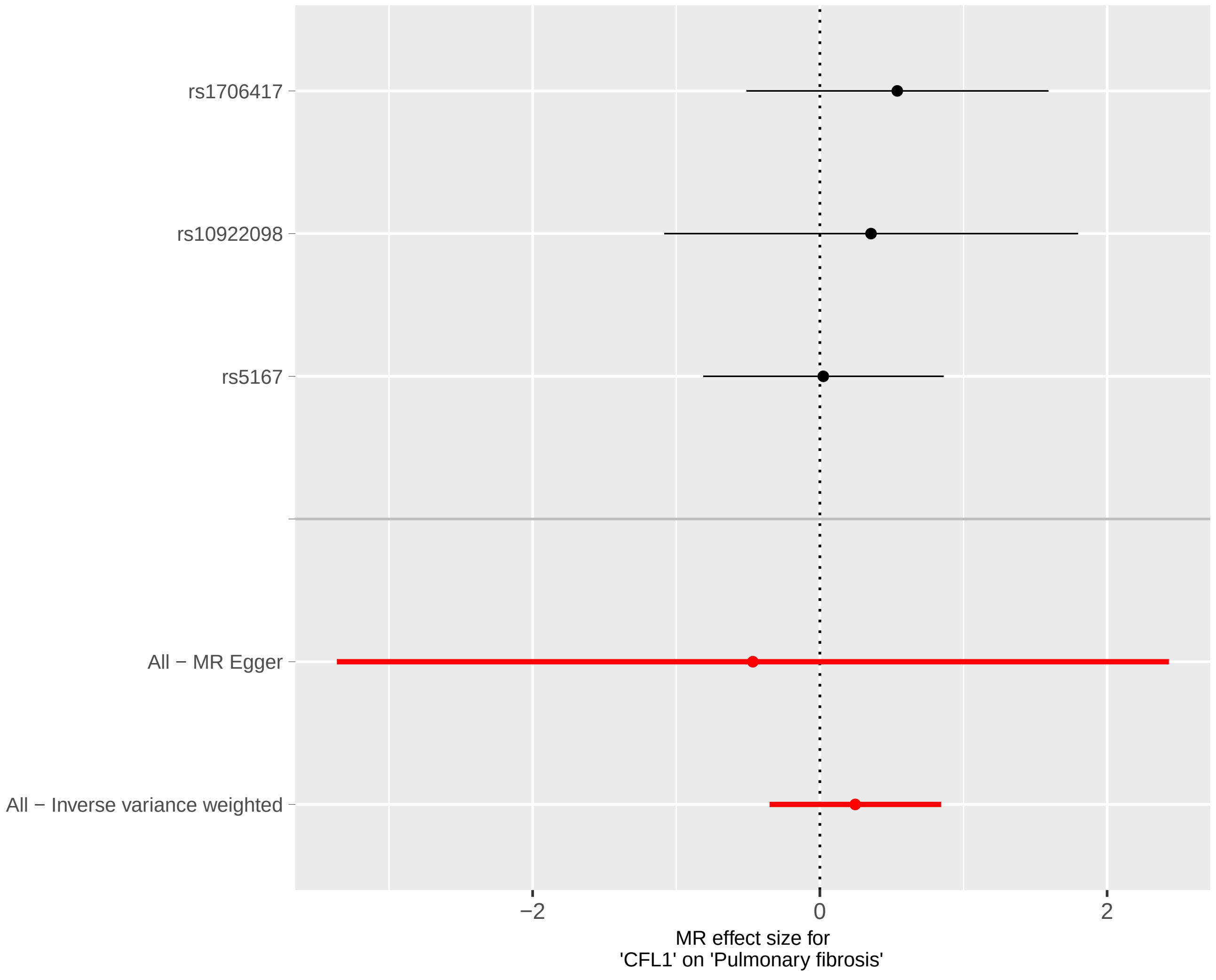
rs1706417
rs10922098
rs5167
All − MR Egger
All − Inverse variance weighted
−2
0
2
MR effect size for
'CFL1' on 'Pulmonary fibrosis'

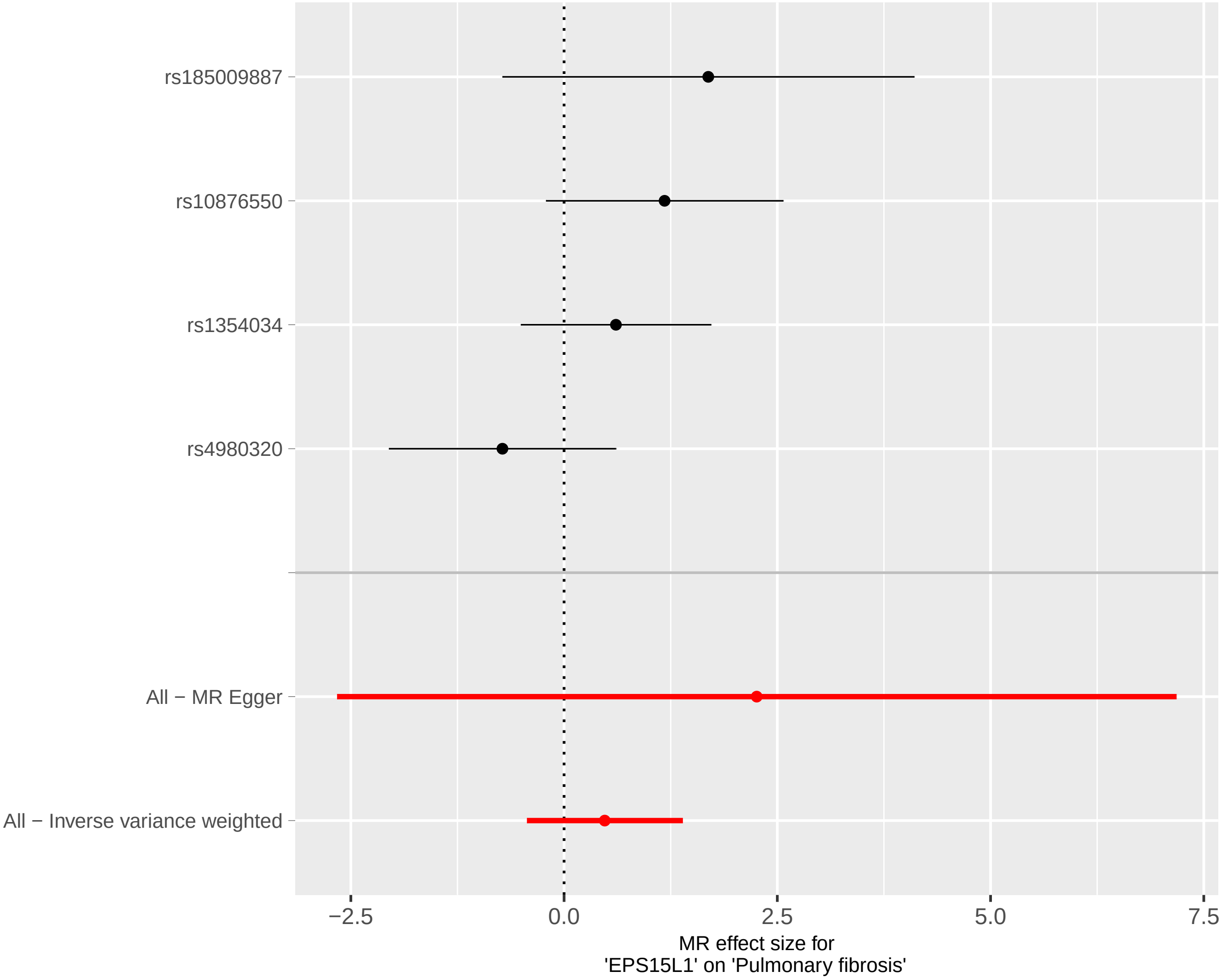

rs185009887
rs10876550
rs1354034
rs4980320
All − MR Egger
All − Inverse variance weighted
−2.5
0.0
2.5
5.0
7.5
MR effect size for
'EPS15L1' on 'Pulmonary fibrosis'

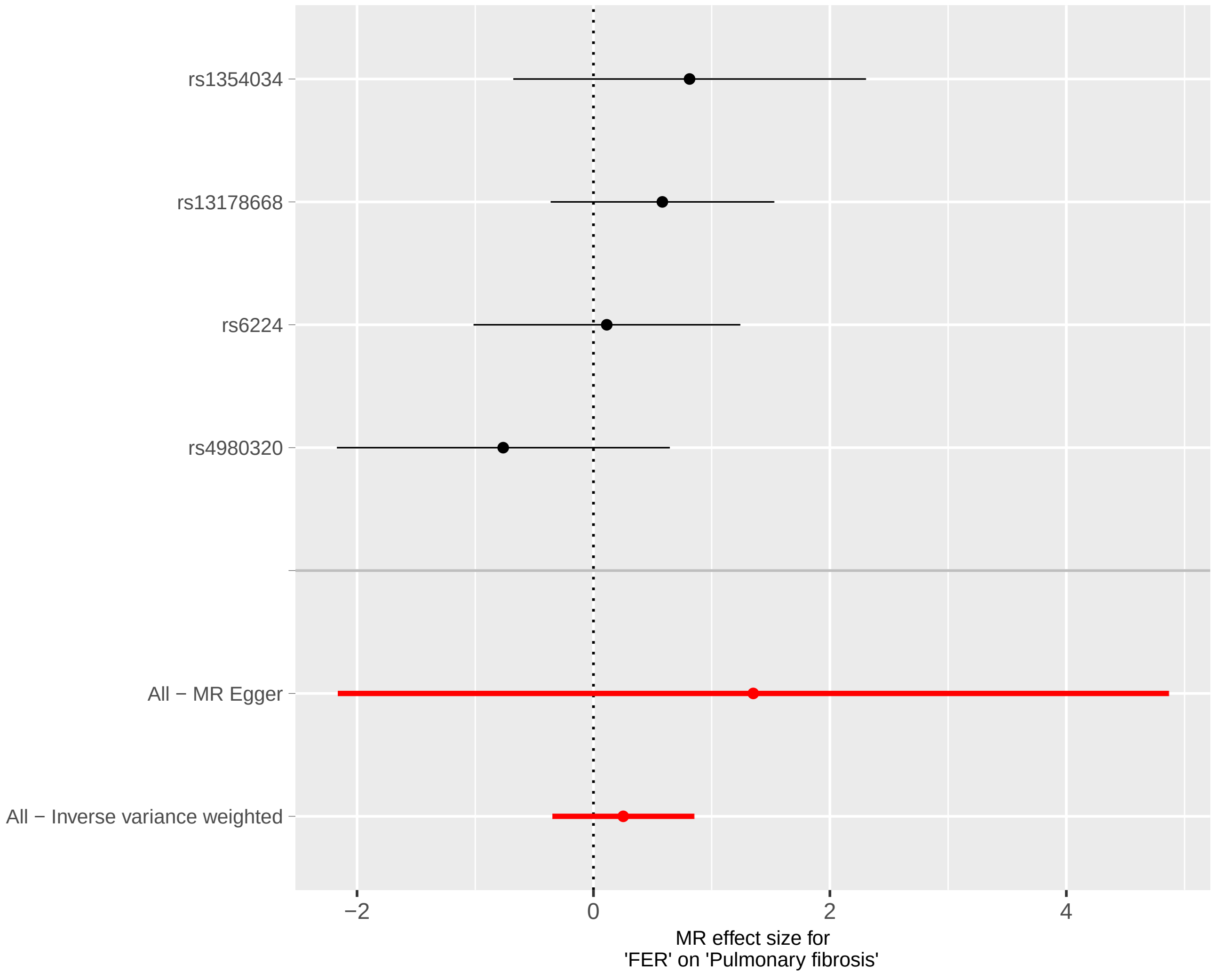
rs1354034
rs13178668
rs6224
rs4980320
All − MR Egger
All − Inverse variance weighted
−2
0
2
4
MR effect size for
'FER' on 'Pulmonary fibrosis'

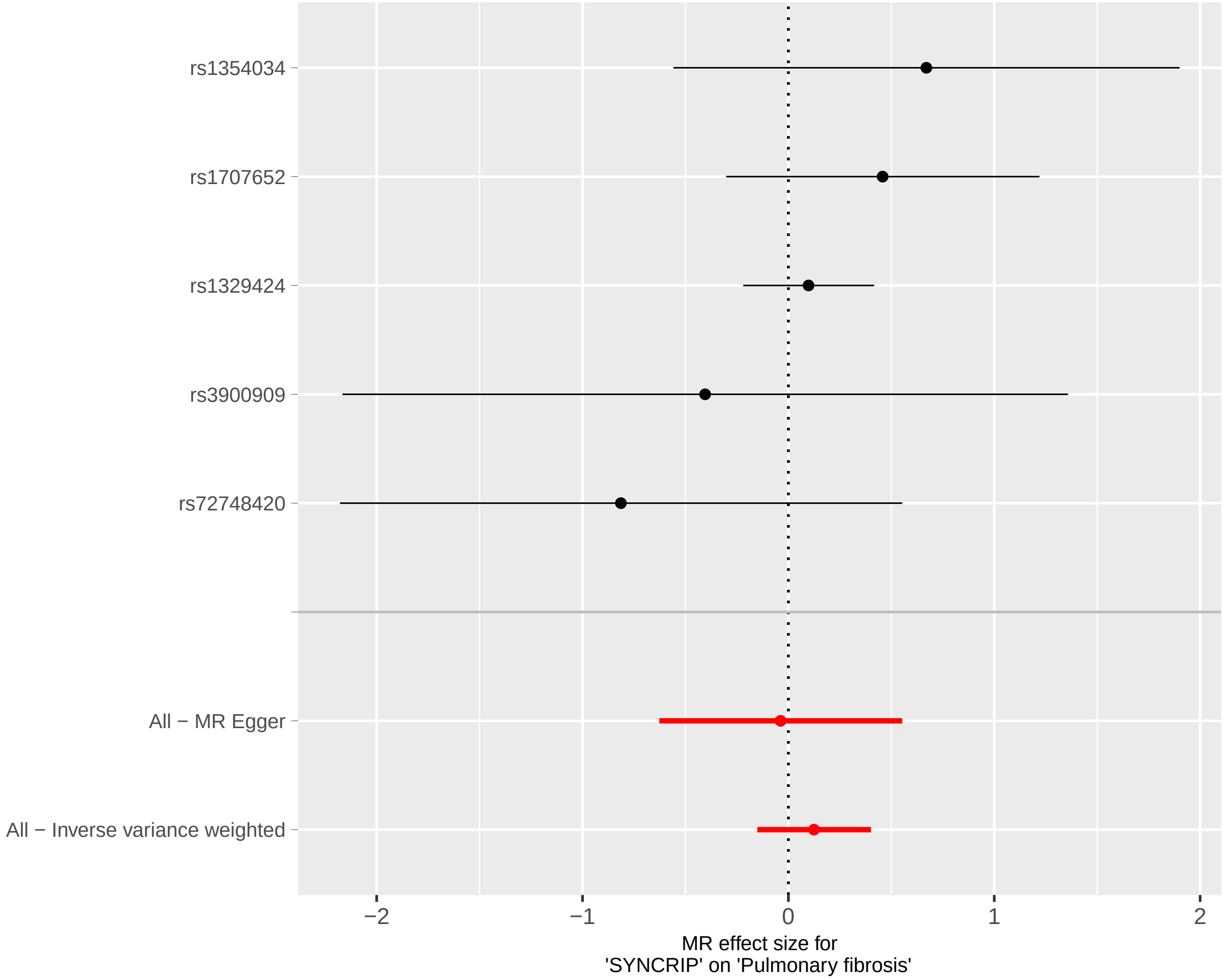

rs1354034
rs1707652
rs1329424
rs3900909
rs72748420
All − MR Egger
All − Inverse variance weighted
−2
−1
0
1
2
MR effect size for
'SYNCRIP' on 'Pulmonary fibrosis'

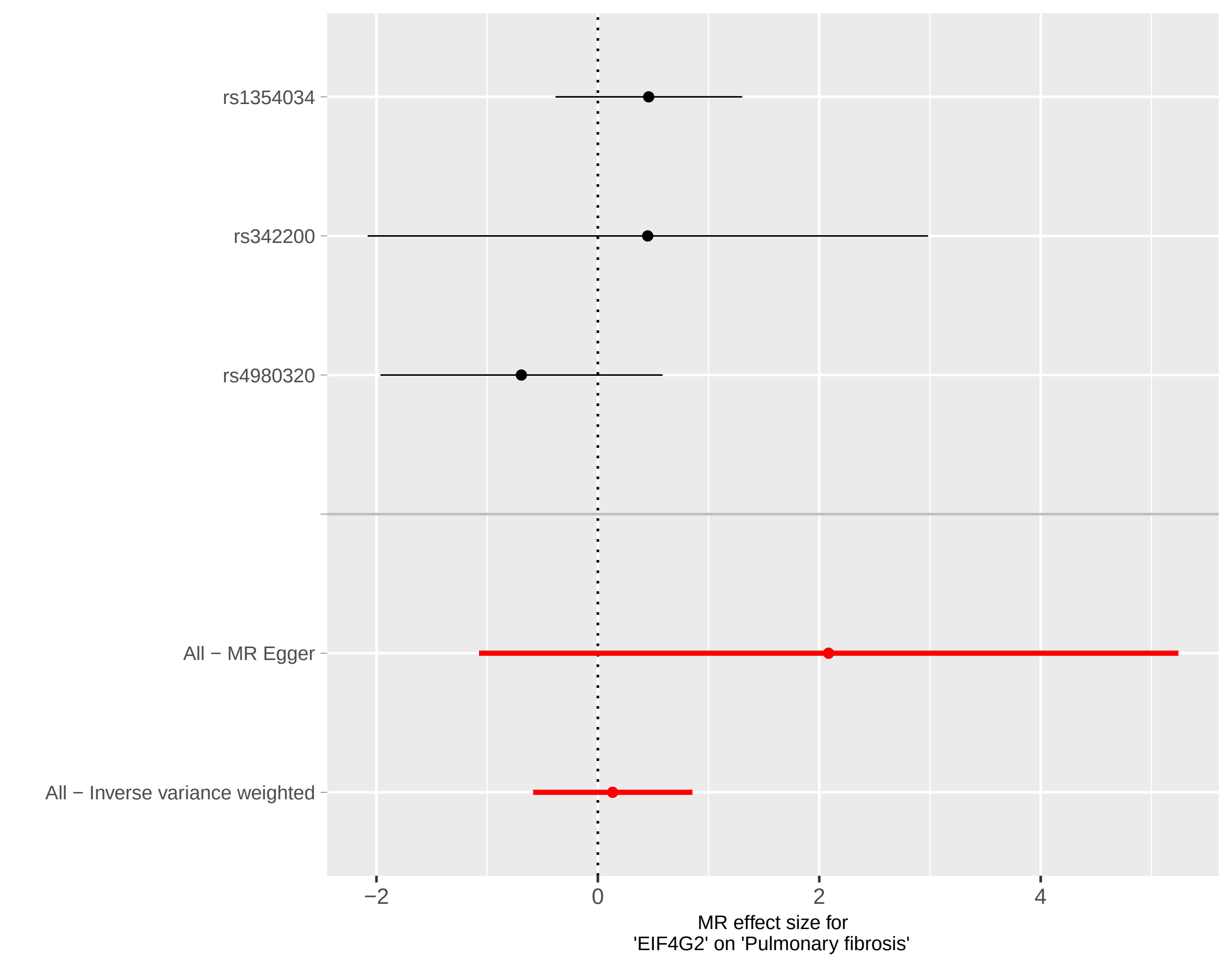
rs1354034
rs342200
rs4980320
All − MR Egger
All − Inverse variance weighted
−2
0
2
4
MR effect size for
'EIF4G2' on 'Pulmonary fibrosis'

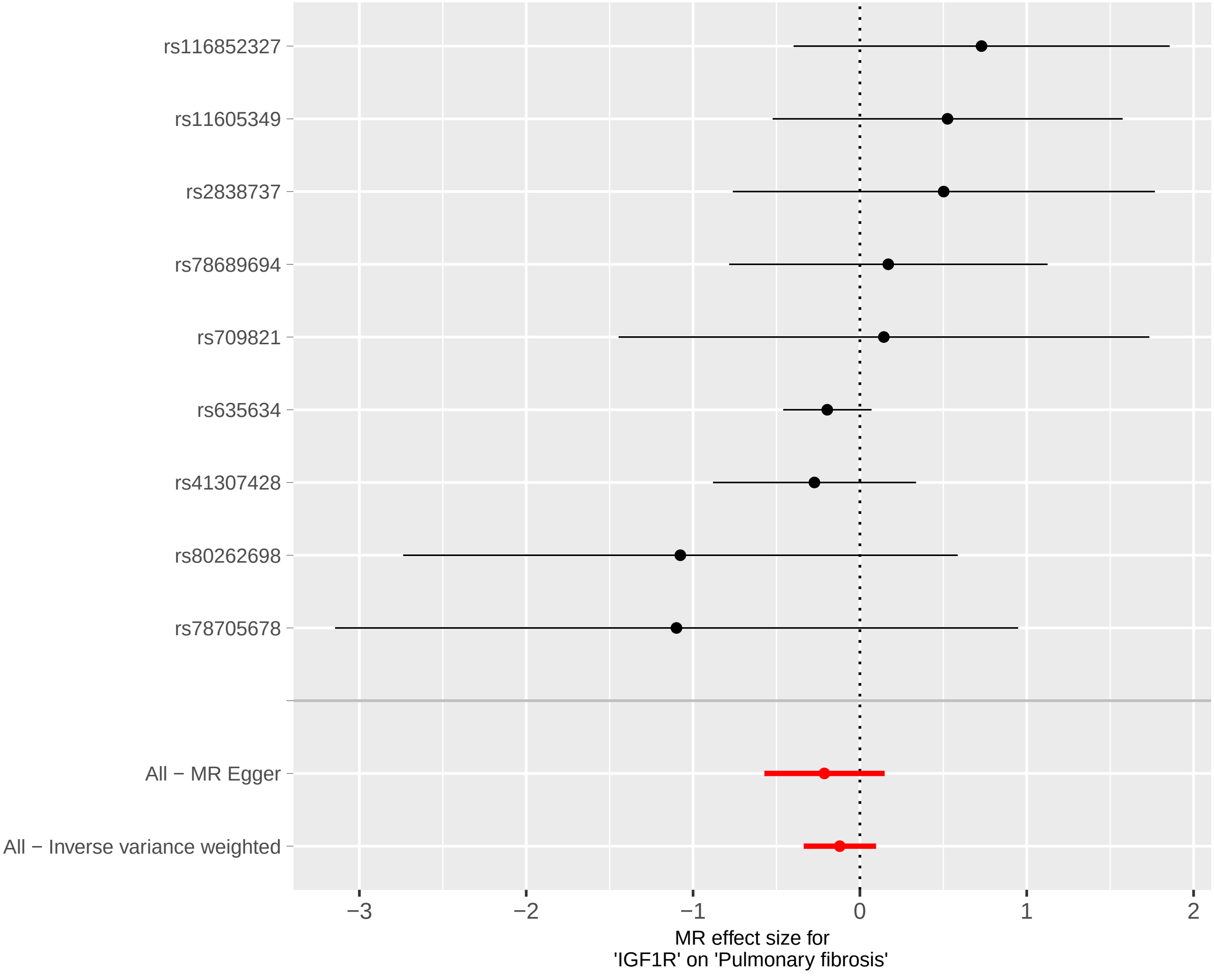
rs116852327
rs11605349
rs2838737
rs78689694
rs709821
rs635634
rs41307428
rs80262698
rs78705678
All − MR Egger
All − Inverse variance weighted
−3
−2
−1
0
1
2
MR effect size for
'IGF1R' on 'Pulmonary fibrosis'

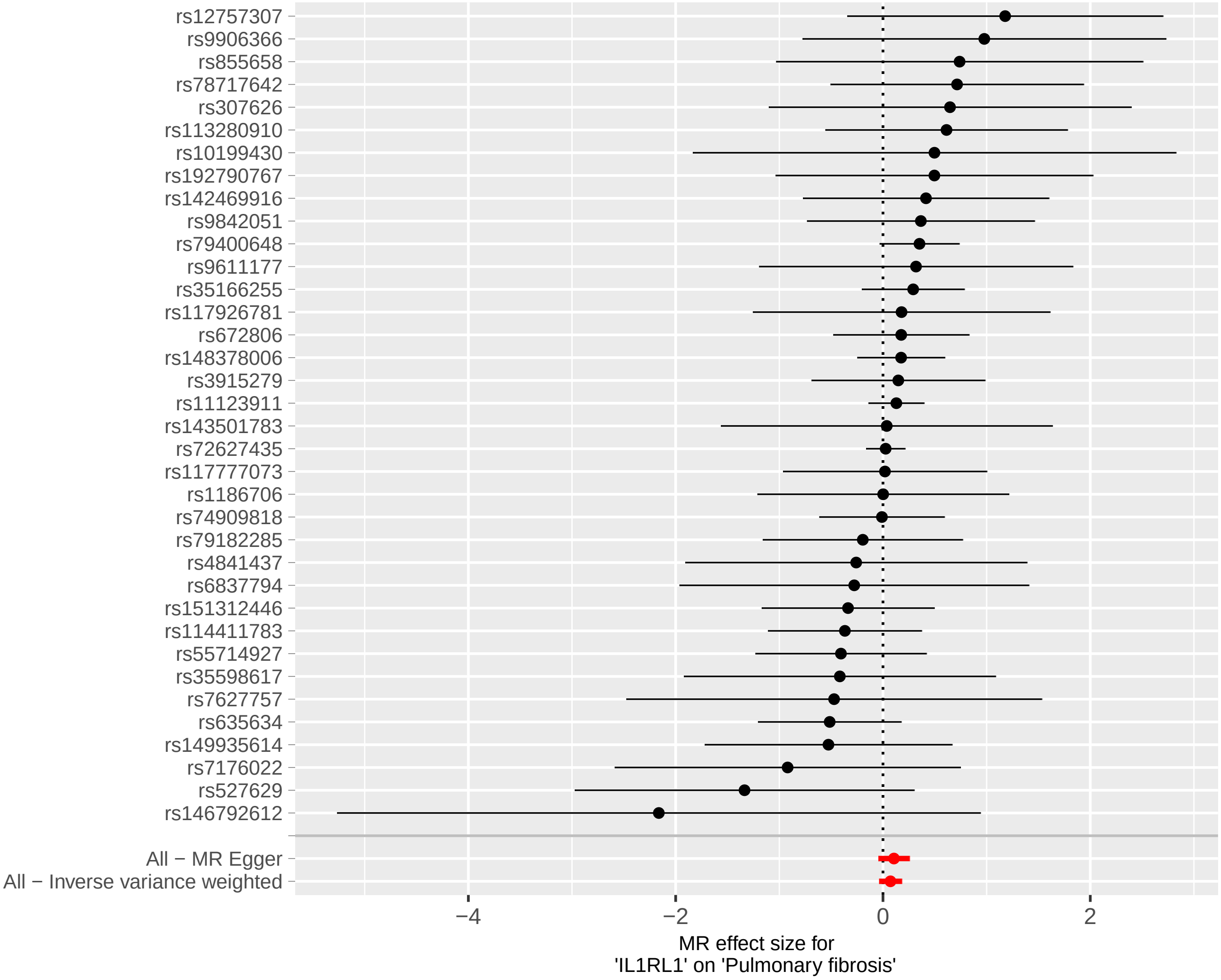

rs12757307
rs9906366
rs855658
rs78717642
rs307626
rs113280910
rs10199430
rs192790767
rs142469916
rs9842051
rs79400648
rs9611177
rs35166255
rs117926781
rs672806
rs148378006
rs3915279
rs11123911
rs143501783
rs72627435
rs117777073
rs1186706
rs74909818
rs79182285
rs4841437
rs6837794
rs151312446
rs114411783
rs55714927
rs35598617
rs7627757
rs635634
rs149935614
rs7176022
rs527629
rs146792612
All − MR Egger
All − Inverse variance weighted
−4
−2
0
2
MR effect size for
'IL1RL1' on 'Pulmonary fibrosis'

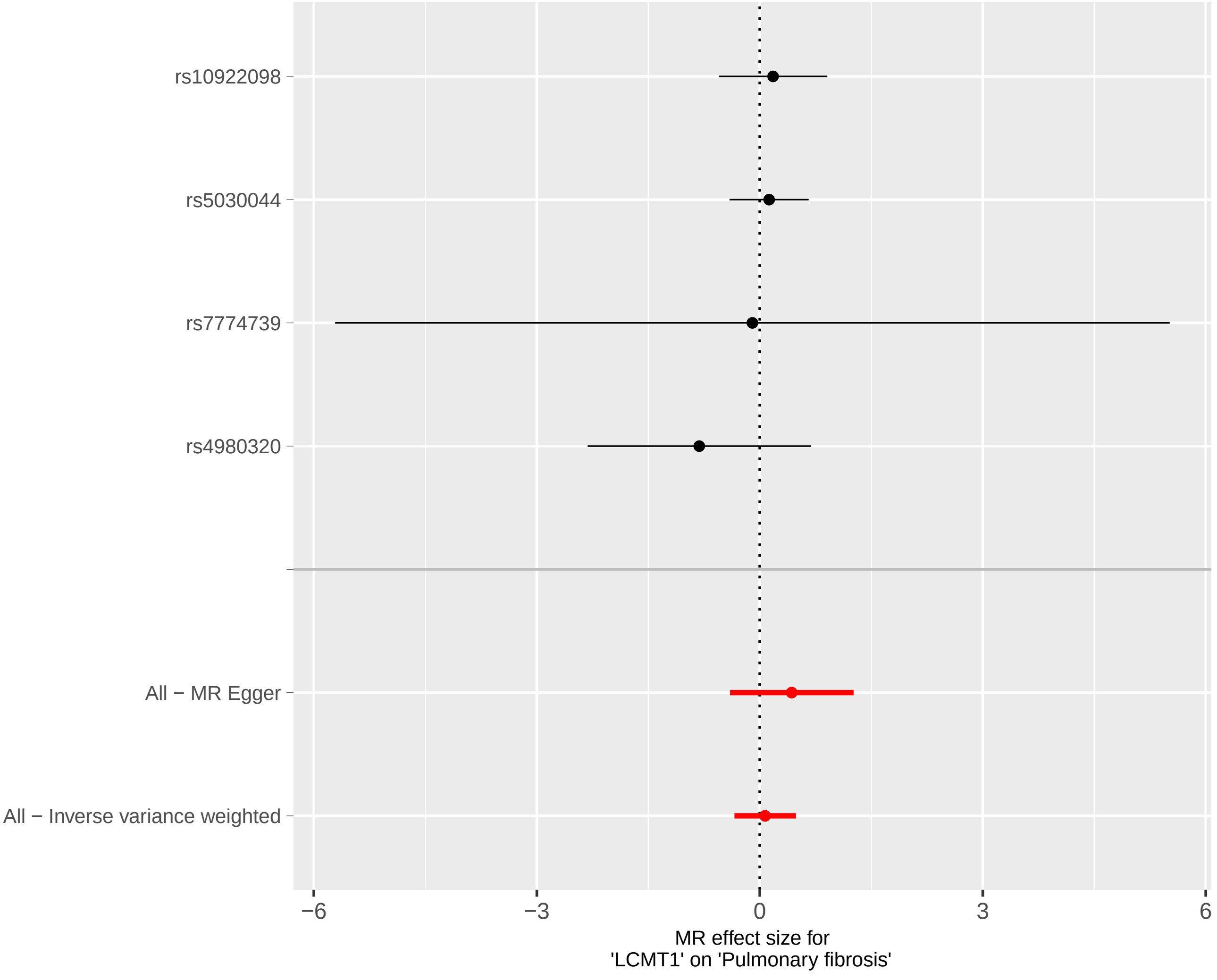
rs10922098
rs5030044
rs7774739
rs4980320
All − MR Egger
All − Inverse variance weighted
−6
−3
0
3
6
MR effect size for
'LCMT1' on 'Pulmonary fibrosis'

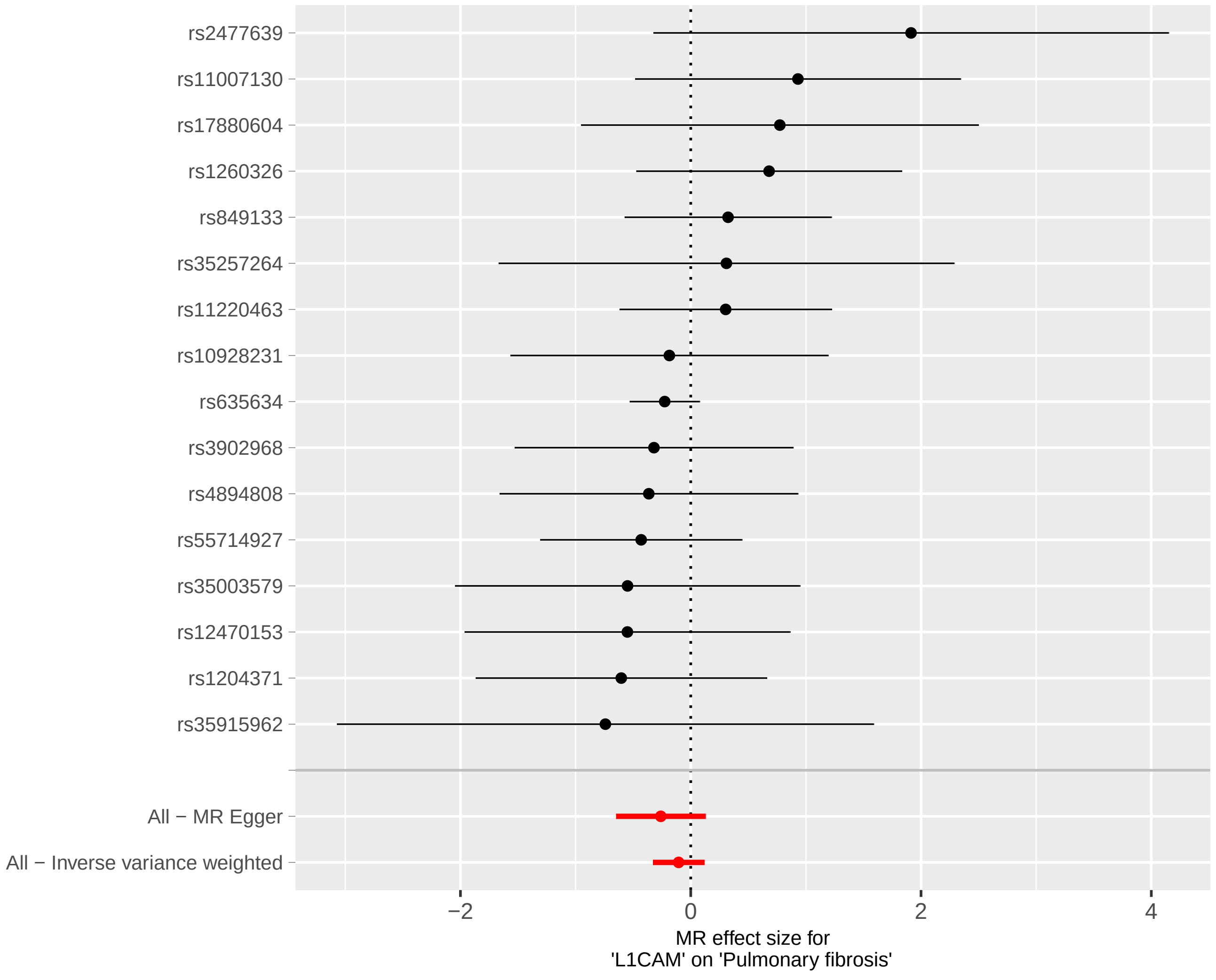

rs2477639
rs11007130
rs17880604
rs1260326
rs849133
rs35257264
rs11220463
rs10928231
rs635634
rs3902968
rs4894808
rs55714927
rs35003579
rs12470153
rs1204371
rs35915962
All − MR Egger
All − Inverse variance weighted
−2
0
2
4
MR effect size for
'L1CAM' on 'Pulmonary fibrosis'

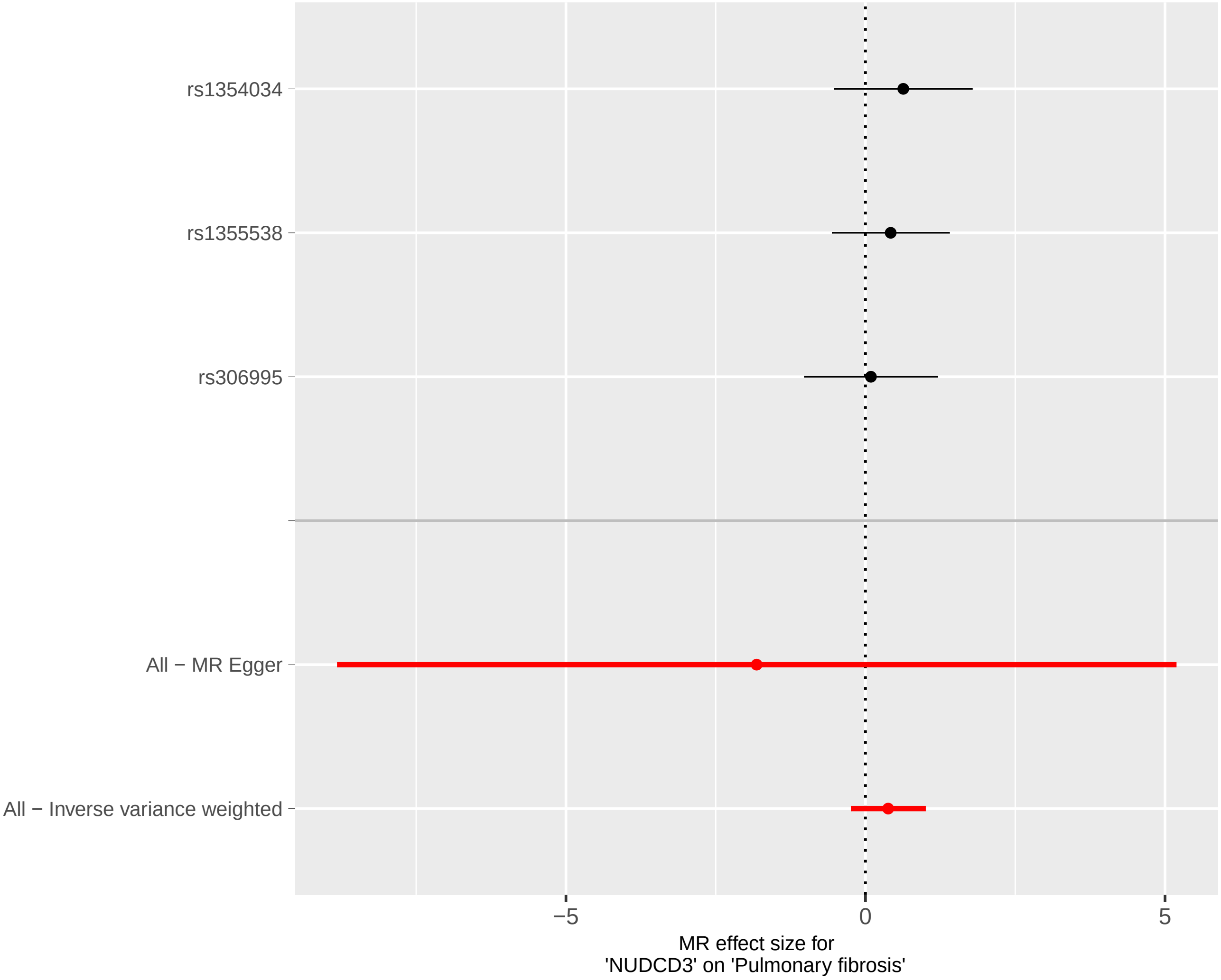

rs1354034
rs1355538
rs306995
All − MR Egger
All − Inverse variance weighted
−5
0
5
MR effect size for
'NUDCD3' on 'Pulmonary fibrosis'

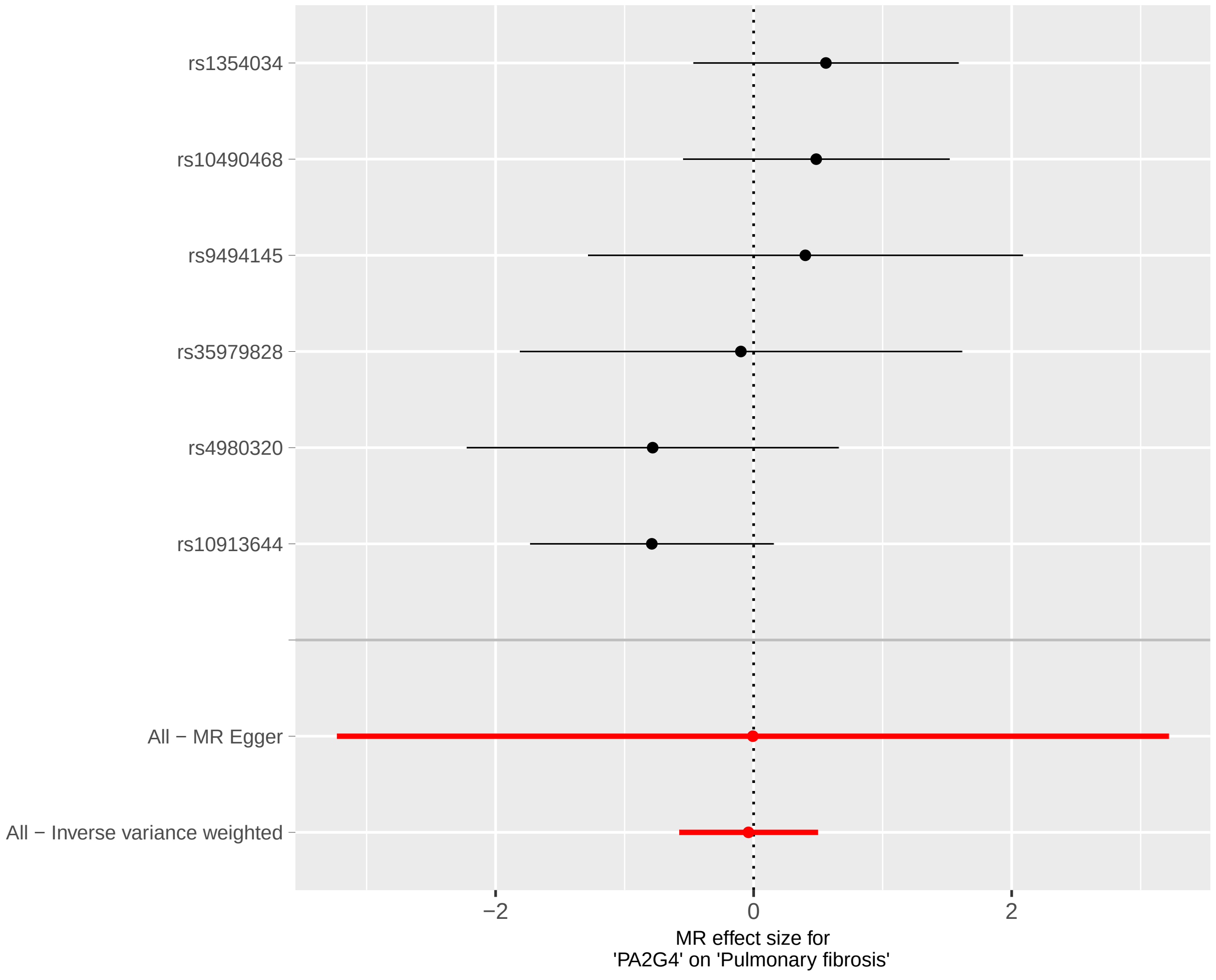
rs1354034
rs10490468
rs9494145
rs35979828
rs4980320
rs10913644
All − MR Egger
All − Inverse variance weighted
−2
0
2
MR effect size for
'PA2G4' on 'Pulmonary fibrosis'

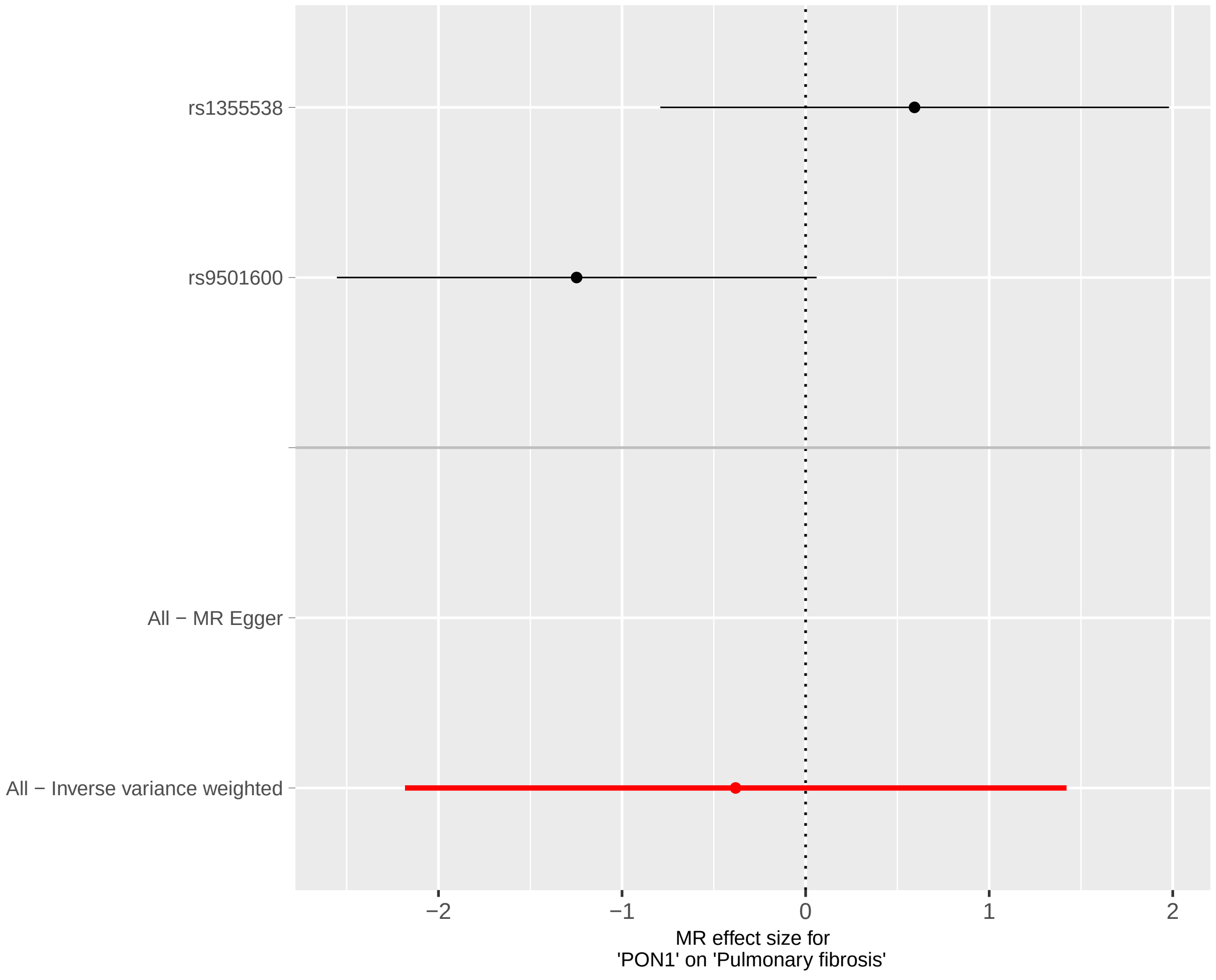
rs1355538
rs9501600
All − MR Egger
All − Inverse variance weighted
−2
−1
0
1
2
MR effect size for
'PON1' on 'Pulmonary fibrosis'

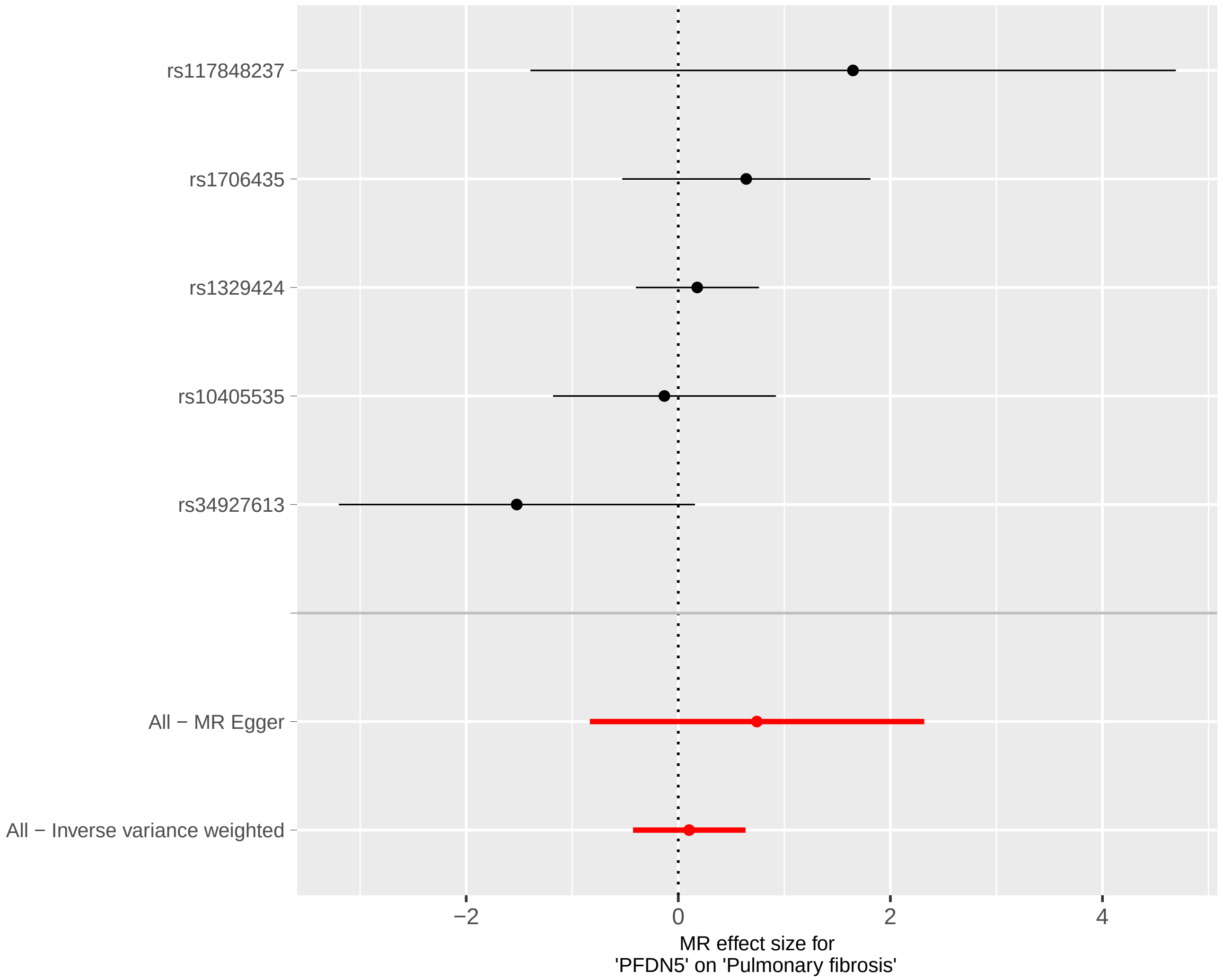

rs117848237
rs1706435
rs1329424
rs10405535
rs34927613
All − MR Egger
All − Inverse variance weighted
−2
0
2
4
MR effect size for
'PFDN5' on 'Pulmonary fibrosis'

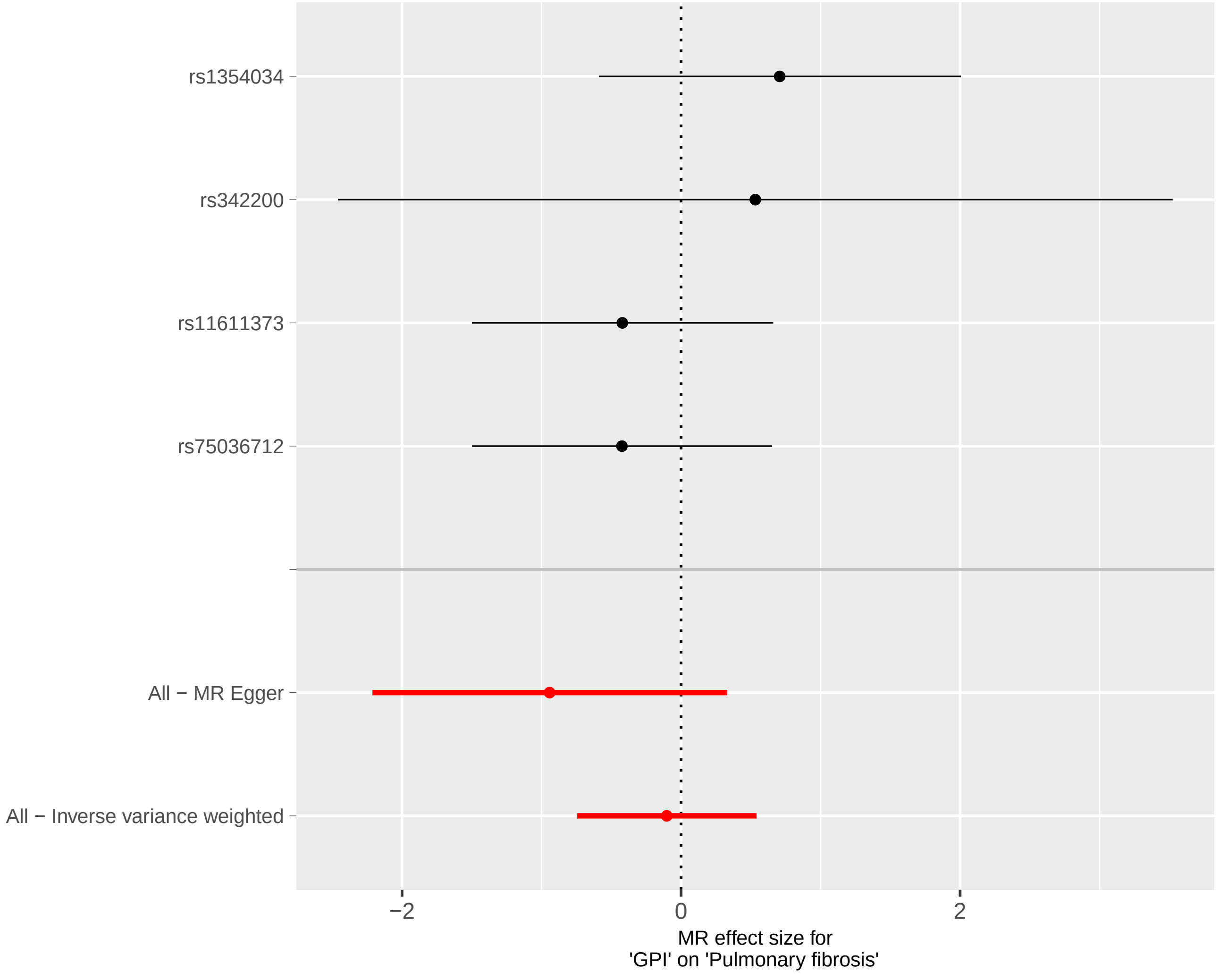
rs1354034
rs342200
rs11611373
rs75036712
All − MR Egger
All − Inverse variance weighted
−2
0
2
MR effect size for
'GPI' on 'Pulmonary fibrosis'

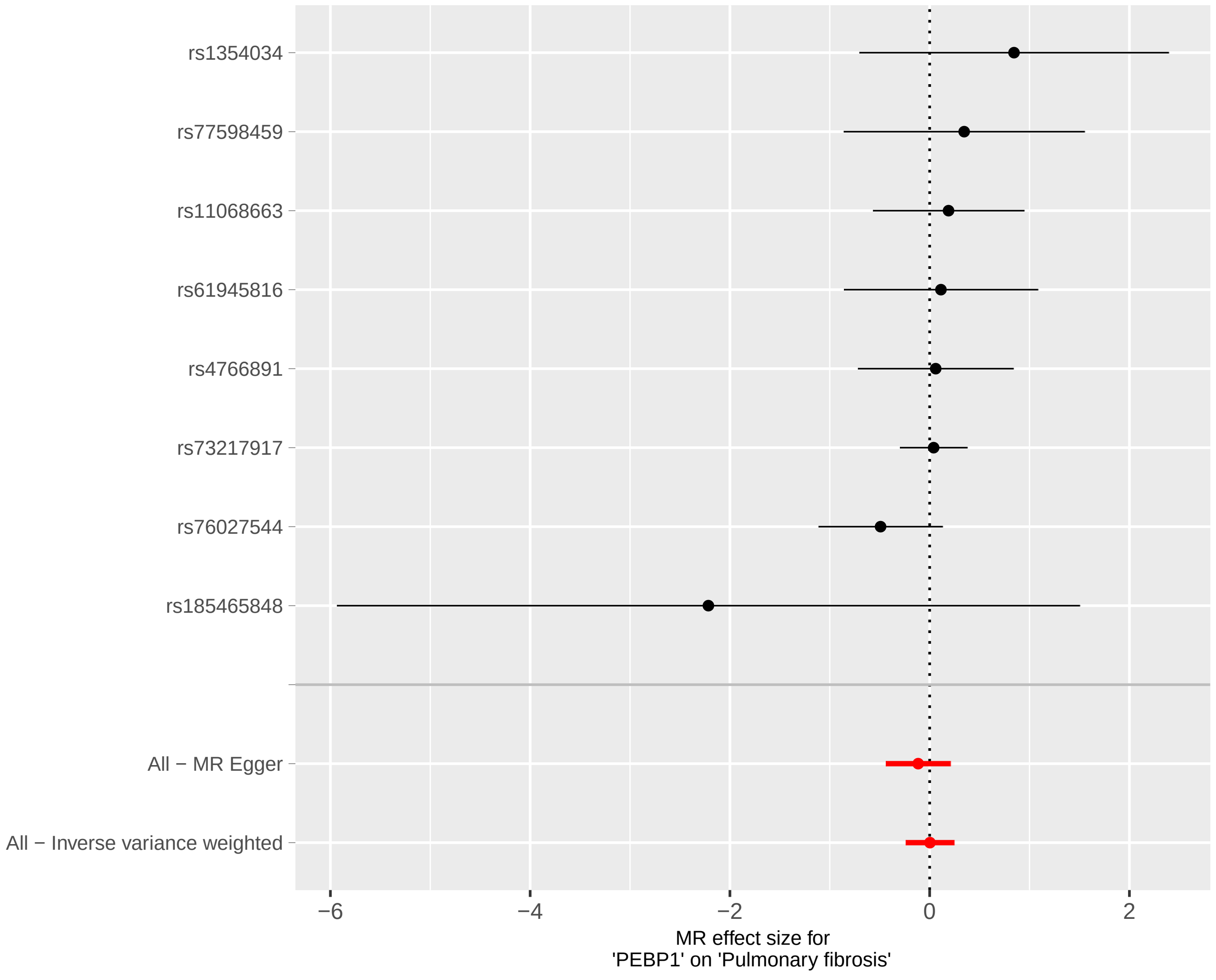
rs1354034
rs77598459
rs11068663
rs61945816
rs4766891
rs73217917
rs76027544
rs185465848
All − MR Egger
All − Inverse variance weighted
−6
−4
−2
0
2
MR effect size for
'PEBP1' on 'Pulmonary fibrosis'

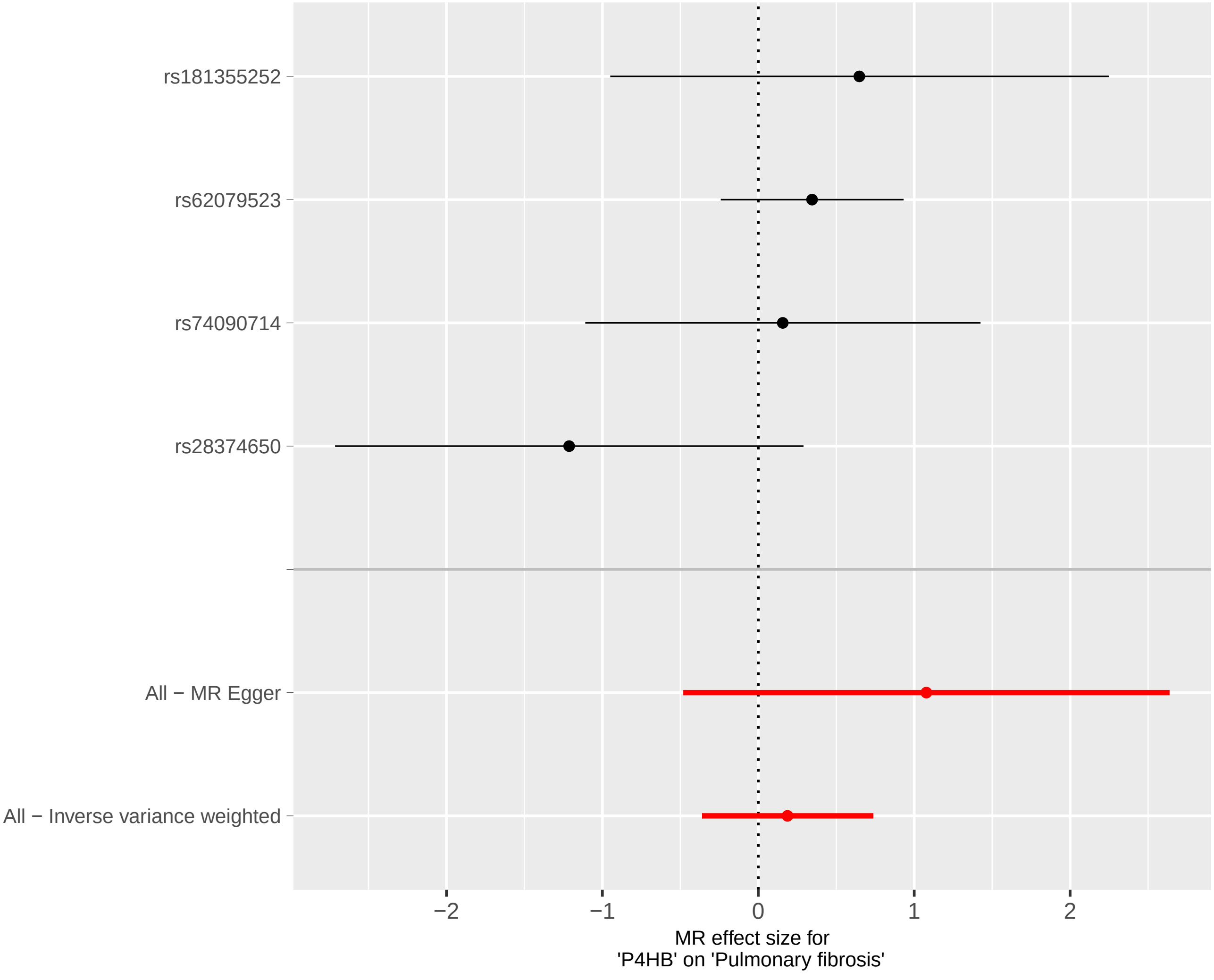

rs181355252
rs62079523
rs74090714
rs28374650
All − MR Egger
All − Inverse variance weighted
−2
−1
0
1
2
MR effect size for
'P4HB' on 'Pulmonary fibrosis'

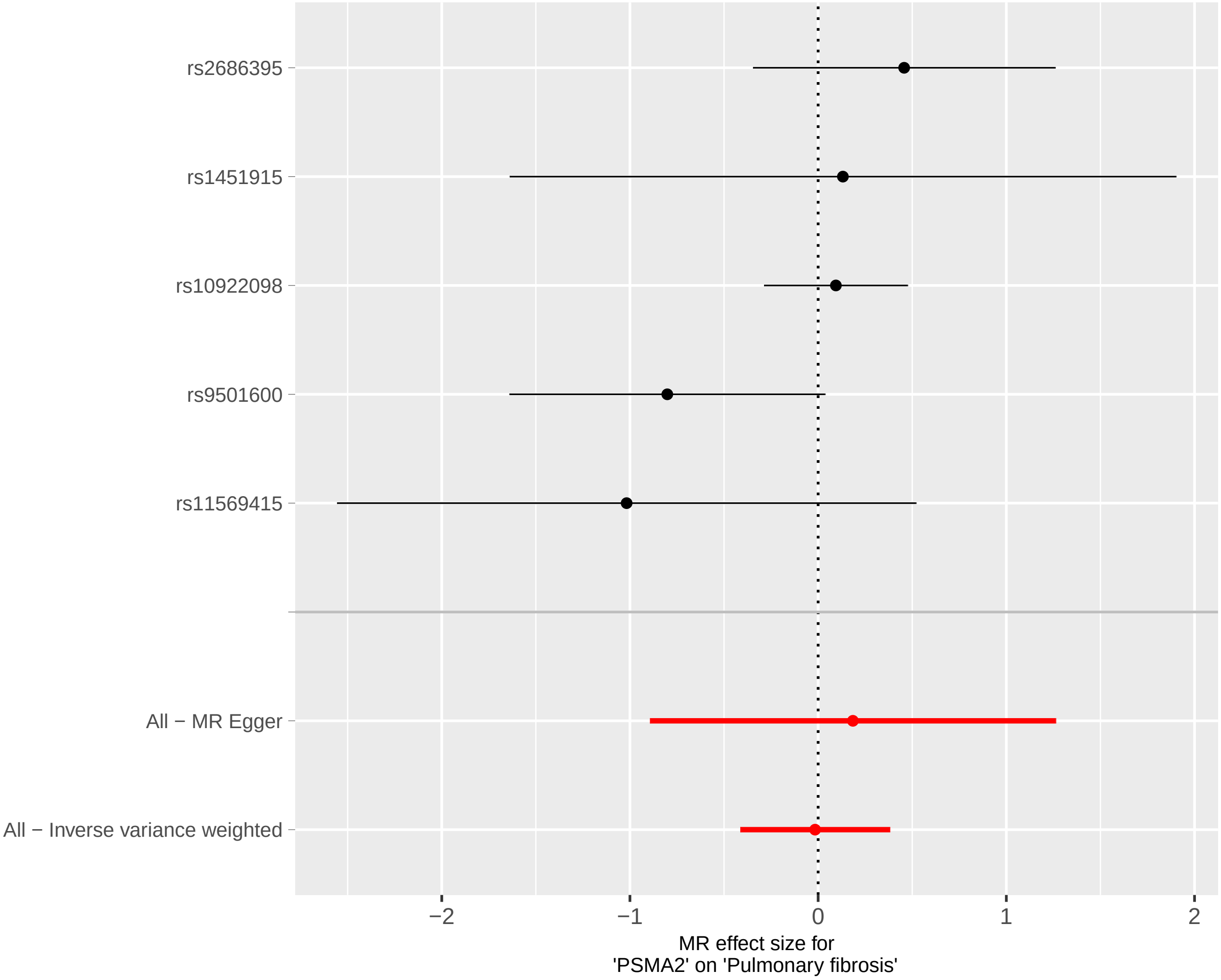
rs2686395
rs1451915
rs10922098
rs9501600
rs11569415
All − MR Egger
All − Inverse variance weighted
−2
−1
0
1
2
MR effect size for
'PSMA2' on 'Pulmonary fibrosis'

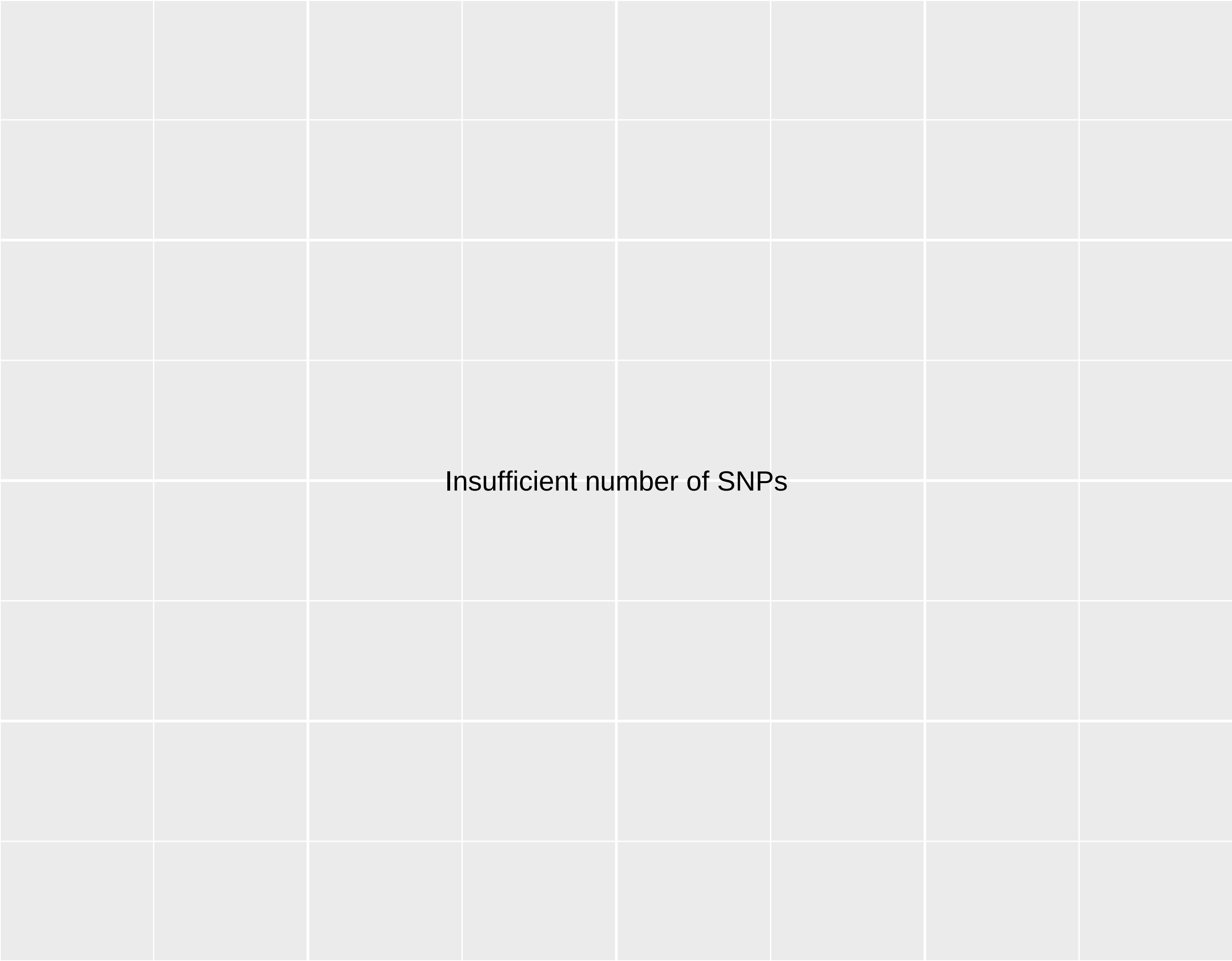
Insufficient number of SNPs

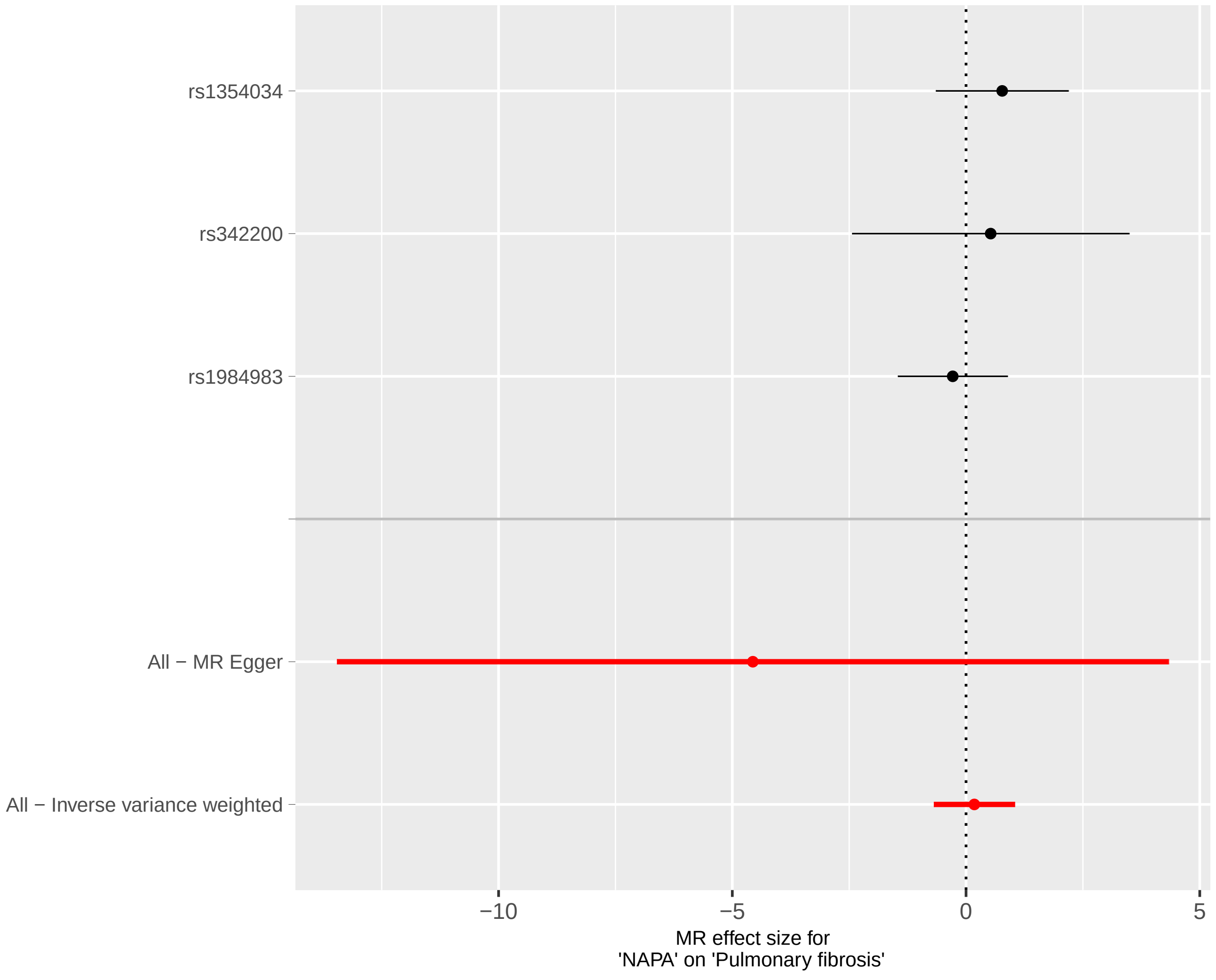
rs1354034
rs342200
rs1984983
All − MR Egger
All − Inverse variance weighted
−10
−5
0
5
MR effect size for
'NAPA' on 'Pulmonary fibrosis'

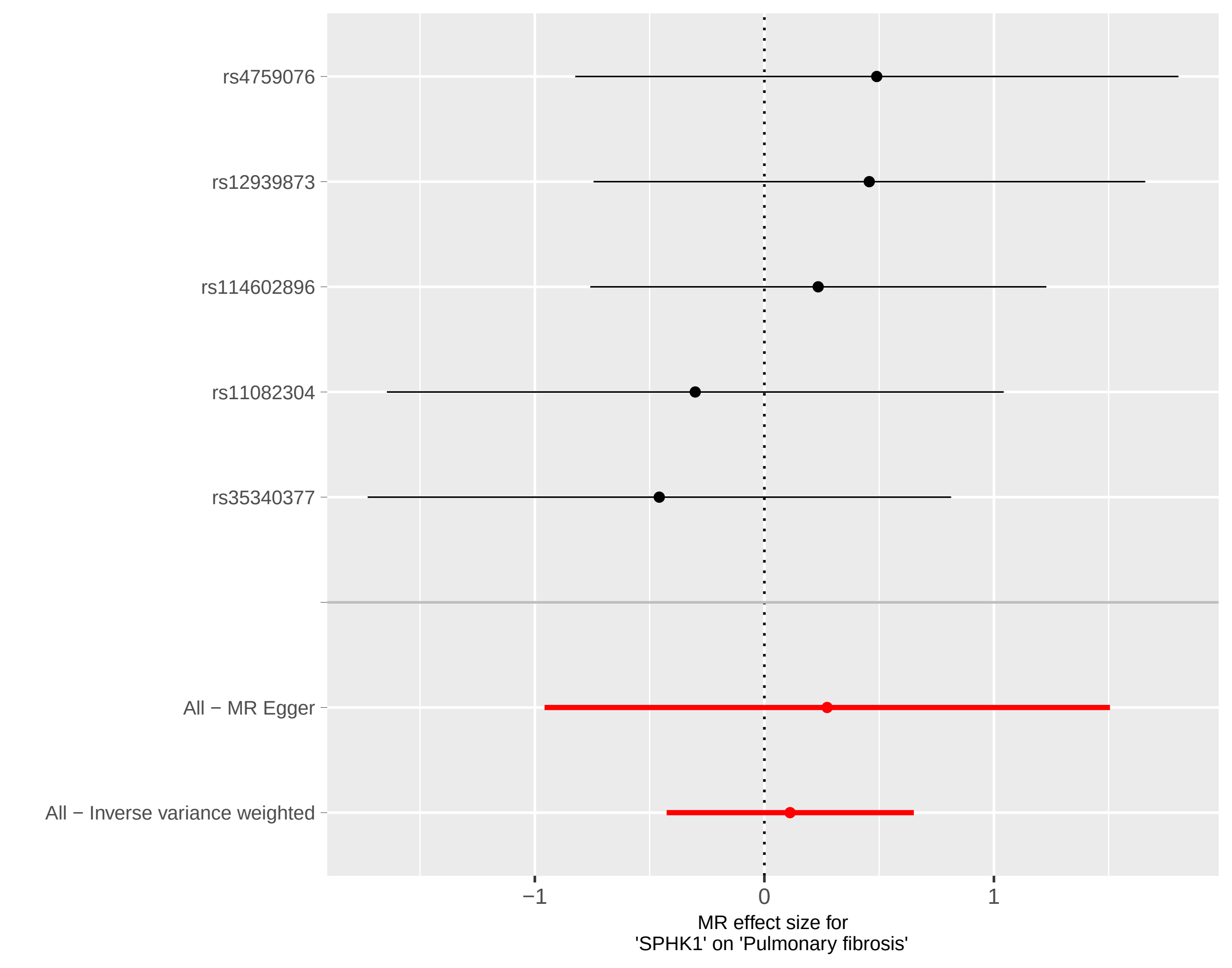
rs4759076
rs12939873
rs114602896
rs11082304
rs35340377
All − MR Egger
All − Inverse variance weighted
−1
0
1
MR effect size for
'SPHK1' on 'Pulmonary fibrosis'

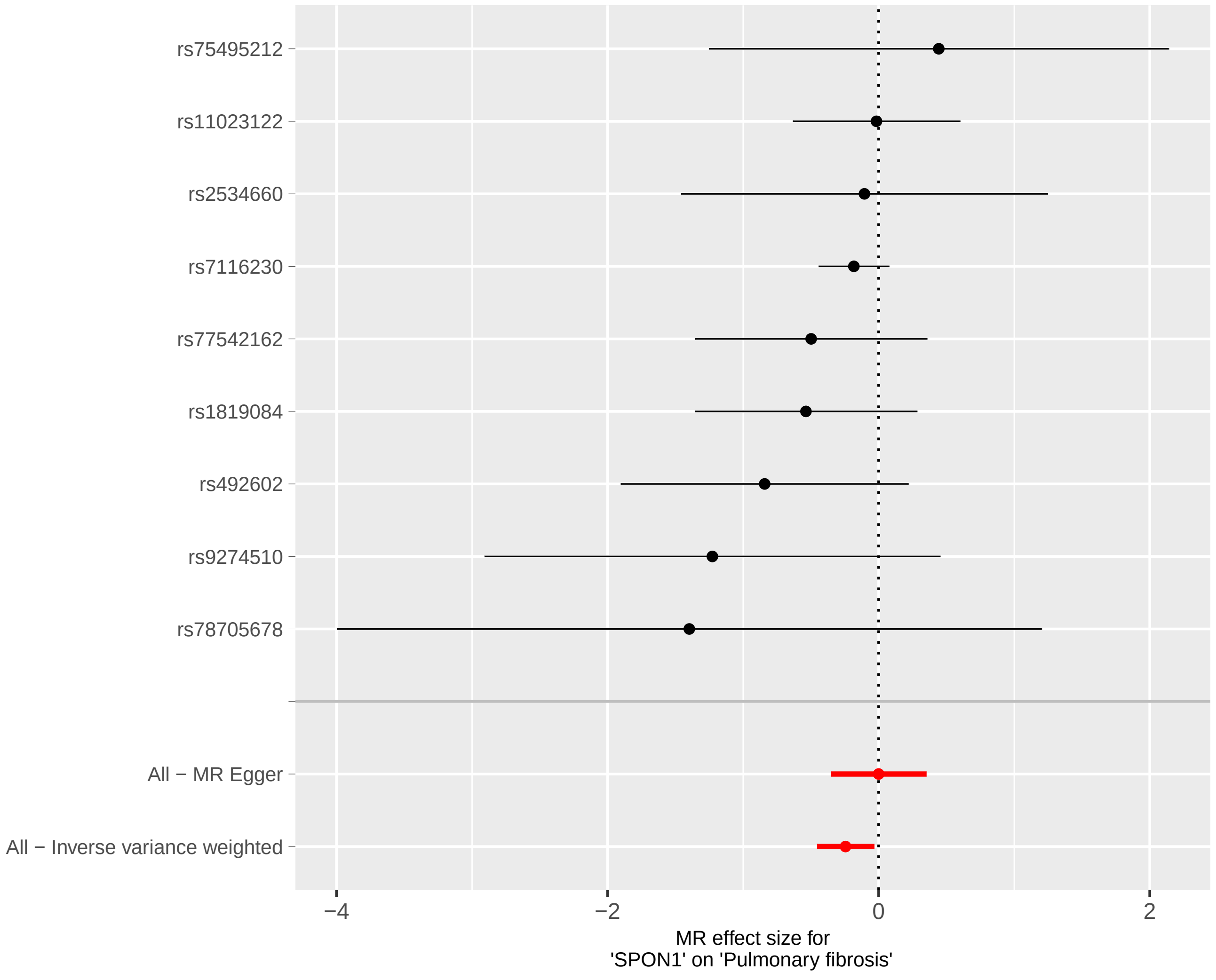

rs75495212
rs11023122
rs2534660
rs7116230
rs77542162
rs1819084
rs492602
rs9274510
rs78705678
All − MR Egger
All − Inverse variance weighted
−4
−2
0
2
MR effect size for
'SPON1' on 'Pulmonary fibrosis'

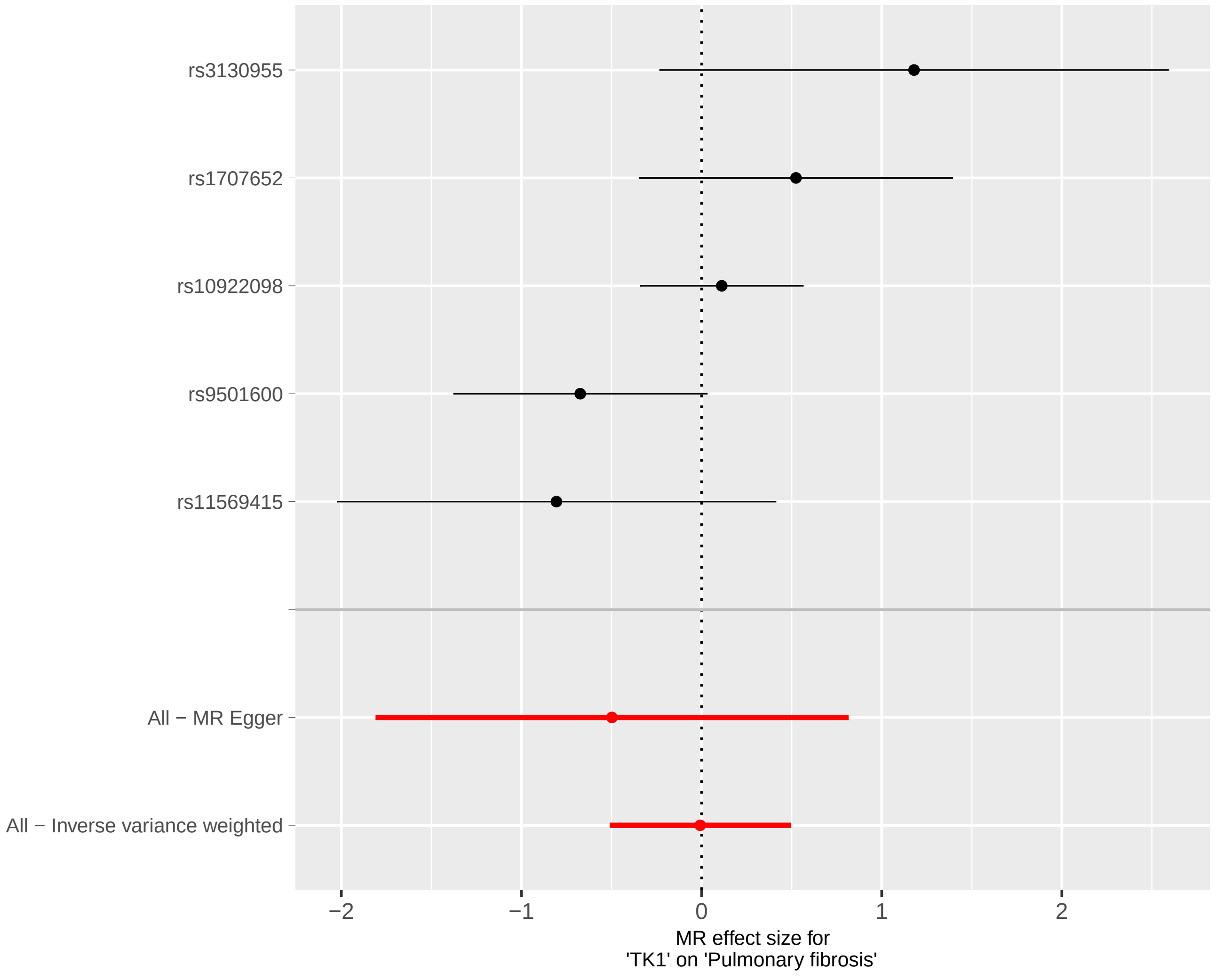
rs3130955
rs1707652
rs10922098
rs9501600
rs11569415
All − MR Egger
All − Inverse variance weighted
−2
−1
0
1
2
MR effect size for
'TK1' on 'Pulmonary fibrosis'

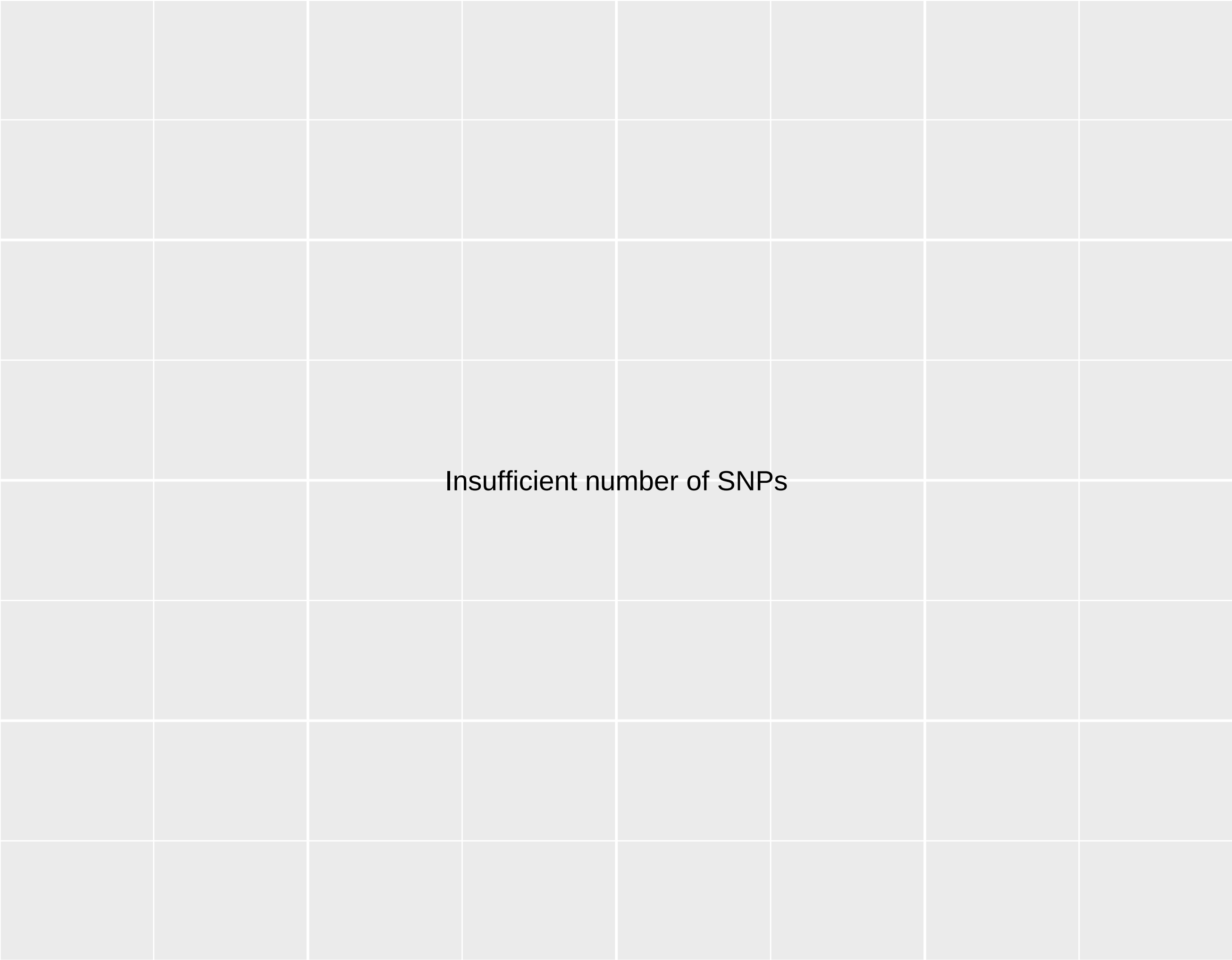
Insufficient number of SNPs

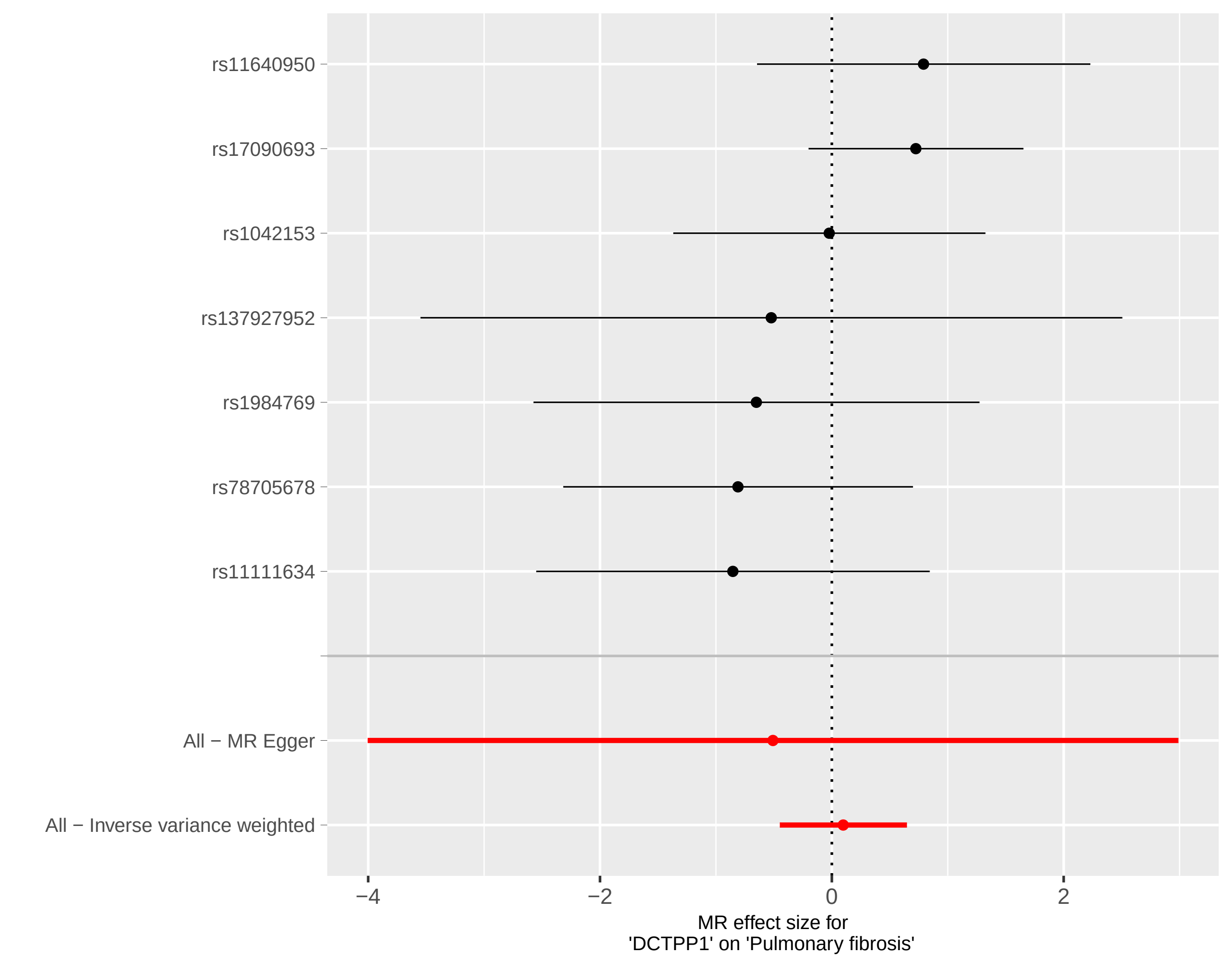
rs11640950
rs17090693
rs1042153
rs137927952
rs1984769
rs78705678
rs11111634
All − MR Egger
All − Inverse variance weighted
−4
−2
0
2
MR effect size for
'DCTPP1' on 'Pulmonary fibrosis'

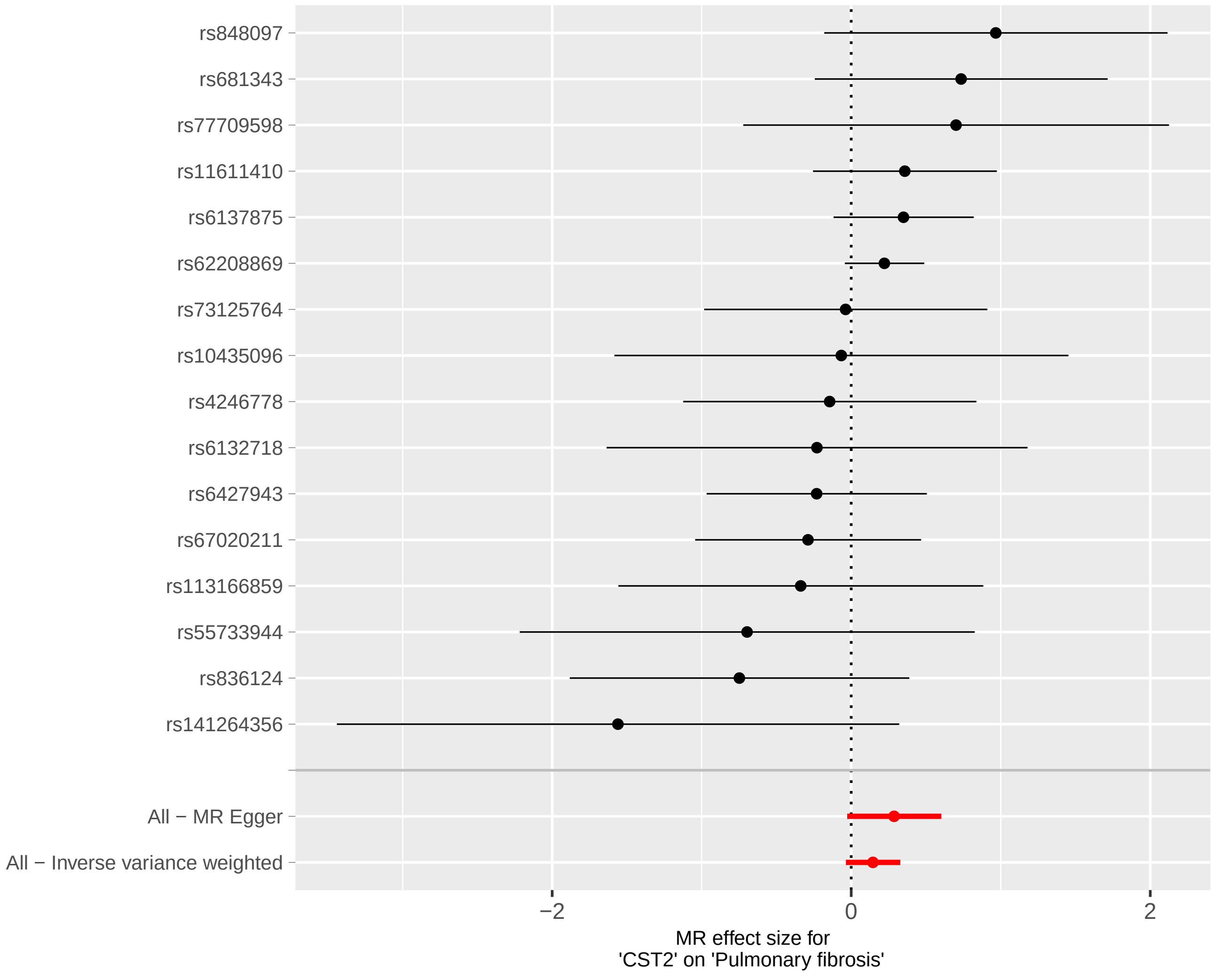
rs848097
rs681343
rs77709598
rs11611410
rs6137875
rs62208869
rs73125764
rs10435096
rs4246778
rs6132718
rs6427943
rs67020211
rs113166859
rs55733944
rs836124
rs141264356
All − MR Egger
All − Inverse variance weighted
−2
0
2
MR effect size for
'CST2' on 'Pulmonary fibrosis'

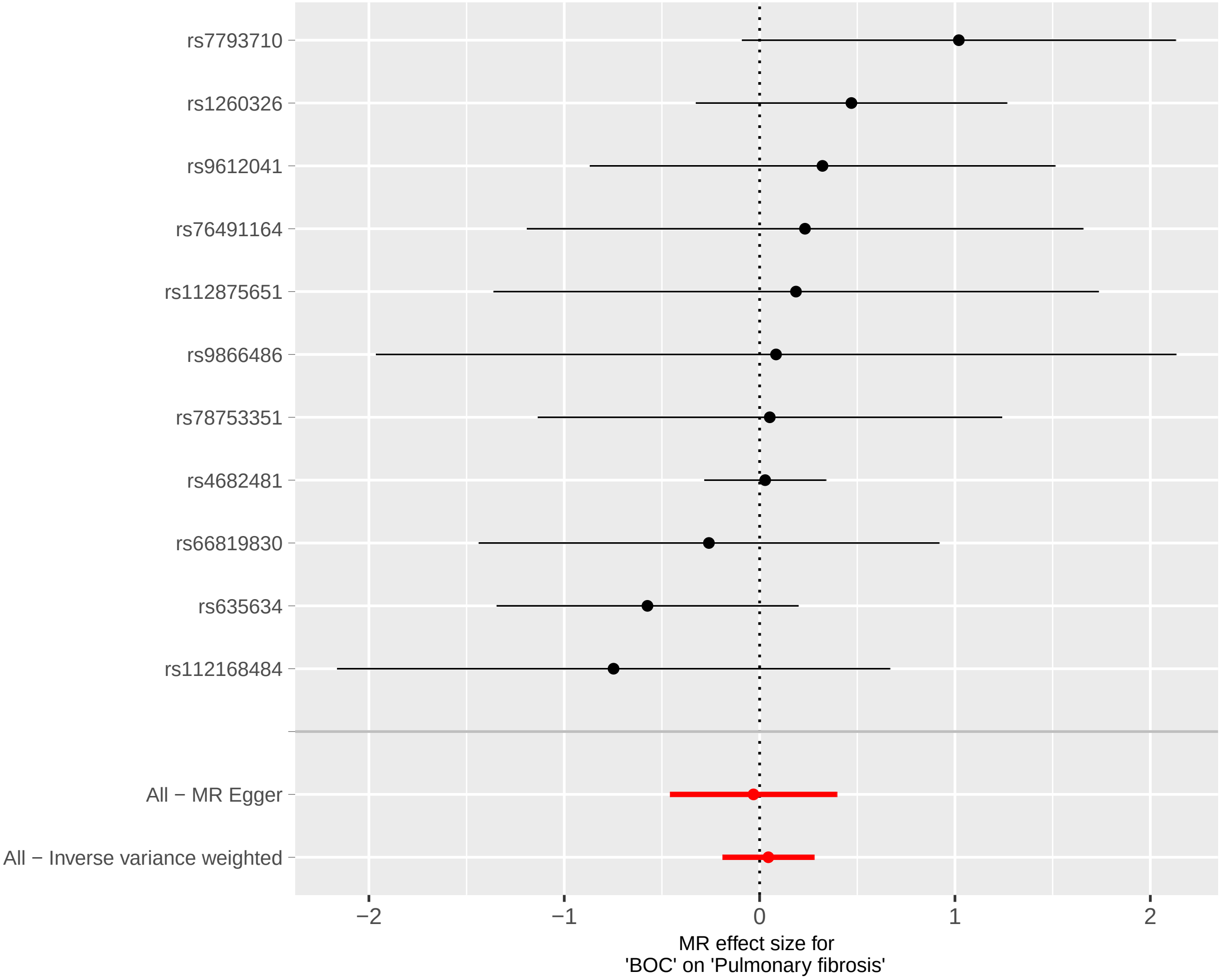

rs7793710
rs1260326
rs9612041
rs76491164
rs112875651
rs9866486
rs78753351
rs4682481
rs66819830
rs635634
rs112168484
All − MR Egger
All − Inverse variance weighted
−2
−1
0
1
2
MR effect size for
'BOC' on 'Pulmonary fibrosis'

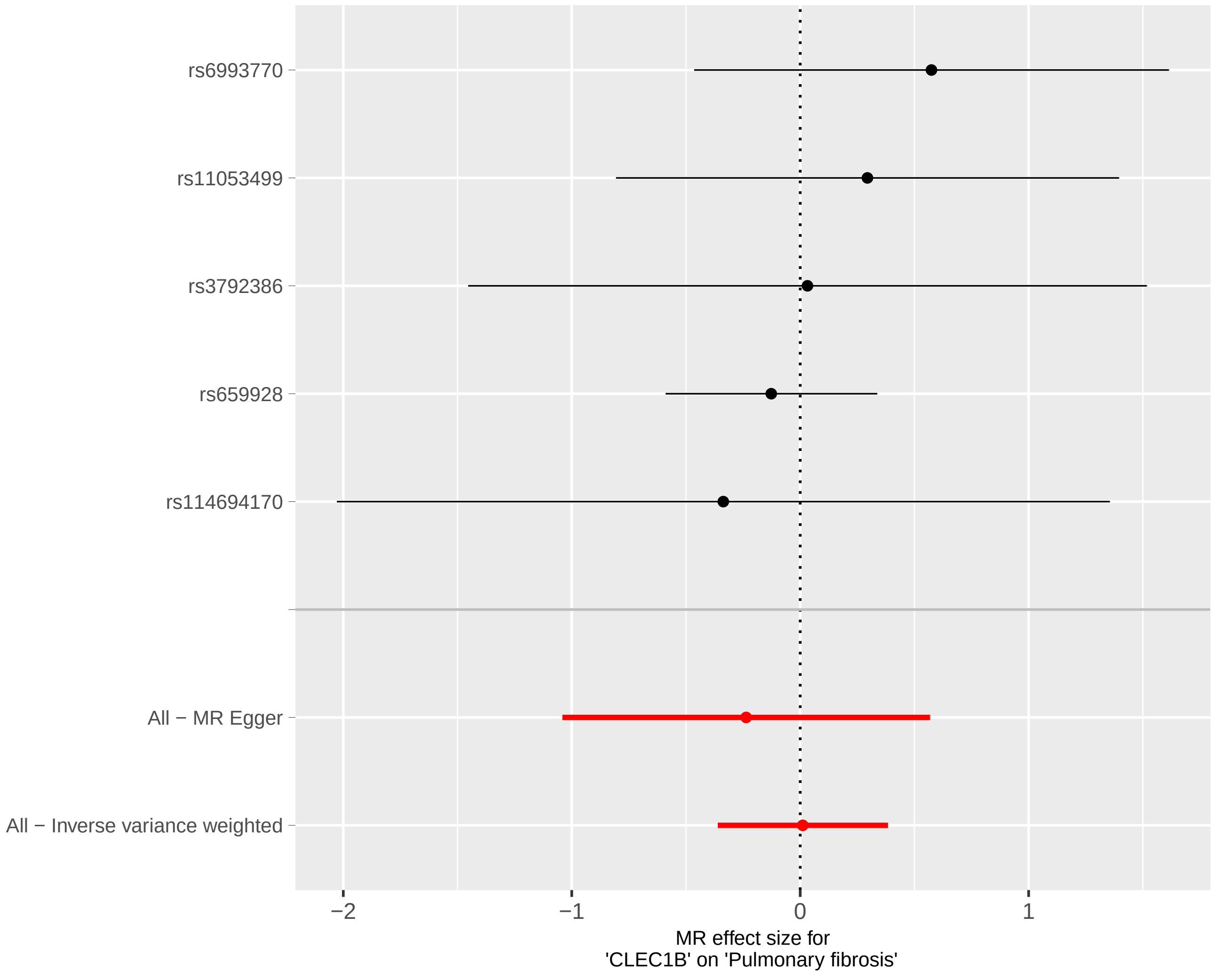

rs6993770
rs11053499
rs3792386
rs659928
rs114694170
All − MR Egger
All − Inverse variance weighted
−2
−1
0
1
MR effect size for
'CLEC1B' on 'Pulmonary fibrosis'

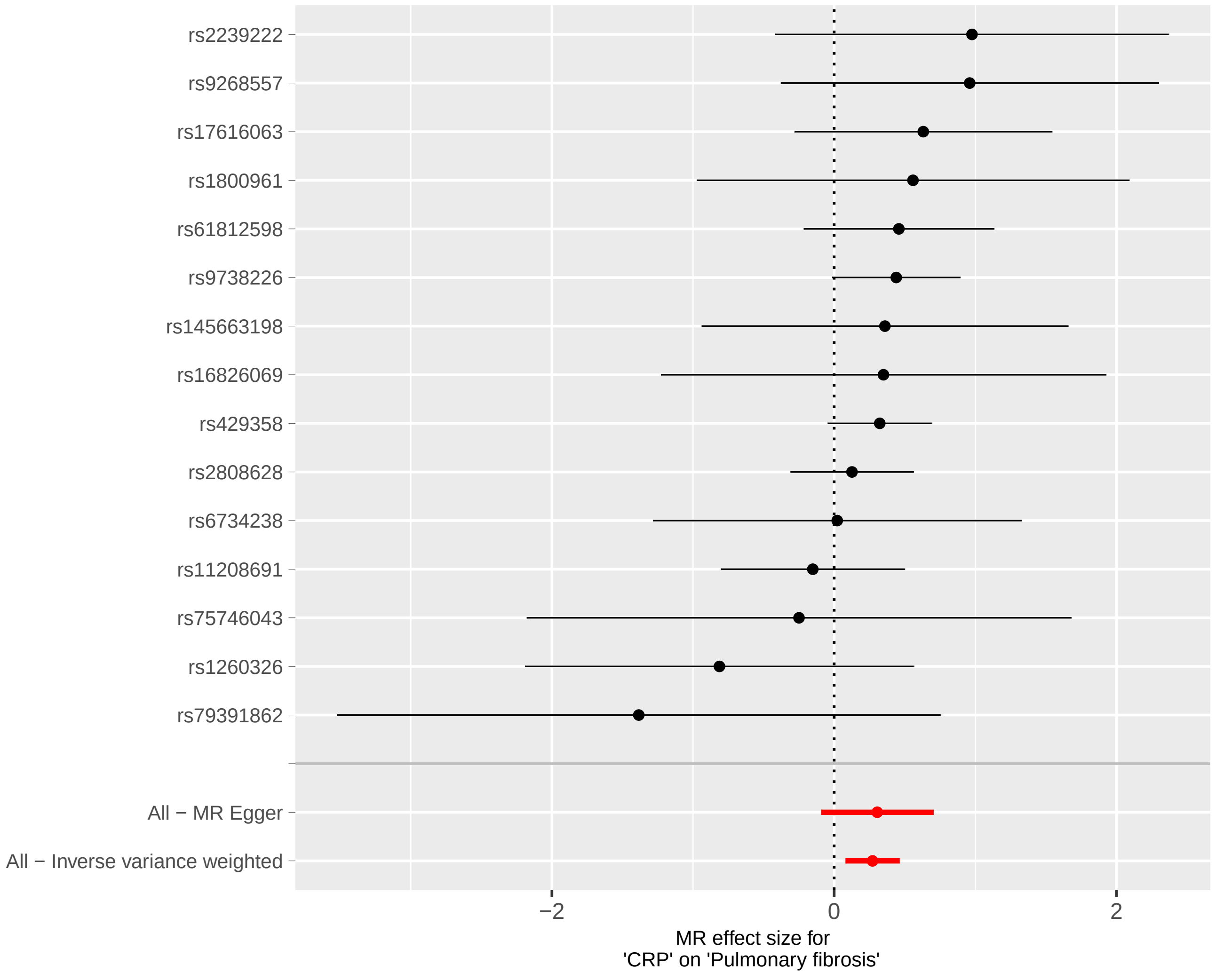
rs2239222
rs9268557
rs17616063
rs1800961
rs61812598
rs9738226
rs145663198
rs16826069
rs429358
rs2808628
rs6734238
rs11208691
rs75746043
rs1260326
rs79391862
All − MR Egger
All − Inverse variance weighted
−2
0
2
MR effect size for
'CRP' on 'Pulmonary fibrosis'

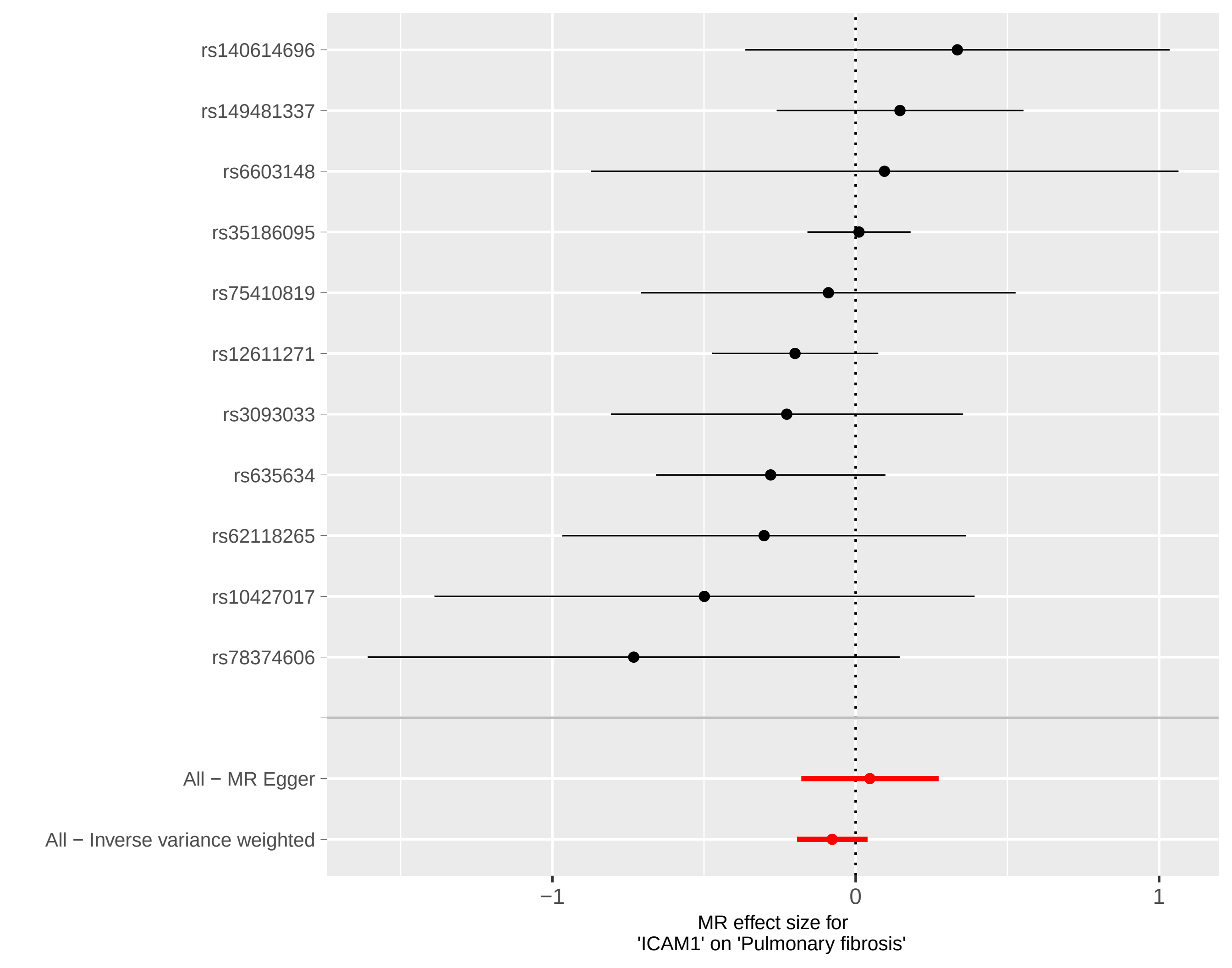
rs140614696
rs149481337
rs6603148
rs35186095
rs75410819
rs12611271
rs3093033
rs635634
rs62118265
rs10427017
rs78374606
All − MR Egger
All − Inverse variance weighted
−1
0
1
MR effect size for
'ICAM1' on 'Pulmonary fibrosis'

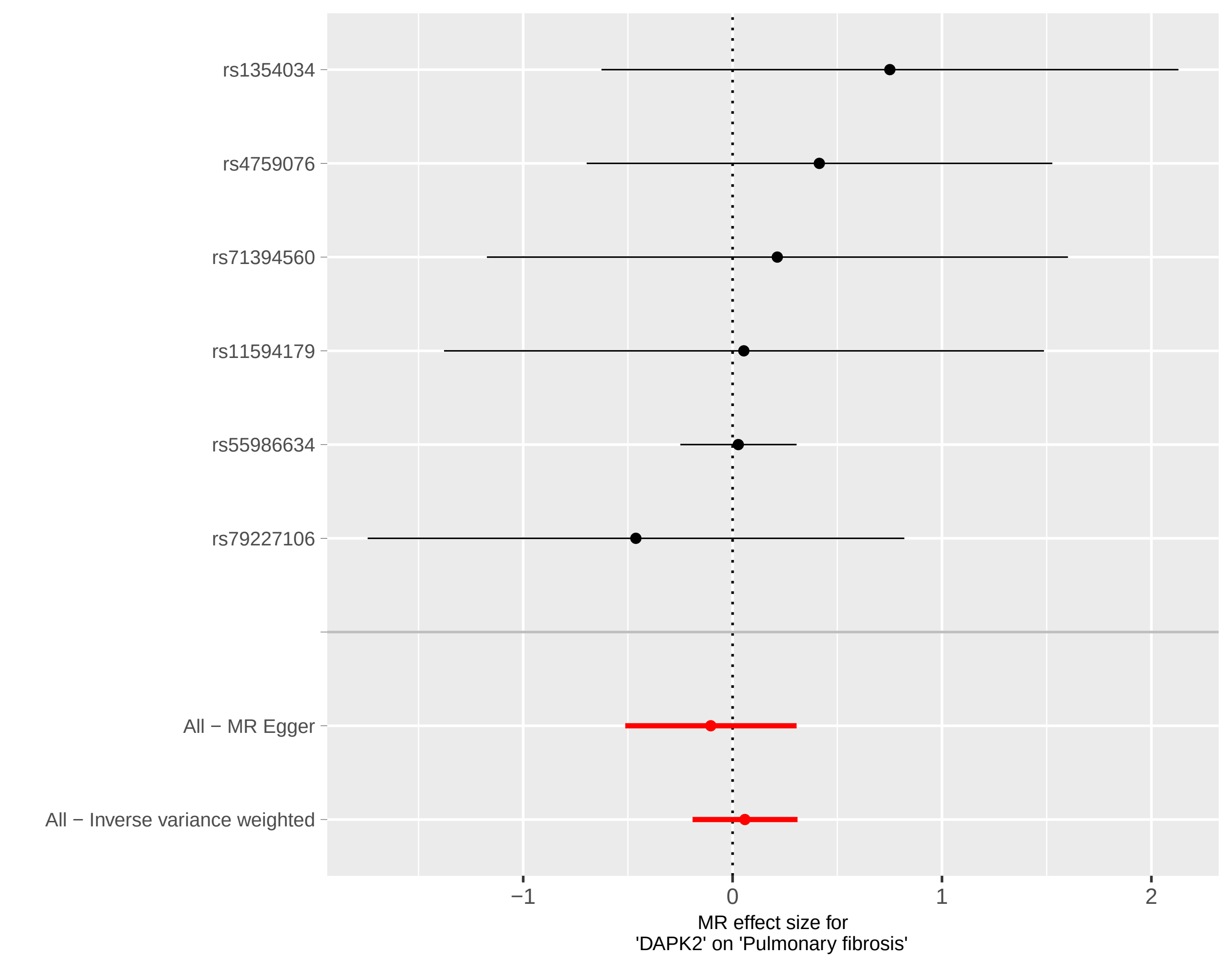

rs1354034
rs4759076
rs71394560
rs11594179
rs55986634
rs79227106
All − MR Egger
All − Inverse variance weighted
−1
0
1
2
MR effect size for
'DAPK2' on 'Pulmonary fibrosis'

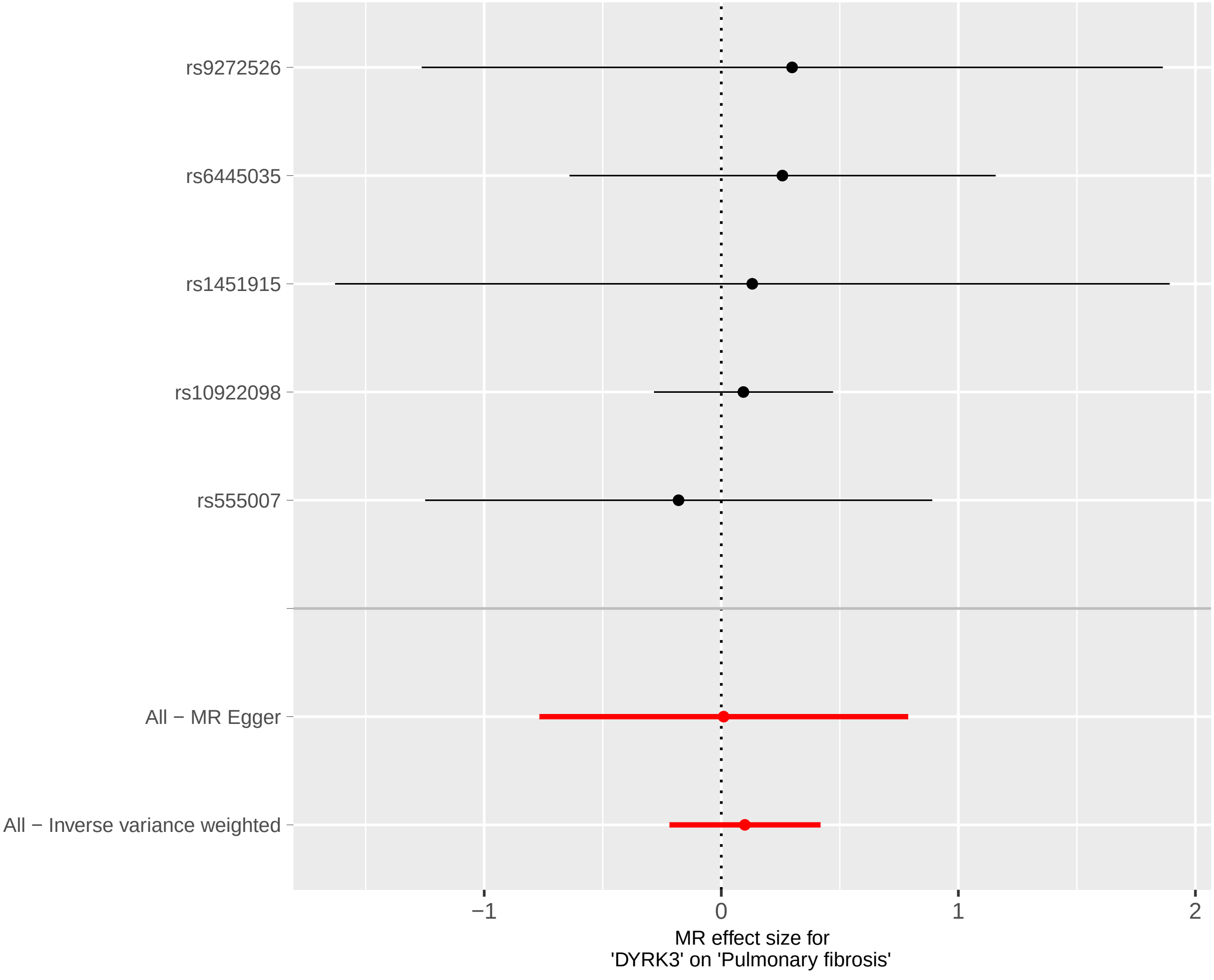

rs9272526
rs6445035
rs1451915
rs10922098
rs555007
All − MR Egger
All − Inverse variance weighted
−1
0
1
2
MR effect size for
'DYRK3' on 'Pulmonary fibrosis'

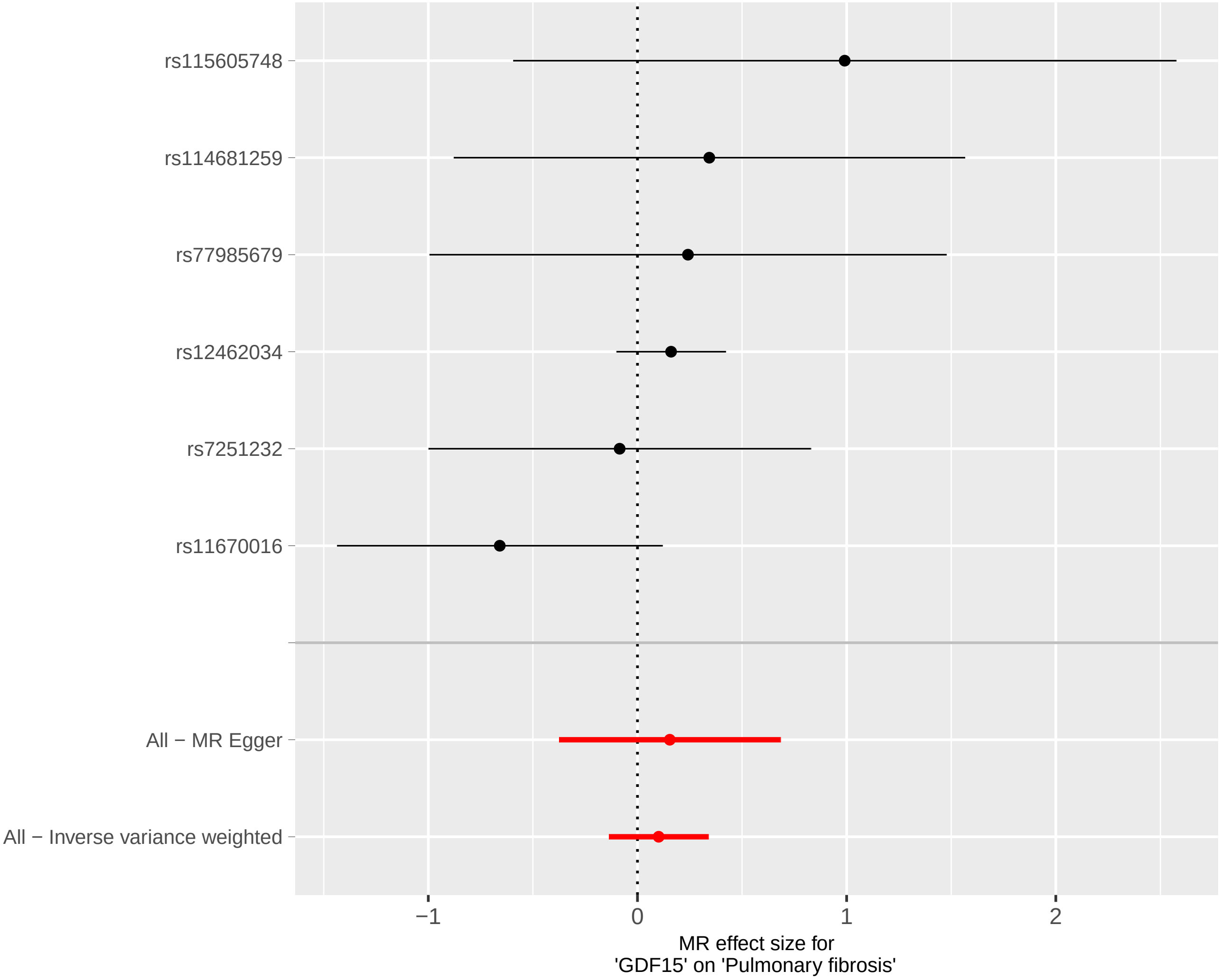

rs115605748
rs114681259
rs77985679
rs12462034
rs7251232
rs11670016
All − MR Egger
All − Inverse variance weighted
−1
0
1
2
MR effect size for
'GDF15' on 'Pulmonary fibrosis'

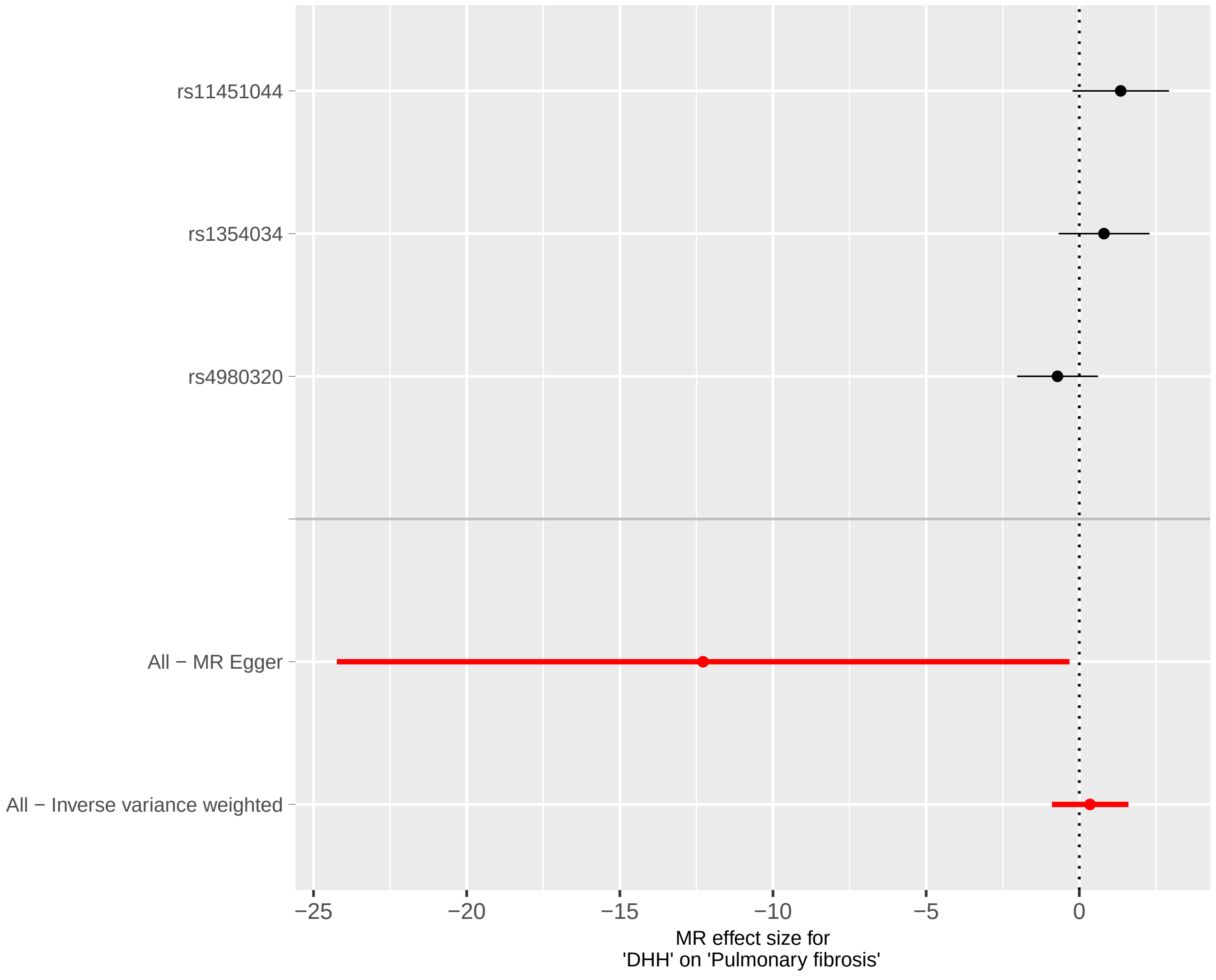

rs11451044
rs1354034
rs4980320
All − MR Egger
All − Inverse variance weighted
−25
−20
−15
−10
−5
0
MR effect size for
'DHH' on 'Pulmonary fibrosis'

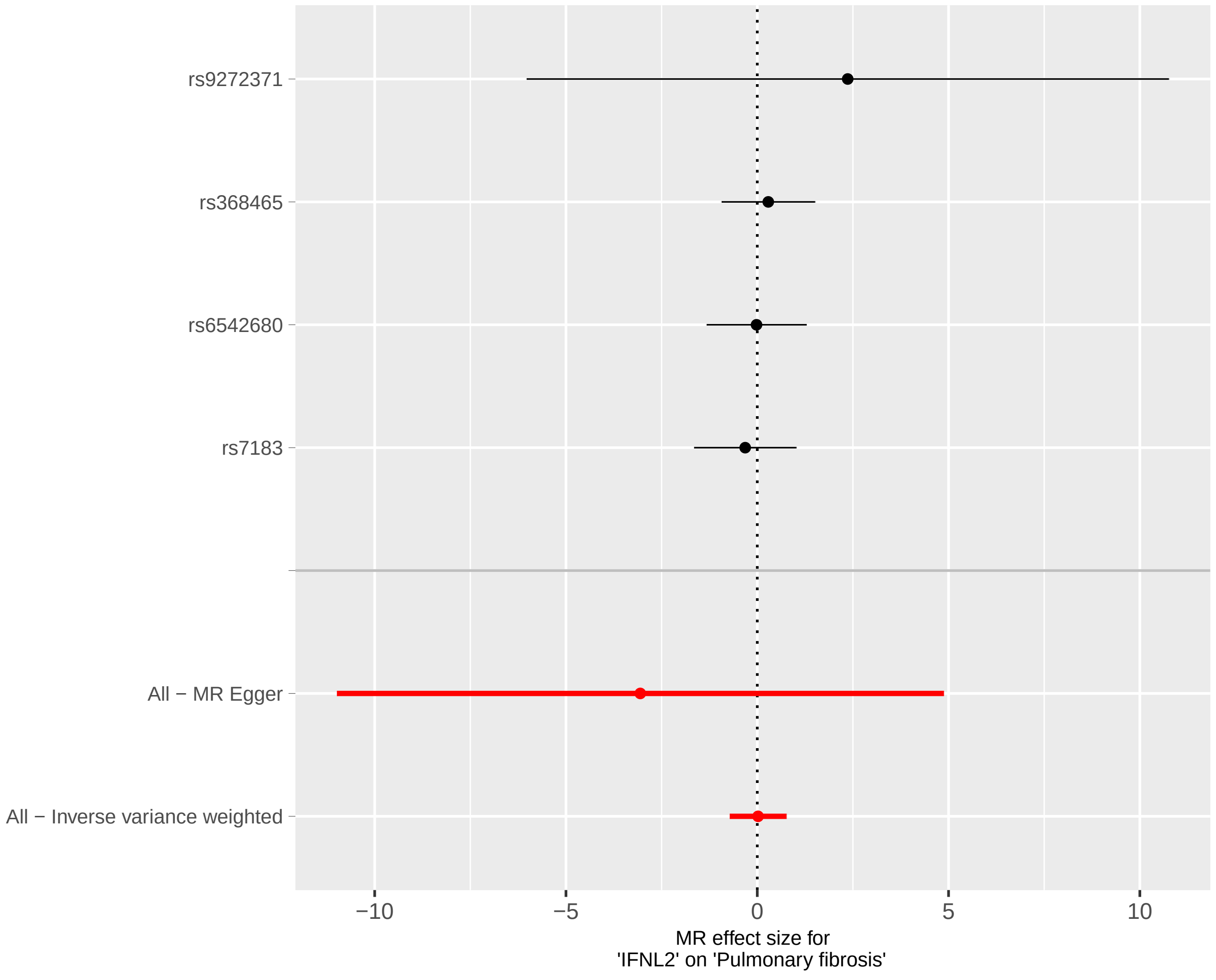

rs9272371
rs368465
rs6542680
rs7183
All − MR Egger
All − Inverse variance weighted
−10
−5
0
5
10
MR effect size for
'IFNL2' on 'Pulmonary fibrosis'

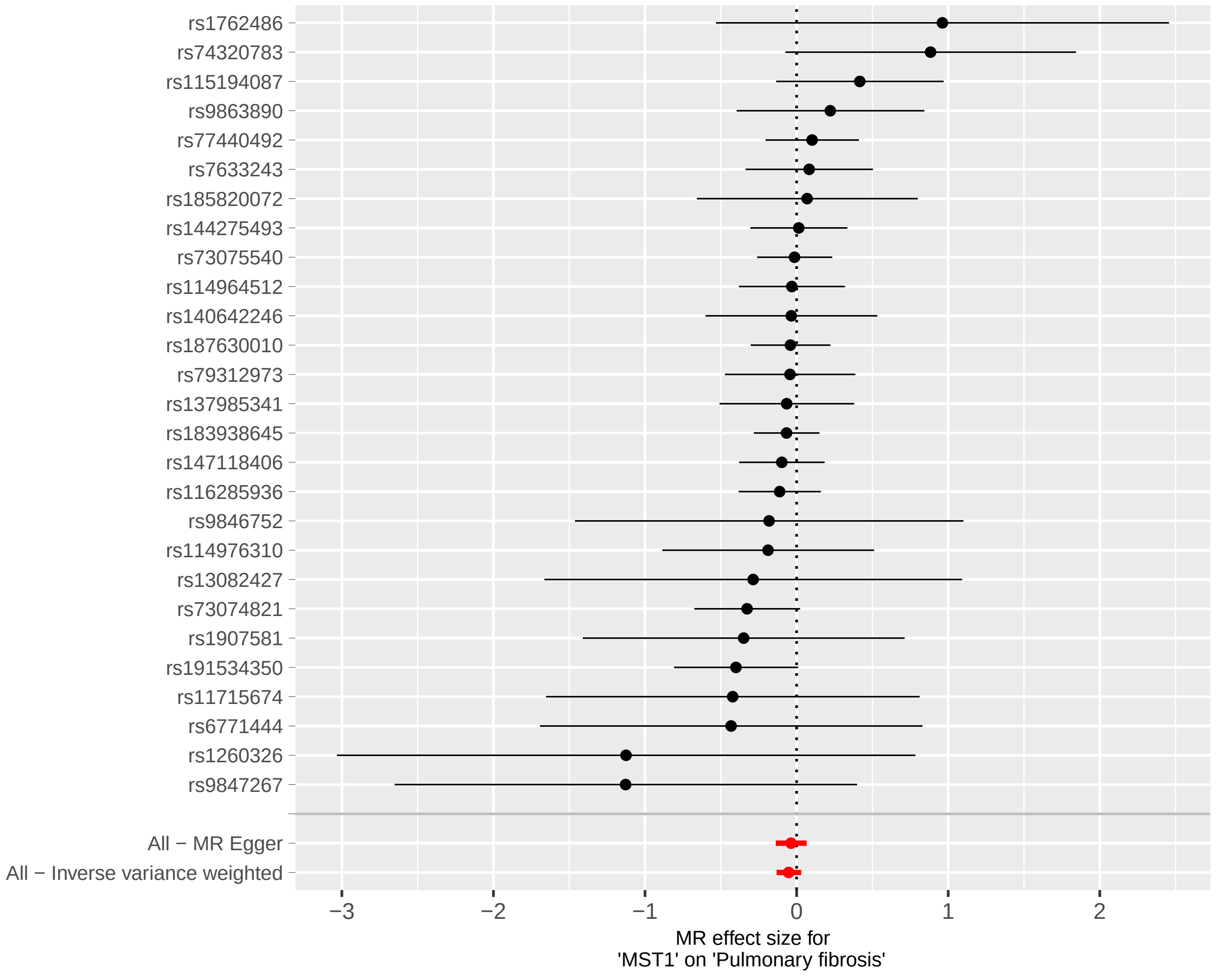

rs1762486
rs74320783
rs115194087
rs9863890
rs77440492
rs7633243
rs185820072
rs144275493
rs73075540
rs114964512
rs140642246
rs187630010
rs79312973
rs137985341
rs183938645
rs147118406
rs116285936
rs9846752
rs114976310
rs13082427
rs73074821
rs1907581
rs191534350
rs11715674
rs6771444
rs1260326
rs9847267
All − MR Egger
All − Inverse variance weighted
−3
−2
−1
0
1
2
MR effect size for
'MST1' on 'Pulmonary fibrosis'

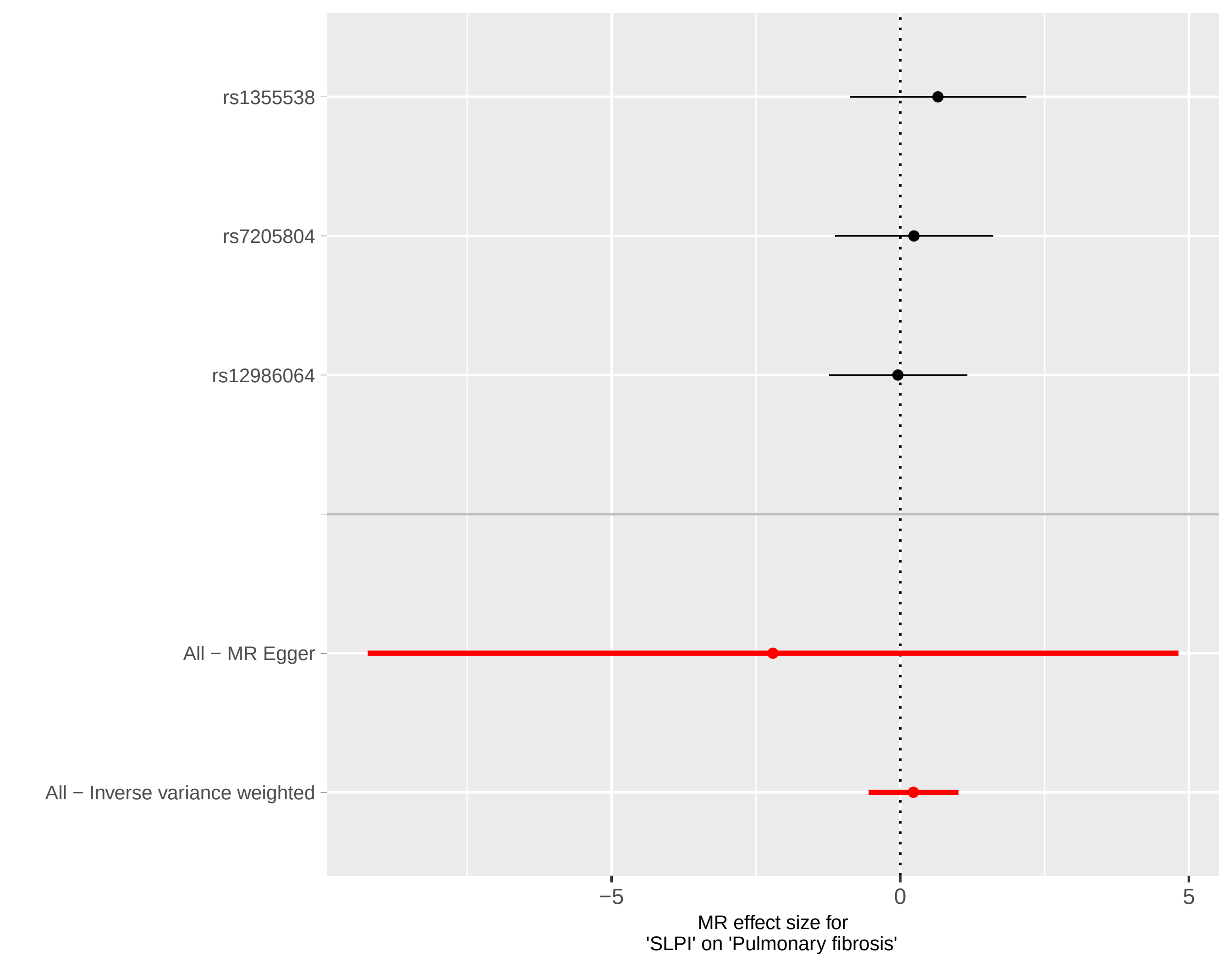

rs1355538
rs7205804
rs12986064
All − MR Egger
All − Inverse variance weighted
−5
0
5
MR effect size for
'SLPI' on 'Pulmonary fibrosis'

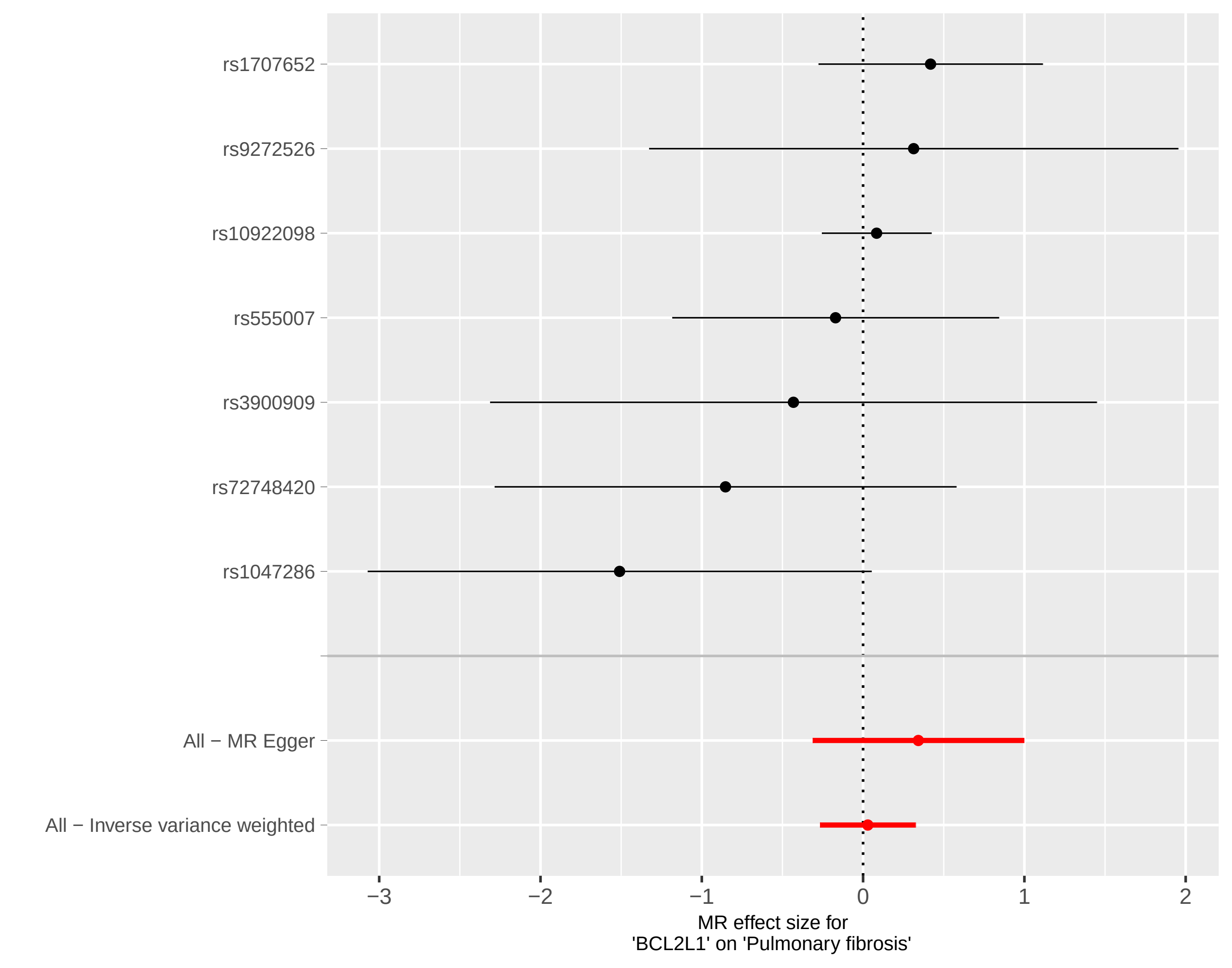

rs1707652
rs9272526
rs10922098
rs555007
rs3900909
rs72748420
rs1047286
All − MR Egger
All − Inverse variance weighted
−3
−2
−1
0
1
2
MR effect size for
'BCL2L1' on 'Pulmonary fibrosis'

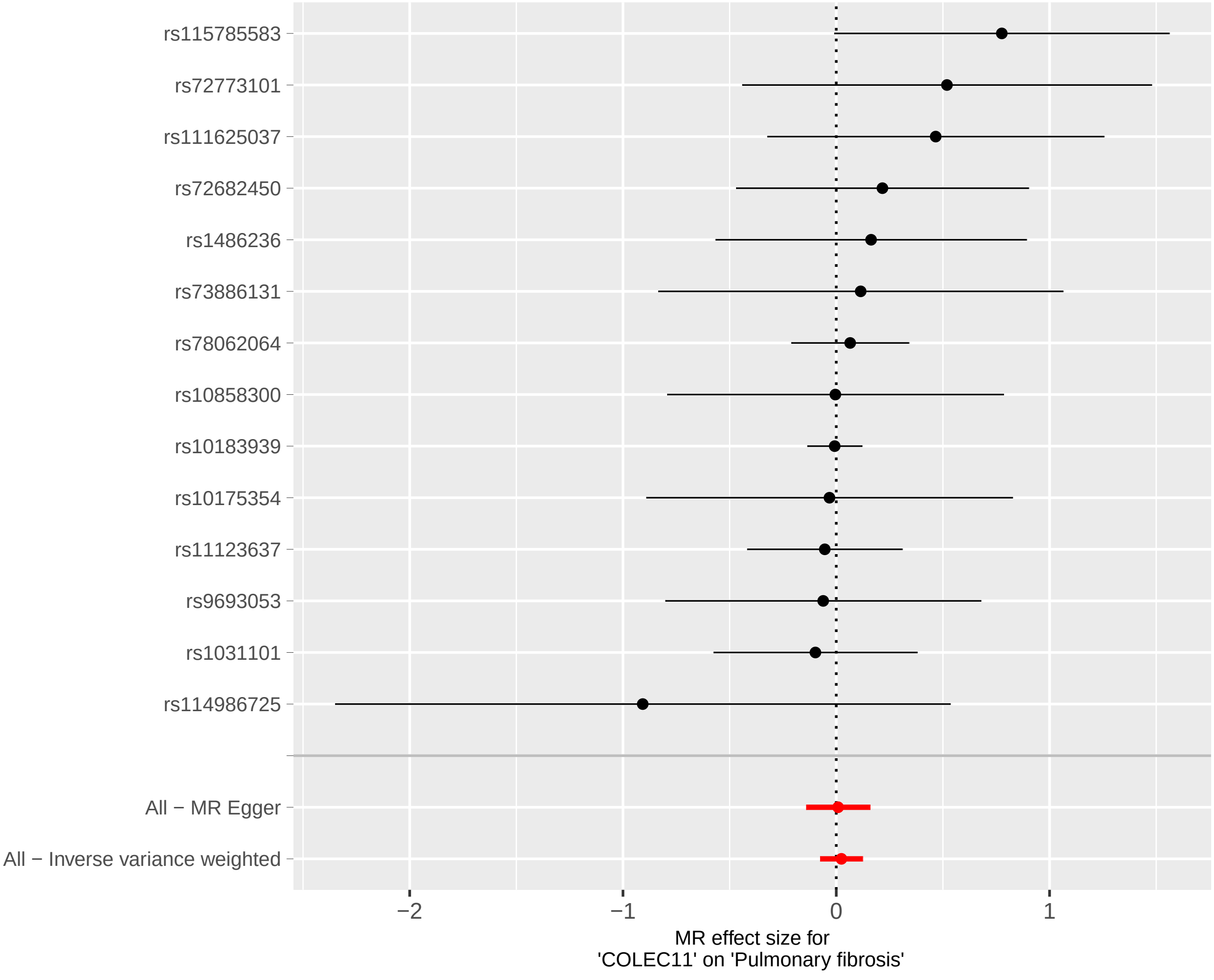

rs115785583
rs72773101
rs111625037
rs72682450
rs1486236
rs73886131
rs78062064
rs10858300
rs10183939
rs10175354
rs11123637
rs9693053
rs1031101
rs114986725
All − MR Egger
All − Inverse variance weighted
−2
−1
0
1
MR effect size for
'COLEC11' on 'Pulmonary fibrosis'

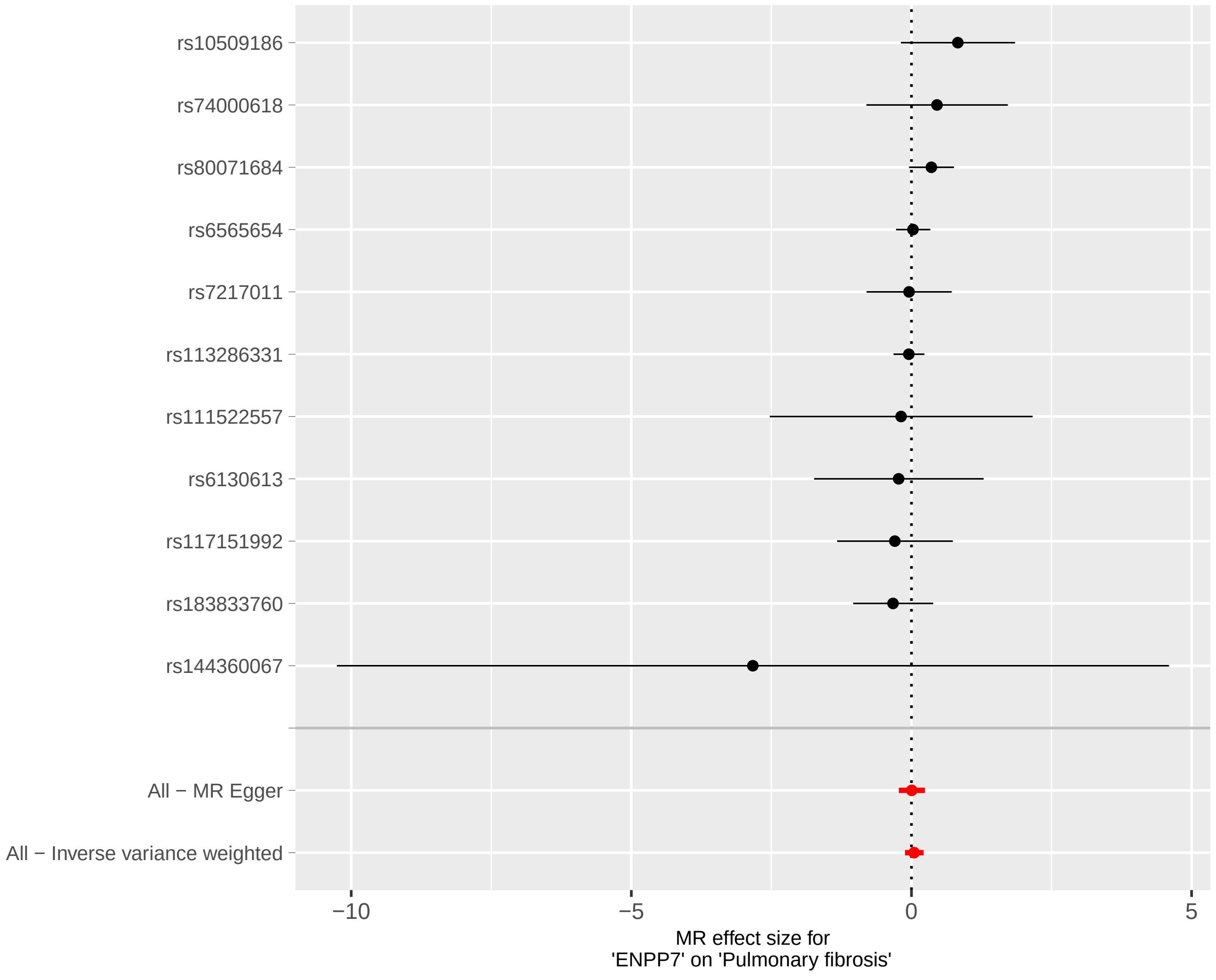

rs10509186
rs74000618
rs80071684
rs6565654
rs7217011
rs113286331
rs111522557
rs6130613
rs117151992
rs183833760
rs144360067
All − MR Egger
All − Inverse variance weighted
−10
−5
0
5
MR effect size for
'ENPP7' on 'Pulmonary fibrosis'

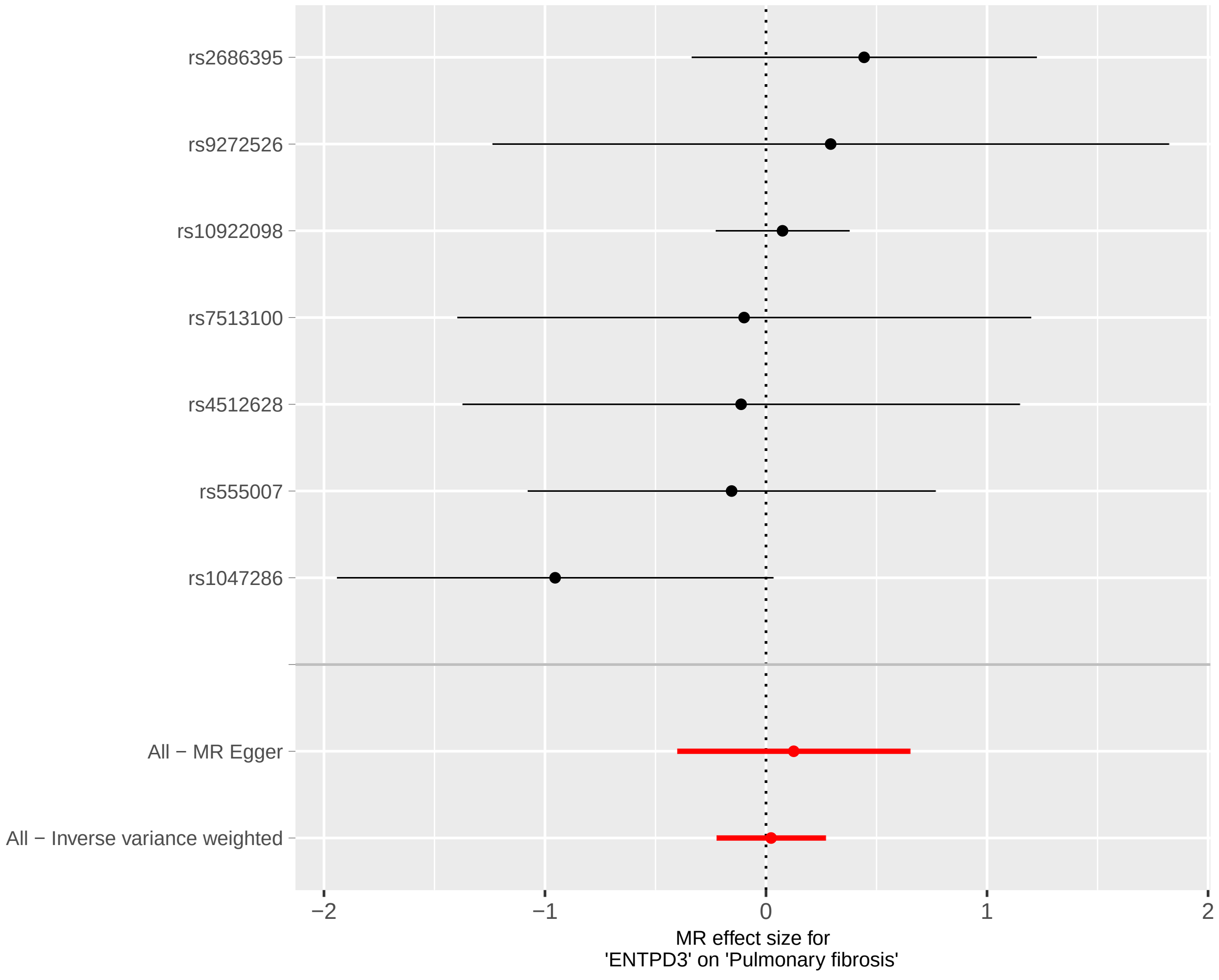

rs2686395
rs9272526
rs10922098
rs7513100
rs4512628
rs555007
rs1047286
All − MR Egger
All − Inverse variance weighted
−2
−1
0
1
2
MR effect size for
'ENTPD3' on 'Pulmonary fibrosis'

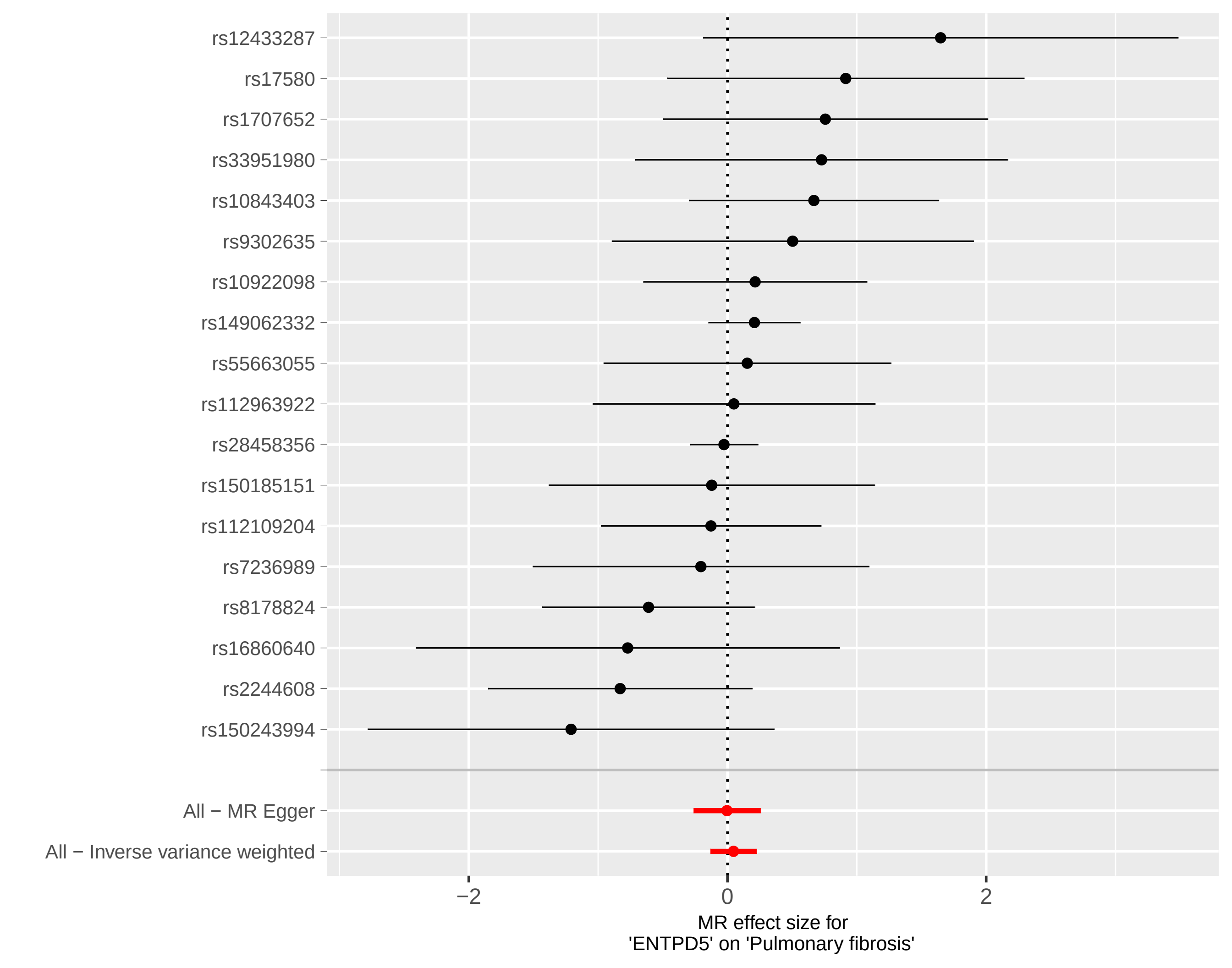

rs12433287
rs17580
rs1707652
rs33951980
rs10843403
rs9302635
rs10922098
rs149062332
rs55663055
rs112963922
rs28458356
rs150185151
rs112109204
rs7236989
rs8178824
rs16860640
rs2244608
rs150243994
All − MR Egger
All − Inverse variance weighted
−2
0
2
MR effect size for
'ENTPD5' on 'Pulmonary fibrosis'

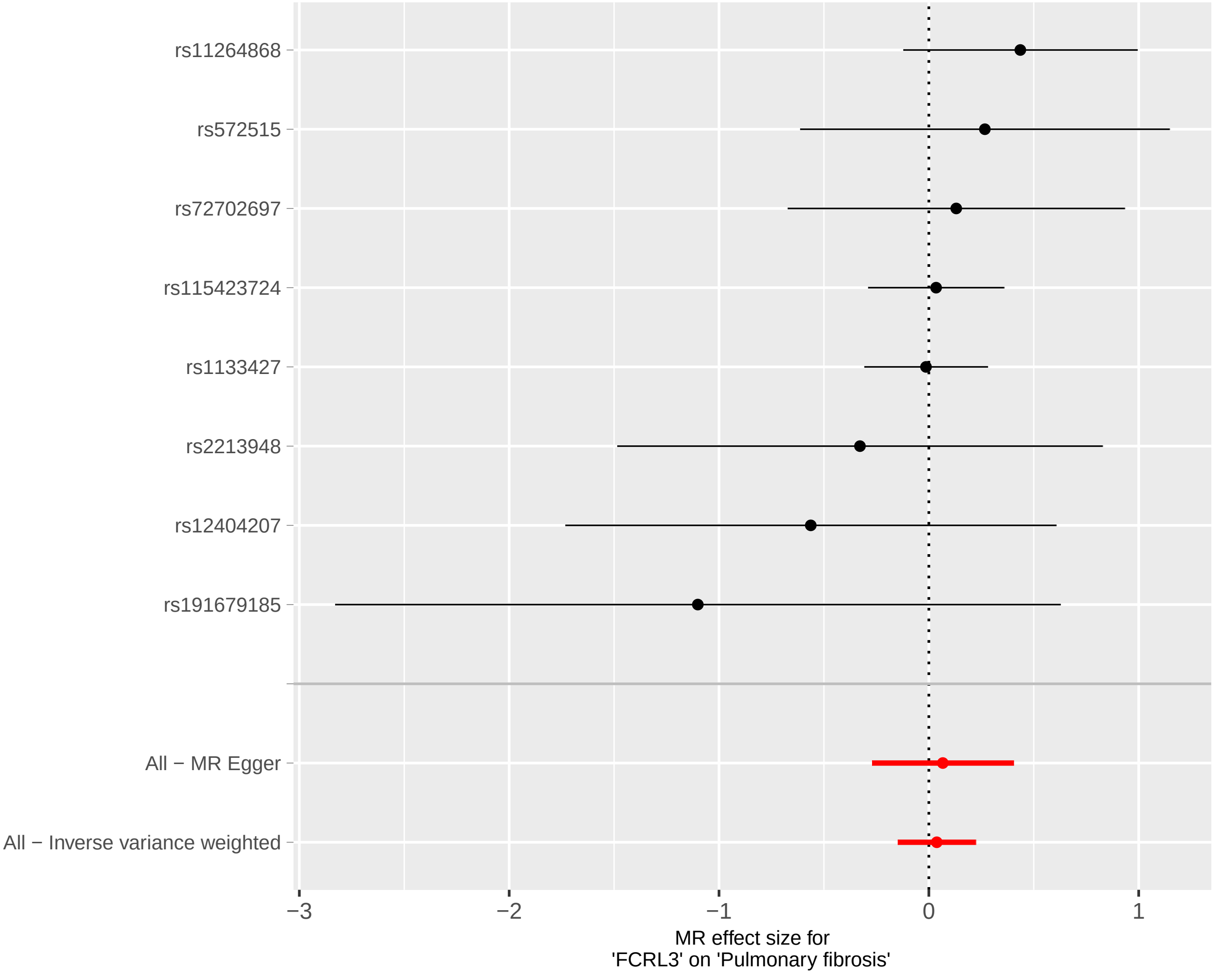

rs11264868
rs572515
rs72702697
rs115423724
rs1133427
rs2213948
rs12404207
rs191679185
All − MR Egger
All − Inverse variance weighted
−3
−2
−1
0
1
MR effect size for
'FCRL3' on 'Pulmonary fibrosis'

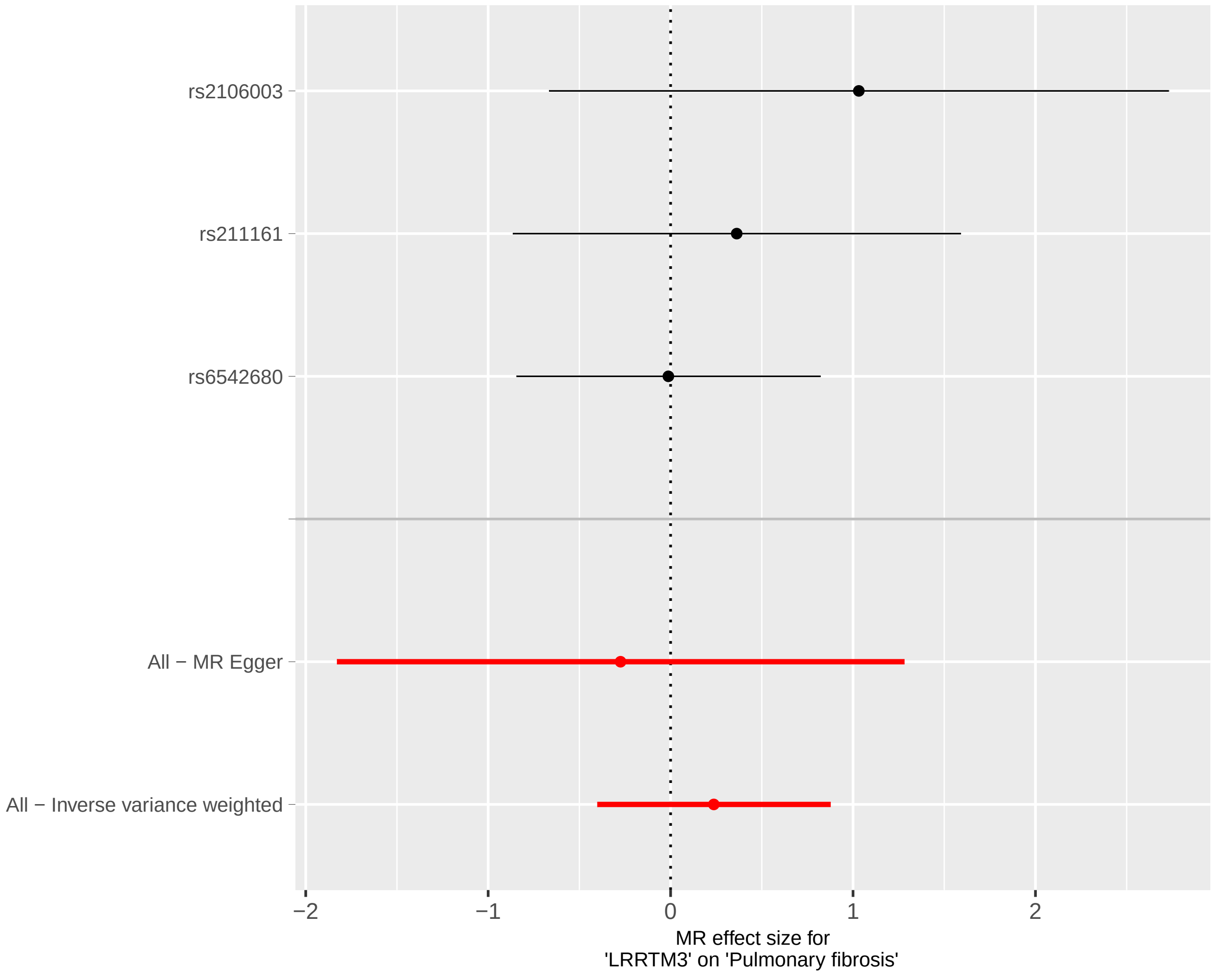

rs2106003
rs211161
rs6542680
All − MR Egger
All − Inverse variance weighted
−2
−1
0
1
2
MR effect size for
'LRRTM3' on 'Pulmonary fibrosis'

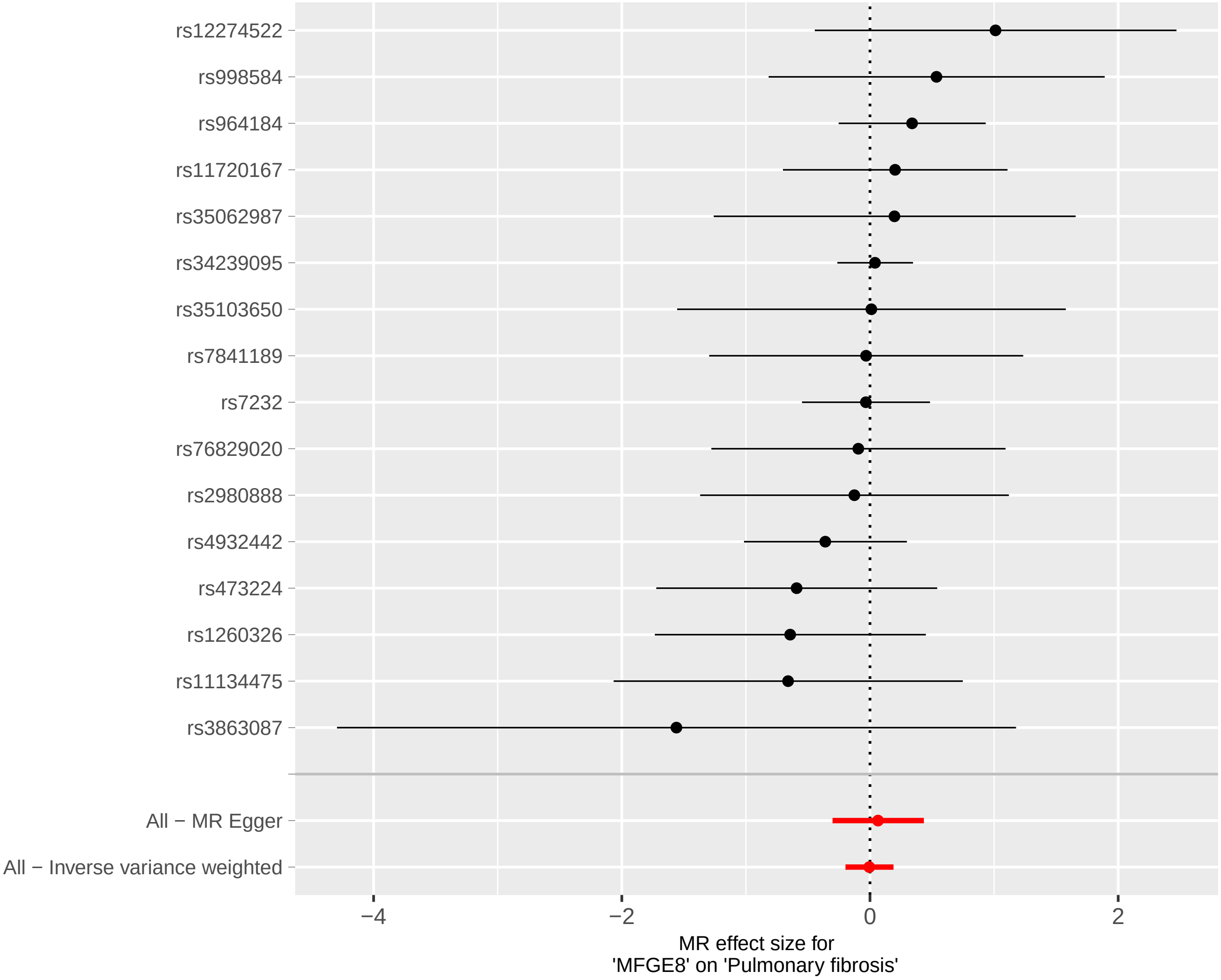
rs12274522
rs998584
rs964184
rs11720167
rs35062987
rs34239095
rs35103650
rs7841189
rs7232
rs76829020
rs2980888
rs4932442
rs473224
rs1260326
rs11134475
rs3863087
All − MR Egger
All − Inverse variance weighted
−4
−2
0
2
MR effect size for
'MFGE8' on 'Pulmonary fibrosis'

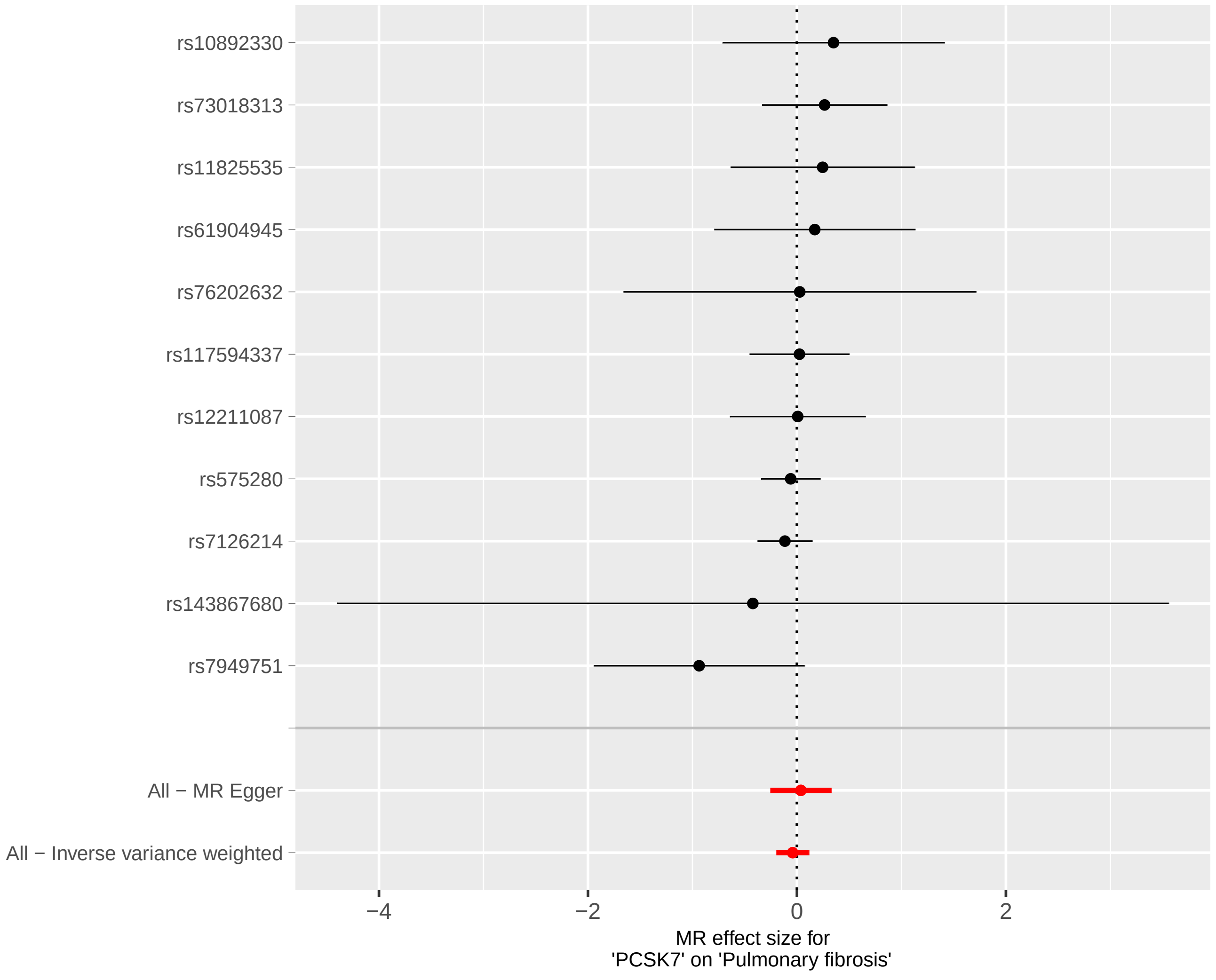

rs10892330
rs73018313
rs11825535
rs61904945
rs76202632
rs117594337
rs12211087
rs575280
rs7126214
rs143867680
rs7949751
All − MR Egger
All − Inverse variance weighted
−4
−2
0
2
MR effect size for
'PCSK7' on 'Pulmonary fibrosis'

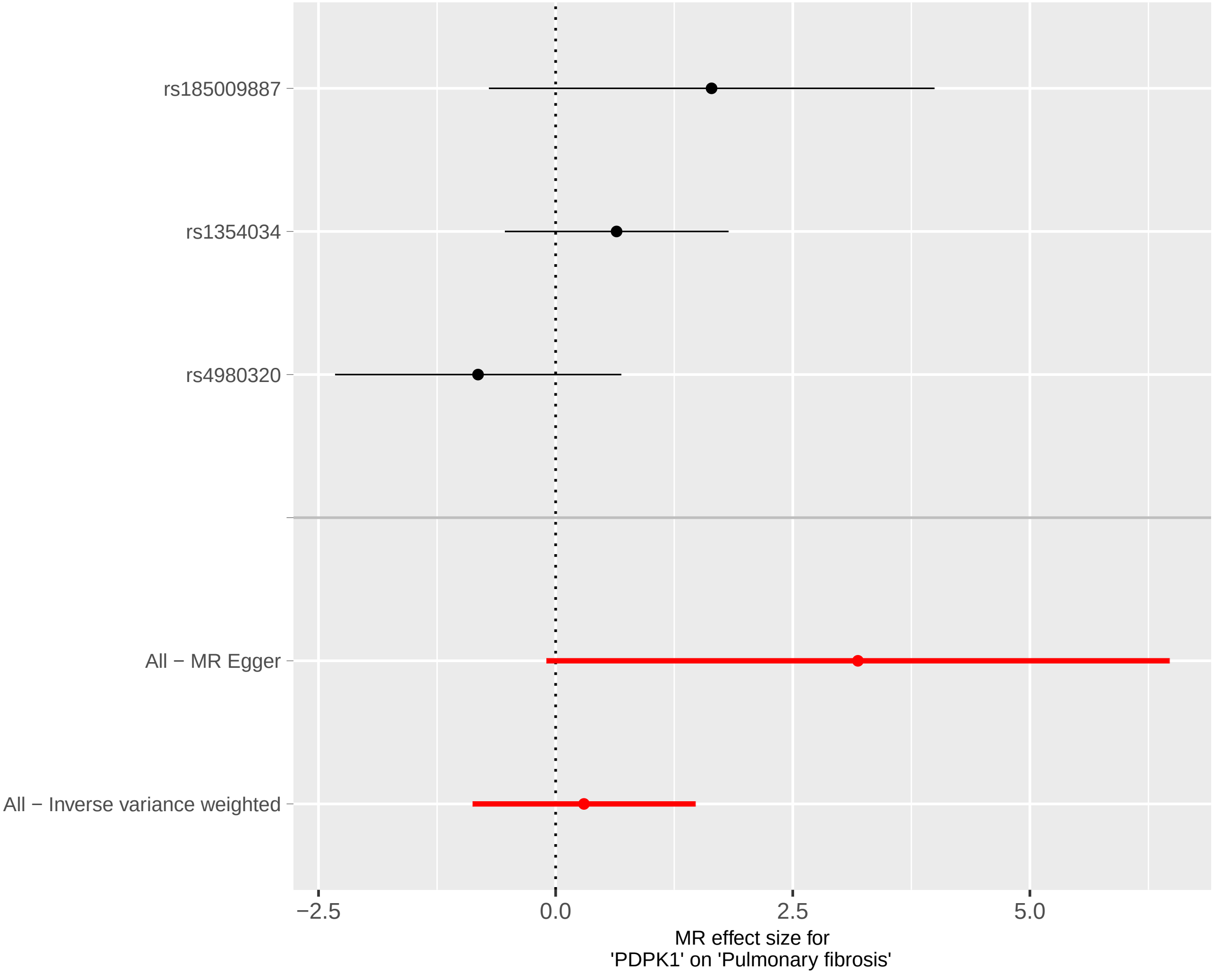

rs185009887
rs1354034
rs4980320
All − MR Egger
All − Inverse variance weighted
−2.5
0.0
2.5
5.0
MR effect size for
'PDPK1' on 'Pulmonary fibrosis'

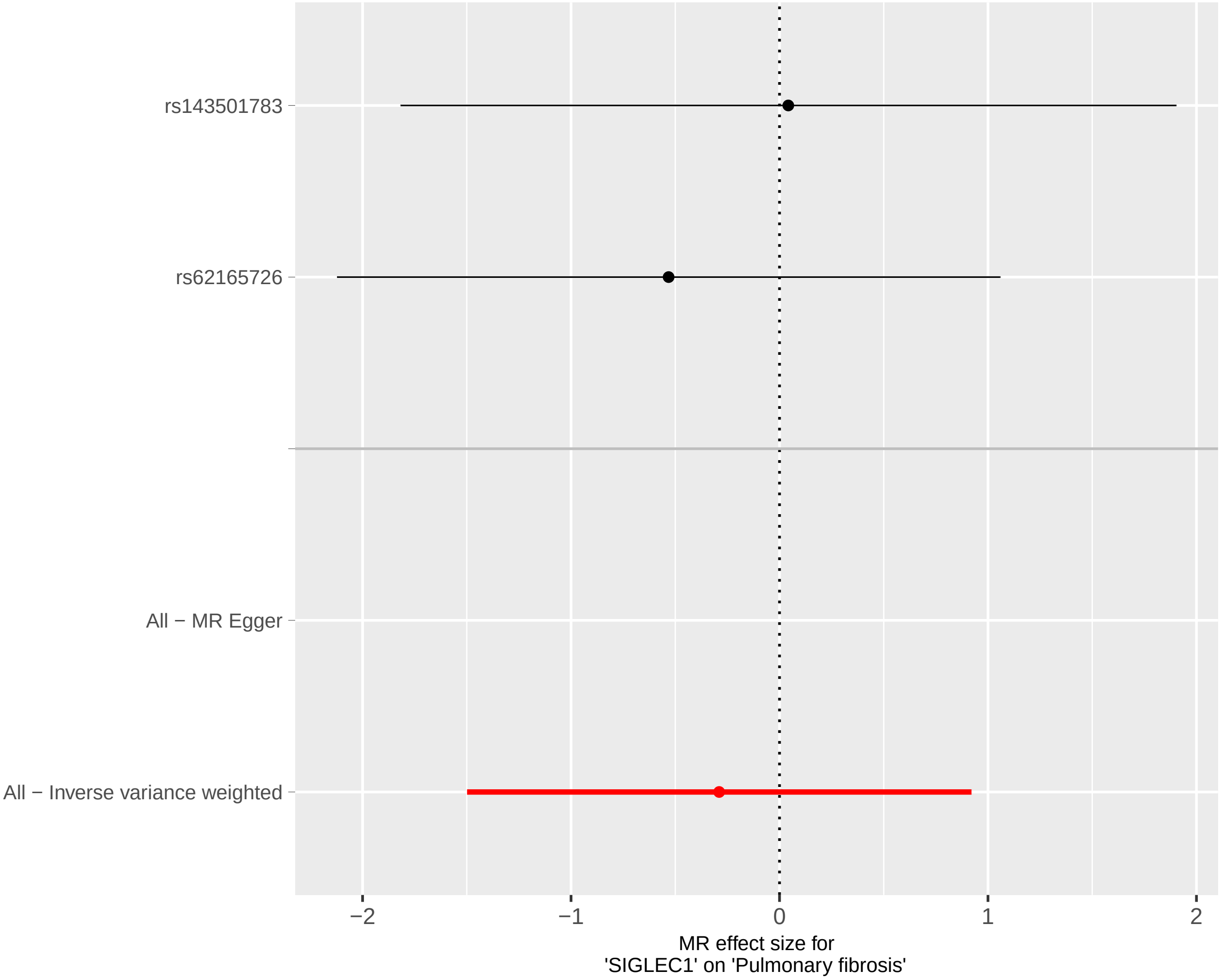
rs143501783
rs62165726
All − MR Egger
All − Inverse variance weighted
−2
−1
0
1
2
MR effect size for
'SIGLEC1' on 'Pulmonary fibrosis'

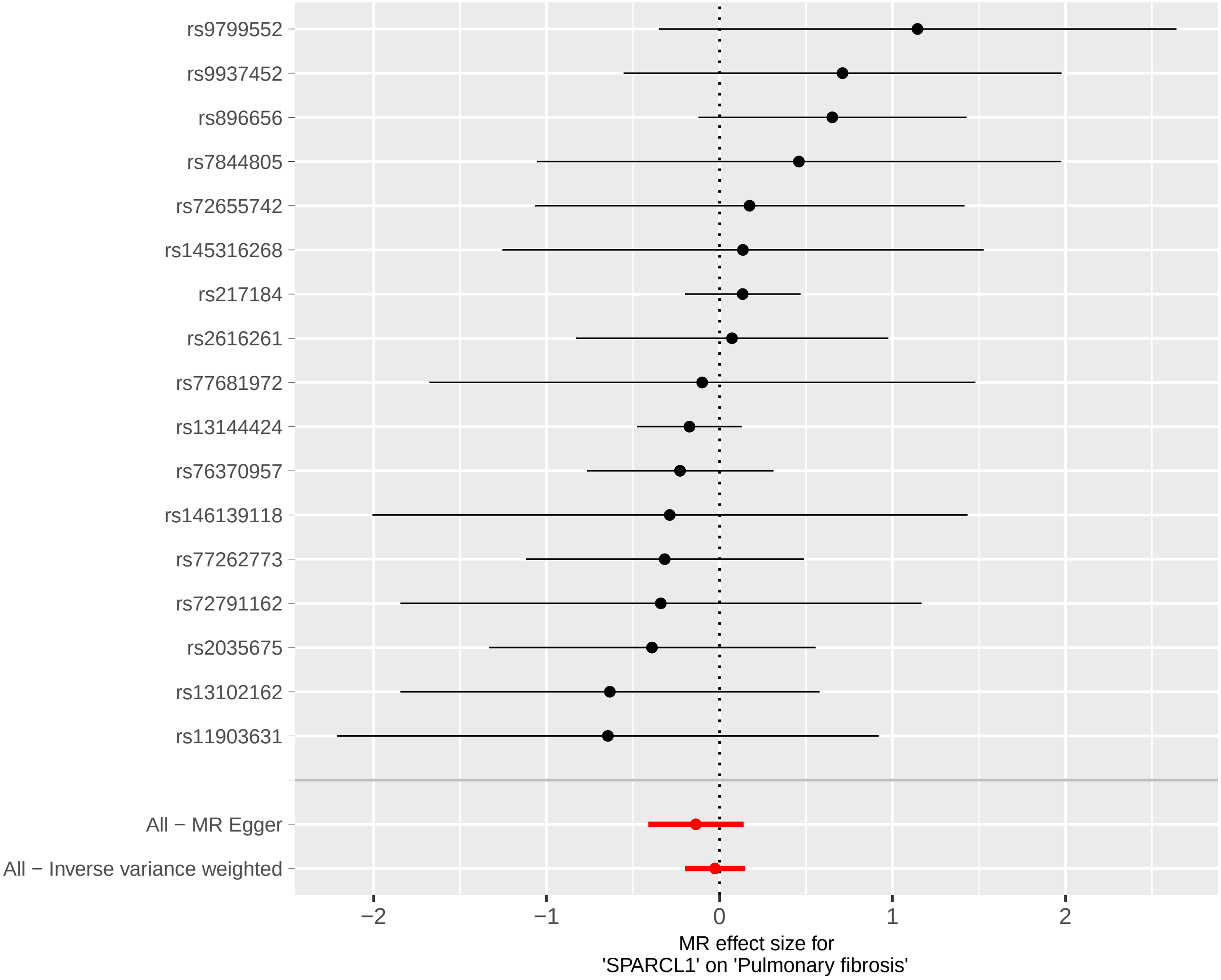

rs9799552
rs9937452
rs896656
rs7844805
rs72655742
rs145316268
rs217184
rs2616261
rs77681972
rs13144424
rs76370957
rs146139118
rs77262773
rs72791162
rs2035675
rs13102162
rs11903631
All − MR Egger
All − Inverse variance weighted
−2
−1
0
1
2
MR effect size for
'SPARCL1' on 'Pulmonary fibrosis'

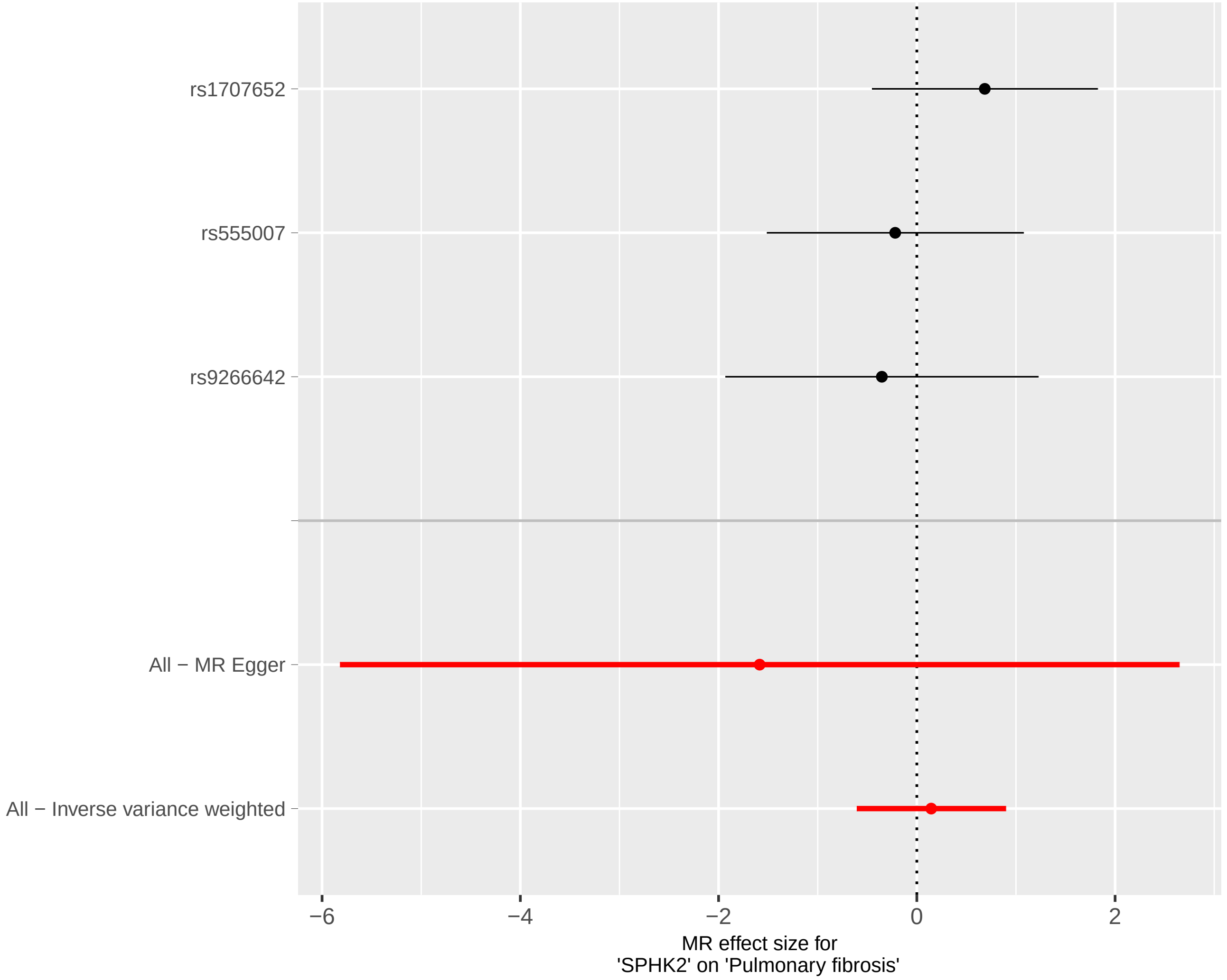
rs1707652
rs555007
rs9266642
All − MR Egger
All − Inverse variance weighted
−6
−4
−2
0
2
MR effect size for
'SPHK2' on 'Pulmonary fibrosis'

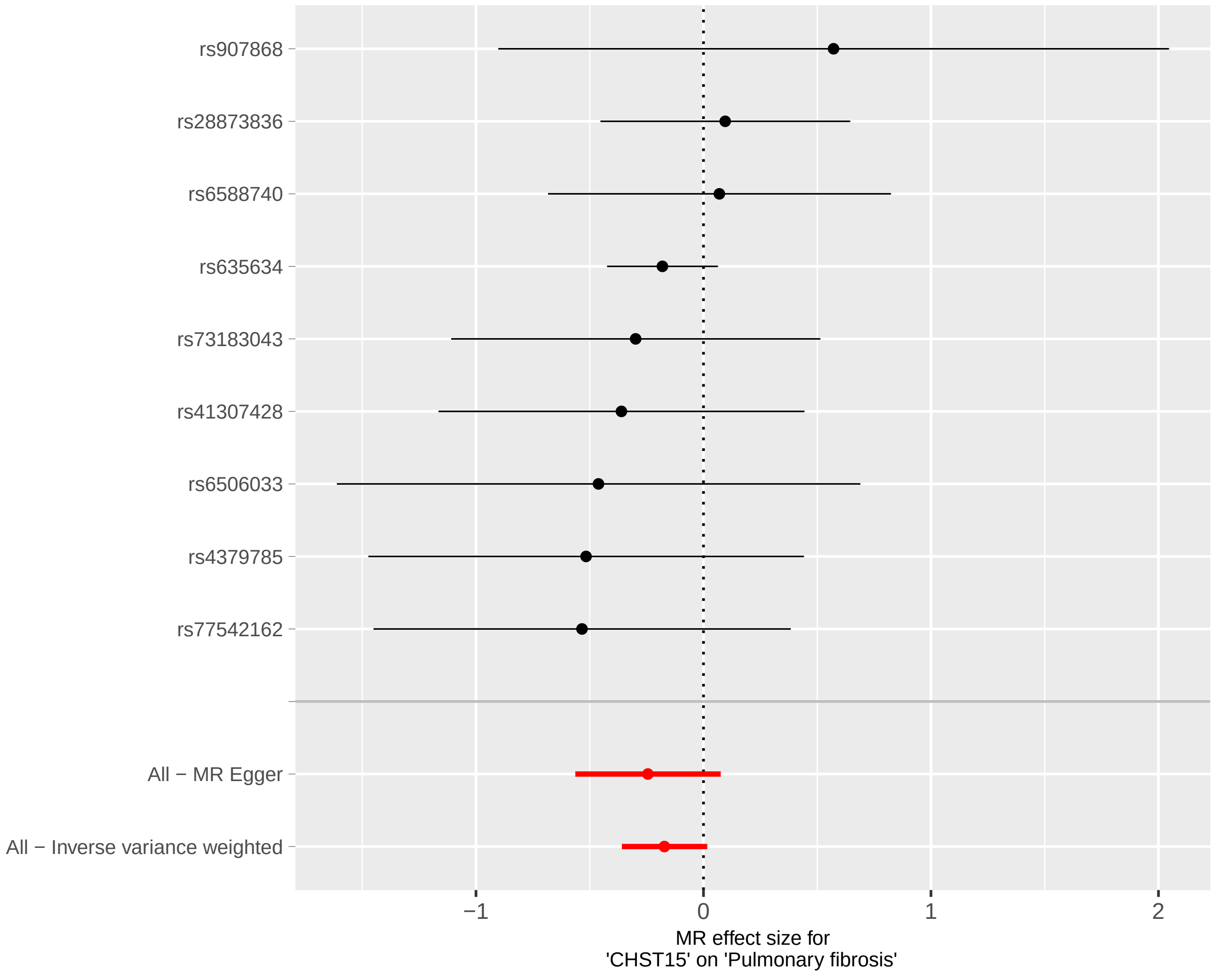
rs907868
rs28873836
rs6588740
rs635634
rs73183043
rs41307428
rs6506033
rs4379785
rs77542162
All − MR Egger
All − Inverse variance weighted
−1
0
1
2
MR effect size for
'CHST15' on 'Pulmonary fibrosis'

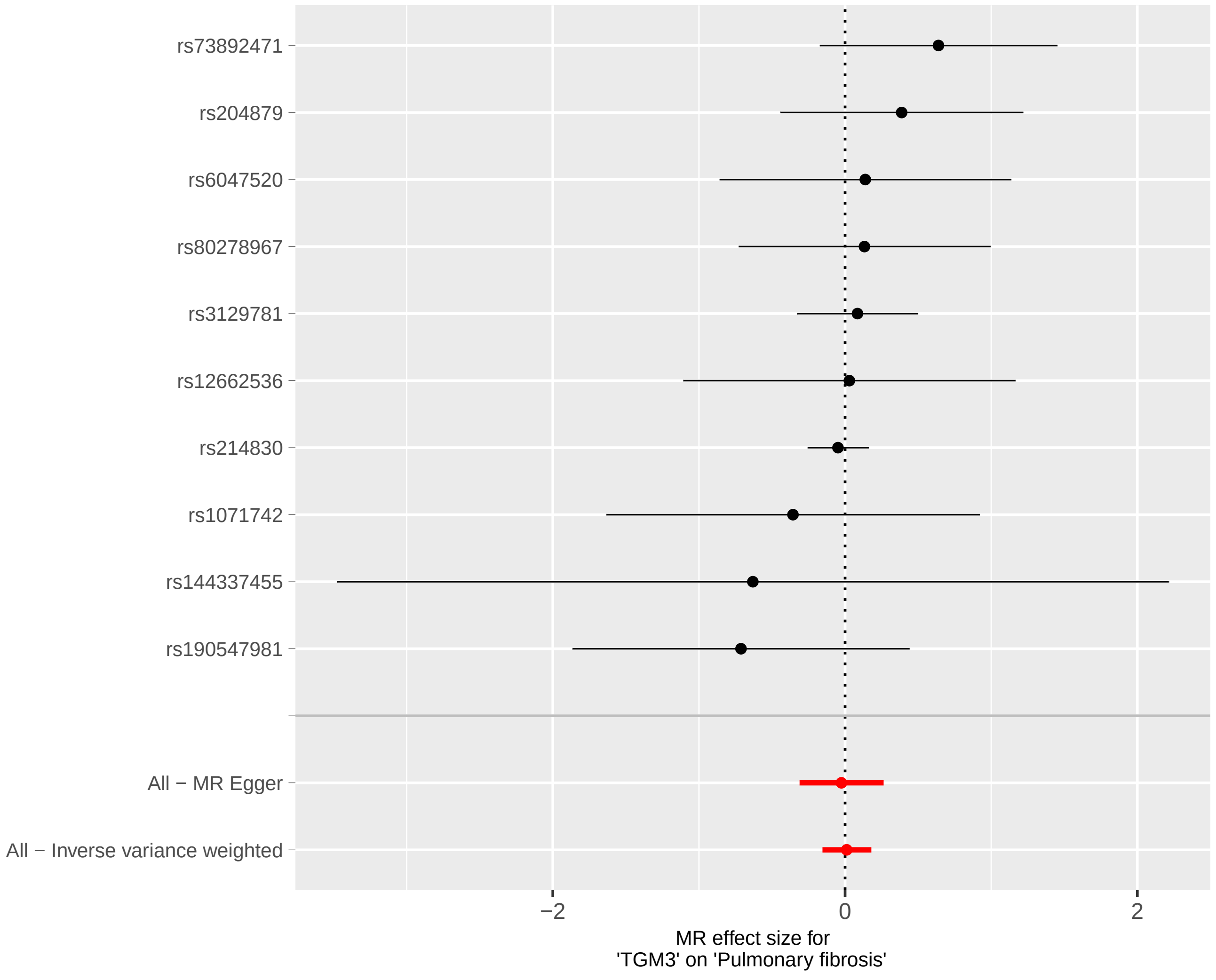
rs73892471
rs204879
rs6047520
rs80278967
rs3129781
rs12662536
rs214830
rs1071742
rs144337455
rs190547981
All − MR Egger
All − Inverse variance weighted
−2
0
2
MR effect size for
'TGM3' on 'Pulmonary fibrosis'

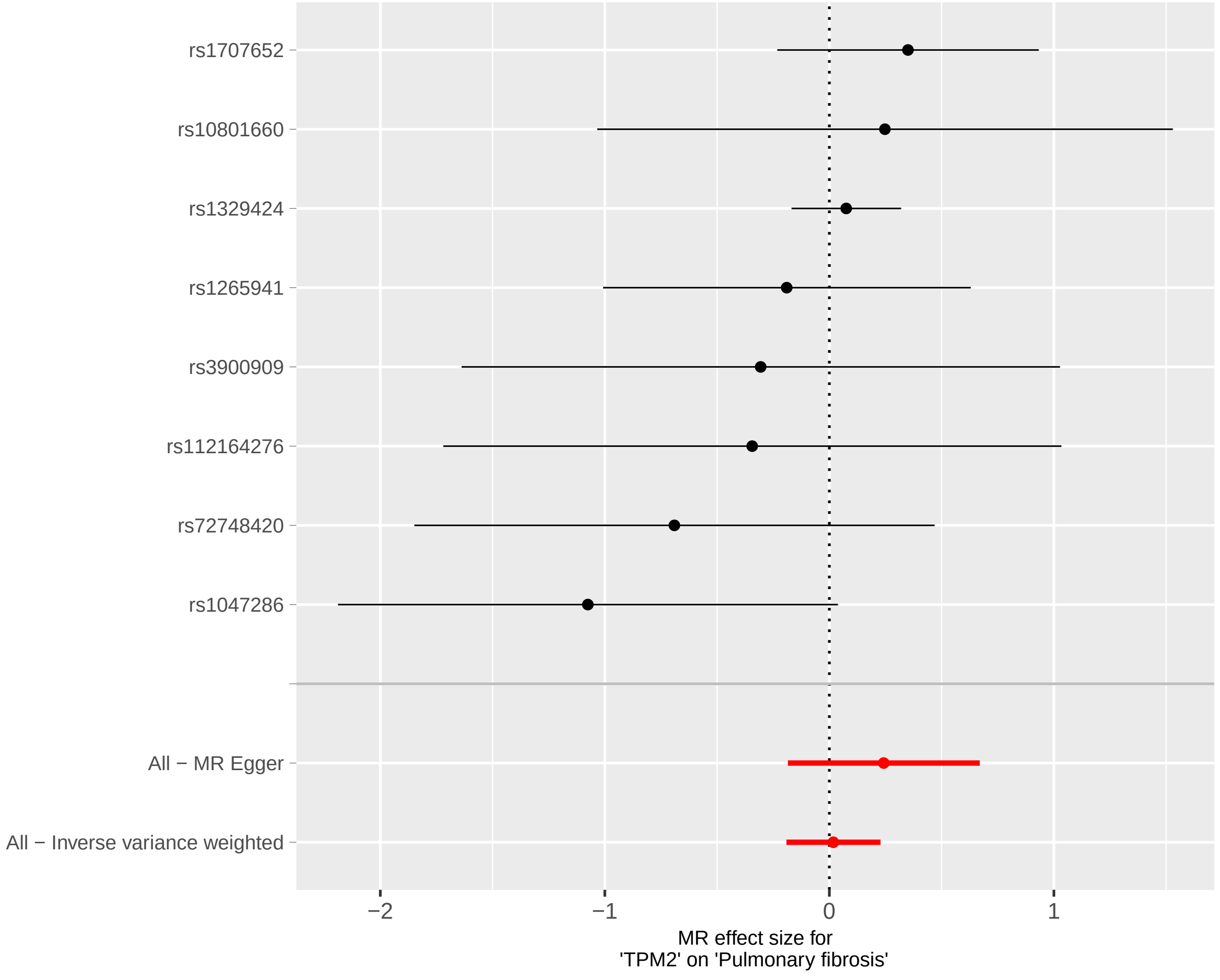

rs1707652
rs10801660
rs1329424
rs1265941
rs3900909
rs112164276
rs72748420
rs1047286
All − MR Egger
All − Inverse variance weighted
−2
−1
0
1
MR effect size for
'TPM2' on 'Pulmonary fibrosis'

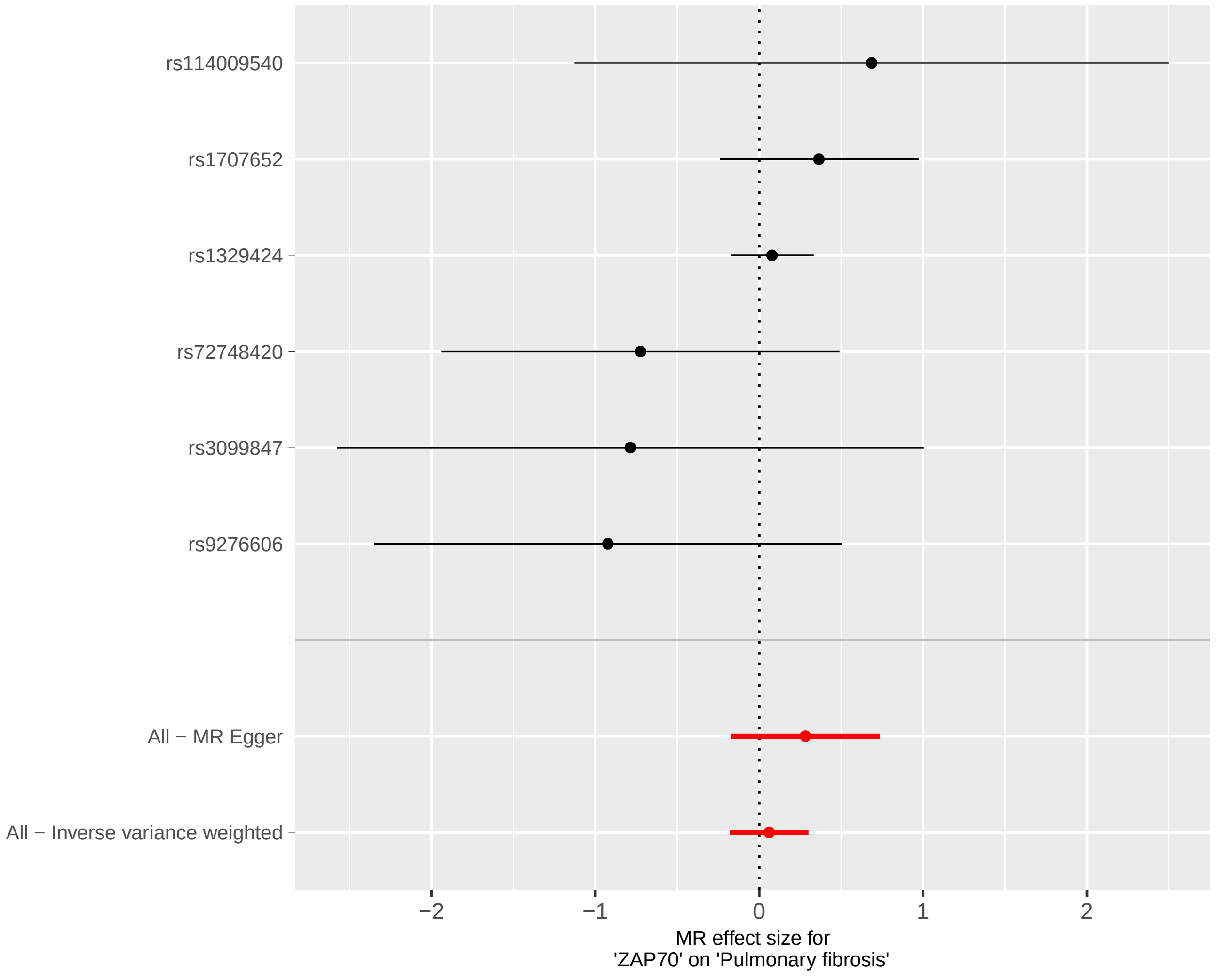

rs114009540
rs1707652
rs1329424
rs72748420
rs3099847
rs9276606
All − MR Egger
All − Inverse variance weighted
−2
−1
0
1
2
MR effect size for
'ZAP70' on 'Pulmonary fibrosis'

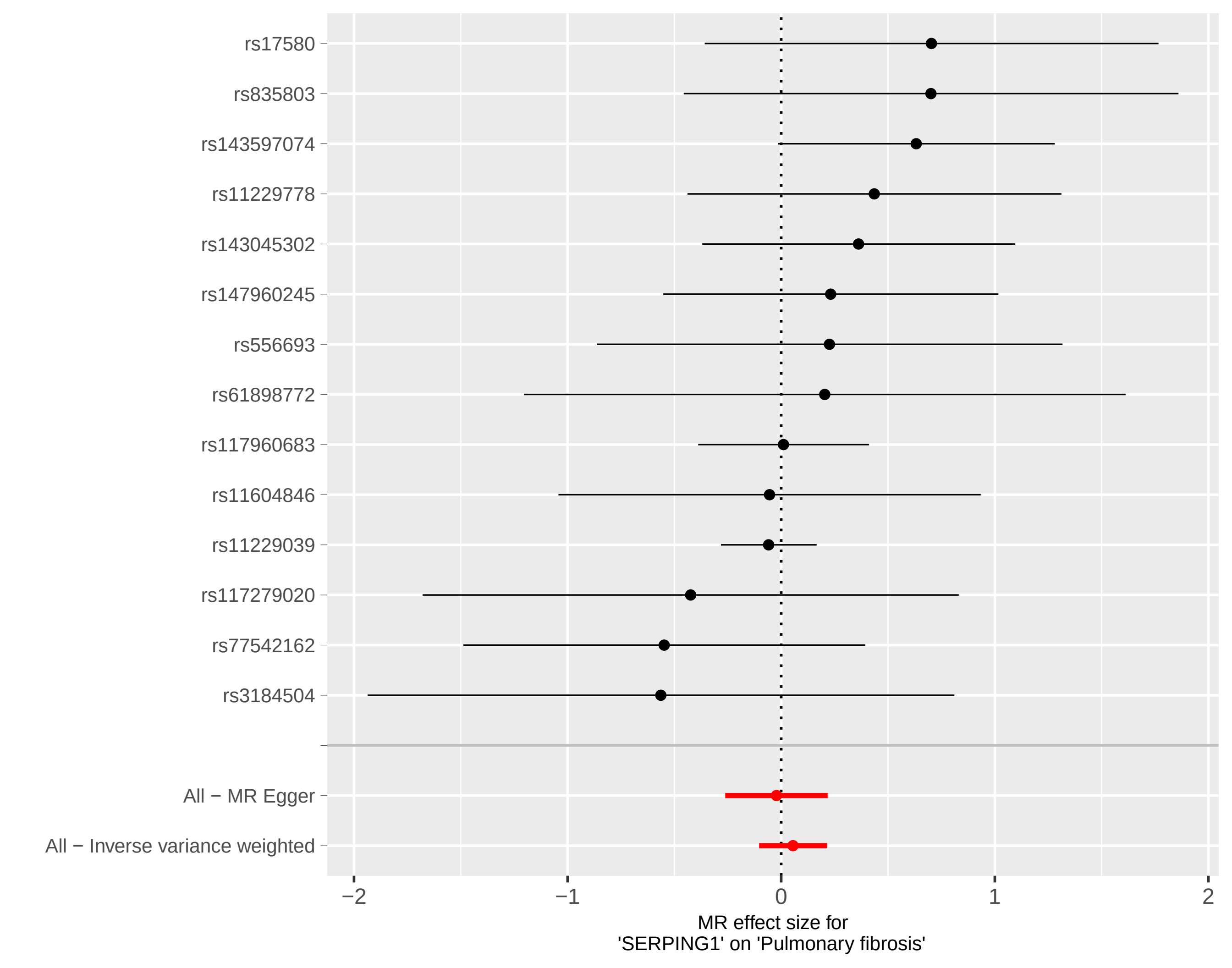

rs17580
rs835803
rs143597074
rs11229778
rs143045302
rs147960245
rs556693
rs61898772
rs117960683
rs11604846
rs11229039
rs117279020
rs77542162
rs3184504
All − MR Egger
All − Inverse variance weighted
−2
−1
0
1
2
MR effect size for
'SERPING1' on 'Pulmonary fibrosis'

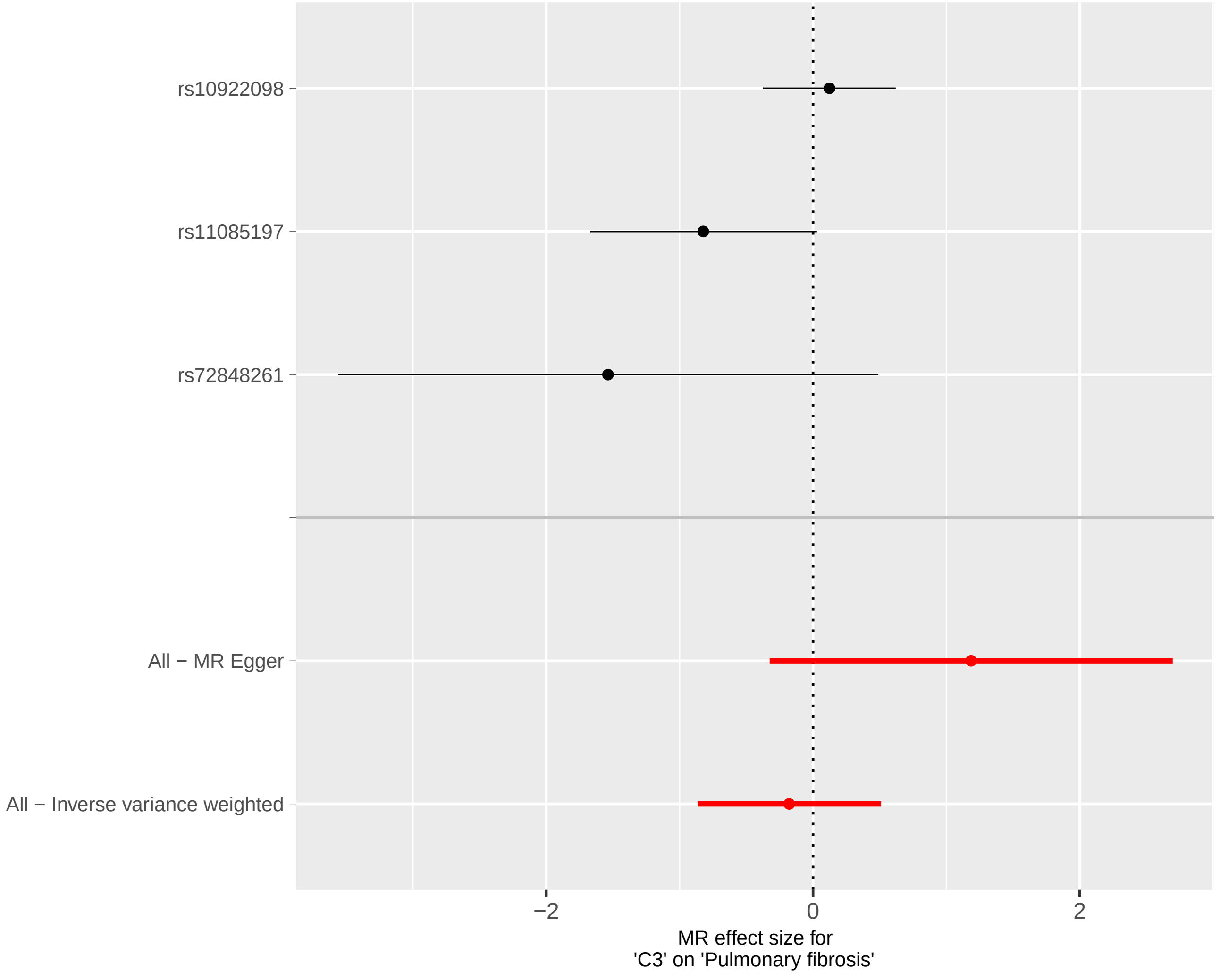
rs10922098
rs11085197
rs72848261
All − MR Egger
All − Inverse variance weighted
−2
0
2
MR effect size for
'C3' on 'Pulmonary fibrosis'

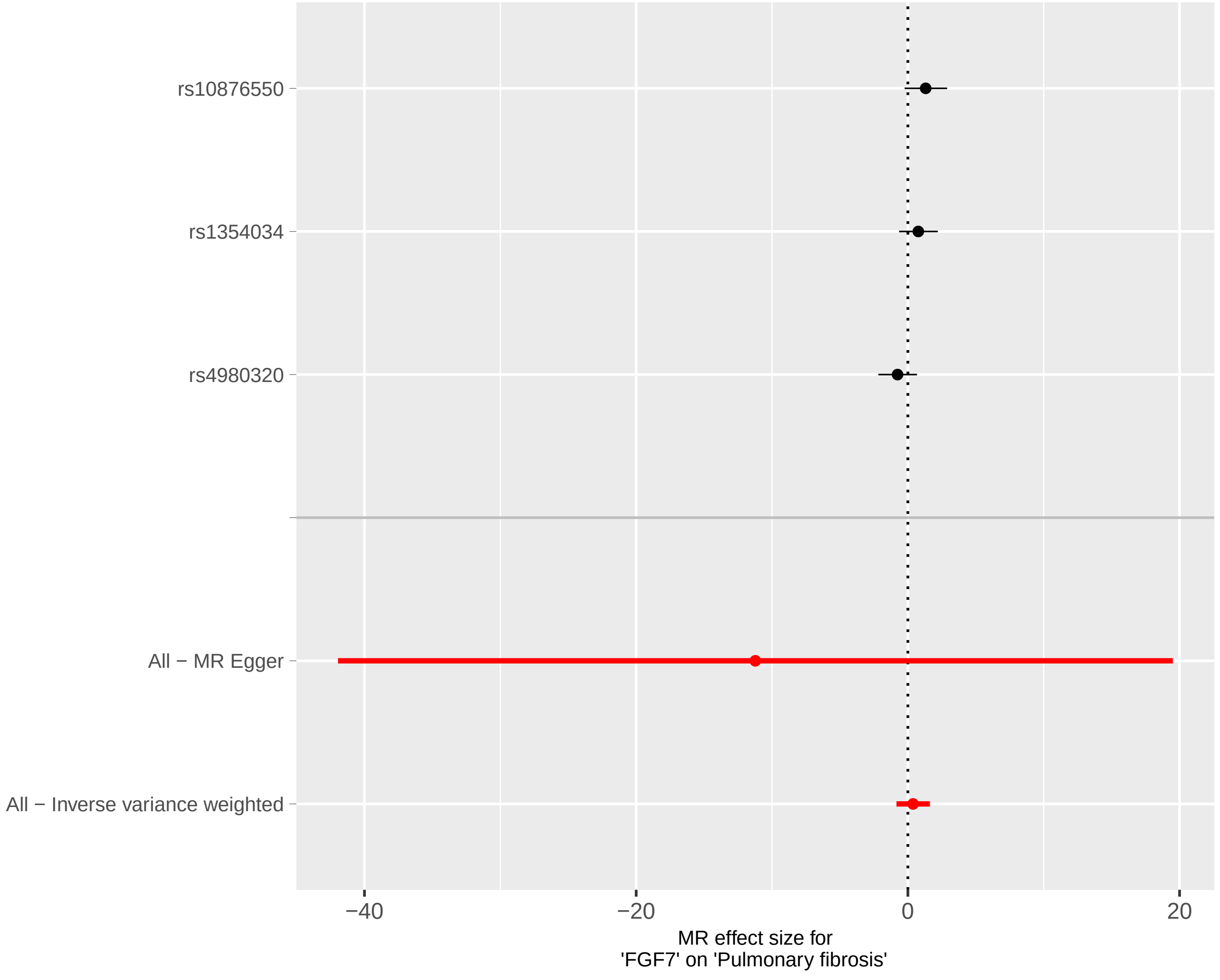

rs10876550
rs1354034
rs4980320
All − MR Egger
All − Inverse variance weighted
−40
−20
0
20
MR effect size for
'FGF7' on 'Pulmonary fibrosis'

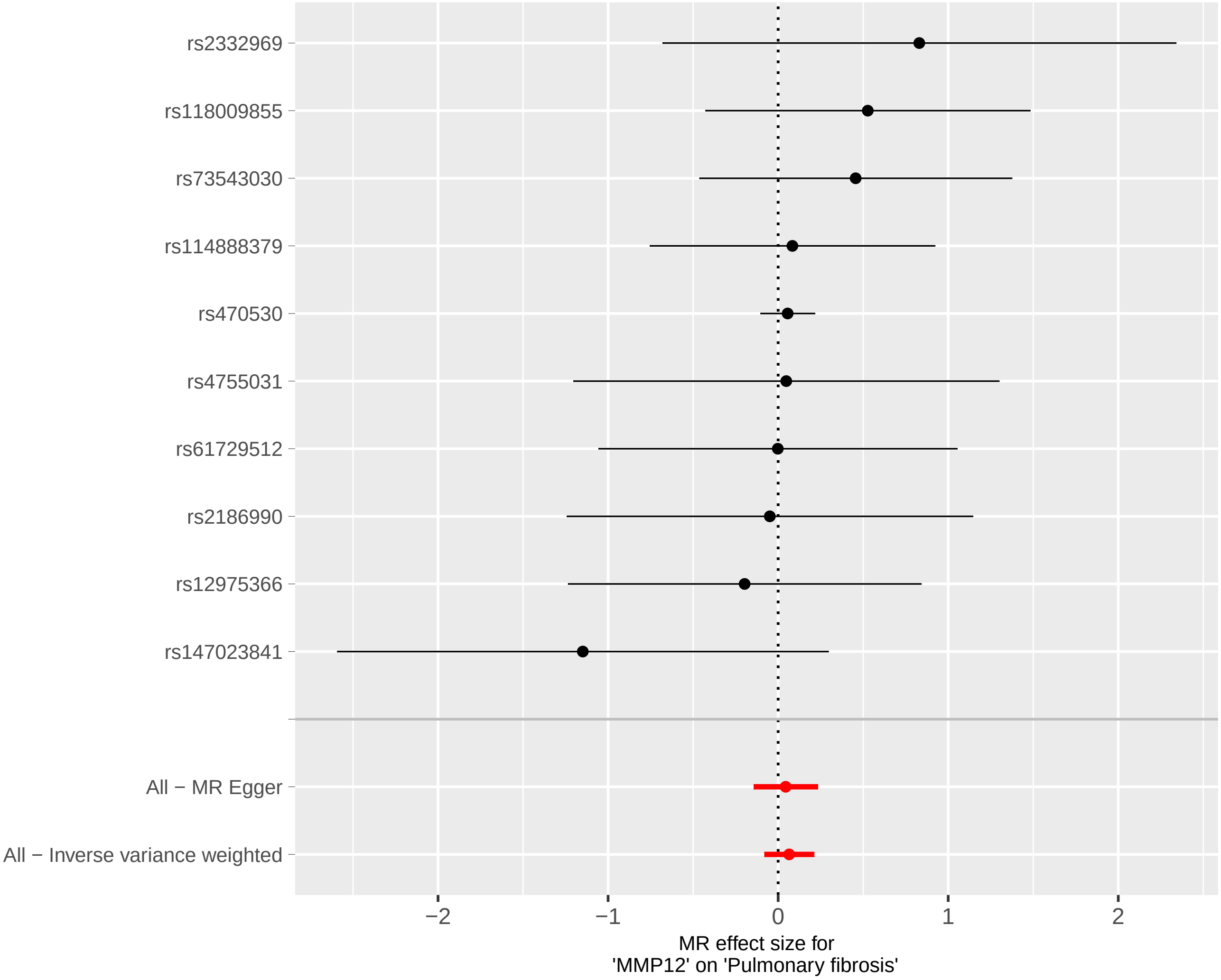

rs2332969
rs118009855
rs73543030
rs114888379
rs470530
rs4755031
rs61729512
rs2186990
rs12975366
rs147023841
All − MR Egger
All − Inverse variance weighted
−2
−1
0
1
2
MR effect size for
'MMP12' on 'Pulmonary fibrosis'

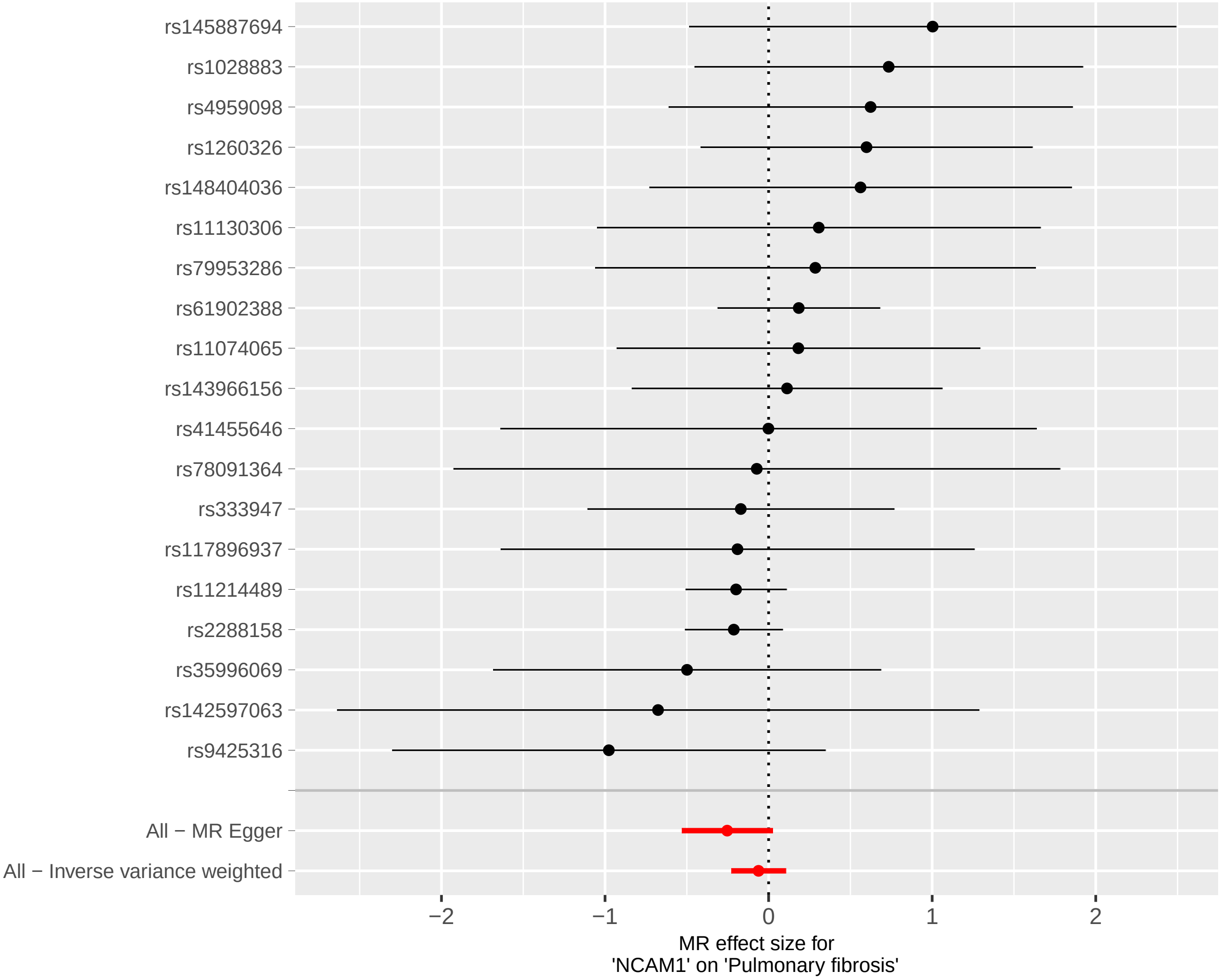
rs145887694
rs1028883
rs4959098
rs1260326
rs148404036
rs11130306
rs79953286
rs61902388
rs11074065
rs143966156
rs41455646
rs78091364
rs333947
rs117896937
rs11214489
rs2288158
rs35996069
rs142597063
rs9425316
All − MR Egger
All − Inverse variance weighted
−2
−1
0
1
2
MR effect size for
'NCAM1' on 'Pulmonary fibrosis'

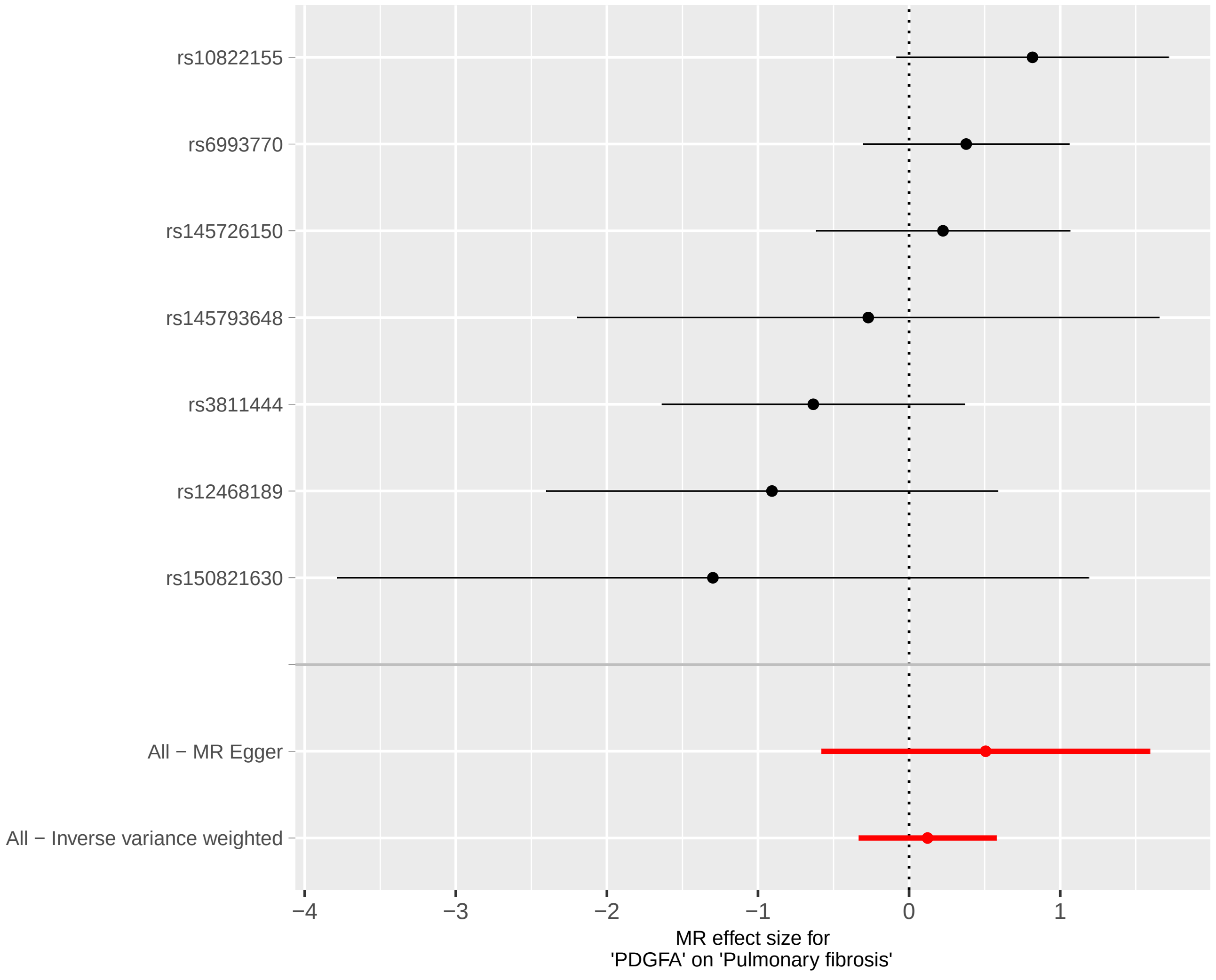
rs10822155
rs6993770
rs145726150
rs145793648
rs3811444
rs12468189
rs150821630
All − MR Egger
All − Inverse variance weighted
−4
−3
−2
−1
0
1
MR effect size for
'PDGFA' on 'Pulmonary fibrosis'

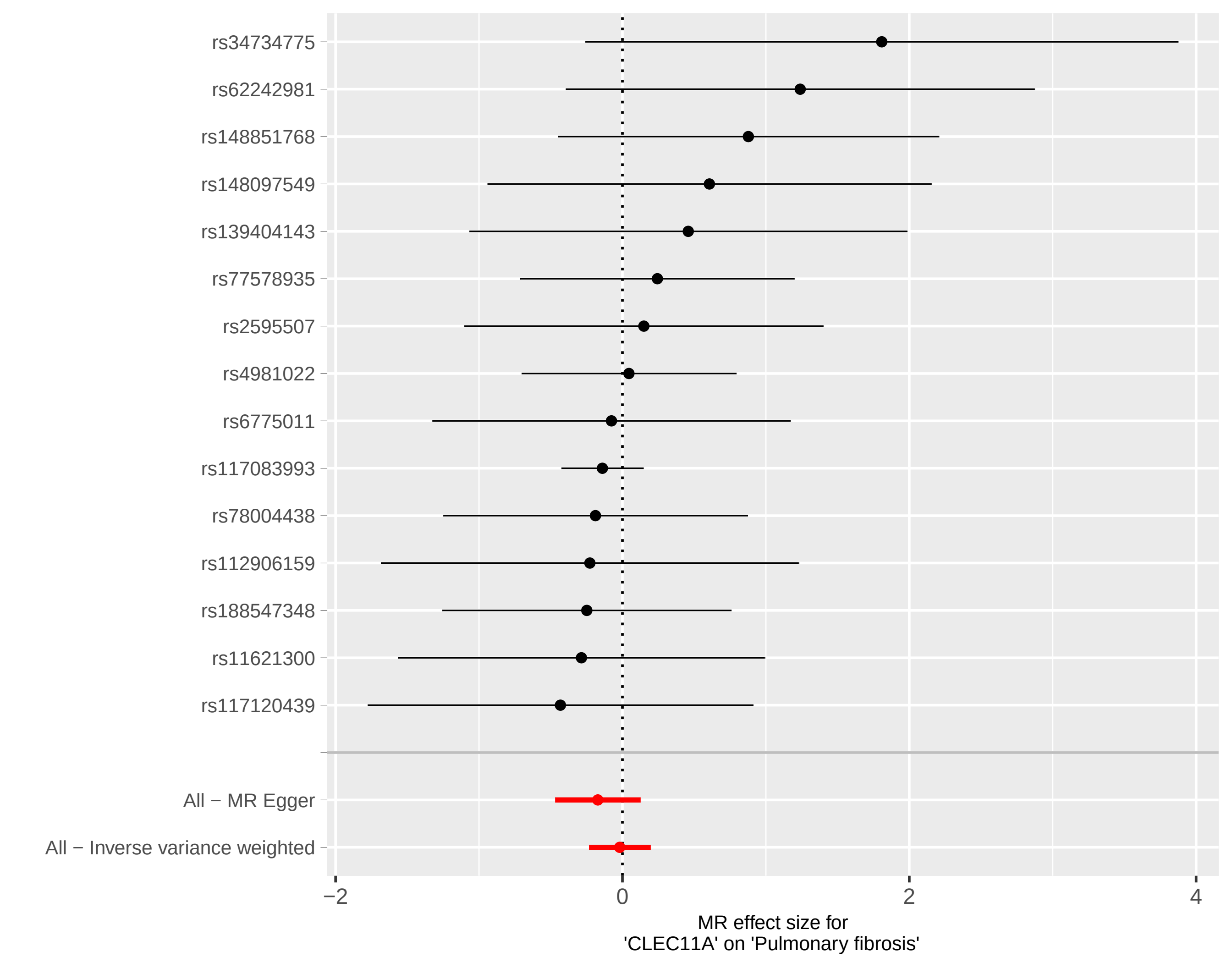
rs34734775
rs62242981
rs148851768
rs148097549
rs139404143
rs77578935
rs2595507
rs4981022
rs6775011
rs117083993
rs78004438
rs112906159
rs188547348
rs11621300
rs117120439
All − MR Egger
All − Inverse variance weighted
−2
0
2
4
MR effect size for
'CLEC11A' on 'Pulmonary fibrosis'

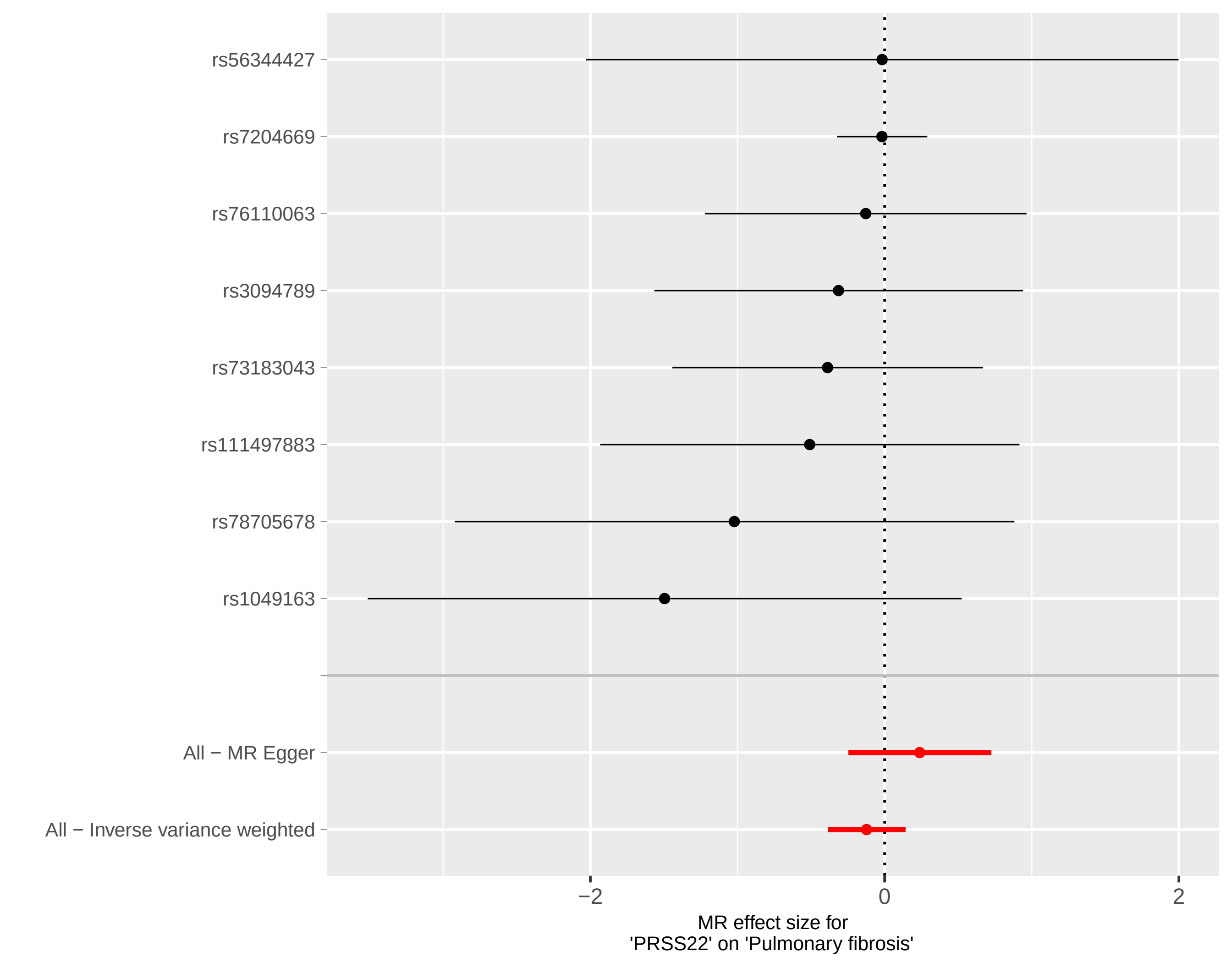

rs56344427
rs7204669
rs76110063
rs3094789
rs73183043
rs111497883
rs78705678
rs1049163
All − MR Egger
All − Inverse variance weighted
−2
0
2
MR effect size for
'PRSS22' on 'Pulmonary fibrosis'

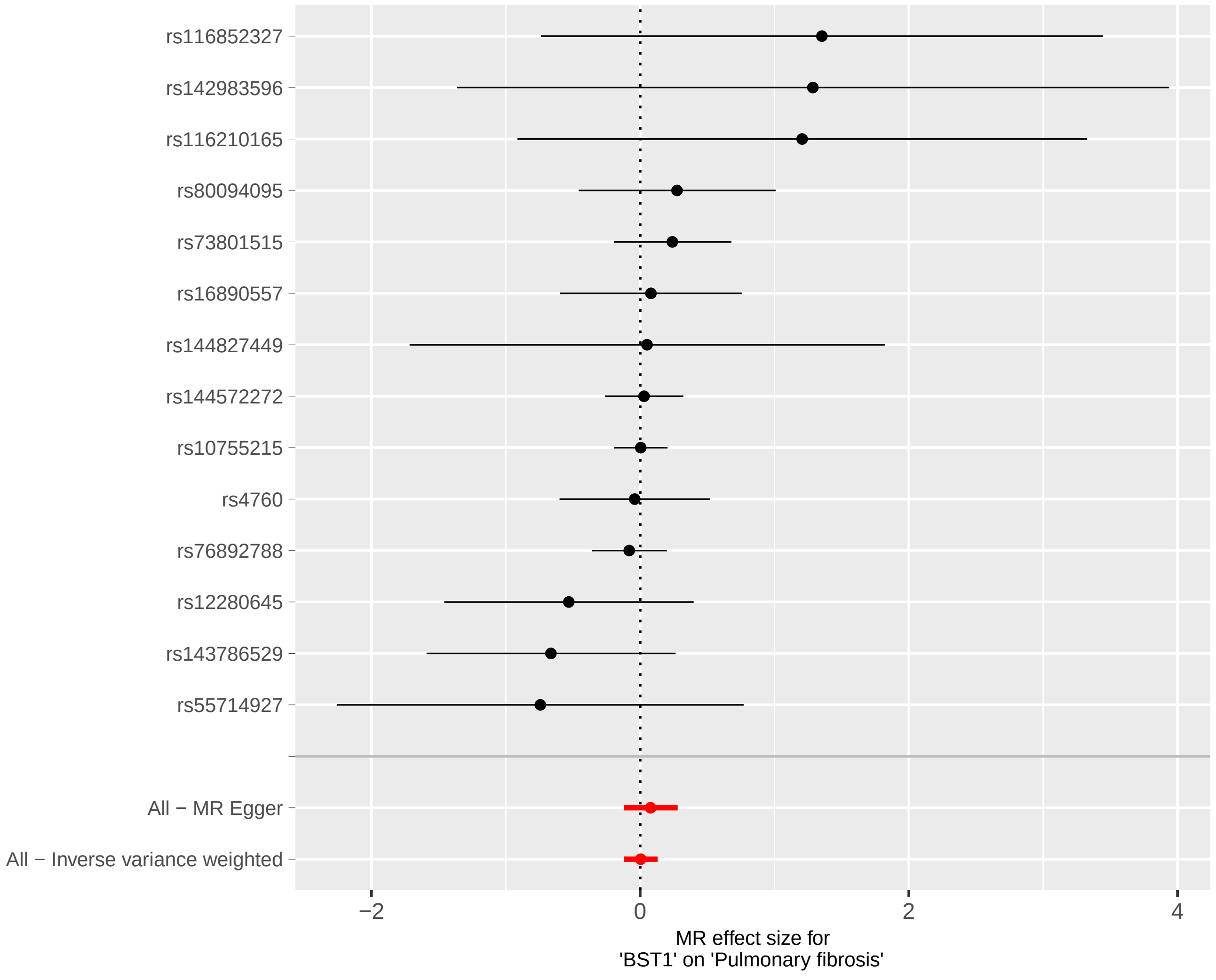

rs116852327
rs142983596
rs116210165
rs80094095
rs73801515
rs16890557
rs144827449
rs144572272
rs10755215
rs4760
rs76892788
rs12280645
rs143786529
rs55714927
All − MR Egger
All − Inverse variance weighted
−2
0
2
4
MR effect size for
'BST1' on 'Pulmonary fibrosis'

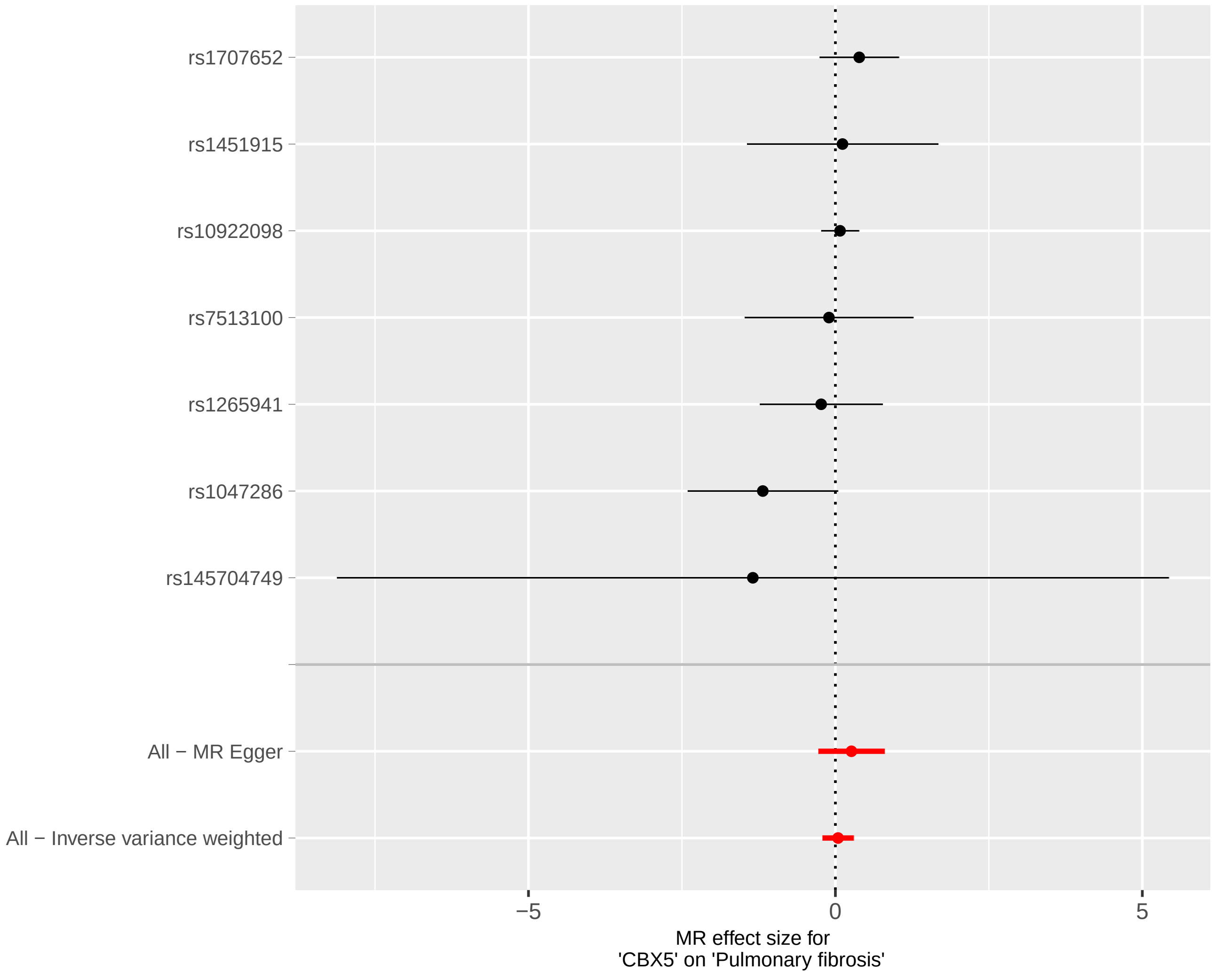
rs1707652
rs1451915
rs10922098
rs7513100
rs1265941
rs1047286
rs145704749
All − MR Egger
All − Inverse variance weighted
−5
0
5
MR effect size for
'CBX5' on 'Pulmonary fibrosis'

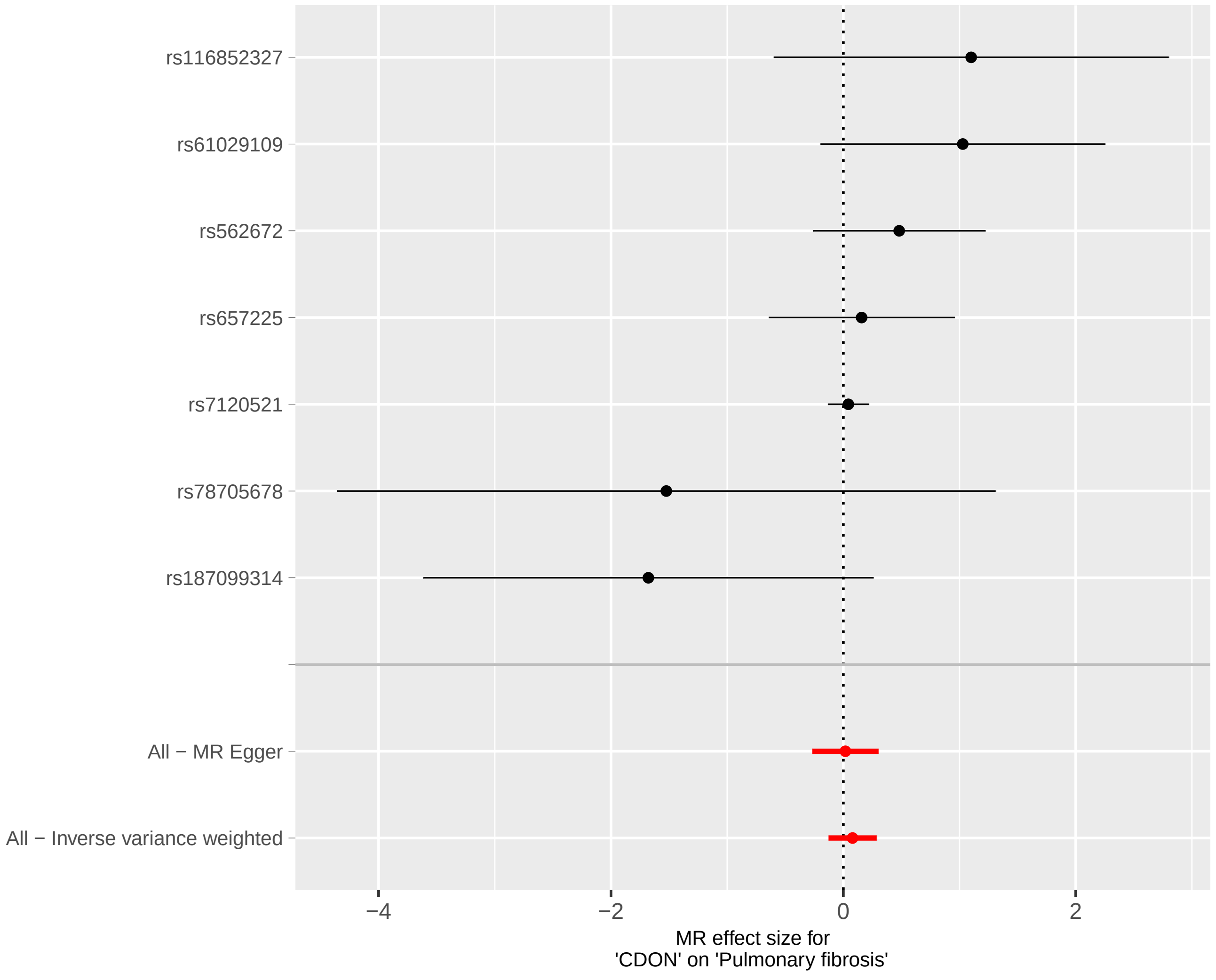

rs116852327
rs61029109
rs562672
rs657225
rs7120521
rs78705678
rs187099314
All − MR Egger
All − Inverse variance weighted
−4
−2
0
2
MR effect size for
'CDON' on 'Pulmonary fibrosis'

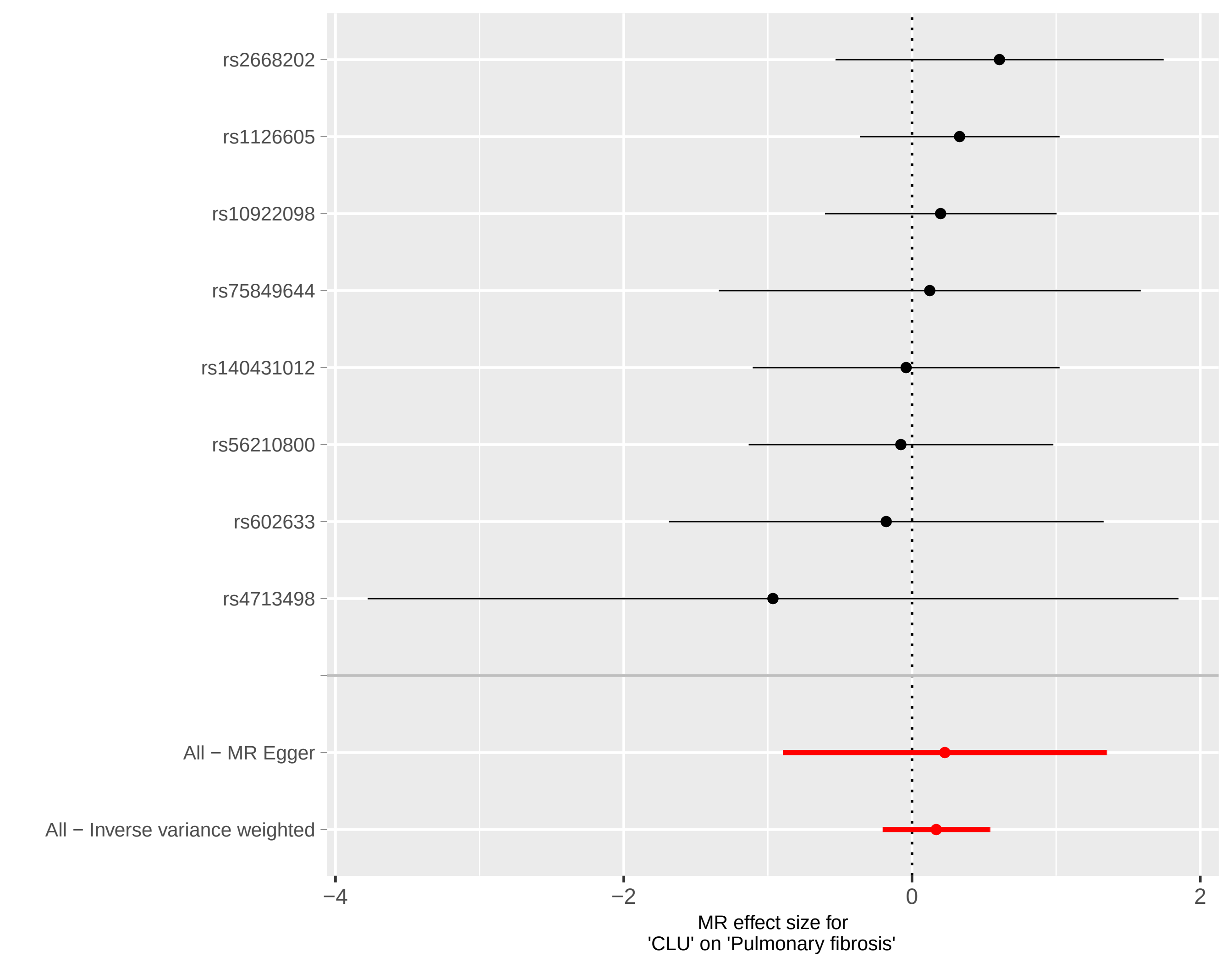

rs2668202
rs1126605
rs10922098
rs75849644
rs140431012
rs56210800
rs602633
rs4713498
All − MR Egger
All − Inverse variance weighted
−4
−2
0
2
MR effect size for
'CLU' on 'Pulmonary fibrosis'

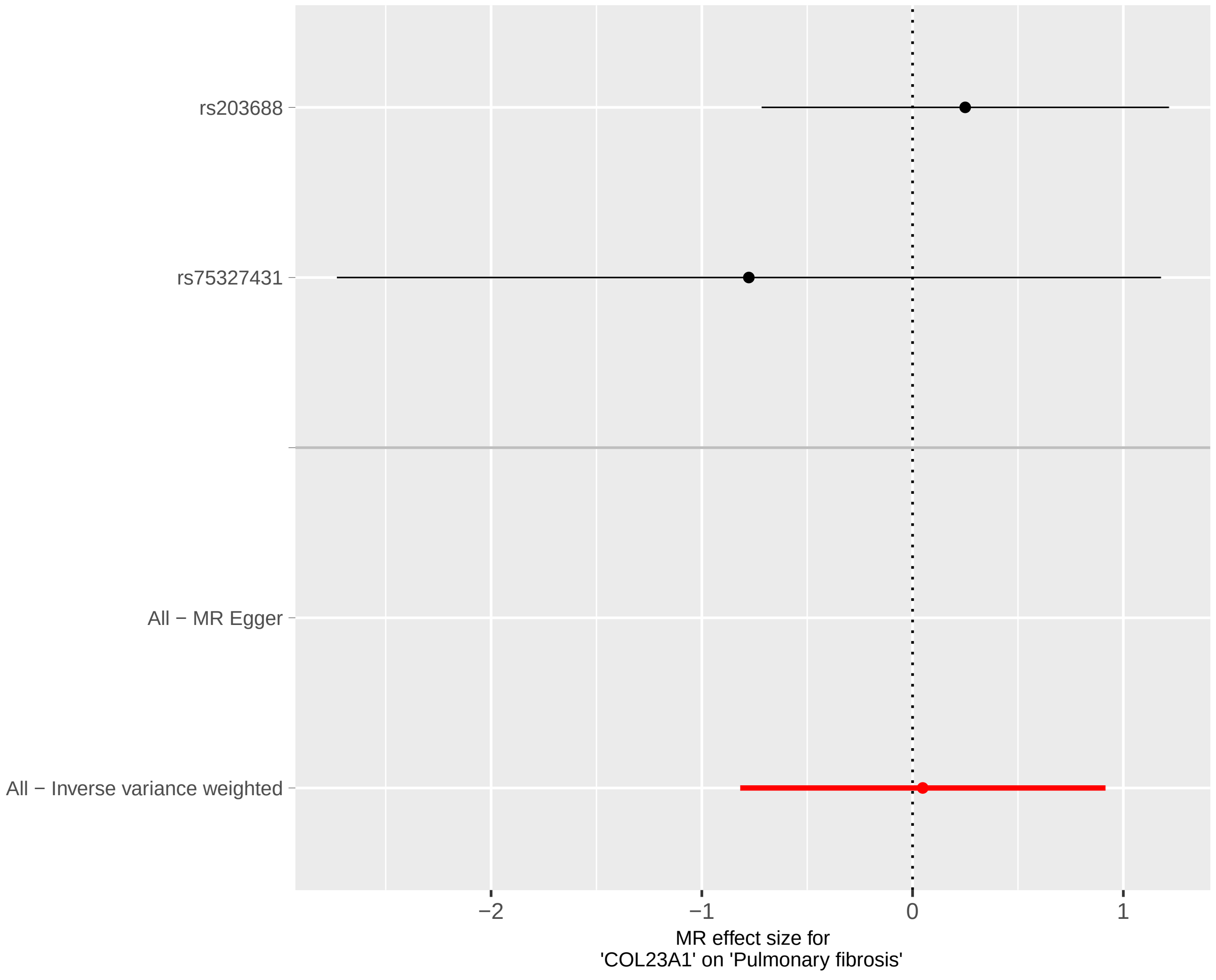
rs203688
rs75327431
All − MR Egger
All − Inverse variance weighted
−2
−1
0
1
MR effect size for
'COL23A1' on 'Pulmonary fibrosis'

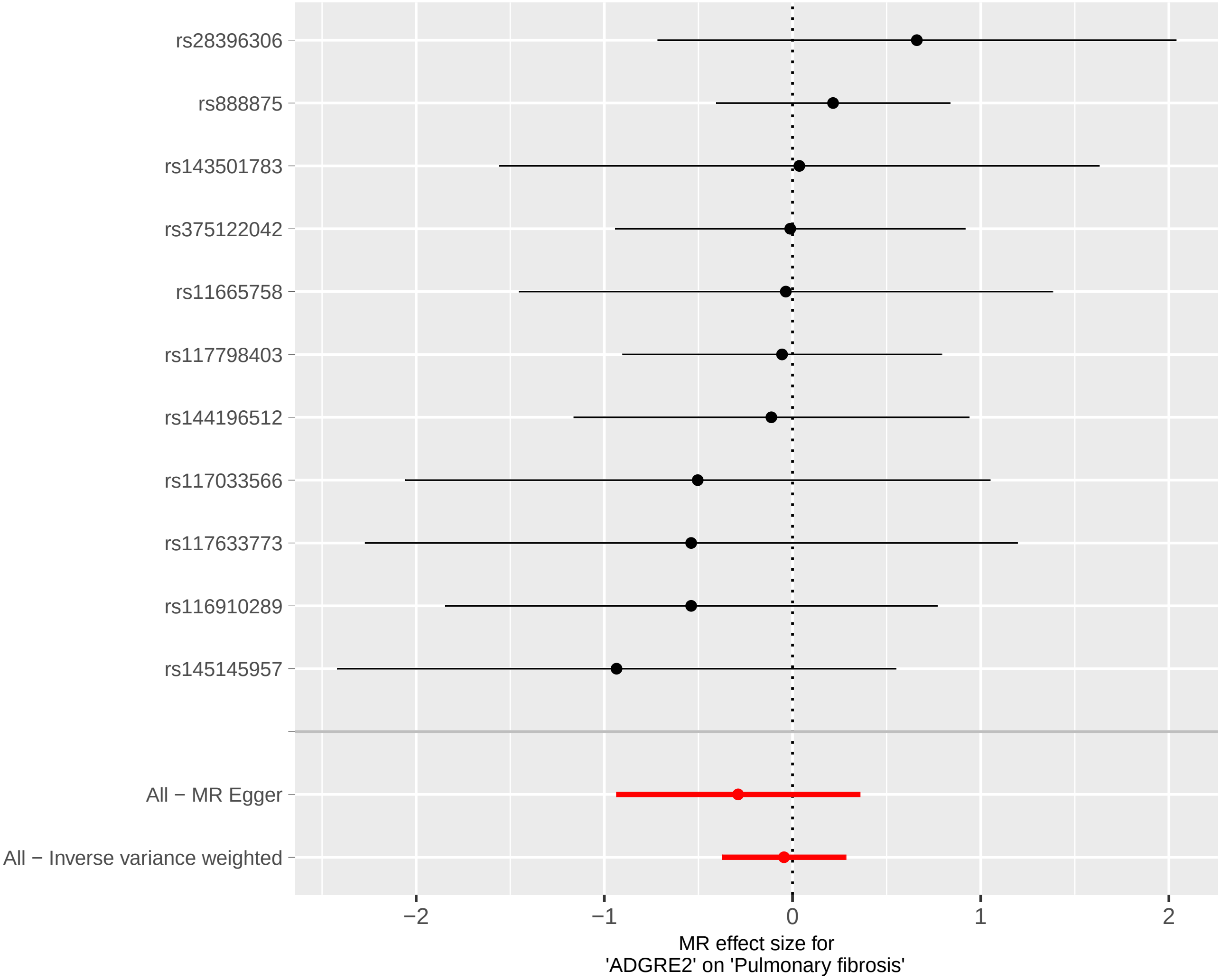

rs28396306
rs888875
rs143501783
rs375122042
rs11665758
rs117798403
rs144196512
rs117033566
rs117633773
rs116910289
rs145145957
All − MR Egger
All − Inverse variance weighted
−2
−1
0
1
2
MR effect size for
'ADGRE2' on 'Pulmonary fibrosis'

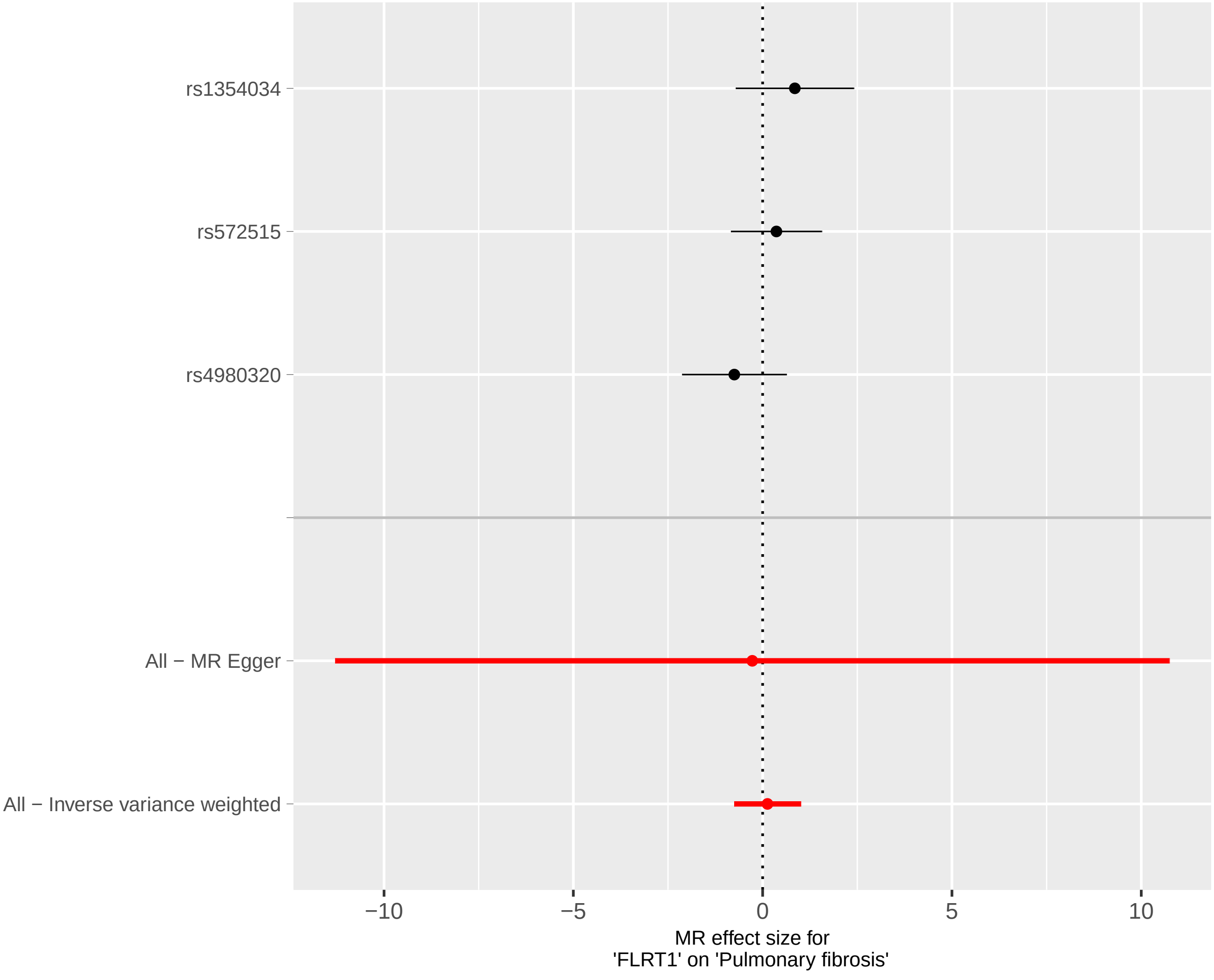

rs1354034
rs572515
rs4980320
All − MR Egger
All − Inverse variance weighted
−10
−5
0
5
10
MR effect size for
'FLRT1' on 'Pulmonary fibrosis'

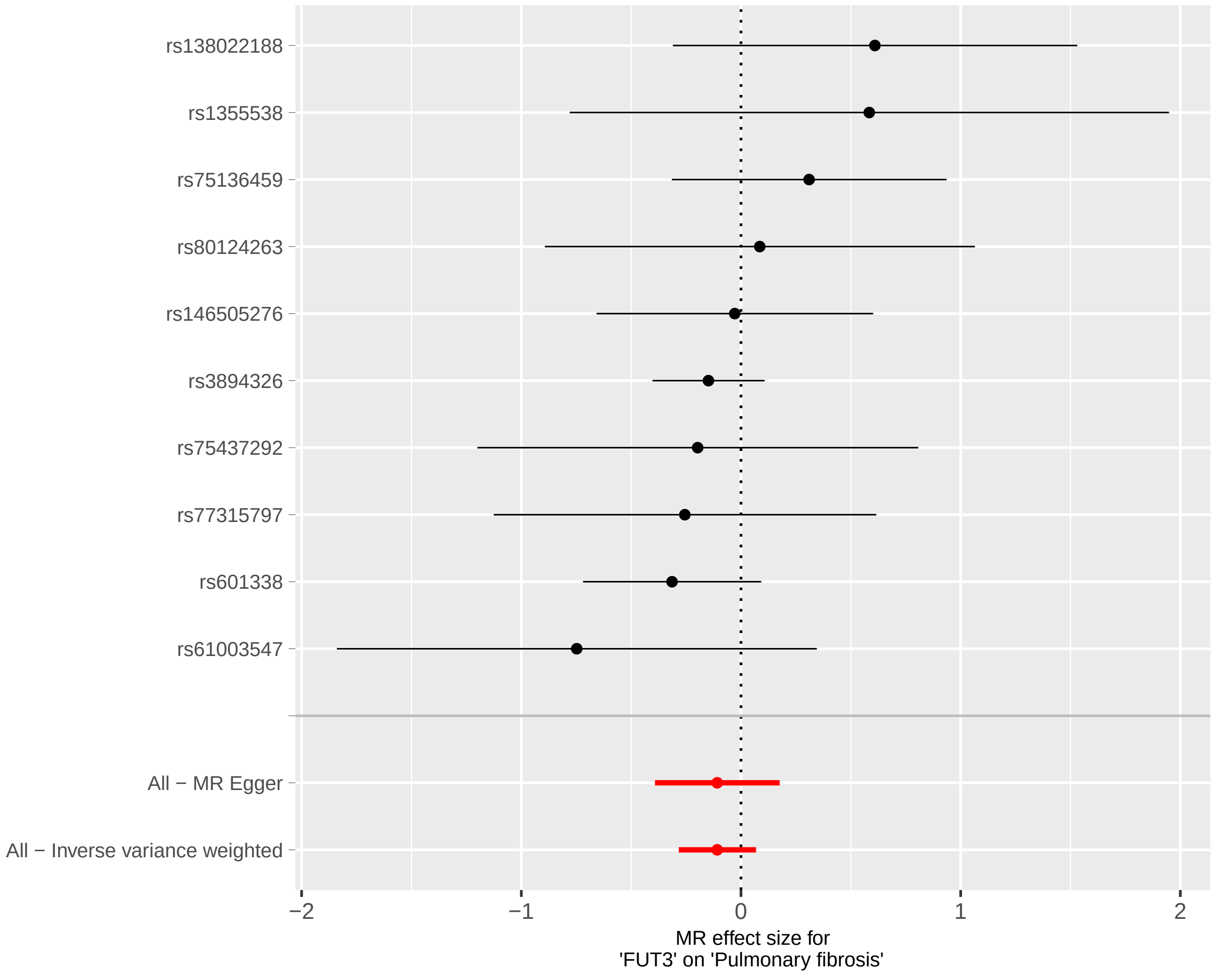

rs138022188
rs1355538
rs75136459
rs80124263
rs146505276
rs3894326
rs75437292
rs77315797
rs601338
rs61003547
All − MR Egger
All − Inverse variance weighted
−2
−1
0
1
2
MR effect size for
'FUT3' on 'Pulmonary fibrosis'

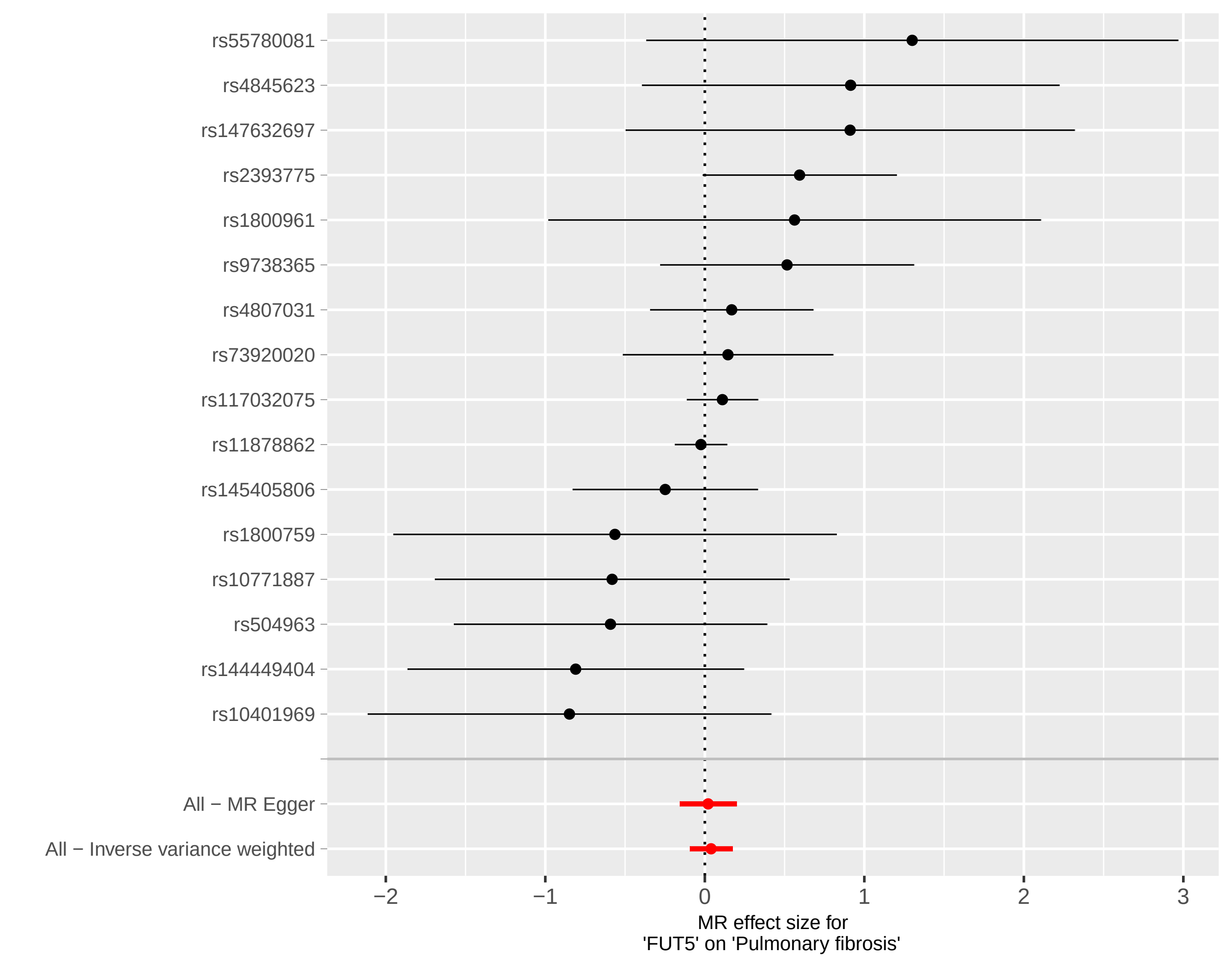

rs55780081
rs4845623
rs147632697
rs2393775
rs1800961
rs9738365
rs4807031
rs73920020
rs117032075
rs11878862
rs145405806
rs1800759
rs10771887
rs504963
rs144449404
rs10401969
All − MR Egger
All − Inverse variance weighted
−2
−1
0
1
2
3
MR effect size for
'FUT5' on 'Pulmonary fibrosis'

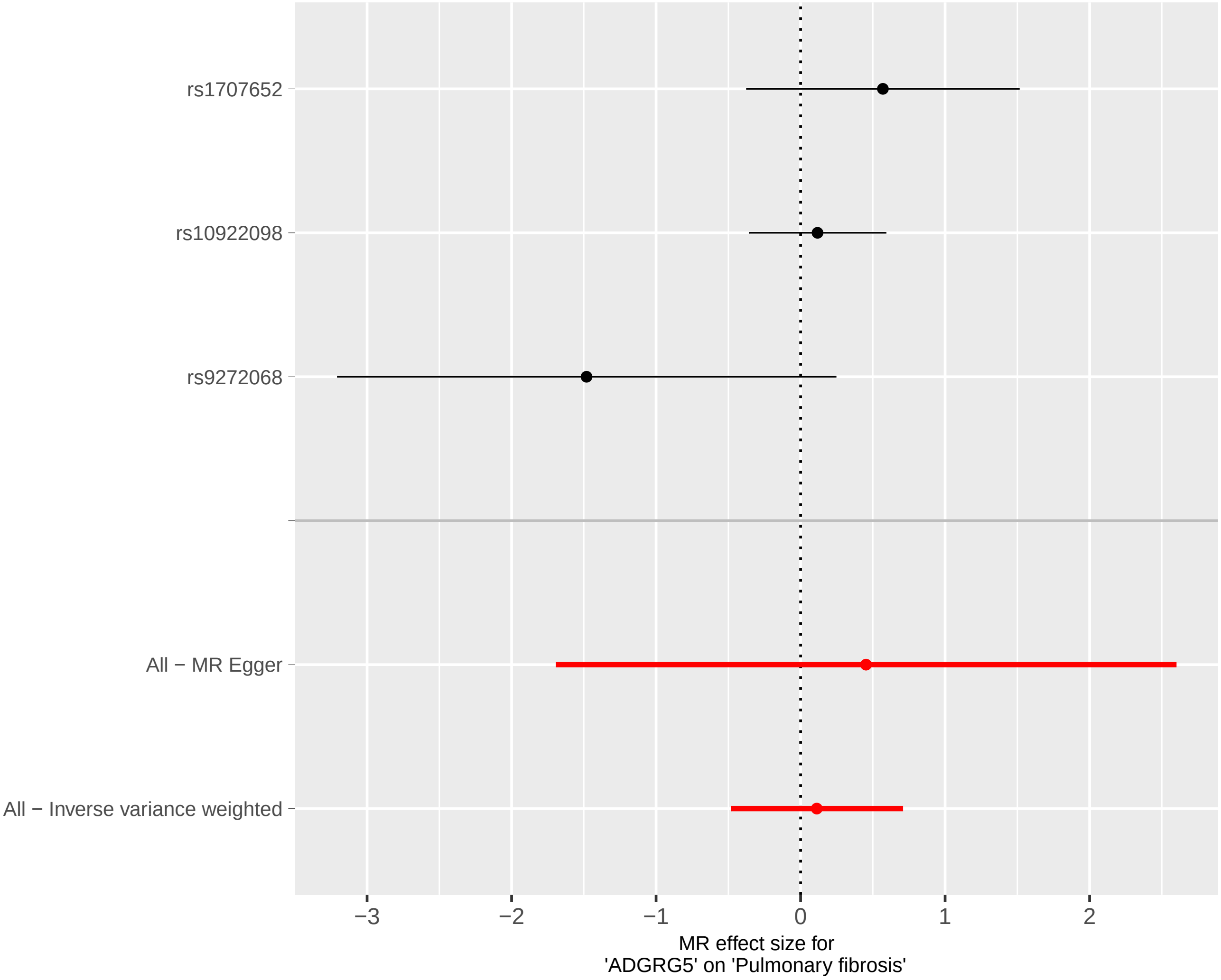
rs1707652
rs10922098
rs9272068
All − MR Egger
All − Inverse variance weighted
−3
−2
−1
0
1
2
MR effect size for
'ADGRG5' on 'Pulmonary fibrosis'

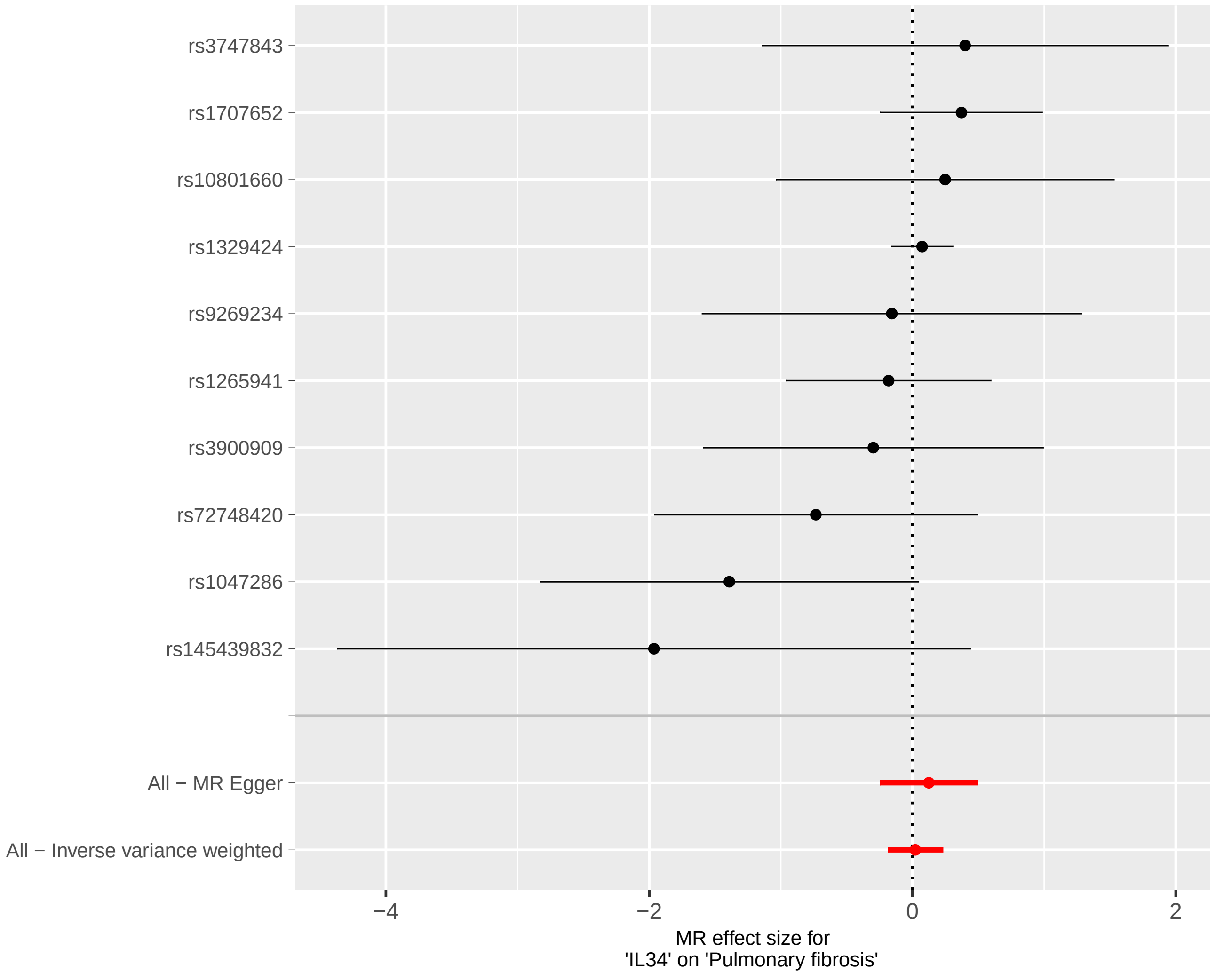
rs3747843
rs1707652
rs10801660
rs1329424
rs9269234
rs1265941
rs3900909
rs72748420
rs1047286
rs145439832
All − MR Egger
All − Inverse variance weighted
−4
−2
0
2
MR effect size for
'IL34' on 'Pulmonary fibrosis'

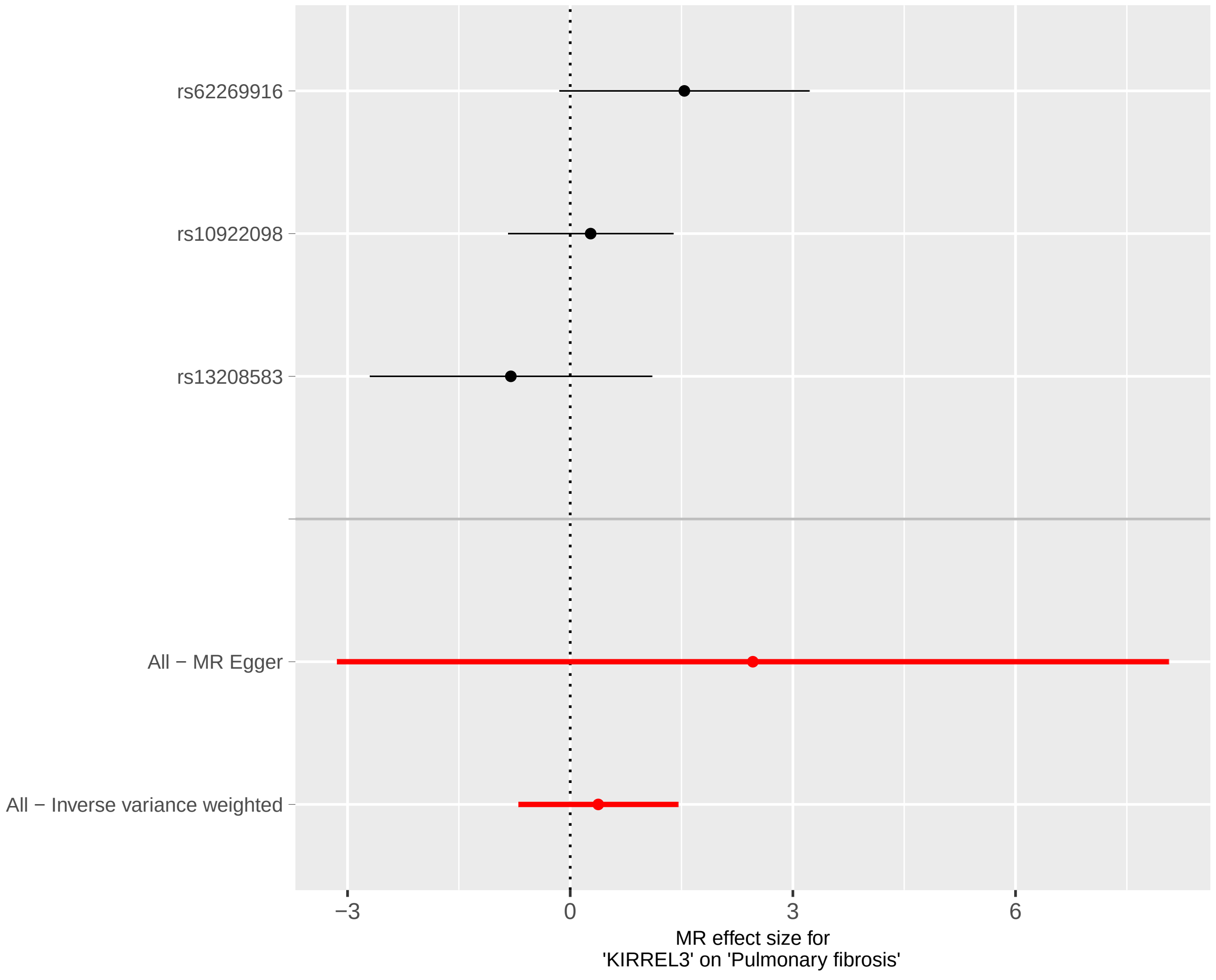

rs62269916
rs10922098
rs13208583
All − MR Egger
All − Inverse variance weighted
−3
0
3
6
MR effect size for
'KIRREL3' on 'Pulmonary fibrosis'

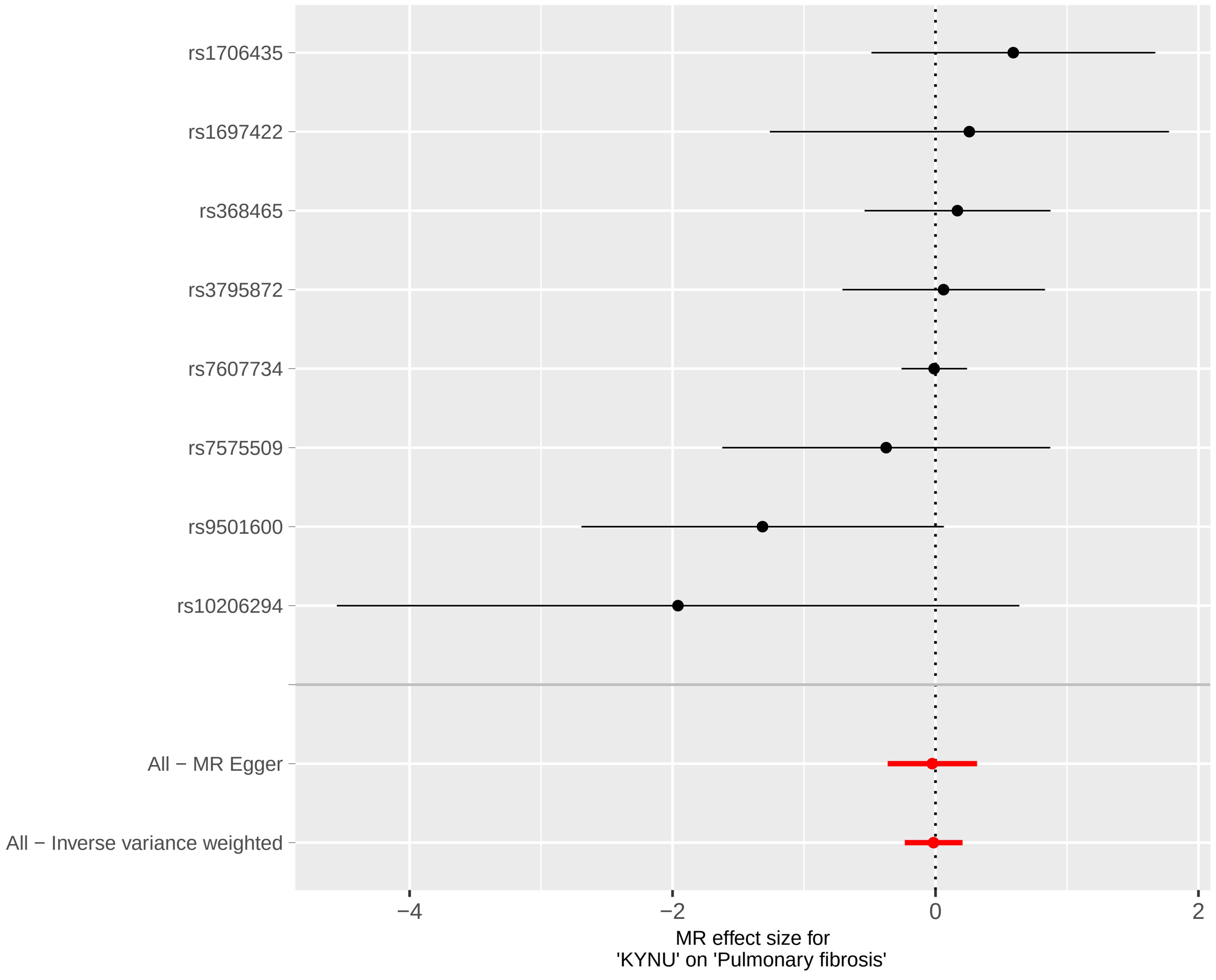
rs1706435
rs1697422
rs368465
rs3795872
rs7607734
rs7575509
rs9501600
rs10206294
All − MR Egger
All − Inverse variance weighted
−4
−2
0
2
MR effect size for
'KYNU' on 'Pulmonary fibrosis'

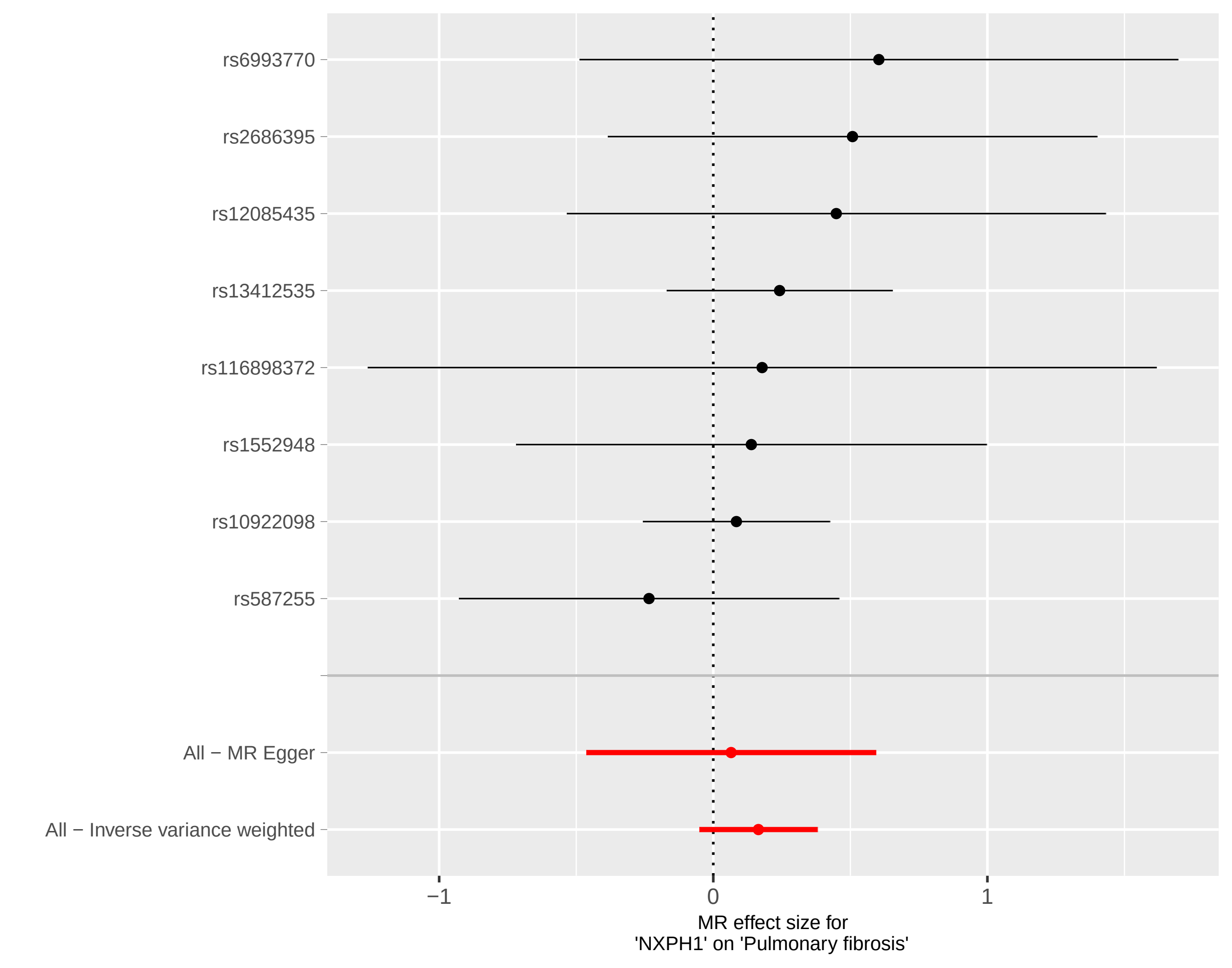
rs6993770
rs2686395
rs12085435
rs13412535
rs116898372
rs1552948
rs10922098
rs587255
All − MR Egger
All − Inverse variance weighted
−1
0
1
MR effect size for
'NXPH1' on 'Pulmonary fibrosis'

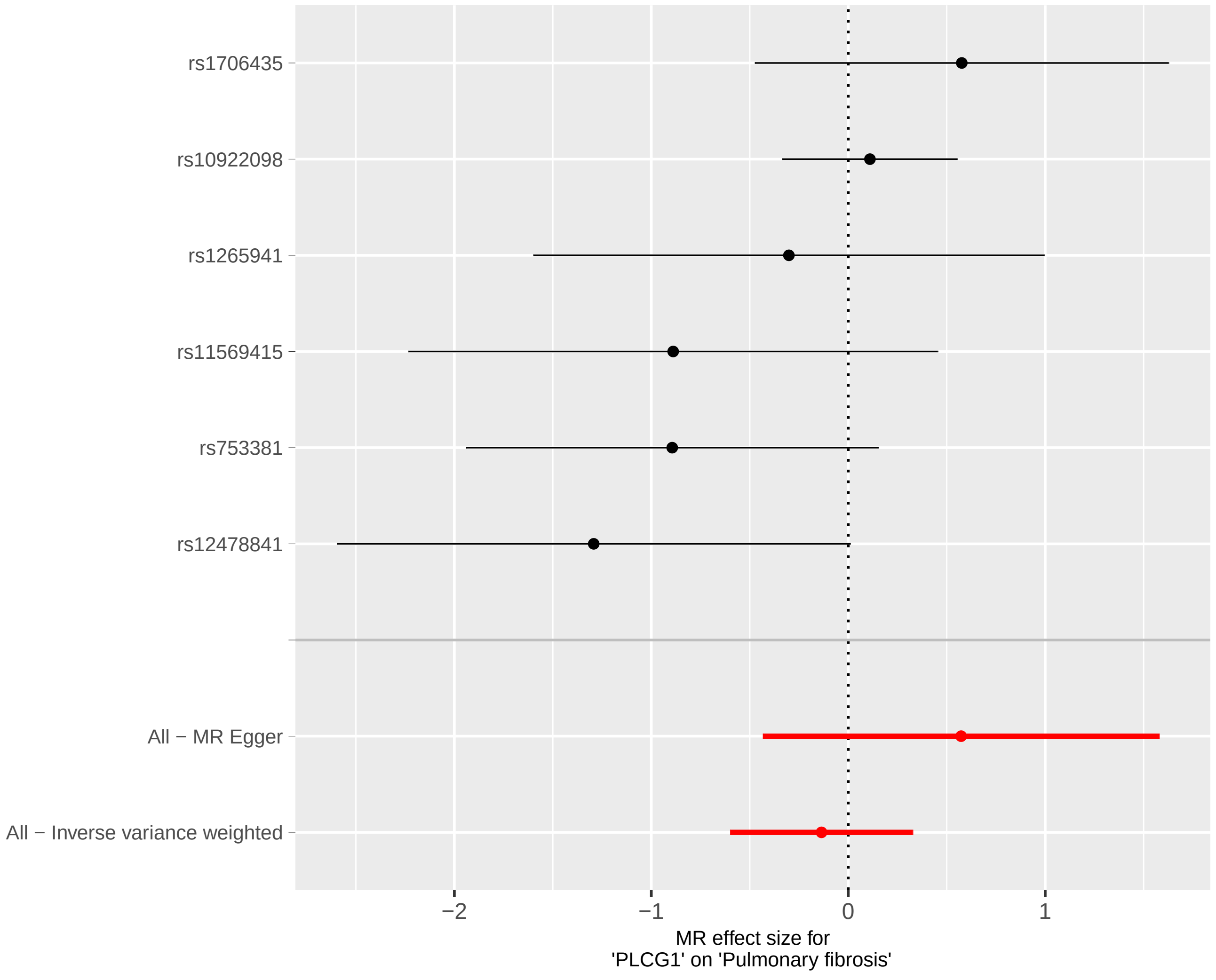
rs1706435
rs10922098
rs1265941
rs11569415
rs753381
rs12478841
All − MR Egger
All − Inverse variance weighted
−2
−1
0
1
MR effect size for
'PLCG1' on 'Pulmonary fibrosis'

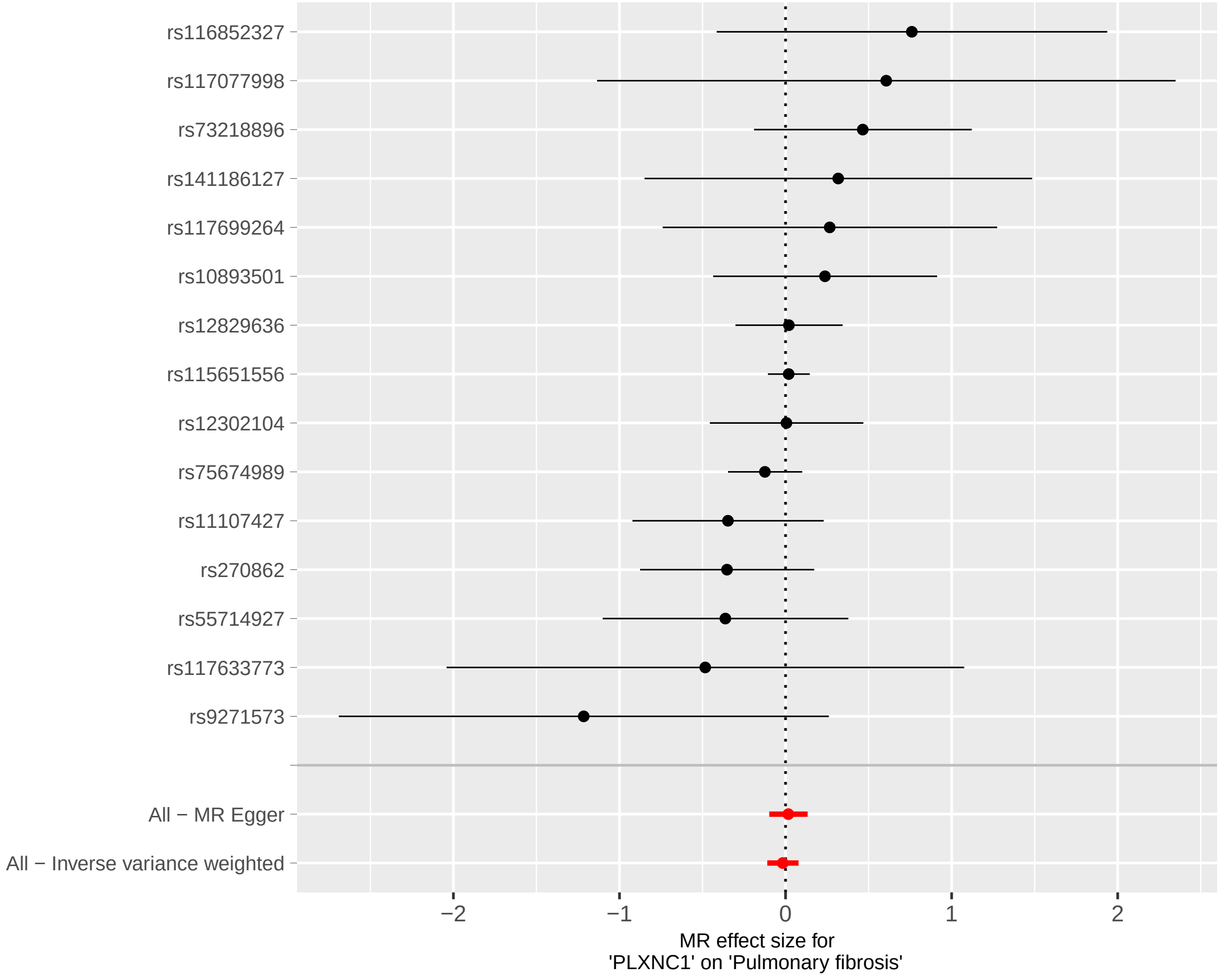

rs116852327
rs117077998
rs73218896
rs141186127
rs117699264
rs10893501
rs12829636
rs115651556
rs12302104
rs75674989
rs11107427
rs270862
rs55714927
rs117633773
rs9271573
All − MR Egger
All − Inverse variance weighted
−2
−1
0
1
2
MR effect size for
'PLXNC1' on 'Pulmonary fibrosis'

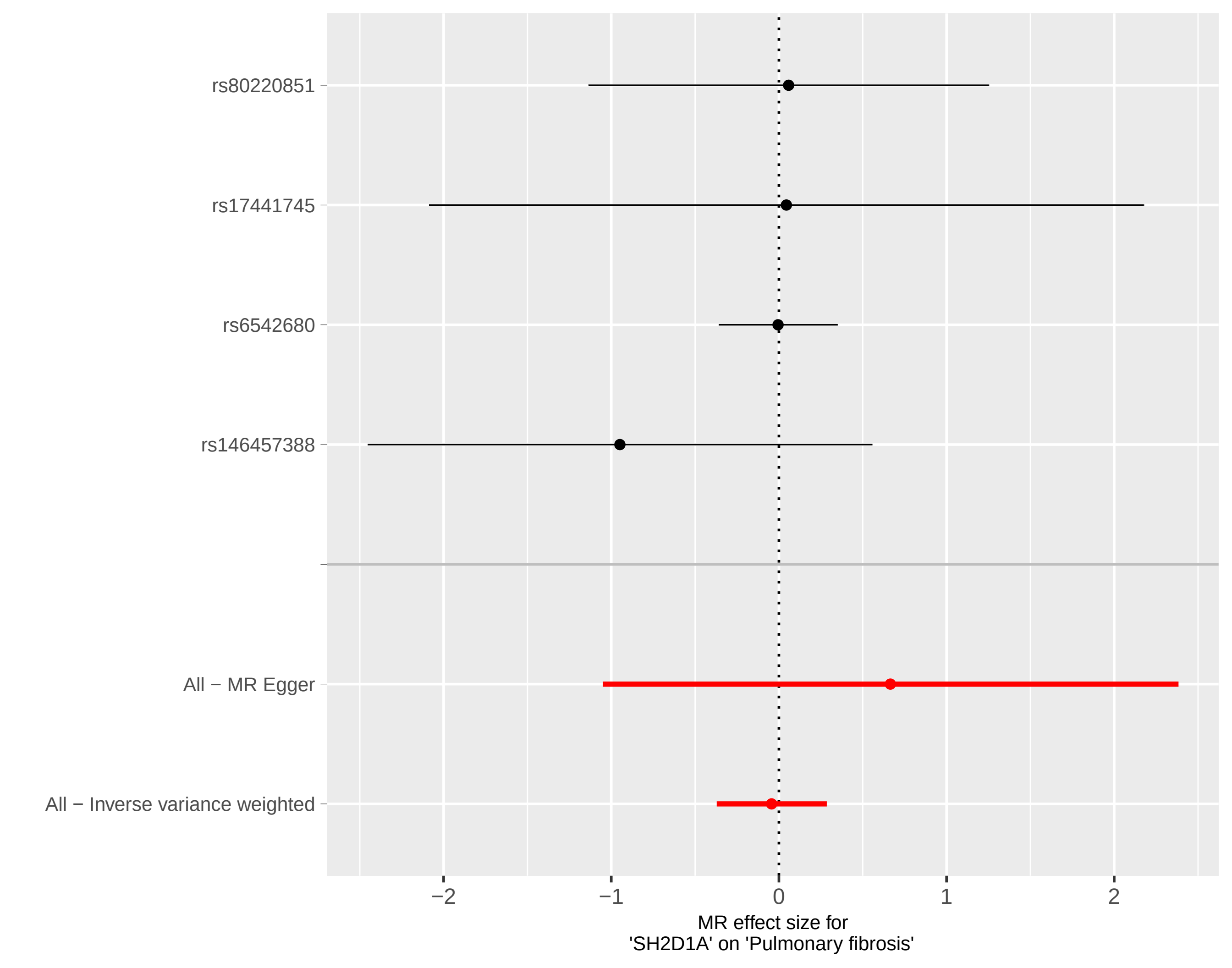
rs80220851
rs17441745
rs6542680
rs146457388
All − MR Egger
All − Inverse variance weighted
−2
−1
0
1
2
MR effect size for
'SH2D1A' on 'Pulmonary fibrosis'

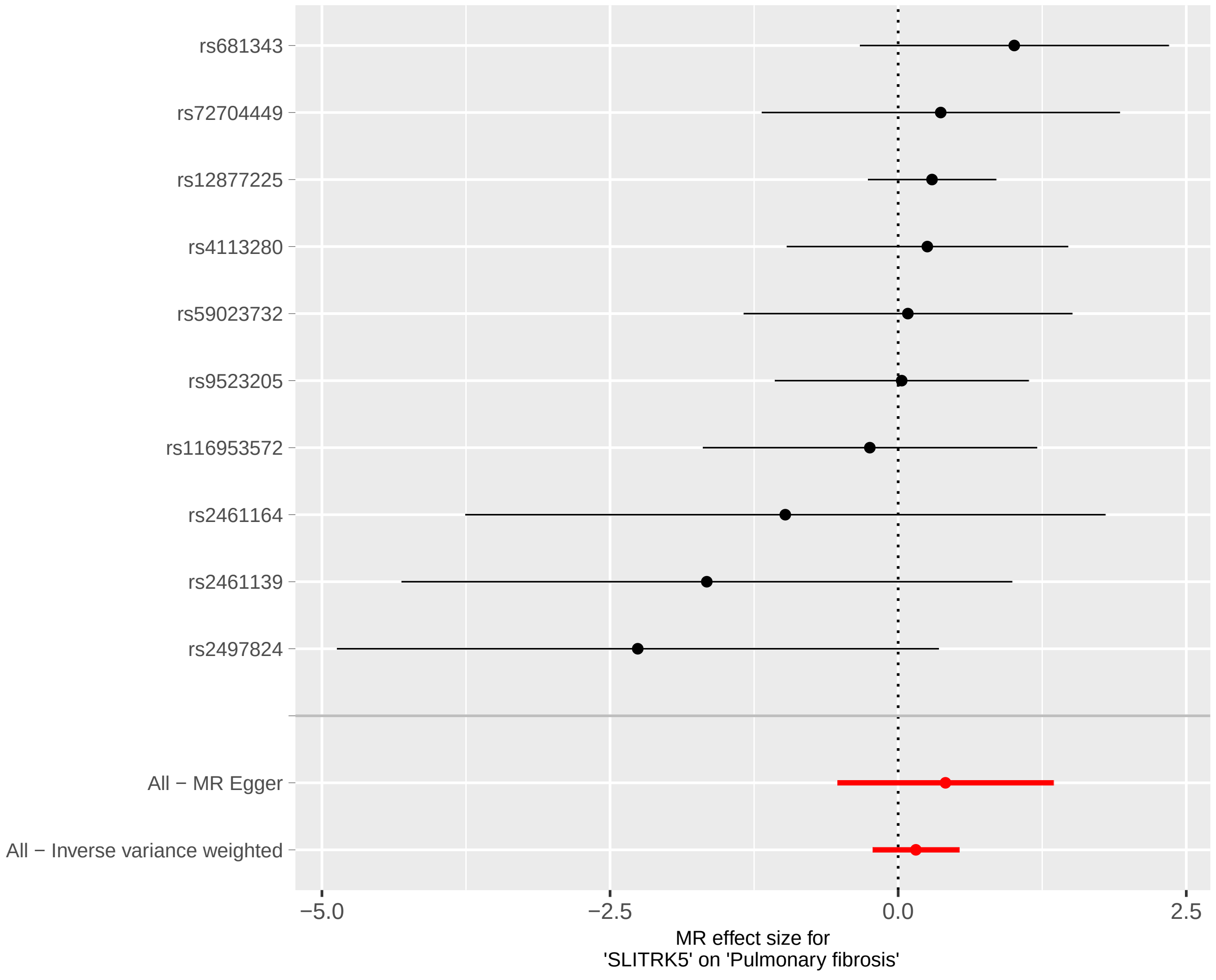

rs681343
rs72704449
rs12877225
rs4113280
rs59023732
rs9523205
rs116953572
rs2461164
rs2461139
rs2497824
All − MR Egger
All − Inverse variance weighted
−5.0
−2.5
0.0
2.5
MR effect size for
'SLITRK5' on 'Pulmonary fibrosis'

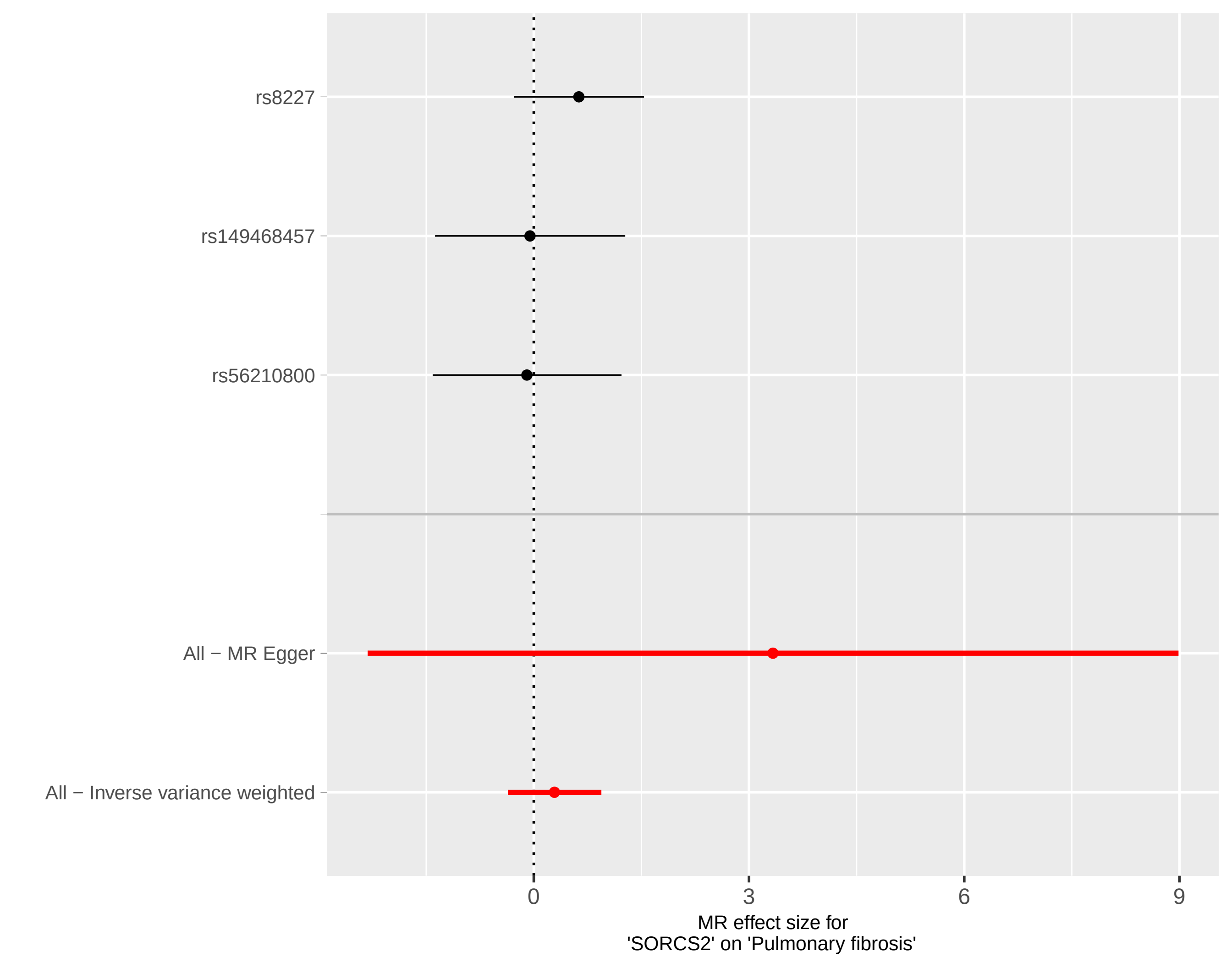

rs8227
rs149468457
rs56210800
All − MR Egger
All − Inverse variance weighted
0
3
6
9
MR effect size for
'SORCS2' on 'Pulmonary fibrosis'

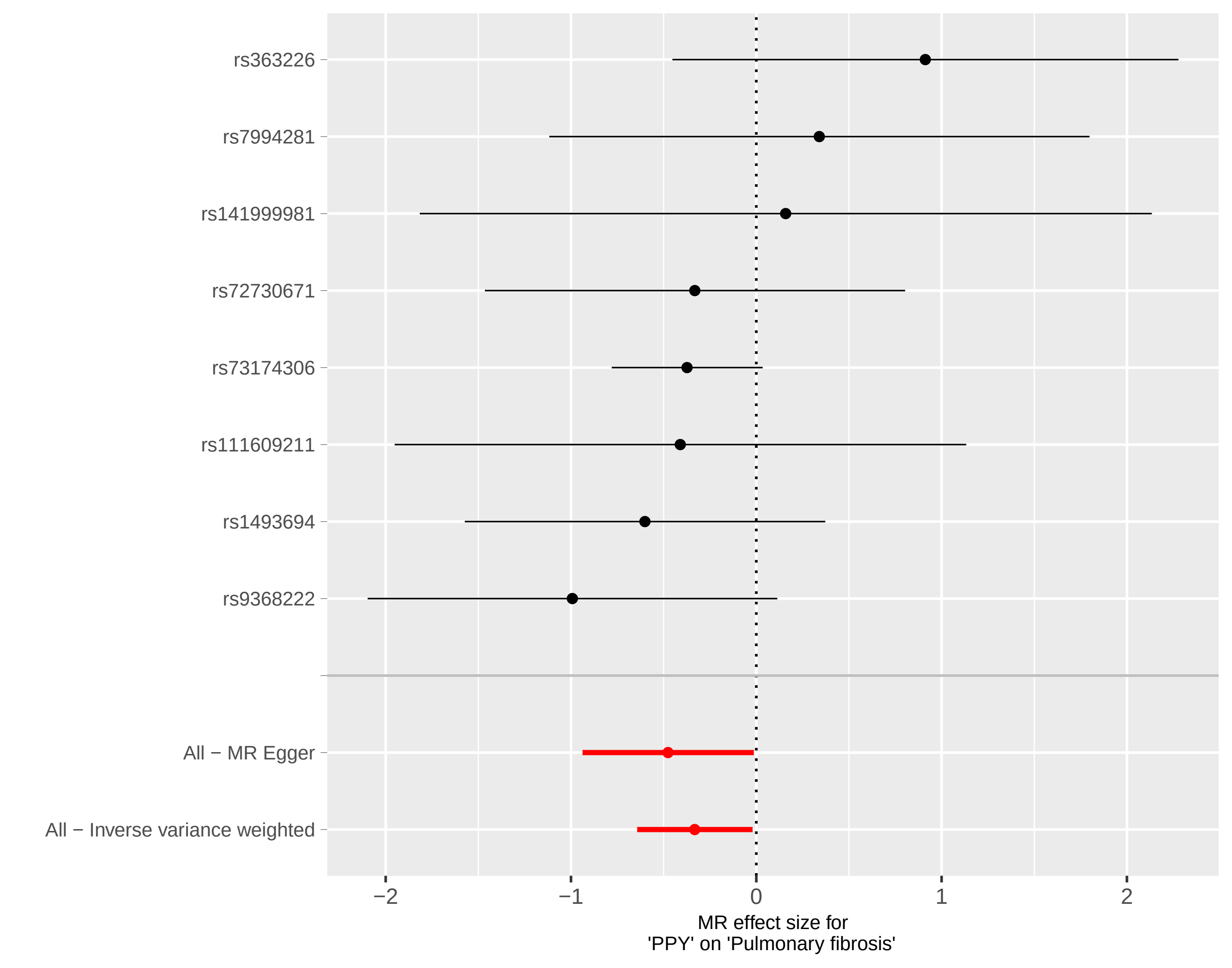

rs363226
rs7994281
rs141999981
rs72730671
rs73174306
rs111609211
rs1493694
rs9368222
All − MR Egger
All − Inverse variance weighted
−2
−1
0
1
2
MR effect size for
'PPY' on 'Pulmonary fibrosis'

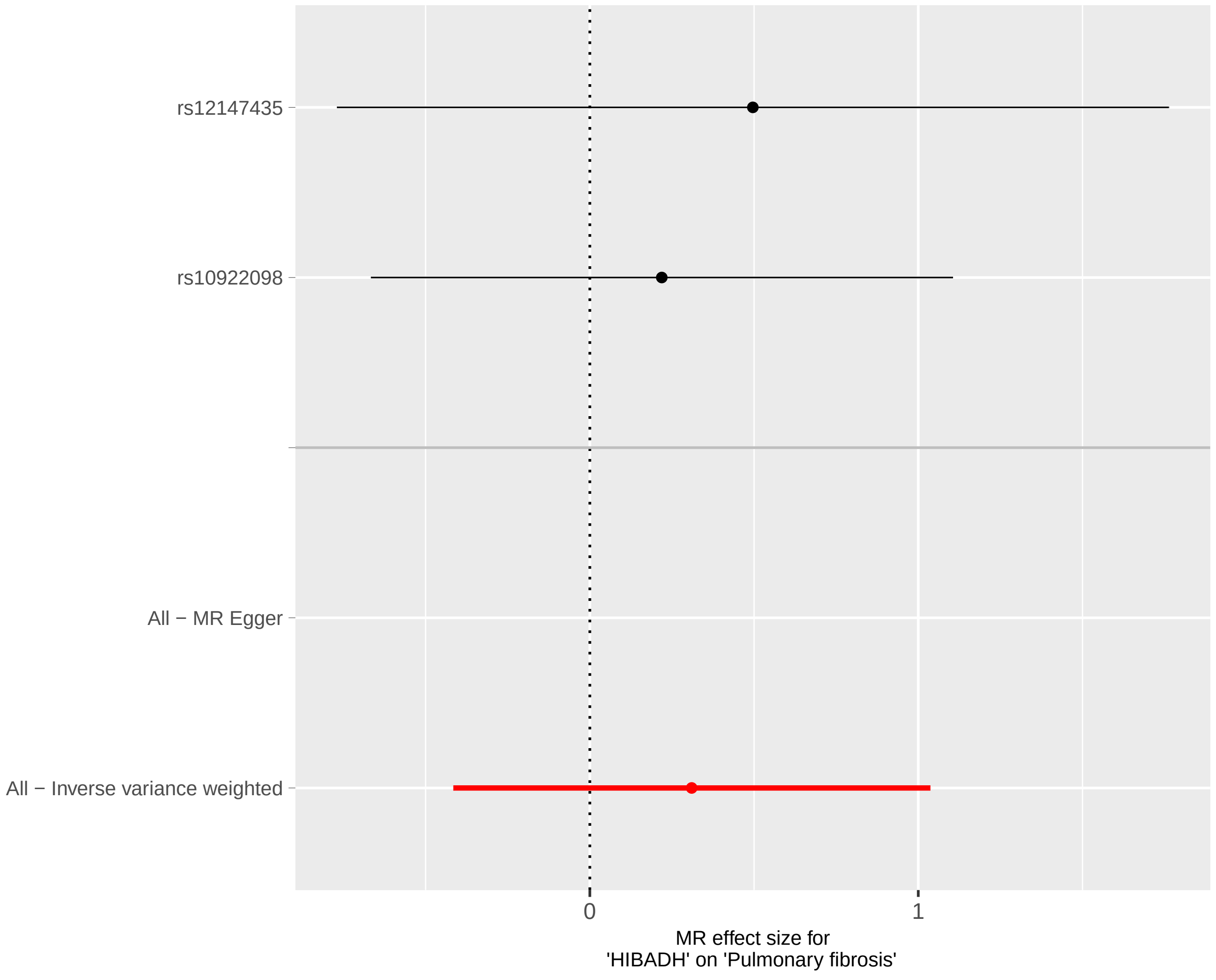
rs12147435
rs10922098
All − MR Egger
All − Inverse variance weighted
0
1
MR effect size for
'HIBADH' on 'Pulmonary fibrosis'

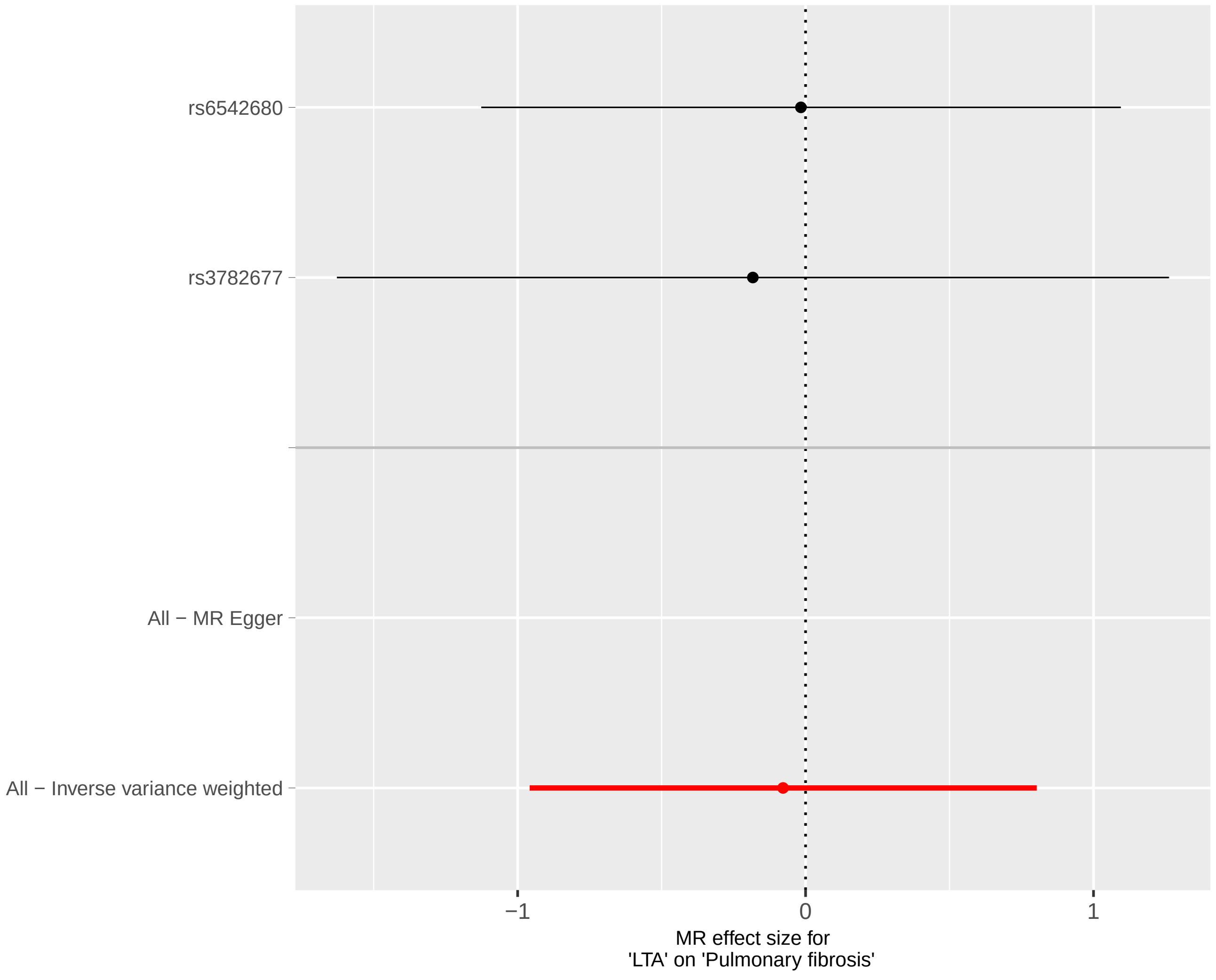
rs6542680
rs3782677
All − MR Egger
All − Inverse variance weighted
−1
0
1
MR effect size for
'LTA' on 'Pulmonary fibrosis'

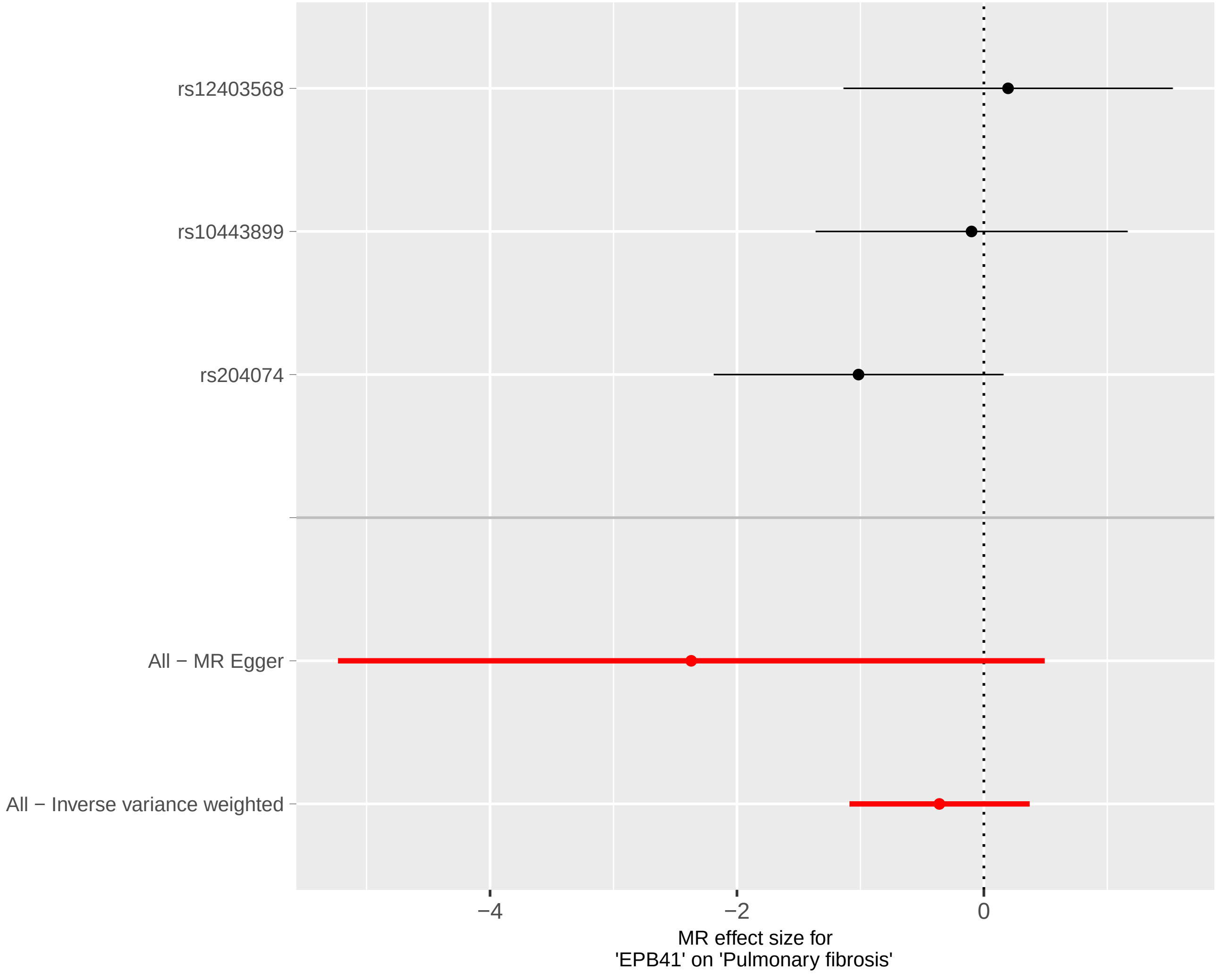

rs12403568
rs10443899
rs204074
All − MR Egger
All − Inverse variance weighted
−4
−2
0
MR effect size for
'EPB41' on 'Pulmonary fibrosis'

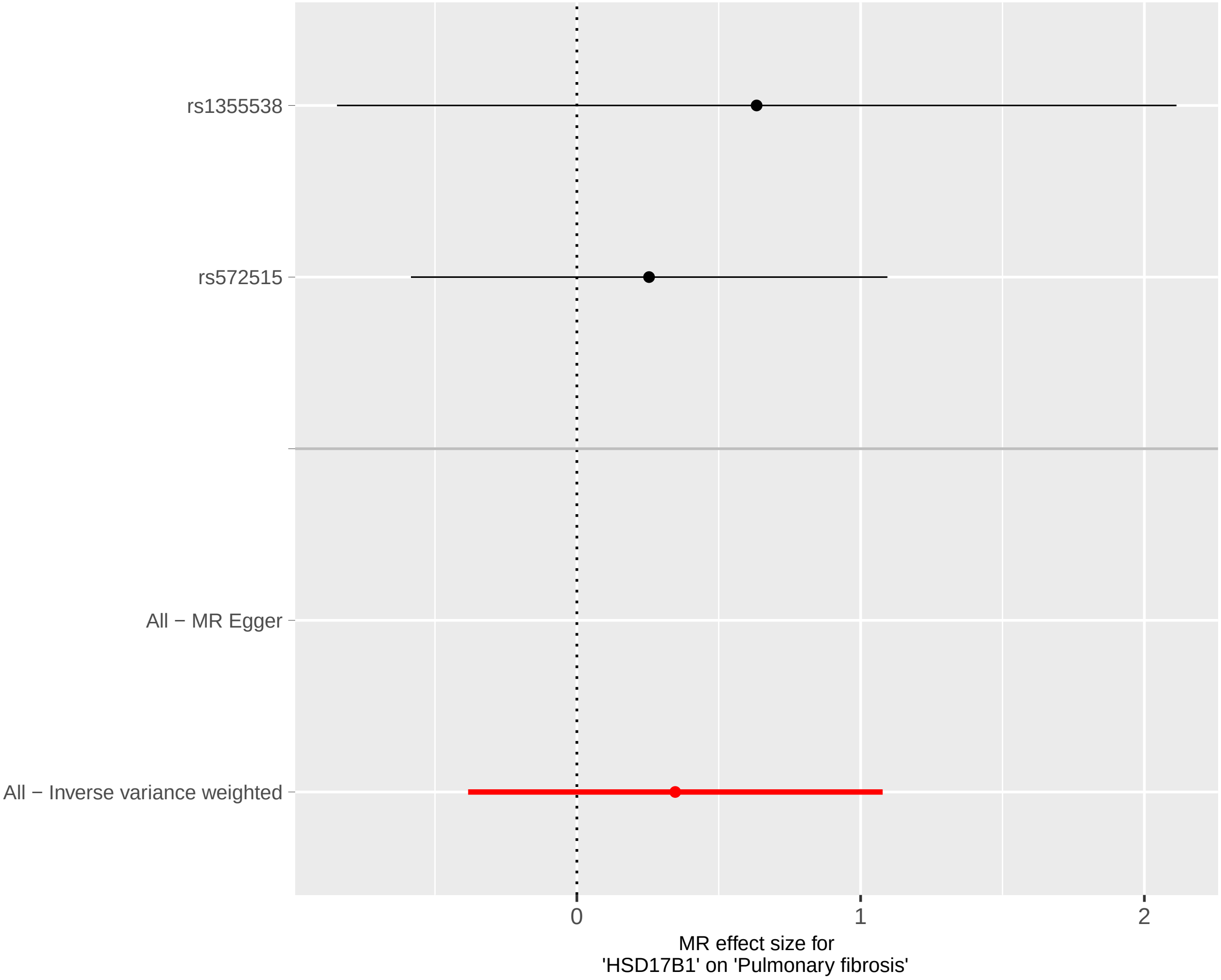
rs1355538
rs572515
All − MR Egger
All − Inverse variance weighted
0
1
2
MR effect size for
'HSD17B1' on 'Pulmonary fibrosis'

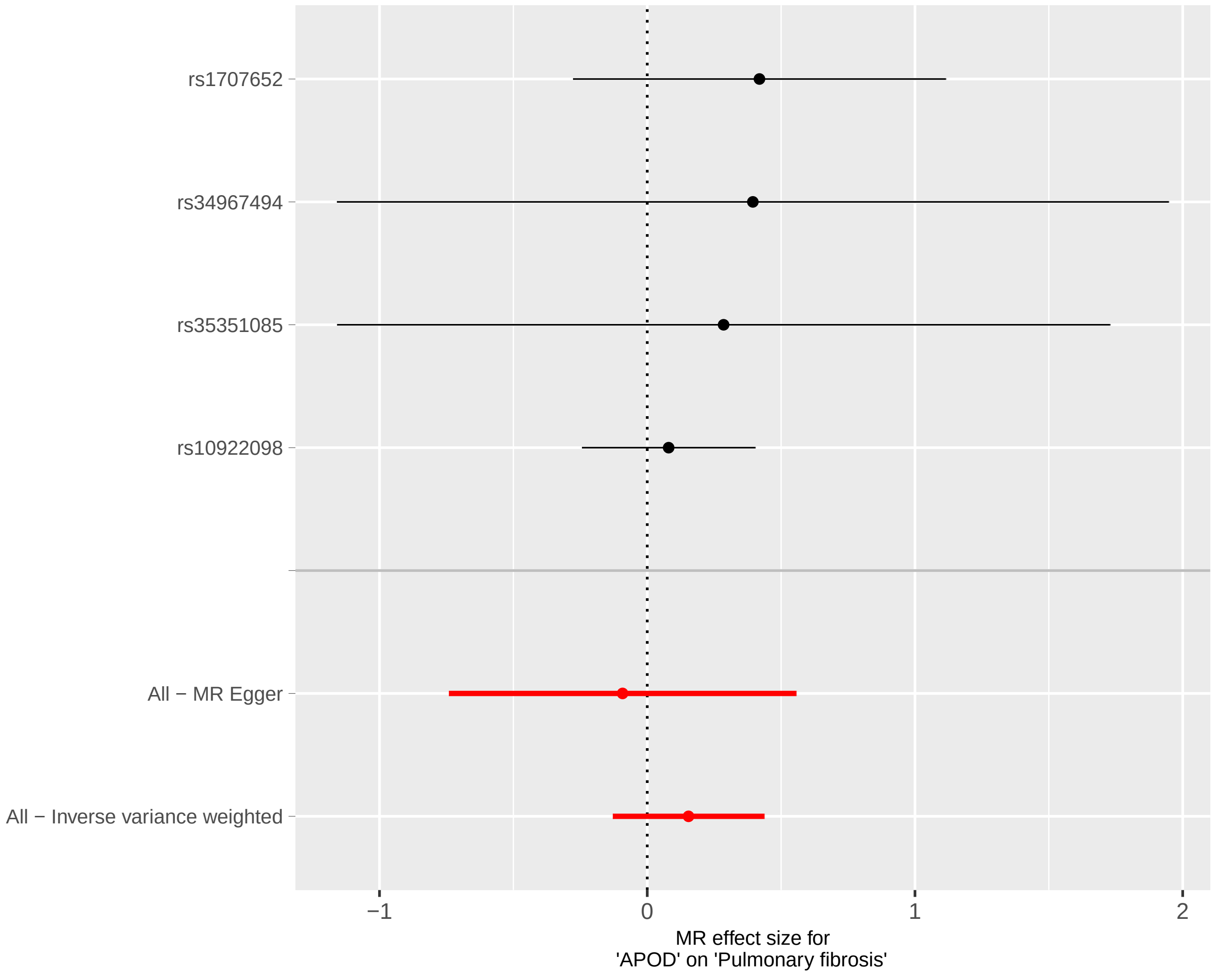

rs1707652
rs34967494
rs35351085
rs10922098
All − MR Egger
All − Inverse variance weighted
−1
0
1
2
MR effect size for
'APOD' on 'Pulmonary fibrosis'

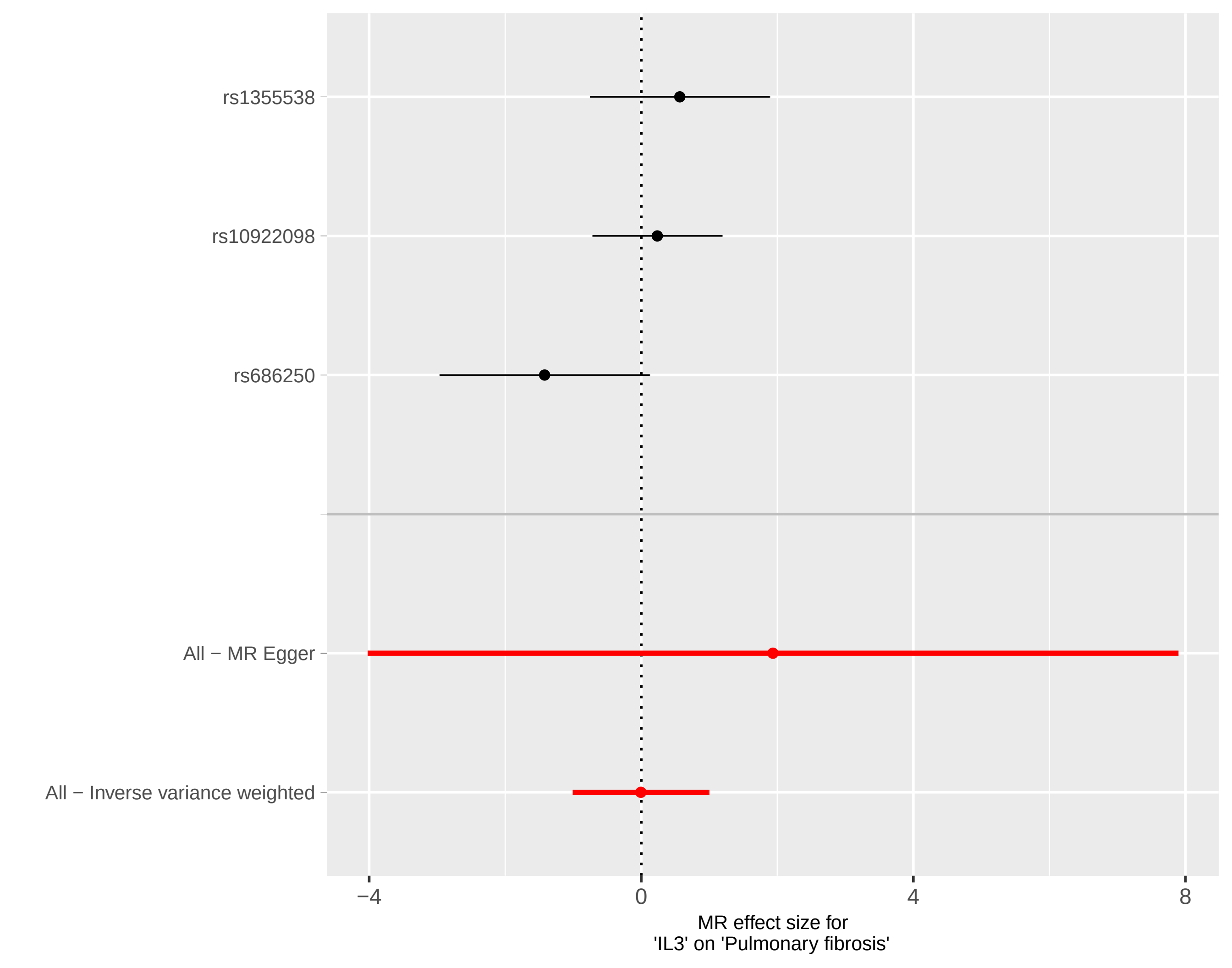
rs1355538
rs10922098
rs686250
All − MR Egger
All − Inverse variance weighted
−4
0
4
8
MR effect size for
'IL3' on 'Pulmonary fibrosis'

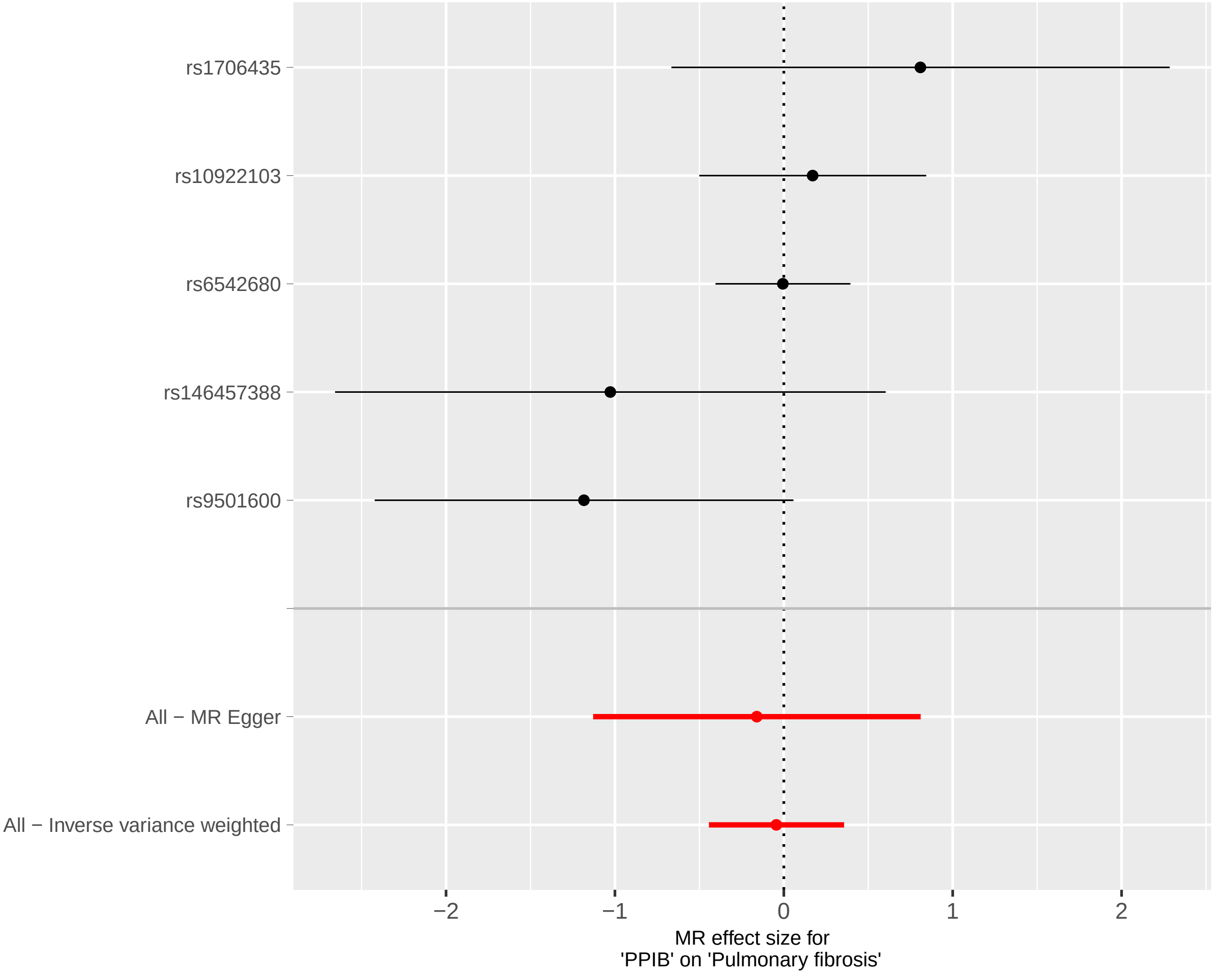

rs1706435
rs10922103
rs6542680
rs146457388
rs9501600
All − MR Egger
All − Inverse variance weighted
−2
−1
0
1
2
MR effect size for
'PPIB' on 'Pulmonary fibrosis'

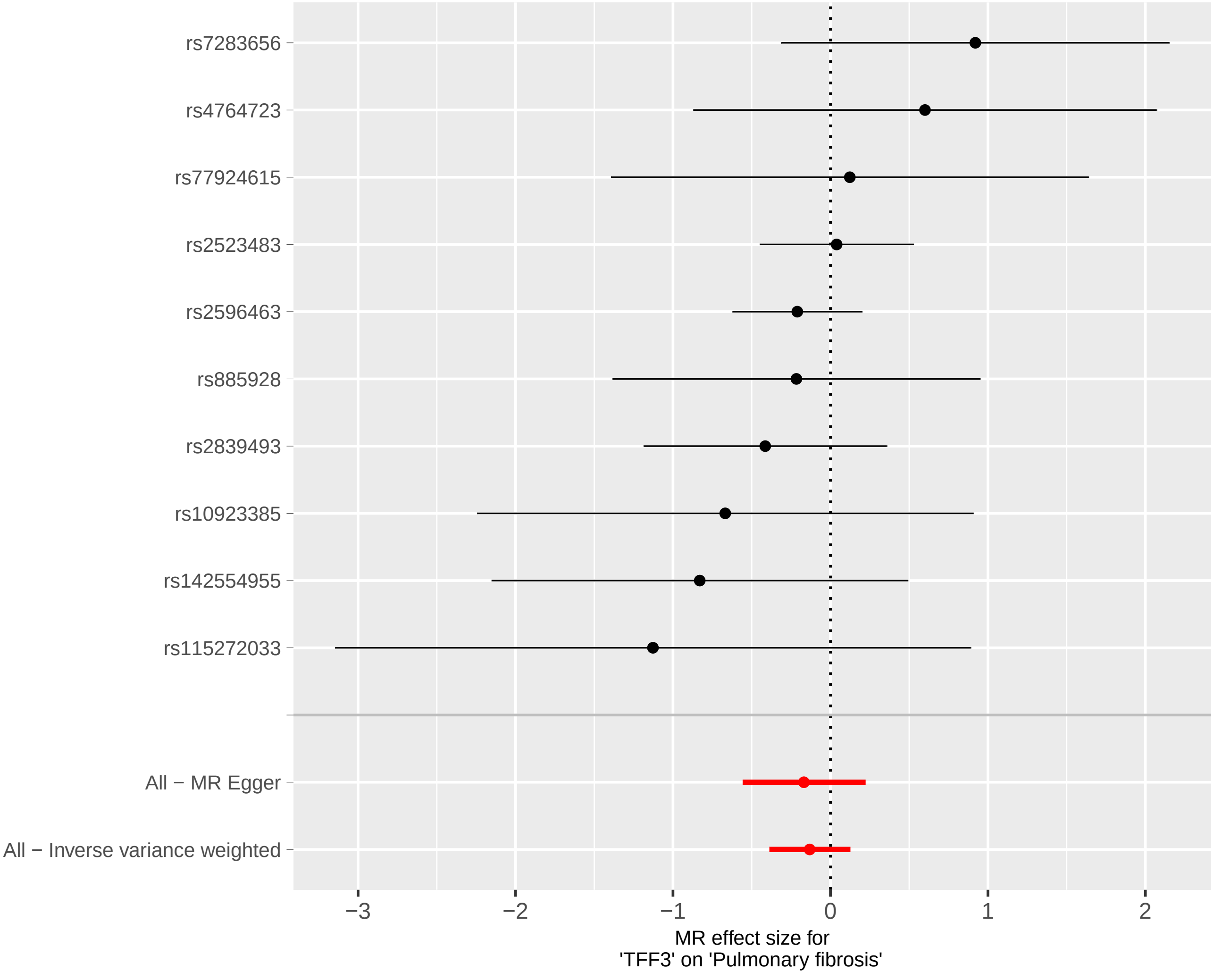

rs7283656
rs4764723
rs77924615
rs2523483
rs2596463
rs885928
rs2839493
rs10923385
rs142554955
rs115272033
All − MR Egger
All − Inverse variance weighted
−3
−2
−1
0
1
2
MR effect size for
'TFF3' on 'Pulmonary fibrosis'

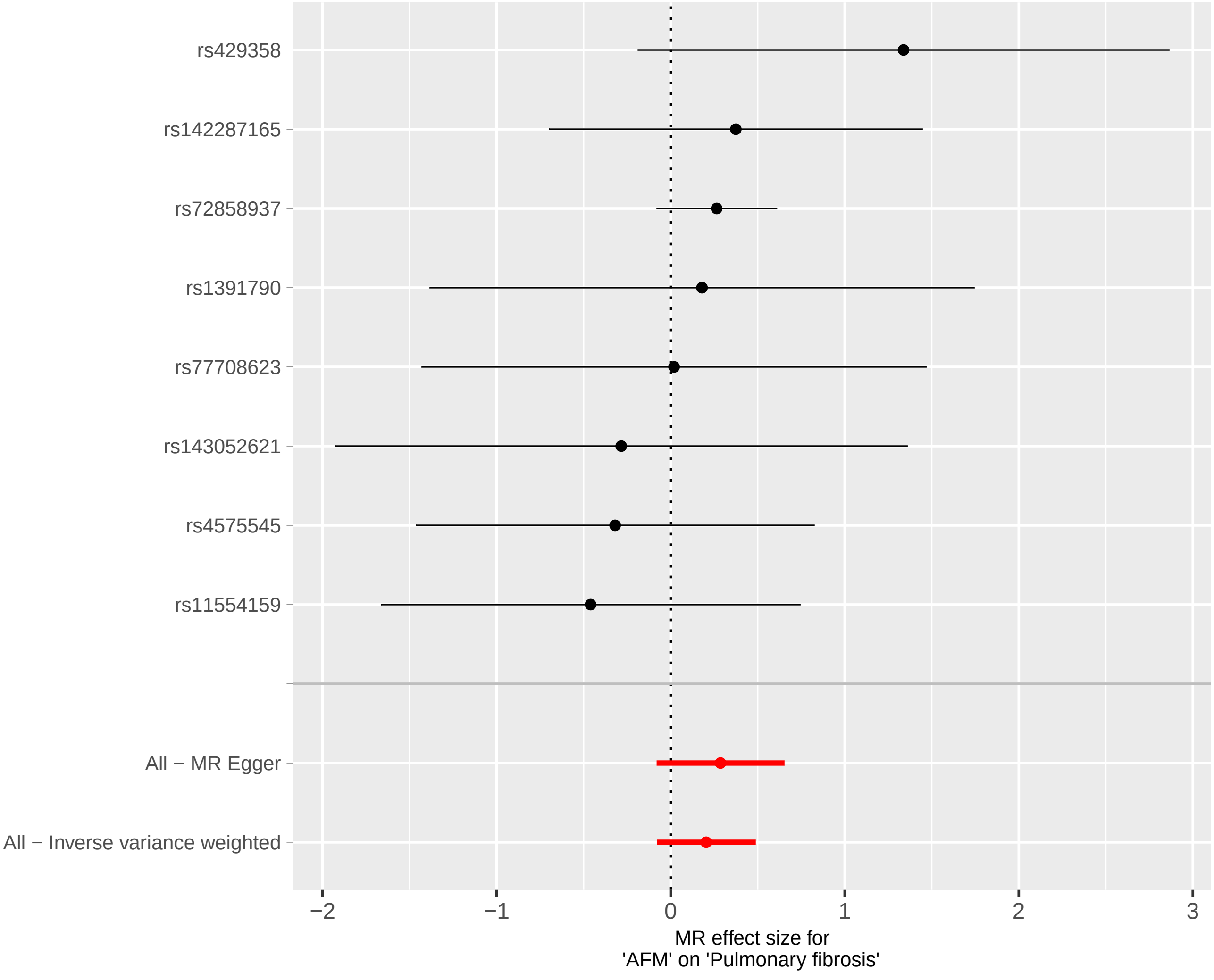

rs429358
rs142287165
rs72858937
rs1391790
rs77708623
rs143052621
rs4575545
rs11554159
All − MR Egger
All − Inverse variance weighted
−2
−1
0
1
2
3
MR effect size for
'AFM' on 'Pulmonary fibrosis'

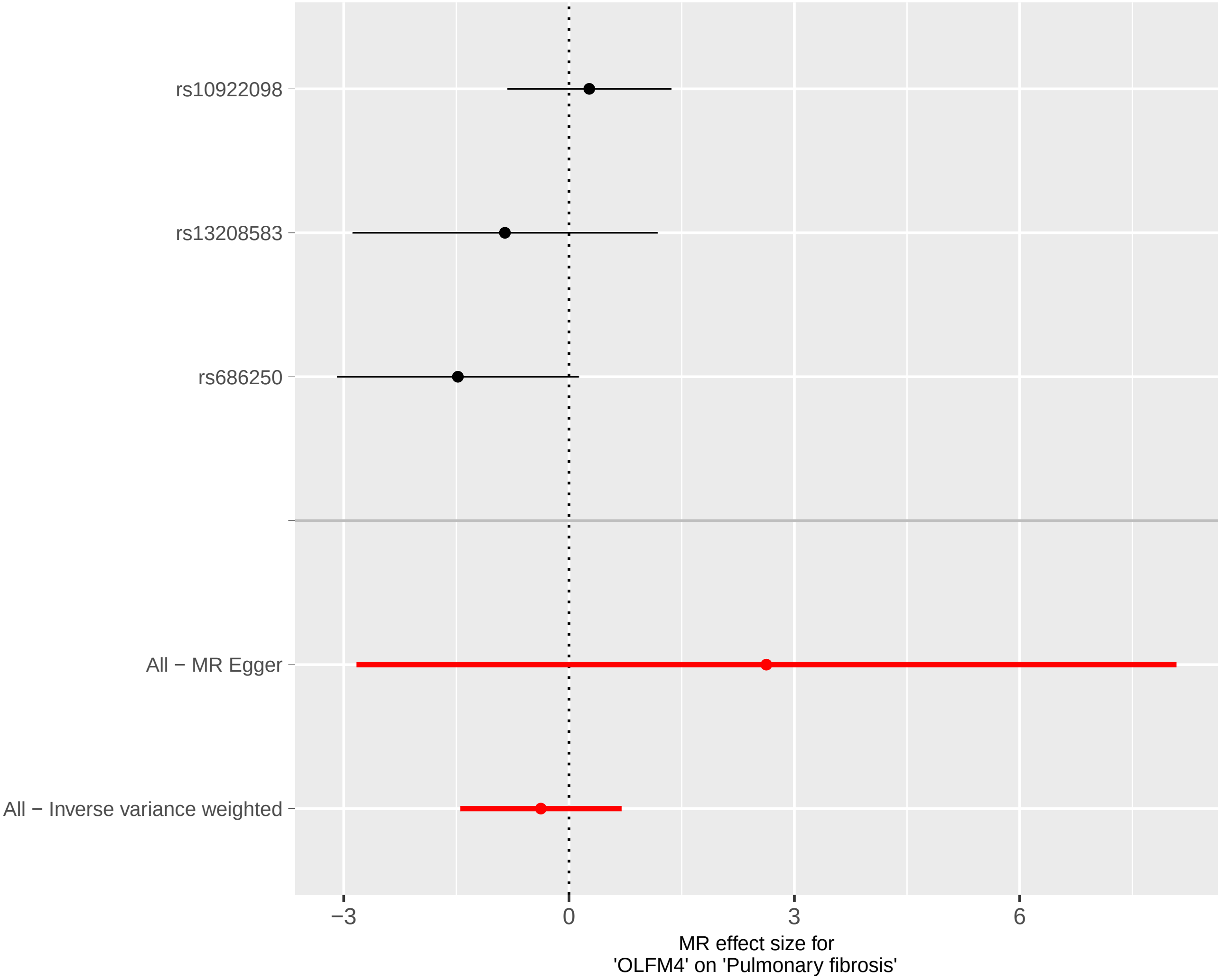
rs10922098
rs13208583
rs686250
All − MR Egger
All − Inverse variance weighted
−3
0
3
6
MR effect size for
'OLFM4' on 'Pulmonary fibrosis'

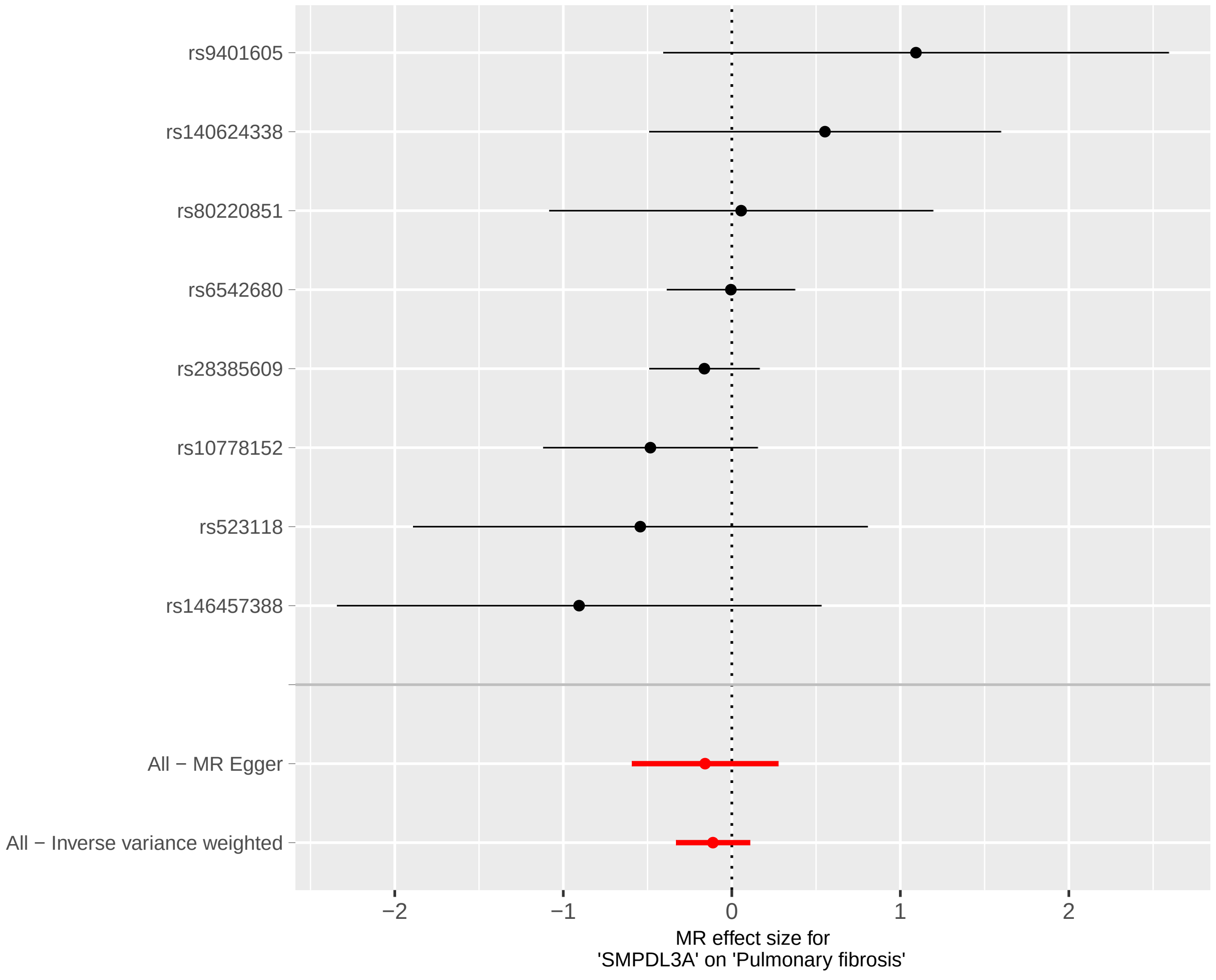

rs9401605
rs140624338
rs80220851
rs6542680
rs28385609
rs10778152
rs523118
rs146457388
All − MR Egger
All − Inverse variance weighted
−2
−1
0
1
2
MR effect size for
'SMPDL3A' on 'Pulmonary fibrosis'

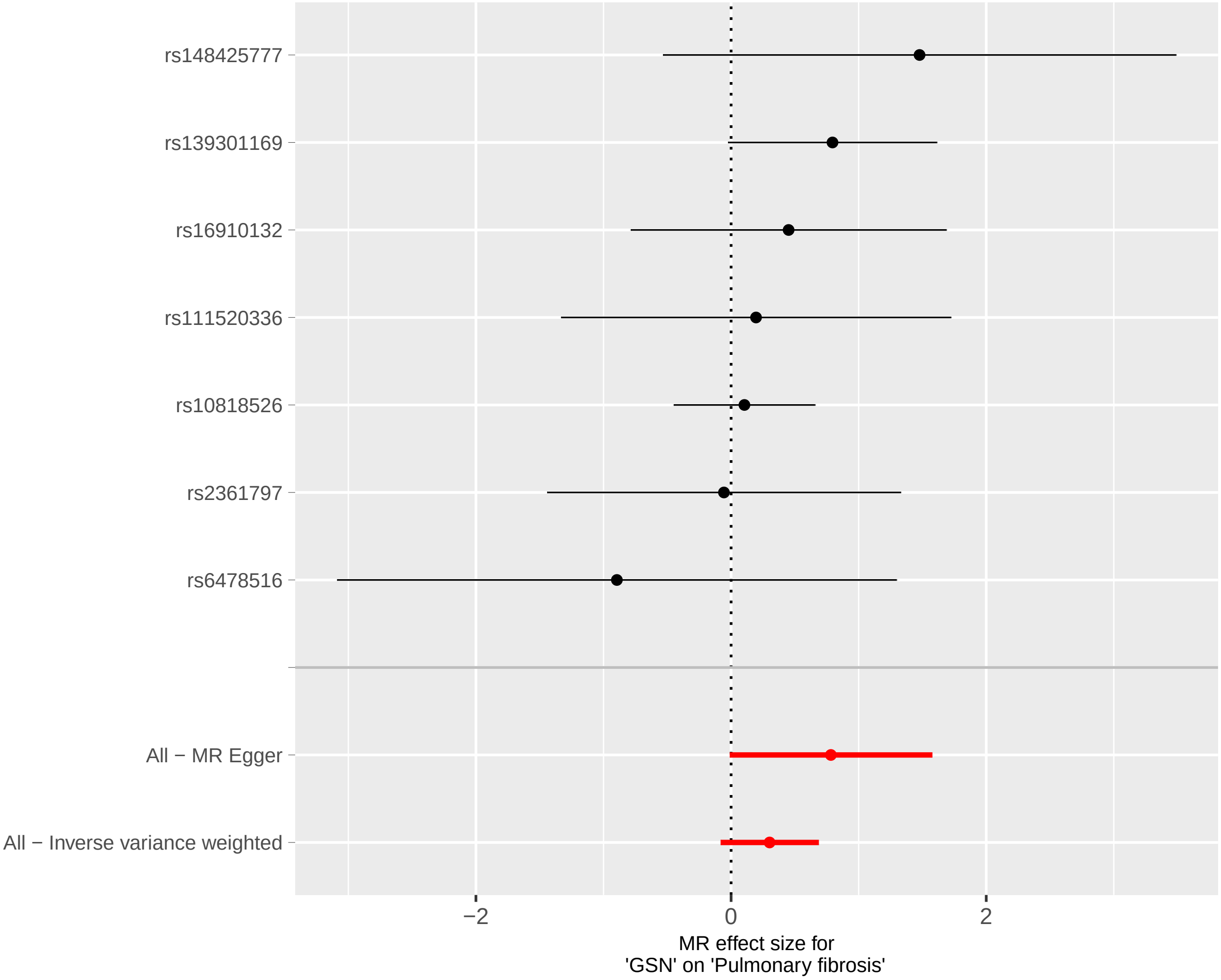
rs148425777
rs139301169
rs16910132
rs111520336
rs10818526
rs2361797
rs6478516
All − MR Egger
All − Inverse variance weighted
−2
0
2
MR effect size for
'GSN' on 'Pulmonary fibrosis'

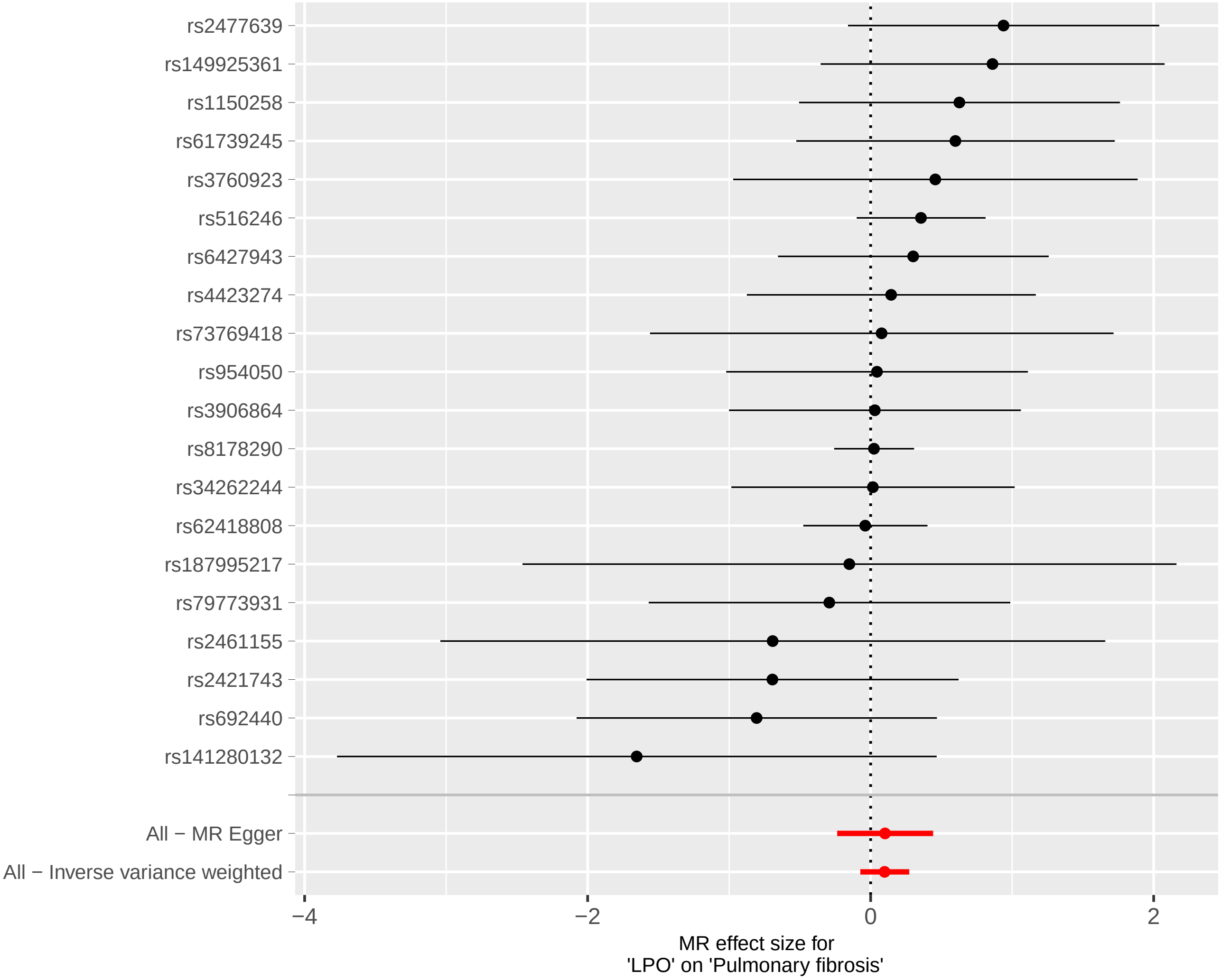
rs2477639
rs149925361
rs1150258
rs61739245
rs3760923
rs516246
rs6427943
rs4423274
rs73769418
rs954050
rs3906864
rs8178290
rs34262244
rs62418808
rs187995217
rs79773931
rs2461155
rs2421743
rs692440
rs141280132
All − MR Egger
All − Inverse variance weighted
−4
−2
0
2
MR effect size for
'LPO' on 'Pulmonary fibrosis'

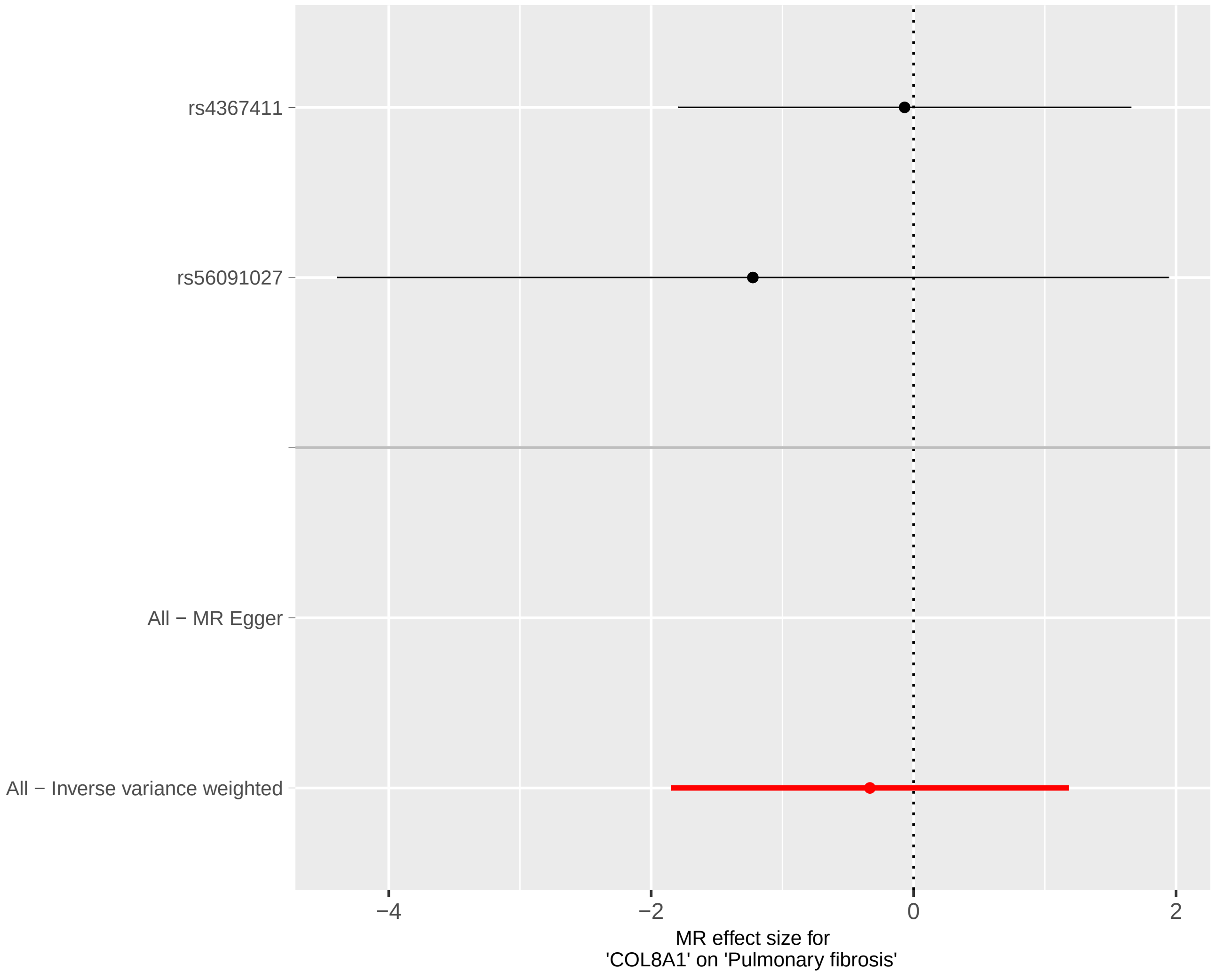
rs4367411
rs56091027
All − MR Egger
All − Inverse variance weighted
−4
−2
0
2
MR effect size for
'COL8A1' on 'Pulmonary fibrosis'

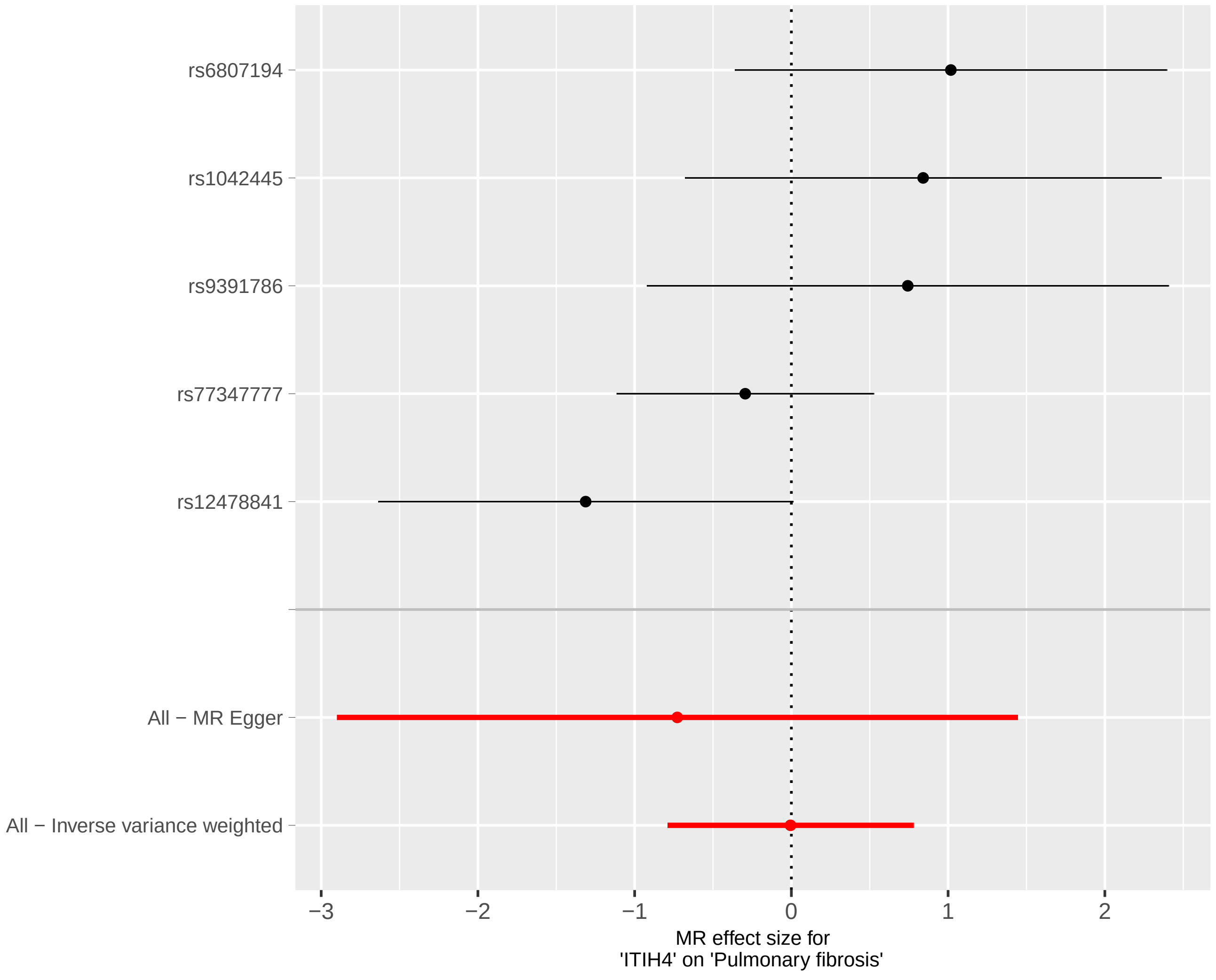

rs6807194
rs1042445
rs9391786
rs77347777
rs12478841
All − MR Egger
All − Inverse variance weighted
−3
−2
−1
0
1
2
MR effect size for
'ITIH4' on 'Pulmonary fibrosis'

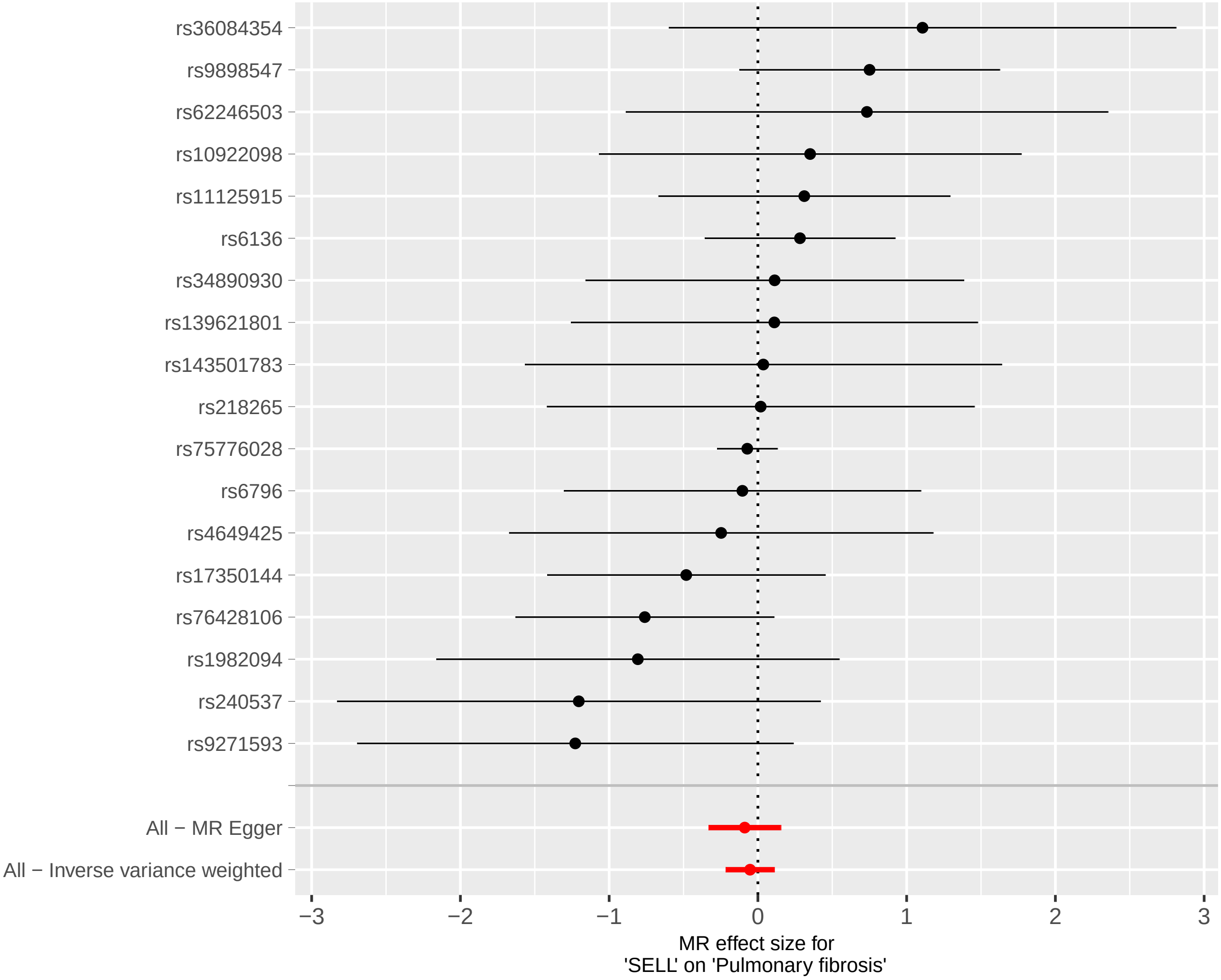
rs36084354
rs9898547
rs62246503
rs10922098
rs11125915
rs6136
rs34890930
rs139621801
rs143501783
rs218265
rs75776028
rs6796
rs4649425
rs17350144
rs76428106
rs1982094
rs240537
rs9271593
All − MR Egger
All − Inverse variance weighted
−3
−2
−1
0
1
2
3
MR effect size for
'SELL' on 'Pulmonary fibrosis'

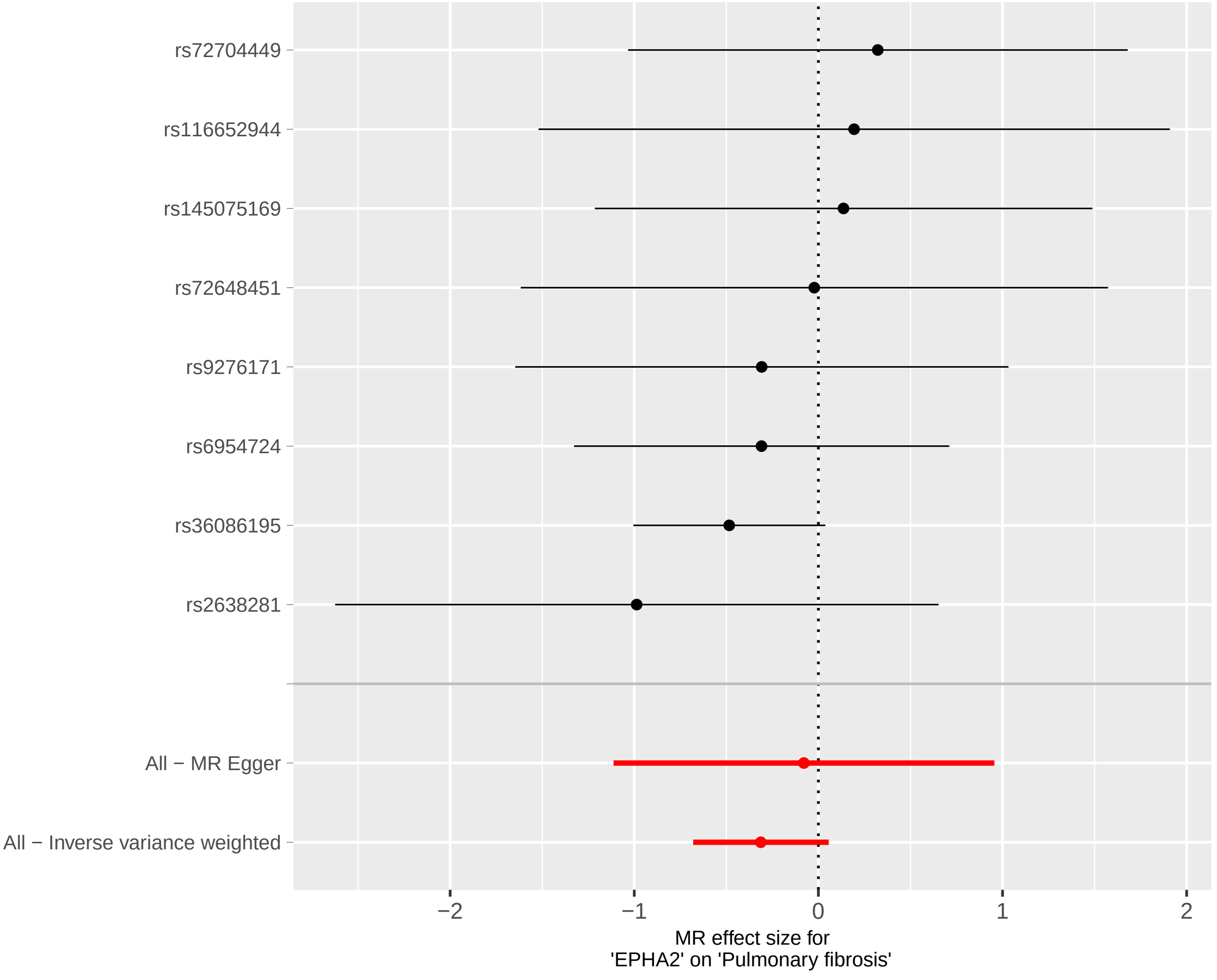
rs72704449
rs116652944
rs145075169
rs72648451
rs9276171
rs6954724
rs36086195
rs2638281
All − MR Egger
All − Inverse variance weighted
−2
−1
0
1
2
MR effect size for
'EPHA2' on 'Pulmonary fibrosis'

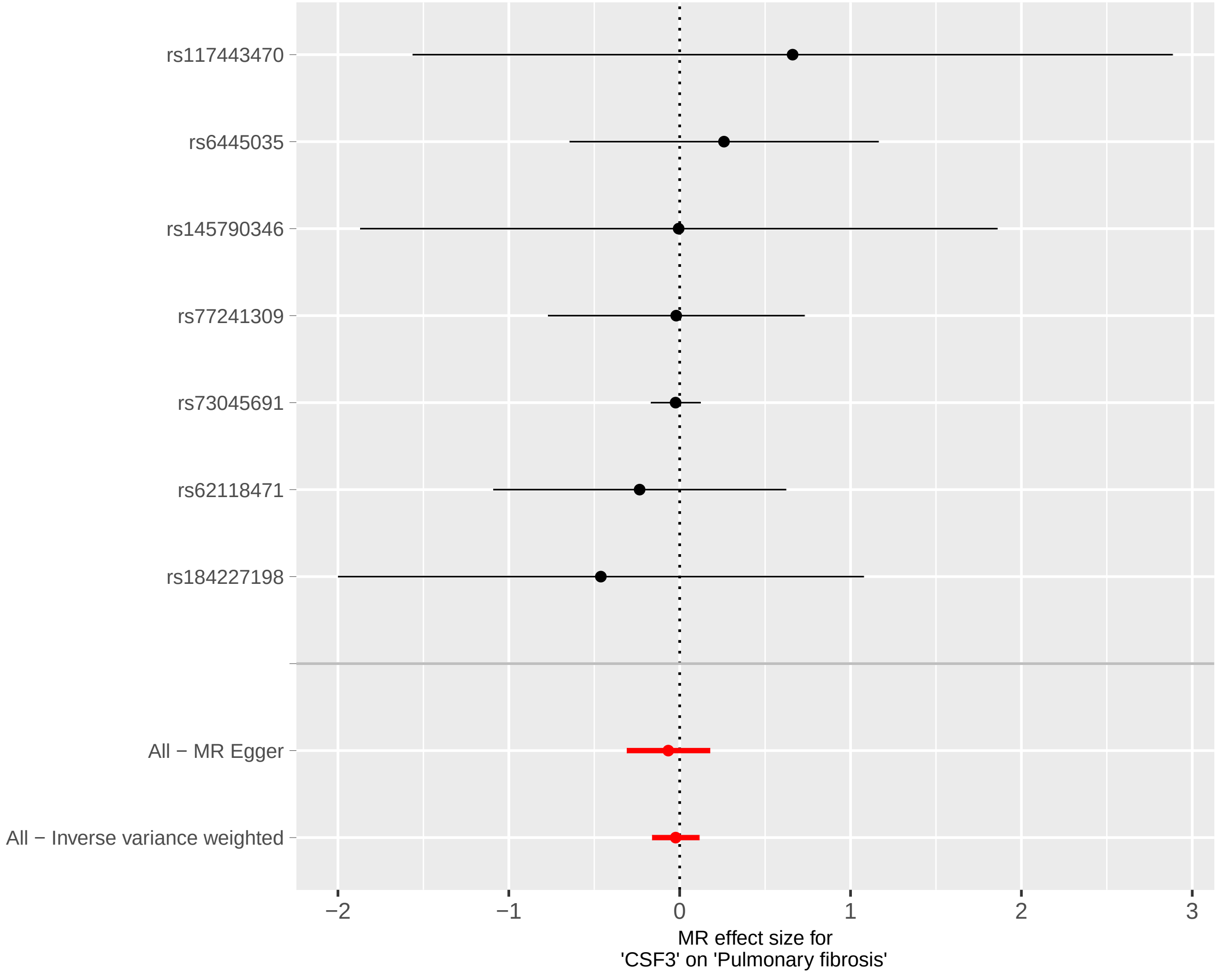

rs117443470
rs6445035
rs145790346
rs77241309
rs73045691
rs62118471
rs184227198
All − MR Egger
All − Inverse variance weighted
−2
−1
0
1
2
3
MR effect size for
'CSF3' on 'Pulmonary fibrosis'

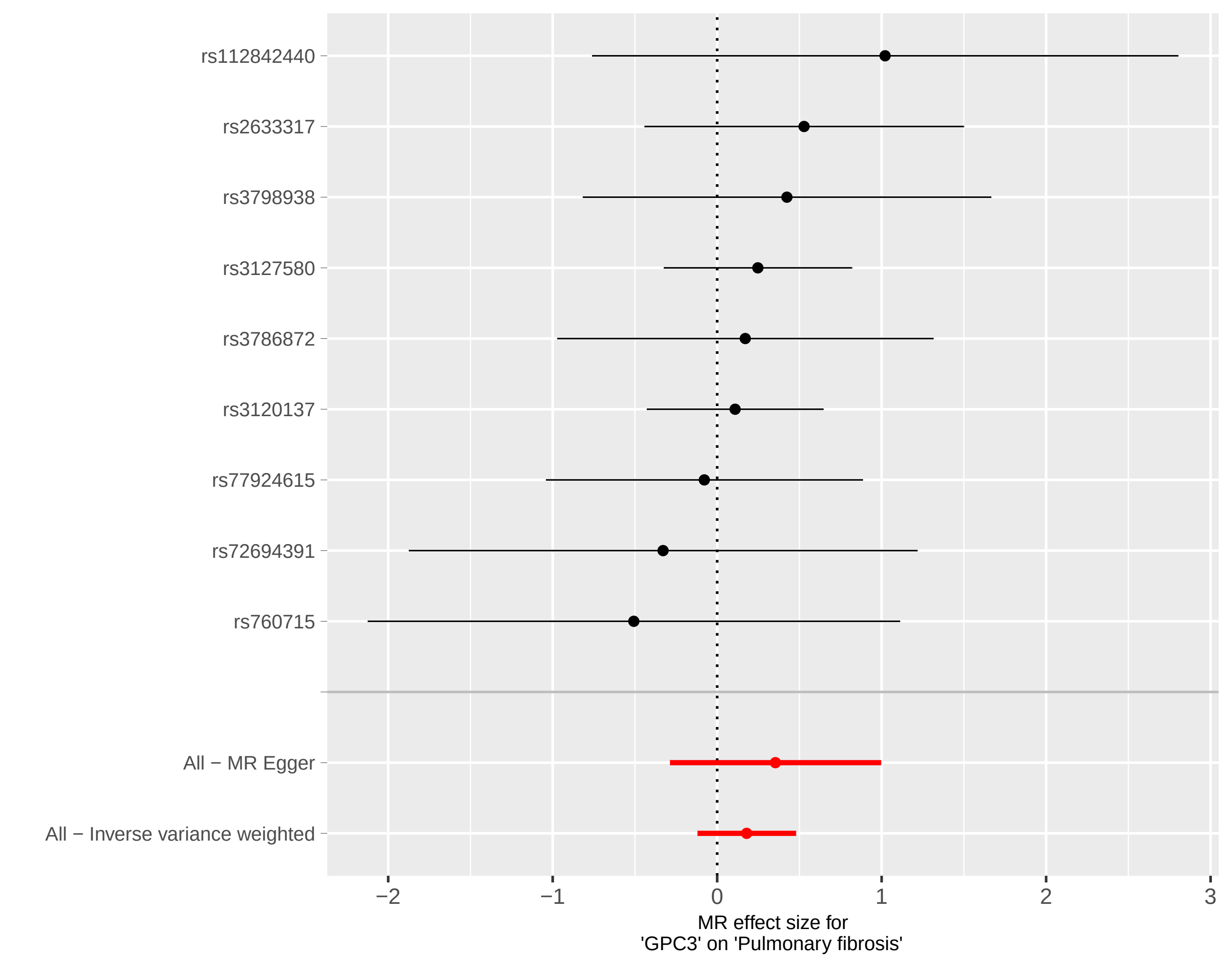

rs112842440
rs2633317
rs3798938
rs3127580
rs3786872
rs3120137
rs77924615
rs72694391
rs760715
All − MR Egger
All − Inverse variance weighted
−2
−1
0
1
2
3
MR effect size for
'GPC3' on 'Pulmonary fibrosis'

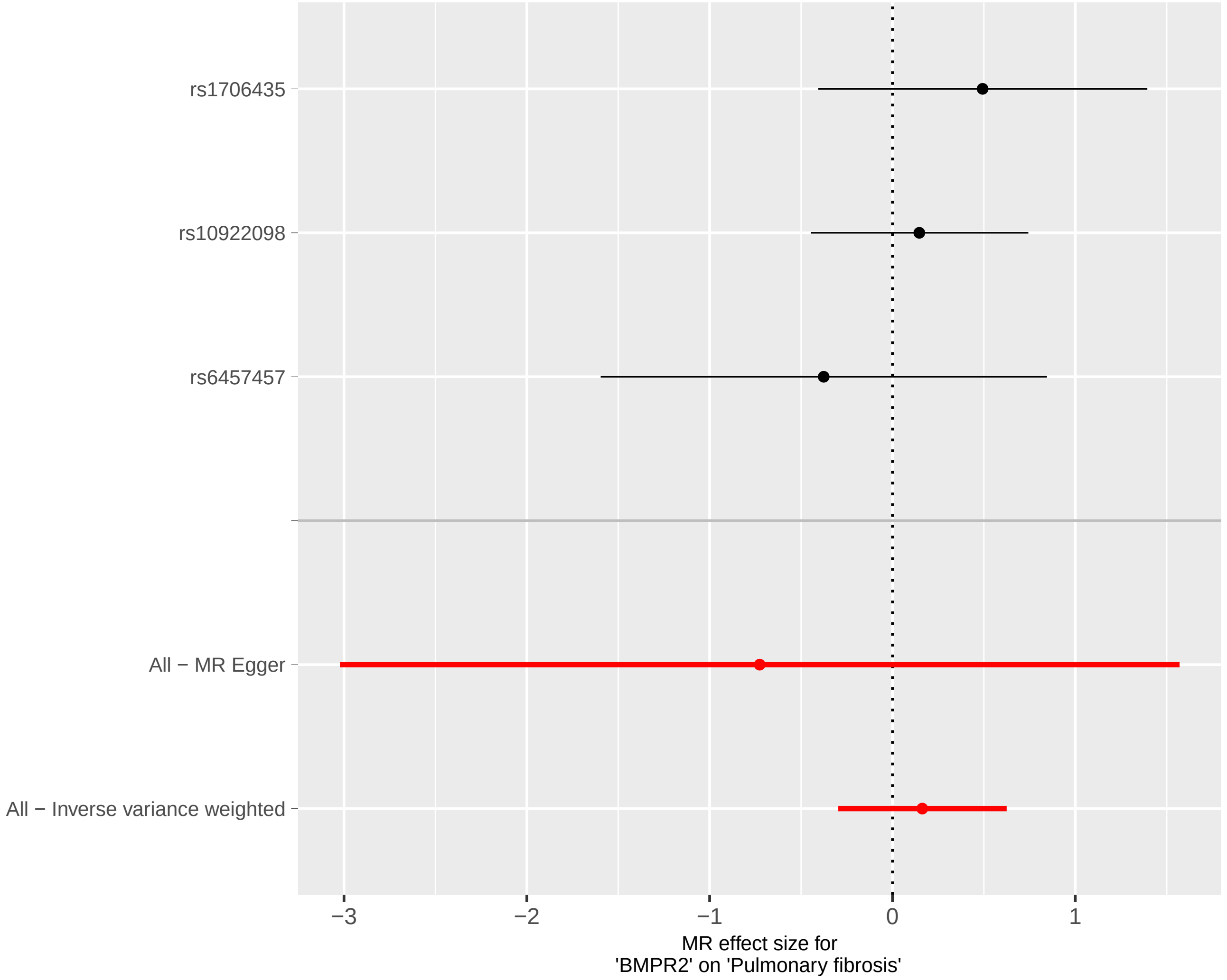

rs1706435
rs10922098
rs6457457
All − MR Egger
All − Inverse variance weighted
−3
−2
−1
0
1
MR effect size for
'BMPR2' on 'Pulmonary fibrosis'

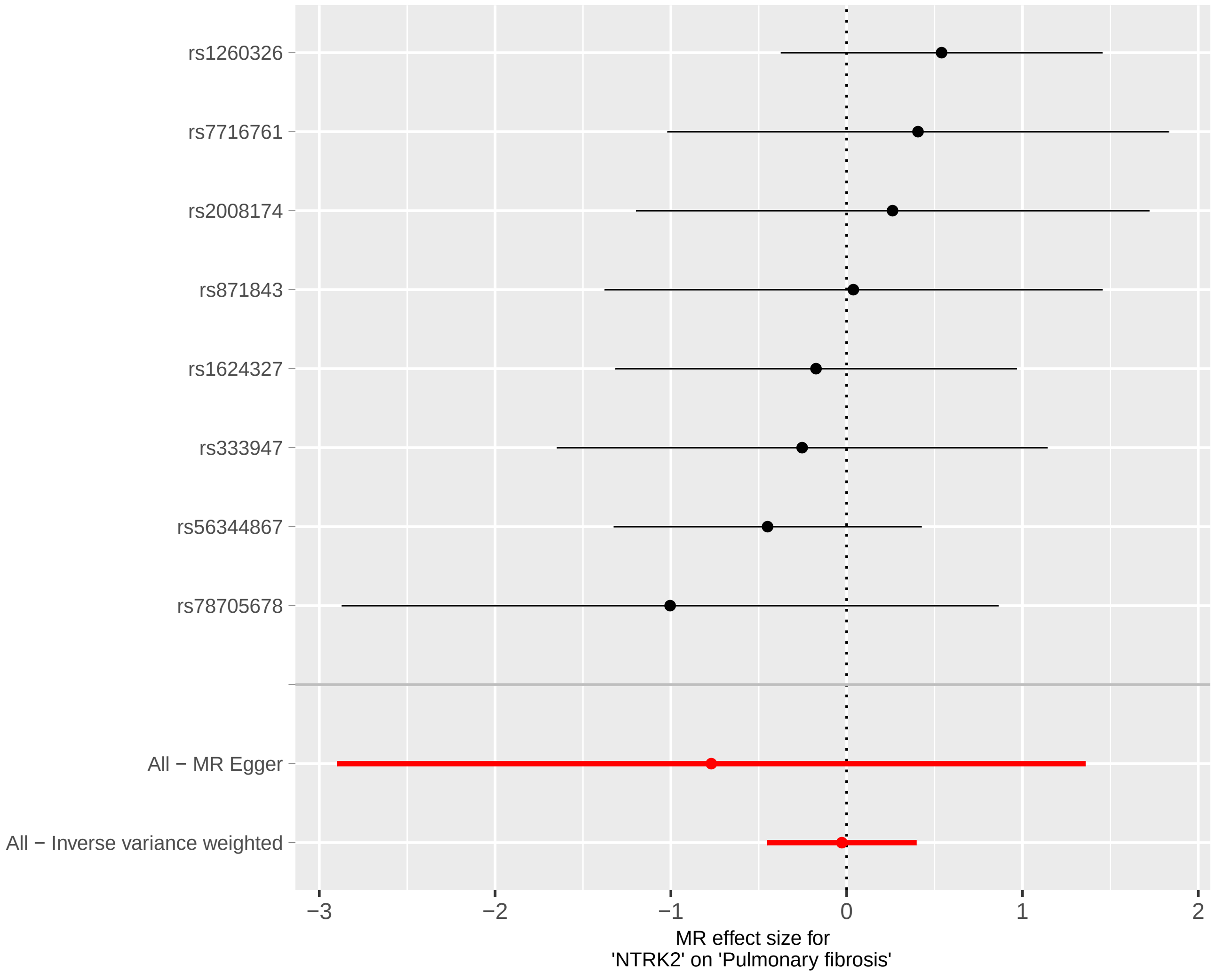

rs1260326
rs7716761
rs2008174
rs871843
rs1624327
rs333947
rs56344867
rs78705678
All − MR Egger
All − Inverse variance weighted
−3
−2
−1
0
1
2
MR effect size for
'NTRK2' on 'Pulmonary fibrosis'

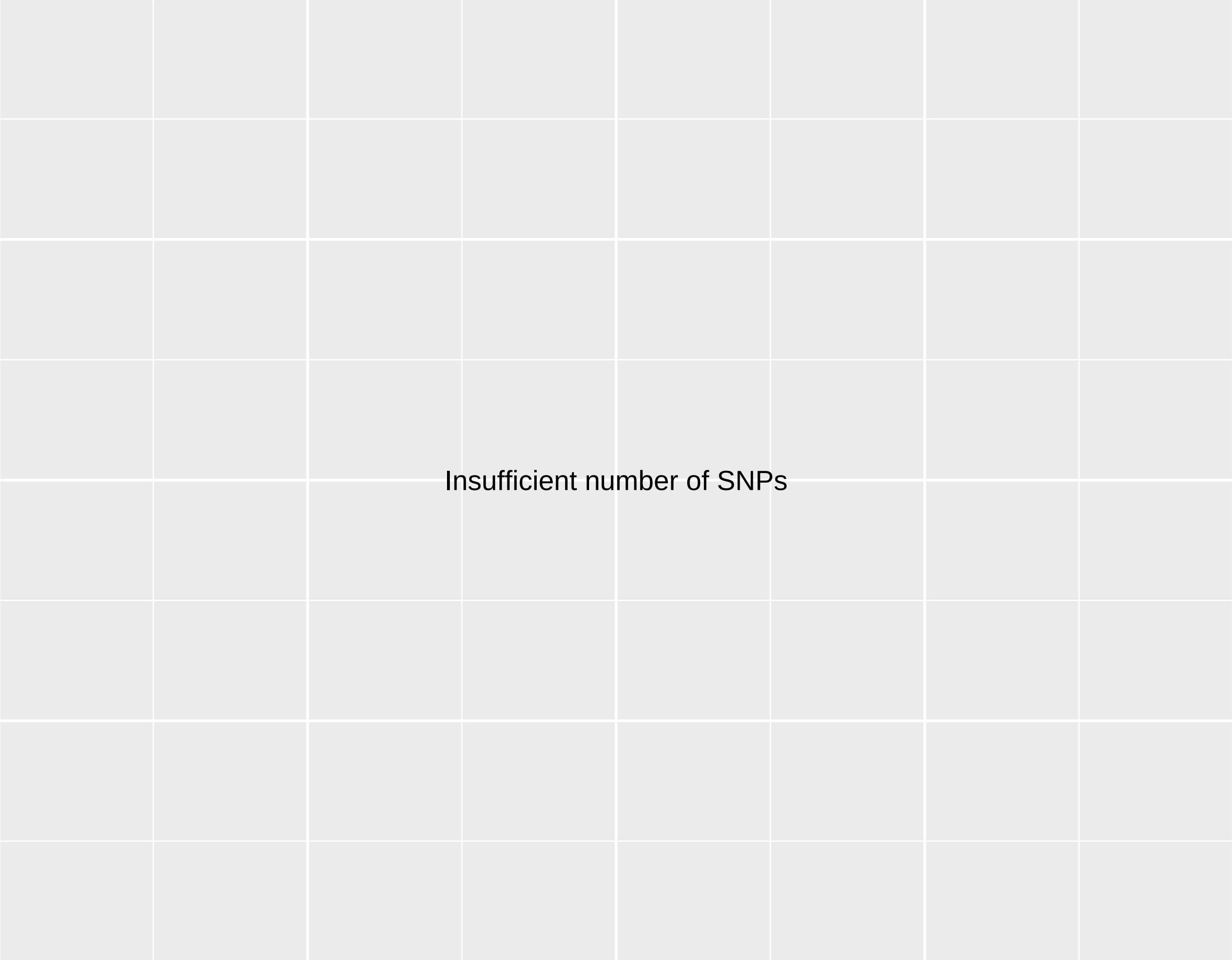
Insufficient number of SNPs

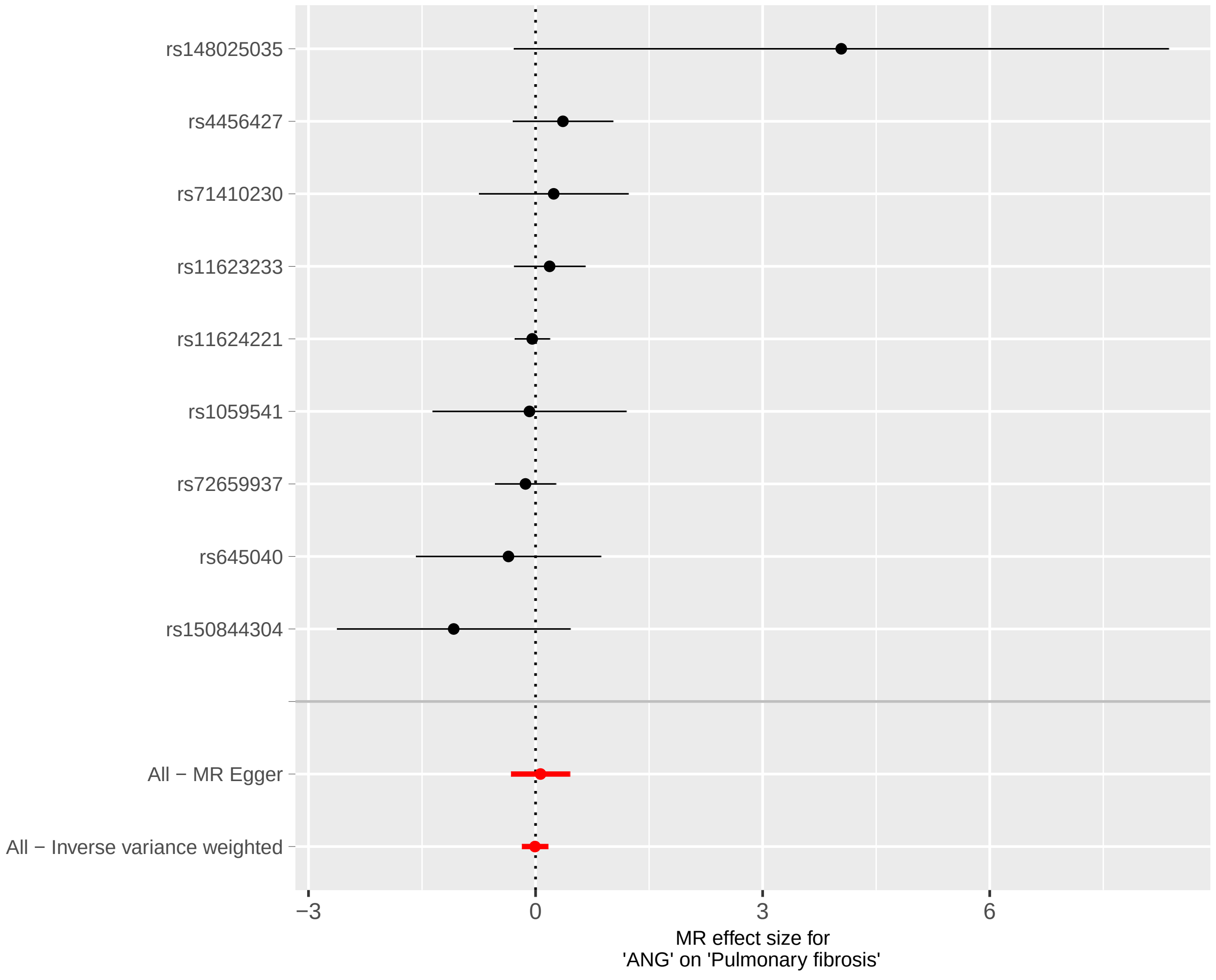
rs148025035
rs4456427
rs71410230
rs11623233
rs11624221
rs1059541
rs72659937
rs645040
rs150844304
All − MR Egger
All − Inverse variance weighted
−3
0
3
6
MR effect size for
'ANG' on 'Pulmonary fibrosis'

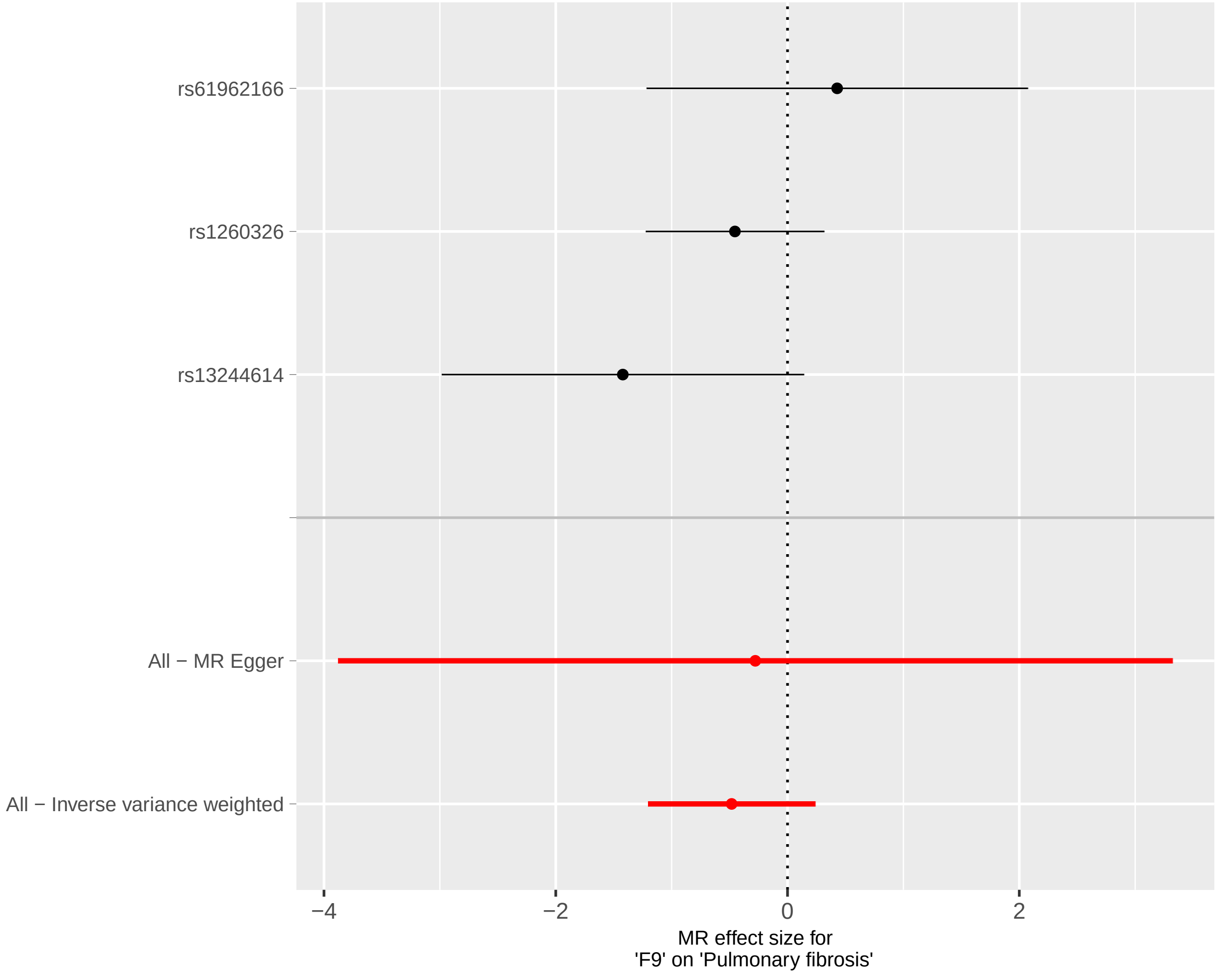

rs61962166
rs1260326
rs13244614
All − MR Egger
All − Inverse variance weighted
−4
−2
0
2
MR effect size for
'F9' on 'Pulmonary fibrosis'

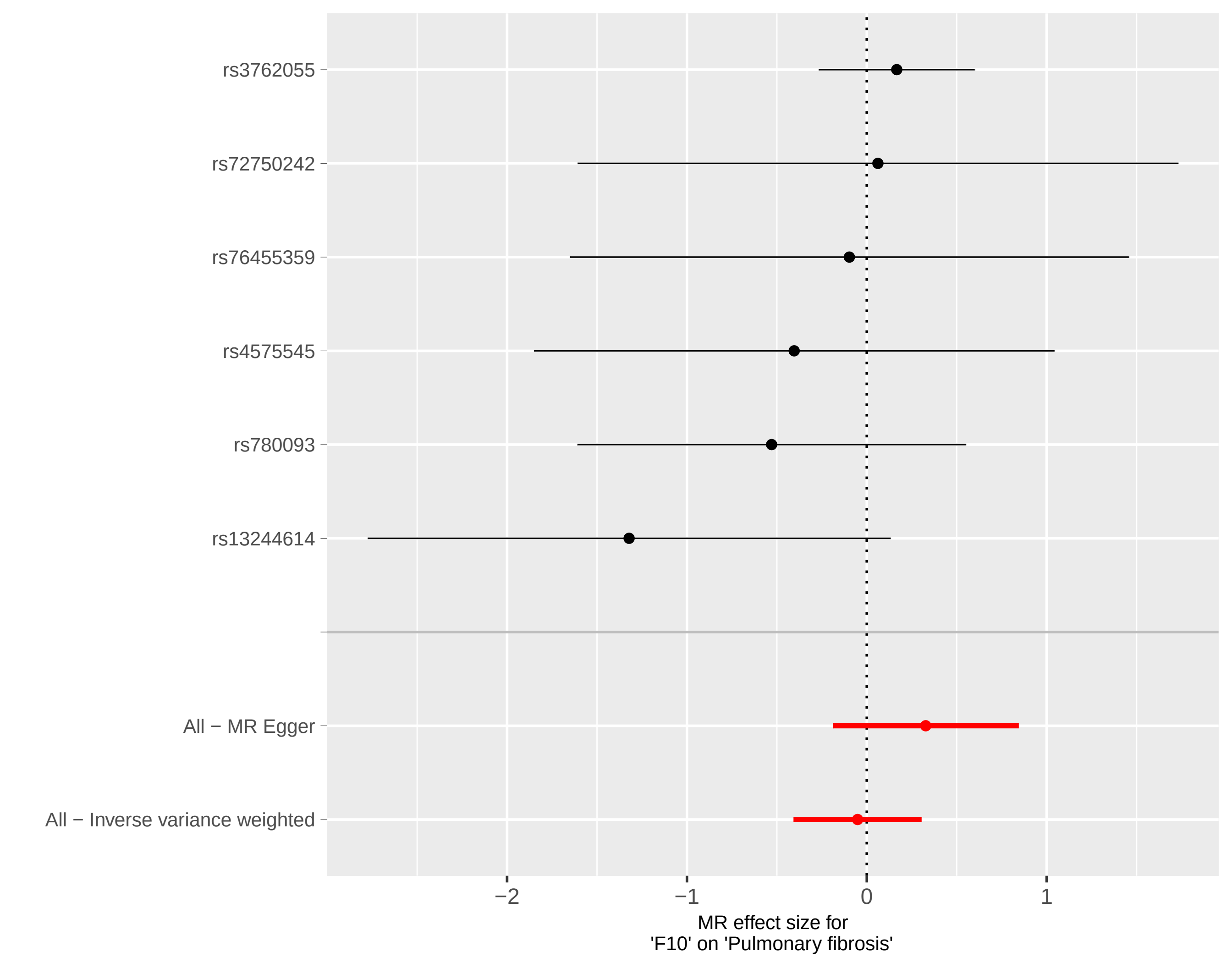

rs3762055
rs72750242
rs76455359
rs4575545
rs780093
rs13244614
All − MR Egger
All − Inverse variance weighted
−2
−1
0
1
MR effect size for
'F10' on 'Pulmonary fibrosis'

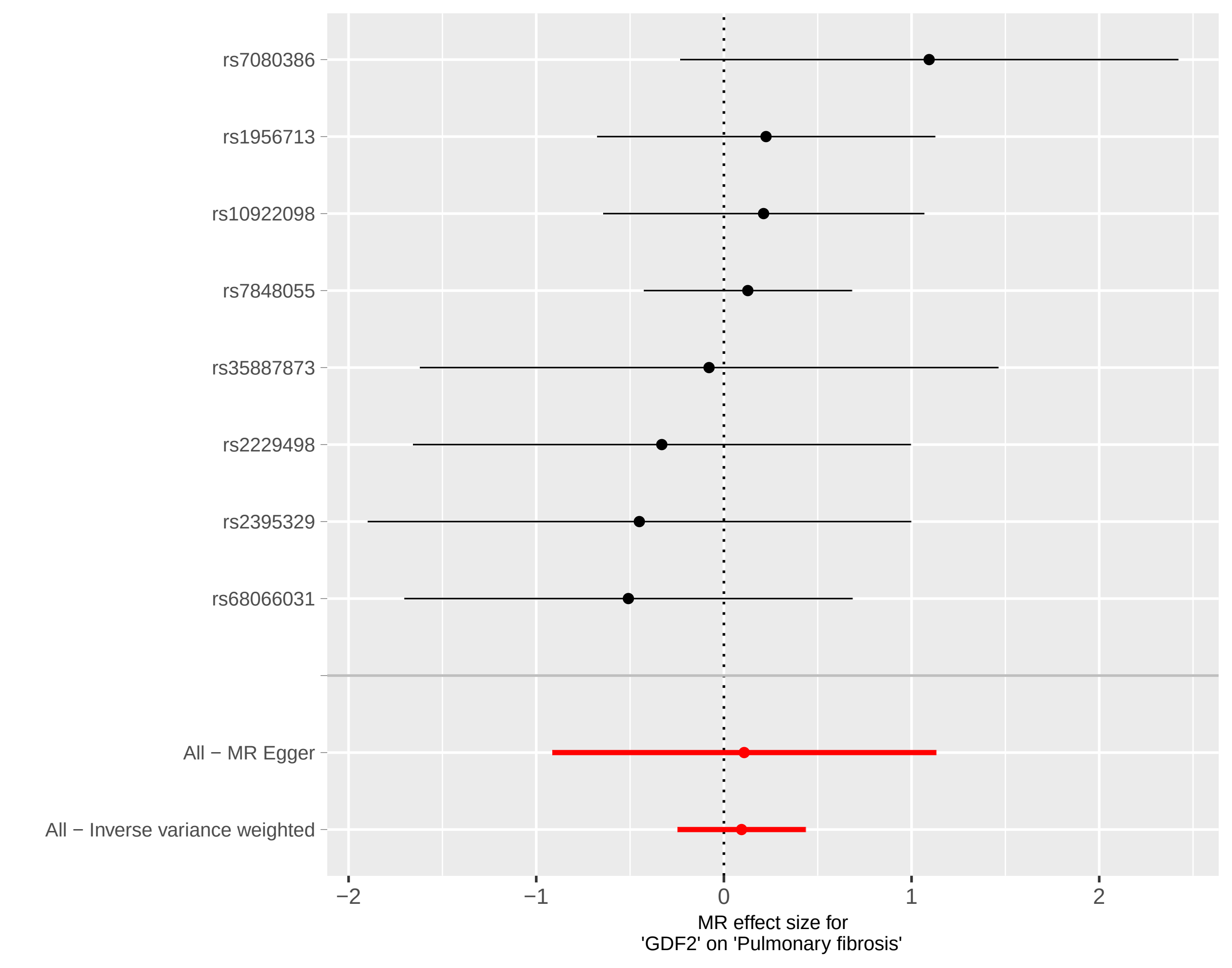
rs7080386
rs1956713
rs10922098
rs7848055
rs35887873
rs2229498
rs2395329
rs68066031
All − MR Egger
All − Inverse variance weighted
−2
−1
0
1
2
MR effect size for
'GDF2' on 'Pulmonary fibrosis'

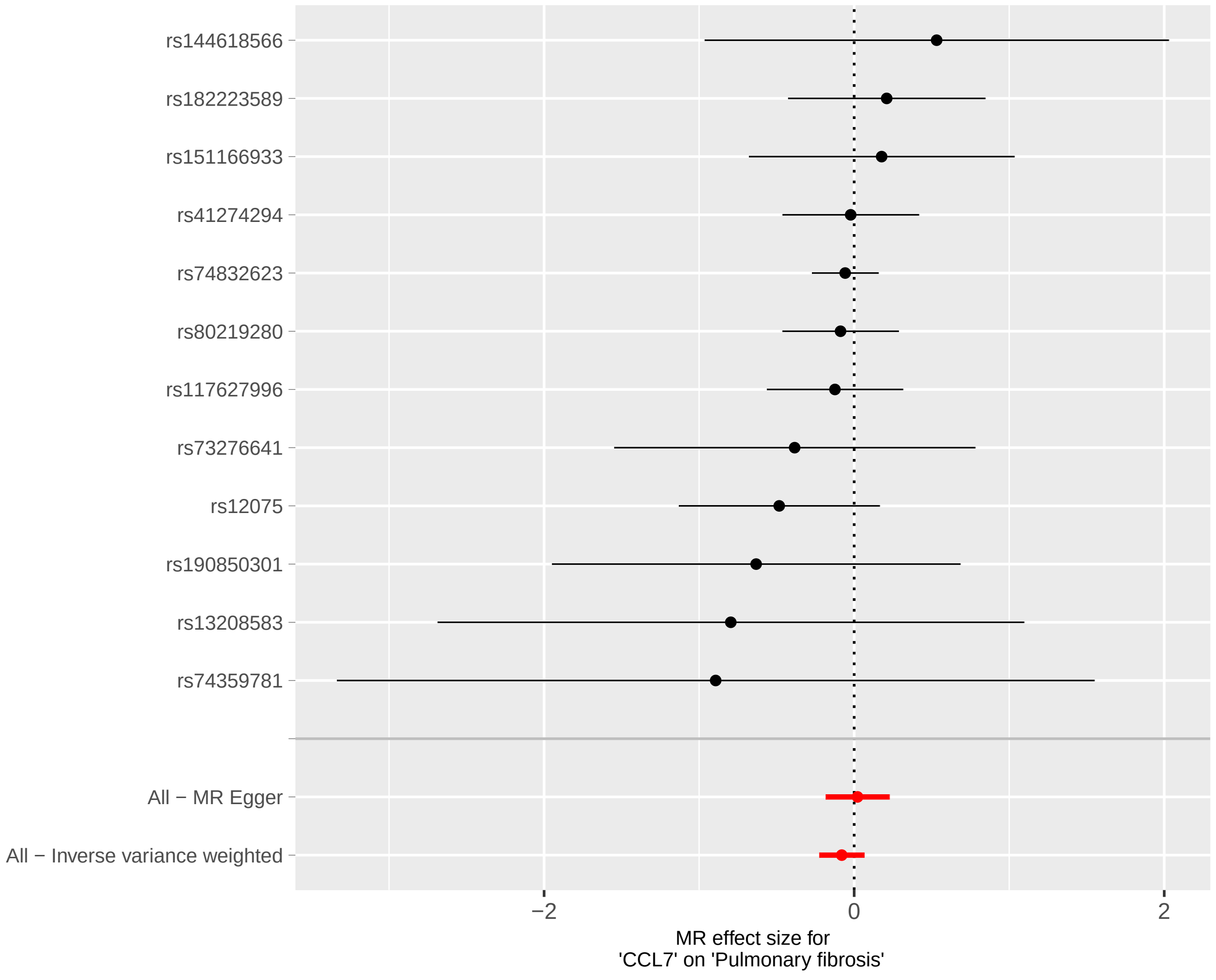
rs144618566
rs182223589
rs151166933
rs41274294
rs74832623
rs80219280
rs117627996
rs73276641
rs12075
rs190850301
rs13208583
rs74359781
All − MR Egger
All − Inverse variance weighted
−2
0
2
MR effect size for
'CCL7' on 'Pulmonary fibrosis'

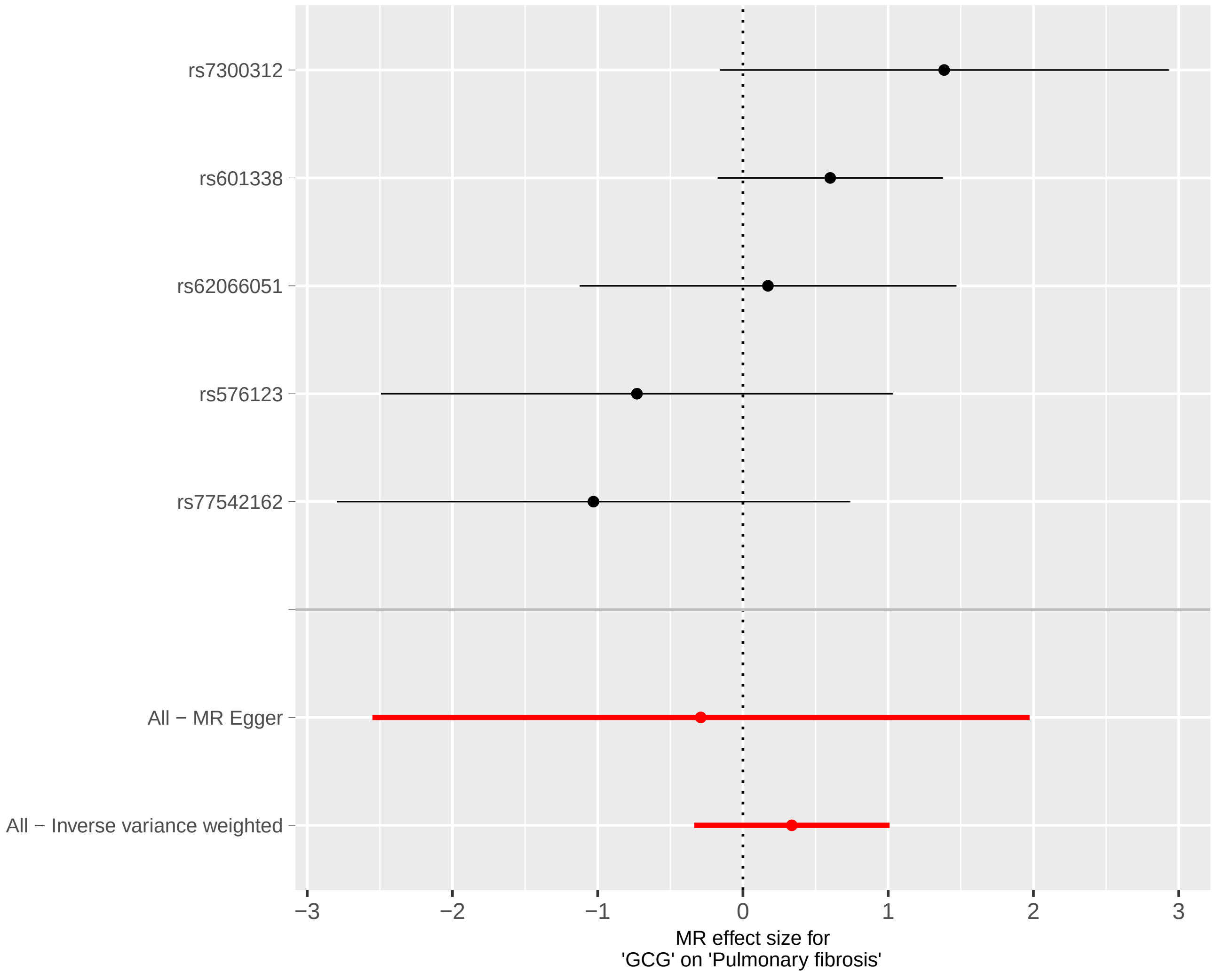

rs7300312
rs601338
rs62066051
rs576123
rs77542162
All − MR Egger
All − Inverse variance weighted
−3
−2
−1
0
1
2
3
MR effect size for
'GCG' on 'Pulmonary fibrosis'

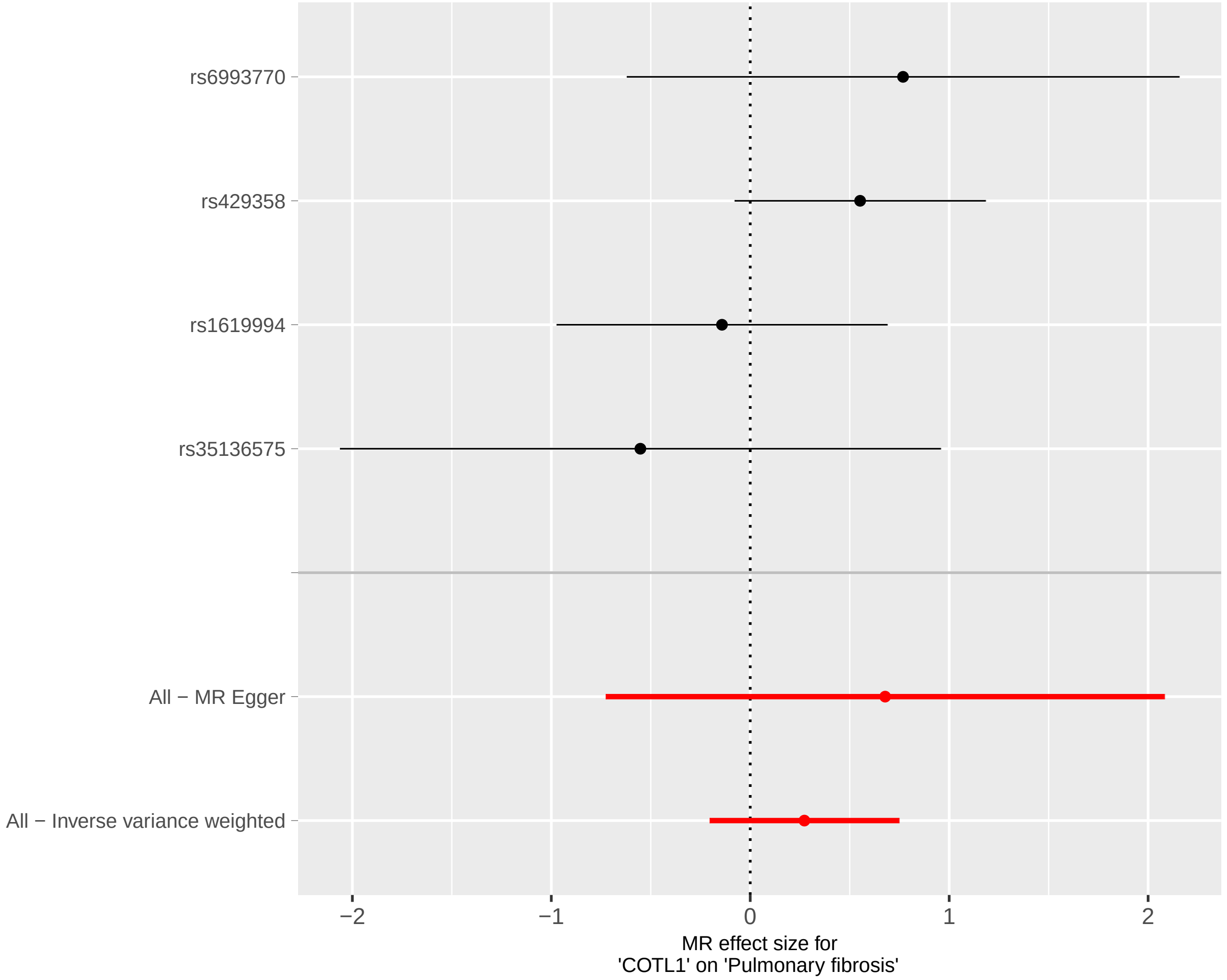

rs6993770
rs429358
rs1619994
rs35136575
All − MR Egger
All − Inverse variance weighted
−2
−1
0
1
2
MR effect size for
'COTL1' on 'Pulmonary fibrosis'

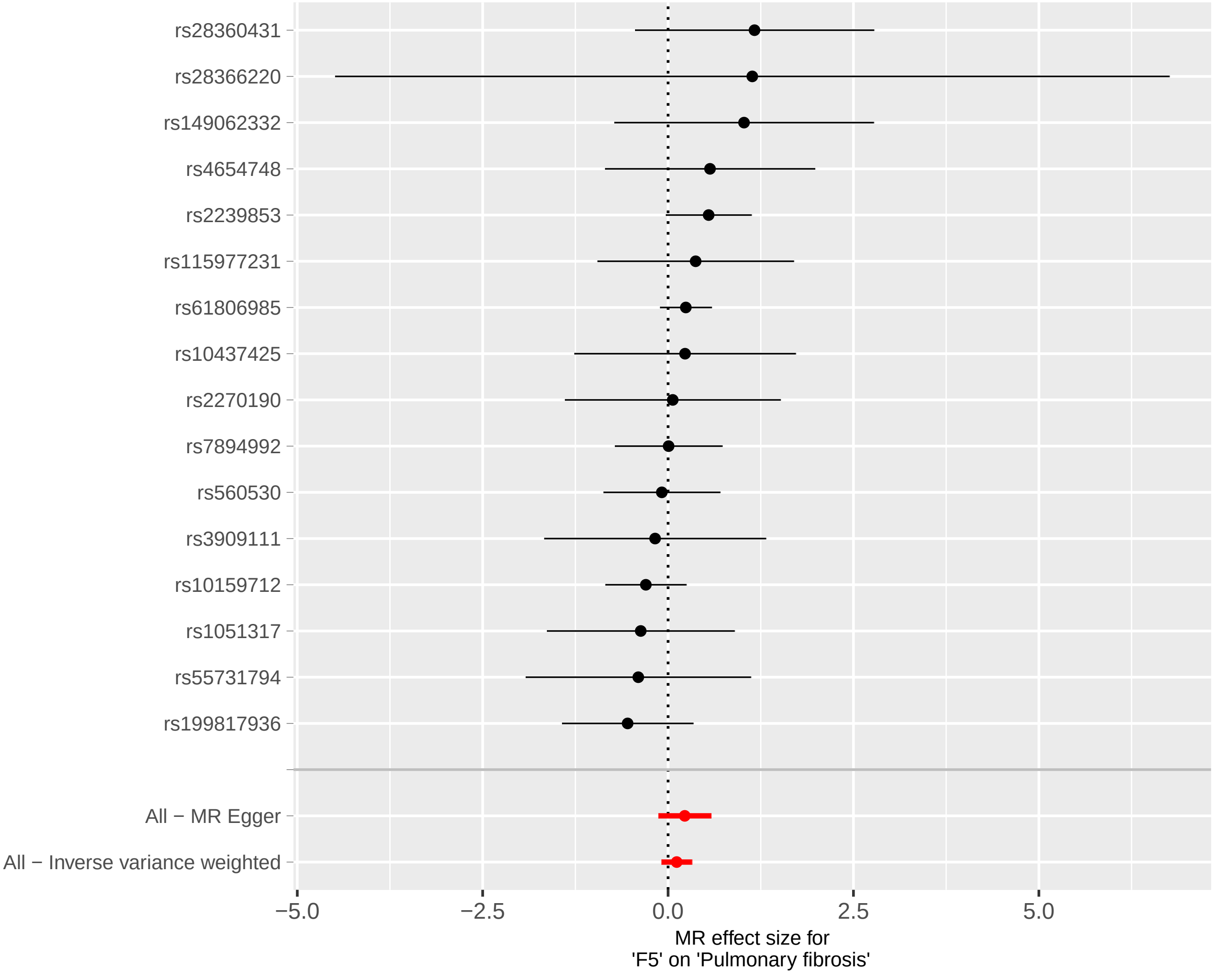
rs28360431
rs28366220
rs149062332
rs4654748
rs2239853
rs115977231
rs61806985
rs10437425
rs2270190
rs7894992
rs560530
rs3909111
rs10159712
rs1051317
rs55731794
rs199817936
All − MR Egger
All − Inverse variance weighted
−5.0
−2.5
0.0
2.5
5.0
MR effect size for
'F5' on 'Pulmonary fibrosis'

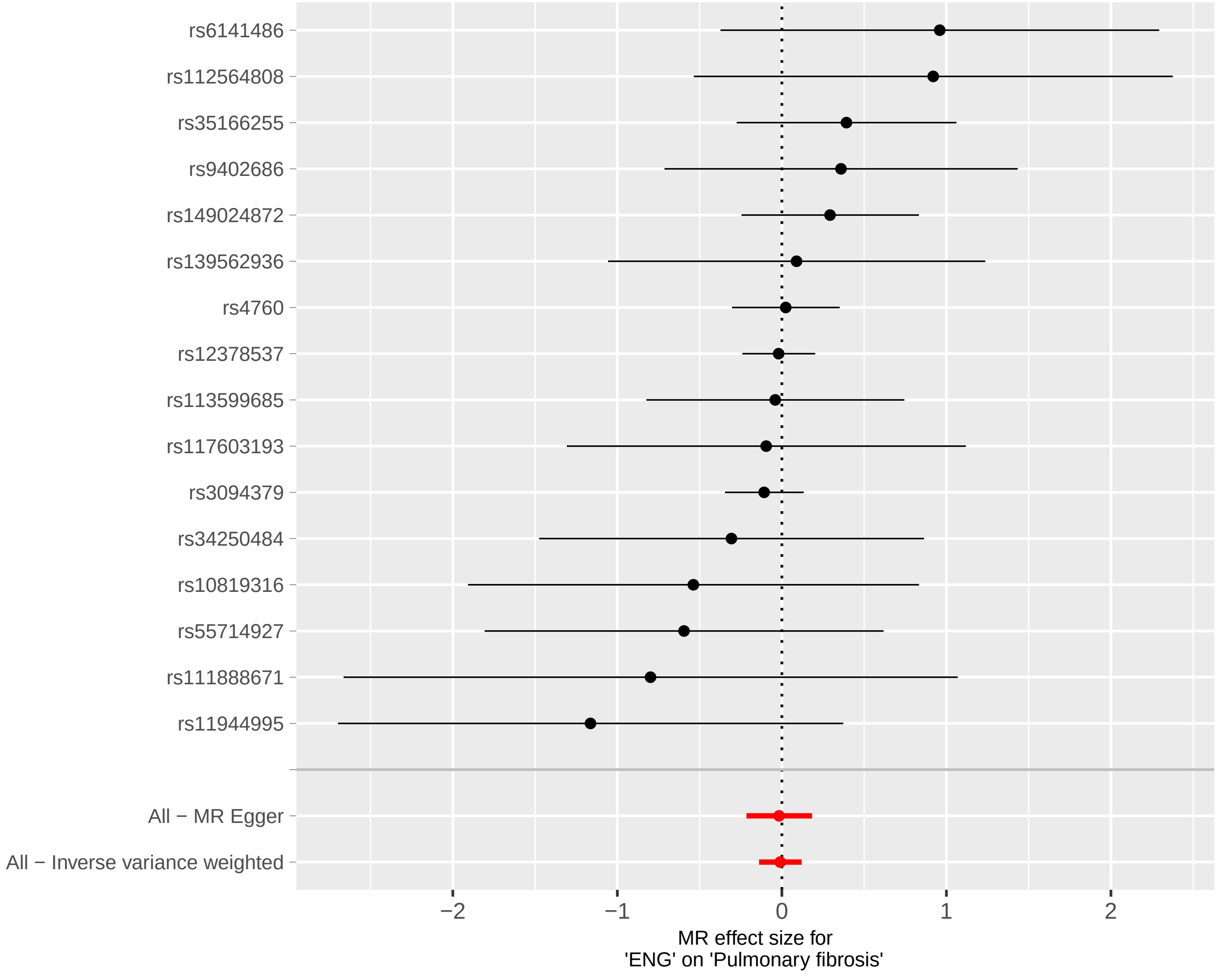

rs6141486
rs112564808
rs35166255
rs9402686
rs149024872
rs139562936
rs4760
rs12378537
rs113599685
rs117603193
rs3094379
rs34250484
rs10819316
rs55714927
rs111888671
rs11944995
All − MR Egger
All − Inverse variance weighted
−2
−1
0
1
2
MR effect size for
'ENG' on 'Pulmonary fibrosis'

Insufficient number of SNPs

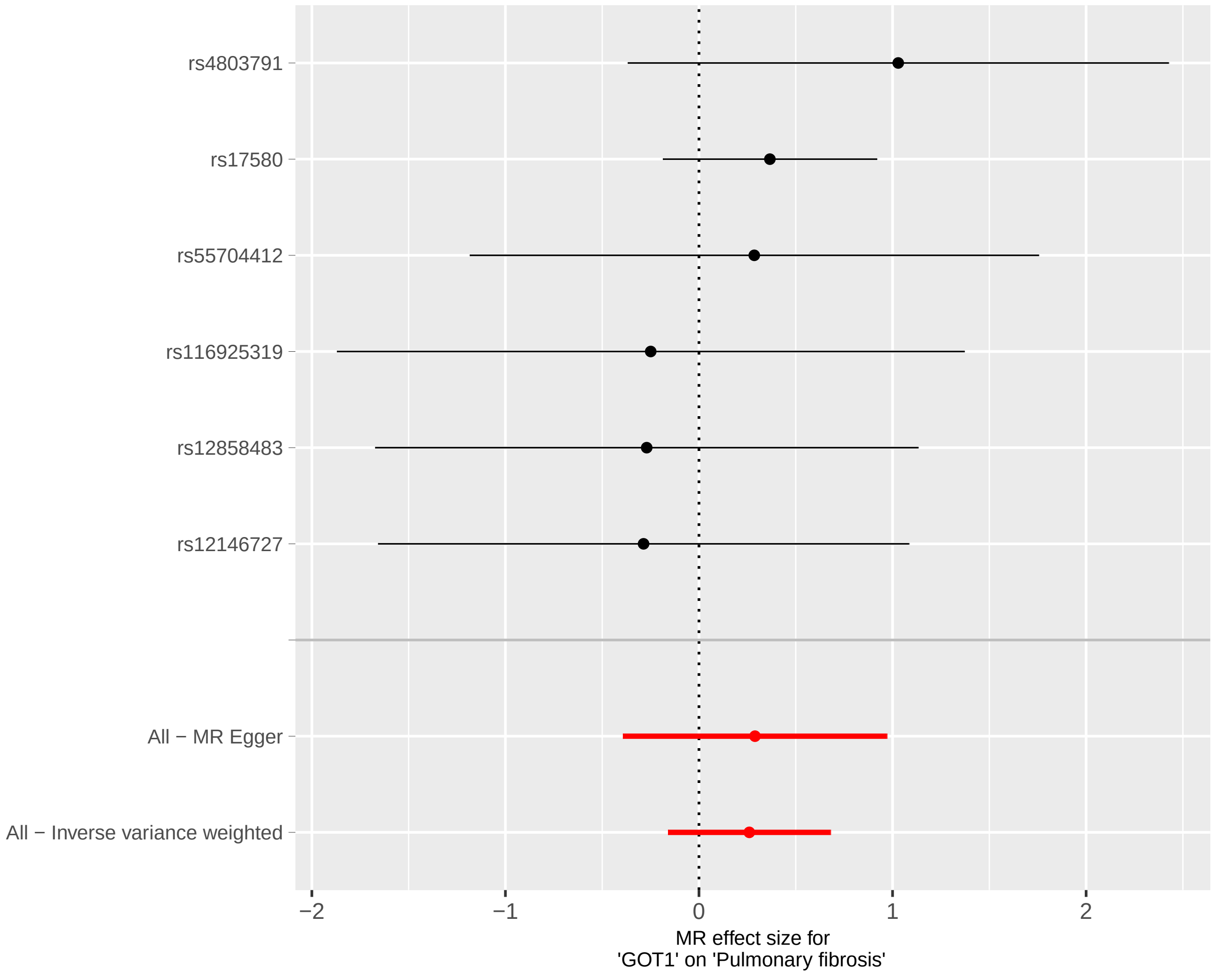

rs4803791
rs17580
rs55704412
rs116925319
rs12858483
rs12146727
All − MR Egger
All − Inverse variance weighted
−2
−1
0
1
2
MR effect size for
'GOT1' on 'Pulmonary fibrosis'

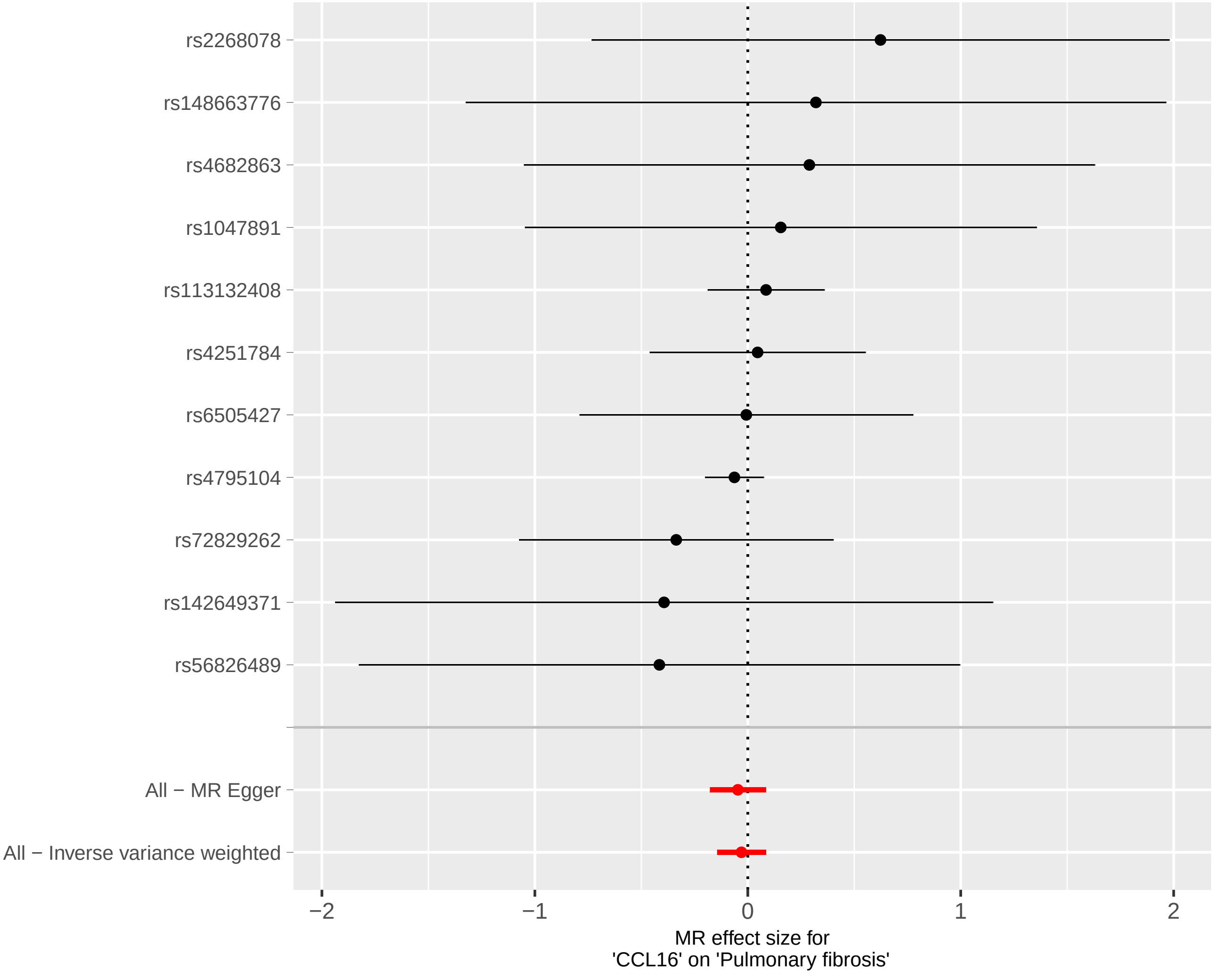
rs2268078
rs148663776
rs4682863
rs1047891
rs113132408
rs4251784
rs6505427
rs4795104
rs72829262
rs142649371
rs56826489
All − MR Egger
All − Inverse variance weighted
−2
−1
0
1
2
MR effect size for
'CCL16' on 'Pulmonary fibrosis'

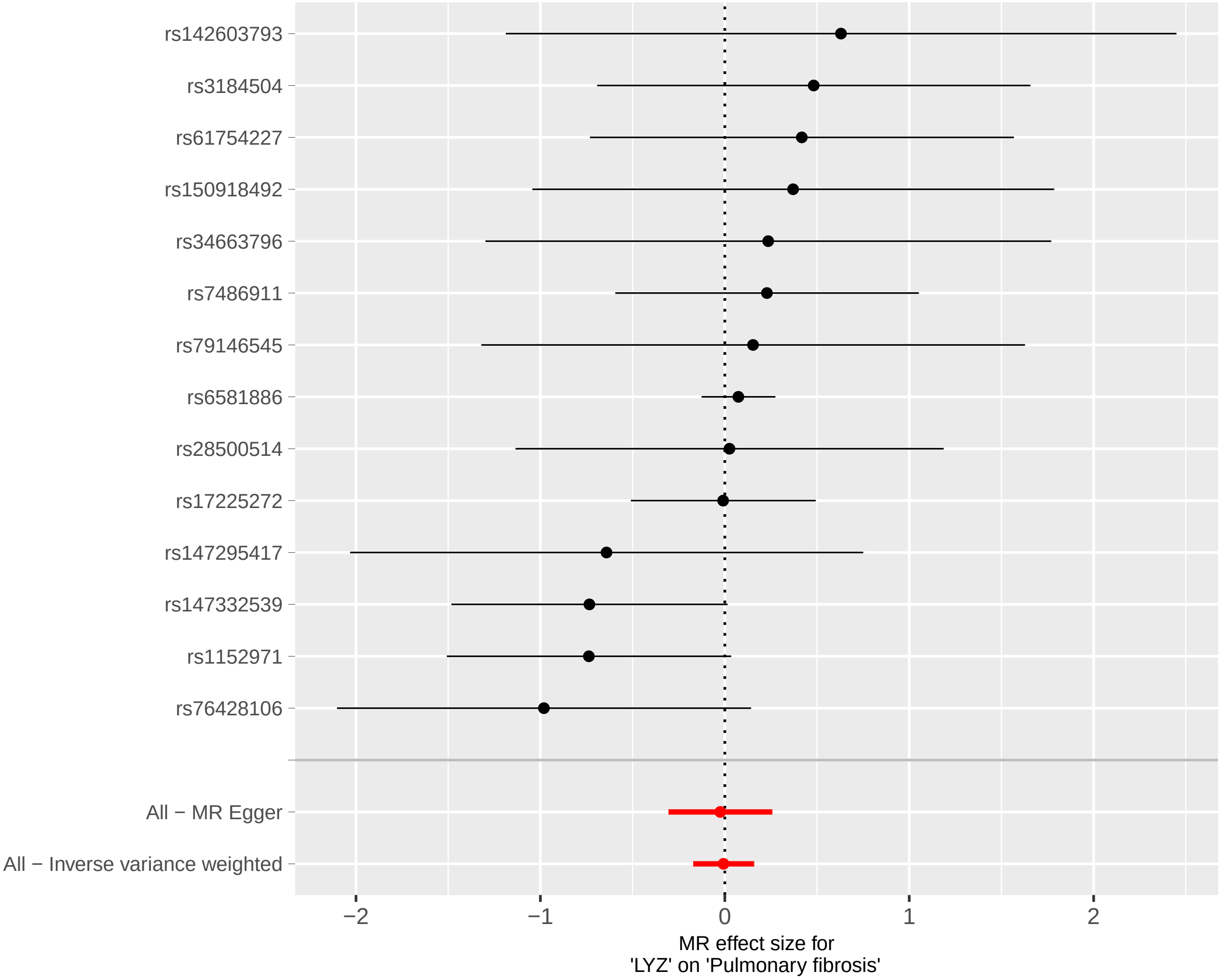

rs142603793
rs3184504
rs61754227
rs150918492
rs34663796
rs7486911
rs79146545
rs6581886
rs28500514
rs17225272
rs147295417
rs147332539
rs1152971
rs76428106
All − MR Egger
All − Inverse variance weighted
−2
−1
0
1
2
MR effect size for
'LYZ' on 'Pulmonary fibrosis'

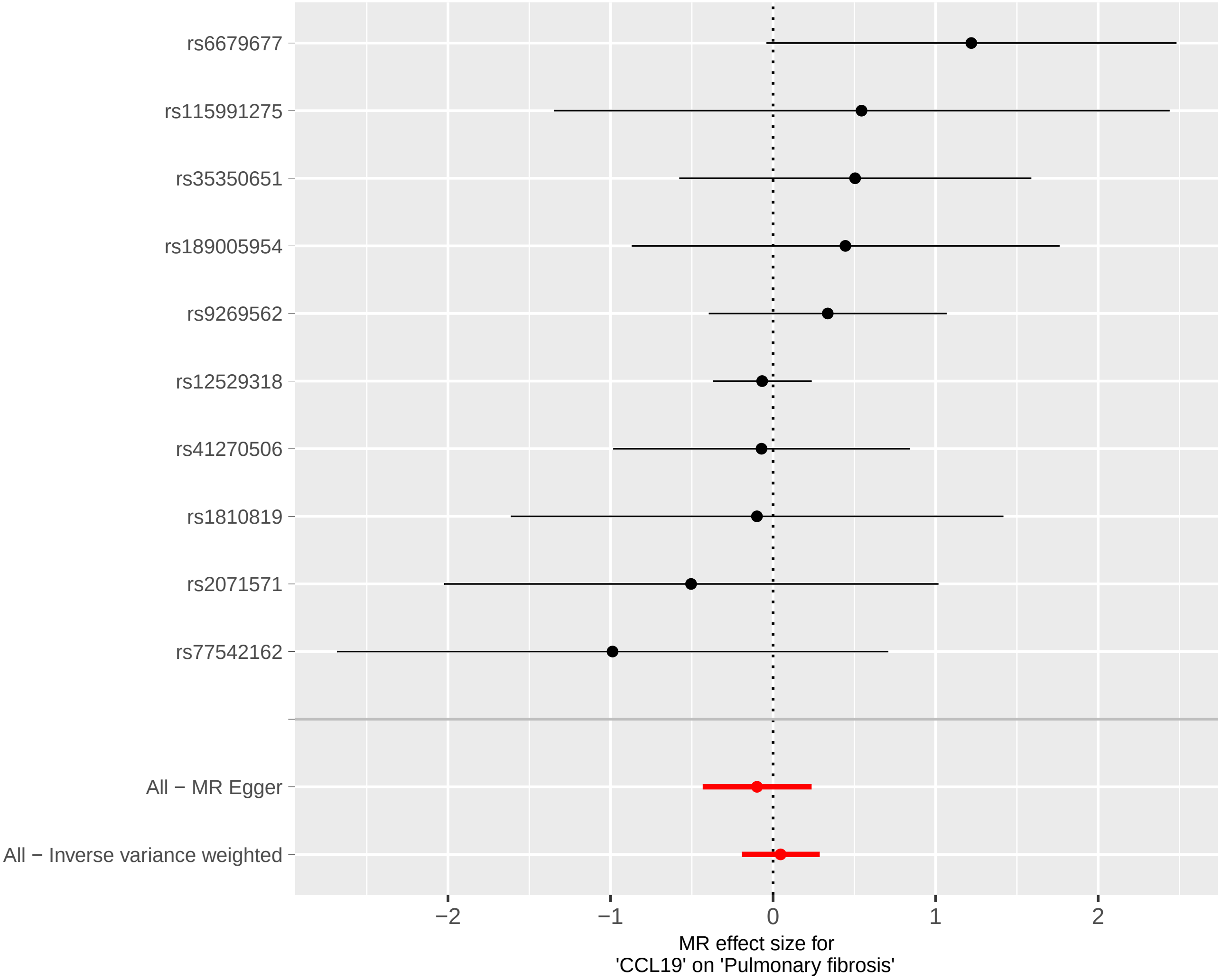

rs6679677
rs115991275
rs35350651
rs189005954
rs9269562
rs12529318
rs41270506
rs1810819
rs2071571
rs77542162
All − MR Egger
All − Inverse variance weighted
−2
−1
0
1
2
MR effect size for
'CCL19' on 'Pulmonary fibrosis'

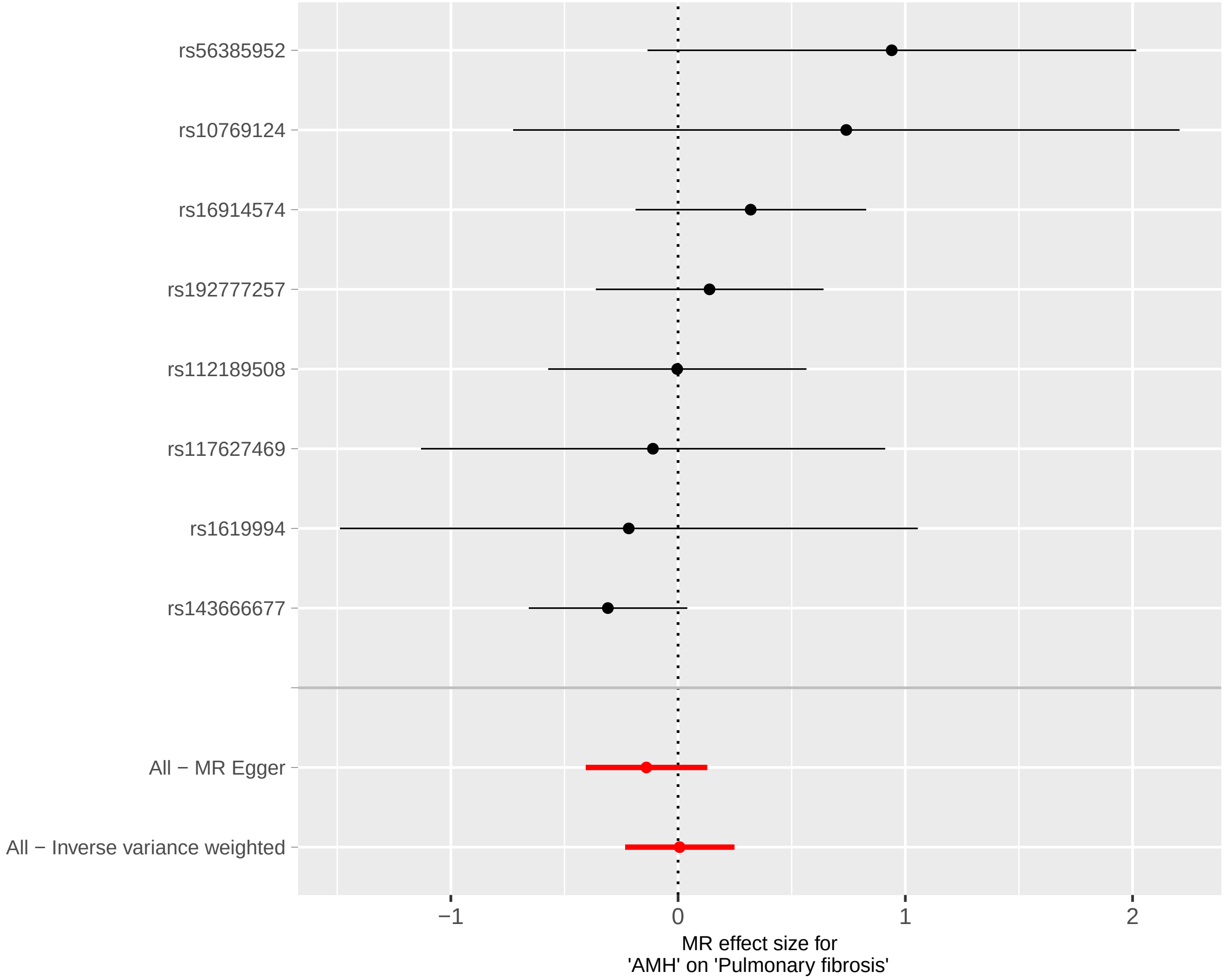
rs56385952
rs10769124
rs16914574
rs192777257
rs112189508
rs117627469
rs1619994
rs143666677
All − MR Egger
All − Inverse variance weighted
−1
0
1
2
MR effect size for
'AMH' on 'Pulmonary fibrosis'

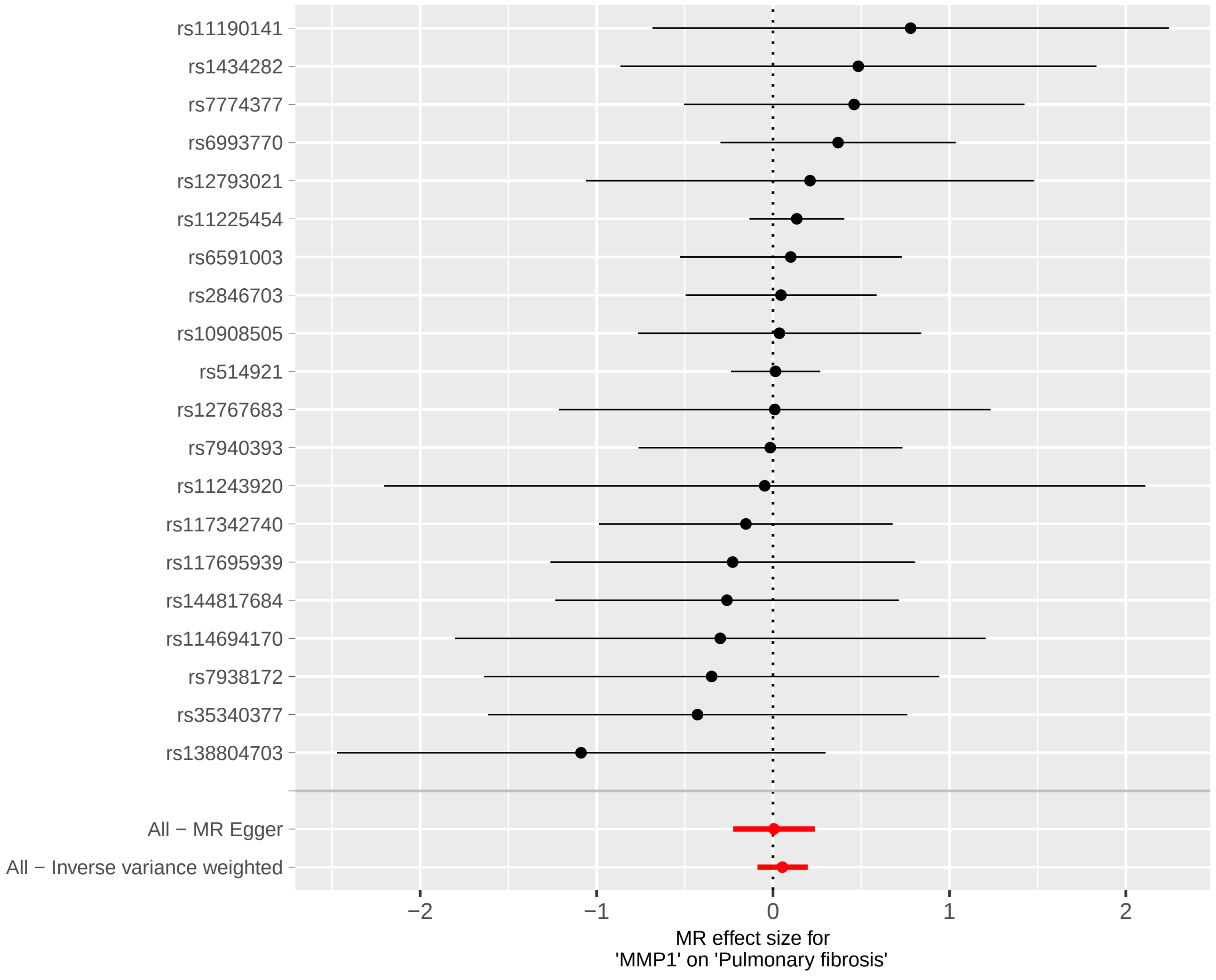
rs11190141
rs1434282
rs7774377
rs6993770
rs12793021
rs11225454
rs6591003
rs2846703
rs10908505
rs514921
rs12767683
rs7940393
rs11243920
rs117342740
rs117695939
rs144817684
rs114694170
rs7938172
rs35340377
rs138804703
All − MR Egger
All − Inverse variance weighted
−2
−1
0
1
2
MR effect size for
'MMP1' on 'Pulmonary fibrosis'

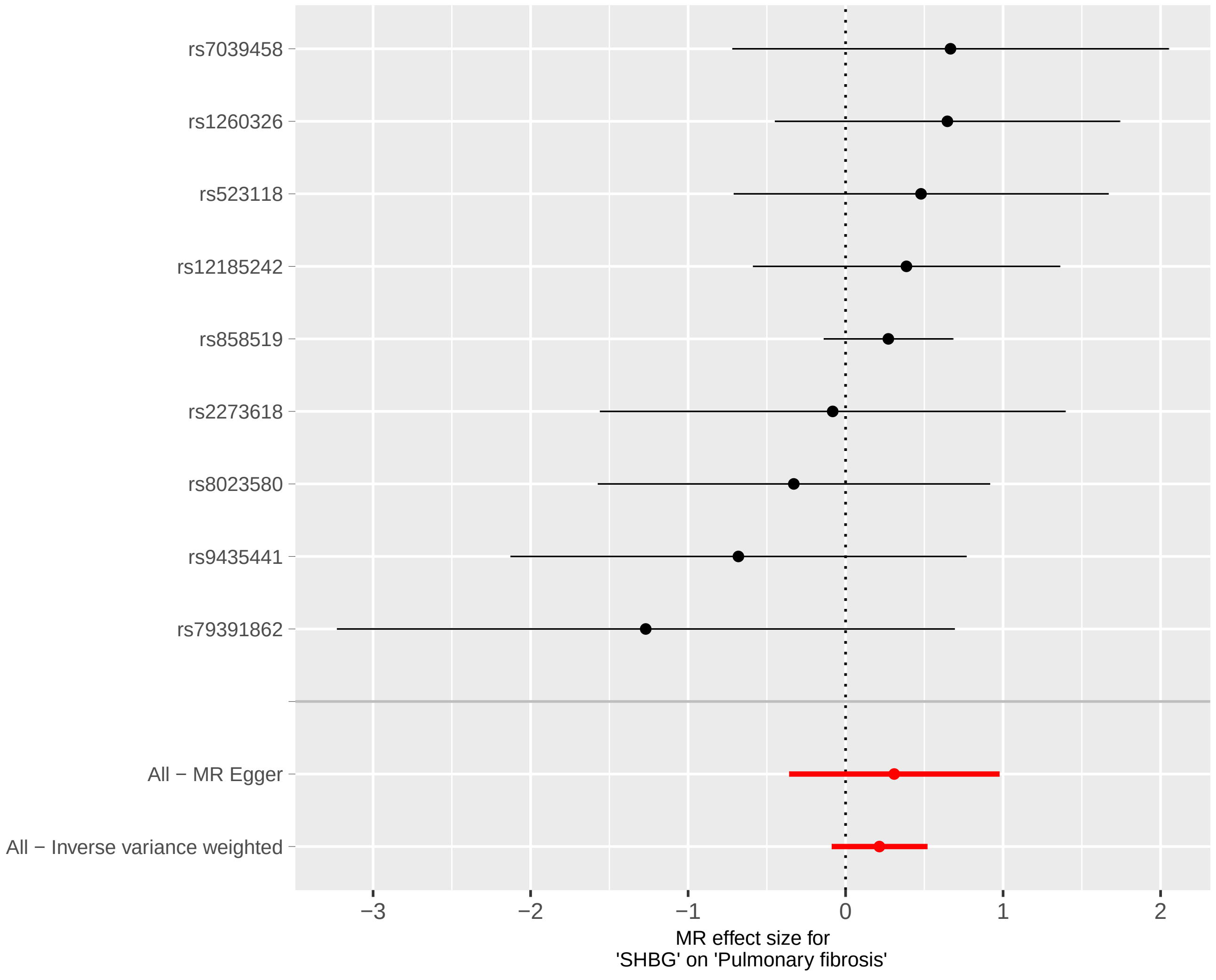

rs7039458
rs1260326
rs523118
rs12185242
rs858519
rs2273618
rs8023580
rs9435441
rs79391862
All − MR Egger
All − Inverse variance weighted
−3
−2
−1
0
1
2
MR effect size for
'SHBG' on 'Pulmonary fibrosis'

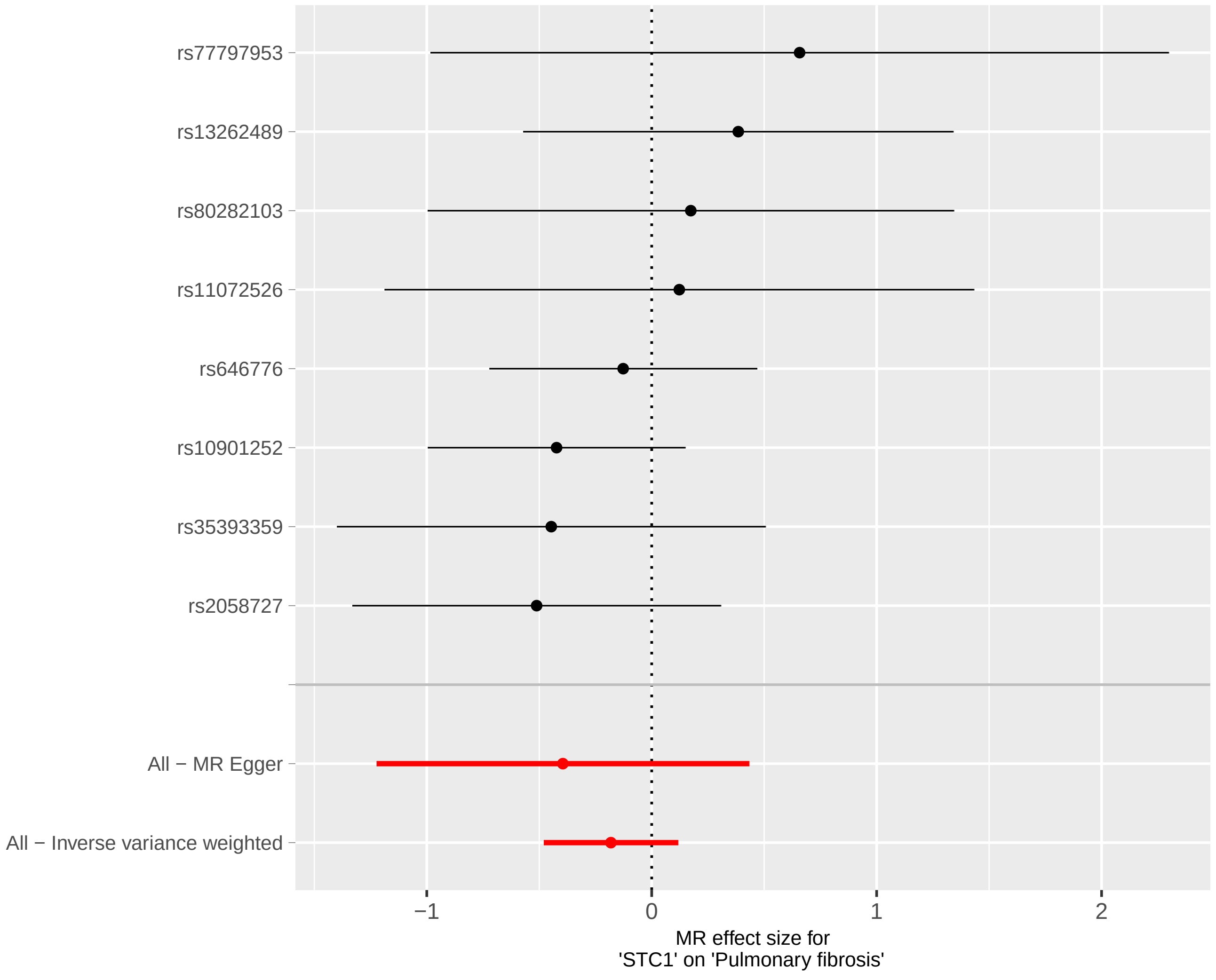
rs77797953
rs13262489
rs80282103
rs11072526
rs646776
rs10901252
rs35393359
rs2058727
All − MR Egger
All − Inverse variance weighted
−1
0
1
2
MR effect size for
'STC1' on 'Pulmonary fibrosis'

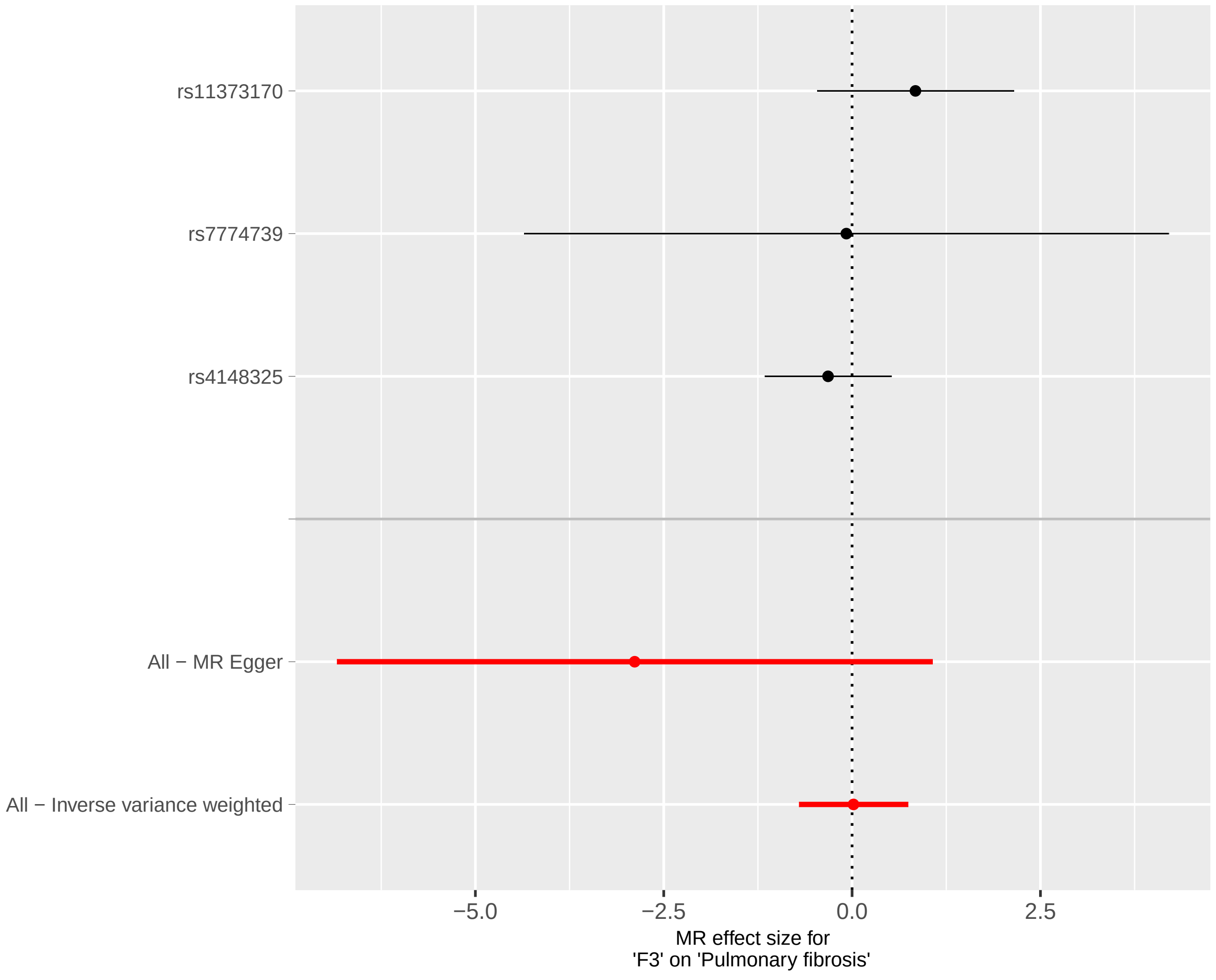

rs11373170
rs7774739
rs4148325
All − MR Egger
All − Inverse variance weighted
−5.0
−2.5
0.0
2.5
MR effect size for
'F3' on 'Pulmonary fibrosis'

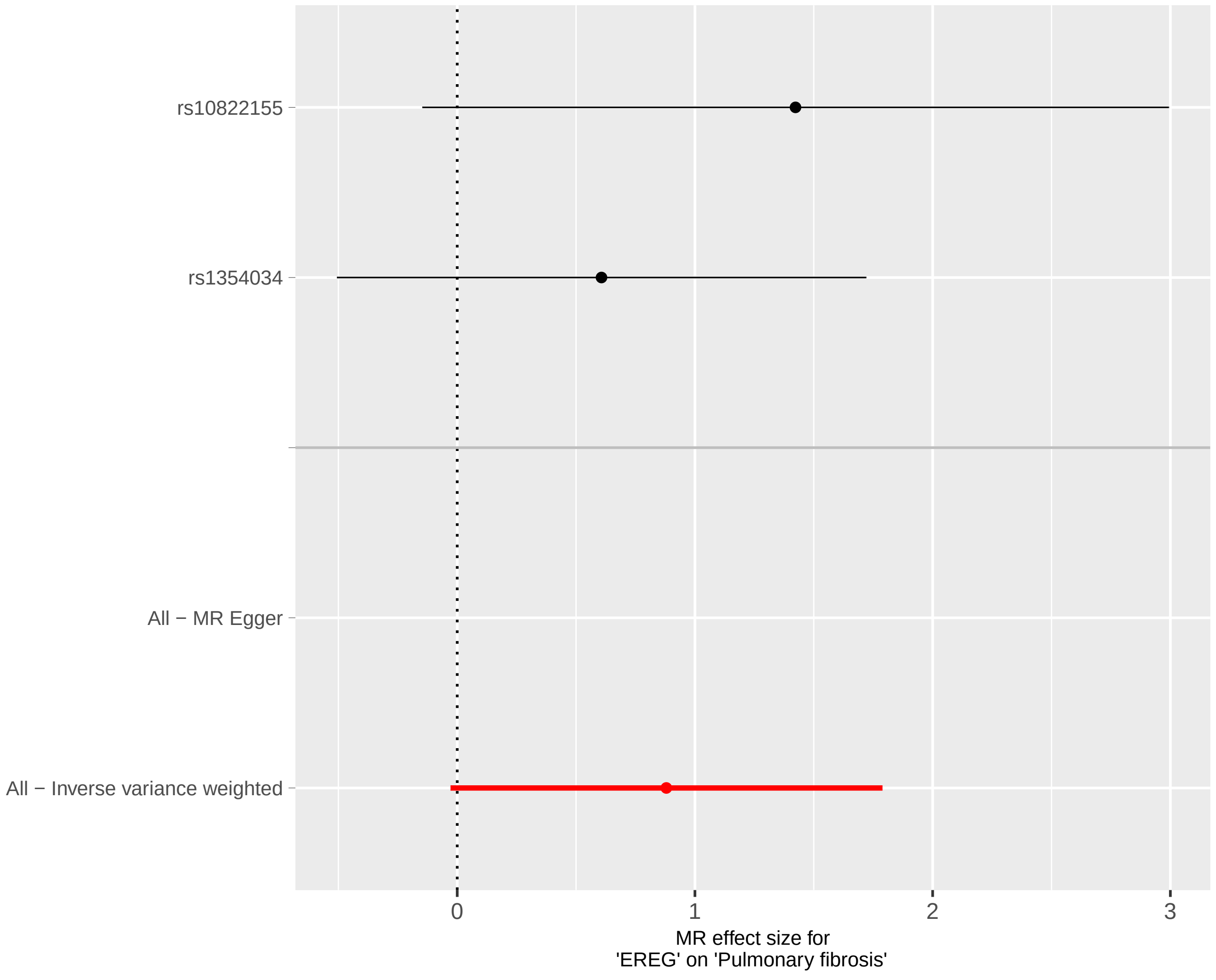
rs10822155
rs1354034
All − MR Egger
All − Inverse variance weighted
0
1
2
3
MR effect size for
'EREG' on 'Pulmonary fibrosis'

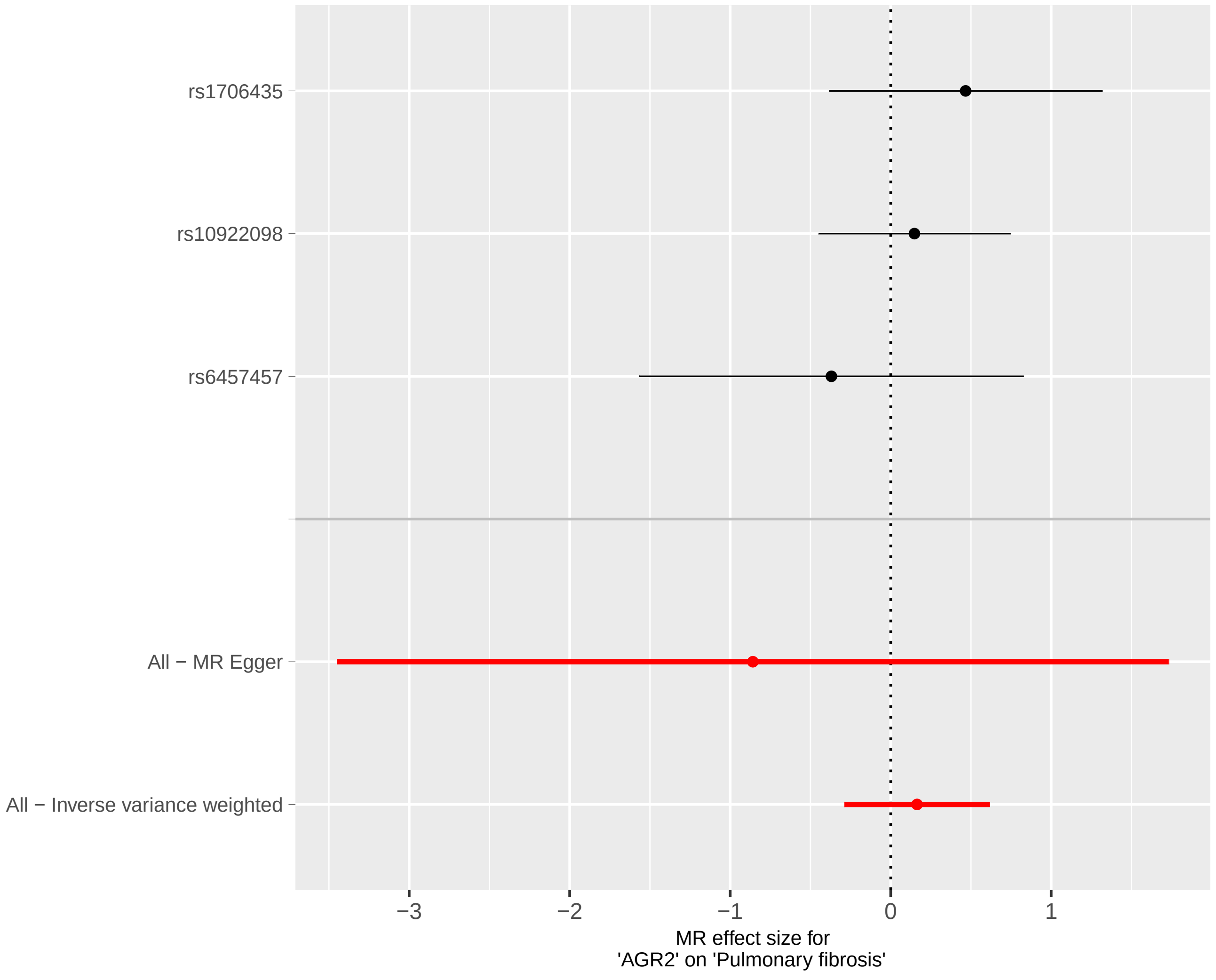

rs1706435
rs10922098
rs6457457
All − MR Egger
All − Inverse variance weighted
−3
−2
−1
0
1
MR effect size for
'AGR2' on 'Pulmonary fibrosis'

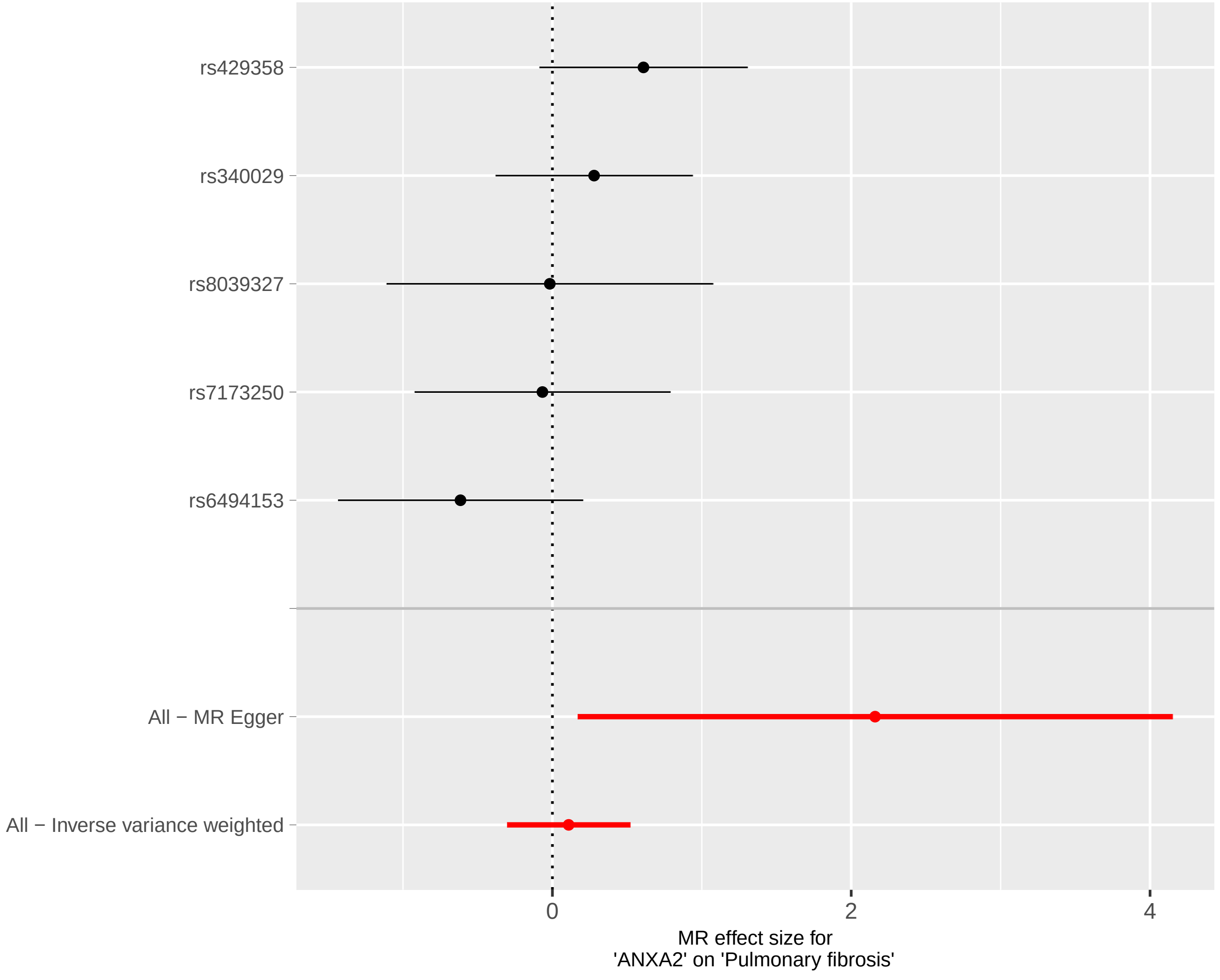

rs429358
rs340029
rs8039327
rs7173250
rs6494153
All − MR Egger
All − Inverse variance weighted
0
2
4
MR effect size for
'ANXA2' on 'Pulmonary fibrosis'

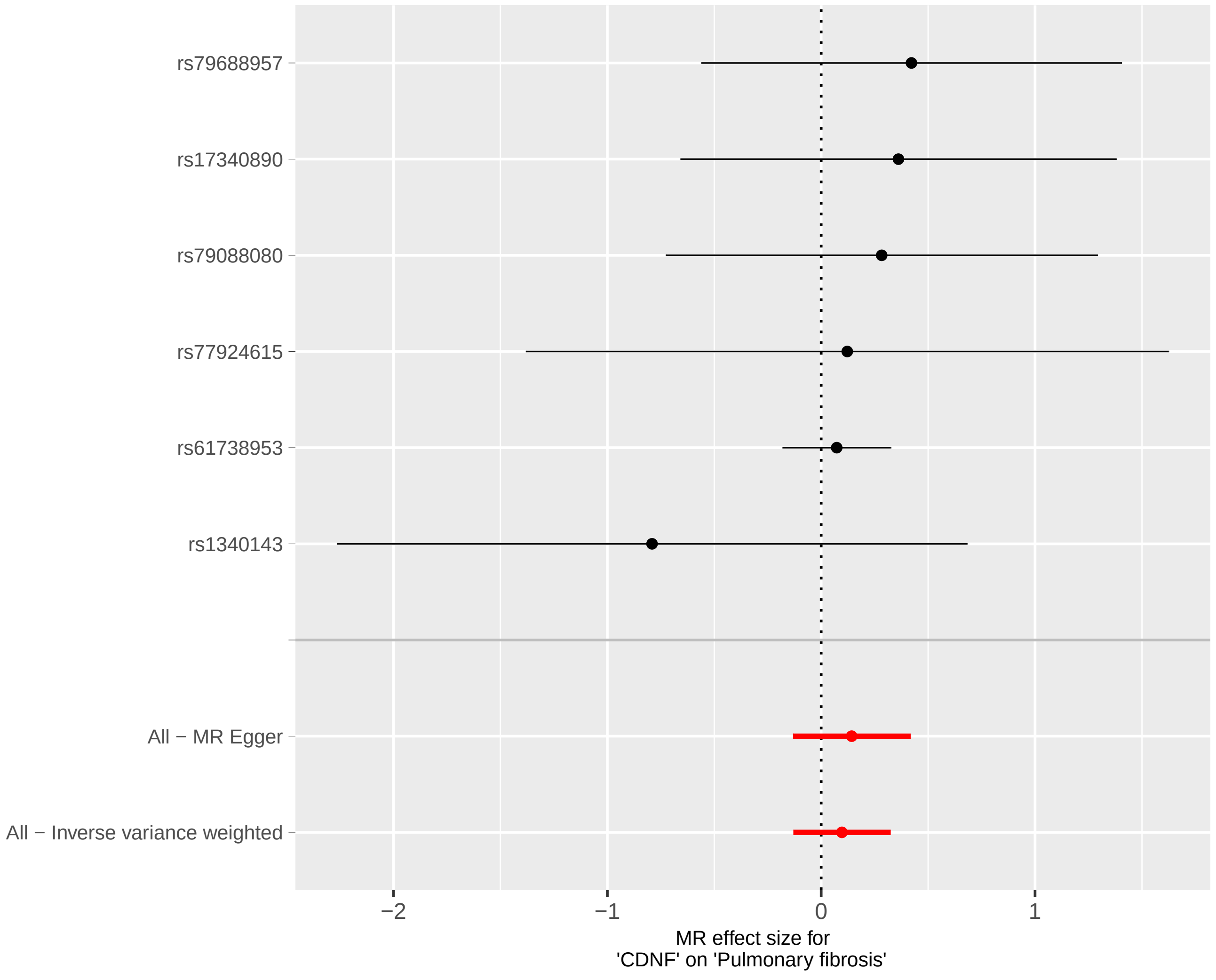
rs79688957
rs17340890
rs79088080
rs77924615
rs61738953
rs1340143
All − MR Egger
All − Inverse variance weighted
−2
−1
0
1
MR effect size for
'CDNF' on 'Pulmonary fibrosis'

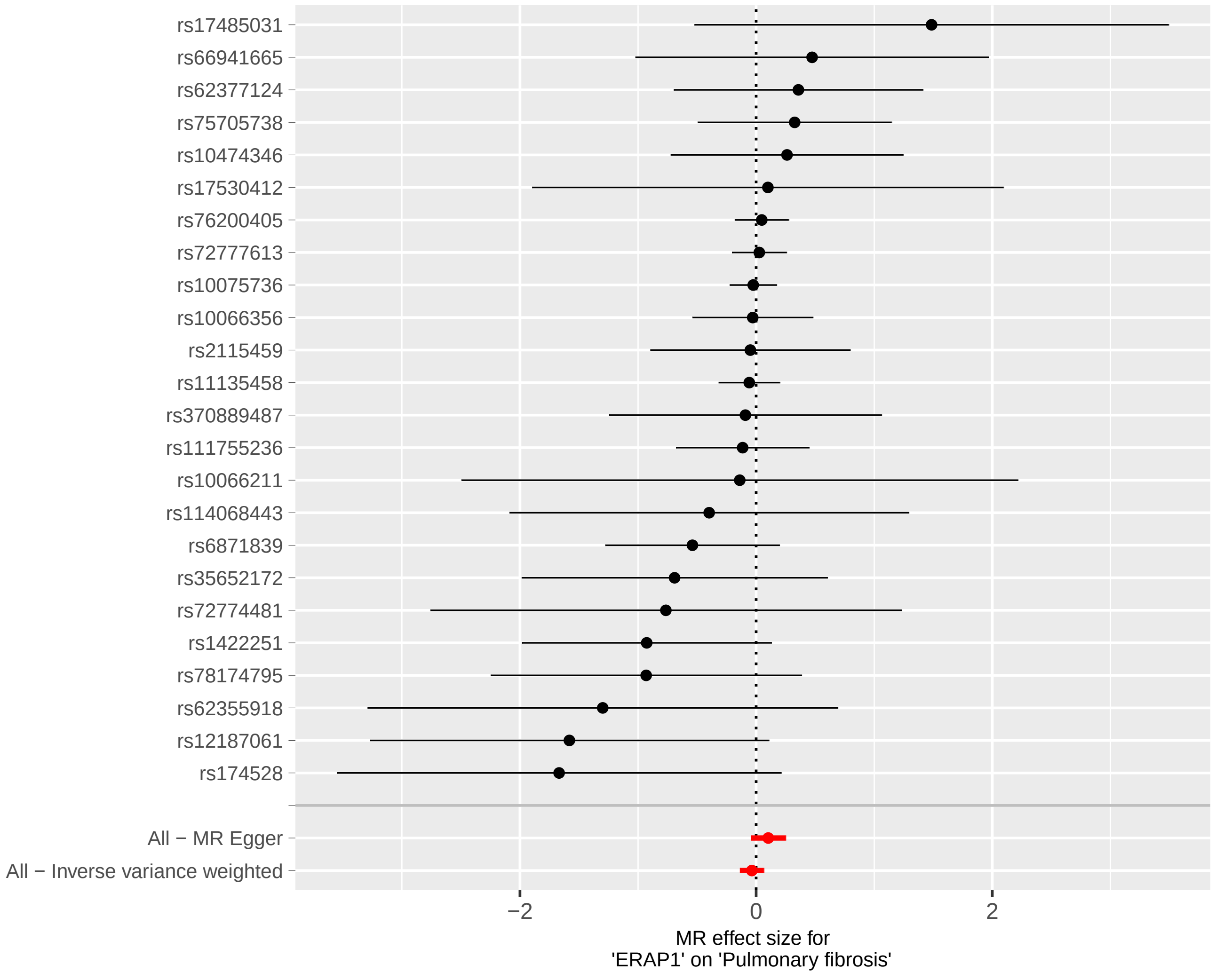
rs17485031
rs66941665
rs62377124
rs75705738
rs10474346
rs17530412
rs76200405
rs72777613
rs10075736
rs10066356
rs2115459
rs11135458
rs370889487
rs111755236
rs10066211
rs114068443
rs6871839
rs35652172
rs72774481
rs1422251
rs78174795
rs62355918
rs12187061
rs174528
All − MR Egger
All − Inverse variance weighted
−2
0
2
MR effect size for
'ERAP1' on 'Pulmonary fibrosis'

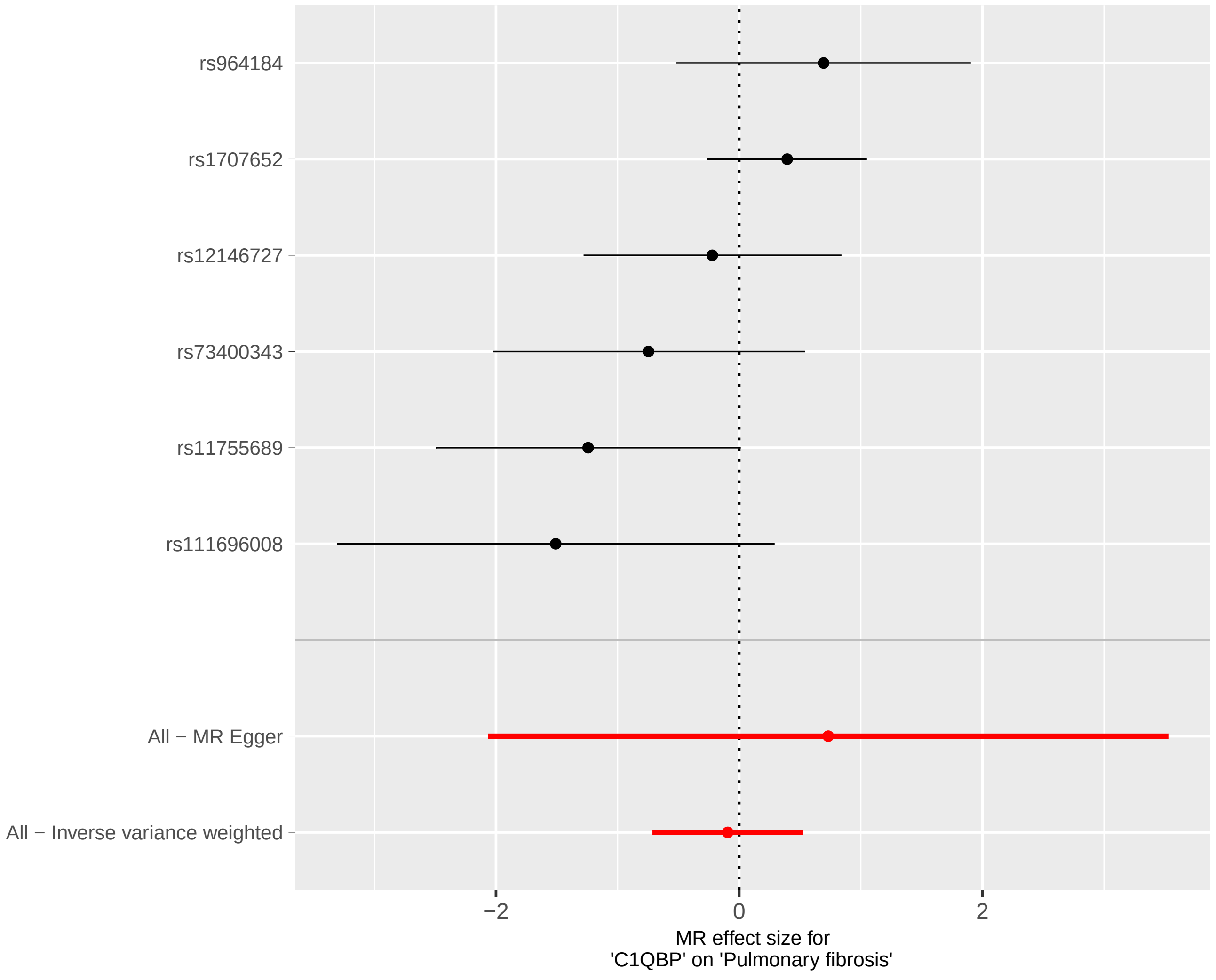

rs964184
rs1707652
rs12146727
rs73400343
rs11755689
rs111696008
All − MR Egger
All − Inverse variance weighted
−2
0
2
MR effect size for
'C1QBP' on 'Pulmonary fibrosis'

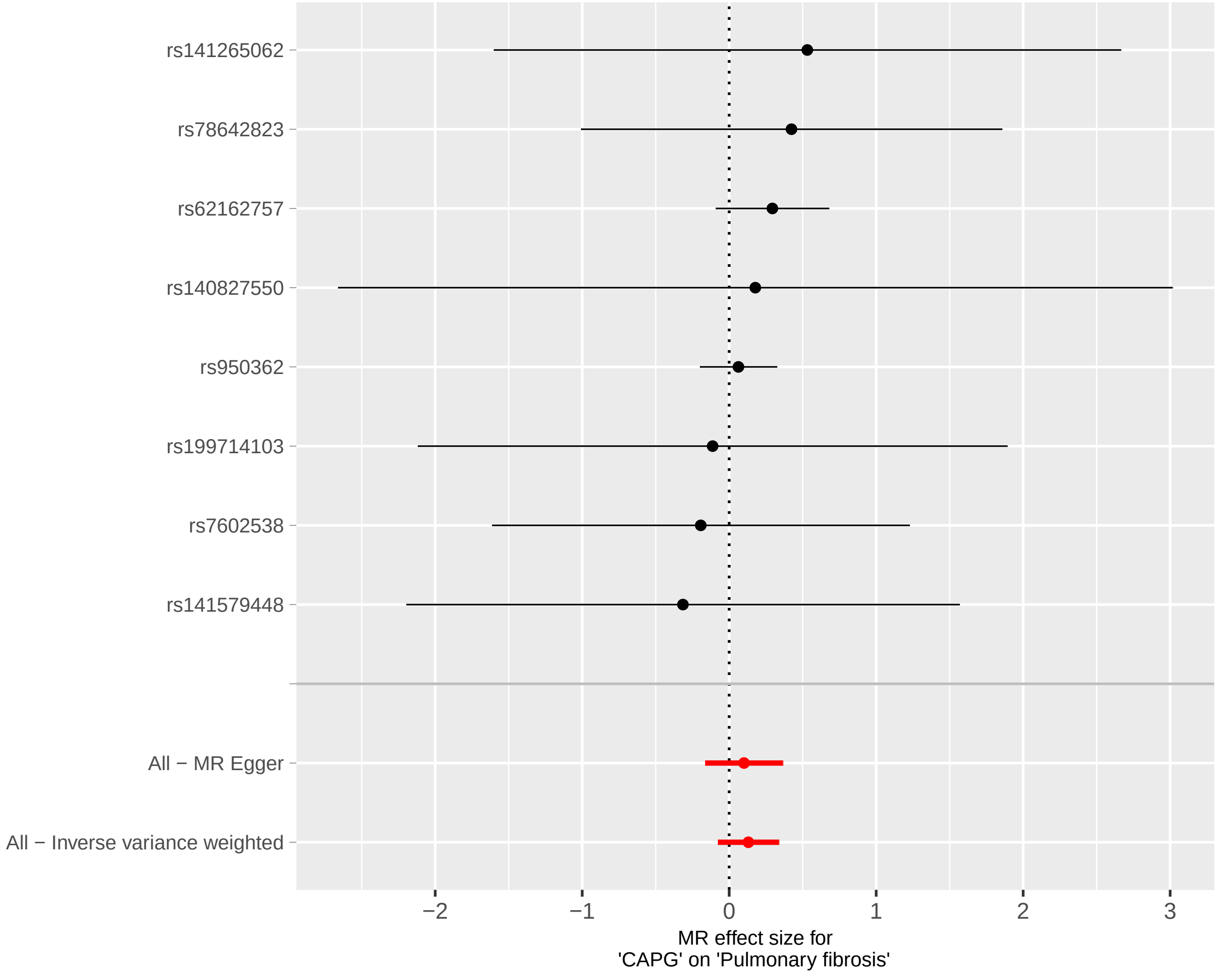

rs141265062
rs78642823
rs62162757
rs140827550
rs950362
rs199714103
rs7602538
rs141579448
All − MR Egger
All − Inverse variance weighted
−2
−1
0
1
2
3
MR effect size for
'CAPG' on 'Pulmonary fibrosis'

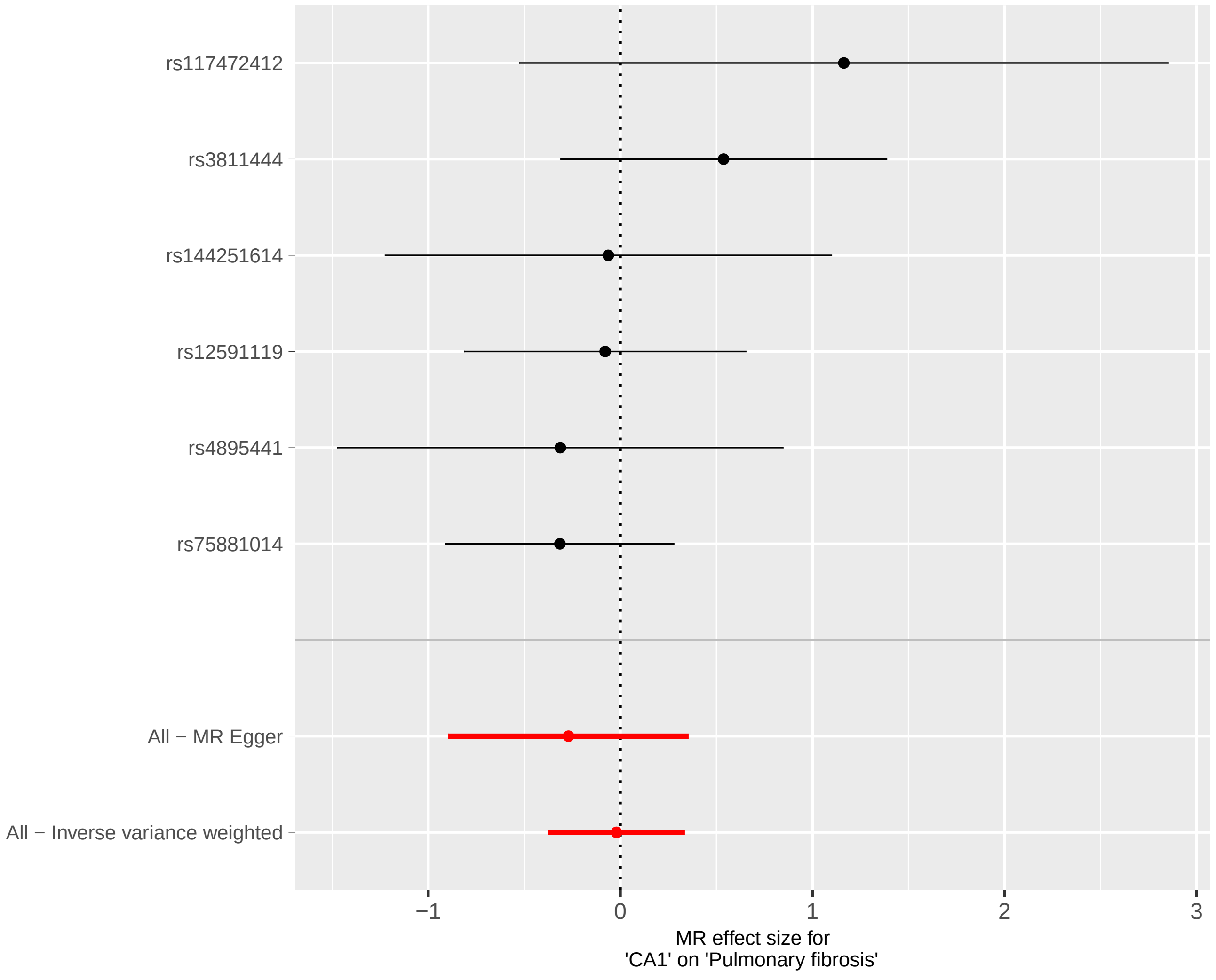

rs117472412
rs3811444
rs144251614
rs12591119
rs4895441
rs75881014
All − MR Egger
All − Inverse variance weighted
−1
0
1
2
3
MR effect size for
'CA1' on 'Pulmonary fibrosis'

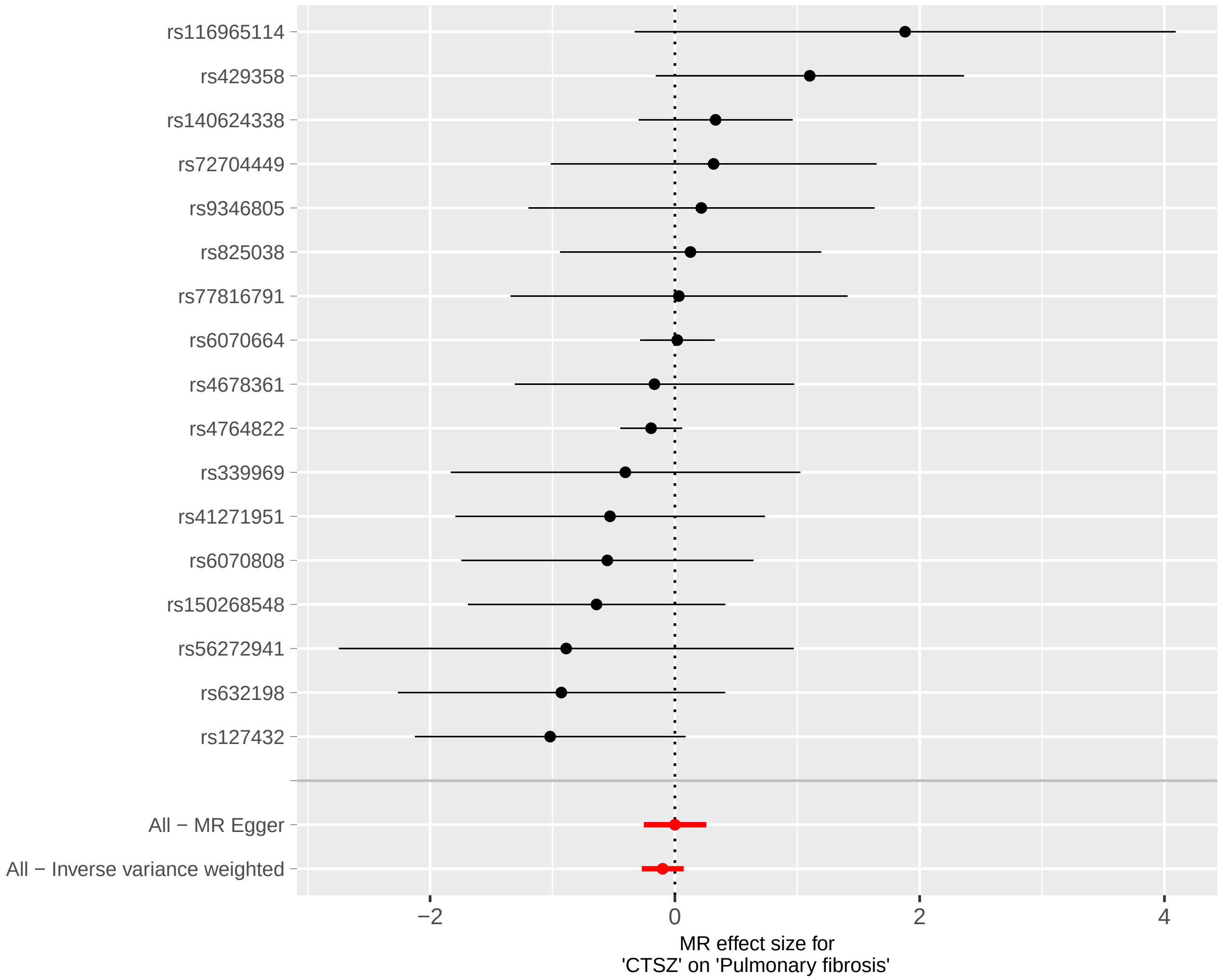
rs116965114
rs429358
rs140624338
rs72704449
rs9346805
rs825038
rs77816791
rs6070664
rs4678361
rs4764822
rs339969
rs41271951
rs6070808
rs150268548
rs56272941
rs632198
rs127432
All − MR Egger
All − Inverse variance weighted
−2
0
2
4
MR effect size for
'CTSZ' on 'Pulmonary fibrosis'

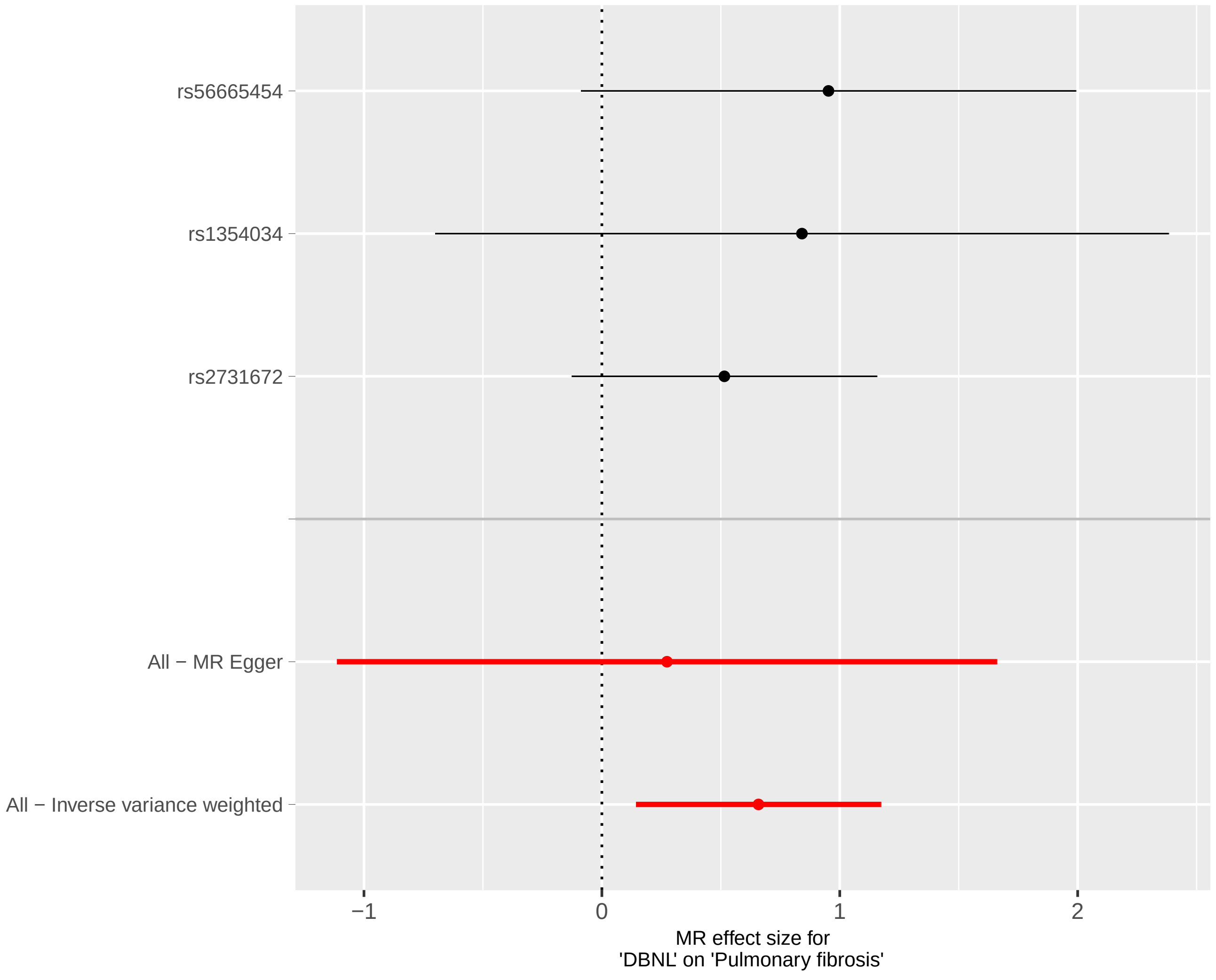

rs56665454
rs1354034
rs2731672
All − MR Egger
All − Inverse variance weighted
−1
0
1
2
MR effect size for
'DBNL' on 'Pulmonary fibrosis'

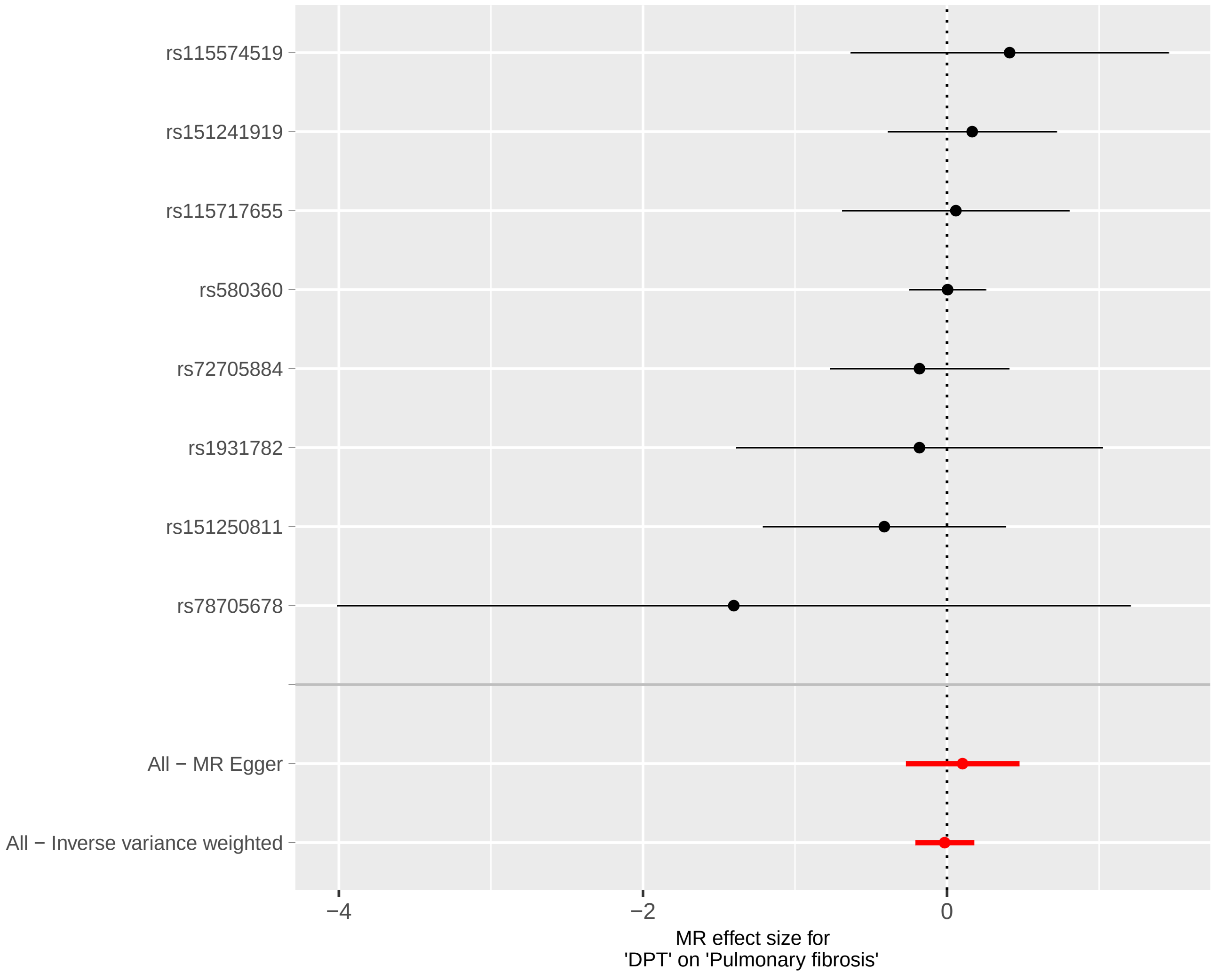

rs115574519
rs151241919
rs115717655
rs580360
rs72705884
rs1931782
rs151250811
rs78705678
All − MR Egger
All − Inverse variance weighted
−4
−2
0
MR effect size for
'DPT' on 'Pulmonary fibrosis'

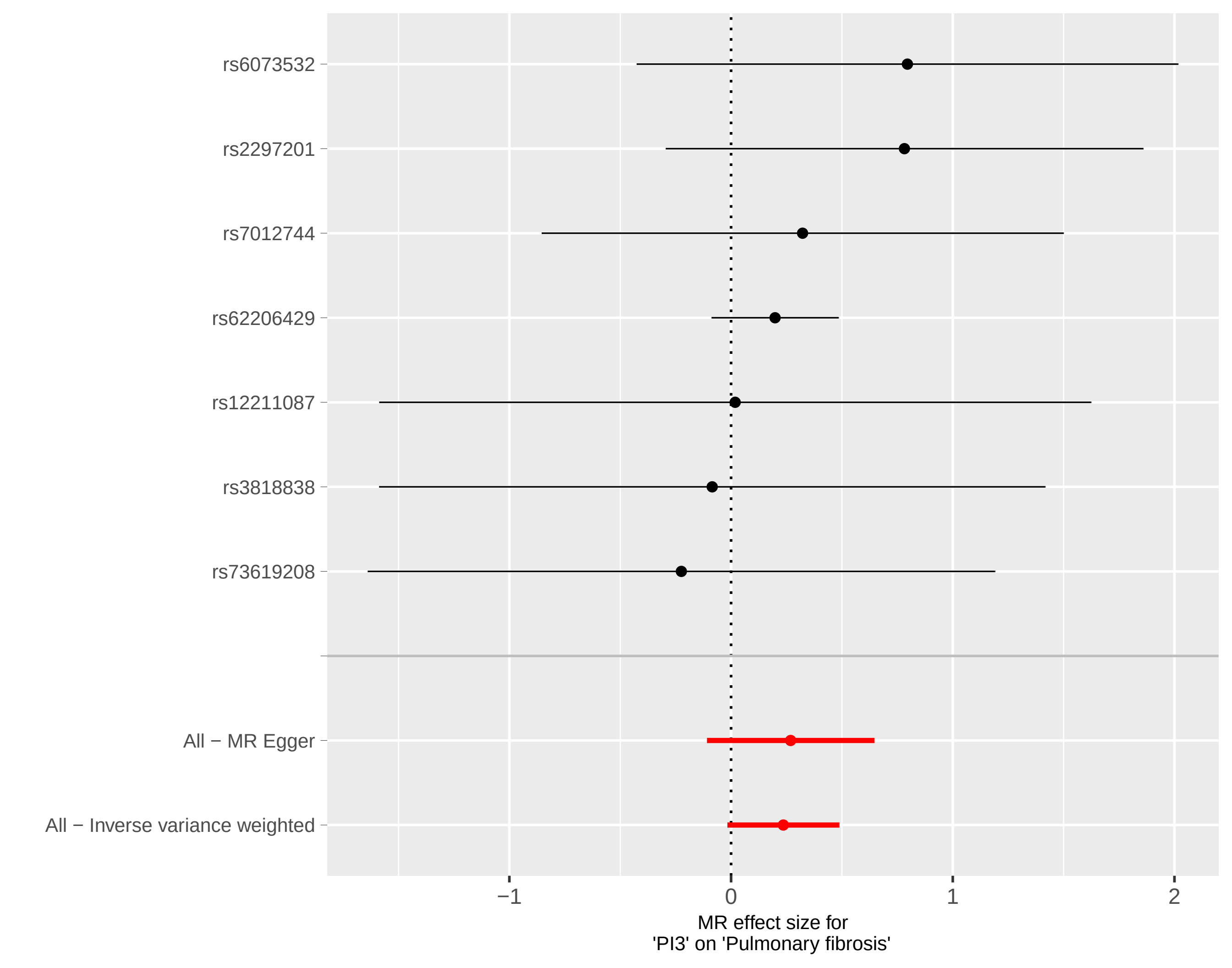
rs6073532
rs2297201
rs7012744
rs62206429
rs12211087
rs3818838
rs73619208
All − MR Egger
All − Inverse variance weighted
−1
0
1
2
MR effect size for
'PI3' on 'Pulmonary fibrosis'

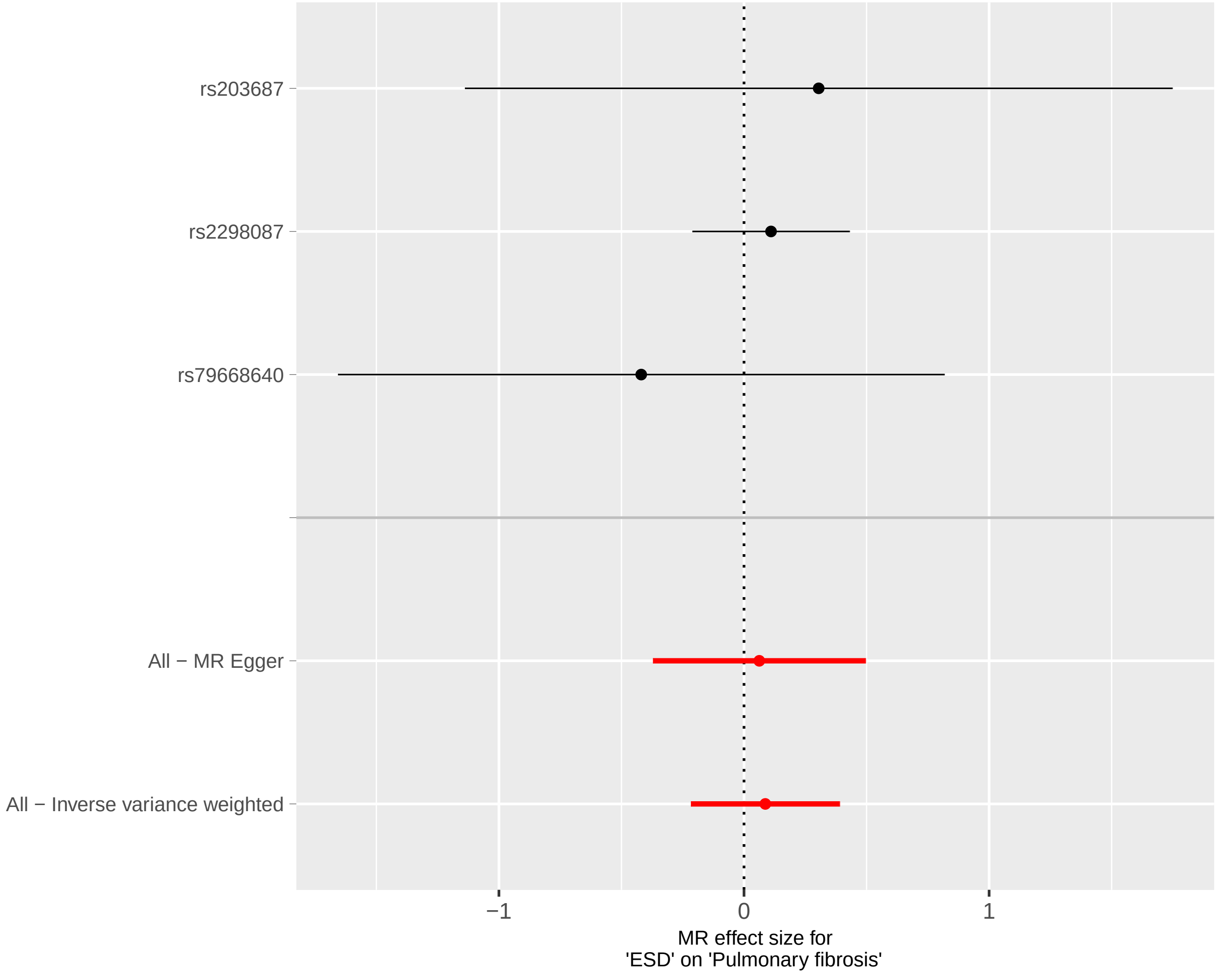
rs203687
rs2298087
rs79668640
All − MR Egger
All − Inverse variance weighted
−1
0
1
MR effect size for
'ESD' on 'Pulmonary fibrosis'

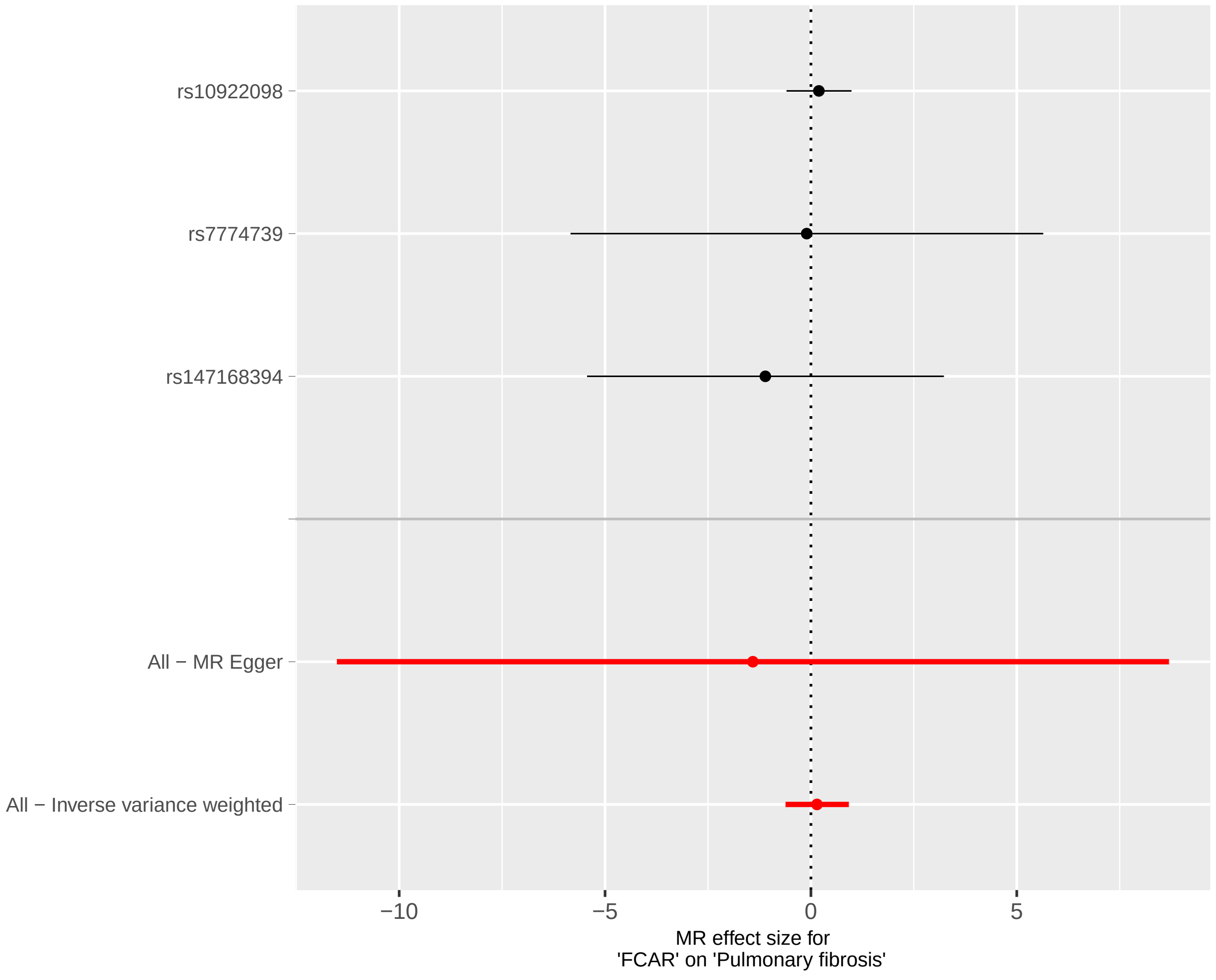
rs10922098
rs7774739
rs147168394
All − MR Egger
All − Inverse variance weighted
−10
−5
0
5
MR effect size for
'FCAR' on 'Pulmonary fibrosis'

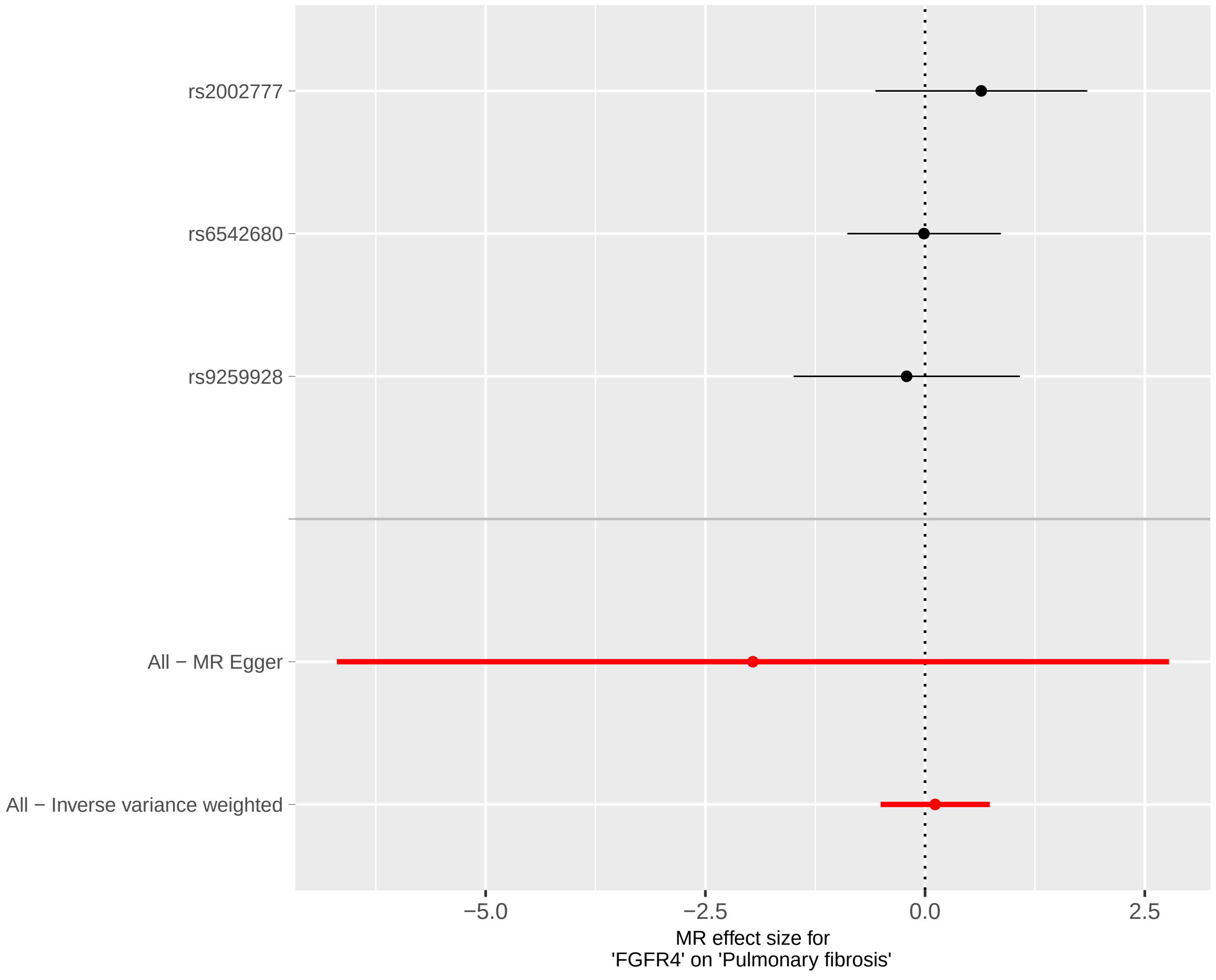
rs2002777
rs6542680
rs9259928
All − MR Egger
All − Inverse variance weighted
−5.0
−2.5
0.0
2.5
MR effect size for
'FGFR4' on 'Pulmonary fibrosis'

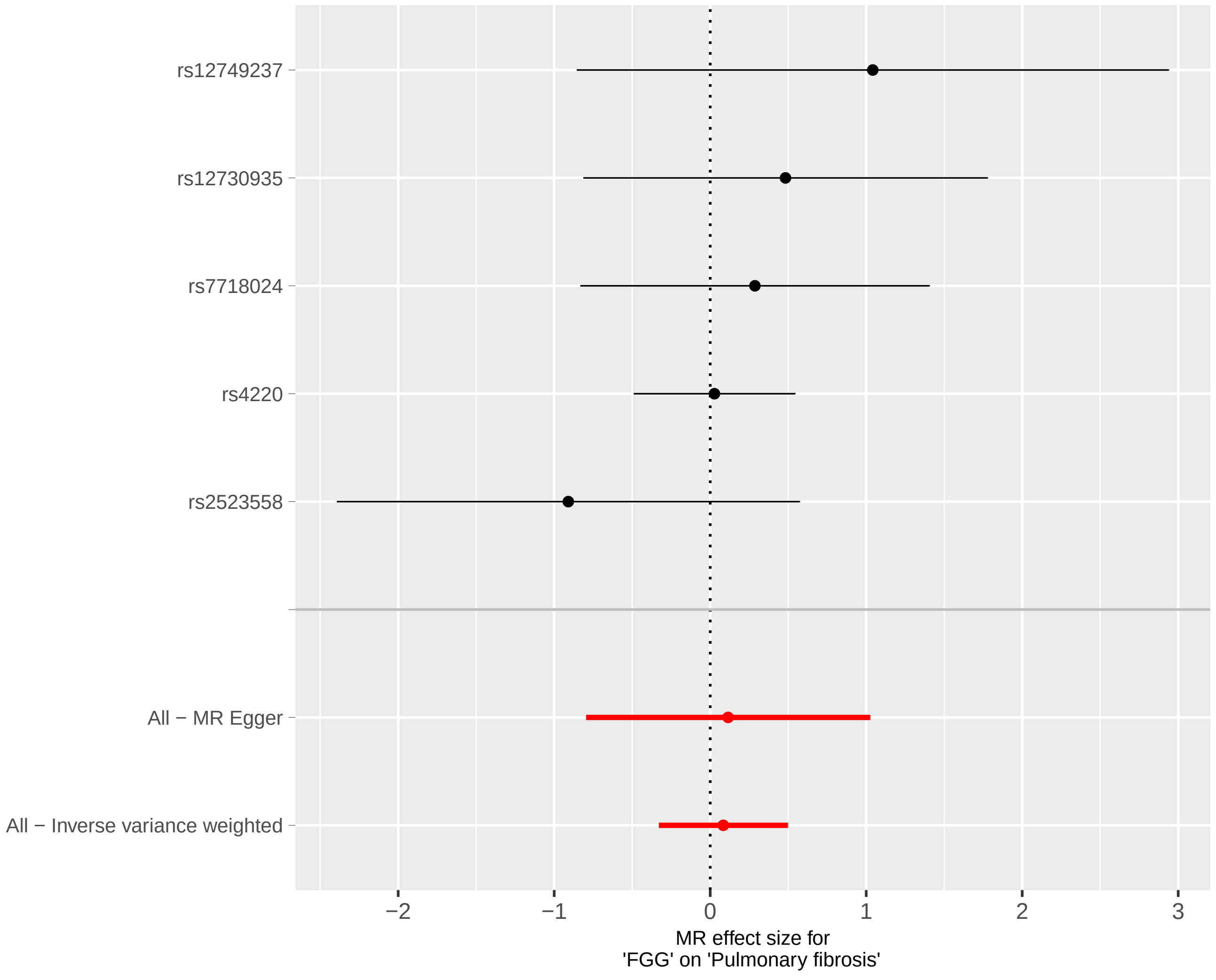

rs12749237
rs12730935
rs7718024
rs4220
rs2523558
All − MR Egger
All − Inverse variance weighted
−2
−1
0
1
2
3
MR effect size for
'FGG' on 'Pulmonary fibrosis'

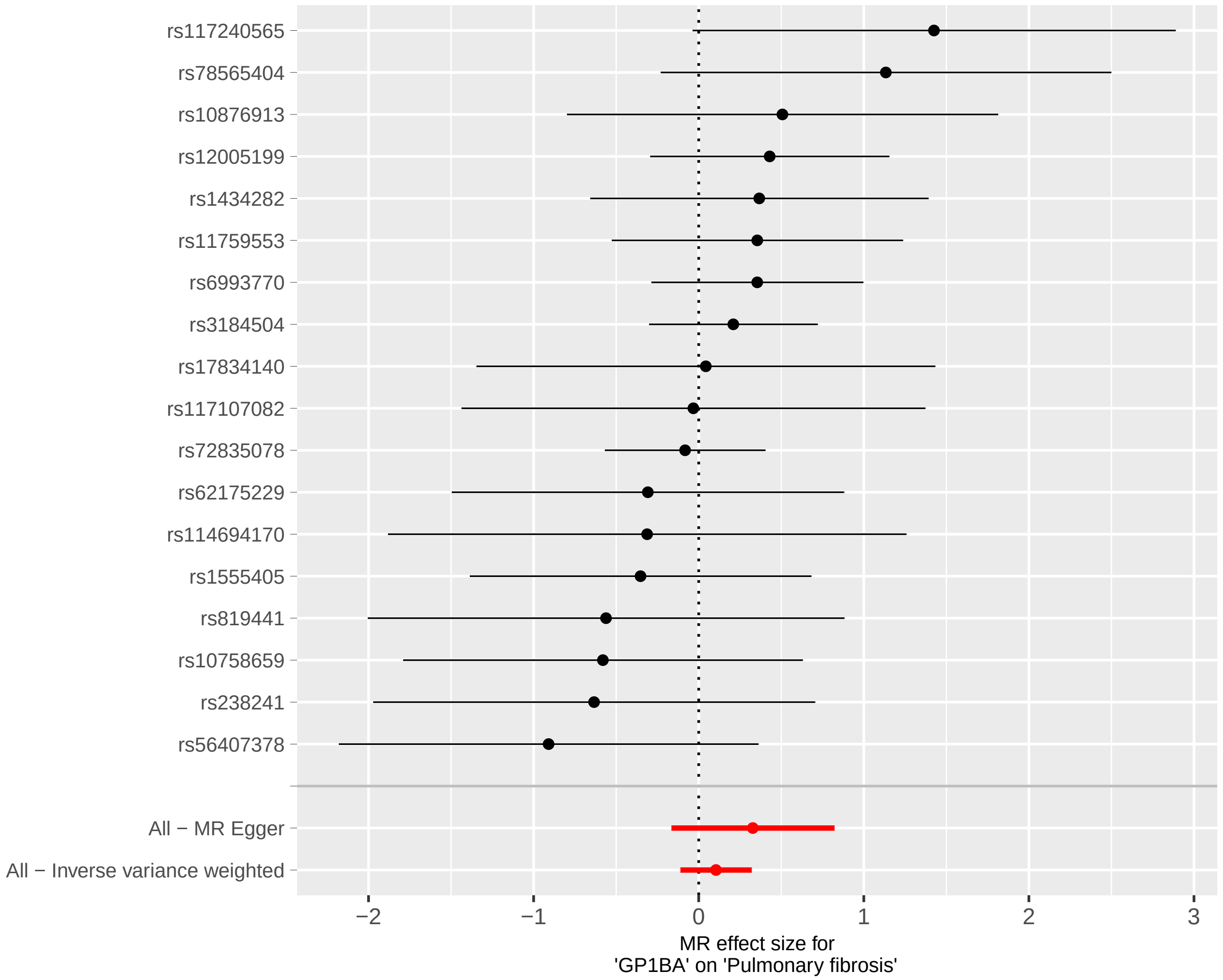

rs117240565
rs78565404
rs10876913
rs12005199
rs1434282
rs11759553
rs6993770
rs3184504
rs17834140
rs117107082
rs72835078
rs62175229
rs114694170
rs1555405
rs819441
rs10758659
rs238241
rs56407378
All − MR Egger
All − Inverse variance weighted
−2
−1
0
1
2
3
MR effect size for
'GP1BA' on 'Pulmonary fibrosis'

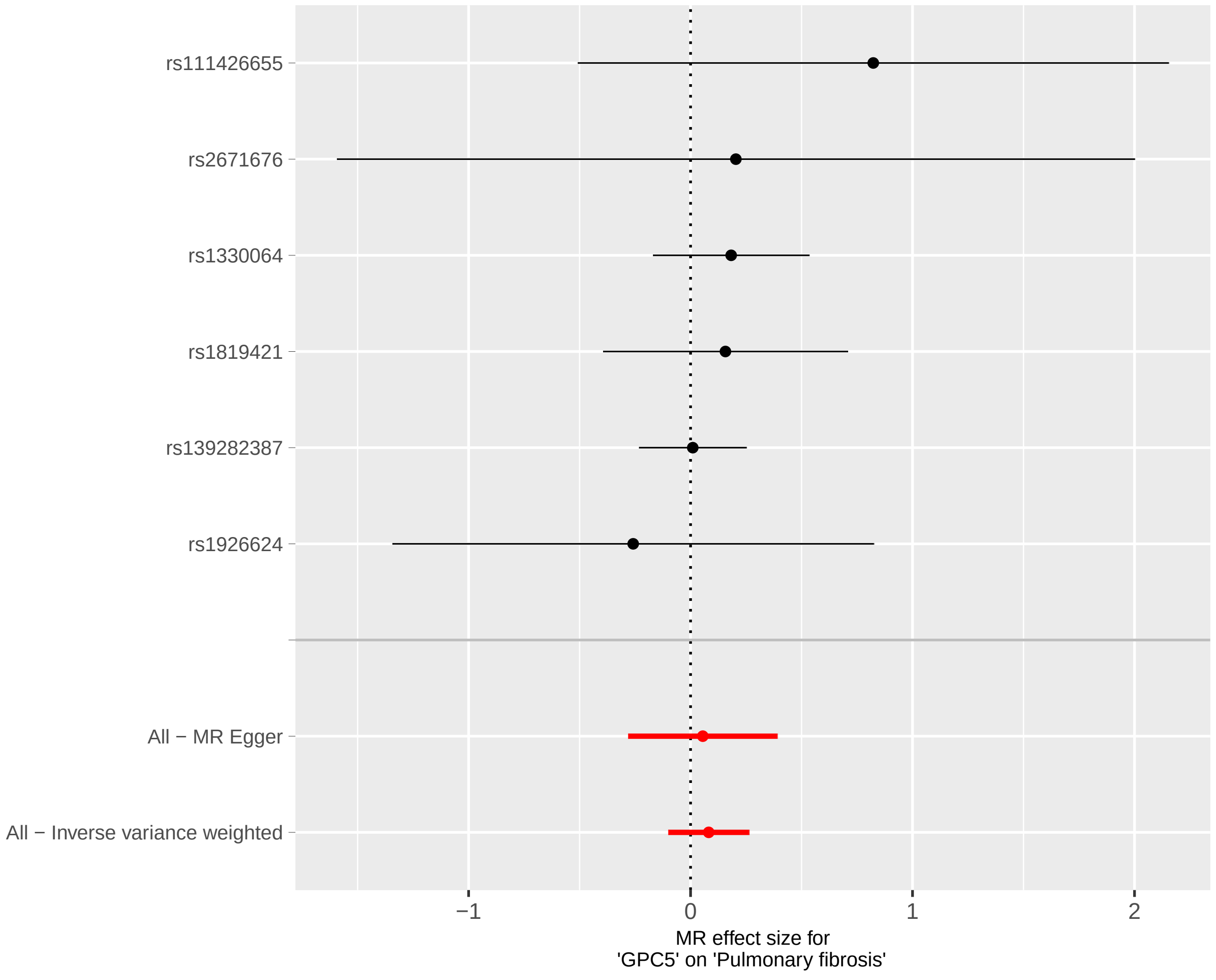

rs111426655
rs2671676
rs1330064
rs1819421
rs139282387
rs1926624
All − MR Egger
All − Inverse variance weighted
−1
0
1
2
MR effect size for
'GPC5' on 'Pulmonary fibrosis'

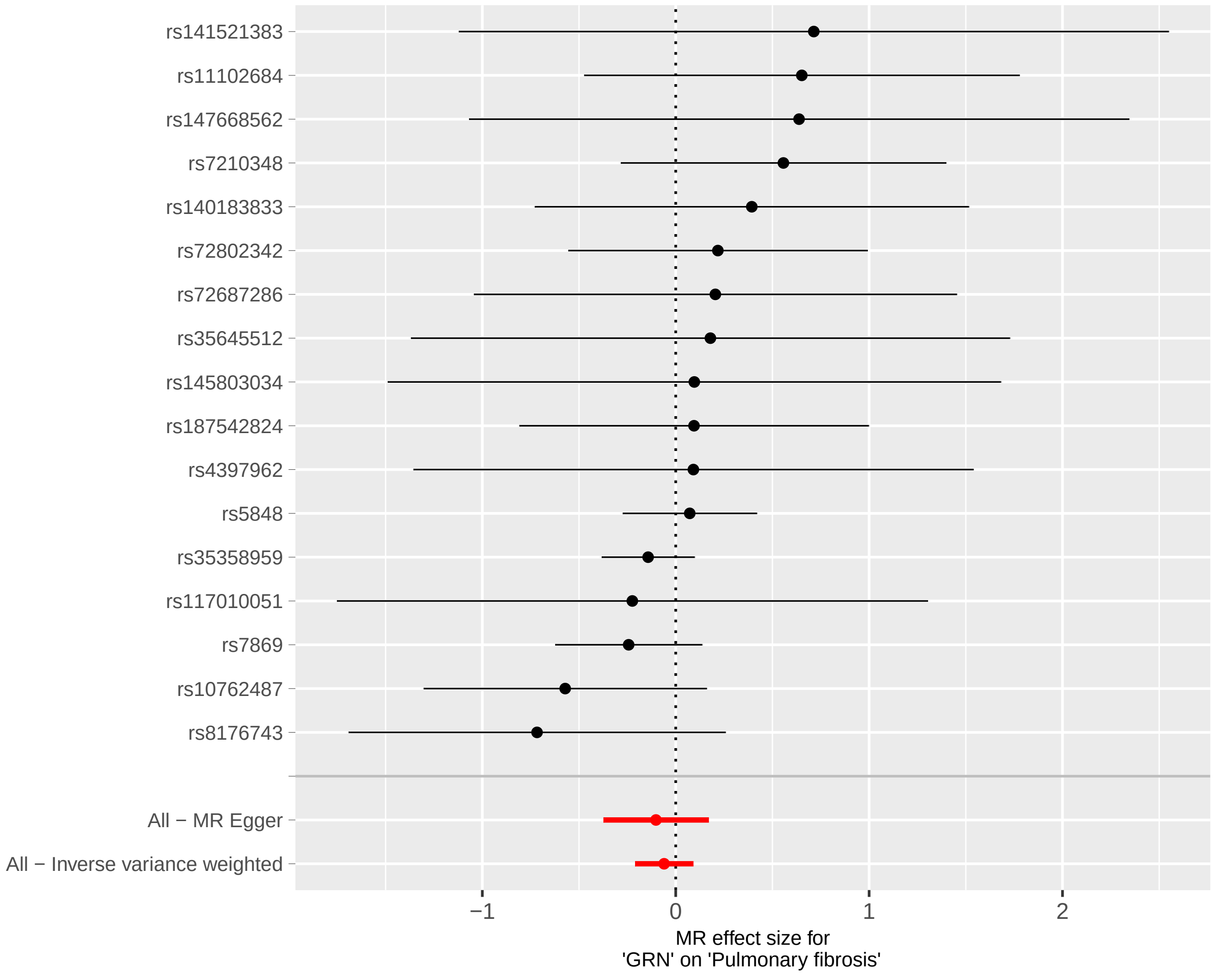
rs141521383
rs11102684
rs147668562
rs7210348
rs140183833
rs72802342
rs72687286
rs35645512
rs145803034
rs187542824
rs4397962
rs5848
rs35358959
rs117010051
rs7869
rs10762487
rs8176743
All − MR Egger
All − Inverse variance weighted
−1
0
1
2
MR effect size for
'GRN' on 'Pulmonary fibrosis'

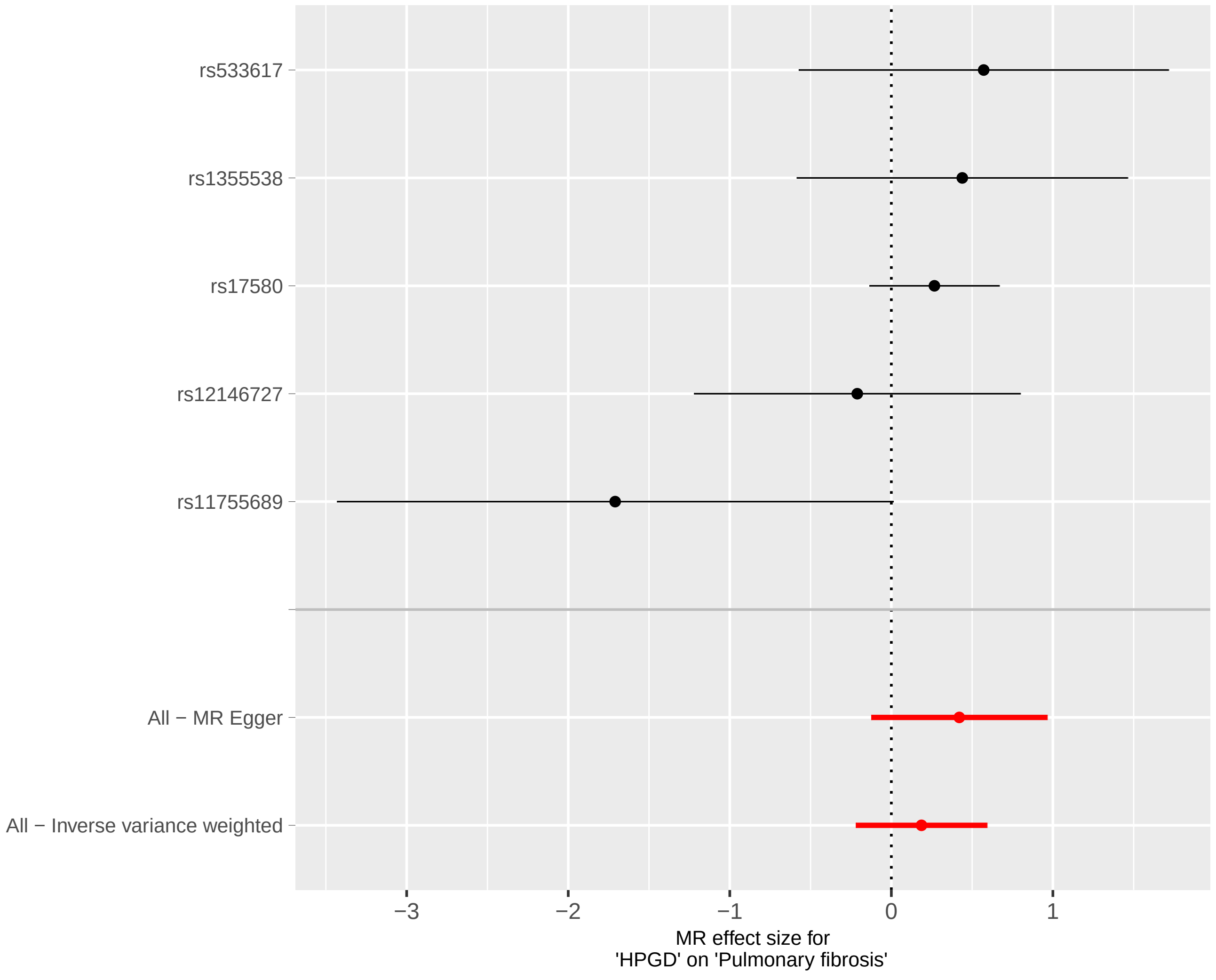

rs533617
rs1355538
rs17580
rs12146727
rs11755689
All − MR Egger
All − Inverse variance weighted
−3
−2
−1
0
1
MR effect size for
'HPGD' on 'Pulmonary fibrosis'

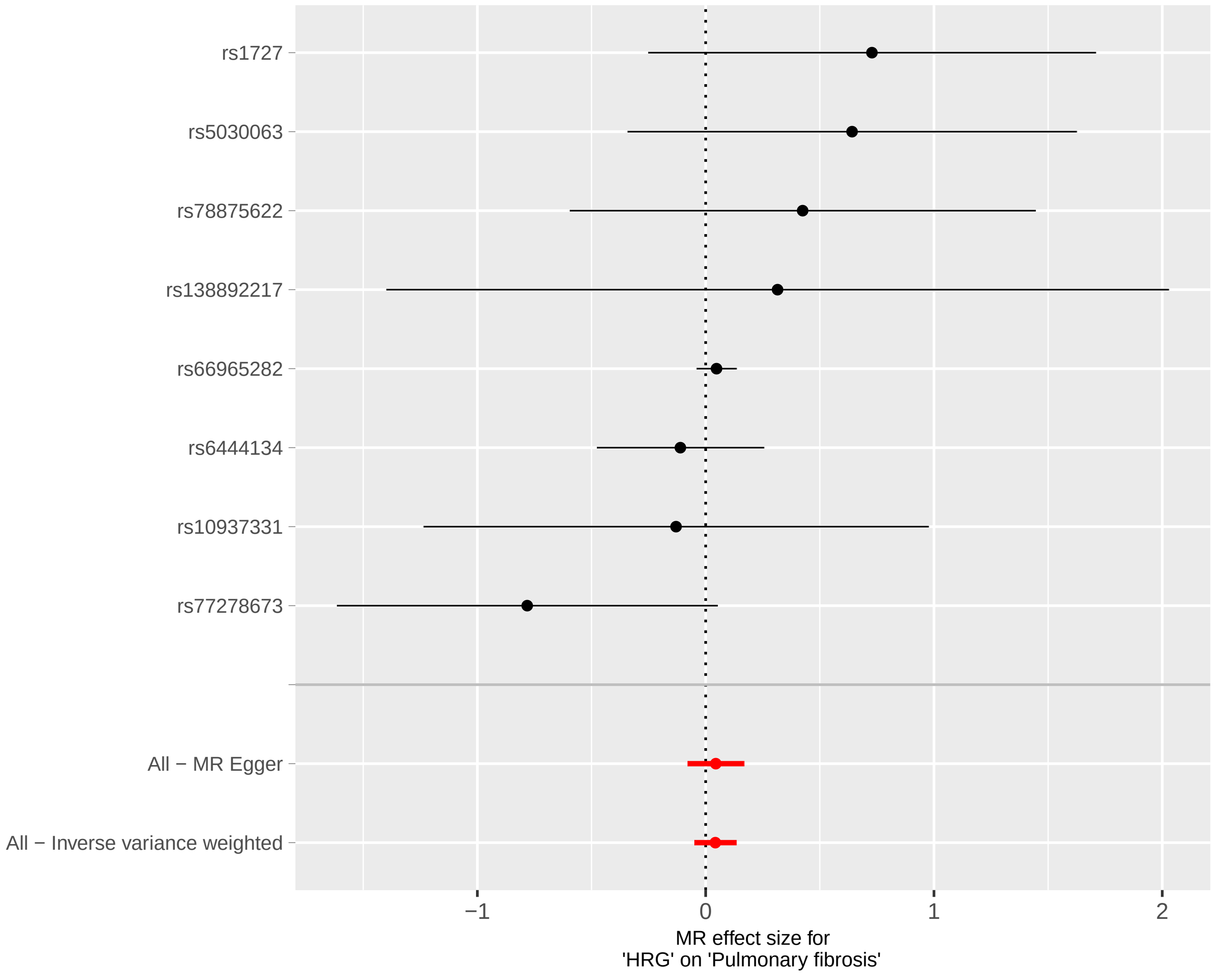
rs1727
rs5030063
rs78875622
rs138892217
rs66965282
rs6444134
rs10937331
rs77278673
All − MR Egger
All − Inverse variance weighted
−1
0
1
2
MR effect size for
'HRG' on 'Pulmonary fibrosis'

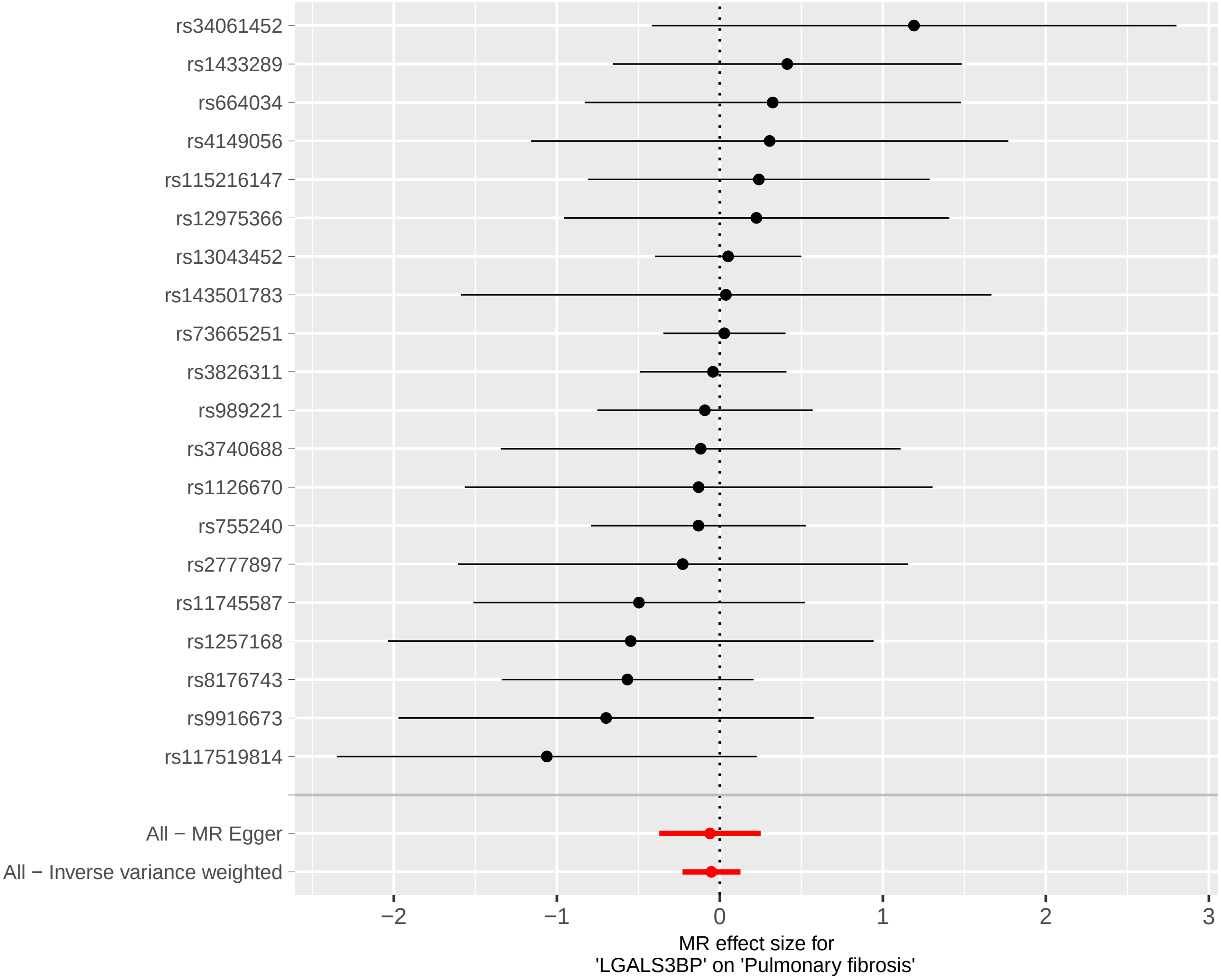
rs34061452
rs1433289
rs664034
rs4149056
rs115216147
rs12975366
rs13043452
rs143501783
rs73665251
rs3826311
rs989221
rs3740688
rs1126670
rs755240
rs2777897
rs11745587
rs1257168
rs8176743
rs9916673
rs117519814
All − MR Egger
All − Inverse variance weighted
−2
−1
0
1
2
3
MR effect size for
'LGALS3BP' on 'Pulmonary fibrosis'

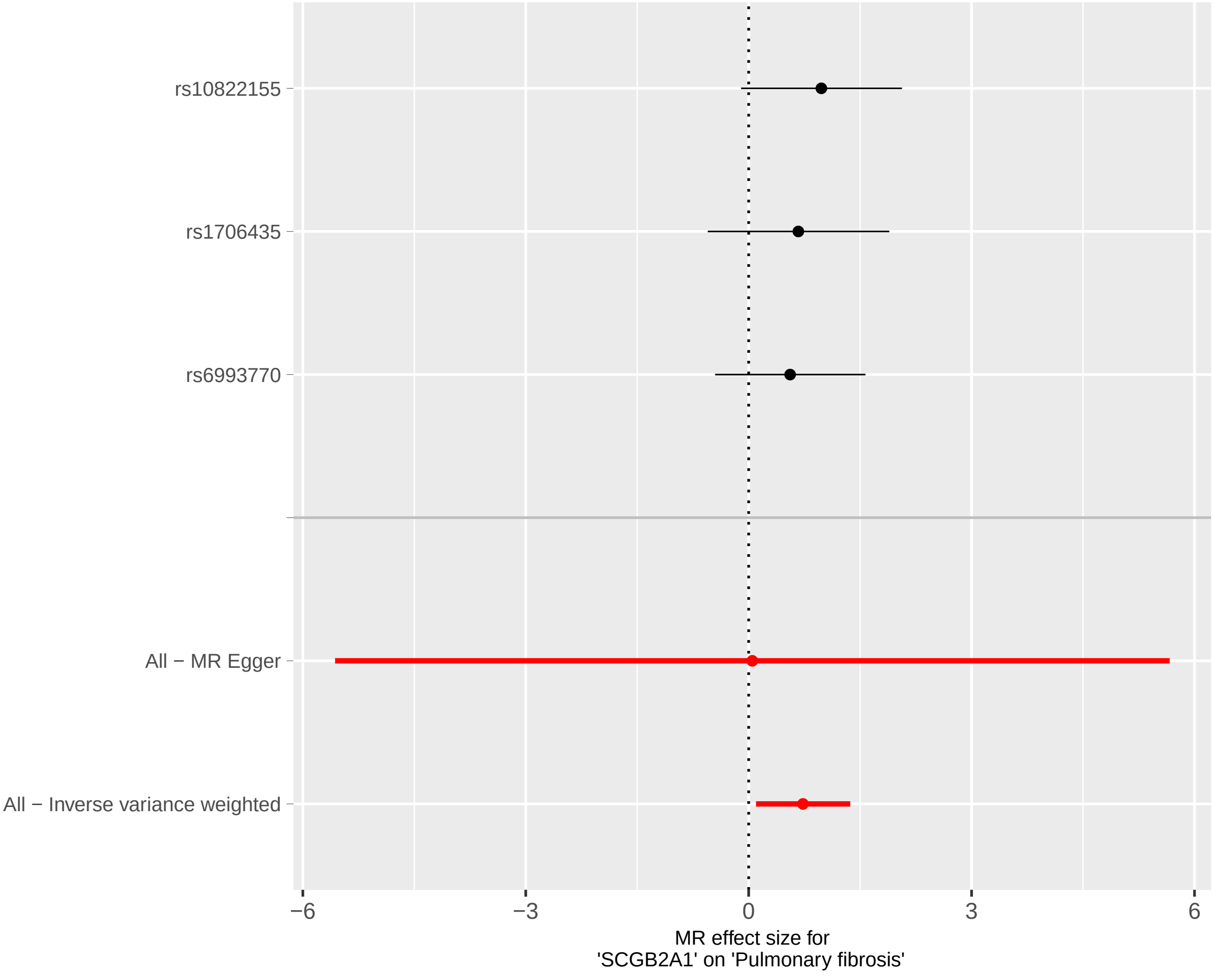

rs10822155
rs1706435
rs6993770
All − MR Egger
All − Inverse variance weighted
−6
−3
0
3
6
MR effect size for
'SCGB2A1' on 'Pulmonary fibrosis'

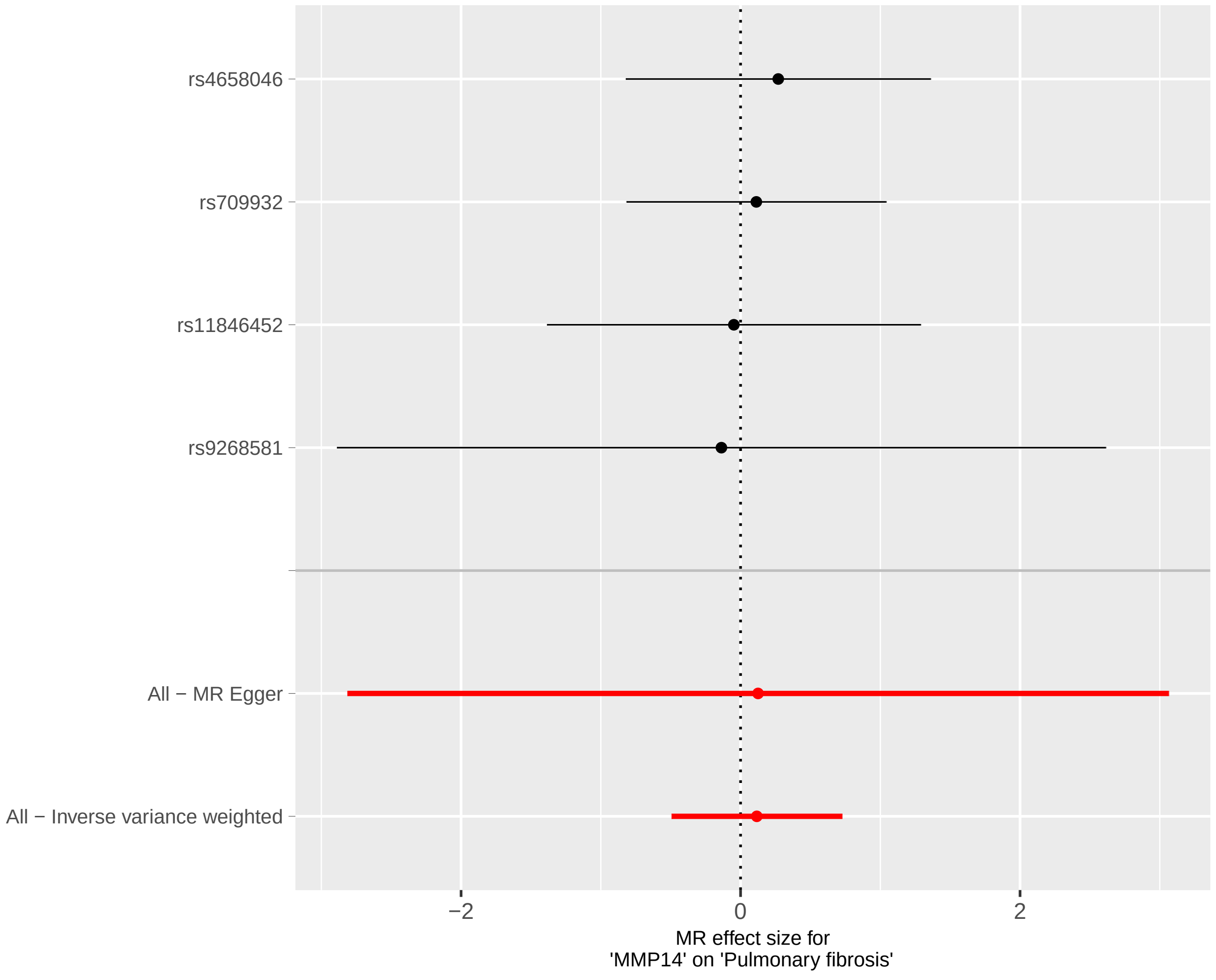

rs4658046
rs709932
rs11846452
rs9268581
All − MR Egger
All − Inverse variance weighted
−2
0
2
MR effect size for
'MMP14' on 'Pulmonary fibrosis'

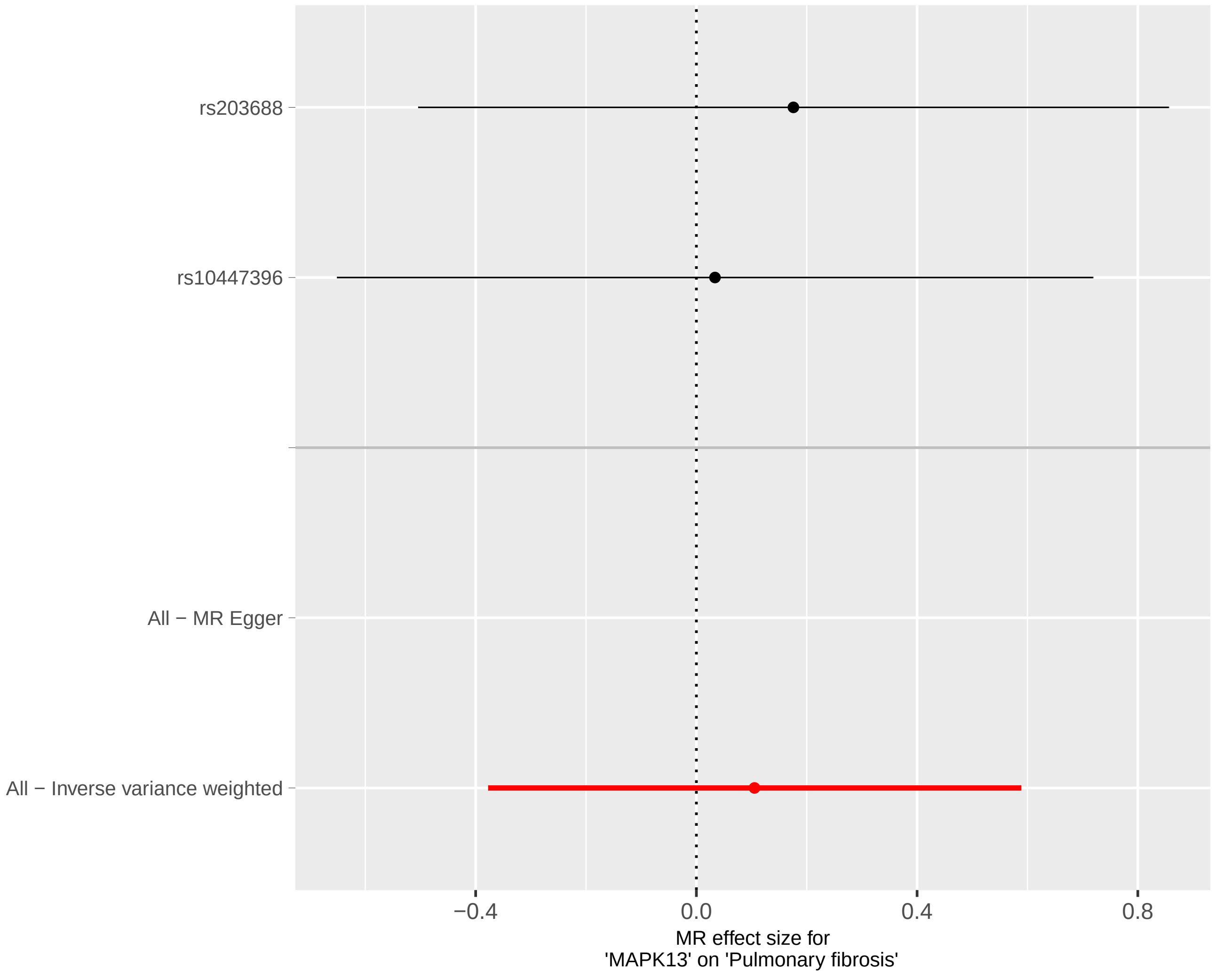
rs203688
rs10447396
All − MR Egger
All − Inverse variance weighted
−0.4
0.0
0.4
0.8
MR effect size for
'MAPK13' on 'Pulmonary fibrosis'

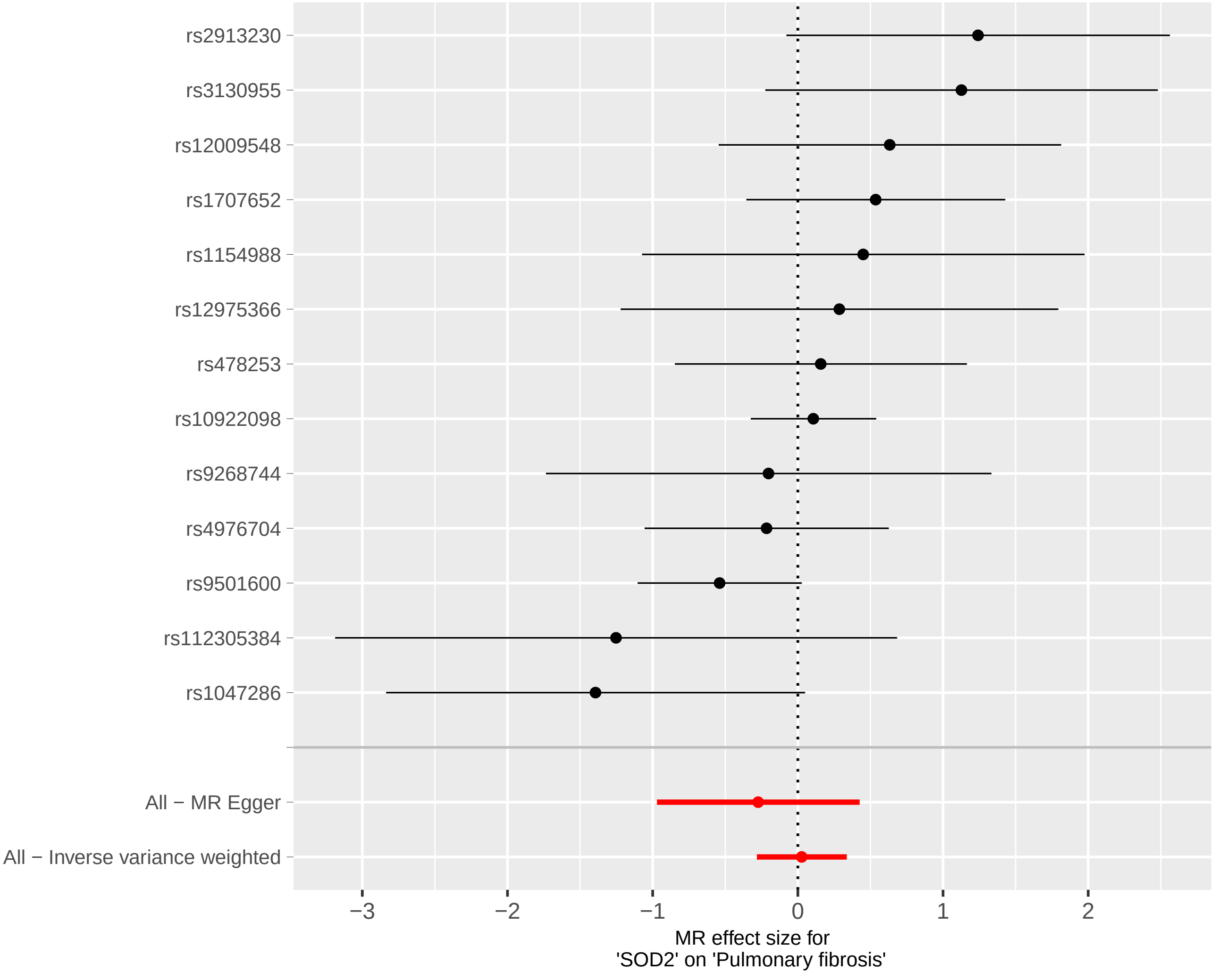

rs2913230
rs3130955
rs12009548
rs1707652
rs1154988
rs12975366
rs478253
rs10922098
rs9268744
rs4976704
rs9501600
rs112305384
rs1047286
All − MR Egger
All − Inverse variance weighted
−3
−2
−1
0
1
2
MR effect size for
'SOD2' on 'Pulmonary fibrosis'

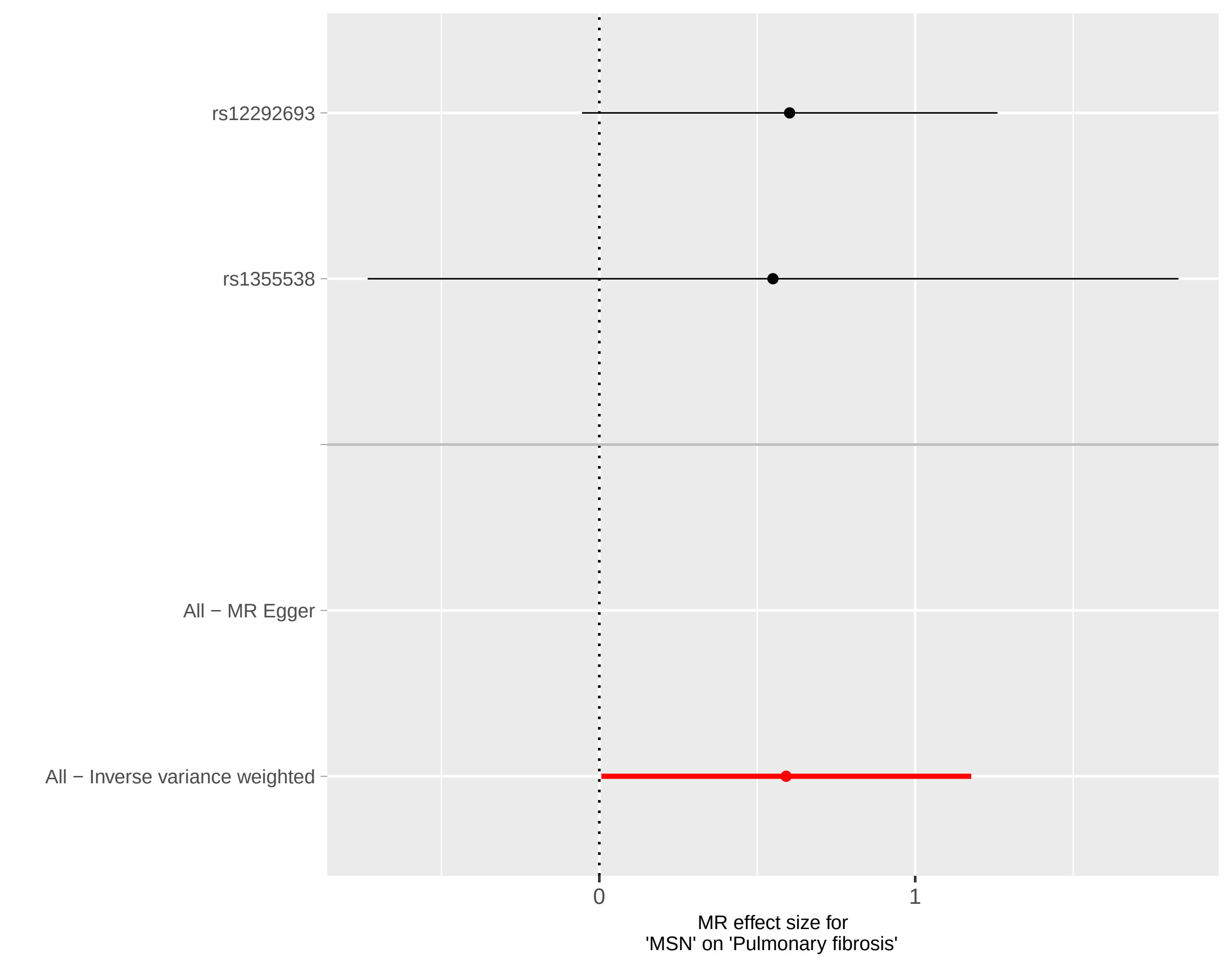
rs12292693
rs1355538
All − MR Egger
All − Inverse variance weighted
0
1
MR effect size for
'MSN' on 'Pulmonary fibrosis'

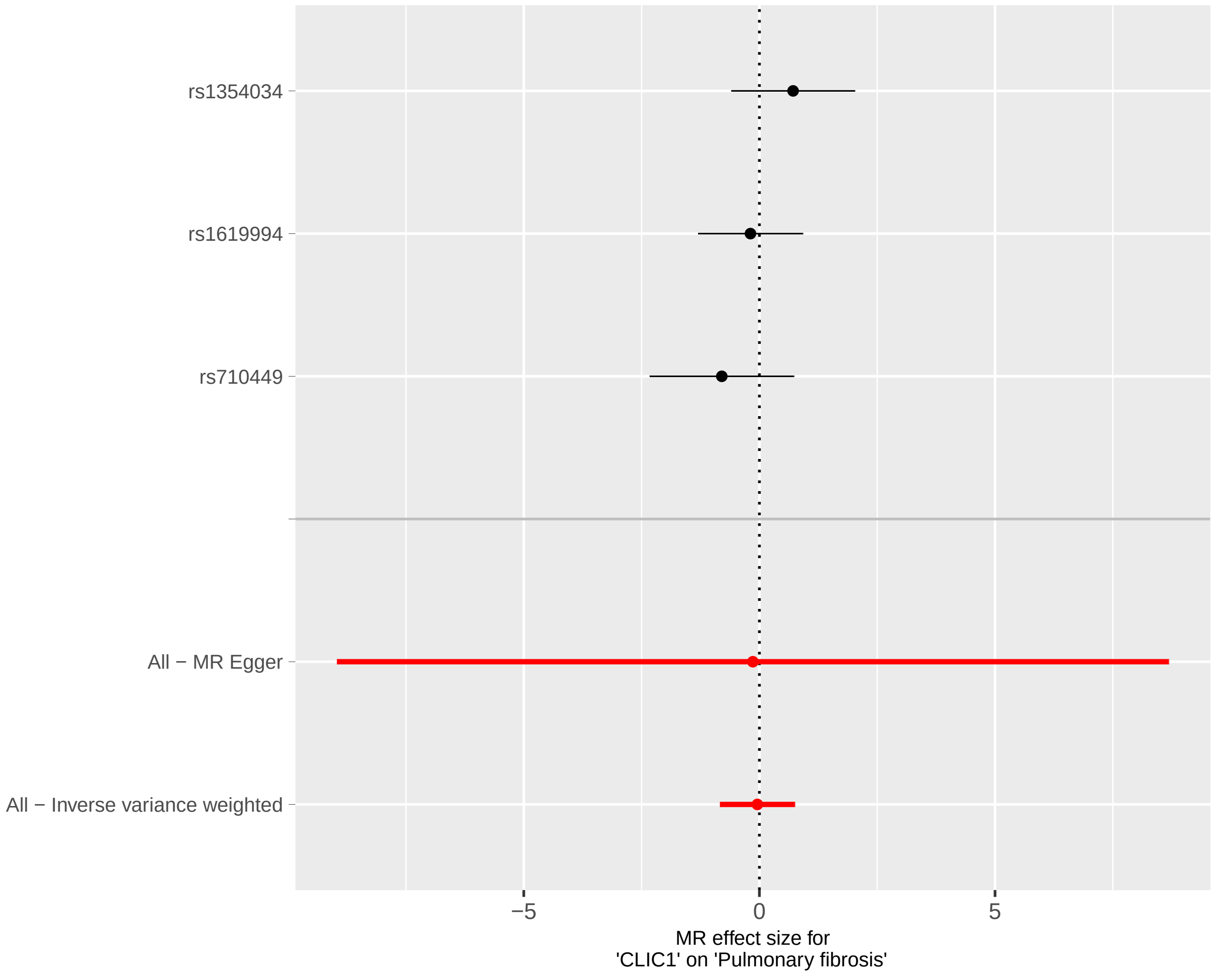

rs1354034
rs1619994
rs710449
All − MR Egger
All − Inverse variance weighted
−5
0
5
MR effect size for
'CLIC1' on 'Pulmonary fibrosis'

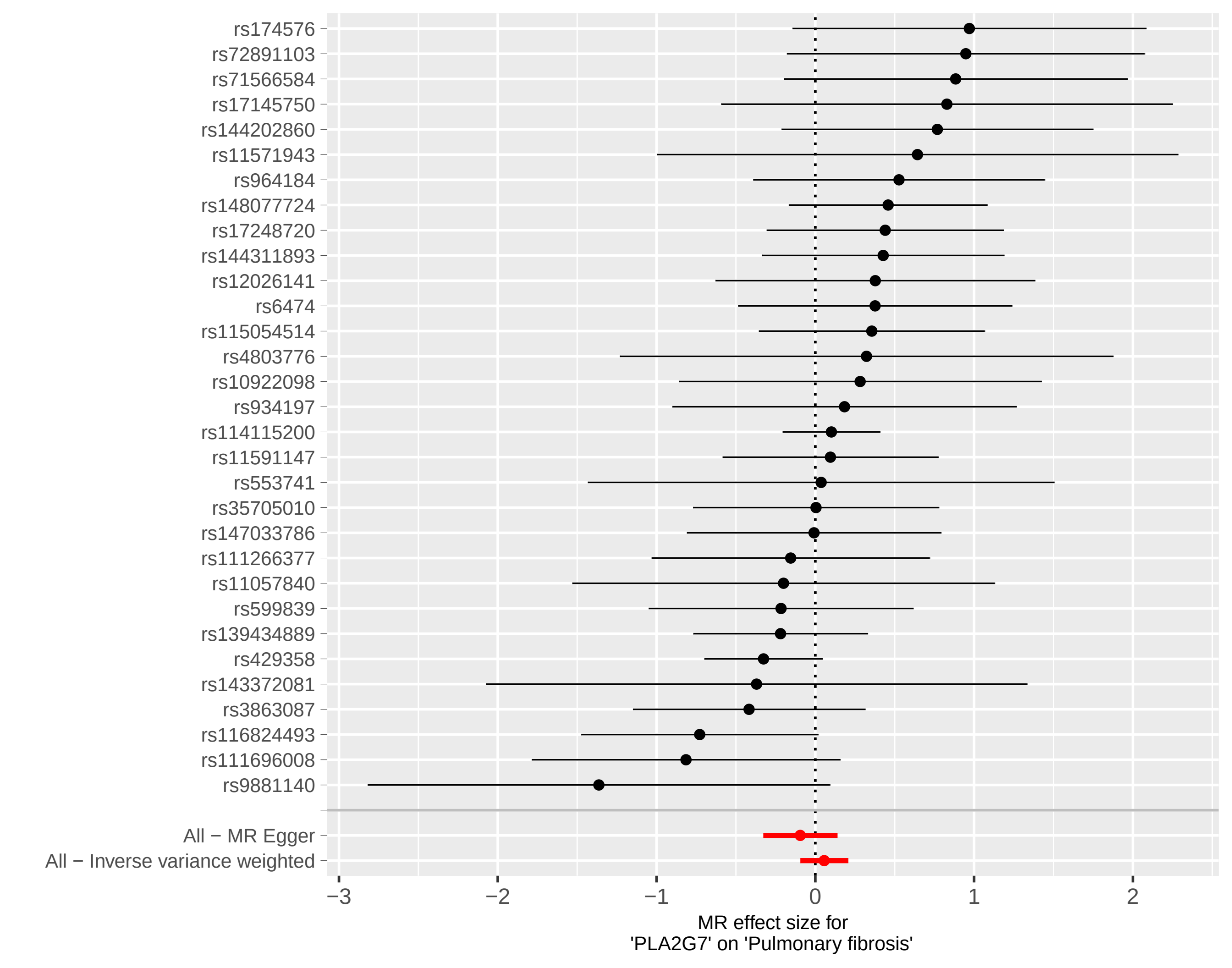
rs174576
rs72891103
rs71566584
rs17145750
rs144202860
rs11571943
rs964184
rs148077724
rs17248720
rs144311893
rs12026141
rs6474
rs115054514
rs4803776
rs10922098
rs934197
rs114115200
rs11591147
rs553741
rs35705010
rs147033786
rs111266377
rs11057840
rs599839
rs139434889
rs429358
rs143372081
rs3863087
rs116824493
rs111696008
rs9881140
All − MR Egger
All − Inverse variance weighted
−3
−2
−1
0
1
2
MR effect size for
'PLA2G7' on 'Pulmonary fibrosis'

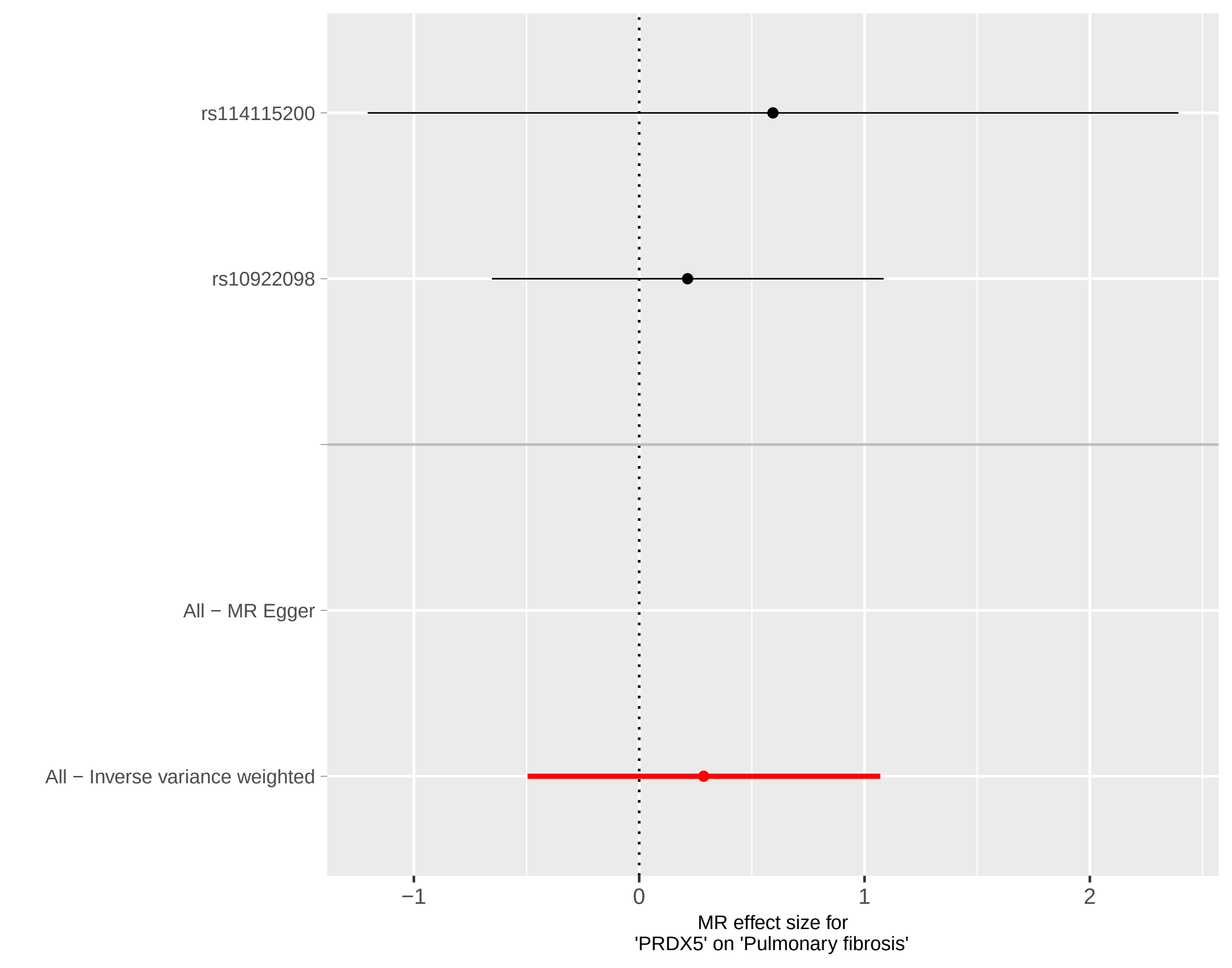

rs114115200
rs10922098
All − MR Egger
All − Inverse variance weighted
−1
0
1
2
MR effect size for
'PRDX5' on 'Pulmonary fibrosis'

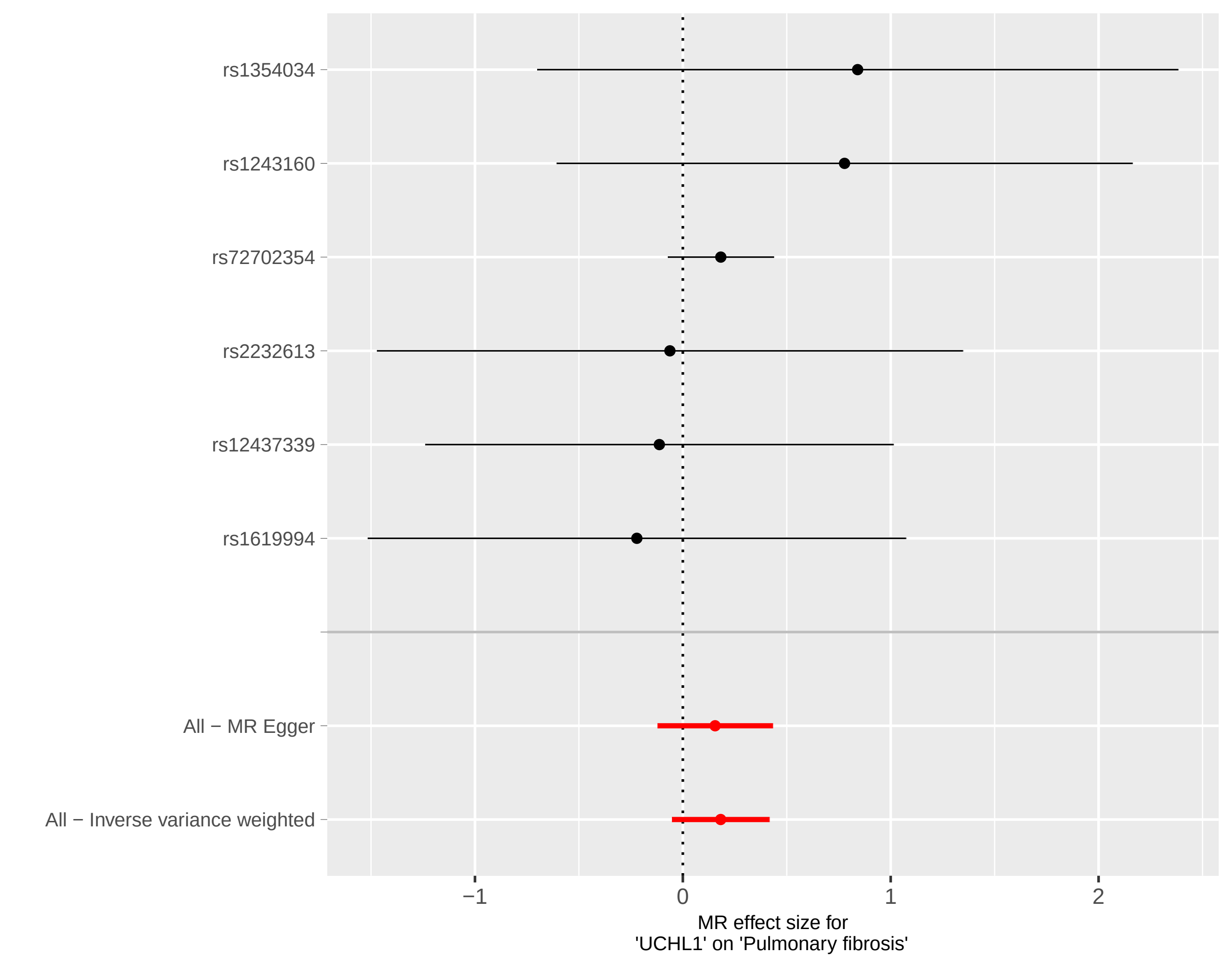
rs1354034
rs1243160
rs72702354
rs2232613
rs12437339
rs1619994
All − MR Egger
All − Inverse variance weighted
−1
0
1
2
MR effect size for
'UCHL1' on 'Pulmonary fibrosis'

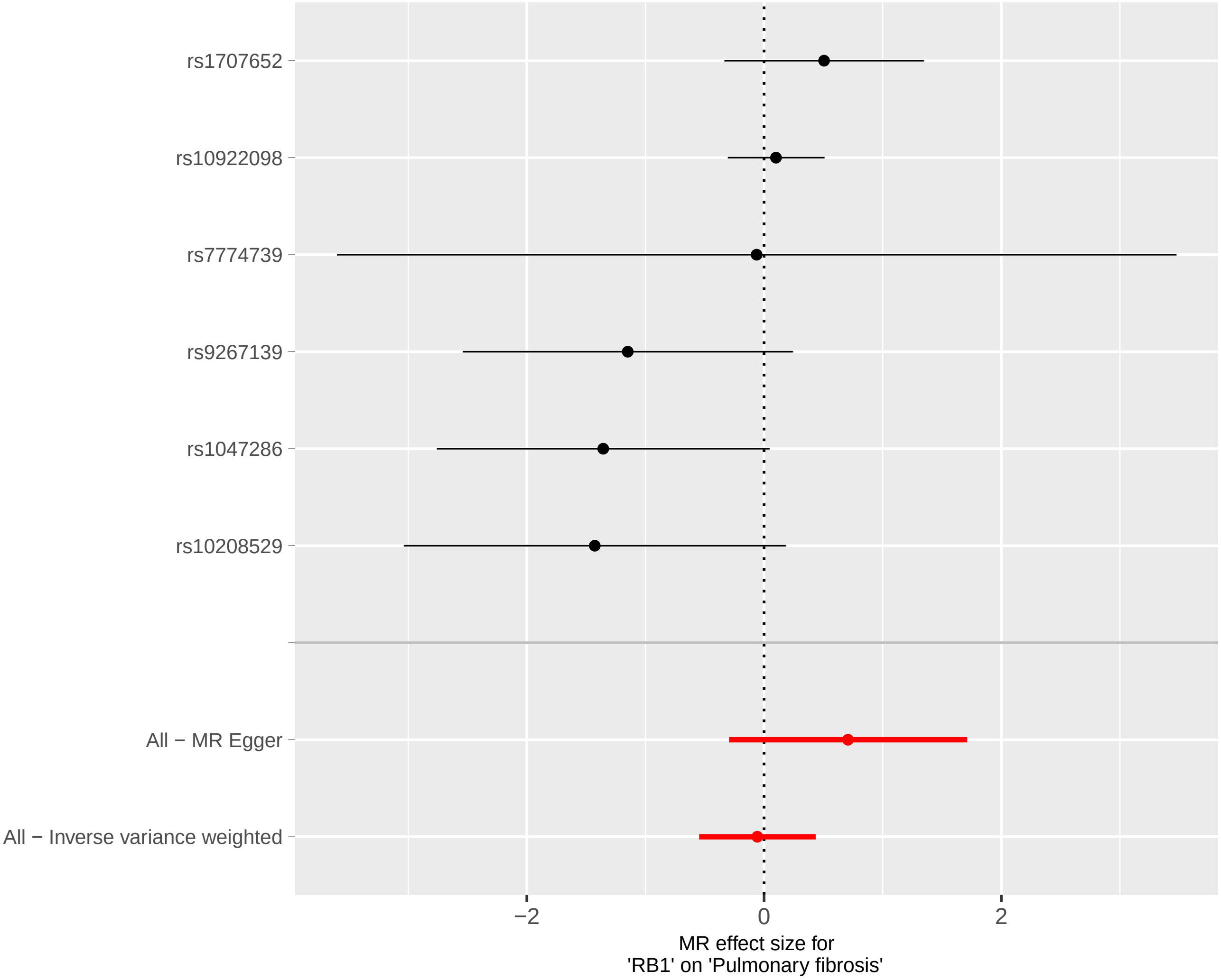

rs1707652
rs10922098
rs7774739
rs9267139
rs1047286
rs10208529
All − MR Egger
All − Inverse variance weighted
−2
0
2
MR effect size for
'RB1' on 'Pulmonary fibrosis'

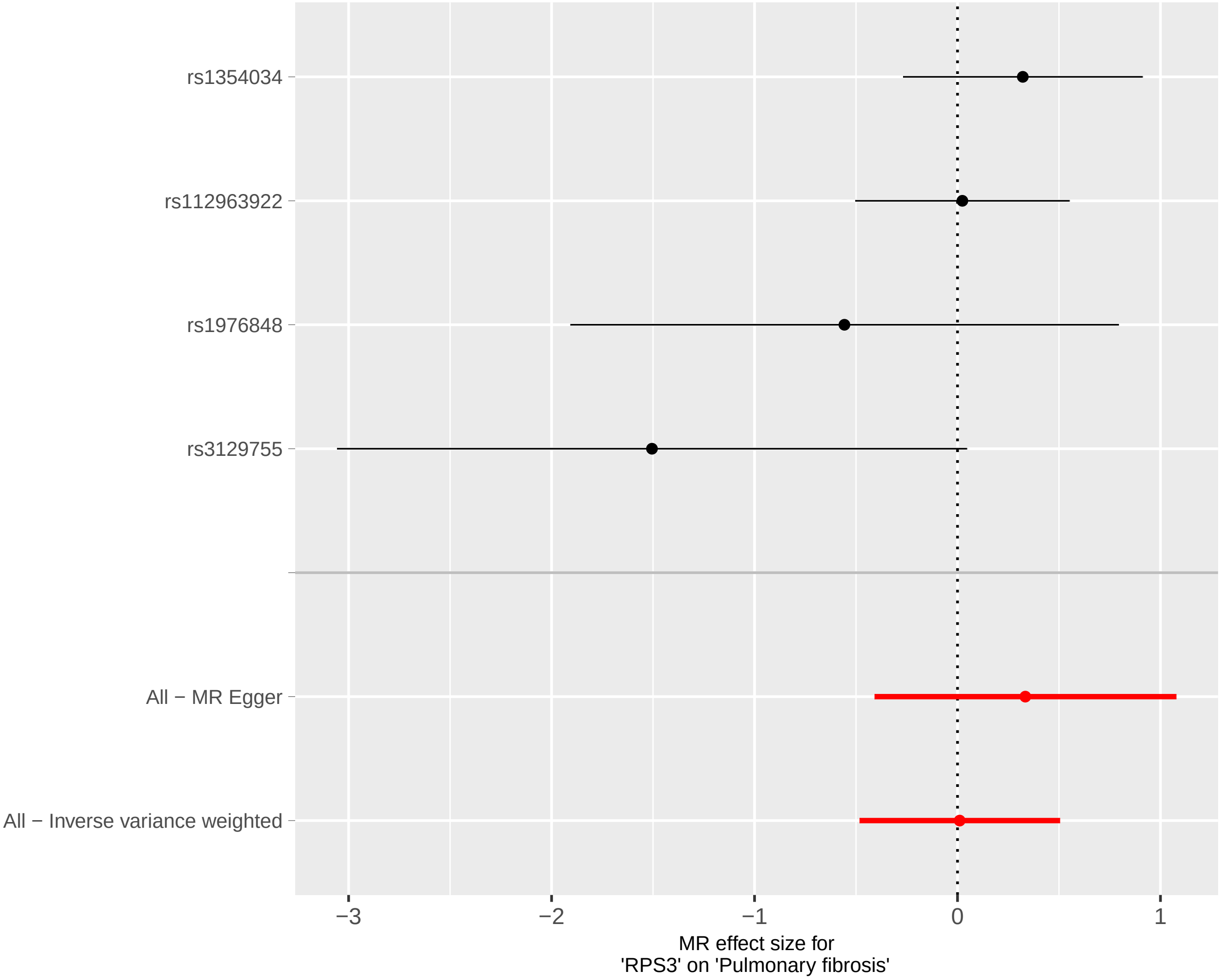
rs1354034
rs112963922
rs1976848
rs3129755
All − MR Egger
All − Inverse variance weighted
−3
−2
−1
0
1
MR effect size for
'RPS3' on 'Pulmonary fibrosis'

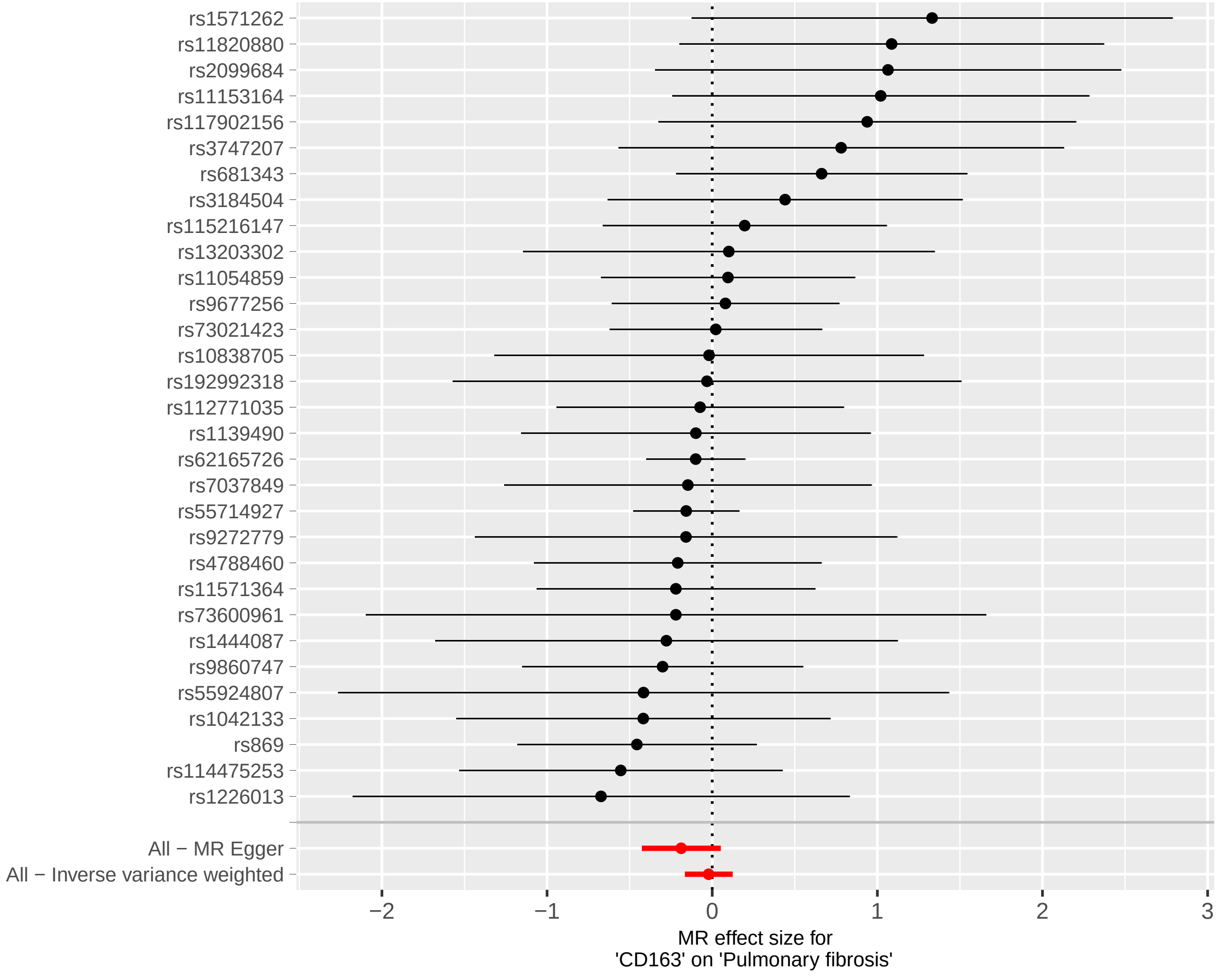

rs1571262
rs11820880
rs2099684
rs11153164
rs117902156
rs3747207
rs681343
rs3184504
rs115216147
rs13203302
rs11054859
rs9677256
rs73021423
rs10838705
rs192992318
rs112771035
rs1139490
rs62165726
rs7037849
rs55714927
rs9272779
rs4788460
rs11571364
rs73600961
rs1444087
rs9860747
rs55924807
rs1042133
rs869
rs114475253
rs1226013
All − MR Egger
All − Inverse variance weighted
−2
−1
0
1
2
3
MR effect size for
'CD163' on 'Pulmonary fibrosis'

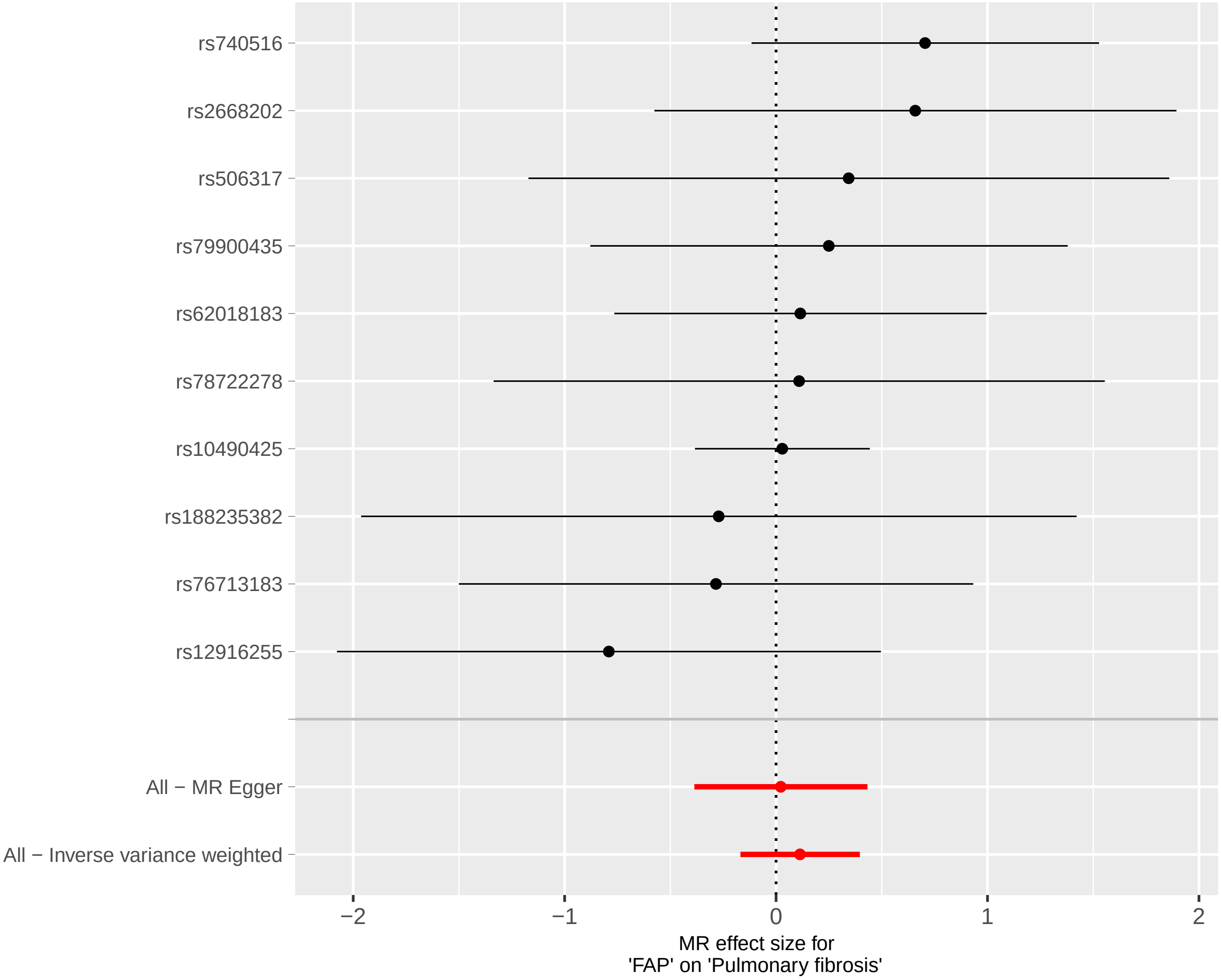

rs740516
rs2668202
rs506317
rs79900435
rs62018183
rs78722278
rs10490425
rs188235382
rs76713183
rs12916255
All − MR Egger
All − Inverse variance weighted
−2
−1
0
1
2
MR effect size for
'FAP' on 'Pulmonary fibrosis'

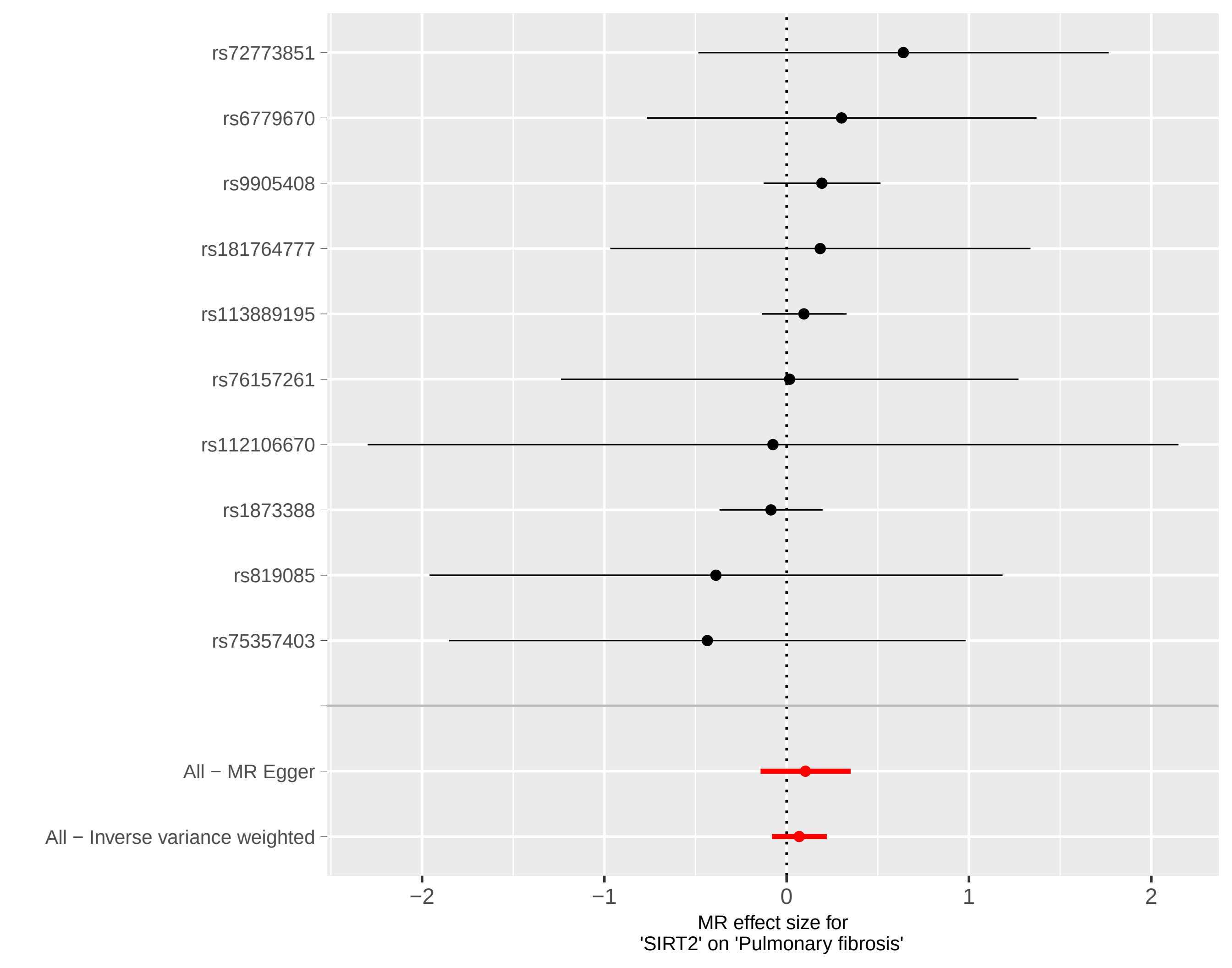

rs72773851
rs6779670
rs9905408
rs181764777
rs113889195
rs76157261
rs112106670
rs1873388
rs819085
rs75357403
All − MR Egger
All − Inverse variance weighted
−2
−1
0
1
2
MR effect size for
'SIRT2' on 'Pulmonary fibrosis'

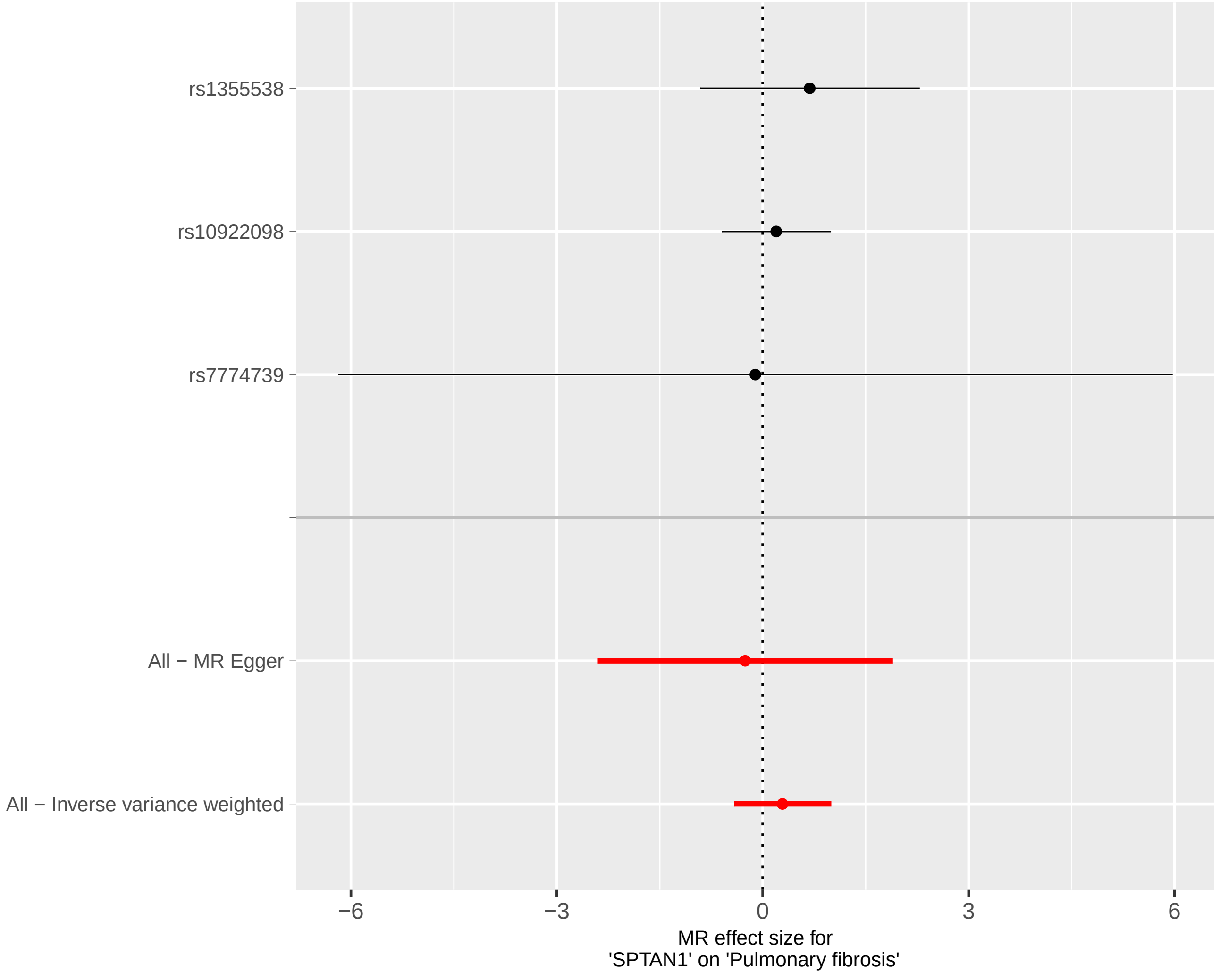

rs1355538
rs10922098
rs7774739
All − MR Egger
All − Inverse variance weighted
−6
−3
0
3
6
MR effect size for
'SPTAN1' on 'Pulmonary fibrosis'

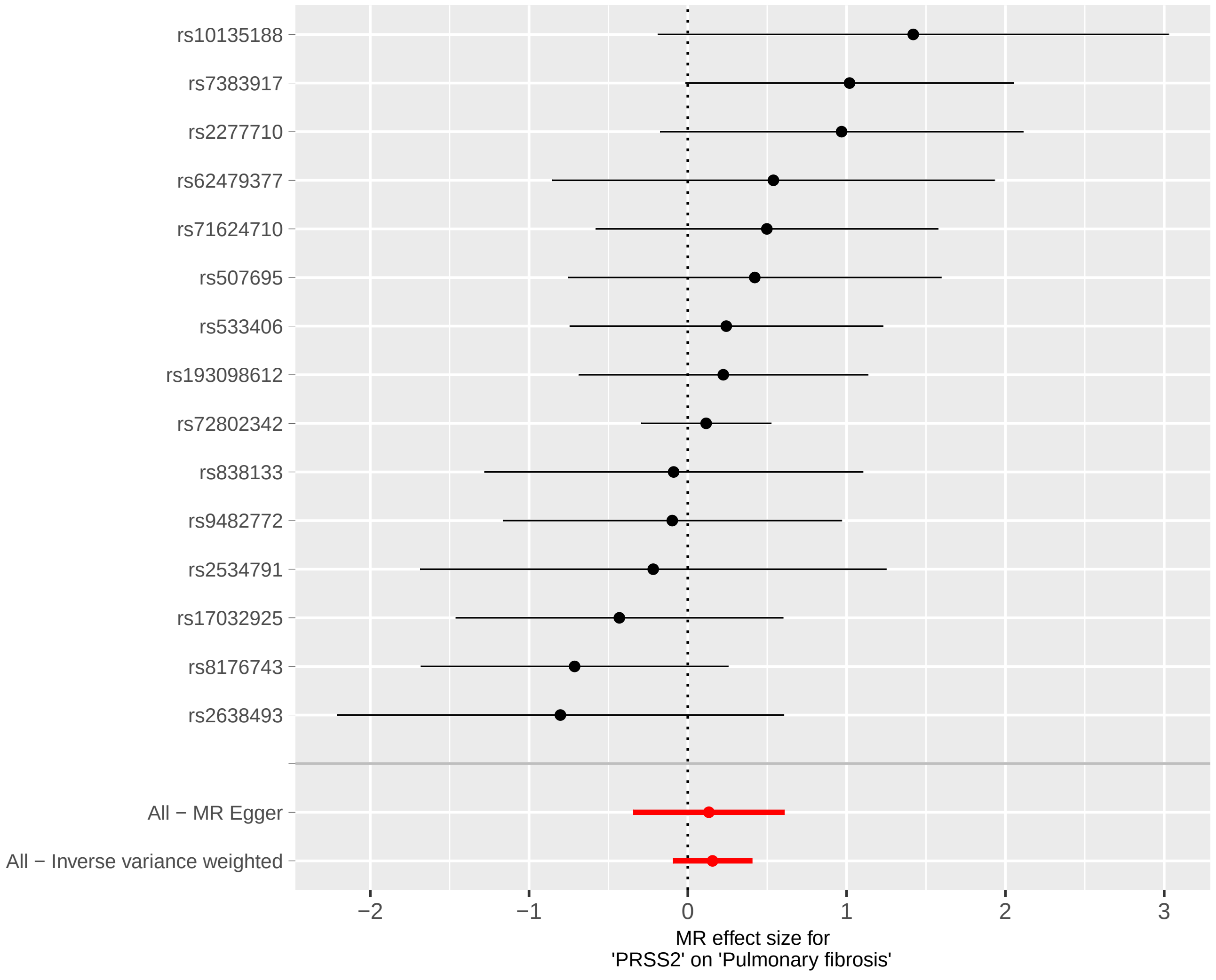

rs10135188
rs7383917
rs2277710
rs62479377
rs71624710
rs507695
rs533406
rs193098612
rs72802342
rs838133
rs9482772
rs2534791
rs17032925
rs8176743
rs2638493
All − MR Egger
All − Inverse variance weighted
−2
−1
0
1
2
3
MR effect size for
'PRSS2' on 'Pulmonary fibrosis'

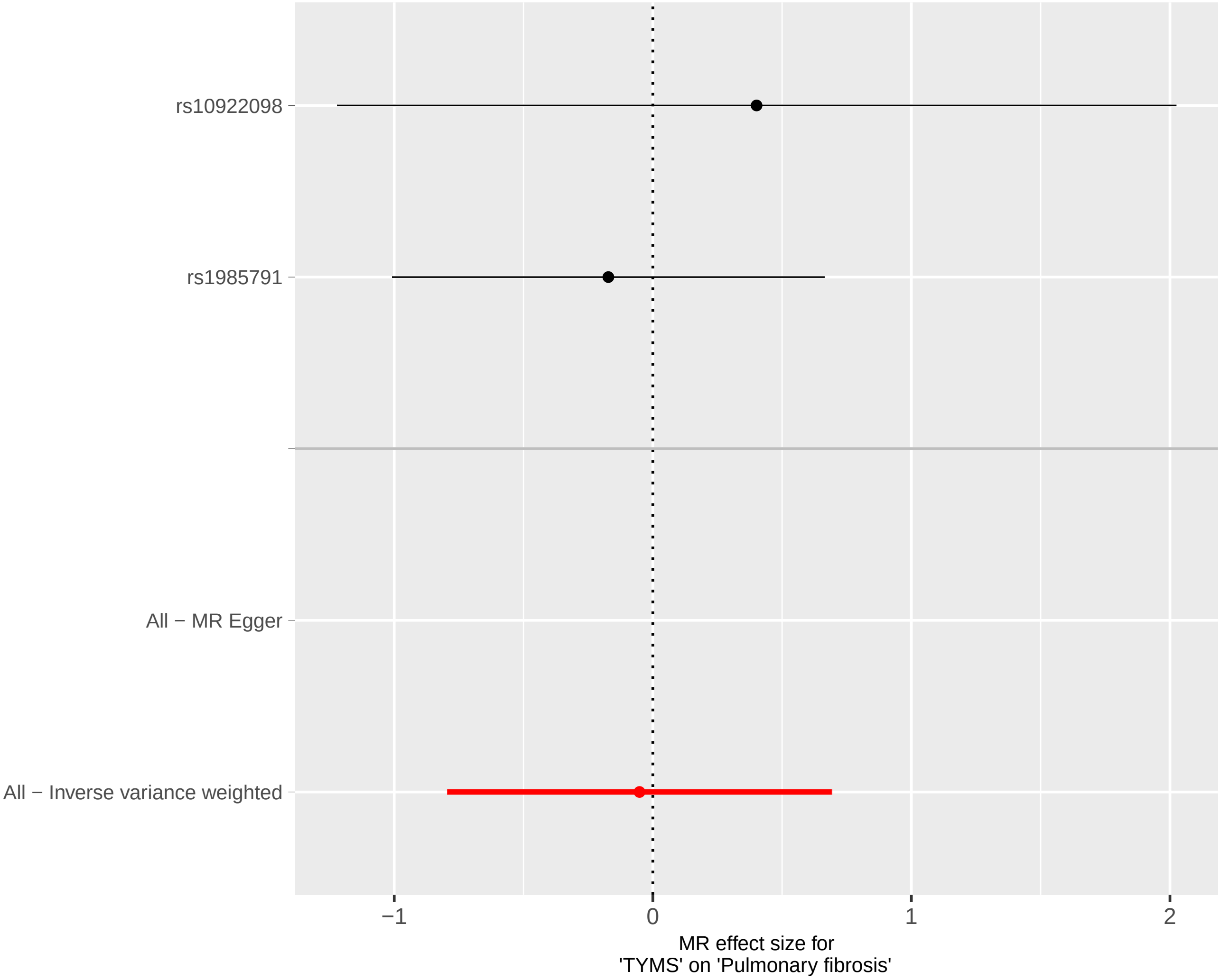

rs10922098
rs1985791
All − MR Egger
All − Inverse variance weighted
−1
0
1
2
MR effect size for
'TYMS' on 'Pulmonary fibrosis'

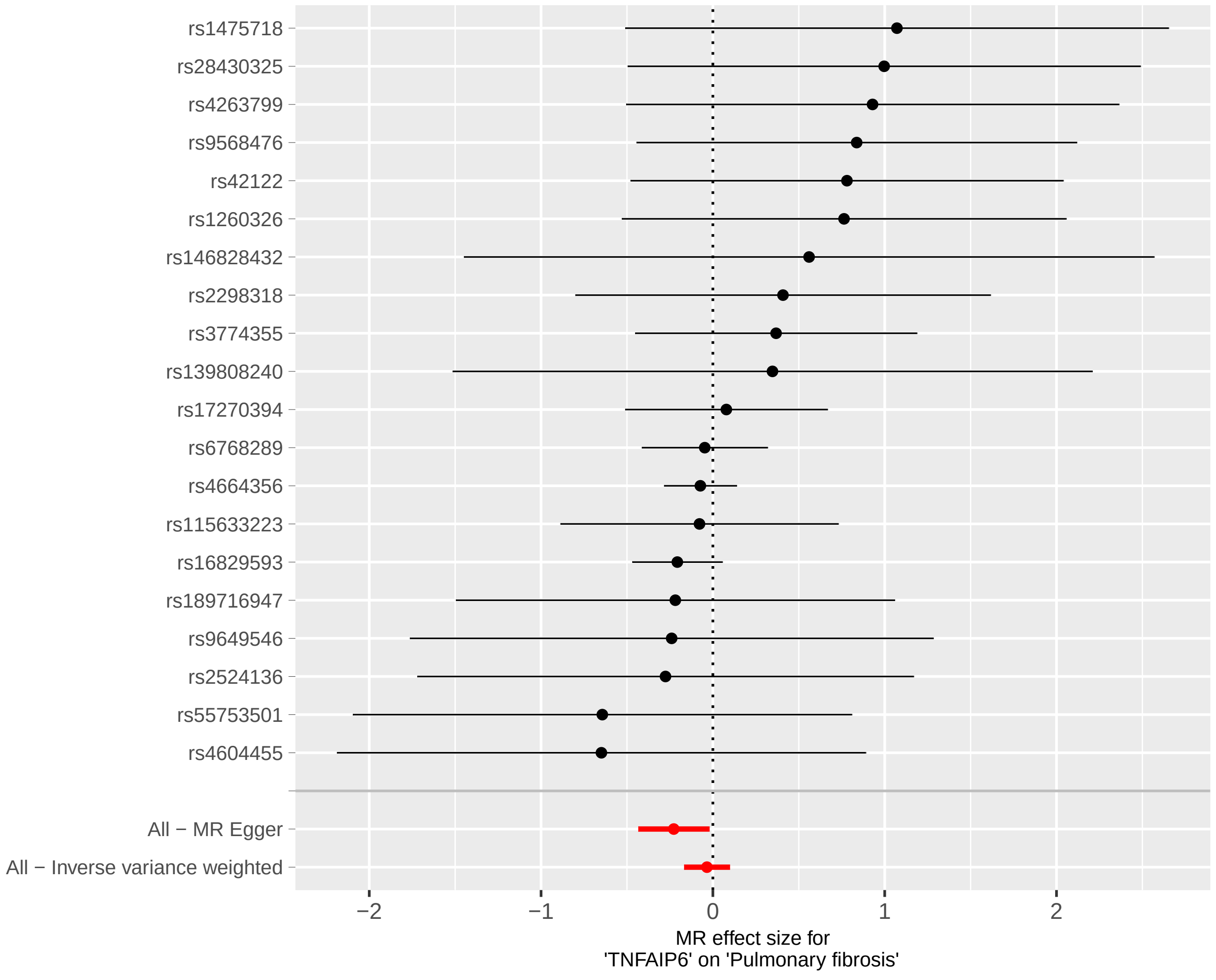
rs1475718
rs28430325
rs4263799
rs9568476
rs42122
rs1260326
rs146828432
rs2298318
rs3774355
rs139808240
rs17270394
rs6768289
rs4664356
rs115633223
rs16829593
rs189716947
rs9649546
rs2524136
rs55753501
rs4604455
All − MR Egger
All − Inverse variance weighted
−2
−1
0
1
2
MR effect size for
'TNFAIP6' on 'Pulmonary fibrosis'

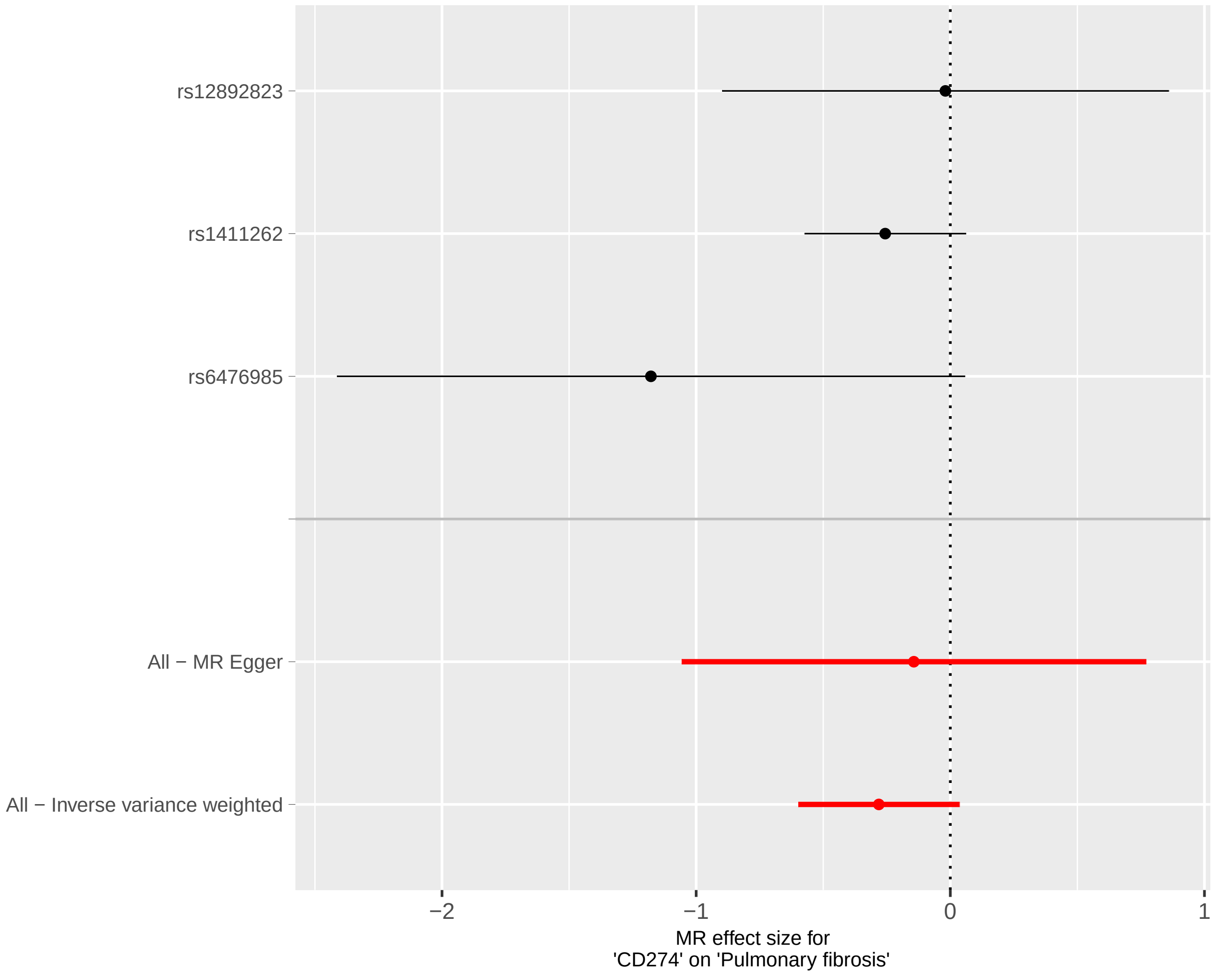
rs12892823
rs1411262
rs6476985
All − MR Egger
All − Inverse variance weighted
−2
−1
0
1
MR effect size for
'CD274' on 'Pulmonary fibrosis'

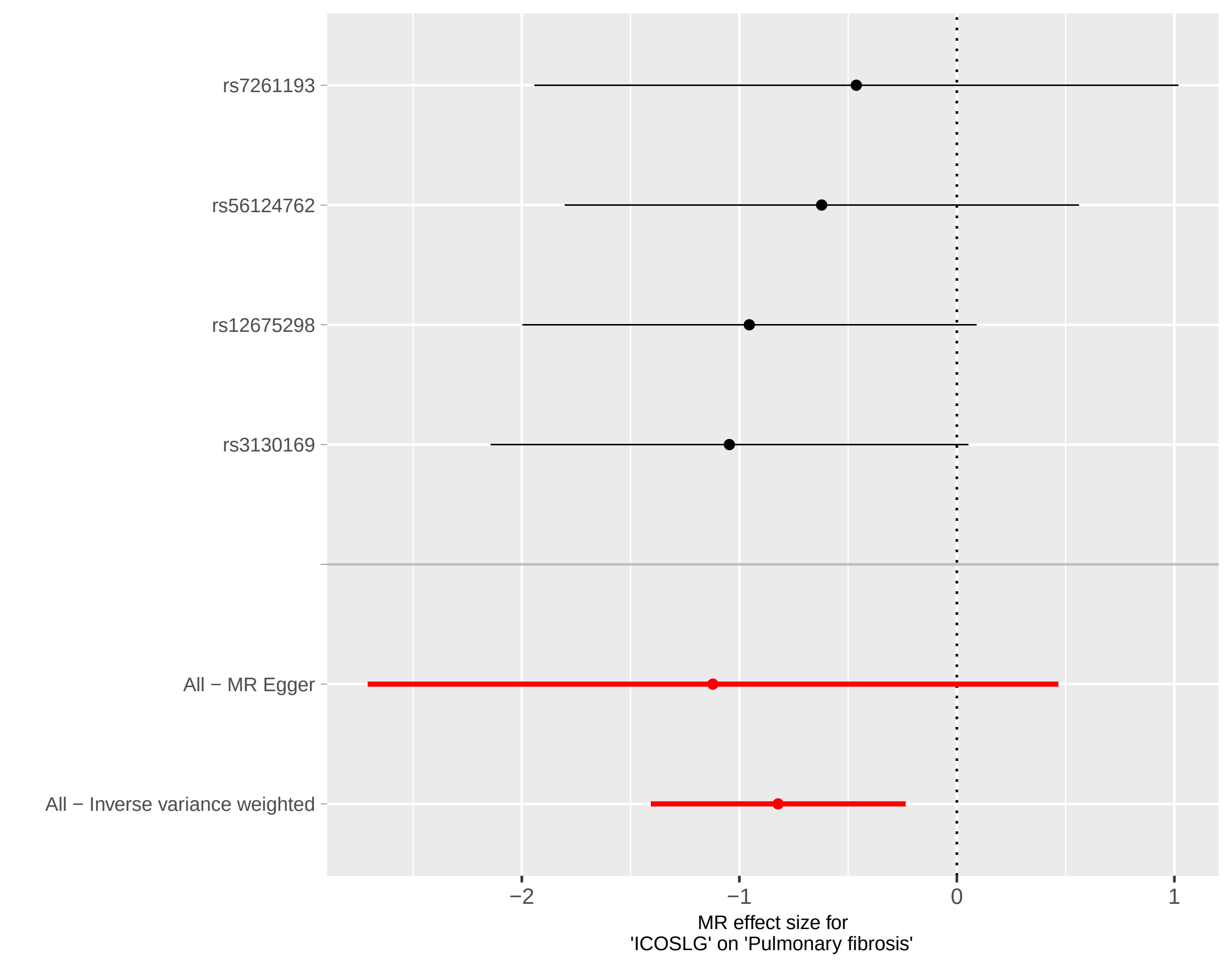

rs7261193
rs56124762
rs12675298
rs3130169
All − MR Egger
All − Inverse variance weighted
−2
−1
0
1
MR effect size for
'ICOSLG' on 'Pulmonary fibrosis'

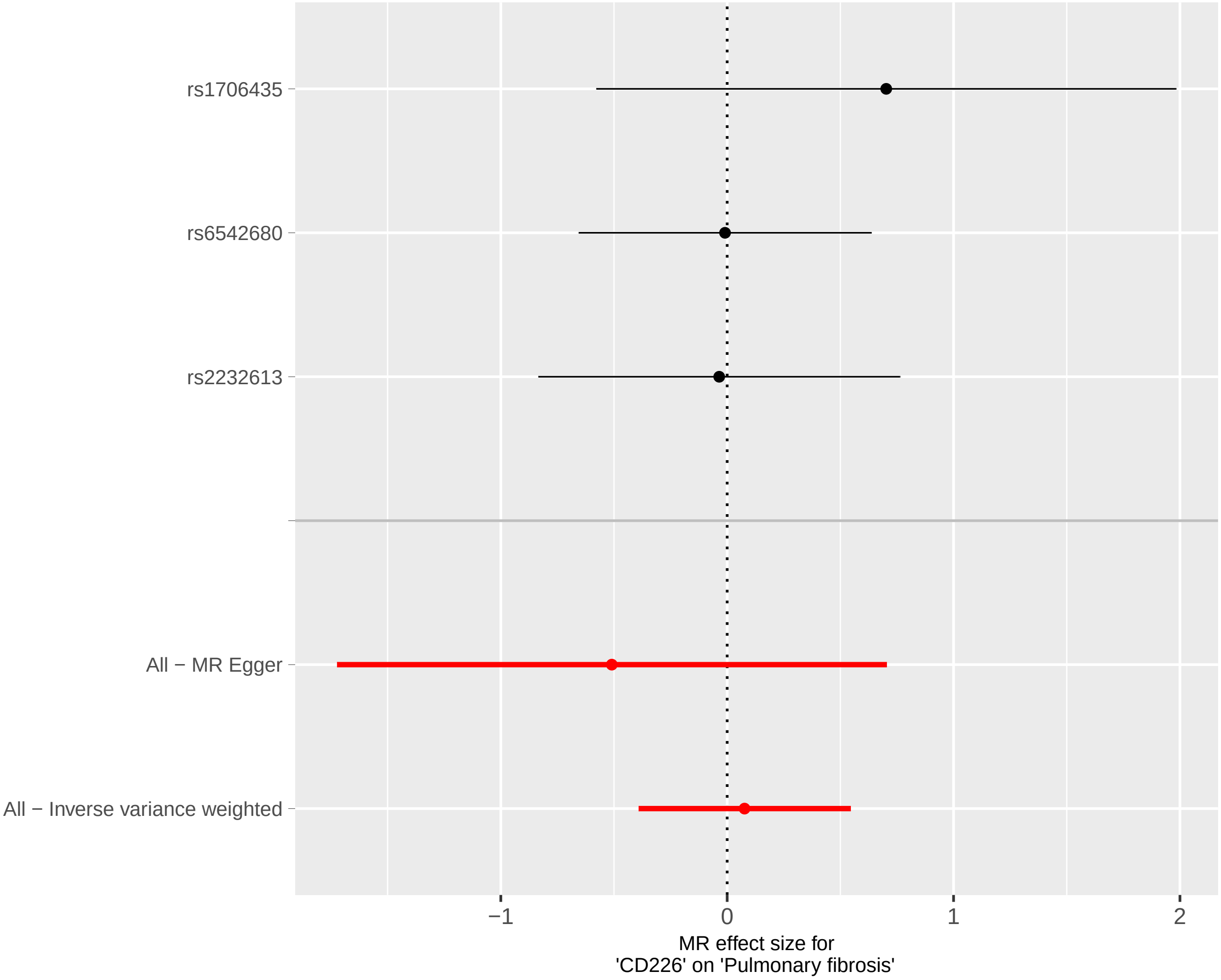
rs1706435
rs6542680
rs2232613
All − MR Egger
All − Inverse variance weighted
−1
0
1
2
MR effect size for
'CD226' on 'Pulmonary fibrosis'

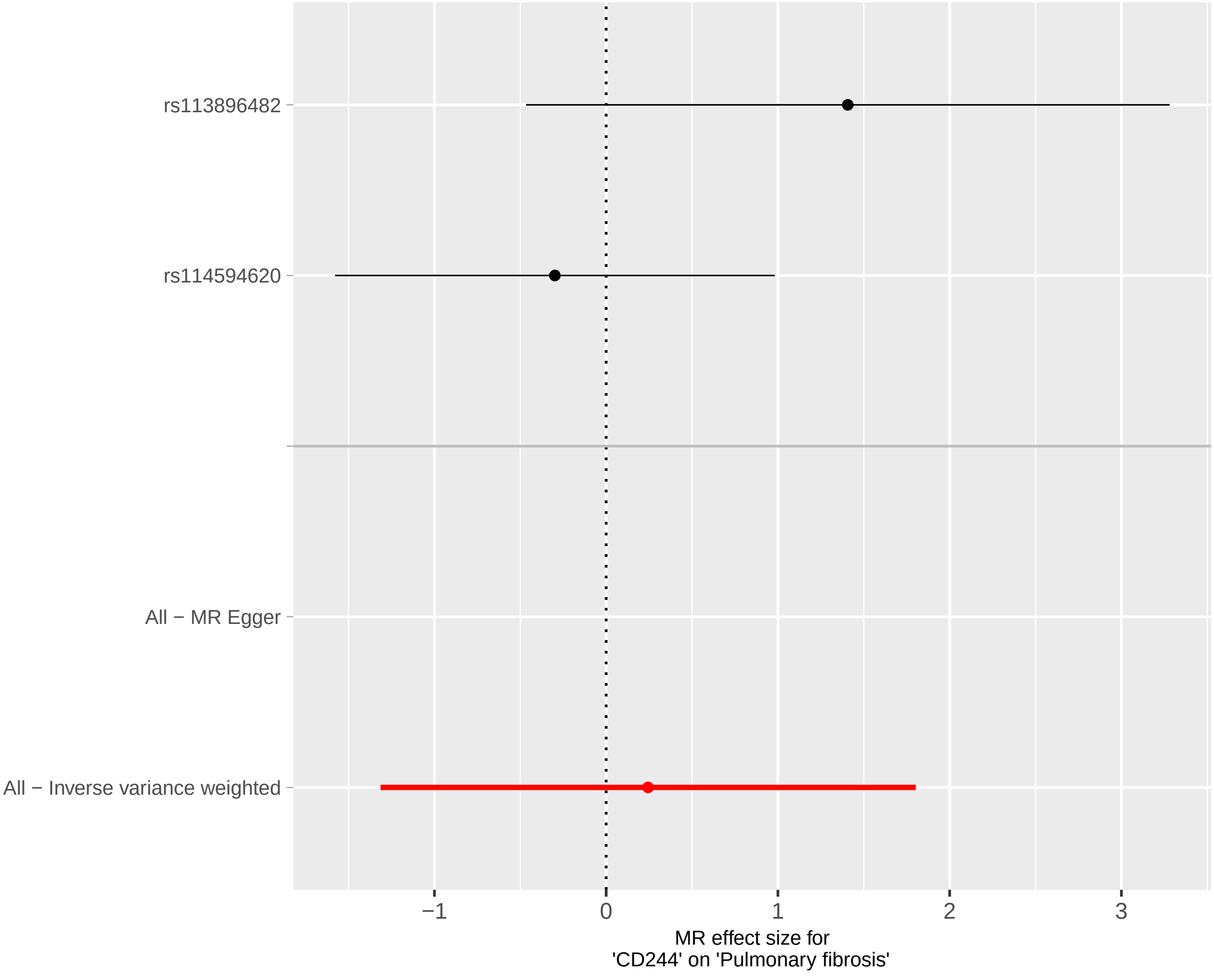

rs113896482
rs114594620
All − MR Egger
All − Inverse variance weighted
−1
0
1
2
3
MR effect size for
'CD244' on 'Pulmonary fibrosis'

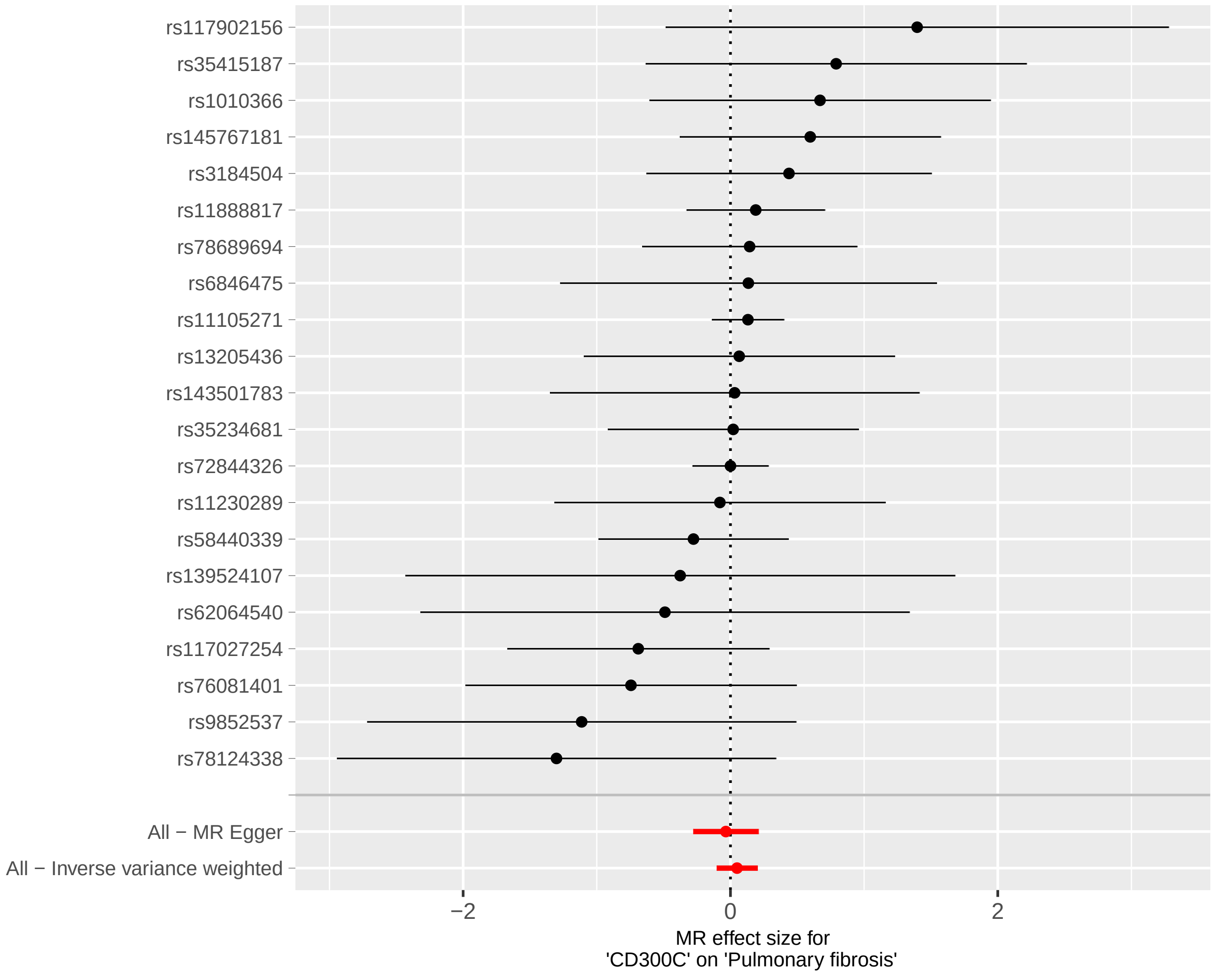

rs117902156
rs35415187
rs1010366
rs145767181
rs3184504
rs11888817
rs78689694
rs6846475
rs11105271
rs13205436
rs143501783
rs35234681
rs72844326
rs11230289
rs58440339
rs139524107
rs62064540
rs117027254
rs76081401
rs9852537
rs78124338
All − MR Egger
All − Inverse variance weighted
−2
0
2
MR effect size for
'CD300C' on 'Pulmonary fibrosis'

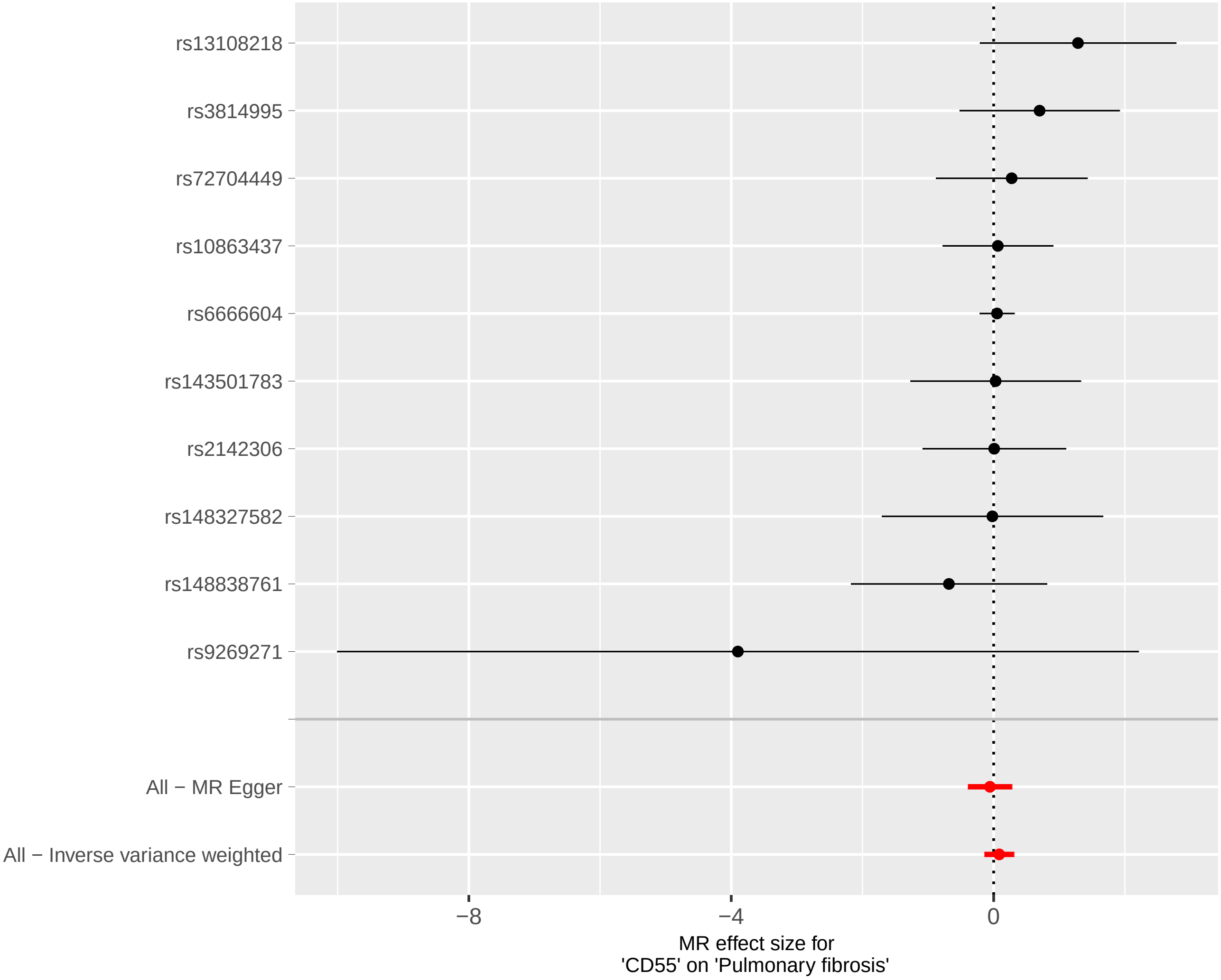
rs13108218
rs3814995
rs72704449
rs10863437
rs6666604
rs143501783
rs2142306
rs148327582
rs148838761
rs9269271
All − MR Egger
All − Inverse variance weighted
−8
−4
0
MR effect size for
'CD55' on 'Pulmonary fibrosis'

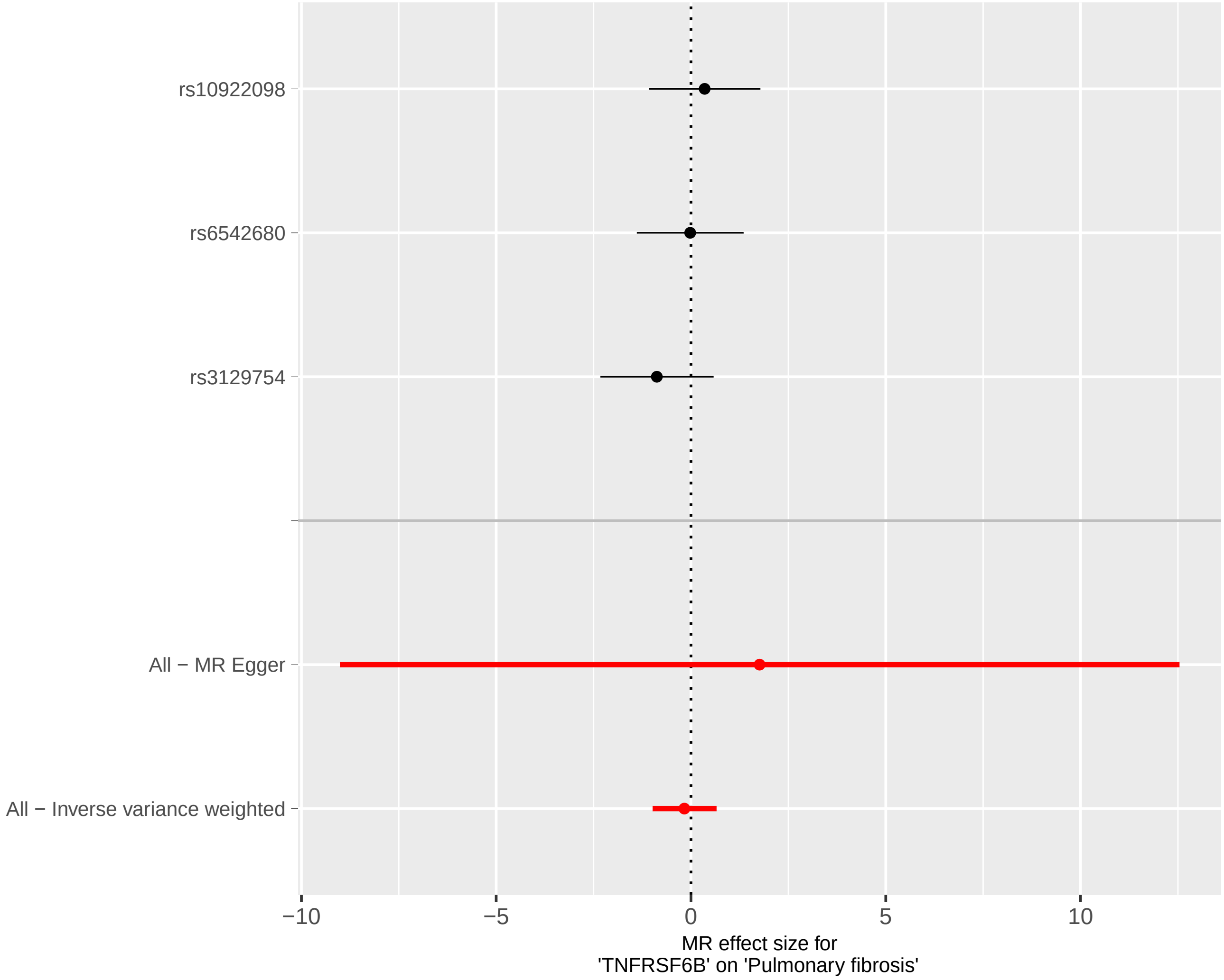

rs10922098
rs6542680
rs3129754
All − MR Egger
All − Inverse variance weighted
−10
−5
0
5
10
MR effect size for
'TNFRSF6B' on 'Pulmonary fibrosis'

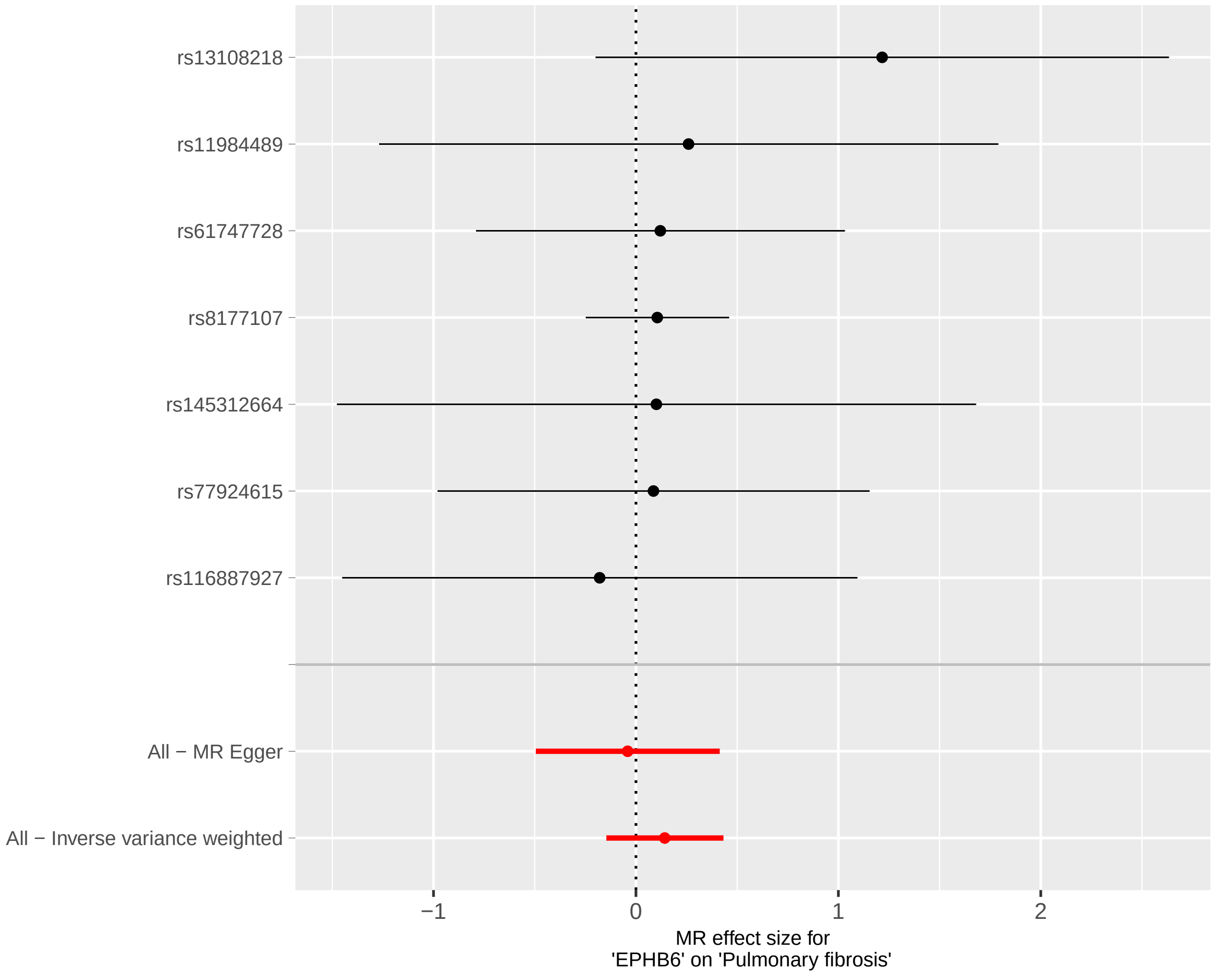
rs13108218
rs11984489
rs61747728
rs8177107
rs145312664
rs77924615
rs116887927
All − MR Egger
All − Inverse variance weighted
−1
0
1
2
MR effect size for
'EPHB6' on 'Pulmonary fibrosis'

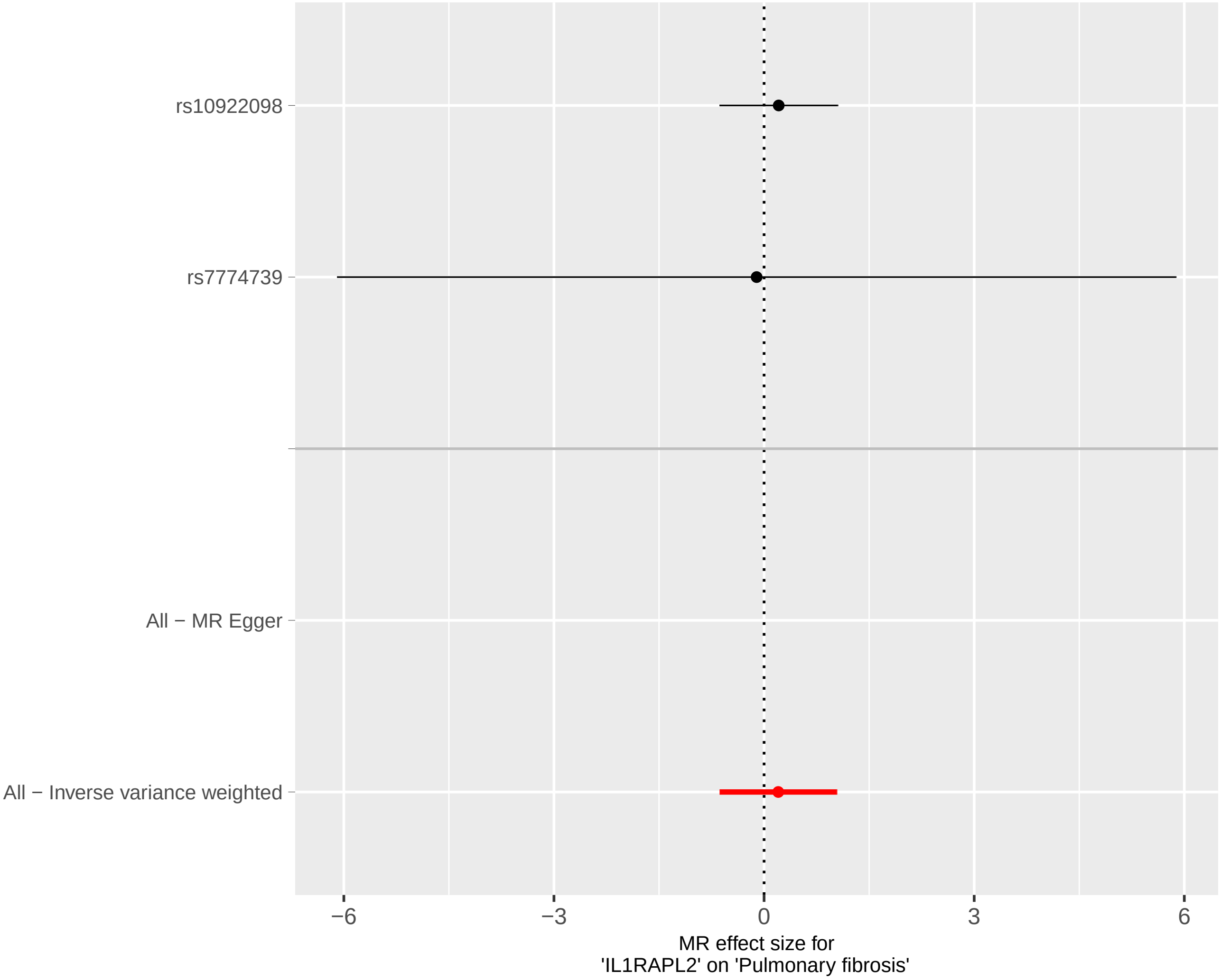

rs10922098
rs7774739
All − MR Egger
All − Inverse variance weighted
−6
−3
0
3
6
MR effect size for
'IL1RAPL2' on 'Pulmonary fibrosis'

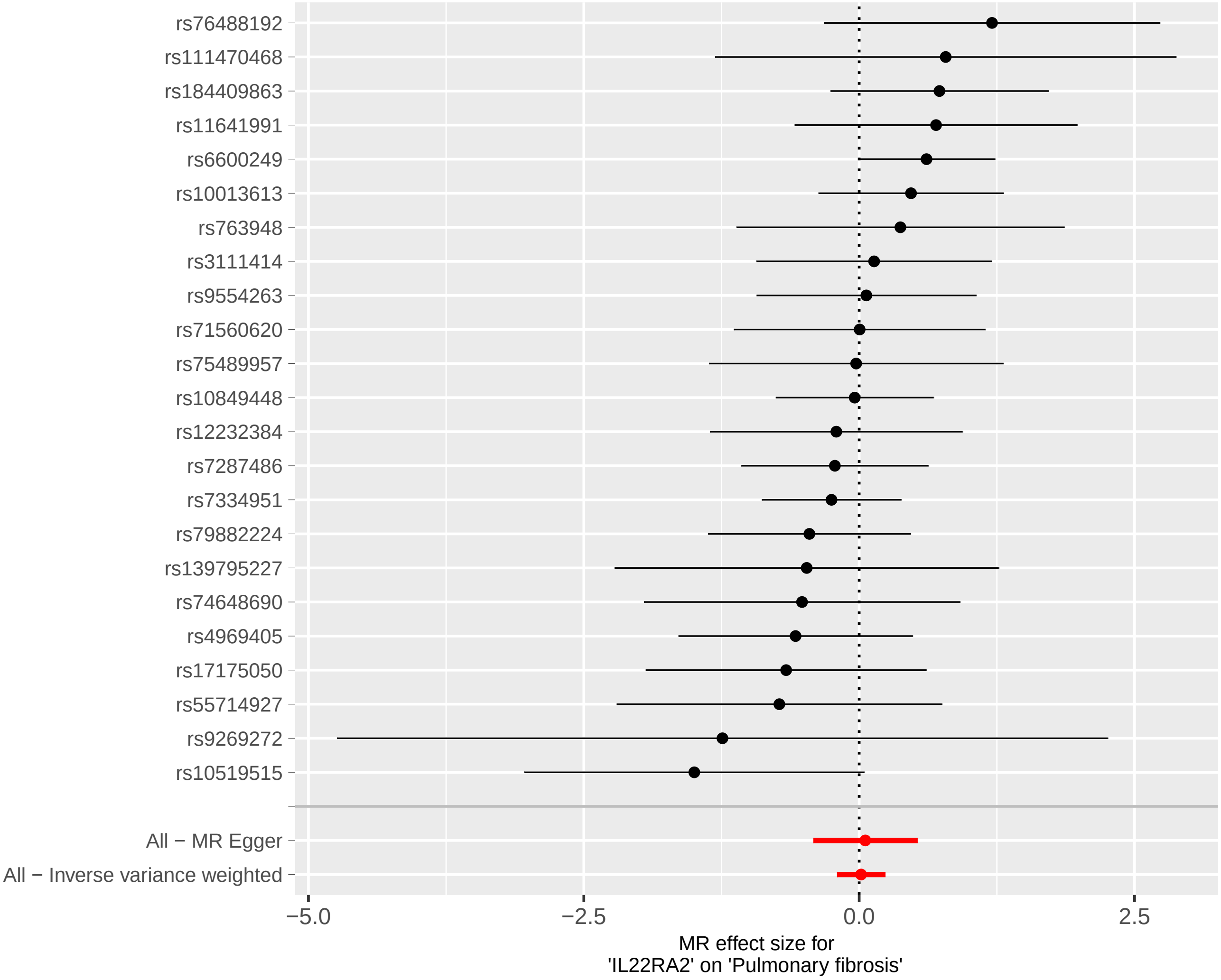

rs76488192
rs111470468
rs184409863
rs11641991
rs6600249
rs10013613
rs763948
rs3111414
rs9554263
rs71560620
rs75489957
rs10849448
rs12232384
rs7287486
rs7334951
rs79882224
rs139795227
rs74648690
rs4969405
rs17175050
rs55714927
rs9269272
rs10519515
All − MR Egger
All − Inverse variance weighted
−5.0
−2.5
0.0
2.5
MR effect size for
'IL22RA2' on 'Pulmonary fibrosis'

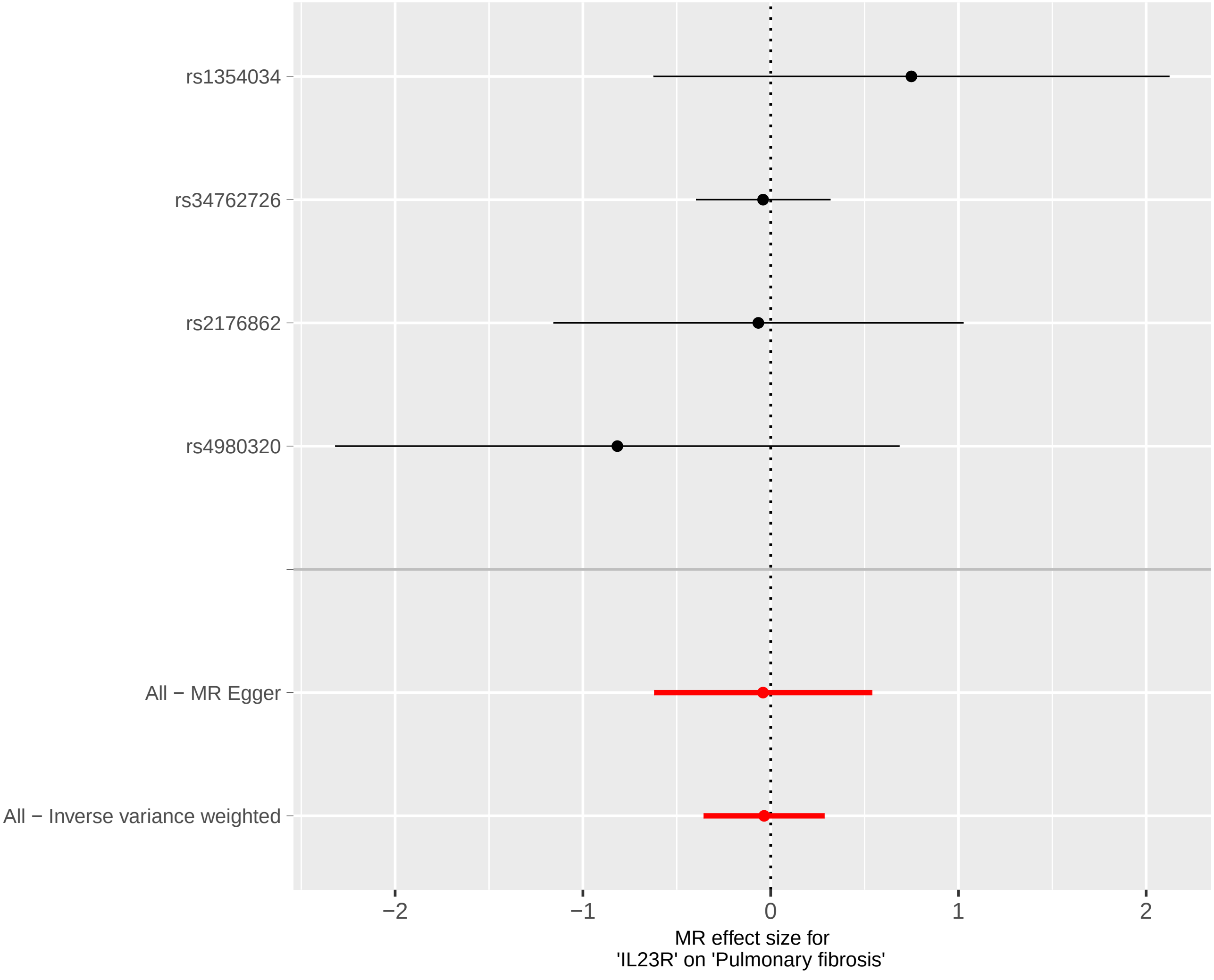

rs1354034
rs34762726
rs2176862
rs4980320
All − MR Egger
All − Inverse variance weighted
−2
−1
0
1
2
MR effect size for
'IL23R' on 'Pulmonary fibrosis'

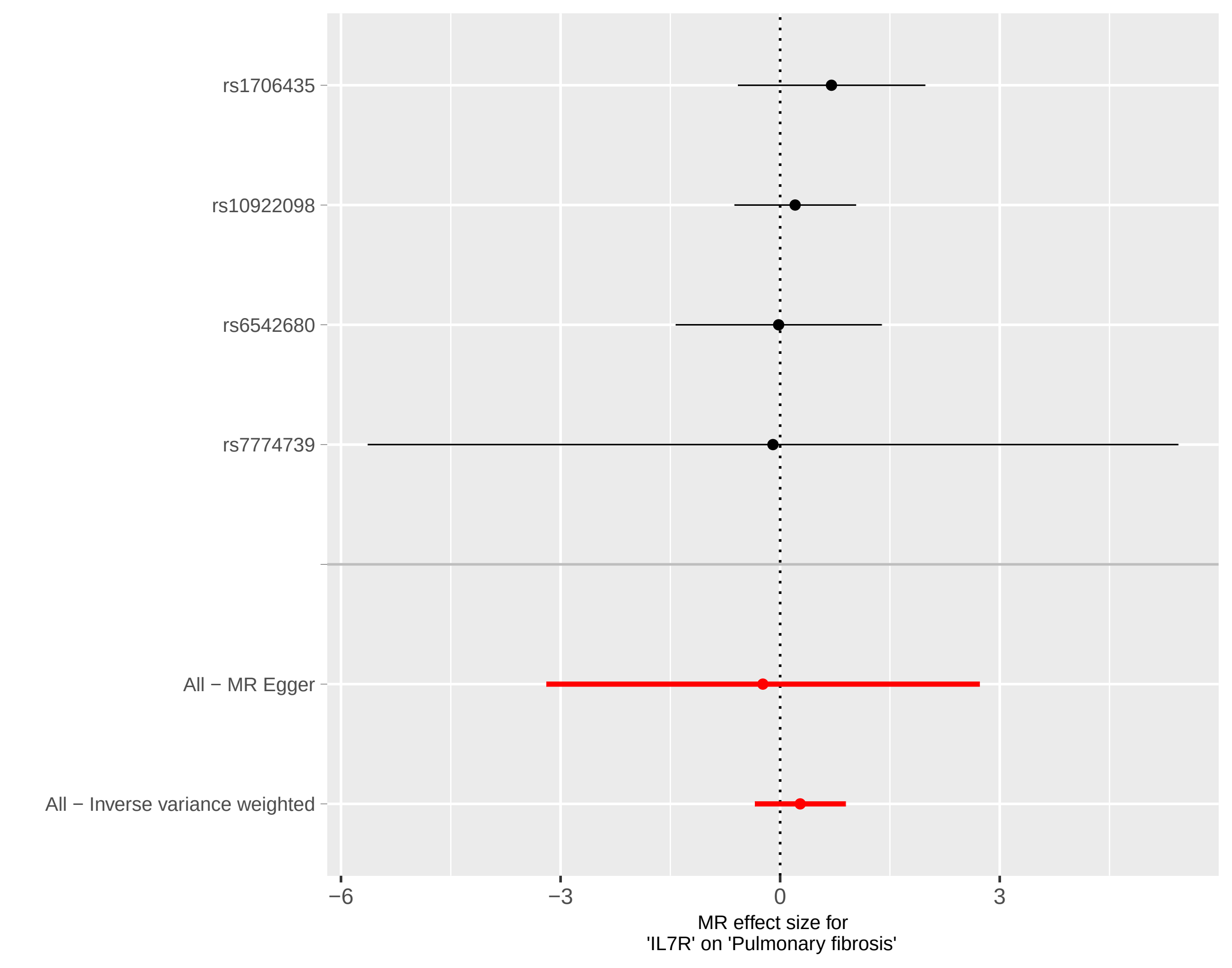

rs1706435
rs10922098
rs6542680
rs7774739
All − MR Egger
All − Inverse variance weighted
−6
−3
0
3
MR effect size for
'IL7R' on 'Pulmonary fibrosis'

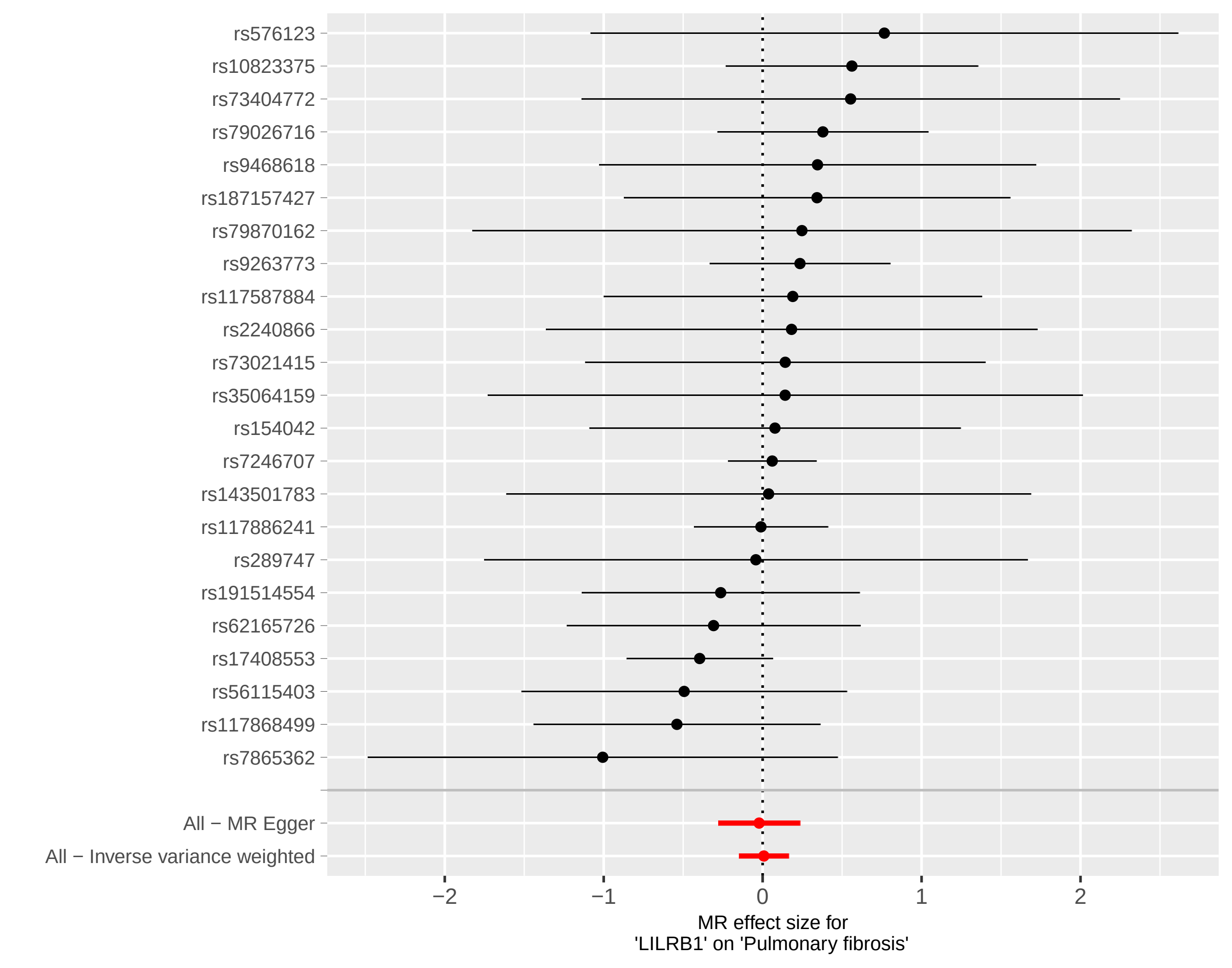

rs576123
rs10823375
rs73404772
rs79026716
rs9468618
rs187157427
rs79870162
rs9263773
rs117587884
rs2240866
rs73021415
rs35064159
rs154042
rs7246707
rs143501783
rs117886241
rs289747
rs191514554
rs62165726
rs17408553
rs56115403
rs117868499
rs7865362
All − MR Egger
All − Inverse variance weighted
−2
−1
0
1
2
MR effect size for
'LILRB1' on 'Pulmonary fibrosis'

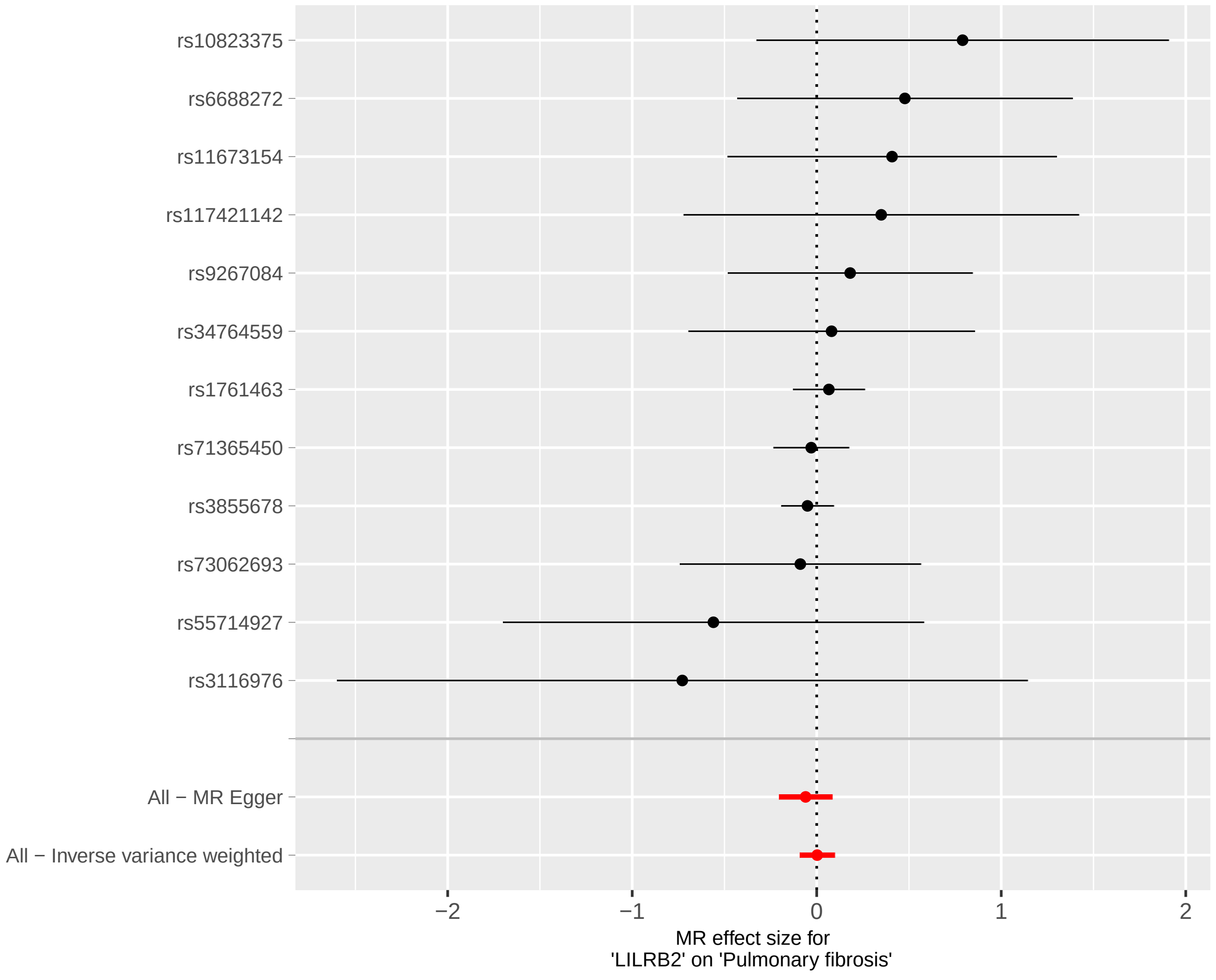
rs10823375
rs6688272
rs11673154
rs117421142
rs9267084
rs34764559
rs1761463
rs71365450
rs3855678
rs73062693
rs55714927
rs3116976
All − MR Egger
All − Inverse variance weighted
−2
−1
0
1
2
MR effect size for
'LILRB2' on 'Pulmonary fibrosis'

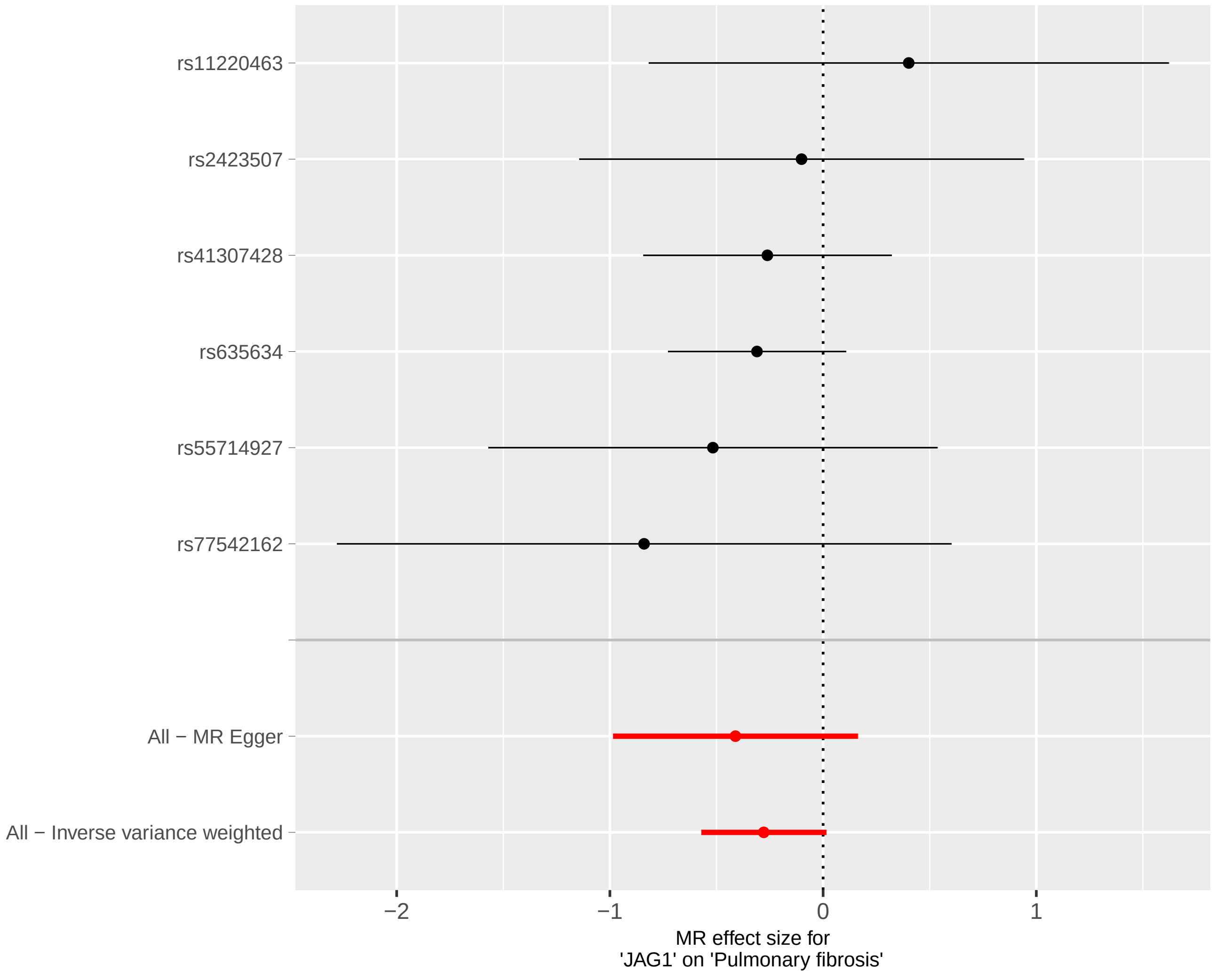

rs11220463
rs2423507
rs41307428
rs635634
rs55714927
rs77542162
All − MR Egger
All − Inverse variance weighted
−2
−1
0
1
MR effect size for
'JAG1' on 'Pulmonary fibrosis'

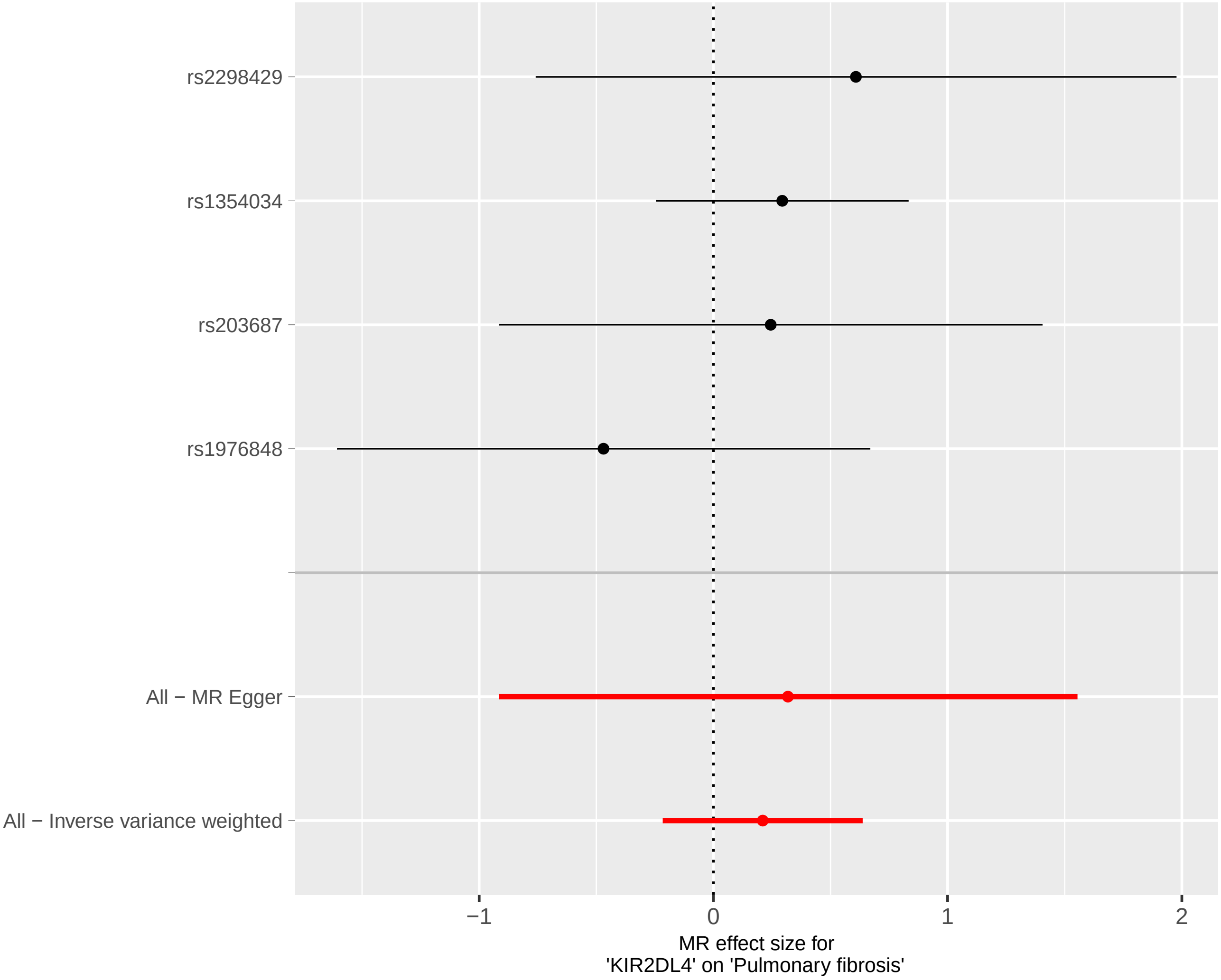

rs2298429
rs1354034
rs203687
rs1976848
All − MR Egger
All − Inverse variance weighted
−1
0
1
2
MR effect size for
'KIR2DL4' on 'Pulmonary fibrosis'

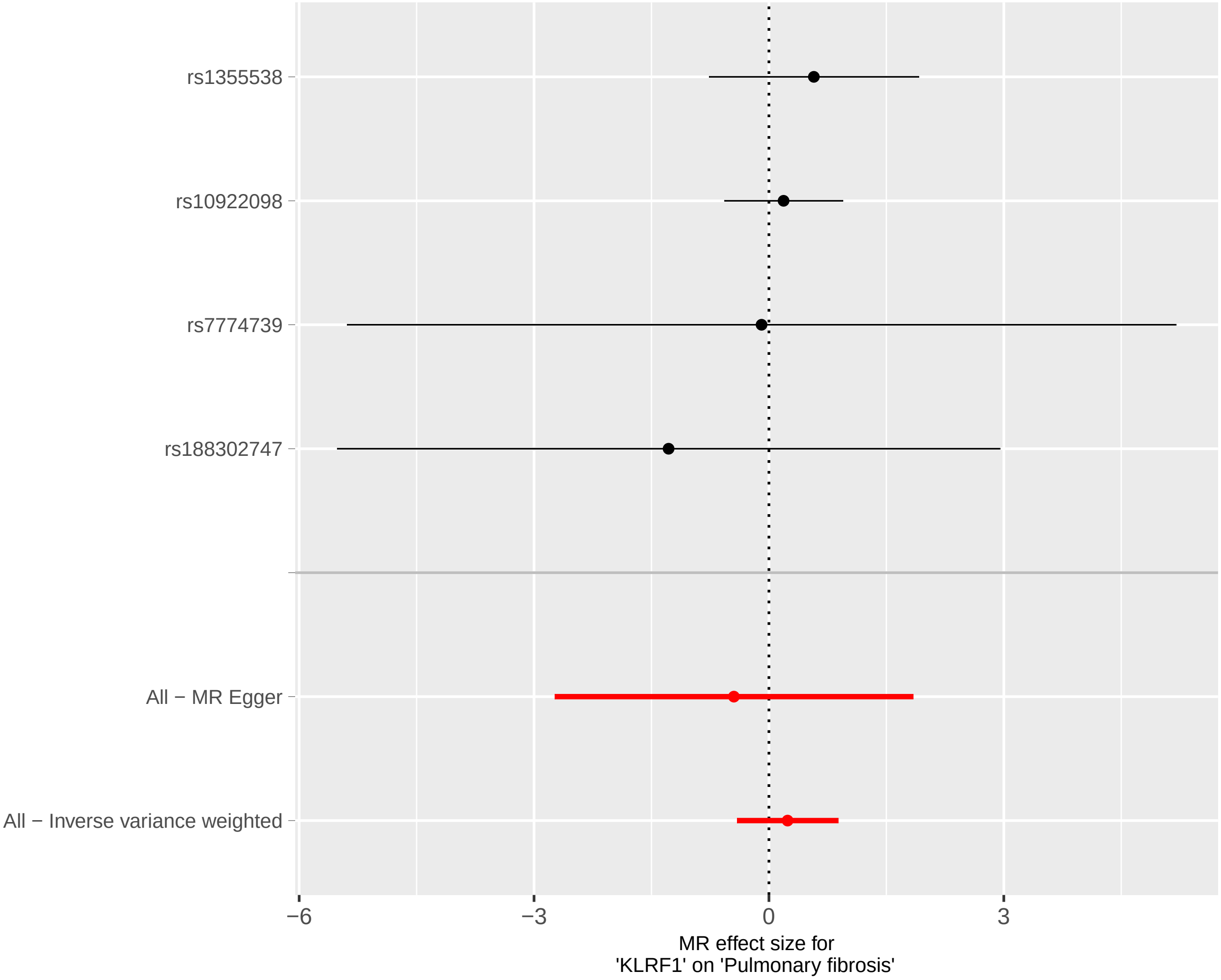

rs1355538
rs10922098
rs7774739
rs188302747
All − MR Egger
All − Inverse variance weighted
−6
−3
0
3
MR effect size for
'KLRF1' on 'Pulmonary fibrosis'

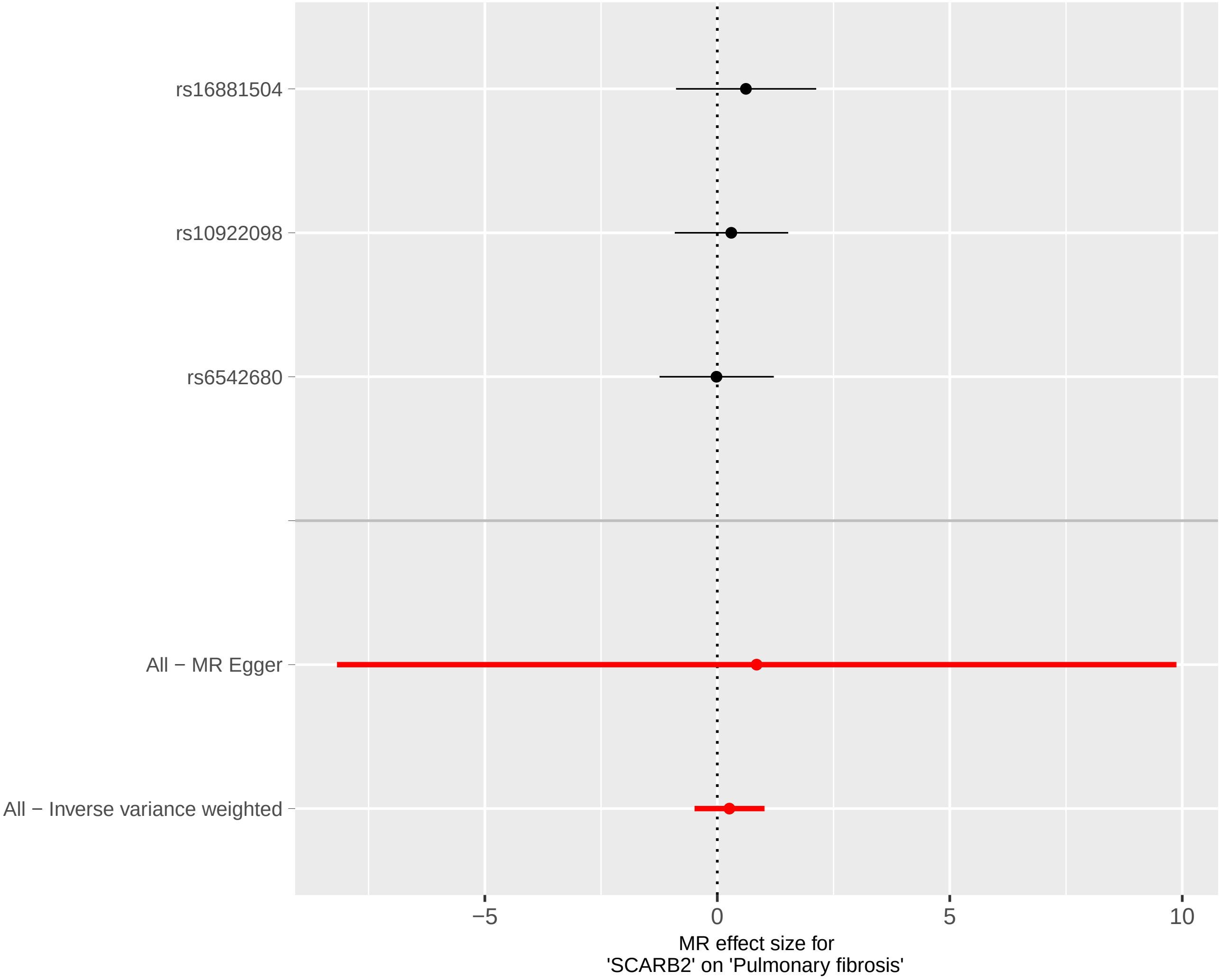
rs16881504
rs10922098
rs6542680
All − MR Egger
All − Inverse variance weighted
−5
0
5
10
MR effect size for
'SCARB2' on 'Pulmonary fibrosis'

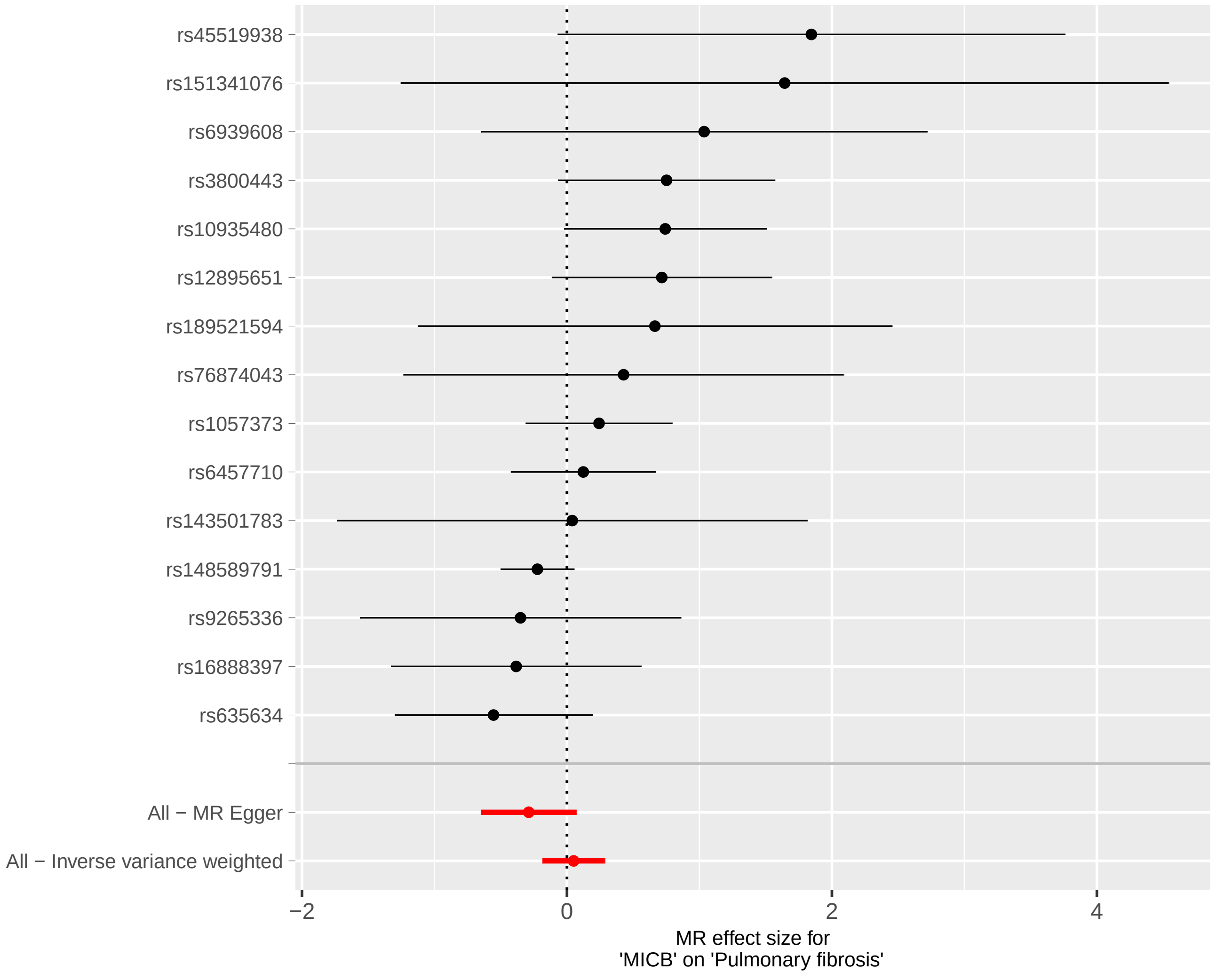
rs45519938
rs151341076
rs6939608
rs3800443
rs10935480
rs12895651
rs189521594
rs76874043
rs1057373
rs6457710
rs143501783
rs148589791
rs9265336
rs16888397
rs635634
All − MR Egger
All − Inverse variance weighted
−2
0
2
4
MR effect size for
'MICB' on 'Pulmonary fibrosis'

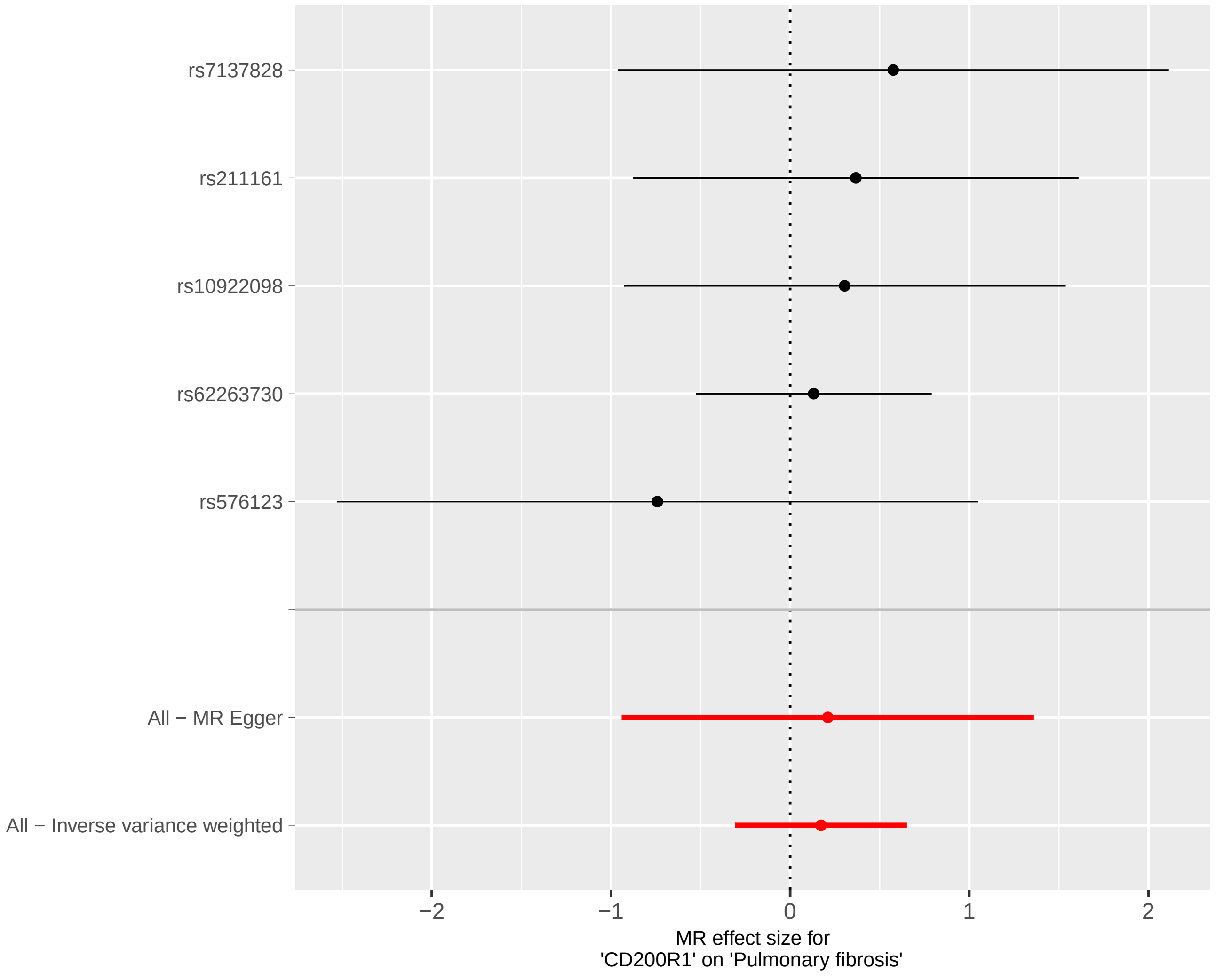

rs7137828
rs211161
rs10922098
rs62263730
rs576123
All − MR Egger
All − Inverse variance weighted
−2
−1
0
1
2
MR effect size for
'CD200R1' on 'Pulmonary fibrosis'

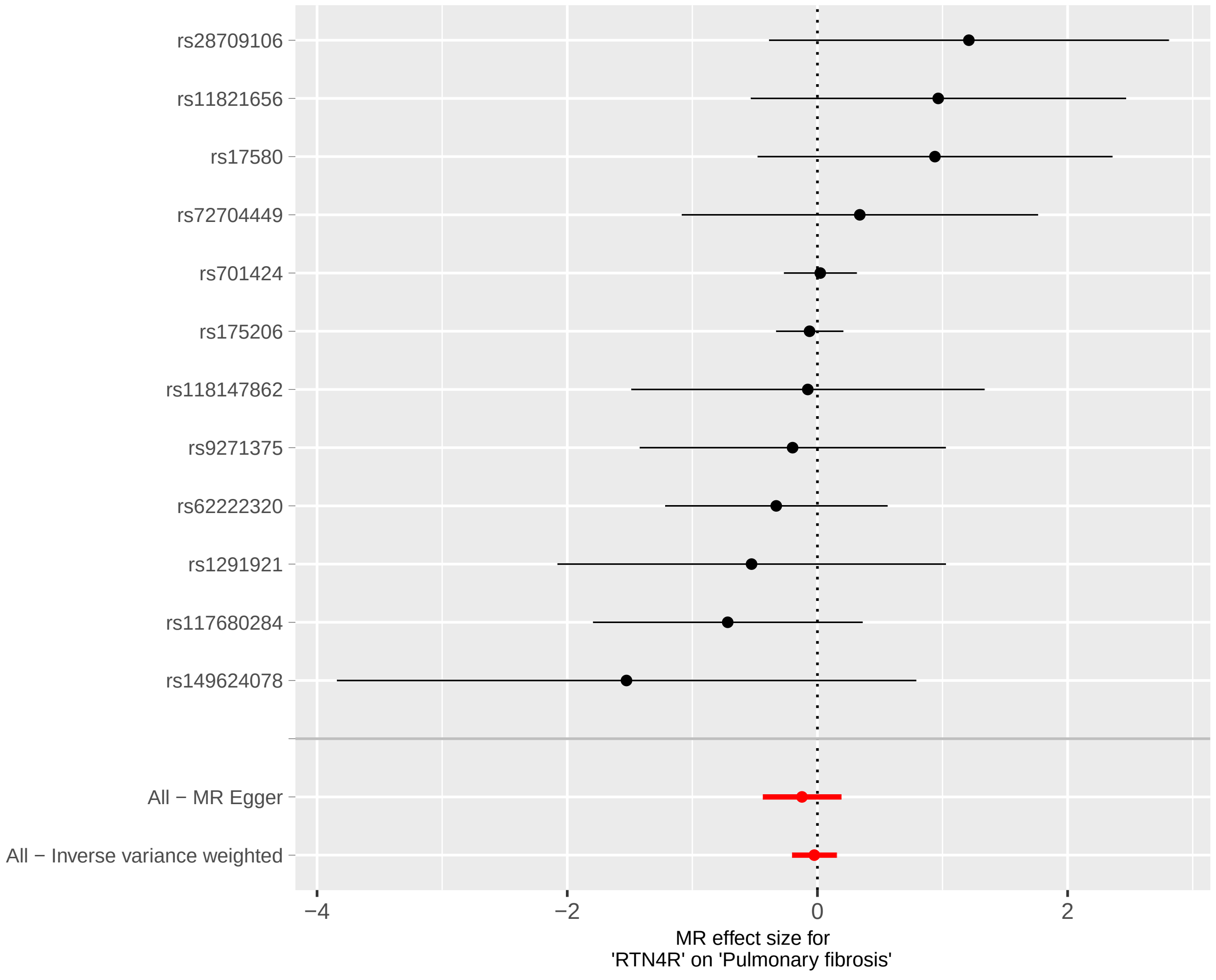
rs28709106
rs11821656
rs17580
rs72704449
rs701424
rs175206
rs118147862
rs9271375
rs62222320
rs1291921
rs117680284
rs149624078
All − MR Egger
All − Inverse variance weighted
−4
−2
0
2
MR effect size for
'RTN4R' on 'Pulmonary fibrosis'

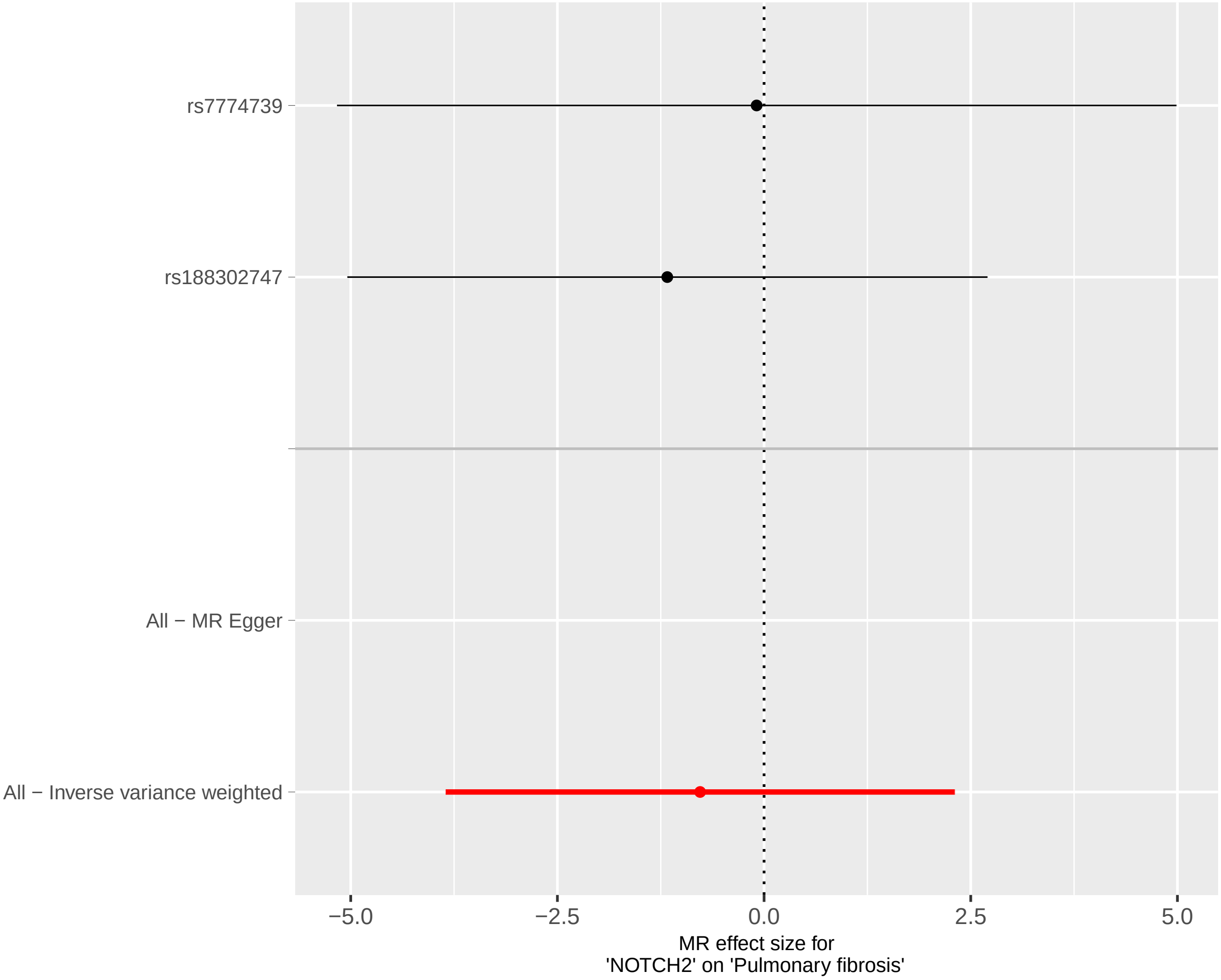

rs7774739
rs188302747
All − MR Egger
All − Inverse variance weighted
−5.0
−2.5
0.0
2.5
5.0
MR effect size for
'NOTCH2' on 'Pulmonary fibrosis'

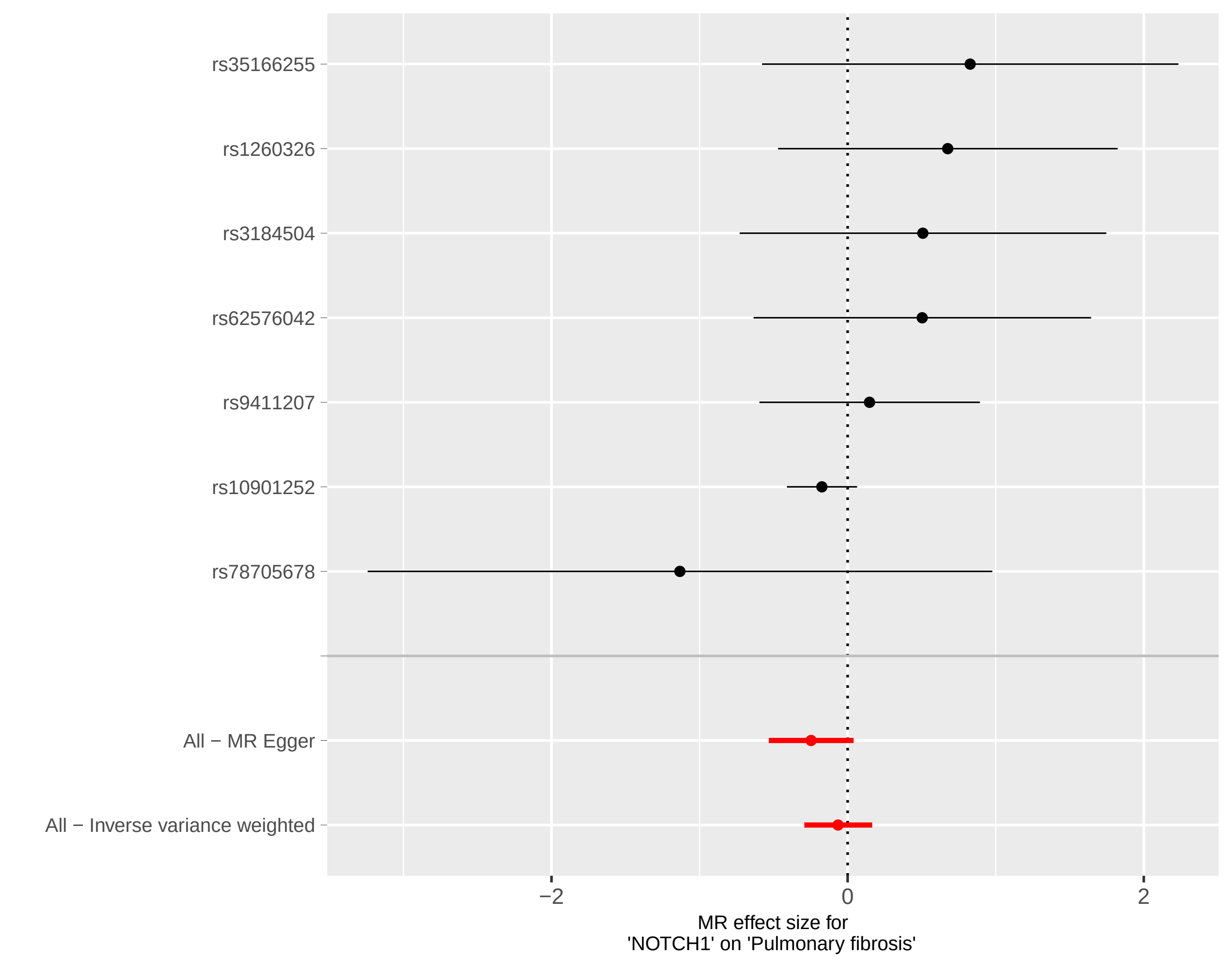
rs35166255
rs1260326
rs3184504
rs62576042
rs9411207
rs10901252
rs78705678
All − MR Egger
All − Inverse variance weighted
−2
0
2
MR effect size for
'NOTCH1' on 'Pulmonary fibrosis'

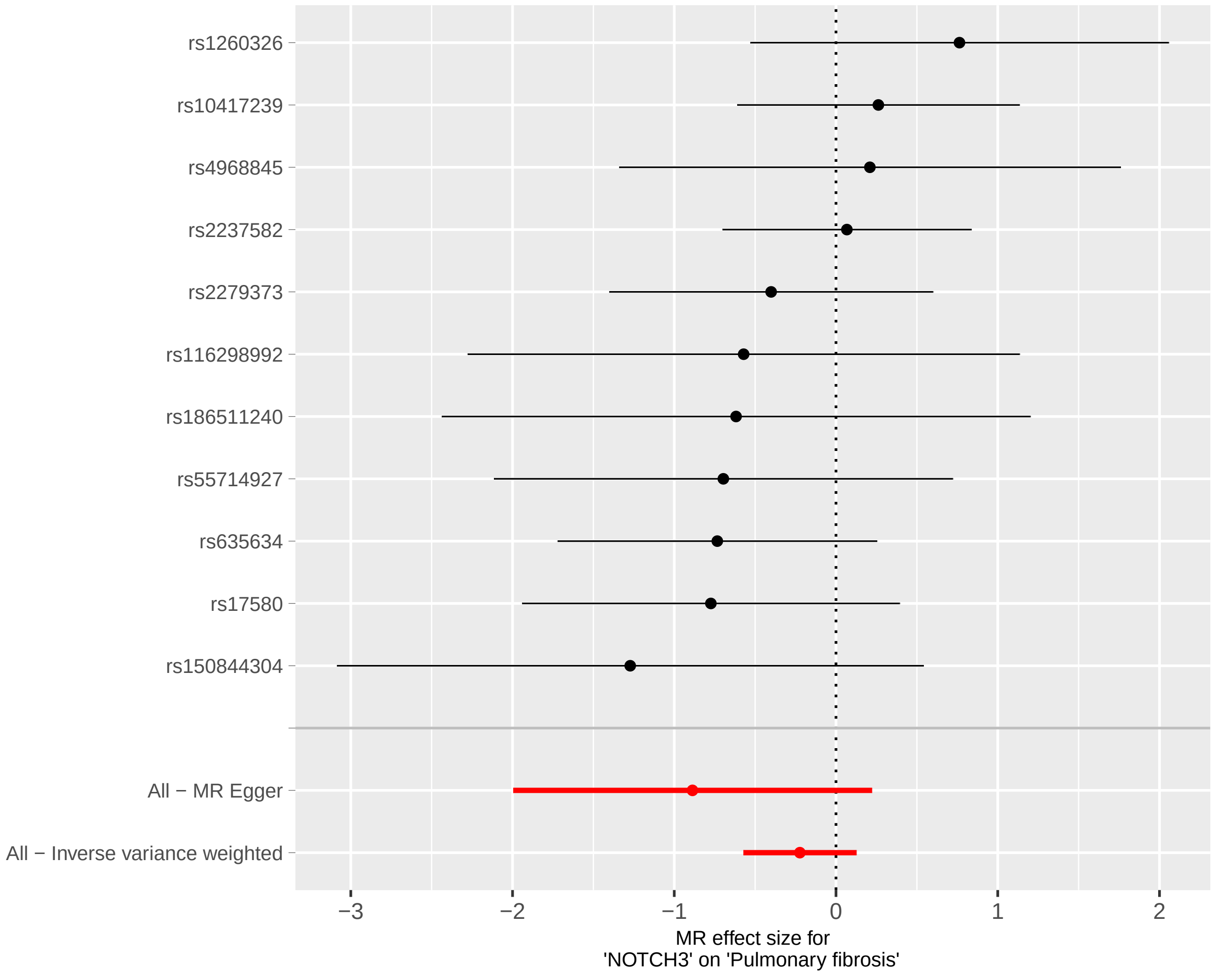

rs1260326
rs10417239
rs4968845
rs2237582
rs2279373
rs116298992
rs186511240
rs55714927
rs635634
rs17580
rs150844304
All − MR Egger
All − Inverse variance weighted
−3
−2
−1
0
1
2
MR effect size for
'NOTCH3' on 'Pulmonary fibrosis'

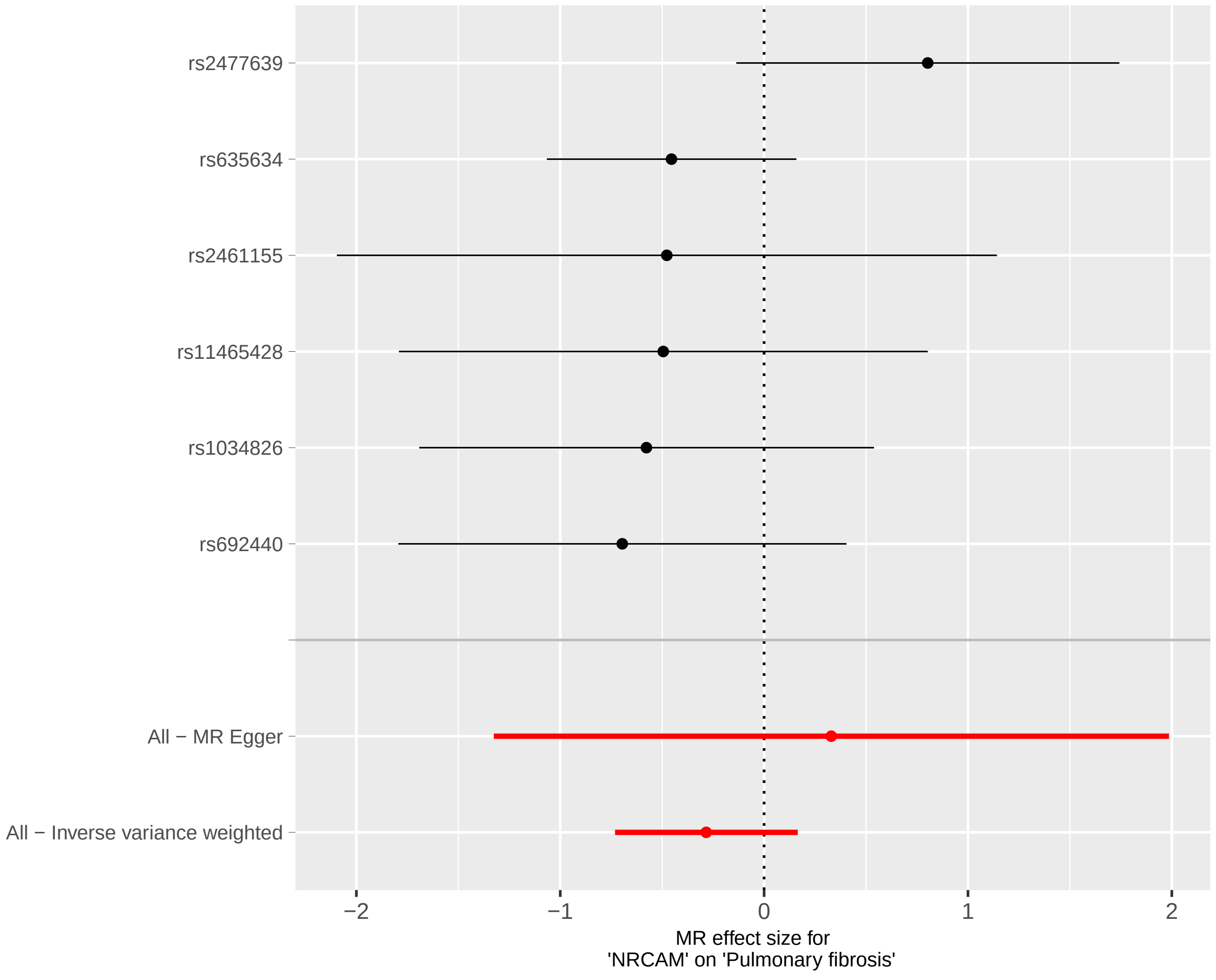
rs2477639
rs635634
rs2461155
rs11465428
rs1034826
rs692440
All − MR Egger
All − Inverse variance weighted
−2
−1
0
1
2
MR effect size for
'NRCAM' on 'Pulmonary fibrosis'

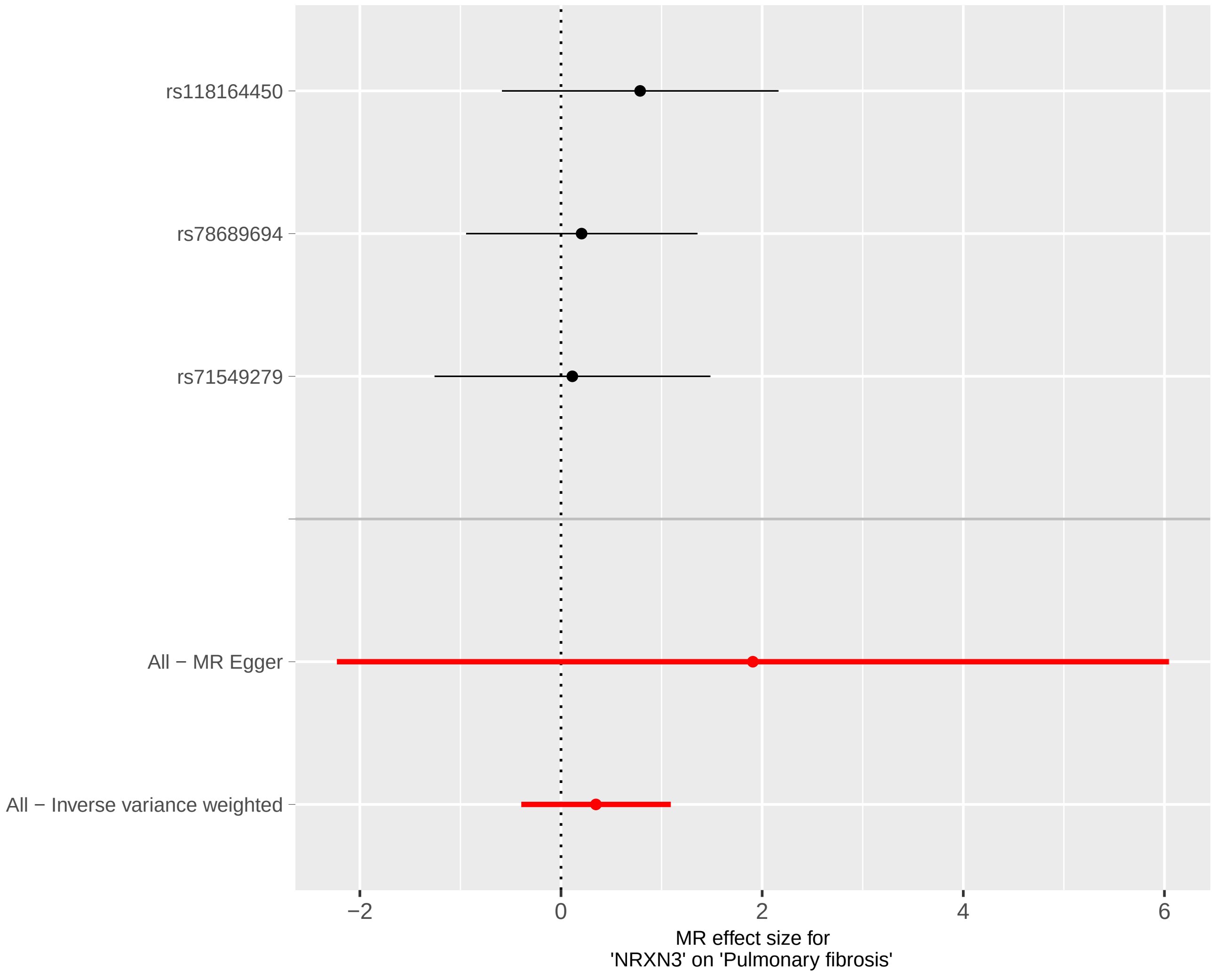
rs118164450
rs78689694
rs71549279
All − MR Egger
All − Inverse variance weighted
−2
0
2
4
6
MR effect size for
'NRXN3' on 'Pulmonary fibrosis'

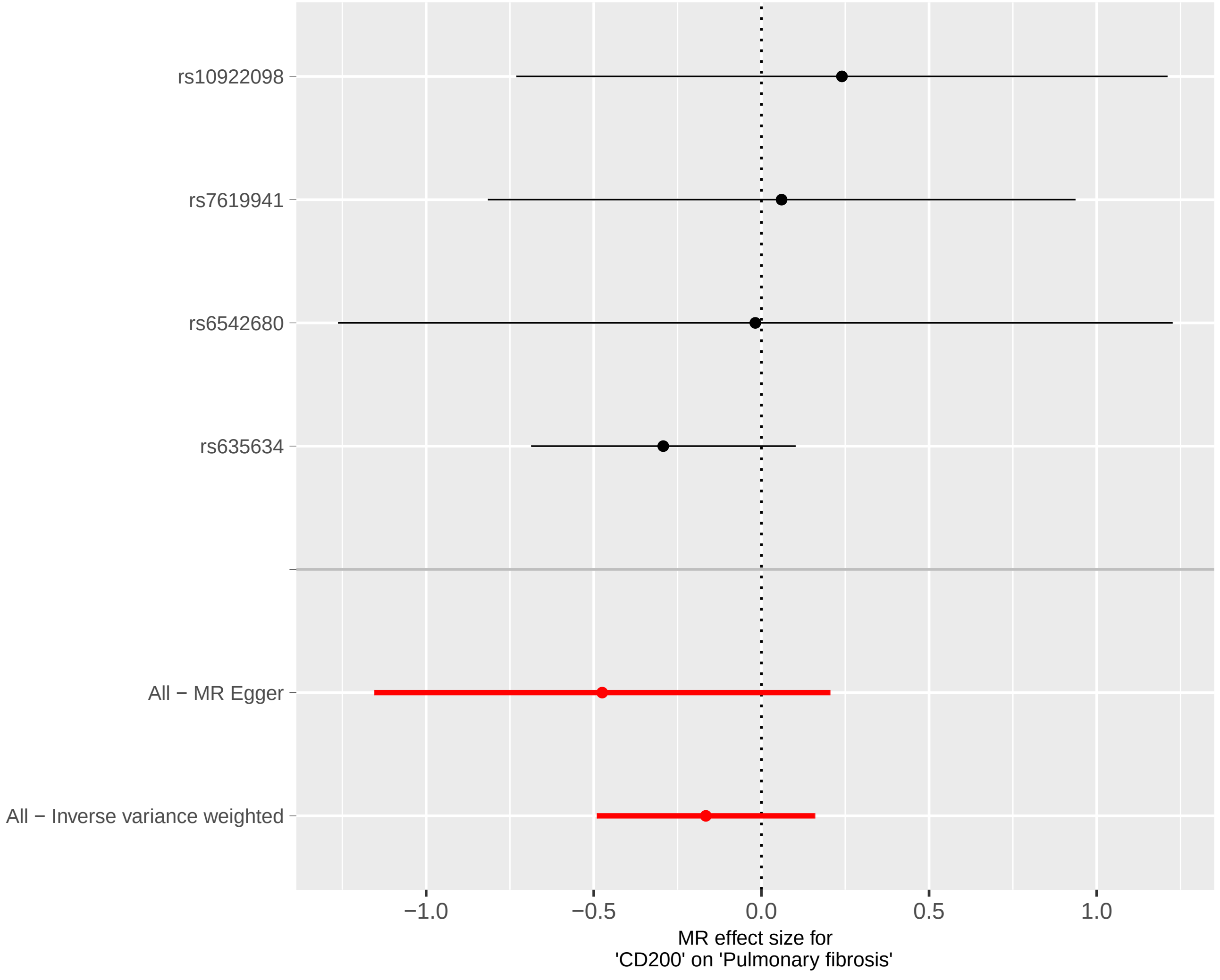

rs10922098
rs7619941
rs6542680
rs635634
All − MR Egger
All − Inverse variance weighted
−1.0
−0.5
0.0
0.5
1.0
MR effect size for
'CD200' on 'Pulmonary fibrosis'

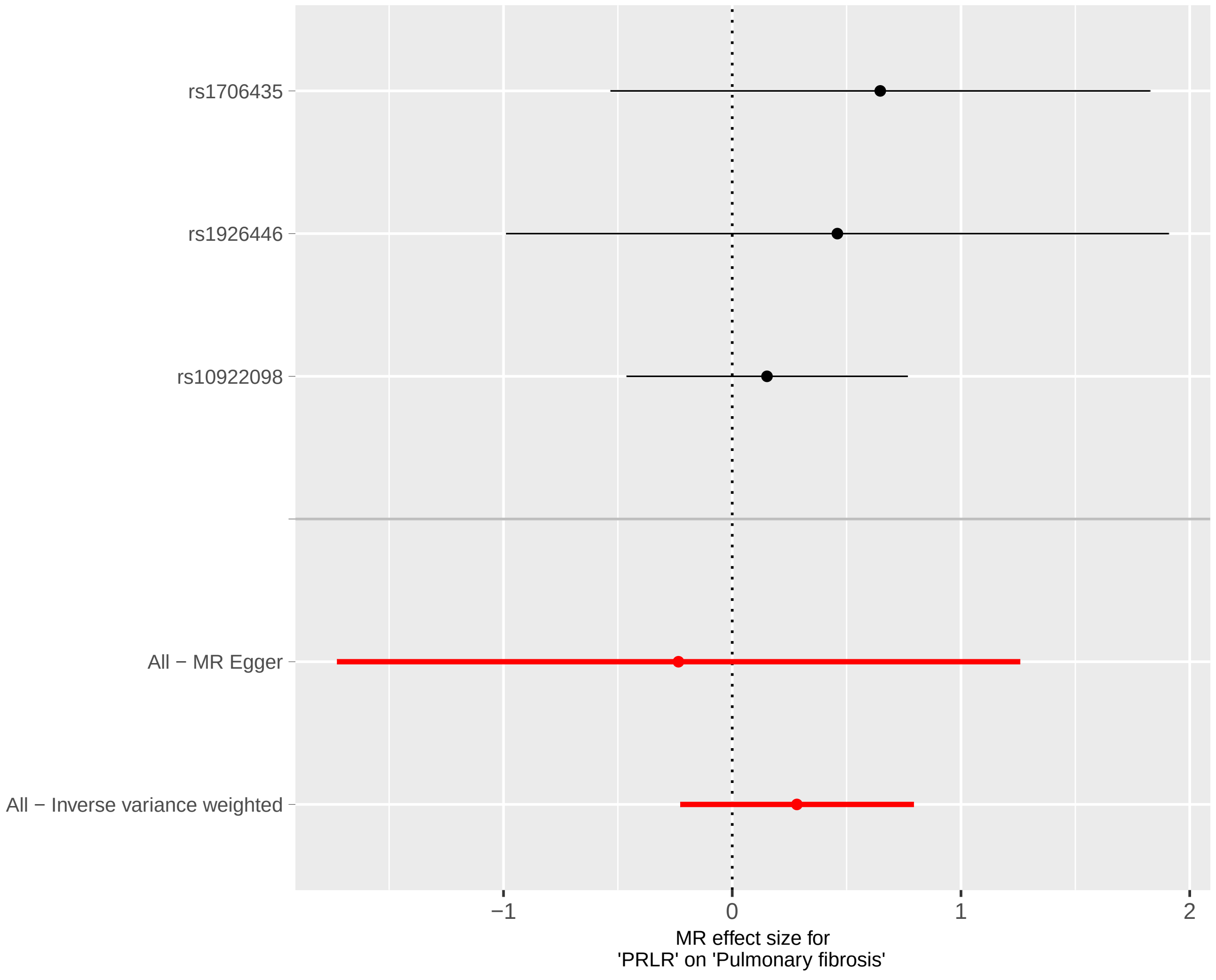

rs1706435
rs1926446
rs10922098
All − MR Egger
All − Inverse variance weighted
−1
0
1
2
MR effect size for
'PRLR' on 'Pulmonary fibrosis'

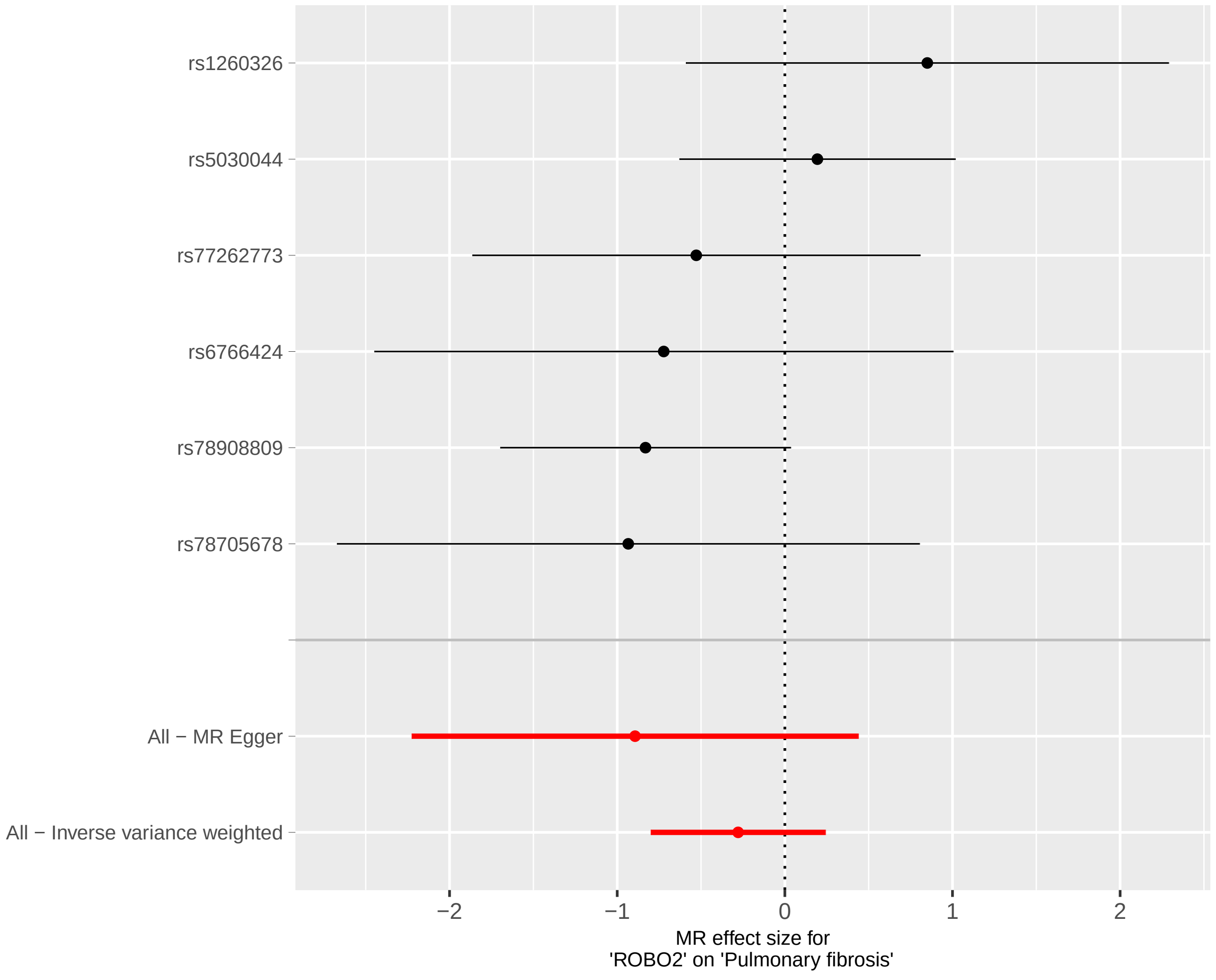

rs1260326
rs5030044
rs77262773
rs6766424
rs78908809
rs78705678
All − MR Egger
All − Inverse variance weighted
−2
−1
0
1
2
MR effect size for
'ROBO2' on 'Pulmonary fibrosis'

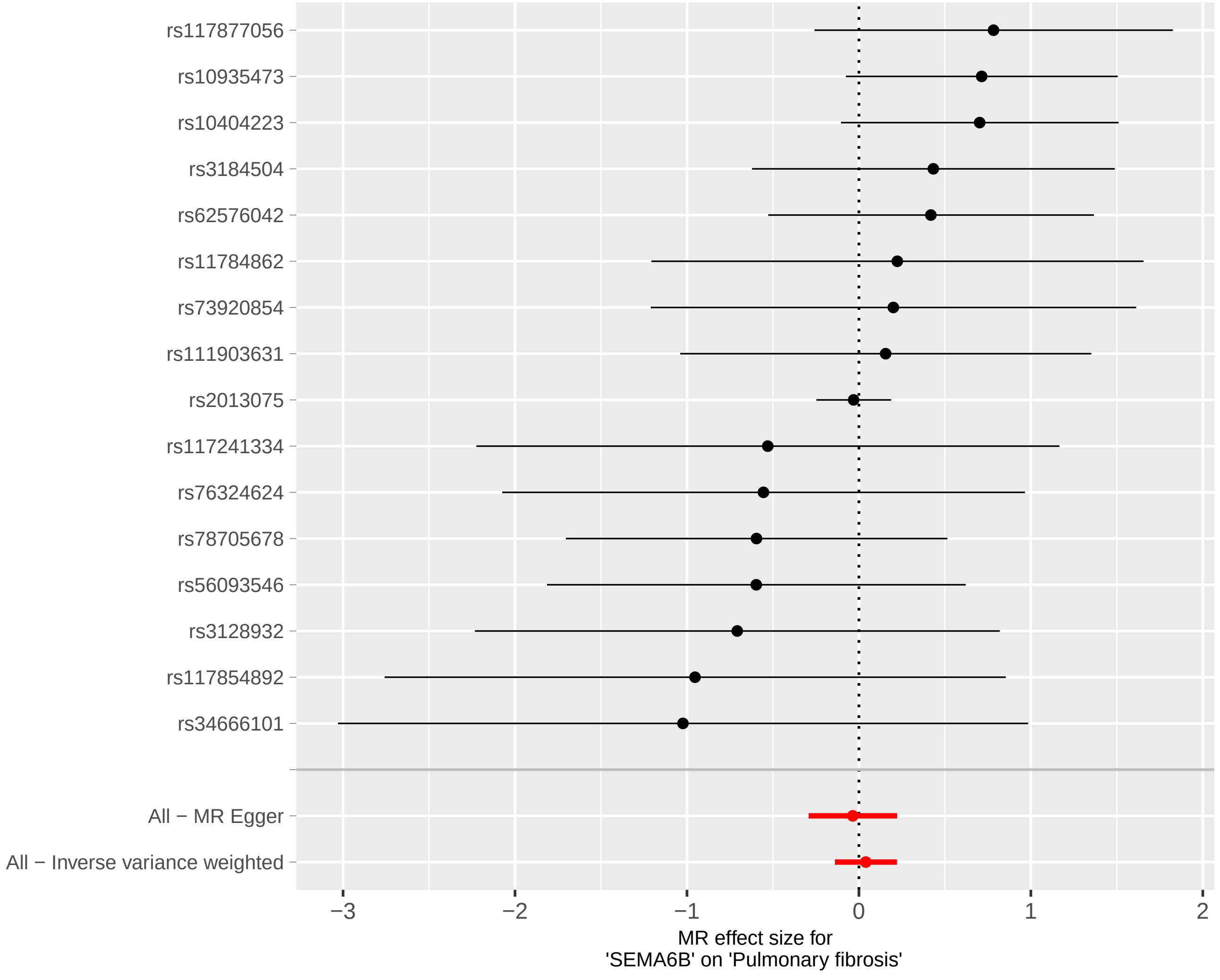
rs117877056
rs10935473
rs10404223
rs3184504
rs62576042
rs11784862
rs73920854
rs111903631
rs2013075
rs117241334
rs76324624
rs78705678
rs56093546
rs3128932
rs117854892
rs34666101
All − MR Egger
All − Inverse variance weighted
−3
−2
−1
0
1
2
MR effect size for
'SEMA6B' on 'Pulmonary fibrosis'

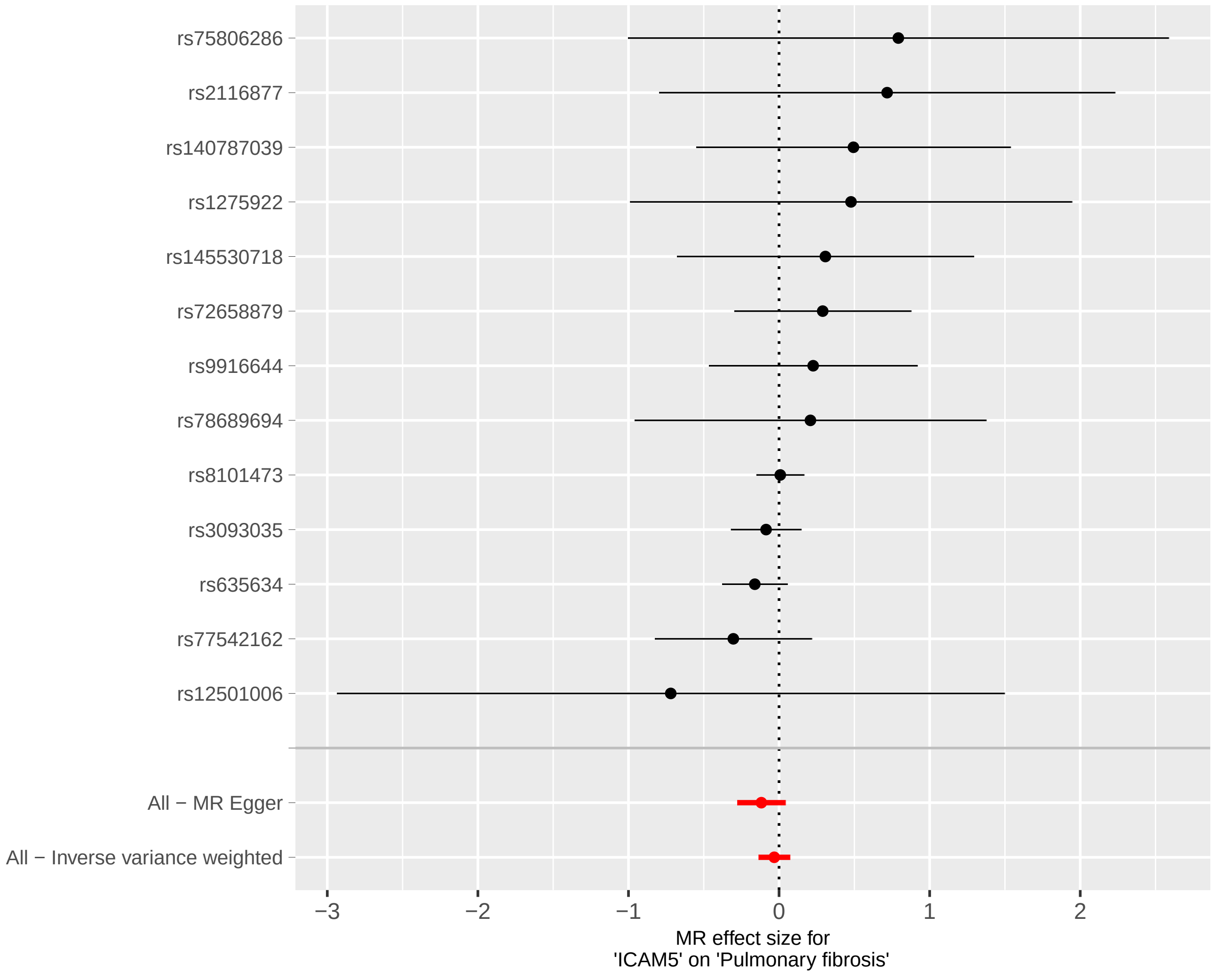
rs75806286
rs2116877
rs140787039
rs1275922
rs145530718
rs72658879
rs9916644
rs78689694
rs8101473
rs3093035
rs635634
rs77542162
rs12501006
All − MR Egger
All − Inverse variance weighted
−3
−2
−1
0
1
2
MR effect size for
'ICAM5' on 'Pulmonary fibrosis'

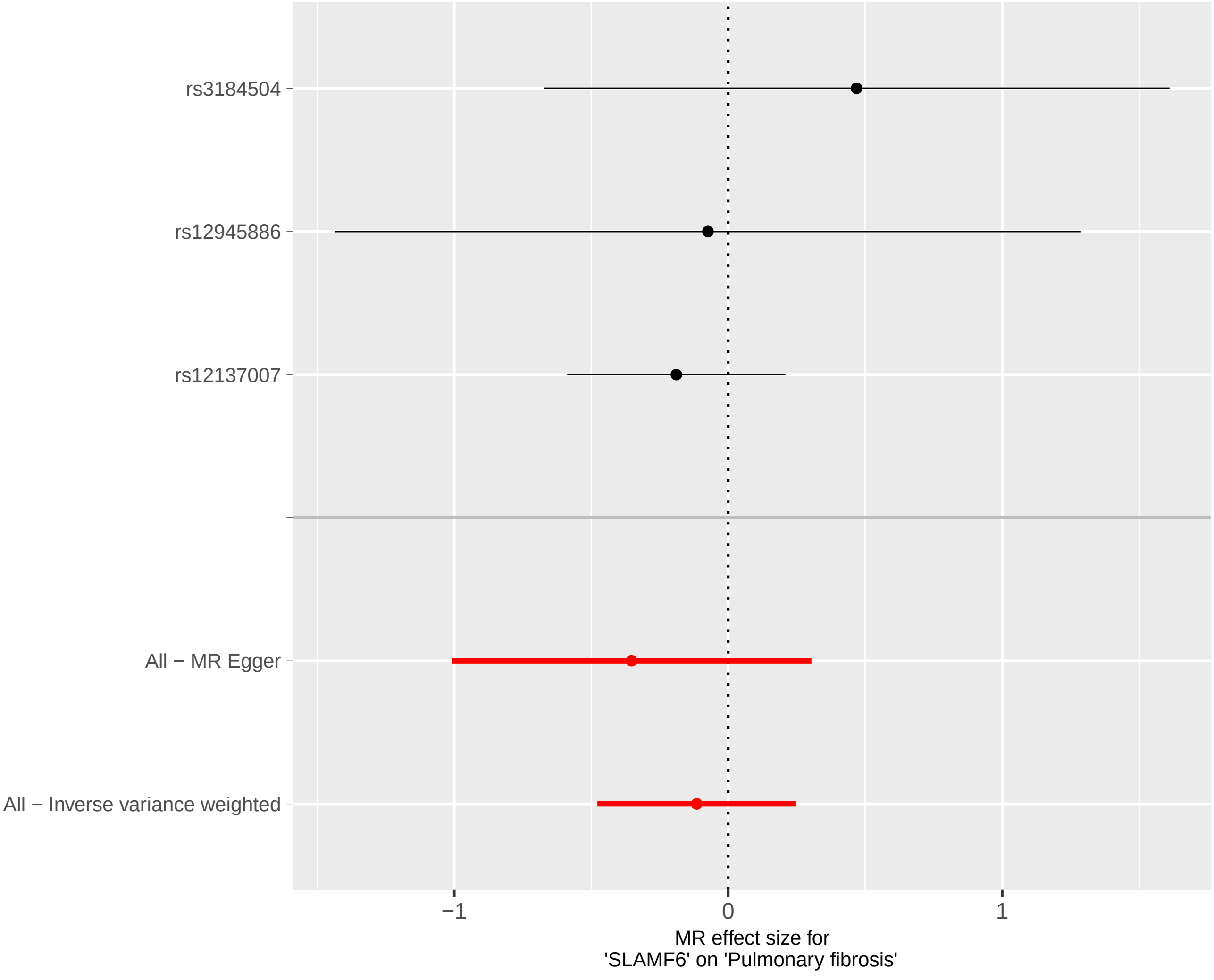

rs3184504
rs12945886
rs12137007
All − MR Egger
All − Inverse variance weighted
−1
0
1
MR effect size for
'SLAMF6' on 'Pulmonary fibrosis'

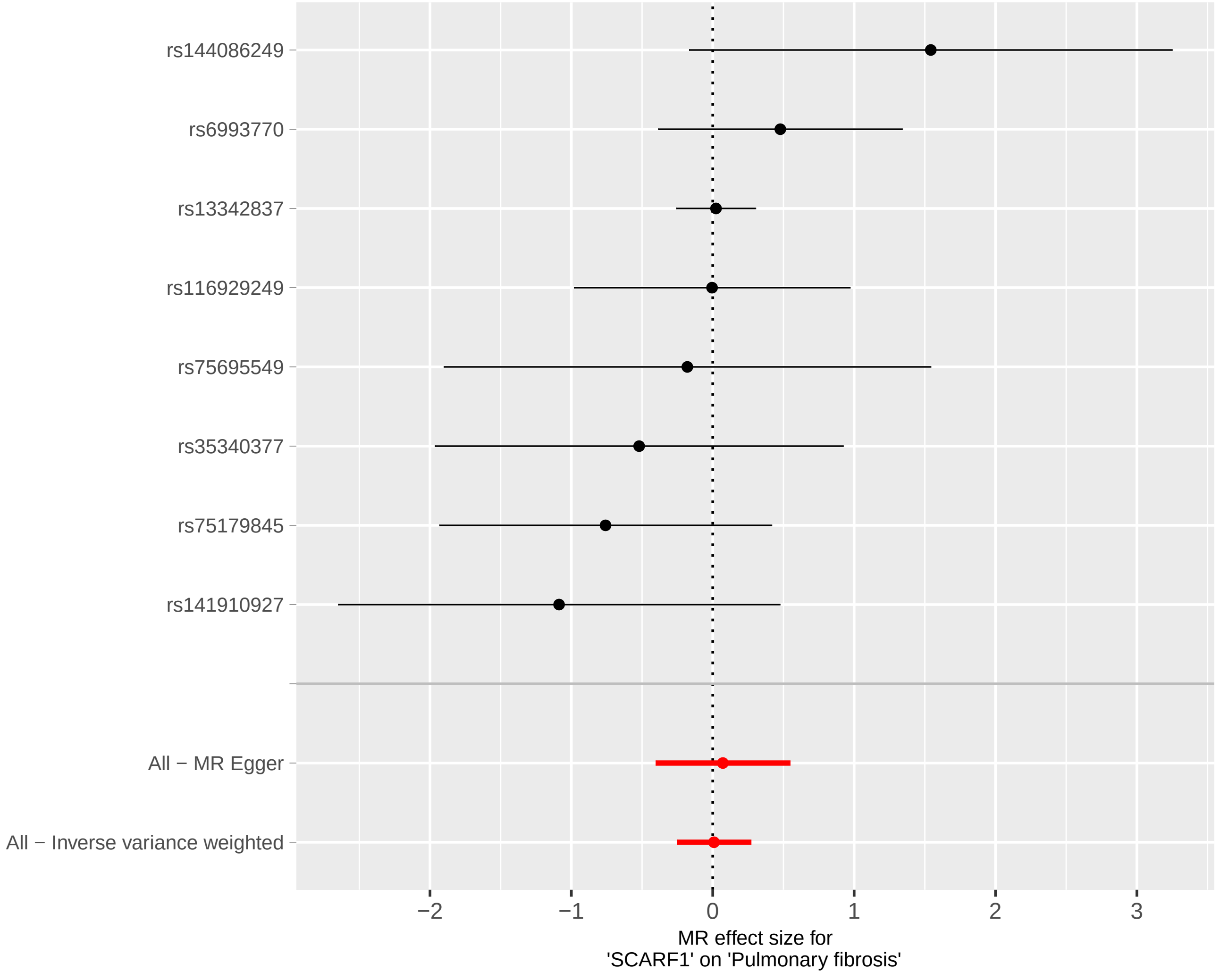

rs144086249
rs6993770
rs13342837
rs116929249
rs75695549
rs35340377
rs75179845
rs141910927
All − MR Egger
All − Inverse variance weighted
−2
−1
0
1
2
3
MR effect size for
'SCARF1' on 'Pulmonary fibrosis'

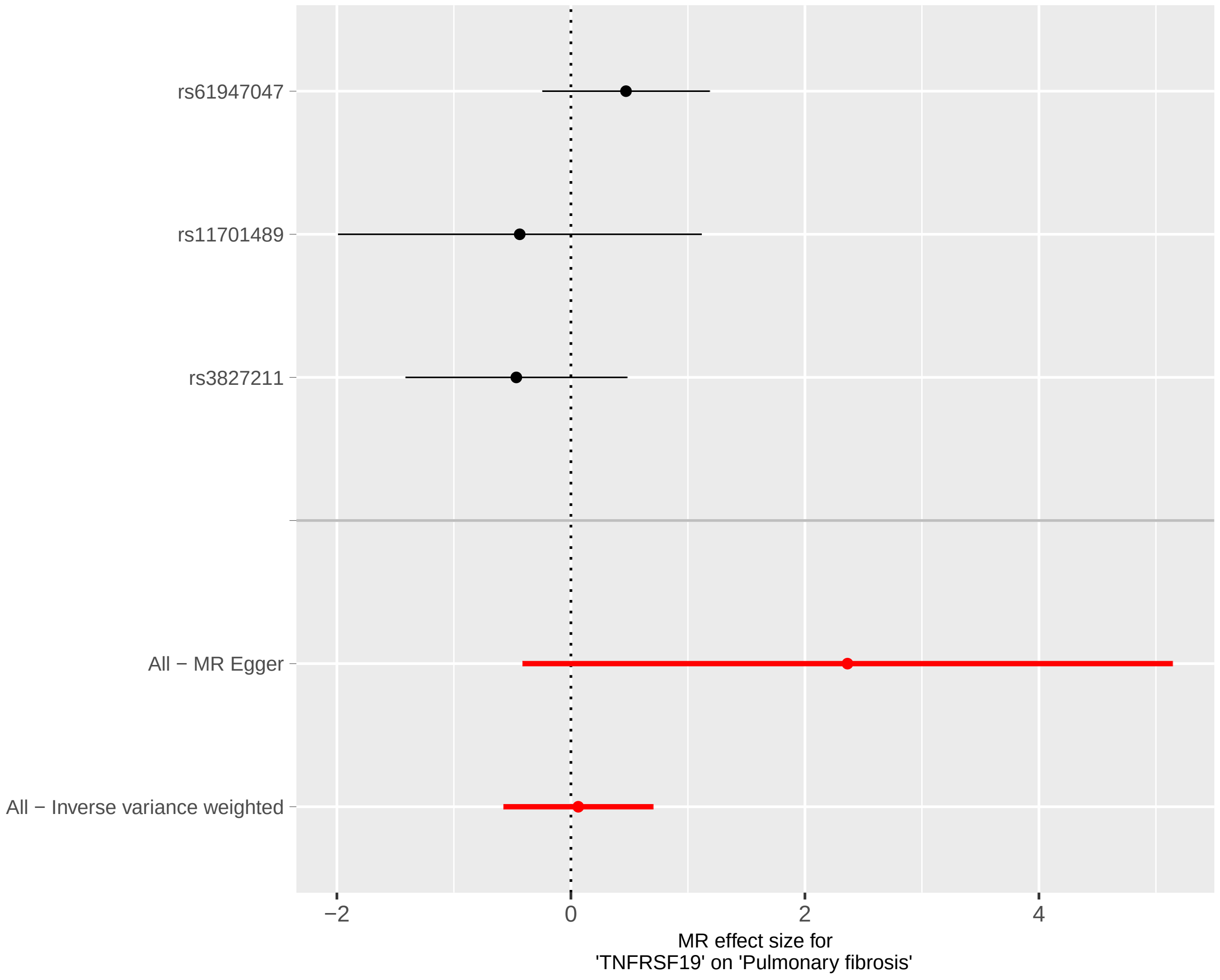

rs61947047
rs11701489
rs3827211
All − MR Egger
All − Inverse variance weighted
−2
0
2
4
MR effect size for
'TNFRSF19' on 'Pulmonary fibrosis'

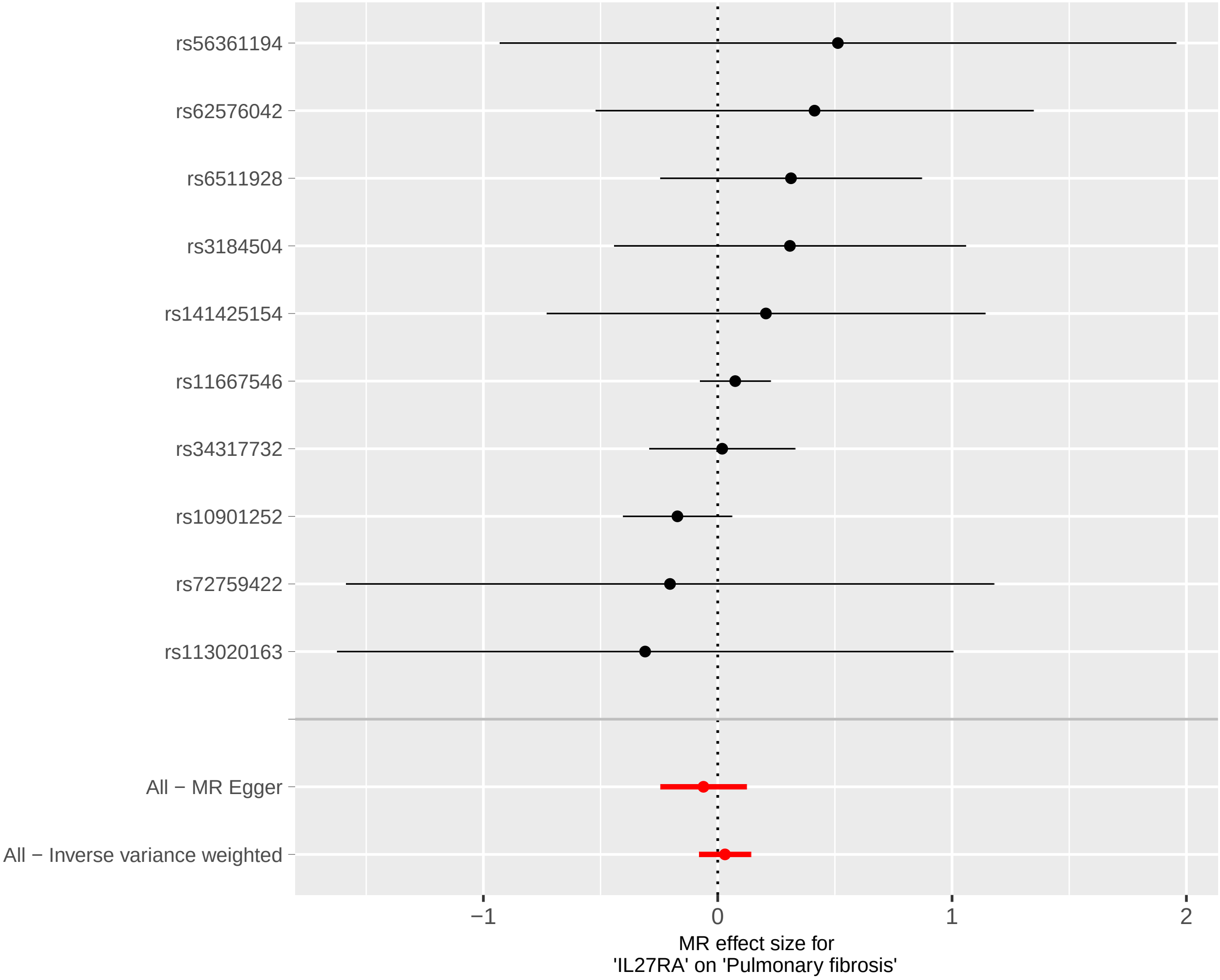
rs56361194
rs62576042
rs6511928
rs3184504
rs141425154
rs11667546
rs34317732
rs10901252
rs72759422
rs113020163
All − MR Egger
All − Inverse variance weighted
−1
0
1
2
MR effect size for
'IL27RA' on 'Pulmonary fibrosis'

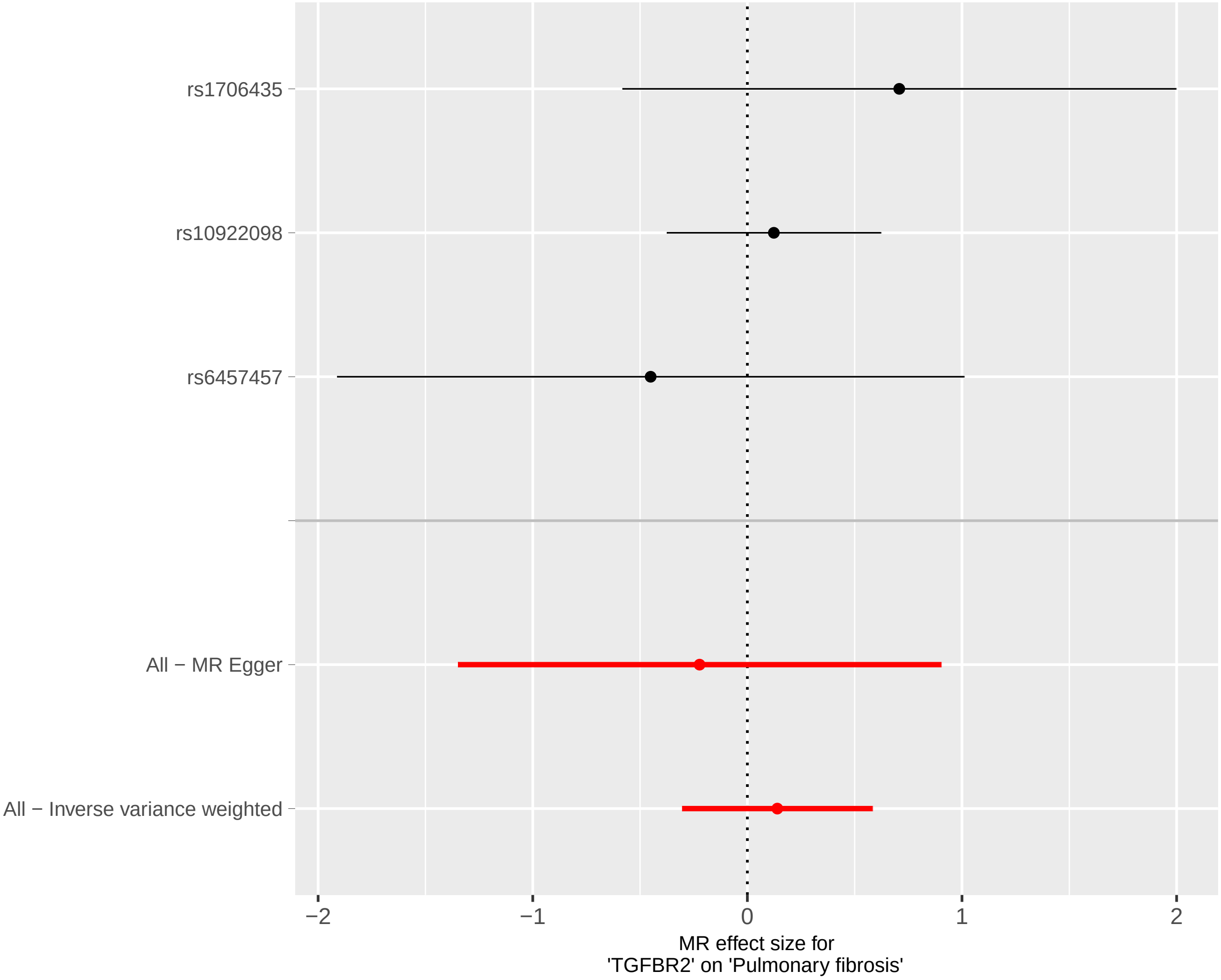

rs1706435
rs10922098
rs6457457
All − MR Egger
All − Inverse variance weighted
−2
−1
0
1
2
MR effect size for
'TGFBR2' on 'Pulmonary fibrosis'

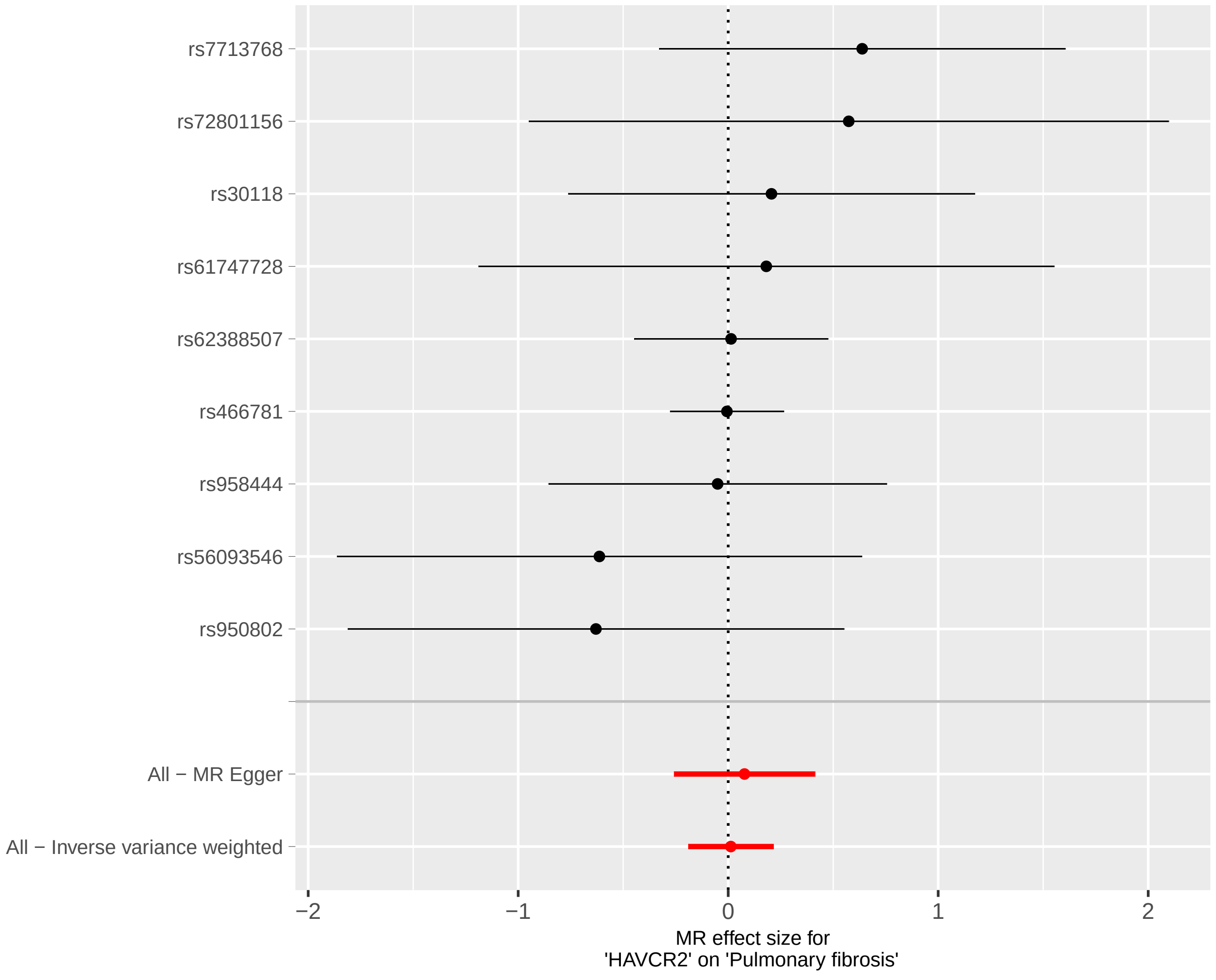
rs7713768
rs72801156
rs30118
rs61747728
rs62388507
rs466781
rs958444
rs56093546
rs950802
All − MR Egger
All − Inverse variance weighted
−2
−1
0
1
2
MR effect size for
'HAVCR2' on 'Pulmonary fibrosis'

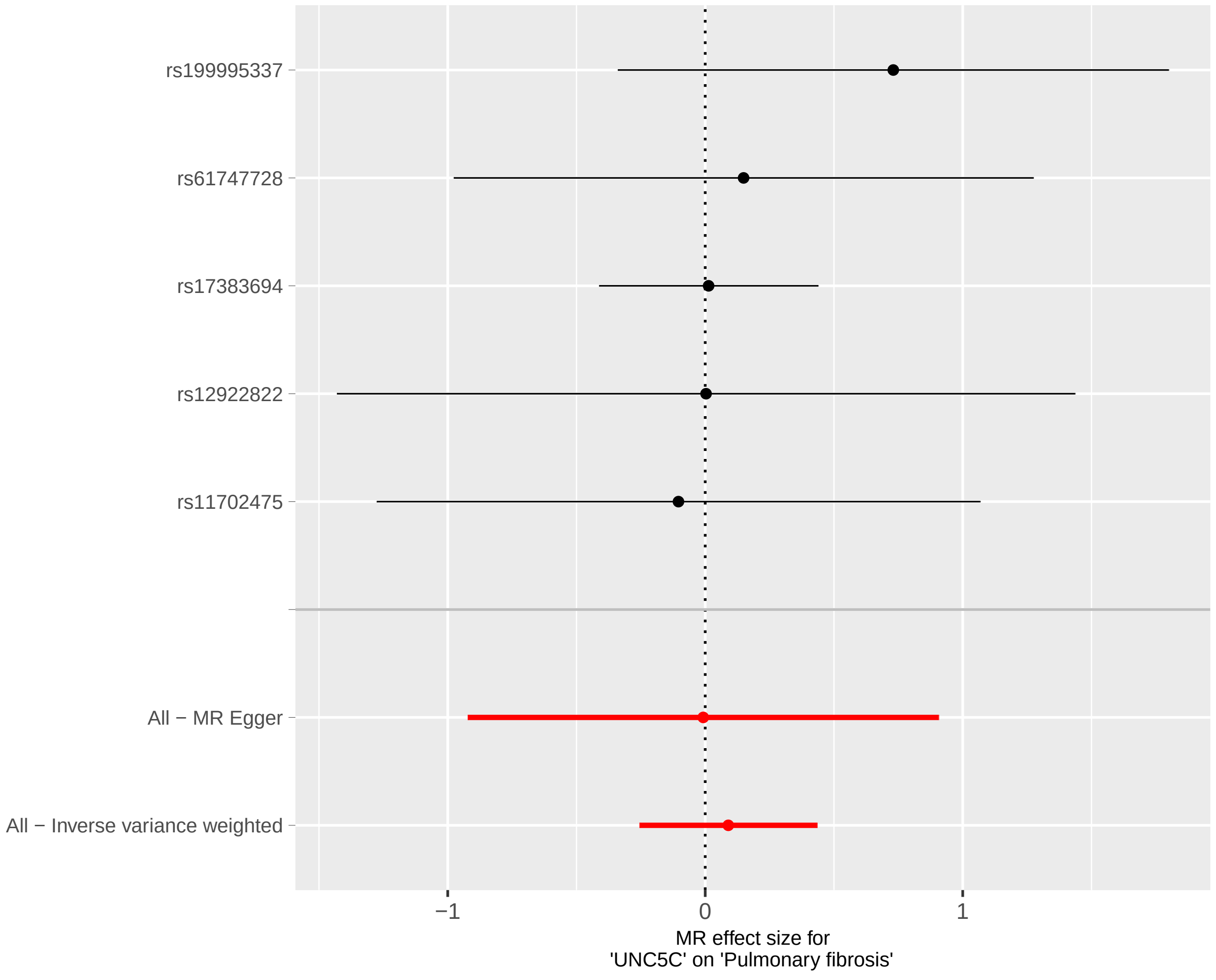
rs199995337
rs61747728
rs17383694
rs12922822
rs11702475
All − MR Egger
All − Inverse variance weighted
−1
0
1
MR effect size for
'UNC5C' on 'Pulmonary fibrosis'

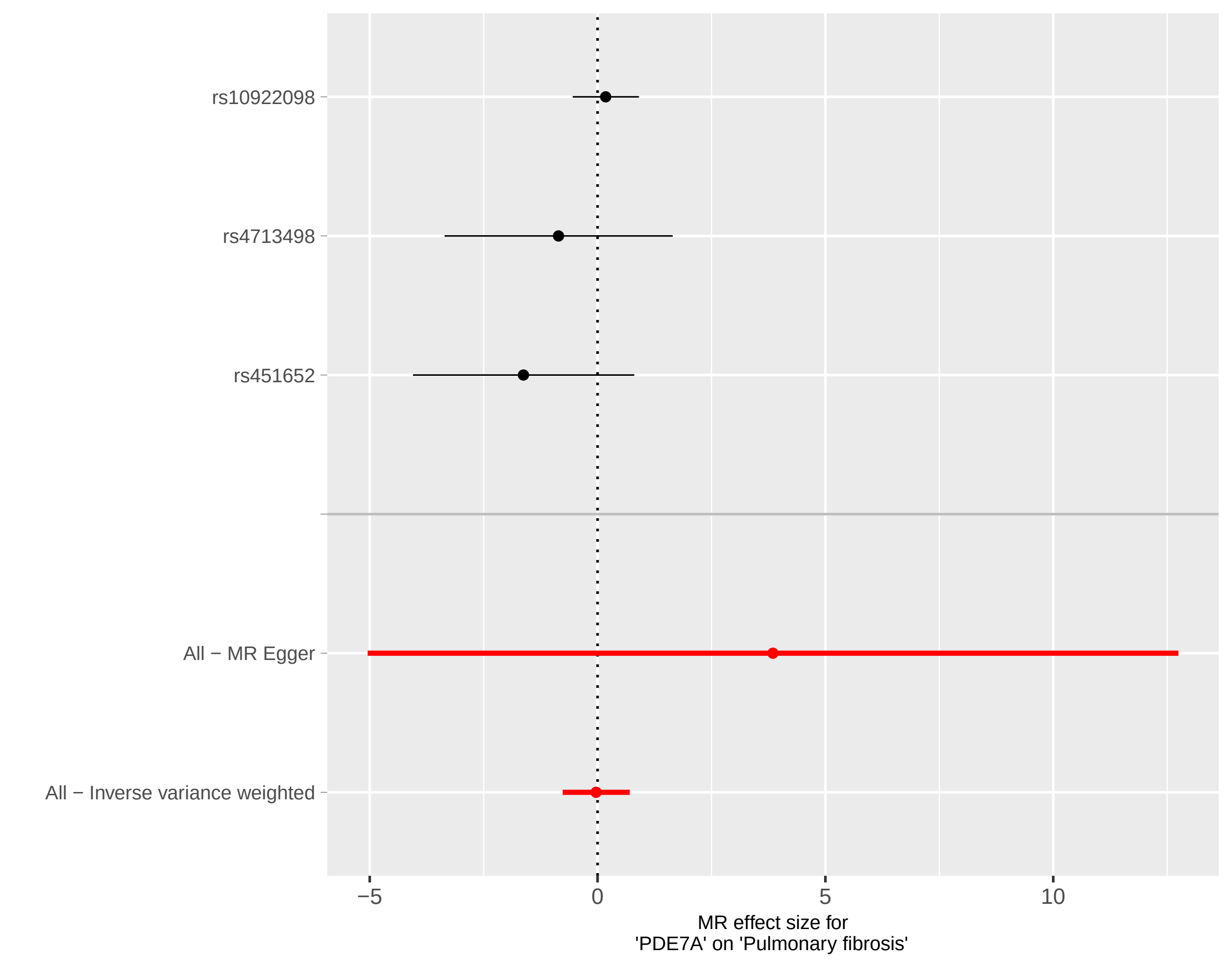

rs10922098
rs4713498
rs451652
All − MR Egger
All − Inverse variance weighted
−5
0
5
10
MR effect size for
'PDE7A' on 'Pulmonary fibrosis'

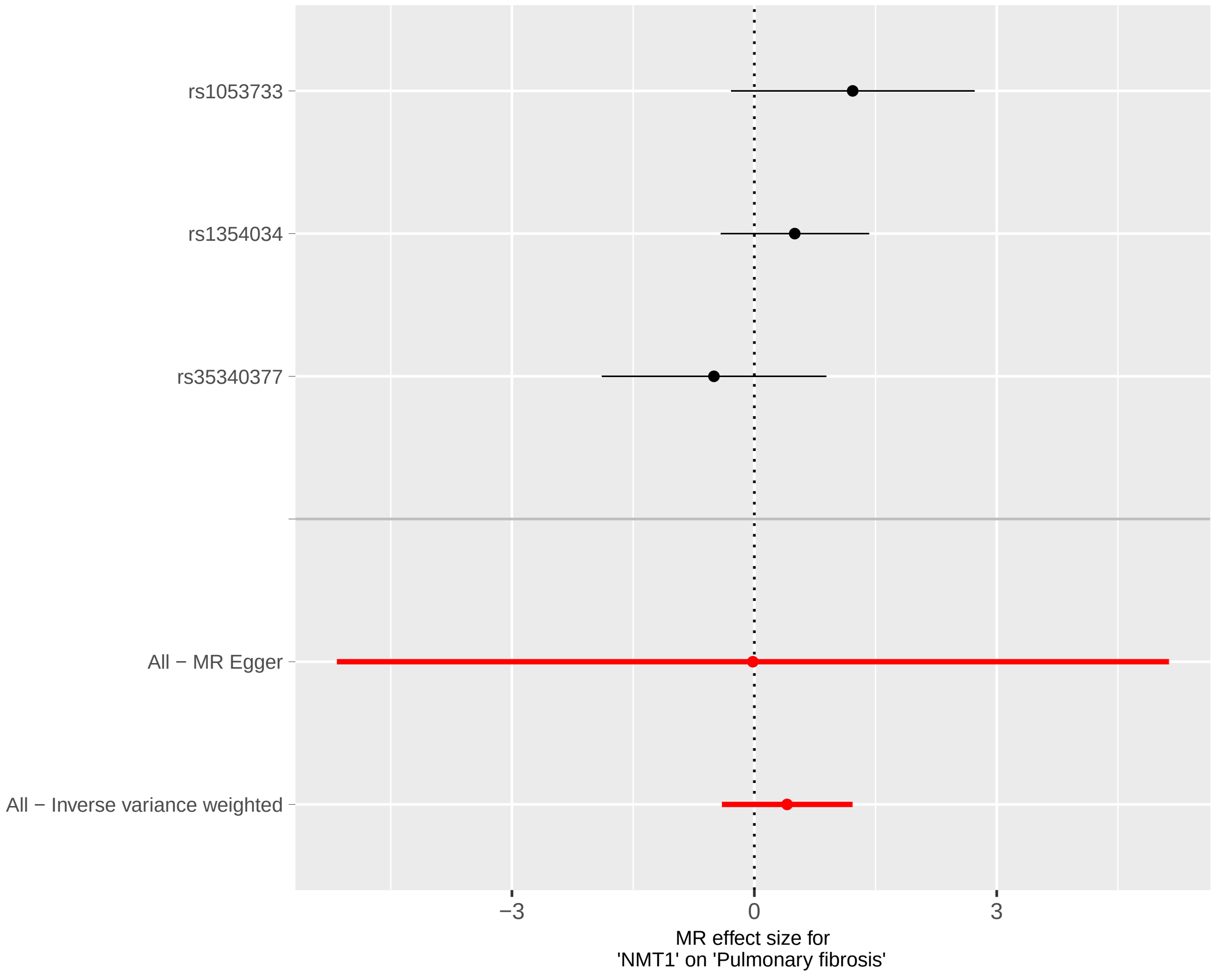

rs1053733
rs1354034
rs35340377
All − MR Egger
All − Inverse variance weighted
−3
0
3
MR effect size for
'NMT1' on 'Pulmonary fibrosis'

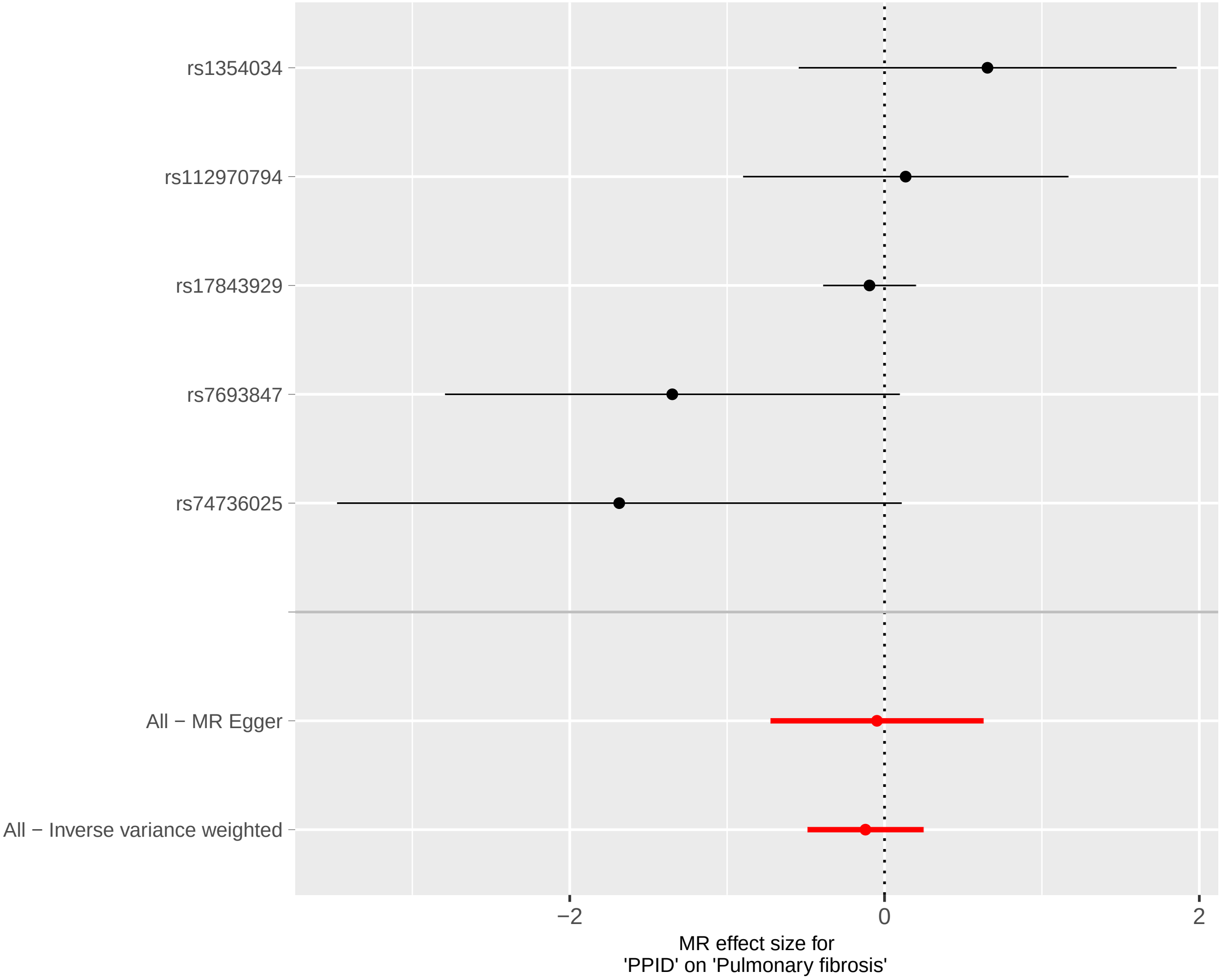
rs1354034
rs112970794
rs17843929
rs7693847
rs74736025
All − MR Egger
All − Inverse variance weighted
−2
0
2
MR effect size for
'PPID' on 'Pulmonary fibrosis'

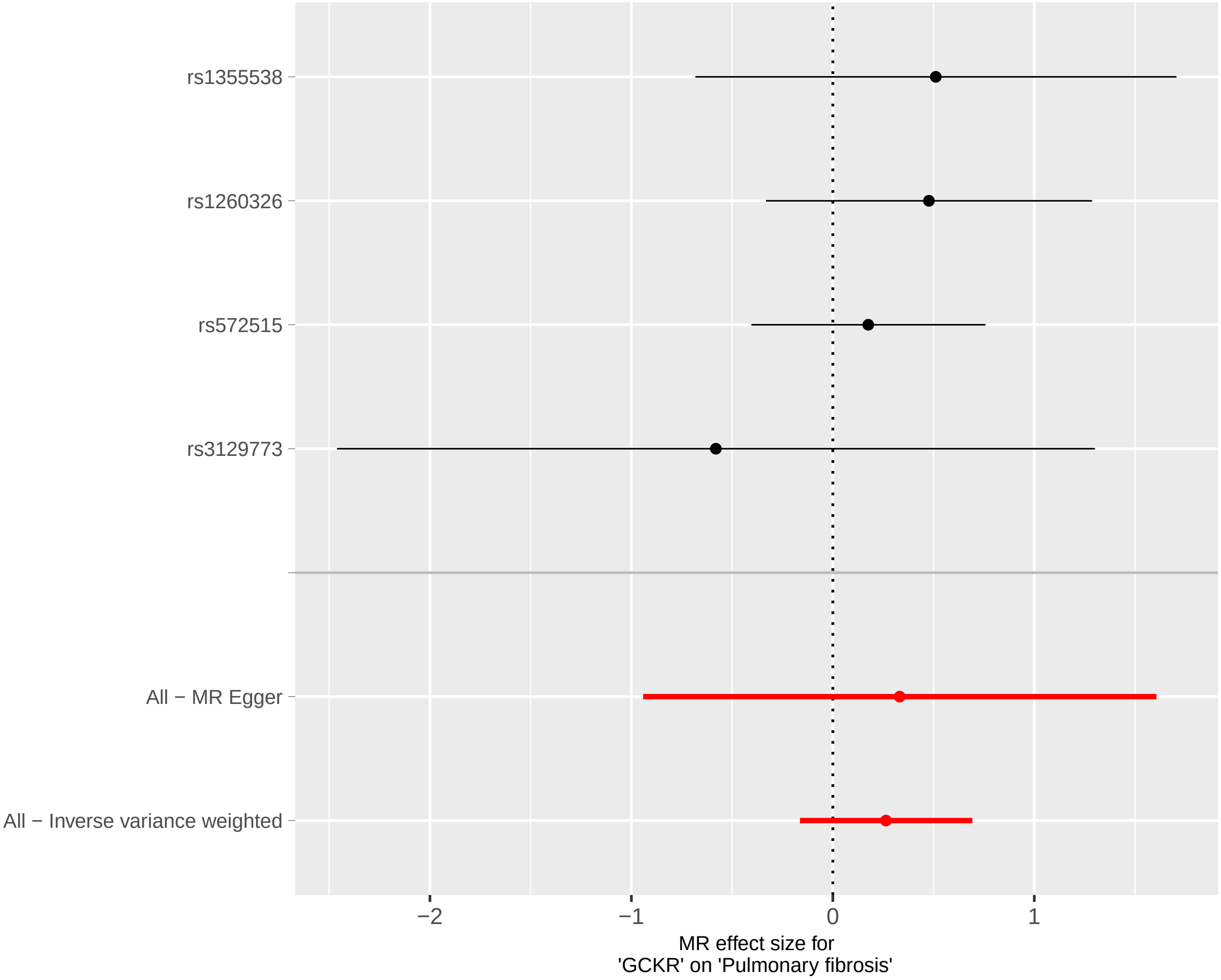
rs1355538
rs1260326
rs572515
rs3129773
All − MR Egger
All − Inverse variance weighted
−2
−1
0
1
MR effect size for
'GCKR' on 'Pulmonary fibrosis'

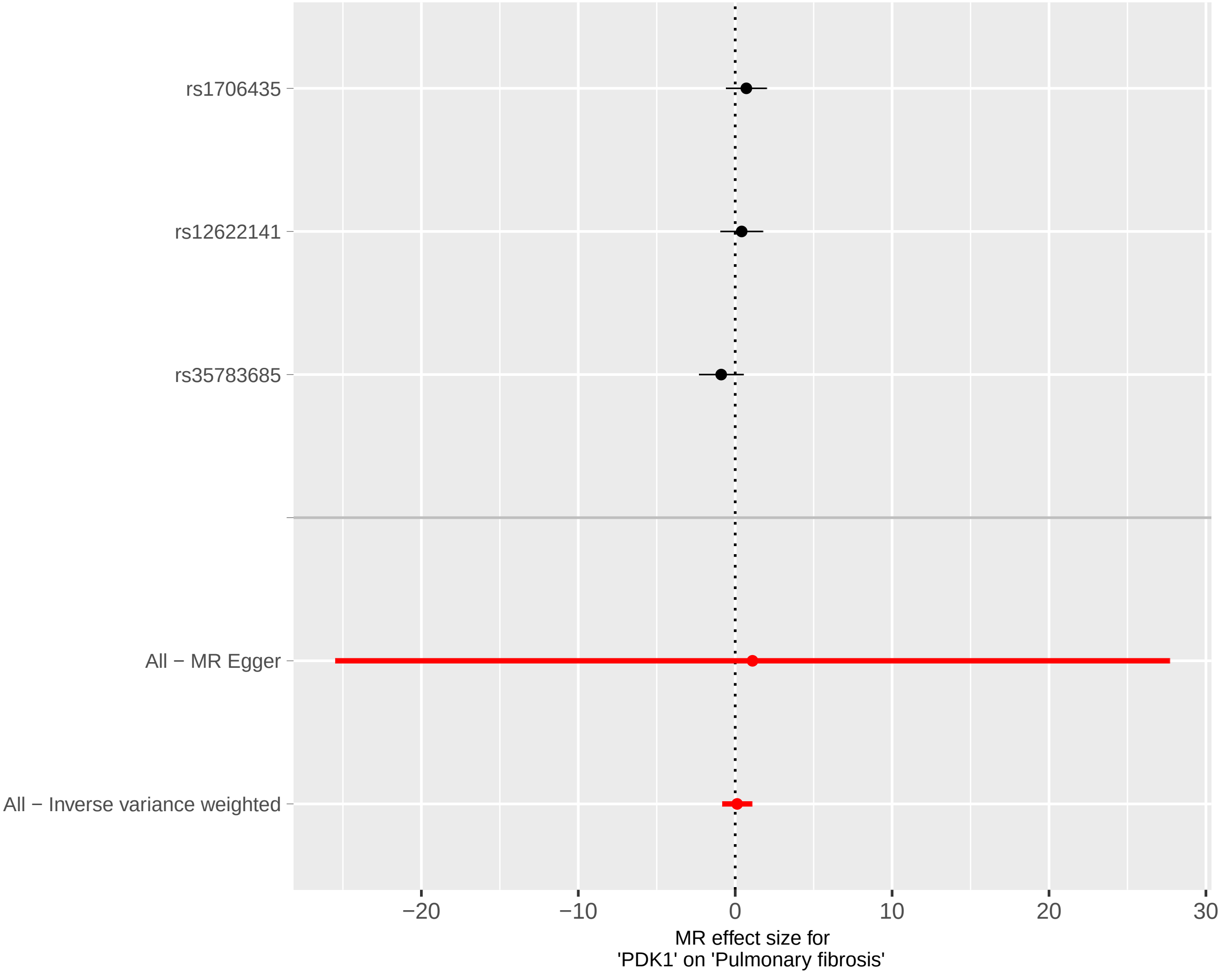
rs1706435
rs12622141
rs35783685
All − MR Egger
All − Inverse variance weighted
−20
−10
0
10
20
30
MR effect size for
'PDK1' on 'Pulmonary fibrosis'

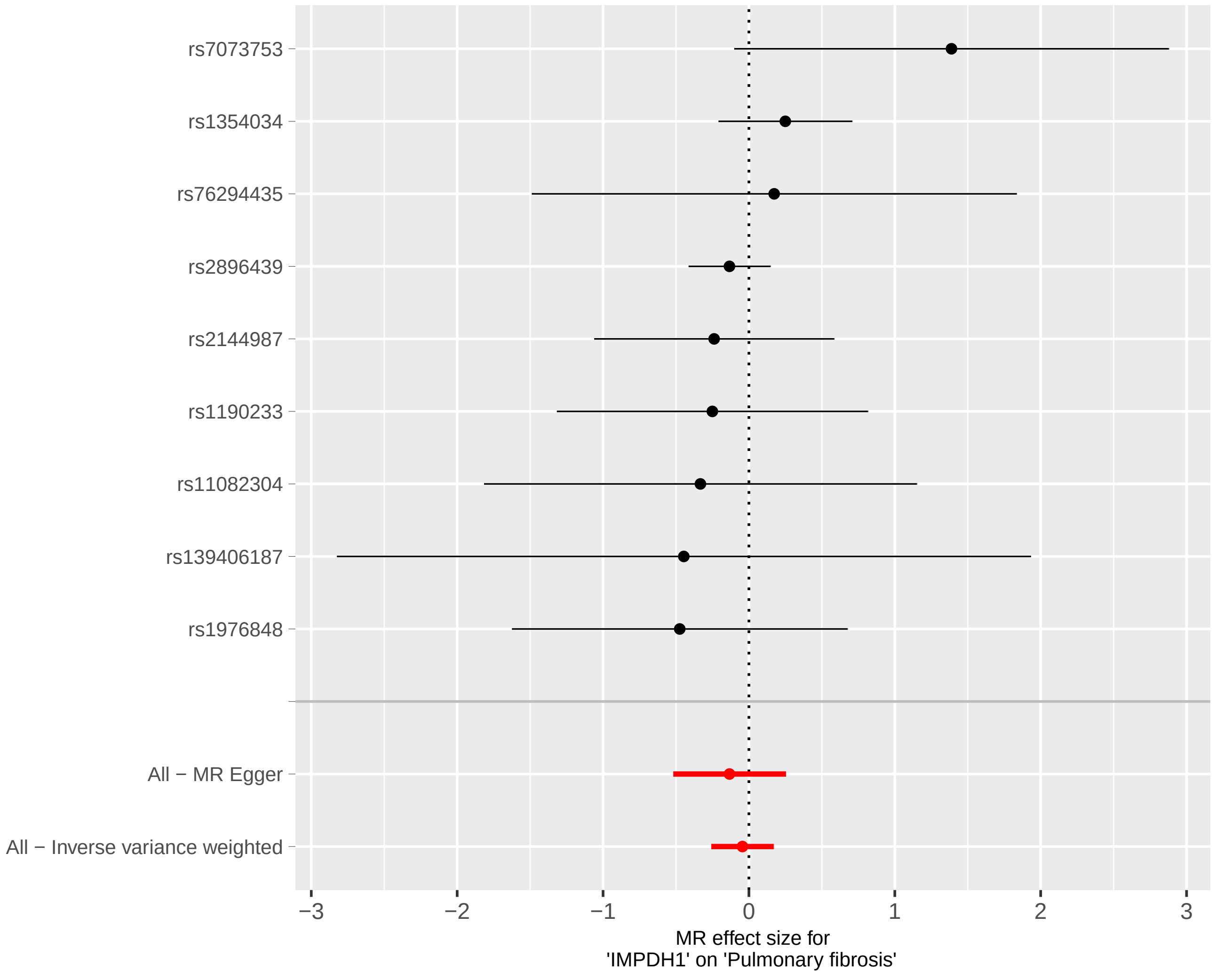

rs7073753
rs1354034
rs76294435
rs2896439
rs2144987
rs1190233
rs11082304
rs139406187
rs1976848
All − MR Egger
All − Inverse variance weighted
−3
−2
−1
0
1
2
3
MR effect size for
'IMPDH1' on 'Pulmonary fibrosis'

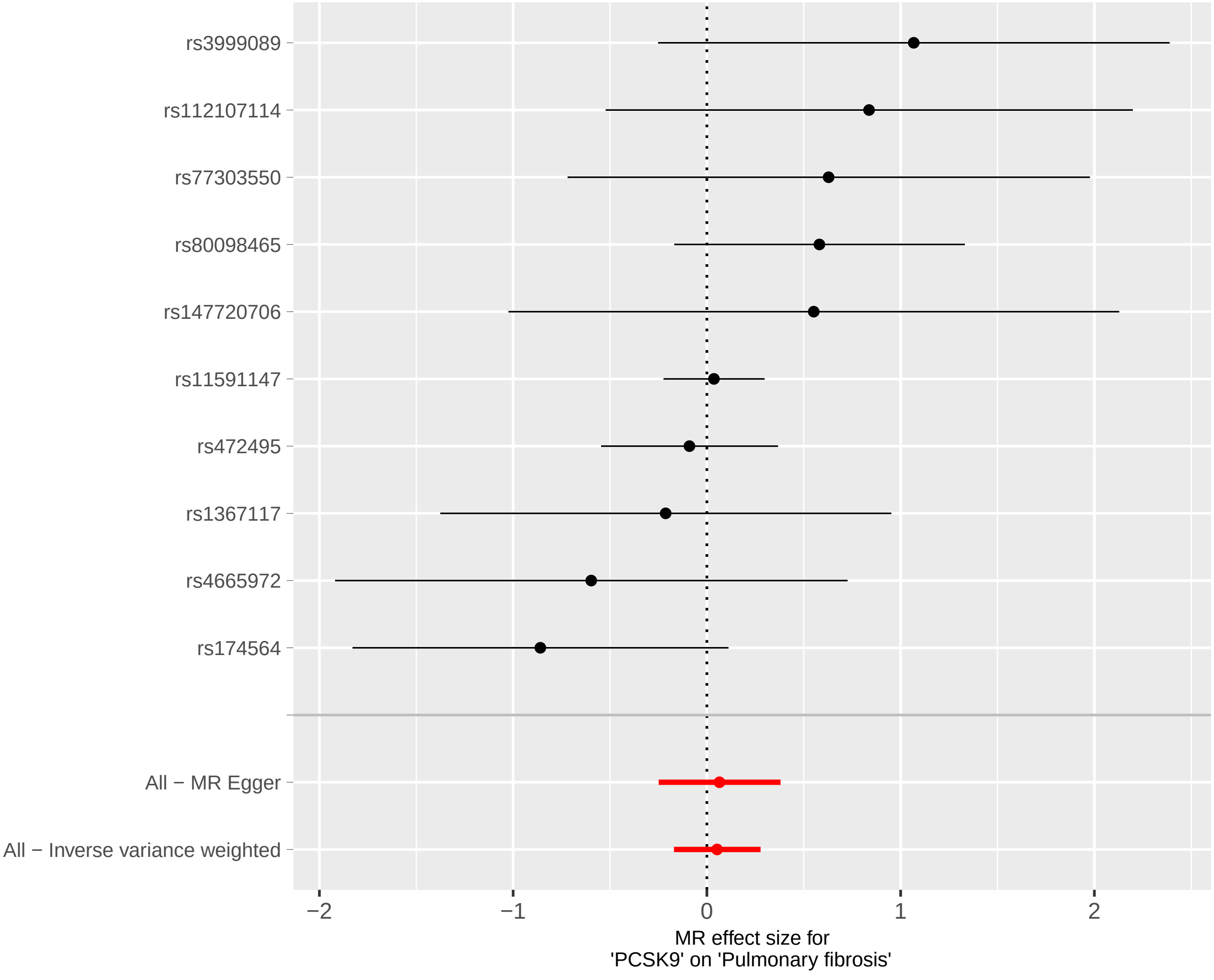

rs3999089
rs112107114
rs77303550
rs80098465
rs147720706
rs11591147
rs472495
rs1367117
rs4665972
rs174564
All − MR Egger
All − Inverse variance weighted
−2
−1
0
1
2
MR effect size for
'PCSK9' on 'Pulmonary fibrosis'

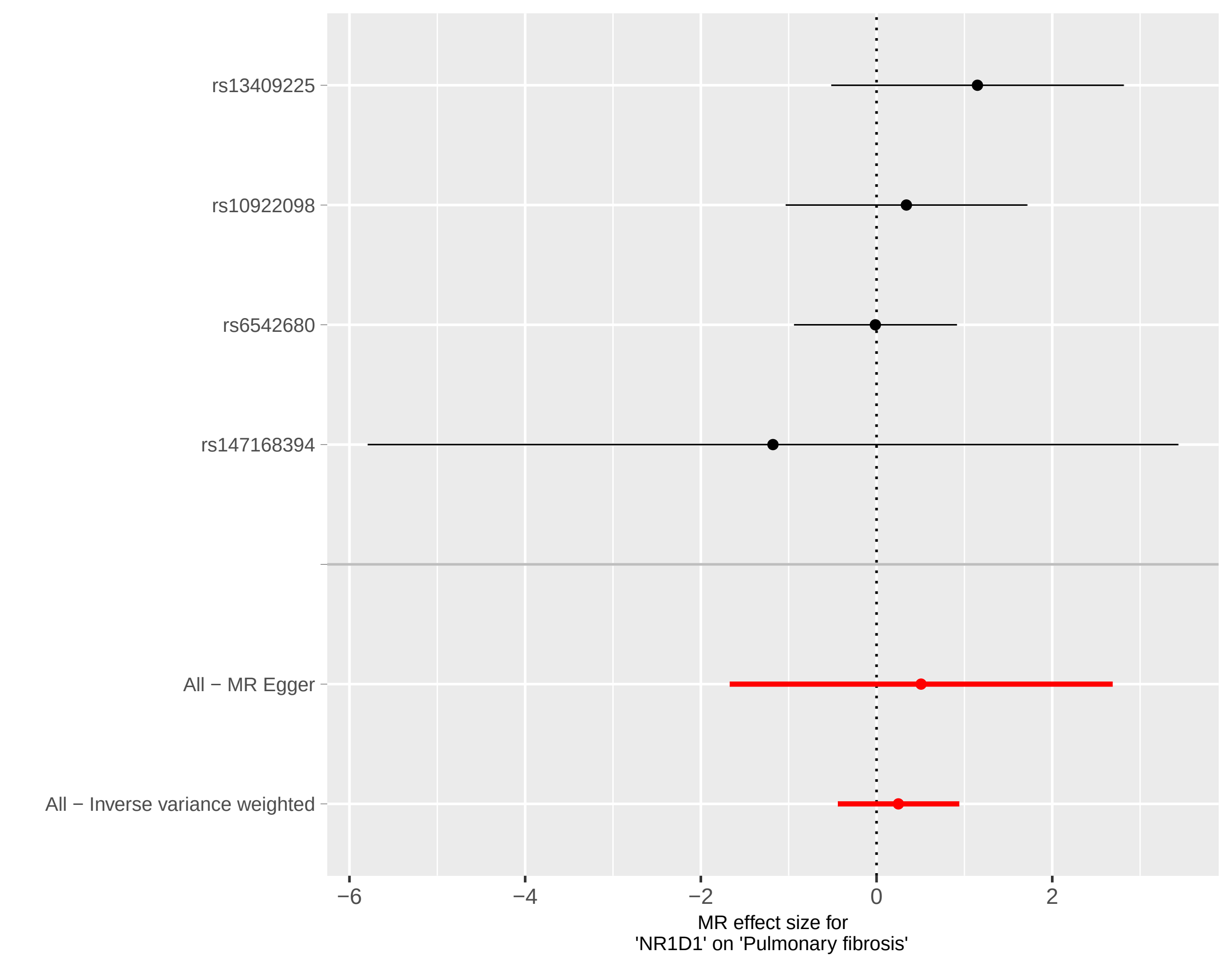

rs13409225
rs10922098
rs6542680
rs147168394
All − MR Egger
All − Inverse variance weighted
−6
−4
−2
0
2
MR effect size for
'NR1D1' on 'Pulmonary fibrosis'

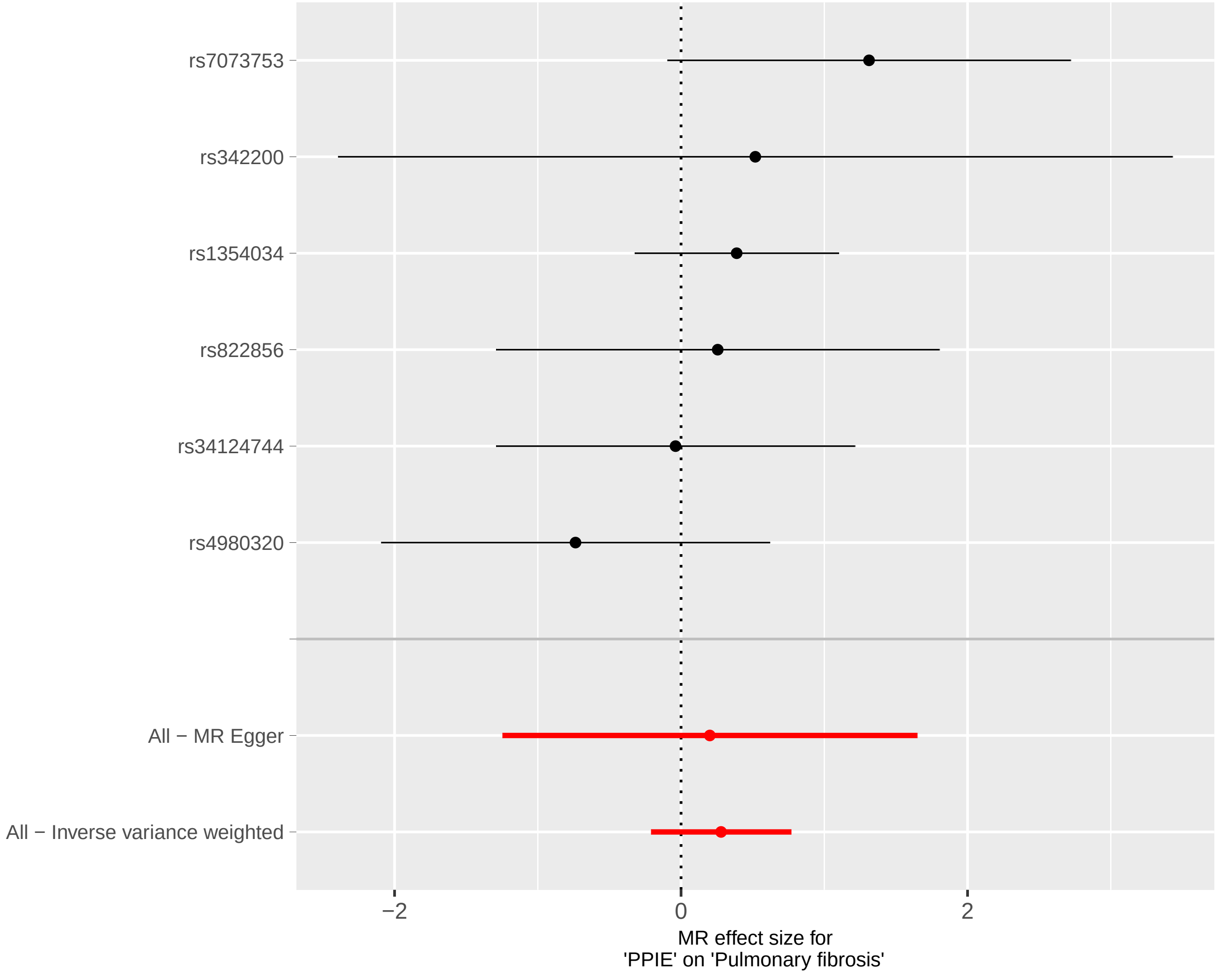
rs7073753
rs342200
rs1354034
rs822856
rs34124744
rs4980320
All − MR Egger
All − Inverse variance weighted
−2
0
2
MR effect size for
'PPIE' on 'Pulmonary fibrosis'

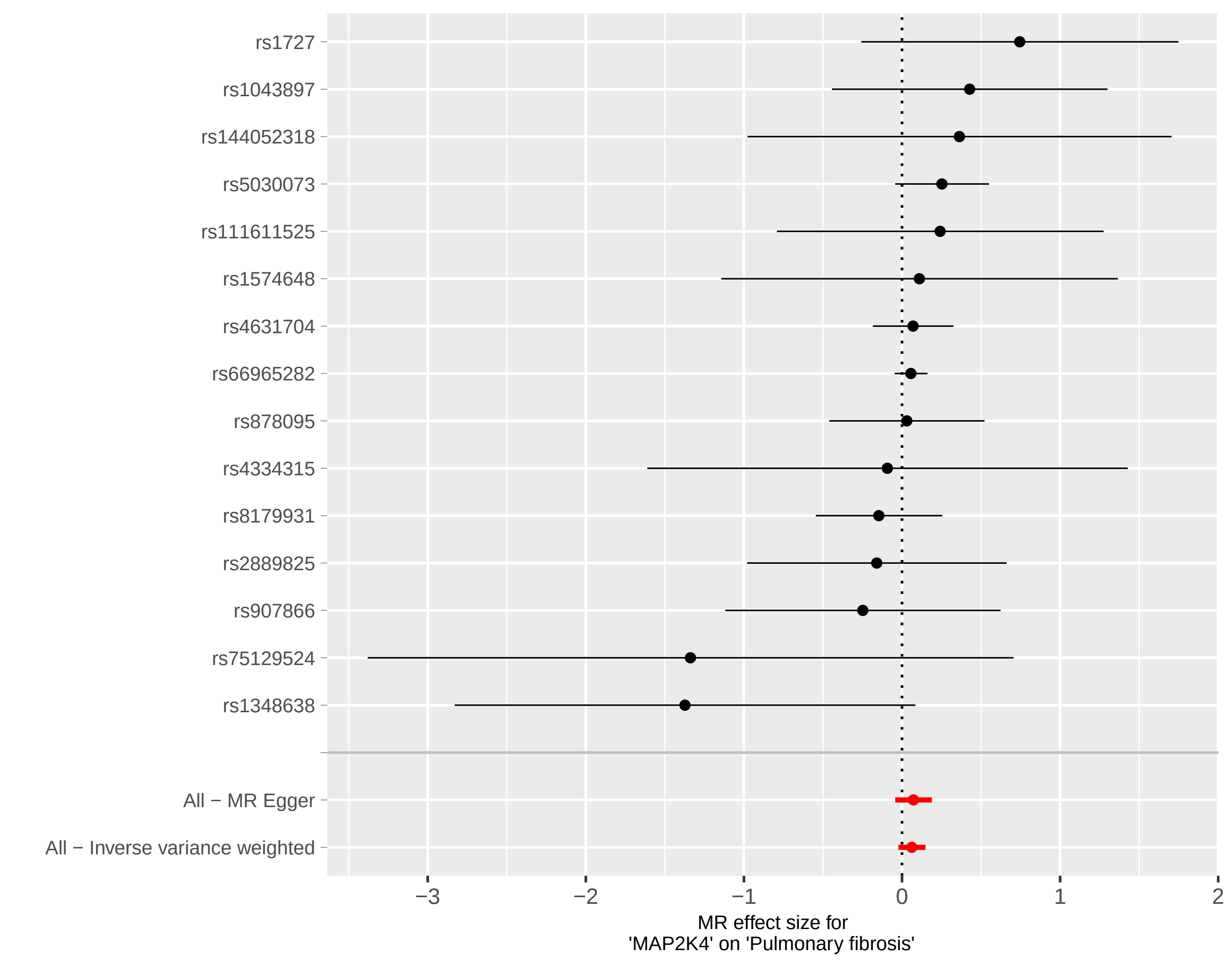
rs1727
rs1043897
rs144052318
rs5030073
rs111611525
rs1574648
rs4631704
rs66965282
rs878095
rs4334315
rs8179931
rs2889825
rs907866
rs75129524
rs1348638
All − MR Egger
All − Inverse variance weighted
−3
−2
−1
0
1
2
MR effect size for
'MAP2K4' on 'Pulmonary fibrosis'

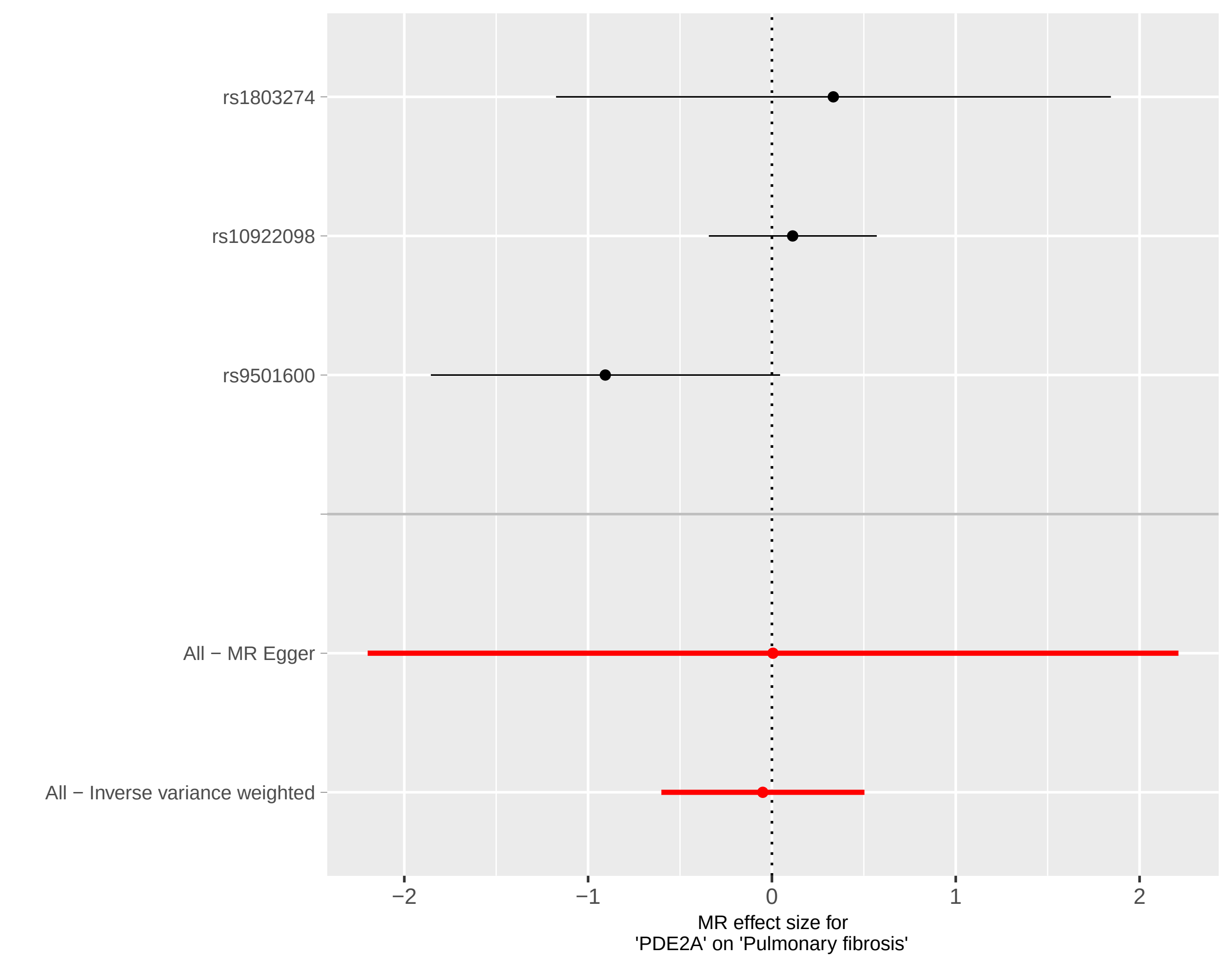
rs1803274
rs10922098
rs9501600
All − MR Egger
All − Inverse variance weighted
−2
−1
0
1
2
MR effect size for
'PDE2A' on 'Pulmonary fibrosis'

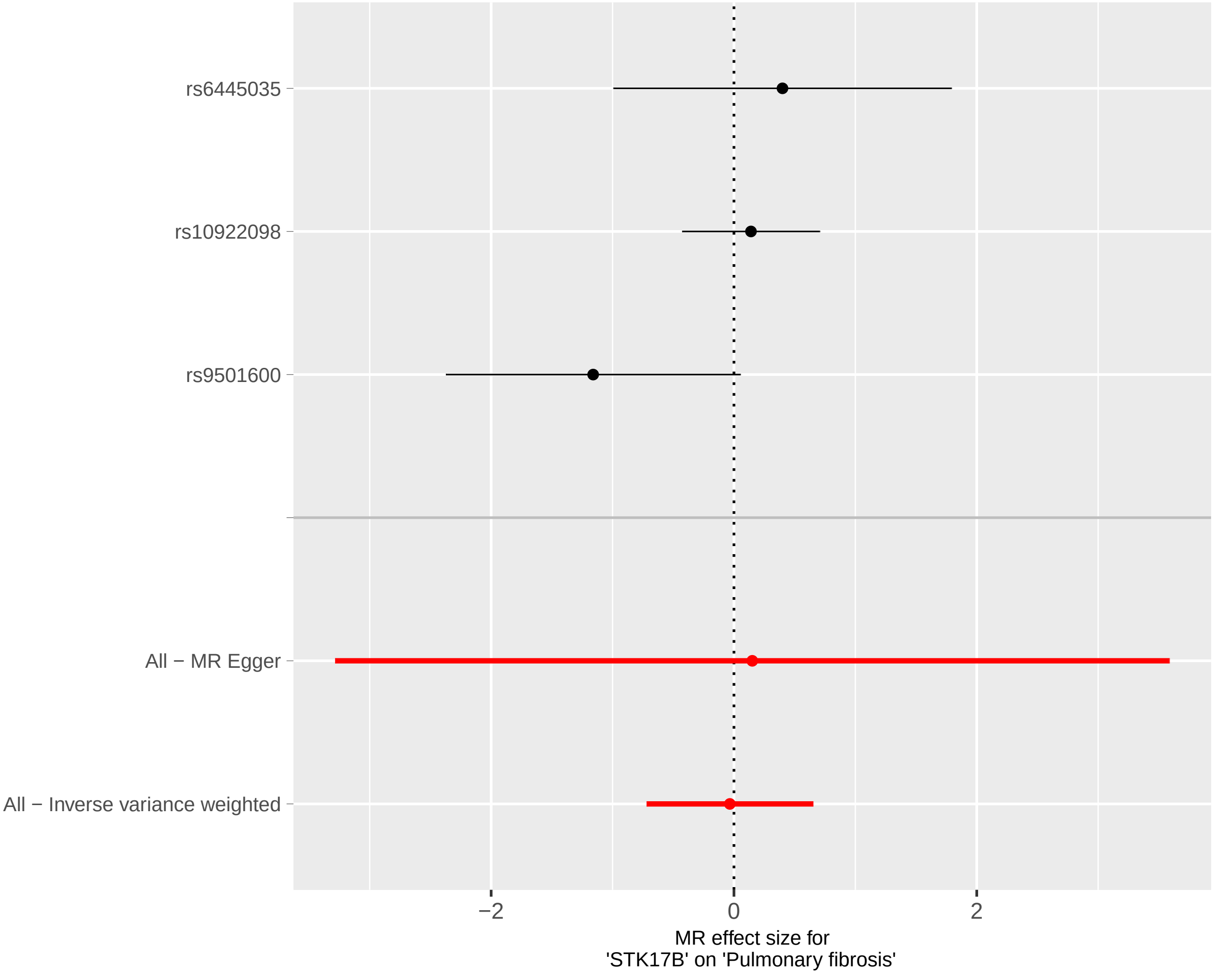
rs6445035
rs10922098
rs9501600
All − MR Egger
All − Inverse variance weighted
−2
0
2
MR effect size for
'STK17B' on 'Pulmonary fibrosis'

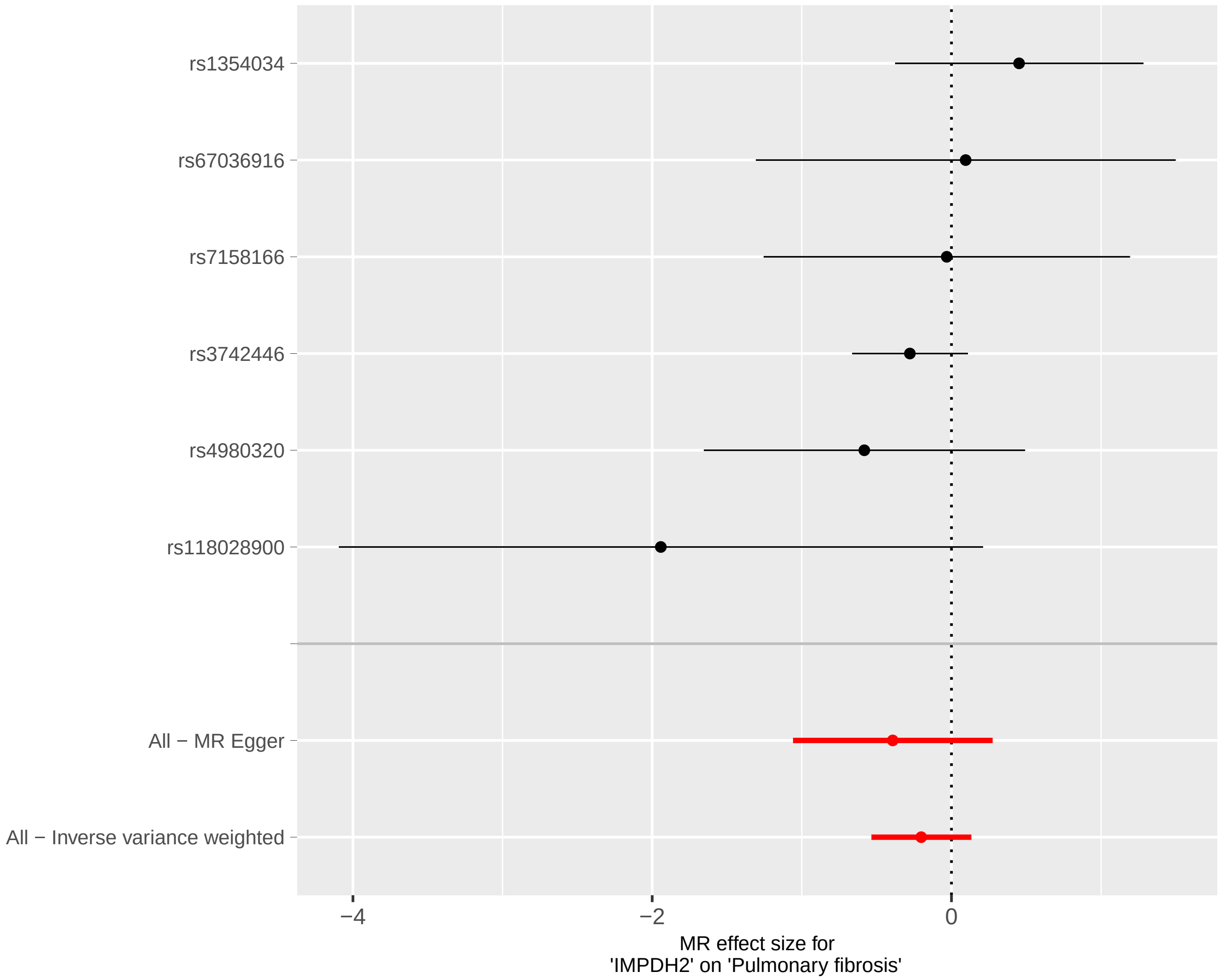
rs1354034
rs67036916
rs7158166
rs3742446
rs4980320
rs118028900
All − MR Egger
All − Inverse variance weighted
−4
−2
0
MR effect size for
'IMPDH2' on 'Pulmonary fibrosis'

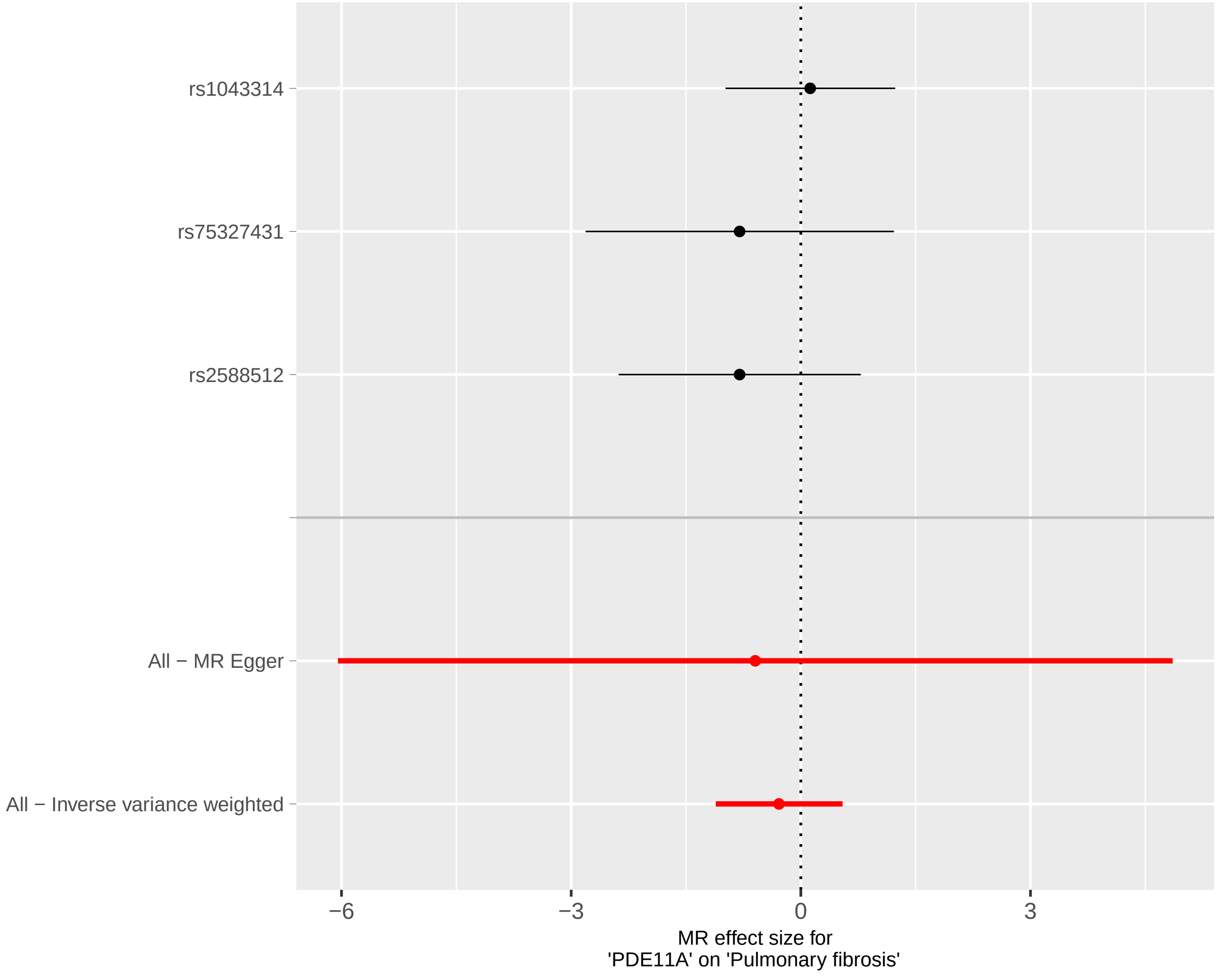
rs1043314
rs75327431
rs2588512
All − MR Egger
All − Inverse variance weighted
−6
−3
0
3
MR effect size for
'PDE11A' on 'Pulmonary fibrosis'

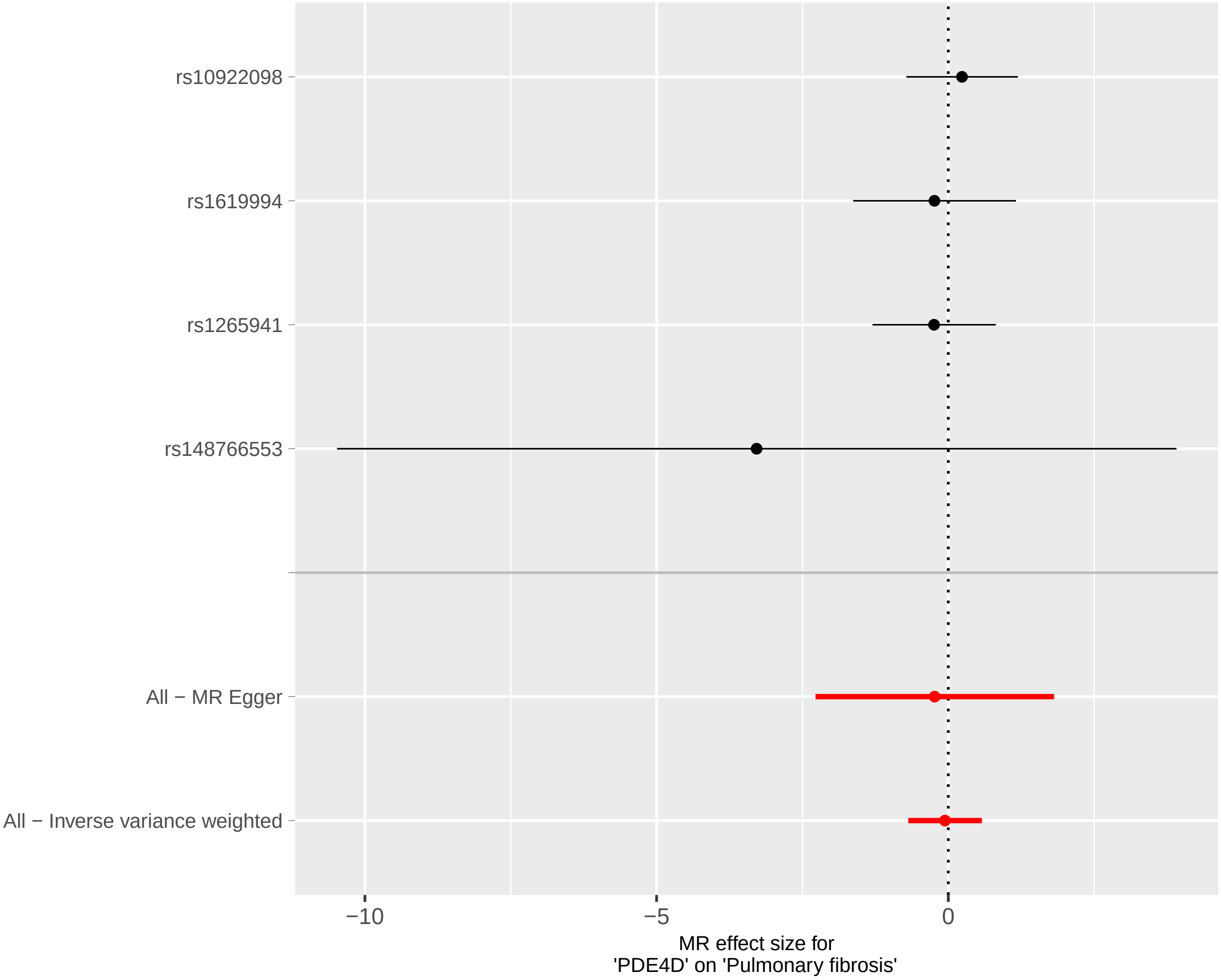

rs10922098
rs1619994
rs1265941
rs148766553
All − MR Egger
All − Inverse variance weighted
−10
−5
0
MR effect size for
'PDE4D' on 'Pulmonary fibrosis'

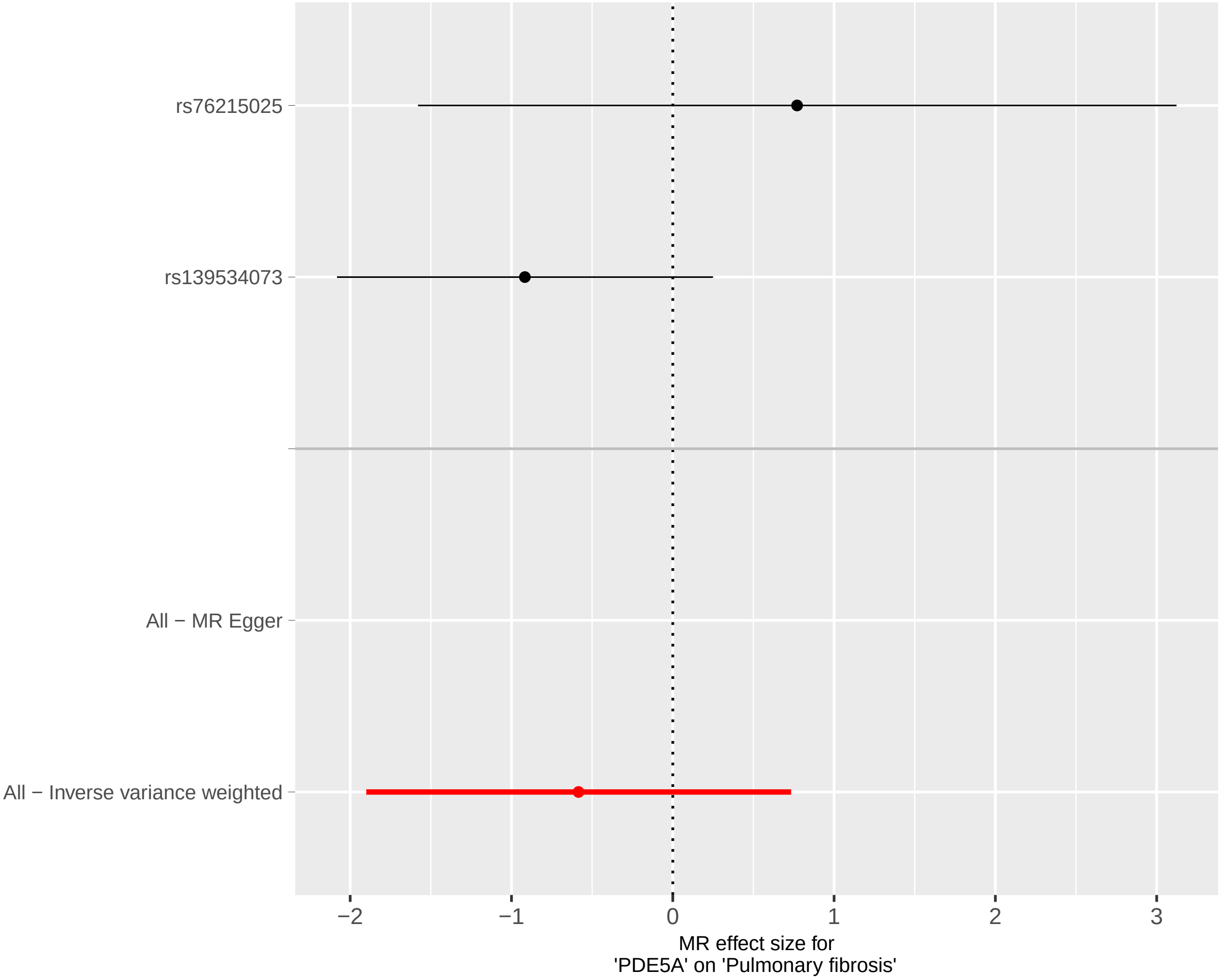
rs76215025
rs139534073
All − MR Egger
All − Inverse variance weighted
−2
−1
0
1
2
3
MR effect size for
'PDE5A' on 'Pulmonary fibrosis'

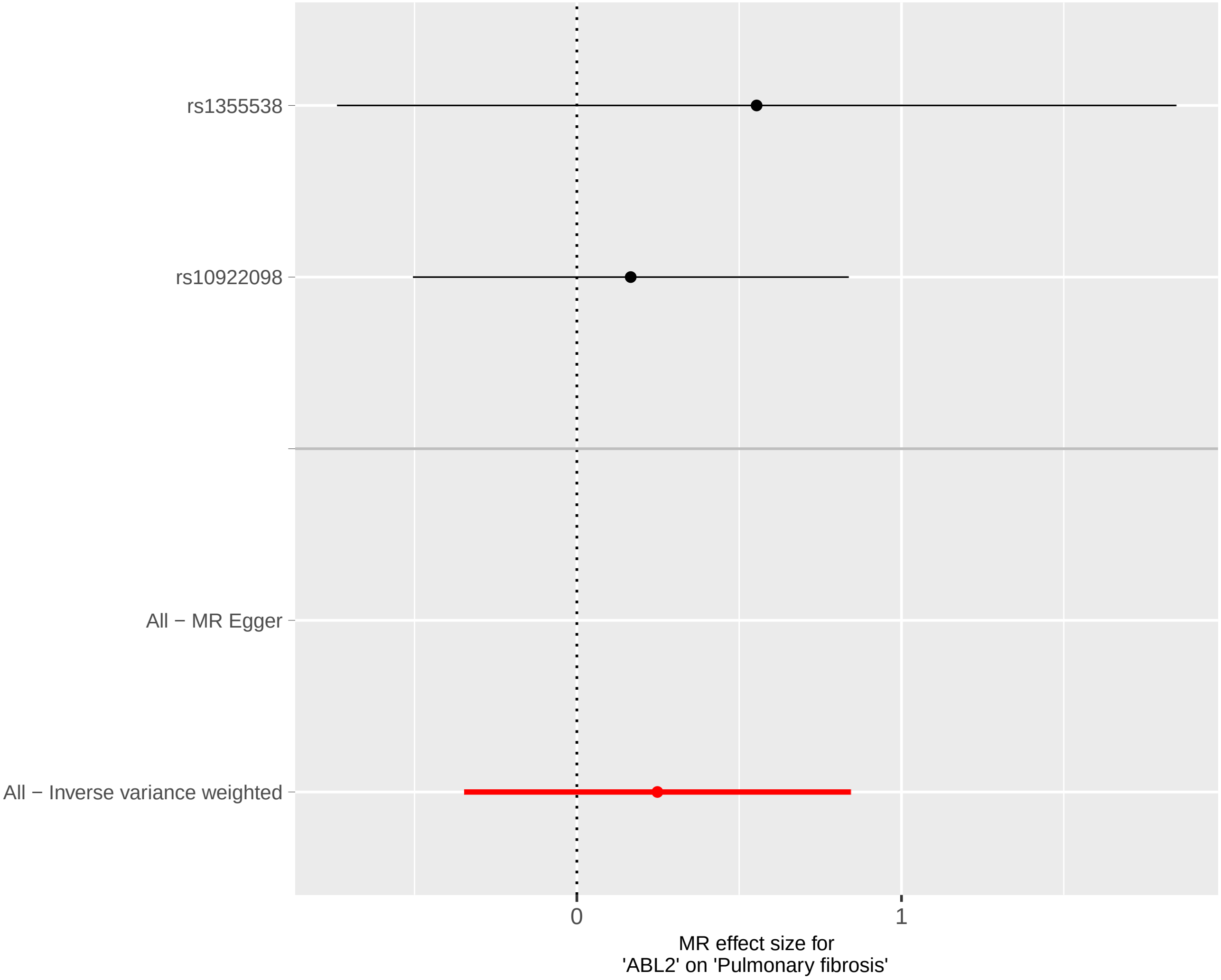

rs1355538
rs10922098
All − MR Egger
All − Inverse variance weighted
0
1
MR effect size for
'ABL2' on 'Pulmonary fibrosis'

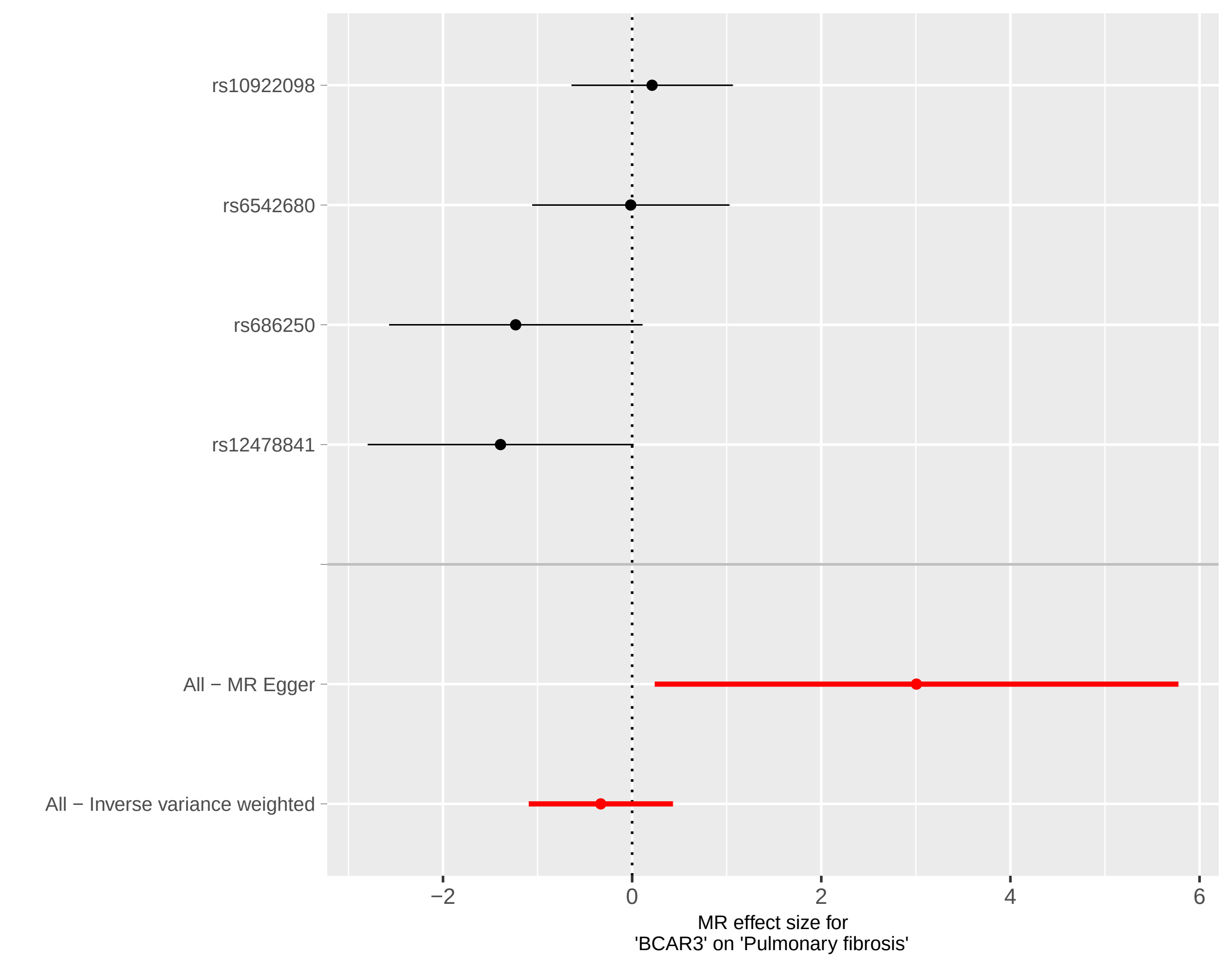

rs10922098
rs6542680
rs686250
rs12478841
All − MR Egger
All − Inverse variance weighted
−2
0
2
4
6
MR effect size for
'BCAR3' on 'Pulmonary fibrosis'

Insufficient number of SNPs

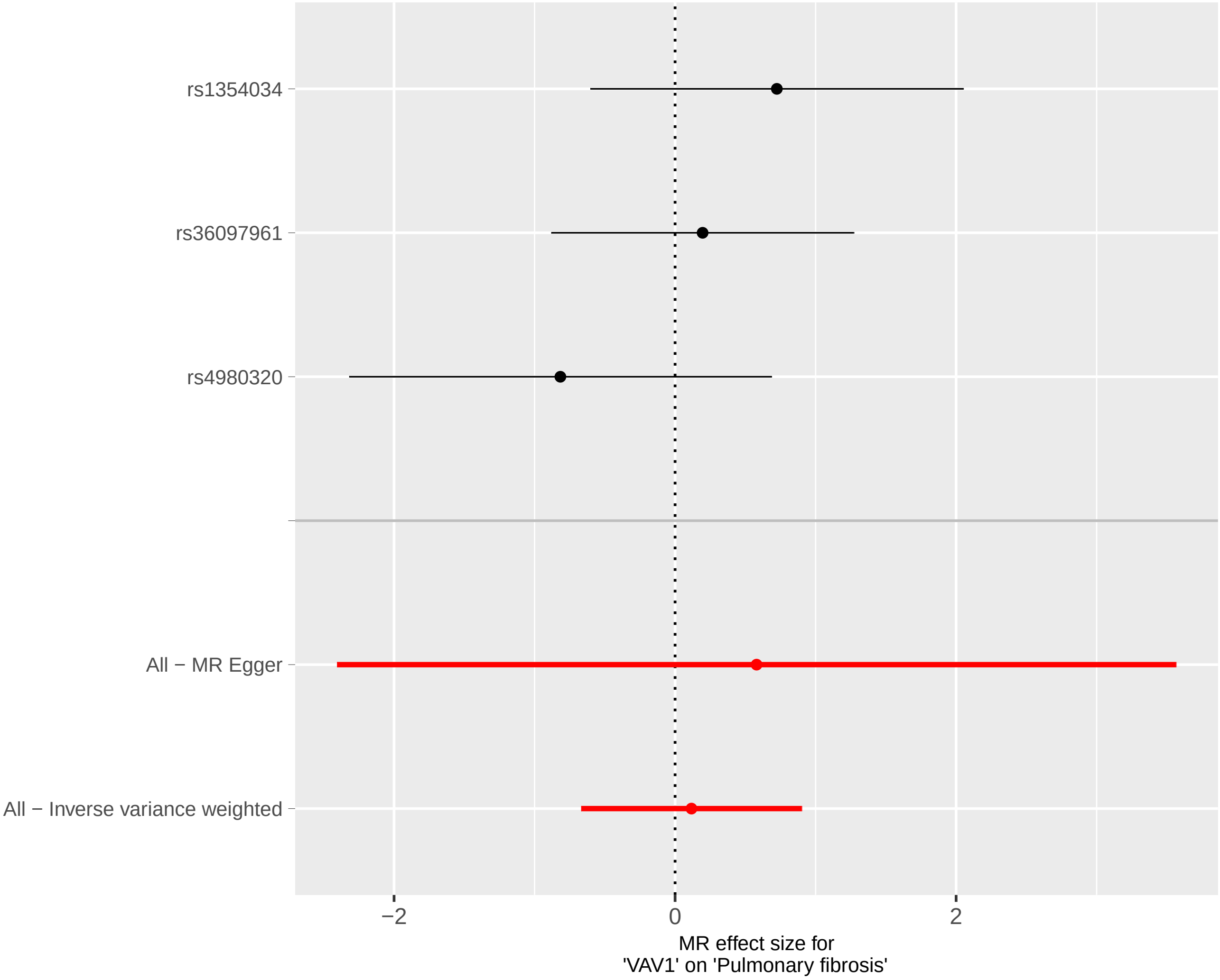
rs1354034
rs36097961
rs4980320
All − MR Egger
All − Inverse variance weighted
−2
0
2
MR effect size for
'VAV1' on 'Pulmonary fibrosis'

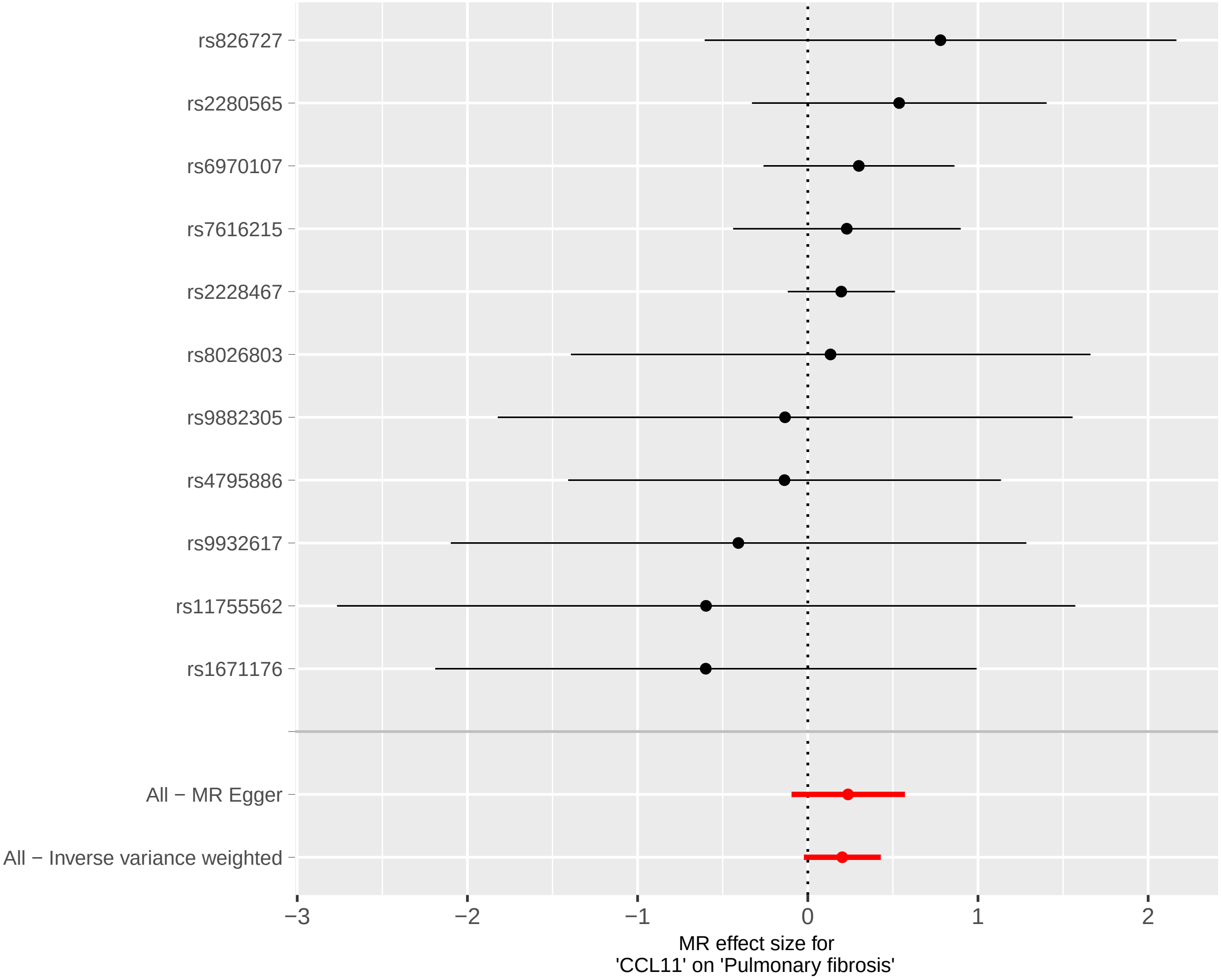

rs826727
rs2280565
rs6970107
rs7616215
rs2228467
rs8026803
rs9882305
rs4795886
rs9932617
rs11755562
rs1671176
All − MR Egger
All − Inverse variance weighted
−3
−2
−1
0
1
2
MR effect size for
'CCL11' on 'Pulmonary fibrosis'

Insufficient number of SNPs

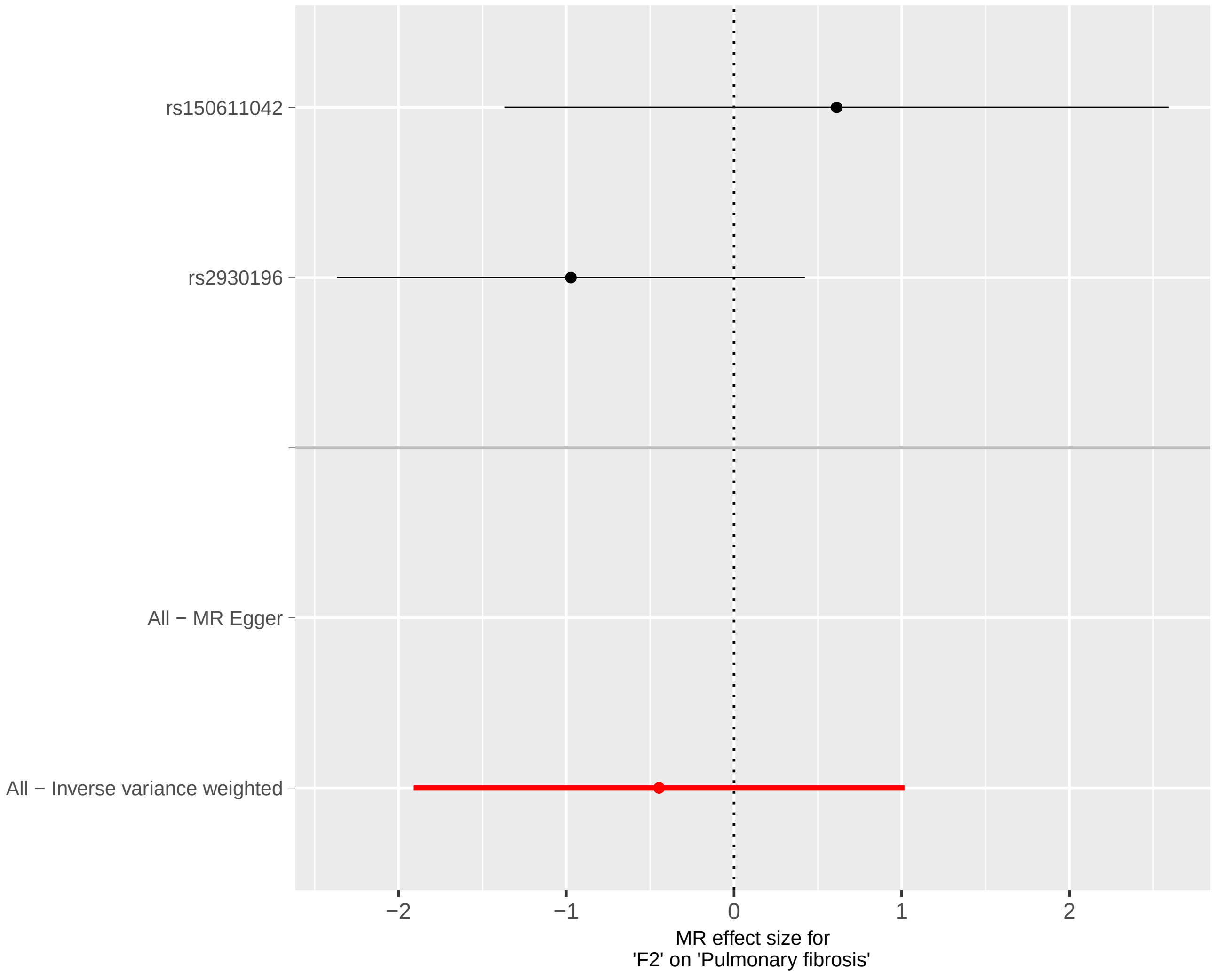
rs150611042
rs2930196
All − MR Egger
All − Inverse variance weighted
−2
−1
0
1
2
MR effect size for
'F2' on 'Pulmonary fibrosis'

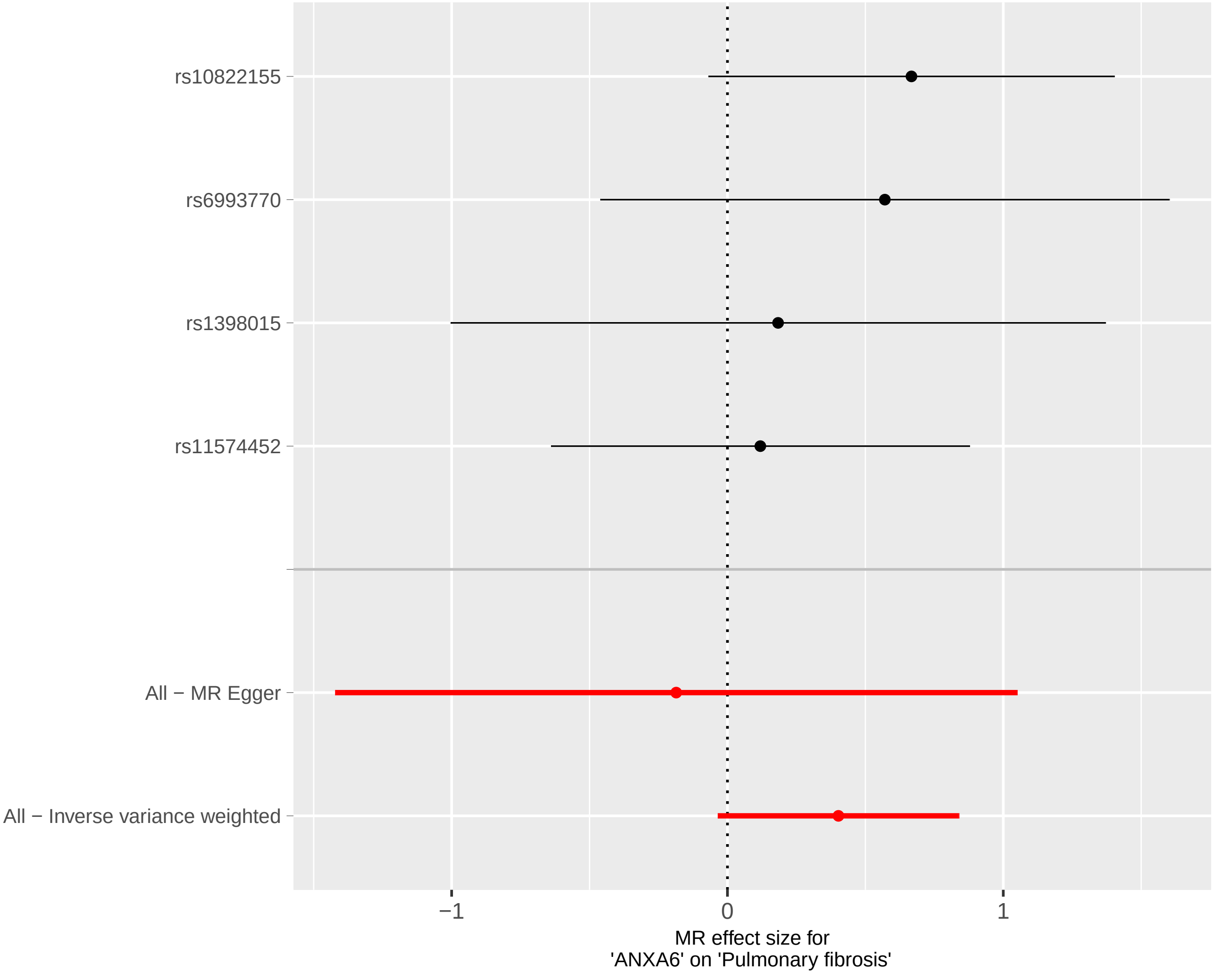
rs10822155
rs6993770
rs1398015
rs11574452
All − MR Egger
All − Inverse variance weighted
−1
0
1
MR effect size for
'ANXA6' on 'Pulmonary fibrosis'

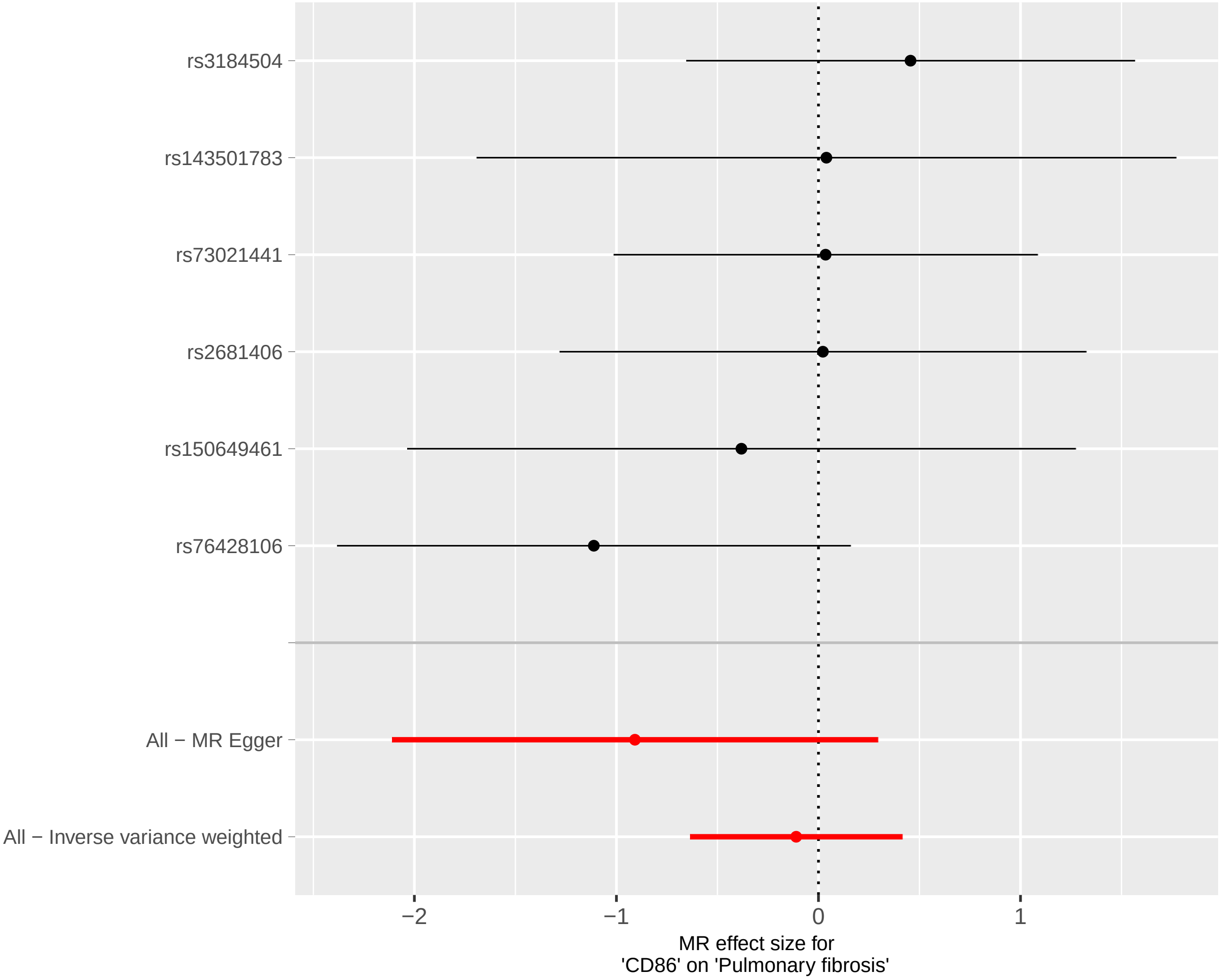

rs3184504
rs143501783
rs73021441
rs2681406
rs150649461
rs76428106
All − MR Egger
All − Inverse variance weighted
−2
−1
0
1
MR effect size for
'CD86' on 'Pulmonary fibrosis'

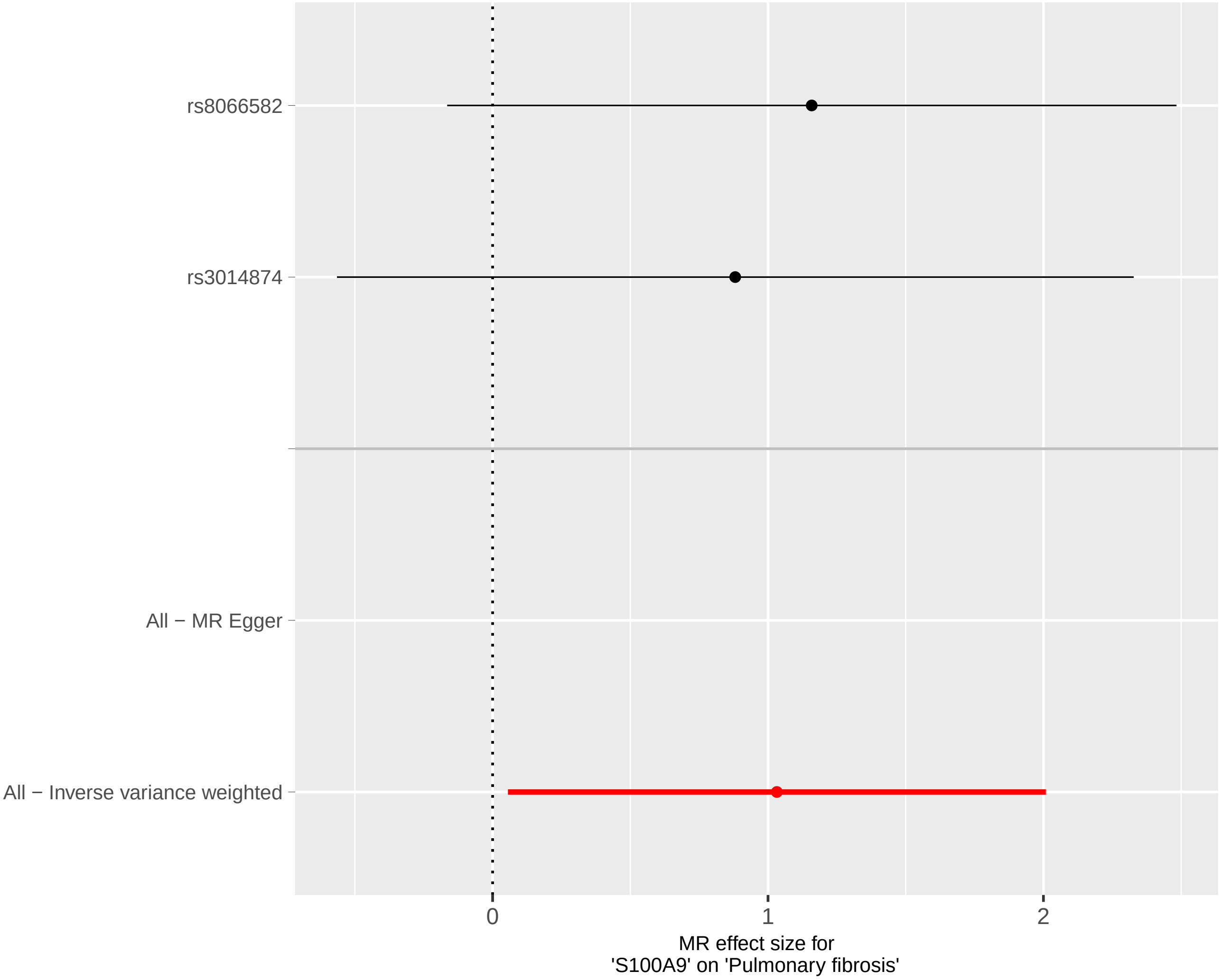
rs8066582
rs3014874
All − MR Egger
All − Inverse variance weighted
0
1
2
MR effect size for
'S100A9' on 'Pulmonary fibrosis'

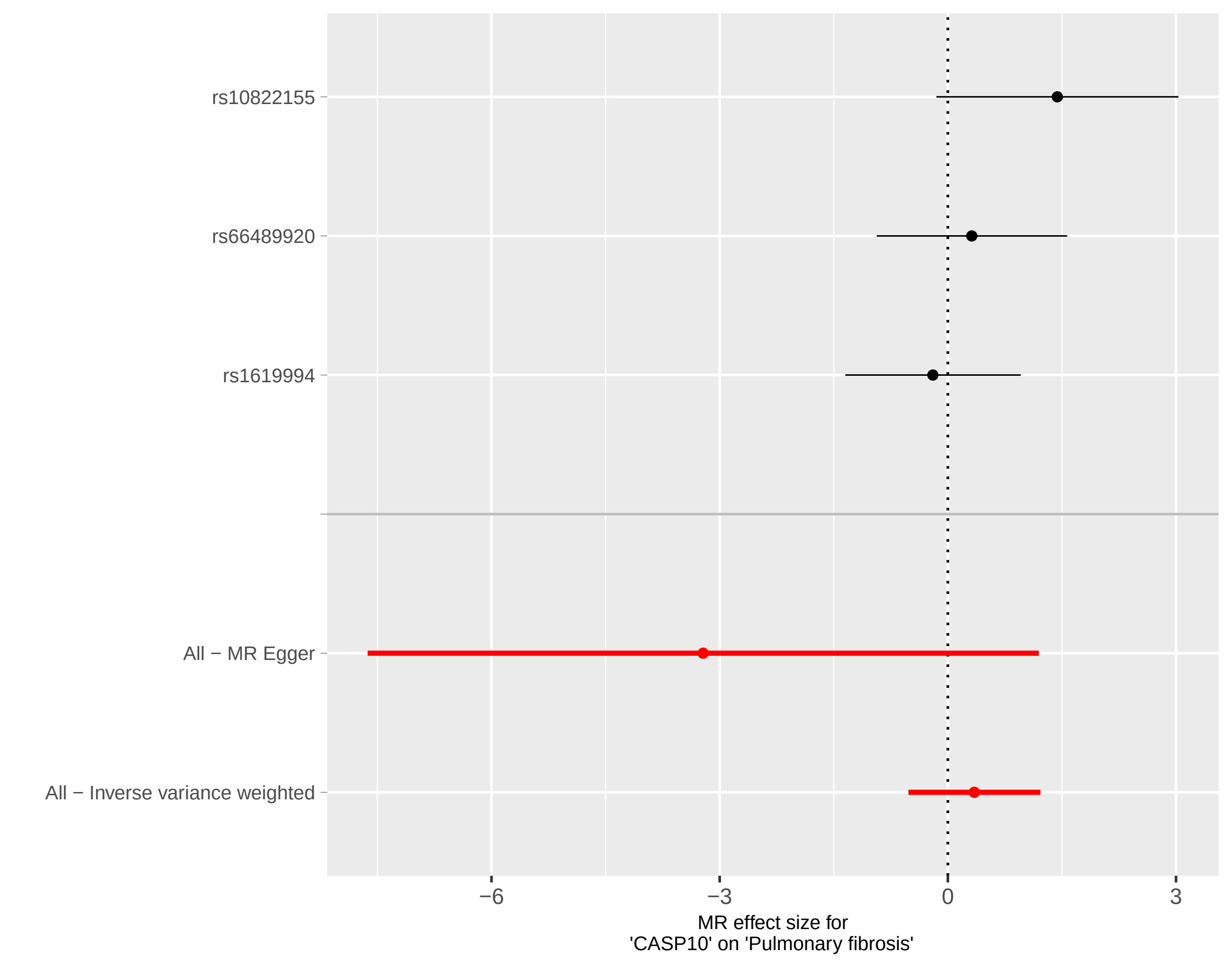
rs10822155
rs66489920
rs1619994
All − MR Egger
All − Inverse variance weighted
−6
−3
0
3
MR effect size for
'CASP10' on 'Pulmonary fibrosis'

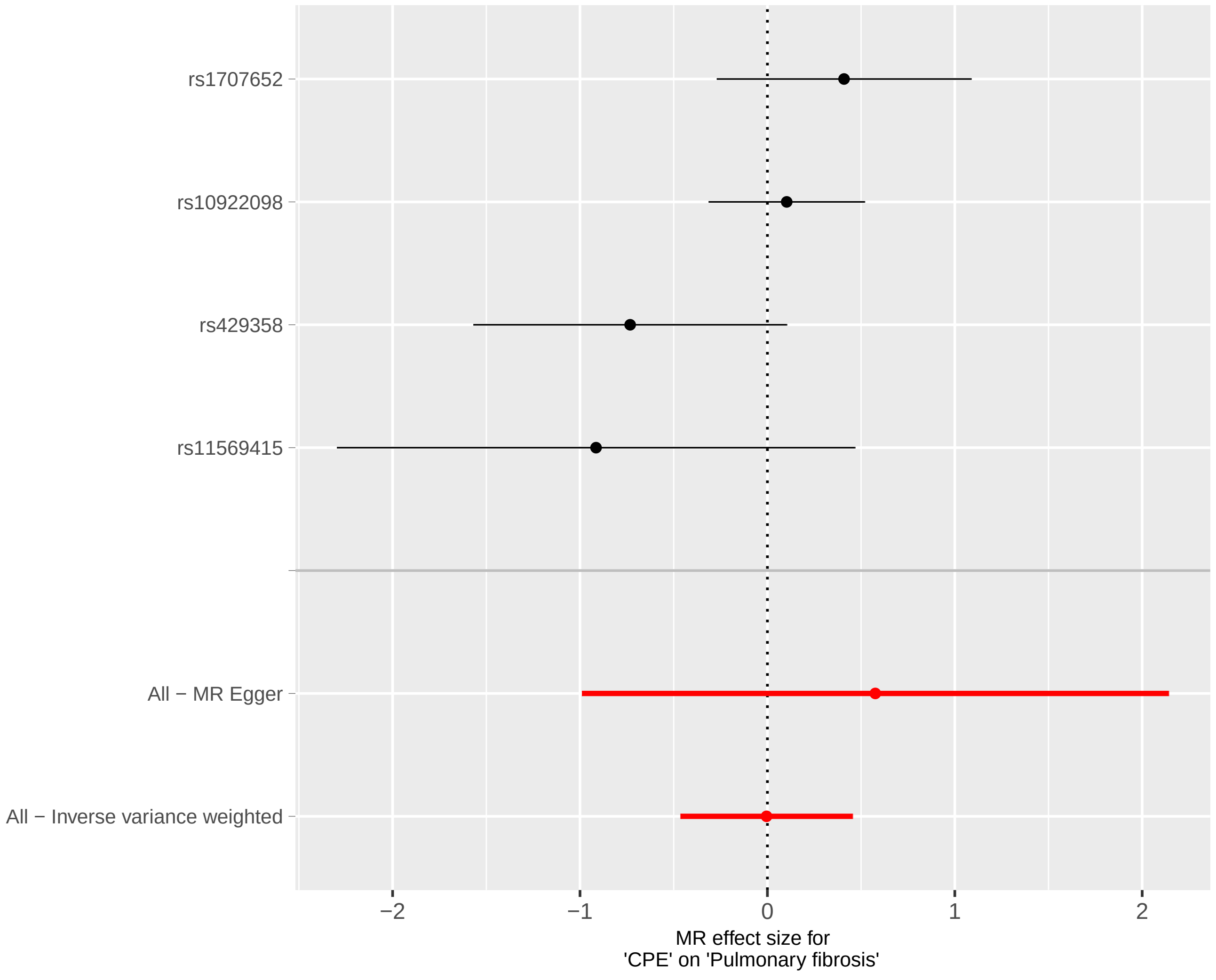

rs1707652
rs10922098
rs429358
rs11569415
All − MR Egger
All − Inverse variance weighted
−2
−1
0
1
2
MR effect size for
'CPE' on 'Pulmonary fibrosis'

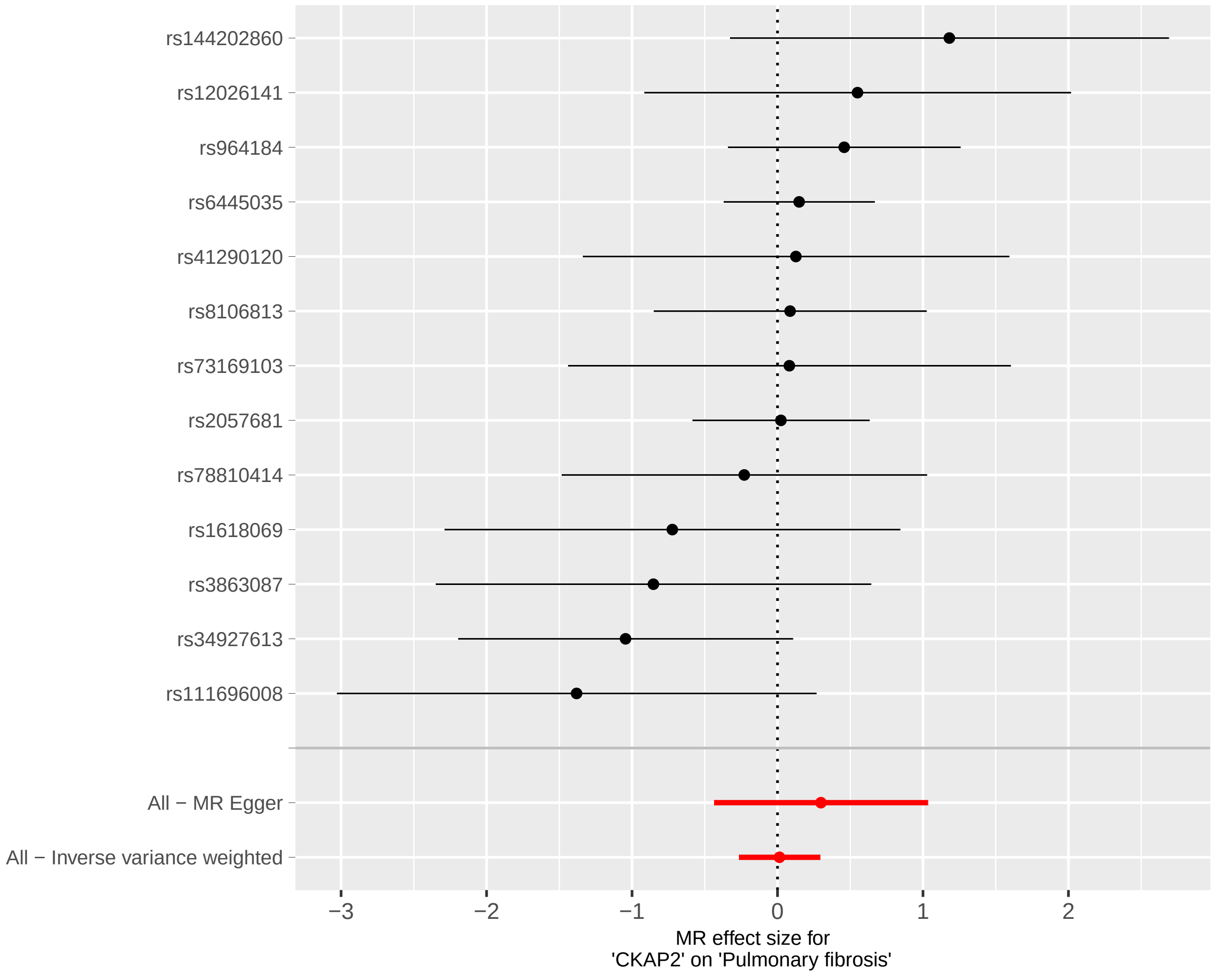
rs144202860
rs12026141
rs964184
rs6445035
rs41290120
rs8106813
rs73169103
rs2057681
rs78810414
rs1618069
rs3863087
rs34927613
rs111696008
All − MR Egger
All − Inverse variance weighted
−3
−2
−1
0
1
2
MR effect size for
'CKAP2' on 'Pulmonary fibrosis'

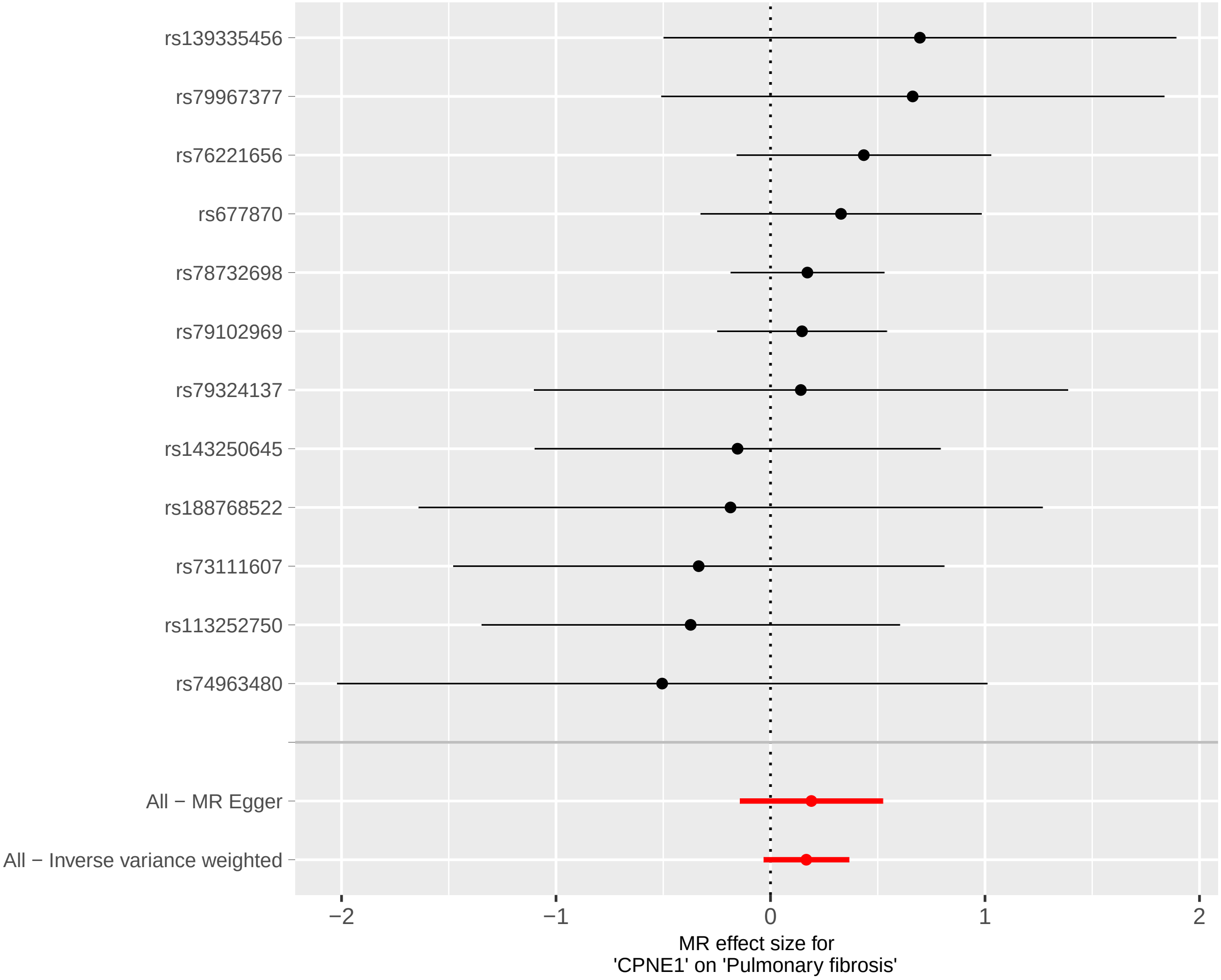
rs139335456
rs79967377
rs76221656
rs677870
rs78732698
rs79102969
rs79324137
rs143250645
rs188768522
rs73111607
rs113252750
rs74963480
All − MR Egger
All − Inverse variance weighted
−2
−1
0
1
2
MR effect size for
'CPNE1' on 'Pulmonary fibrosis'

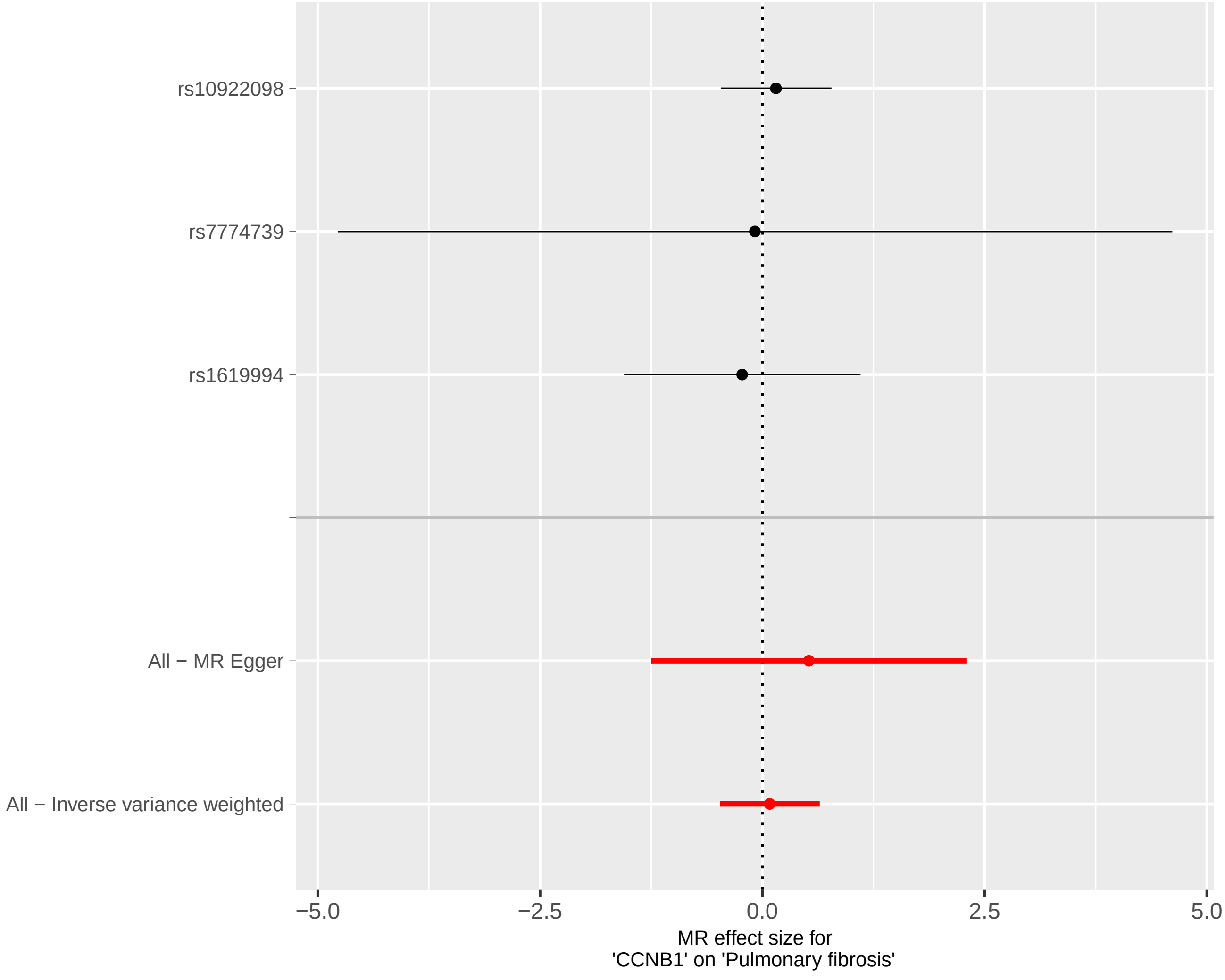
rs10922098
rs7774739
rs1619994
All − MR Egger
All − Inverse variance weighted
−5.0
−2.5
0.0
2.5
5.0
MR effect size for
'CCNB1' on 'Pulmonary fibrosis'

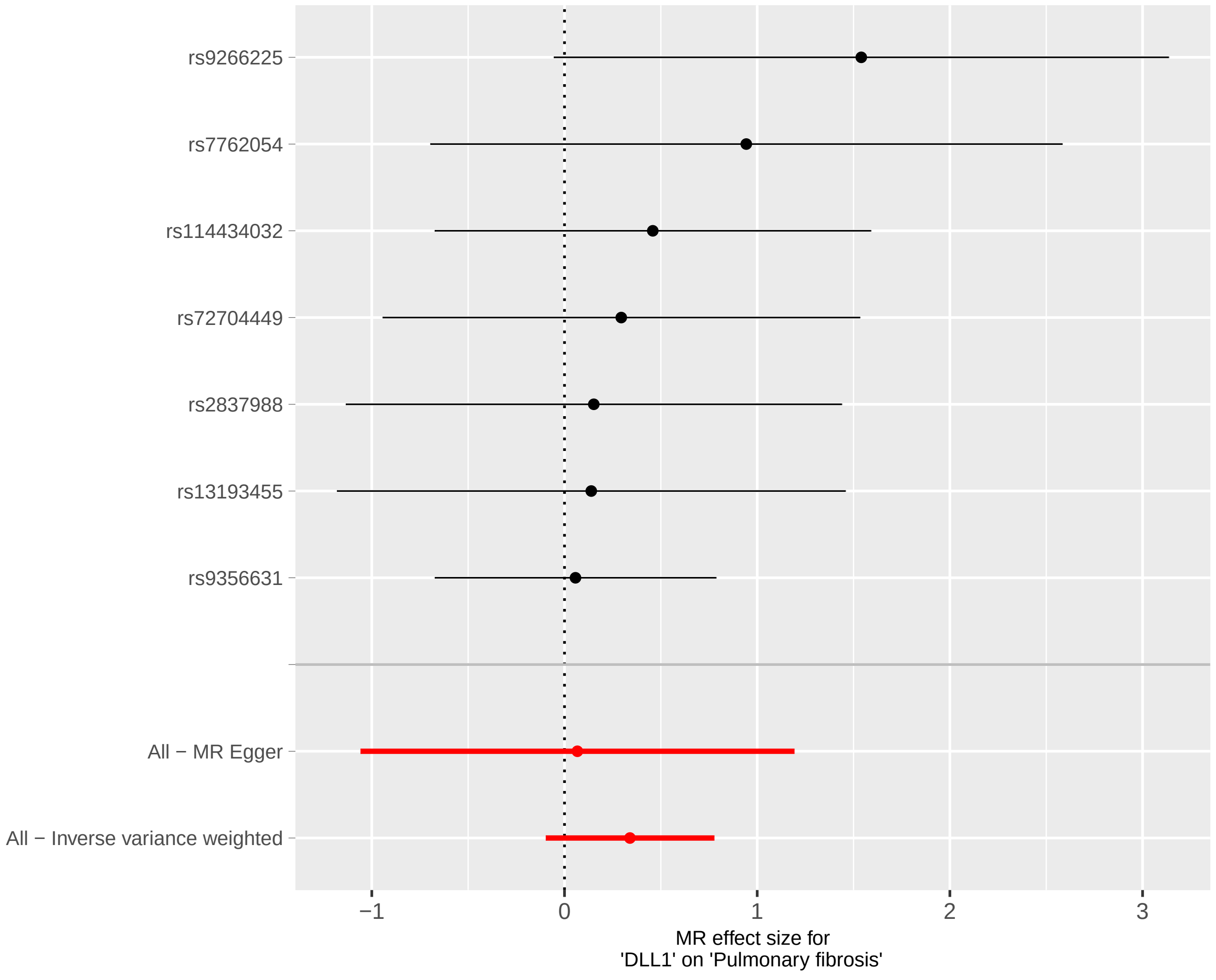
rs9266225
rs7762054
rs114434032
rs72704449
rs2837988
rs13193455
rs9356631
All − MR Egger
All − Inverse variance weighted
−1
0
1
2
3
MR effect size for
'DLL1' on 'Pulmonary fibrosis'

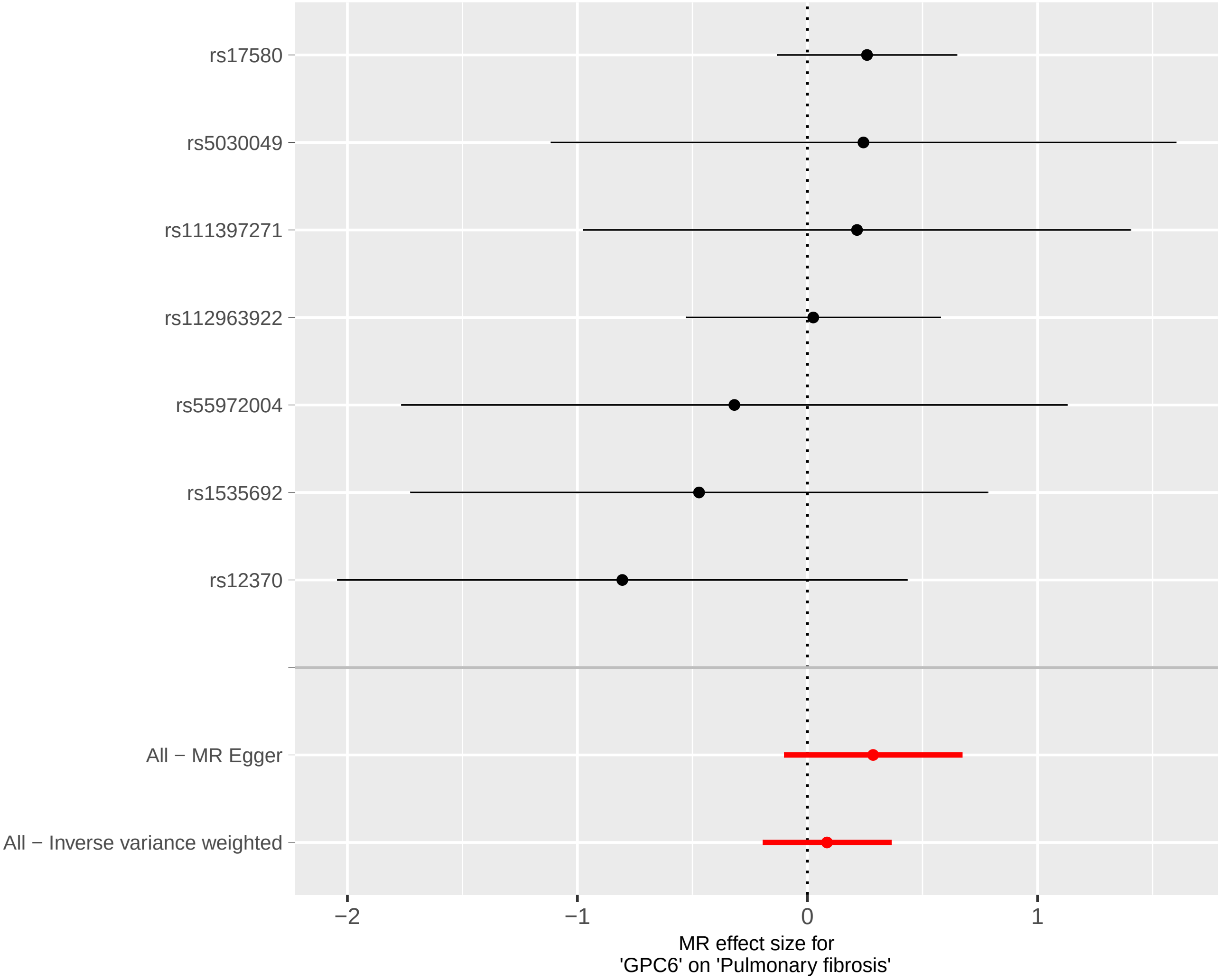

rs17580
rs5030049
rs111397271
rs112963922
rs55972004
rs1535692
rs12370
All − MR Egger
All − Inverse variance weighted
−2
−1
0
1
MR effect size for
'GPC6' on 'Pulmonary fibrosis'

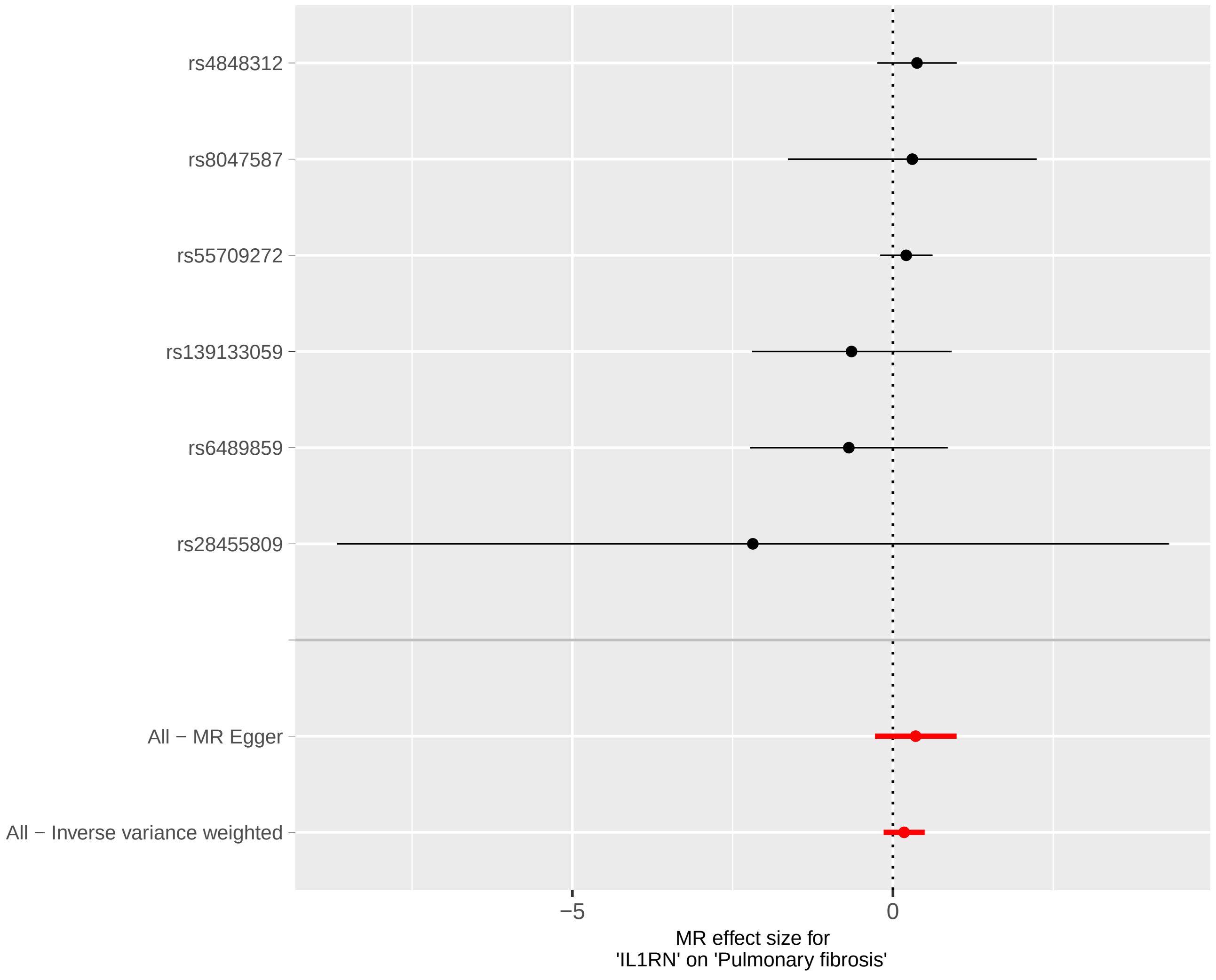
rs4848312
rs8047587
rs55709272
rs139133059
rs6489859
rs28455809
All − MR Egger
All − Inverse variance weighted
−5
0
MR effect size for
'IL1RN' on 'Pulmonary fibrosis'

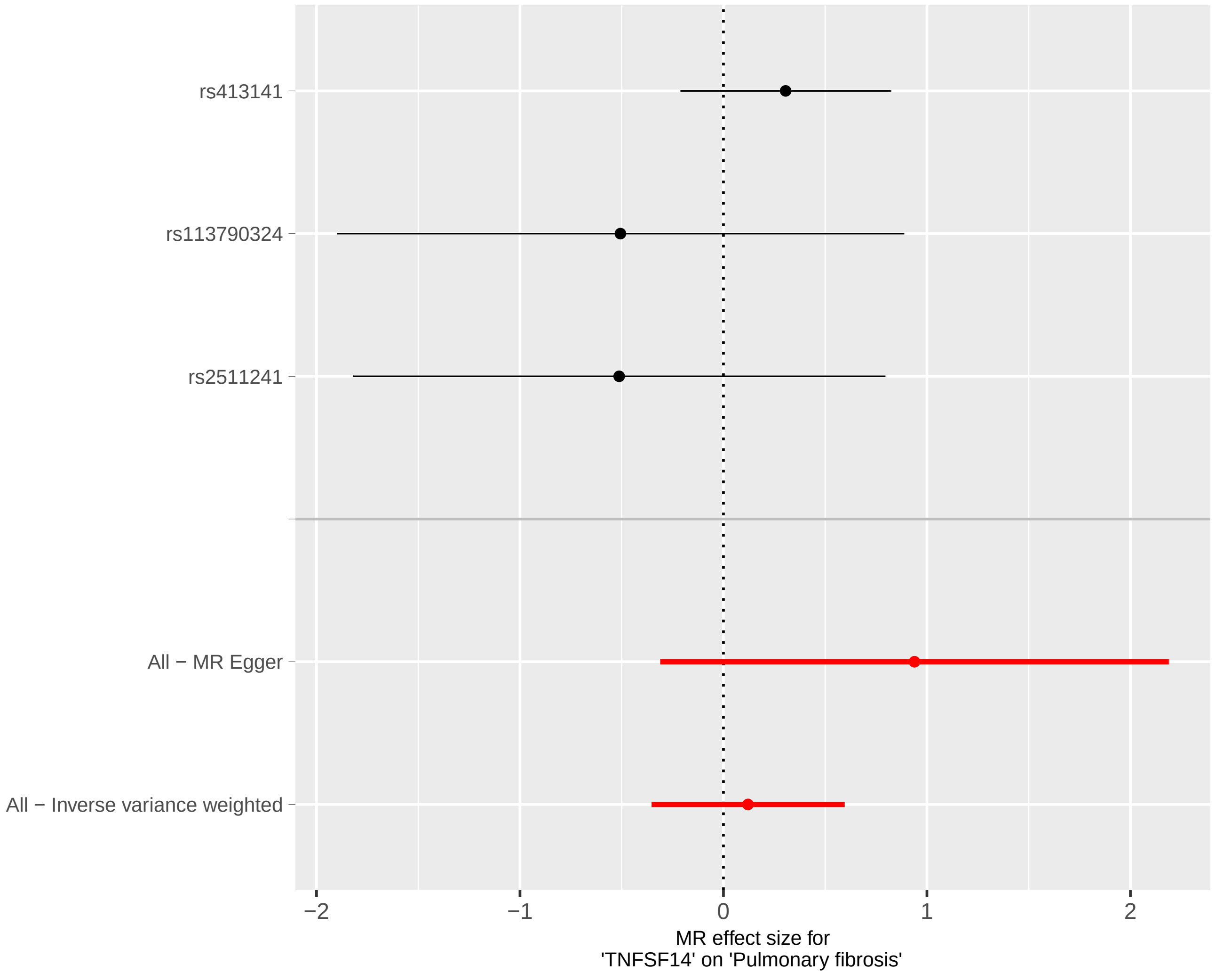

rs413141
rs113790324
rs2511241
All − MR Egger
All − Inverse variance weighted
−2
−1
0
1
2
MR effect size for
'TNFSF14' on 'Pulmonary fibrosis'

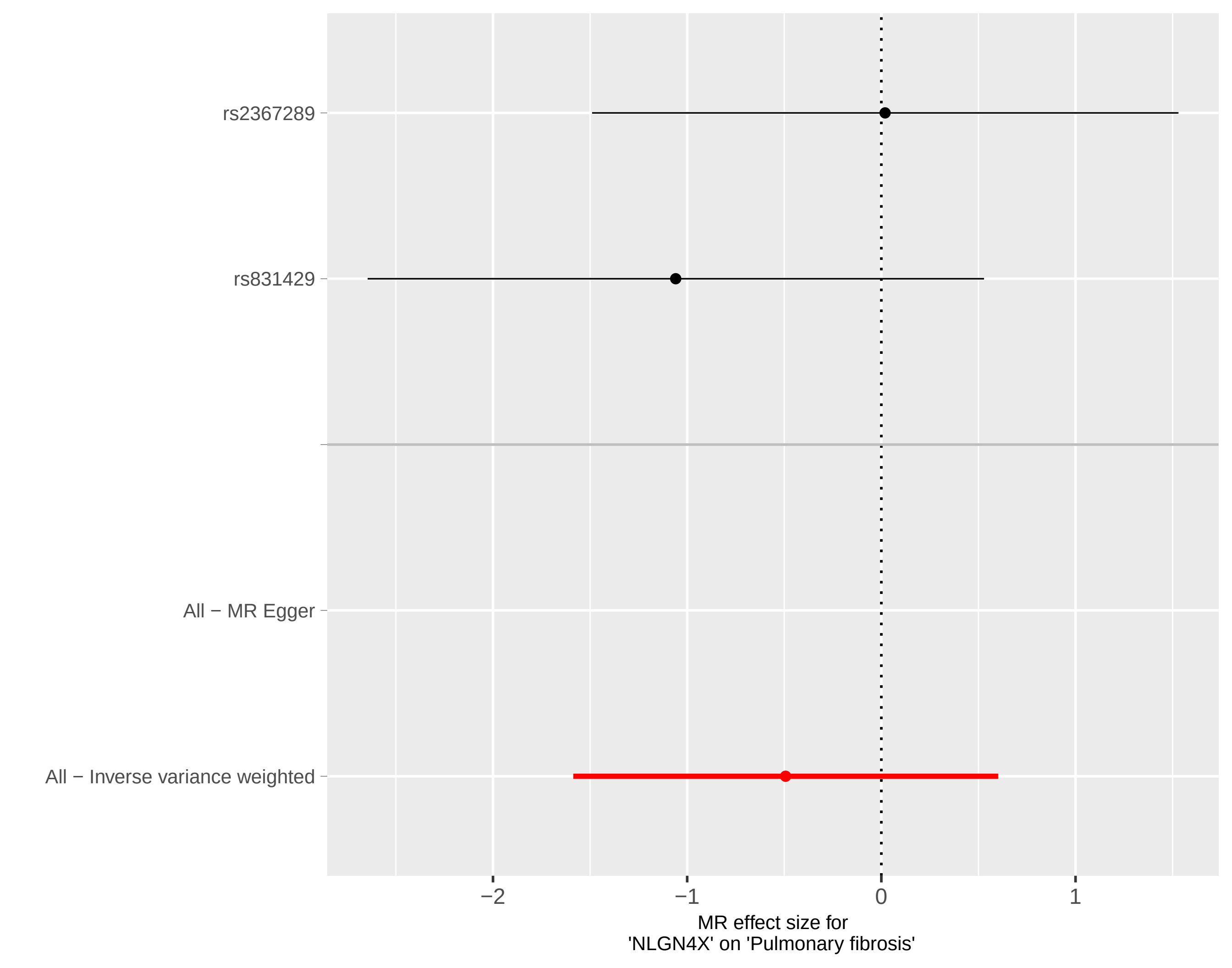

rs2367289
rs831429
All − MR Egger
All − Inverse variance weighted
−2
−1
0
1
MR effect size for
'NLGN4X' on 'Pulmonary fibrosis'

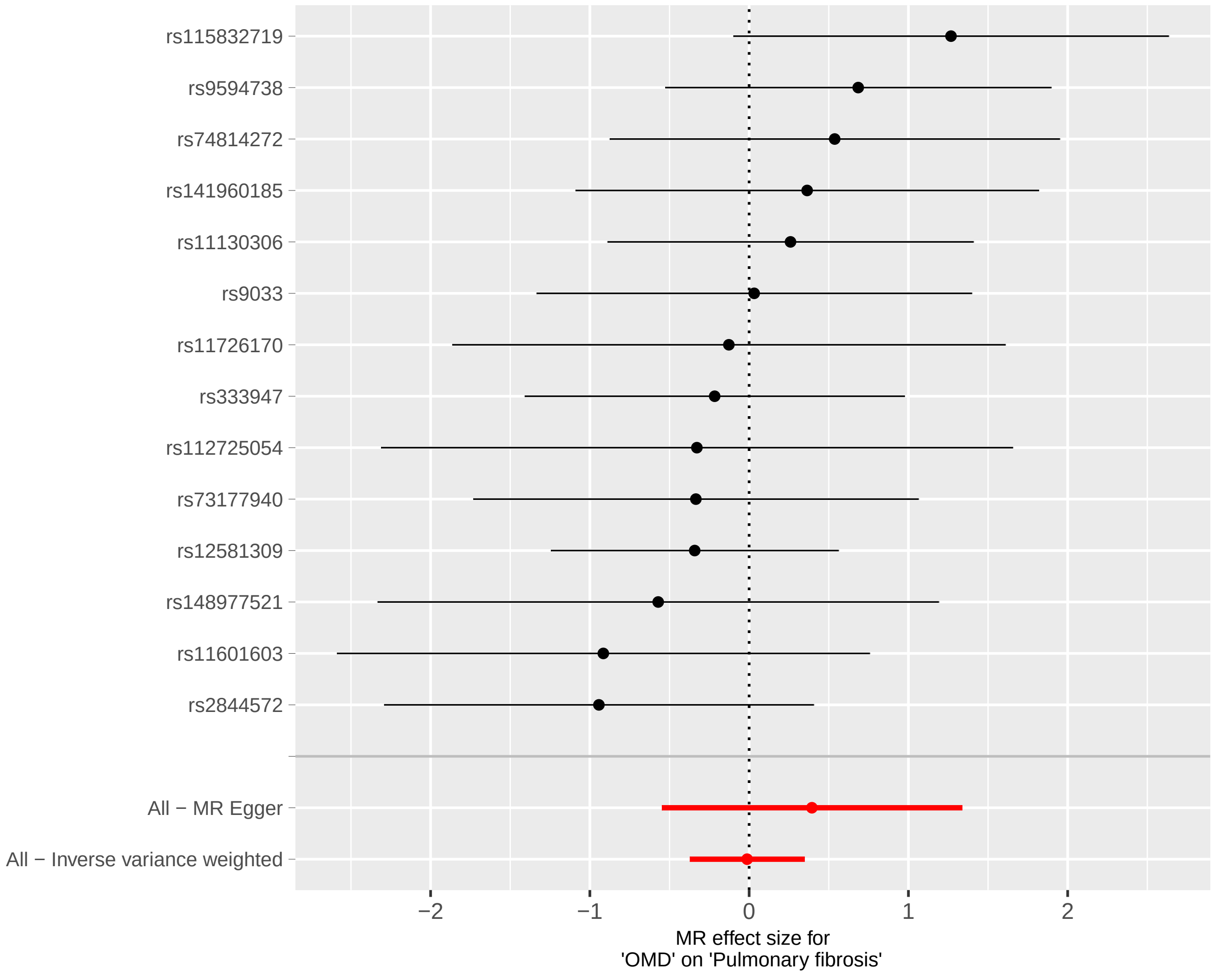

rs115832719
rs9594738
rs74814272
rs141960185
rs11130306
rs9033
rs11726170
rs333947
rs112725054
rs73177940
rs12581309
rs148977521
rs11601603
rs2844572
All − MR Egger
All − Inverse variance weighted
−2
−1
0
1
2
MR effect size for
'OMD' on 'Pulmonary fibrosis'

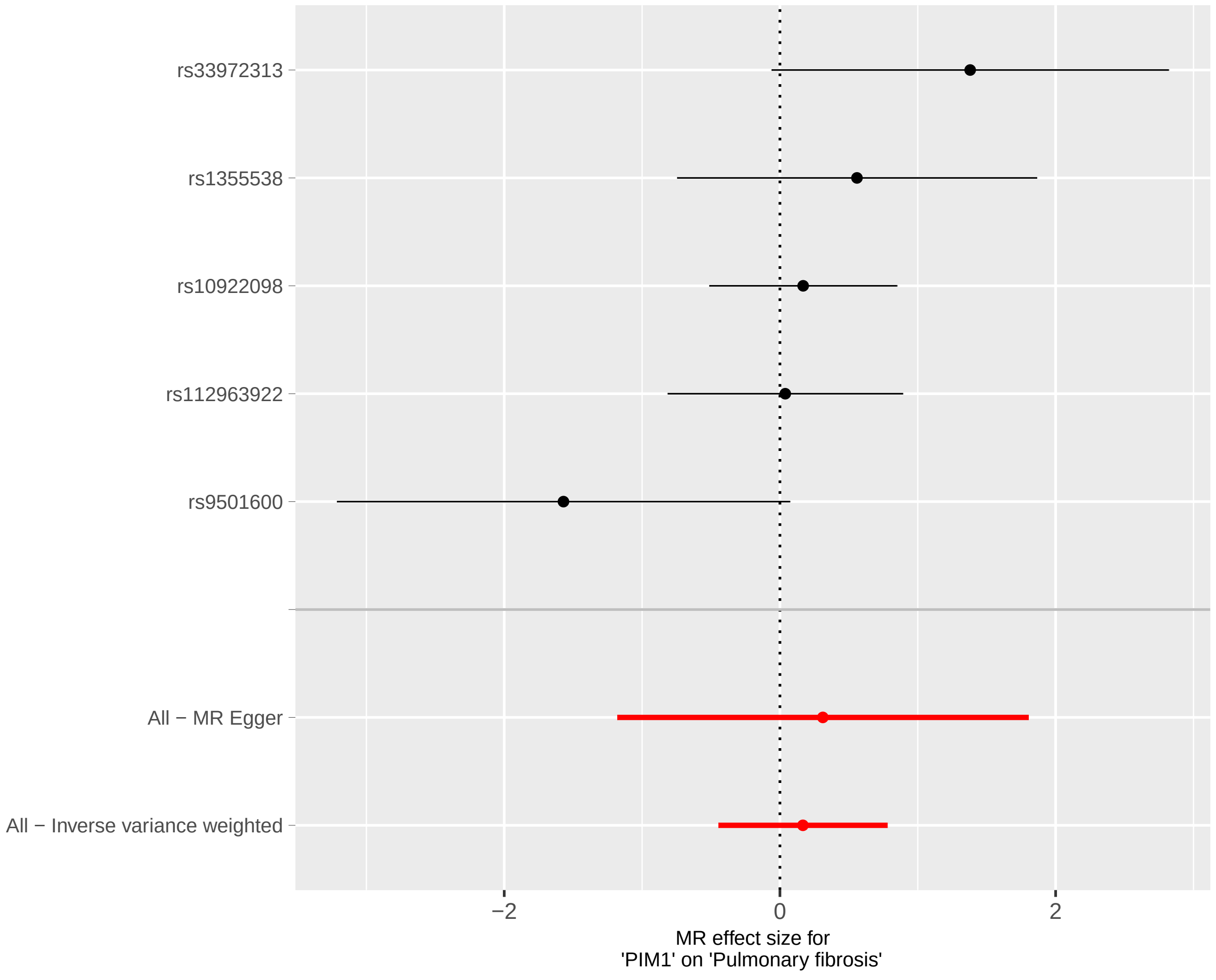
rs33972313
rs1355538
rs10922098
rs112963922
rs9501600
All − MR Egger
All − Inverse variance weighted
−2
0
2
MR effect size for
'PIM1' on 'Pulmonary fibrosis'

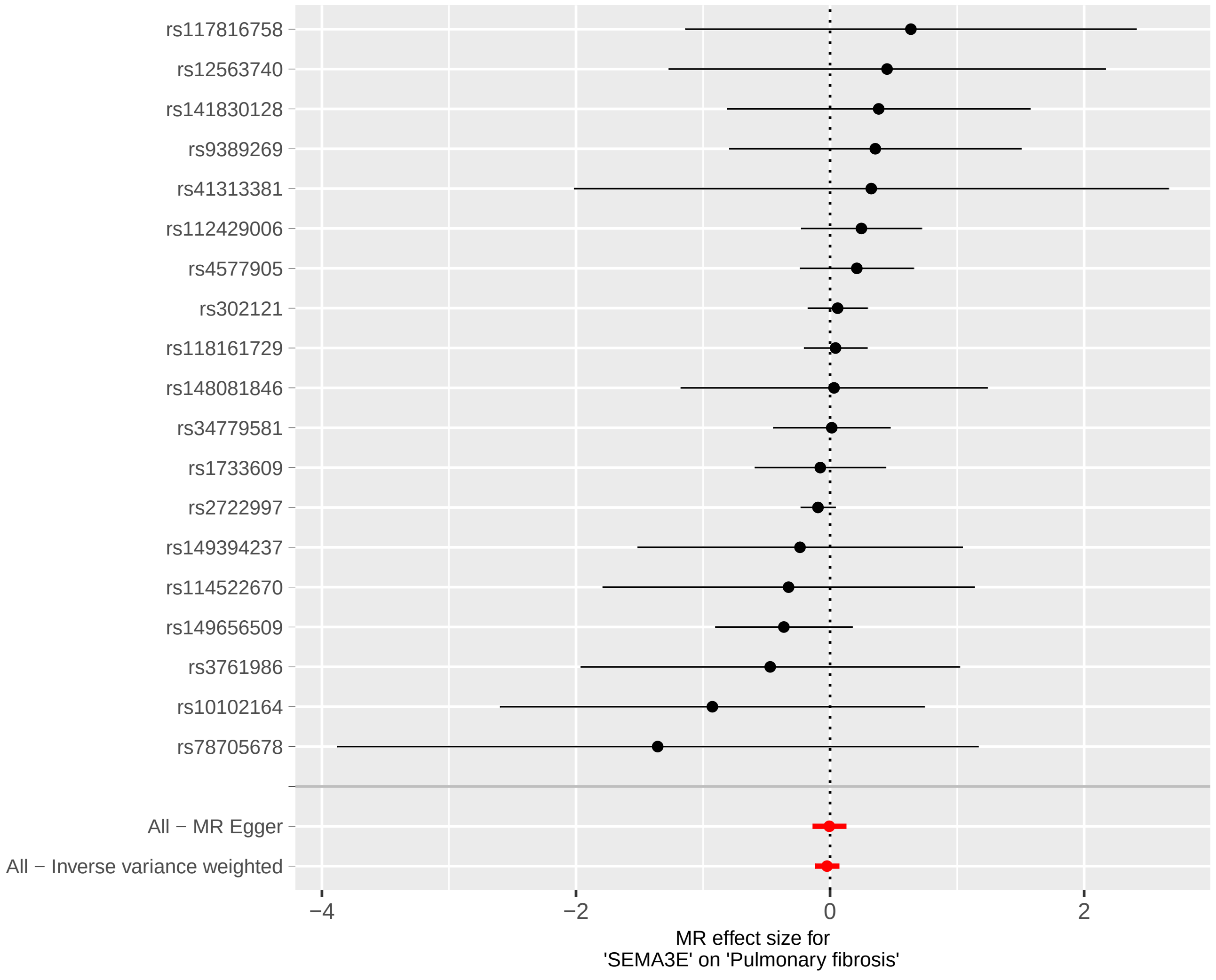
rs117816758
rs12563740
rs141830128
rs9389269
rs41313381
rs112429006
rs4577905
rs302121
rs118161729
rs148081846
rs34779581
rs1733609
rs2722997
rs149394237
rs114522670
rs149656509
rs3761986
rs10102164
rs78705678
All − MR Egger
All − Inverse variance weighted
−4
−2
0
2
MR effect size for
'SEMA3E' on 'Pulmonary fibrosis'

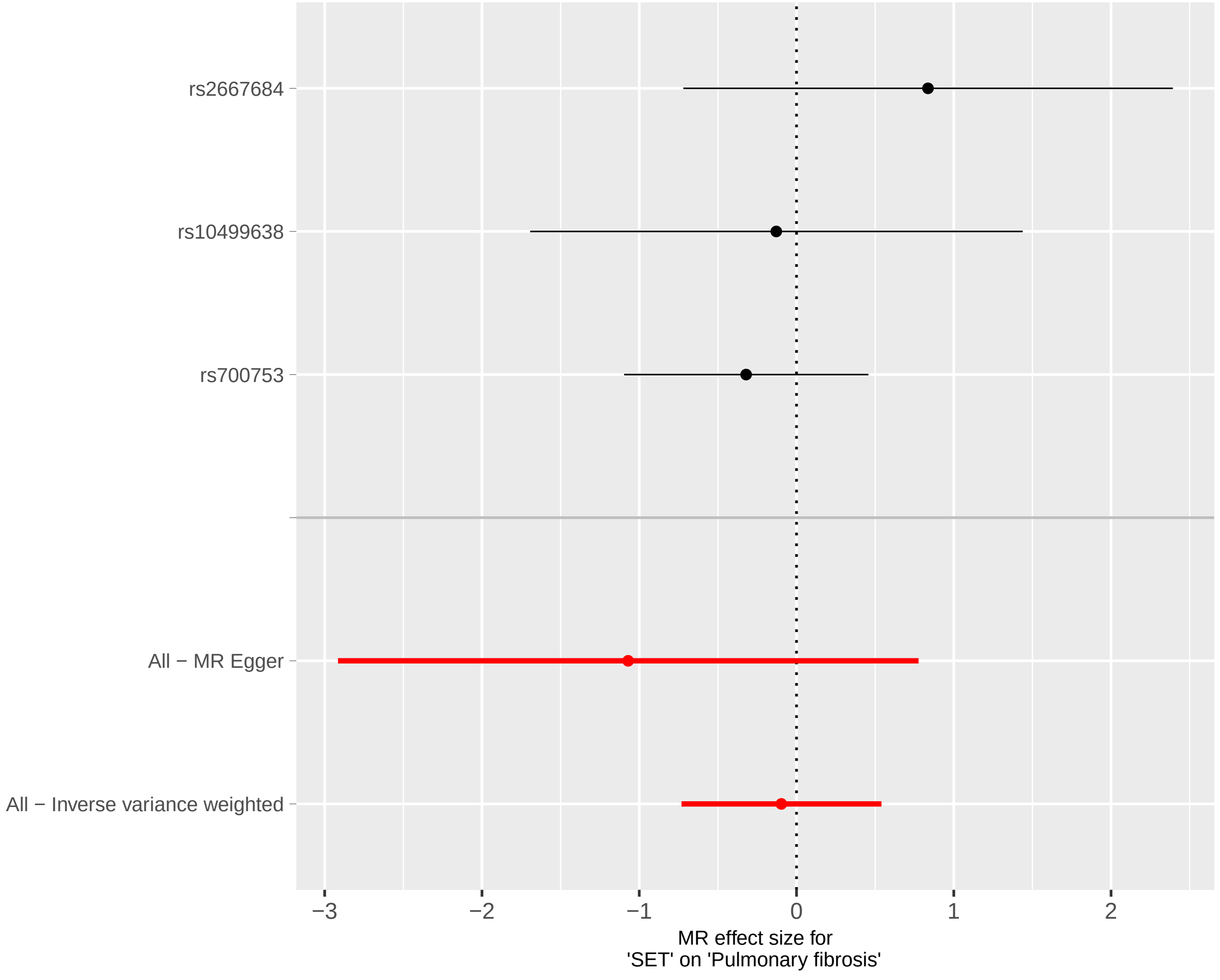

rs2667684
rs10499638
rs700753
All − MR Egger
All − Inverse variance weighted
−3
−2
−1
0
1
2
MR effect size for
'SET' on 'Pulmonary fibrosis'

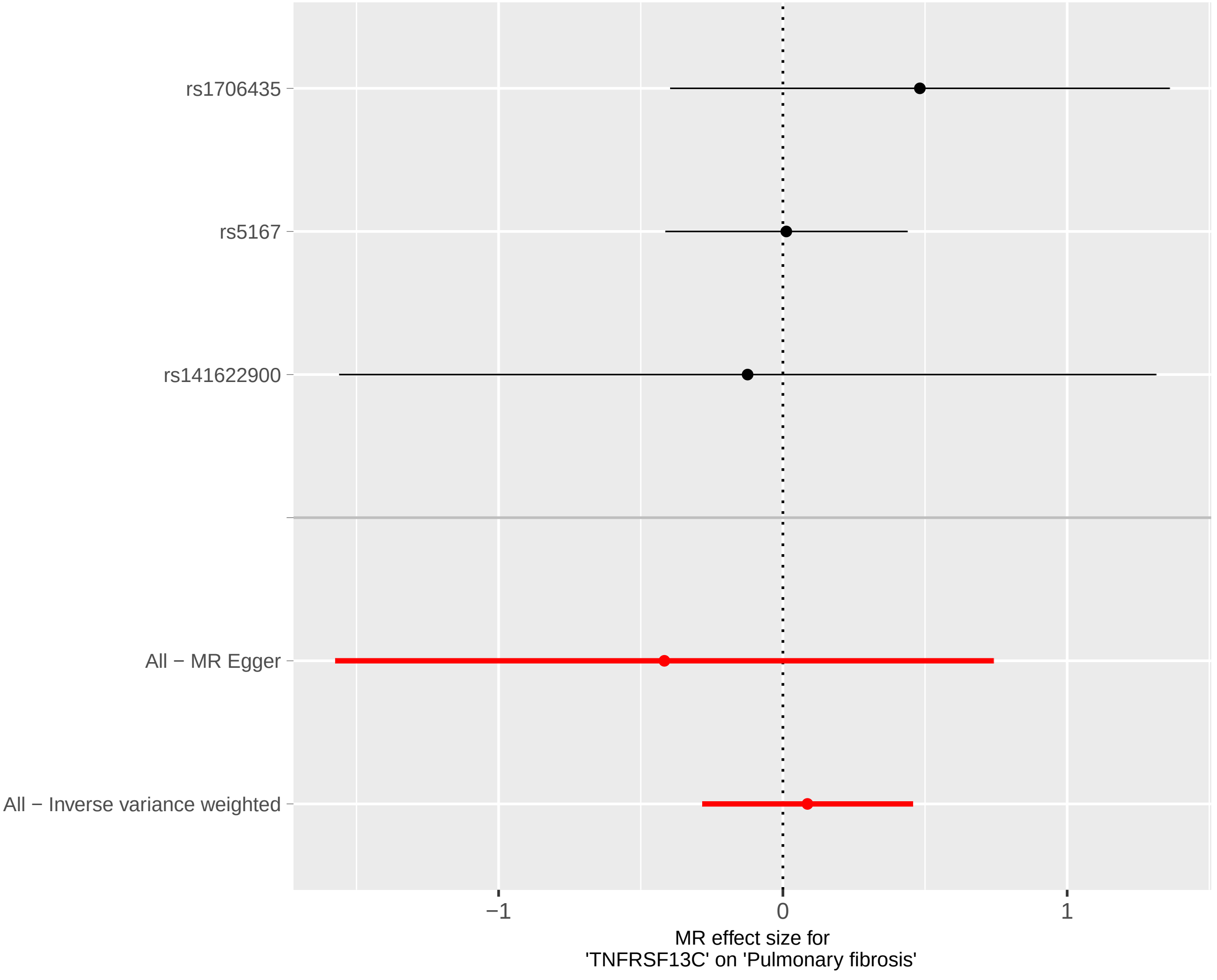

rs1706435
rs5167
rs141622900
All − MR Egger
All − Inverse variance weighted
−1
0
1
MR effect size for
'TNFRSF13C' on 'Pulmonary fibrosis'

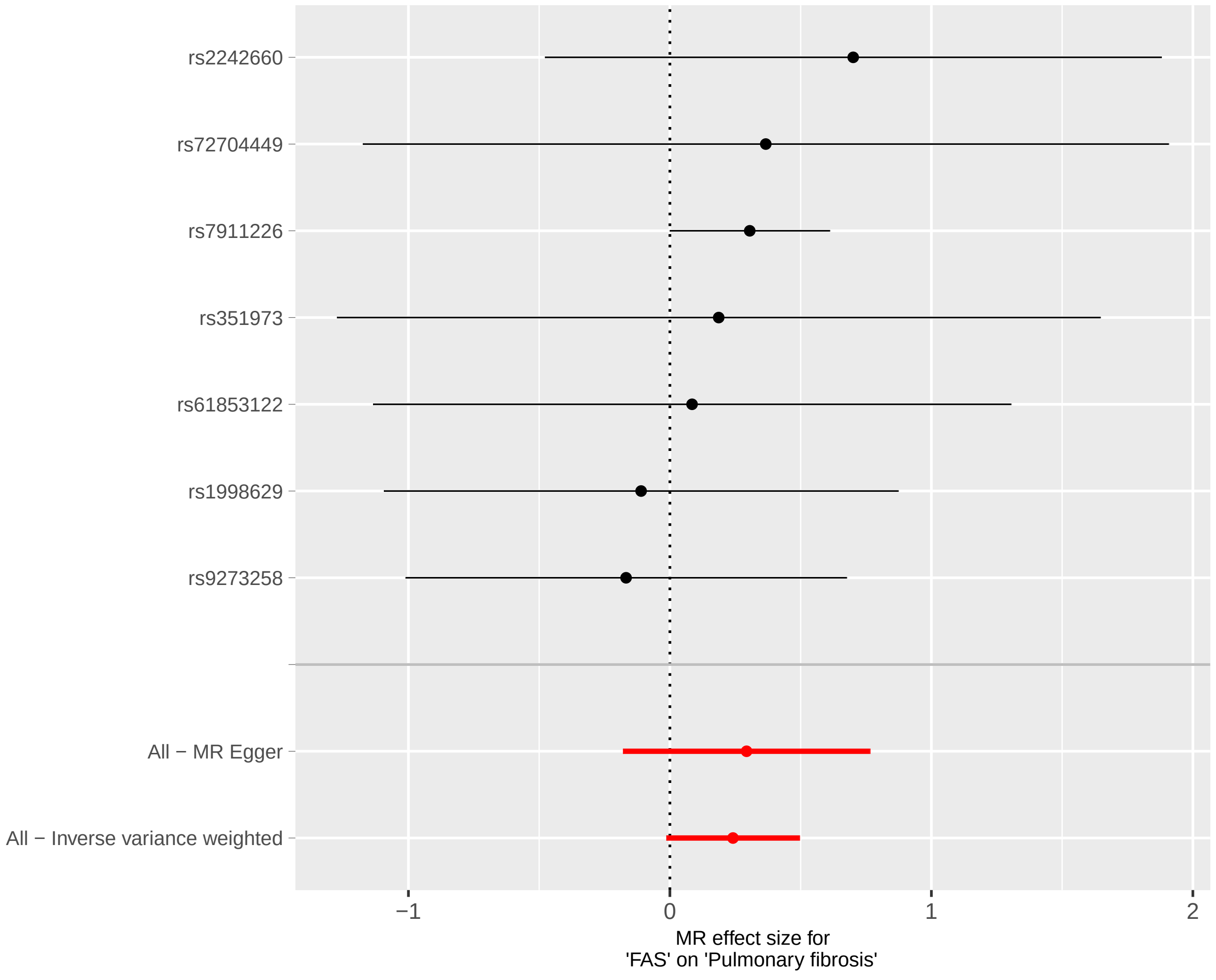
rs2242660
rs72704449
rs7911226
rs351973
rs61853122
rs1998629
rs9273258
All − MR Egger
All − Inverse variance weighted
−1
0
1
2
MR effect size for
'FAS' on 'Pulmonary fibrosis'

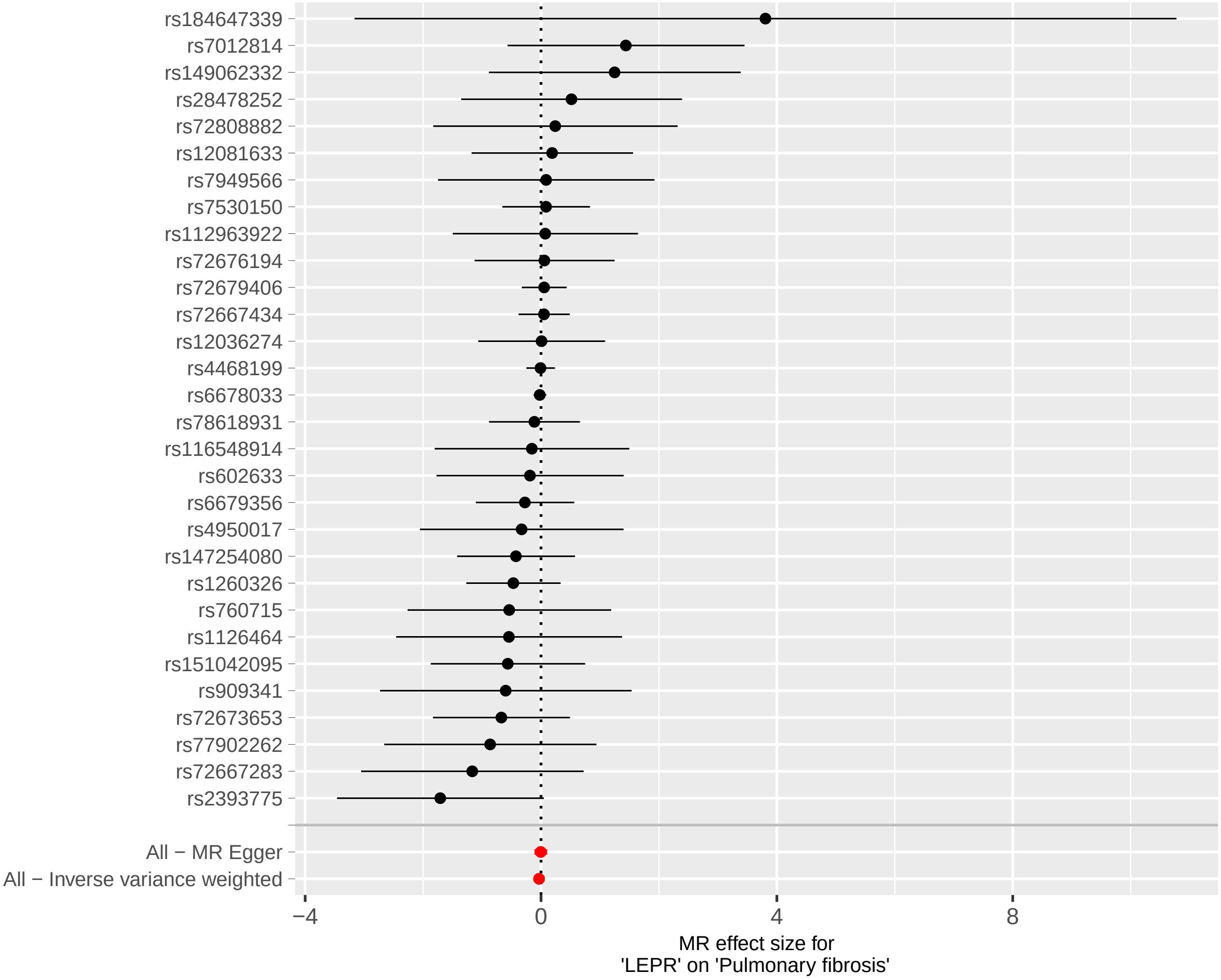

rs184647339
rs7012814
rs149062332
rs28478252
rs72808882
rs12081633
rs7949566
rs7530150
rs112963922
rs72676194
rs72679406
rs72667434
rs12036274
rs4468199
rs6678033
rs78618931
rs116548914
rs602633
rs6679356
rs4950017
rs147254080
rs1260326
rs760715
rs1126464
rs151042095
rs909341
rs72673653
rs77902262
rs72667283
rs2393775
All − MR Egger
All − Inverse variance weighted
−4
0
4
8
MR effect size for
'LEPR' on 'Pulmonary fibrosis'

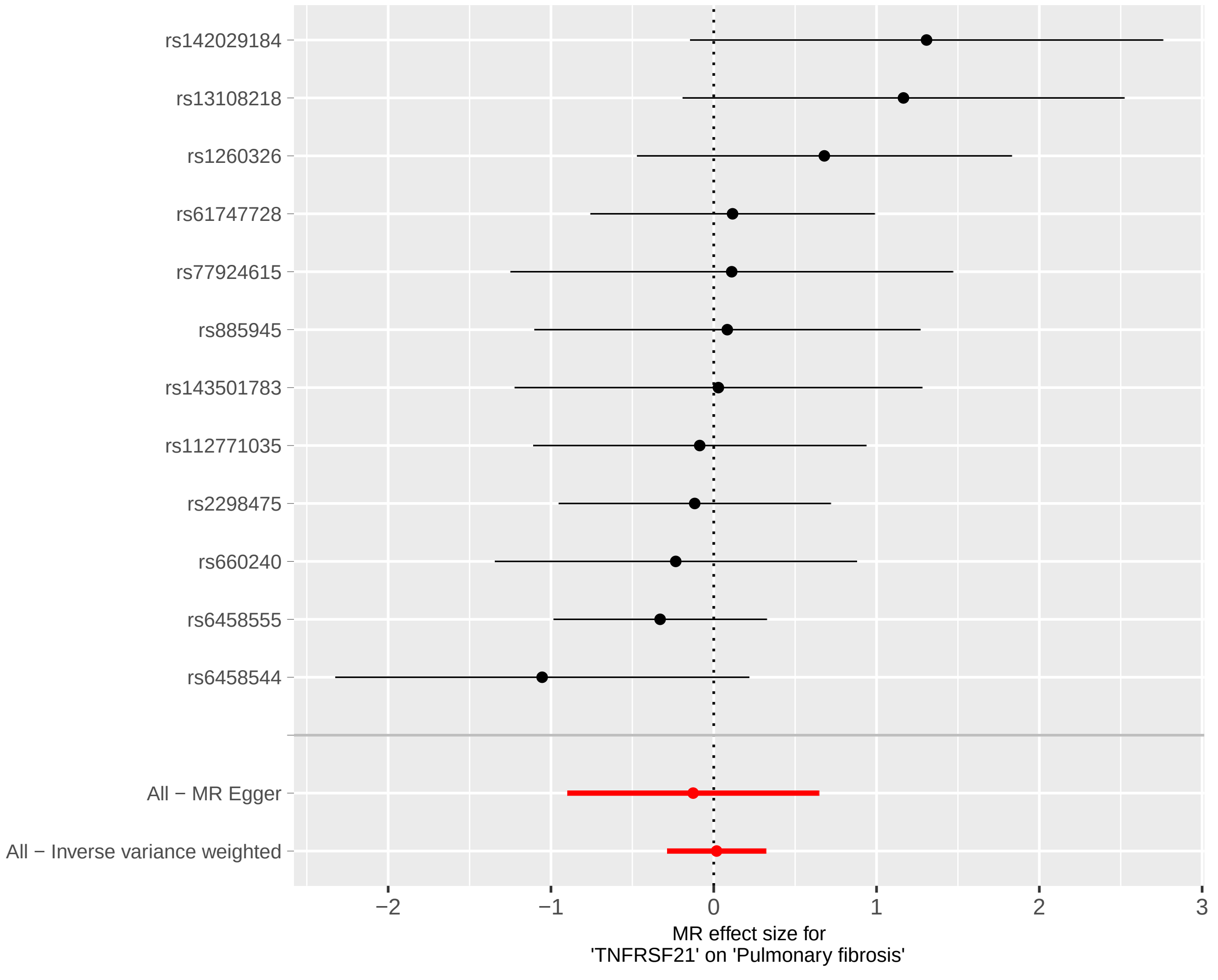

rs142029184
rs13108218
rs1260326
rs61747728
rs77924615
rs885945
rs143501783
rs112771035
rs2298475
rs660240
rs6458555
rs6458544
All − MR Egger
All − Inverse variance weighted
−2
−1
0
1
2
3
MR effect size for
'TNFRSF21' on 'Pulmonary fibrosis'

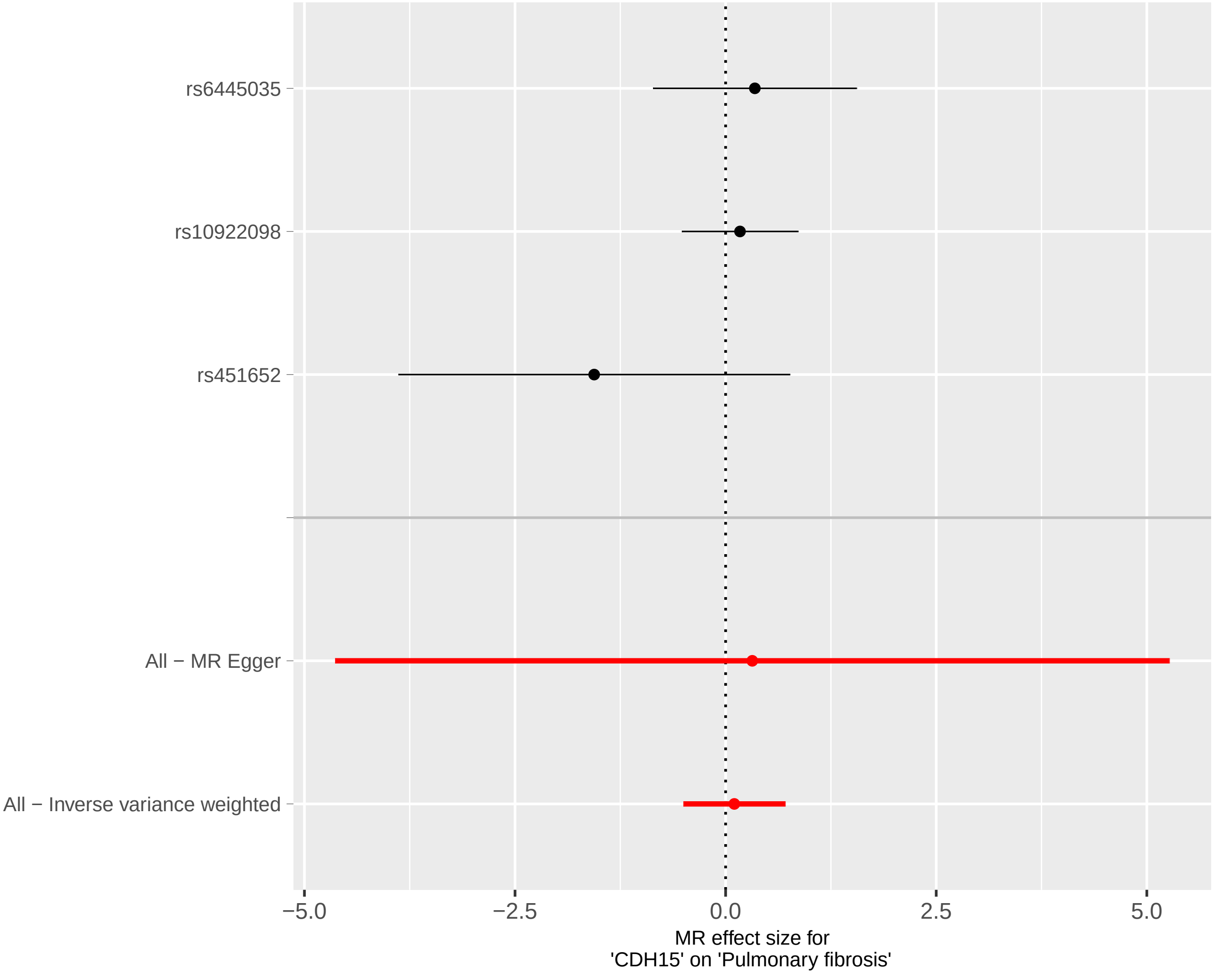
rs6445035
rs10922098
rs451652
All − MR Egger
All − Inverse variance weighted
−5.0
−2.5
0.0
2.5
5.0
MR effect size for
'CDH15' on 'Pulmonary fibrosis'

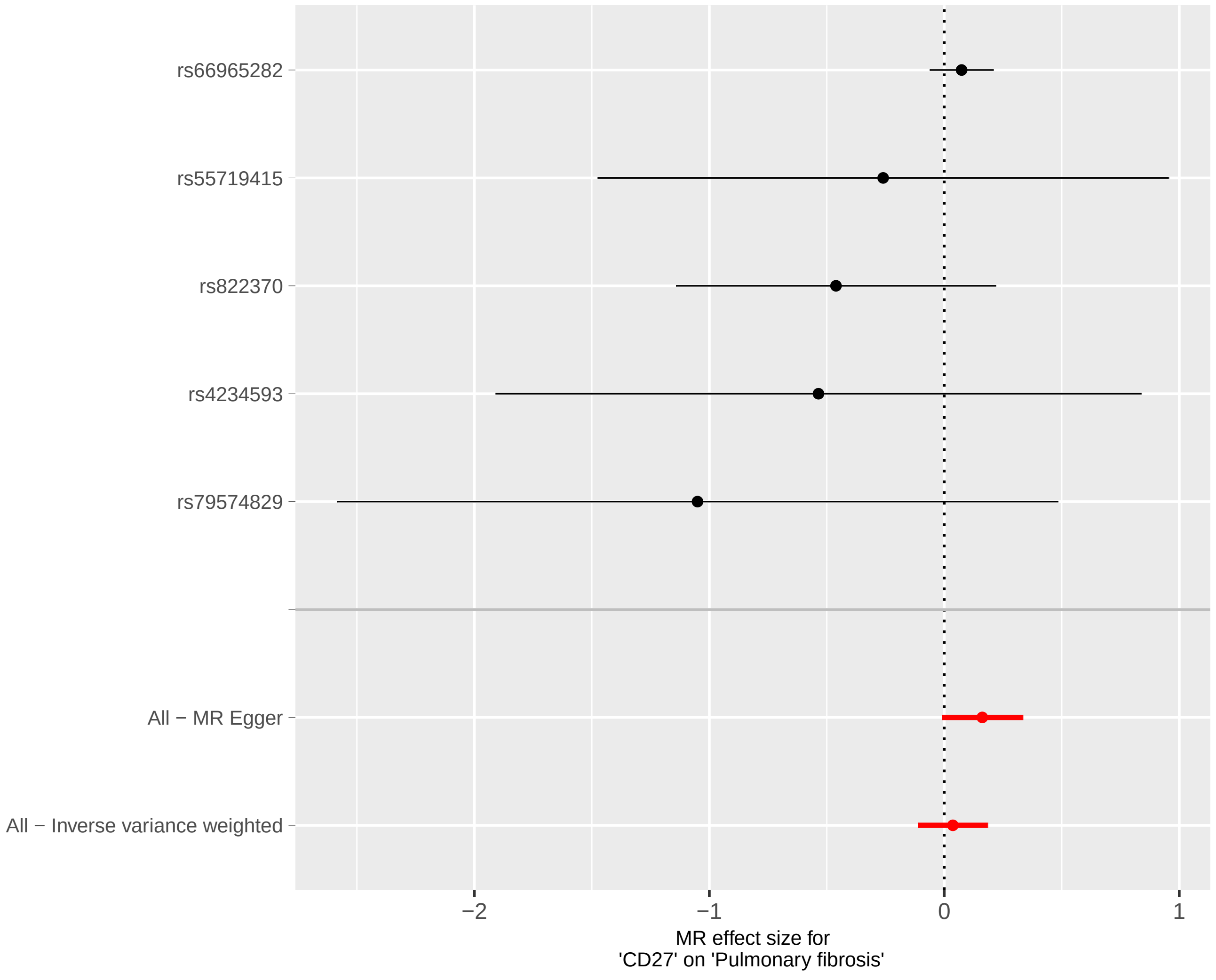
rs66965282
rs55719415
rs822370
rs4234593
rs79574829
All − MR Egger
All − Inverse variance weighted
−2
−1
0
1
MR effect size for
'CD27' on 'Pulmonary fibrosis'

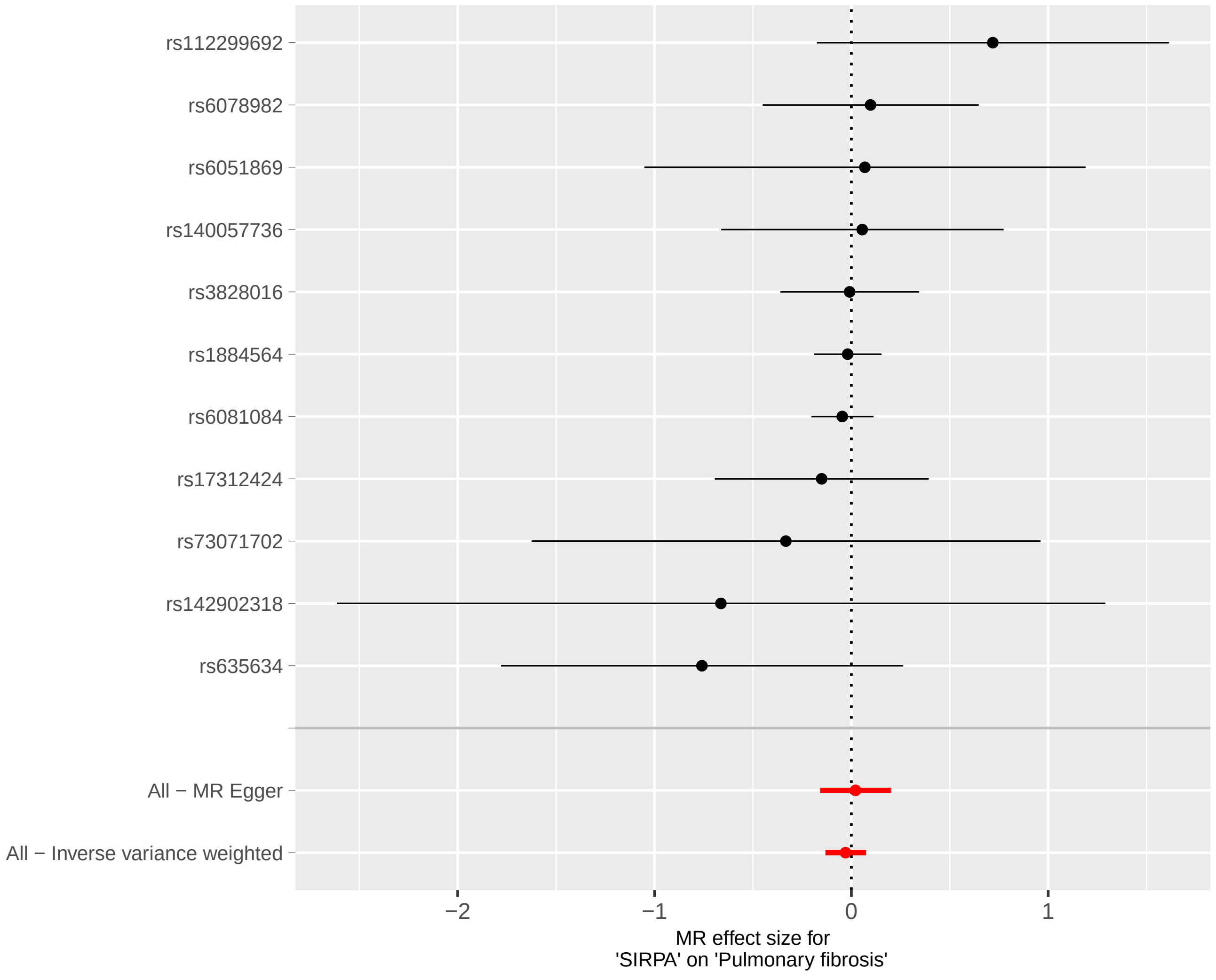
rs112299692
rs6078982
rs6051869
rs140057736
rs3828016
rs1884564
rs6081084
rs17312424
rs73071702
rs142902318
rs635634
All − MR Egger
All − Inverse variance weighted
−2
−1
0
1
MR effect size for
'SIRPA' on 'Pulmonary fibrosis'

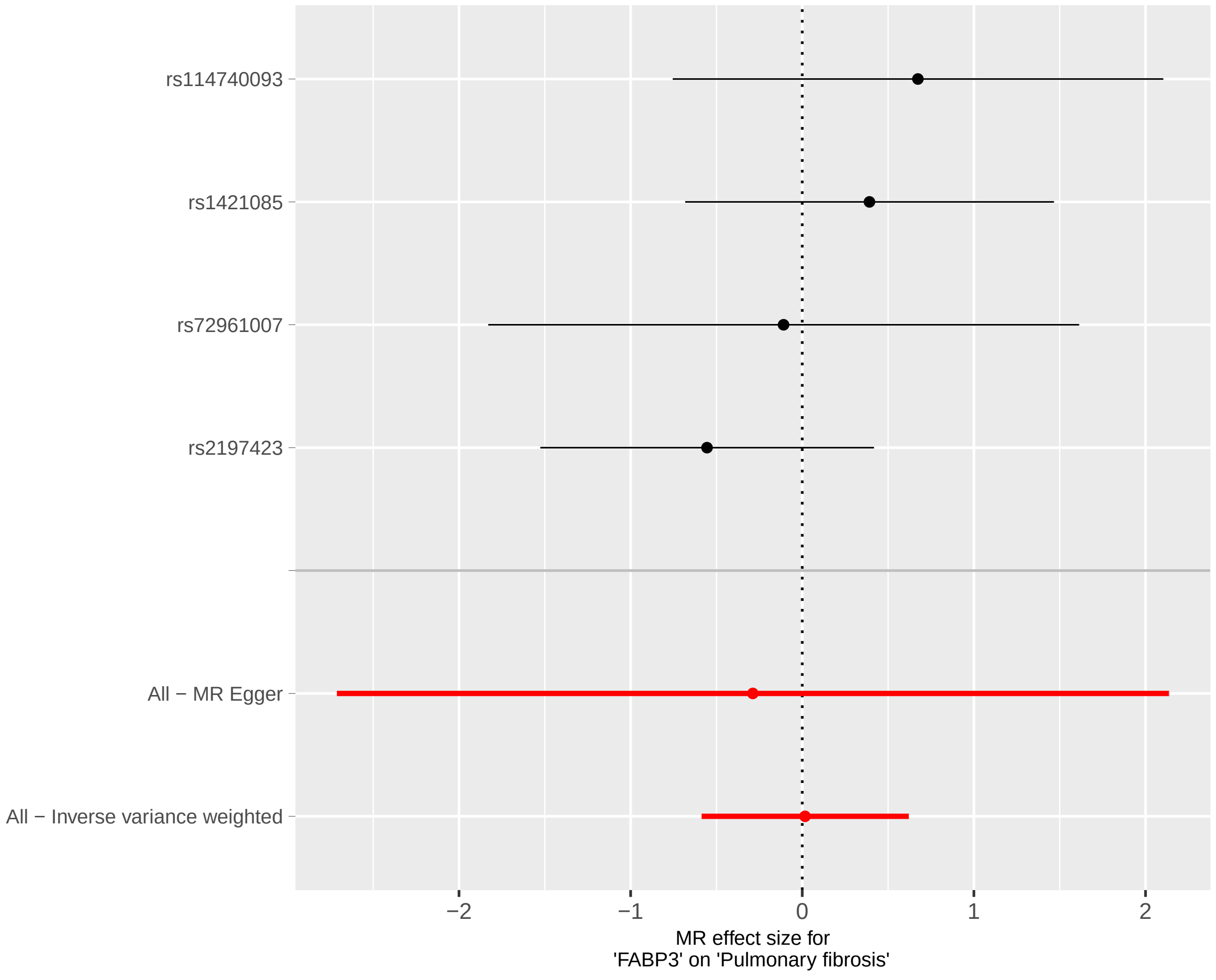
rs114740093
rs1421085
rs72961007
rs2197423
All − MR Egger
All − Inverse variance weighted
−2
−1
0
1
2
MR effect size for
'FABP3' on 'Pulmonary fibrosis'

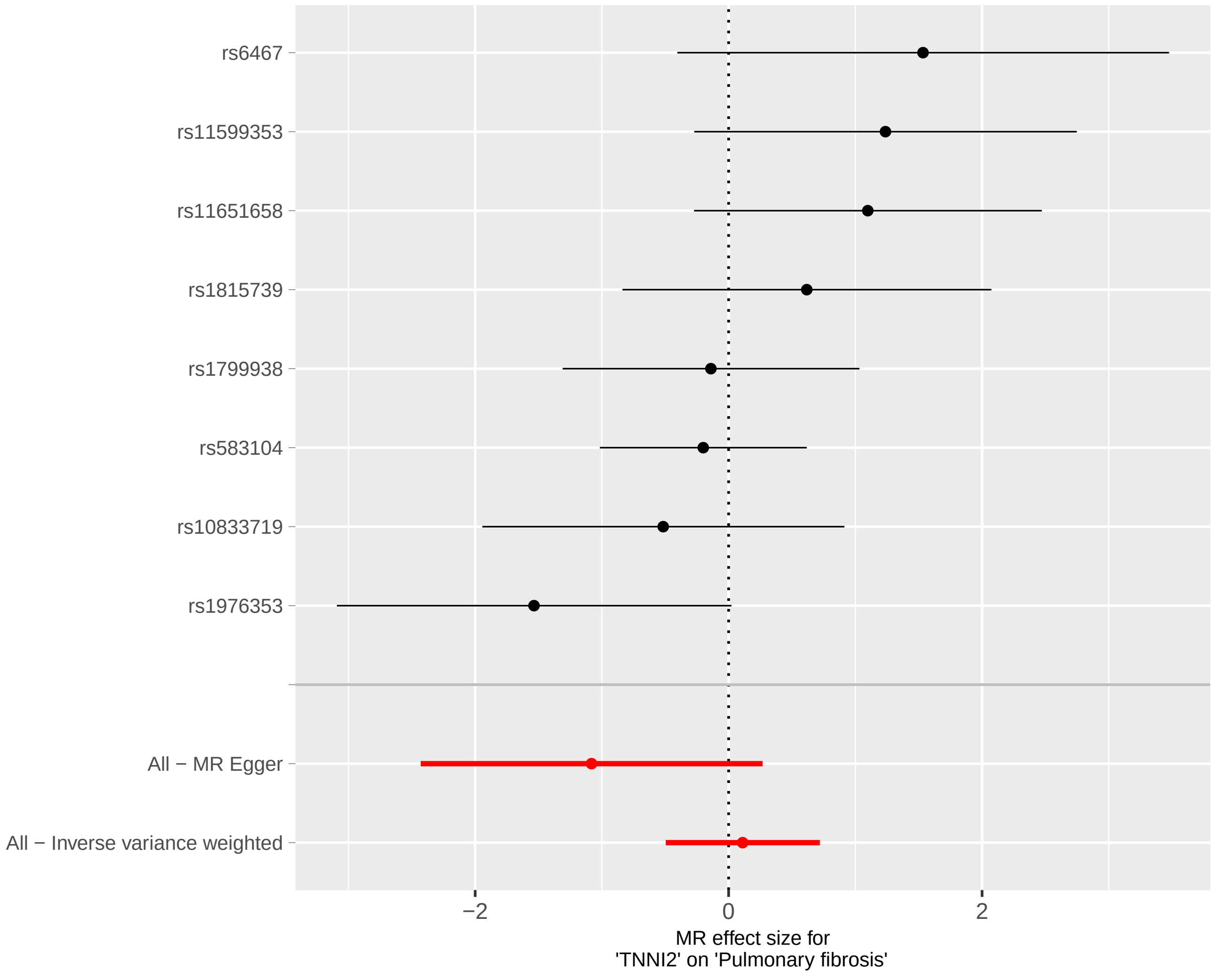
rs6467
rs11599353
rs11651658
rs1815739
rs1799938
rs583104
rs10833719
rs1976353
All − MR Egger
All − Inverse variance weighted
−2
0
2
MR effect size for
'TNNI2' on 'Pulmonary fibrosis'

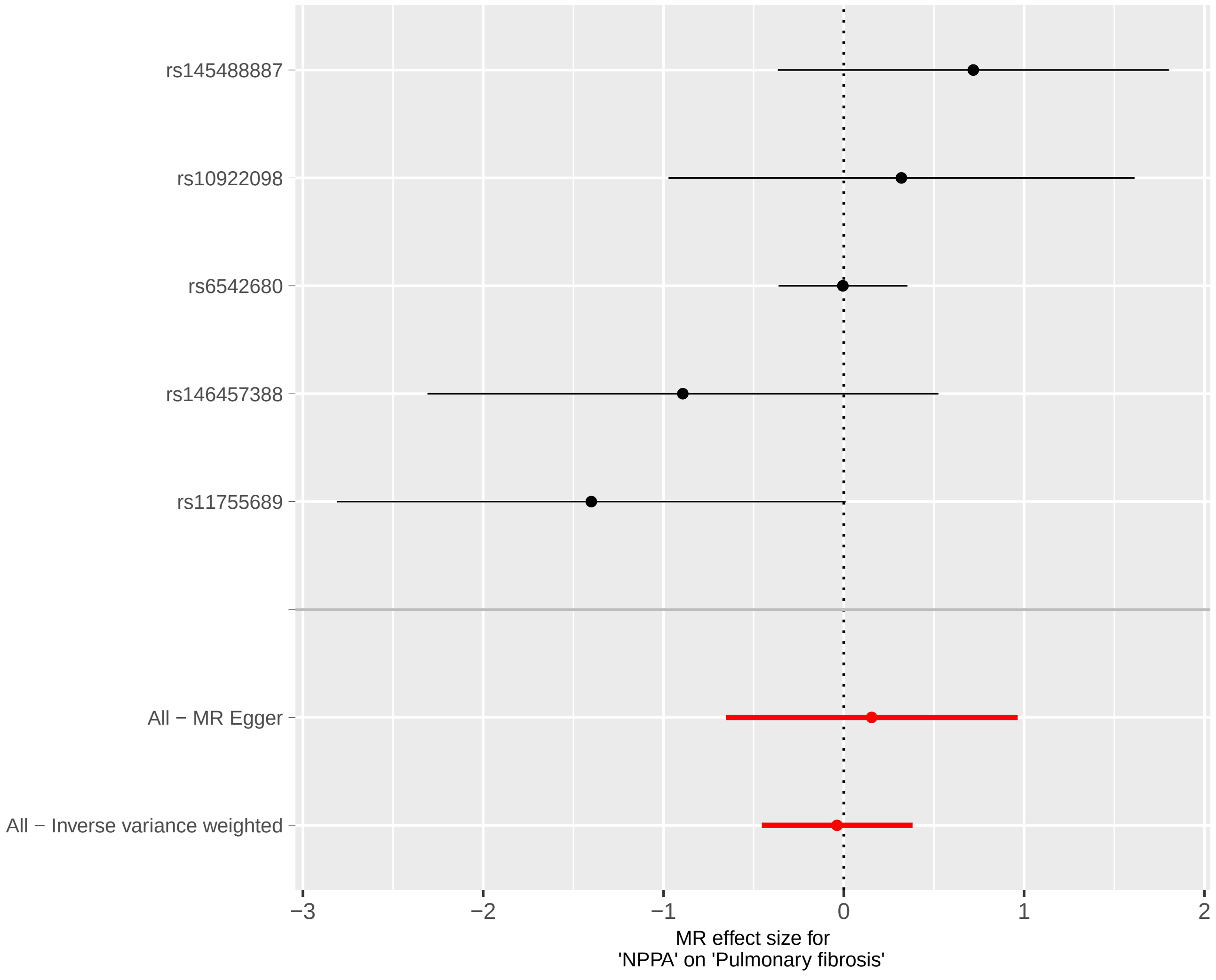

rs145488887
rs10922098
rs6542680
rs146457388
rs11755689
All − MR Egger
All − Inverse variance weighted
−3
−2
−1
0
1
2
MR effect size for
'NPPA' on 'Pulmonary fibrosis'

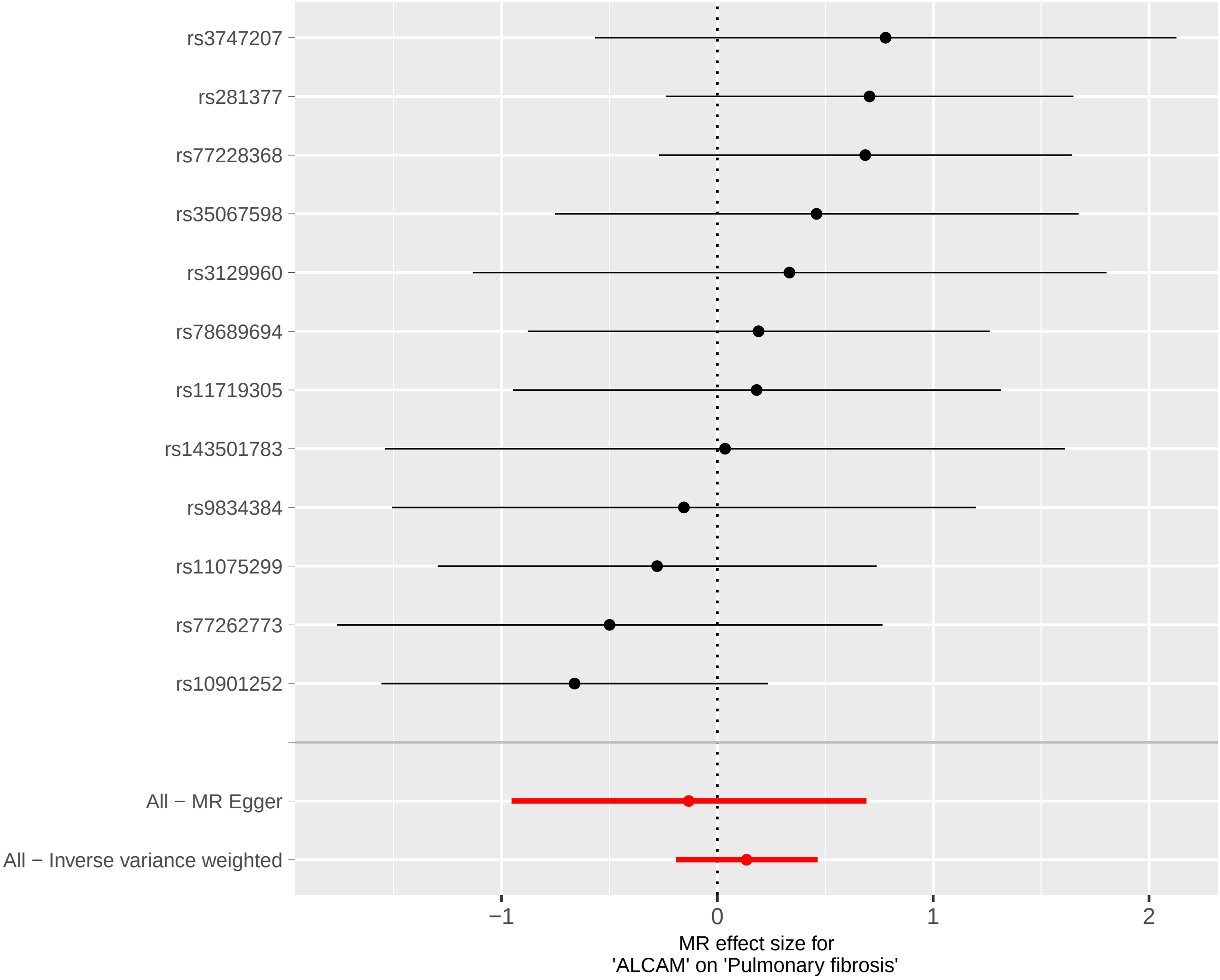

rs3747207
rs281377
rs77228368
rs35067598
rs3129960
rs78689694
rs11719305
rs143501783
rs9834384
rs11075299
rs77262773
rs10901252
All − MR Egger
All − Inverse variance weighted
−1
0
1
2
MR effect size for
'ALCAM' on 'Pulmonary fibrosis'

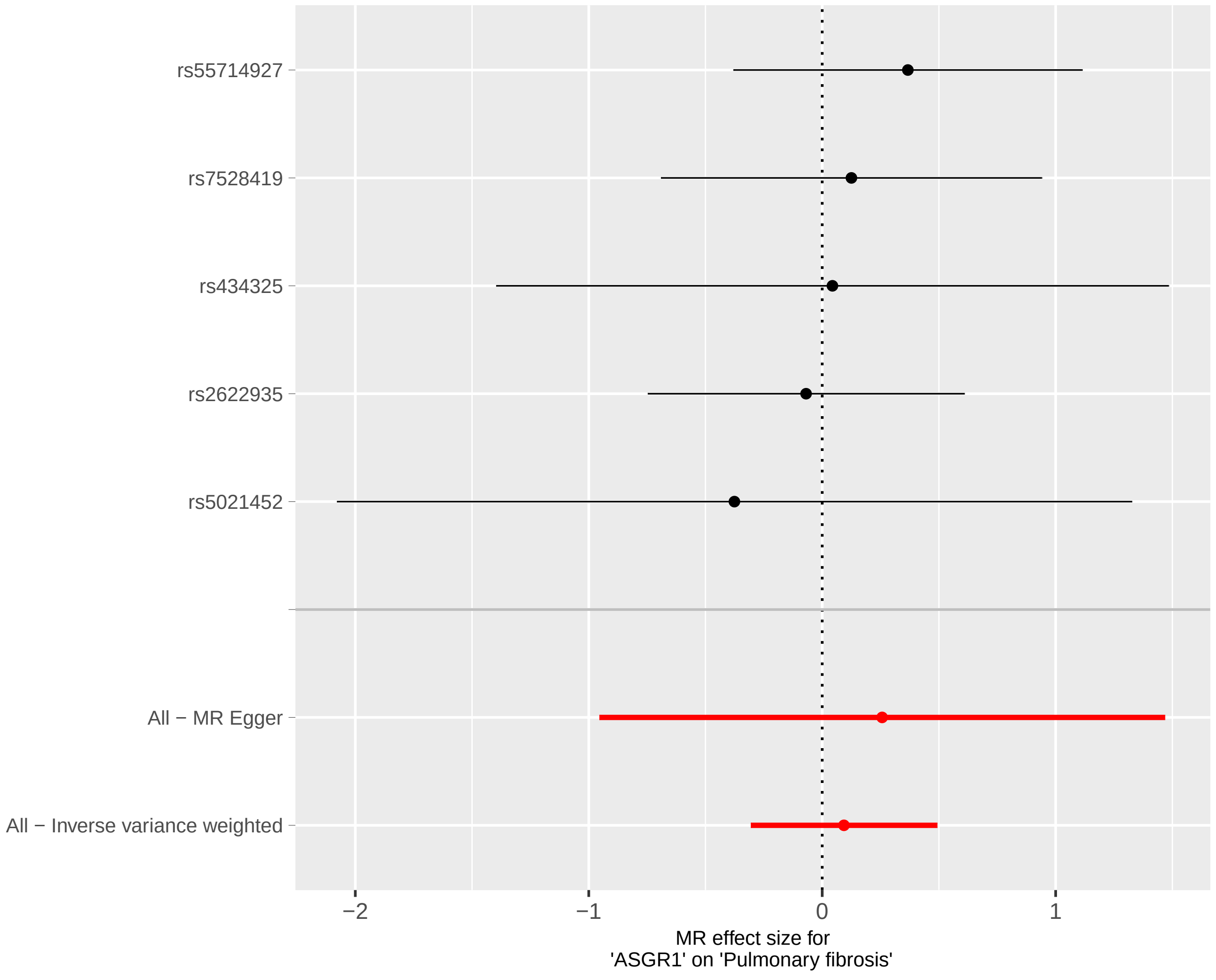
rs55714927
rs7528419
rs434325
rs2622935
rs5021452
All − MR Egger
All − Inverse variance weighted
−2
−1
0
1
MR effect size for
'ASGR1' on 'Pulmonary fibrosis'

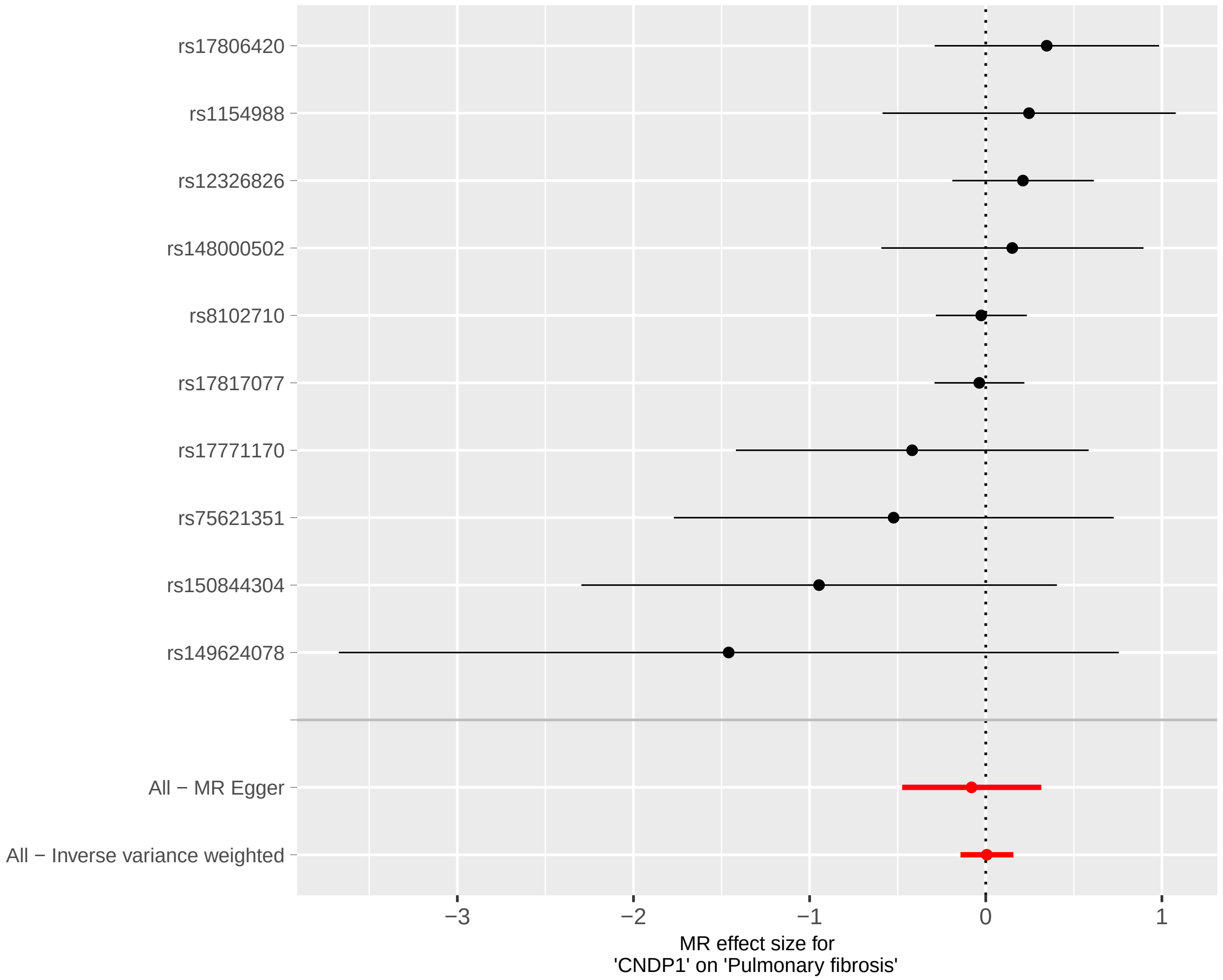
rs17806420
rs1154988
rs12326826
rs148000502
rs8102710
rs17817077
rs17771170
rs75621351
rs150844304
rs149624078
All − MR Egger
All − Inverse variance weighted
−3
−2
−1
0
1
MR effect size for
'CNDP1' on 'Pulmonary fibrosis'

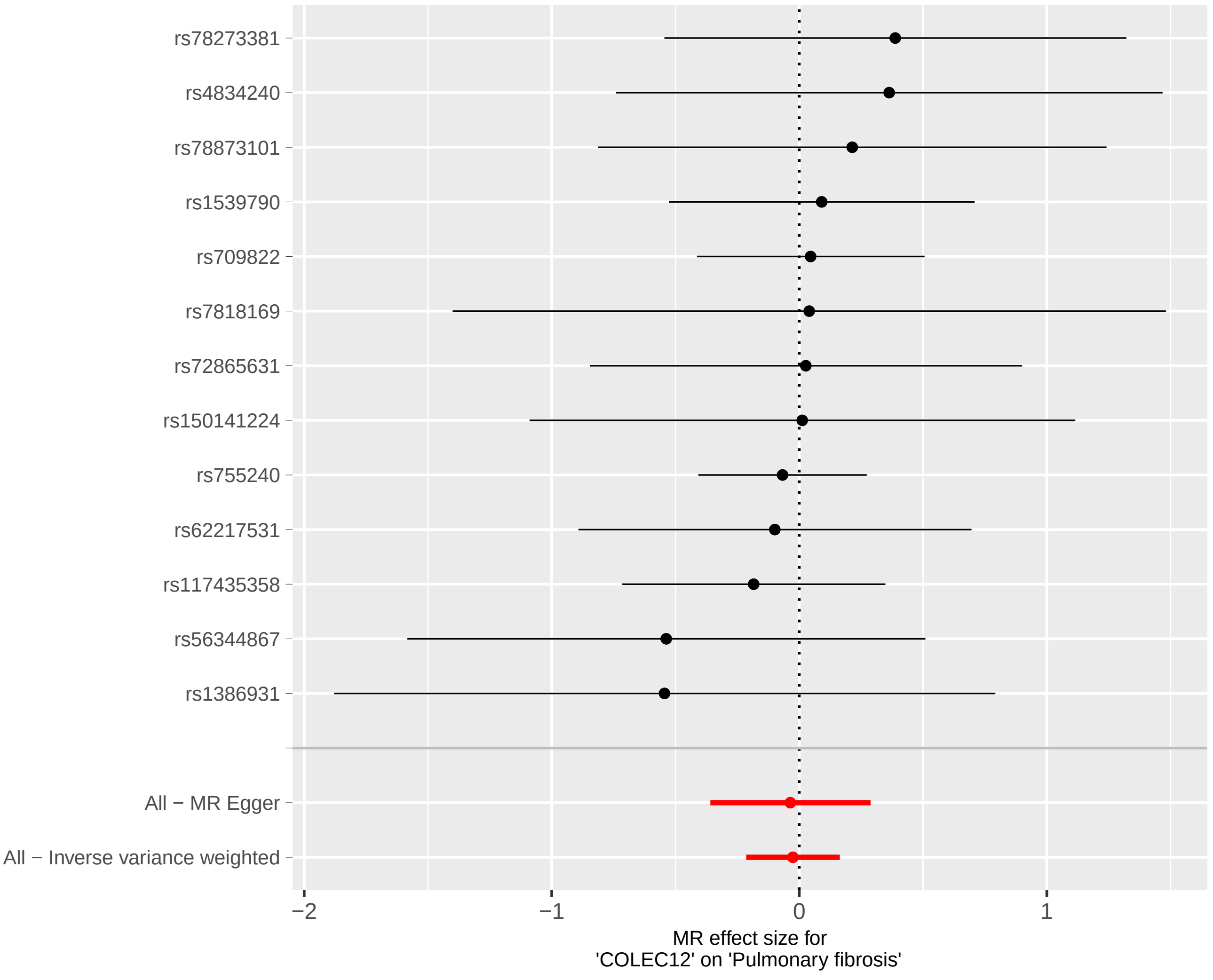

rs78273381
rs4834240
rs78873101
rs1539790
rs709822
rs7818169
rs72865631
rs150141224
rs755240
rs62217531
rs117435358
rs56344867
rs1386931
All − MR Egger
All − Inverse variance weighted
−2
−1
0
1
MR effect size for
'COLEC12' on 'Pulmonary fibrosis'

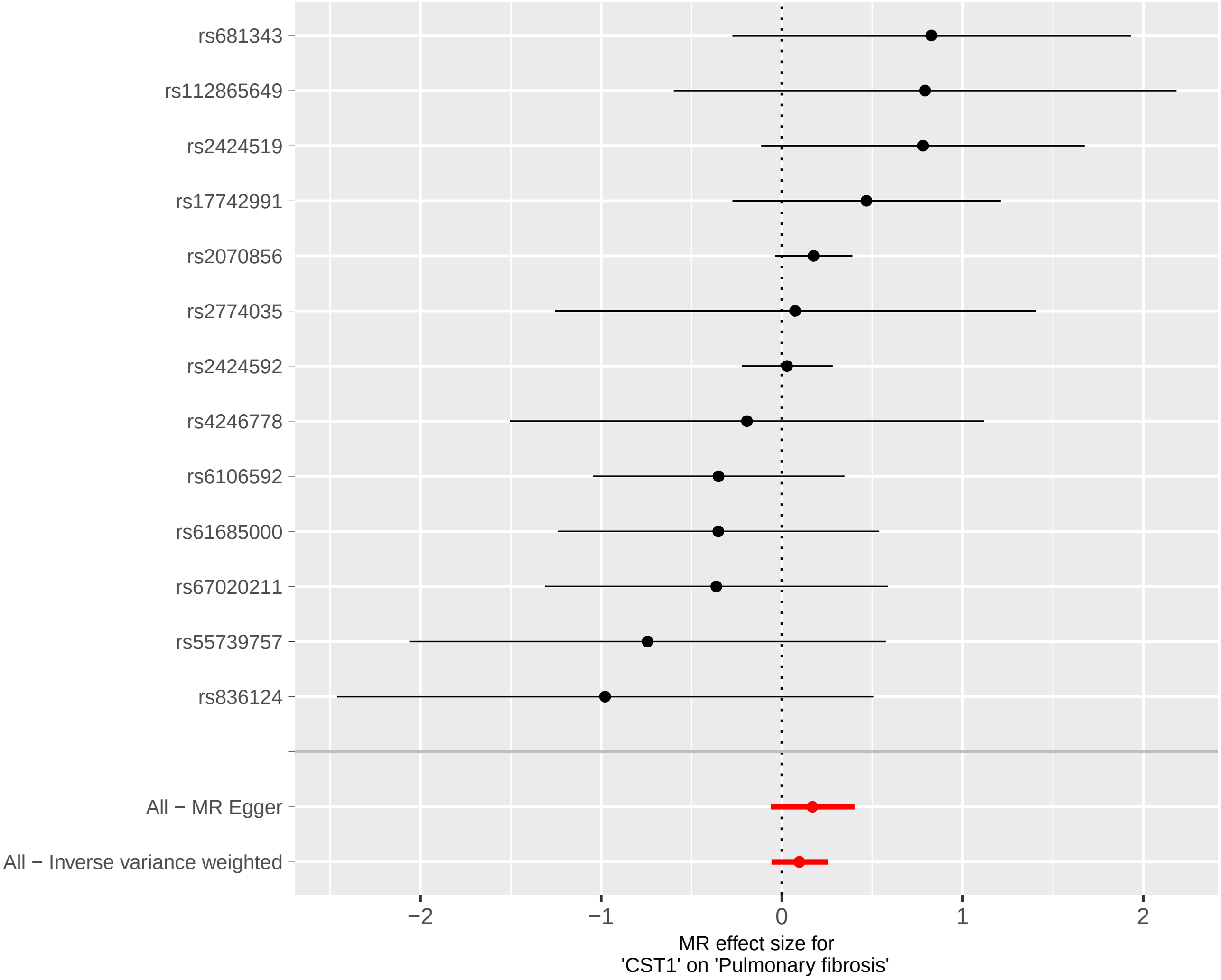
rs681343
rs112865649
rs2424519
rs17742991
rs2070856
rs2774035
rs2424592
rs4246778
rs6106592
rs61685000
rs67020211
rs55739757
rs836124
All − MR Egger
All − Inverse variance weighted
−2
−1
0
1
2
MR effect size for
'CST1' on 'Pulmonary fibrosis'

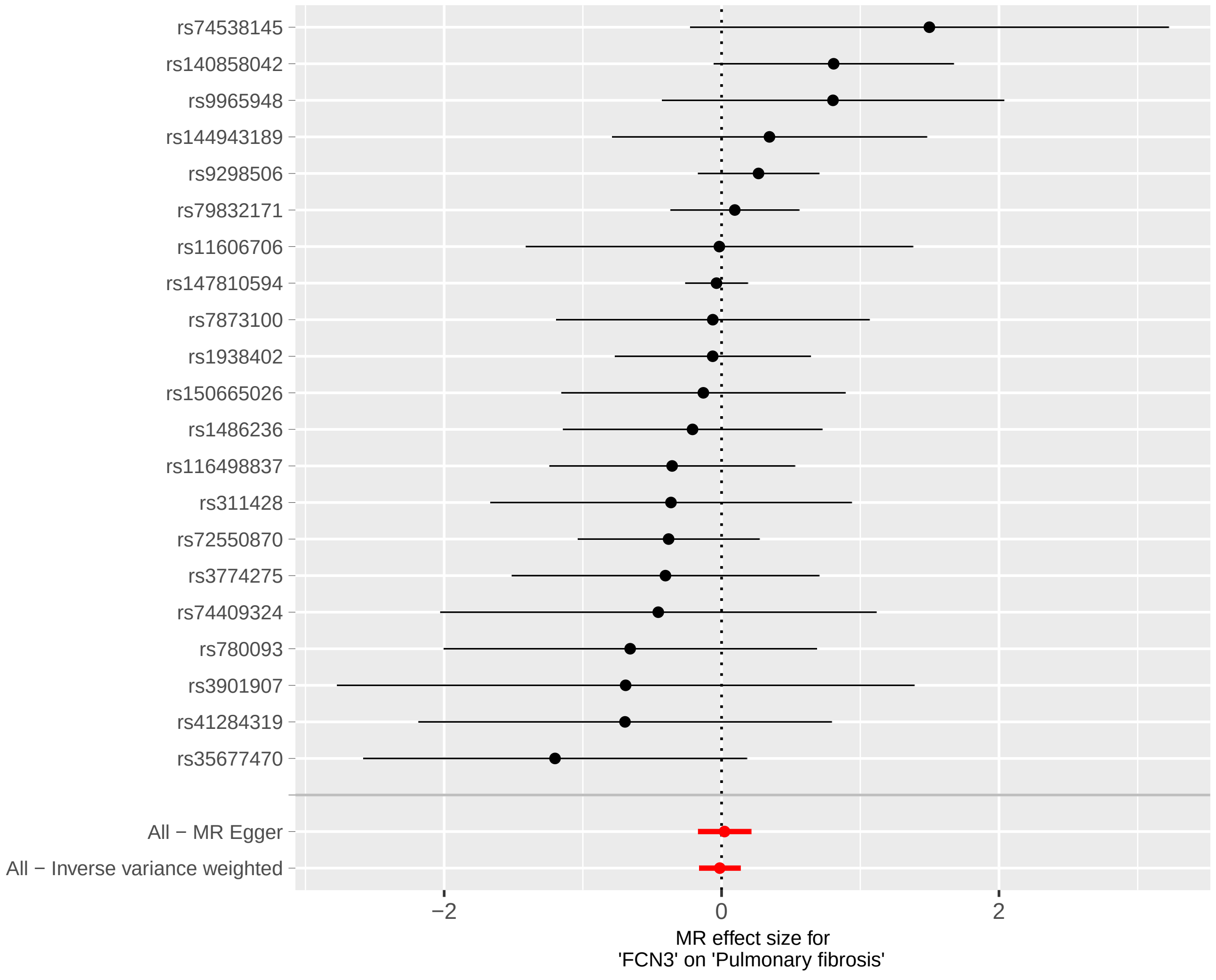
rs74538145
rs140858042
rs9965948
rs144943189
rs9298506
rs79832171
rs11606706
rs147810594
rs7873100
rs1938402
rs150665026
rs1486236
rs116498837
rs311428
rs72550870
rs3774275
rs74409324
rs780093
rs3901907
rs41284319
rs35677470
All − MR Egger
All − Inverse variance weighted
−2
0
2
MR effect size for
'FCN3' on 'Pulmonary fibrosis'

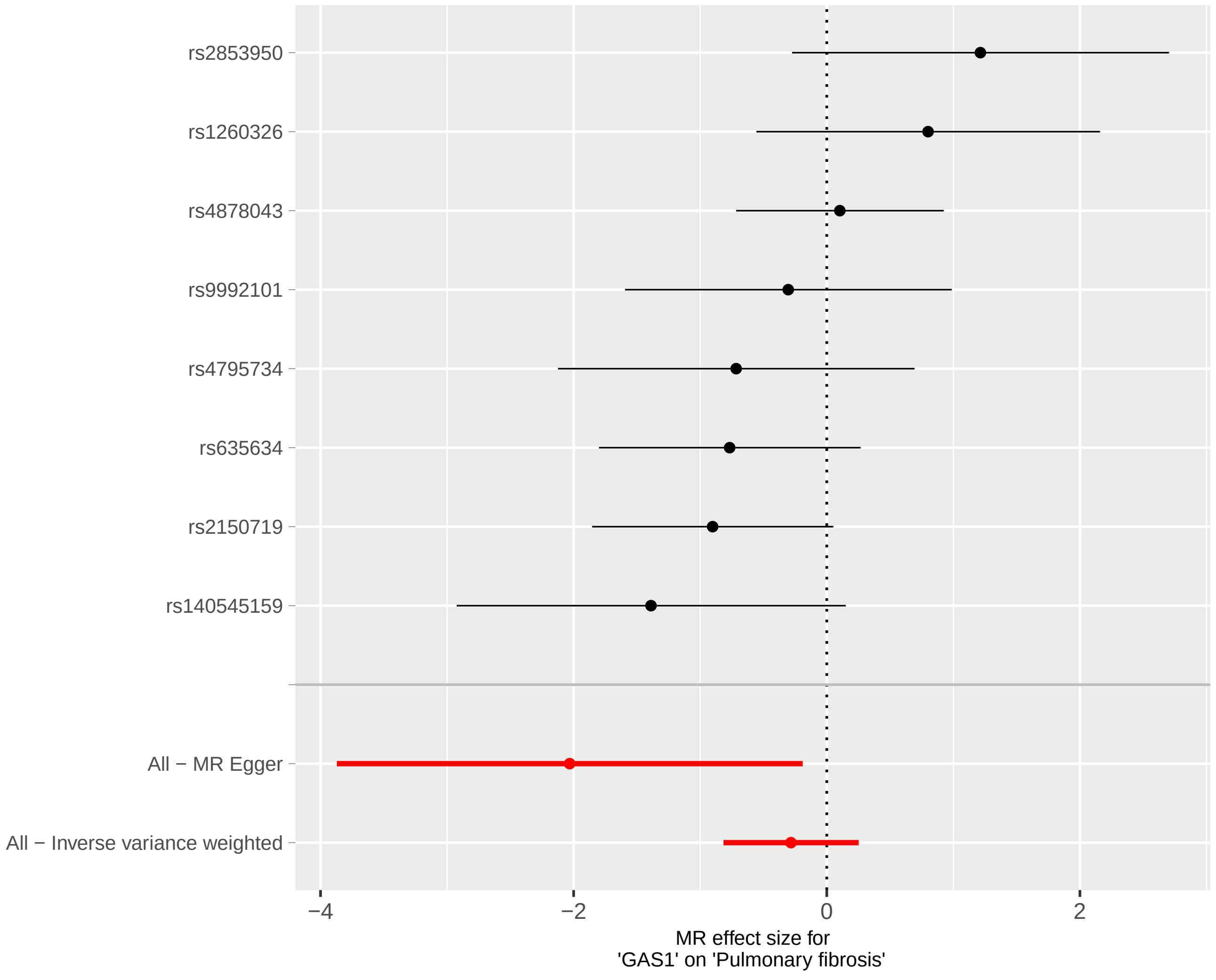
rs2853950
rs1260326
rs4878043
rs9992101
rs4795734
rs635634
rs2150719
rs140545159
All − MR Egger
All − Inverse variance weighted
−4
−2
0
2
MR effect size for
'GAS1' on 'Pulmonary fibrosis'

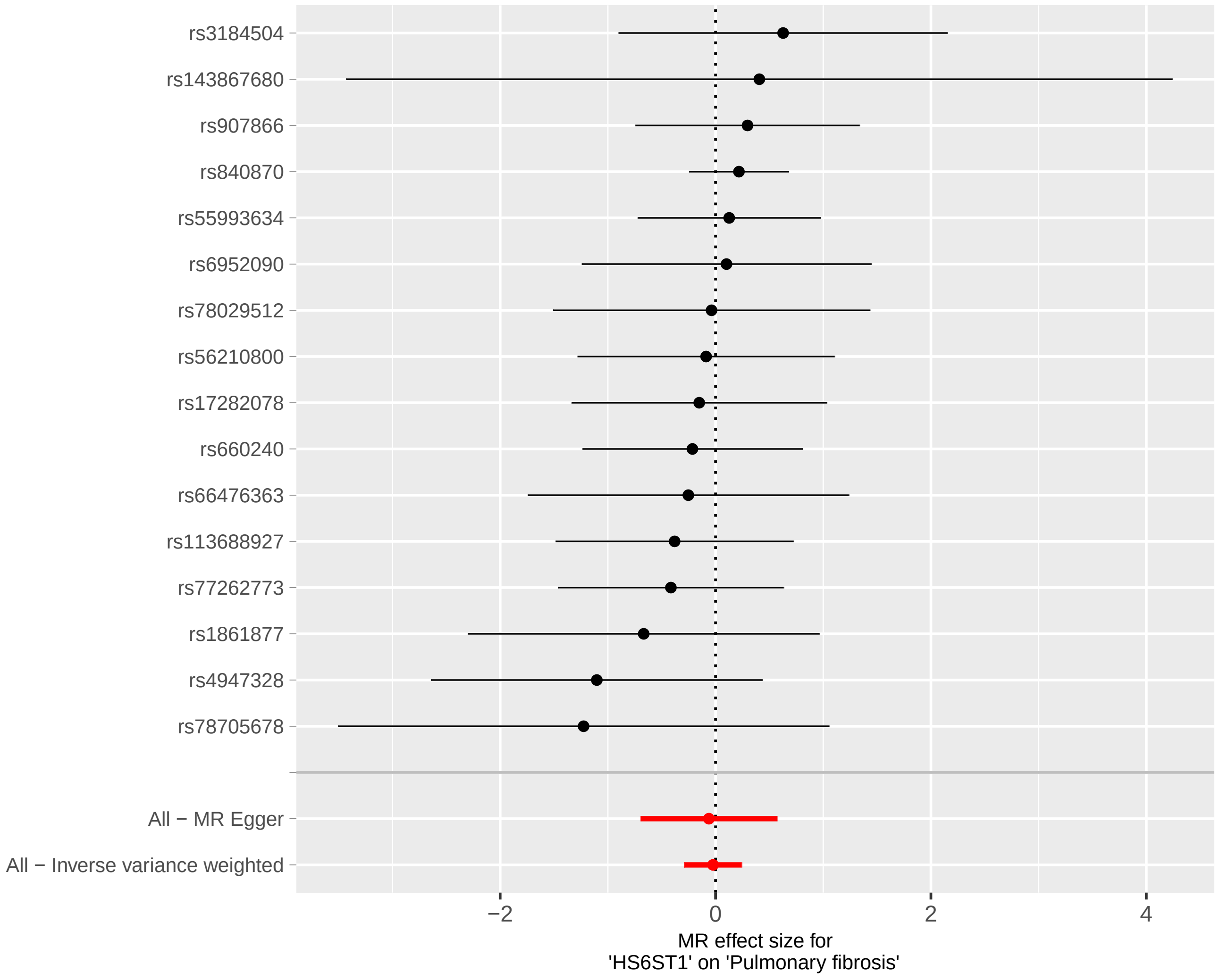
rs3184504
rs143867680
rs907866
rs840870
rs55993634
rs6952090
rs78029512
rs56210800
rs17282078
rs660240
rs66476363
rs113688927
rs77262773
rs1861877
rs4947328
rs78705678
All − MR Egger
All − Inverse variance weighted
−2
0
2
4
MR effect size for
'HS6ST1' on 'Pulmonary fibrosis'

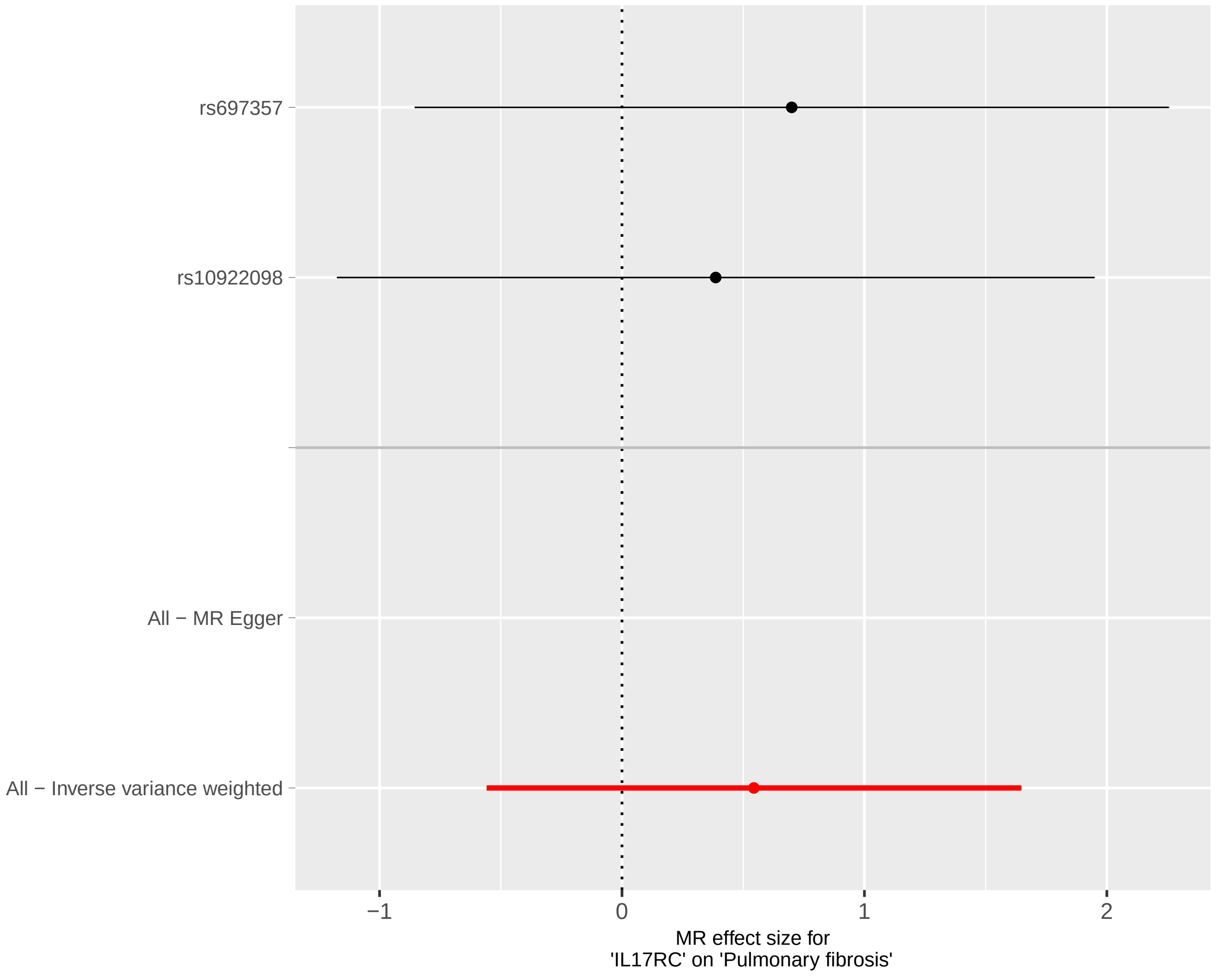
rs697357
rs10922098
All − MR Egger
All − Inverse variance weighted
−1
0
1
2
MR effect size for
'IL17RC' on 'Pulmonary fibrosis'

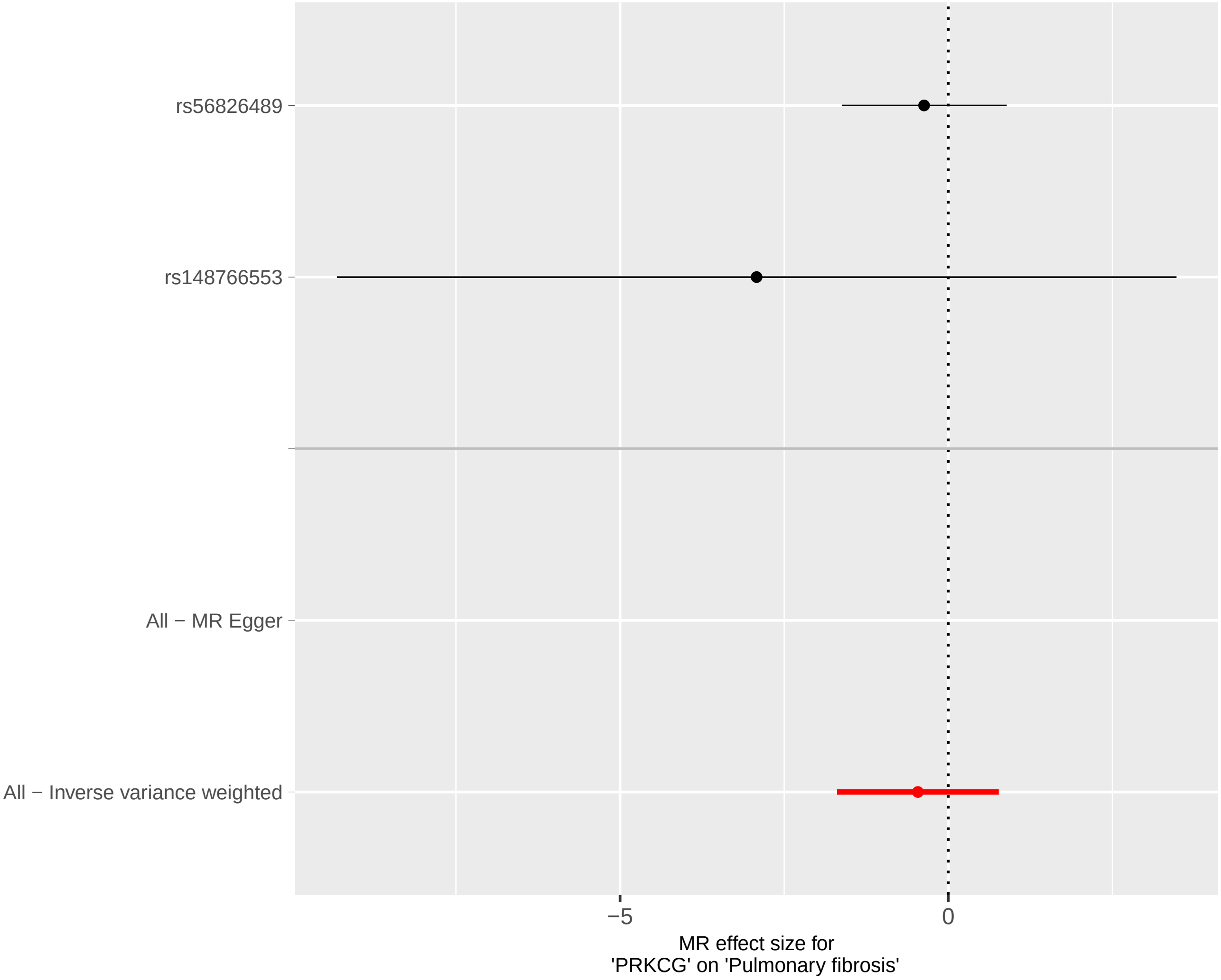
rs56826489
rs148766553
All − MR Egger
All − Inverse variance weighted
−5
0
MR effect size for
'PRKCG' on 'Pulmonary fibrosis'

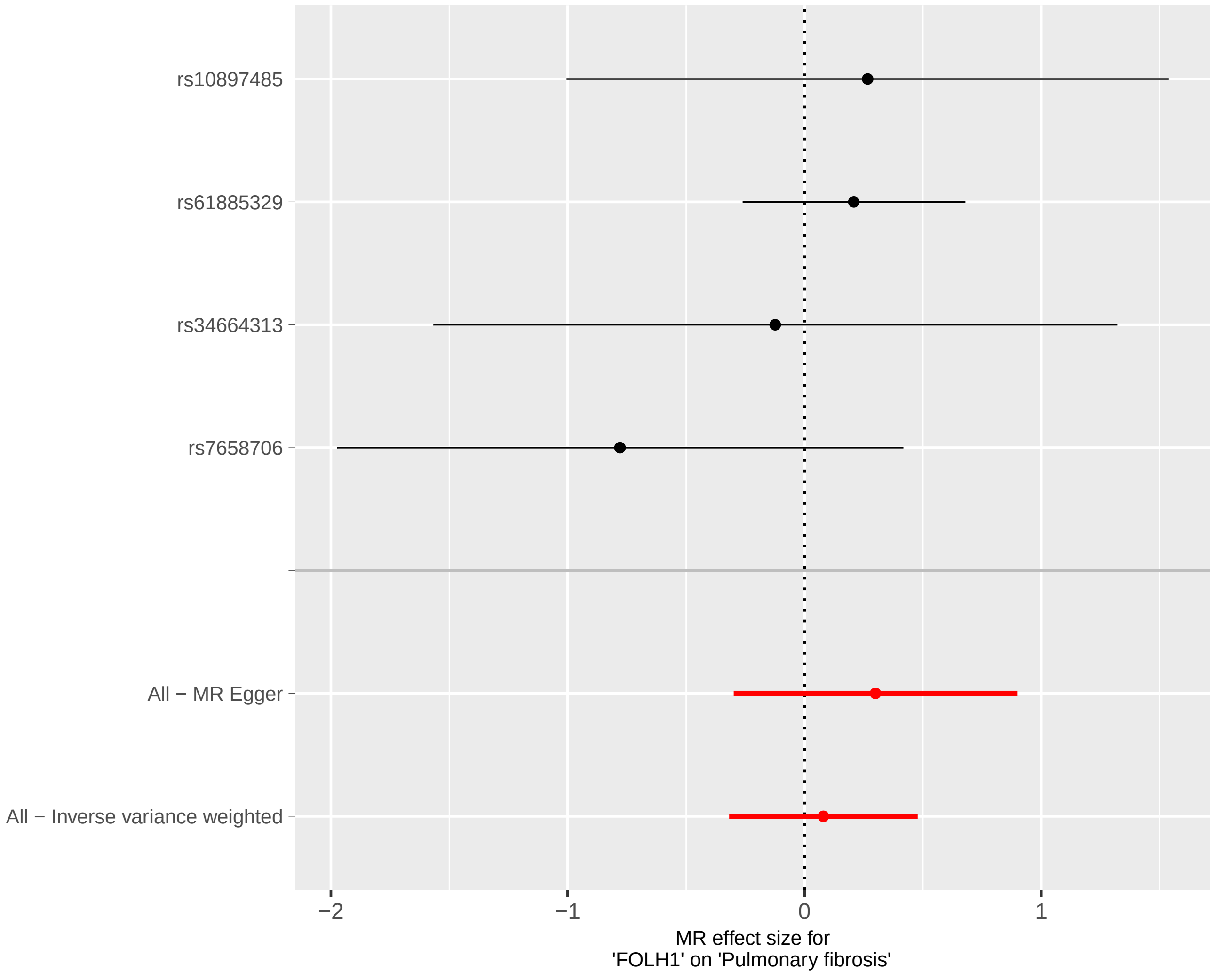

rs10897485
rs61885329
rs34664313
rs7658706
All − MR Egger
All − Inverse variance weighted
−2
−1
0
1
MR effect size for
'FOLH1' on 'Pulmonary fibrosis'

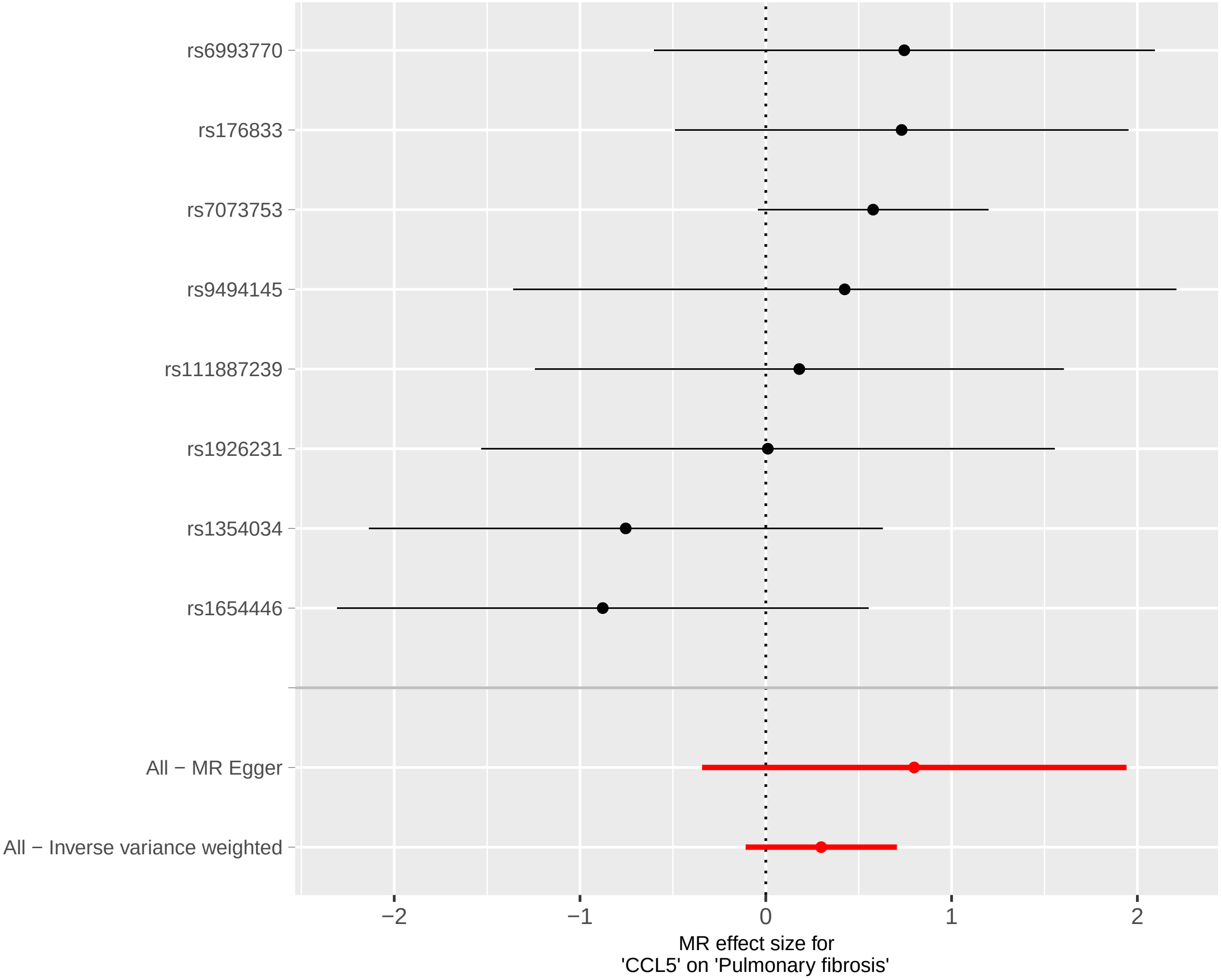

rs6993770
rs176833
rs7073753
rs9494145
rs111887239
rs1926231
rs1354034
rs1654446
All − MR Egger
All − Inverse variance weighted
−2
−1
0
1
2
MR effect size for
'CCL5' on 'Pulmonary fibrosis'

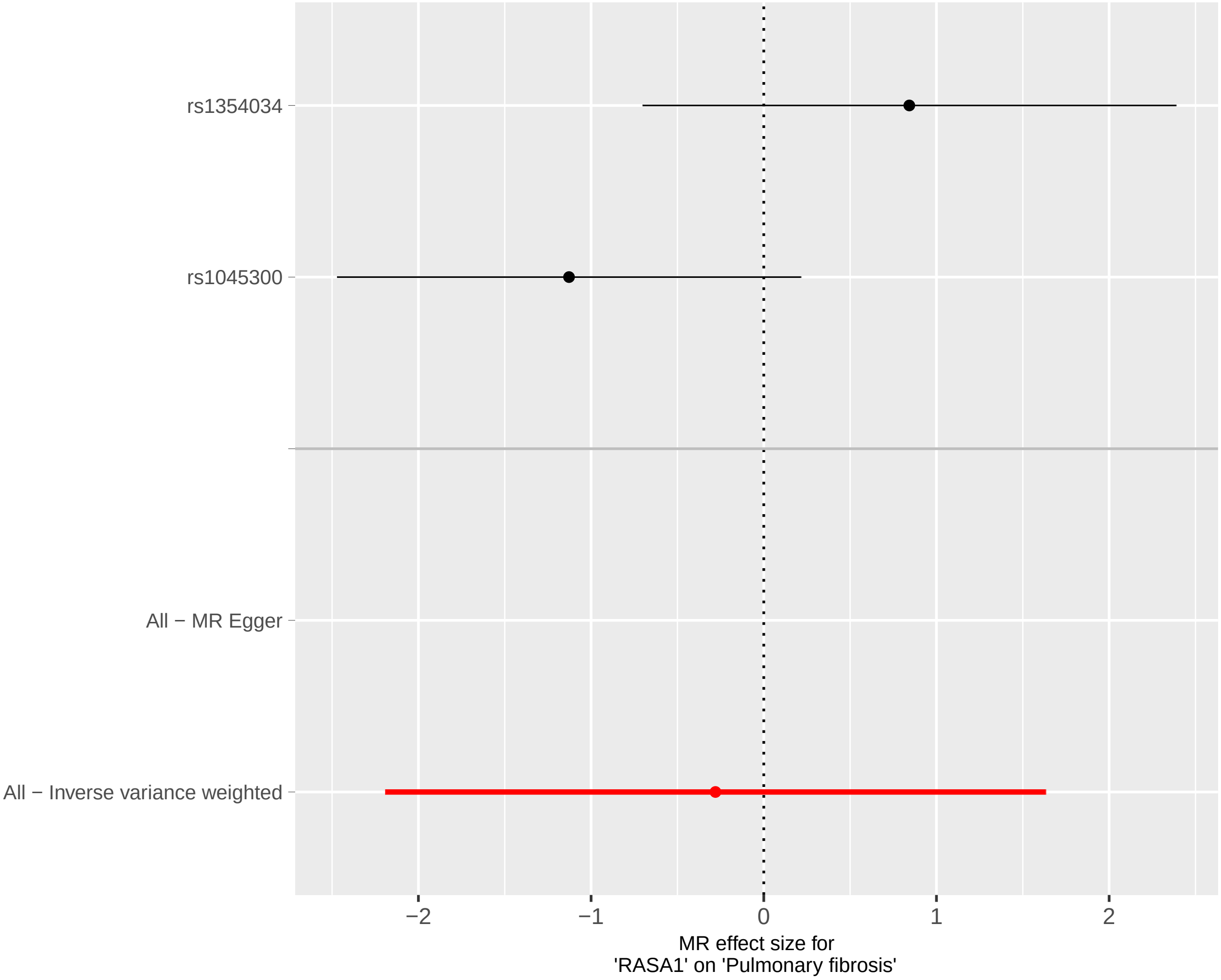
rs1354034
rs1045300
All − MR Egger
All − Inverse variance weighted
−2
−1
0
1
2
MR effect size for
'RASA1' on 'Pulmonary fibrosis'

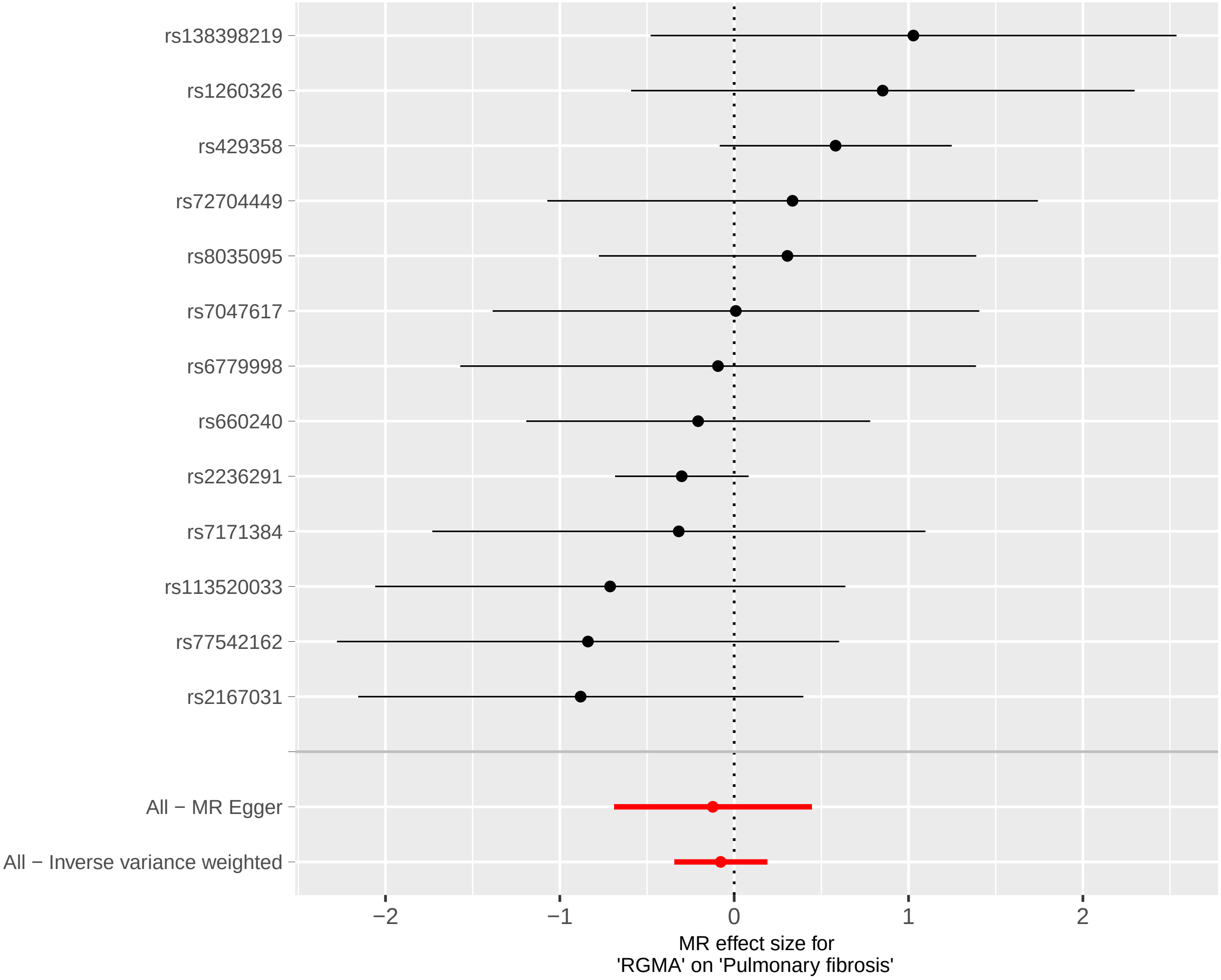

rs138398219
rs1260326
rs429358
rs72704449
rs8035095
rs7047617
rs6779998
rs660240
rs2236291
rs7171384
rs113520033
rs77542162
rs2167031
All − MR Egger
All − Inverse variance weighted
−2
−1
0
1
2
MR effect size for
'RGMA' on 'Pulmonary fibrosis'

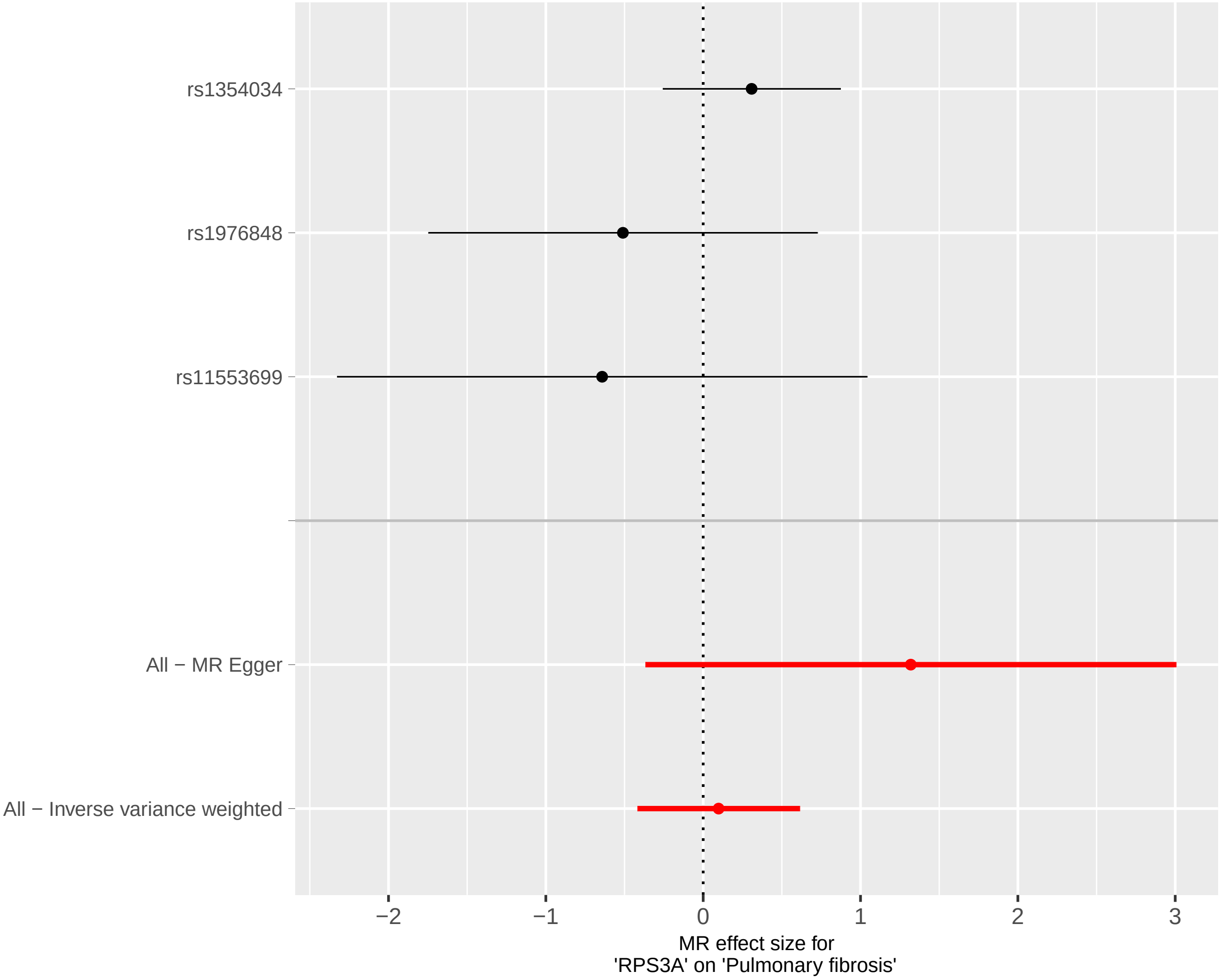

rs1354034
rs1976848
rs11553699
All − MR Egger
All − Inverse variance weighted
−2
−1
0
1
2
3
MR effect size for
'RPS3A' on 'Pulmonary fibrosis'

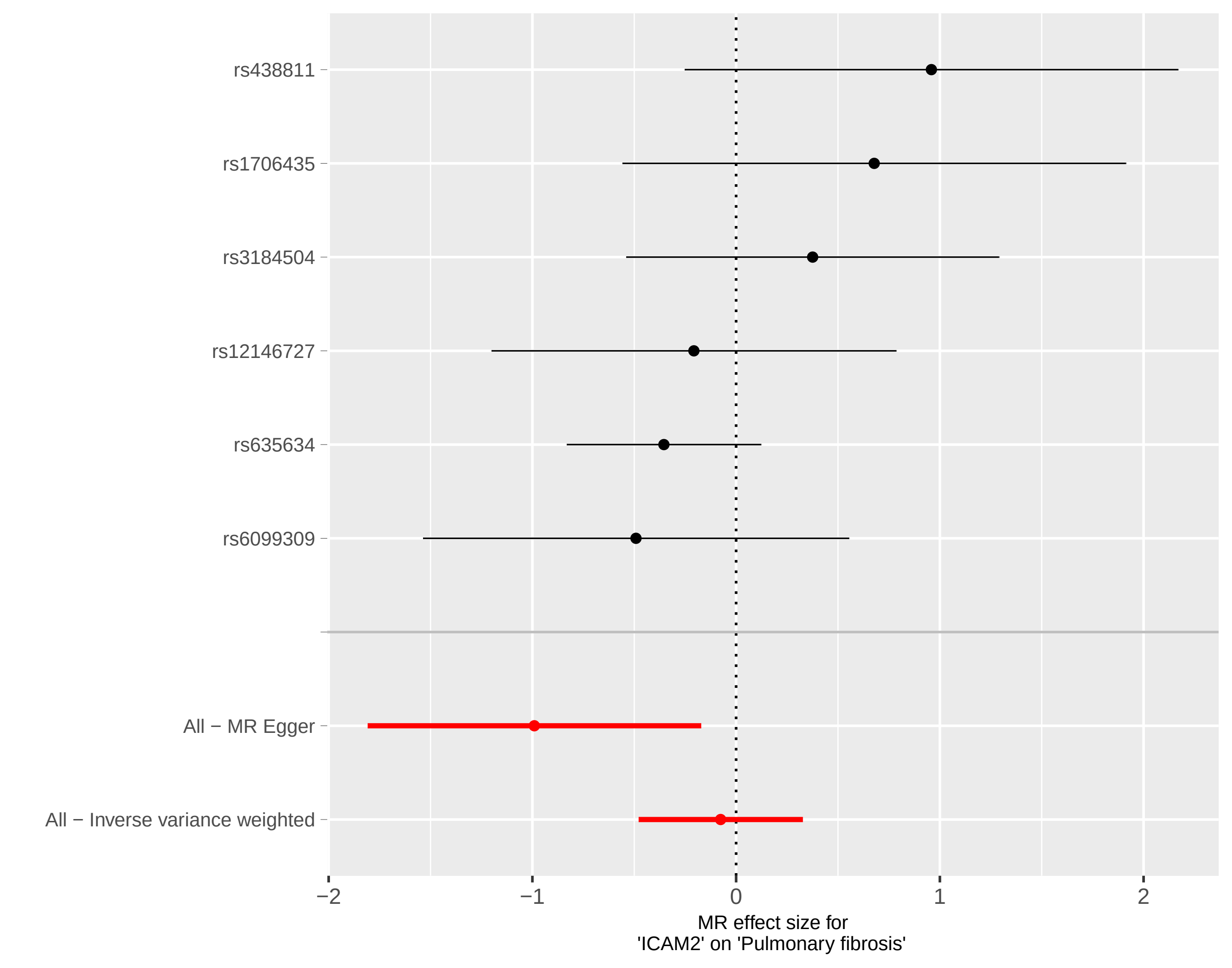
rs438811
rs1706435
rs3184504
rs12146727
rs635634
rs6099309
All − MR Egger
All − Inverse variance weighted
−2
−1
0
1
2
MR effect size for
'ICAM2' on 'Pulmonary fibrosis'

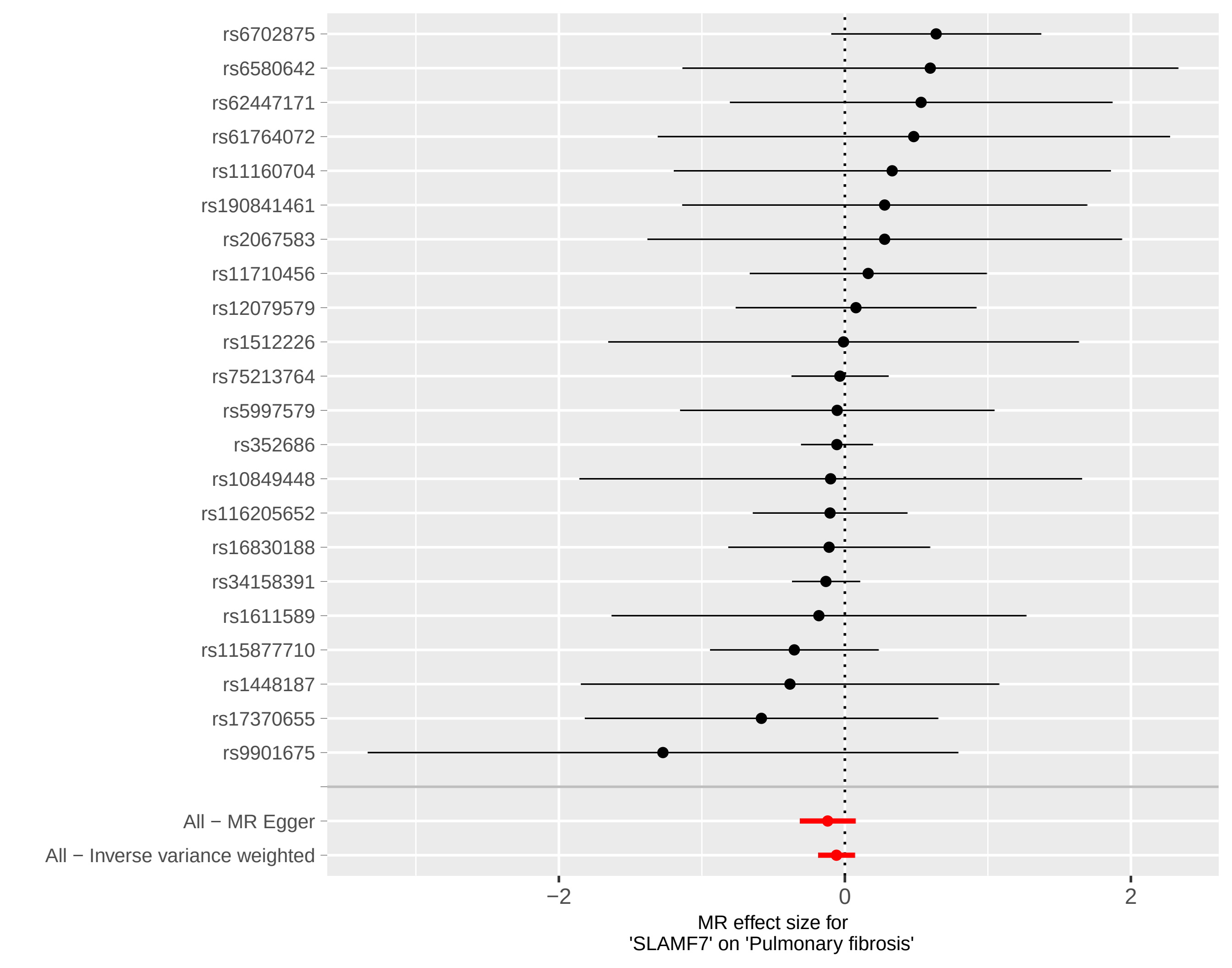

rs6702875
rs6580642
rs62447171
rs61764072
rs11160704
rs190841461
rs2067583
rs11710456
rs12079579
rs1512226
rs75213764
rs5997579
rs352686
rs10849448
rs116205652
rs16830188
rs34158391
rs1611589
rs115877710
rs1448187
rs17370655
rs9901675
All − MR Egger
All − Inverse variance weighted
−2
0
2
MR effect size for
'SLAMF7' on 'Pulmonary fibrosis'

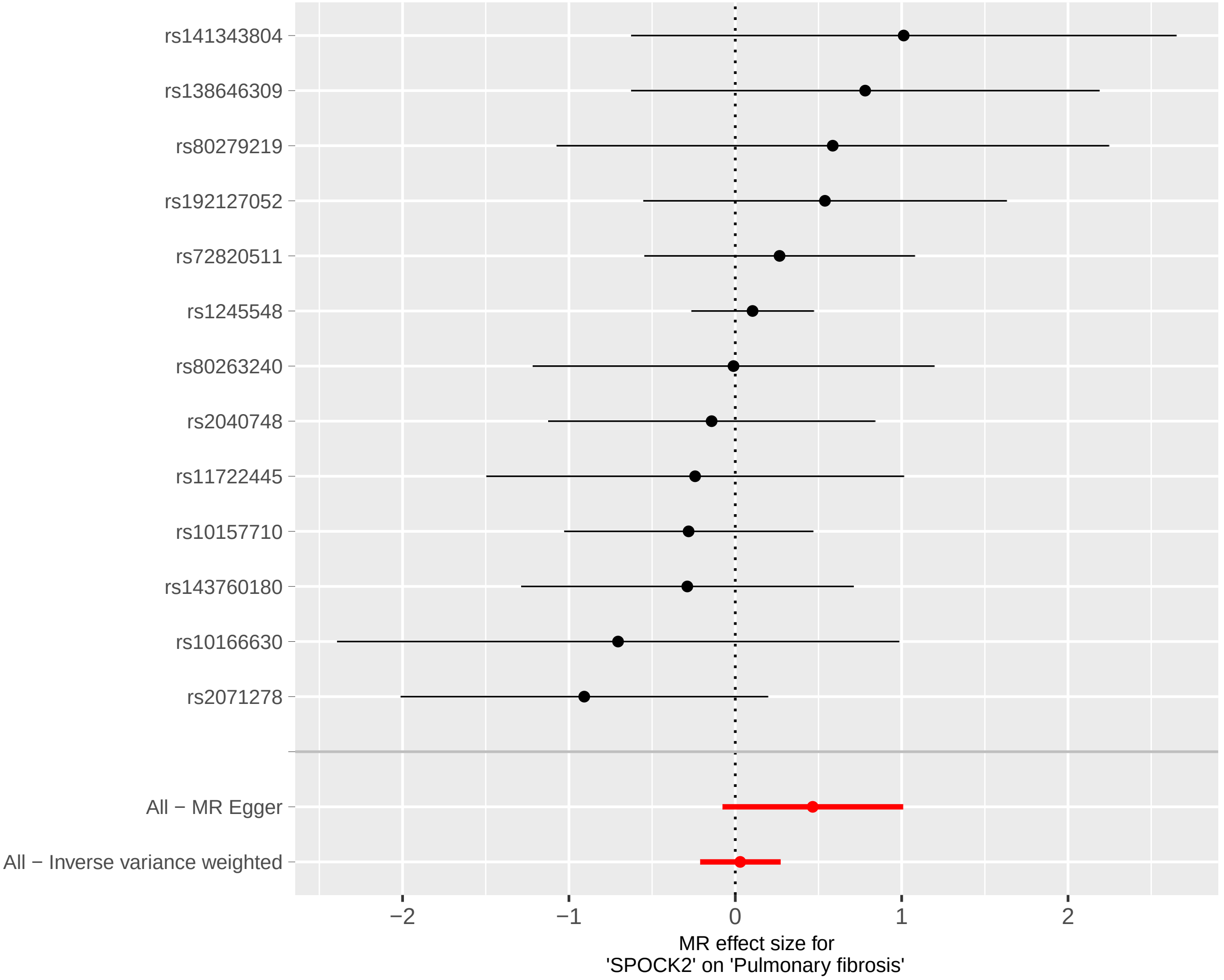

rs141343804
rs138646309
rs80279219
rs192127052
rs72820511
rs1245548
rs80263240
rs2040748
rs11722445
rs10157710
rs143760180
rs10166630
rs2071278
All − MR Egger
All − Inverse variance weighted
−2
−1
0
1
2
MR effect size for
'SPOCK2' on 'Pulmonary fibrosis'

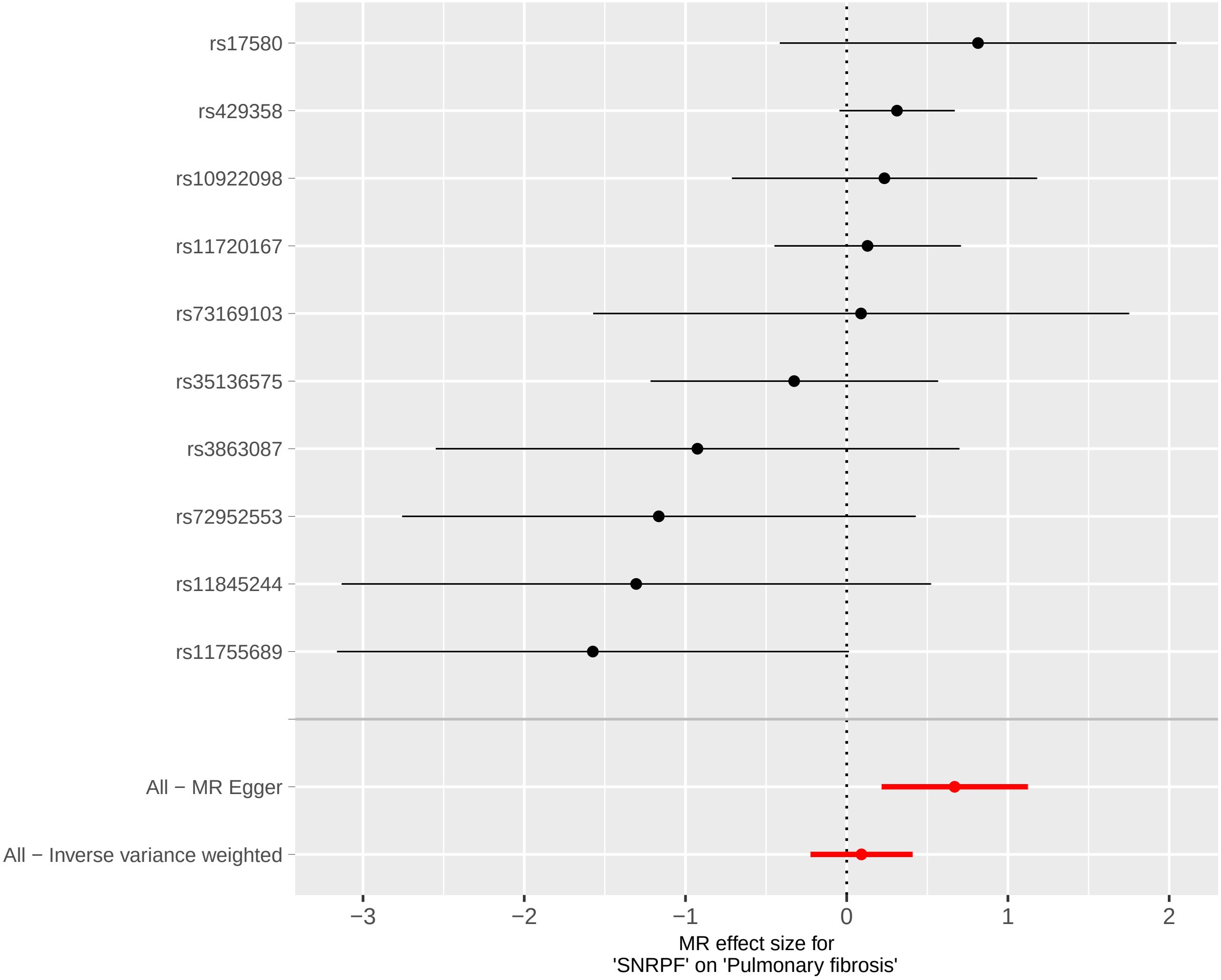

rs17580
rs429358
rs10922098
rs11720167
rs73169103
rs35136575
rs3863087
rs72952553
rs11845244
rs11755689
All − MR Egger
All − Inverse variance weighted
−3
−2
−1
0
1
2
MR effect size for
'SNRPF' on 'Pulmonary fibrosis'

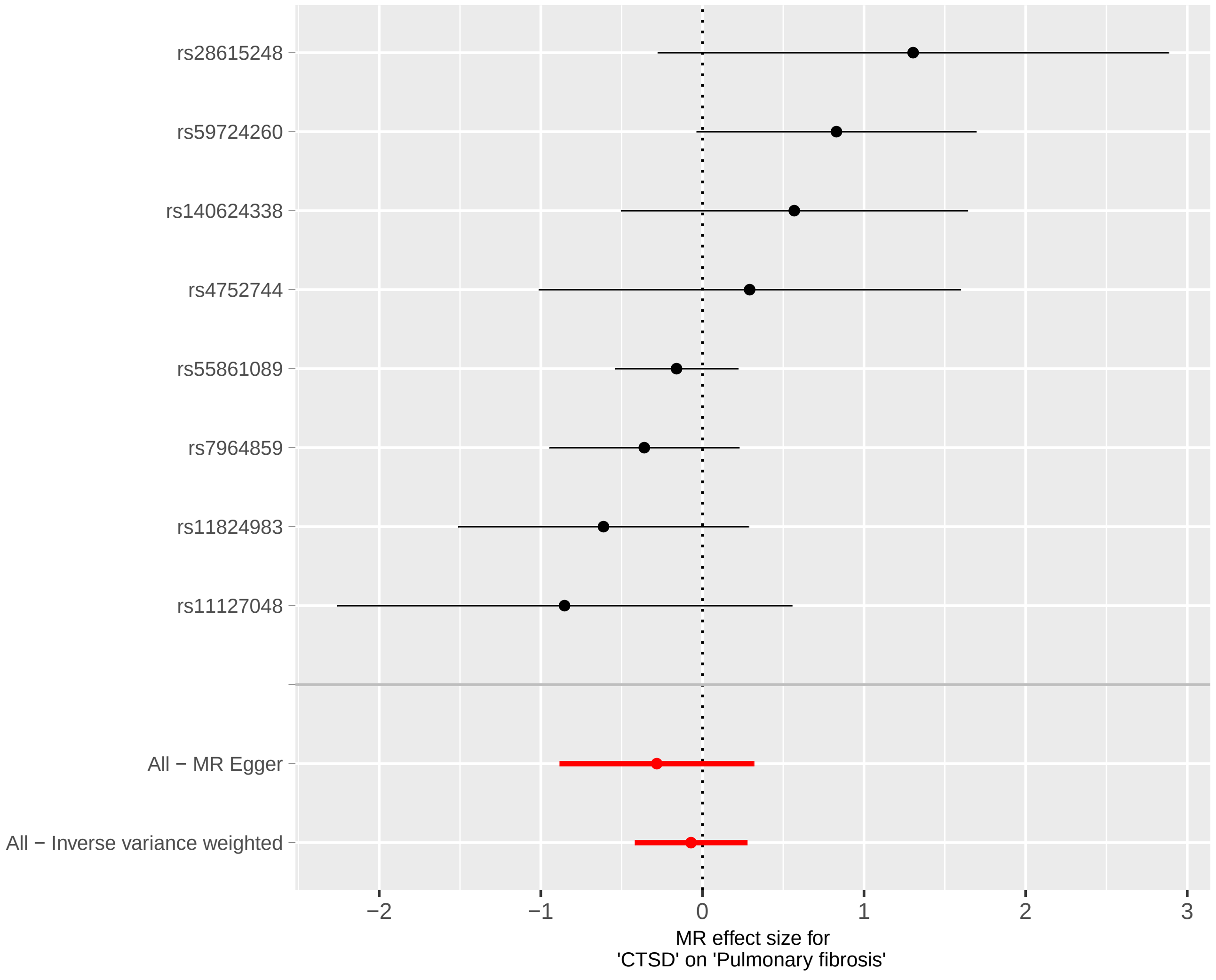
rs28615248
rs59724260
rs140624338
rs4752744
rs55861089
rs7964859
rs11824983
rs11127048
All − MR Egger
All − Inverse variance weighted
−2
−1
0
1
2
3
MR effect size for
'CTSD' on 'Pulmonary fibrosis'

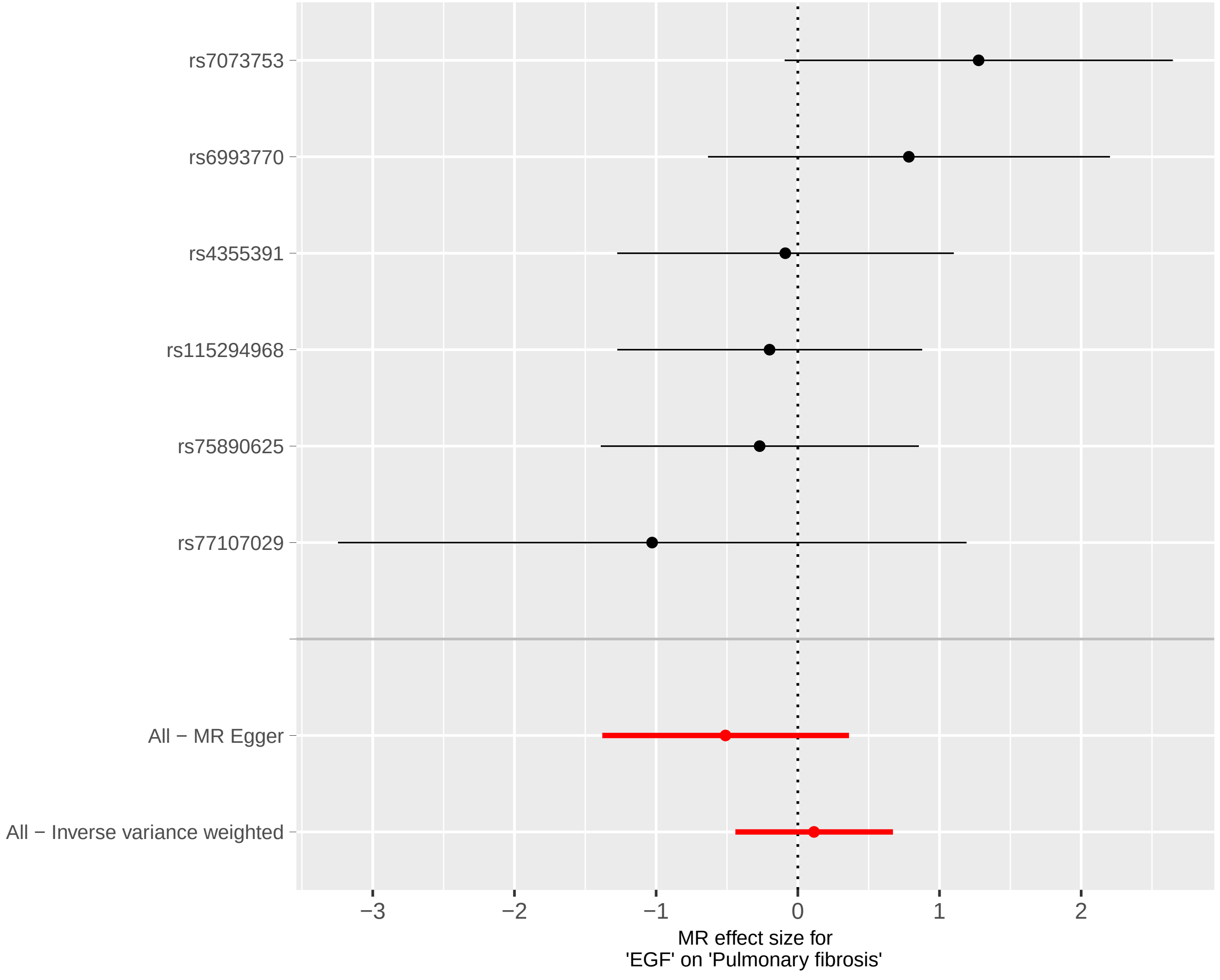

rs7073753
rs6993770
rs4355391
rs115294968
rs75890625
rs77107029
All − MR Egger
All − Inverse variance weighted
−3
−2
−1
0
1
2
MR effect size for
'EGF' on 'Pulmonary fibrosis'

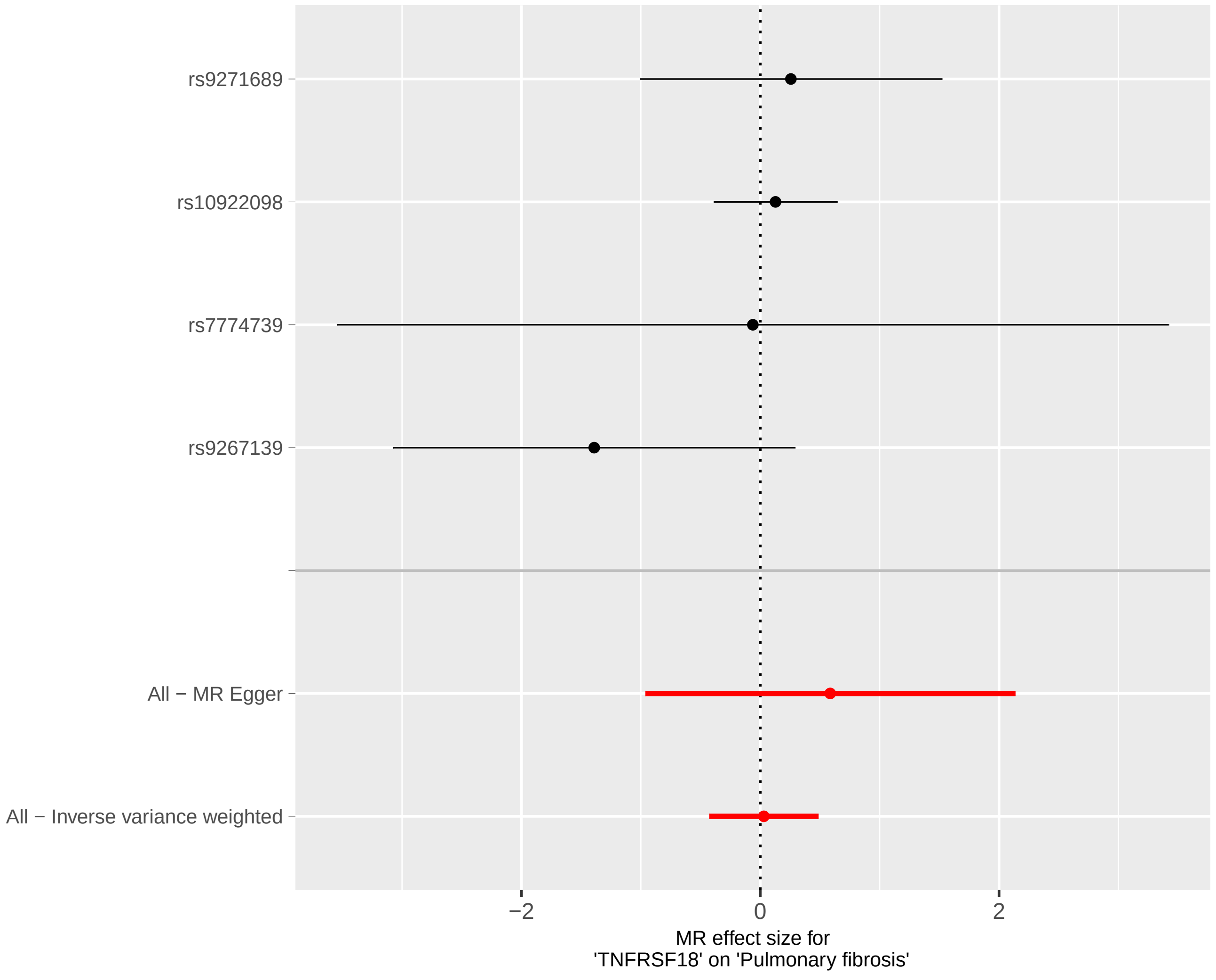

rs9271689
rs10922098
rs7774739
rs9267139
All − MR Egger
All − Inverse variance weighted
−2
0
2
MR effect size for
'TNFRSF18' on 'Pulmonary fibrosis'

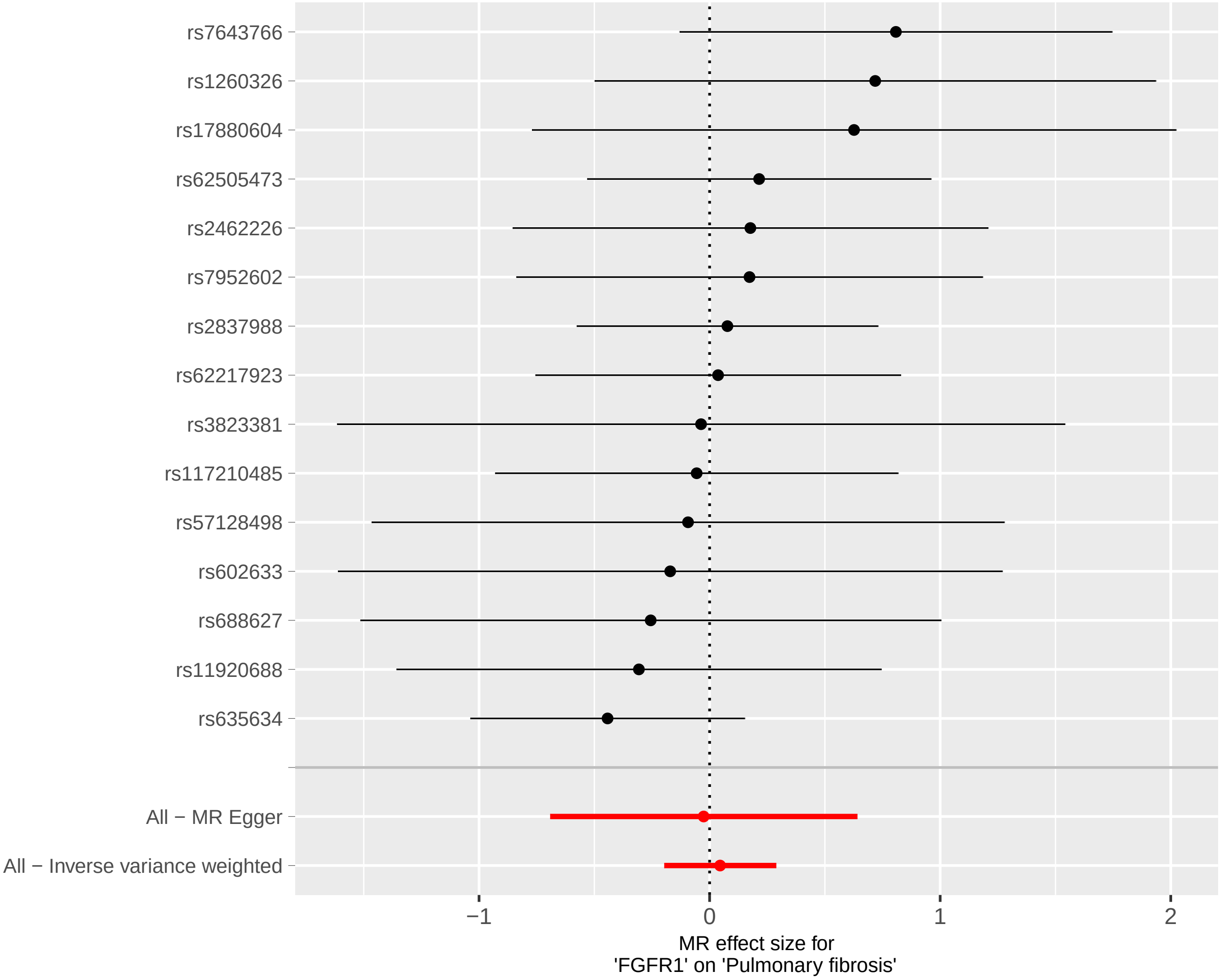
rs7643766
rs1260326
rs17880604
rs62505473
rs2462226
rs7952602
rs2837988
rs62217923
rs3823381
rs117210485
rs57128498
rs602633
rs688627
rs11920688
rs635634
All − MR Egger
All − Inverse variance weighted
−1
0
1
2
MR effect size for
'FGFR1' on 'Pulmonary fibrosis'

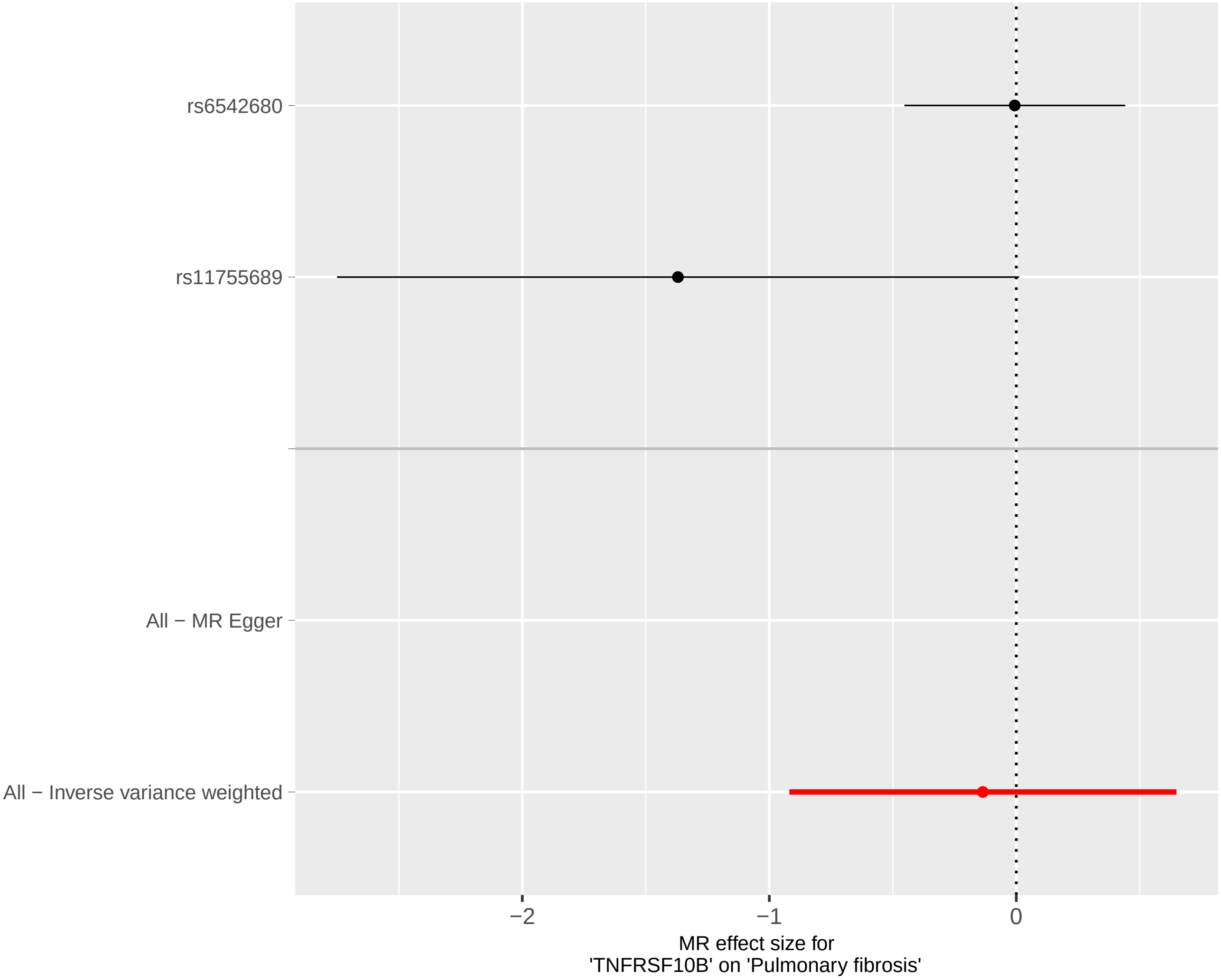
rs6542680
rs11755689
All − MR Egger
All − Inverse variance weighted
−2
−1
0
MR effect size for
'TNFRSF10B' on 'Pulmonary fibrosis'

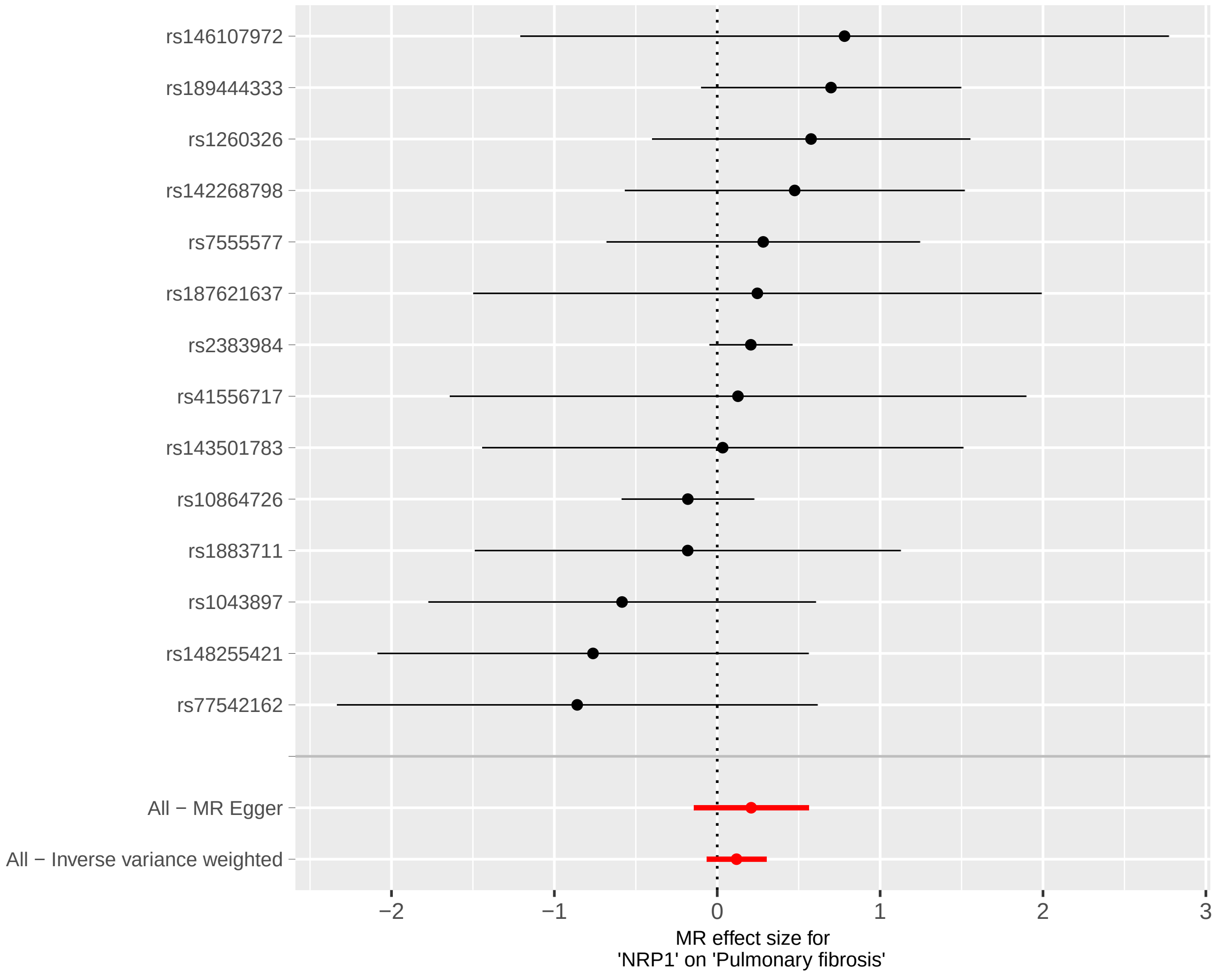

rs146107972
rs189444333
rs1260326
rs142268798
rs7555577
rs187621637
rs2383984
rs41556717
rs143501783
rs10864726
rs1883711
rs1043897
rs148255421
rs77542162
All − MR Egger
All − Inverse variance weighted
−2
−1
0
1
2
3
MR effect size for
'NRP1' on 'Pulmonary fibrosis'

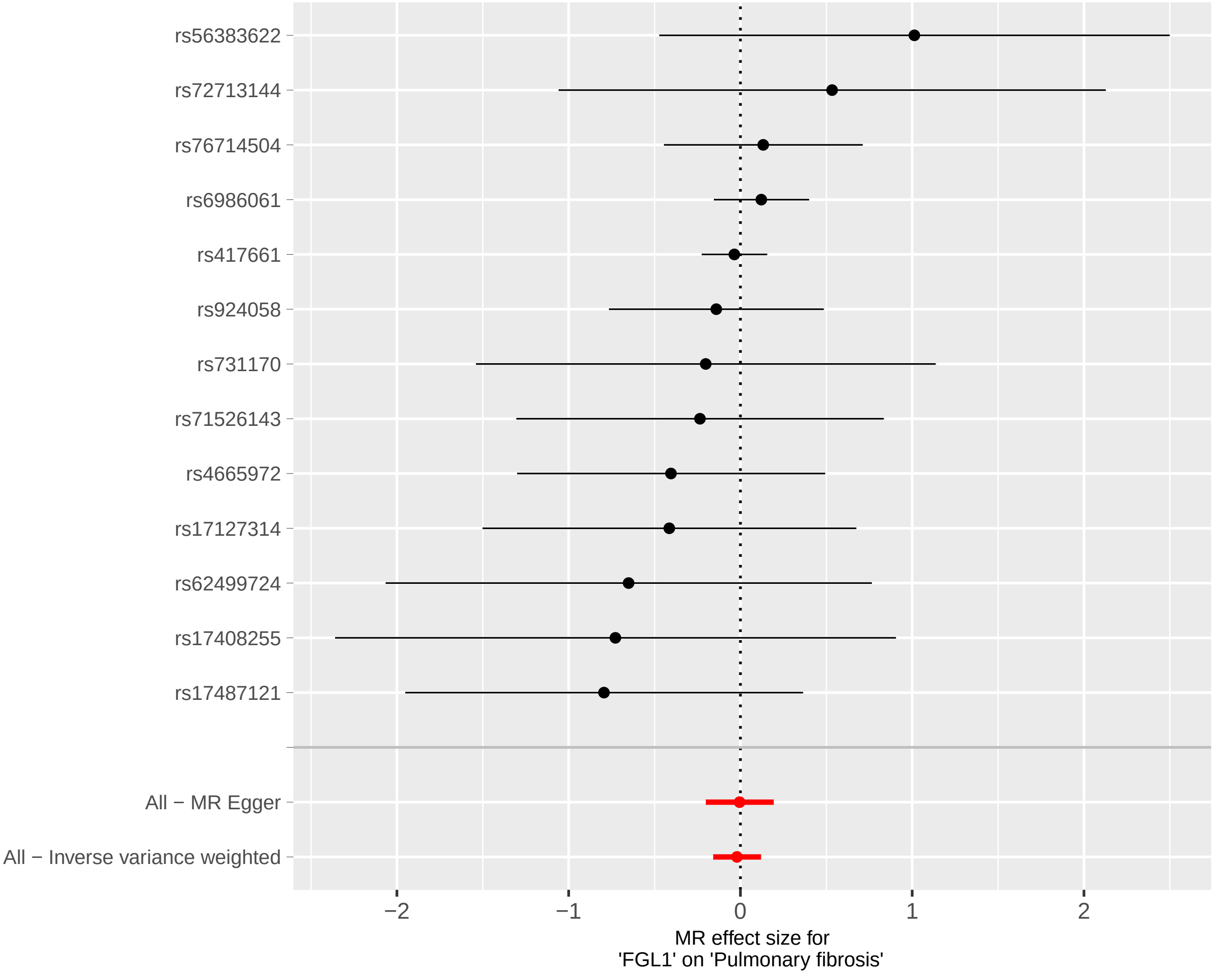

rs56383622
rs72713144
rs76714504
rs6986061
rs417661
rs924058
rs731170
rs71526143
rs4665972
rs17127314
rs62499724
rs17408255
rs17487121
All − MR Egger
All − Inverse variance weighted
−2
−1
0
1
2
MR effect size for
'FGL1' on 'Pulmonary fibrosis'

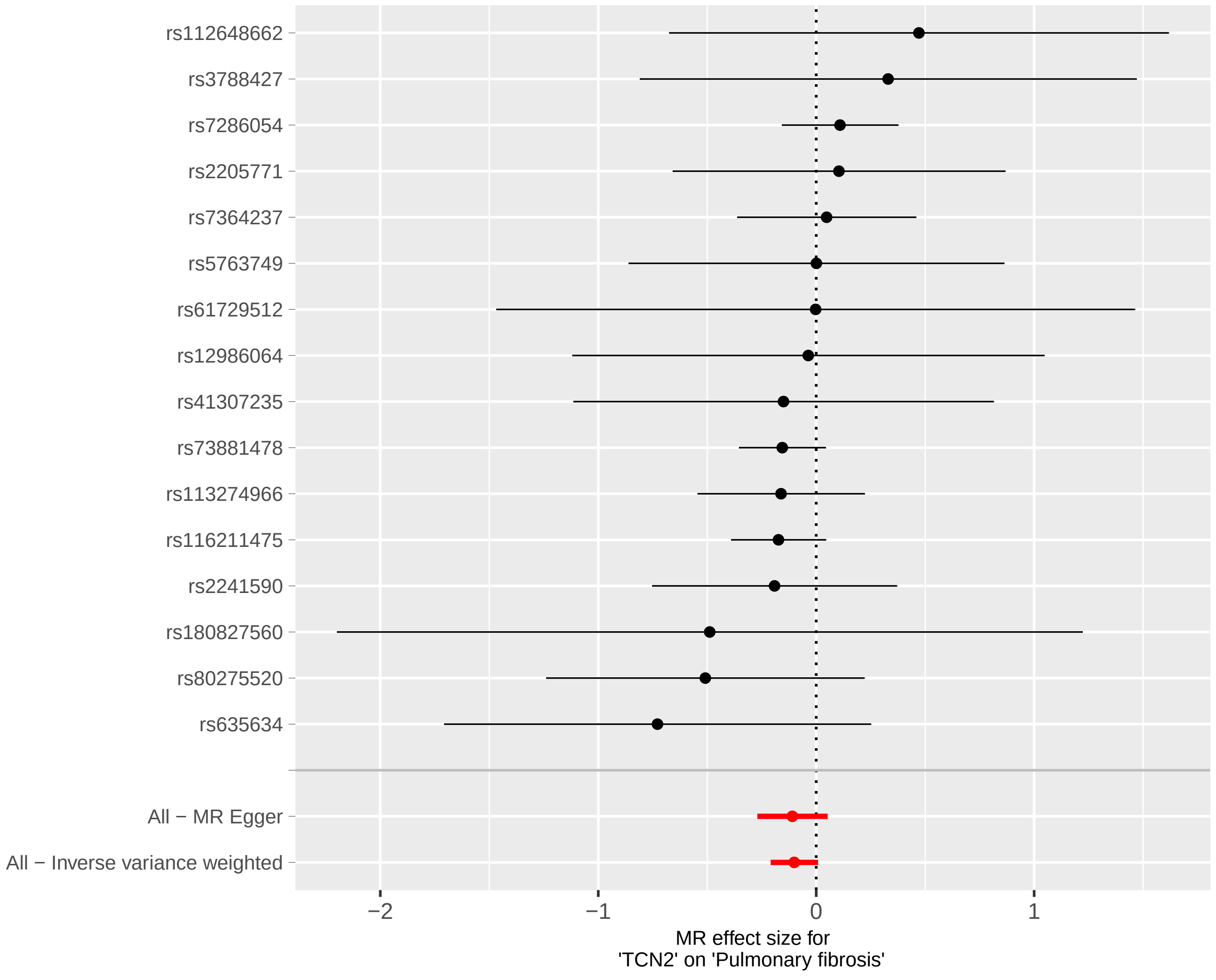
rs112648662
rs3788427
rs7286054
rs2205771
rs7364237
rs5763749
rs61729512
rs12986064
rs41307235
rs73881478
rs113274966
rs116211475
rs2241590
rs180827560
rs80275520
rs635634
All − MR Egger
All − Inverse variance weighted
−2
−1
0
1
MR effect size for
'TCN2' on 'Pulmonary fibrosis'

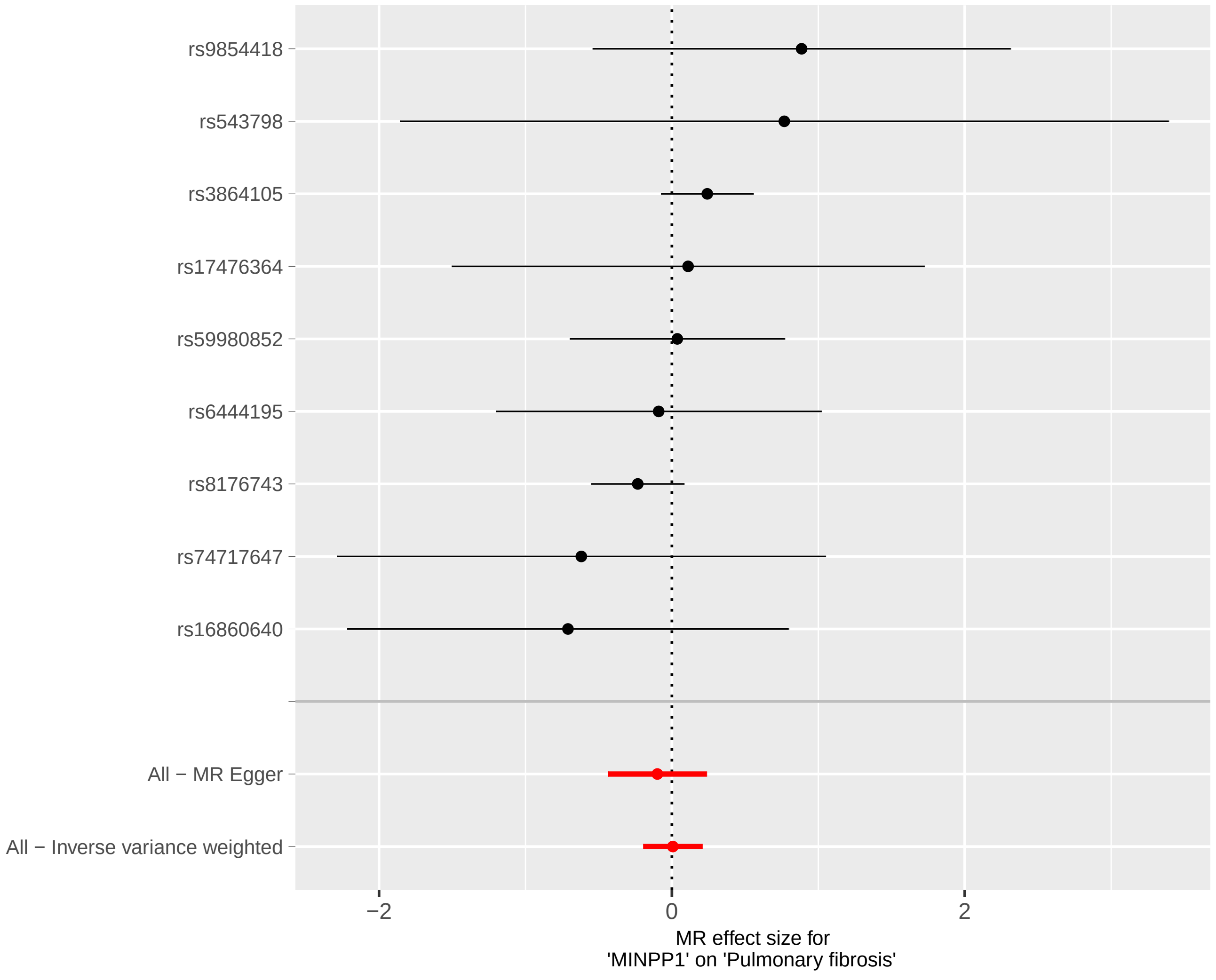
rs9854418
rs543798
rs3864105
rs17476364
rs59980852
rs6444195
rs8176743
rs74717647
rs16860640
All − MR Egger
All − Inverse variance weighted
−2
0
2
MR effect size for
'MINPP1' on 'Pulmonary fibrosis'

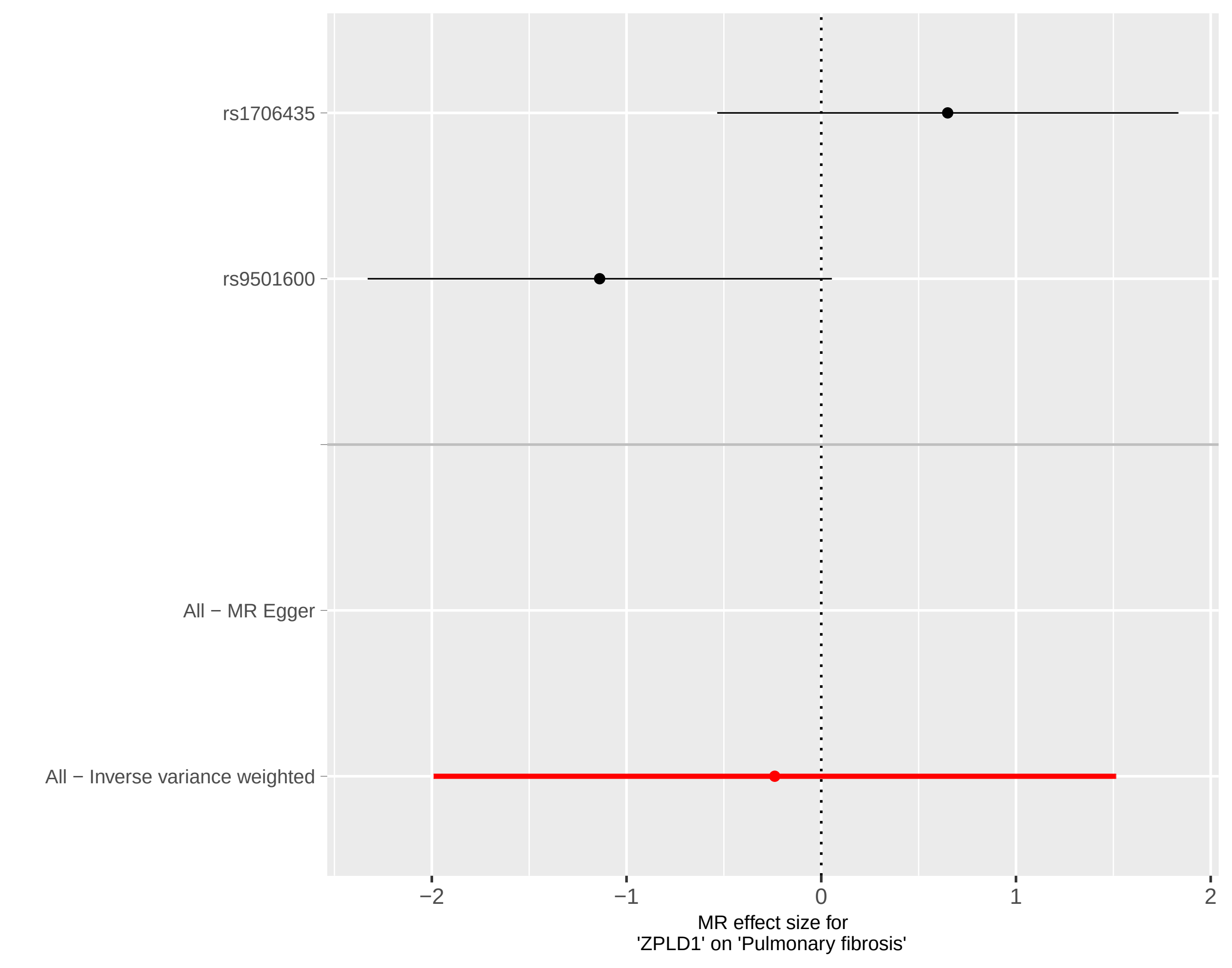
rs1706435
rs9501600
All − MR Egger
All − Inverse variance weighted
−2
−1
0
1
2
MR effect size for
'ZPLD1' on 'Pulmonary fibrosis'

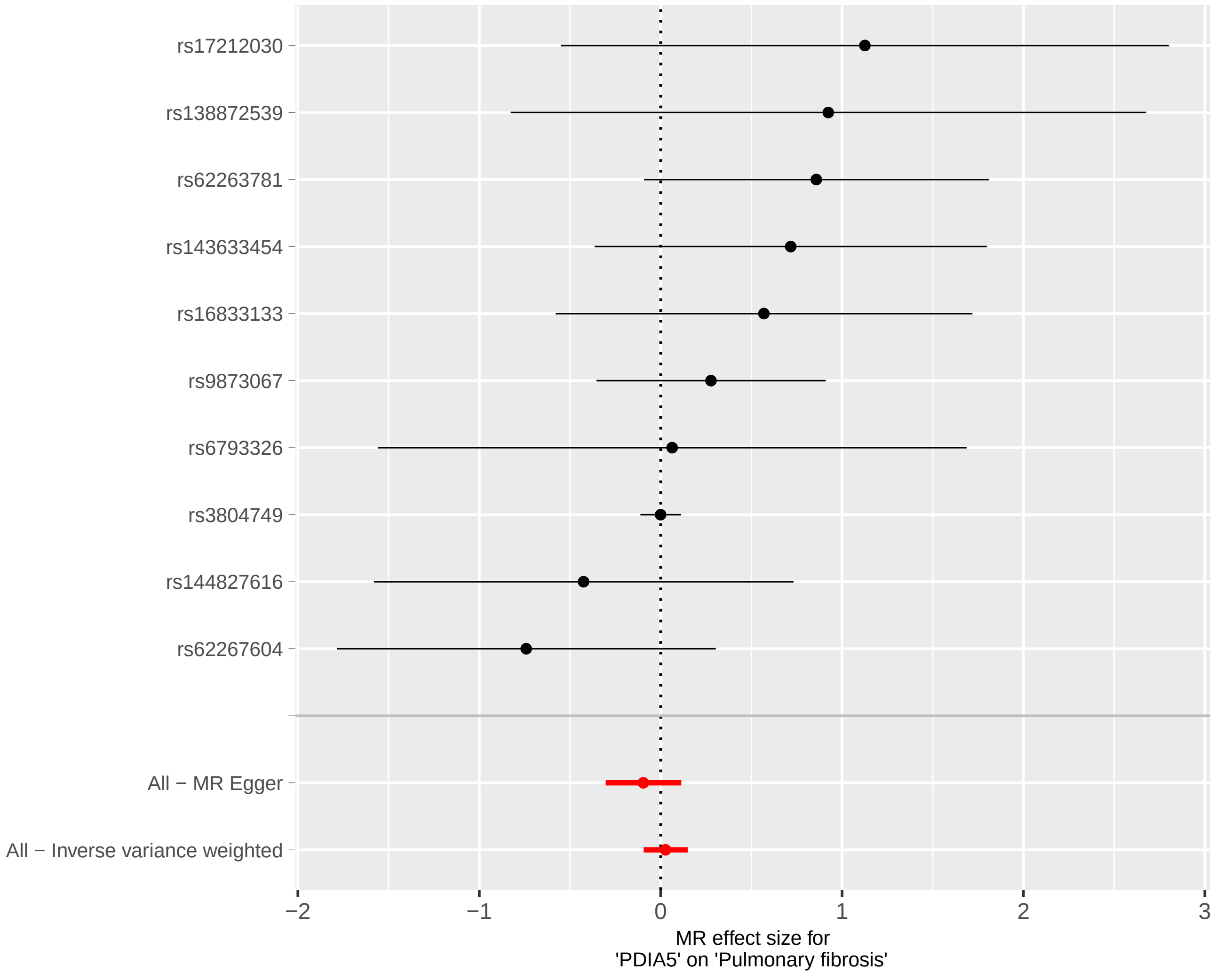
rs17212030
rs138872539
rs62263781
rs143633454
rs16833133
rs9873067
rs6793326
rs3804749
rs144827616
rs62267604
All − MR Egger
All − Inverse variance weighted
−2
−1
0
1
2
3
MR effect size for
'PDIA5' on 'Pulmonary fibrosis'

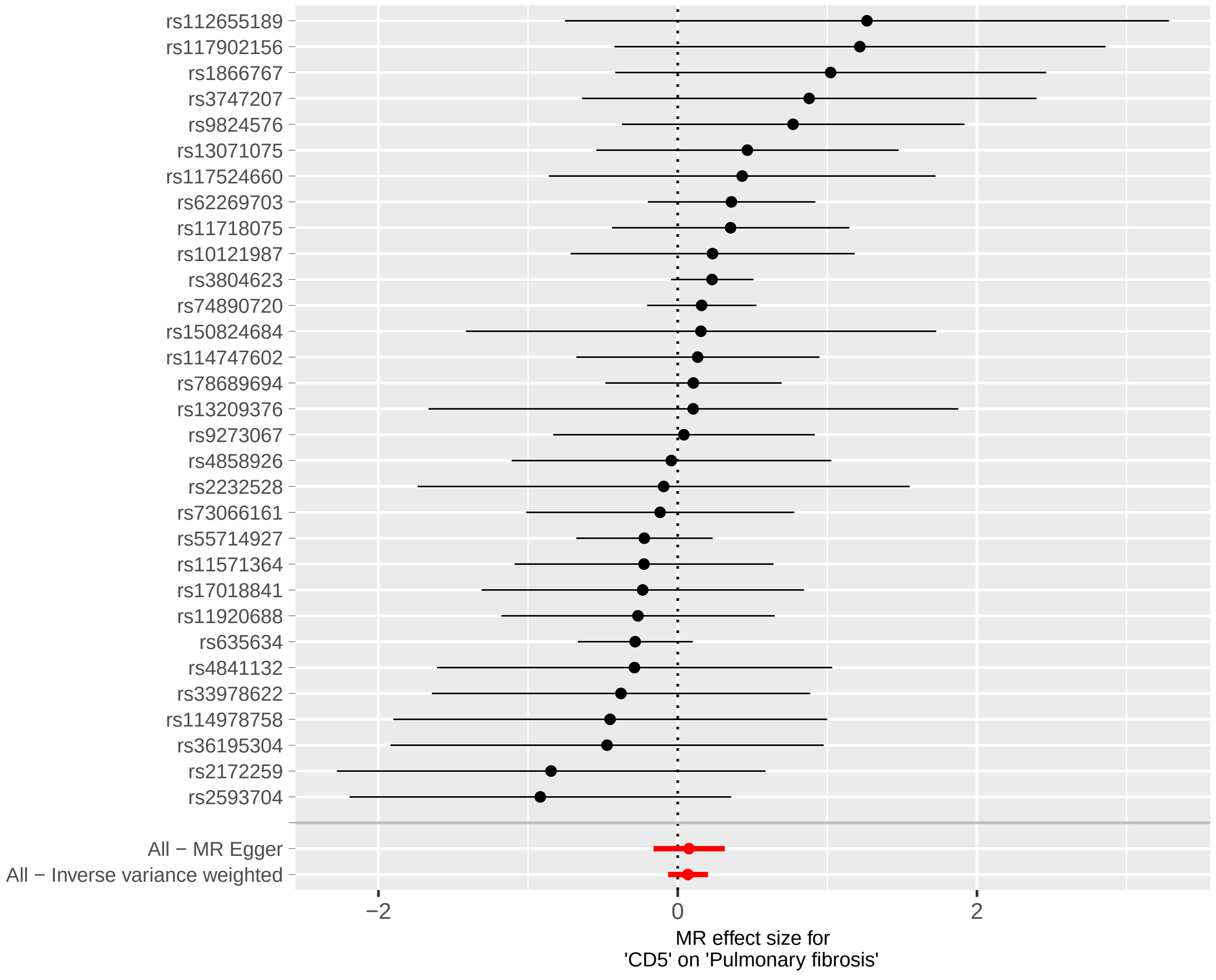

rs112655189
rs117902156
rs1866767
rs3747207
rs9824576
rs13071075
rs117524660
rs62269703
rs11718075
rs10121987
rs3804623
rs74890720
rs150824684
rs114747602
rs78689694
rs13209376
rs9273067
rs4858926
rs2232528
rs73066161
rs55714927
rs11571364
rs17018841
rs11920688
rs635634
rs4841132
rs33978622
rs114978758
rs36195304
rs2172259
rs2593704
All − MR Egger
All − Inverse variance weighted
−2
0
2
MR effect size for
'CD5' on 'Pulmonary fibrosis'

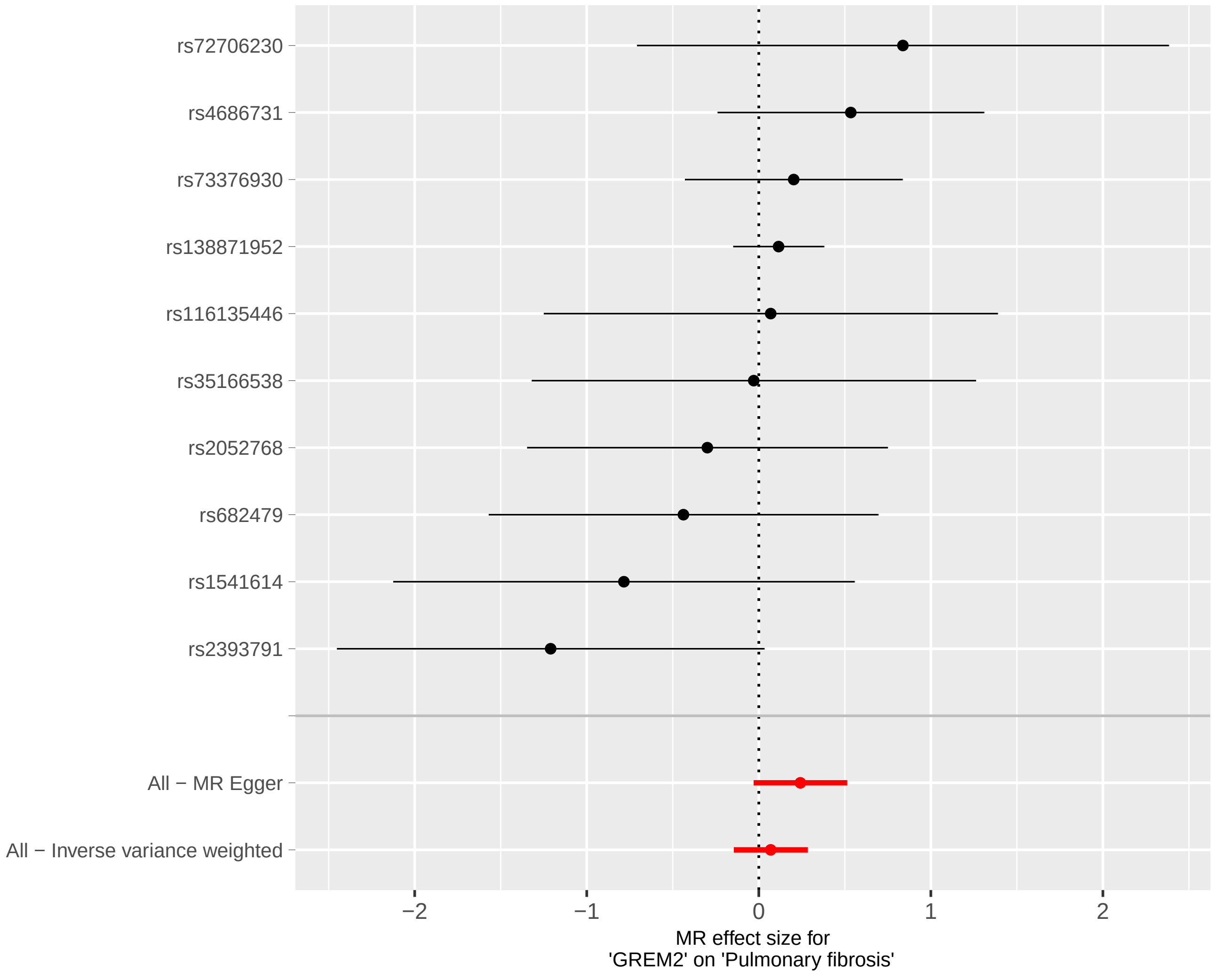
rs72706230
rs4686731
rs73376930
rs138871952
rs116135446
rs35166538
rs2052768
rs682479
rs1541614
rs2393791
All − MR Egger
All − Inverse variance weighted
−2
−1
0
1
2
MR effect size for
'GREM2' on 'Pulmonary fibrosis'

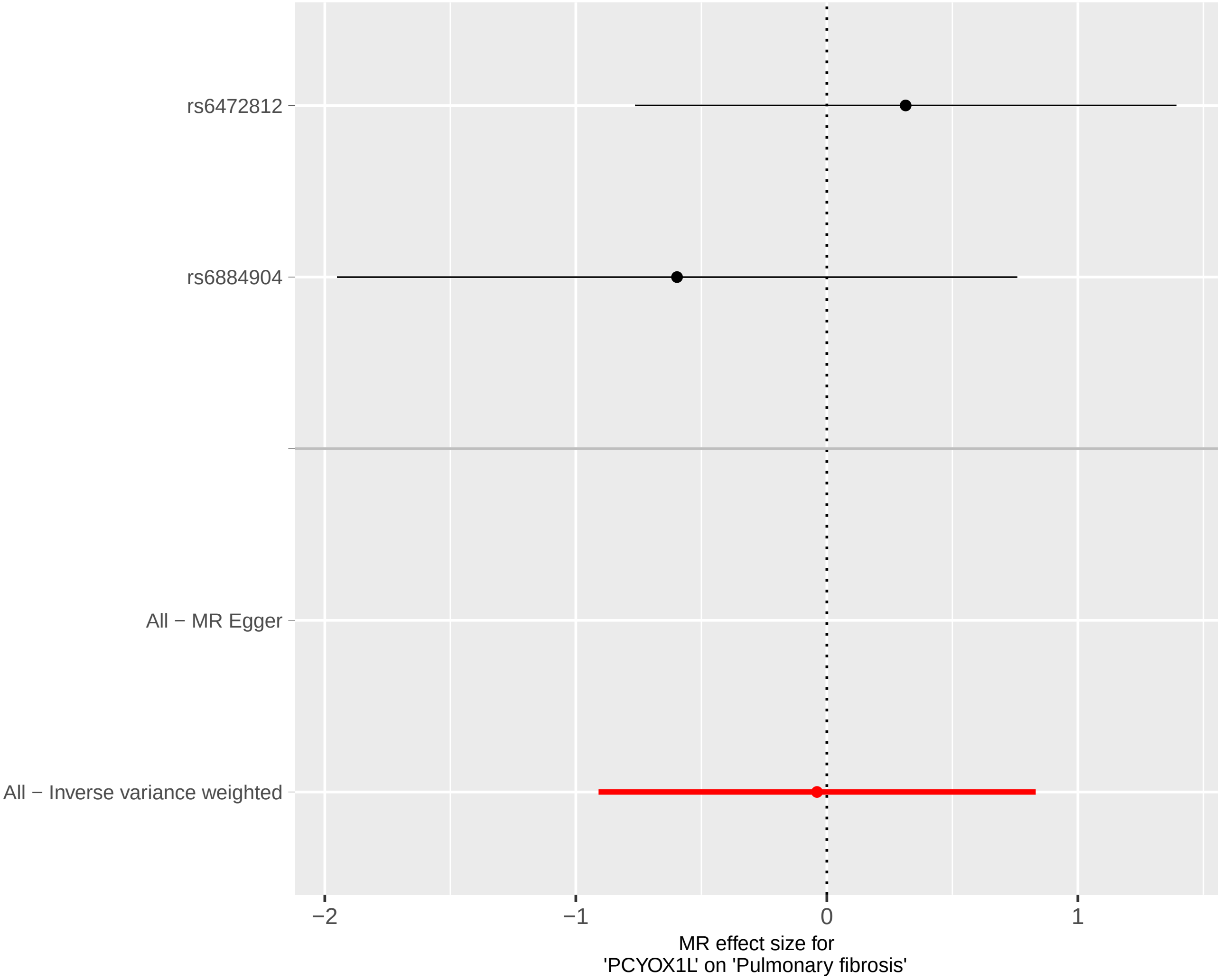

rs6472812
rs6884904
All − MR Egger
All − Inverse variance weighted
−2
−1
0
1
MR effect size for
'PCYOX1L' on 'Pulmonary fibrosis'

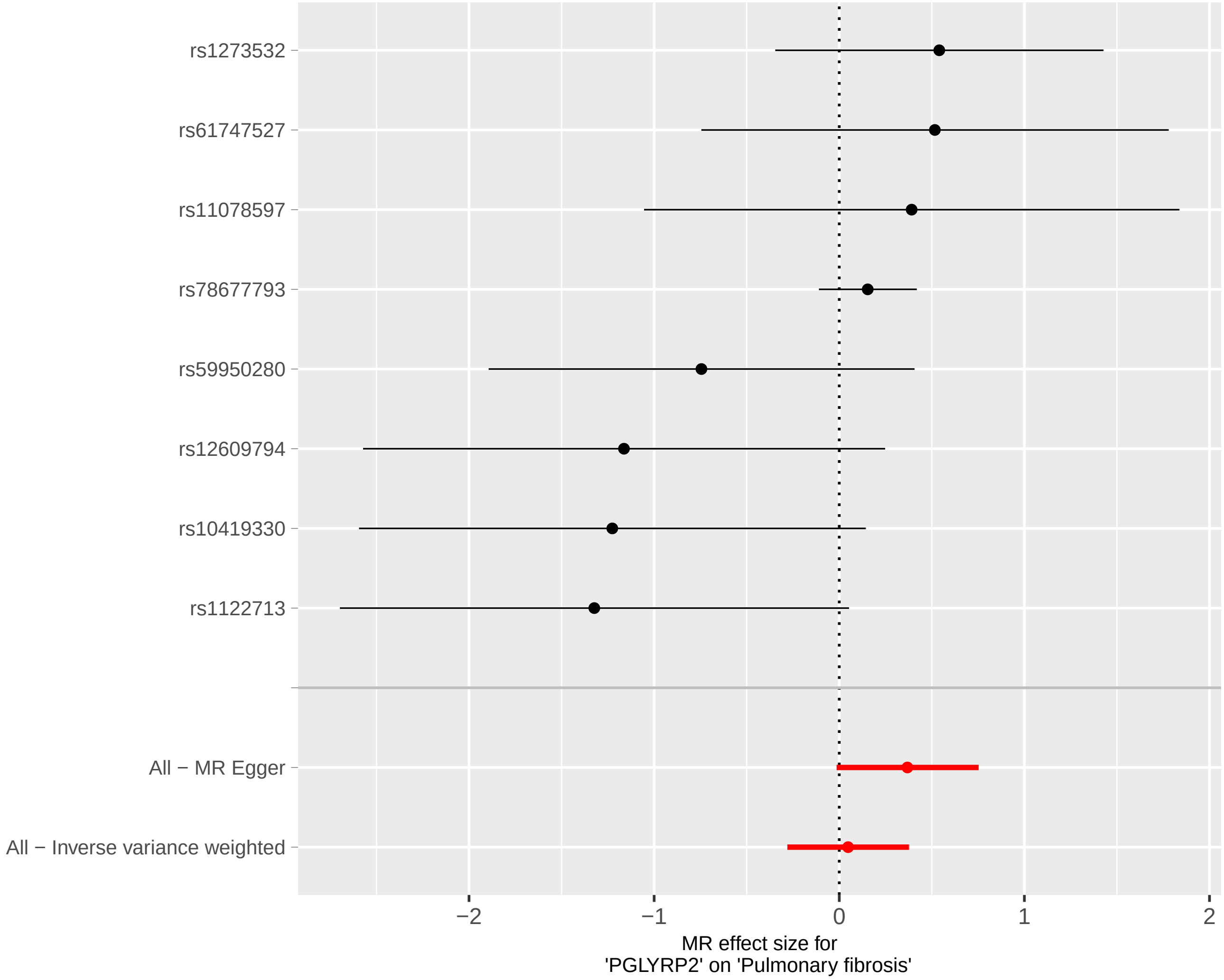
rs1273532
rs61747527
rs11078597
rs78677793
rs59950280
rs12609794
rs10419330
rs1122713
All − MR Egger
All − Inverse variance weighted
−2
−1
0
1
2
MR effect size for
'PGLYRP2' on 'Pulmonary fibrosis'

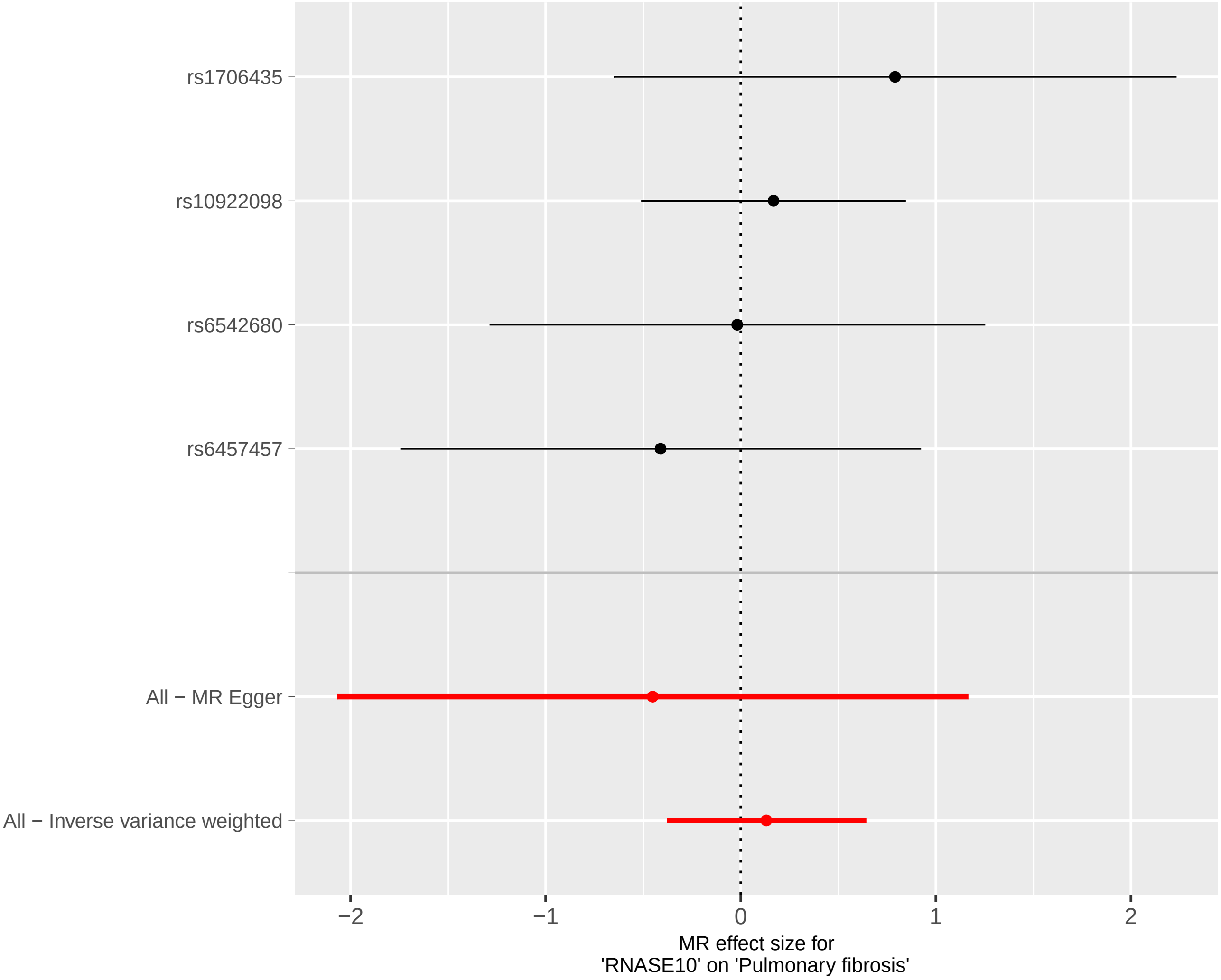
rs1706435
rs10922098
rs6542680
rs6457457
All − MR Egger
All − Inverse variance weighted
−2
−1
0
1
2
MR effect size for
'RNASE10' on 'Pulmonary fibrosis'

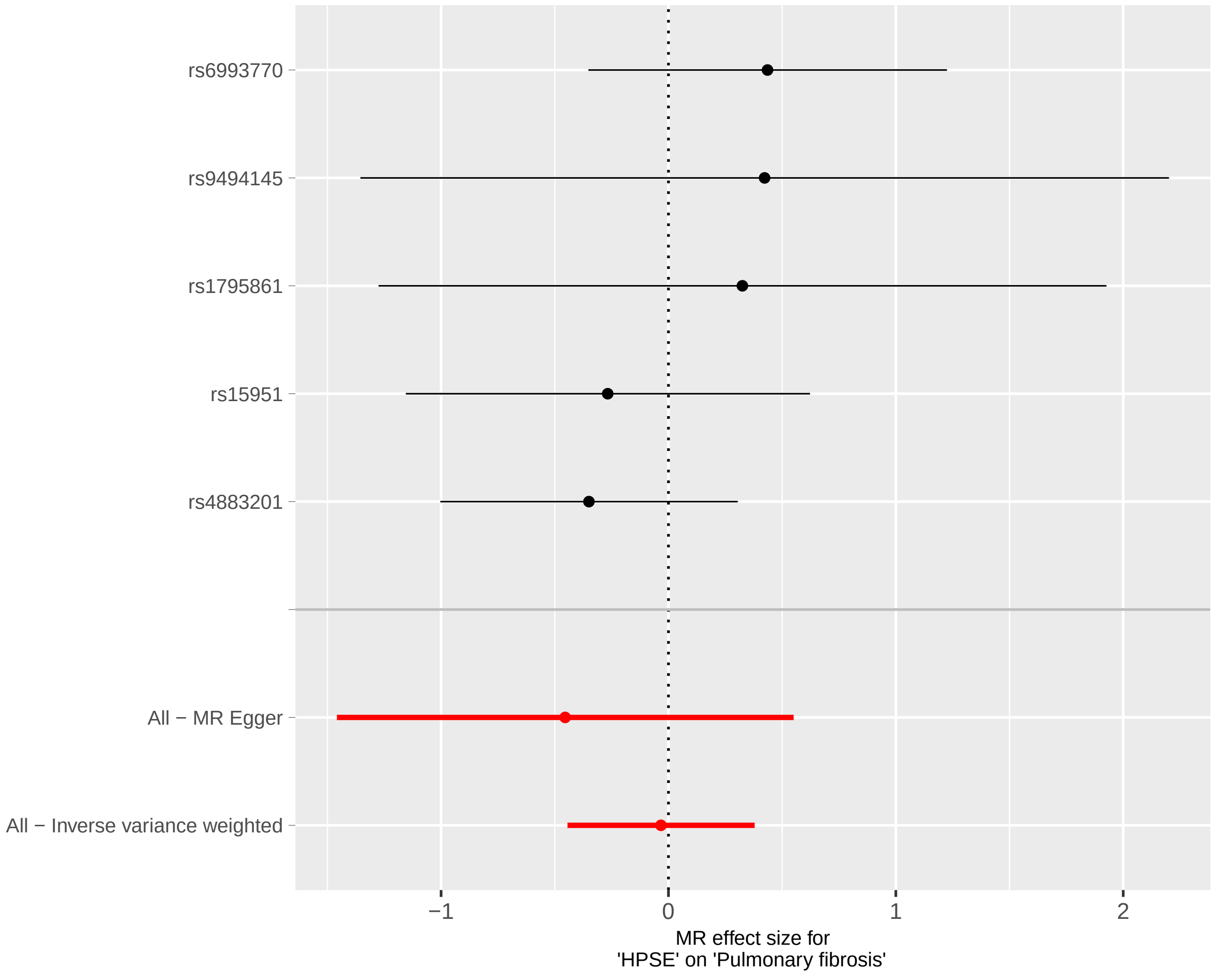
rs6993770
rs9494145
rs1795861
rs15951
rs4883201
All − MR Egger
All − Inverse variance weighted
−1
0
1
2
MR effect size for
'HPSE' on 'Pulmonary fibrosis'

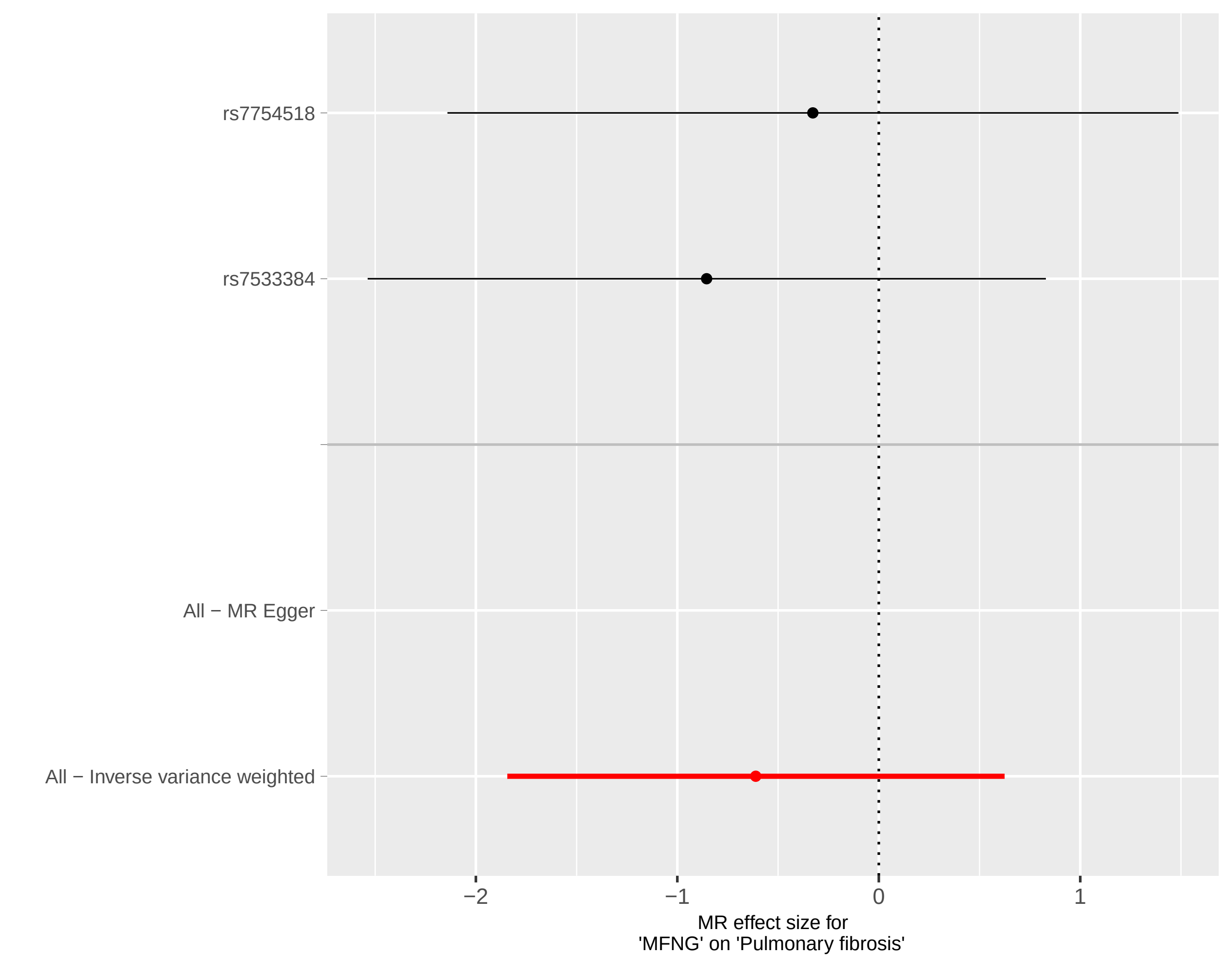

rs7754518
rs7533384
All − MR Egger
All − Inverse variance weighted
−2
−1
0
1
MR effect size for
'MFNG' on 'Pulmonary fibrosis'

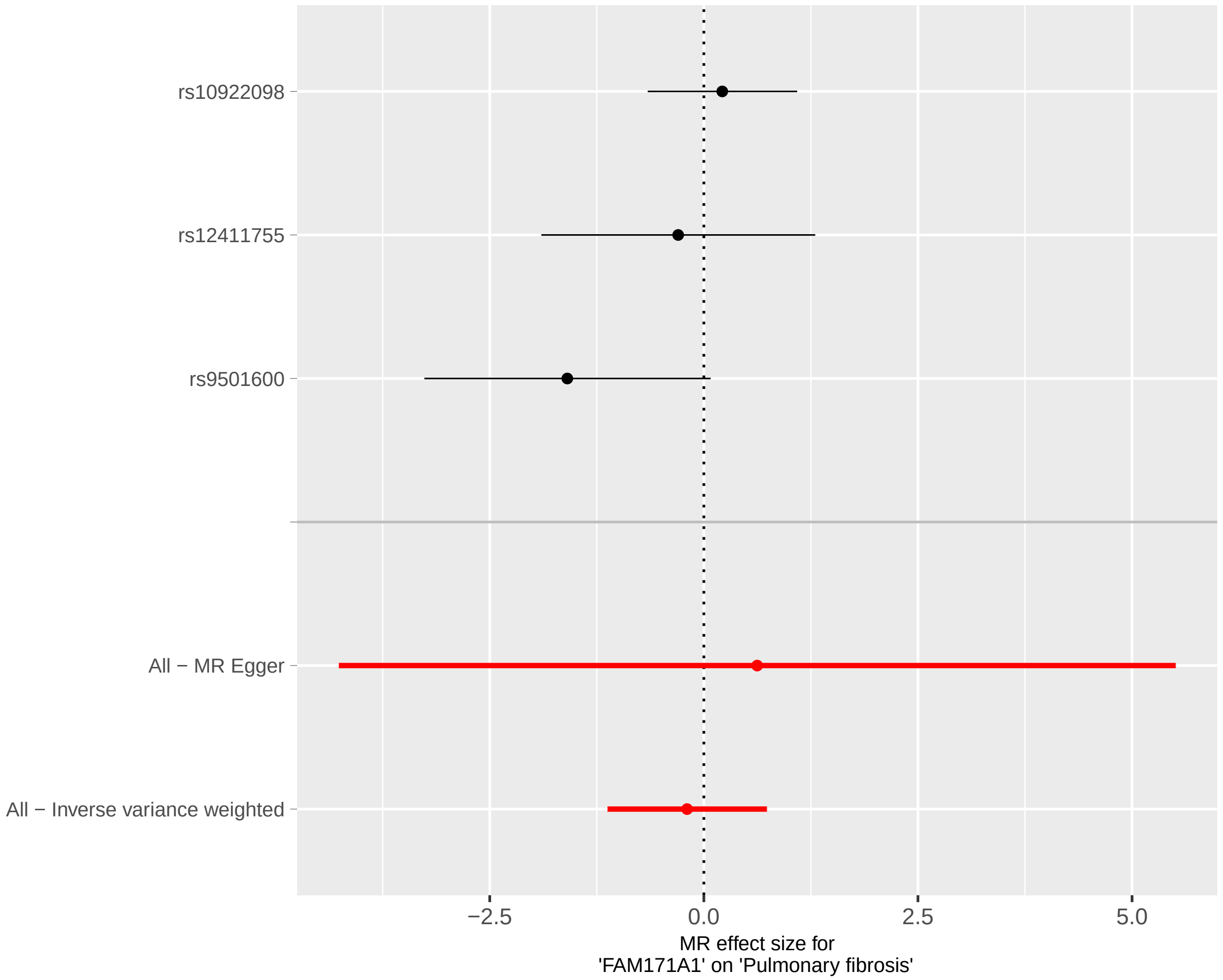

rs10922098
rs12411755
rs9501600
All − MR Egger
All − Inverse variance weighted
−2.5
0.0
2.5
5.0
MR effect size for
'FAM171A1' on 'Pulmonary fibrosis'

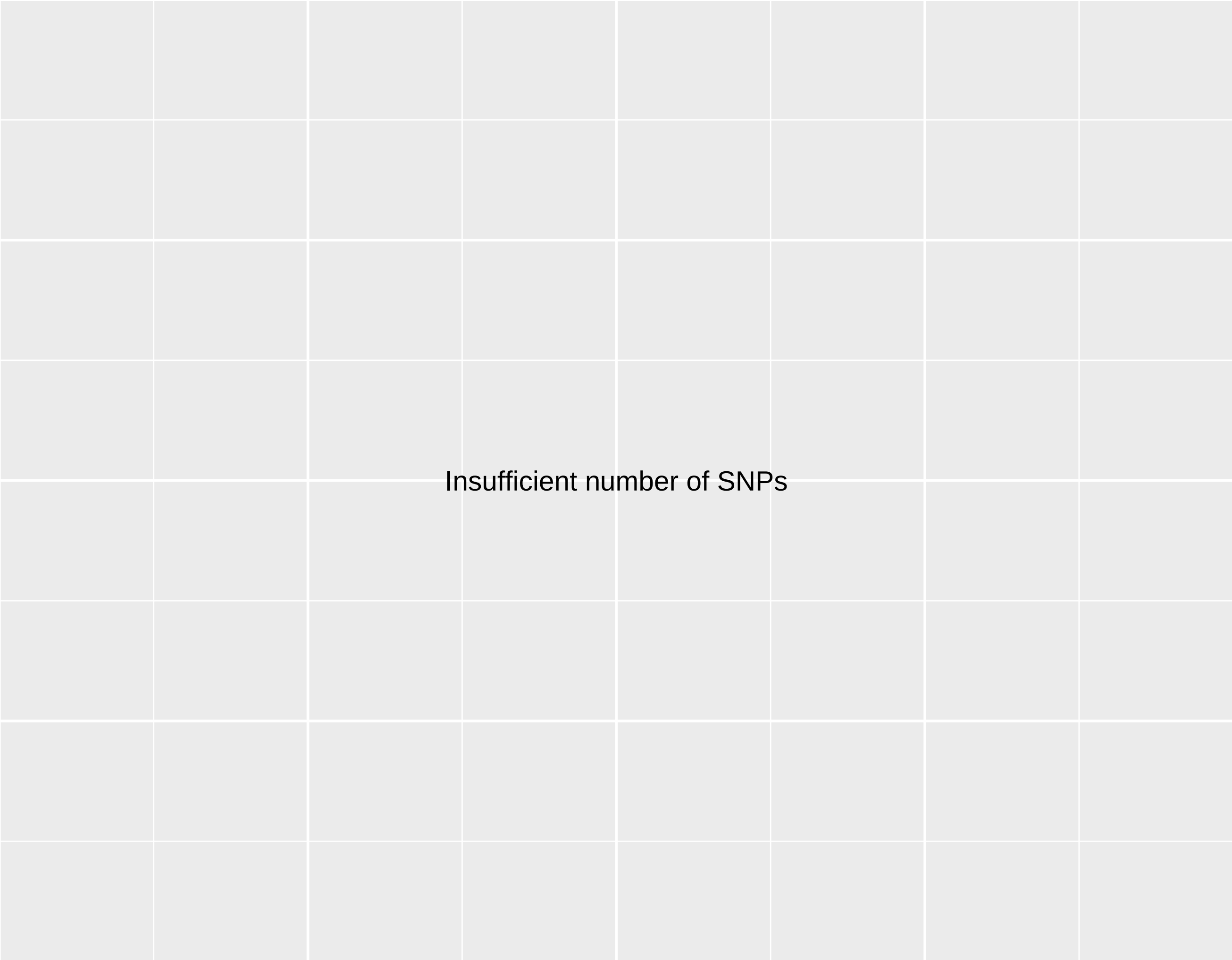
Insufficient number of SNPs

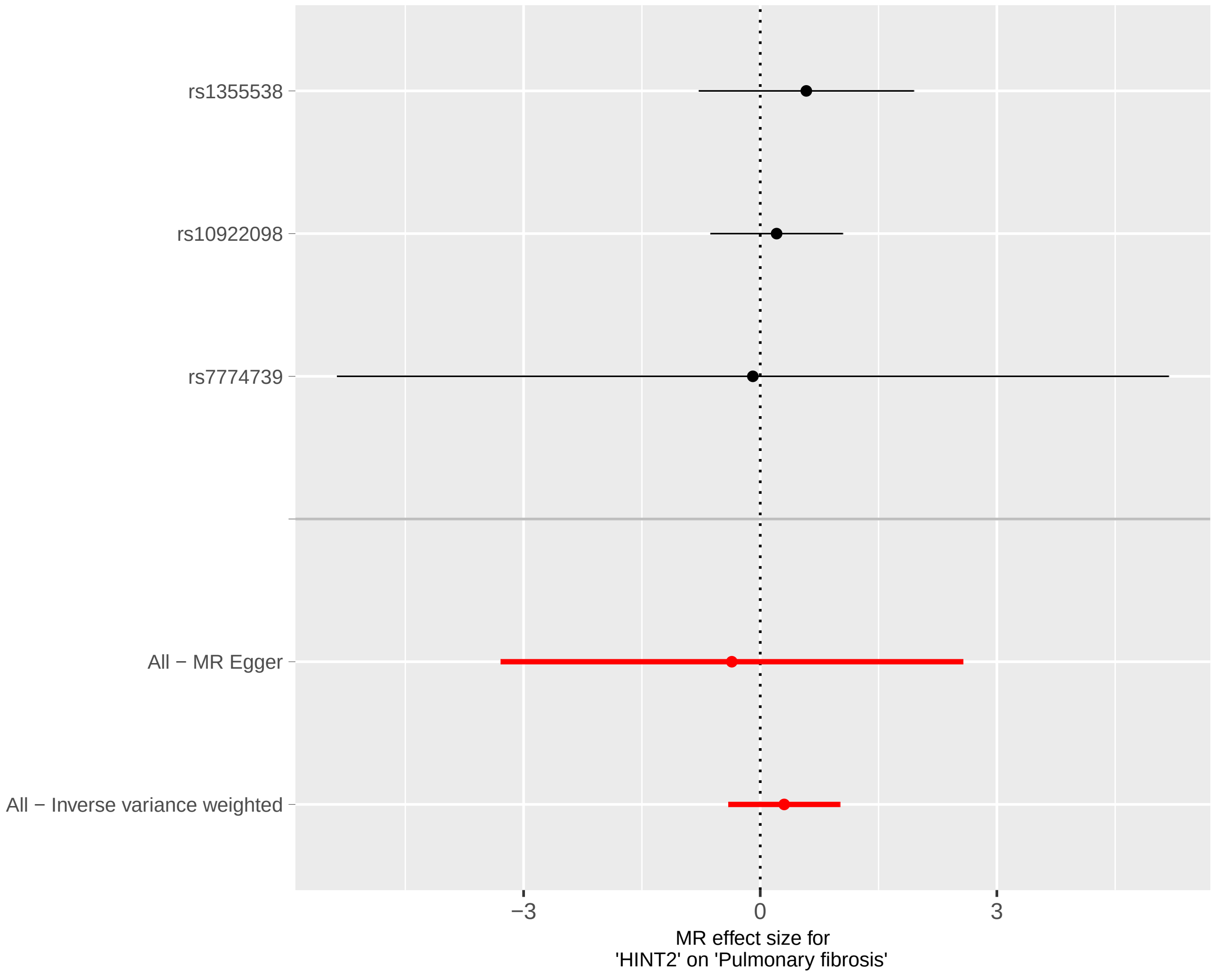
rs1355538
rs10922098
rs7774739
All − MR Egger
All − Inverse variance weighted
−3
0
3
MR effect size for
'HINT2' on 'Pulmonary fibrosis'

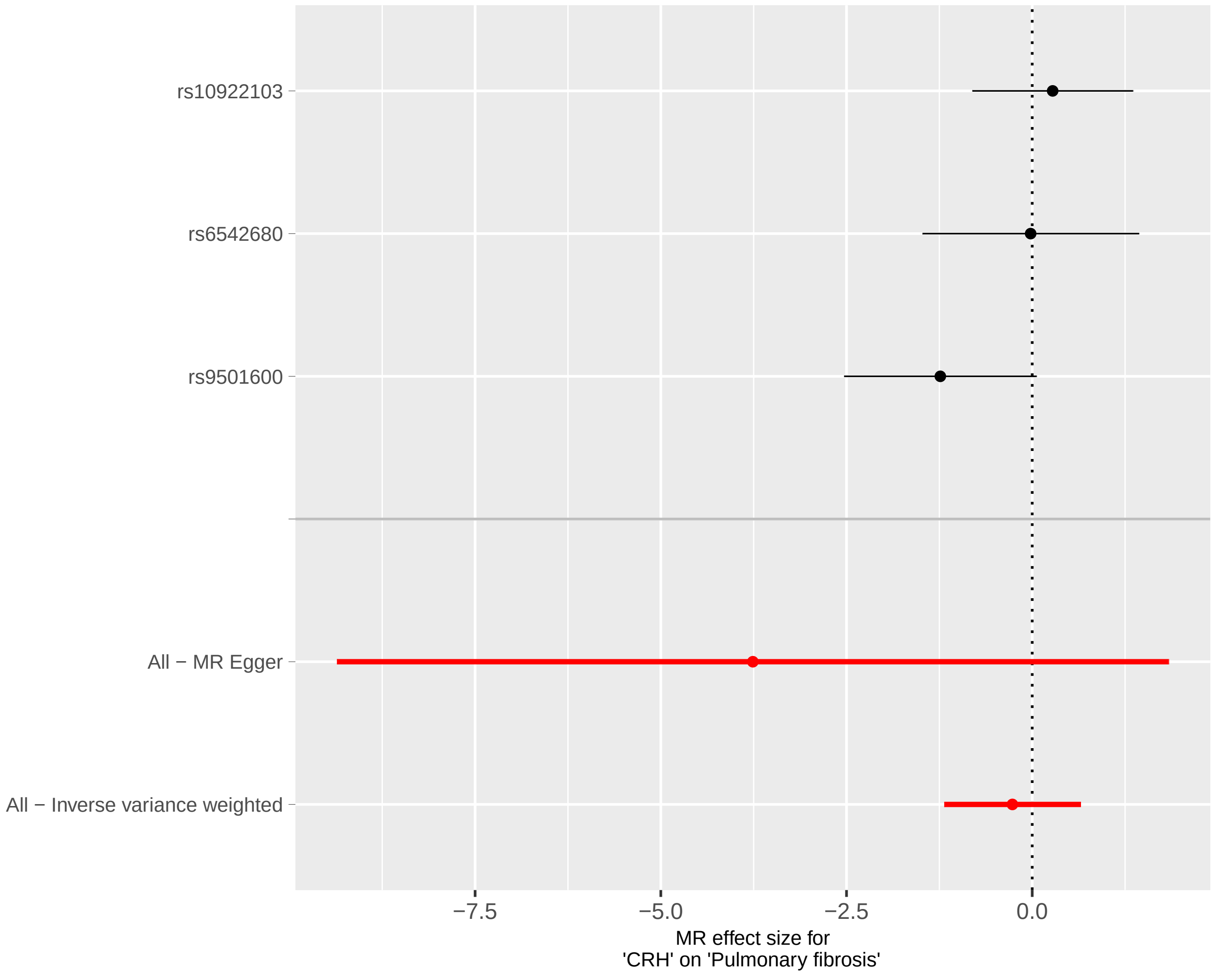
rs10922103
rs6542680
rs9501600
All − MR Egger
All − Inverse variance weighted
−7.5
−5.0
−2.5
0.0
MR effect size for
'CRH' on 'Pulmonary fibrosis'

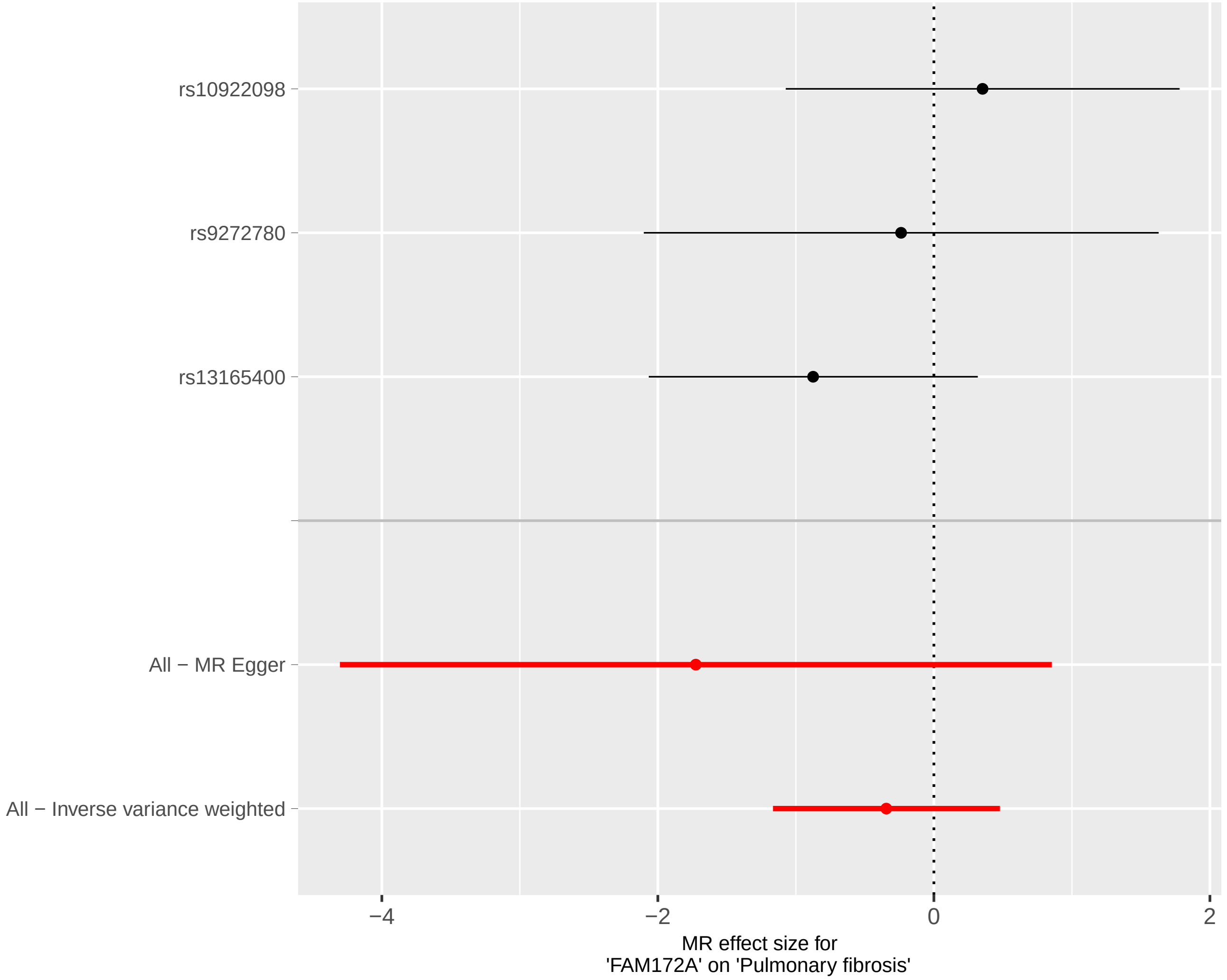

rs10922098
rs9272780
rs13165400
All − MR Egger
All − Inverse variance weighted
−4
−2
0
2
MR effect size for
'FAM172A' on 'Pulmonary fibrosis'

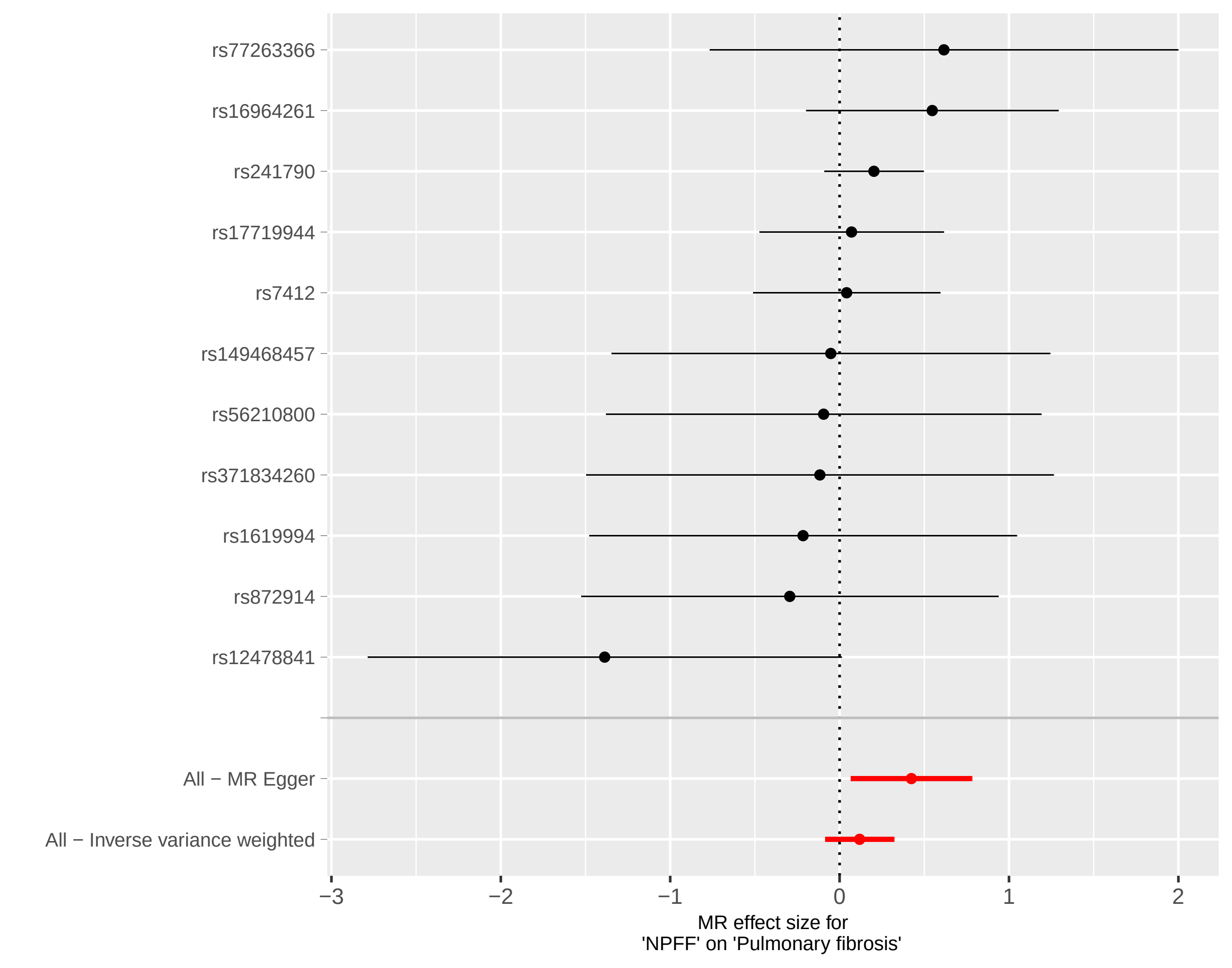
rs77263366
rs16964261
rs241790
rs17719944
rs7412
rs149468457
rs56210800
rs371834260
rs1619994
rs872914
rs12478841
All − MR Egger
All − Inverse variance weighted
−3
−2
−1
0
1
2
MR effect size for
'NPFF' on 'Pulmonary fibrosis'

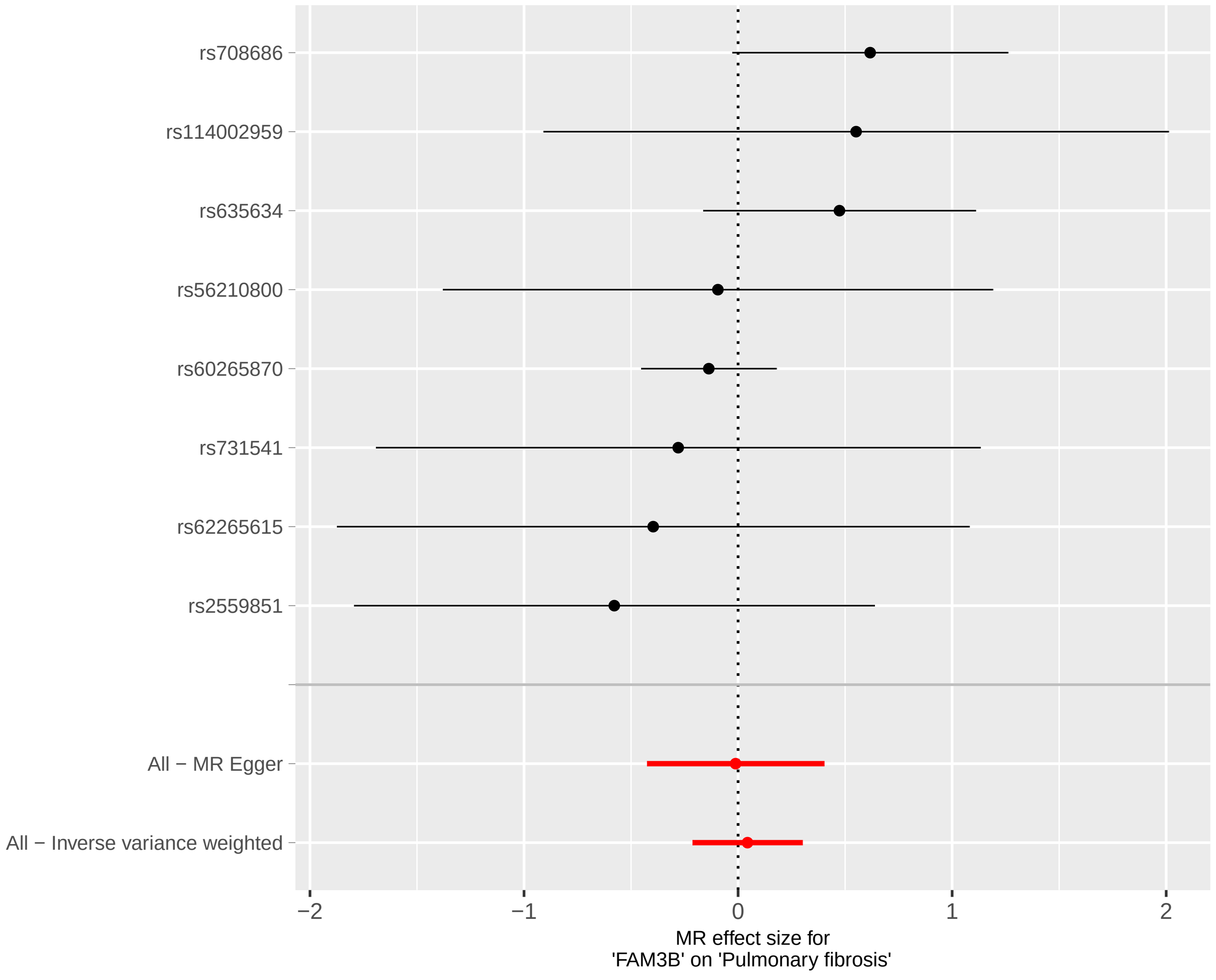
rs708686
rs114002959
rs635634
rs56210800
rs60265870
rs731541
rs62265615
rs2559851
All − MR Egger
All − Inverse variance weighted
−2
−1
0
1
2
MR effect size for
'FAM3B' on 'Pulmonary fibrosis'

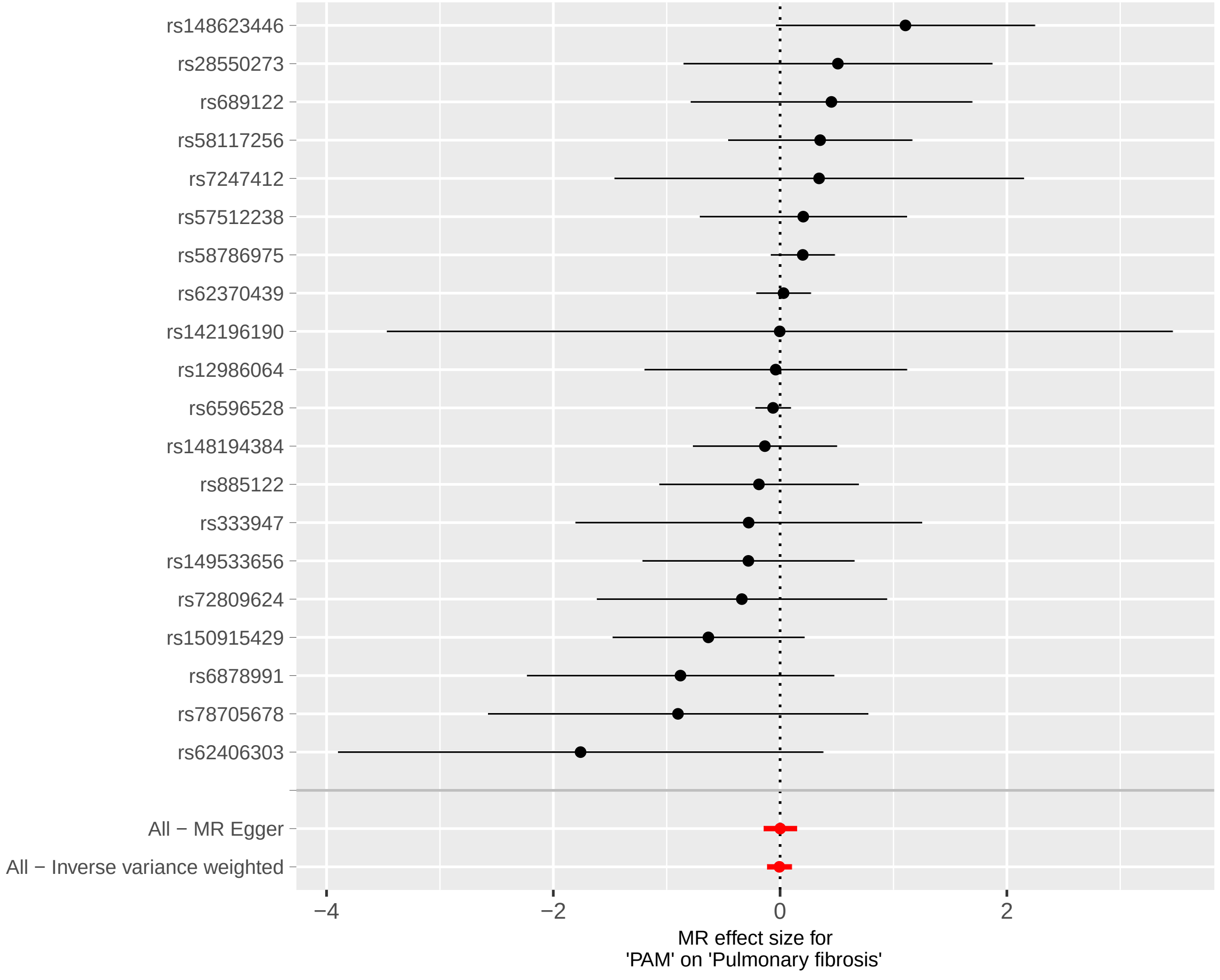
rs148623446
rs28550273
rs689122
rs58117256
rs7247412
rs57512238
rs58786975
rs62370439
rs142196190
rs12986064
rs6596528
rs148194384
rs885122
rs333947
rs149533656
rs72809624
rs150915429
rs6878991
rs78705678
rs62406303
All − MR Egger
All − Inverse variance weighted
−4
−2
0
2
MR effect size for
'PAM' on 'Pulmonary fibrosis'

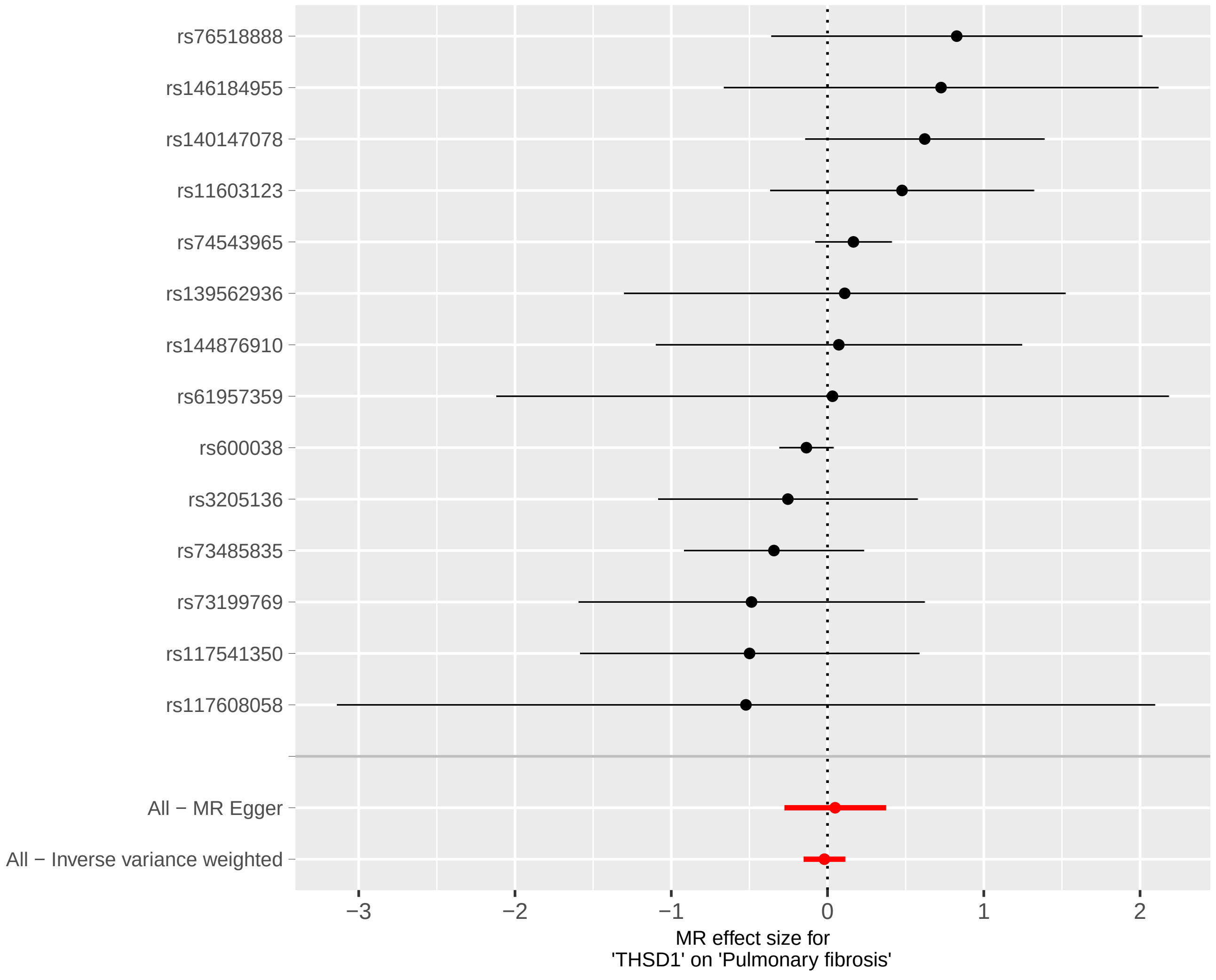

rs76518888
rs146184955
rs140147078
rs11603123
rs74543965
rs139562936
rs144876910
rs61957359
rs600038
rs3205136
rs73485835
rs73199769
rs117541350
rs117608058
All − MR Egger
All − Inverse variance weighted
−3
−2
−1
0
1
2
MR effect size for
'THSD1' on 'Pulmonary fibrosis'

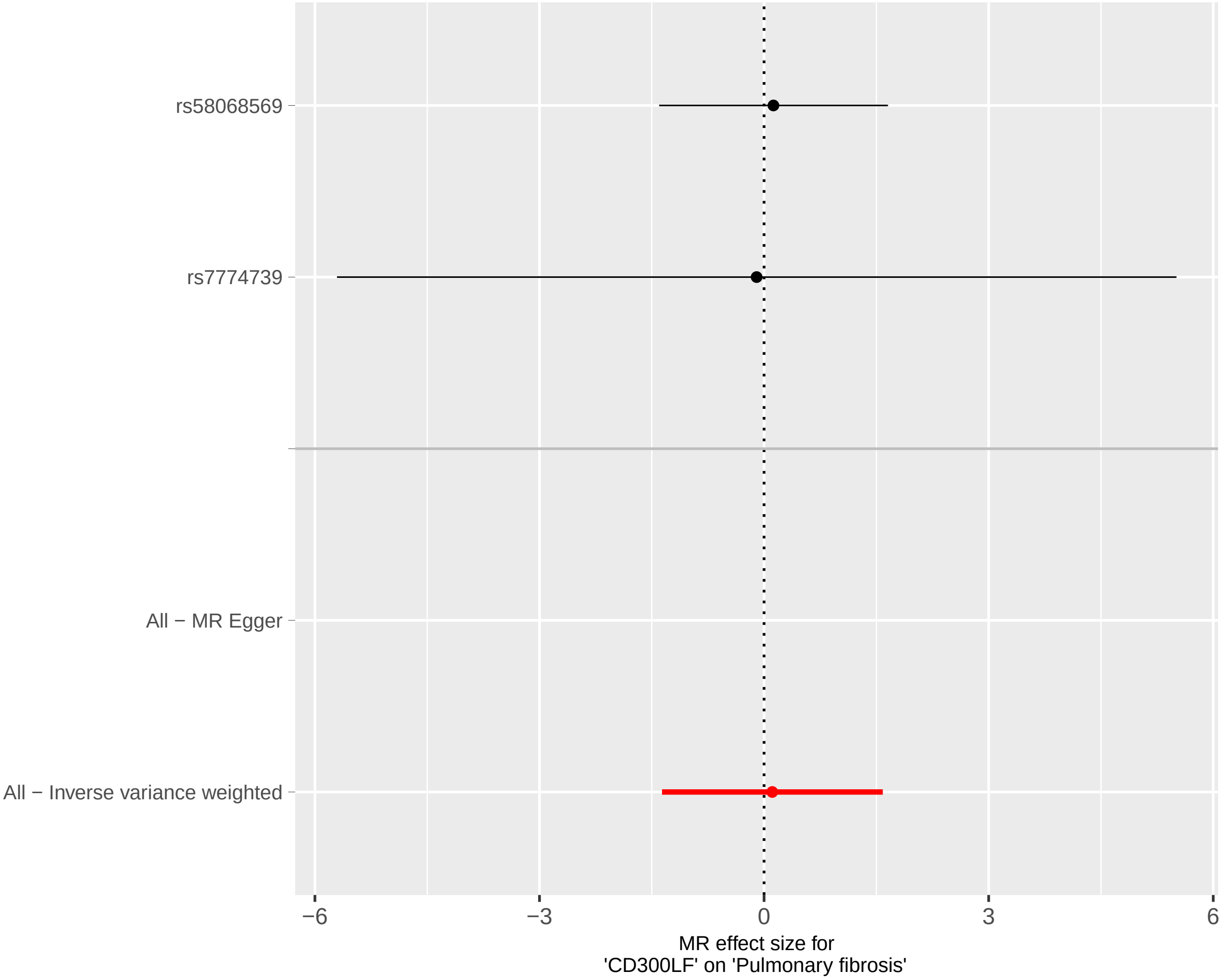

rs58068569
rs7774739
All − MR Egger
All − Inverse variance weighted
−6
−3
0
3
6
MR effect size for
'CD300LF' on 'Pulmonary fibrosis'

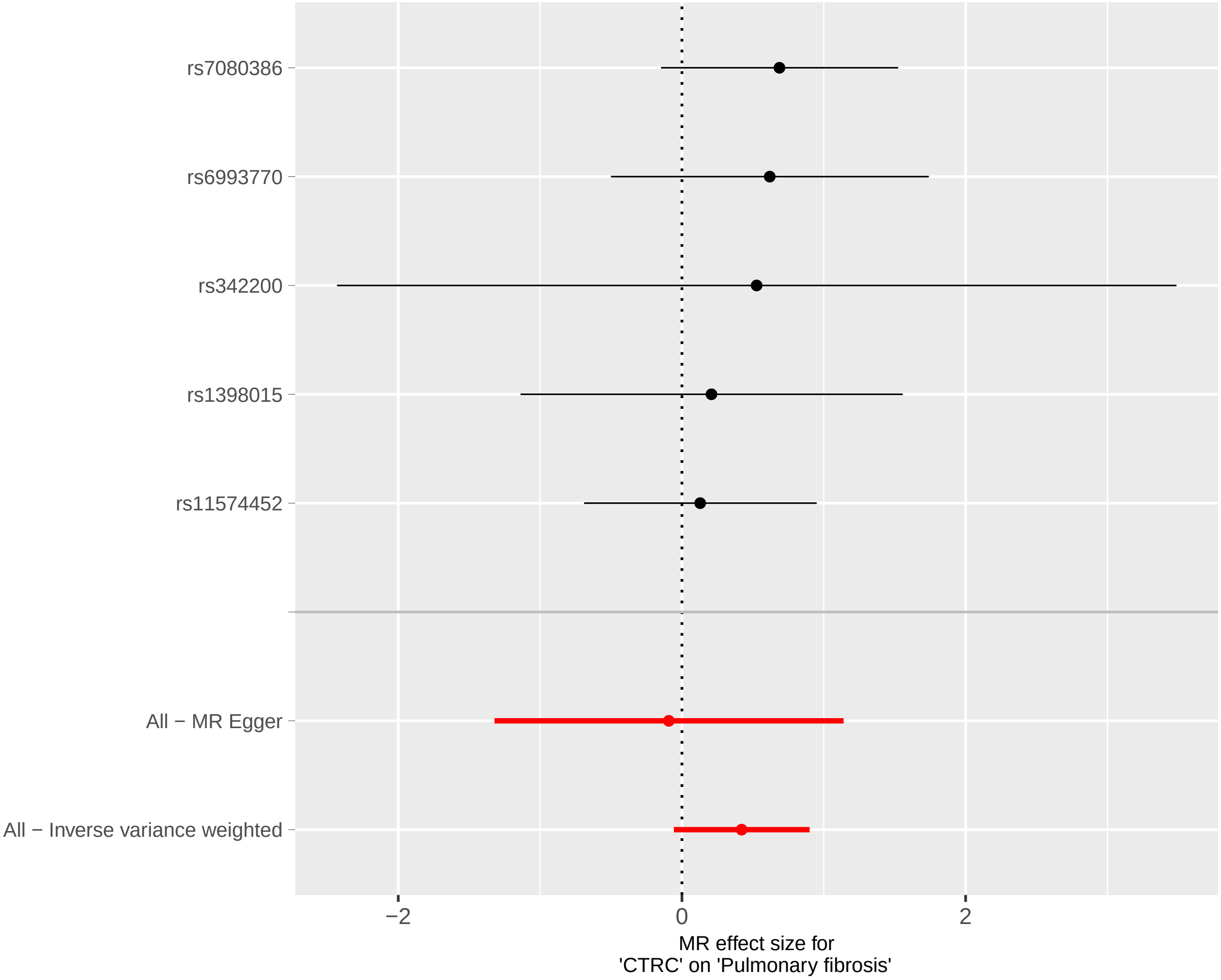

rs7080386
rs6993770
rs342200
rs1398015
rs11574452
All − MR Egger
All − Inverse variance weighted
−2
0
2
MR effect size for
'CTRC' on 'Pulmonary fibrosis'

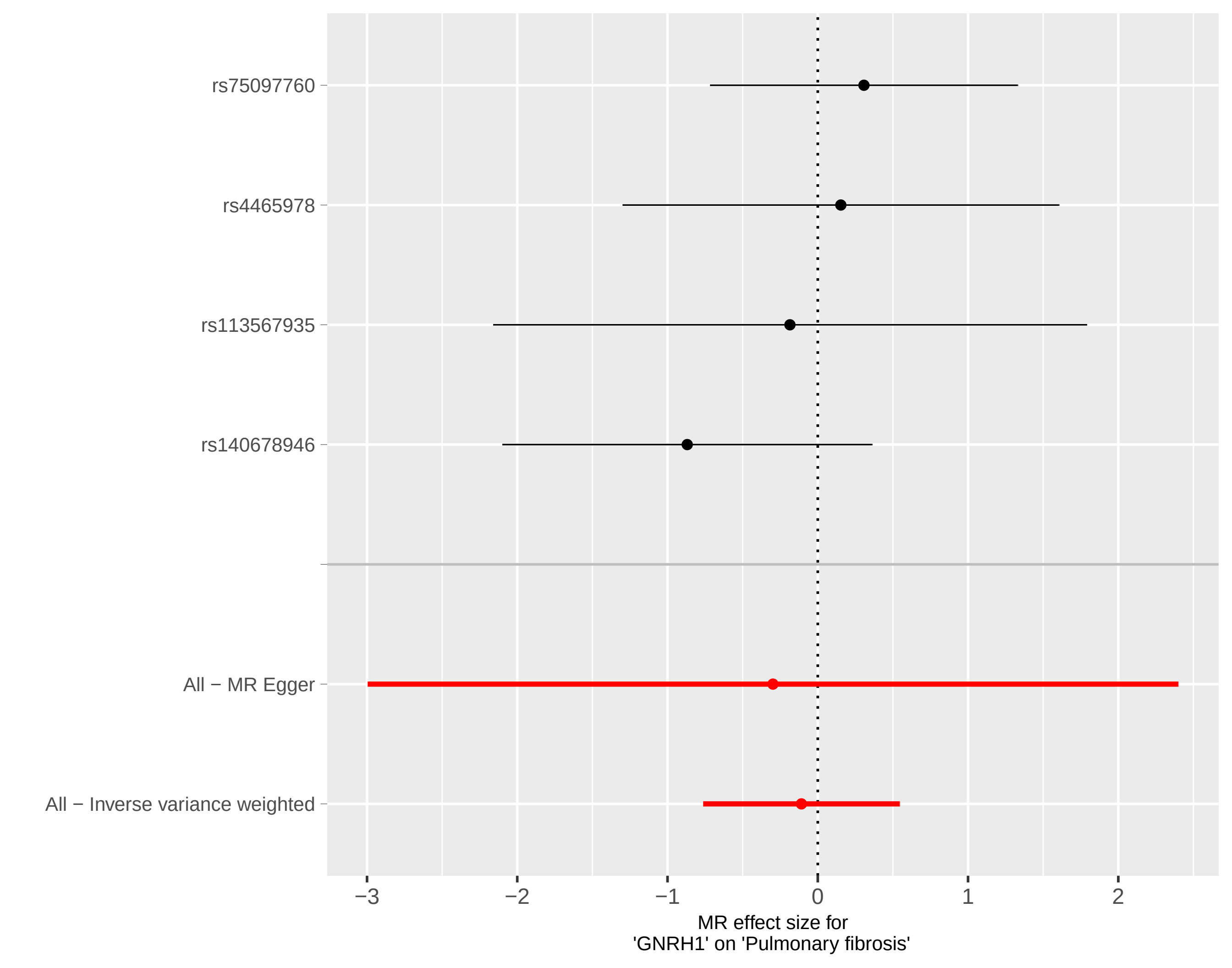
rs75097760
rs4465978
rs113567935
rs140678946
All − MR Egger
All − Inverse variance weighted
−3
−2
−1
0
1
2
MR effect size for
'GNRH1' on 'Pulmonary fibrosis'

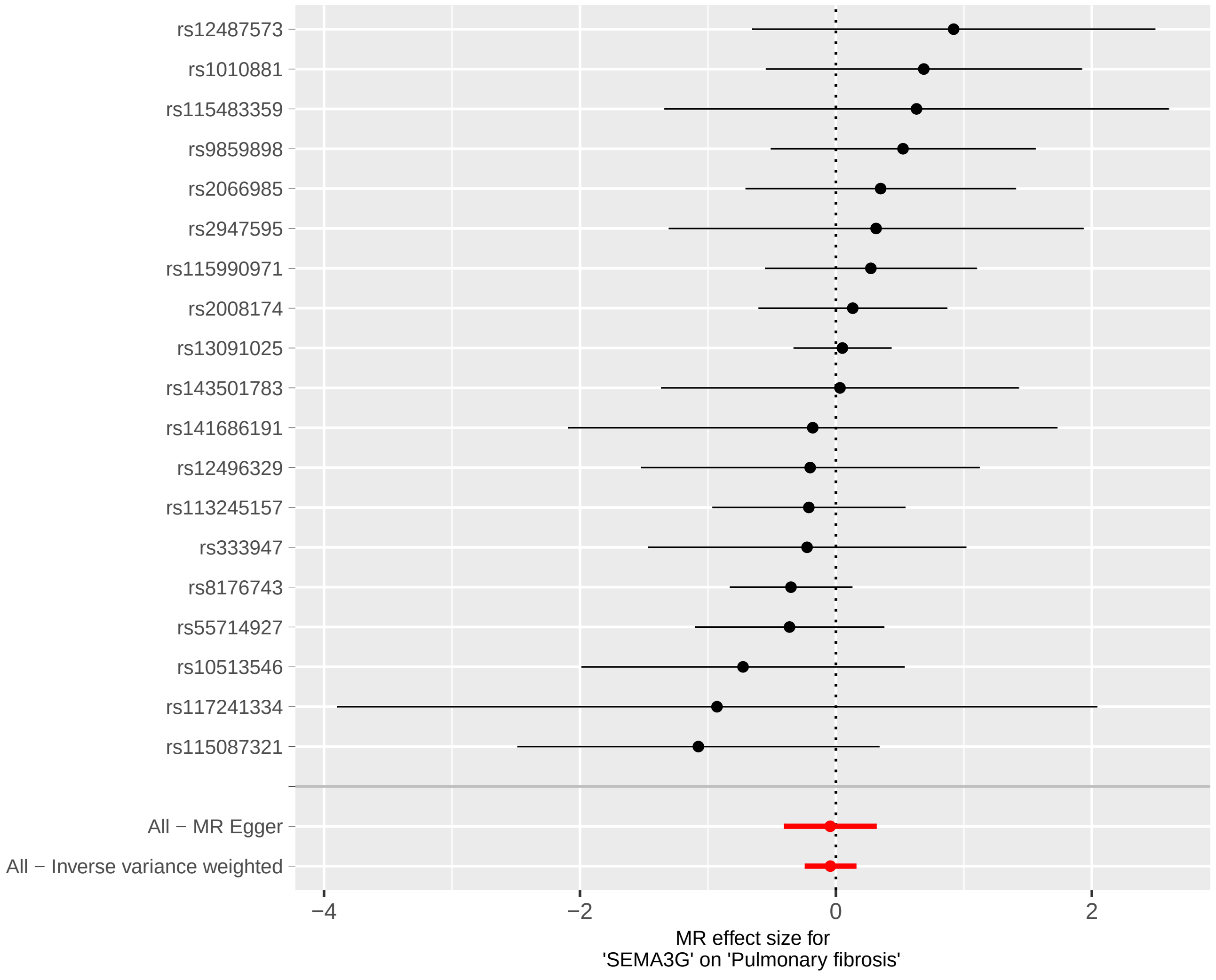
rs12487573
rs1010881
rs115483359
rs9859898
rs2066985
rs2947595
rs115990971
rs2008174
rs13091025
rs143501783
rs141686191
rs12496329
rs113245157
rs333947
rs8176743
rs55714927
rs10513546
rs117241334
rs115087321
All − MR Egger
All − Inverse variance weighted
−4
−2
0
2
MR effect size for
'SEMA3G' on 'Pulmonary fibrosis'

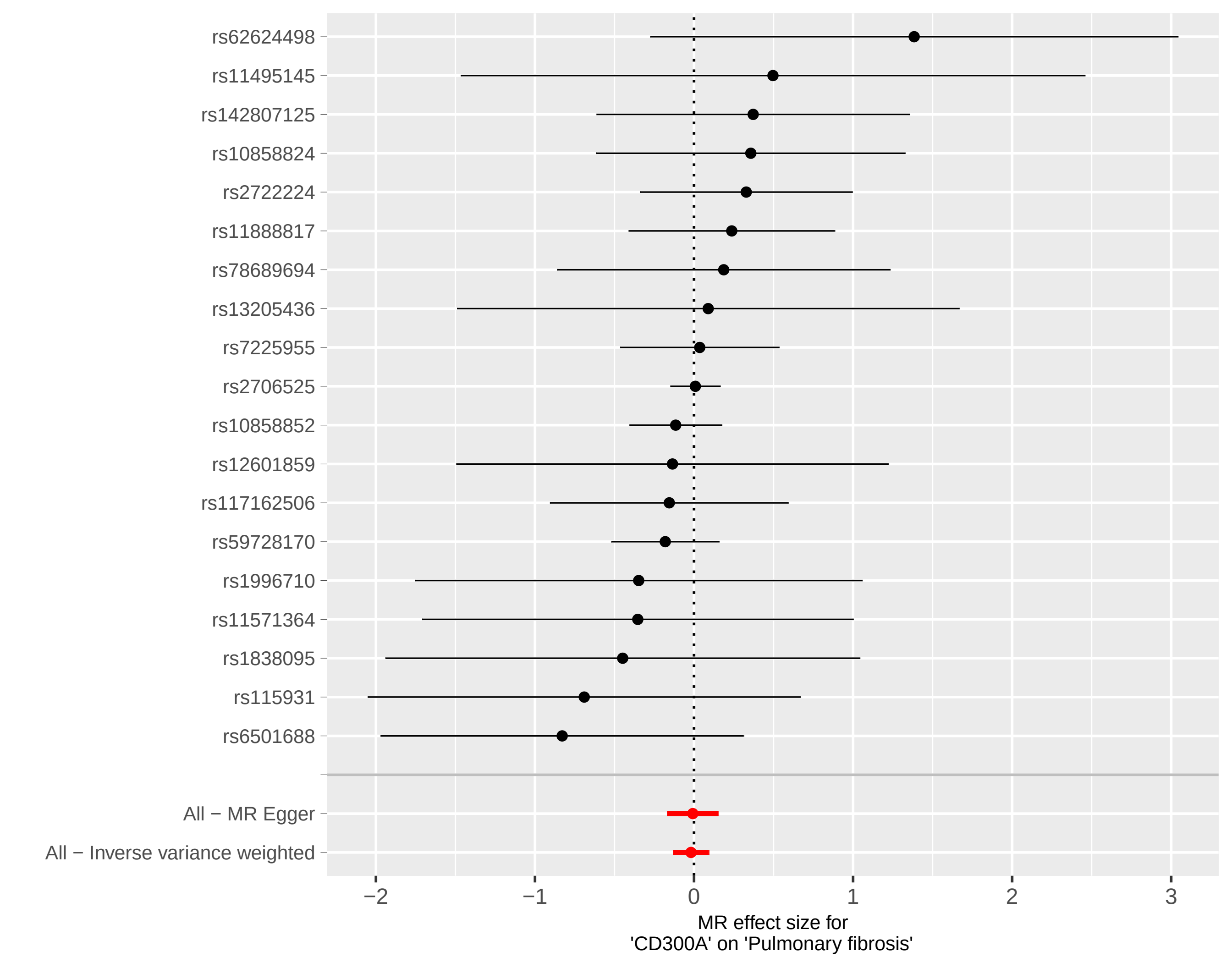

rs62624498
rs11495145
rs142807125
rs10858824
rs2722224
rs11888817
rs78689694
rs13205436
rs7225955
rs2706525
rs10858852
rs12601859
rs117162506
rs59728170
rs1996710
rs11571364
rs1838095
rs115931
rs6501688
All − MR Egger
All − Inverse variance weighted
−2
−1
0
1
2
3
MR effect size for
'CD300A' on 'Pulmonary fibrosis'

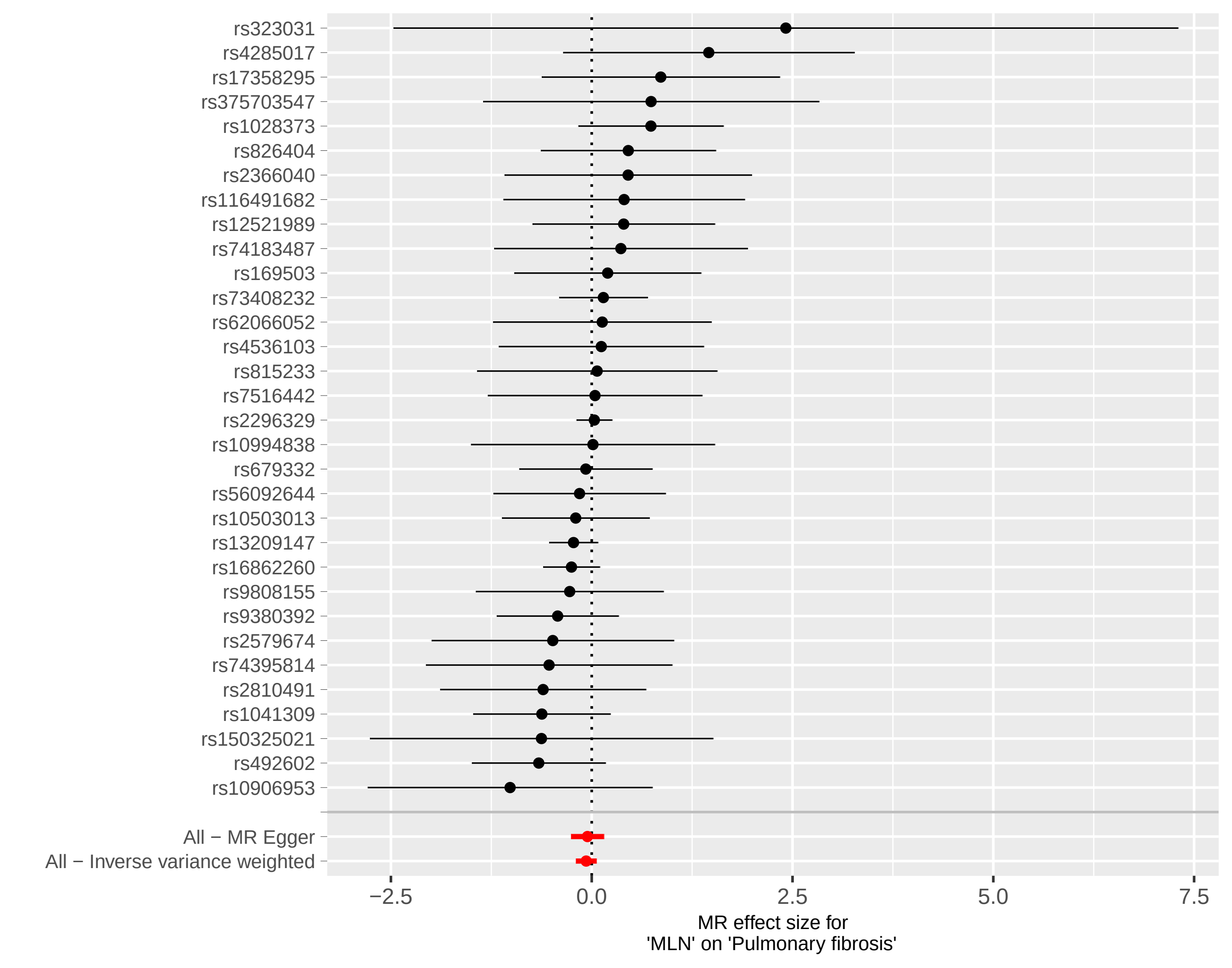

rs323031
rs4285017
rs17358295
rs375703547
rs1028373
rs826404
rs2366040
rs116491682
rs12521989
rs74183487
rs169503
rs73408232
rs62066052
rs4536103
rs815233
rs7516442
rs2296329
rs10994838
rs679332
rs56092644
rs10503013
rs13209147
rs16862260
rs9808155
rs9380392
rs2579674
rs74395814
rs2810491
rs1041309
rs150325021
rs492602
rs10906953
All − MR Egger
All − Inverse variance weighted
−2.5
0.0
2.5
5.0
7.5
MR effect size for
'MLN' on 'Pulmonary fibrosis'

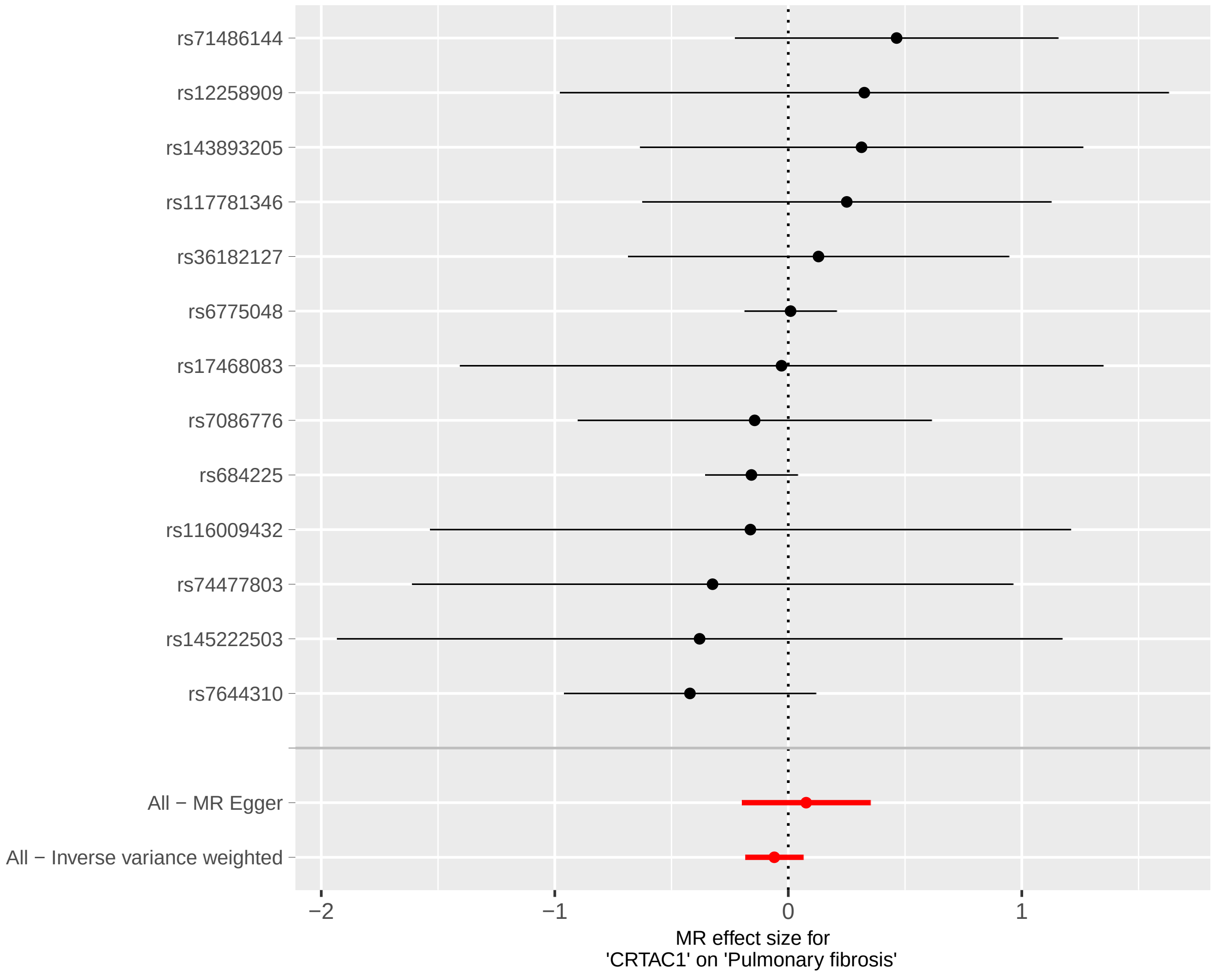

rs71486144
rs12258909
rs143893205
rs117781346
rs36182127
rs6775048
rs17468083
rs7086776
rs684225
rs116009432
rs74477803
rs145222503
rs7644310
All − MR Egger
All − Inverse variance weighted
−2
−1
0
1
MR effect size for
'CRTAC1' on 'Pulmonary fibrosis'

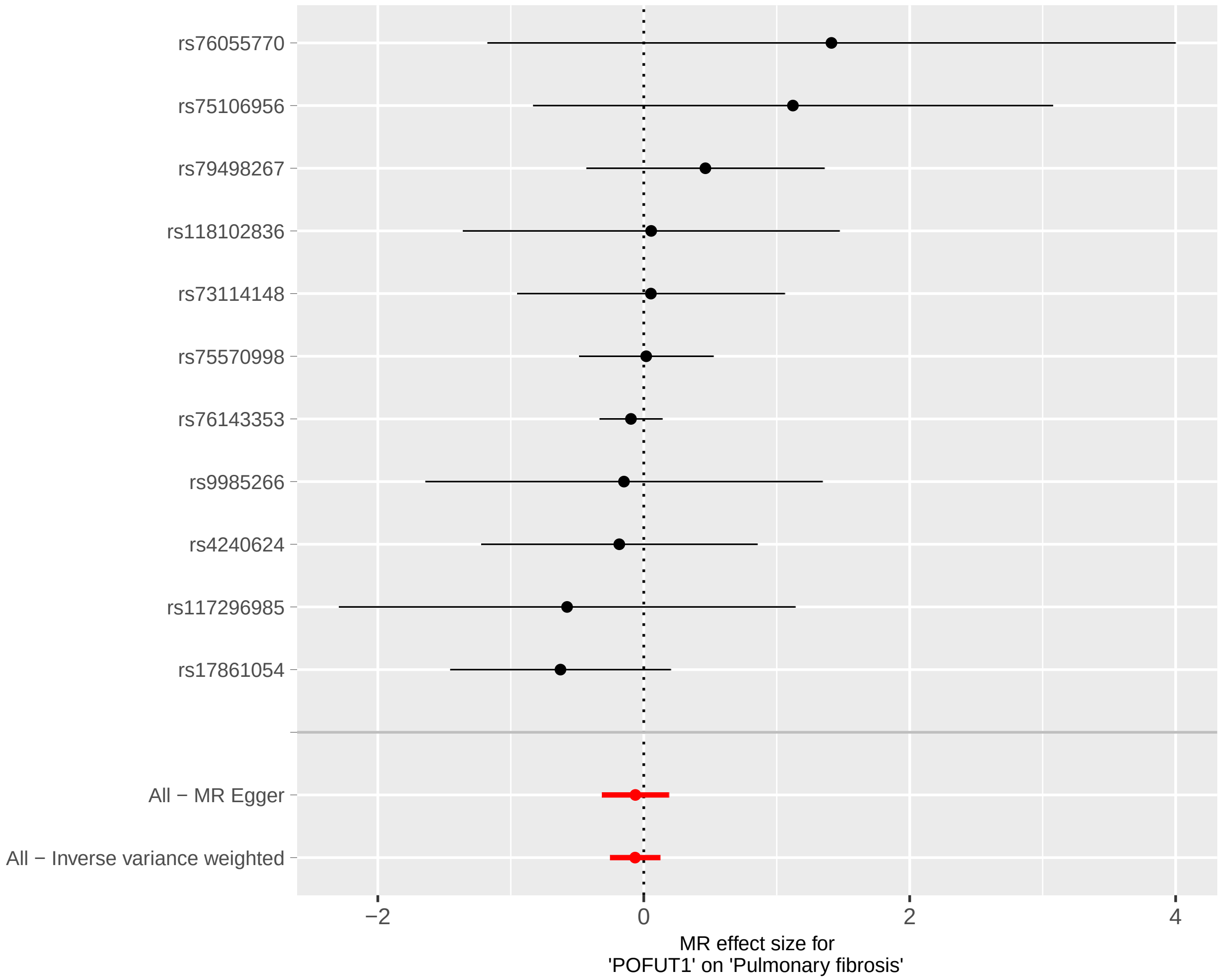
rs76055770
rs75106956
rs79498267
rs118102836
rs73114148
rs75570998
rs76143353
rs9985266
rs4240624
rs117296985
rs17861054
All − MR Egger
All − Inverse variance weighted
−2
0
2
4
MR effect size for
'POFUT1' on 'Pulmonary fibrosis'

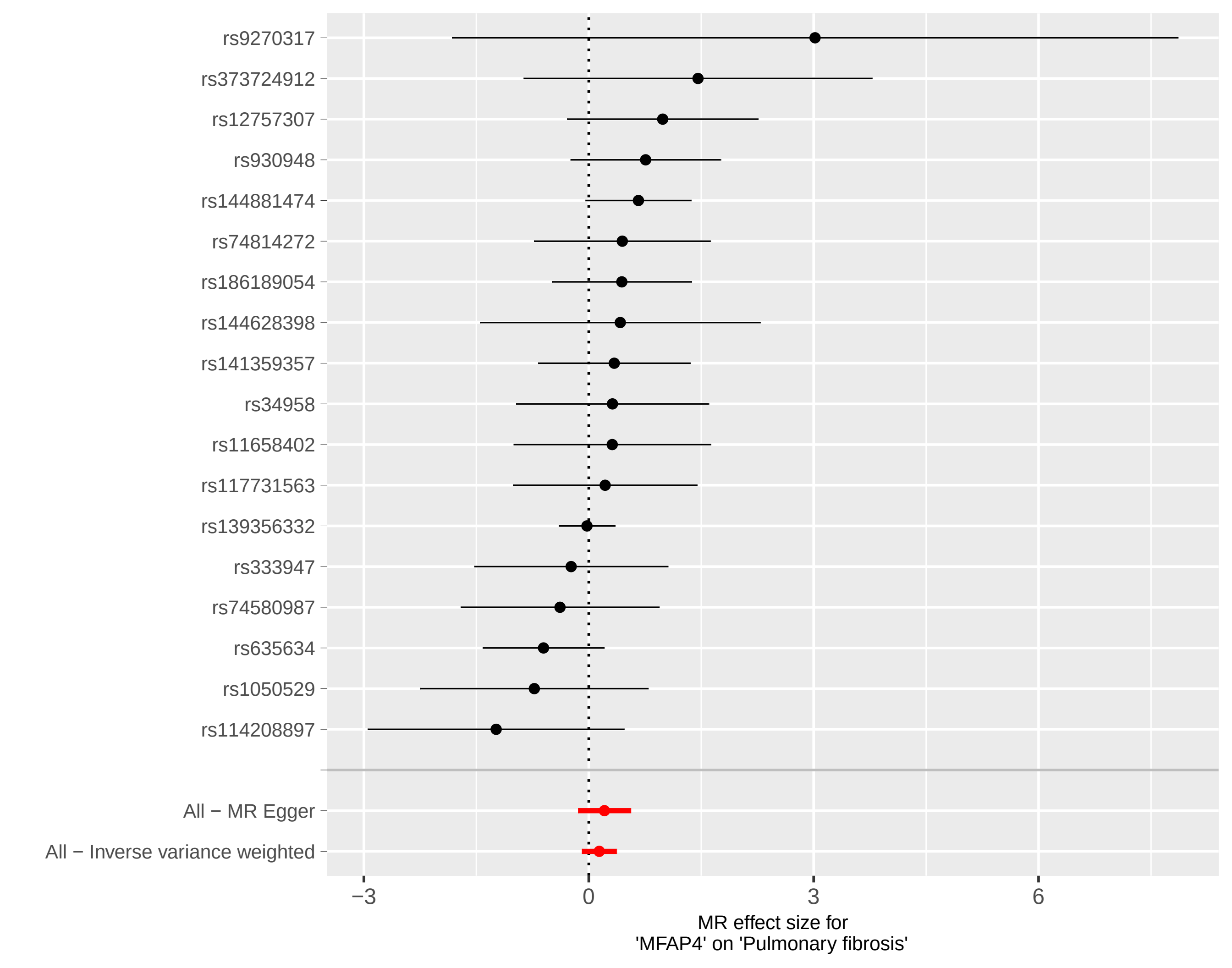
rs9270317
rs373724912
rs12757307
rs930948
rs144881474
rs74814272
rs186189054
rs144628398
rs141359357
rs34958
rs11658402
rs117731563
rs139356332
rs333947
rs74580987
rs635634
rs1050529
rs114208897
All − MR Egger
All − Inverse variance weighted
−3
0
3
6
MR effect size for
'MFAP4' on 'Pulmonary fibrosis'

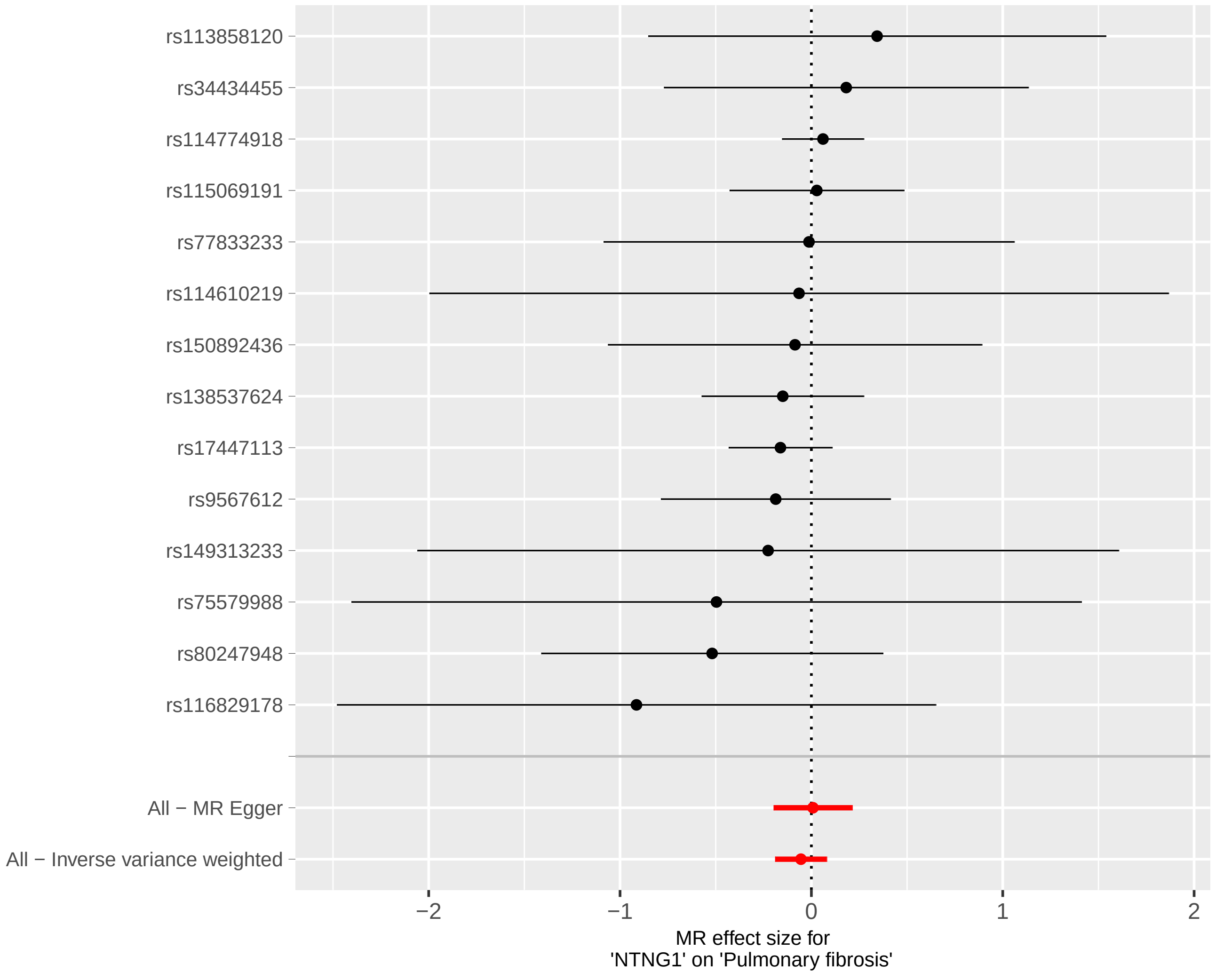

rs113858120
rs34434455
rs114774918
rs115069191
rs77833233
rs114610219
rs150892436
rs138537624
rs17447113
rs9567612
rs149313233
rs75579988
rs80247948
rs116829178
All − MR Egger
All − Inverse variance weighted
−2
−1
0
1
2
MR effect size for
'NTNG1' on 'Pulmonary fibrosis'

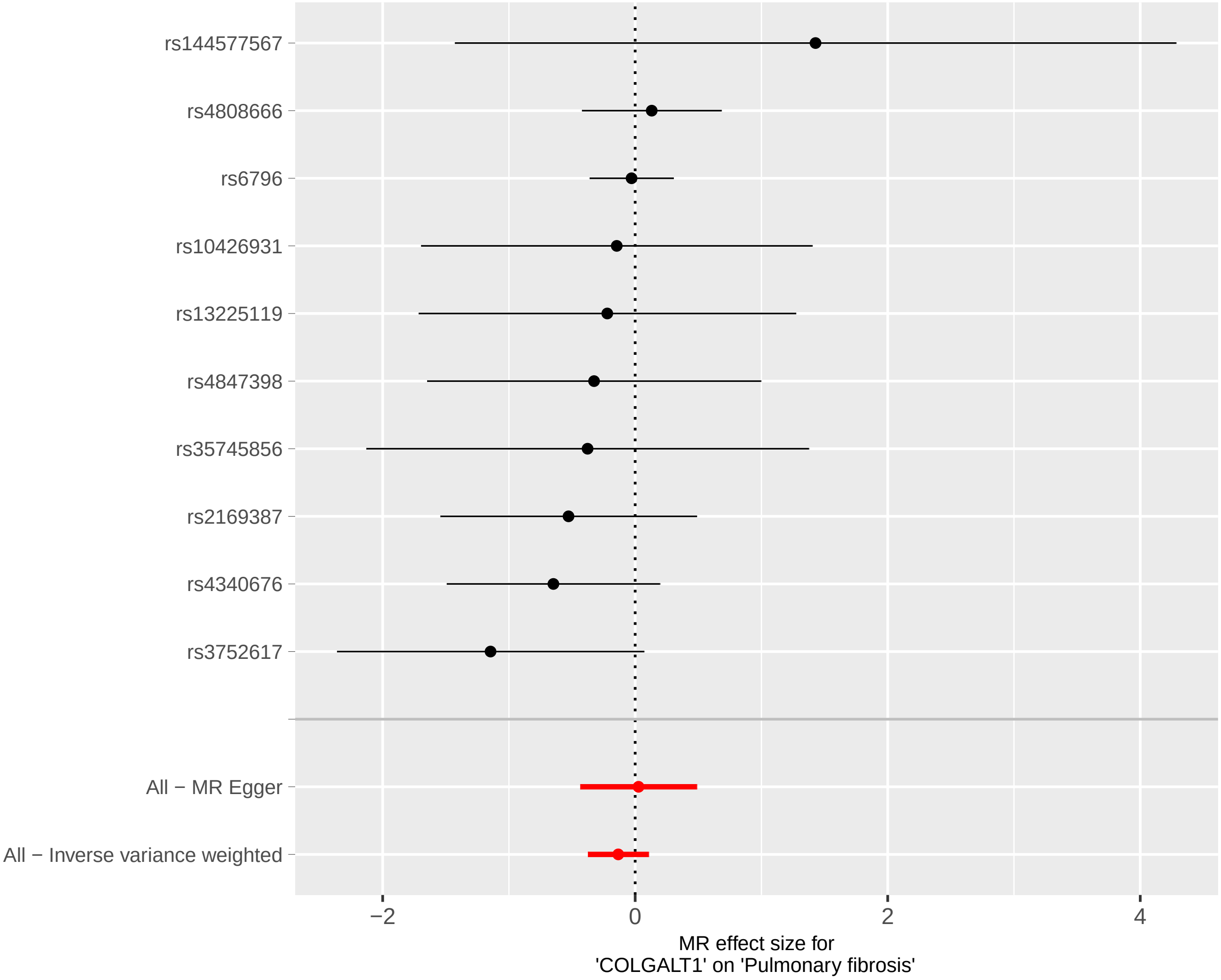
rs144577567
rs4808666
rs6796
rs10426931
rs13225119
rs4847398
rs35745856
rs2169387
rs4340676
rs3752617
All − MR Egger
All − Inverse variance weighted
−2
0
2
4
MR effect size for
'COLGALT1' on 'Pulmonary fibrosis'

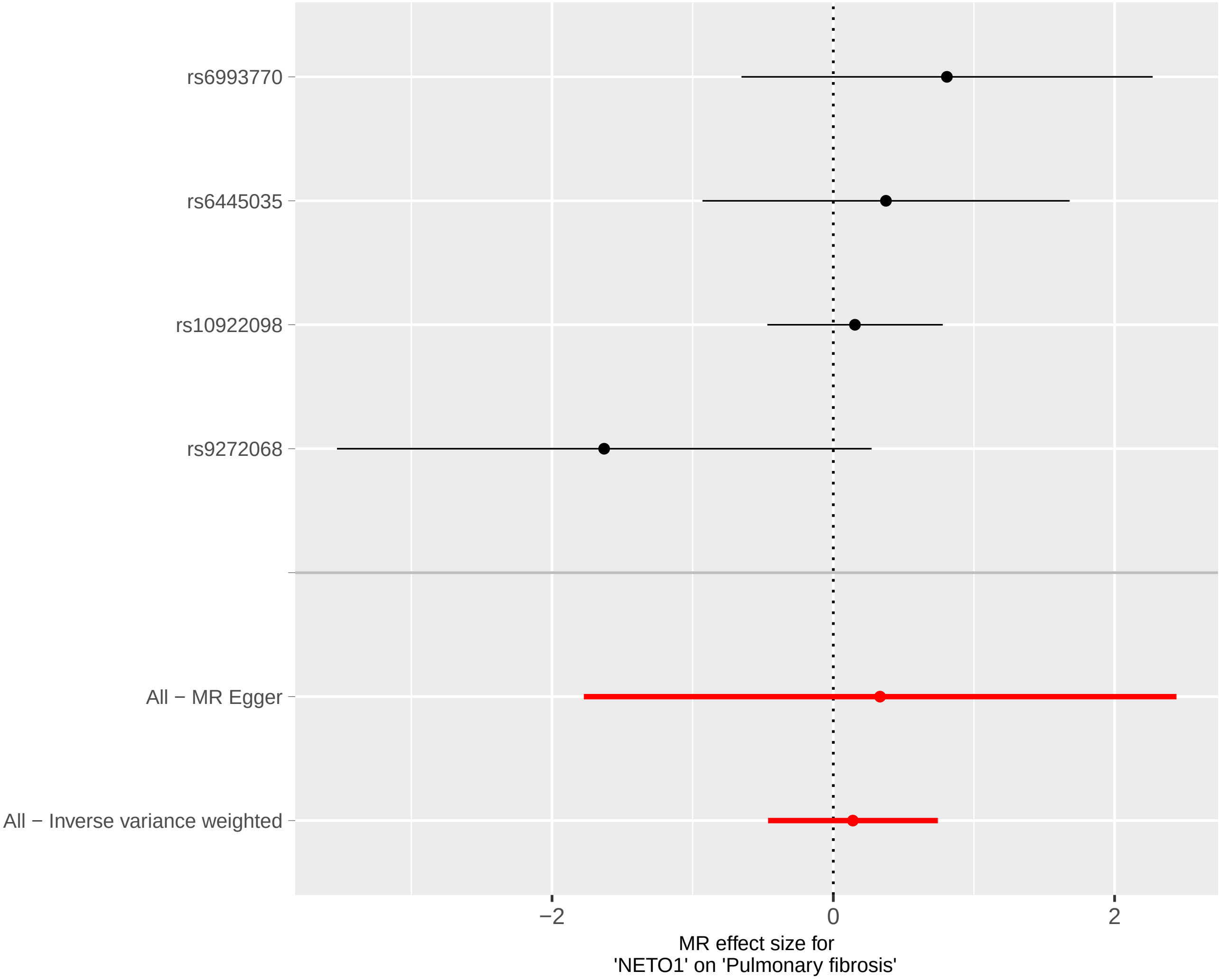

rs6993770
rs6445035
rs10922098
rs9272068
All − MR Egger
All − Inverse variance weighted
−2
0
2
MR effect size for
'NETO1' on 'Pulmonary fibrosis'

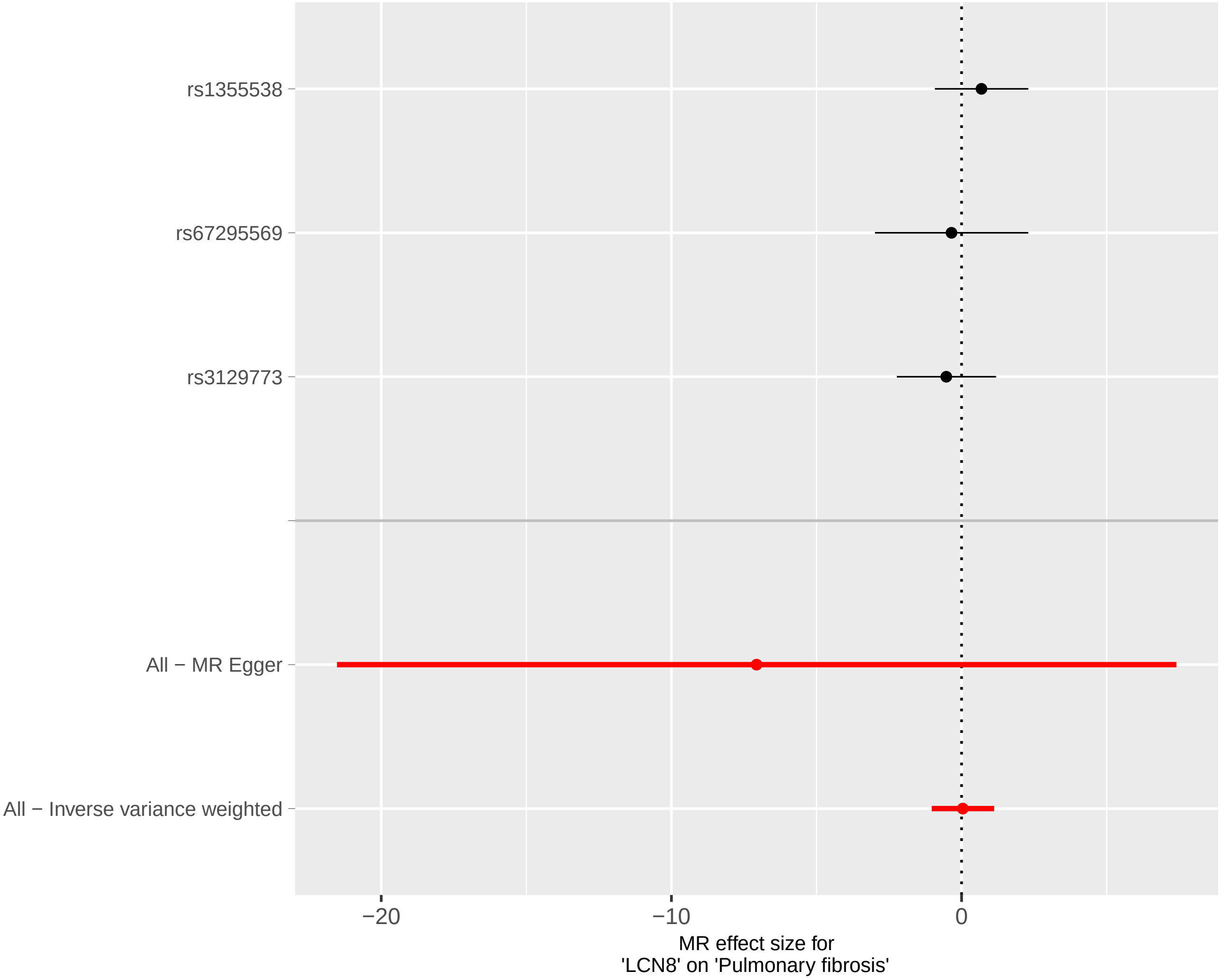
rs1355538
rs67295569
rs3129773
All − MR Egger
All − Inverse variance weighted
−20
−10
0
MR effect size for
'LCN8' on 'Pulmonary fibrosis'

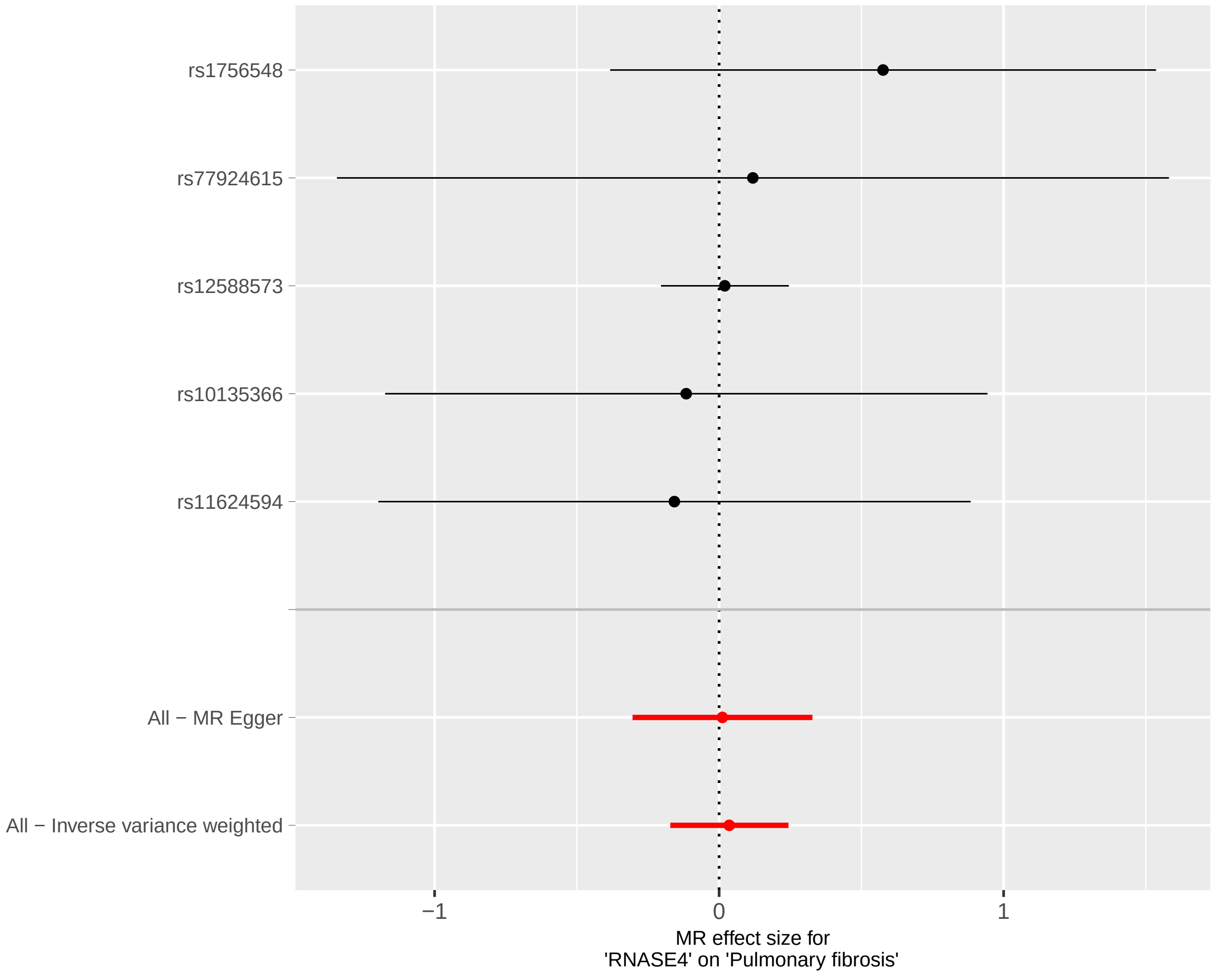
rs1756548
rs77924615
rs12588573
rs10135366
rs11624594
All − MR Egger
All − Inverse variance weighted
−1
0
1
MR effect size for
'RNASE4' on 'Pulmonary fibrosis'

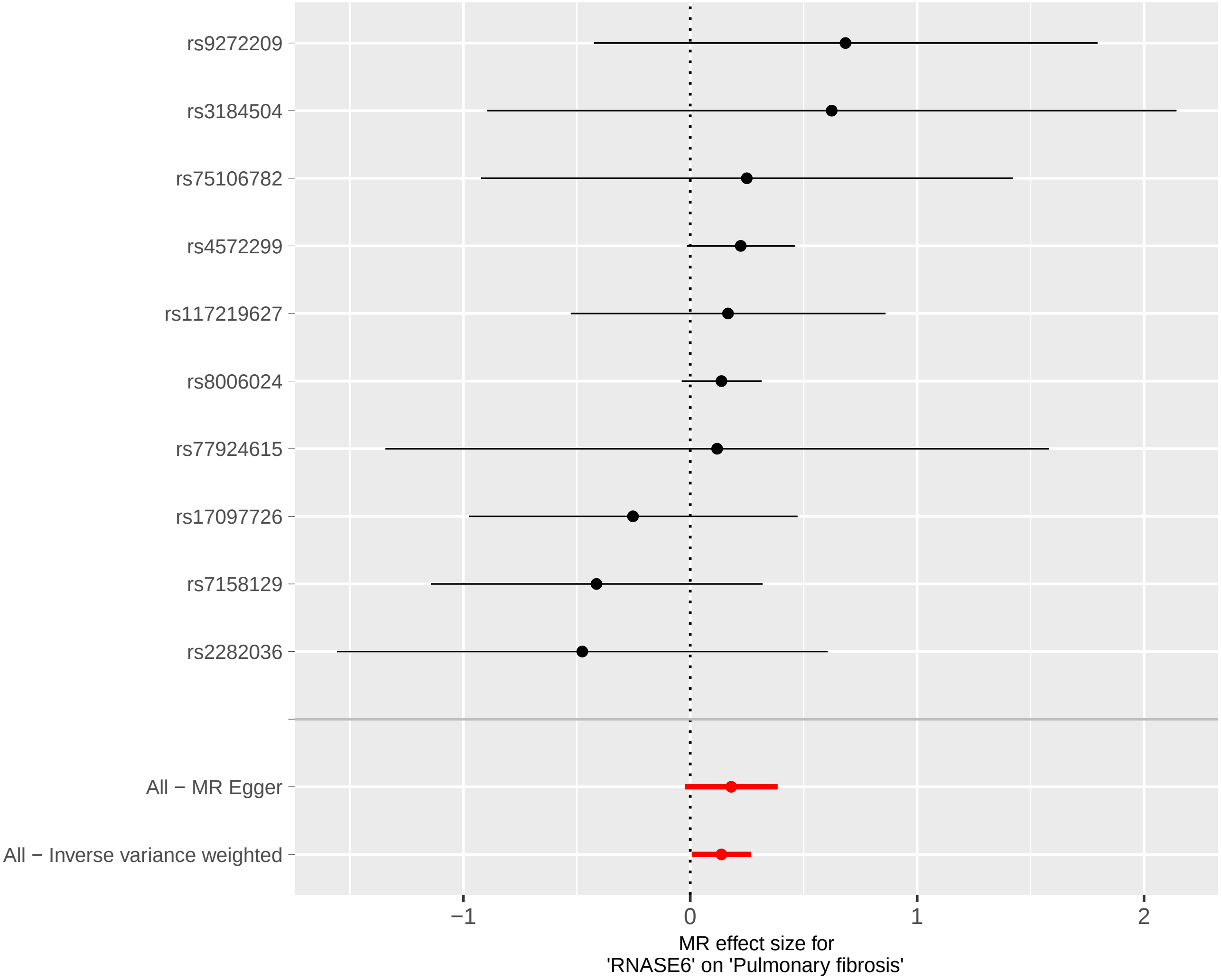
rs9272209
rs3184504
rs75106782
rs4572299
rs117219627
rs8006024
rs77924615
rs17097726
rs7158129
rs2282036
All − MR Egger
All − Inverse variance weighted
−1
0
1
2
MR effect size for
'RNASE6' on 'Pulmonary fibrosis'

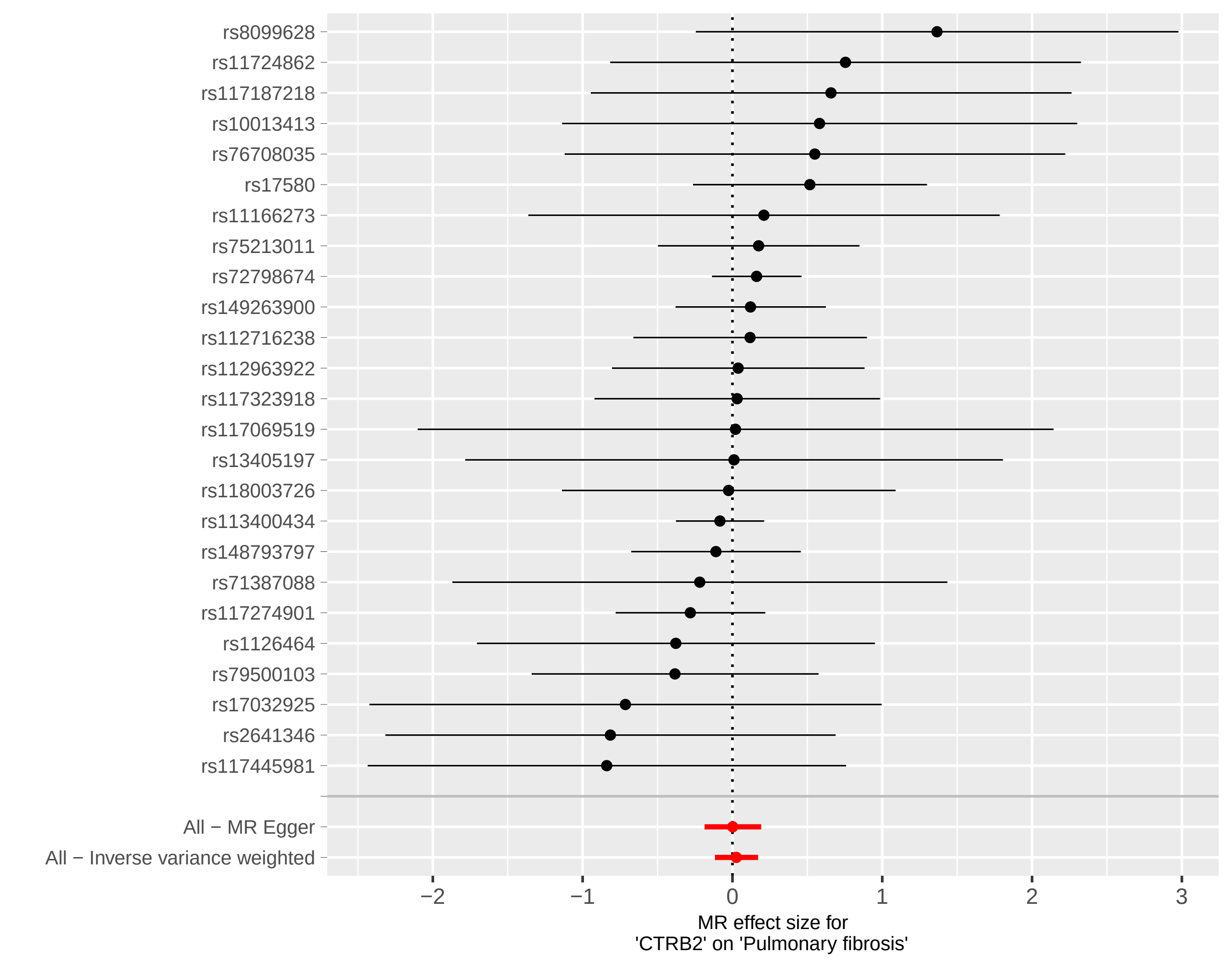
rs8099628
rs11724862
rs117187218
rs10013413
rs76708035
rs17580
rs11166273
rs75213011
rs72798674
rs149263900
rs112716238
rs112963922
rs117323918
rs117069519
rs13405197
rs118003726
rs113400434
rs148793797
rs71387088
rs117274901
rs1126464
rs79500103
rs17032925
rs2641346
rs117445981
All − MR Egger
All − Inverse variance weighted
−2
−1
0
1
2
3
MR effect size for
'CTRB2' on 'Pulmonary fibrosis'

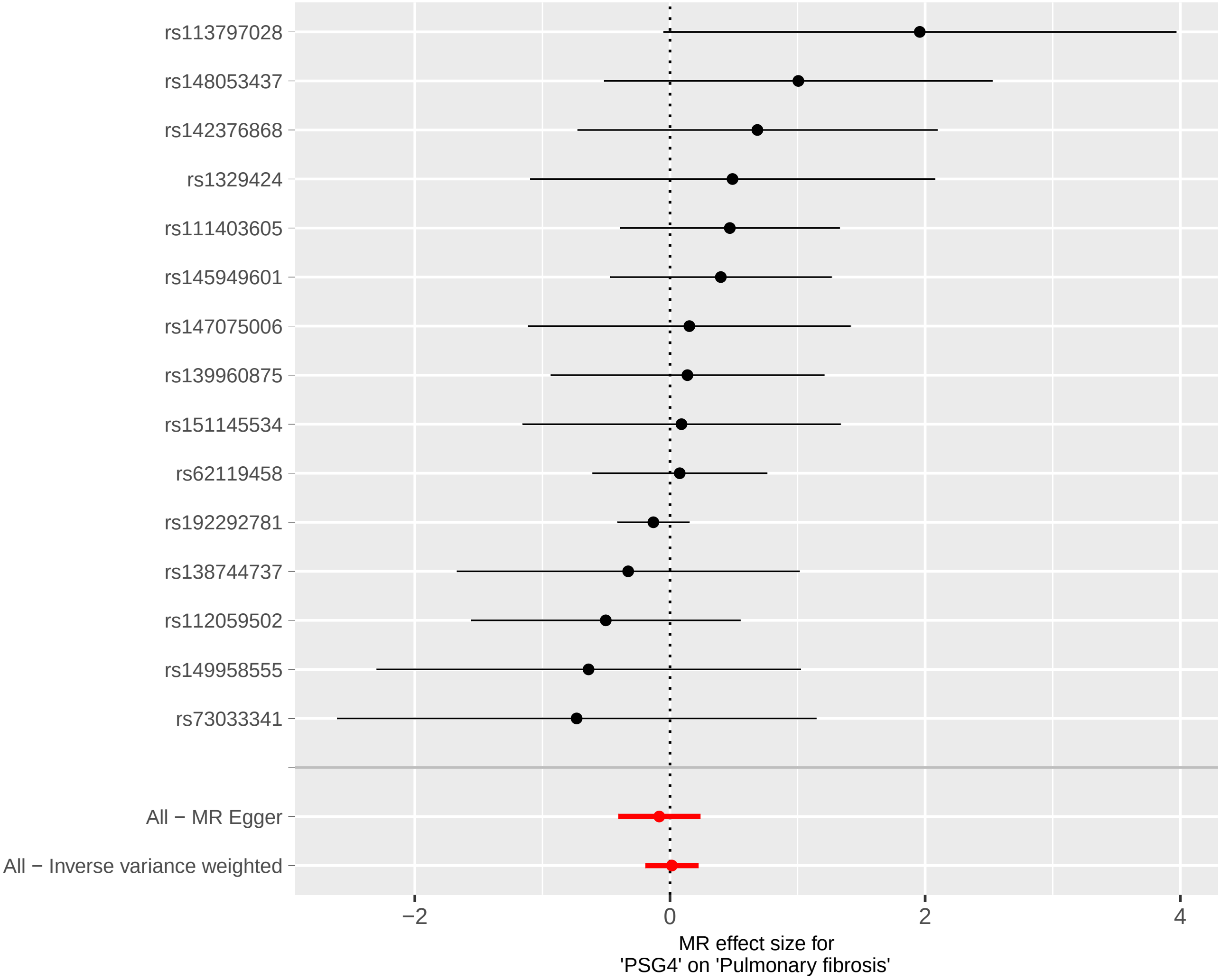
rs113797028
rs148053437
rs142376868
rs1329424
rs111403605
rs145949601
rs147075006
rs139960875
rs151145534
rs62119458
rs192292781
rs138744737
rs112059502
rs149958555
rs73033341
All − MR Egger
All − Inverse variance weighted
−2
0
2
4
MR effect size for
'PSG4' on 'Pulmonary fibrosis'

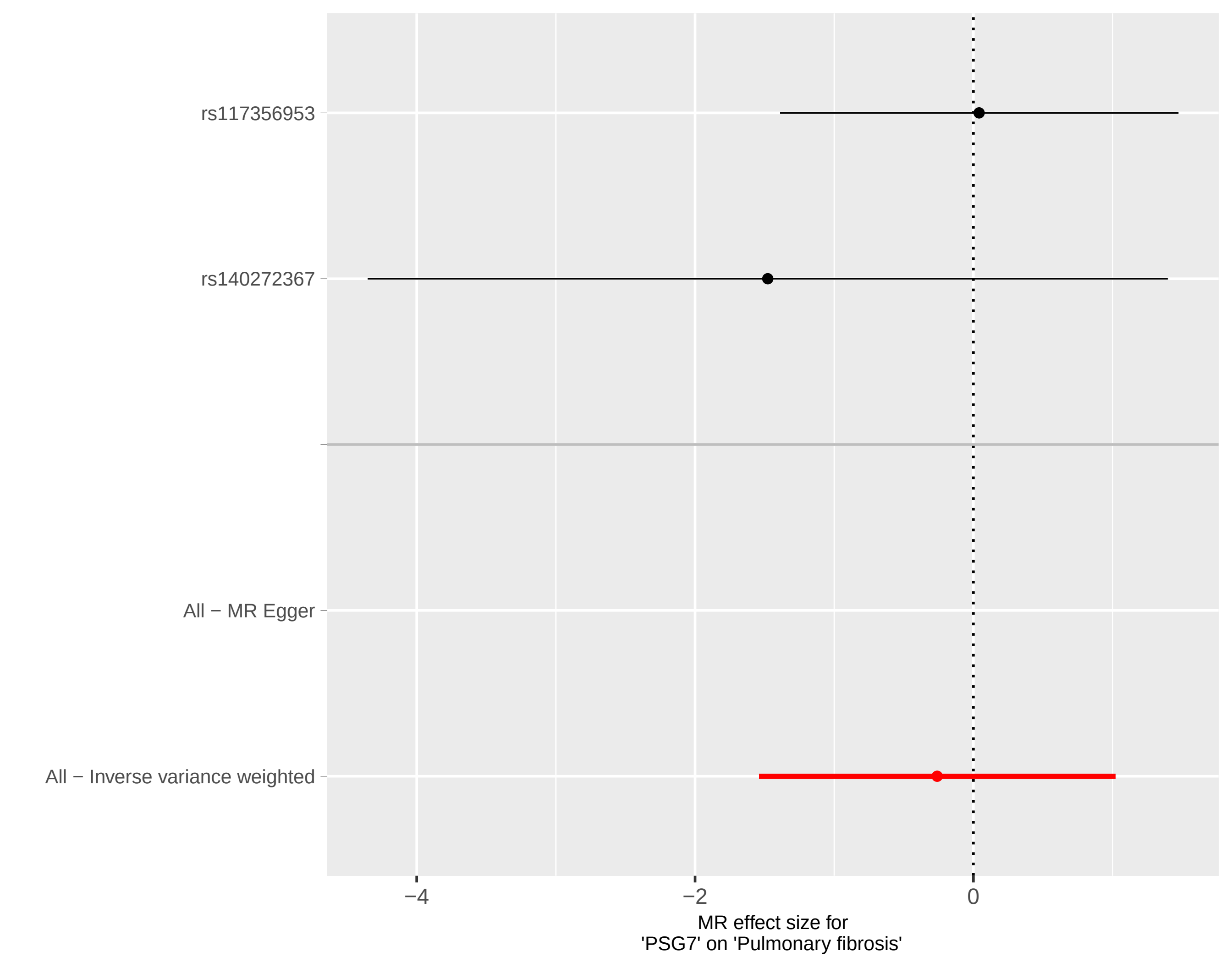
rs117356953
rs140272367
All − MR Egger
All − Inverse variance weighted
−4
−2
0
MR effect size for
'PSG7' on 'Pulmonary fibrosis'

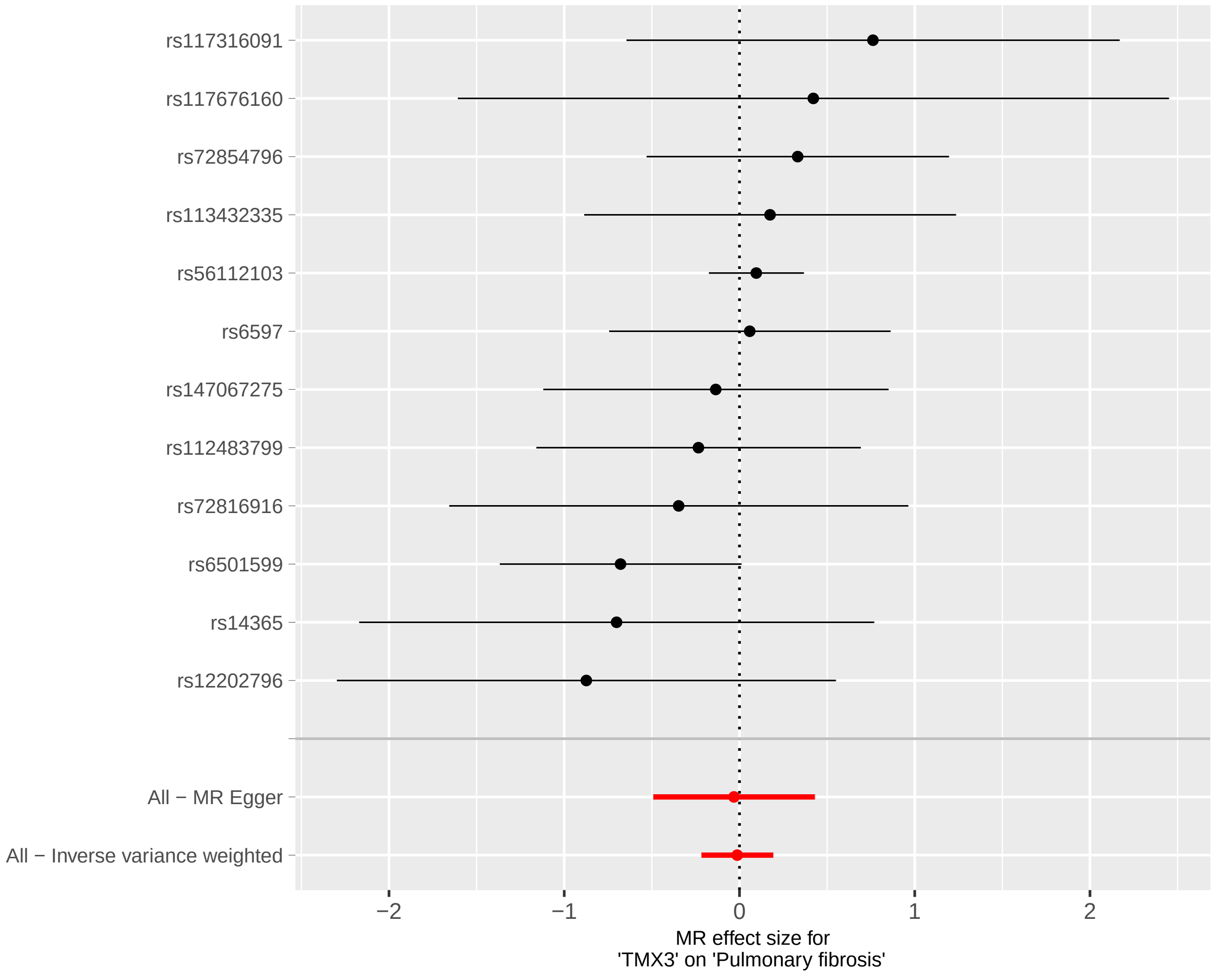

rs117316091
rs117676160
rs72854796
rs113432335
rs56112103
rs6597
rs147067275
rs112483799
rs72816916
rs6501599
rs14365
rs12202796
All − MR Egger
All − Inverse variance weighted
−2
−1
0
1
2
MR effect size for
'TMX3' on 'Pulmonary fibrosis'

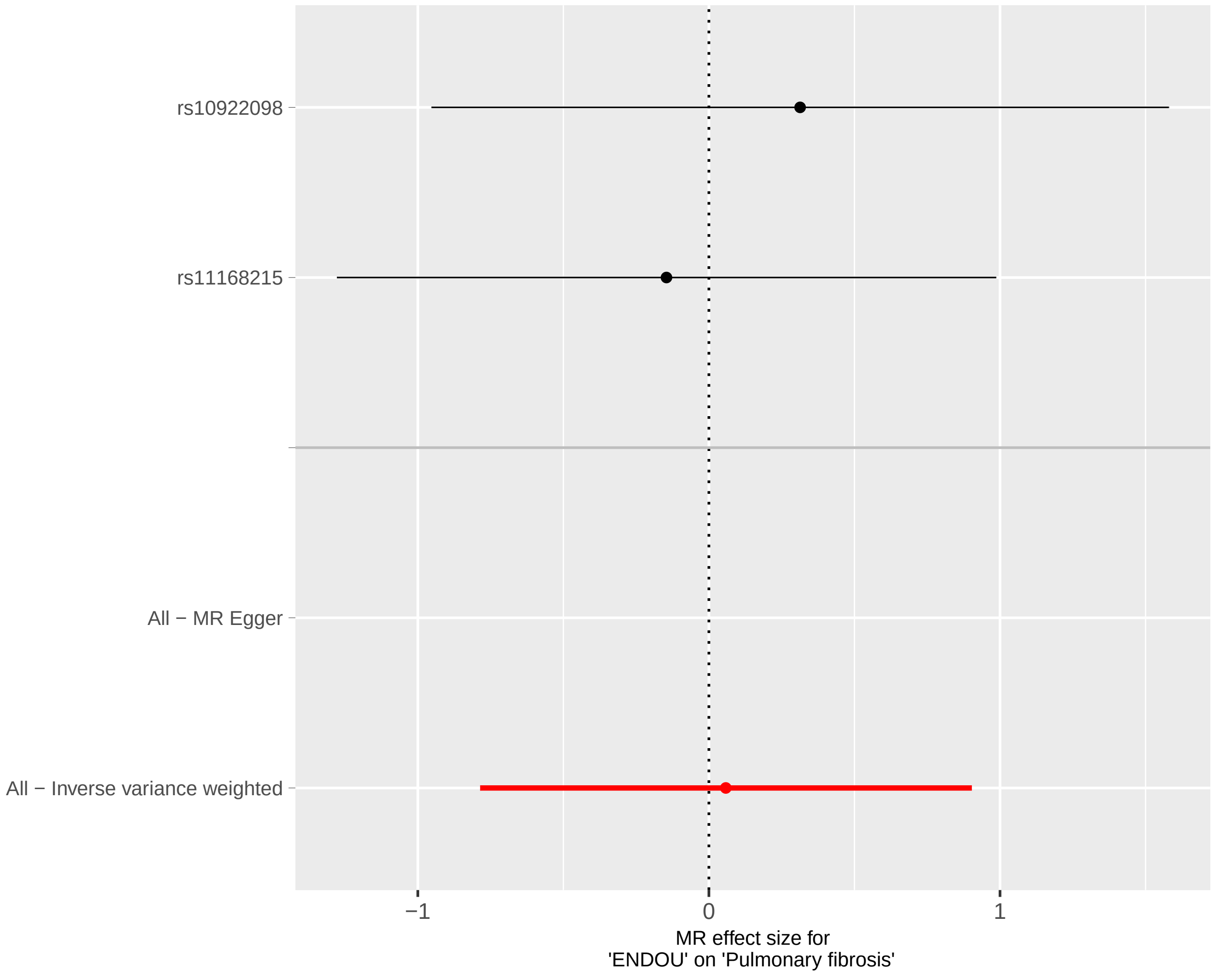
rs10922098
rs11168215
All − MR Egger
All − Inverse variance weighted
−1
0
1
MR effect size for
'ENDOU' on 'Pulmonary fibrosis'

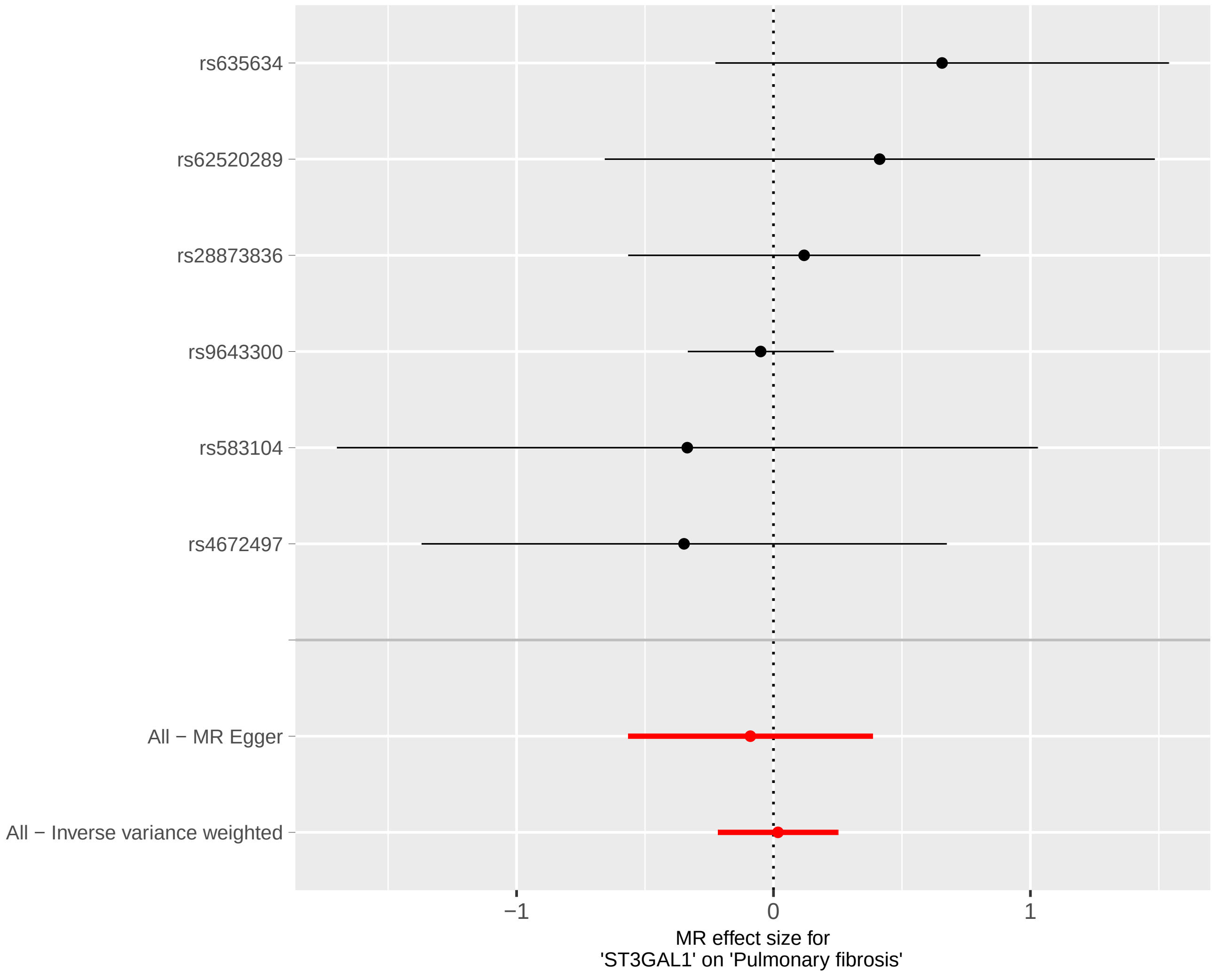
rs635634
rs62520289
rs28873836
rs9643300
rs583104
rs4672497
All − MR Egger
All − Inverse variance weighted
−1
0
1
MR effect size for
'ST3GAL1' on 'Pulmonary fibrosis'

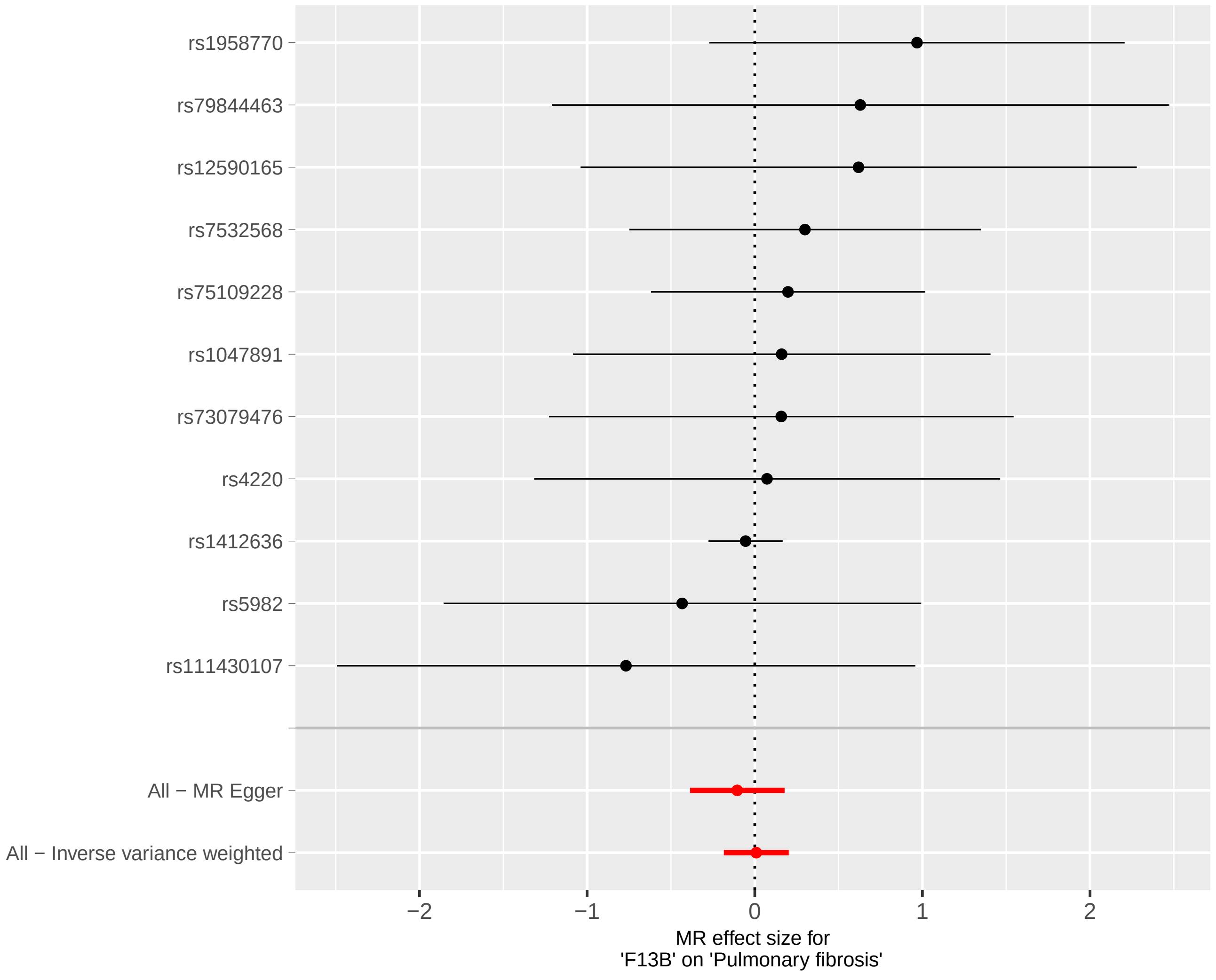

rs1958770
rs79844463
rs12590165
rs7532568
rs75109228
rs1047891
rs73079476
rs4220
rs1412636
rs5982
rs111430107
All − MR Egger
All − Inverse variance weighted
−2
−1
0
1
2
MR effect size for
'F13B' on 'Pulmonary fibrosis'

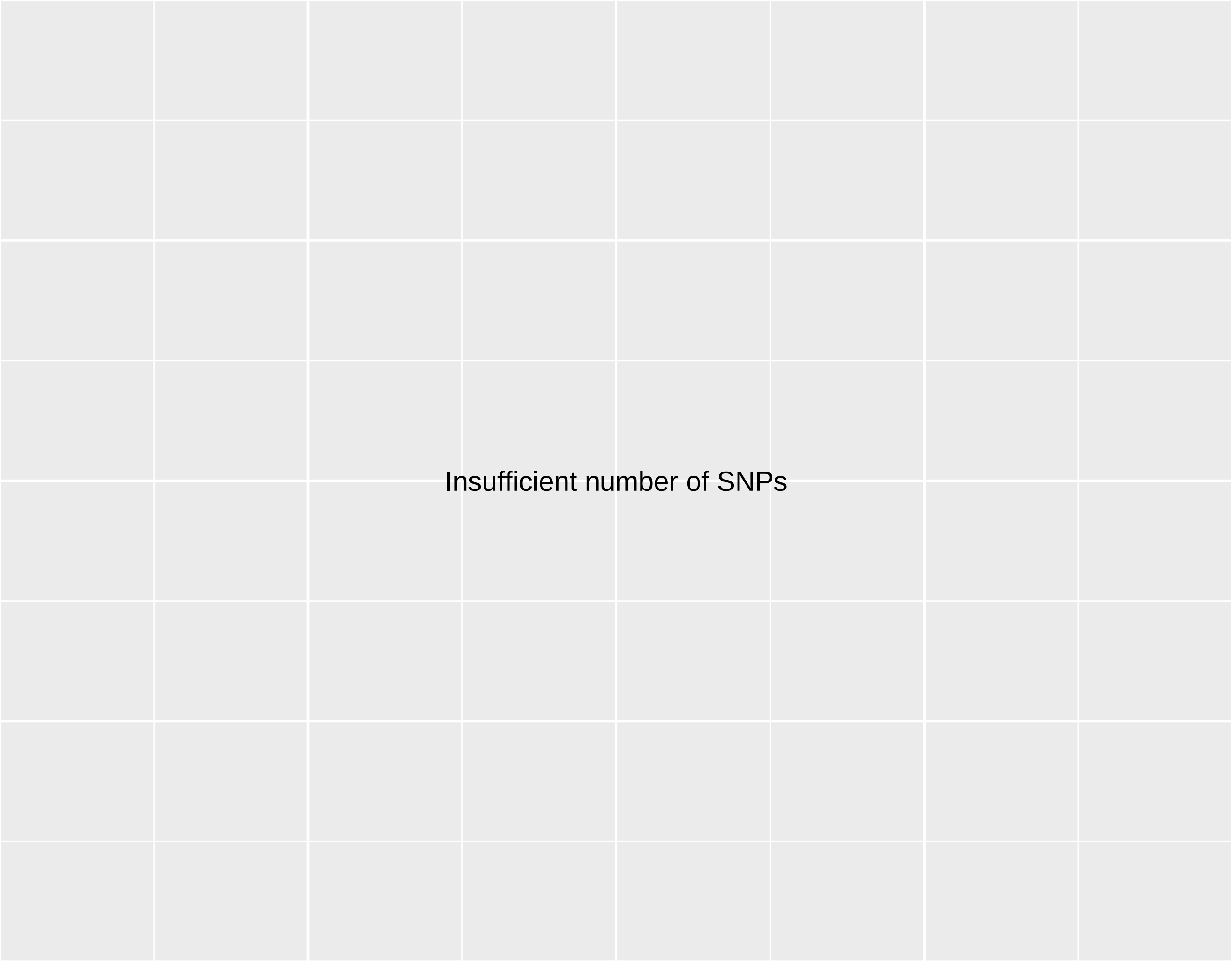
Insufficient number of SNPs

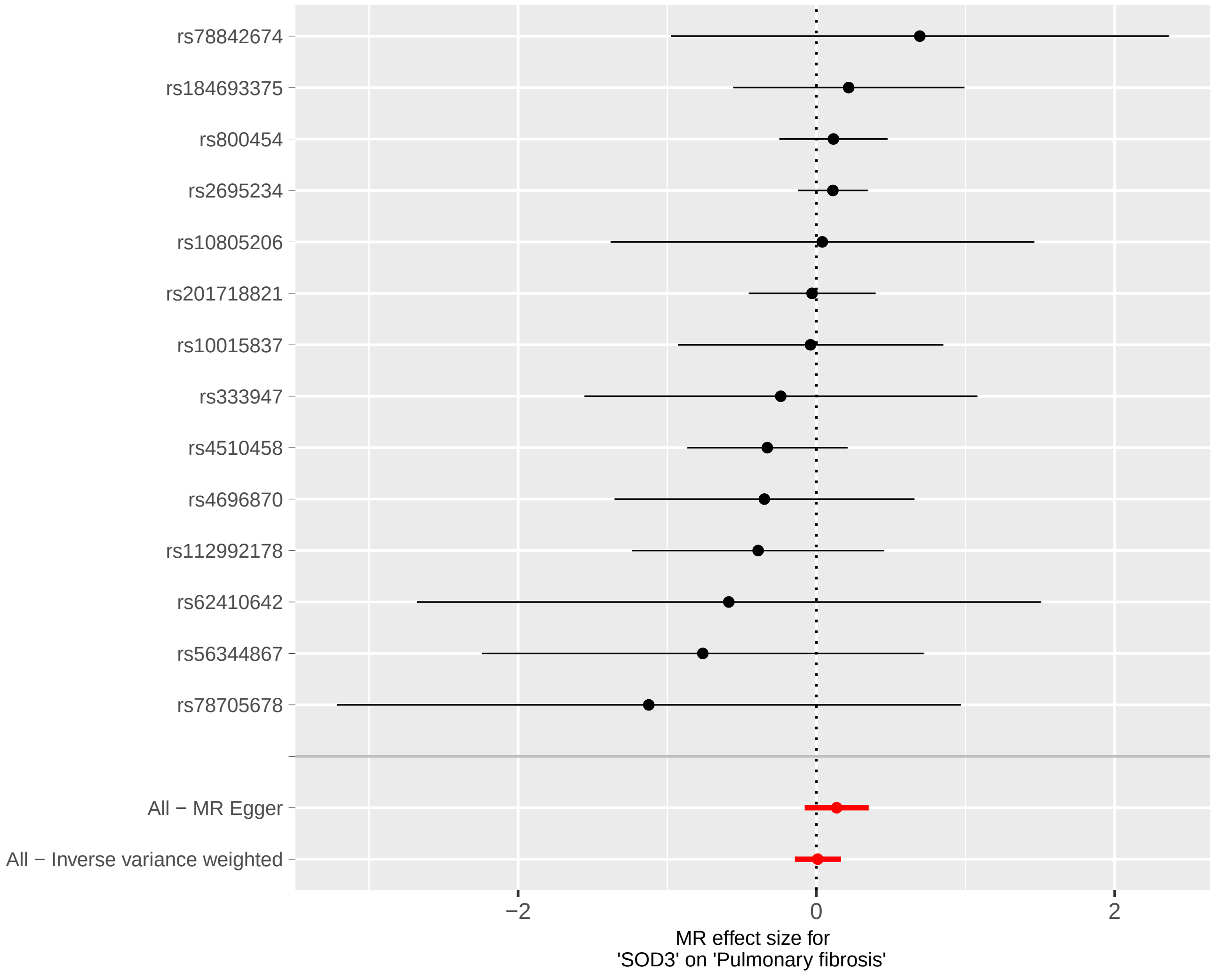

rs78842674
rs184693375
rs800454
rs2695234
rs10805206
rs201718821
rs10015837
rs333947
rs4510458
rs4696870
rs112992178
rs62410642
rs56344867
rs78705678
All − MR Egger
All − Inverse variance weighted
−2
0
2
MR effect size for
'SOD3' on 'Pulmonary fibrosis'

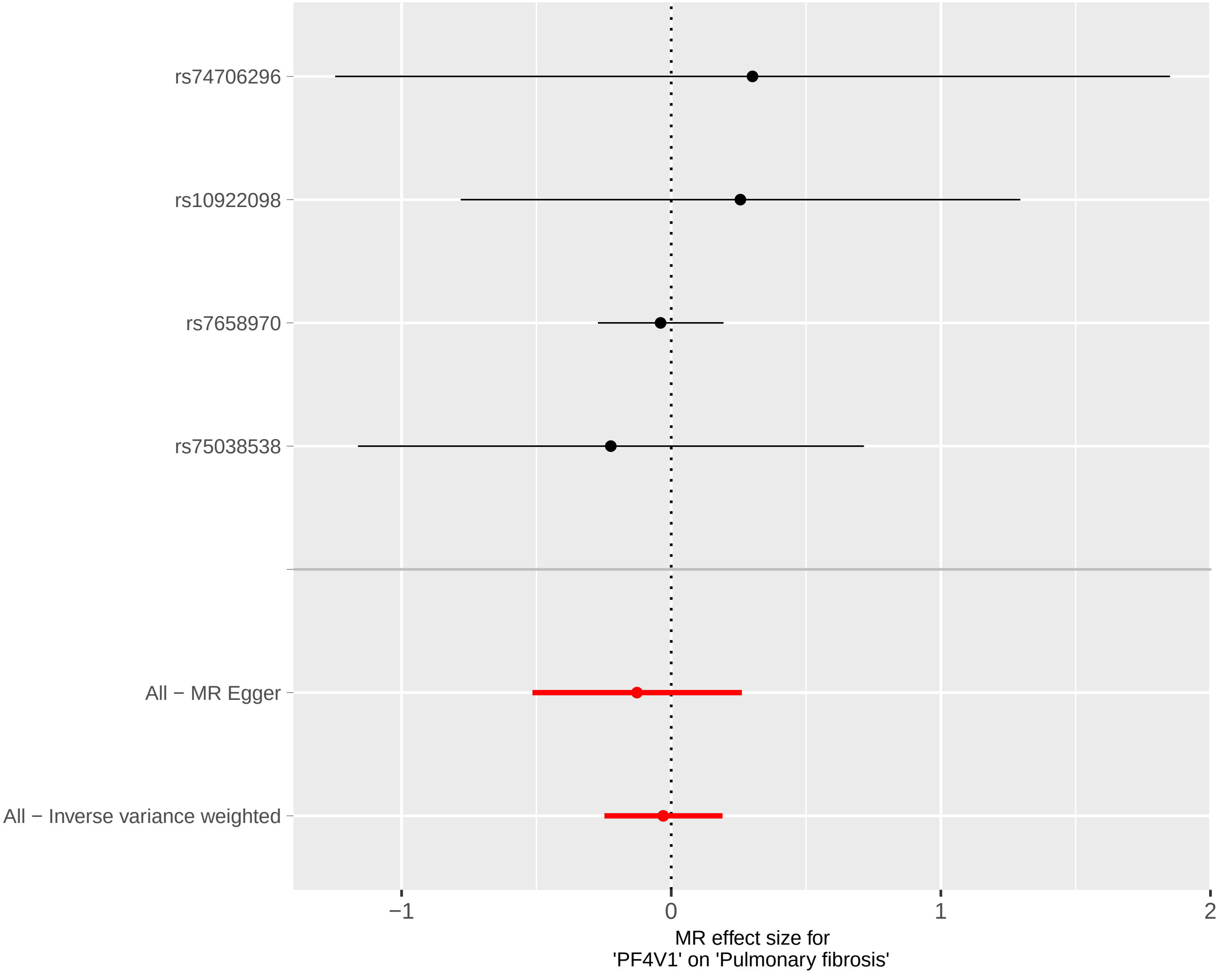

rs74706296
rs10922098
rs7658970
rs75038538
All − MR Egger
All − Inverse variance weighted
−1
0
1
2
MR effect size for
'PF4V1' on 'Pulmonary fibrosis'

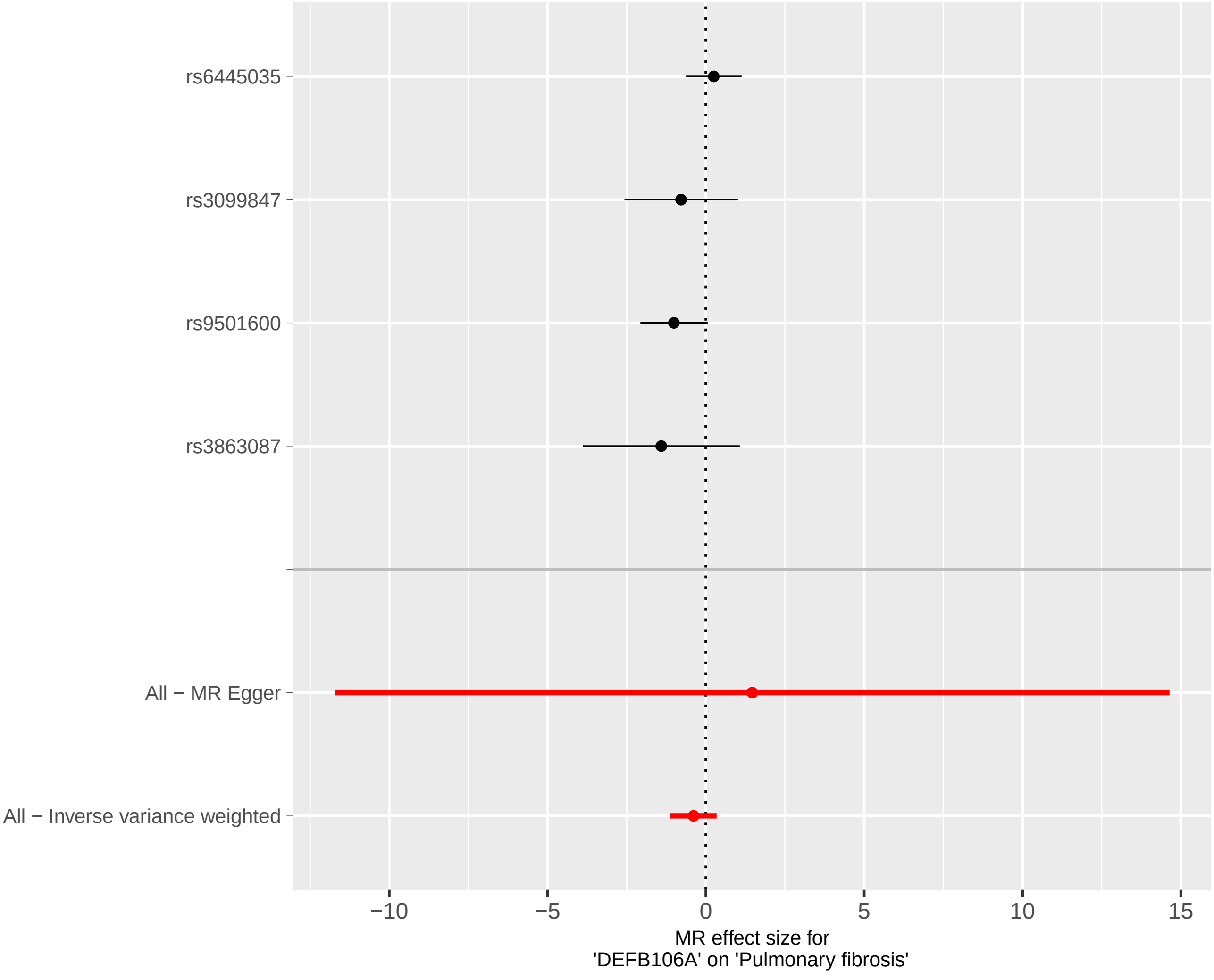

rs6445035
rs3099847
rs9501600
rs3863087
All − MR Egger
All − Inverse variance weighted
−10
−5
0
5
10
15
MR effect size for
'DEFB106A' on 'Pulmonary fibrosis'

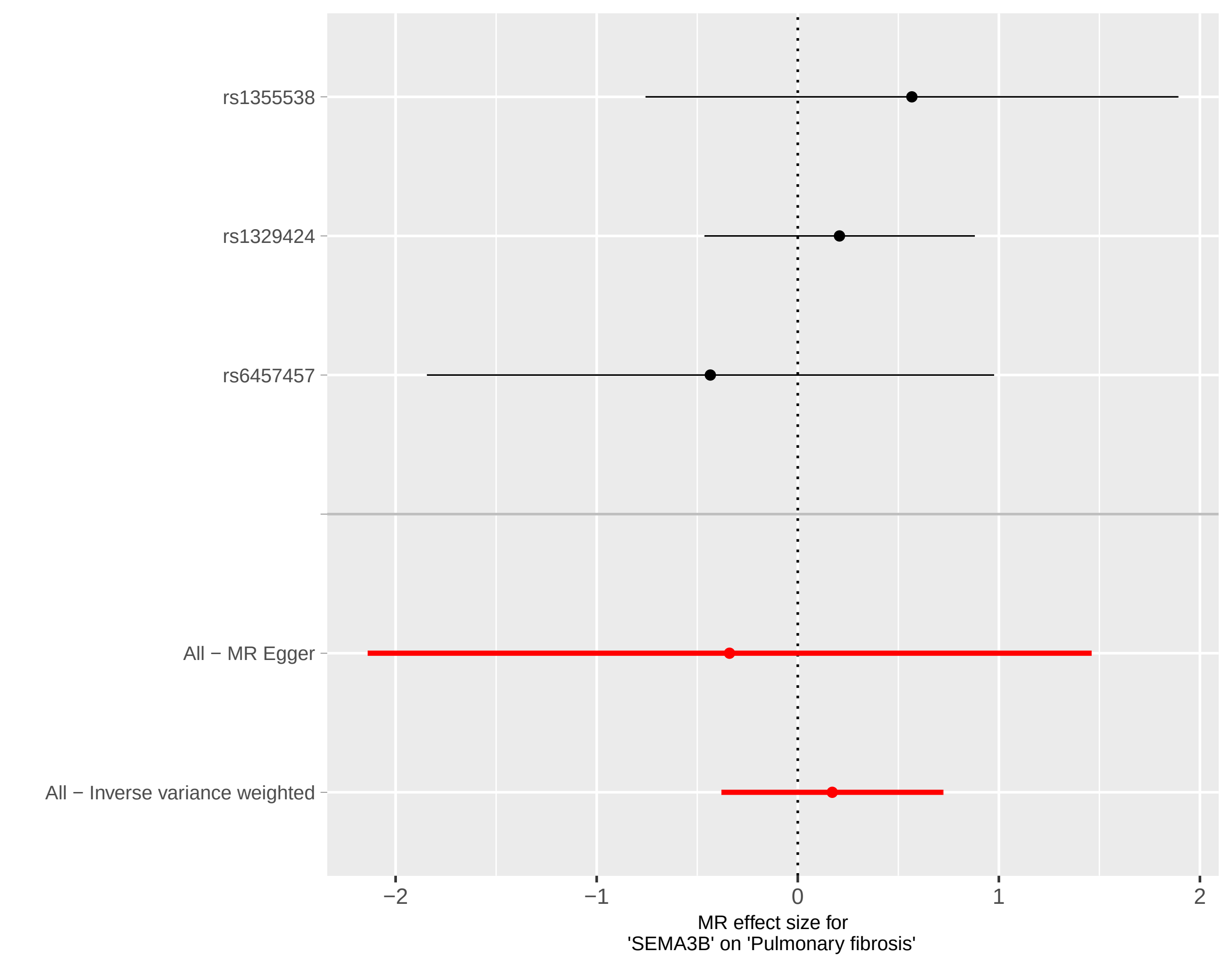
rs1355538
rs1329424
rs6457457
All − MR Egger
All − Inverse variance weighted
−2
−1
0
1
2
MR effect size for
'SEMA3B' on 'Pulmonary fibrosis'

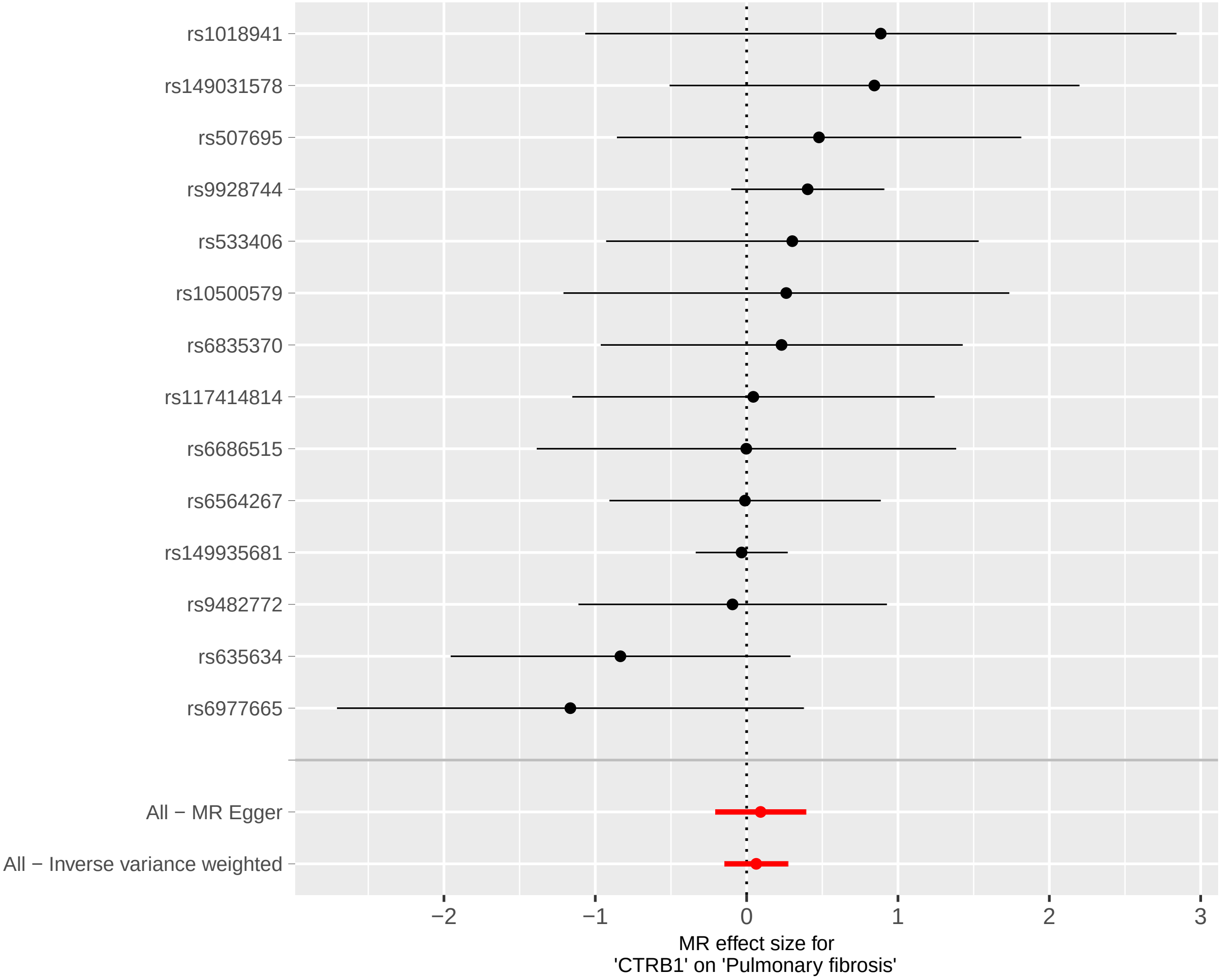
rs1018941
rs149031578
rs507695
rs9928744
rs533406
rs10500579
rs6835370
rs117414814
rs6686515
rs6564267
rs149935681
rs9482772
rs635634
rs6977665
All − MR Egger
All − Inverse variance weighted
−2
−1
0
1
2
3
MR effect size for
'CTRB1' on 'Pulmonary fibrosis'

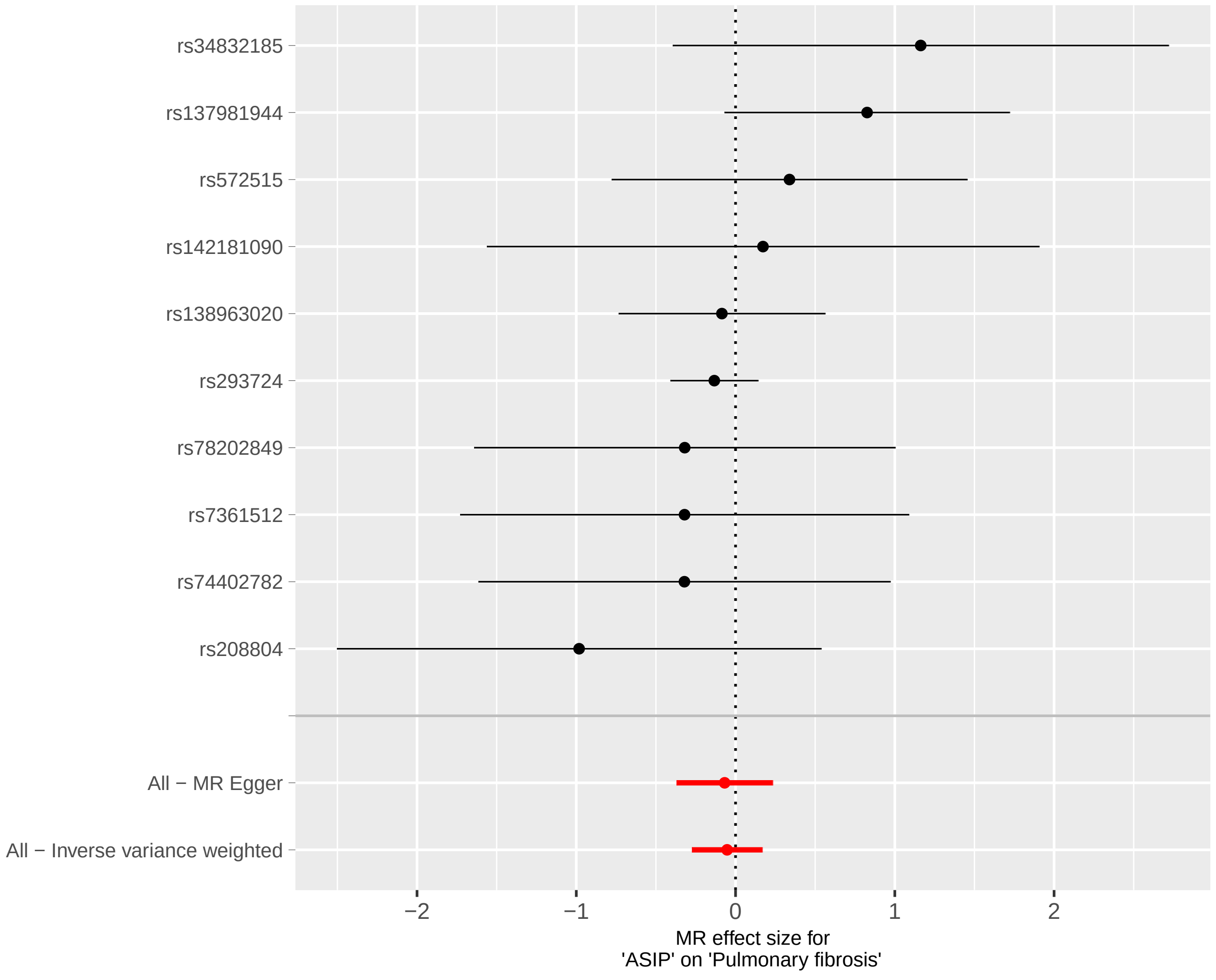
rs34832185
rs137981944
rs572515
rs142181090
rs138963020
rs293724
rs78202849
rs7361512
rs74402782
rs208804
All − MR Egger
All − Inverse variance weighted
−2
−1
0
1
2
MR effect size for
'ASIP' on 'Pulmonary fibrosis'

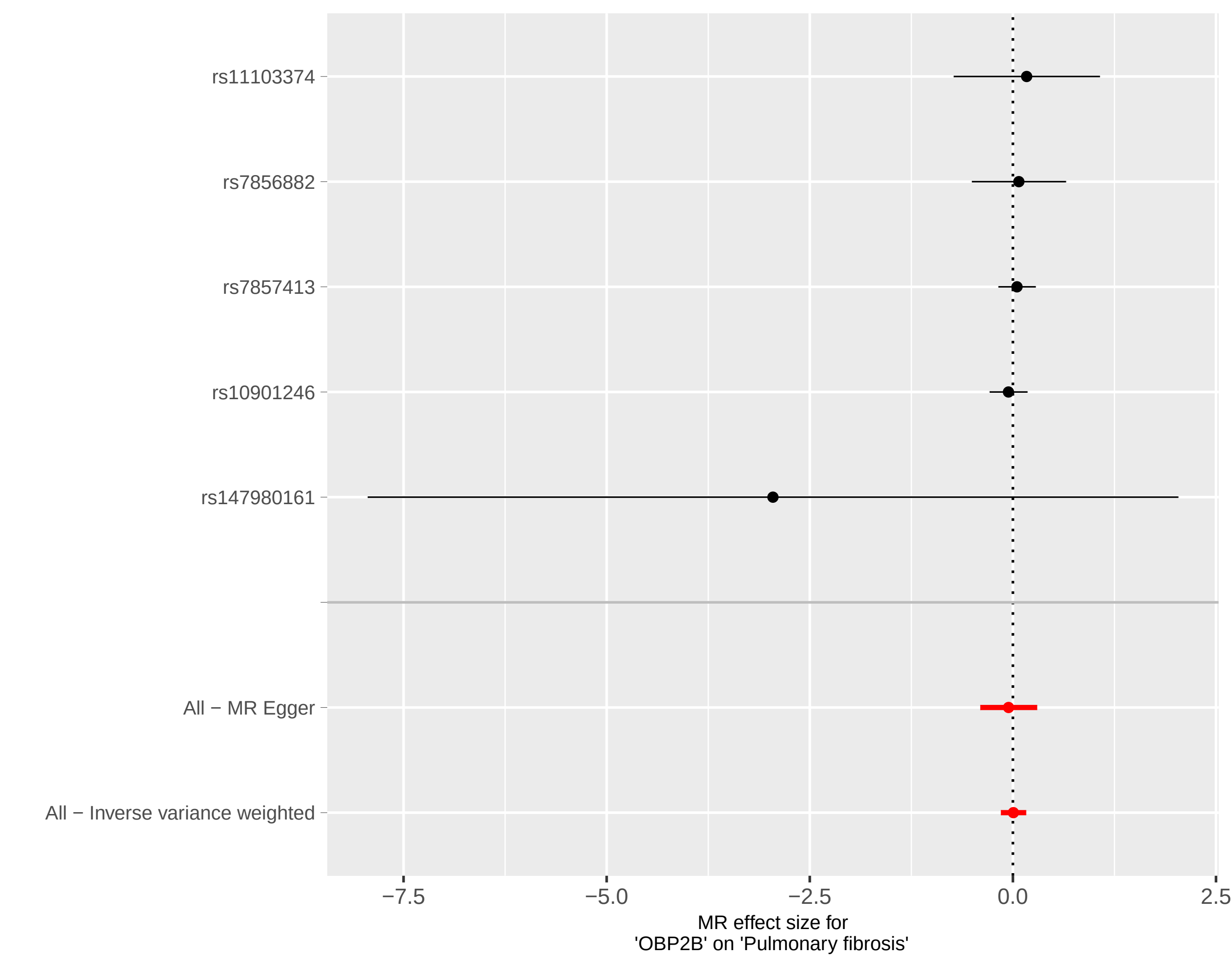

rs11103374
rs7856882
rs7857413
rs10901246
rs147980161
All − MR Egger
All − Inverse variance weighted
−7.5
−5.0
−2.5
0.0
2.5
MR effect size for
'OBP2B' on 'Pulmonary fibrosis'

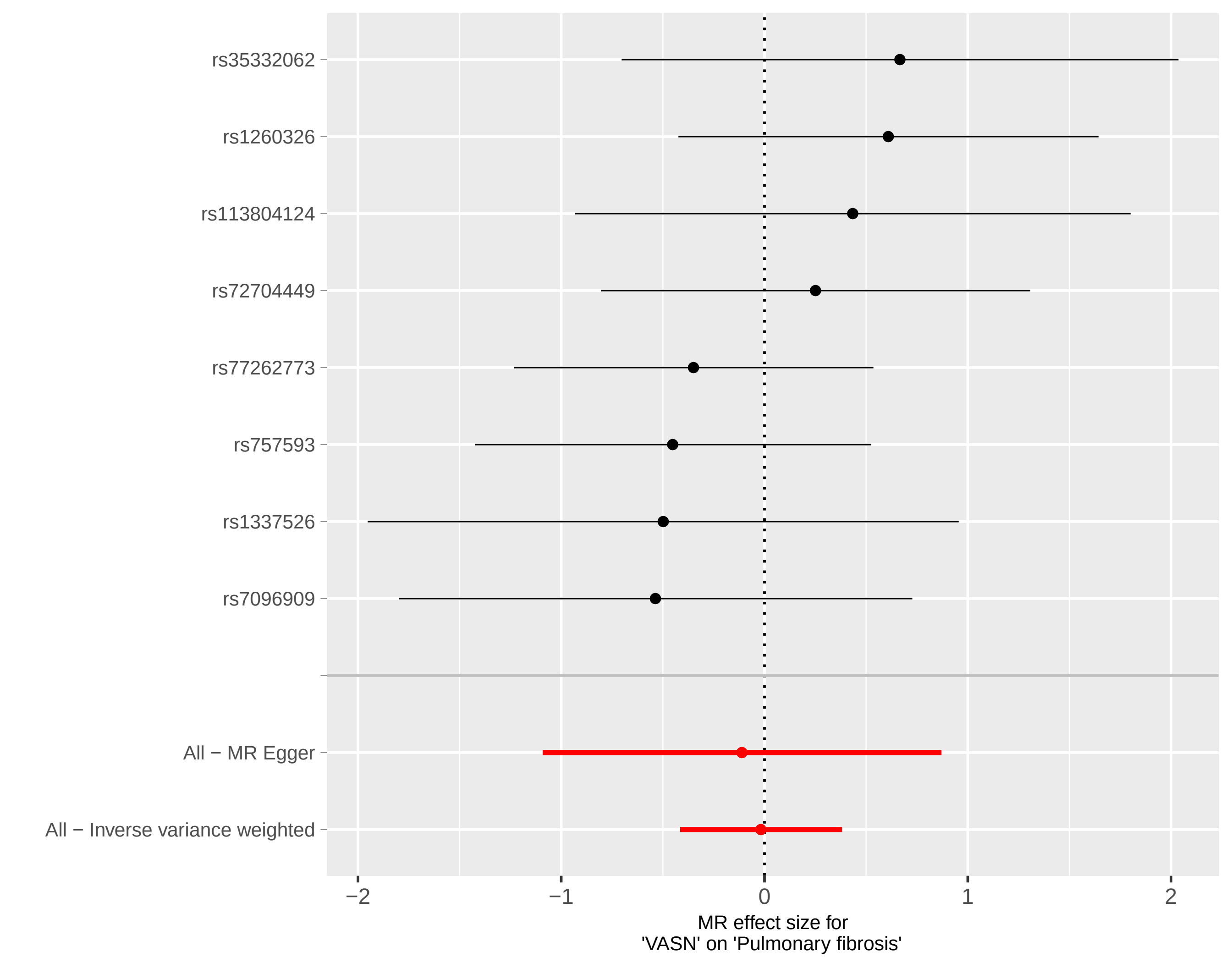

rs35332062
rs1260326
rs113804124
rs72704449
rs77262773
rs757593
rs1337526
rs7096909
All − MR Egger
All − Inverse variance weighted
−2
−1
0
1
2
MR effect size for
'VASN' on 'Pulmonary fibrosis'

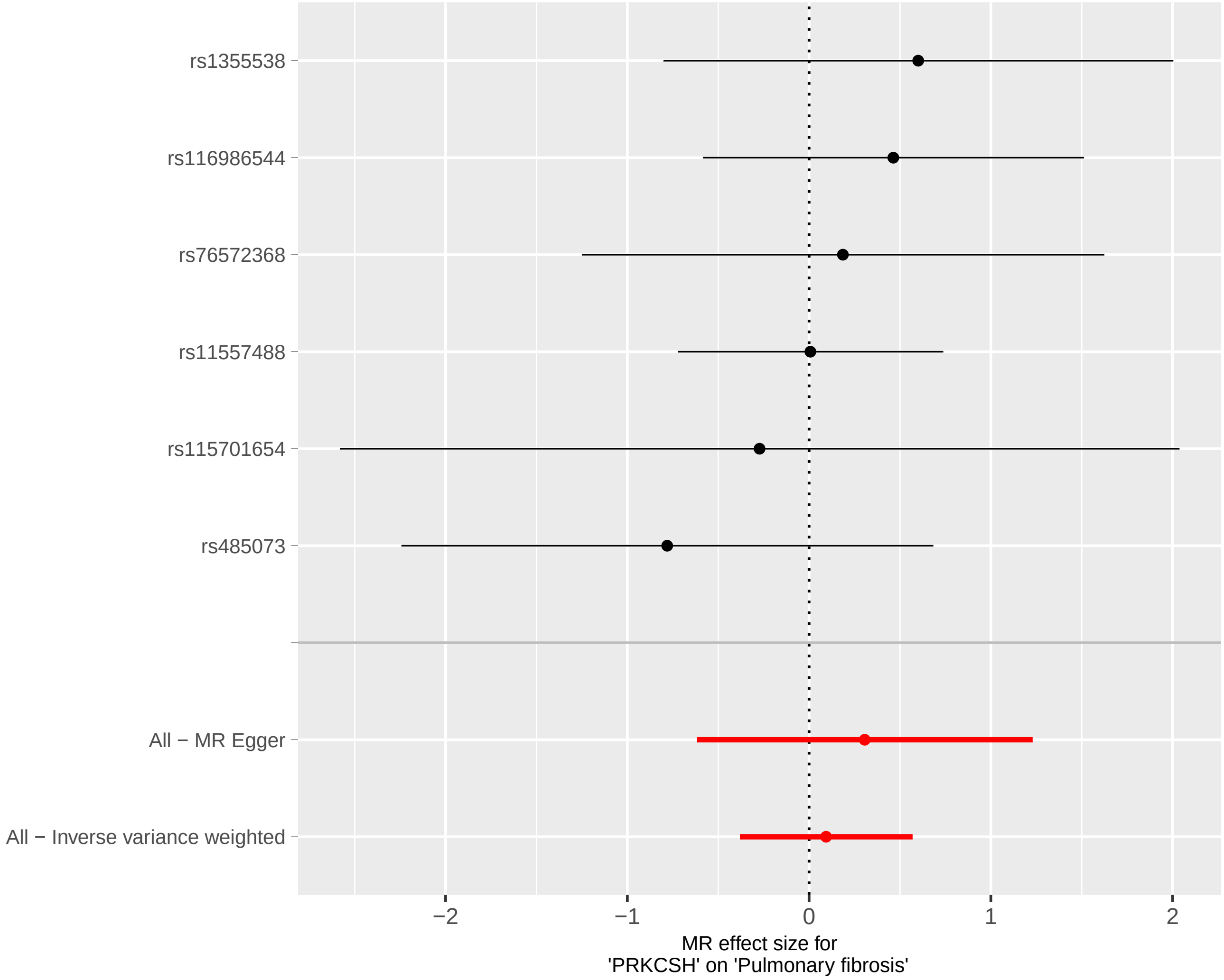
rs1355538
rs116986544
rs76572368
rs11557488
rs115701654
rs485073
All − MR Egger
All − Inverse variance weighted
−2
−1
0
1
2
MR effect size for
'PRKCSH' on 'Pulmonary fibrosis'

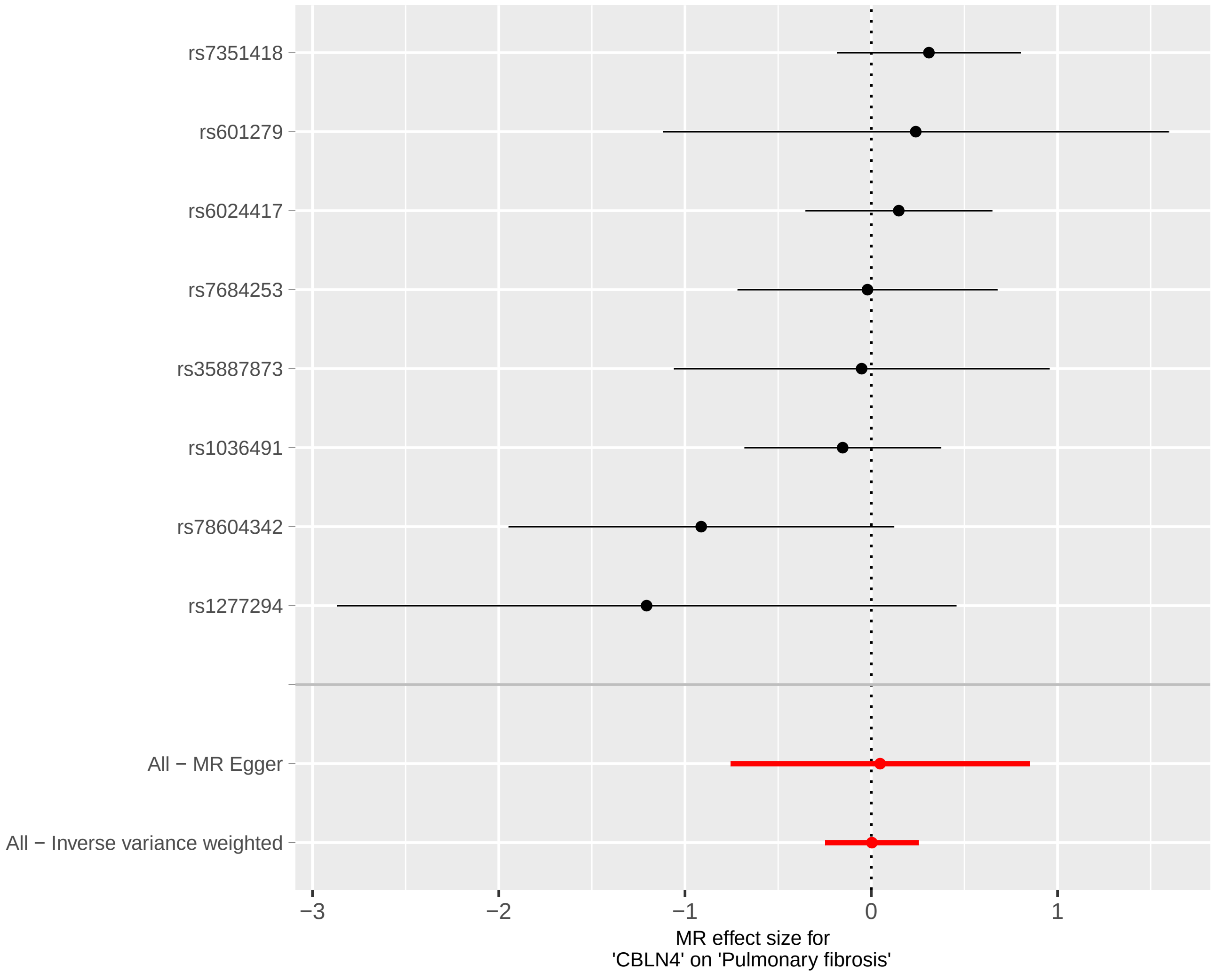
rs7351418
rs601279
rs6024417
rs7684253
rs35887873
rs1036491
rs78604342
rs1277294
All − MR Egger
All − Inverse variance weighted
−3
−2
−1
0
1
MR effect size for
'CBLN4' on 'Pulmonary fibrosis'

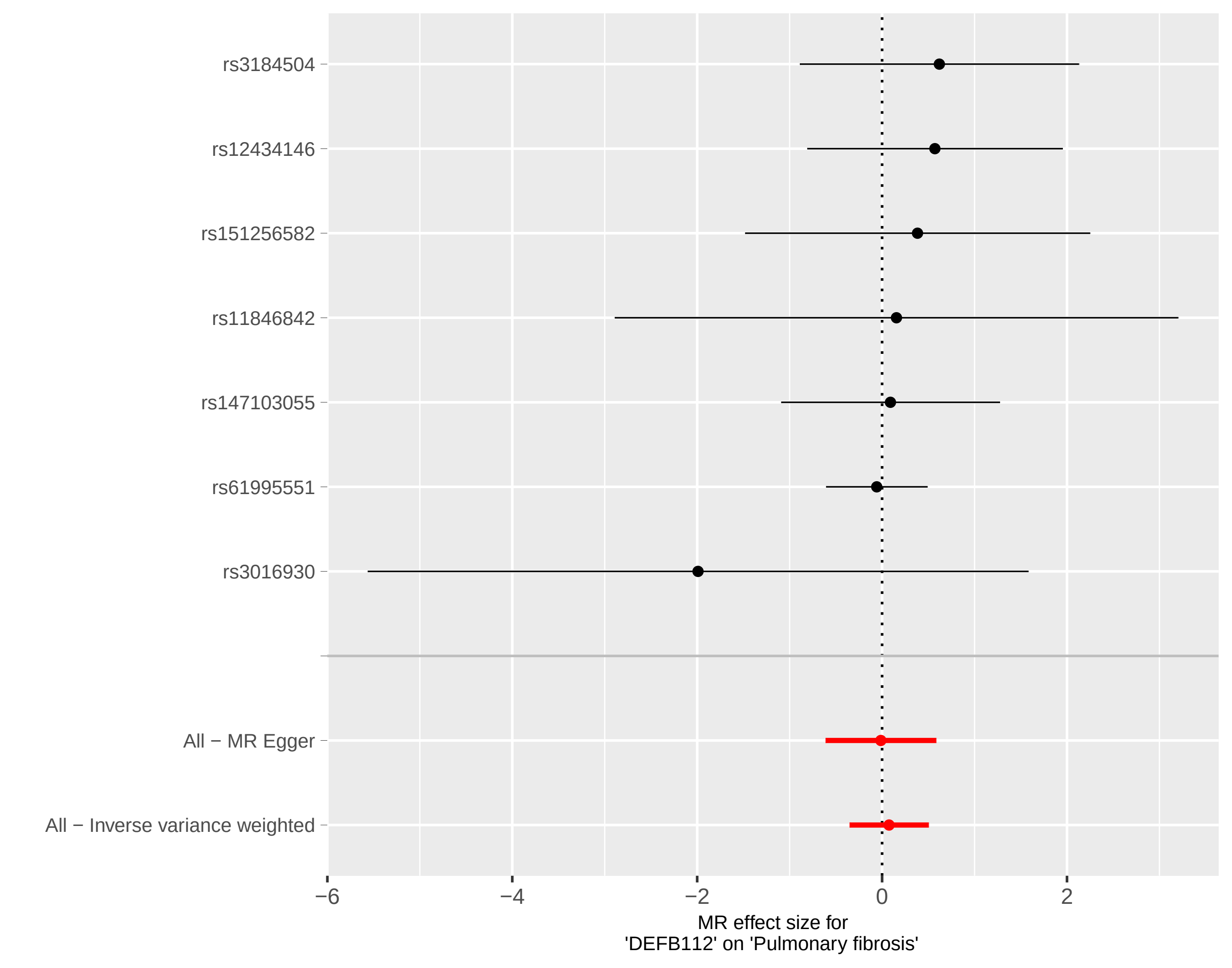
rs3184504
rs12434146
rs151256582
rs11846842
rs147103055
rs61995551
rs3016930
All − MR Egger
All − Inverse variance weighted
−6
−4
−2
0
2
MR effect size for
'DEFB112' on 'Pulmonary fibrosis'

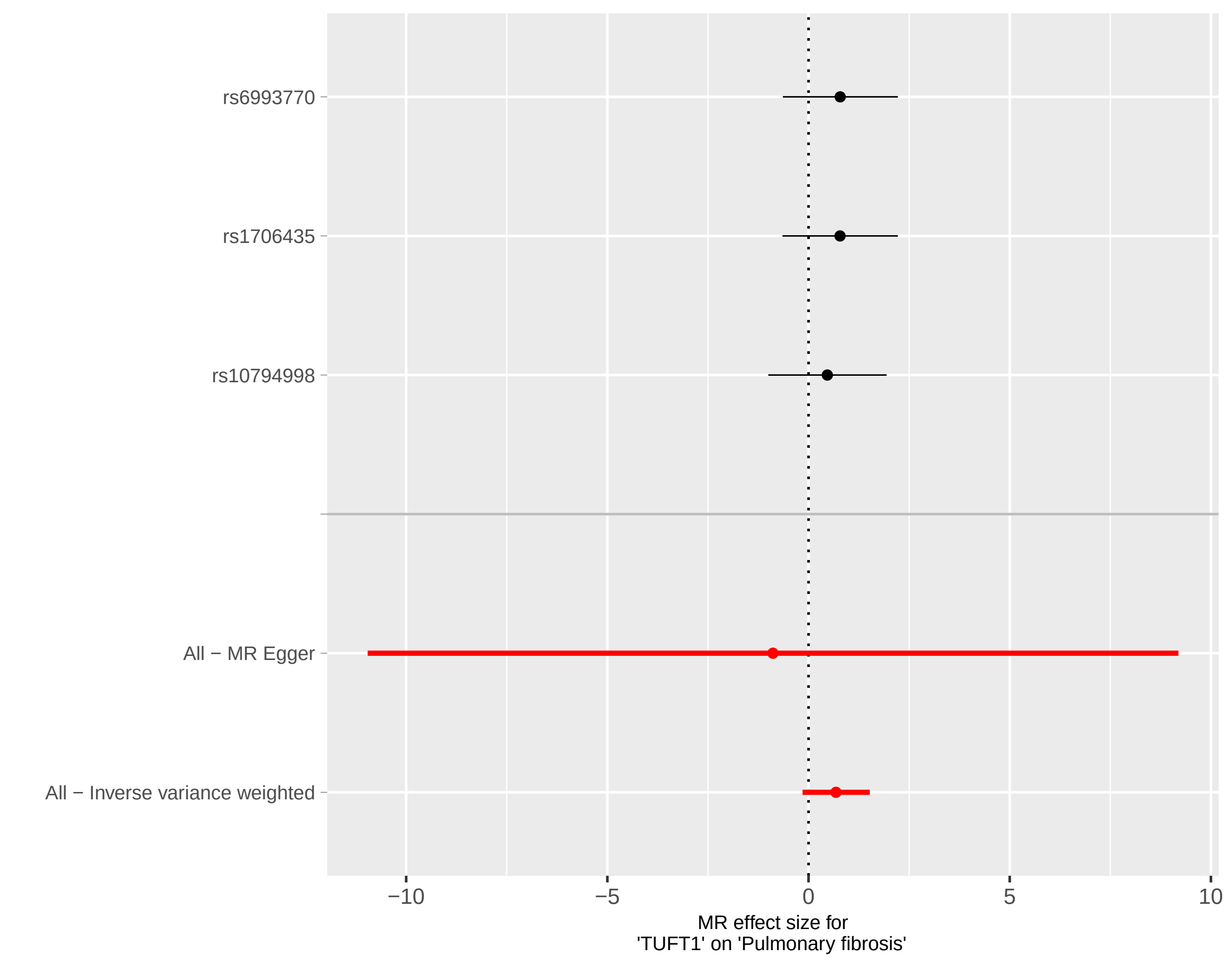

rs6993770
rs1706435
rs10794998
All − MR Egger
All − Inverse variance weighted
−10
−5
0
5
10
MR effect size for
'TUFT1' on 'Pulmonary fibrosis'

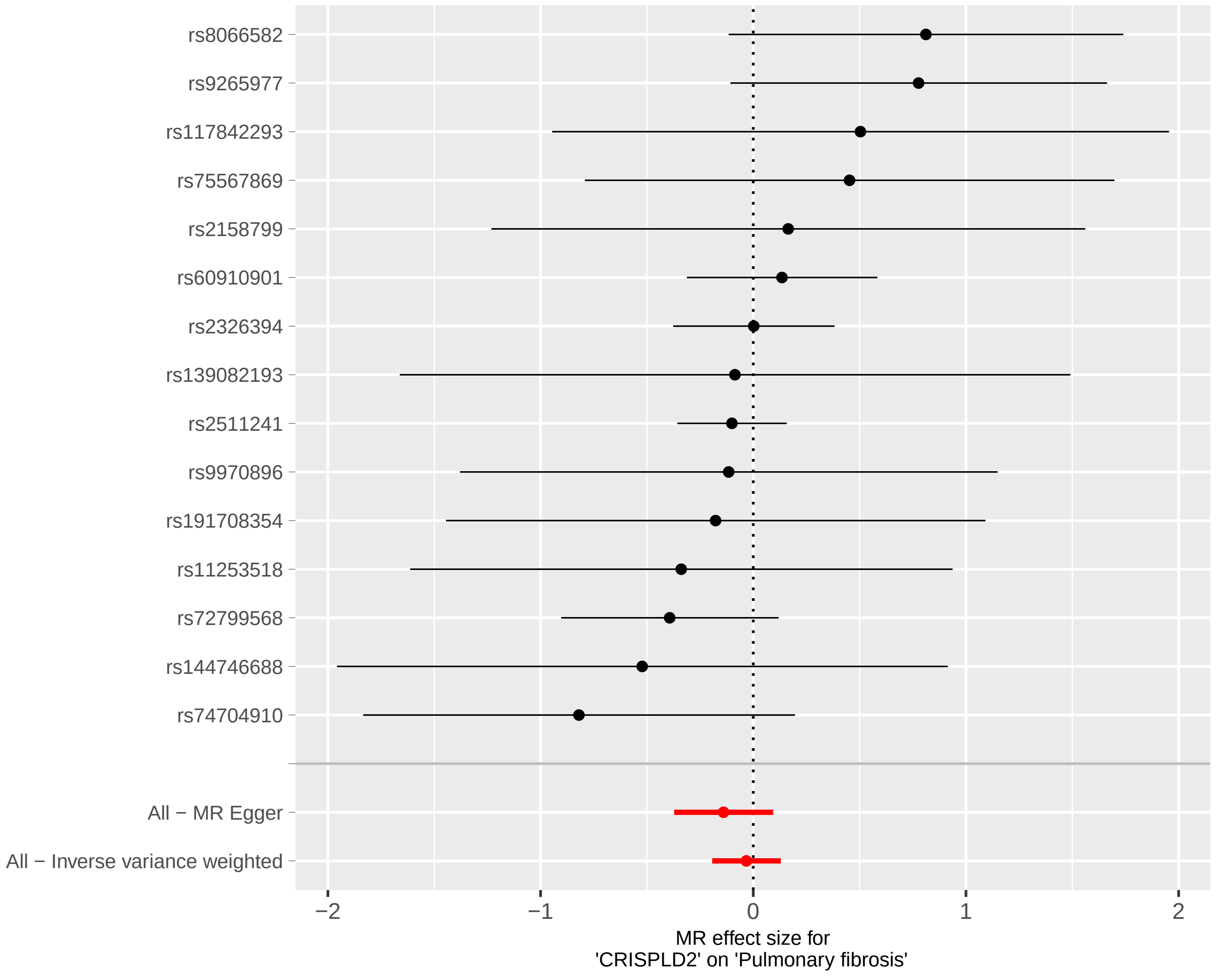
rs8066582
rs9265977
rs117842293
rs75567869
rs2158799
rs60910901
rs2326394
rs139082193
rs2511241
rs9970896
rs191708354
rs11253518
rs72799568
rs144746688
rs74704910
All − MR Egger
All − Inverse variance weighted
−2
−1
0
1
2
MR effect size for
'CRISPLD2' on 'Pulmonary fibrosis'

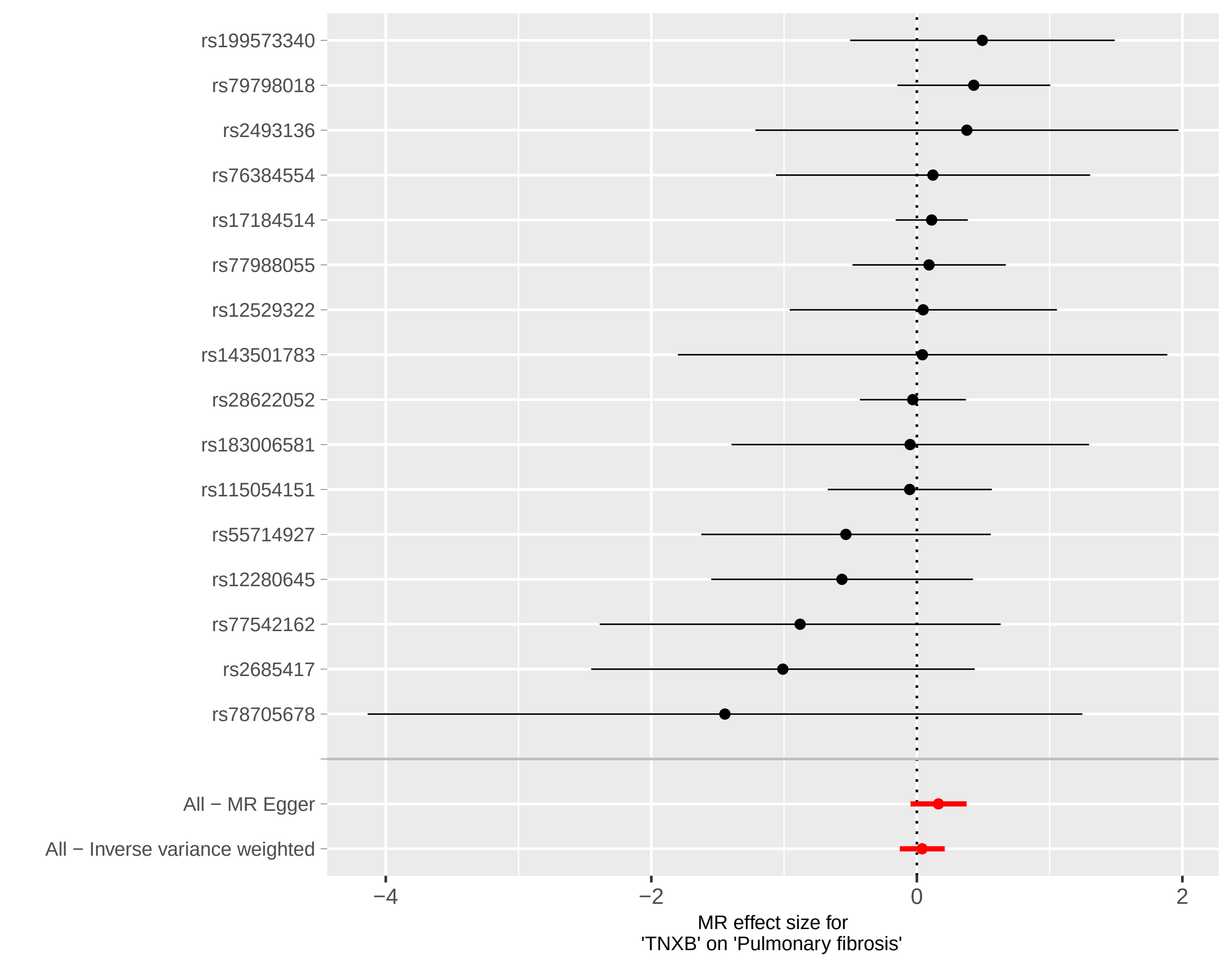
rs199573340
rs79798018
rs2493136
rs76384554
rs17184514
rs77988055
rs12529322
rs143501783
rs28622052
rs183006581
rs115054151
rs55714927
rs12280645
rs77542162
rs2685417
rs78705678
All − MR Egger
All − Inverse variance weighted
−4
−2
0
2
MR effect size for
'TNXB' on 'Pulmonary fibrosis'

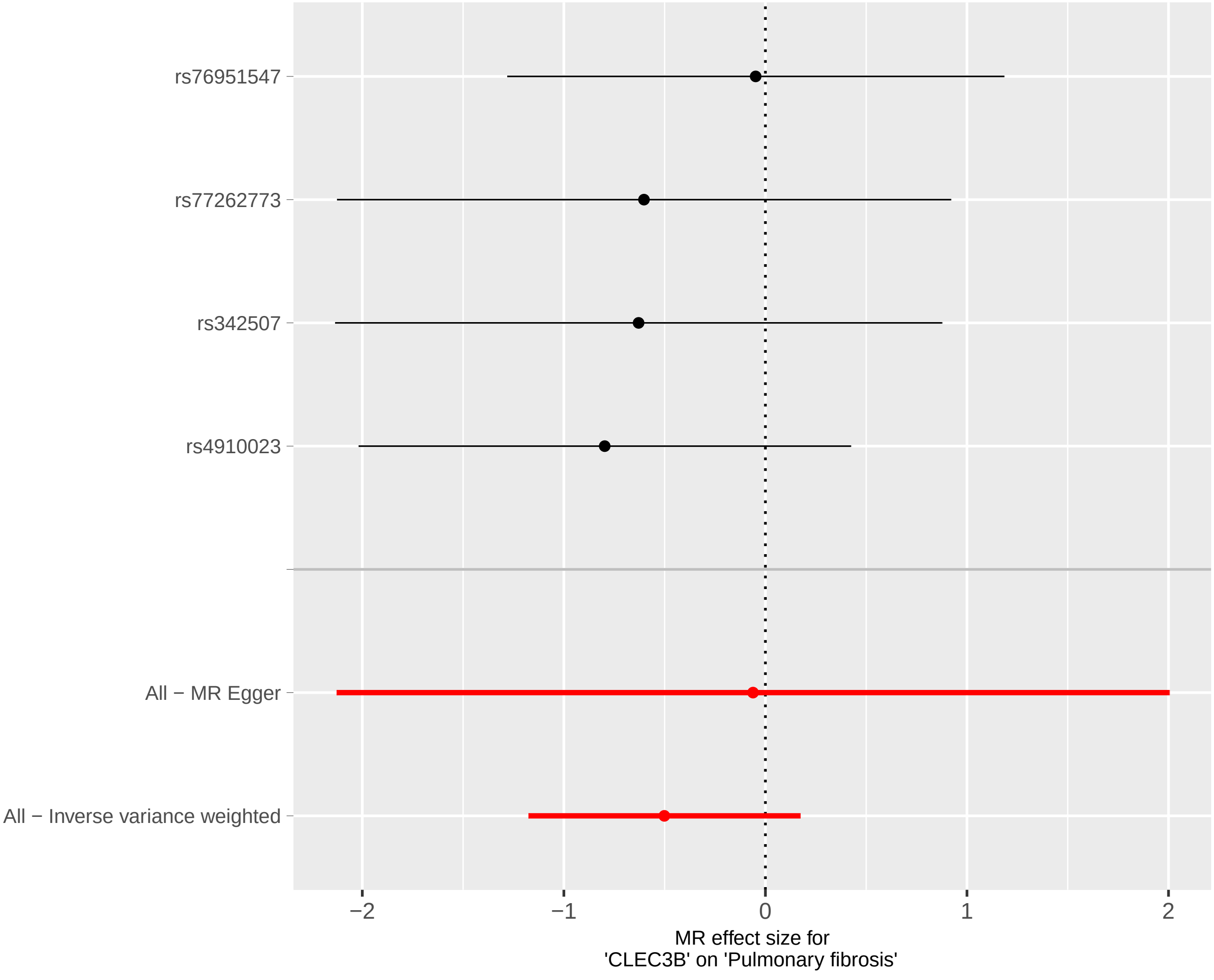
rs76951547
rs77262773
rs342507
rs4910023
All − MR Egger
All − Inverse variance weighted
−2
−1
0
1
2
MR effect size for
'CLEC3B' on 'Pulmonary fibrosis'

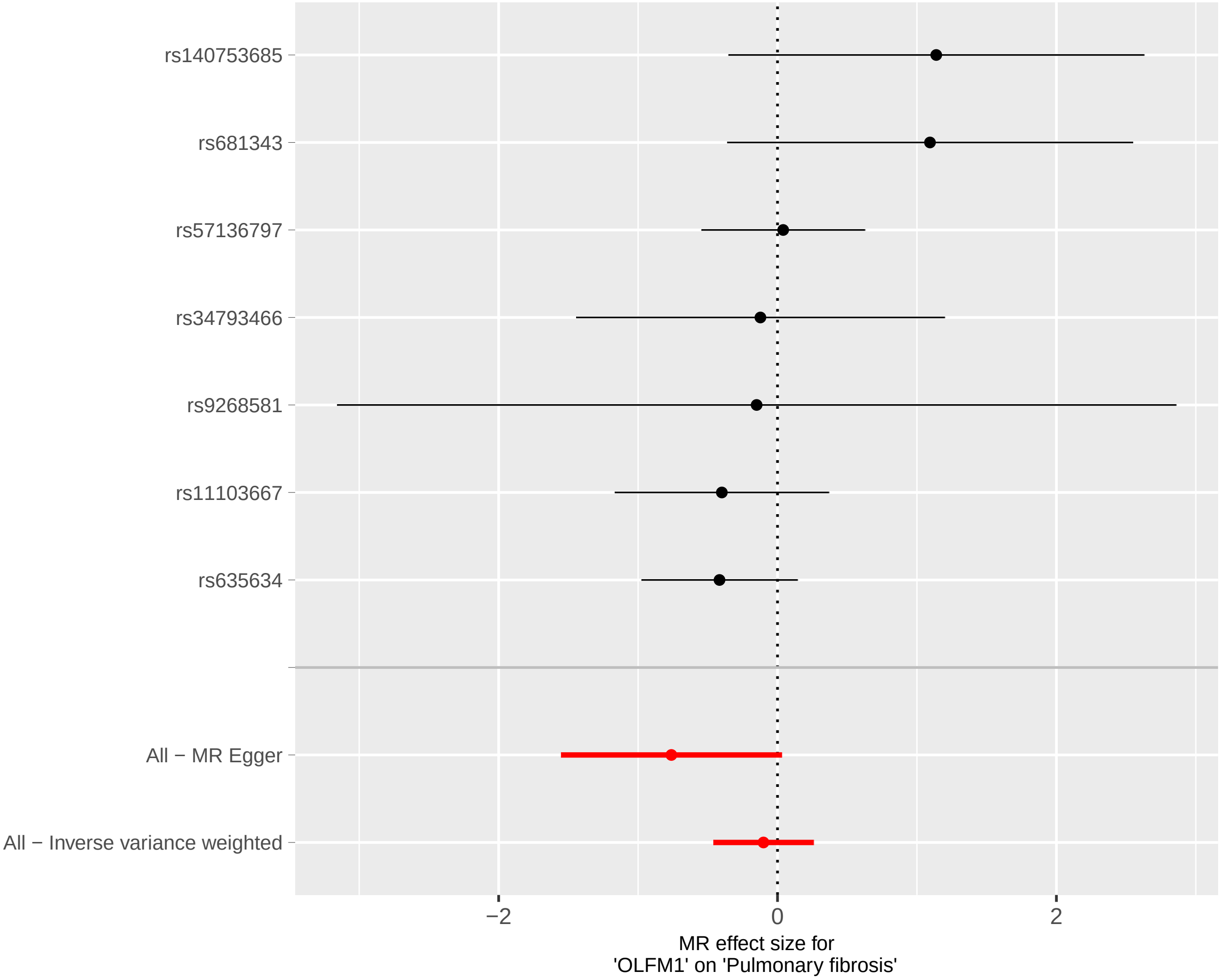
rs140753685
rs681343
rs57136797
rs34793466
rs9268581
rs11103667
rs635634
All − MR Egger
All − Inverse variance weighted
−2
0
2
MR effect size for
'OLFM1' on 'Pulmonary fibrosis'

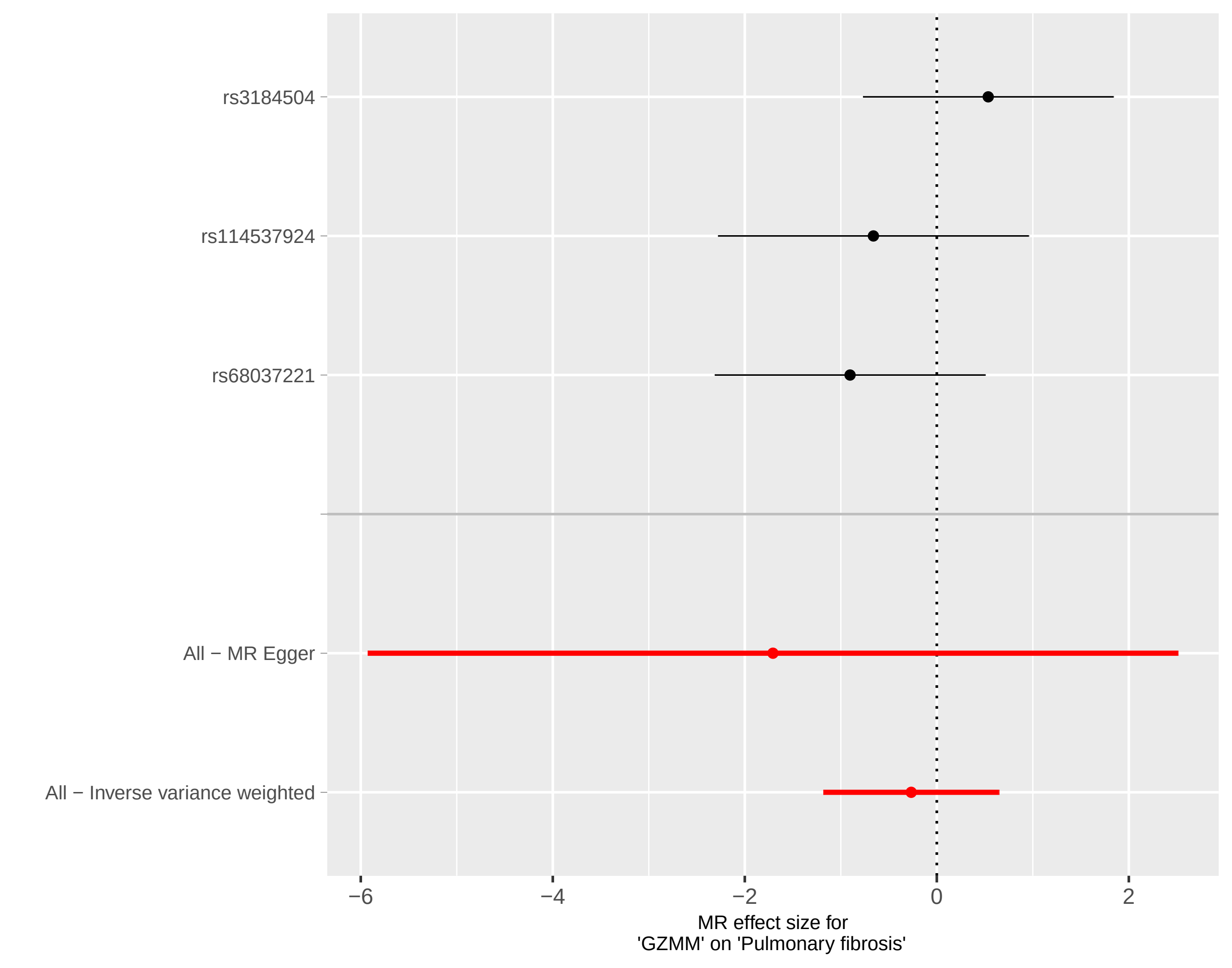
rs3184504
rs114537924
rs68037221
All − MR Egger
All − Inverse variance weighted
−6
−4
−2
0
2
MR effect size for
'GZMM' on 'Pulmonary fibrosis'

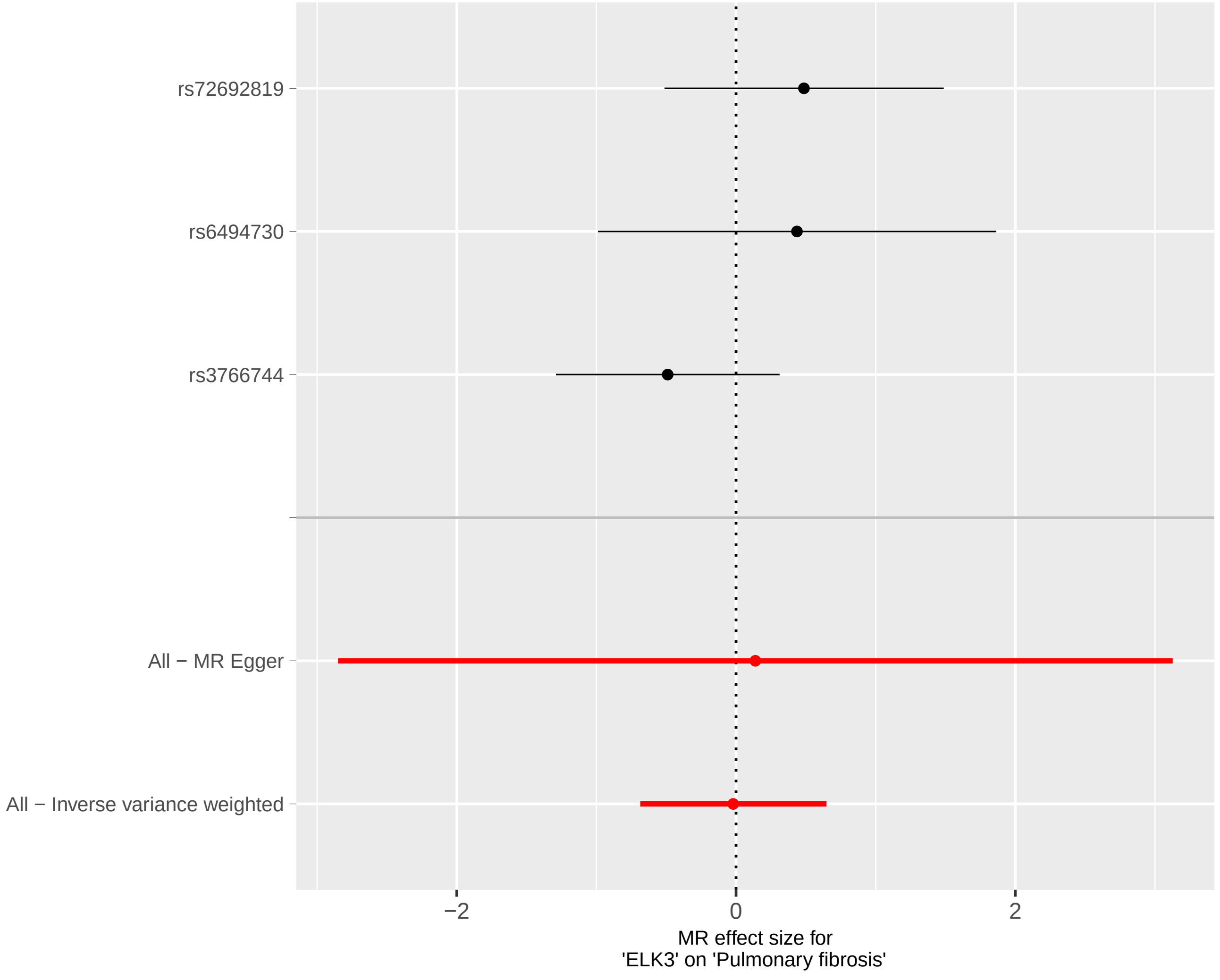
rs72692819
rs6494730
rs3766744
All − MR Egger
All − Inverse variance weighted
−2
0
2
MR effect size for
'ELK3' on 'Pulmonary fibrosis'

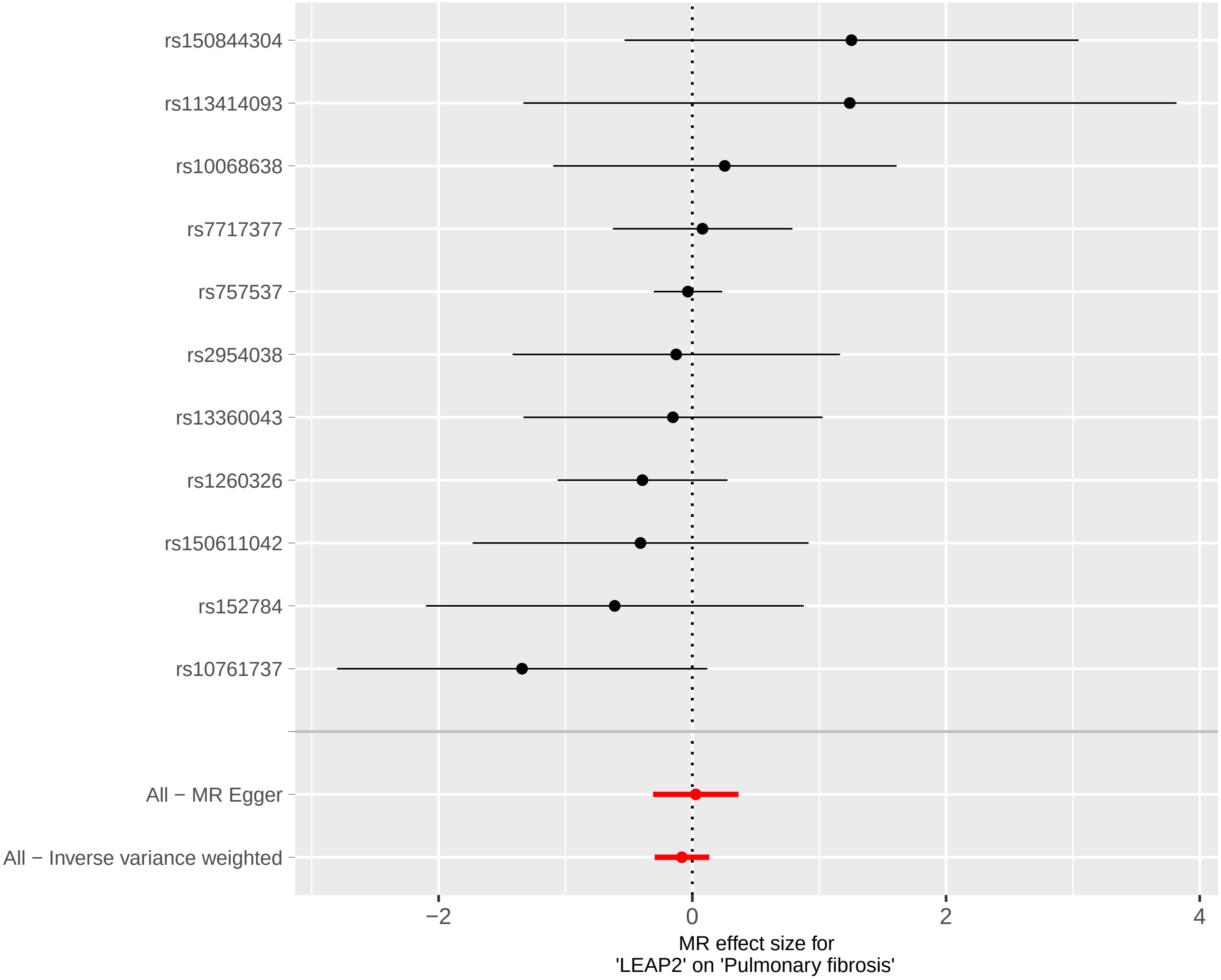
rs150844304
rs113414093
rs10068638
rs7717377
rs757537
rs2954038
rs13360043
rs1260326
rs150611042
rs152784
rs10761737
All − MR Egger
All − Inverse variance weighted
−2
0
2
4
MR effect size for
'LEAP2' on 'Pulmonary fibrosis'

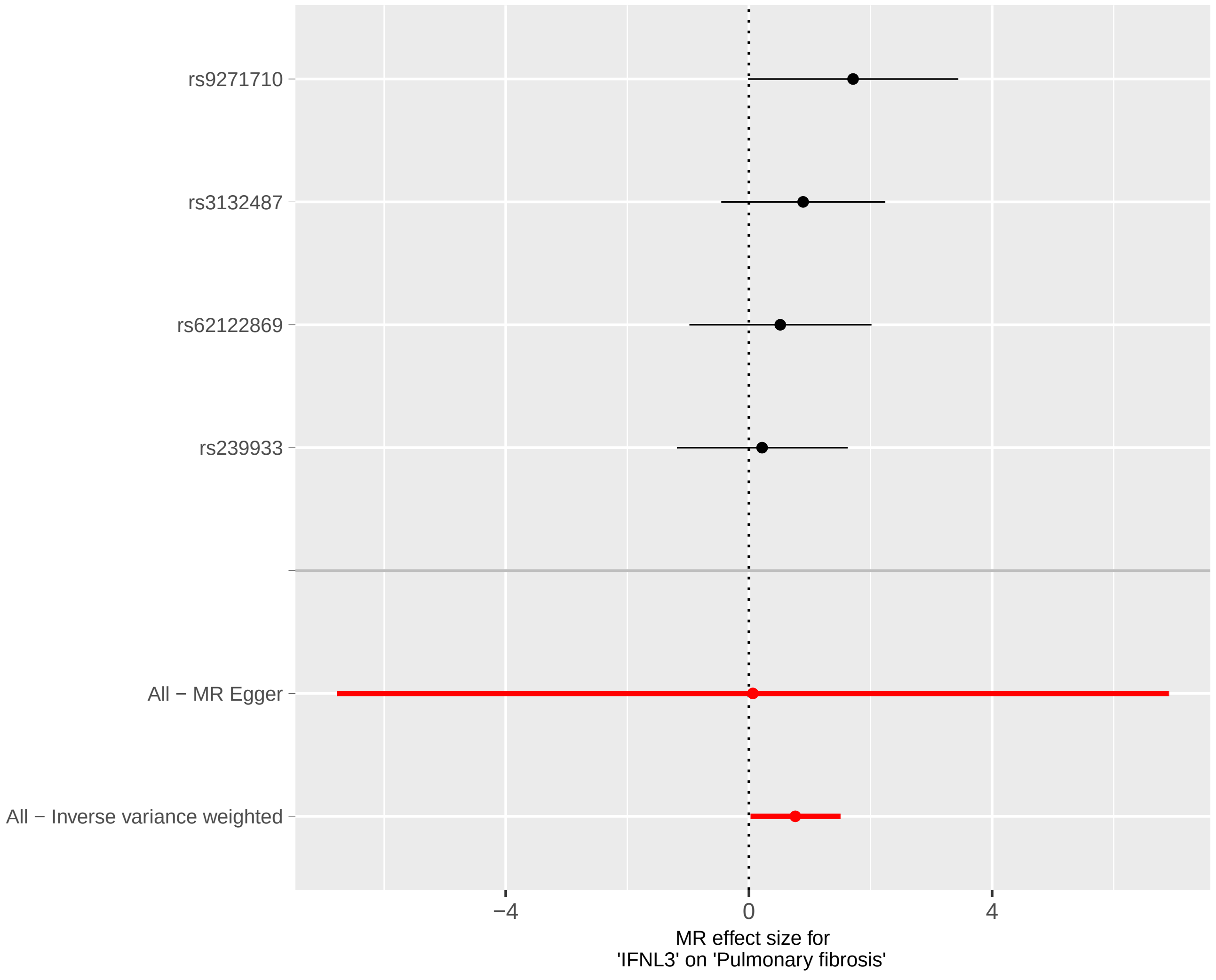
rs9271710
rs3132487
rs62122869
rs239933
All − MR Egger
All − Inverse variance weighted
−4
0
4
MR effect size for
'IFNL3' on 'Pulmonary fibrosis'

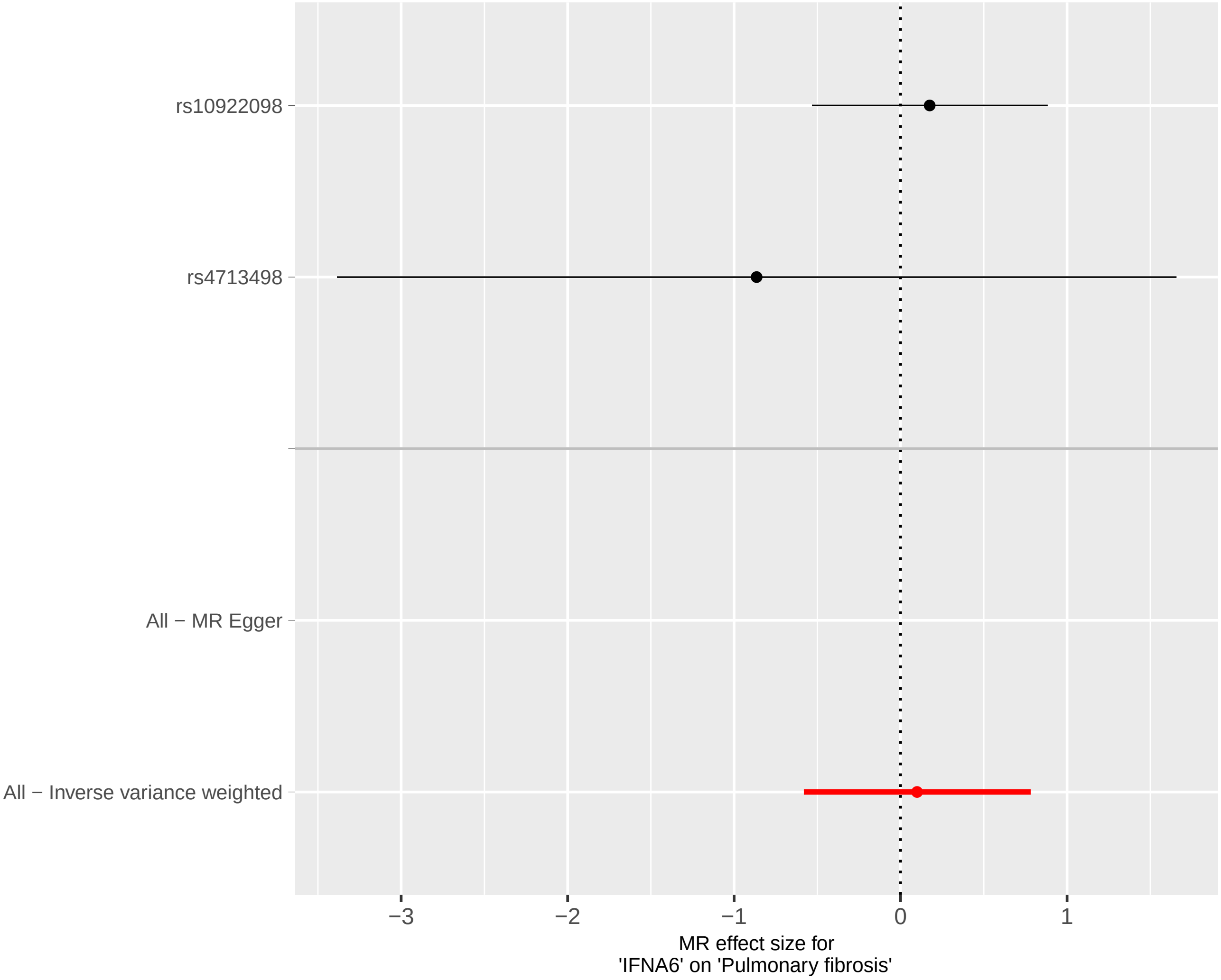
rs10922098
rs4713498
All − MR Egger
All − Inverse variance weighted
−3
−2
−1
0
1
MR effect size for
'IFNA6' on 'Pulmonary fibrosis'

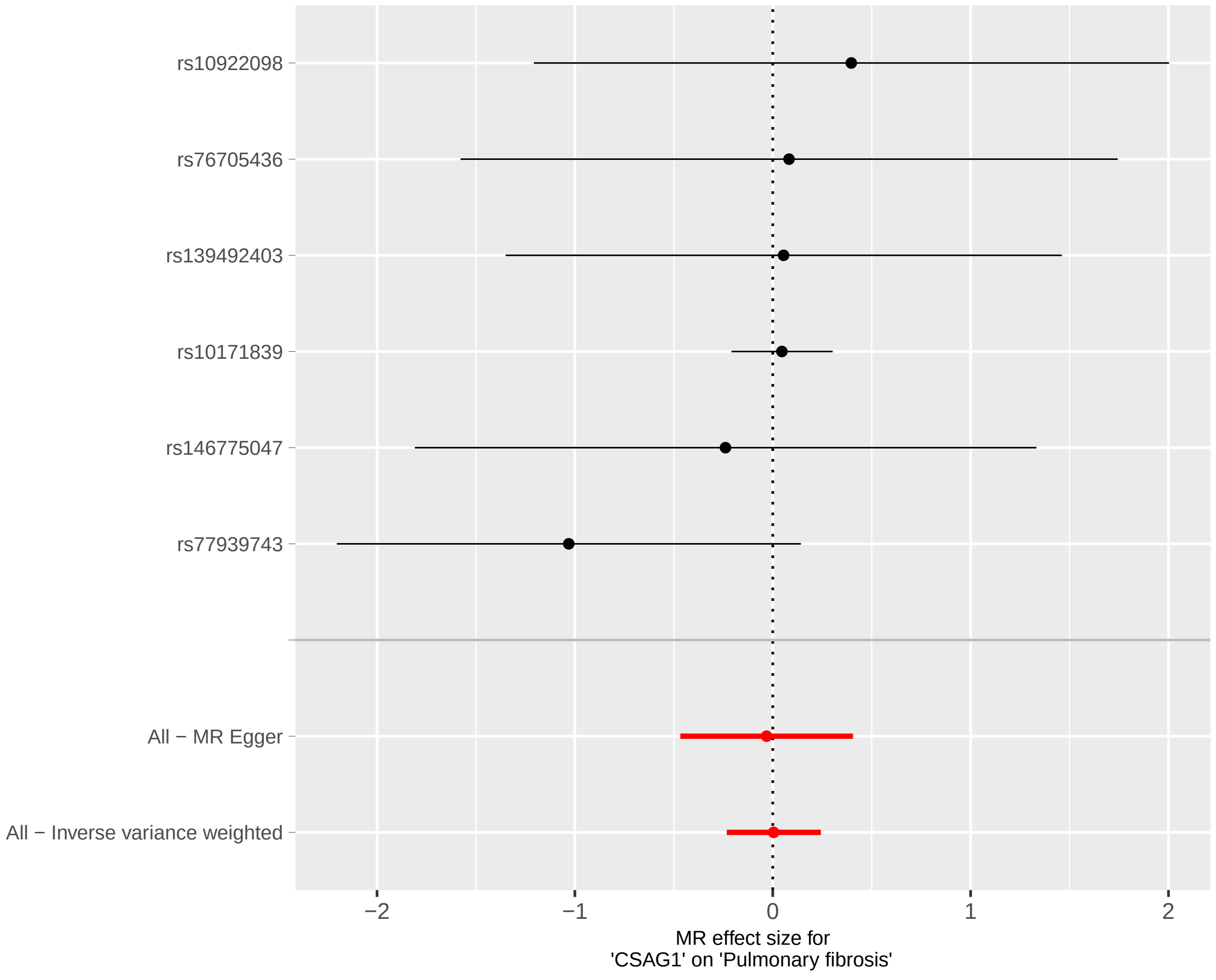
rs10922098
rs76705436
rs139492403
rs10171839
rs146775047
rs77939743
All − MR Egger
All − Inverse variance weighted
−2
−1
0
1
2
MR effect size for
'CSAG1' on 'Pulmonary fibrosis'

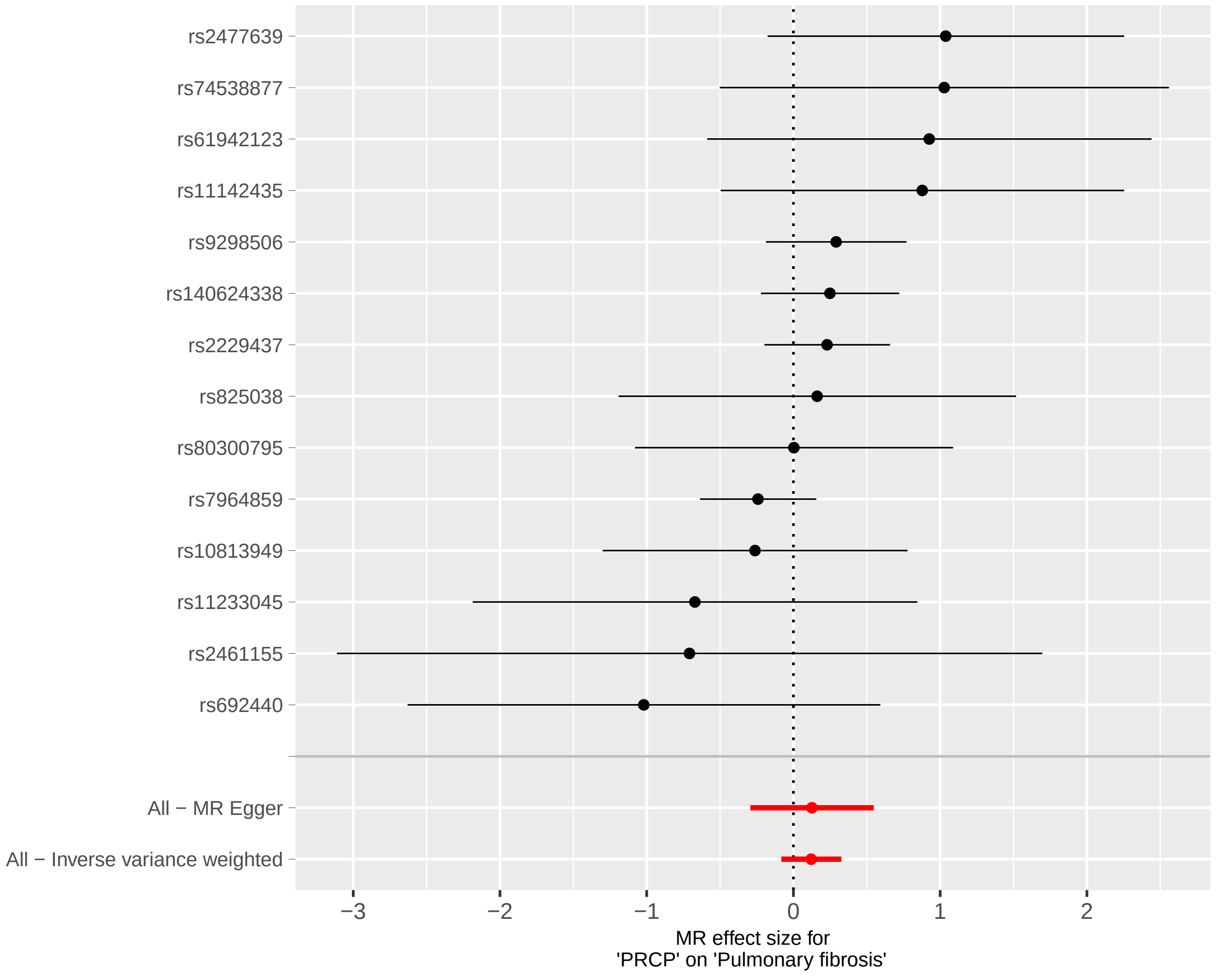

rs2477639
rs74538877
rs61942123
rs11142435
rs9298506
rs140624338
rs2229437
rs825038
rs80300795
rs7964859
rs10813949
rs11233045
rs2461155
rs692440
All − MR Egger
All − Inverse variance weighted
−3
−2
−1
0
1
2
MR effect size for
'PRCP' on 'Pulmonary fibrosis'

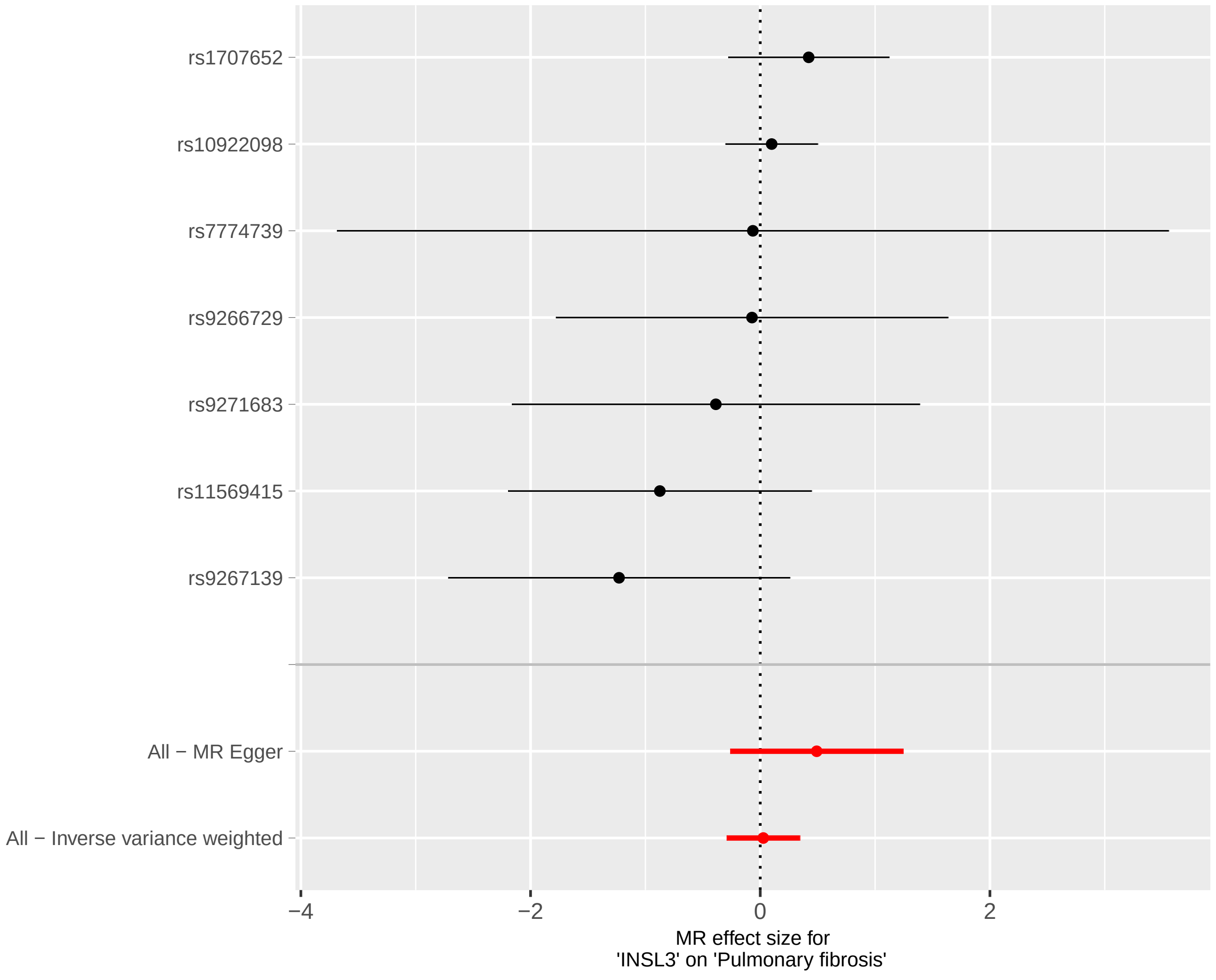

rs1707652
rs10922098
rs7774739
rs9266729
rs9271683
rs11569415
rs9267139
All − MR Egger
All − Inverse variance weighted
−4
−2
0
2
MR effect size for
'INSL3' on 'Pulmonary fibrosis'

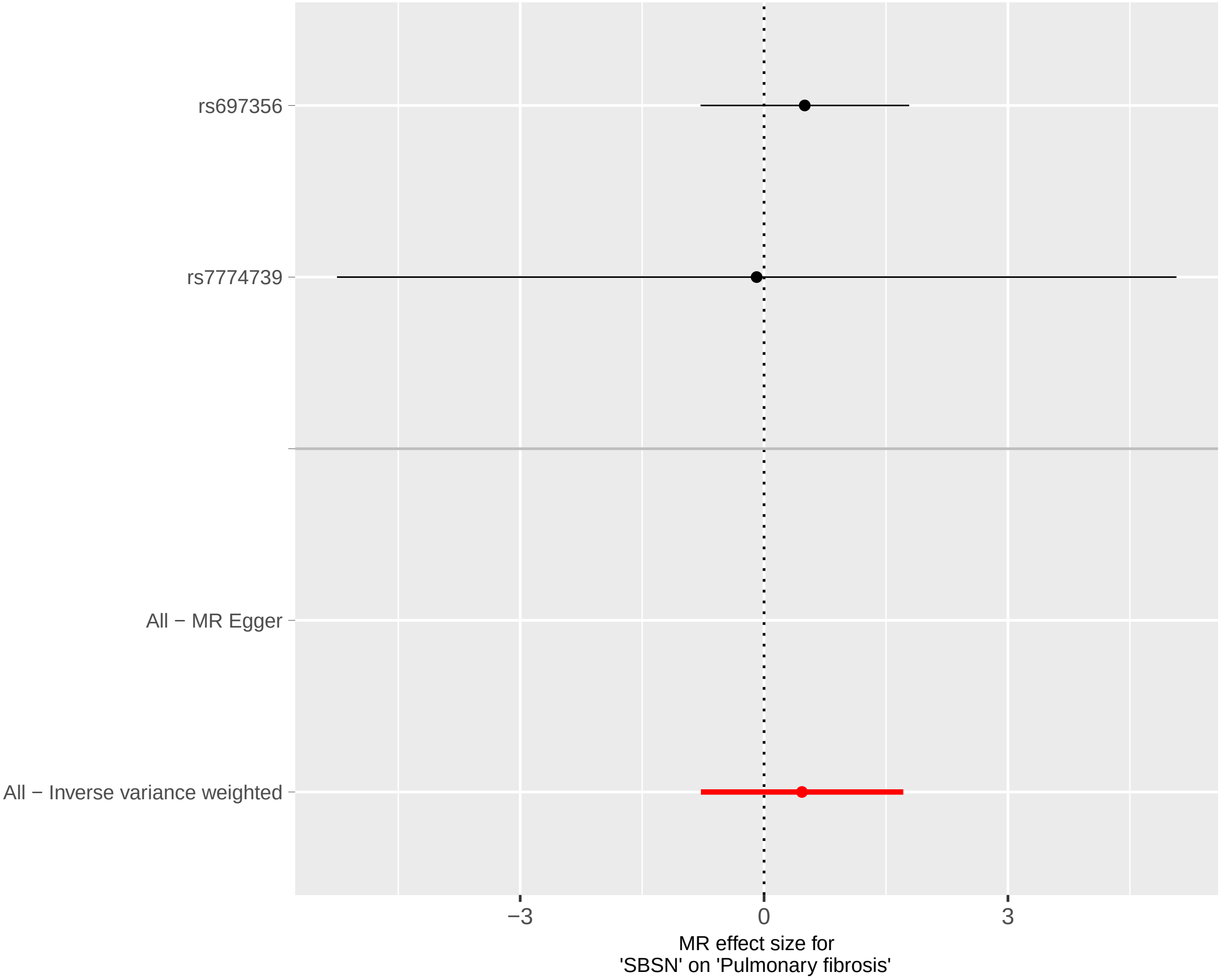

rs697356
rs7774739
All − MR Egger
All − Inverse variance weighted
−3
0
3
MR effect size for
'SBSN' on 'Pulmonary fibrosis'

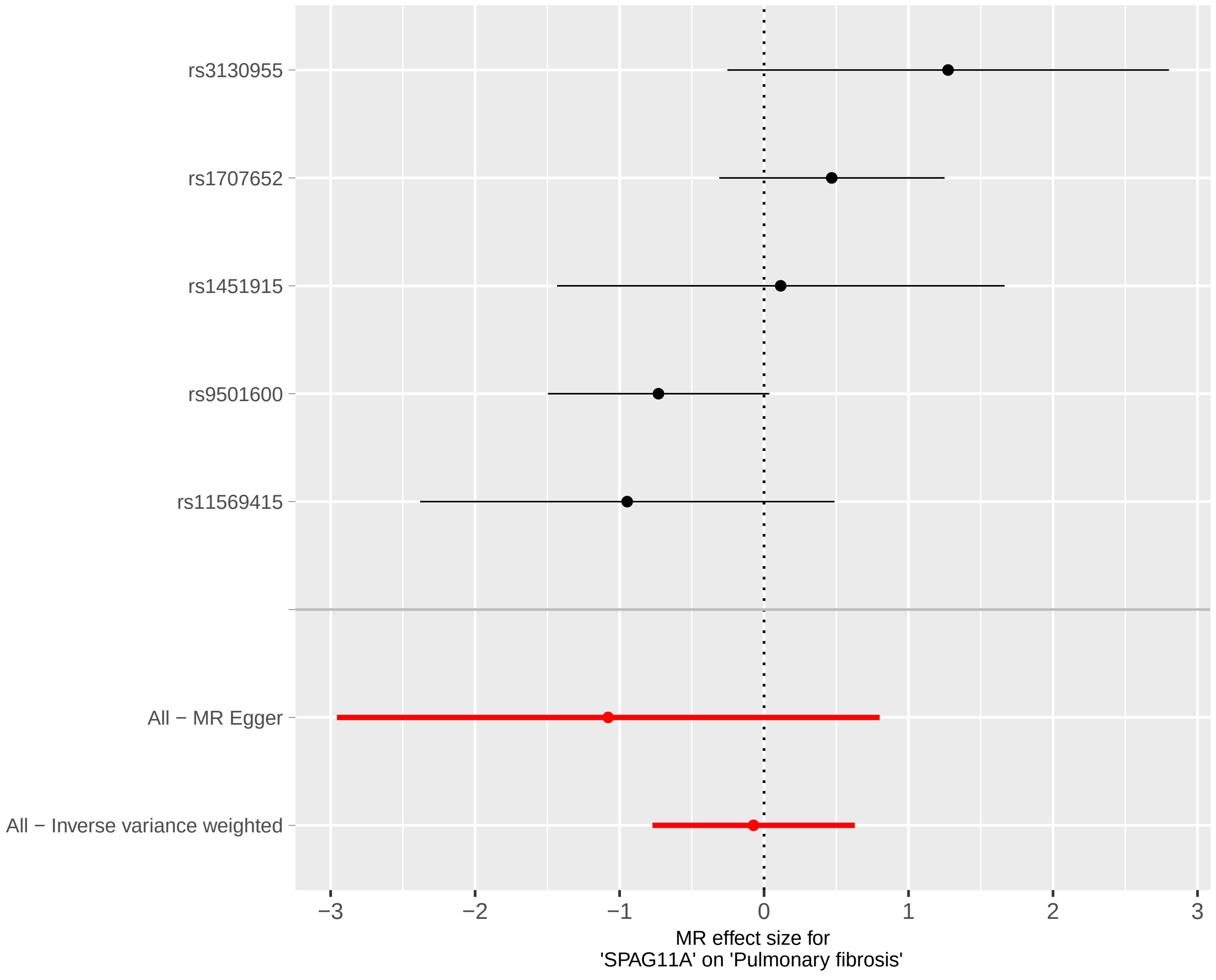

rs3130955
rs1707652
rs1451915
rs9501600
rs11569415
All − MR Egger
All − Inverse variance weighted
−3
−2
−1
0
1
2
3
MR effect size for
'SPAG11A' on 'Pulmonary fibrosis'

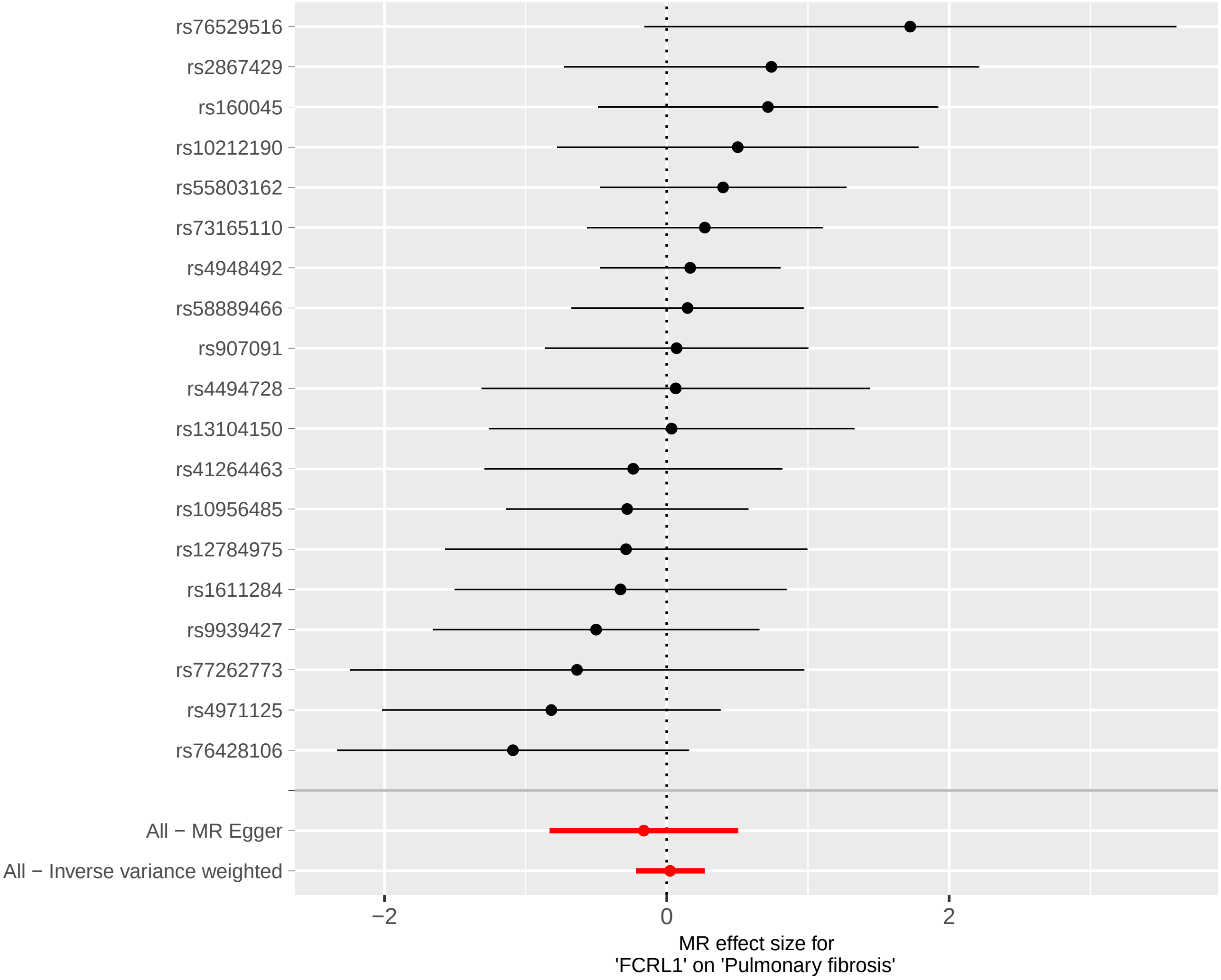

rs76529516
rs2867429
rs160045
rs10212190
rs55803162
rs73165110
rs4948492
rs58889466
rs907091
rs4494728
rs13104150
rs41264463
rs10956485
rs12784975
rs1611284
rs9939427
rs77262773
rs4971125
rs76428106
All − MR Egger
All − Inverse variance weighted
−2
0
2
MR effect size for
'FCRL1' on 'Pulmonary fibrosis'

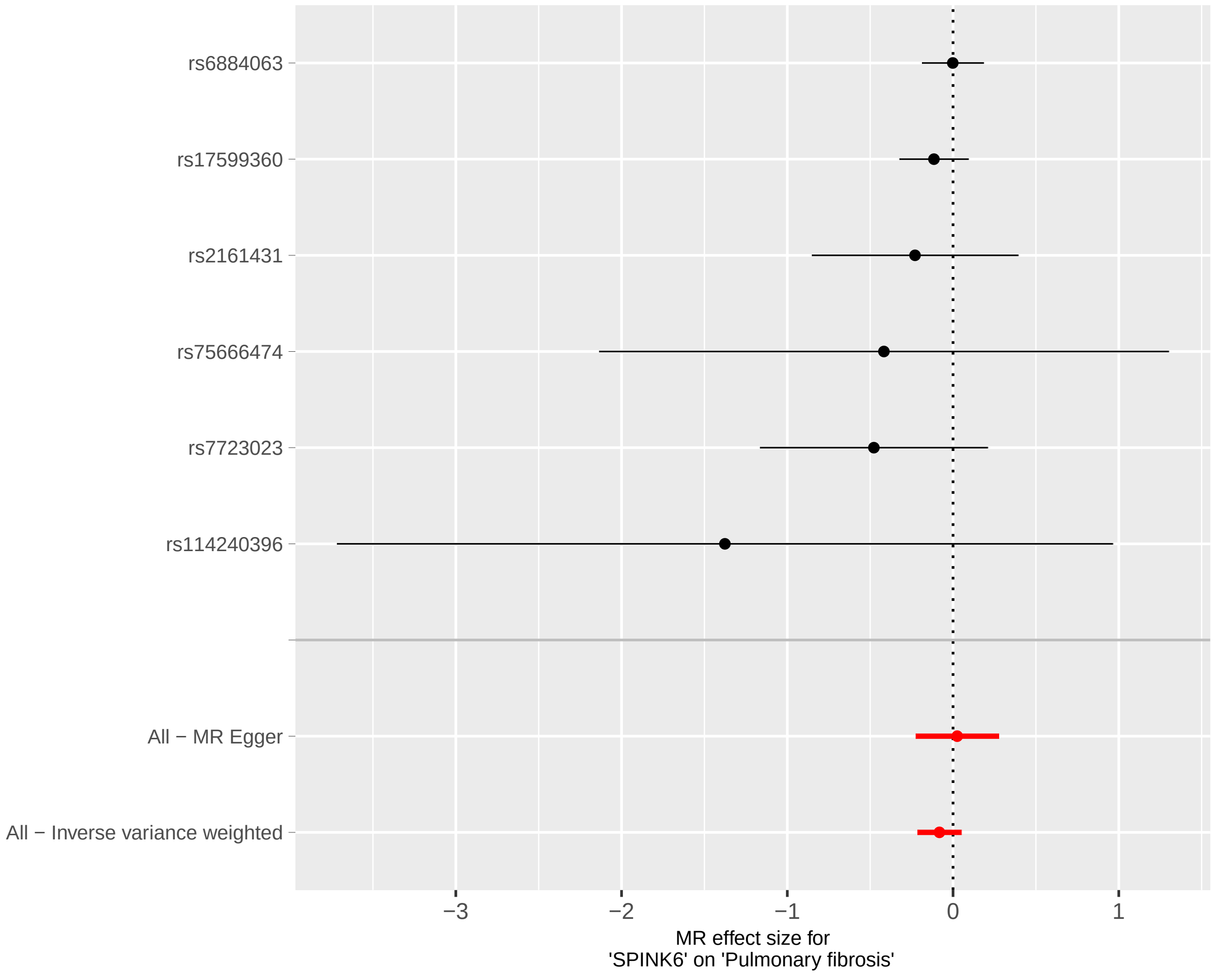
rs6884063
rs17599360
rs2161431
rs75666474
rs7723023
rs114240396
All − MR Egger
All − Inverse variance weighted
−3
−2
−1
0
1
MR effect size for
'SPINK6' on 'Pulmonary fibrosis'

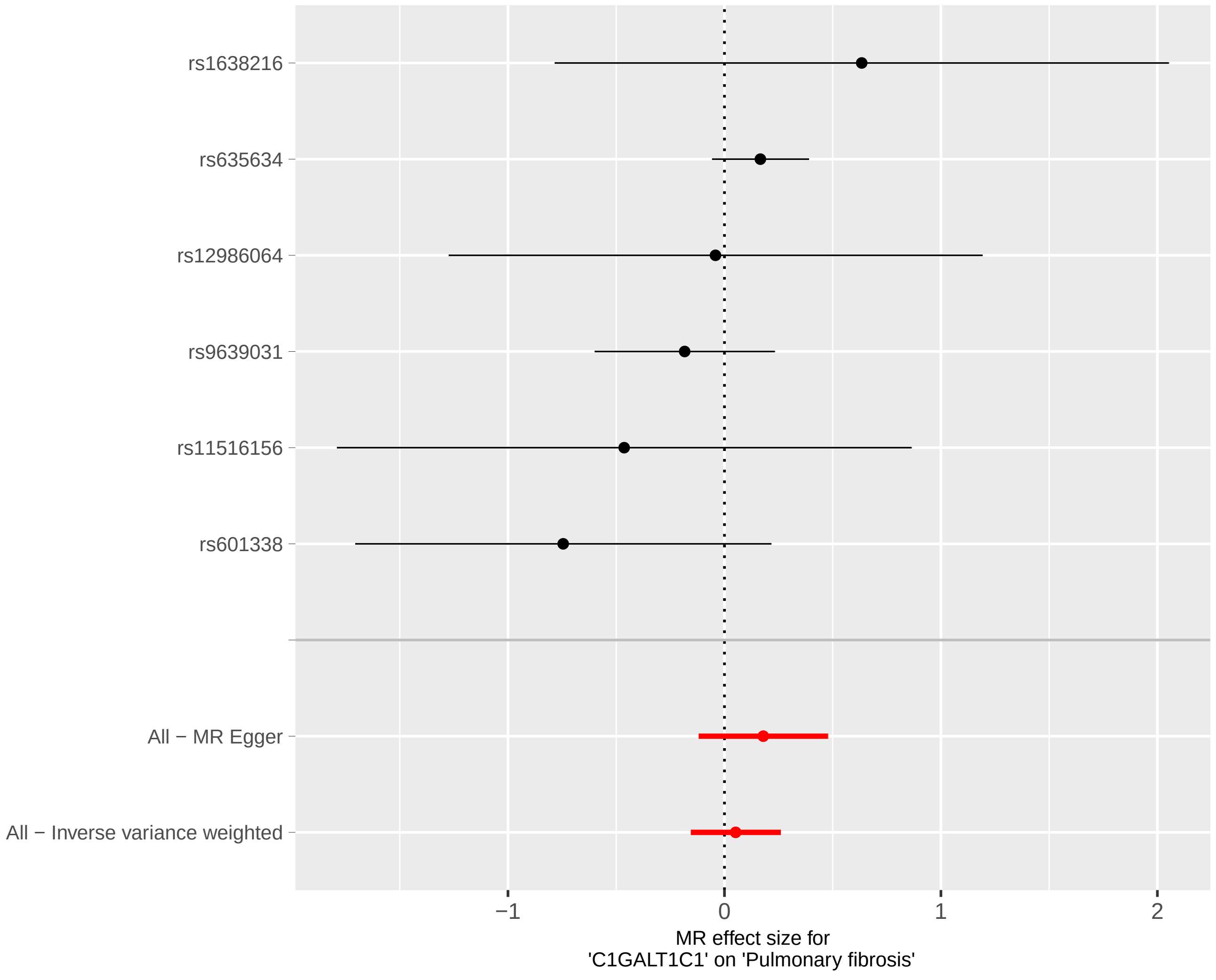

rs1638216
rs635634
rs12986064
rs9639031
rs11516156
rs601338
All − MR Egger
All − Inverse variance weighted
−1
0
1
2
MR effect size for
'C1GALT1C1' on 'Pulmonary fibrosis'

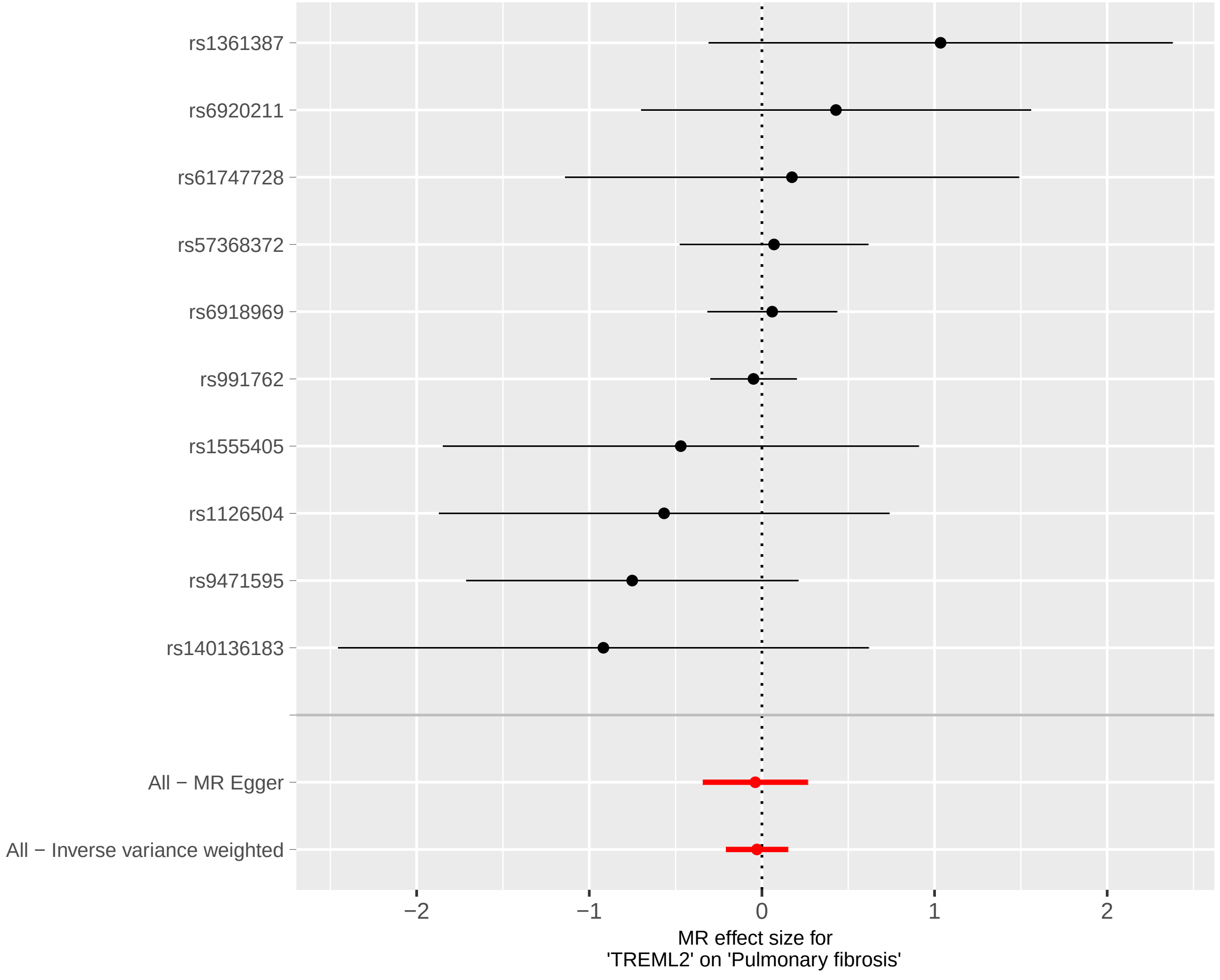
rs1361387
rs6920211
rs61747728
rs57368372
rs6918969
rs991762
rs1555405
rs1126504
rs9471595
rs140136183
All − MR Egger
All − Inverse variance weighted
−2
−1
0
1
2
MR effect size for
'TREML2' on 'Pulmonary fibrosis'

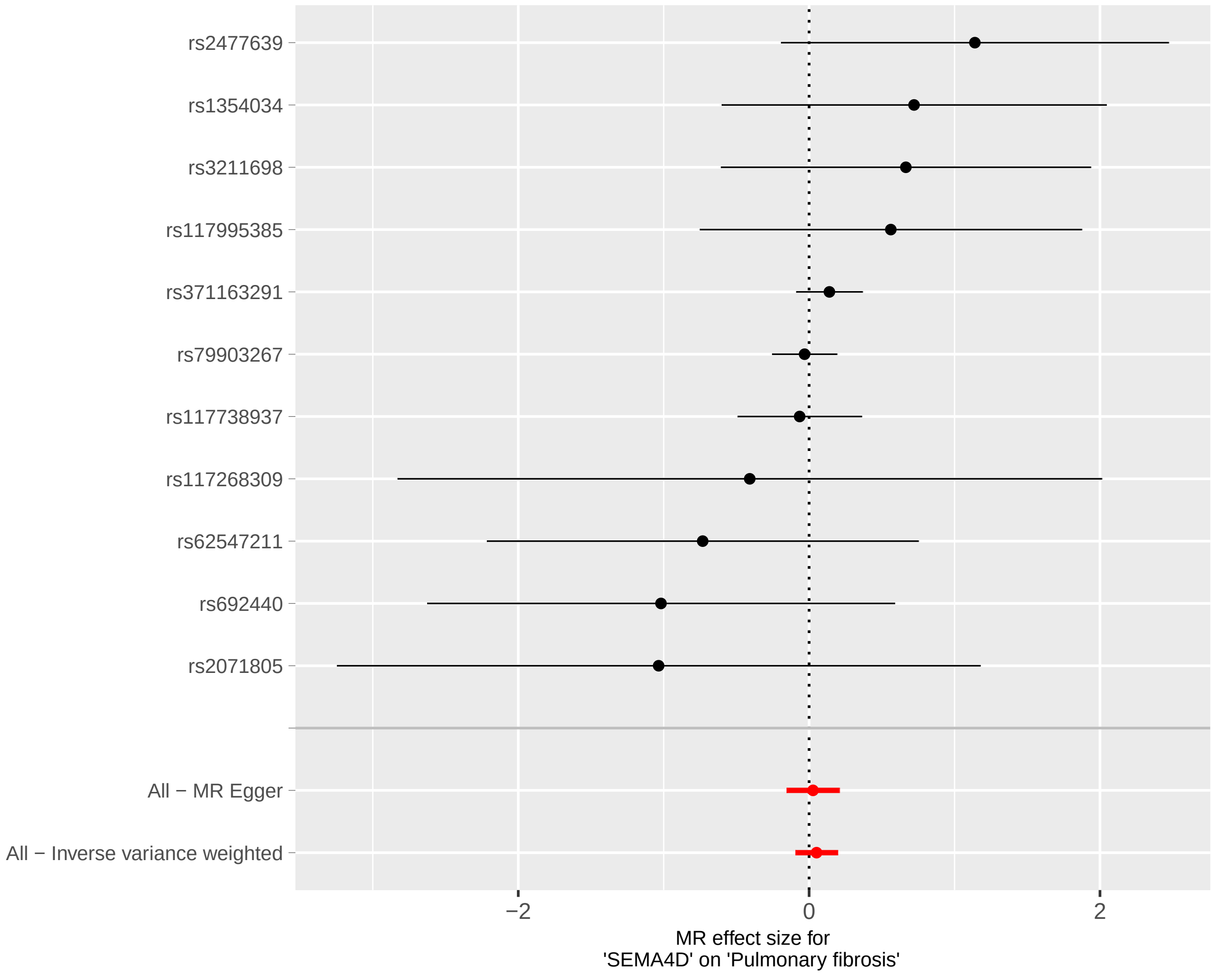

rs2477639
rs1354034
rs3211698
rs117995385
rs371163291
rs79903267
rs117738937
rs117268309
rs62547211
rs692440
rs2071805
All − MR Egger
All − Inverse variance weighted
−2
0
2
MR effect size for
'SEMA4D' on 'Pulmonary fibrosis'

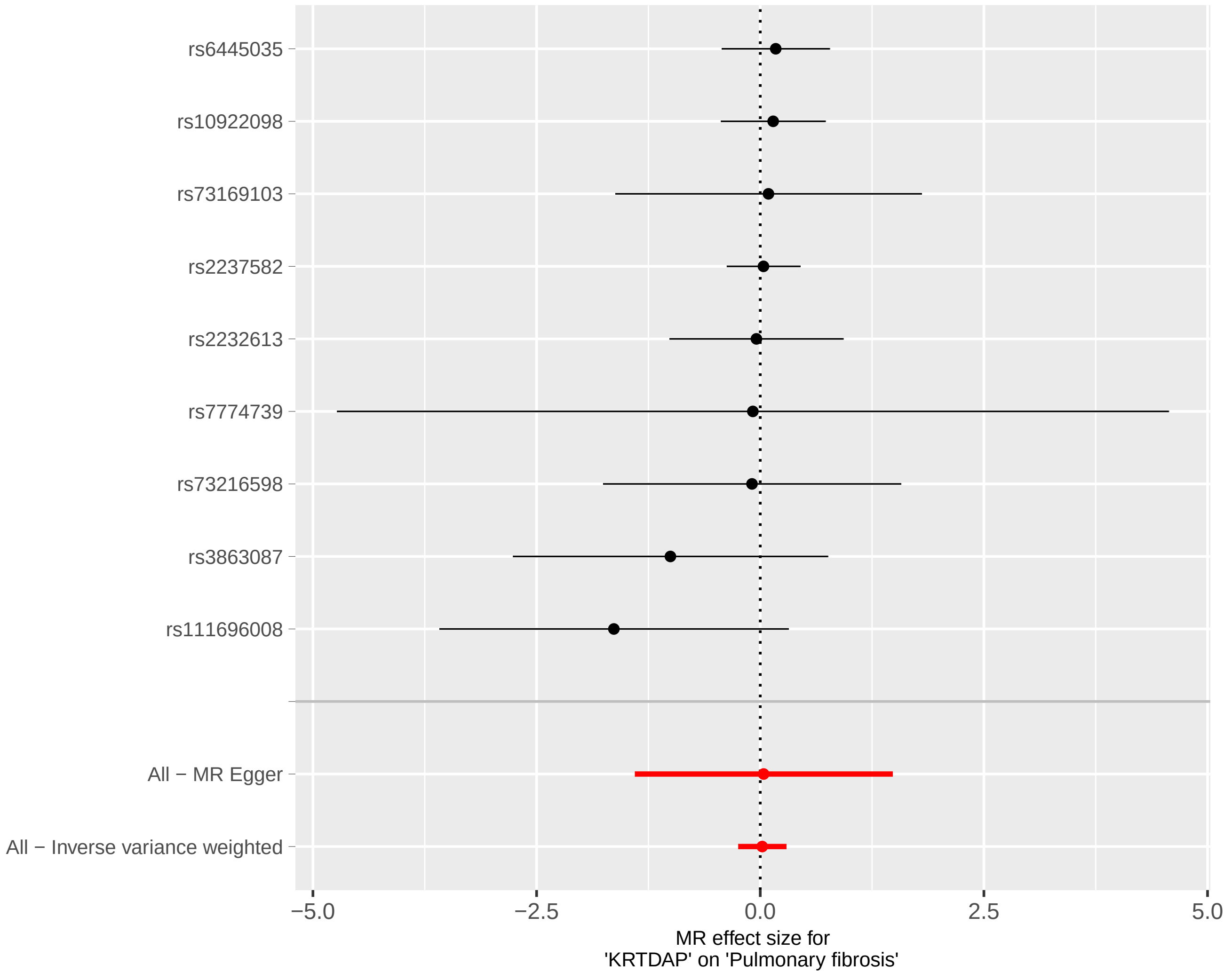

rs6445035
rs10922098
rs73169103
rs2237582
rs2232613
rs7774739
rs73216598
rs3863087
rs111696008
All − MR Egger
All − Inverse variance weighted
−5.0
−2.5
0.0
2.5
5.0
MR effect size for
'KRTDAP' on 'Pulmonary fibrosis'

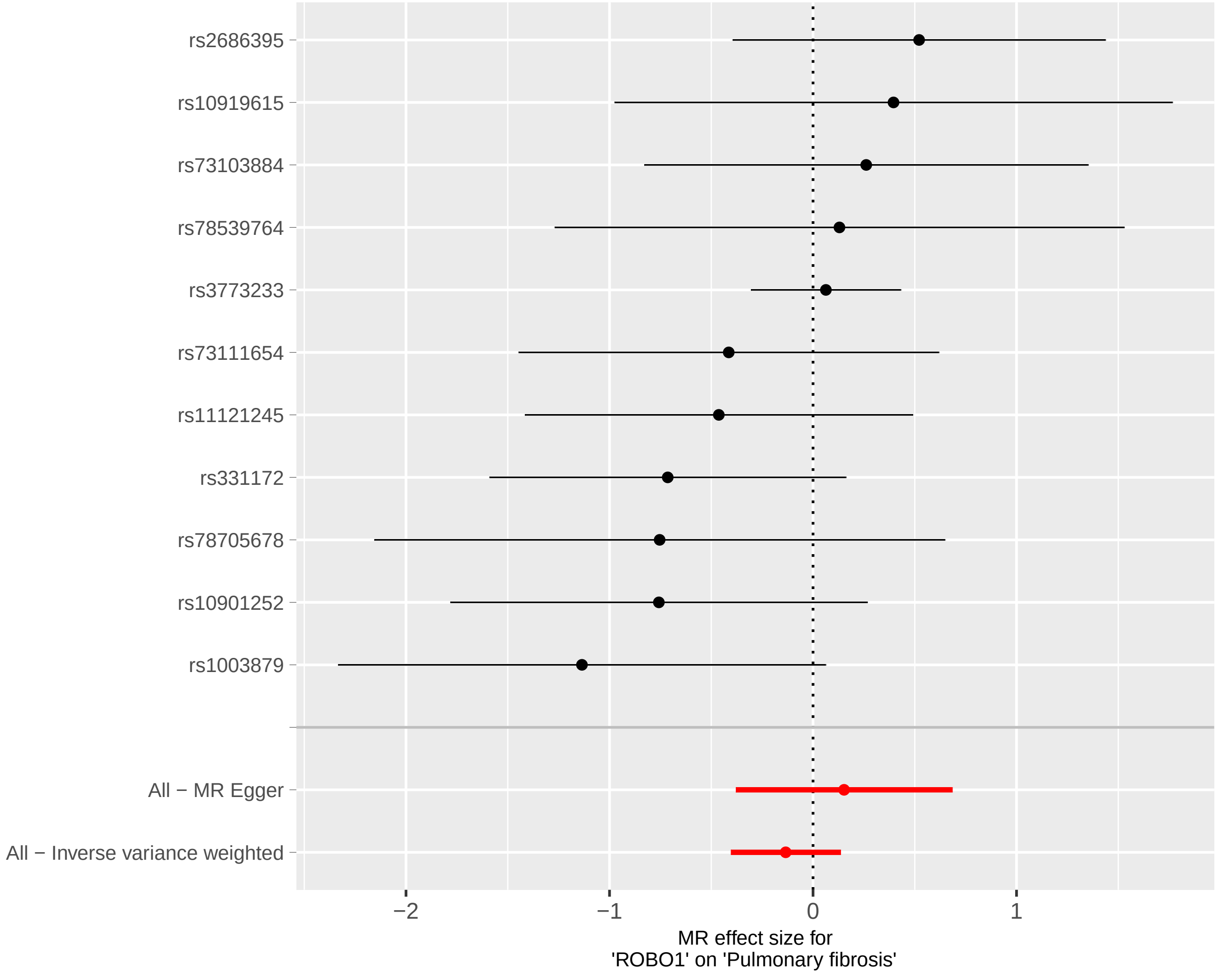

rs2686395
rs10919615
rs73103884
rs78539764
rs3773233
rs73111654
rs11121245
rs331172
rs78705678
rs10901252
rs1003879
All − MR Egger
All − Inverse variance weighted
−2
−1
0
1
MR effect size for
'ROBO1' on 'Pulmonary fibrosis'

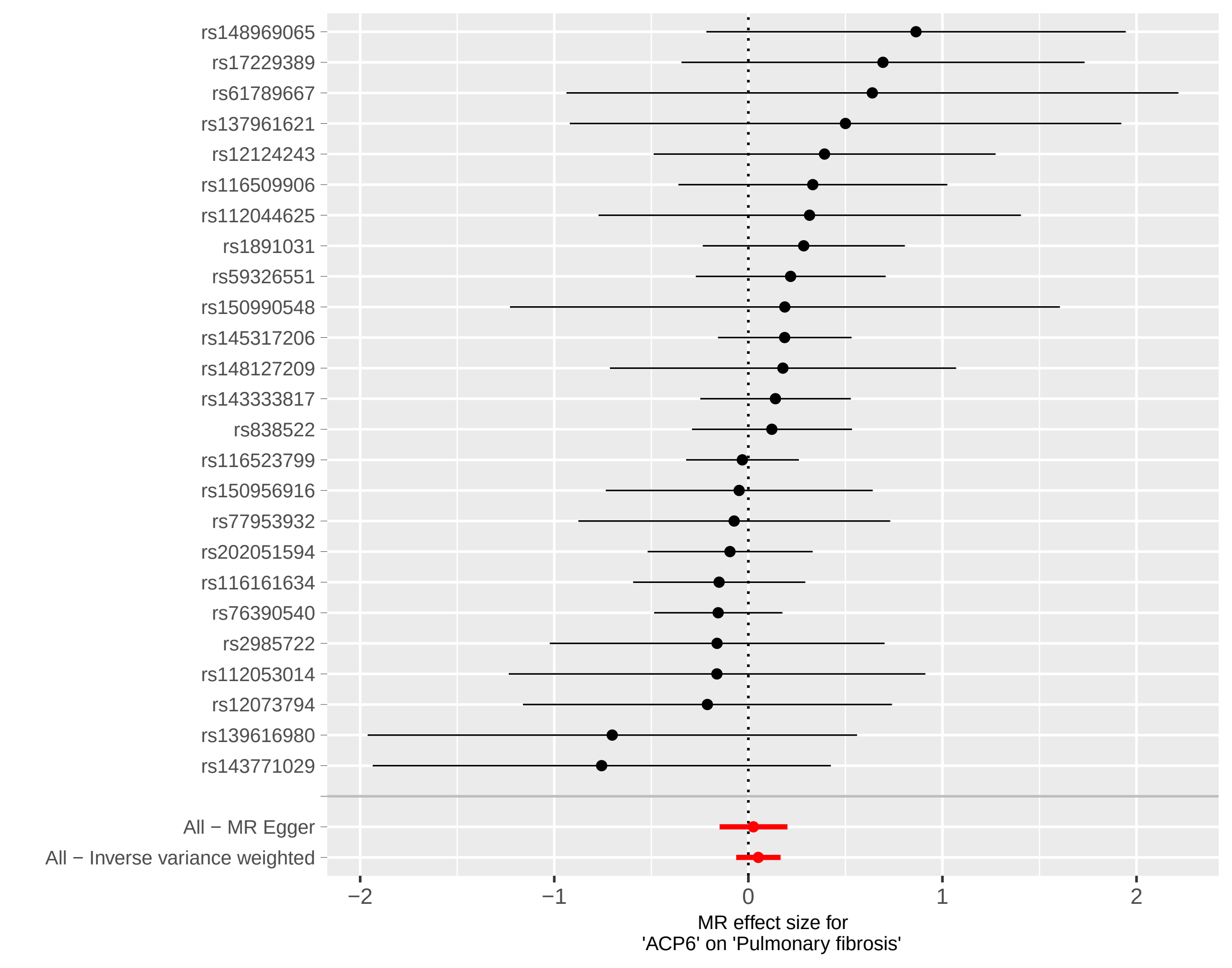

rs148969065
rs17229389
rs61789667
rs137961621
rs12124243
rs116509906
rs112044625
rs1891031
rs59326551
rs150990548
rs145317206
rs148127209
rs143333817
rs838522
rs116523799
rs150956916
rs77953932
rs202051594
rs116161634
rs76390540
rs2985722
rs112053014
rs12073794
rs139616980
rs143771029
All − MR Egger
All − Inverse variance weighted
−2
−1
0
1
2
MR effect size for
'ACP6' on 'Pulmonary fibrosis'

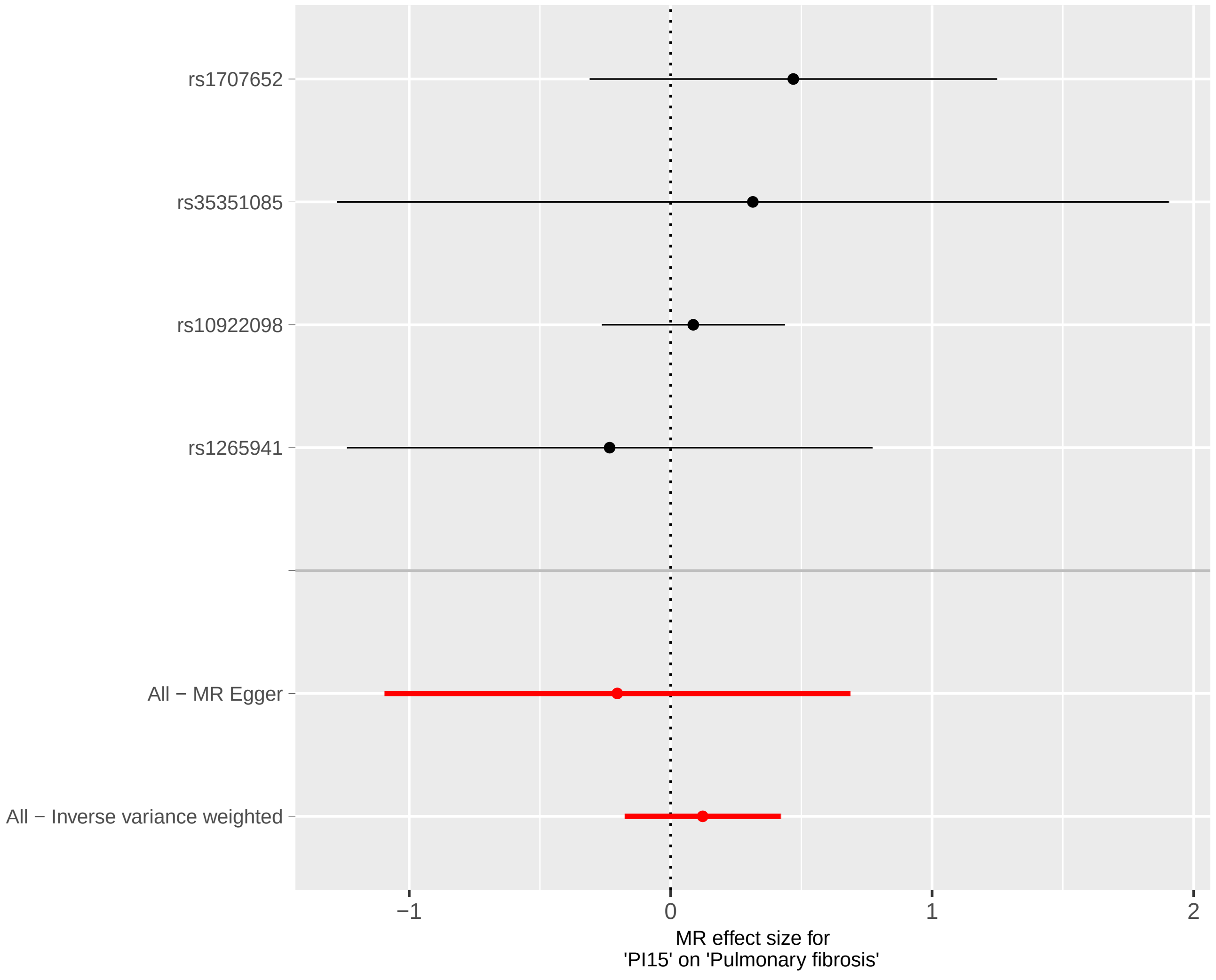
rs1707652
rs35351085
rs10922098
rs1265941
All − MR Egger
All − Inverse variance weighted
−1
0
1
2
MR effect size for
'PI15' on 'Pulmonary fibrosis'

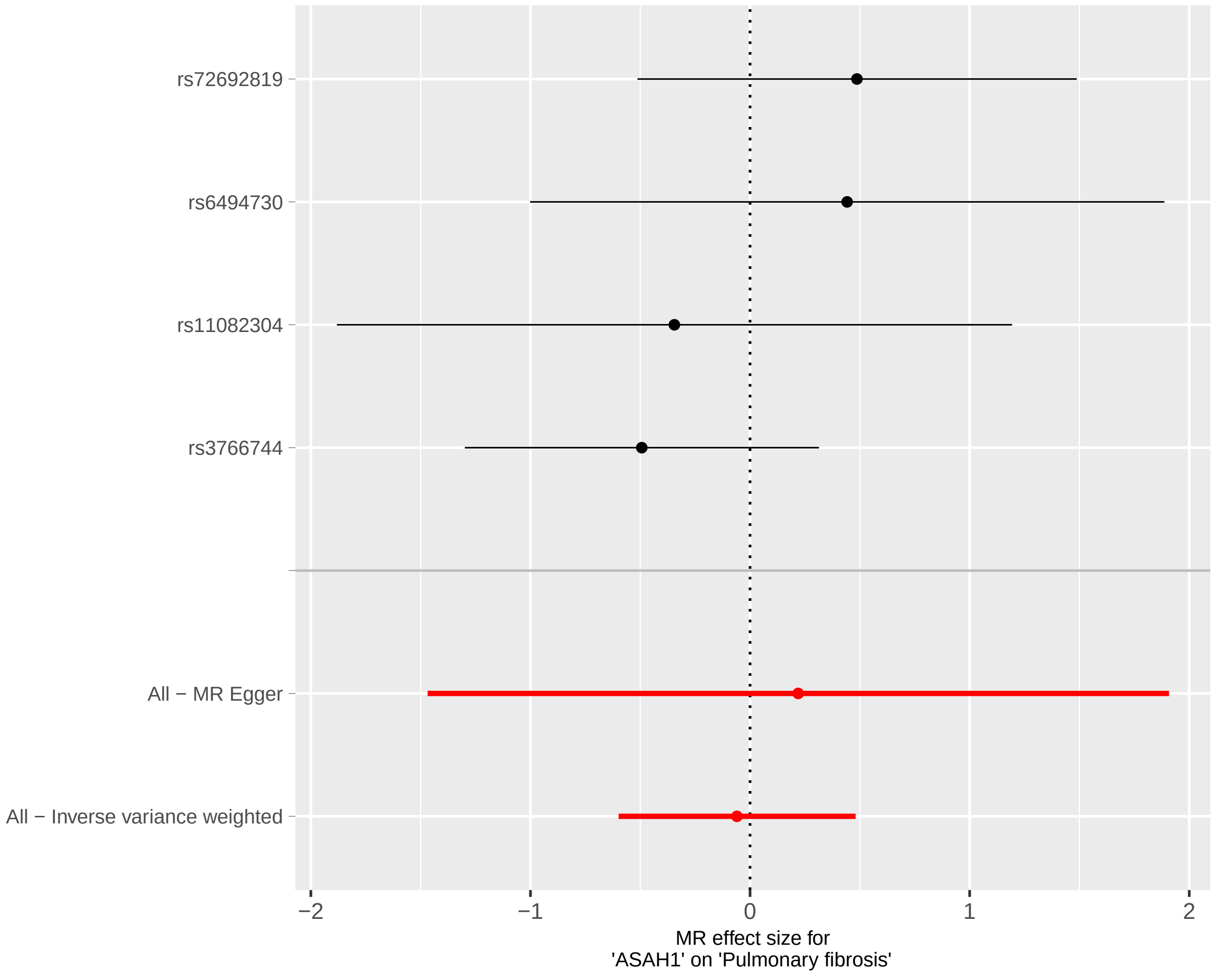
rs72692819
rs6494730
rs11082304
rs3766744
All − MR Egger
All − Inverse variance weighted
−2
−1
0
1
2
MR effect size for
'ASAH1' on 'Pulmonary fibrosis'

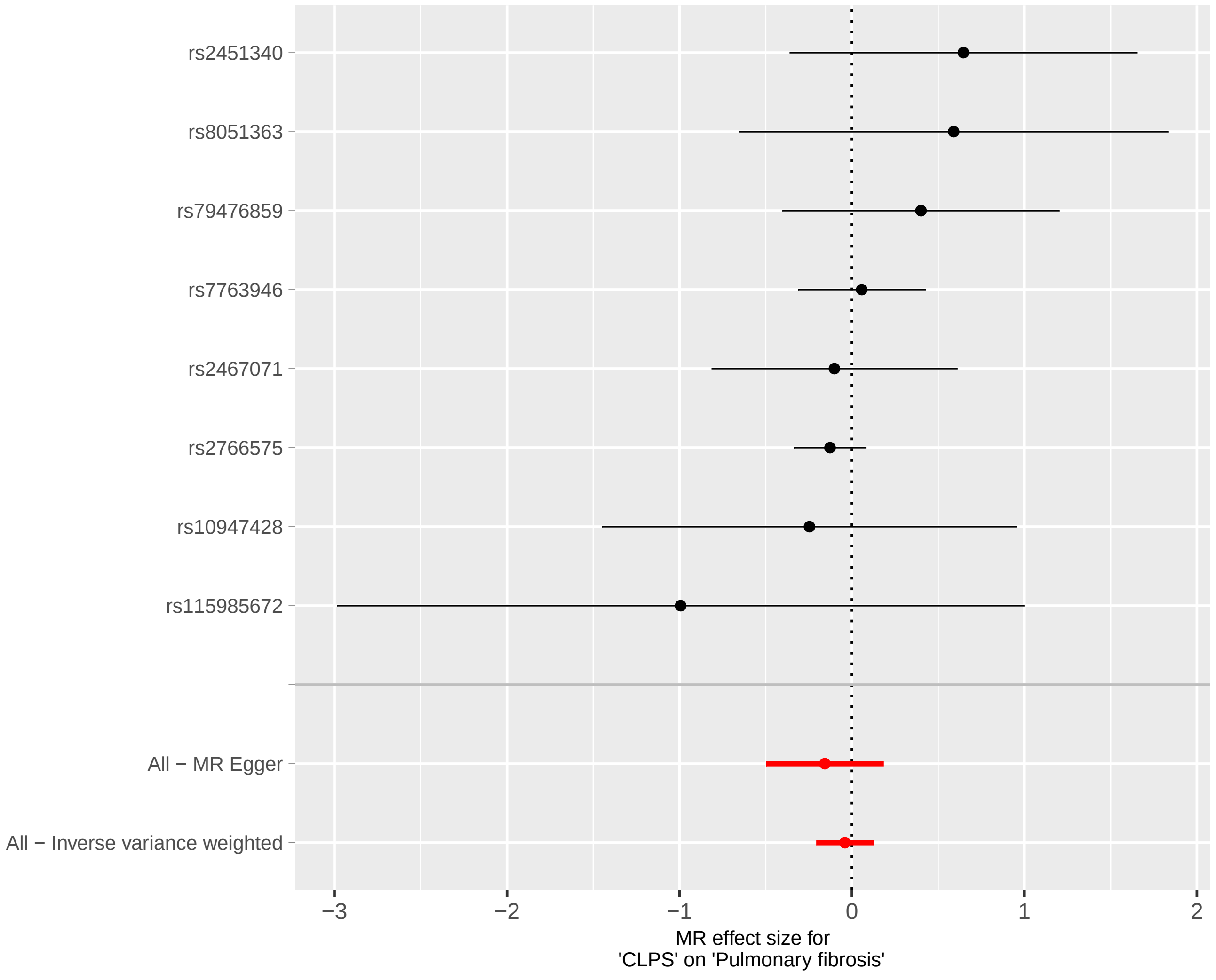
rs2451340
rs8051363
rs79476859
rs7763946
rs2467071
rs2766575
rs10947428
rs115985672
All − MR Egger
All − Inverse variance weighted
−3
−2
−1
0
1
2
MR effect size for
'CLPS' on 'Pulmonary fibrosis'

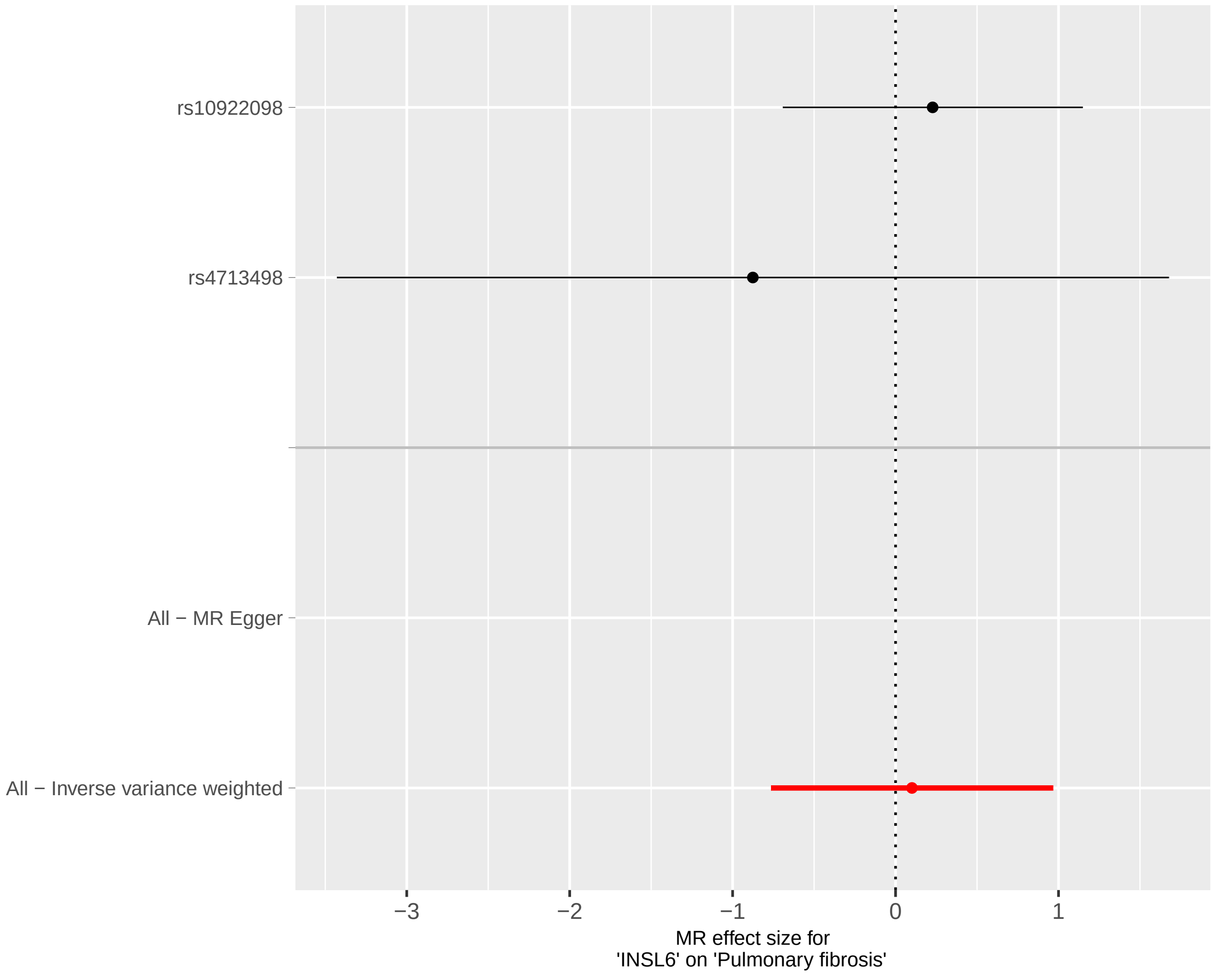

rs10922098
rs4713498
All − MR Egger
All − Inverse variance weighted
−3
−2
−1
0
1
MR effect size for
'INSL6' on 'Pulmonary fibrosis'

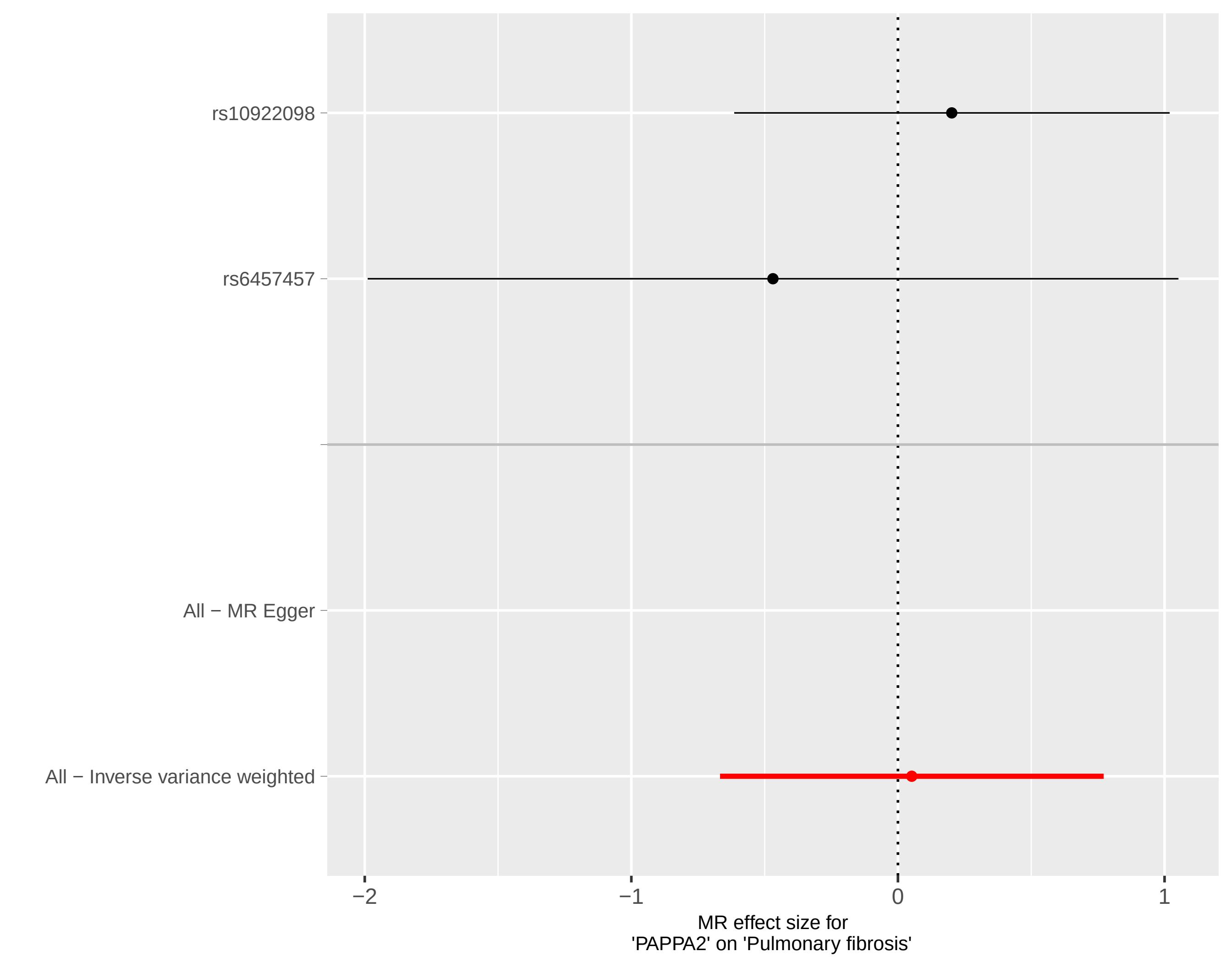
rs10922098
rs6457457
All − MR Egger
All − Inverse variance weighted
−2
−1
0
1
MR effect size for
'PAPPA2' on 'Pulmonary fibrosis'

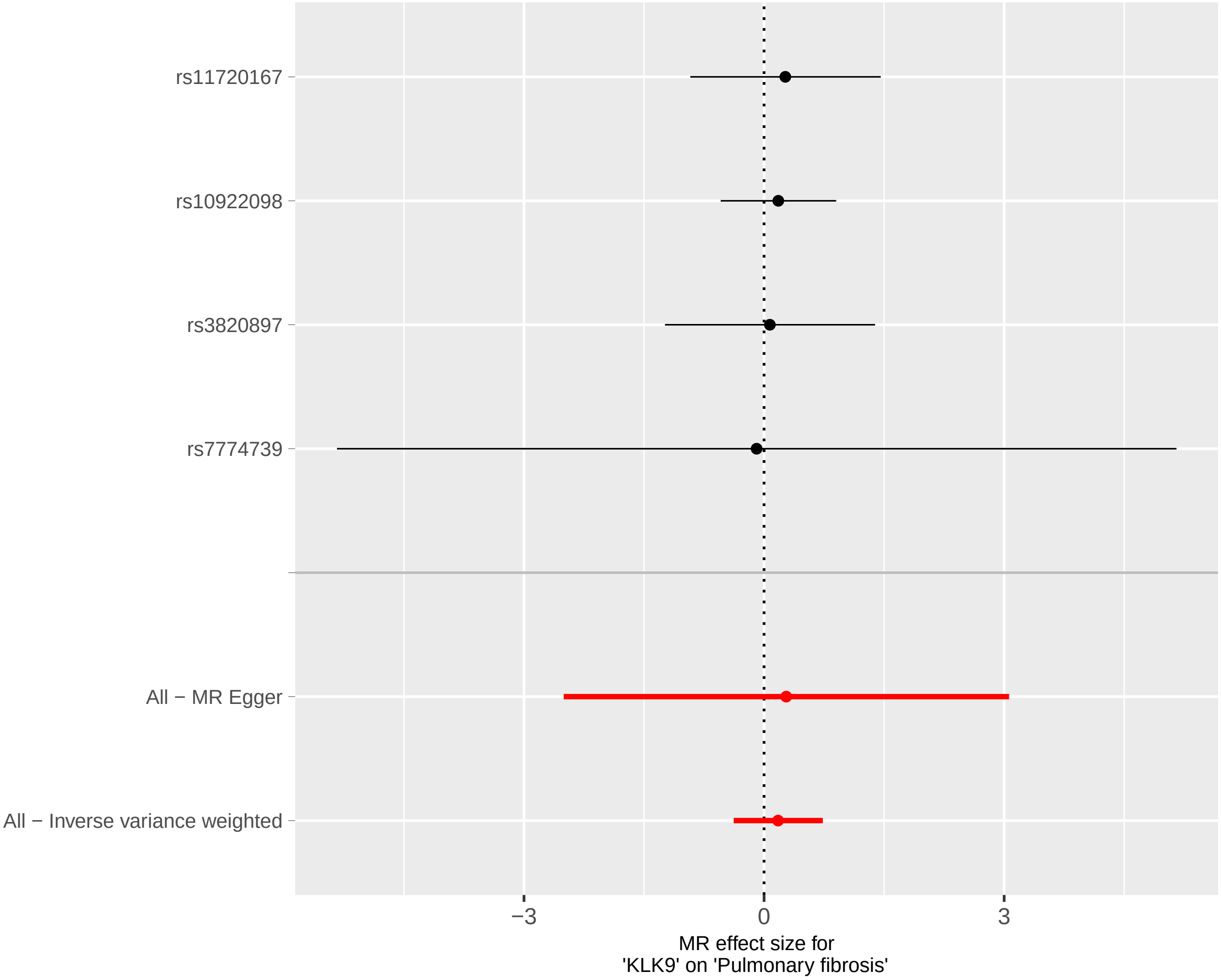
rs11720167
rs10922098
rs3820897
rs7774739
All − MR Egger
All − Inverse variance weighted
−3
0
3
MR effect size for
'KLK9' on 'Pulmonary fibrosis'

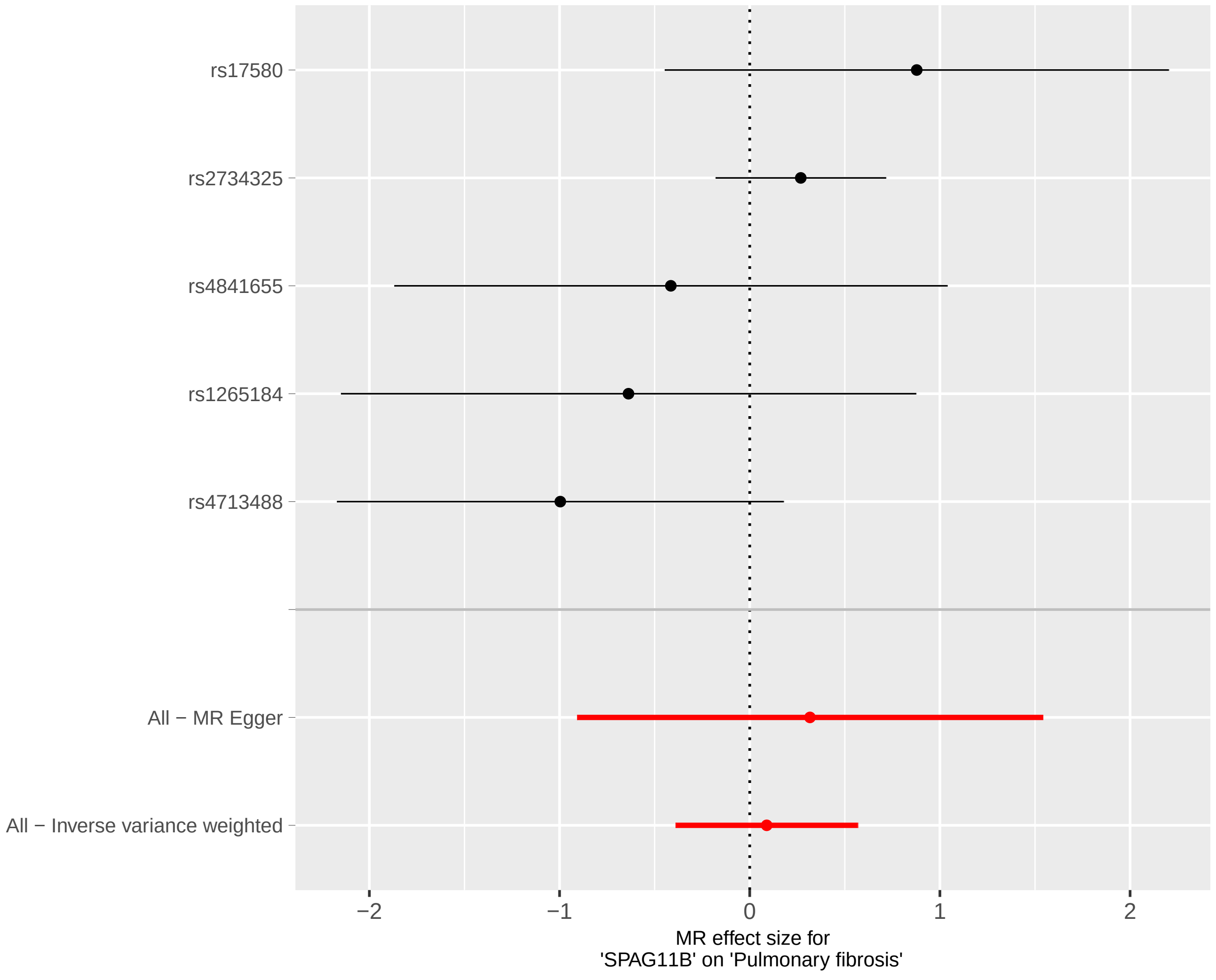

rs17580
rs2734325
rs4841655
rs1265184
rs4713488
All − MR Egger
All − Inverse variance weighted
−2
−1
0
1
2
MR effect size for
'SPAG11B' on 'Pulmonary fibrosis'

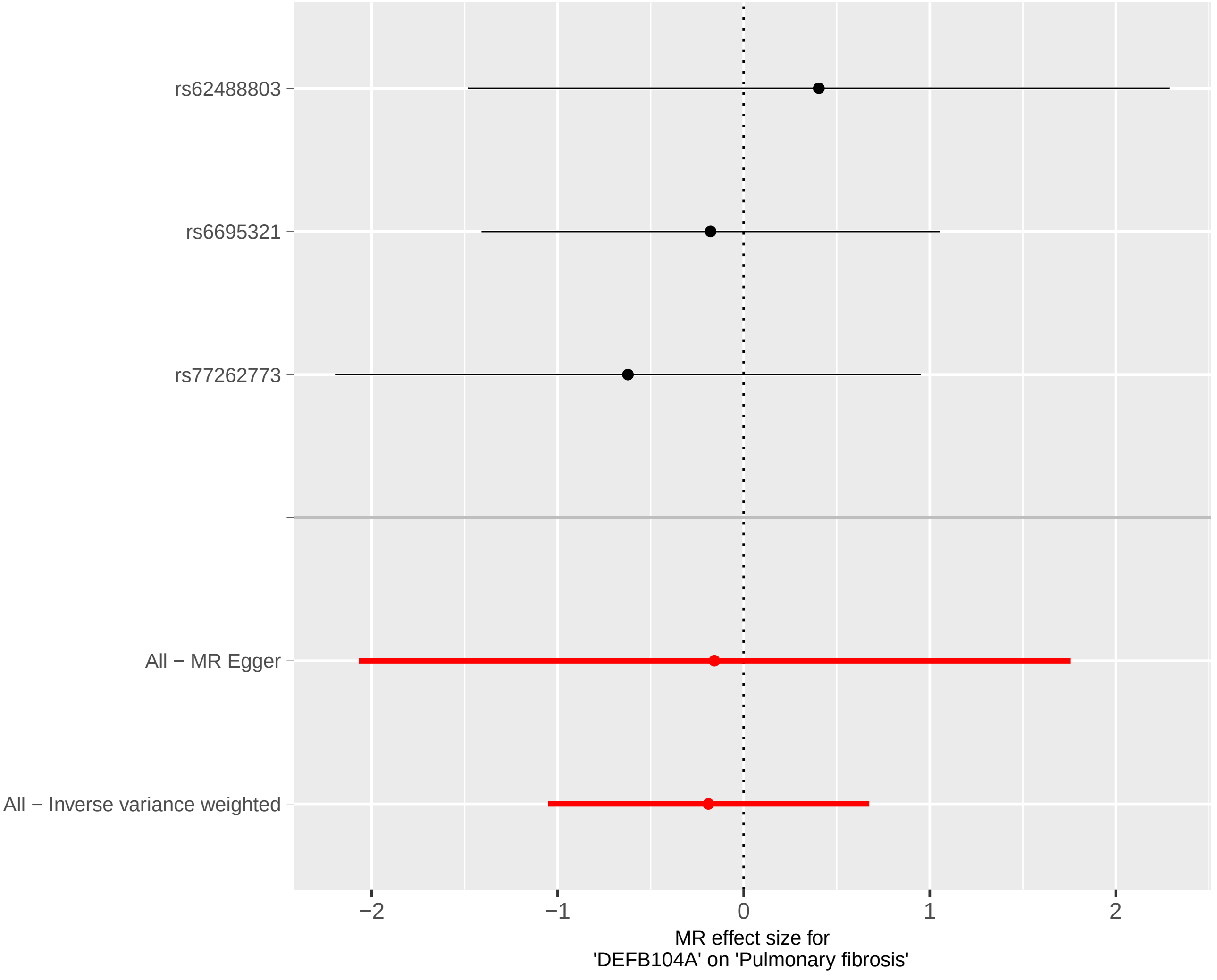

rs62488803
rs6695321
rs77262773
All − MR Egger
All − Inverse variance weighted
−2
−1
0
1
2
MR effect size for
'DEFB104A' on 'Pulmonary fibrosis'

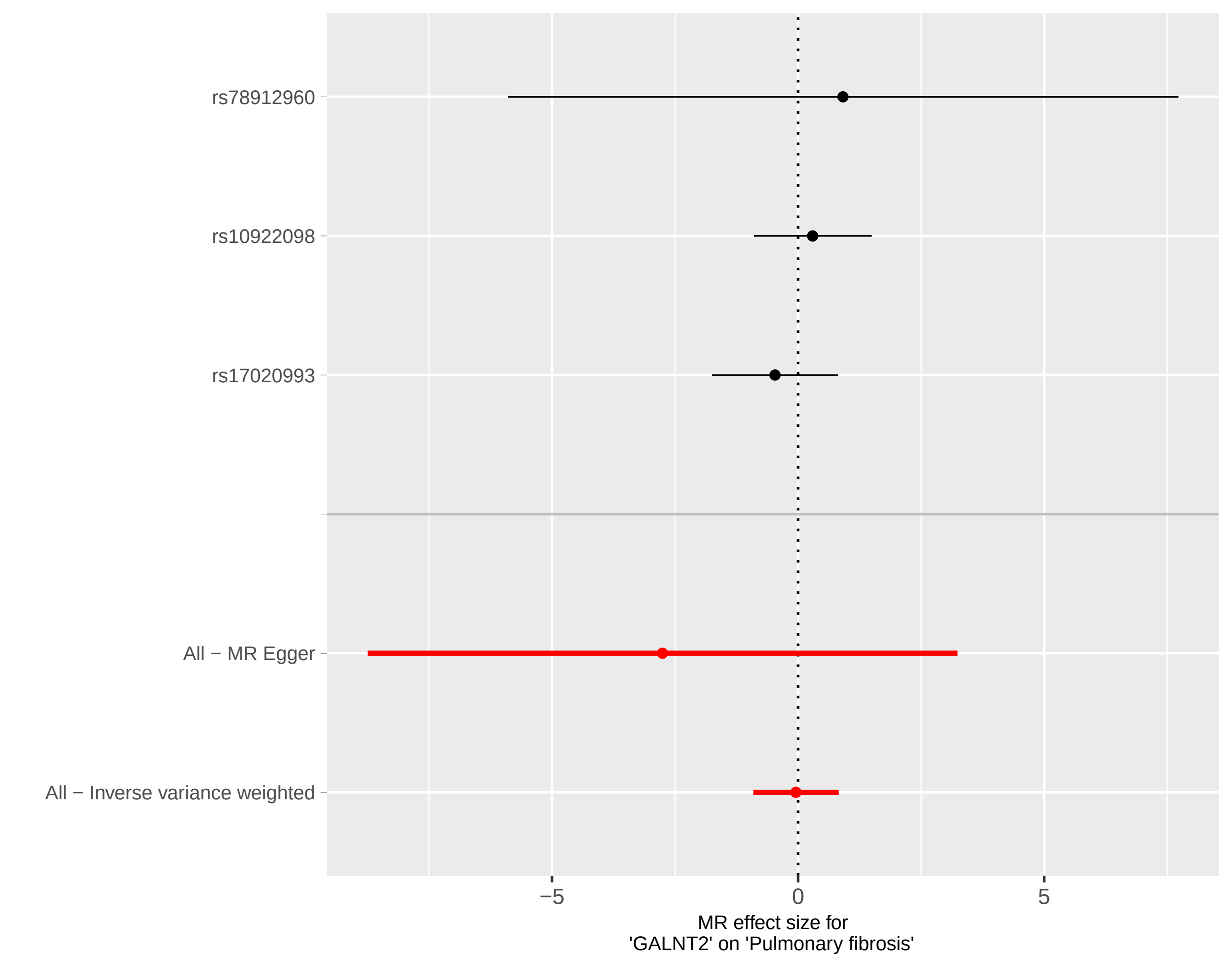

rs78912960
rs10922098
rs17020993
All − MR Egger
All − Inverse variance weighted
−5
0
5
MR effect size for
'GALNT2' on 'Pulmonary fibrosis'

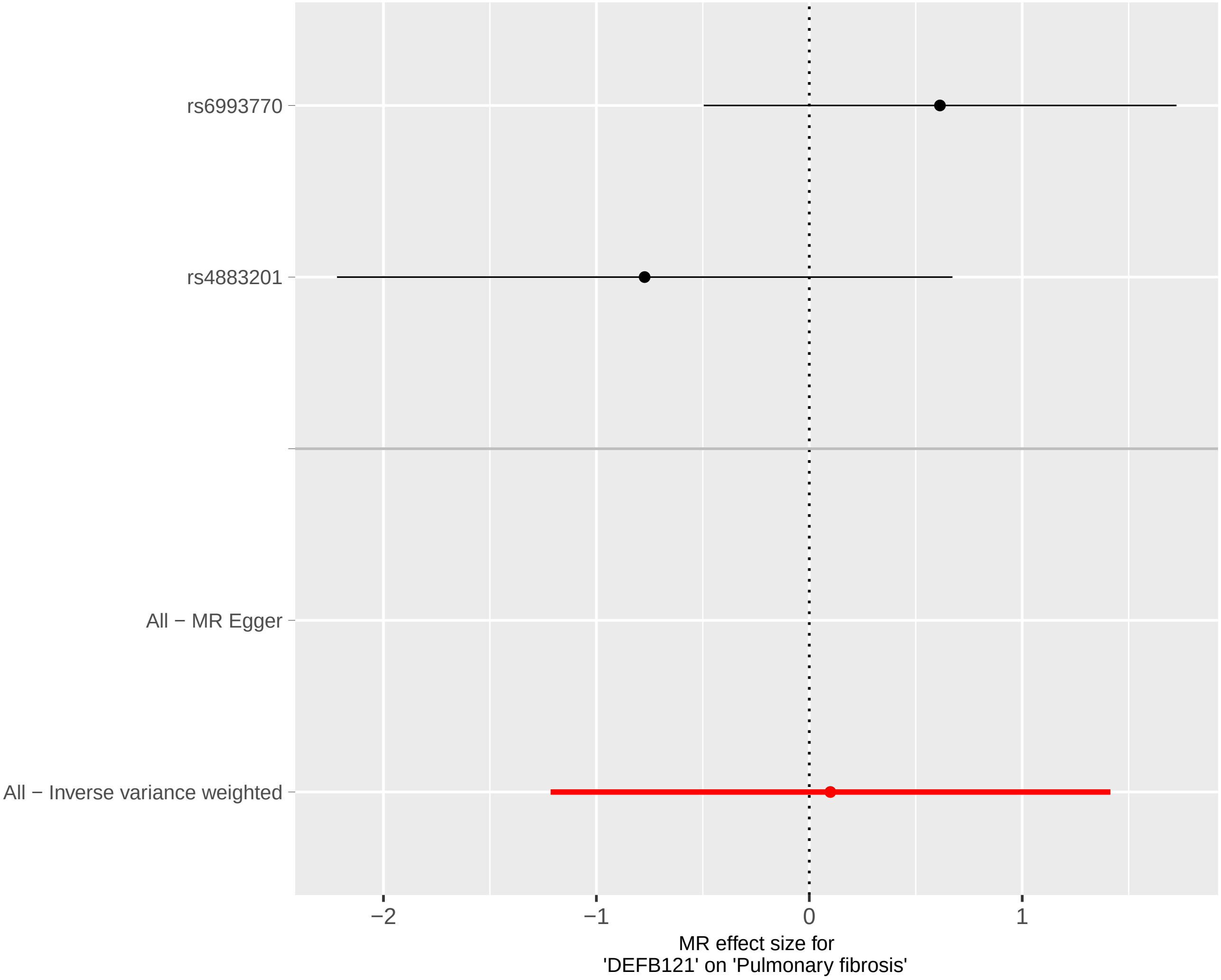

rs6993770
rs4883201
All − MR Egger
All − Inverse variance weighted
−2
−1
0
1
MR effect size for
'DEFB121' on 'Pulmonary fibrosis'

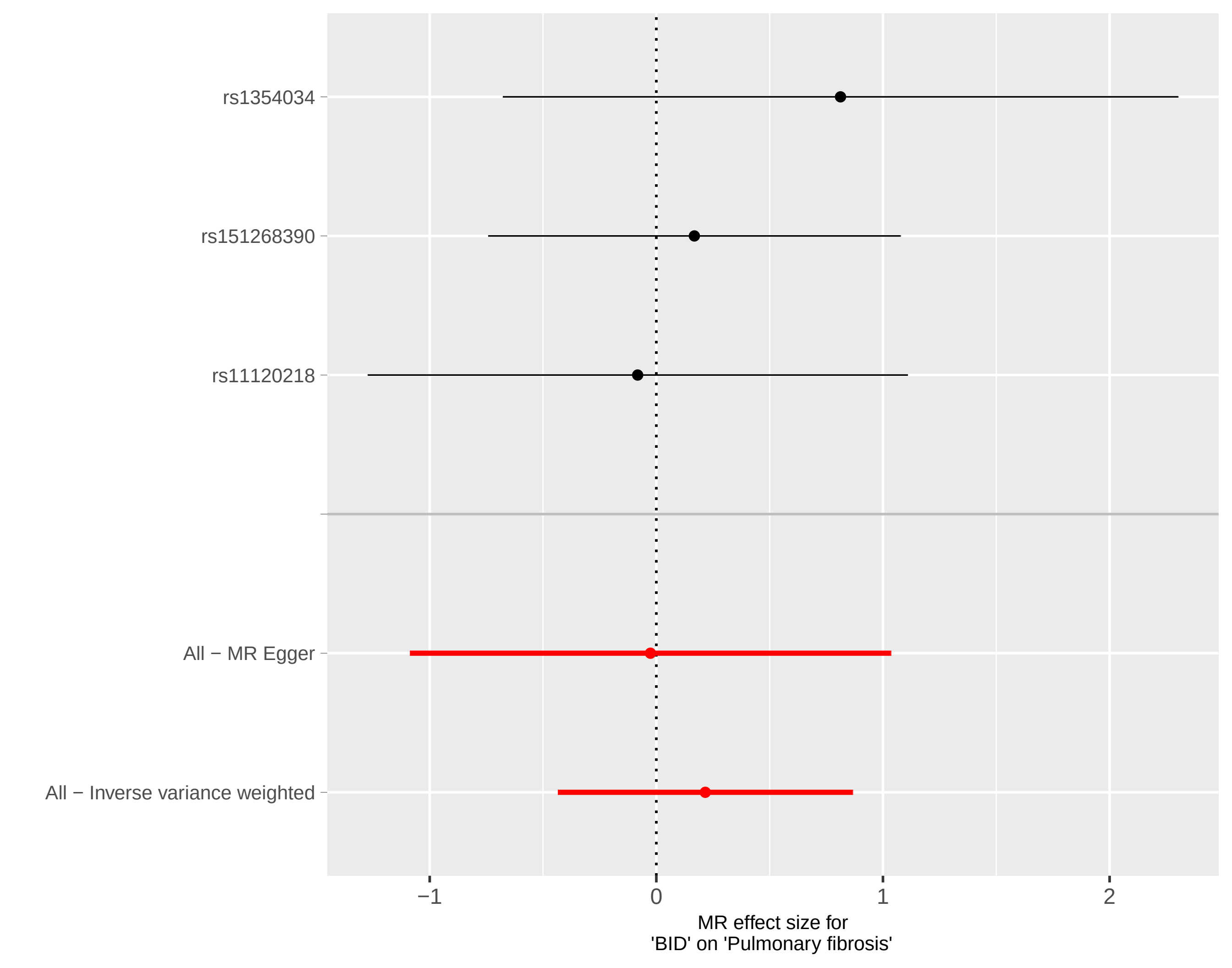

rs1354034
rs151268390
rs11120218
All − MR Egger
All − Inverse variance weighted
−1
0
1
2
MR effect size for
'BID' on 'Pulmonary fibrosis'

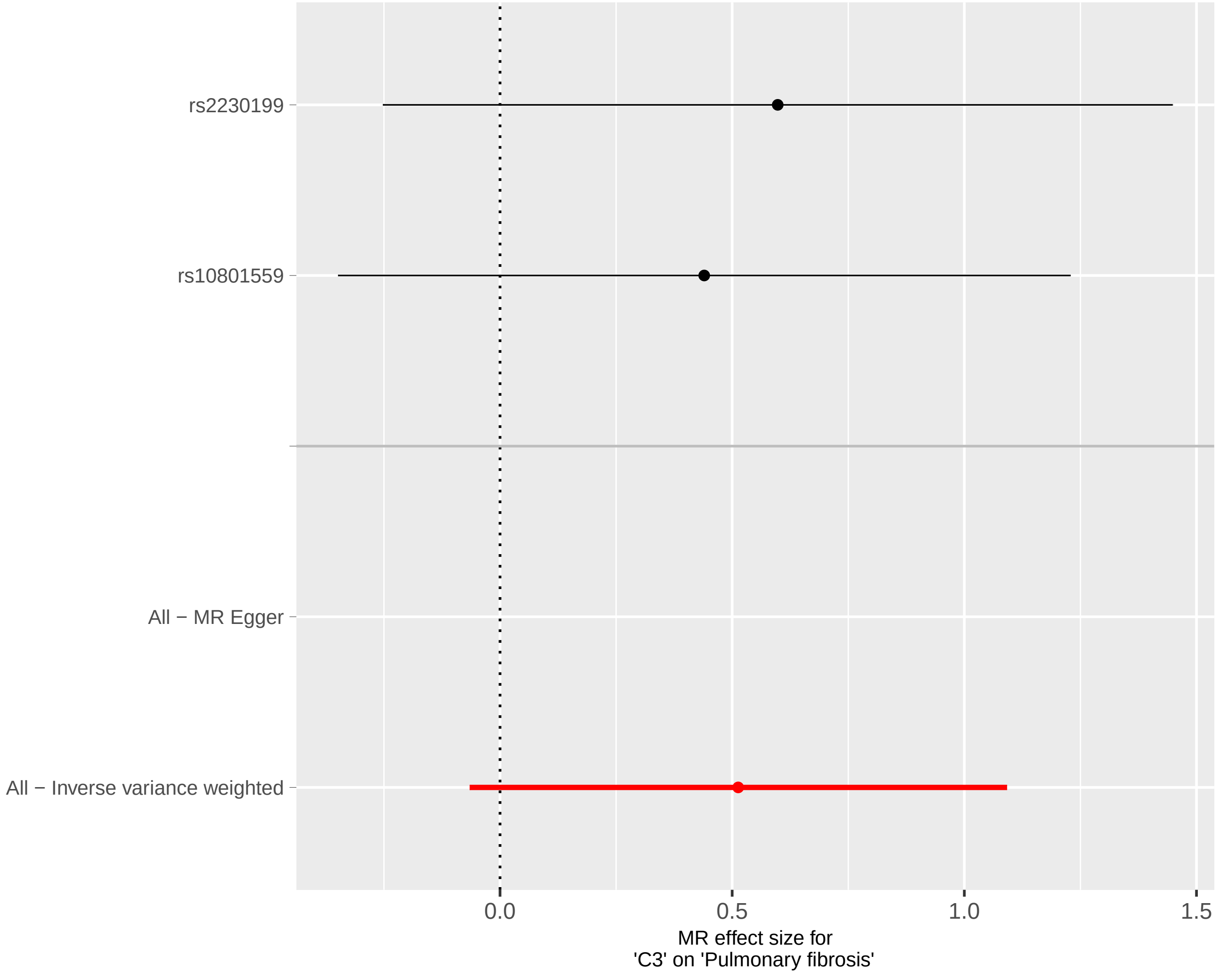
rs2230199
rs10801559
All − MR Egger
All − Inverse variance weighted
0.0
0.5
1.0
1.5
MR effect size for
'C3' on 'Pulmonary fibrosis'

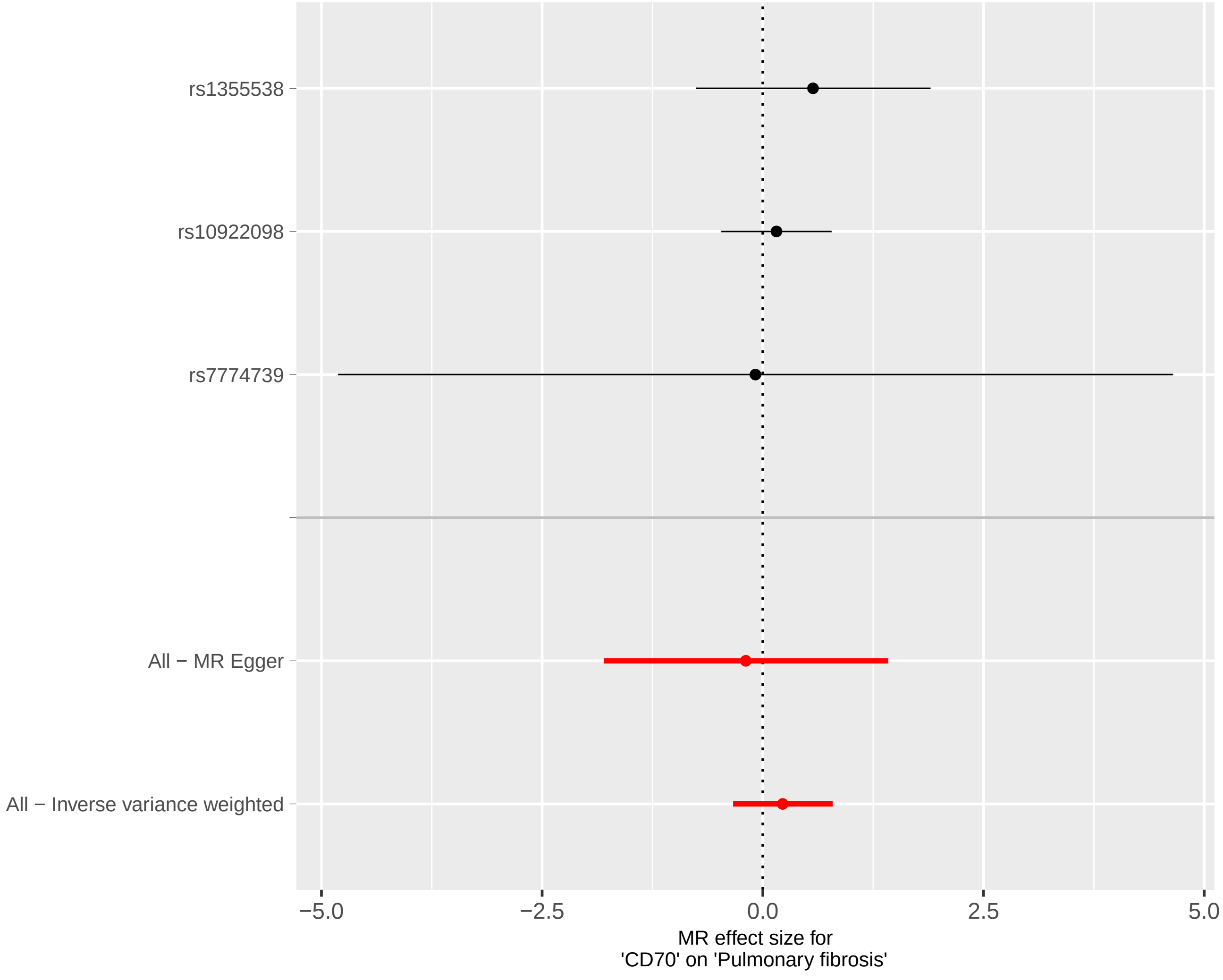
rs1355538
rs10922098
rs7774739
All − MR Egger
All − Inverse variance weighted
−5.0
−2.5
0.0
2.5
5.0
MR effect size for
'CD70' on 'Pulmonary fibrosis'

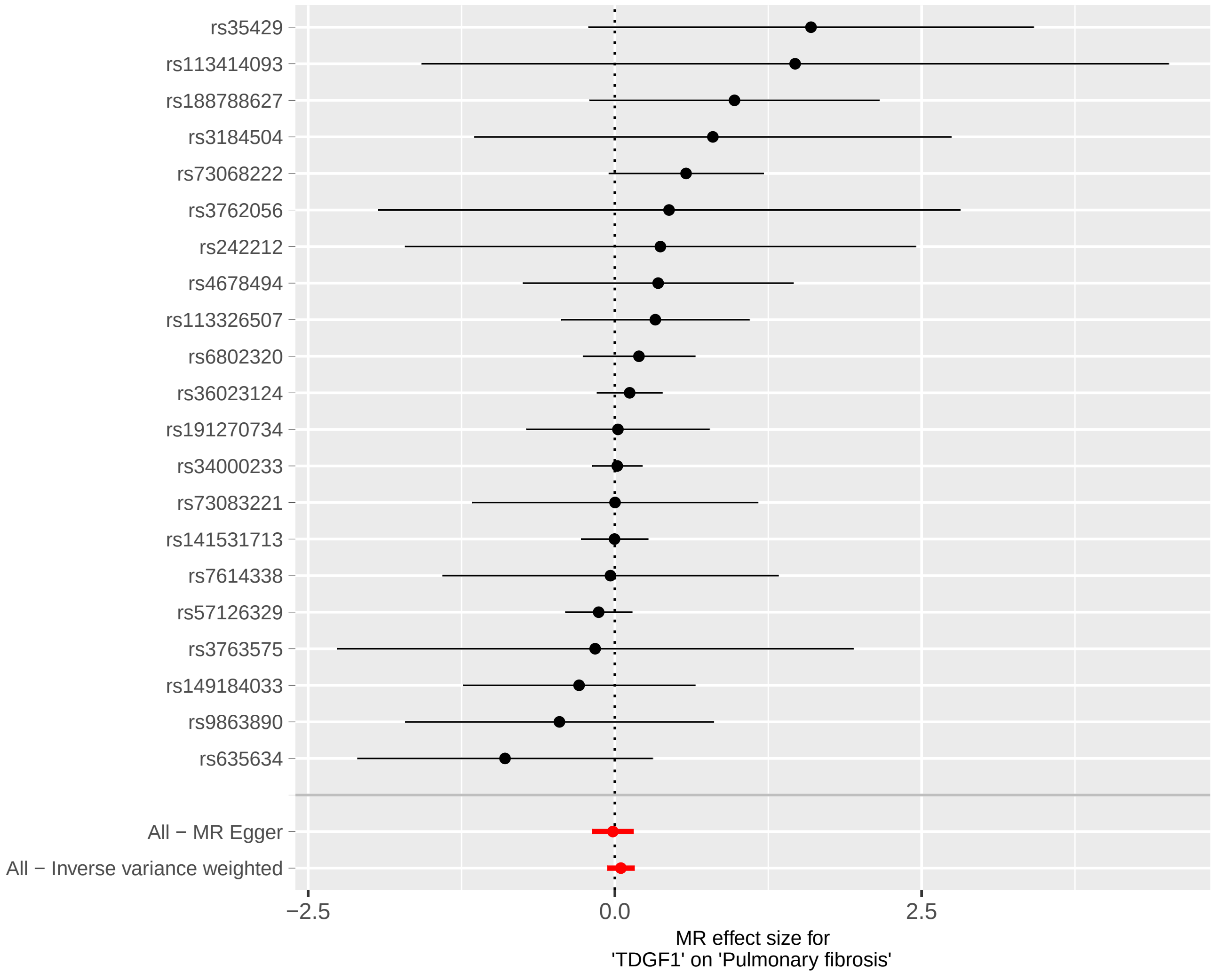

rs35429
rs113414093
rs188788627
rs3184504
rs73068222
rs3762056
rs242212
rs4678494
rs113326507
rs6802320
rs36023124
rs191270734
rs34000233
rs73083221
rs141531713
rs7614338
rs57126329
rs3763575
rs149184033
rs9863890
rs635634
All − MR Egger
All − Inverse variance weighted
−2.5
0.0
2.5
MR effect size for
'TDGF1' on 'Pulmonary fibrosis'

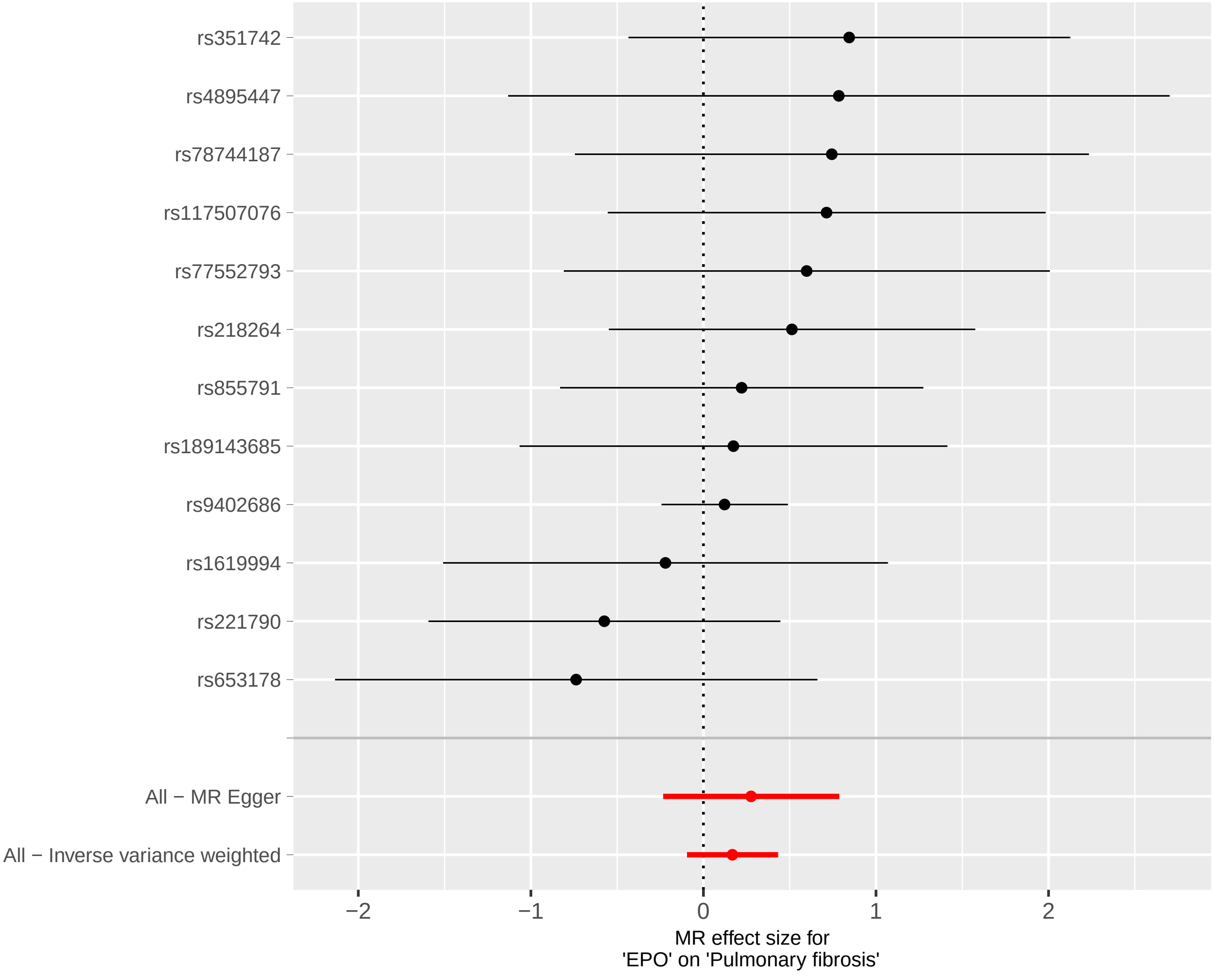

rs351742
rs4895447
rs78744187
rs117507076
rs77552793
rs218264
rs855791
rs189143685
rs9402686
rs1619994
rs221790
rs653178
All − MR Egger
All − Inverse variance weighted
−2
−1
0
1
2
MR effect size for
'EPO' on 'Pulmonary fibrosis'

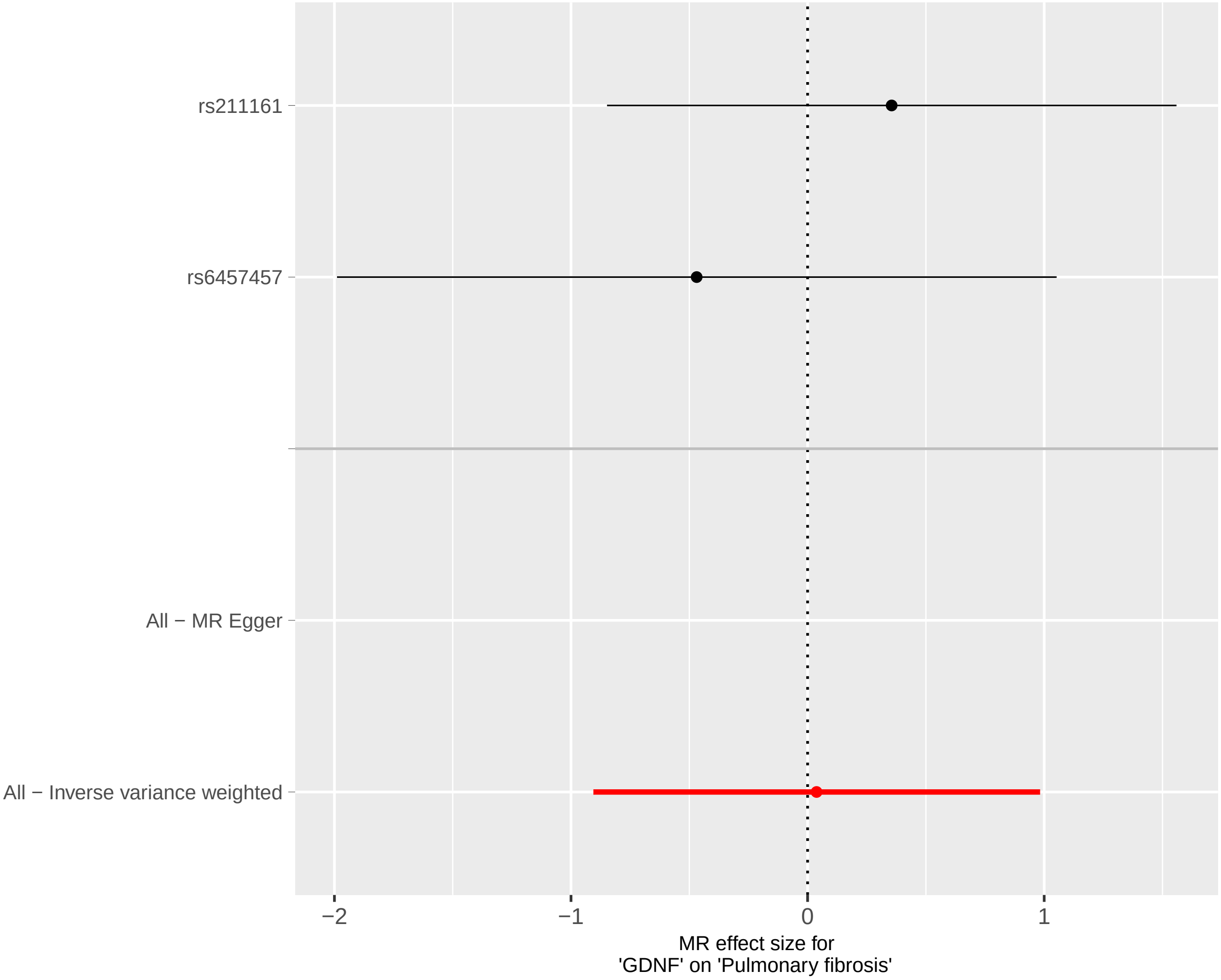

rs211161
rs6457457
All − MR Egger
All − Inverse variance weighted
−2
−1
0
1
MR effect size for
'GDNF' on 'Pulmonary fibrosis'

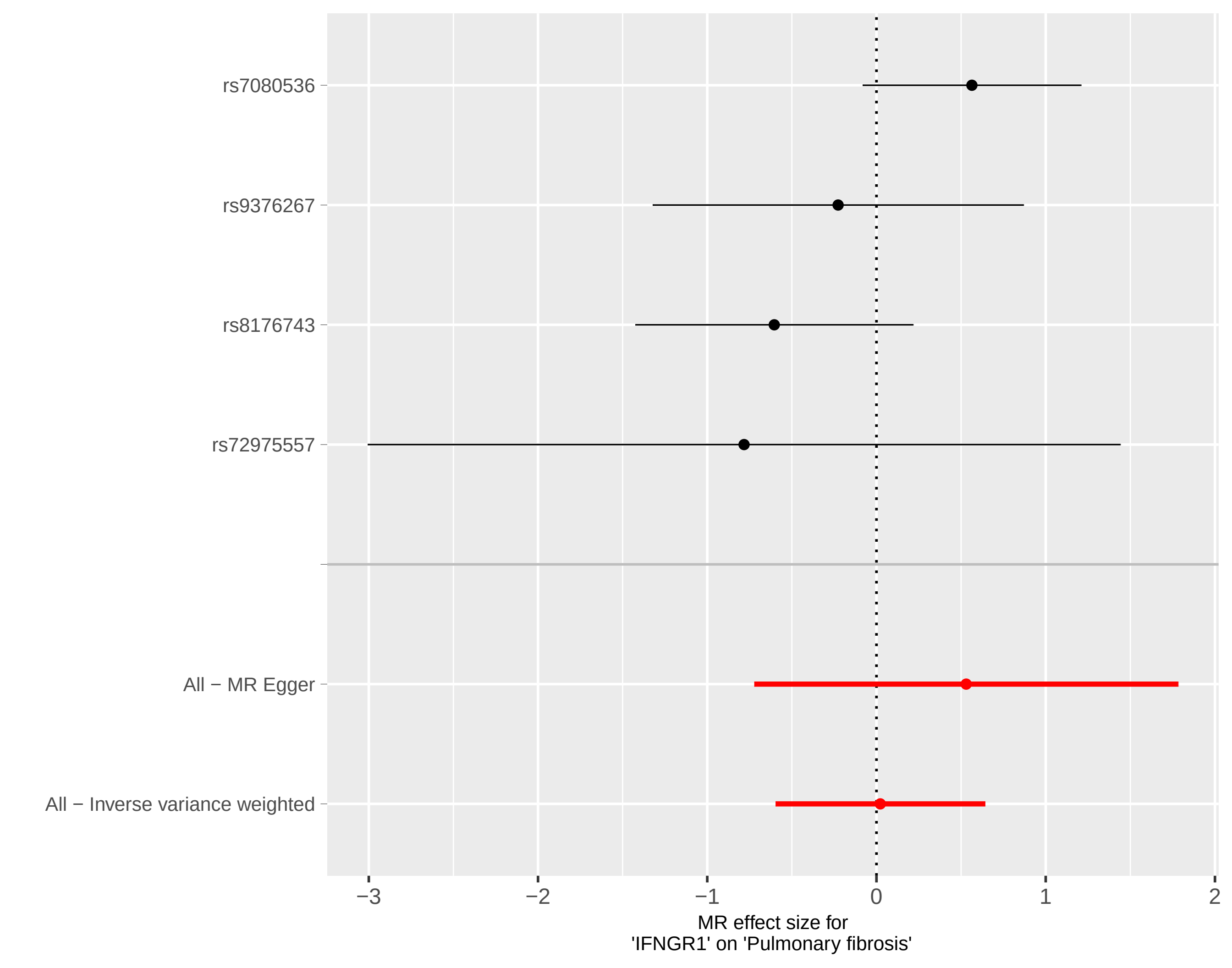

rs7080536
rs9376267
rs8176743
rs72975557
All − MR Egger
All − Inverse variance weighted
−3
−2
−1
0
1
2
MR effect size for
'IFNGR1' on 'Pulmonary fibrosis'

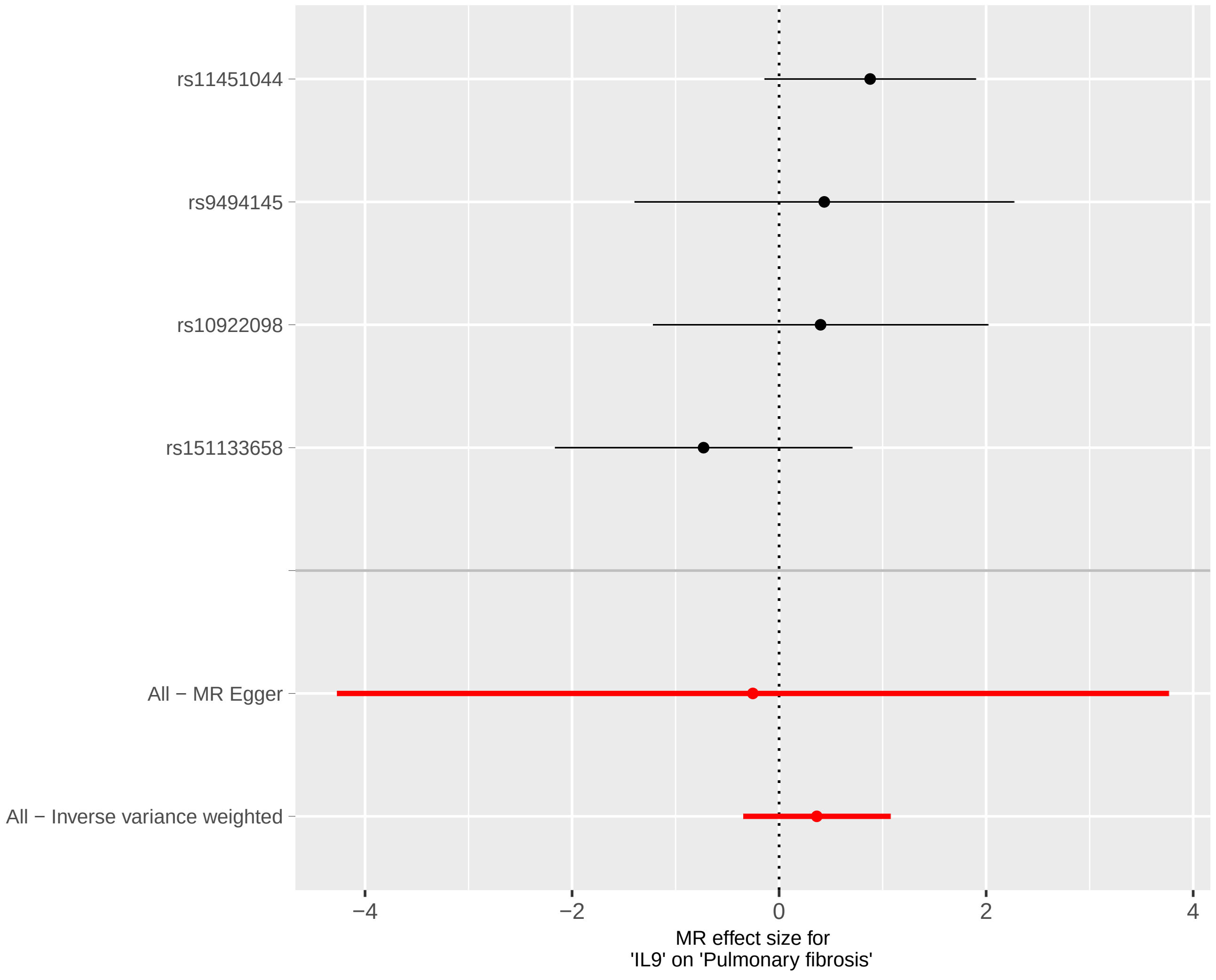

rs11451044
rs9494145
rs10922098
rs151133658
All − MR Egger
All − Inverse variance weighted
−4
−2
0
2
4
MR effect size for
'IL9' on 'Pulmonary fibrosis'

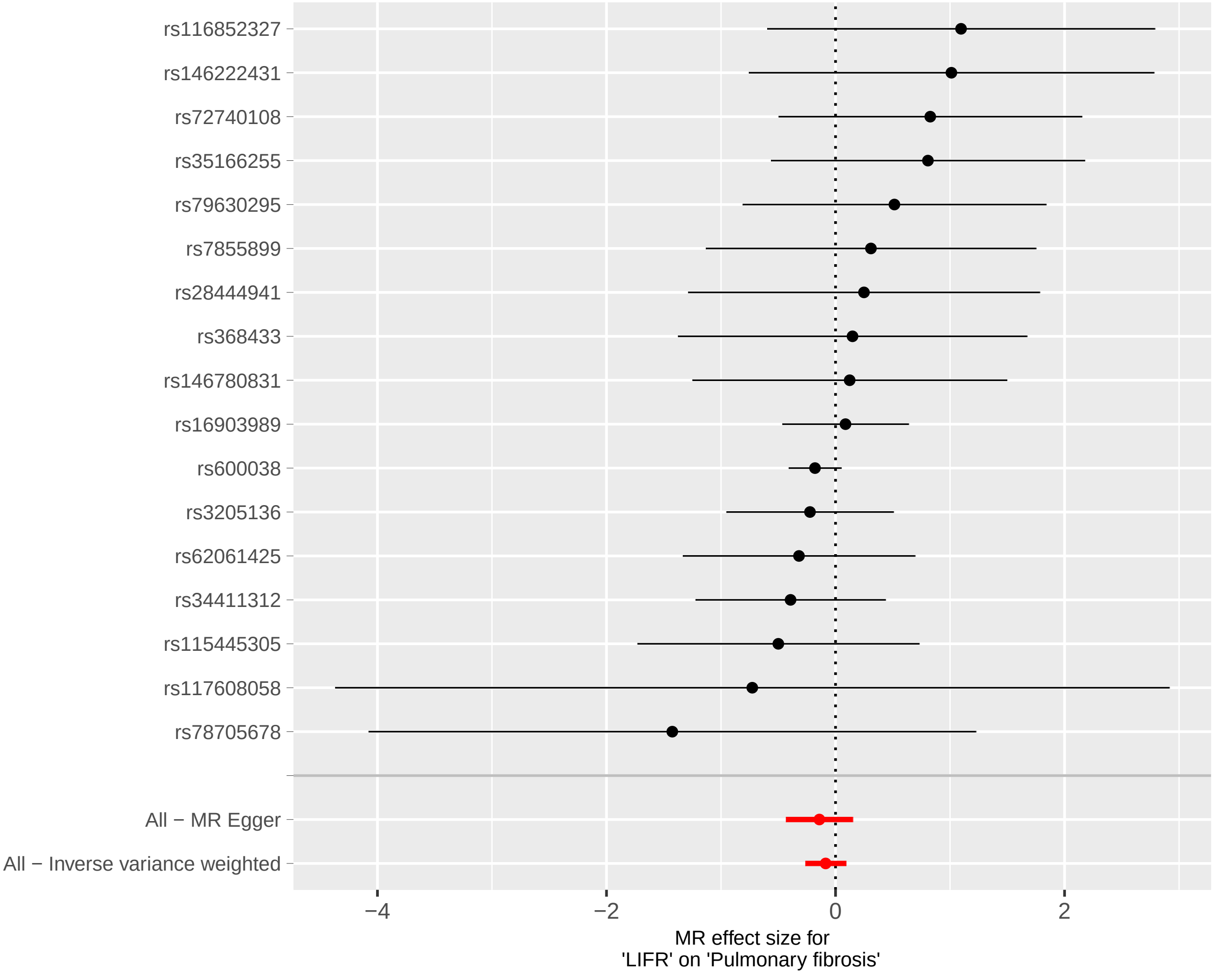

rs116852327
rs146222431
rs72740108
rs35166255
rs79630295
rs7855899
rs28444941
rs368433
rs146780831
rs16903989
rs600038
rs3205136
rs62061425
rs34411312
rs115445305
rs117608058
rs78705678
All − MR Egger
All − Inverse variance weighted
−4
−2
0
2
MR effect size for
'LIFR' on 'Pulmonary fibrosis'

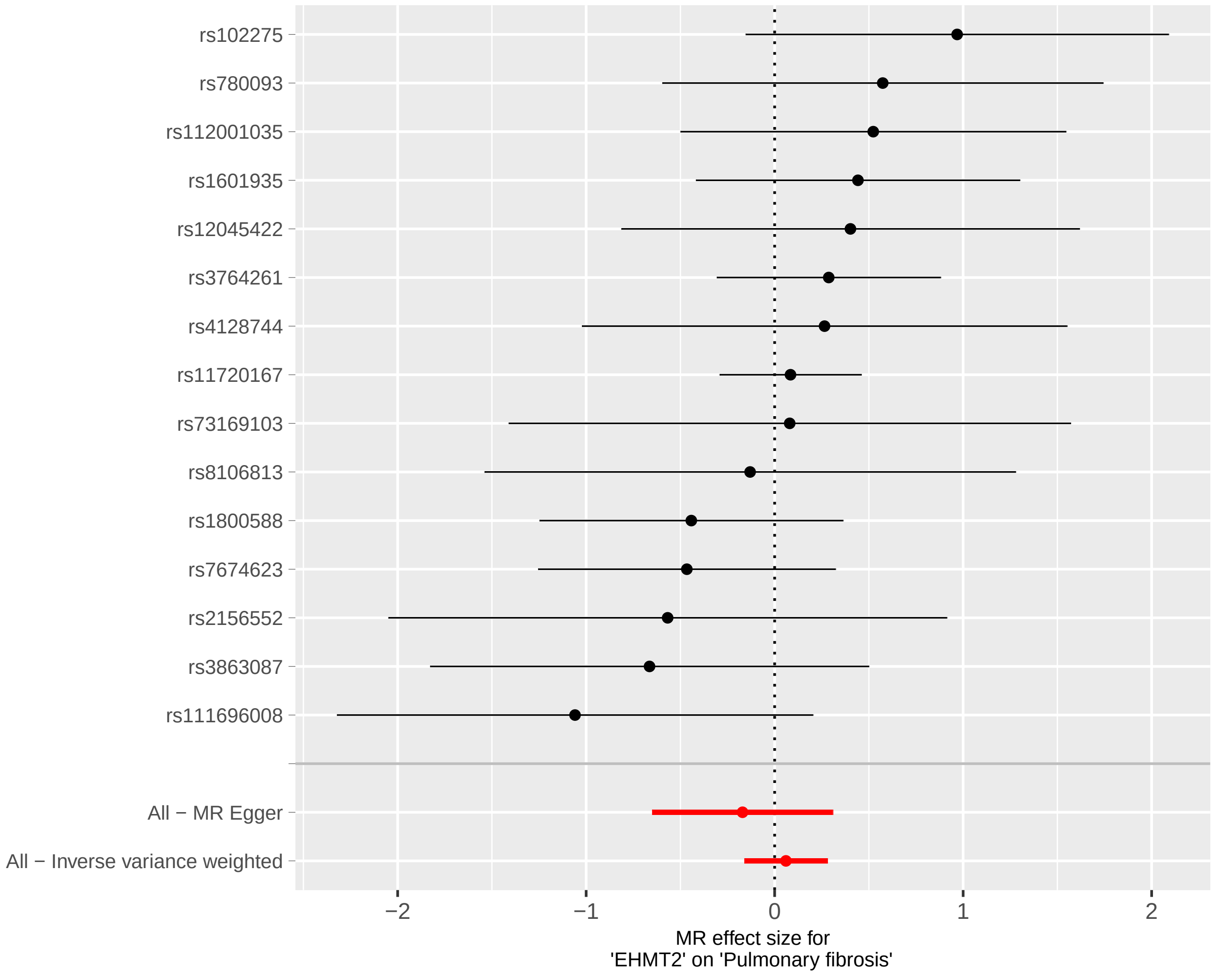
rs102275
rs780093
rs112001035
rs1601935
rs12045422
rs3764261
rs4128744
rs11720167
rs73169103
rs8106813
rs1800588
rs7674623
rs2156552
rs3863087
rs111696008
All − MR Egger
All − Inverse variance weighted
−2
−1
0
1
2
MR effect size for
'EHMT2' on 'Pulmonary fibrosis'

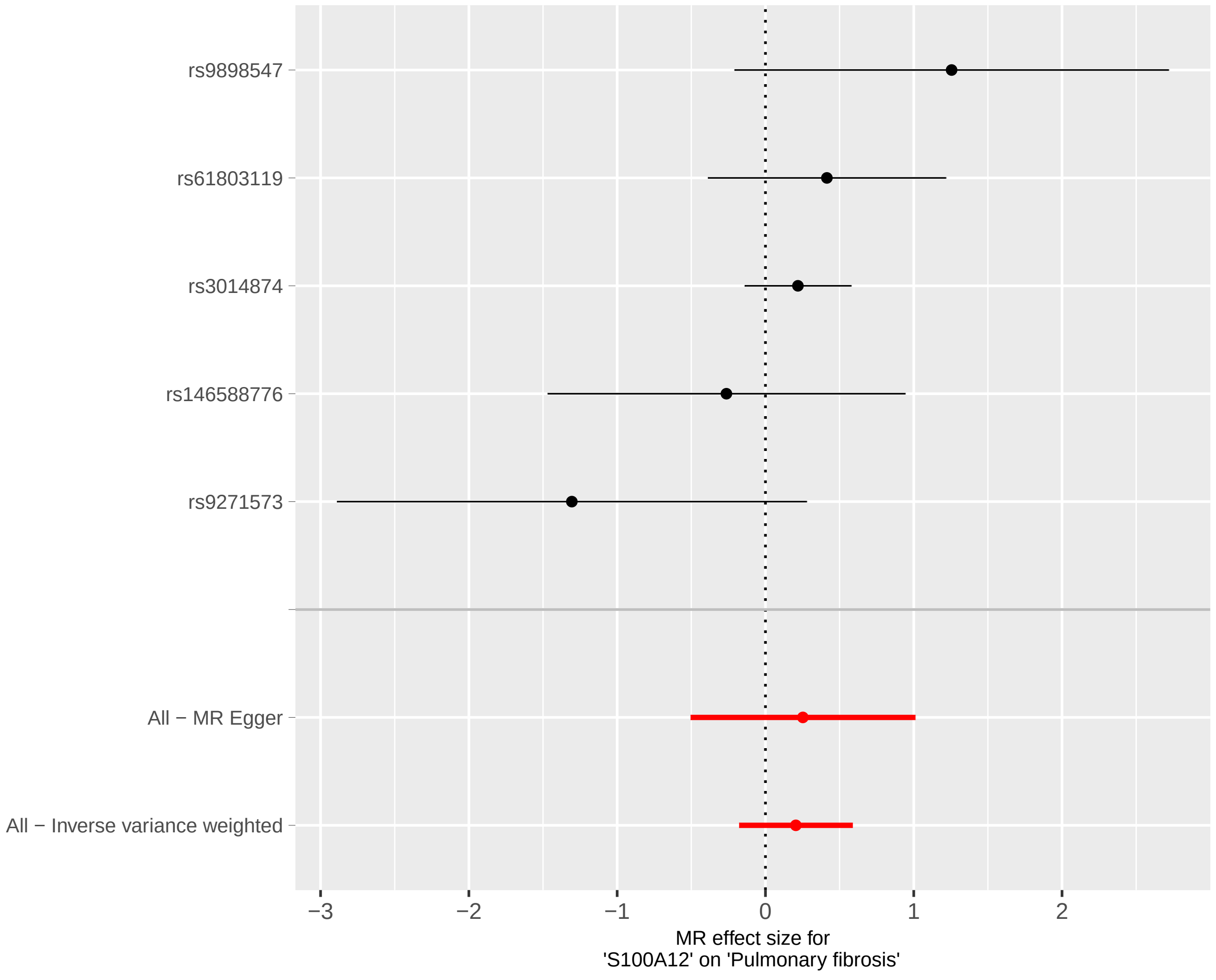

rs9898547
rs61803119
rs3014874
rs146588776
rs9271573
All − MR Egger
All − Inverse variance weighted
−3
−2
−1
0
1
2
MR effect size for
'S100A12' on 'Pulmonary fibrosis'

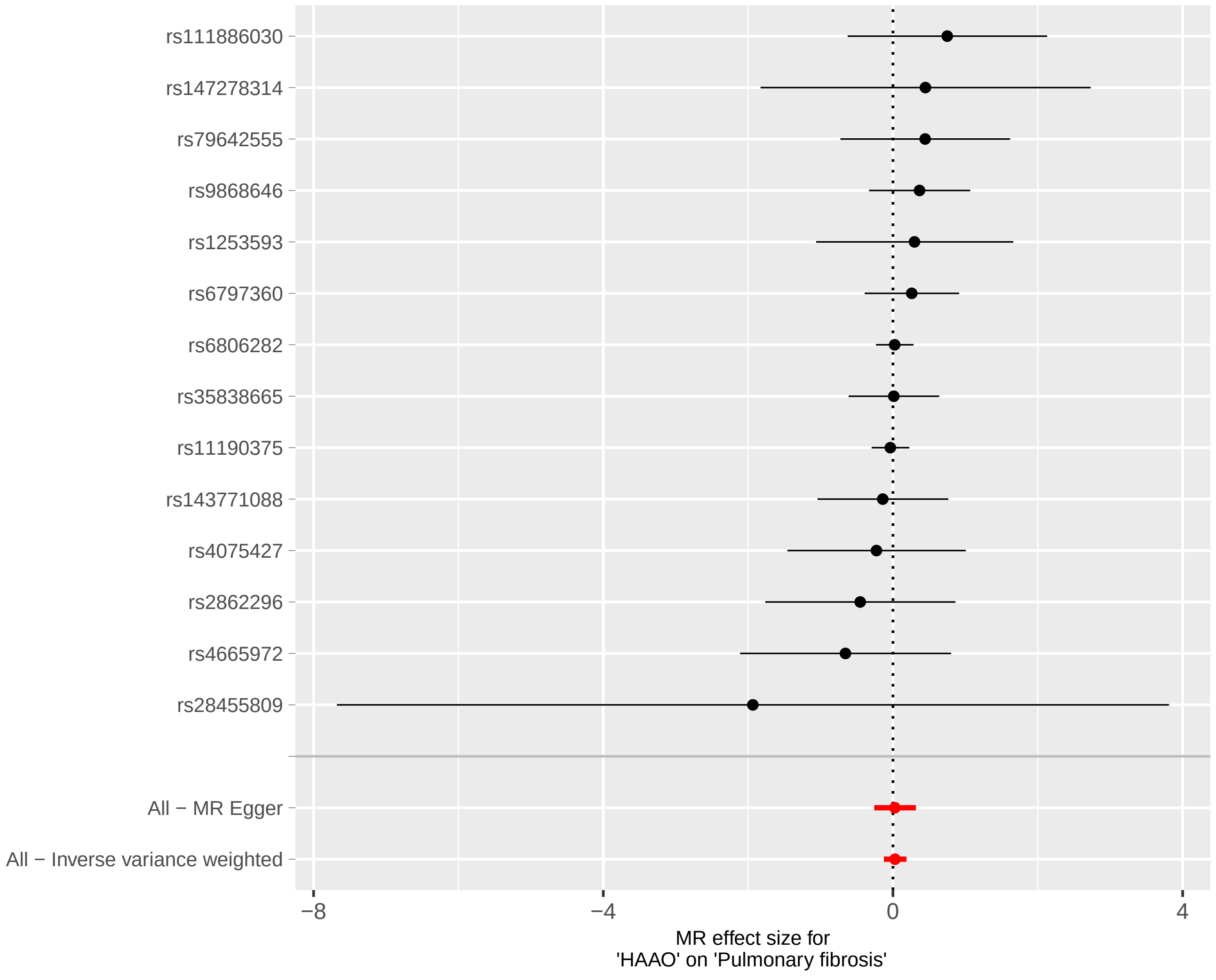

rs111886030
rs147278314
rs79642555
rs9868646
rs1253593
rs6797360
rs6806282
rs35838665
rs11190375
rs143771088
rs4075427
rs2862296
rs4665972
rs28455809
All − MR Egger
All − Inverse variance weighted
−8
−4
0
4
MR effect size for
'HAAO' on 'Pulmonary fibrosis'

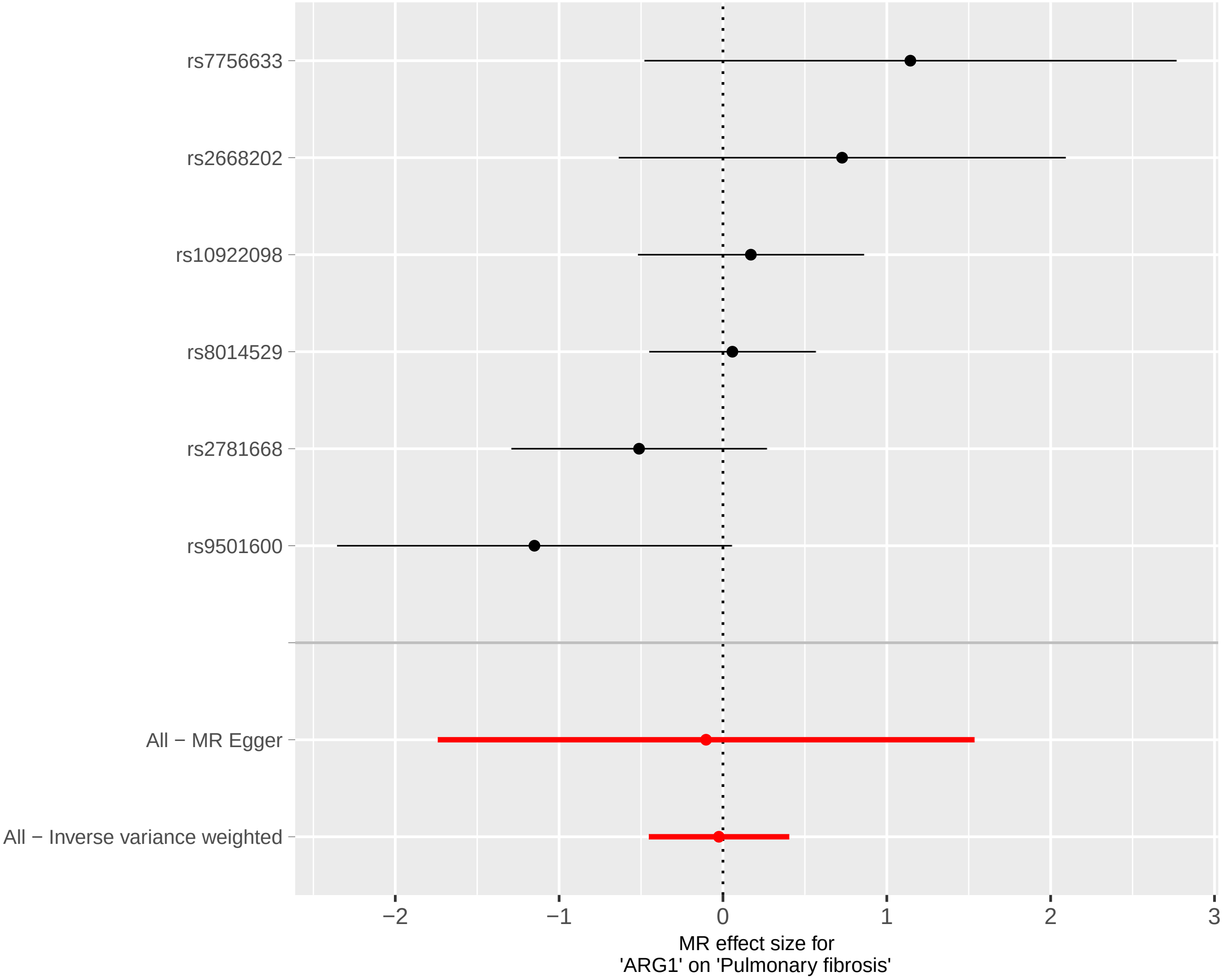

rs7756633
rs2668202
rs10922098
rs8014529
rs2781668
rs9501600
All − MR Egger
All − Inverse variance weighted
−2
−1
0
1
2
3
MR effect size for
'ARG1' on 'Pulmonary fibrosis'

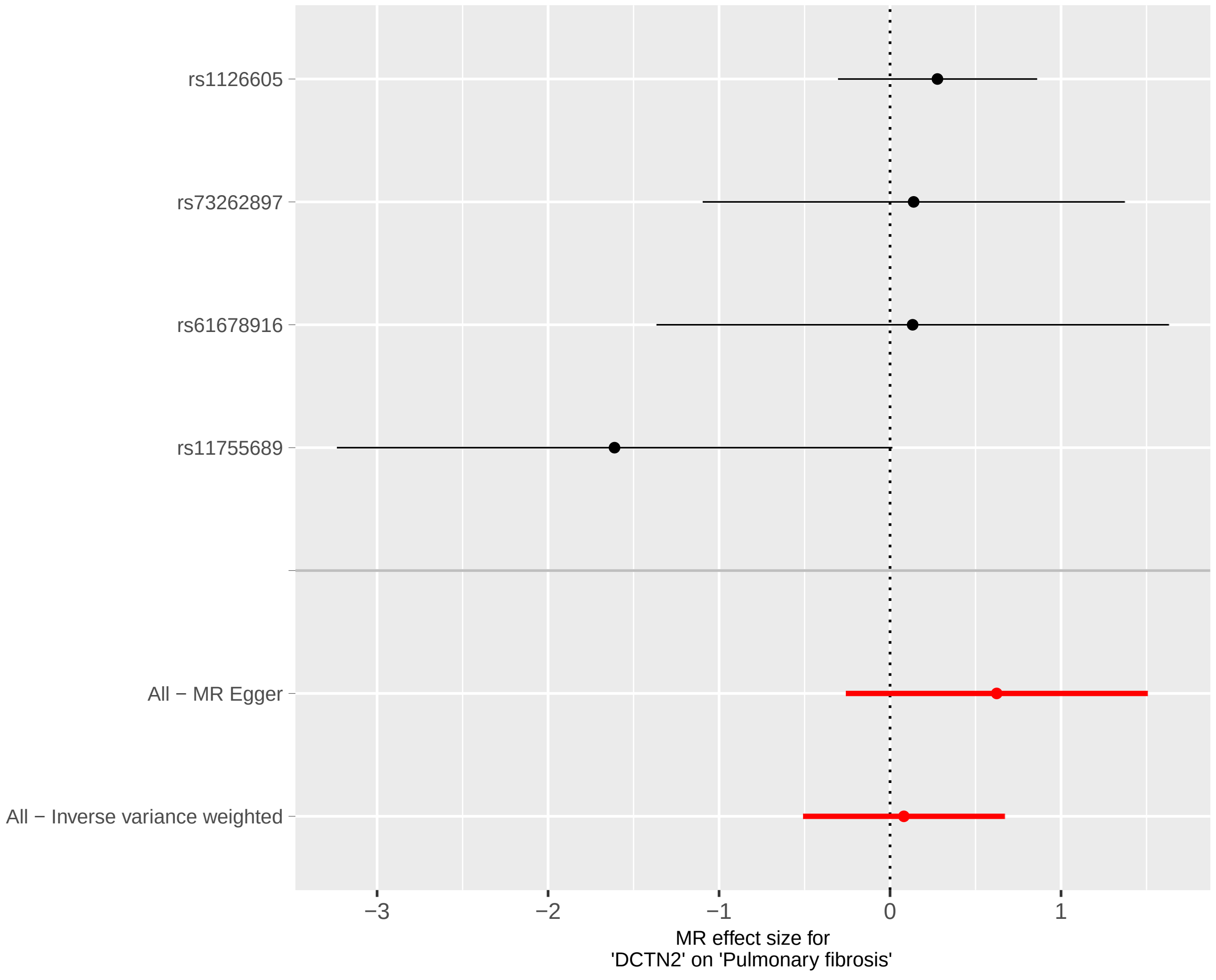

rs1126605
rs73262897
rs61678916
rs11755689
All − MR Egger
All − Inverse variance weighted
−3
−2
−1
0
1
MR effect size for
'DCTN2' on 'Pulmonary fibrosis'

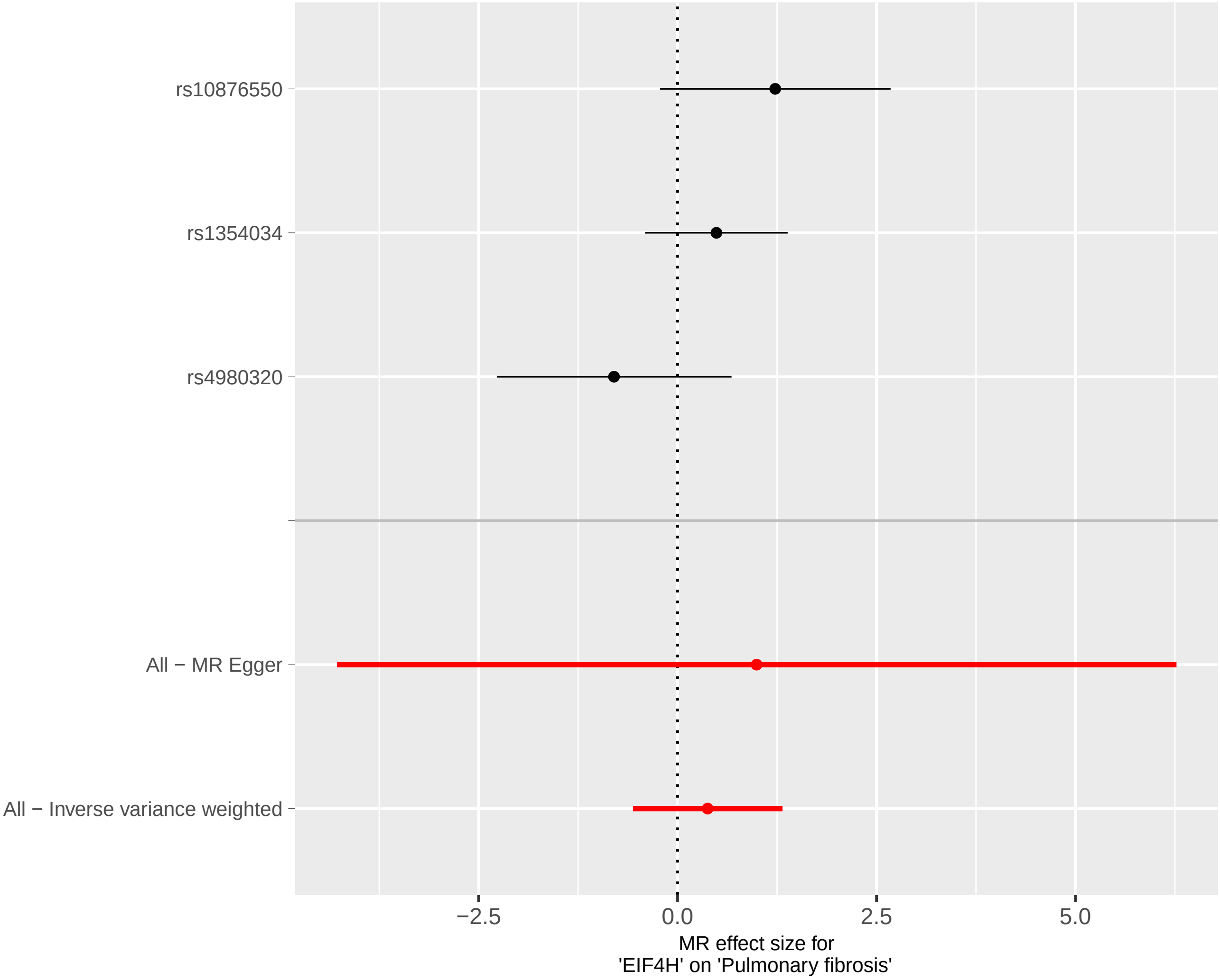
rs10876550
rs1354034
rs4980320
All − MR Egger
All − Inverse variance weighted
−2.5
0.0
2.5
5.0
MR effect size for
'EIF4H' on 'Pulmonary fibrosis'

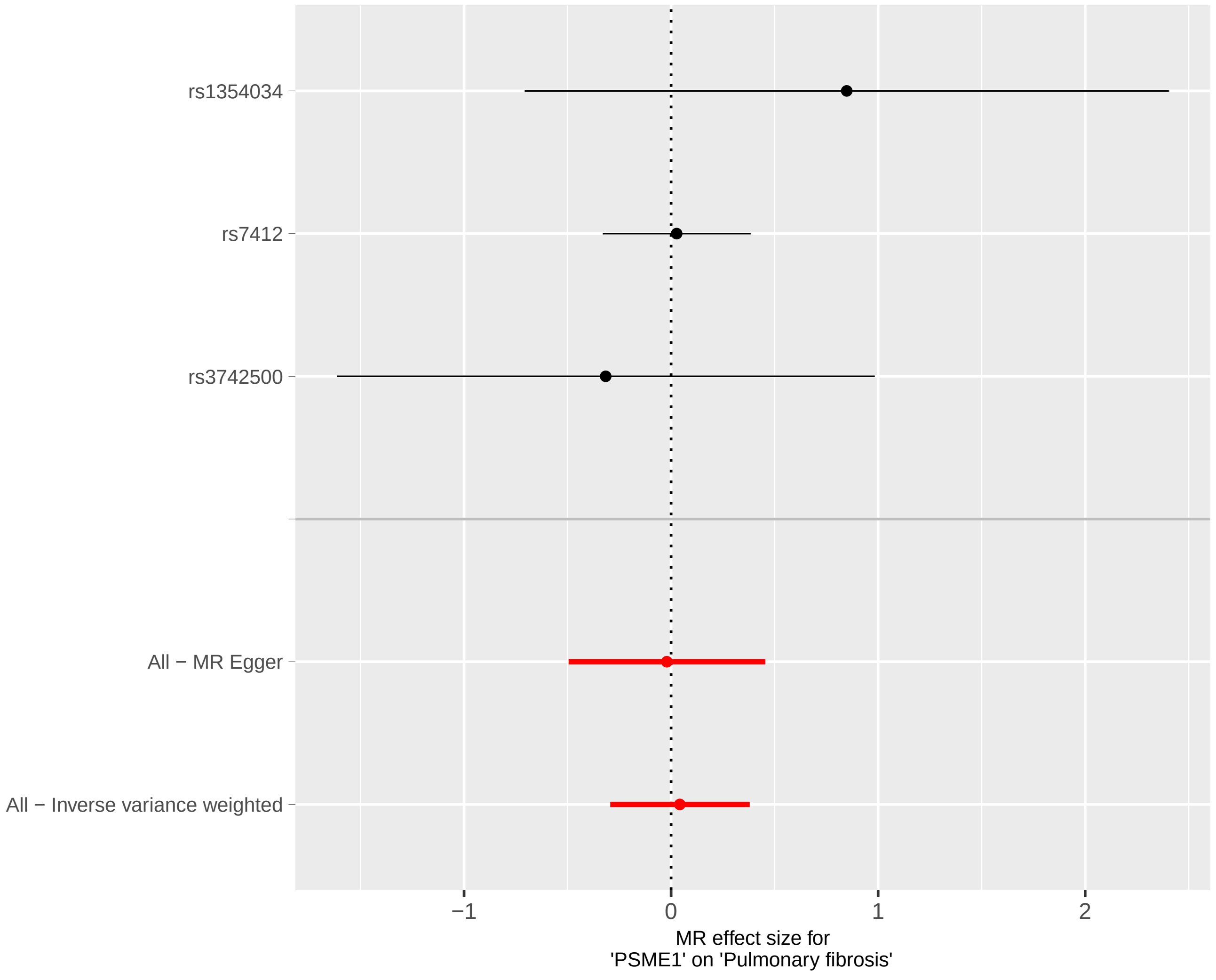

rs1354034
rs7412
rs3742500
All − MR Egger
All − Inverse variance weighted
−1
0
1
2
MR effect size for
'PSME1' on 'Pulmonary fibrosis'

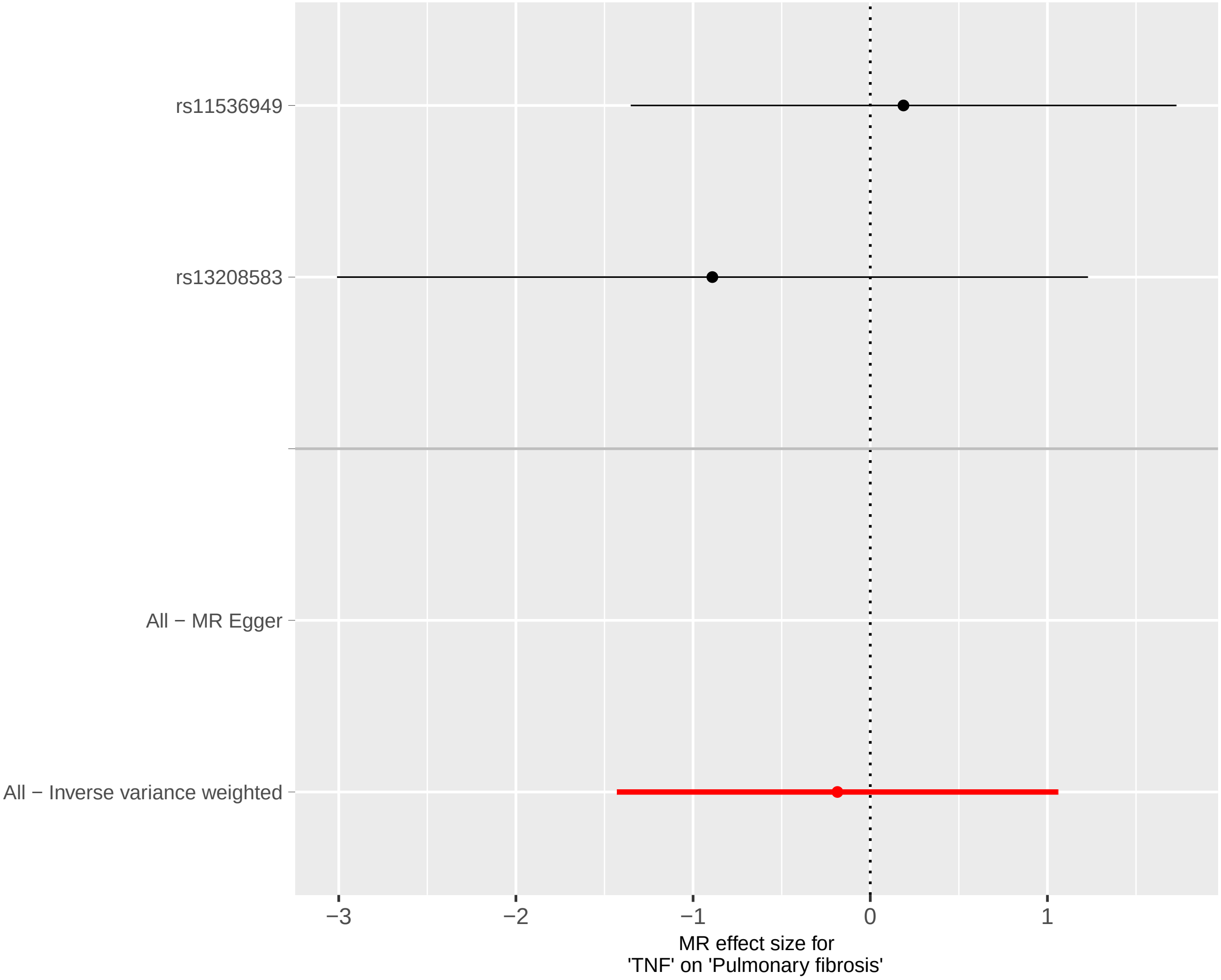

rs11536949
rs13208583
All − MR Egger
All − Inverse variance weighted
−3
−2
−1
0
1
MR effect size for
'TNF' on 'Pulmonary fibrosis'

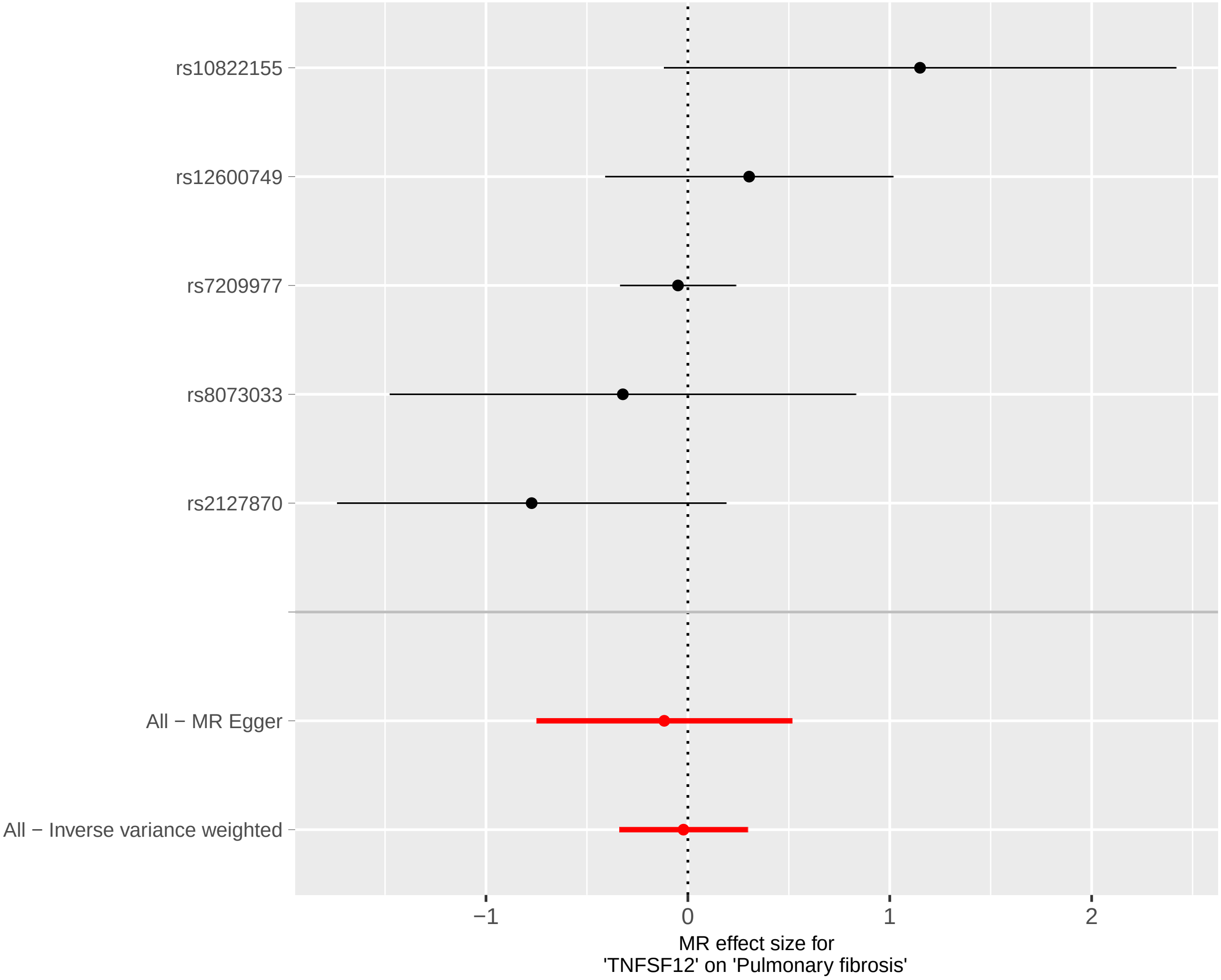
rs10822155
rs12600749
rs7209977
rs8073033
rs2127870
All − MR Egger
All − Inverse variance weighted
−1
0
1
2
MR effect size for
'TNFSF12' on 'Pulmonary fibrosis'

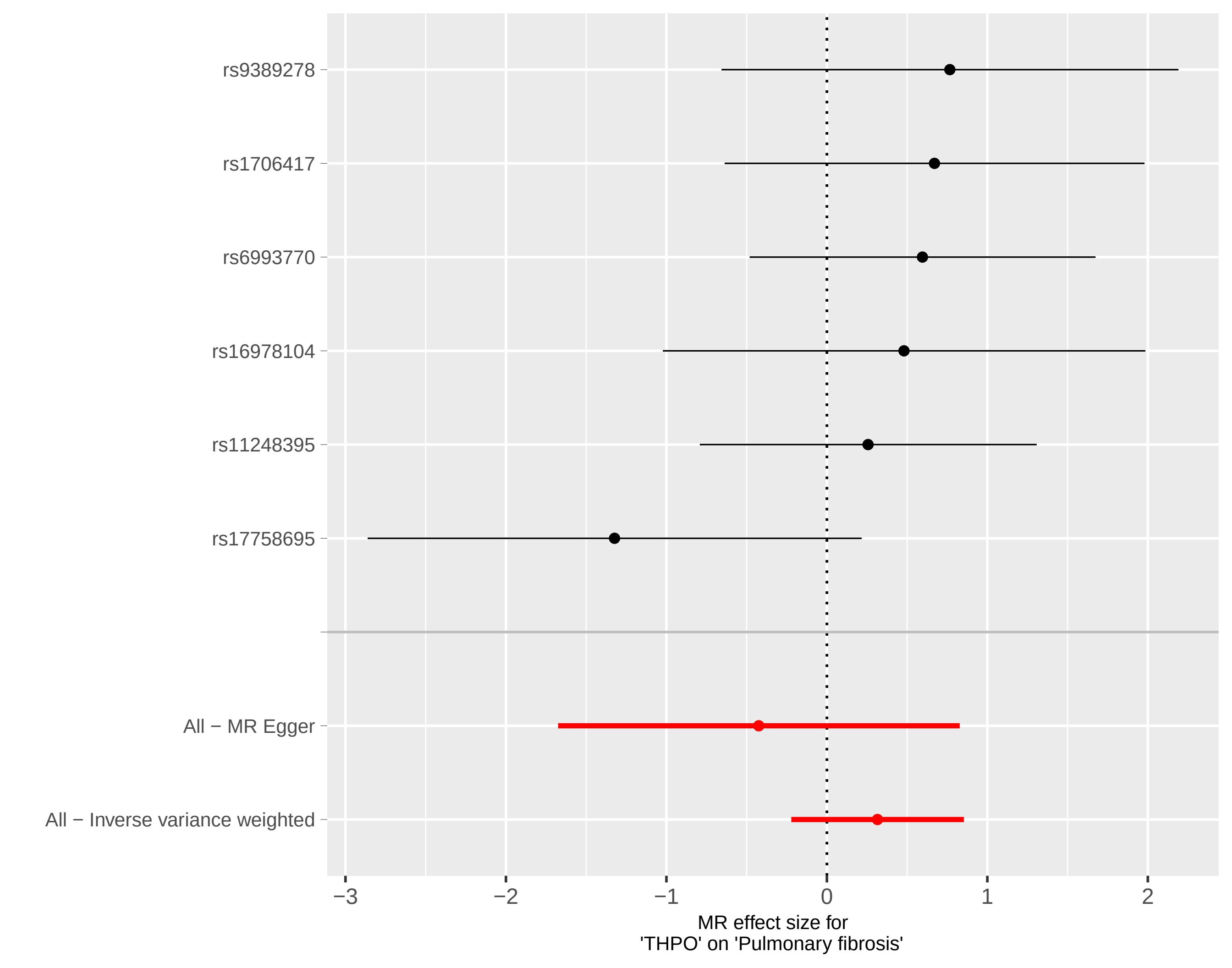
rs9389278
rs1706417
rs6993770
rs16978104
rs11248395
rs17758695
All − MR Egger
All − Inverse variance weighted
−3
−2
−1
0
1
2
MR effect size for
'THPO' on 'Pulmonary fibrosis'

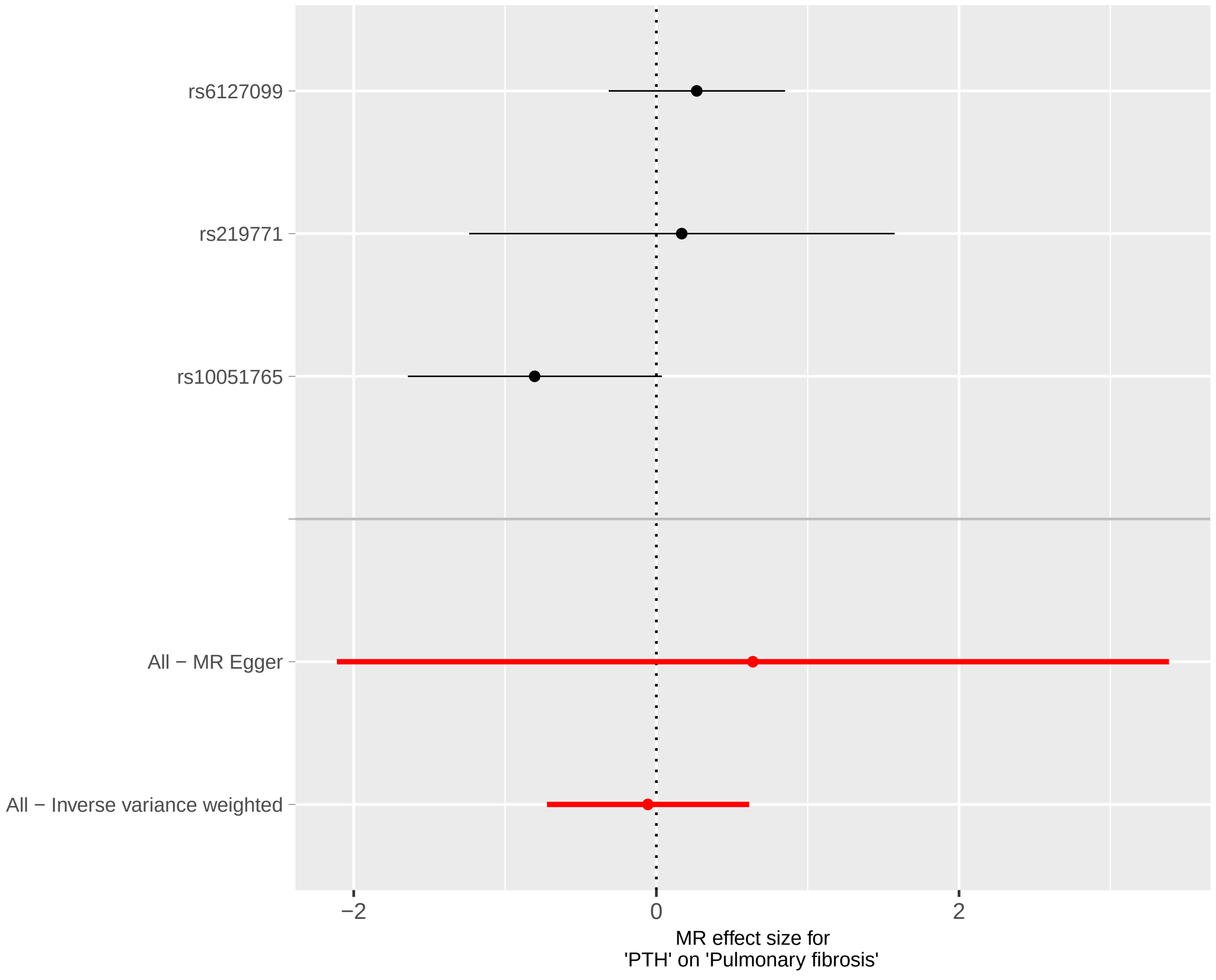
rs6127099
rs219771
rs10051765
All − MR Egger
All − Inverse variance weighted
−2
0
2
MR effect size for
'PTH' on 'Pulmonary fibrosis'

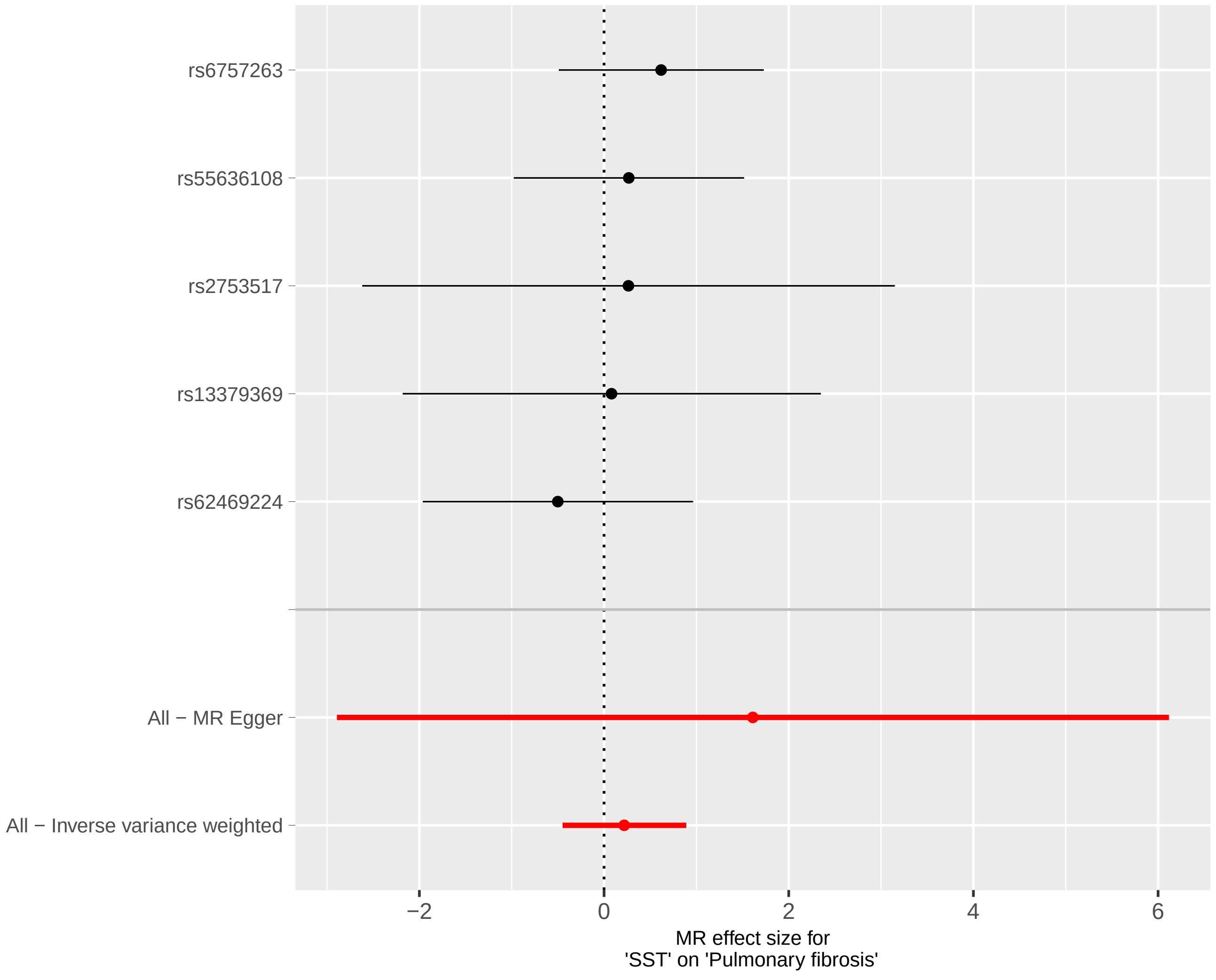
rs6757263
rs55636108
rs2753517
rs13379369
rs62469224
All − MR Egger
All − Inverse variance weighted
−2
0
2
4
6
MR effect size for
'SST' on 'Pulmonary fibrosis'

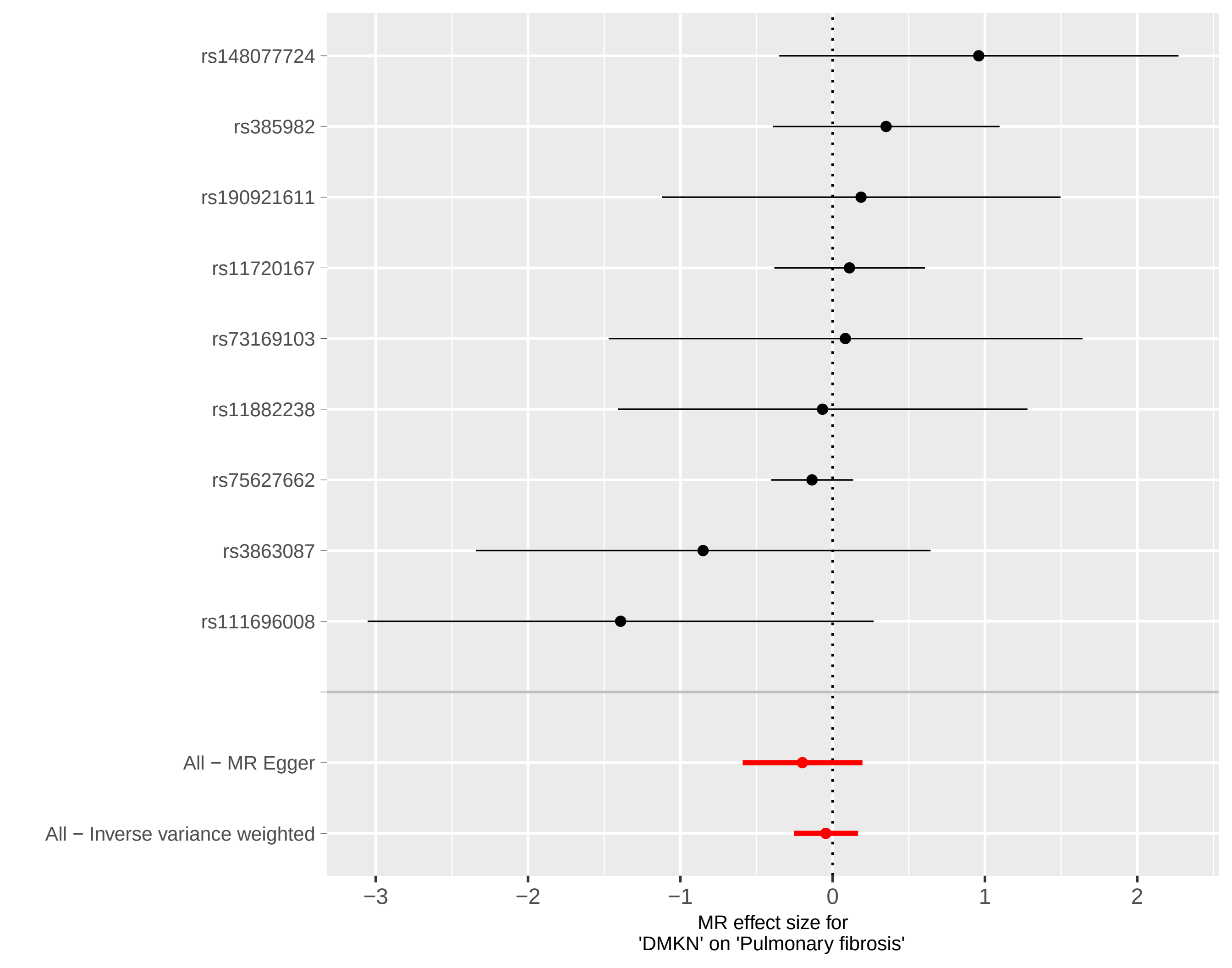

rs148077724
rs385982
rs190921611
rs11720167
rs73169103
rs11882238
rs75627662
rs3863087
rs111696008
All − MR Egger
All − Inverse variance weighted
−3
−2
−1
0
1
2
MR effect size for
'DMKN' on 'Pulmonary fibrosis'

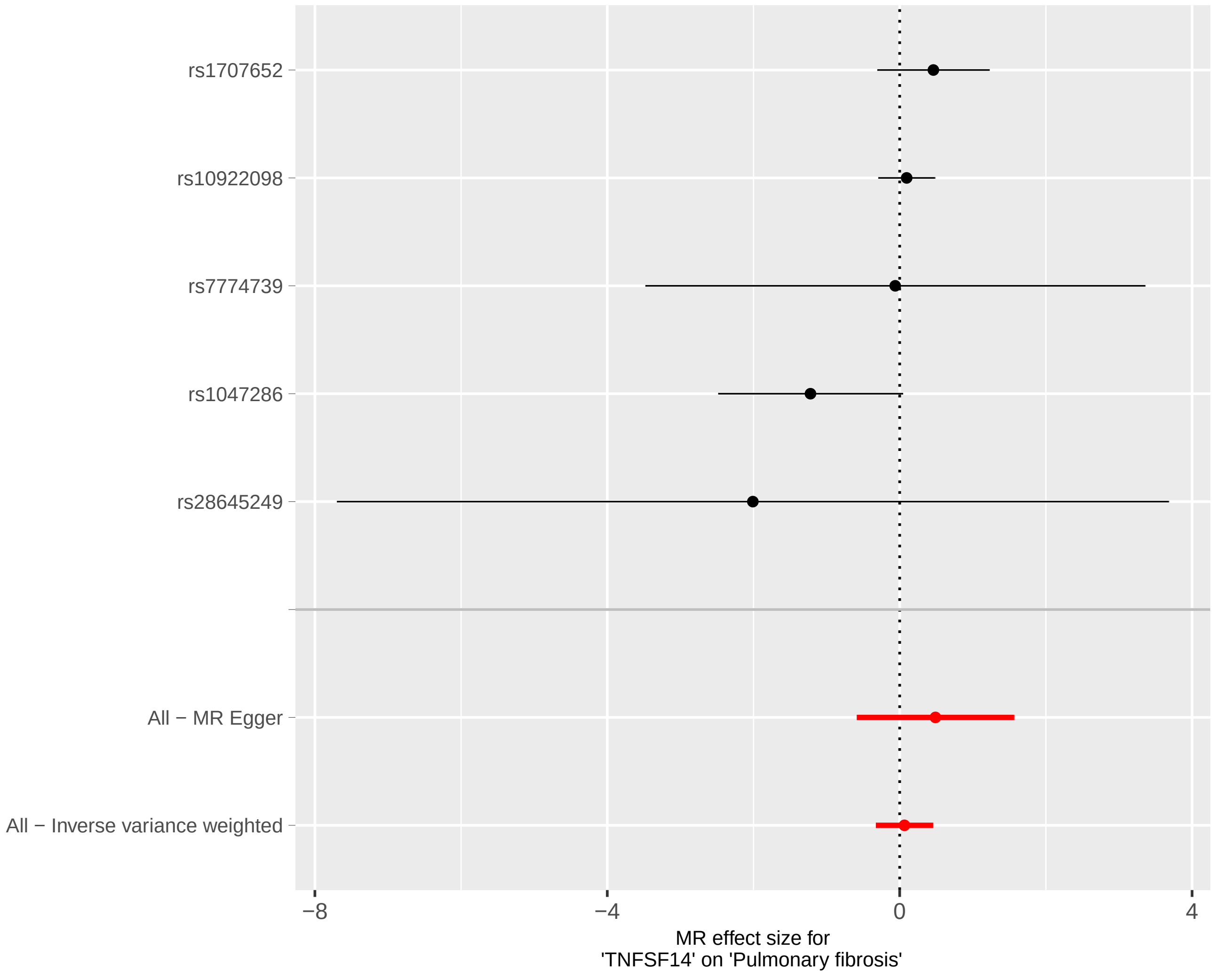

rs1707652
rs10922098
rs7774739
rs1047286
rs28645249
All − MR Egger
All − Inverse variance weighted
−8
−4
0
4
MR effect size for
'TNFSF14' on 'Pulmonary fibrosis'

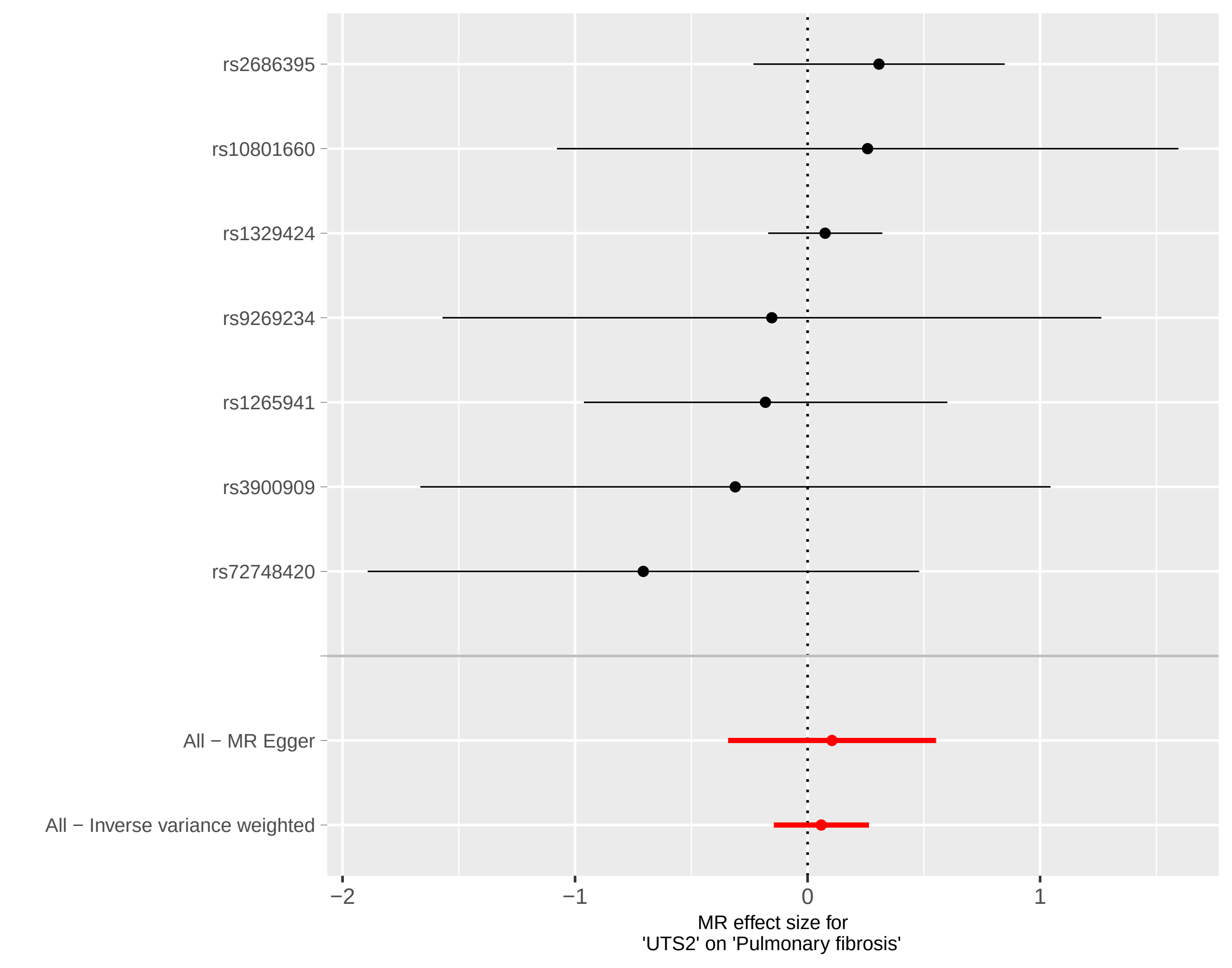

rs2686395
rs10801660
rs1329424
rs9269234
rs1265941
rs3900909
rs72748420
All − MR Egger
All − Inverse variance weighted
−2
−1
0
1
MR effect size for
'UTS2' on 'Pulmonary fibrosis'

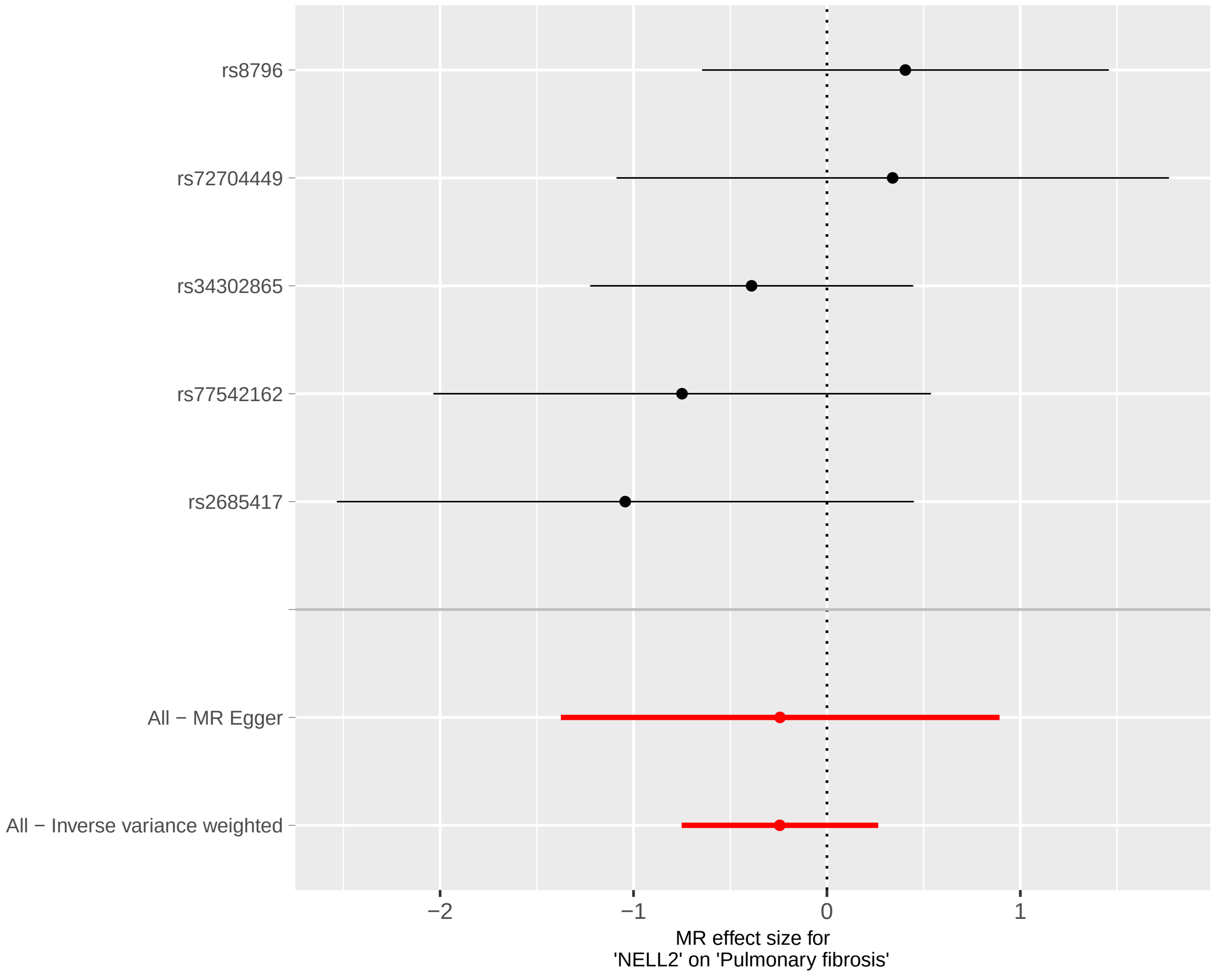

rs8796
rs72704449
rs34302865
rs77542162
rs2685417
All − MR Egger
All − Inverse variance weighted
−2
−1
0
1
MR effect size for
'NELL2' on 'Pulmonary fibrosis'

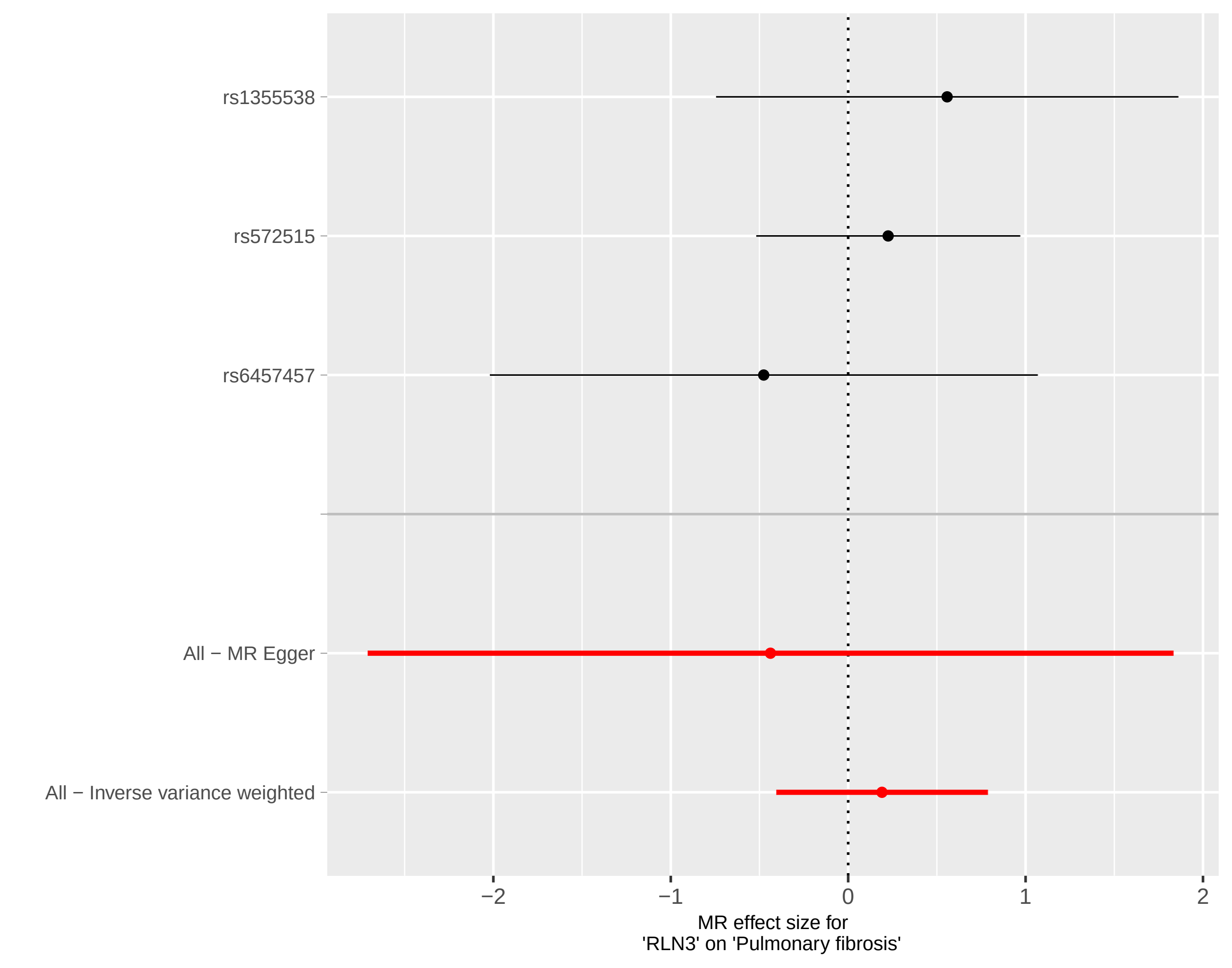
rs1355538
rs572515
rs6457457
All − MR Egger
All − Inverse variance weighted
−2
−1
0
1
2
MR effect size for
'RLN3' on 'Pulmonary fibrosis'

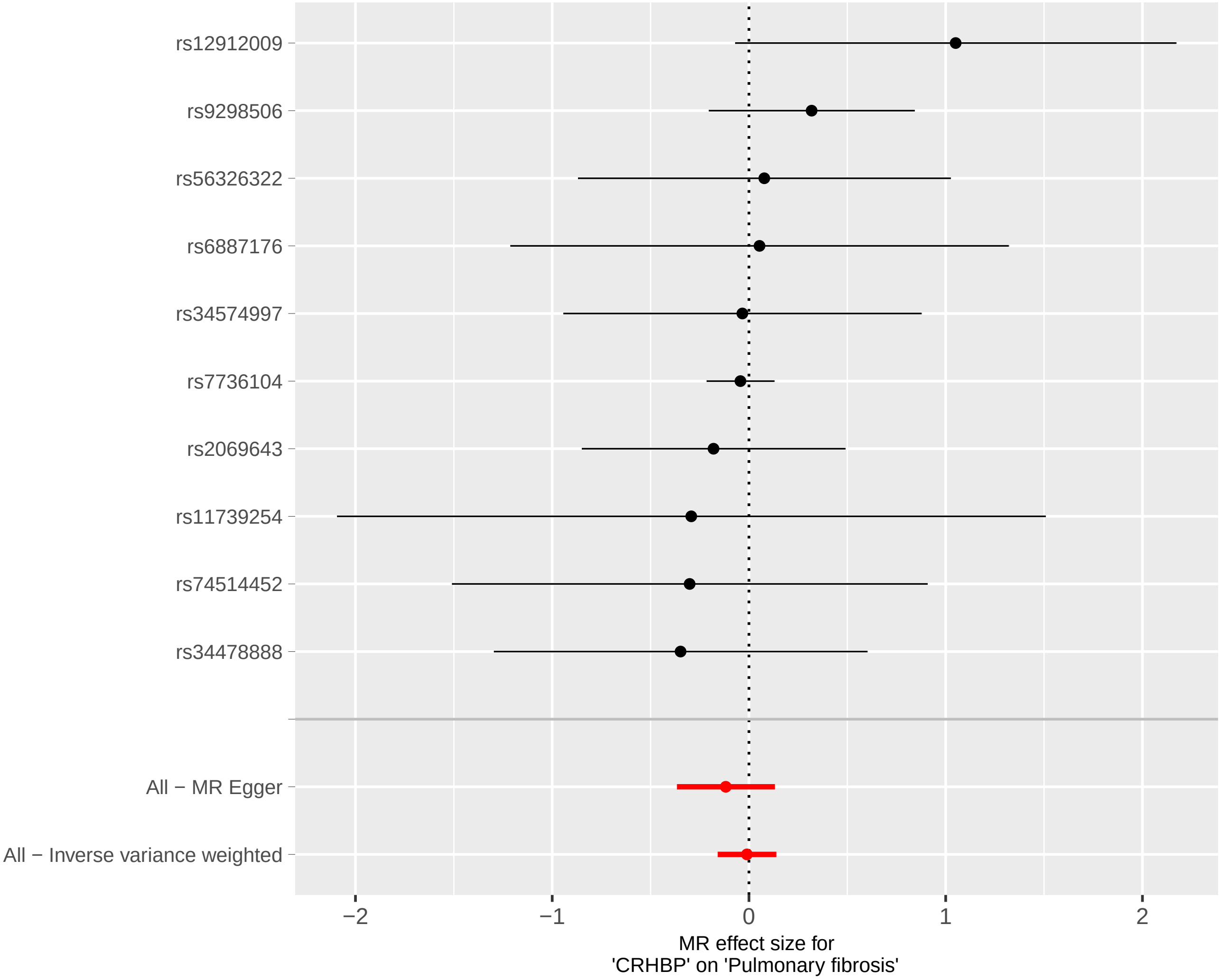
rs12912009
rs9298506
rs56326322
rs6887176
rs34574997
rs7736104
rs2069643
rs11739254
rs74514452
rs34478888
All − MR Egger
All − Inverse variance weighted
−2
−1
0
1
2
MR effect size for
'CRHBP' on 'Pulmonary fibrosis'

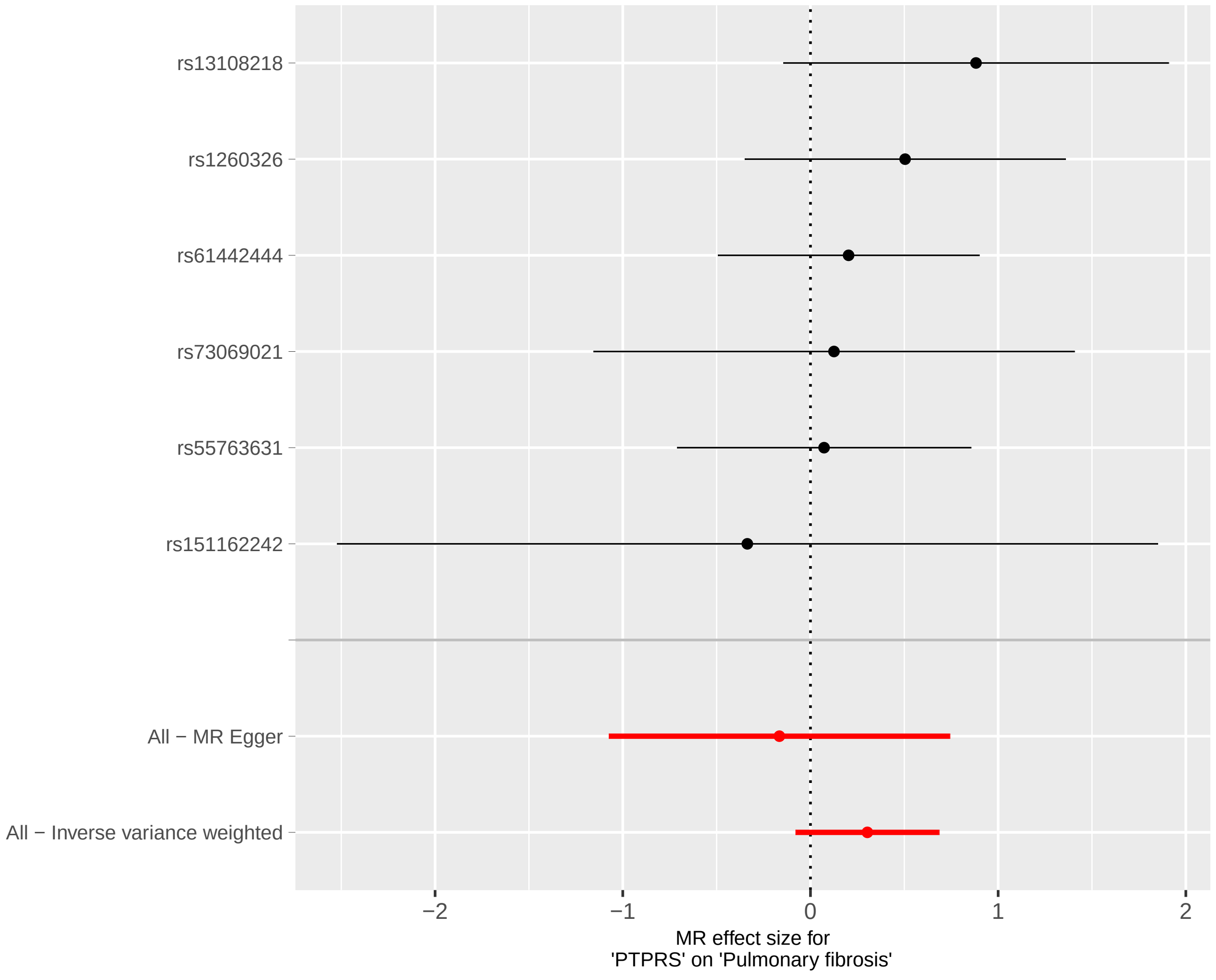

rs13108218
rs1260326
rs61442444
rs73069021
rs55763631
rs151162242
All − MR Egger
All − Inverse variance weighted
−2
−1
0
1
2
MR effect size for
'PTPRS' on 'Pulmonary fibrosis'

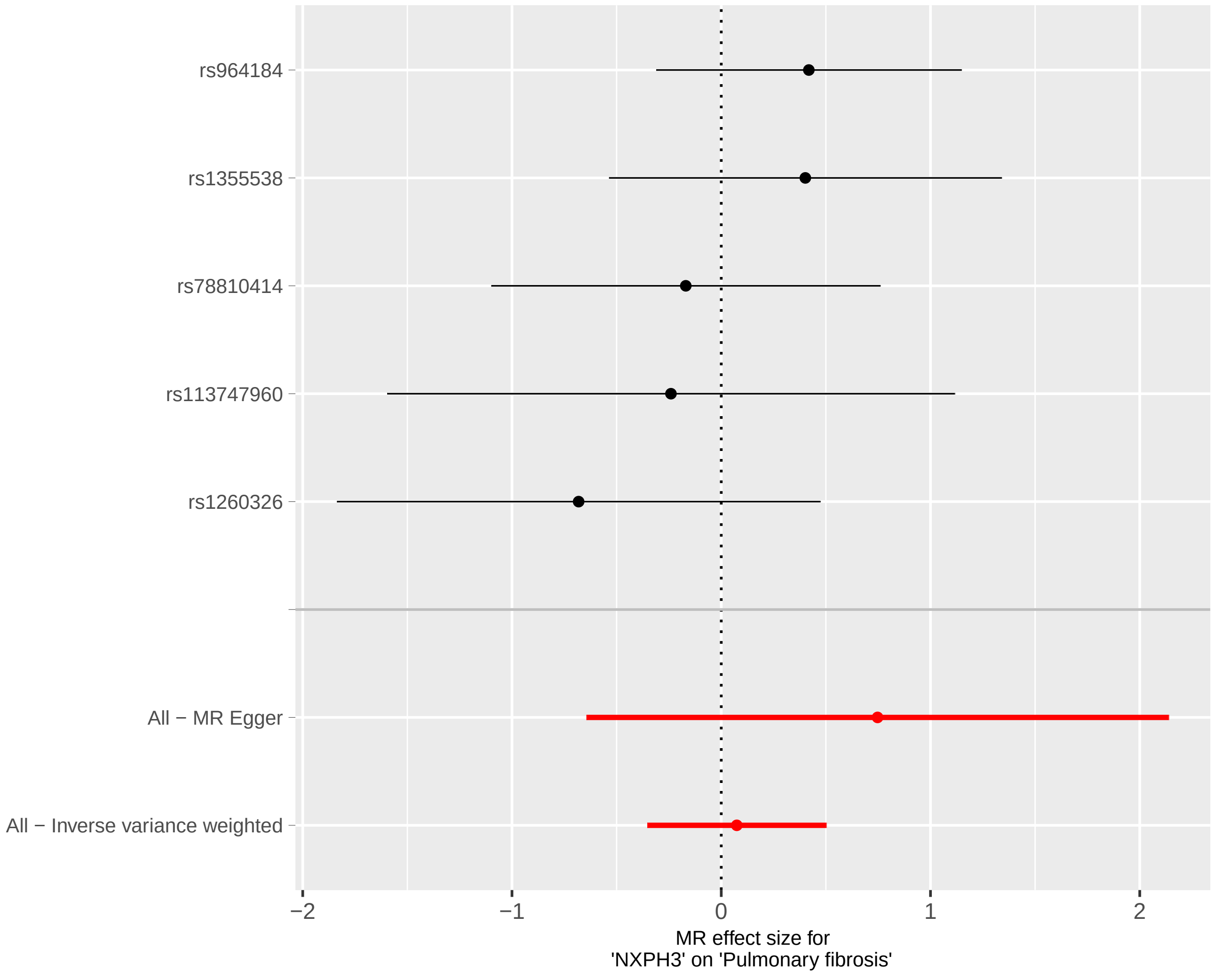
rs964184
rs1355538
rs78810414
rs113747960
rs1260326
All − MR Egger
All − Inverse variance weighted
−2
−1
0
1
2
MR effect size for
'NXPH3' on 'Pulmonary fibrosis'

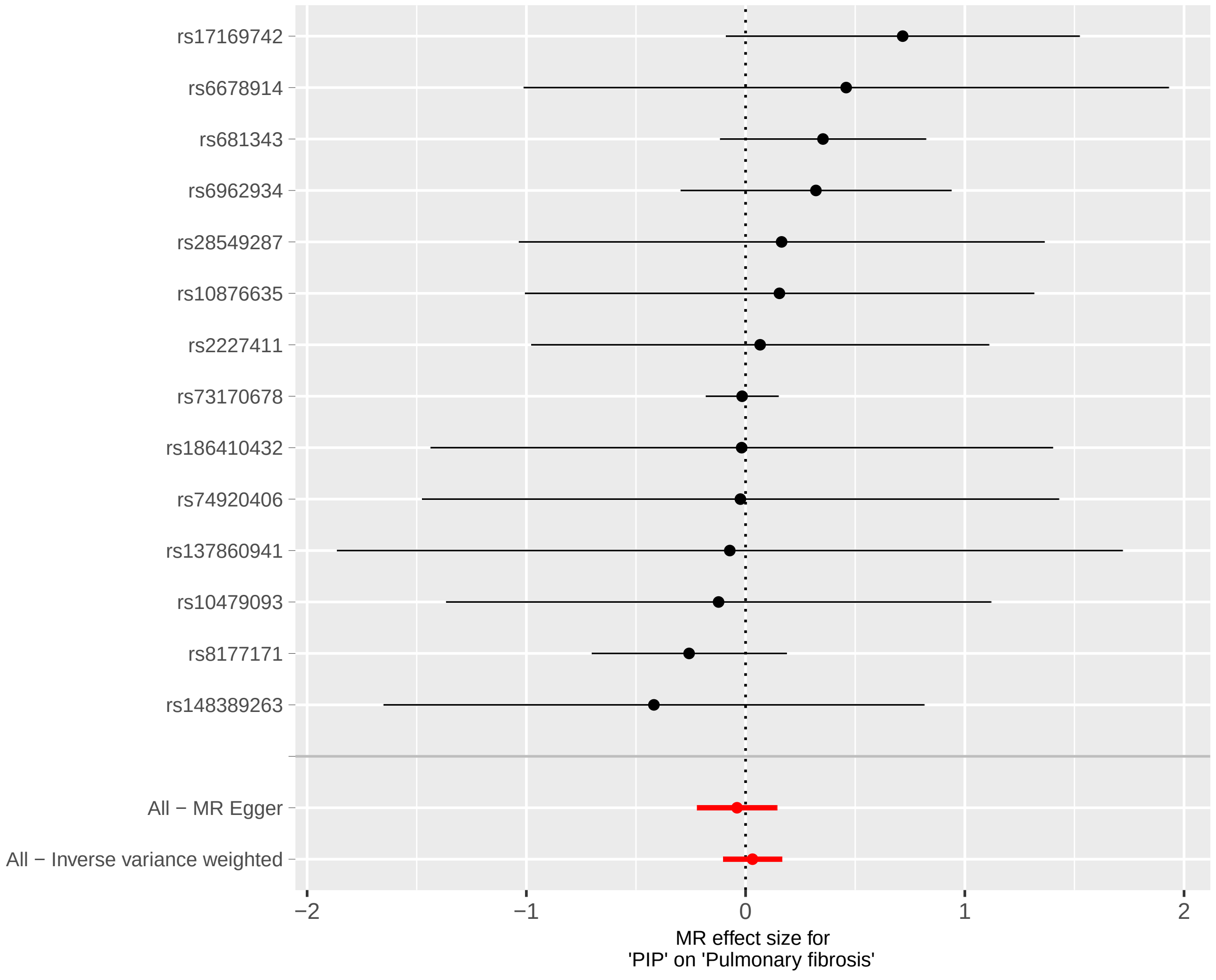

rs17169742
rs6678914
rs681343
rs6962934
rs28549287
rs10876635
rs2227411
rs73170678
rs186410432
rs74920406
rs137860941
rs10479093
rs8177171
rs148389263
All − MR Egger
All − Inverse variance weighted
−2
−1
0
1
2
MR effect size for
'PIP' on 'Pulmonary fibrosis'

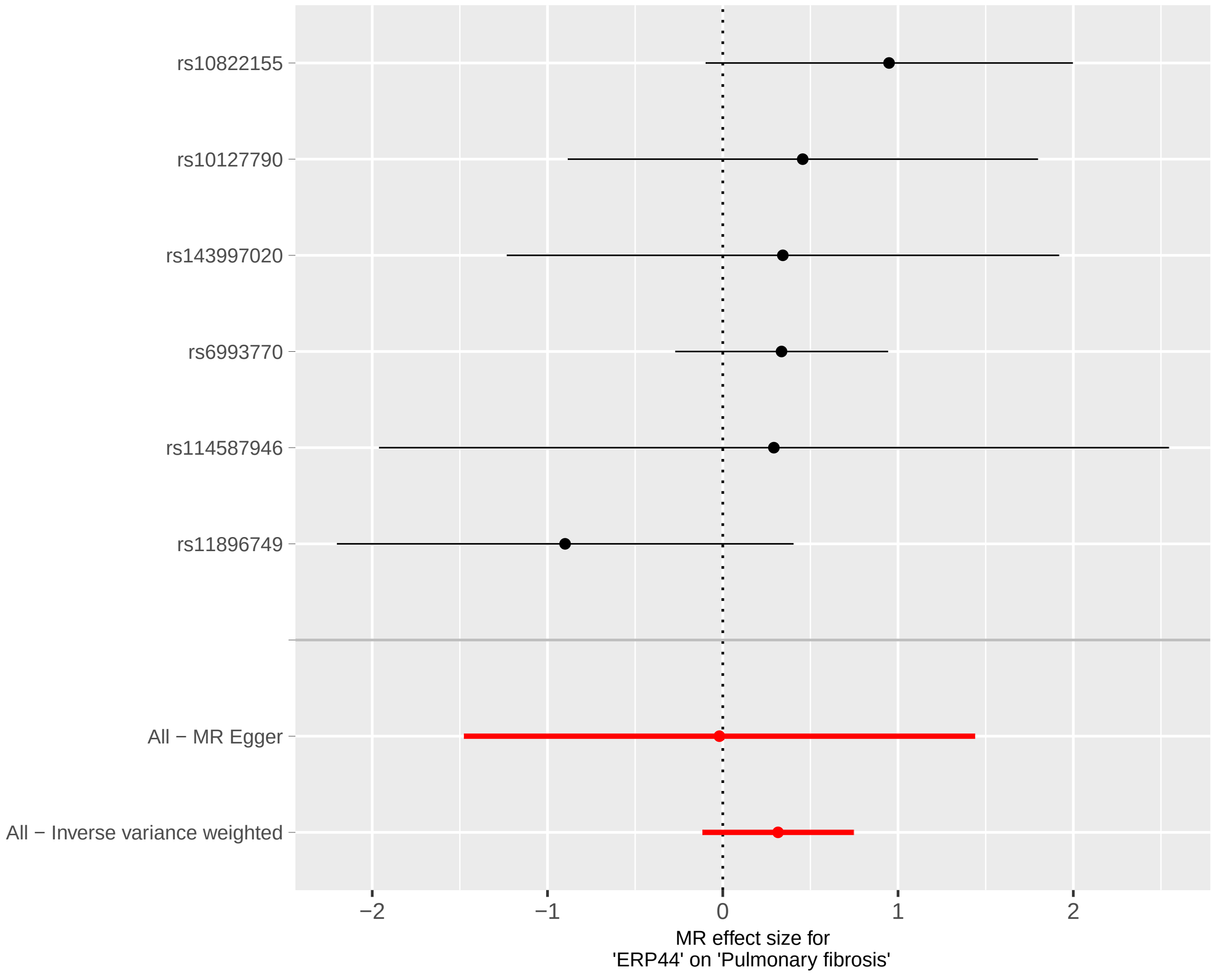
rs10822155
rs10127790
rs143997020
rs6993770
rs114587946
rs11896749
All − MR Egger
All − Inverse variance weighted
−2
−1
0
1
2
MR effect size for
'ERP44' on 'Pulmonary fibrosis'

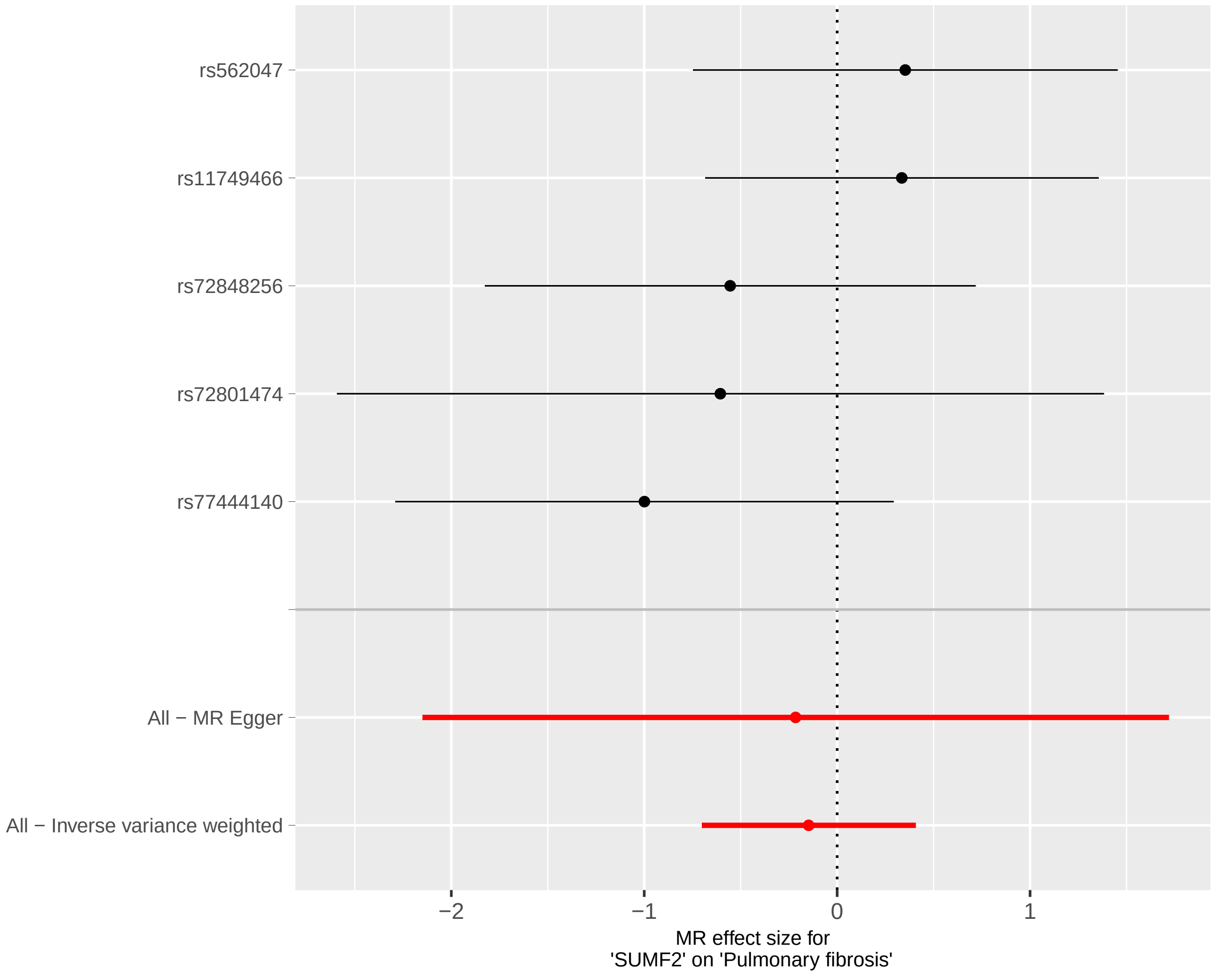
rs562047
rs11749466
rs72848256
rs72801474
rs77444140
All − MR Egger
All − Inverse variance weighted
−2
−1
0
1
MR effect size for
'SUMF2' on 'Pulmonary fibrosis'

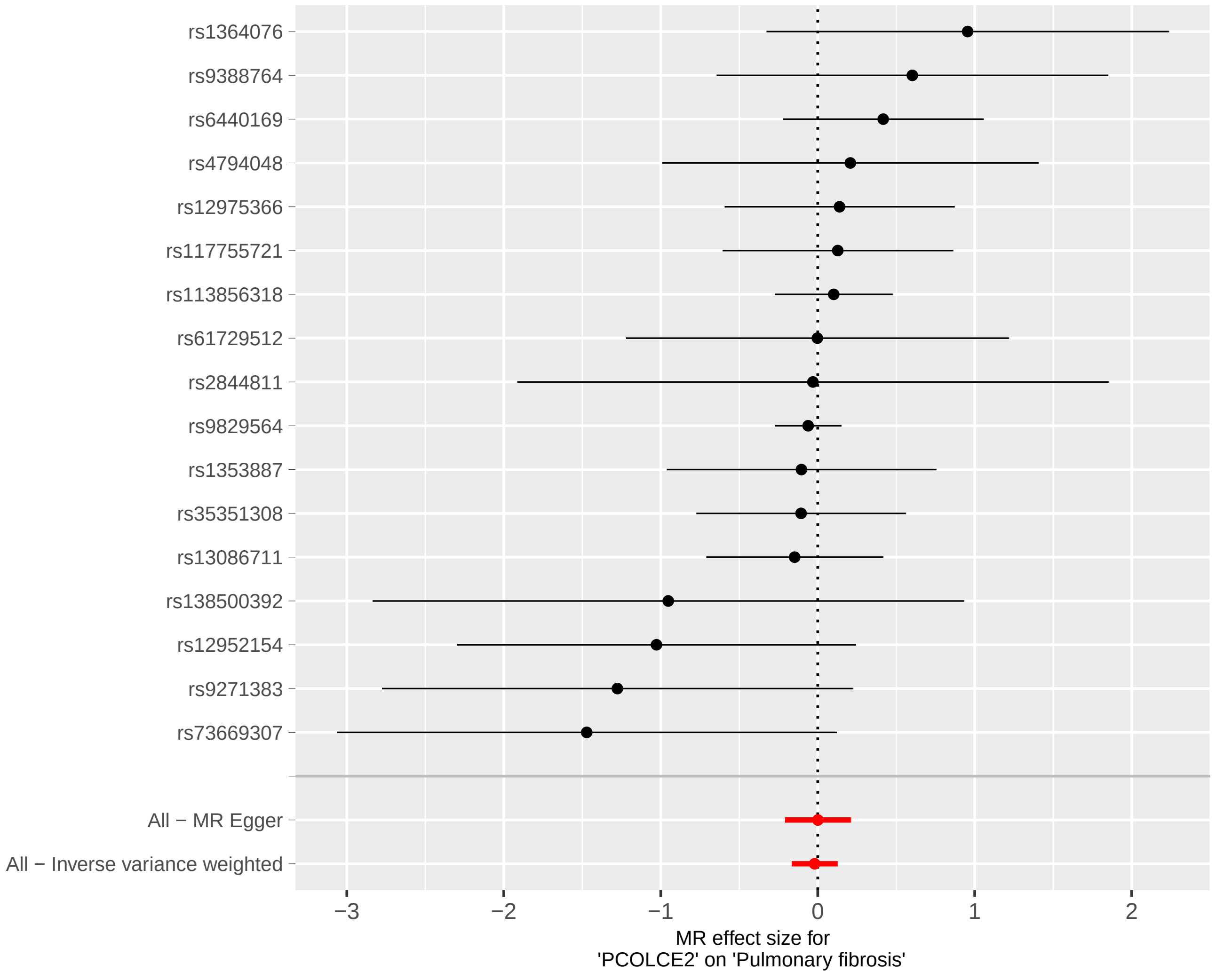
rs1364076
rs9388764
rs6440169
rs4794048
rs12975366
rs117755721
rs113856318
rs61729512
rs2844811
rs9829564
rs1353887
rs35351308
rs13086711
rs138500392
rs12952154
rs9271383
rs73669307
All − MR Egger
All − Inverse variance weighted
−3
−2
−1
0
1
2
MR effect size for
'PCOLCE2' on 'Pulmonary fibrosis'

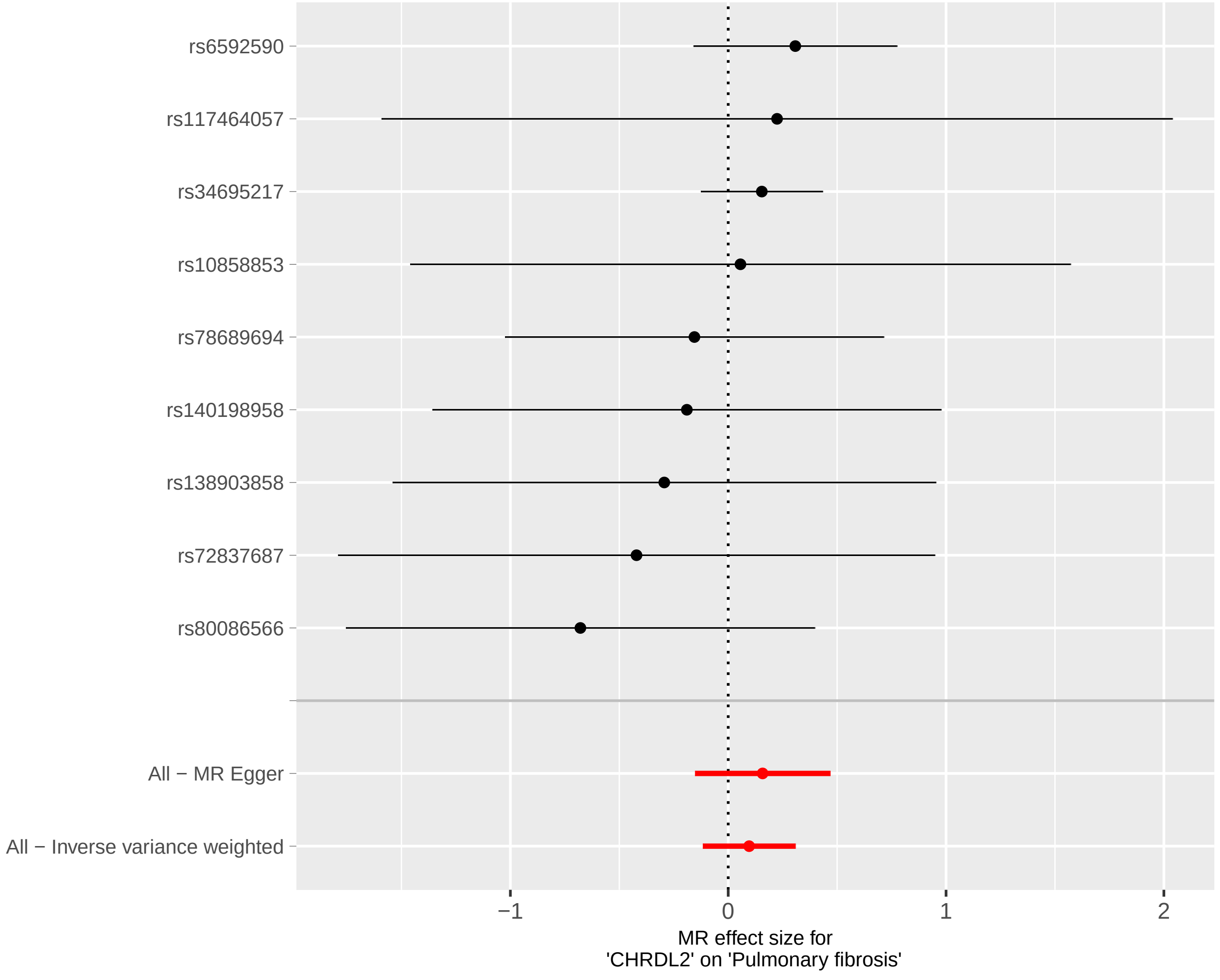

rs6592590
rs117464057
rs34695217
rs10858853
rs78689694
rs140198958
rs138903858
rs72837687
rs80086566
All − MR Egger
All − Inverse variance weighted
−1
0
1
2
MR effect size for
'CHRDL2' on 'Pulmonary fibrosis'

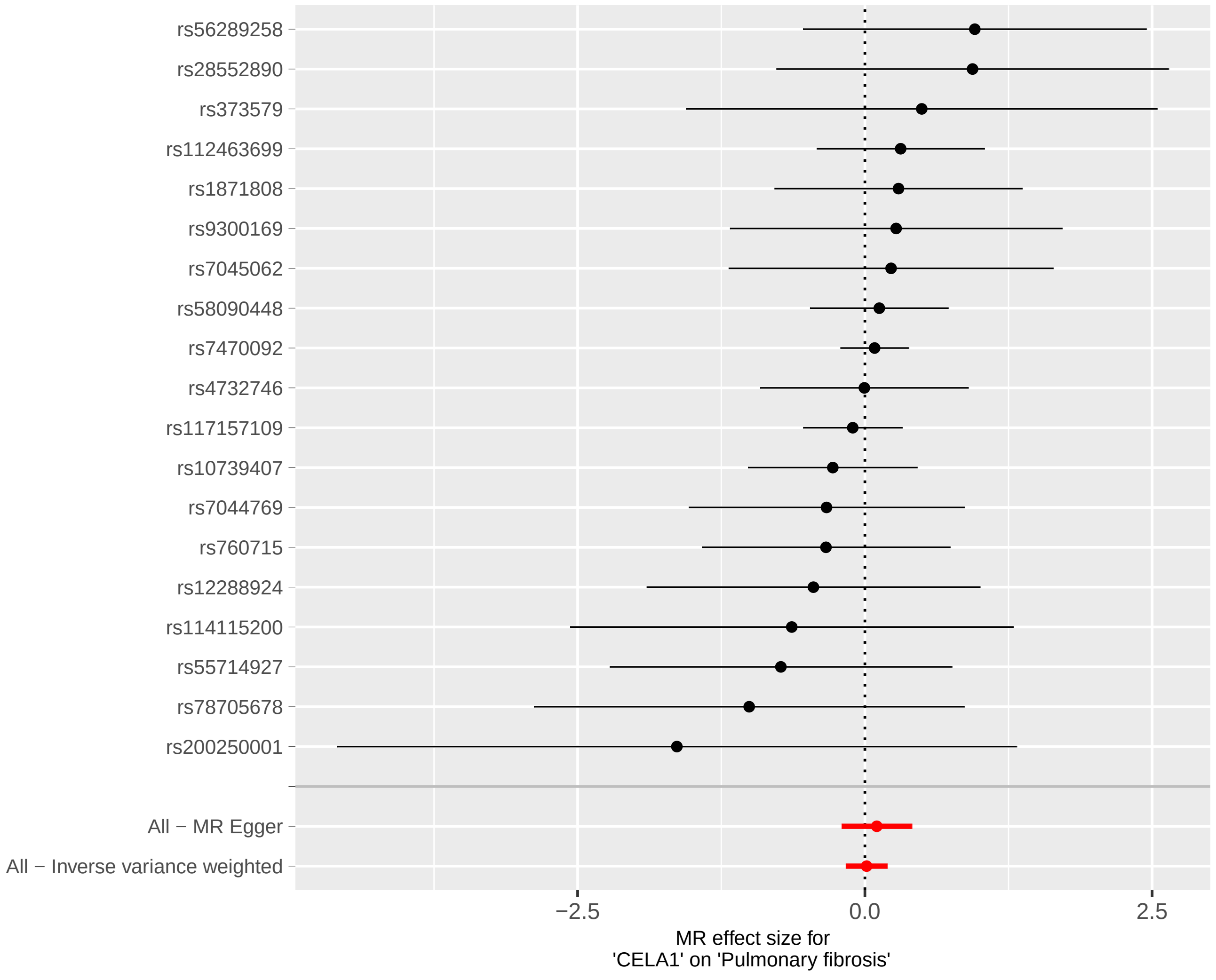
rs56289258
rs28552890
rs373579
rs112463699
rs1871808
rs9300169
rs7045062
rs58090448
rs7470092
rs4732746
rs117157109
rs10739407
rs7044769
rs760715
rs12288924
rs114115200
rs55714927
rs78705678
rs200250001
All − MR Egger
All − Inverse variance weighted
−2.5
0.0
2.5
MR effect size for
'CELA1' on 'Pulmonary fibrosis'

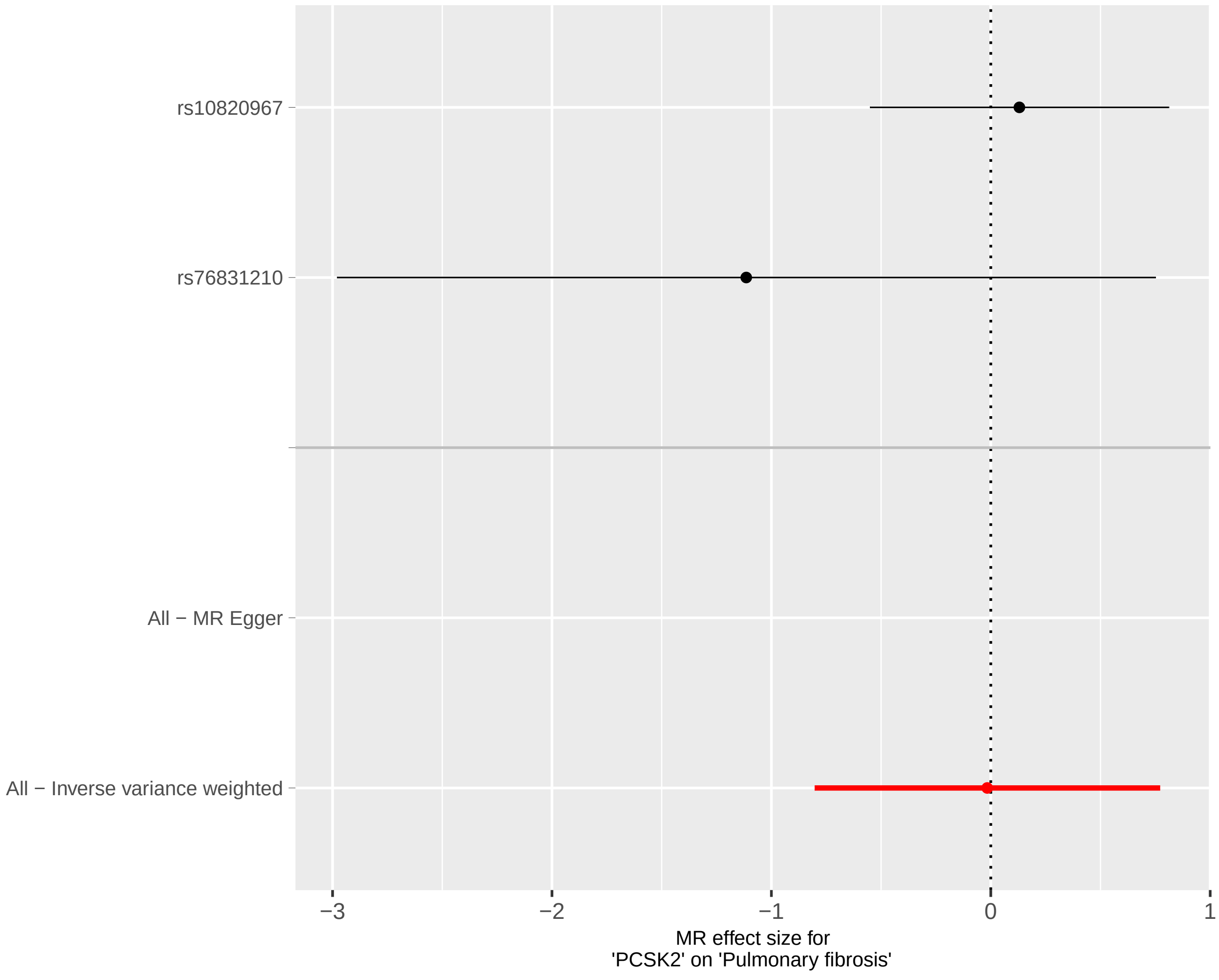

rs10820967
rs76831210
All − MR Egger
All − Inverse variance weighted
−3
−2
−1
0
1
MR effect size for
'PCSK2' on 'Pulmonary fibrosis'

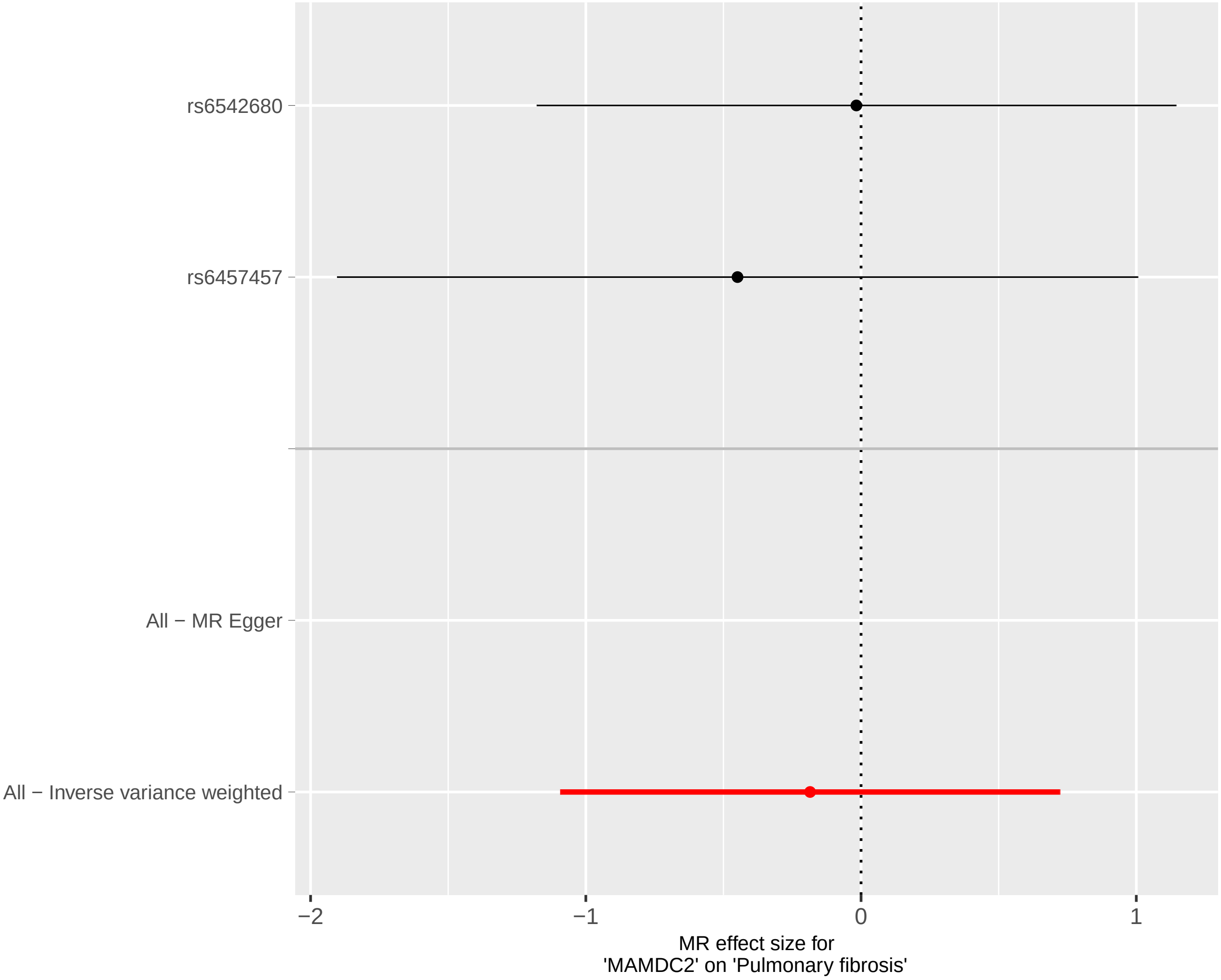

rs6542680
rs6457457
All − MR Egger
All − Inverse variance weighted
−2
−1
0
1
MR effect size for
'MAMDC2' on 'Pulmonary fibrosis'

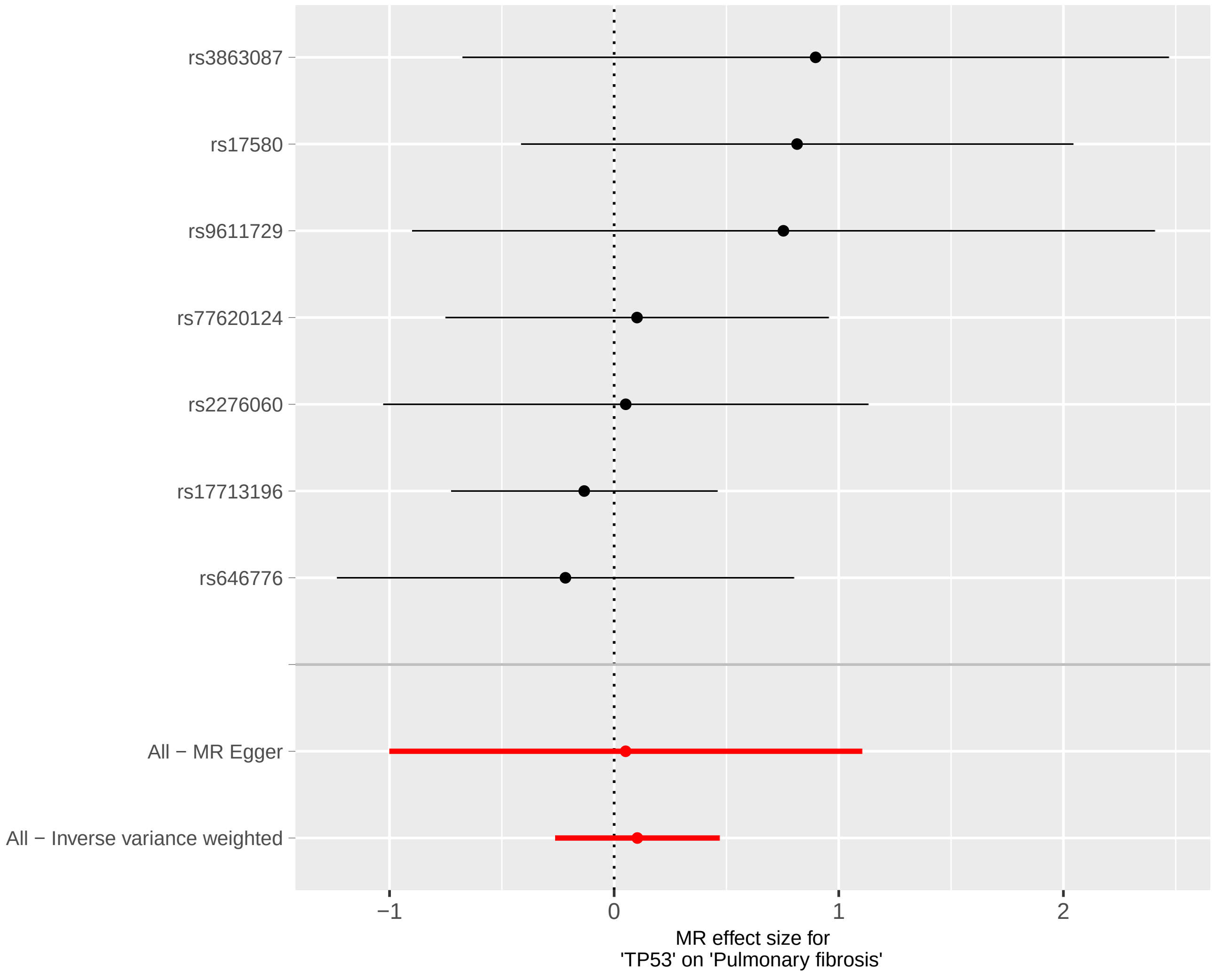

rs3863087
rs17580
rs9611729
rs77620124
rs2276060
rs17713196
rs646776
All − MR Egger
All − Inverse variance weighted
−1
0
1
2
MR effect size for
'TP53' on 'Pulmonary fibrosis'

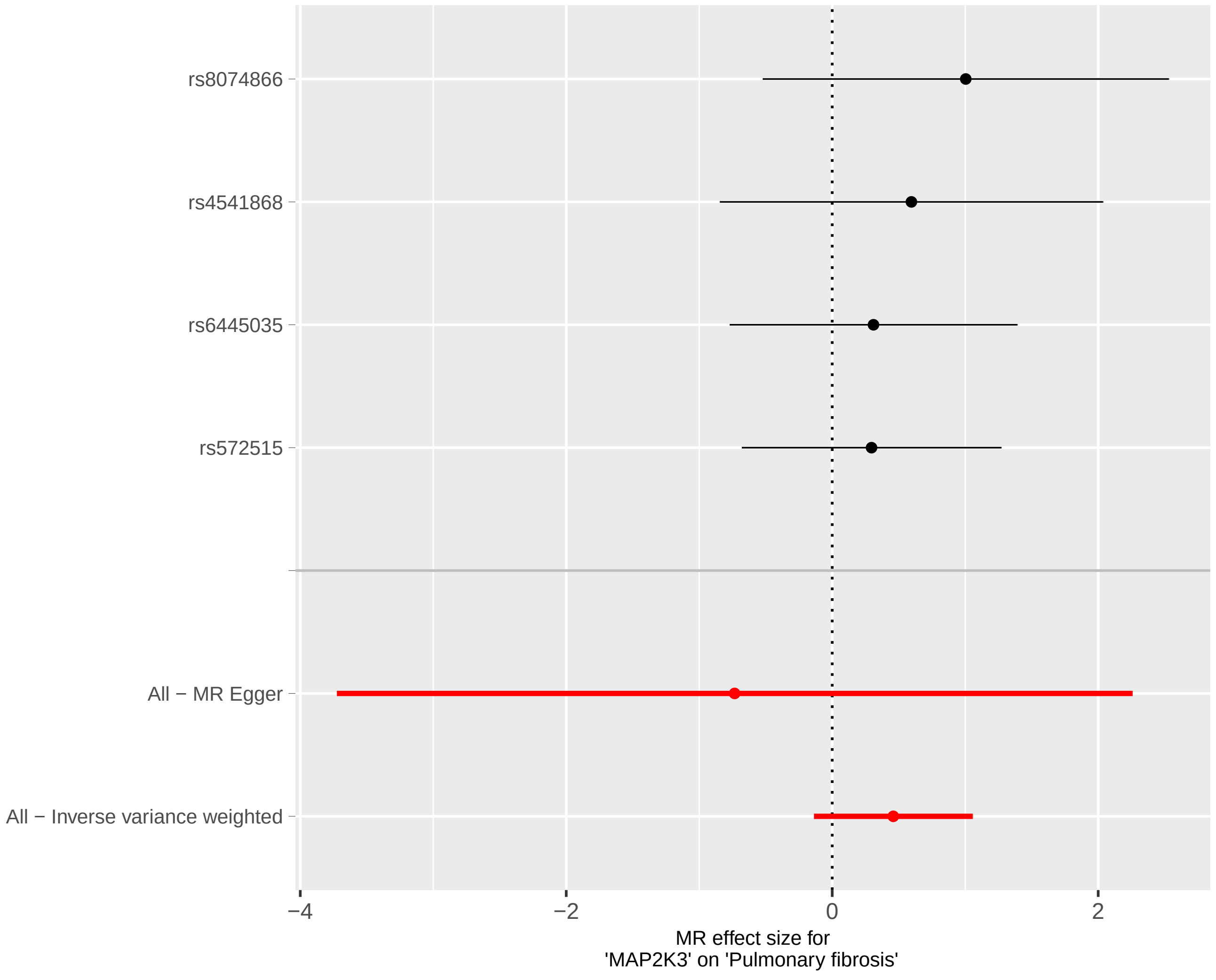
rs8074866
rs4541868
rs6445035
rs572515
All − MR Egger
All − Inverse variance weighted
−4
−2
0
2
MR effect size for
'MAP2K3' on 'Pulmonary fibrosis'

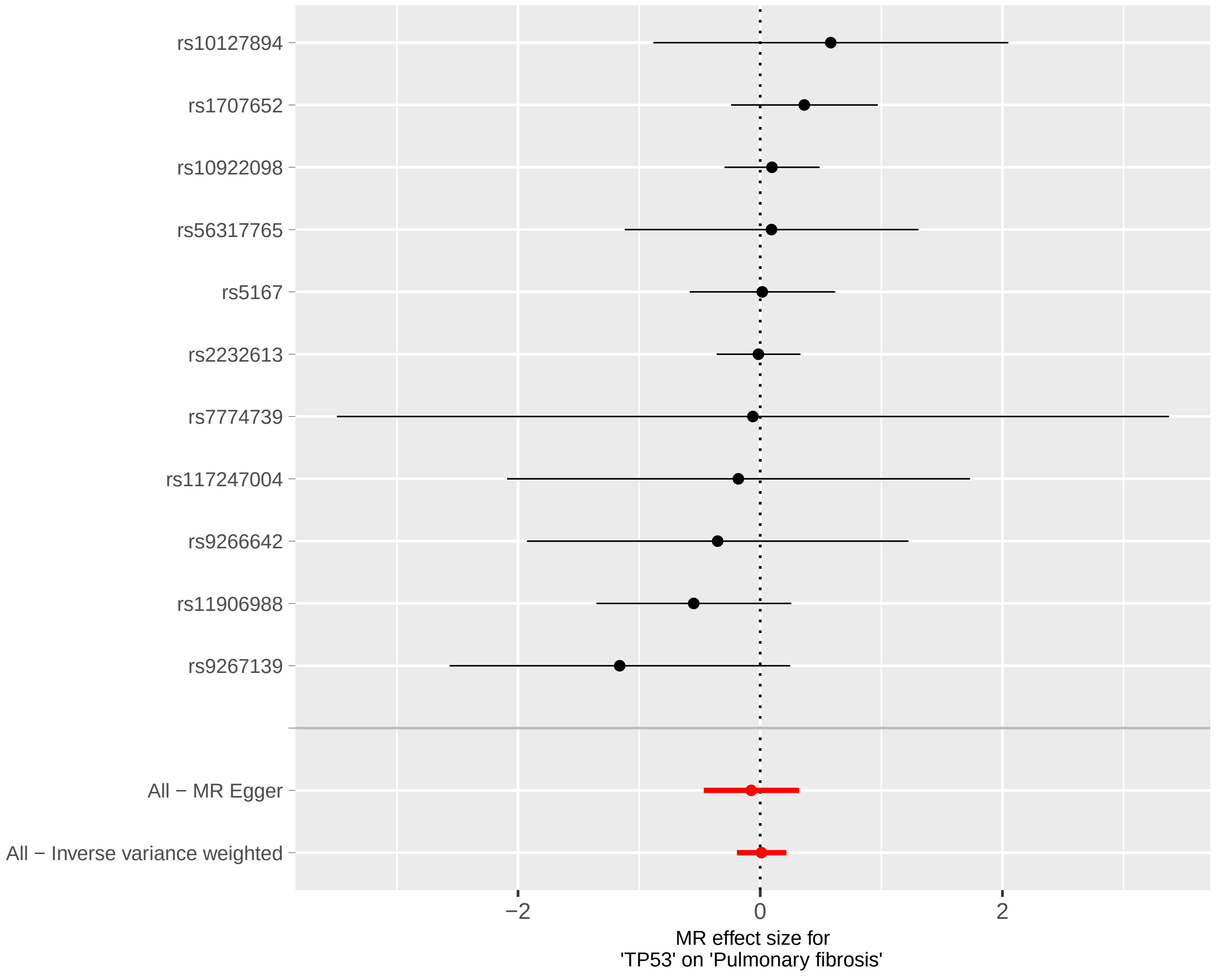

rs10127894
rs1707652
rs10922098
rs56317765
rs5167
rs2232613
rs7774739
rs117247004
rs9266642
rs11906988
rs9267139
All − MR Egger
All − Inverse variance weighted
−2
0
2
MR effect size for
'TP53' on 'Pulmonary fibrosis'

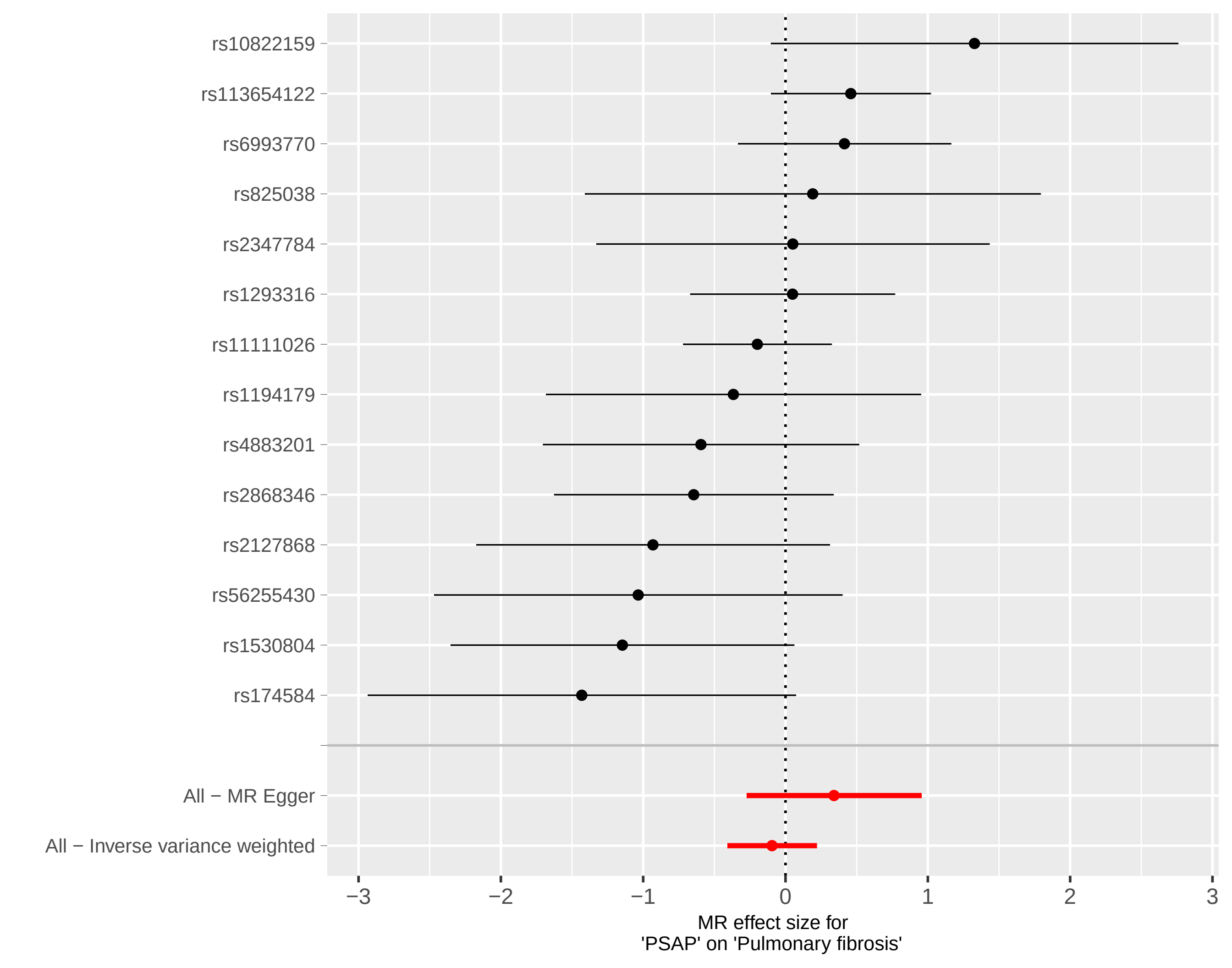

rs10822159
rs113654122
rs6993770
rs825038
rs2347784
rs1293316
rs11111026
rs1194179
rs4883201
rs2868346
rs2127868
rs56255430
rs1530804
rs174584
All − MR Egger
All − Inverse variance weighted
−3
−2
−1
0
1
2
3
MR effect size for
'PSAP' on 'Pulmonary fibrosis'

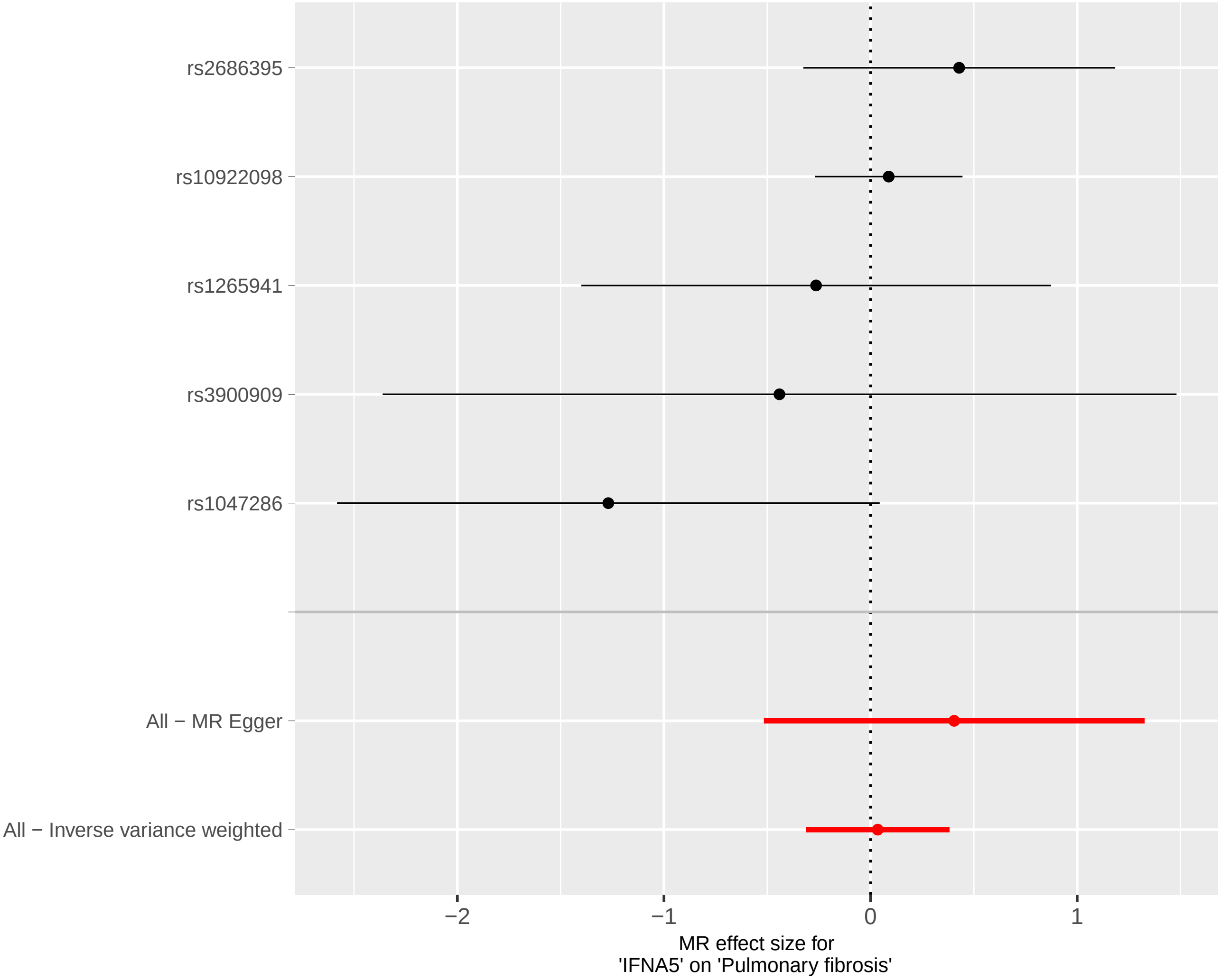
rs2686395
rs10922098
rs1265941
rs3900909
rs1047286
All − MR Egger
All − Inverse variance weighted
−2
−1
0
1
MR effect size for
'IFNA5' on 'Pulmonary fibrosis'

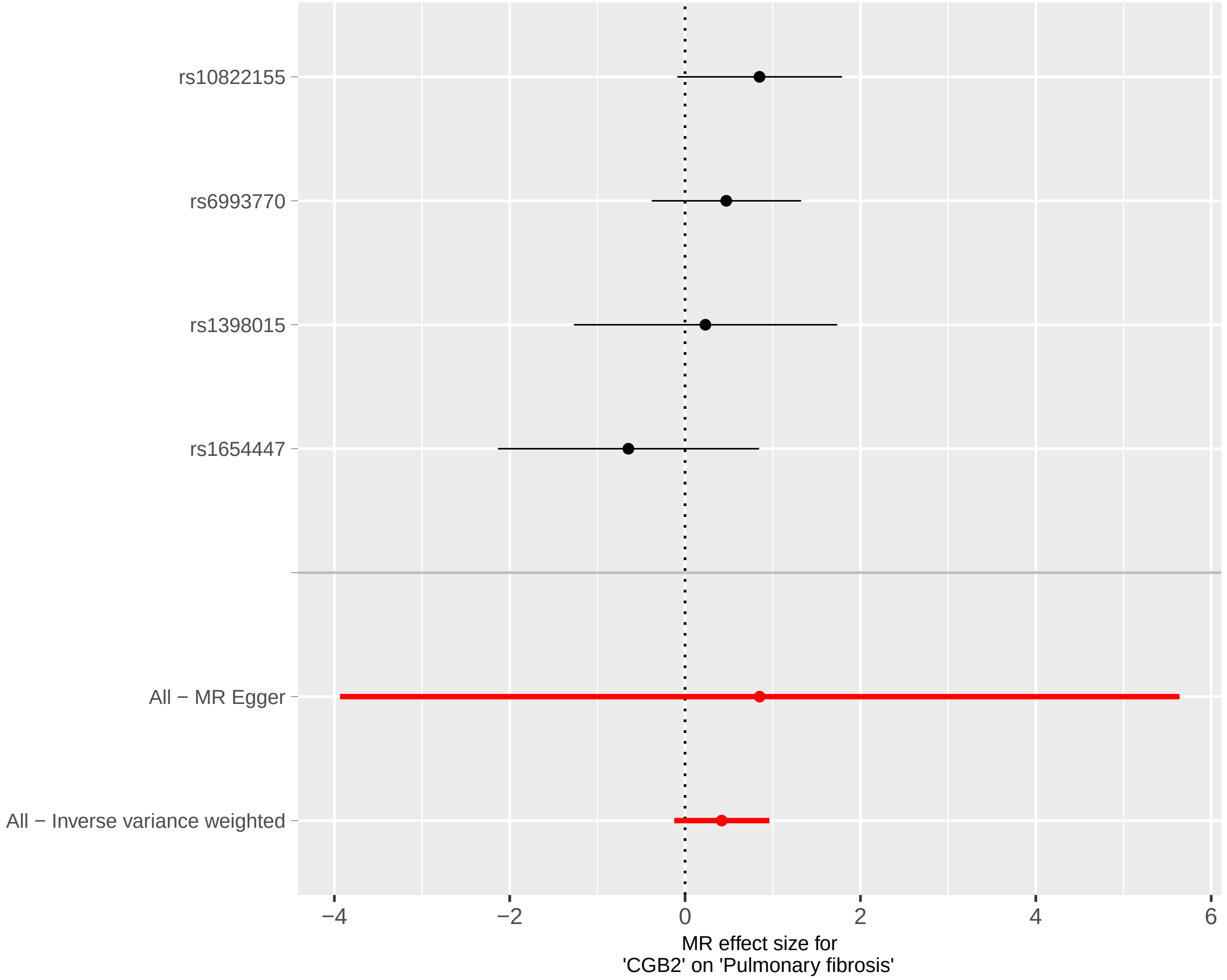

rs10822155
rs6993770
rs1398015
rs1654447
All − MR Egger
All − Inverse variance weighted
−4
−2
0
2
4
6
MR effect size for
'CGB2' on 'Pulmonary fibrosis'

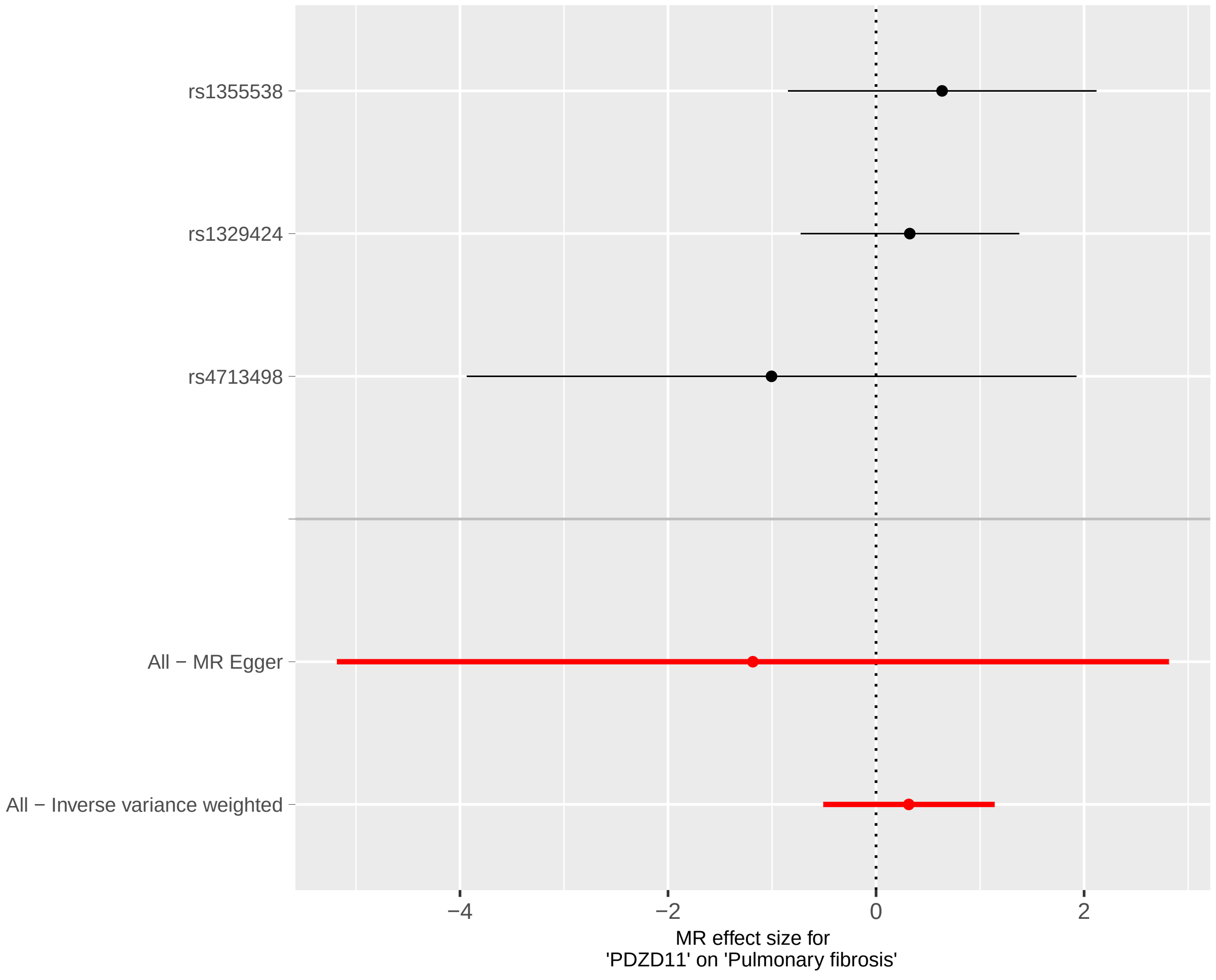
rs1355538
rs1329424
rs4713498
All − MR Egger
All − Inverse variance weighted
−4
−2
0
2
MR effect size for
'PDZD11' on 'Pulmonary fibrosis'

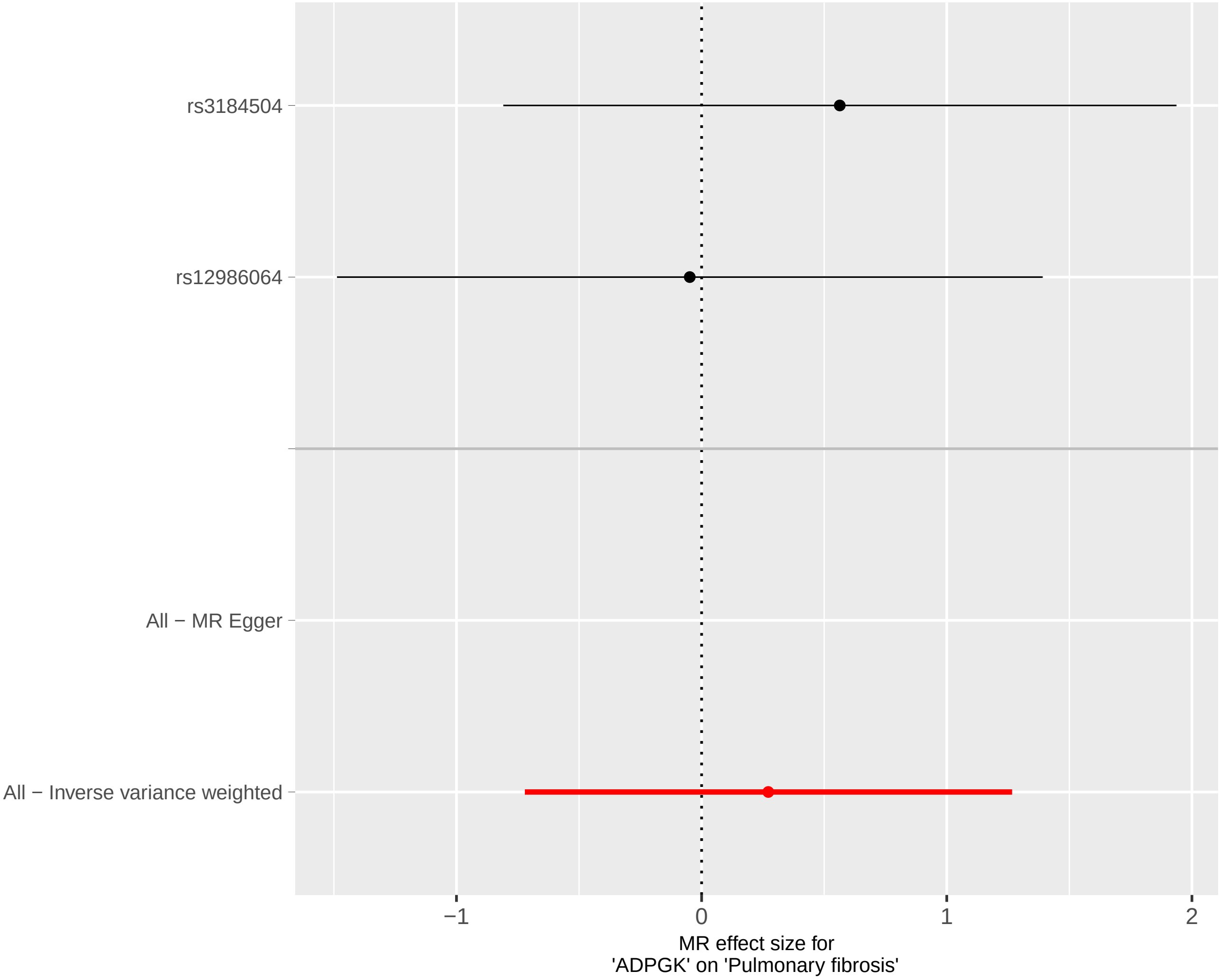

rs3184504
rs12986064
All − MR Egger
All − Inverse variance weighted
−1
0
1
2
MR effect size for
'ADPGK' on 'Pulmonary fibrosis'

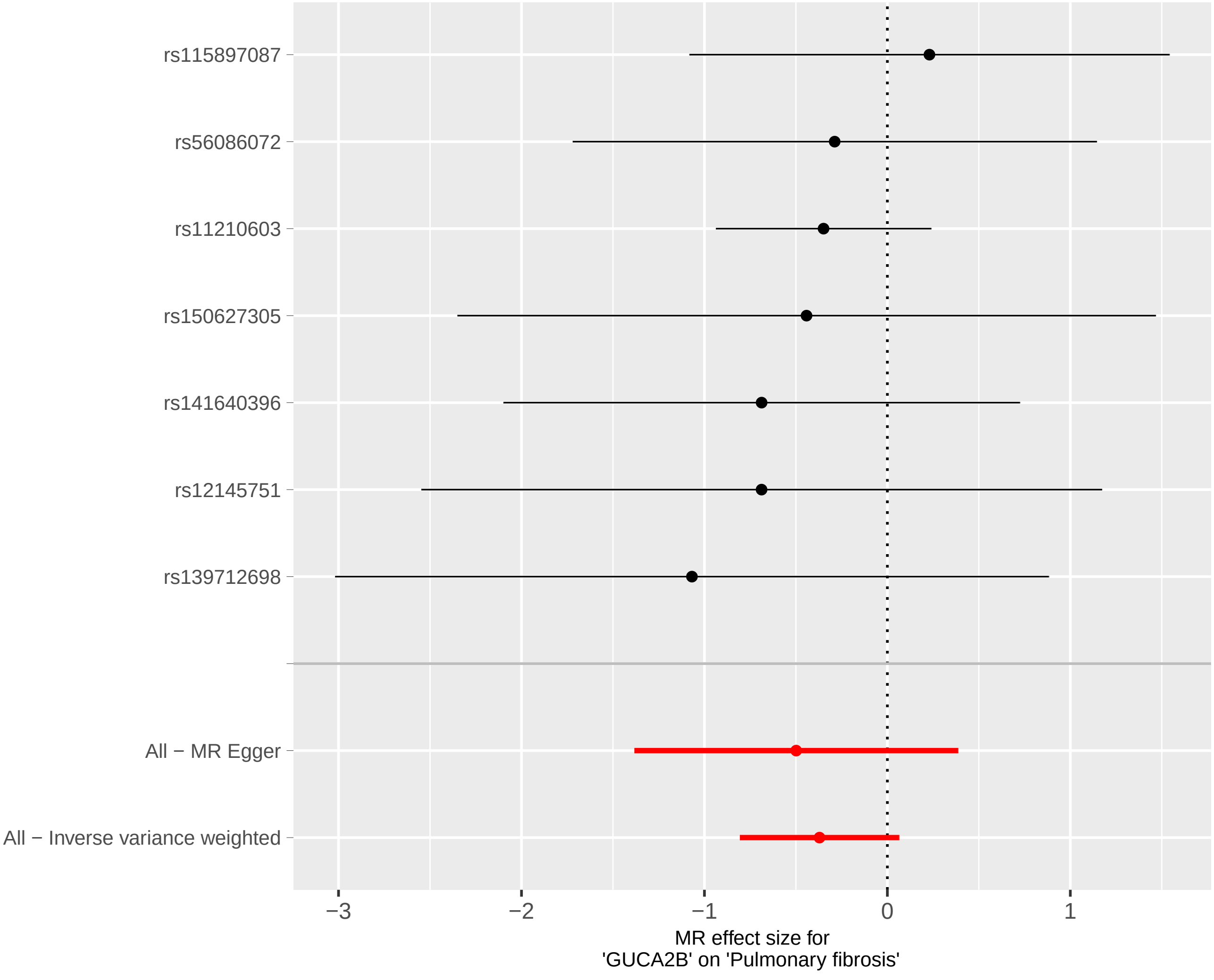
rs115897087
rs56086072
rs11210603
rs150627305
rs141640396
rs12145751
rs139712698
All − MR Egger
All − Inverse variance weighted
−3
−2
−1
0
1
MR effect size for
'GUCA2B' on 'Pulmonary fibrosis'

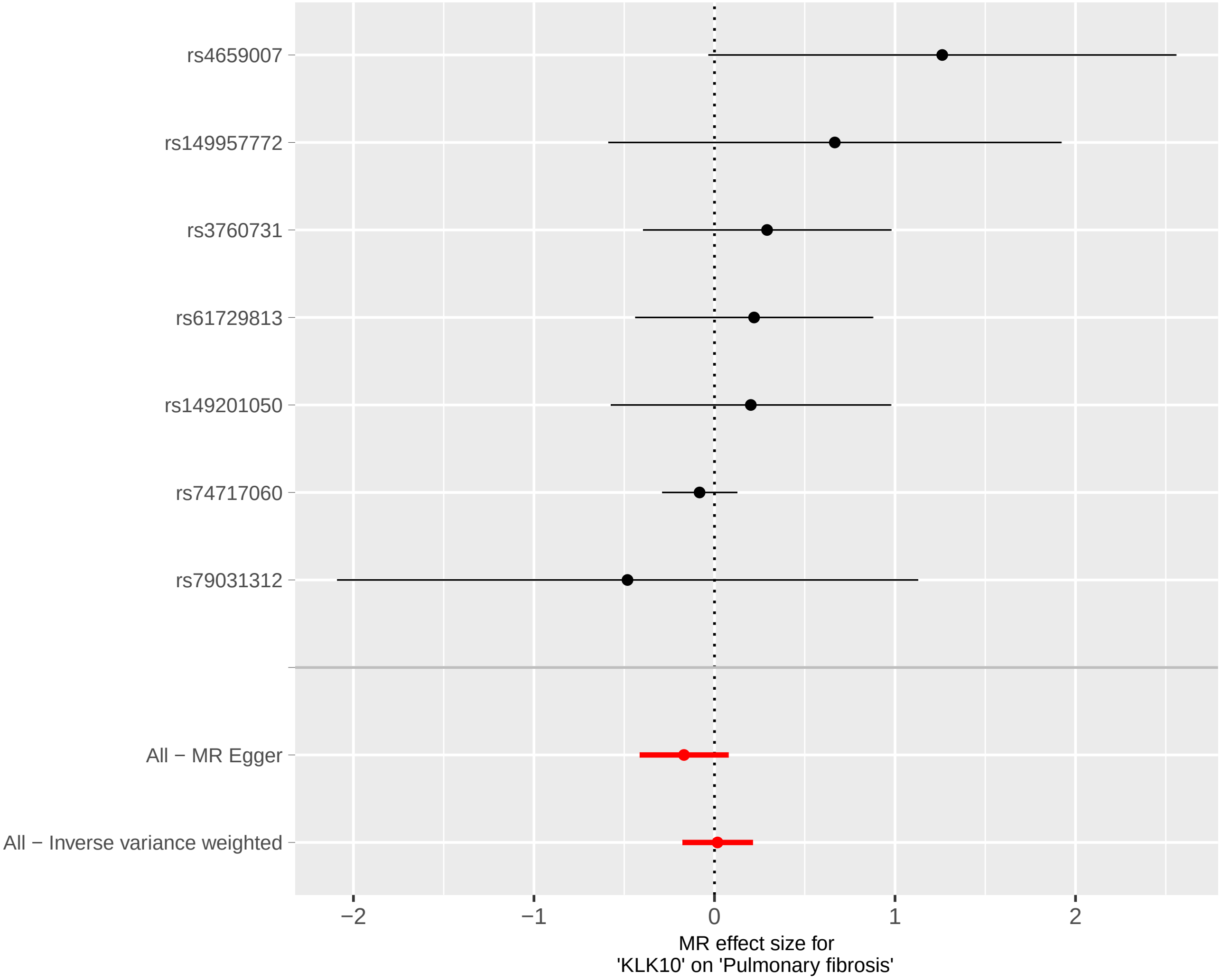

rs4659007
rs149957772
rs3760731
rs61729813
rs149201050
rs74717060
rs79031312
All − MR Egger
All − Inverse variance weighted
−2
−1
0
1
2
MR effect size for
'KLK10' on 'Pulmonary fibrosis'

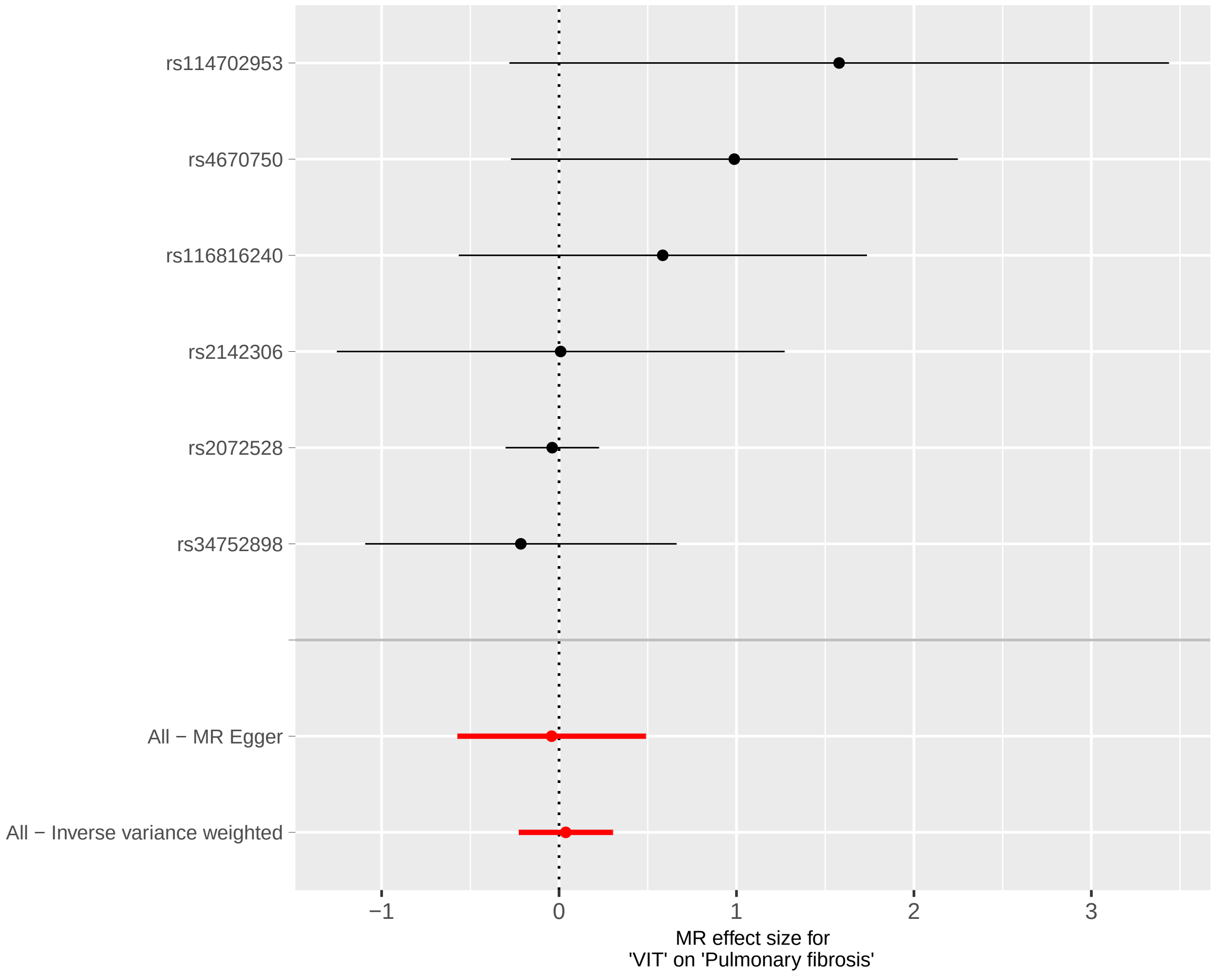

rs114702953
rs4670750
rs116816240
rs2142306
rs2072528
rs34752898
All − MR Egger
All − Inverse variance weighted
−1
0
1
2
3
MR effect size for
'VIT' on 'Pulmonary fibrosis'

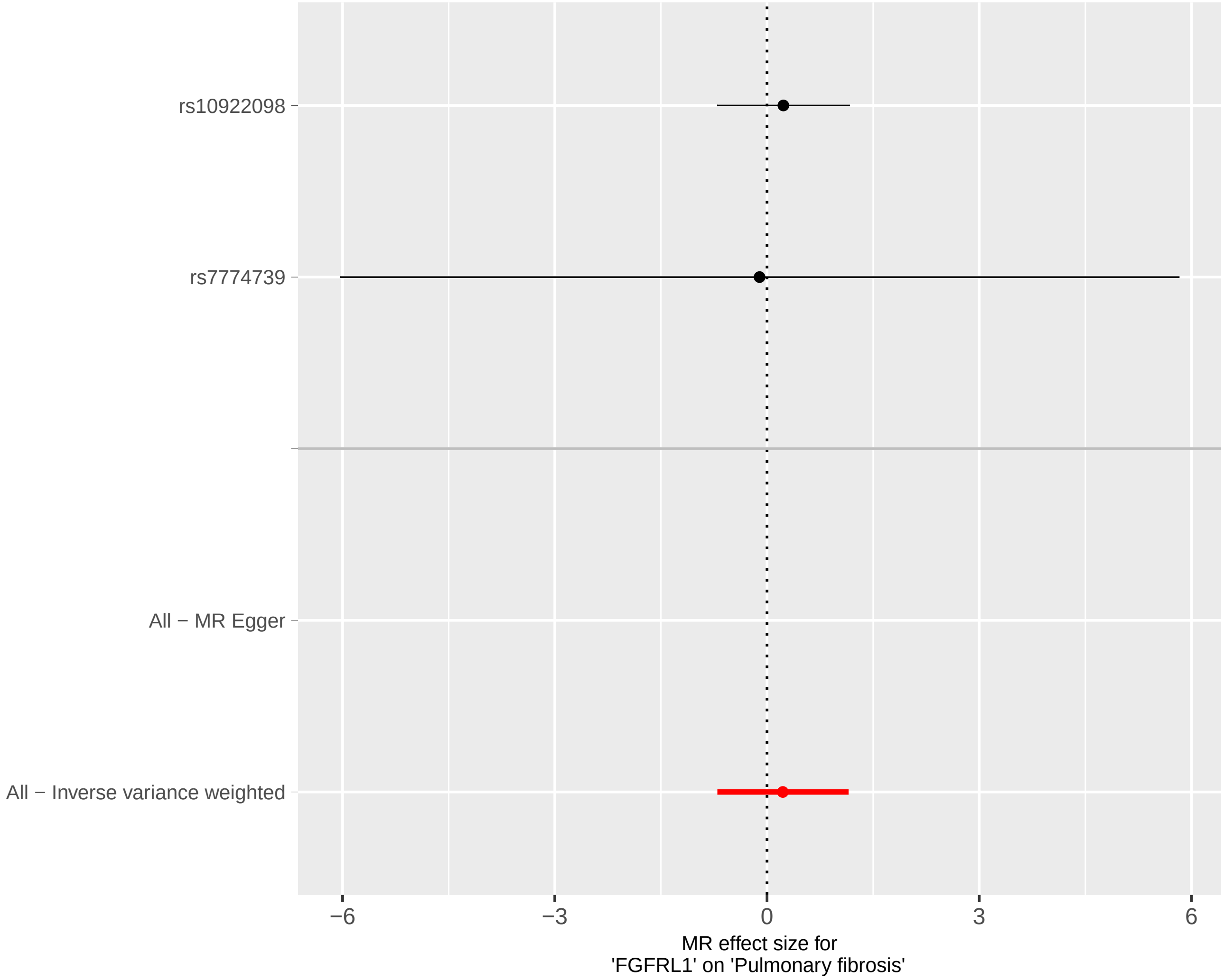

rs10922098
rs7774739
All − MR Egger
All − Inverse variance weighted
−6
−3
0
3
6
MR effect size for
'FGFRL1' on 'Pulmonary fibrosis'

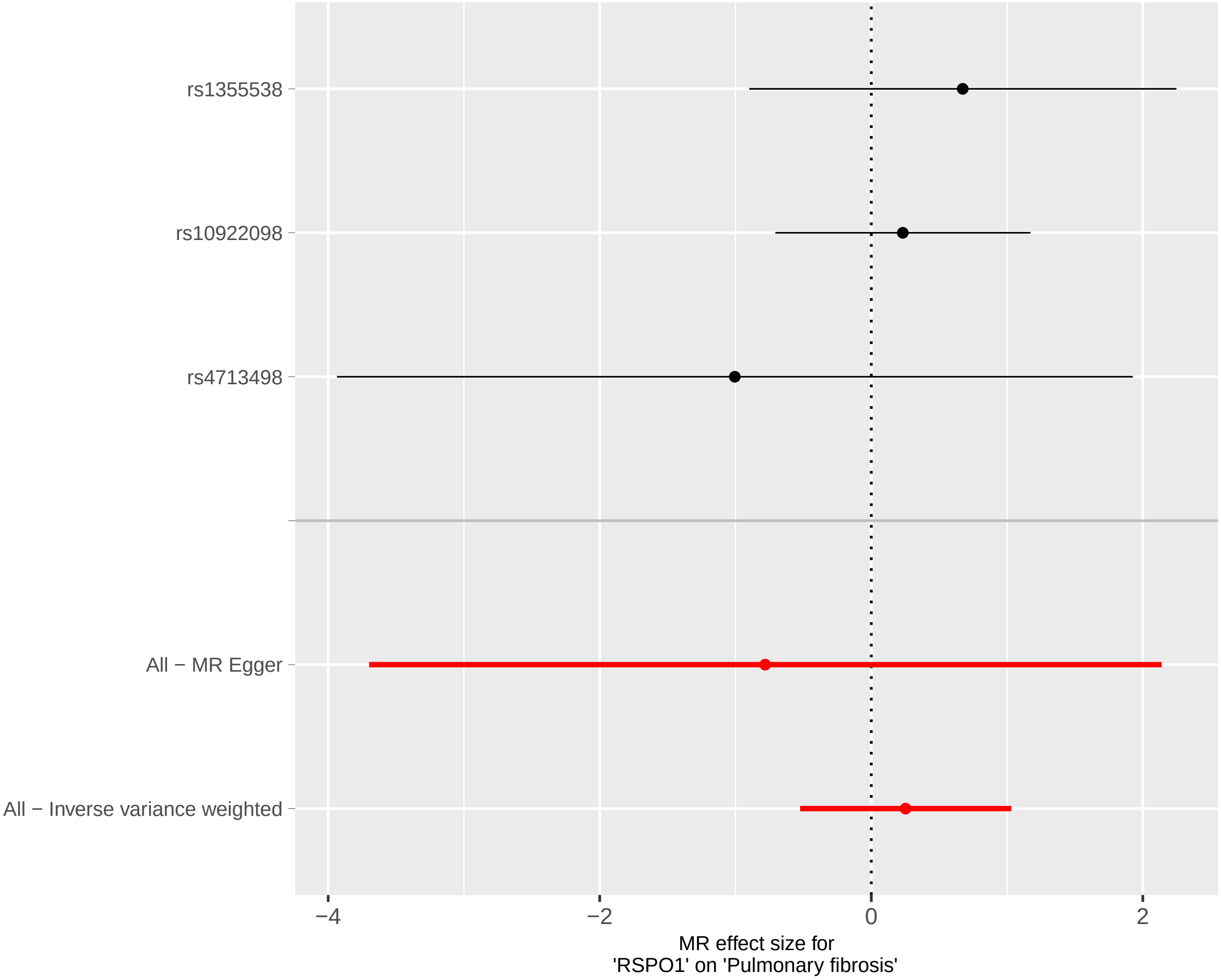
rs1355538
rs10922098
rs4713498
All − MR Egger
All − Inverse variance weighted
−4
−2
0
2
MR effect size for
'RSPO1' on 'Pulmonary fibrosis'

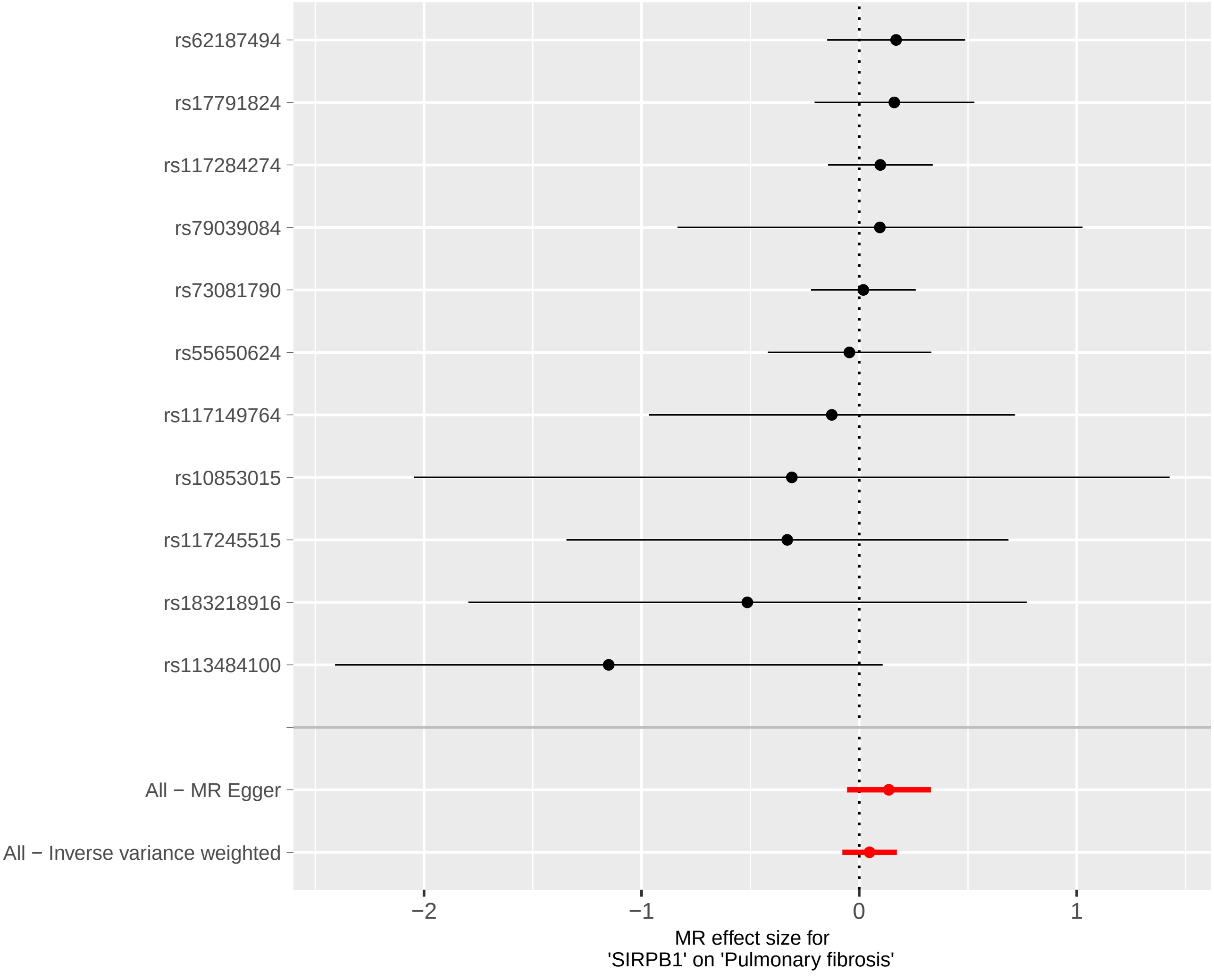

rs62187494
rs17791824
rs117284274
rs79039084
rs73081790
rs55650624
rs117149764
rs10853015
rs117245515
rs183218916
rs113484100
All − MR Egger
All − Inverse variance weighted
−2
−1
0
1
MR effect size for
'SIRPB1' on 'Pulmonary fibrosis'

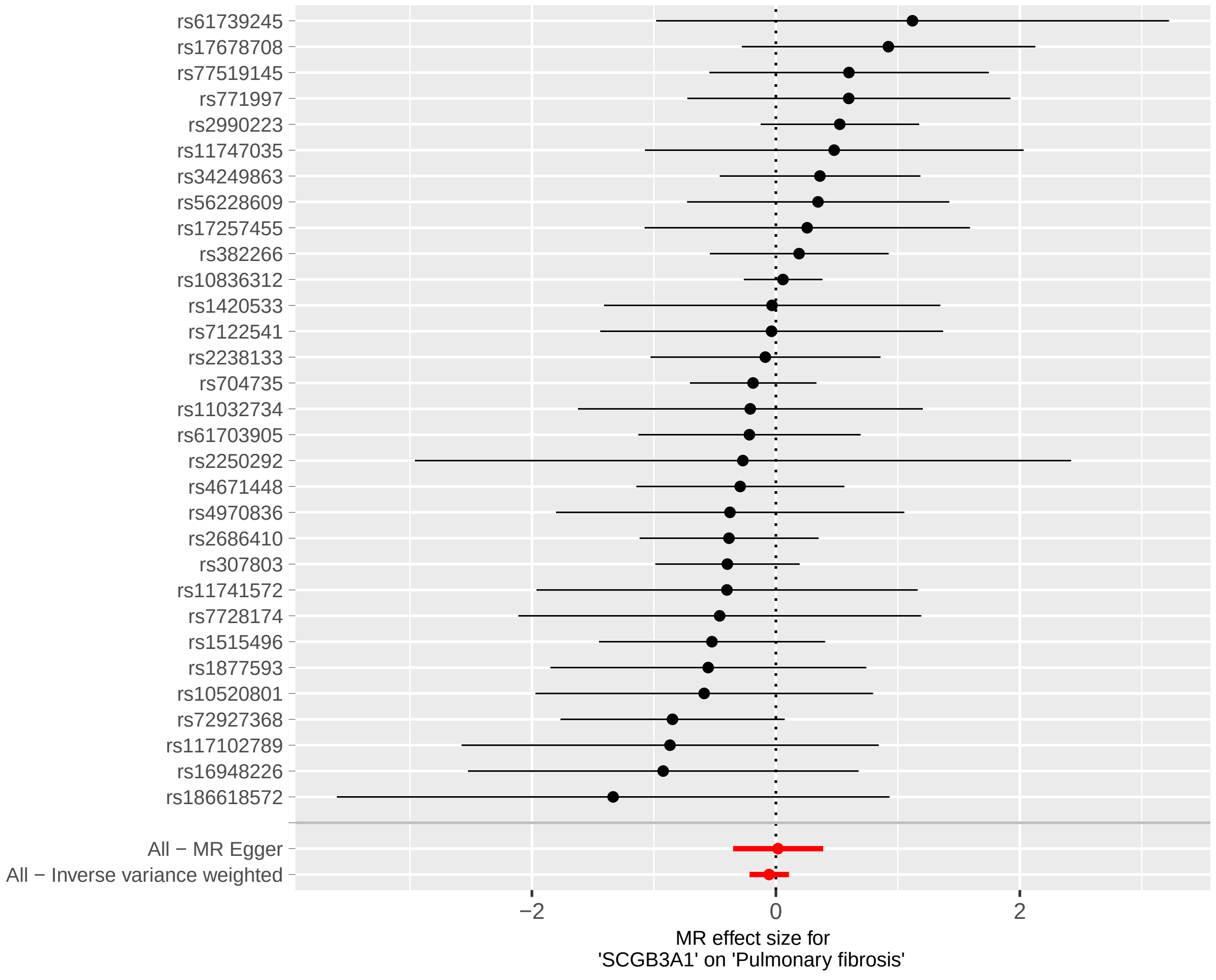

rs61739245
rs17678708
rs77519145
rs771997
rs2990223
rs11747035
rs34249863
rs56228609
rs17257455
rs382266
rs10836312
rs1420533
rs7122541
rs2238133
rs704735
rs11032734
rs61703905
rs2250292
rs4671448
rs4970836
rs2686410
rs307803
rs11741572
rs7728174
rs1515496
rs1877593
rs10520801
rs72927368
rs117102789
rs16948226
rs186618572
All − MR Egger
All − Inverse variance weighted
−2
0
2
MR effect size for
'SCGB3A1' on 'Pulmonary fibrosis'

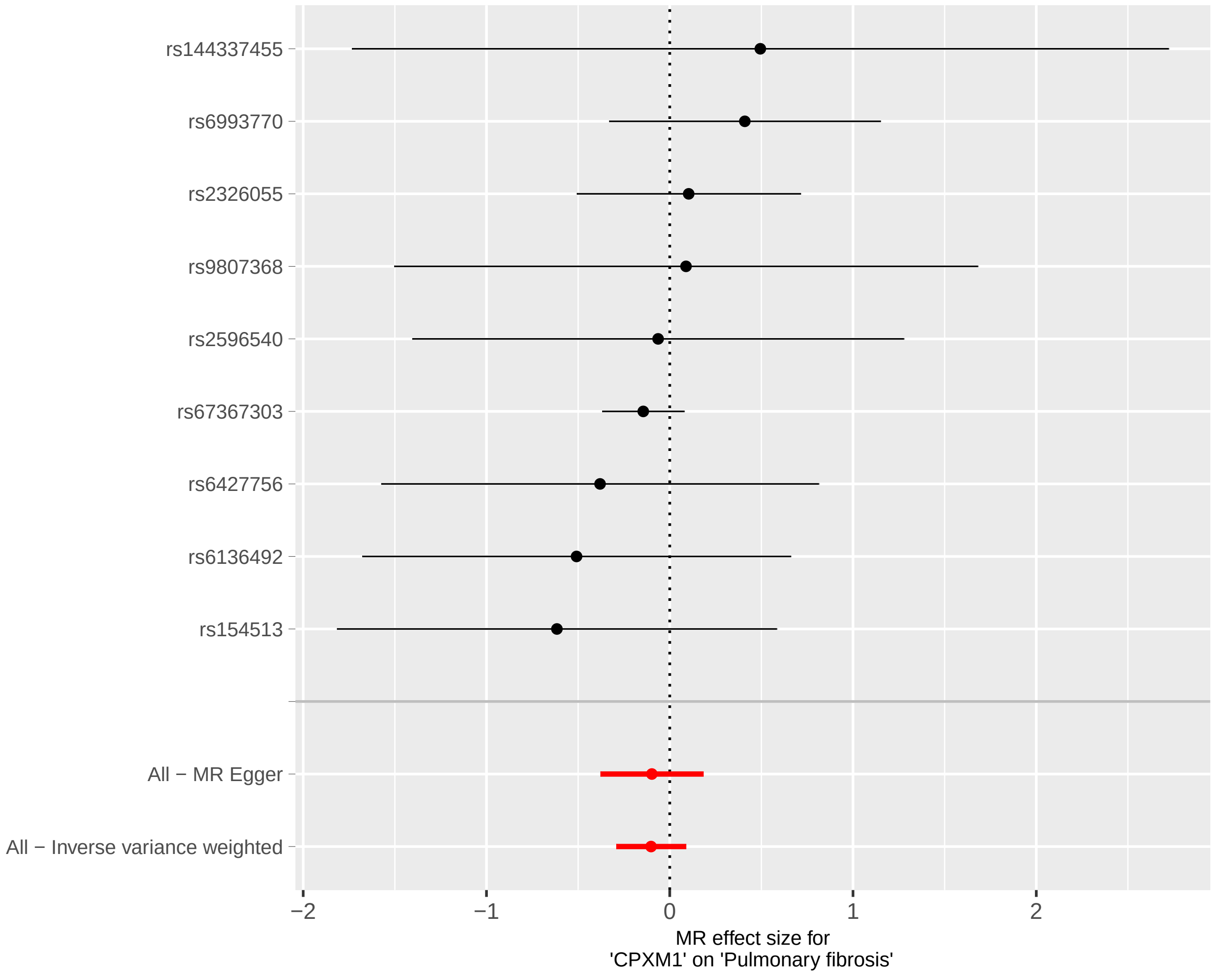
rs144337455
rs6993770
rs2326055
rs9807368
rs2596540
rs67367303
rs6427756
rs6136492
rs154513
All − MR Egger
All − Inverse variance weighted
−2
−1
0
1
2
MR effect size for
'CPXM1' on 'Pulmonary fibrosis'

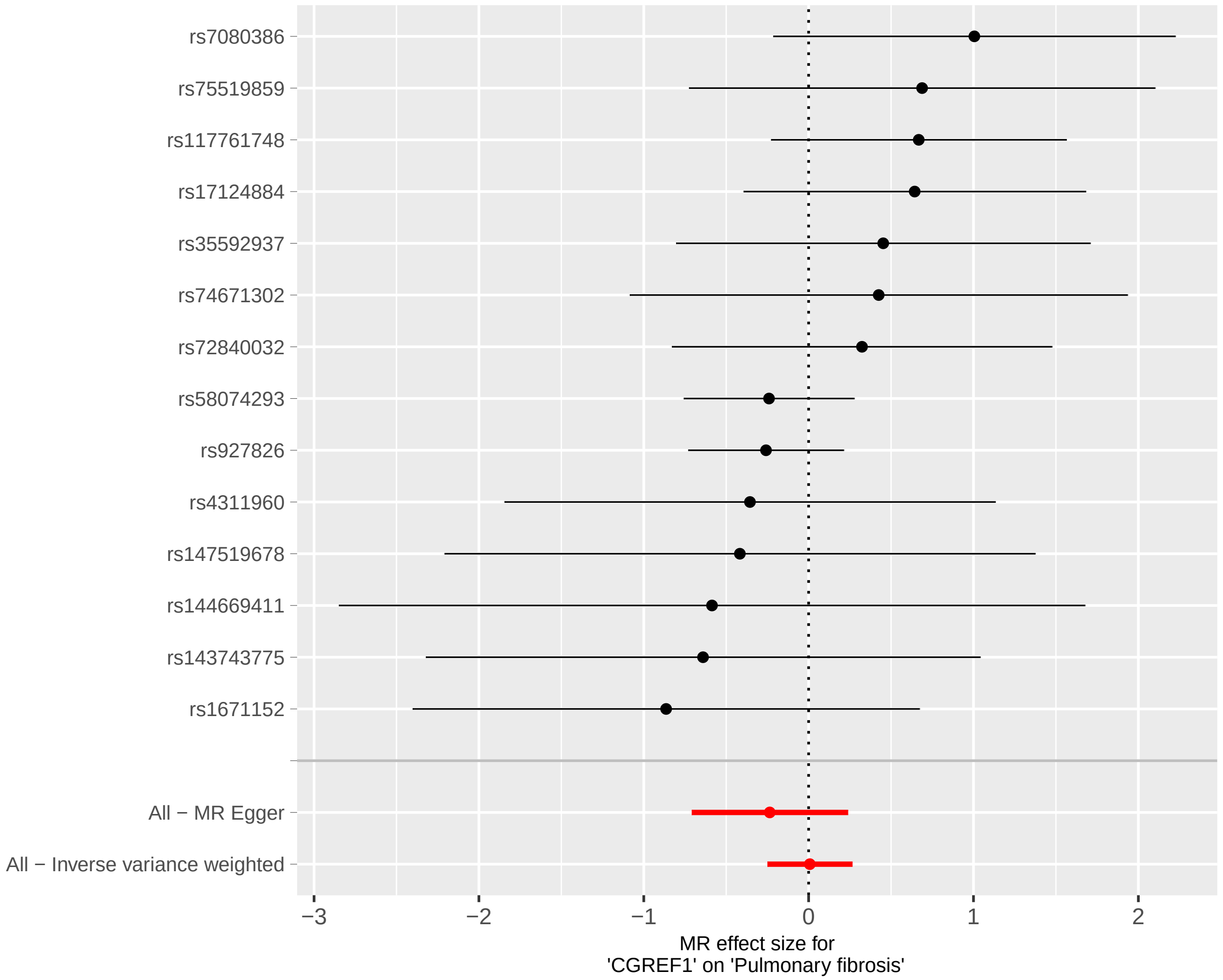
rs7080386
rs75519859
rs117761748
rs17124884
rs35592937
rs74671302
rs72840032
rs58074293
rs927826
rs4311960
rs147519678
rs144669411
rs143743775
rs1671152
All − MR Egger
All − Inverse variance weighted
−3
−2
−1
0
1
2
MR effect size for
'CGREF1' on 'Pulmonary fibrosis'

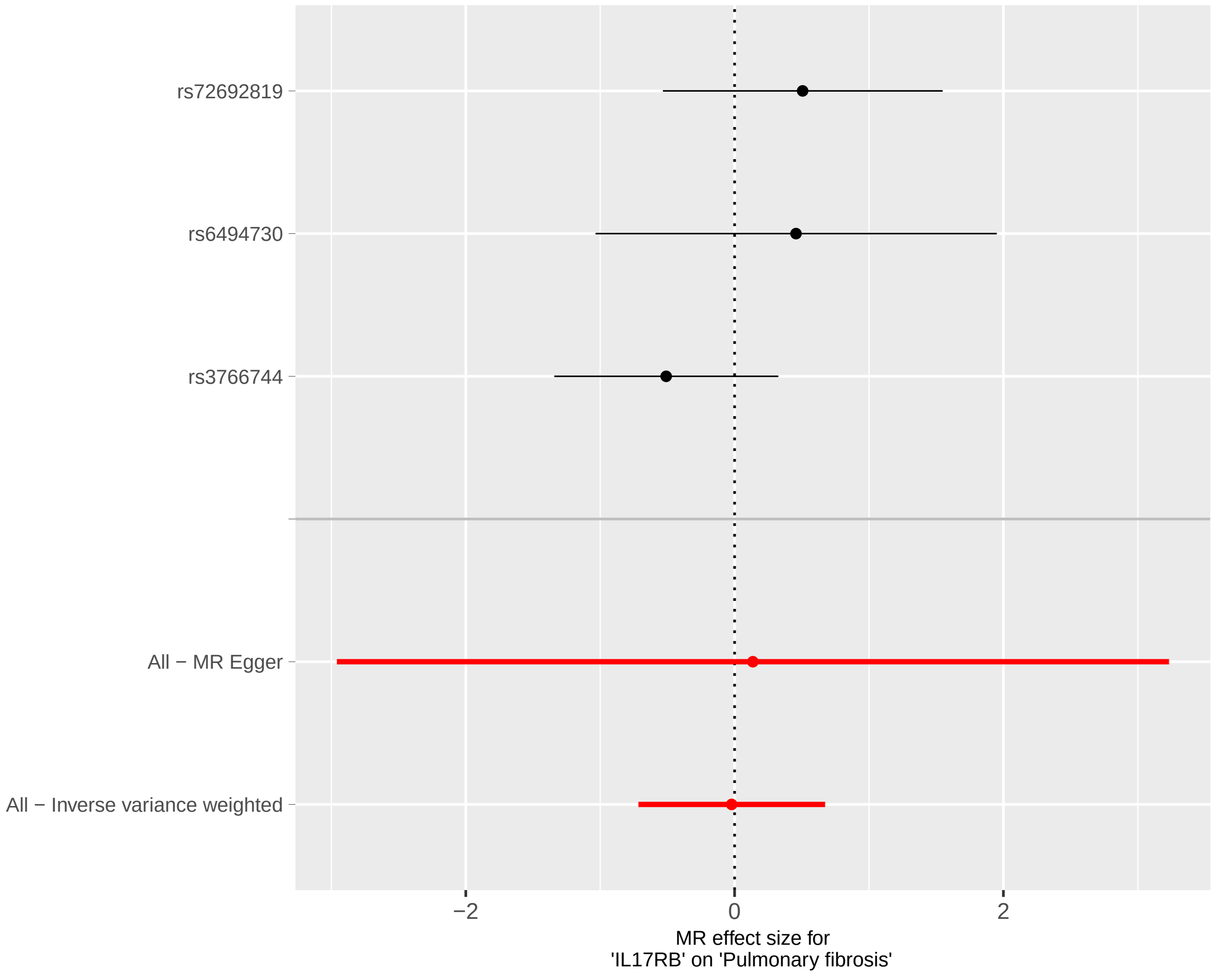

rs72692819
rs6494730
rs3766744
All − MR Egger
All − Inverse variance weighted
−2
0
2
MR effect size for
'IL17RB' on 'Pulmonary fibrosis'

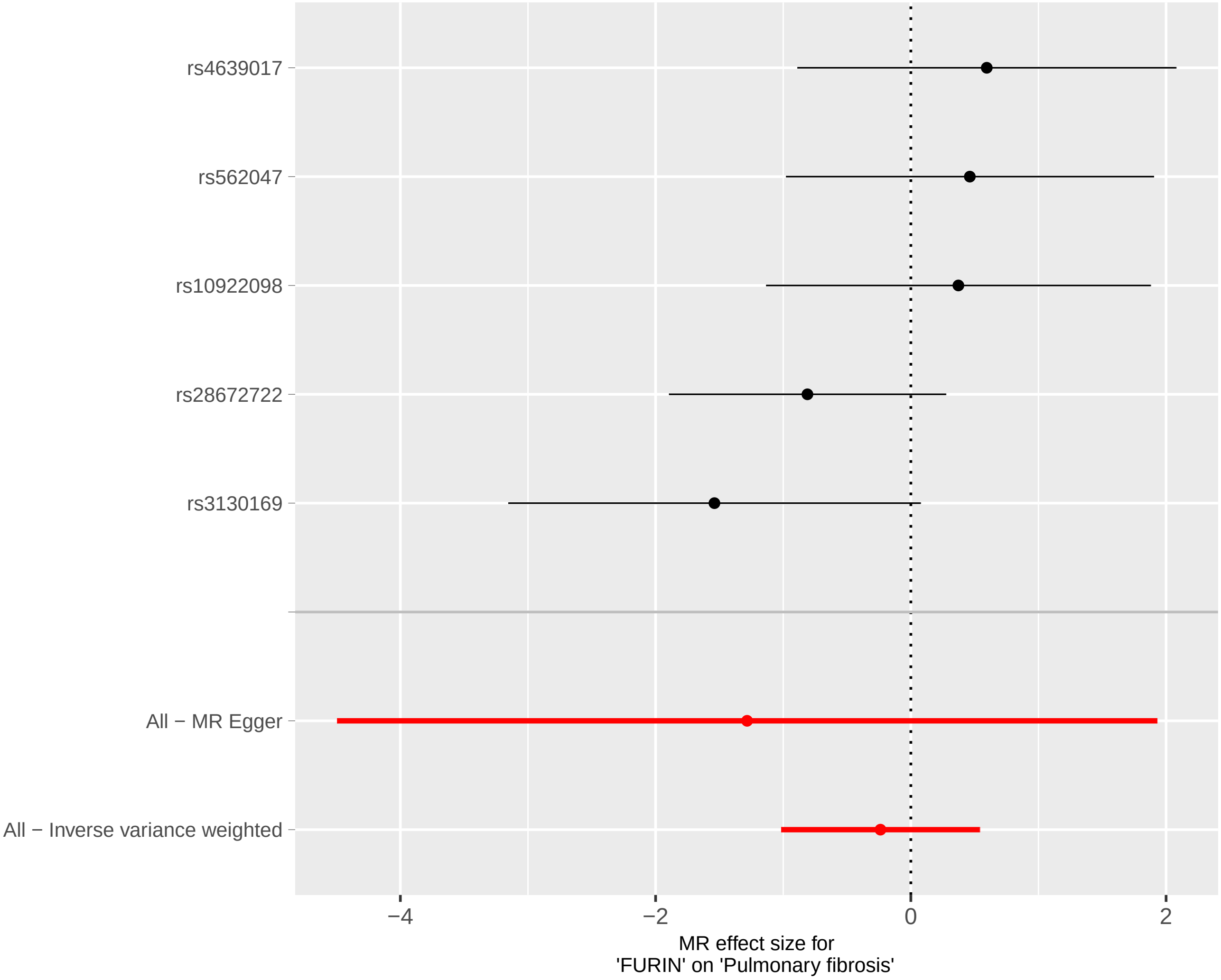

rs4639017
rs562047
rs10922098
rs28672722
rs3130169
All − MR Egger
All − Inverse variance weighted
−4
−2
0
2
MR effect size for
'FURIN' on 'Pulmonary fibrosis'

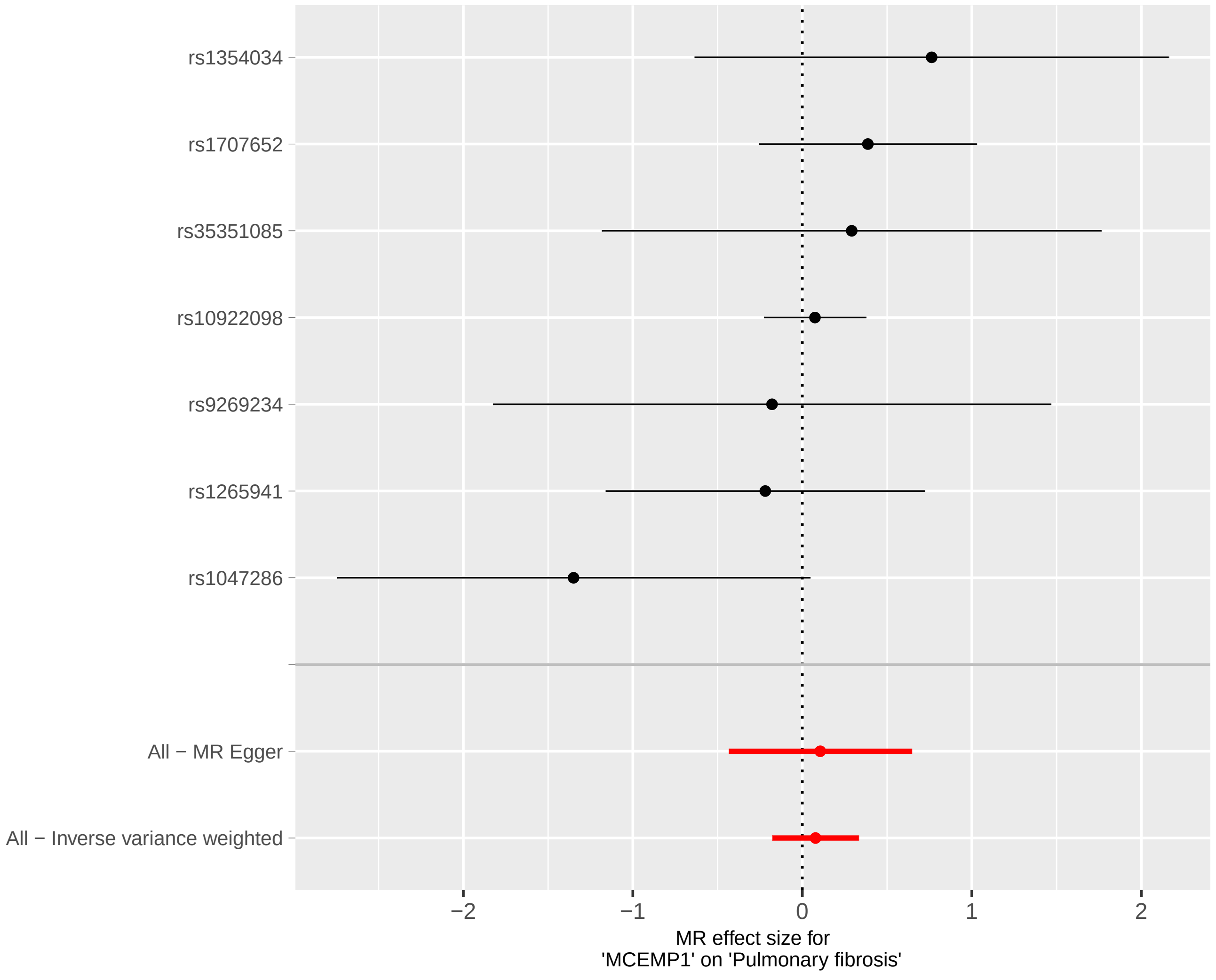
rs1354034
rs1707652
rs35351085
rs10922098
rs9269234
rs1265941
rs1047286
All − MR Egger
All − Inverse variance weighted
−2
−1
0
1
2
MR effect size for
'MCEMP1' on 'Pulmonary fibrosis'

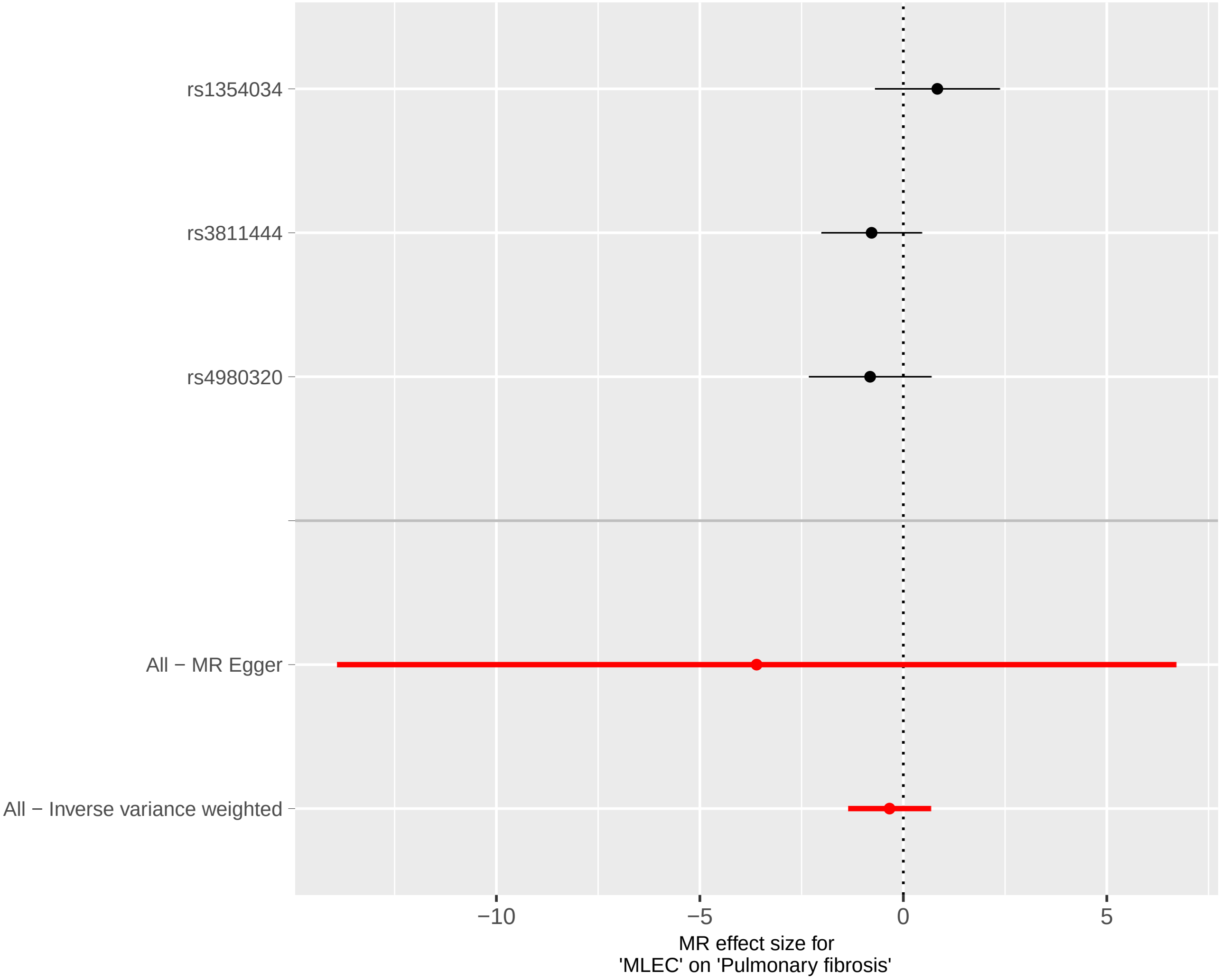
rs1354034
rs3811444
rs4980320
All − MR Egger
All − Inverse variance weighted
−10
−5
0
5
MR effect size for
'MLEC' on 'Pulmonary fibrosis'

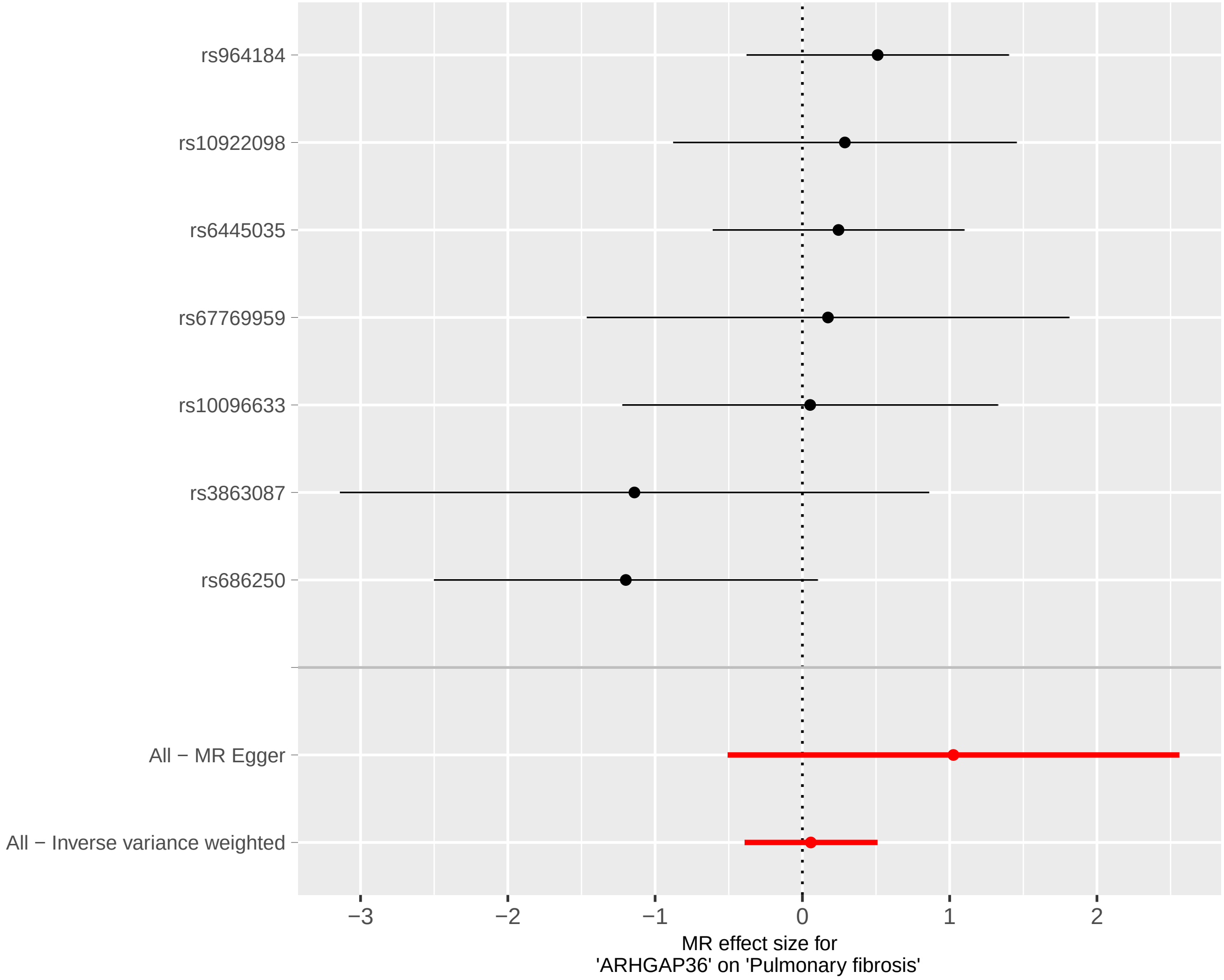

rs964184
rs10922098
rs6445035
rs67769959
rs10096633
rs3863087
rs686250
All − MR Egger
All − Inverse variance weighted
−3
−2
−1
0
1
2
MR effect size for
'ARHGAP36' on 'Pulmonary fibrosis'

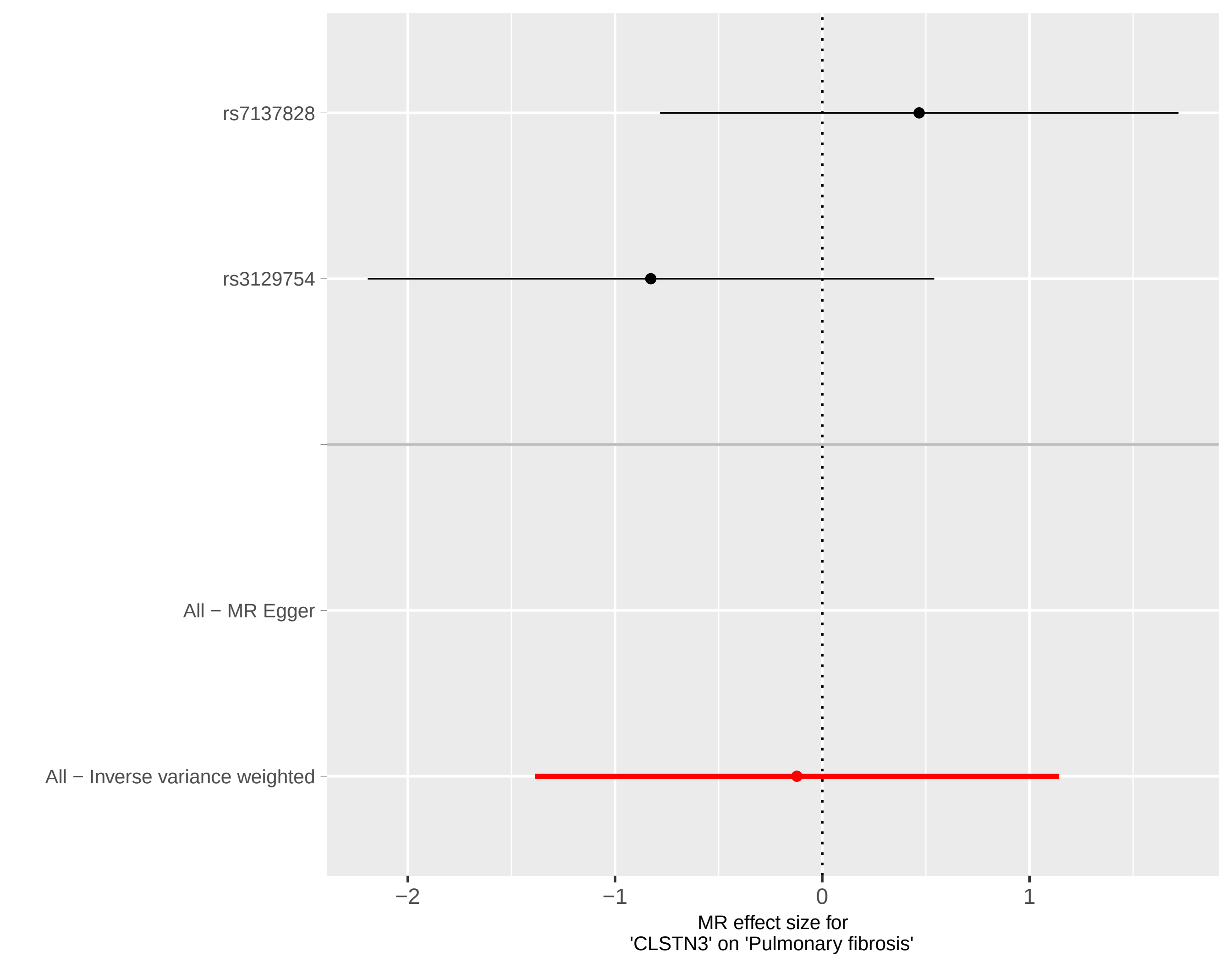

rs7137828
rs3129754
All − MR Egger
All − Inverse variance weighted
−2
−1
0
1
MR effect size for
'CLSTN3' on 'Pulmonary fibrosis'

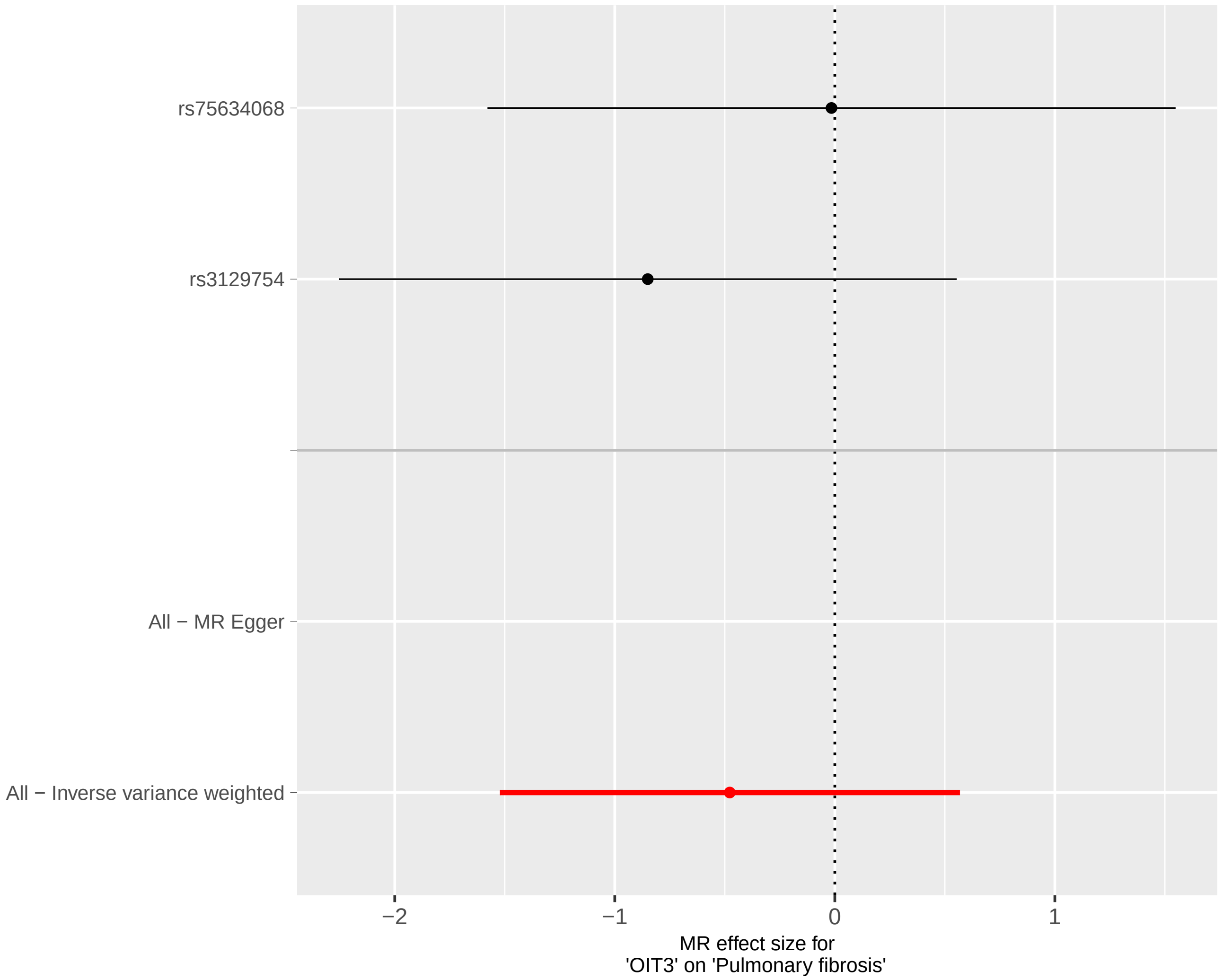
rs75634068
rs3129754
All − MR Egger
All − Inverse variance weighted
−2
−1
0
1
MR effect size for
'OIT3' on 'Pulmonary fibrosis'

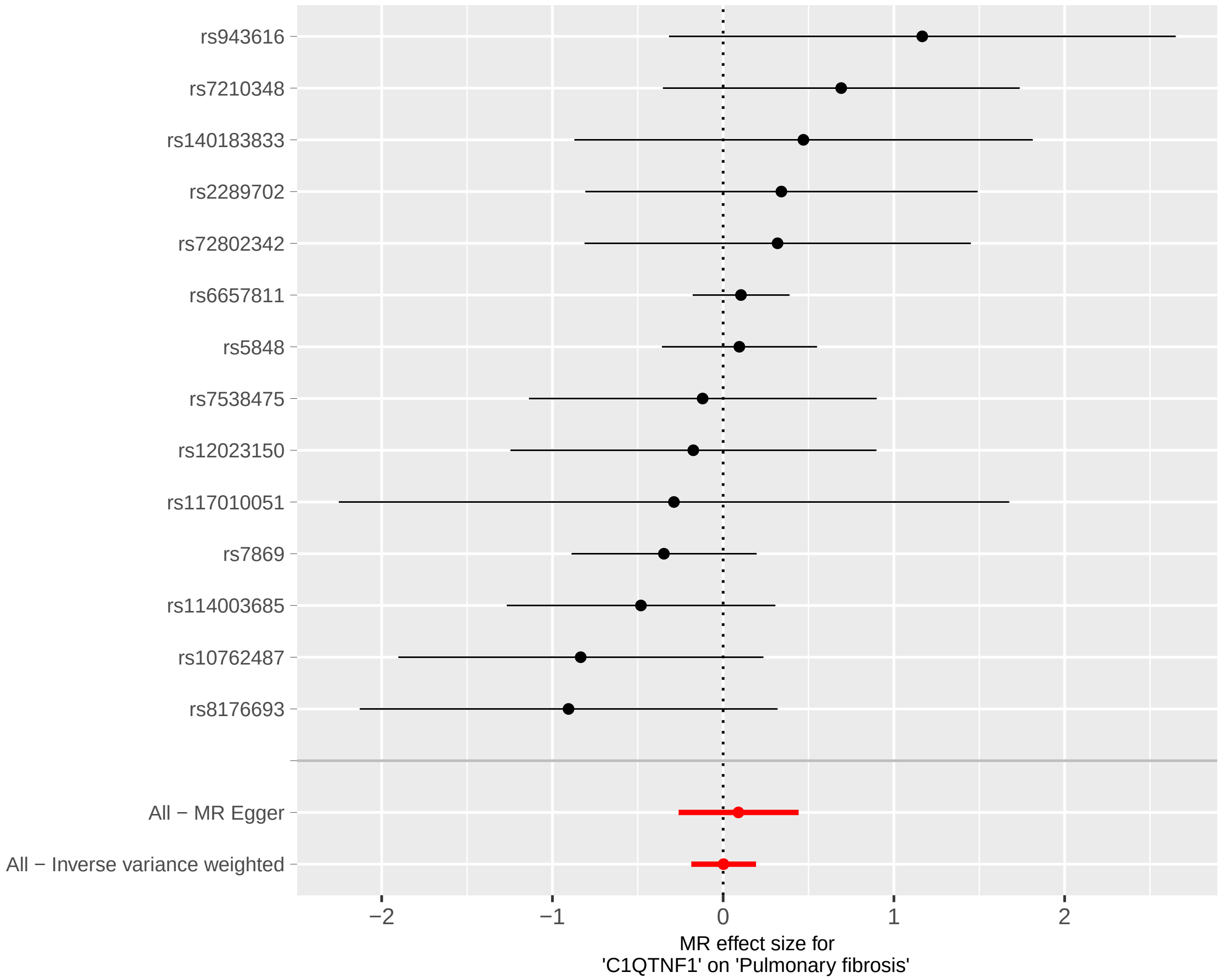
rs943616
rs7210348
rs140183833
rs2289702
rs72802342
rs6657811
rs5848
rs7538475
rs12023150
rs117010051
rs7869
rs114003685
rs10762487
rs8176693
All − MR Egger
All − Inverse variance weighted
−2
−1
0
1
2
MR effect size for
'C1QTNF1' on 'Pulmonary fibrosis'

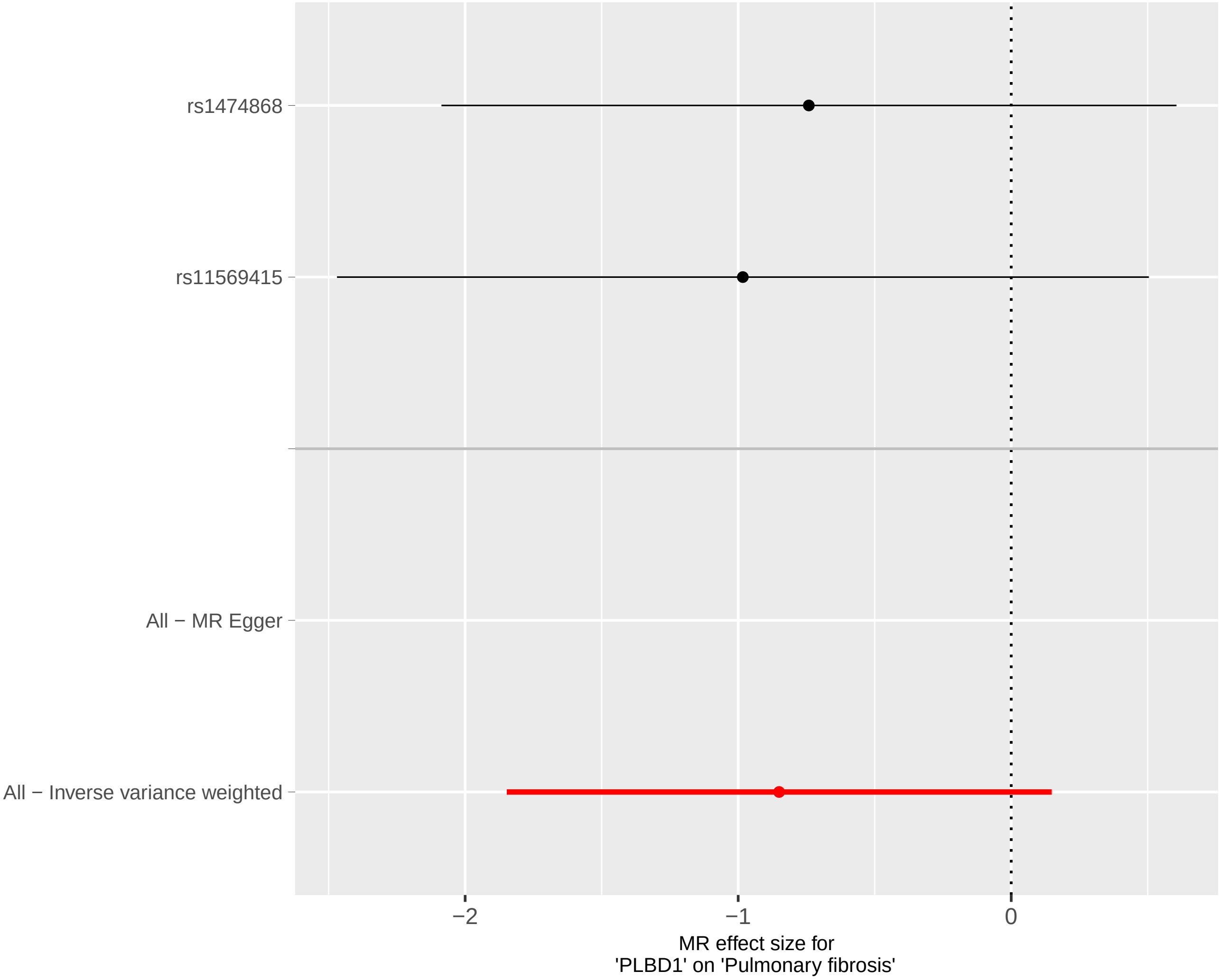

rs1474868
rs11569415
All − MR Egger
All − Inverse variance weighted
−2
−1
0
MR effect size for
'PLBD1' on 'Pulmonary fibrosis'

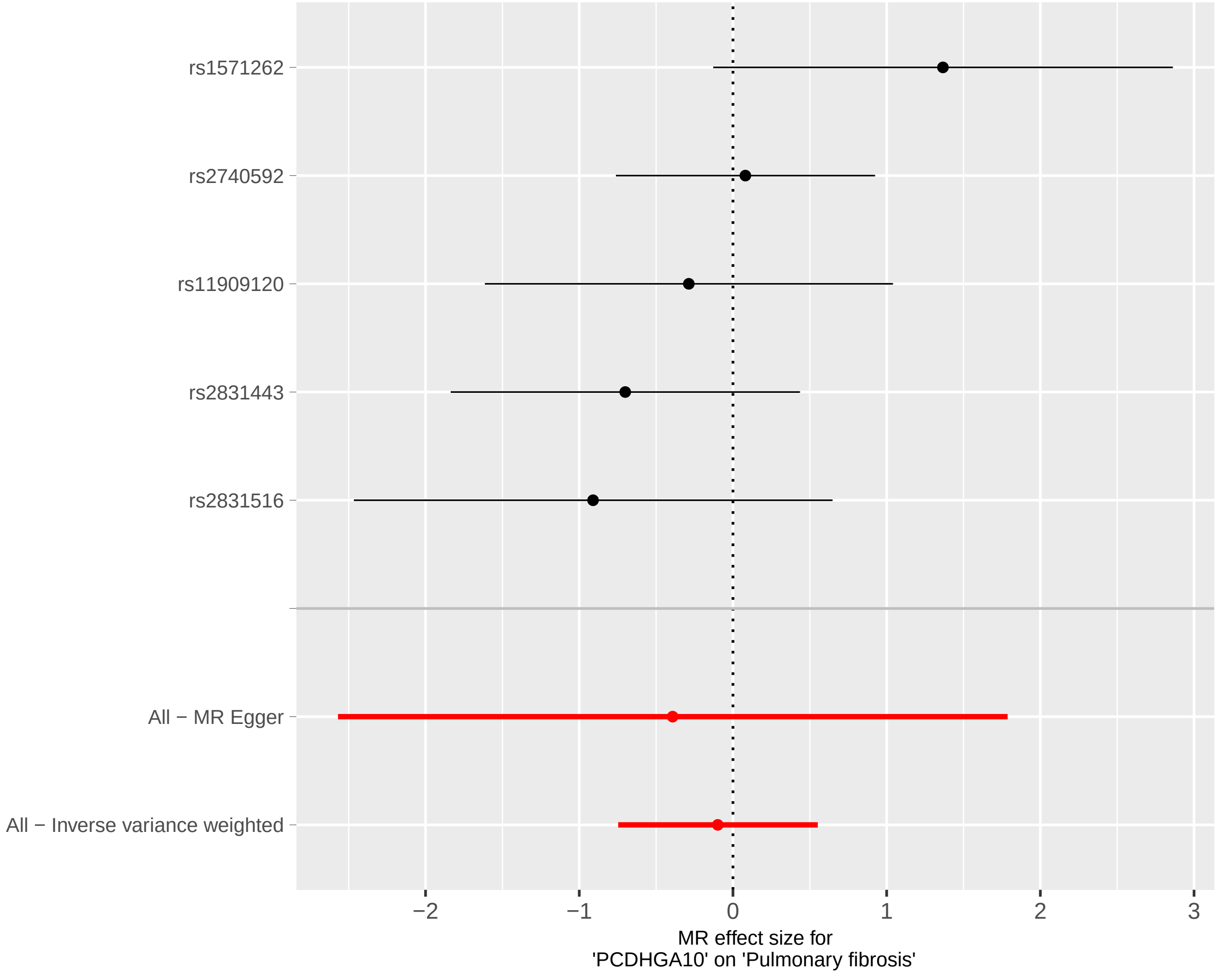

rs1571262
rs2740592
rs11909120
rs2831443
rs2831516
All − MR Egger
All − Inverse variance weighted
−2
−1
0
1
2
3
MR effect size for
'PCDHGA10' on 'Pulmonary fibrosis'

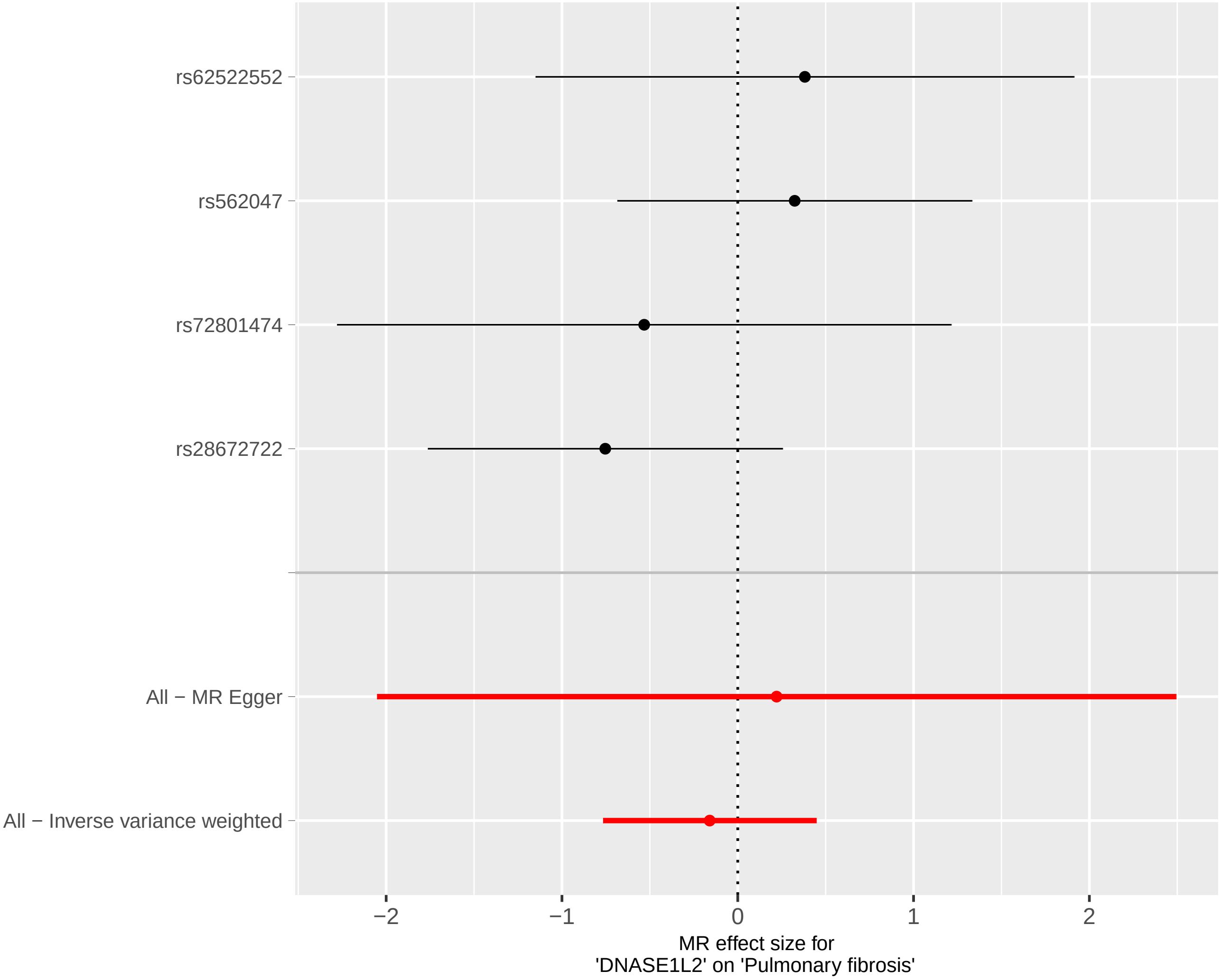

rs62522552
rs562047
rs72801474
rs28672722
All − MR Egger
All − Inverse variance weighted
−2
−1
0
1
2
MR effect size for
'DNASE1L2' on 'Pulmonary fibrosis'

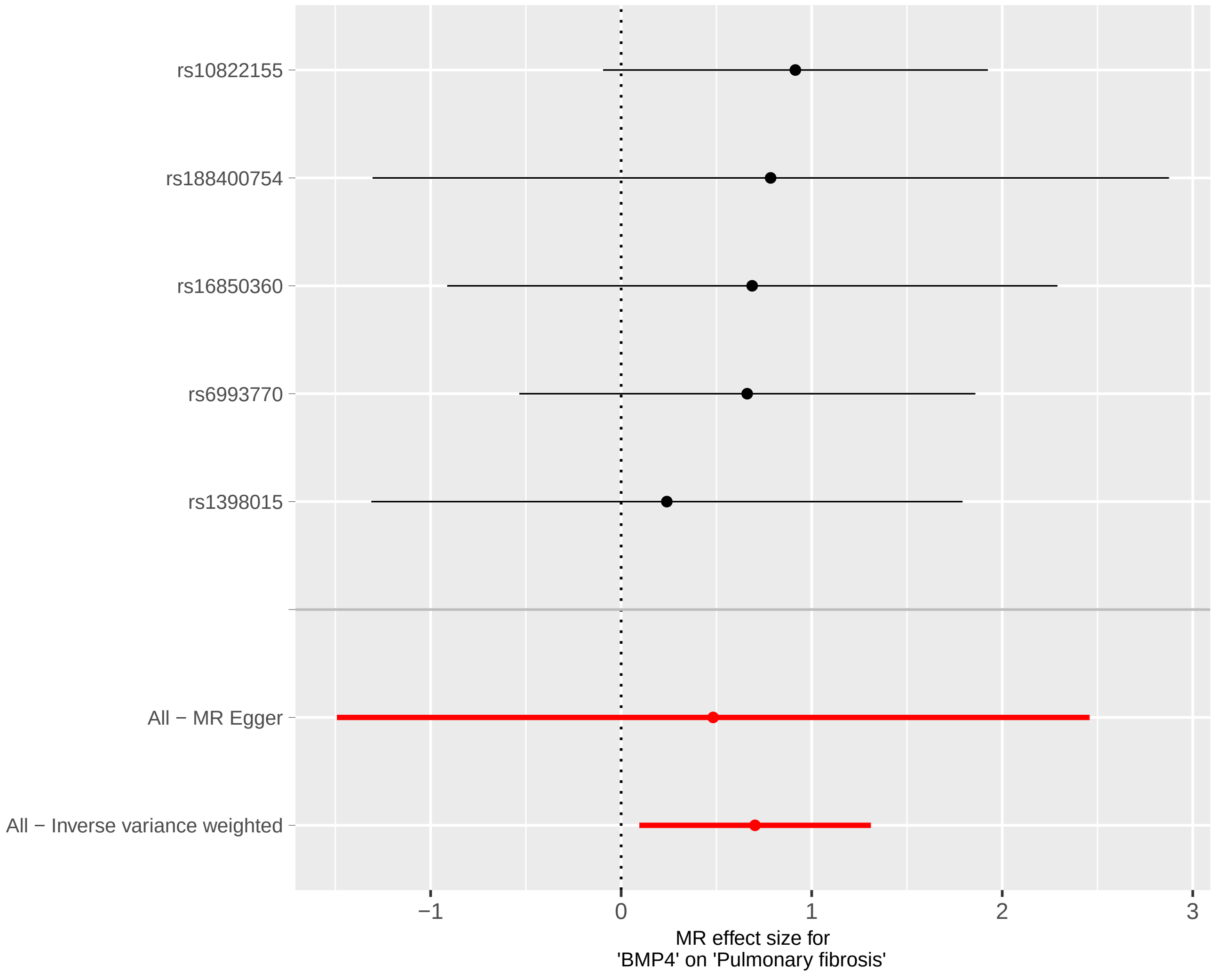
rs10822155
rs188400754
rs16850360
rs6993770
rs1398015
All − MR Egger
All − Inverse variance weighted
−1
0
1
2
3
MR effect size for
'BMP4' on 'Pulmonary fibrosis'

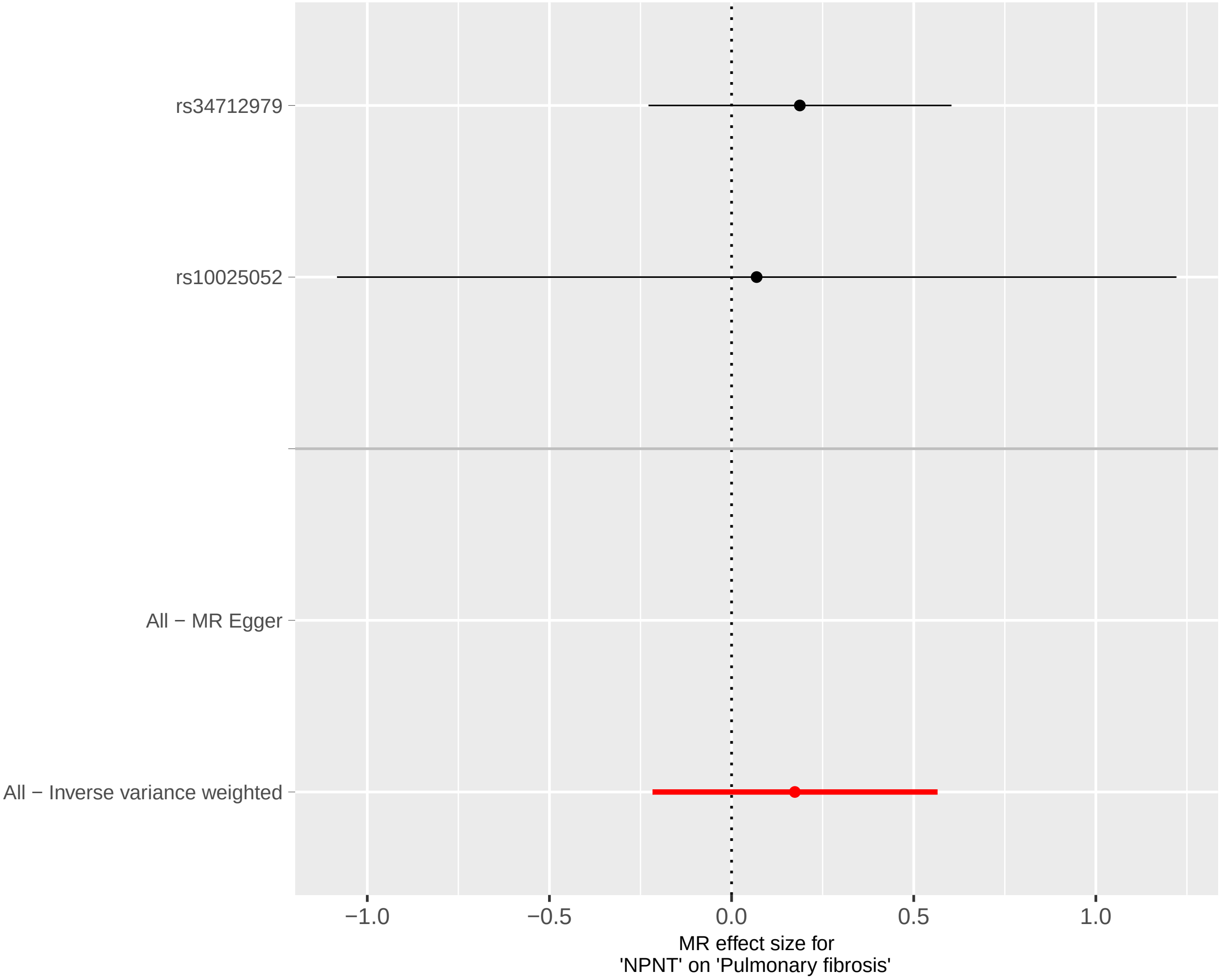
rs34712979
rs10025052
All − MR Egger
All − Inverse variance weighted
−1.0
−0.5
0.0
0.5
1.0
MR effect size for
'NPNT' on 'Pulmonary fibrosis'

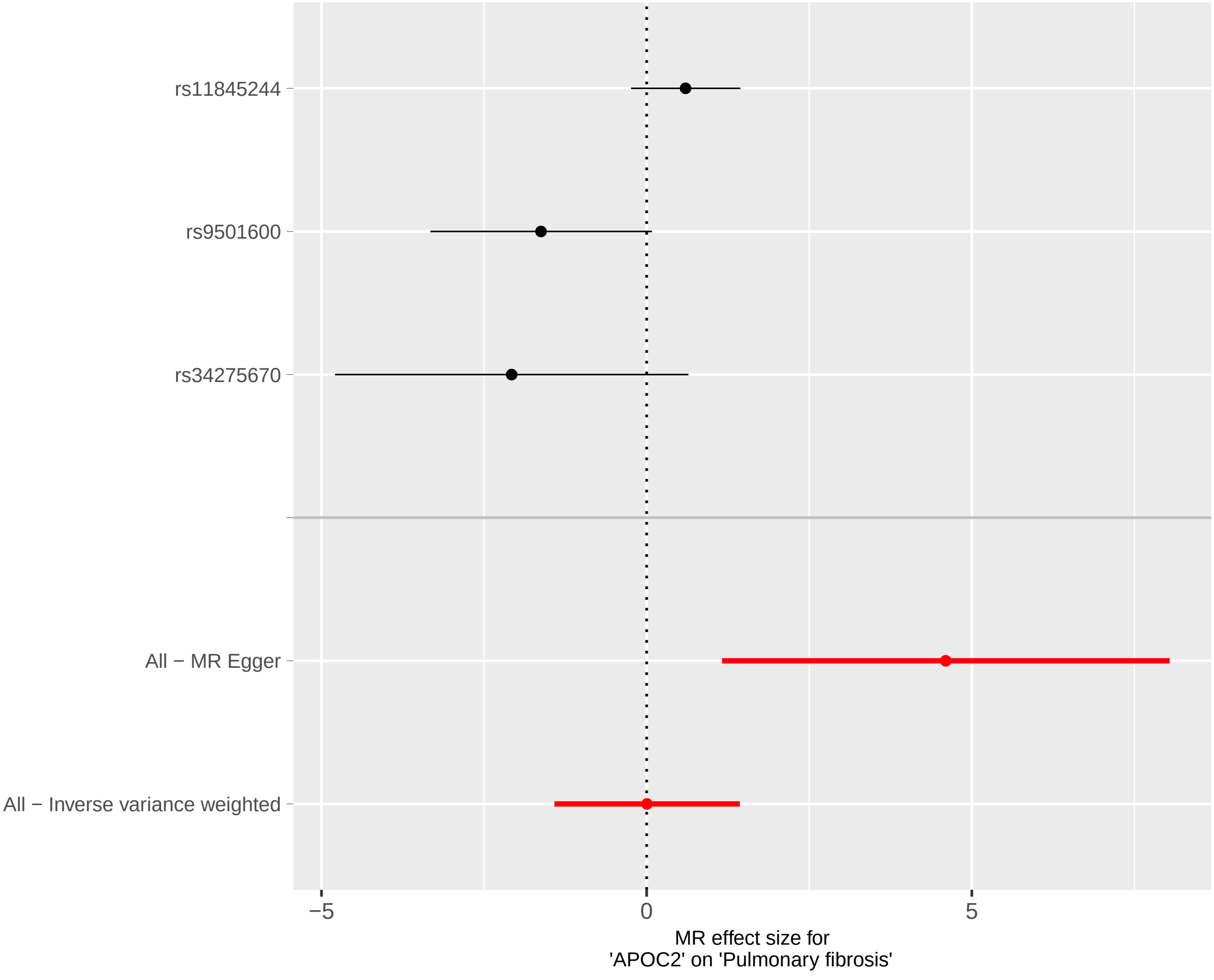
rs11845244
rs9501600
rs34275670
All − MR Egger
All − Inverse variance weighted
−5
0
5
MR effect size for
'APOC2' on 'Pulmonary fibrosis'

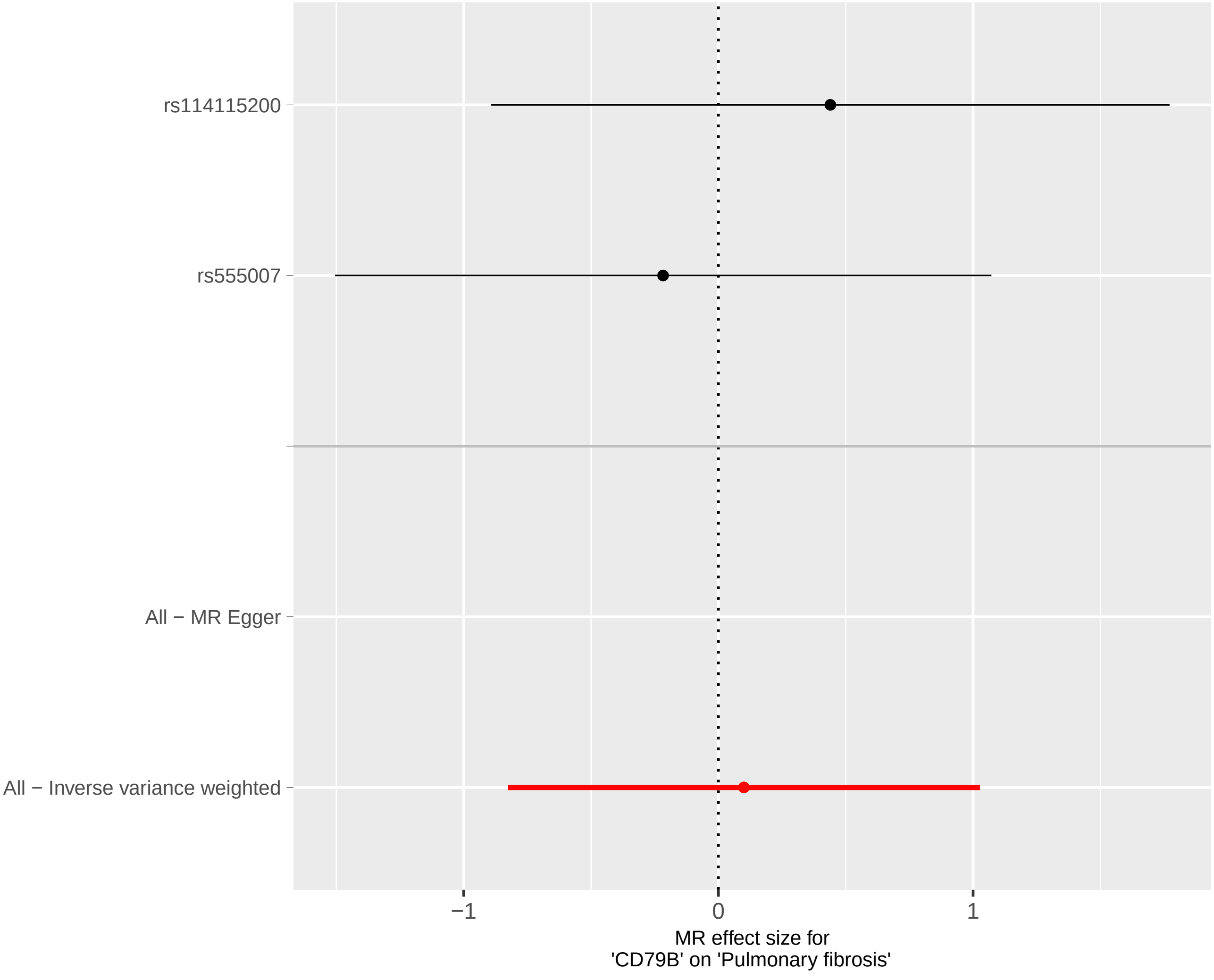
rs114115200
rs555007
All − MR Egger
All − Inverse variance weighted
−1
0
1
MR effect size for
'CD79B' on 'Pulmonary fibrosis'

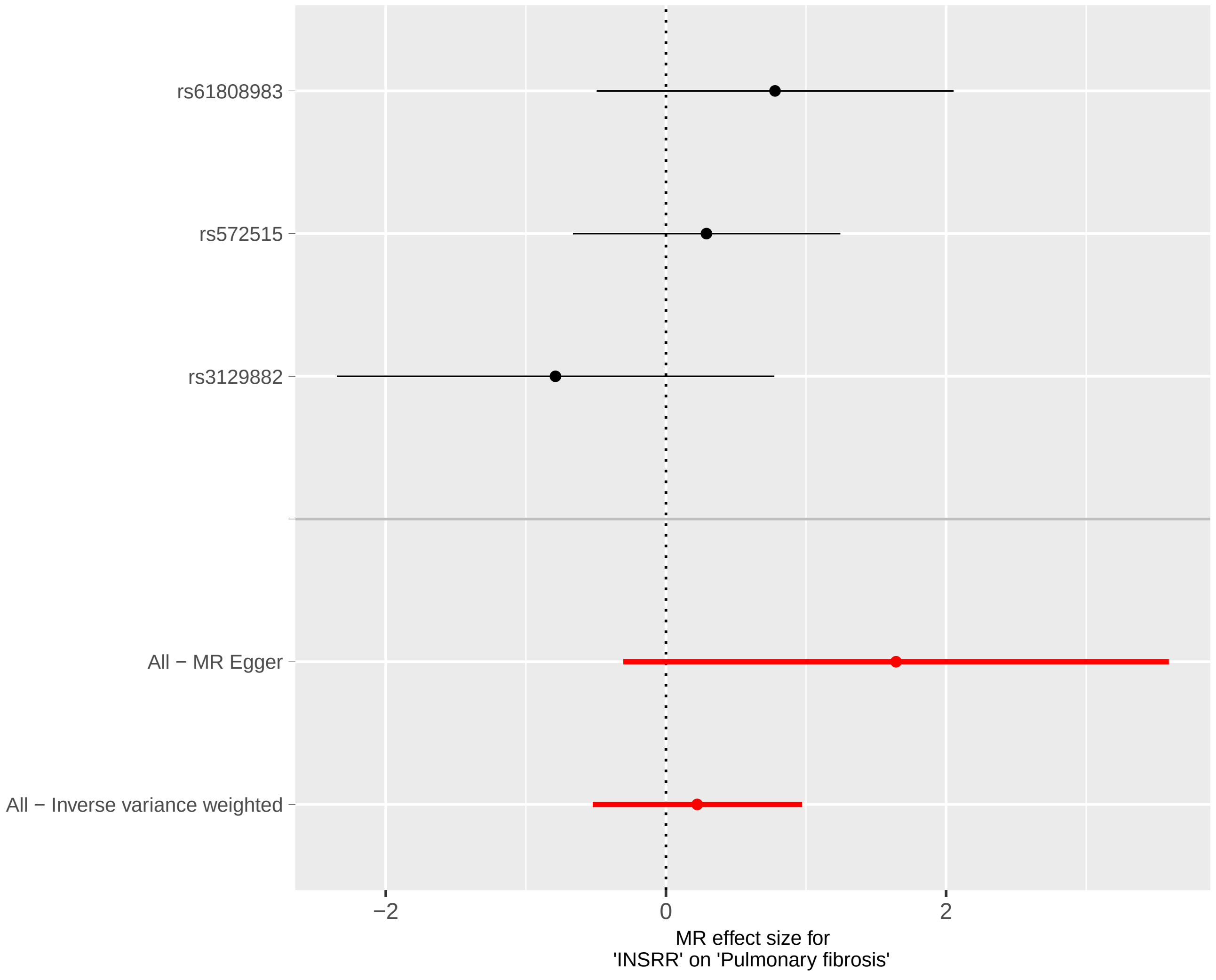
rs61808983
rs572515
rs3129882
All − MR Egger
All − Inverse variance weighted
−2
0
2
MR effect size for
'INSRR' on 'Pulmonary fibrosis'

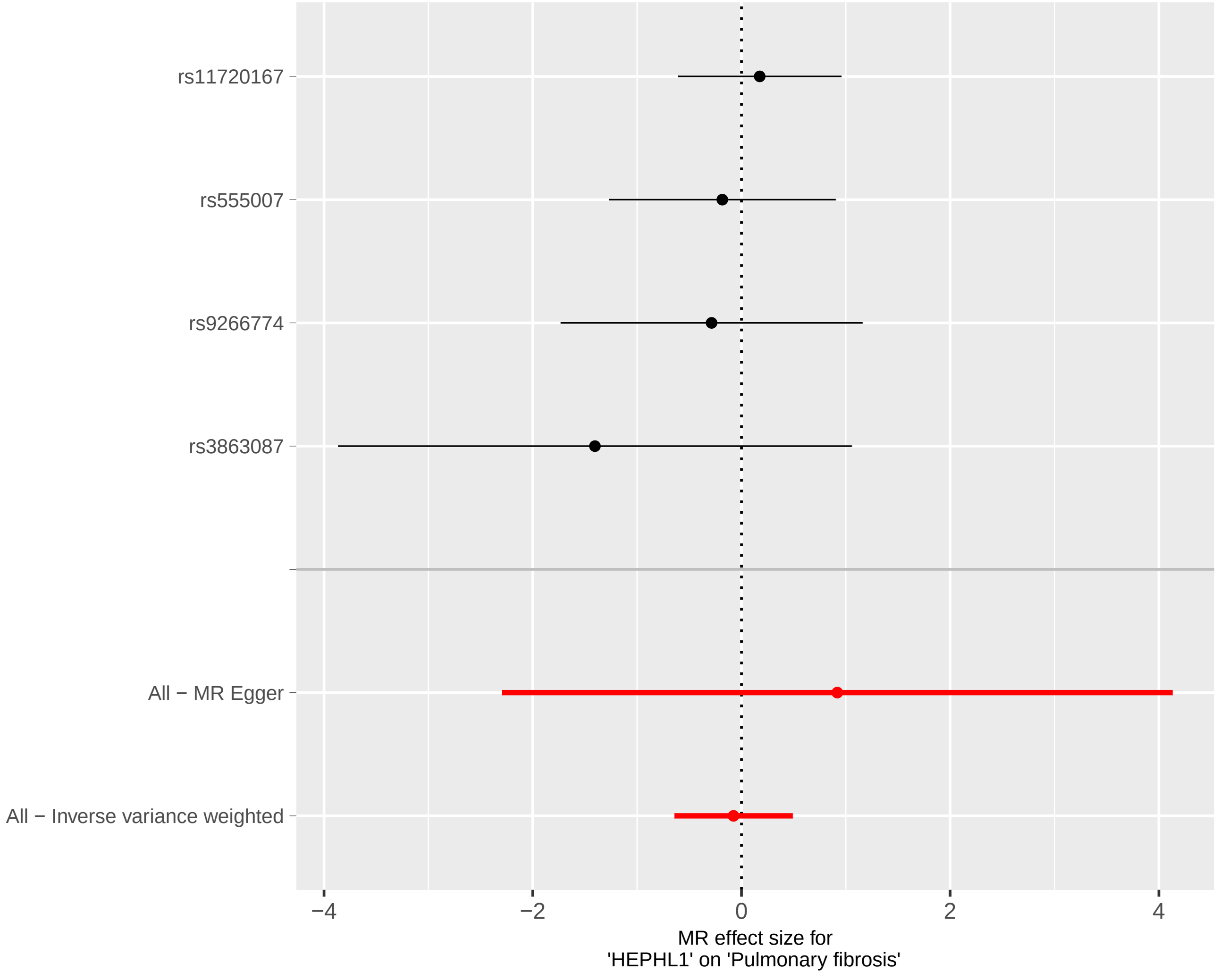

rs11720167
rs555007
rs9266774
rs3863087
All − MR Egger
All − Inverse variance weighted
−4
−2
0
2
4
MR effect size for
'HEPHL1' on 'Pulmonary fibrosis'

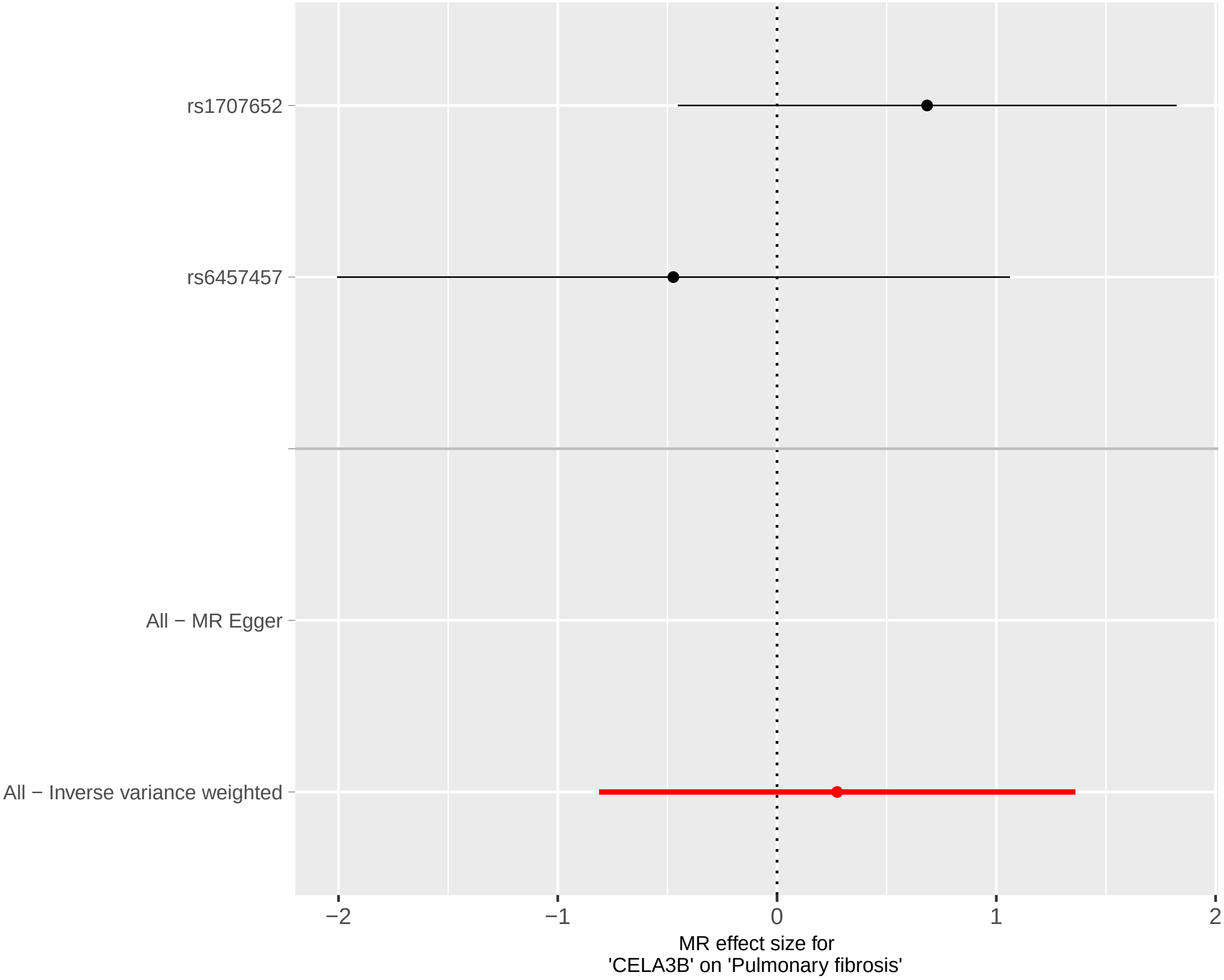

rs1707652
rs6457457
All − MR Egger
All − Inverse variance weighted
−2
−1
0
1
2
MR effect size for
'CELA3B' on 'Pulmonary fibrosis'

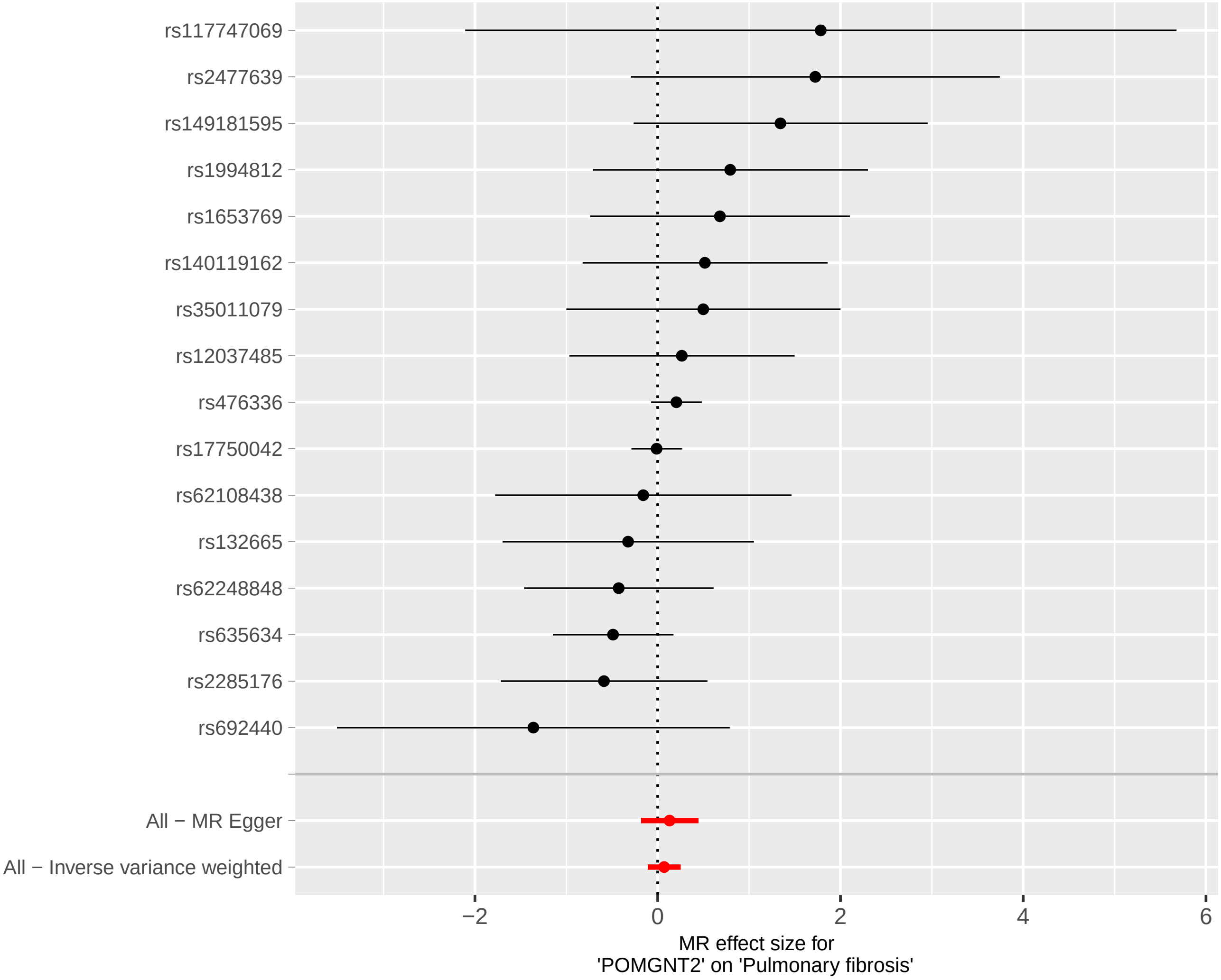

rs117747069
rs2477639
rs149181595
rs1994812
rs1653769
rs140119162
rs35011079
rs12037485
rs476336
rs17750042
rs62108438
rs132665
rs62248848
rs635634
rs2285176
rs692440
All − MR Egger
All − Inverse variance weighted
−2
0
2
4
6
MR effect size for
'POMGNT2' on 'Pulmonary fibrosis'

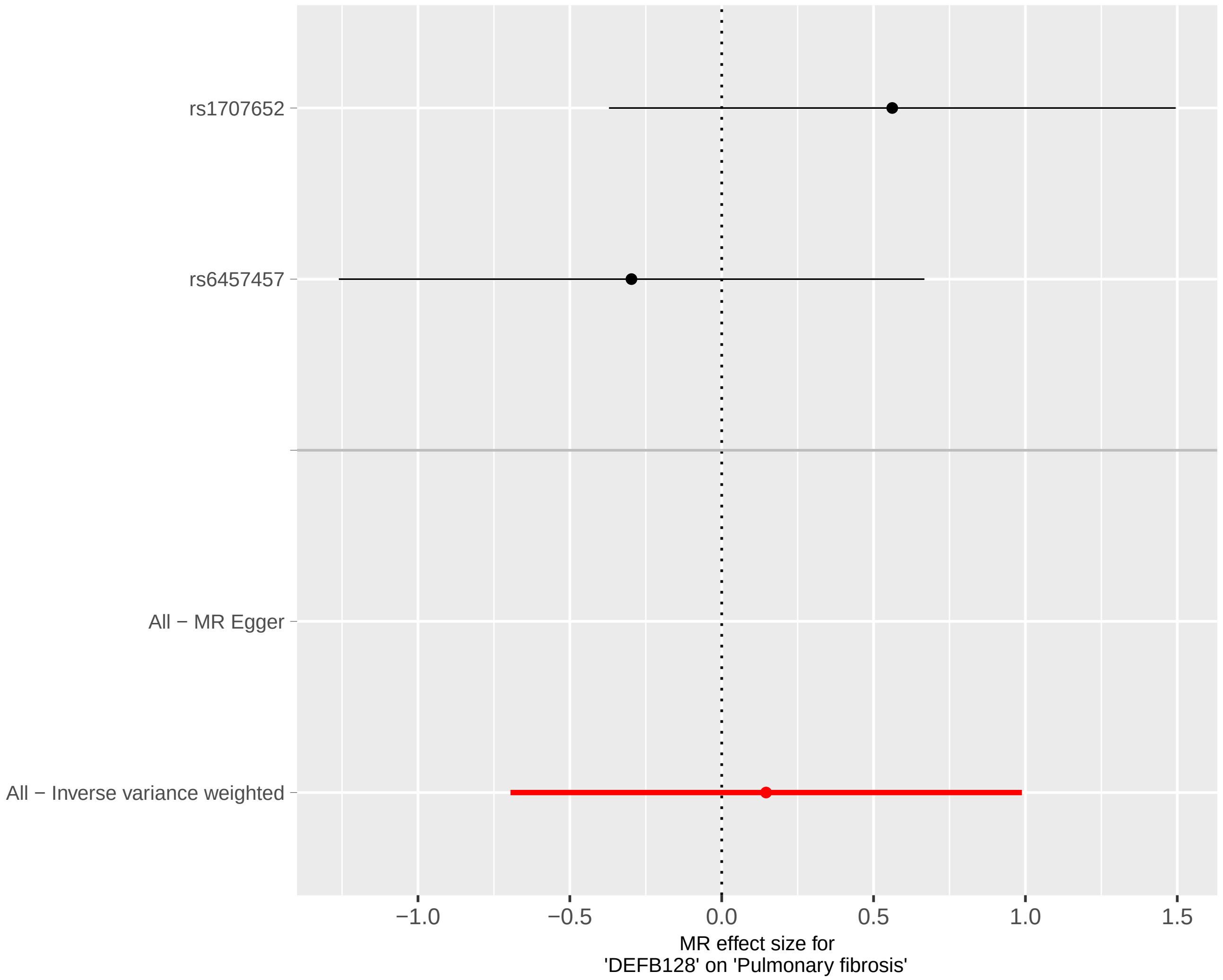
rs1707652
rs6457457
All − MR Egger
All − Inverse variance weighted
−1.0
−0.5
0.0
0.5
1.0
1.5
MR effect size for
'DEFB128' on 'Pulmonary fibrosis'

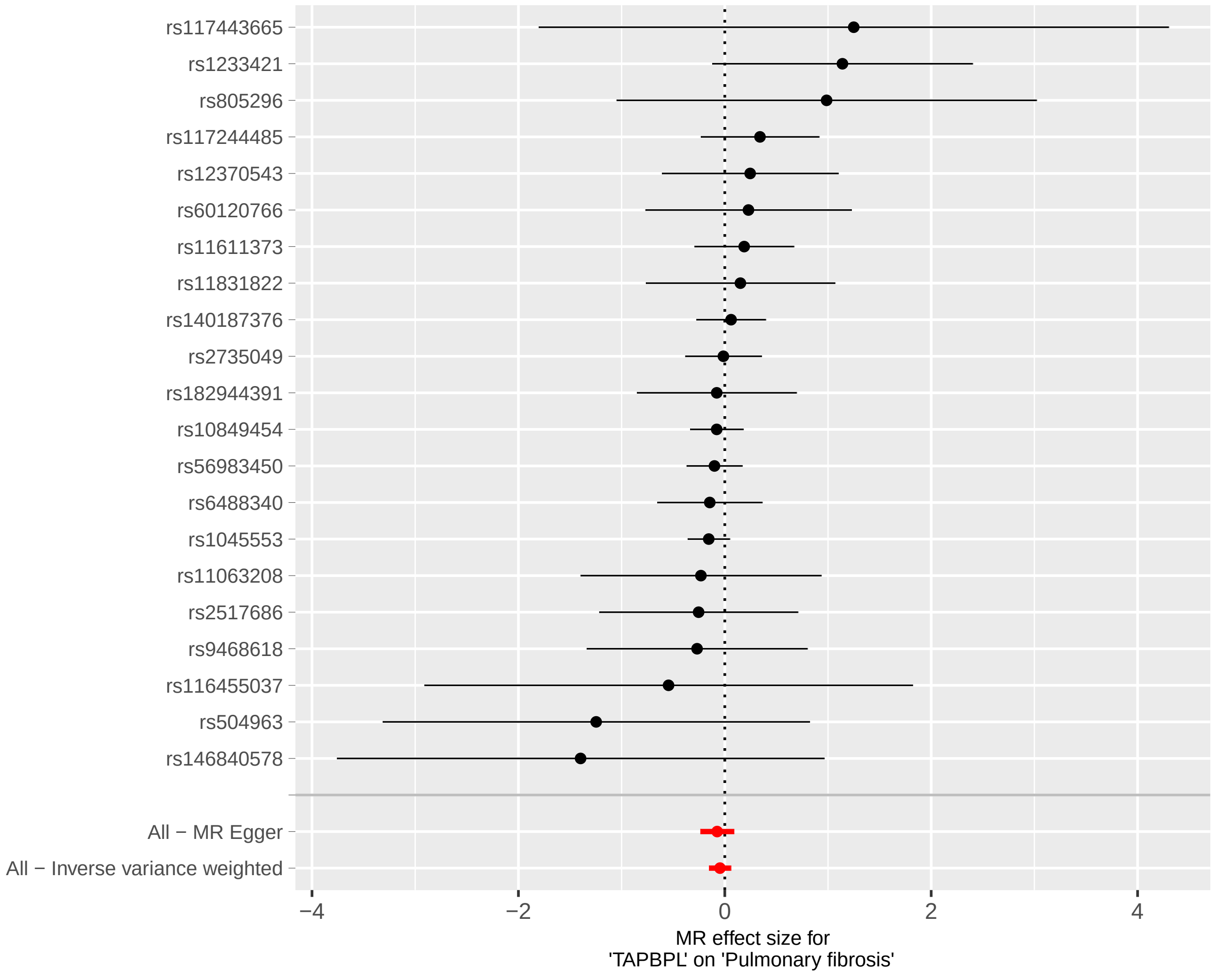

rs117443665
rs1233421
rs805296
rs117244485
rs12370543
rs60120766
rs11611373
rs11831822
rs140187376
rs2735049
rs182944391
rs10849454
rs56983450
rs6488340
rs1045553
rs11063208
rs2517686
rs9468618
rs116455037
rs504963
rs146840578
All − MR Egger
All − Inverse variance weighted
−4
−2
0
2
4
MR effect size for
'TAPBPL' on 'Pulmonary fibrosis'

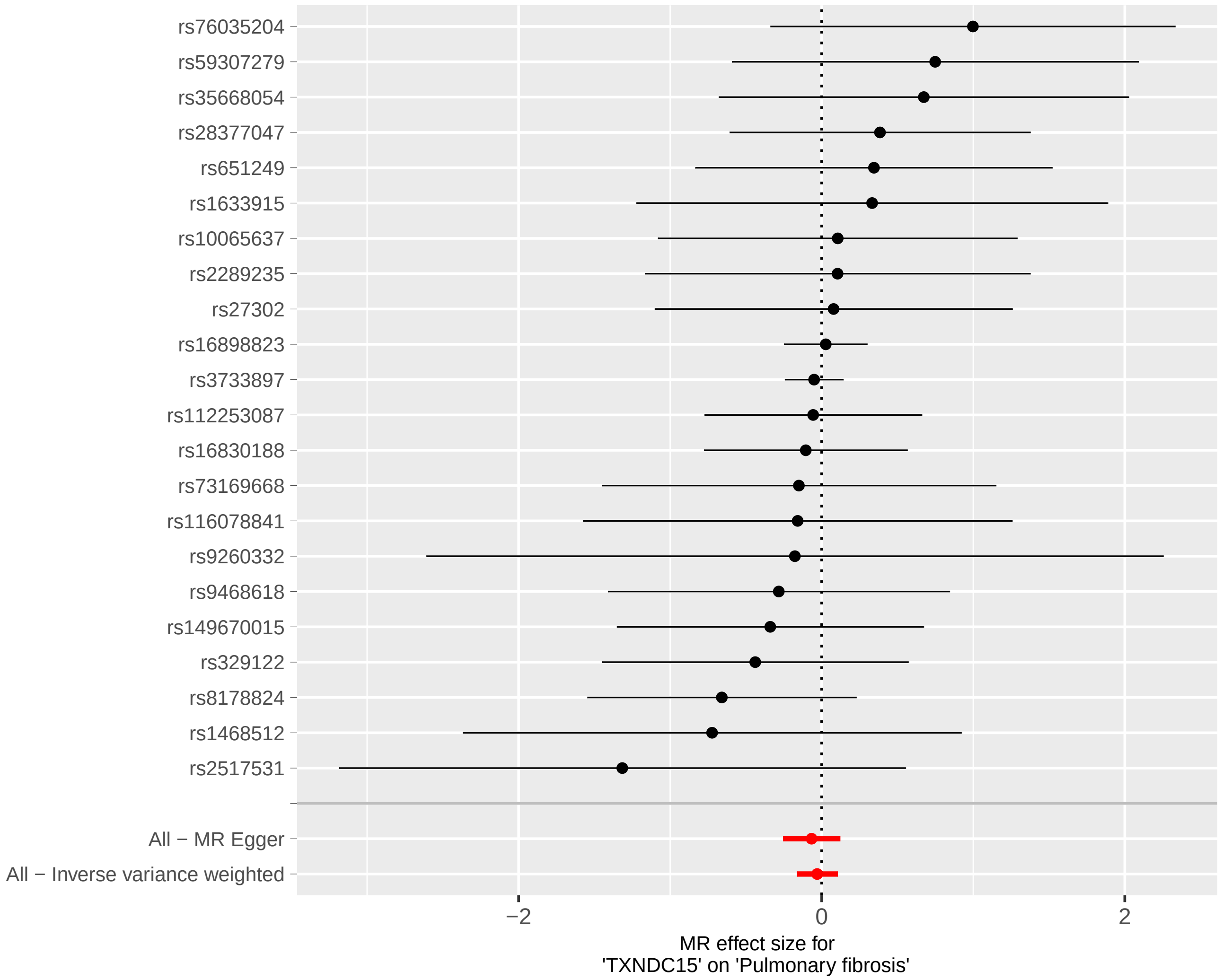

rs76035204
rs59307279
rs35668054
rs28377047
rs651249
rs1633915
rs10065637
rs2289235
rs27302
rs16898823
rs3733897
rs112253087
rs16830188
rs73169668
rs116078841
rs9260332
rs9468618
rs149670015
rs329122
rs8178824
rs1468512
rs2517531
All − MR Egger
All − Inverse variance weighted
−2
0
2
MR effect size for
'TXNDC15' on 'Pulmonary fibrosis'

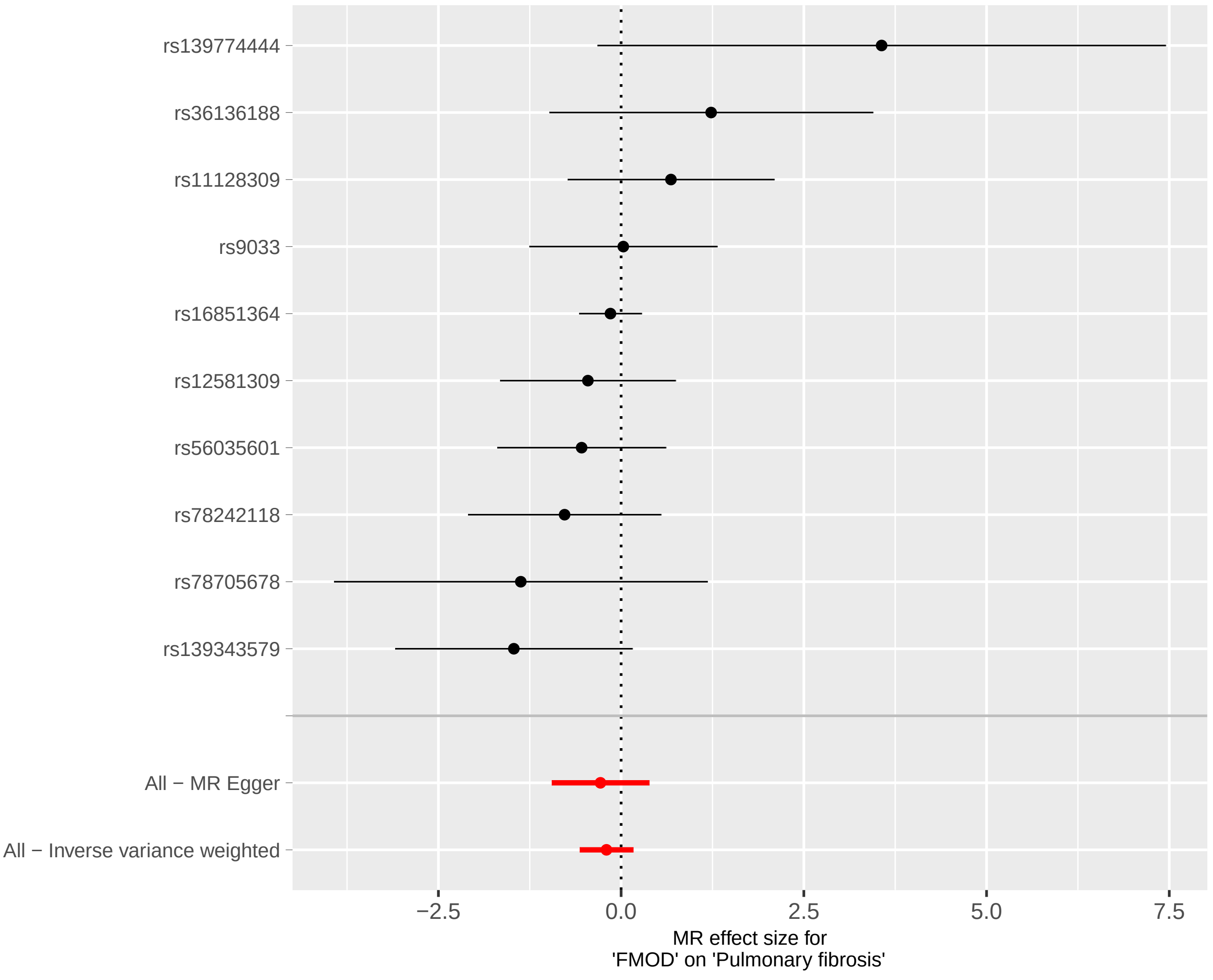
rs139774444
rs36136188
rs11128309
rs9033
rs16851364
rs12581309
rs56035601
rs78242118
rs78705678
rs139343579
All − MR Egger
All − Inverse variance weighted
−2.5
0.0
2.5
5.0
7.5
MR effect size for
'FMOD' on 'Pulmonary fibrosis'

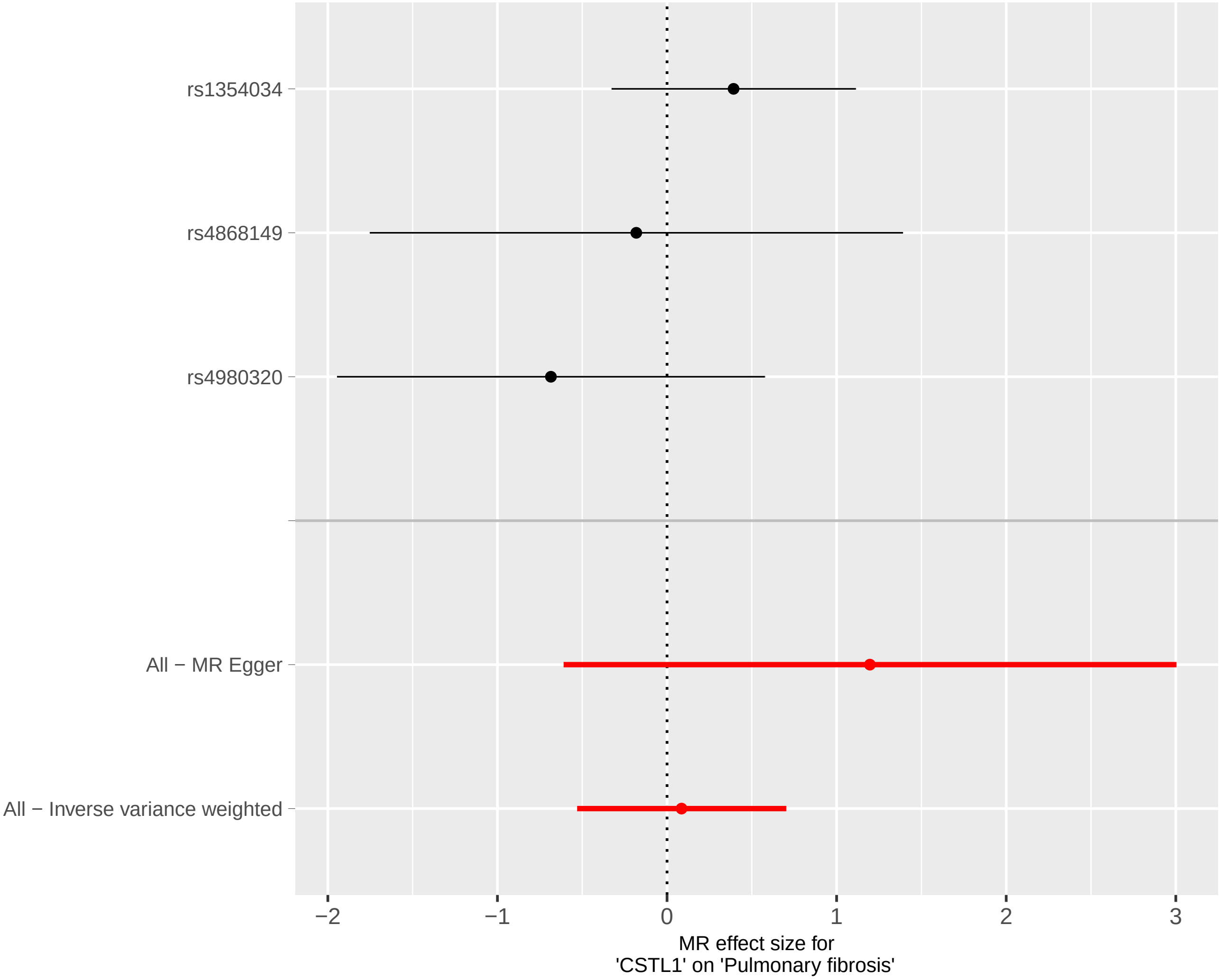
rs1354034
rs4868149
rs4980320
All − MR Egger
All − Inverse variance weighted
−2
−1
0
1
2
3
MR effect size for
'CSTL1' on 'Pulmonary fibrosis'

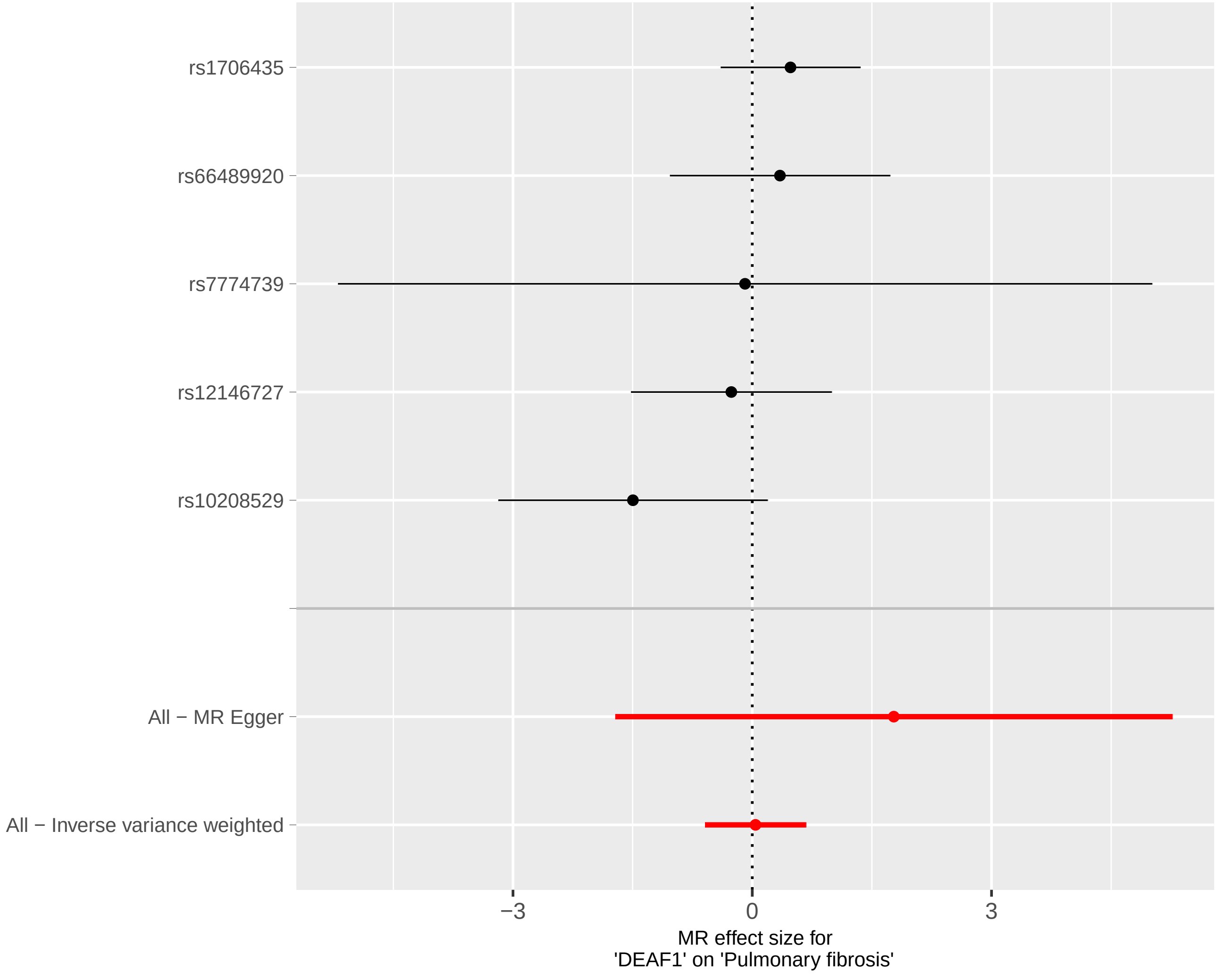

rs1706435
rs66489920
rs7774739
rs12146727
rs10208529
All − MR Egger
All − Inverse variance weighted
−3
0
3
MR effect size for
'DEAF1' on 'Pulmonary fibrosis'

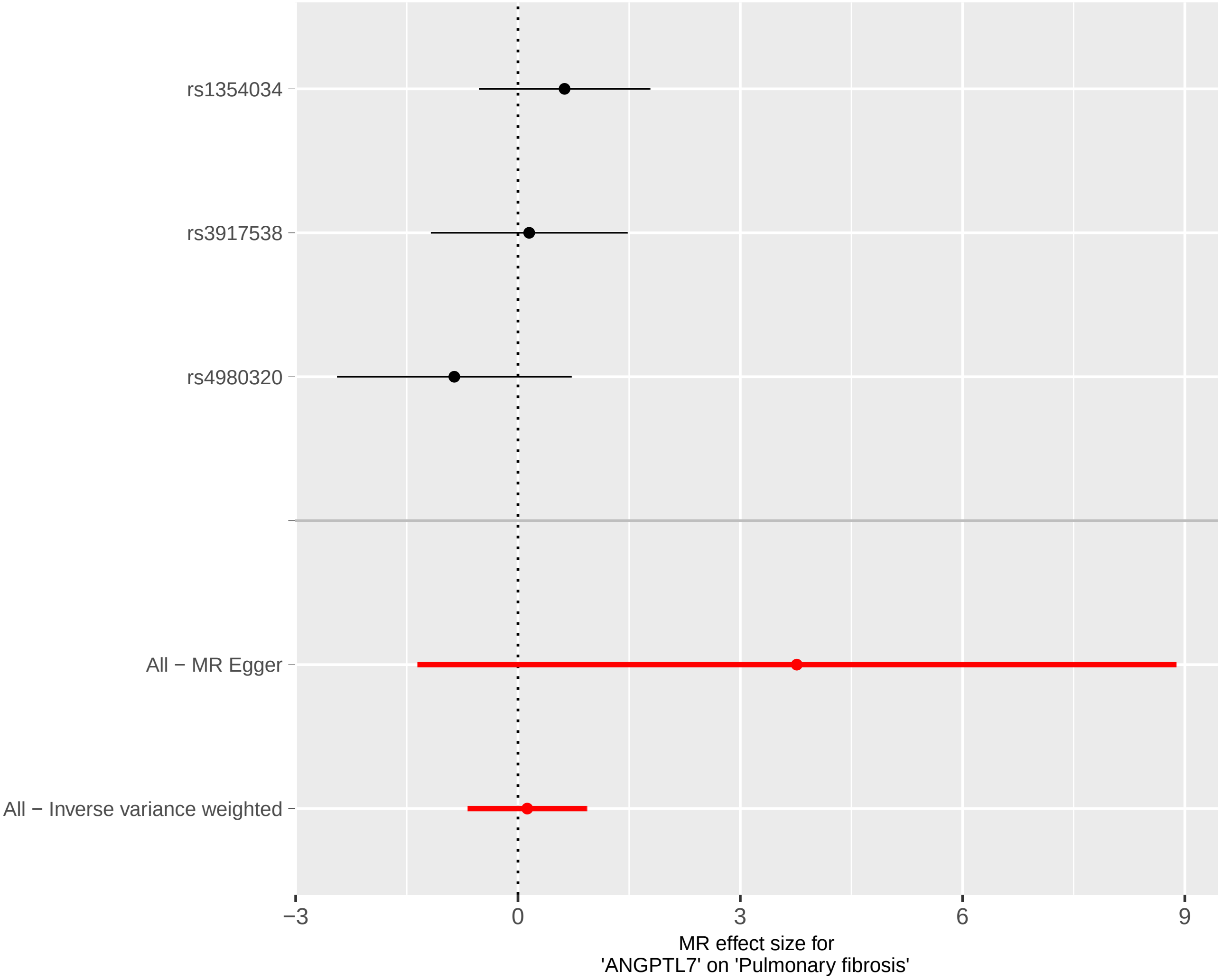
rs1354034
rs3917538
rs4980320
All − MR Egger
All − Inverse variance weighted
−3
0
3
6
9
MR effect size for
'ANGPTL7' on 'Pulmonary fibrosis'

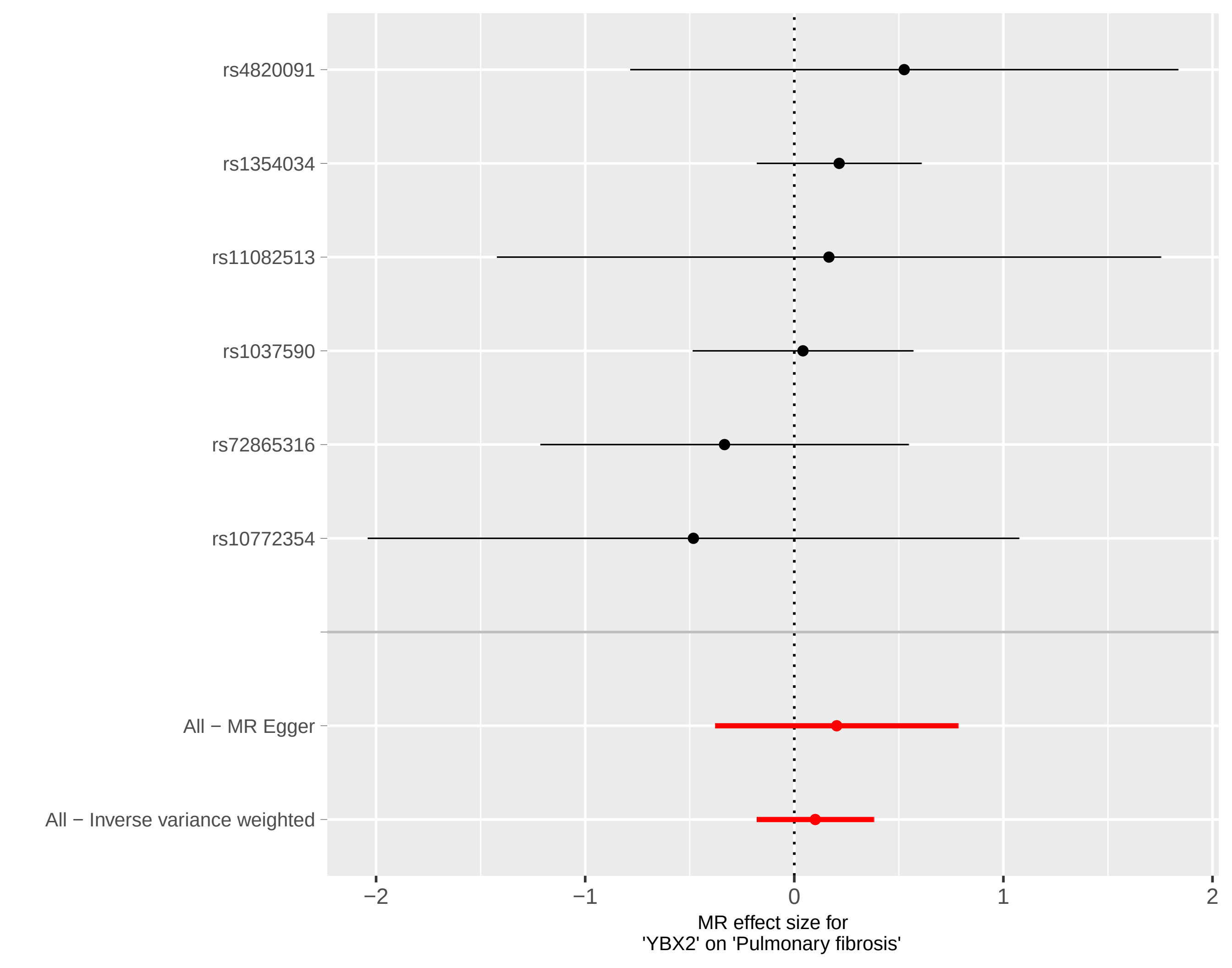
rs4820091
rs1354034
rs11082513
rs1037590
rs72865316
rs10772354
All − MR Egger
All − Inverse variance weighted
−2
−1
0
1
2
MR effect size for
'YBX2' on 'Pulmonary fibrosis'

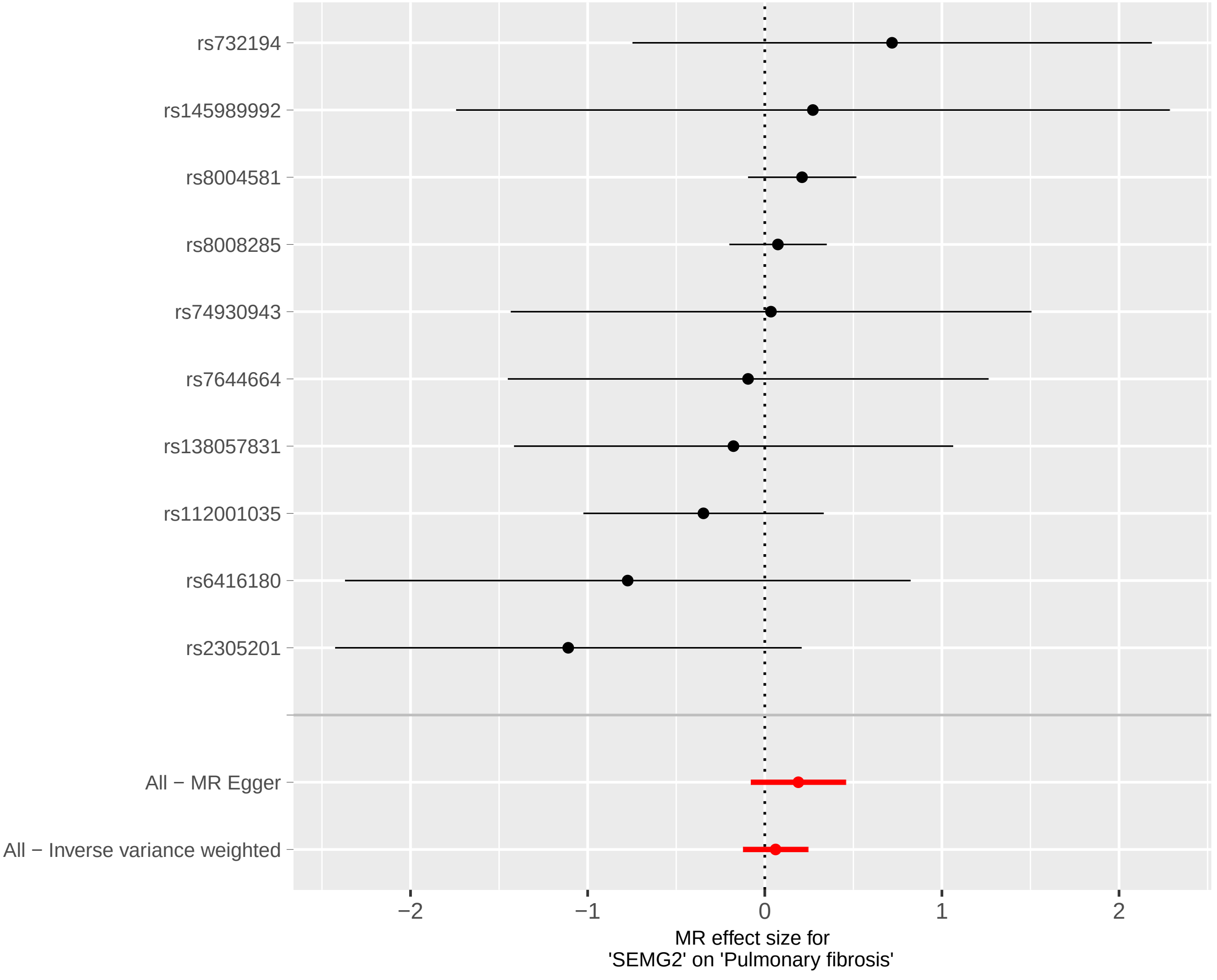
rs732194
rs145989992
rs8004581
rs8008285
rs74930943
rs7644664
rs138057831
rs112001035
rs6416180
rs2305201
All − MR Egger
All − Inverse variance weighted
−2
−1
0
1
2
MR effect size for
'SEMG2' on 'Pulmonary fibrosis'

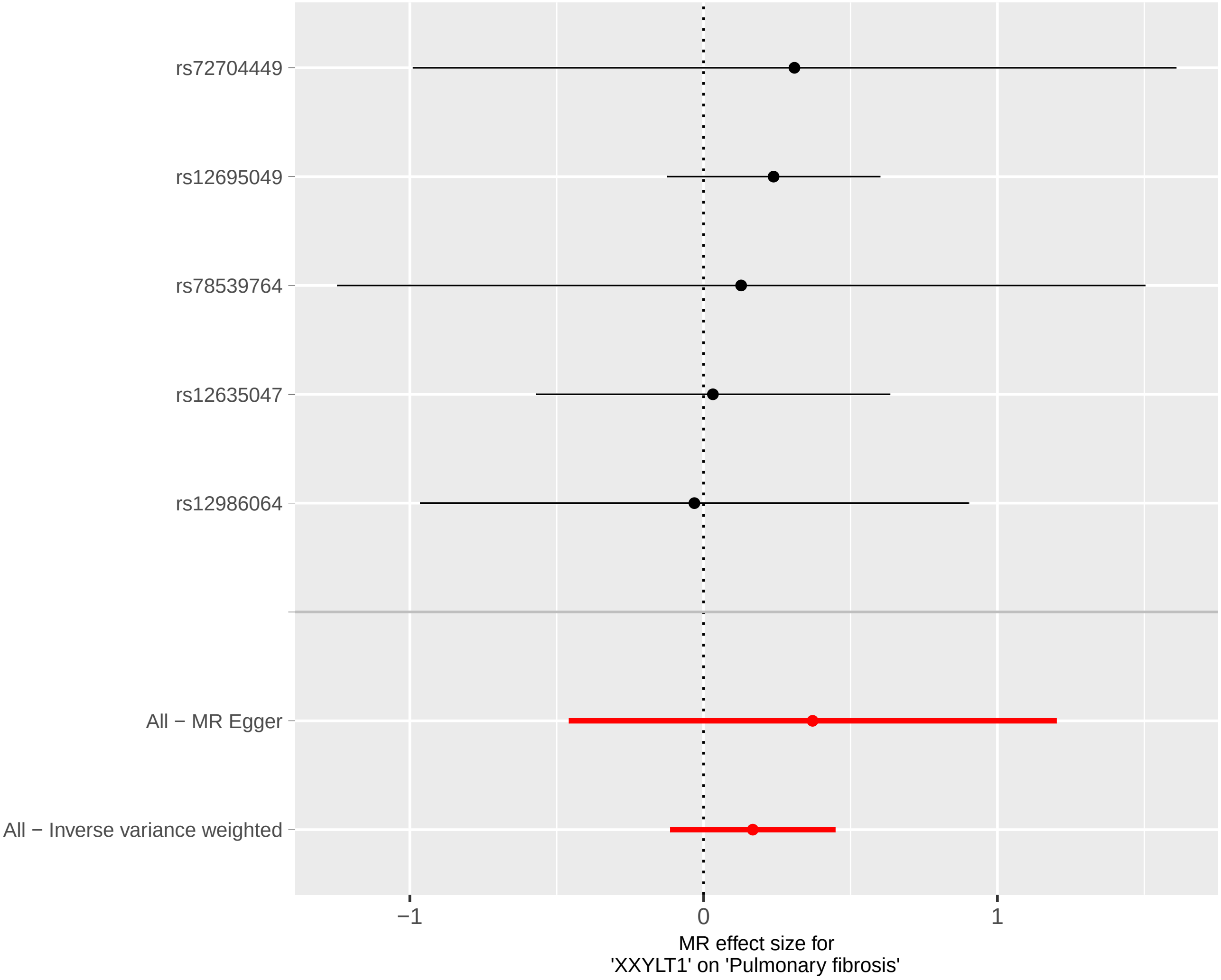

rs72704449
rs12695049
rs78539764
rs12635047
rs12986064
All − MR Egger
All − Inverse variance weighted
−1
0
1
MR effect size for
'XXYLT1' on 'Pulmonary fibrosis'

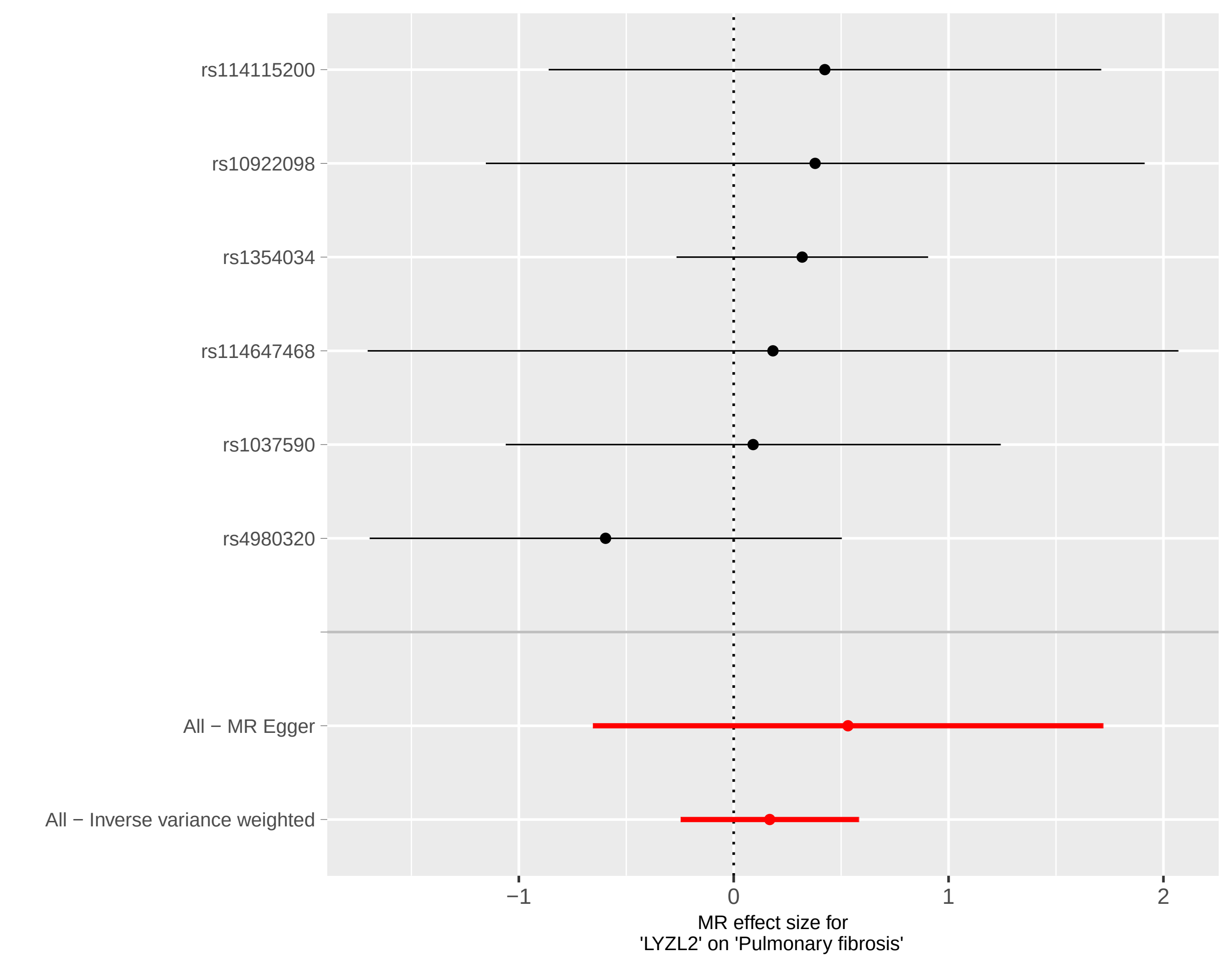

rs114115200
rs10922098
rs1354034
rs114647468
rs1037590
rs4980320
All − MR Egger
All − Inverse variance weighted
−1
0
1
2
MR effect size for
'LYZL2' on 'Pulmonary fibrosis'

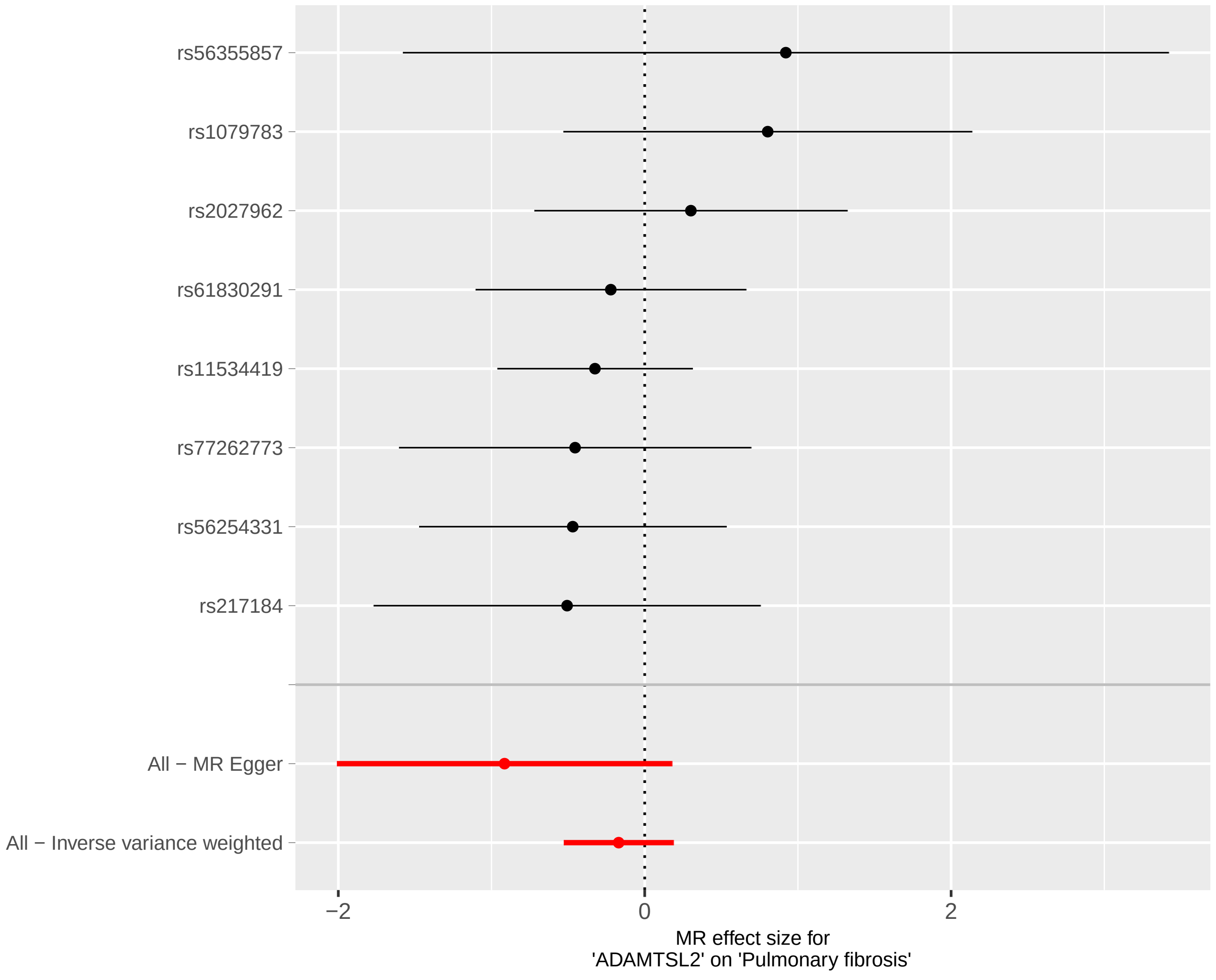
rs56355857
rs1079783
rs2027962
rs61830291
rs11534419
rs77262773
rs56254331
rs217184
All − MR Egger
All − Inverse variance weighted
−2
0
2
MR effect size for
'ADAMTSL2' on 'Pulmonary fibrosis'

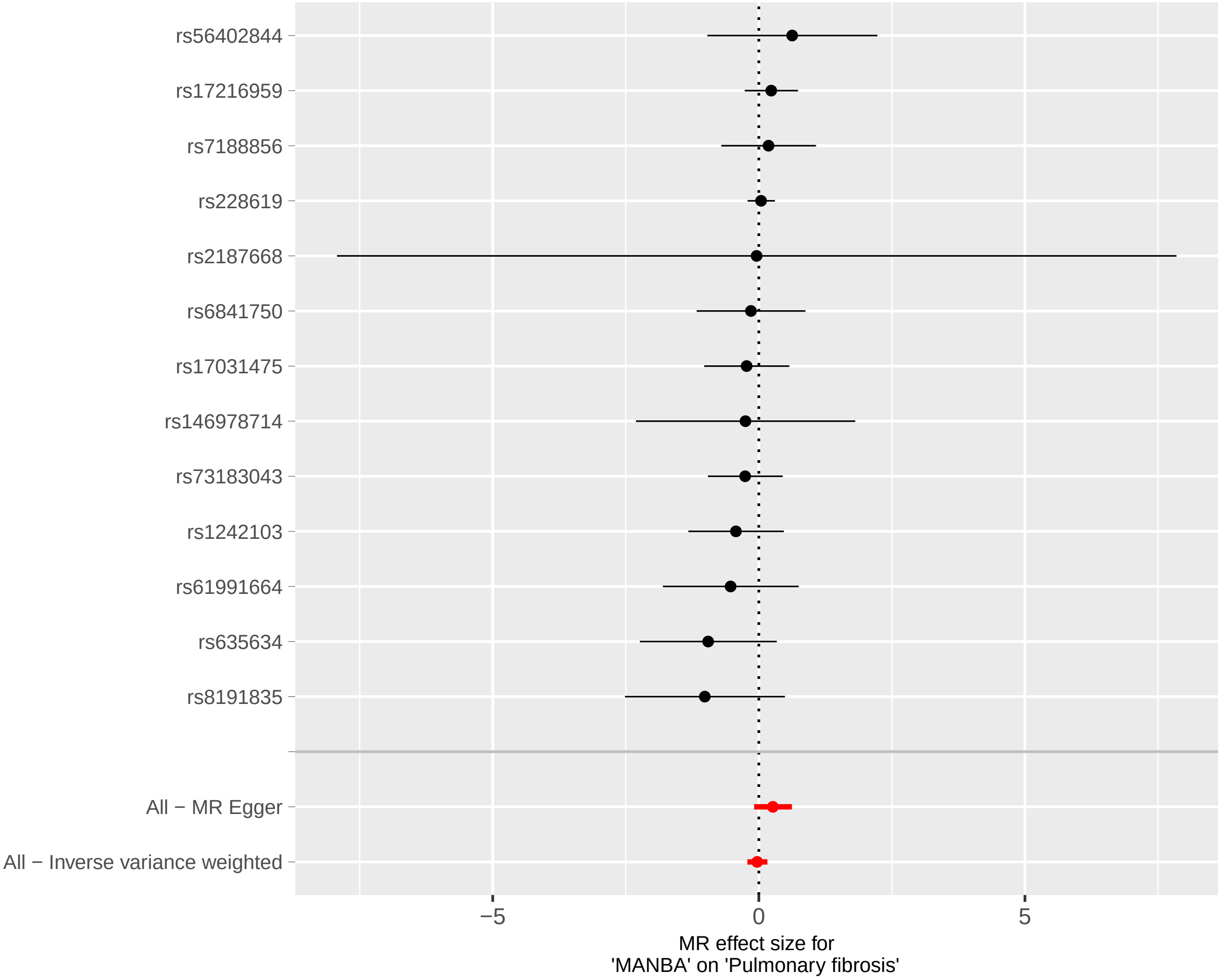

rs56402844
rs17216959
rs7188856
rs228619
rs2187668
rs6841750
rs17031475
rs146978714
rs73183043
rs1242103
rs61991664
rs635634
rs8191835
All − MR Egger
All − Inverse variance weighted
−5
0
5
MR effect size for
'MANBA' on 'Pulmonary fibrosis'

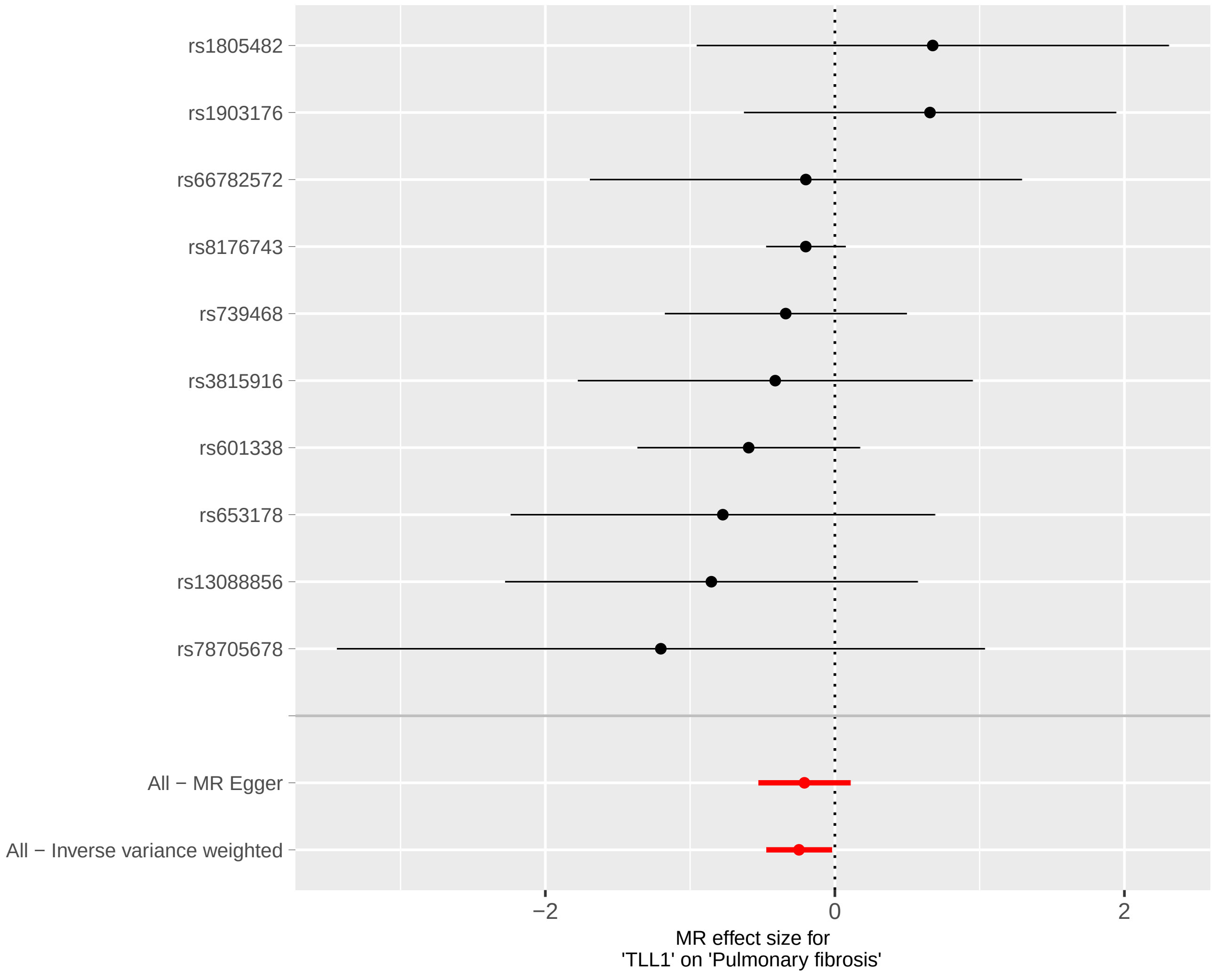

rs1805482
rs1903176
rs66782572
rs8176743
rs739468
rs3815916
rs601338
rs653178
rs13088856
rs78705678
All − MR Egger
All − Inverse variance weighted
−2
0
2
MR effect size for
'TLL1' on 'Pulmonary fibrosis'

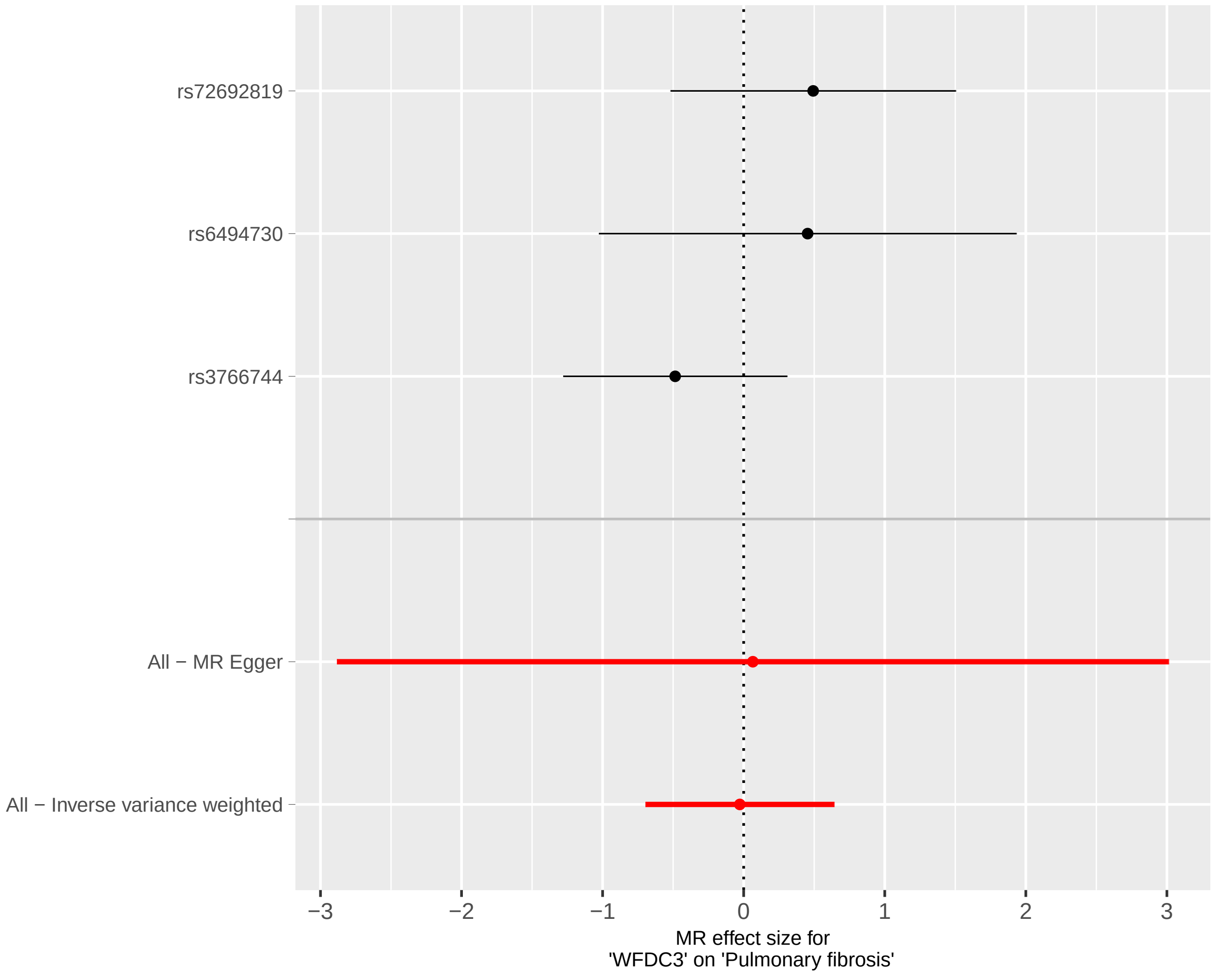
rs72692819
rs6494730
rs3766744
All − MR Egger
All − Inverse variance weighted
−3
−2
−1
0
1
2
3
MR effect size for
'WFDC3' on 'Pulmonary fibrosis'

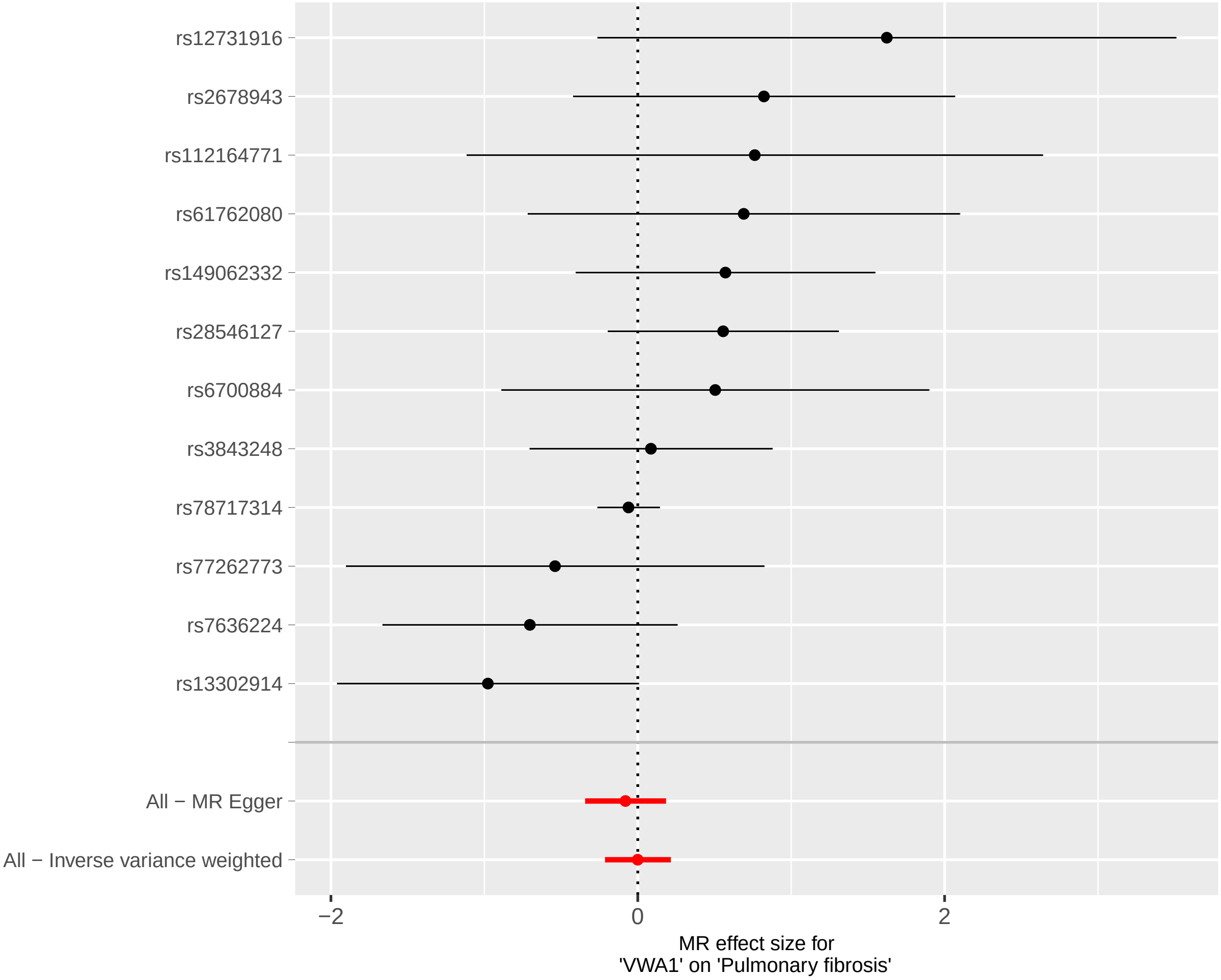

rs12731916
rs2678943
rs112164771
rs61762080
rs149062332
rs28546127
rs6700884
rs3843248
rs78717314
rs77262773
rs7636224
rs13302914
All − MR Egger
All − Inverse variance weighted
−2
0
2
MR effect size for
'VWA1' on 'Pulmonary fibrosis'

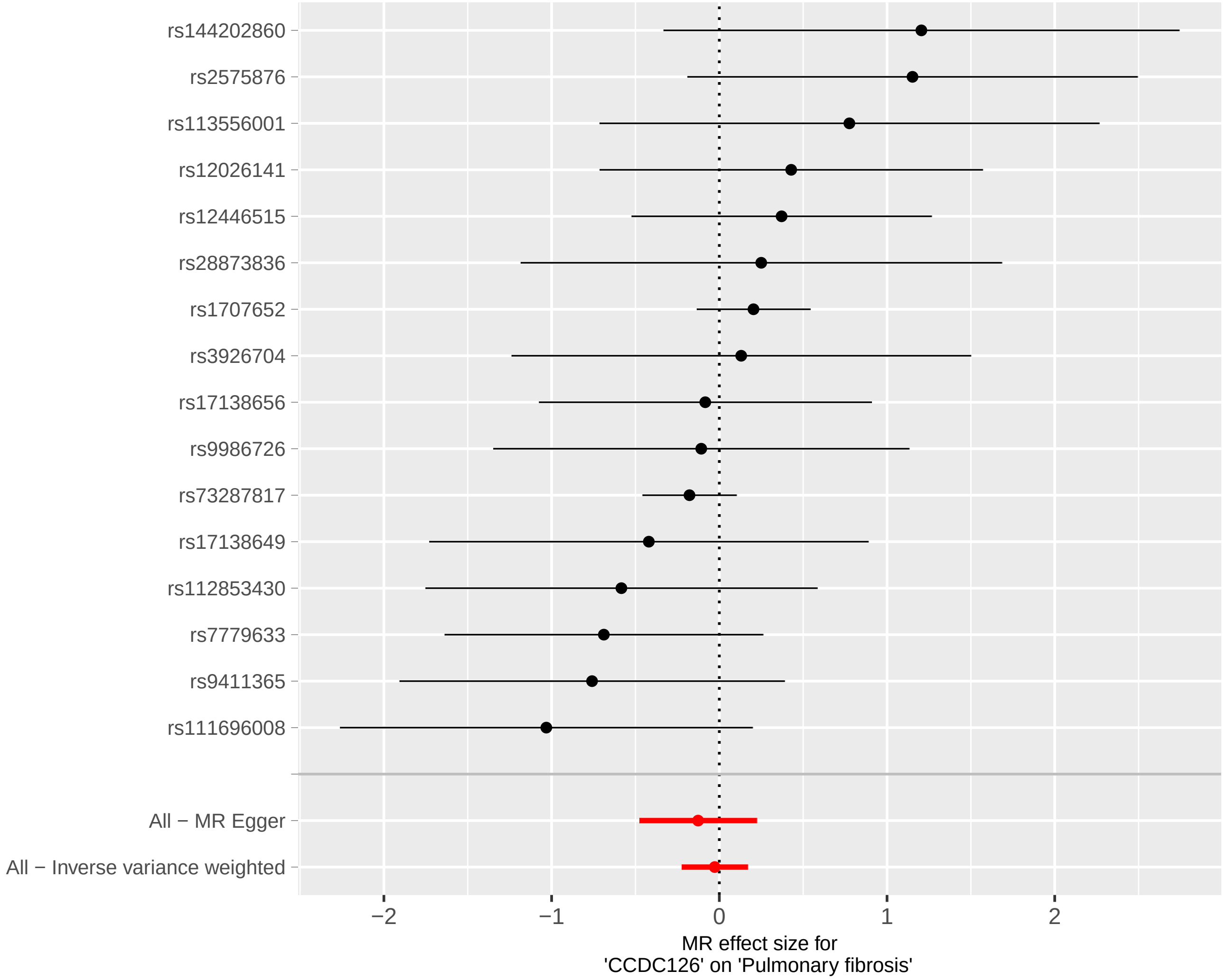
rs144202860
rs2575876
rs113556001
rs12026141
rs12446515
rs28873836
rs1707652
rs3926704
rs17138656
rs9986726
rs73287817
rs17138649
rs112853430
rs7779633
rs9411365
rs111696008
All − MR Egger
All − Inverse variance weighted
−2
−1
0
1
2
MR effect size for
'CCDC126' on 'Pulmonary fibrosis'

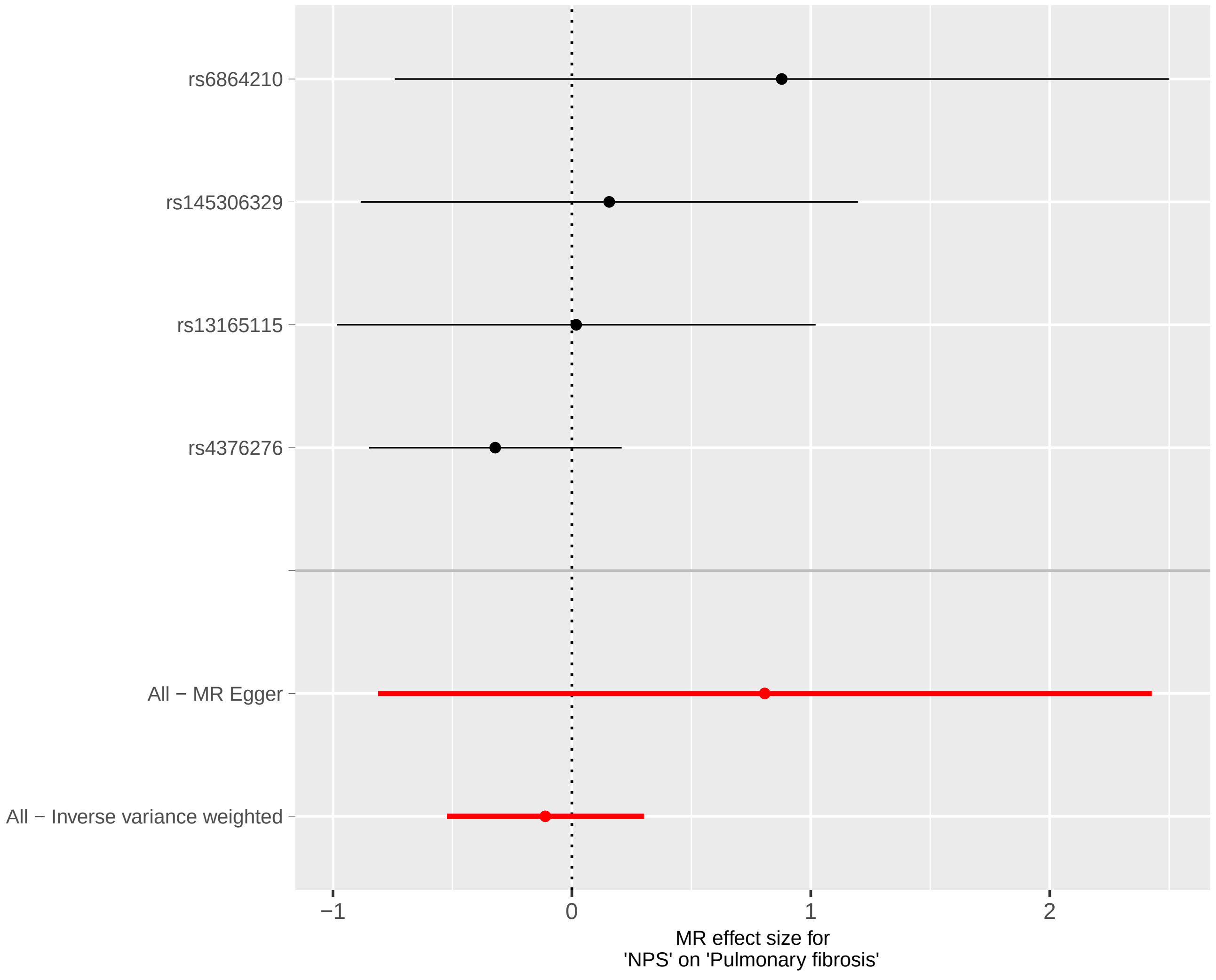

rs6864210
rs145306329
rs13165115
rs4376276
All − MR Egger
All − Inverse variance weighted
−1
0
1
2
MR effect size for
'NPS' on 'Pulmonary fibrosis'

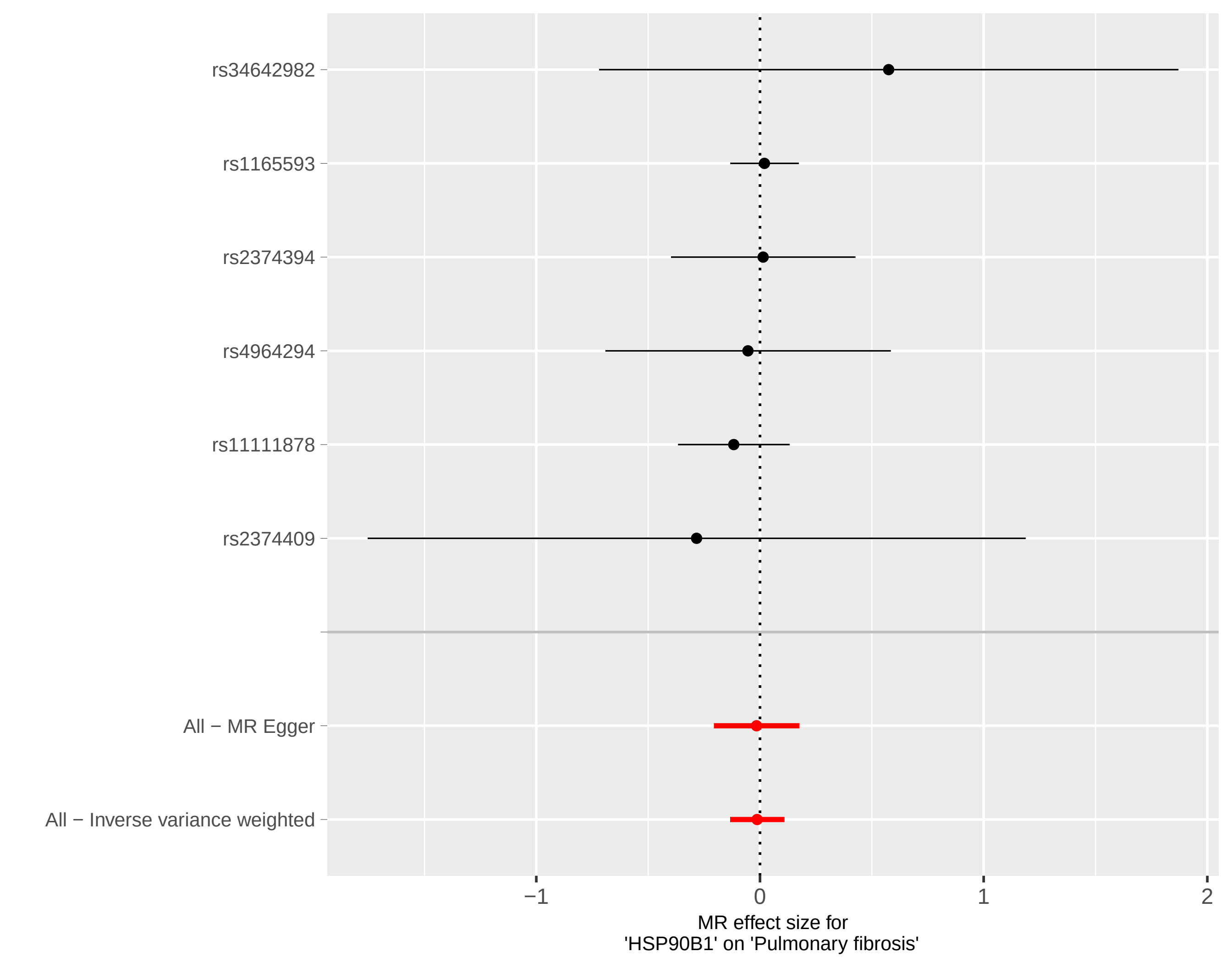
rs34642982
rs1165593
rs2374394
rs4964294
rs11111878
rs2374409
All − MR Egger
All − Inverse variance weighted
−1
0
1
2
MR effect size for
'HSP90B1' on 'Pulmonary fibrosis'

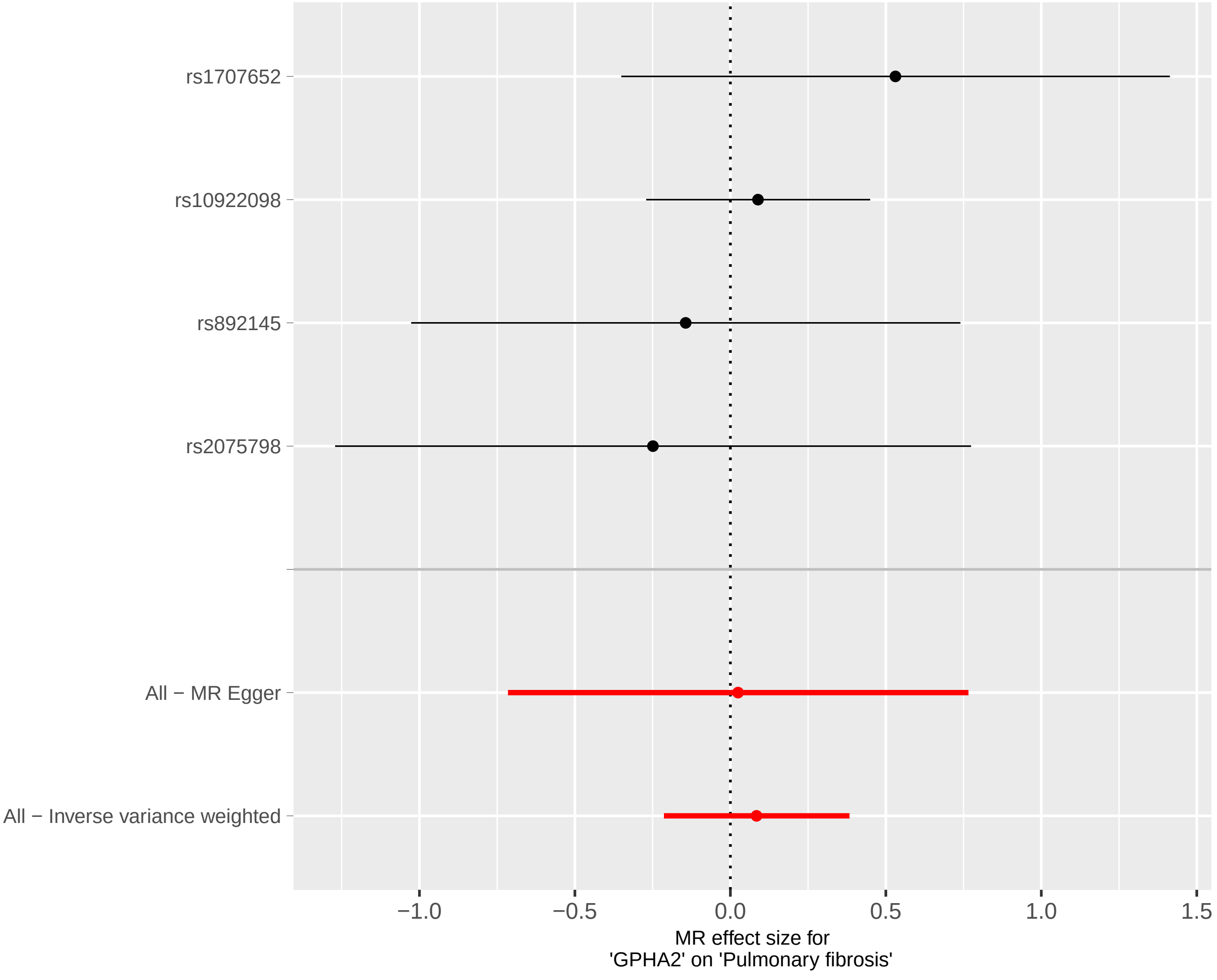

rs1707652
rs10922098
rs892145
rs2075798
All − MR Egger
All − Inverse variance weighted
−1.0
−0.5
0.0
0.5
1.0
1.5
MR effect size for
'GPHA2' on 'Pulmonary fibrosis'

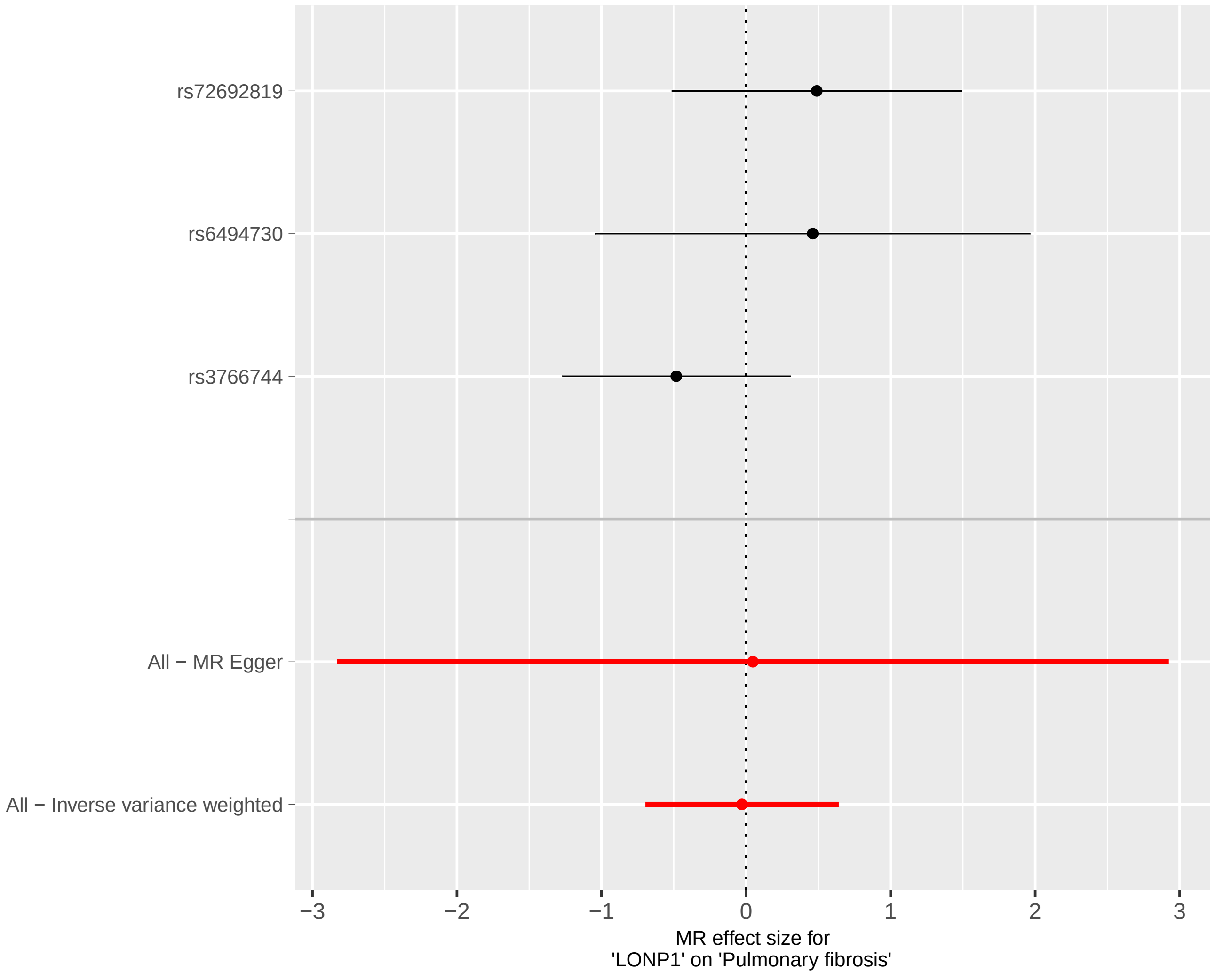
rs72692819
rs6494730
rs3766744
All − MR Egger
All − Inverse variance weighted
−3
−2
−1
0
1
2
3
MR effect size for
'LONP1' on 'Pulmonary fibrosis'

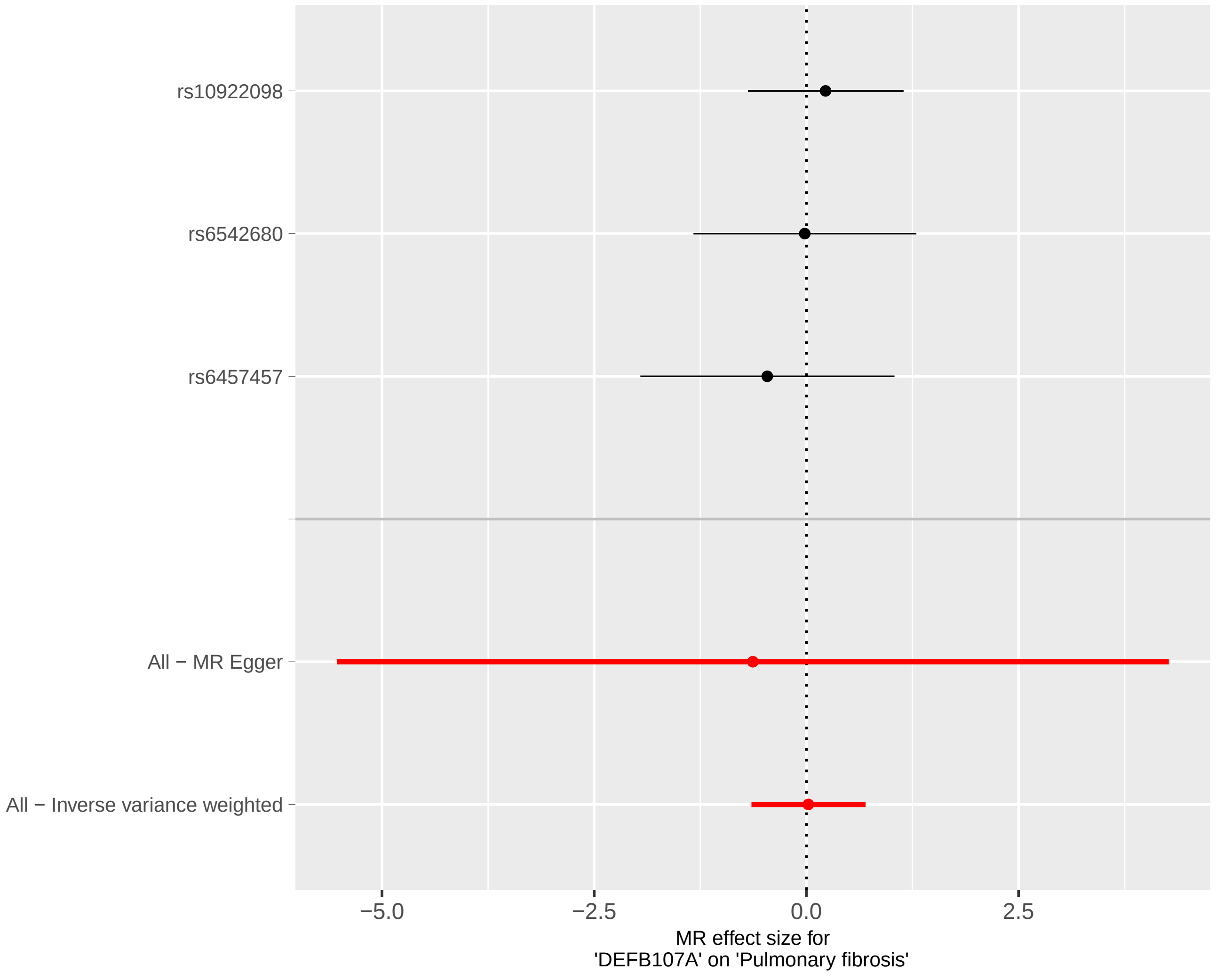
rs10922098
rs6542680
rs6457457
All − MR Egger
All − Inverse variance weighted
−5.0
−2.5
0.0
2.5
MR effect size for
'DEFB107A' on 'Pulmonary fibrosis'

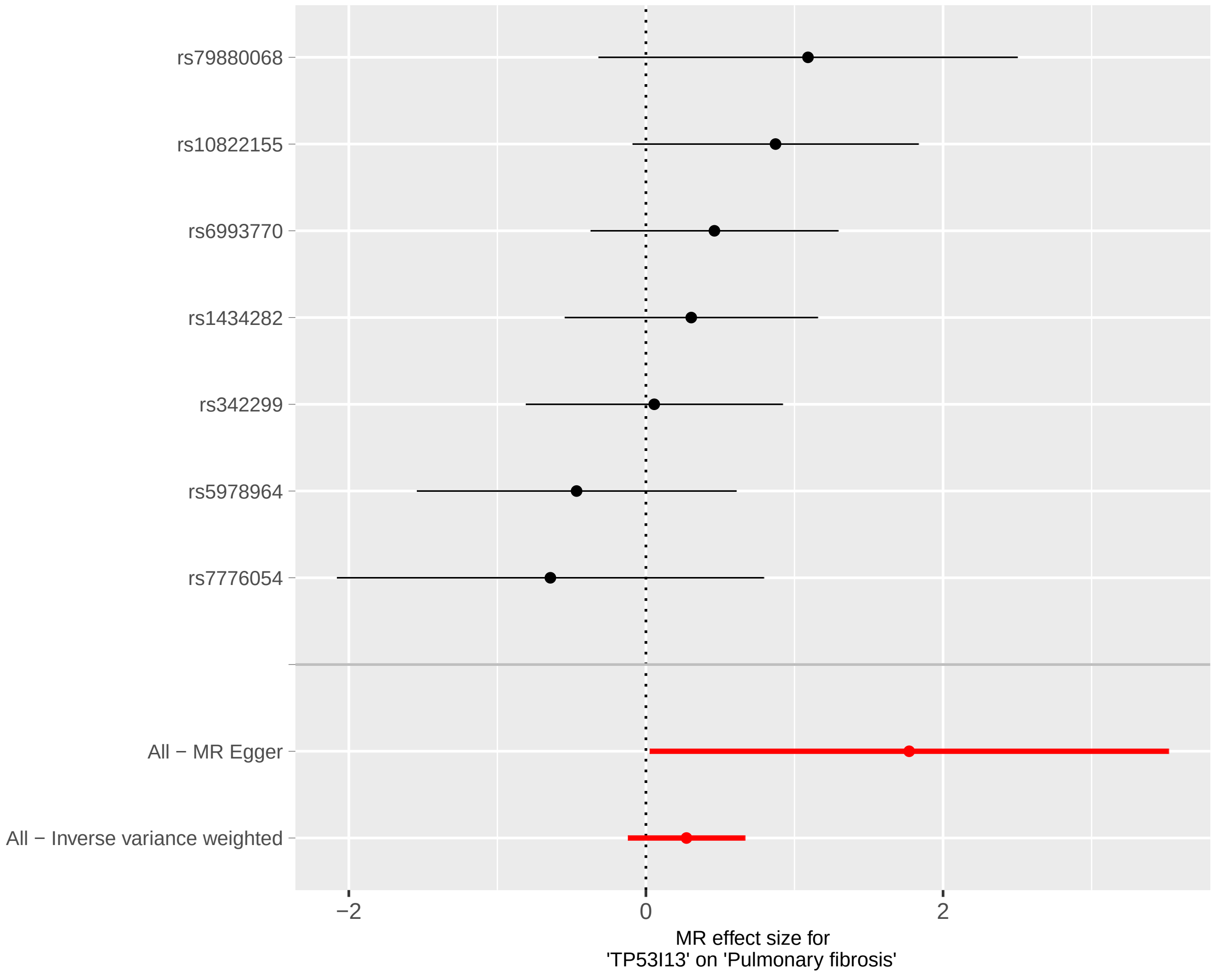
rs79880068
rs10822155
rs6993770
rs1434282
rs342299
rs5978964
rs7776054
All − MR Egger
All − Inverse variance weighted
−2
0
2
MR effect size for
'TP53I13' on 'Pulmonary fibrosis'

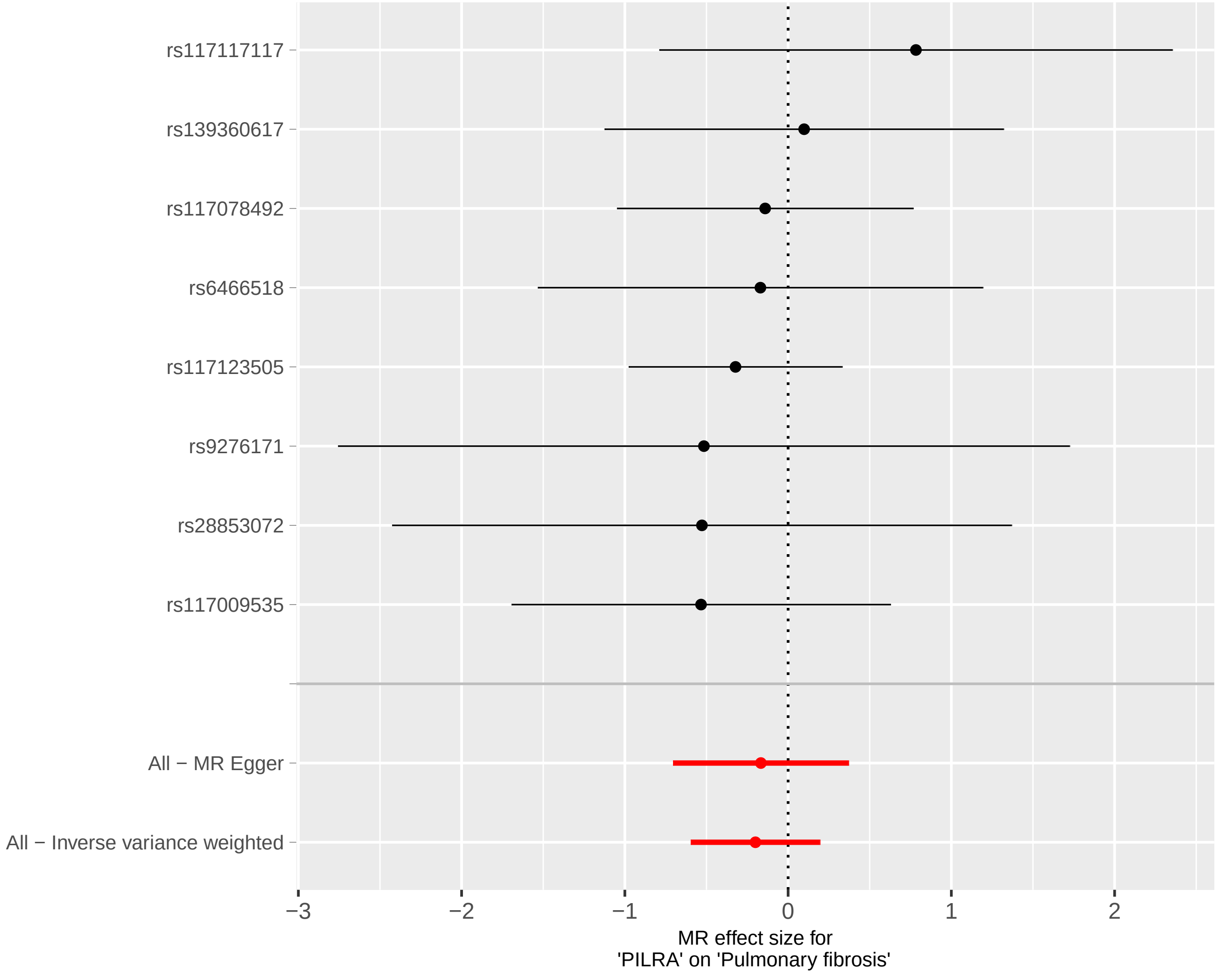

rs117117117
rs139360617
rs117078492
rs6466518
rs117123505
rs9276171
rs28853072
rs117009535
All − MR Egger
All − Inverse variance weighted
−3
−2
−1
0
1
2
MR effect size for
'PILRA' on 'Pulmonary fibrosis'

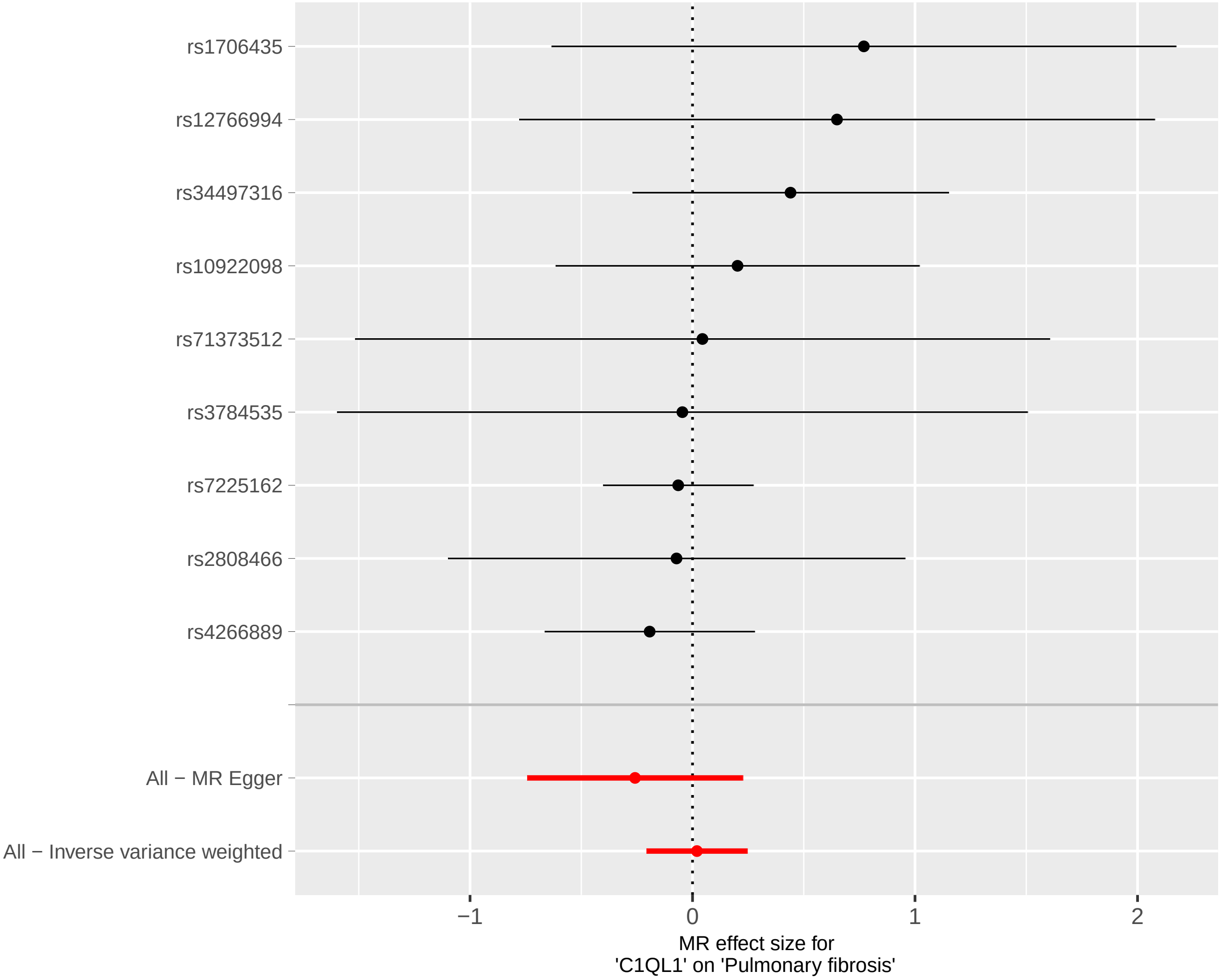

rs1706435
rs12766994
rs34497316
rs10922098
rs71373512
rs3784535
rs7225162
rs2808466
rs4266889
All − MR Egger
All − Inverse variance weighted
−1
0
1
2
MR effect size for
'C1QL1' on 'Pulmonary fibrosis'

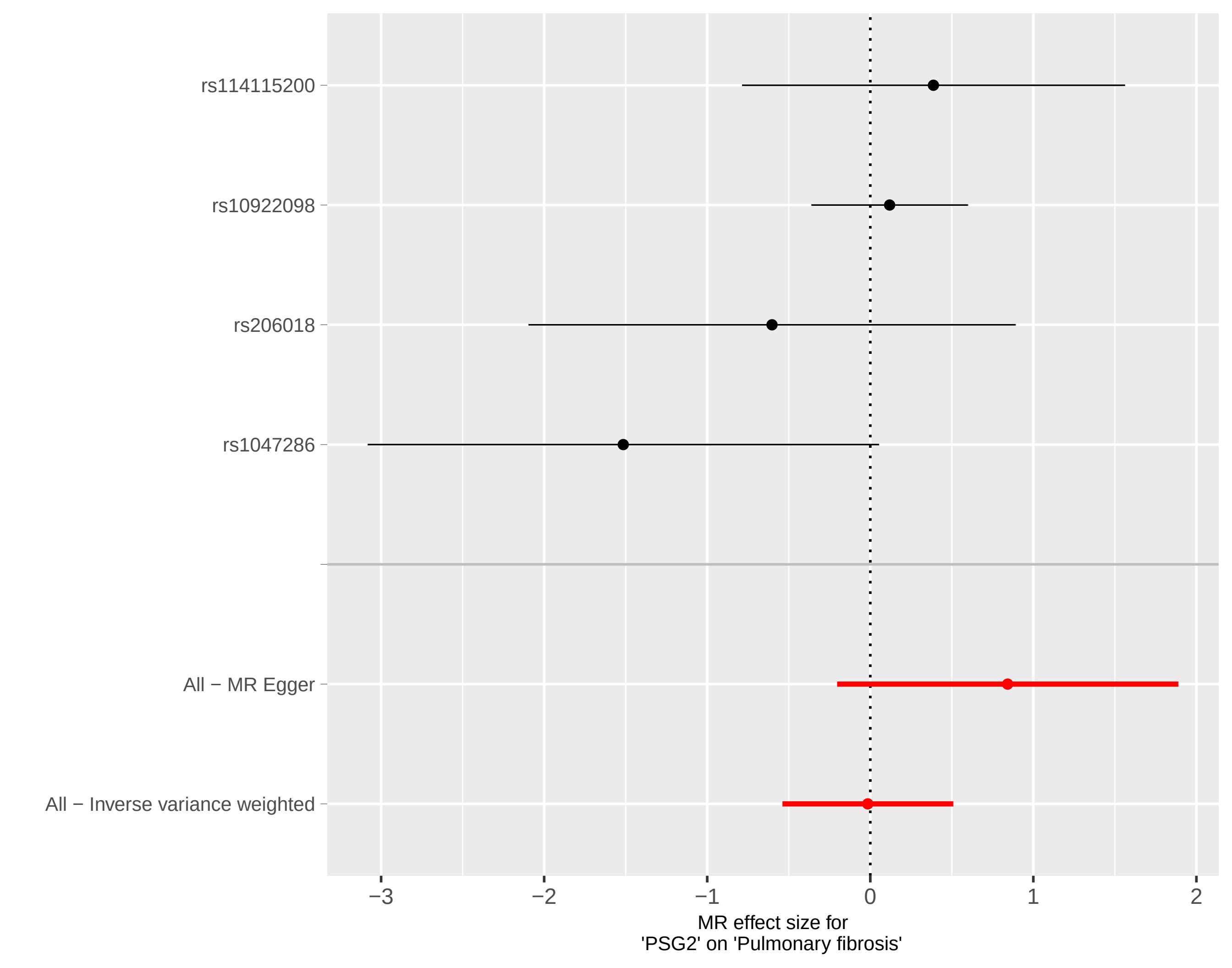
rs114115200
rs10922098
rs206018
rs1047286
All − MR Egger
All − Inverse variance weighted
−3
−2
−1
0
1
2
MR effect size for
'PSG2' on 'Pulmonary fibrosis'

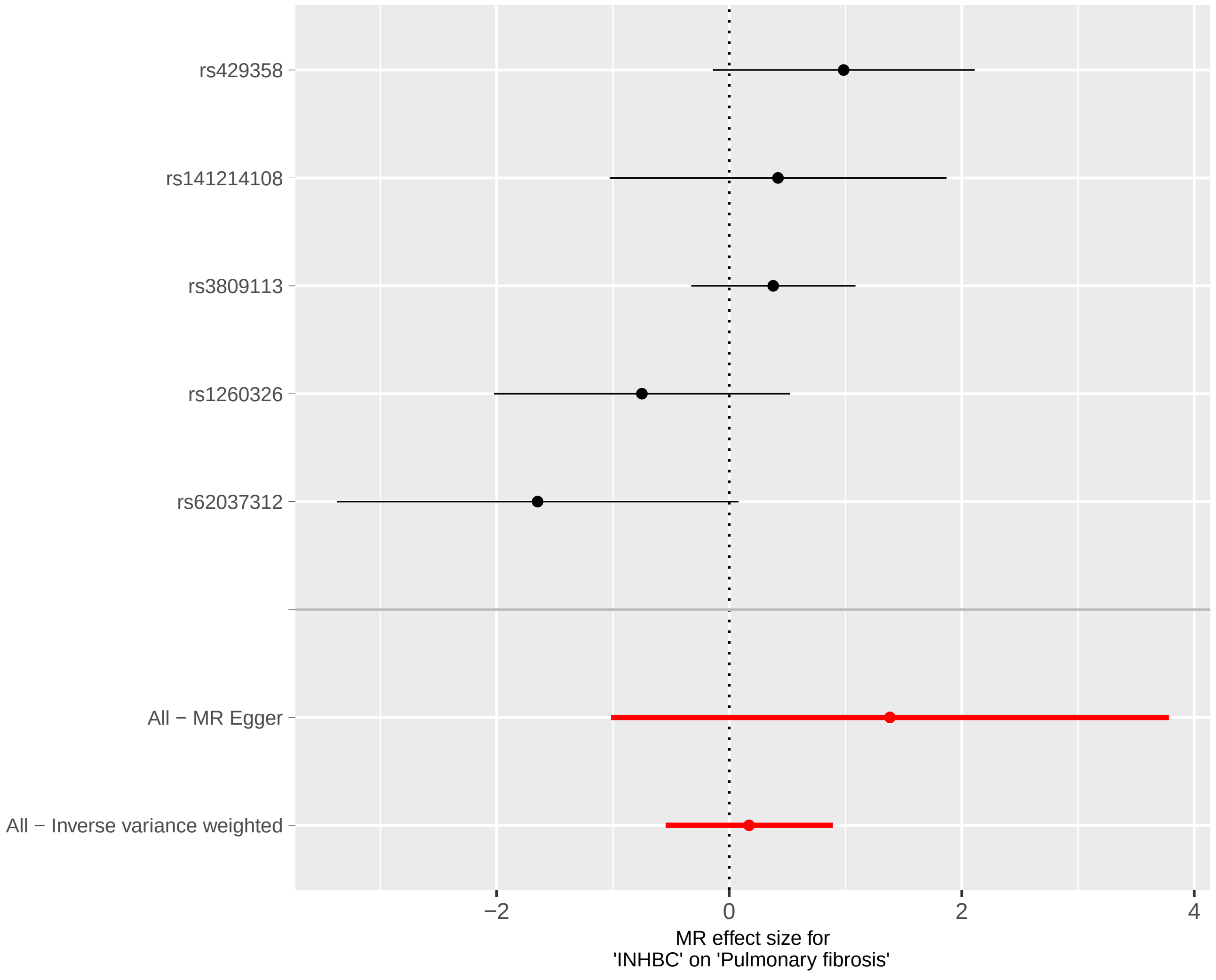
rs429358
rs141214108
rs3809113
rs1260326
rs62037312
All − MR Egger
All − Inverse variance weighted
−2
0
2
4
MR effect size for
'INHBC' on 'Pulmonary fibrosis'

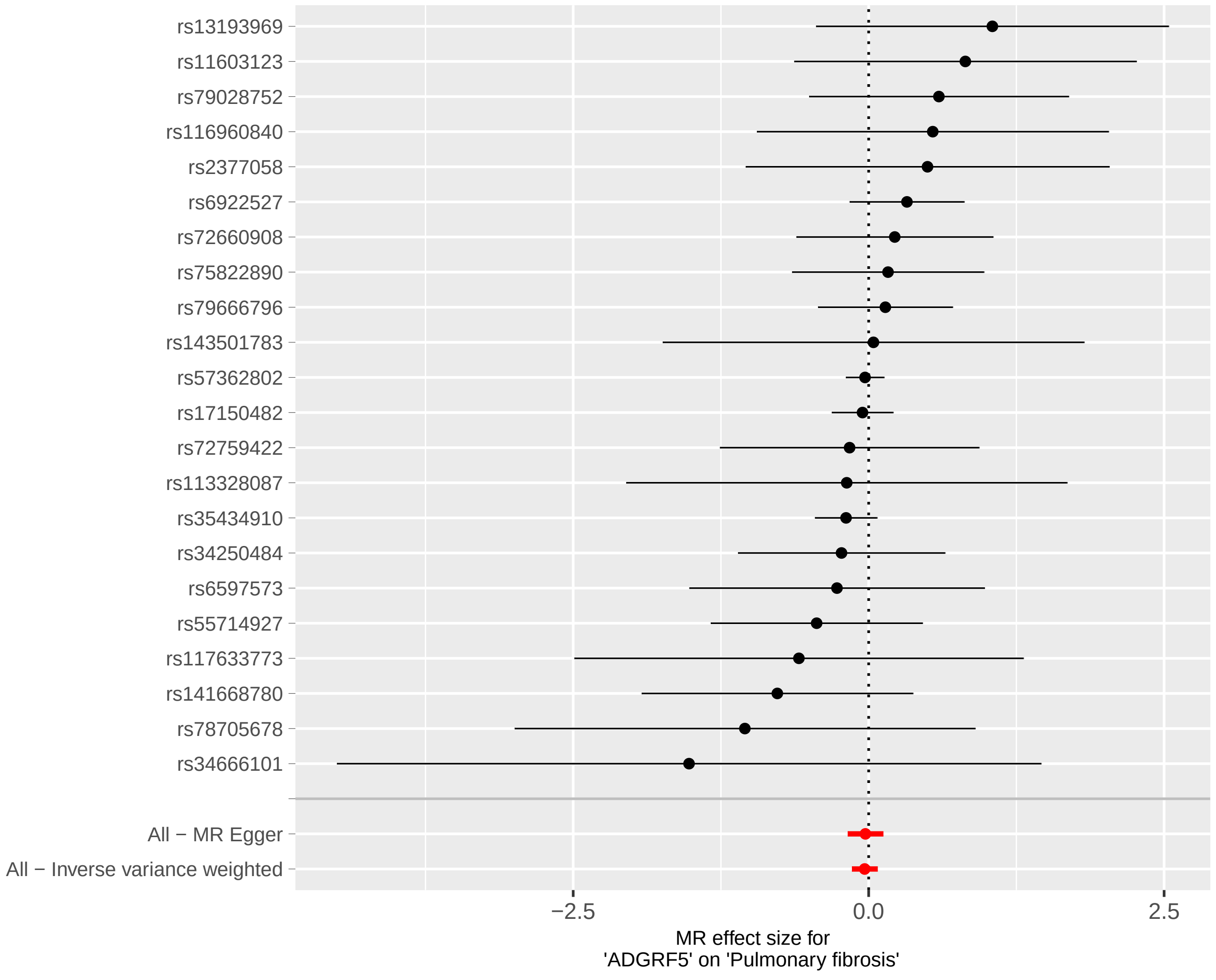
rs13193969
rs11603123
rs79028752
rs116960840
rs2377058
rs6922527
rs72660908
rs75822890
rs79666796
rs143501783
rs57362802
rs17150482
rs72759422
rs113328087
rs35434910
rs34250484
rs6597573
rs55714927
rs117633773
rs141668780
rs78705678
rs34666101
All − MR Egger
All − Inverse variance weighted
−2.5
0.0
2.5
MR effect size for
'ADGRF5' on 'Pulmonary fibrosis'

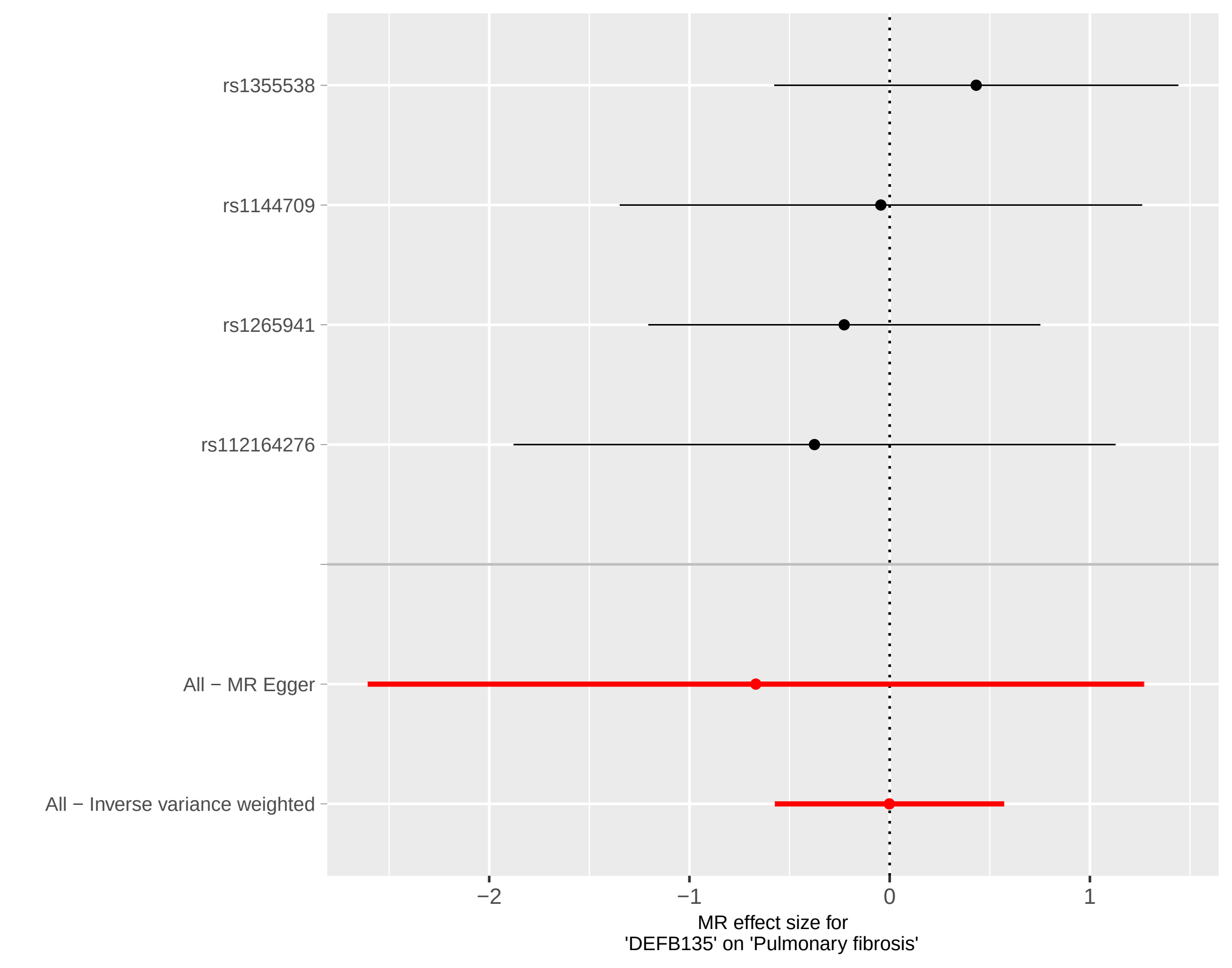
rs1355538
rs1144709
rs1265941
rs112164276
All − MR Egger
All − Inverse variance weighted
−2
−1
0
1
MR effect size for
'DEFB135' on 'Pulmonary fibrosis'

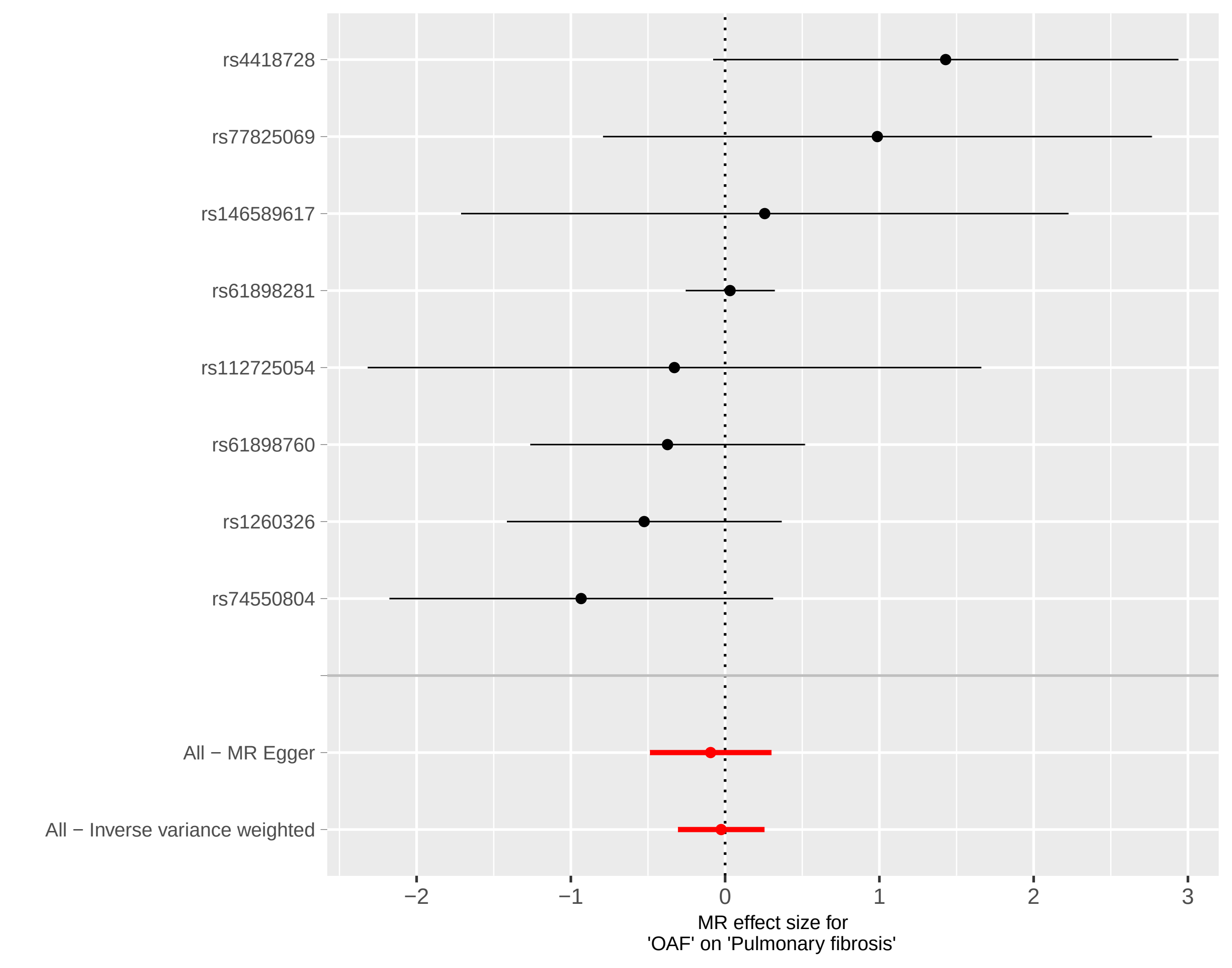
rs4418728
rs77825069
rs146589617
rs61898281
rs112725054
rs61898760
rs1260326
rs74550804
All − MR Egger
All − Inverse variance weighted
−2
−1
0
1
2
3
MR effect size for
'OAF' on 'Pulmonary fibrosis'

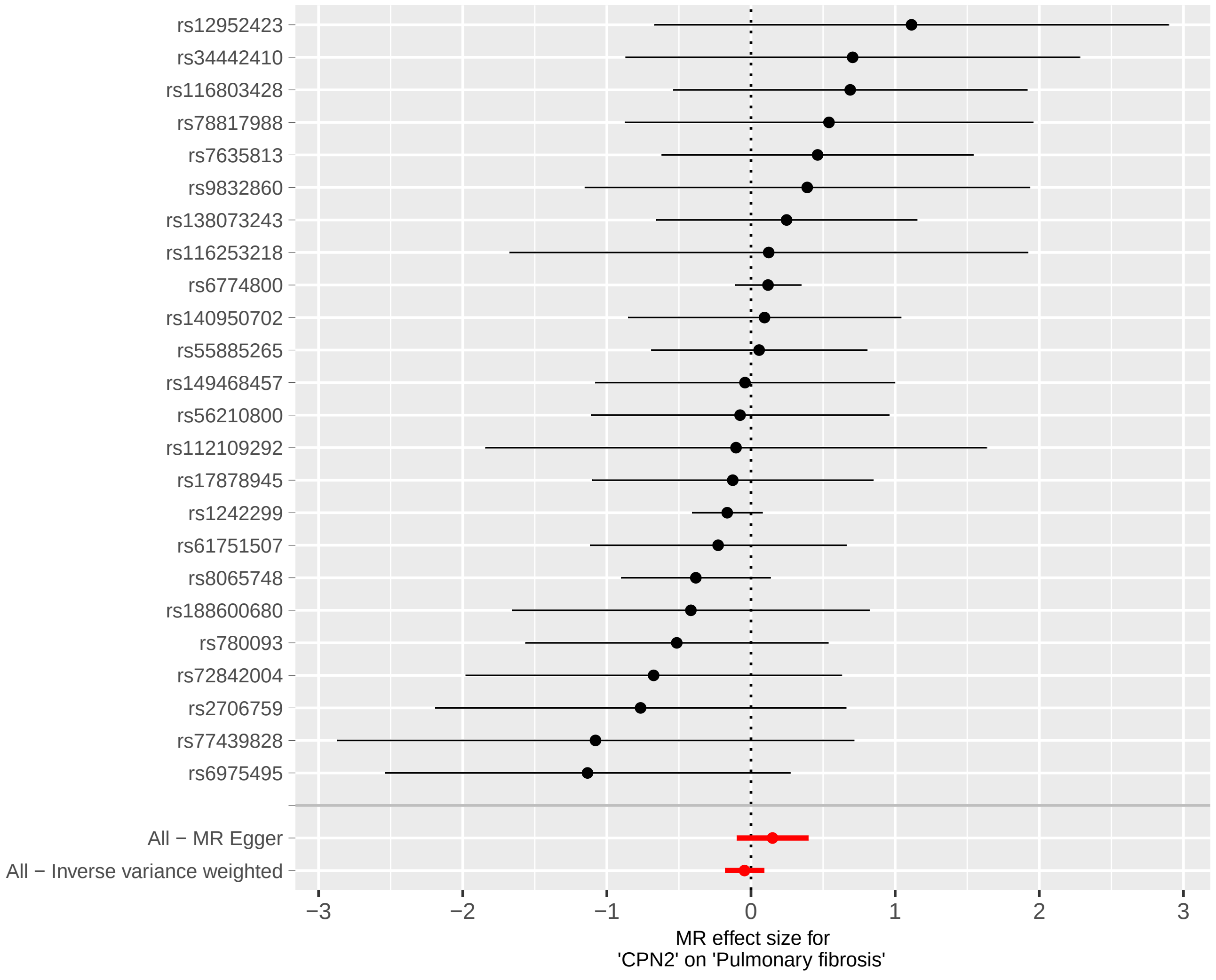
rs12952423
rs34442410
rs116803428
rs78817988
rs7635813
rs9832860
rs138073243
rs116253218
rs6774800
rs140950702
rs55885265
rs149468457
rs56210800
rs112109292
rs17878945
rs1242299
rs61751507
rs8065748
rs188600680
rs780093
rs72842004
rs2706759
rs77439828
rs6975495
All − MR Egger
All − Inverse variance weighted
−3
−2
−1
0
1
2
3
MR effect size for
'CPN2' on 'Pulmonary fibrosis'

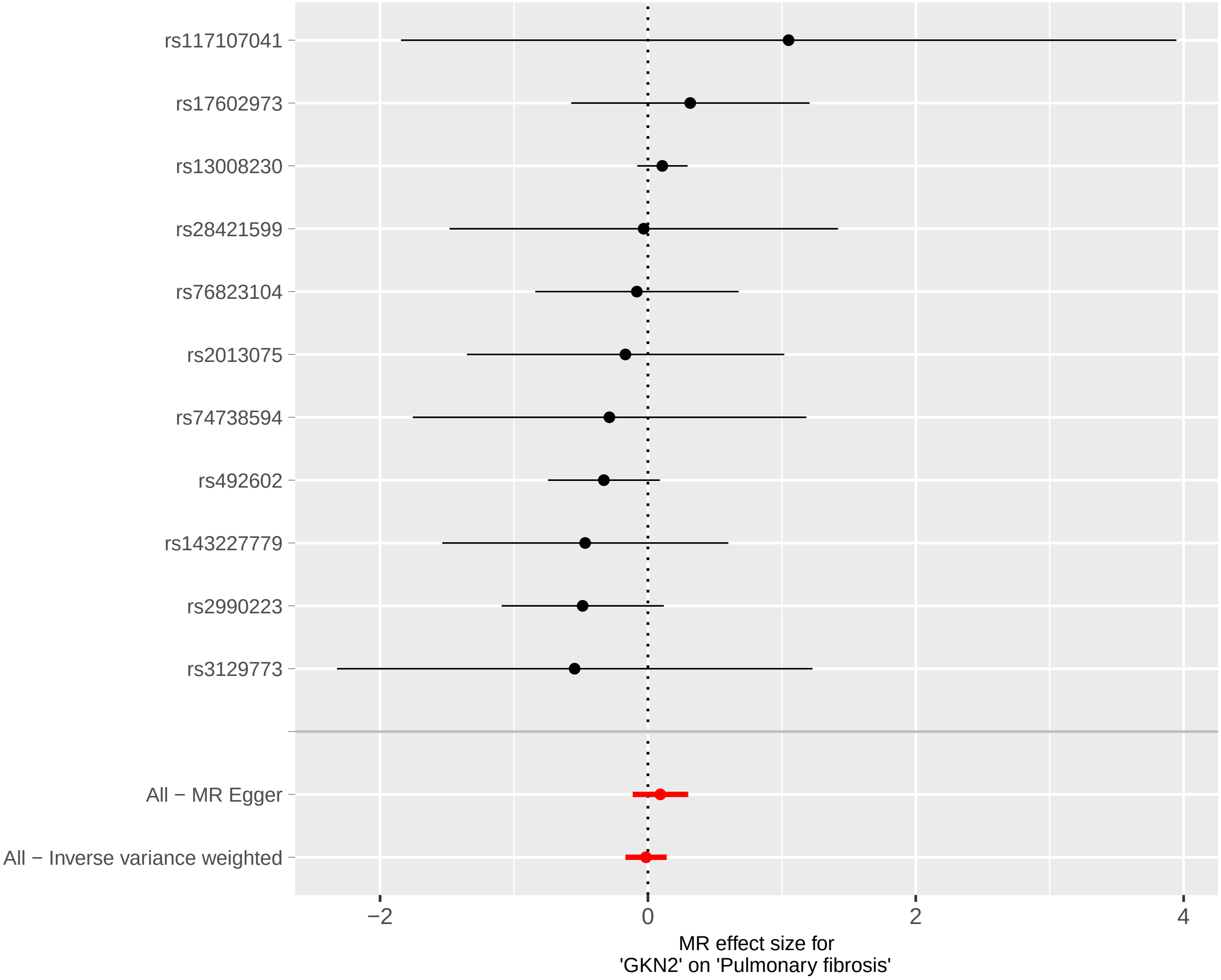

rs117107041
rs17602973
rs13008230
rs28421599
rs76823104
rs2013075
rs74738594
rs492602
rs143227779
rs2990223
rs3129773
All − MR Egger
All − Inverse variance weighted
−2
0
2
4
MR effect size for
'GKN2' on 'Pulmonary fibrosis'

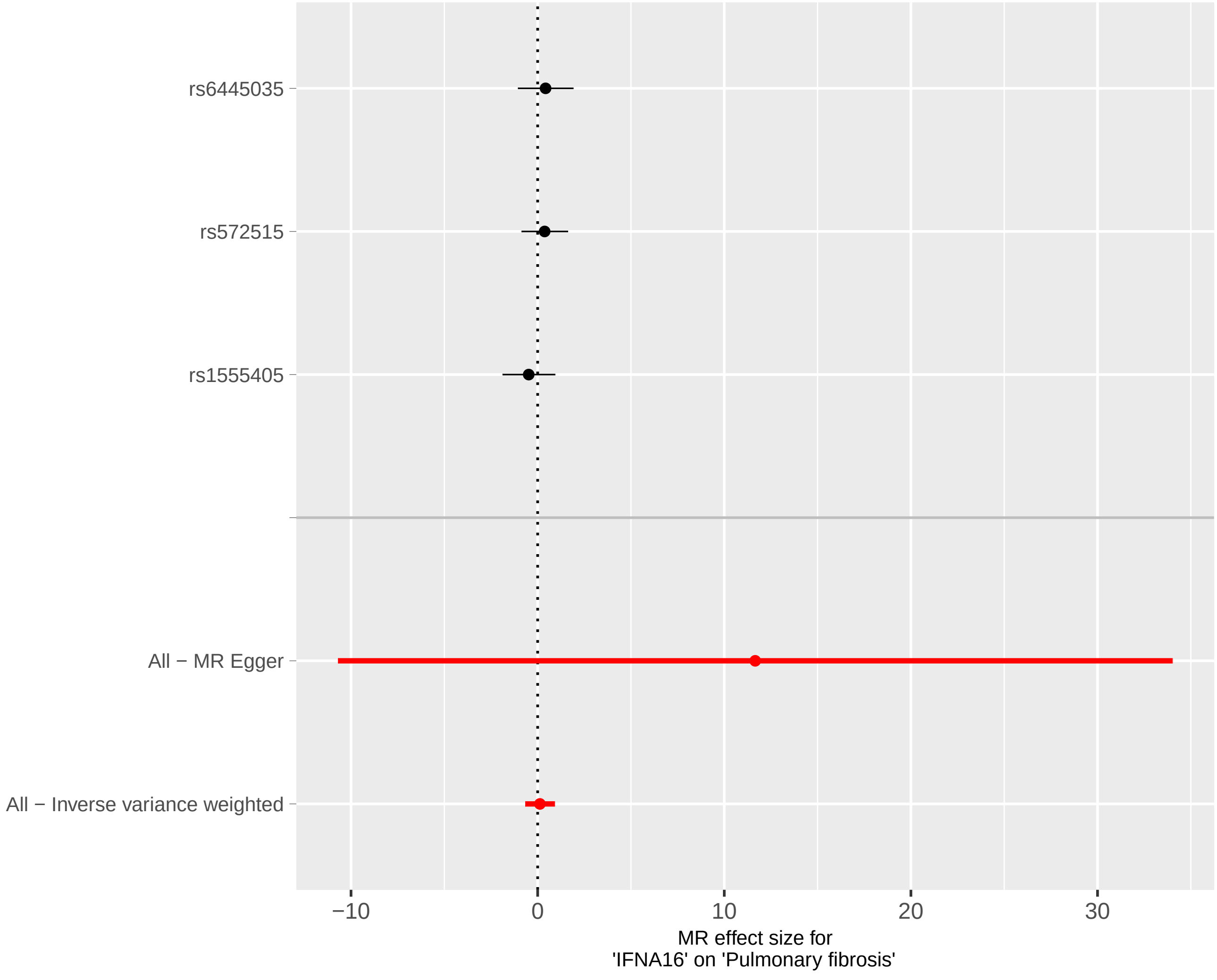

rs6445035
rs572515
rs1555405
All − MR Egger
All − Inverse variance weighted
−10
0
10
20
30
MR effect size for
'IFNA16' on 'Pulmonary fibrosis'

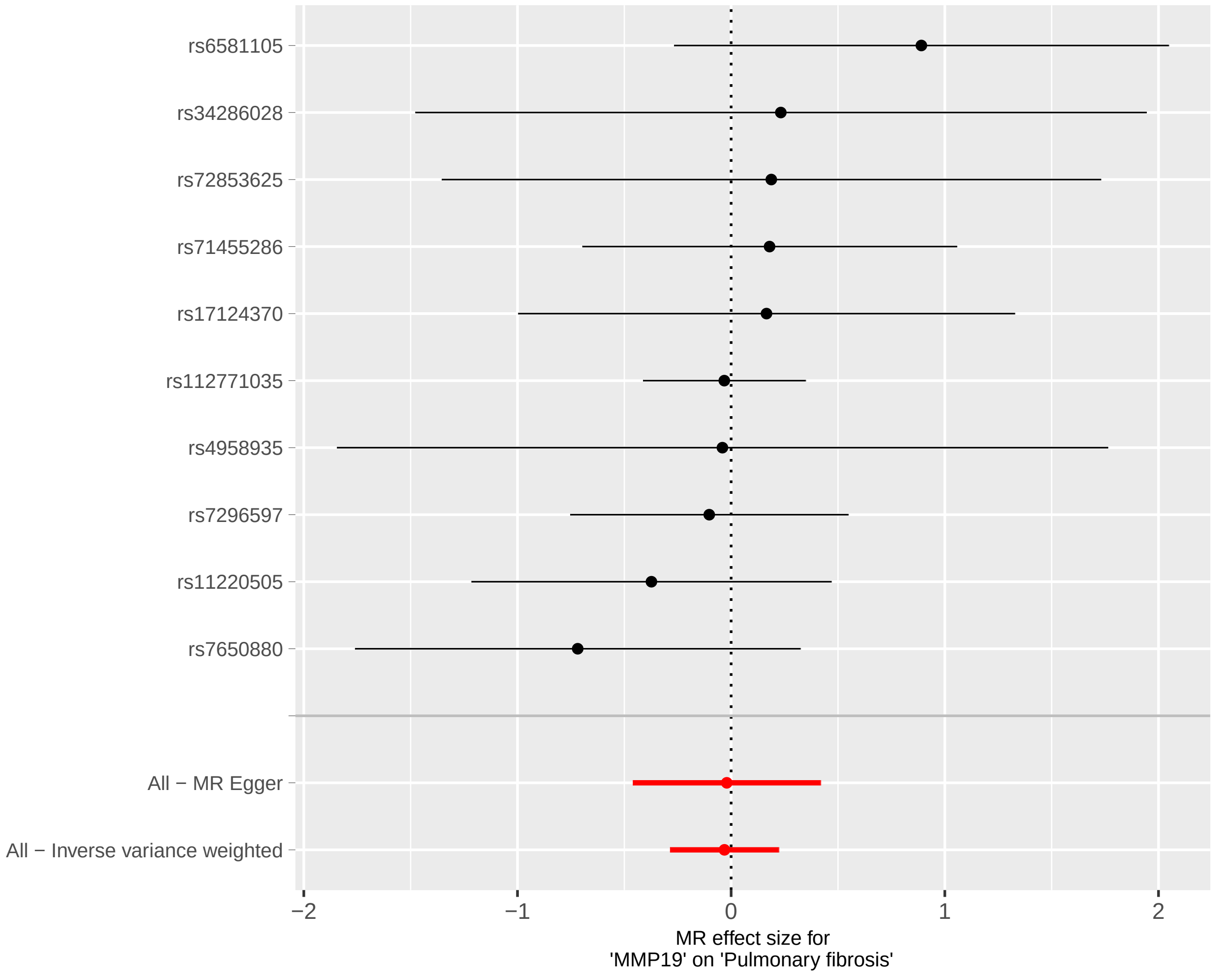

rs6581105
rs34286028
rs72853625
rs71455286
rs17124370
rs112771035
rs4958935
rs7296597
rs11220505
rs7650880
All − MR Egger
All − Inverse variance weighted
−2
−1
0
1
2
MR effect size for
'MMP19' on 'Pulmonary fibrosis'

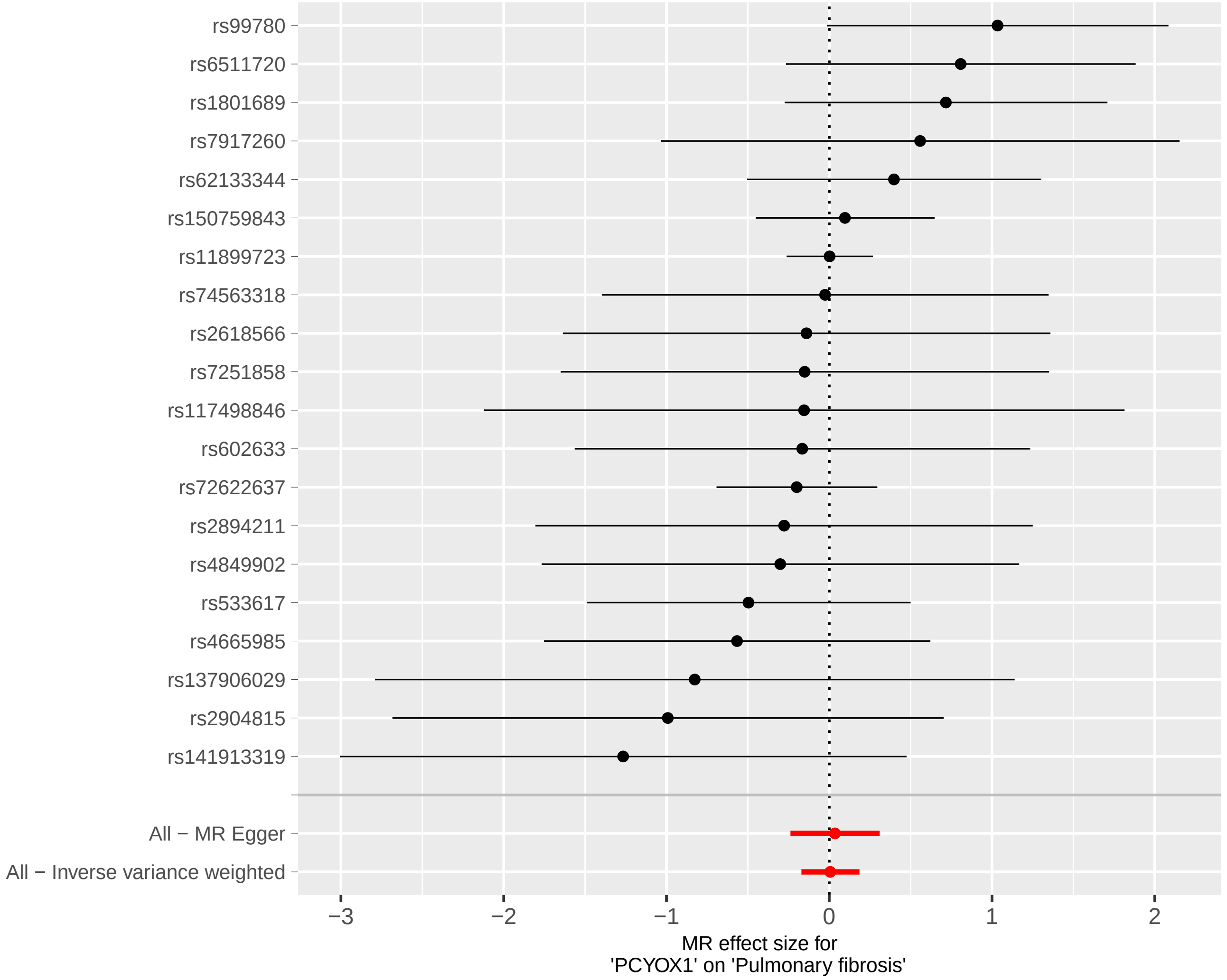
rs99780
rs6511720
rs1801689
rs7917260
rs62133344
rs150759843
rs11899723
rs74563318
rs2618566
rs7251858
rs117498846
rs602633
rs72622637
rs2894211
rs4849902
rs533617
rs4665985
rs137906029
rs2904815
rs141913319
All − MR Egger
All − Inverse variance weighted
−3
−2
−1
0
1
2
MR effect size for
'PCYOX1' on 'Pulmonary fibrosis'

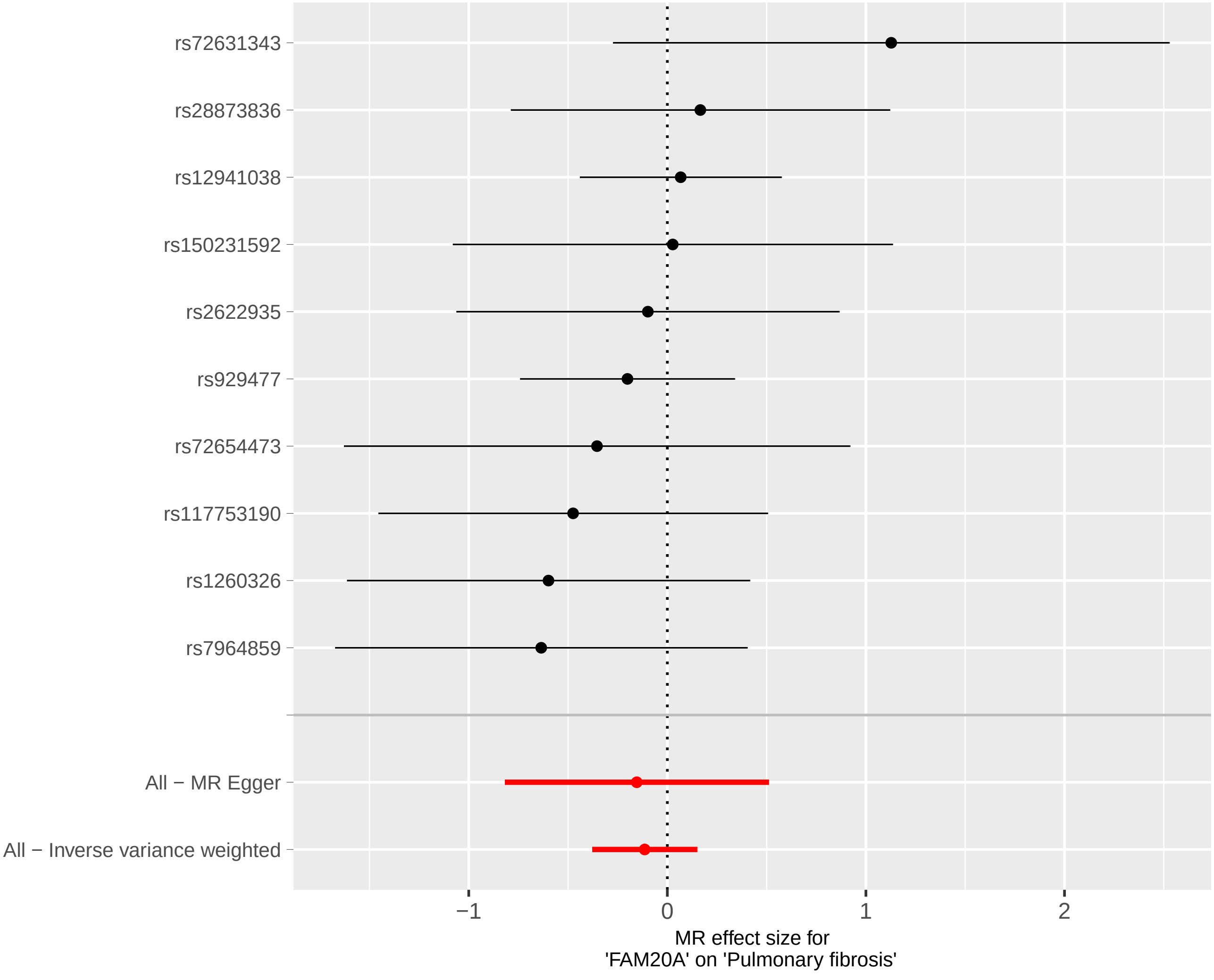
rs72631343
rs28873836
rs12941038
rs150231592
rs2622935
rs929477
rs72654473
rs117753190
rs1260326
rs7964859
All − MR Egger
All − Inverse variance weighted
−1
0
1
2
MR effect size for
'FAM20A' on 'Pulmonary fibrosis'

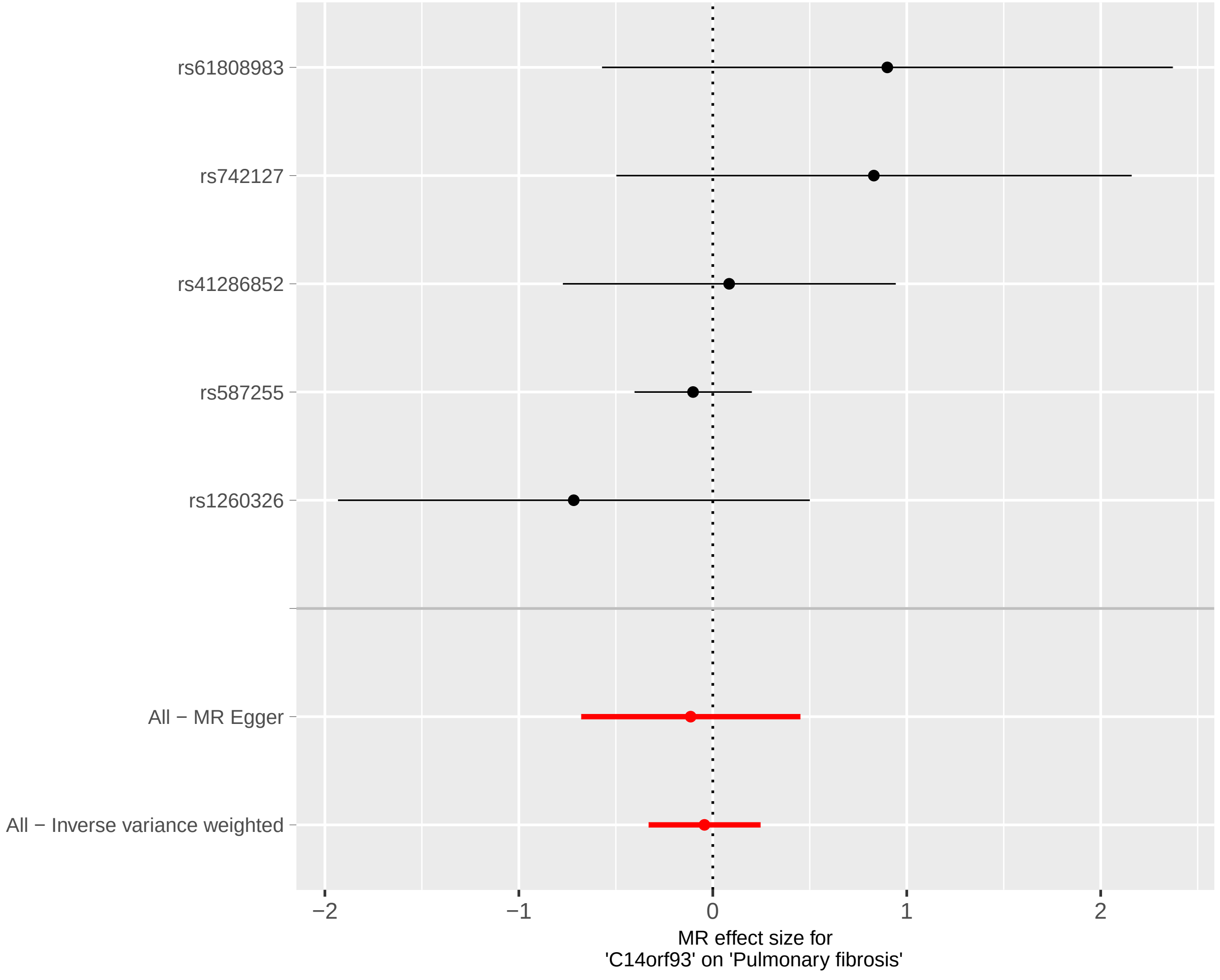

rs61808983
rs742127
rs41286852
rs587255
rs1260326
All − MR Egger
All − Inverse variance weighted
−2
−1
0
1
2
MR effect size for
'C14orf93' on 'Pulmonary fibrosis'

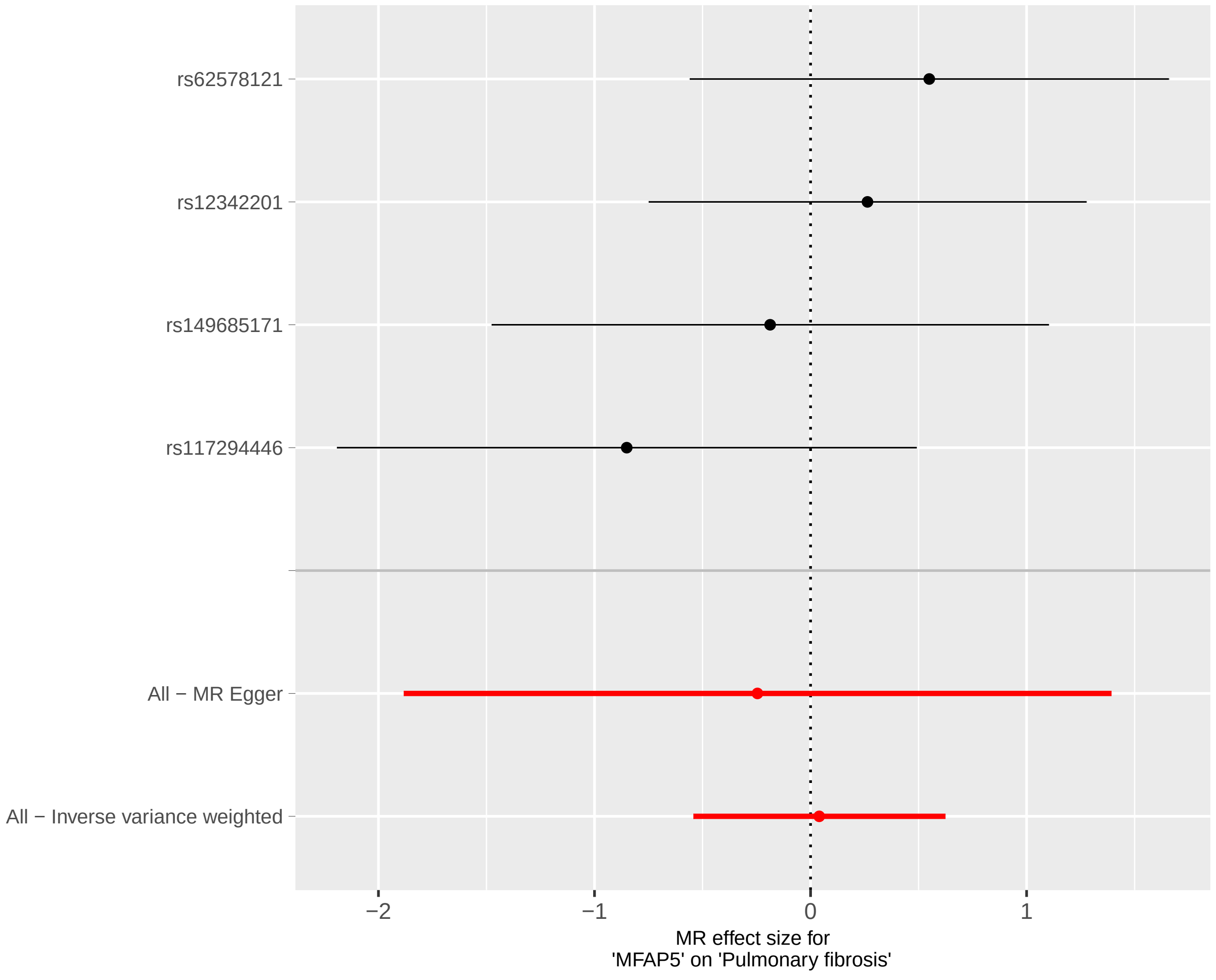
rs62578121
rs12342201
rs149685171
rs117294446
All − MR Egger
All − Inverse variance weighted
−2
−1
0
1
MR effect size for
'MFAP5' on 'Pulmonary fibrosis'

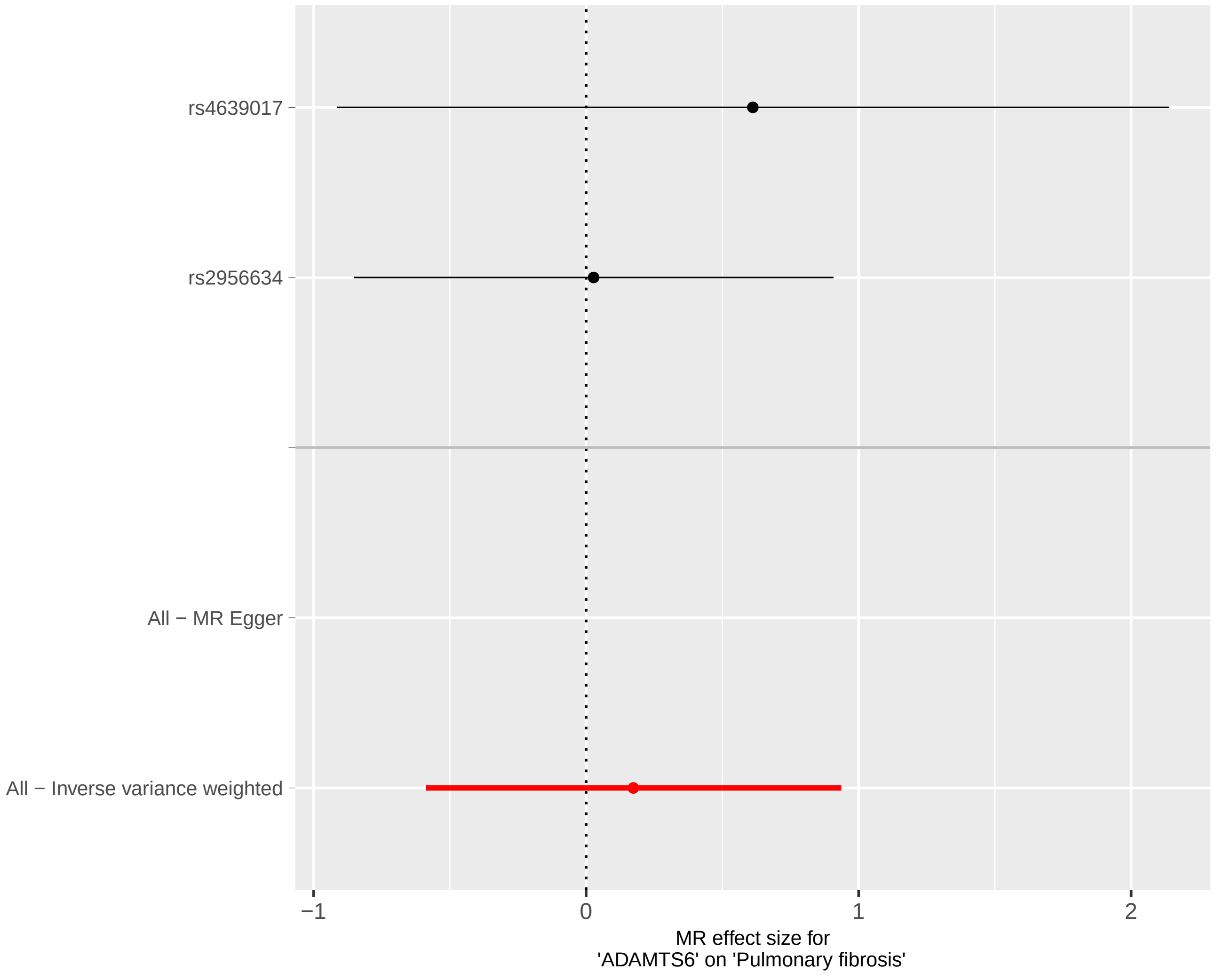

rs4639017
rs2956634
All − MR Egger
All − Inverse variance weighted
−1
0
1
2
MR effect size for
'ADAMTS6' on 'Pulmonary fibrosis'

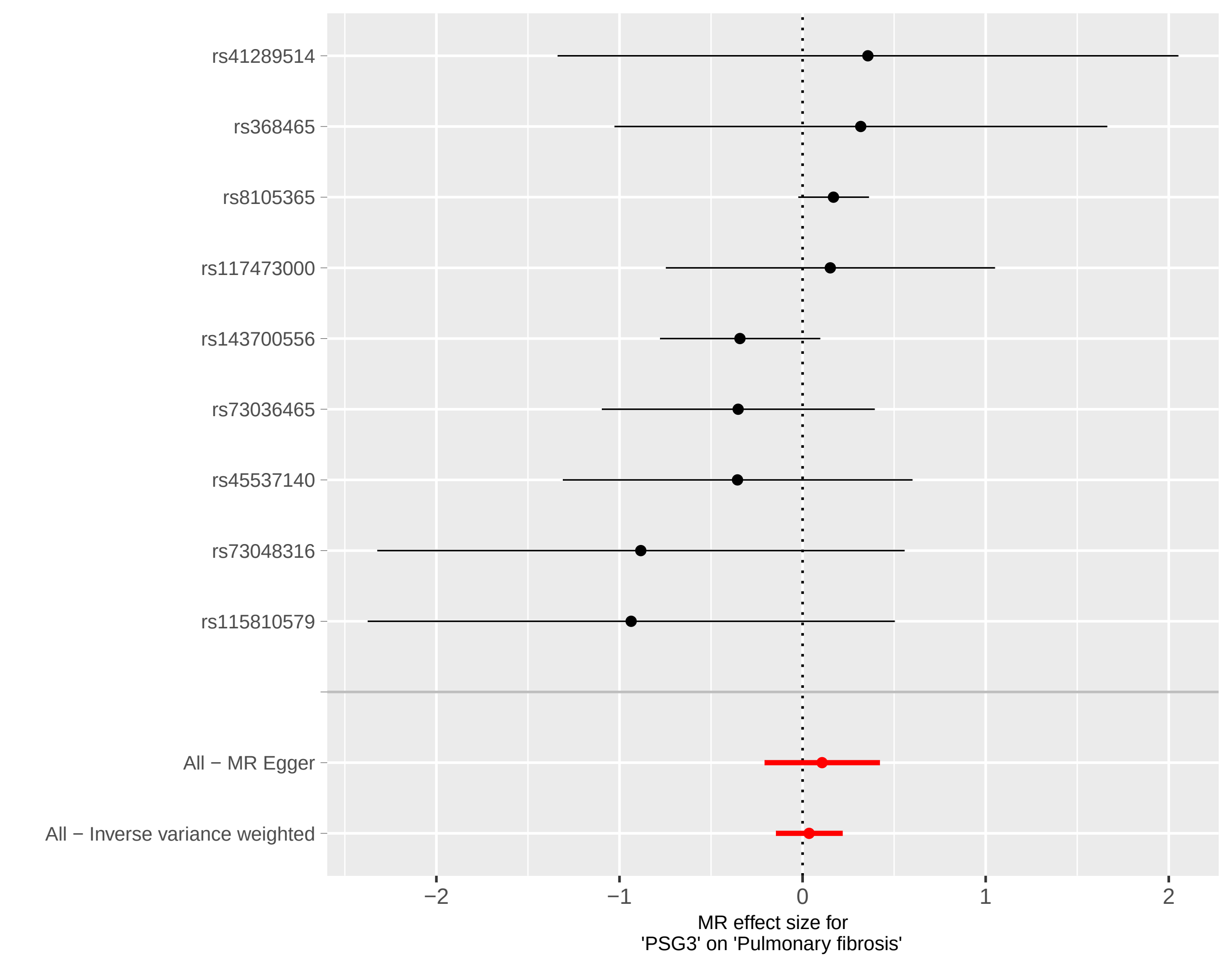
rs41289514
rs368465
rs8105365
rs117473000
rs143700556
rs73036465
rs45537140
rs73048316
rs115810579
All − MR Egger
All − Inverse variance weighted
−2
−1
0
1
2
MR effect size for
'PSG3' on 'Pulmonary fibrosis'

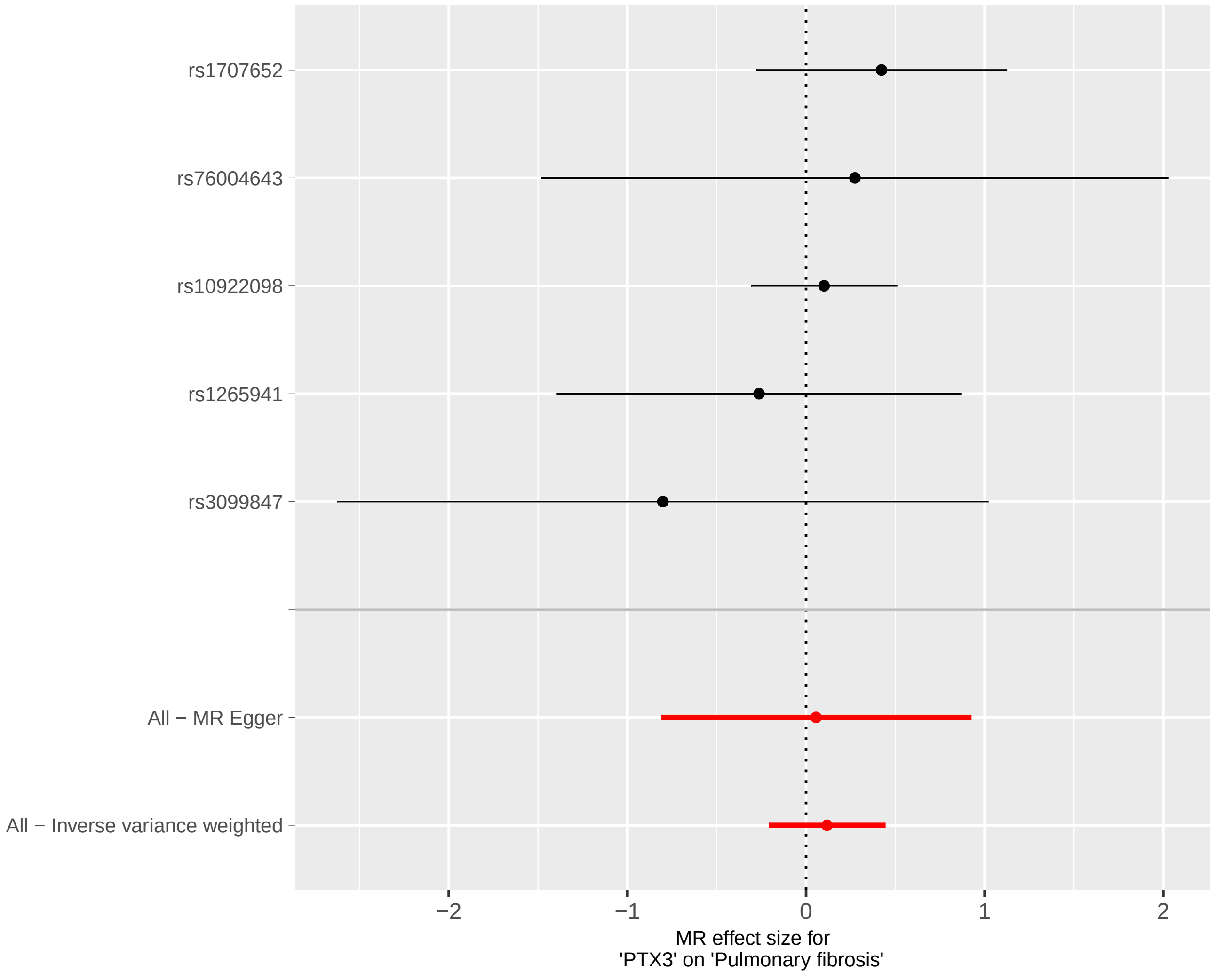
rs1707652
rs76004643
rs10922098
rs1265941
rs3099847
All − MR Egger
All − Inverse variance weighted
−2
−1
0
1
2
MR effect size for
'PTX3' on 'Pulmonary fibrosis'

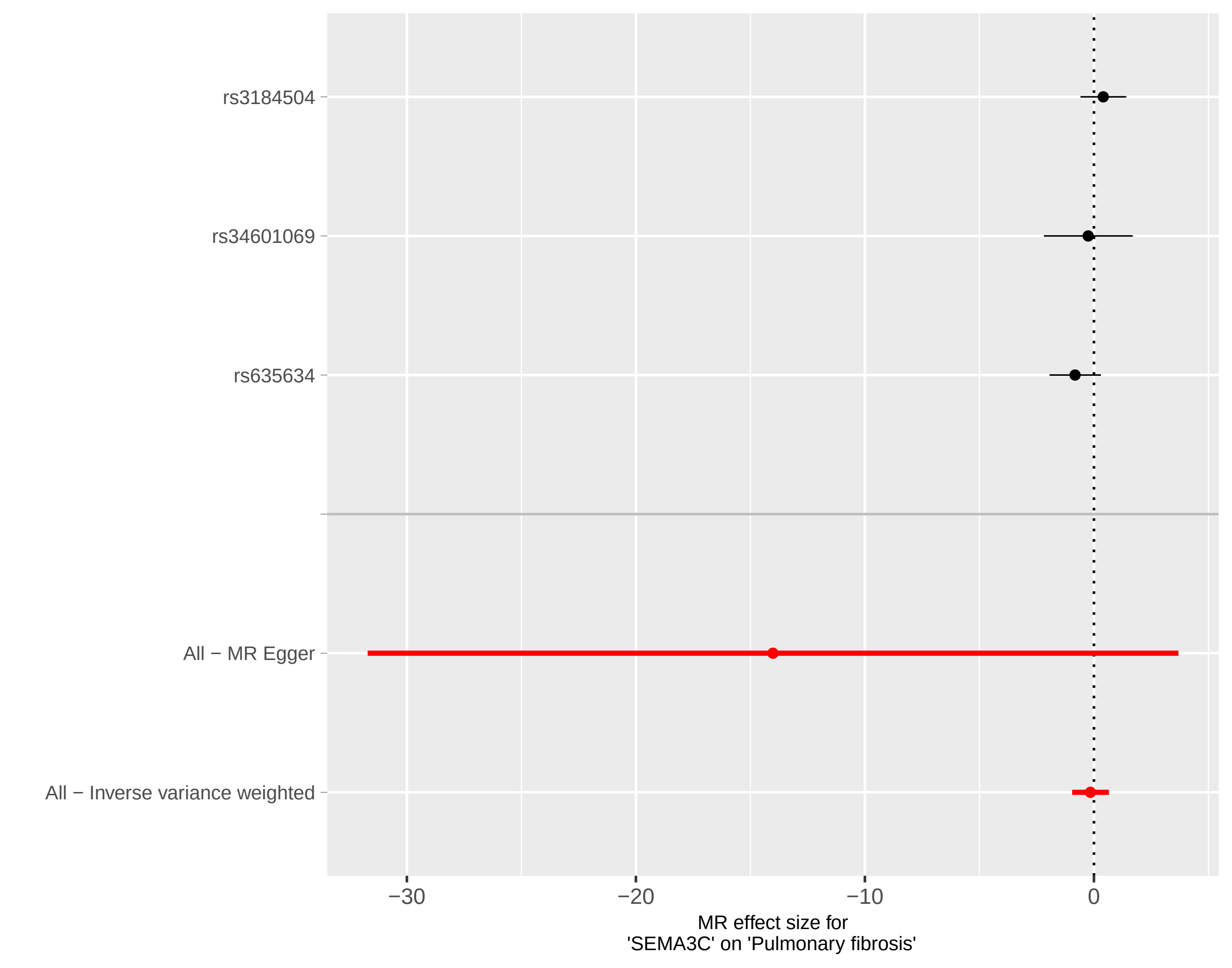

rs3184504
rs34601069
rs635634
All − MR Egger
All − Inverse variance weighted
−30
−20
−10
0
MR effect size for
'SEMA3C' on 'Pulmonary fibrosis'

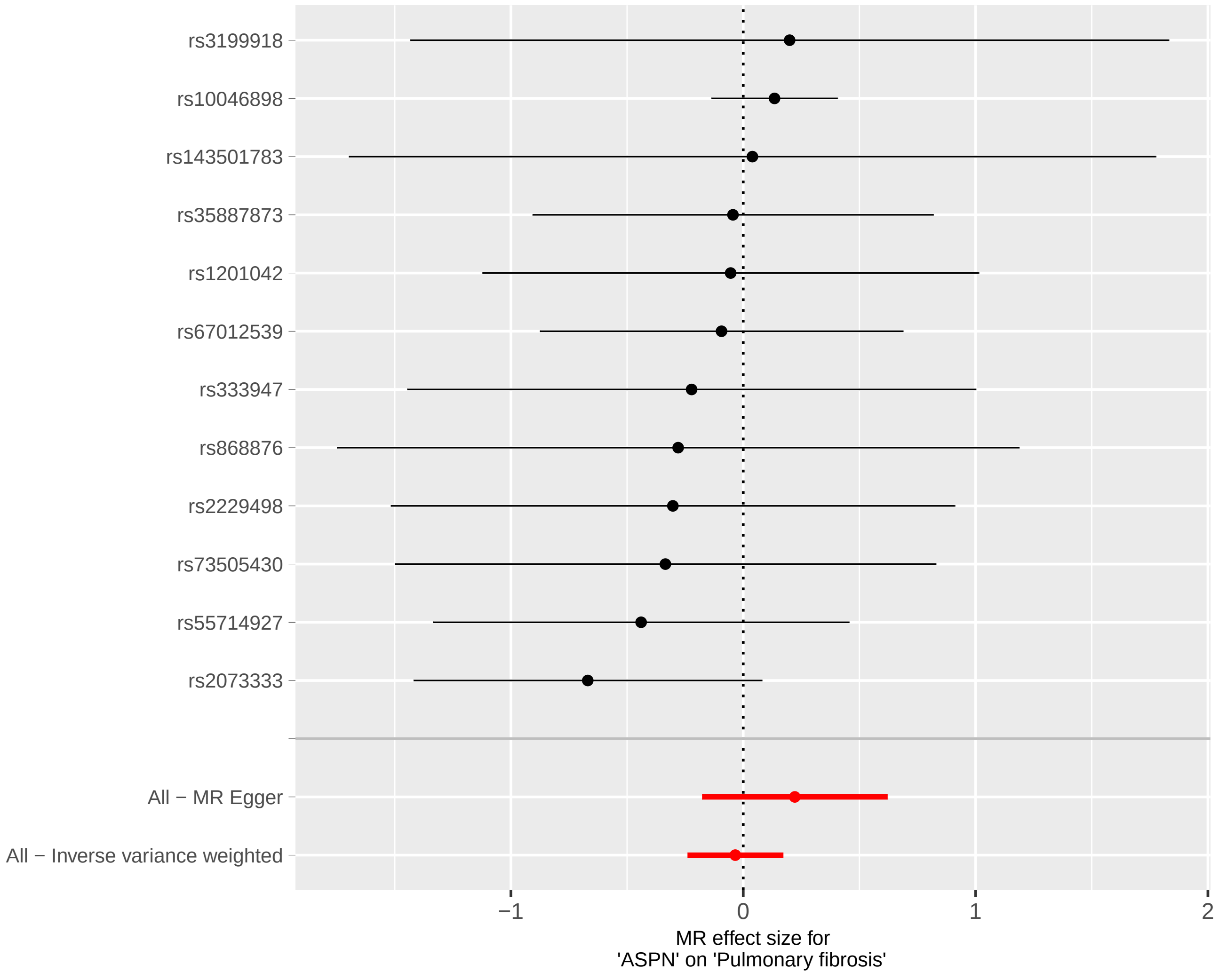
rs3199918
rs10046898
rs143501783
rs35887873
rs1201042
rs67012539
rs333947
rs868876
rs2229498
rs73505430
rs55714927
rs2073333
All − MR Egger
All − Inverse variance weighted
−1
0
1
2
MR effect size for
'ASPN' on 'Pulmonary fibrosis'

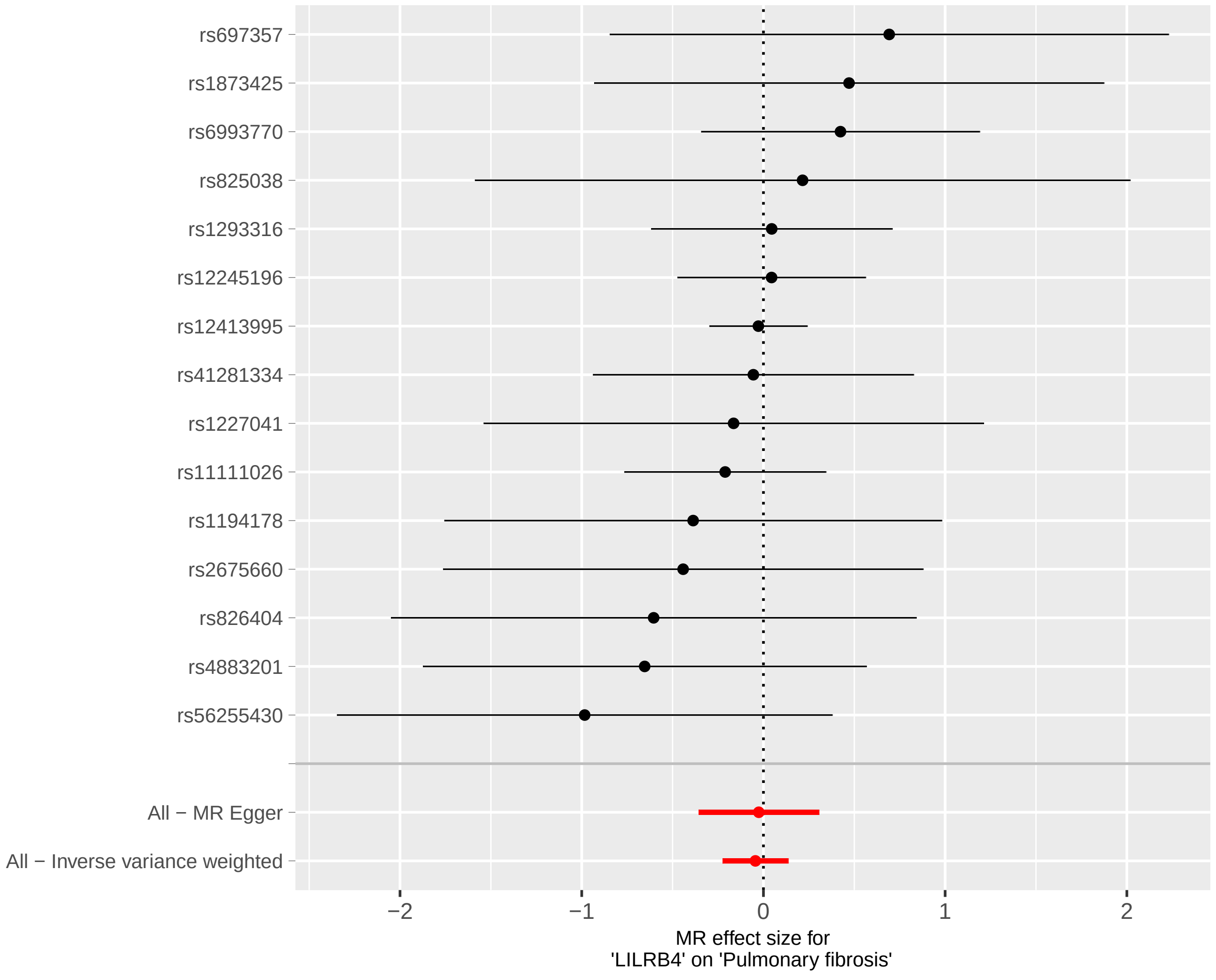

rs697357
rs1873425
rs6993770
rs825038
rs1293316
rs12245196
rs12413995
rs41281334
rs1227041
rs11111026
rs1194178
rs2675660
rs826404
rs4883201
rs56255430
All − MR Egger
All − Inverse variance weighted
−2
−1
0
1
2
MR effect size for
'LILRB4' on 'Pulmonary fibrosis'

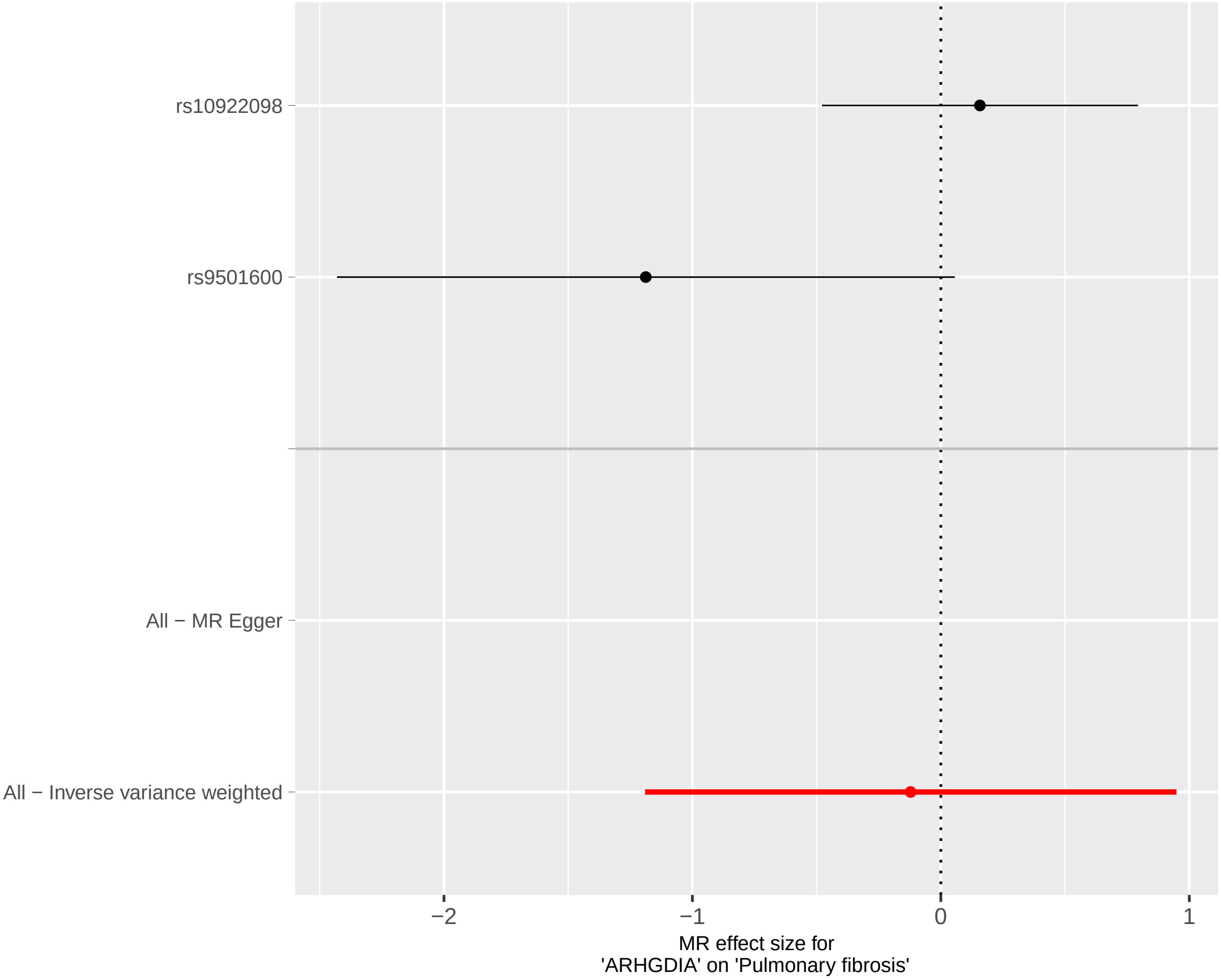

rs10922098
rs9501600
All − MR Egger
All − Inverse variance weighted
−2
−1
0
1
MR effect size for
'ARHGDIA' on 'Pulmonary fibrosis'

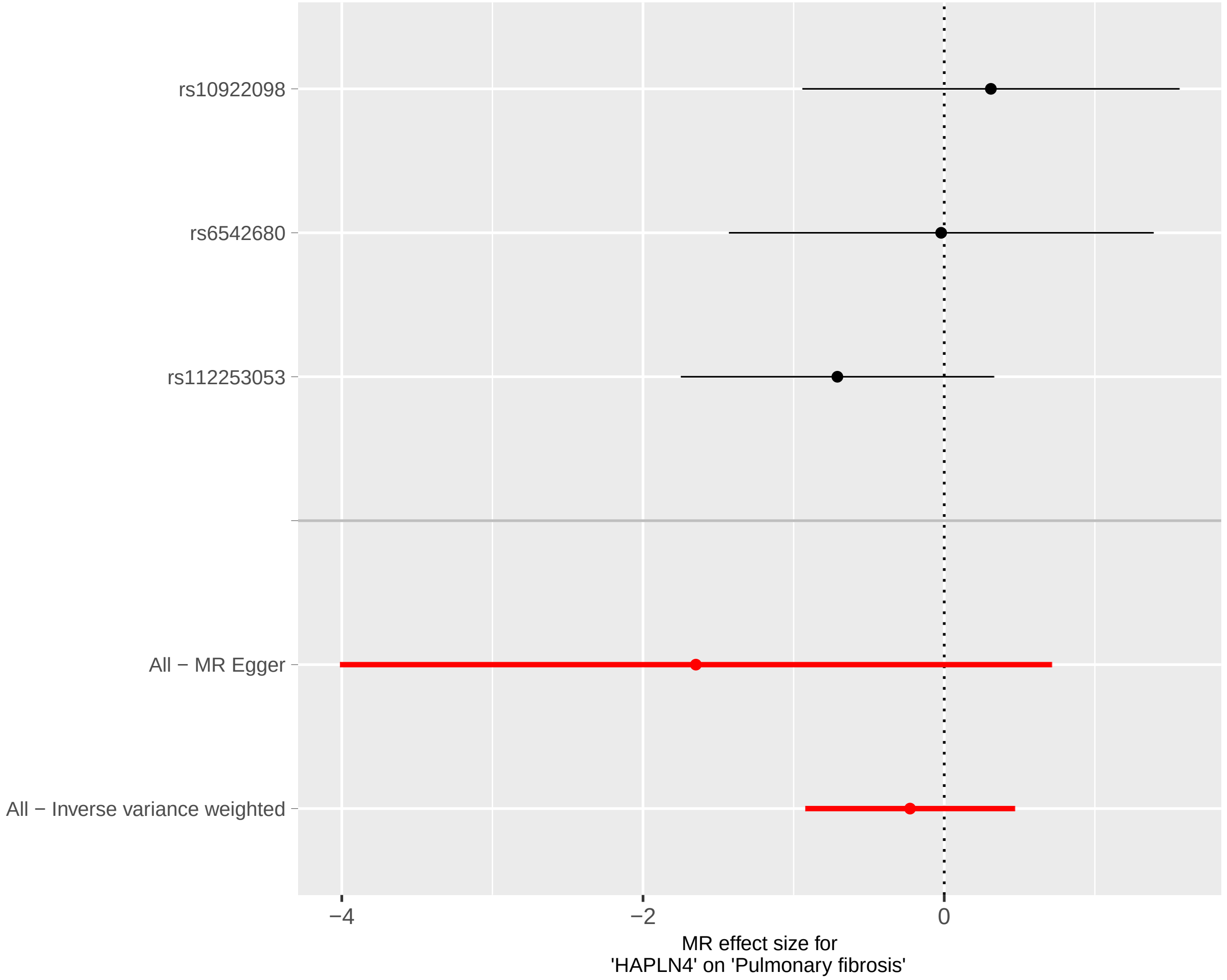

rs10922098
rs6542680
rs112253053
All − MR Egger
All − Inverse variance weighted
−4
−2
0
MR effect size for
'HAPLN4' on 'Pulmonary fibrosis'

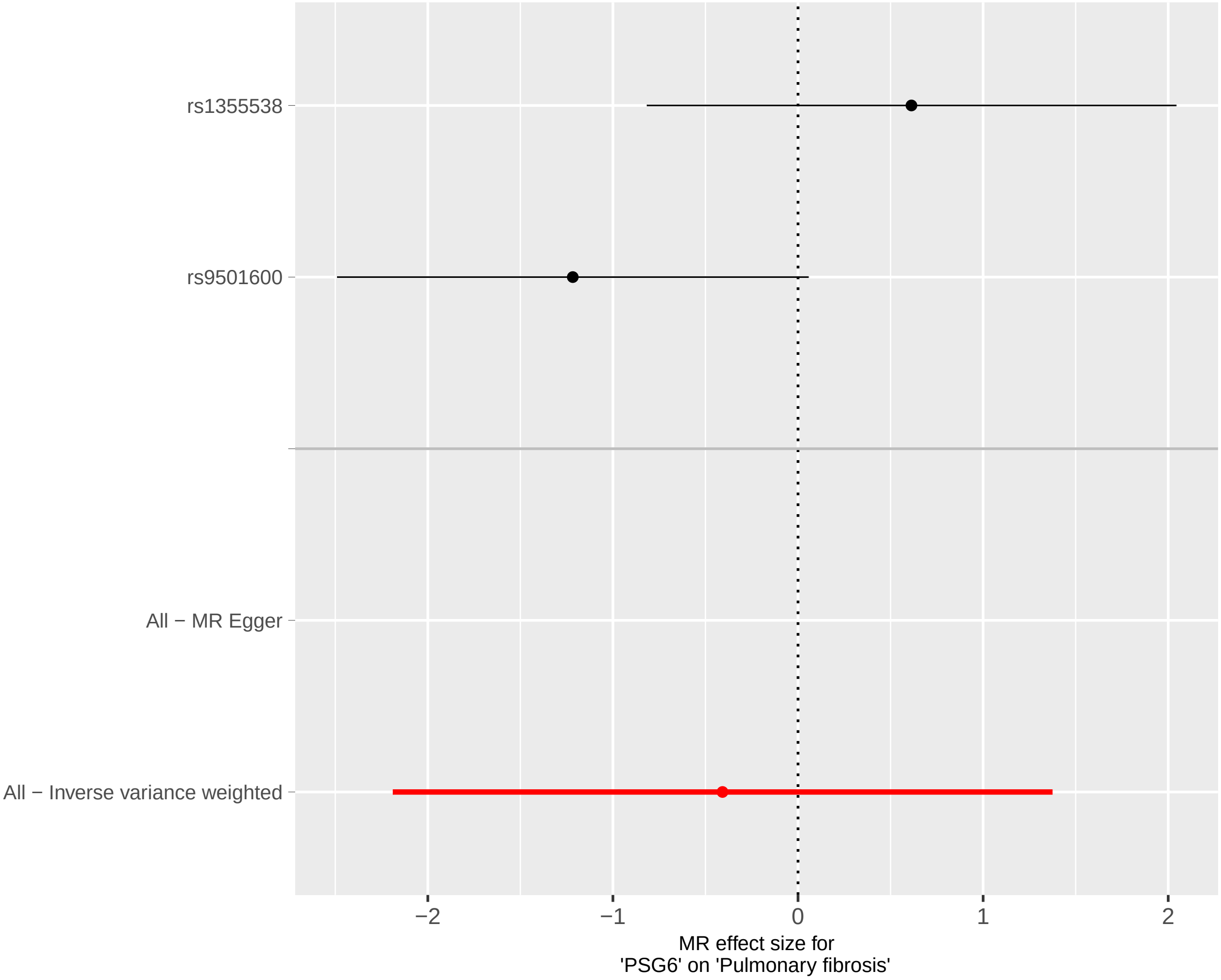

rs1355538
rs9501600
All − MR Egger
All − Inverse variance weighted
−2
−1
0
1
2
MR effect size for
'PSG6' on 'Pulmonary fibrosis'

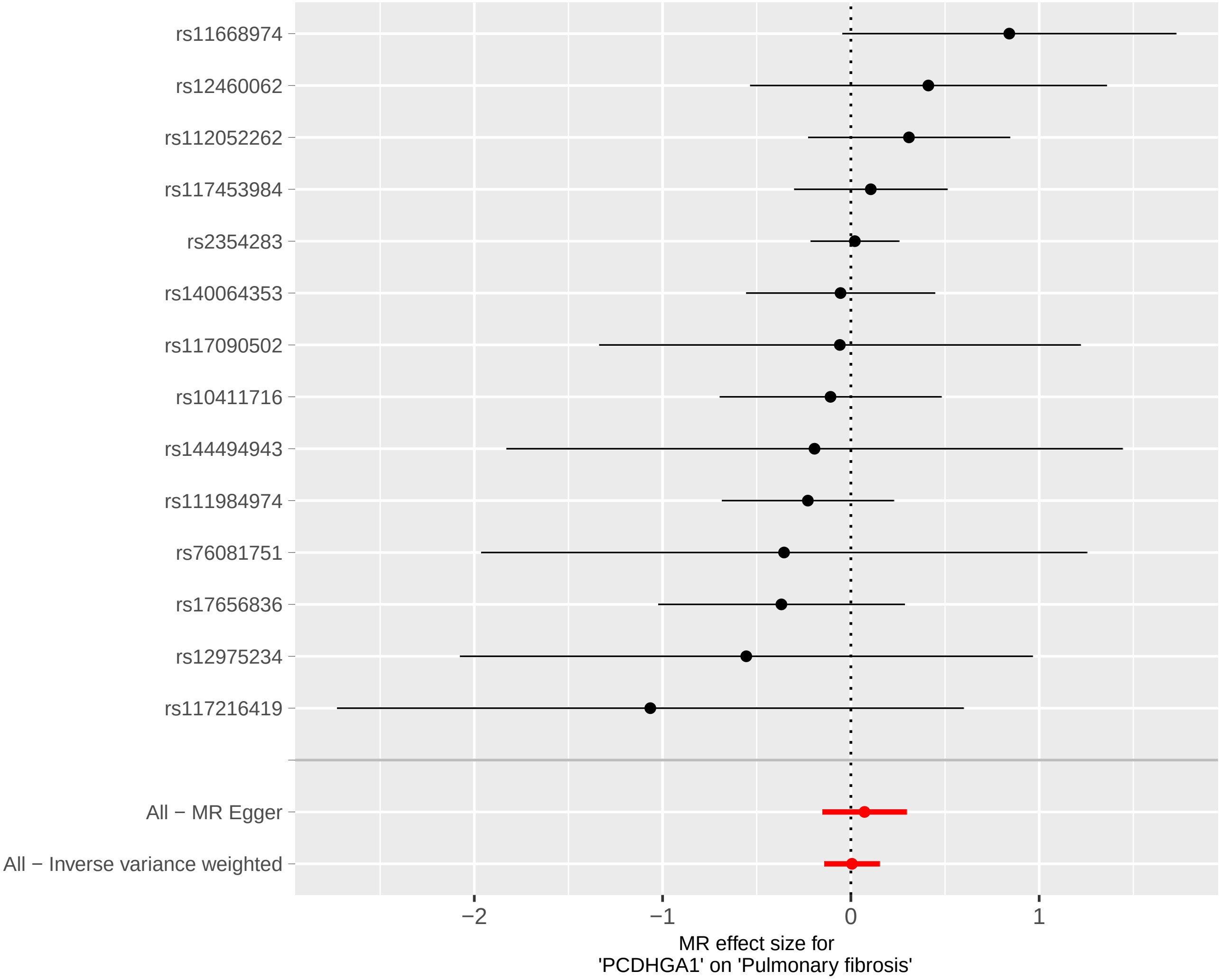

rs11668974
rs12460062
rs112052262
rs117453984
rs2354283
rs140064353
rs117090502
rs10411716
rs144494943
rs111984974
rs76081751
rs17656836
rs12975234
rs117216419
All − MR Egger
All − Inverse variance weighted
−2
−1
0
1
MR effect size for
'PCDHGA1' on 'Pulmonary fibrosis'

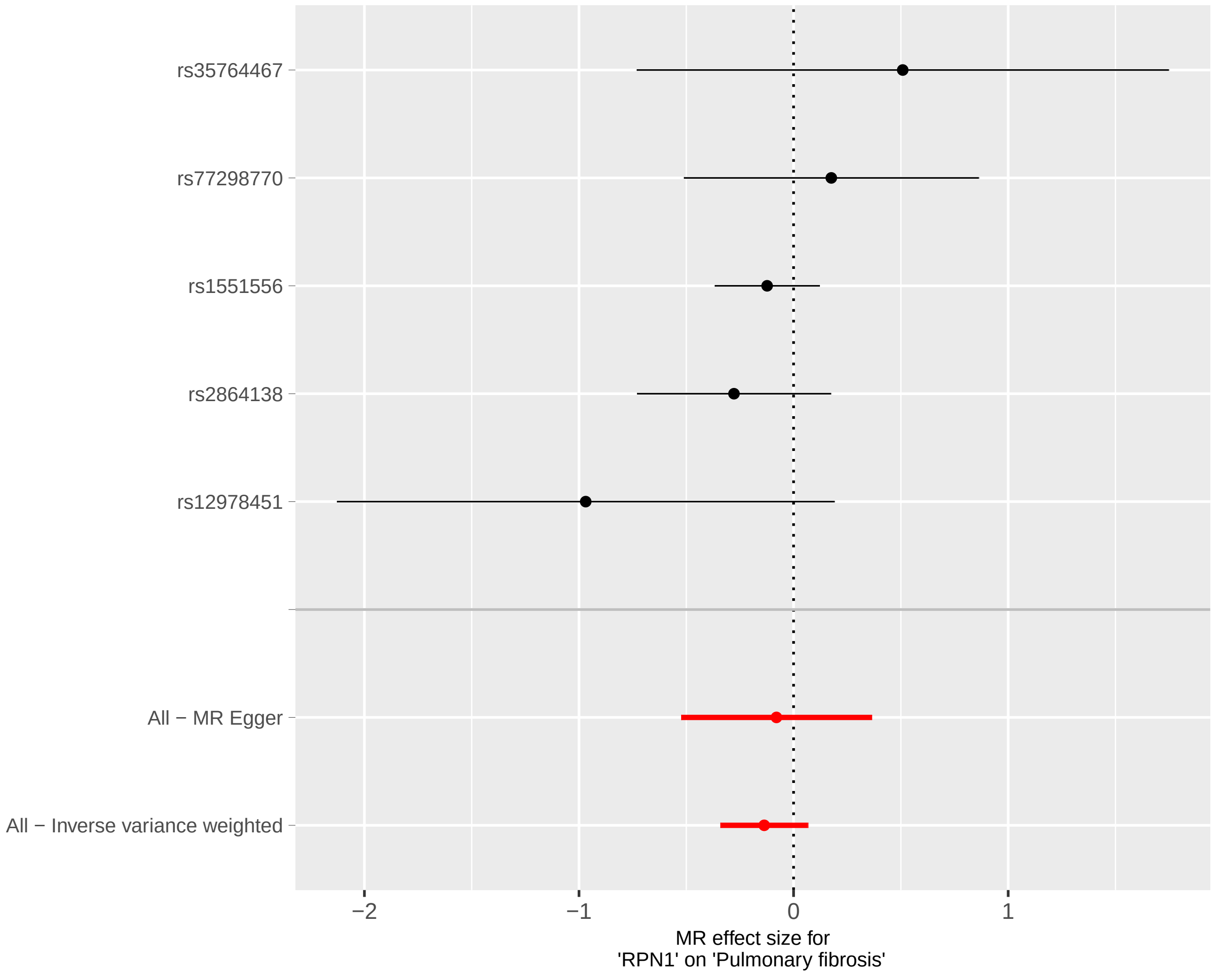

rs35764467
rs77298770
rs1551556
rs2864138
rs12978451
All − MR Egger
All − Inverse variance weighted
−2
−1
0
1
MR effect size for
'RPN1' on 'Pulmonary fibrosis'

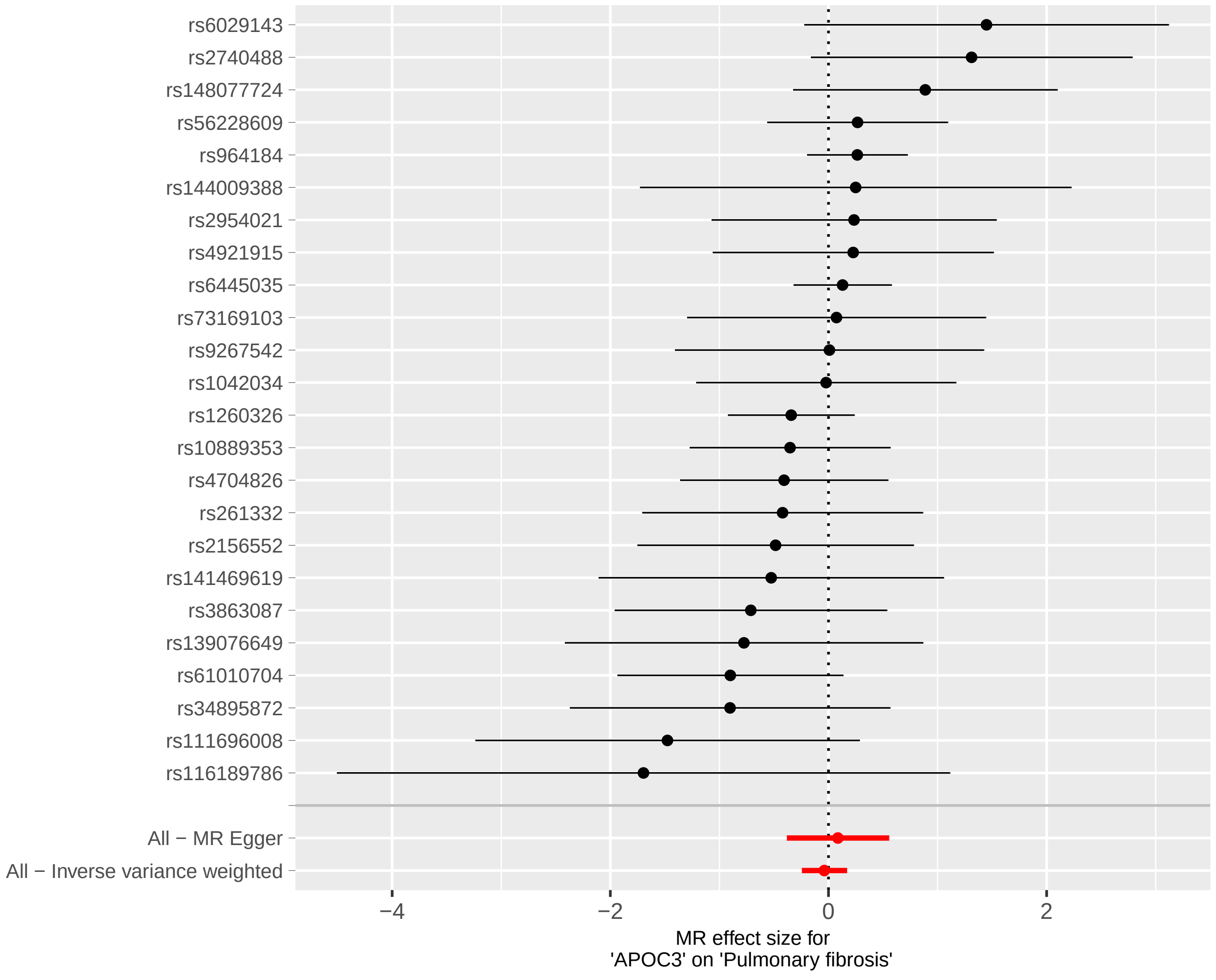

rs6029143
rs2740488
rs148077724
rs56228609
rs964184
rs144009388
rs2954021
rs4921915
rs6445035
rs73169103
rs9267542
rs1042034
rs1260326
rs10889353
rs4704826
rs261332
rs2156552
rs141469619
rs3863087
rs139076649
rs61010704
rs34895872
rs111696008
rs116189786
All − MR Egger
All − Inverse variance weighted
−4
−2
0
2
MR effect size for
'APOC3' on 'Pulmonary fibrosis'

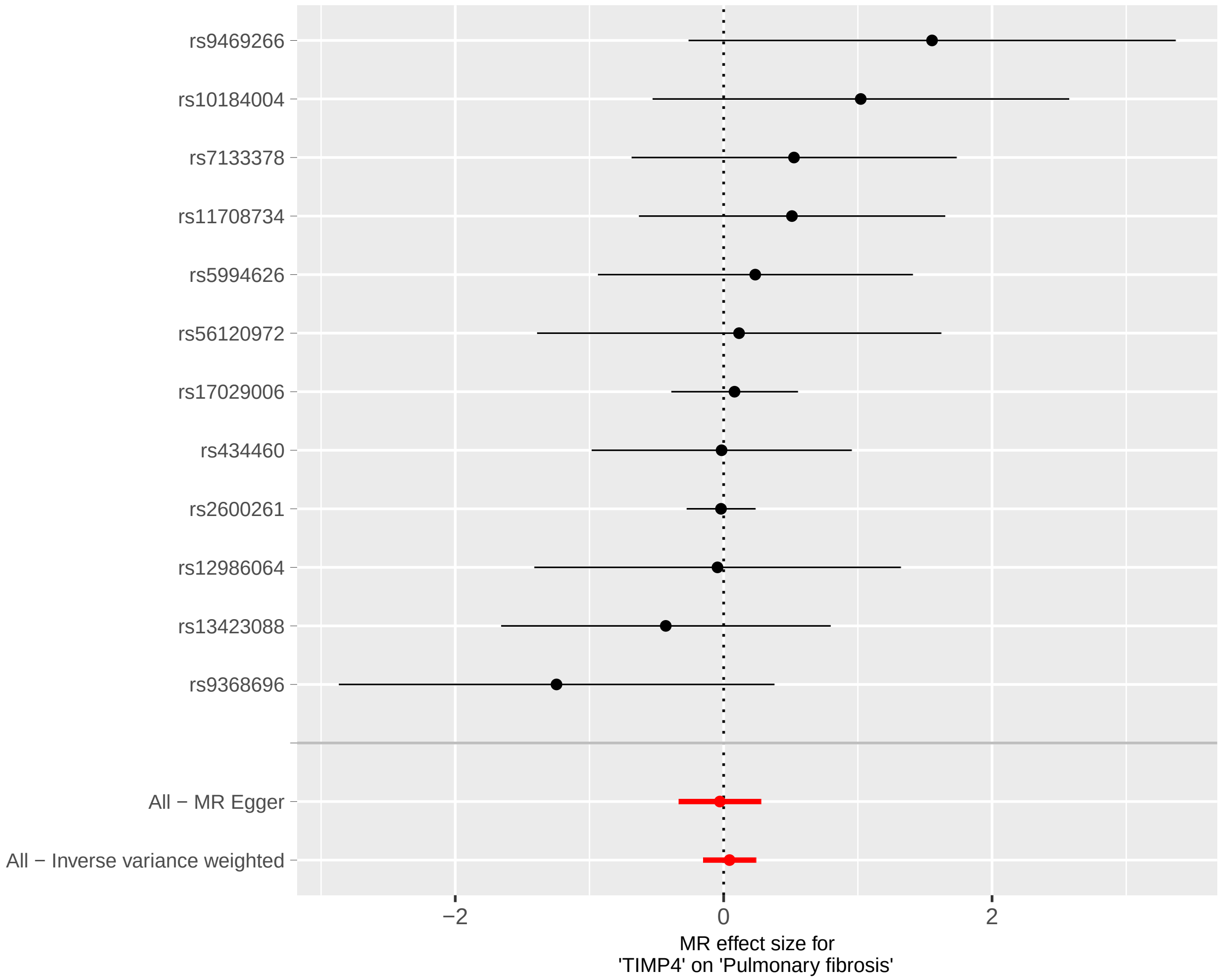
rs9469266
rs10184004
rs7133378
rs11708734
rs5994626
rs56120972
rs17029006
rs434460
rs2600261
rs12986064
rs13423088
rs9368696
All − MR Egger
All − Inverse variance weighted
−2
0
2
MR effect size for
'TIMP4' on 'Pulmonary fibrosis'

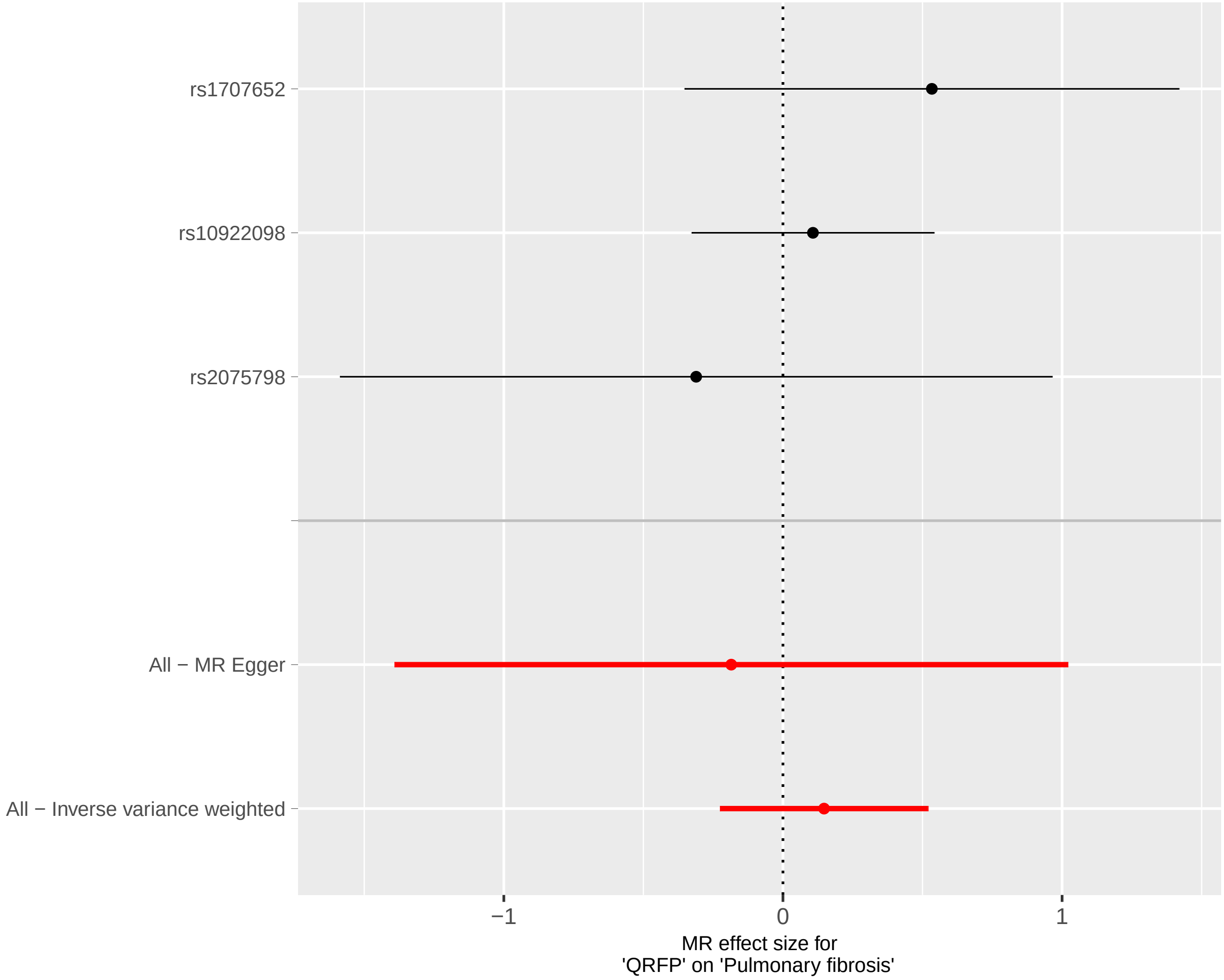

rs1707652
rs10922098
rs2075798
All − MR Egger
All − Inverse variance weighted
−1
0
1
MR effect size for
'QRFP' on 'Pulmonary fibrosis'

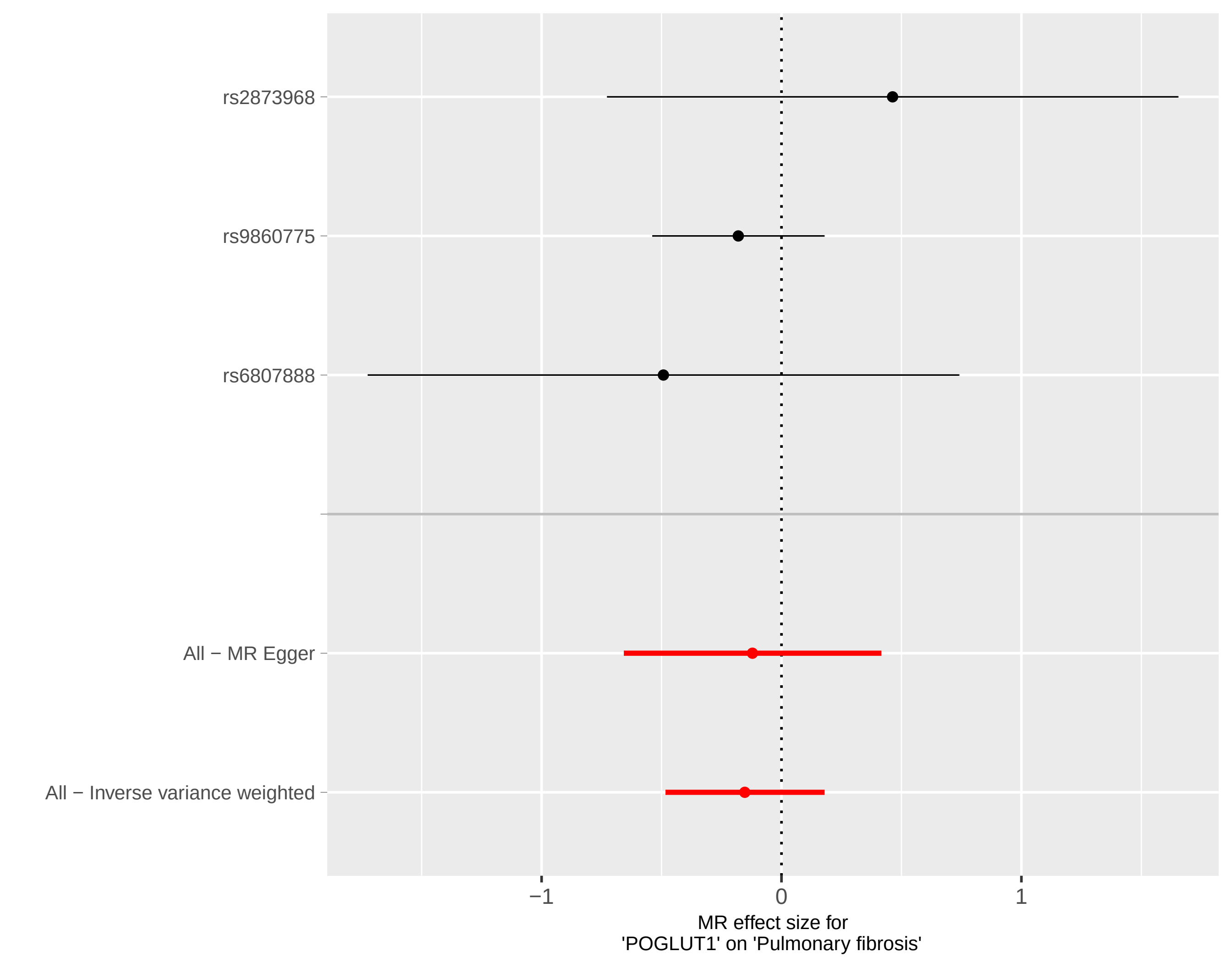

rs2873968
rs9860775
rs6807888
All − MR Egger
All − Inverse variance weighted
−1
0
1
MR effect size for
'POGLUT1' on 'Pulmonary fibrosis'

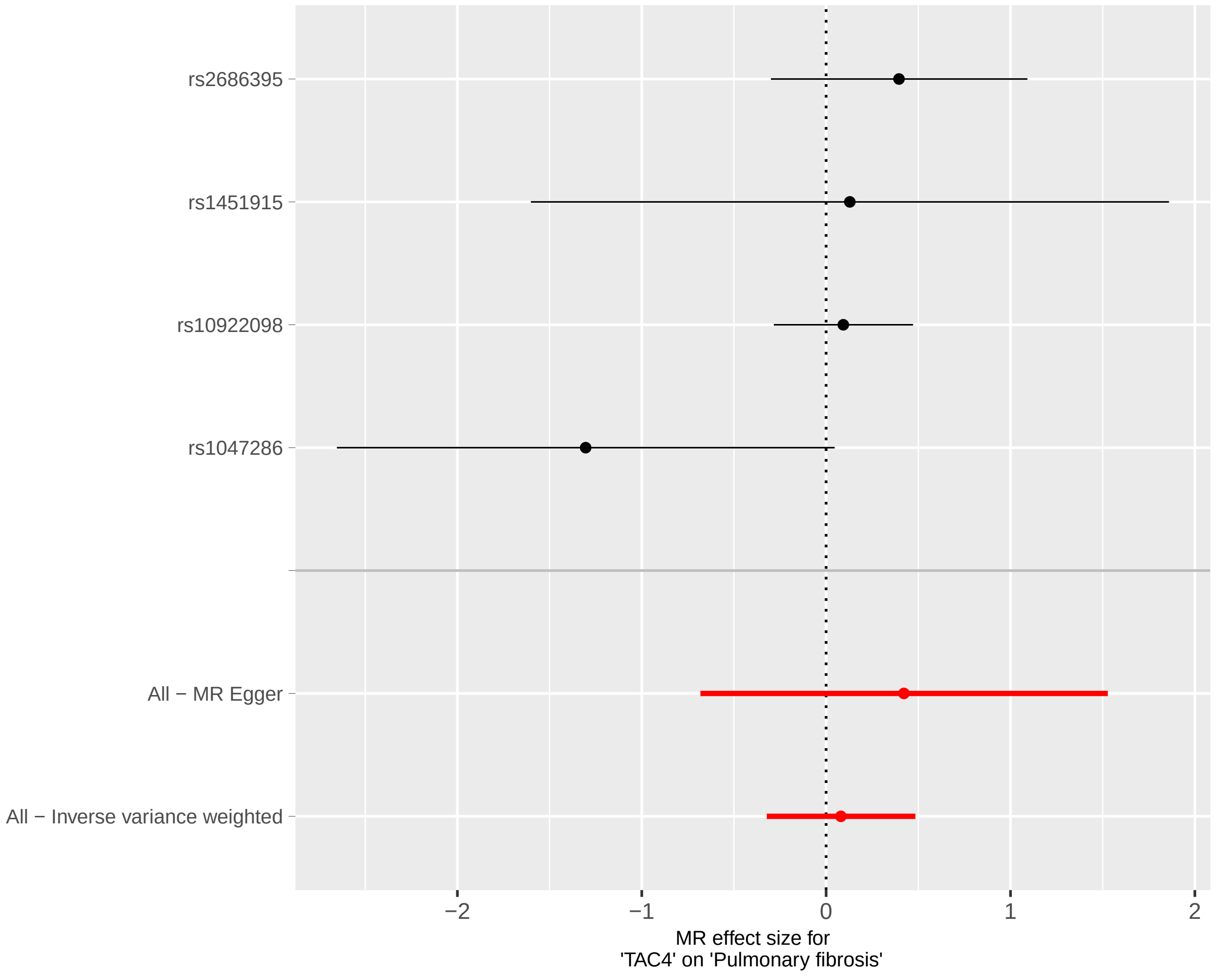

rs2686395
rs1451915
rs10922098
rs1047286
All − MR Egger
All − Inverse variance weighted
−2
−1
0
1
2
MR effect size for
'TAC4' on 'Pulmonary fibrosis'

Insufficient number of SNPs

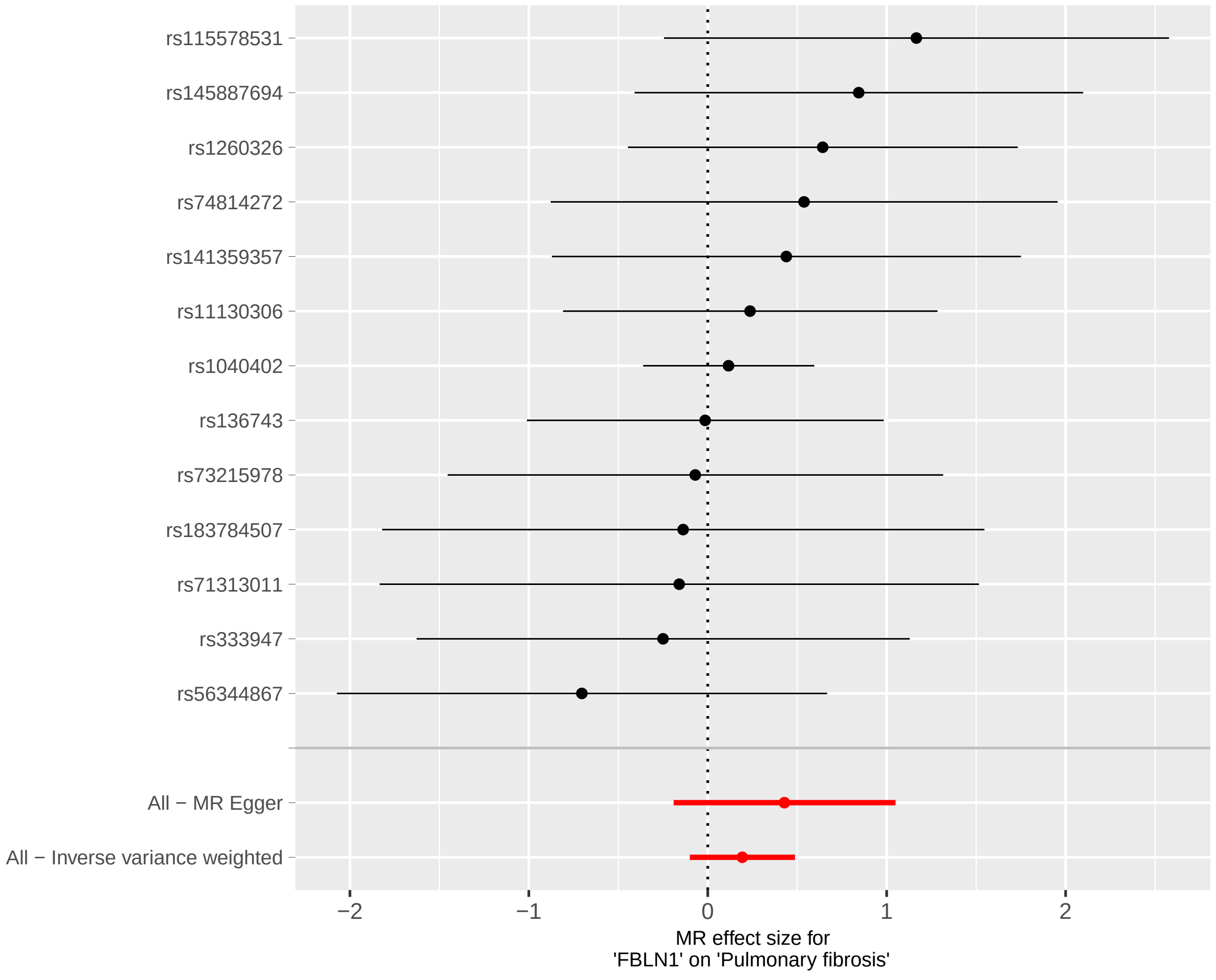
rs115578531
rs145887694
rs1260326
rs74814272
rs141359357
rs11130306
rs1040402
rs136743
rs73215978
rs183784507
rs71313011
rs333947
rs56344867
All − MR Egger
All − Inverse variance weighted
−2
−1
0
1
2
MR effect size for
'FBLN1' on 'Pulmonary fibrosis'

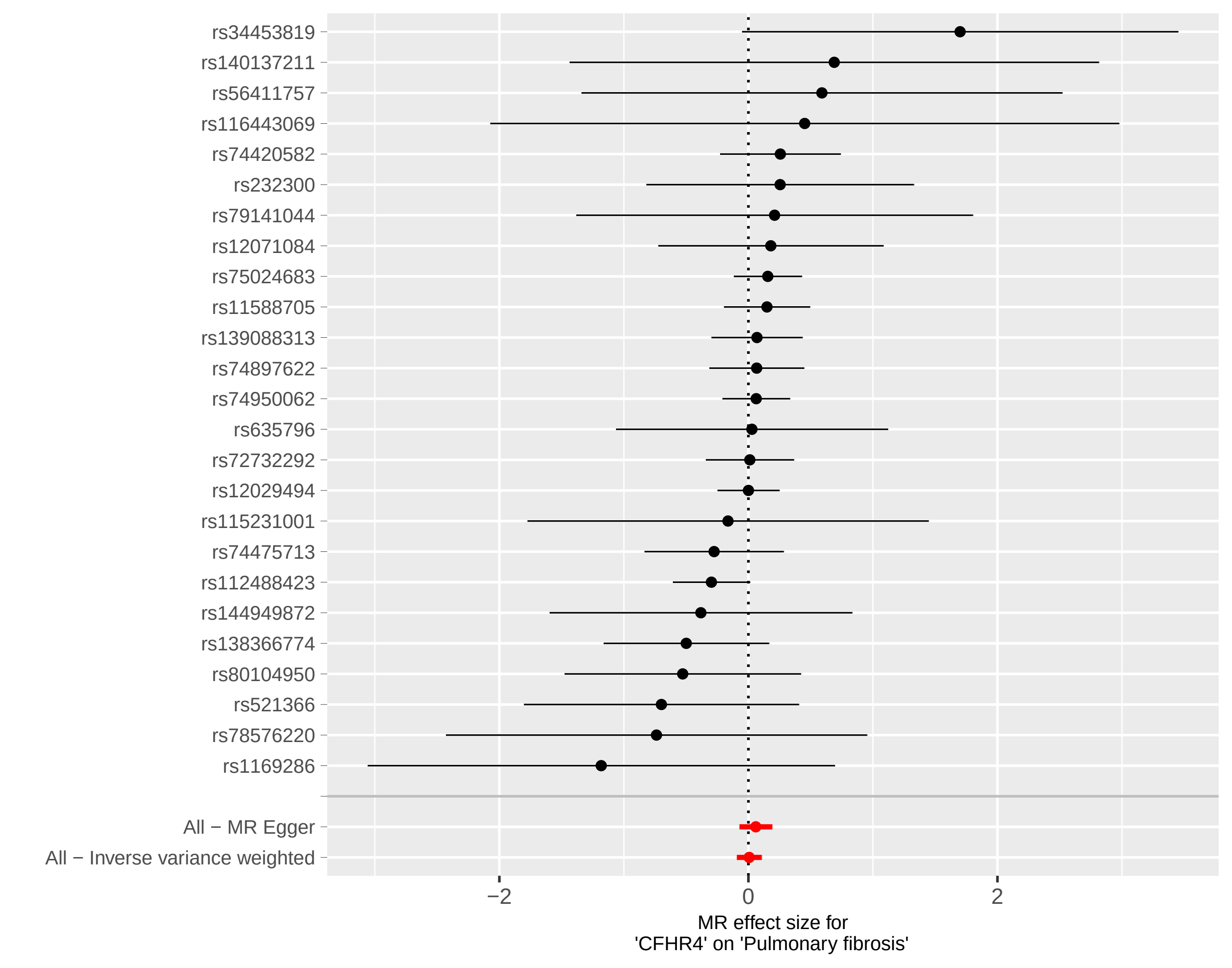
rs34453819
rs140137211
rs56411757
rs116443069
rs74420582
rs232300
rs79141044
rs12071084
rs75024683
rs11588705
rs139088313
rs74897622
rs74950062
rs635796
rs72732292
rs12029494
rs115231001
rs74475713
rs112488423
rs144949872
rs138366774
rs80104950
rs521366
rs78576220
rs1169286
All − MR Egger
All − Inverse variance weighted
−2
0
2
MR effect size for
'CFHR4' on 'Pulmonary fibrosis'

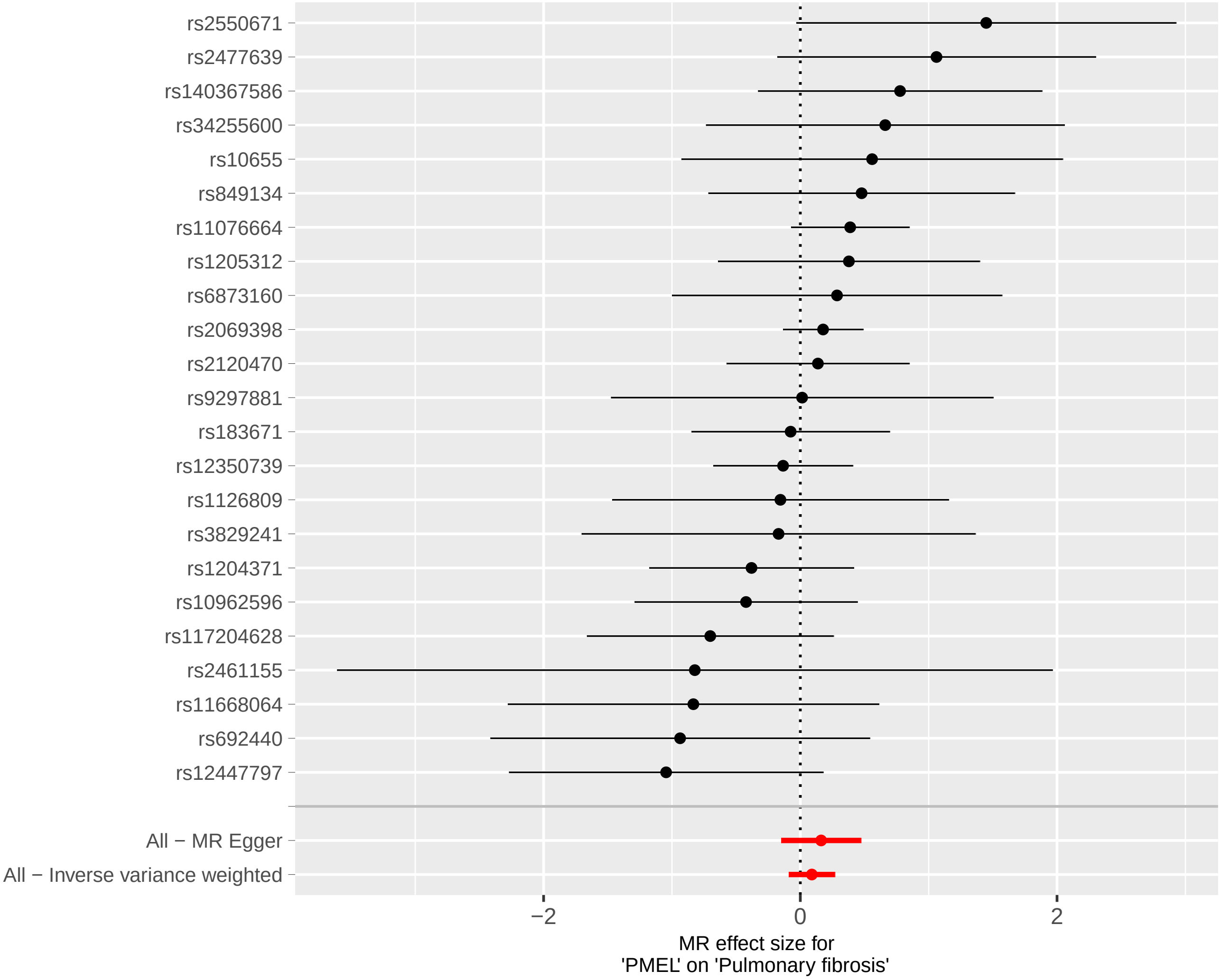
rs2550671
rs2477639
rs140367586
rs34255600
rs10655
rs849134
rs11076664
rs1205312
rs6873160
rs2069398
rs2120470
rs9297881
rs183671
rs12350739
rs1126809
rs3829241
rs1204371
rs10962596
rs117204628
rs2461155
rs11668064
rs692440
rs12447797
All − MR Egger
All − Inverse variance weighted
−2
0
2
MR effect size for
'PMEL' on 'Pulmonary fibrosis'

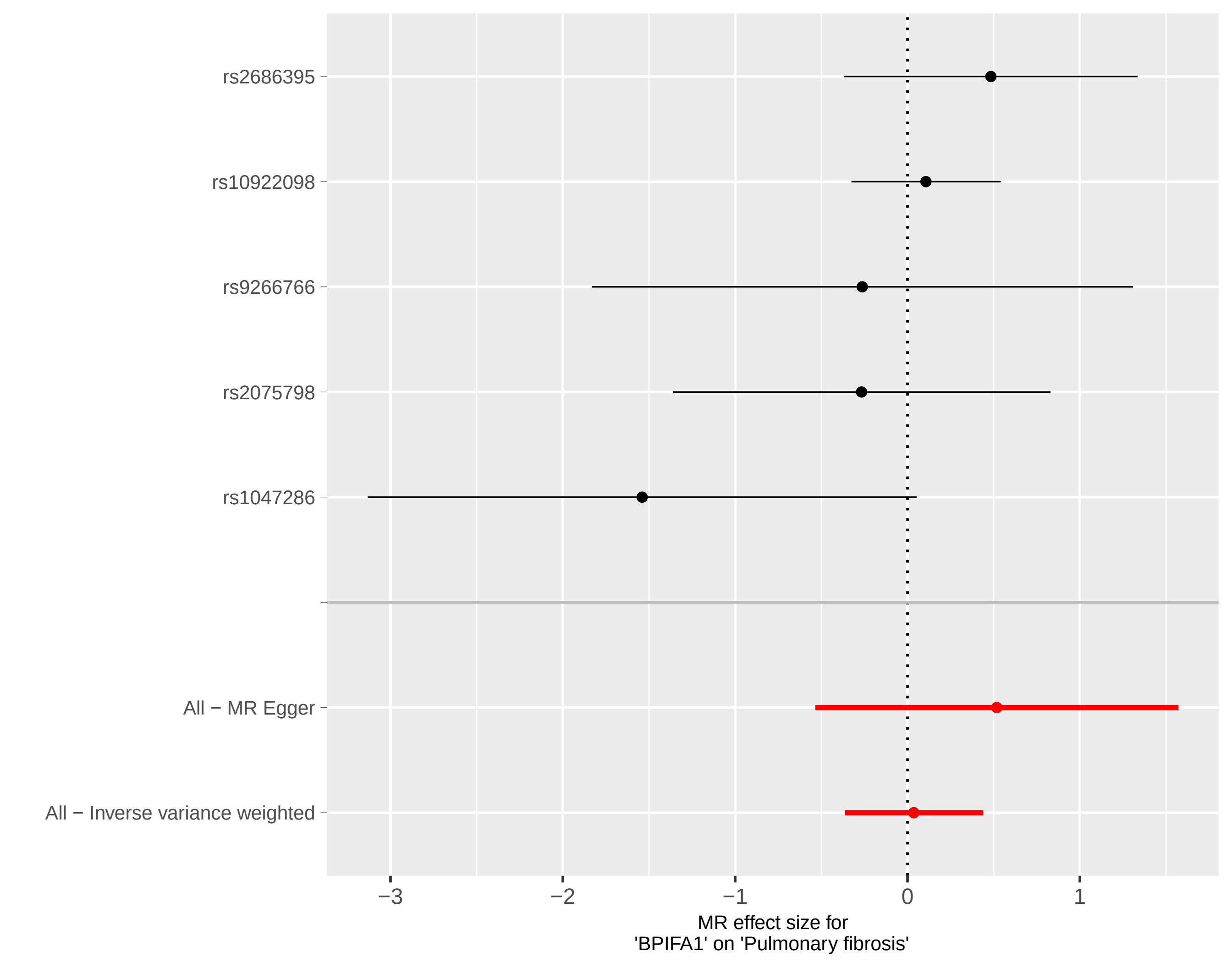
rs2686395
rs10922098
rs9266766
rs2075798
rs1047286
All − MR Egger
All − Inverse variance weighted
−3
−2
−1
0
1
MR effect size for
'BPIFA1' on 'Pulmonary fibrosis'

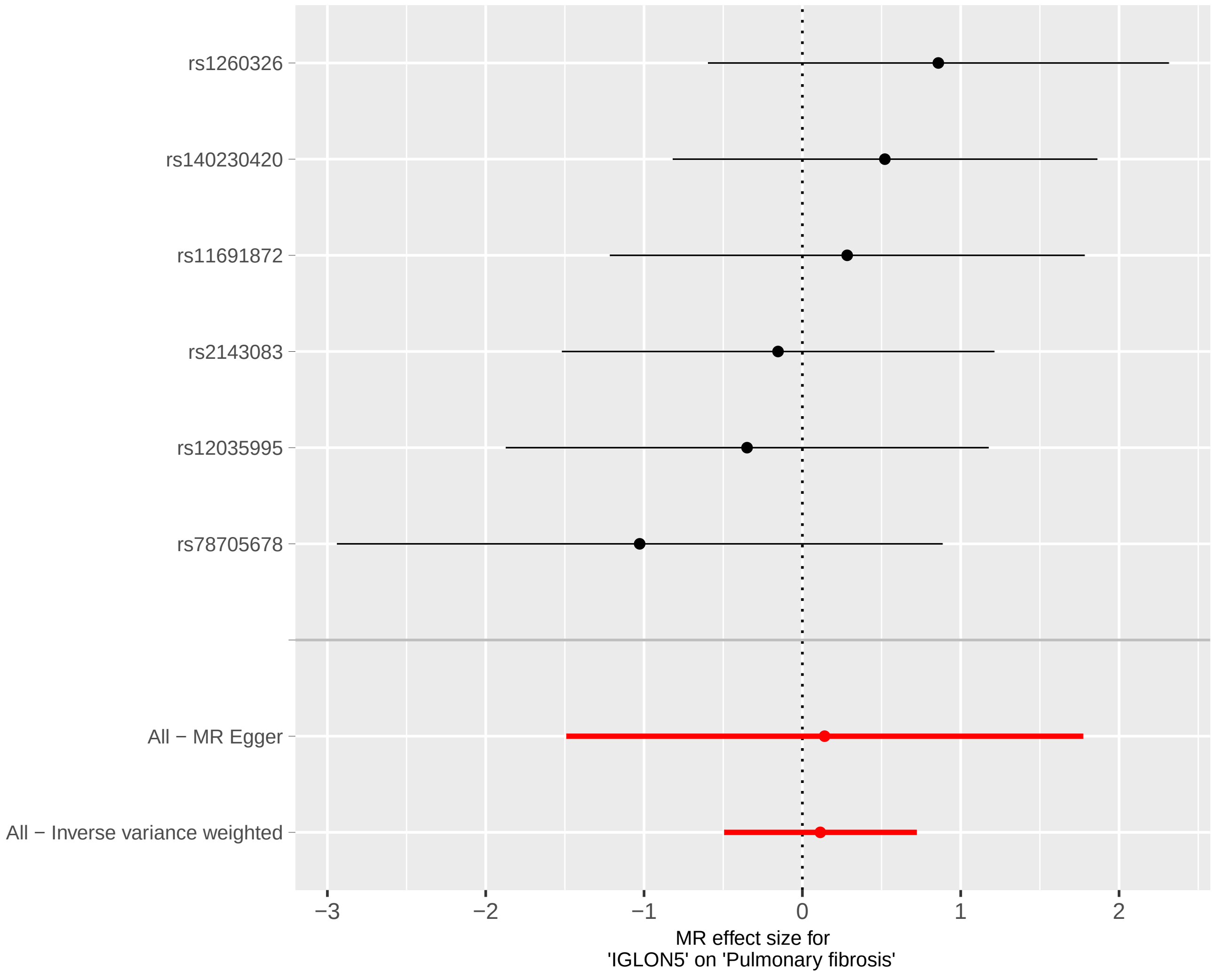
rs1260326
rs140230420
rs11691872
rs2143083
rs12035995
rs78705678
All − MR Egger
All − Inverse variance weighted
−3
−2
−1
0
1
2
MR effect size for
'IGLON5' on 'Pulmonary fibrosis'

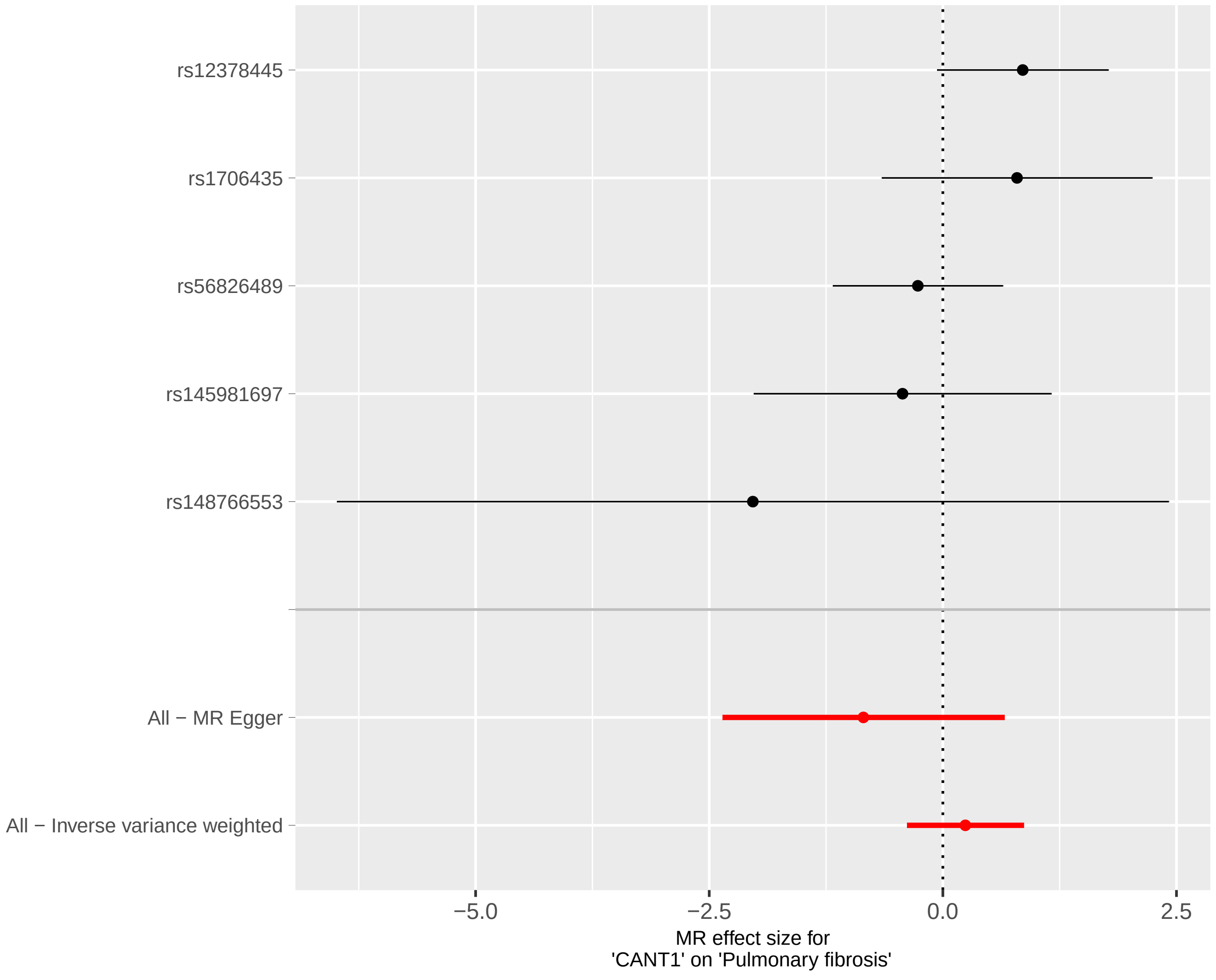
rs12378445
rs1706435
rs56826489
rs145981697
rs148766553
All − MR Egger
All − Inverse variance weighted
−5.0
−2.5
0.0
2.5
MR effect size for
'CANT1' on 'Pulmonary fibrosis'

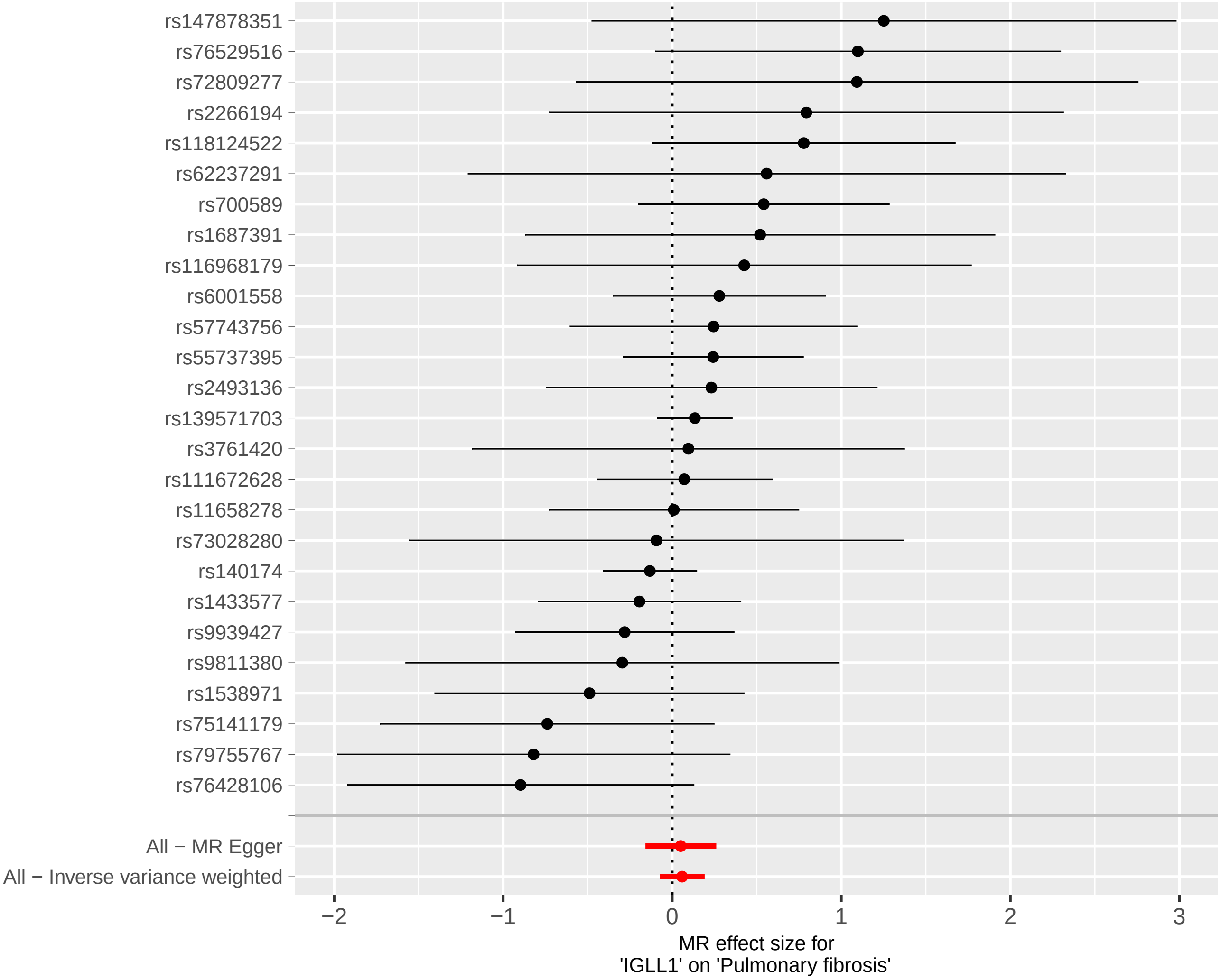

rs147878351
rs76529516
rs72809277
rs2266194
rs118124522
rs62237291
rs700589
rs1687391
rs116968179
rs6001558
rs57743756
rs55737395
rs2493136
rs139571703
rs3761420
rs111672628
rs11658278
rs73028280
rs140174
rs1433577
rs9939427
rs9811380
rs1538971
rs75141179
rs79755767
rs76428106
All − MR Egger
All − Inverse variance weighted
−2
−1
0
1
2
3
MR effect size for
'IGLL1' on 'Pulmonary fibrosis'

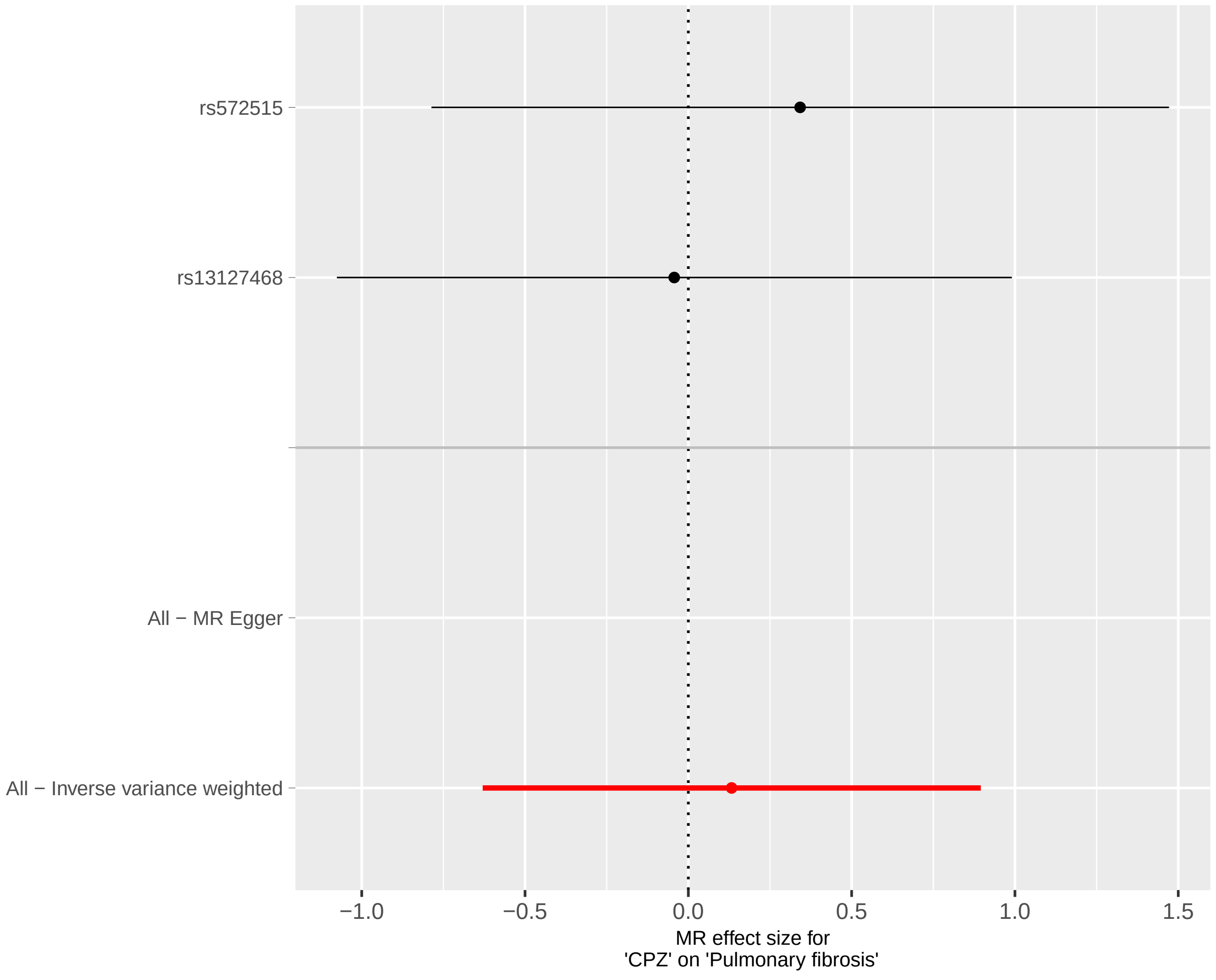
rs572515
rs13127468
All − MR Egger
All − Inverse variance weighted
−1.0
−0.5
0.0
0.5
1.0
1.5
MR effect size for
'CPZ' on 'Pulmonary fibrosis'

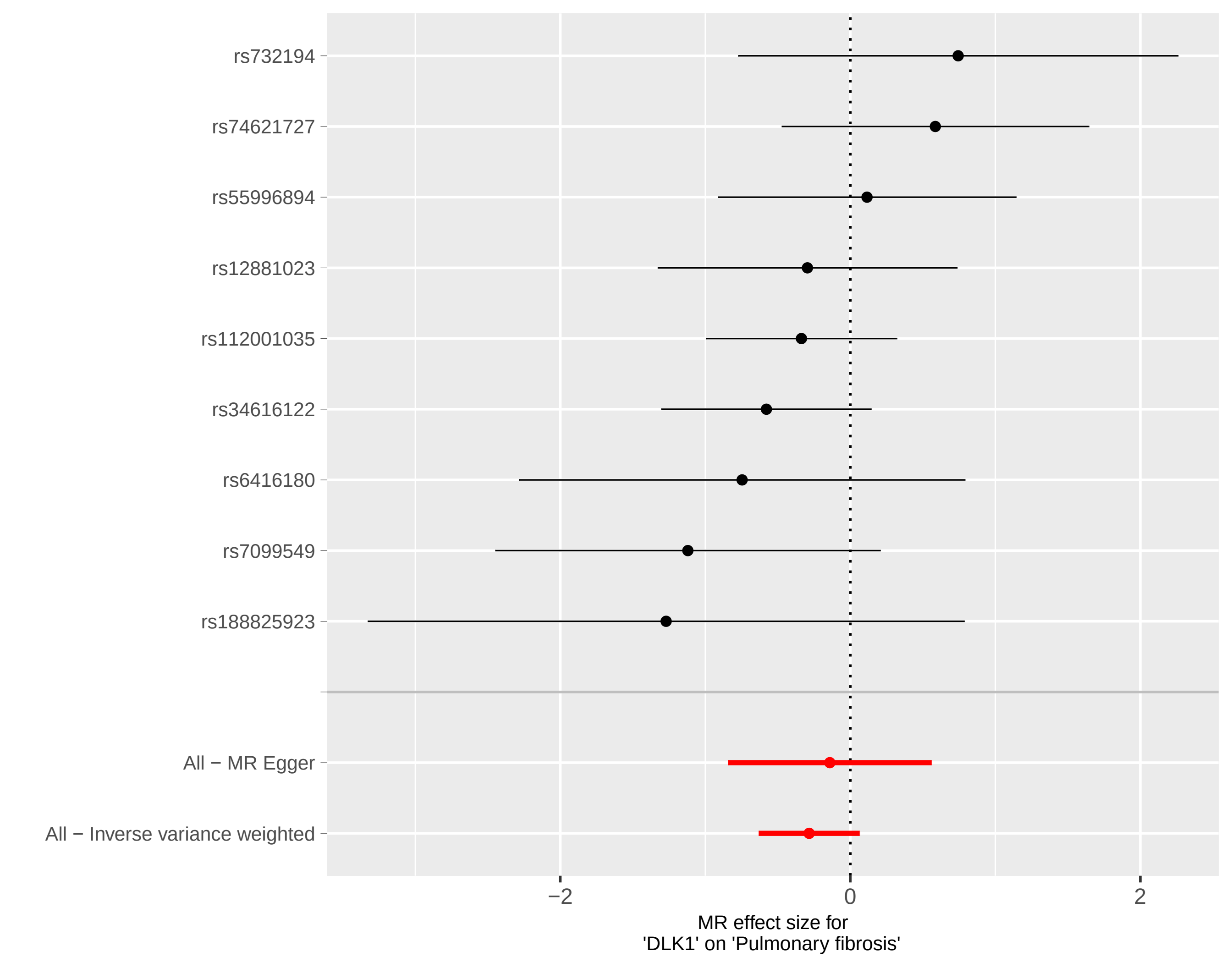

rs732194
rs74621727
rs55996894
rs12881023
rs112001035
rs34616122
rs6416180
rs7099549
rs188825923
All − MR Egger
All − Inverse variance weighted
−2
0
2
MR effect size for
'DLK1' on 'Pulmonary fibrosis'

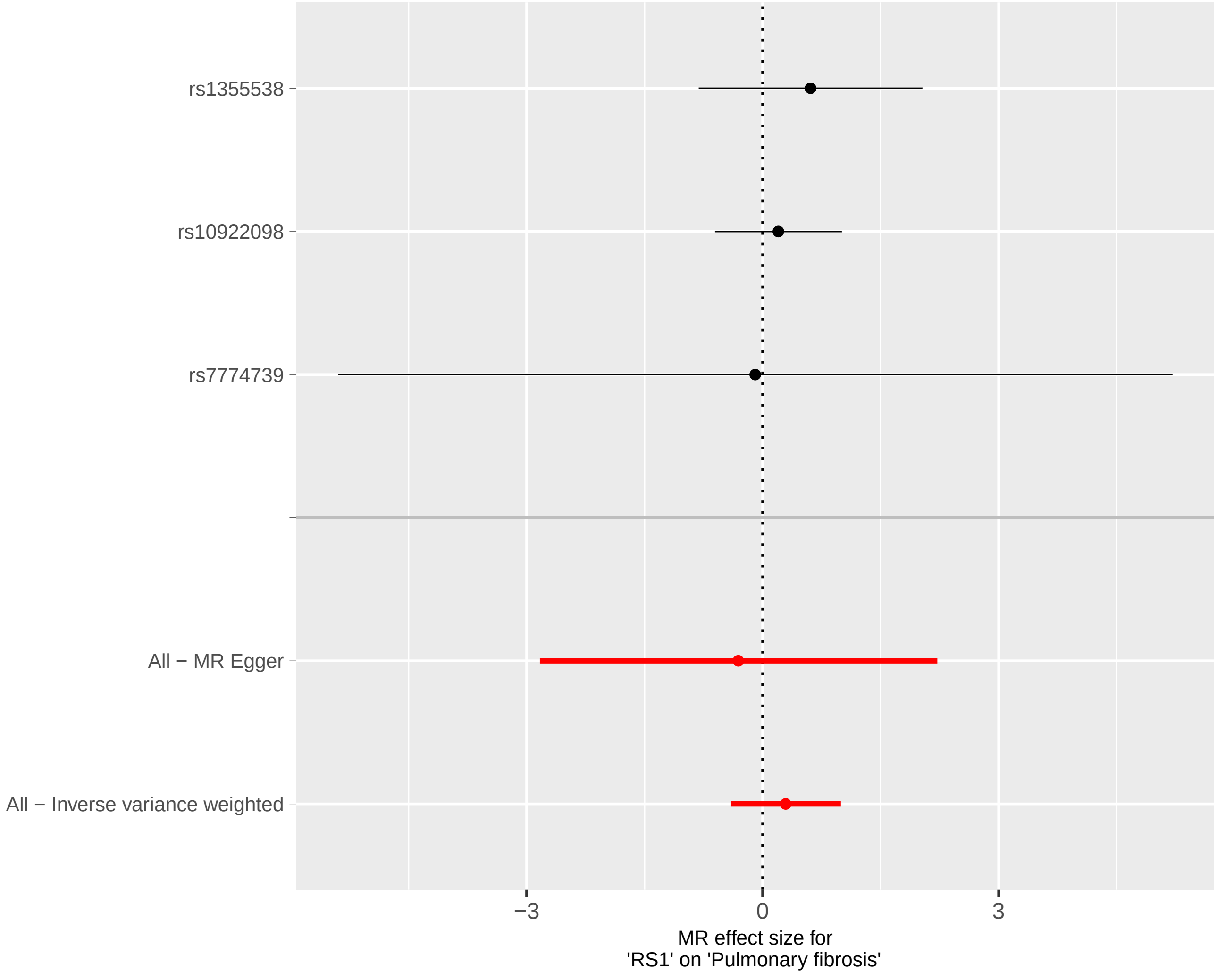

rs1355538
rs10922098
rs7774739
All − MR Egger
All − Inverse variance weighted
−3
0
3
MR effect size for
'RS1' on 'Pulmonary fibrosis'

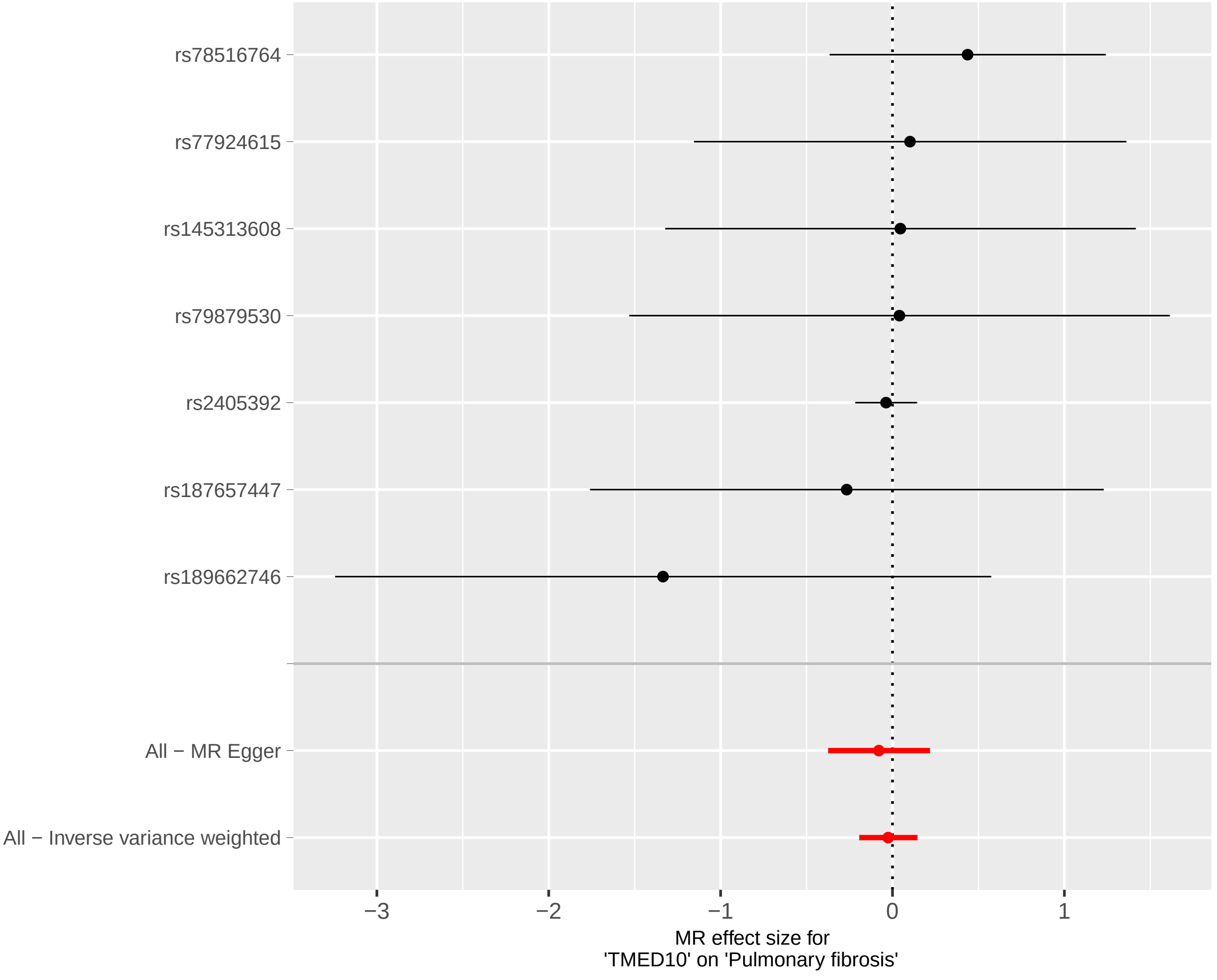

rs78516764
rs77924615
rs145313608
rs79879530
rs2405392
rs187657447
rs189662746
All − MR Egger
All − Inverse variance weighted
−3
−2
−1
0
1
MR effect size for
'TMED10' on 'Pulmonary fibrosis'

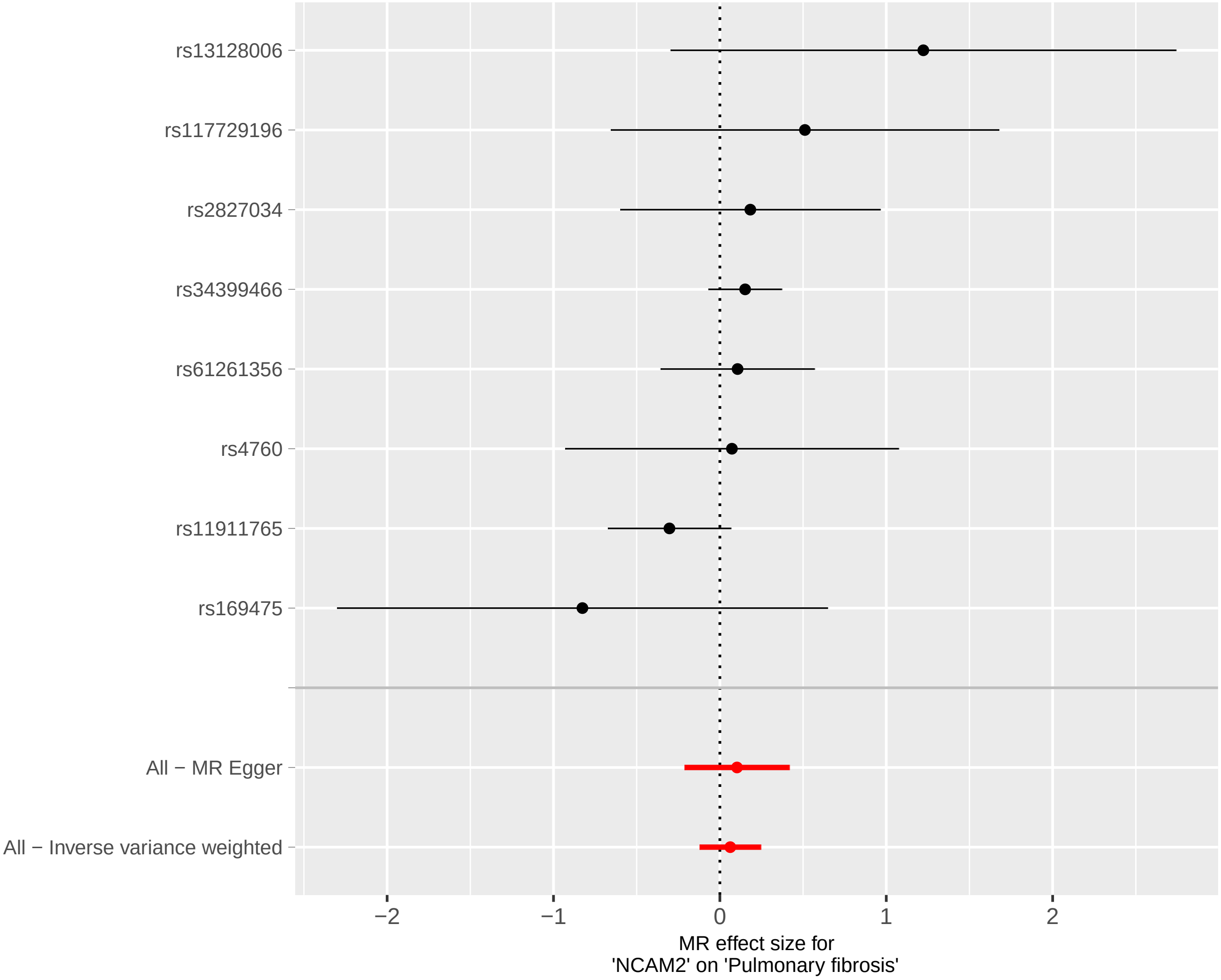

rs13128006
rs117729196
rs2827034
rs34399466
rs61261356
rs4760
rs11911765
rs169475
All − MR Egger
All − Inverse variance weighted
−2
−1
0
1
2
MR effect size for
'NCAM2' on 'Pulmonary fibrosis'

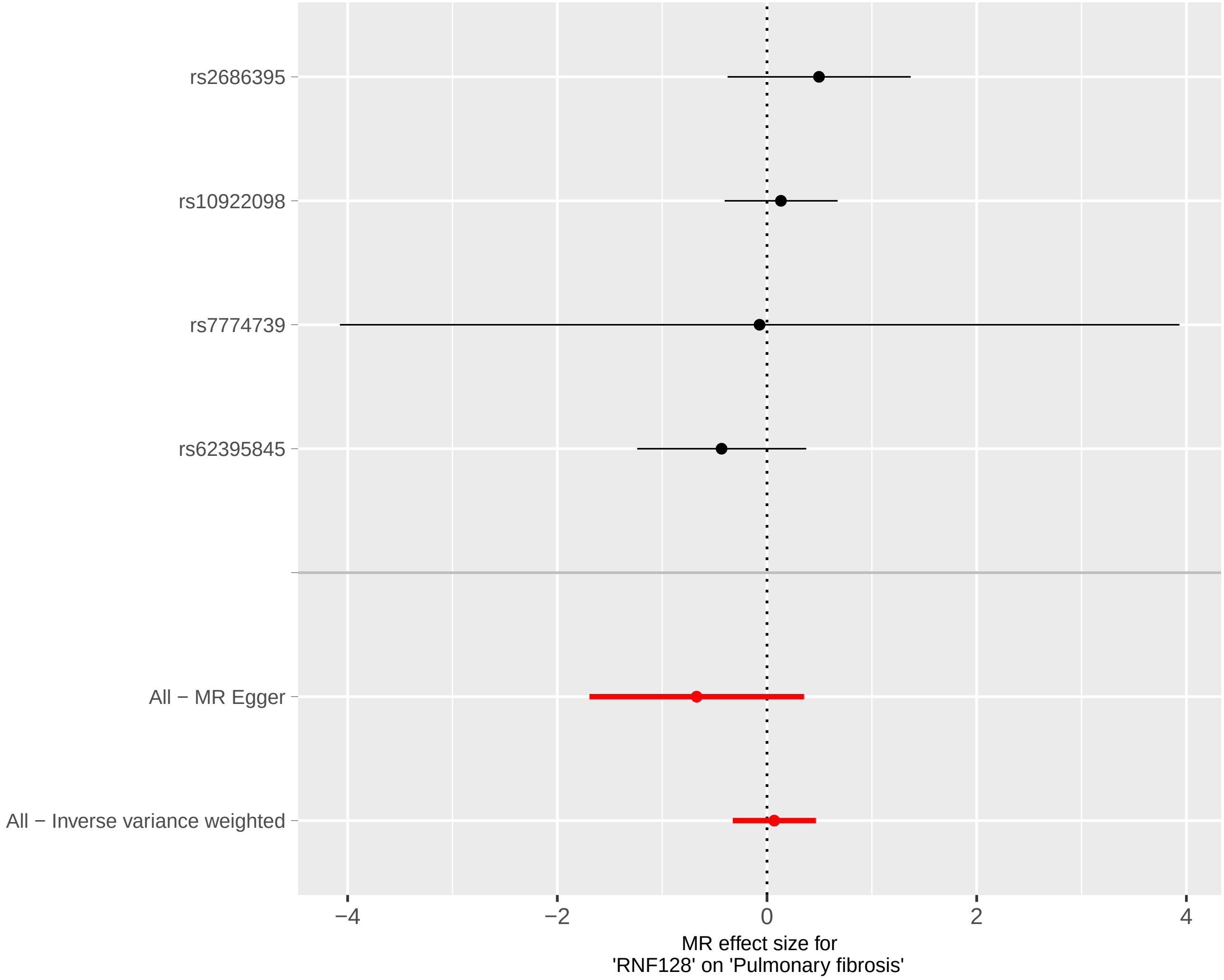

rs2686395
rs10922098
rs7774739
rs62395845
All − MR Egger
All − Inverse variance weighted
−4
−2
0
2
4
MR effect size for
'RNF128' on 'Pulmonary fibrosis'

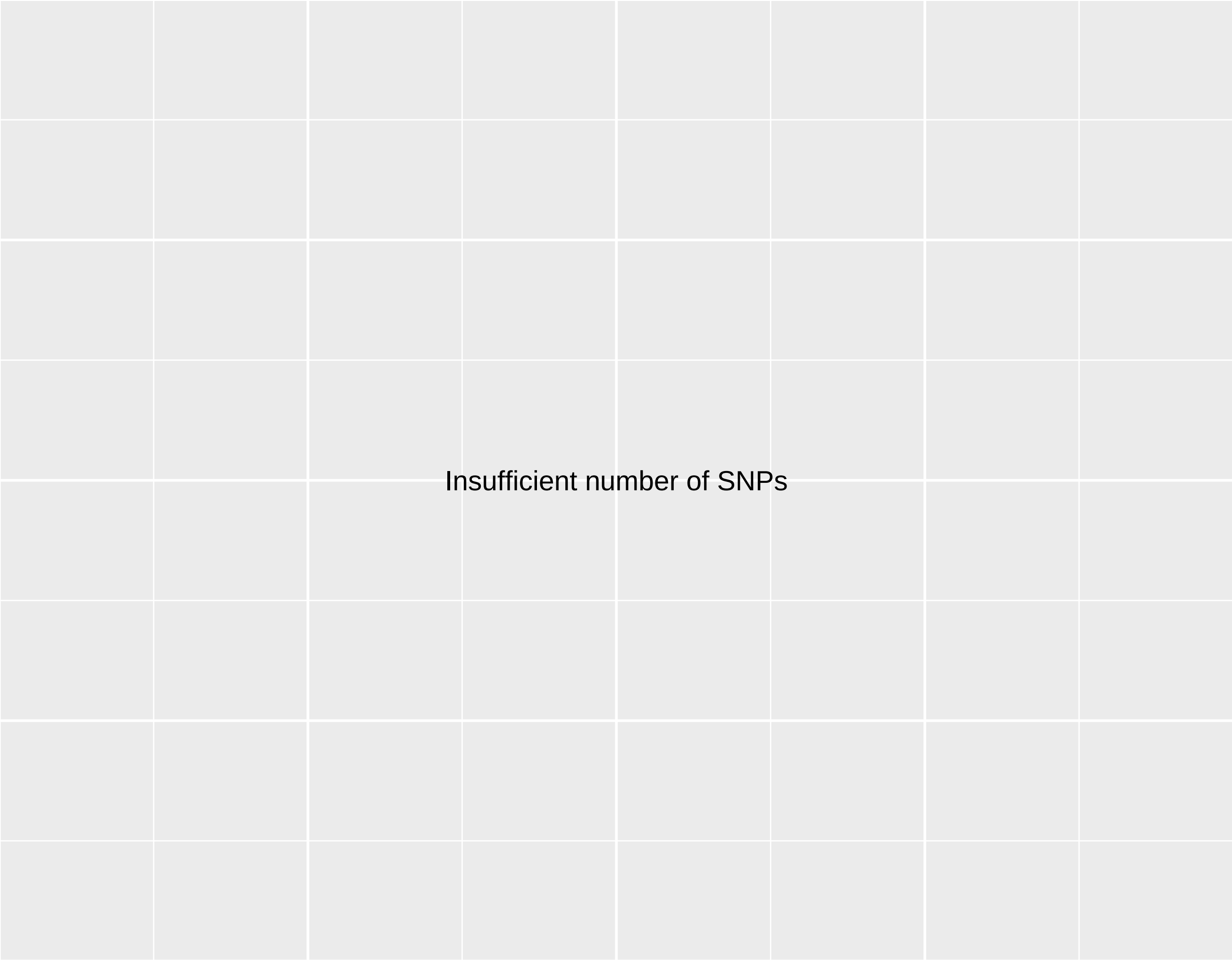
Insufficient number of SNPs

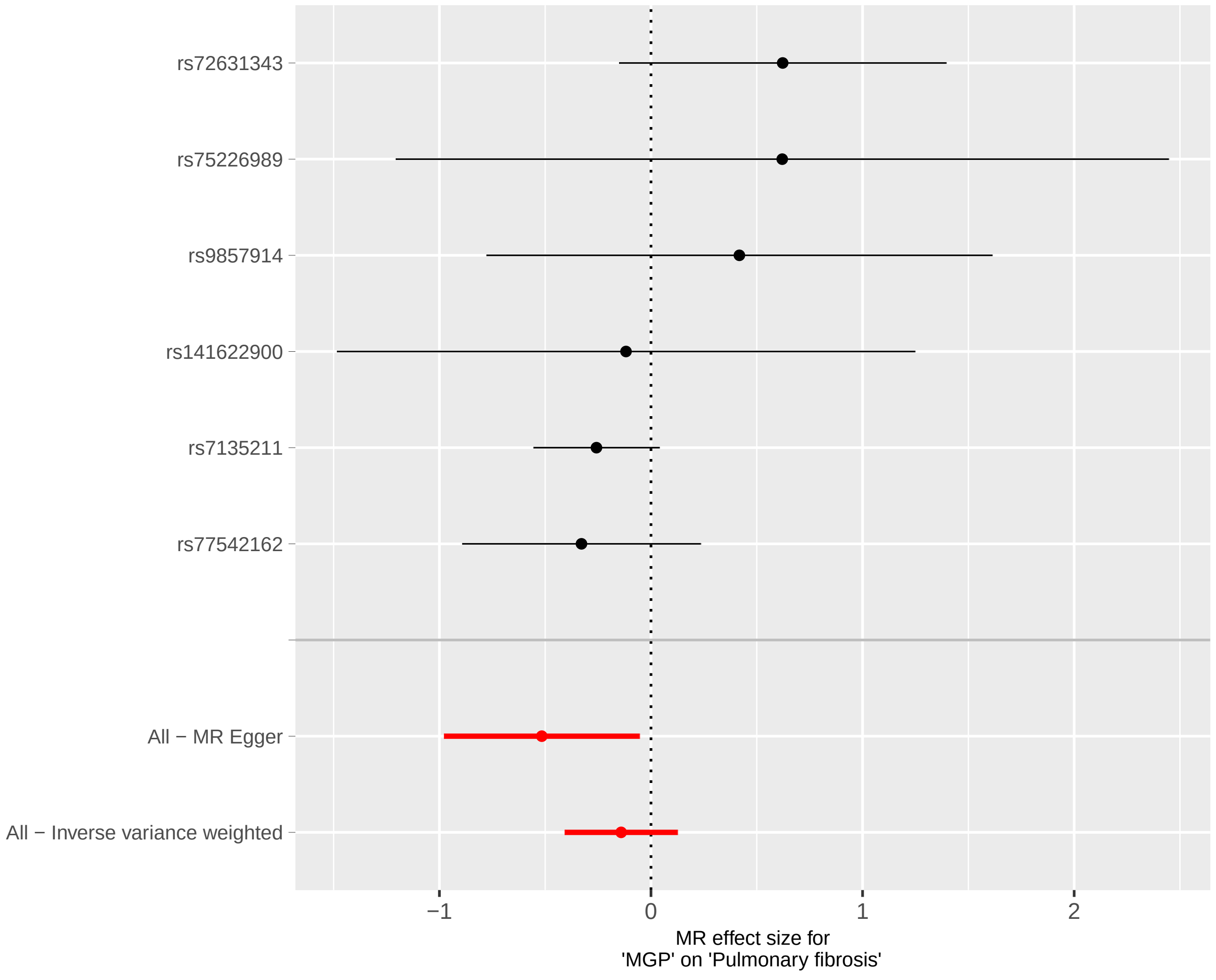

rs72631343
rs75226989
rs9857914
rs141622900
rs7135211
rs77542162
All − MR Egger
All − Inverse variance weighted
−1
0
1
2
MR effect size for
'MGP' on 'Pulmonary fibrosis'

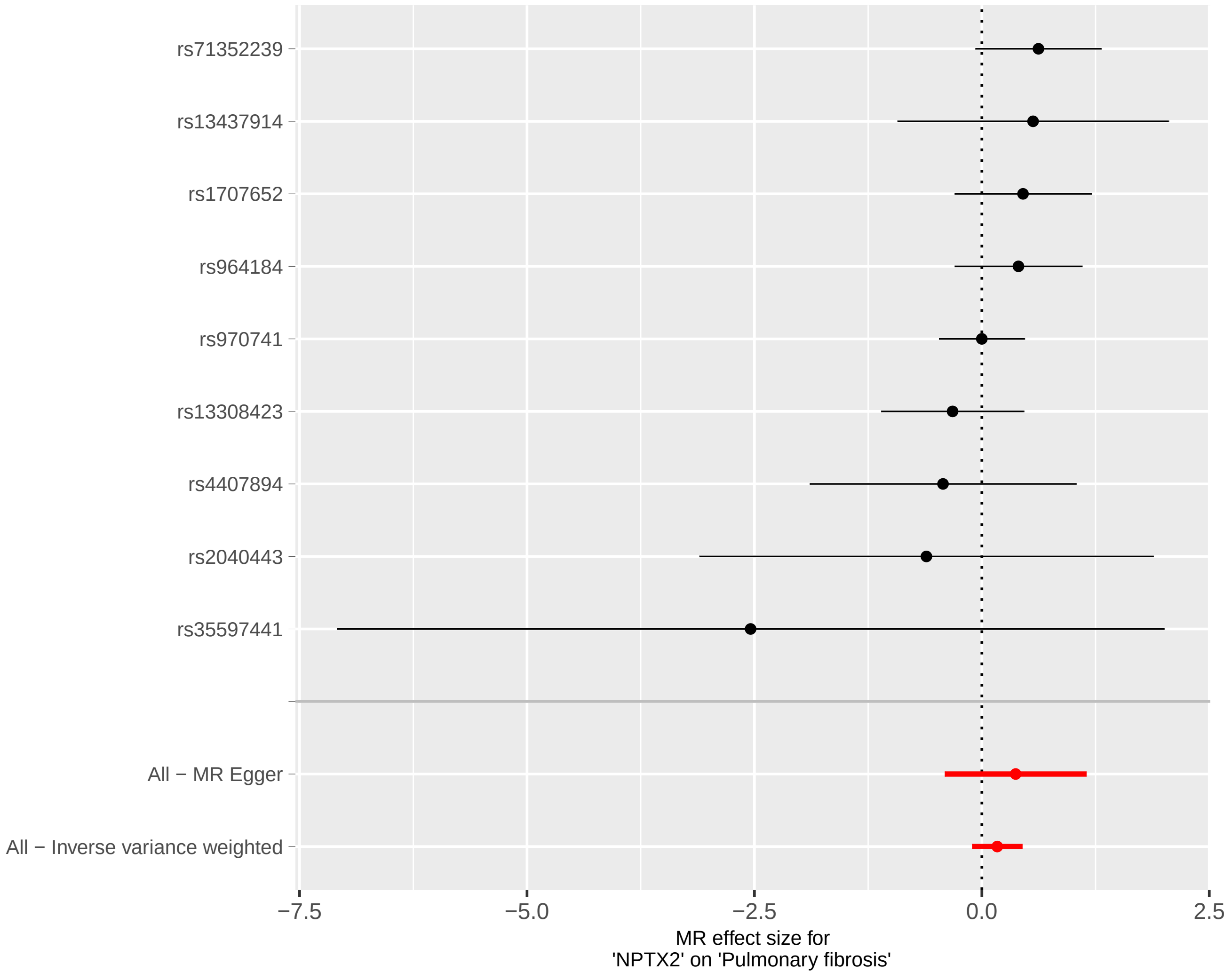
rs71352239
rs13437914
rs1707652
rs964184
rs970741
rs13308423
rs4407894
rs2040443
rs35597441
All − MR Egger
All − Inverse variance weighted
−7.5
−5.0
−2.5
0.0
2.5
MR effect size for
'NPTX2' on 'Pulmonary fibrosis'

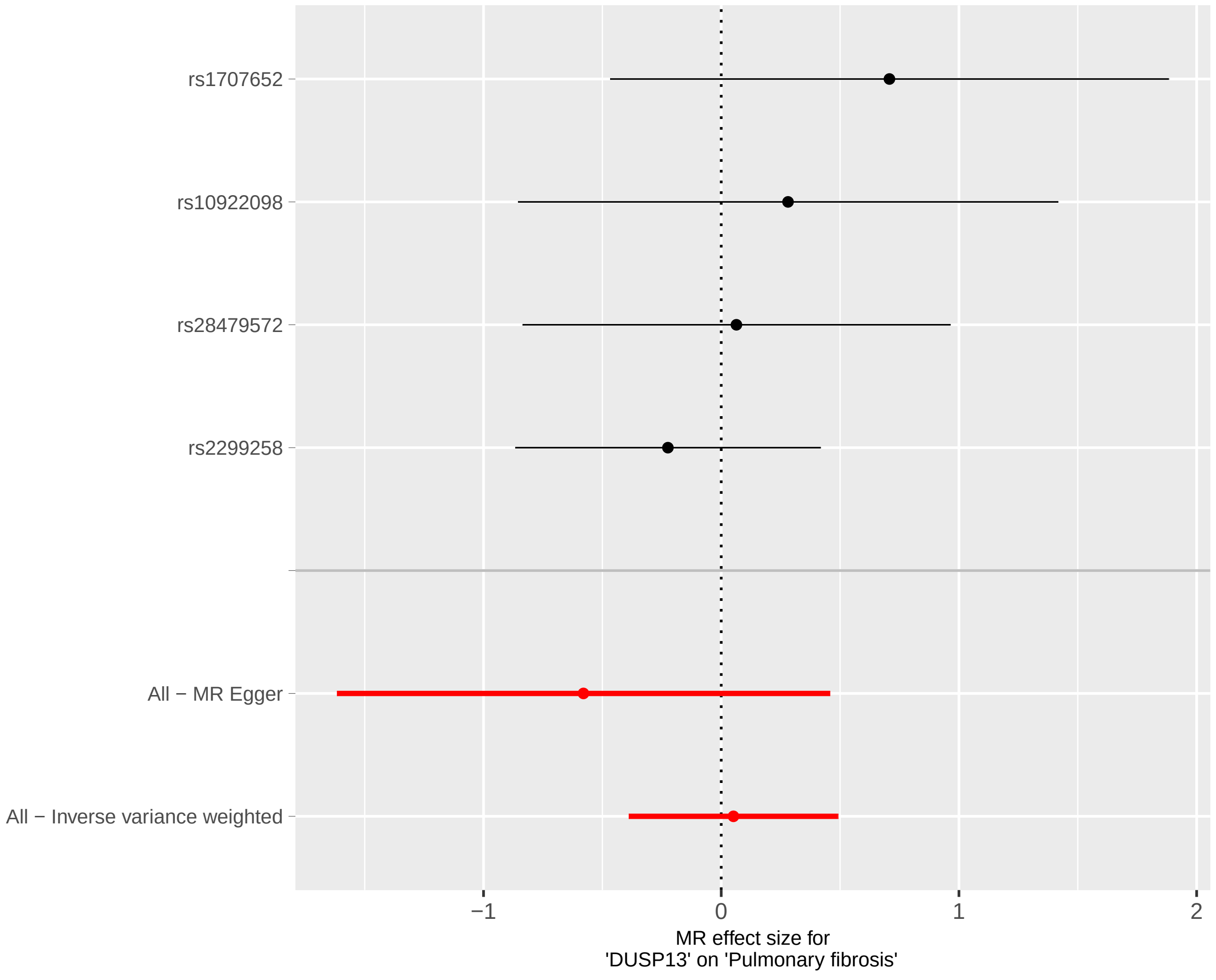

rs1707652
rs10922098
rs28479572
rs2299258
All − MR Egger
All − Inverse variance weighted
−1
0
1
2
MR effect size for
'DUSP13' on 'Pulmonary fibrosis'

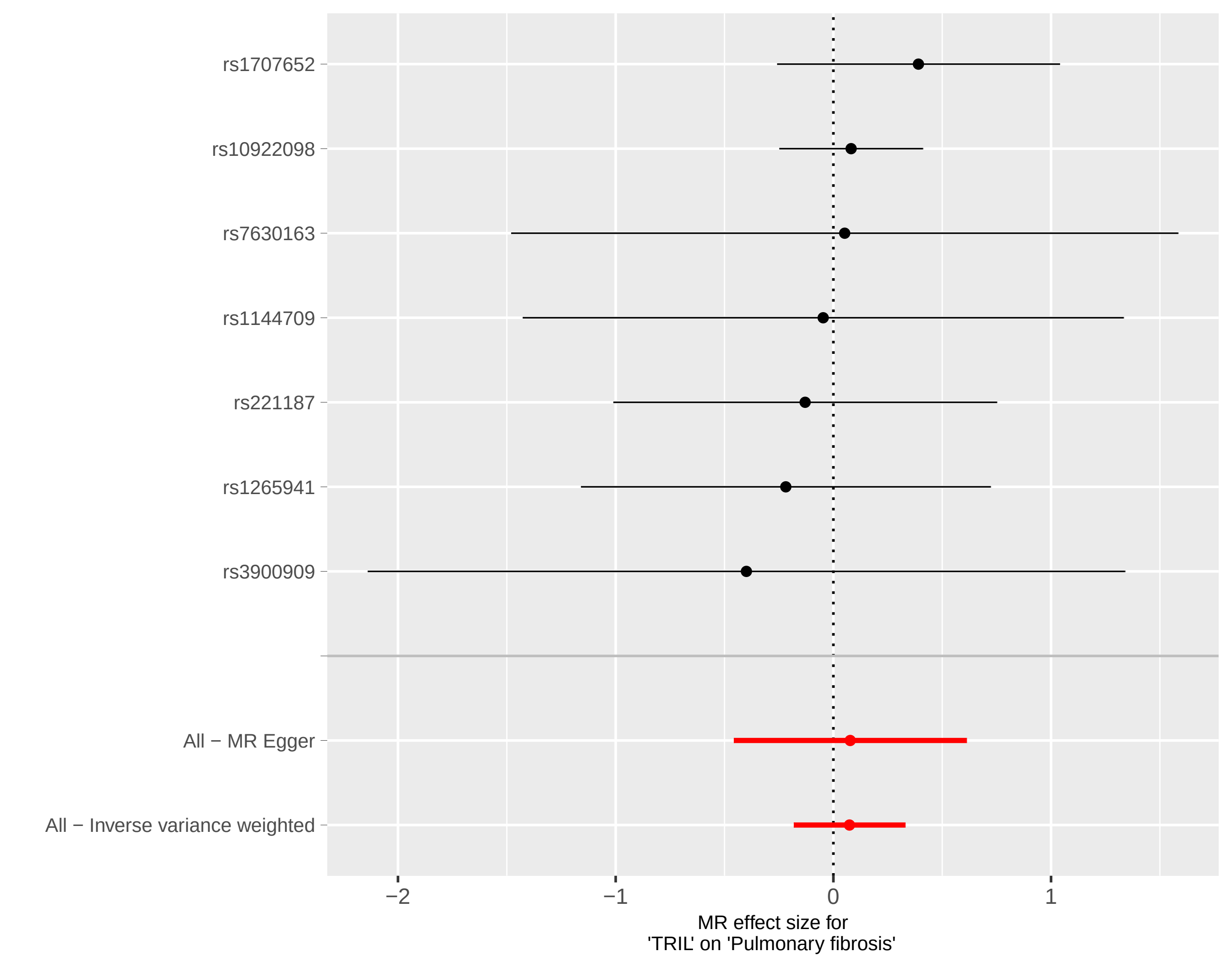
rs1707652
rs10922098
rs7630163
rs1144709
rs221187
rs1265941
rs3900909
All − MR Egger
All − Inverse variance weighted
−2
−1
0
1
MR effect size for
'TRIL' on 'Pulmonary fibrosis'

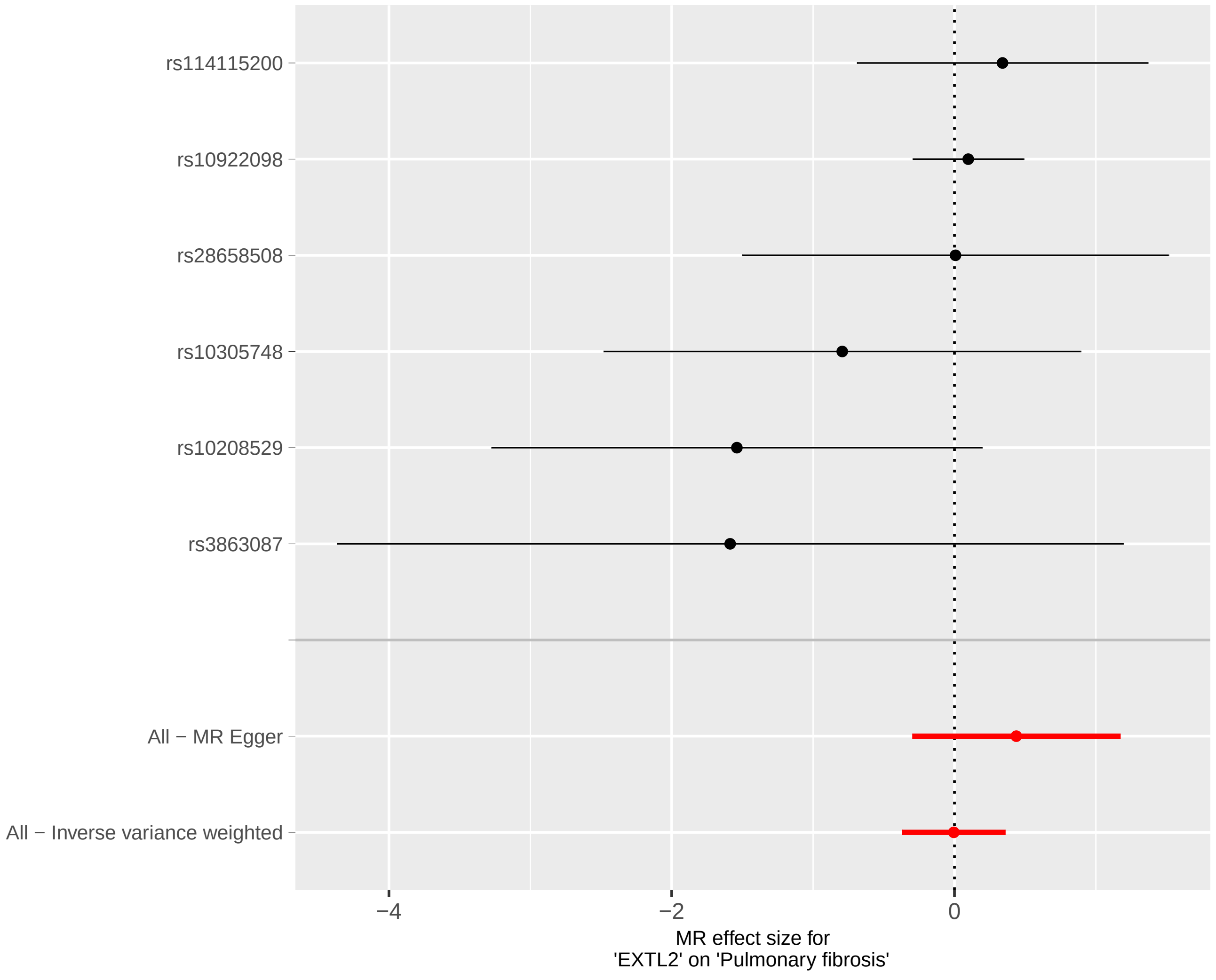

rs114115200
rs10922098
rs28658508
rs10305748
rs10208529
rs3863087
All − MR Egger
All − Inverse variance weighted
−4
−2
0
MR effect size for
'EXTL2' on 'Pulmonary fibrosis'

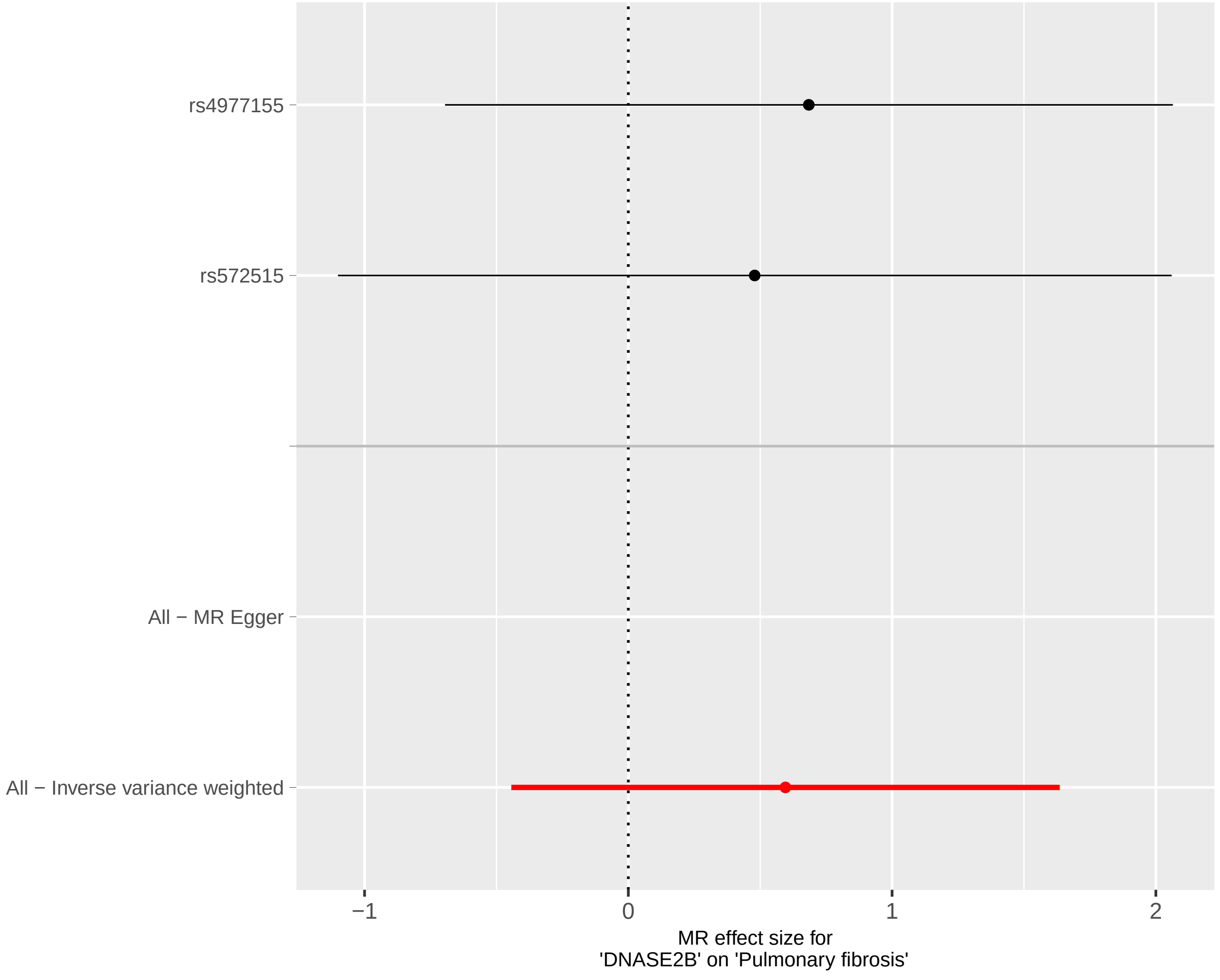
rs4977155
rs572515
All − MR Egger
All − Inverse variance weighted
−1
0
1
2
MR effect size for
'DNASE2B' on 'Pulmonary fibrosis'

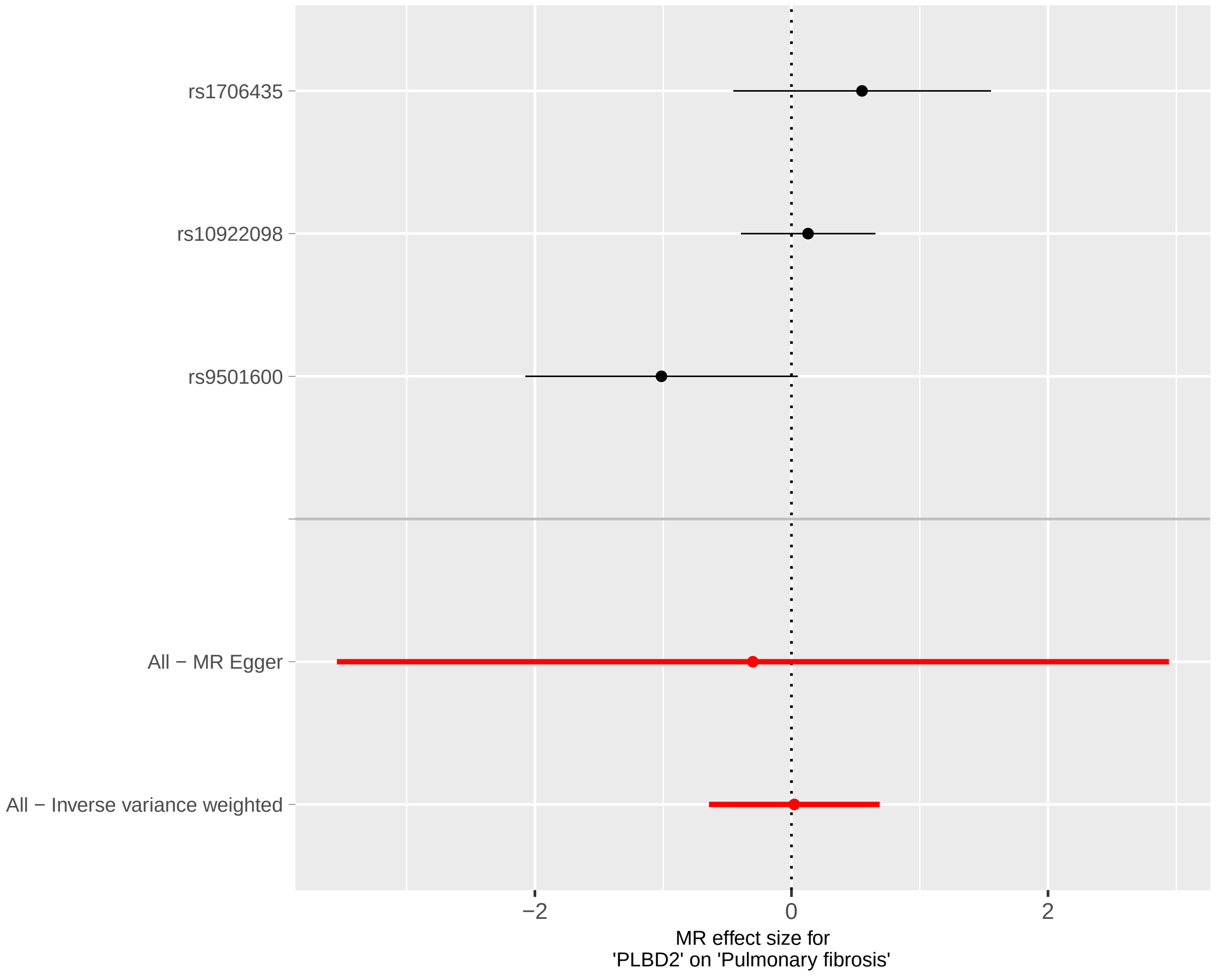
rs1706435
rs10922098
rs9501600
All − MR Egger
All − Inverse variance weighted
−2
0
2
MR effect size for
'PLBD2' on 'Pulmonary fibrosis'

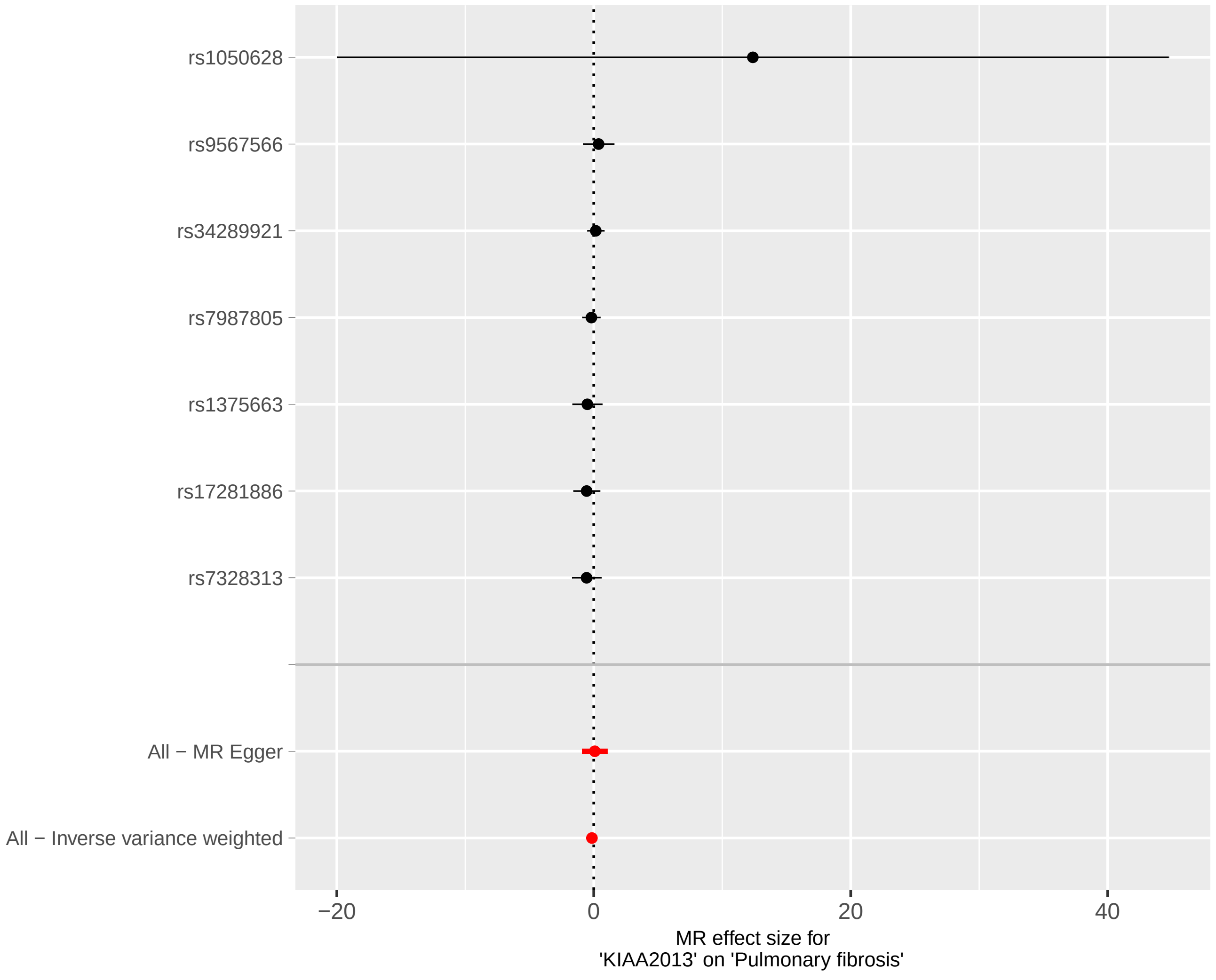
rs1050628
rs9567566
rs34289921
rs7987805
rs1375663
rs17281886
rs7328313
All − MR Egger
All − Inverse variance weighted
−20
0
20
40
MR effect size for
'KIAA2013' on 'Pulmonary fibrosis'

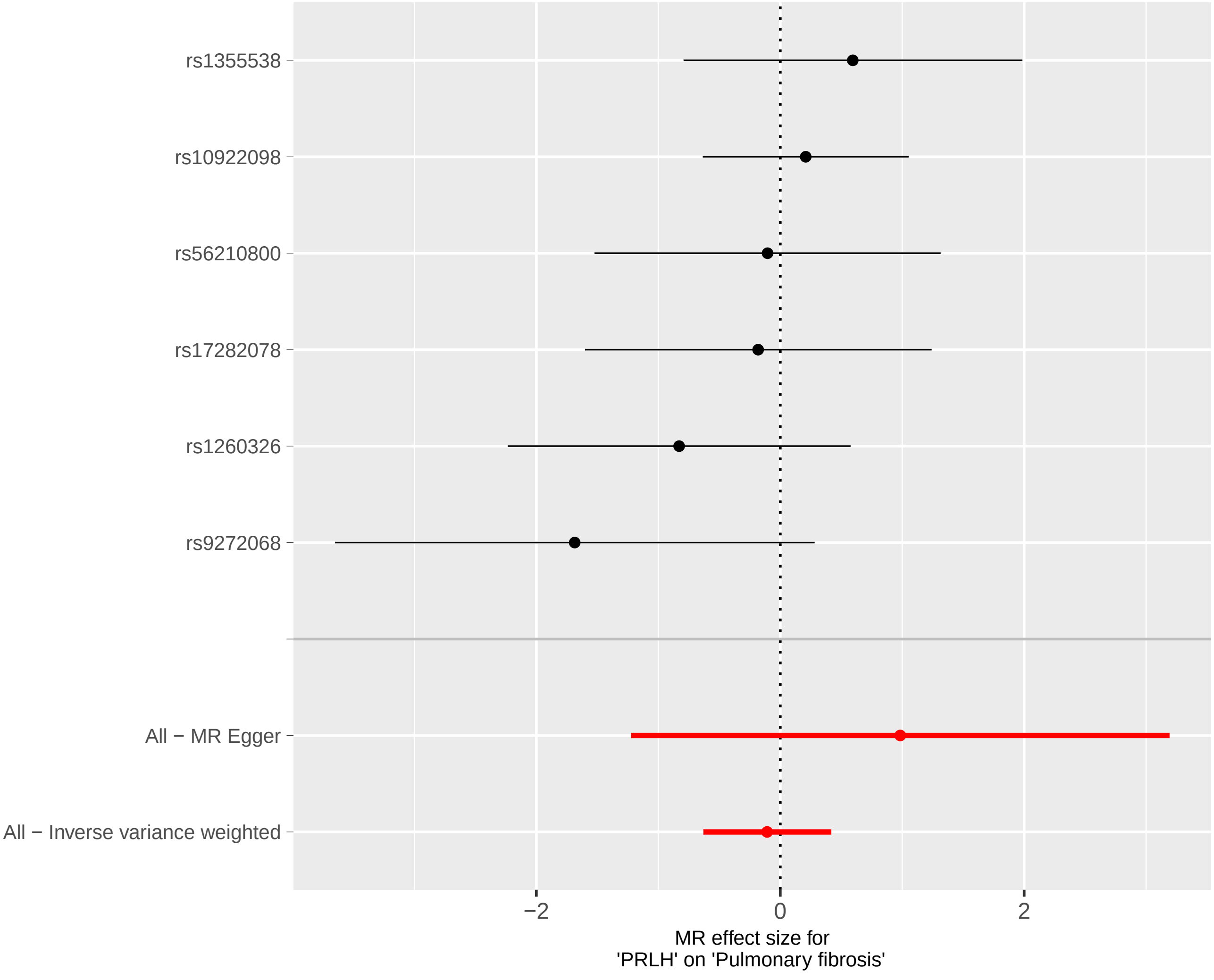
rs1355538
rs10922098
rs56210800
rs17282078
rs1260326
rs9272068
All − MR Egger
All − Inverse variance weighted
−2
0
2
MR effect size for
'PRLH' on 'Pulmonary fibrosis'

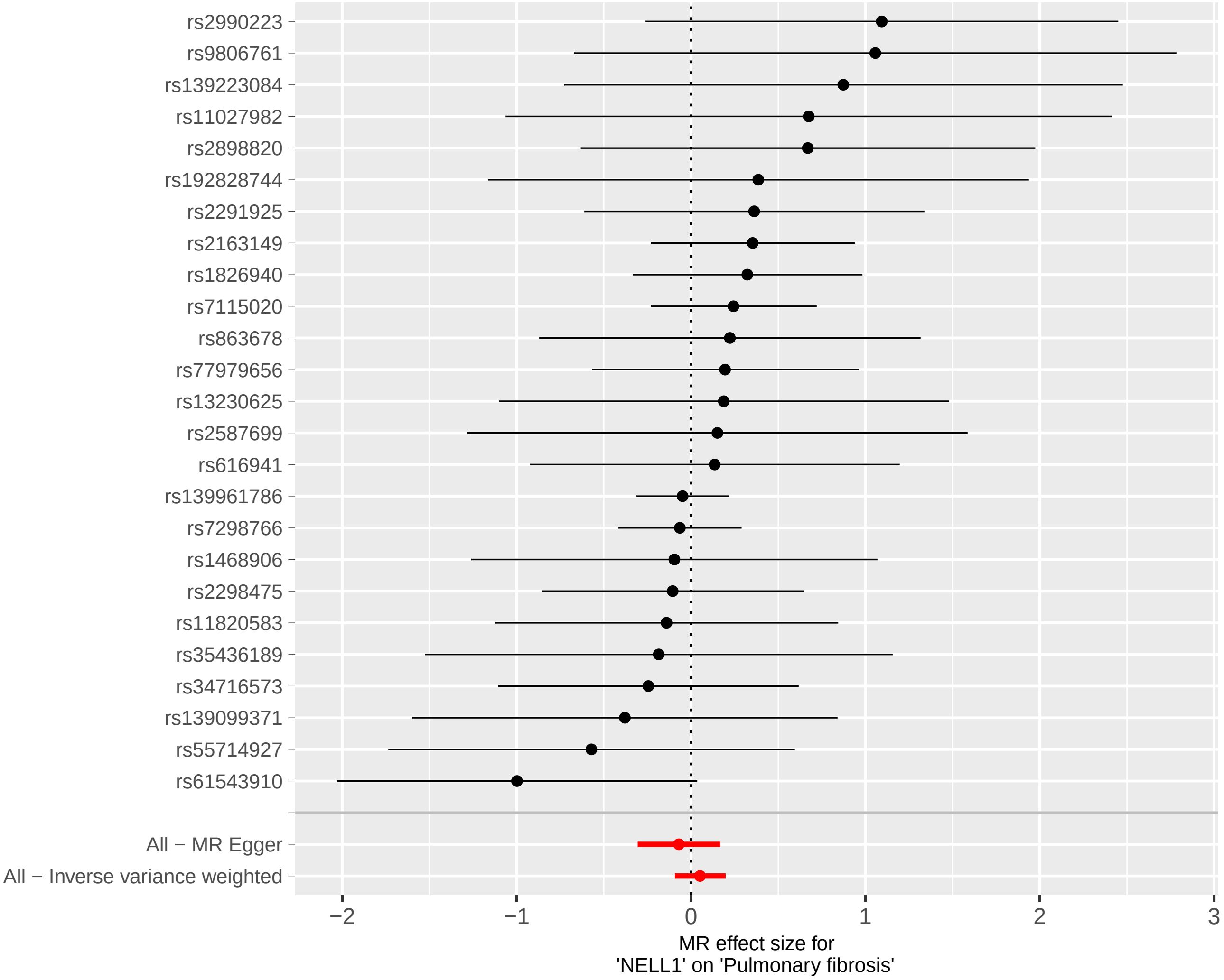

rs2990223
rs9806761
rs139223084
rs11027982
rs2898820
rs192828744
rs2291925
rs2163149
rs1826940
rs7115020
rs863678
rs77979656
rs13230625
rs2587699
rs616941
rs139961786
rs7298766
rs1468906
rs2298475
rs11820583
rs35436189
rs34716573
rs139099371
rs55714927
rs61543910
All − MR Egger
All − Inverse variance weighted
−2
−1
0
1
2
3
MR effect size for
'NELL1' on 'Pulmonary fibrosis'

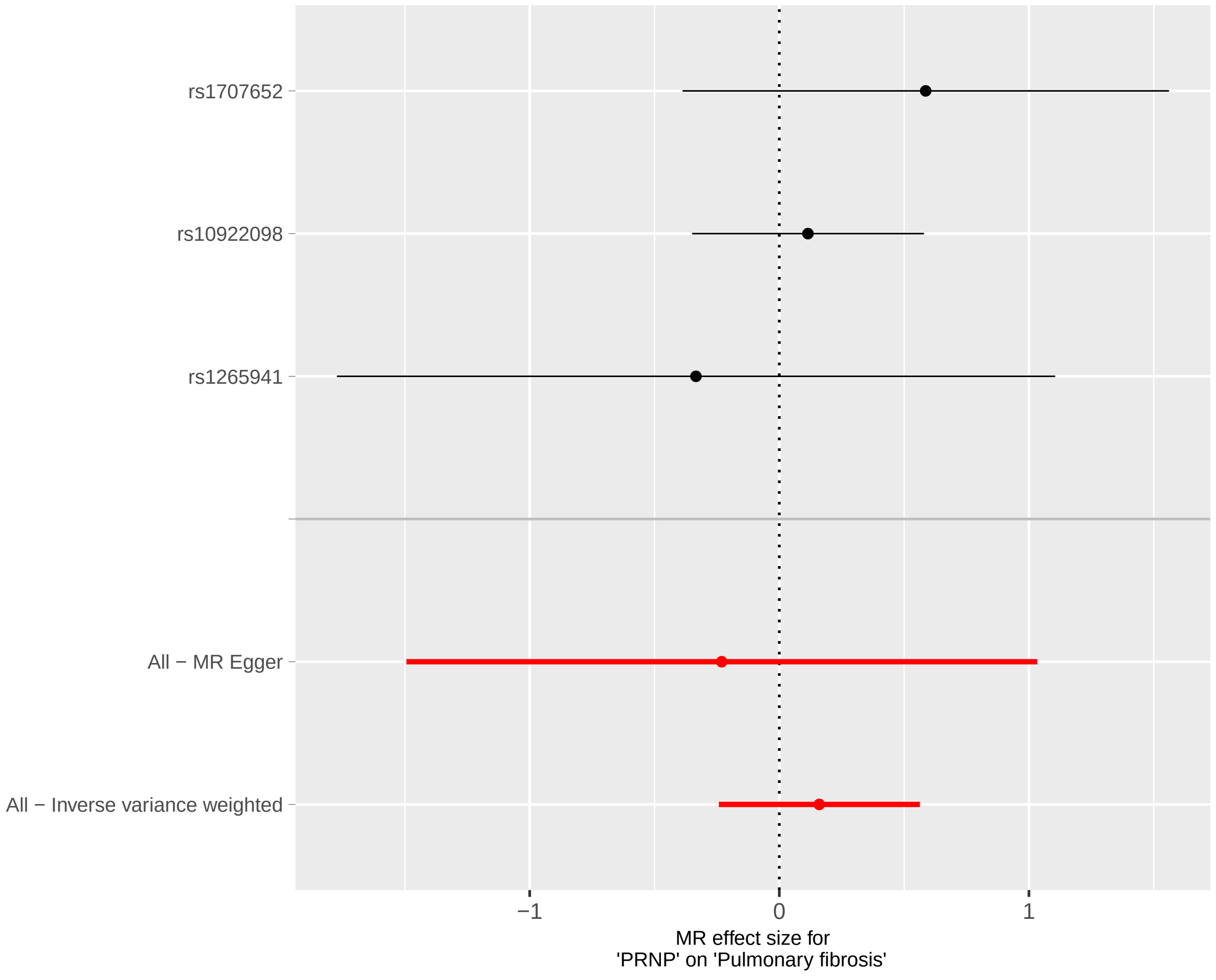
rs1707652
rs10922098
rs1265941
All − MR Egger
All − Inverse variance weighted
−1
0
1
MR effect size for
'PRNP' on 'Pulmonary fibrosis'

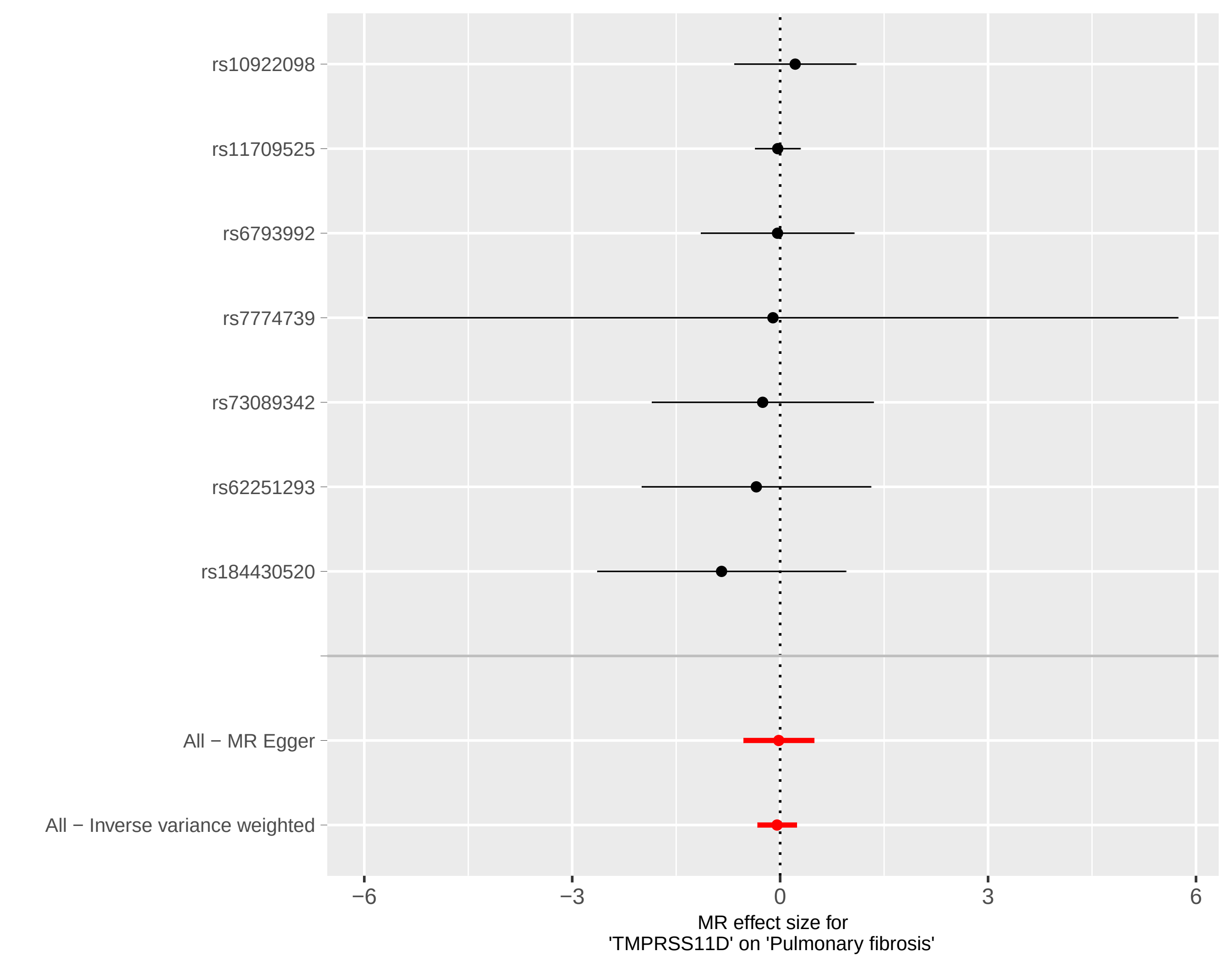

rs10922098
rs11709525
rs6793992
rs7774739
rs73089342
rs62251293
rs184430520
All − MR Egger
All − Inverse variance weighted
−6
−3
0
3
6
MR effect size for
'TMPRSS11D' on 'Pulmonary fibrosis'

Insufficient number of SNPs

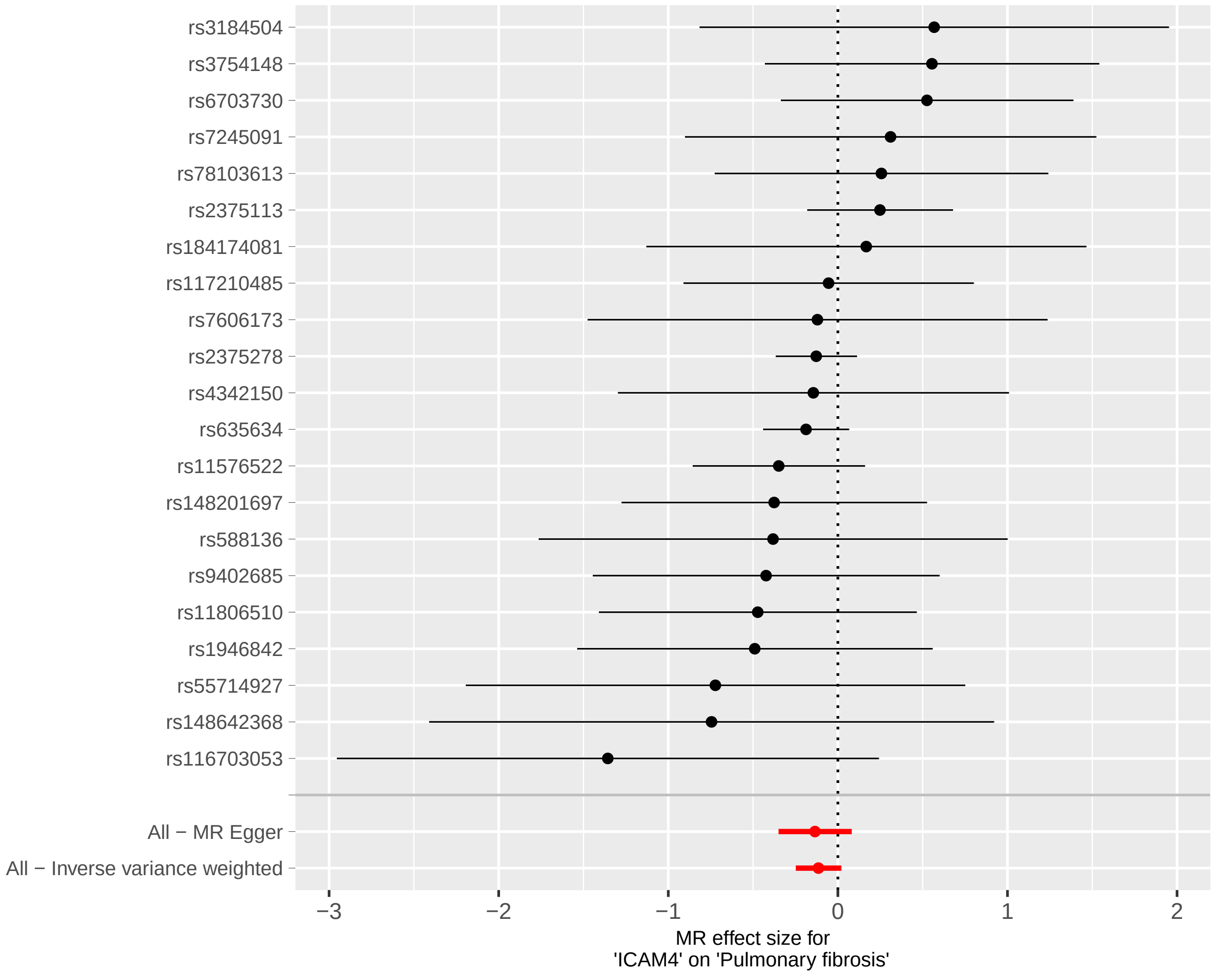

rs3184504
rs3754148
rs6703730
rs7245091
rs78103613
rs2375113
rs184174081
rs117210485
rs7606173
rs2375278
rs4342150
rs635634
rs11576522
rs148201697
rs588136
rs9402685
rs11806510
rs1946842
rs55714927
rs148642368
rs116703053
All − MR Egger
All − Inverse variance weighted
−3
−2
−1
0
1
2
MR effect size for
'ICAM4' on 'Pulmonary fibrosis'

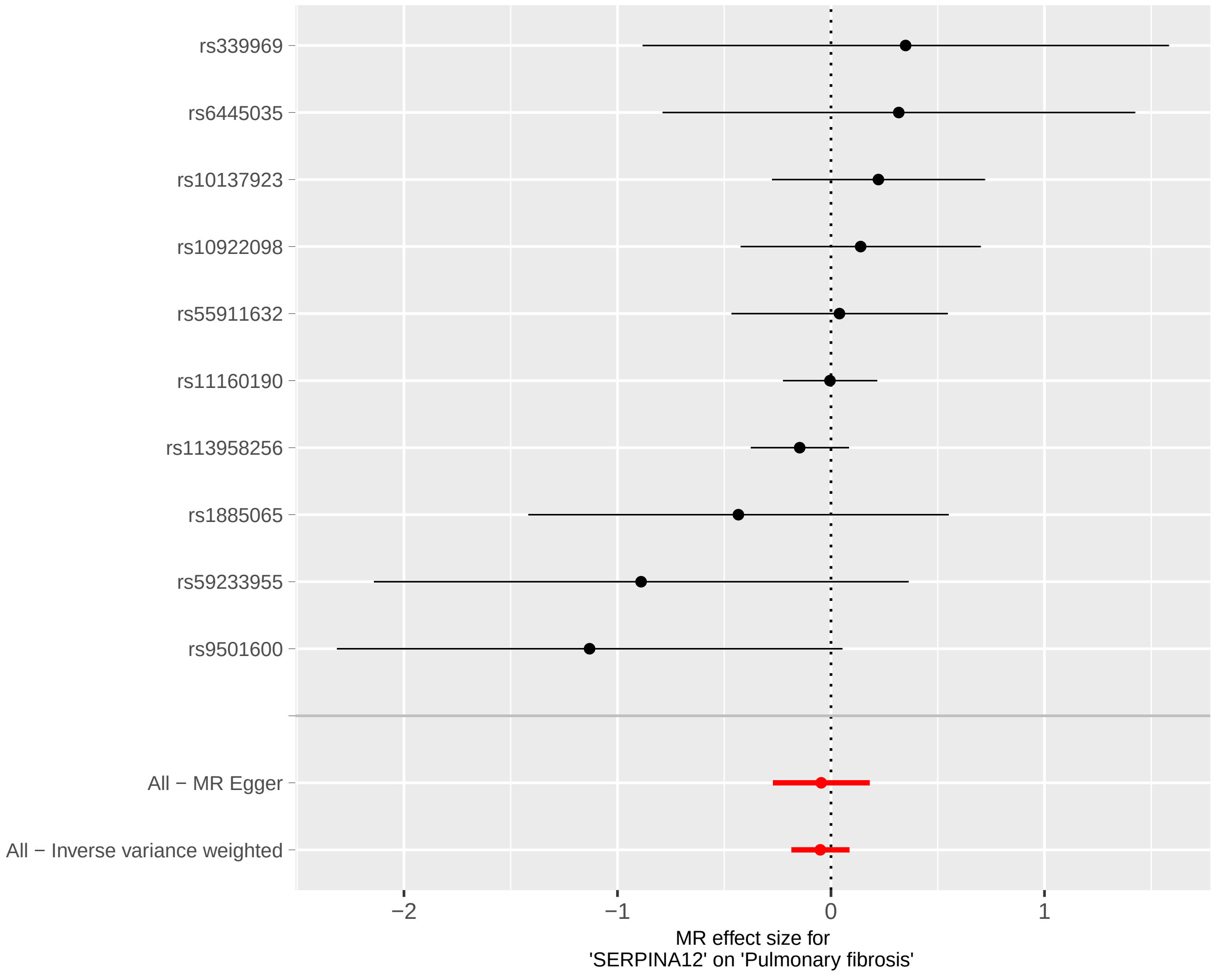
rs339969
rs6445035
rs10137923
rs10922098
rs55911632
rs11160190
rs113958256
rs1885065
rs59233955
rs9501600
All − MR Egger
All − Inverse variance weighted
−2
−1
0
1
MR effect size for
'SERPINA12' on 'Pulmonary fibrosis'

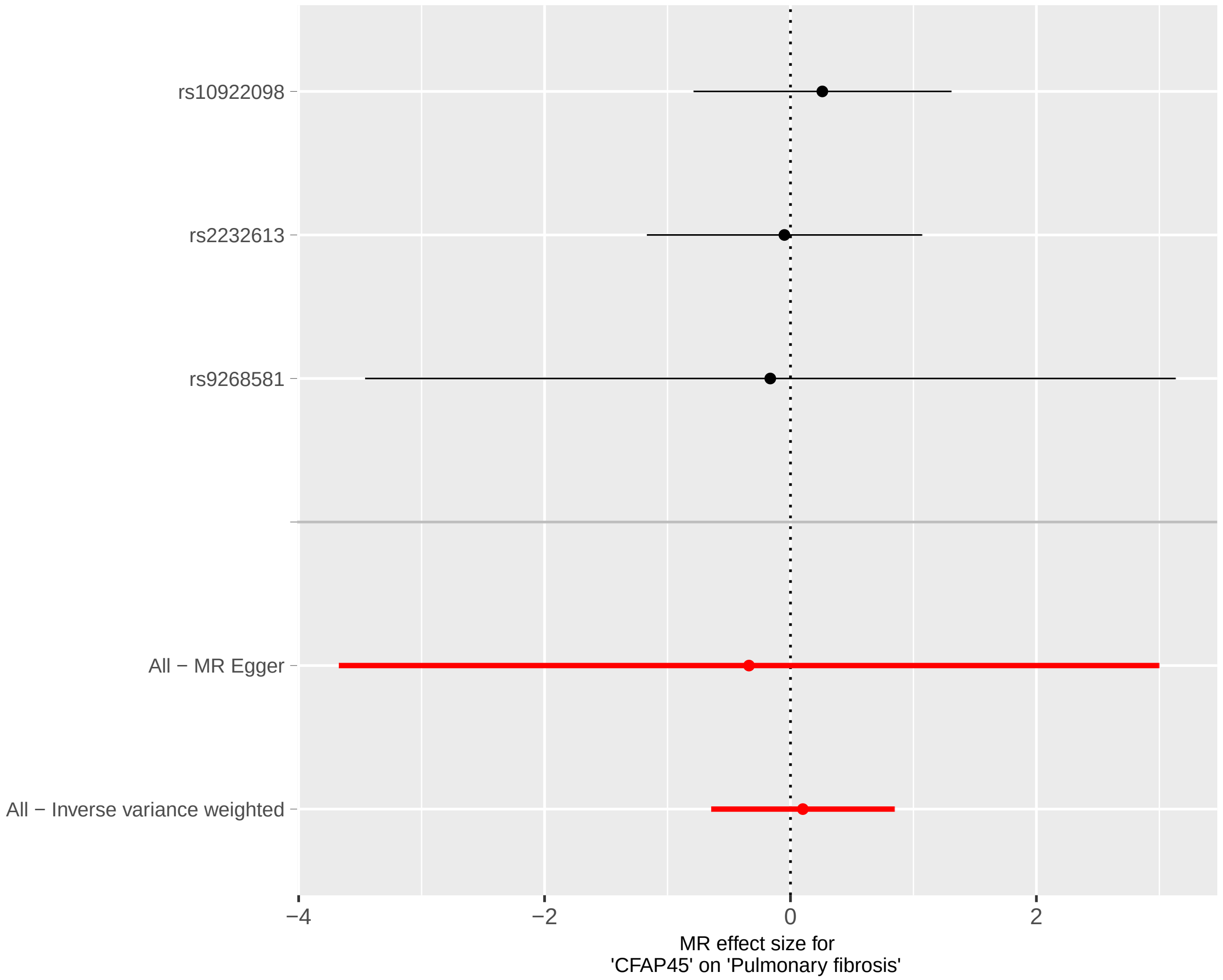
rs10922098
rs2232613
rs9268581
All − MR Egger
All − Inverse variance weighted
−4
−2
0
2
MR effect size for
'CFAP45' on 'Pulmonary fibrosis'

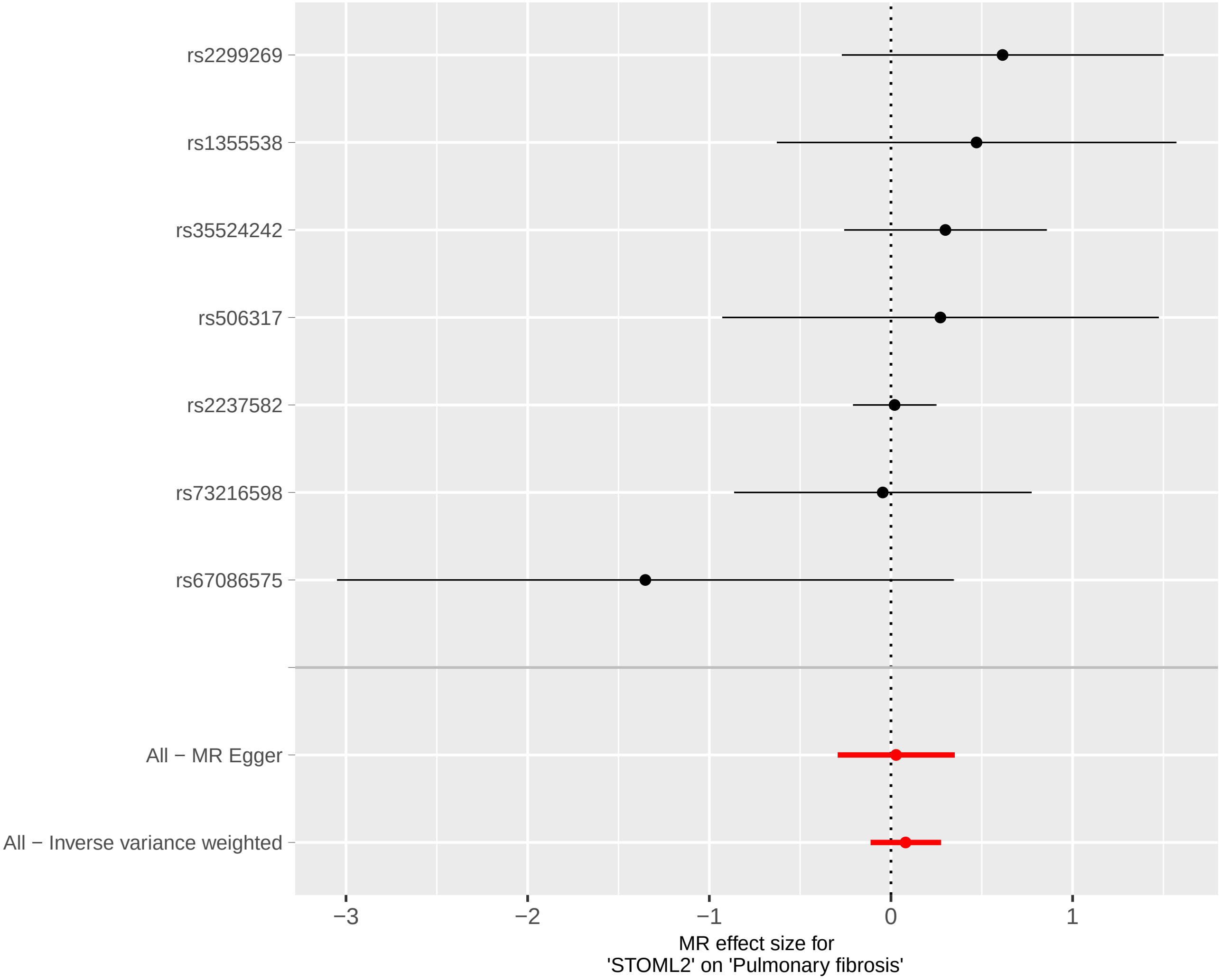

rs2299269
rs1355538
rs35524242
rs506317
rs2237582
rs73216598
rs67086575
All − MR Egger
All − Inverse variance weighted
−3
−2
−1
0
1
MR effect size for
'STOML2' on 'Pulmonary fibrosis'

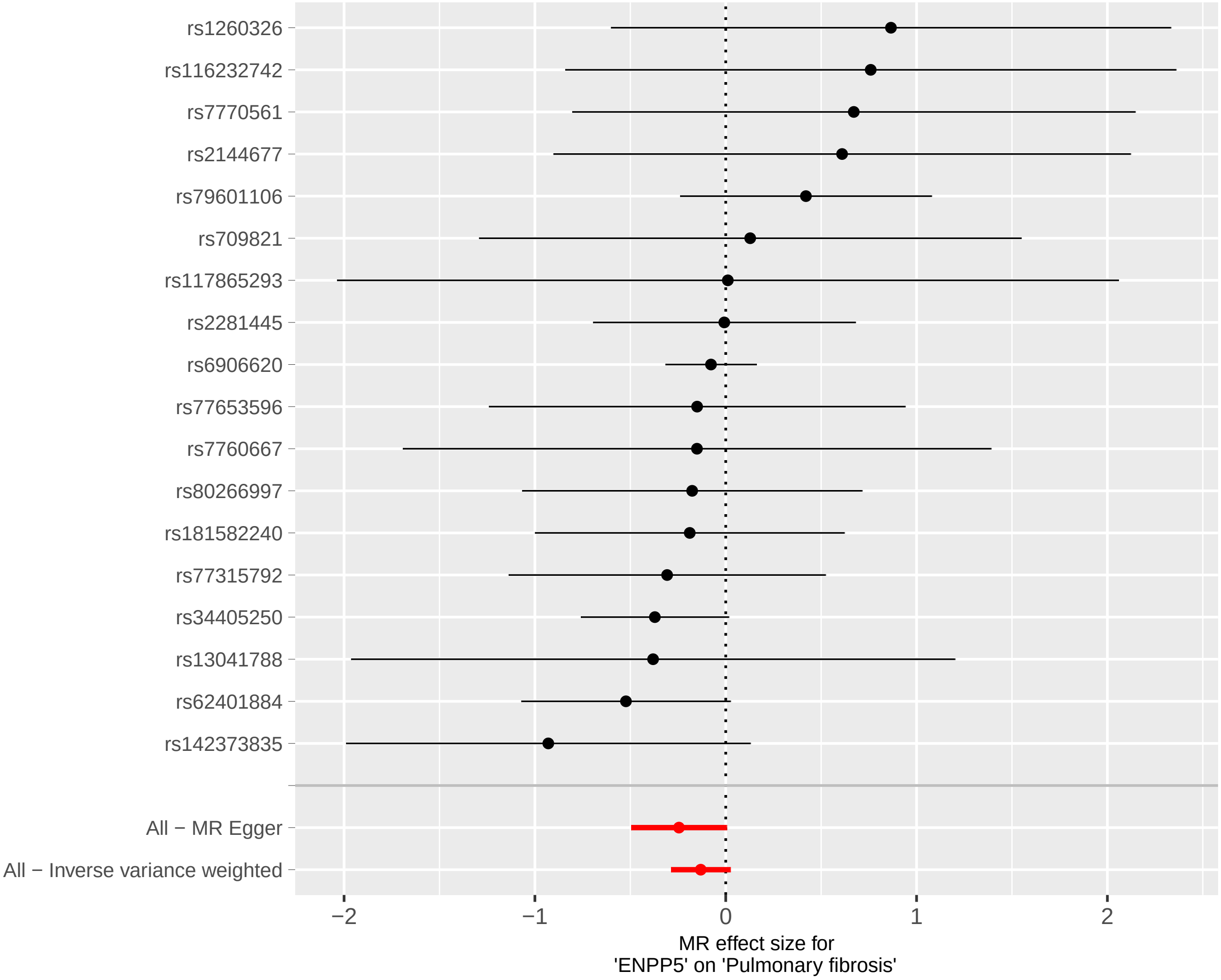
rs1260326
rs116232742
rs7770561
rs2144677
rs79601106
rs709821
rs117865293
rs2281445
rs6906620
rs77653596
rs7760667
rs80266997
rs181582240
rs77315792
rs34405250
rs13041788
rs62401884
rs142373835
All − MR Egger
All − Inverse variance weighted
−2
−1
0
1
2
MR effect size for
'ENPP5' on 'Pulmonary fibrosis'

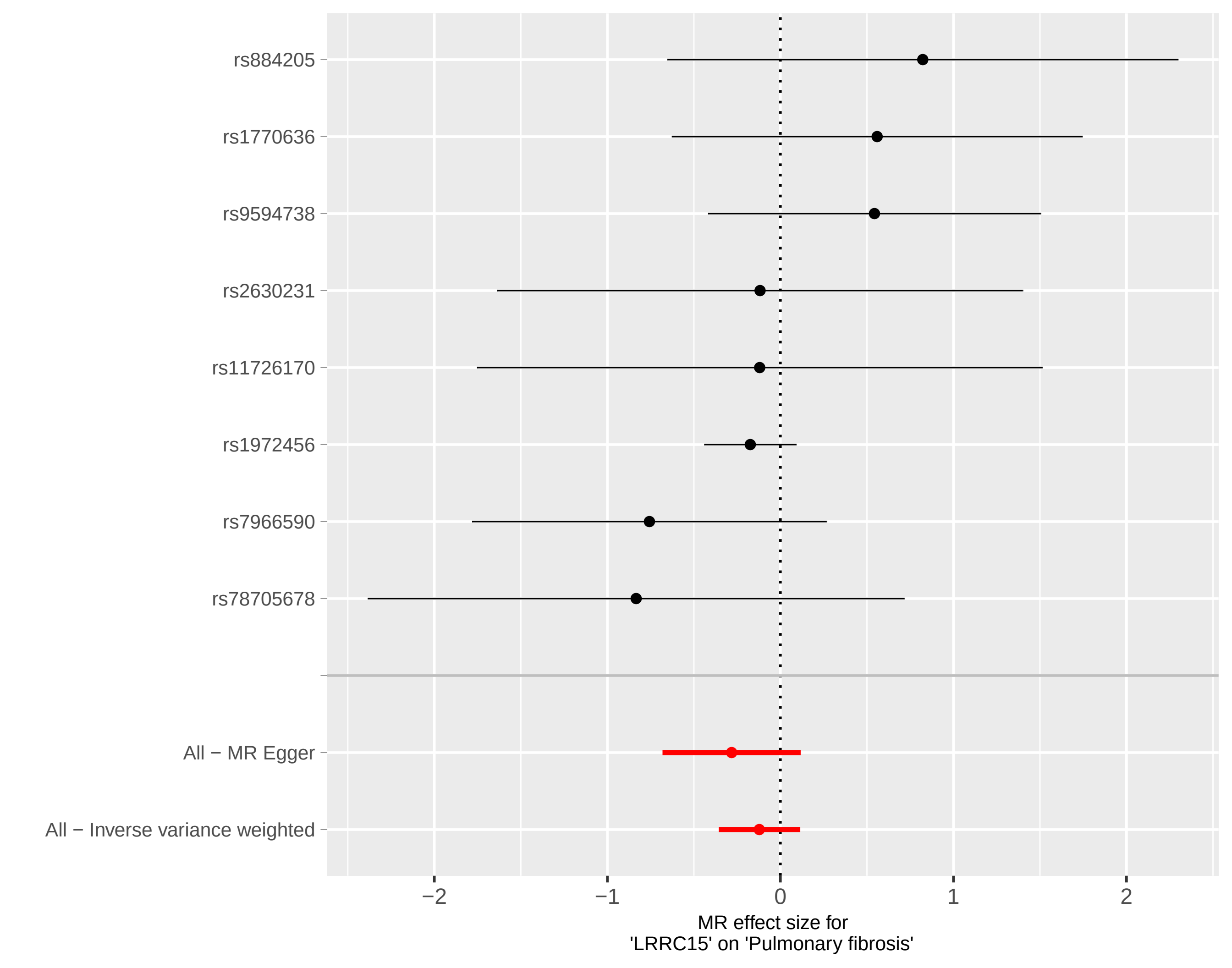

rs884205
rs1770636
rs9594738
rs2630231
rs11726170
rs1972456
rs7966590
rs78705678
All − MR Egger
All − Inverse variance weighted
−2
−1
0
1
2
MR effect size for
'LRRC15' on 'Pulmonary fibrosis'

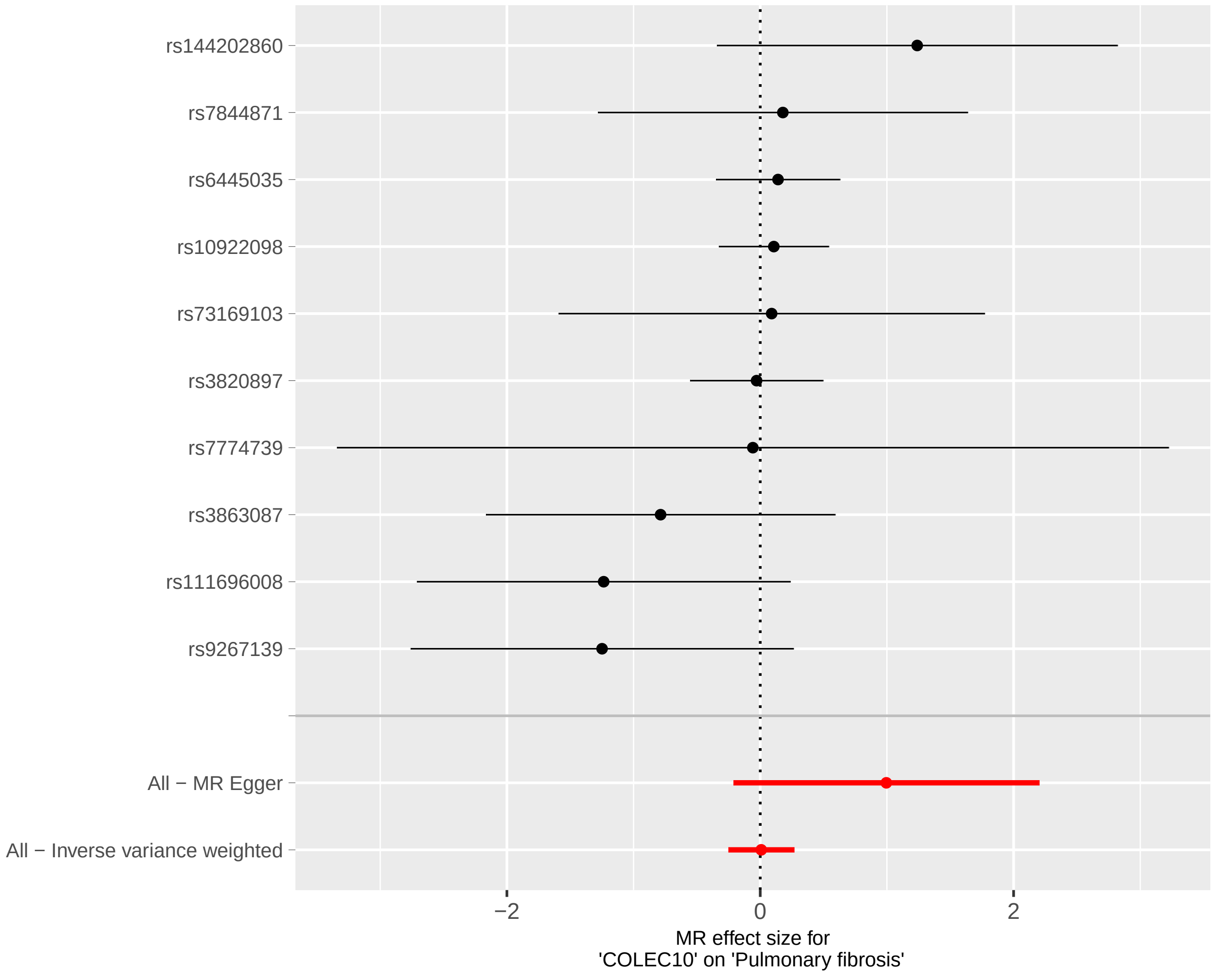
rs144202860
rs7844871
rs6445035
rs10922098
rs73169103
rs3820897
rs7774739
rs3863087
rs111696008
rs9267139
All − MR Egger
All − Inverse variance weighted
−2
0
2
MR effect size for
'COLEC10' on 'Pulmonary fibrosis'

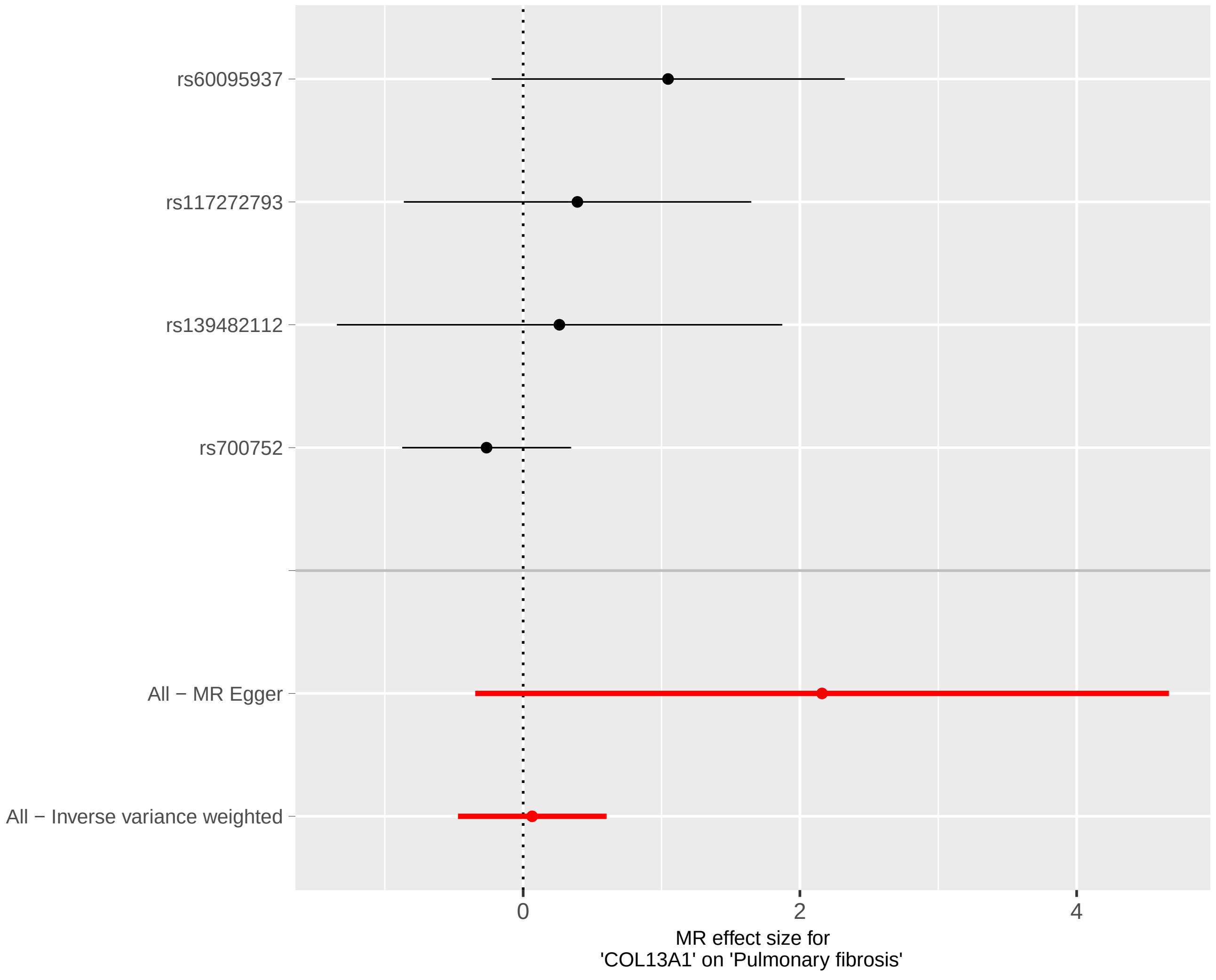

rs60095937
rs117272793
rs139482112
rs700752
All − MR Egger
All − Inverse variance weighted
0
2
4
MR effect size for
'COL13A1' on 'Pulmonary fibrosis'

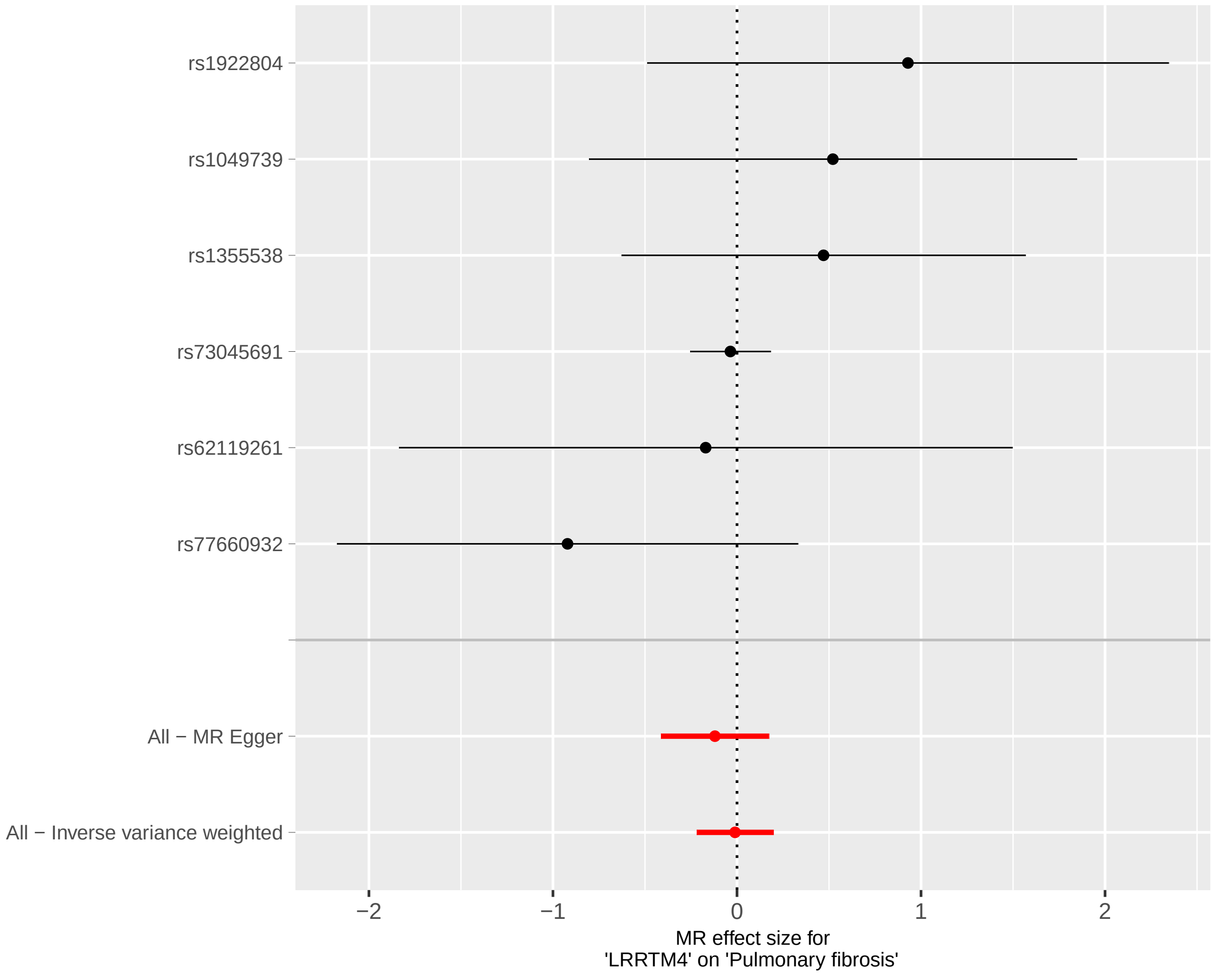

rs1922804
rs1049739
rs1355538
rs73045691
rs62119261
rs77660932
All − MR Egger
All − Inverse variance weighted
−2
−1
0
1
2
MR effect size for
'LRRTM4' on 'Pulmonary fibrosis'

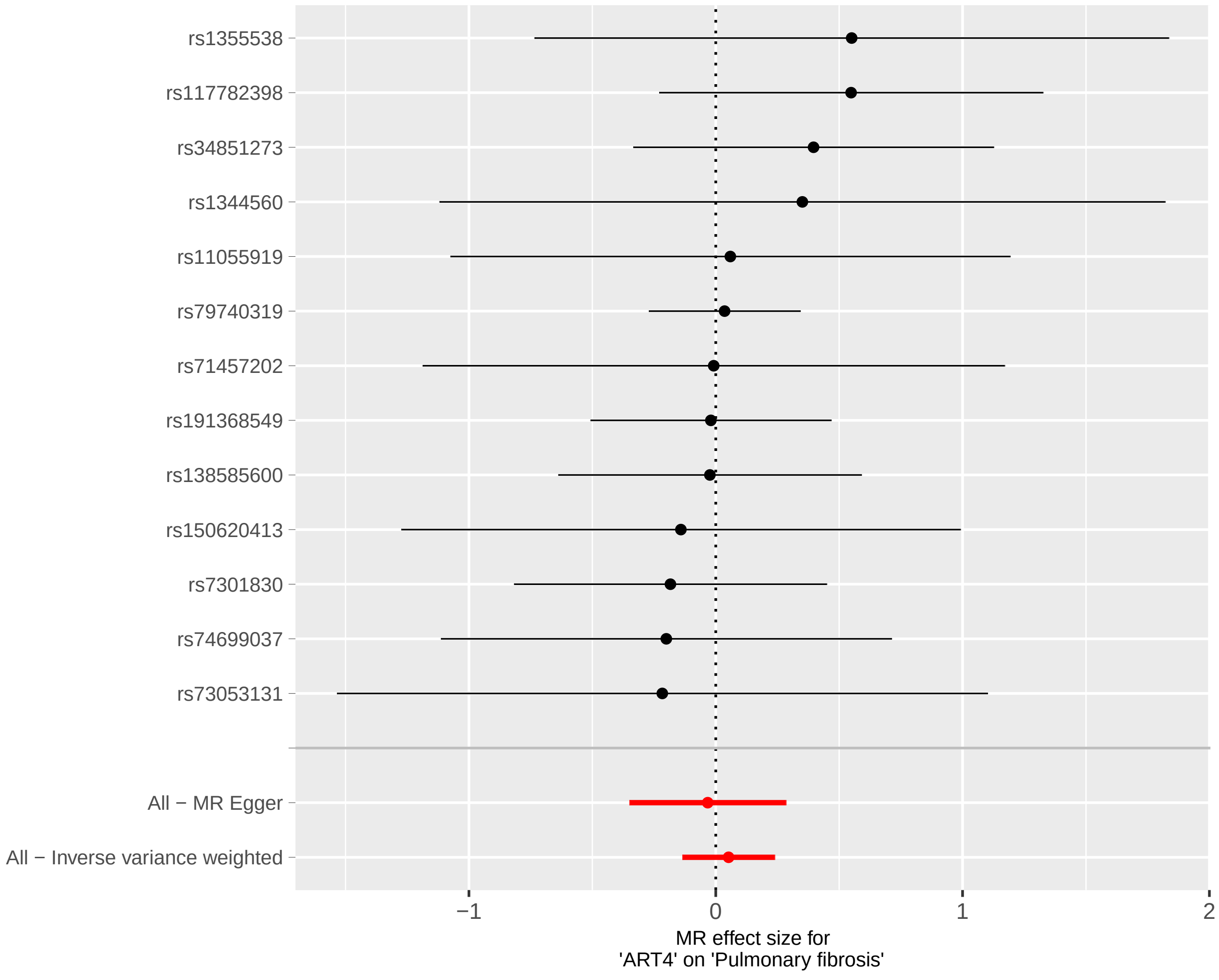

rs1355538
rs117782398
rs34851273
rs1344560
rs11055919
rs79740319
rs71457202
rs191368549
rs138585600
rs150620413
rs7301830
rs74699037
rs73053131
All − MR Egger
All − Inverse variance weighted
−1
0
1
2
MR effect size for
'ART4' on 'Pulmonary fibrosis'

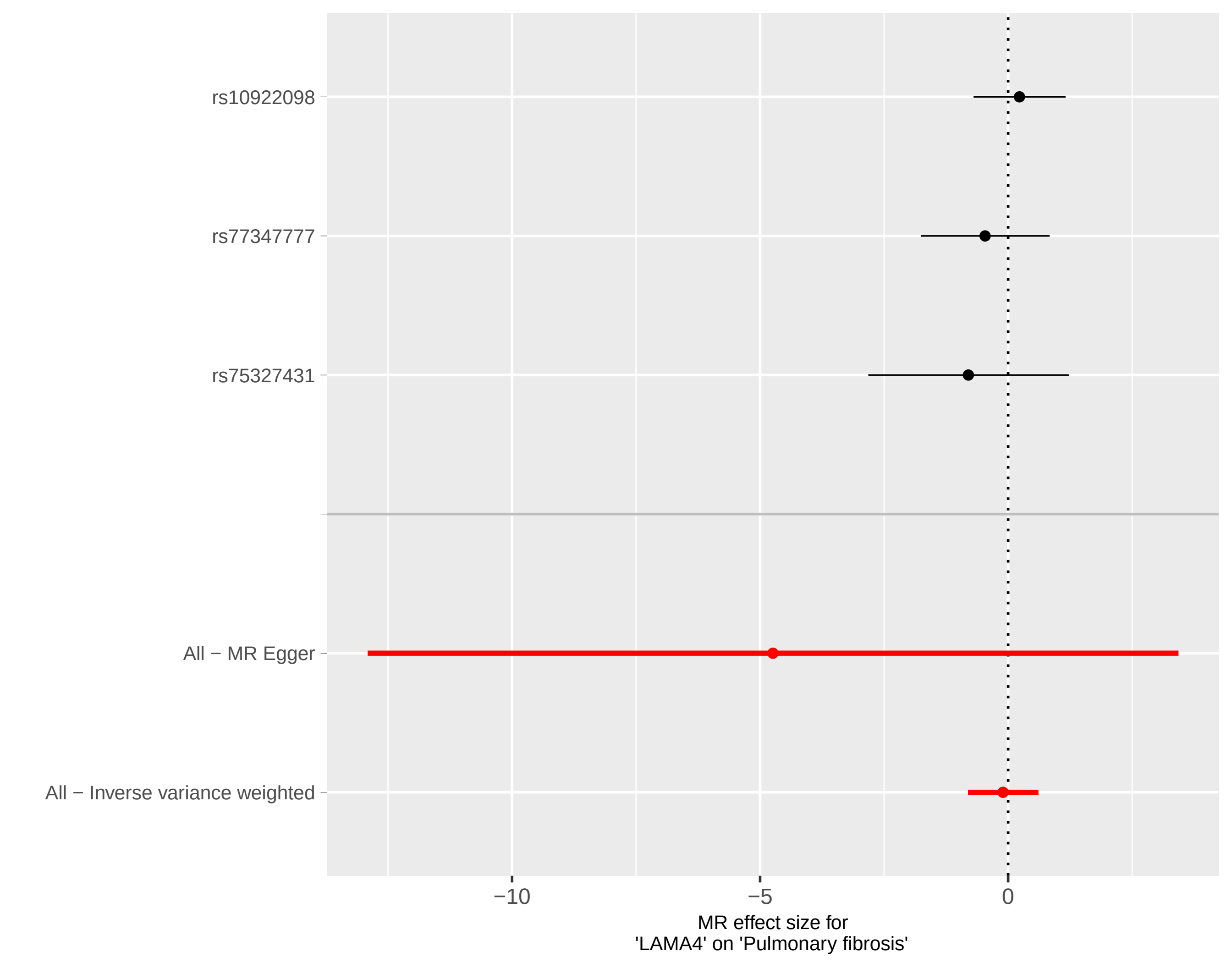
rs10922098
rs77347777
rs75327431
All − MR Egger
All − Inverse variance weighted
−10
−5
0
MR effect size for
'LAMA4' on 'Pulmonary fibrosis'

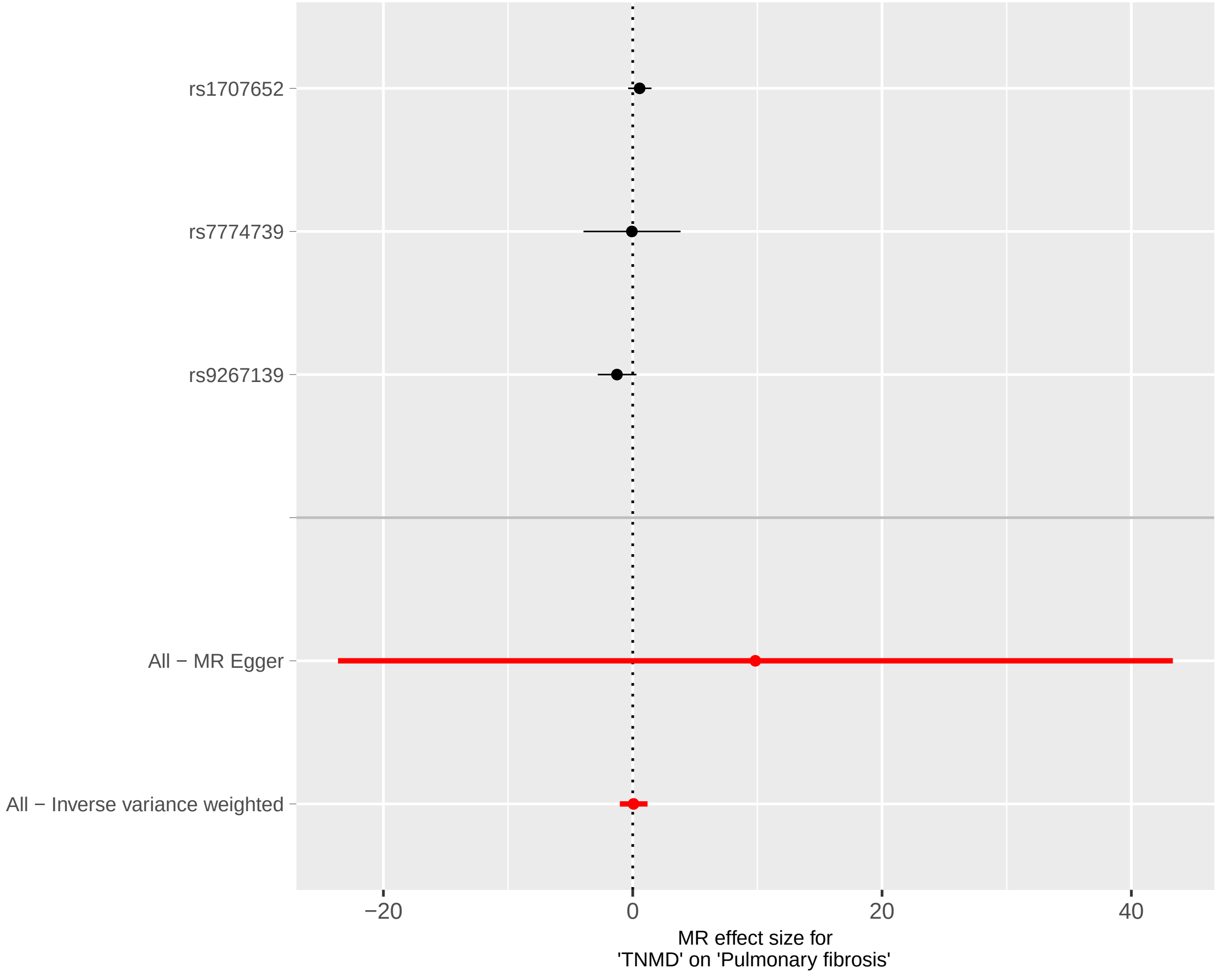

rs1707652
rs7774739
rs9267139
All − MR Egger
All − Inverse variance weighted
−20
0
20
40
MR effect size for
'TNMD' on 'Pulmonary fibrosis'

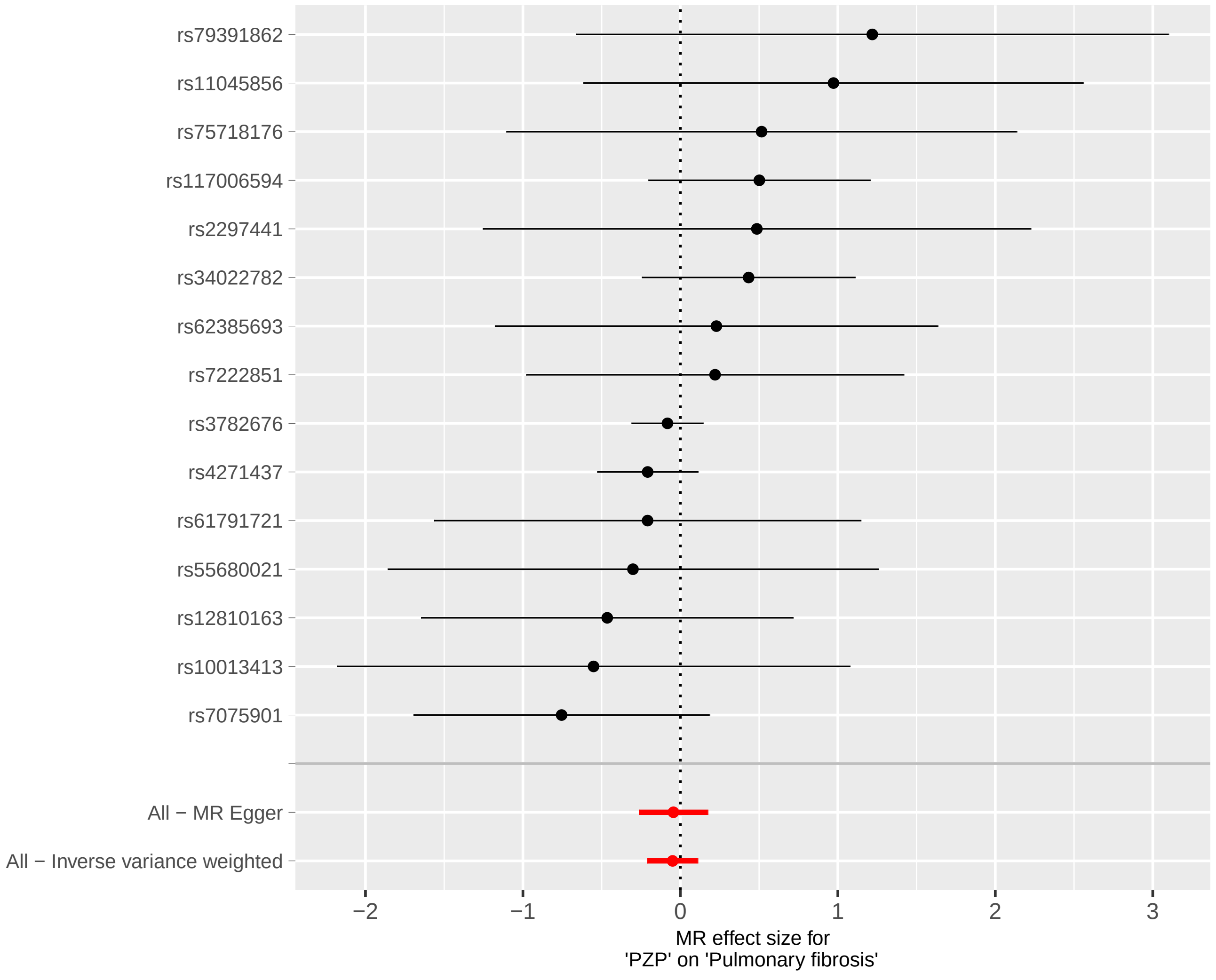

rs79391862
rs11045856
rs75718176
rs117006594
rs2297441
rs34022782
rs62385693
rs7222851
rs3782676
rs4271437
rs61791721
rs55680021
rs12810163
rs10013413
rs7075901
All − MR Egger
All − Inverse variance weighted
−2
−1
0
1
2
3
MR effect size for
'PZP' on 'Pulmonary fibrosis'

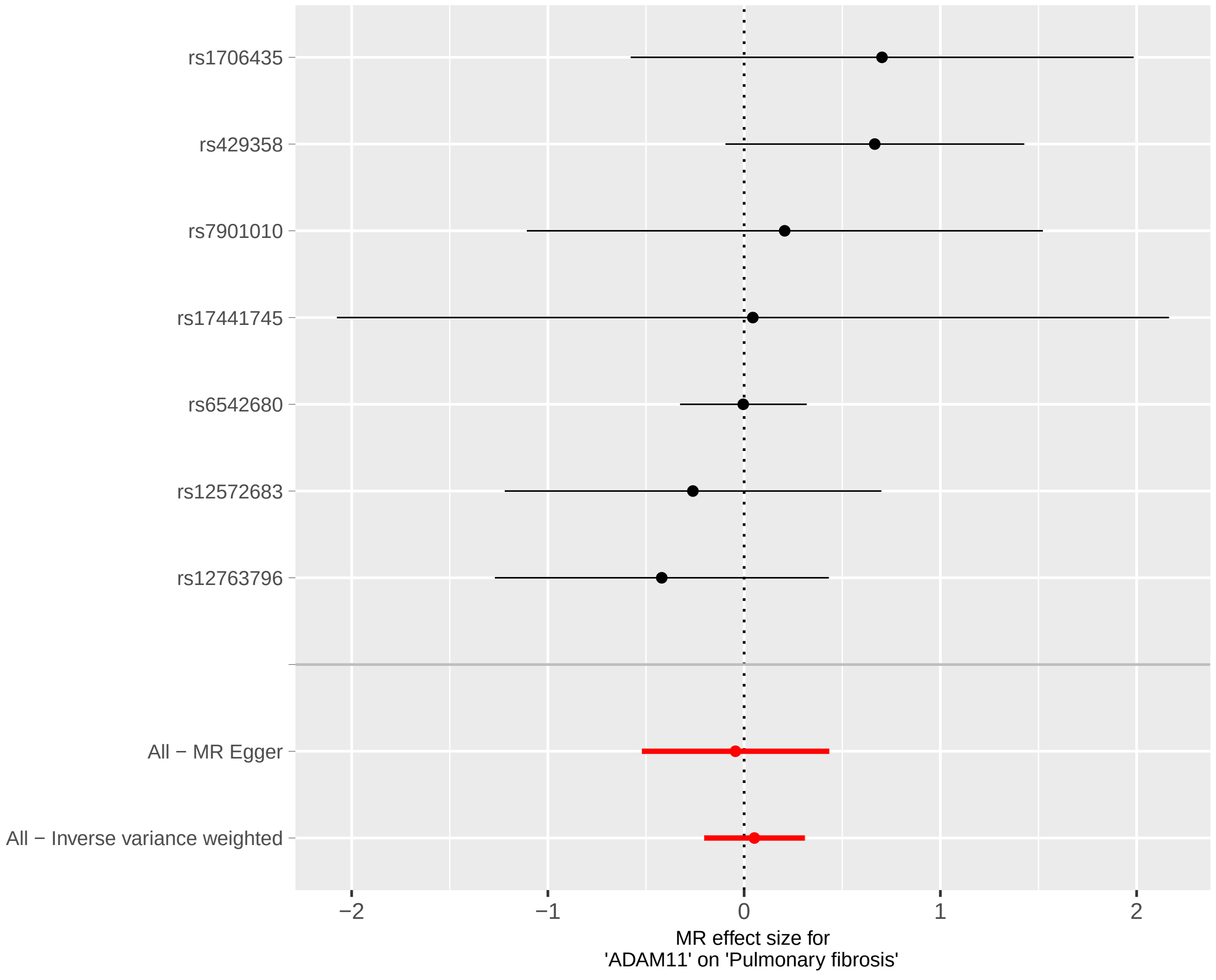

rs1706435
rs429358
rs7901010
rs17441745
rs6542680
rs12572683
rs12763796
All − MR Egger
All − Inverse variance weighted
−2
−1
0
1
2
MR effect size for
'ADAM11' on 'Pulmonary fibrosis'

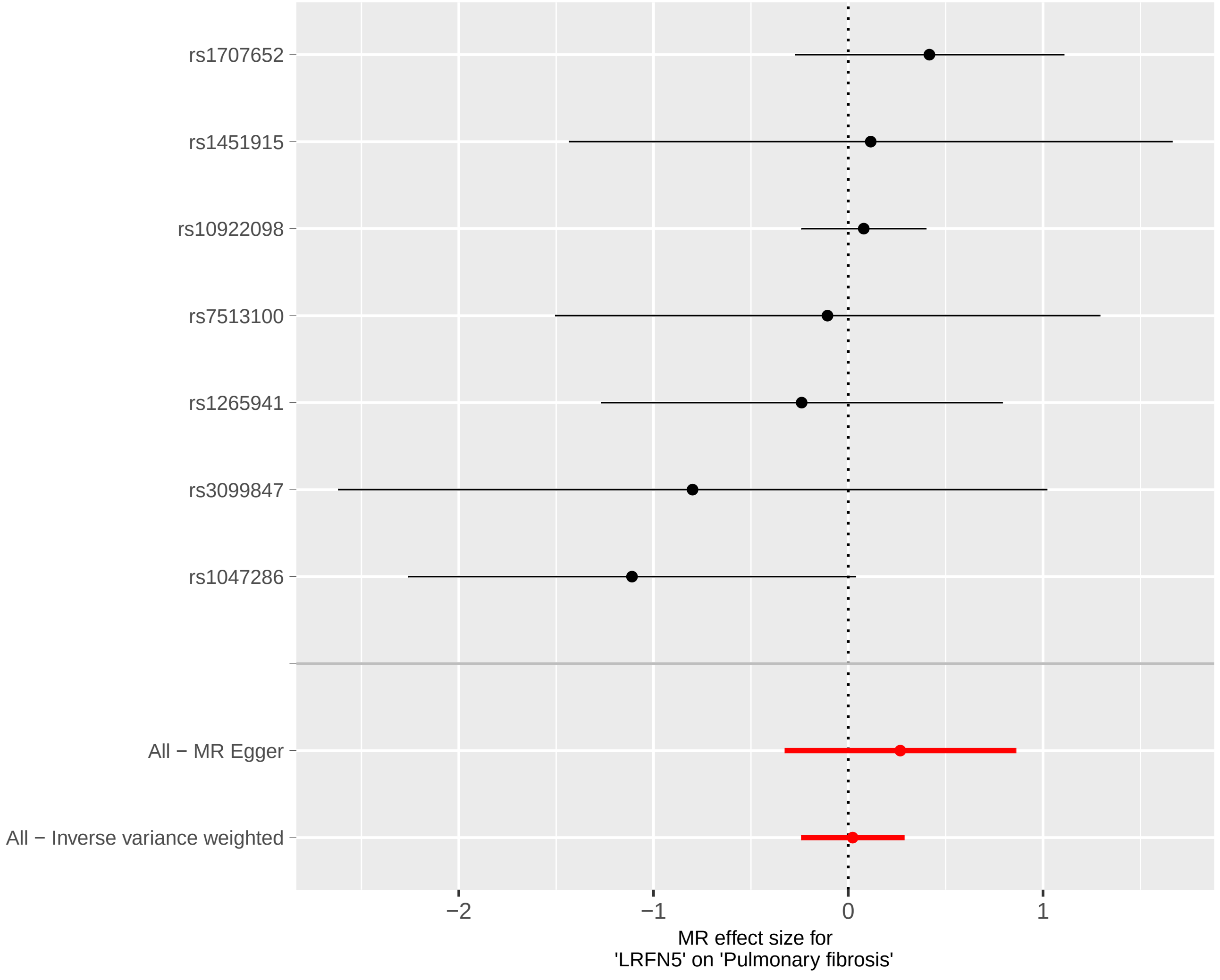

rs1707652
rs1451915
rs10922098
rs7513100
rs1265941
rs3099847
rs1047286
All − MR Egger
All − Inverse variance weighted
−2
−1
0
1
MR effect size for
'LRFN5' on 'Pulmonary fibrosis'

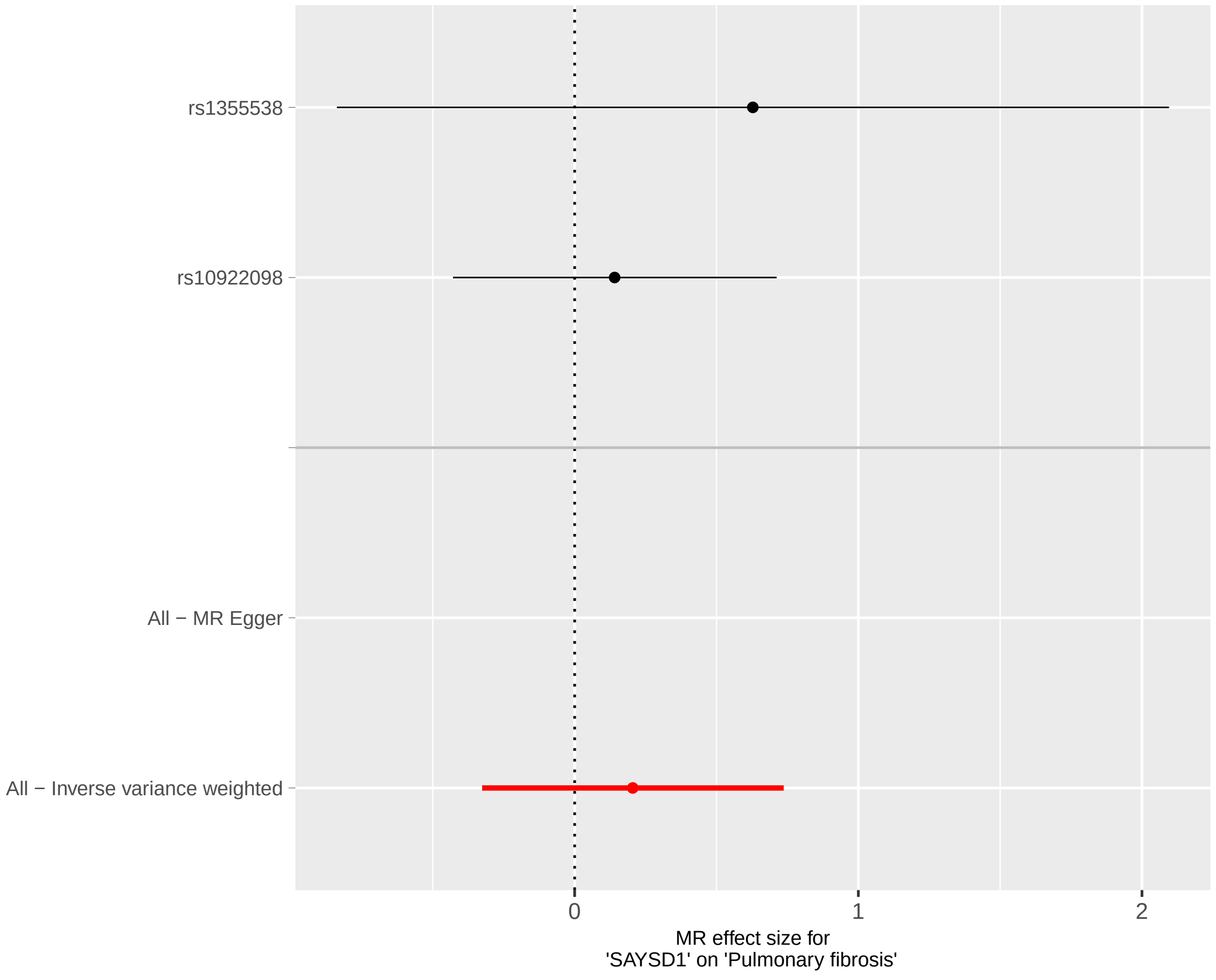

rs1355538
rs10922098
All − MR Egger
All − Inverse variance weighted
0
1
2
MR effect size for
'SAYSD1' on 'Pulmonary fibrosis'

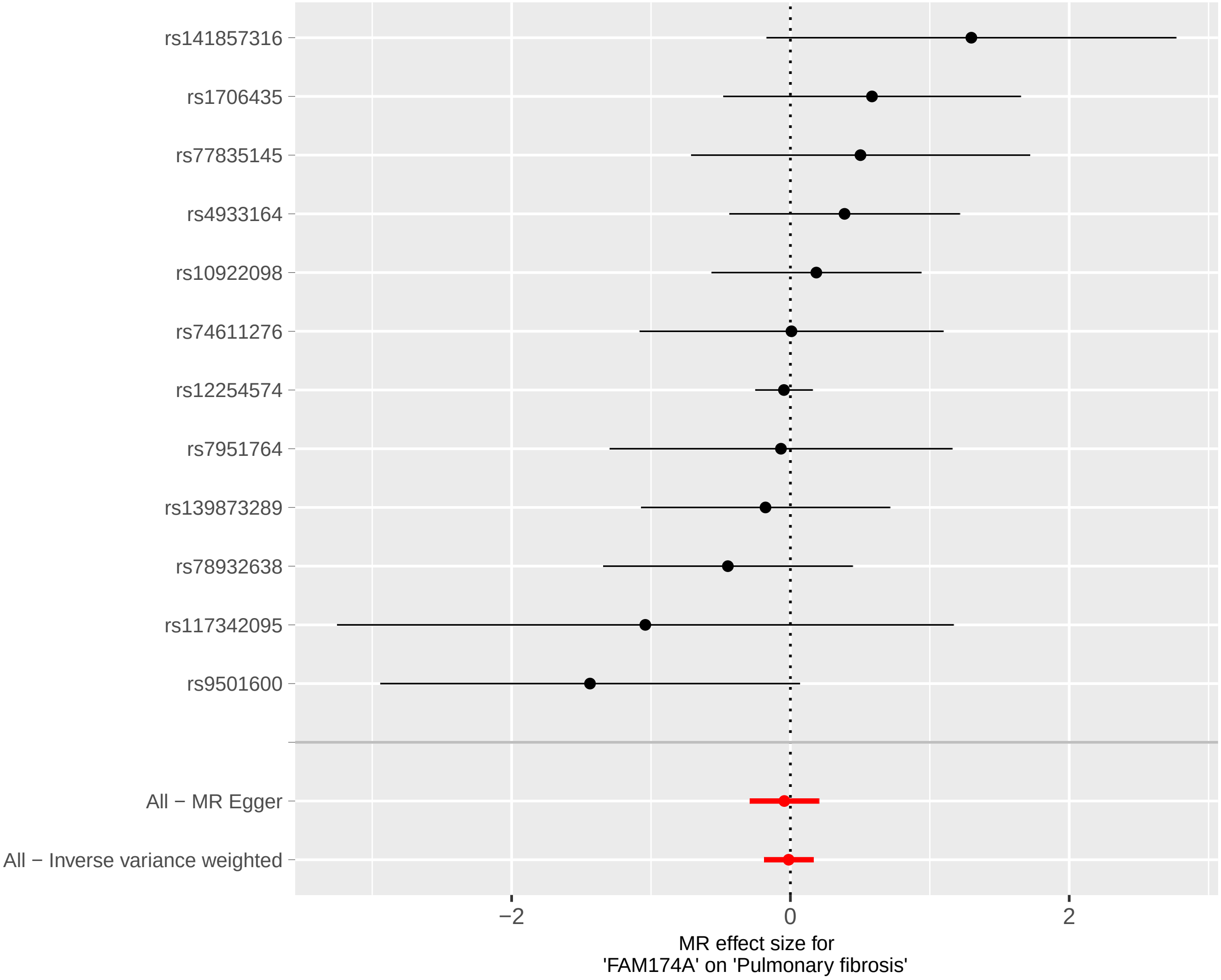

rs141857316
rs1706435
rs77835145
rs4933164
rs10922098
rs74611276
rs12254574
rs7951764
rs139873289
rs78932638
rs117342095
rs9501600
All − MR Egger
All − Inverse variance weighted
−2
0
2
MR effect size for
'FAM174A' on 'Pulmonary fibrosis'

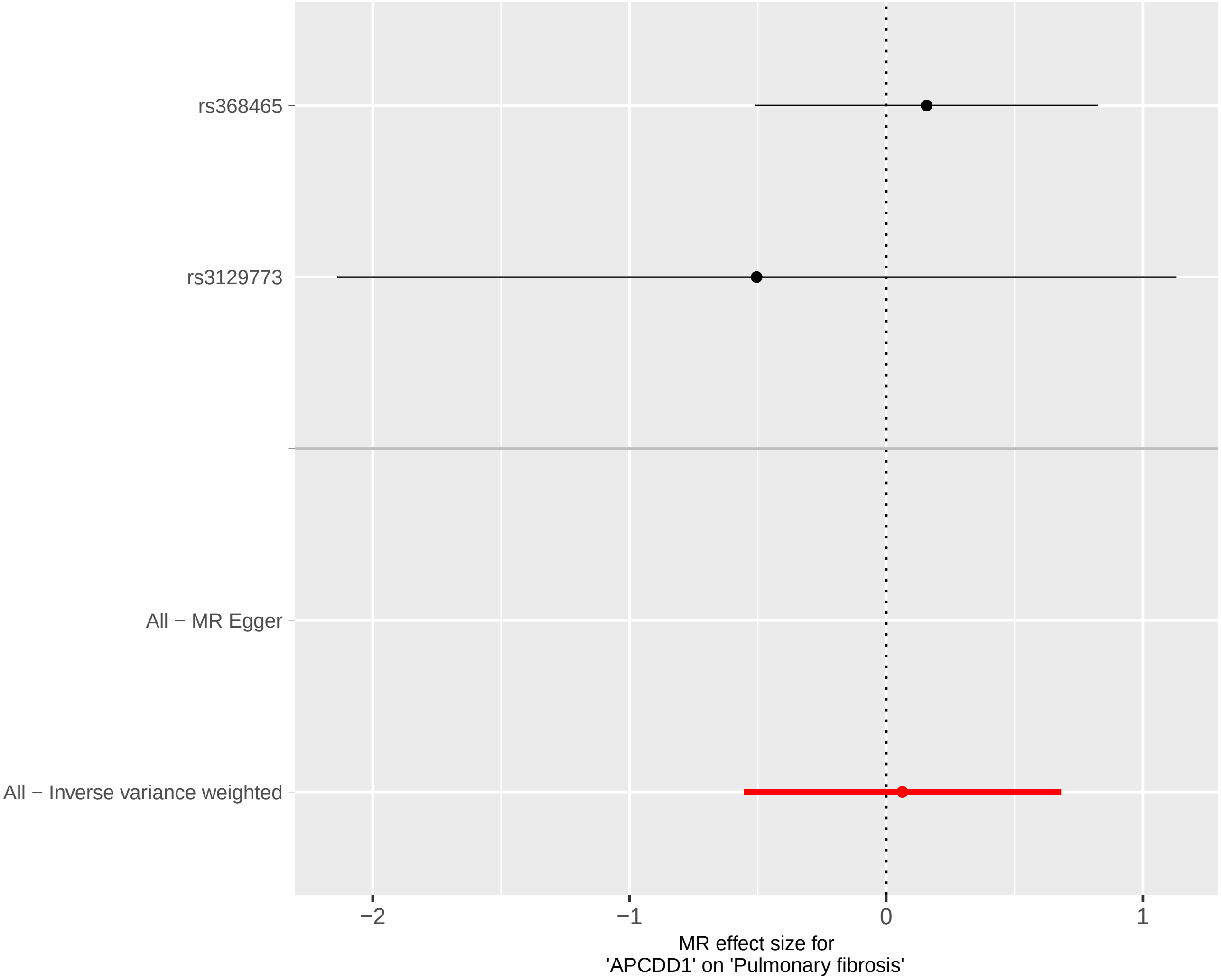
rs368465
rs3129773
All − MR Egger
All − Inverse variance weighted
−2
−1
0
1
MR effect size for
'APCDD1' on 'Pulmonary fibrosis'

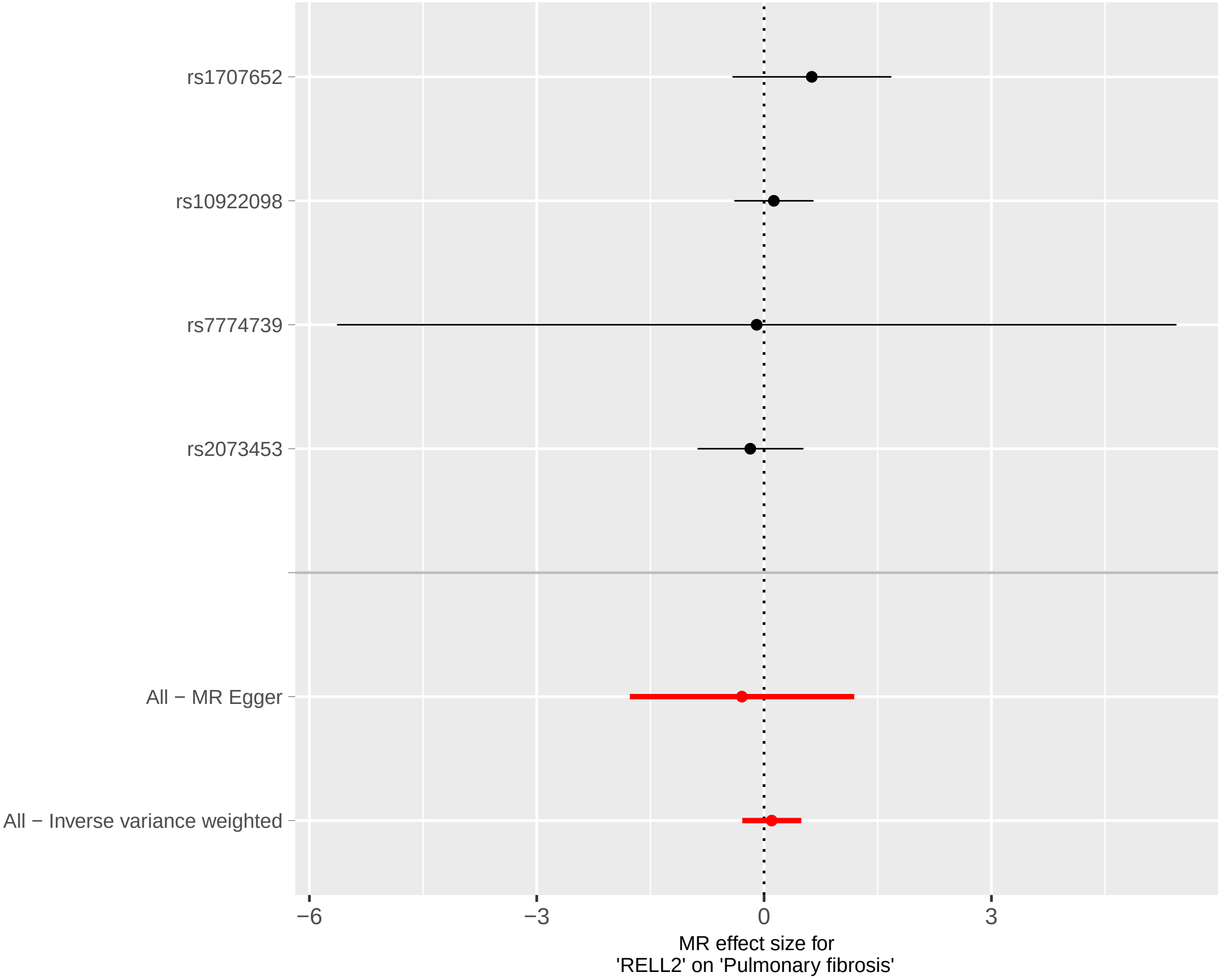

rs1707652
rs10922098
rs7774739
rs2073453
All − MR Egger
All − Inverse variance weighted
−6
−3
0
3
MR effect size for
'RELL2' on 'Pulmonary fibrosis'

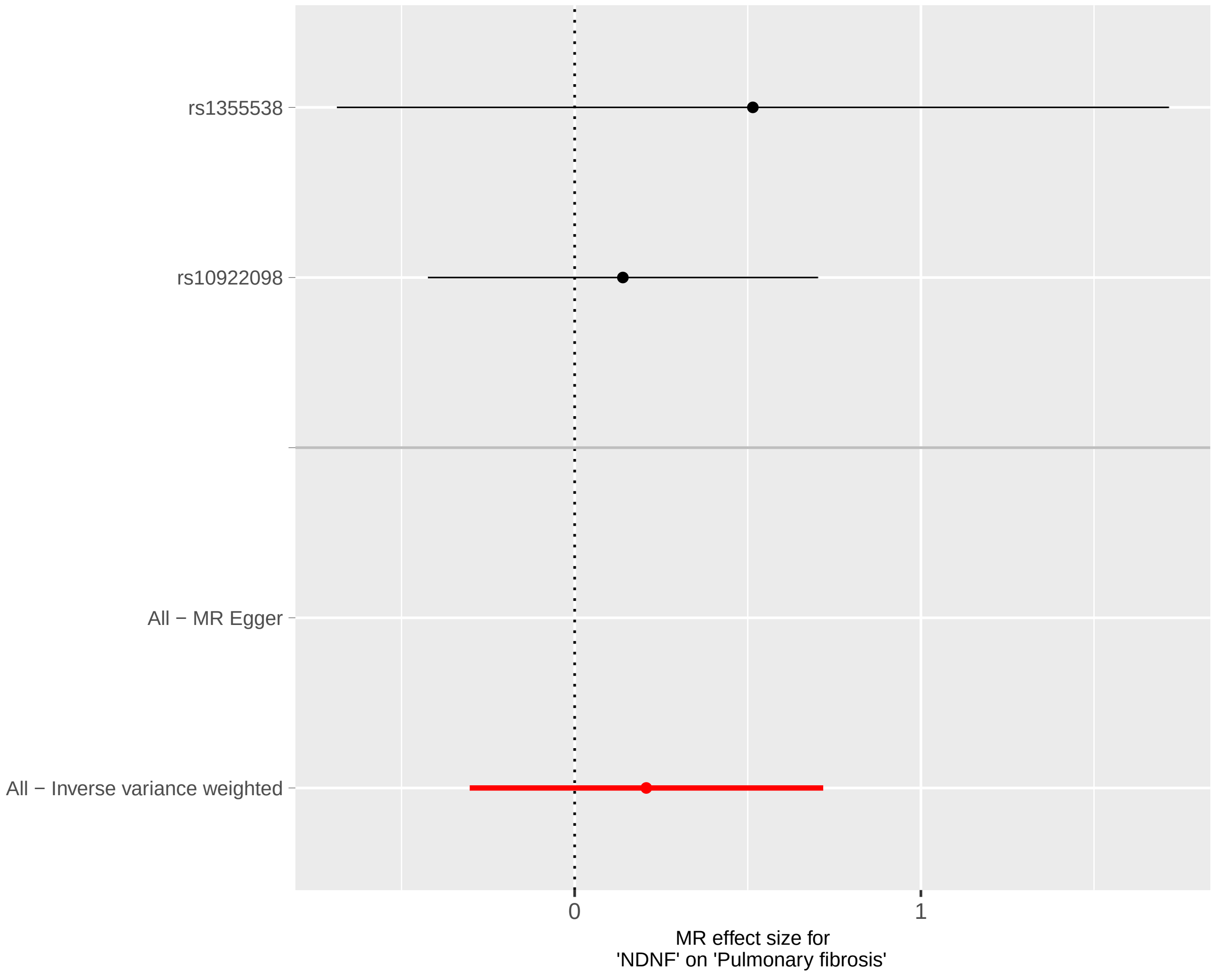
rs1355538
rs10922098
All − MR Egger
All − Inverse variance weighted
0
1
MR effect size for
'NDNF' on 'Pulmonary fibrosis'

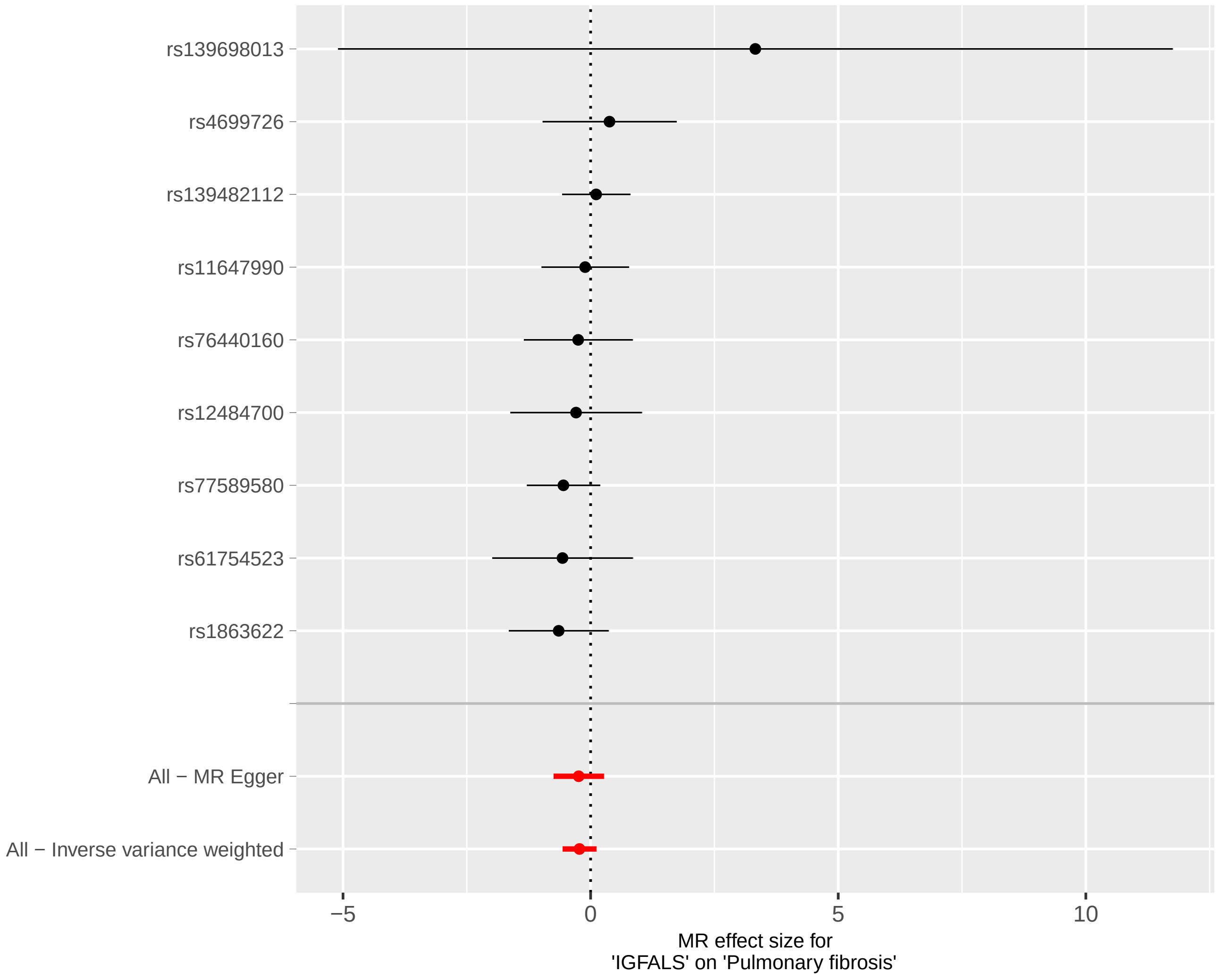

rs139698013
rs4699726
rs139482112
rs11647990
rs76440160
rs12484700
rs77589580
rs61754523
rs1863622
All − MR Egger
All − Inverse variance weighted
−5
0
5
10
MR effect size for
'IGFALS' on 'Pulmonary fibrosis'

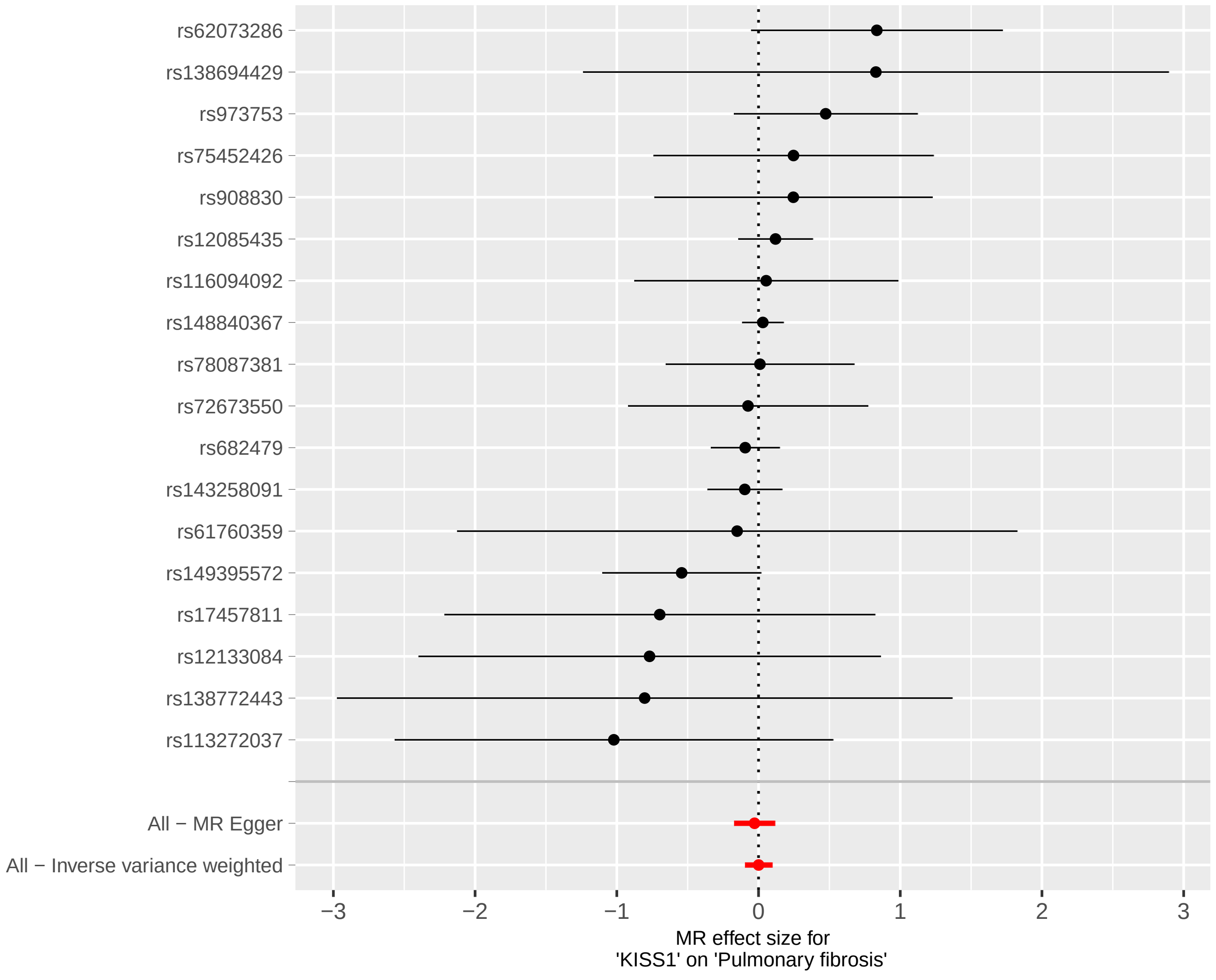

rs62073286
rs138694429
rs973753
rs75452426
rs908830
rs12085435
rs116094092
rs148840367
rs78087381
rs72673550
rs682479
rs143258091
rs61760359
rs149395572
rs17457811
rs12133084
rs138772443
rs113272037
All − MR Egger
All − Inverse variance weighted
−3
−2
−1
0
1
2
3
MR effect size for
'KISS1' on 'Pulmonary fibrosis'

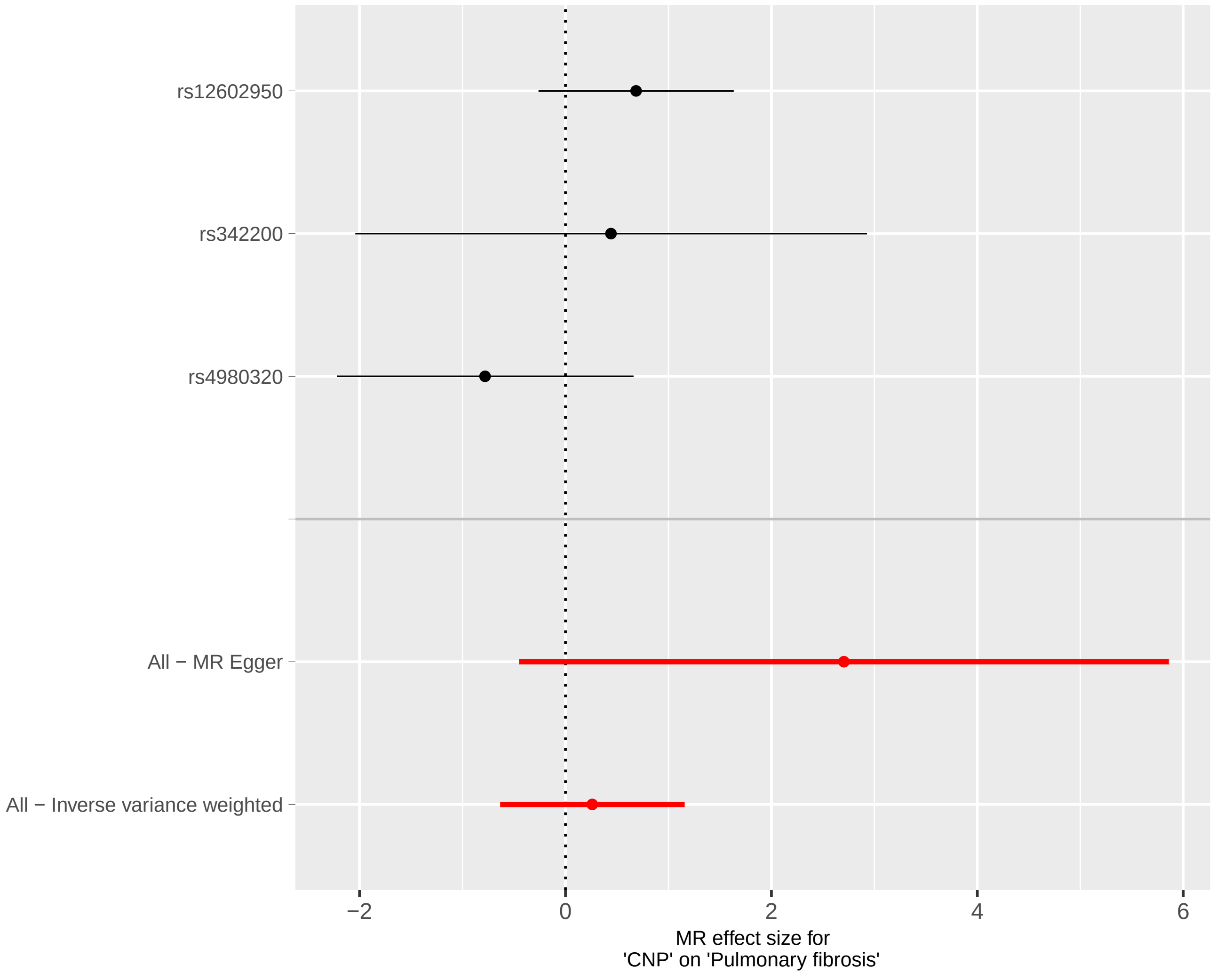
rs12602950
rs342200
rs4980320
All − MR Egger
All − Inverse variance weighted
−2
0
2
4
6
MR effect size for
'CNP' on 'Pulmonary fibrosis'

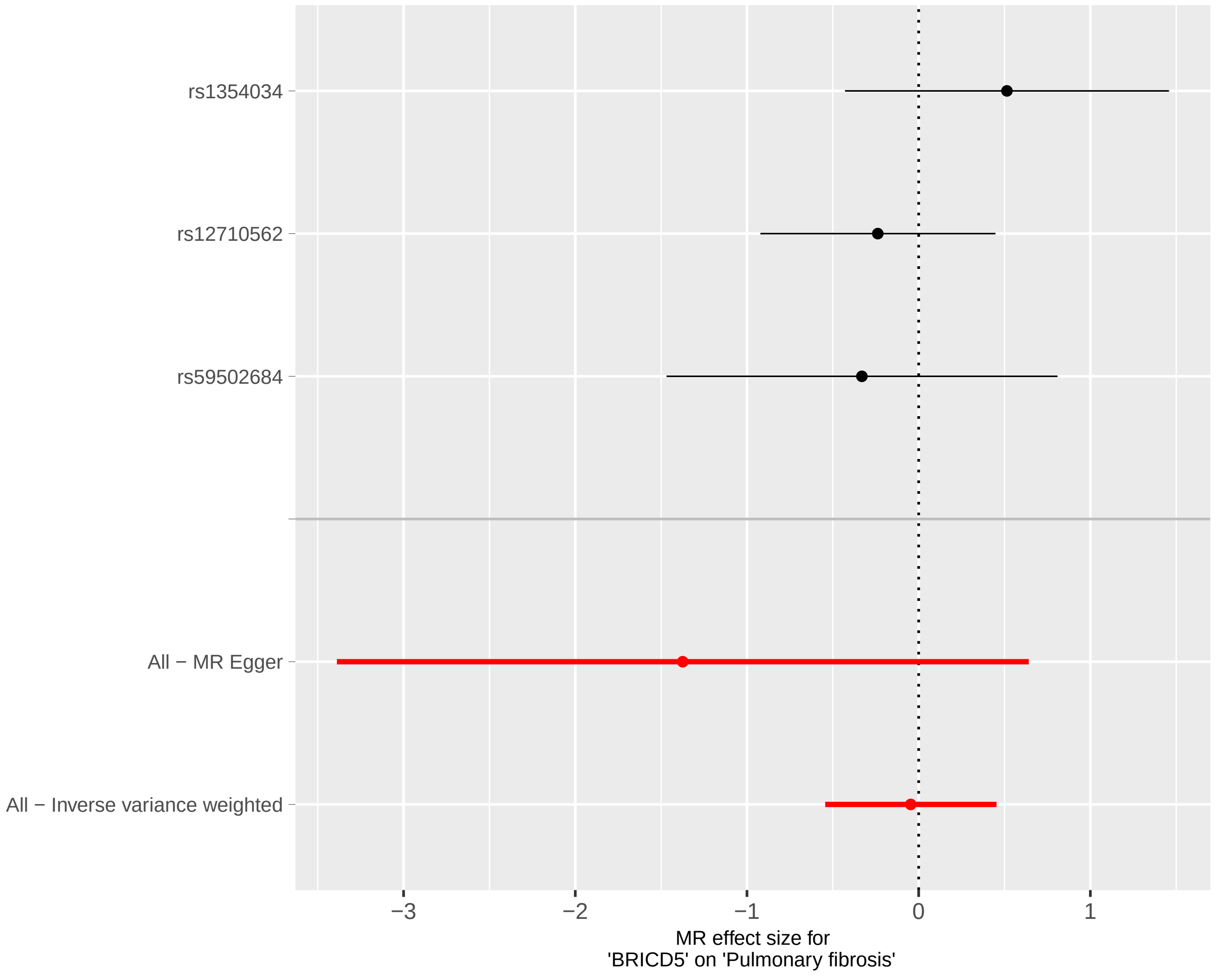
rs1354034
rs12710562
rs59502684
All − MR Egger
All − Inverse variance weighted
−3
−2
−1
0
1
MR effect size for
'BRICD5' on 'Pulmonary fibrosis'

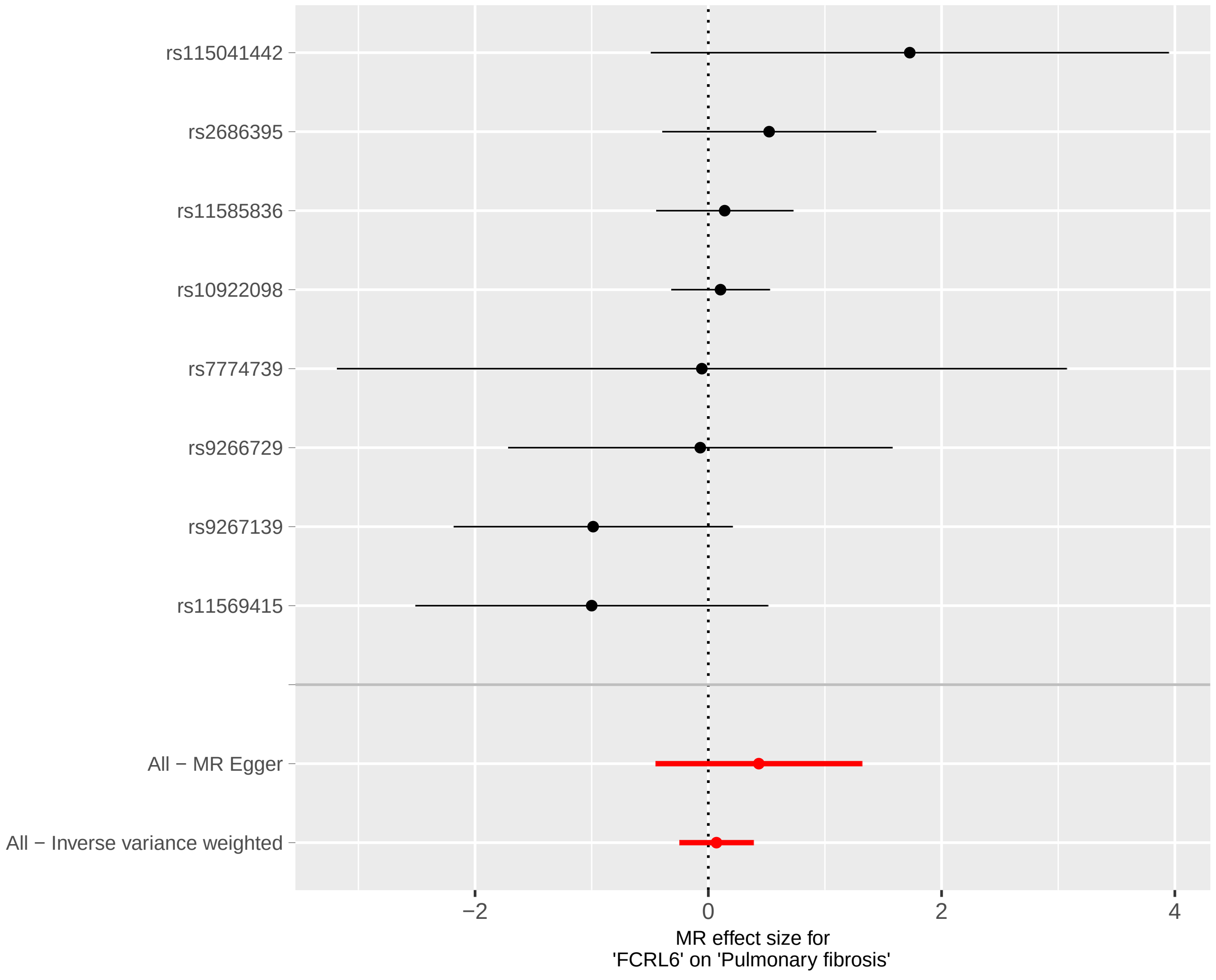
rs115041442
rs2686395
rs11585836
rs10922098
rs7774739
rs9266729
rs9267139
rs11569415
All − MR Egger
All − Inverse variance weighted
−2
0
2
4
MR effect size for
'FCRL6' on 'Pulmonary fibrosis'

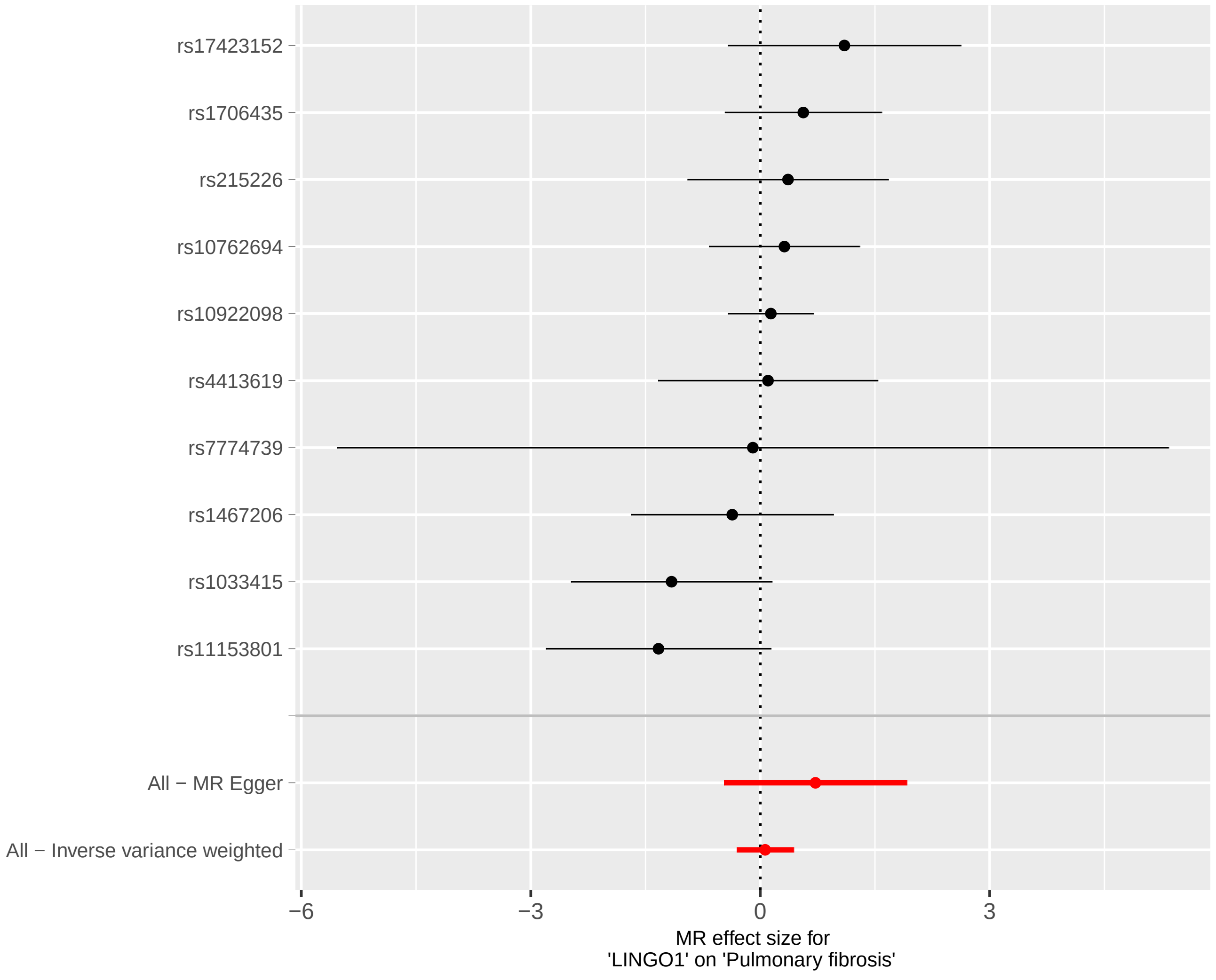
rs17423152
rs1706435
rs215226
rs10762694
rs10922098
rs4413619
rs7774739
rs1467206
rs1033415
rs11153801
All − MR Egger
All − Inverse variance weighted
−6
−3
0
3
MR effect size for
'LINGO1' on 'Pulmonary fibrosis'

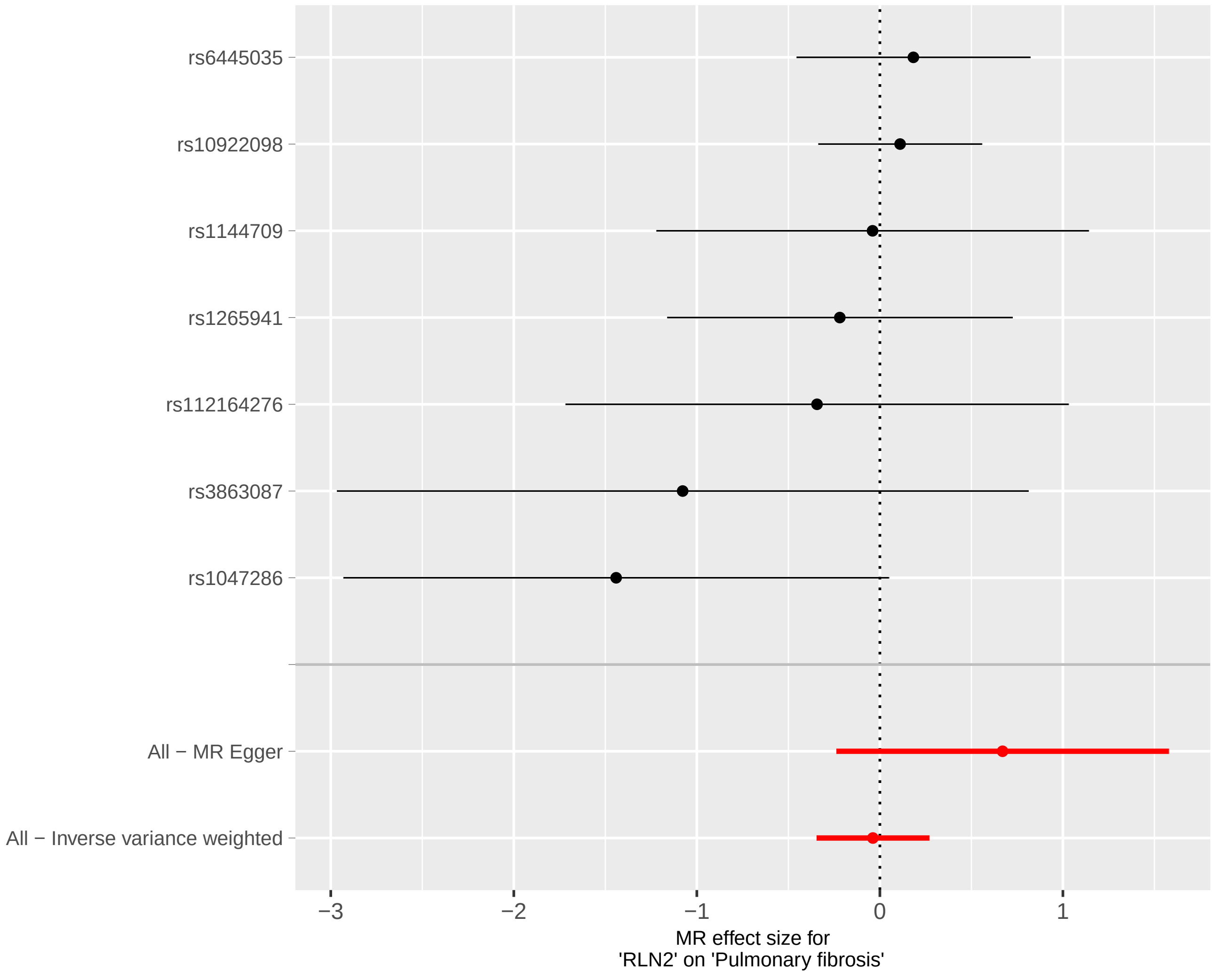

rs6445035
rs10922098
rs1144709
rs1265941
rs112164276
rs3863087
rs1047286
All − MR Egger
All − Inverse variance weighted
−3
−2
−1
0
1
MR effect size for
'RLN2' on 'Pulmonary fibrosis'

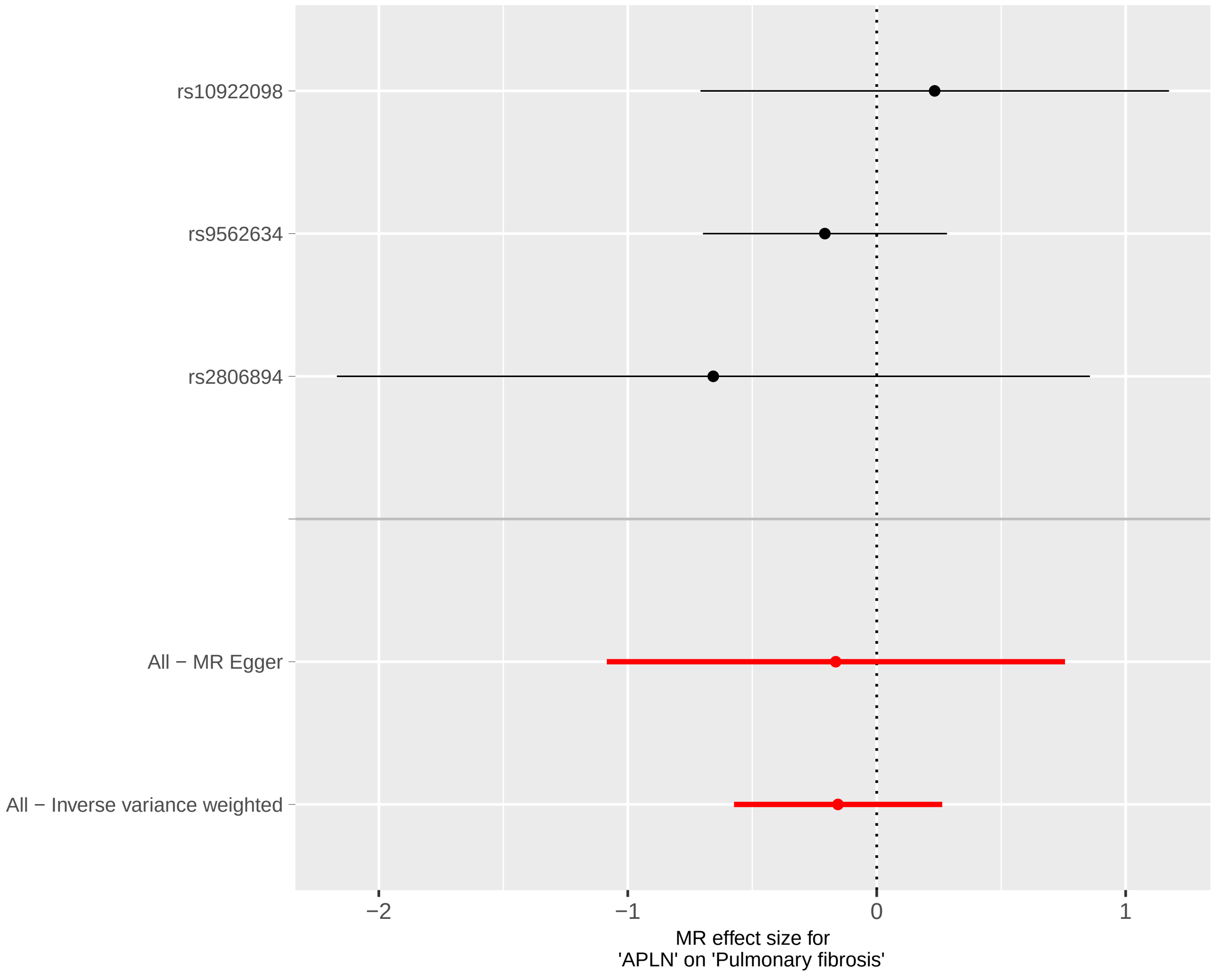

rs10922098
rs9562634
rs2806894
All − MR Egger
All − Inverse variance weighted
−2
−1
0
1
MR effect size for
'APLN' on 'Pulmonary fibrosis'

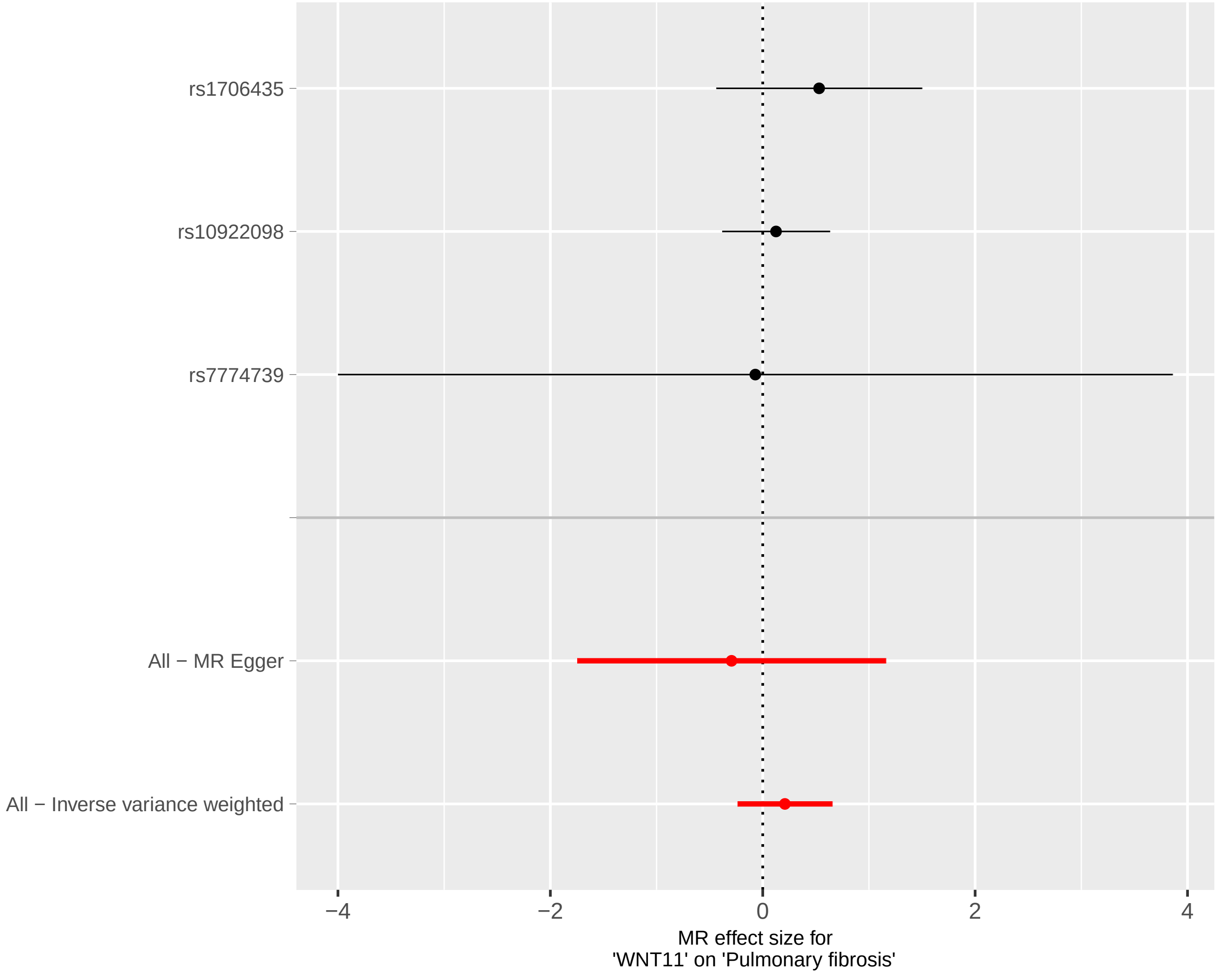

rs1706435
rs10922098
rs7774739
All − MR Egger
All − Inverse variance weighted
−4
−2
0
2
4
MR effect size for
'WNT11' on 'Pulmonary fibrosis'

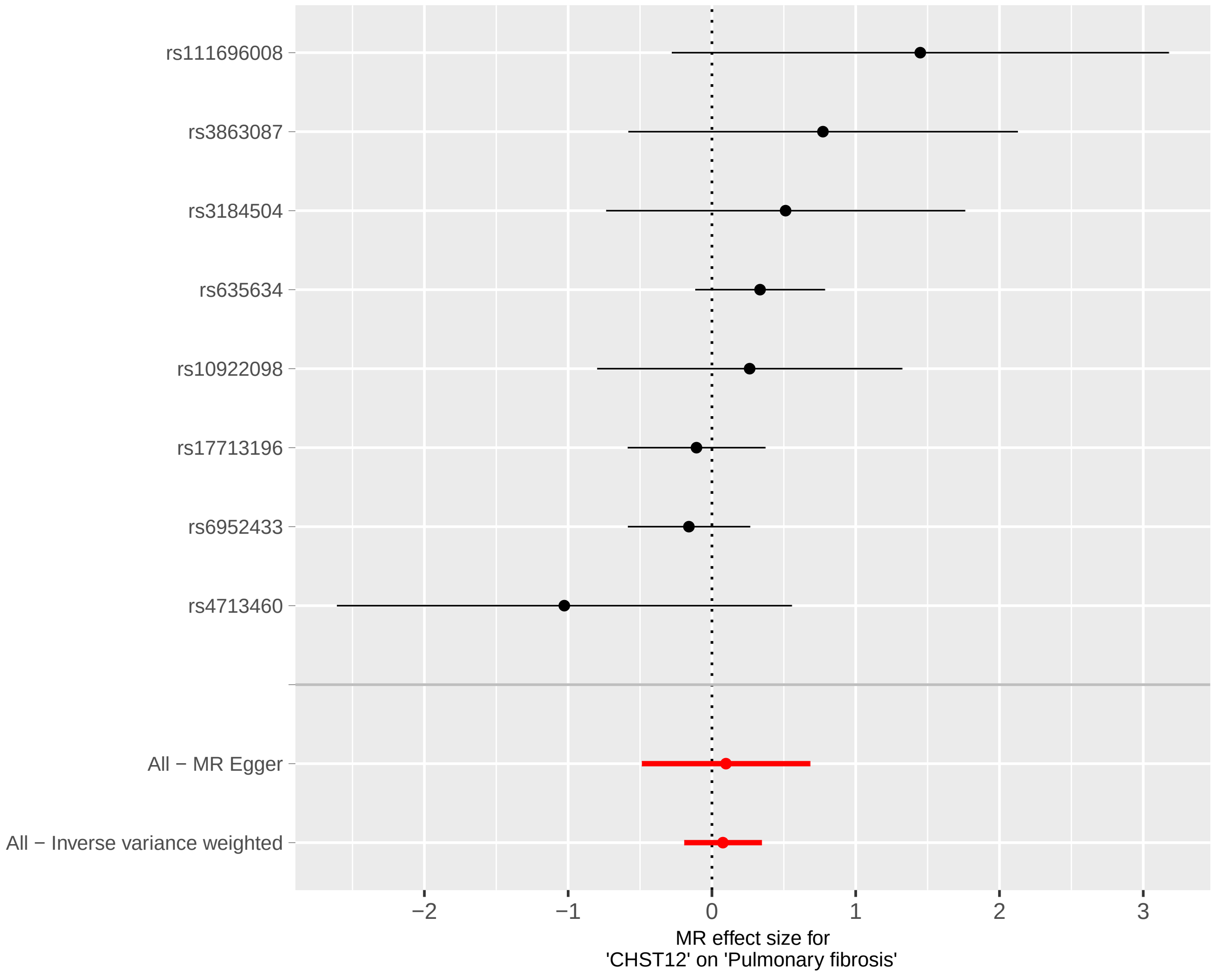

rs111696008
rs3863087
rs3184504
rs635634
rs10922098
rs17713196
rs6952433
rs4713460
All − MR Egger
All − Inverse variance weighted
−2
−1
0
1
2
3
MR effect size for
'CHST12' on 'Pulmonary fibrosis'

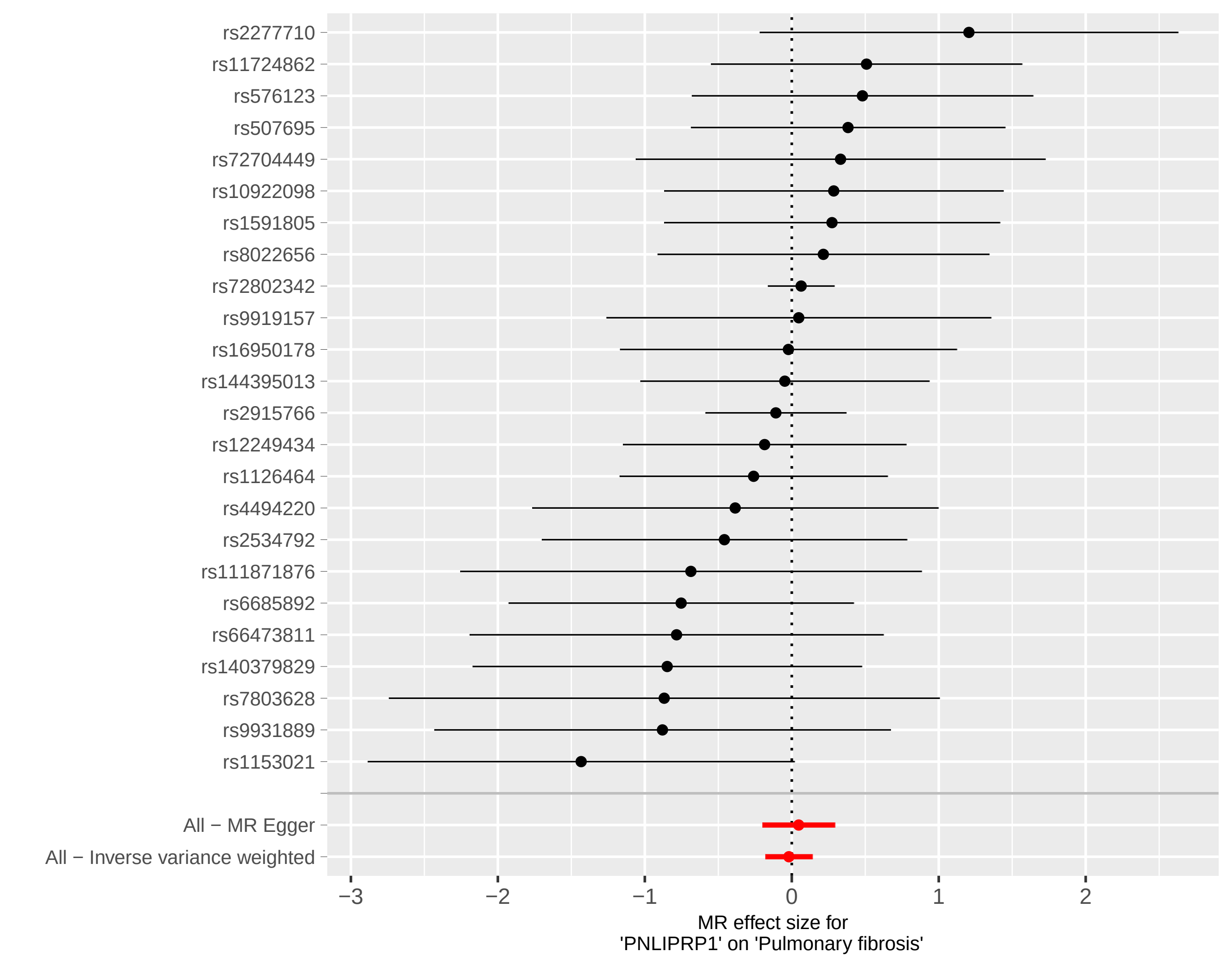

rs2277710
rs11724862
rs576123
rs507695
rs72704449
rs10922098
rs1591805
rs8022656
rs72802342
rs9919157
rs16950178
rs144395013
rs2915766
rs12249434
rs1126464
rs4494220
rs2534792
rs111871876
rs6685892
rs66473811
rs140379829
rs7803628
rs9931889
rs1153021
All − MR Egger
All − Inverse variance weighted
−3
−2
−1
0
1
2
MR effect size for
'PNLIPRP1' on 'Pulmonary fibrosis'

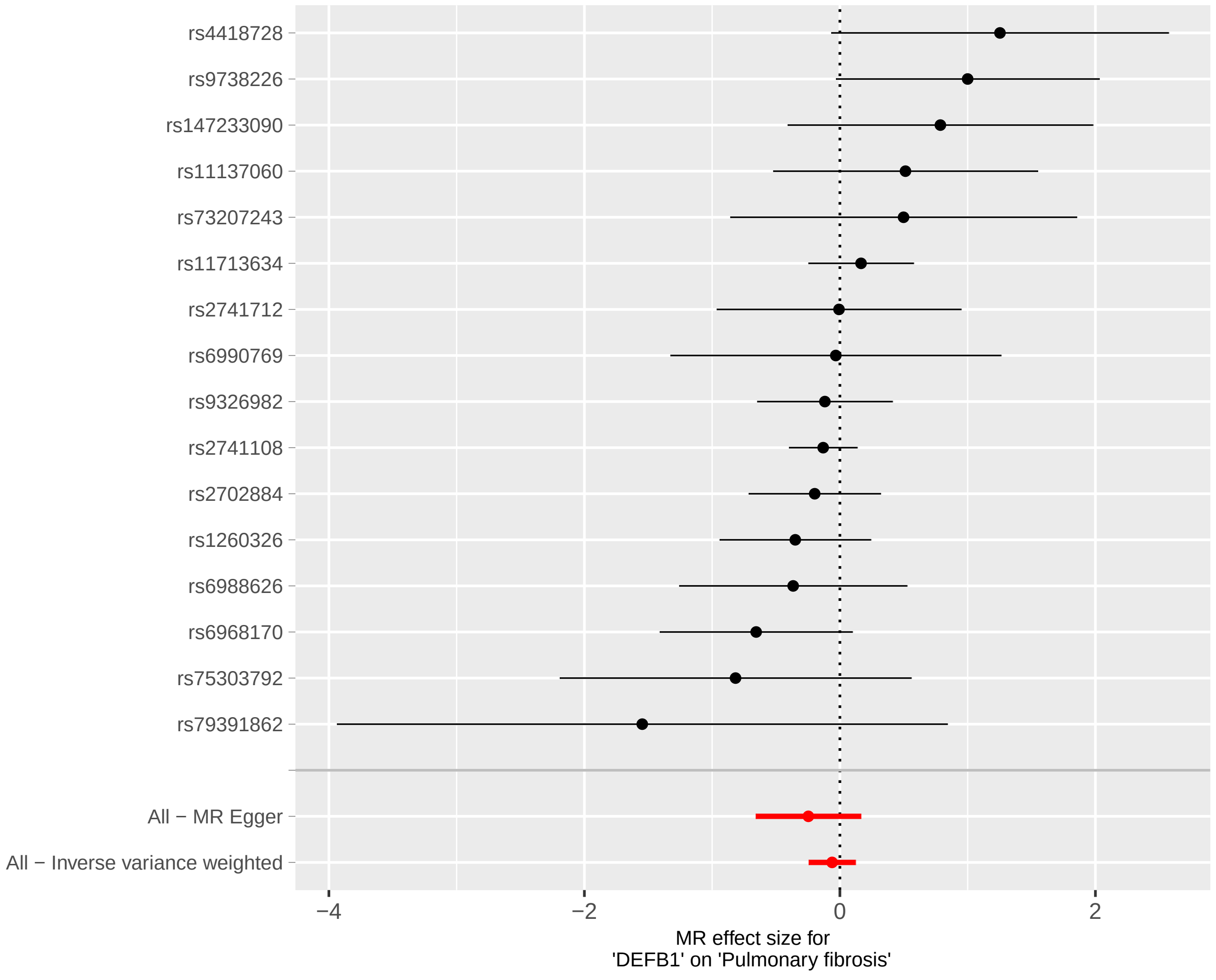
rs4418728
rs9738226
rs147233090
rs11137060
rs73207243
rs11713634
rs2741712
rs6990769
rs9326982
rs2741108
rs2702884
rs1260326
rs6988626
rs6968170
rs75303792
rs79391862
All − MR Egger
All − Inverse variance weighted
−4
−2
0
2
MR effect size for
'DEFB1' on 'Pulmonary fibrosis'

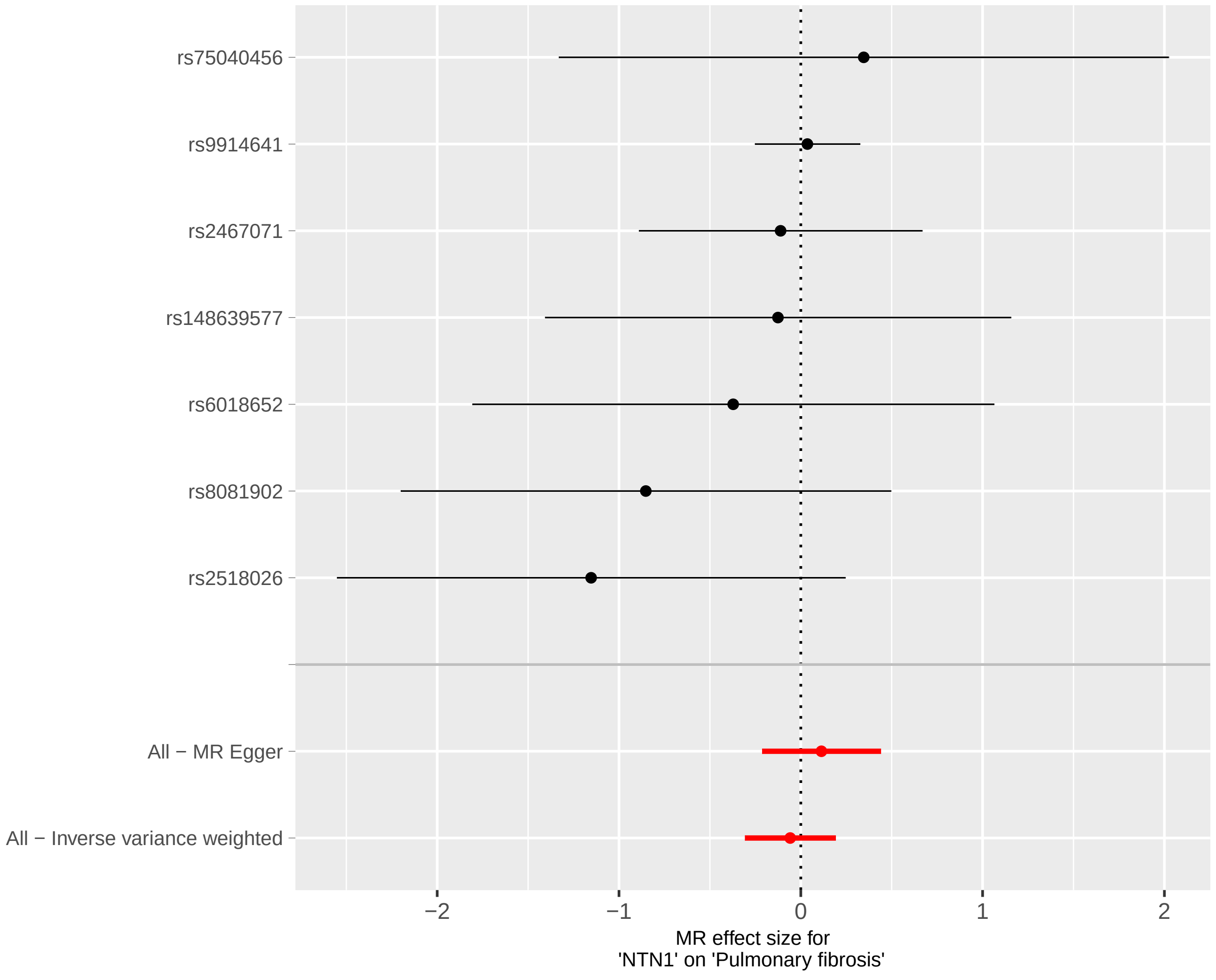
rs75040456
rs9914641
rs2467071
rs148639577
rs6018652
rs8081902
rs2518026
All − MR Egger
All − Inverse variance weighted
−2
−1
0
1
2
MR effect size for
'NTN1' on 'Pulmonary fibrosis'

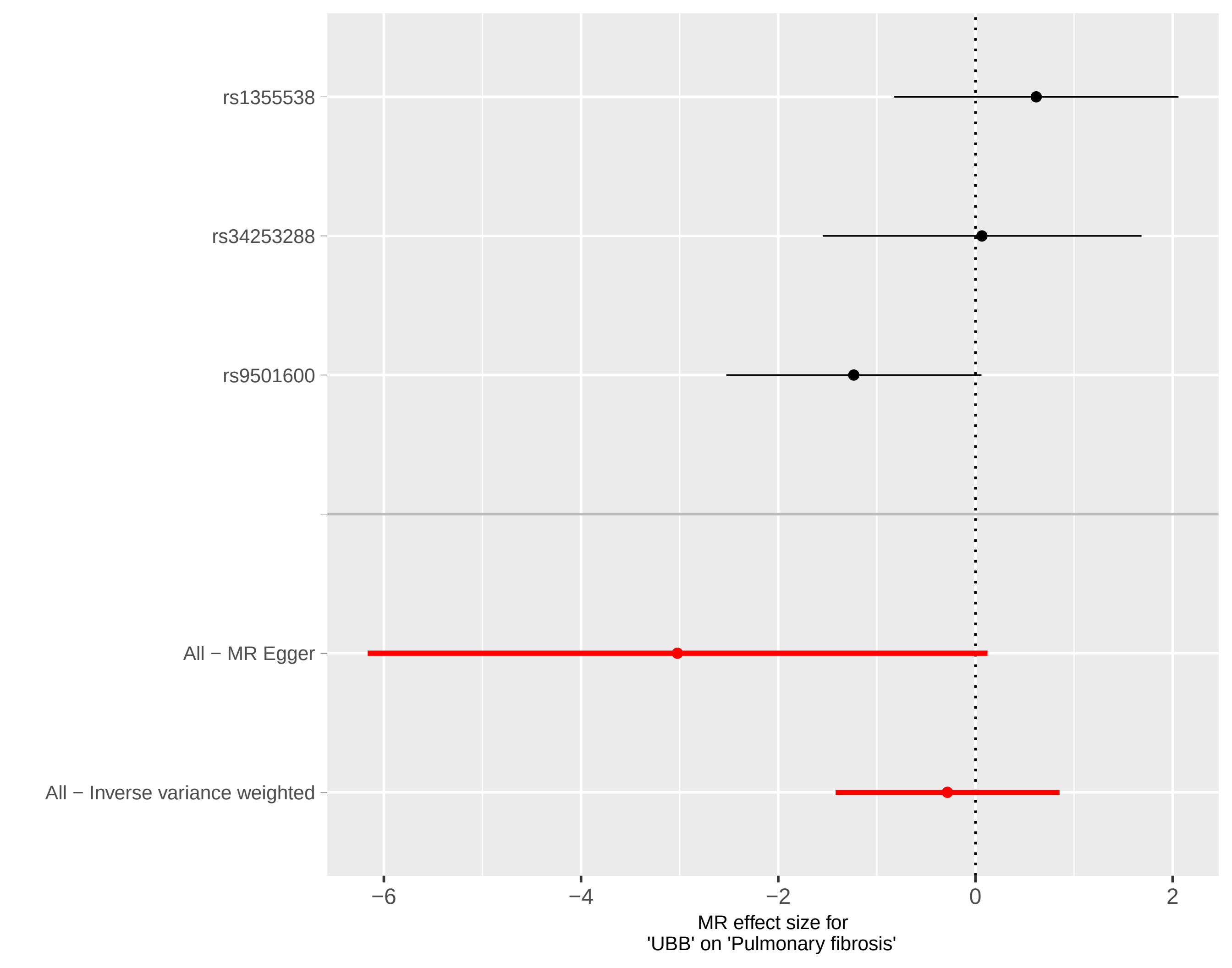

rs1355538
rs34253288
rs9501600
All − MR Egger
All − Inverse variance weighted
−6
−4
−2
0
2
MR effect size for
'UBB' on 'Pulmonary fibrosis'

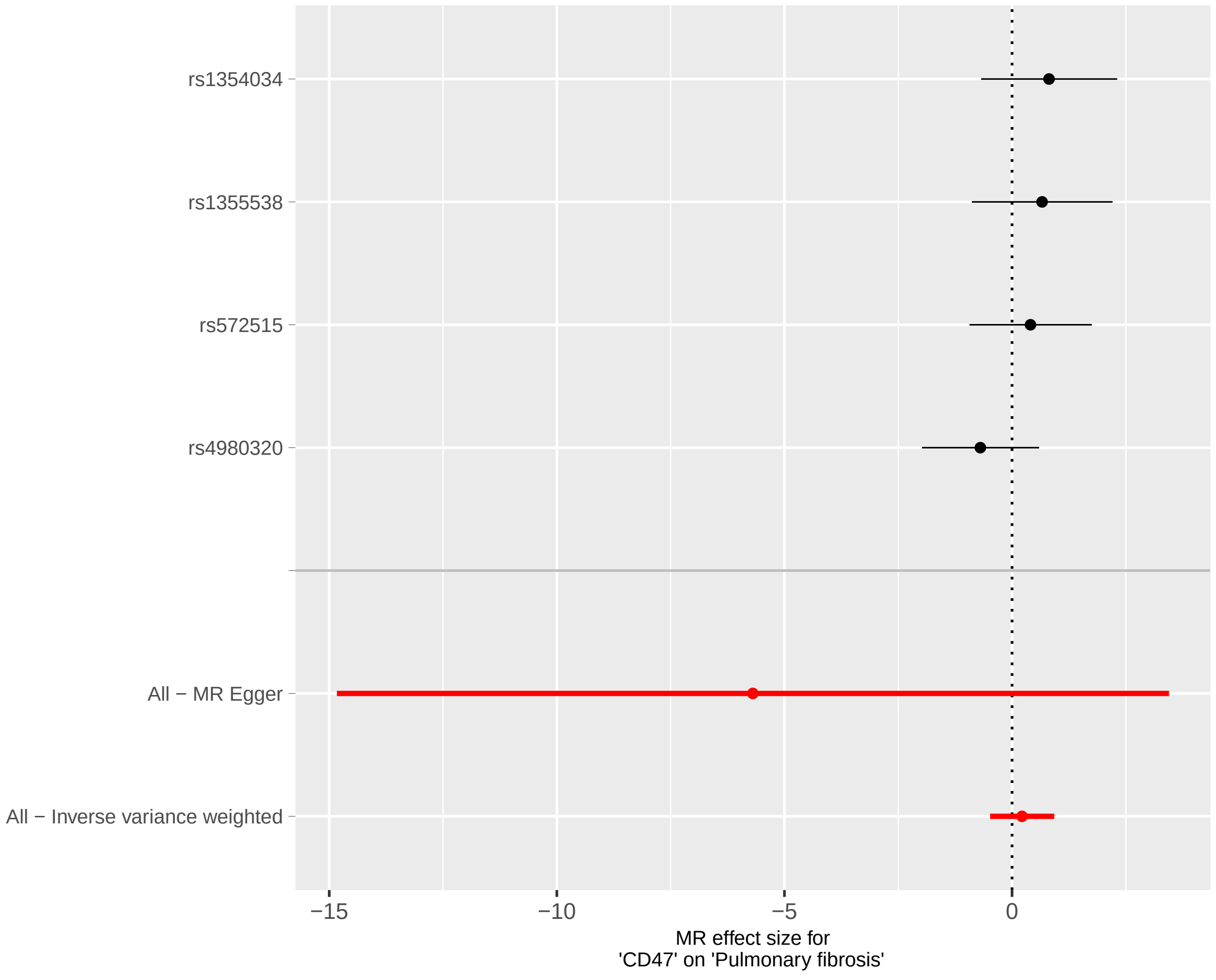
rs1354034
rs1355538
rs572515
rs4980320
All − MR Egger
All − Inverse variance weighted
−15
−10
−5
0
MR effect size for
'CD47' on 'Pulmonary fibrosis'

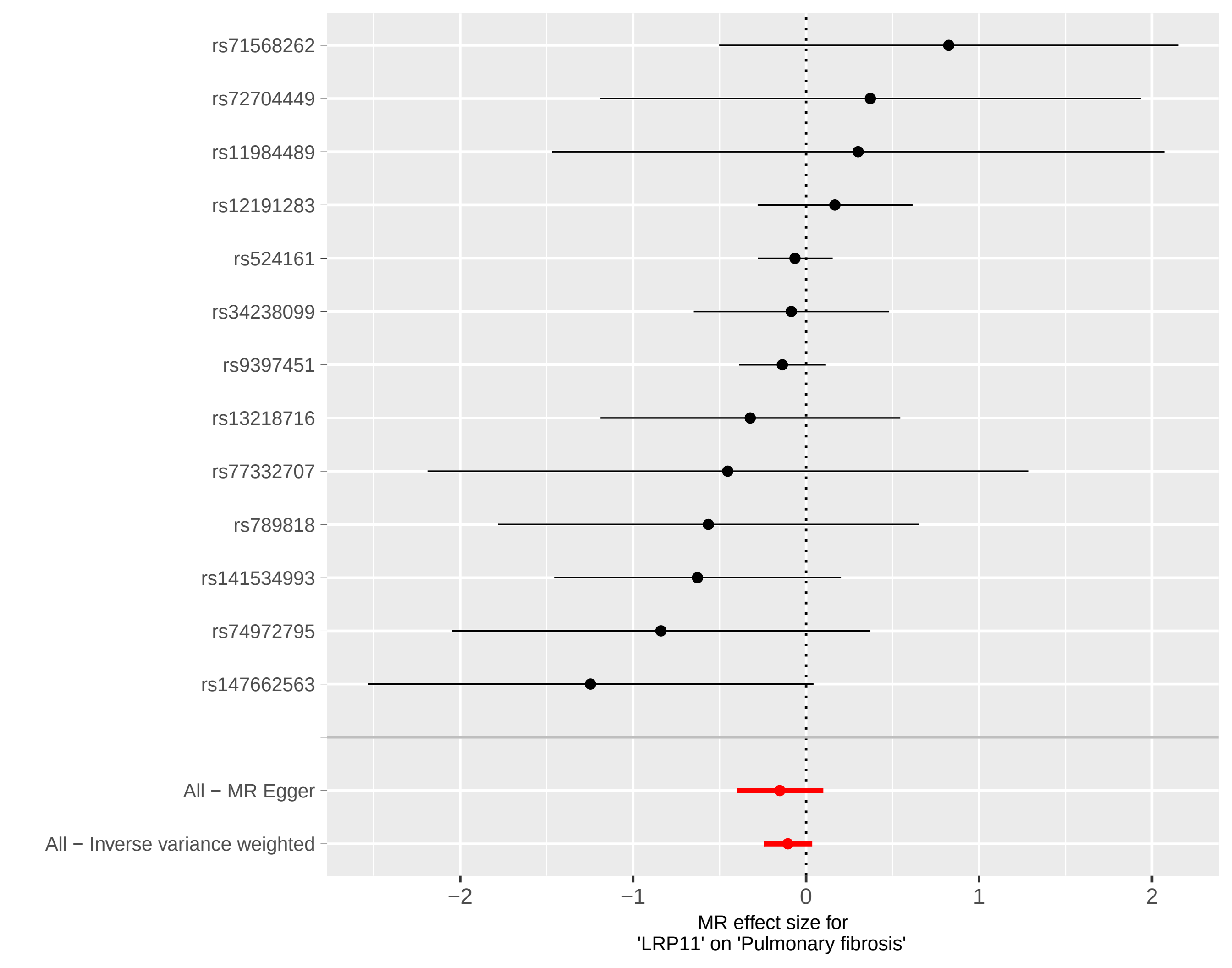

rs71568262
rs72704449
rs11984489
rs12191283
rs524161
rs34238099
rs9397451
rs13218716
rs77332707
rs789818
rs141534993
rs74972795
rs147662563
All − MR Egger
All − Inverse variance weighted
−2
−1
0
1
2
MR effect size for
'LRP11' on 'Pulmonary fibrosis'

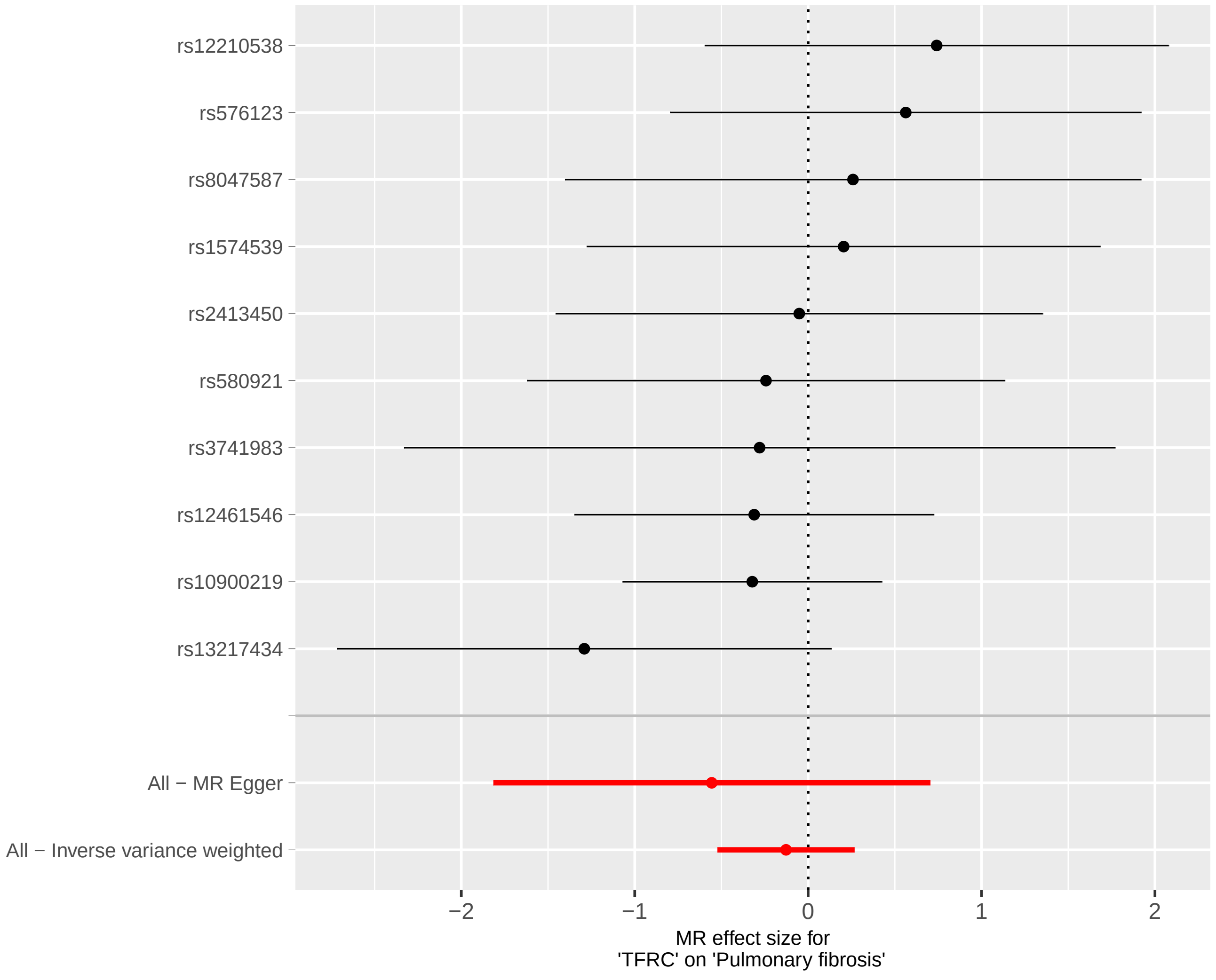

rs12210538
rs576123
rs8047587
rs1574539
rs2413450
rs580921
rs3741983
rs12461546
rs10900219
rs13217434
All − MR Egger
All − Inverse variance weighted
−2
−1
0
1
2
MR effect size for
'TFRC' on 'Pulmonary fibrosis'

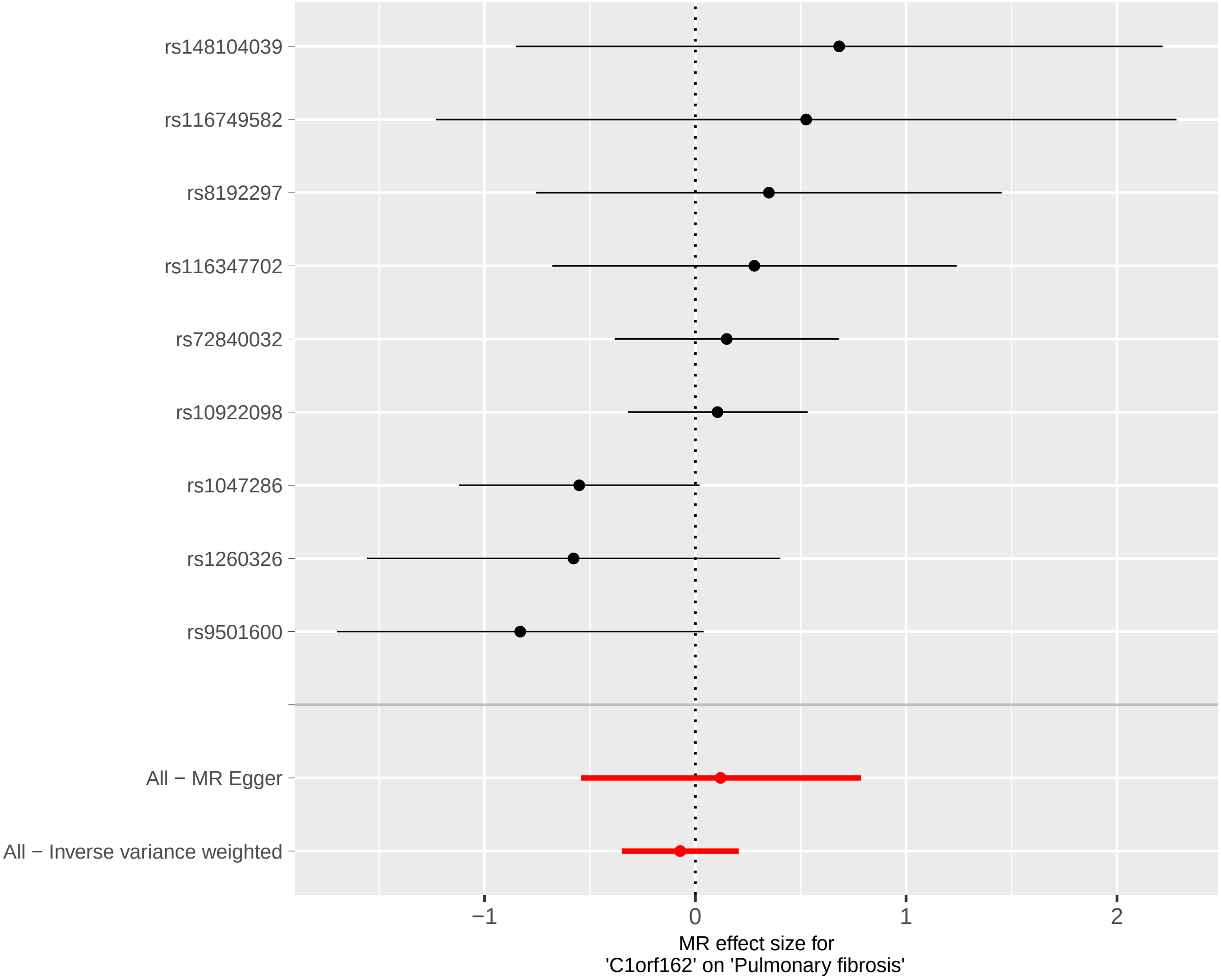

rs148104039
rs116749582
rs8192297
rs116347702
rs72840032
rs10922098
rs1047286
rs1260326
rs9501600
All − MR Egger
All − Inverse variance weighted
−1
0
1
2
MR effect size for
'C1orf162' on 'Pulmonary fibrosis'

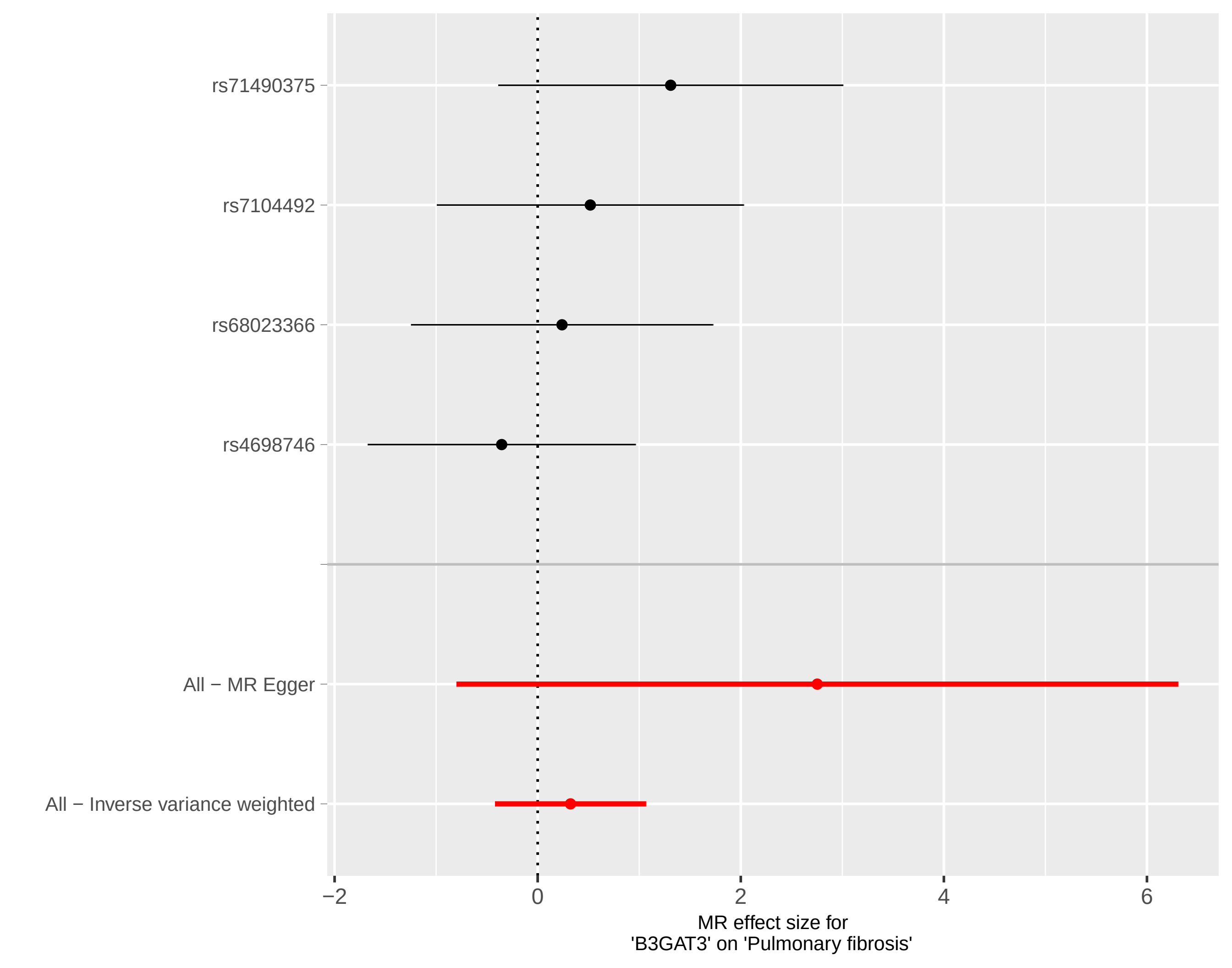

rs71490375
rs7104492
rs68023366
rs4698746
All − MR Egger
All − Inverse variance weighted
−2
0
2
4
6
MR effect size for
'B3GAT3' on 'Pulmonary fibrosis'

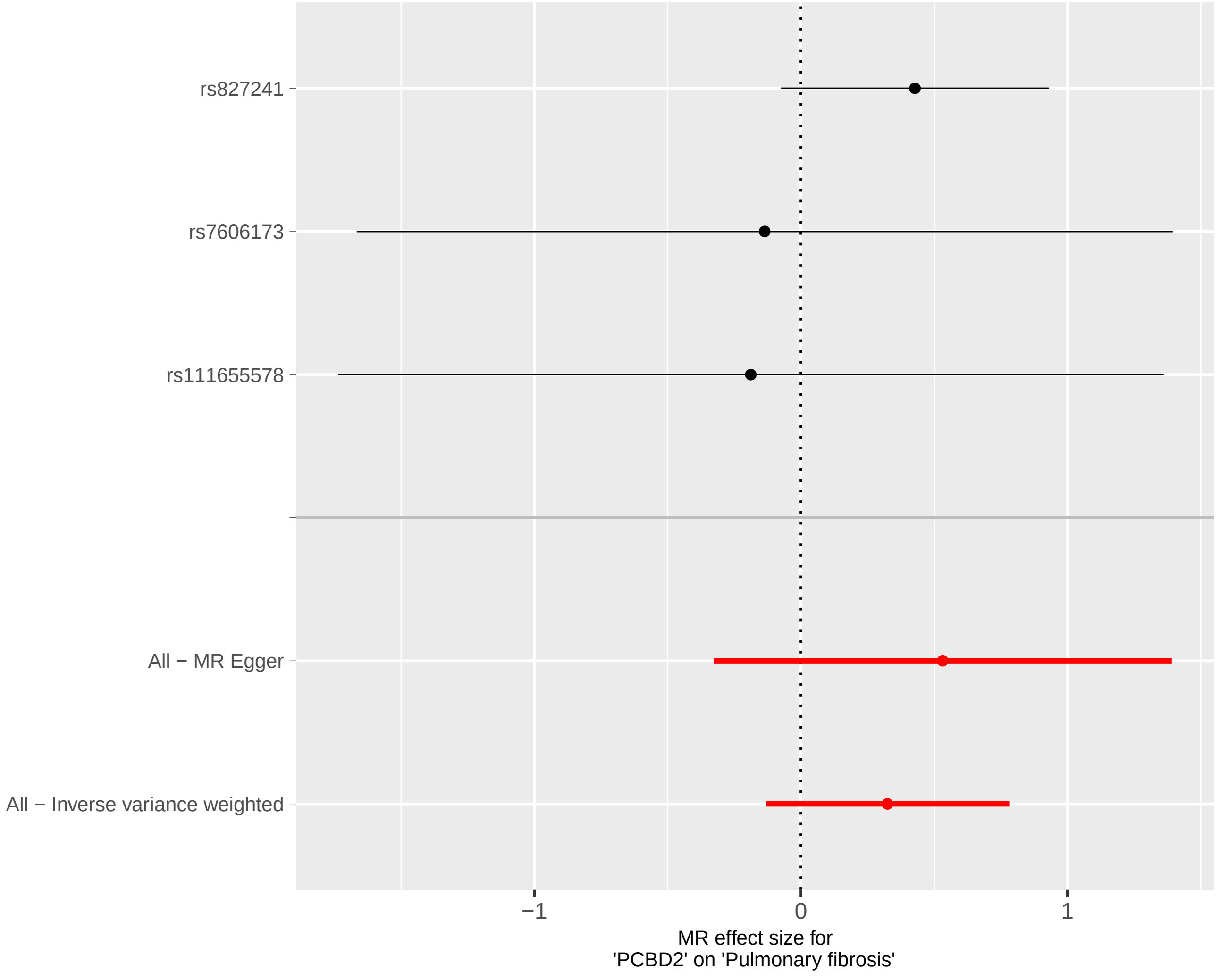

rs827241
rs7606173
rs111655578
All − MR Egger
All − Inverse variance weighted
−1
0
1
MR effect size for
'PCBD2' on 'Pulmonary fibrosis'

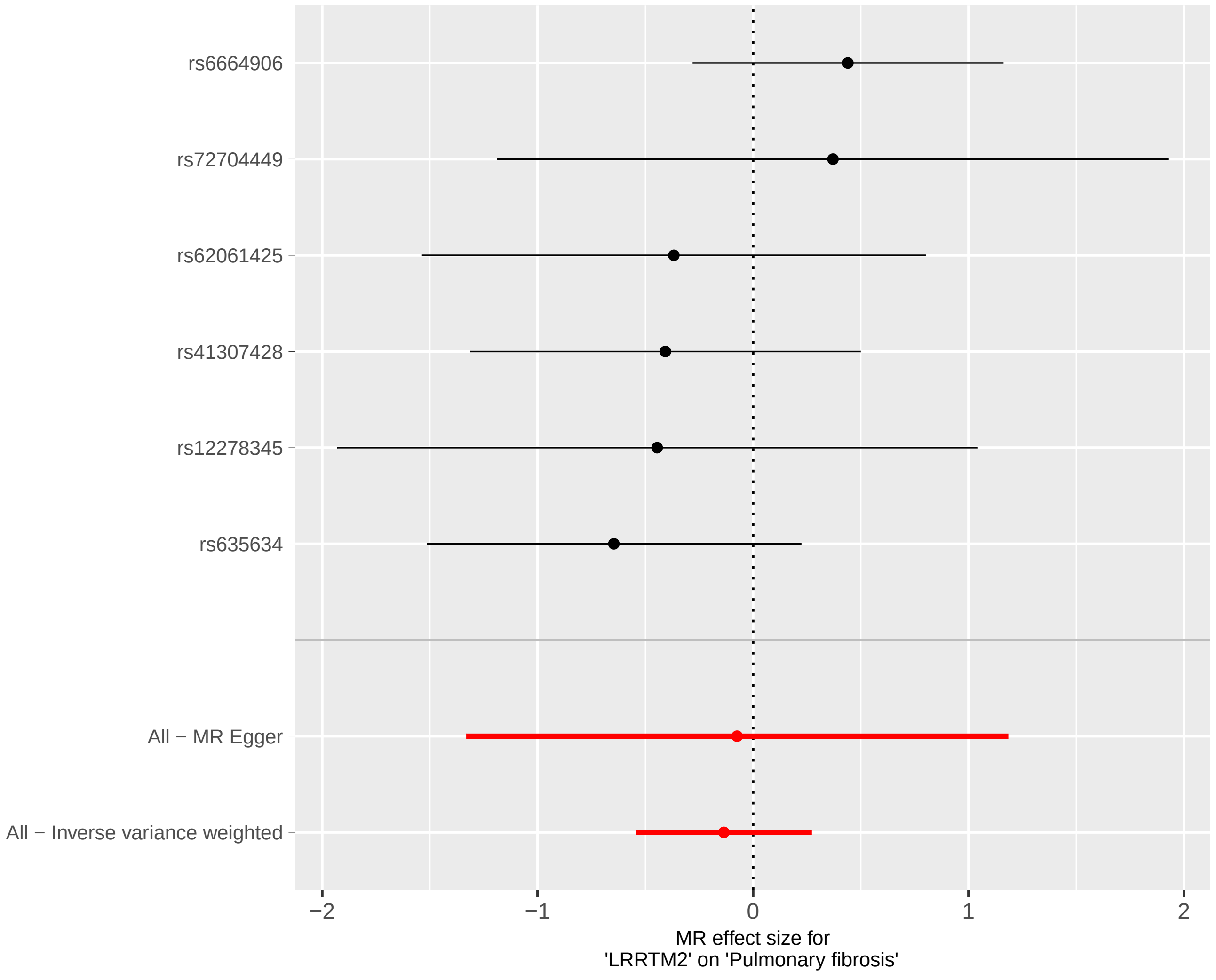
rs6664906
rs72704449
rs62061425
rs41307428
rs12278345
rs635634
All − MR Egger
All − Inverse variance weighted
−2
−1
0
1
2
MR effect size for
'LRRTM2' on 'Pulmonary fibrosis'

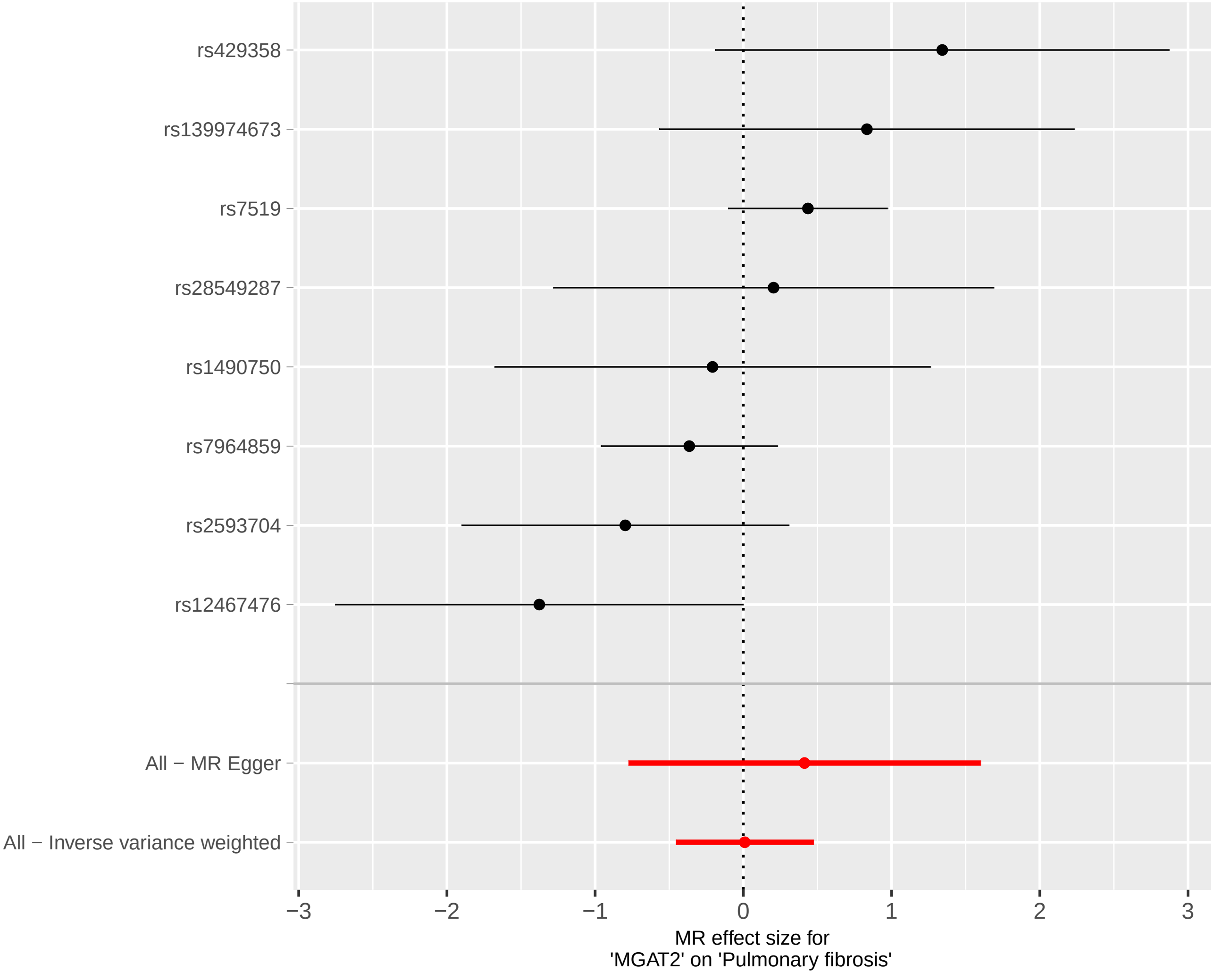

rs429358
rs139974673
rs7519
rs28549287
rs1490750
rs7964859
rs2593704
rs12467476
All − MR Egger
All − Inverse variance weighted
−3
−2
−1
0
1
2
3
MR effect size for
'MGAT2' on 'Pulmonary fibrosis'

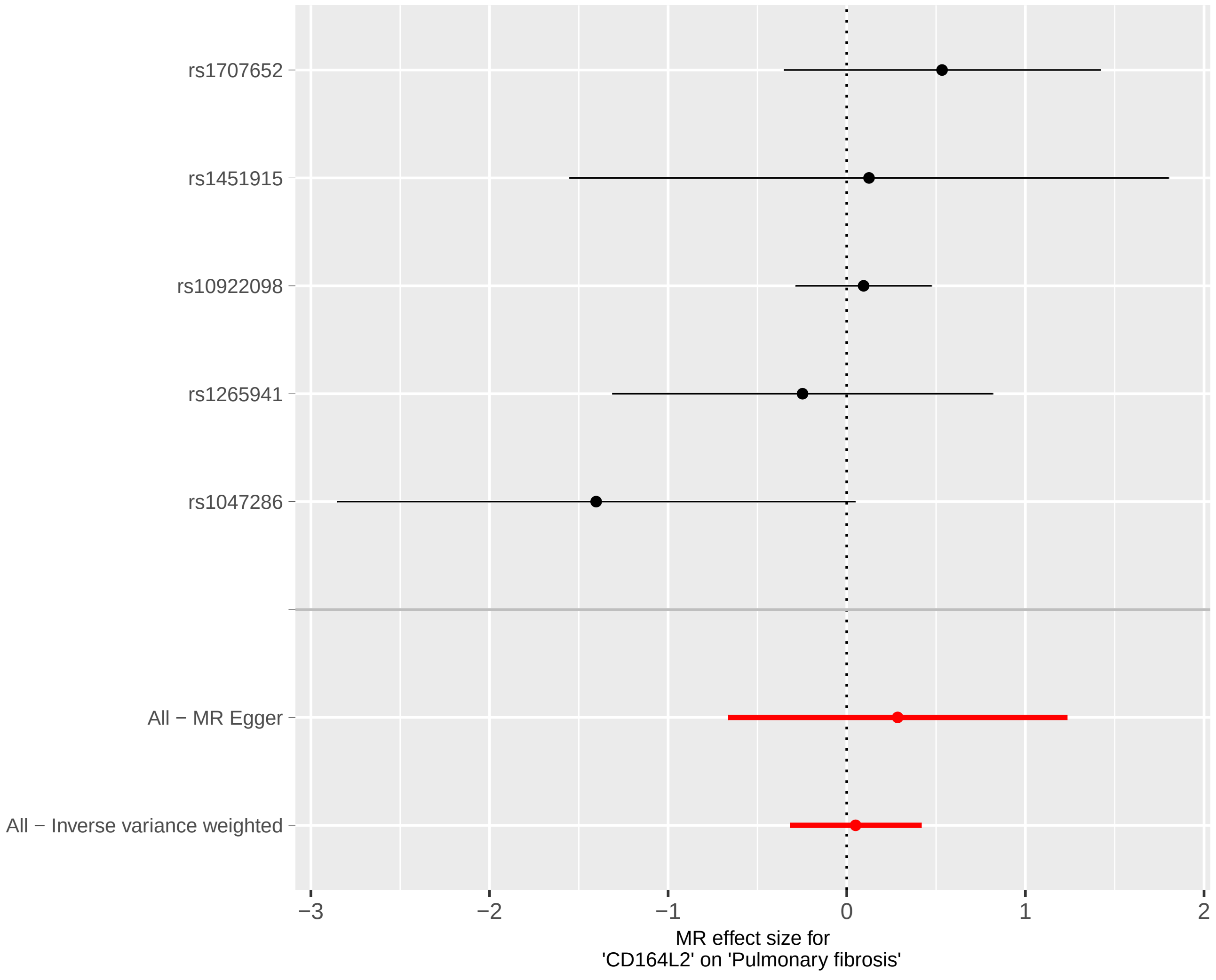

rs1707652
rs1451915
rs10922098
rs1265941
rs1047286
All − MR Egger
All − Inverse variance weighted
−3
−2
−1
0
1
2
MR effect size for
'CD164L2' on 'Pulmonary fibrosis'

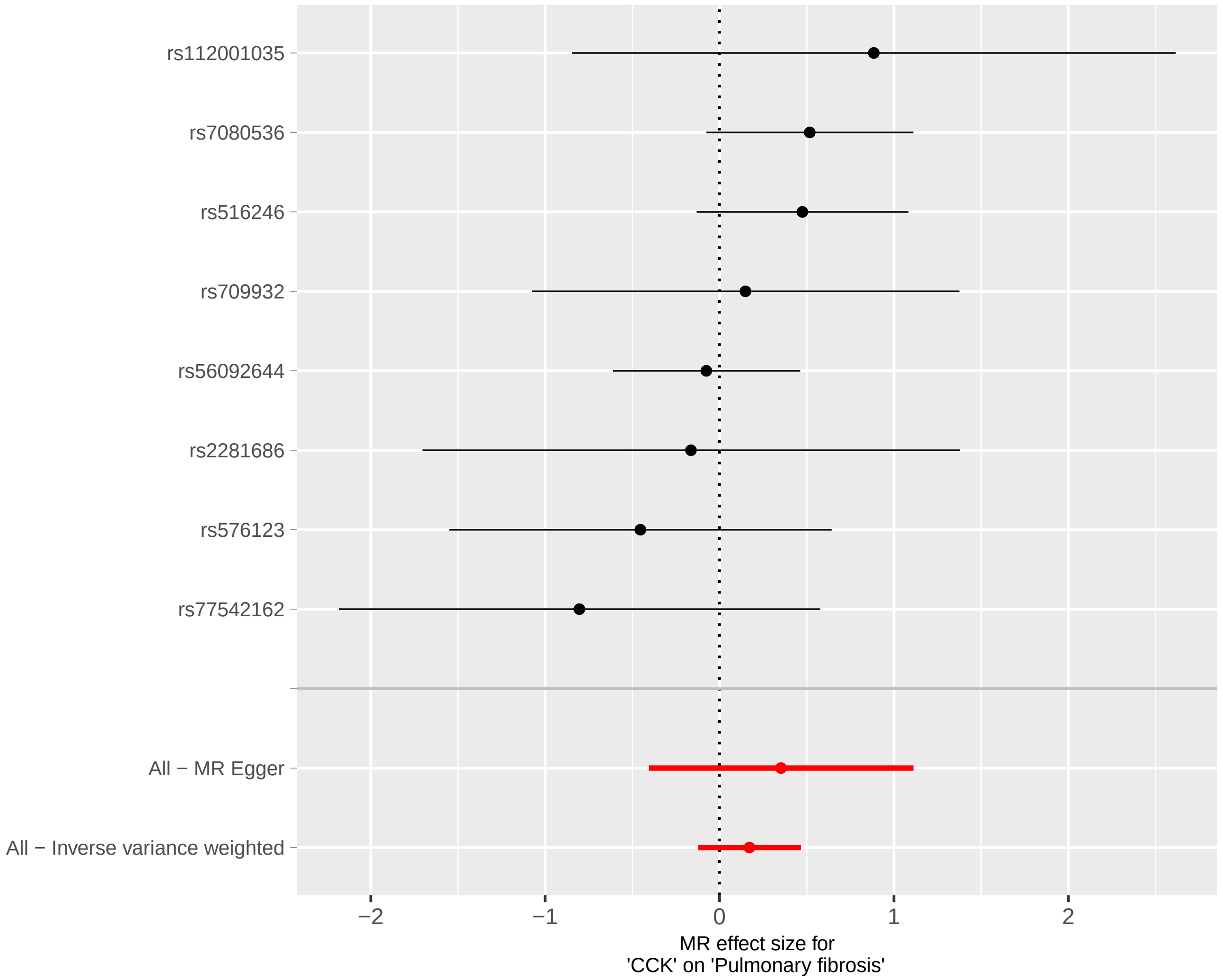

rs112001035
rs7080536
rs516246
rs709932
rs56092644
rs2281686
rs576123
rs77542162
All − MR Egger
All − Inverse variance weighted
−2
−1
0
1
2
MR effect size for
'CCK' on 'Pulmonary fibrosis'

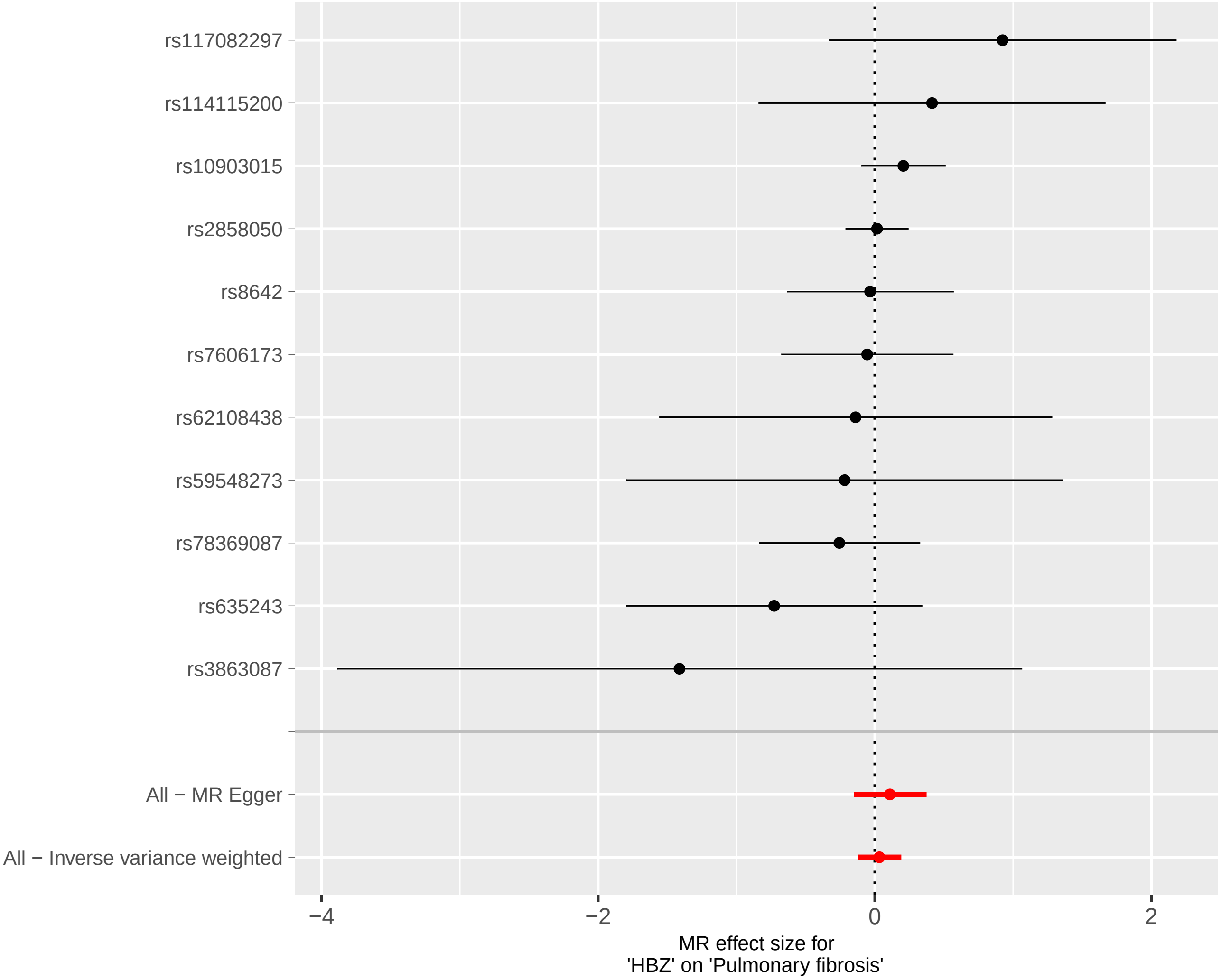

rs117082297
rs114115200
rs10903015
rs2858050
rs8642
rs7606173
rs62108438
rs59548273
rs78369087
rs635243
rs3863087
All − MR Egger
All − Inverse variance weighted
−4
−2
0
2
MR effect size for
'HBZ' on 'Pulmonary fibrosis'

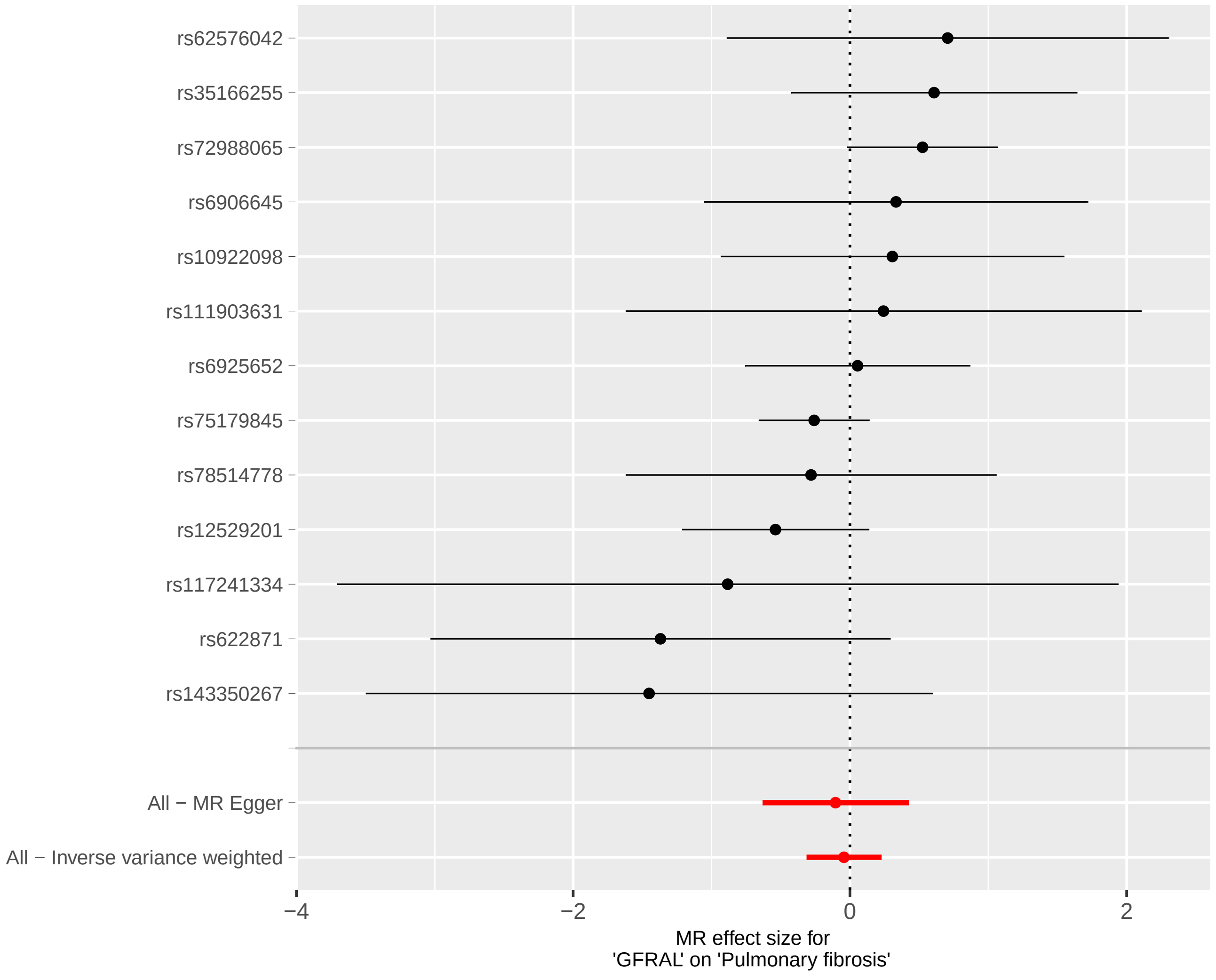

rs62576042
rs35166255
rs72988065
rs6906645
rs10922098
rs111903631
rs6925652
rs75179845
rs78514778
rs12529201
rs117241334
rs622871
rs143350267
All − MR Egger
All − Inverse variance weighted
−4
−2
0
2
MR effect size for
'GFRAL' on 'Pulmonary fibrosis'

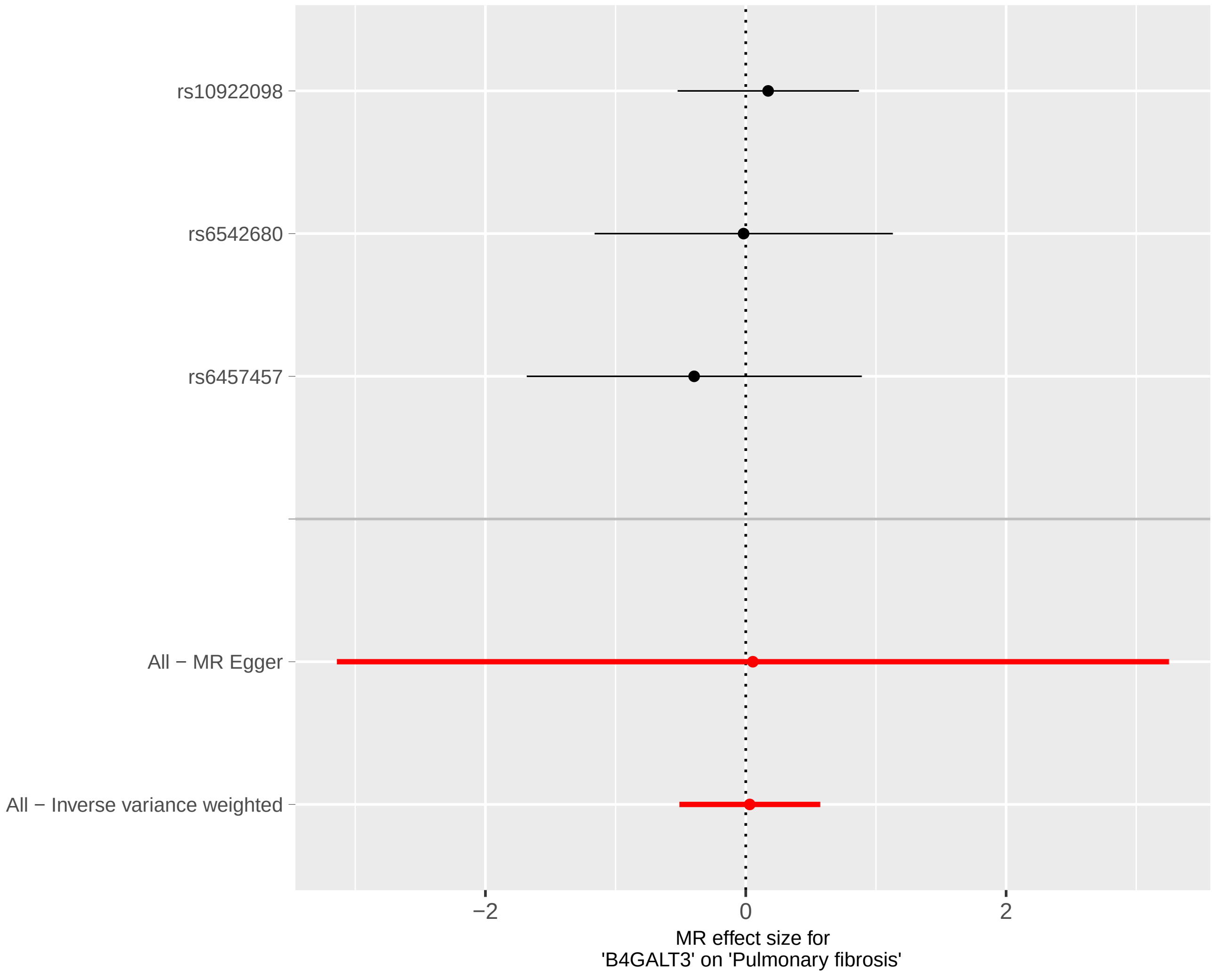
rs10922098
rs6542680
rs6457457
All − MR Egger
All − Inverse variance weighted
−2
0
2
MR effect size for
'B4GALT3' on 'Pulmonary fibrosis'

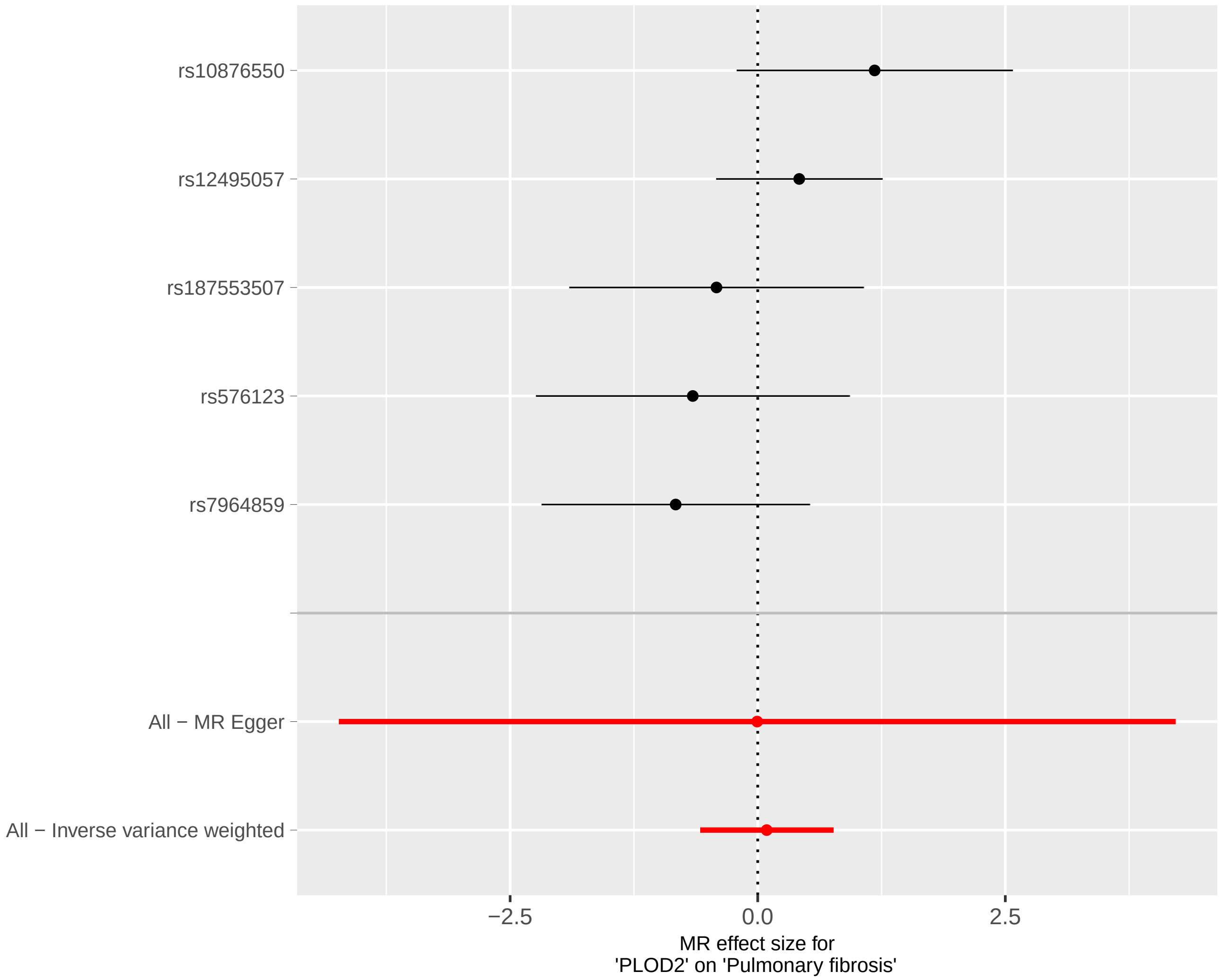
rs10876550
rs12495057
rs187553507
rs576123
rs7964859
All − MR Egger
All − Inverse variance weighted
−2.5
0.0
2.5
MR effect size for
'PLOD2' on 'Pulmonary fibrosis'

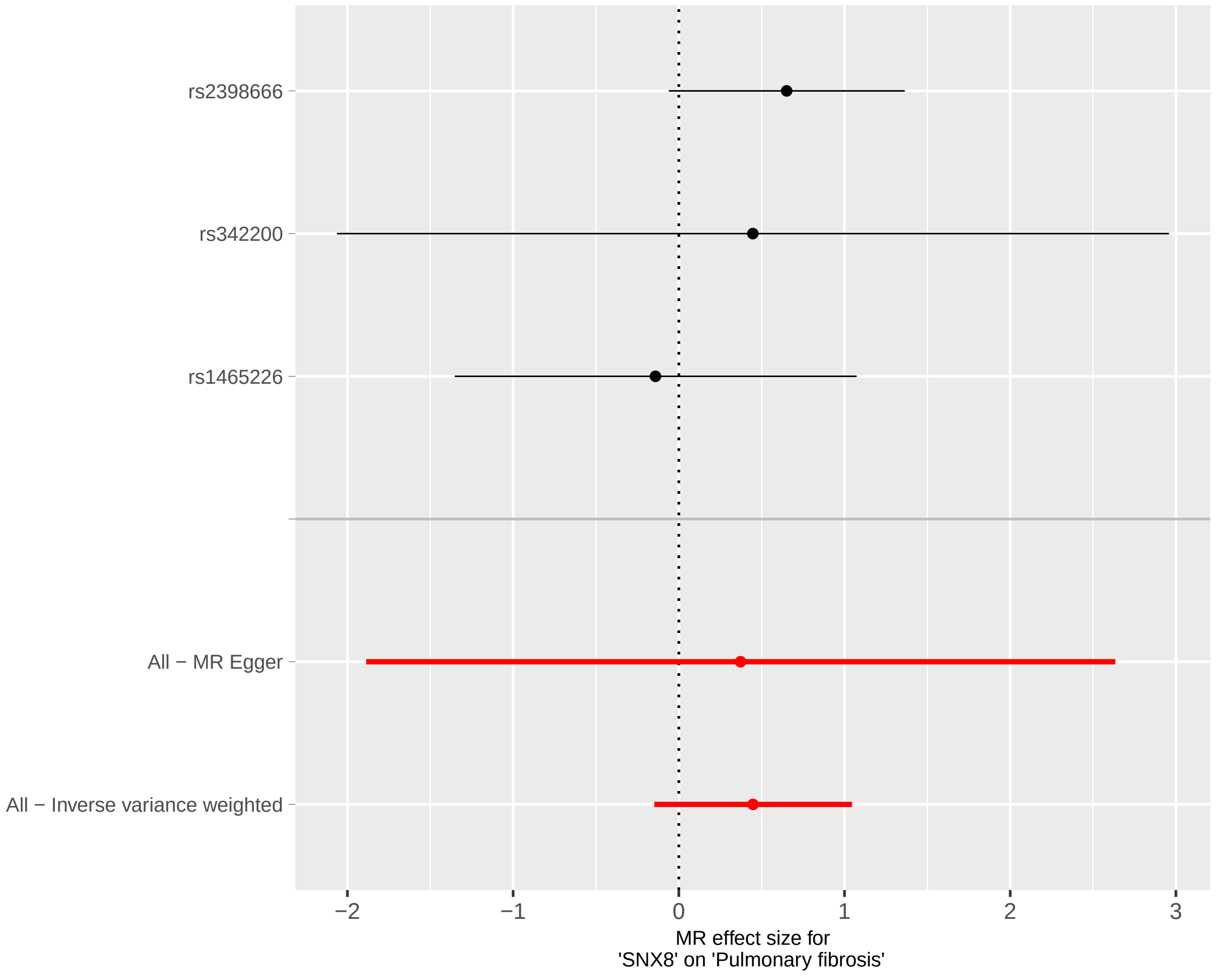

rs2398666
rs342200
rs1465226
All − MR Egger
All − Inverse variance weighted
−2
−1
0
1
2
3
MR effect size for
'SNX8' on 'Pulmonary fibrosis'

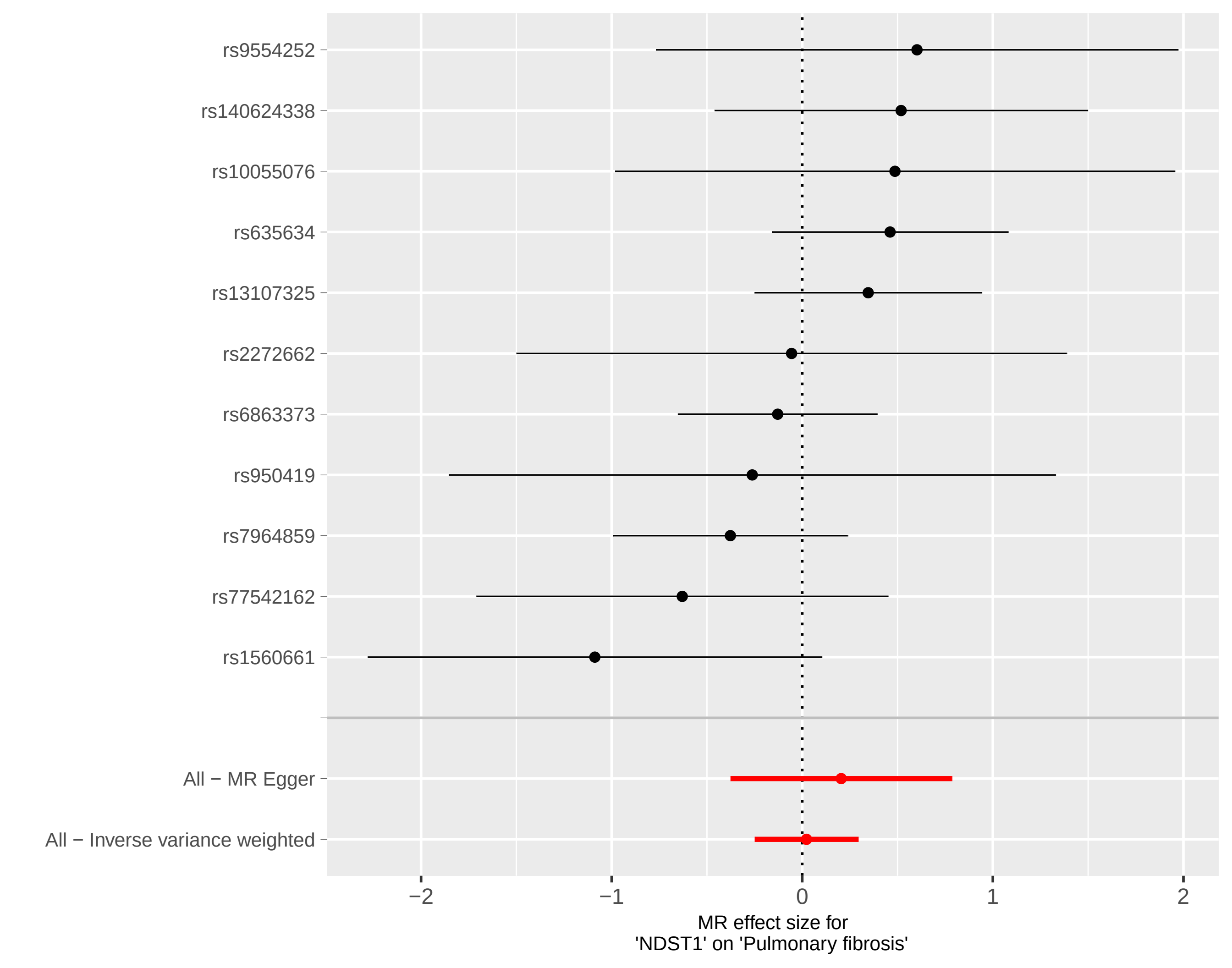
rs9554252
rs140624338
rs10055076
rs635634
rs13107325
rs2272662
rs6863373
rs950419
rs7964859
rs77542162
rs1560661
All − MR Egger
All − Inverse variance weighted
−2
−1
0
1
2
MR effect size for
'NDST1' on 'Pulmonary fibrosis'

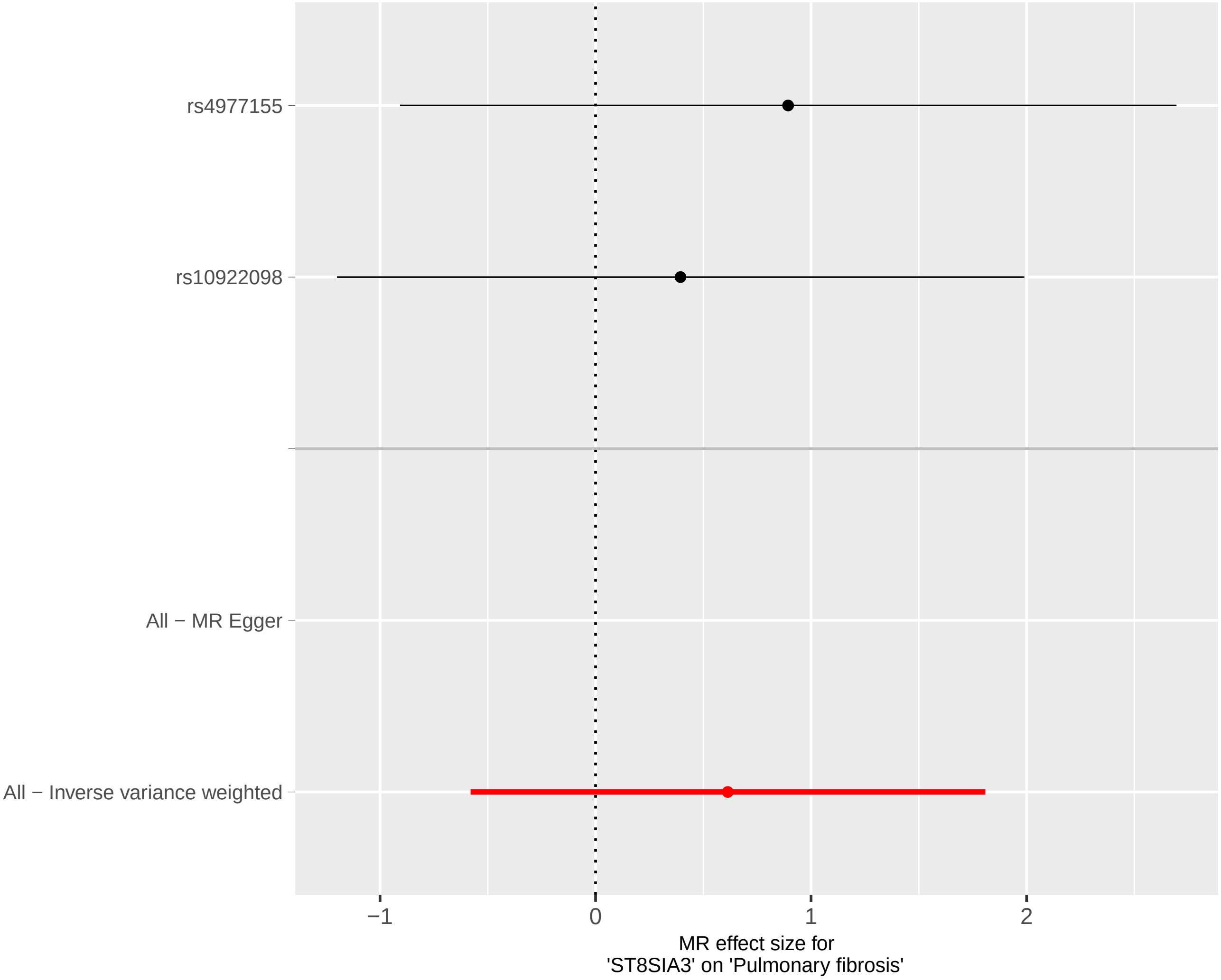

rs4977155
rs10922098
All − MR Egger
All − Inverse variance weighted
−1
0
1
2
MR effect size for
'ST8SIA3' on 'Pulmonary fibrosis'

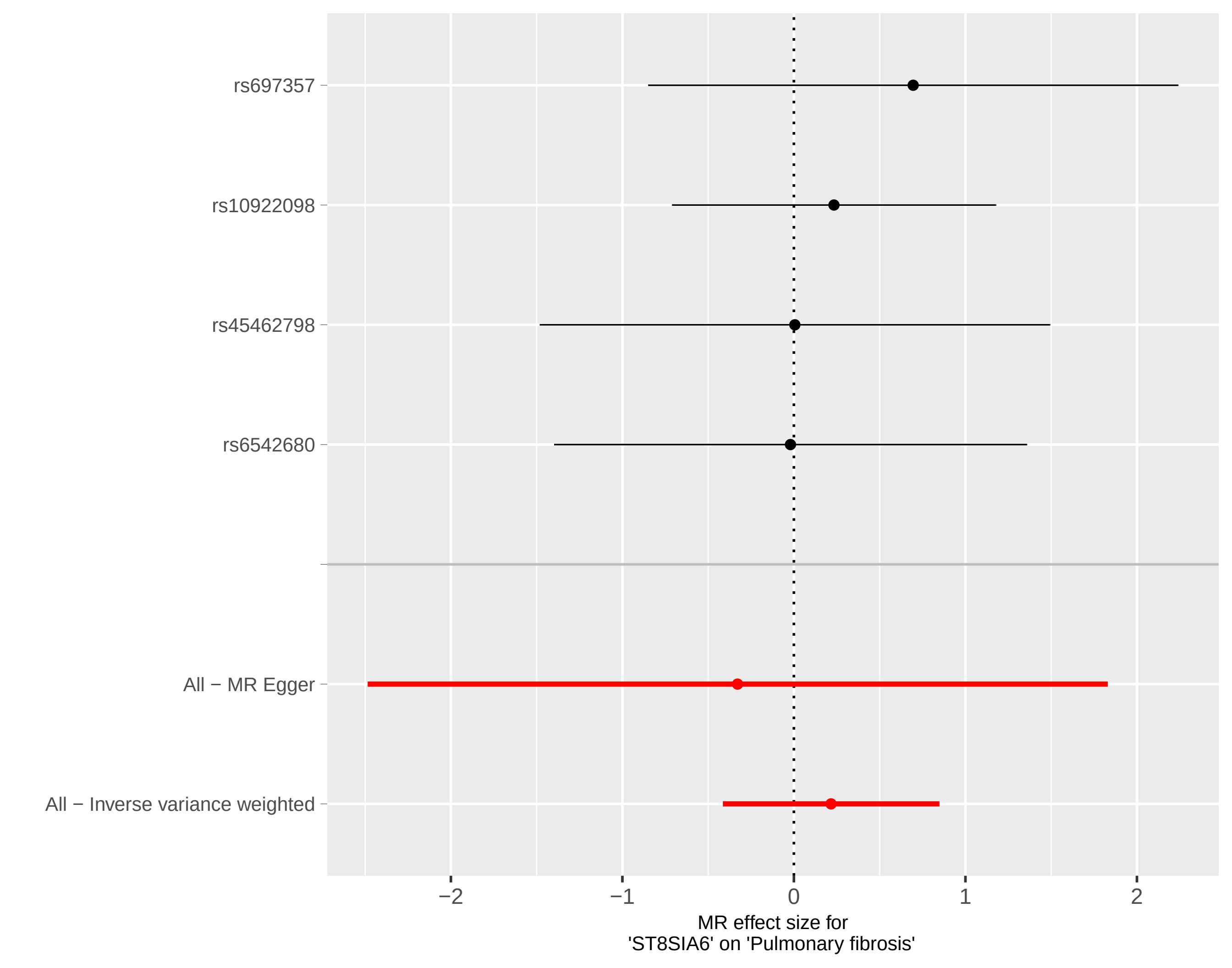

rs697357
rs10922098
rs45462798
rs6542680
All − MR Egger
All − Inverse variance weighted
−2
−1
0
1
2
MR effect size for
'ST8SIA6' on 'Pulmonary fibrosis'

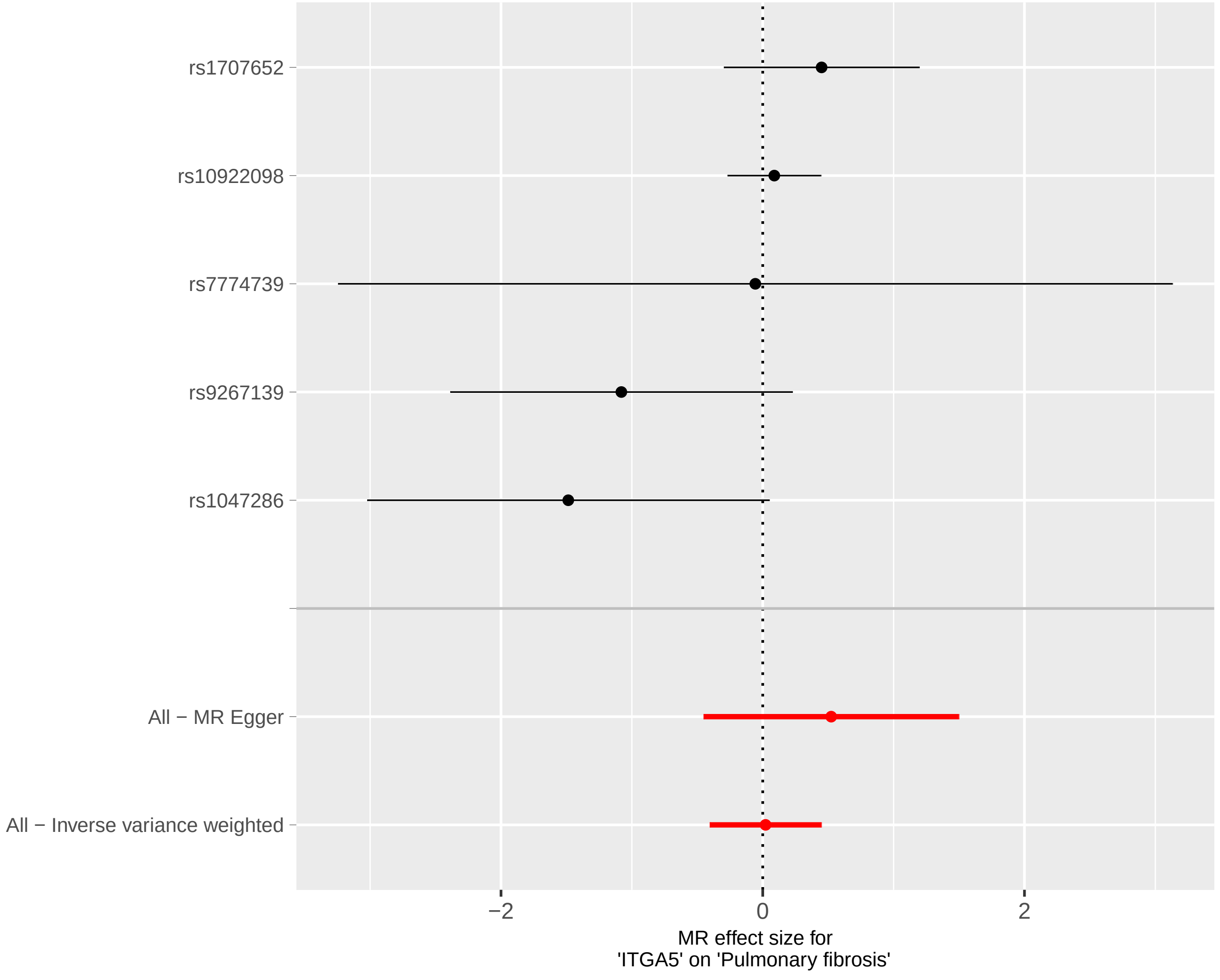
rs1707652
rs10922098
rs7774739
rs9267139
rs1047286
All − MR Egger
All − Inverse variance weighted
−2
0
2
MR effect size for
'ITGA5' on 'Pulmonary fibrosis'

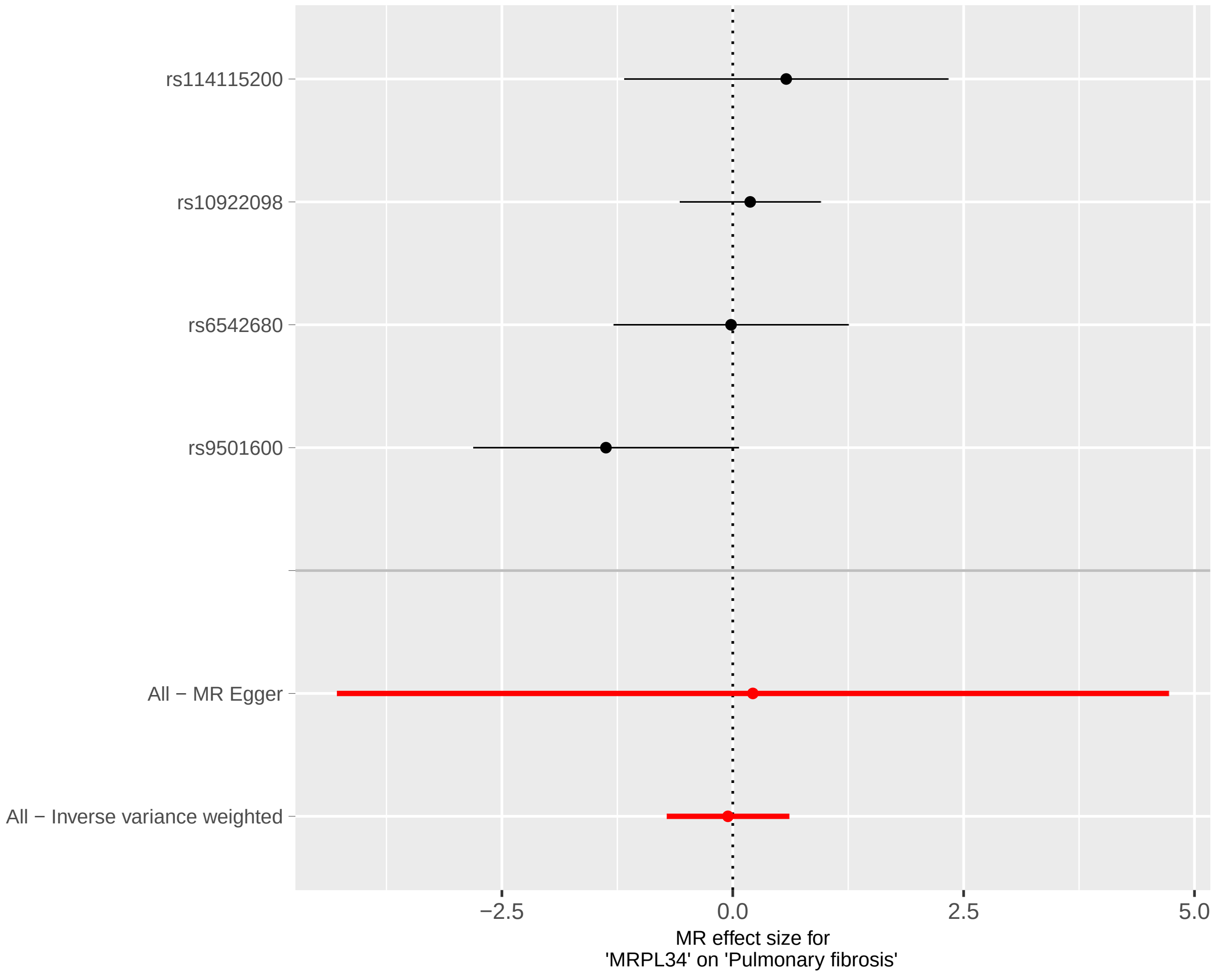

rs114115200
rs10922098
rs6542680
rs9501600
All − MR Egger
All − Inverse variance weighted
−2.5
0.0
2.5
5.0
MR effect size for
'MRPL34' on 'Pulmonary fibrosis'

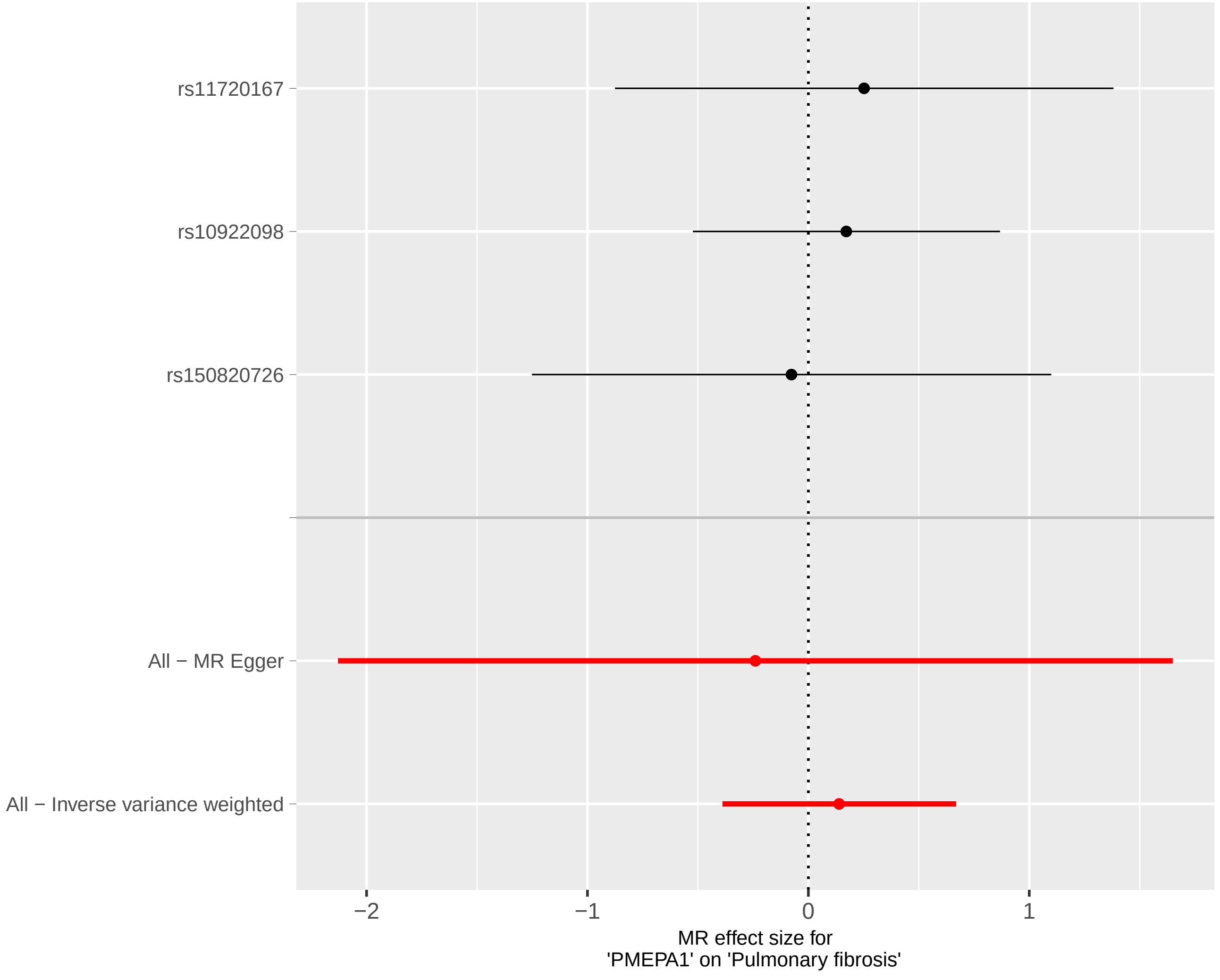

rs11720167
rs10922098
rs150820726
All − MR Egger
All − Inverse variance weighted
−2
−1
0
1
MR effect size for
'PMEPA1' on 'Pulmonary fibrosis'

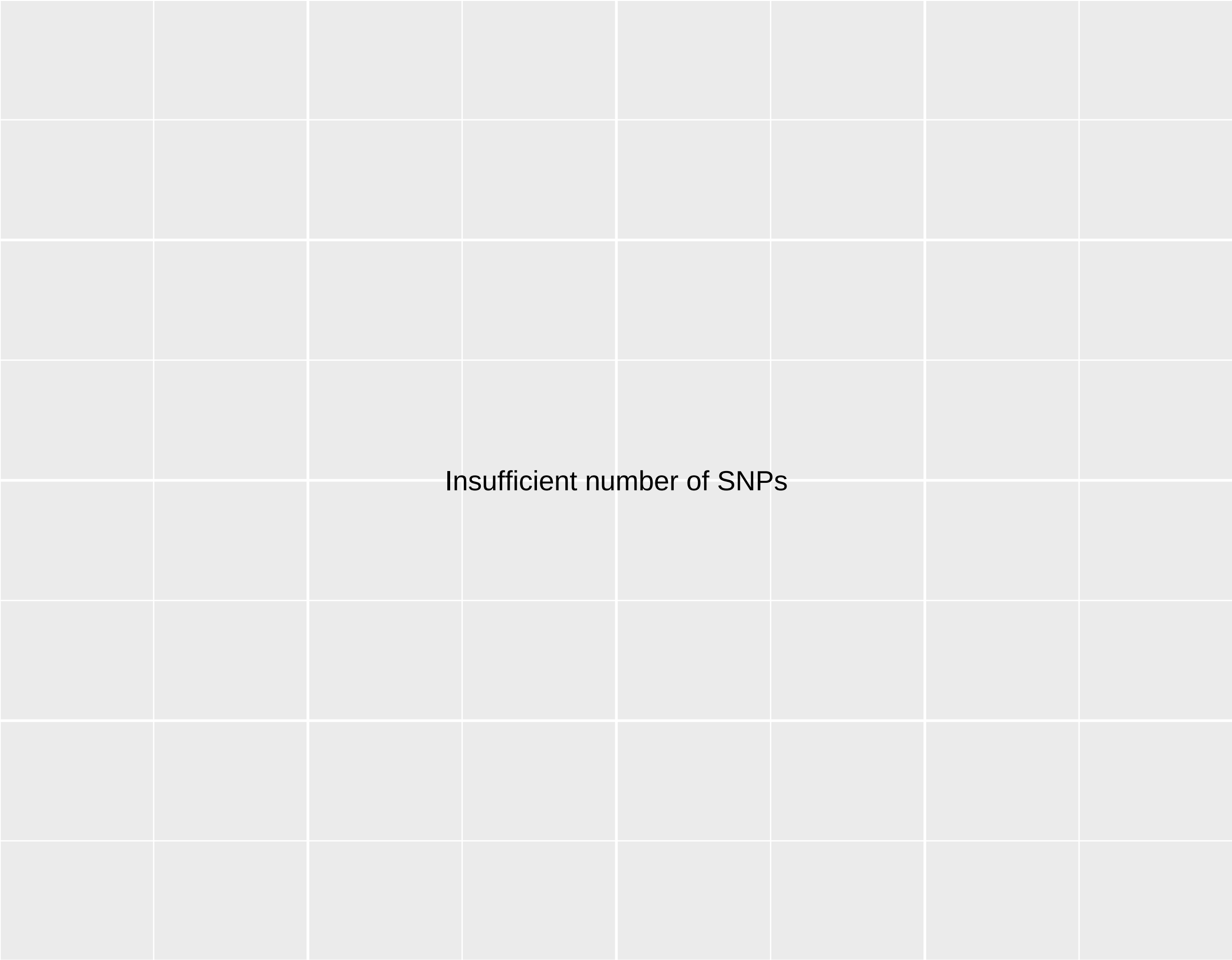
Insufficient number of SNPs

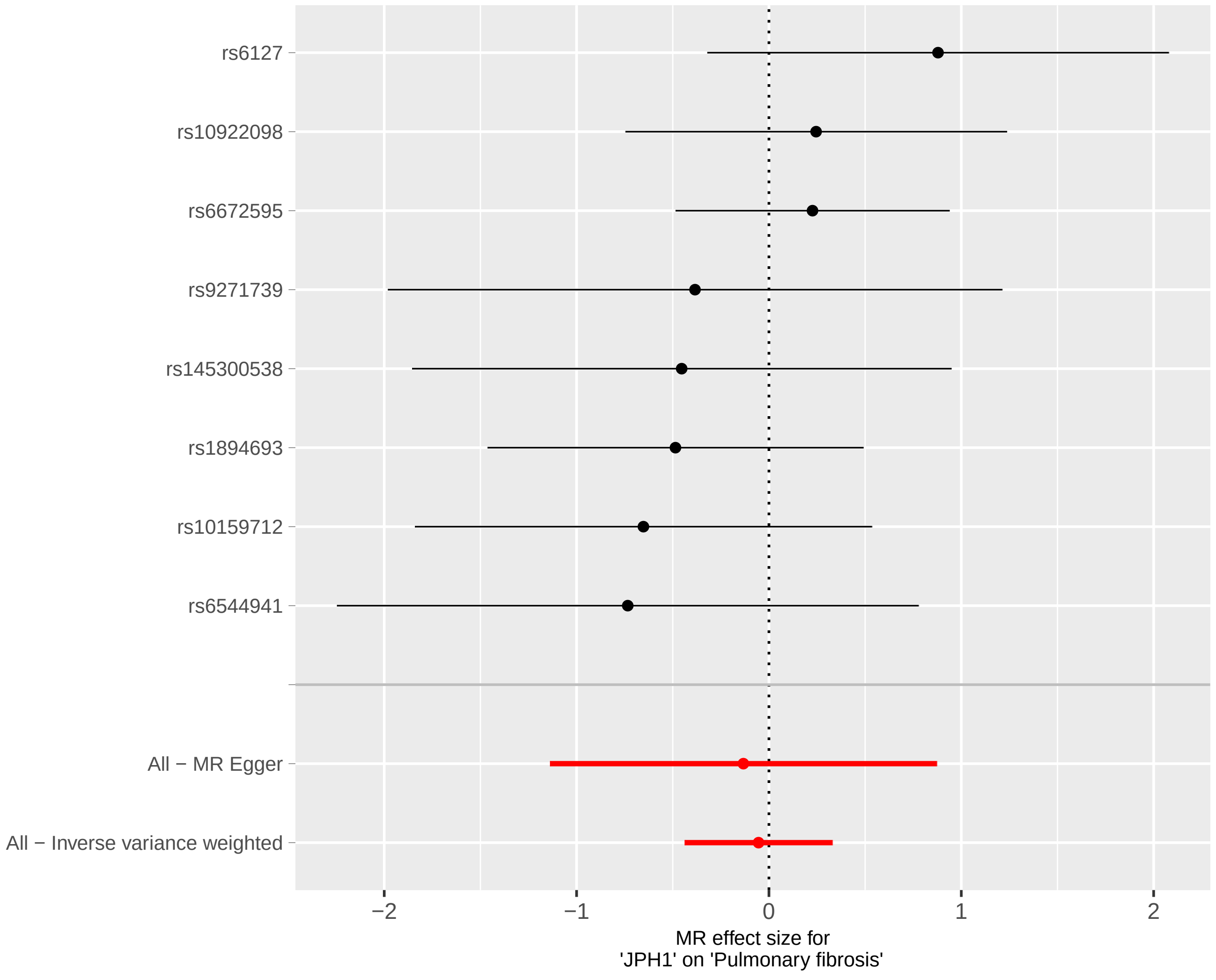
rs6127
rs10922098
rs6672595
rs9271739
rs145300538
rs1894693
rs10159712
rs6544941
All − MR Egger
All − Inverse variance weighted
−2
−1
0
1
2
MR effect size for
'JPH1' on 'Pulmonary fibrosis'

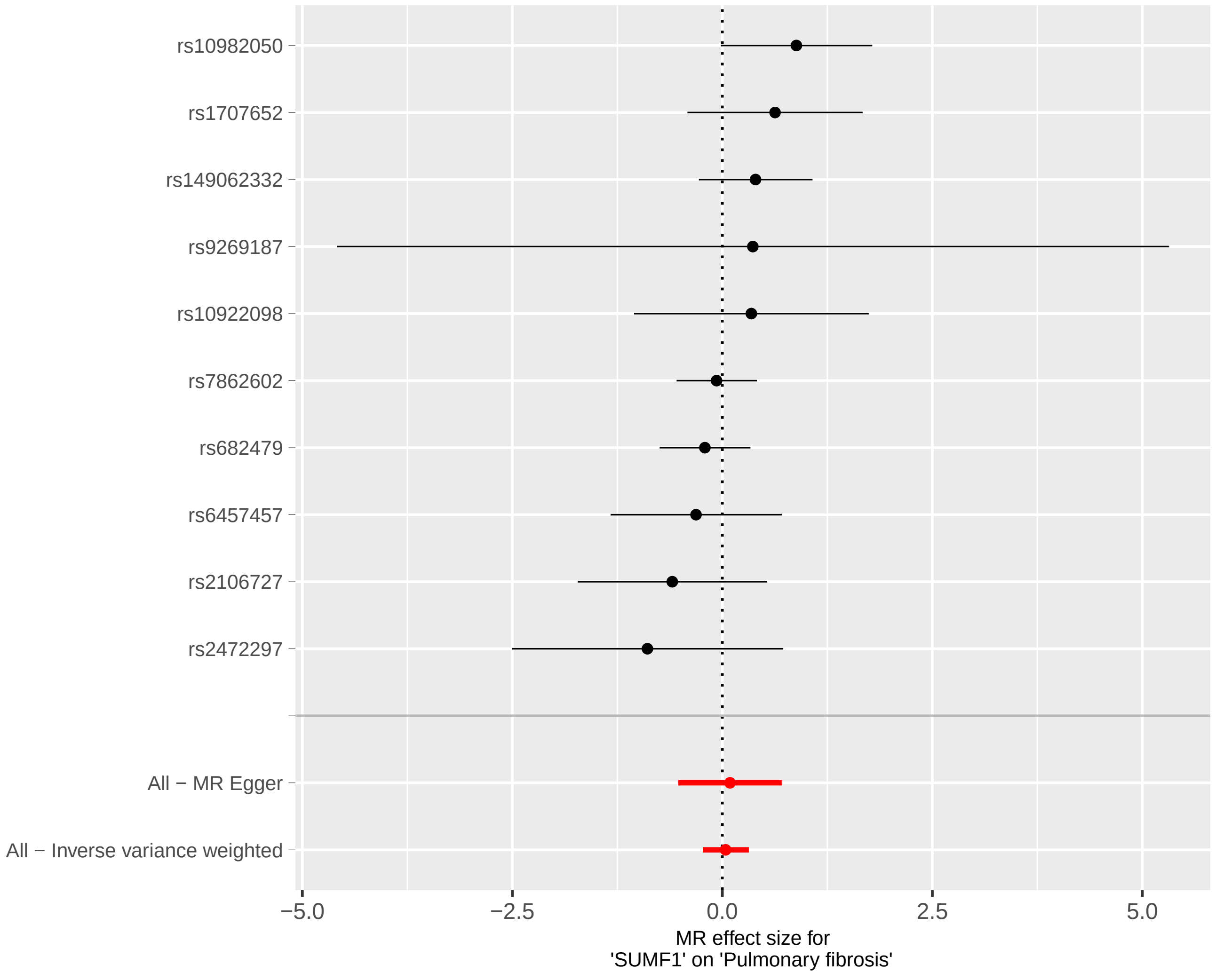

rs10982050
rs1707652
rs149062332
rs9269187
rs10922098
rs7862602
rs682479
rs6457457
rs2106727
rs2472297
All − MR Egger
All − Inverse variance weighted
−5.0
−2.5
0.0
2.5
5.0
MR effect size for
'SUMF1' on 'Pulmonary fibrosis'

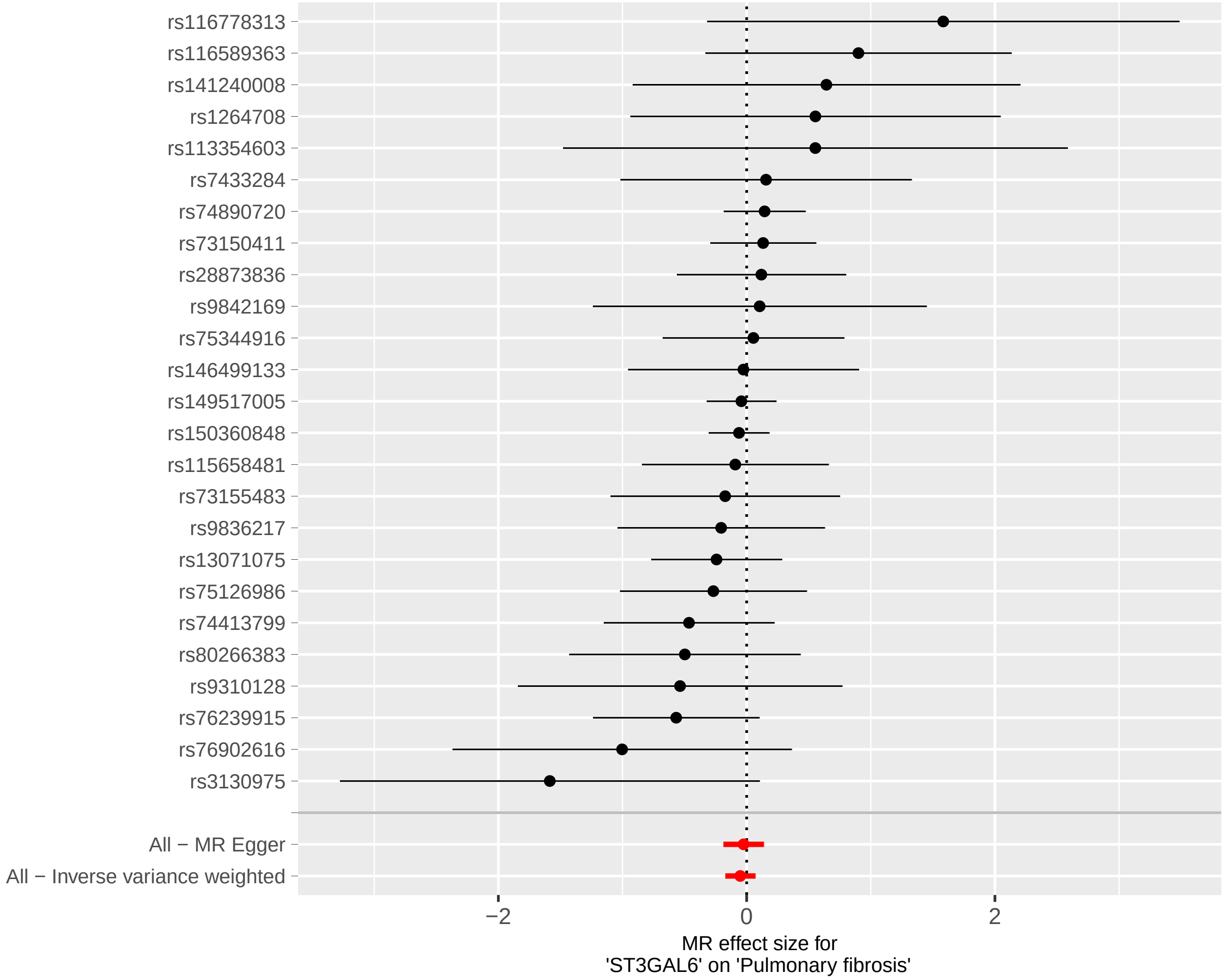

rs116778313
rs116589363
rs141240008
rs1264708
rs113354603
rs7433284
rs74890720
rs73150411
rs28873836
rs9842169
rs75344916
rs146499133
rs149517005
rs150360848
rs115658481
rs73155483
rs9836217
rs13071075
rs75126986
rs74413799
rs80266383
rs9310128
rs76239915
rs76902616
rs3130975
All − MR Egger
All − Inverse variance weighted
−2
0
2
MR effect size for
'ST3GAL6' on 'Pulmonary fibrosis'

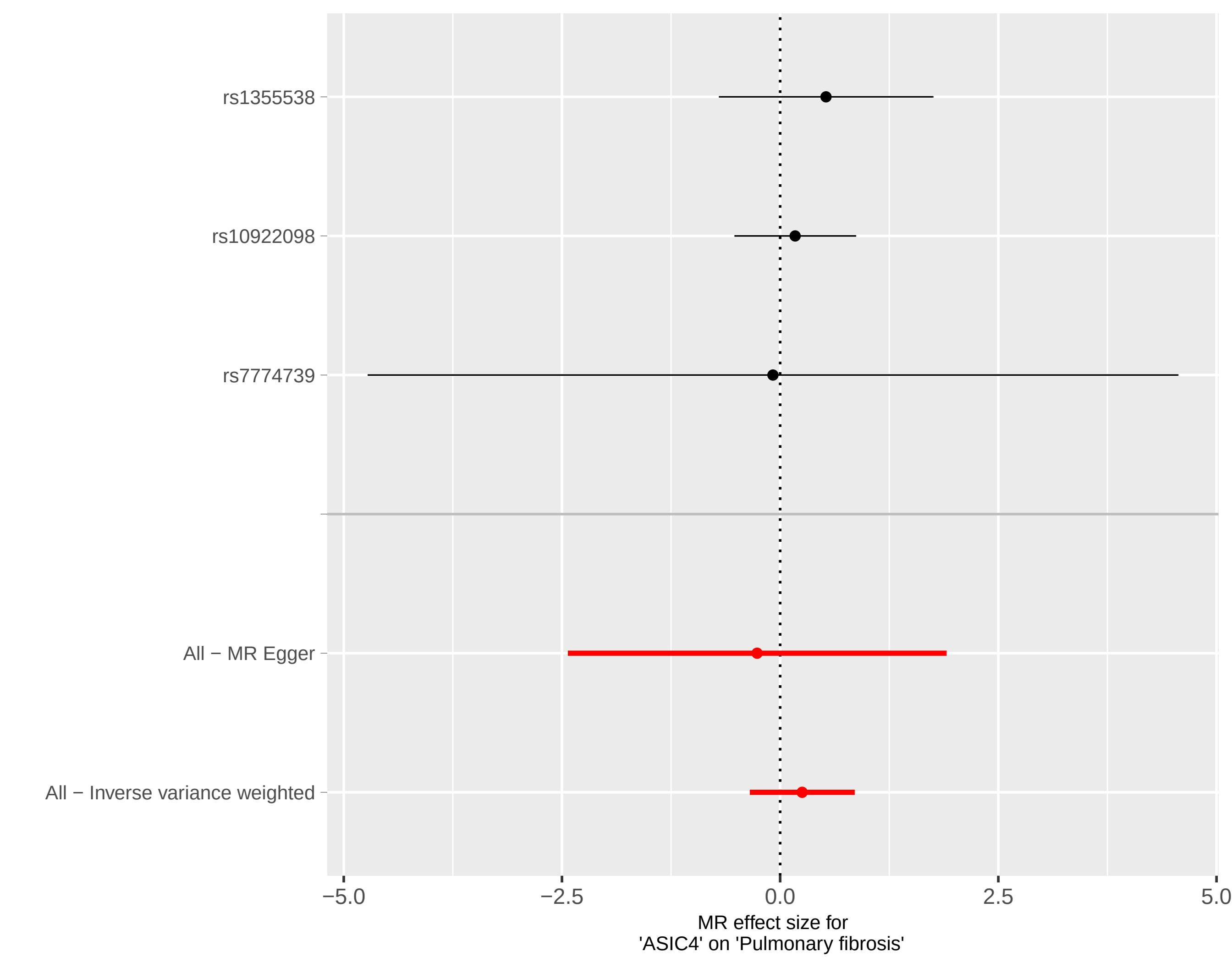
rs1355538
rs10922098
rs7774739
All − MR Egger
All − Inverse variance weighted
−5.0
−2.5
0.0
2.5
5.0
MR effect size for
'ASIC4' on 'Pulmonary fibrosis'

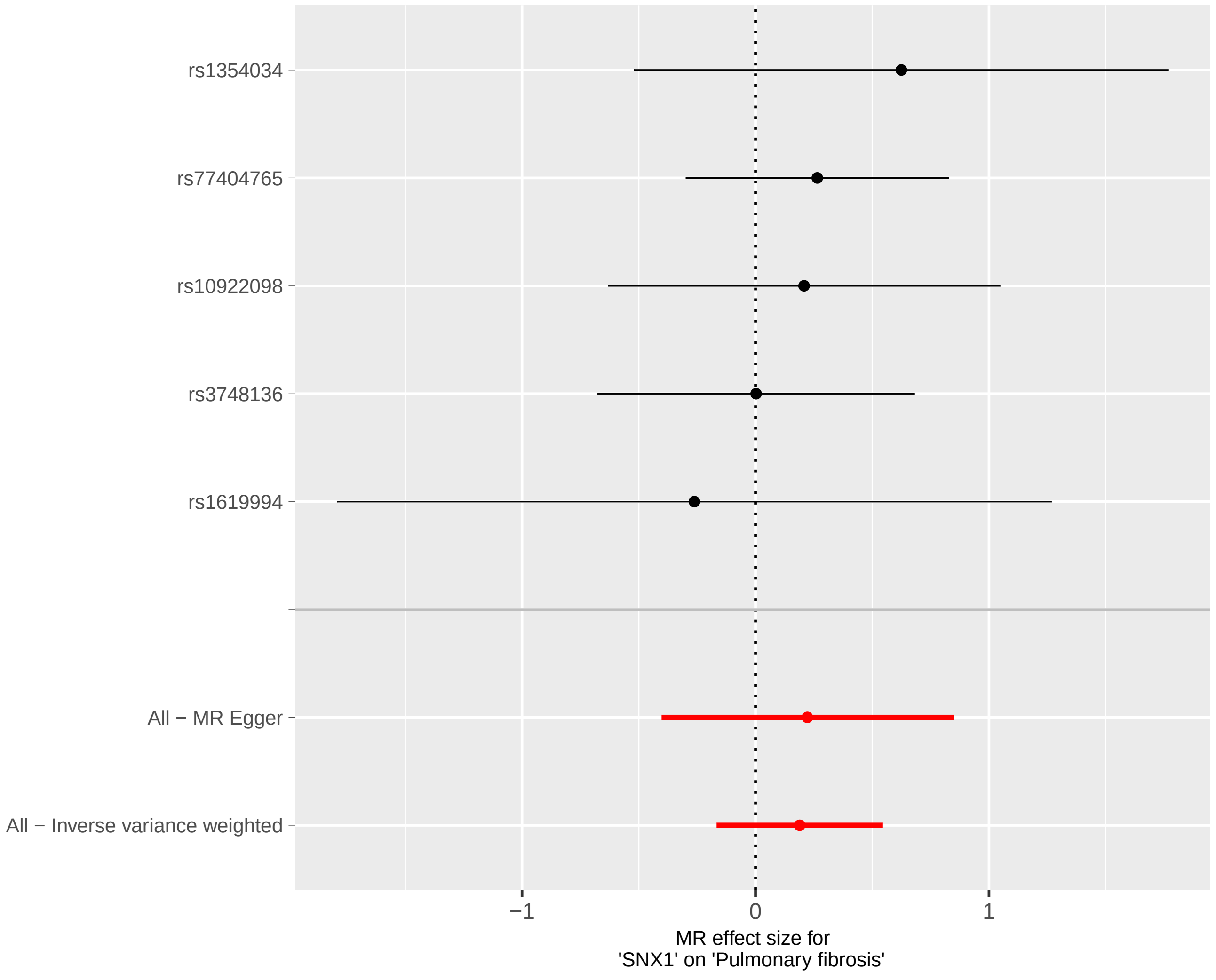
rs1354034
rs77404765
rs10922098
rs3748136
rs1619994
All − MR Egger
All − Inverse variance weighted
−1
0
1
MR effect size for
'SNX1' on 'Pulmonary fibrosis'

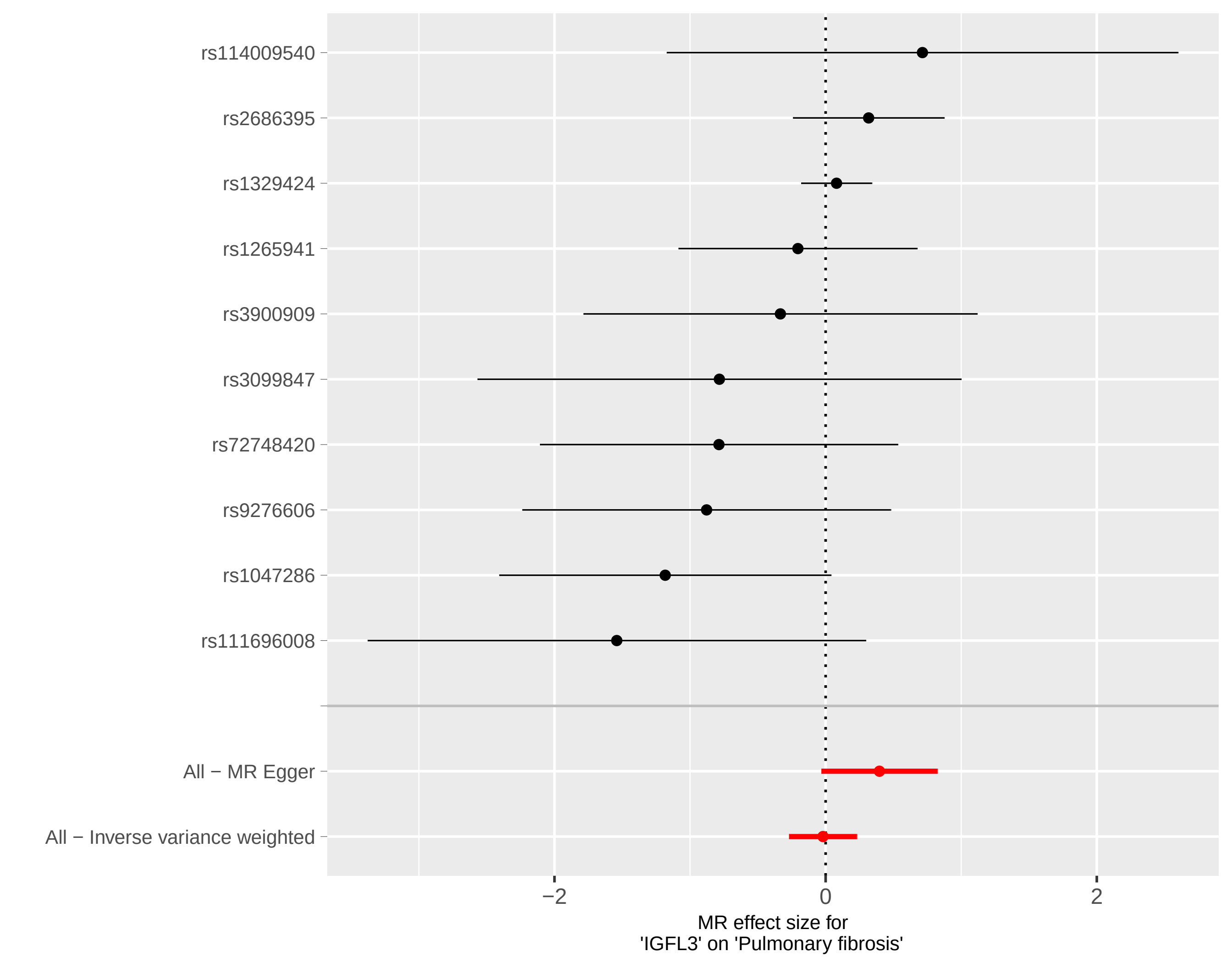

rs114009540
rs2686395
rs1329424
rs1265941
rs3900909
rs3099847
rs72748420
rs9276606
rs1047286
rs111696008
All − MR Egger
All − Inverse variance weighted
−2
0
2
MR effect size for
'IGFL3' on 'Pulmonary fibrosis'

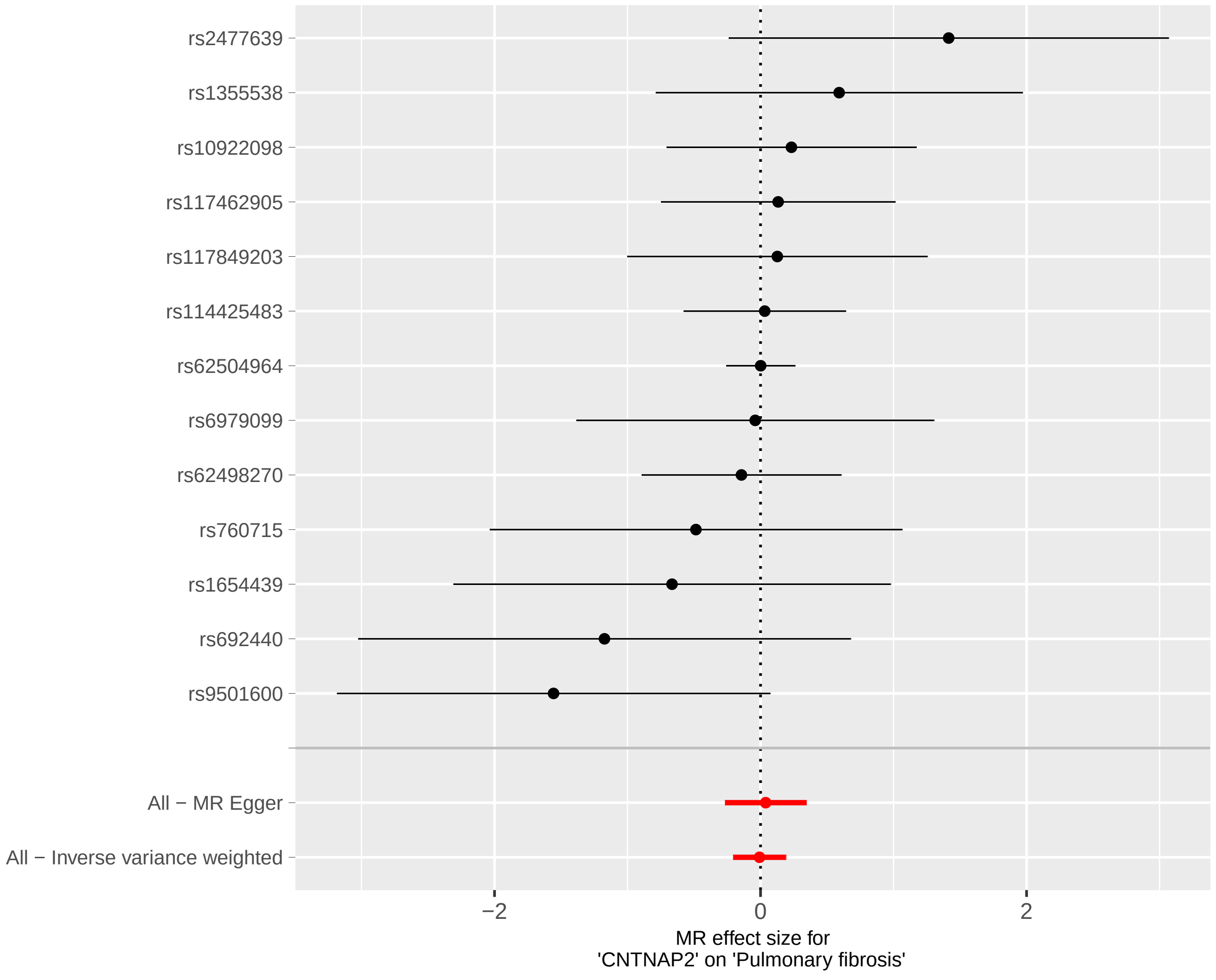

rs2477639
rs1355538
rs10922098
rs117462905
rs117849203
rs114425483
rs62504964
rs6979099
rs62498270
rs760715
rs1654439
rs692440
rs9501600
All − MR Egger
All − Inverse variance weighted
−2
0
2
MR effect size for
'CNTNAP2' on 'Pulmonary fibrosis'

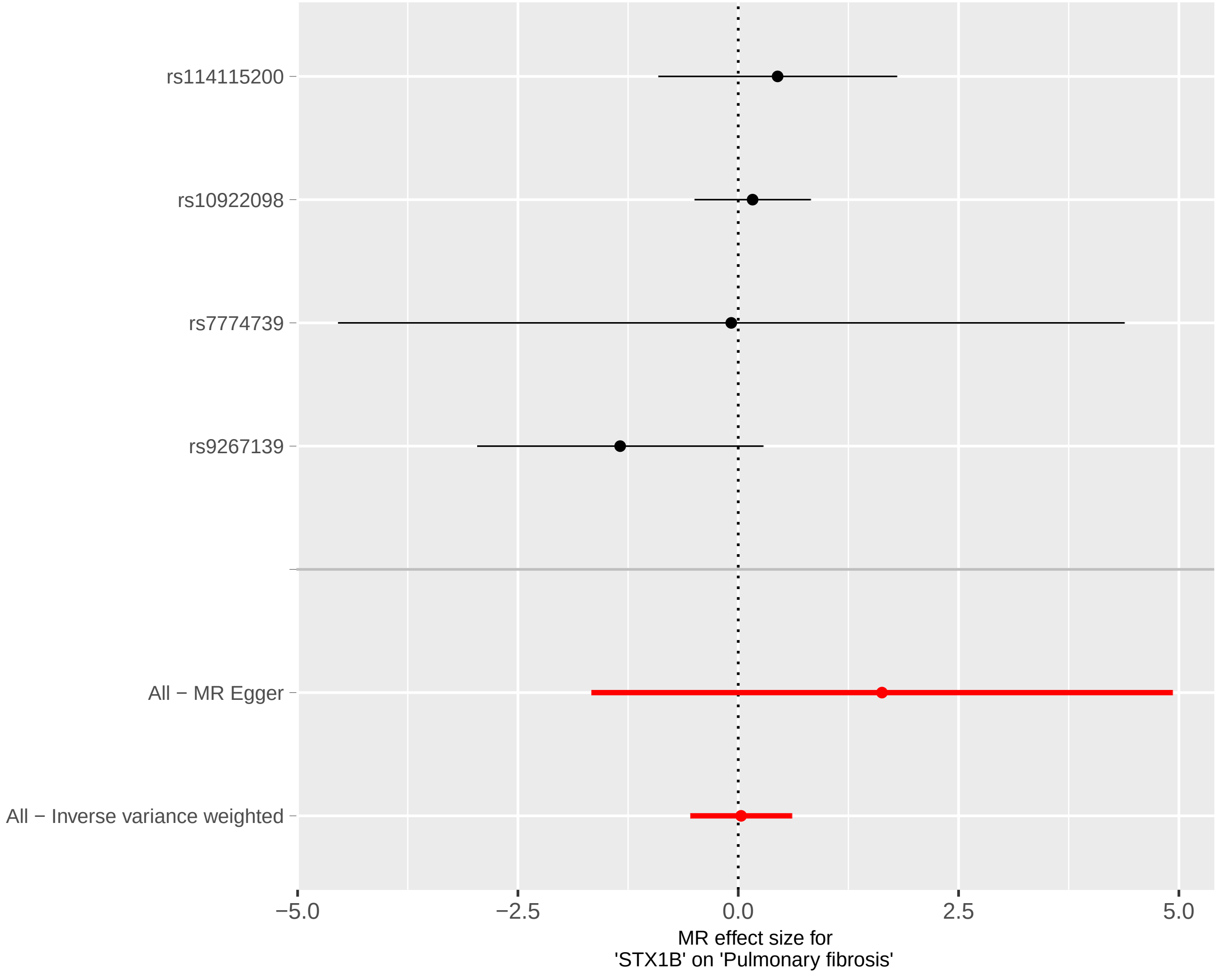

rs114115200
rs10922098
rs7774739
rs9267139
All − MR Egger
All − Inverse variance weighted
−5.0
−2.5
0.0
2.5
5.0
MR effect size for
'STX1B' on 'Pulmonary fibrosis'

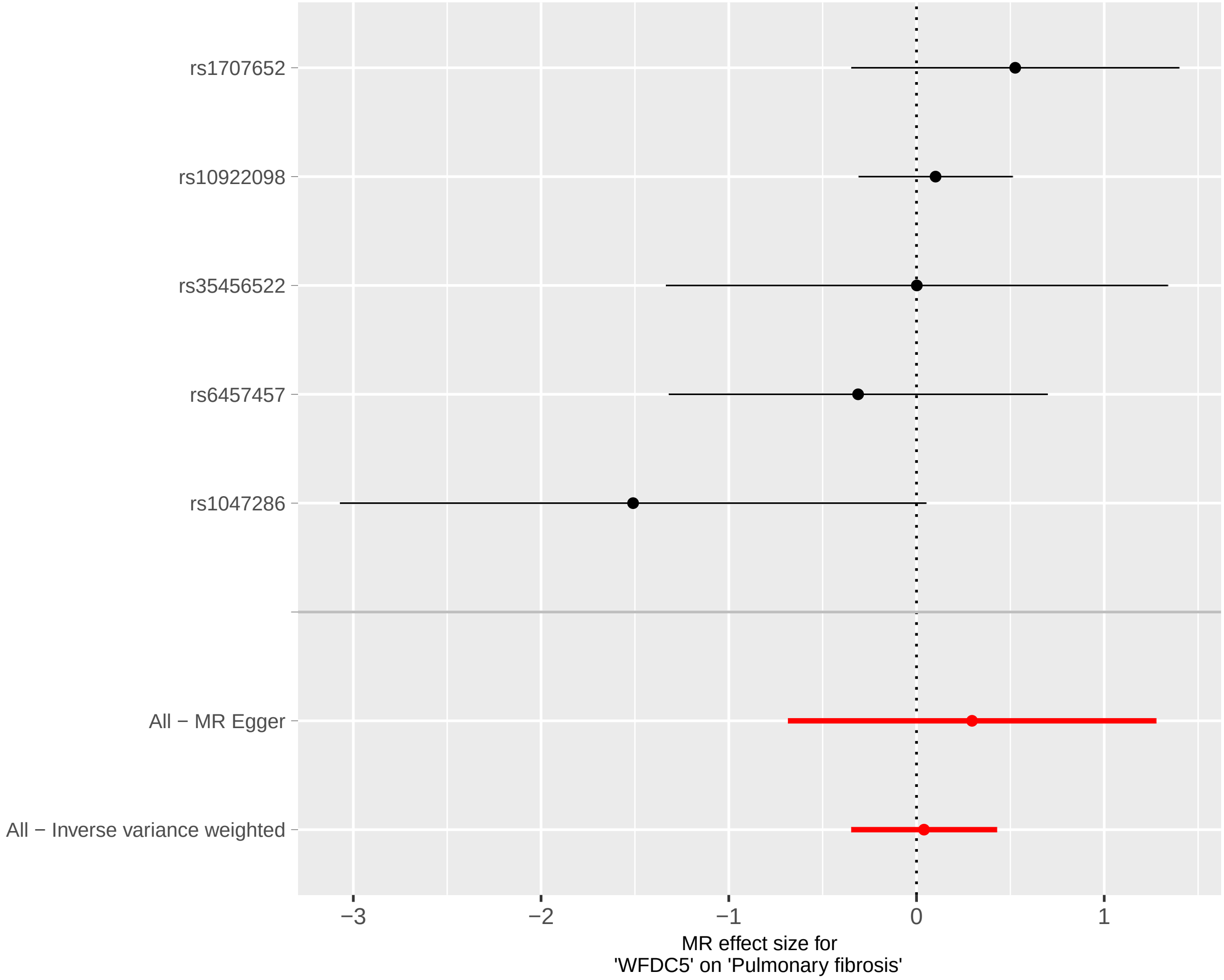

rs1707652
rs10922098
rs35456522
rs6457457
rs1047286
All − MR Egger
All − Inverse variance weighted
−3
−2
−1
0
1
MR effect size for
'WFDC5' on 'Pulmonary fibrosis'

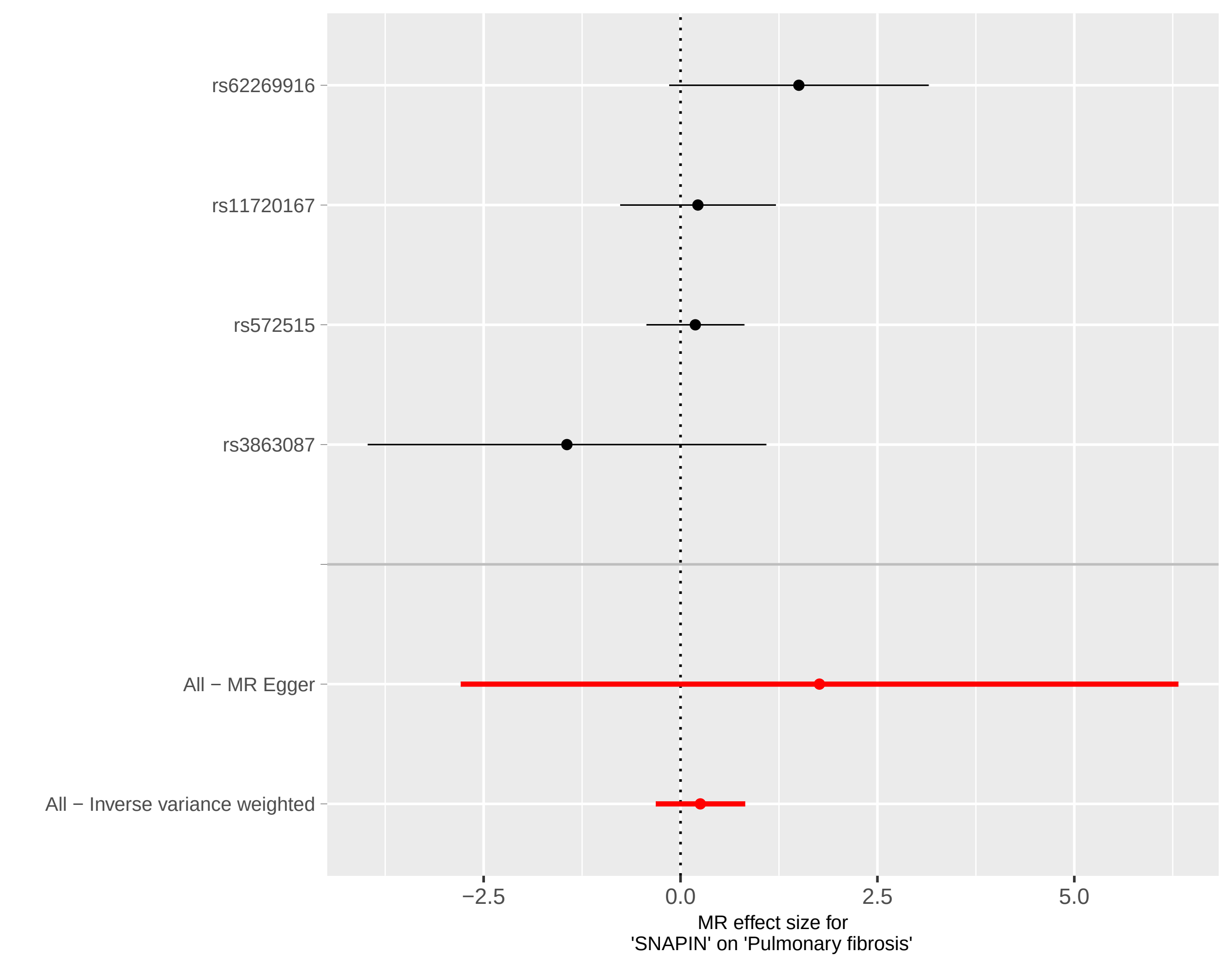

rs62269916
rs11720167
rs572515
rs3863087
All − MR Egger
All − Inverse variance weighted
−2.5
0.0
2.5
5.0
MR effect size for
'SNAPIN' on 'Pulmonary fibrosis'

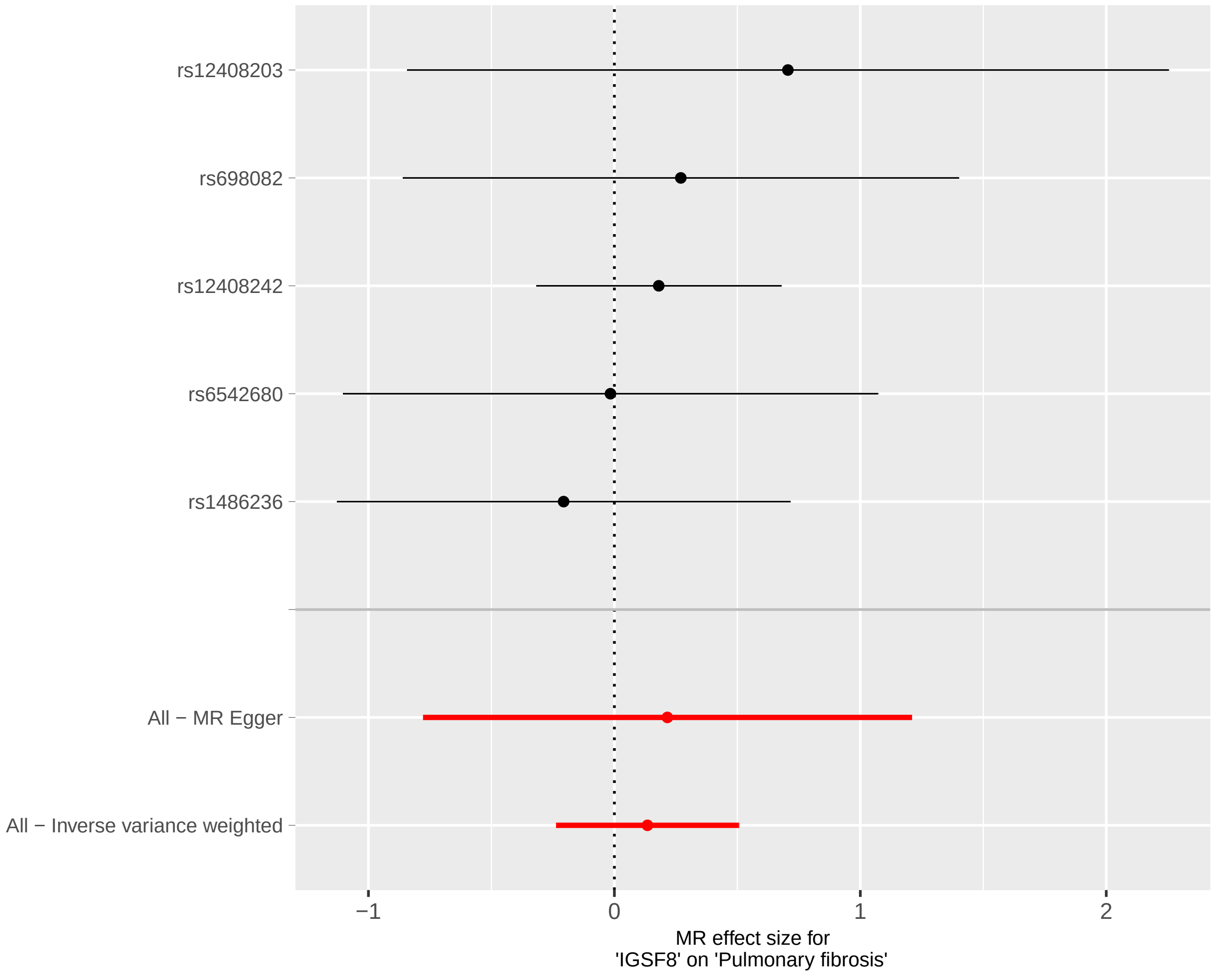
rs12408203
rs698082
rs12408242
rs6542680
rs1486236
All − MR Egger
All − Inverse variance weighted
−1
0
1
2
MR effect size for
'IGSF8' on 'Pulmonary fibrosis'

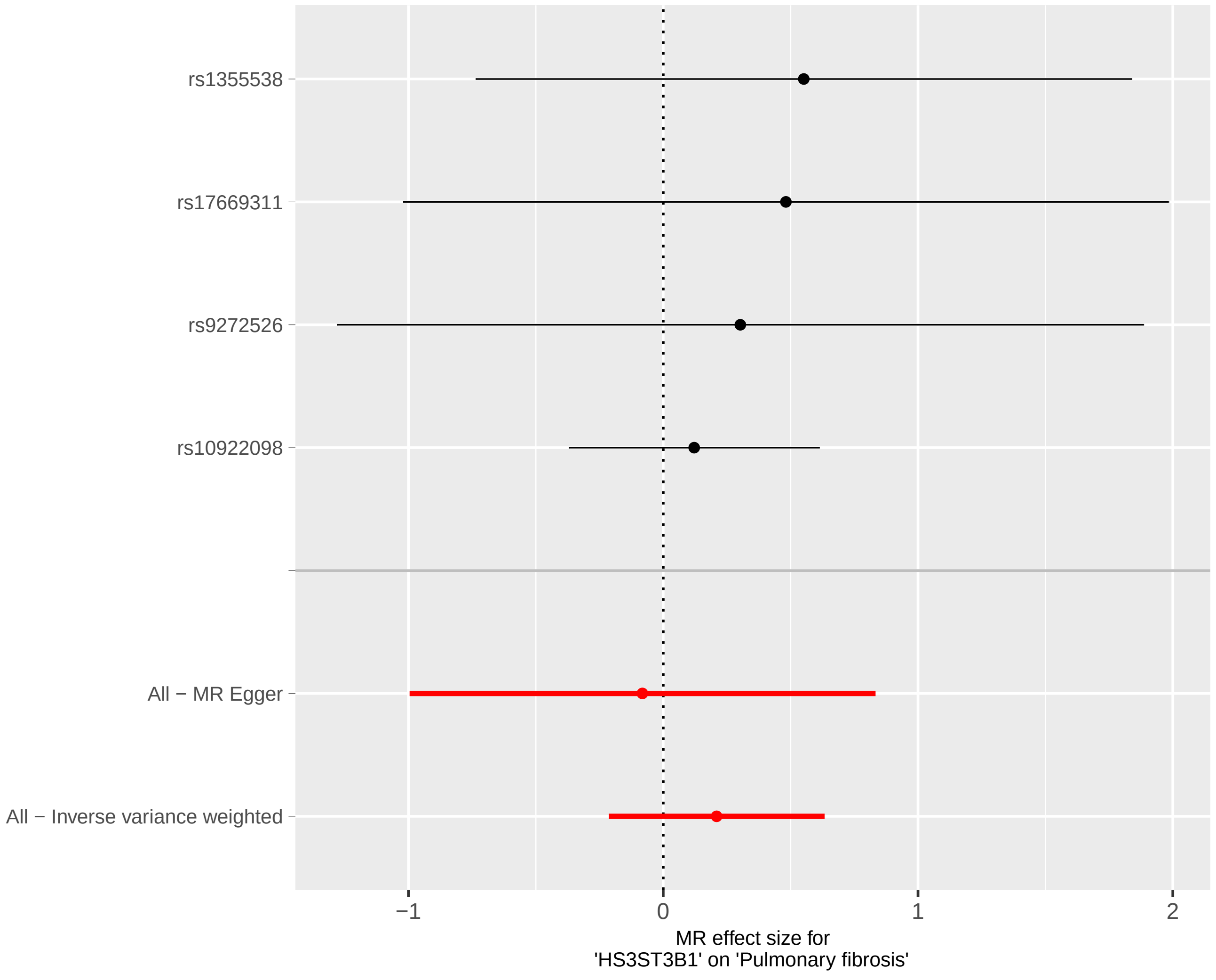

rs1355538
rs17669311
rs9272526
rs10922098
All − MR Egger
All − Inverse variance weighted
−1
0
1
2
MR effect size for
'HS3ST3B1' on 'Pulmonary fibrosis'

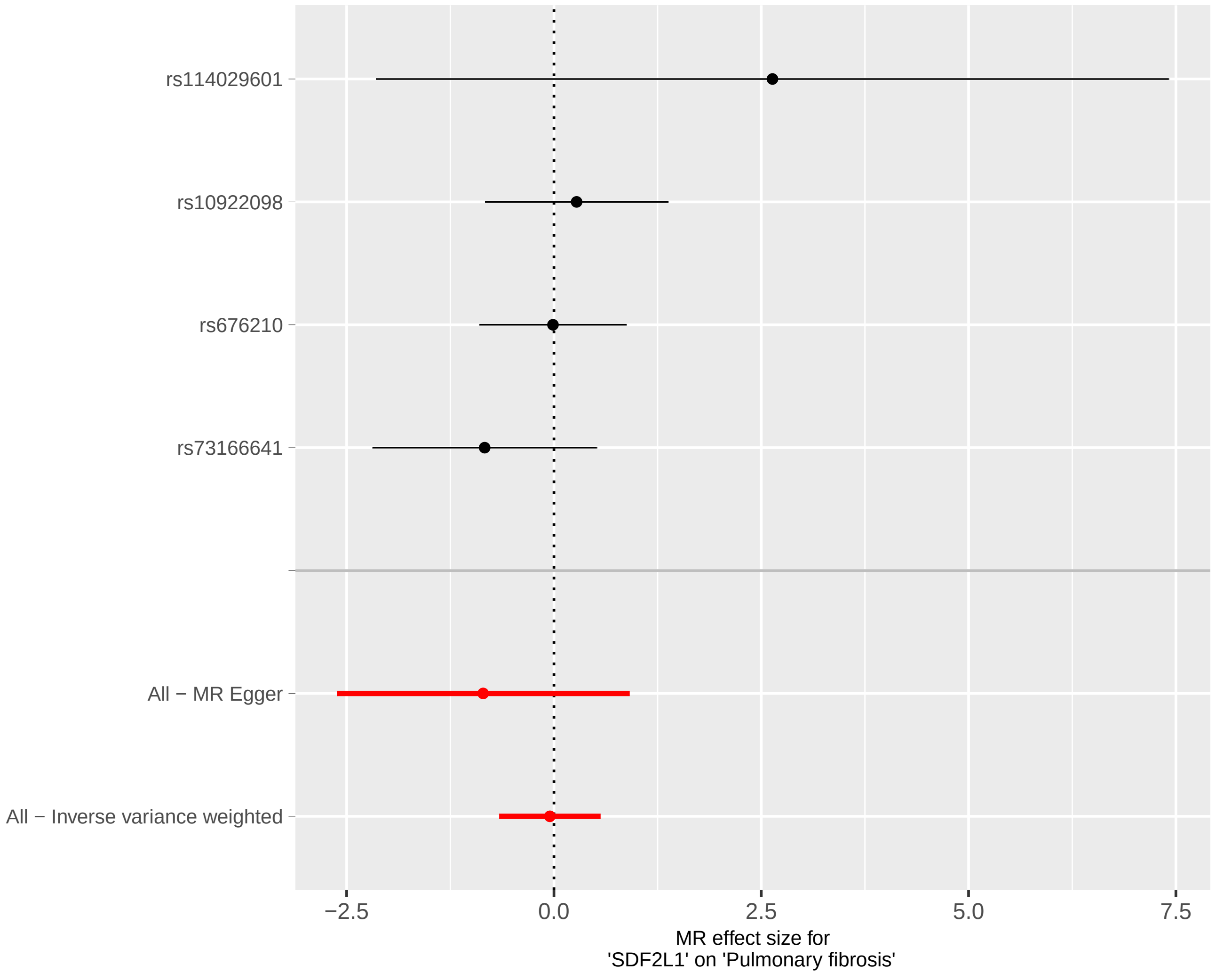

rs114029601
rs10922098
rs676210
rs73166641
All − MR Egger
All − Inverse variance weighted
−2.5
0.0
2.5
5.0
7.5
MR effect size for
'SDF2L1' on 'Pulmonary fibrosis'

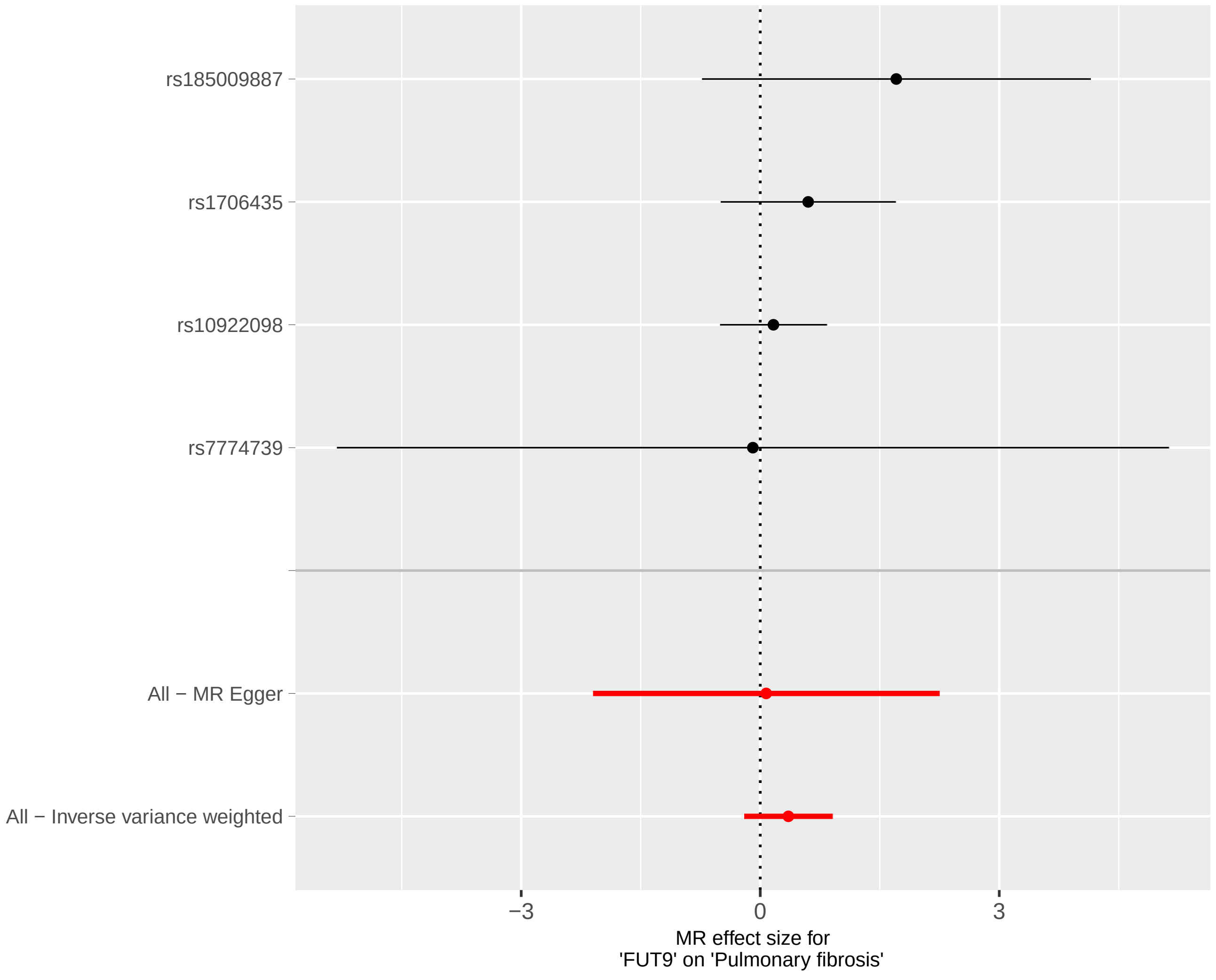
rs185009887
rs1706435
rs10922098
rs7774739
All − MR Egger
All − Inverse variance weighted
−3
0
3
MR effect size for
'FUT9' on 'Pulmonary fibrosis'

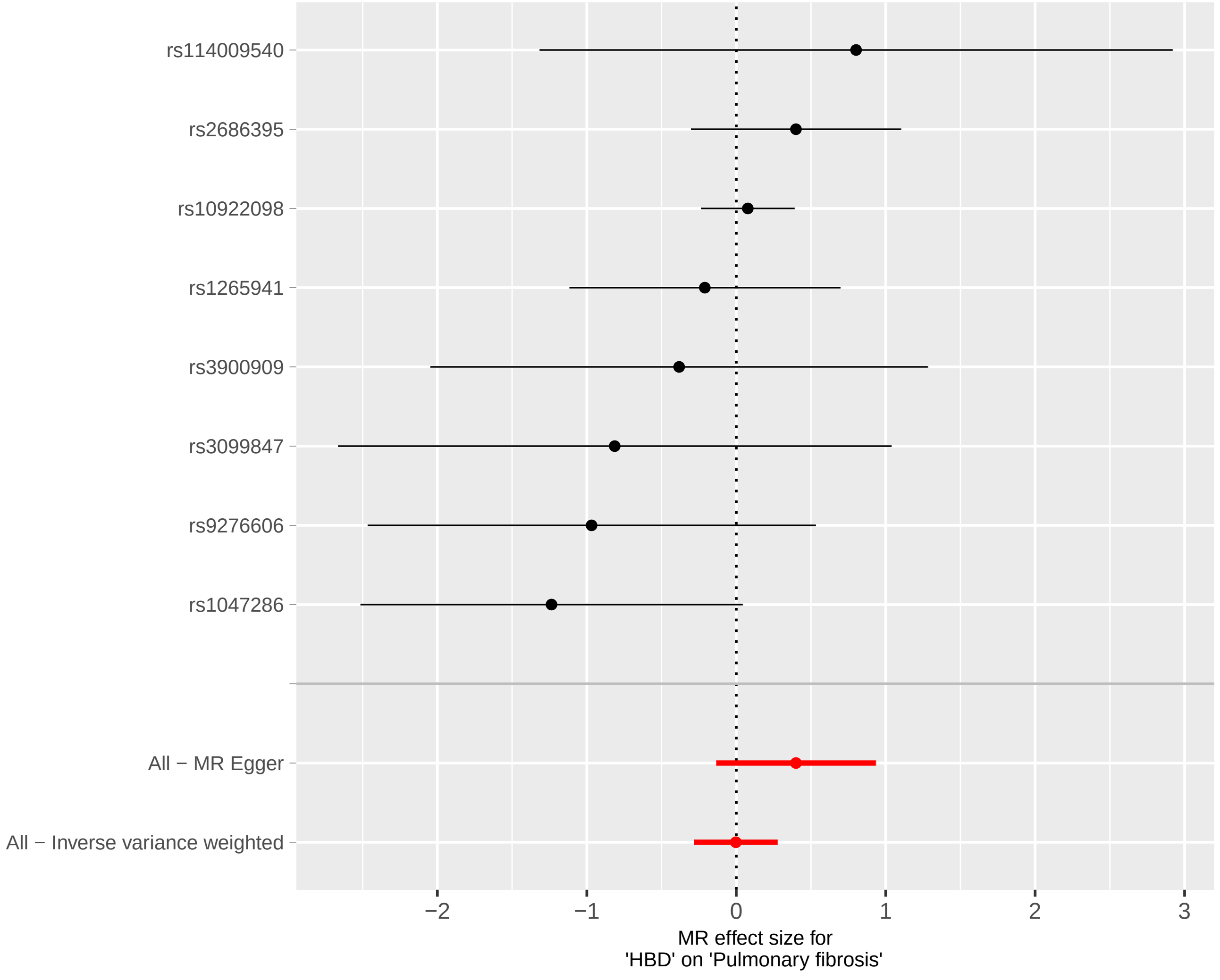
rs114009540
rs2686395
rs10922098
rs1265941
rs3900909
rs3099847
rs9276606
rs1047286
All − MR Egger
All − Inverse variance weighted
−2
−1
0
1
2
3
MR effect size for
'HBD' on 'Pulmonary fibrosis'

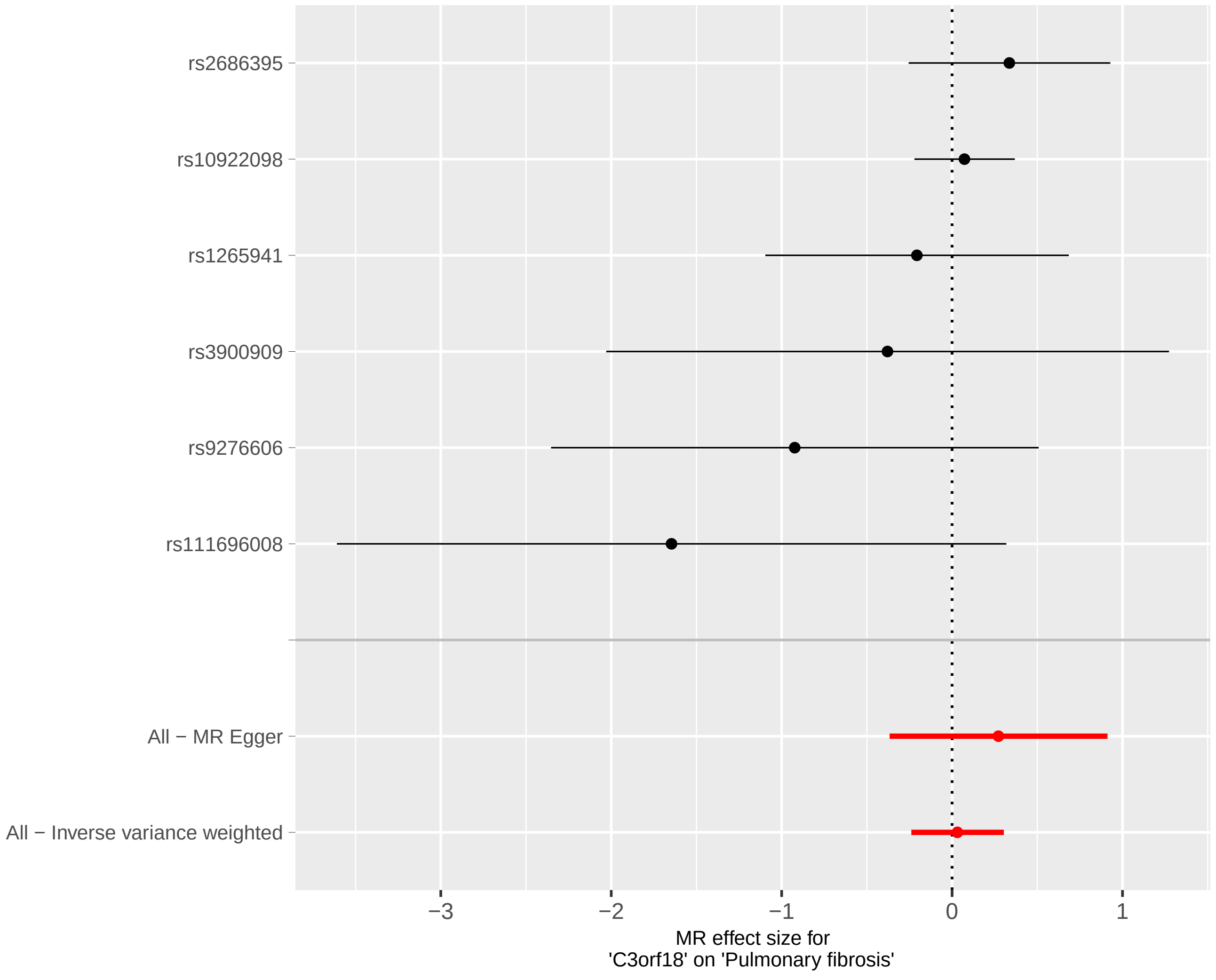

rs2686395
rs10922098
rs1265941
rs3900909
rs9276606
rs111696008
All − MR Egger
All − Inverse variance weighted
−3
−2
−1
0
1
MR effect size for
'C3orf18' on 'Pulmonary fibrosis'

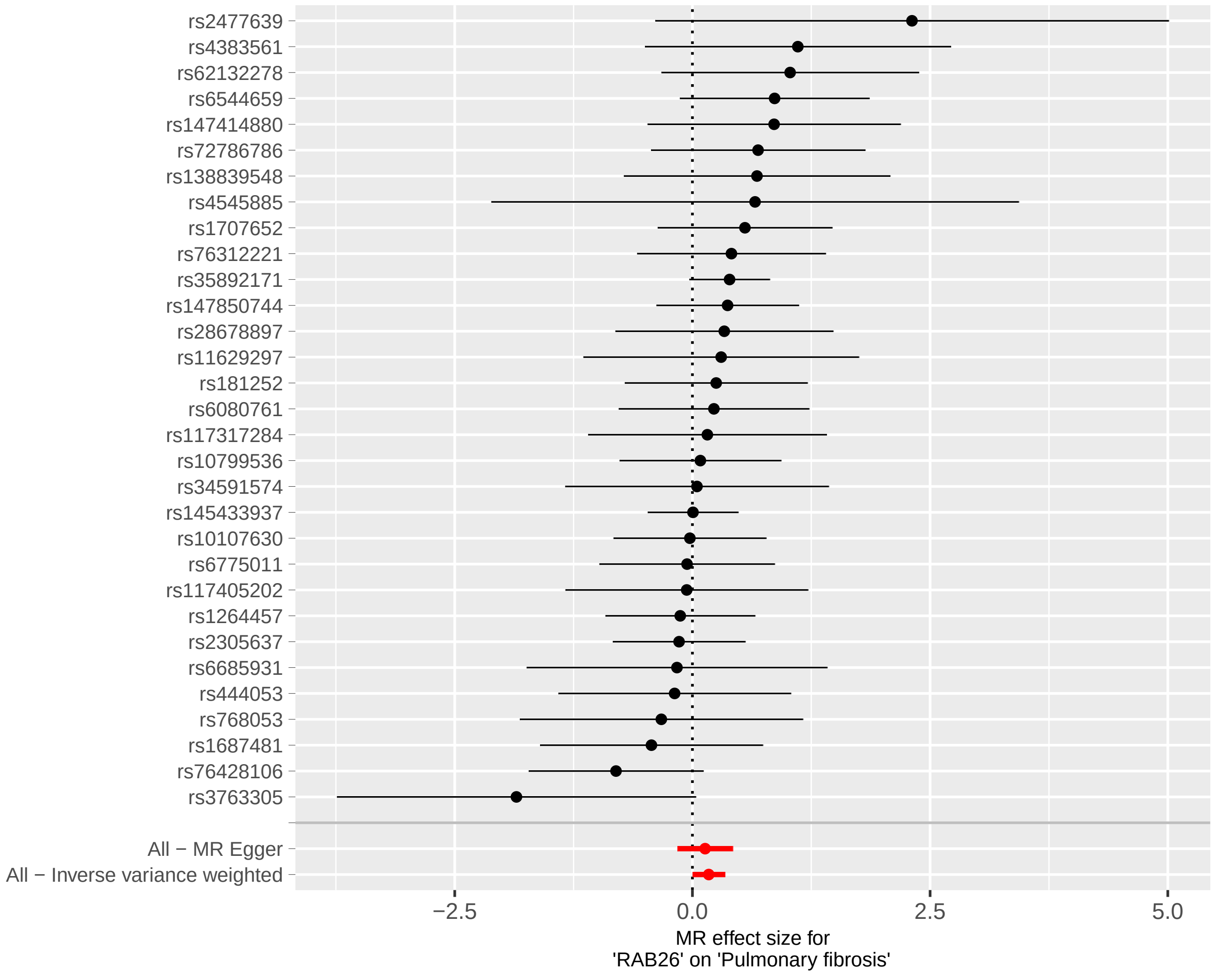
rs2477639
rs4383561
rs62132278
rs6544659
rs147414880
rs72786786
rs138839548
rs4545885
rs1707652
rs76312221
rs35892171
rs147850744
rs28678897
rs11629297
rs181252
rs6080761
rs117317284
rs10799536
rs34591574
rs145433937
rs10107630
rs6775011
rs117405202
rs1264457
rs2305637
rs6685931
rs444053
rs768053
rs1687481
rs76428106
rs3763305
All − MR Egger
All − Inverse variance weighted
−2.5
0.0
2.5
5.0
MR effect size for
'RAB26' on 'Pulmonary fibrosis'

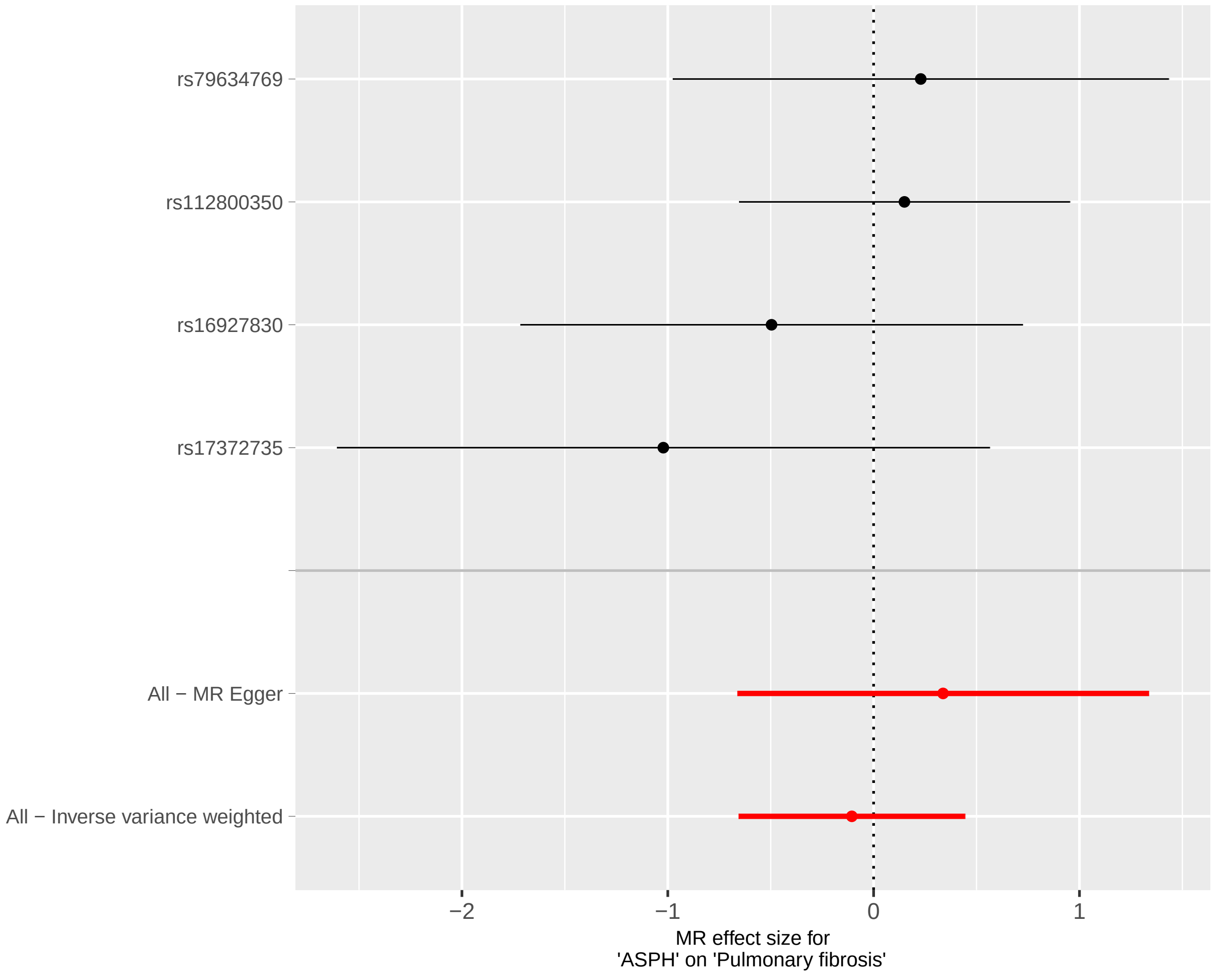

rs79634769
rs112800350
rs16927830
rs17372735
All − MR Egger
All − Inverse variance weighted
−2
−1
0
1
MR effect size for
'ASPH' on 'Pulmonary fibrosis'

Insufficient number of SNPs

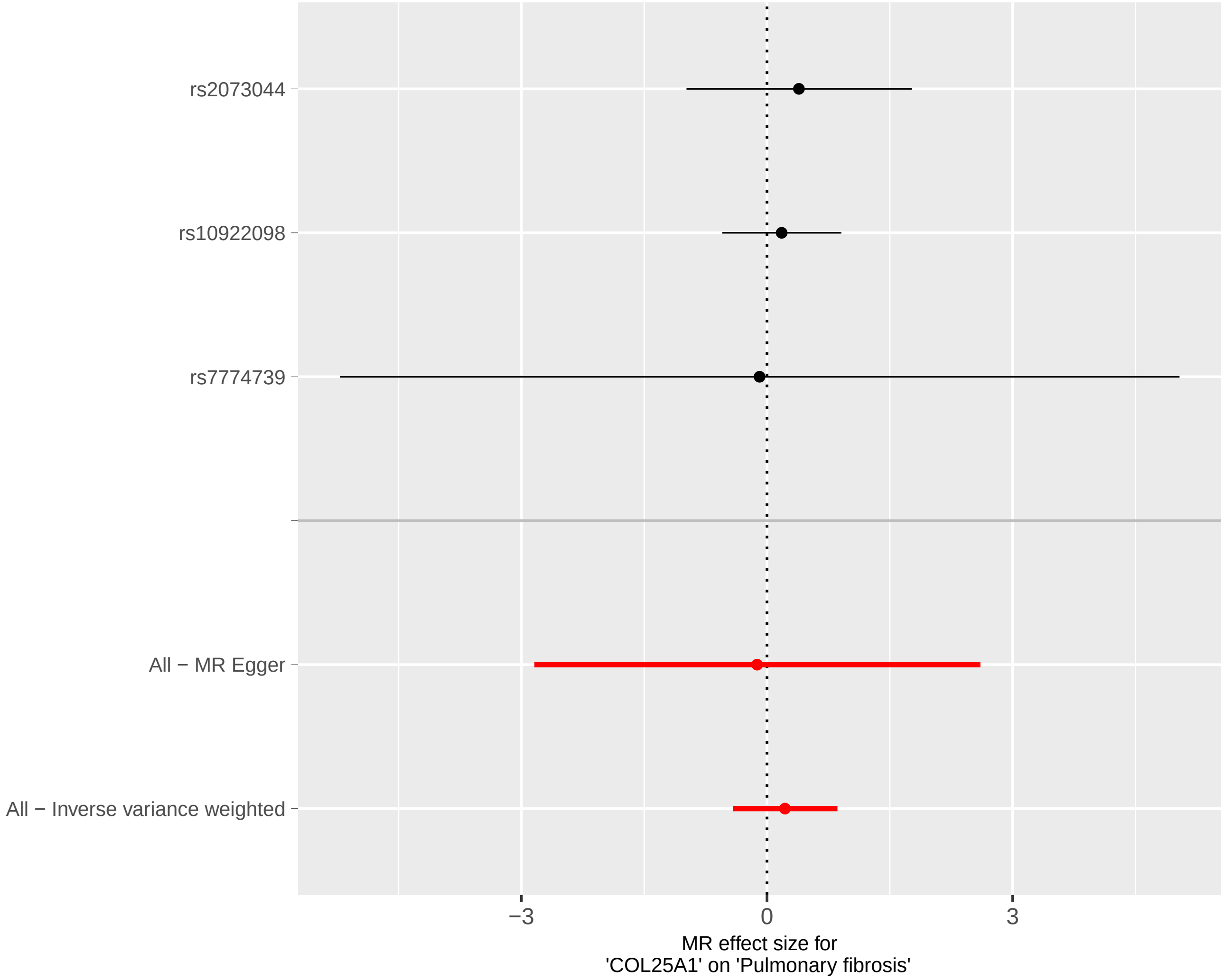

rs2073044
rs10922098
rs7774739
All − MR Egger
All − Inverse variance weighted
−3
0
3
MR effect size for
'COL25A1' on 'Pulmonary fibrosis'

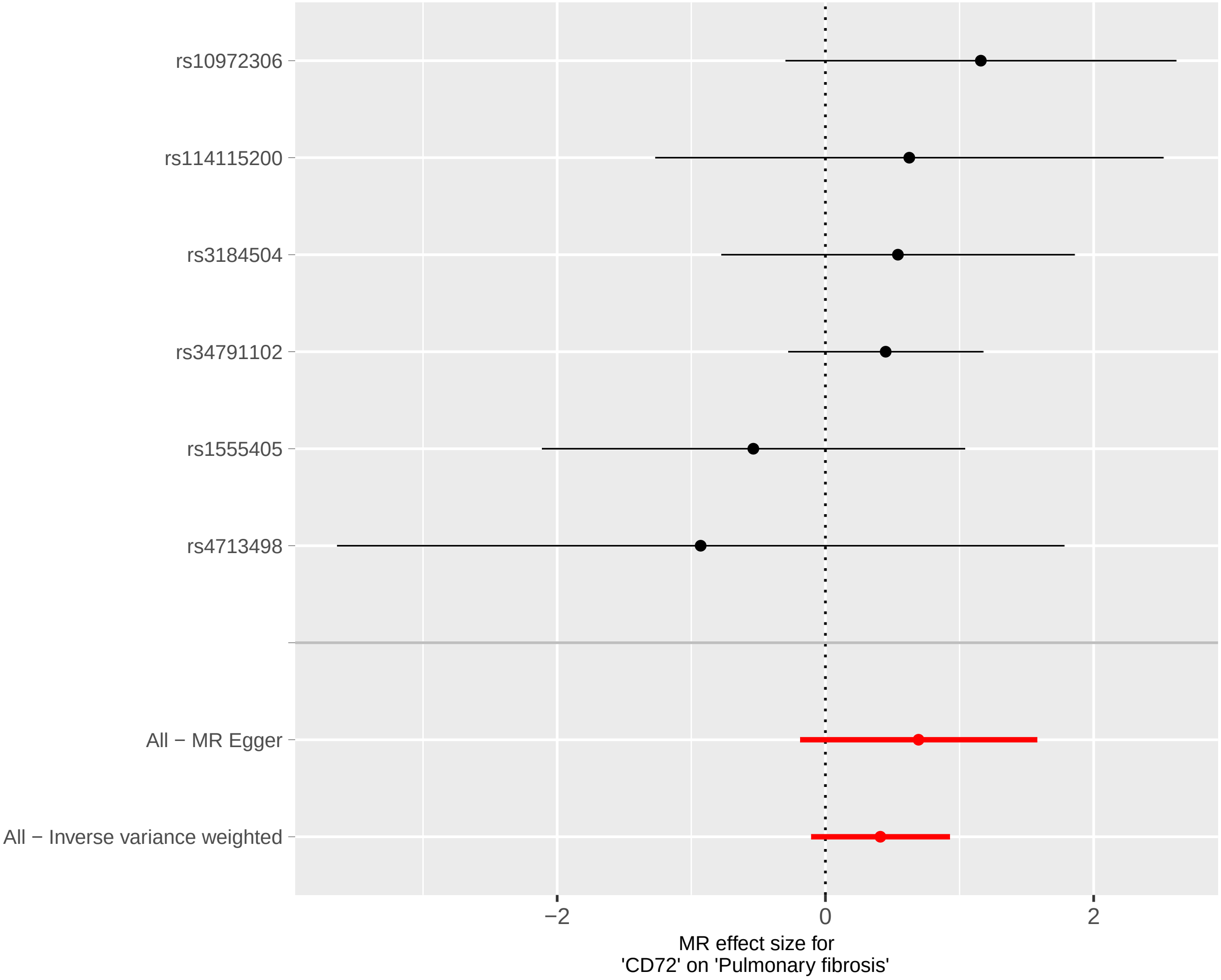
rs10972306
rs114115200
rs3184504
rs34791102
rs1555405
rs4713498
All − MR Egger
All − Inverse variance weighted
−2
0
2
MR effect size for
'CD72' on 'Pulmonary fibrosis'

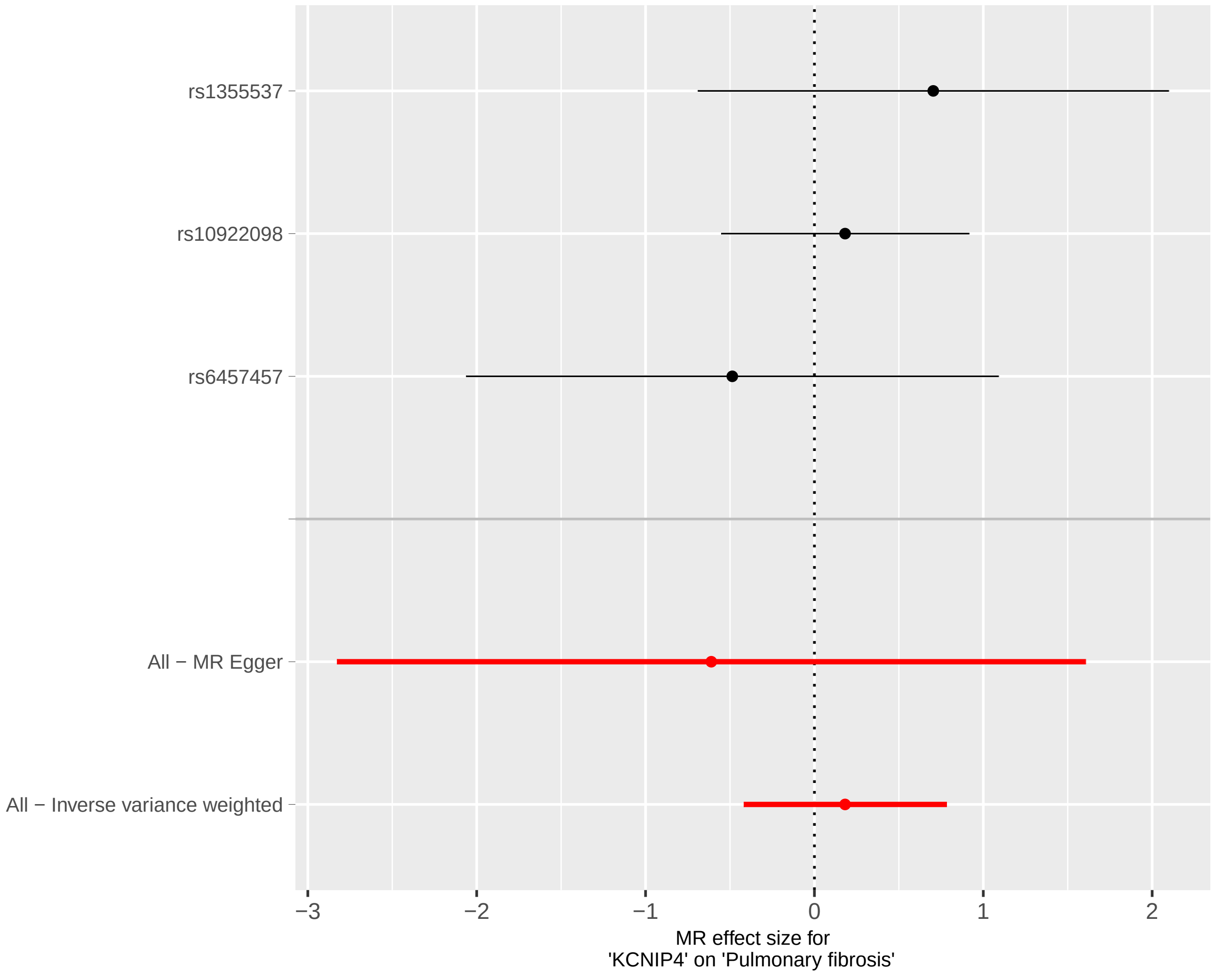

rs1355537
rs10922098
rs6457457
All − MR Egger
All − Inverse variance weighted
−3
−2
−1
0
1
2
MR effect size for
'KCNIP4' on 'Pulmonary fibrosis'

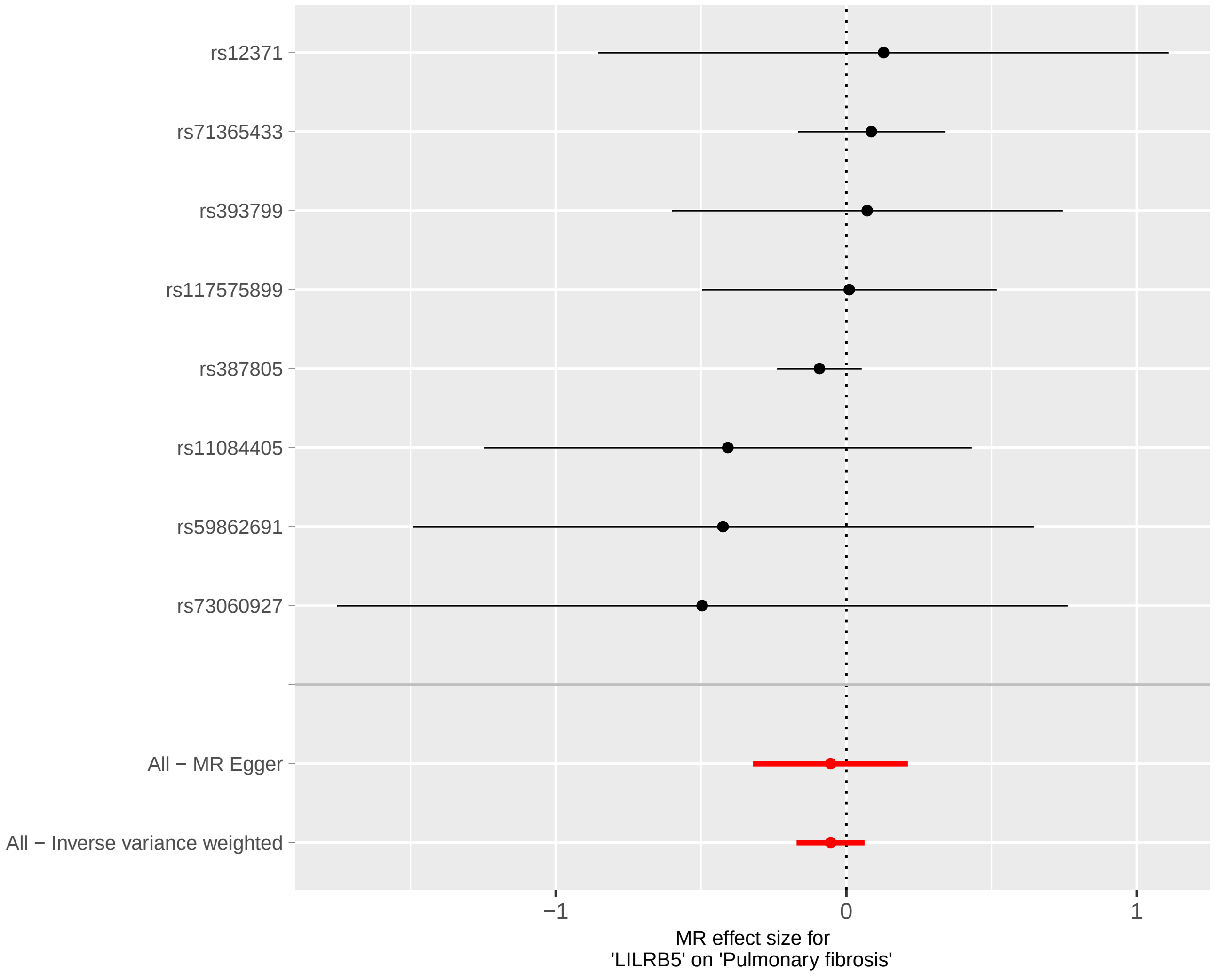
rs12371
rs71365433
rs393799
rs117575899
rs387805
rs11084405
rs59862691
rs73060927
All − MR Egger
All − Inverse variance weighted
−1
0
1
MR effect size for
'LILRB5' on 'Pulmonary fibrosis'

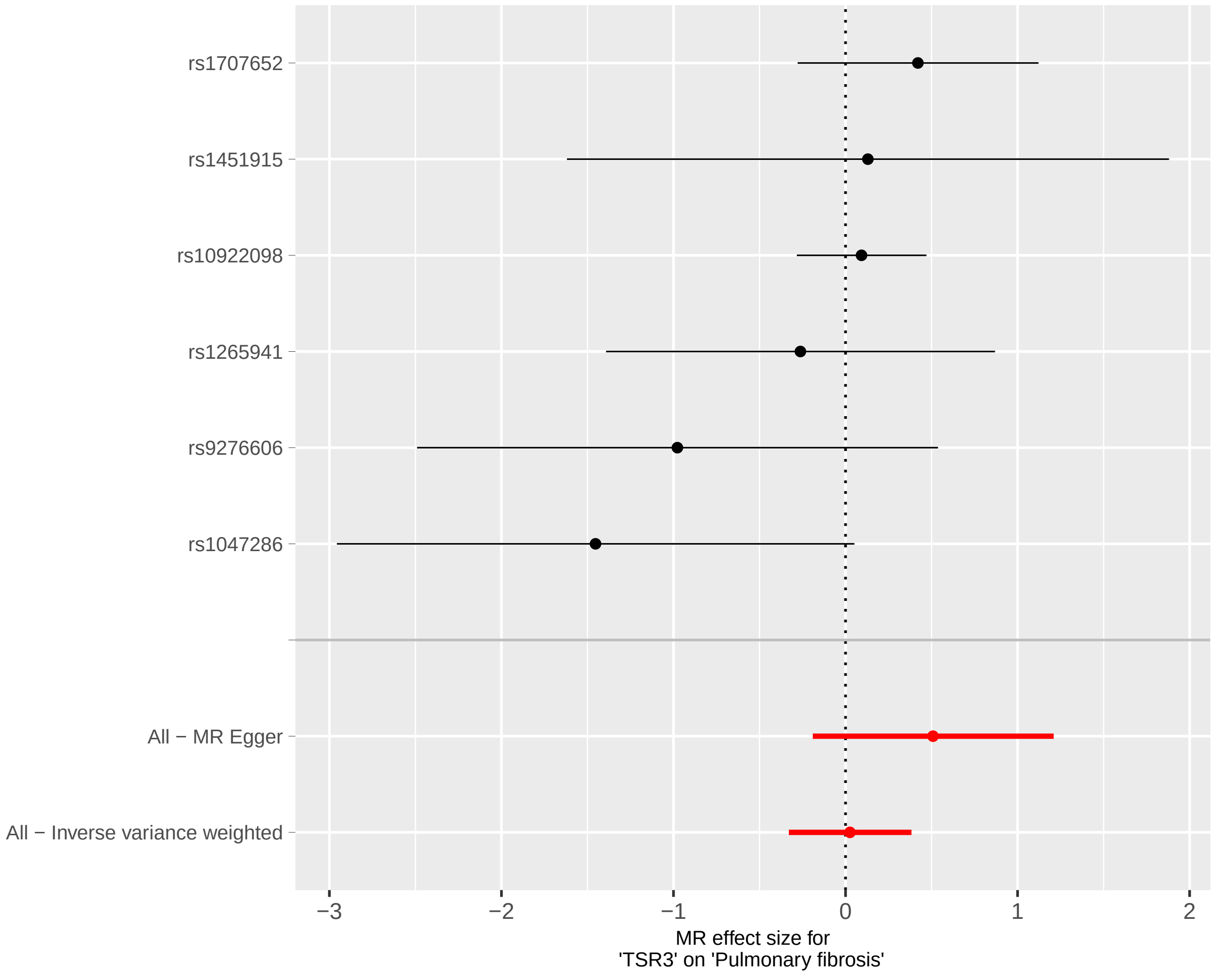
rs1707652
rs1451915
rs10922098
rs1265941
rs9276606
rs1047286
All − MR Egger
All − Inverse variance weighted
−3
−2
−1
0
1
2
MR effect size for
'TSR3' on 'Pulmonary fibrosis'

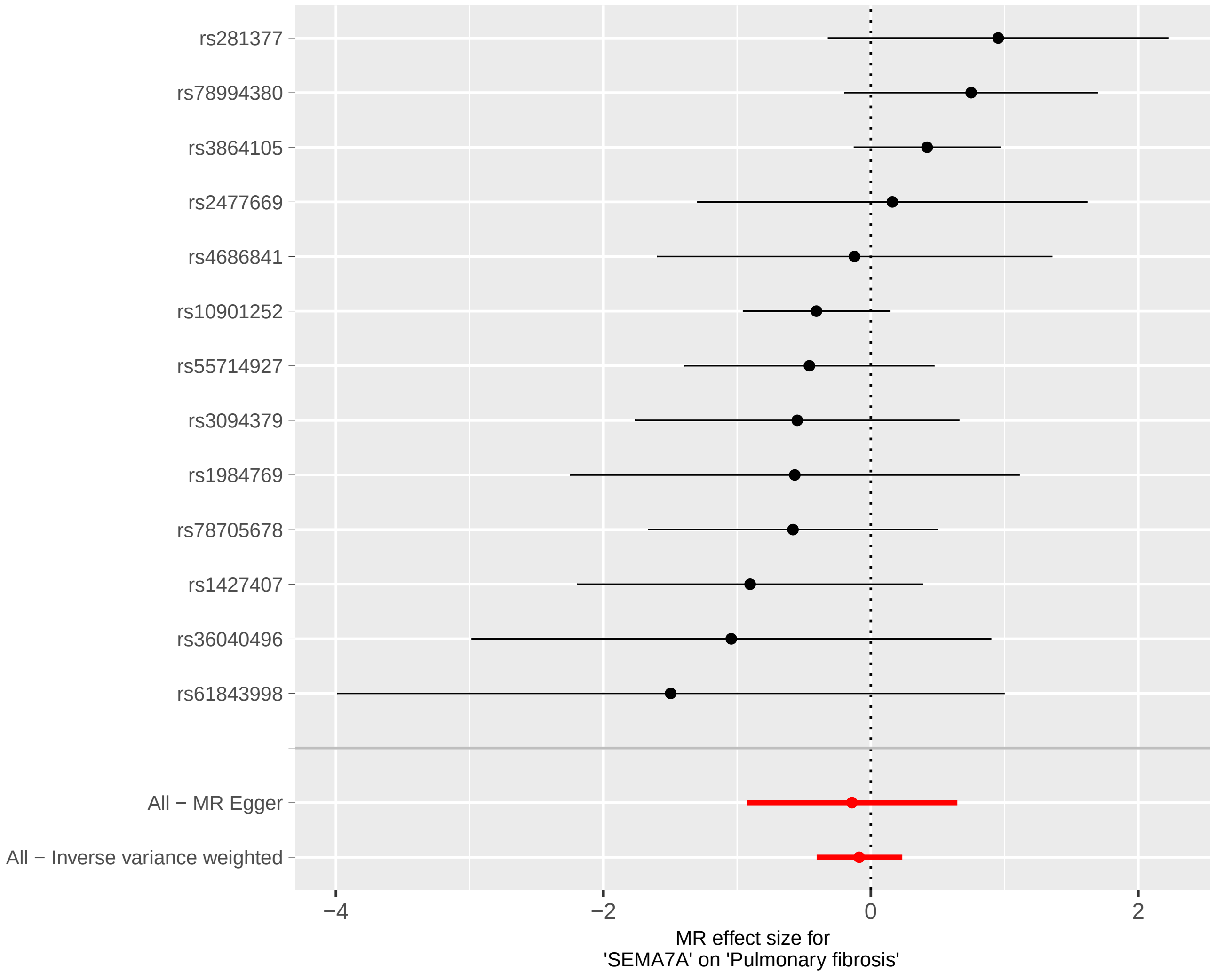

rs281377
rs78994380
rs3864105
rs2477669
rs4686841
rs10901252
rs55714927
rs3094379
rs1984769
rs78705678
rs1427407
rs36040496
rs61843998
All − MR Egger
All − Inverse variance weighted
−4
−2
0
2
MR effect size for
'SEMA7A' on 'Pulmonary fibrosis'

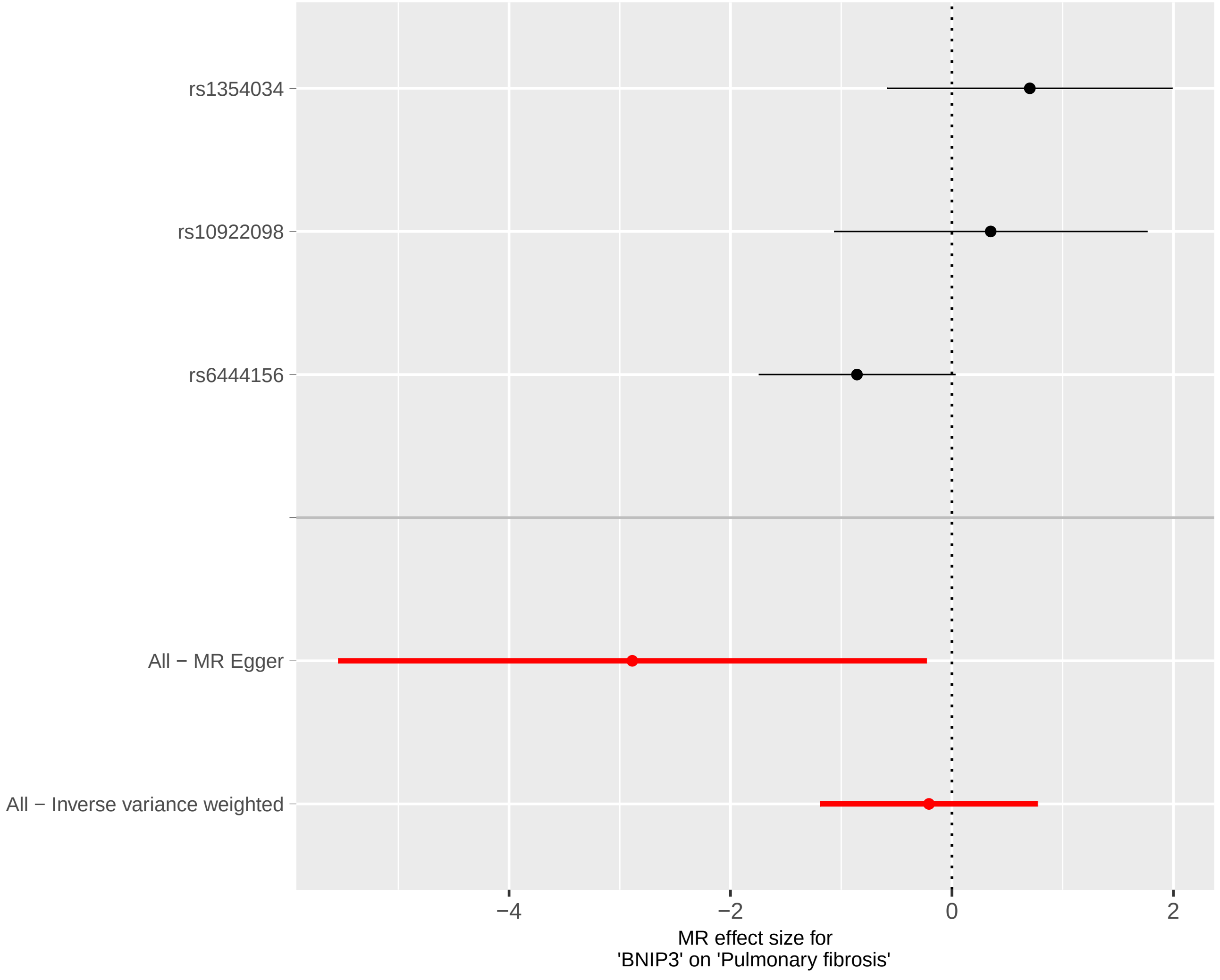
rs1354034
rs10922098
rs6444156
All − MR Egger
All − Inverse variance weighted
−4
−2
0
2
MR effect size for
'BNIP3' on 'Pulmonary fibrosis'

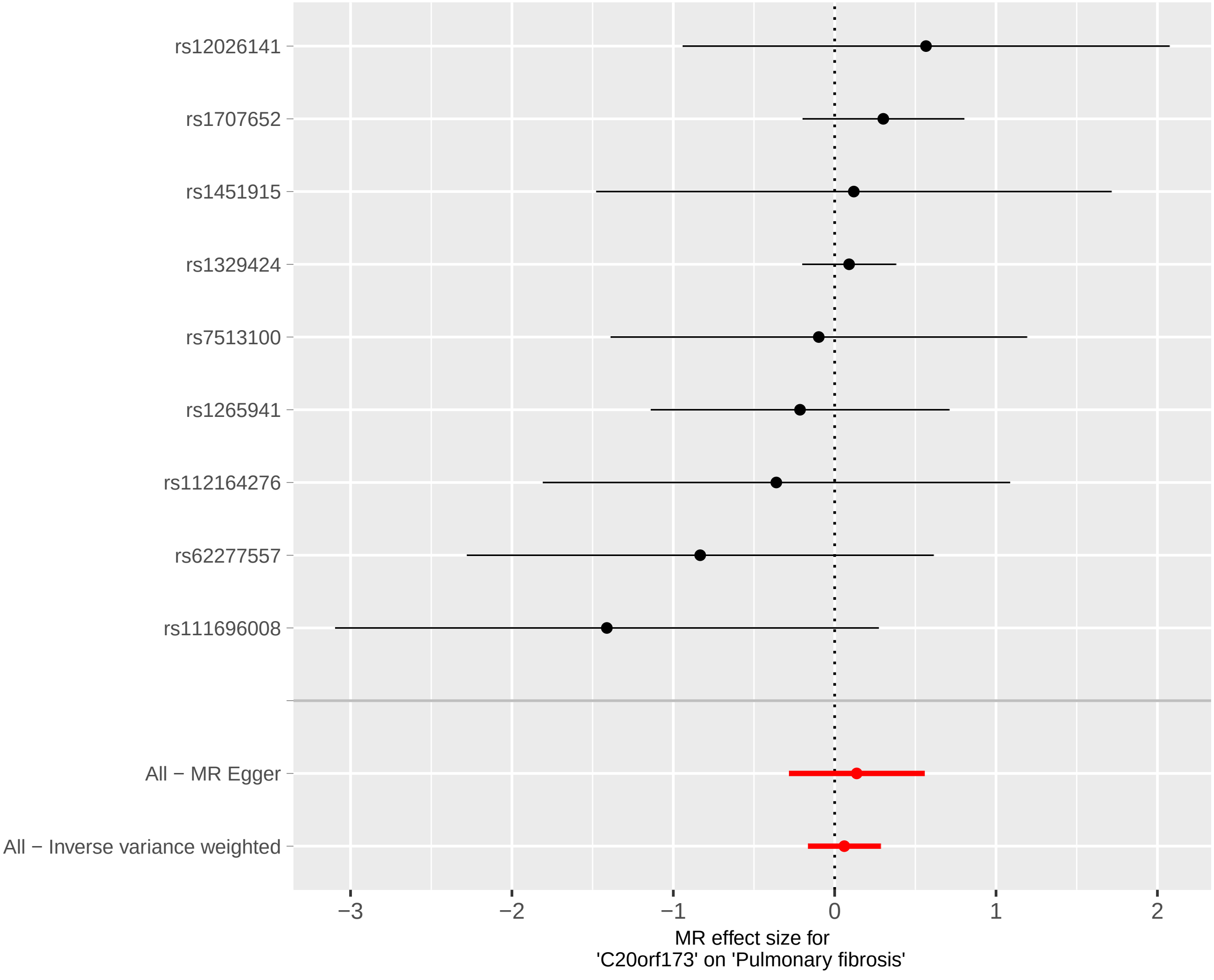
rs12026141
rs1707652
rs1451915
rs1329424
rs7513100
rs1265941
rs112164276
rs62277557
rs111696008
All − MR Egger
All − Inverse variance weighted
−3
−2
−1
0
1
2
MR effect size for
'C20orf173' on 'Pulmonary fibrosis'

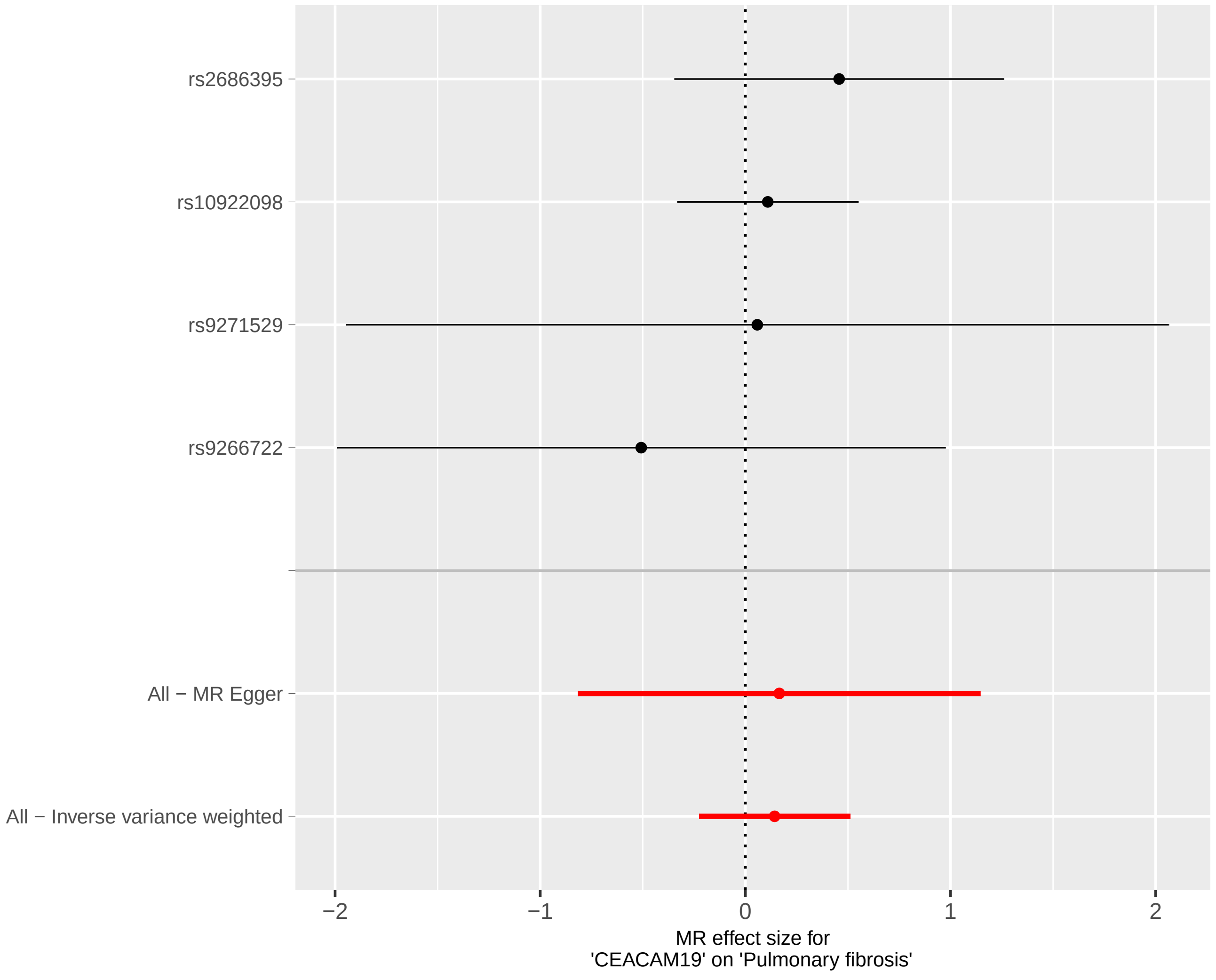
rs2686395
rs10922098
rs9271529
rs9266722
All − MR Egger
All − Inverse variance weighted
−2
−1
0
1
2
MR effect size for
'CEACAM19' on 'Pulmonary fibrosis'

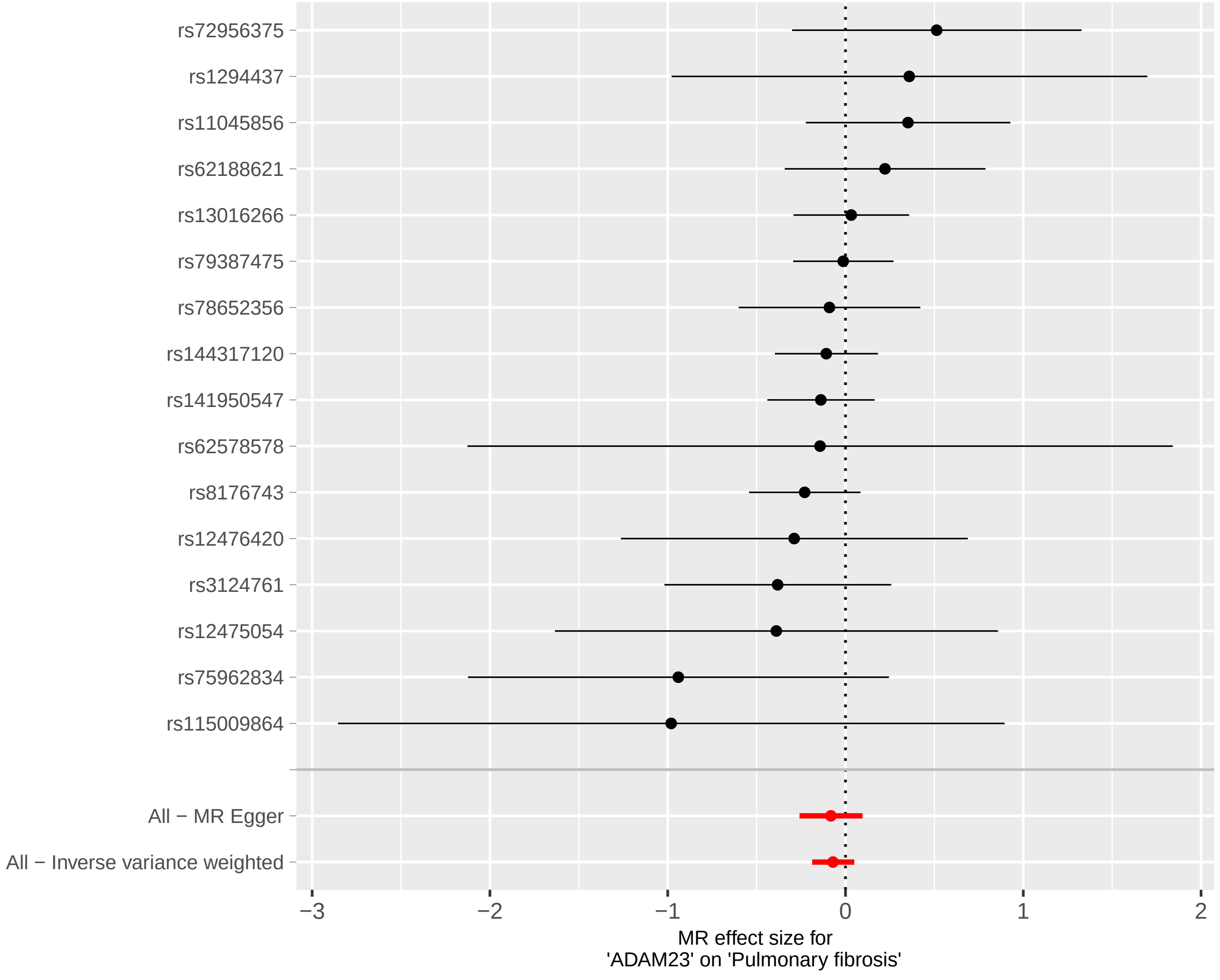
rs72956375
rs1294437
rs11045856
rs62188621
rs13016266
rs79387475
rs78652356
rs144317120
rs141950547
rs62578578
rs8176743
rs12476420
rs3124761
rs12475054
rs75962834
rs115009864
All − MR Egger
All − Inverse variance weighted
−3
−2
−1
0
1
2
MR effect size for
'ADAM23' on 'Pulmonary fibrosis'

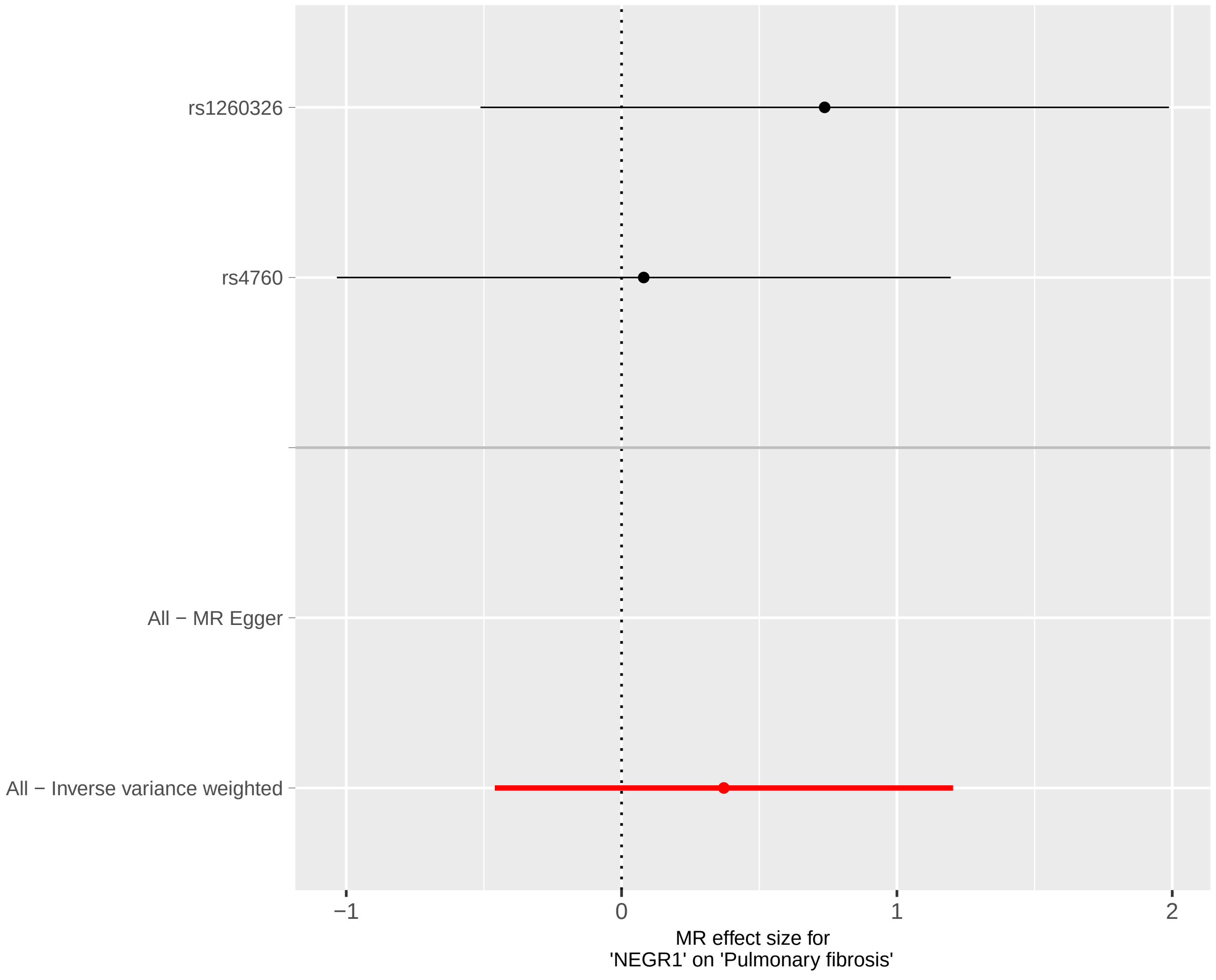
rs1260326
rs4760
All − MR Egger
All − Inverse variance weighted
−1
0
1
2
MR effect size for
'NEGR1' on 'Pulmonary fibrosis'

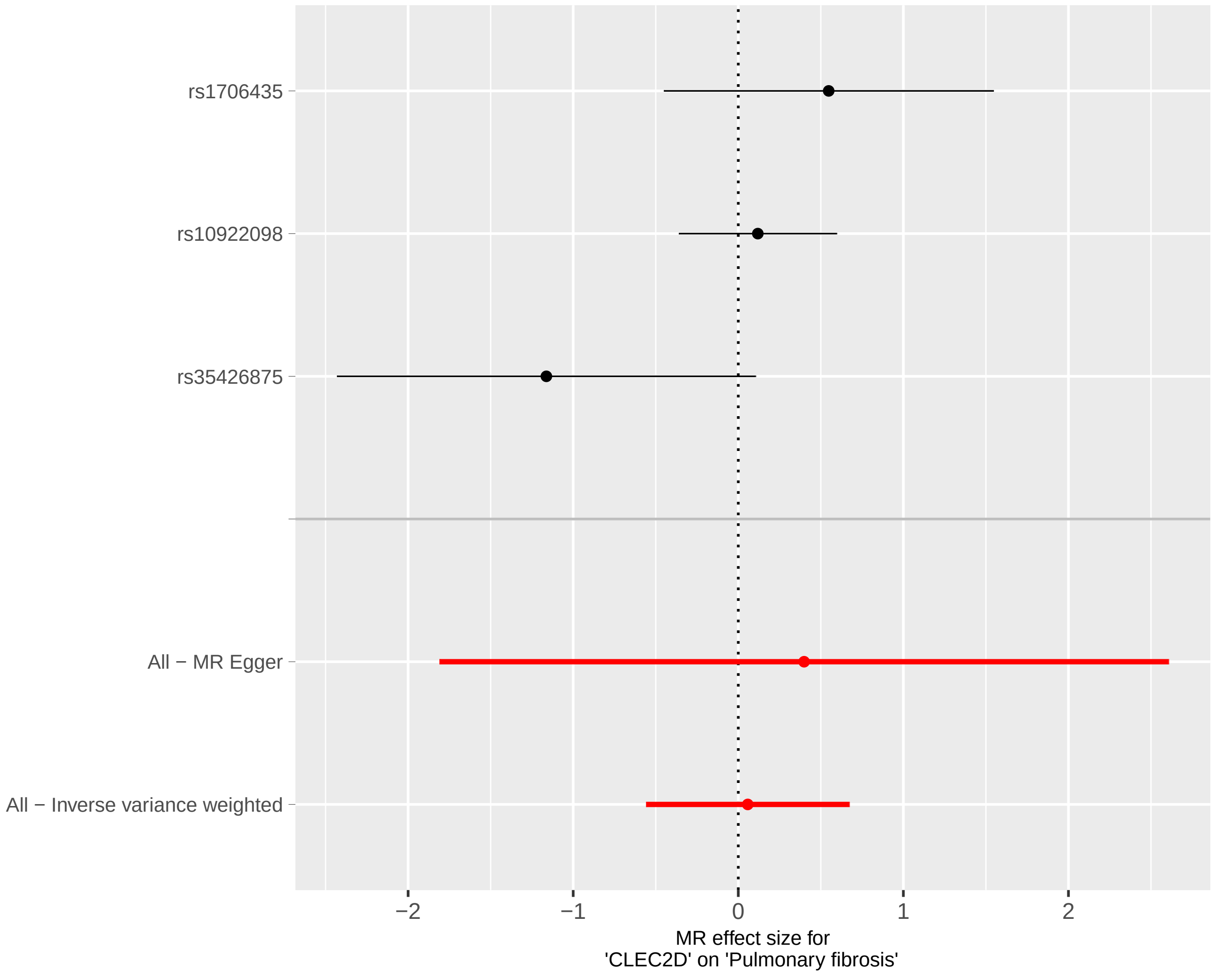
rs1706435
rs10922098
rs35426875
All − MR Egger
All − Inverse variance weighted
−2
−1
0
1
2
MR effect size for
'CLEC2D' on 'Pulmonary fibrosis'

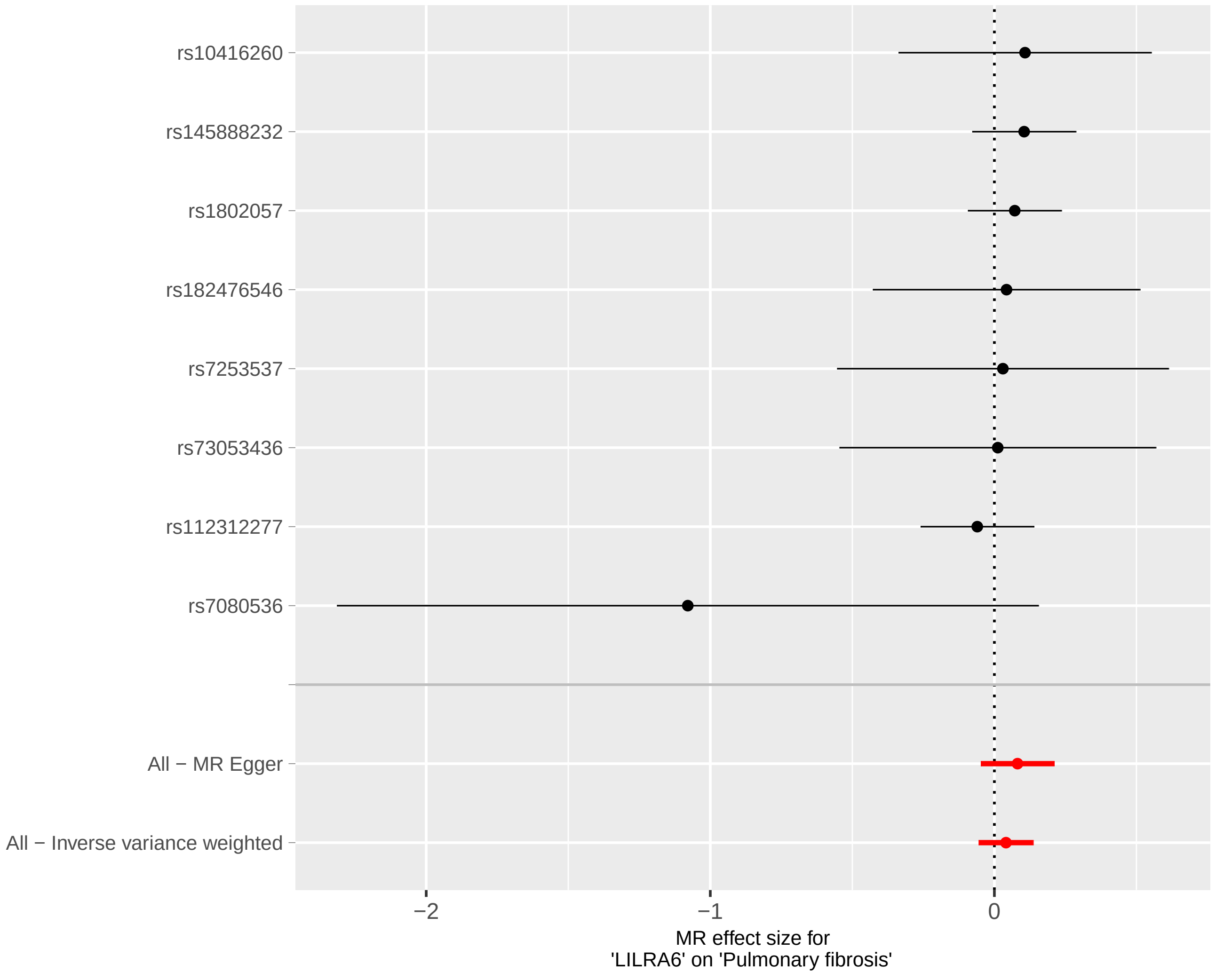
rs10416260
rs145888232
rs1802057
rs182476546
rs7253537
rs73053436
rs112312277
rs7080536
All − MR Egger
All − Inverse variance weighted
−2
−1
0
MR effect size for
'LILRA6' on 'Pulmonary fibrosis'

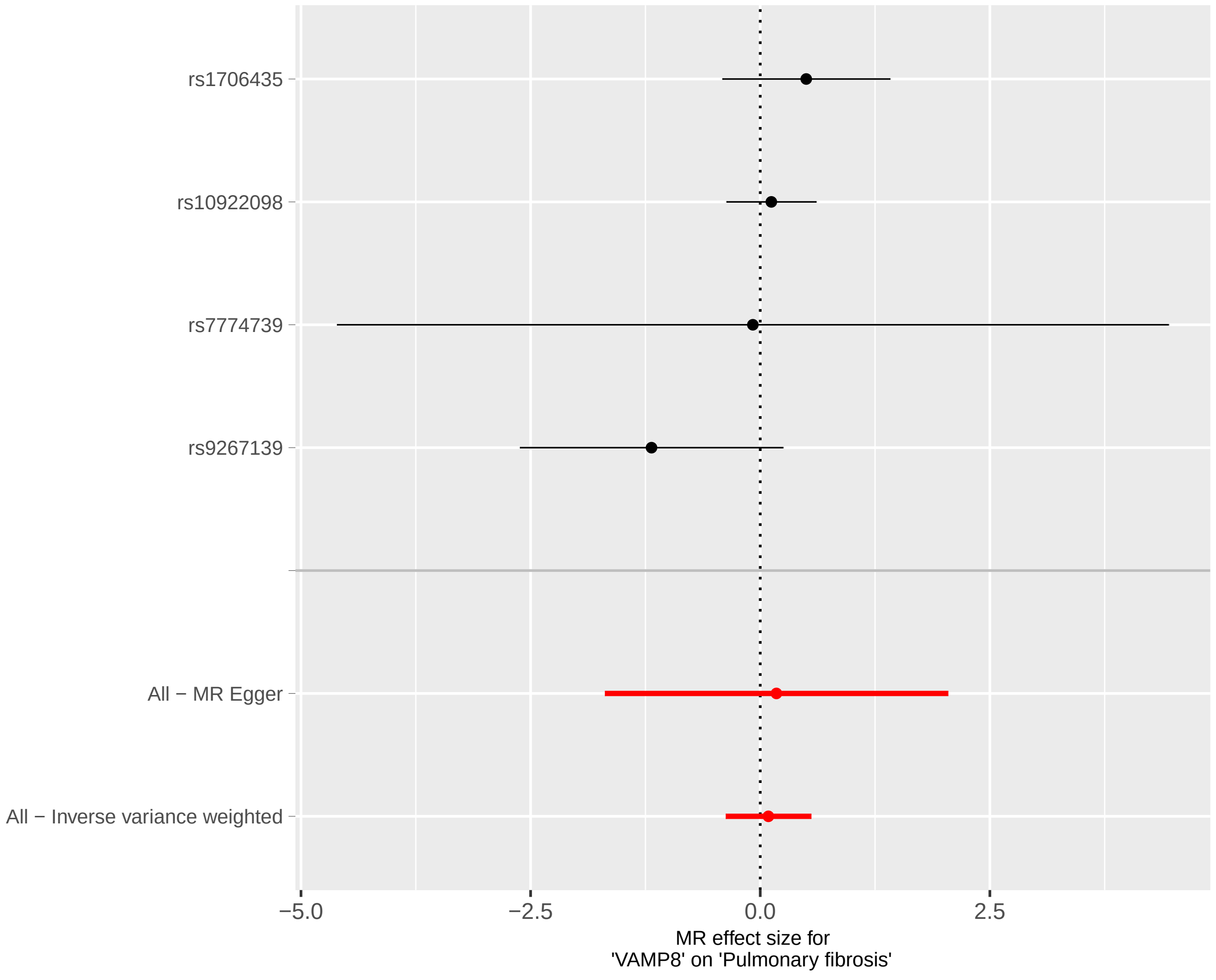
rs1706435
rs10922098
rs7774739
rs9267139
All − MR Egger
All − Inverse variance weighted
−5.0
−2.5
0.0
2.5
MR effect size for
'VAMP8' on 'Pulmonary fibrosis'

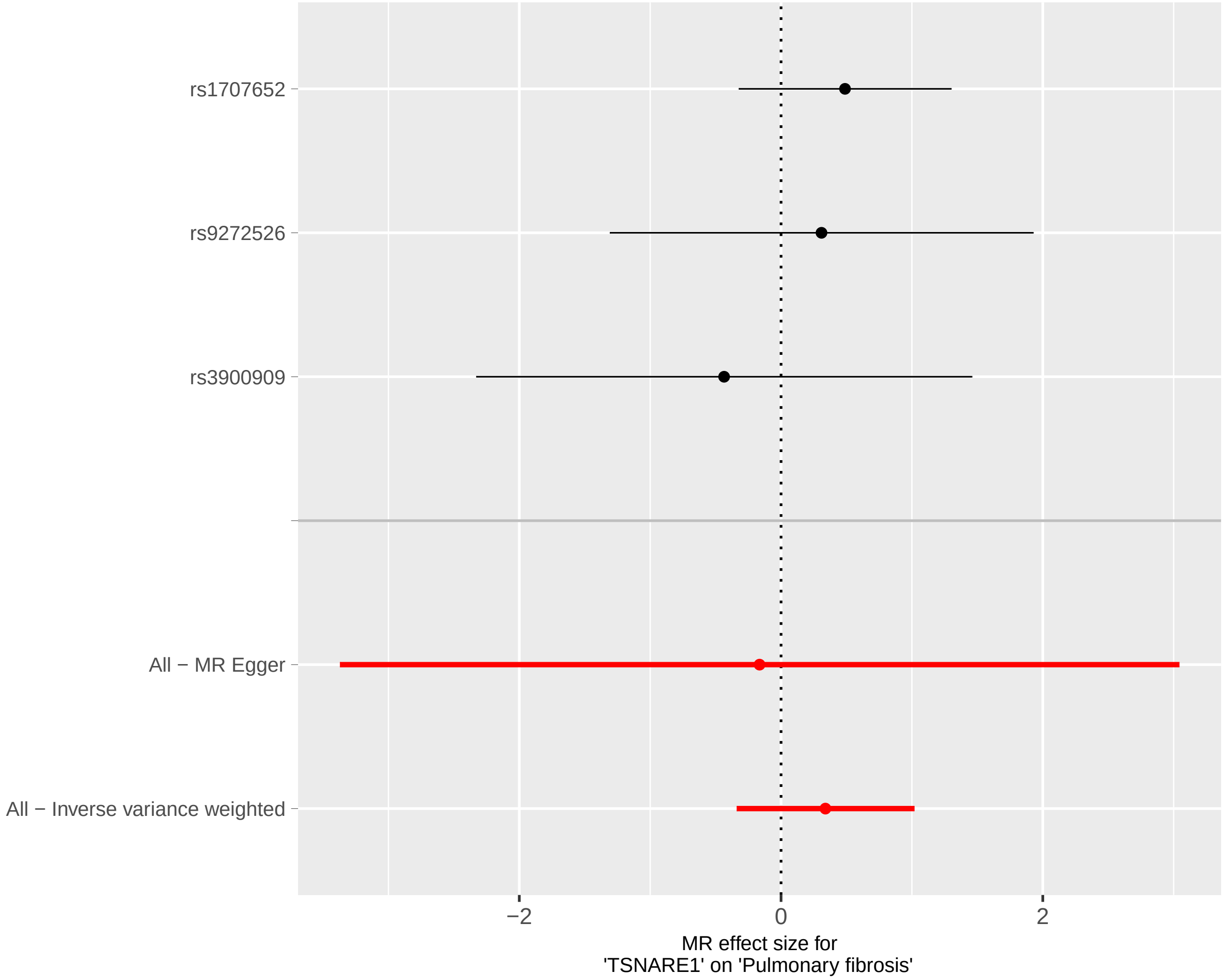
rs1707652
rs9272526
rs3900909
All − MR Egger
All − Inverse variance weighted
−2
0
2
MR effect size for
'TSNARE1' on 'Pulmonary fibrosis'

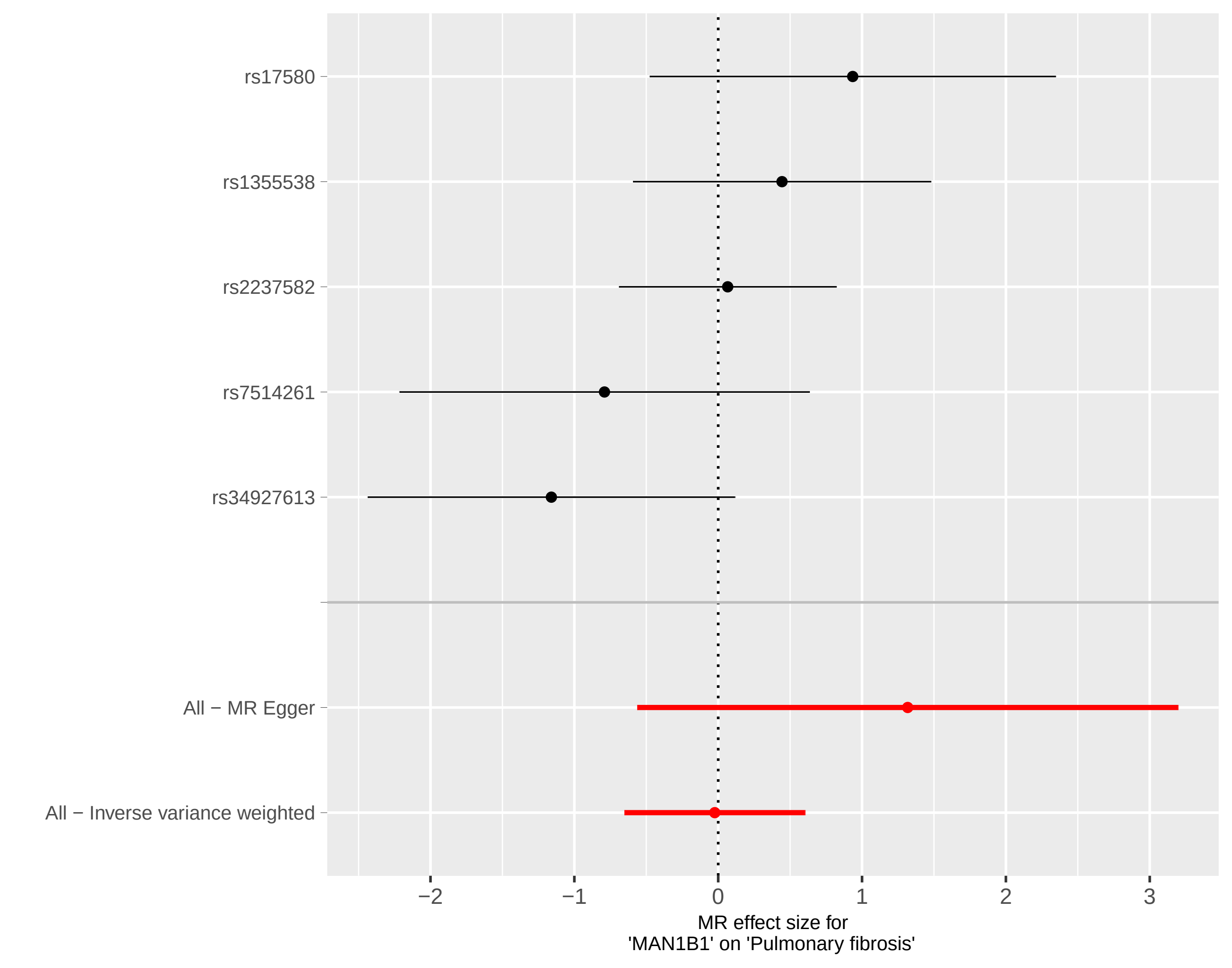
rs17580
rs1355538
rs2237582
rs7514261
rs34927613
All − MR Egger
All − Inverse variance weighted
−2
−1
0
1
2
3
MR effect size for
'MAN1B1' on 'Pulmonary fibrosis'

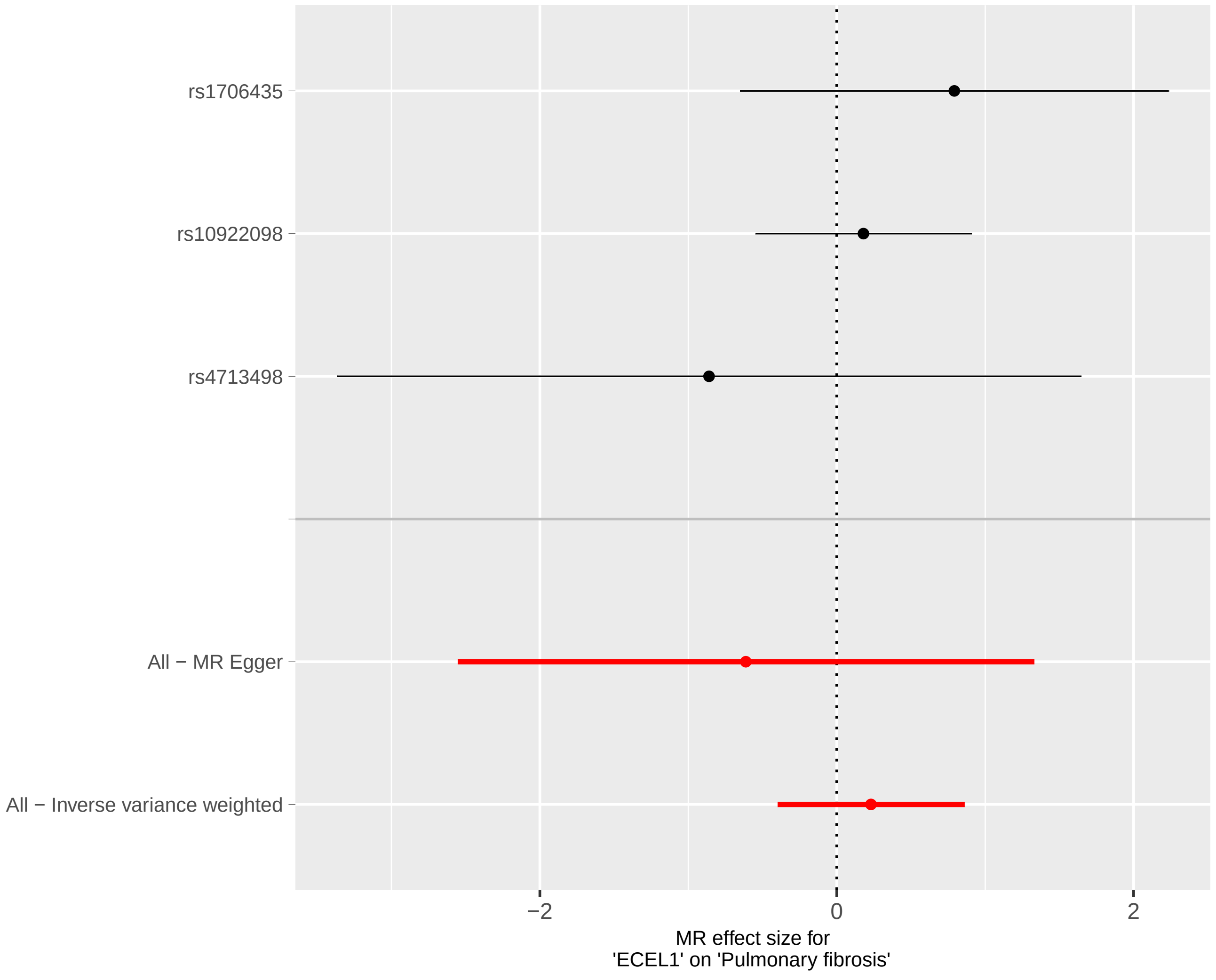

rs1706435
rs10922098
rs4713498
All − MR Egger
All − Inverse variance weighted
−2
0
2
MR effect size for
'ECEL1' on 'Pulmonary fibrosis'

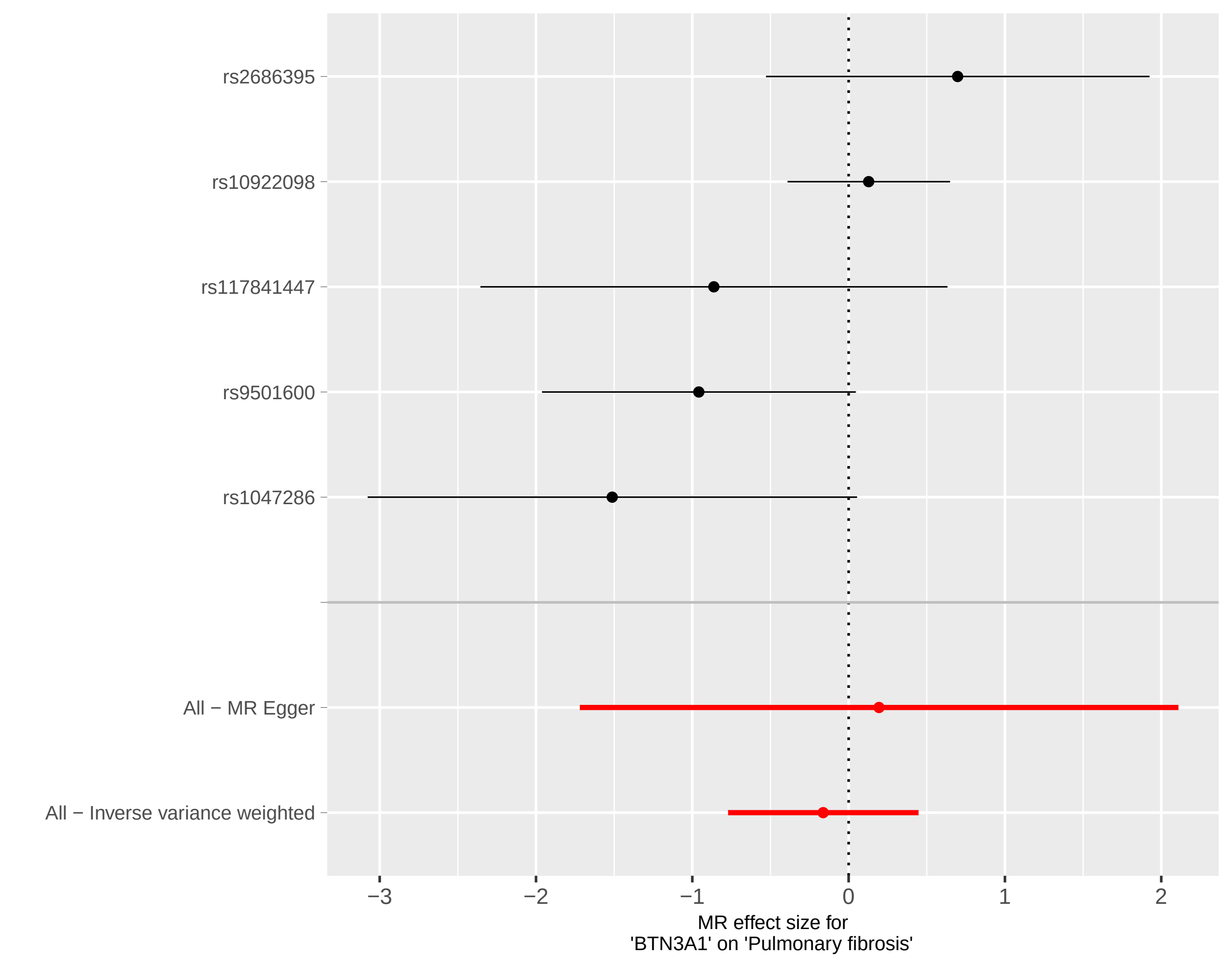
rs2686395
rs10922098
rs117841447
rs9501600
rs1047286
All − MR Egger
All − Inverse variance weighted
−3
−2
−1
0
1
2
MR effect size for
'BTN3A1' on 'Pulmonary fibrosis'

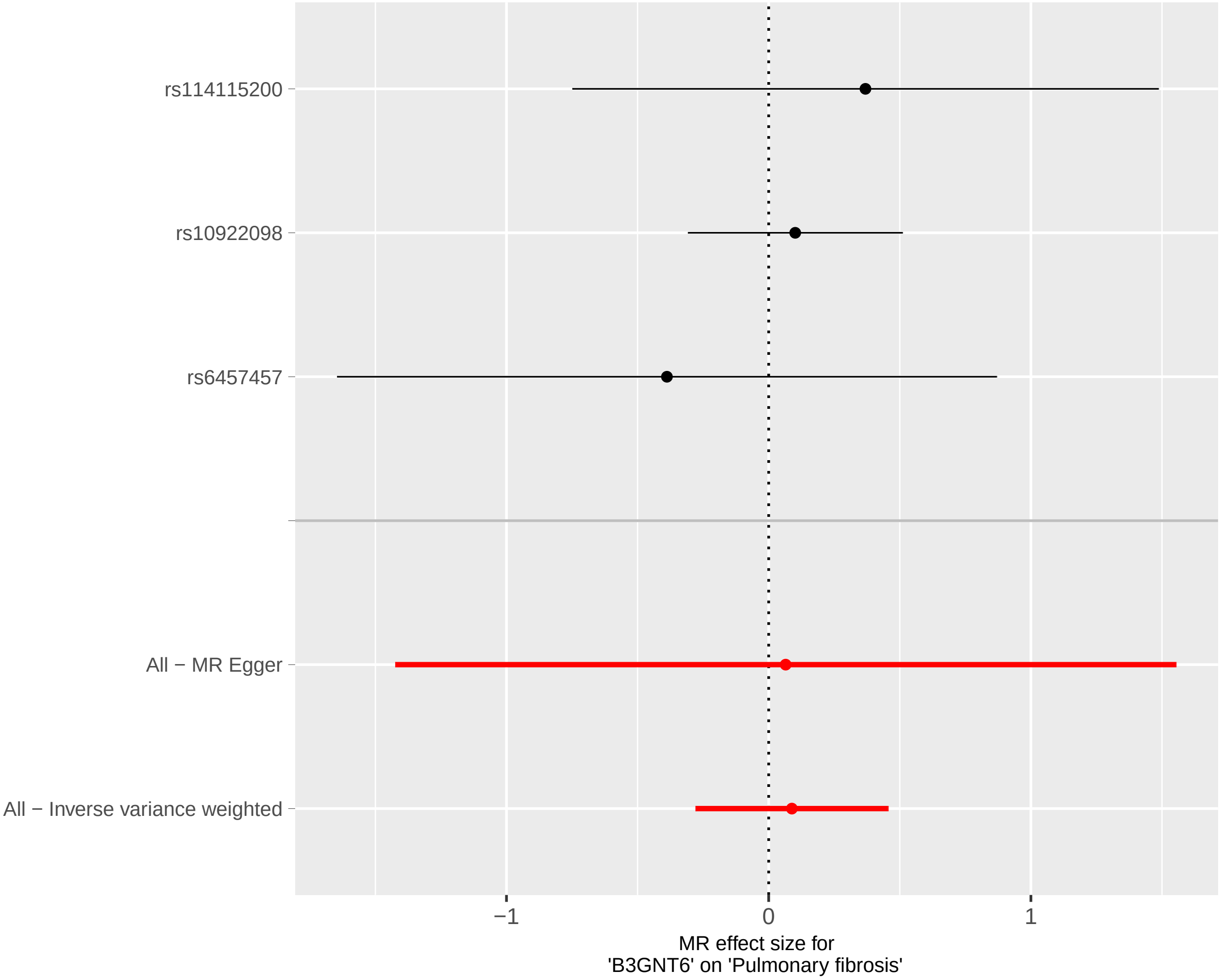
rs114115200
rs10922098
rs6457457
All − MR Egger
All − Inverse variance weighted
−1
0
1
MR effect size for
'B3GNT6' on 'Pulmonary fibrosis'

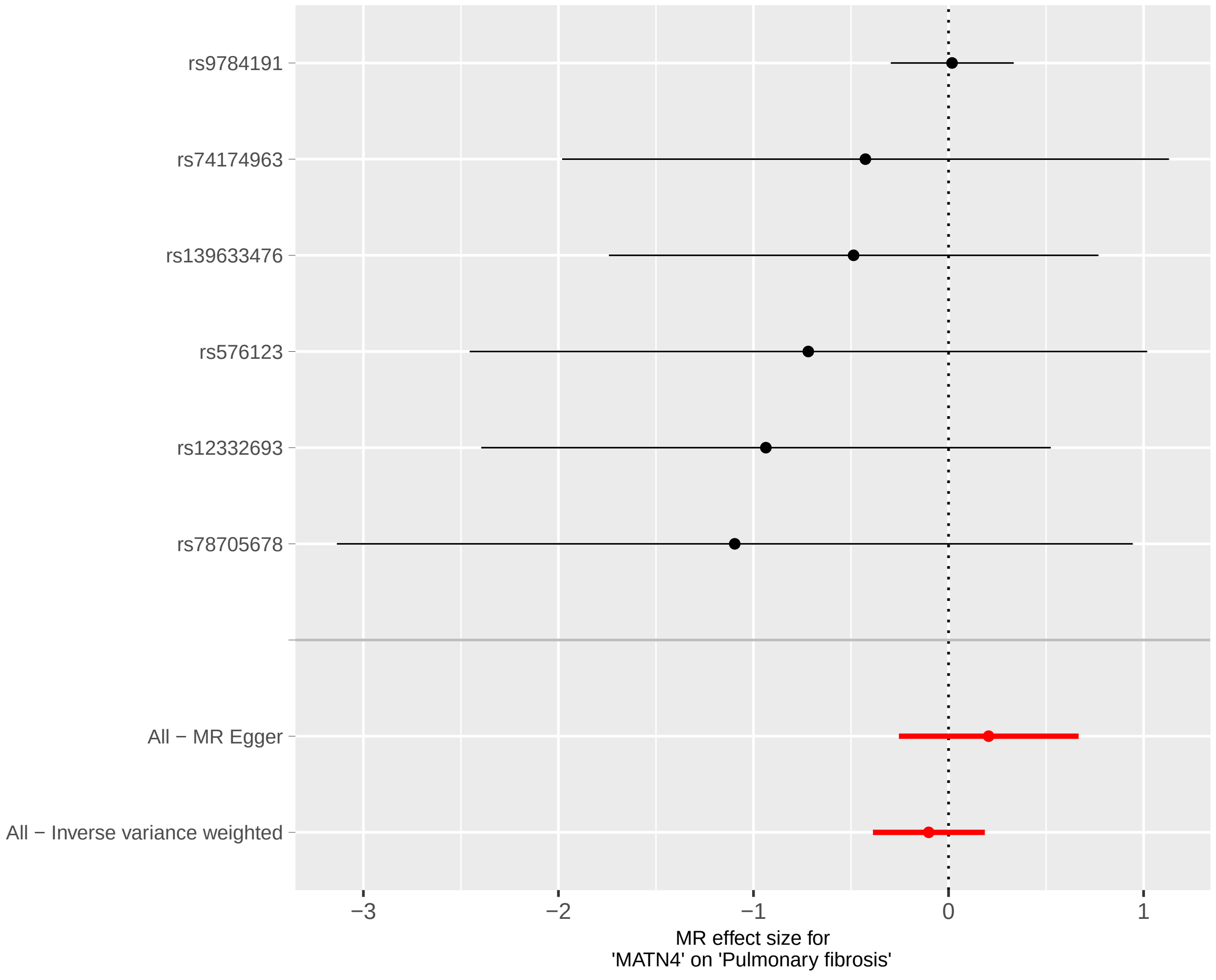

rs9784191
rs74174963
rs139633476
rs576123
rs12332693
rs78705678
All − MR Egger
All − Inverse variance weighted
−3
−2
−1
0
1
MR effect size for
'MATN4' on 'Pulmonary fibrosis'

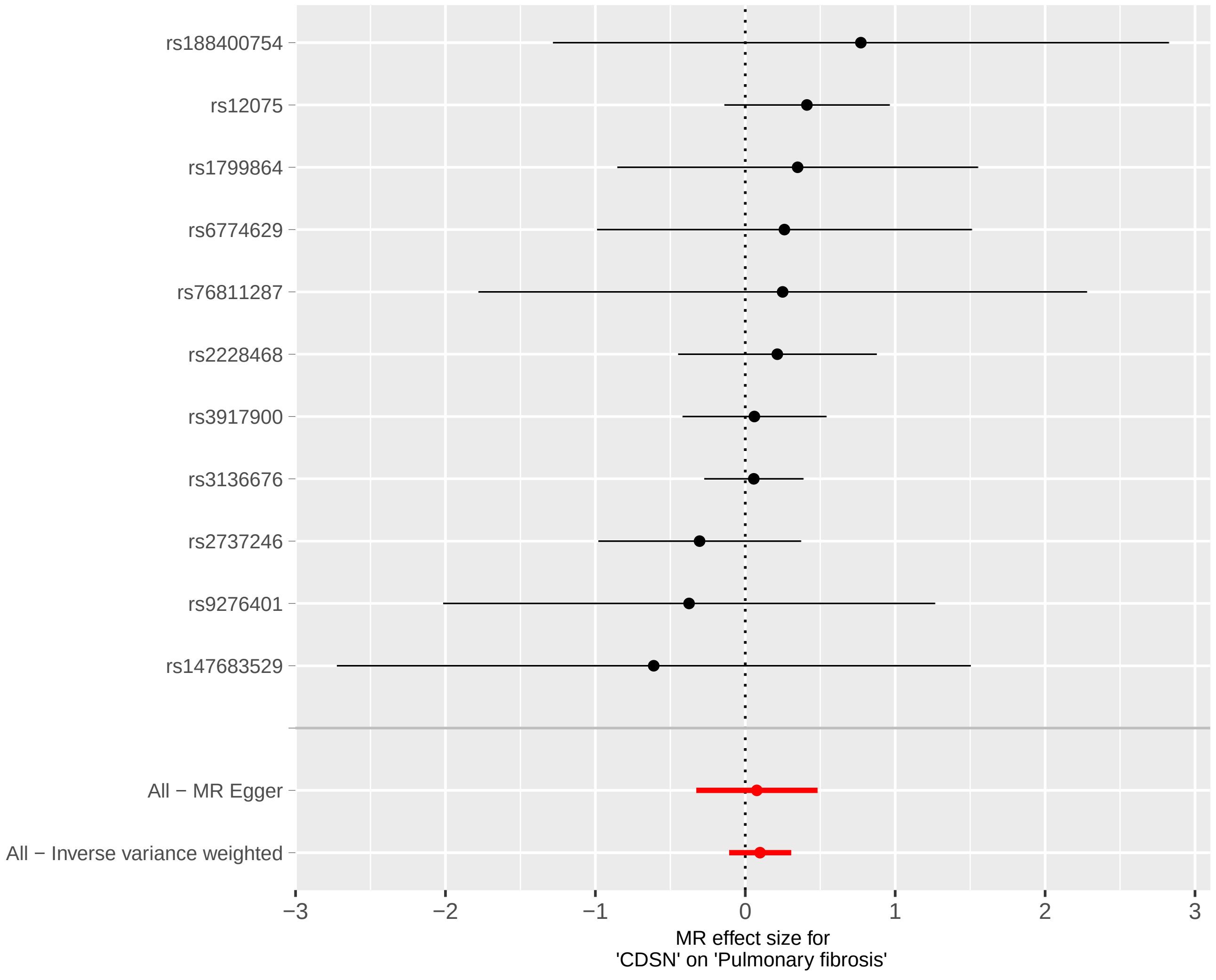

rs188400754
rs12075
rs1799864
rs6774629
rs76811287
rs2228468
rs3917900
rs3136676
rs2737246
rs9276401
rs147683529
All − MR Egger
All − Inverse variance weighted
−3
−2
−1
0
1
2
3
MR effect size for
'CDSN' on 'Pulmonary fibrosis'

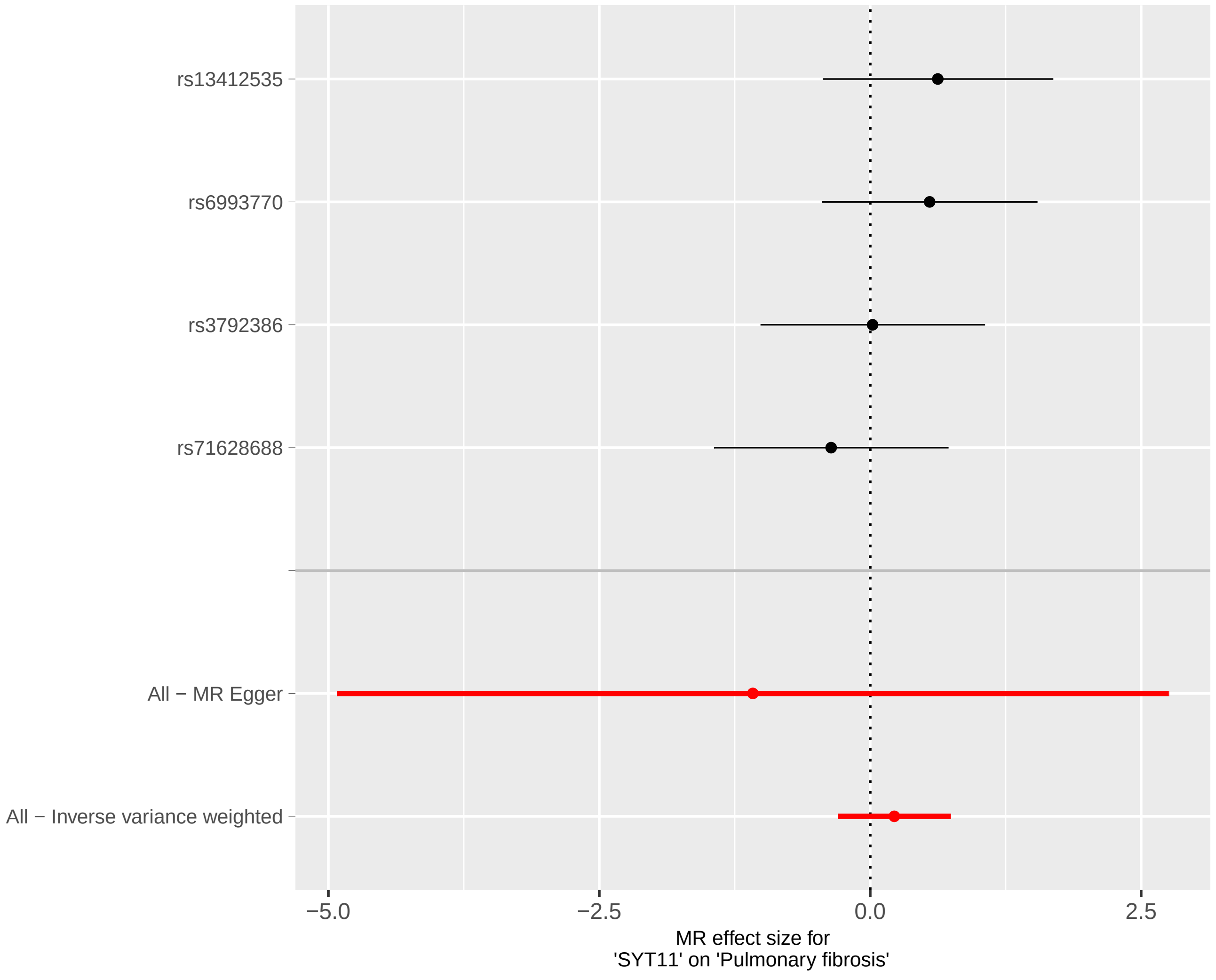
rs13412535
rs6993770
rs3792386
rs71628688
All − MR Egger
All − Inverse variance weighted
−5.0
−2.5
0.0
2.5
MR effect size for
'SYT11' on 'Pulmonary fibrosis'

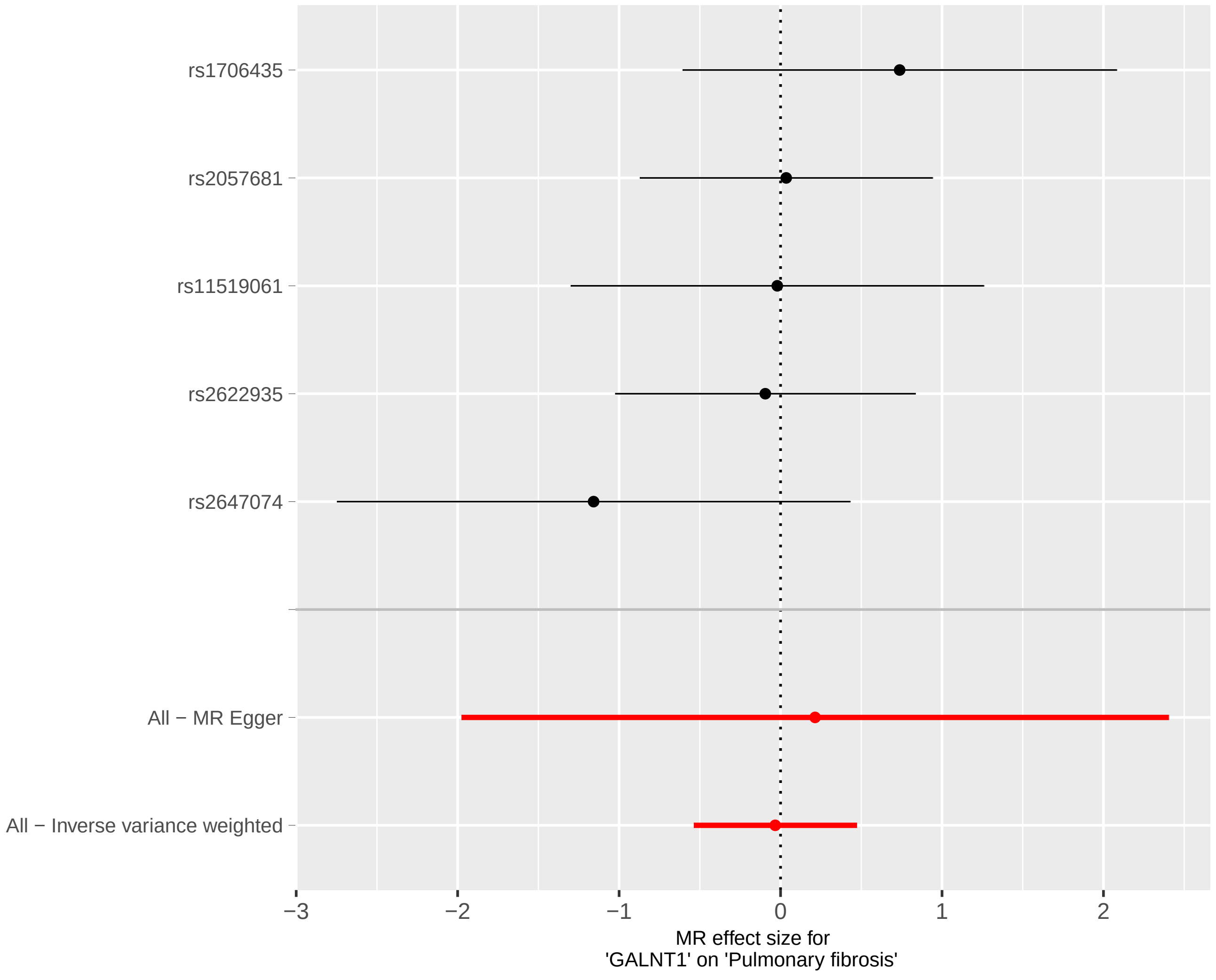
rs1706435
rs2057681
rs11519061
rs2622935
rs2647074
All − MR Egger
All − Inverse variance weighted
−3
−2
−1
0
1
2
MR effect size for
'GALNT1' on 'Pulmonary fibrosis'

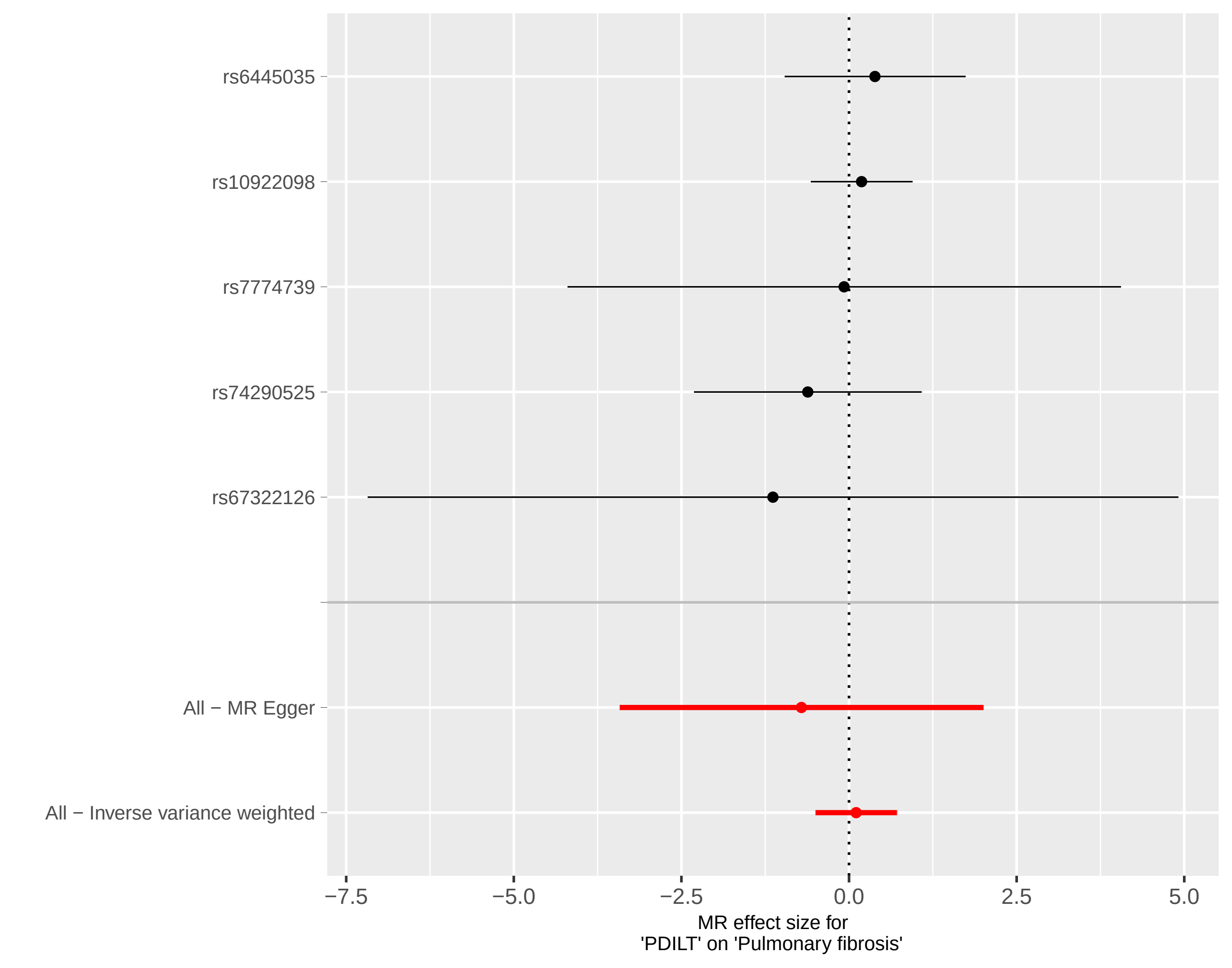

rs6445035
rs10922098
rs7774739
rs74290525
rs67322126
All − MR Egger
All − Inverse variance weighted
−7.5
−5.0
−2.5
0.0
2.5
5.0
MR effect size for
'PDILT' on 'Pulmonary fibrosis'

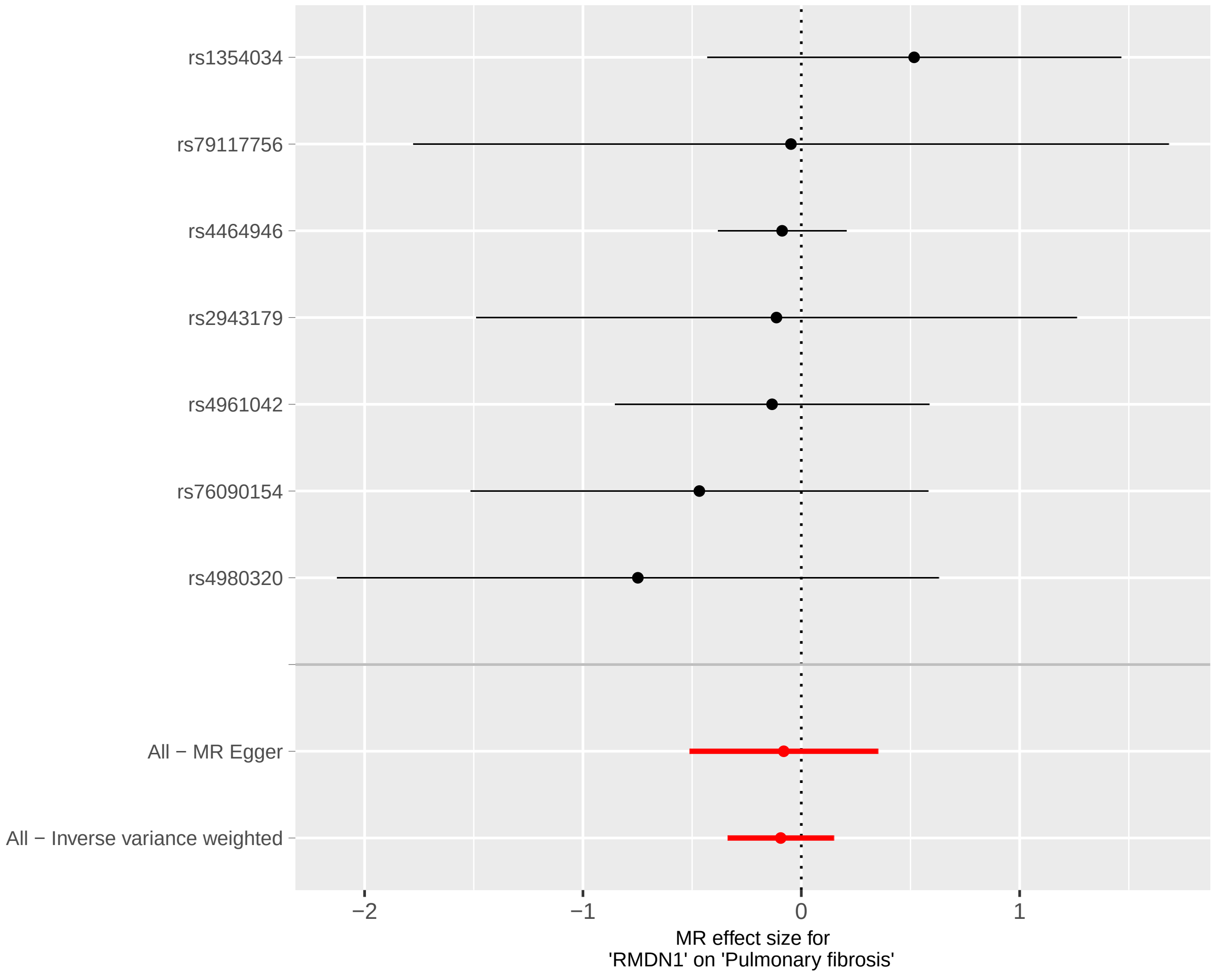
rs1354034
rs79117756
rs4464946
rs2943179
rs4961042
rs76090154
rs4980320
All − MR Egger
All − Inverse variance weighted
−2
−1
0
1
MR effect size for
'RMDN1' on 'Pulmonary fibrosis'

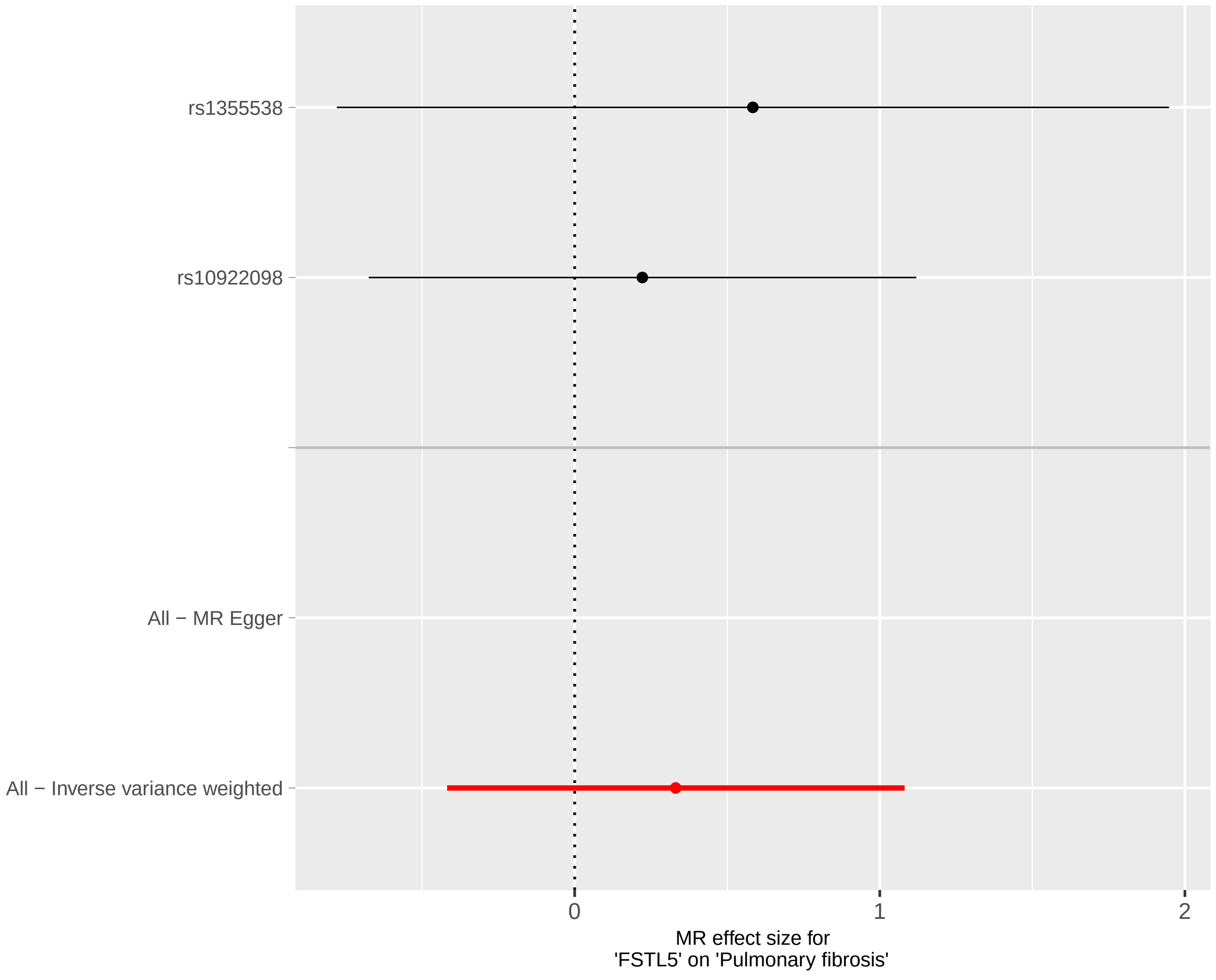
rs1355538
rs10922098
All − MR Egger
All − Inverse variance weighted
0
1
2
MR effect size for
'FSTL5' on 'Pulmonary fibrosis'

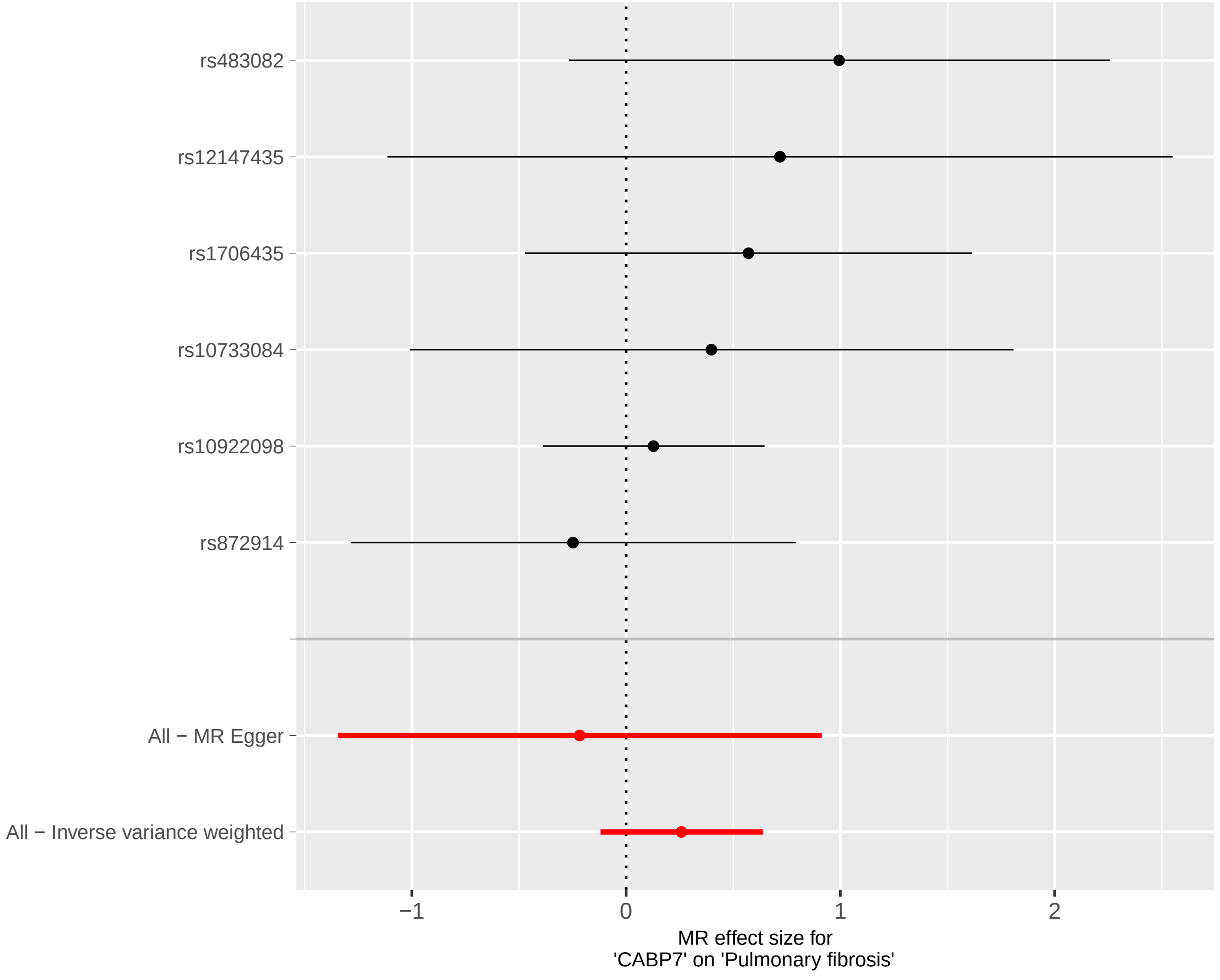

rs483082
rs12147435
rs1706435
rs10733084
rs10922098
rs872914
All − MR Egger
All − Inverse variance weighted
−1
0
1
2
MR effect size for
'CABP7' on 'Pulmonary fibrosis'

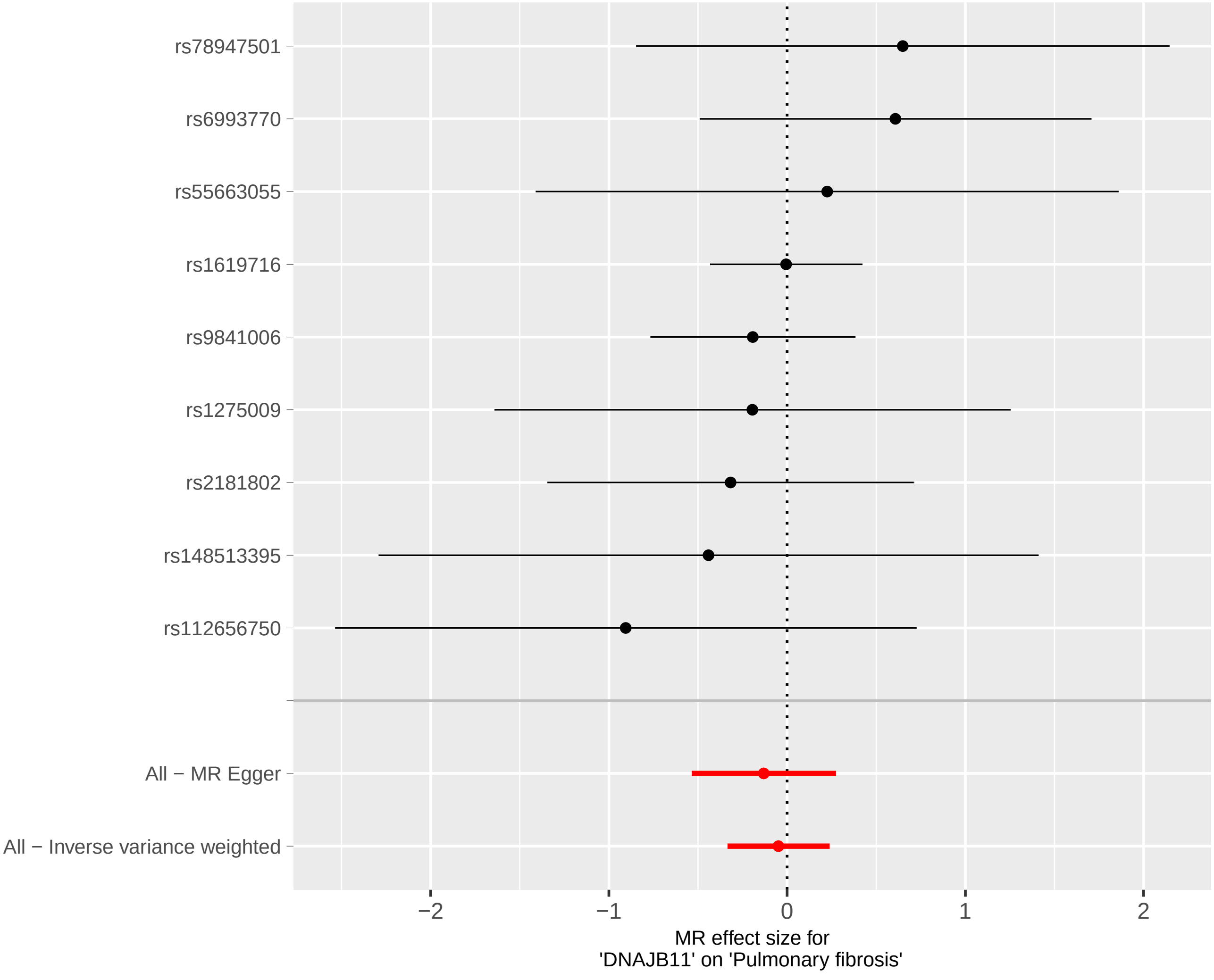

rs78947501
rs6993770
rs55663055
rs1619716
rs9841006
rs1275009
rs2181802
rs148513395
rs112656750
All − MR Egger
All − Inverse variance weighted
−2
−1
0
1
2
MR effect size for
'DNAJB11' on 'Pulmonary fibrosis'

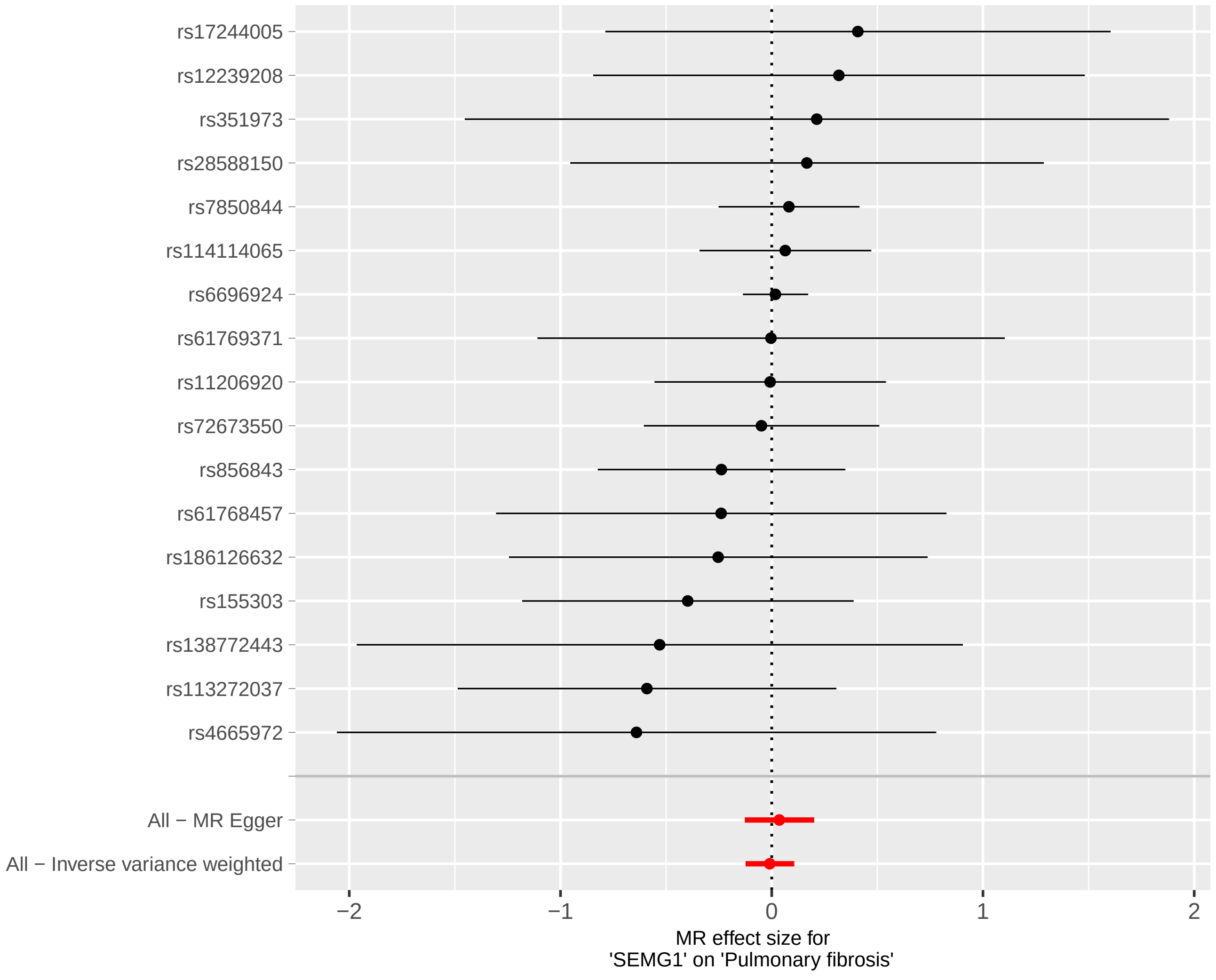

rs17244005
rs12239208
rs351973
rs28588150
rs7850844
rs114114065
rs6696924
rs61769371
rs11206920
rs72673550
rs856843
rs61768457
rs186126632
rs155303
rs138772443
rs113272037
rs4665972
All − MR Egger
All − Inverse variance weighted
−2
−1
0
1
2
MR effect size for
'SEMG1' on 'Pulmonary fibrosis'

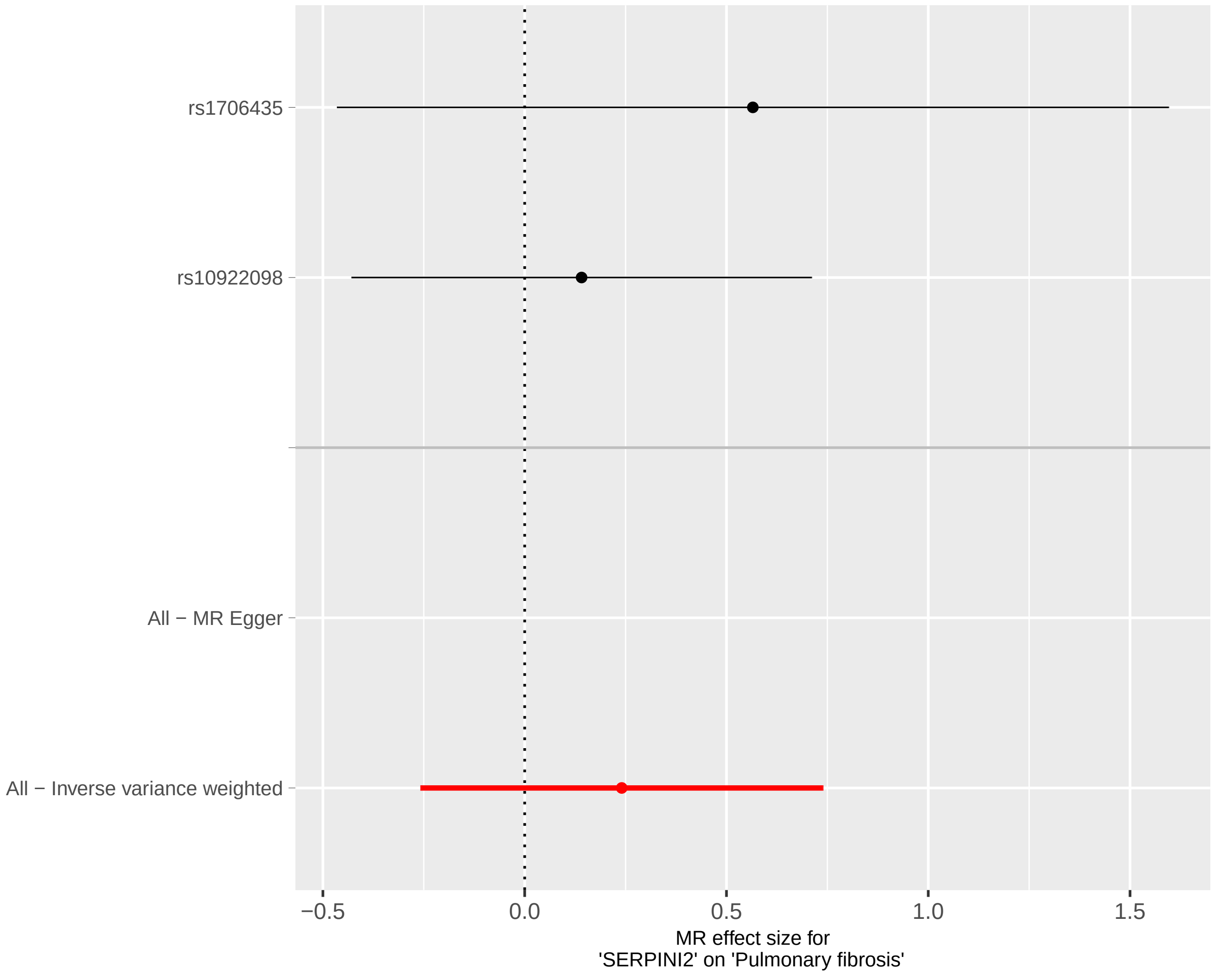
rs1706435
rs10922098
All − MR Egger
All − Inverse variance weighted
−0.5
0.0
0.5
1.0
1.5
MR effect size for
'SERPINI2' on 'Pulmonary fibrosis'

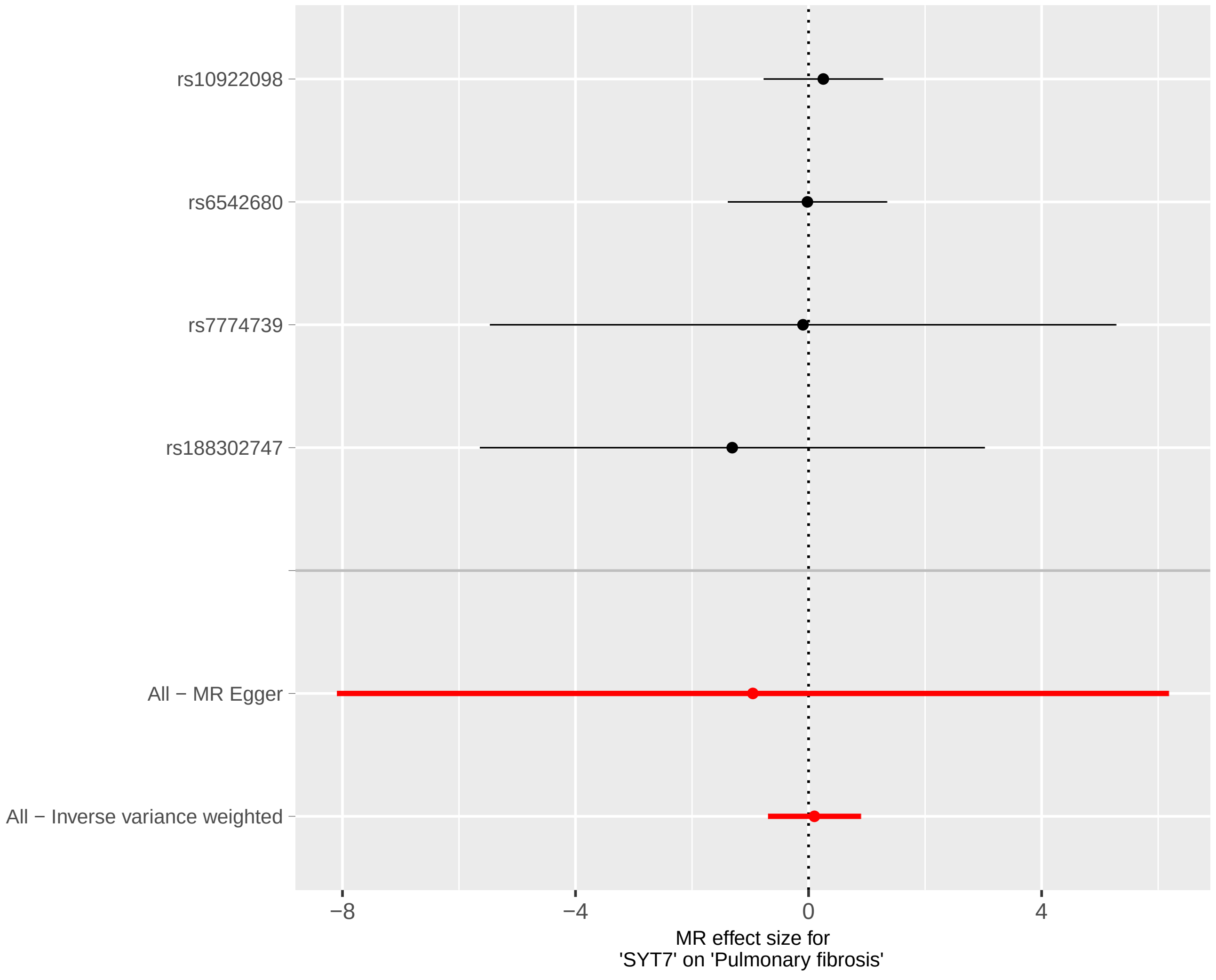
rs10922098
rs6542680
rs7774739
rs188302747
All − MR Egger
All − Inverse variance weighted
−8
−4
0
4
MR effect size for
'SYT7' on 'Pulmonary fibrosis'

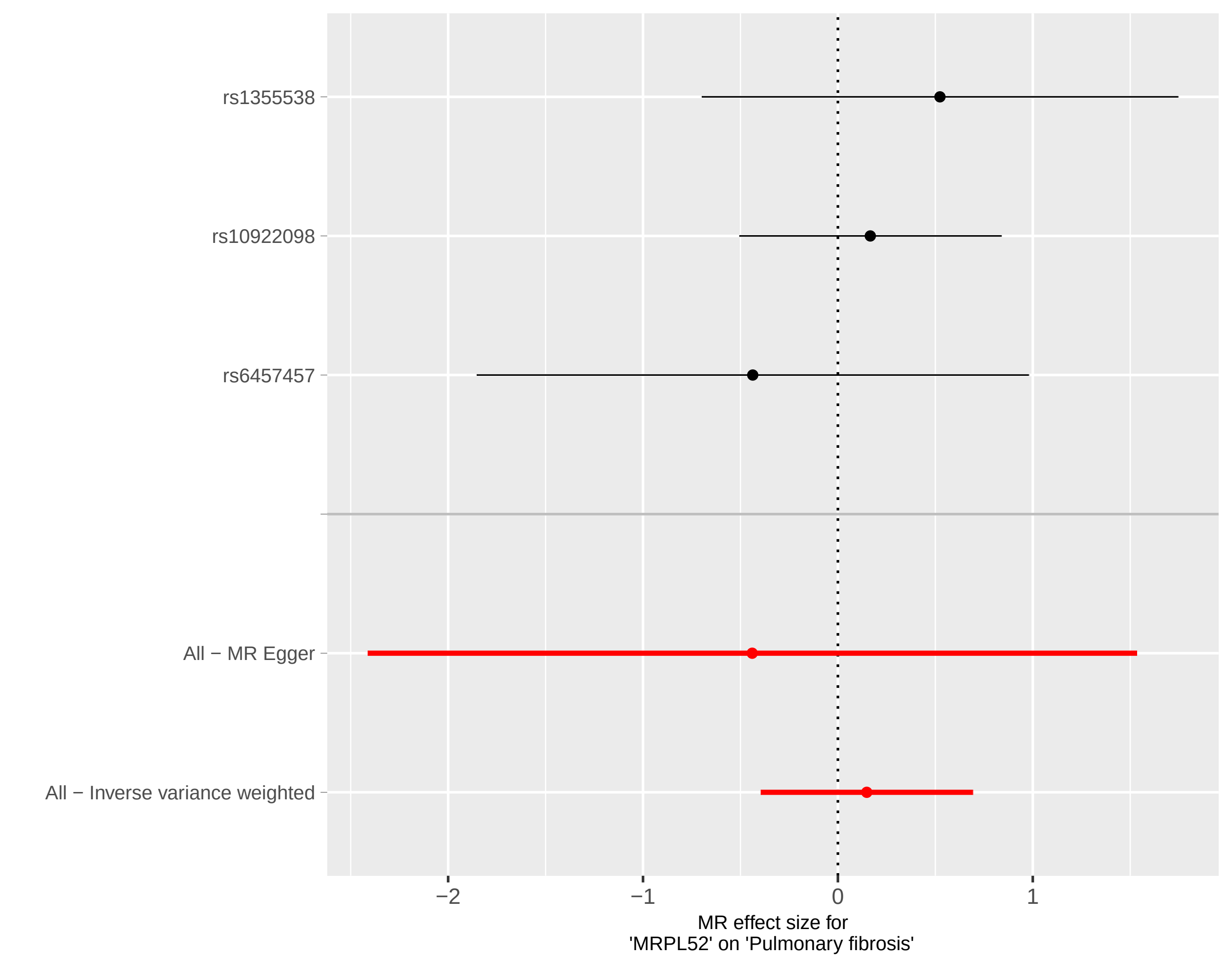
rs1355538
rs10922098
rs6457457
All − MR Egger
All − Inverse variance weighted
−2
−1
0
1
MR effect size for
'MRPL52' on 'Pulmonary fibrosis'

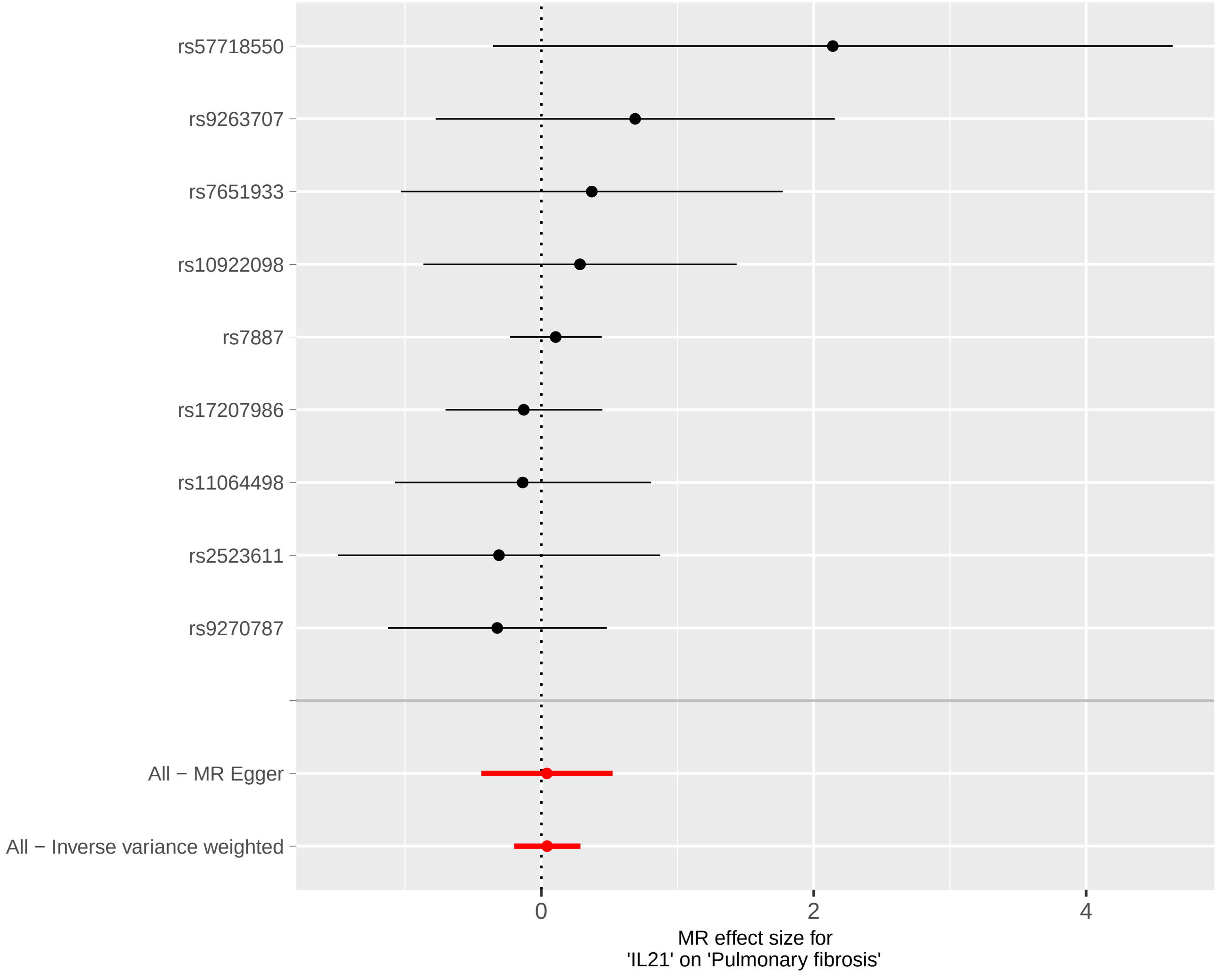

rs57718550
rs9263707
rs7651933
rs10922098
rs7887
rs17207986
rs11064498
rs2523611
rs9270787
All − MR Egger
All − Inverse variance weighted
0
2
4
MR effect size for
'IL21' on 'Pulmonary fibrosis'

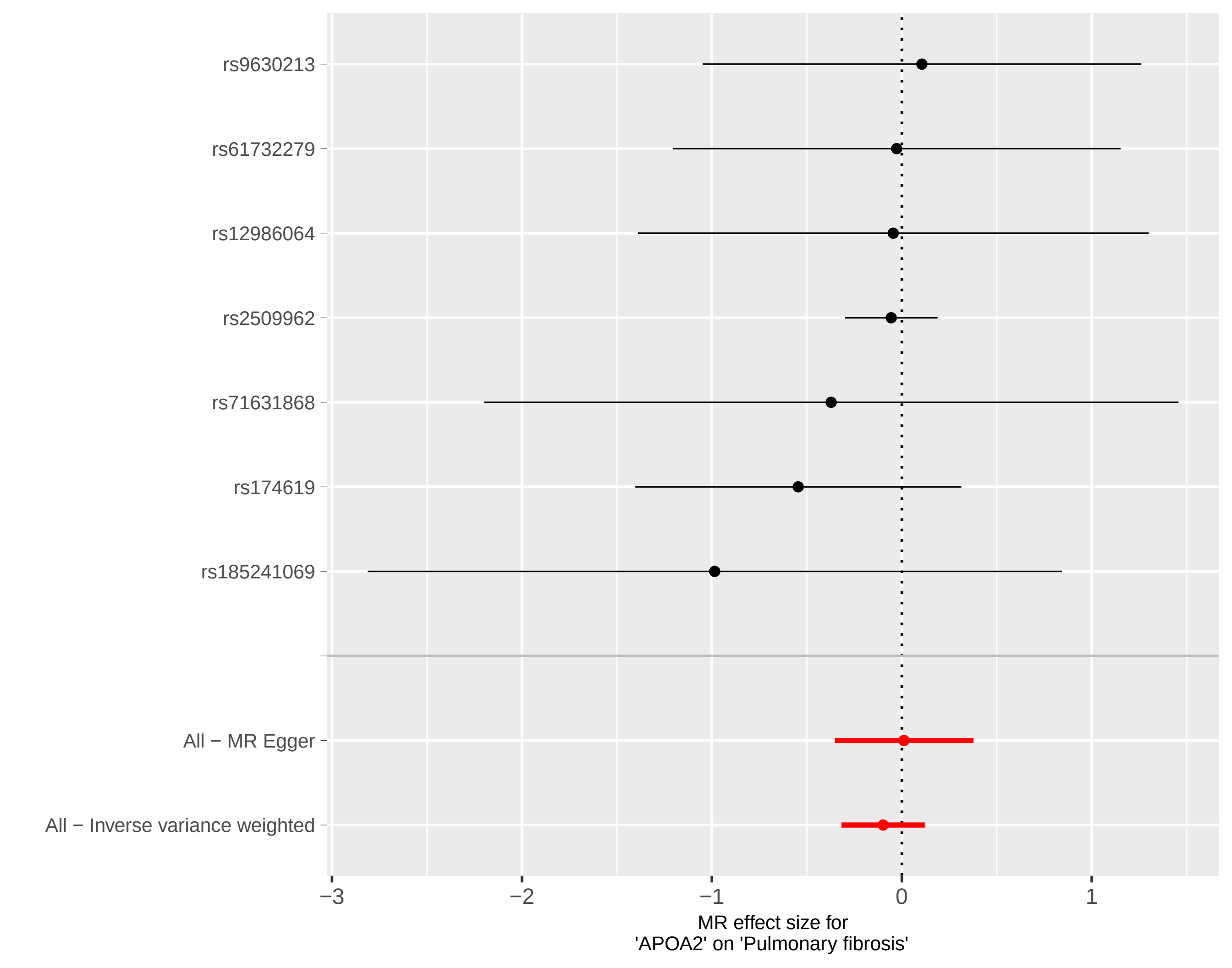
rs9630213
rs61732279
rs12986064
rs2509962
rs71631868
rs174619
rs185241069
All − MR Egger
All − Inverse variance weighted
−3
−2
−1
0
1
MR effect size for
'APOA2' on 'Pulmonary fibrosis'

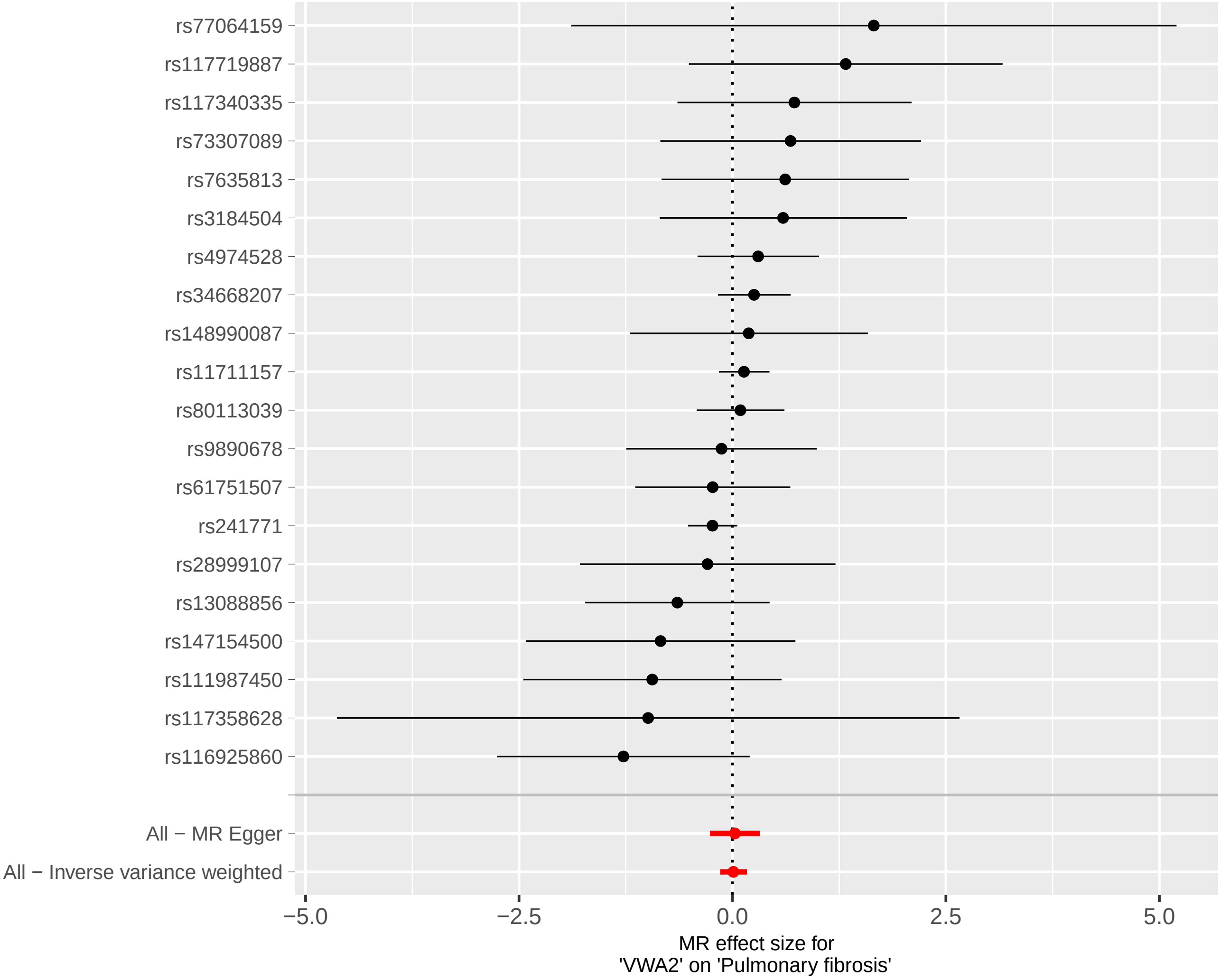
rs77064159
rs117719887
rs117340335
rs73307089
rs7635813
rs3184504
rs4974528
rs34668207
rs148990087
rs11711157
rs80113039
rs9890678
rs61751507
rs241771
rs28999107
rs13088856
rs147154500
rs111987450
rs117358628
rs116925860
All − MR Egger
All − Inverse variance weighted
−5.0
−2.5
0.0
2.5
5.0
MR effect size for
'VWA2' on 'Pulmonary fibrosis'

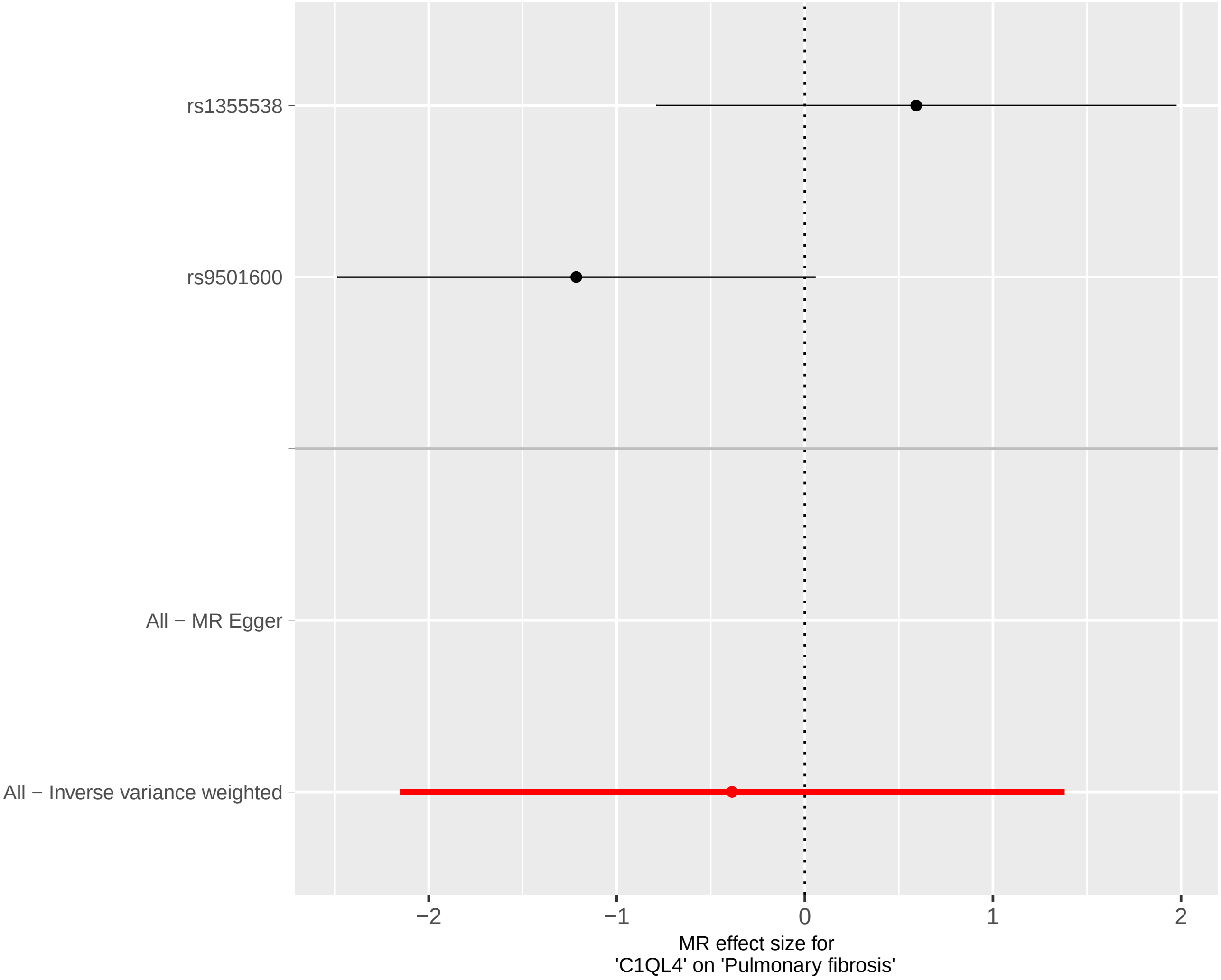
rs1355538
rs9501600
All − MR Egger
All − Inverse variance weighted
−2
−1
0
1
2
MR effect size for
'C1QL4' on 'Pulmonary fibrosis'

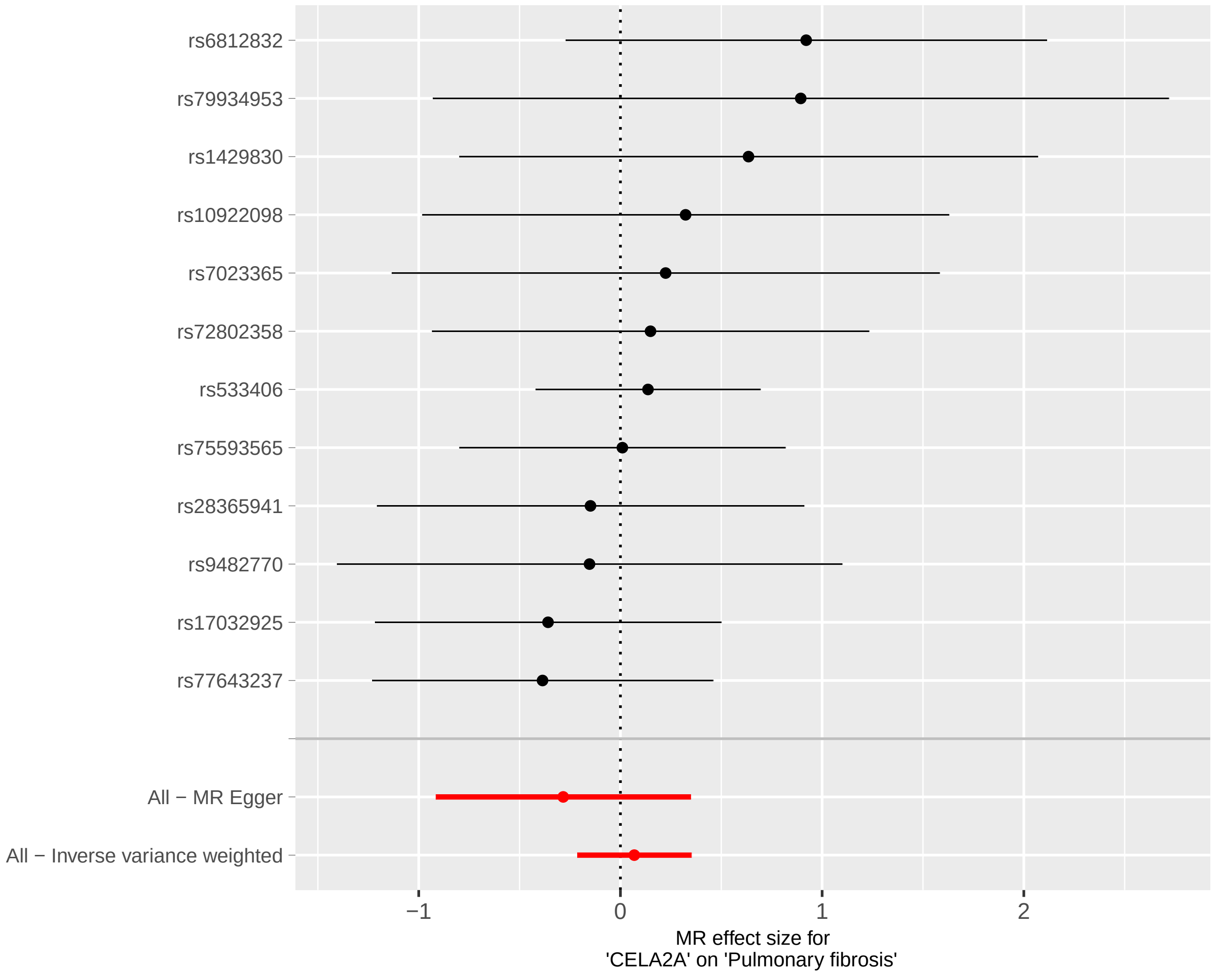

rs6812832
rs79934953
rs1429830
rs10922098
rs7023365
rs72802358
rs533406
rs75593565
rs28365941
rs9482770
rs17032925
rs77643237
All − MR Egger
All − Inverse variance weighted
−1
0
1
2
MR effect size for
'CELA2A' on 'Pulmonary fibrosis'

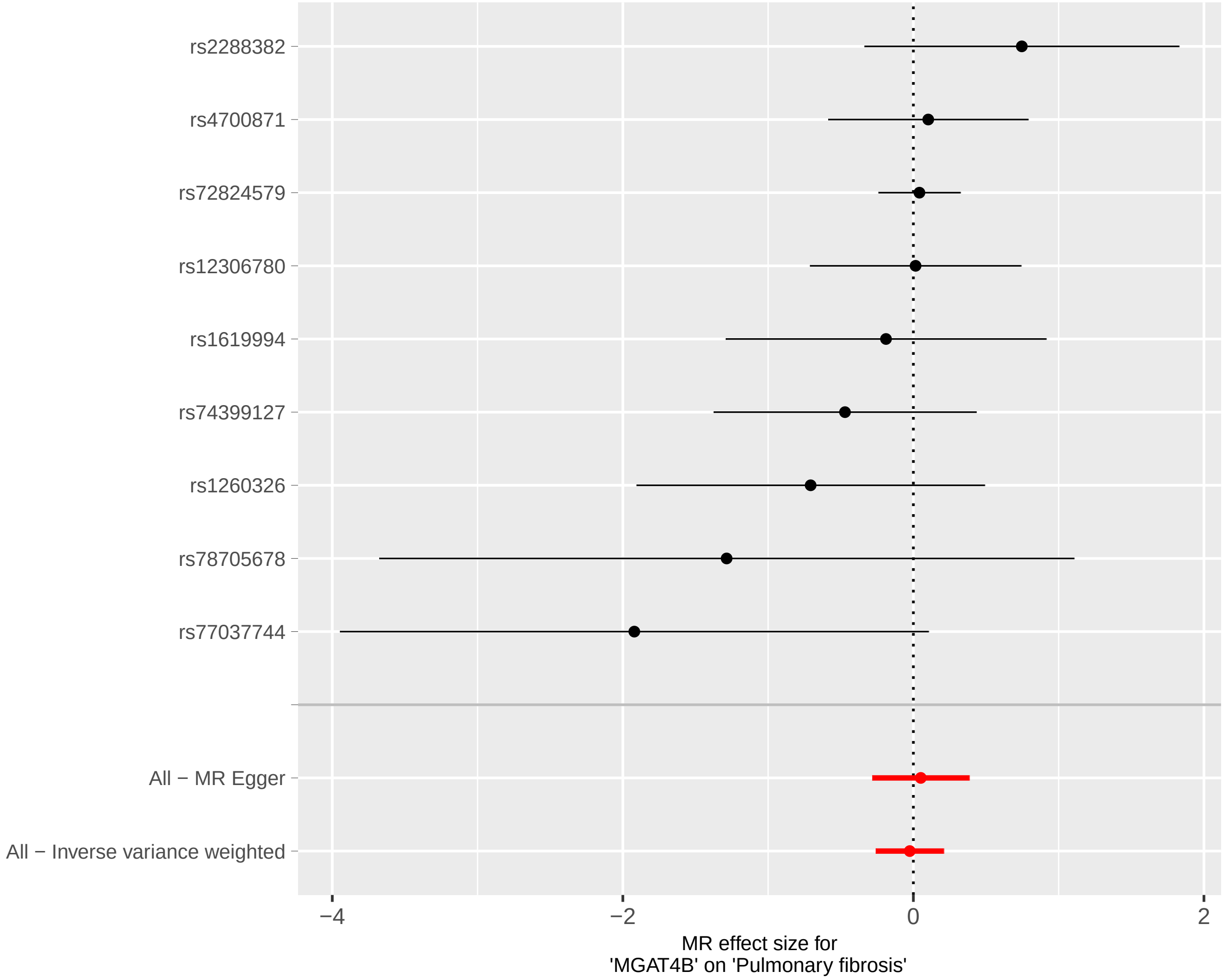

rs2288382
rs4700871
rs72824579
rs12306780
rs1619994
rs74399127
rs1260326
rs78705678
rs77037744
All − MR Egger
All − Inverse variance weighted
−4
−2
0
2
MR effect size for
'MGAT4B' on 'Pulmonary fibrosis'

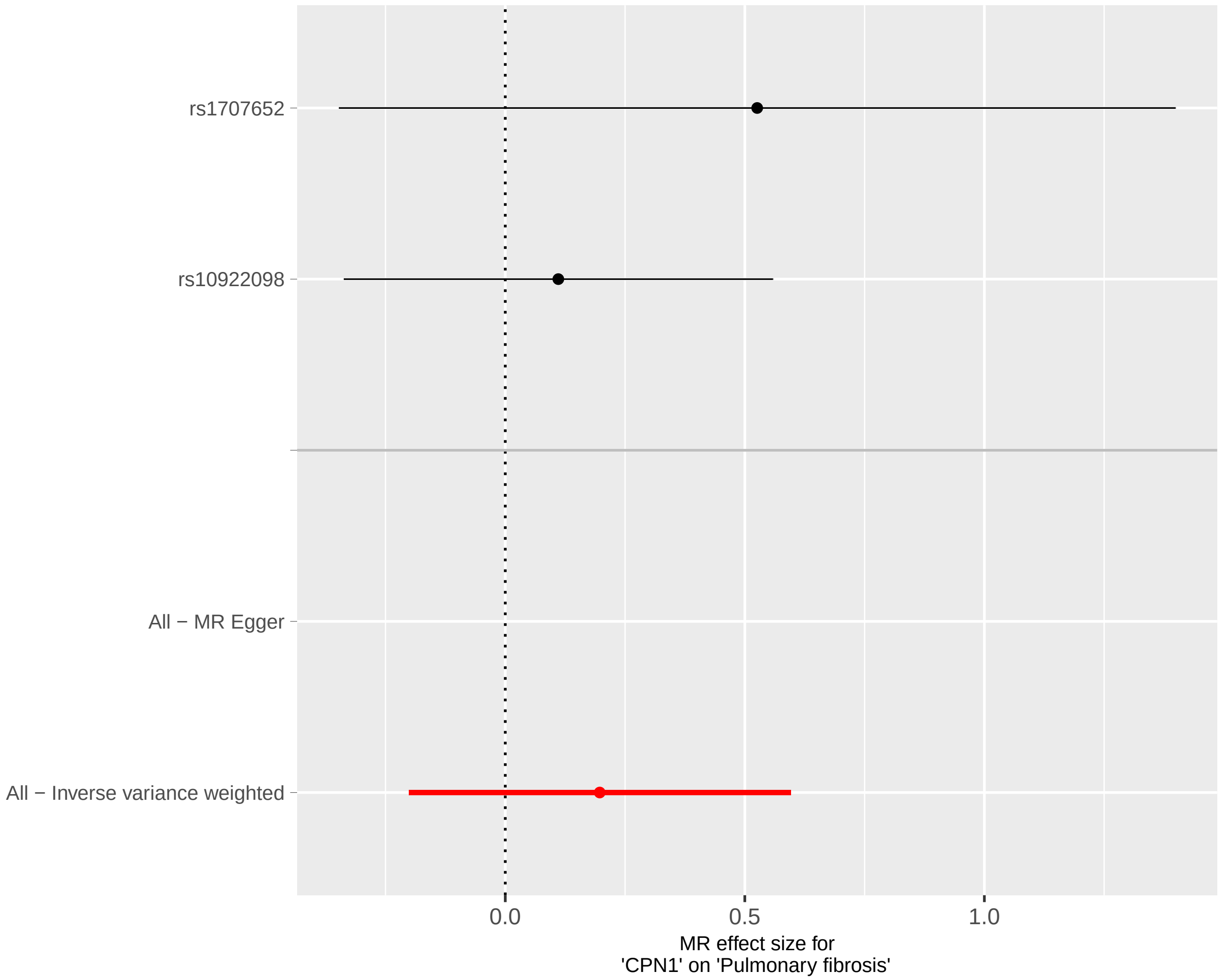
rs1707652
rs10922098
All − MR Egger
All − Inverse variance weighted
0.0
0.5
1.0
MR effect size for
'CPN1' on 'Pulmonary fibrosis'

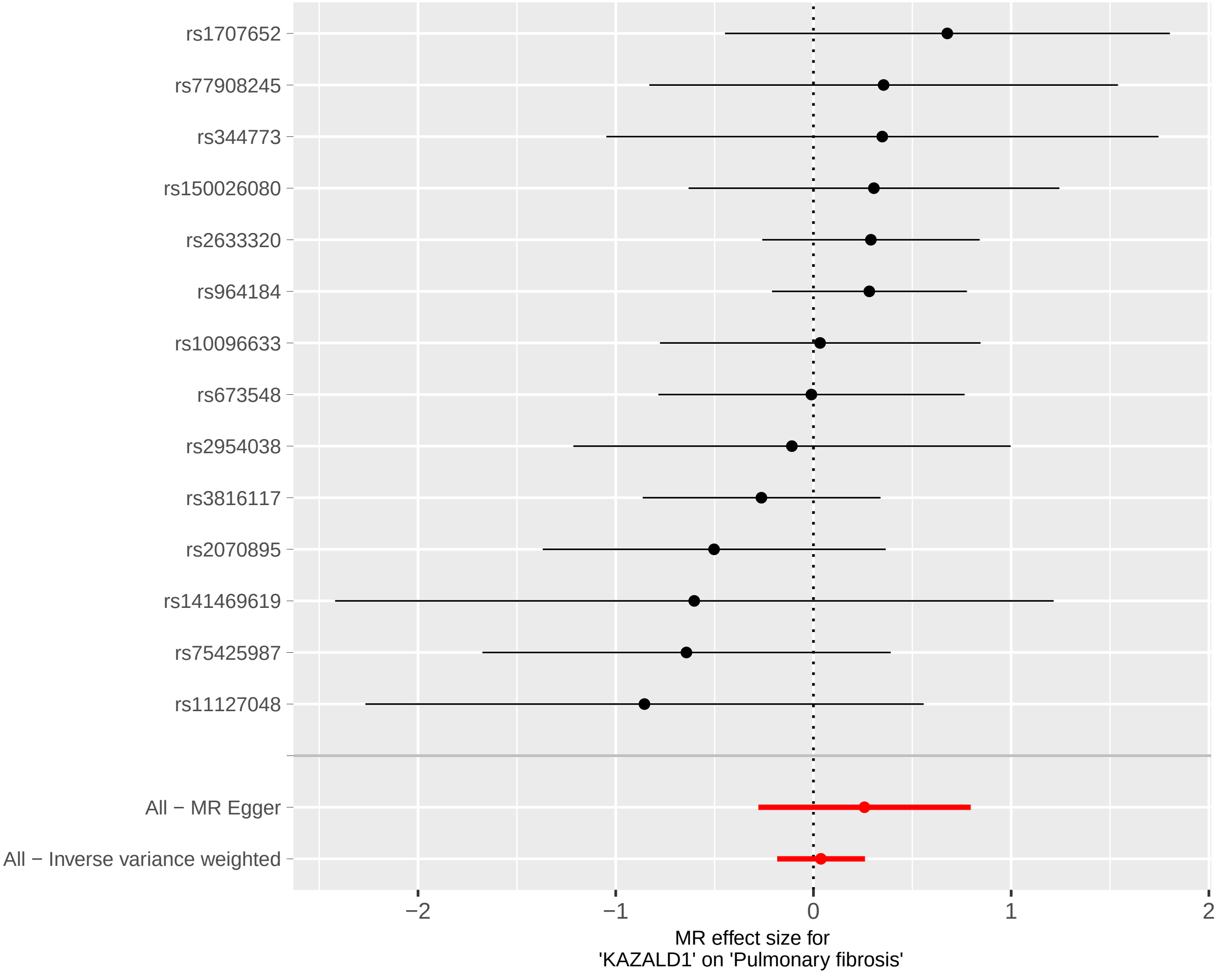

rs1707652
rs77908245
rs344773
rs150026080
rs2633320
rs964184
rs10096633
rs673548
rs2954038
rs3816117
rs2070895
rs141469619
rs75425987
rs11127048
All − MR Egger
All − Inverse variance weighted
−2
−1
0
1
2
MR effect size for
'KAZALD1' on 'Pulmonary fibrosis'

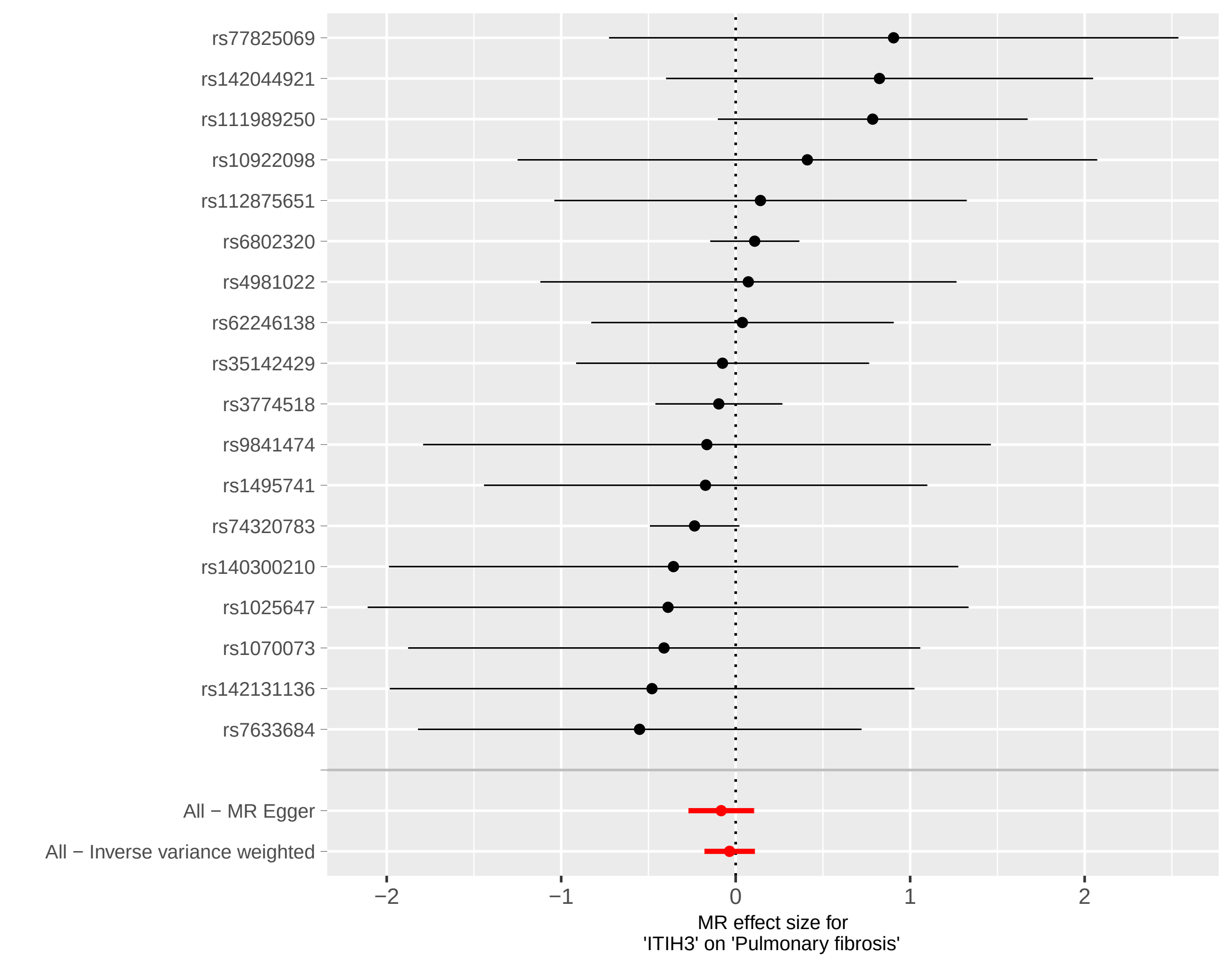
rs77825069
rs142044921
rs111989250
rs10922098
rs112875651
rs6802320
rs4981022
rs62246138
rs35142429
rs3774518
rs9841474
rs1495741
rs74320783
rs140300210
rs1025647
rs1070073
rs142131136
rs7633684
All − MR Egger
All − Inverse variance weighted
−2
−1
0
1
2
MR effect size for
'ITIH3' on 'Pulmonary fibrosis'

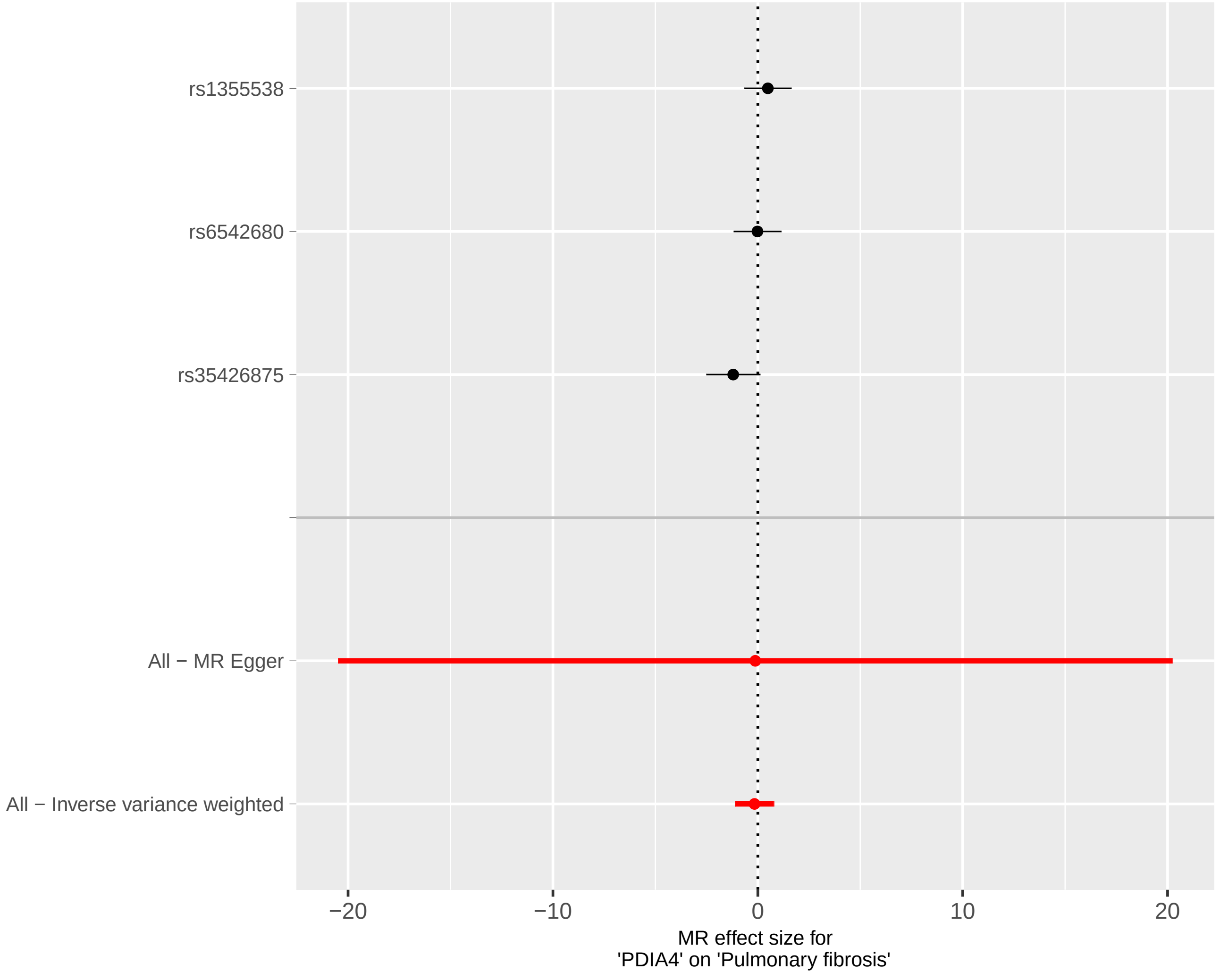
rs1355538
rs6542680
rs35426875
All − MR Egger
All − Inverse variance weighted
−20
−10
0
10
20
MR effect size for
'PDIA4' on 'Pulmonary fibrosis'

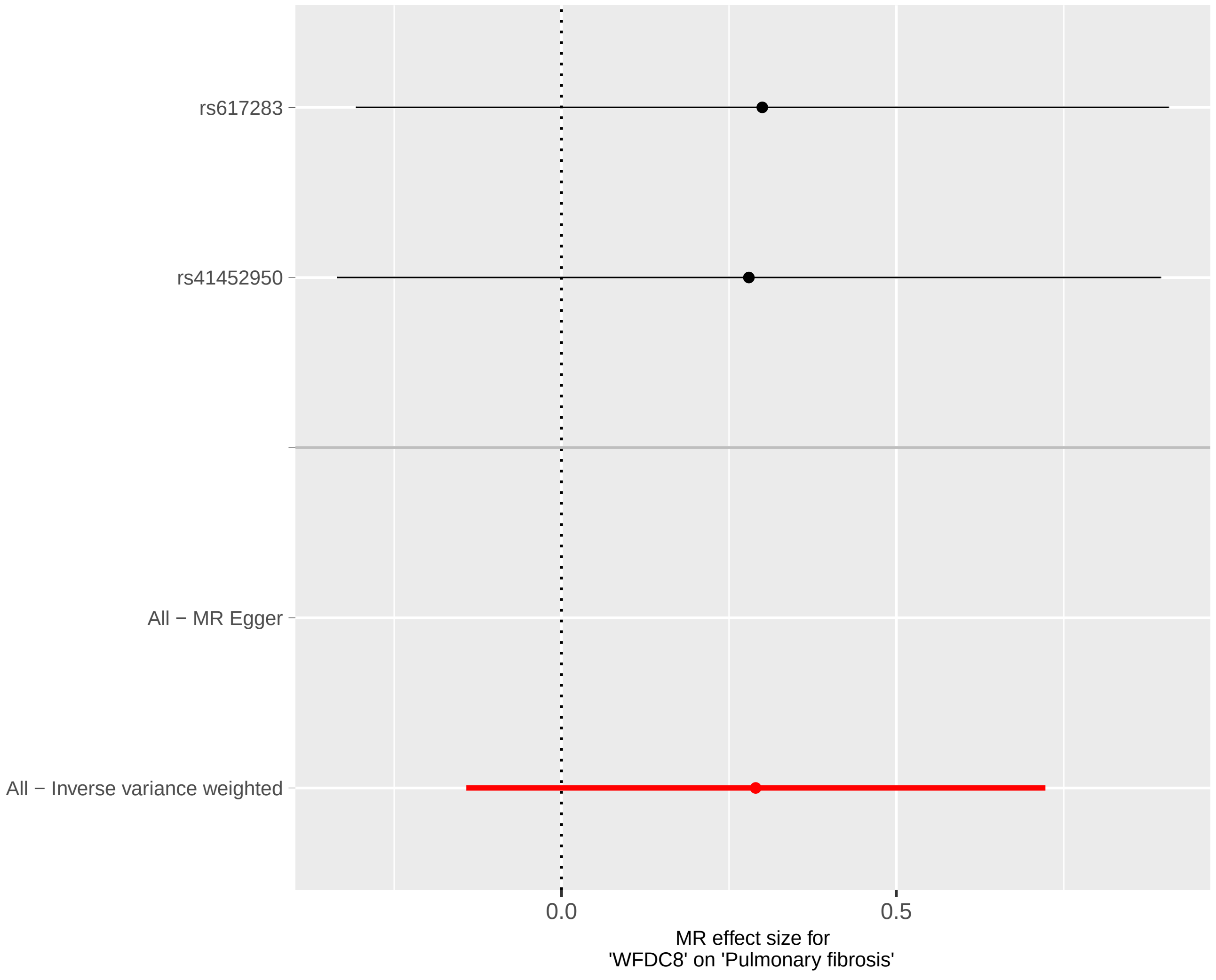
rs617283
rs41452950
All − MR Egger
All − Inverse variance weighted
0.0
0.5
MR effect size for
'WFDC8' on 'Pulmonary fibrosis'

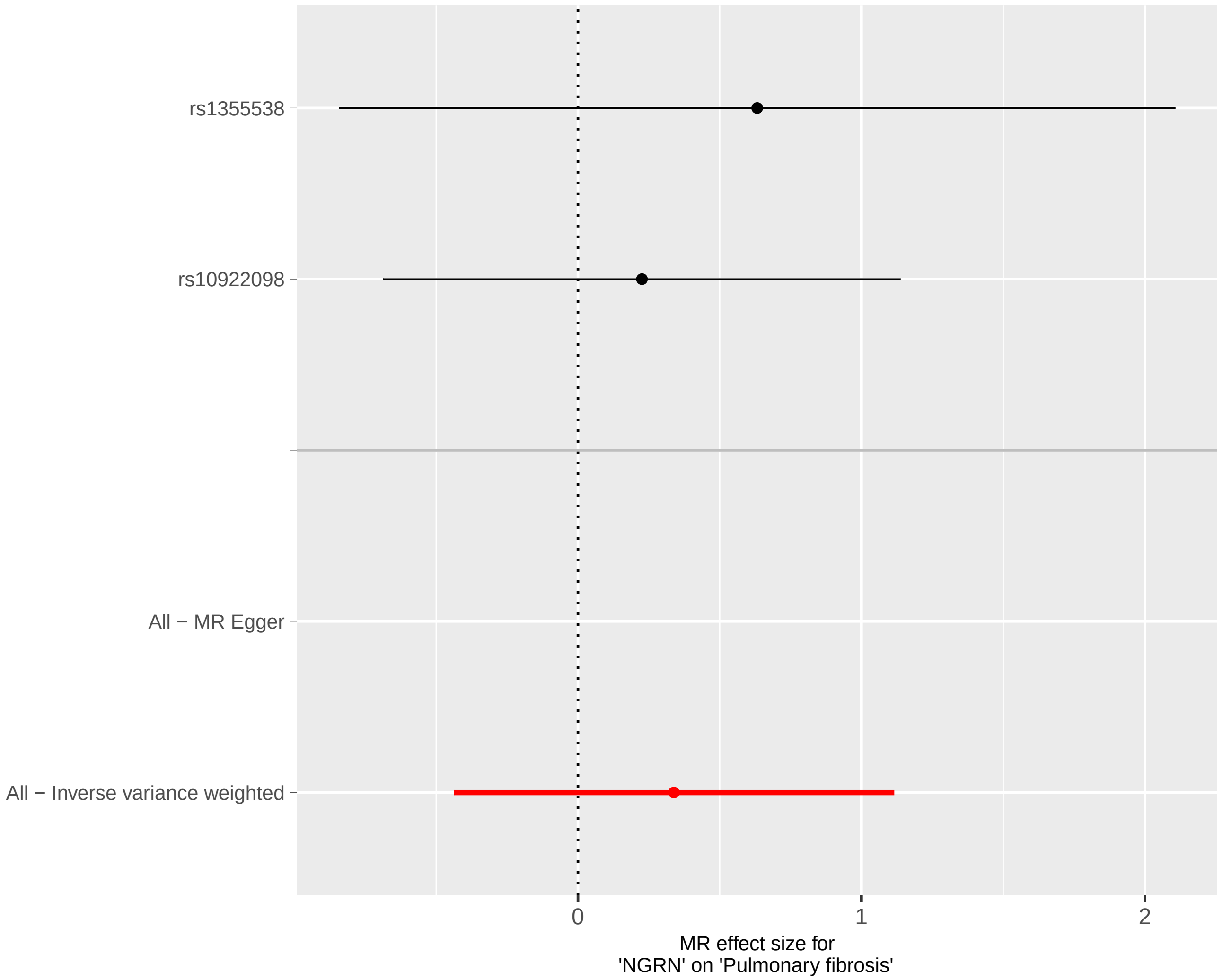
rs1355538
rs10922098
All − MR Egger
All − Inverse variance weighted
0
1
2
MR effect size for
'NGRN' on 'Pulmonary fibrosis'

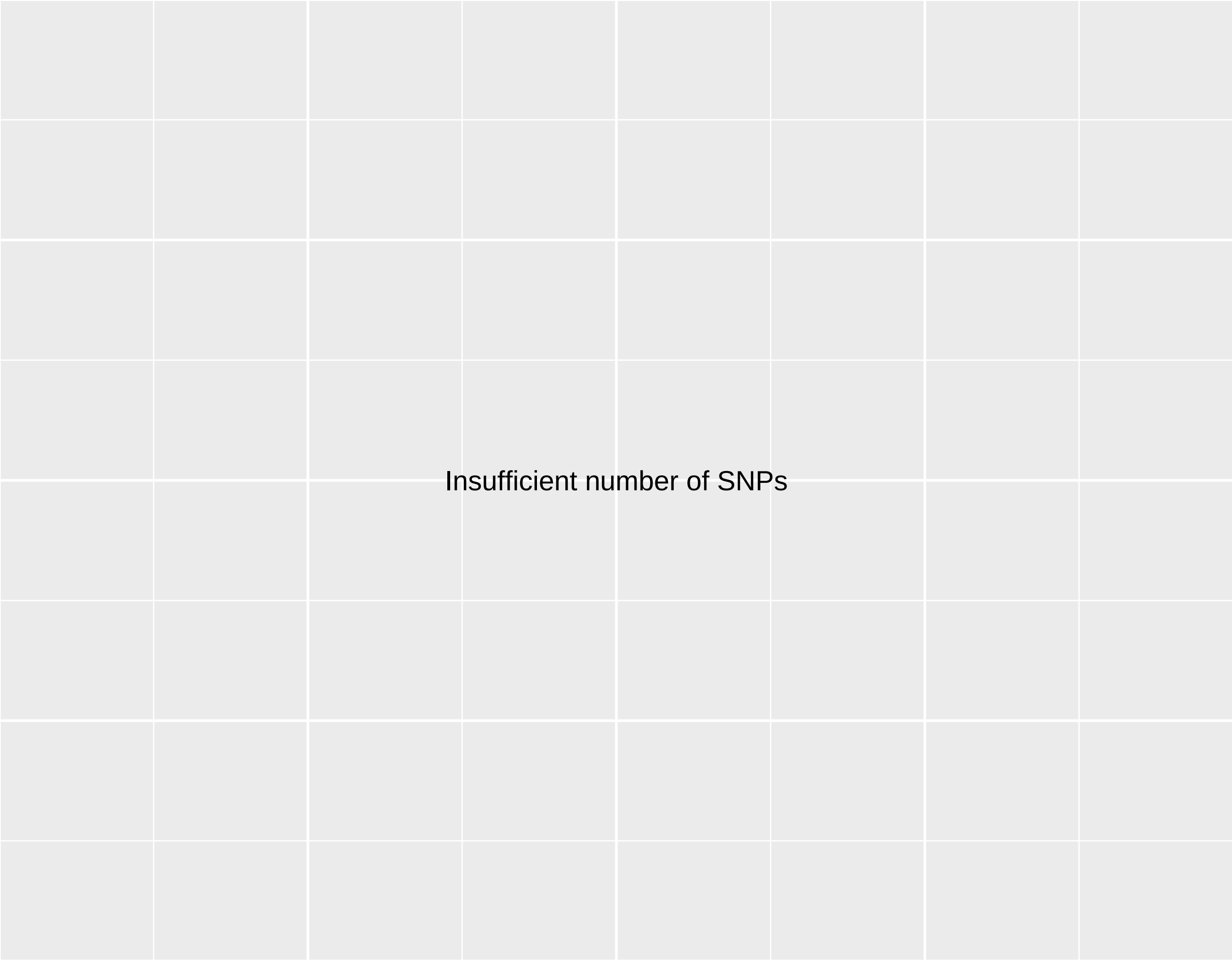
Insufficient number of SNPs

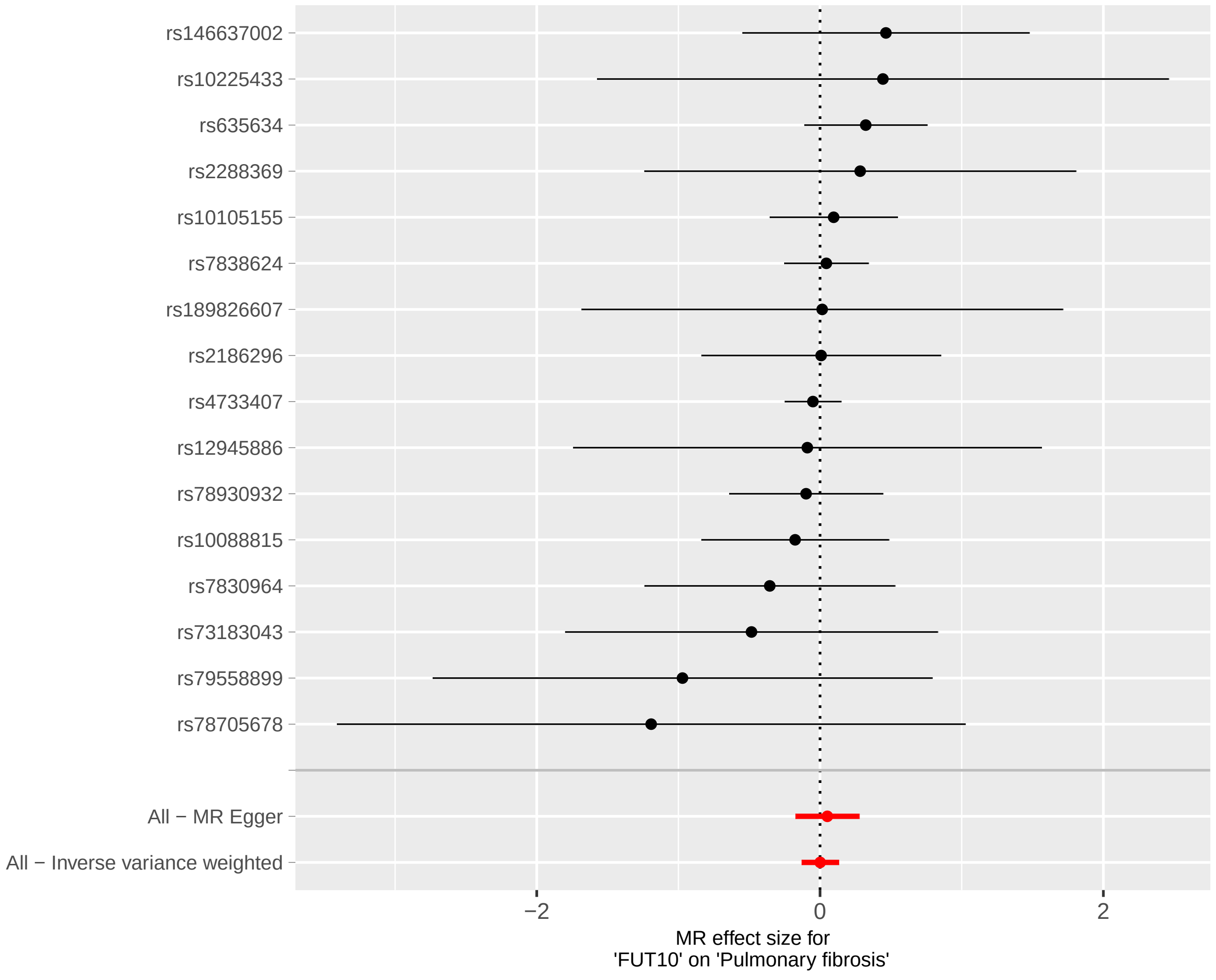

rs146637002
rs10225433
rs635634
rs2288369
rs10105155
rs7838624
rs189826607
rs2186296
rs4733407
rs12945886
rs78930932
rs10088815
rs7830964
rs73183043
rs79558899
rs78705678
All − MR Egger
All − Inverse variance weighted
−2
0
2
MR effect size for
'FUT10' on 'Pulmonary fibrosis'

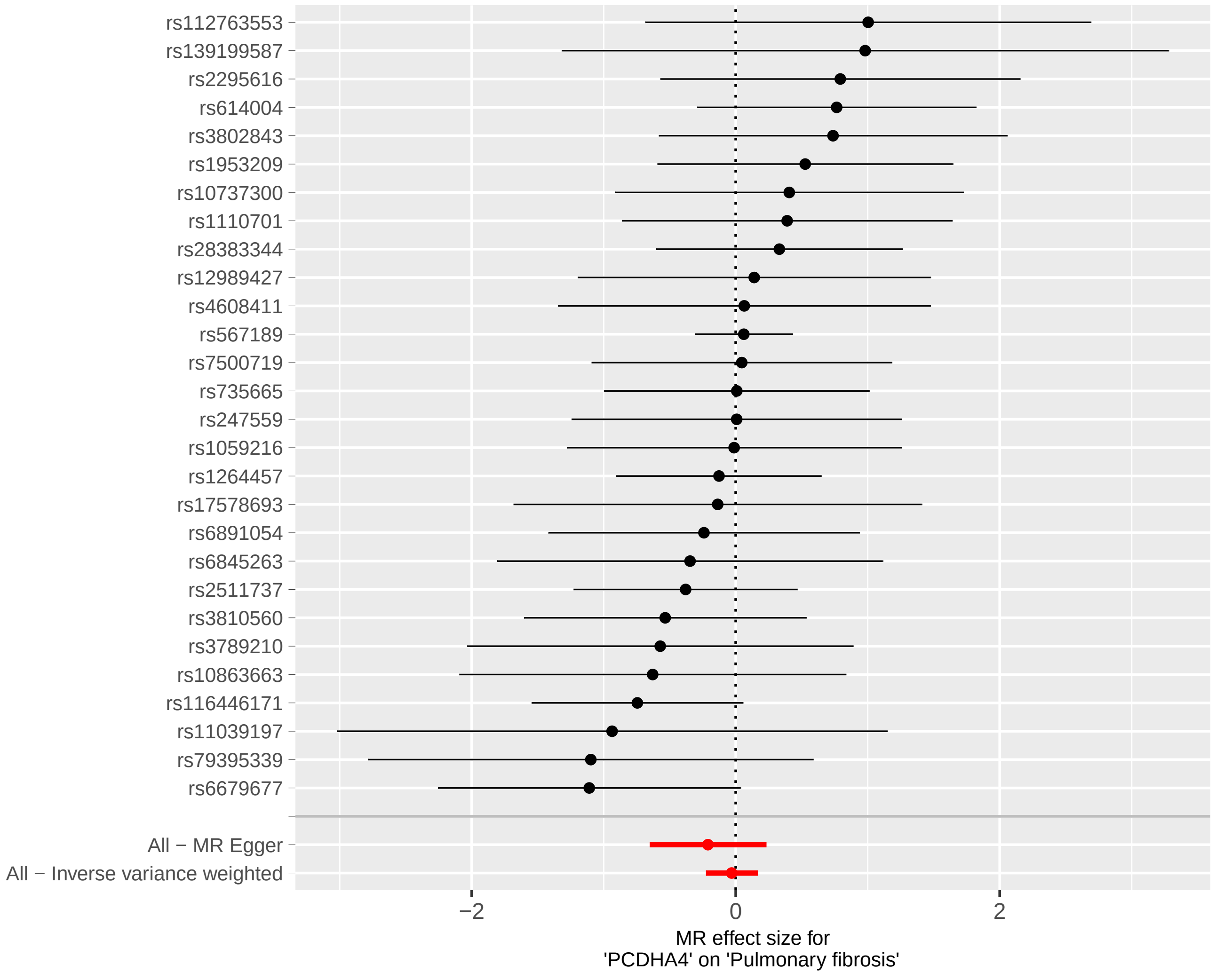
rs112763553
rs139199587
rs2295616
rs614004
rs3802843
rs1953209
rs10737300
rs1110701
rs28383344
rs12989427
rs4608411
rs567189
rs7500719
rs735665
rs247559
rs1059216
rs1264457
rs17578693
rs6891054
rs6845263
rs2511737
rs3810560
rs3789210
rs10863663
rs116446171
rs11039197
rs79395339
rs6679677
All − MR Egger
All − Inverse variance weighted
−2
0
2
MR effect size for
'PCDHA4' on 'Pulmonary fibrosis'

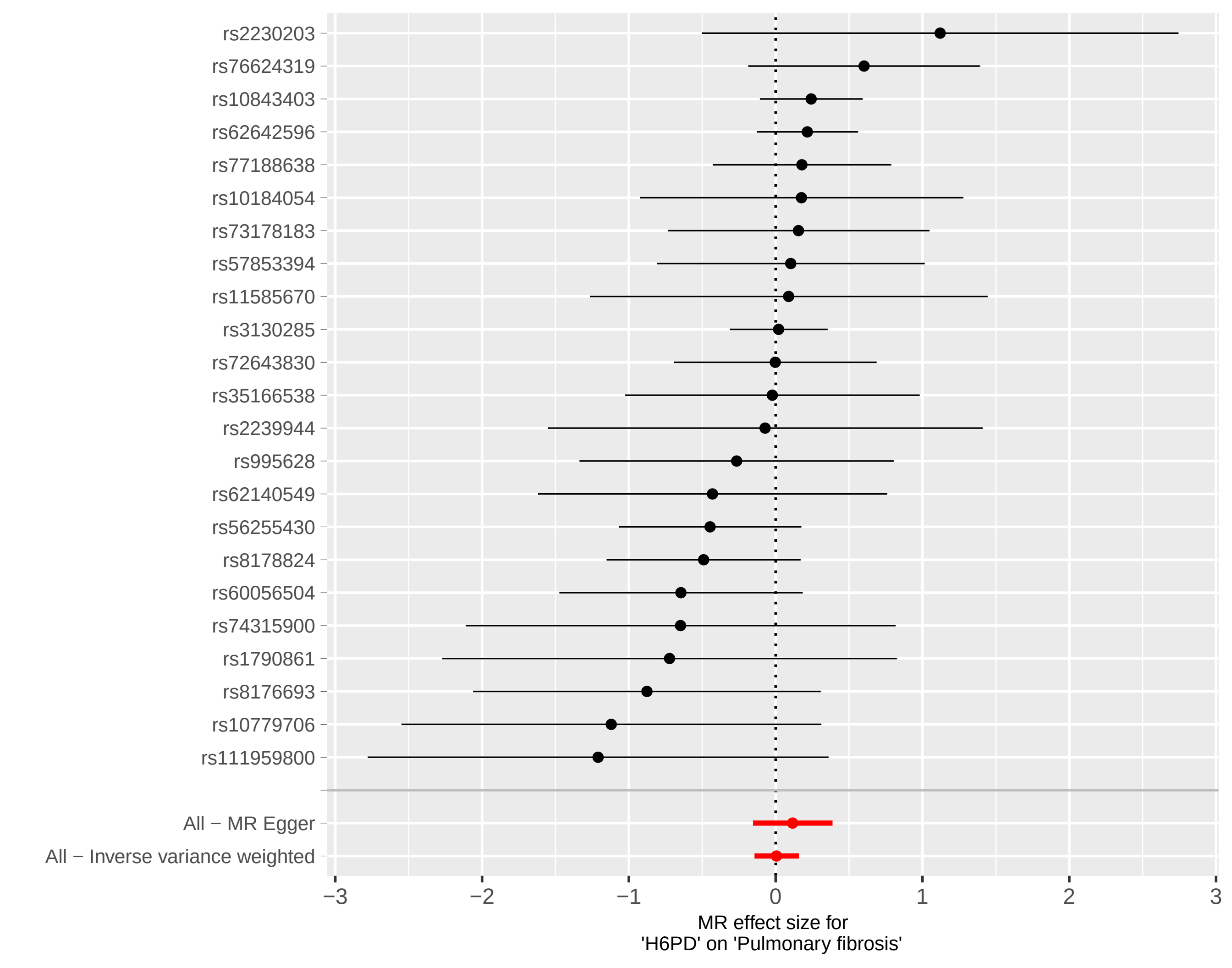
rs2230203
rs76624319
rs10843403
rs62642596
rs77188638
rs10184054
rs73178183
rs57853394
rs11585670
rs3130285
rs72643830
rs35166538
rs2239944
rs995628
rs62140549
rs56255430
rs8178824
rs60056504
rs74315900
rs1790861
rs8176693
rs10779706
rs111959800
All − MR Egger
All − Inverse variance weighted
−3
−2
−1
0
1
2
3
MR effect size for
'H6PD' on 'Pulmonary fibrosis'

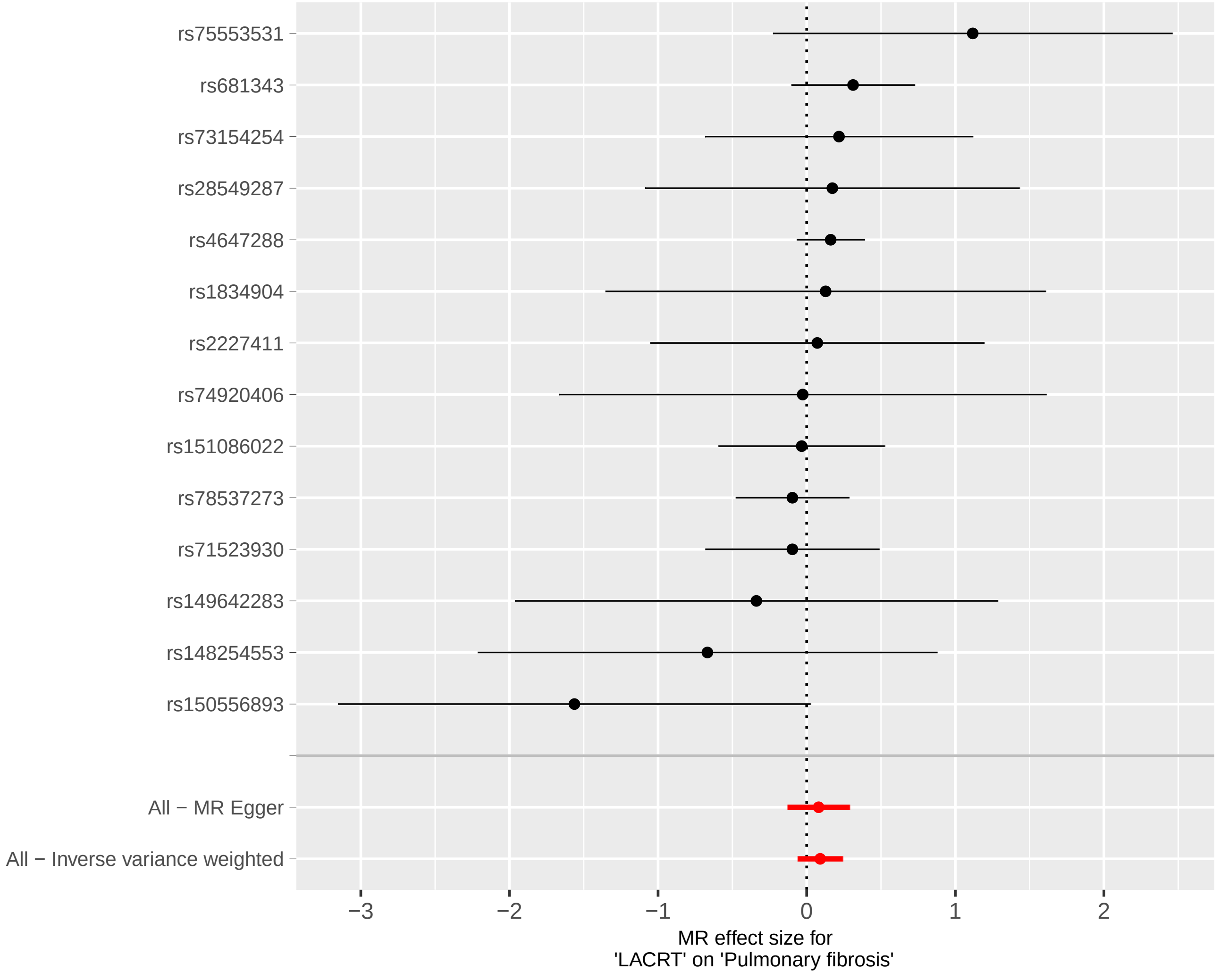
rs75553531
rs681343
rs73154254
rs28549287
rs4647288
rs1834904
rs2227411
rs74920406
rs151086022
rs78537273
rs71523930
rs149642283
rs148254553
rs150556893
All − MR Egger
All − Inverse variance weighted
−3
−2
−1
0
1
2
MR effect size for
'LACRT' on 'Pulmonary fibrosis'

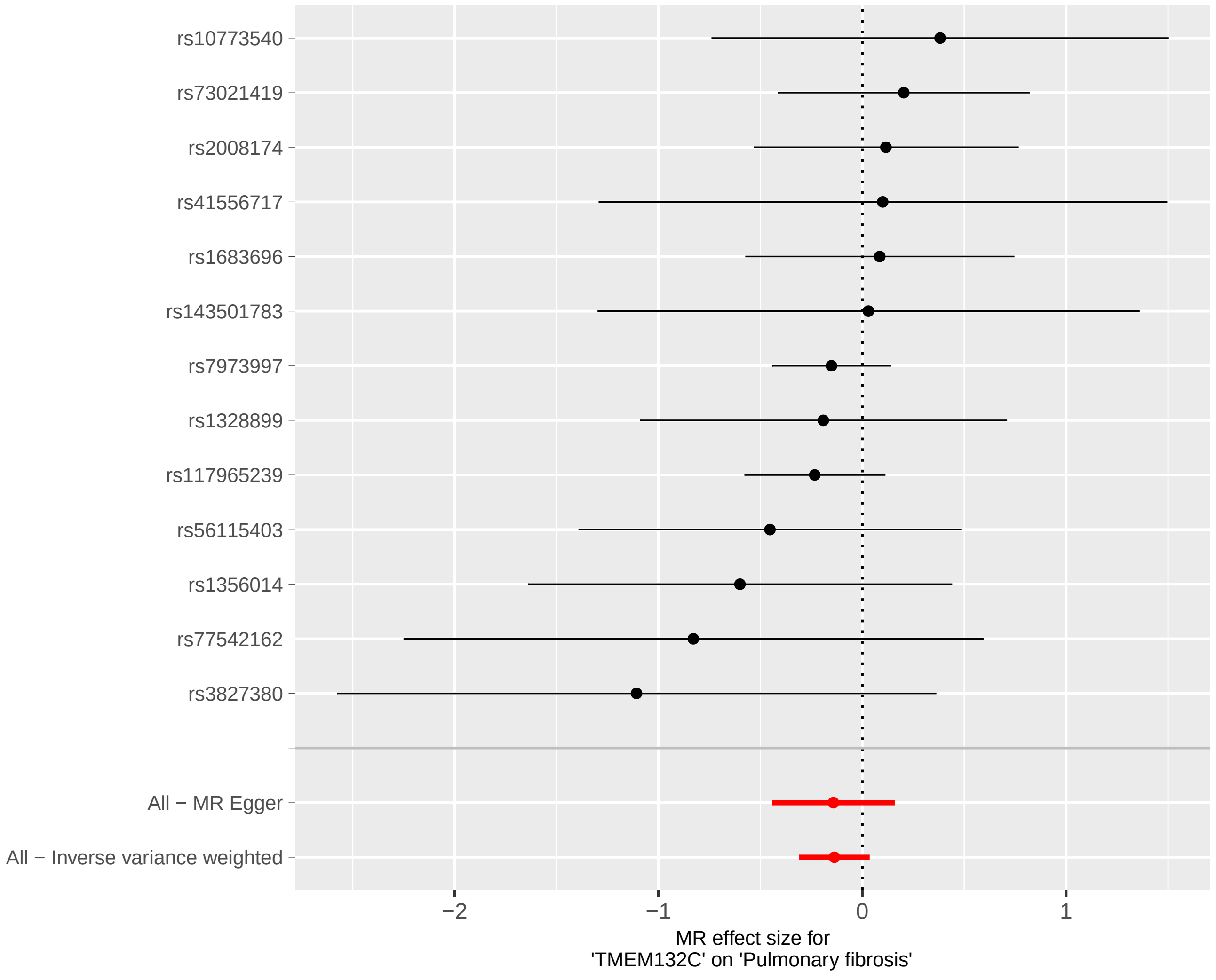
rs10773540
rs73021419
rs2008174
rs41556717
rs1683696
rs143501783
rs7973997
rs1328899
rs117965239
rs56115403
rs1356014
rs77542162
rs3827380
All − MR Egger
All − Inverse variance weighted
−2
−1
0
1
MR effect size for
'TMEM132C' on 'Pulmonary fibrosis'

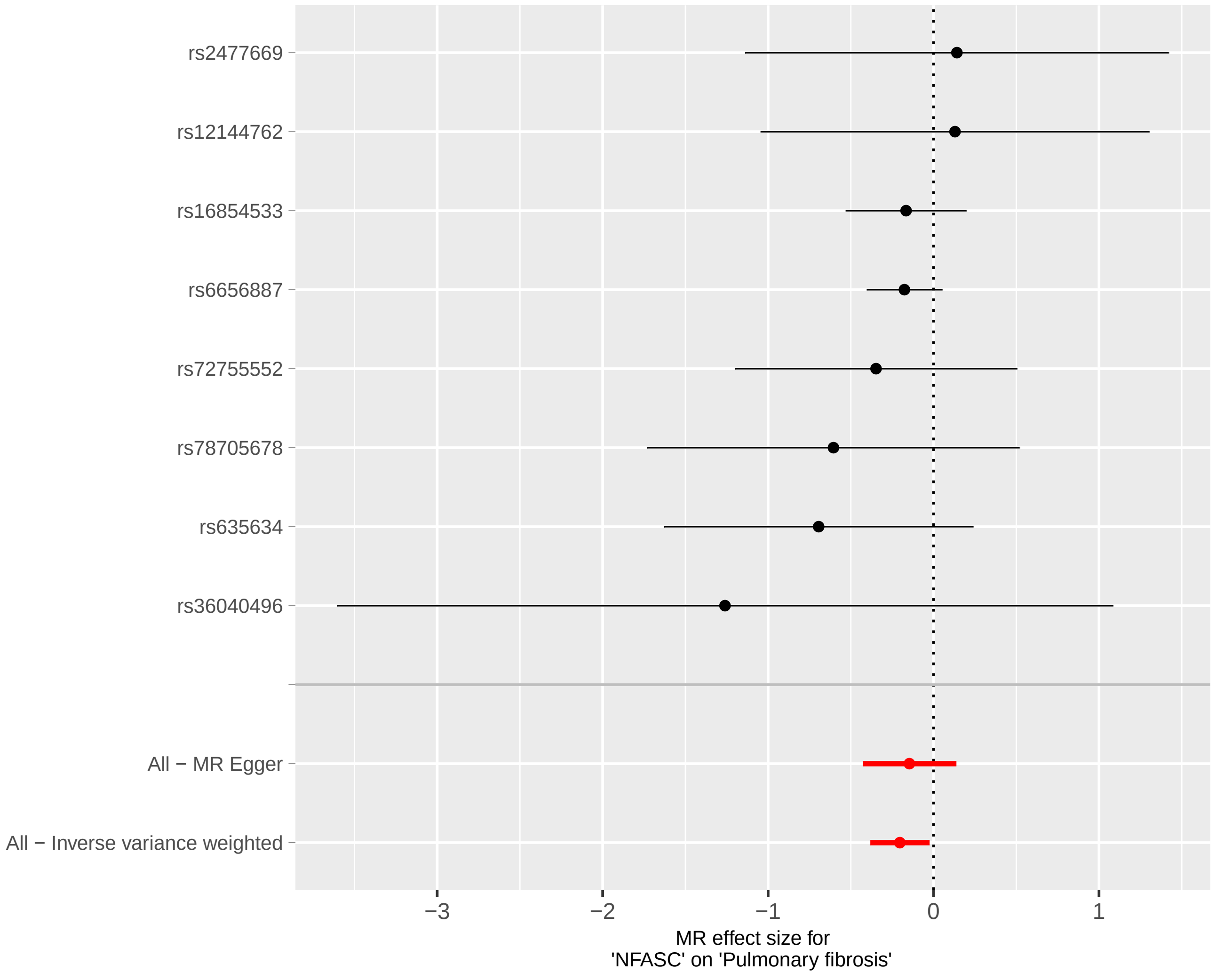

rs2477669
rs12144762
rs16854533
rs6656887
rs72755552
rs78705678
rs635634
rs36040496
All − MR Egger
All − Inverse variance weighted
−3
−2
−1
0
1
MR effect size for
'NFASC' on 'Pulmonary fibrosis'

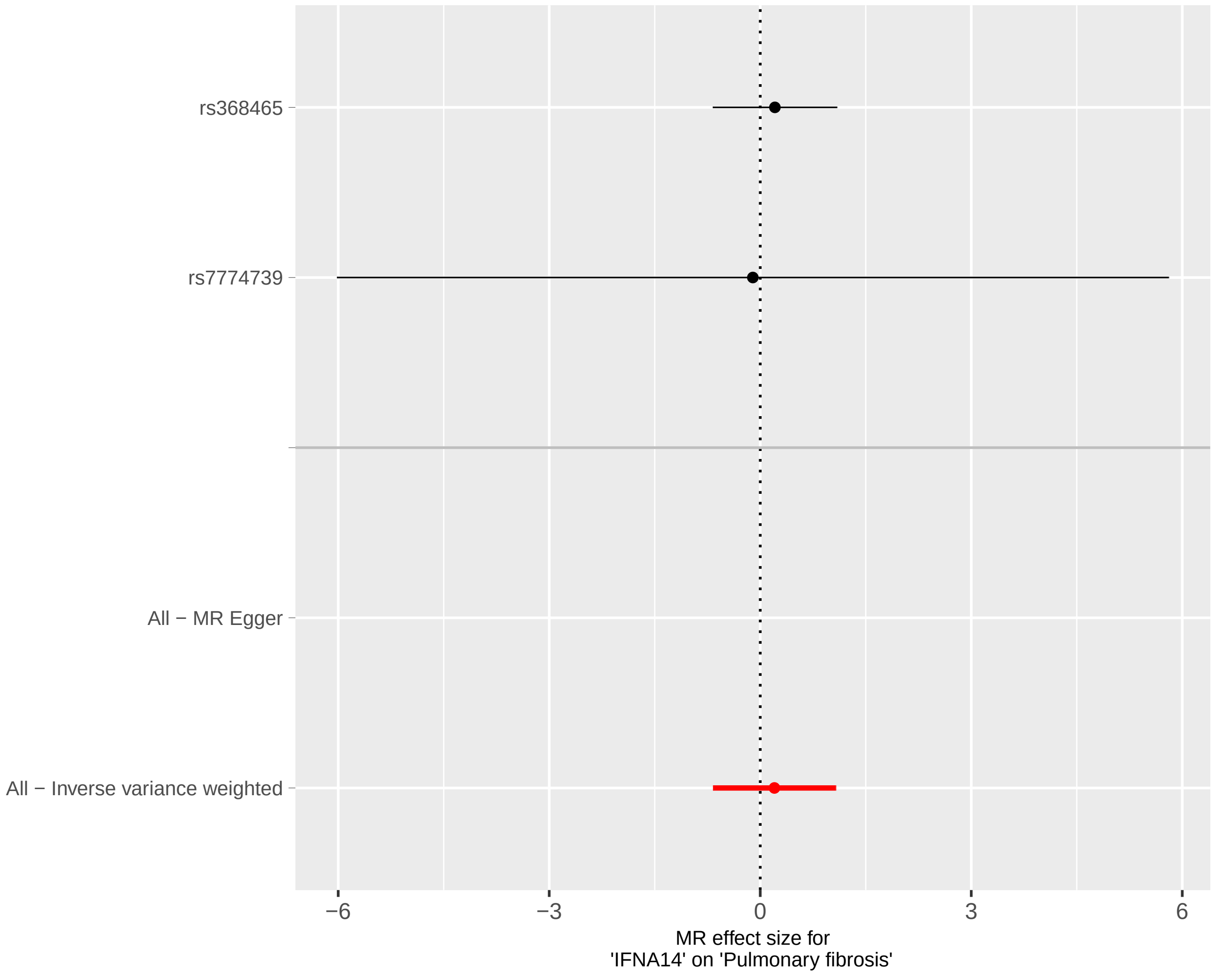

rs368465
rs7774739
All − MR Egger
All − Inverse variance weighted
−6
−3
0
3
6
MR effect size for
'IFNA14' on 'Pulmonary fibrosis'

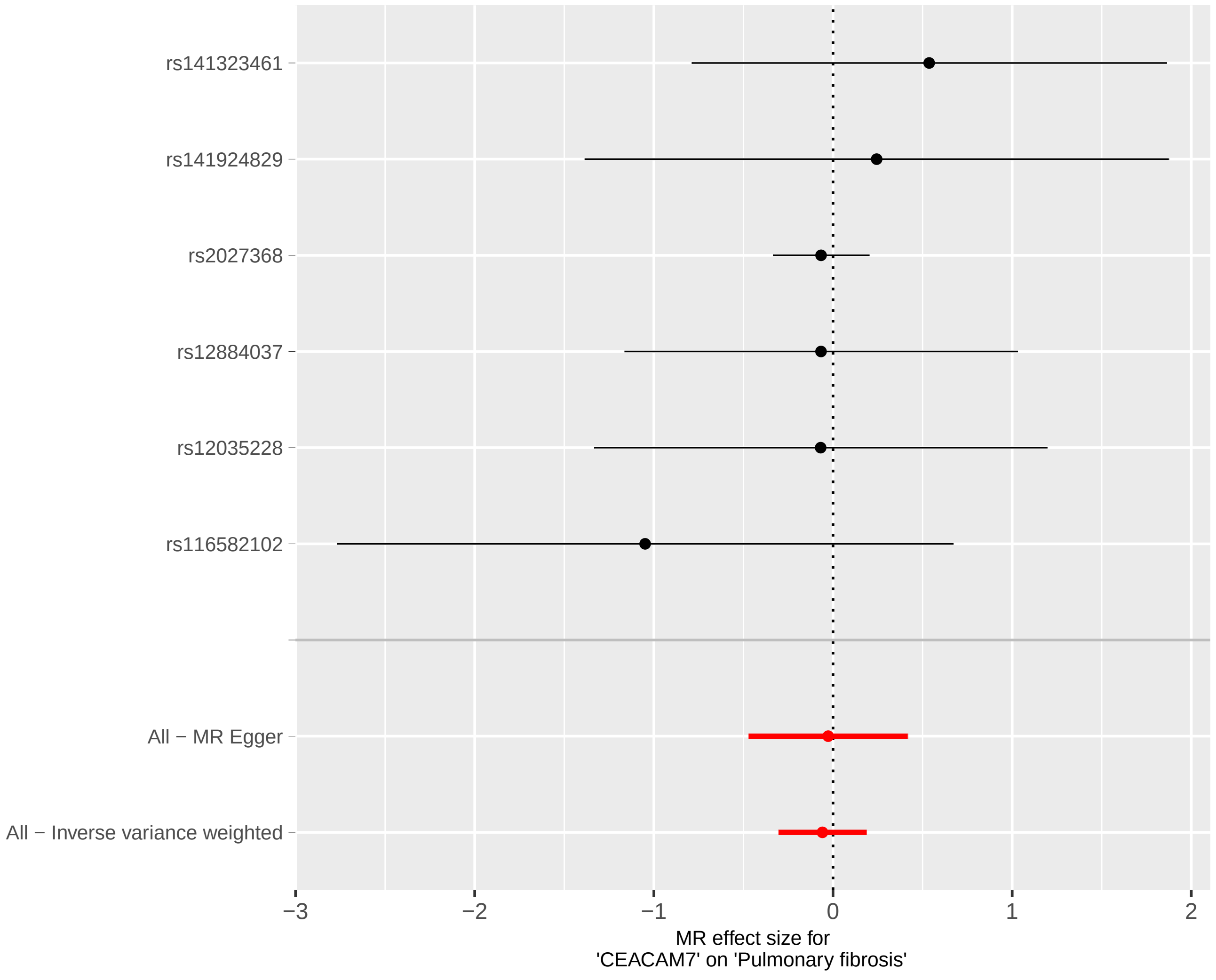

rs141323461
rs141924829
rs2027368
rs12884037
rs12035228
rs116582102
All − MR Egger
All − Inverse variance weighted
−3
−2
−1
0
1
2
MR effect size for
'CEACAM7' on 'Pulmonary fibrosis'

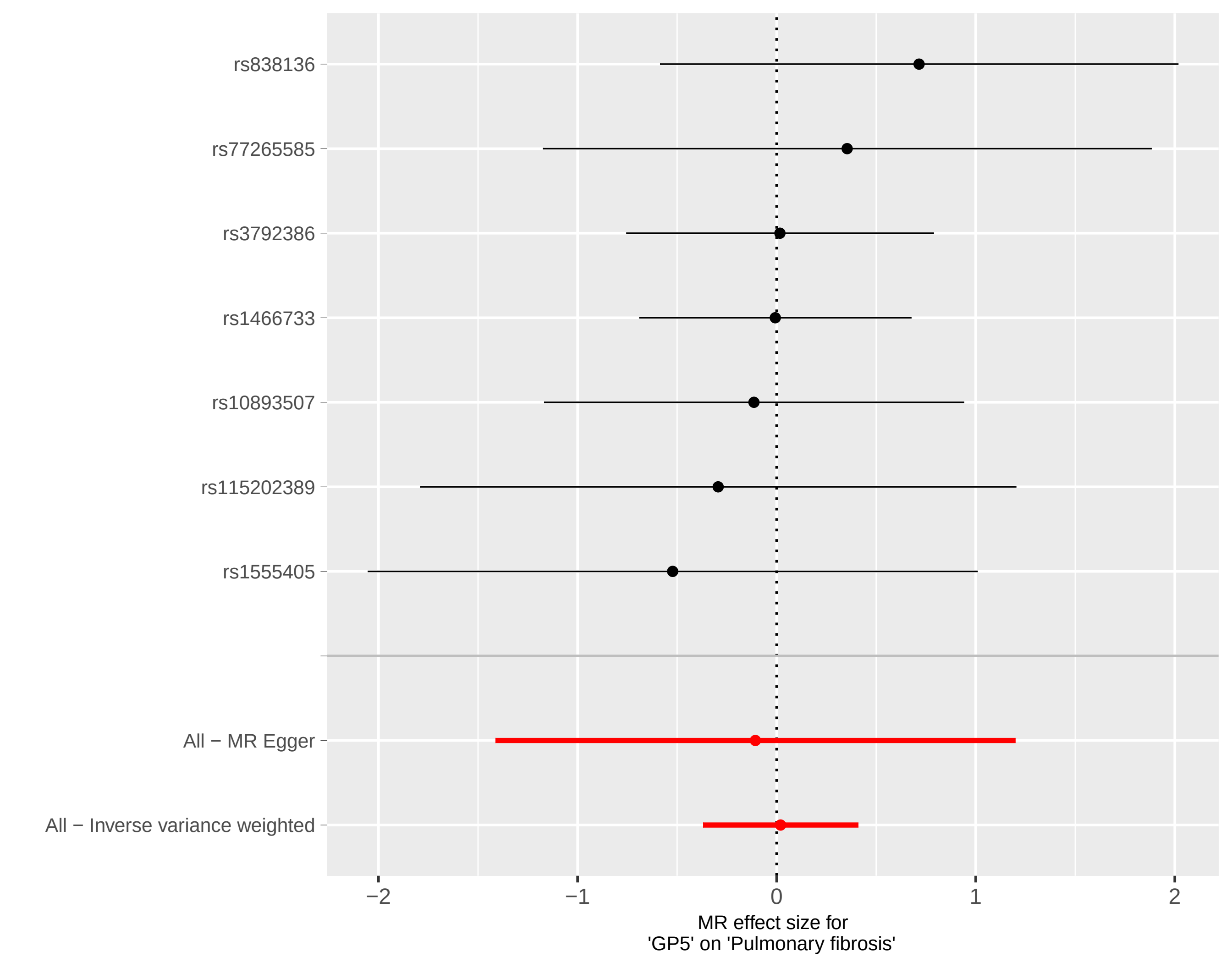

rs838136
rs77265585
rs3792386
rs1466733
rs10893507
rs115202389
rs1555405
All − MR Egger
All − Inverse variance weighted
−2
−1
0
1
2
MR effect size for
'GP5' on 'Pulmonary fibrosis'

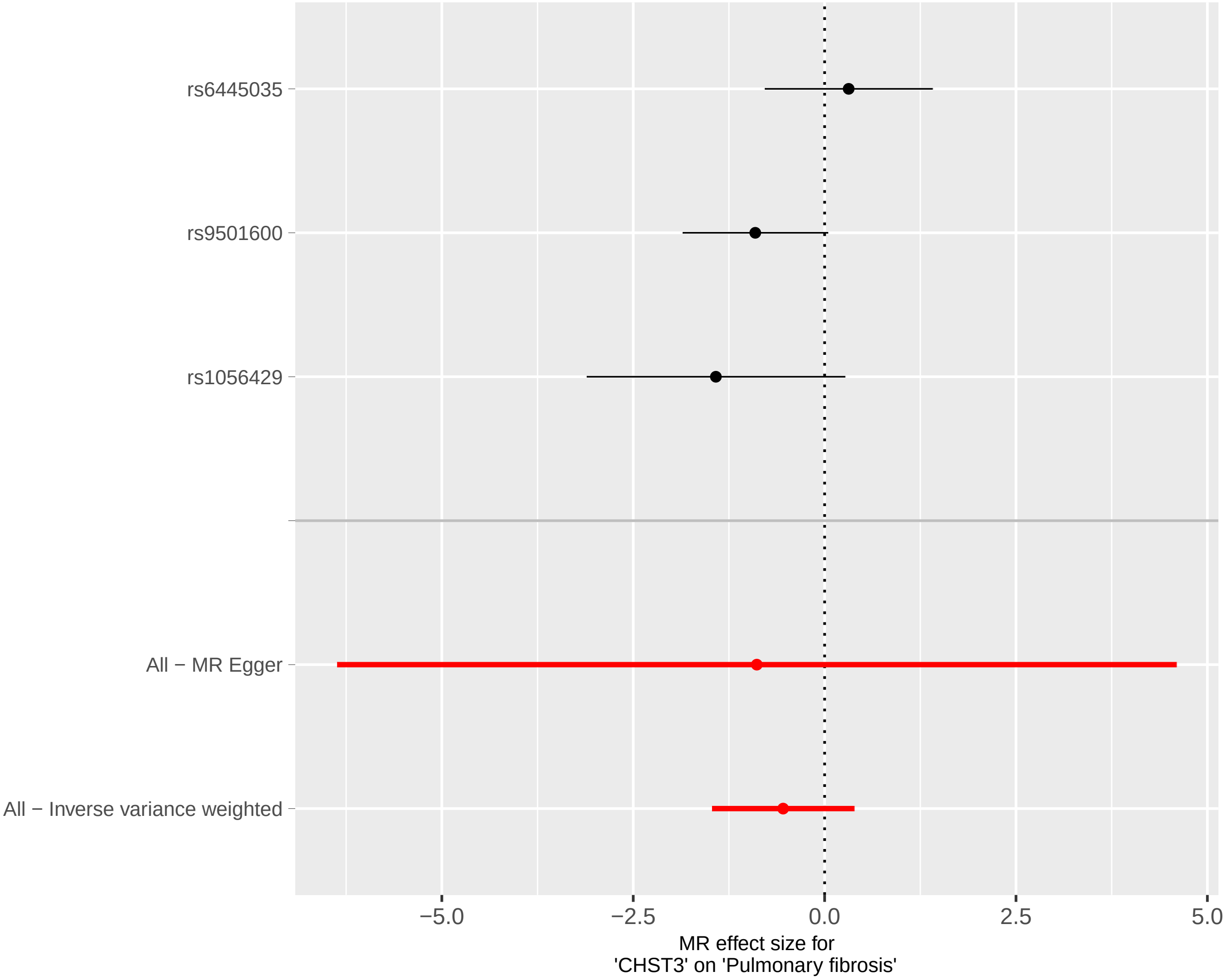

rs6445035
rs9501600
rs1056429
All − MR Egger
All − Inverse variance weighted
−5.0
−2.5
0.0
2.5
5.0
MR effect size for
'CHST3' on 'Pulmonary fibrosis'

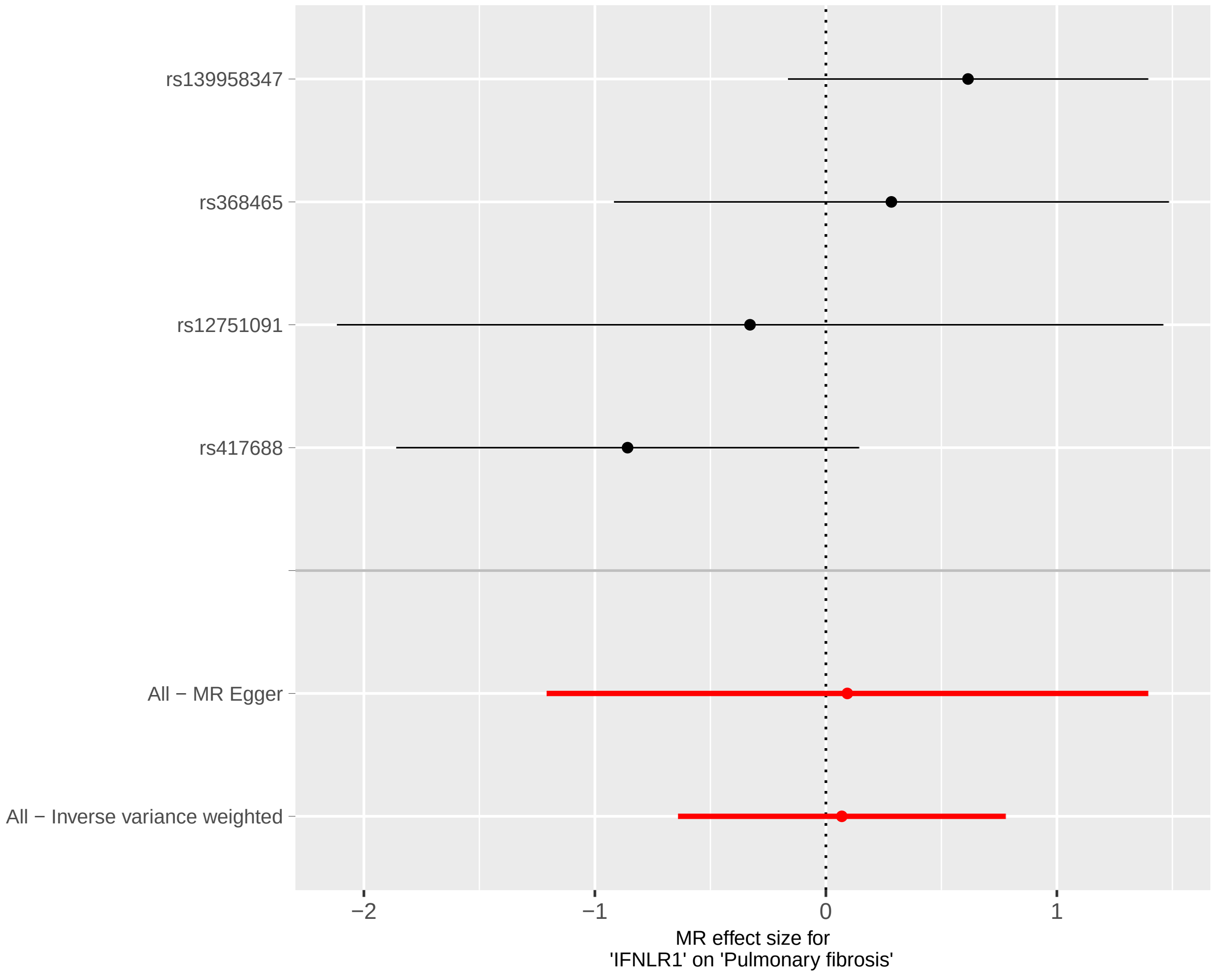

rs139958347
rs368465
rs12751091
rs417688
All − MR Egger
All − Inverse variance weighted
−2
−1
0
1
MR effect size for
'IFNLR1' on 'Pulmonary fibrosis'

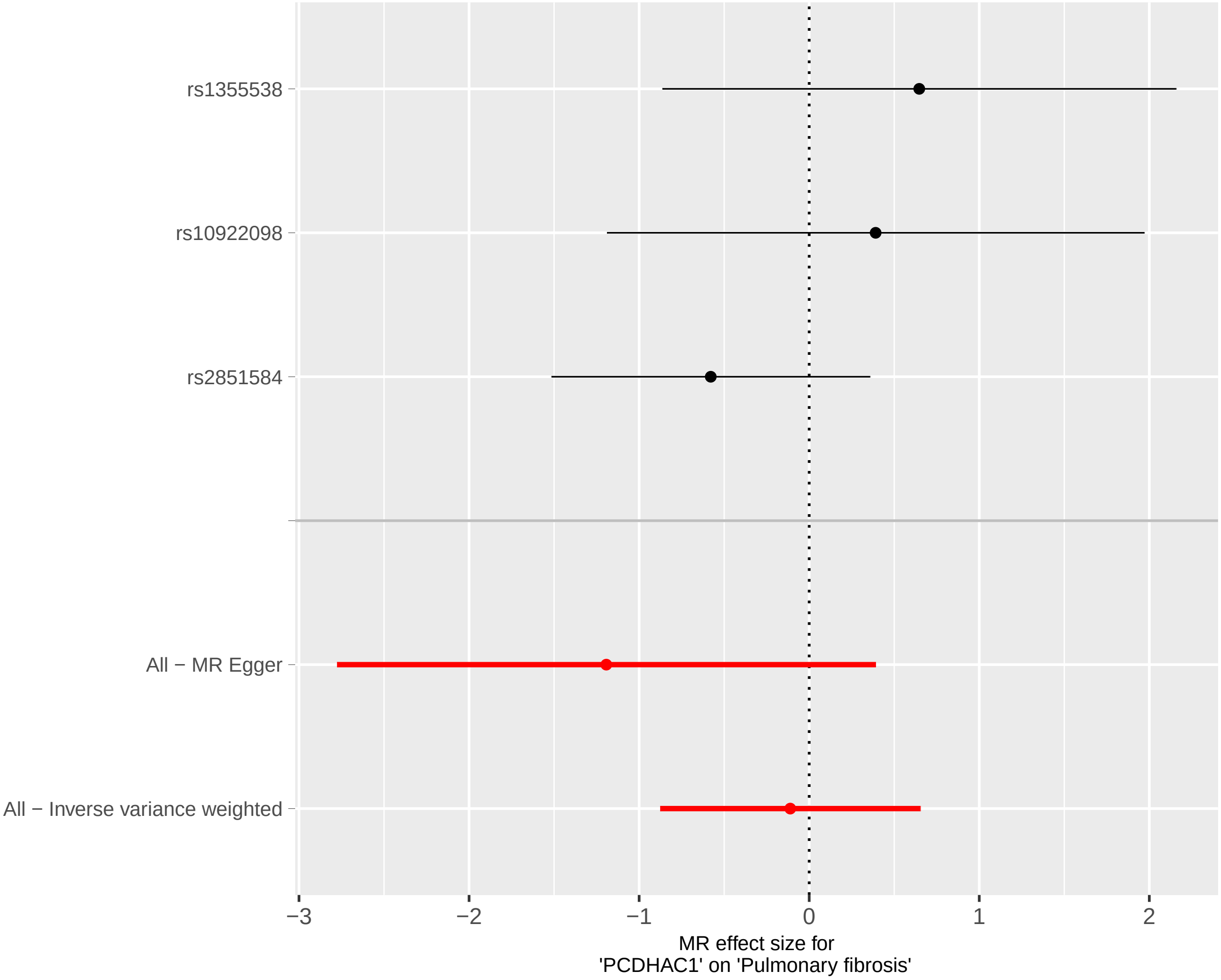

rs1355538
rs10922098
rs2851584
All − MR Egger
All − Inverse variance weighted
−3
−2
−1
0
1
2
MR effect size for
'PCDHAC1' on 'Pulmonary fibrosis'

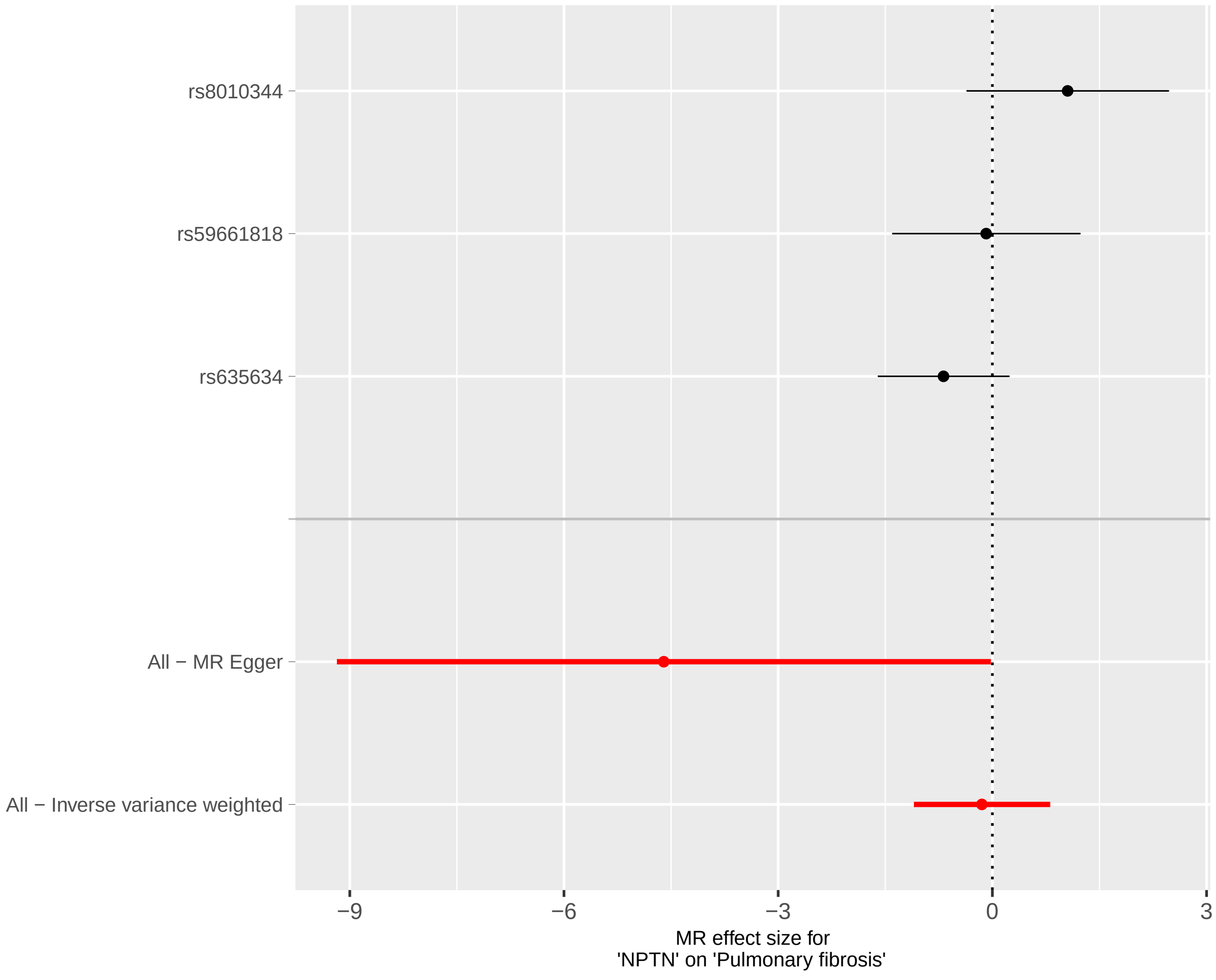
rs8010344
rs59661818
rs635634
All − MR Egger
All − Inverse variance weighted
−9
−6
−3
0
3
MR effect size for
'NPTN' on 'Pulmonary fibrosis'

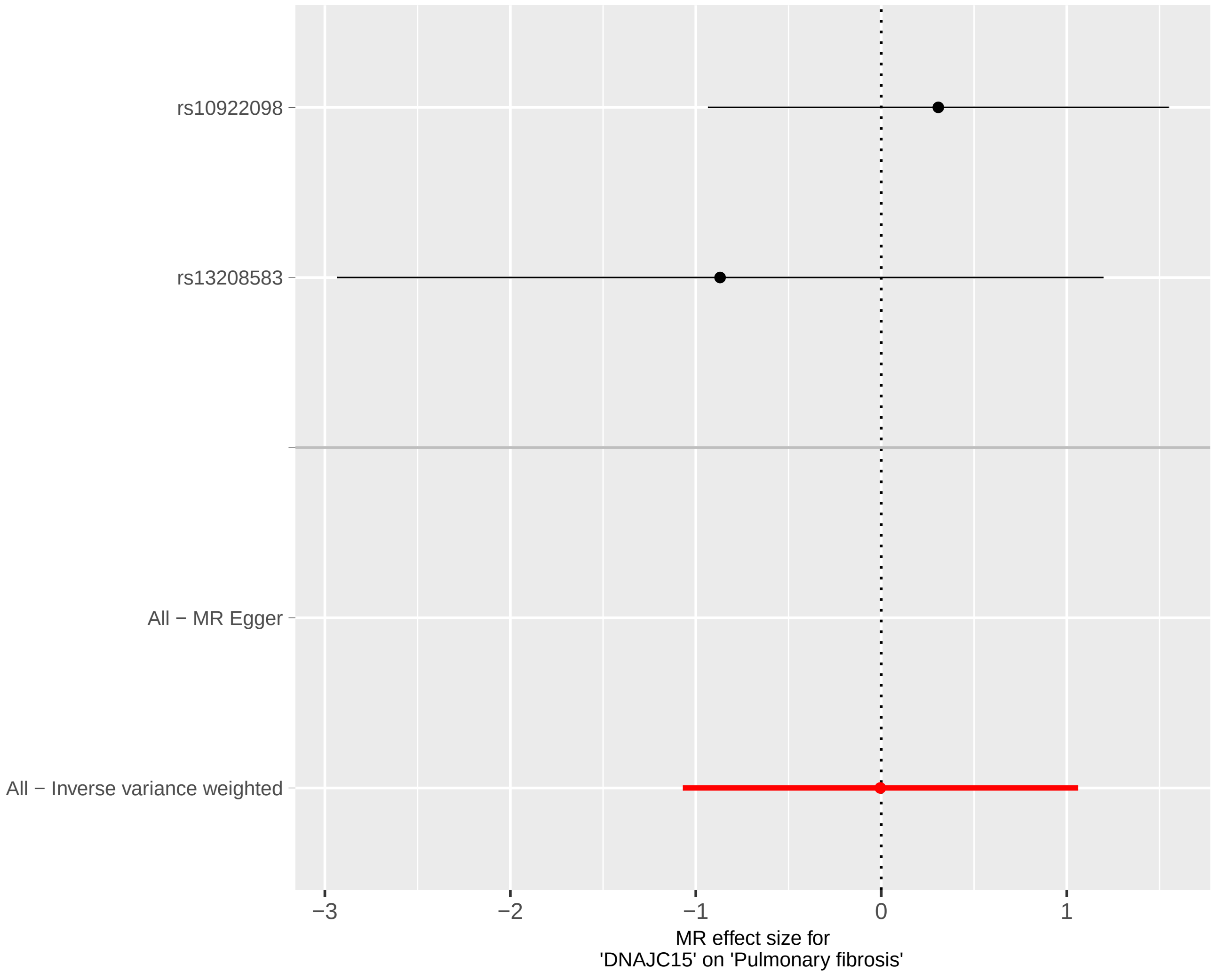

rs10922098
rs13208583
All − MR Egger
All − Inverse variance weighted
−3
−2
−1
0
1
MR effect size for
'DNAJC15' on 'Pulmonary fibrosis'

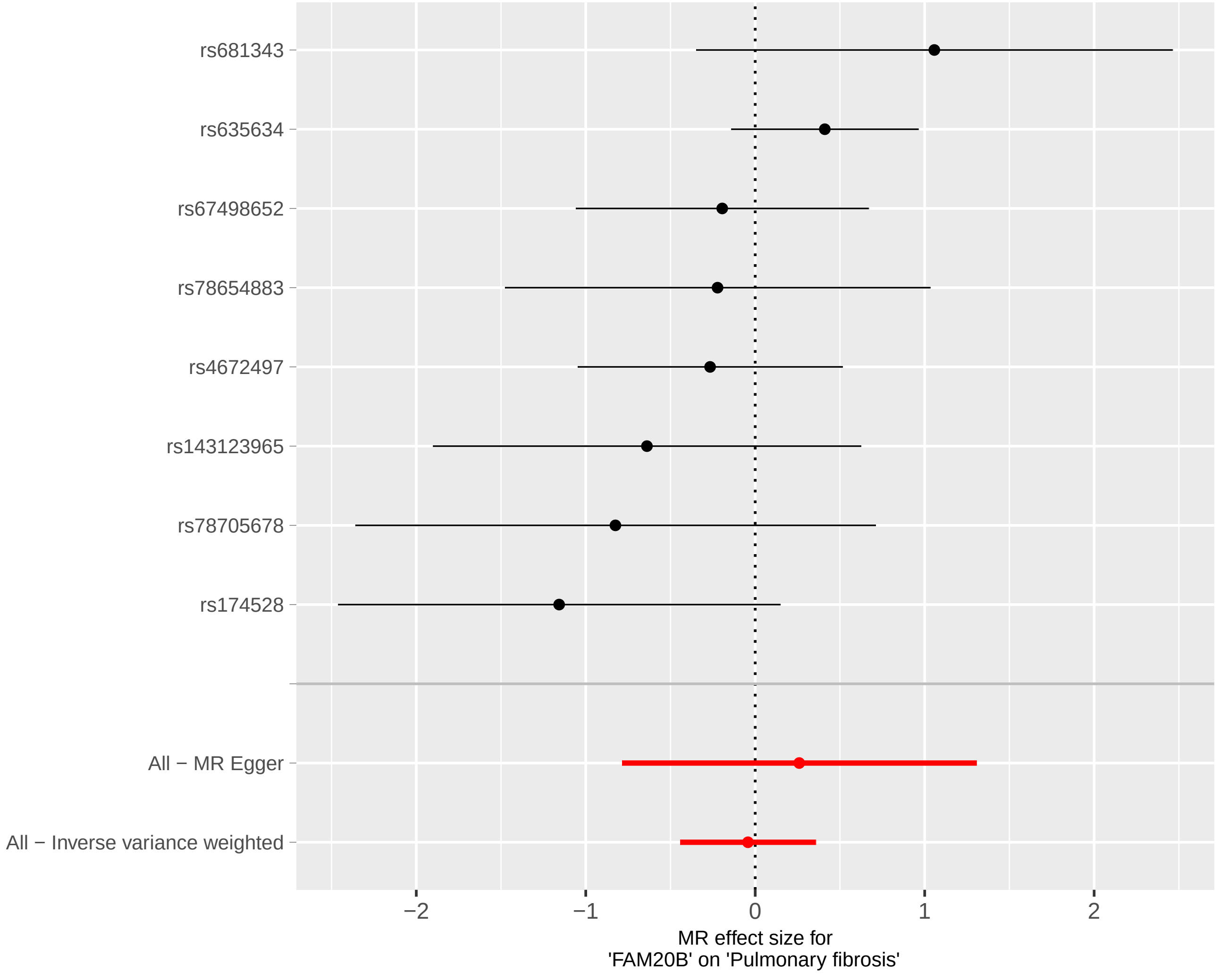
rs681343
rs635634
rs67498652
rs78654883
rs4672497
rs143123965
rs78705678
rs174528
All − MR Egger
All − Inverse variance weighted
−2
−1
0
1
2
MR effect size for
'FAM20B' on 'Pulmonary fibrosis'

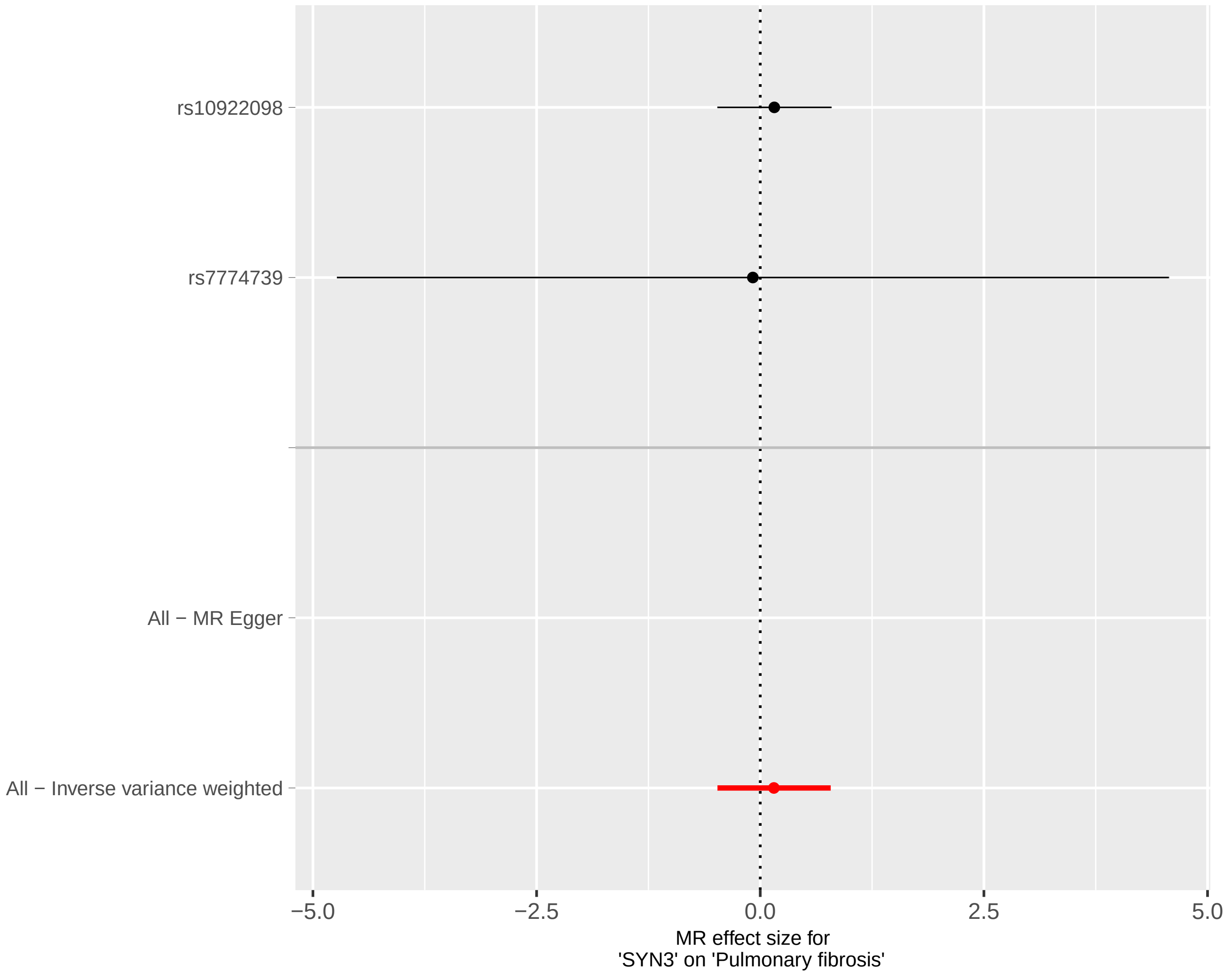

rs10922098
rs7774739
All − MR Egger
All − Inverse variance weighted
−5.0
−2.5
0.0
2.5
5.0
MR effect size for
'SYN3' on 'Pulmonary fibrosis'

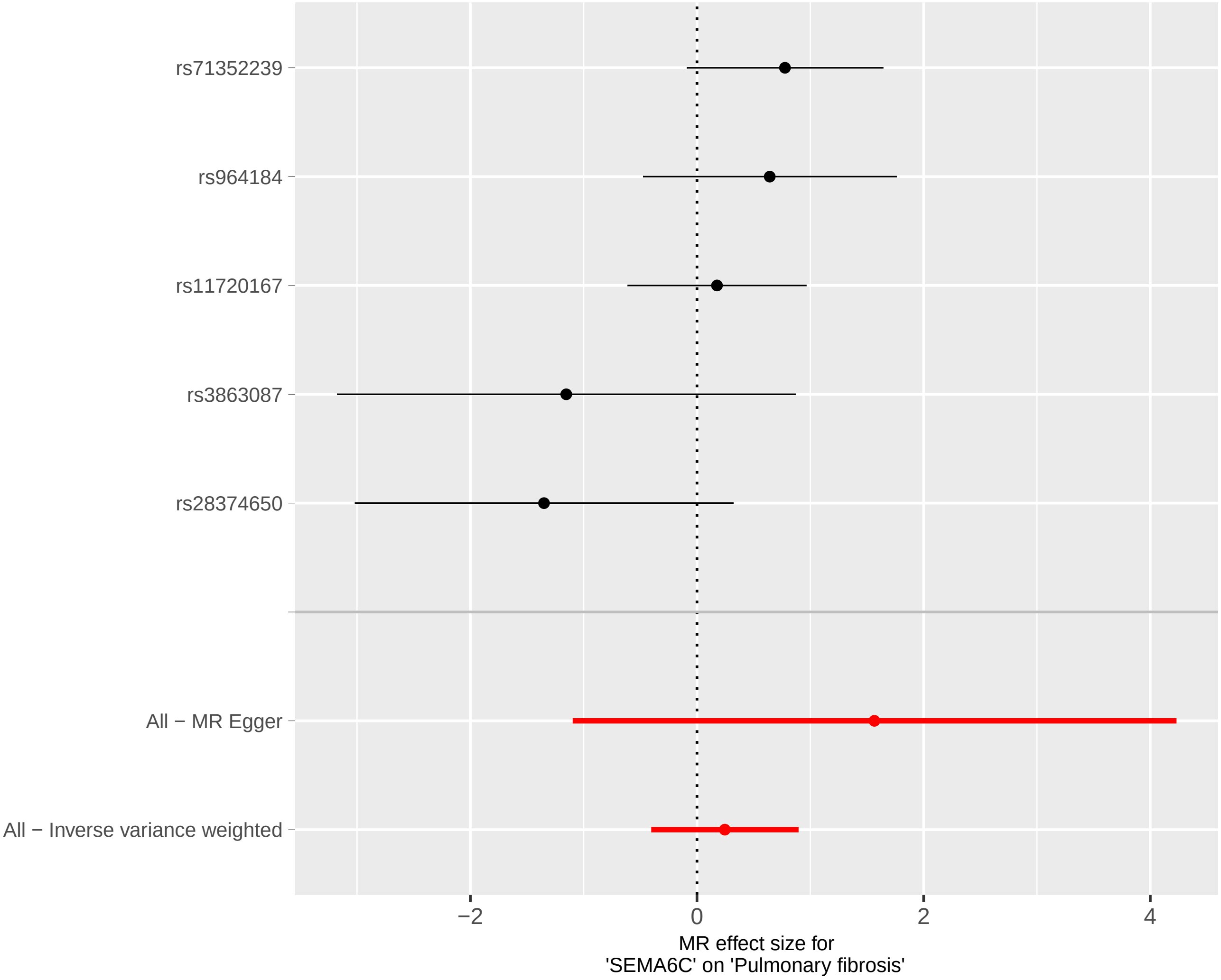
rs71352239
rs964184
rs11720167
rs3863087
rs28374650
All − MR Egger
All − Inverse variance weighted
−2
0
2
4
MR effect size for
'SEMA6C' on 'Pulmonary fibrosis'

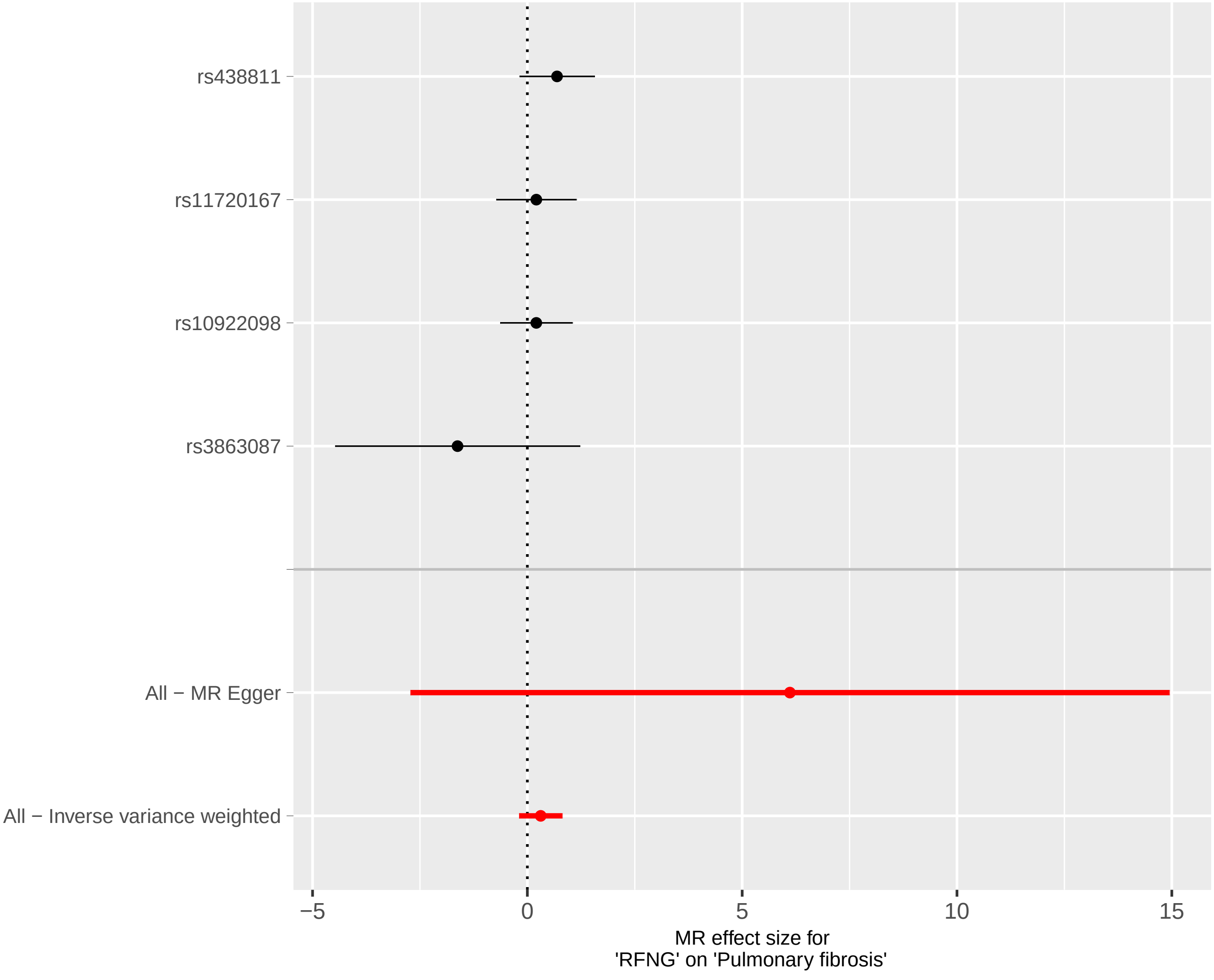
rs438811
rs11720167
rs10922098
rs3863087
All − MR Egger
All − Inverse variance weighted
−5
0
5
10
15
MR effect size for
'RFNG' on 'Pulmonary fibrosis'

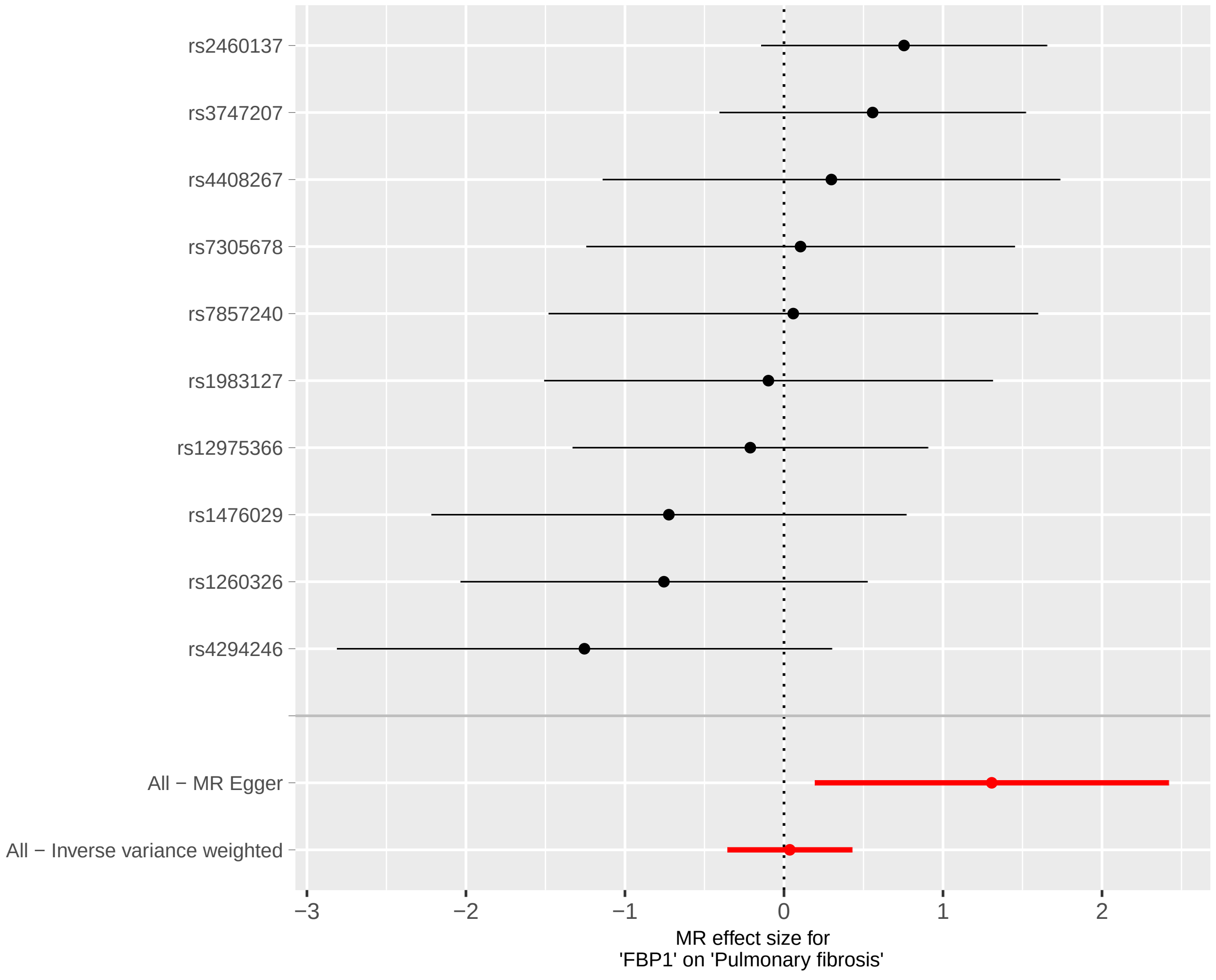

rs2460137
rs3747207
rs4408267
rs7305678
rs7857240
rs1983127
rs12975366
rs1476029
rs1260326
rs4294246
All − MR Egger
All − Inverse variance weighted
−3
−2
−1
0
1
2
MR effect size for
'FBP1' on 'Pulmonary fibrosis'

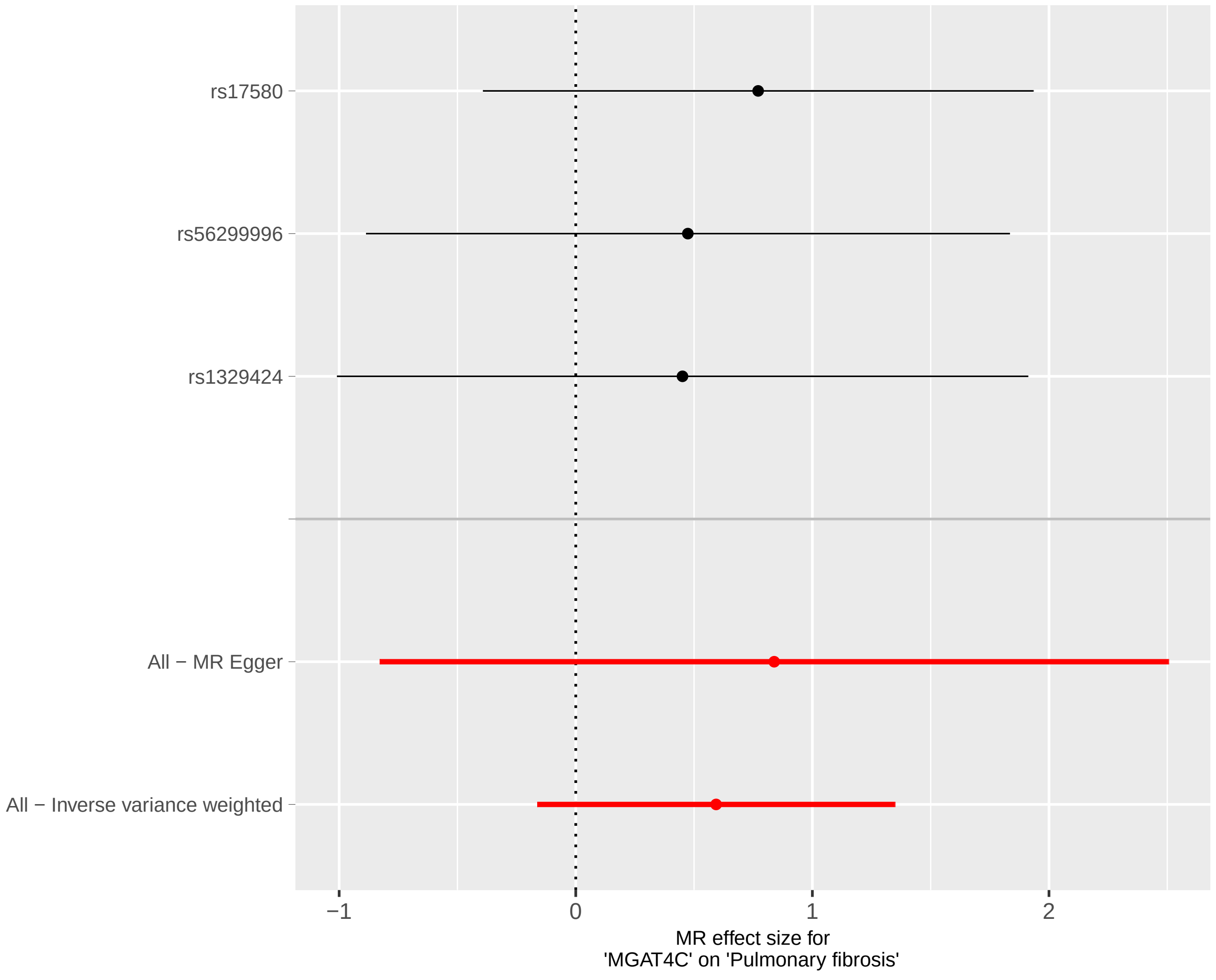
rs17580
rs56299996
rs1329424
All − MR Egger
All − Inverse variance weighted
−1
0
1
2
MR effect size for
'MGAT4C' on 'Pulmonary fibrosis'

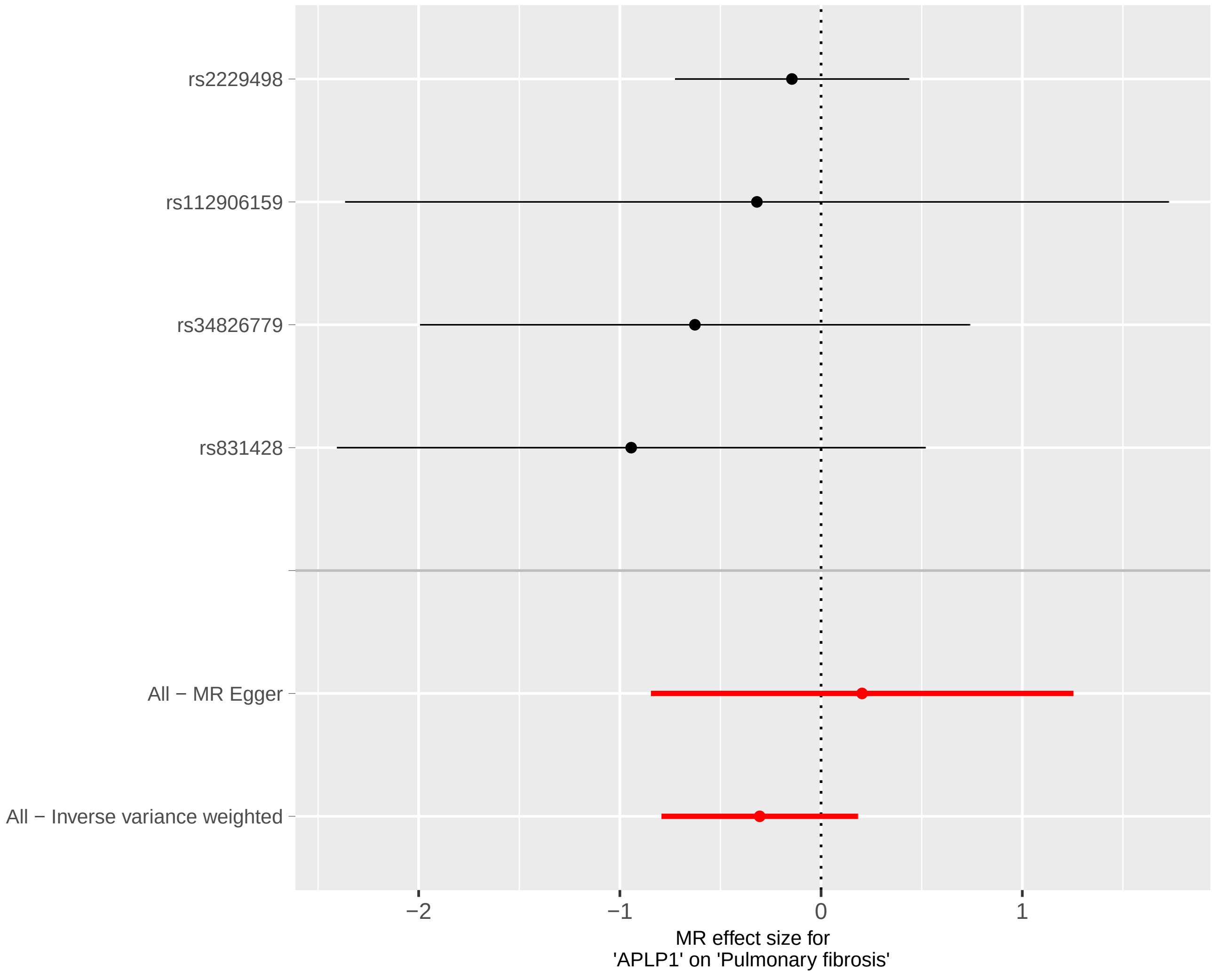
rs2229498
rs112906159
rs34826779
rs831428
All − MR Egger
All − Inverse variance weighted
−2
−1
0
1
MR effect size for
'APLP1' on 'Pulmonary fibrosis'

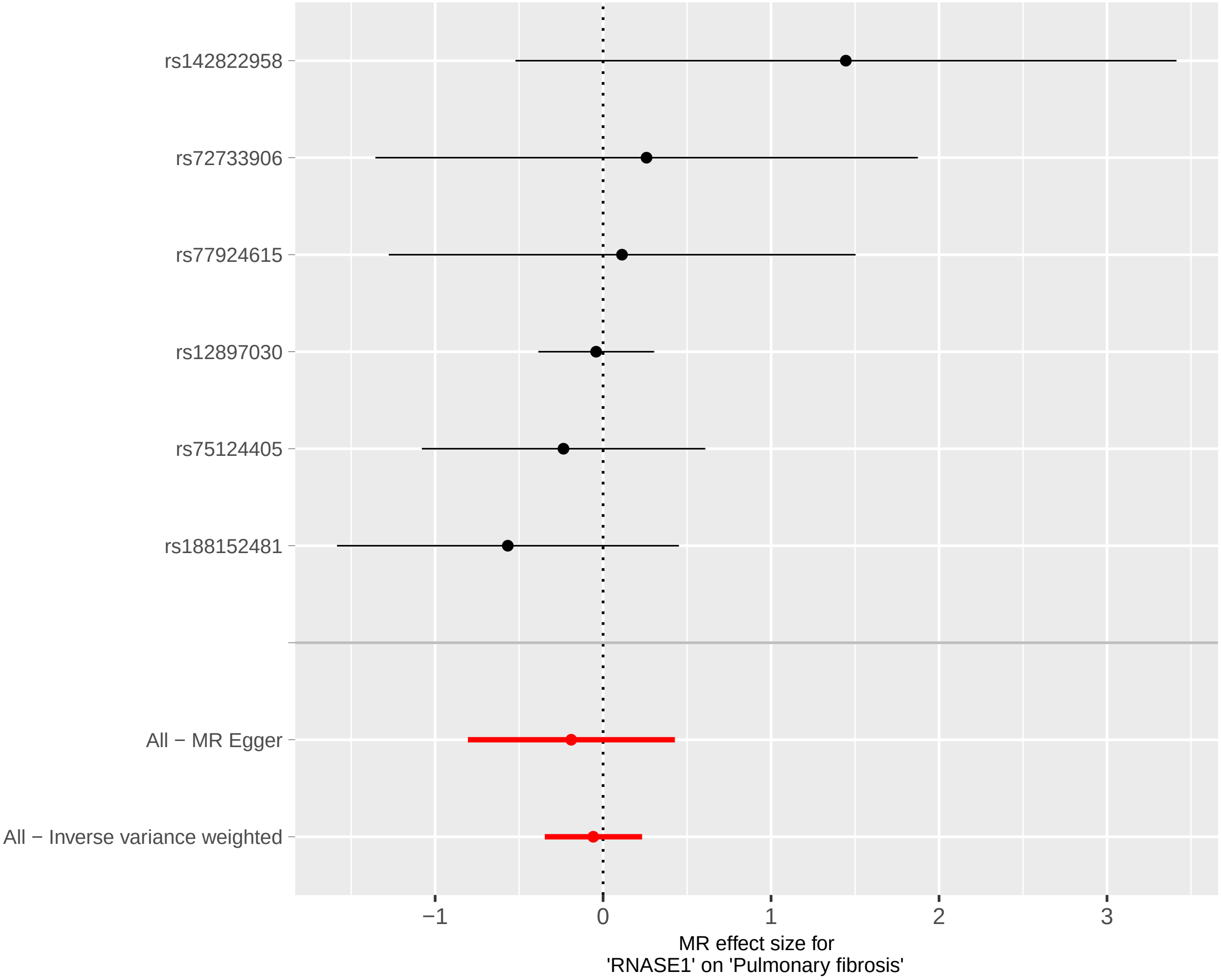

rs142822958
rs72733906
rs77924615
rs12897030
rs75124405
rs188152481
All − MR Egger
All − Inverse variance weighted
−1
0
1
2
3
MR effect size for
'RNASE1' on 'Pulmonary fibrosis'

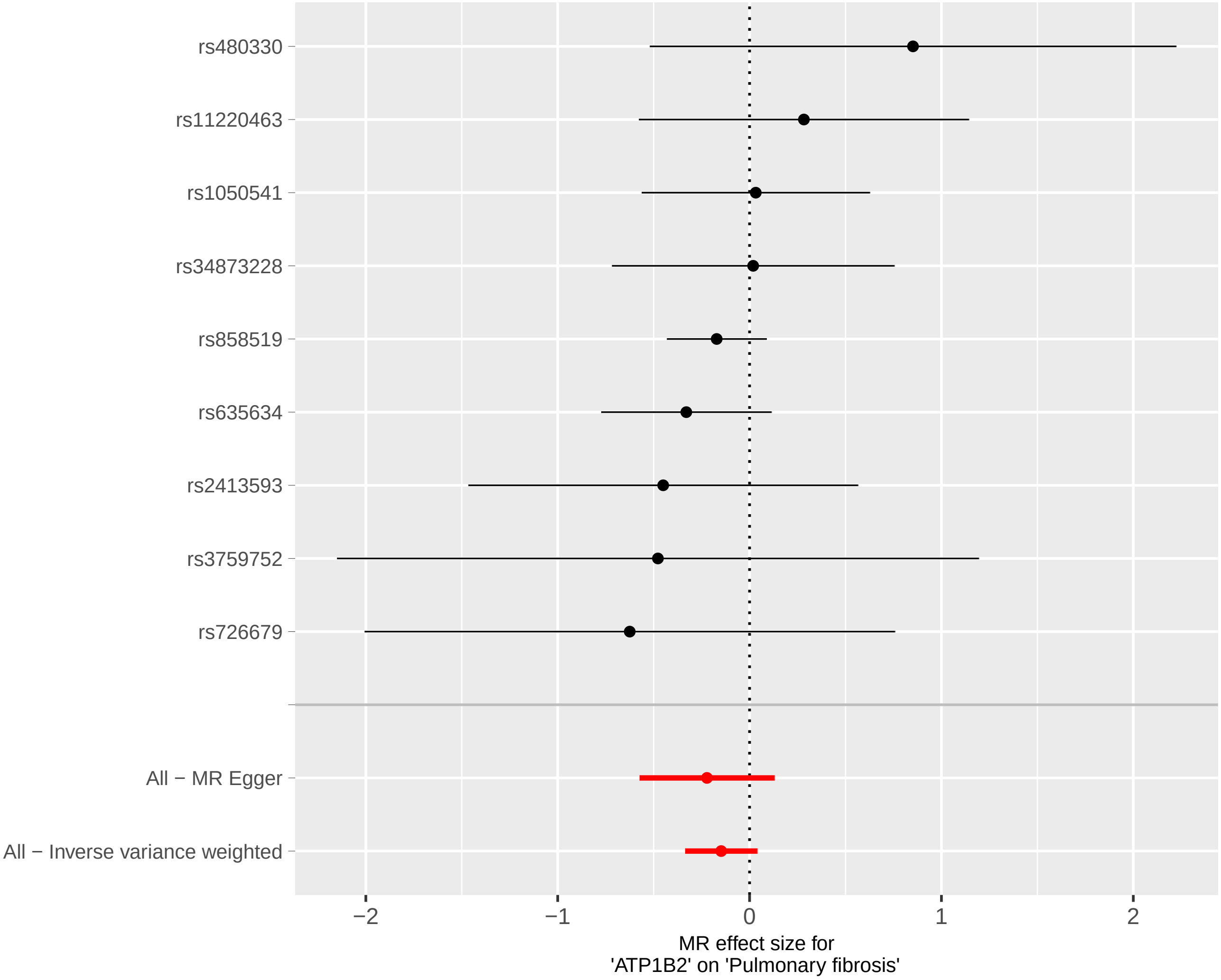

rs480330
rs11220463
rs1050541
rs34873228
rs858519
rs635634
rs2413593
rs3759752
rs726679
All − MR Egger
All − Inverse variance weighted
−2
−1
0
1
2
MR effect size for
'ATP1B2' on 'Pulmonary fibrosis'

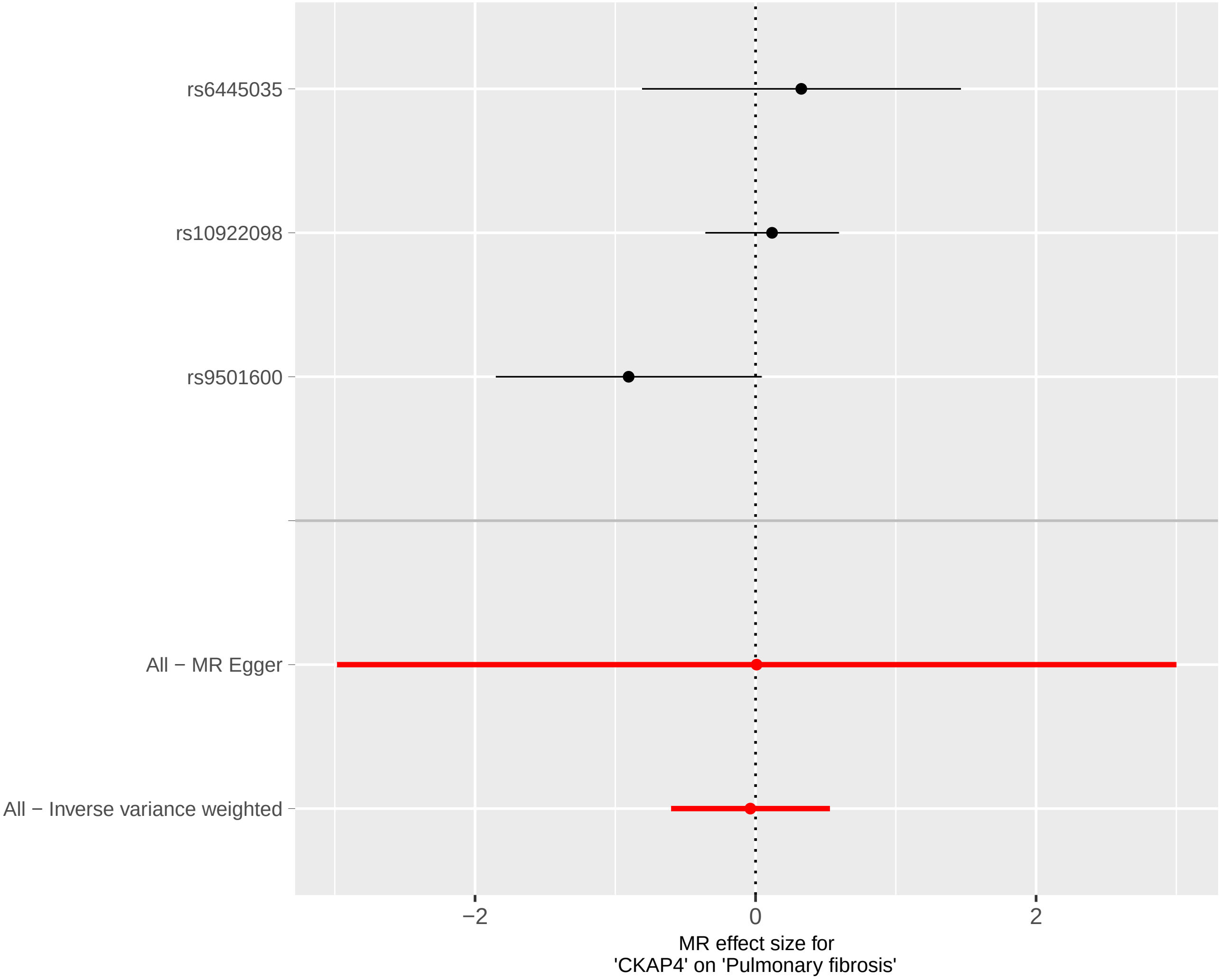
rs6445035
rs10922098
rs9501600
All − MR Egger
All − Inverse variance weighted
−2
0
2
MR effect size for
'CKAP4' on 'Pulmonary fibrosis'

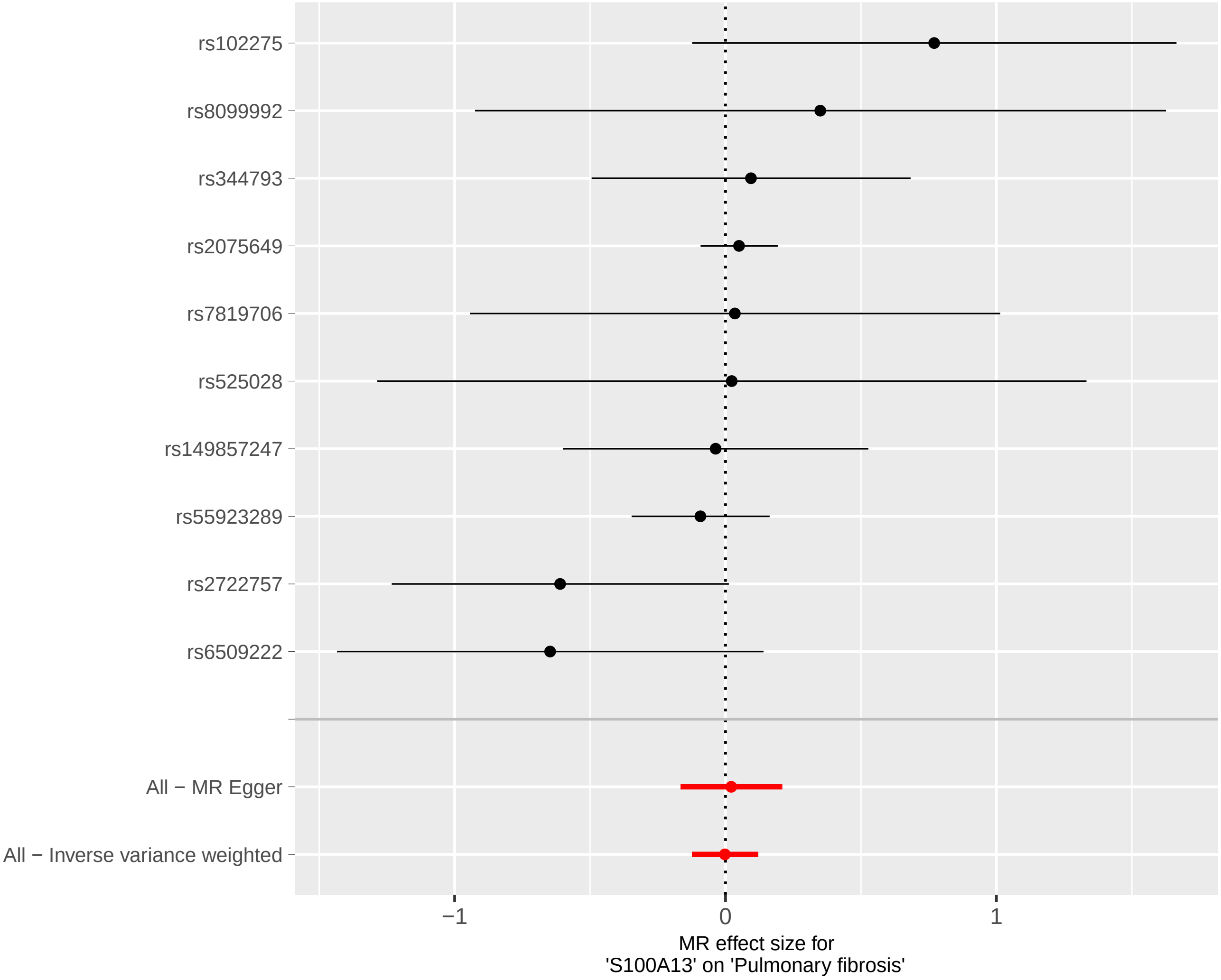

rs102275
rs8099992
rs344793
rs2075649
rs7819706
rs525028
rs149857247
rs55923289
rs2722757
rs6509222
All − MR Egger
All − Inverse variance weighted
−1
0
1
MR effect size for
'S100A13' on 'Pulmonary fibrosis'

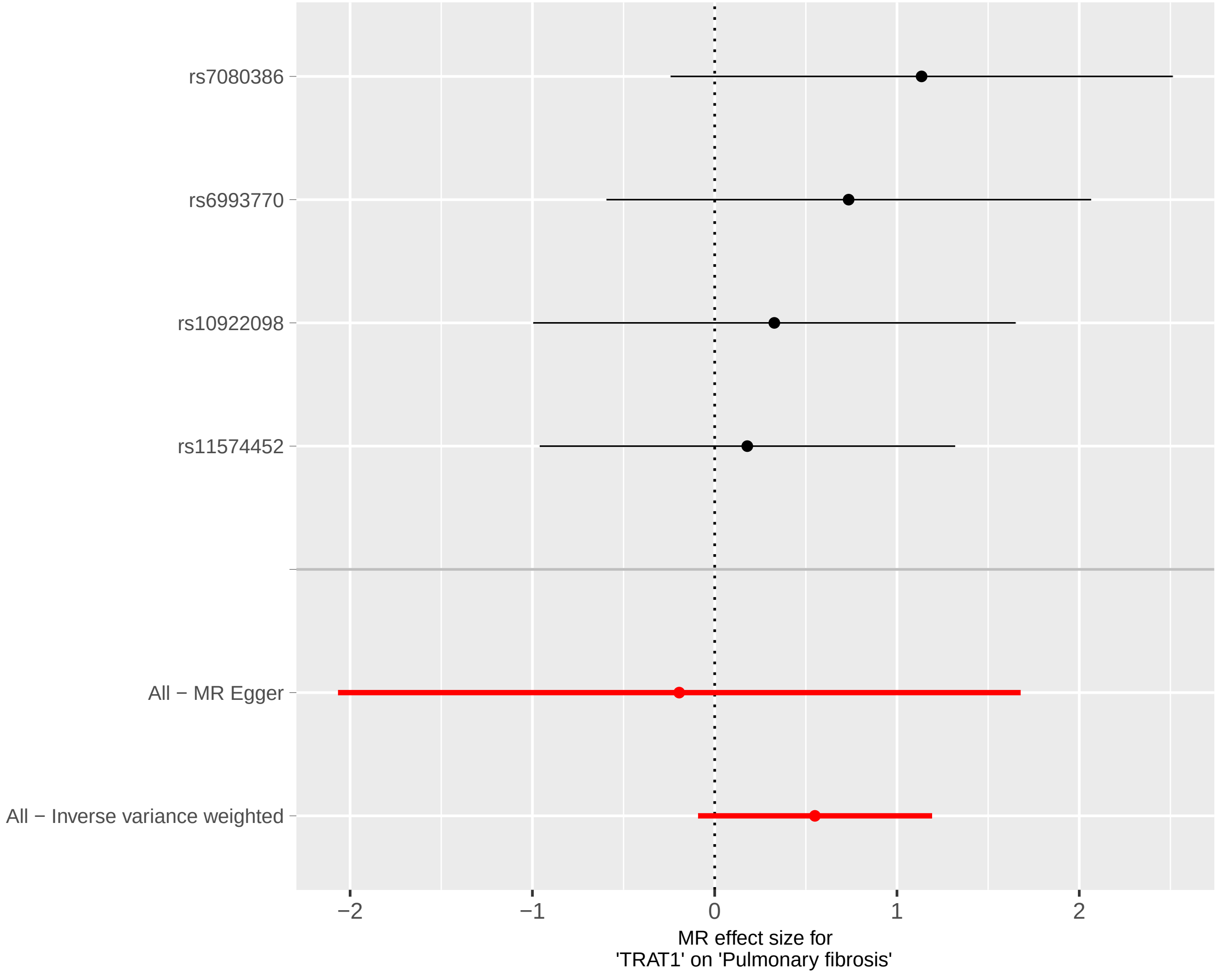

rs7080386
rs6993770
rs10922098
rs11574452
All − MR Egger
All − Inverse variance weighted
−2
−1
0
1
2
MR effect size for
'TRAT1' on 'Pulmonary fibrosis'

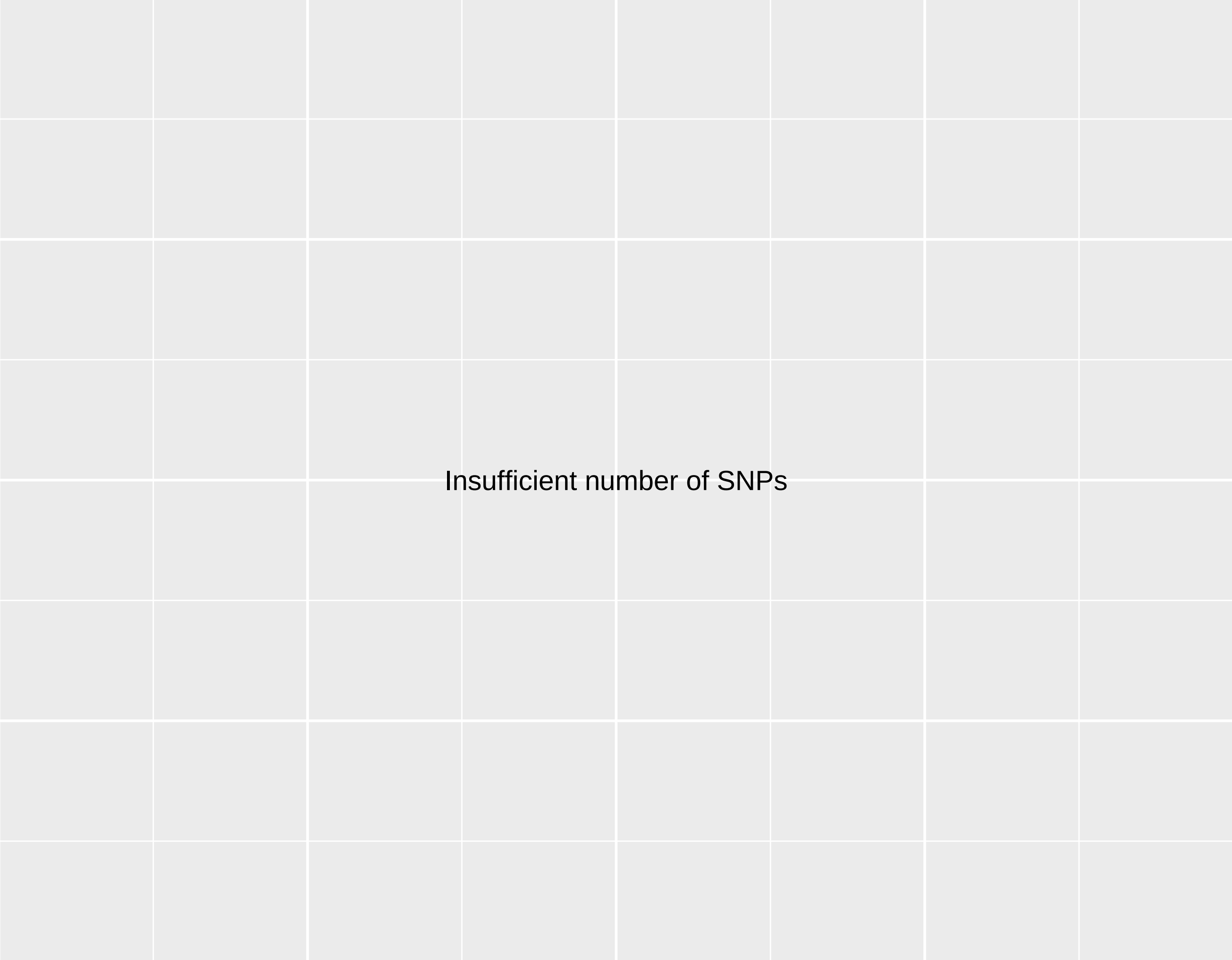
Insufficient number of SNPs

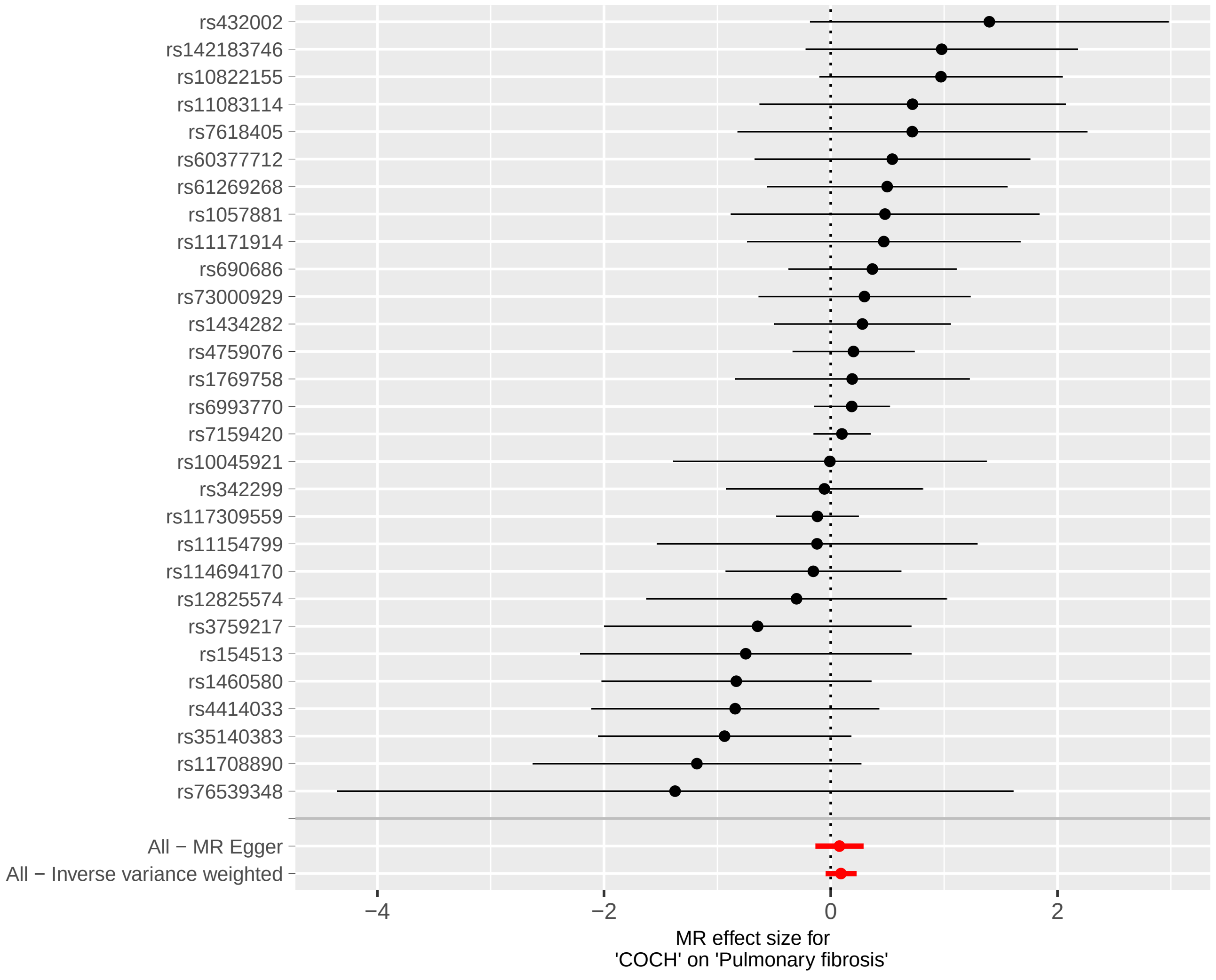

rs432002
rs142183746
rs10822155
rs11083114
rs7618405
rs60377712
rs61269268
rs1057881
rs11171914
rs690686
rs73000929
rs1434282
rs4759076
rs1769758
rs6993770
rs7159420
rs10045921
rs342299
rs117309559
rs11154799
rs114694170
rs12825574
rs3759217
rs154513
rs1460580
rs4414033
rs35140383
rs11708890
rs76539348
All − MR Egger
All − Inverse variance weighted
−4
−2
0
2
MR effect size for
'COCH' on 'Pulmonary fibrosis'

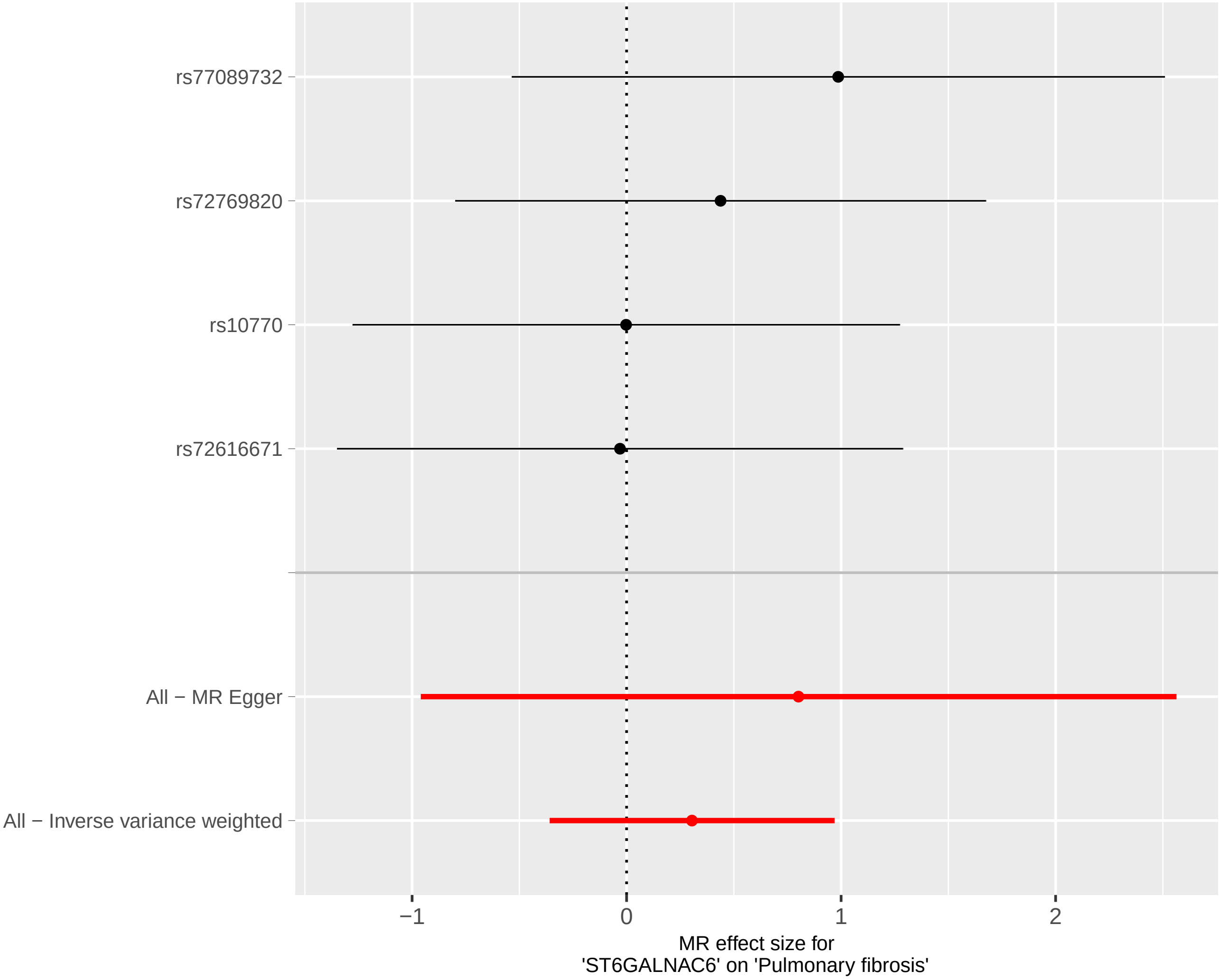

rs77089732
rs72769820
rs10770
rs72616671
All − MR Egger
All − Inverse variance weighted
−1
0
1
2
MR effect size for
'ST6GALNAC6' on 'Pulmonary fibrosis'

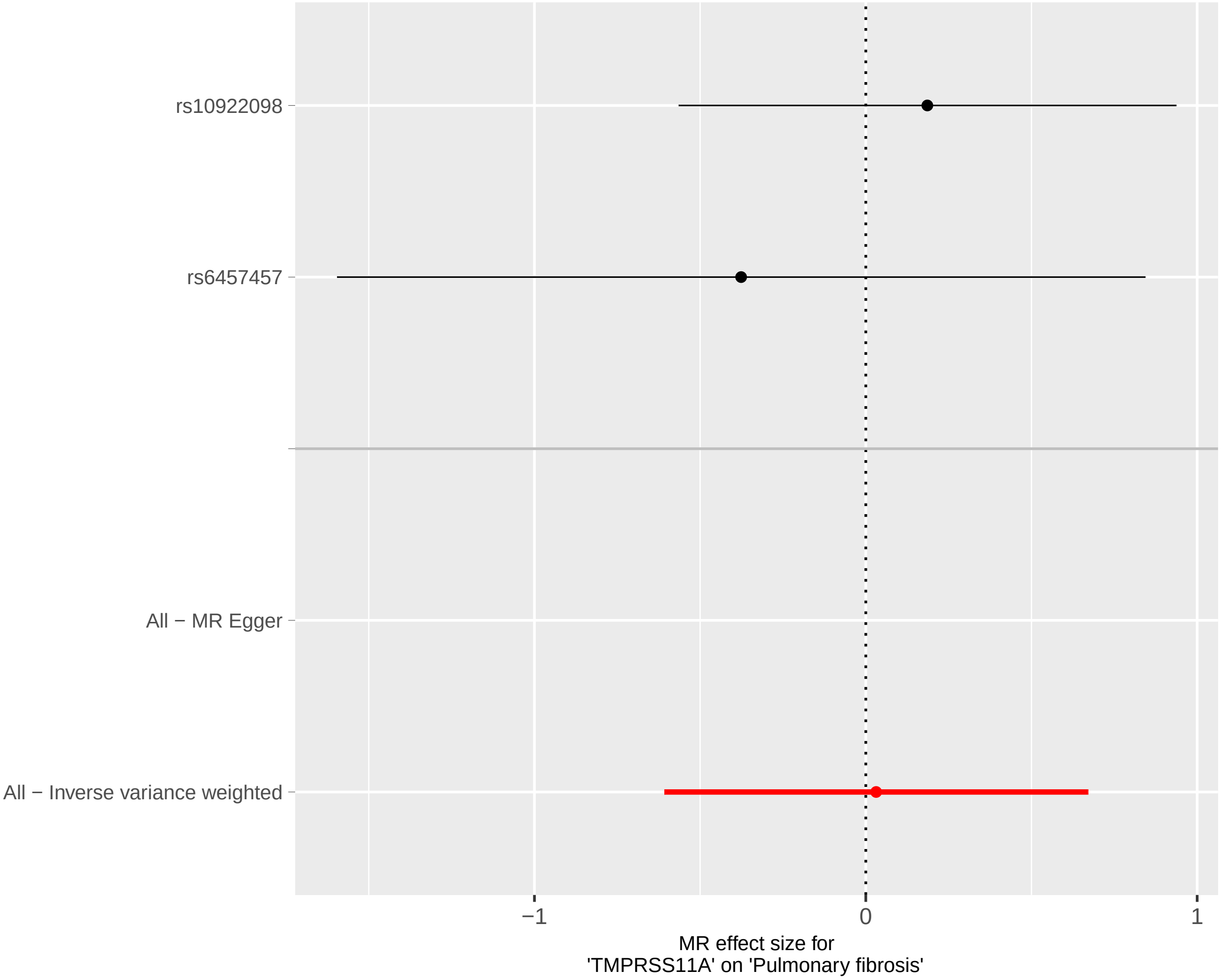
rs10922098
rs6457457
All − MR Egger
All − Inverse variance weighted
−1
0
1
MR effect size for
'TMPRSS11A' on 'Pulmonary fibrosis'

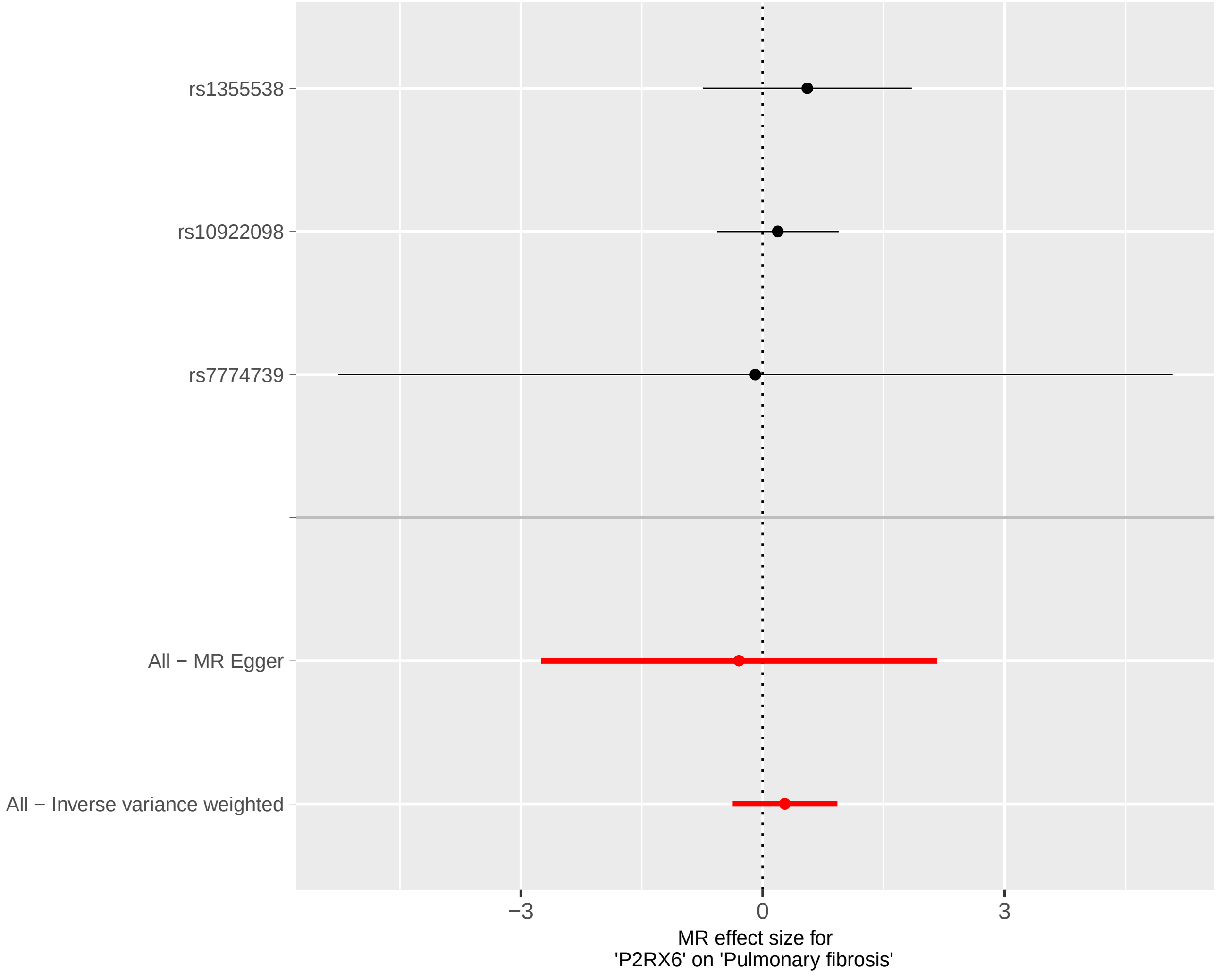

rs1355538
rs10922098
rs7774739
All − MR Egger
All − Inverse variance weighted
−3
0
3
MR effect size for
'P2RX6' on 'Pulmonary fibrosis'

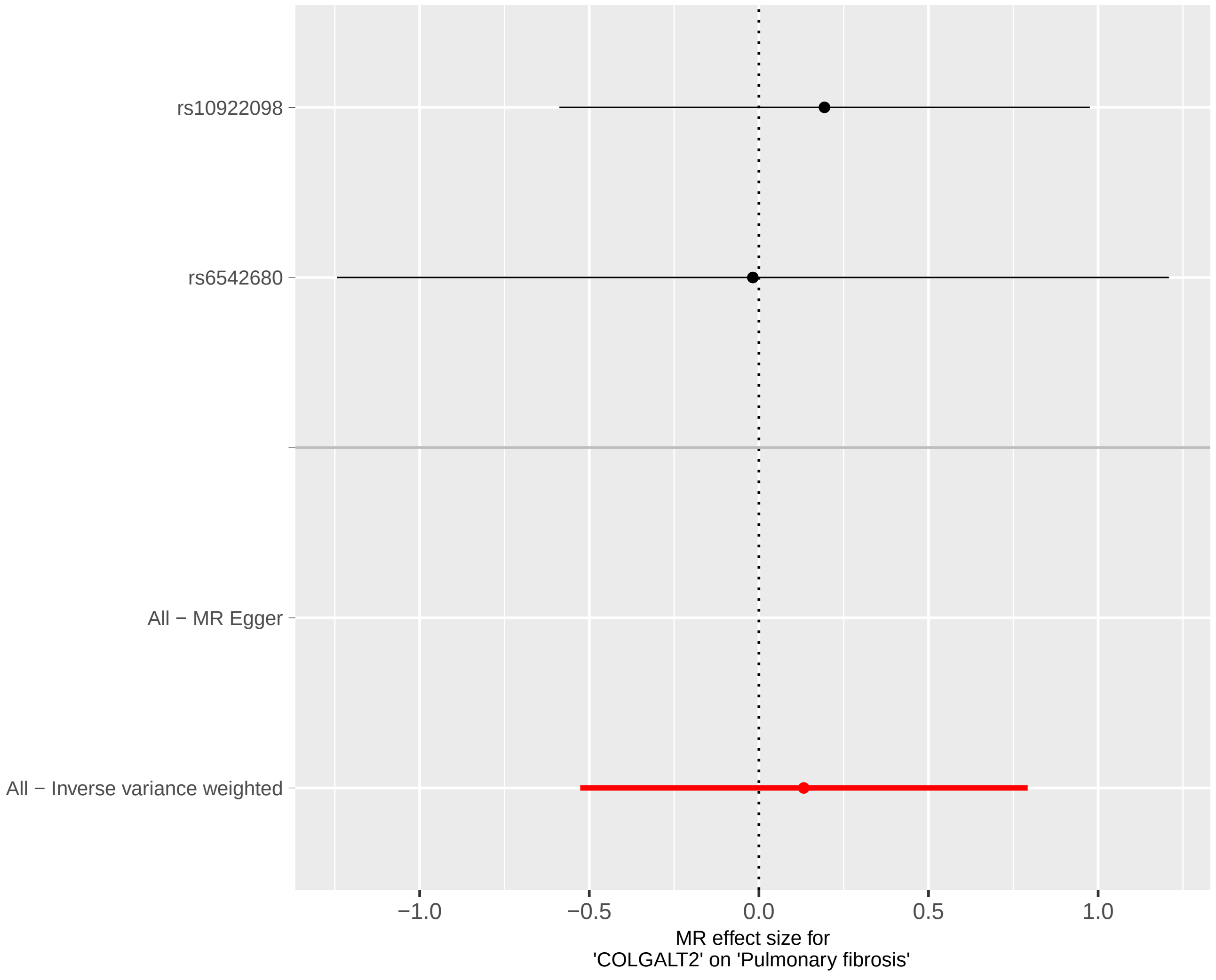
rs10922098
rs6542680
All − MR Egger
All − Inverse variance weighted
−1.0
−0.5
0.0
0.5
1.0
MR effect size for
'COLGALT2' on 'Pulmonary fibrosis'

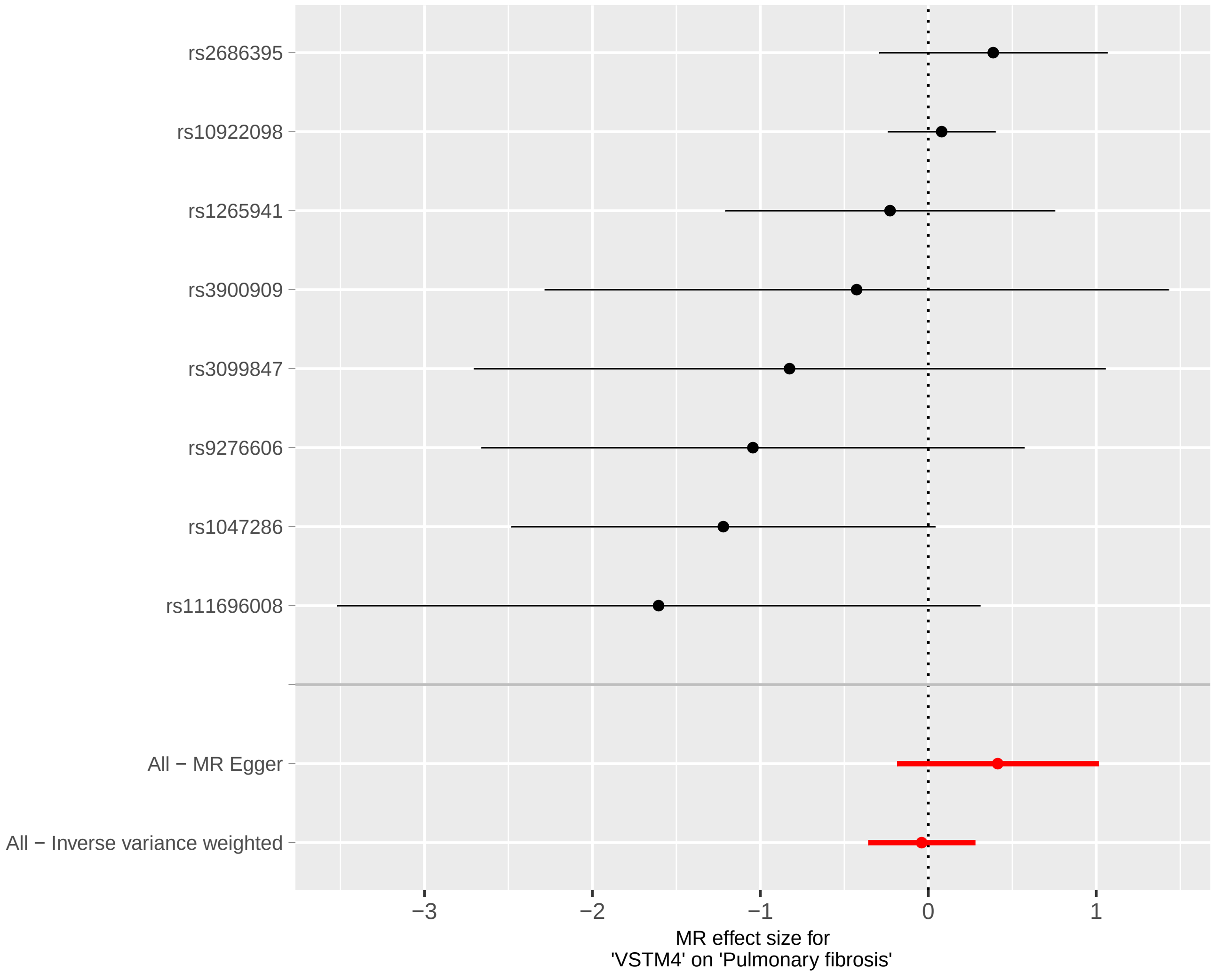

rs2686395
rs10922098
rs1265941
rs3900909
rs3099847
rs9276606
rs1047286
rs111696008
All − MR Egger
All − Inverse variance weighted
−3
−2
−1
0
1
MR effect size for
'VSTM4' on 'Pulmonary fibrosis'

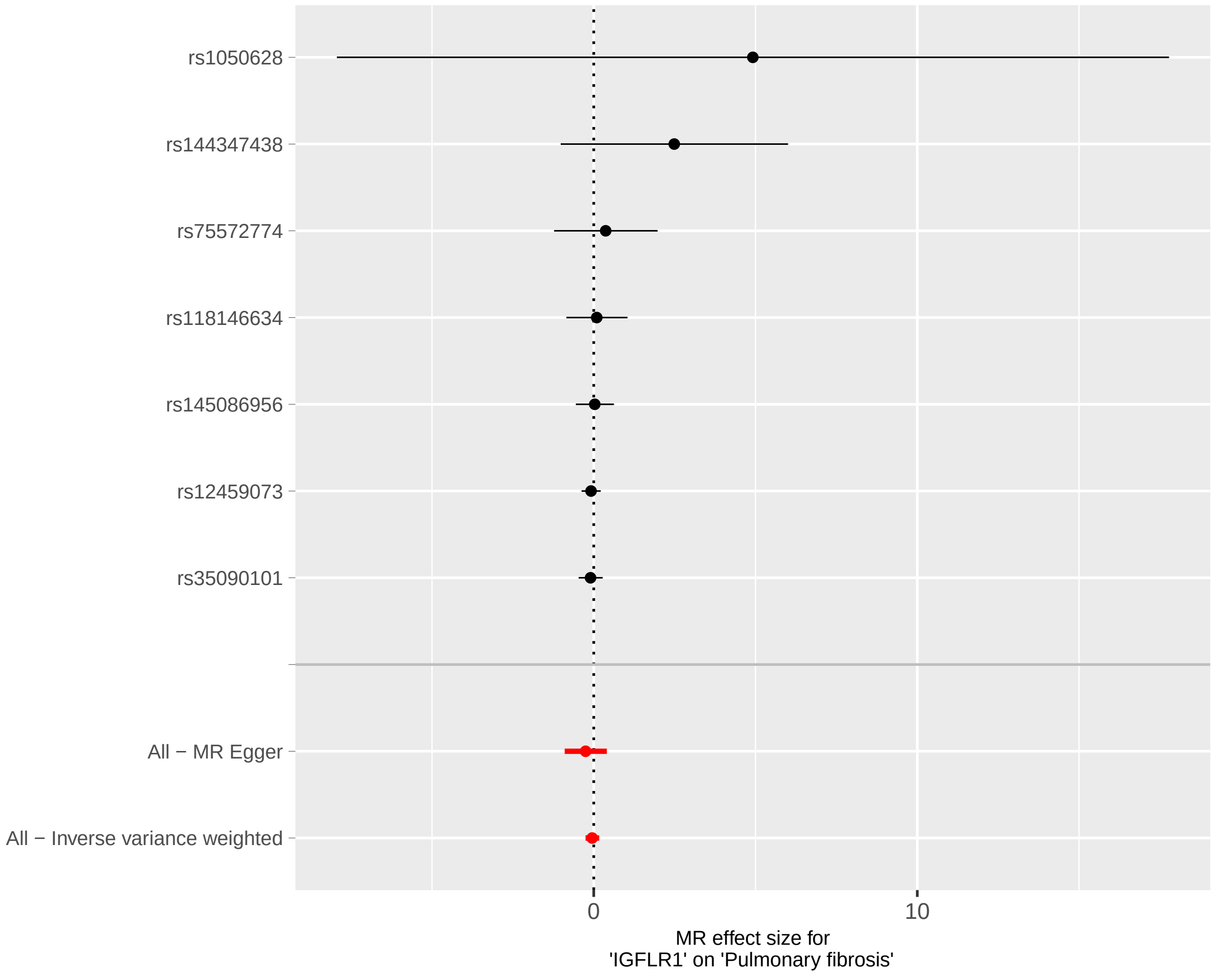
rs1050628
rs144347438
rs75572774
rs118146634
rs145086956
rs12459073
rs35090101
All − MR Egger
All − Inverse variance weighted
0
10
MR effect size for
'IGFLR1' on 'Pulmonary fibrosis'

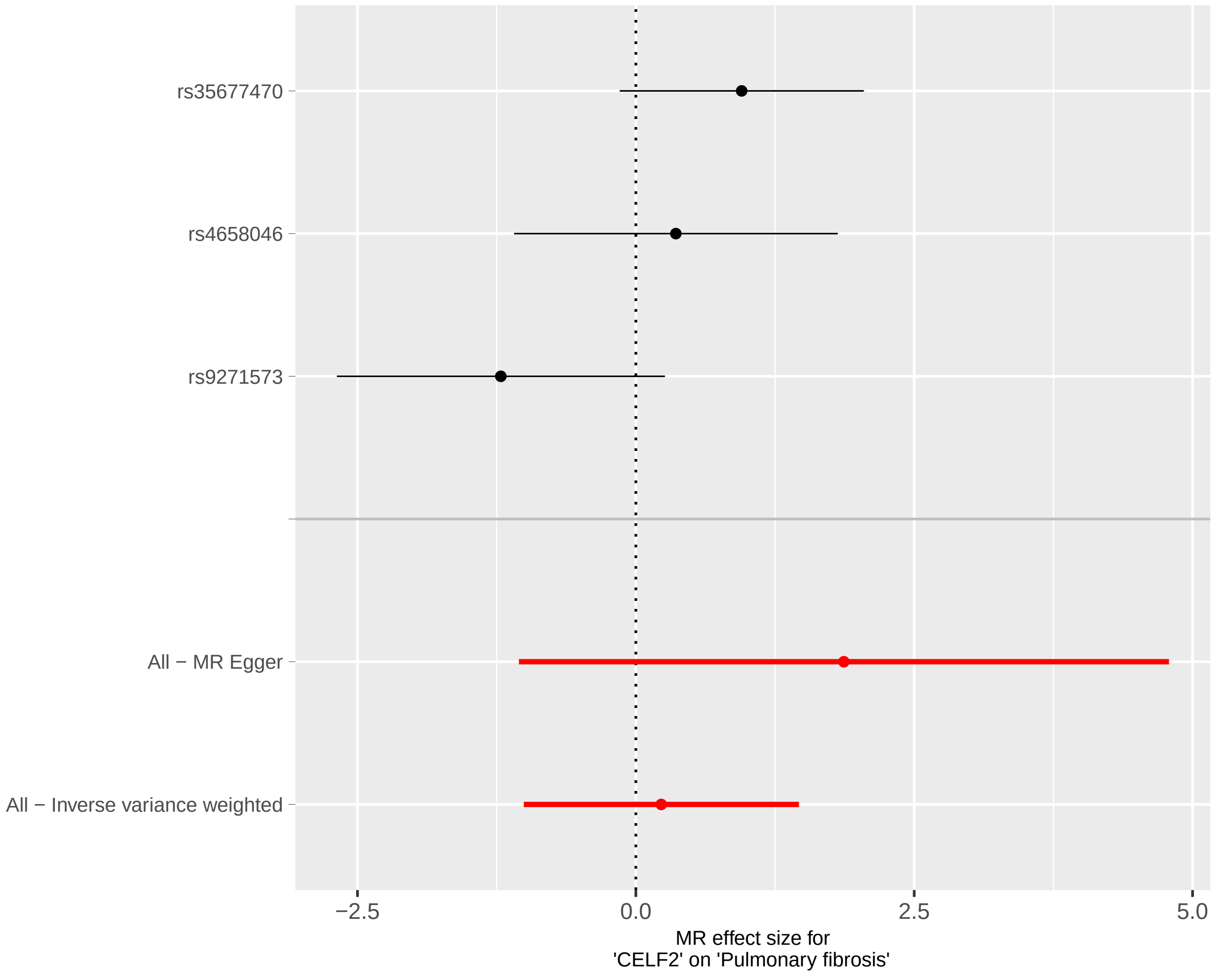

rs35677470
rs4658046
rs9271573
All − MR Egger
All − Inverse variance weighted
−2.5
0.0
2.5
5.0
MR effect size for
'CELF2' on 'Pulmonary fibrosis'

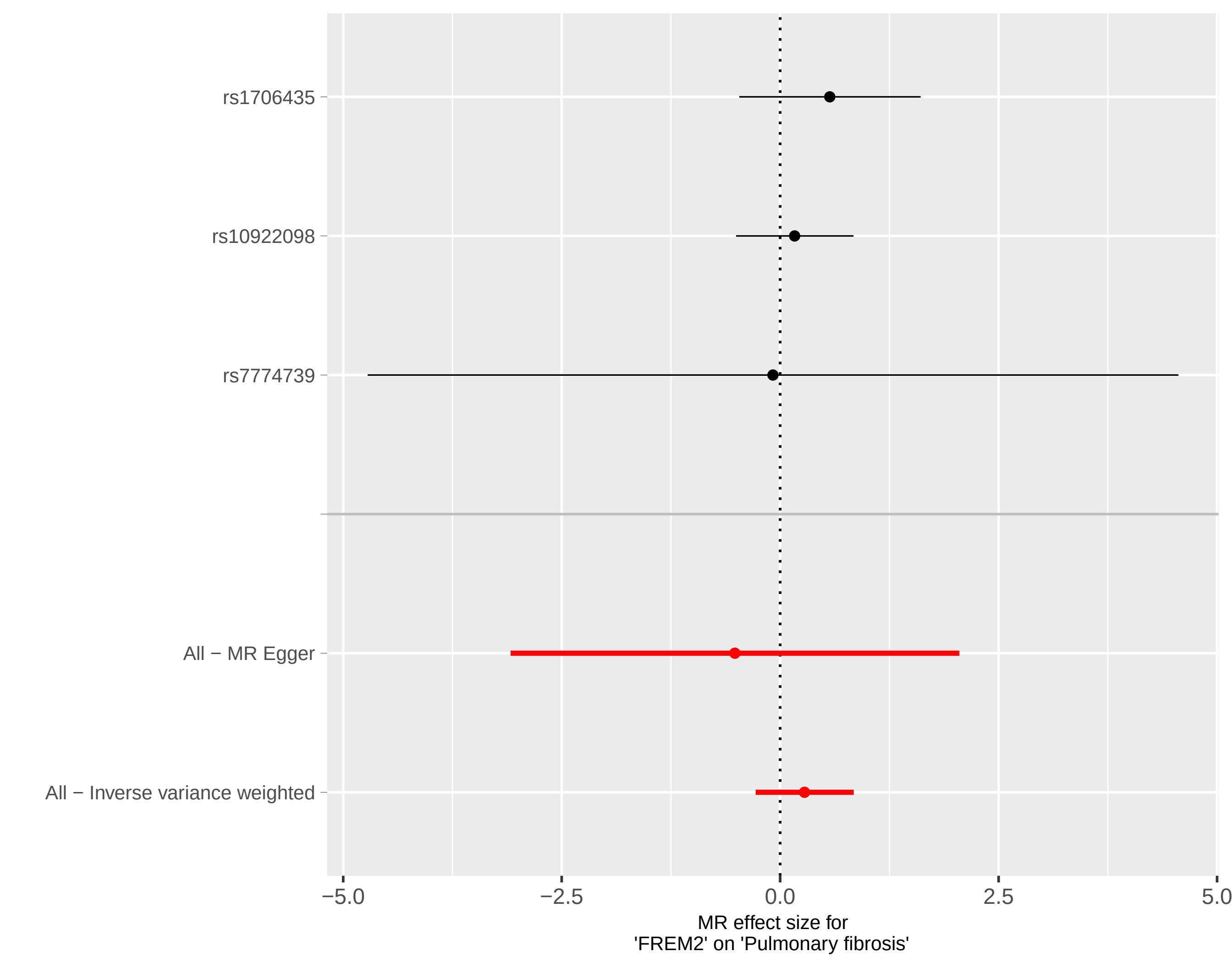

rs1706435
rs10922098
rs7774739
All − MR Egger
All − Inverse variance weighted
−5.0
−2.5
0.0
2.5
5.0
MR effect size for
'FREM2' on 'Pulmonary fibrosis'

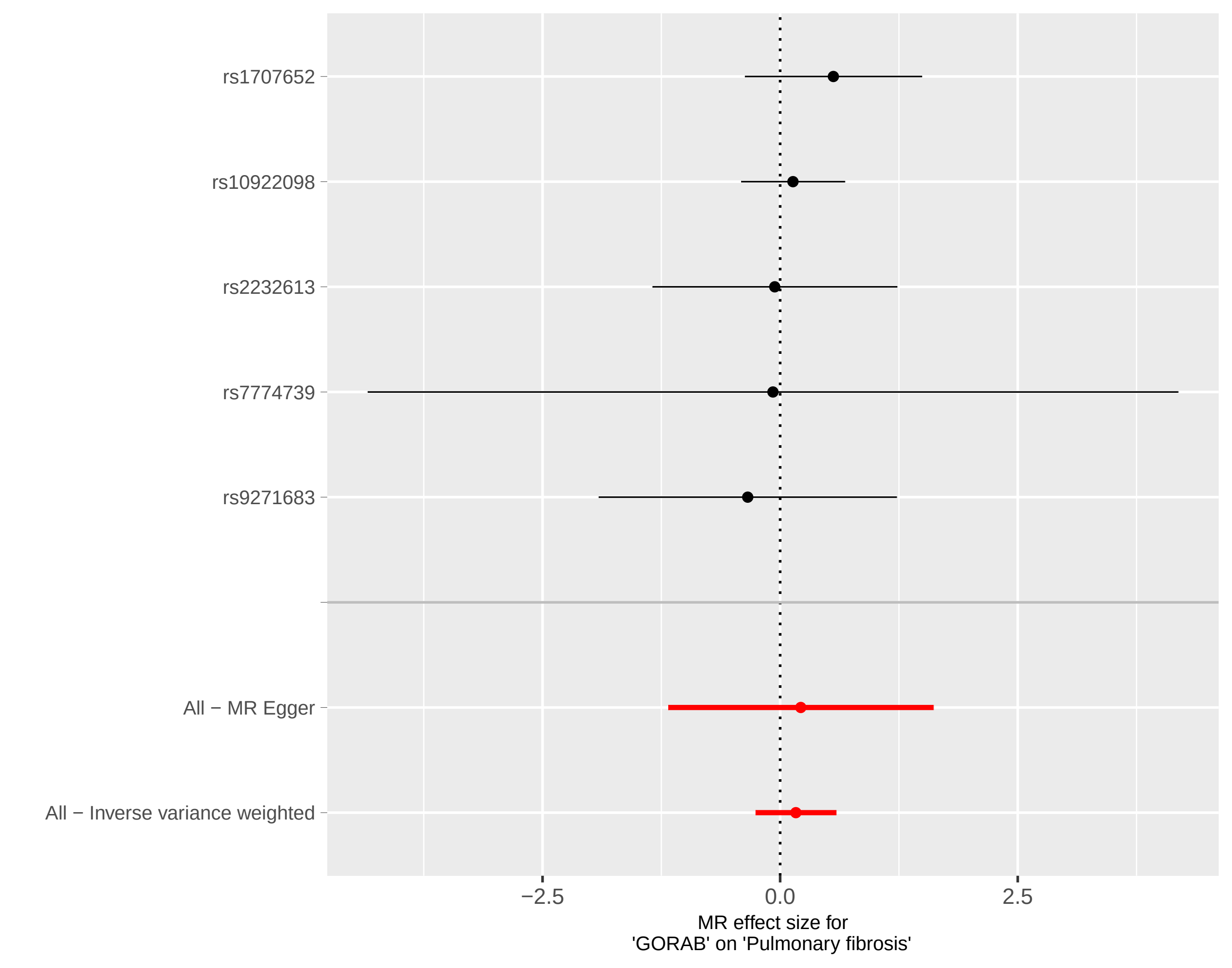
rs1707652
rs10922098
rs2232613
rs7774739
rs9271683
All − MR Egger
All − Inverse variance weighted
−2.5
0.0
2.5
MR effect size for
'GORAB' on 'Pulmonary fibrosis'

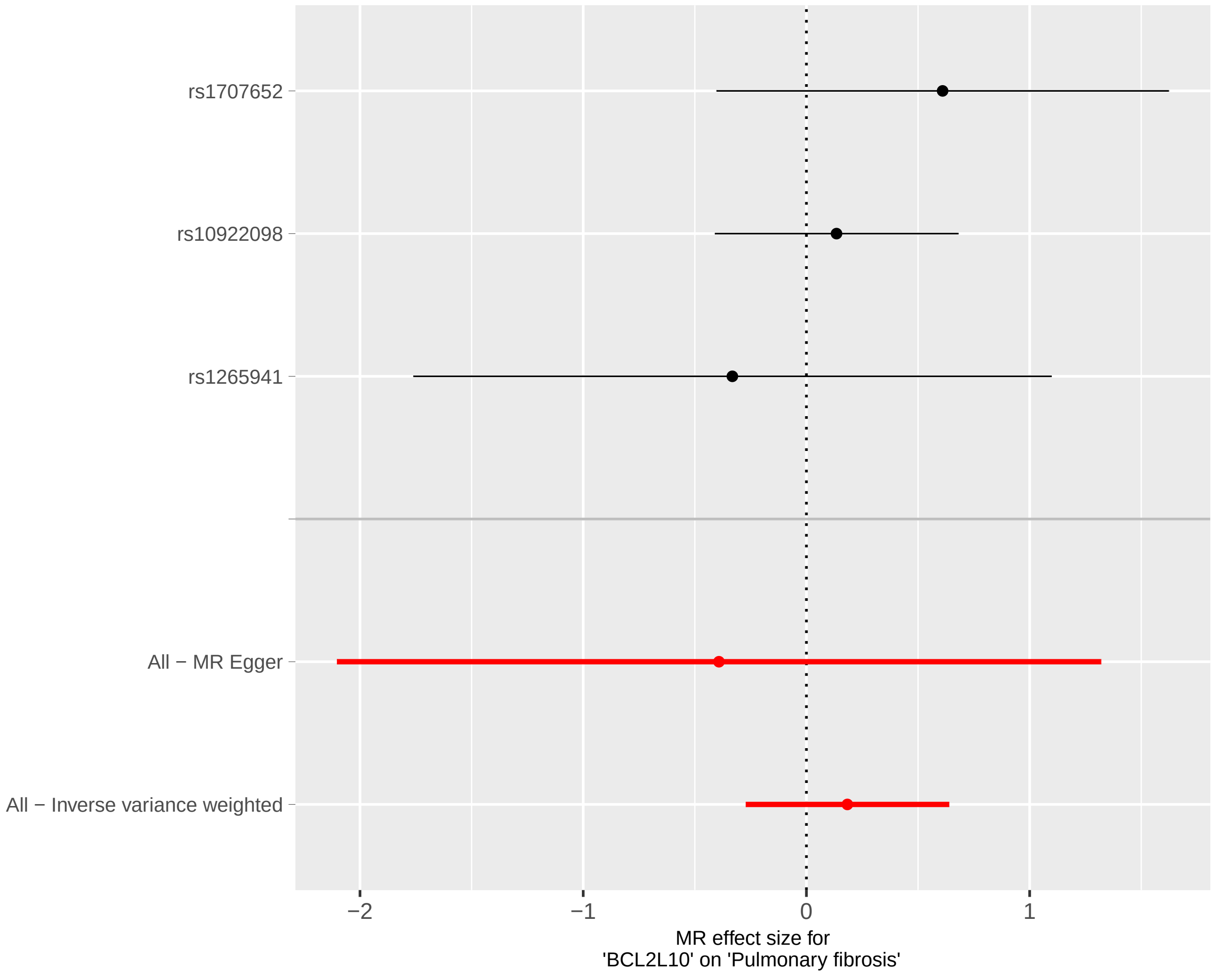
rs1707652
rs10922098
rs1265941
All − MR Egger
All − Inverse variance weighted
−2
−1
0
1
MR effect size for
'BCL2L10' on 'Pulmonary fibrosis'

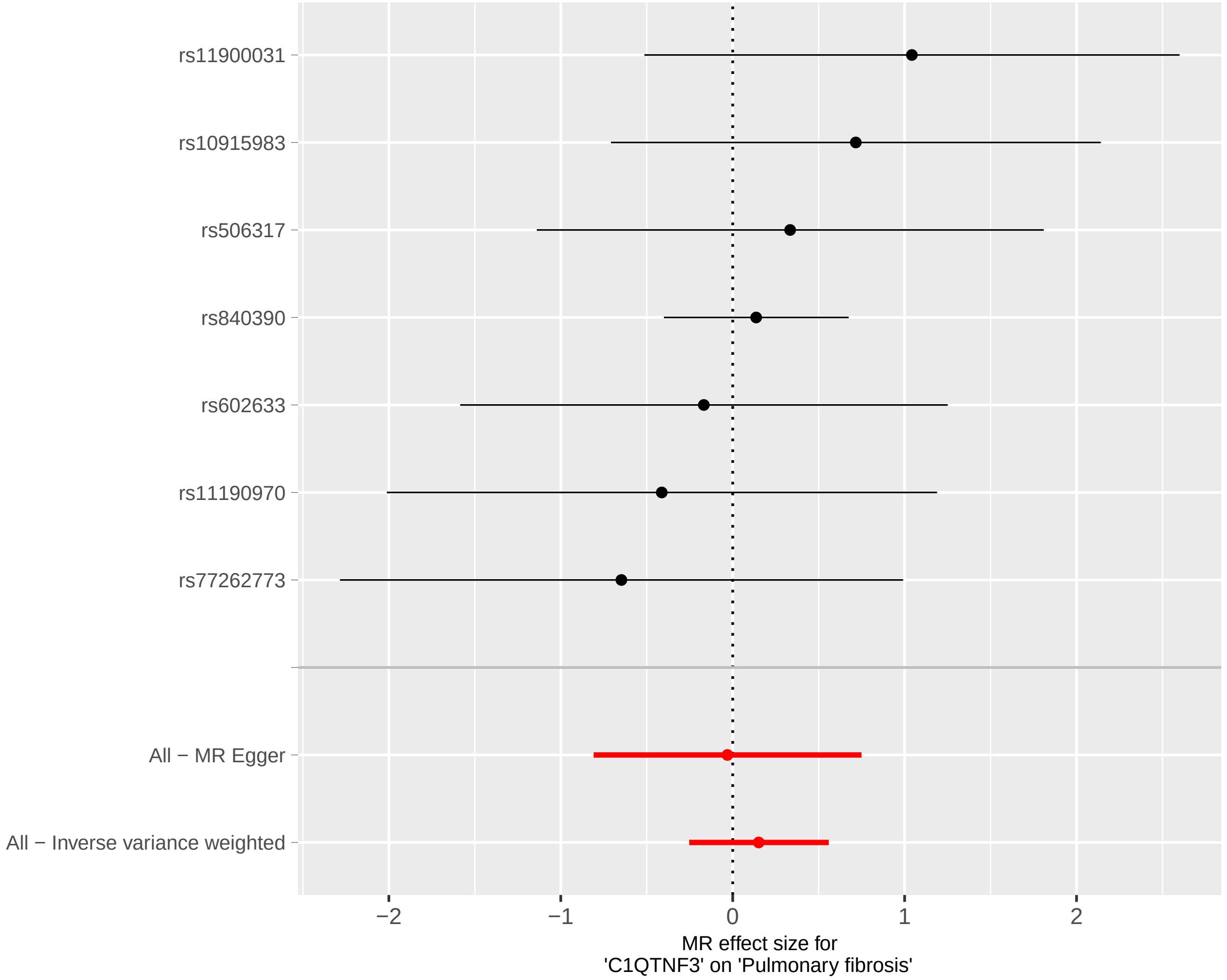
rs11900031
rs10915983
rs506317
rs840390
rs602633
rs11190970
rs77262773
All − MR Egger
All − Inverse variance weighted
−2
−1
0
1
2
MR effect size for
'C1QTNF3' on 'Pulmonary fibrosis'

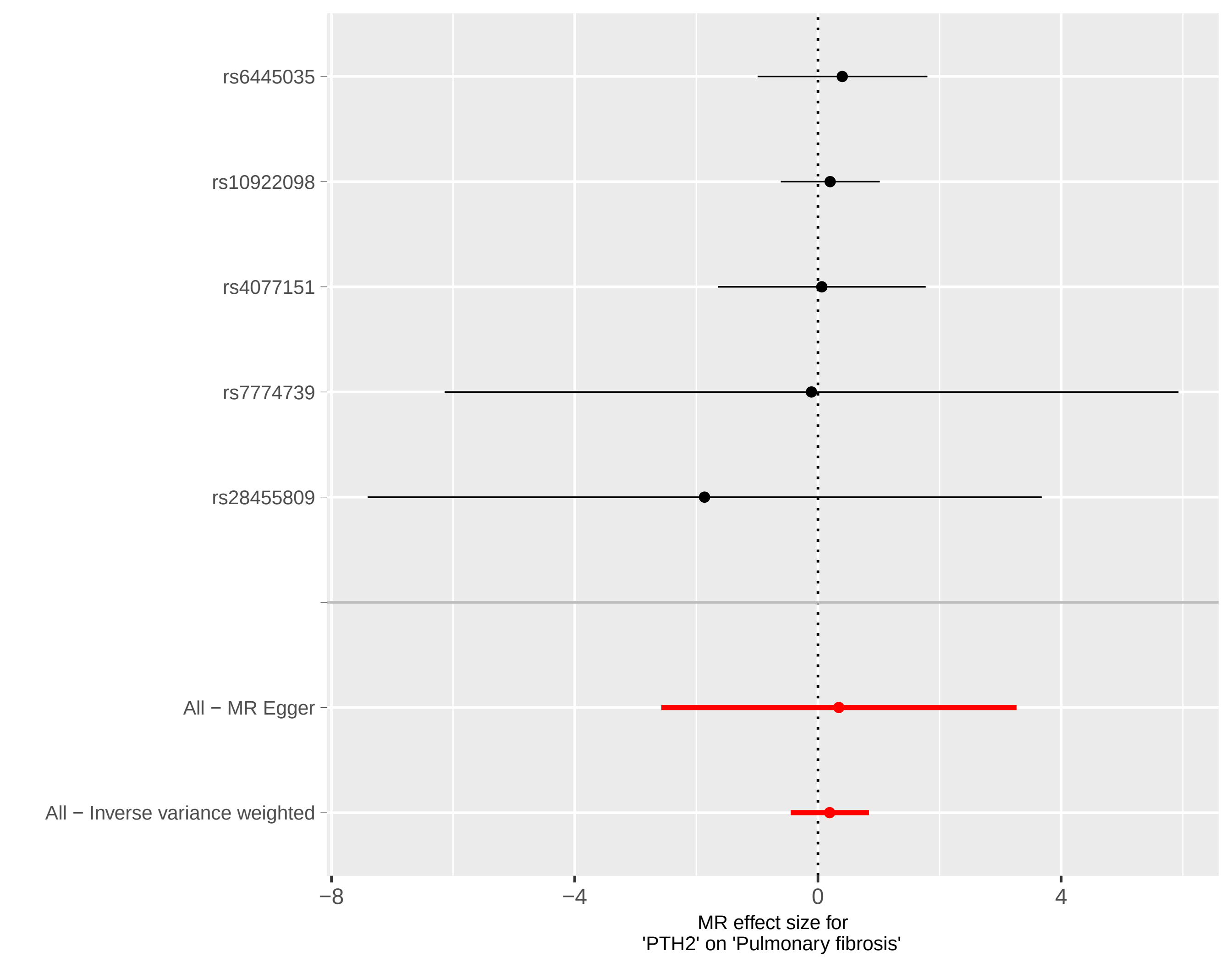

rs6445035
rs10922098
rs4077151
rs7774739
rs28455809
All − MR Egger
All − Inverse variance weighted
−8
−4
0
4
MR effect size for
'PTH2' on 'Pulmonary fibrosis'

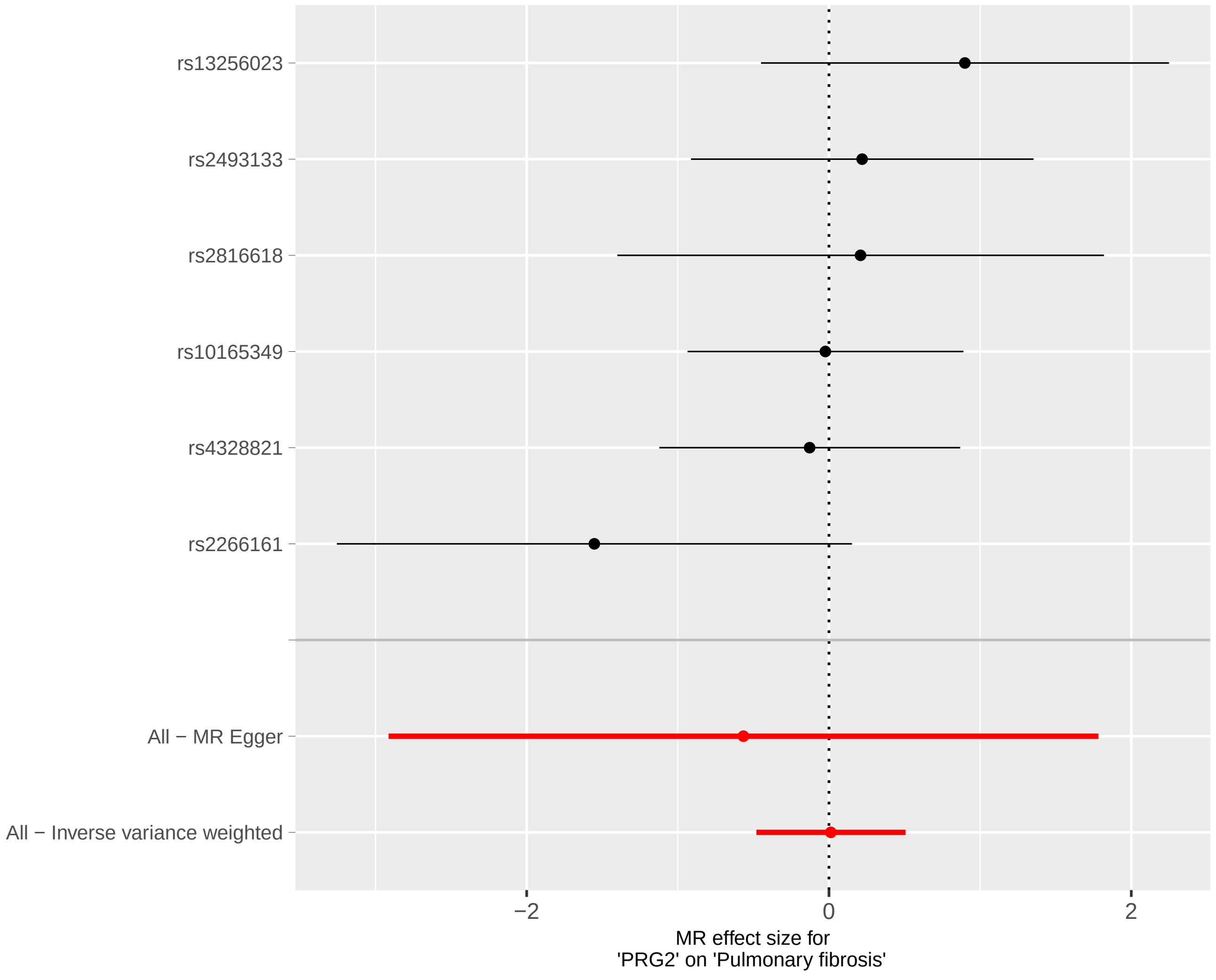
rs13256023
rs2493133
rs2816618
rs10165349
rs4328821
rs2266161
All − MR Egger
All − Inverse variance weighted
−2
0
2
MR effect size for
'PRG2' on 'Pulmonary fibrosis'

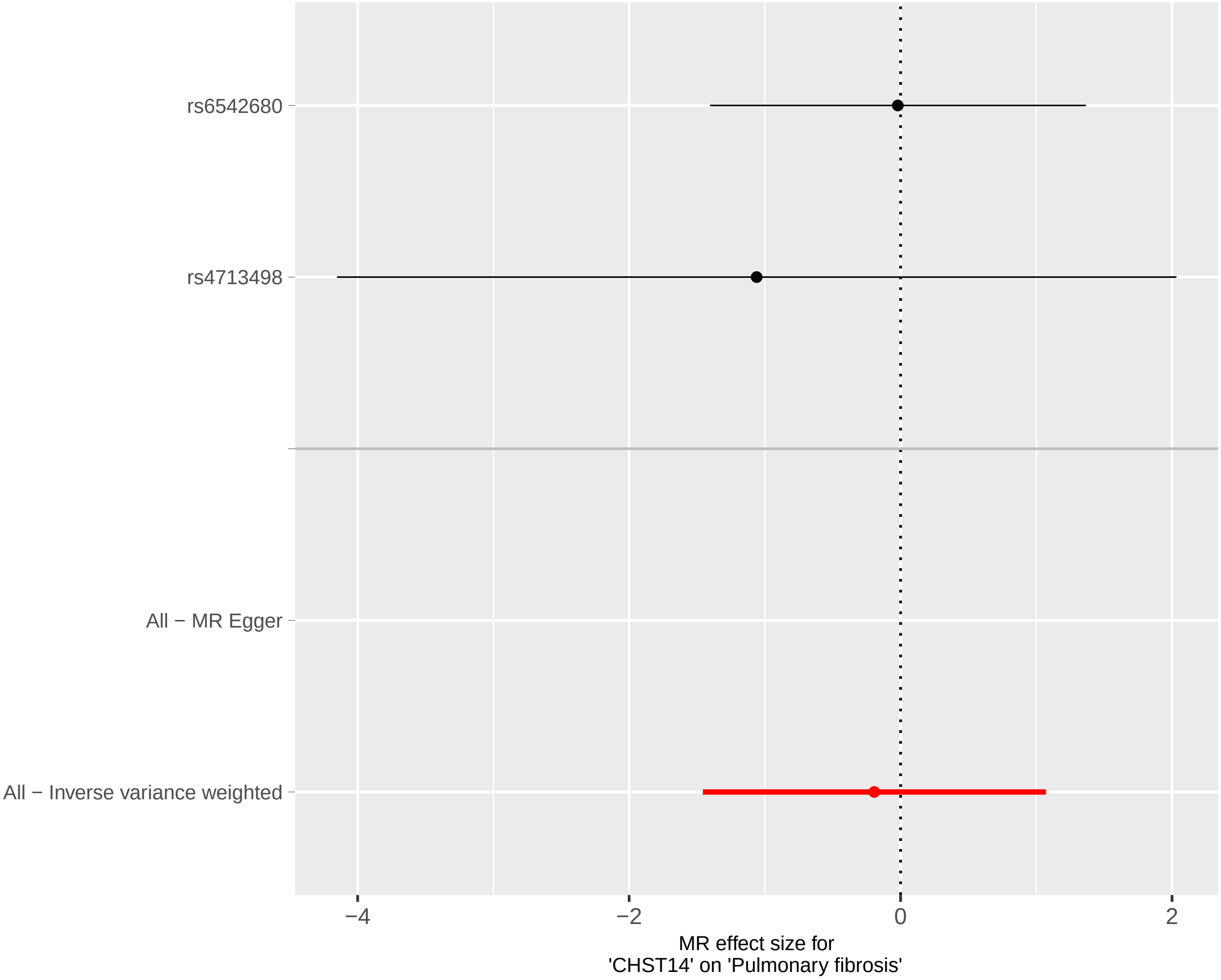
rs6542680
rs4713498
All − MR Egger
All − Inverse variance weighted
−4
−2
0
2
MR effect size for
'CHST14' on 'Pulmonary fibrosis'

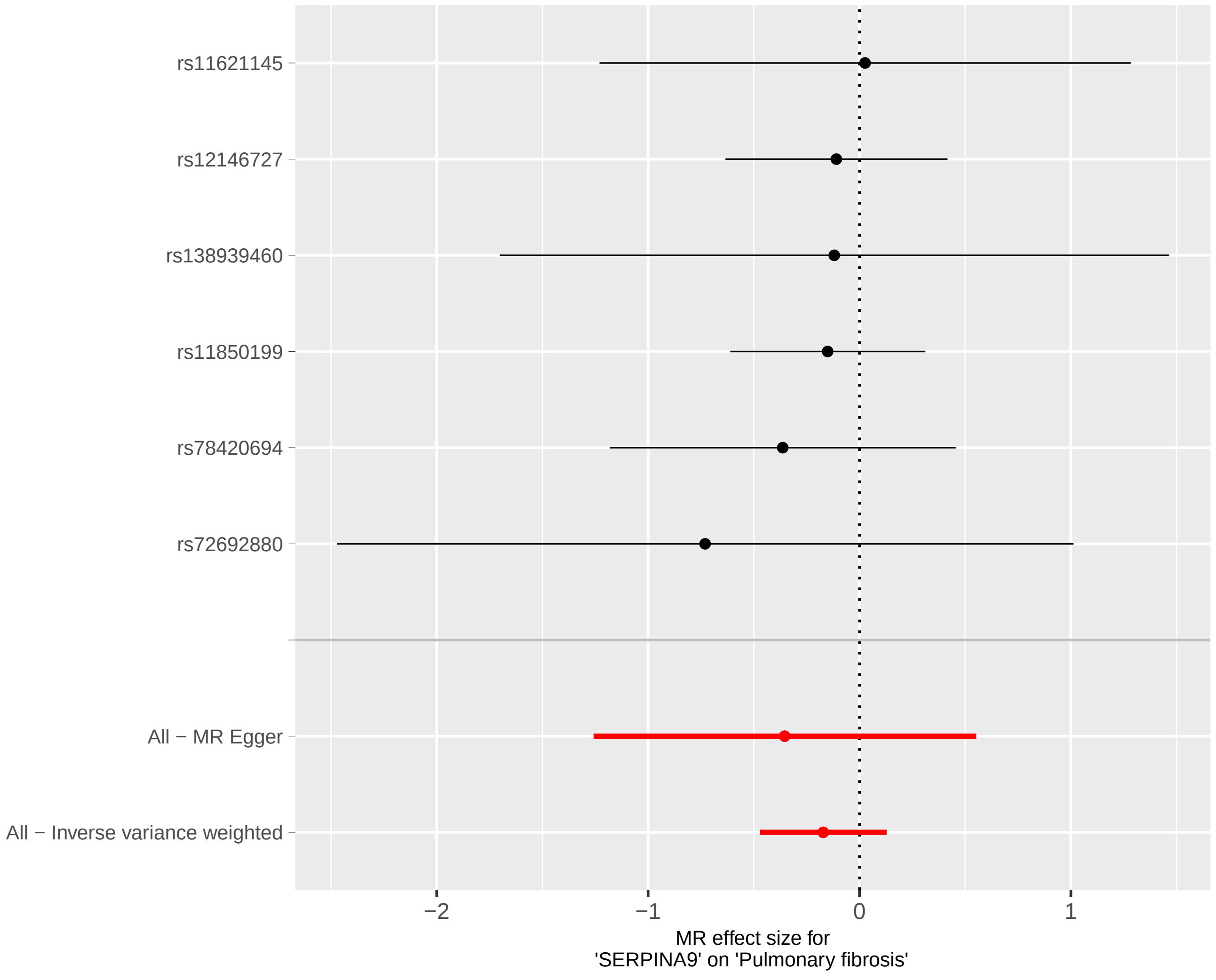

rs11621145
rs12146727
rs138939460
rs11850199
rs78420694
rs72692880
All − MR Egger
All − Inverse variance weighted
−2
−1
0
1
MR effect size for
'SERPINA9' on 'Pulmonary fibrosis'

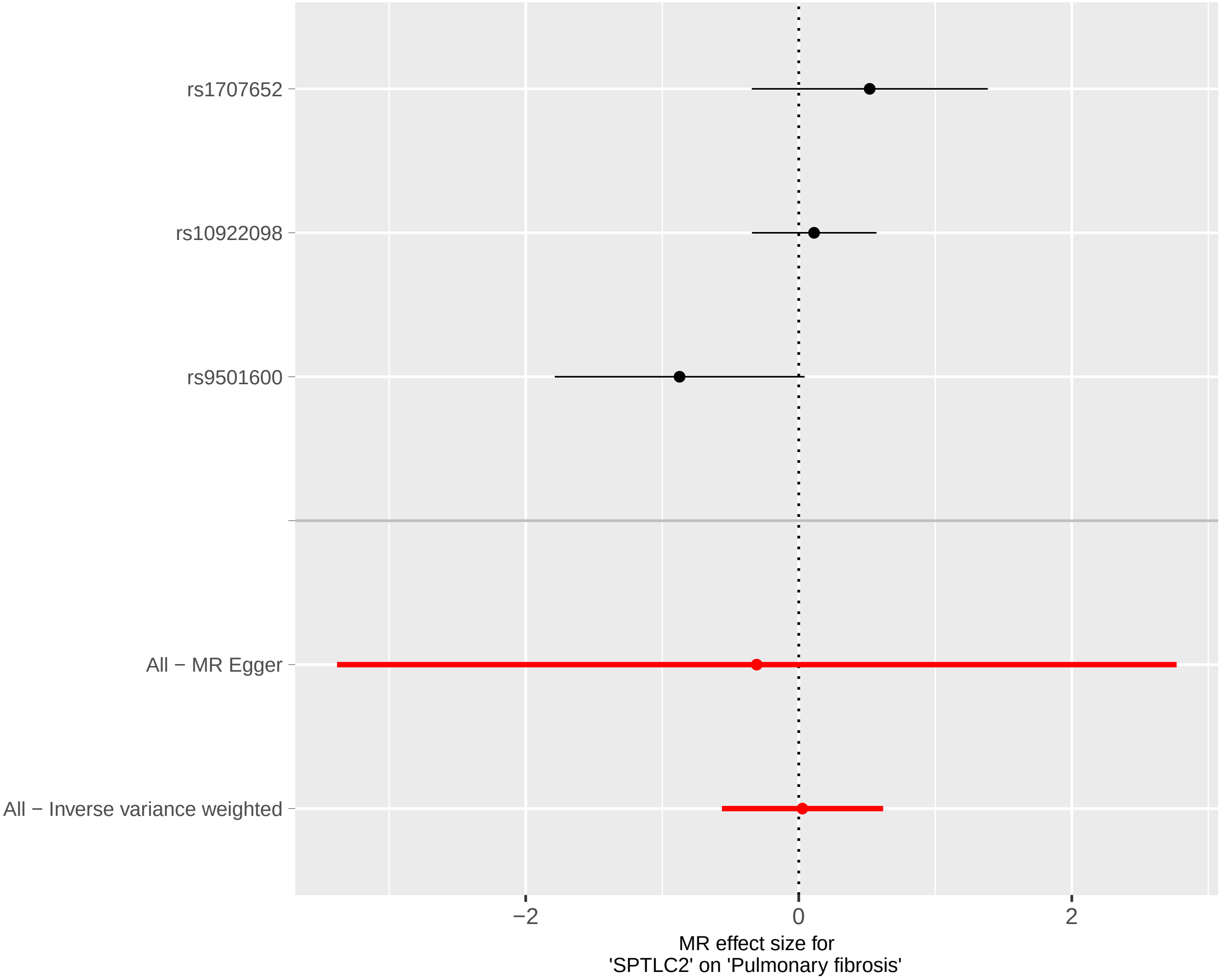

rs1707652
rs10922098
rs9501600
All − MR Egger
All − Inverse variance weighted
−2
0
2
MR effect size for
'SPTLC2' on 'Pulmonary fibrosis'

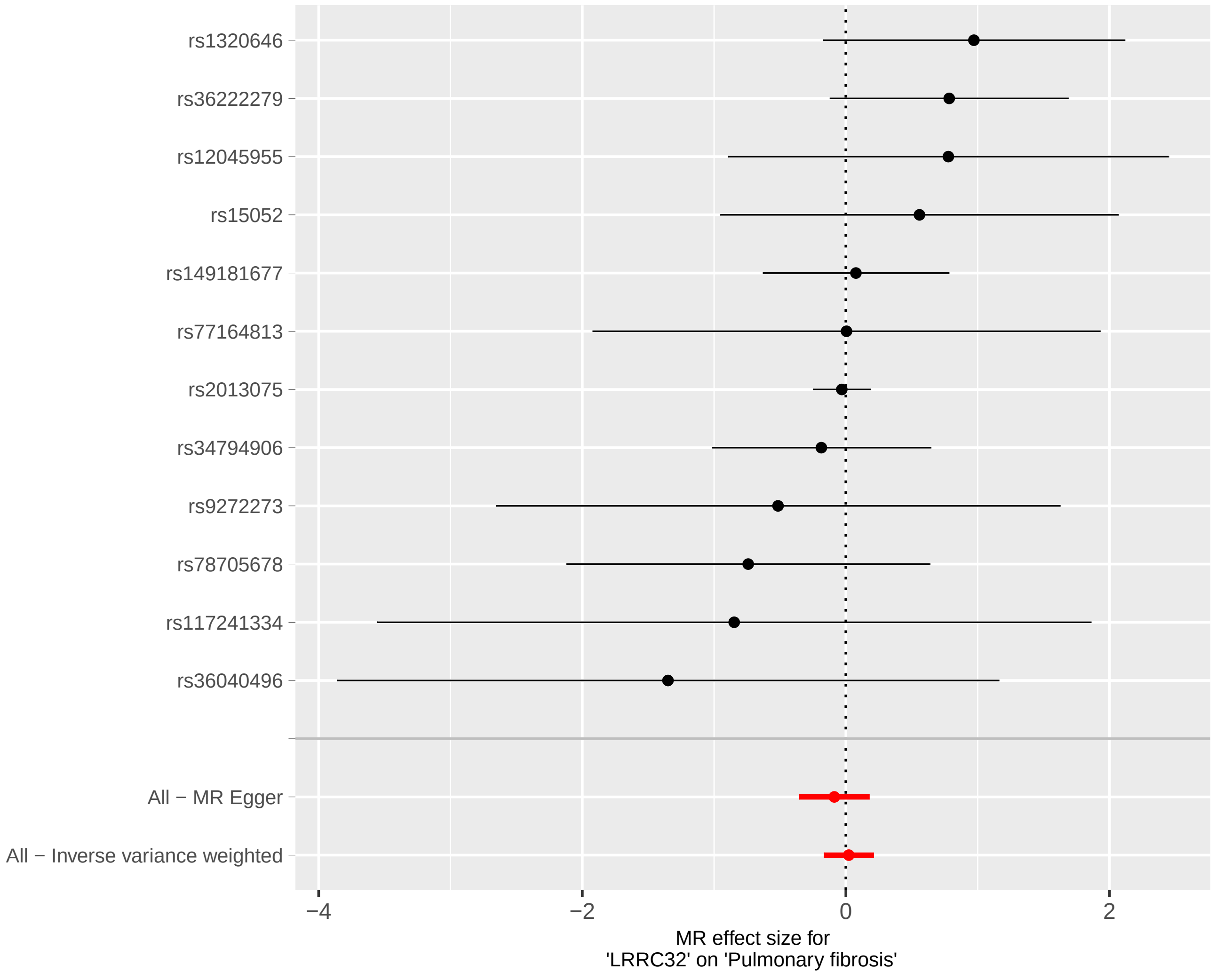

rs1320646
rs36222279
rs12045955
rs15052
rs149181677
rs77164813
rs2013075
rs34794906
rs9272273
rs78705678
rs117241334
rs36040496
All − MR Egger
All − Inverse variance weighted
−4
−2
0
2
MR effect size for
'LRRC32' on 'Pulmonary fibrosis'

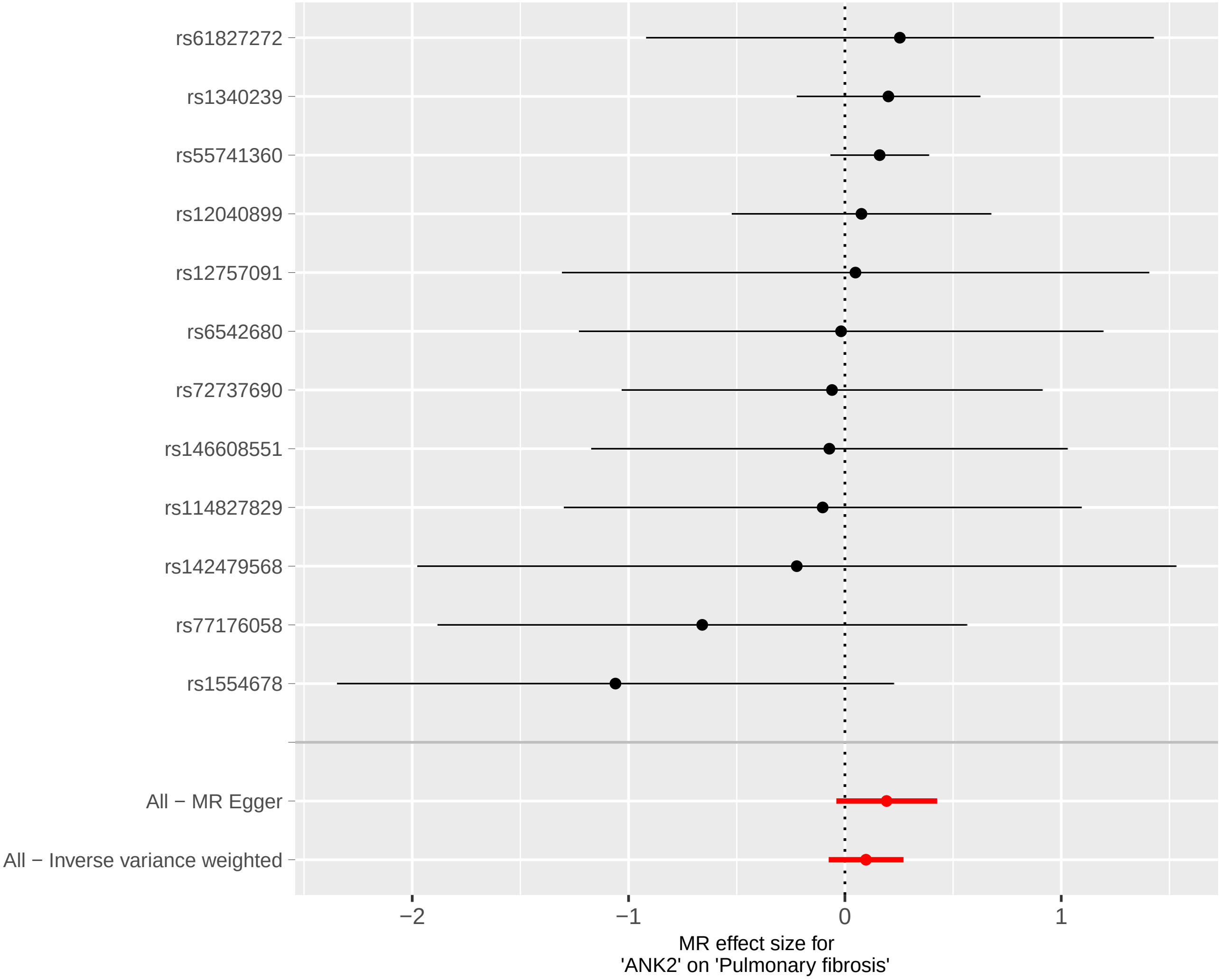
rs61827272
rs1340239
rs55741360
rs12040899
rs12757091
rs6542680
rs72737690
rs146608551
rs114827829
rs142479568
rs77176058
rs1554678
All − MR Egger
All − Inverse variance weighted
−2
−1
0
1
MR effect size for
'ANK2' on 'Pulmonary fibrosis'

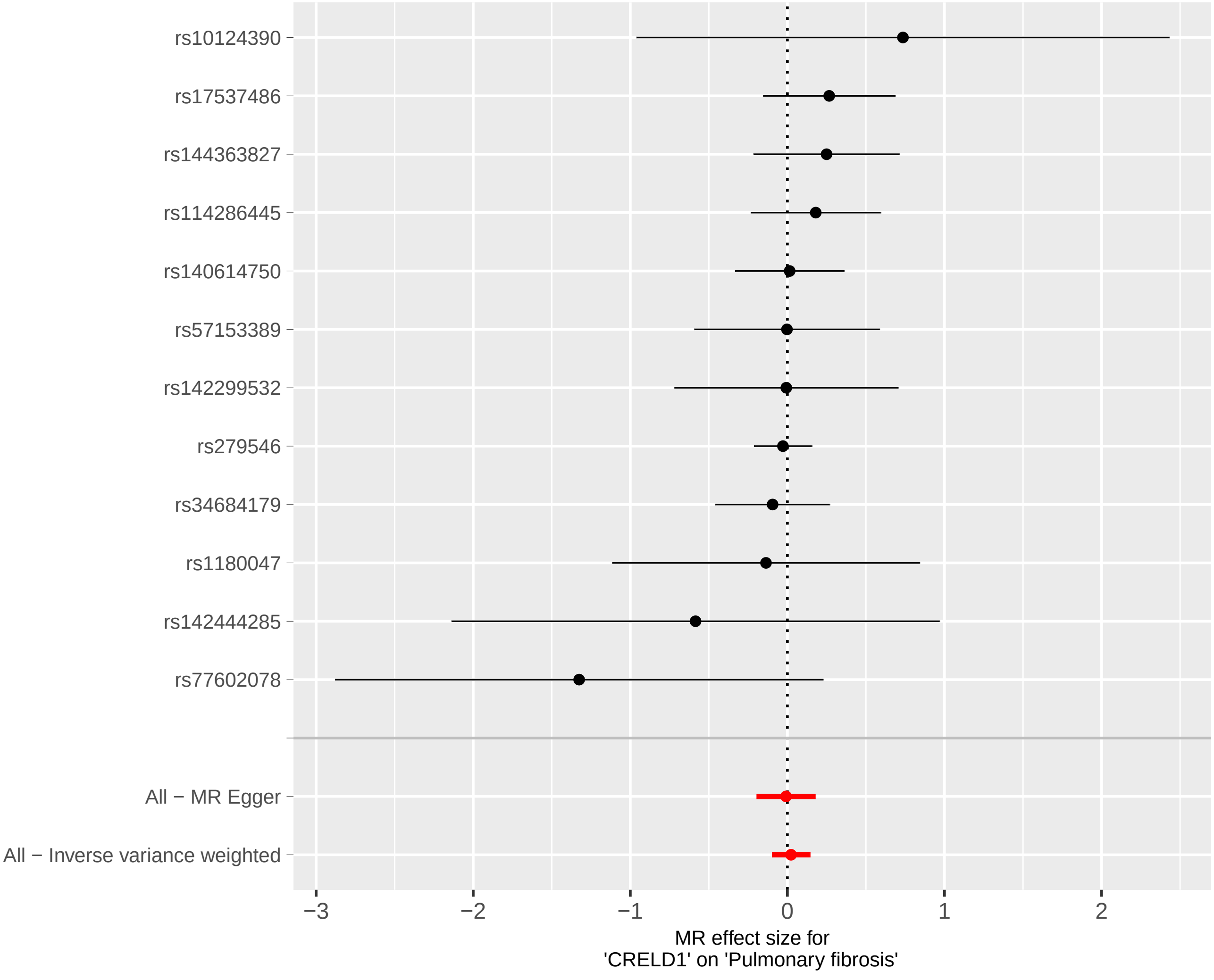

rs10124390
rs17537486
rs144363827
rs114286445
rs140614750
rs57153389
rs142299532
rs279546
rs34684179
rs1180047
rs142444285
rs77602078
All − MR Egger
All − Inverse variance weighted
−3
−2
−1
0
1
2
MR effect size for
'CRELD1' on 'Pulmonary fibrosis'

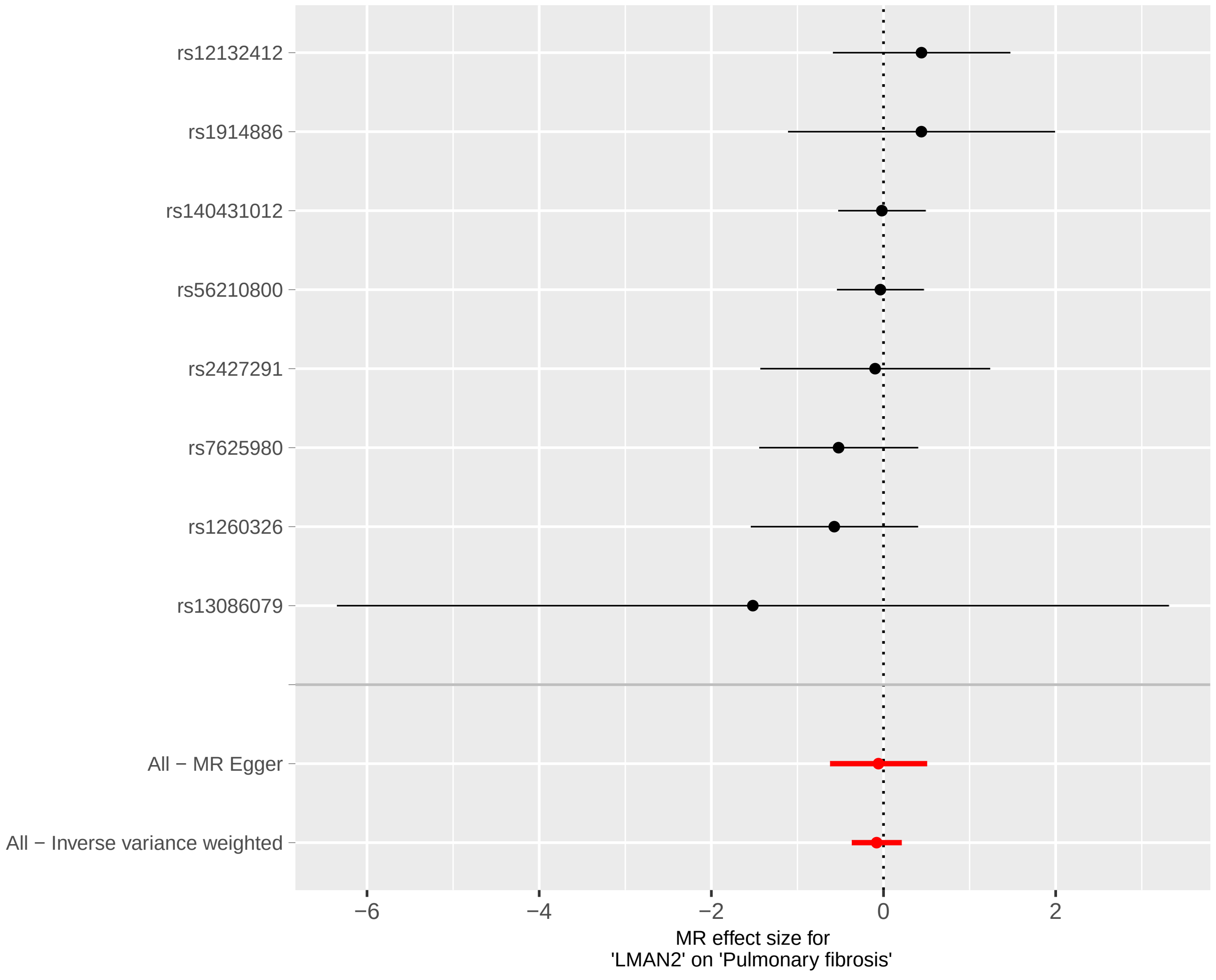

rs12132412
rs1914886
rs140431012
rs56210800
rs2427291
rs7625980
rs1260326
rs13086079
All − MR Egger
All − Inverse variance weighted
−6
−4
−2
0
2
MR effect size for
'LMAN2' on 'Pulmonary fibrosis'

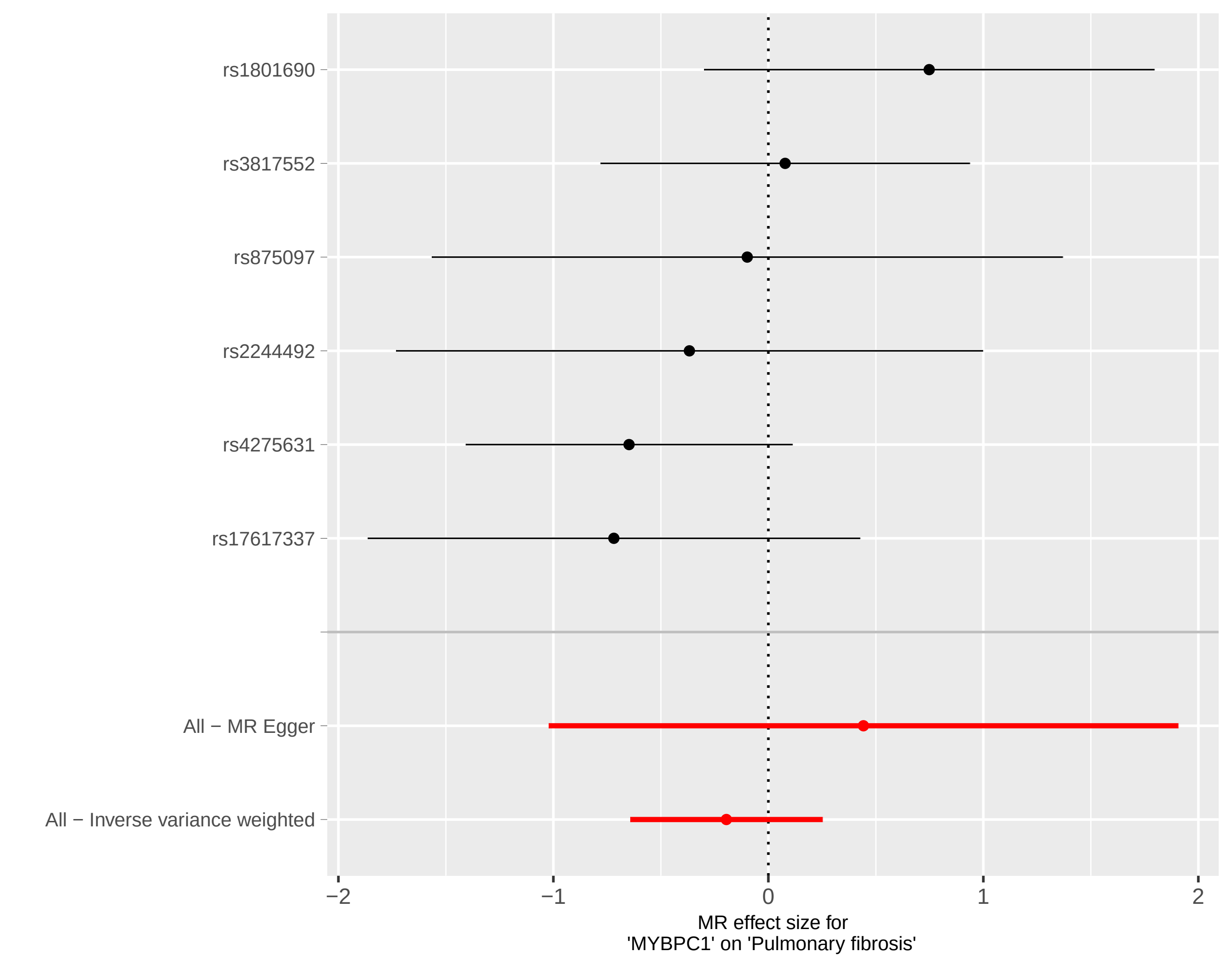
rs1801690
rs3817552
rs875097
rs2244492
rs4275631
rs17617337
All − MR Egger
All − Inverse variance weighted
−2
−1
0
1
2
MR effect size for
'MYBPC1' on 'Pulmonary fibrosis'

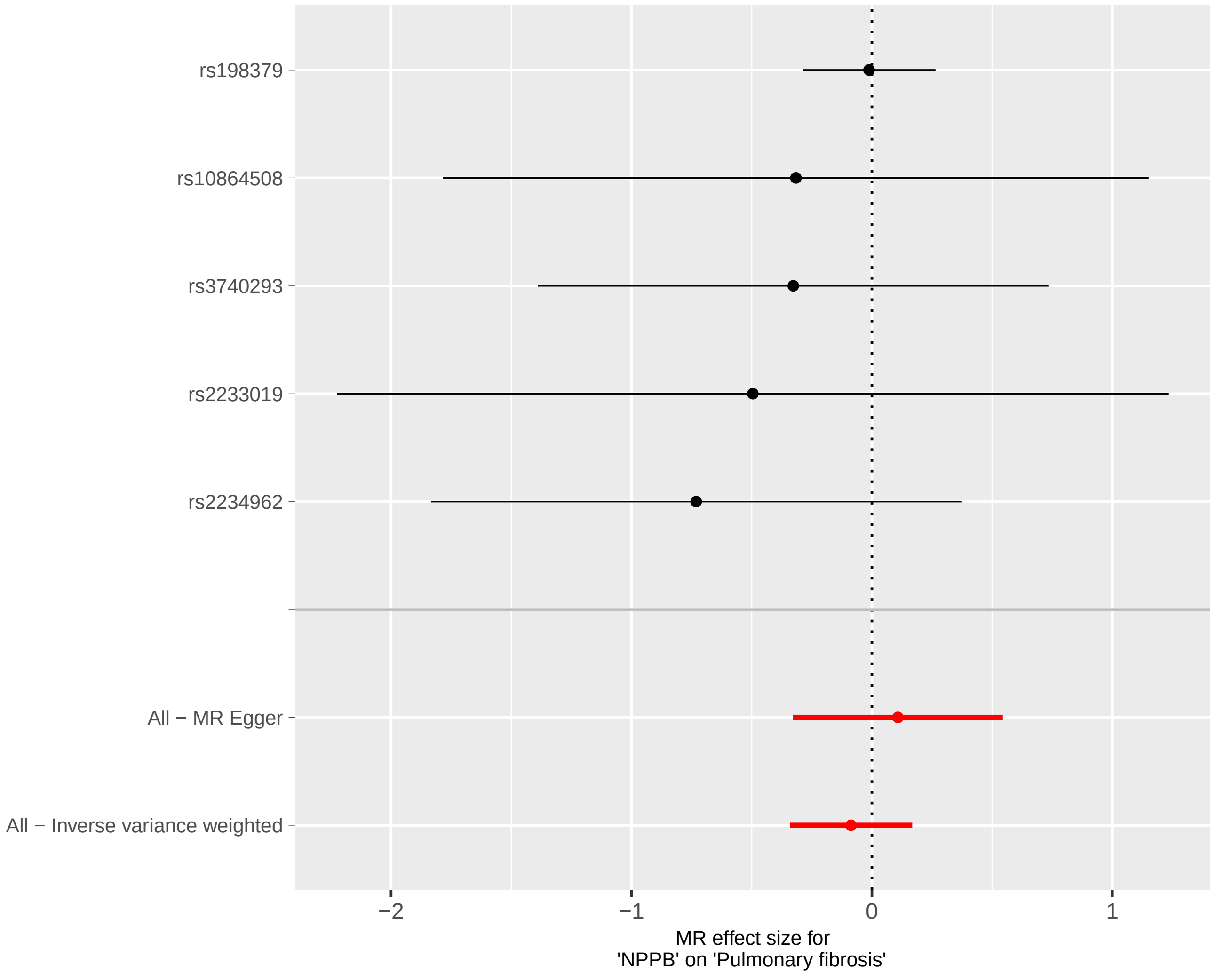
rs198379
rs10864508
rs3740293
rs2233019
rs2234962
All − MR Egger
All − Inverse variance weighted
−2
−1
0
1
MR effect size for
'NPPB' on 'Pulmonary fibrosis'

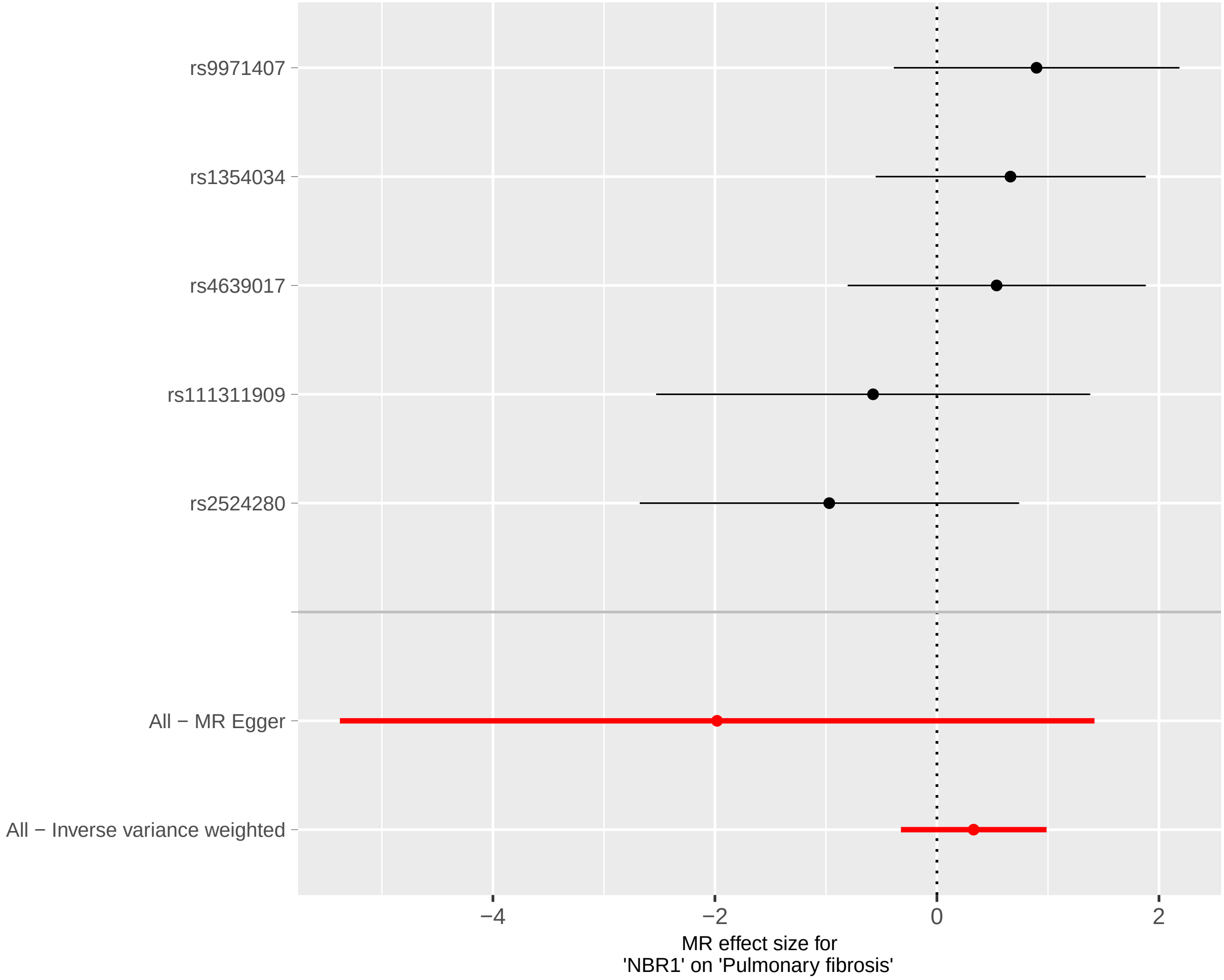

rs9971407
rs1354034
rs4639017
rs111311909
rs2524280
All − MR Egger
All − Inverse variance weighted
−4
−2
0
2
MR effect size for
'NBR1' on 'Pulmonary fibrosis'

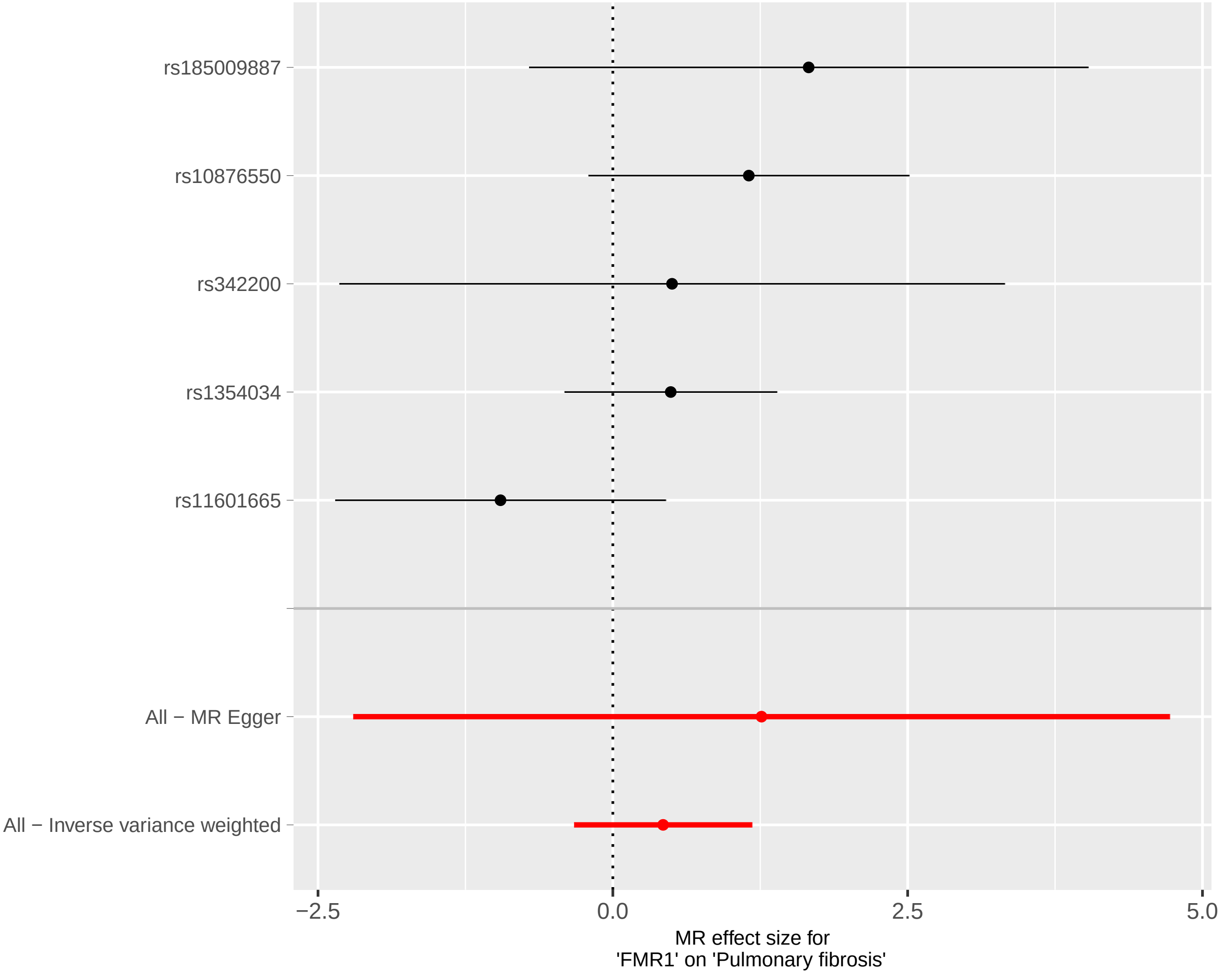

rs185009887
rs10876550
rs342200
rs1354034
rs11601665
All − MR Egger
All − Inverse variance weighted
−2.5
0.0
2.5
5.0
MR effect size for
'FMR1' on 'Pulmonary fibrosis'

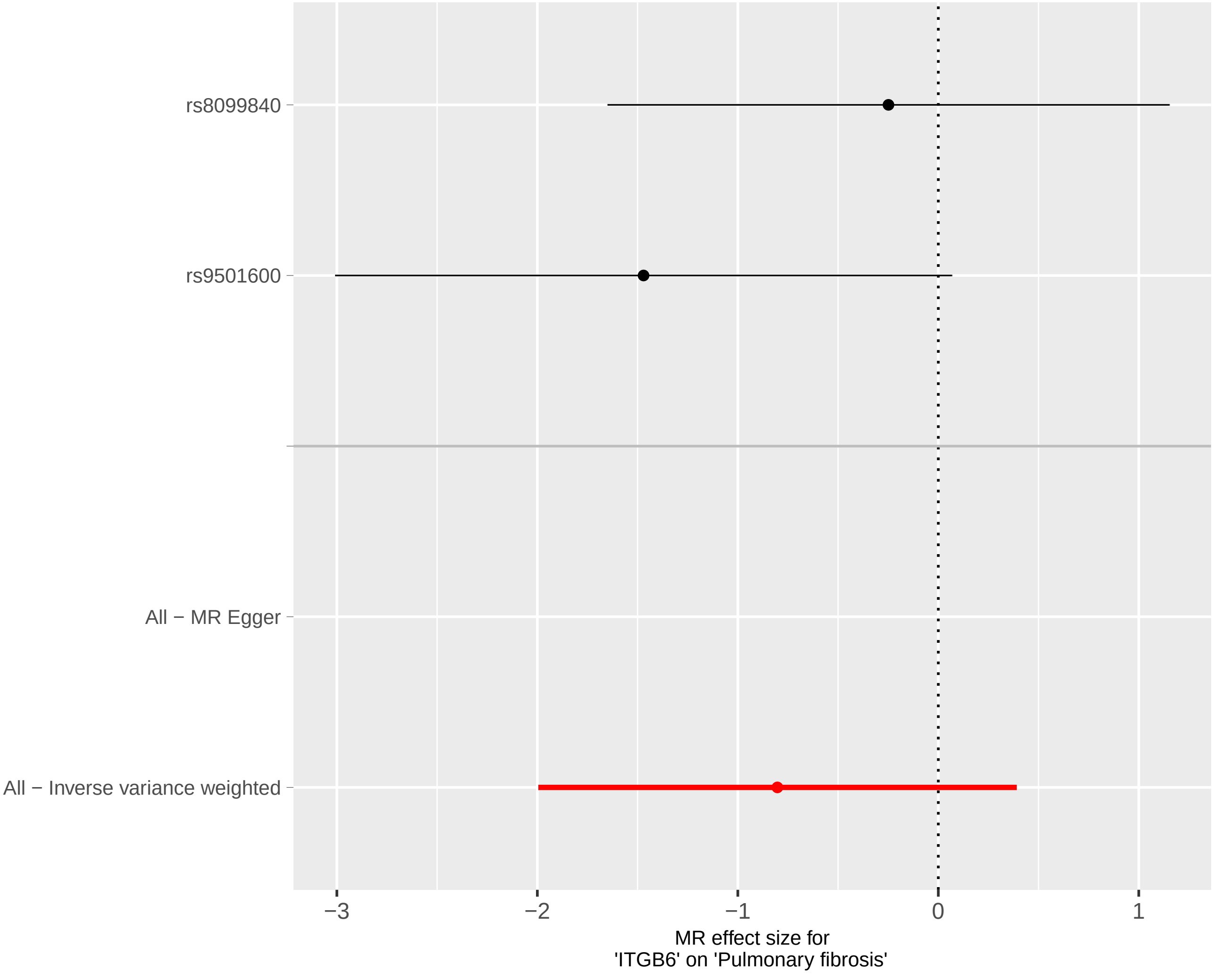

rs8099840
rs9501600
All − MR Egger
All − Inverse variance weighted
−3
−2
−1
0
1
MR effect size for
'ITGB6' on 'Pulmonary fibrosis'

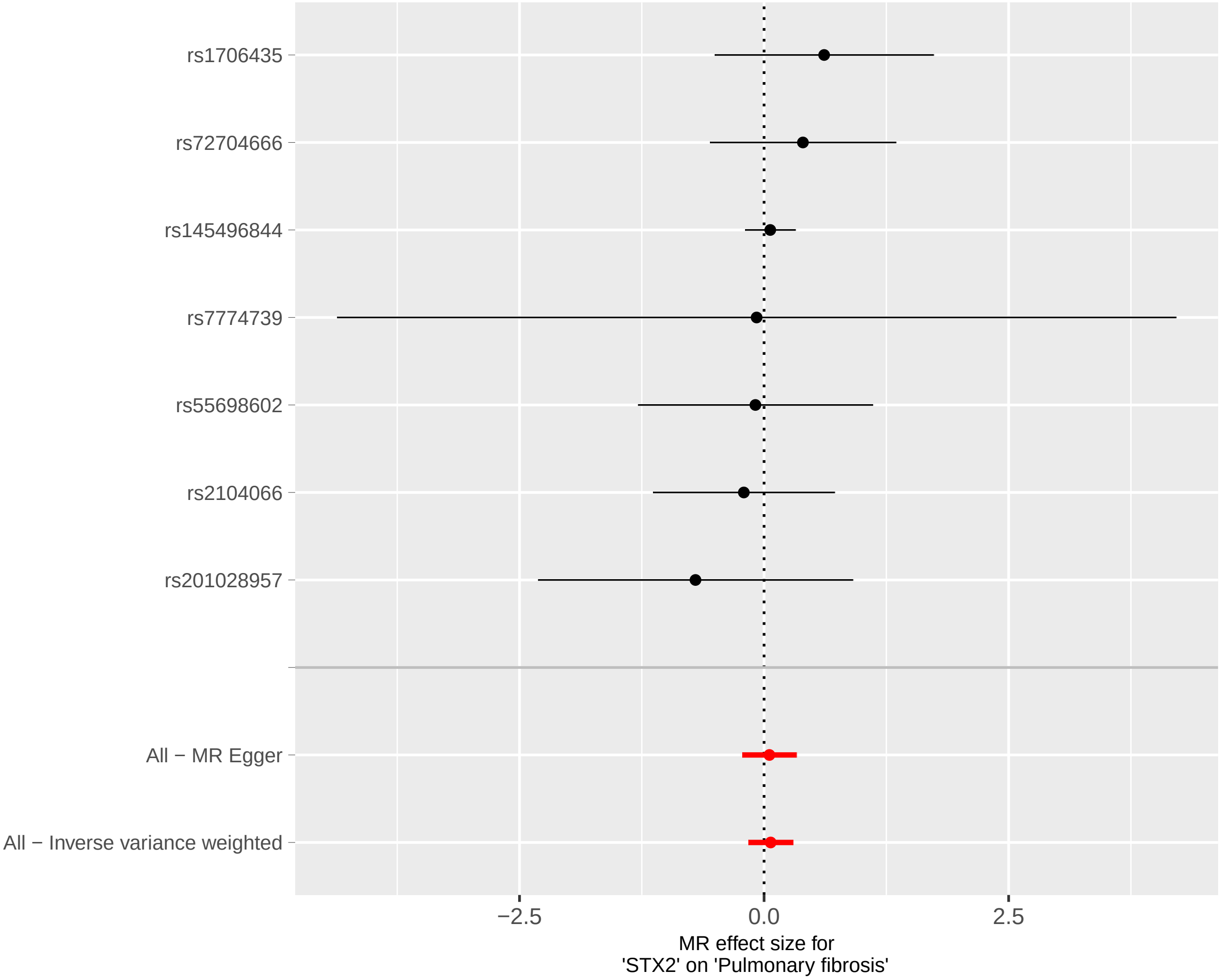

rs1706435
rs72704666
rs145496844
rs7774739
rs55698602
rs2104066
rs201028957
All − MR Egger
All − Inverse variance weighted
−2.5
0.0
2.5
MR effect size for
'STX2' on 'Pulmonary fibrosis'

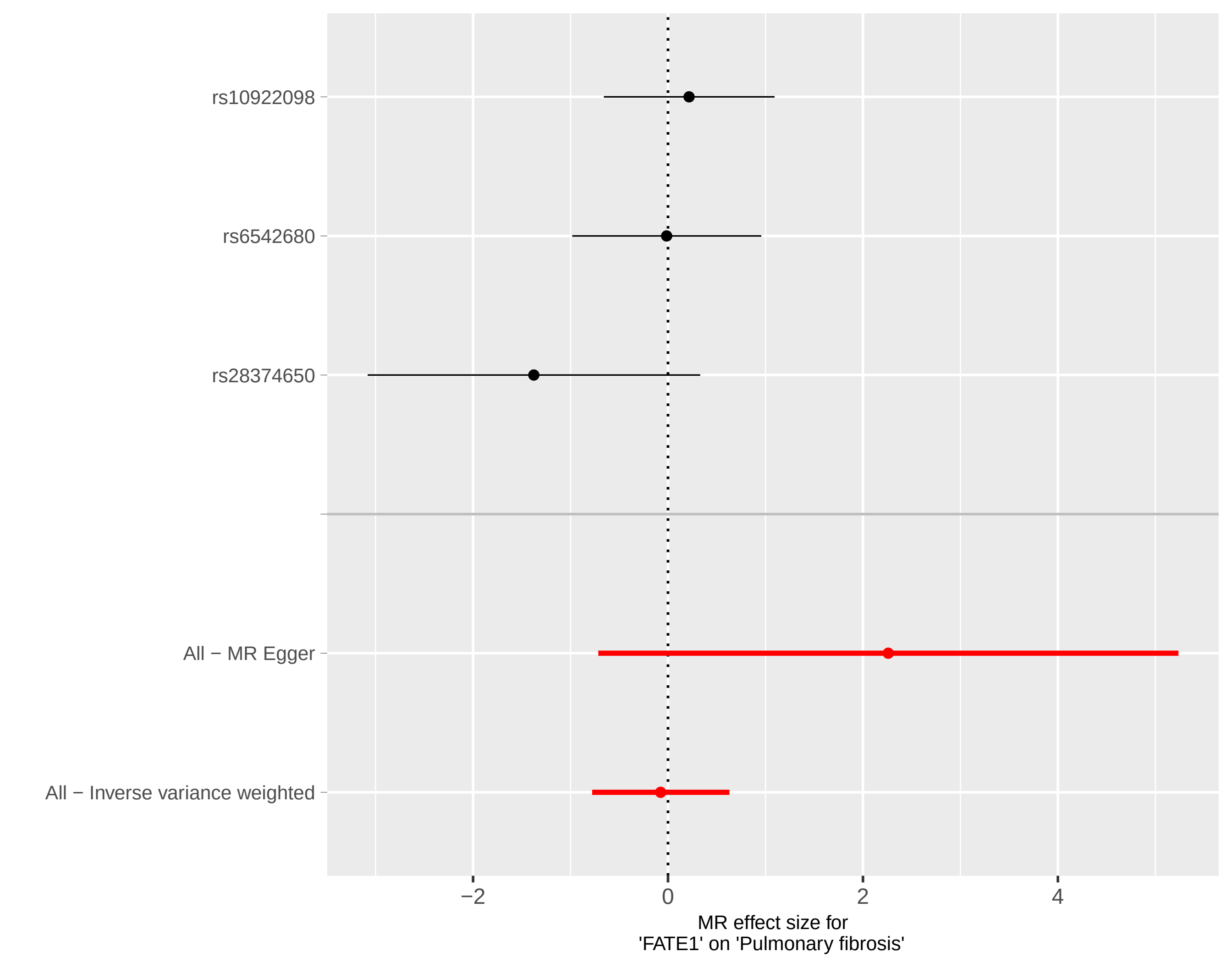

rs10922098
rs6542680
rs28374650
All − MR Egger
All − Inverse variance weighted
−2
0
2
4
MR effect size for
'FATE1' on 'Pulmonary fibrosis'

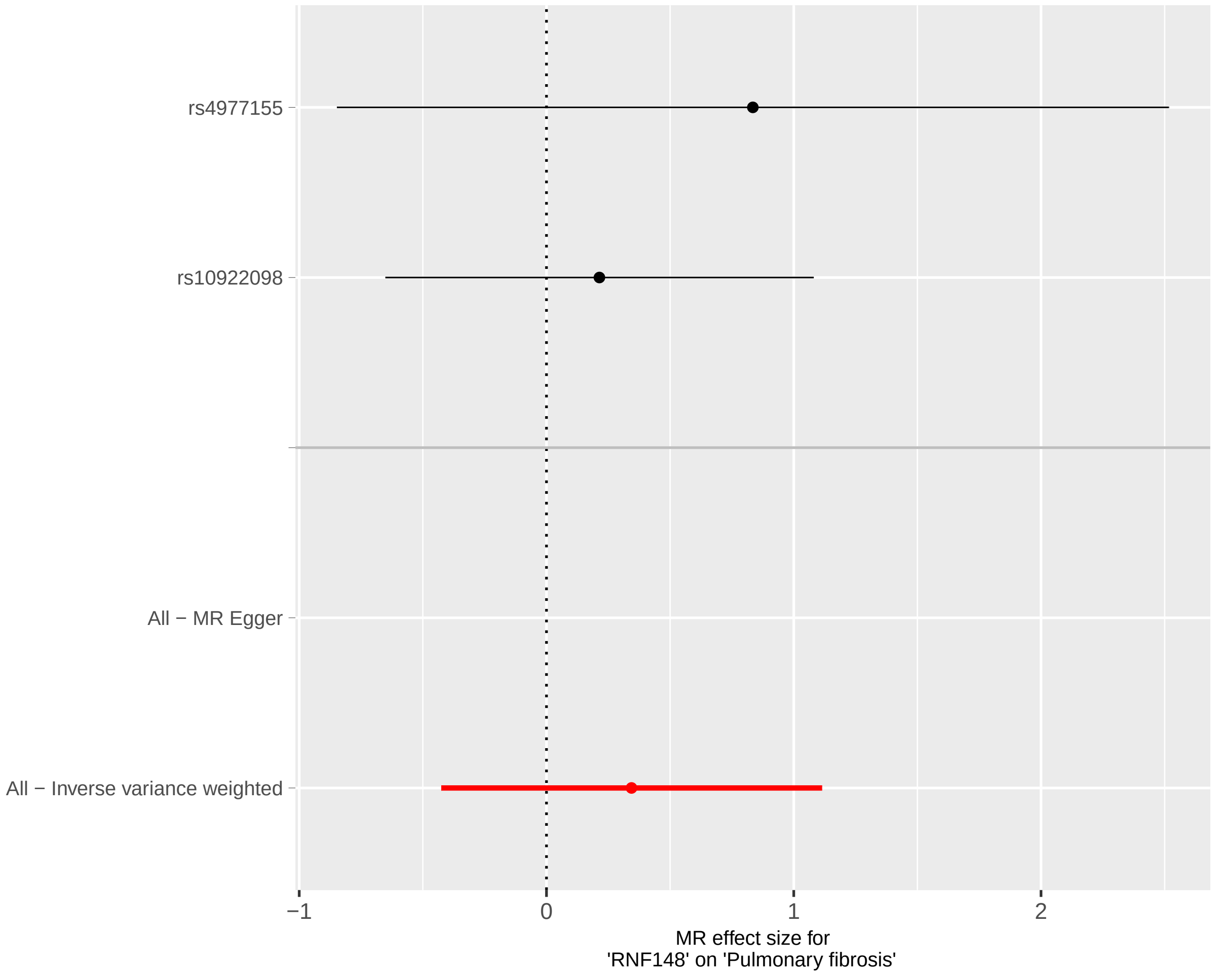

rs4977155
rs10922098
All − MR Egger
All − Inverse variance weighted
−1
0
1
2
MR effect size for
'RNF148' on 'Pulmonary fibrosis'

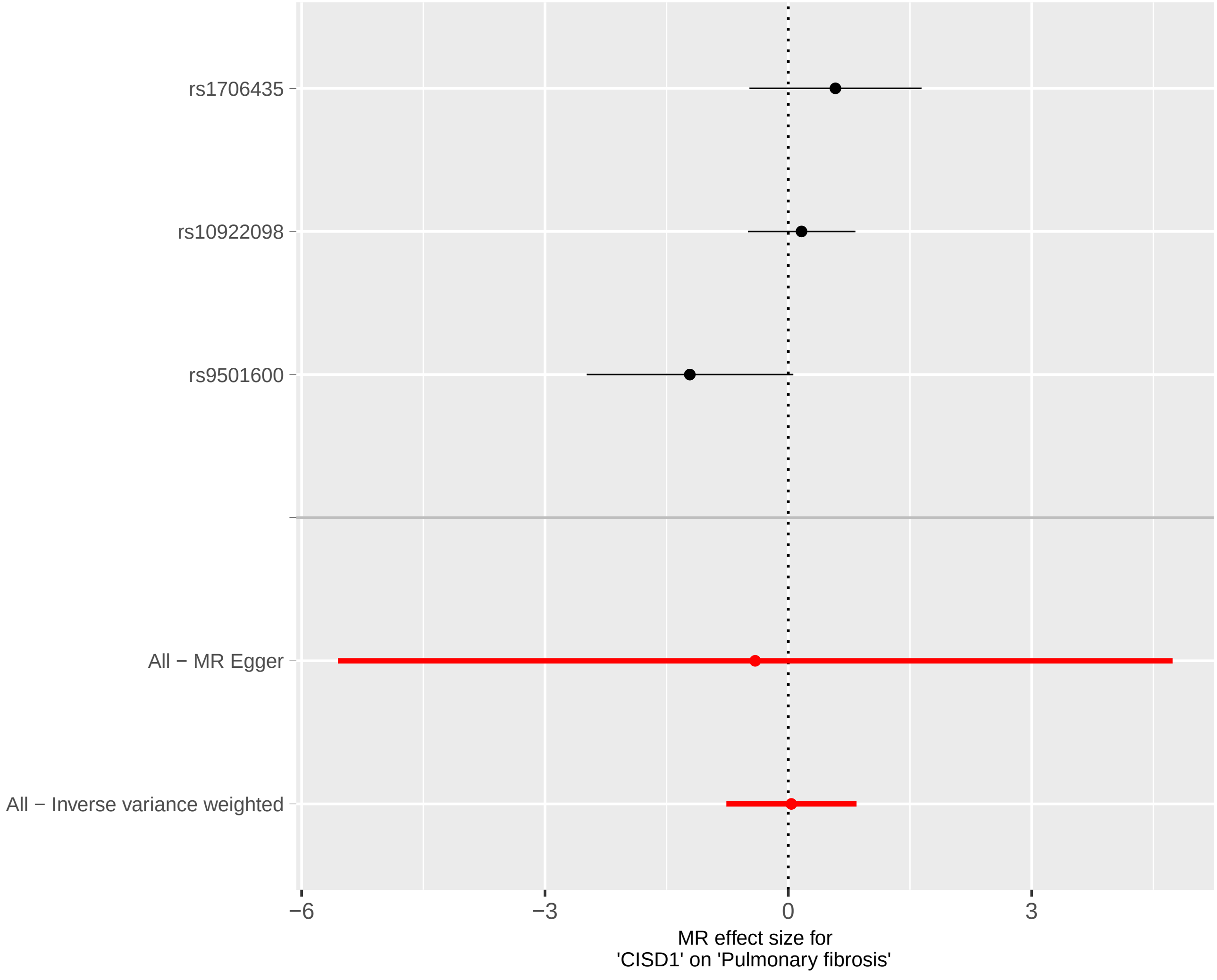

rs1706435
rs10922098
rs9501600
All − MR Egger
All − Inverse variance weighted
−6
−3
0
3
MR effect size for
'CISD1' on 'Pulmonary fibrosis'

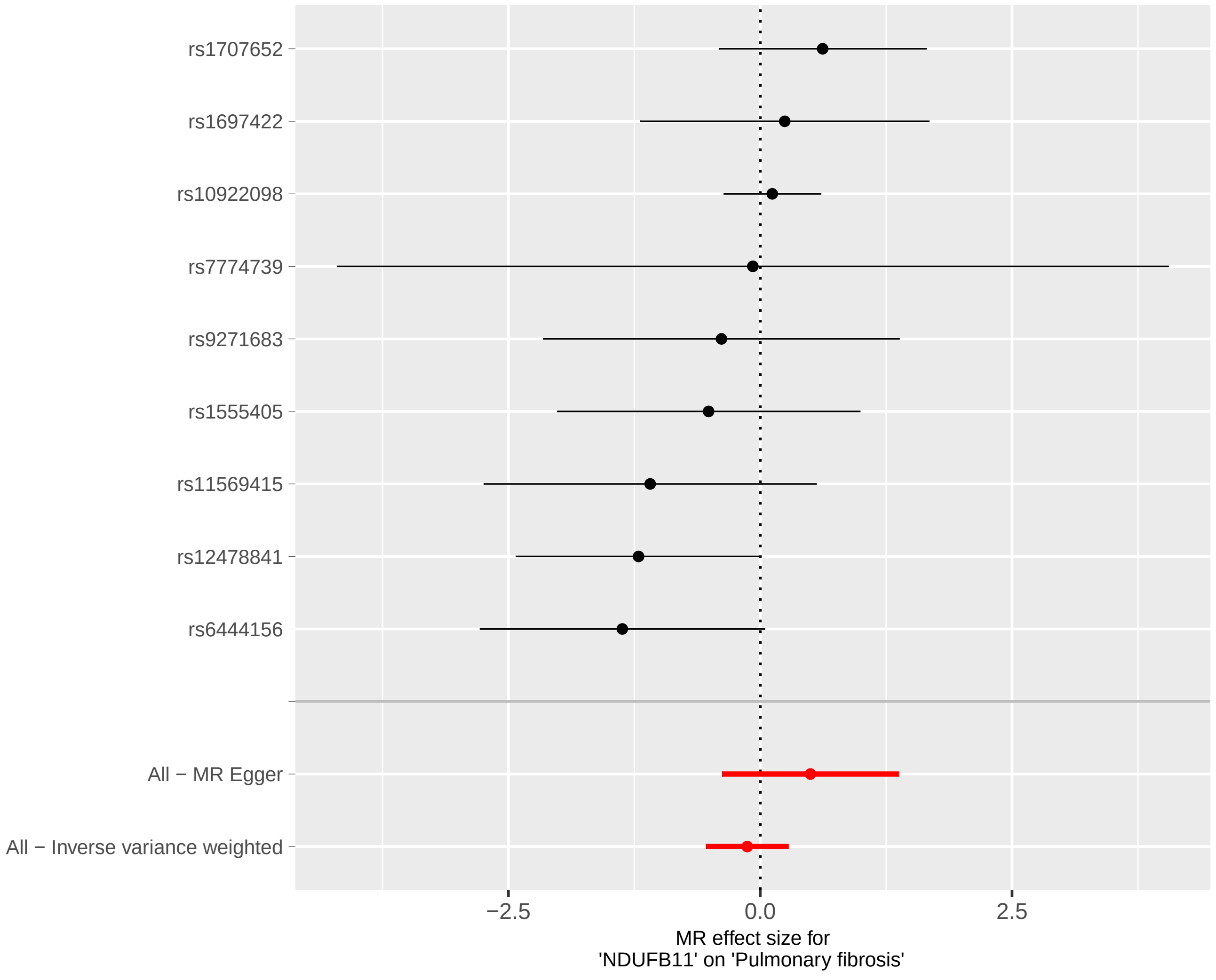
rs1707652
rs1697422
rs10922098
rs7774739
rs9271683
rs1555405
rs11569415
rs12478841
rs6444156
All − MR Egger
All − Inverse variance weighted
−2.5
0.0
2.5
MR effect size for
'NDUFB11' on 'Pulmonary fibrosis'

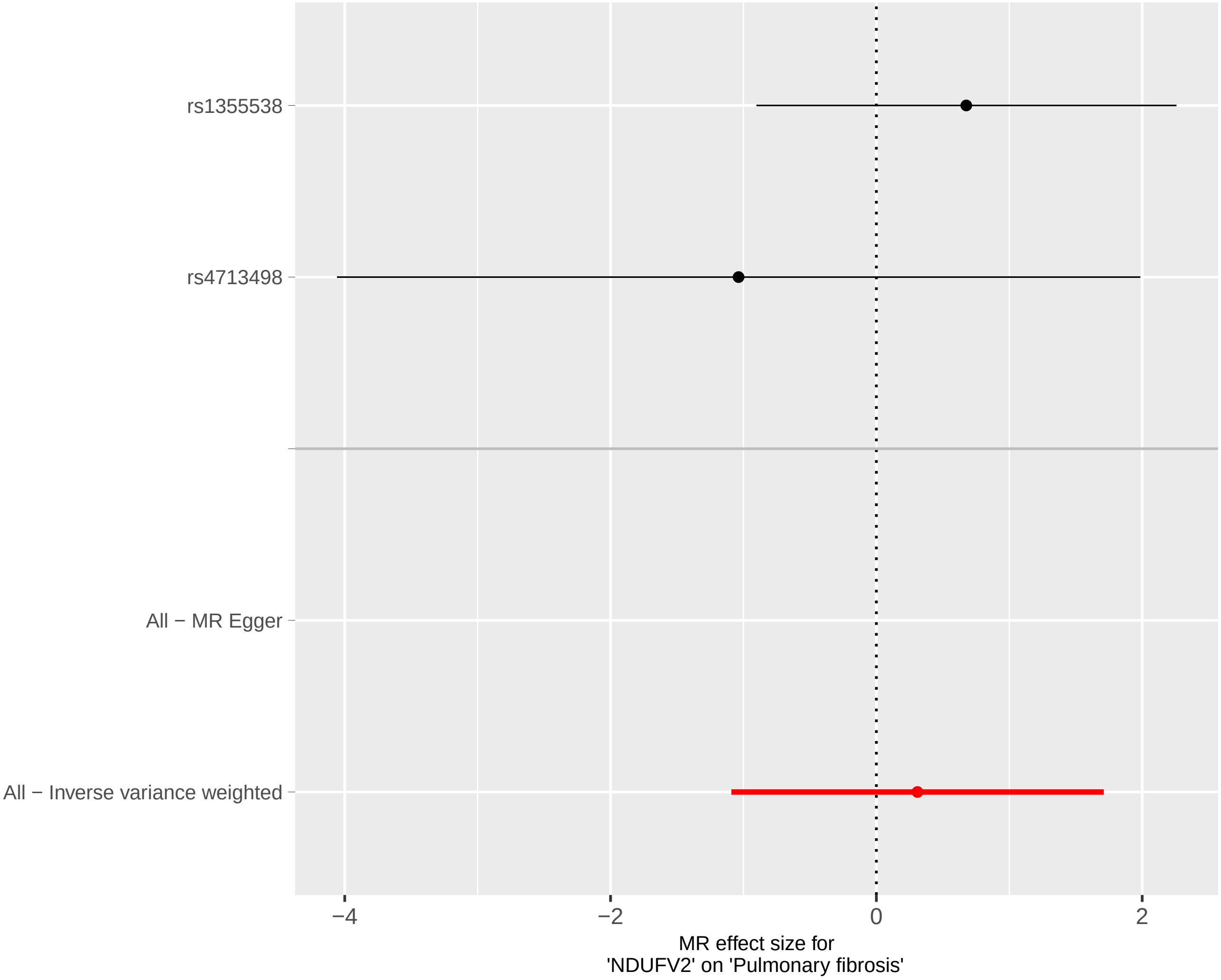
rs1355538
rs4713498
All − MR Egger
All − Inverse variance weighted
−4
−2
0
2
MR effect size for
'NDUFV2' on 'Pulmonary fibrosis'

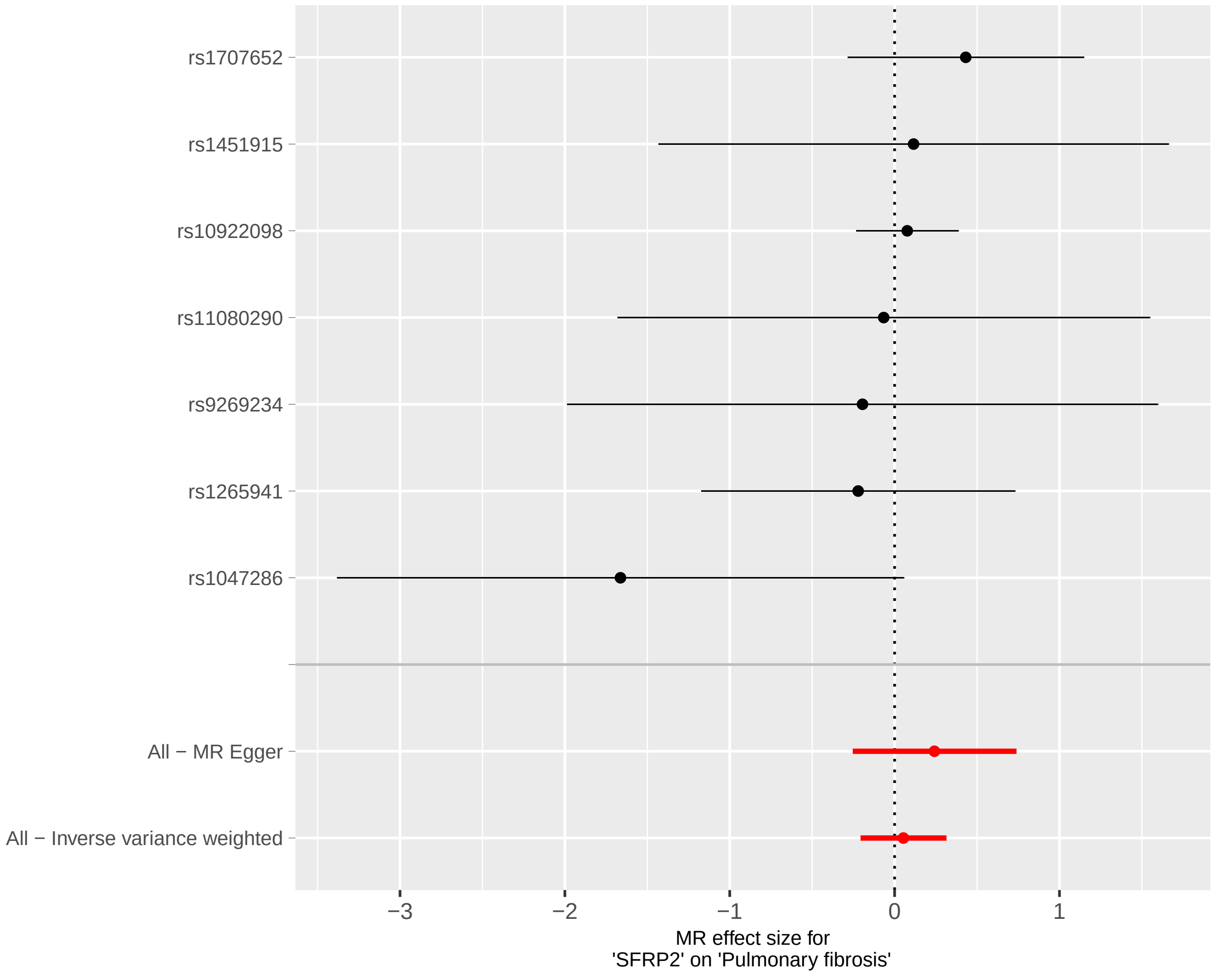

rs1707652
rs1451915
rs10922098
rs11080290
rs9269234
rs1265941
rs1047286
All − MR Egger
All − Inverse variance weighted
−3
−2
−1
0
1
MR effect size for
'SFRP2' on 'Pulmonary fibrosis'

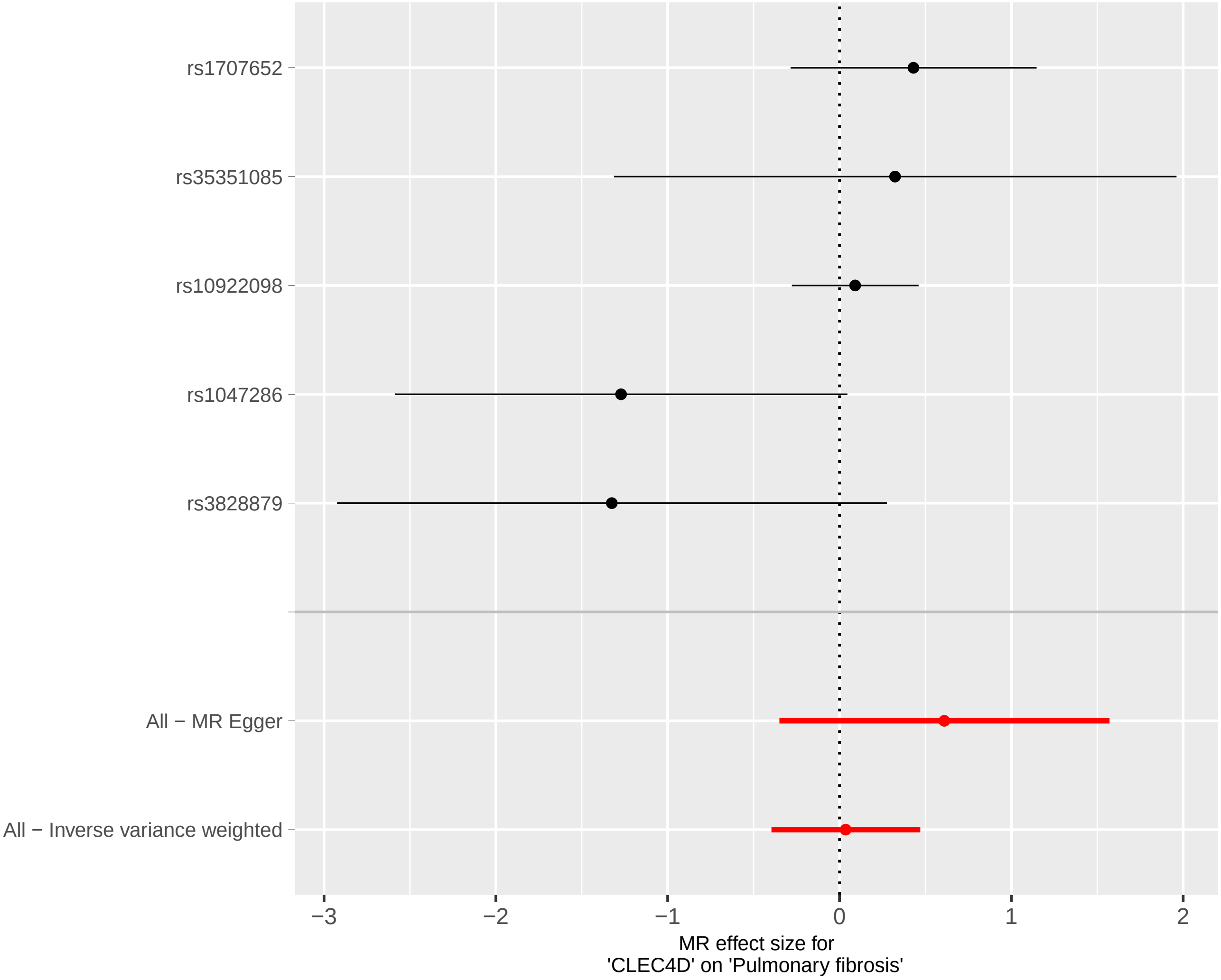
rs1707652
rs35351085
rs10922098
rs1047286
rs3828879
All − MR Egger
All − Inverse variance weighted
−3
−2
−1
0
1
2
MR effect size for
'CLEC4D' on 'Pulmonary fibrosis'

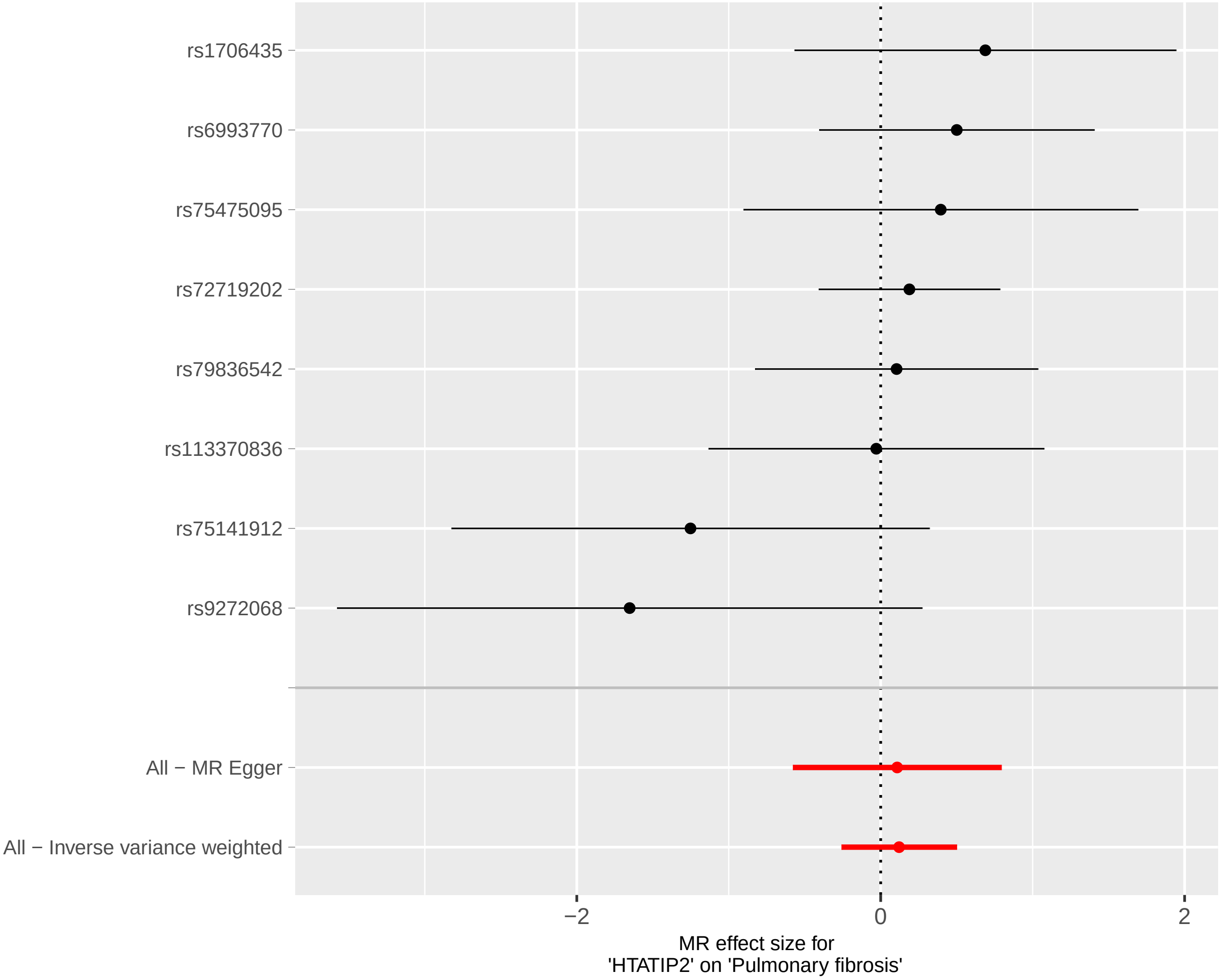
rs1706435
rs6993770
rs75475095
rs72719202
rs79836542
rs113370836
rs75141912
rs9272068
All − MR Egger
All − Inverse variance weighted
−2
0
2
MR effect size for
'HTATIP2' on 'Pulmonary fibrosis'

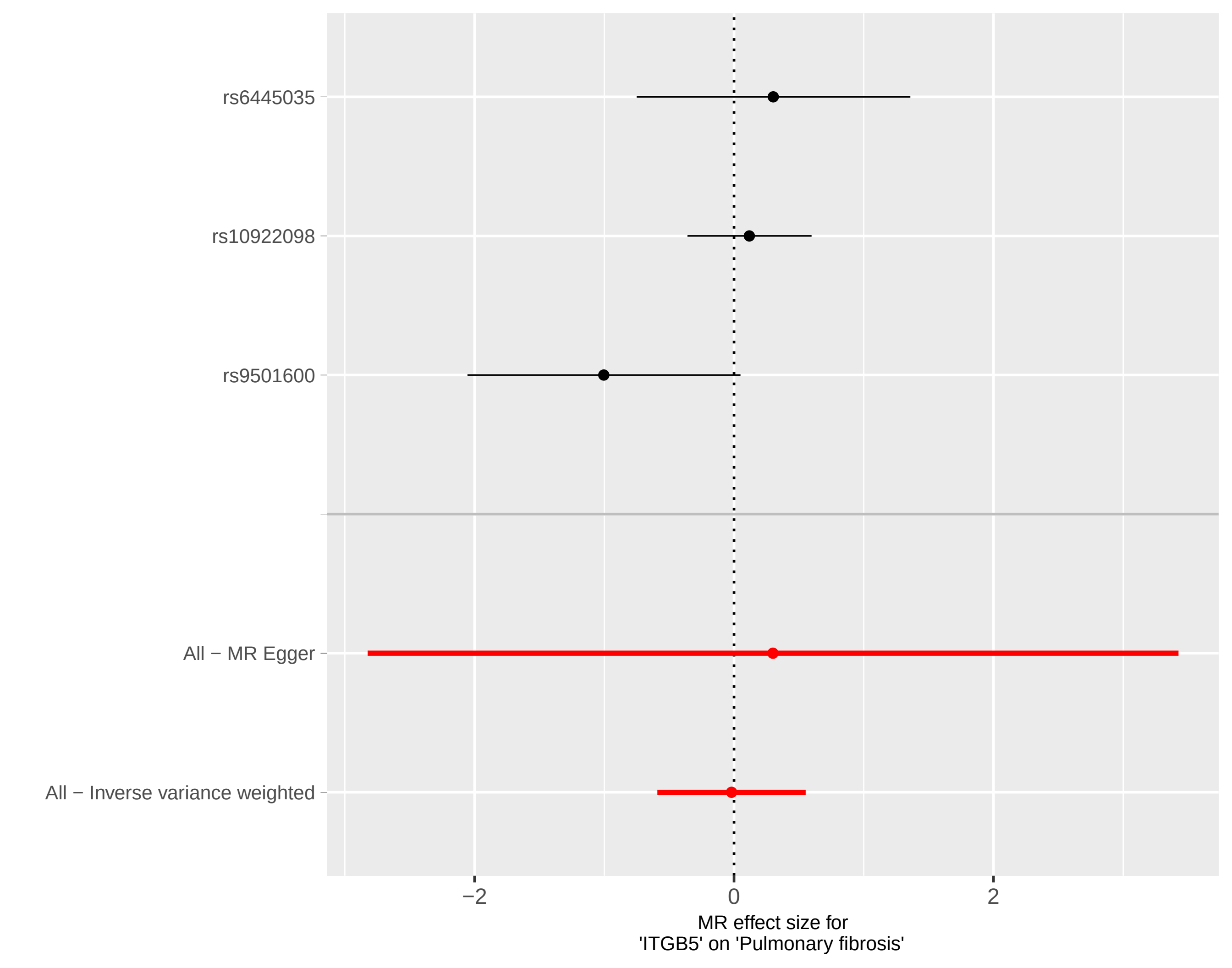

rs6445035
rs10922098
rs9501600
All − MR Egger
All − Inverse variance weighted
−2
0
2
MR effect size for
'ITGB5' on 'Pulmonary fibrosis'

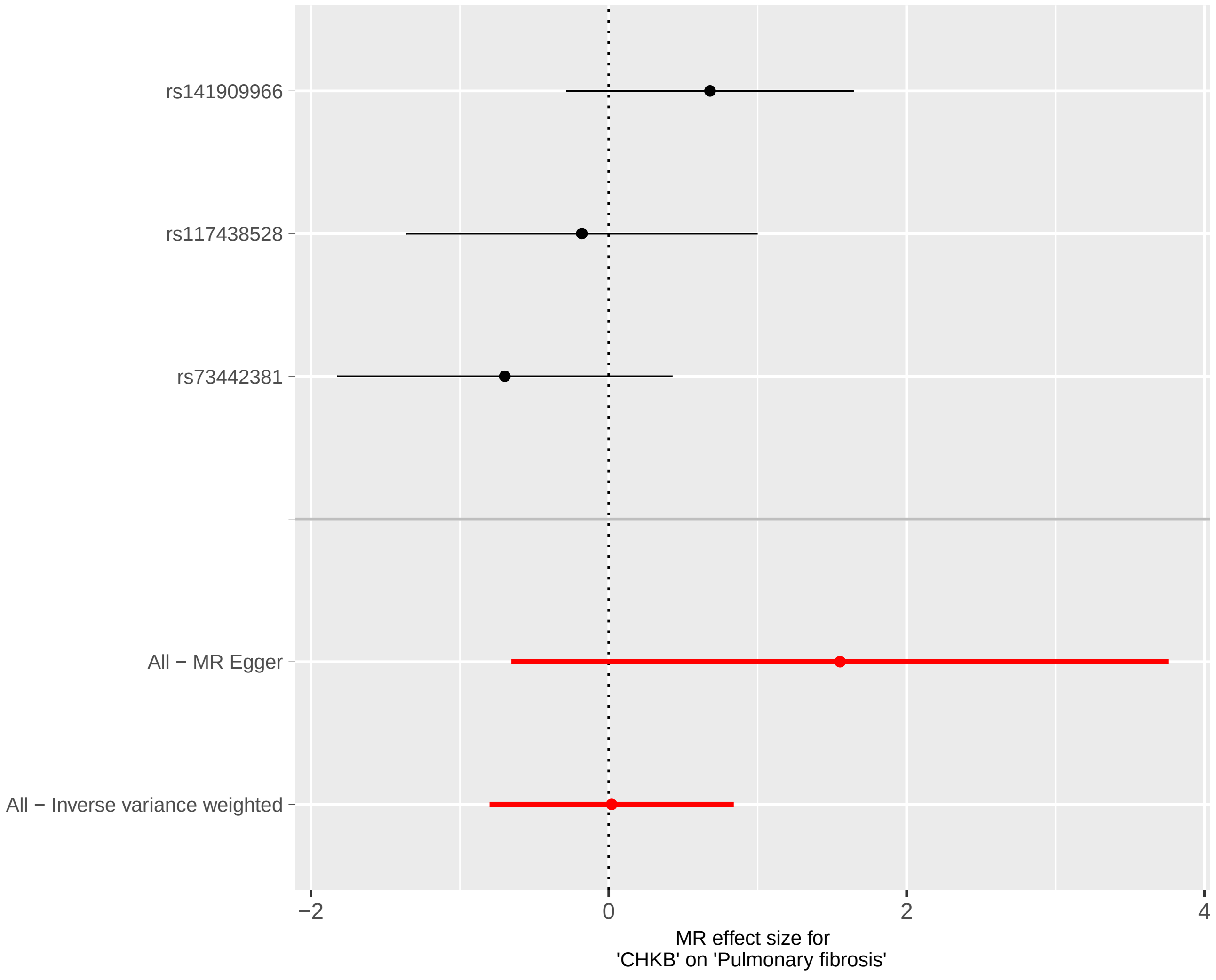

rs141909966
rs117438528
rs73442381
All − MR Egger
All − Inverse variance weighted
−2
0
2
4
MR effect size for
'CHKB' on 'Pulmonary fibrosis'

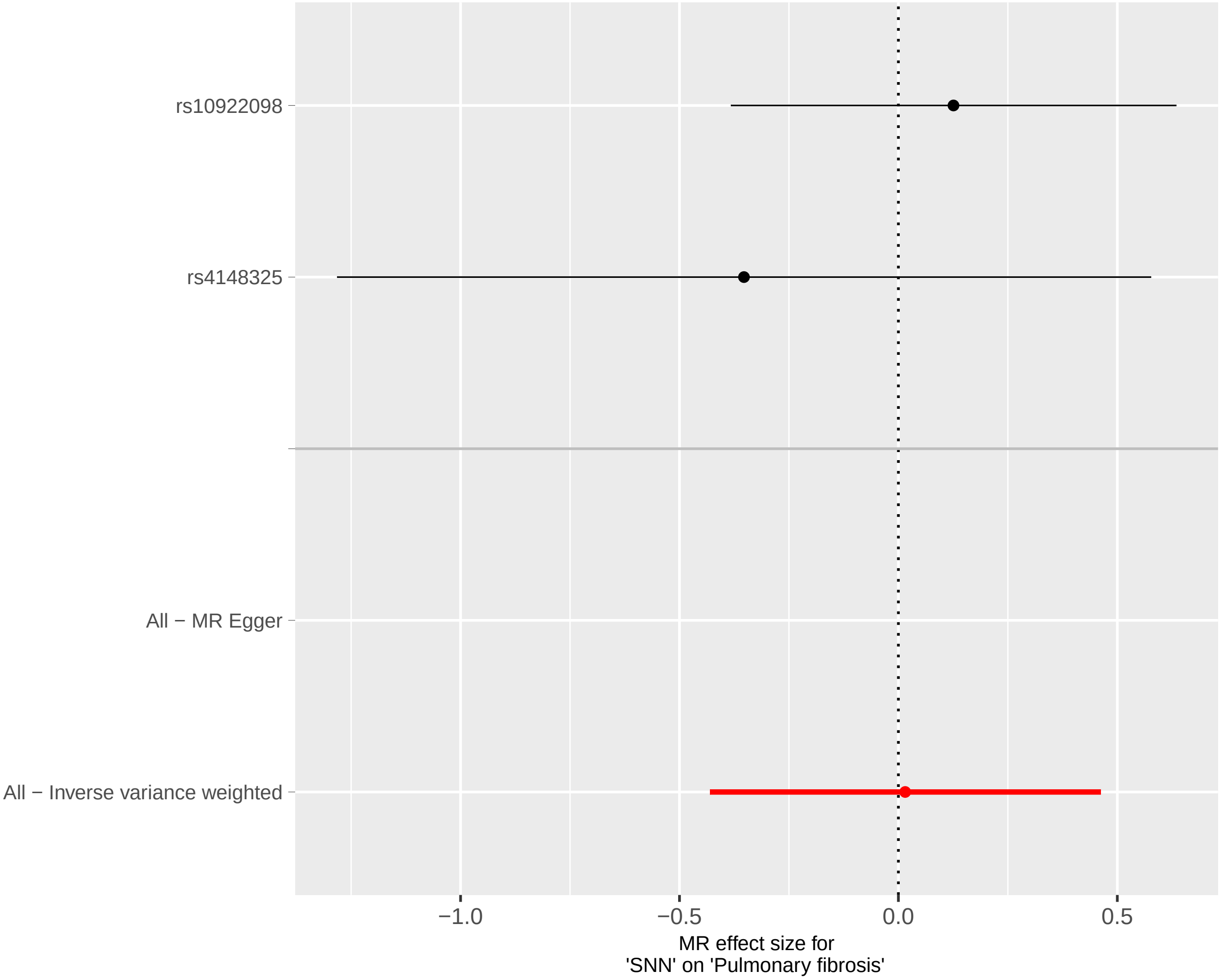

rs10922098
rs4148325
All − MR Egger
All − Inverse variance weighted
−1.0
−0.5
0.0
0.5
MR effect size for
'SNN' on 'Pulmonary fibrosis'

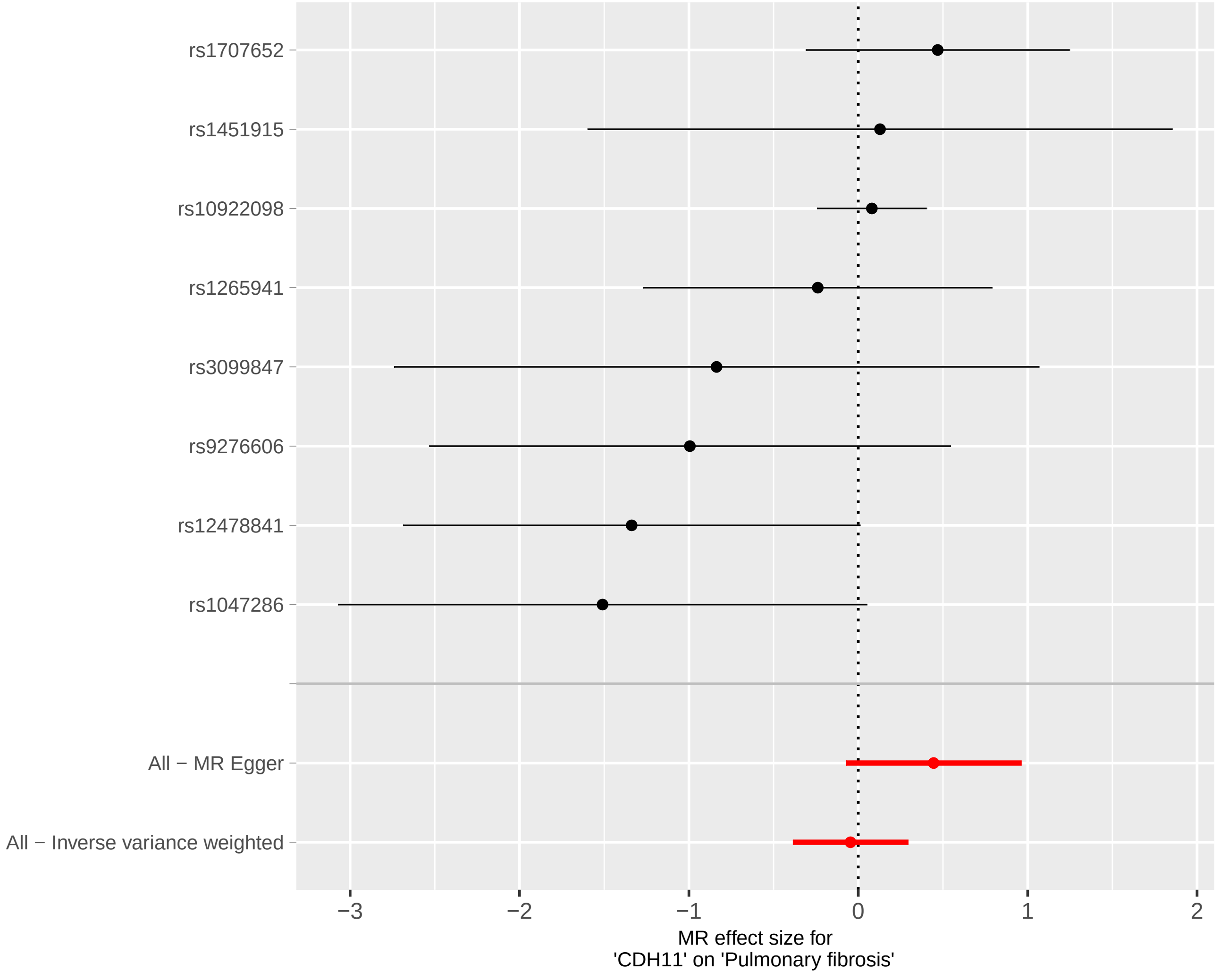
rs1707652
rs1451915
rs10922098
rs1265941
rs3099847
rs9276606
rs12478841
rs1047286
All − MR Egger
All − Inverse variance weighted
−3
−2
−1
0
1
2
MR effect size for
'CDH11' on 'Pulmonary fibrosis'

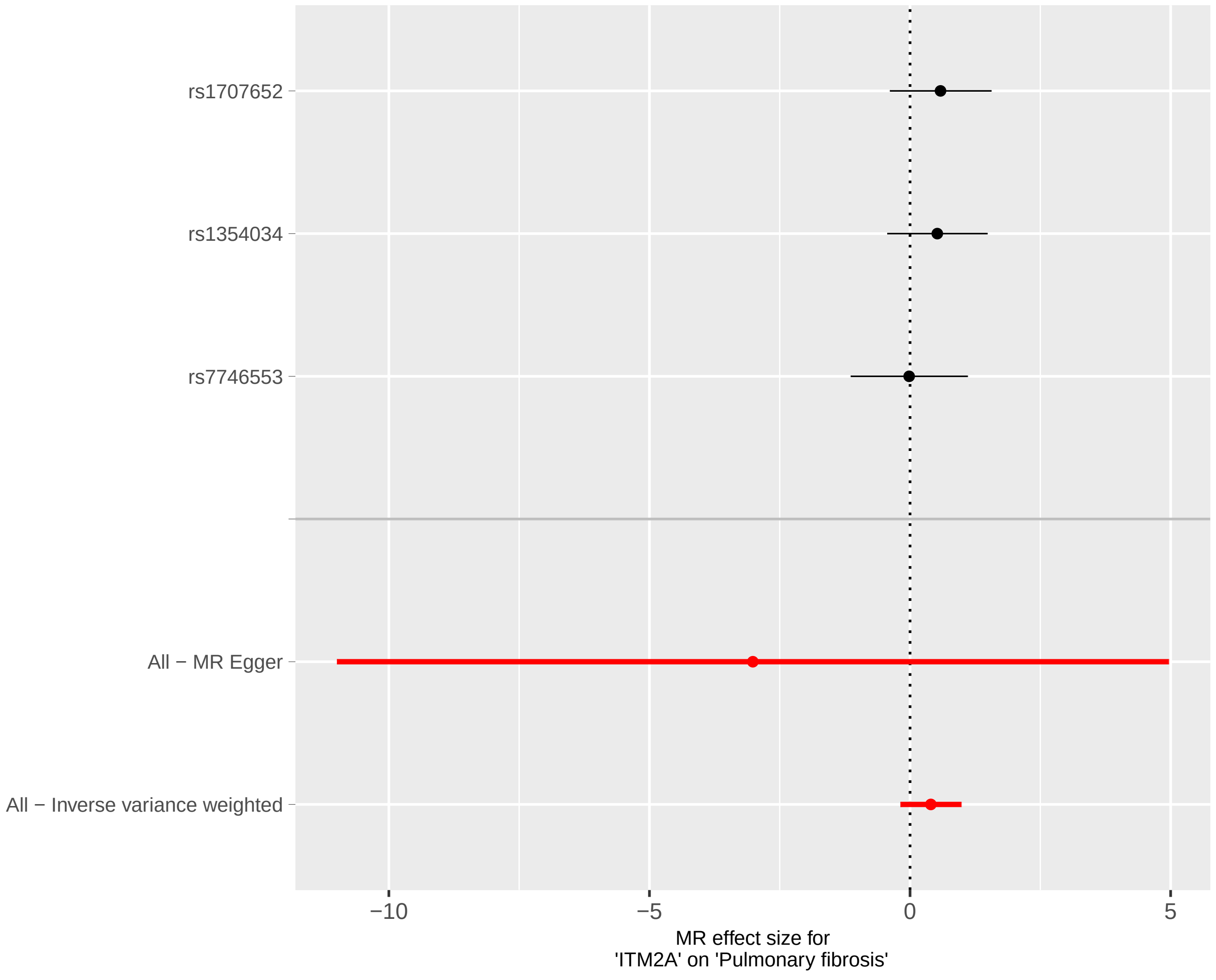
rs1707652
rs1354034
rs7746553
All − MR Egger
All − Inverse variance weighted
−10
−5
0
5
MR effect size for
'ITM2A' on 'Pulmonary fibrosis'

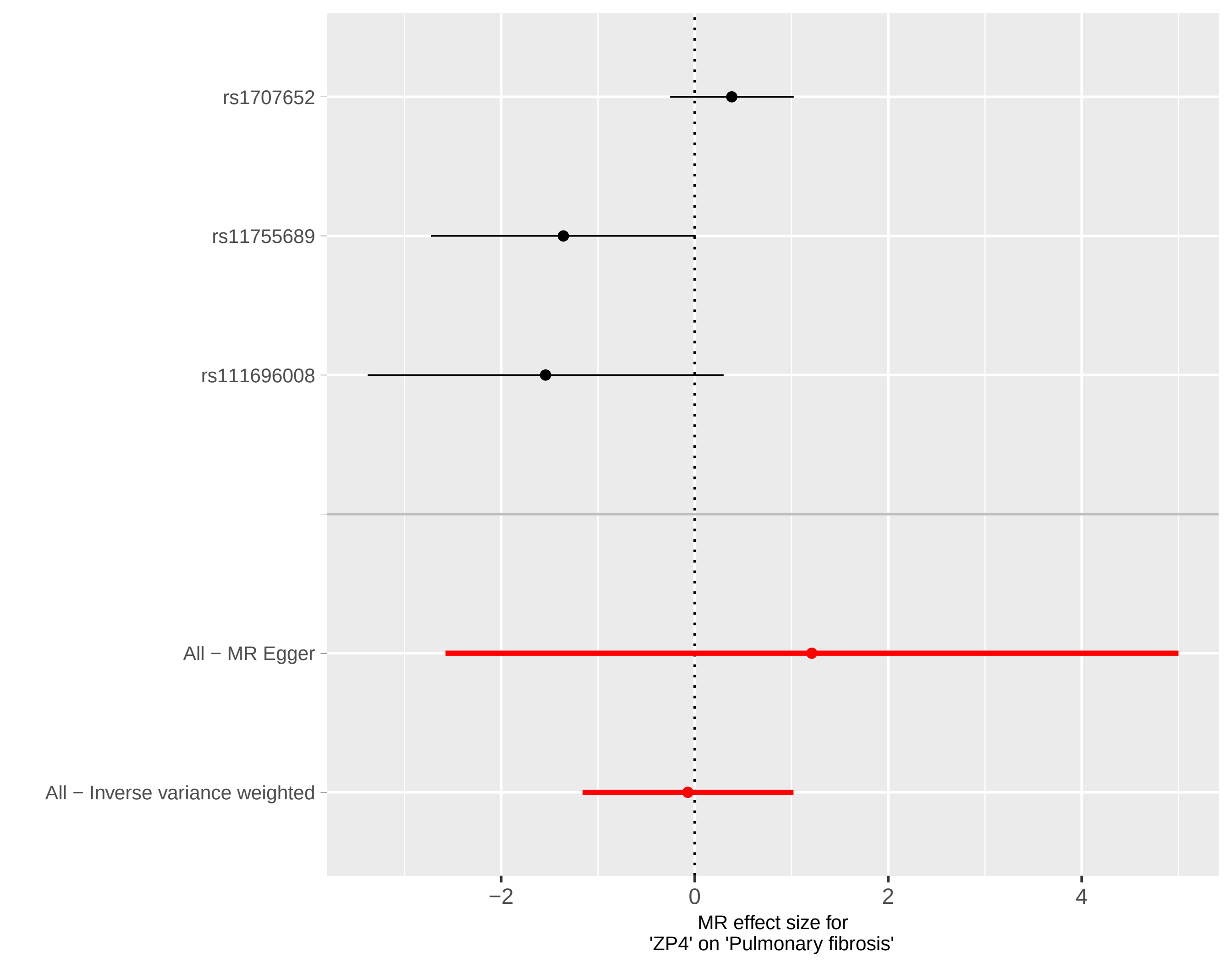

rs1707652
rs11755689
rs111696008
All − MR Egger
All − Inverse variance weighted
−2
0
2
4
MR effect size for
'ZP4' on 'Pulmonary fibrosis'

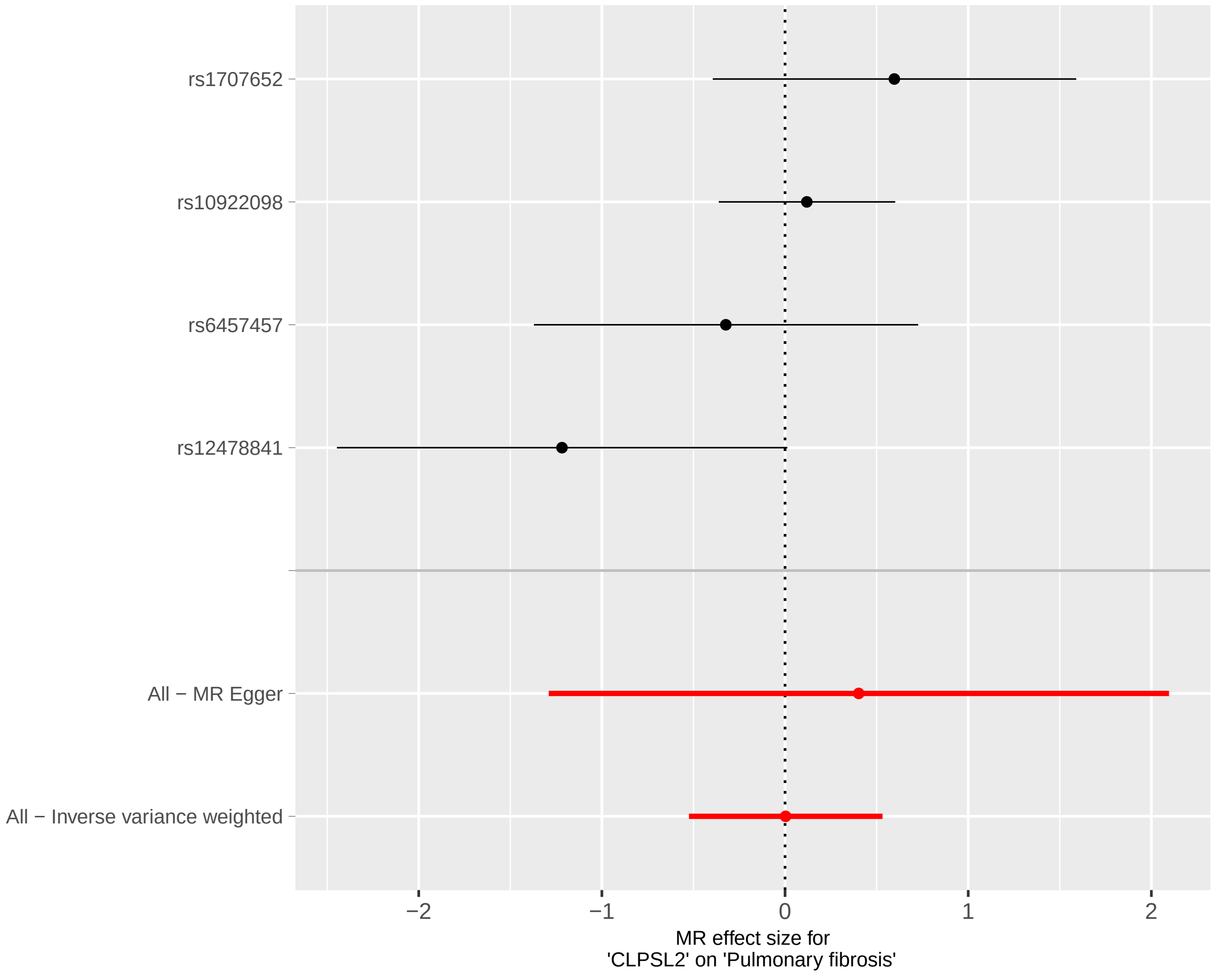

rs1707652
rs10922098
rs6457457
rs12478841
All − MR Egger
All − Inverse variance weighted
−2
−1
0
1
2
MR effect size for
'CLPSL2' on 'Pulmonary fibrosis'

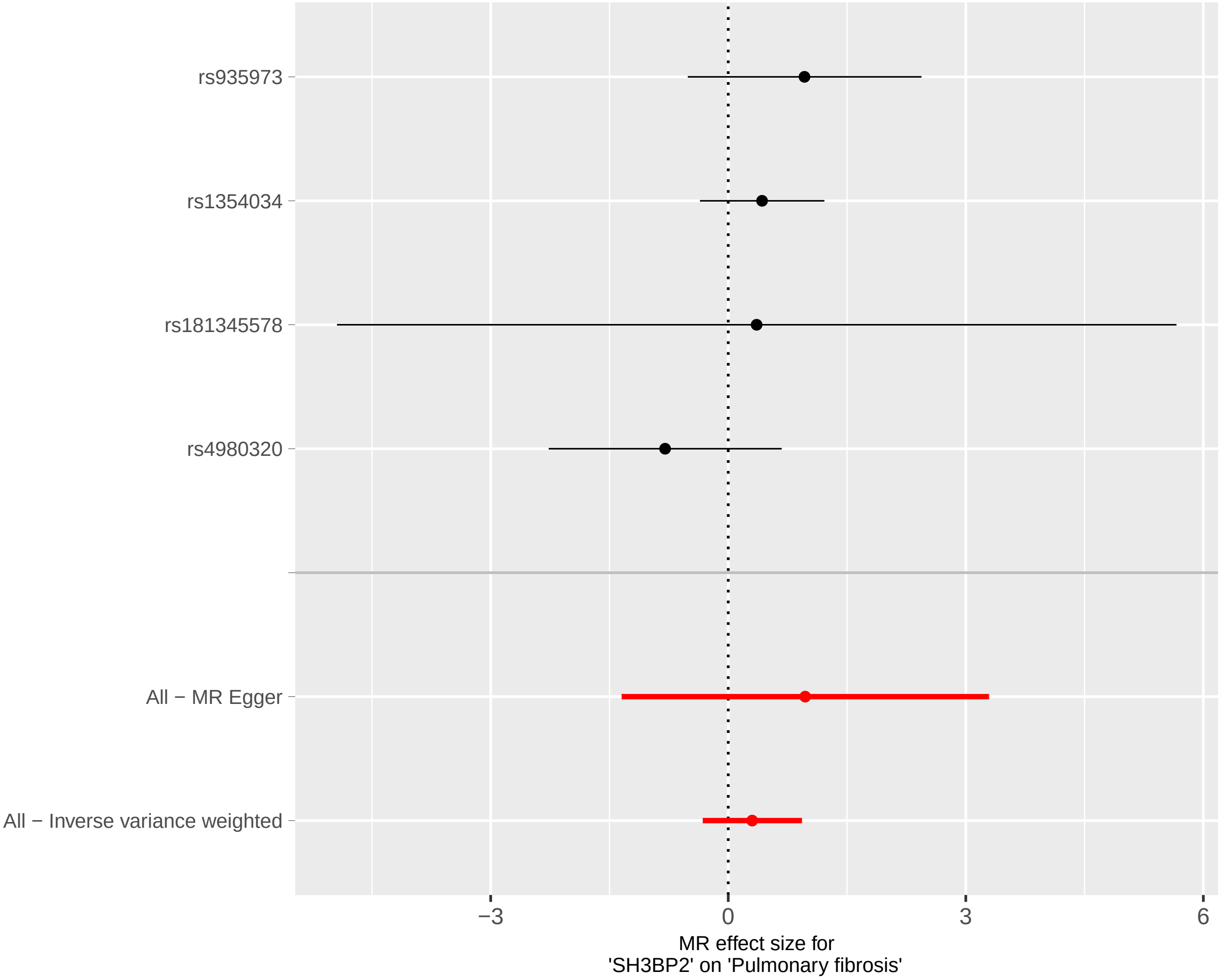

rs935973
rs1354034
rs181345578
rs4980320
All − MR Egger
All − Inverse variance weighted
−3
0
3
6
MR effect size for
'SH3BP2' on 'Pulmonary fibrosis'

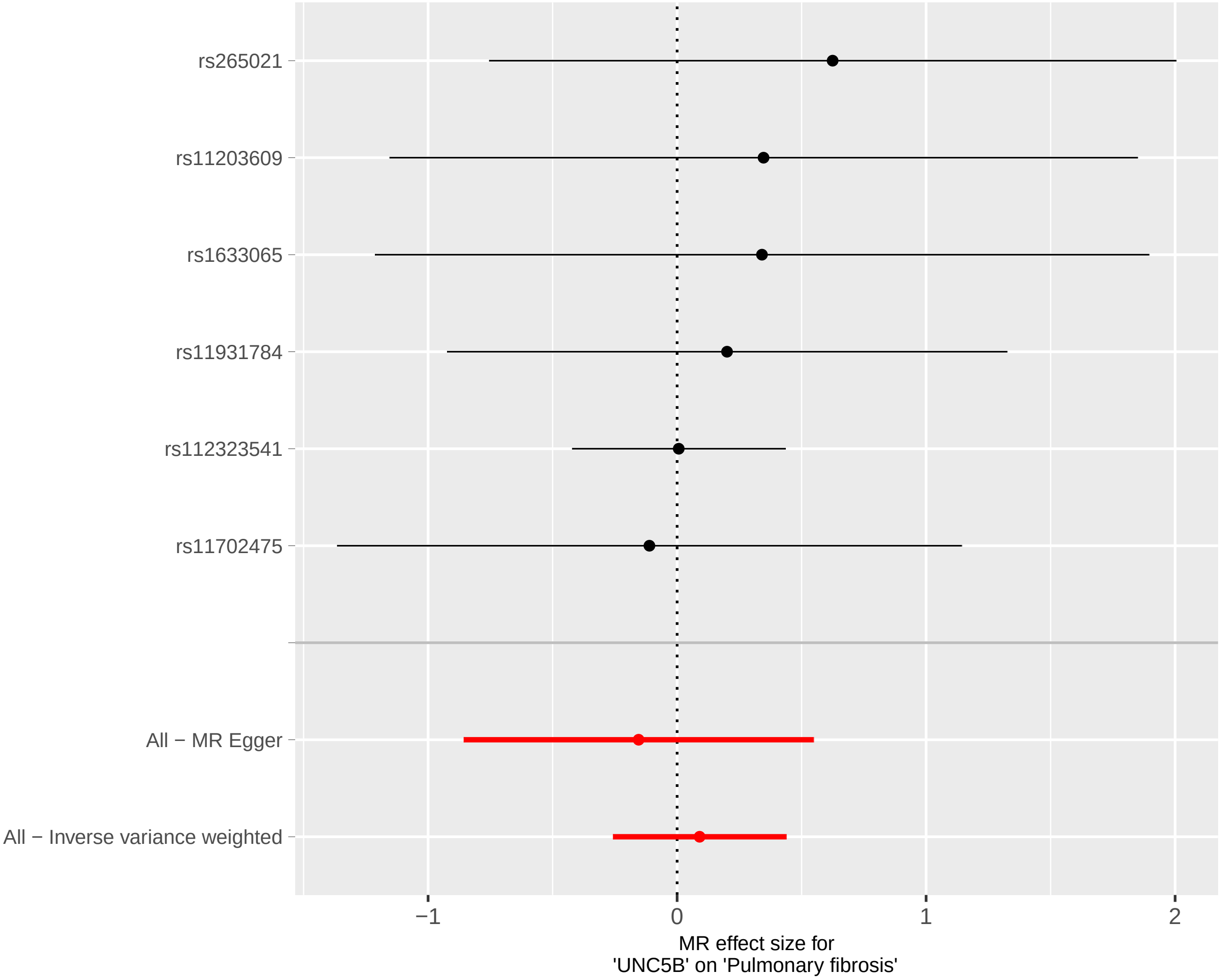

rs265021
rs11203609
rs1633065
rs11931784
rs112323541
rs11702475
All − MR Egger
All − Inverse variance weighted
−1
0
1
2
MR effect size for
'UNC5B' on 'Pulmonary fibrosis'

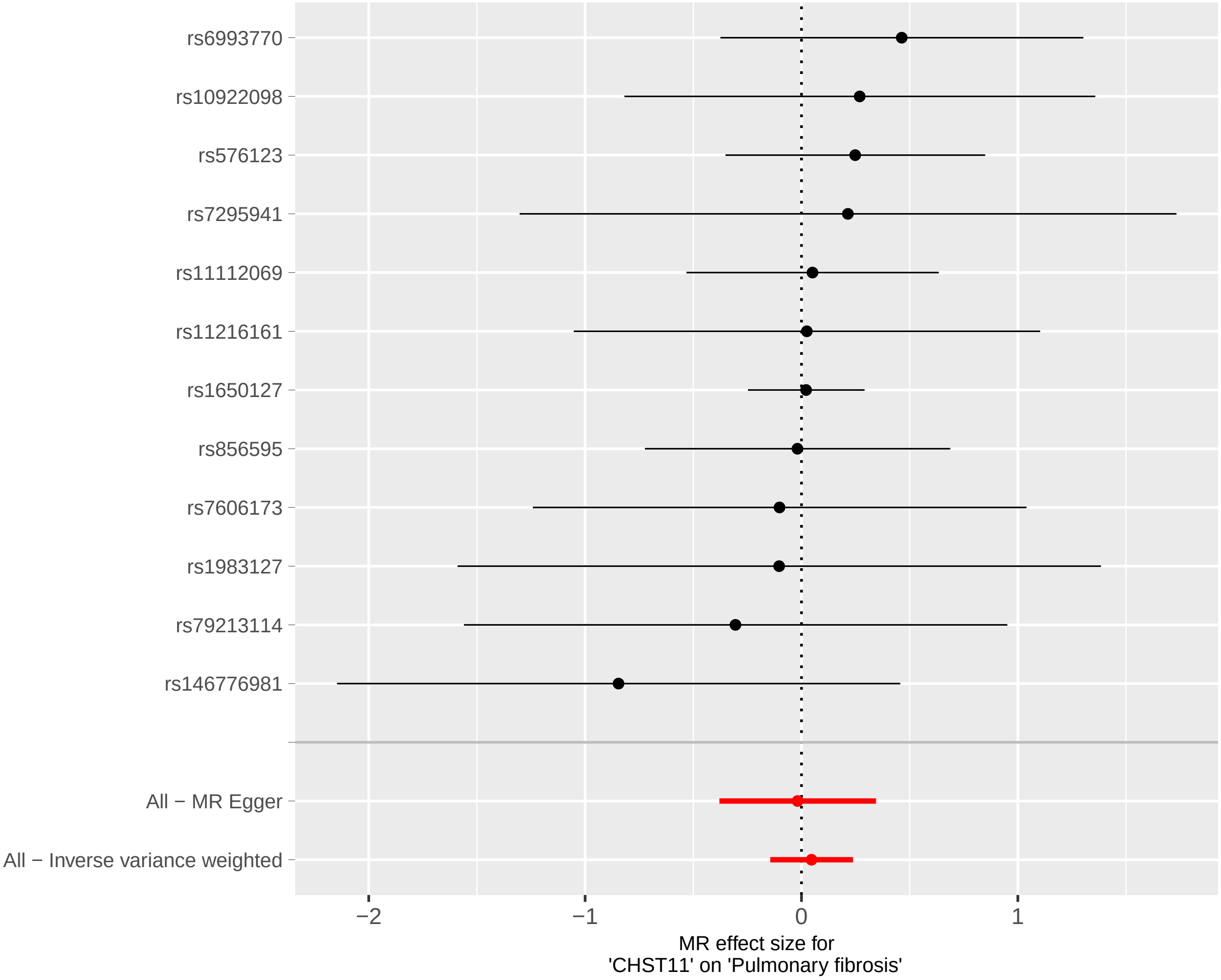
rs6993770
rs10922098
rs576123
rs7295941
rs11112069
rs11216161
rs1650127
rs856595
rs7606173
rs1983127
rs79213114
rs146776981
All − MR Egger
All − Inverse variance weighted
−2
−1
0
1
MR effect size for
'CHST11' on 'Pulmonary fibrosis'

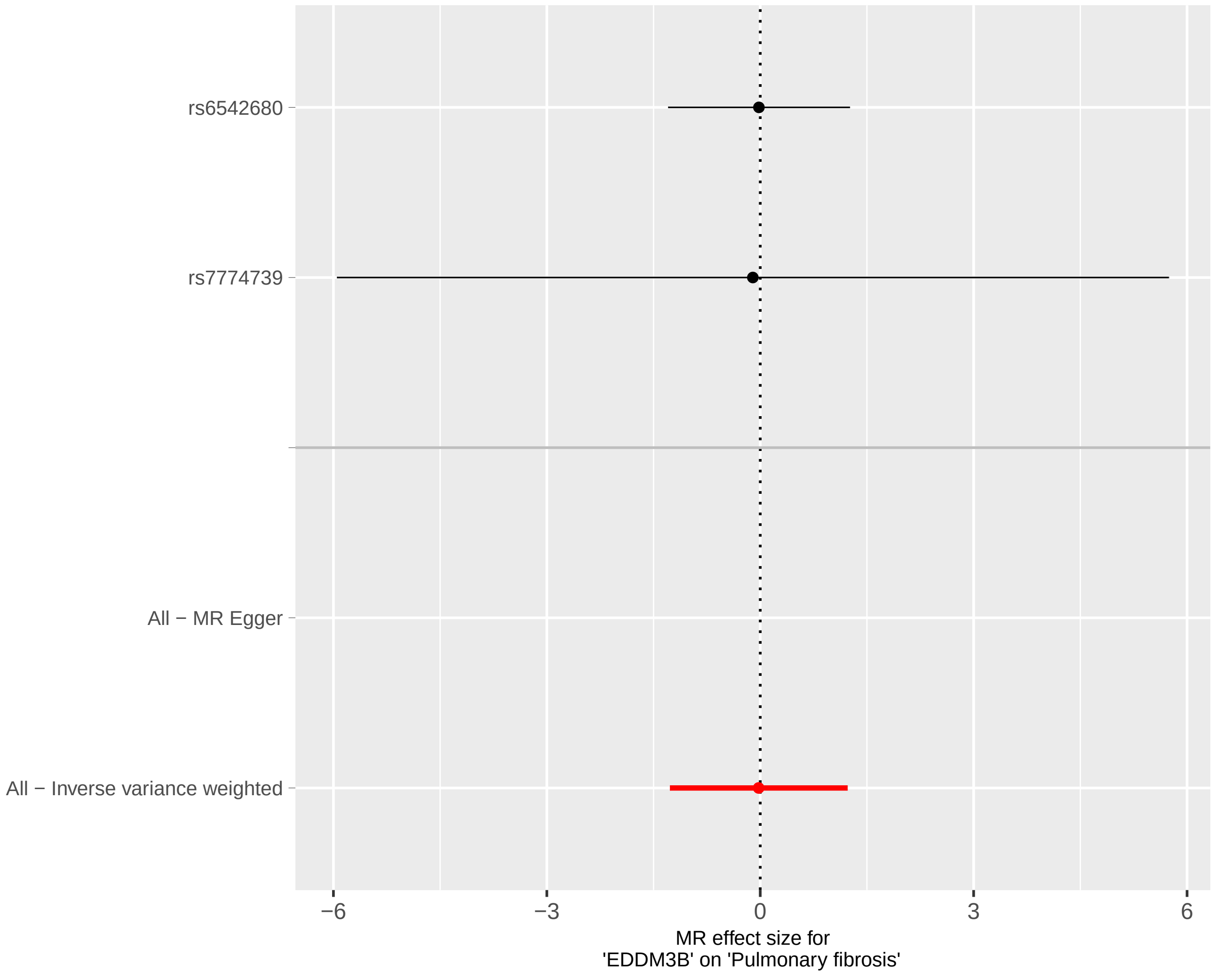

rs6542680
rs7774739
All − MR Egger
All − Inverse variance weighted
−6
−3
0
3
6
MR effect size for
'EDDM3B' on 'Pulmonary fibrosis'

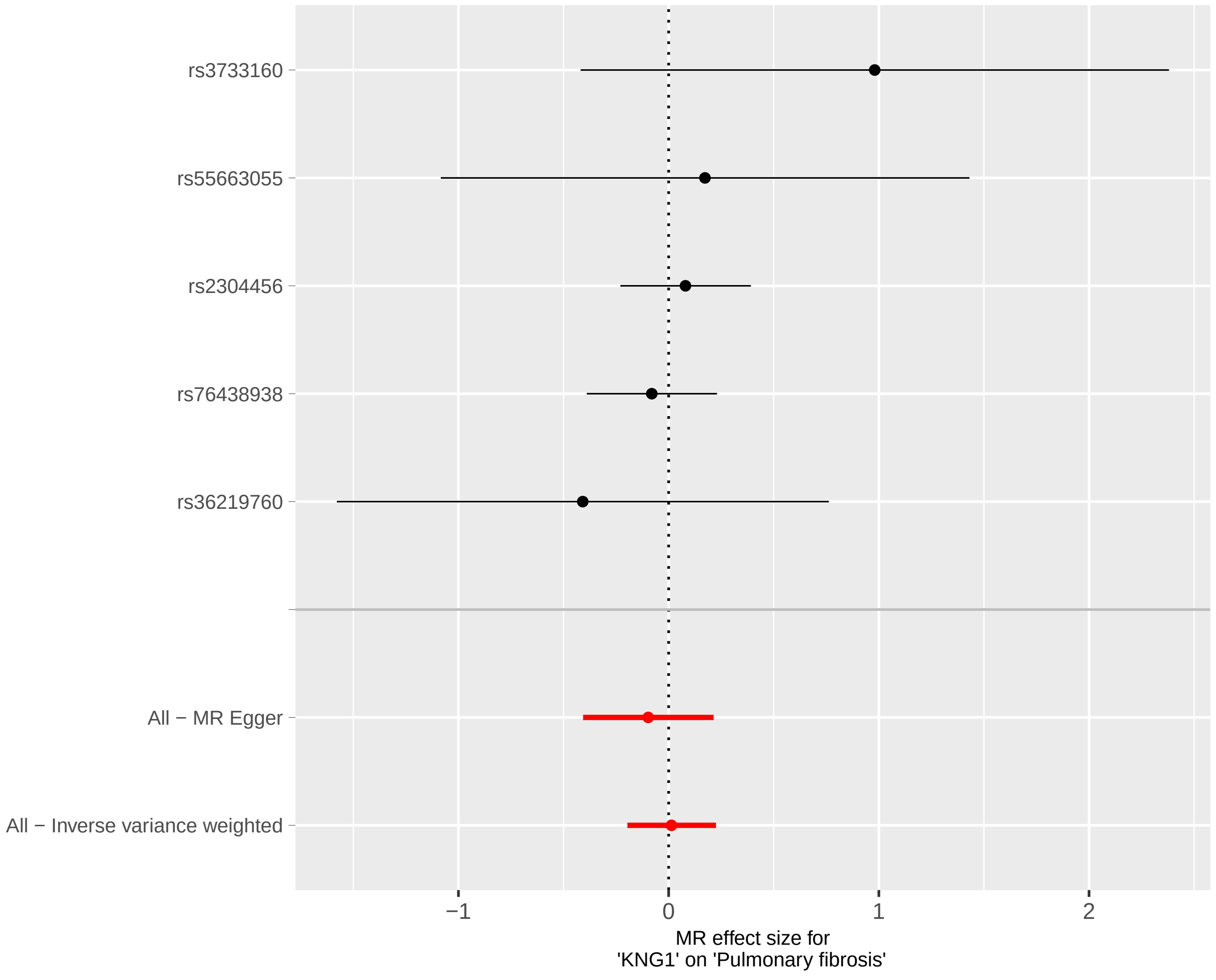

rs3733160
rs55663055
rs2304456
rs76438938
rs36219760
All − MR Egger
All − Inverse variance weighted
−1
0
1
2
MR effect size for
'KNG1' on 'Pulmonary fibrosis'

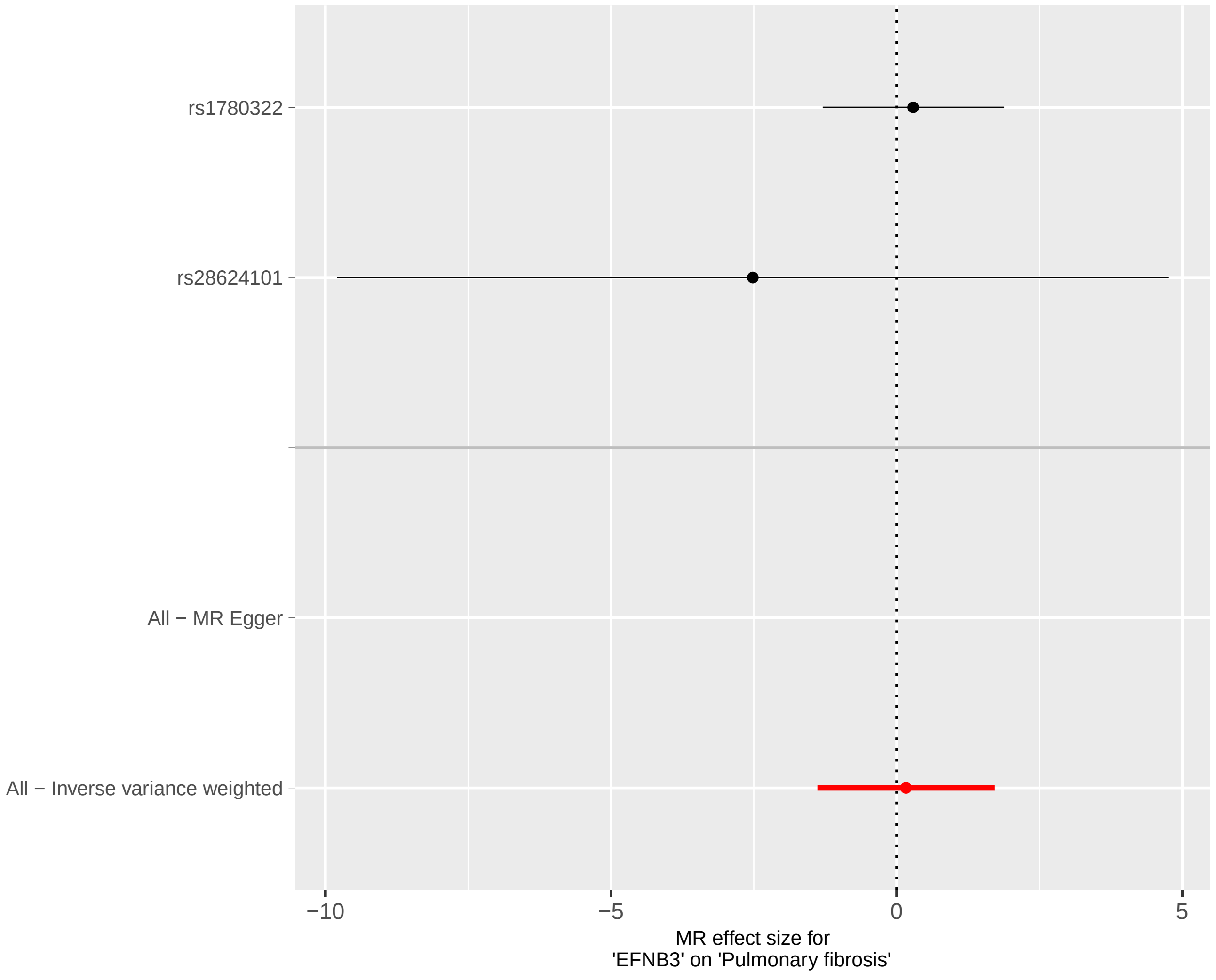

rs1780322
rs28624101
All − MR Egger
All − Inverse variance weighted
−10
−5
0
5
MR effect size for
'EFNB3' on 'Pulmonary fibrosis'

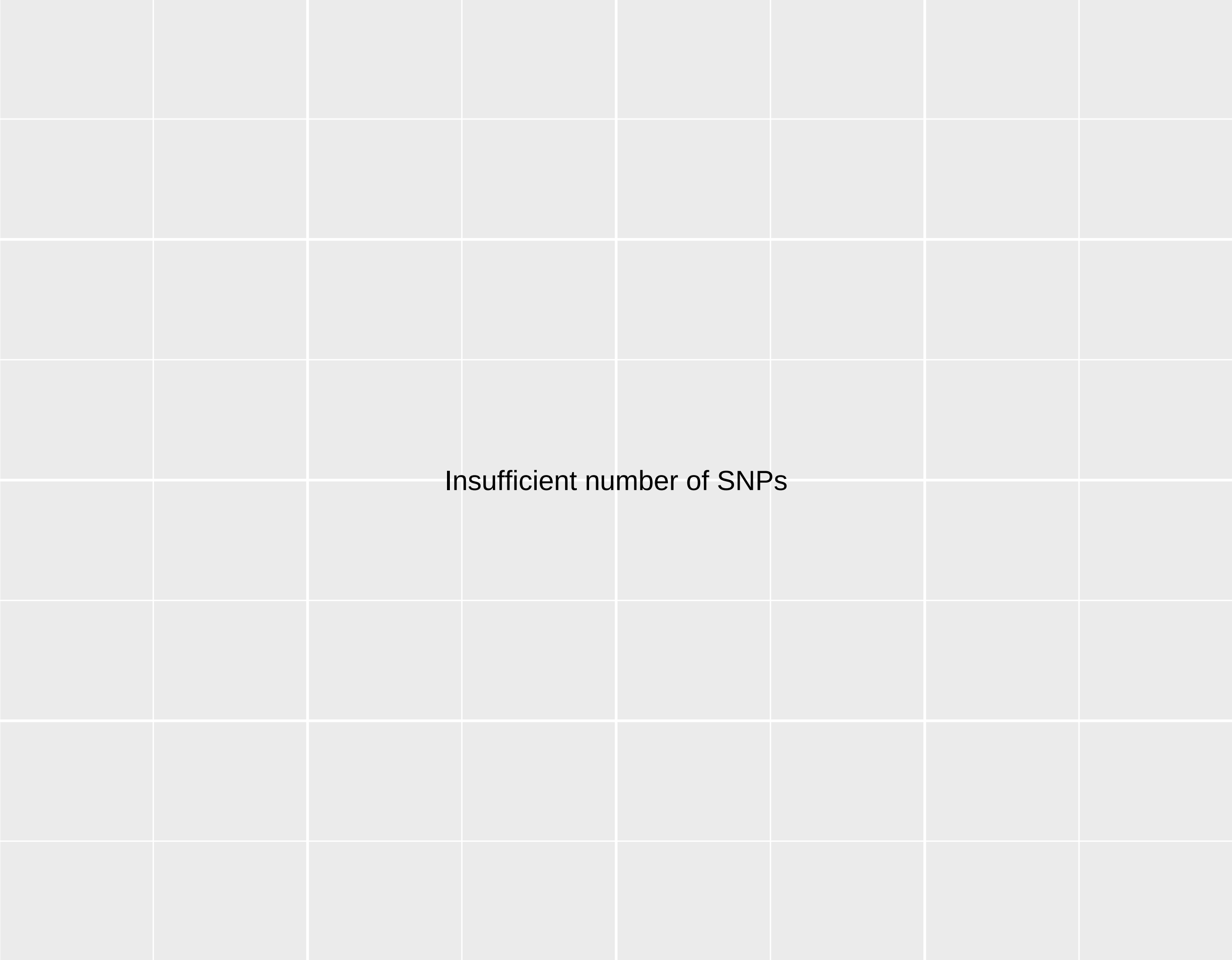
Insufficient number of SNPs

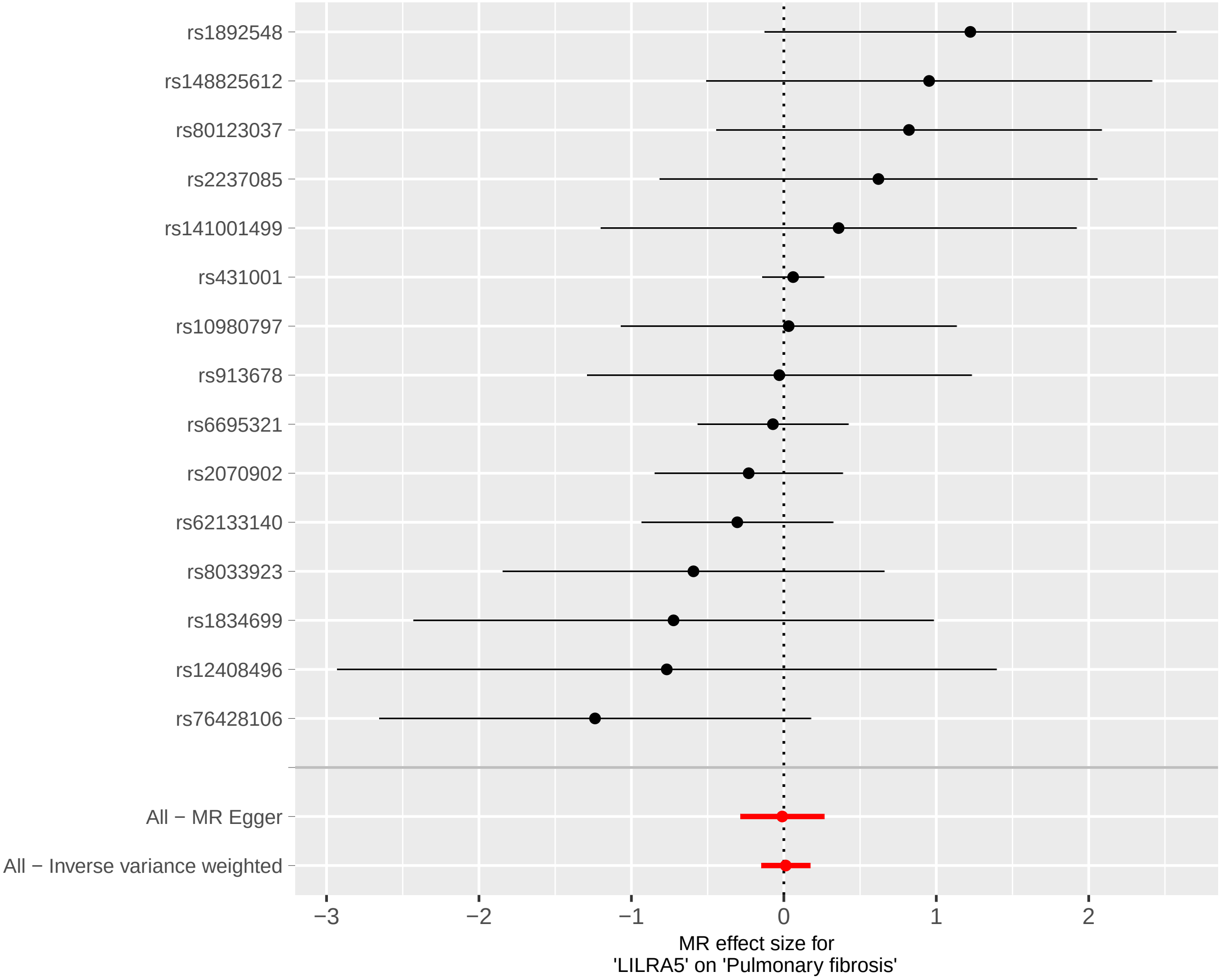

rs1892548
rs148825612
rs80123037
rs2237085
rs141001499
rs431001
rs10980797
rs913678
rs6695321
rs2070902
rs62133140
rs8033923
rs1834699
rs12408496
rs76428106
All − MR Egger
All − Inverse variance weighted
−3
−2
−1
0
1
2
MR effect size for
'LILRA5' on 'Pulmonary fibrosis'

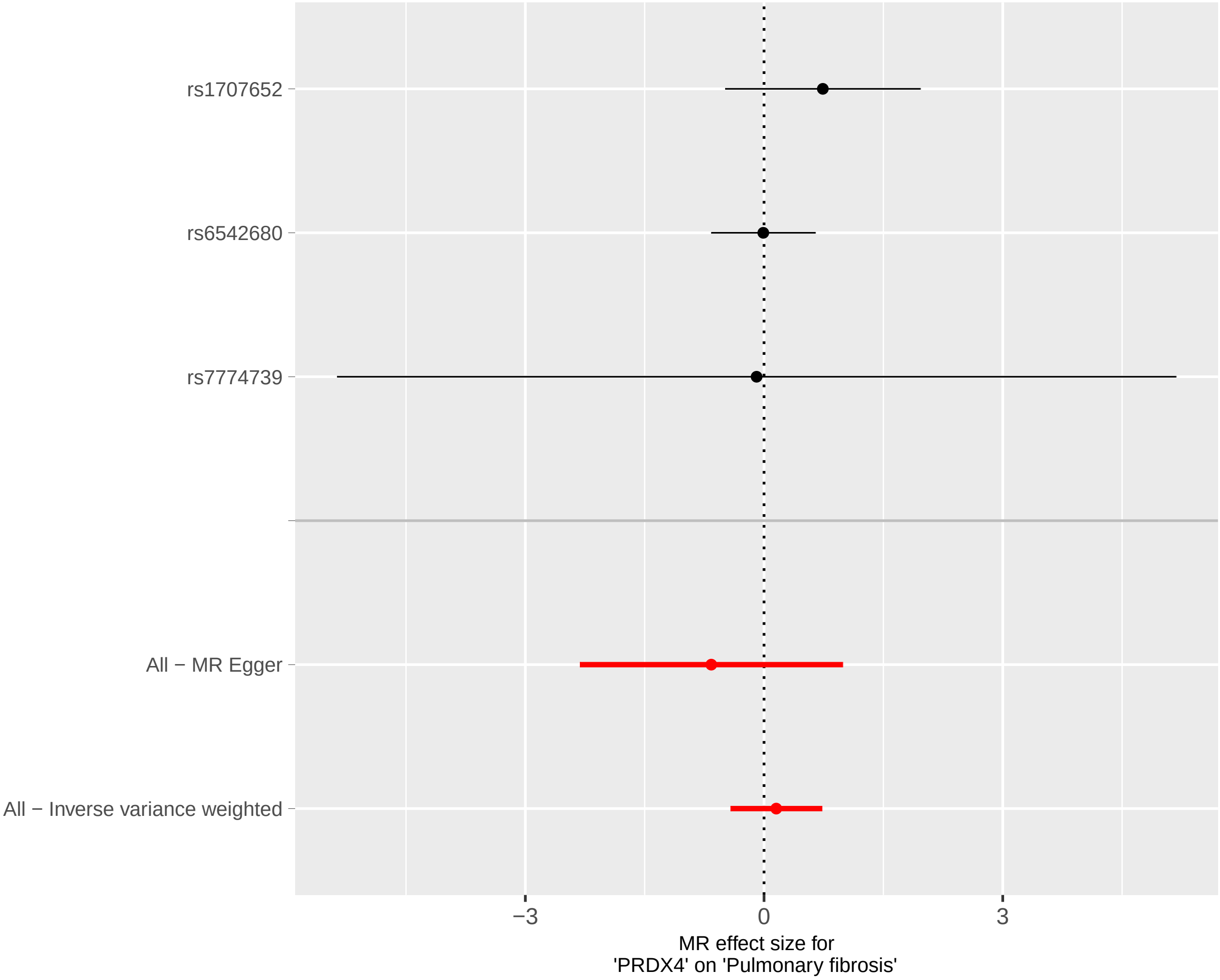
rs1707652
rs6542680
rs7774739
All − MR Egger
All − Inverse variance weighted
−3
0
3
MR effect size for
'PRDX4' on 'Pulmonary fibrosis'

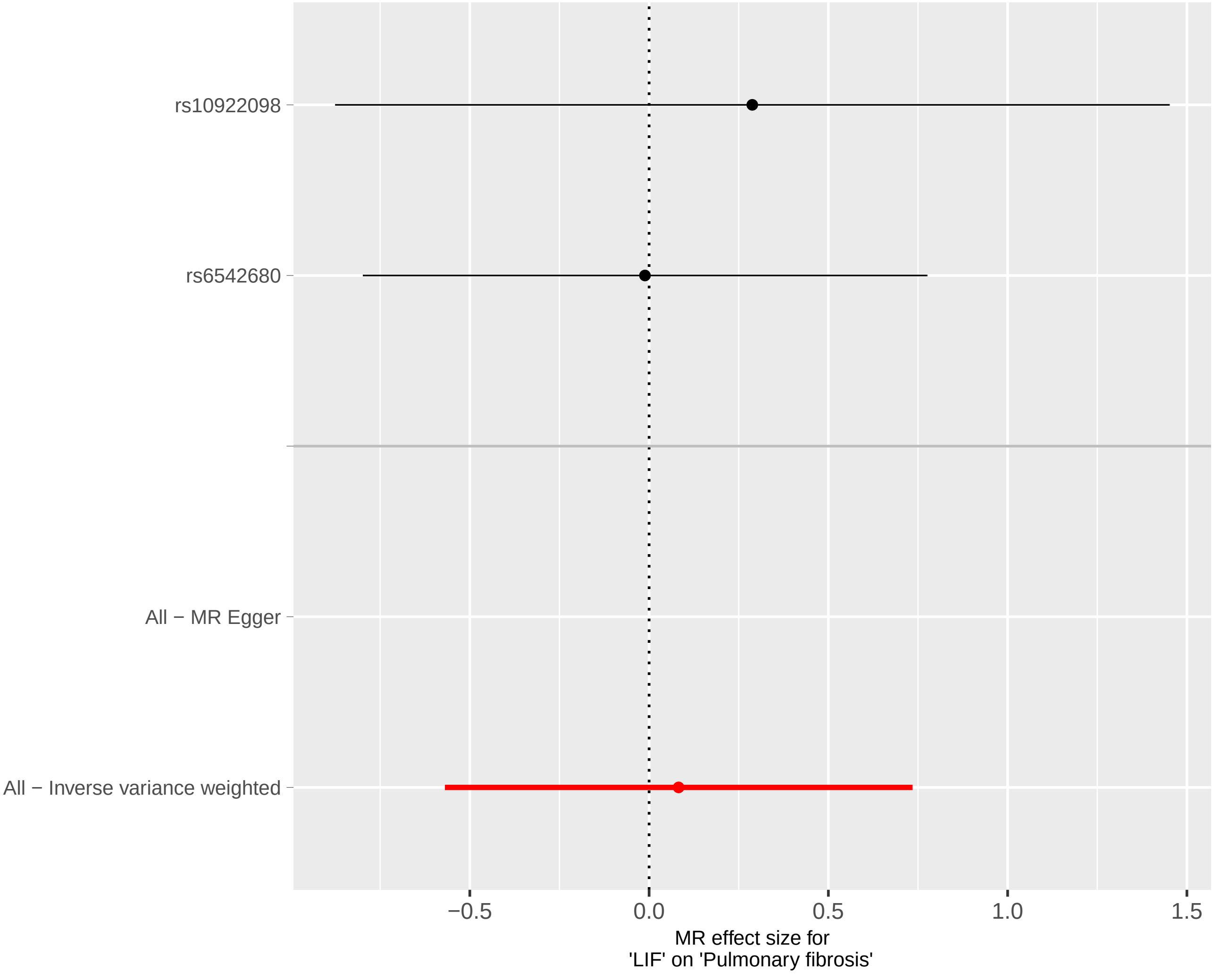

rs10922098
rs6542680
All − MR Egger
All − Inverse variance weighted
−0.5
0.0
0.5
1.0
1.5
MR effect size for
'LIF' on 'Pulmonary fibrosis'

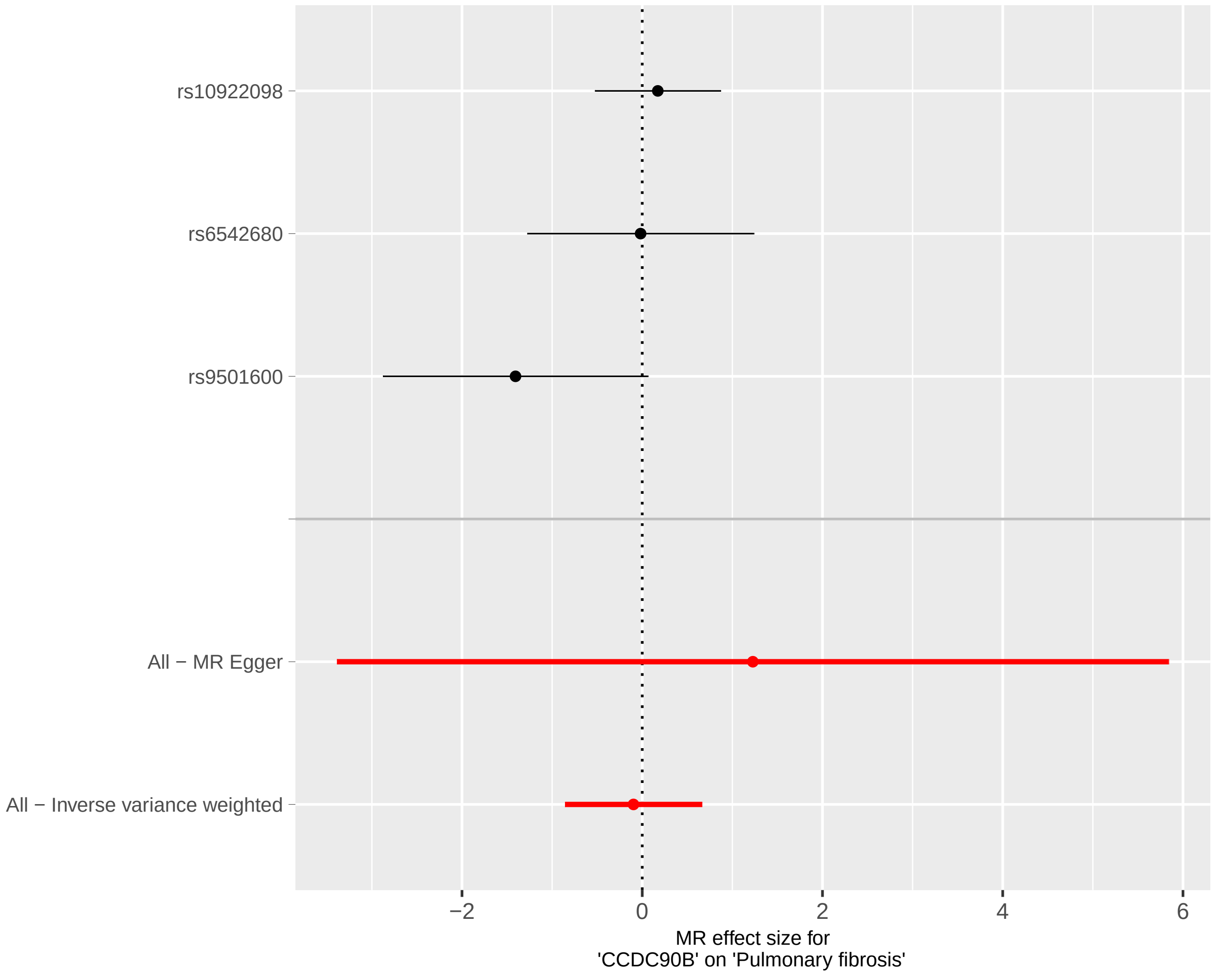

rs10922098
rs6542680
rs9501600
All − MR Egger
All − Inverse variance weighted
−2
0
2
4
6
MR effect size for
'CCDC90B' on 'Pulmonary fibrosis'

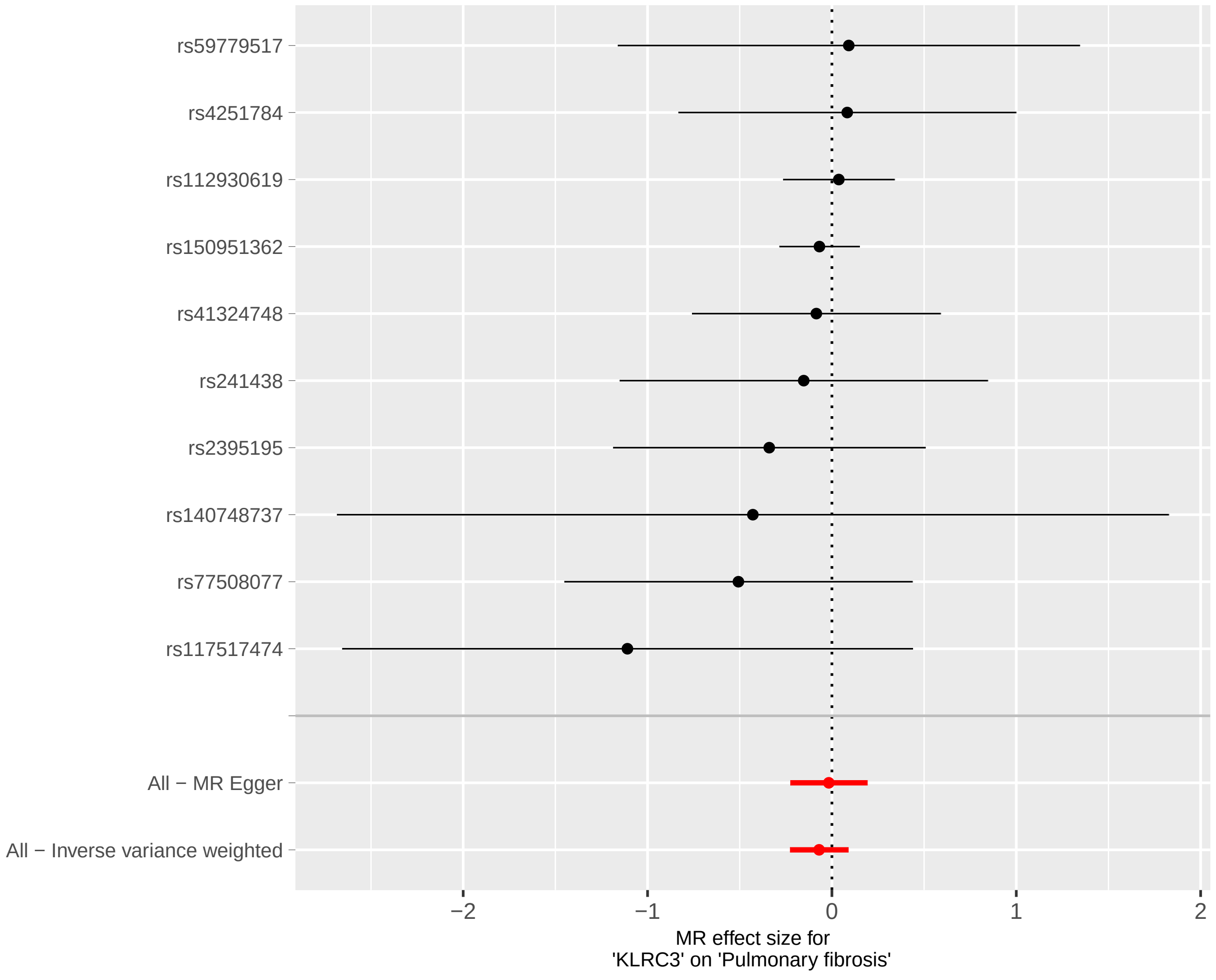

rs59779517
rs4251784
rs112930619
rs150951362
rs41324748
rs241438
rs2395195
rs140748737
rs77508077
rs117517474
All − MR Egger
All − Inverse variance weighted
−2
−1
0
1
2
MR effect size for
'KLRC3' on 'Pulmonary fibrosis'

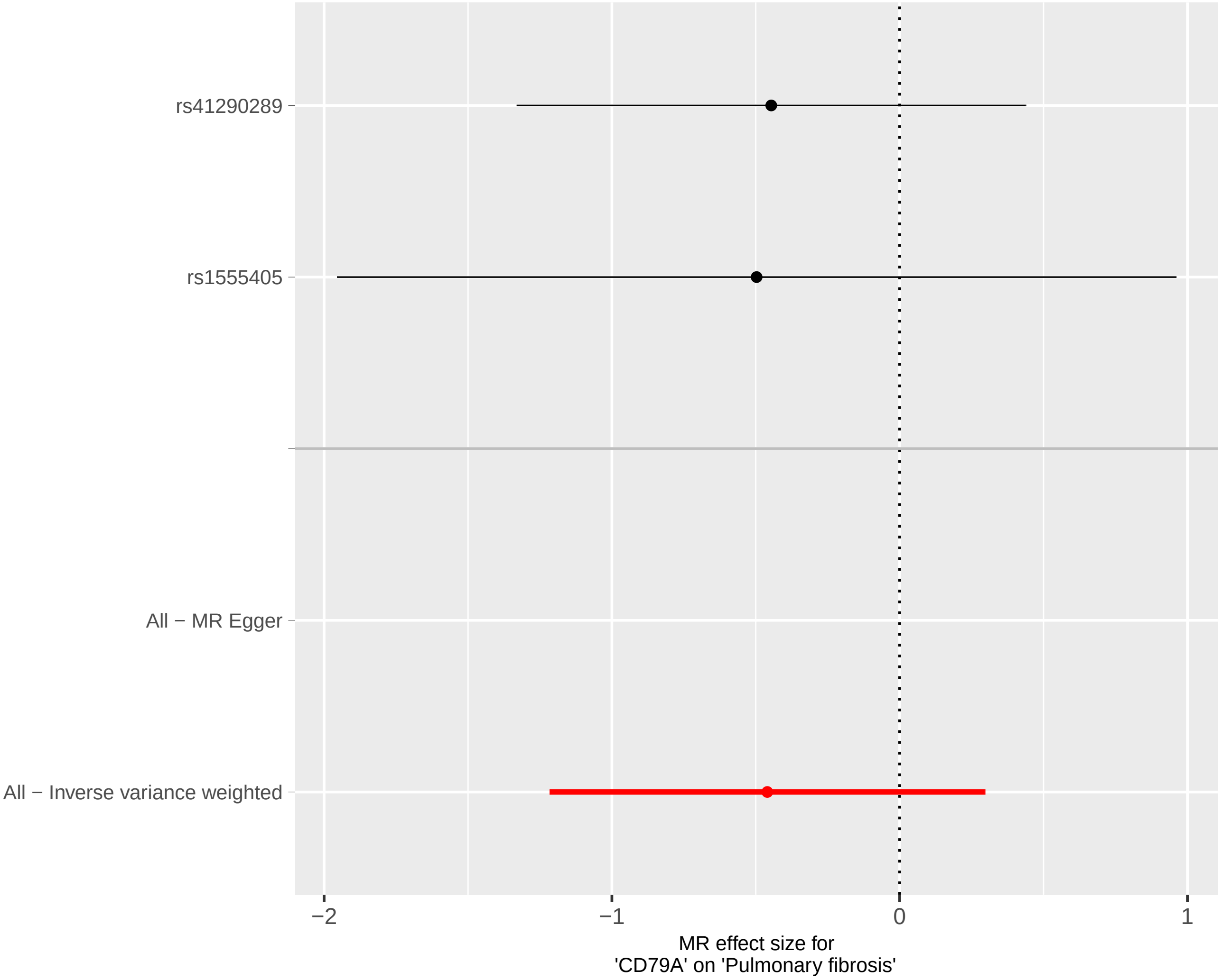
rs41290289
rs1555405
All − MR Egger
All − Inverse variance weighted
−2
−1
0
1
MR effect size for
'CD79A' on 'Pulmonary fibrosis'

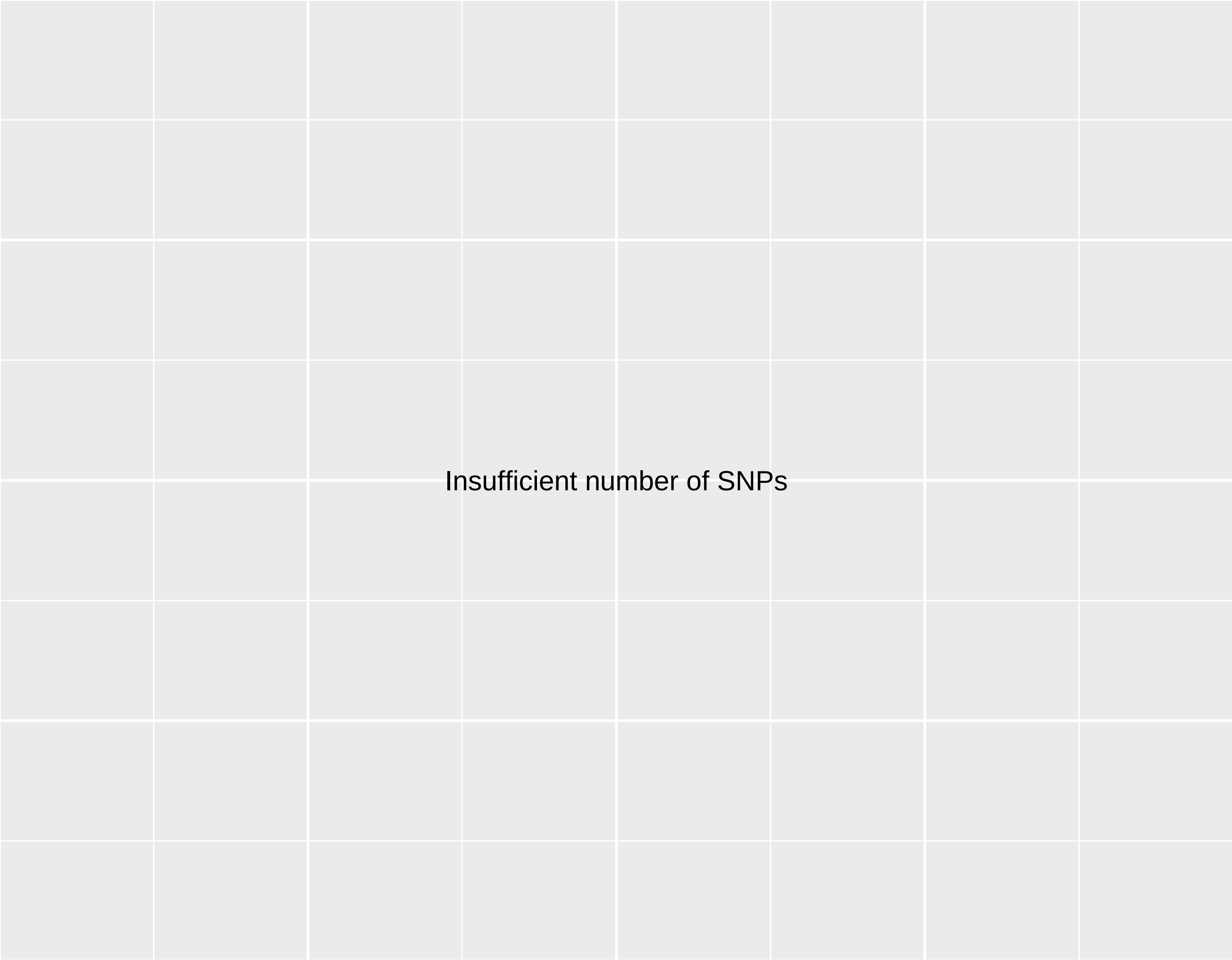
Insufficient number of SNPs

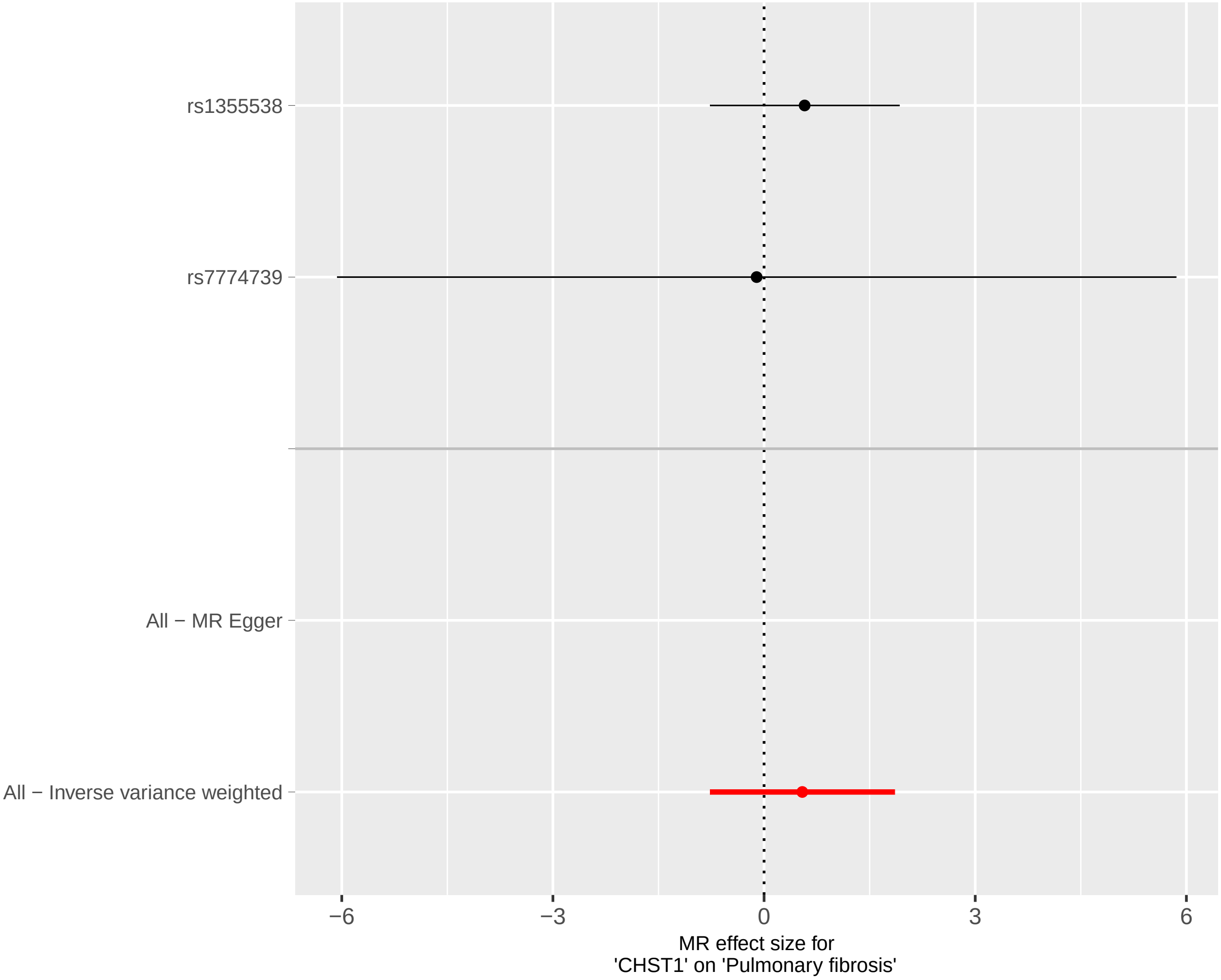
rs1355538
rs7774739
All − MR Egger
All − Inverse variance weighted
−6
−3
0
3
6
MR effect size for
'CHST1' on 'Pulmonary fibrosis'

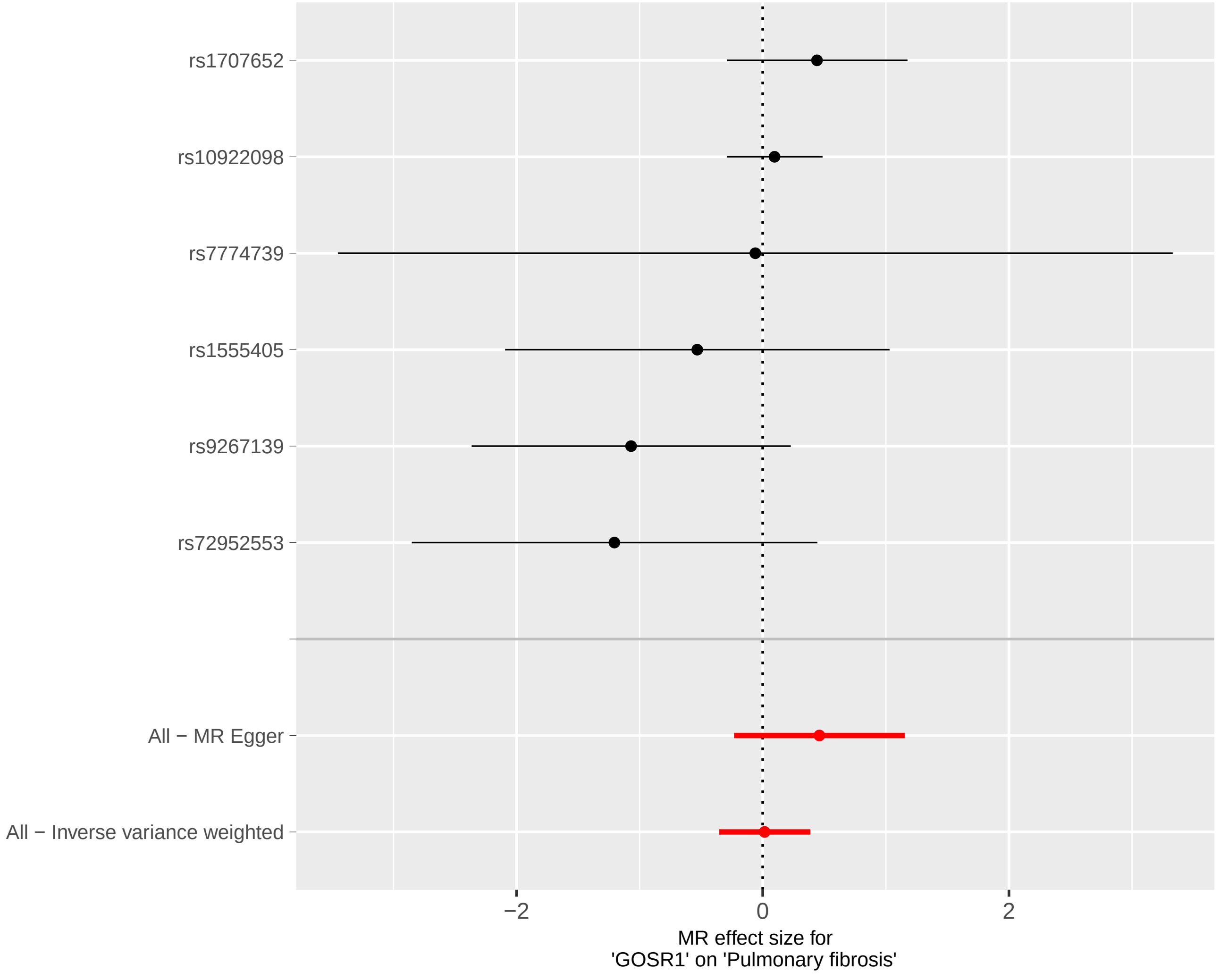

rs1707652
rs10922098
rs7774739
rs1555405
rs9267139
rs72952553
All − MR Egger
All − Inverse variance weighted
−2
0
2
MR effect size for
'GOSR1' on 'Pulmonary fibrosis'

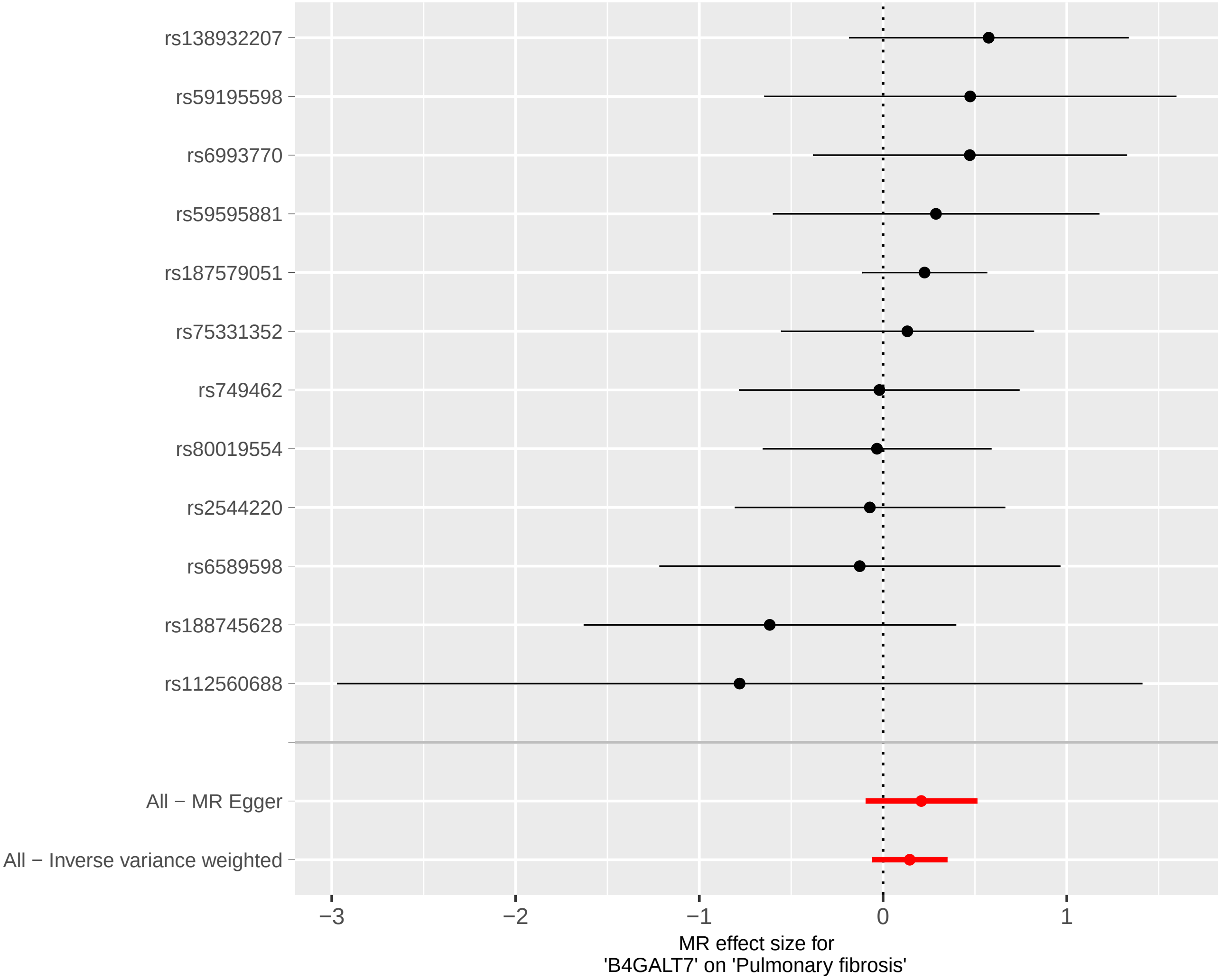
rs138932207
rs59195598
rs6993770
rs59595881
rs187579051
rs75331352
rs749462
rs80019554
rs2544220
rs6589598
rs188745628
rs112560688
All − MR Egger
All − Inverse variance weighted
−3
−2
−1
0
1
MR effect size for
'B4GALT7' on 'Pulmonary fibrosis'

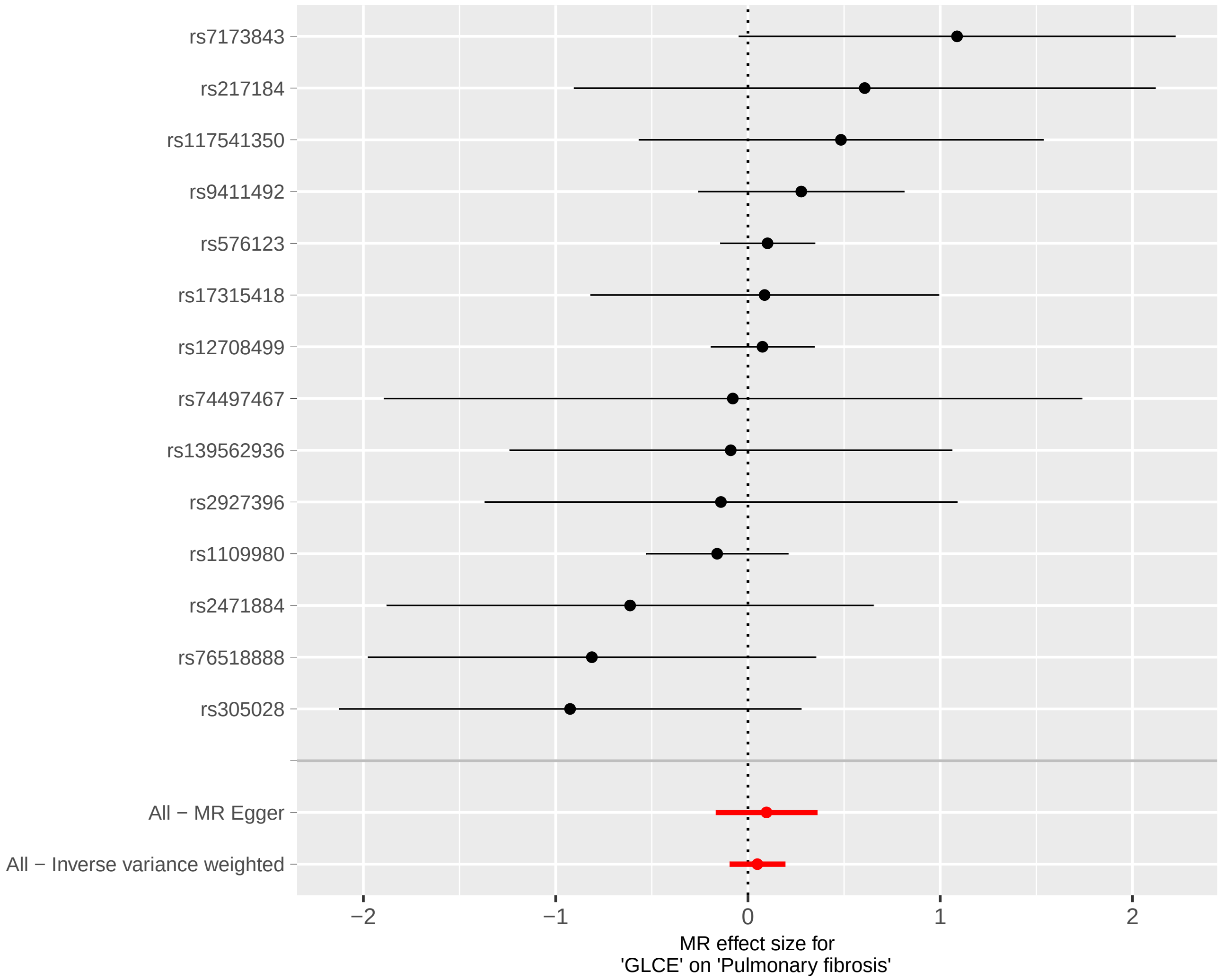
rs7173843
rs217184
rs117541350
rs9411492
rs576123
rs17315418
rs12708499
rs74497467
rs139562936
rs2927396
rs1109980
rs2471884
rs76518888
rs305028
All − MR Egger
All − Inverse variance weighted
−2
−1
0
1
2
MR effect size for
'GLCE' on 'Pulmonary fibrosis'

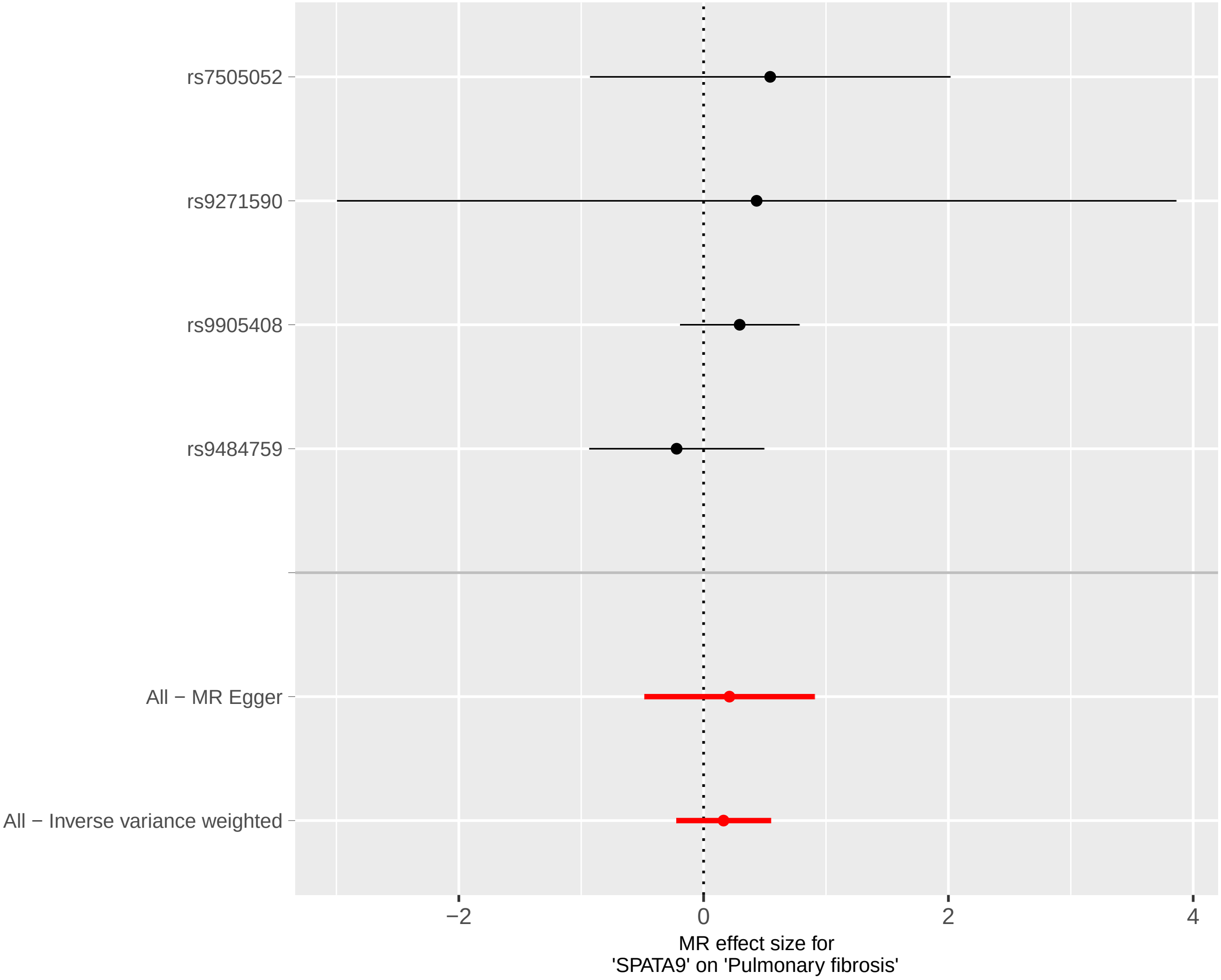

rs7505052
rs9271590
rs9905408
rs9484759
All − MR Egger
All − Inverse variance weighted
−2
0
2
4
MR effect size for
'SPATA9' on 'Pulmonary fibrosis'

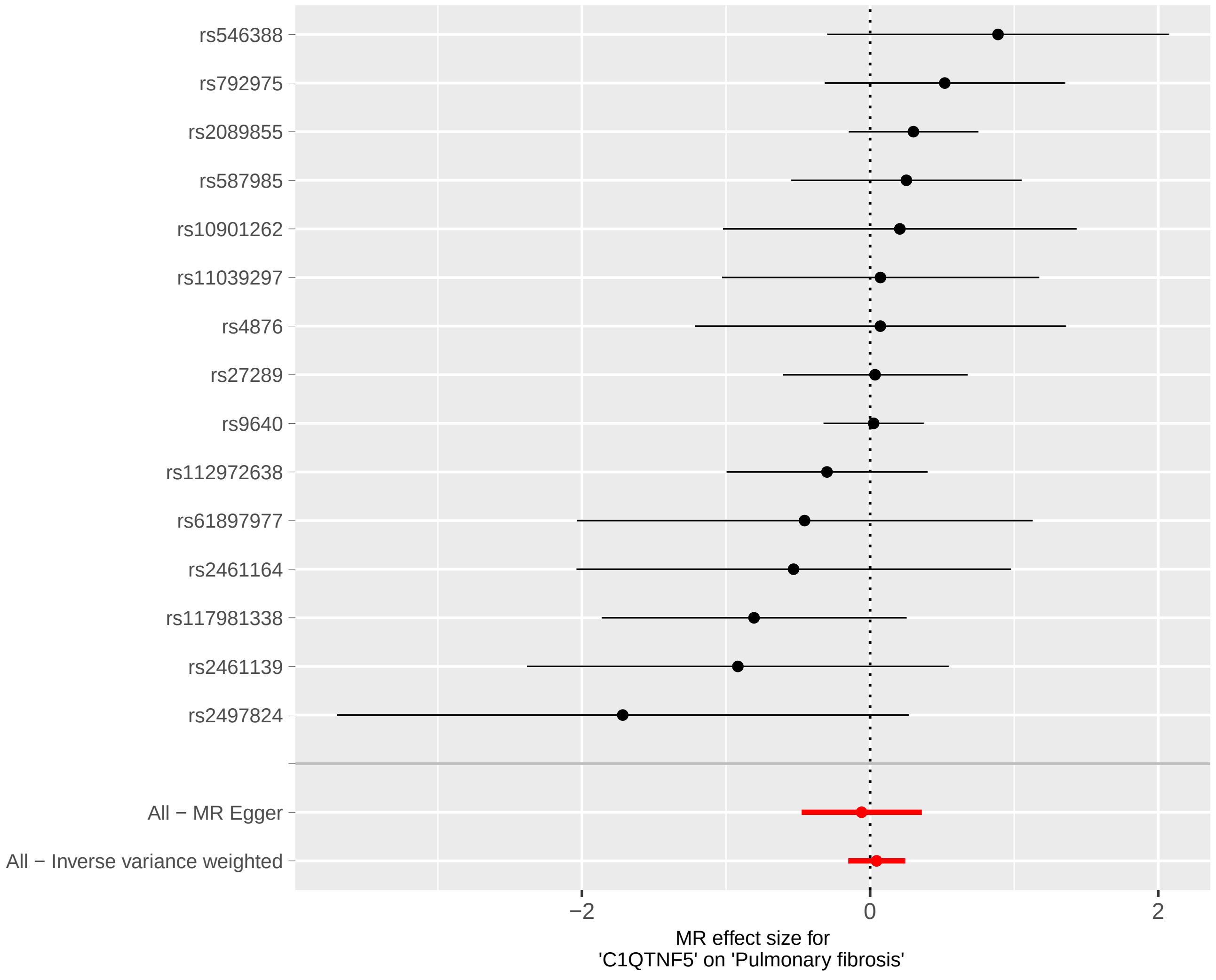
rs546388
rs792975
rs2089855
rs587985
rs10901262
rs11039297
rs4876
rs27289
rs9640
rs112972638
rs61897977
rs2461164
rs117981338
rs2461139
rs2497824
All − MR Egger
All − Inverse variance weighted
−2
0
2
MR effect size for
'C1QTNF5' on 'Pulmonary fibrosis'

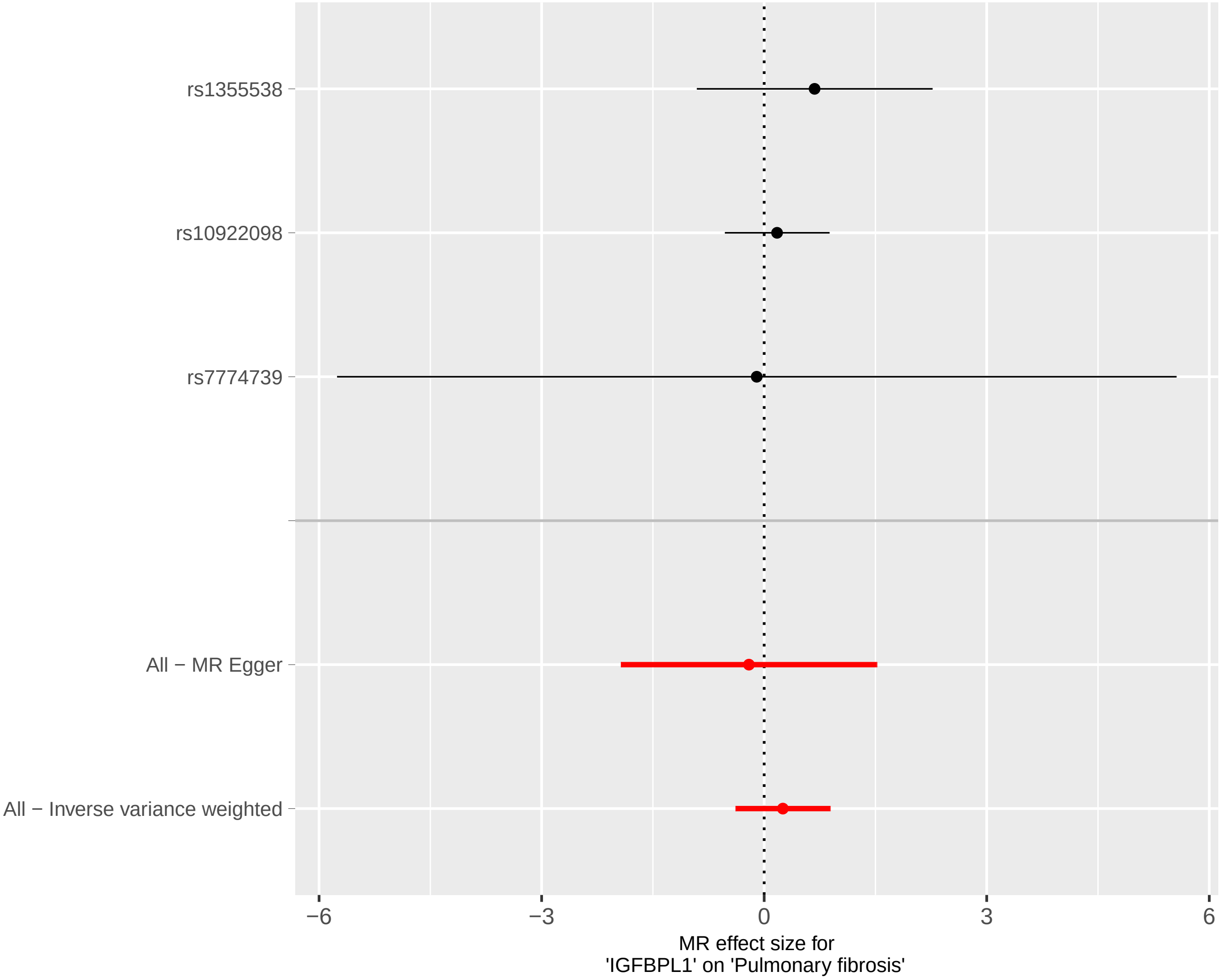
rs1355538
rs10922098
rs7774739
All − MR Egger
All − Inverse variance weighted
−6
−3
0
3
6
MR effect size for
'IGFBPL1' on 'Pulmonary fibrosis'

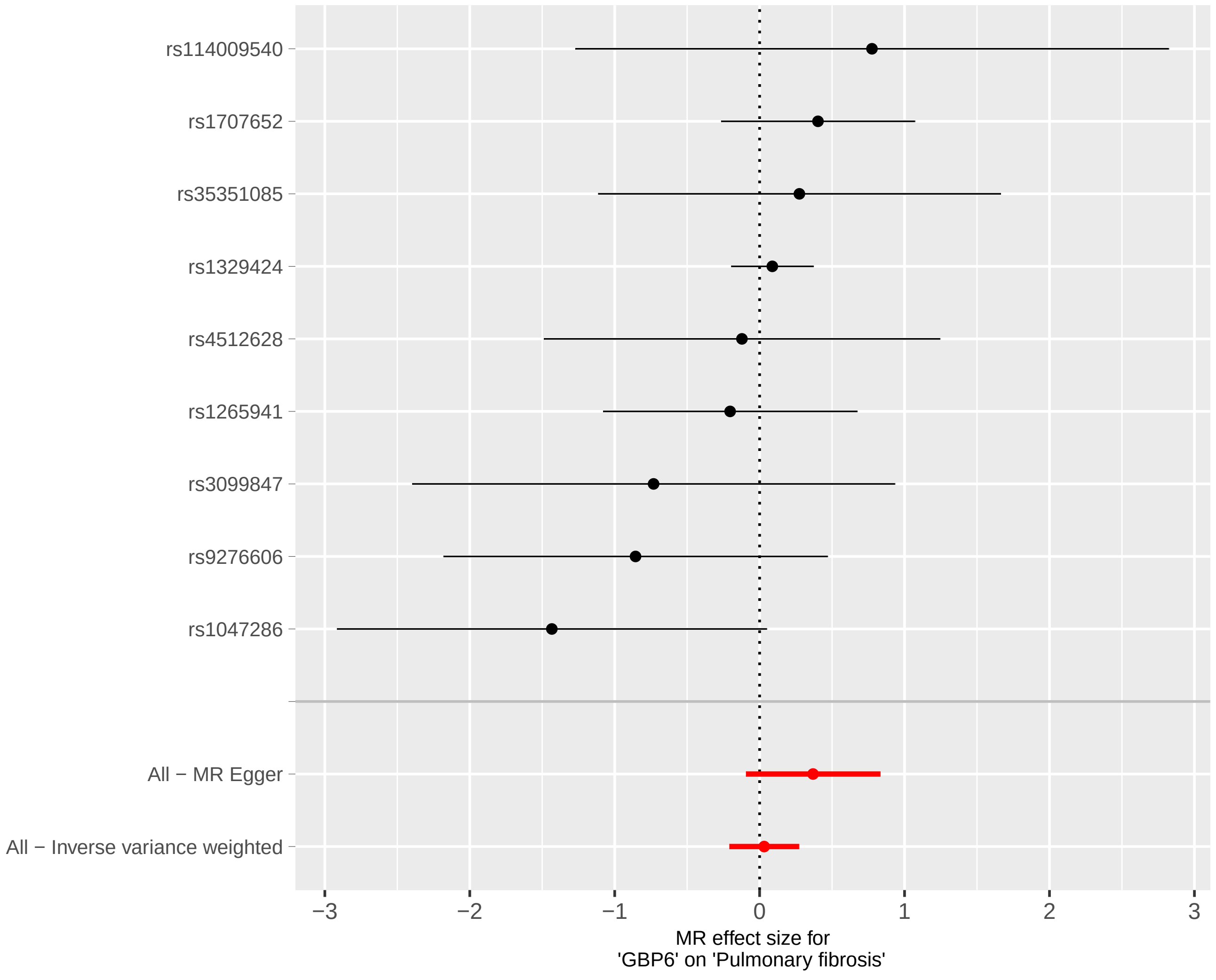
rs114009540
rs1707652
rs35351085
rs1329424
rs4512628
rs1265941
rs3099847
rs9276606
rs1047286
All − MR Egger
All − Inverse variance weighted
−3
−2
−1
0
1
2
3
MR effect size for
'GBP6' on 'Pulmonary fibrosis'

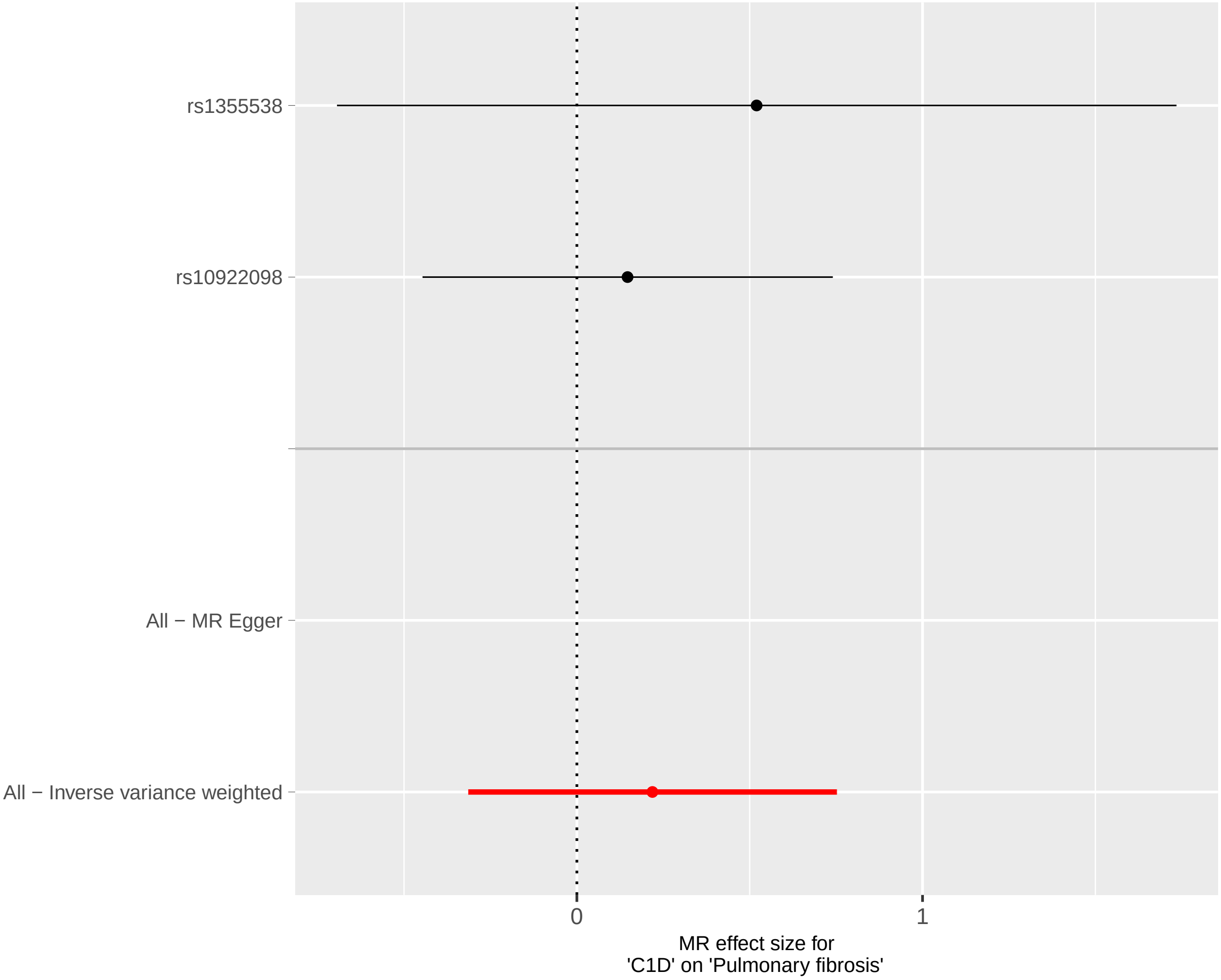

rs1355538
rs10922098
All − MR Egger
All − Inverse variance weighted
0
1
MR effect size for
'C1D' on 'Pulmonary fibrosis'

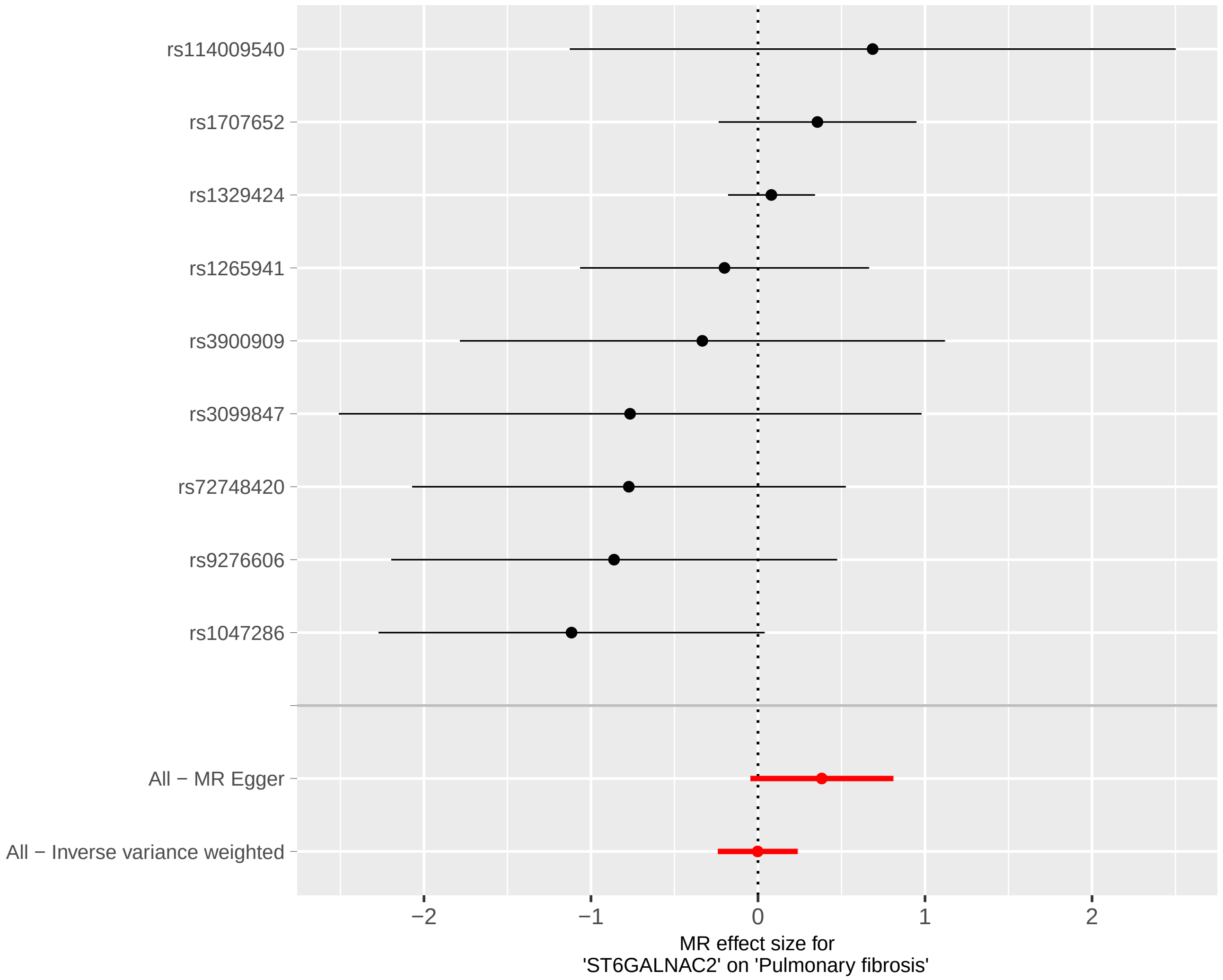

rs114009540
rs1707652
rs1329424
rs1265941
rs3900909
rs3099847
rs72748420
rs9276606
rs1047286
All − MR Egger
All − Inverse variance weighted
−2
−1
0
1
2
MR effect size for
'ST6GALNAC2' on 'Pulmonary fibrosis'

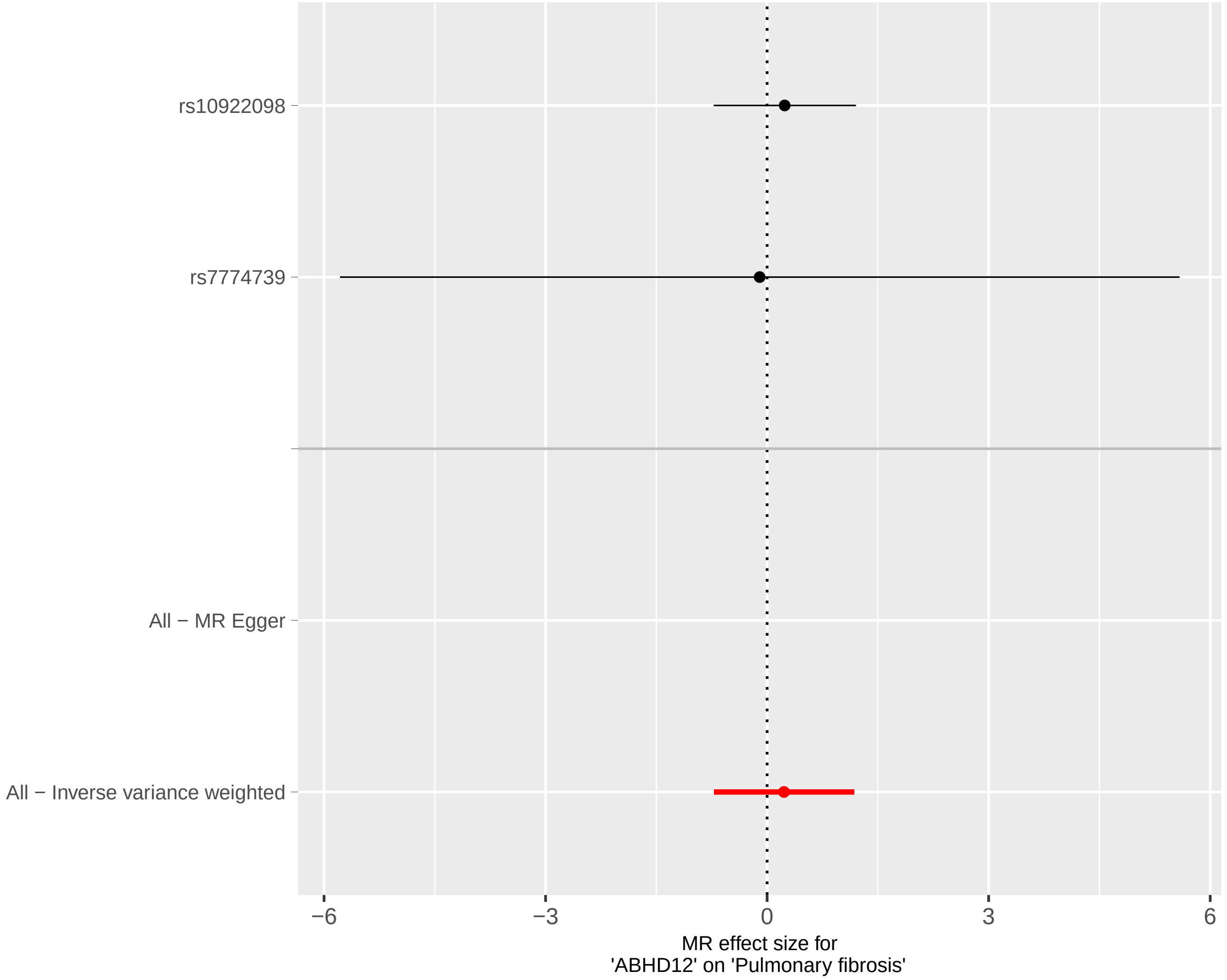

rs10922098
rs7774739
All − MR Egger
All − Inverse variance weighted
−6
−3
0
3
6
MR effect size for
'ABHD12' on 'Pulmonary fibrosis'

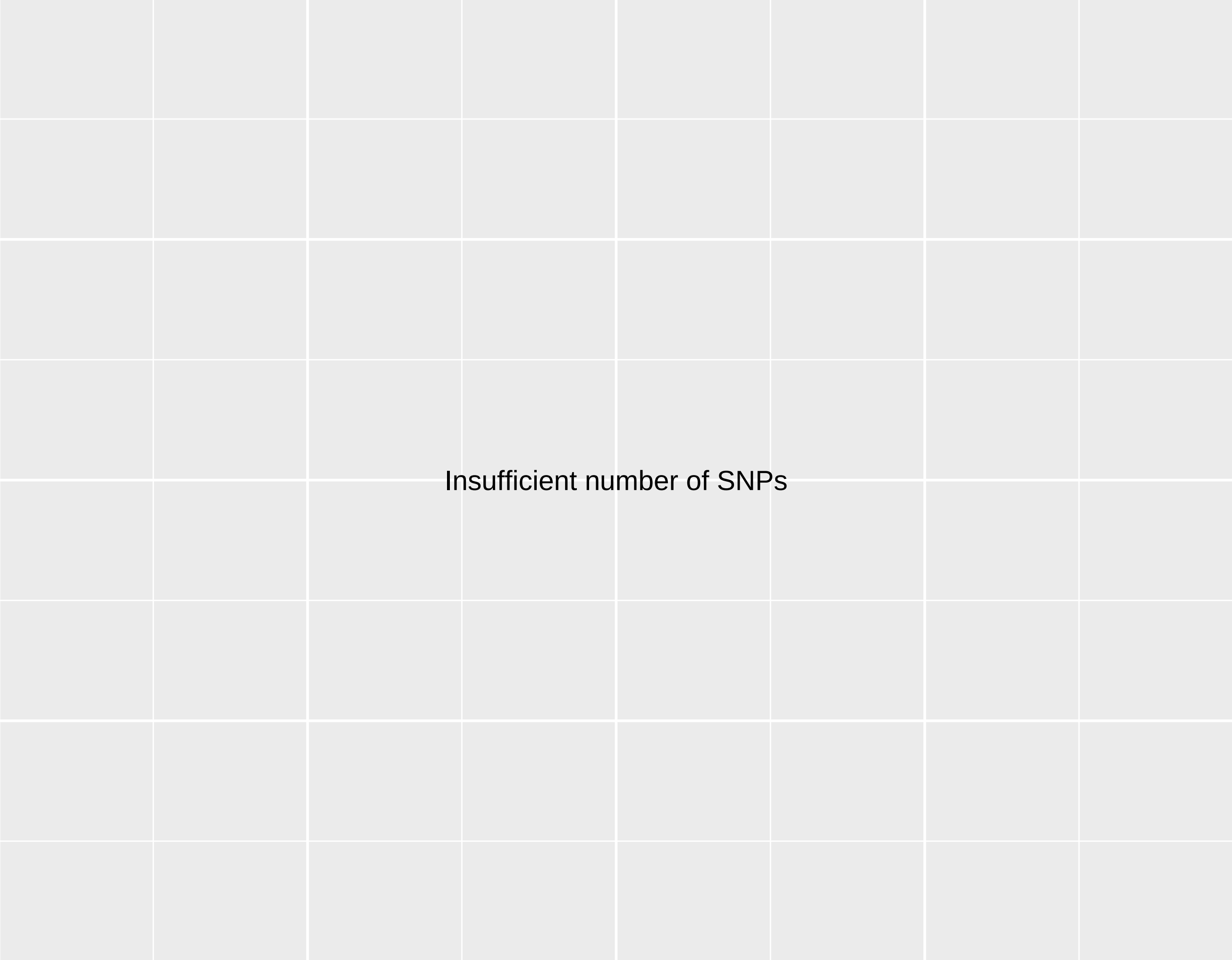
Insufficient number of SNPs

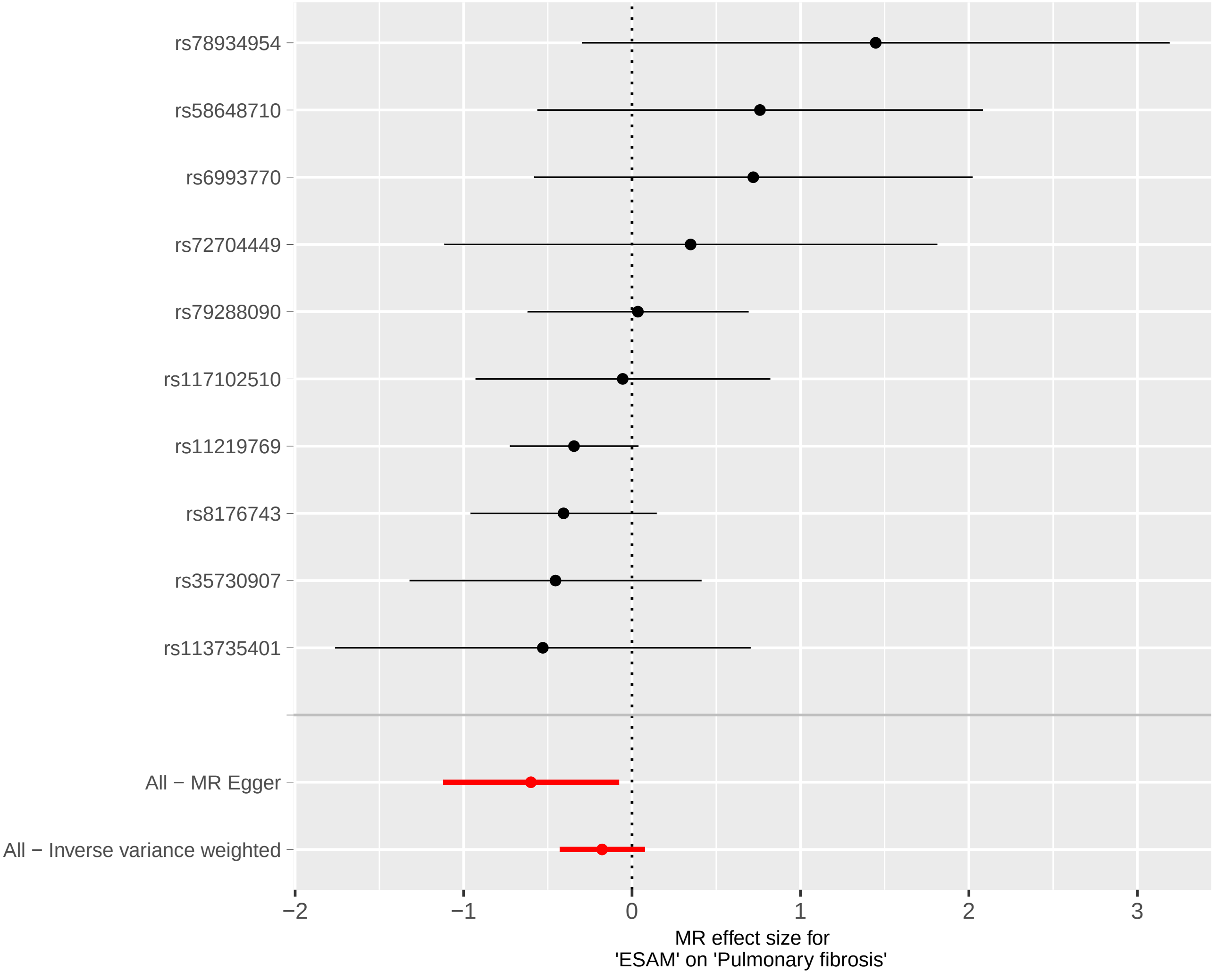

rs78934954
rs58648710
rs6993770
rs72704449
rs79288090
rs117102510
rs11219769
rs8176743
rs35730907
rs113735401
All − MR Egger
All − Inverse variance weighted
−2
−1
0
1
2
3
MR effect size for
'ESAM' on 'Pulmonary fibrosis'

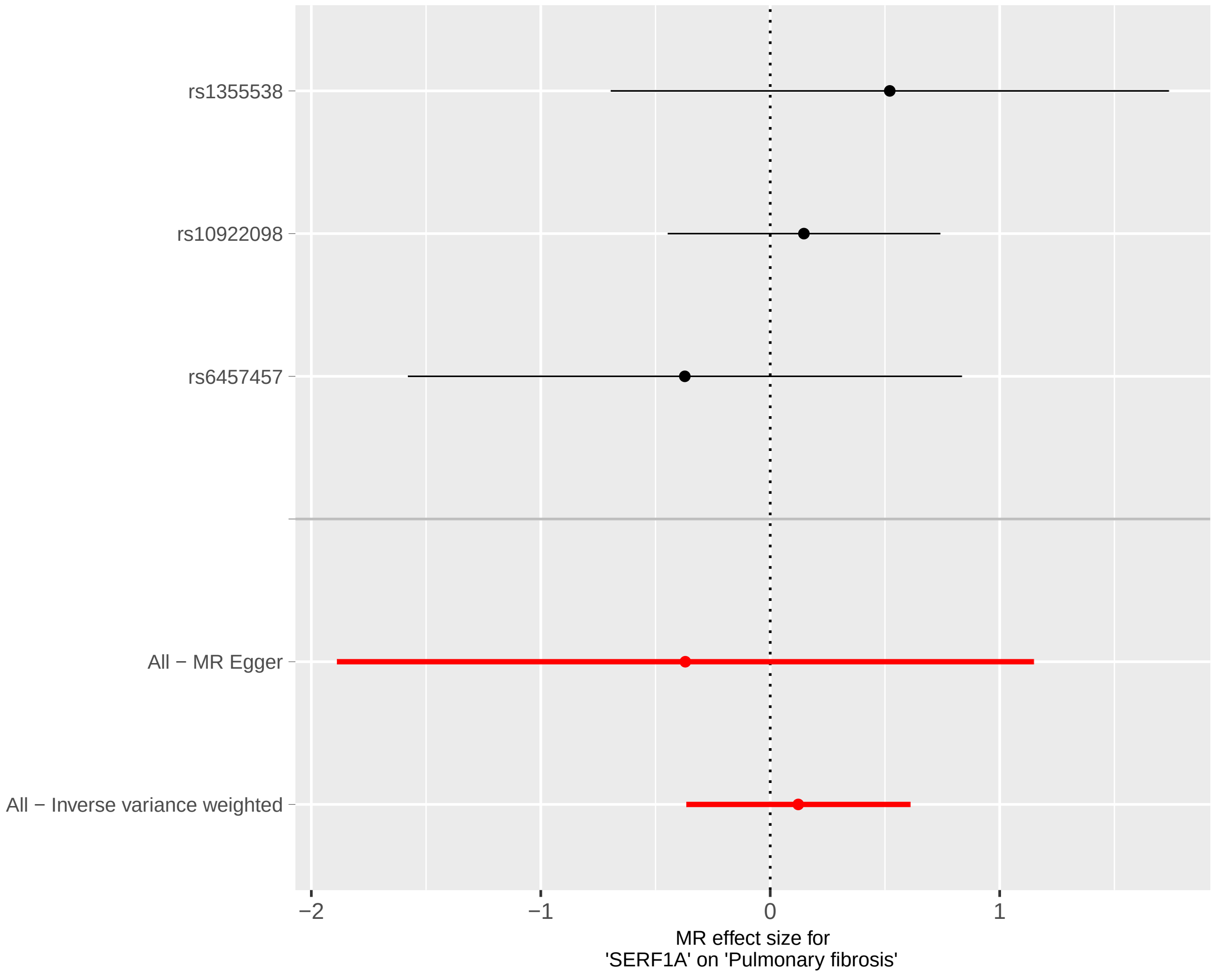

rs1355538
rs10922098
rs6457457
All − MR Egger
All − Inverse variance weighted
−2
−1
0
1
MR effect size for
'SERF1A' on 'Pulmonary fibrosis'

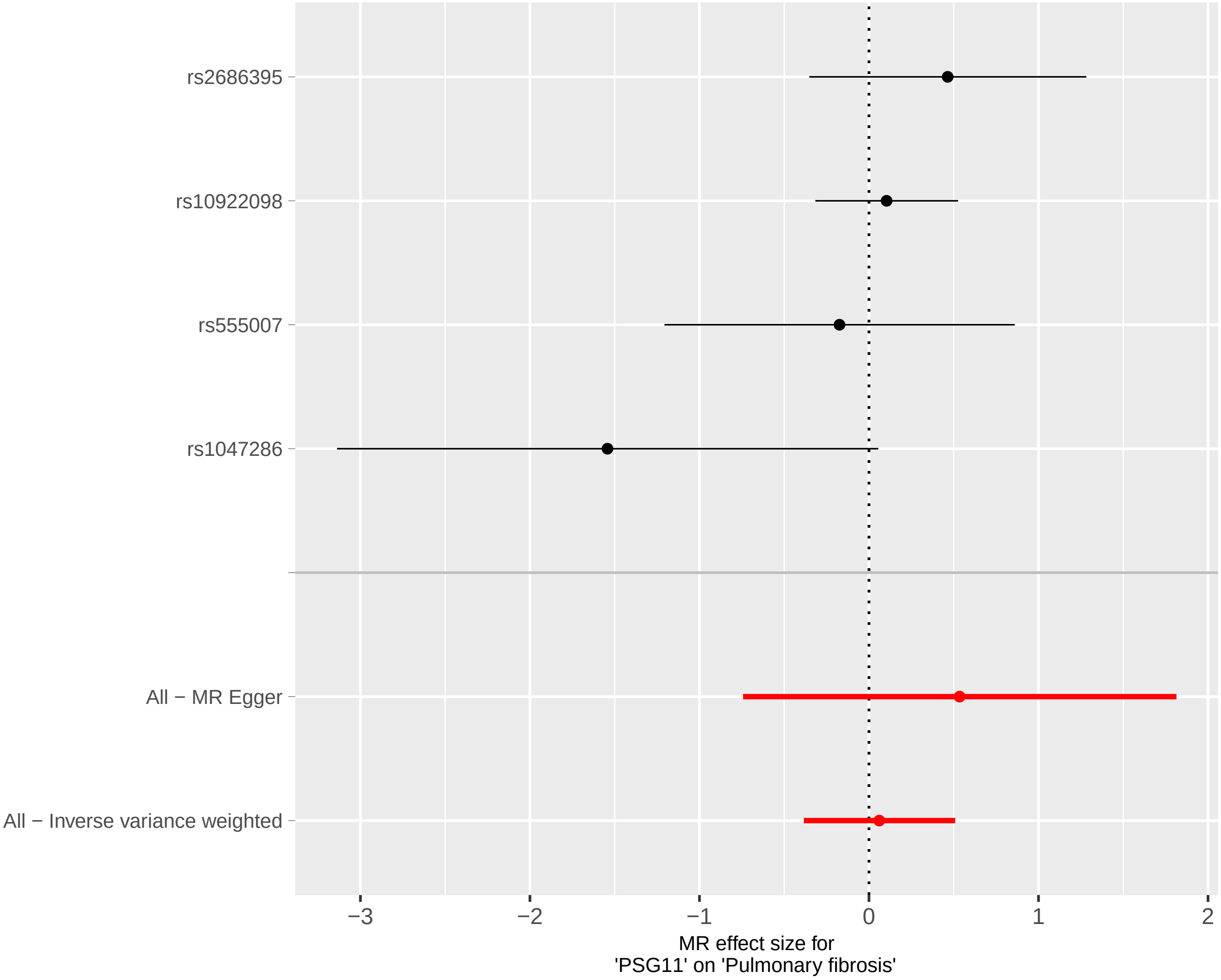
rs2686395
rs10922098
rs555007
rs1047286
All − MR Egger
All − Inverse variance weighted
−3
−2
−1
0
1
2
MR effect size for
'PSG11' on 'Pulmonary fibrosis'

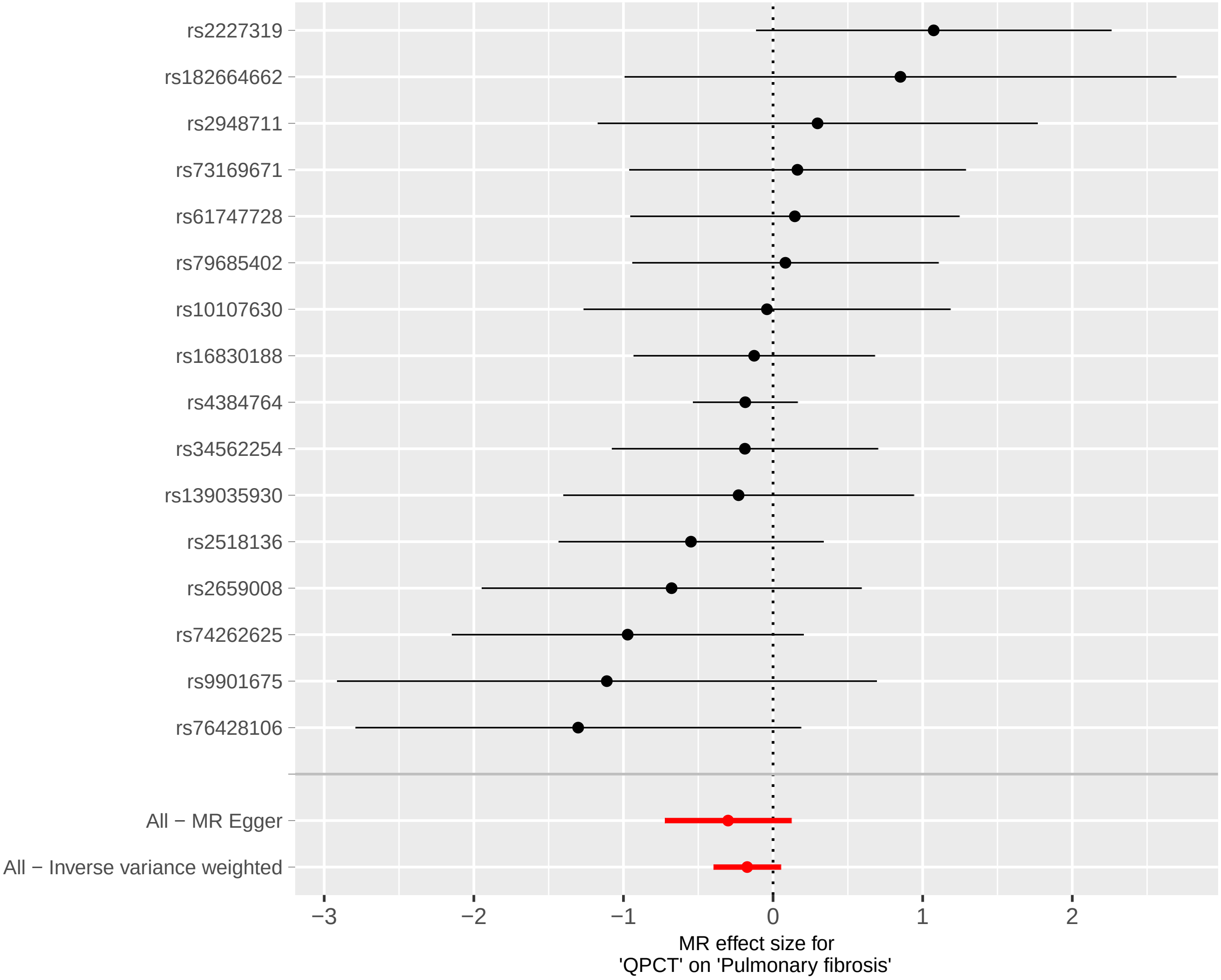
rs2227319
rs182664662
rs2948711
rs73169671
rs61747728
rs79685402
rs10107630
rs16830188
rs4384764
rs34562254
rs139035930
rs2518136
rs2659008
rs74262625
rs9901675
rs76428106
All − MR Egger
All − Inverse variance weighted
−3
−2
−1
0
1
2
MR effect size for
'QPCT' on 'Pulmonary fibrosis'

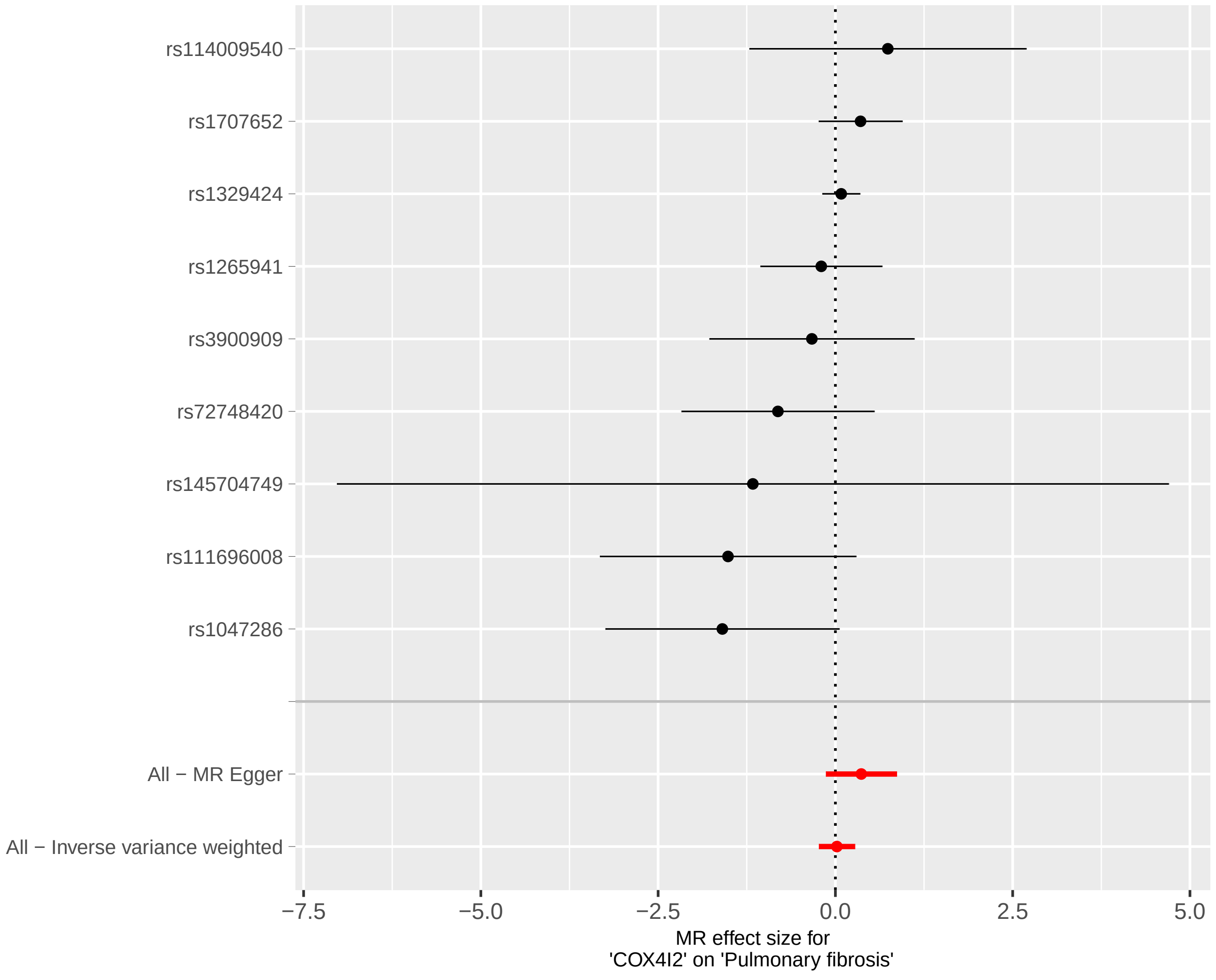
rs114009540
rs1707652
rs1329424
rs1265941
rs3900909
rs72748420
rs145704749
rs111696008
rs1047286
All − MR Egger
All − Inverse variance weighted
−7.5
−5.0
−2.5
0.0
2.5
5.0
MR effect size for
'COX4I2' on 'Pulmonary fibrosis'

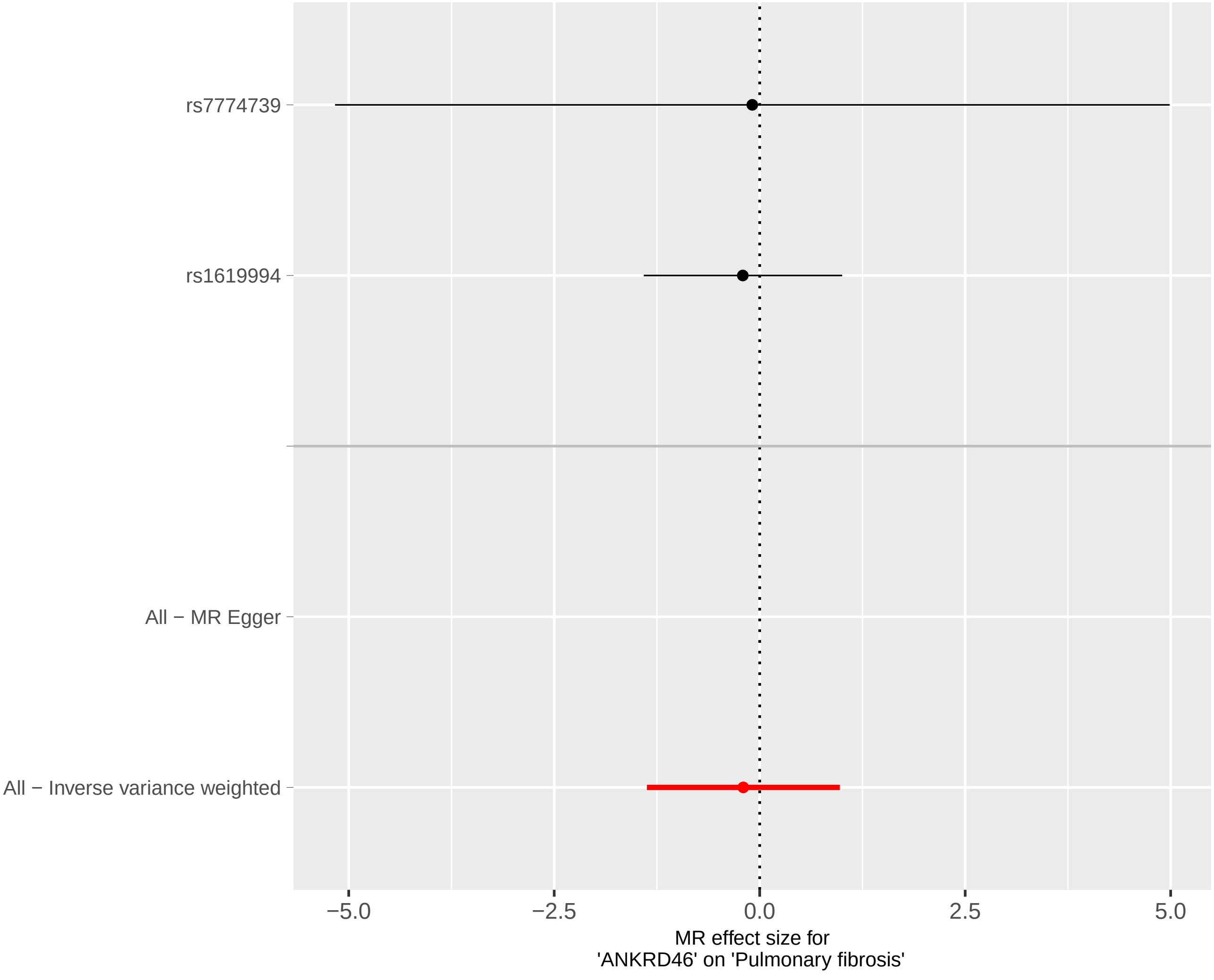
rs7774739
rs1619994
All − MR Egger
All − Inverse variance weighted
−5.0
−2.5
0.0
2.5
5.0
MR effect size for
'ANKRD46' on 'Pulmonary fibrosis'

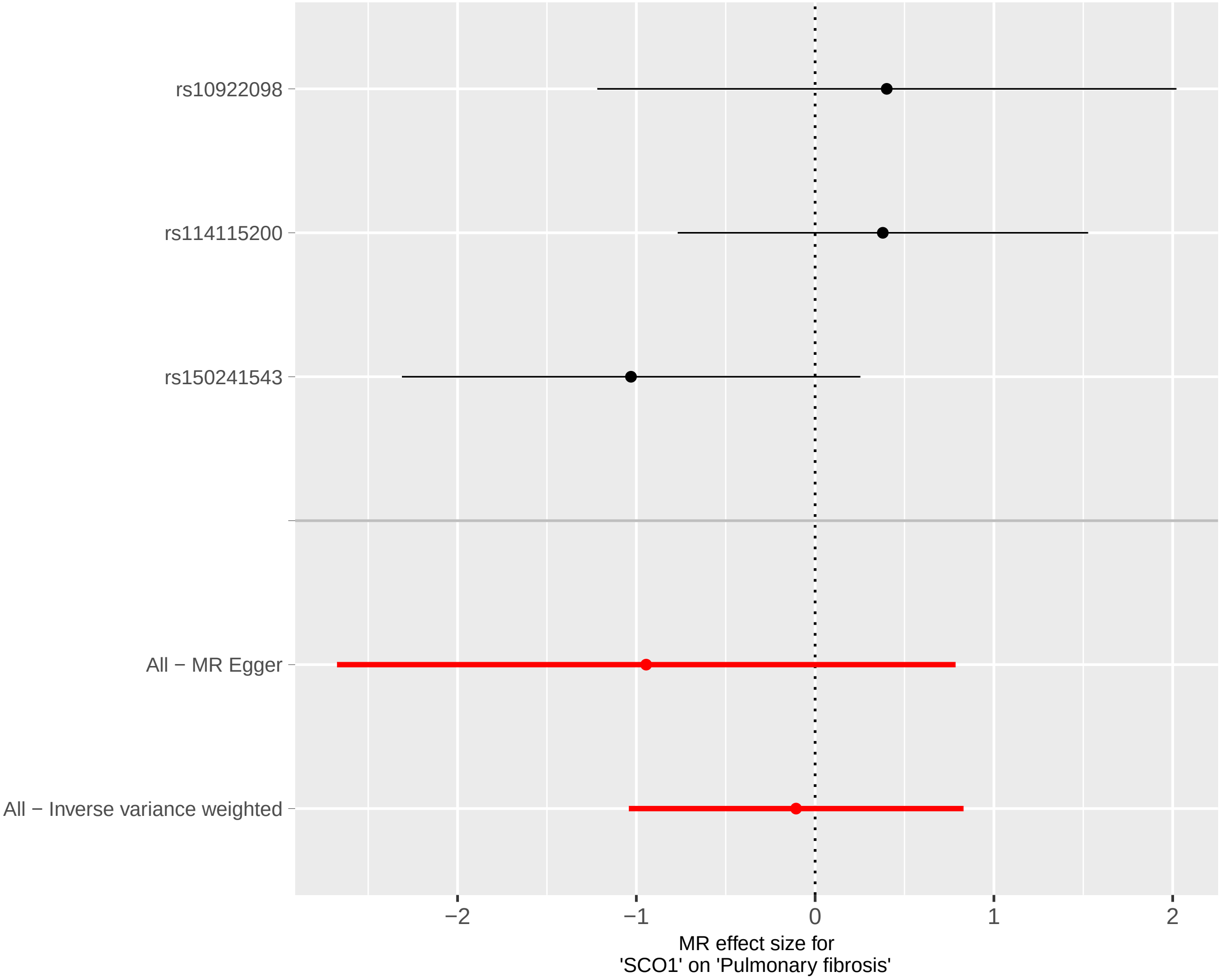

rs10922098
rs114115200
rs150241543
All − MR Egger
All − Inverse variance weighted
−2
−1
0
1
2
MR effect size for
'SCO1' on 'Pulmonary fibrosis'

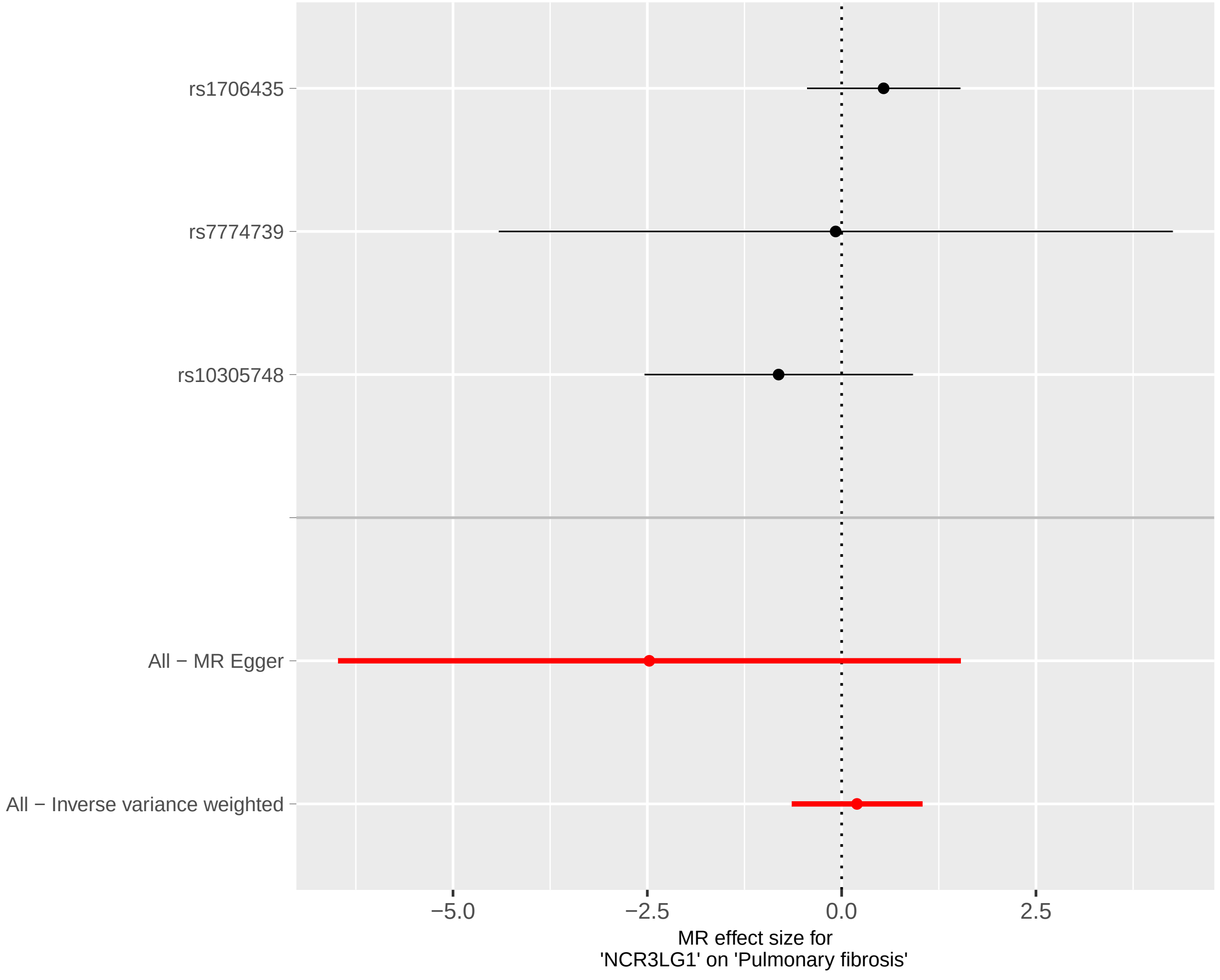

rs1706435
rs7774739
rs10305748
All − MR Egger
All − Inverse variance weighted
−5.0
−2.5
0.0
2.5
MR effect size for
'NCR3LG1' on 'Pulmonary fibrosis'

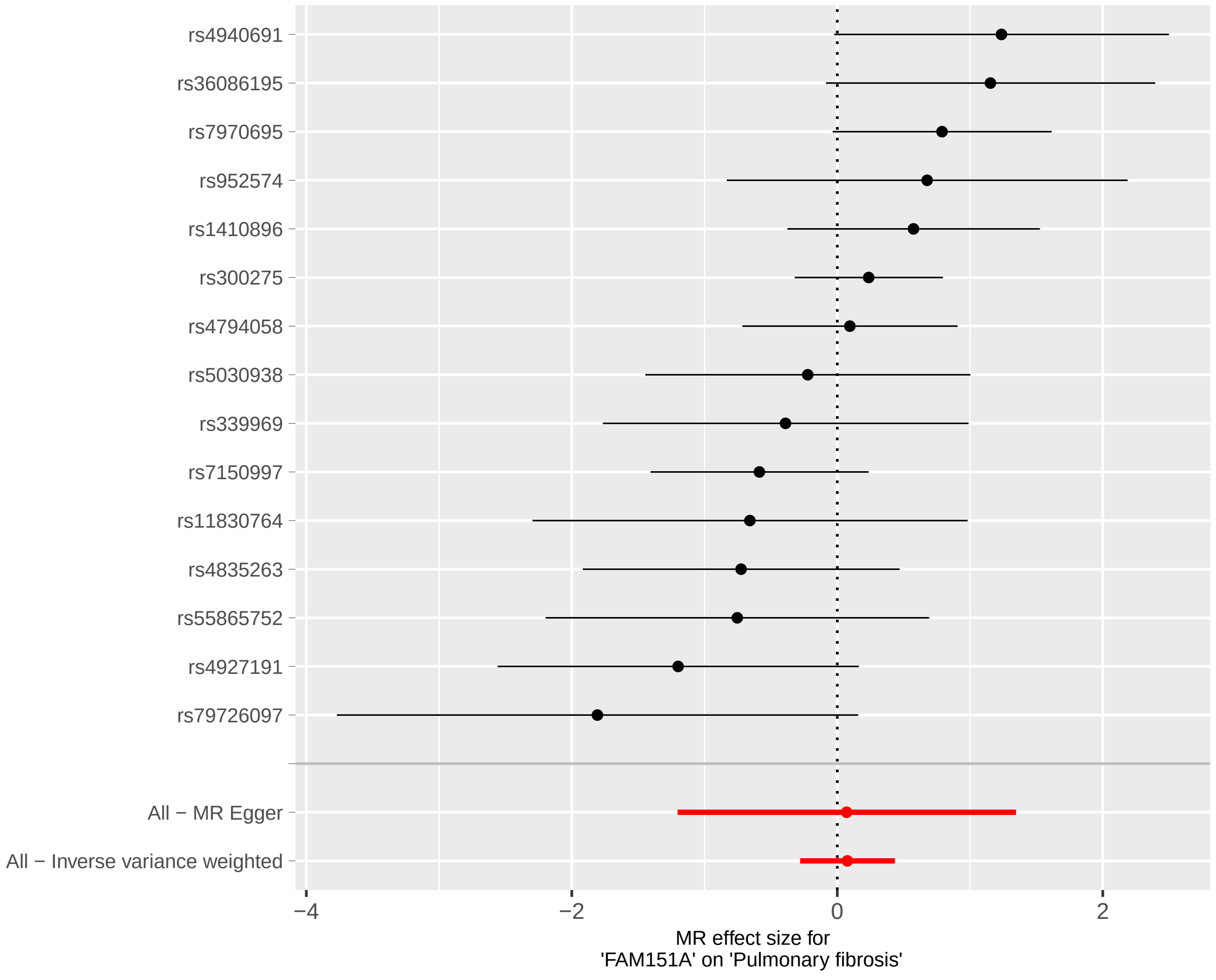
rs4940691
rs36086195
rs7970695
rs952574
rs1410896
rs300275
rs4794058
rs5030938
rs339969
rs7150997
rs11830764
rs4835263
rs55865752
rs4927191
rs79726097
All − MR Egger
All − Inverse variance weighted
−4
−2
0
2
MR effect size for
'FAM151A' on 'Pulmonary fibrosis'

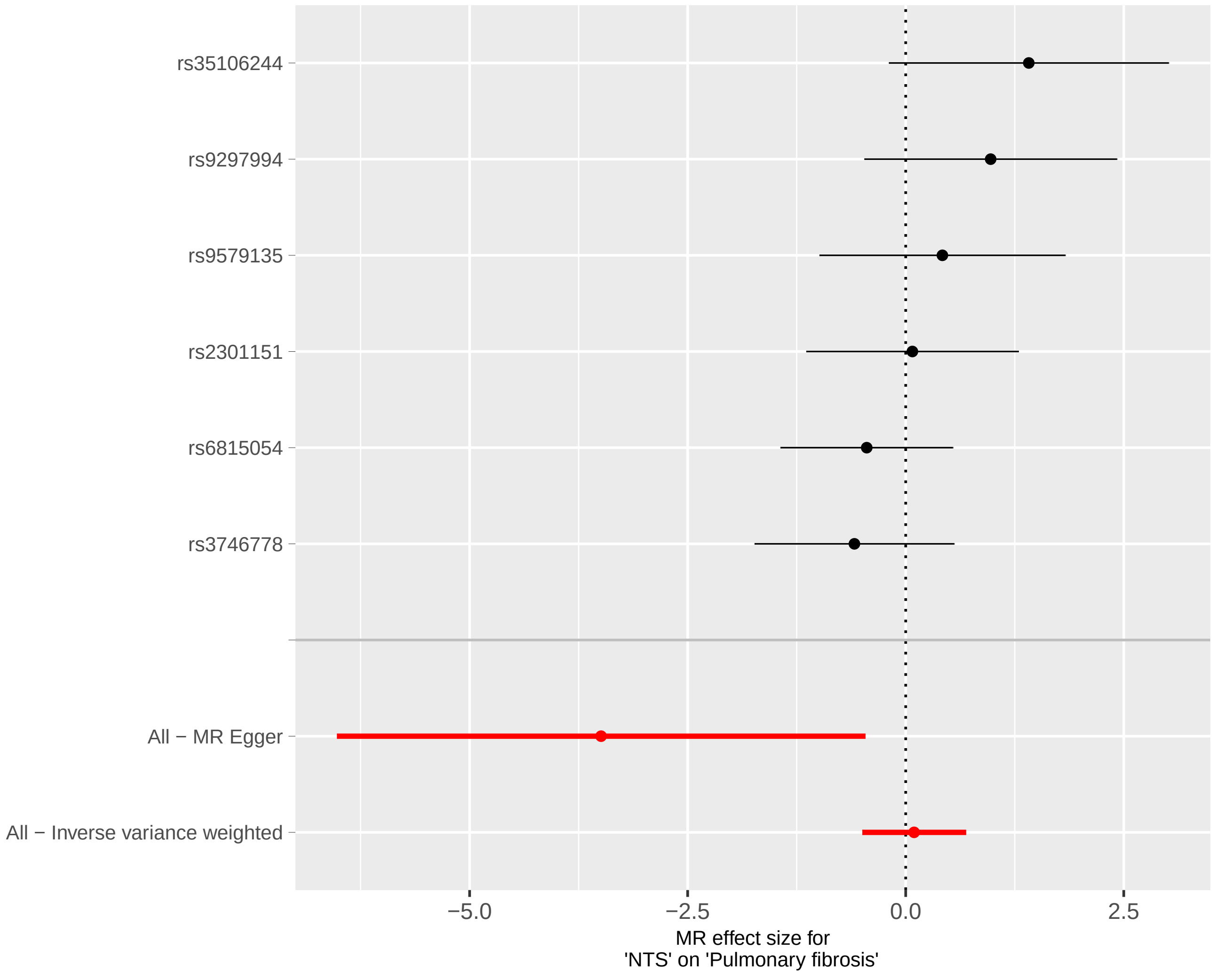

rs35106244
rs9297994
rs9579135
rs2301151
rs6815054
rs3746778
All − MR Egger
All − Inverse variance weighted
−5.0
−2.5
0.0
2.5
MR effect size for
'NTS' on 'Pulmonary fibrosis'

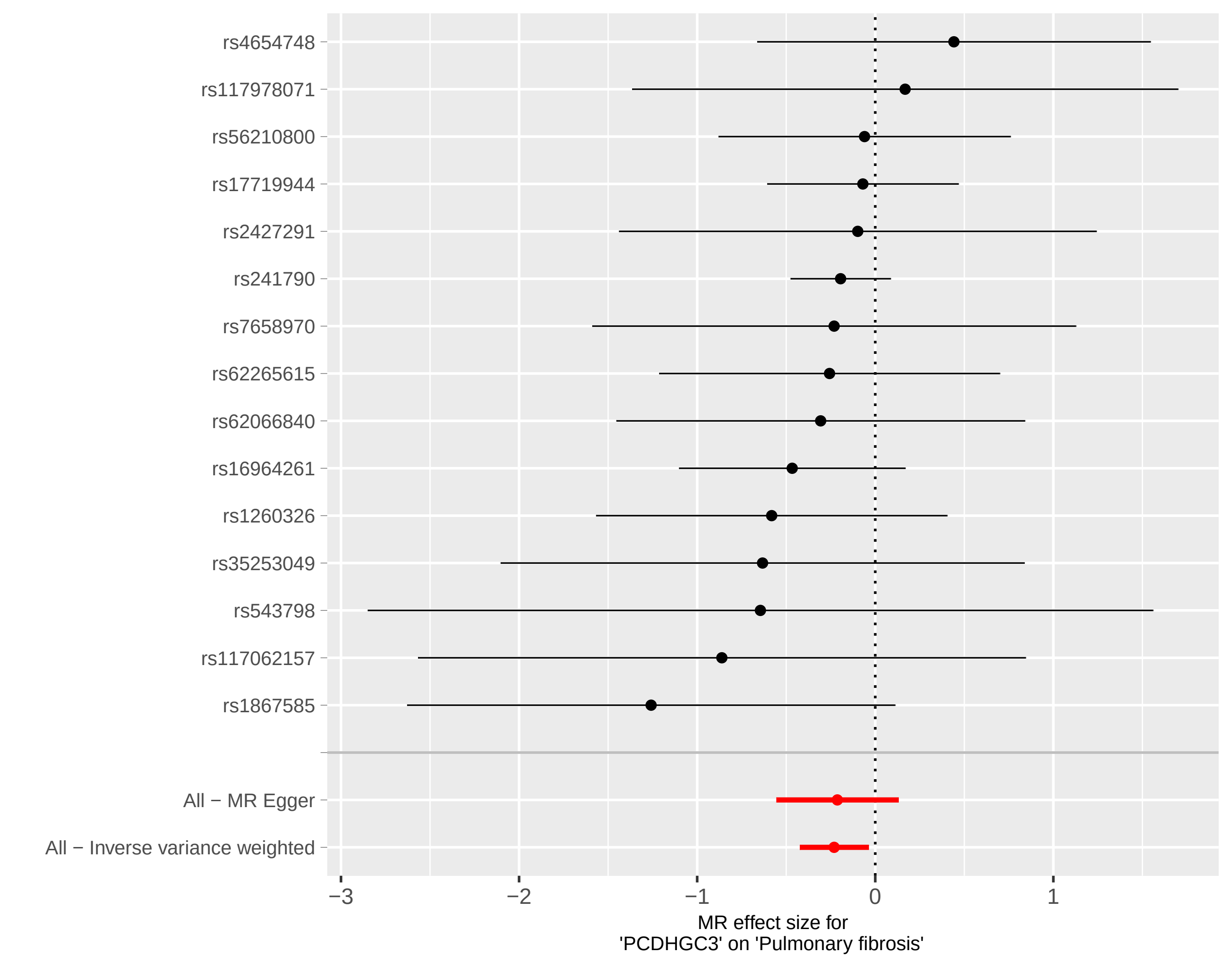
rs4654748
rs117978071
rs56210800
rs17719944
rs2427291
rs241790
rs7658970
rs62265615
rs62066840
rs16964261
rs1260326
rs35253049
rs543798
rs117062157
rs1867585
All − MR Egger
All − Inverse variance weighted
−3
−2
−1
0
1
MR effect size for
'PCDHGC3' on 'Pulmonary fibrosis'

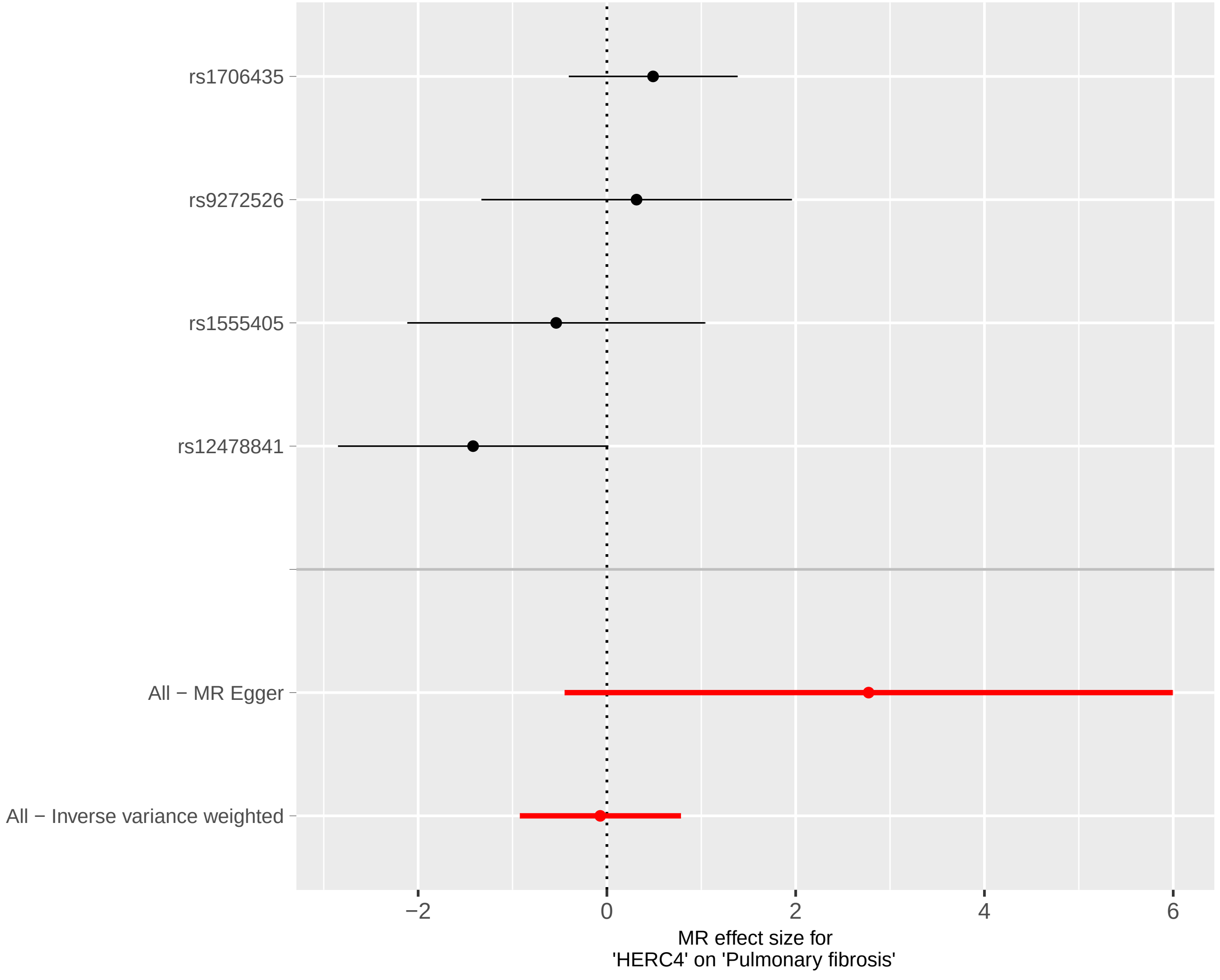

rs1706435
rs9272526
rs1555405
rs12478841
All − MR Egger
All − Inverse variance weighted
−2
0
2
4
6
MR effect size for
'HERC4' on 'Pulmonary fibrosis'

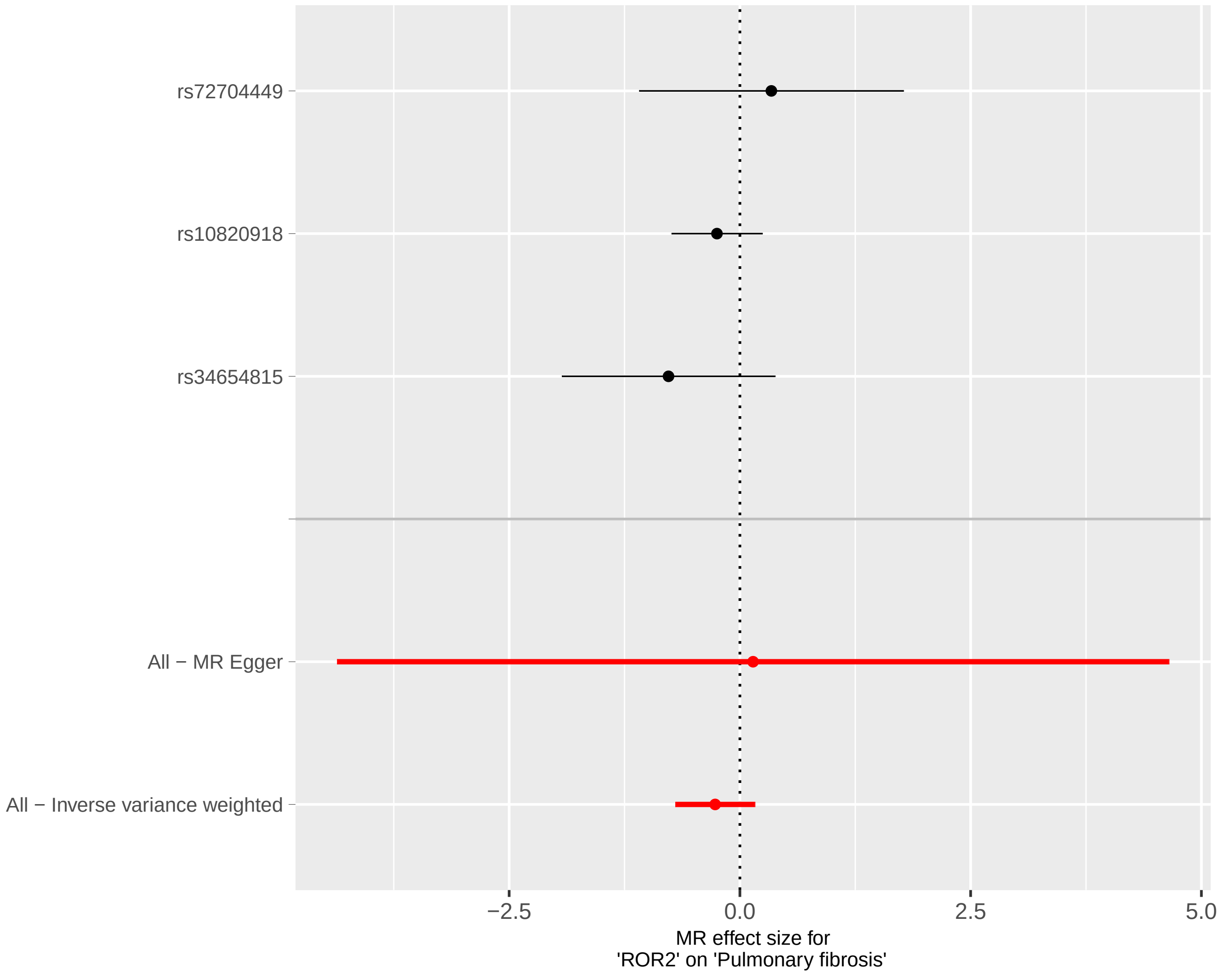
rs72704449
rs10820918
rs34654815
All − MR Egger
All − Inverse variance weighted
−2.5
0.0
2.5
5.0
MR effect size for
'ROR2' on 'Pulmonary fibrosis'

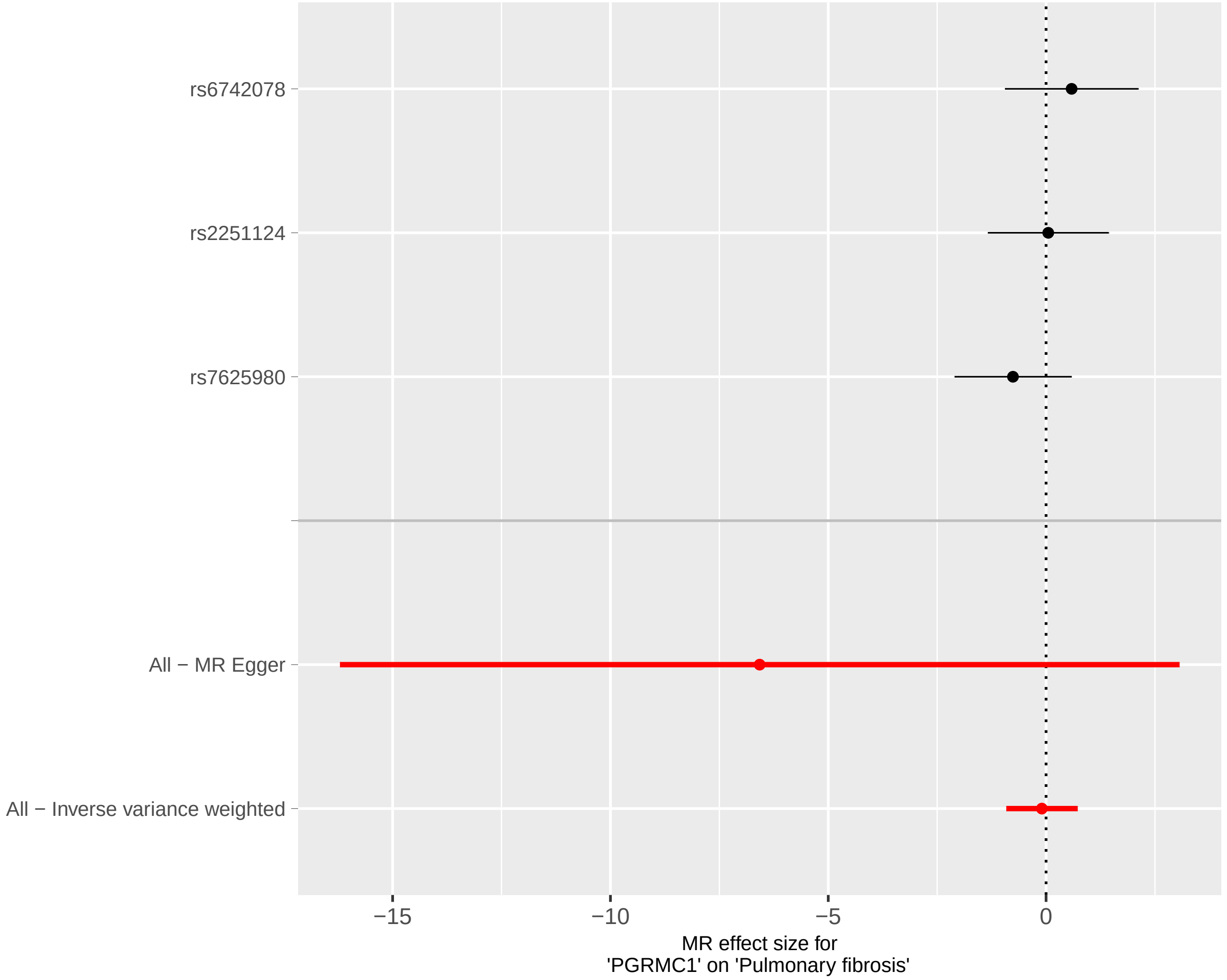

rs6742078
rs2251124
rs7625980
All − MR Egger
All − Inverse variance weighted
−15
−10
−5
0
MR effect size for
'PGRMC1' on 'Pulmonary fibrosis'

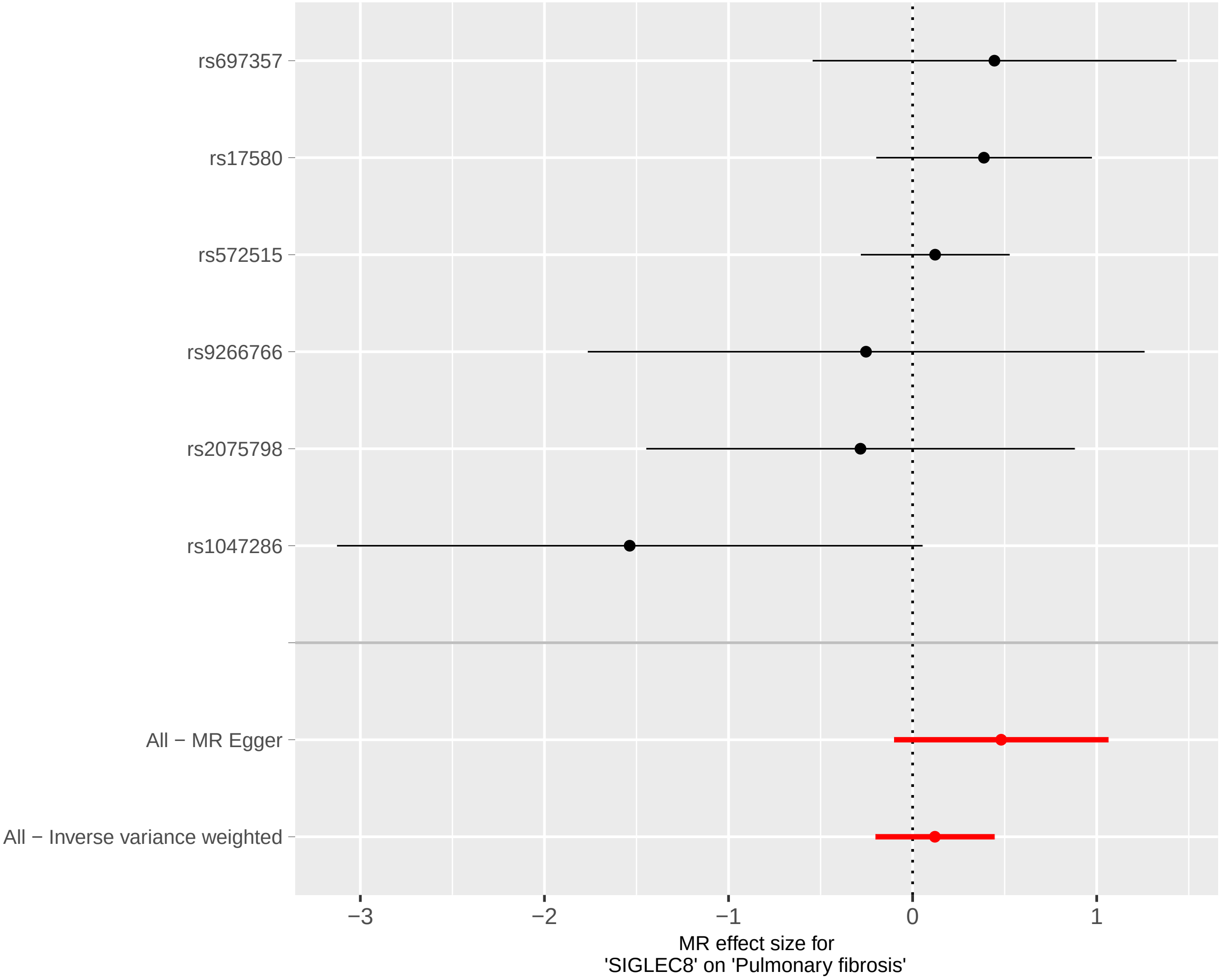

rs697357
rs17580
rs572515
rs9266766
rs2075798
rs1047286
All − MR Egger
All − Inverse variance weighted
−3
−2
−1
0
1
MR effect size for
'SIGLEC8' on 'Pulmonary fibrosis'

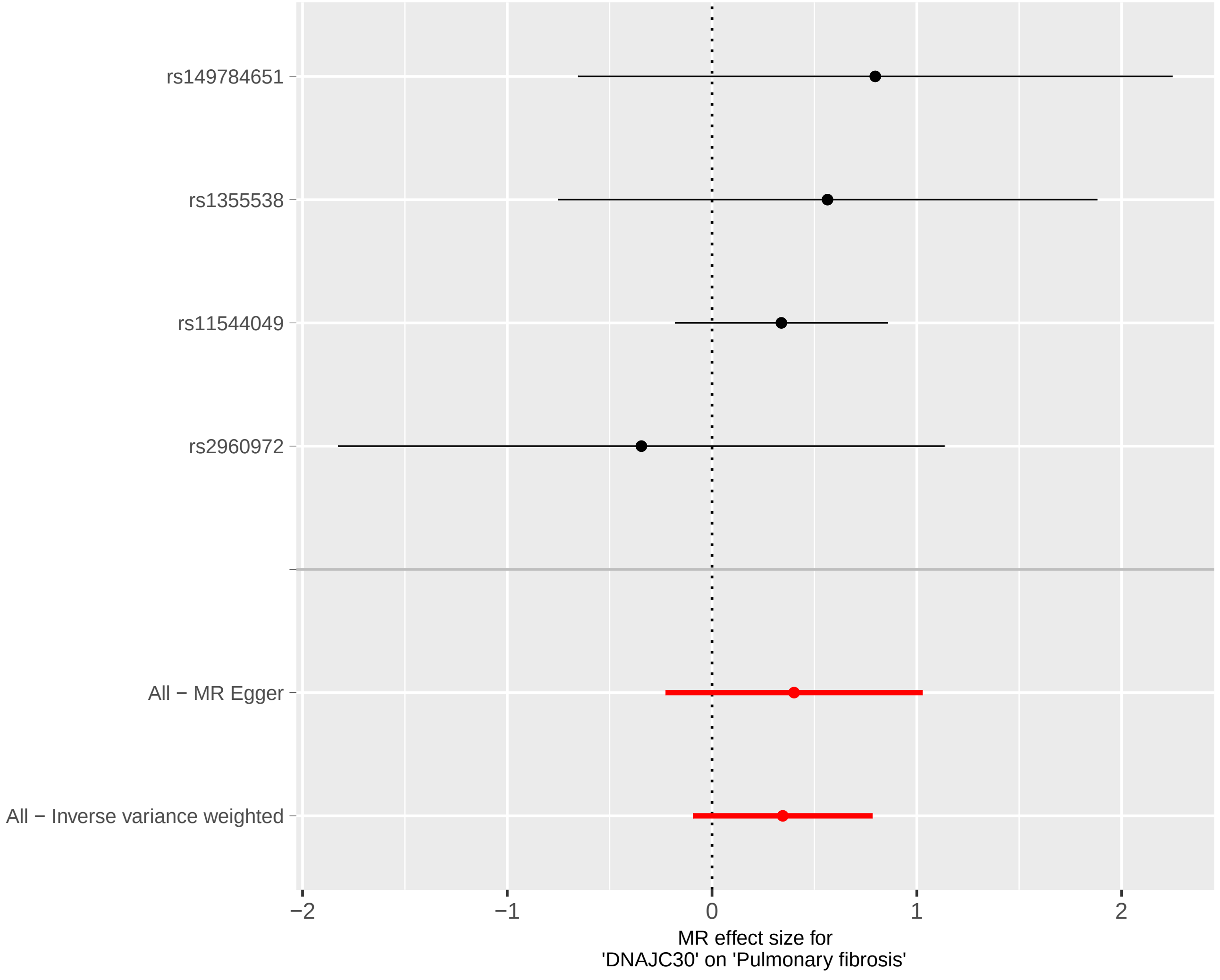

rs149784651
rs1355538
rs11544049
rs2960972
All − MR Egger
All − Inverse variance weighted
−2
−1
0
1
2
MR effect size for
'DNAJC30' on 'Pulmonary fibrosis'

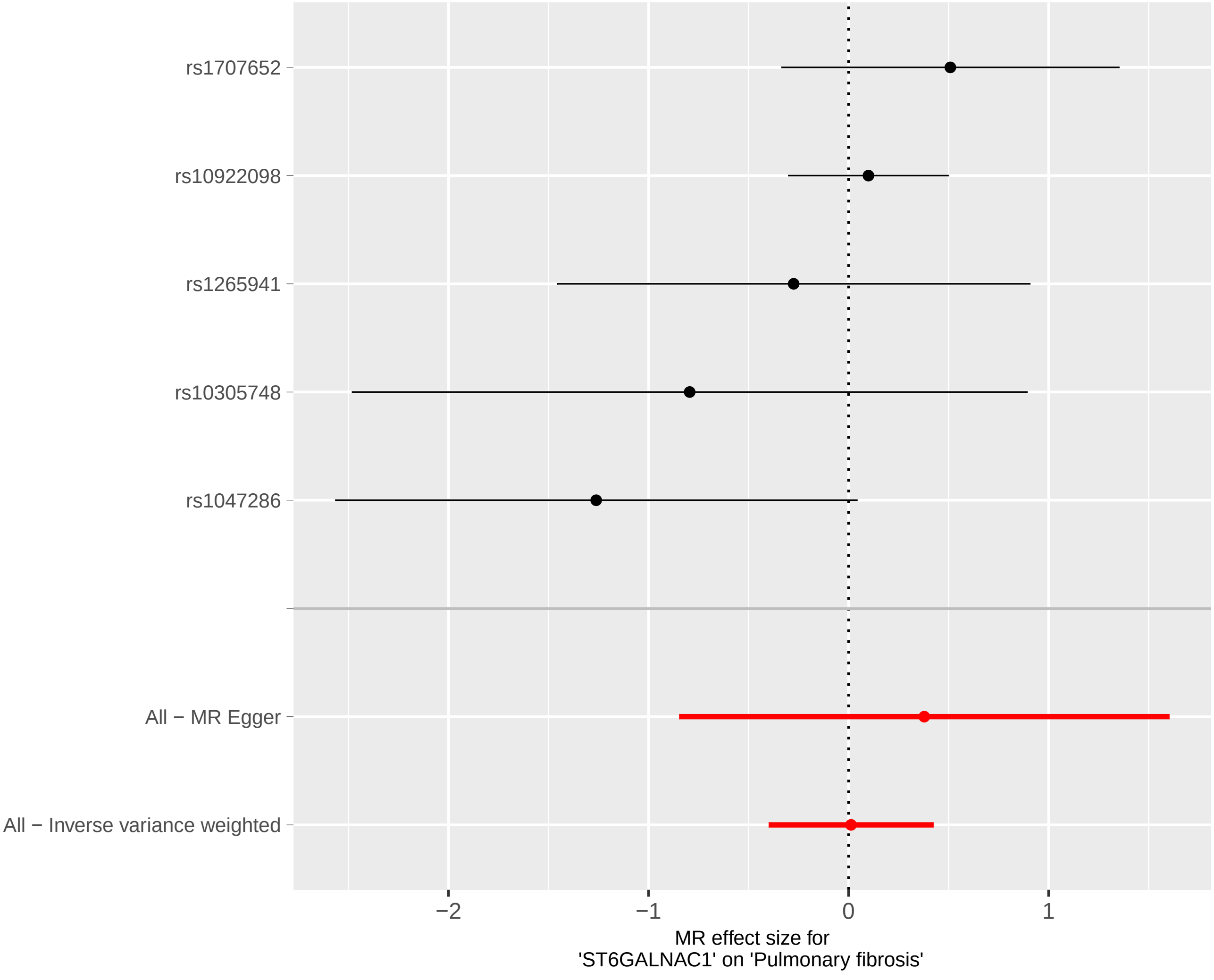
rs1707652
rs10922098
rs1265941
rs10305748
rs1047286
All − MR Egger
All − Inverse variance weighted
−2
−1
0
1
MR effect size for
'ST6GALNAC1' on 'Pulmonary fibrosis'

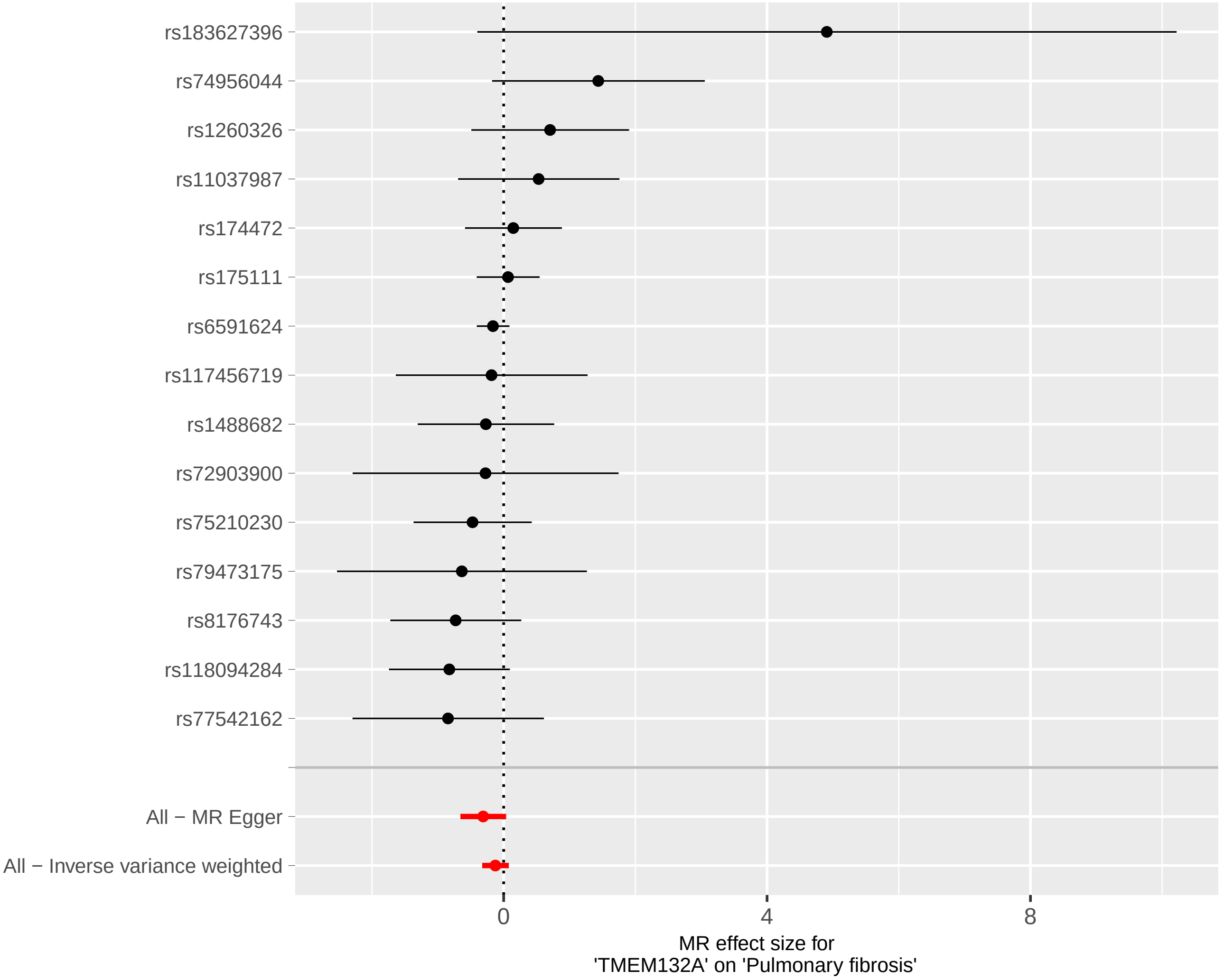
rs183627396
rs74956044
rs1260326
rs11037987
rs174472
rs175111
rs6591624
rs117456719
rs1488682
rs72903900
rs75210230
rs79473175
rs8176743
rs118094284
rs77542162
All − MR Egger
All − Inverse variance weighted
0
4
8
MR effect size for
'TMEM132A' on 'Pulmonary fibrosis'

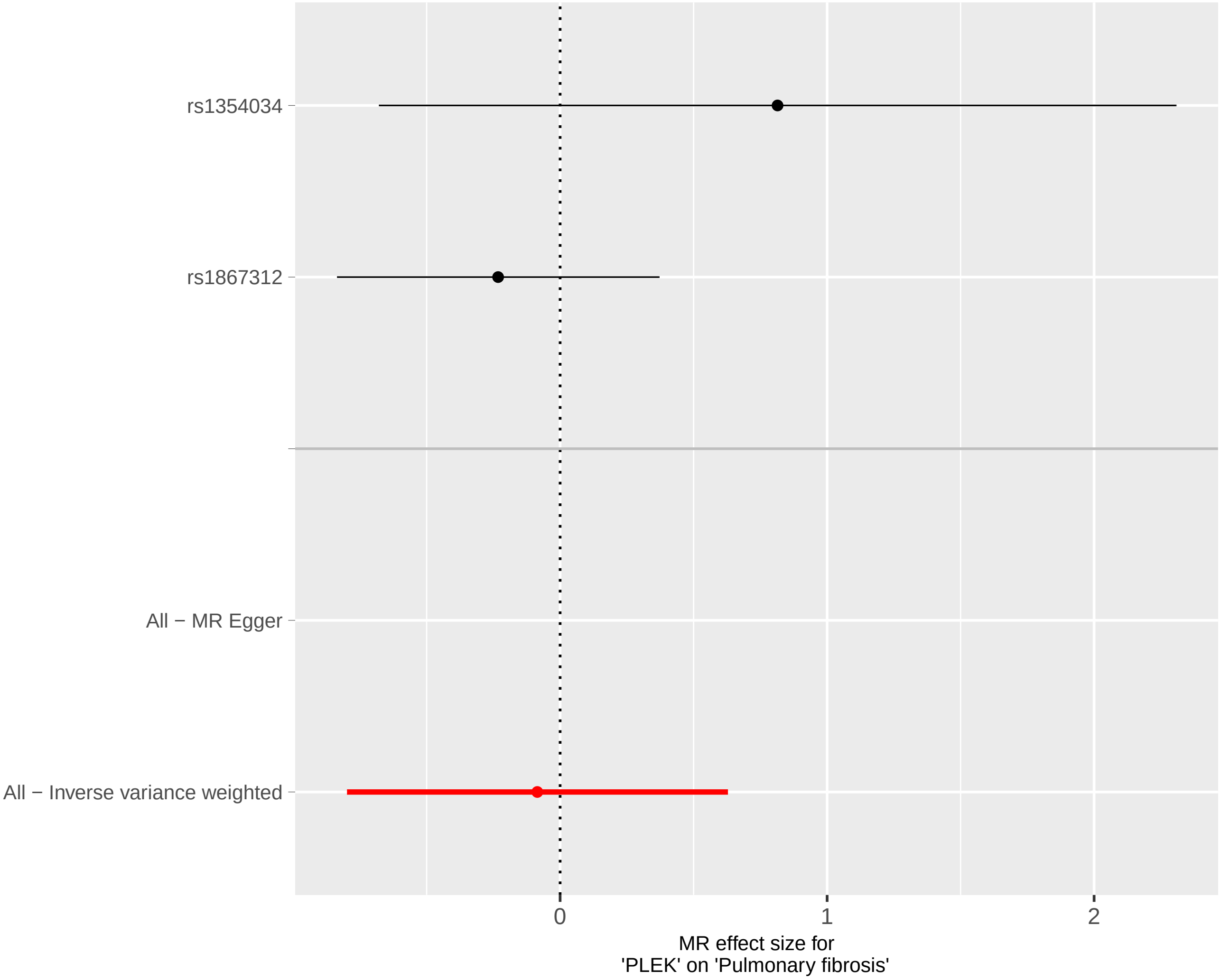
rs1354034
rs1867312
All − MR Egger
All − Inverse variance weighted
0
1
2
MR effect size for
'PLEK' on 'Pulmonary fibrosis'

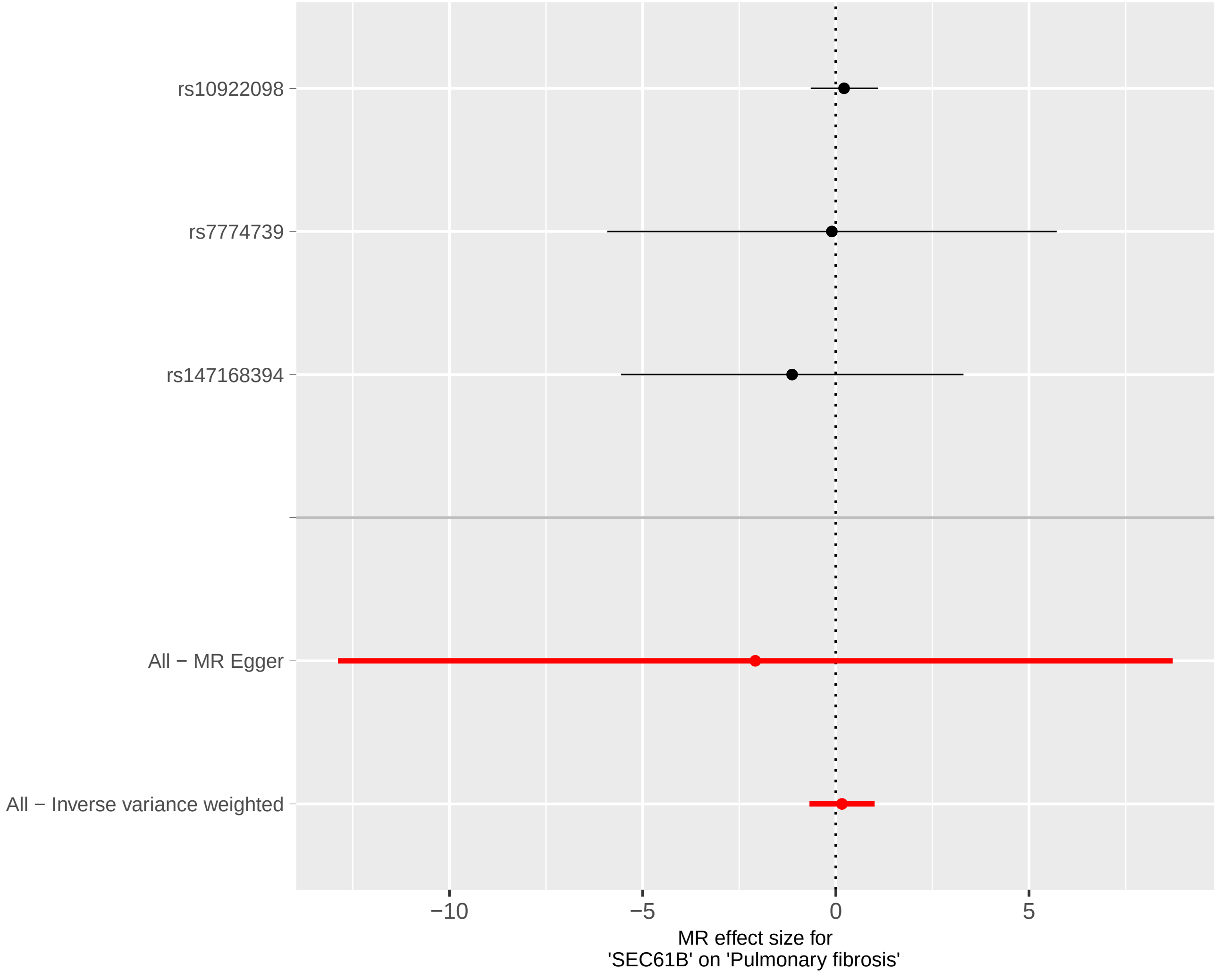
rs10922098
rs7774739
rs147168394
All − MR Egger
All − Inverse variance weighted
−10
−5
0
5
MR effect size for
'SEC61B' on 'Pulmonary fibrosis'

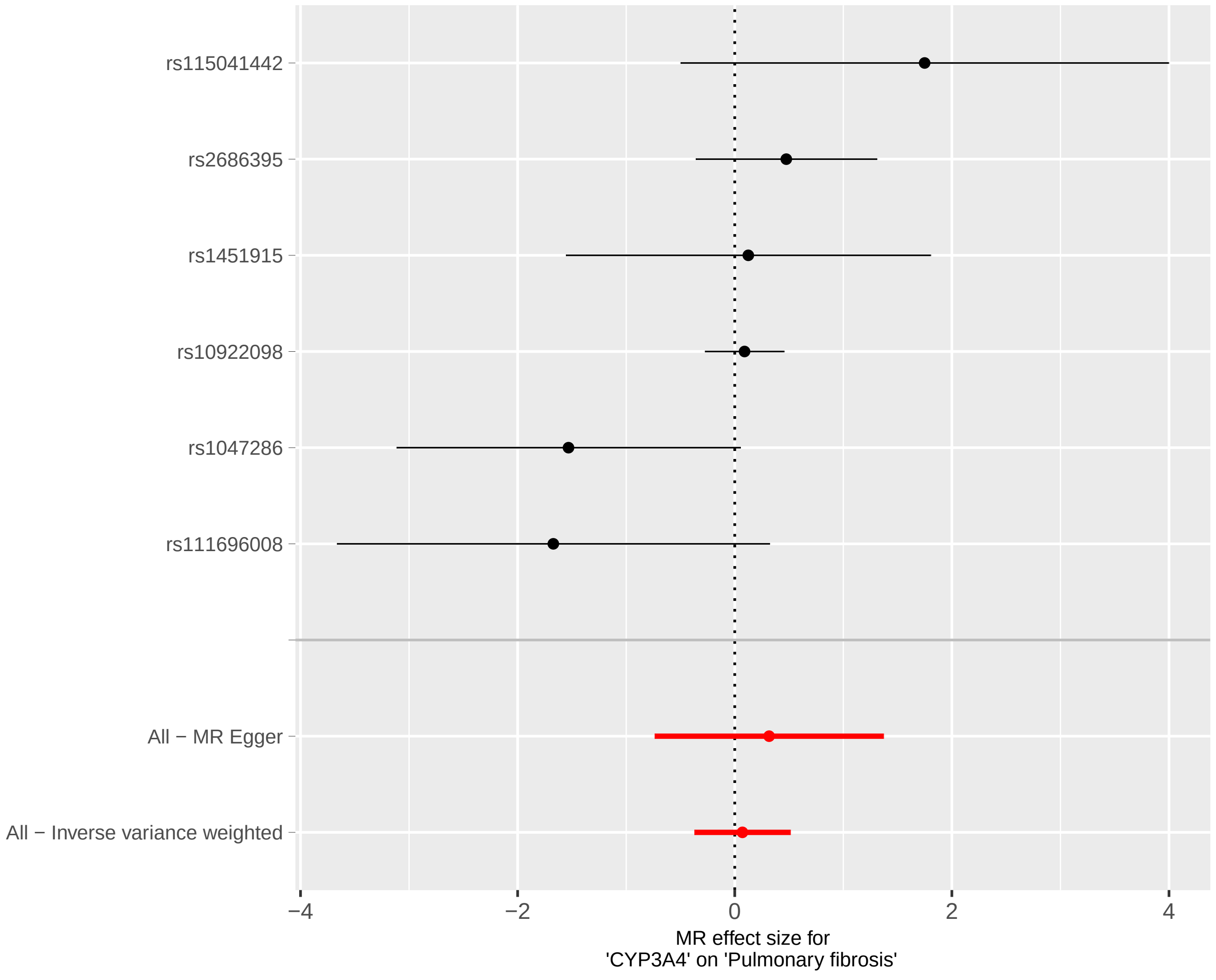
rs115041442
rs2686395
rs1451915
rs10922098
rs1047286
rs111696008
All − MR Egger
All − Inverse variance weighted
−4
−2
0
2
4
MR effect size for
'CYP3A4' on 'Pulmonary fibrosis'

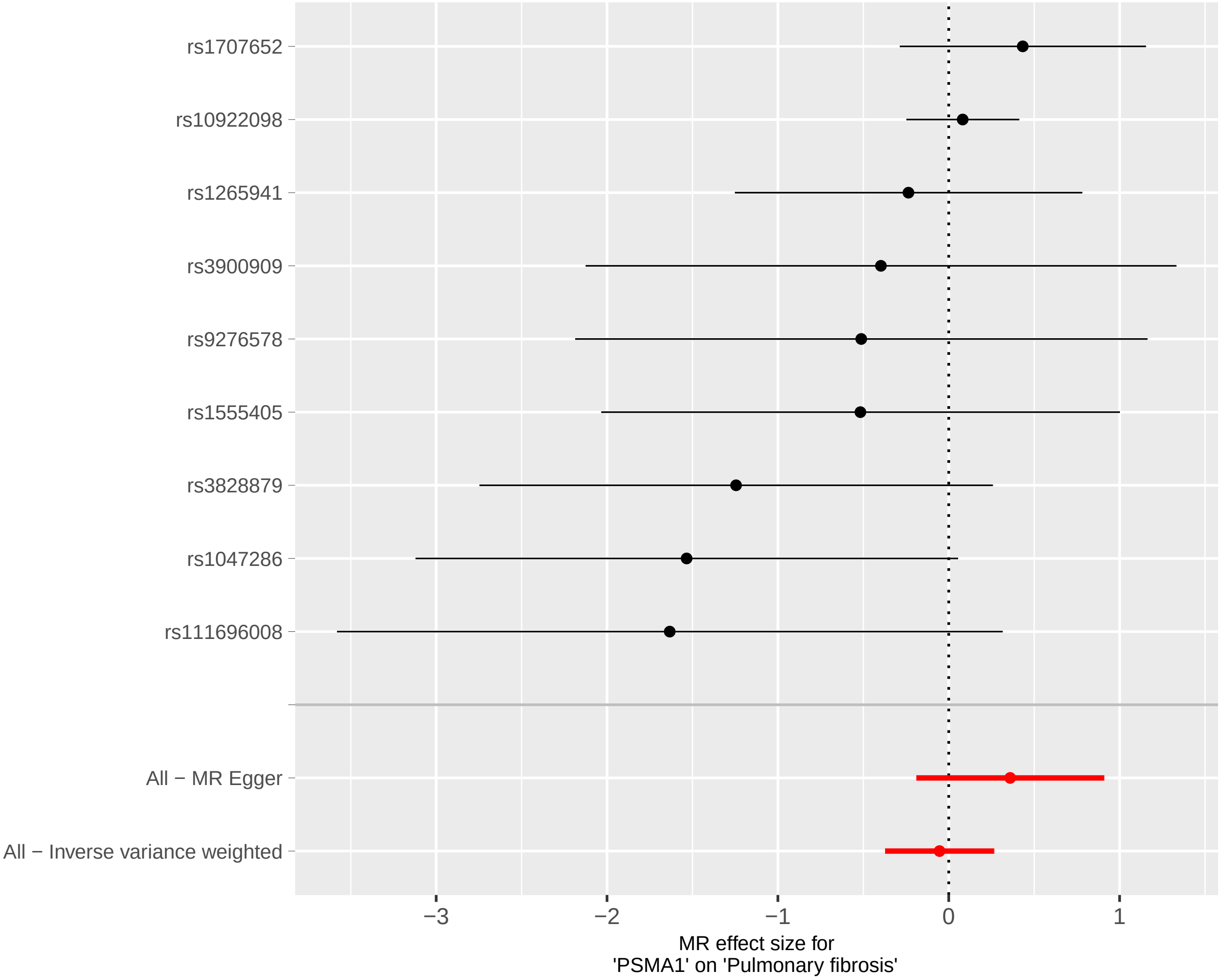
rs1707652
rs10922098
rs1265941
rs3900909
rs9276578
rs1555405
rs3828879
rs1047286
rs111696008
All − MR Egger
All − Inverse variance weighted
−3
−2
−1
0
1
MR effect size for
'PSMA1' on 'Pulmonary fibrosis'

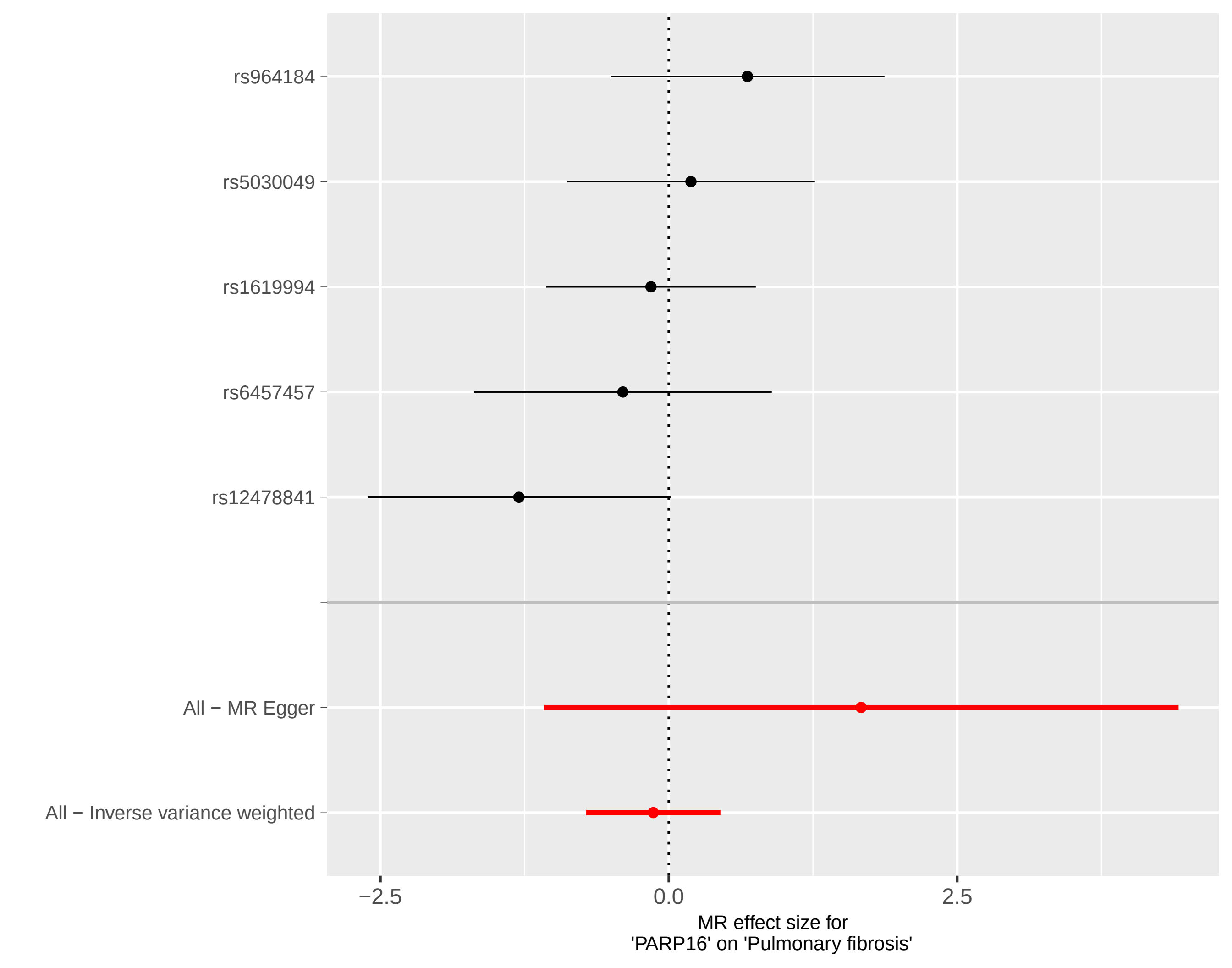

rs964184
rs5030049
rs1619994
rs6457457
rs12478841
All − MR Egger
All − Inverse variance weighted
−2.5
0.0
2.5
MR effect size for
'PARP16' on 'Pulmonary fibrosis'

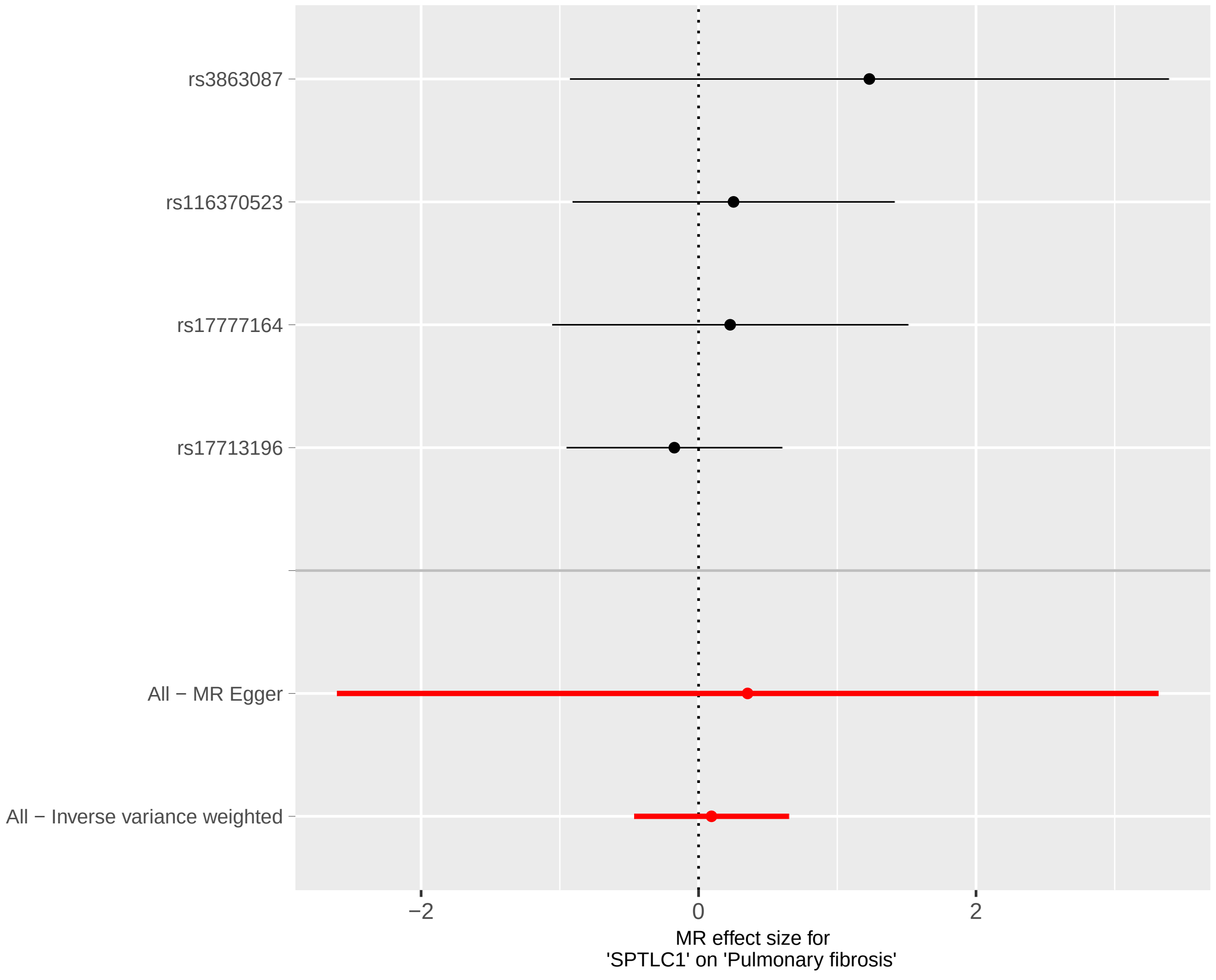
rs3863087
rs116370523
rs17777164
rs17713196
All − MR Egger
All − Inverse variance weighted
−2
0
2
MR effect size for
'SPTLC1' on 'Pulmonary fibrosis'

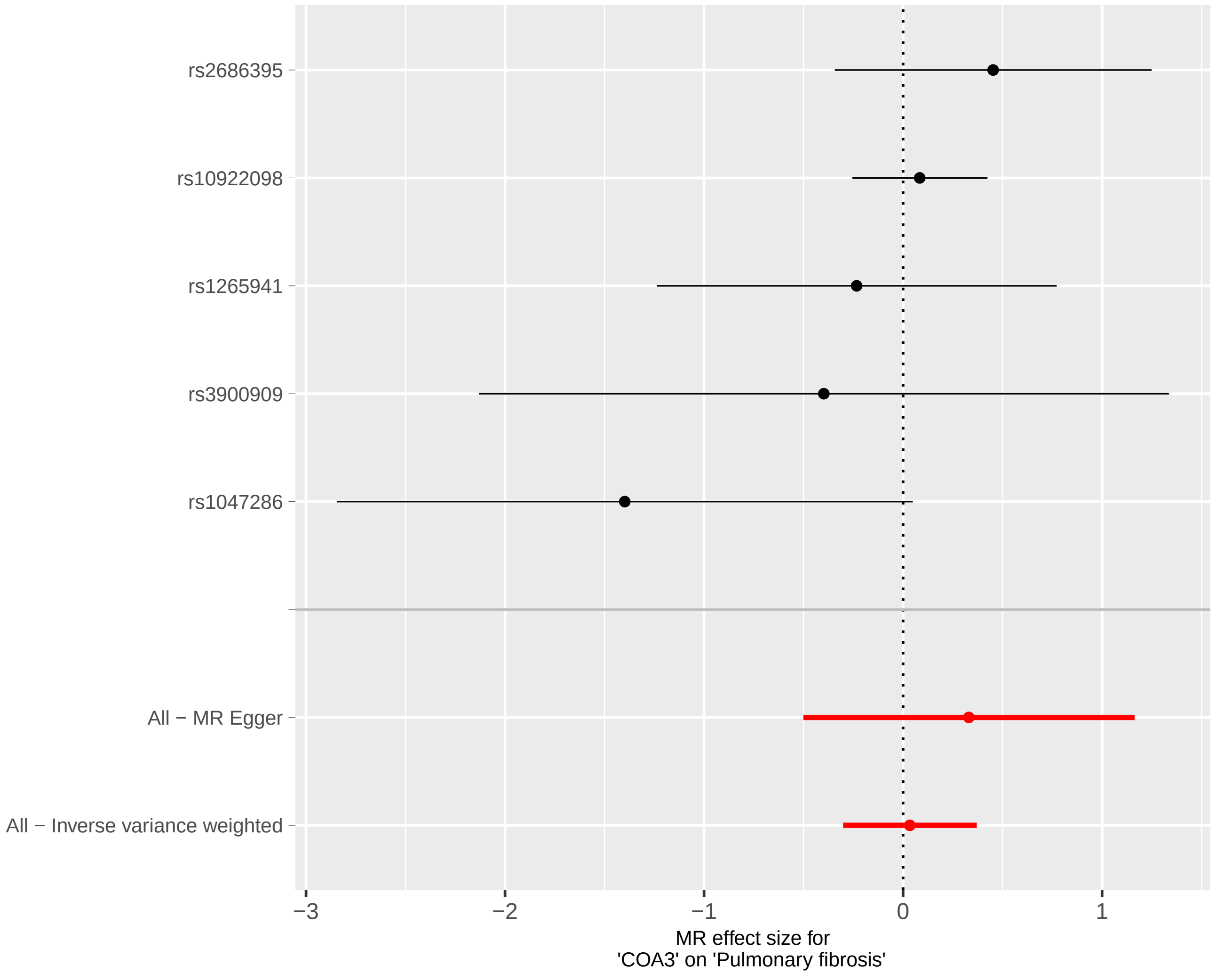

rs2686395
rs10922098
rs1265941
rs3900909
rs1047286
All − MR Egger
All − Inverse variance weighted
−3
−2
−1
0
1
MR effect size for
'COA3' on 'Pulmonary fibrosis'

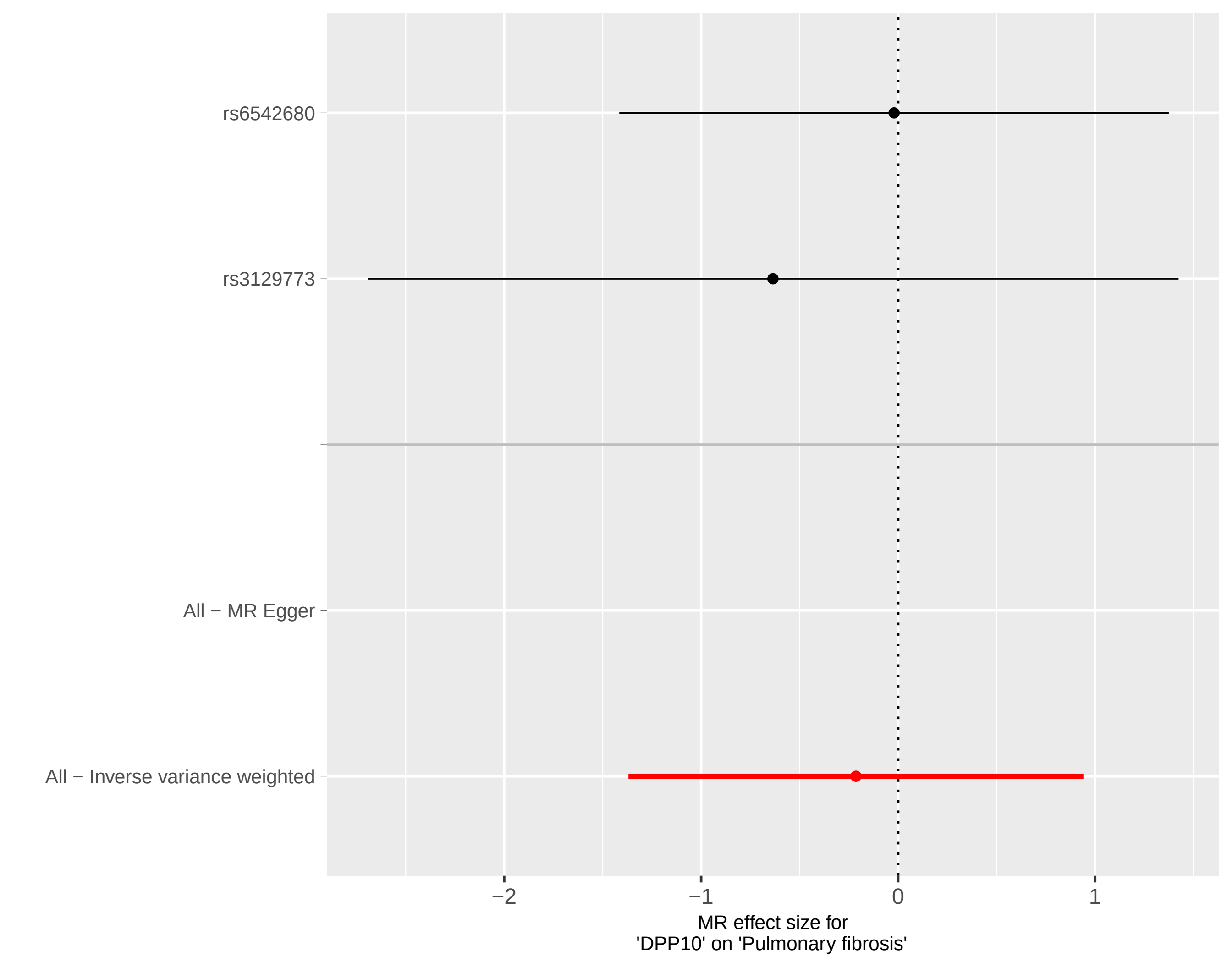
rs6542680
rs3129773
All − MR Egger
All − Inverse variance weighted
−2
−1
0
1
MR effect size for
'DPP10' on 'Pulmonary fibrosis'

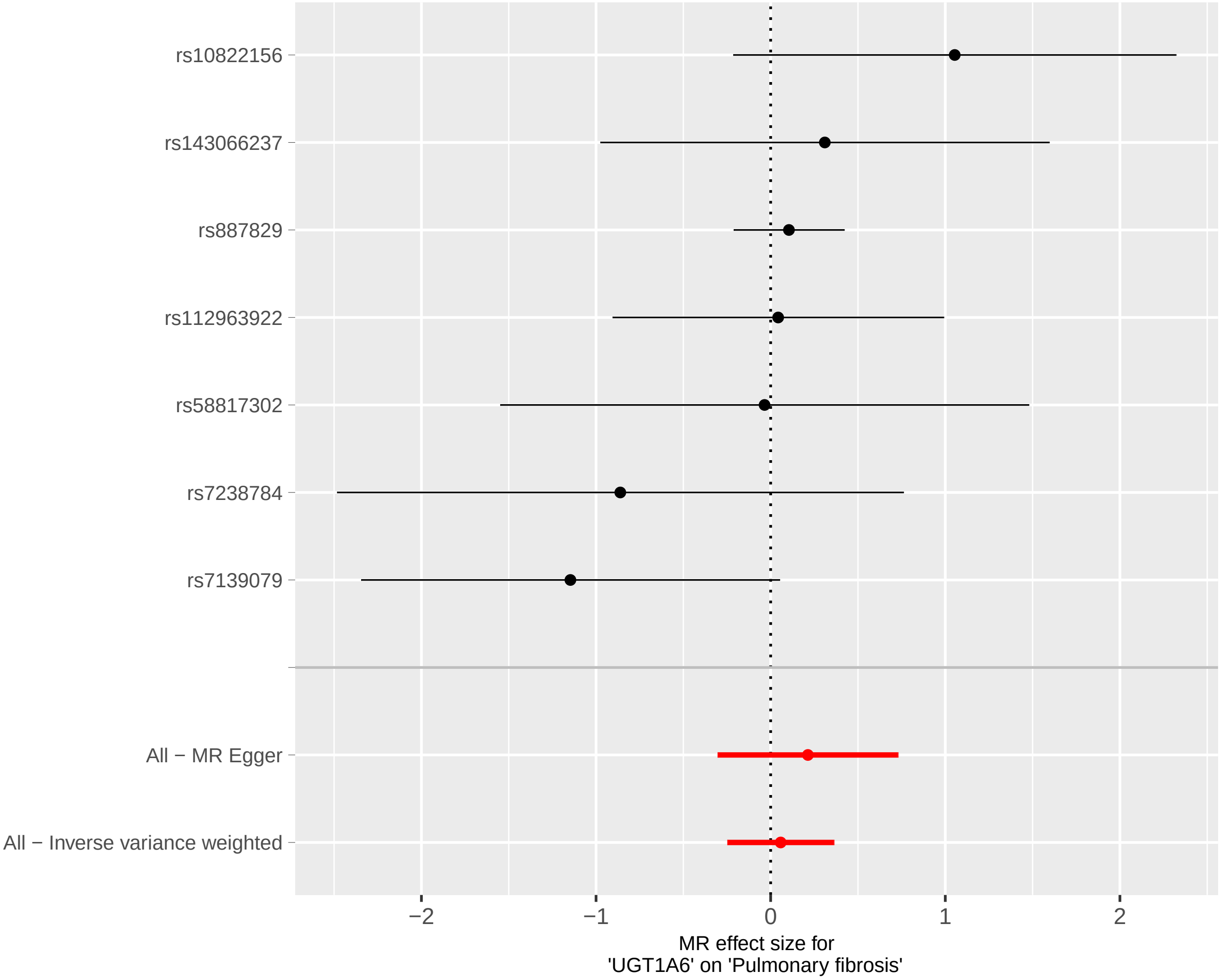
rs10822156
rs143066237
rs887829
rs112963922
rs58817302
rs7238784
rs7139079
All − MR Egger
All − Inverse variance weighted
−2
−1
0
1
2
MR effect size for
'UGT1A6' on 'Pulmonary fibrosis'

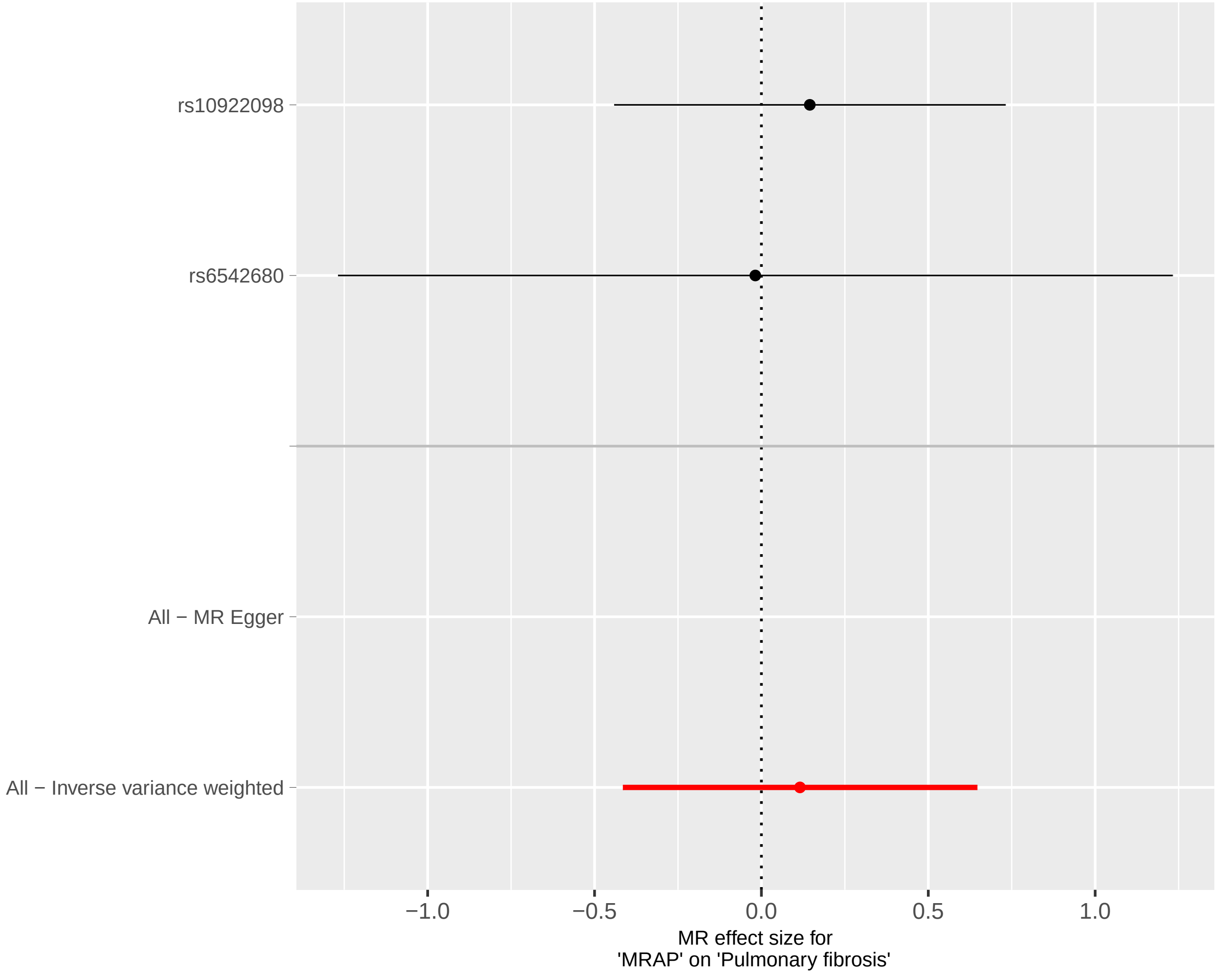

rs10922098
rs6542680
All − MR Egger
All − Inverse variance weighted
−1.0
−0.5
0.0
0.5
1.0
MR effect size for
'MRAP' on 'Pulmonary fibrosis'

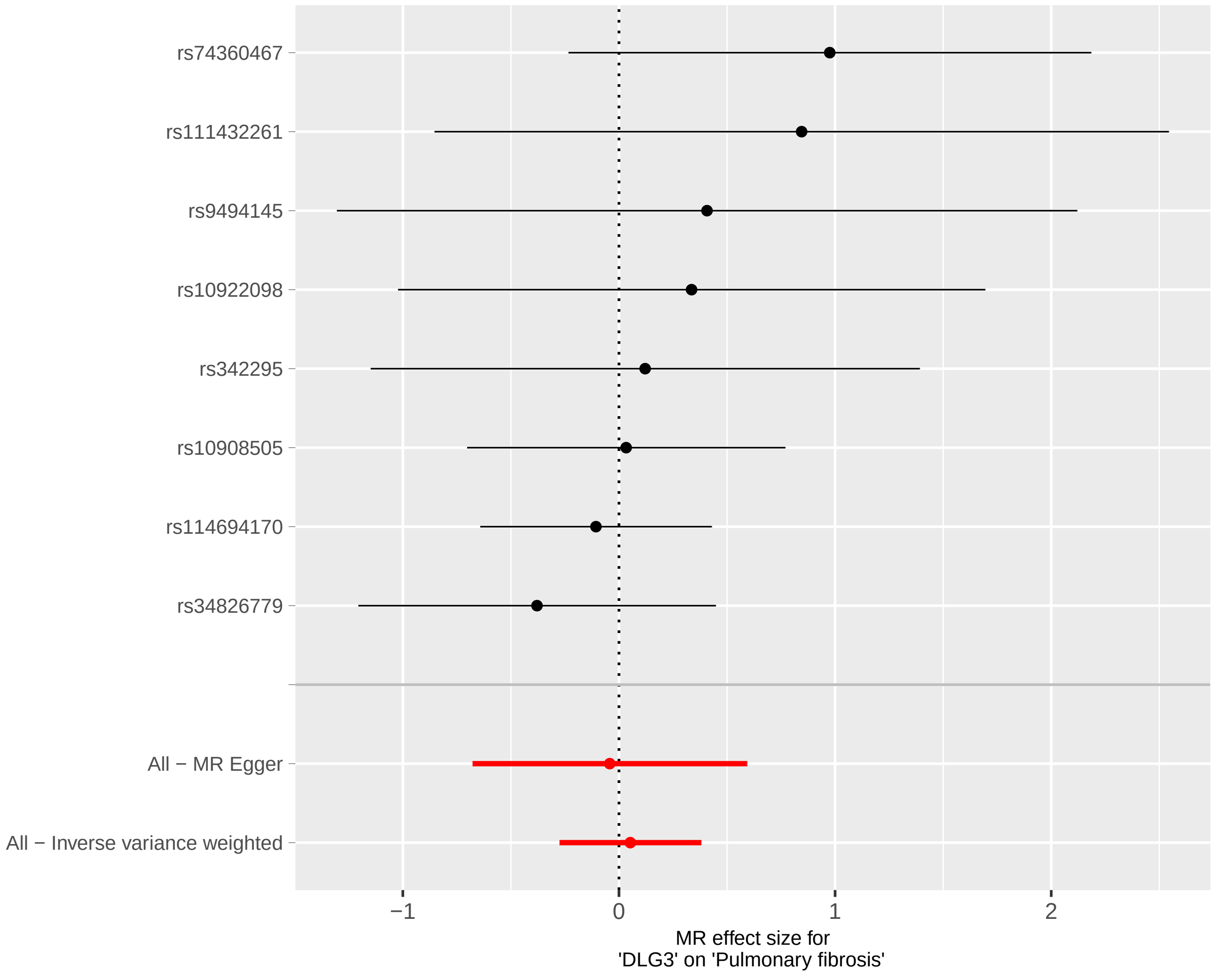
rs74360467
rs111432261
rs9494145
rs10922098
rs342295
rs10908505
rs114694170
rs34826779
All − MR Egger
All − Inverse variance weighted
−1
0
1
2
MR effect size for
'DLG3' on 'Pulmonary fibrosis'

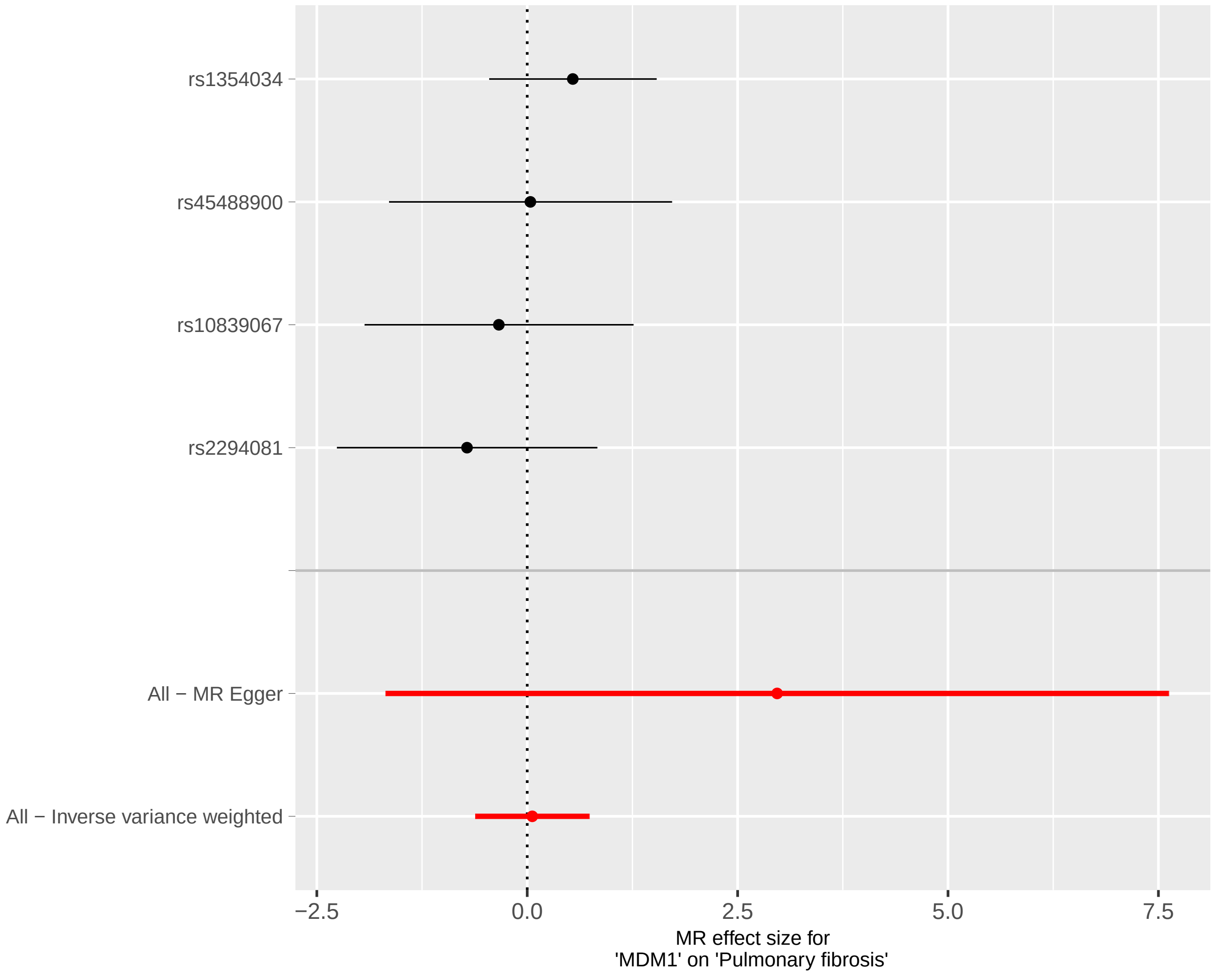

rs1354034
rs45488900
rs10839067
rs2294081
All − MR Egger
All − Inverse variance weighted
−2.5
0.0
2.5
5.0
7.5
MR effect size for
'MDM1' on 'Pulmonary fibrosis'

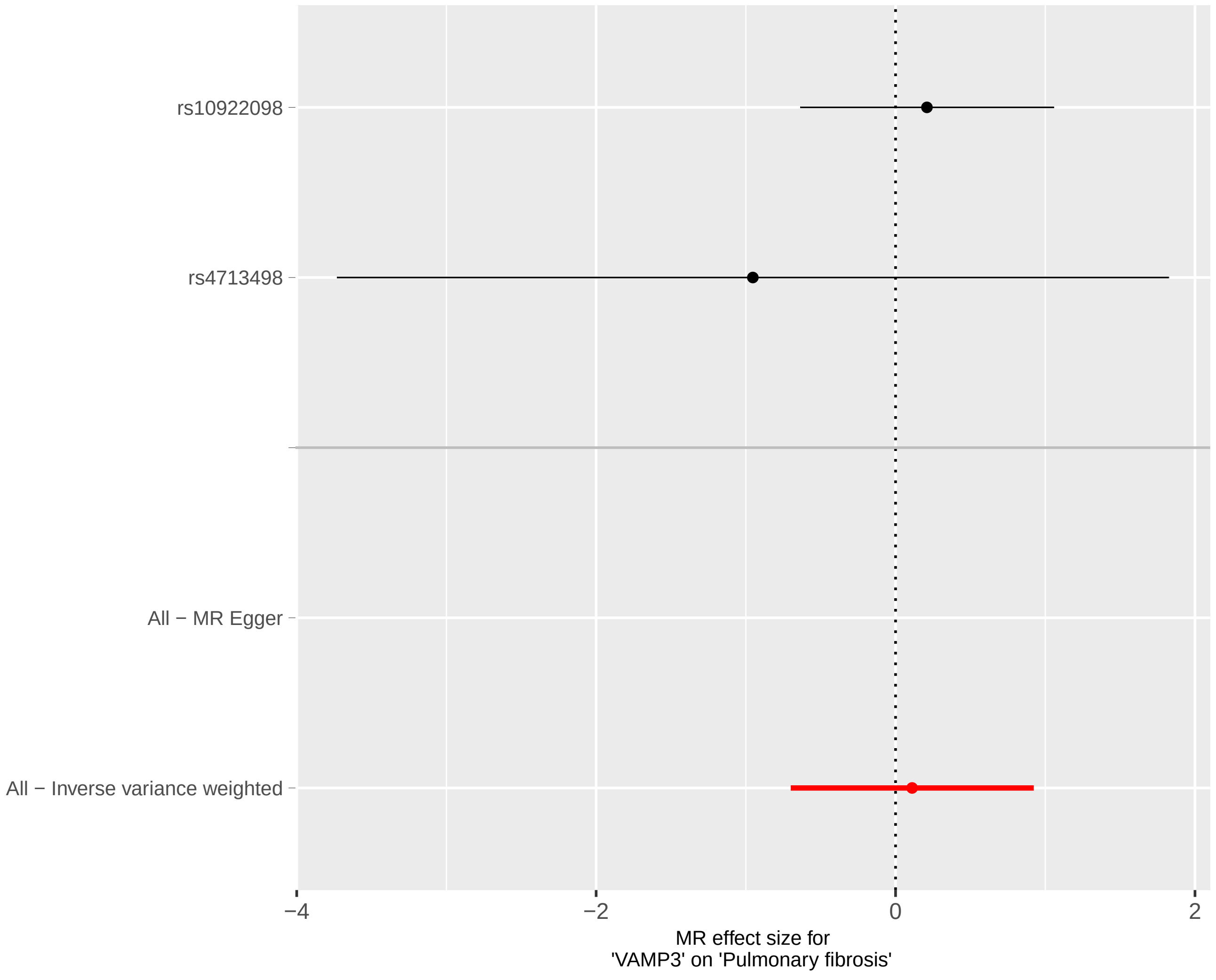
rs10922098
rs4713498
All − MR Egger
All − Inverse variance weighted
−4
−2
0
2
MR effect size for
'VAMP3' on 'Pulmonary fibrosis'

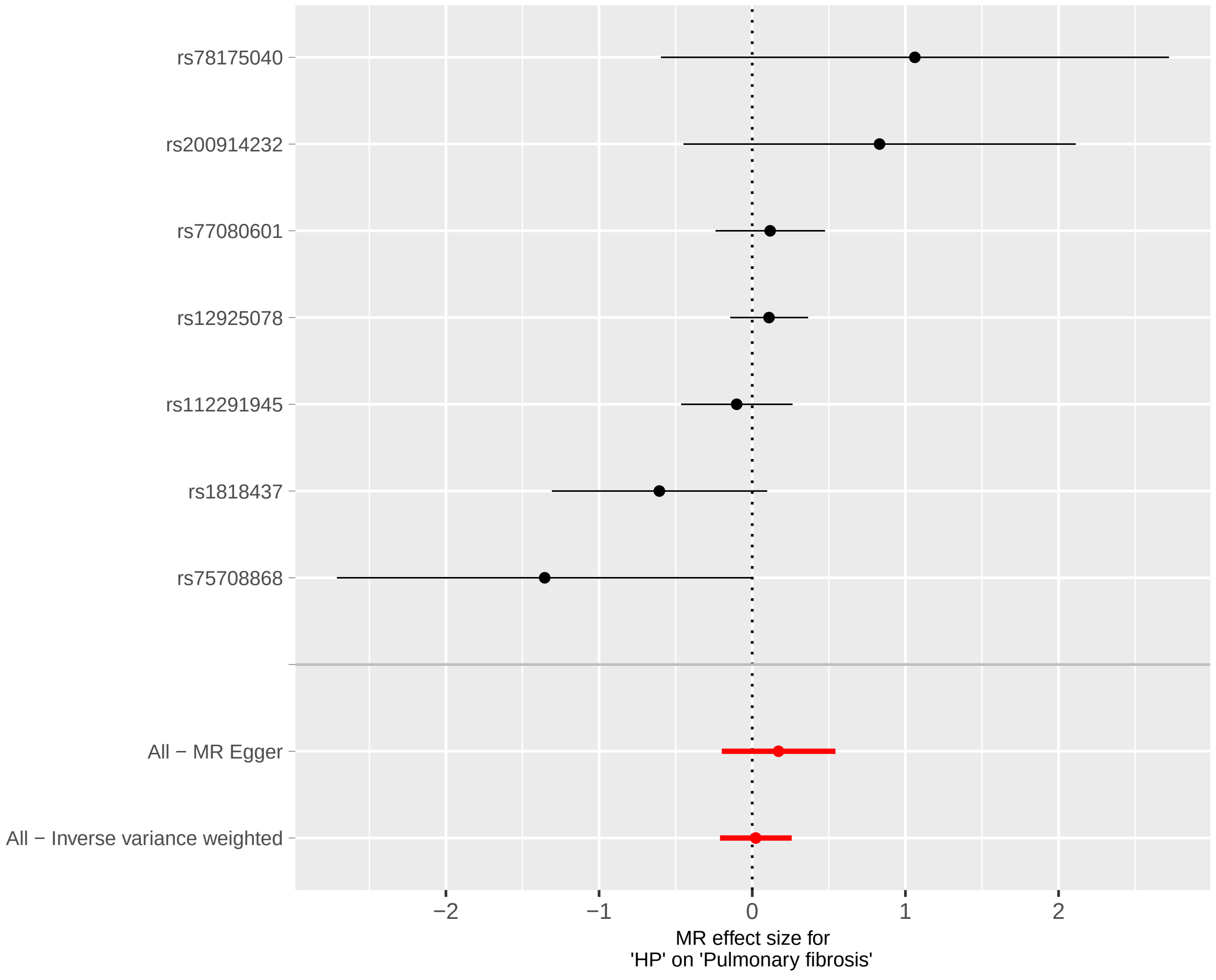
rs78175040
rs200914232
rs77080601
rs12925078
rs112291945
rs1818437
rs75708868
All − MR Egger
All − Inverse variance weighted
−2
−1
0
1
2
MR effect size for
'HP' on 'Pulmonary fibrosis'

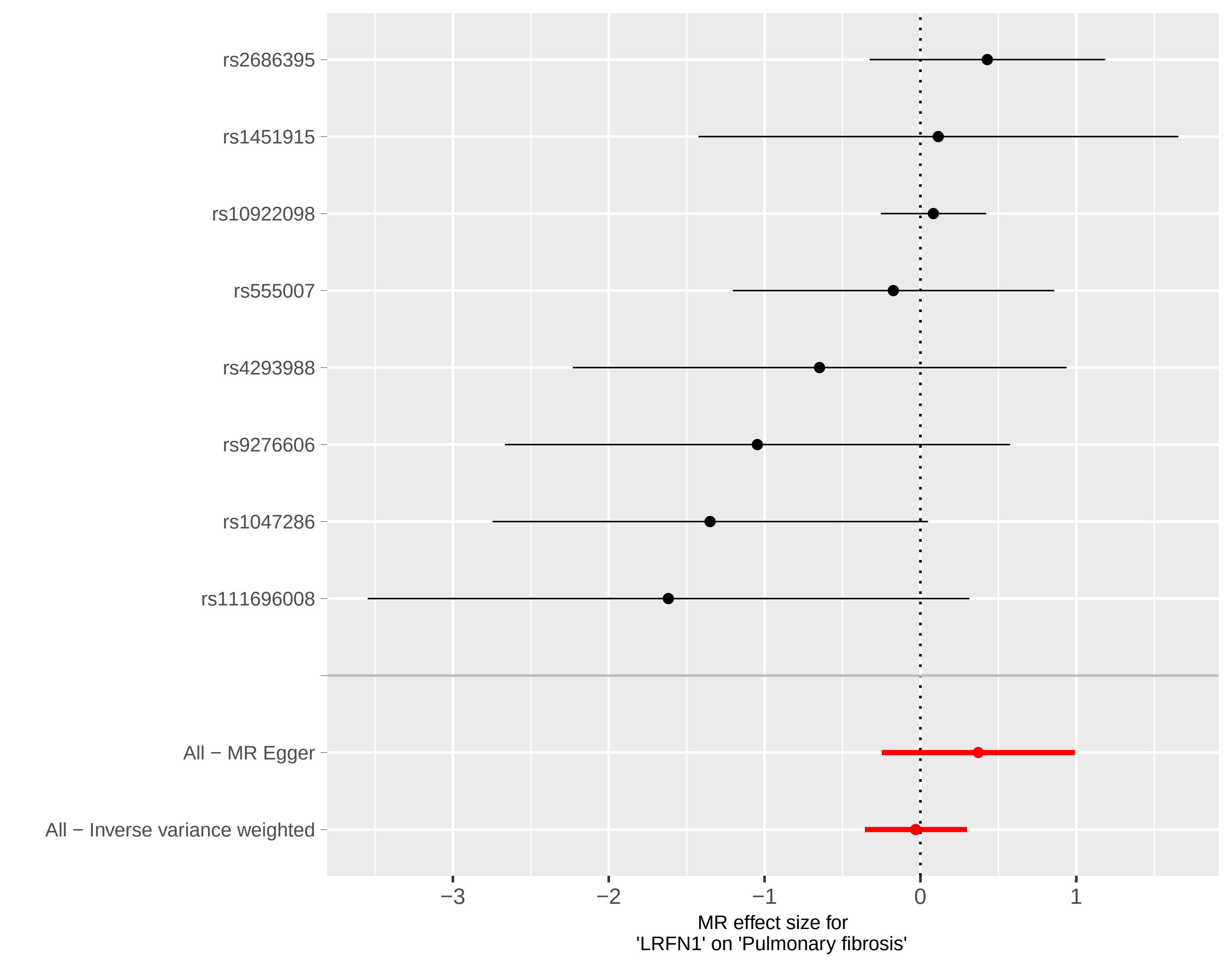
rs2686395
rs1451915
rs10922098
rs555007
rs4293988
rs9276606
rs1047286
rs111696008
All − MR Egger
All − Inverse variance weighted
−3
−2
−1
0
1
MR effect size for
'LRFN1' on 'Pulmonary fibrosis'

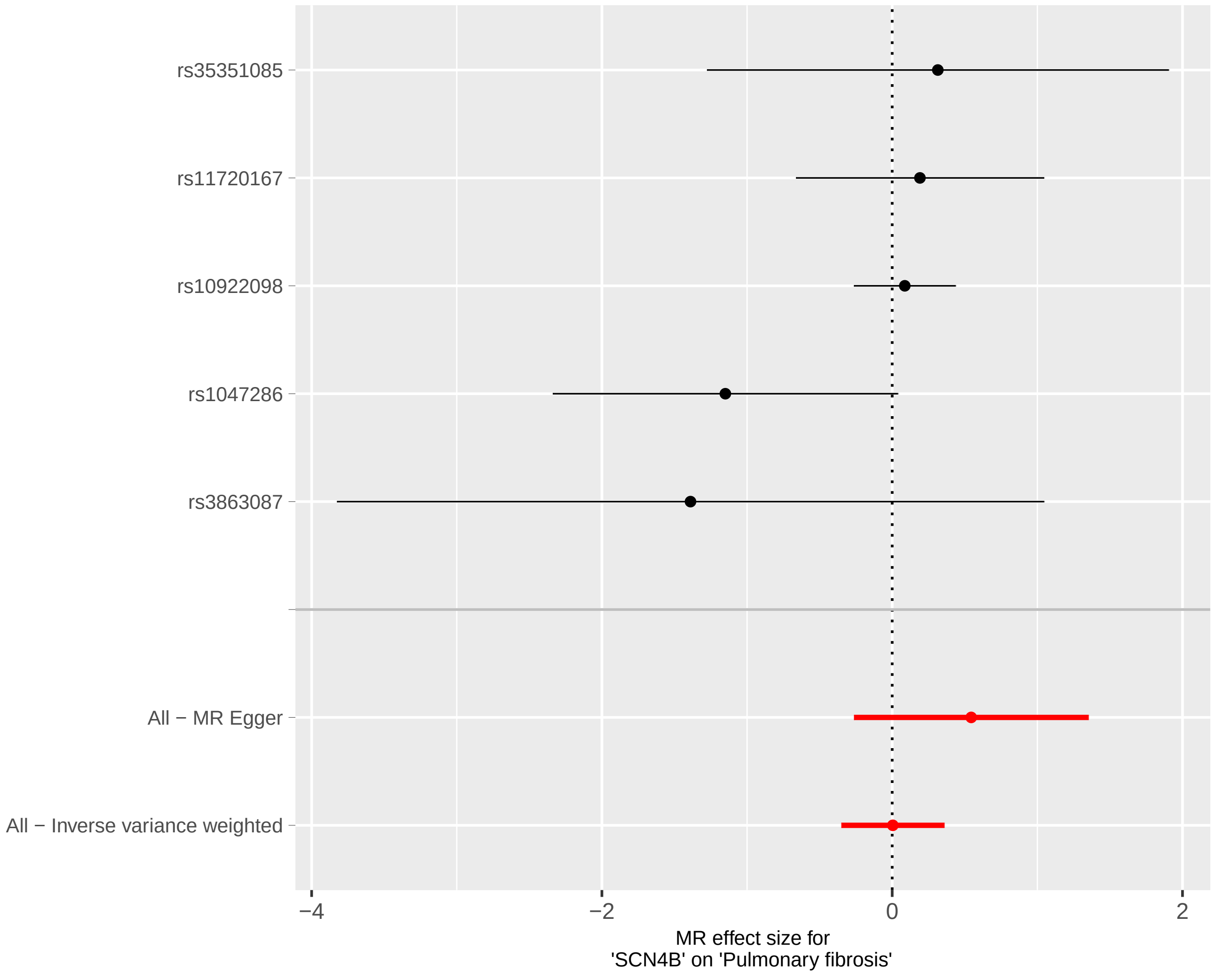

rs35351085
rs11720167
rs10922098
rs1047286
rs3863087
All − MR Egger
All − Inverse variance weighted
−4
−2
0
2
MR effect size for
'SCN4B' on 'Pulmonary fibrosis'

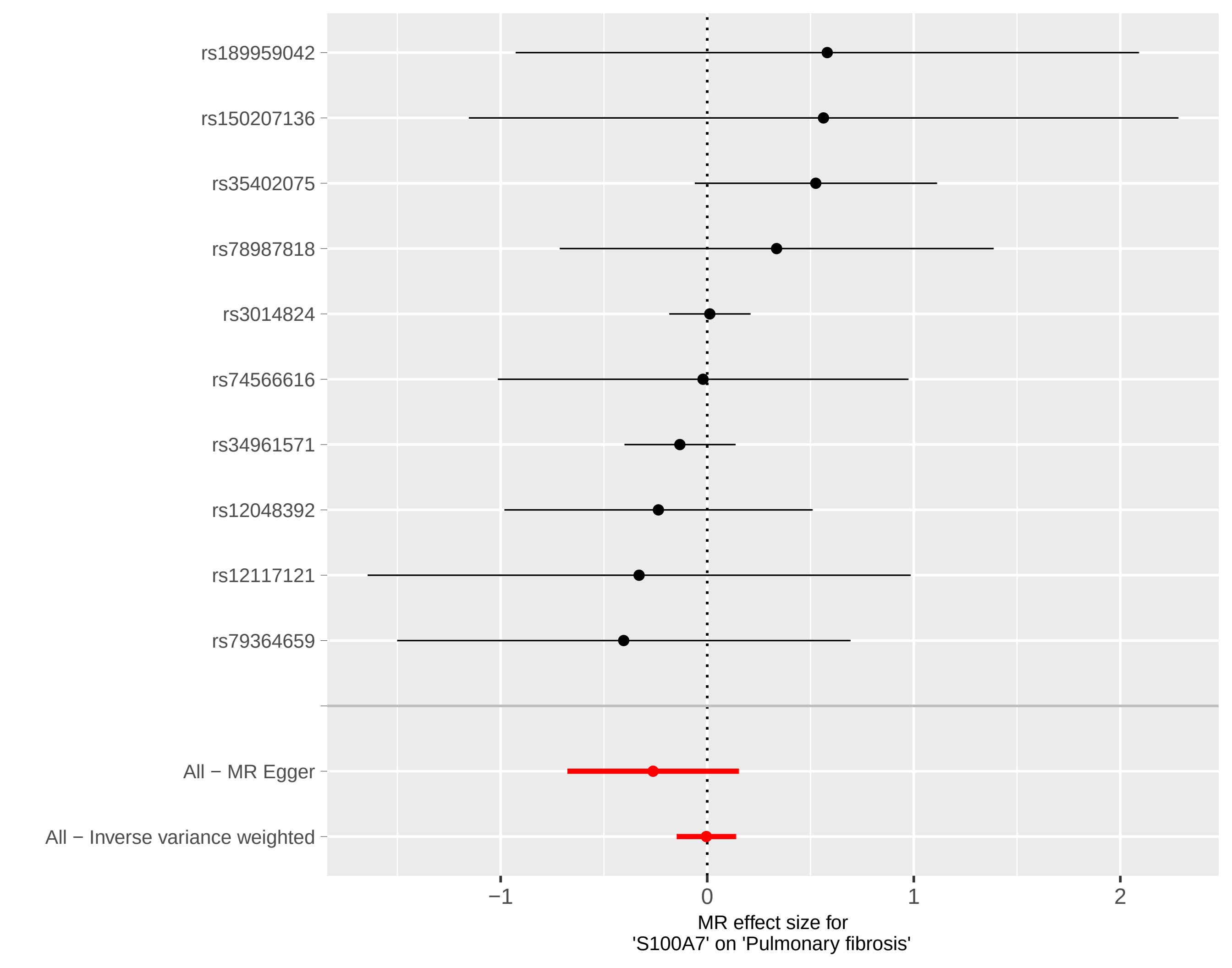
rs189959042
rs150207136
rs35402075
rs78987818
rs3014824
rs74566616
rs34961571
rs12048392
rs12117121
rs79364659
All − MR Egger
All − Inverse variance weighted
−1
0
1
2
MR effect size for
'S100A7' on 'Pulmonary fibrosis'

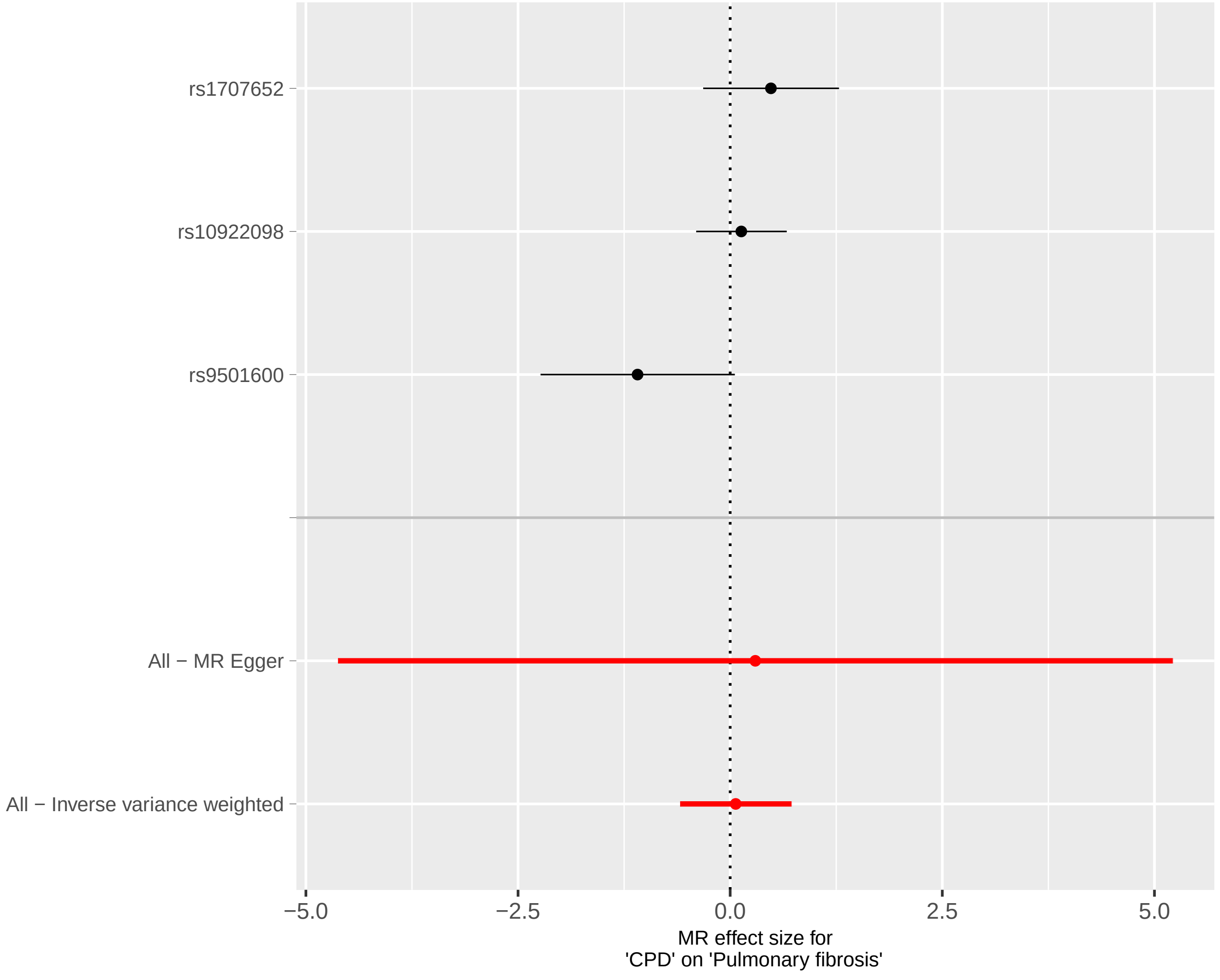

rs1707652
rs10922098
rs9501600
All − MR Egger
All − Inverse variance weighted
−5.0
−2.5
0.0
2.5
5.0
MR effect size for
'CPD' on 'Pulmonary fibrosis'

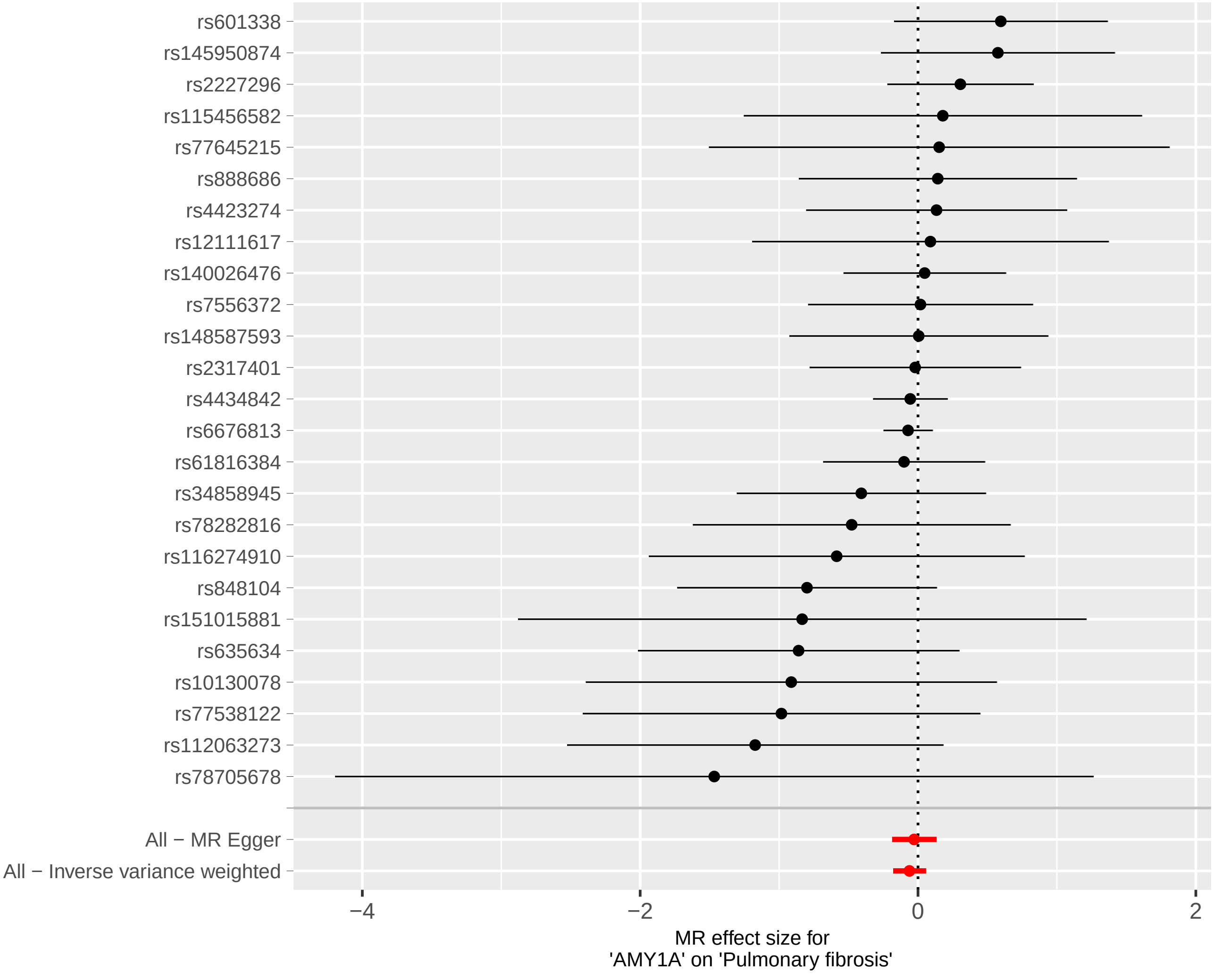

rs601338
rs145950874
rs2227296
rs115456582
rs77645215
rs888686
rs4423274
rs12111617
rs140026476
rs7556372
rs148587593
rs2317401
rs4434842
rs6676813
rs61816384
rs34858945
rs78282816
rs116274910
rs848104
rs151015881
rs635634
rs10130078
rs77538122
rs112063273
rs78705678
All − MR Egger
All − Inverse variance weighted
−4
−2
0
2
MR effect size for
'AMY1A' on 'Pulmonary fibrosis'

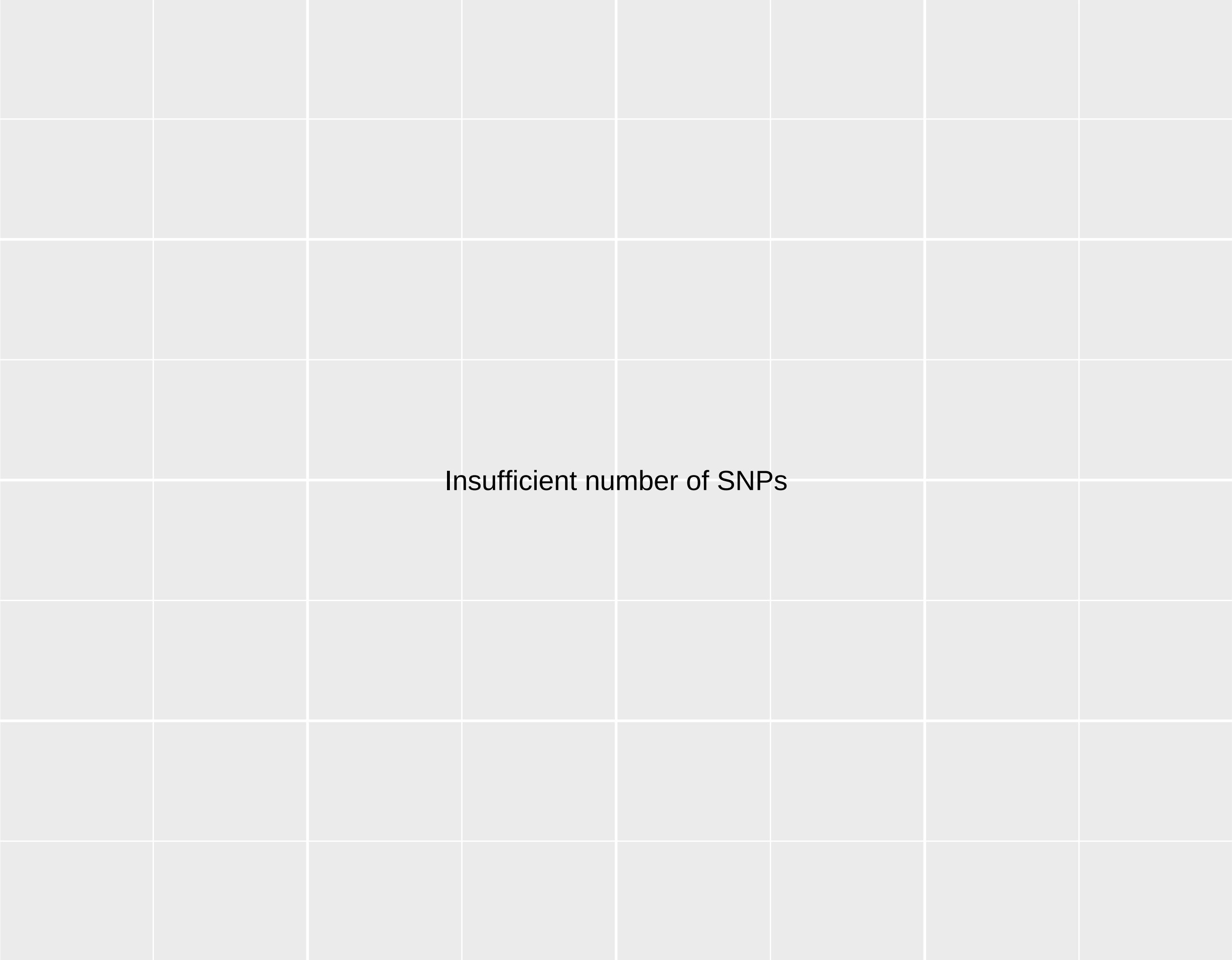
Insufficient number of SNPs

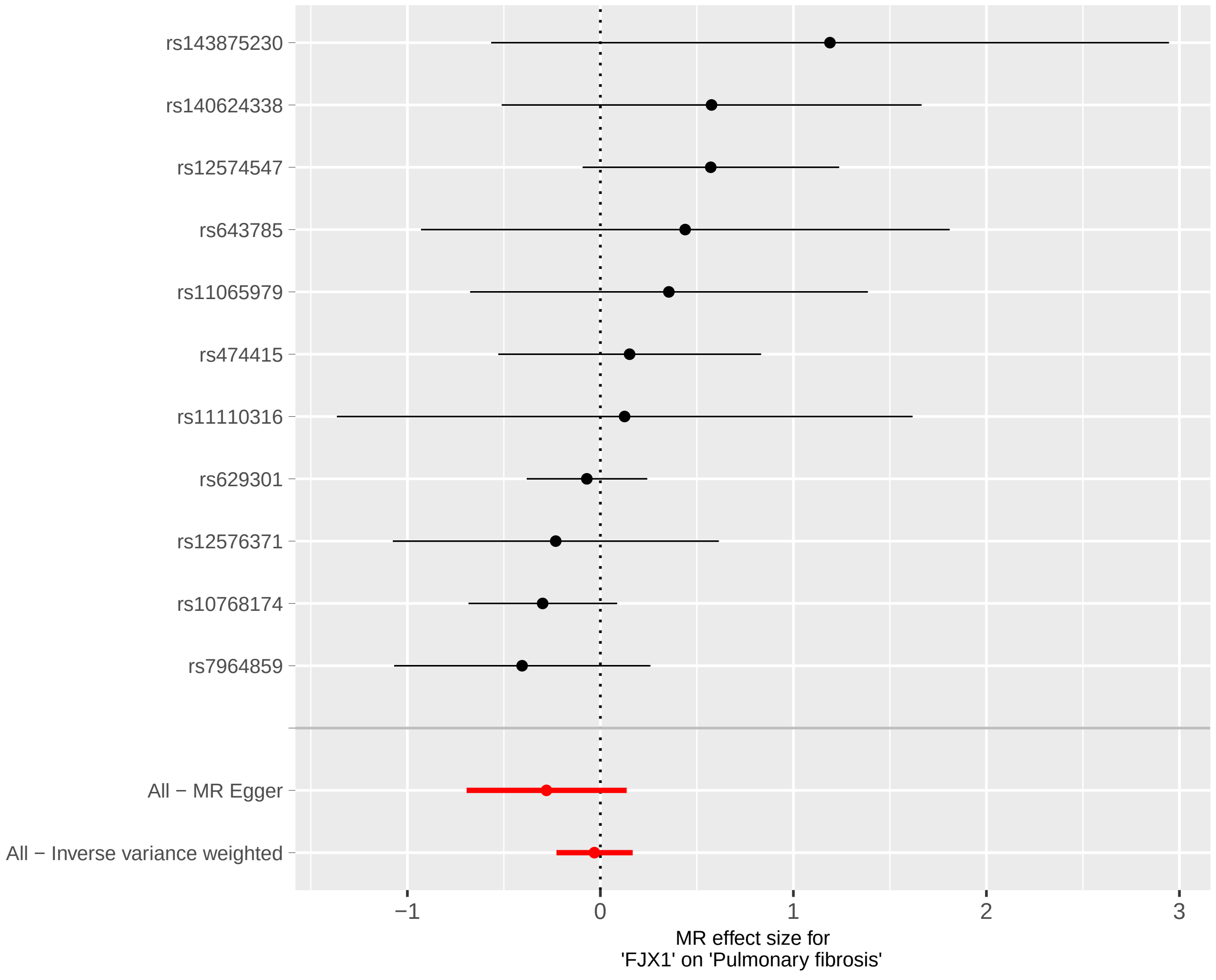
rs143875230
rs140624338
rs12574547
rs643785
rs11065979
rs474415
rs11110316
rs629301
rs12576371
rs10768174
rs7964859
All − MR Egger
All − Inverse variance weighted
−1
0
1
2
3
MR effect size for
'FJX1' on 'Pulmonary fibrosis'

Insufficient number of SNPs

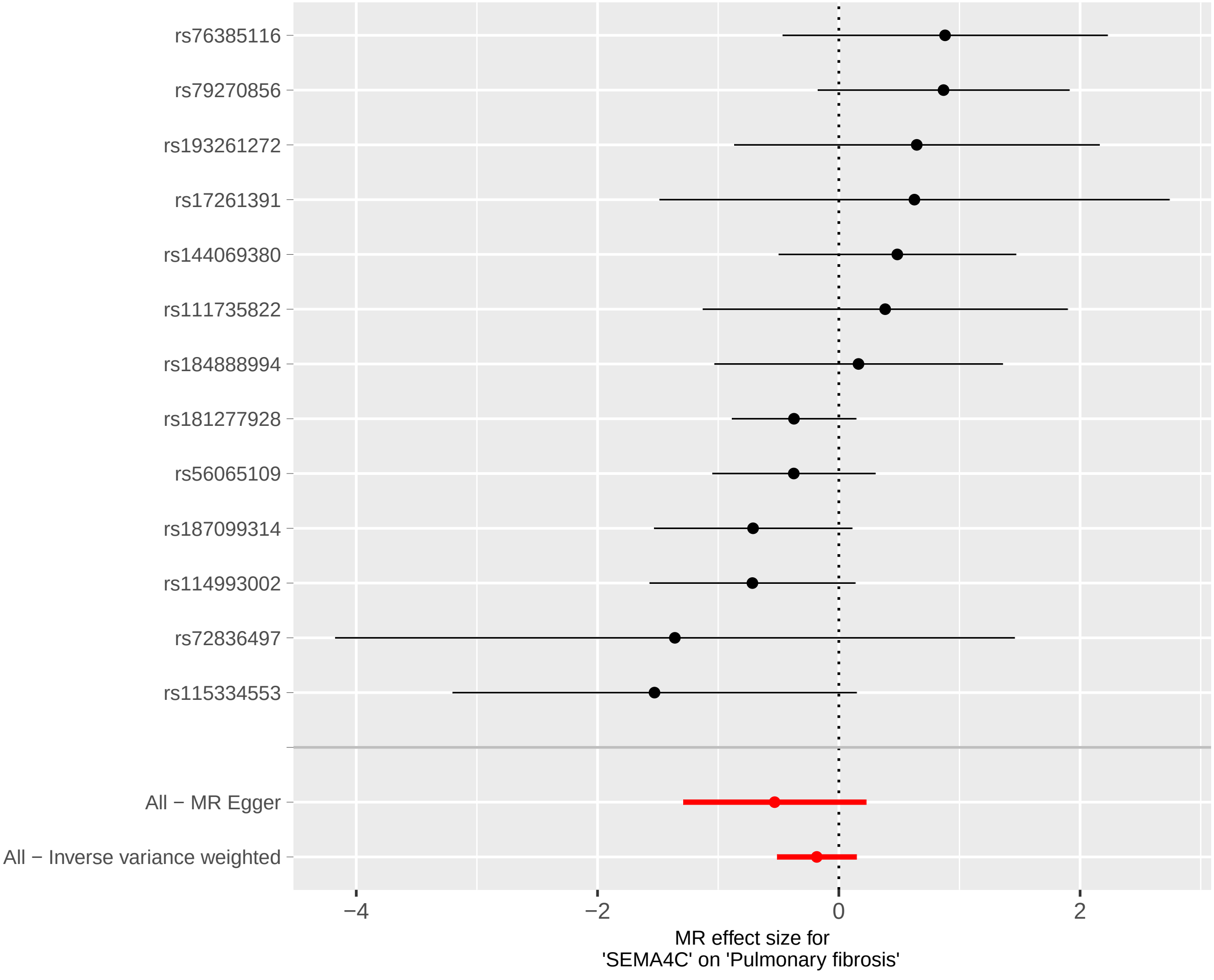

rs76385116
rs79270856
rs193261272
rs17261391
rs144069380
rs111735822
rs184888994
rs181277928
rs56065109
rs187099314
rs114993002
rs72836497
rs115334553
All − MR Egger
All − Inverse variance weighted
−4
−2
0
2
MR effect size for
'SEMA4C' on 'Pulmonary fibrosis'

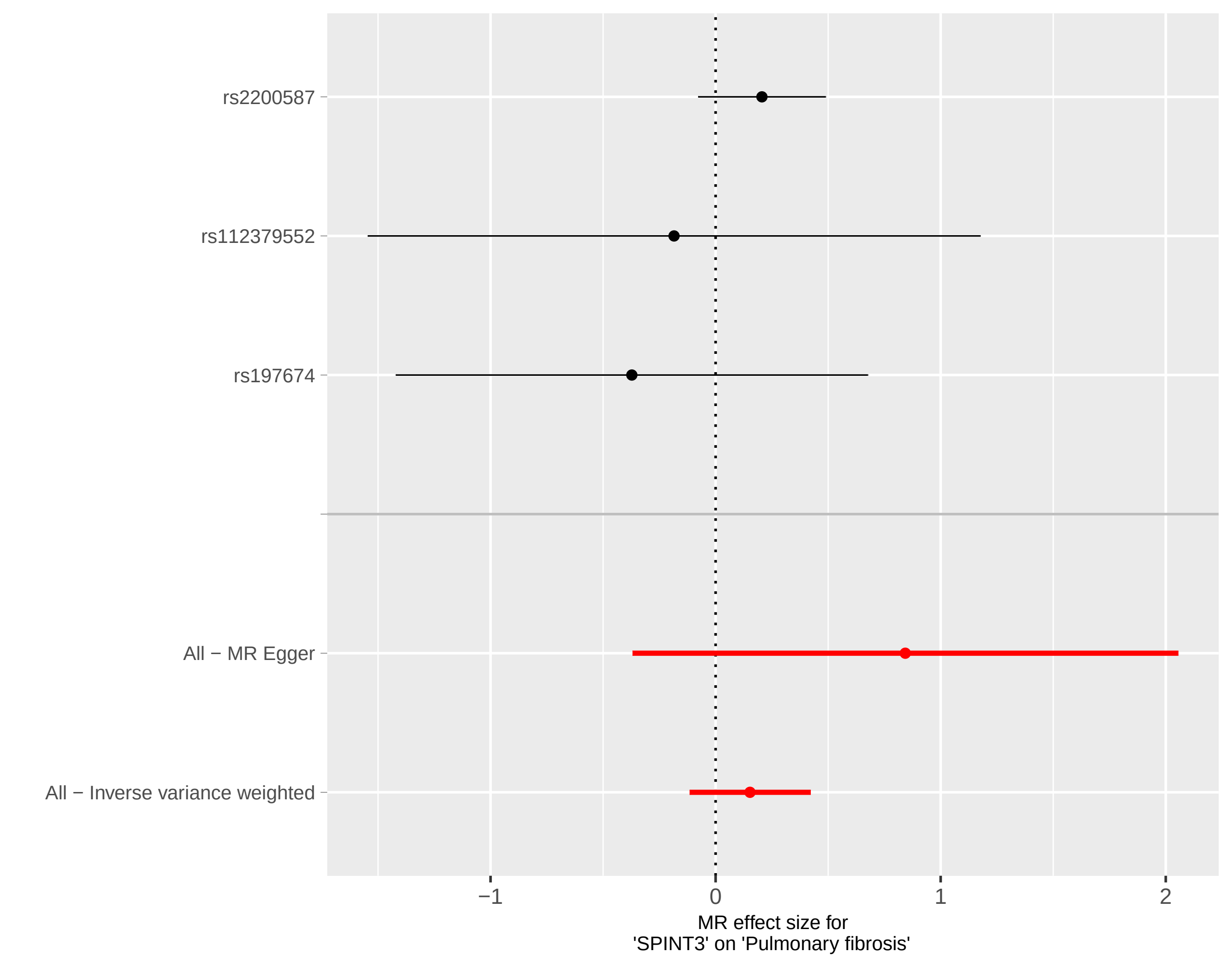
rs2200587
rs112379552
rs197674
All − MR Egger
All − Inverse variance weighted
−1
0
1
2
MR effect size for
'SPINT3' on 'Pulmonary fibrosis'

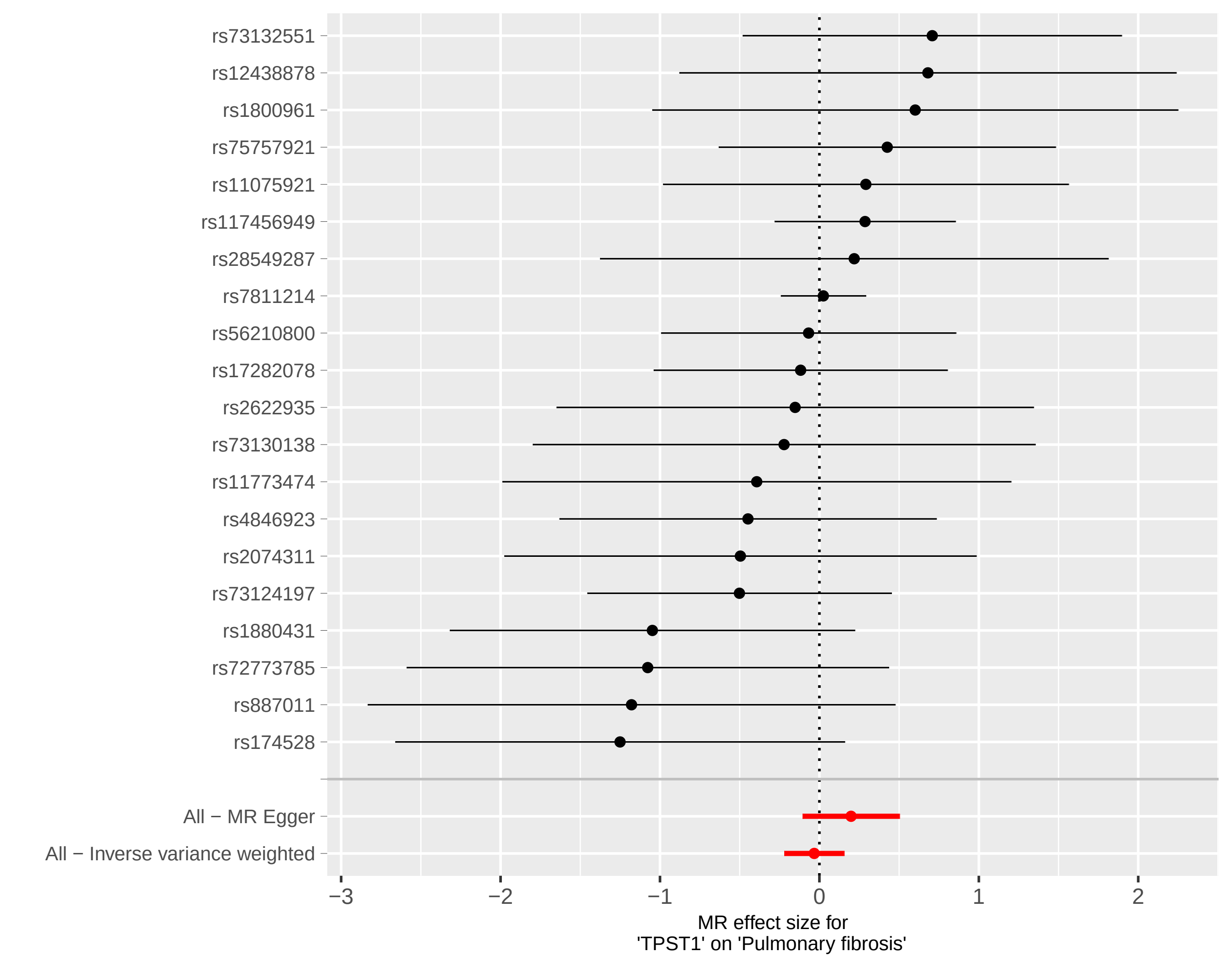
rs73132551
rs12438878
rs1800961
rs75757921
rs11075921
rs117456949
rs28549287
rs7811214
rs56210800
rs17282078
rs2622935
rs73130138
rs11773474
rs4846923
rs2074311
rs73124197
rs1880431
rs72773785
rs887011
rs174528
All − MR Egger
All − Inverse variance weighted
−3
−2
−1
0
1
2
MR effect size for
'TPST1' on 'Pulmonary fibrosis'

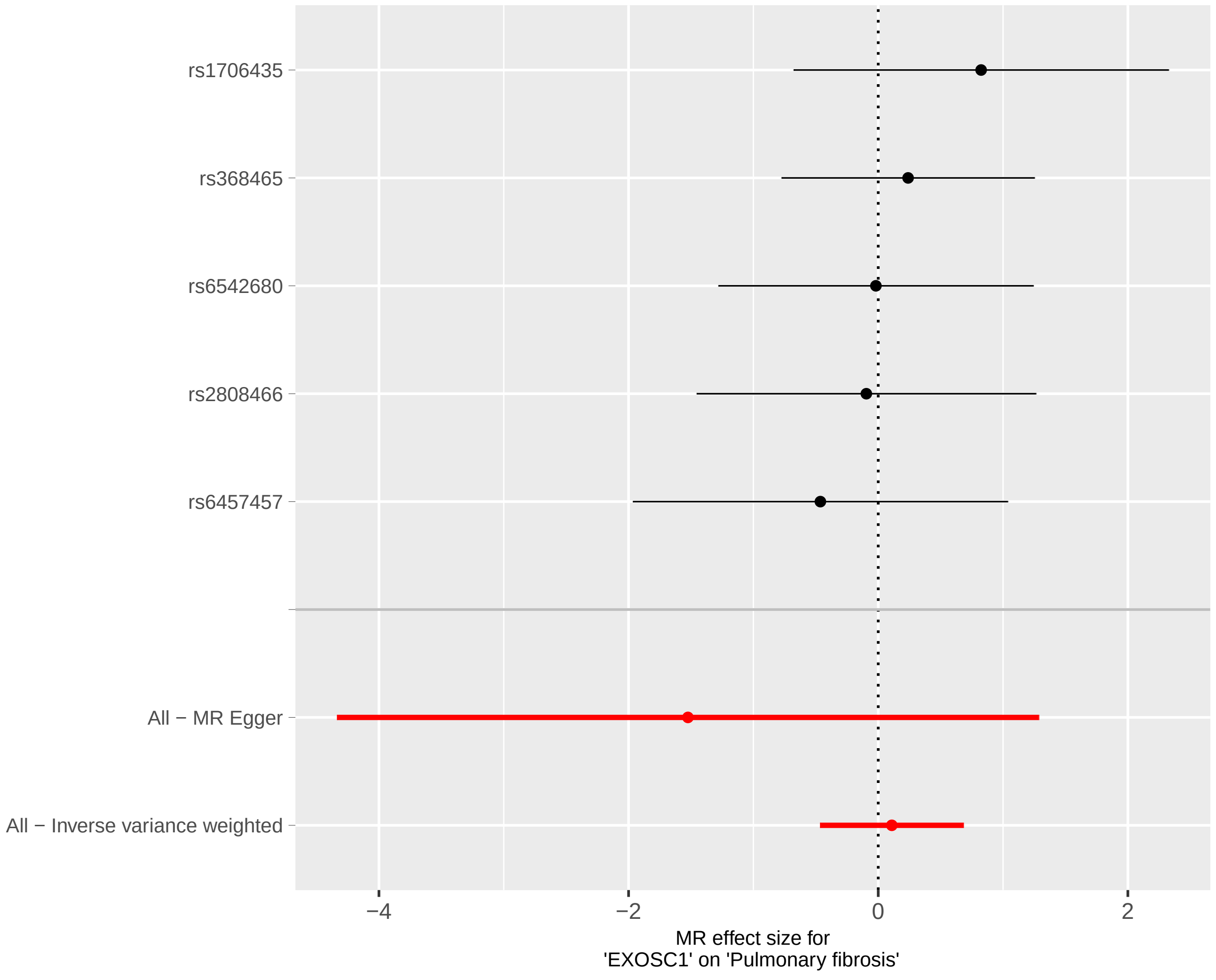
rs1706435
rs368465
rs6542680
rs2808466
rs6457457
All − MR Egger
All − Inverse variance weighted
−4
−2
0
2
MR effect size for
'EXOSC1' on 'Pulmonary fibrosis'

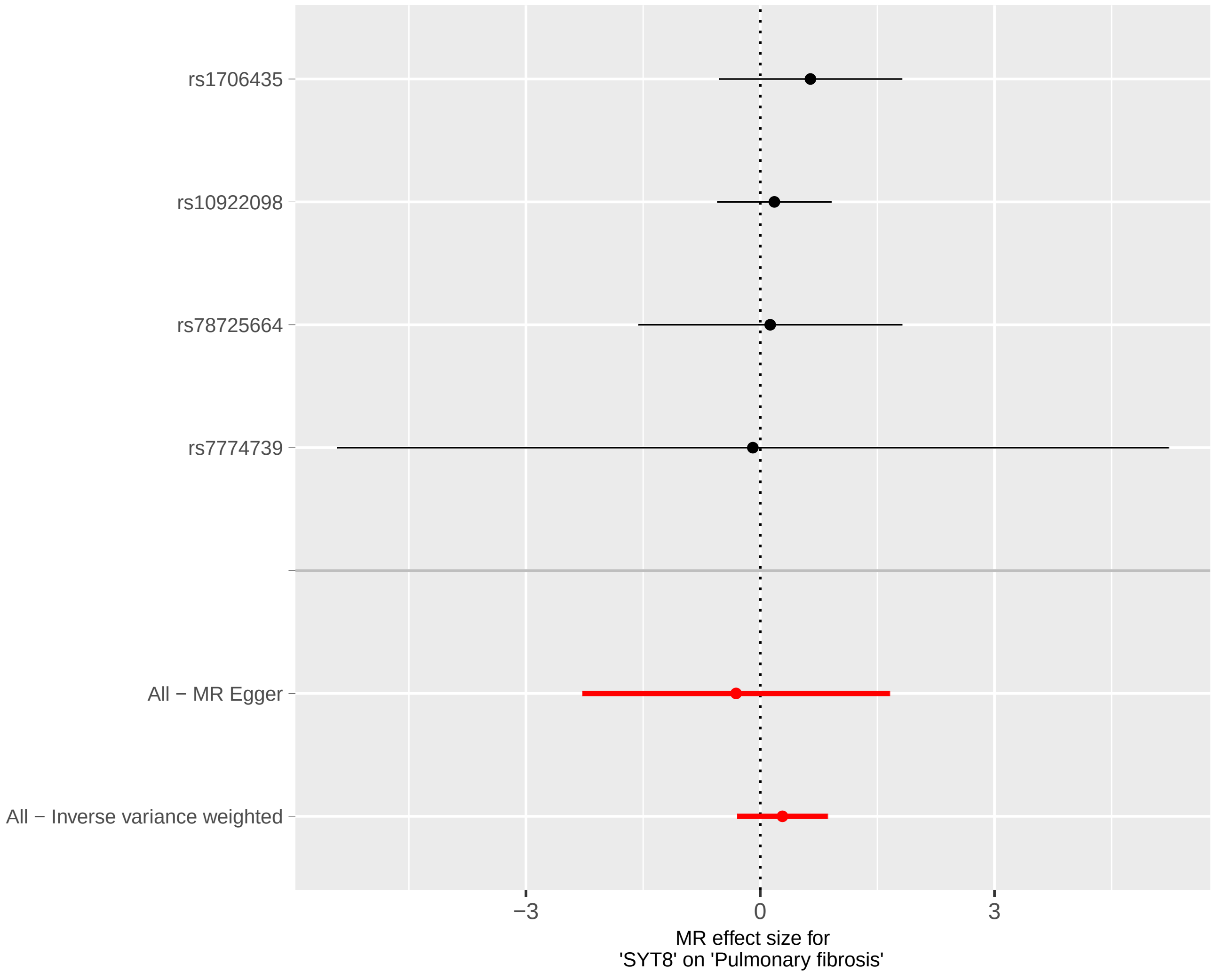

rs1706435
rs10922098
rs78725664
rs7774739
All − MR Egger
All − Inverse variance weighted
−3
0
3
MR effect size for
'SYT8' on 'Pulmonary fibrosis'

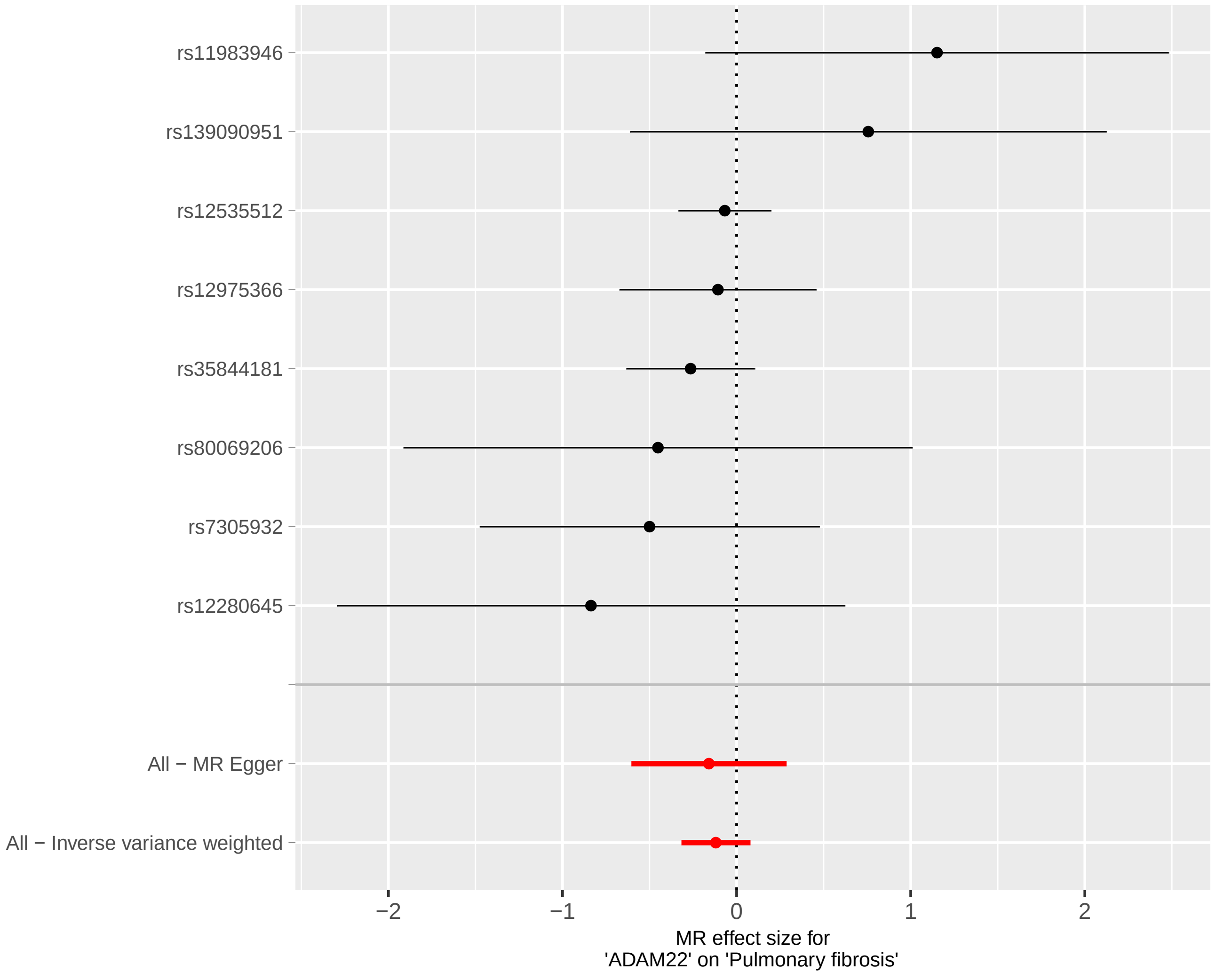

rs11983946
rs139090951
rs12535512
rs12975366
rs35844181
rs80069206
rs7305932
rs12280645
All − MR Egger
All − Inverse variance weighted
−2
−1
0
1
2
MR effect size for
'ADAM22' on 'Pulmonary fibrosis'

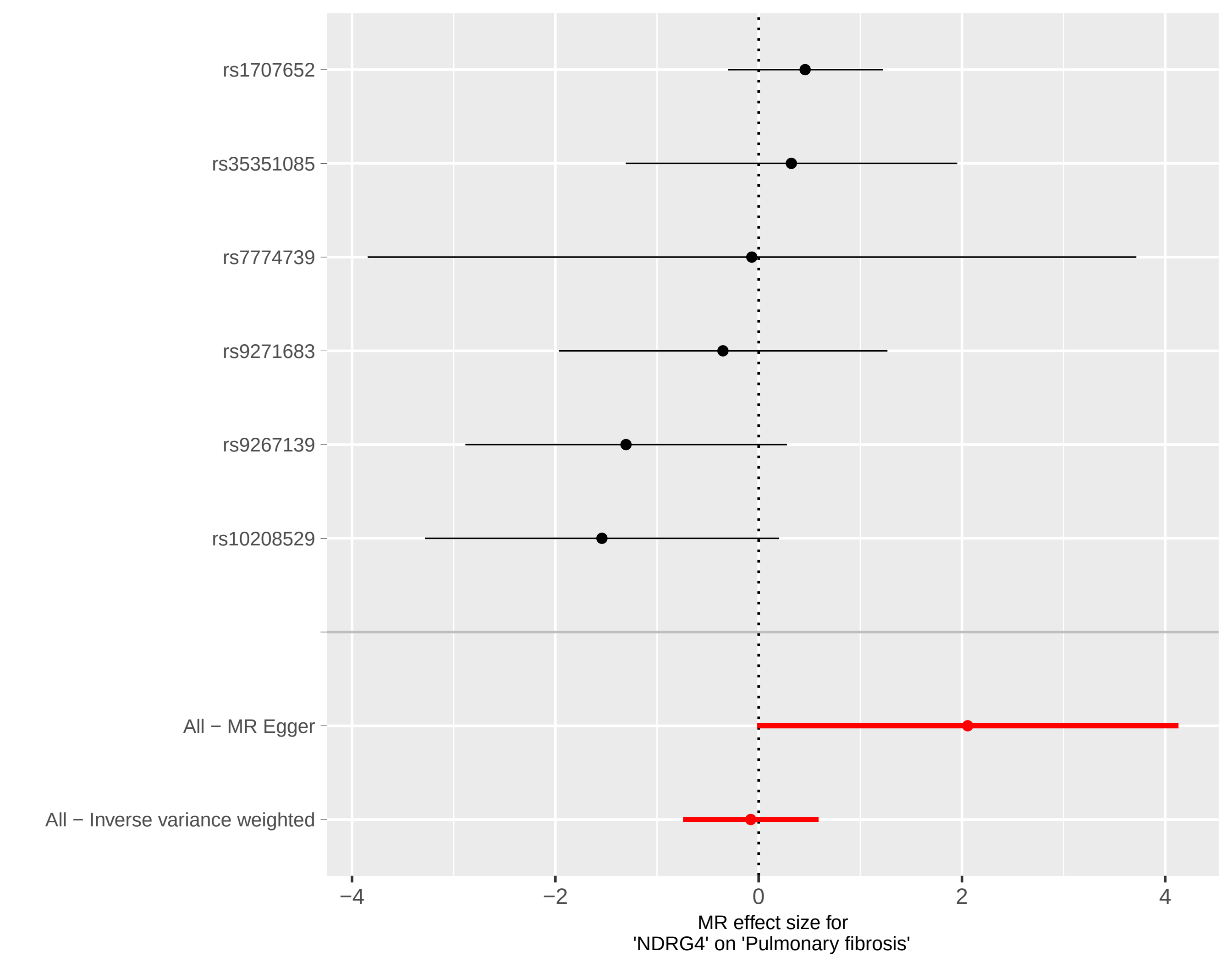

rs1707652
rs35351085
rs7774739
rs9271683
rs9267139
rs10208529
All − MR Egger
All − Inverse variance weighted
−4
−2
0
2
4
MR effect size for
'NDRG4' on 'Pulmonary fibrosis'

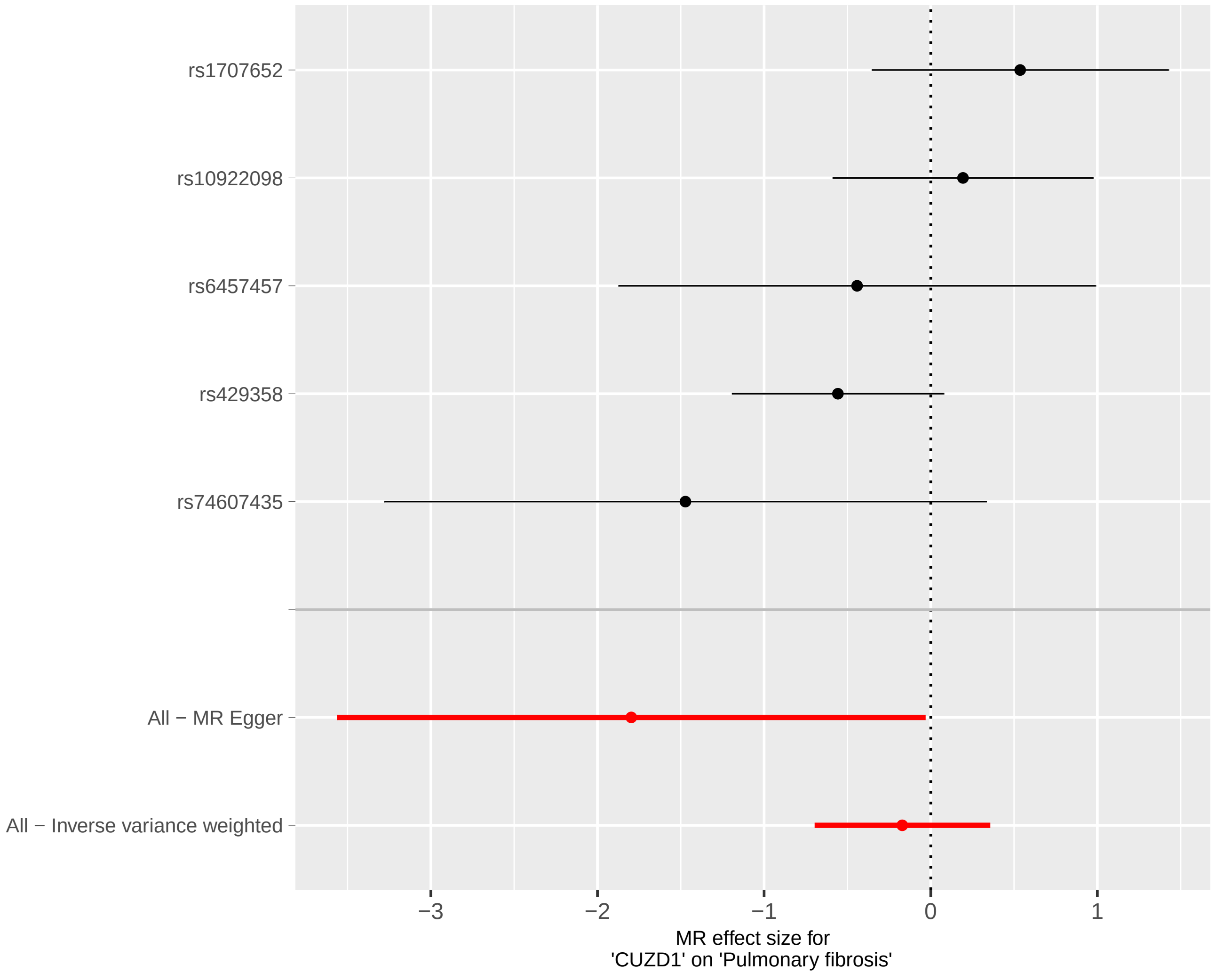

rs1707652
rs10922098
rs6457457
rs429358
rs74607435
All − MR Egger
All − Inverse variance weighted
−3
−2
−1
0
1
MR effect size for
'CUZD1' on 'Pulmonary fibrosis'

Insufficient number of SNPs

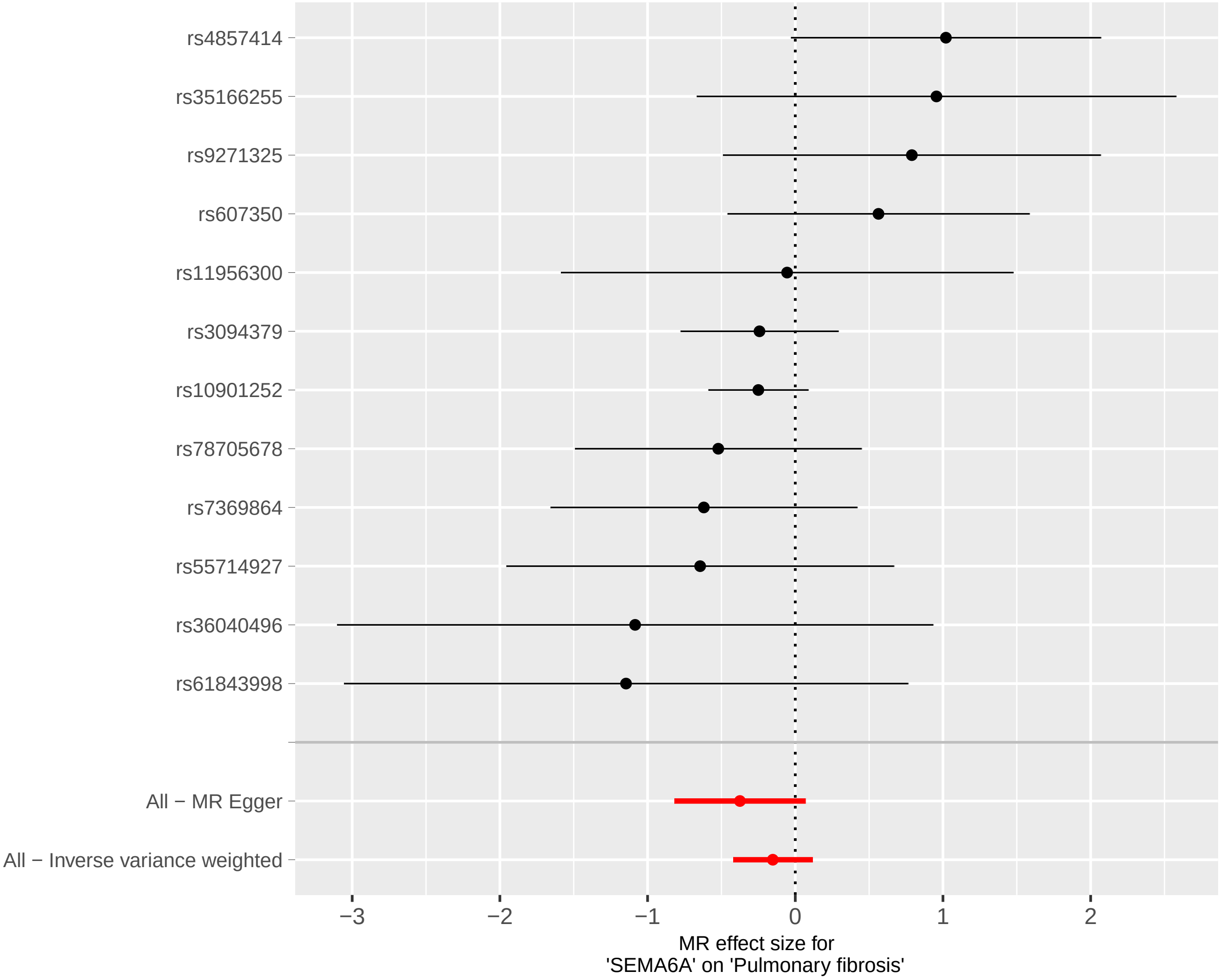
rs4857414
rs35166255
rs9271325
rs607350
rs11956300
rs3094379
rs10901252
rs78705678
rs7369864
rs55714927
rs36040496
rs61843998
All − MR Egger
All − Inverse variance weighted
−3
−2
−1
0
1
2
MR effect size for
'SEMA6A' on 'Pulmonary fibrosis'

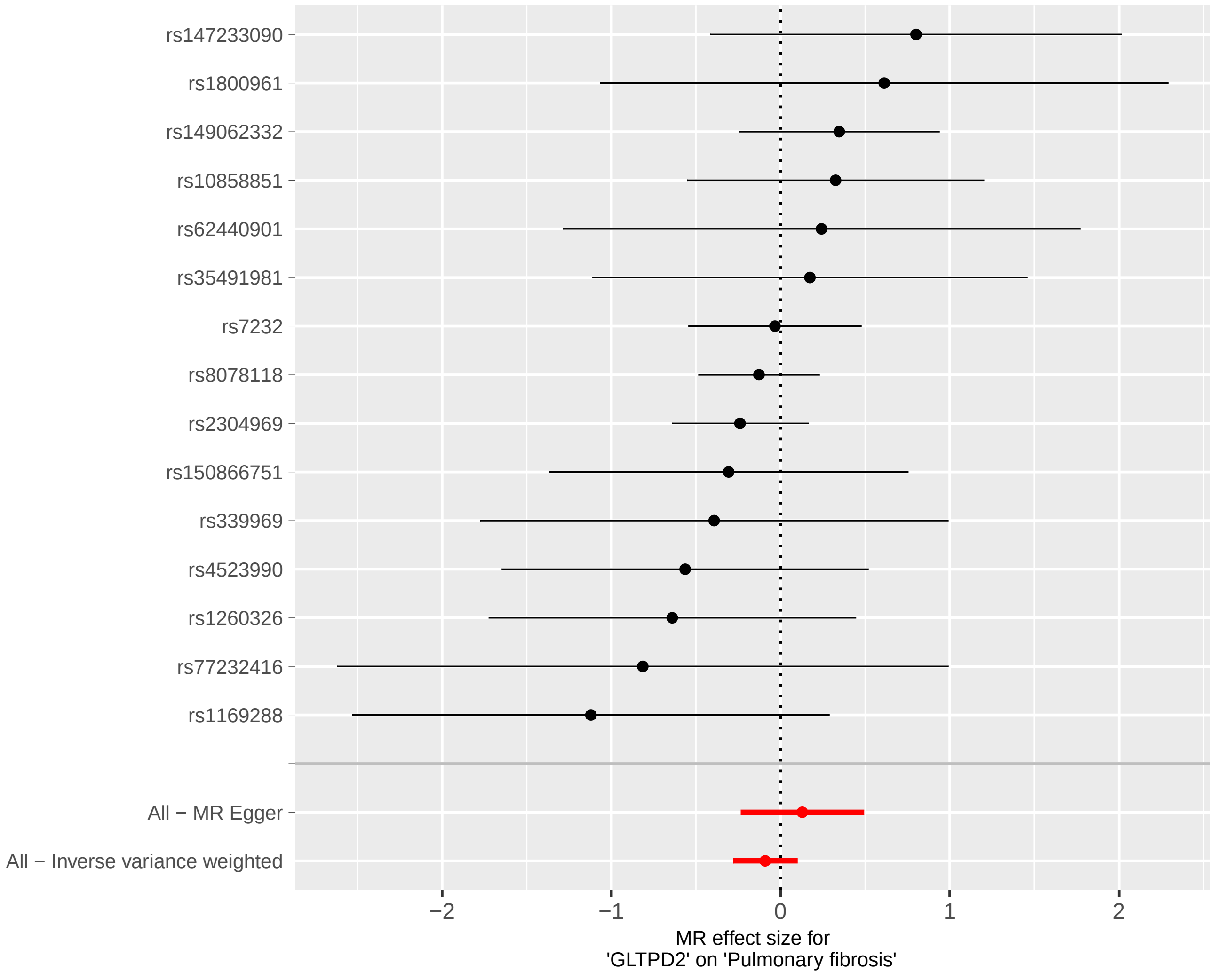
rs147233090
rs1800961
rs149062332
rs10858851
rs62440901
rs35491981
rs7232
rs8078118
rs2304969
rs150866751
rs339969
rs4523990
rs1260326
rs77232416
rs1169288
All − MR Egger
All − Inverse variance weighted
−2
−1
0
1
2
MR effect size for
'GLTPD2' on 'Pulmonary fibrosis'

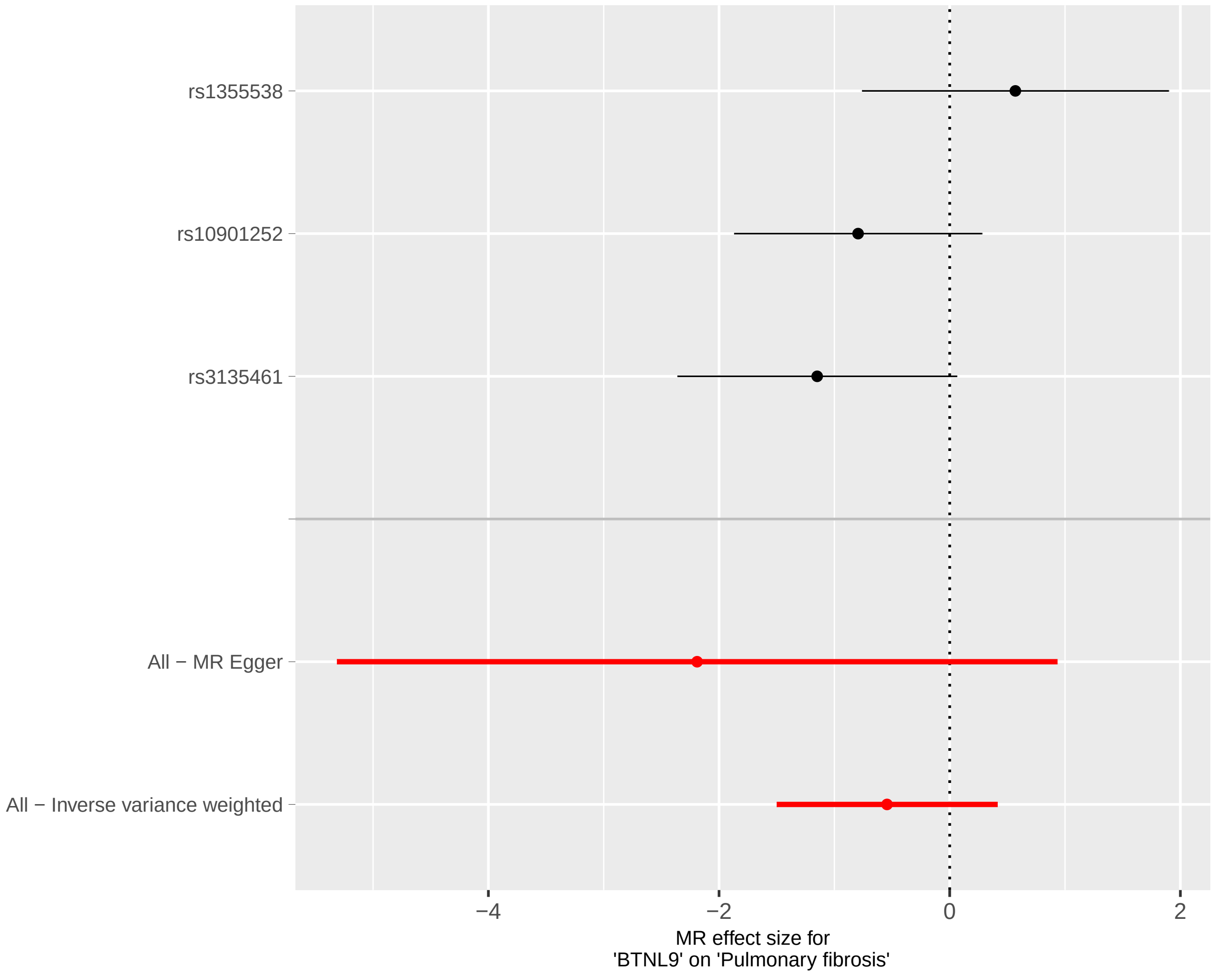

rs1355538
rs10901252
rs3135461
All − MR Egger
All − Inverse variance weighted
−4
−2
0
2
MR effect size for
'BTNL9' on 'Pulmonary fibrosis'

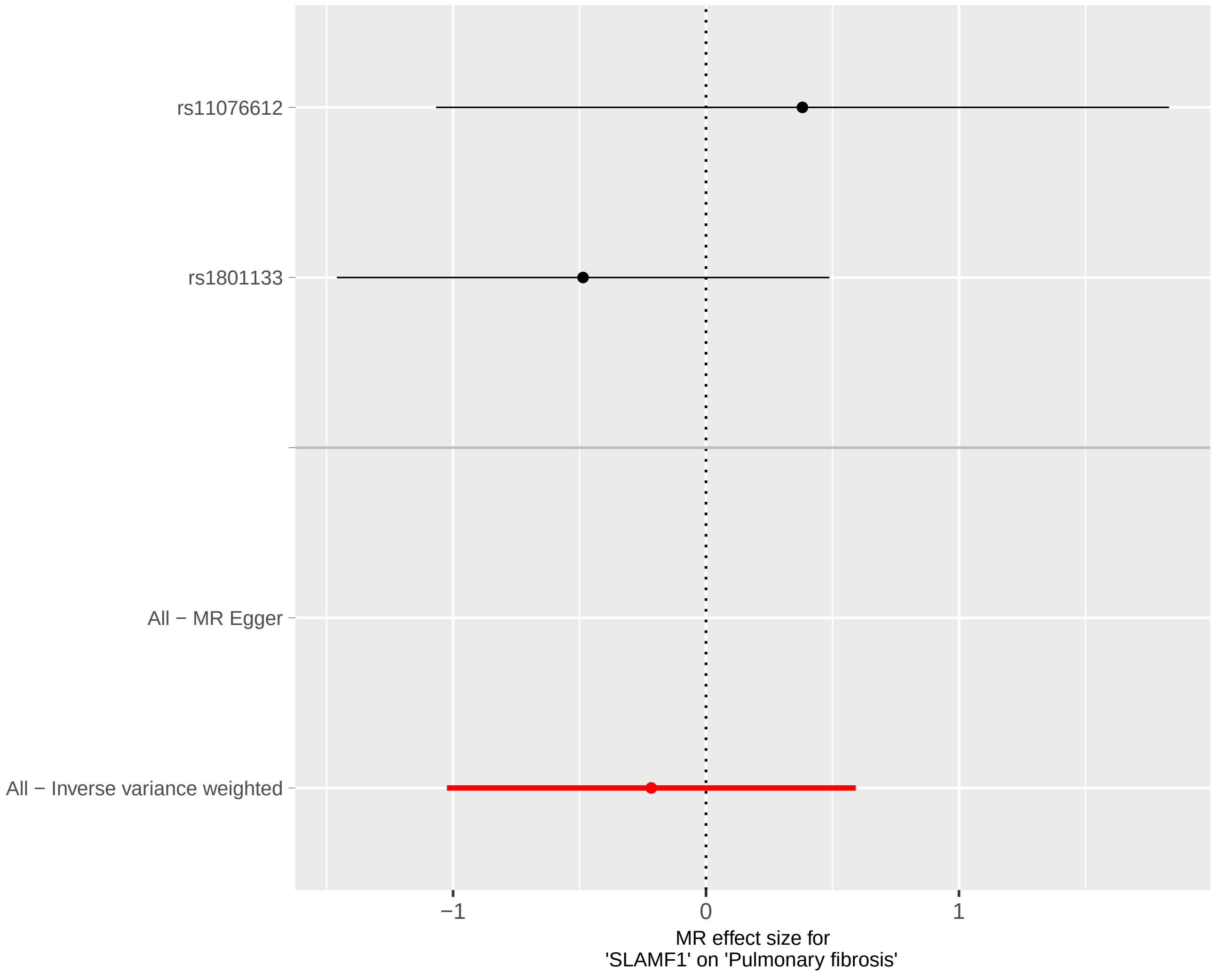
rs11076612
rs1801133
All − MR Egger
All − Inverse variance weighted
−1
0
1
MR effect size for
'SLAMF1' on 'Pulmonary fibrosis'

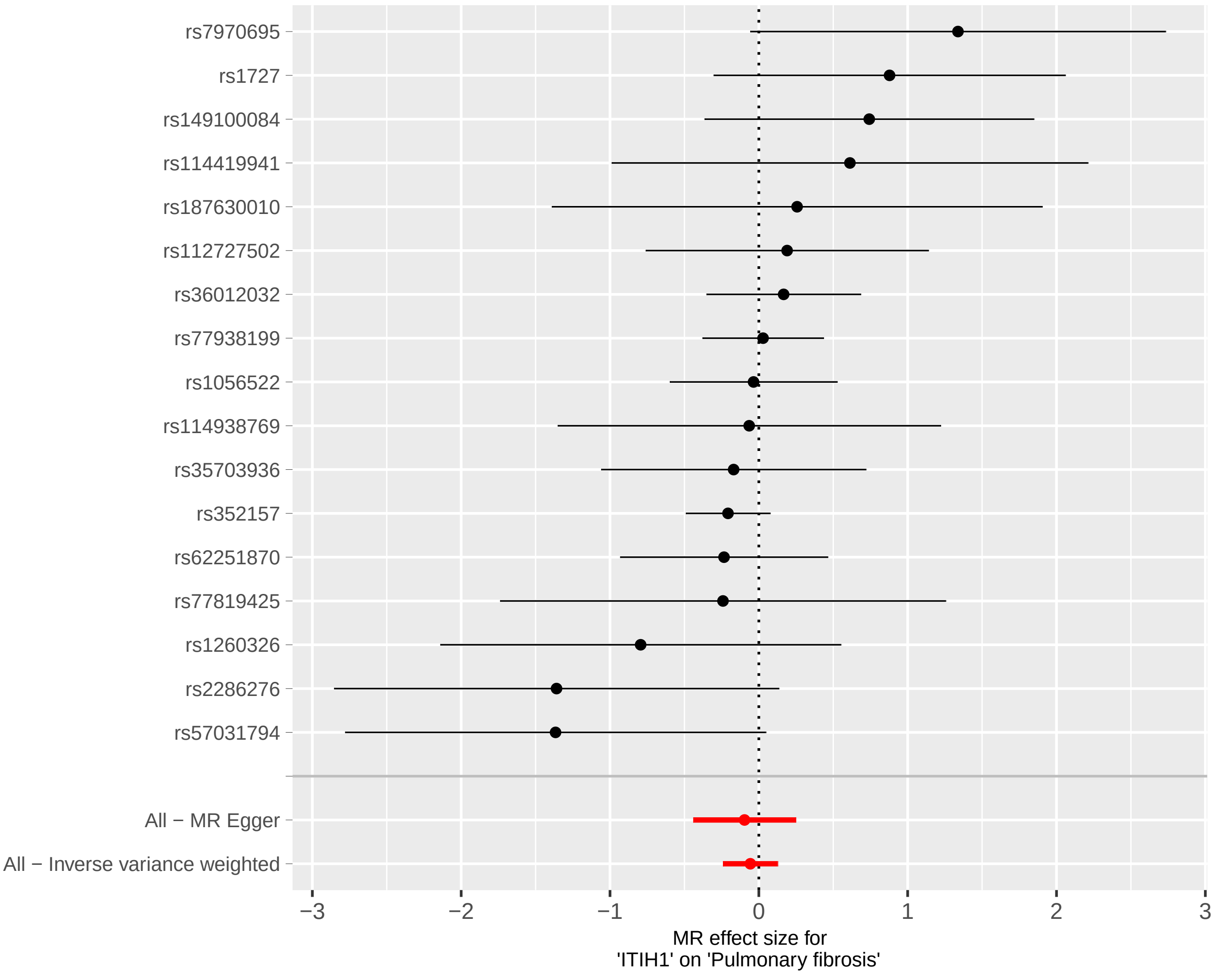

rs7970695
rs1727
rs149100084
rs114419941
rs187630010
rs112727502
rs36012032
rs77938199
rs1056522
rs114938769
rs35703936
rs352157
rs62251870
rs77819425
rs1260326
rs2286276
rs57031794
All − MR Egger
All − Inverse variance weighted
−3
−2
−1
0
1
2
3
MR effect size for
'ITIH1' on 'Pulmonary fibrosis'

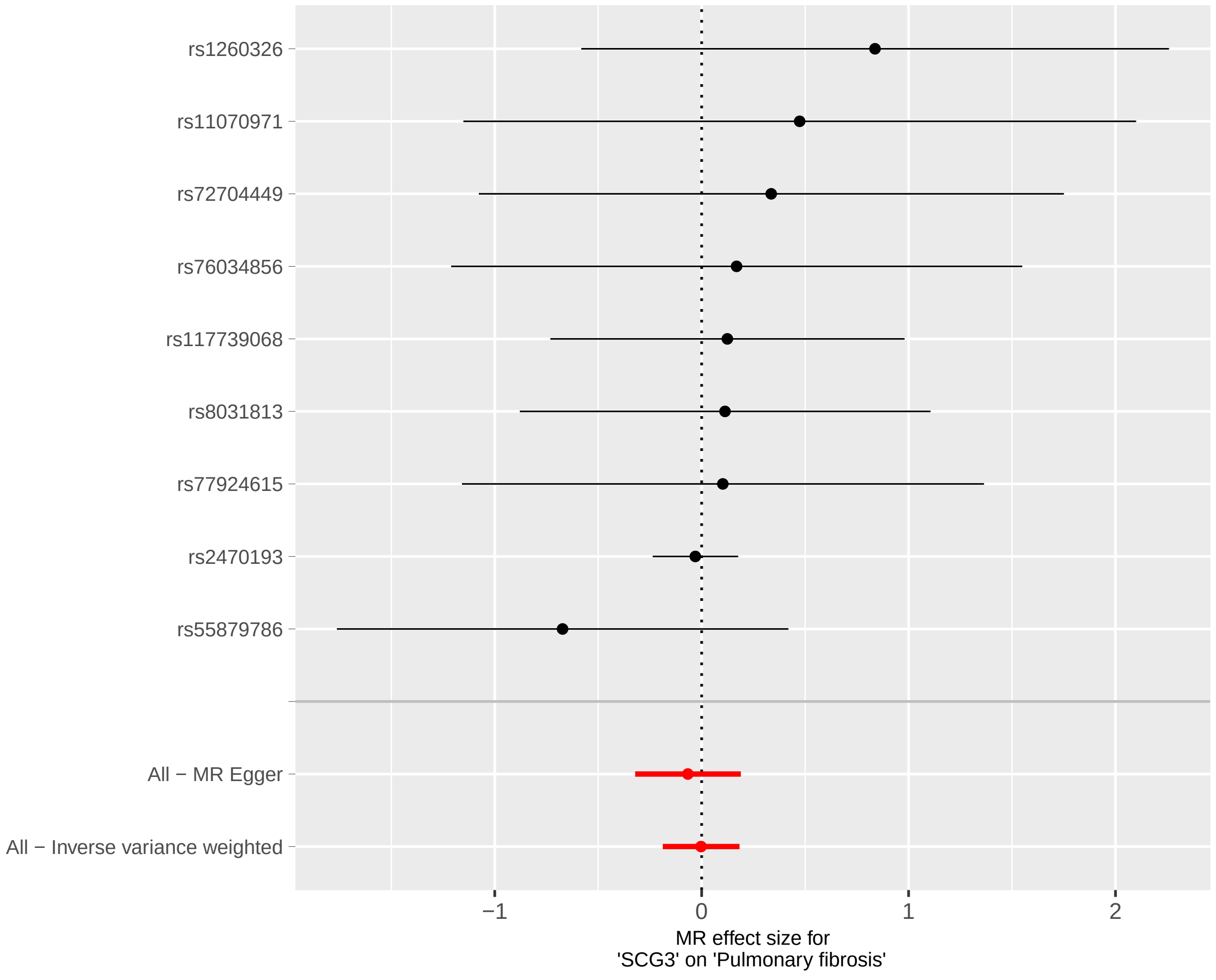
rs1260326
rs11070971
rs72704449
rs76034856
rs117739068
rs8031813
rs77924615
rs2470193
rs55879786
All − MR Egger
All − Inverse variance weighted
−1
0
1
2
MR effect size for
'SCG3' on 'Pulmonary fibrosis'

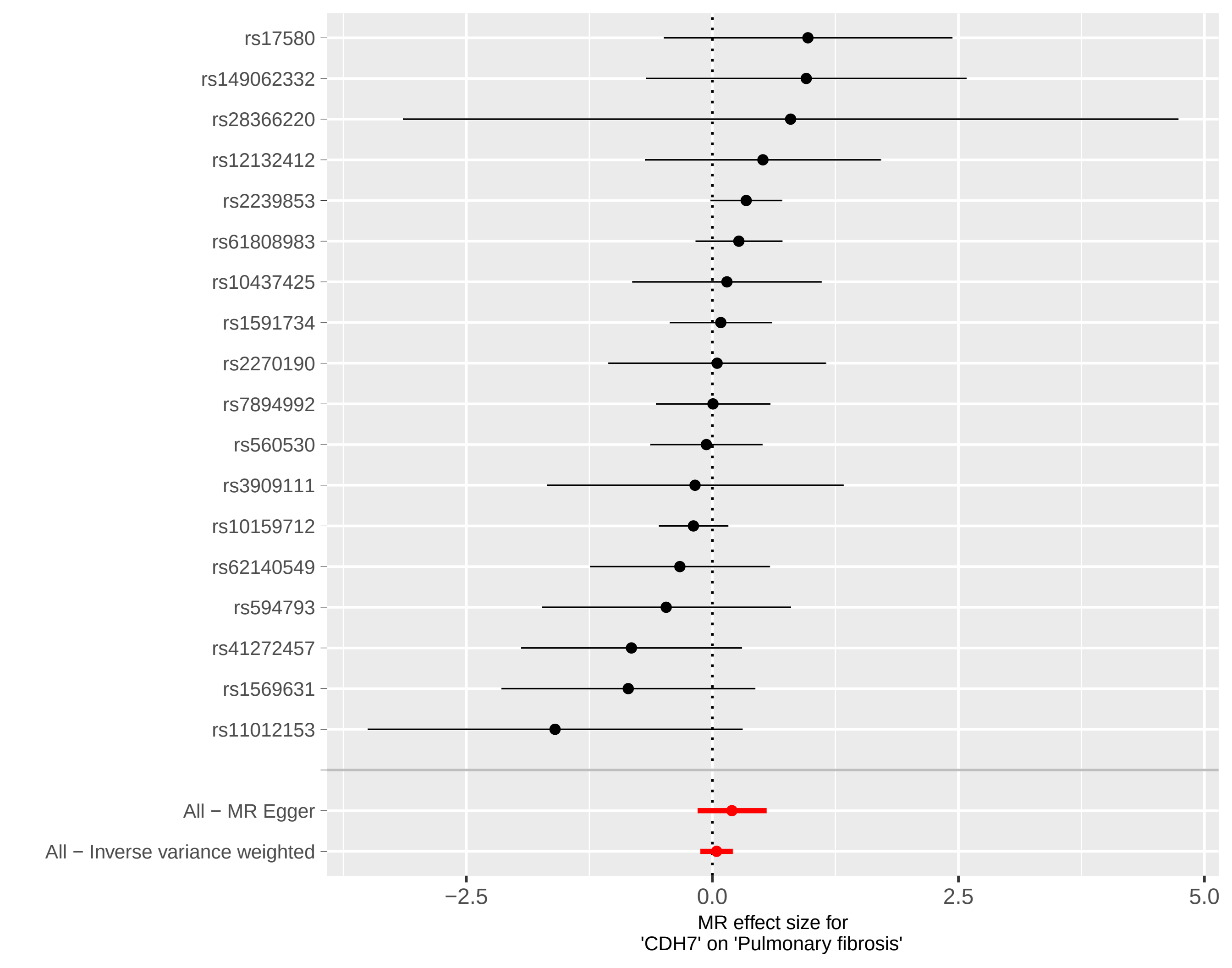
rs17580
rs149062332
rs28366220
rs12132412
rs2239853
rs61808983
rs10437425
rs1591734
rs2270190
rs7894992
rs560530
rs3909111
rs10159712
rs62140549
rs594793
rs41272457
rs1569631
rs11012153
All − MR Egger
All − Inverse variance weighted
−2.5
0.0
2.5
5.0
MR effect size for
'CDH7' on 'Pulmonary fibrosis'

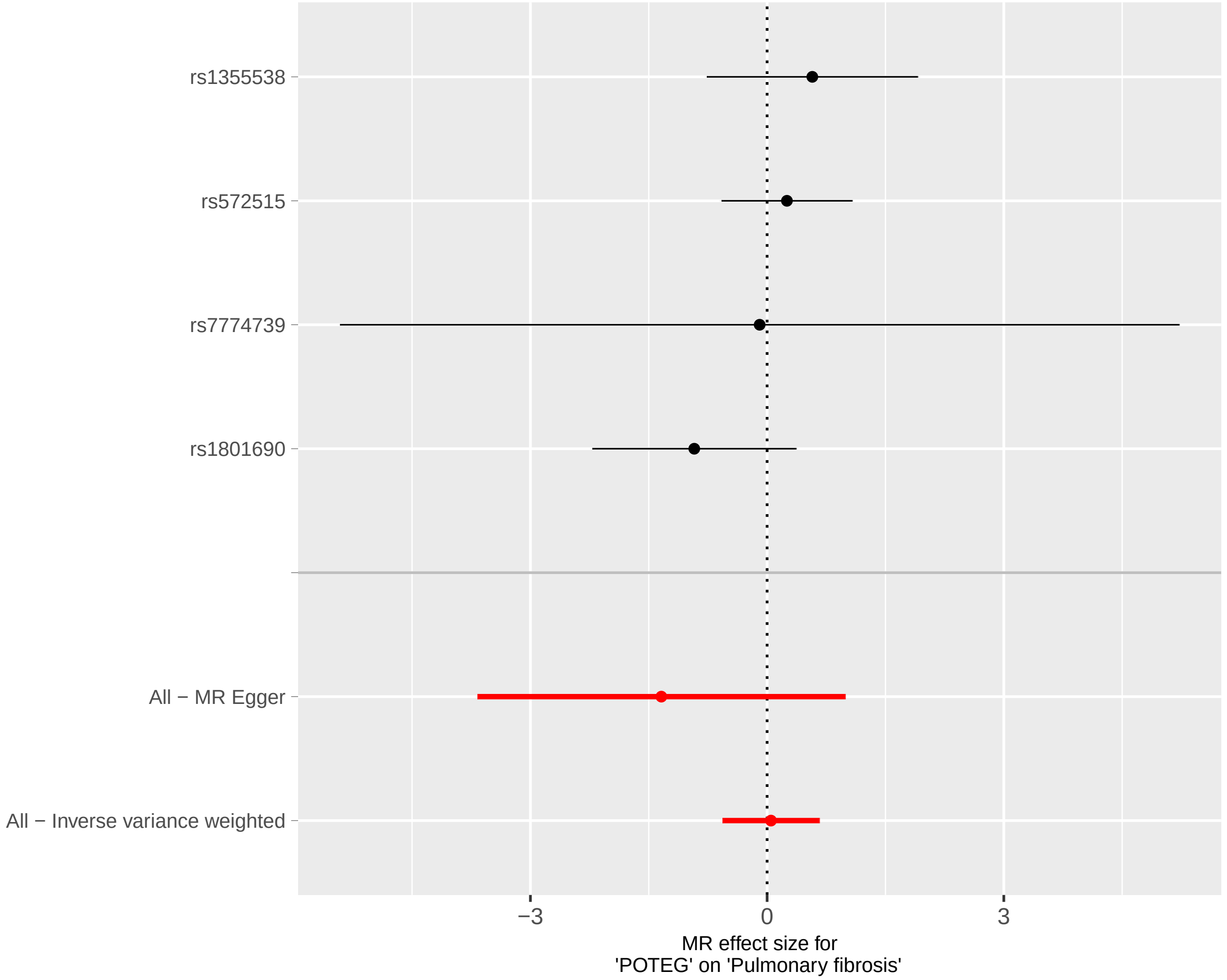
rs1355538
rs572515
rs7774739
rs1801690
All − MR Egger
All − Inverse variance weighted
−3
0
3
MR effect size for
'POTEG' on 'Pulmonary fibrosis'

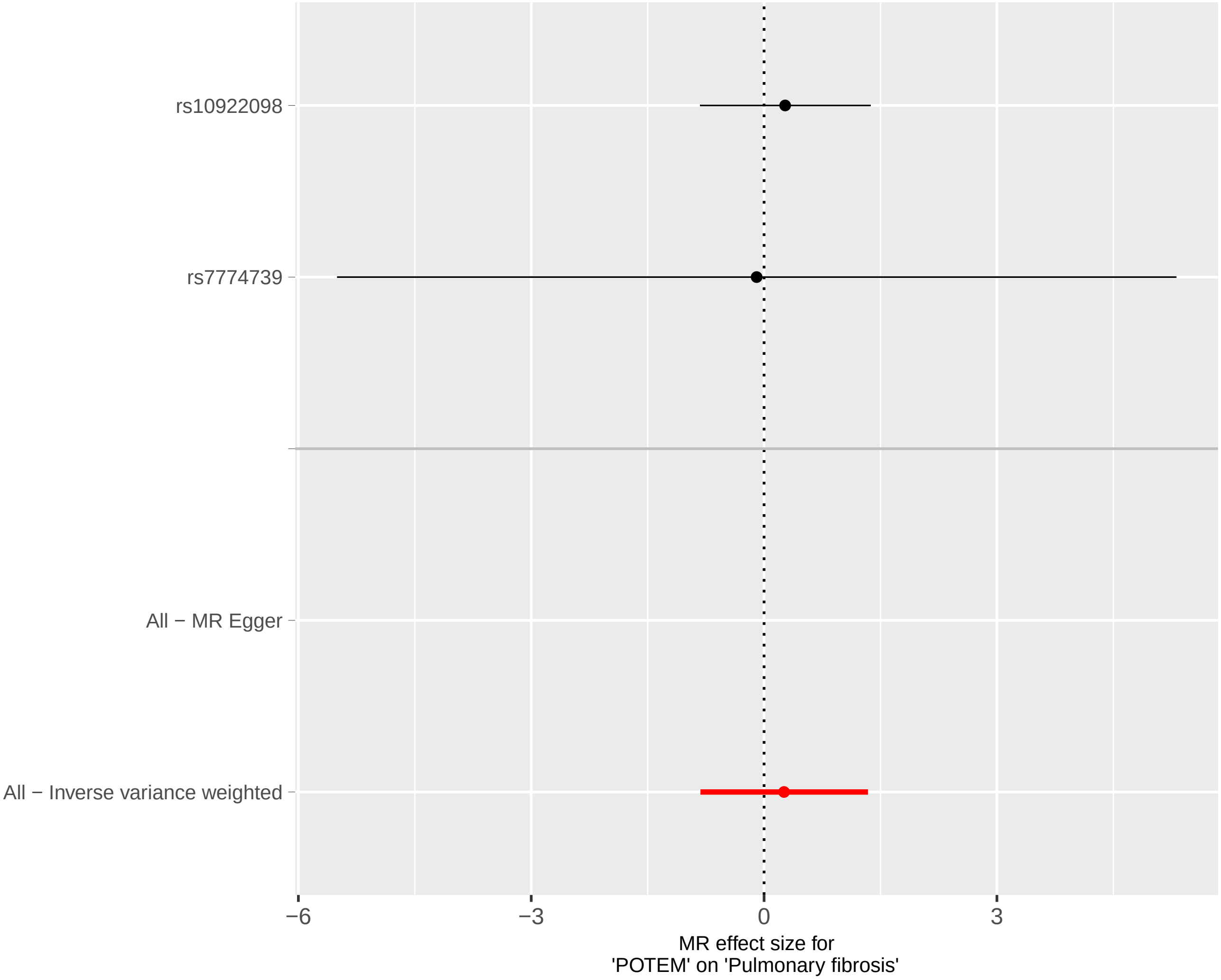

rs10922098
rs7774739
All − MR Egger
All − Inverse variance weighted
−6
−3
0
3
MR effect size for
'POTEM' on 'Pulmonary fibrosis'

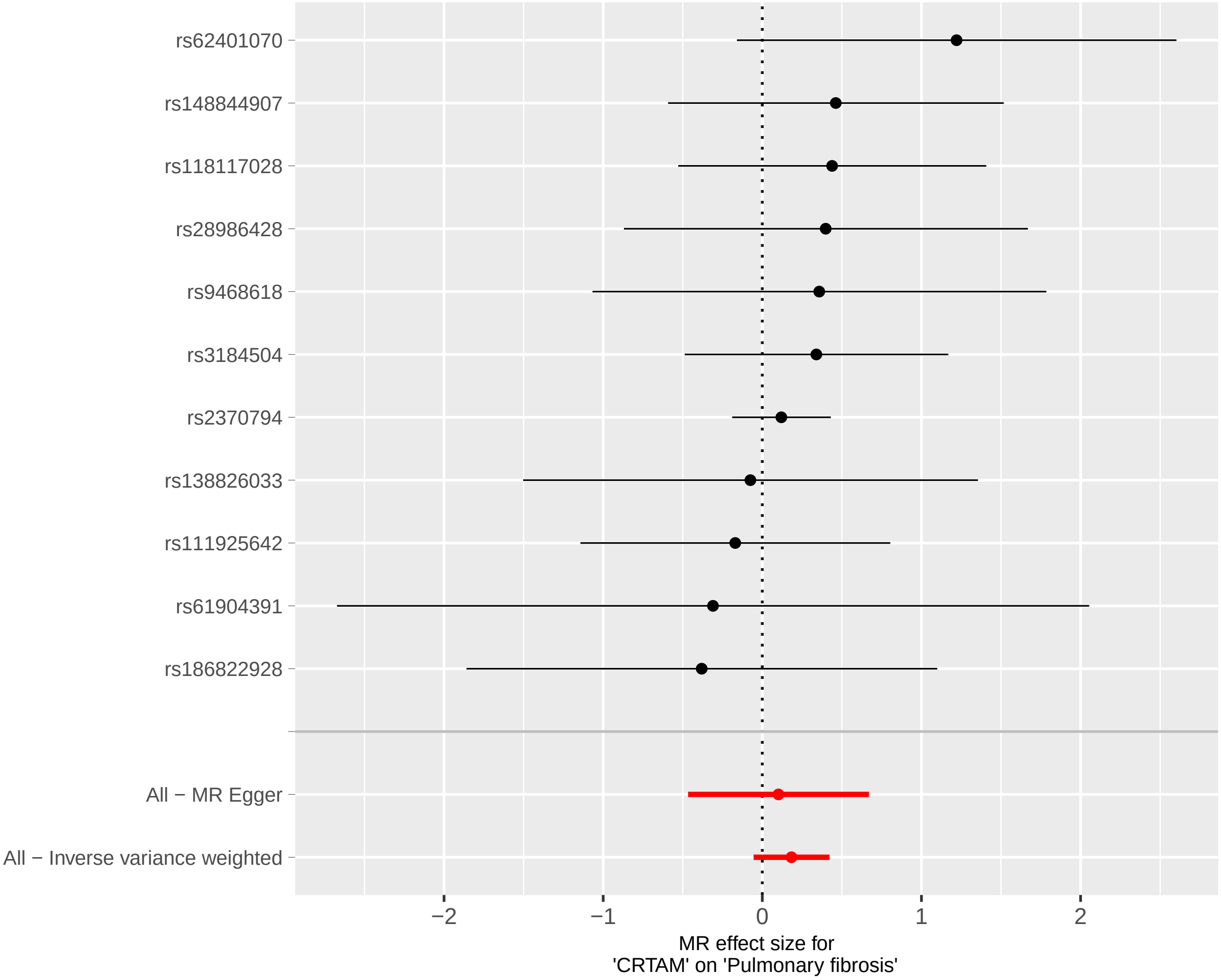

rs62401070
rs148844907
rs118117028
rs28986428
rs9468618
rs3184504
rs2370794
rs138826033
rs111925642
rs61904391
rs186822928
All − MR Egger
All − Inverse variance weighted
−2
−1
0
1
2
MR effect size for
'CRTAM' on 'Pulmonary fibrosis'

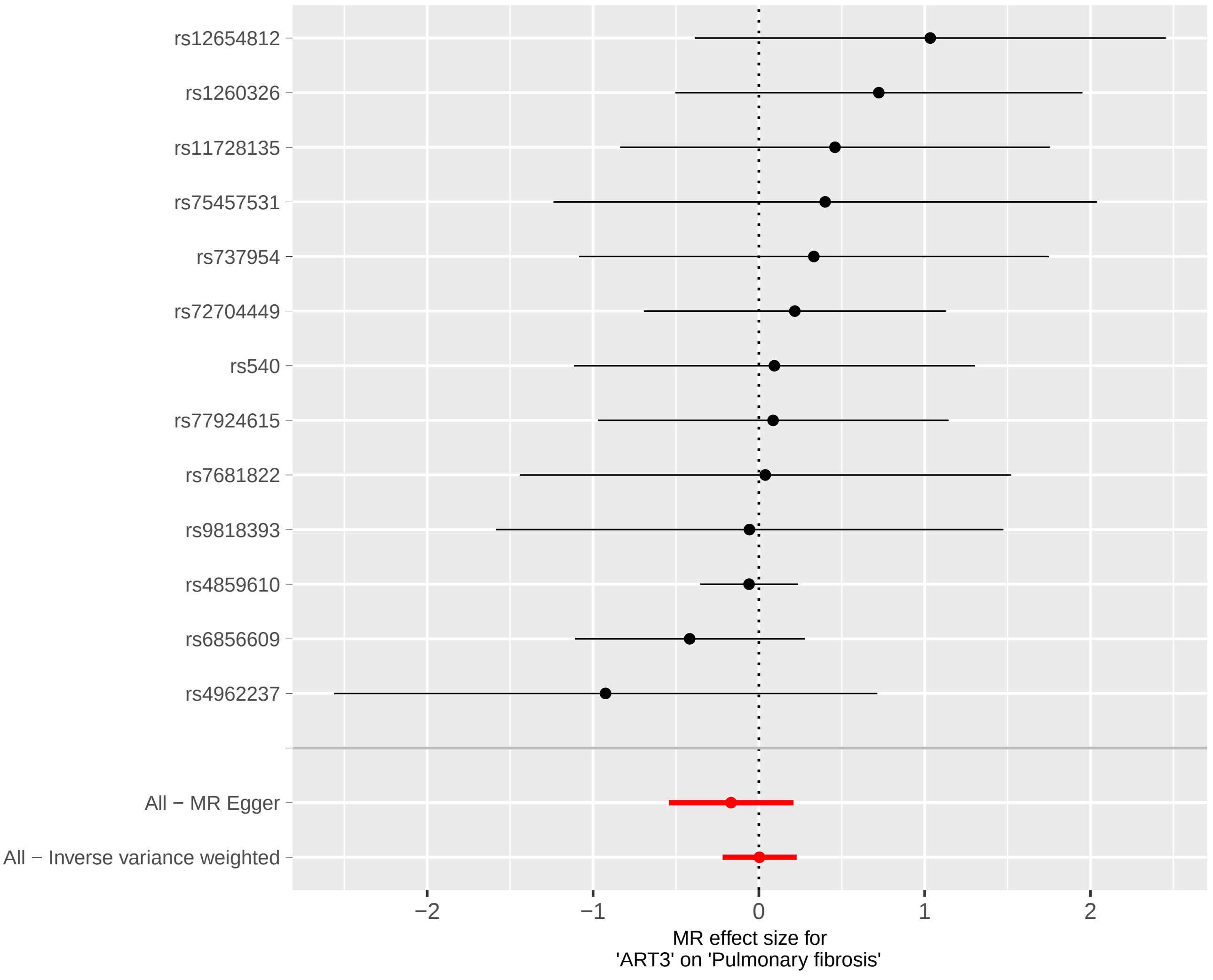
rs12654812
rs1260326
rs11728135
rs75457531
rs737954
rs72704449
rs540
rs77924615
rs7681822
rs9818393
rs4859610
rs6856609
rs4962237
All − MR Egger
All − Inverse variance weighted
−2
−1
0
1
2
MR effect size for
'ART3' on 'Pulmonary fibrosis'

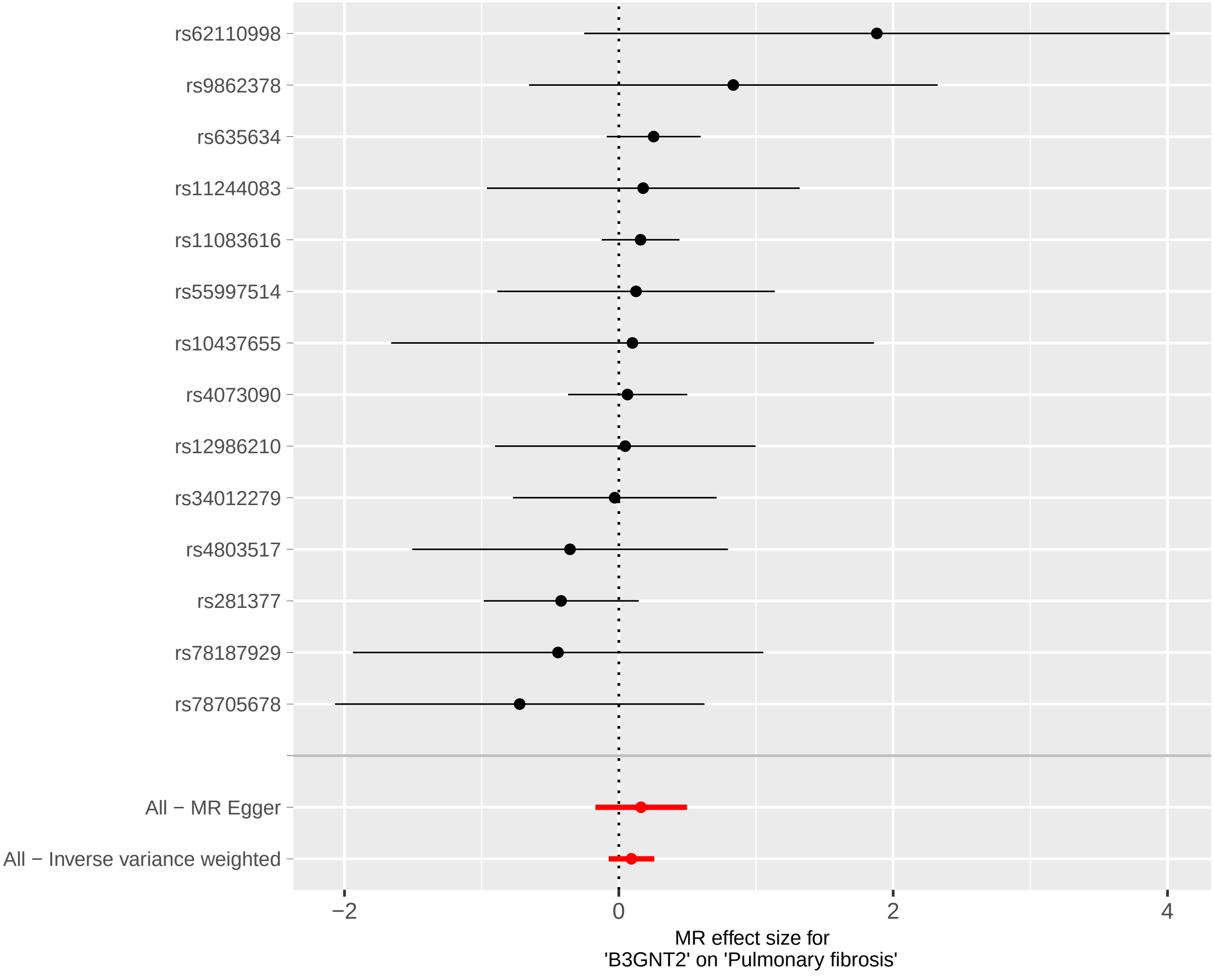

rs62110998
rs9862378
rs635634
rs11244083
rs11083616
rs55997514
rs10437655
rs4073090
rs12986210
rs34012279
rs4803517
rs281377
rs78187929
rs78705678
All − MR Egger
All − Inverse variance weighted
−2
0
2
4
MR effect size for
'B3GNT2' on 'Pulmonary fibrosis'

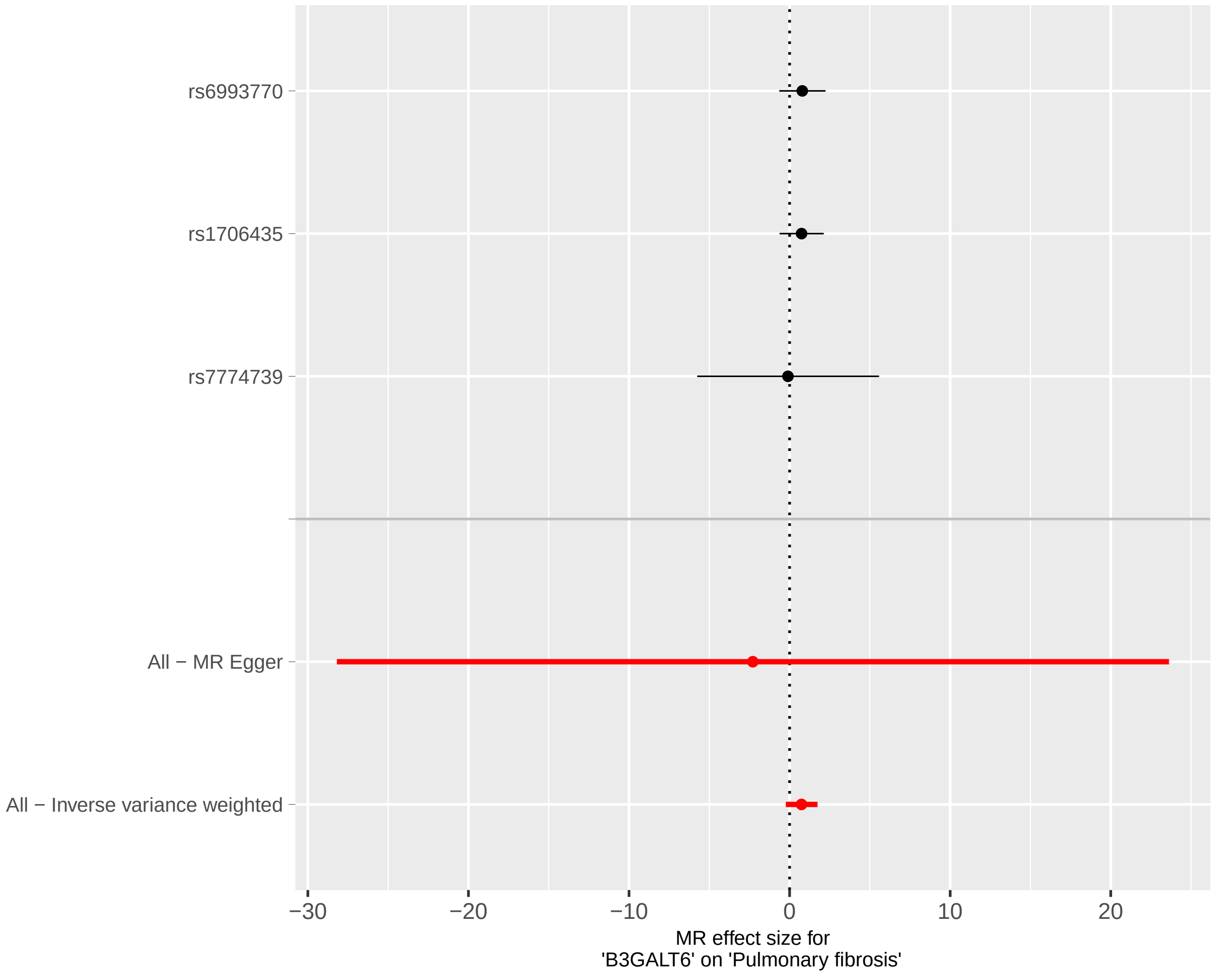
rs6993770
rs1706435
rs7774739
All − MR Egger
All − Inverse variance weighted
−30
−20
−10
0
10
20
MR effect size for
'B3GALT6' on 'Pulmonary fibrosis'

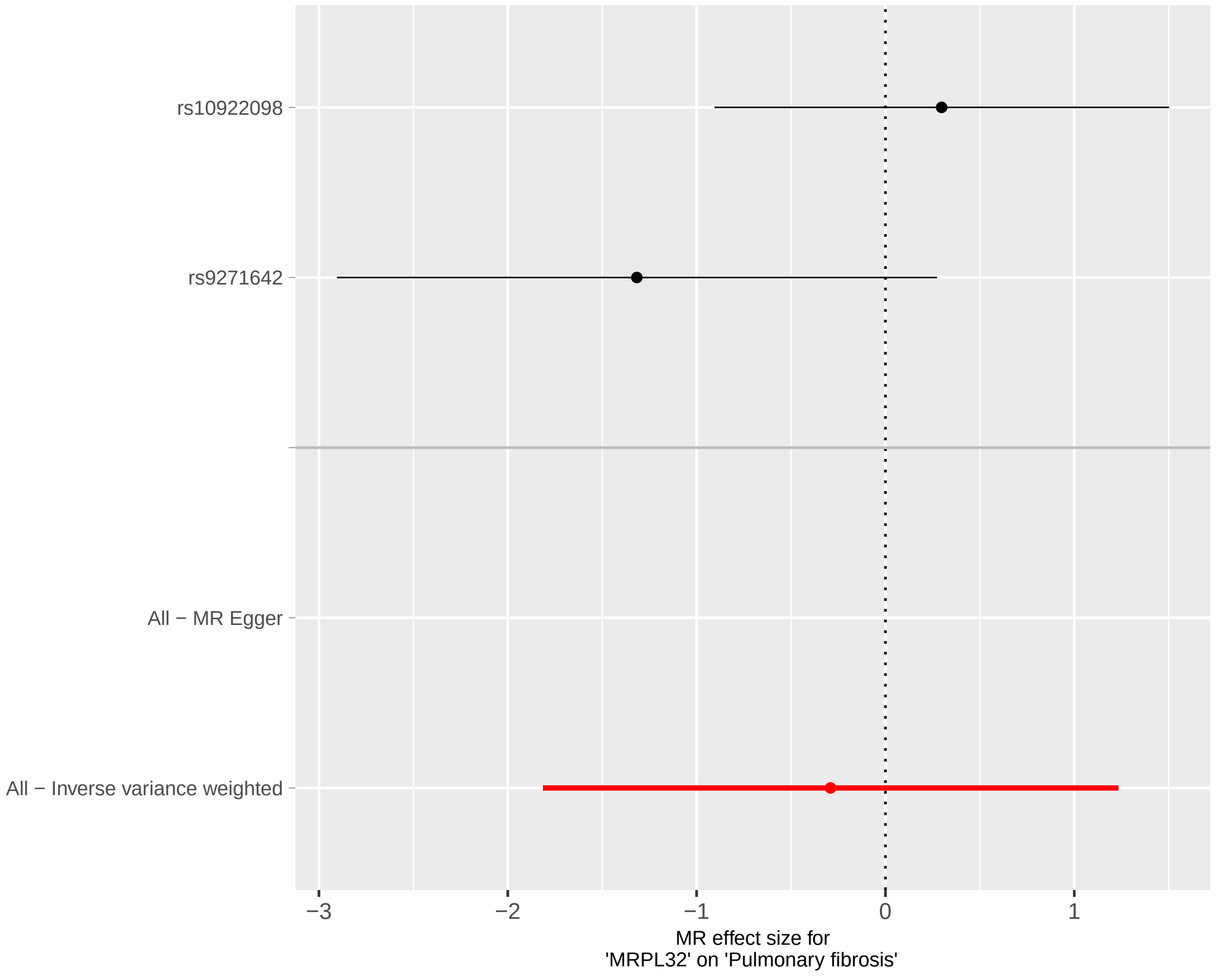

rs10922098
rs9271642
All − MR Egger
All − Inverse variance weighted
−3
−2
−1
0
1
MR effect size for
'MRPL32' on 'Pulmonary fibrosis'

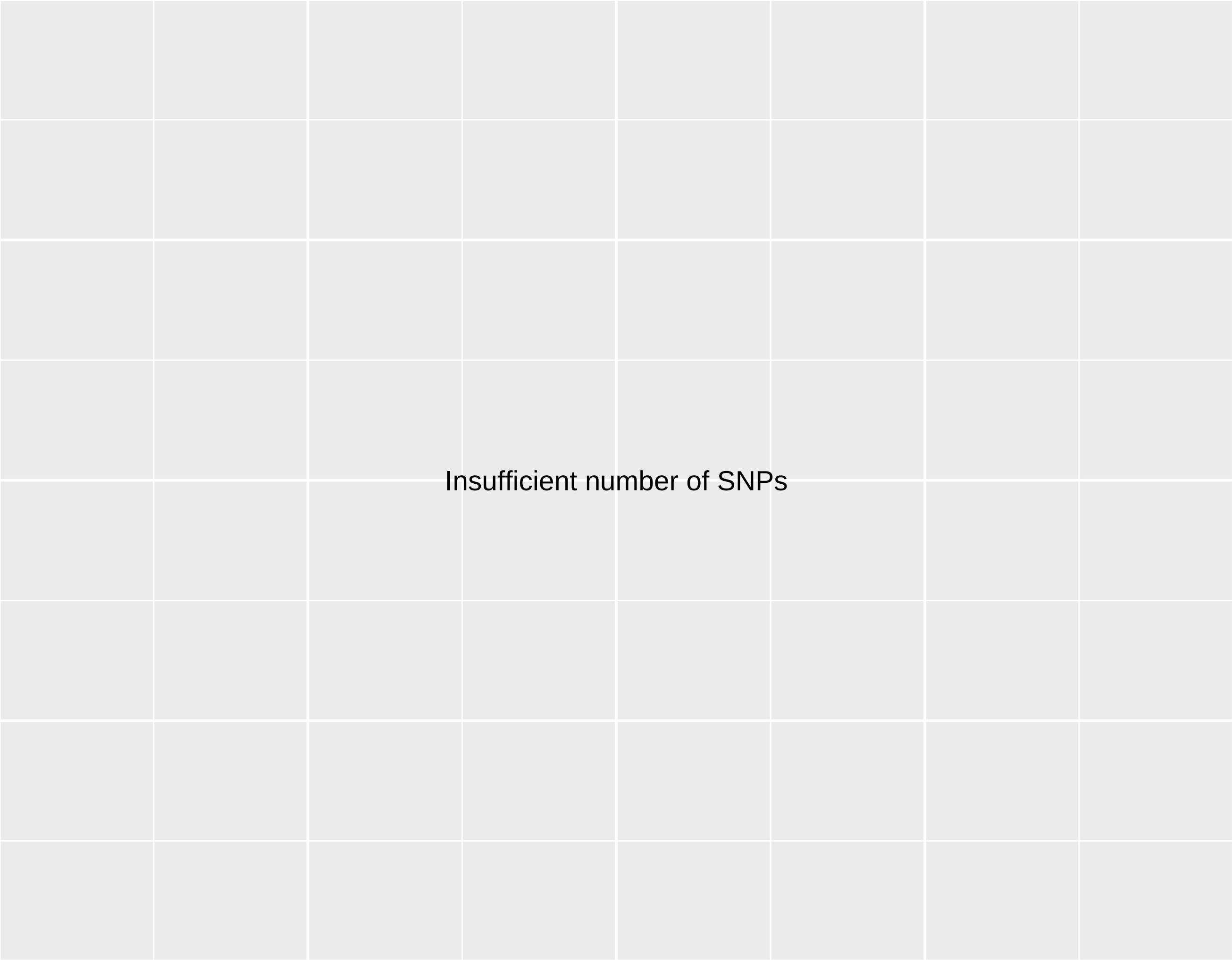
Insufficient number of SNPs

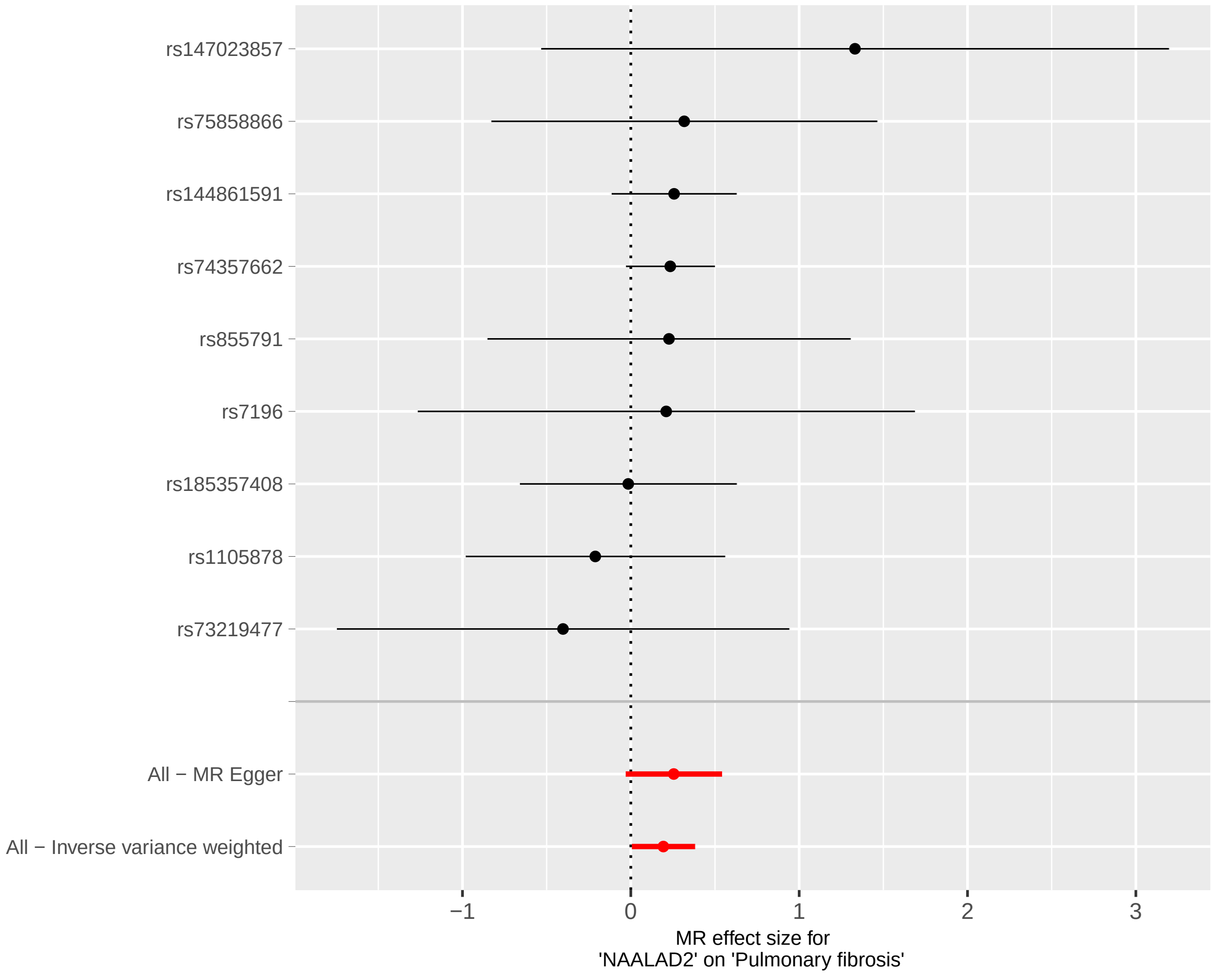
rs147023857
rs75858866
rs144861591
rs74357662
rs855791
rs7196
rs185357408
rs1105878
rs73219477
All − MR Egger
All − Inverse variance weighted
−1
0
1
2
3
MR effect size for
'NAALAD2' on 'Pulmonary fibrosis'

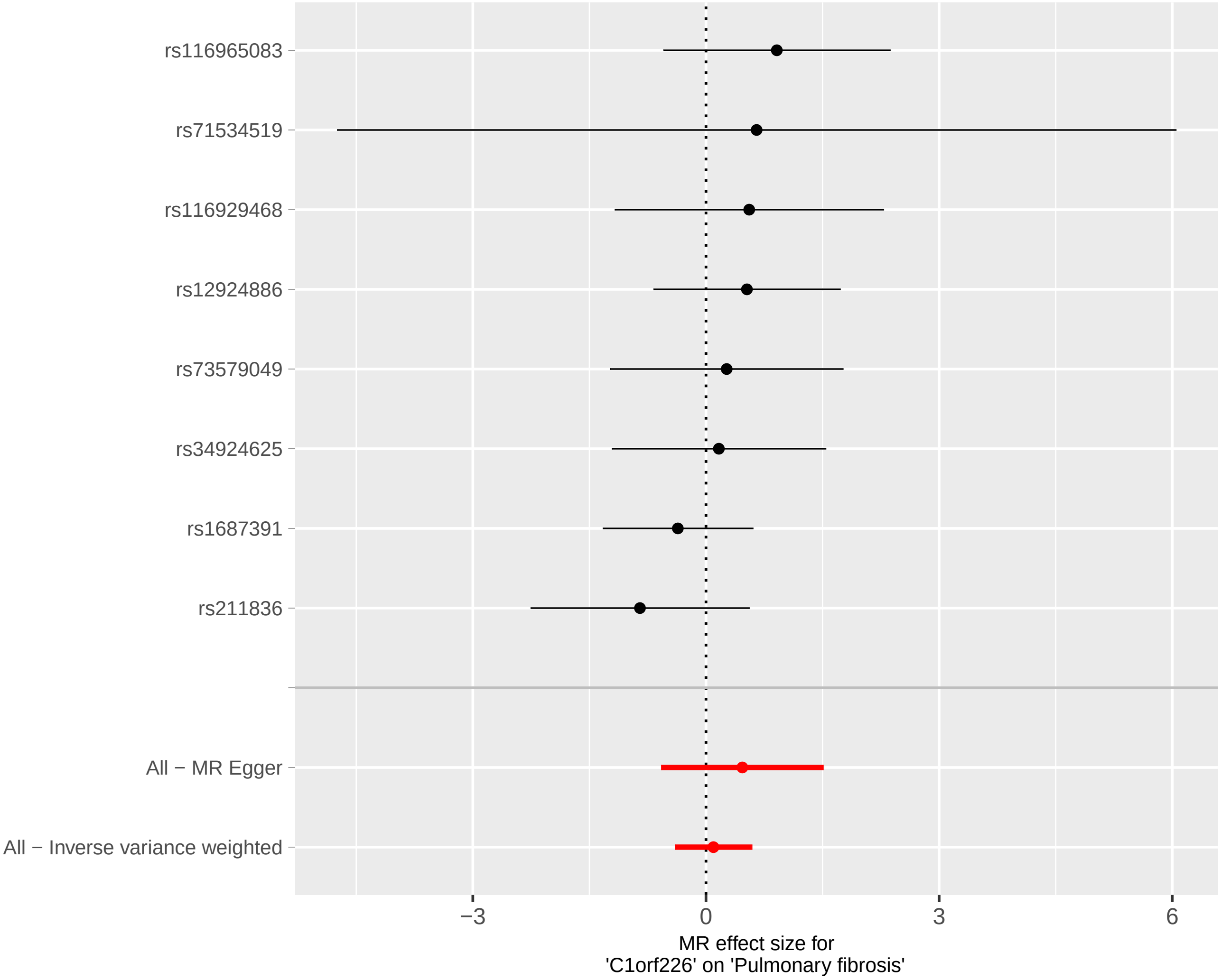

rs116965083
rs71534519
rs116929468
rs12924886
rs73579049
rs34924625
rs1687391
rs211836
All − MR Egger
All − Inverse variance weighted
−3
0
3
6
MR effect size for
'C1orf226' on 'Pulmonary fibrosis'

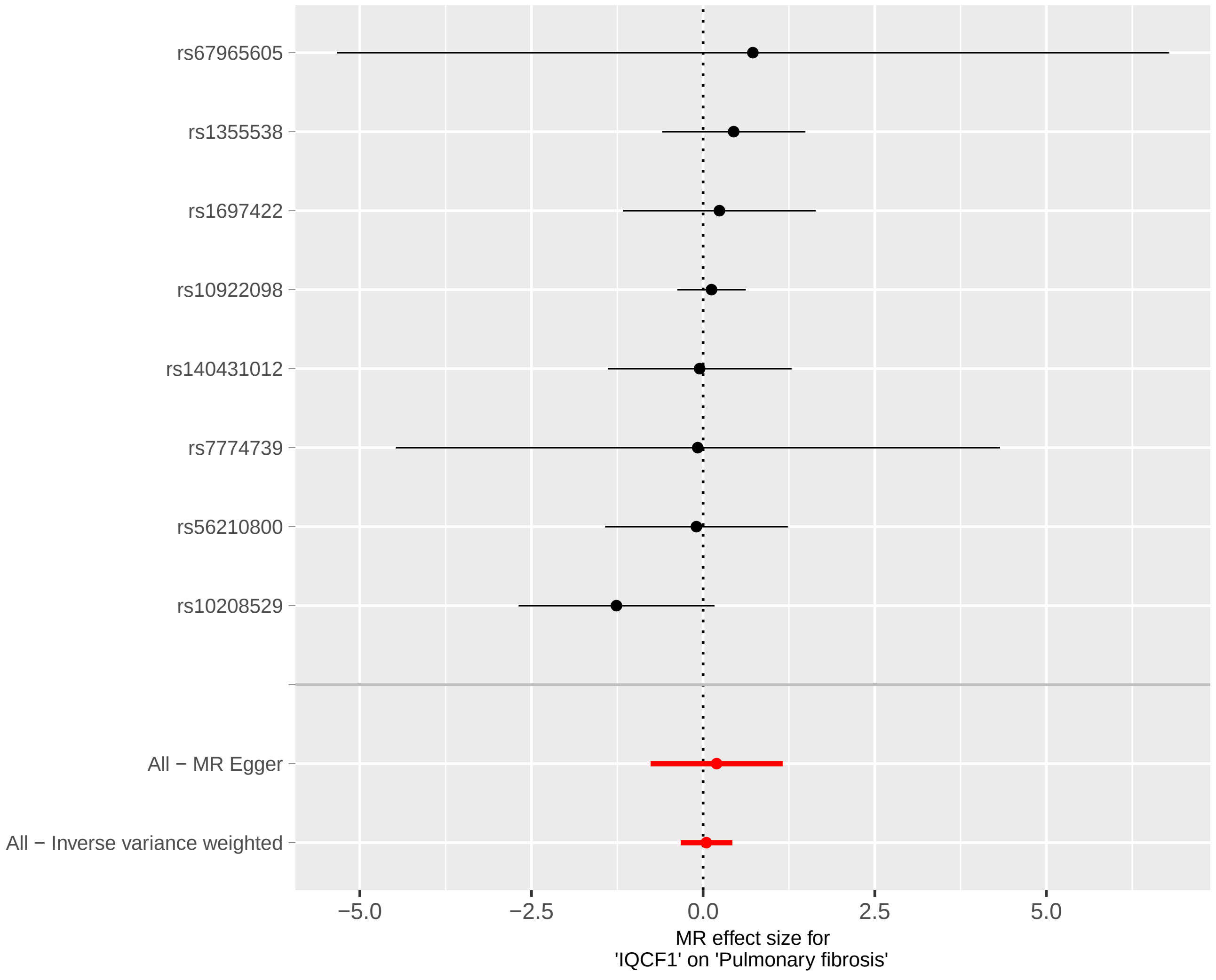

rs67965605
rs1355538
rs1697422
rs10922098
rs140431012
rs7774739
rs56210800
rs10208529
All − MR Egger
All − Inverse variance weighted
−5.0
−2.5
0.0
2.5
5.0
MR effect size for
'IQCF1' on 'Pulmonary fibrosis'

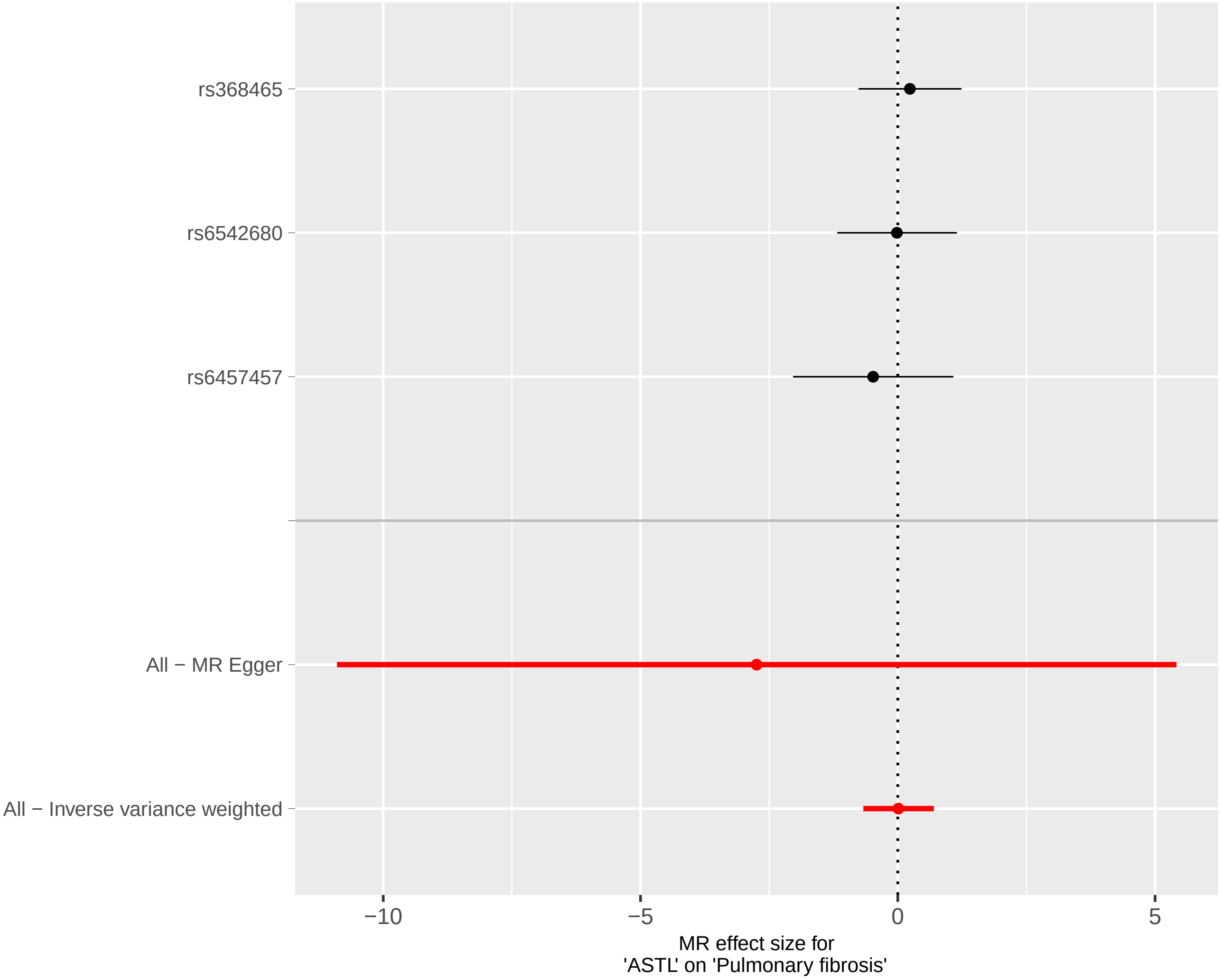

rs368465
rs6542680
rs6457457
All − MR Egger
All − Inverse variance weighted
−10
−5
0
5
MR effect size for
'ASTL' on 'Pulmonary fibrosis'

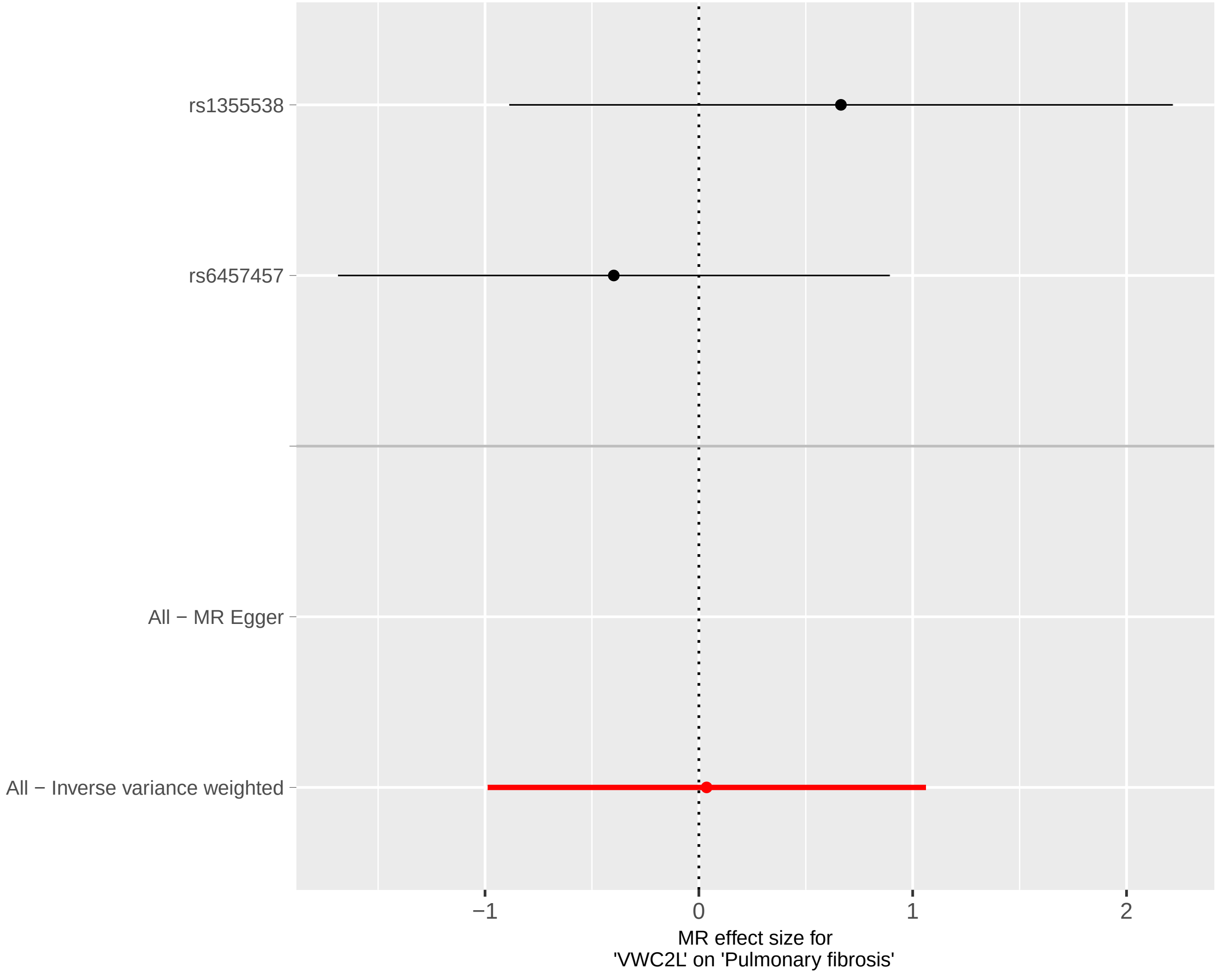
rs1355538
rs6457457
All − MR Egger
All − Inverse variance weighted
−1
0
1
2
MR effect size for
'VWC2L' on 'Pulmonary fibrosis'

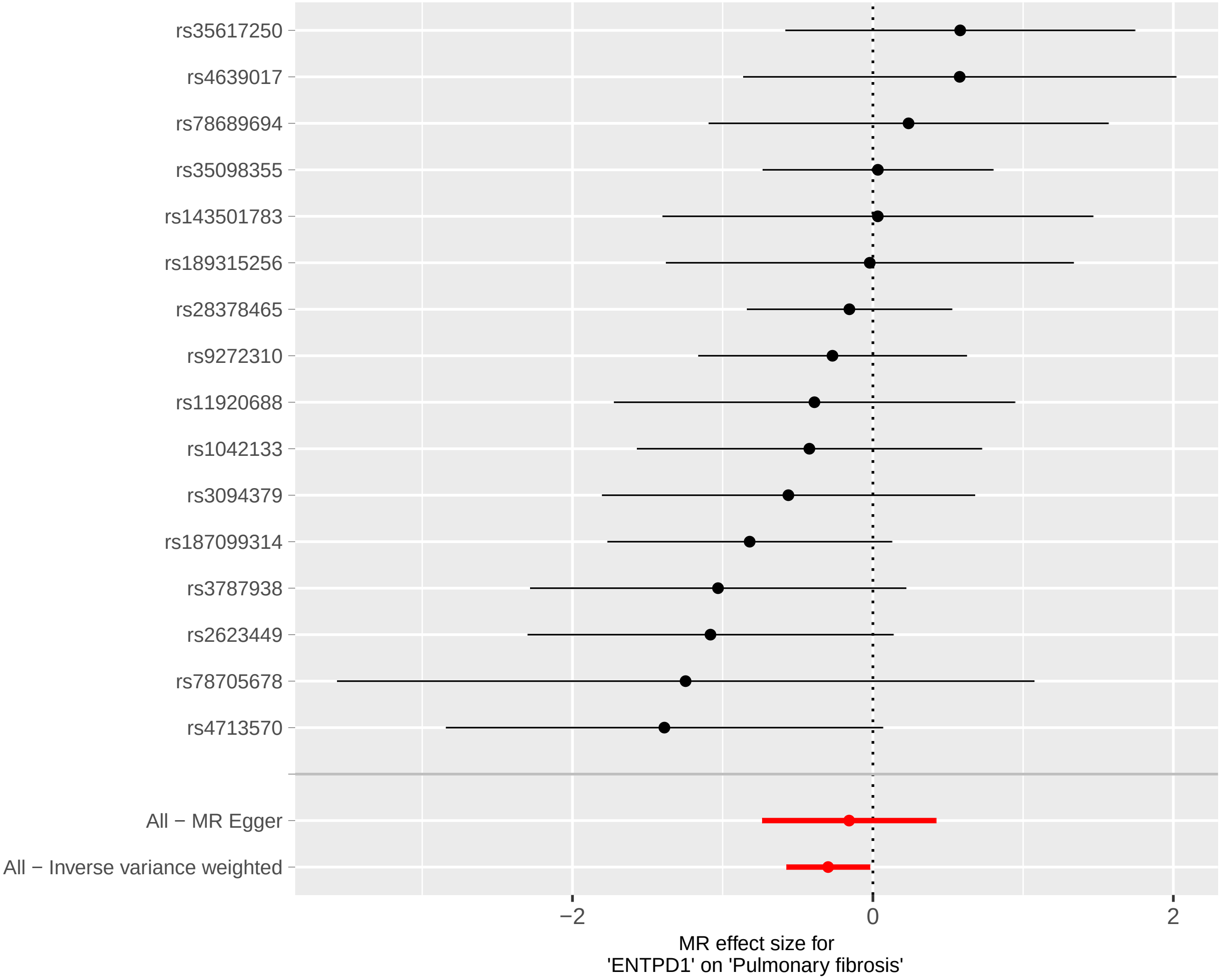
rs35617250
rs4639017
rs78689694
rs35098355
rs143501783
rs189315256
rs28378465
rs9272310
rs11920688
rs1042133
rs3094379
rs187099314
rs3787938
rs2623449
rs78705678
rs4713570
All − MR Egger
All − Inverse variance weighted
−2
0
2
MR effect size for
'ENTPD1' on 'Pulmonary fibrosis'

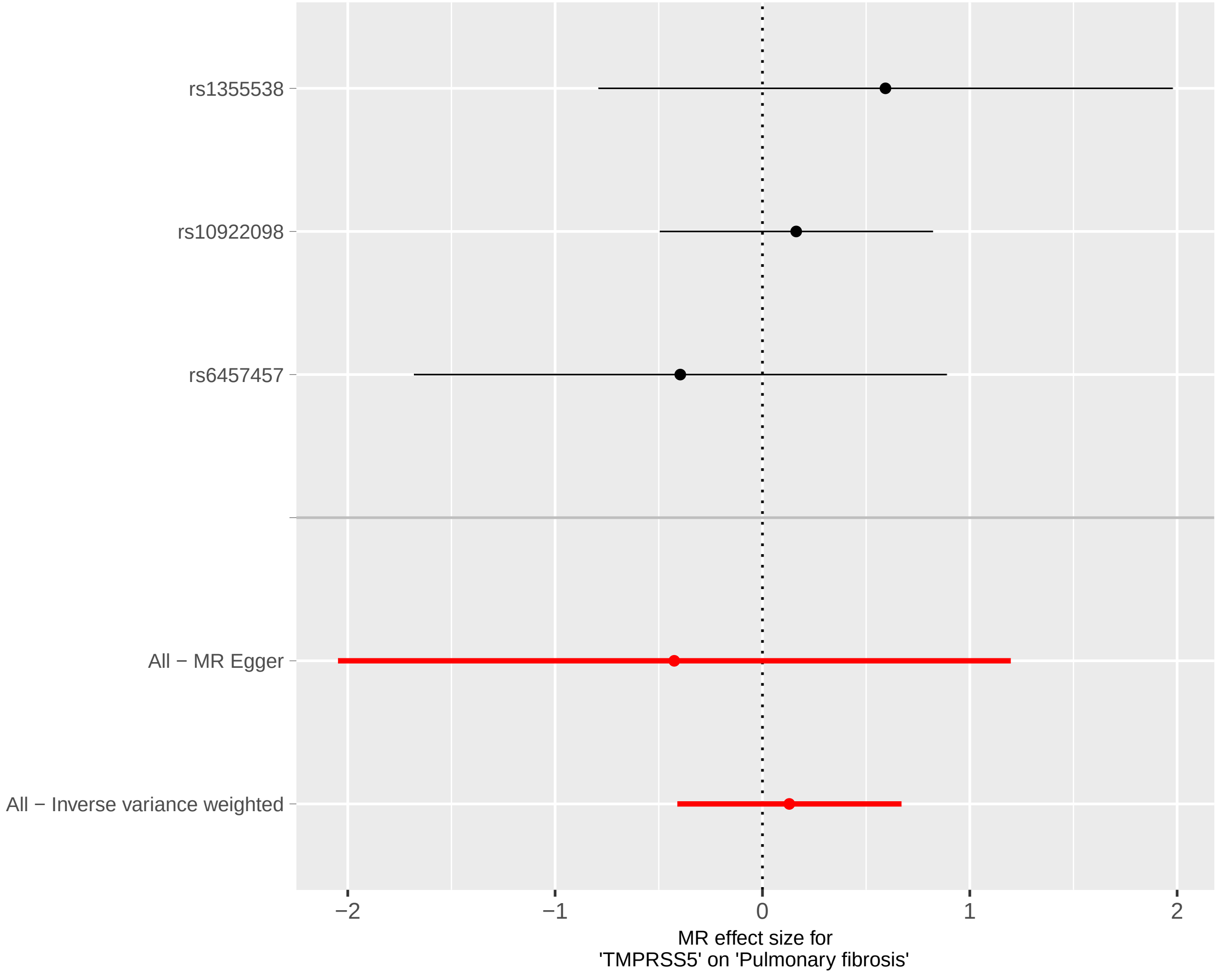
rs1355538
rs10922098
rs6457457
All − MR Egger
All − Inverse variance weighted
−2
−1
0
1
2
MR effect size for
'TMPRSS5' on 'Pulmonary fibrosis'

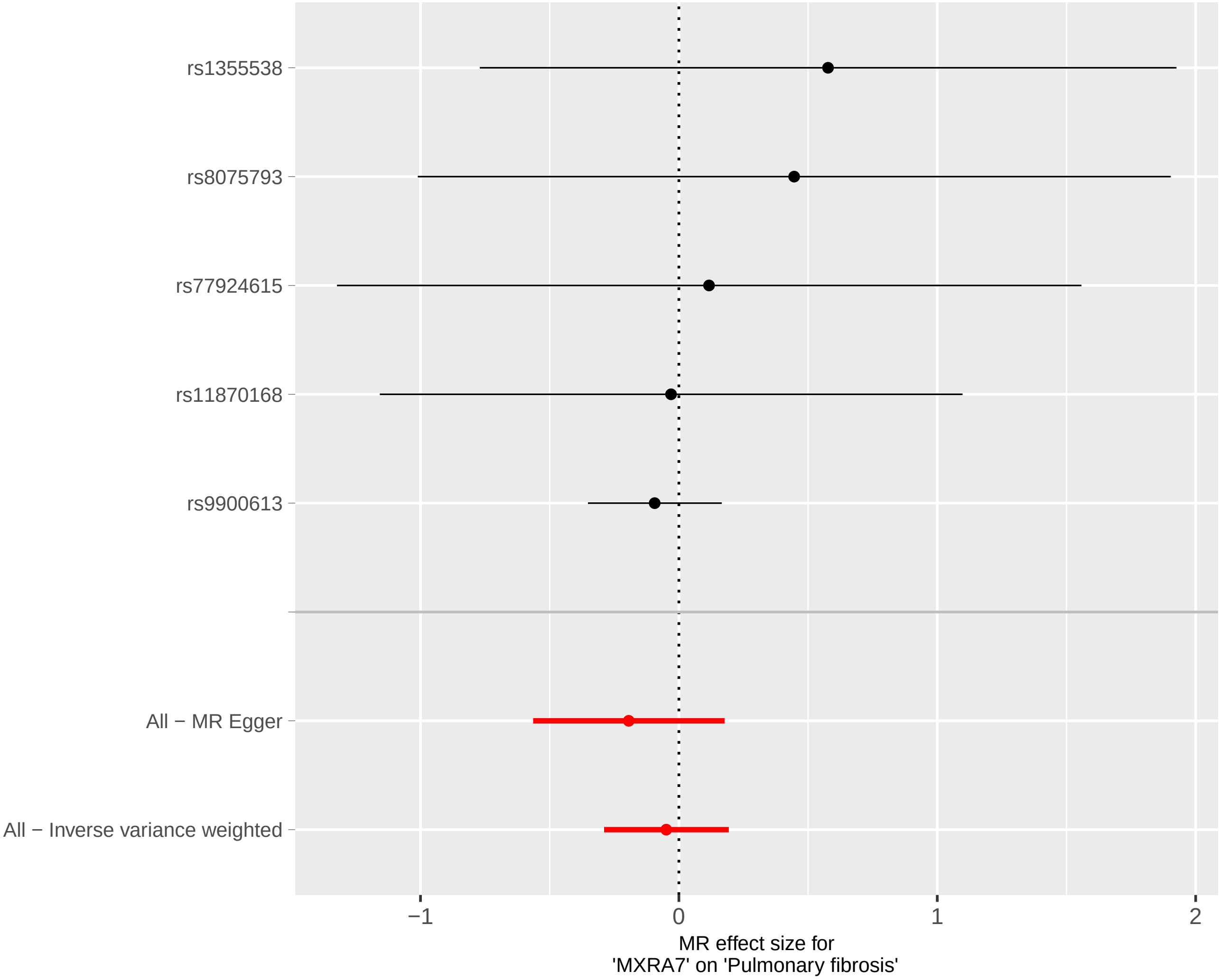
rs1355538
rs8075793
rs77924615
rs11870168
rs9900613
All − MR Egger
All − Inverse variance weighted
−1
0
1
2
MR effect size for
'MXRA7' on 'Pulmonary fibrosis'

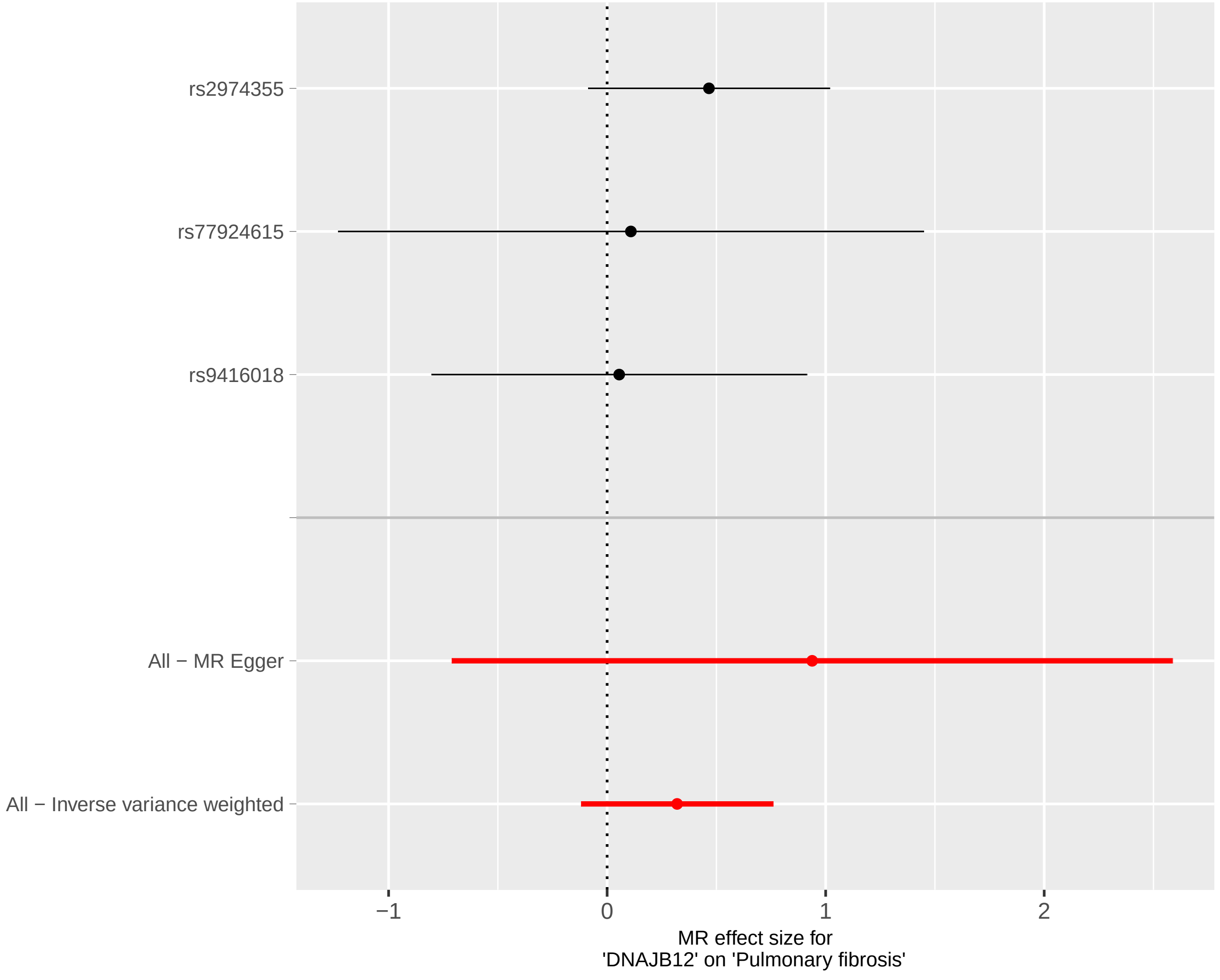
rs2974355
rs77924615
rs9416018
All − MR Egger
All − Inverse variance weighted
−1
0
1
2
MR effect size for
'DNAJB12' on 'Pulmonary fibrosis'

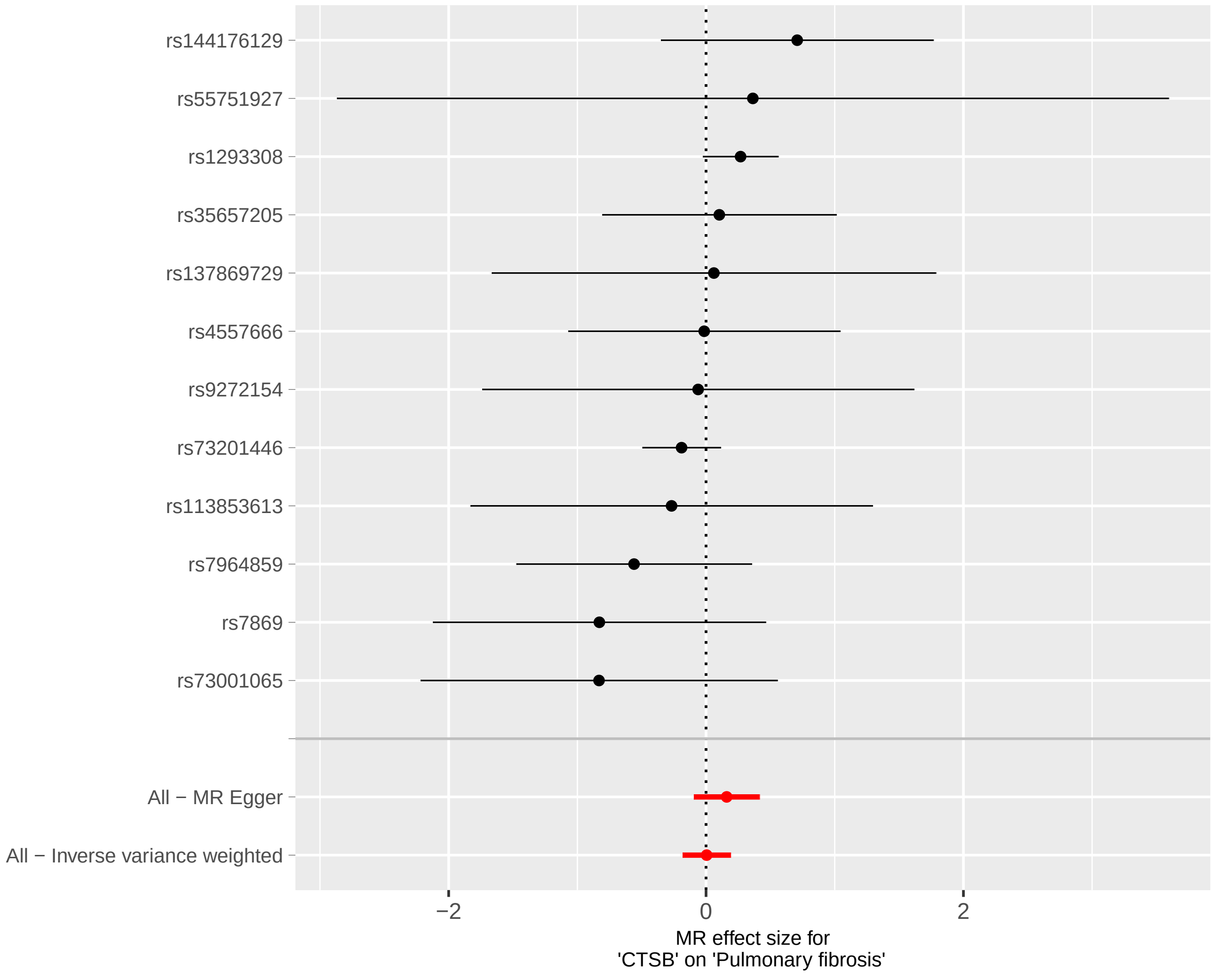

rs144176129
rs55751927
rs1293308
rs35657205
rs137869729
rs4557666
rs9272154
rs73201446
rs113853613
rs7964859
rs7869
rs73001065
All − MR Egger
All − Inverse variance weighted
−2
0
2
MR effect size for
'CTSB' on 'Pulmonary fibrosis'

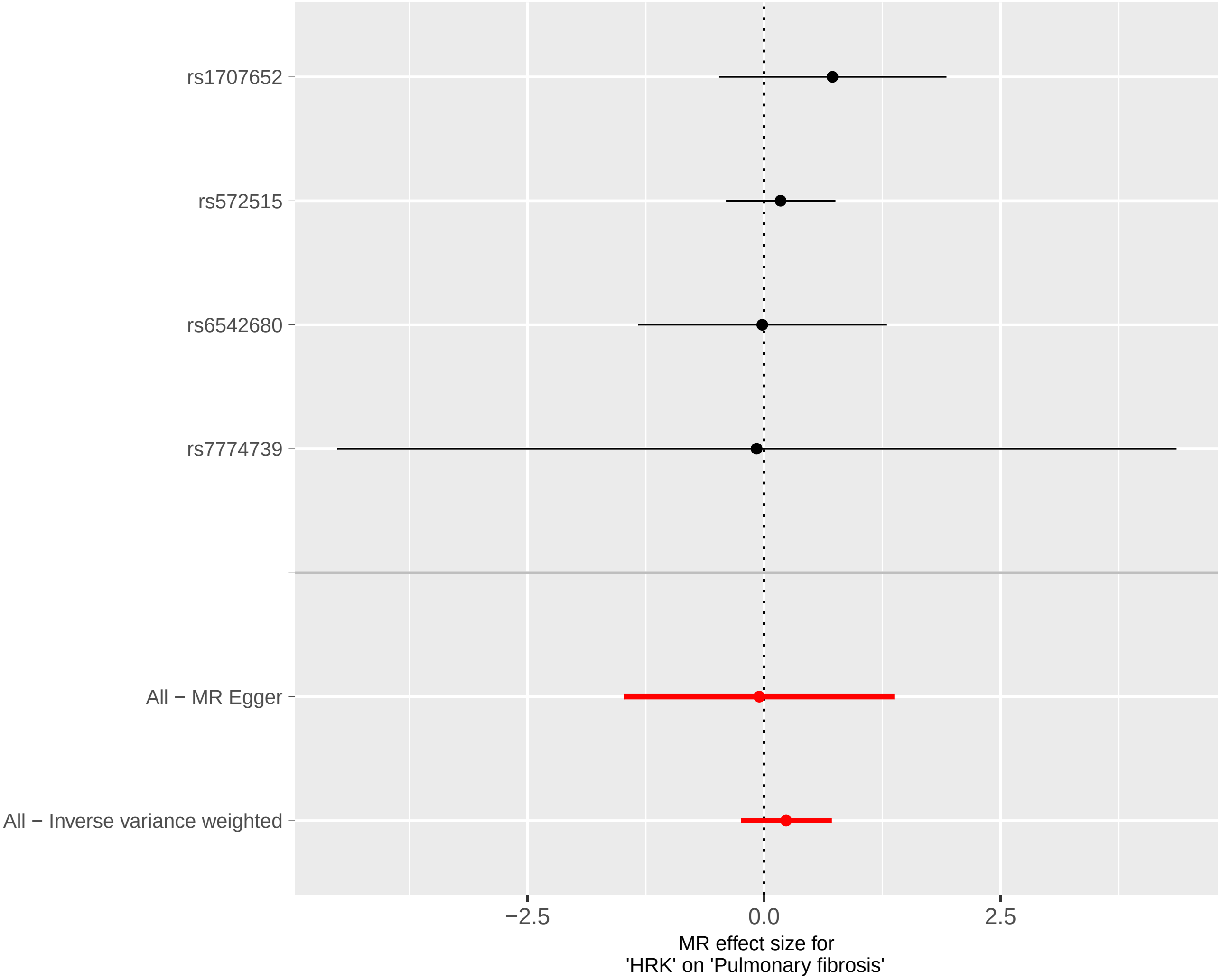
rs1707652
rs572515
rs6542680
rs7774739
All − MR Egger
All − Inverse variance weighted
−2.5
0.0
2.5
MR effect size for
'HRK' on 'Pulmonary fibrosis'

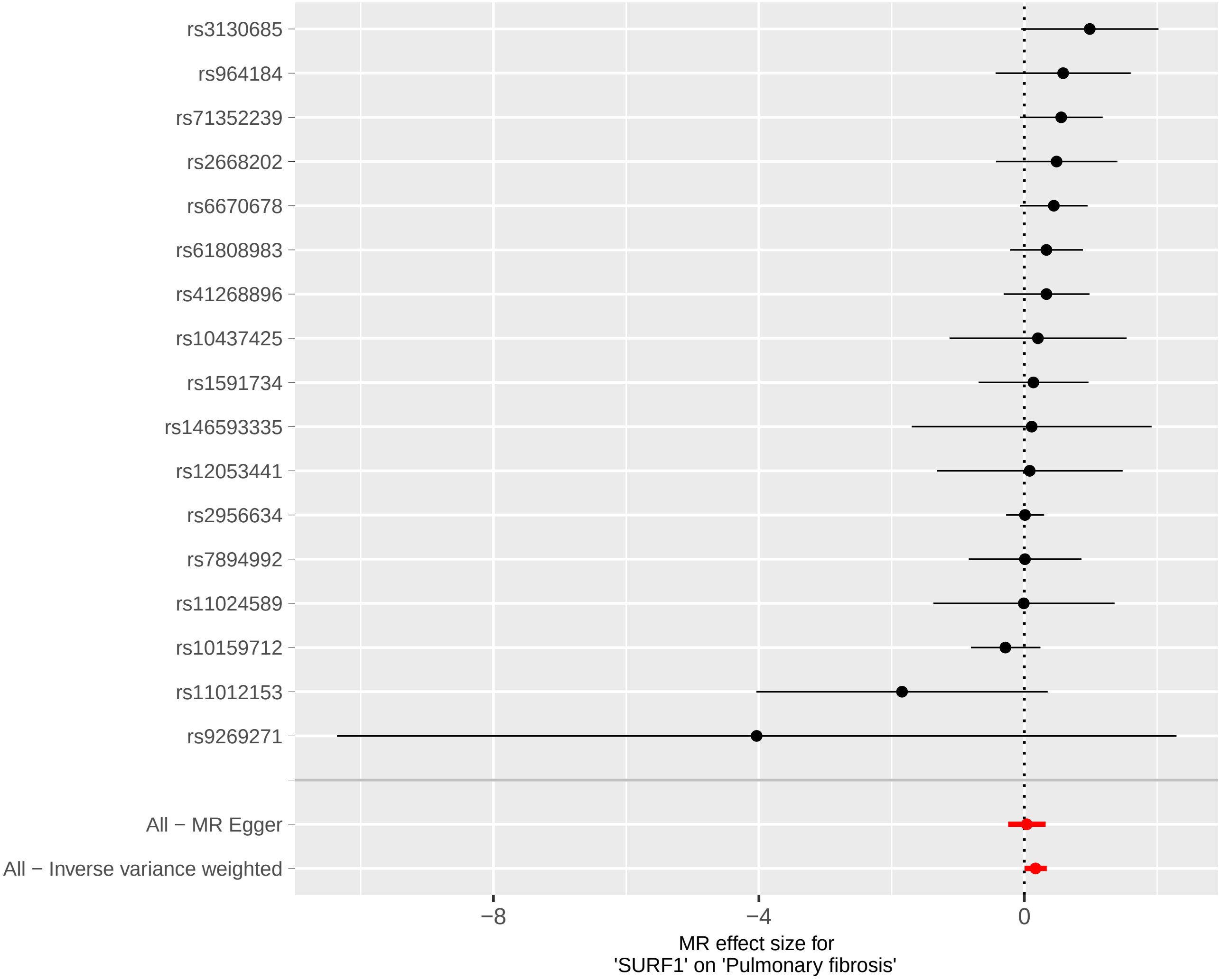
rs3130685
rs964184
rs71352239
rs2668202
rs6670678
rs61808983
rs41268896
rs10437425
rs1591734
rs146593335
rs12053441
rs2956634
rs7894992
rs11024589
rs10159712
rs11012153
rs9269271
All − MR Egger
All − Inverse variance weighted
−8
−4
0
MR effect size for
'SURF1' on 'Pulmonary fibrosis'

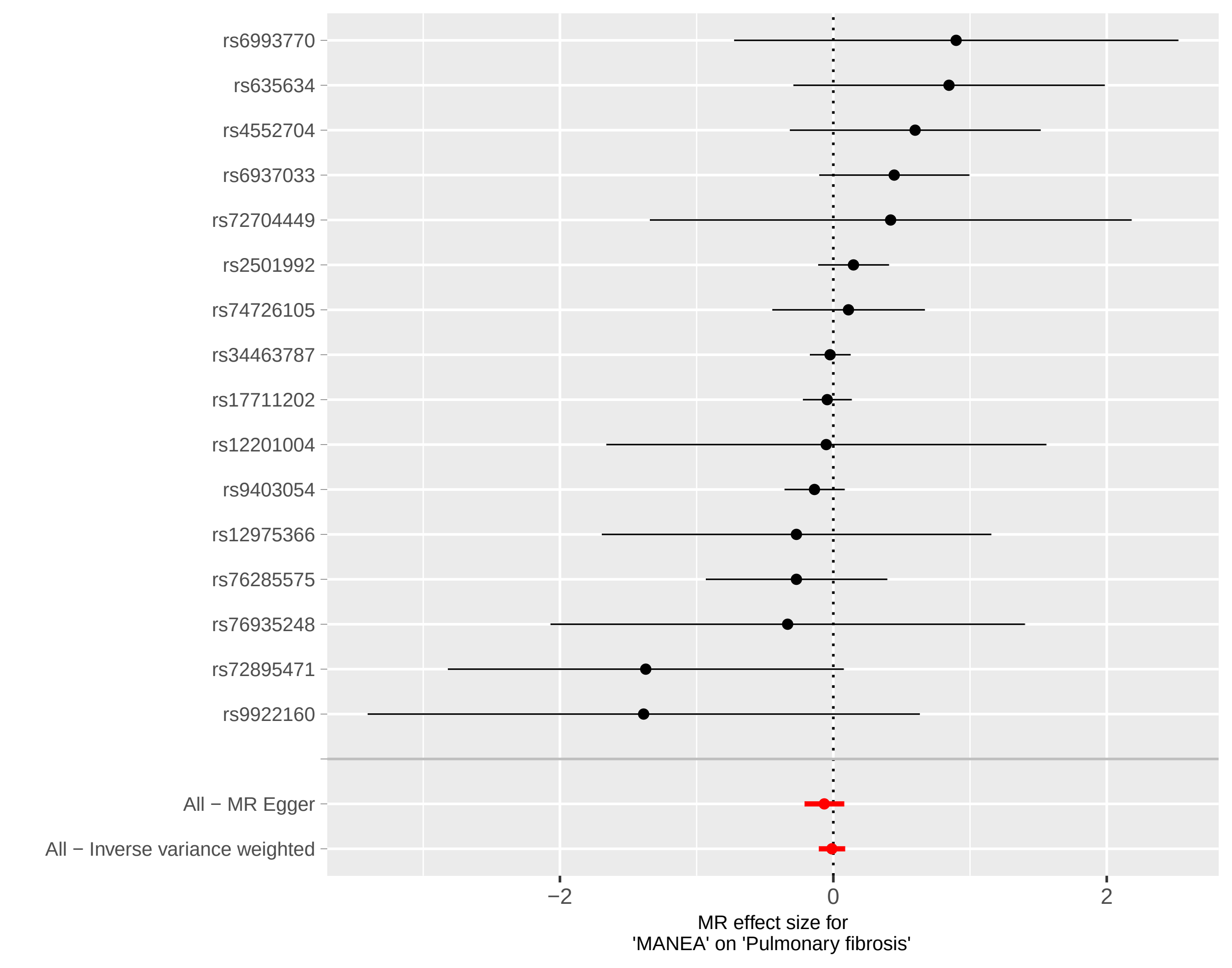
rs6993770
rs635634
rs4552704
rs6937033
rs72704449
rs2501992
rs74726105
rs34463787
rs17711202
rs12201004
rs9403054
rs12975366
rs76285575
rs76935248
rs72895471
rs9922160
All − MR Egger
All − Inverse variance weighted
−2
0
2
MR effect size for
'MANEA' on 'Pulmonary fibrosis'

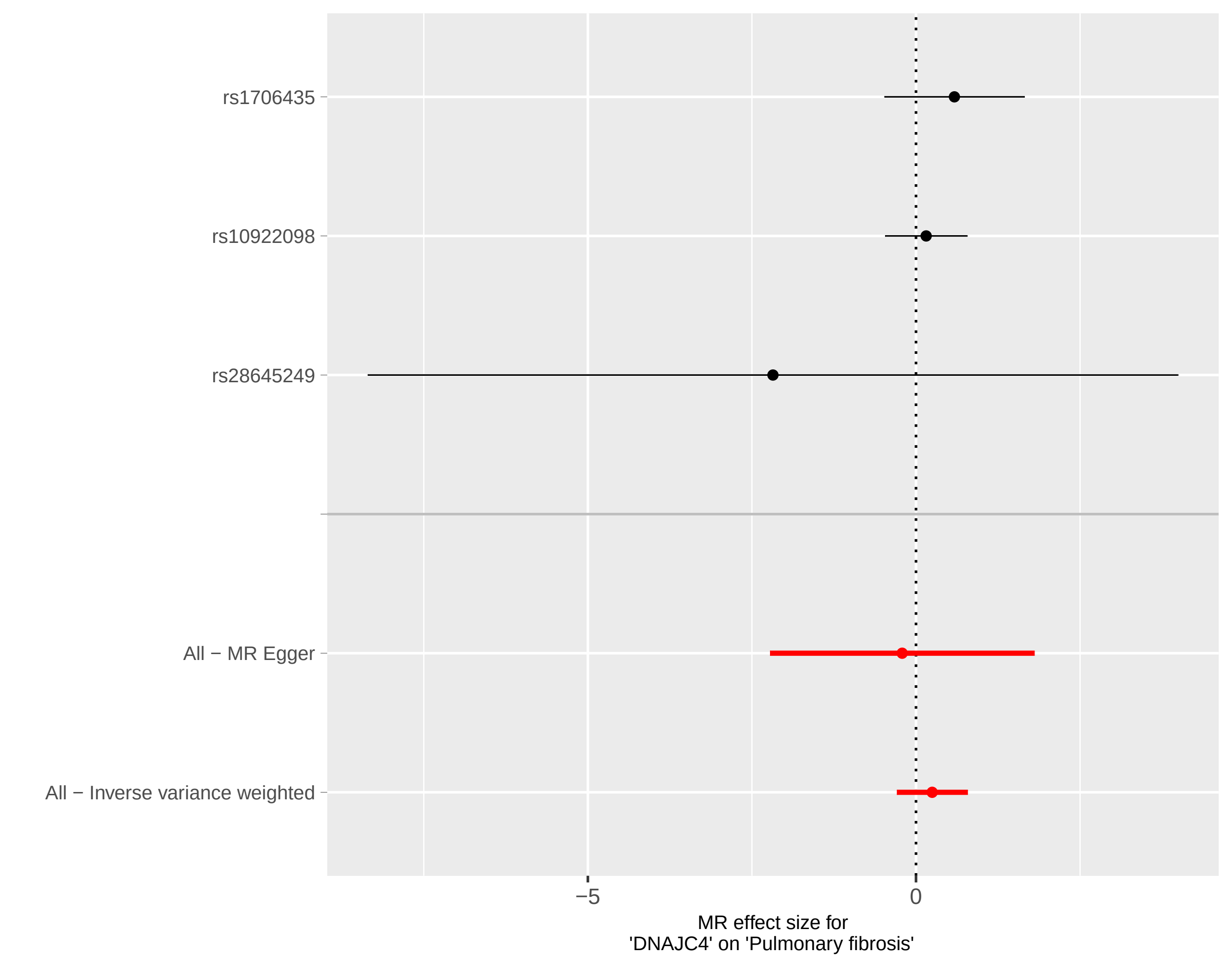
rs1706435
rs10922098
rs28645249
All − MR Egger
All − Inverse variance weighted
−5
0
MR effect size for
'DNAJC4' on 'Pulmonary fibrosis'

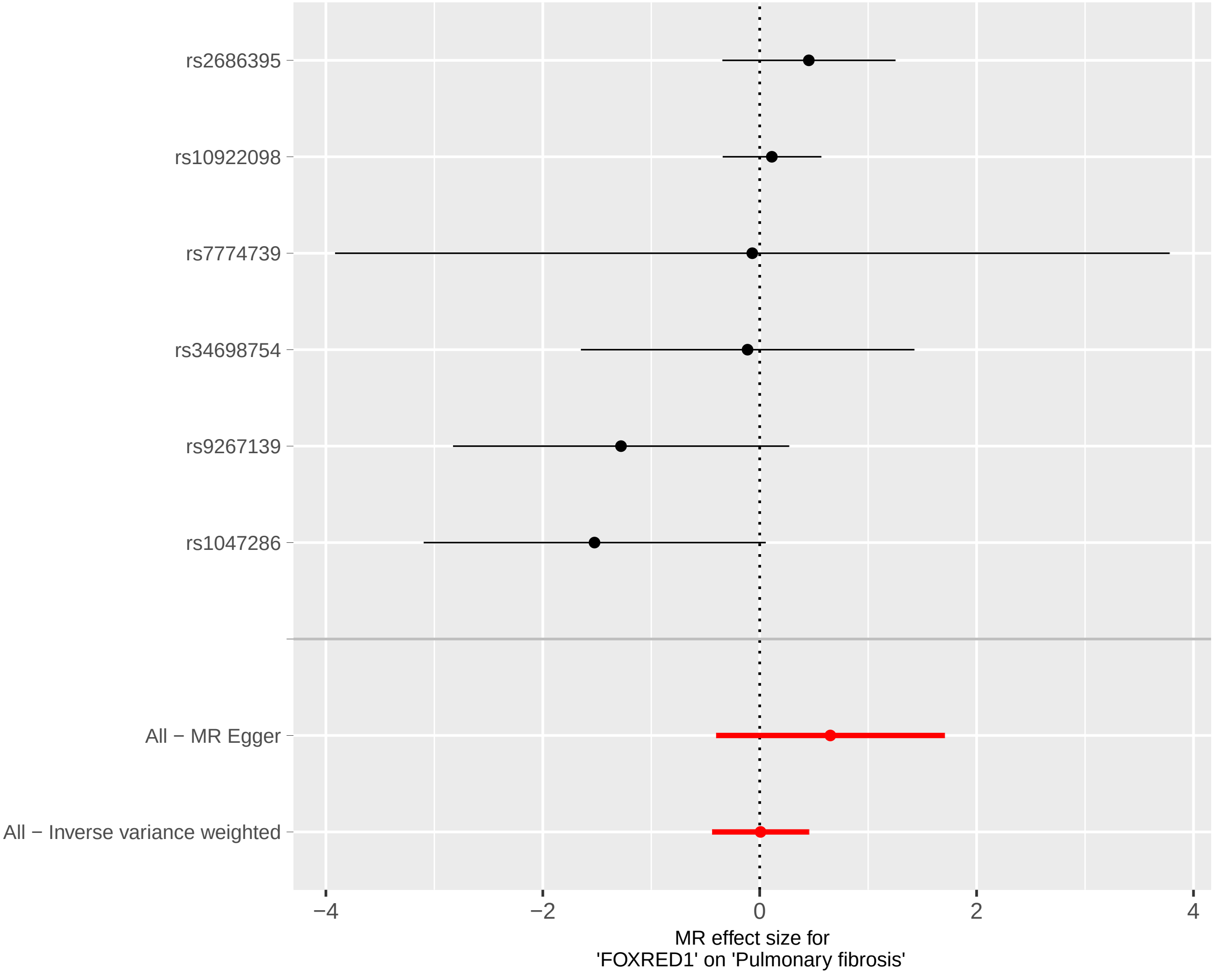
rs2686395
rs10922098
rs7774739
rs34698754
rs9267139
rs1047286
All − MR Egger
All − Inverse variance weighted
−4
−2
0
2
4
MR effect size for
'FOXRED1' on 'Pulmonary fibrosis'

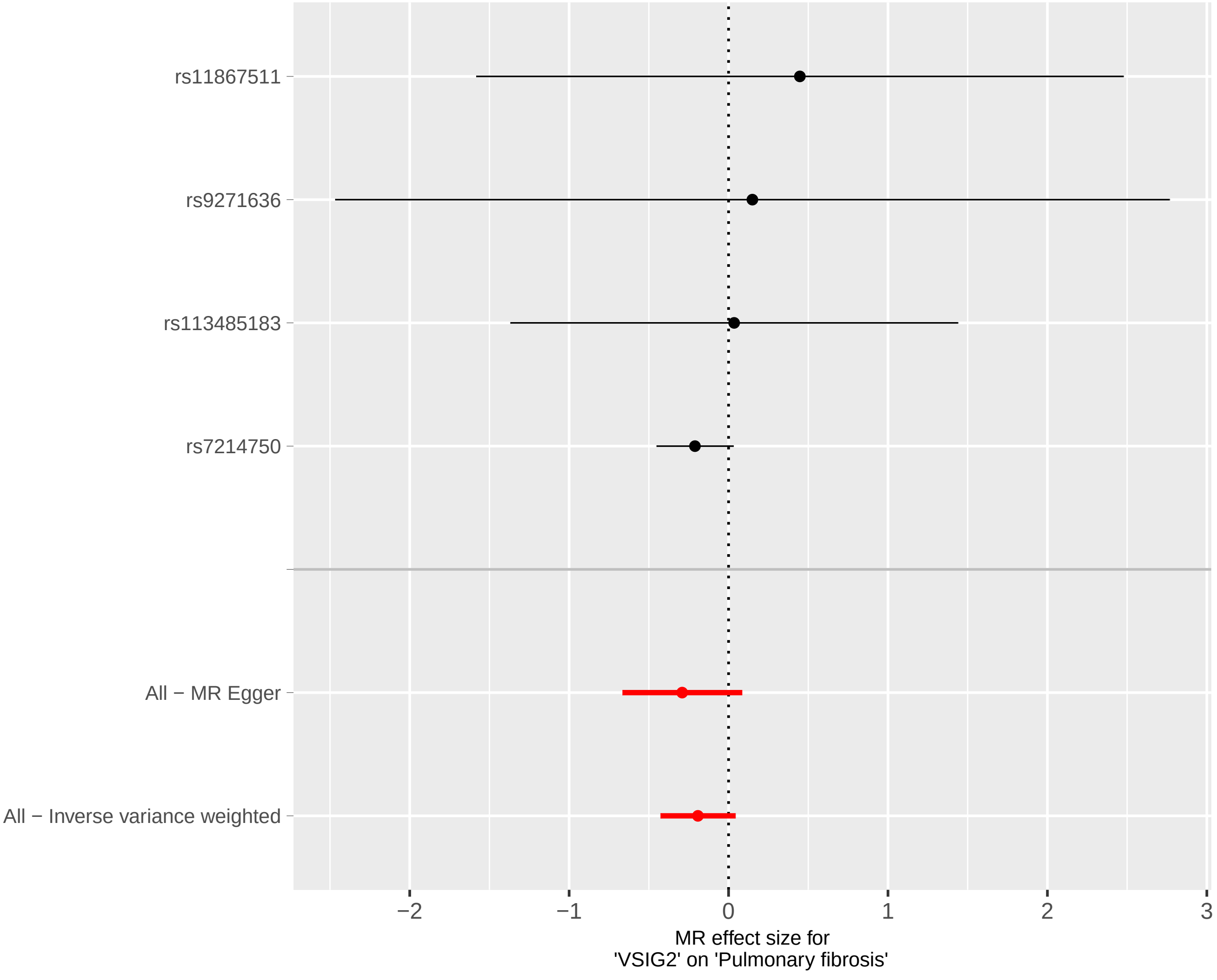
rs11867511
rs9271636
rs113485183
rs7214750
All − MR Egger
All − Inverse variance weighted
−2
−1
0
1
2
3
MR effect size for
'VSIG2' on 'Pulmonary fibrosis'

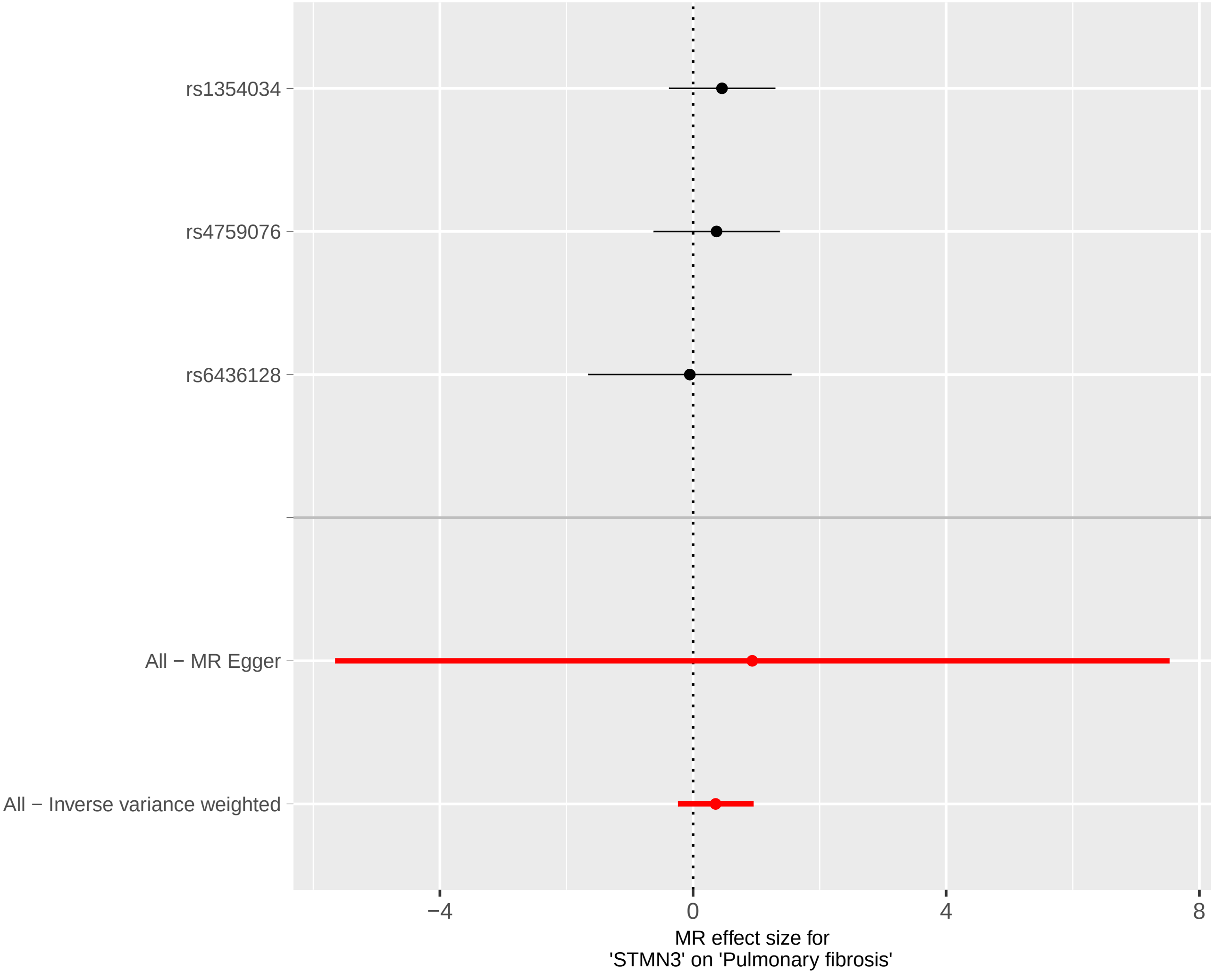

rs1354034
rs4759076
rs6436128
All − MR Egger
All − Inverse variance weighted
−4
0
4
8
MR effect size for
'STMN3' on 'Pulmonary fibrosis'

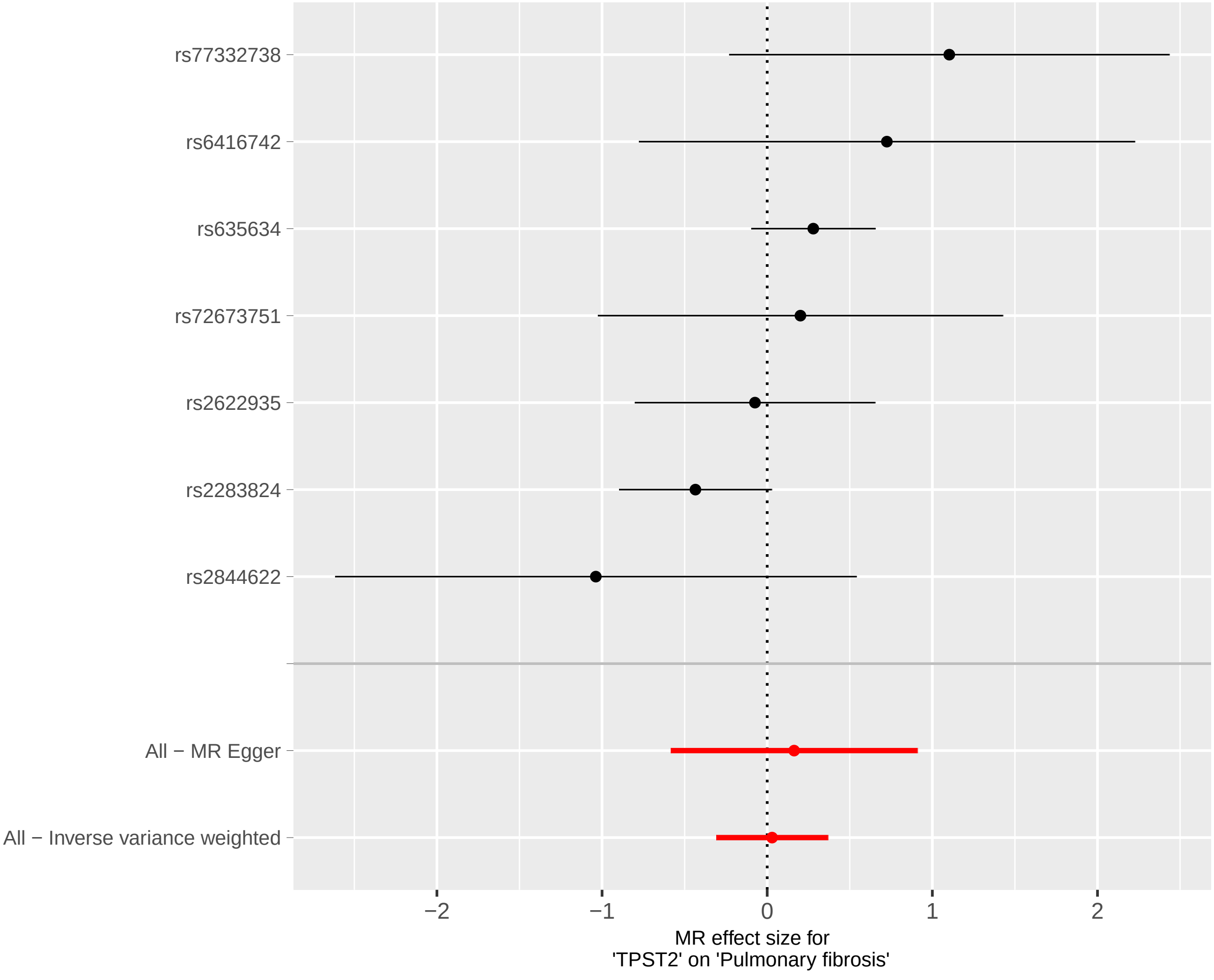

rs77332738
rs6416742
rs635634
rs72673751
rs2622935
rs2283824
rs2844622
All − MR Egger
All − Inverse variance weighted
−2
−1
0
1
2
MR effect size for
'TPST2' on 'Pulmonary fibrosis'

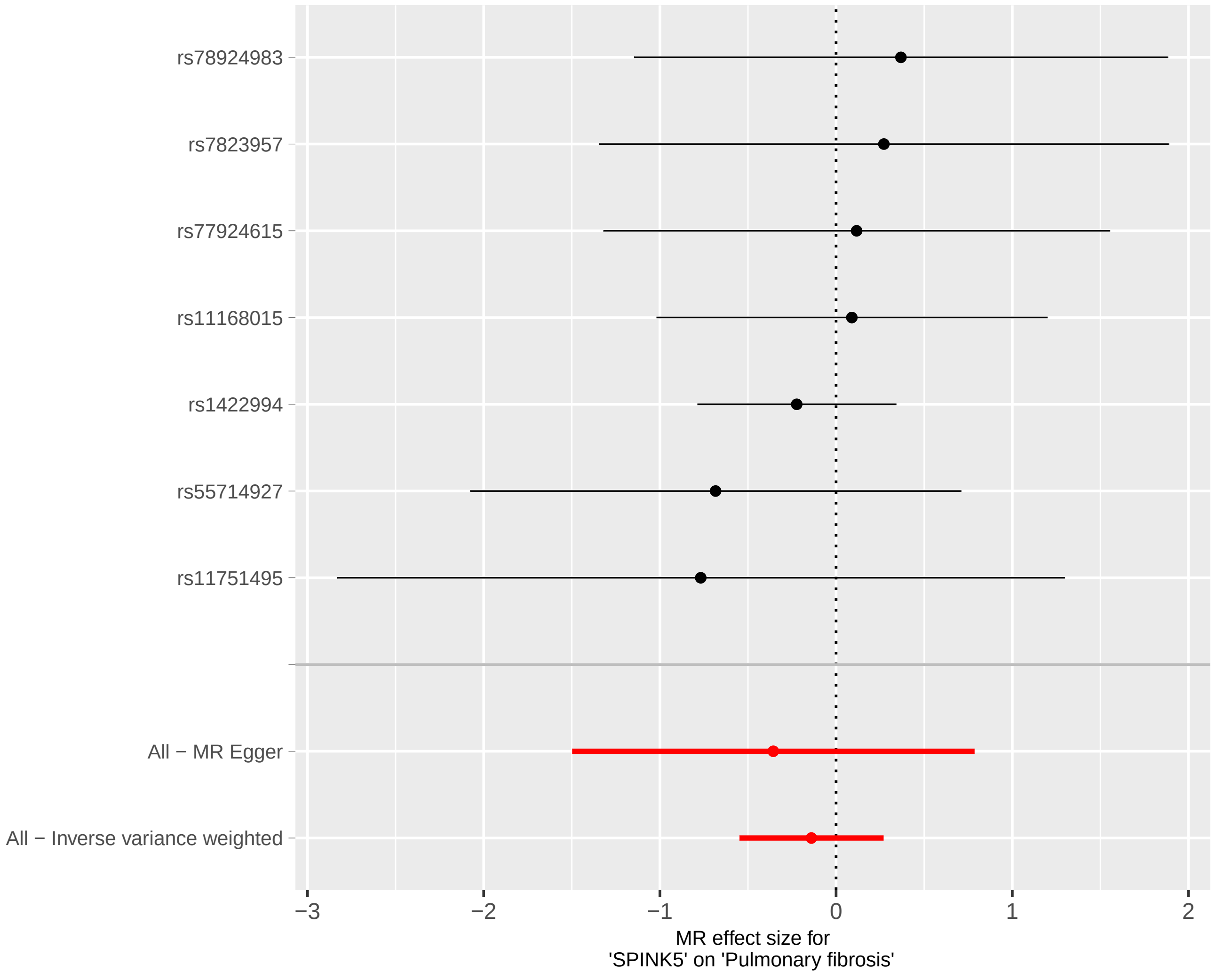
rs78924983
rs7823957
rs77924615
rs11168015
rs1422994
rs55714927
rs11751495
All − MR Egger
All − Inverse variance weighted
−3
−2
−1
0
1
2
MR effect size for
'SPINK5' on 'Pulmonary fibrosis'

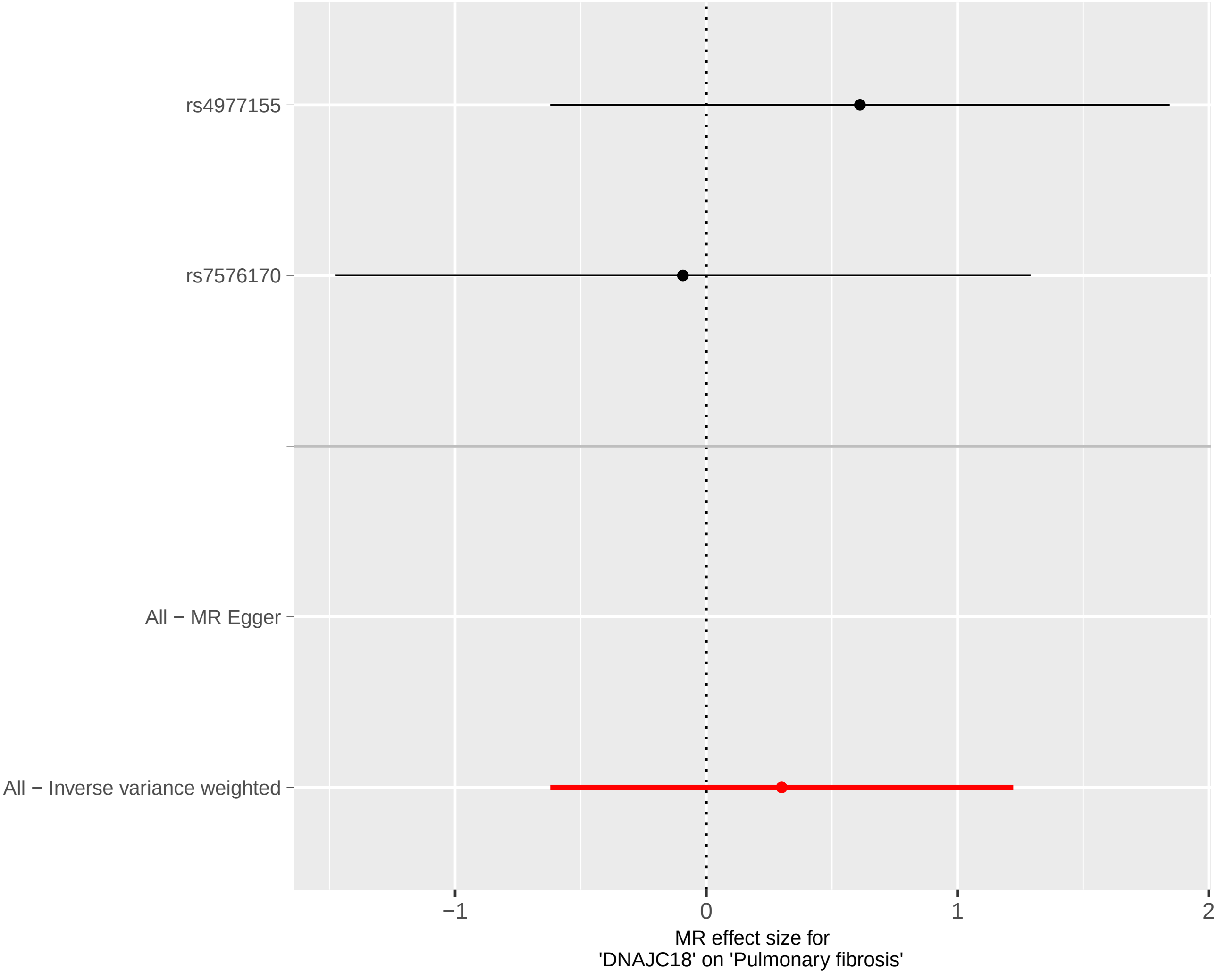

rs4977155
rs7576170
All − MR Egger
All − Inverse variance weighted
−1
0
1
2
MR effect size for
'DNAJC18' on 'Pulmonary fibrosis'

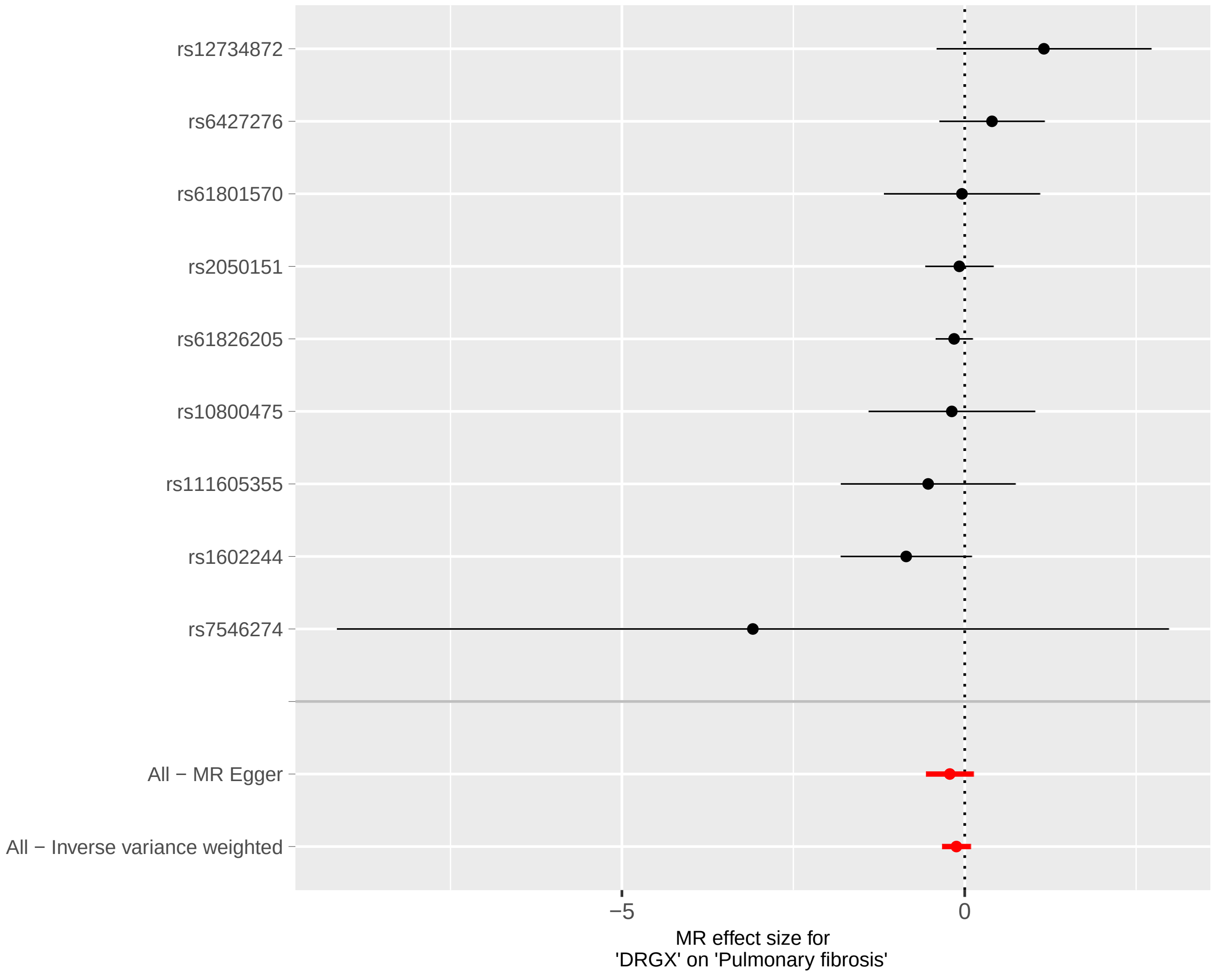

rs12734872
rs6427276
rs61801570
rs2050151
rs61826205
rs10800475
rs111605355
rs1602244
rs7546274
All − MR Egger
All − Inverse variance weighted
−5
0
MR effect size for
'DRGX' on 'Pulmonary fibrosis'

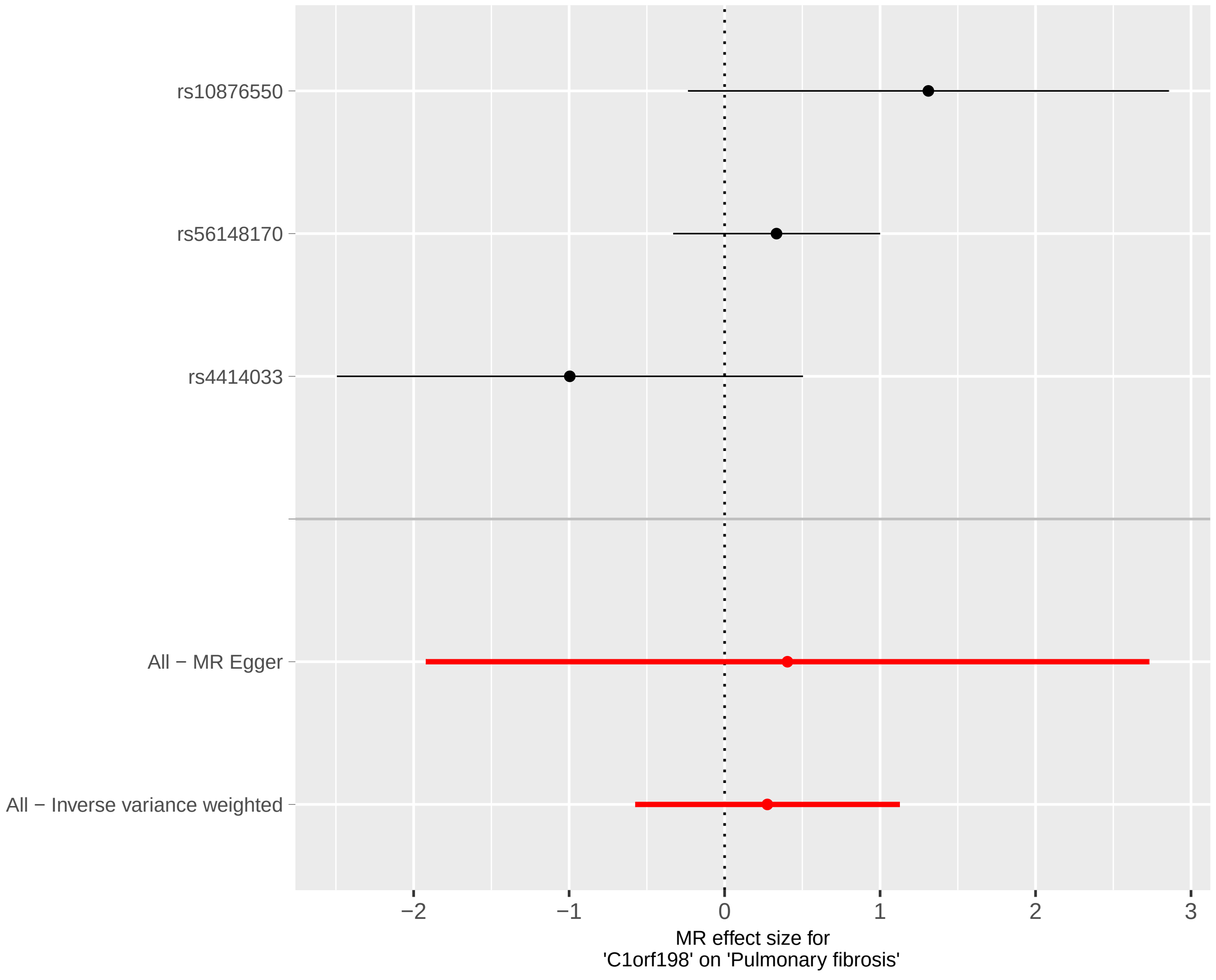
rs10876550
rs56148170
rs4414033
All − MR Egger
All − Inverse variance weighted
−2
−1
0
1
2
3
MR effect size for
'C1orf198' on 'Pulmonary fibrosis'

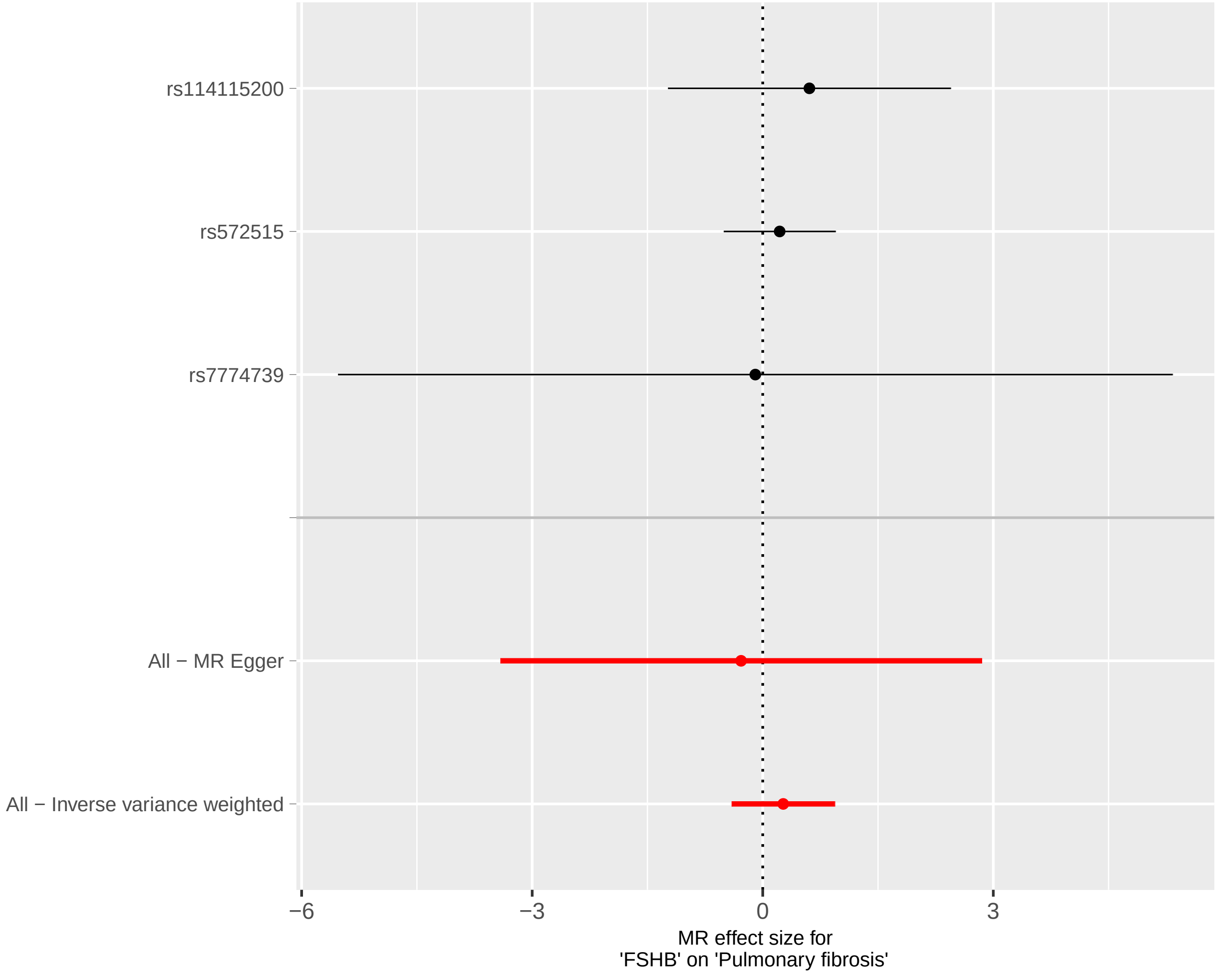

rs114115200
rs572515
rs7774739
All − MR Egger
All − Inverse variance weighted
−6
−3
0
3
MR effect size for
'FSHB' on 'Pulmonary fibrosis'

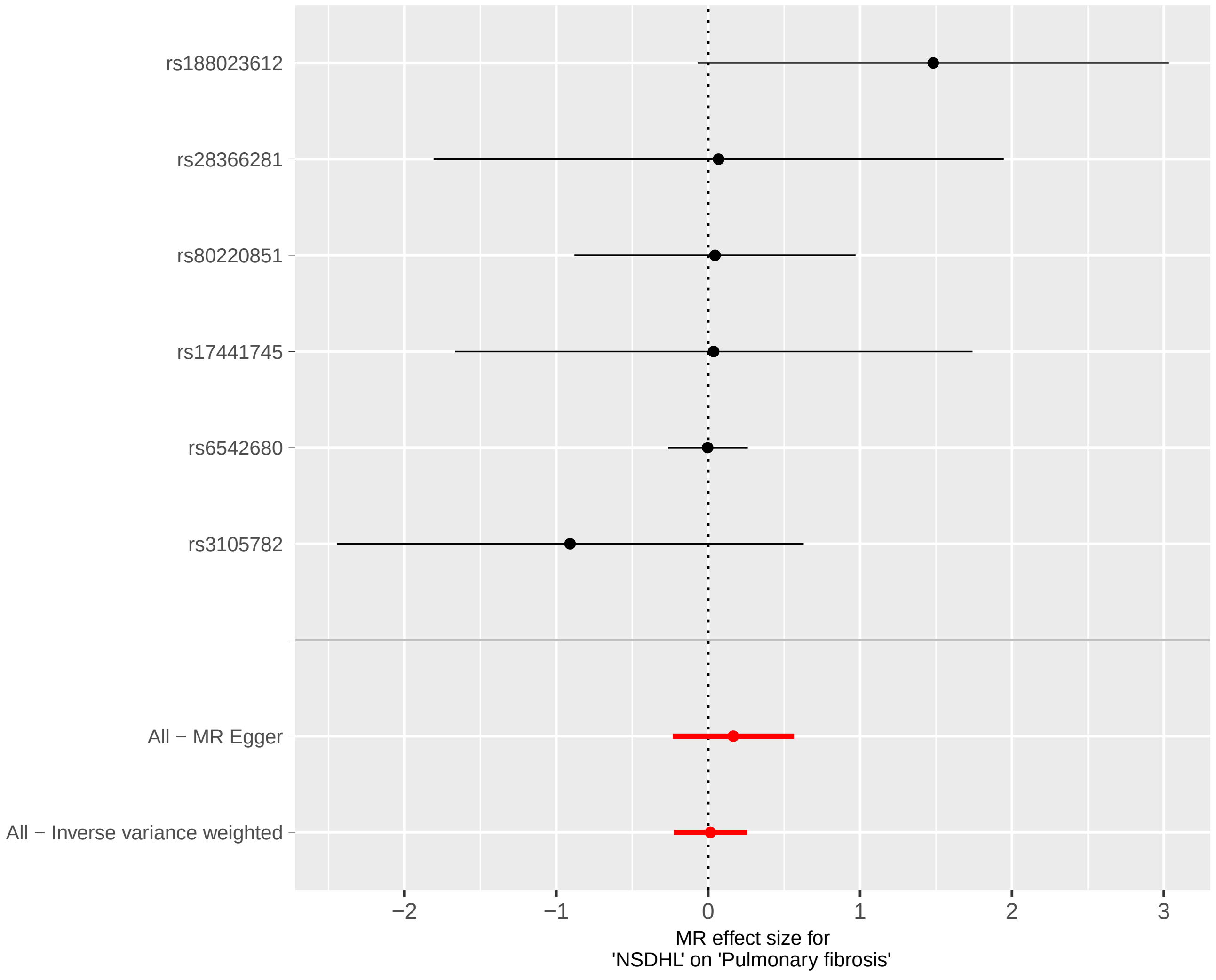
rs188023612
rs28366281
rs80220851
rs17441745
rs6542680
rs3105782
All − MR Egger
All − Inverse variance weighted
−2
−1
0
1
2
3
MR effect size for
'NSDHL' on 'Pulmonary fibrosis'

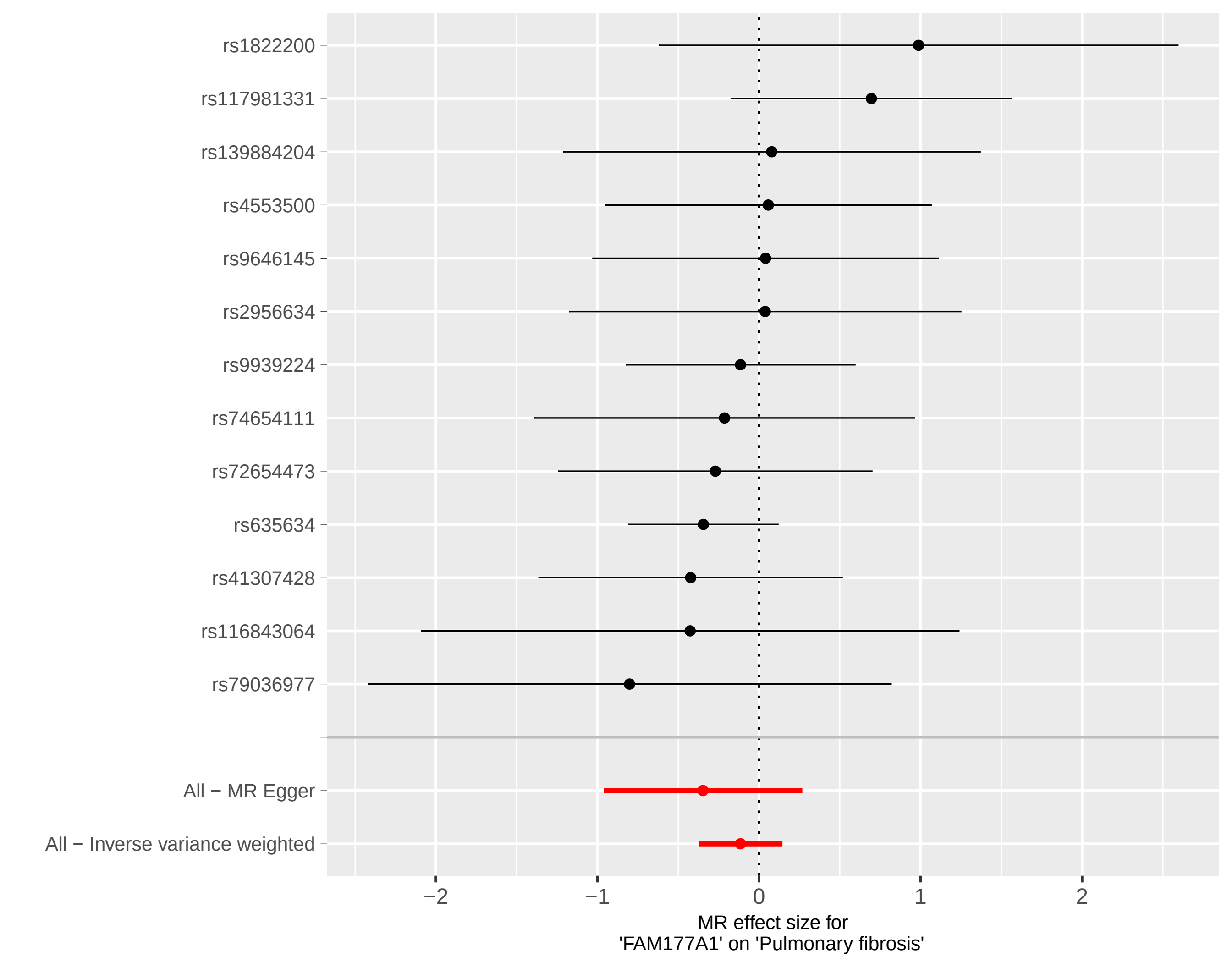

rs1822200
rs117981331
rs139884204
rs4553500
rs9646145
rs2956634
rs9939224
rs74654111
rs72654473
rs635634
rs41307428
rs116843064
rs79036977
All − MR Egger
All − Inverse variance weighted
−2
−1
0
1
2
MR effect size for
'FAM177A1' on 'Pulmonary fibrosis'

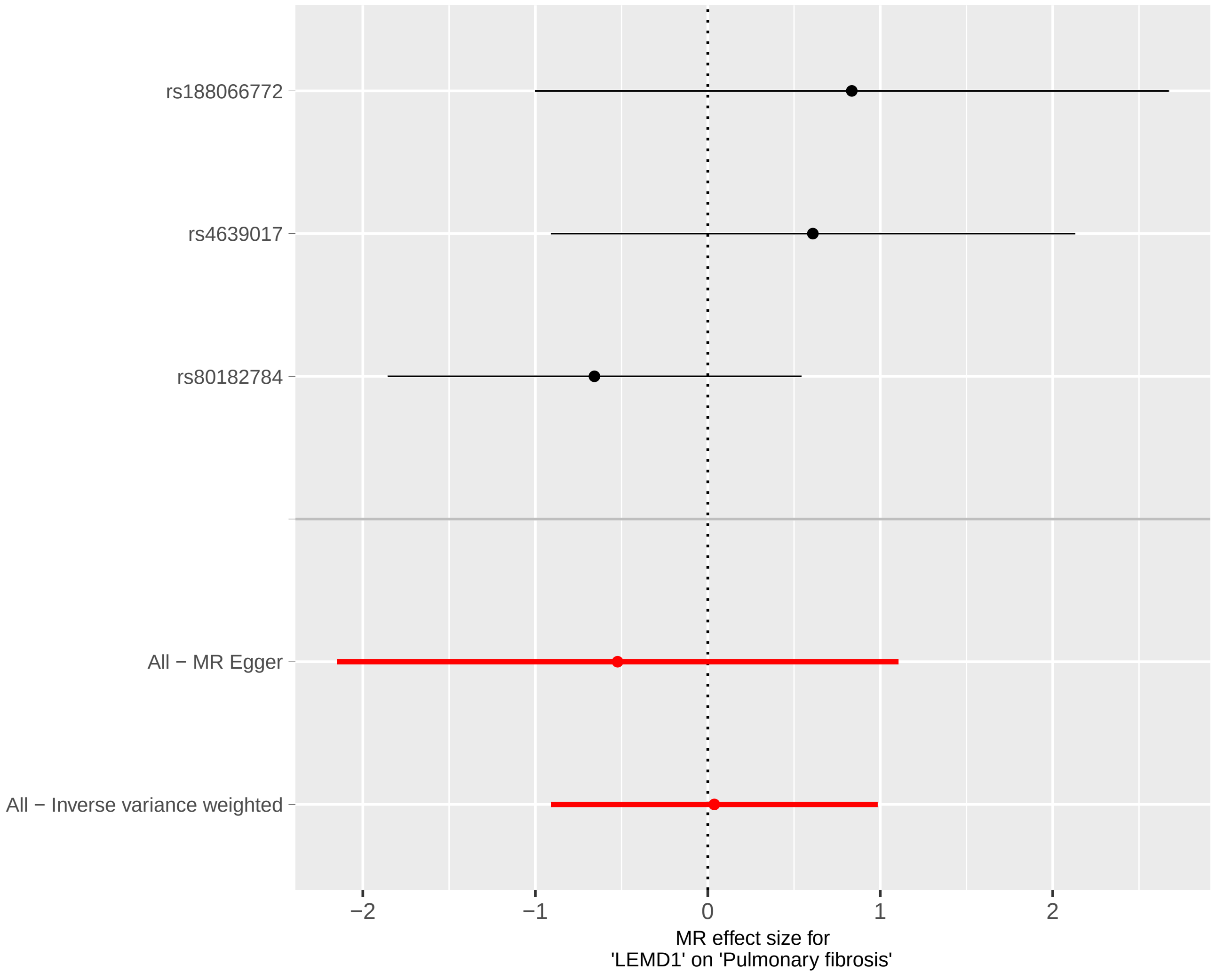
rs188066772
rs4639017
rs80182784
All − MR Egger
All − Inverse variance weighted
−2
−1
0
1
2
MR effect size for
'LEMD1' on 'Pulmonary fibrosis'

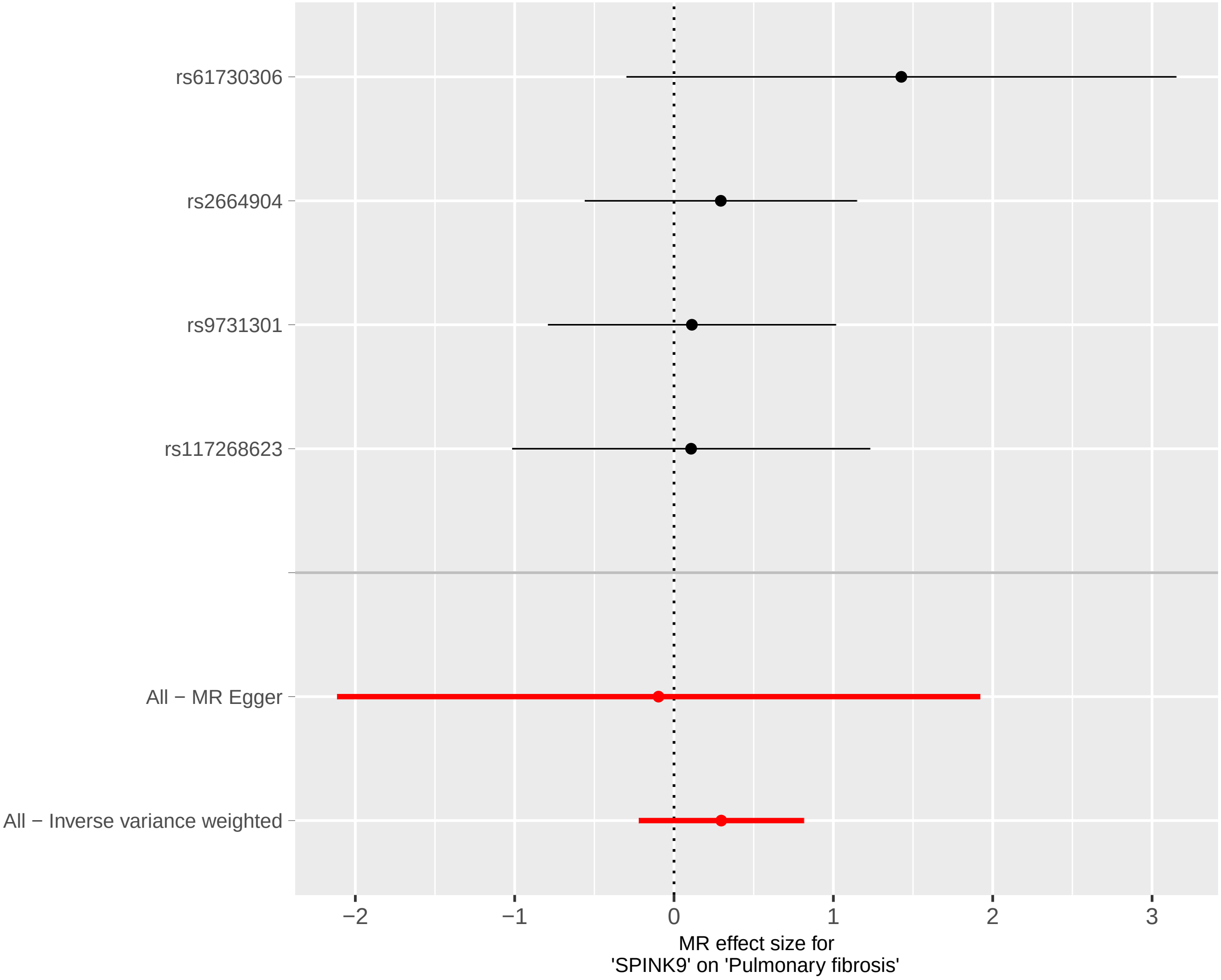
rs61730306
rs2664904
rs9731301
rs117268623
All − MR Egger
All − Inverse variance weighted
−2
−1
0
1
2
3
MR effect size for
'SPINK9' on 'Pulmonary fibrosis'

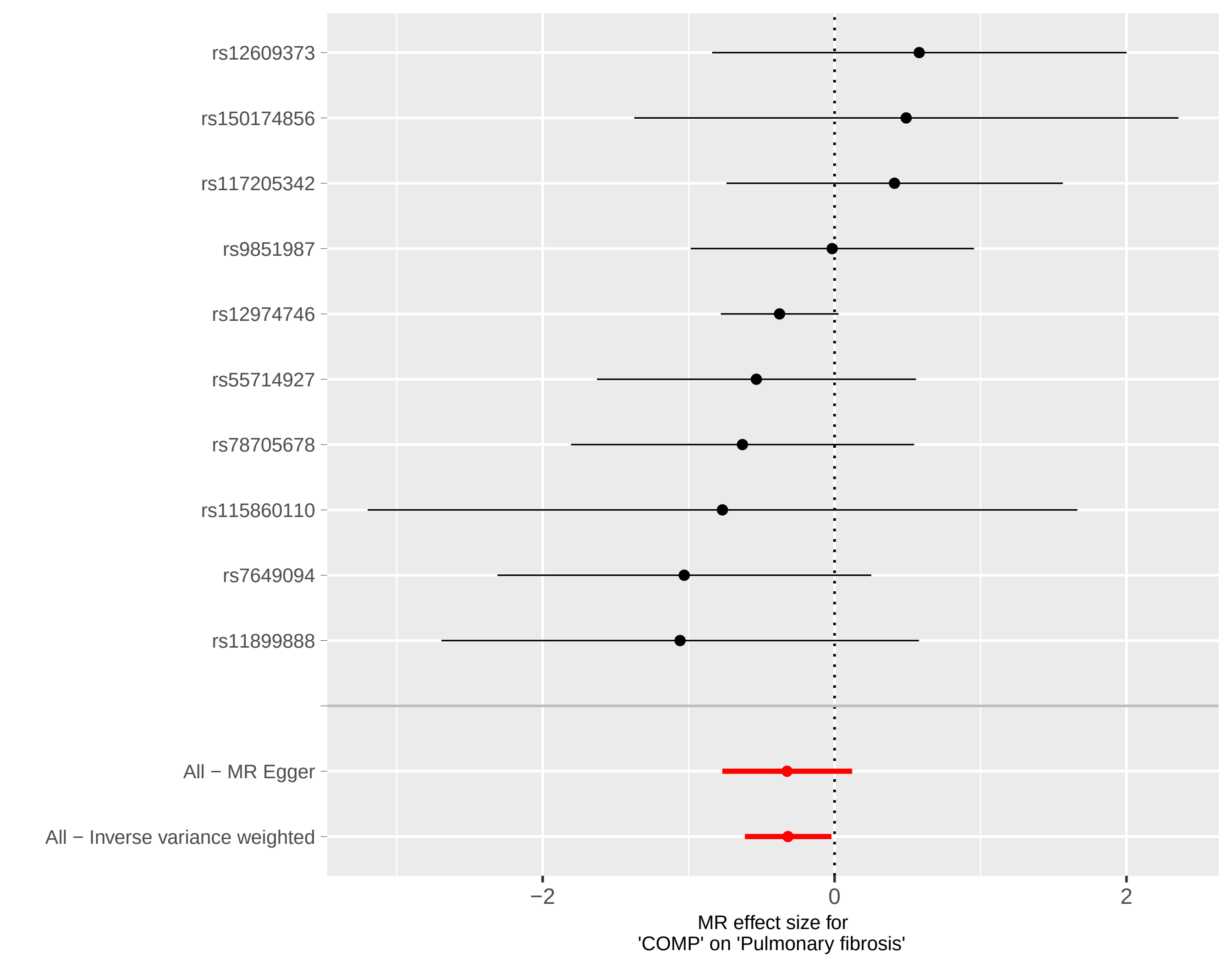
rs12609373
rs150174856
rs117205342
rs9851987
rs12974746
rs55714927
rs78705678
rs115860110
rs7649094
rs11899888
All − MR Egger
All − Inverse variance weighted
−2
0
2
MR effect size for
'COMP' on 'Pulmonary fibrosis'

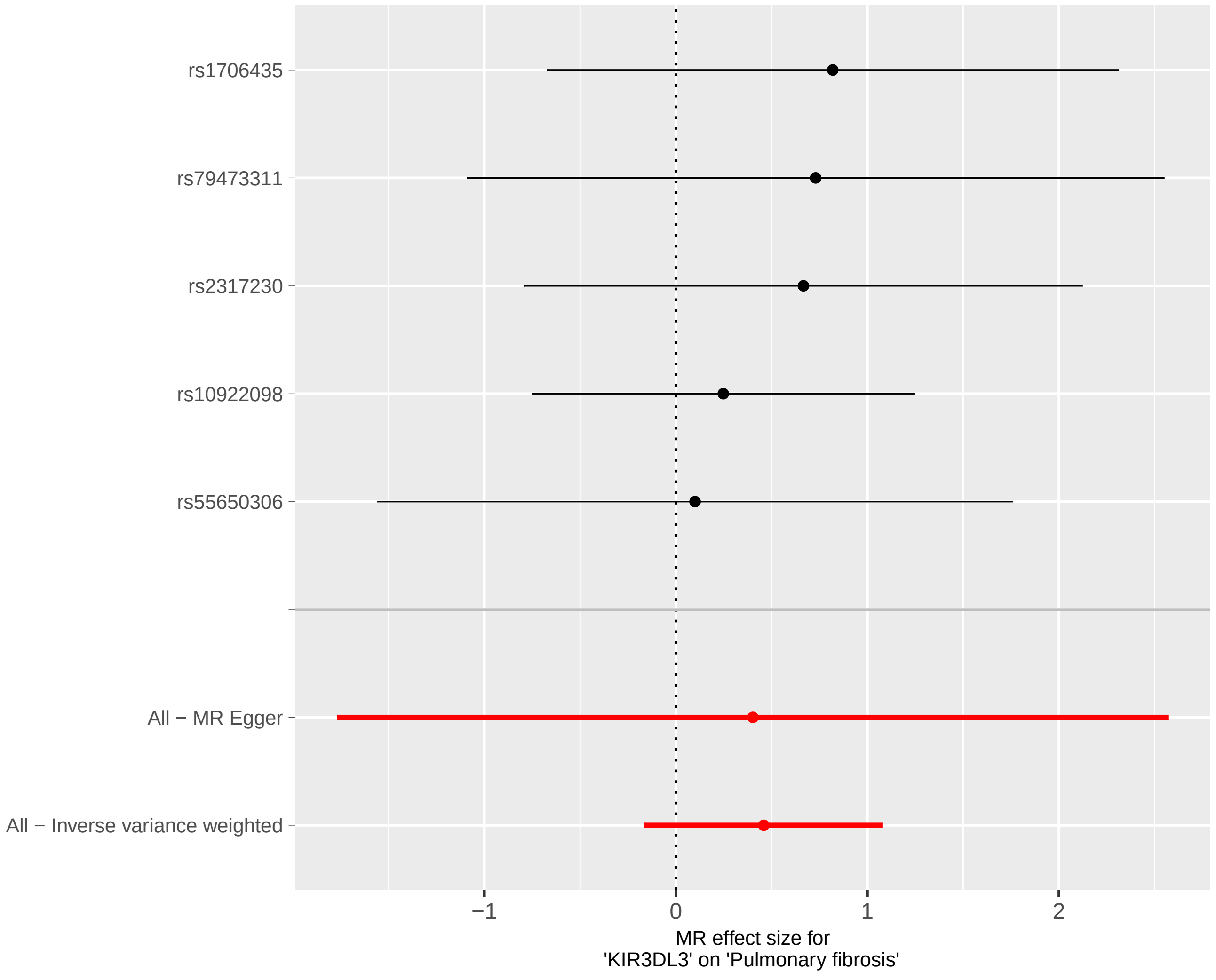
rs1706435
rs79473311
rs2317230
rs10922098
rs55650306
All − MR Egger
All − Inverse variance weighted
−1
0
1
2
MR effect size for
'KIR3DL3' on 'Pulmonary fibrosis'

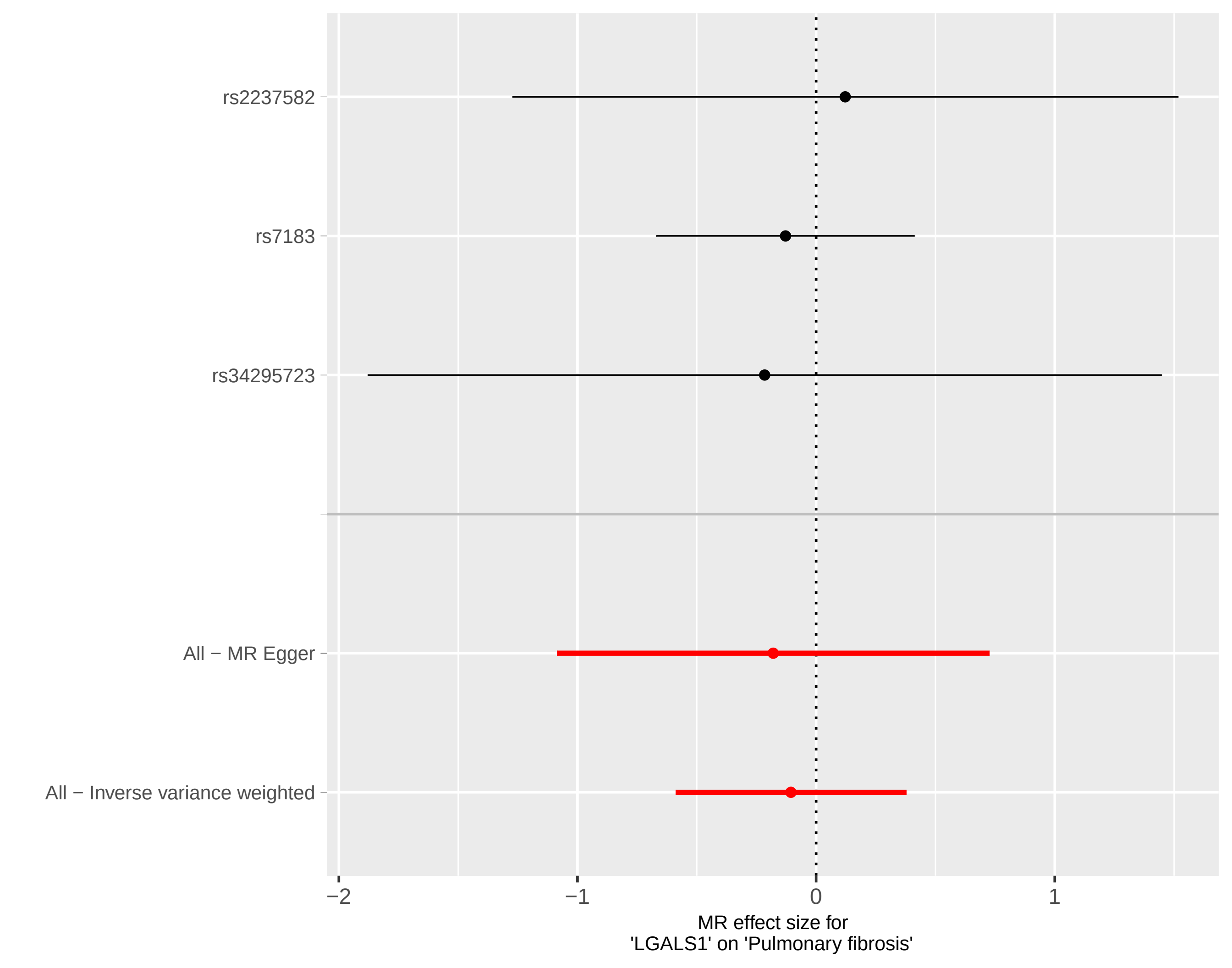
rs2237582
rs7183
rs34295723
All − MR Egger
All − Inverse variance weighted
−2
−1
0
1
MR effect size for
'LGALS1' on 'Pulmonary fibrosis'

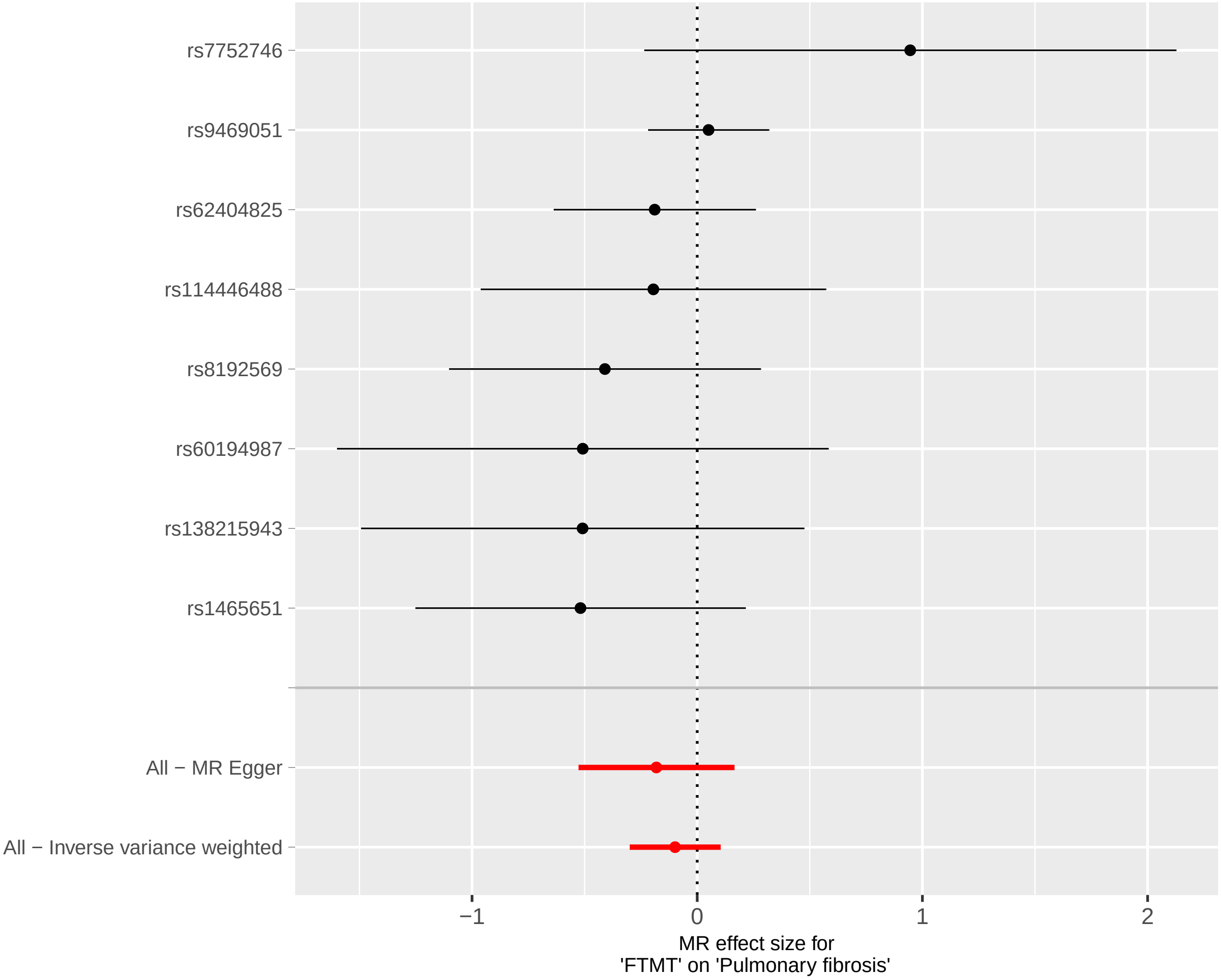
rs7752746
rs9469051
rs62404825
rs114446488
rs8192569
rs60194987
rs138215943
rs1465651
All − MR Egger
All − Inverse variance weighted
−1
0
1
2
MR effect size for
'FTMT' on 'Pulmonary fibrosis'

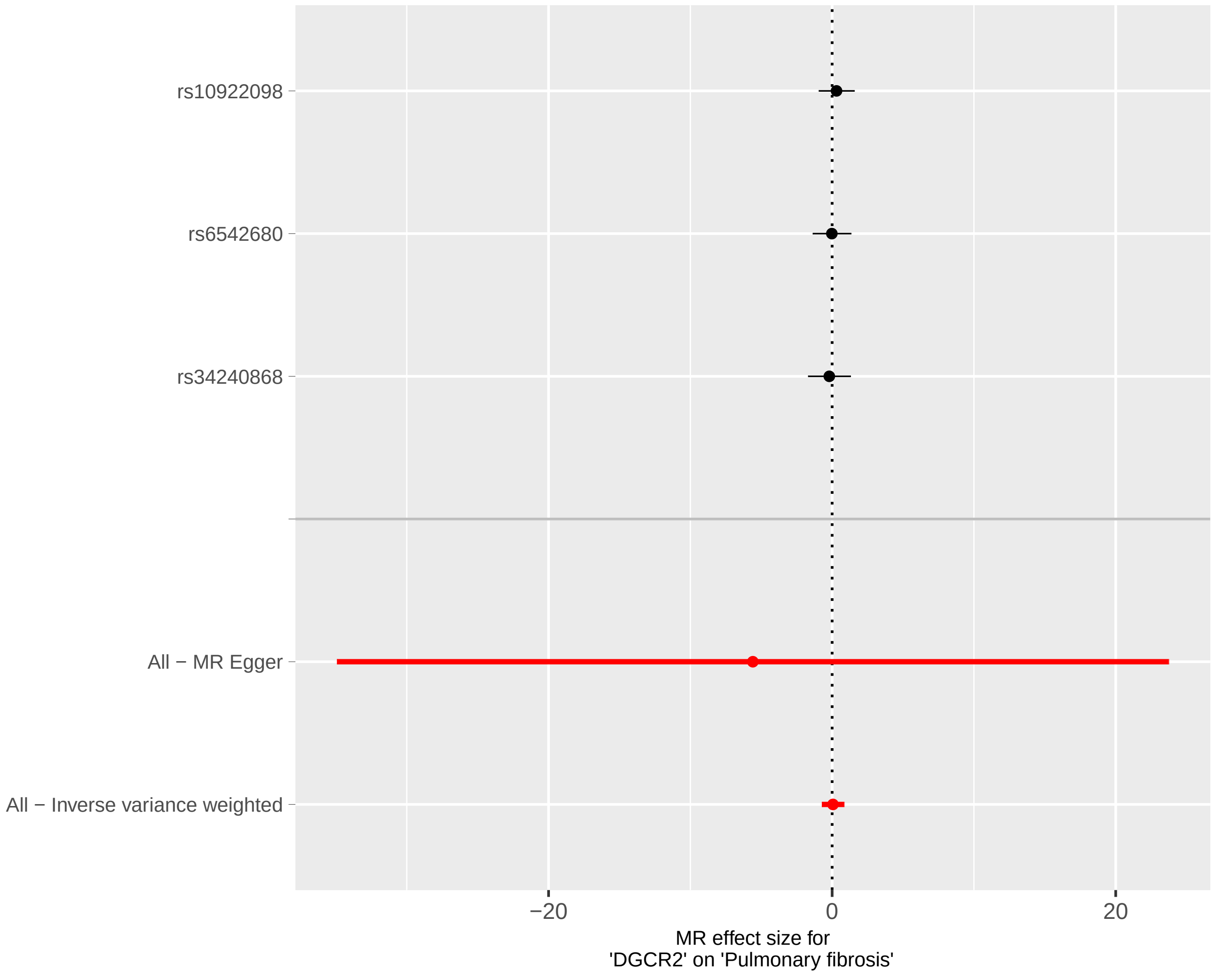

rs10922098
rs6542680
rs34240868
All − MR Egger
All − Inverse variance weighted
−20
0
20
MR effect size for
'DGCR2' on 'Pulmonary fibrosis'

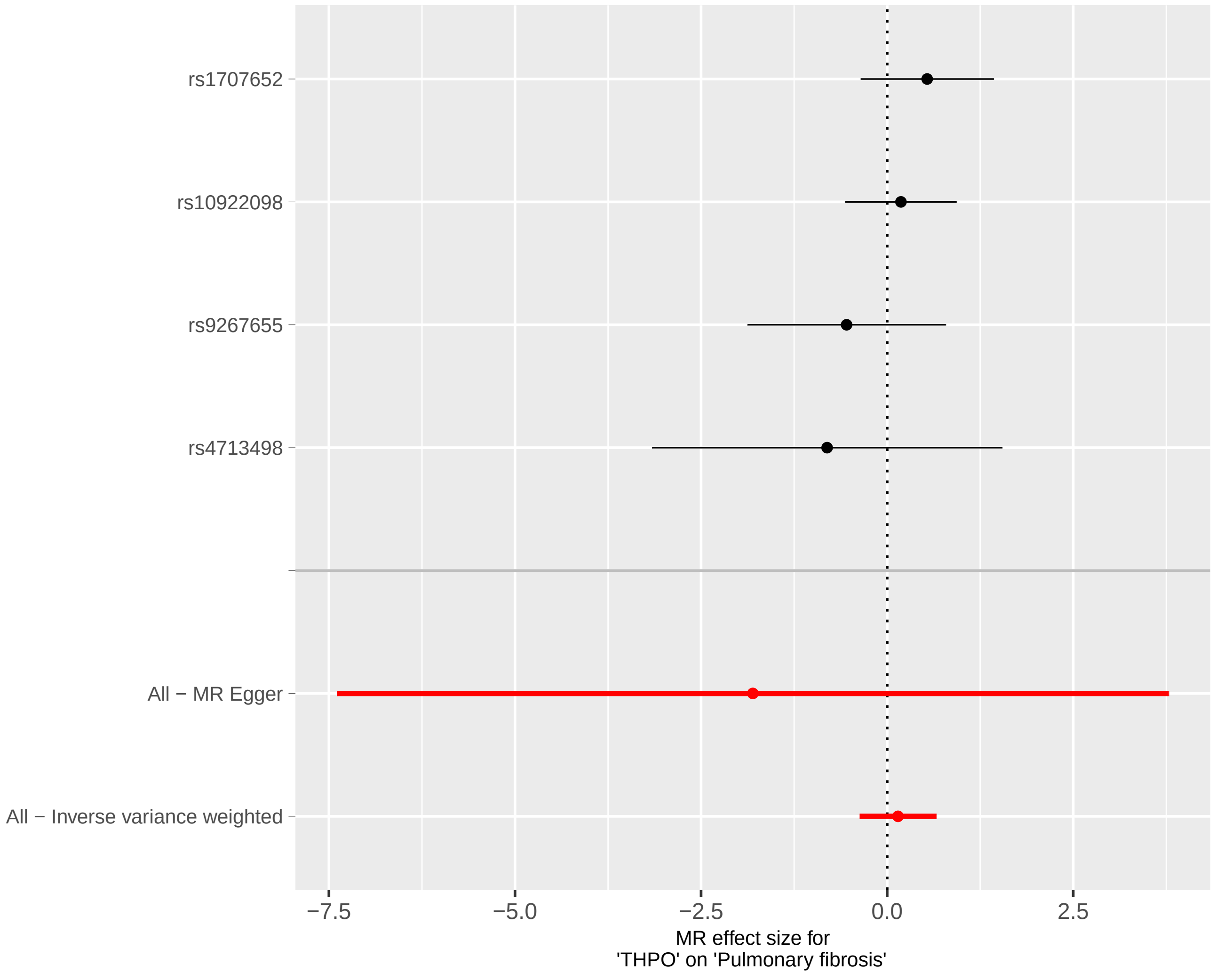
rs1707652
rs10922098
rs9267655
rs4713498
All − MR Egger
All − Inverse variance weighted
−7.5
−5.0
−2.5
0.0
2.5
MR effect size for
'THPO' on 'Pulmonary fibrosis'

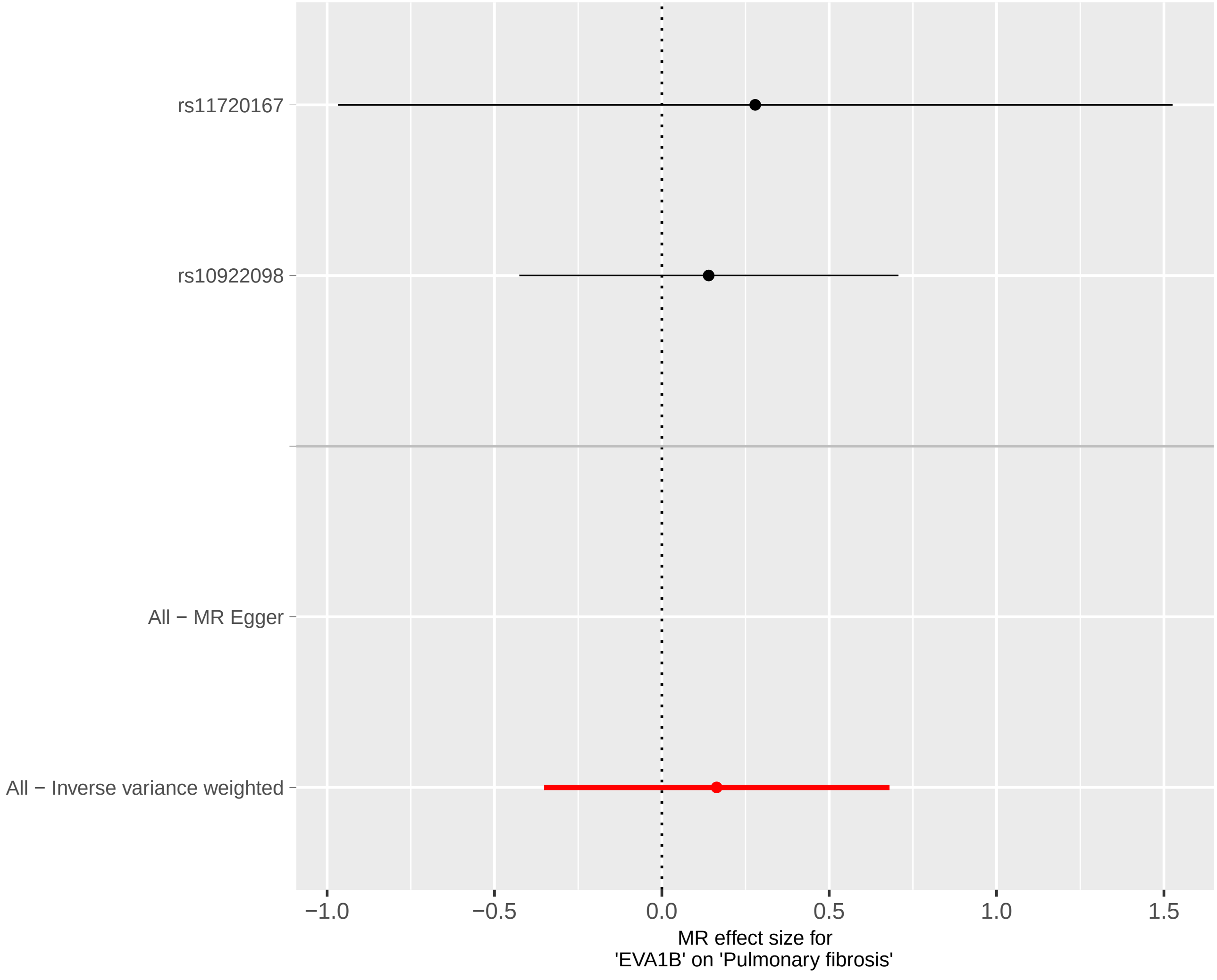
rs11720167
rs10922098
All − MR Egger
All − Inverse variance weighted
−1.0
−0.5
0.0
0.5
1.0
1.5
MR effect size for
'EVA1B' on 'Pulmonary fibrosis'

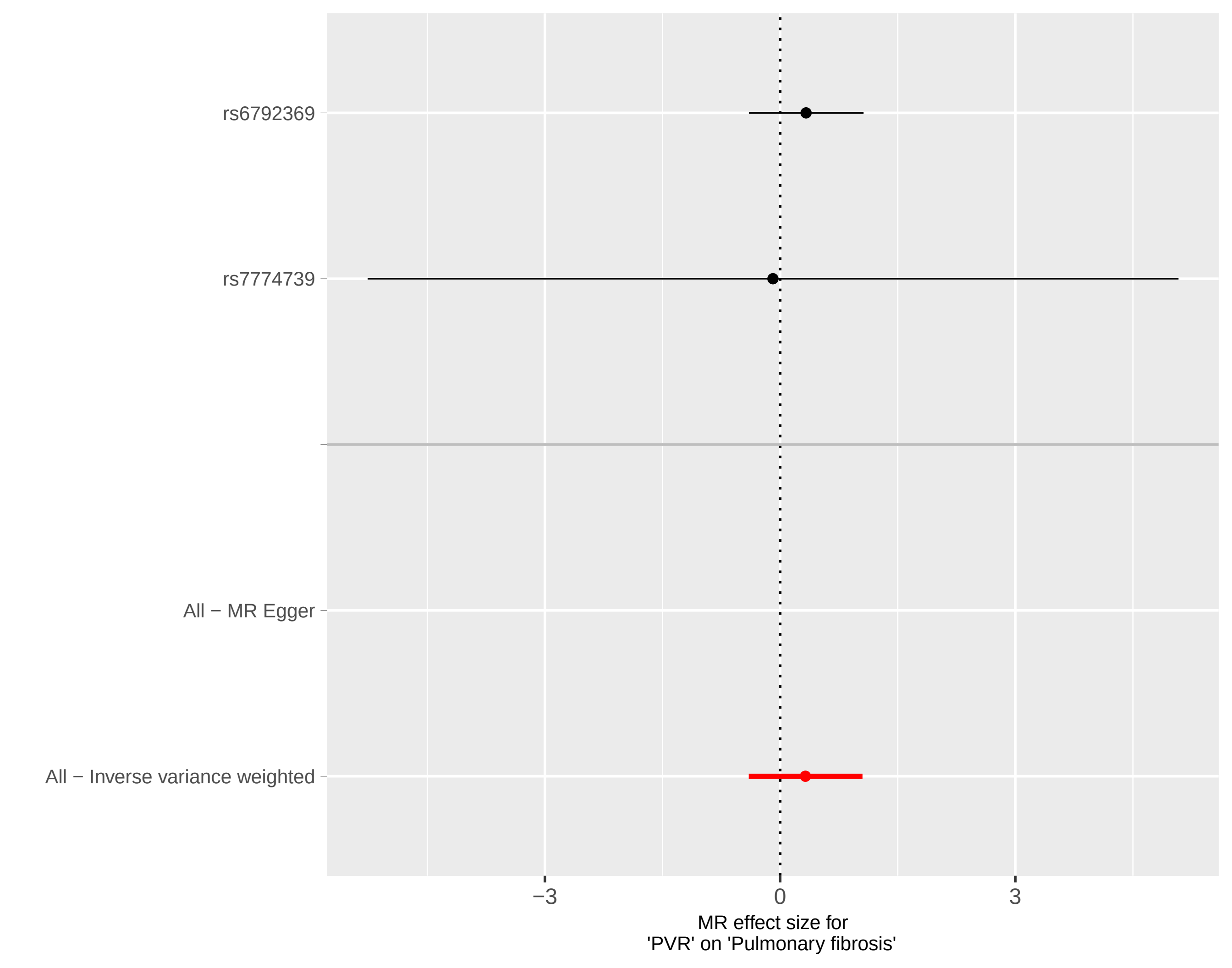
rs6792369
rs7774739
All − MR Egger
All − Inverse variance weighted
−3
0
3
MR effect size for
'PVR' on 'Pulmonary fibrosis'

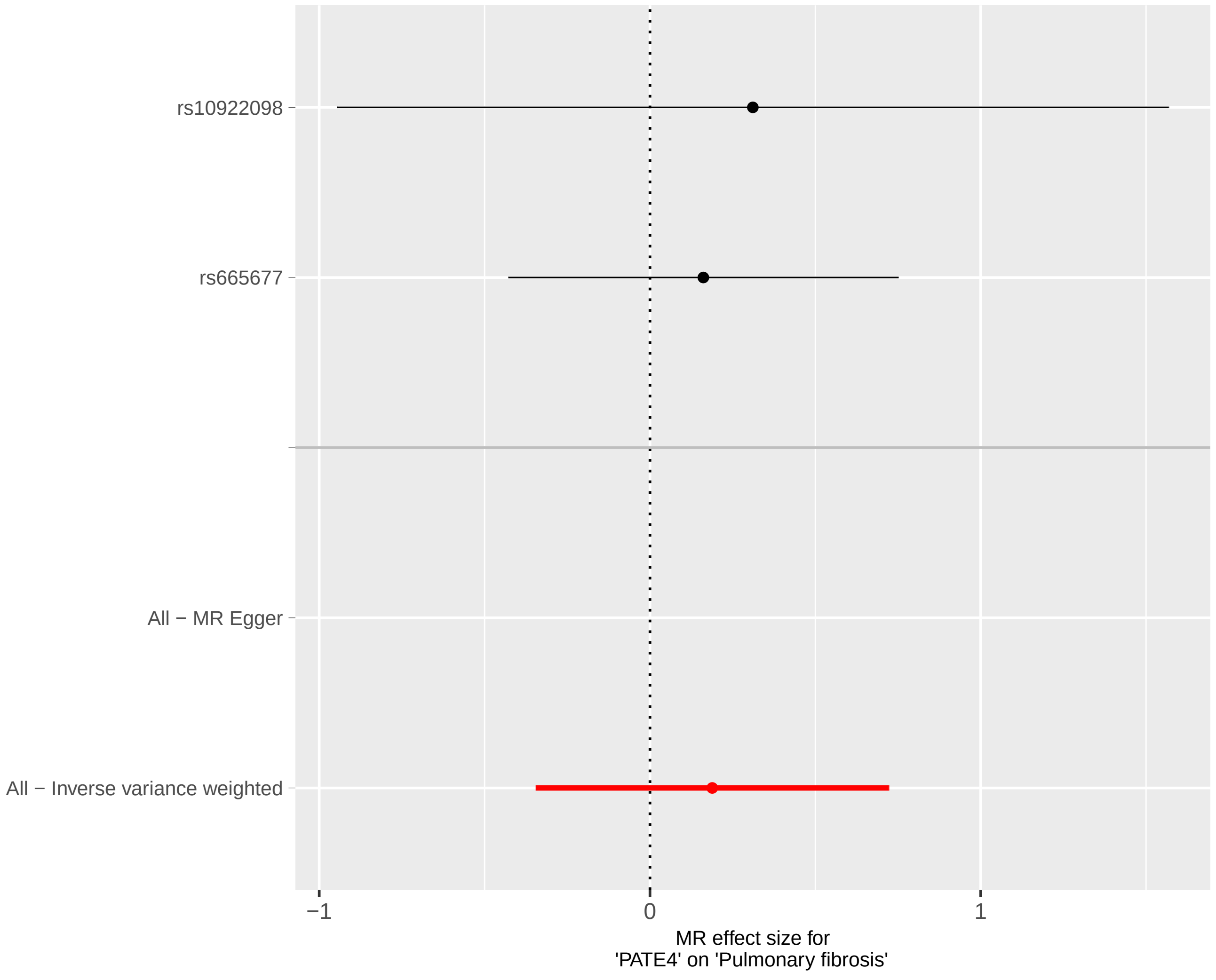
rs10922098
rs665677
All − MR Egger
All − Inverse variance weighted
−1
0
1
MR effect size for
'PATE4' on 'Pulmonary fibrosis'

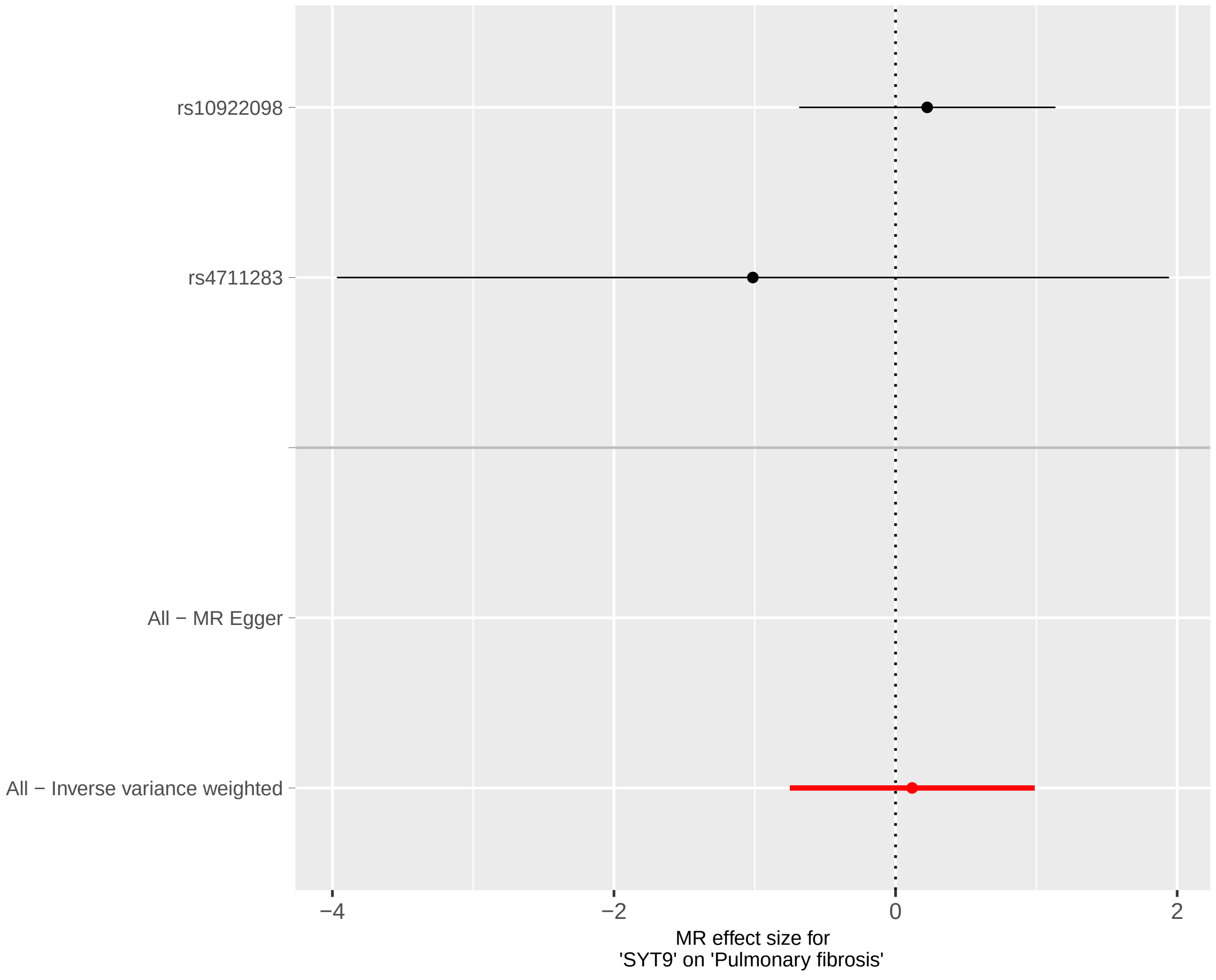

rs10922098
rs4711283
All − MR Egger
All − Inverse variance weighted
−4
−2
0
2
MR effect size for
'SYT9' on 'Pulmonary fibrosis'

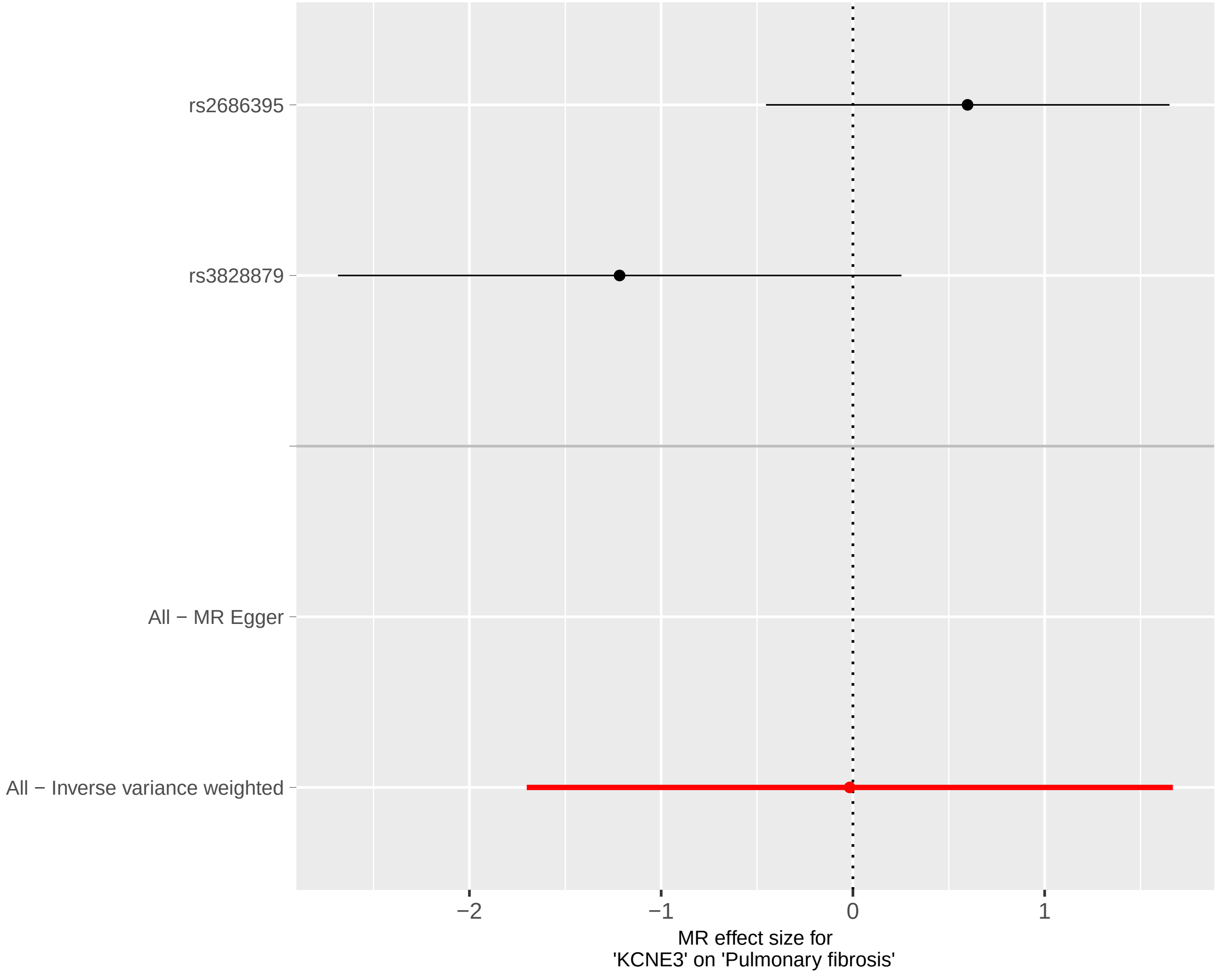
rs2686395
rs3828879
All − MR Egger
All − Inverse variance weighted
−2
−1
0
1
MR effect size for
'KCNE3' on 'Pulmonary fibrosis'

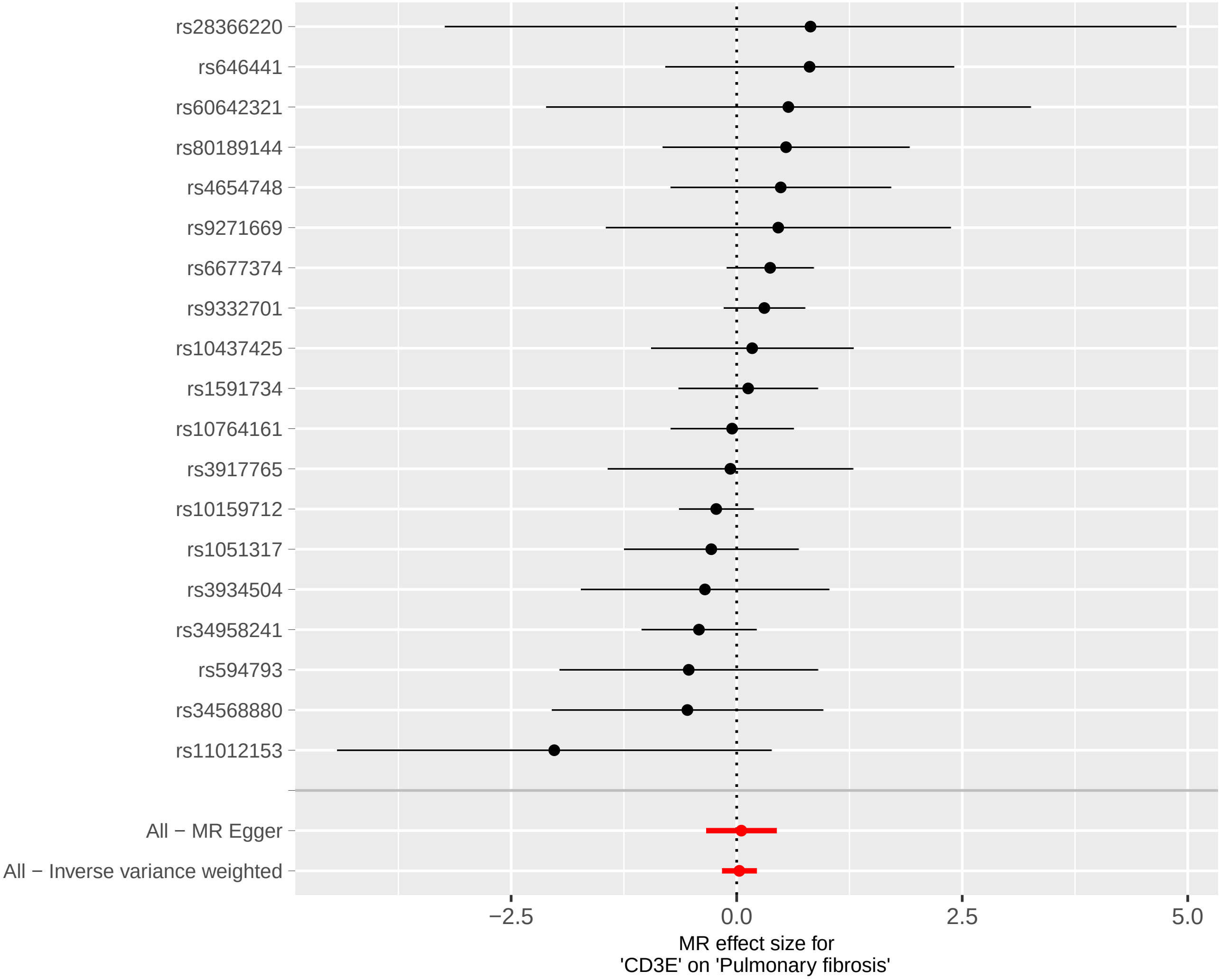
rs28366220
rs646441
rs60642321
rs80189144
rs4654748
rs9271669
rs6677374
rs9332701
rs10437425
rs1591734
rs10764161
rs3917765
rs10159712
rs1051317
rs3934504
rs34958241
rs594793
rs34568880
rs11012153
All − MR Egger
All − Inverse variance weighted
−2.5
0.0
2.5
5.0
MR effect size for
'CD3E' on 'Pulmonary fibrosis'

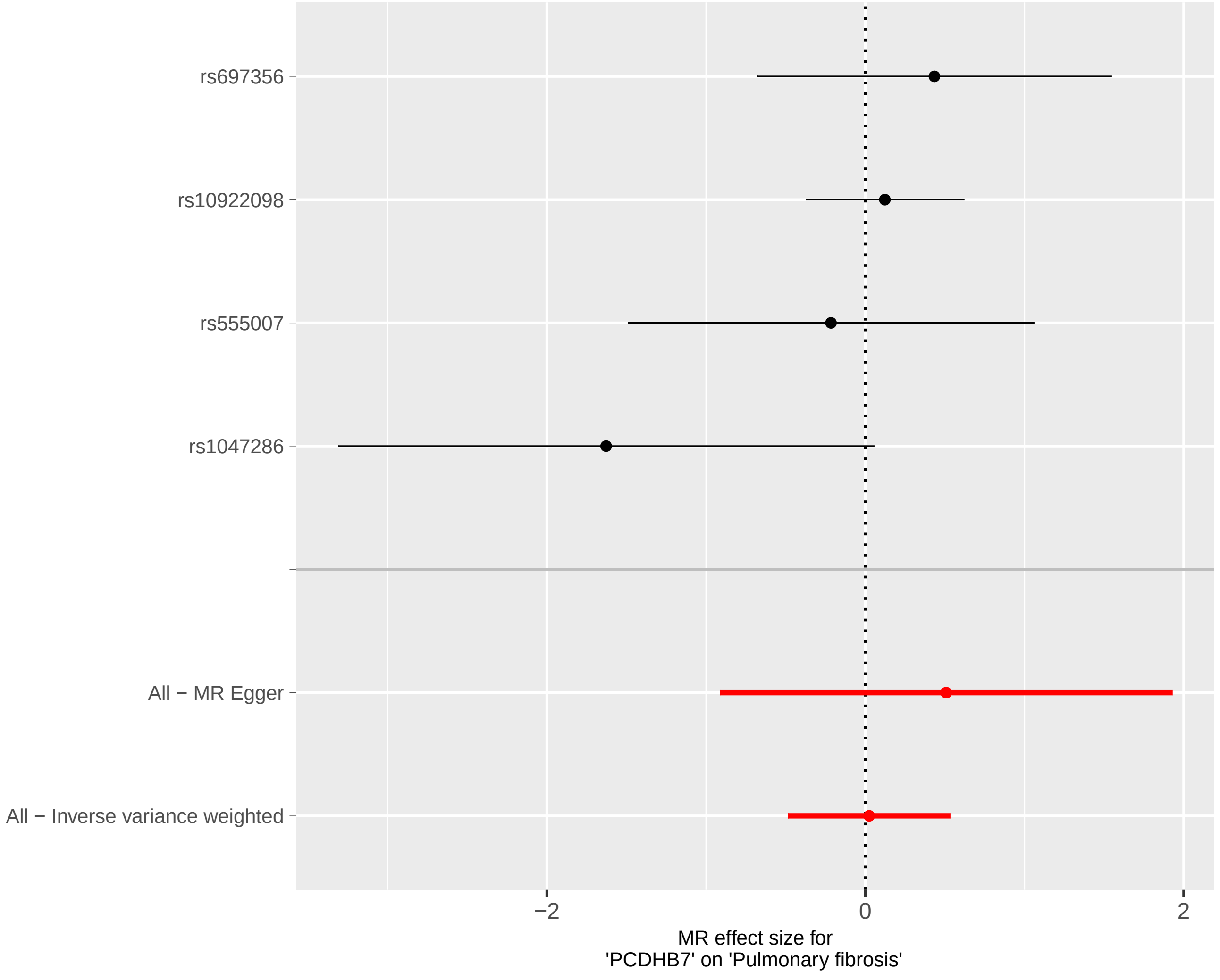

rs697356
rs10922098
rs555007
rs1047286
All − MR Egger
All − Inverse variance weighted
−2
0
2
MR effect size for
'PCDHB7' on 'Pulmonary fibrosis'

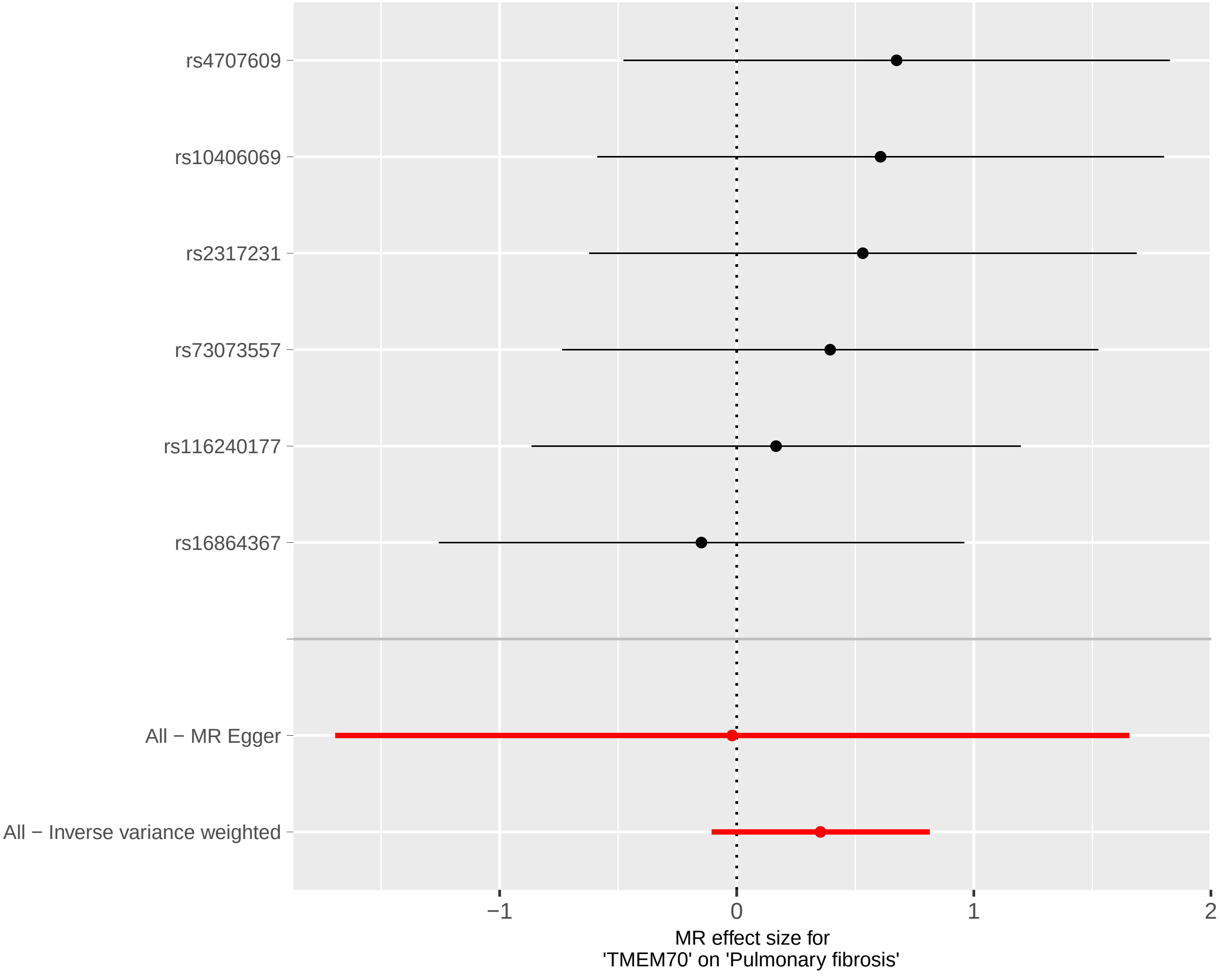

rs4707609
rs10406069
rs2317231
rs73073557
rs116240177
rs16864367
All − MR Egger
All − Inverse variance weighted
−1
0
1
2
MR effect size for
'TMEM70' on 'Pulmonary fibrosis'

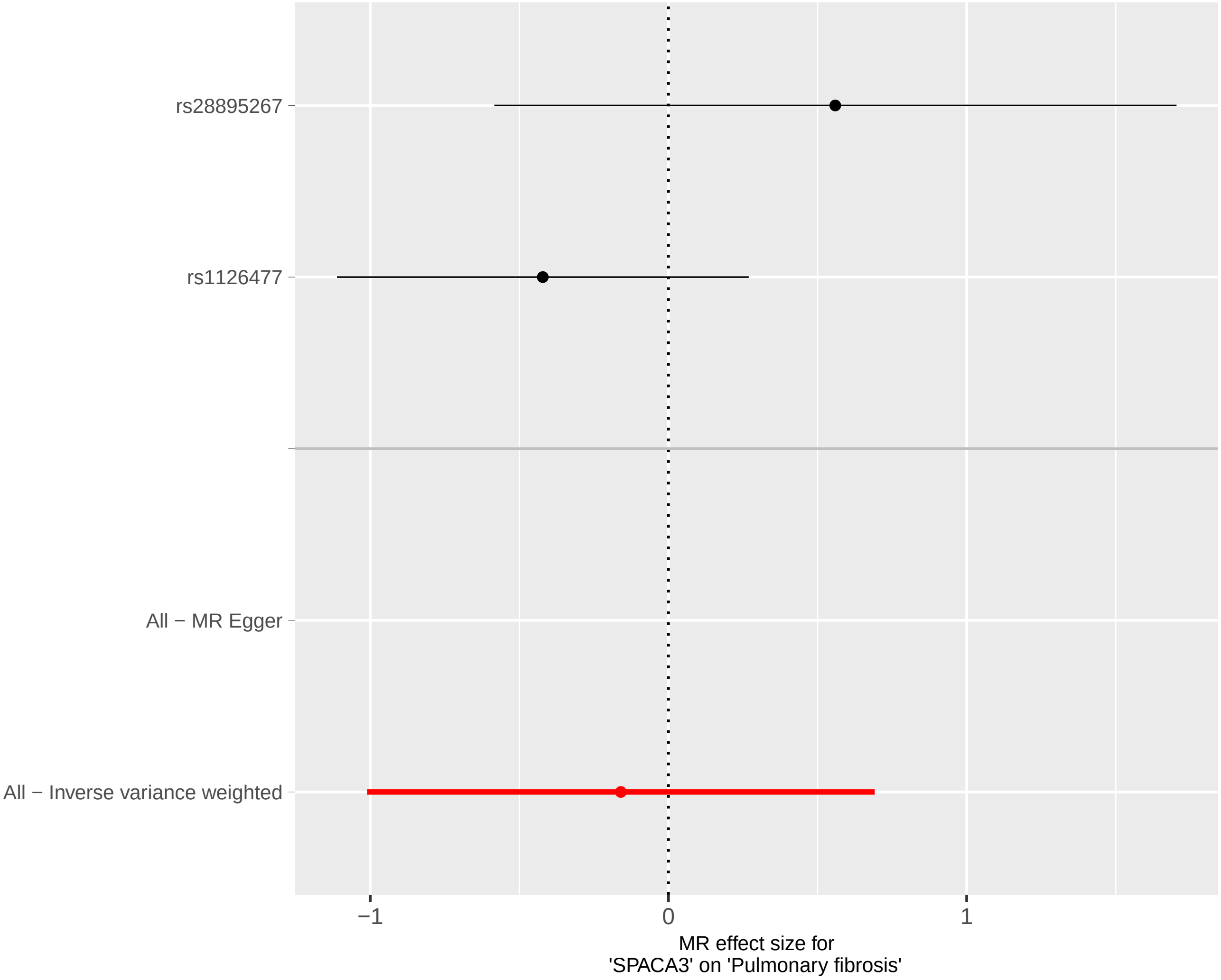
rs28895267
rs1126477
All − MR Egger
All − Inverse variance weighted
−1
0
1
MR effect size for
'SPACA3' on 'Pulmonary fibrosis'

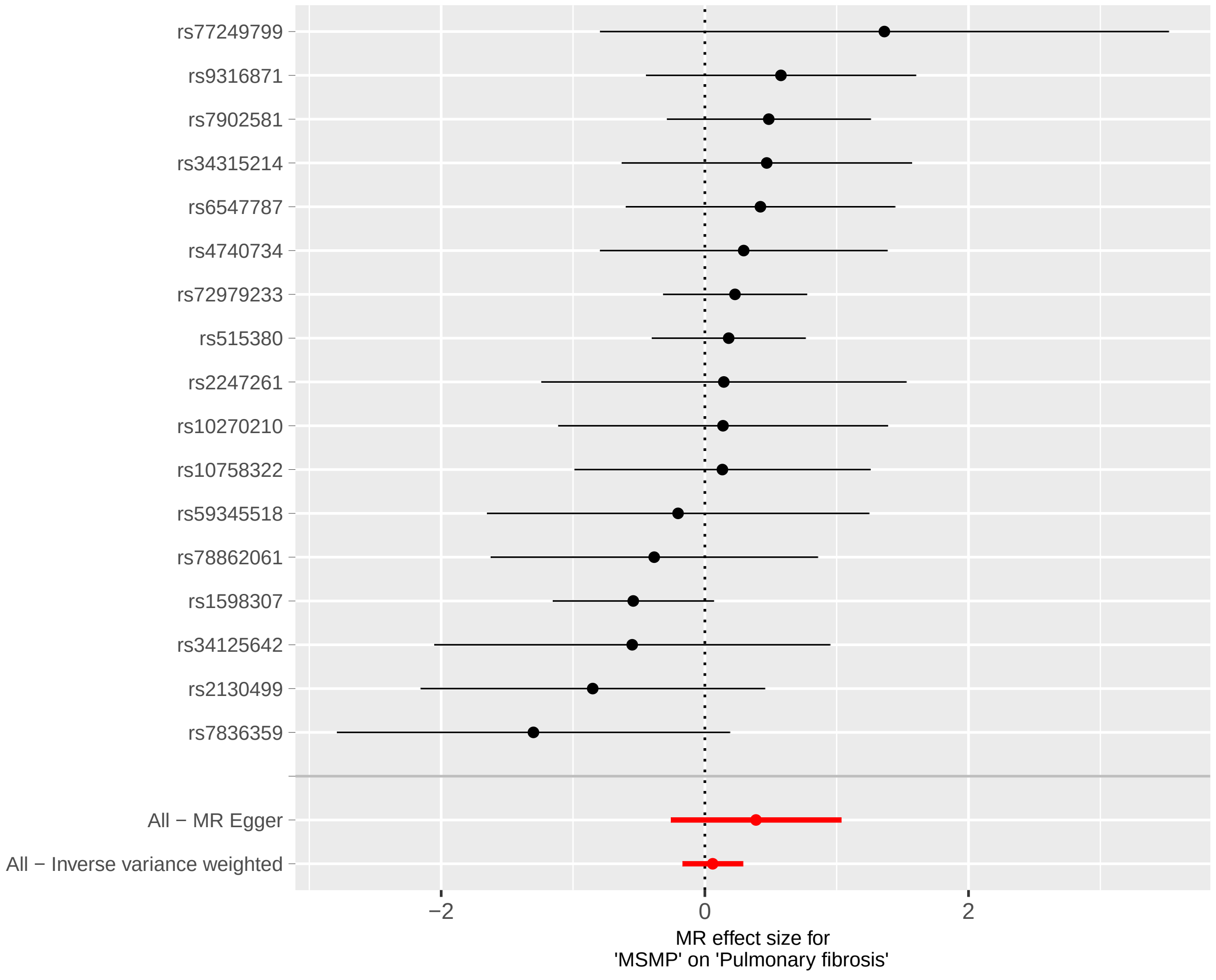

rs77249799
rs9316871
rs7902581
rs34315214
rs6547787
rs4740734
rs72979233
rs515380
rs2247261
rs10270210
rs10758322
rs59345518
rs78862061
rs1598307
rs34125642
rs2130499
rs7836359
All − MR Egger
All − Inverse variance weighted
−2
0
2
MR effect size for
'MSMP' on 'Pulmonary fibrosis'

Insufficient number of SNPs

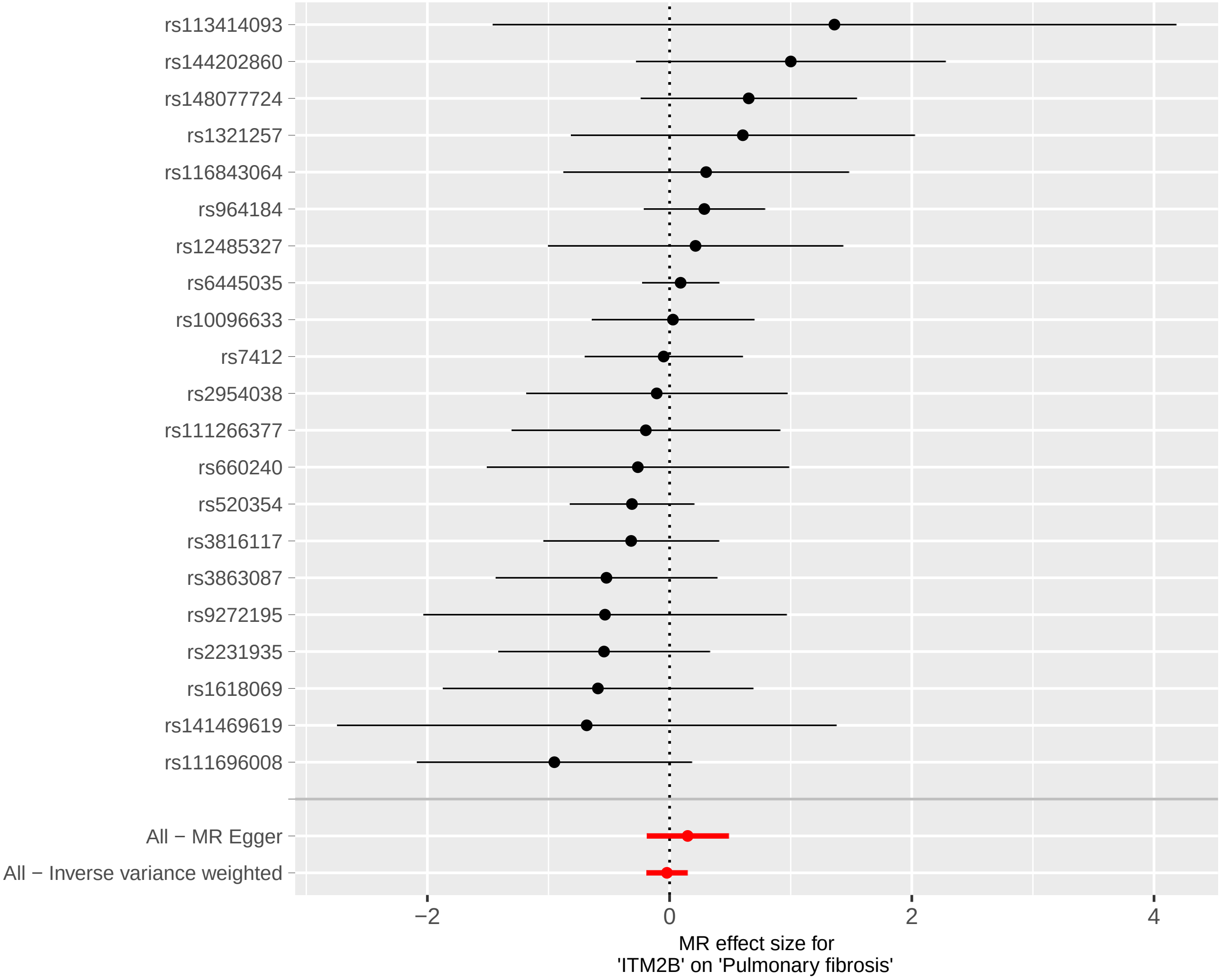
rs113414093
rs144202860
rs148077724
rs1321257
rs116843064
rs964184
rs12485327
rs6445035
rs10096633
rs7412
rs2954038
rs111266377
rs660240
rs520354
rs3816117
rs3863087
rs9272195
rs2231935
rs1618069
rs141469619
rs111696008
All − MR Egger
All − Inverse variance weighted
−2
0
2
4
MR effect size for
'ITM2B' on 'Pulmonary fibrosis'

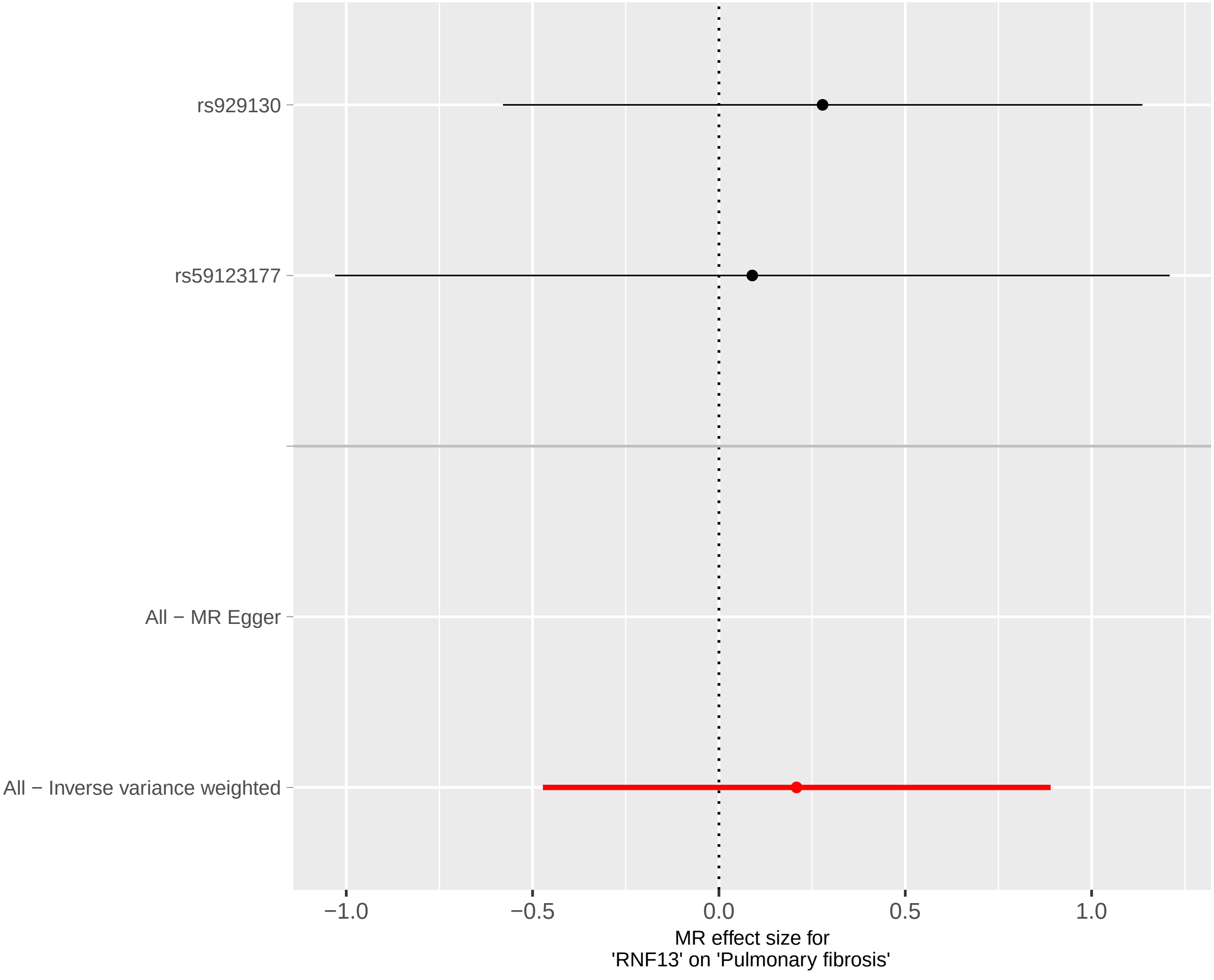

rs929130
rs59123177
All − MR Egger
All − Inverse variance weighted
−1.0
−0.5
0.0
0.5
1.0
MR effect size for
'RNF13' on 'Pulmonary fibrosis'

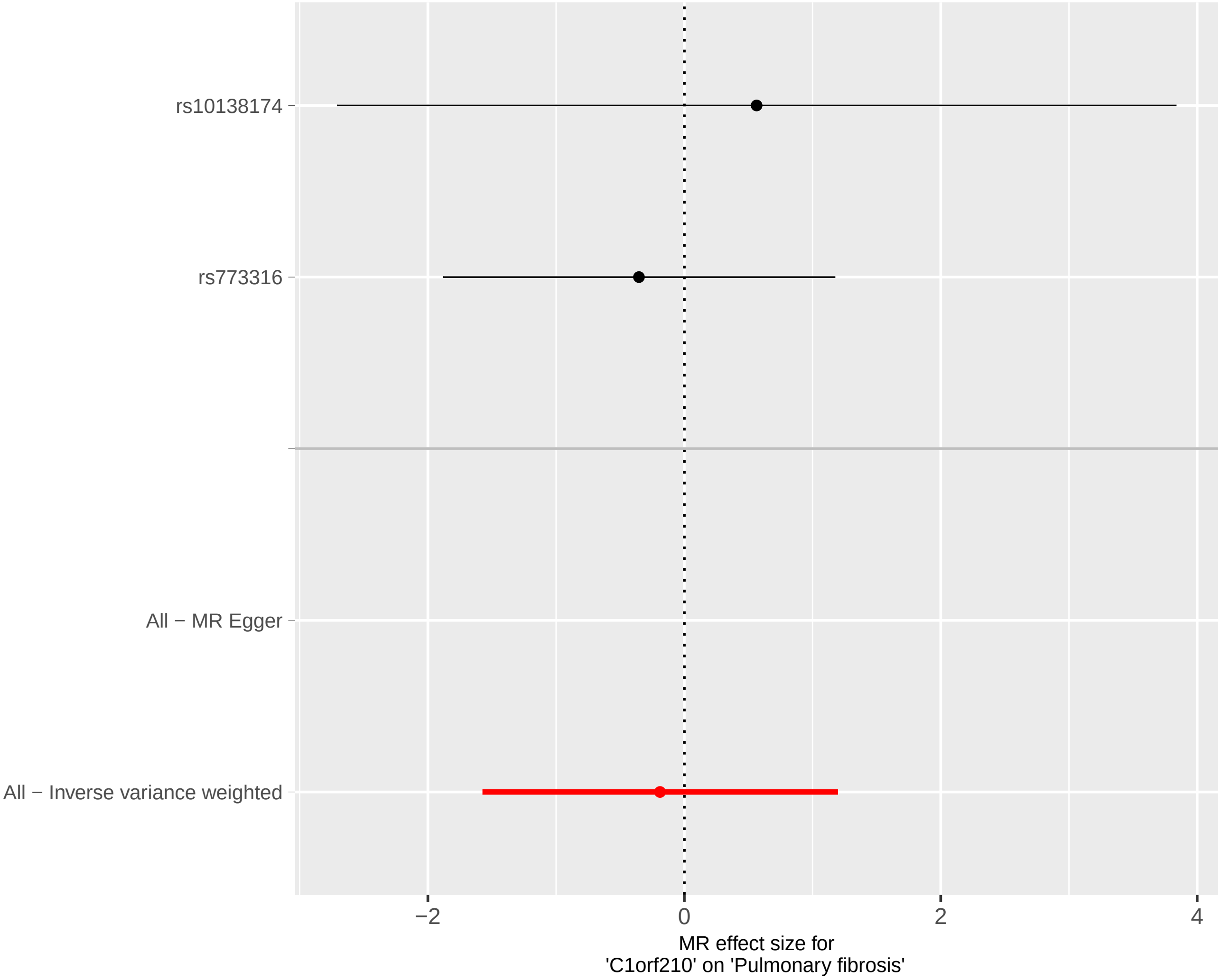
rs10138174
rs773316
All − MR Egger
All − Inverse variance weighted
−2
0
2
4
MR effect size for
'C1orf210' on 'Pulmonary fibrosis'

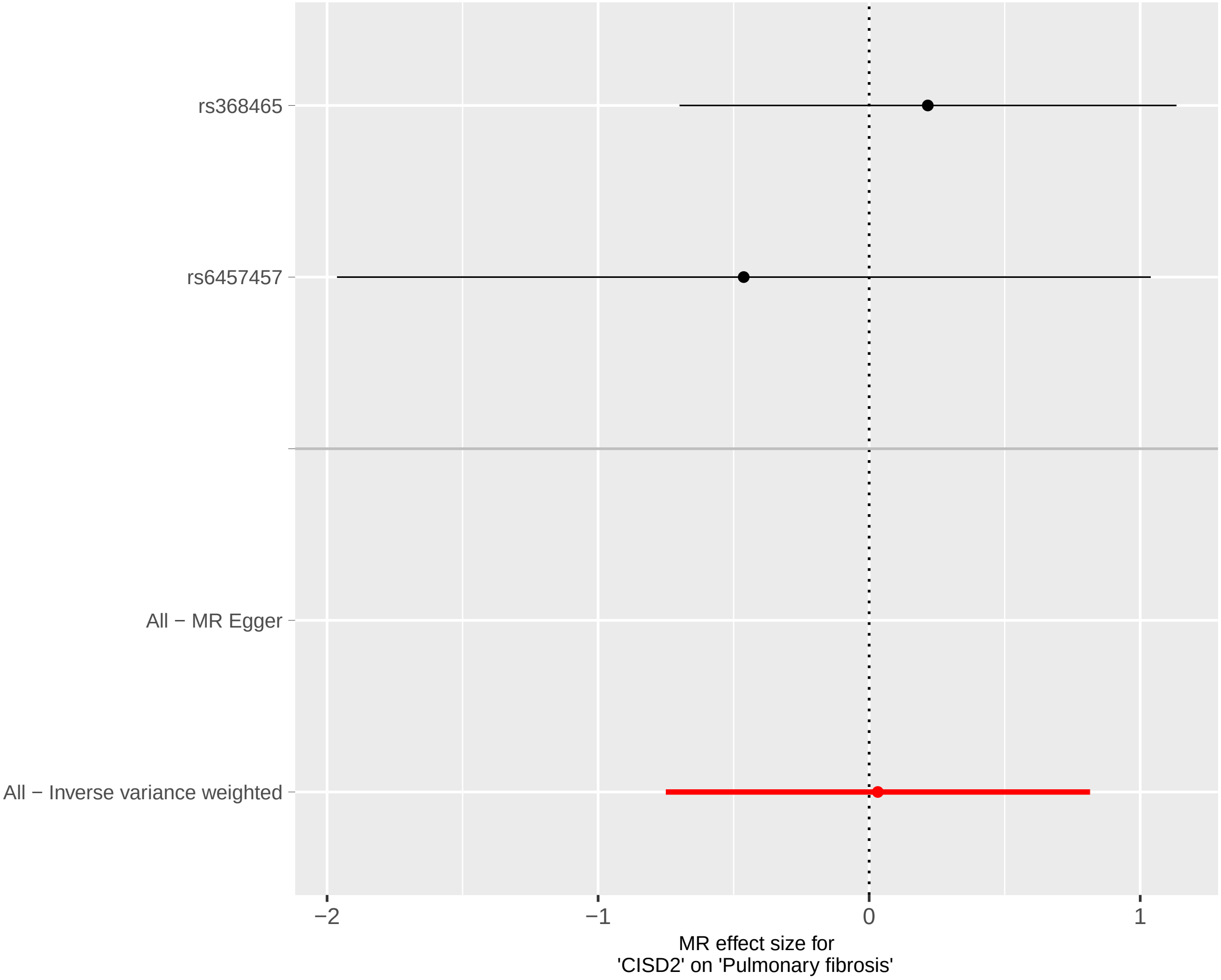

rs368465
rs6457457
All − MR Egger
All − Inverse variance weighted
−2
−1
0
1
MR effect size for
'CISD2' on 'Pulmonary fibrosis'

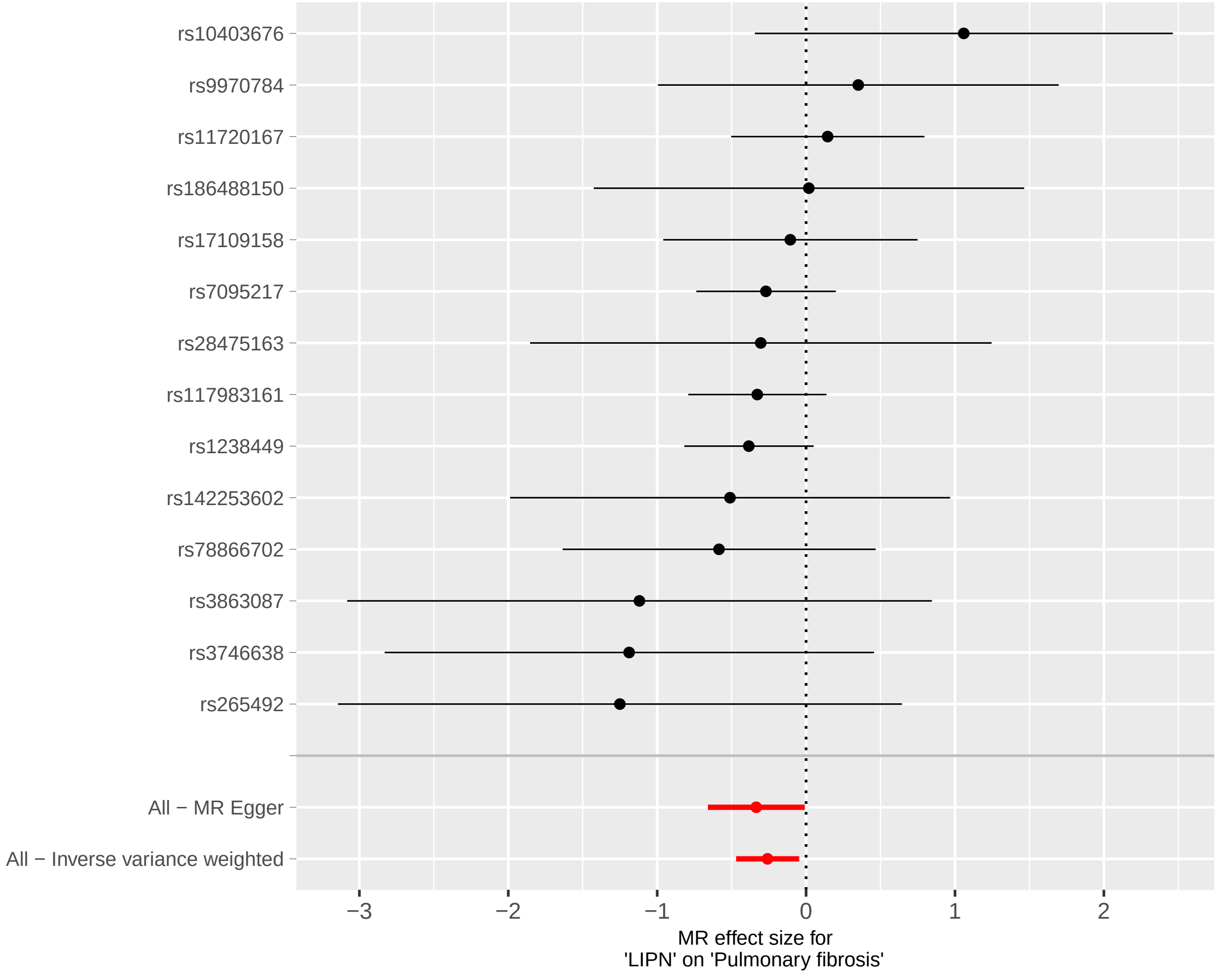
rs10403676
rs9970784
rs11720167
rs186488150
rs17109158
rs7095217
rs28475163
rs117983161
rs1238449
rs142253602
rs78866702
rs3863087
rs3746638
rs265492
All − MR Egger
All − Inverse variance weighted
−3
−2
−1
0
1
2
MR effect size for
'LIPN' on 'Pulmonary fibrosis'

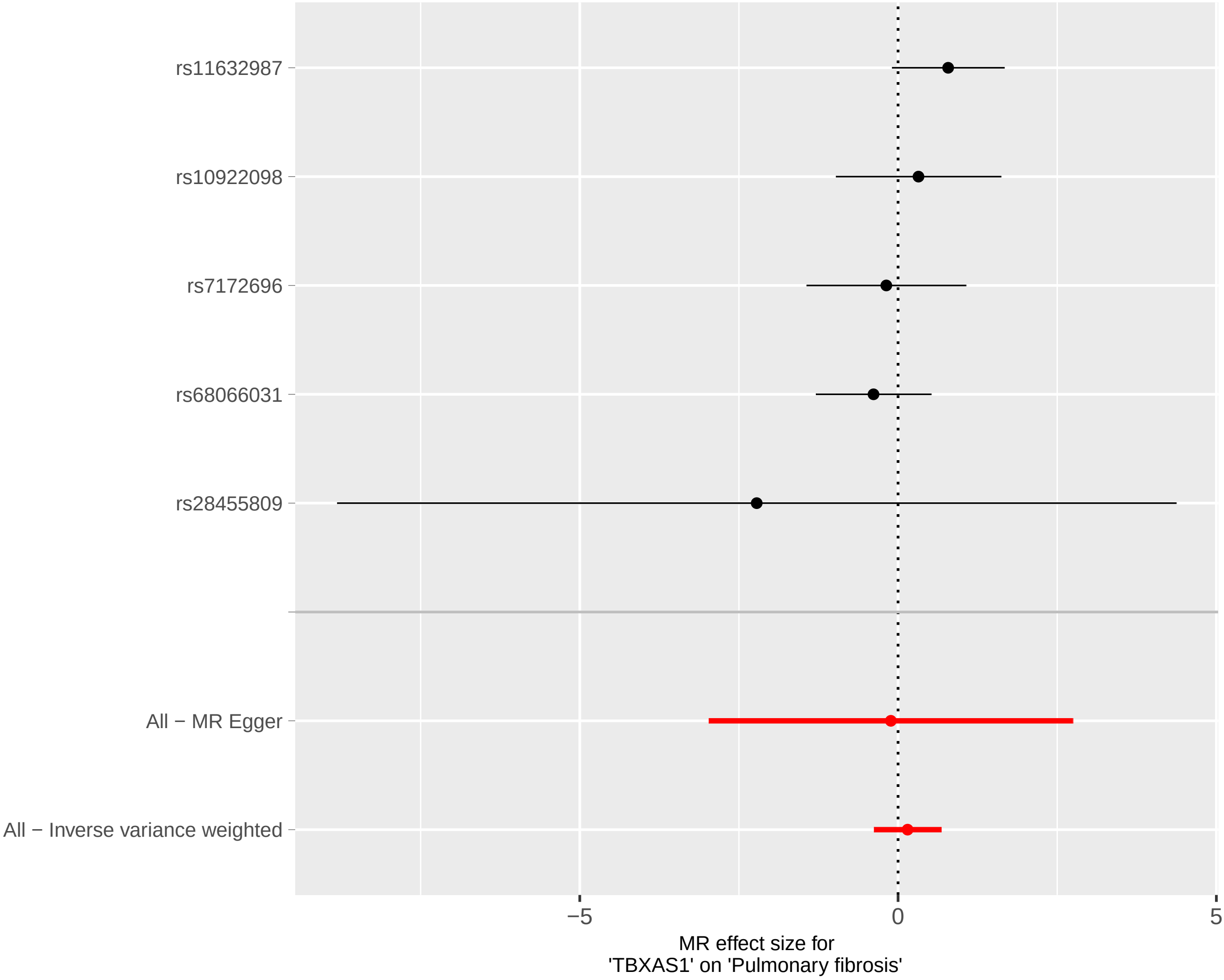

rs11632987
rs10922098
rs7172696
rs68066031
rs28455809
All − MR Egger
All − Inverse variance weighted
−5
0
5
MR effect size for
'TBXAS1' on 'Pulmonary fibrosis'

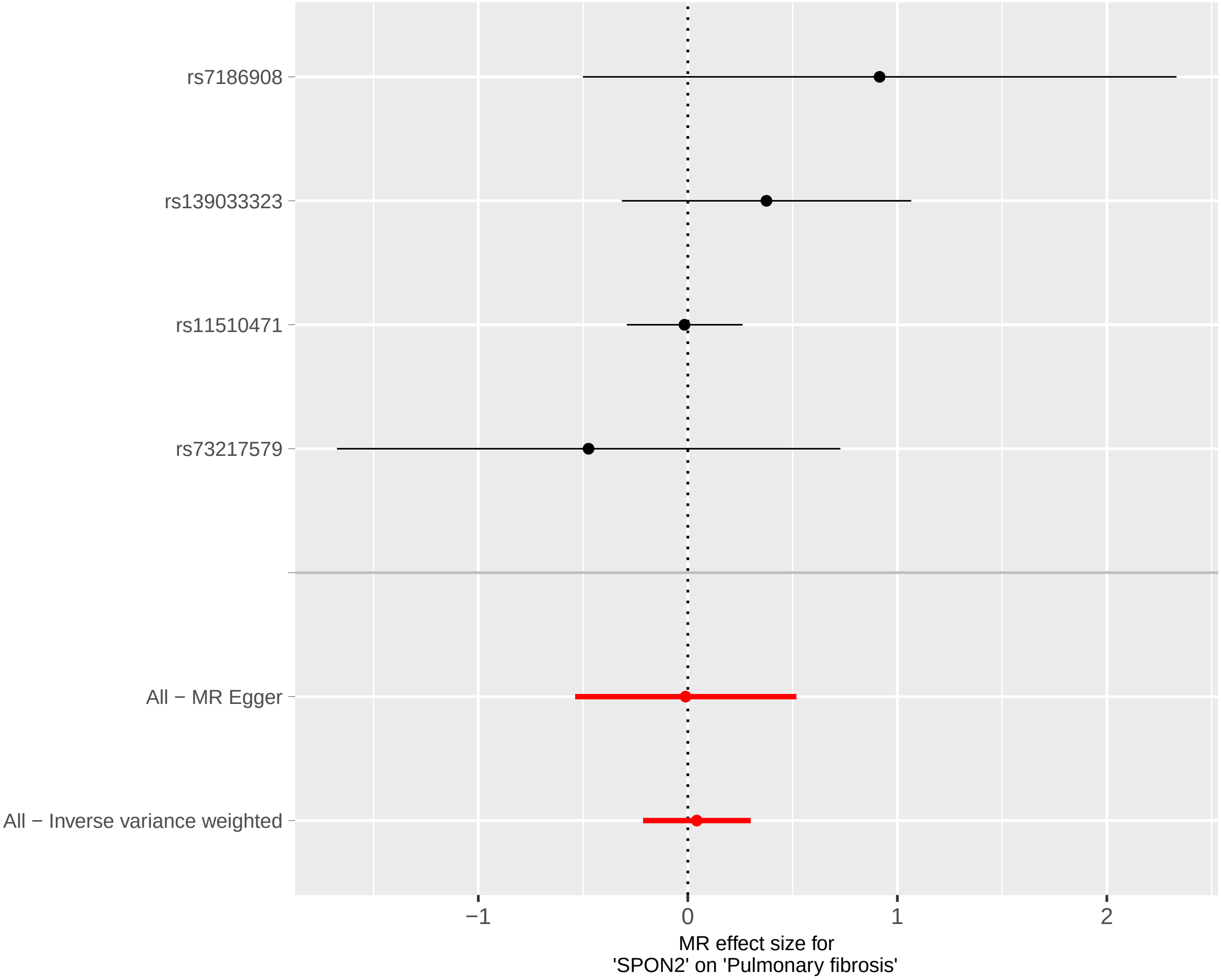
rs7186908
rs139033323
rs11510471
rs73217579
All − MR Egger
All − Inverse variance weighted
−1
0
1
2
MR effect size for
'SPON2' on 'Pulmonary fibrosis'

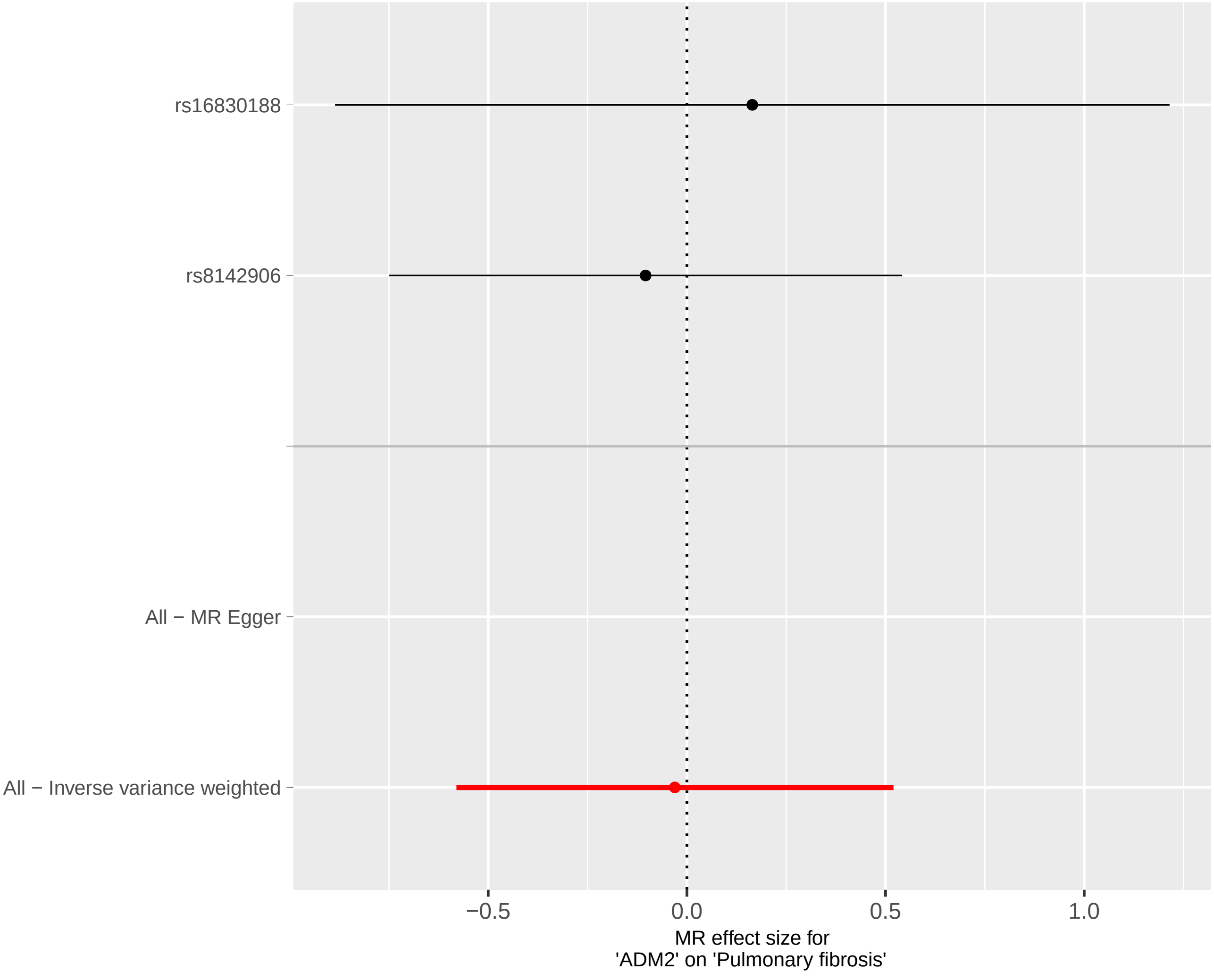

rs16830188
rs8142906
All − MR Egger
All − Inverse variance weighted
−0.5
0.0
0.5
1.0
MR effect size for
'ADM2' on 'Pulmonary fibrosis'

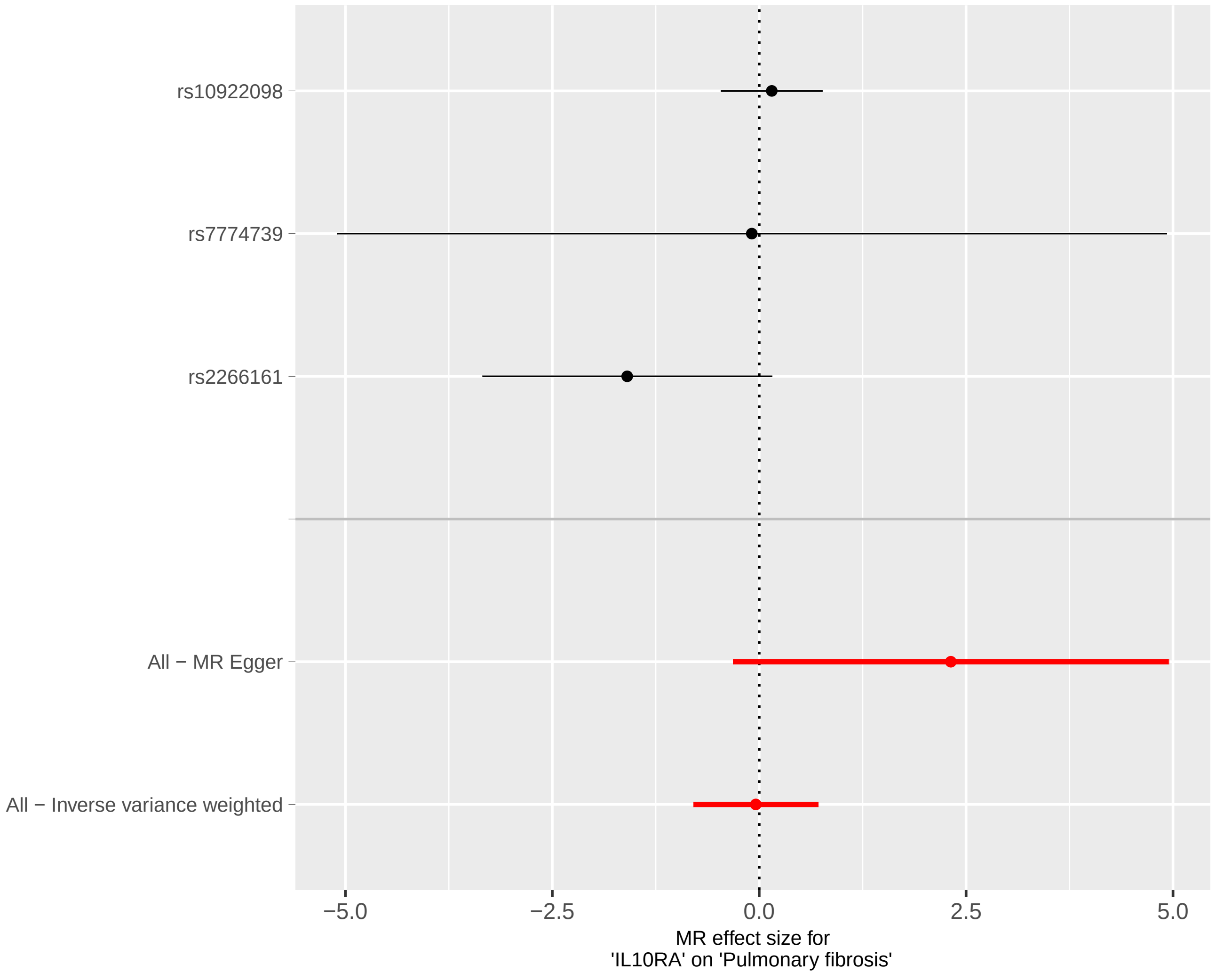

rs10922098
rs7774739
rs2266161
All − MR Egger
All − Inverse variance weighted
−5.0
−2.5
0.0
2.5
5.0
MR effect size for
'IL10RA' on 'Pulmonary fibrosis'

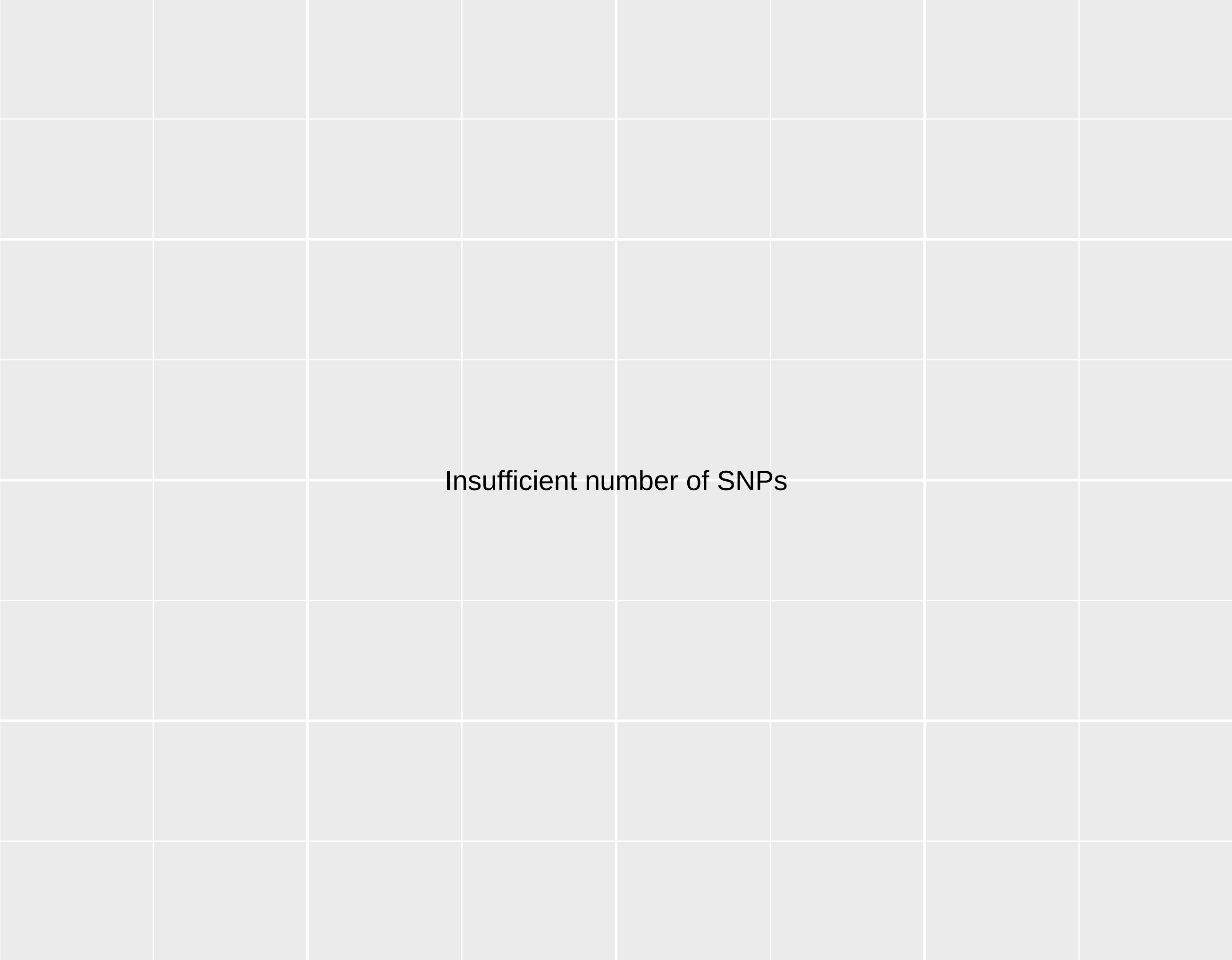
Insufficient number of SNPs

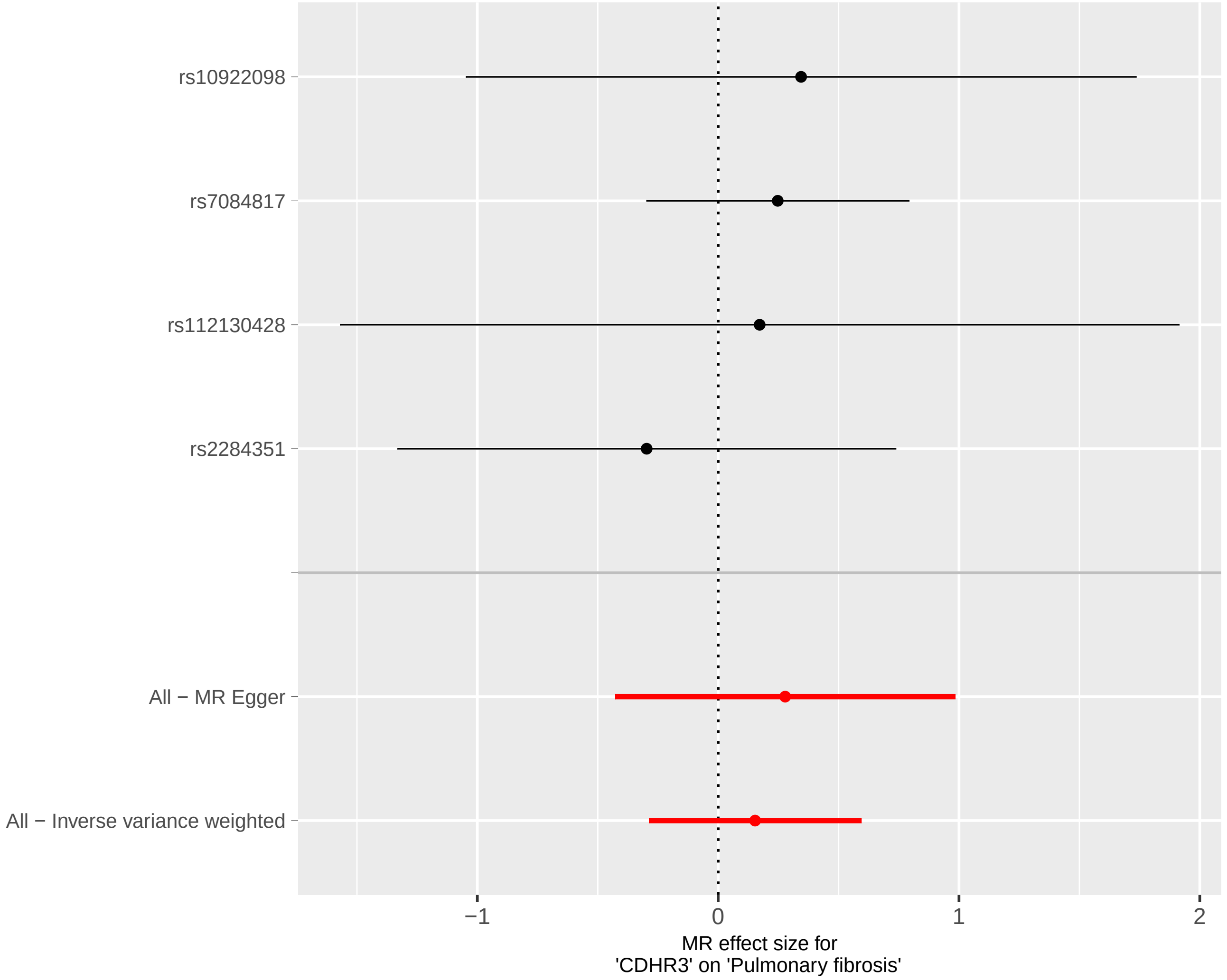
rs10922098
rs7084817
rs112130428
rs2284351
All − MR Egger
All − Inverse variance weighted
−1
0
1
2
MR effect size for
'CDHR3' on 'Pulmonary fibrosis'

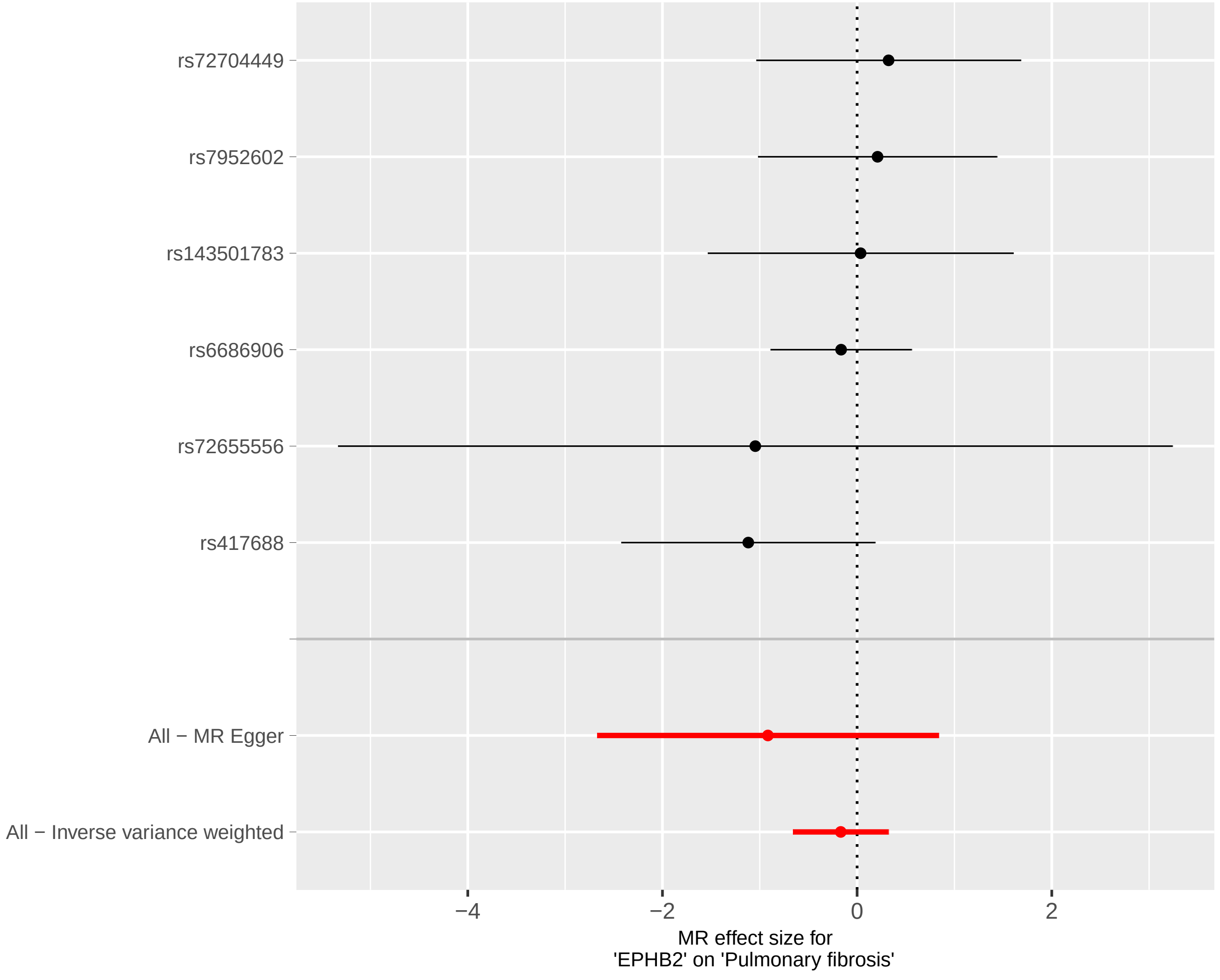
rs72704449
rs7952602
rs143501783
rs6686906
rs72655556
rs417688
All − MR Egger
All − Inverse variance weighted
−4
−2
0
2
MR effect size for
'EPHB2' on 'Pulmonary fibrosis'

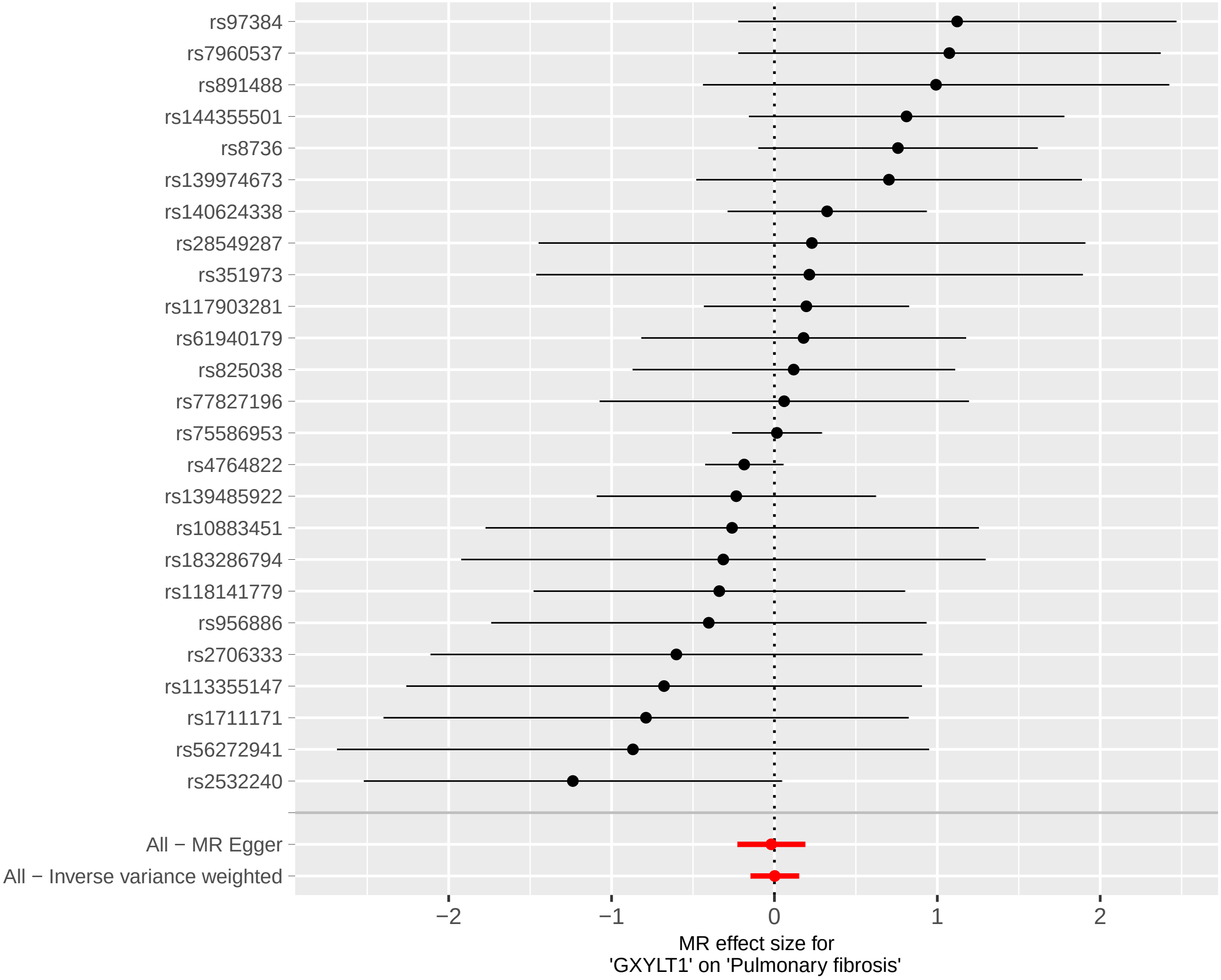
rs97384
rs7960537
rs891488
rs144355501
rs8736
rs139974673
rs140624338
rs28549287
rs351973
rs117903281
rs61940179
rs825038
rs77827196
rs75586953
rs4764822
rs139485922
rs10883451
rs183286794
rs118141779
rs956886
rs2706333
rs113355147
rs1711171
rs56272941
rs2532240
All − MR Egger
All − Inverse variance weighted
−2
−1
0
1
2
MR effect size for
'GXYLT1' on 'Pulmonary fibrosis'

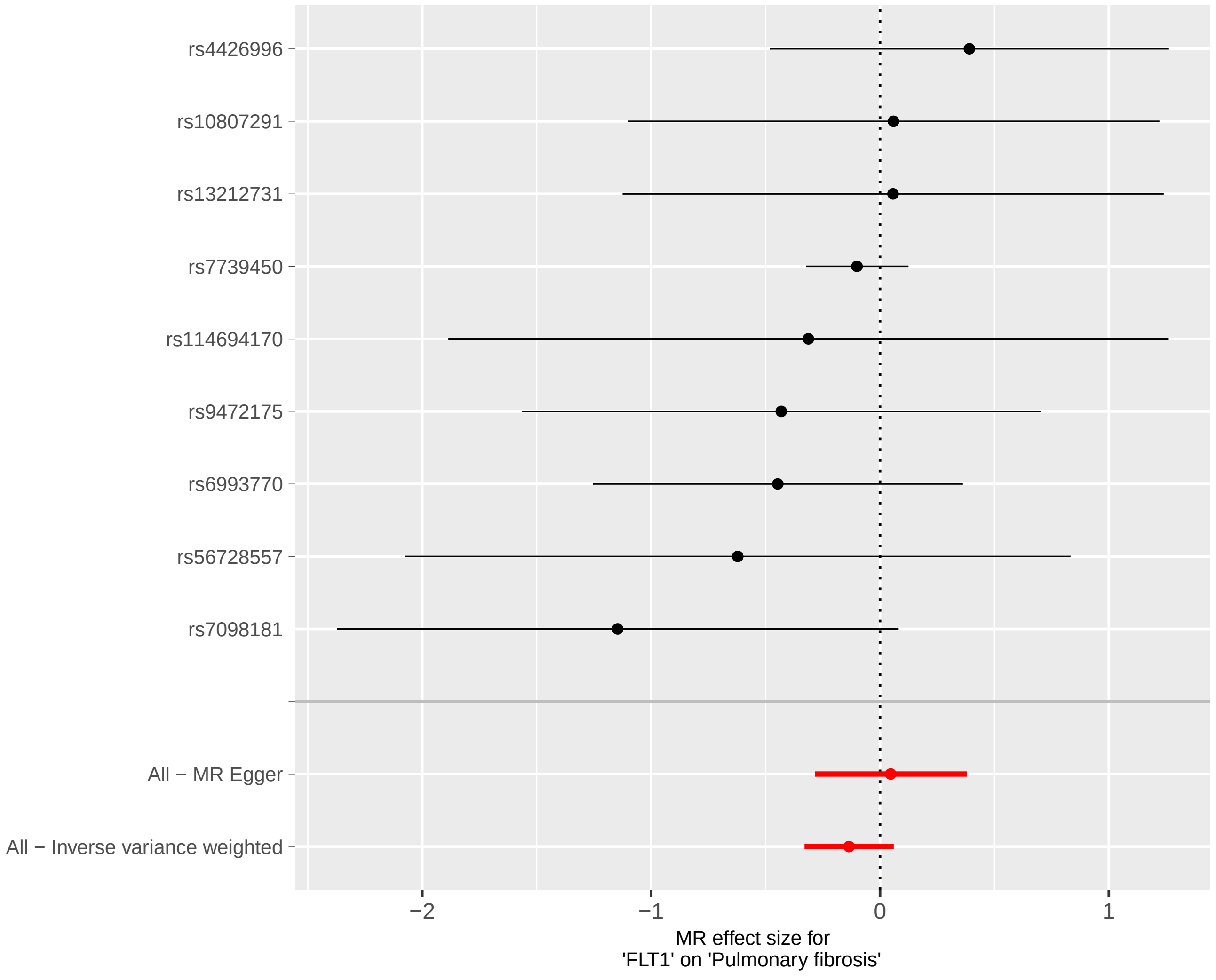

rs4426996
rs10807291
rs13212731
rs7739450
rs114694170
rs9472175
rs6993770
rs56728557
rs7098181
All − MR Egger
All − Inverse variance weighted
−2
−1
0
1
MR effect size for
'FLT1' on 'Pulmonary fibrosis'

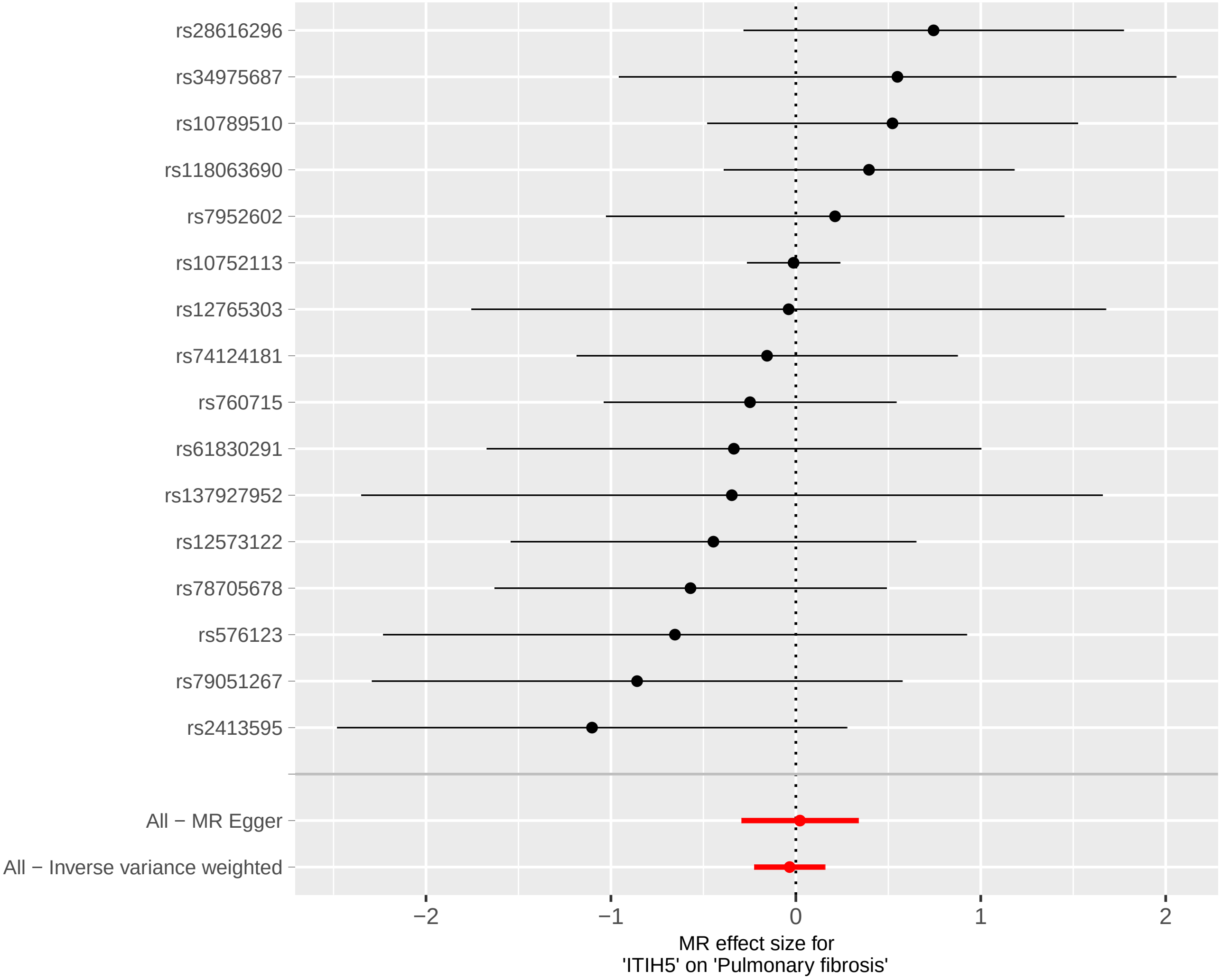
rs28616296
rs34975687
rs10789510
rs118063690
rs7952602
rs10752113
rs12765303
rs74124181
rs760715
rs61830291
rs137927952
rs12573122
rs78705678
rs576123
rs79051267
rs2413595
All − MR Egger
All − Inverse variance weighted
−2
−1
0
1
2
MR effect size for
'ITIH5' on 'Pulmonary fibrosis'

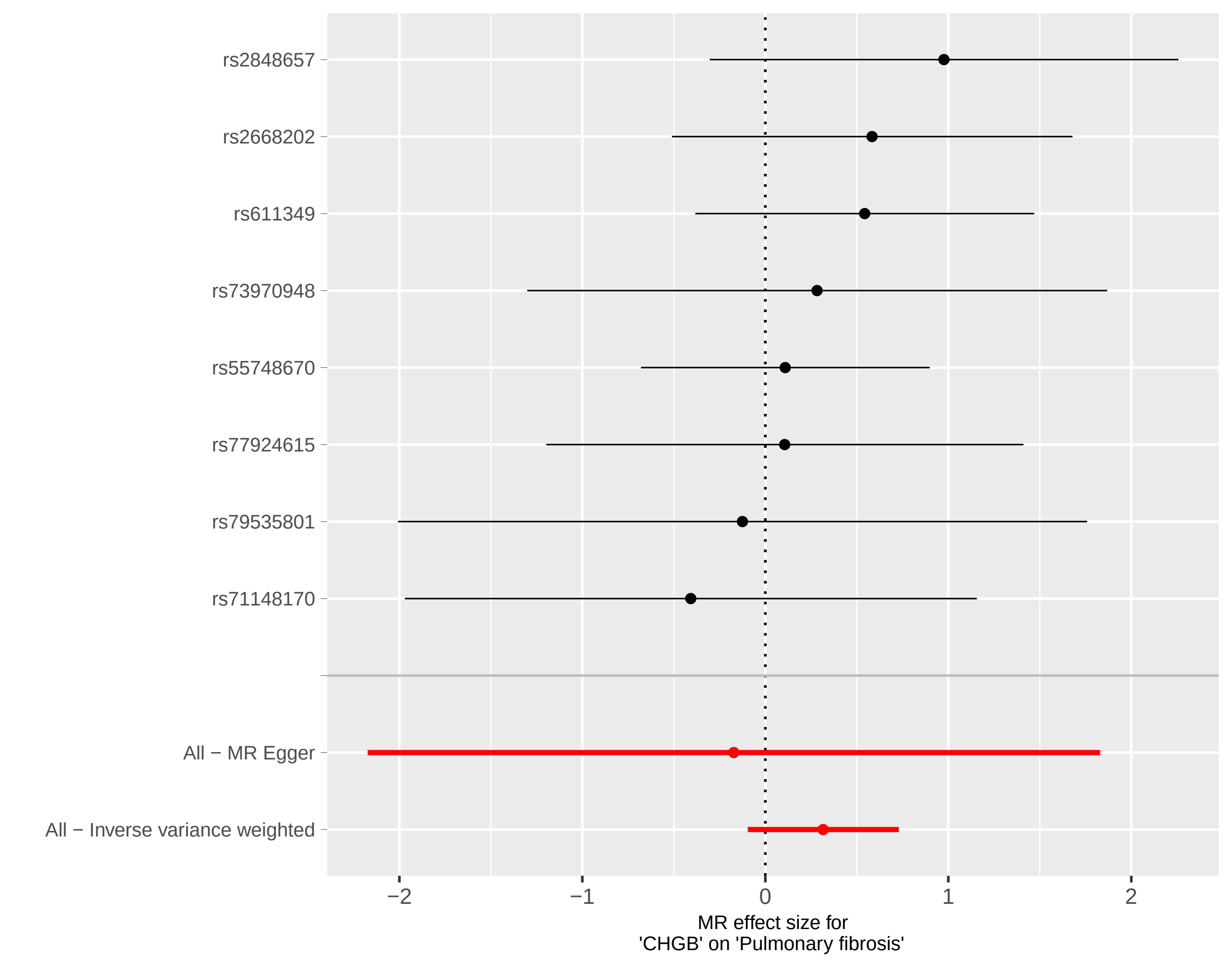

rs2848657
rs2668202
rs611349
rs73970948
rs55748670
rs77924615
rs79535801
rs71148170
All − MR Egger
All − Inverse variance weighted
−2
−1
0
1
2
MR effect size for
'CHGB' on 'Pulmonary fibrosis'

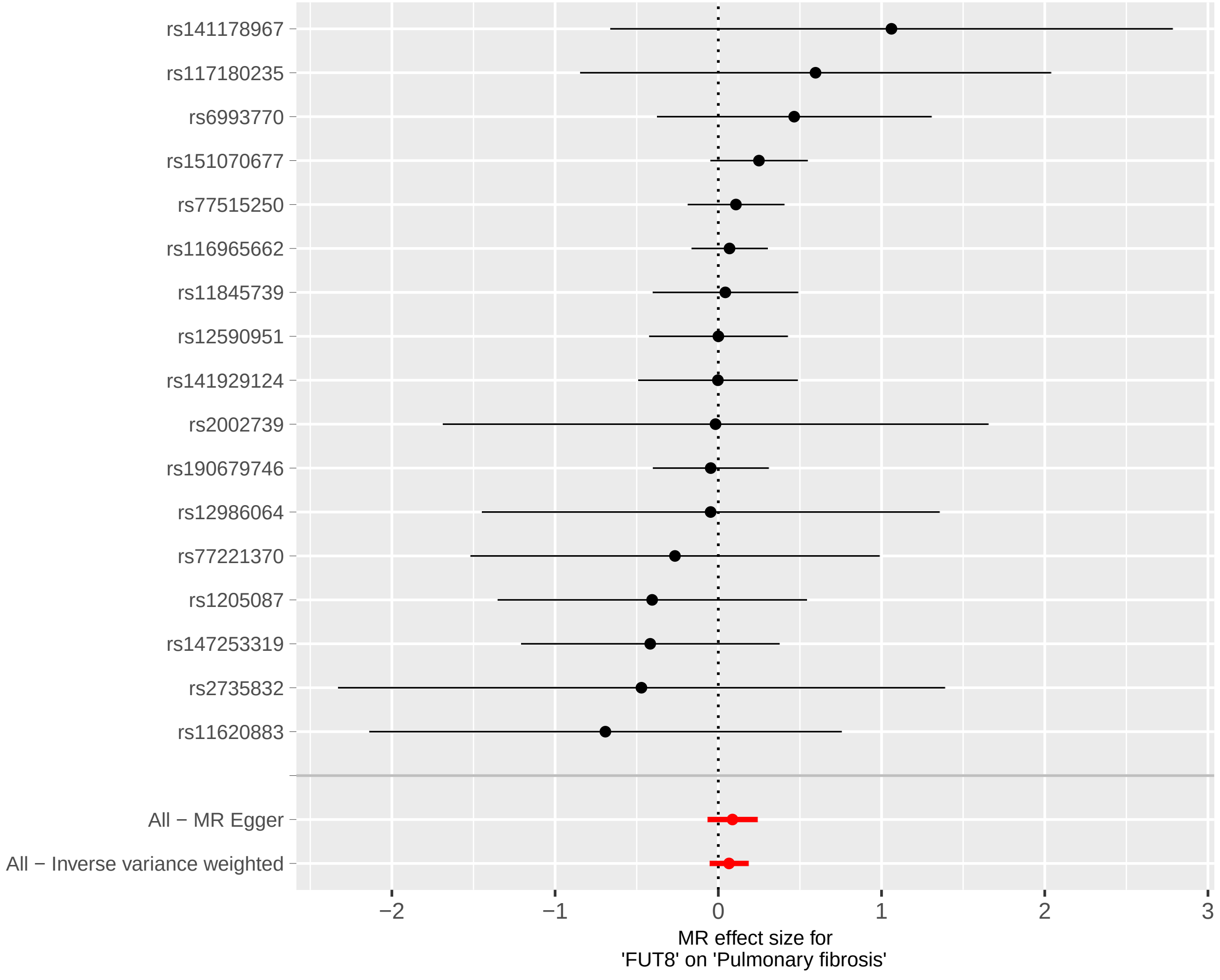

rs141178967
rs117180235
rs6993770
rs151070677
rs77515250
rs116965662
rs11845739
rs12590951
rs141929124
rs2002739
rs190679746
rs12986064
rs77221370
rs1205087
rs147253319
rs2735832
rs11620883
All − MR Egger
All − Inverse variance weighted
−2
−1
0
1
2
3
MR effect size for
'FUT8' on 'Pulmonary fibrosis'

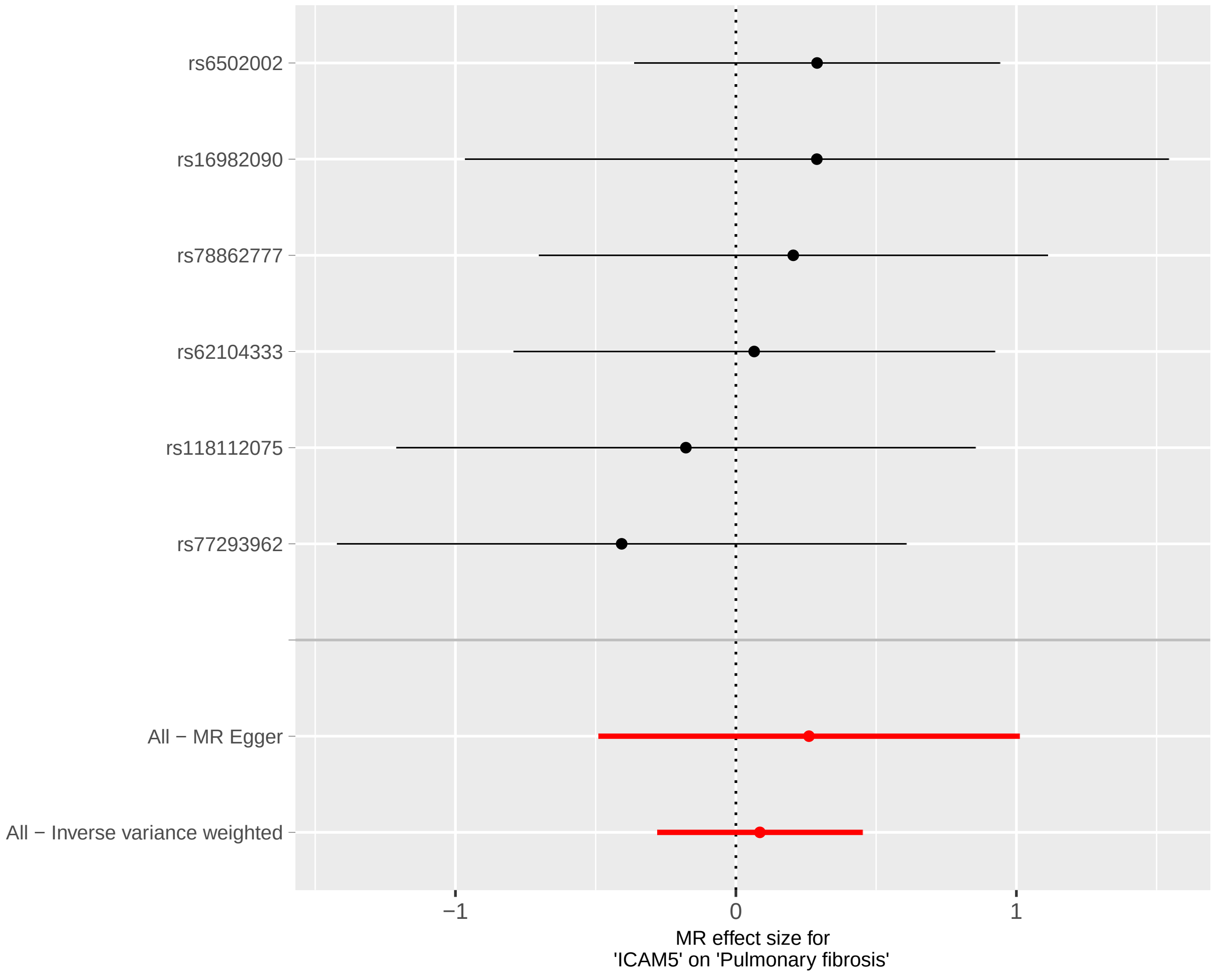
rs6502002
rs16982090
rs78862777
rs62104333
rs118112075
rs77293962
All − MR Egger
All − Inverse variance weighted
−1
0
1
MR effect size for
'ICAM5' on 'Pulmonary fibrosis'

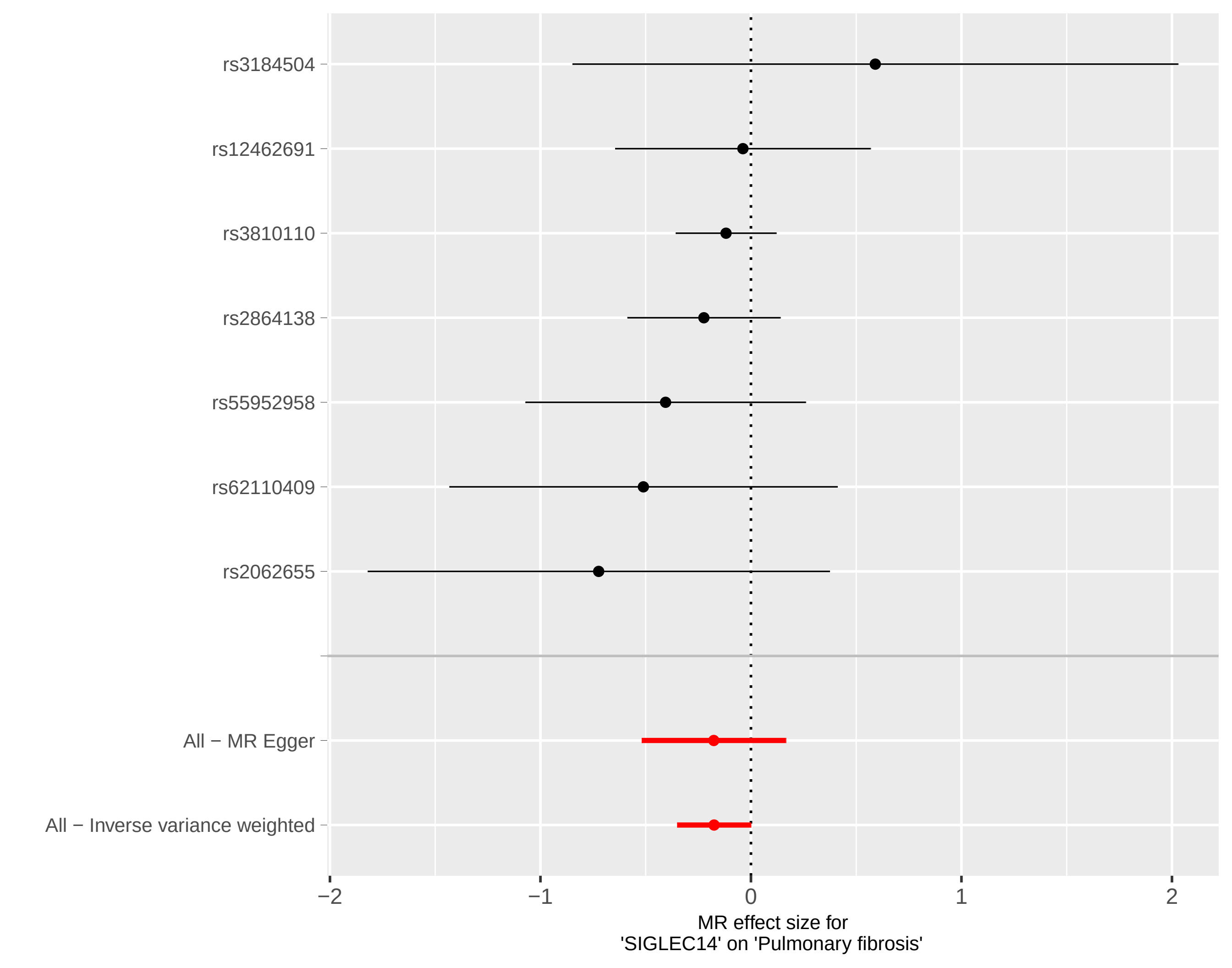

rs3184504
rs12462691
rs3810110
rs2864138
rs55952958
rs62110409
rs2062655
All − MR Egger
All − Inverse variance weighted
−2
−1
0
1
2
MR effect size for
'SIGLEC14' on 'Pulmonary fibrosis'

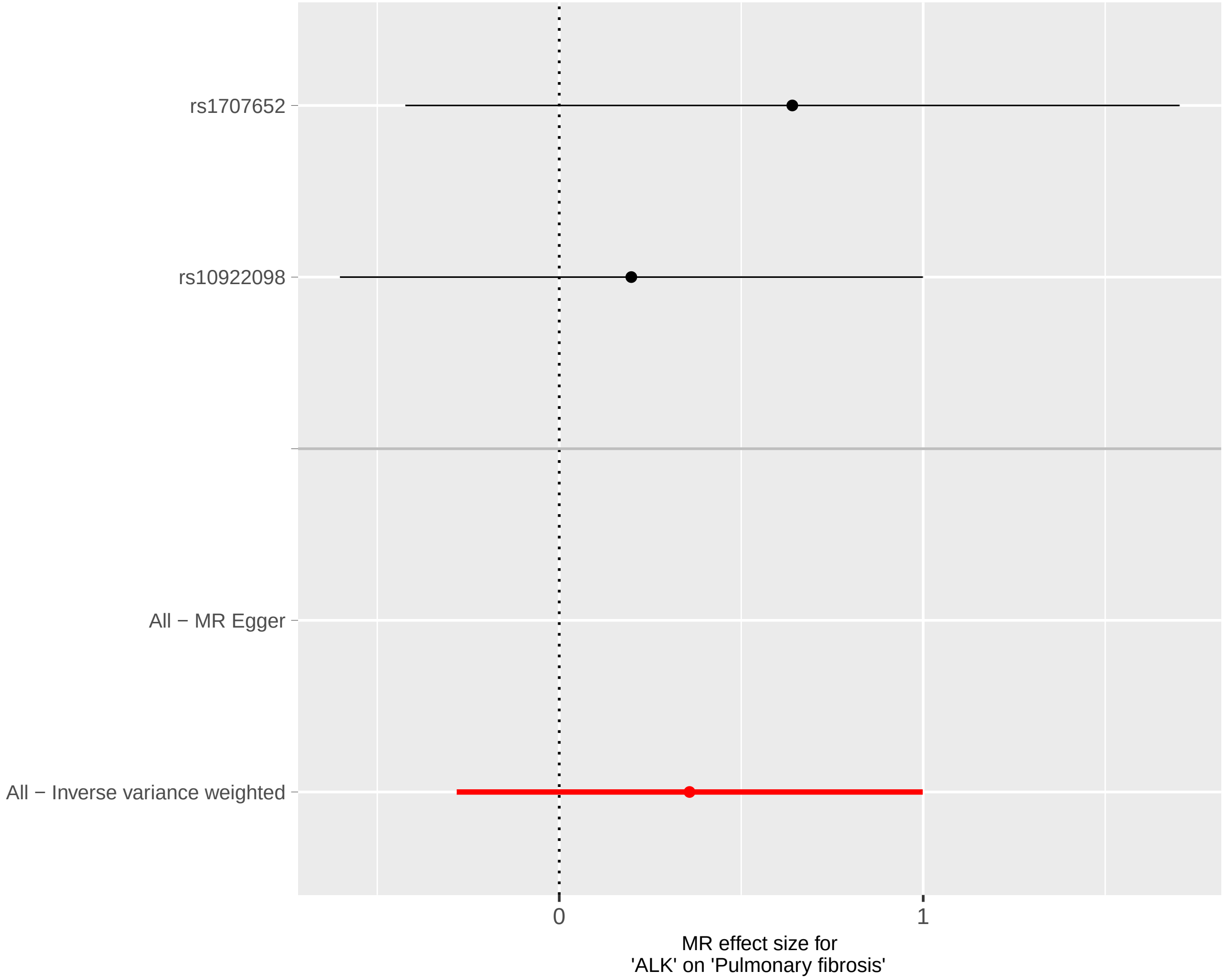
rs1707652
rs10922098
All − MR Egger
All − Inverse variance weighted
0
1
MR effect size for
'ALK' on 'Pulmonary fibrosis'

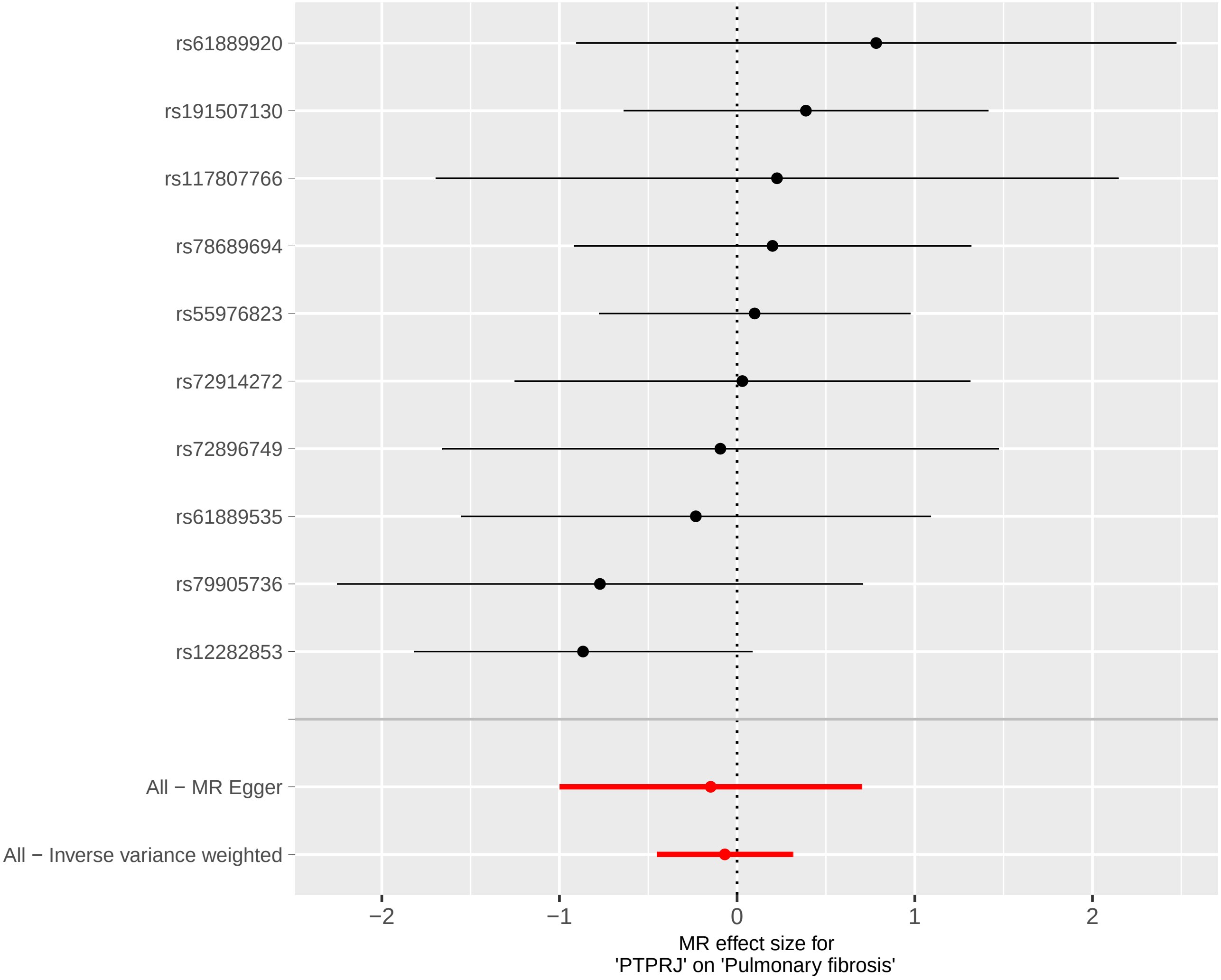

rs61889920
rs191507130
rs117807766
rs78689694
rs55976823
rs72914272
rs72896749
rs61889535
rs79905736
rs12282853
All − MR Egger
All − Inverse variance weighted
−2
−1
0
1
2
MR effect size for
'PTPRJ' on 'Pulmonary fibrosis'

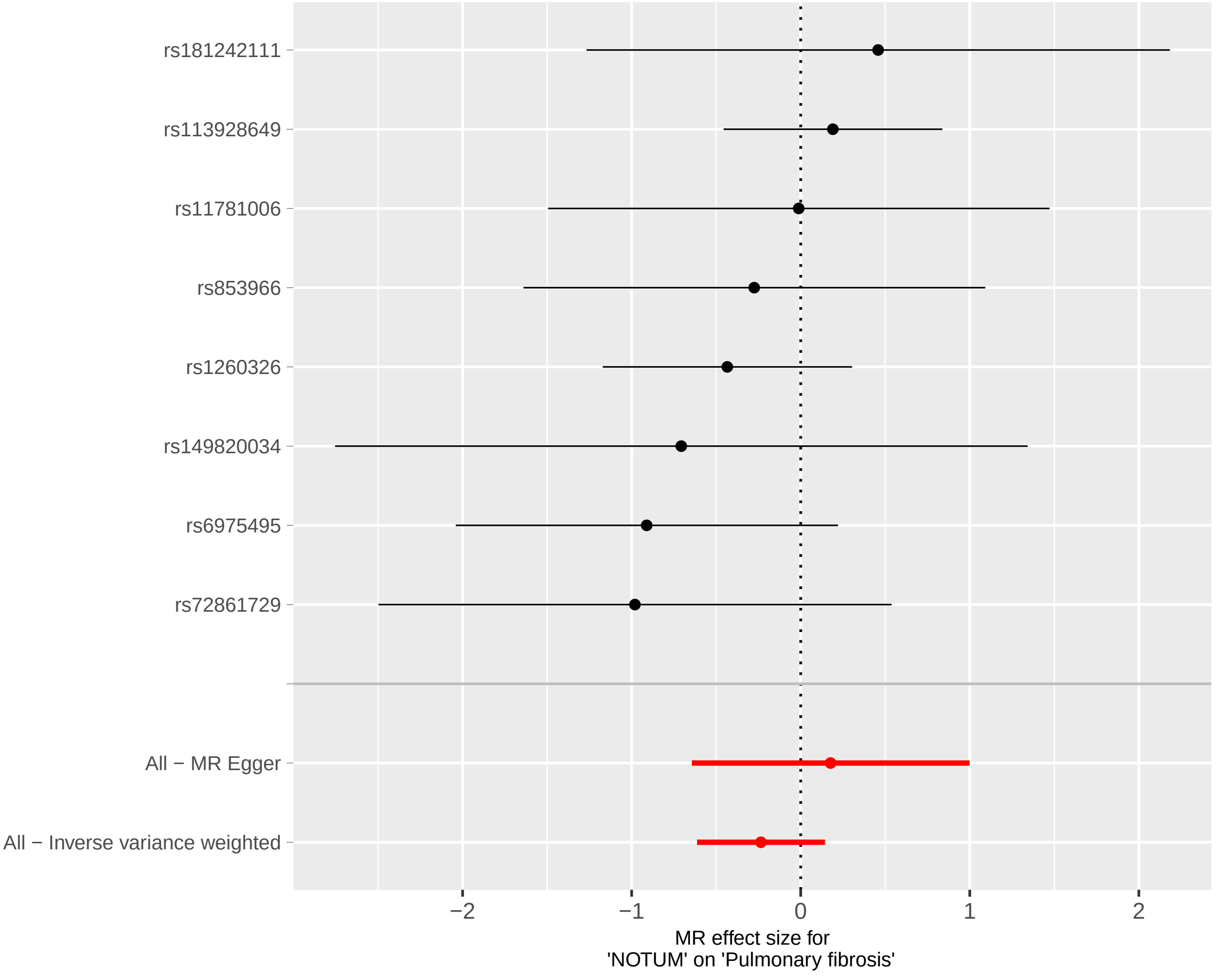
rs181242111
rs113928649
rs11781006
rs853966
rs1260326
rs149820034
rs6975495
rs72861729
All − MR Egger
All − Inverse variance weighted
−2
−1
0
1
2
MR effect size for
'NOTUM' on 'Pulmonary fibrosis'

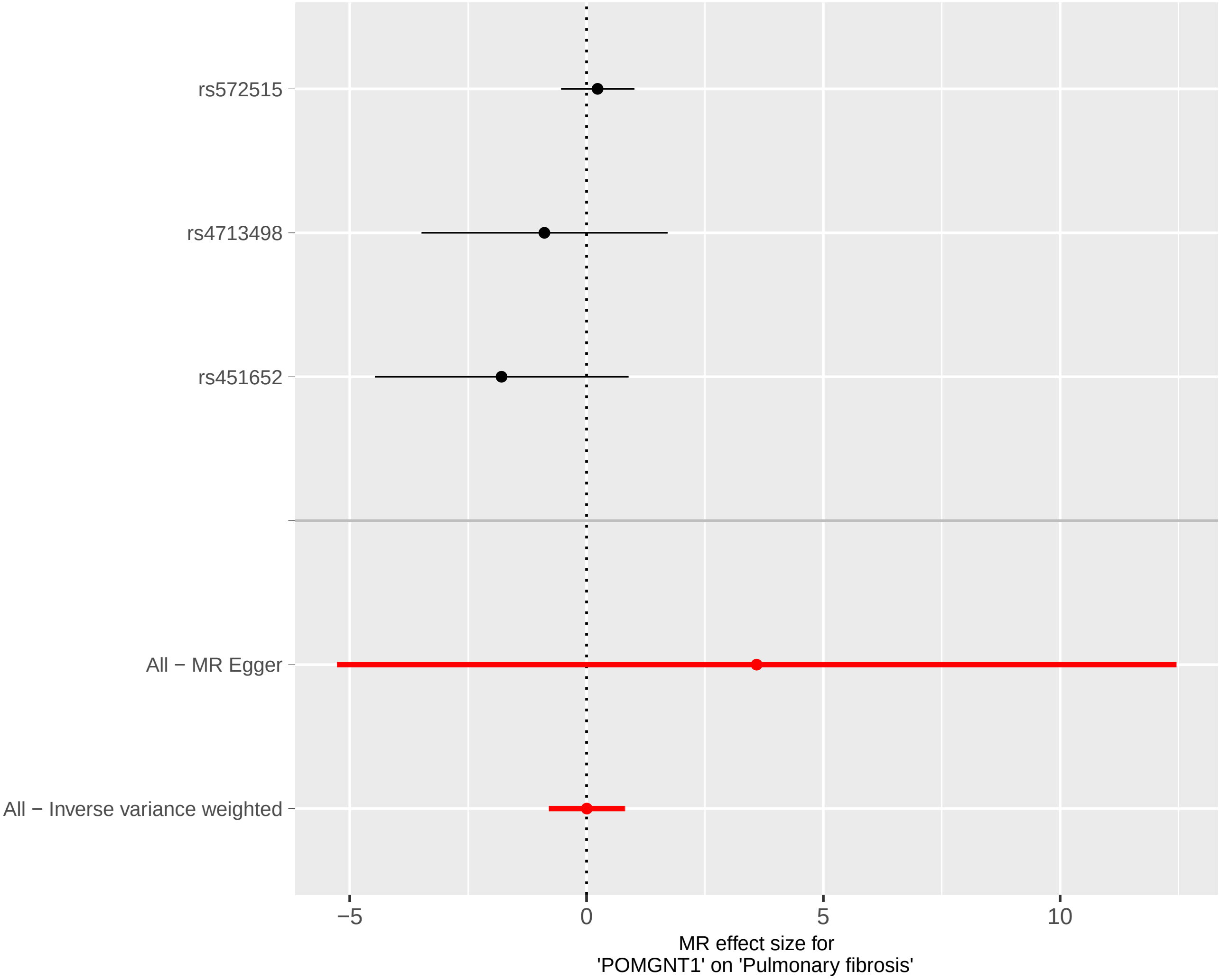
rs572515
rs4713498
rs451652
All − MR Egger
All − Inverse variance weighted
−5
0
5
10
MR effect size for
'POMGNT1' on 'Pulmonary fibrosis'

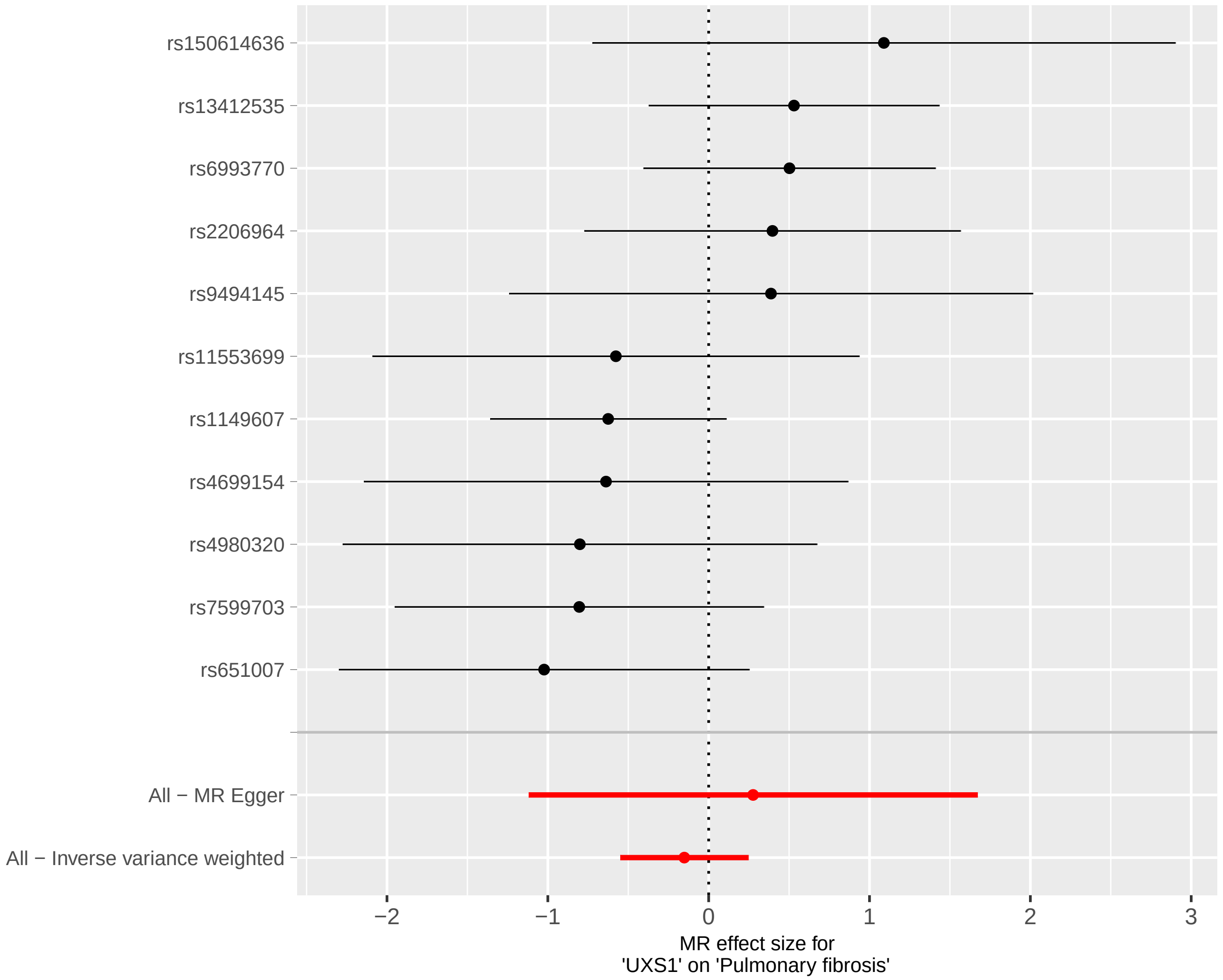

rs150614636
rs13412535
rs6993770
rs2206964
rs9494145
rs11553699
rs1149607
rs4699154
rs4980320
rs7599703
rs651007
All − MR Egger
All − Inverse variance weighted
−2
−1
0
1
2
3
MR effect size for
'UXS1' on 'Pulmonary fibrosis'

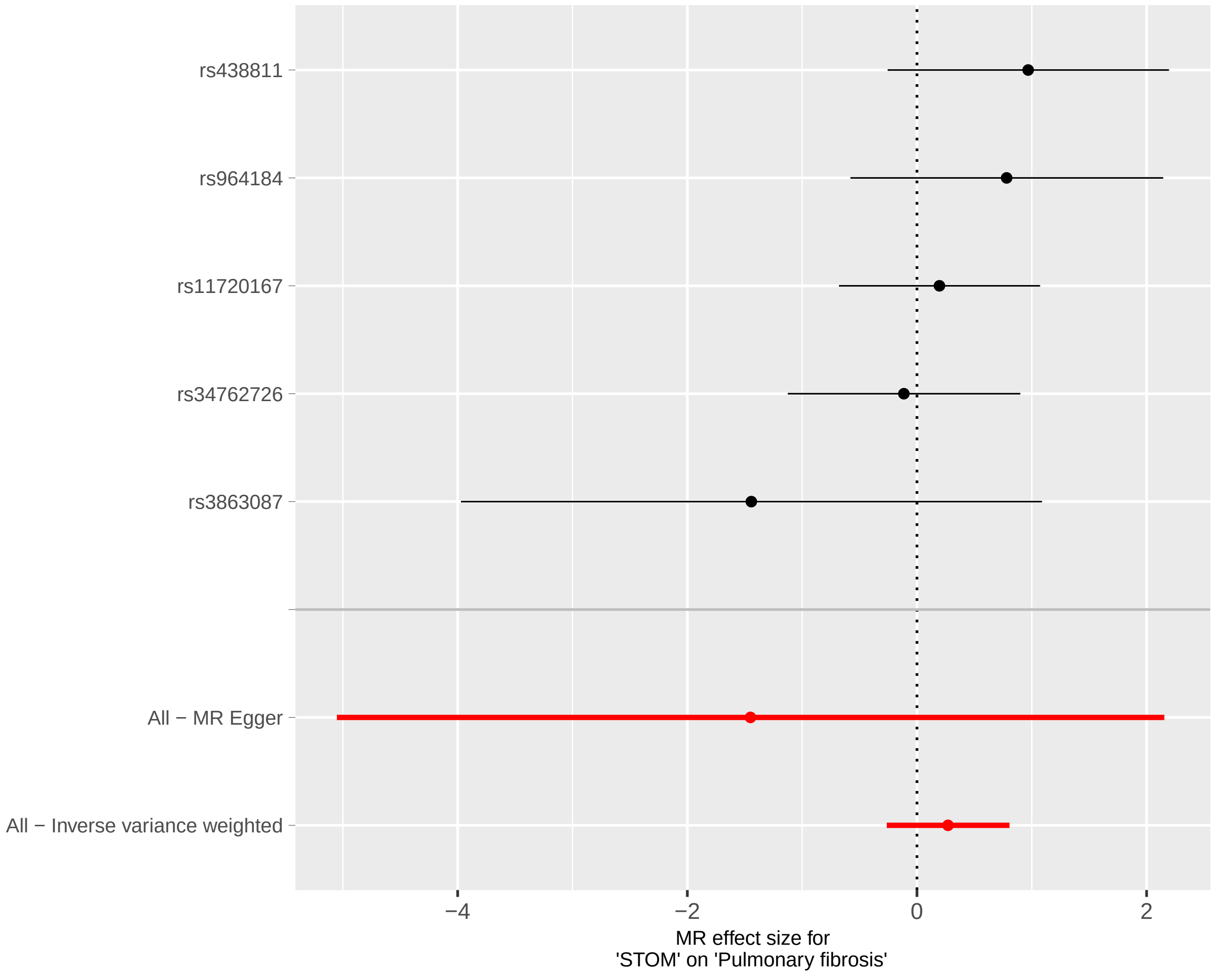

rs438811
rs964184
rs11720167
rs34762726
rs3863087
All − MR Egger
All − Inverse variance weighted
−4
−2
0
2
MR effect size for
'STOM' on 'Pulmonary fibrosis'

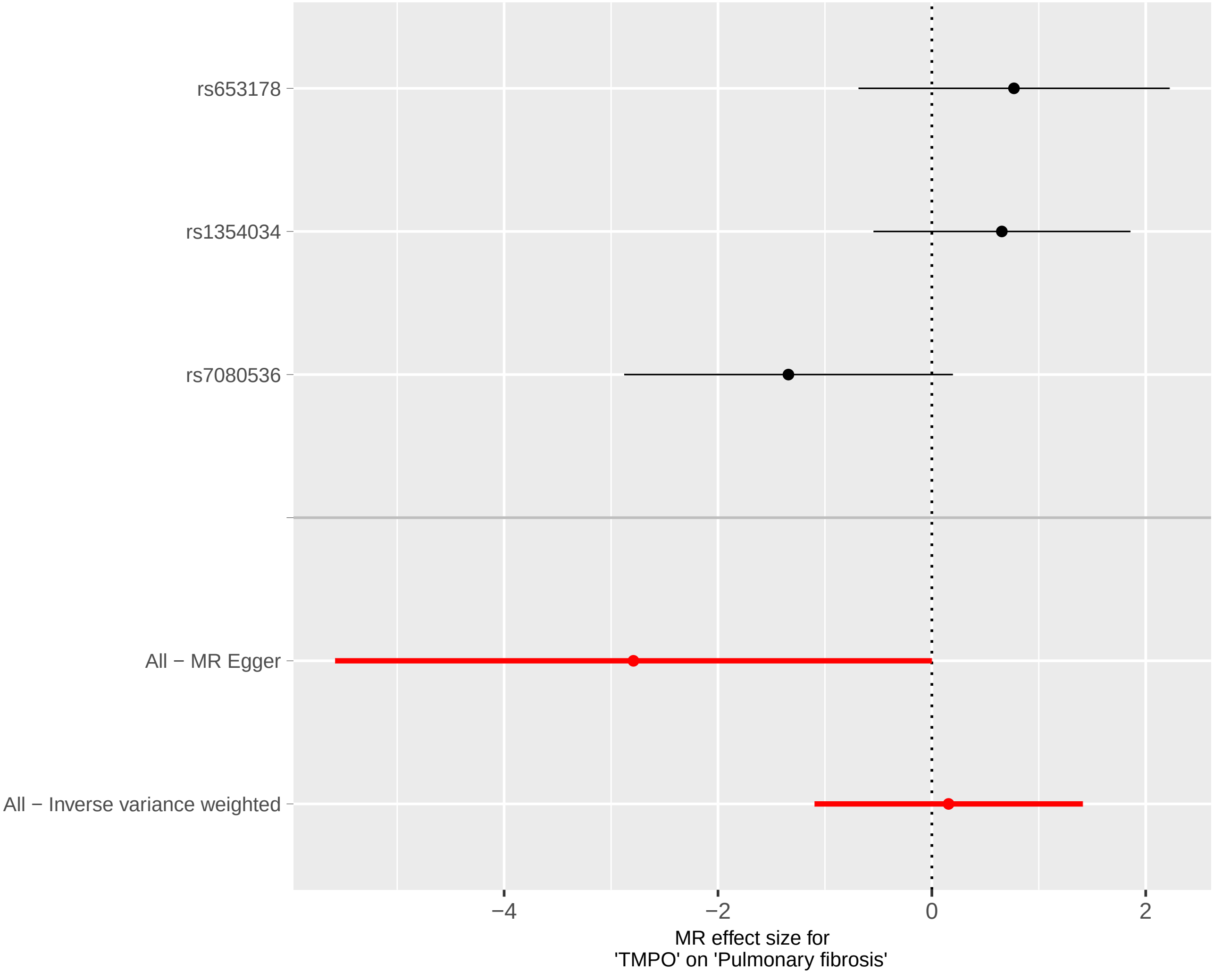
rs653178
rs1354034
rs7080536
All − MR Egger
All − Inverse variance weighted
−4
−2
0
2
MR effect size for
'TMPO' on 'Pulmonary fibrosis'

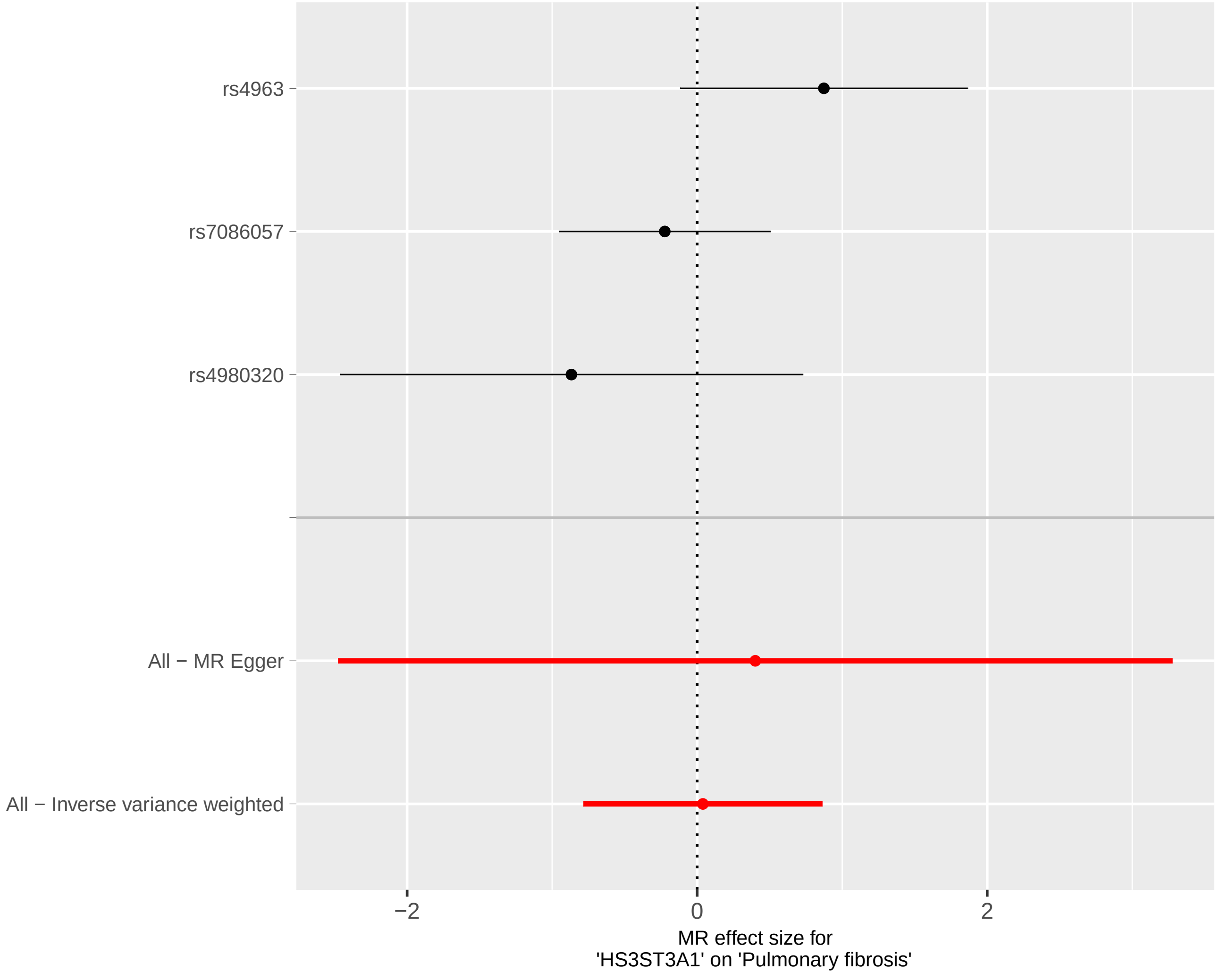

rs4963
rs7086057
rs4980320
All − MR Egger
All − Inverse variance weighted
−2
0
2
MR effect size for
'HS3ST3A1' on 'Pulmonary fibrosis'

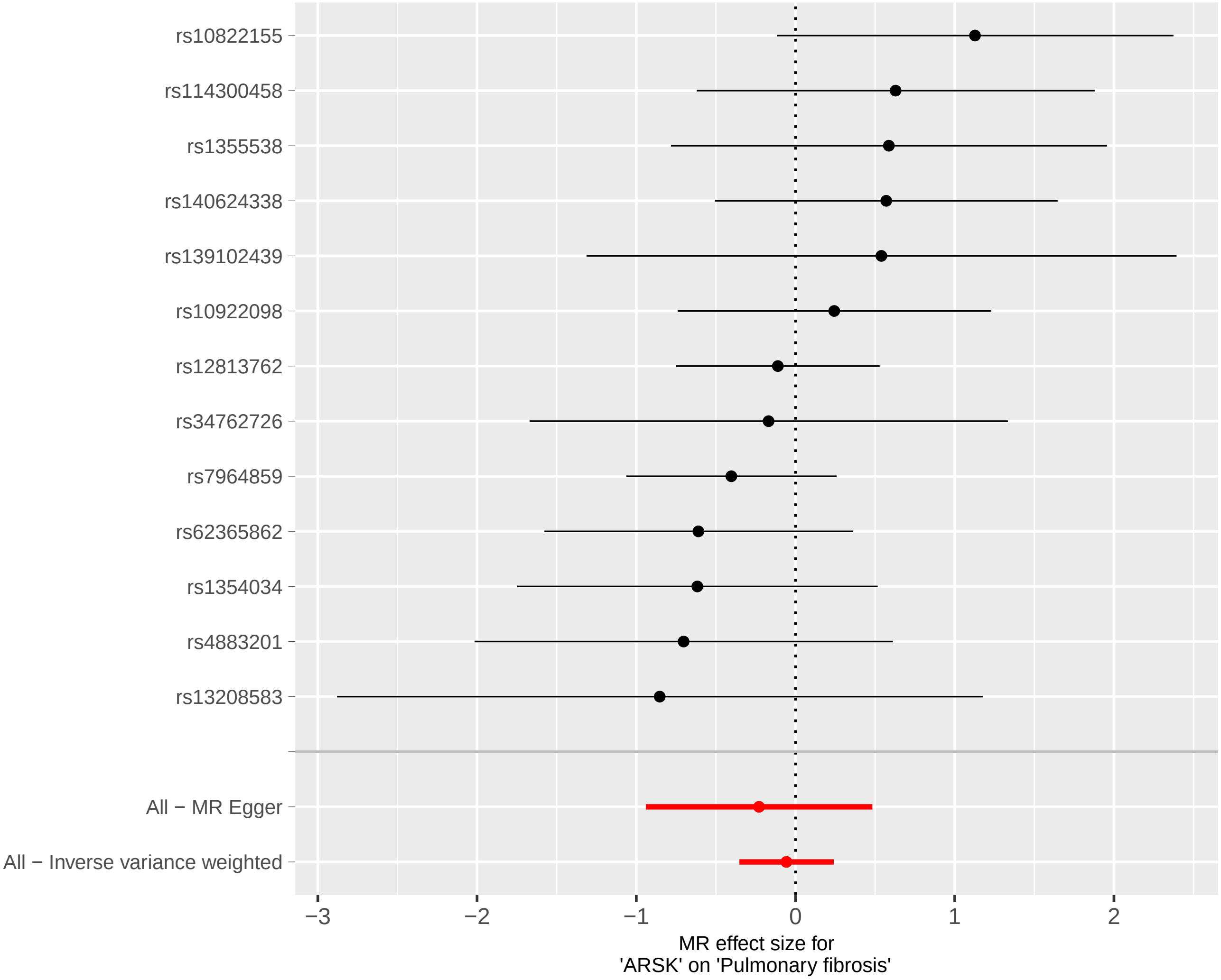

rs10822155
rs114300458
rs1355538
rs140624338
rs139102439
rs10922098
rs12813762
rs34762726
rs7964859
rs62365862
rs1354034
rs4883201
rs13208583
All − MR Egger
All − Inverse variance weighted
−3
−2
−1
0
1
2
MR effect size for
'ARSK' on 'Pulmonary fibrosis'

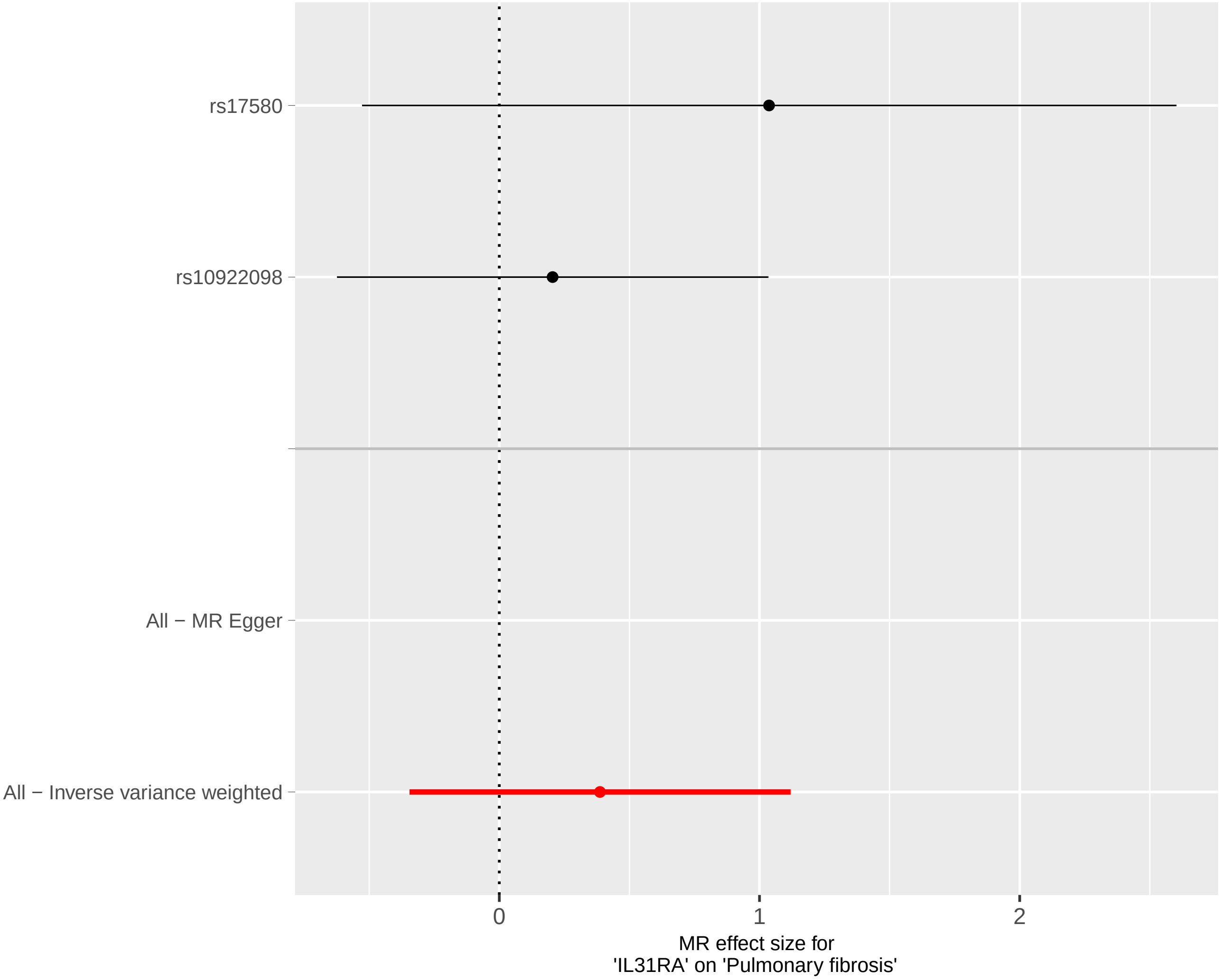
rs17580
rs10922098
All − MR Egger
All − Inverse variance weighted
0
1
2
MR effect size for
'IL31RA' on 'Pulmonary fibrosis'

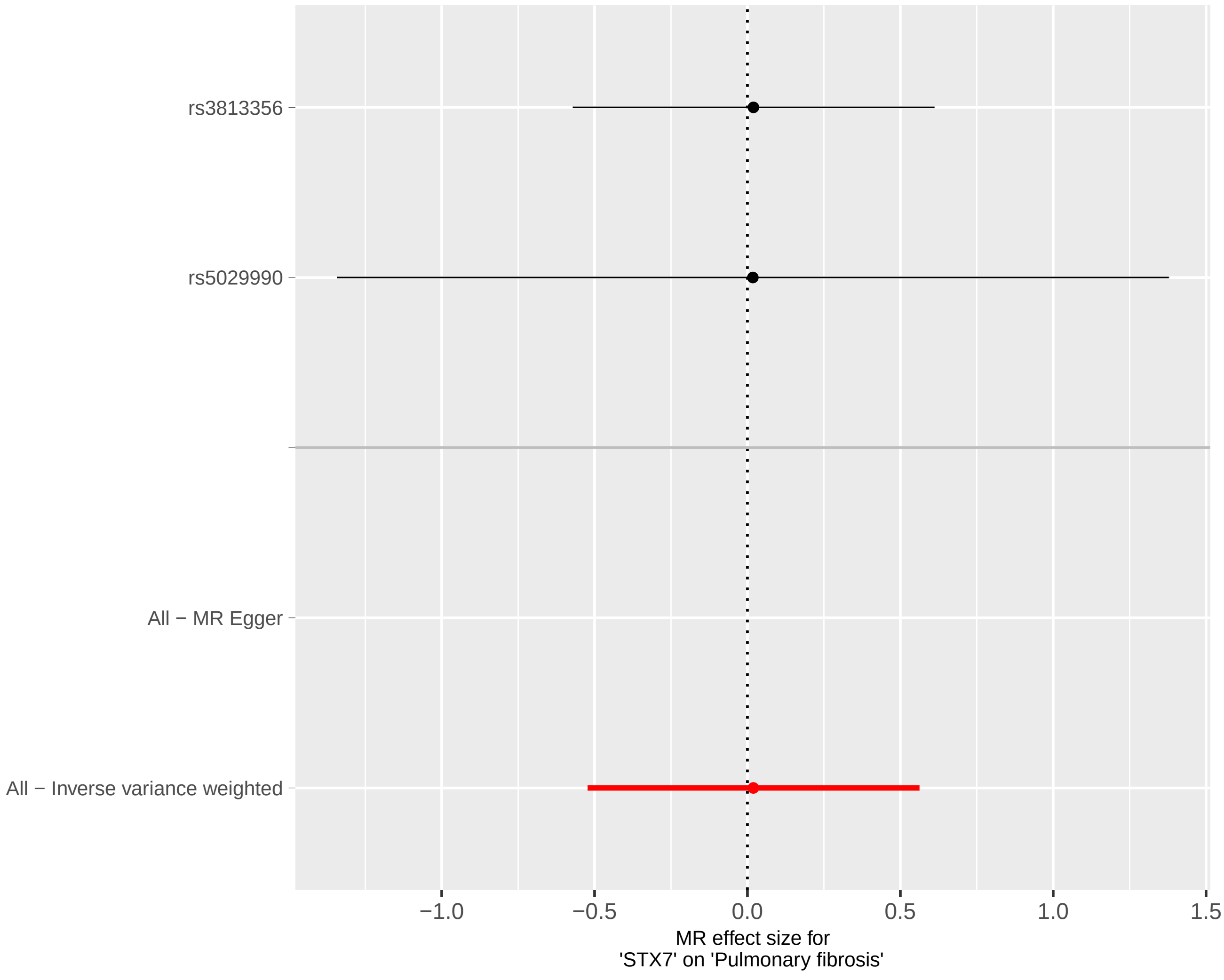
rs3813356
rs5029990
All − MR Egger
All − Inverse variance weighted
−1.0
−0.5
0.0
0.5
1.0
1.5
MR effect size for
'STX7' on 'Pulmonary fibrosis'

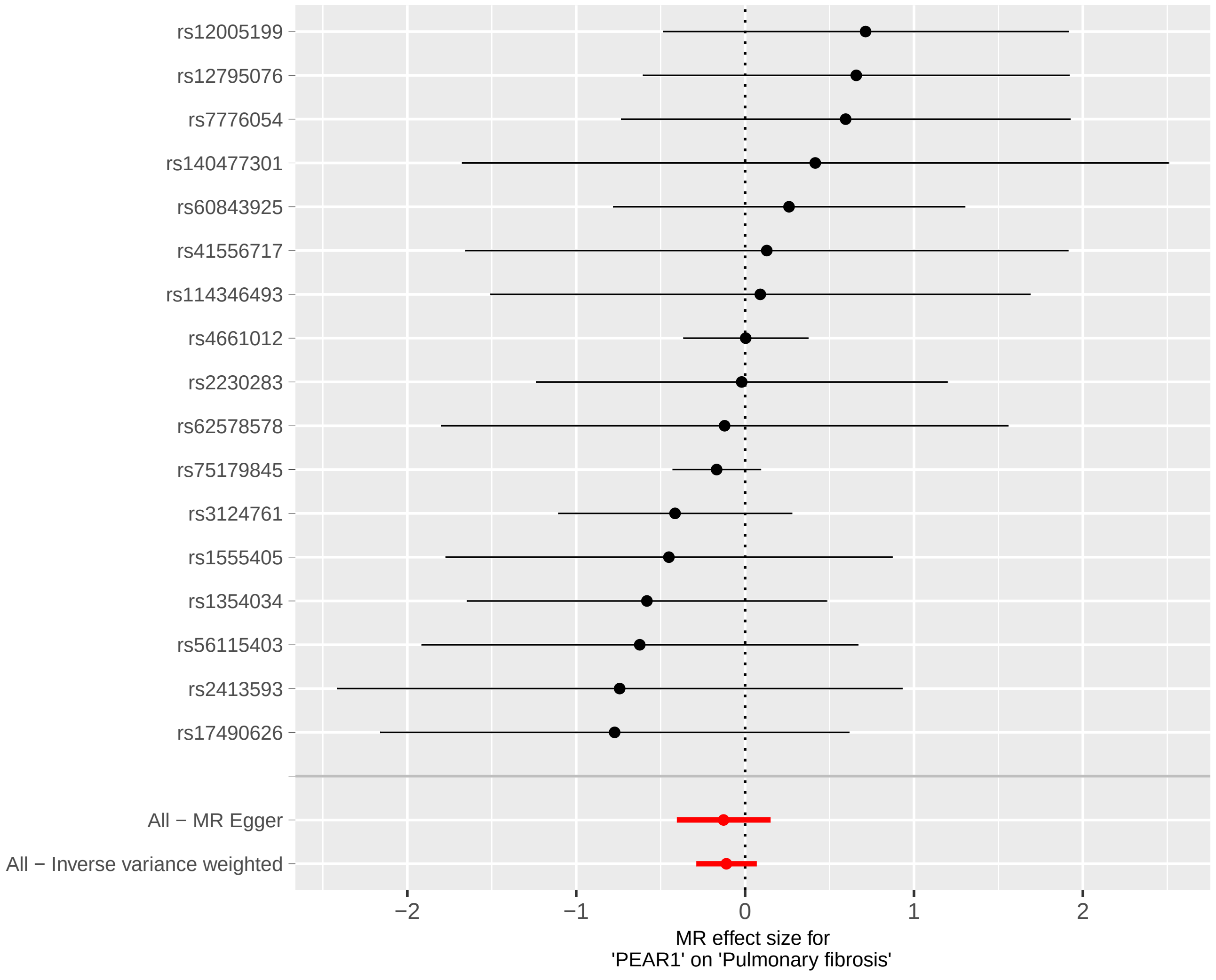
rs12005199
rs12795076
rs7776054
rs140477301
rs60843925
rs41556717
rs114346493
rs4661012
rs2230283
rs62578578
rs75179845
rs3124761
rs1555405
rs1354034
rs56115403
rs2413593
rs17490626
All − MR Egger
All − Inverse variance weighted
−2
−1
0
1
2
MR effect size for
'PEAR1' on 'Pulmonary fibrosis'

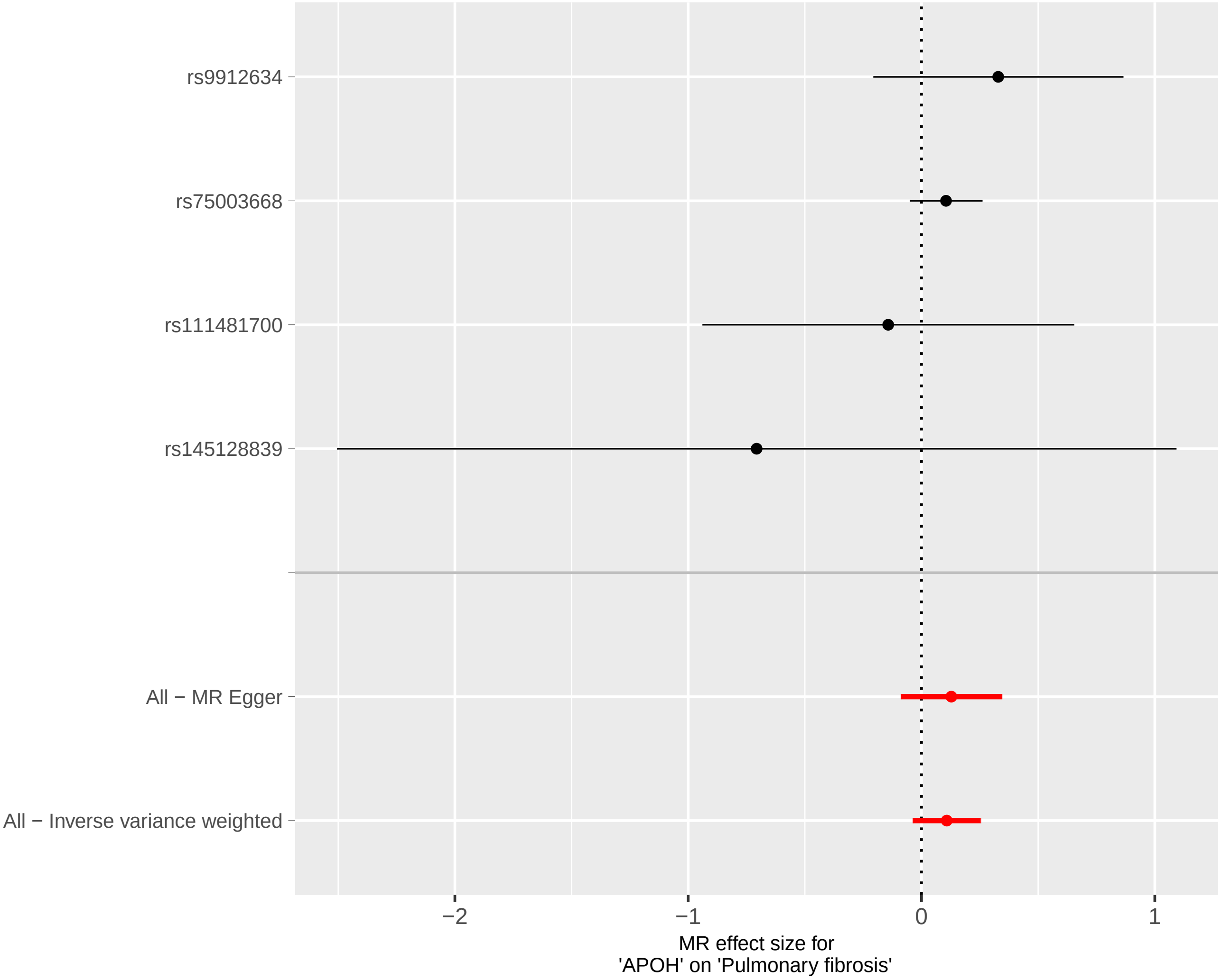
rs9912634
rs75003668
rs111481700
rs145128839
All − MR Egger
All − Inverse variance weighted
−2
−1
0
1
MR effect size for
'APOH' on 'Pulmonary fibrosis'

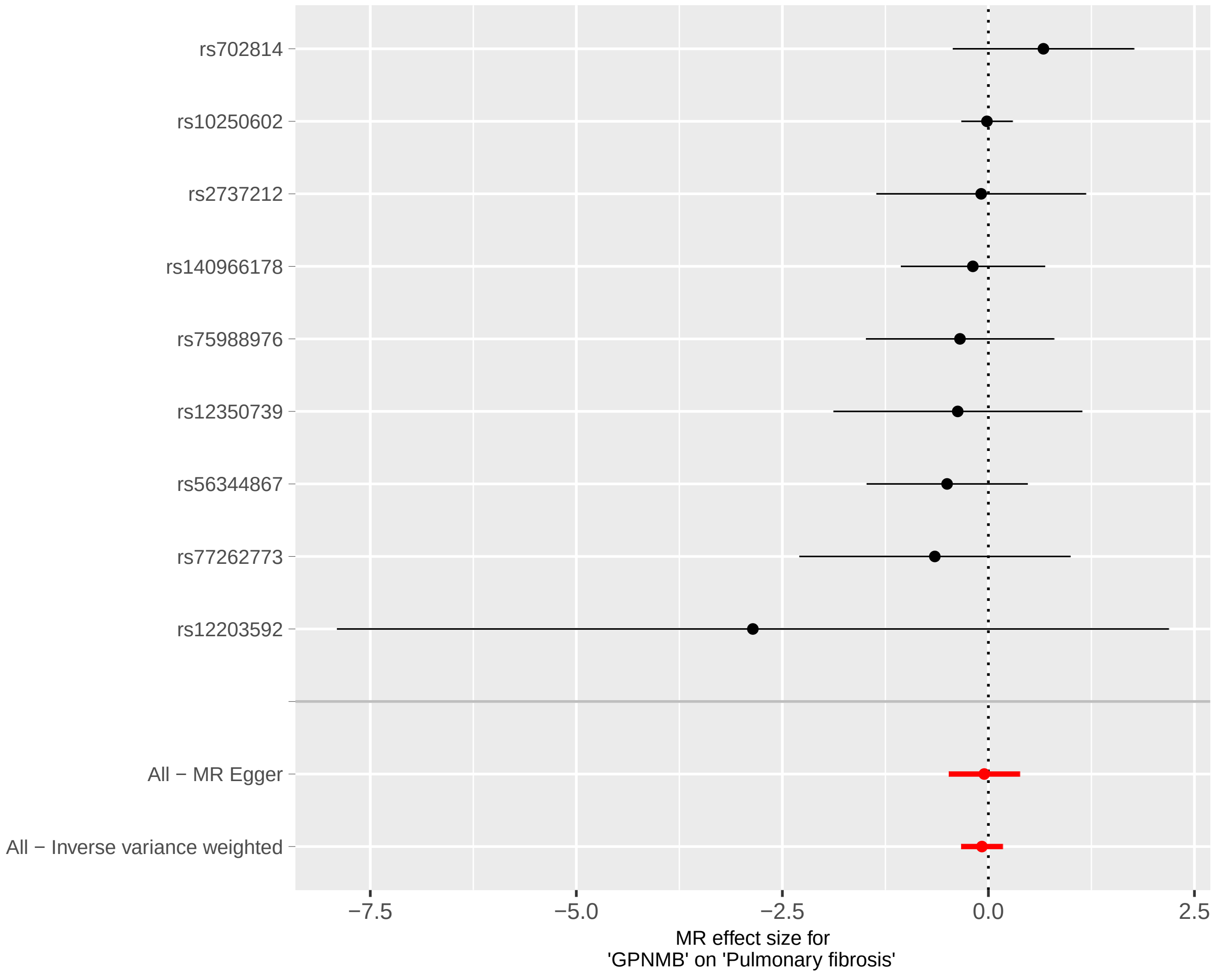
rs702814
rs10250602
rs2737212
rs140966178
rs75988976
rs12350739
rs56344867
rs77262773
rs12203592
All − MR Egger
All − Inverse variance weighted
−7.5
−5.0
−2.5
0.0
2.5
MR effect size for
'GPNMB' on 'Pulmonary fibrosis'

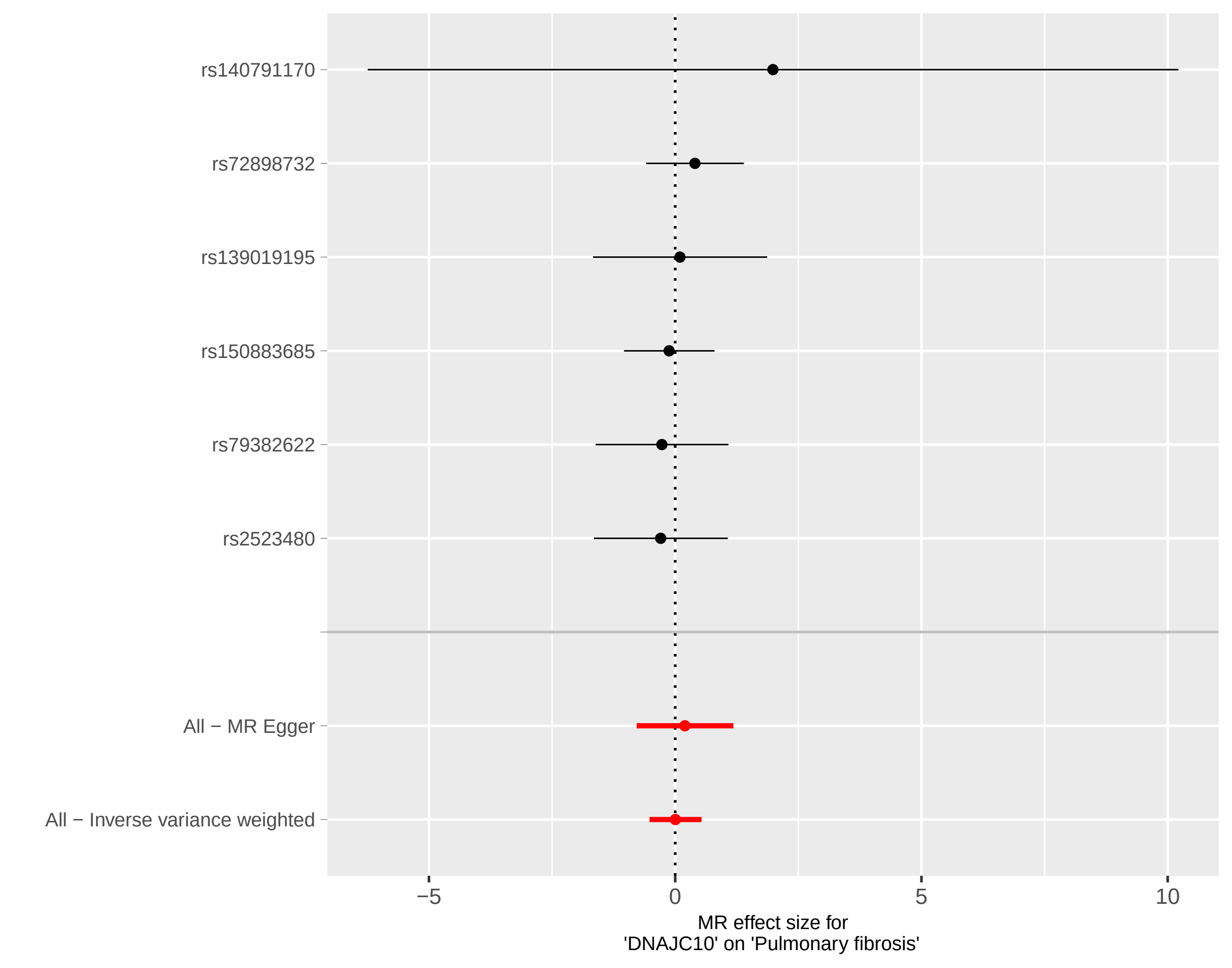

rs140791170
rs72898732
rs139019195
rs150883685
rs79382622
rs2523480
All − MR Egger
All − Inverse variance weighted
−5
0
5
10
MR effect size for
'DNAJC10' on 'Pulmonary fibrosis'

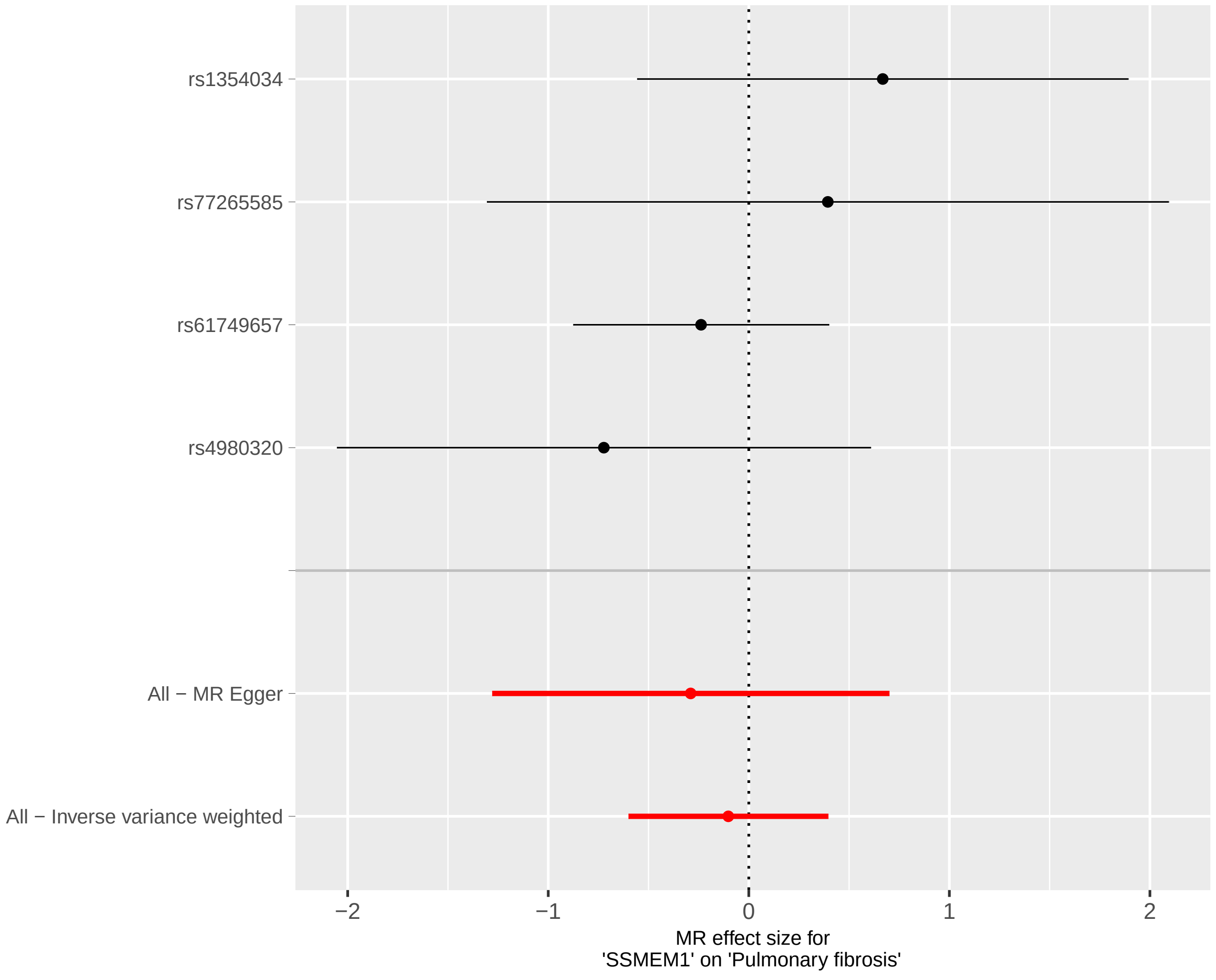

rs1354034
rs77265585
rs61749657
rs4980320
All − MR Egger
All − Inverse variance weighted
−2
−1
0
1
2
MR effect size for
'SSMEM1' on 'Pulmonary fibrosis'

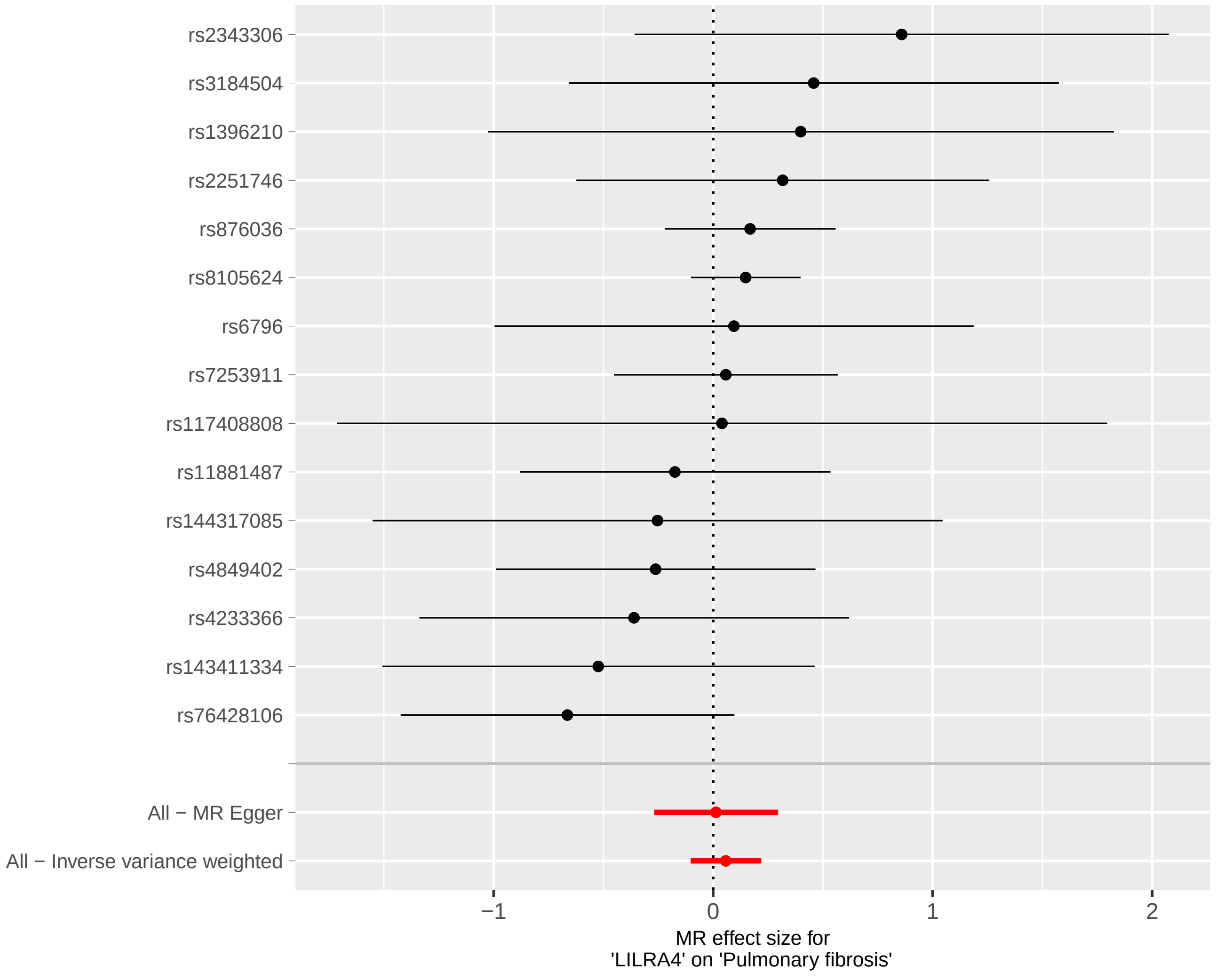
rs2343306
rs3184504
rs1396210
rs2251746
rs876036
rs8105624
rs6796
rs7253911
rs117408808
rs11881487
rs144317085
rs4849402
rs4233366
rs143411334
rs76428106
All − MR Egger
All − Inverse variance weighted
−1
0
1
2
MR effect size for
'LILRA4' on 'Pulmonary fibrosis'

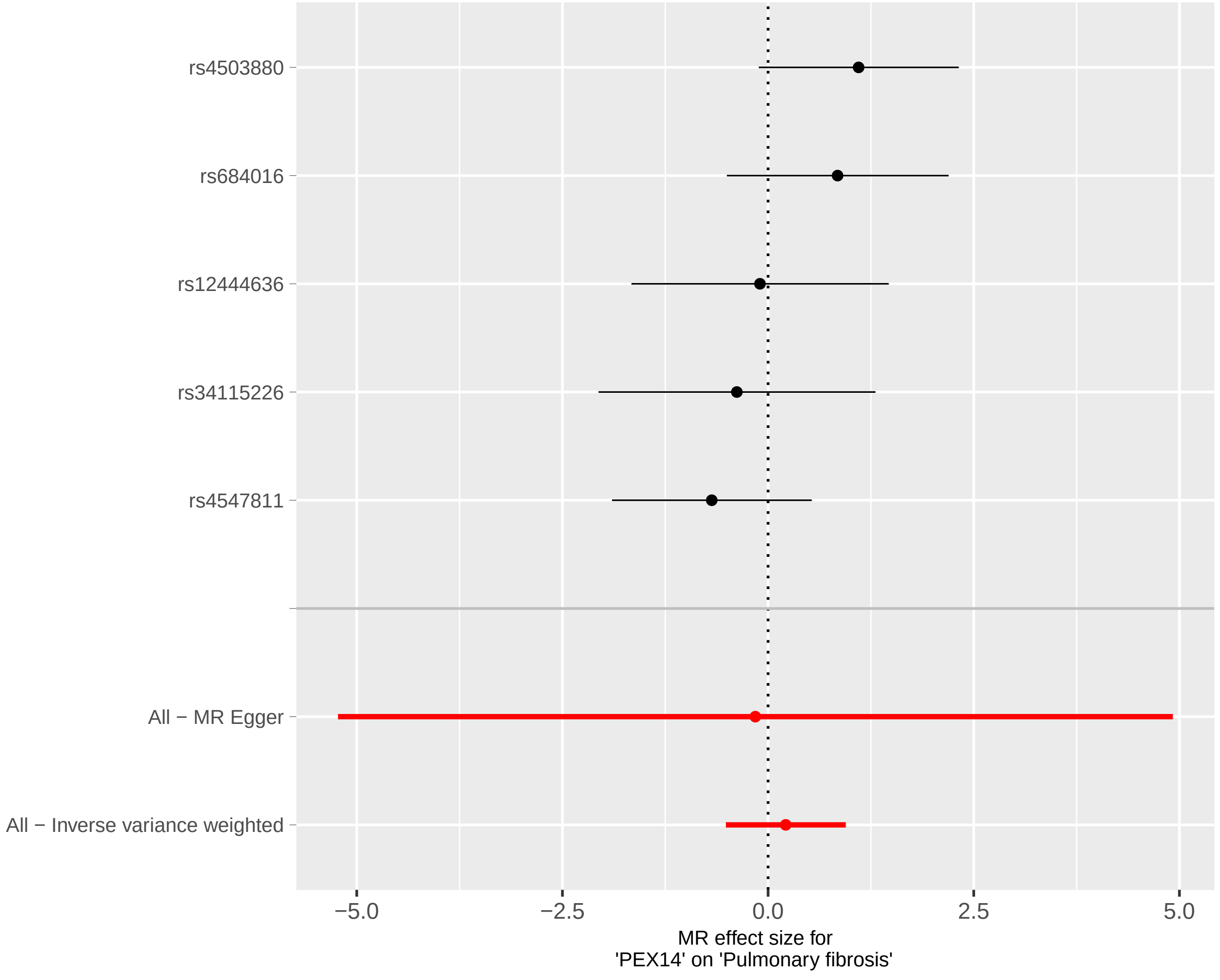
rs4503880
rs684016
rs12444636
rs34115226
rs4547811
All − MR Egger
All − Inverse variance weighted
−5.0
−2.5
0.0
2.5
5.0
MR effect size for
'PEX14' on 'Pulmonary fibrosis'

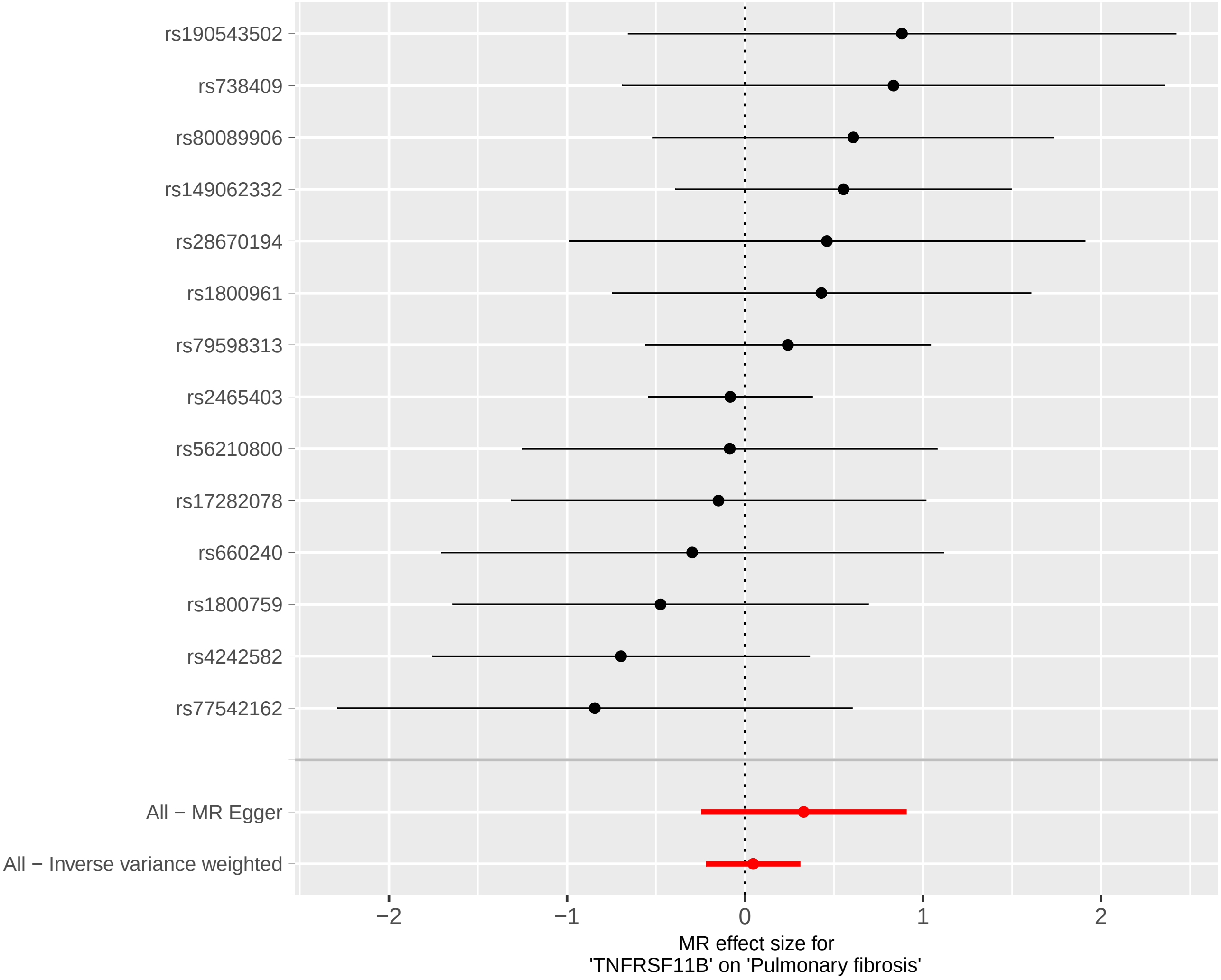
rs190543502
rs738409
rs80089906
rs149062332
rs28670194
rs1800961
rs79598313
rs2465403
rs56210800
rs17282078
rs660240
rs1800759
rs4242582
rs77542162
All − MR Egger
All − Inverse variance weighted
−2
−1
0
1
2
MR effect size for
'TNFRSF11B' on 'Pulmonary fibrosis'

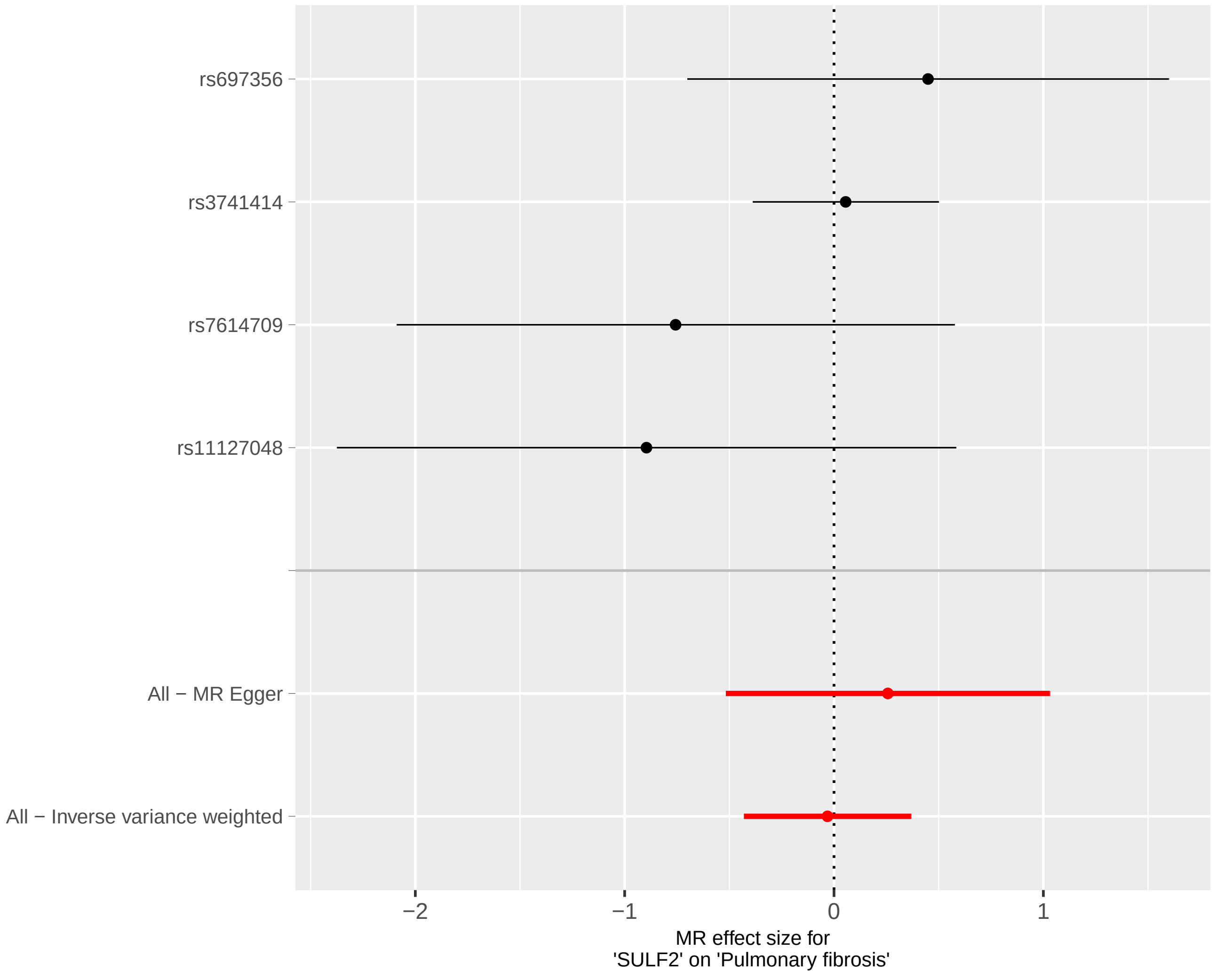

rs697356
rs3741414
rs7614709
rs11127048
All − MR Egger
All − Inverse variance weighted
−2
−1
0
1
MR effect size for
'SULF2' on 'Pulmonary fibrosis'

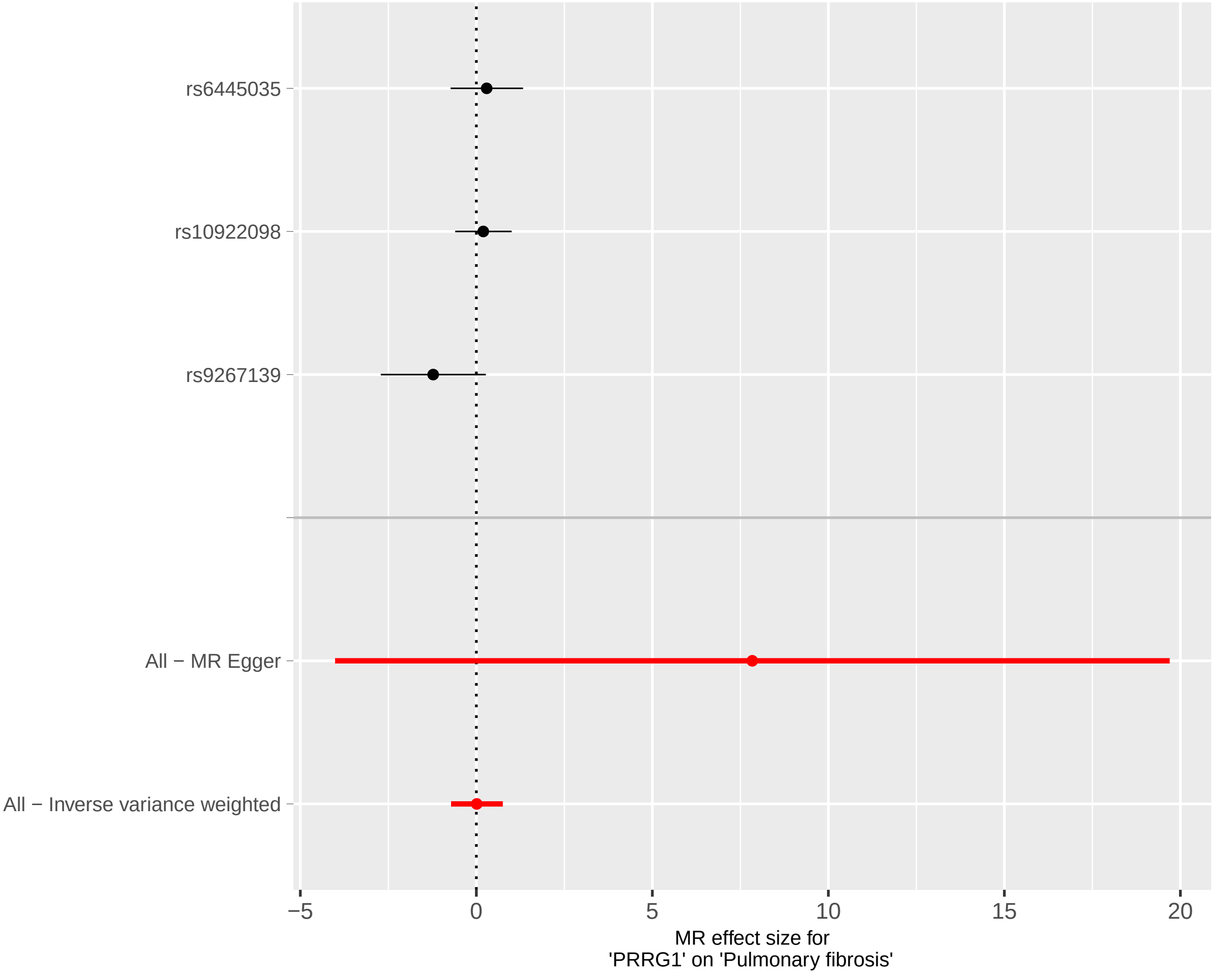

rs6445035
rs10922098
rs9267139
All − MR Egger
All − Inverse variance weighted
−5
0
5
10
15
20
MR effect size for
'PRRG1' on 'Pulmonary fibrosis'

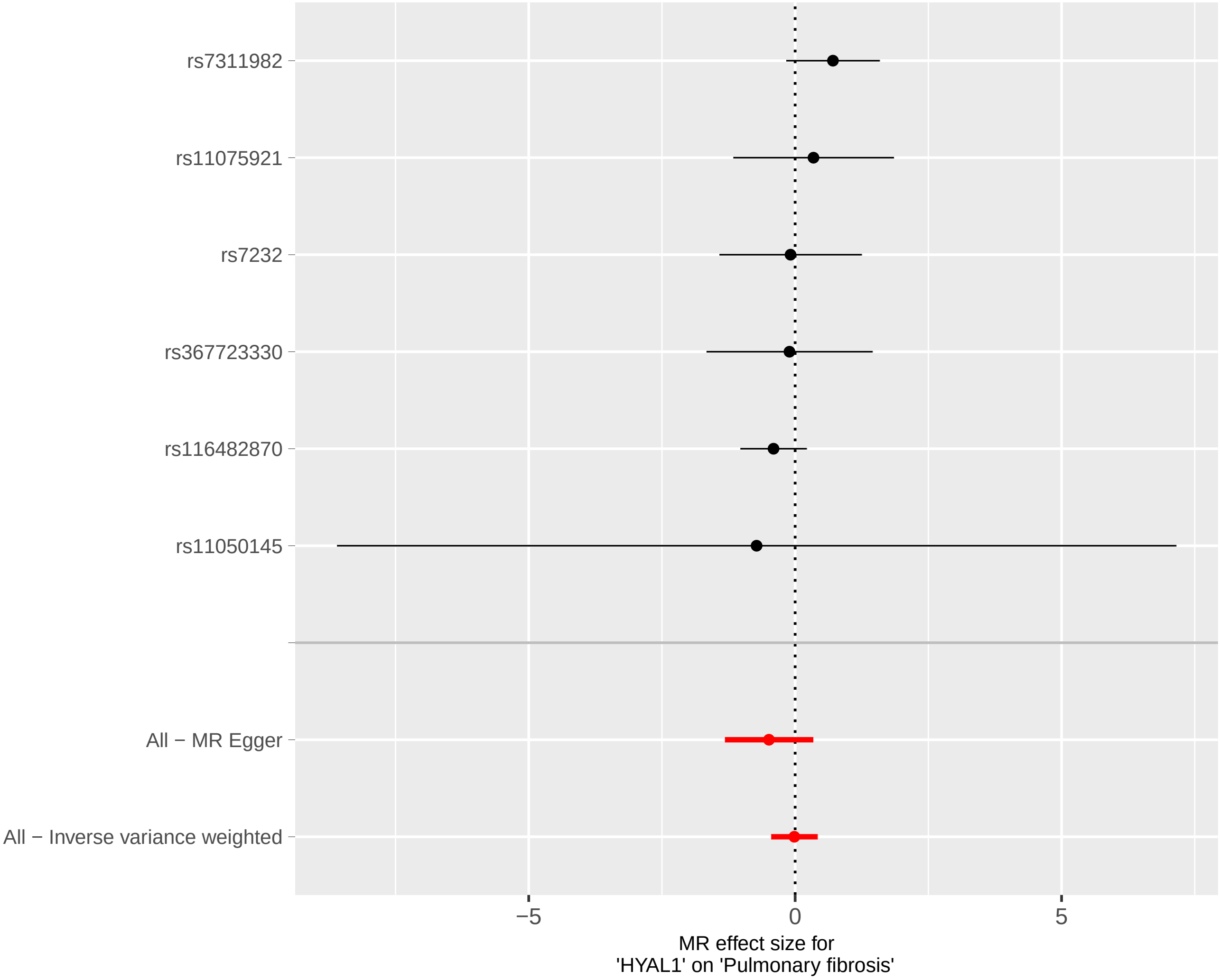
rs7311982
rs11075921
rs7232
rs367723330
rs116482870
rs11050145
All − MR Egger
All − Inverse variance weighted
−5
0
5
MR effect size for
'HYAL1' on 'Pulmonary fibrosis'

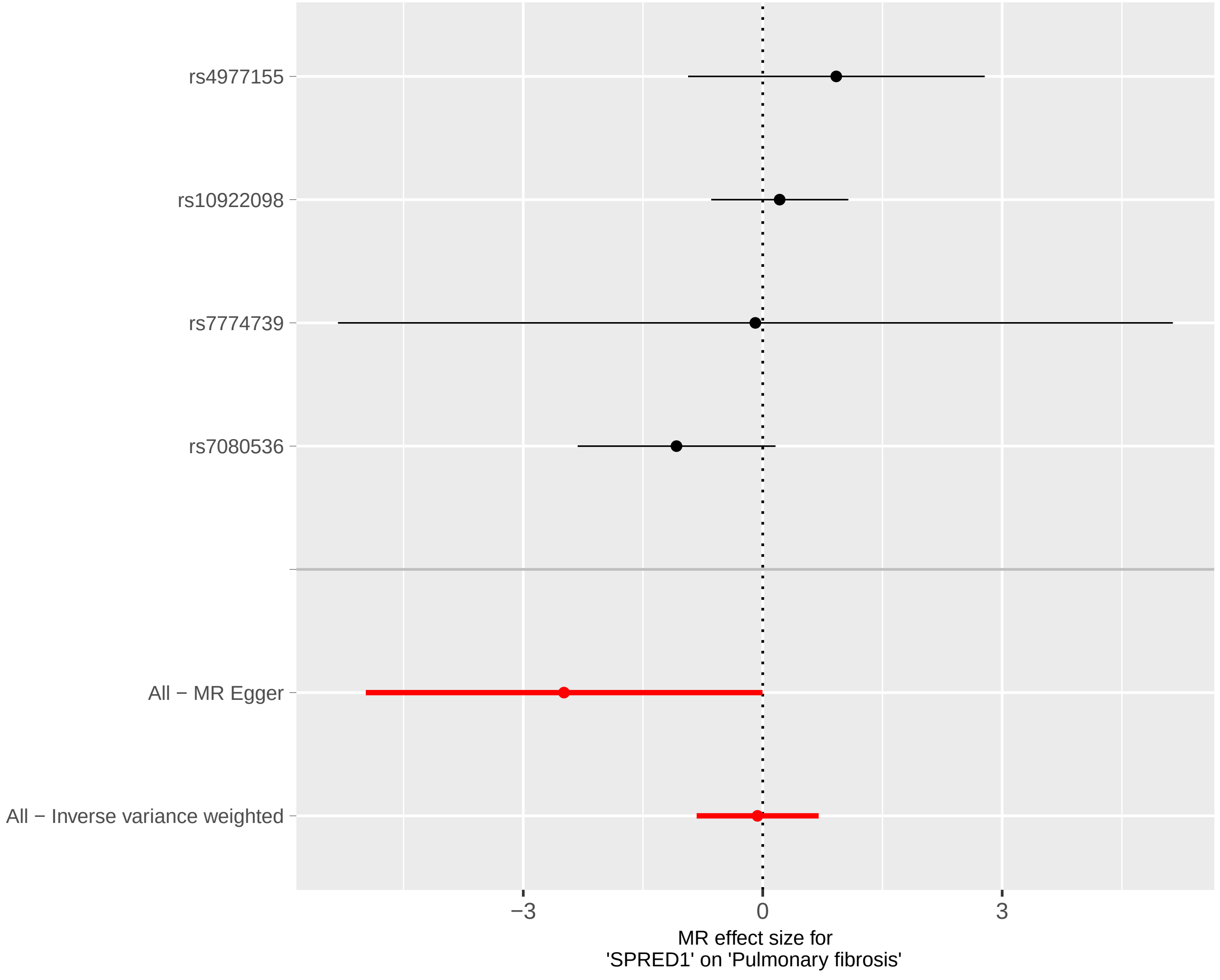

rs4977155
rs10922098
rs7774739
rs7080536
All − MR Egger
All − Inverse variance weighted
−3
0
3
MR effect size for
'SPRED1' on 'Pulmonary fibrosis'

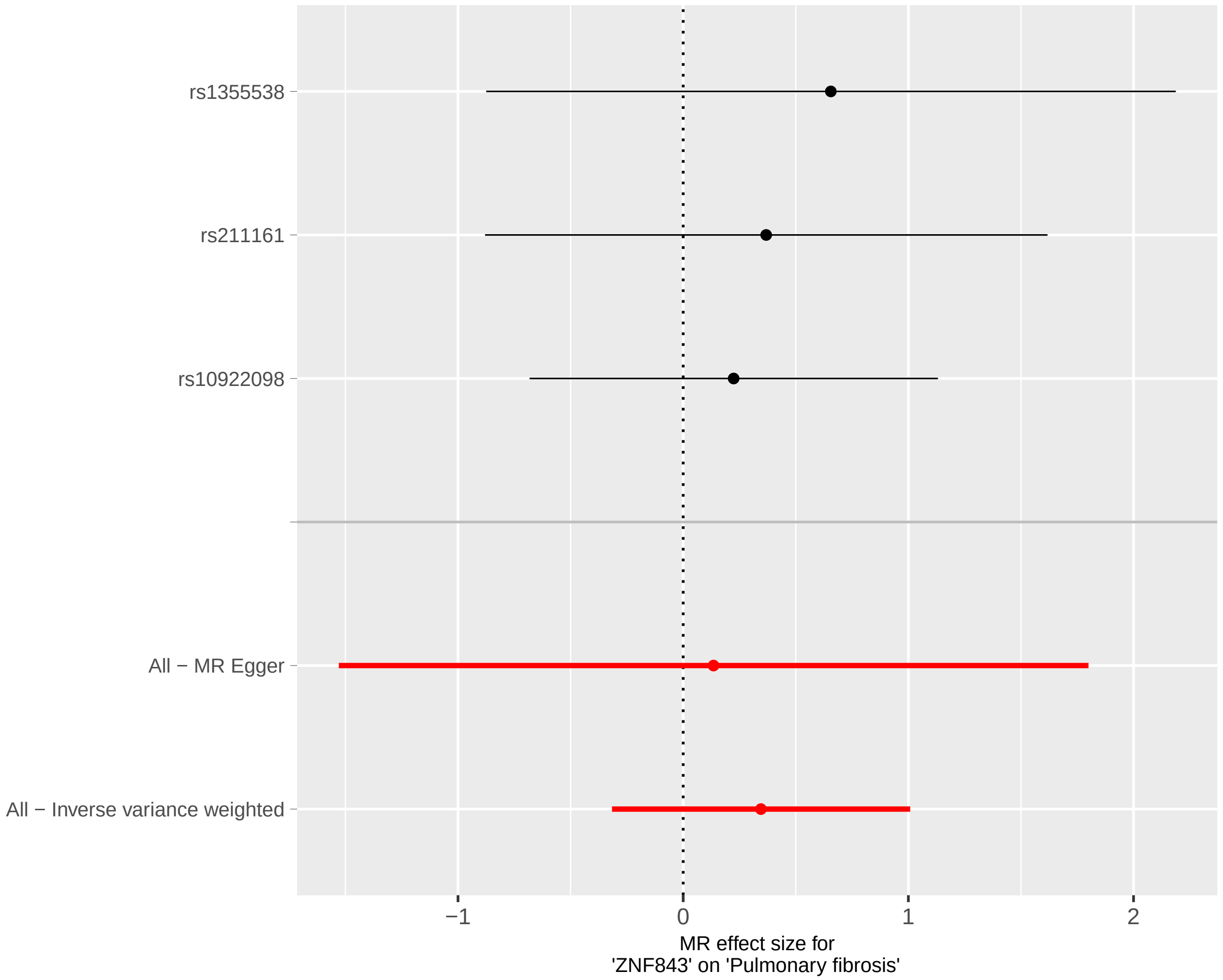
rs1355538
rs211161
rs10922098
All − MR Egger
All − Inverse variance weighted
−1
0
1
2
MR effect size for
'ZNF843' on 'Pulmonary fibrosis'

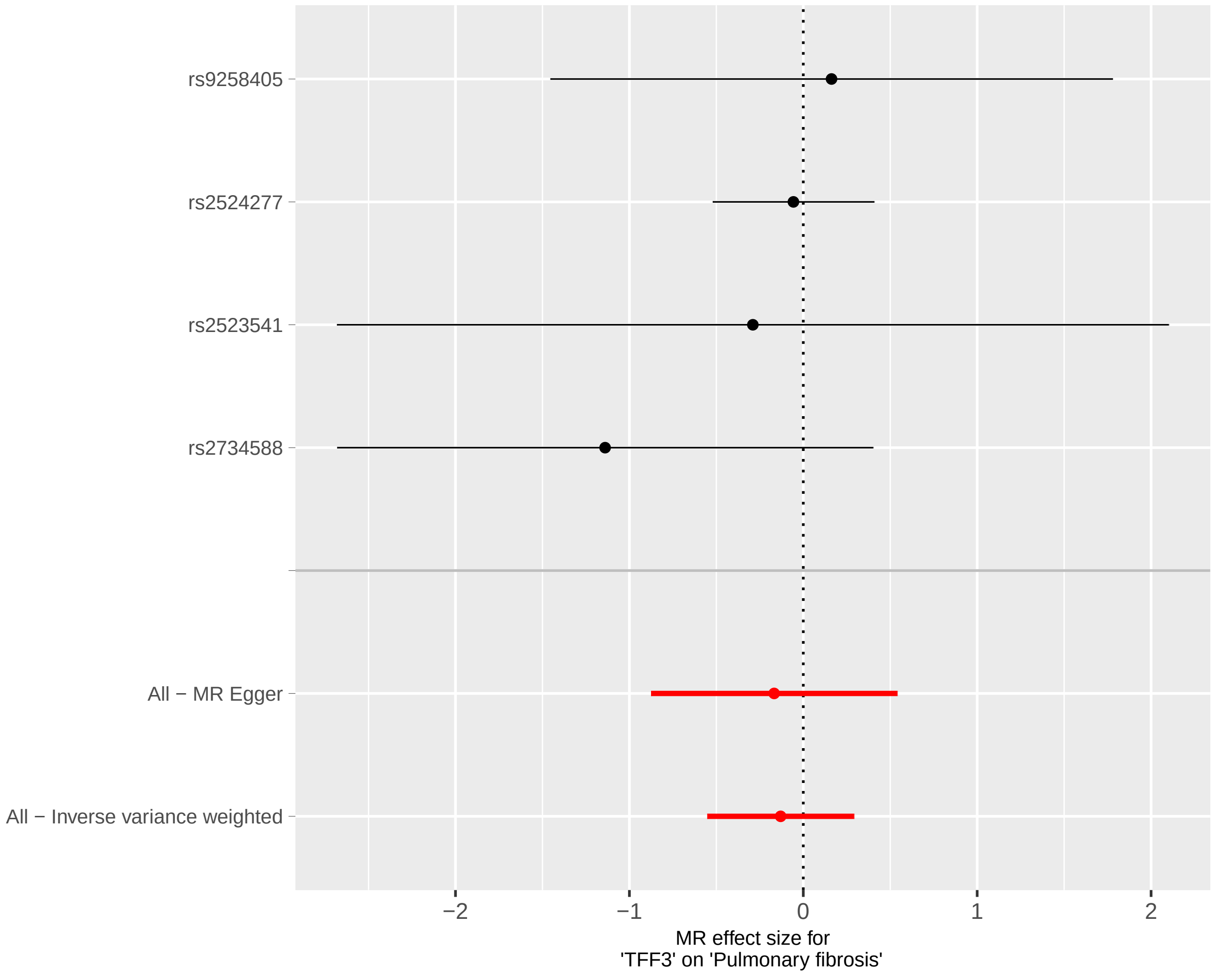
rs9258405
rs2524277
rs2523541
rs2734588
All − MR Egger
All − Inverse variance weighted
−2
−1
0
1
2
MR effect size for
'TFF3' on 'Pulmonary fibrosis'

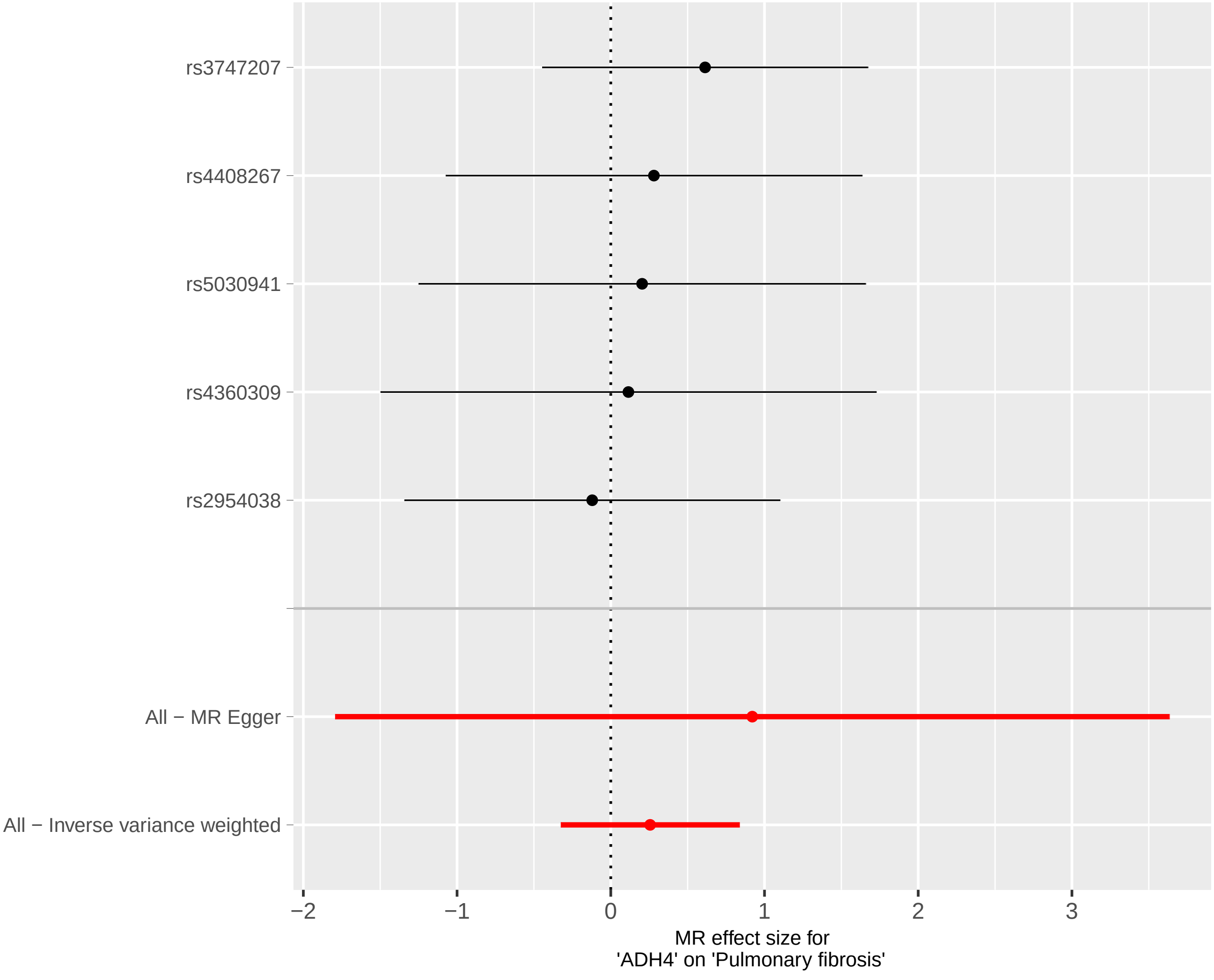

rs3747207
rs4408267
rs5030941
rs4360309
rs2954038
All − MR Egger
All − Inverse variance weighted
−2
−1
0
1
2
3
MR effect size for
'ADH4' on 'Pulmonary fibrosis'

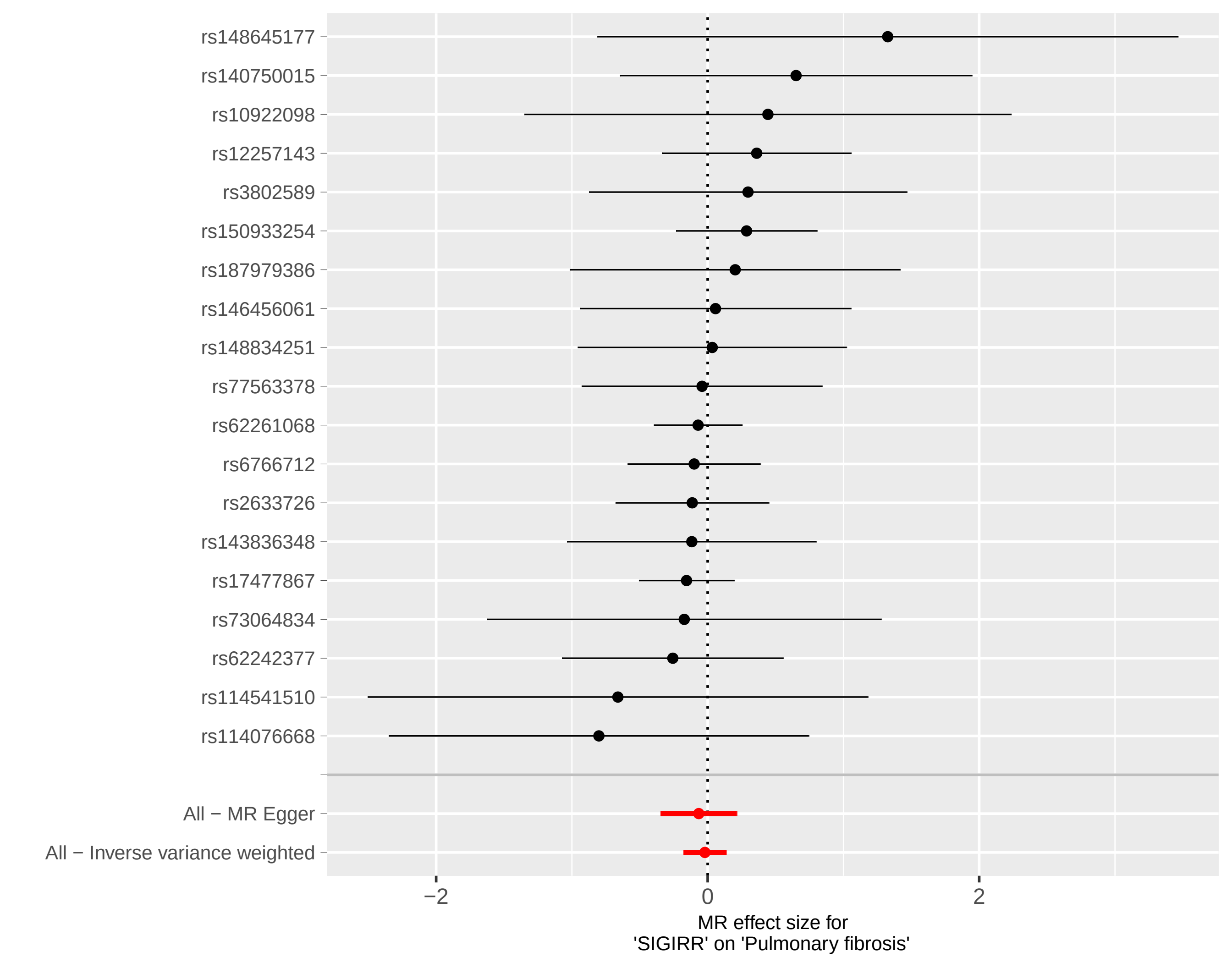

rs148645177
rs140750015
rs10922098
rs12257143
rs3802589
rs150933254
rs187979386
rs146456061
rs148834251
rs77563378
rs62261068
rs6766712
rs2633726
rs143836348
rs17477867
rs73064834
rs62242377
rs114541510
rs114076668
All − MR Egger
All − Inverse variance weighted
−2
0
2
MR effect size for
'SIGIRR' on 'Pulmonary fibrosis'

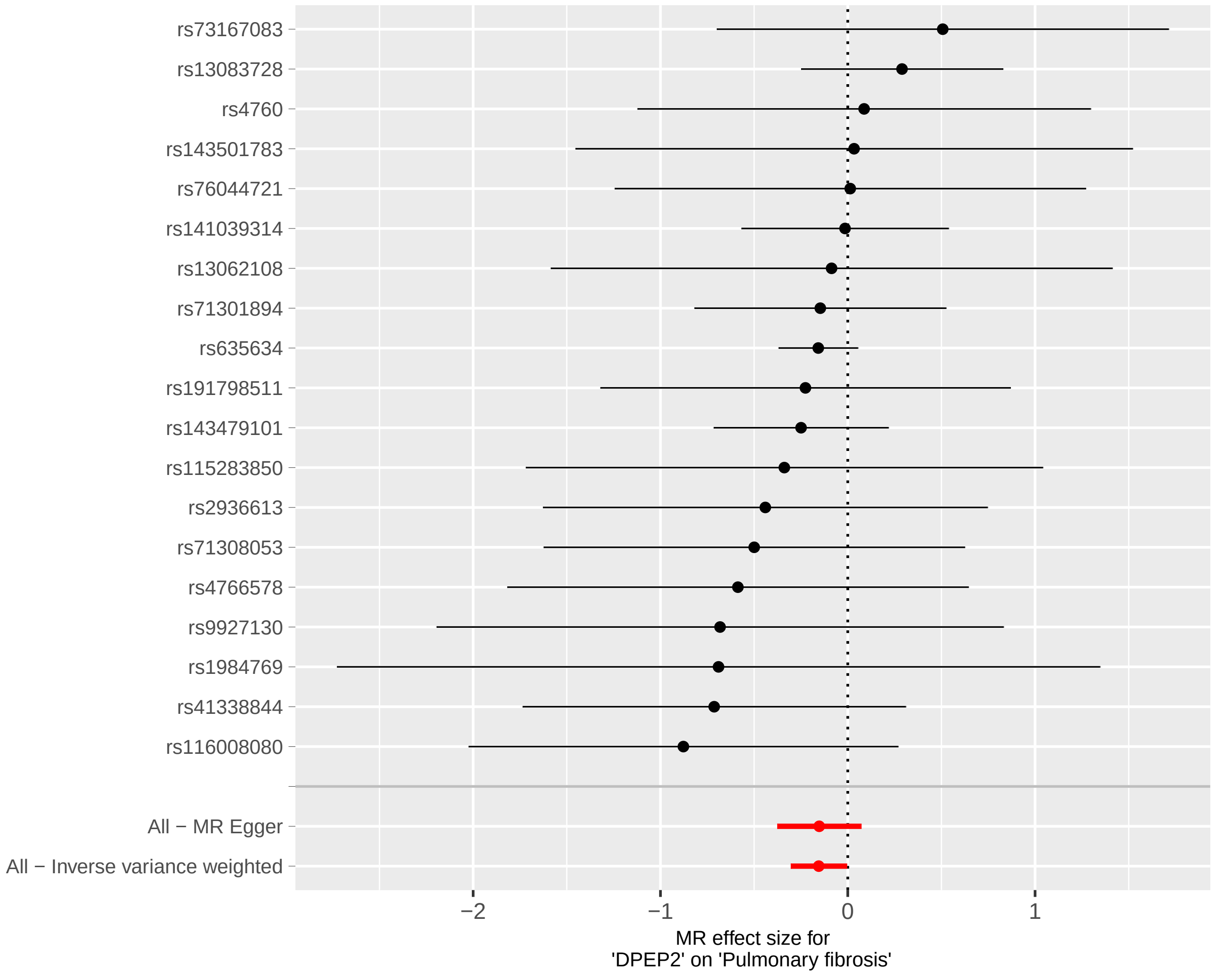
rs73167083
rs13083728
rs4760
rs143501783
rs76044721
rs141039314
rs13062108
rs71301894
rs635634
rs191798511
rs143479101
rs115283850
rs2936613
rs71308053
rs4766578
rs9927130
rs1984769
rs41338844
rs116008080
All − MR Egger
All − Inverse variance weighted
−2
−1
0
1
MR effect size for
'DPEP2' on 'Pulmonary fibrosis'

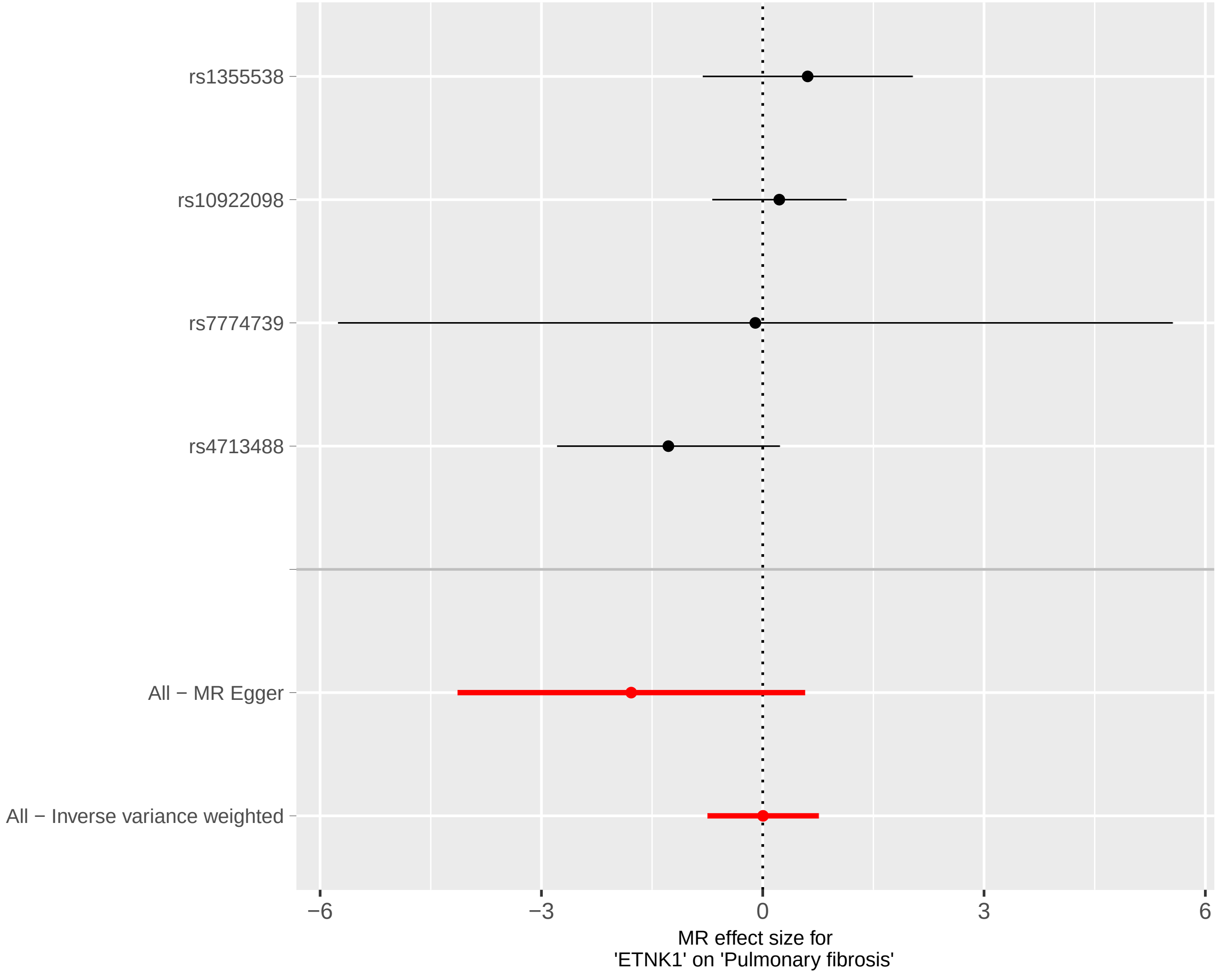
rs1355538
rs10922098
rs7774739
rs4713488
All − MR Egger
All − Inverse variance weighted
−6
−3
0
3
6
MR effect size for
'ETNK1' on 'Pulmonary fibrosis'

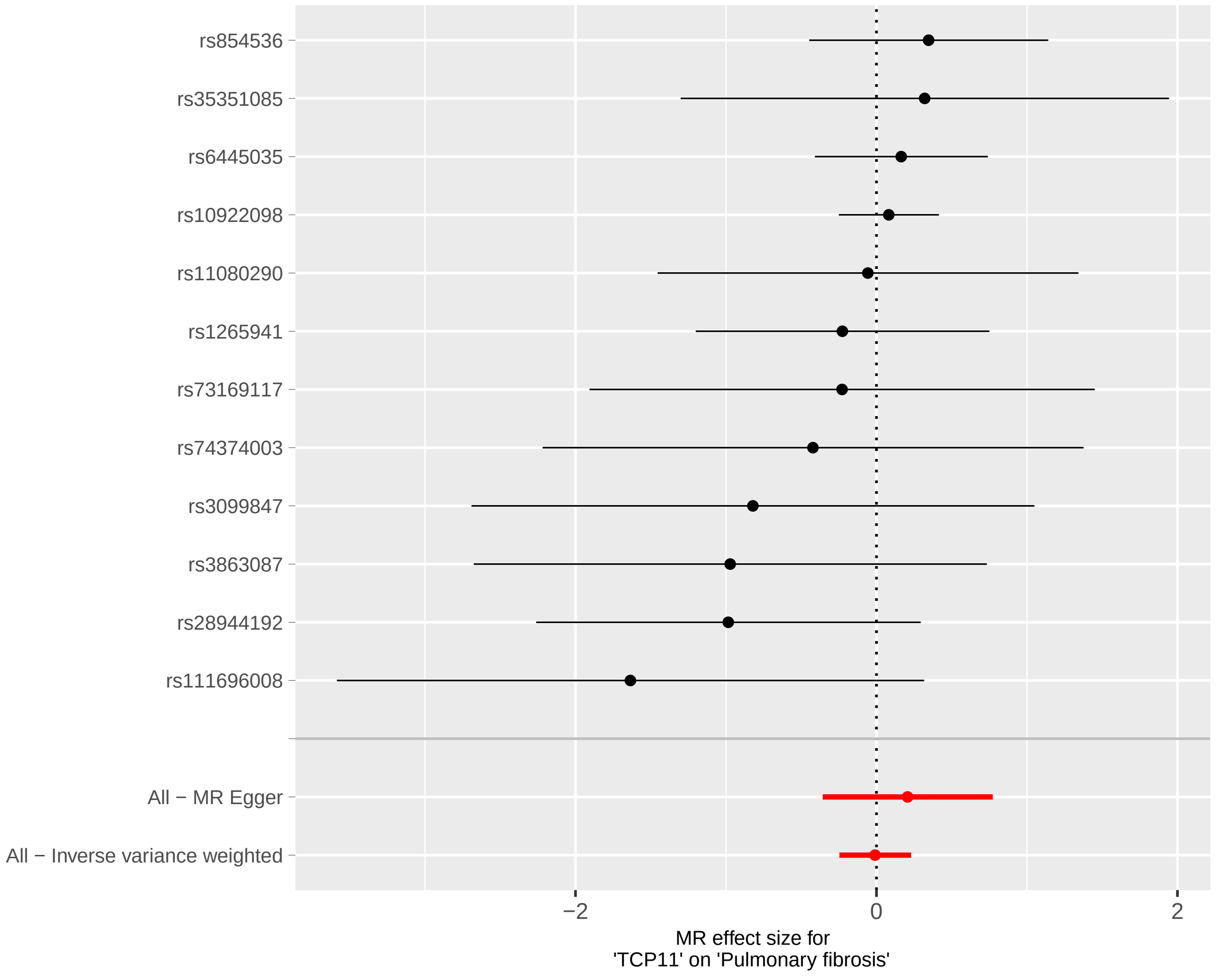

rs854536
rs35351085
rs6445035
rs10922098
rs11080290
rs1265941
rs73169117
rs74374003
rs3099847
rs3863087
rs28944192
rs111696008
All − MR Egger
All − Inverse variance weighted
−2
0
2
MR effect size for
'TCP11' on 'Pulmonary fibrosis'

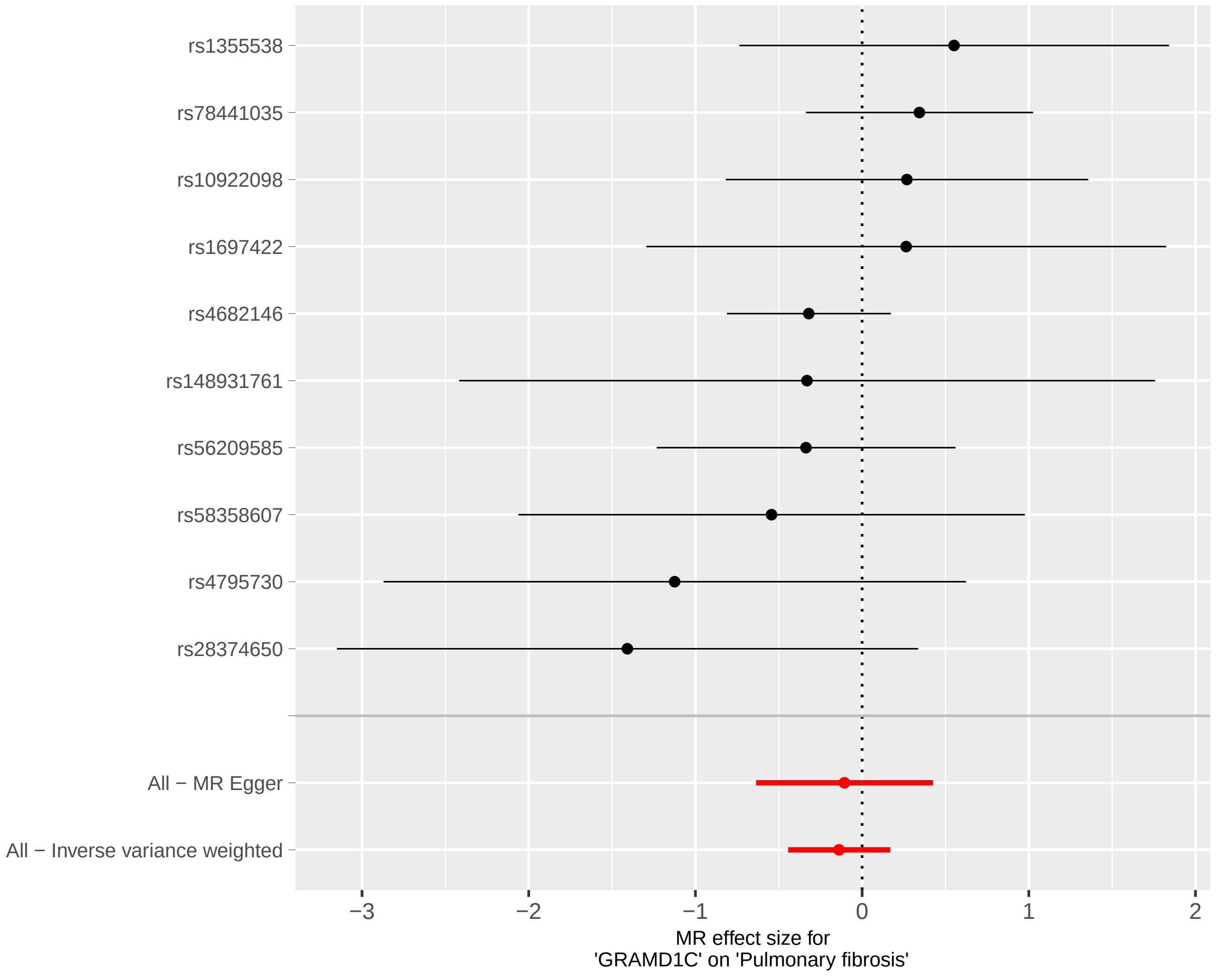
rs1355538
rs78441035
rs10922098
rs1697422
rs4682146
rs148931761
rs56209585
rs58358607
rs4795730
rs28374650
All − MR Egger
All − Inverse variance weighted
−3
−2
−1
0
1
2
MR effect size for
'GRAMD1C' on 'Pulmonary fibrosis'

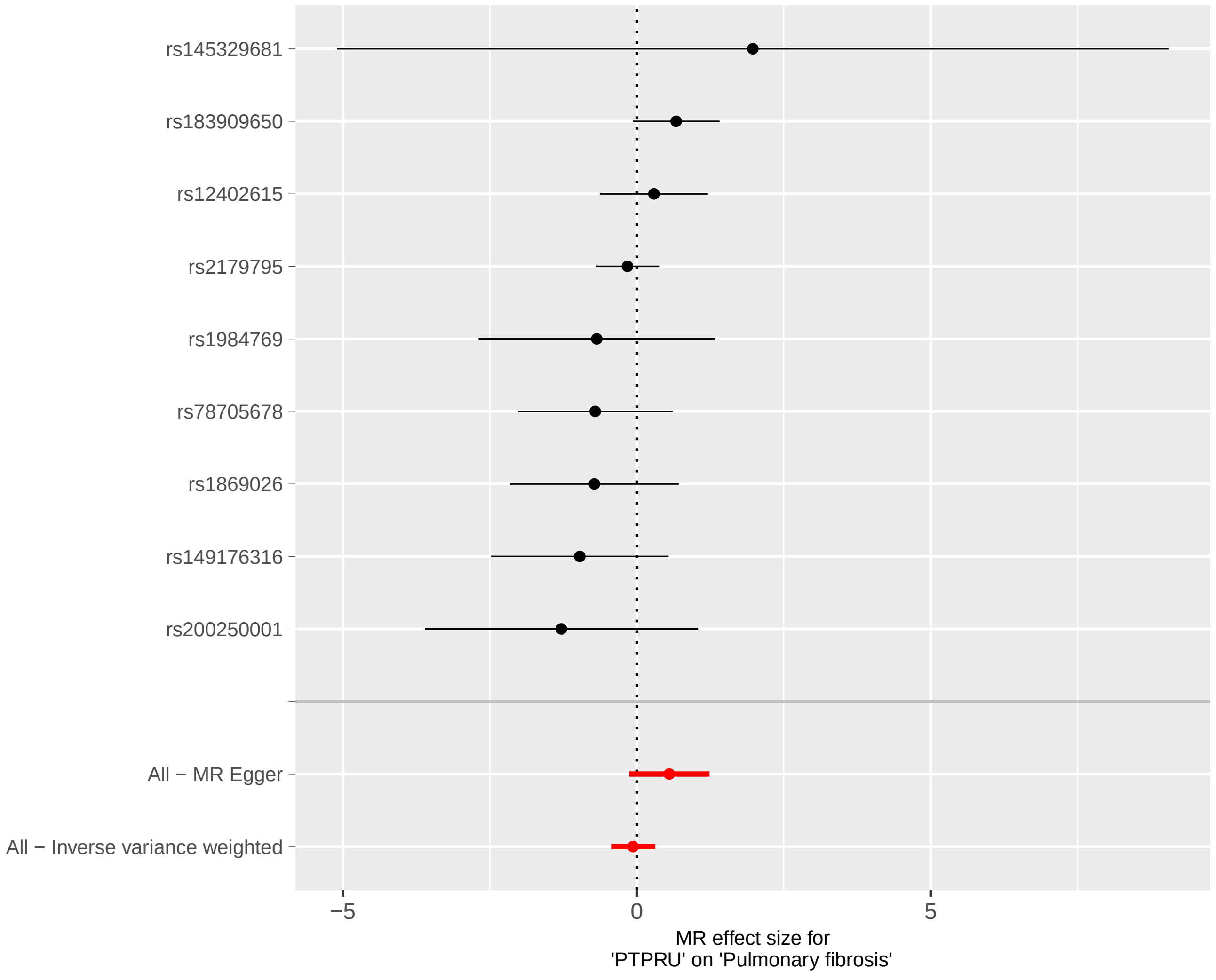

rs145329681
rs183909650
rs12402615
rs2179795
rs1984769
rs78705678
rs1869026
rs149176316
rs200250001
All − MR Egger
All − Inverse variance weighted
−5
0
5
MR effect size for
'PTPRU' on 'Pulmonary fibrosis'

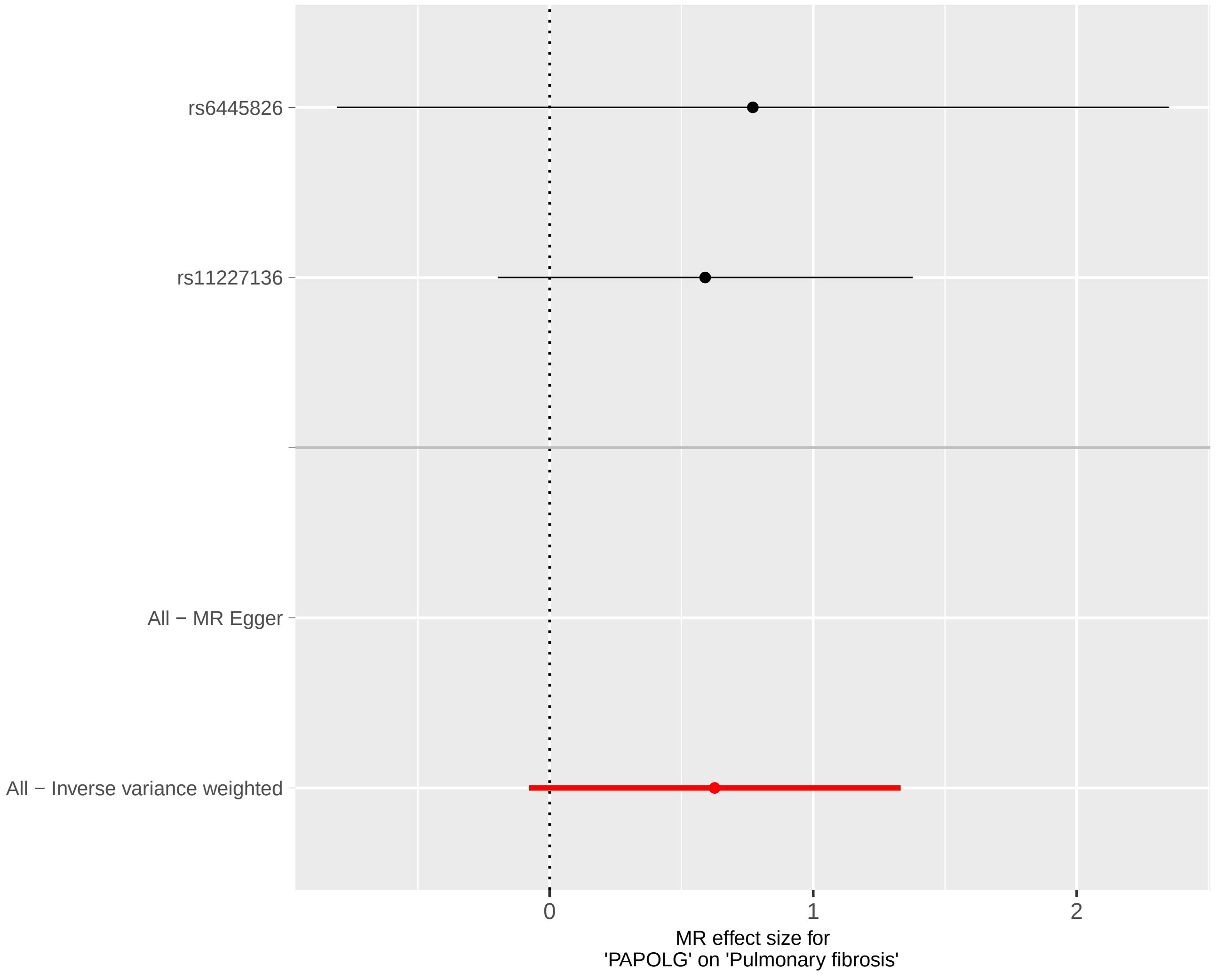

rs6445826
rs11227136
All − MR Egger
All − Inverse variance weighted
0
1
2
MR effect size for
'PAPOLG' on 'Pulmonary fibrosis'

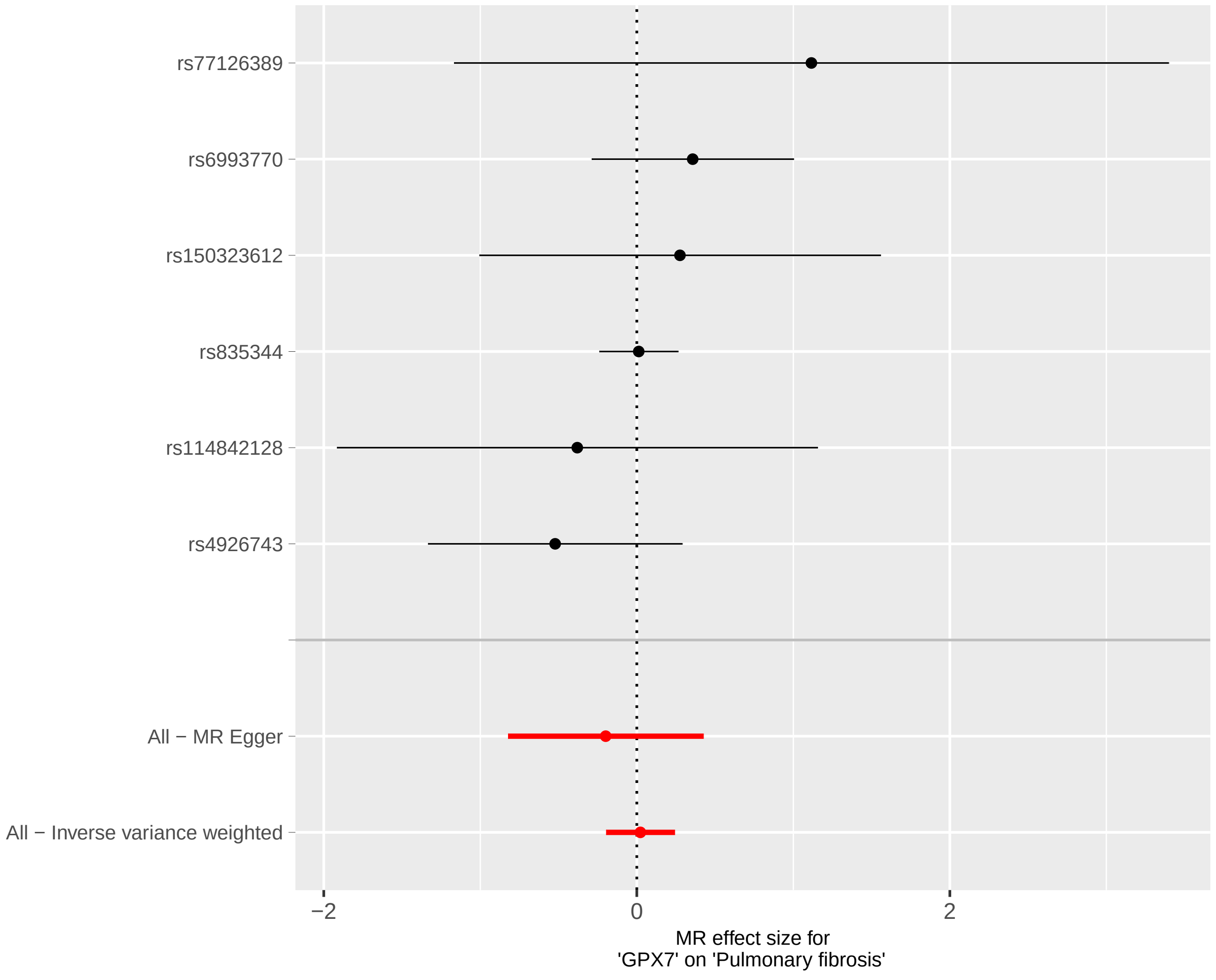
rs77126389
rs6993770
rs150323612
rs835344
rs114842128
rs4926743
All − MR Egger
All − Inverse variance weighted
−2
0
2
MR effect size for
'GPX7' on 'Pulmonary fibrosis'

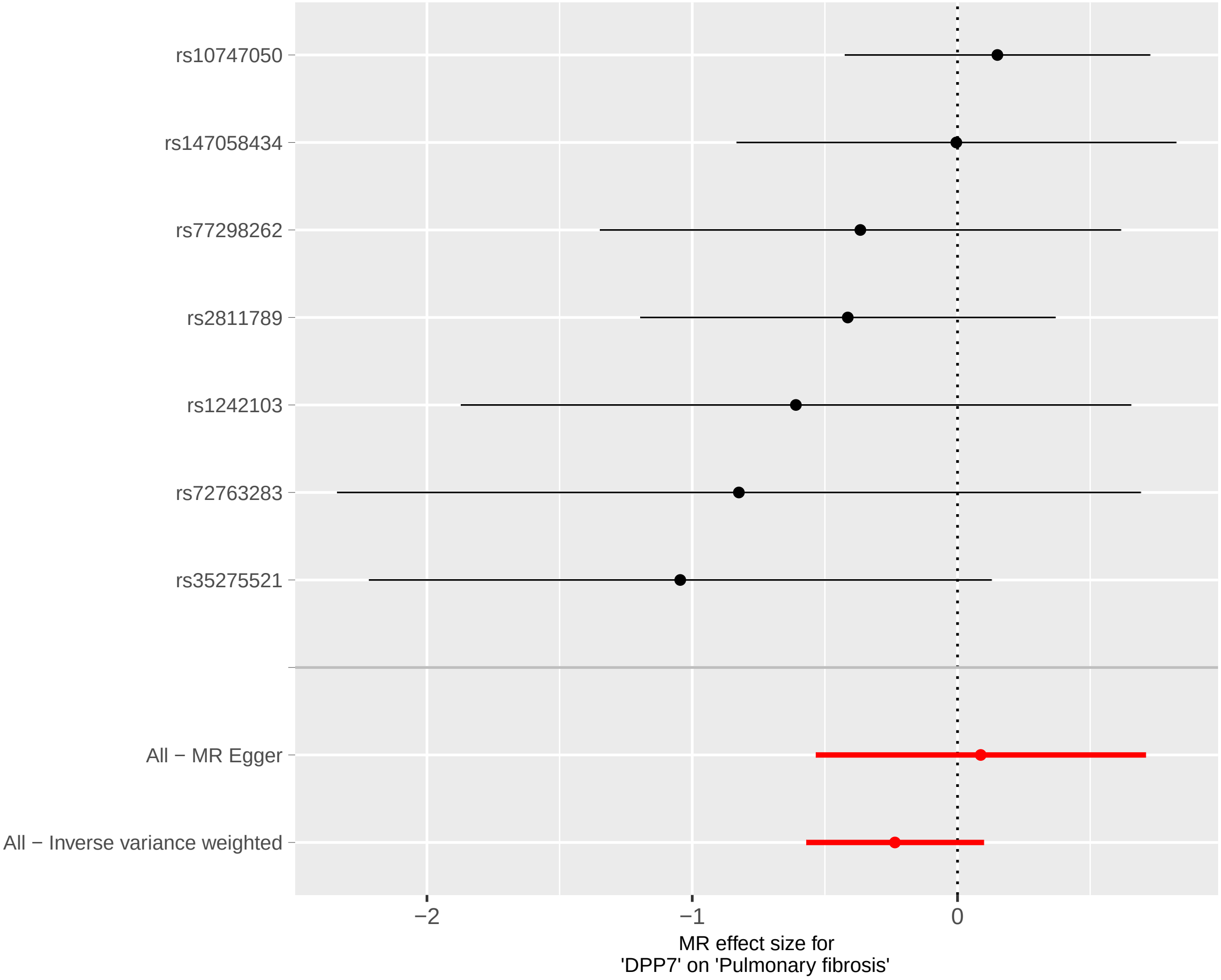
rs10747050
rs147058434
rs77298262
rs2811789
rs1242103
rs72763283
rs35275521
All − MR Egger
All − Inverse variance weighted
−2
−1
0
MR effect size for
'DPP7' on 'Pulmonary fibrosis'

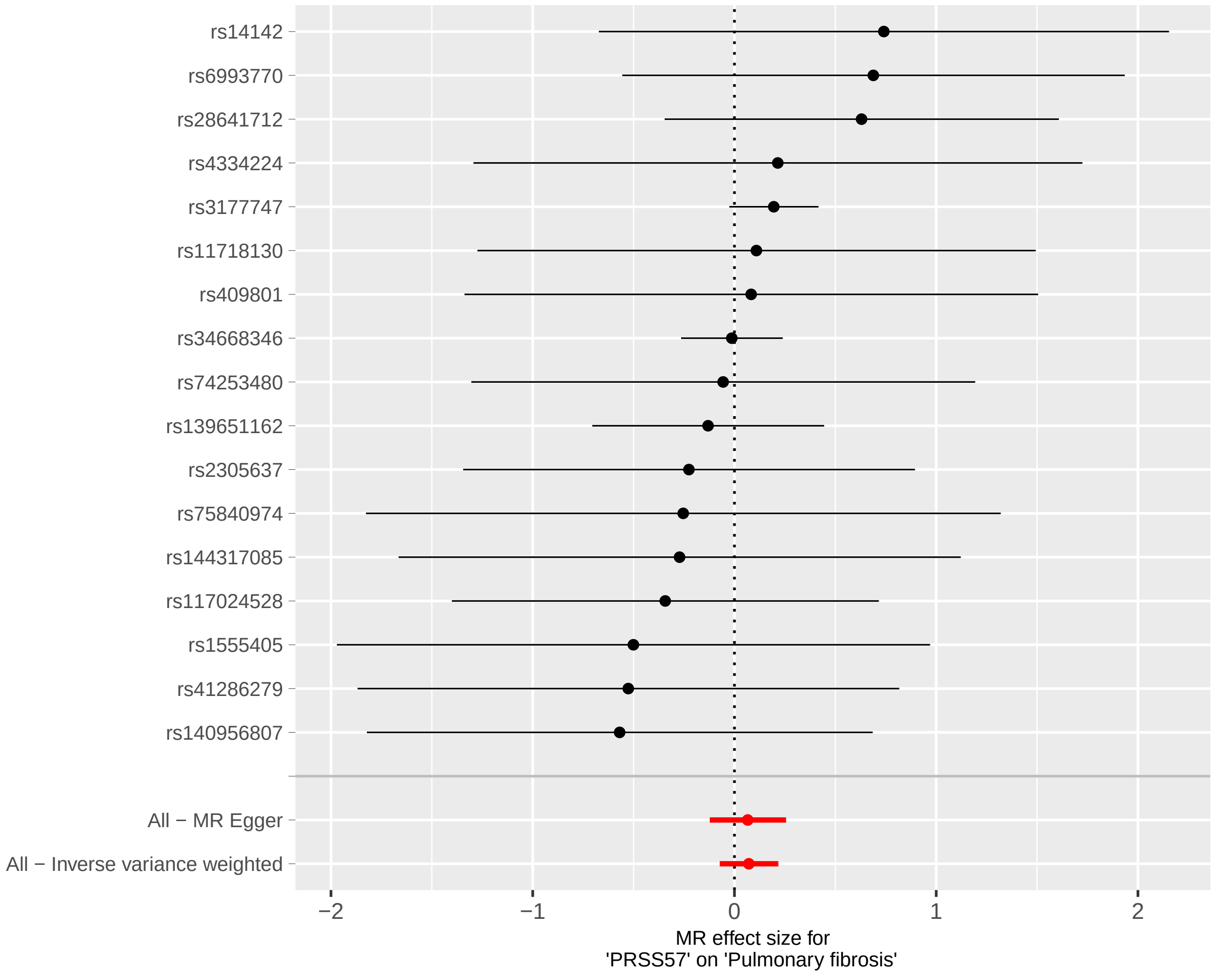

rs14142
rs6993770
rs28641712
rs4334224
rs3177747
rs11718130
rs409801
rs34668346
rs74253480
rs139651162
rs2305637
rs75840974
rs144317085
rs117024528
rs1555405
rs41286279
rs140956807
All − MR Egger
All − Inverse variance weighted
−2
−1
0
1
2
MR effect size for
'PRSS57' on 'Pulmonary fibrosis'

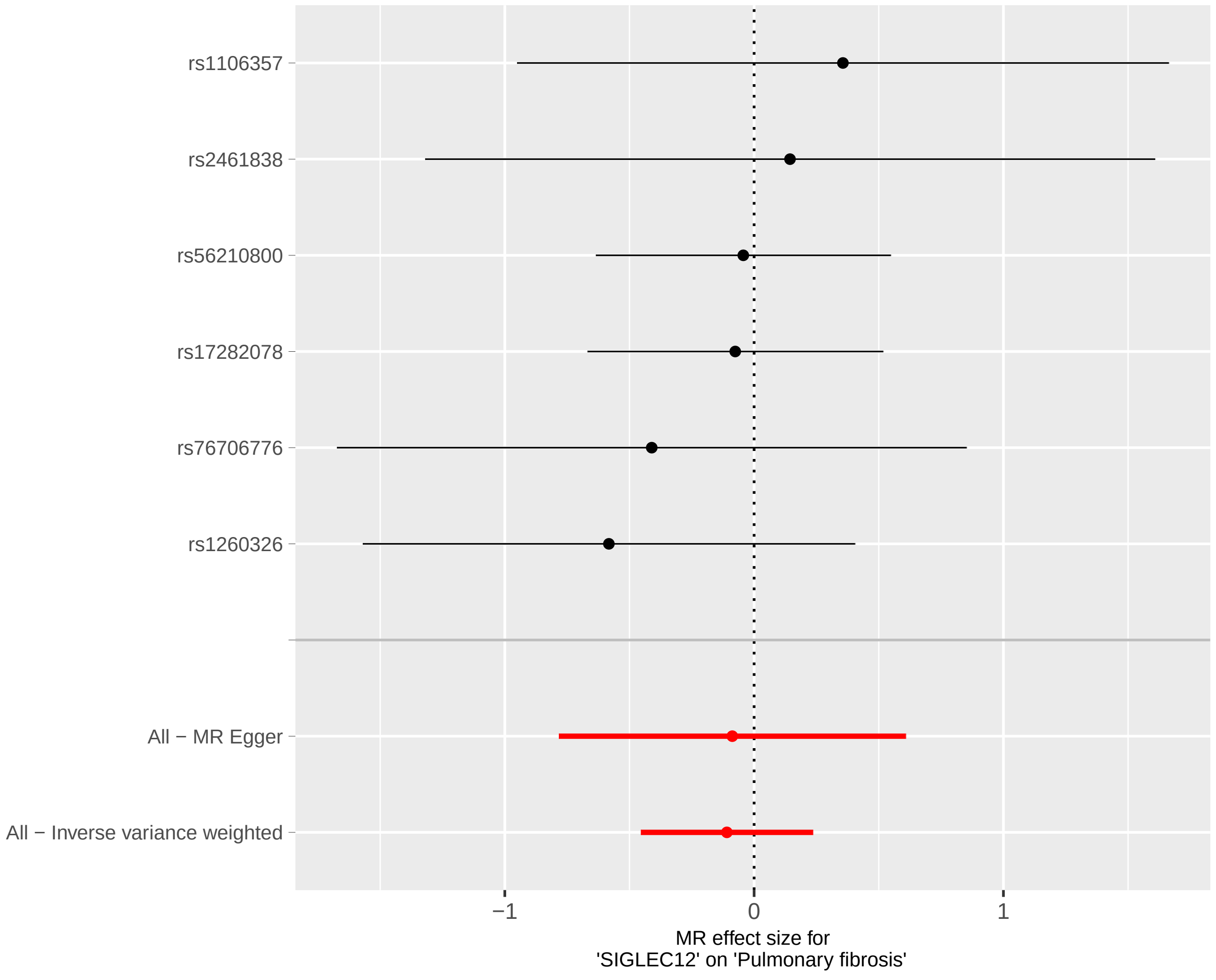
rs1106357
rs2461838
rs56210800
rs17282078
rs76706776
rs1260326
All − MR Egger
All − Inverse variance weighted
−1
0
1
MR effect size for
'SIGLEC12' on 'Pulmonary fibrosis'

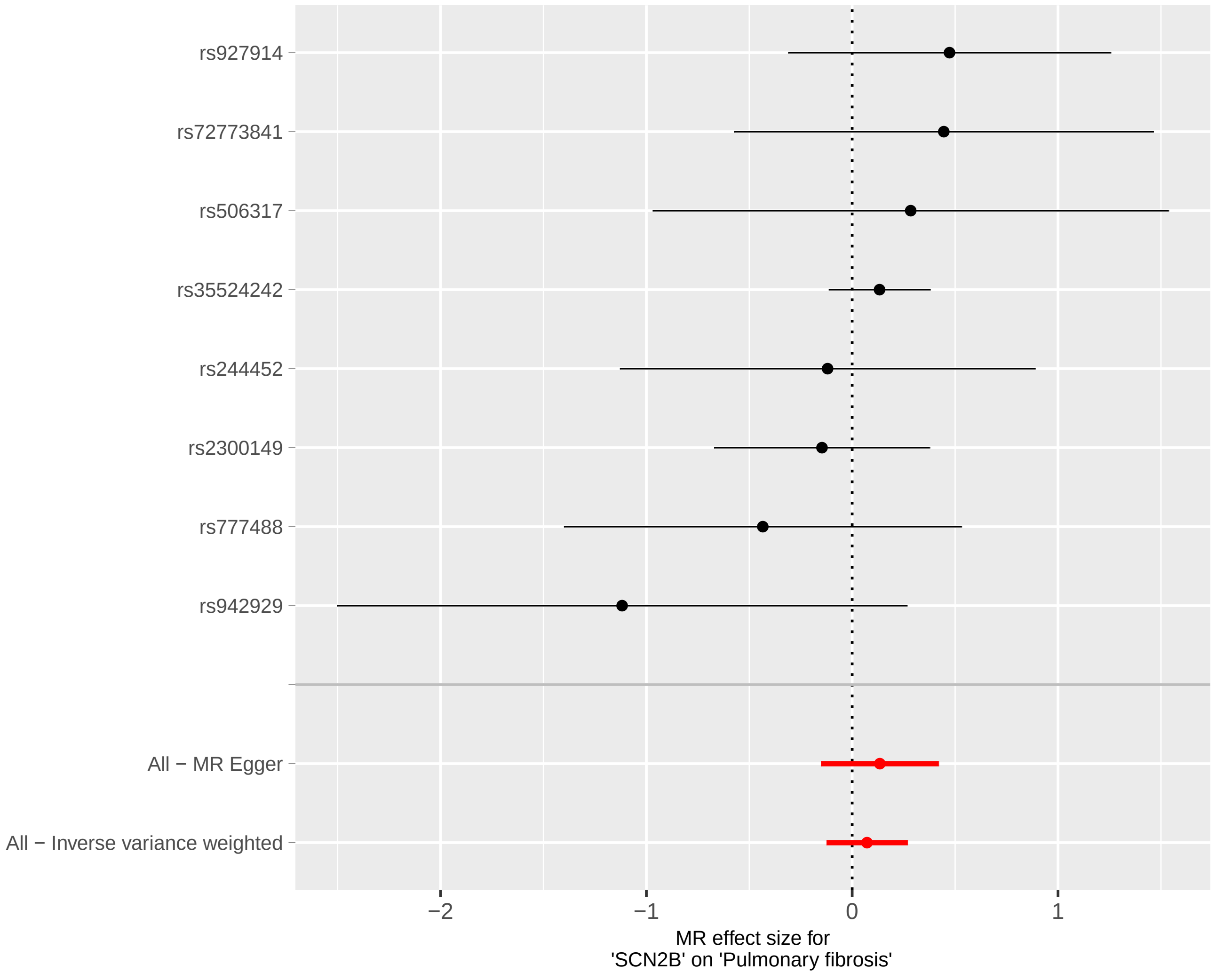
rs927914
rs72773841
rs506317
rs35524242
rs244452
rs2300149
rs777488
rs942929
All − MR Egger
All − Inverse variance weighted
−2
−1
0
1
MR effect size for
'SCN2B' on 'Pulmonary fibrosis'

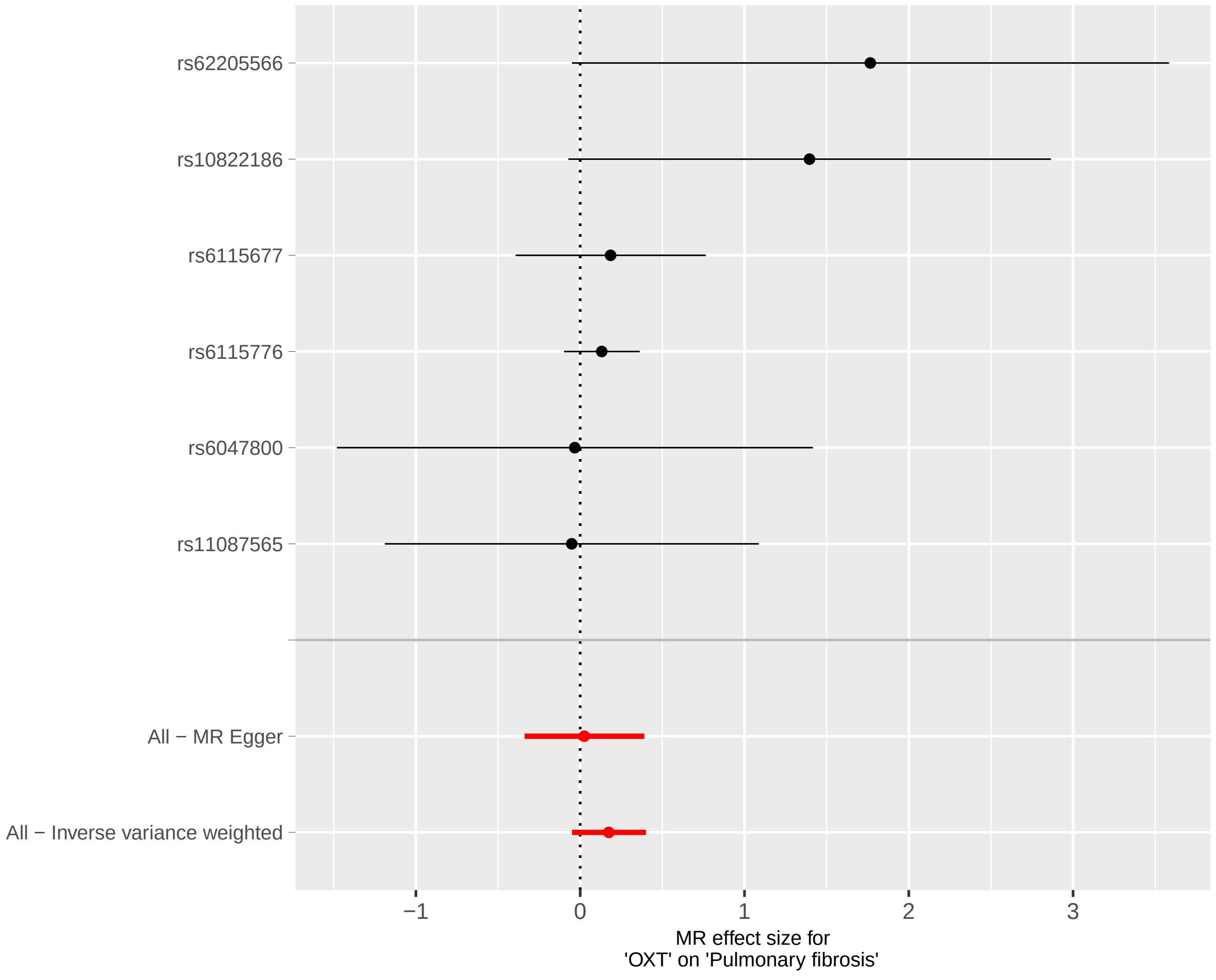
rs62205566
rs10822186
rs6115677
rs6115776
rs6047800
rs11087565
All − MR Egger
All − Inverse variance weighted
−1
0
1
2
3
MR effect size for
'OXT' on 'Pulmonary fibrosis'

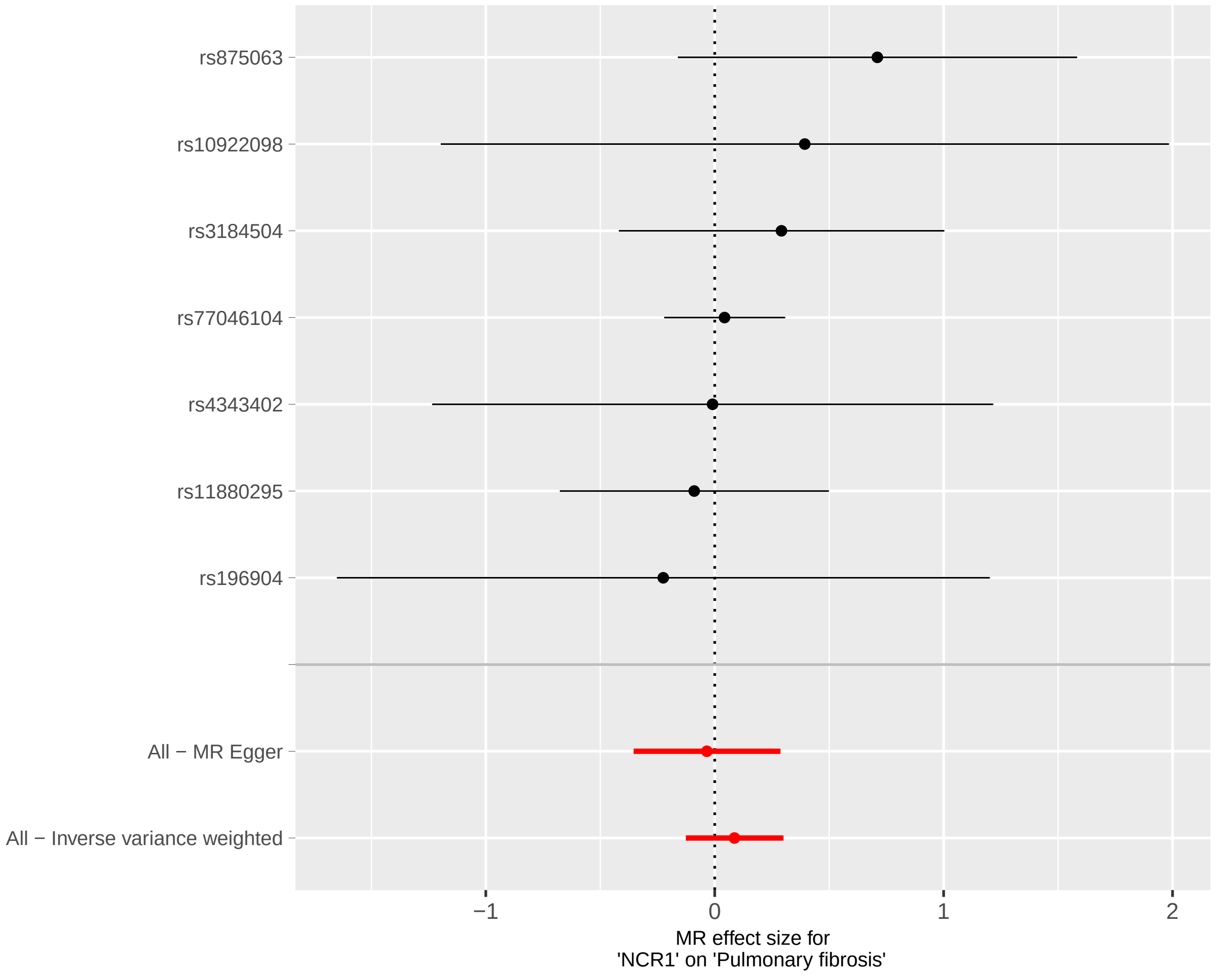
rs875063
rs10922098
rs3184504
rs77046104
rs4343402
rs11880295
rs196904
All − MR Egger
All − Inverse variance weighted
−1
0
1
2
MR effect size for
'NCR1' on 'Pulmonary fibrosis'

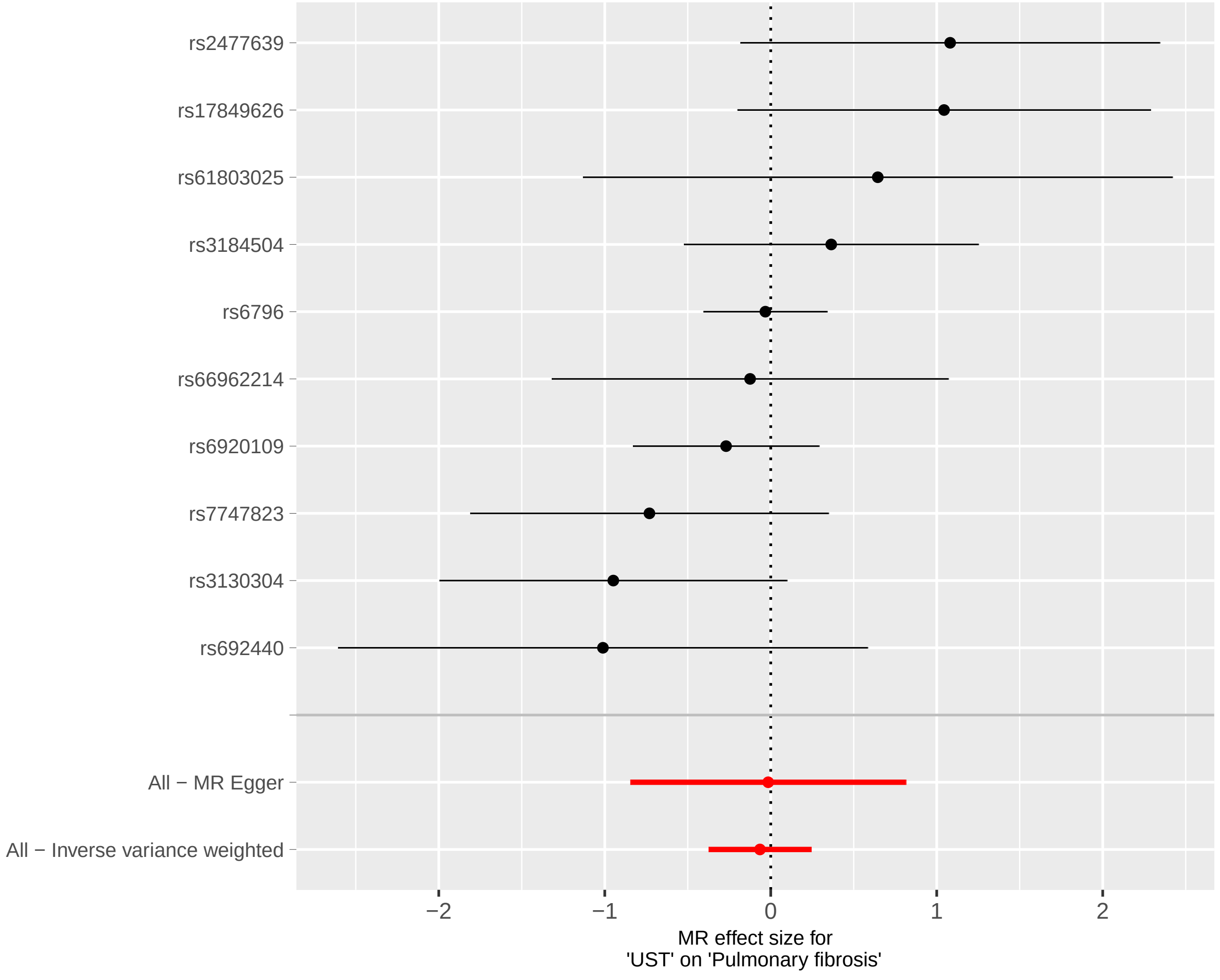

rs2477639
rs17849626
rs61803025
rs3184504
rs6796
rs66962214
rs6920109
rs7747823
rs3130304
rs692440
All − MR Egger
All − Inverse variance weighted
−2
−1
0
1
2
MR effect size for
'UST' on 'Pulmonary fibrosis'

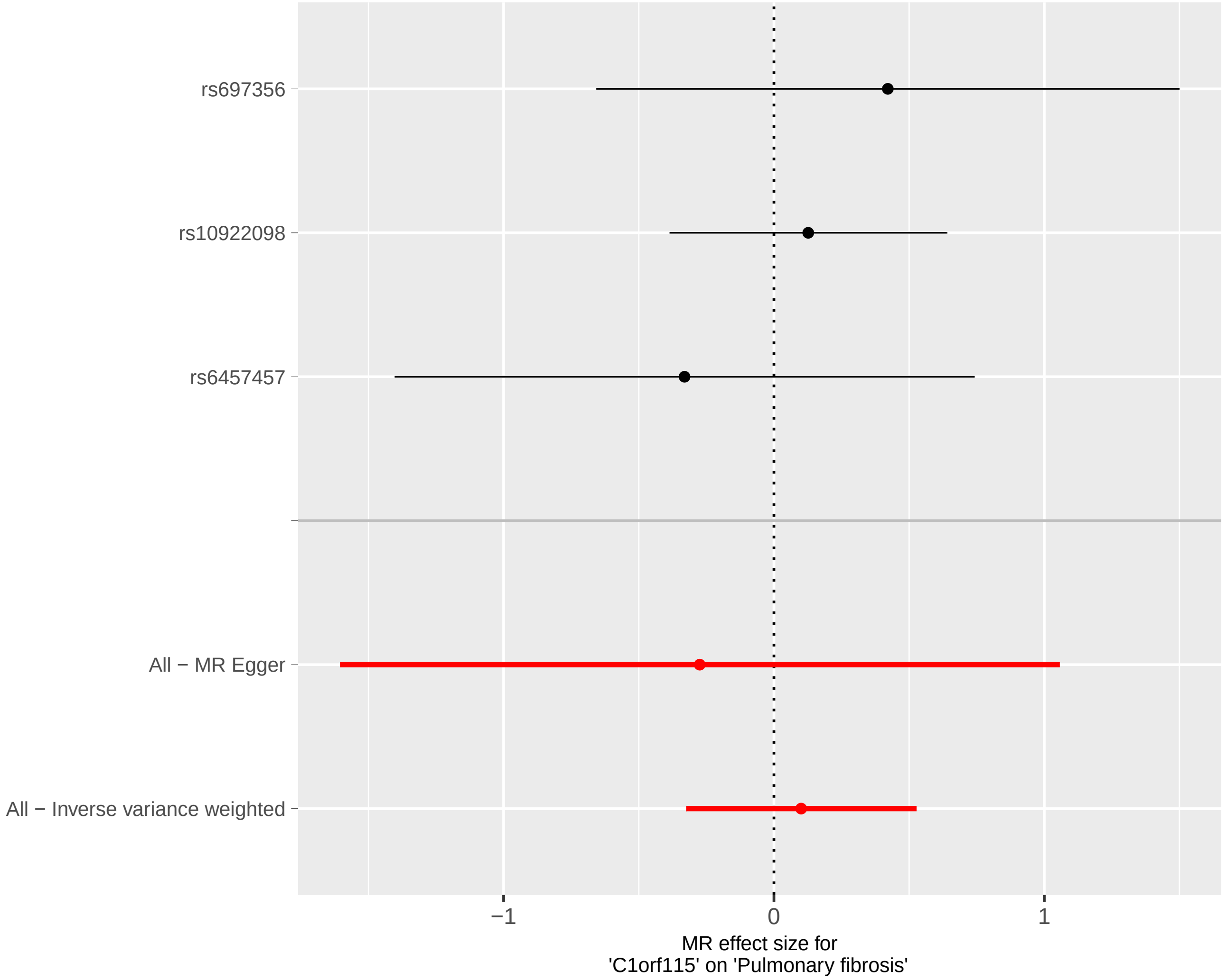
rs697356
rs10922098
rs6457457
All − MR Egger
All − Inverse variance weighted
−1
0
1
MR effect size for
'C1orf115' on 'Pulmonary fibrosis'

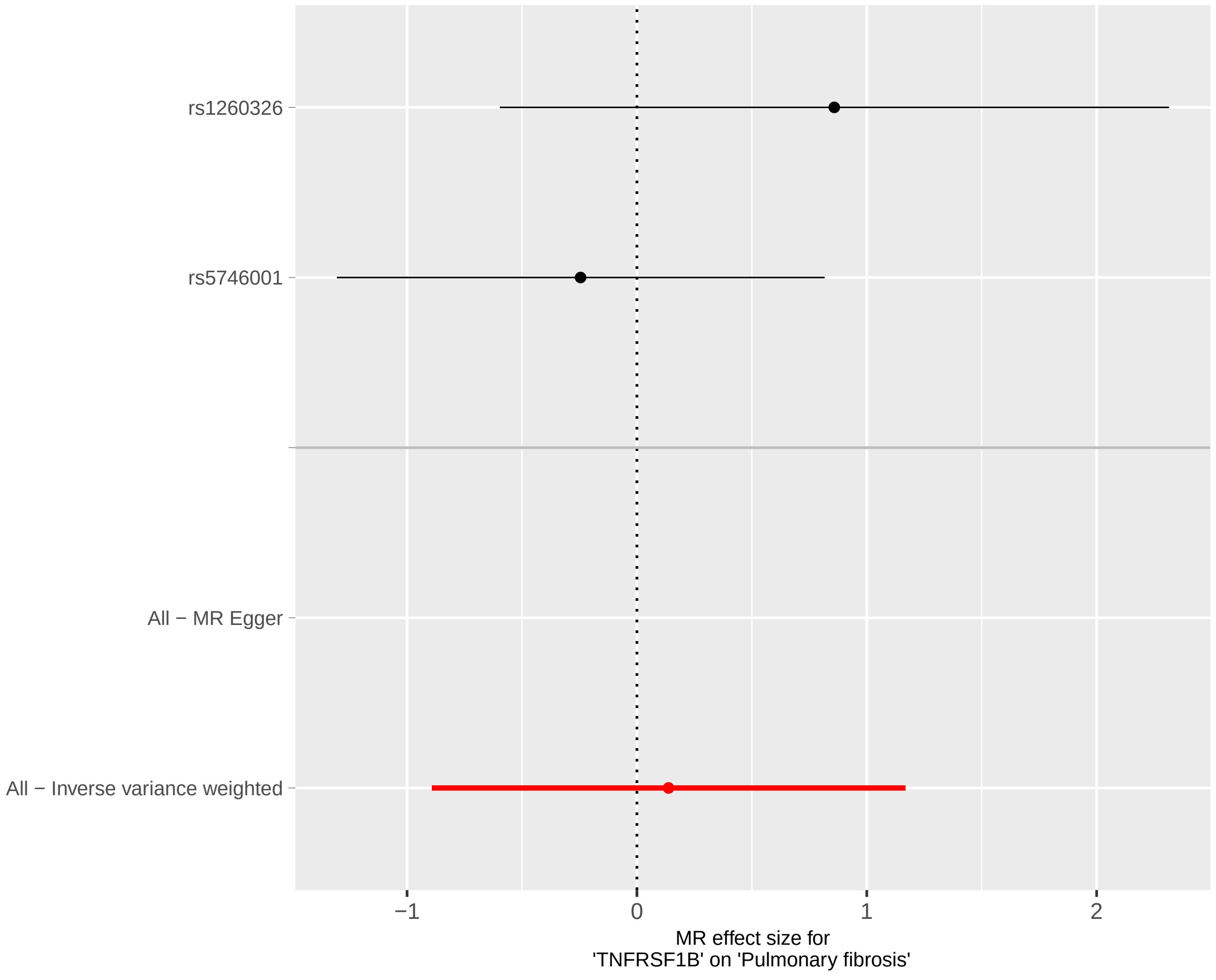
rs1260326
rs5746001
All − MR Egger
All − Inverse variance weighted
−1
0
1
2
MR effect size for
'TNFRSF1B' on 'Pulmonary fibrosis'

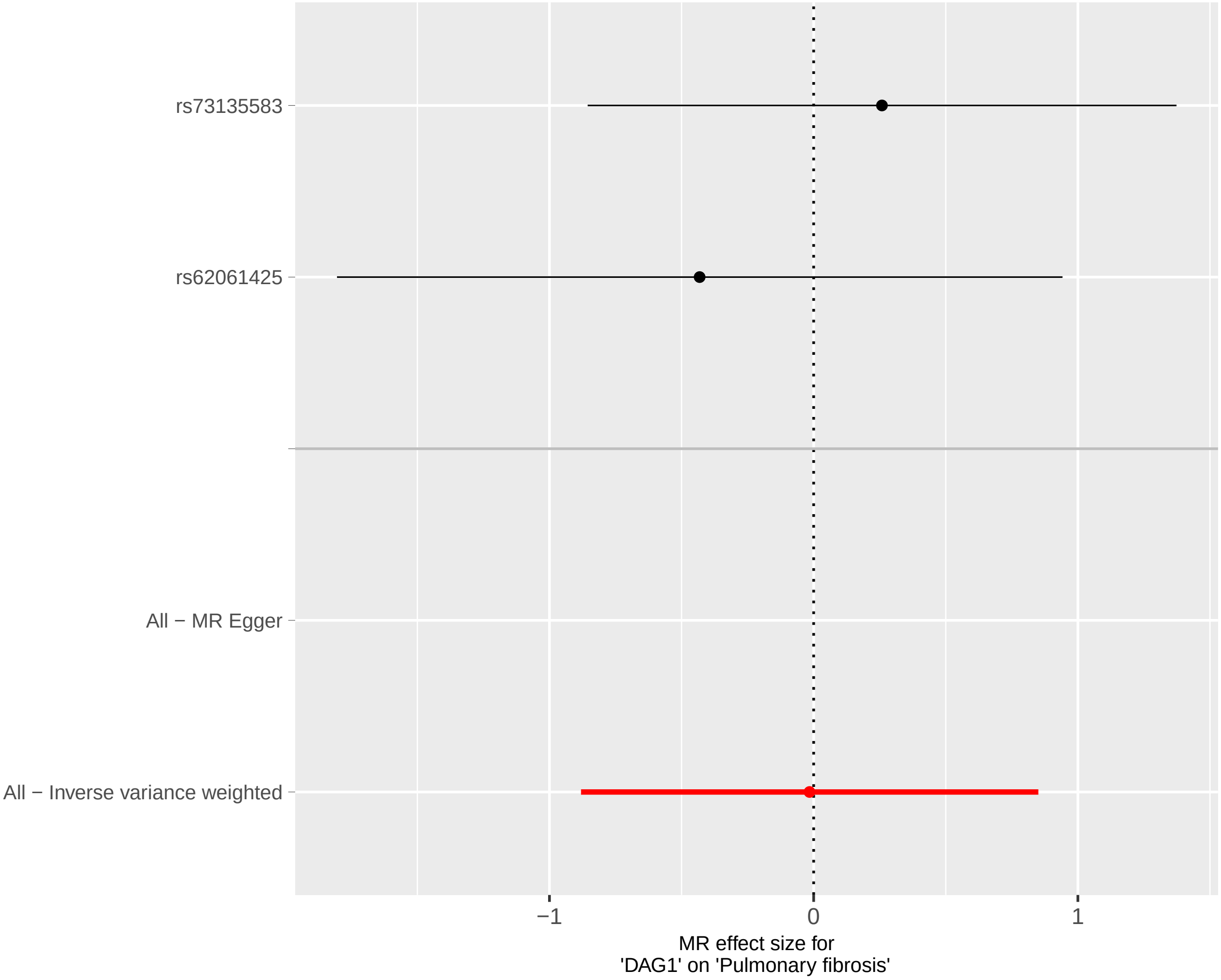

rs73135583
rs62061425
All − MR Egger
All − Inverse variance weighted
−1
0
1
MR effect size for
'DAG1' on 'Pulmonary fibrosis'

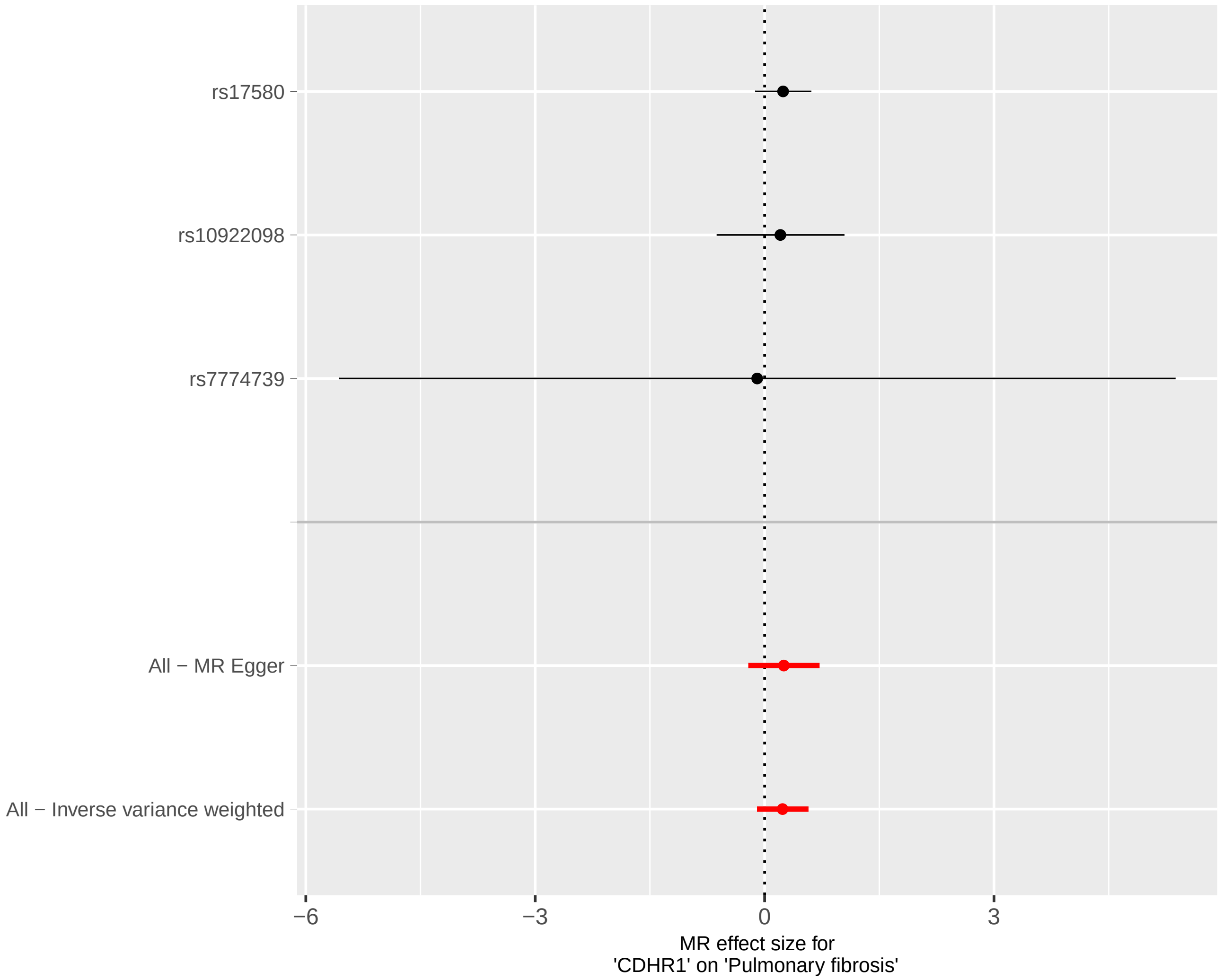

rs17580
rs10922098
rs7774739
All − MR Egger
All − Inverse variance weighted
−6
−3
0
3
MR effect size for
'CDHR1' on 'Pulmonary fibrosis'

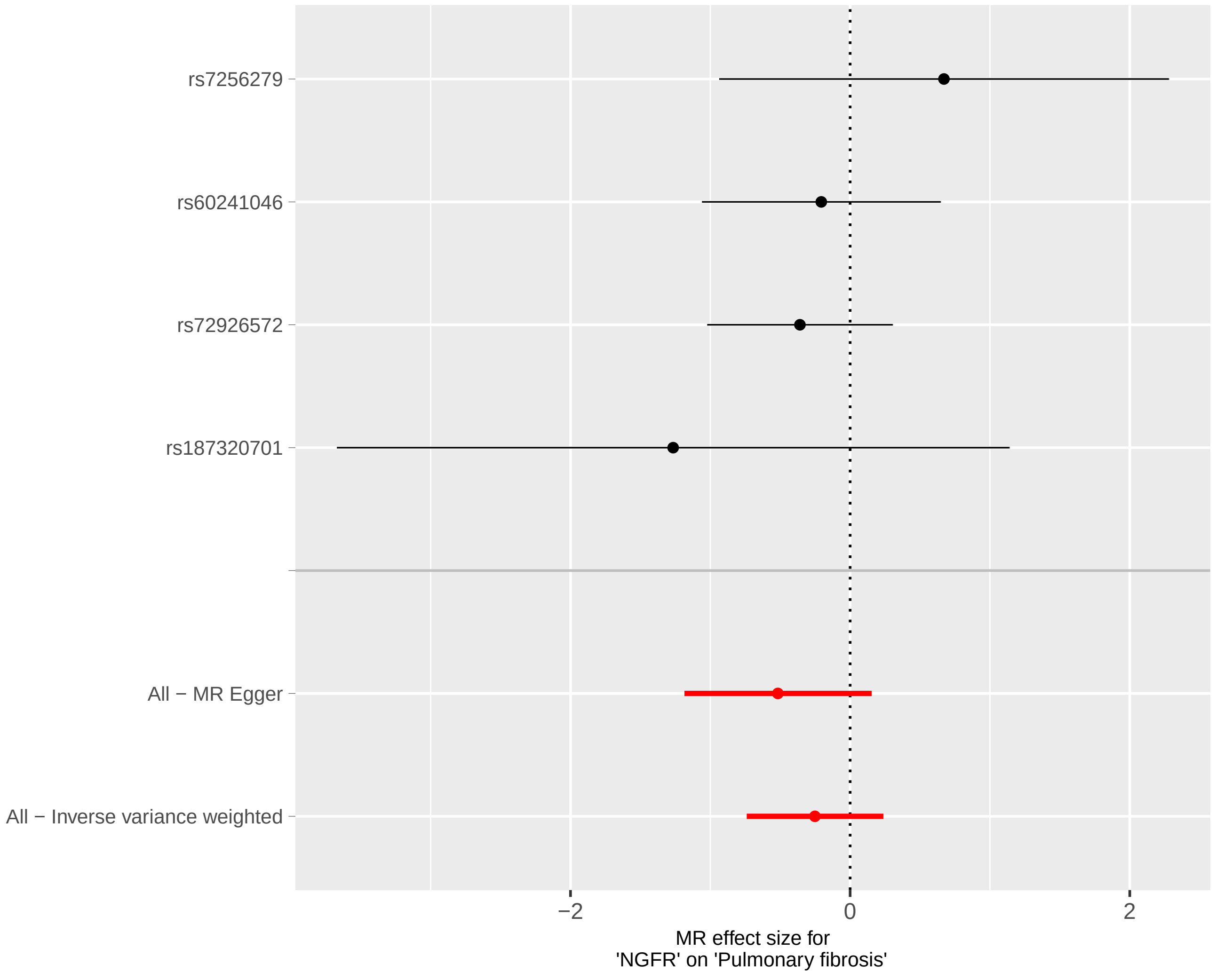
rs7256279
rs60241046
rs72926572
rs187320701
All − MR Egger
All − Inverse variance weighted
−2
0
2
MR effect size for
'NGFR' on 'Pulmonary fibrosis'

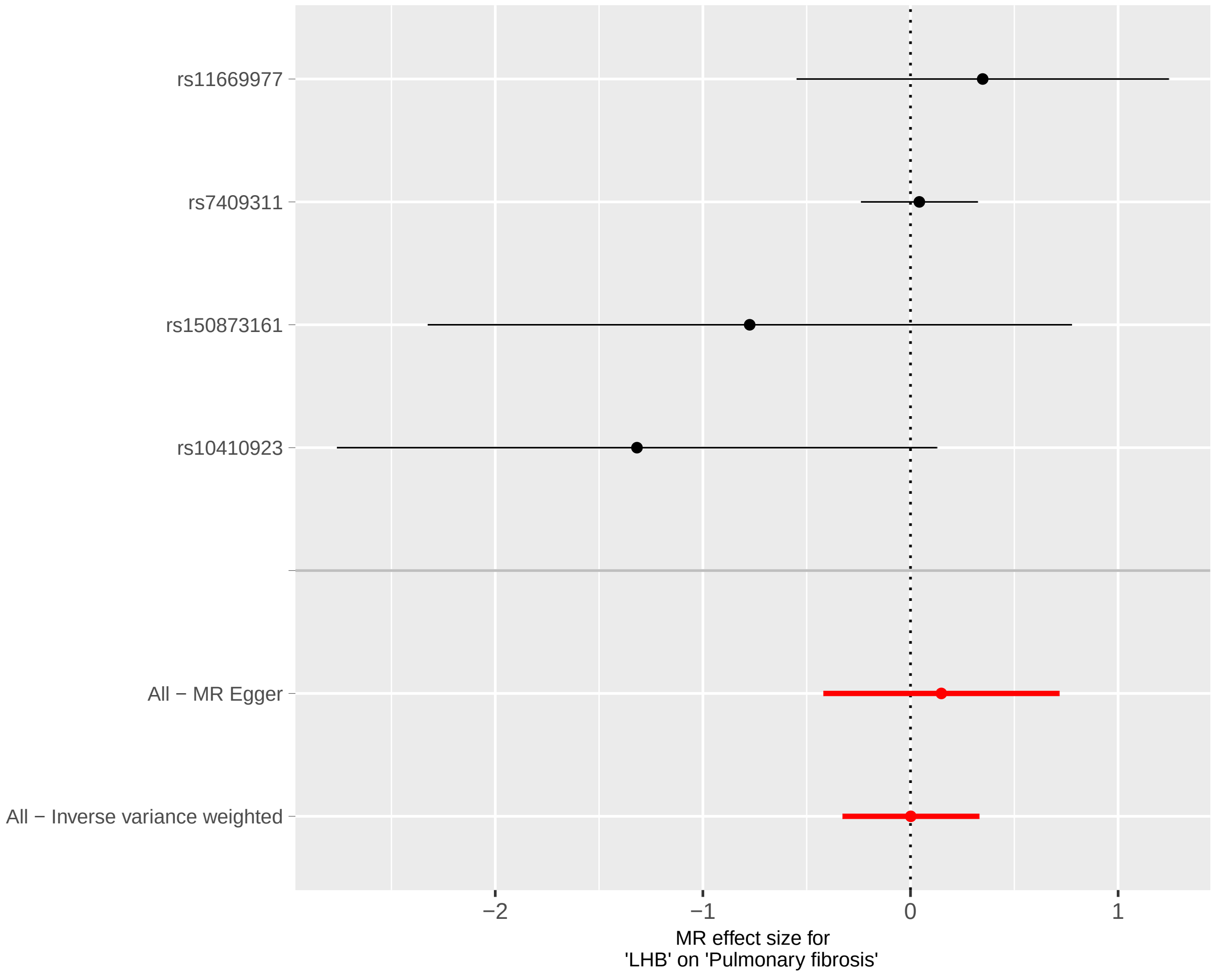

rs11669977
rs7409311
rs150873161
rs10410923
All − MR Egger
All − Inverse variance weighted
−2
−1
0
1
MR effect size for
'LHB' on 'Pulmonary fibrosis'

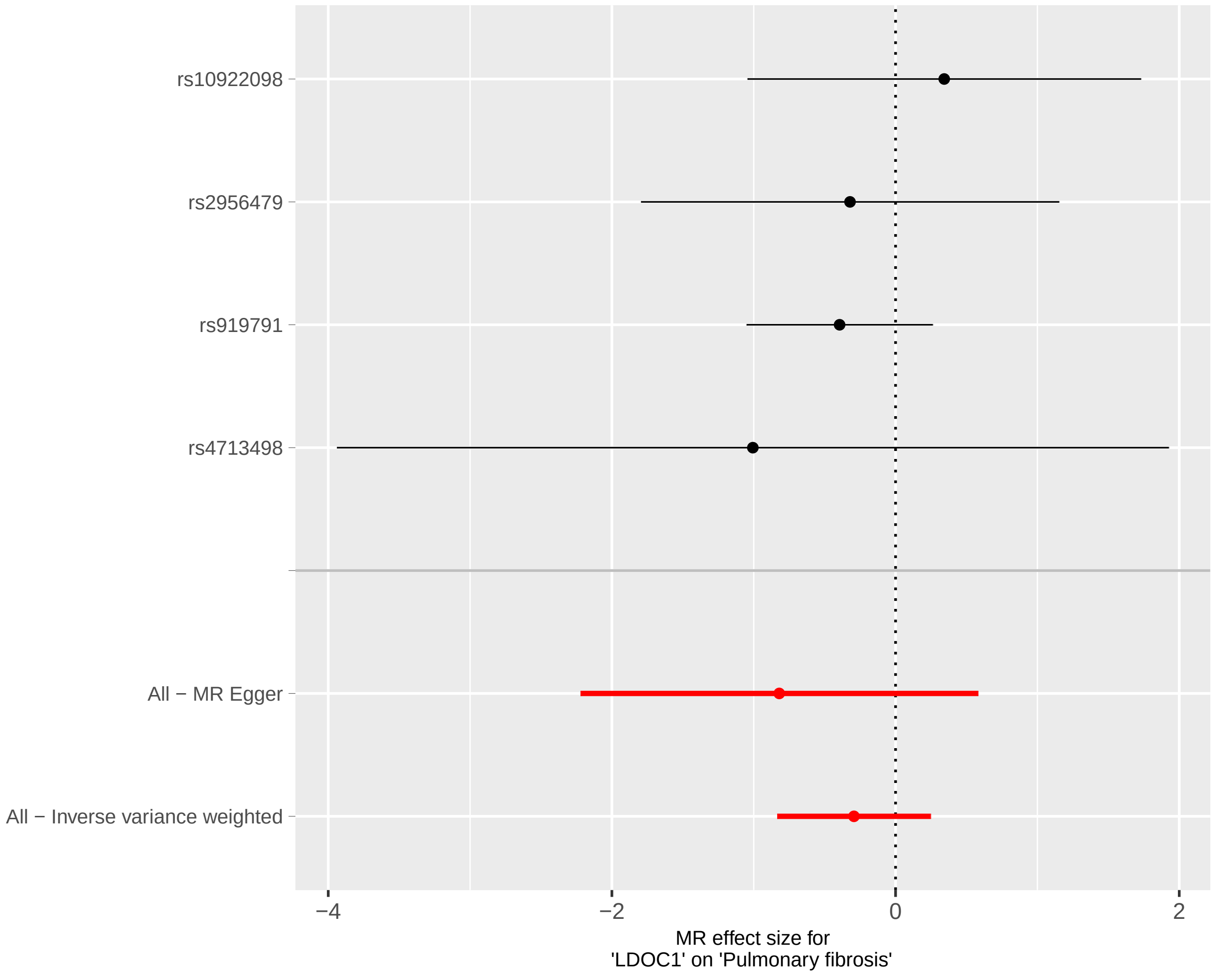
rs10922098
rs2956479
rs919791
rs4713498
All − MR Egger
All − Inverse variance weighted
−4
−2
0
2
MR effect size for
'LDOC1' on 'Pulmonary fibrosis'

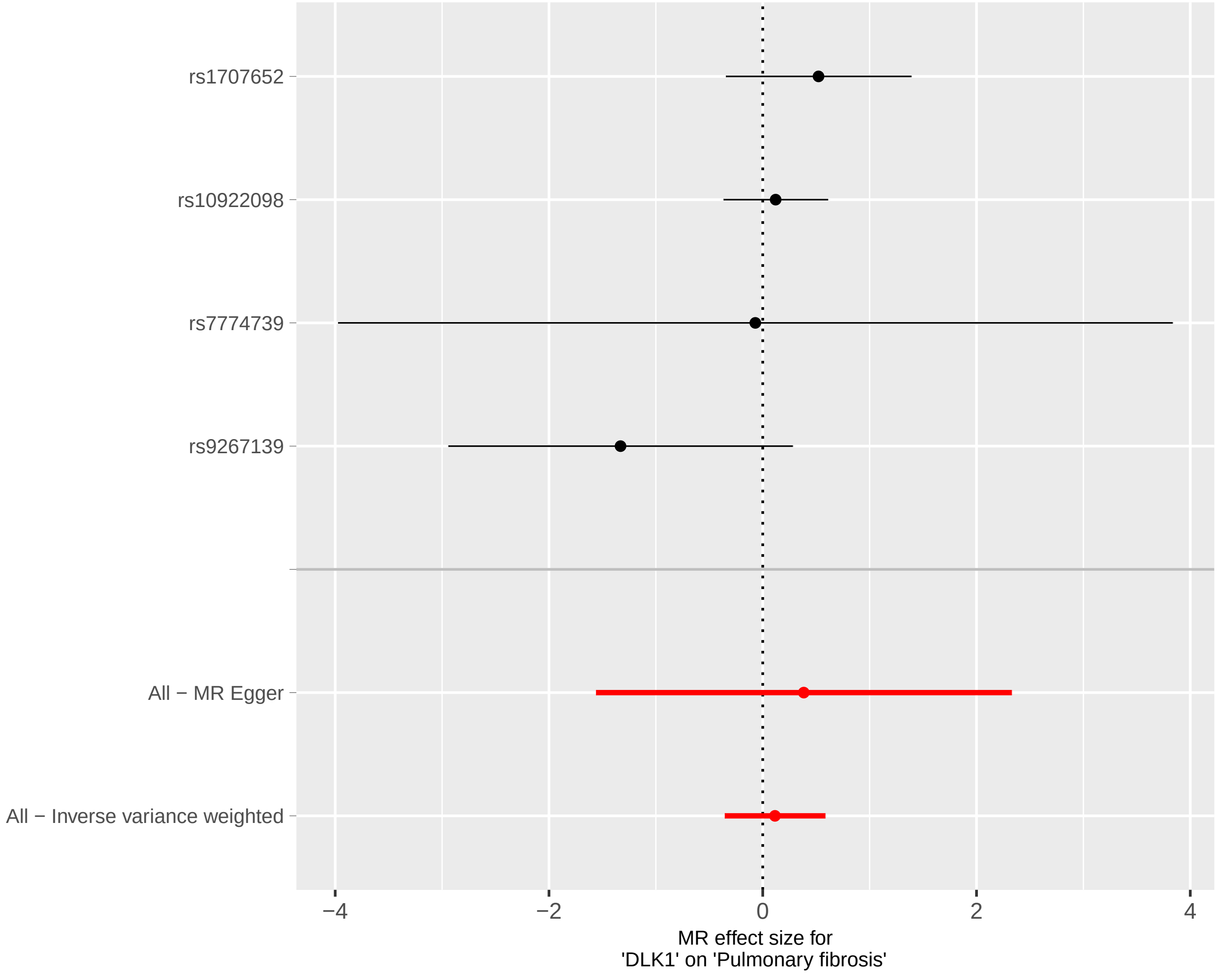

rs1707652
rs10922098
rs7774739
rs9267139
All − MR Egger
All − Inverse variance weighted
−4
−2
0
2
4
MR effect size for
'DLK1' on 'Pulmonary fibrosis'

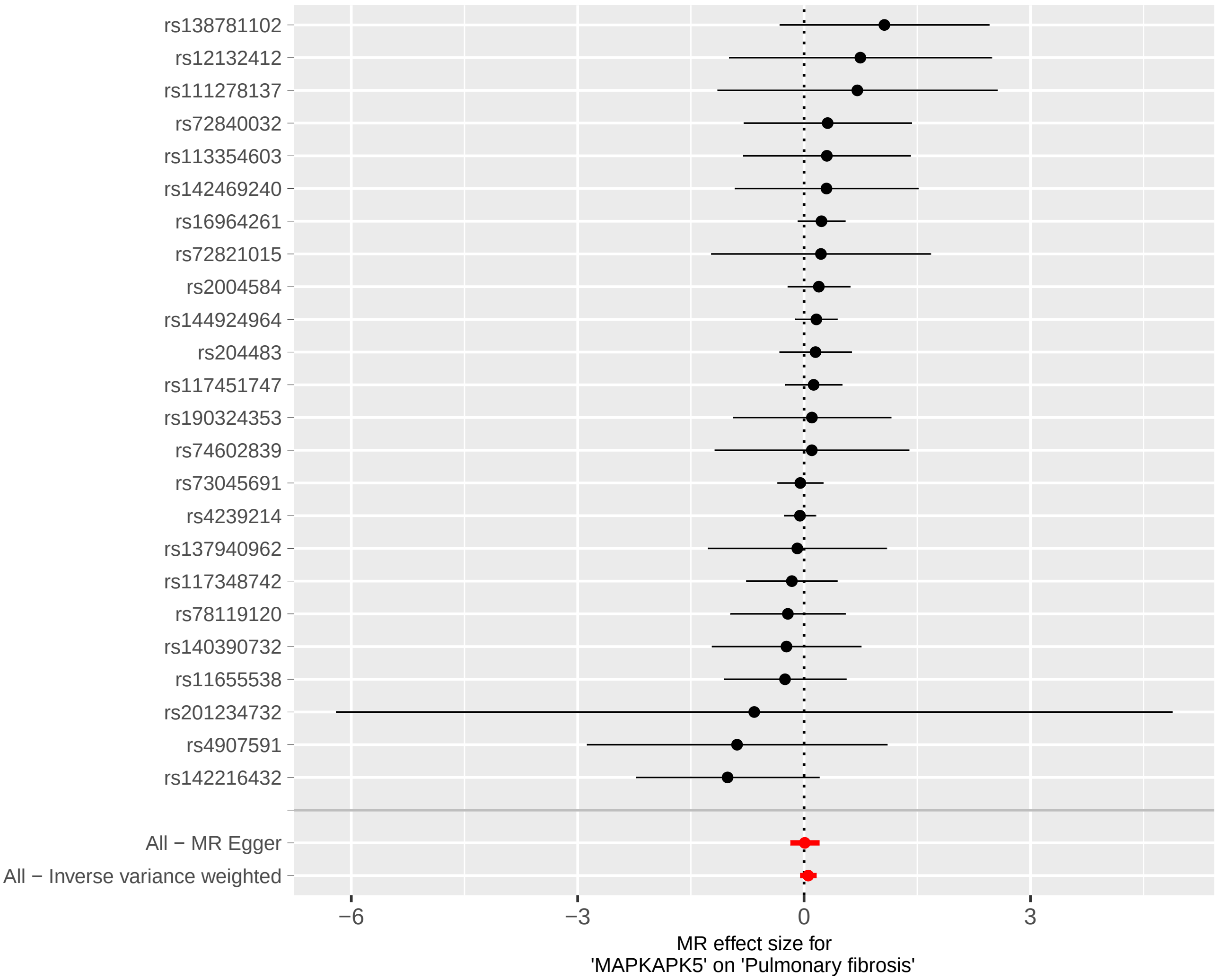

rs138781102
rs12132412
rs111278137
rs72840032
rs113354603
rs142469240
rs16964261
rs72821015
rs2004584
rs144924964
rs204483
rs117451747
rs190324353
rs74602839
rs73045691
rs4239214
rs137940962
rs117348742
rs78119120
rs140390732
rs11655538
rs201234732
rs4907591
rs142216432
All − MR Egger
All − Inverse variance weighted
−6
−3
0
3
MR effect size for
'MAPKAPK5' on 'Pulmonary fibrosis'

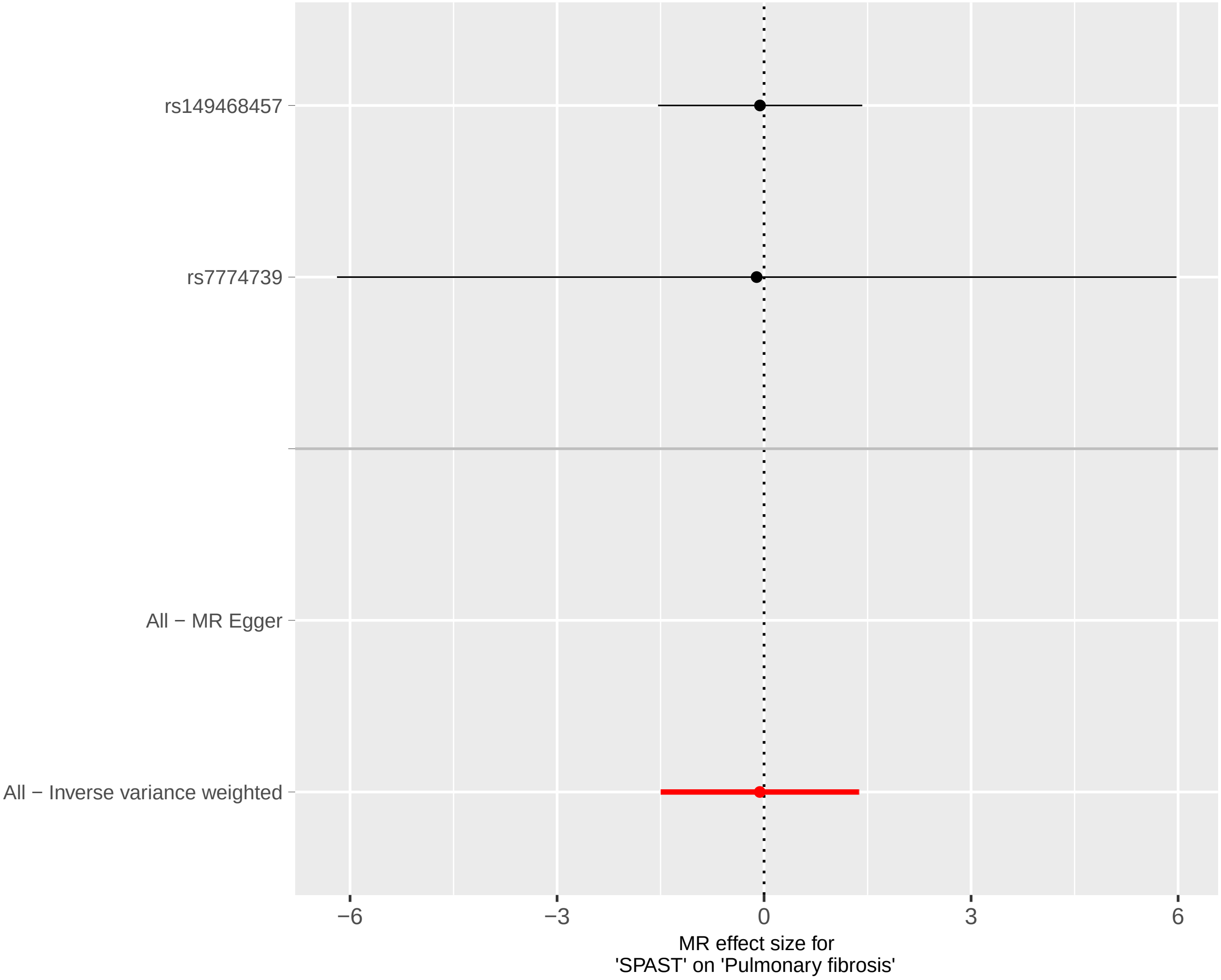

rs149468457
rs7774739
All − MR Egger
All − Inverse variance weighted
−6
−3
0
3
6
MR effect size for
'SPAST' on 'Pulmonary fibrosis'

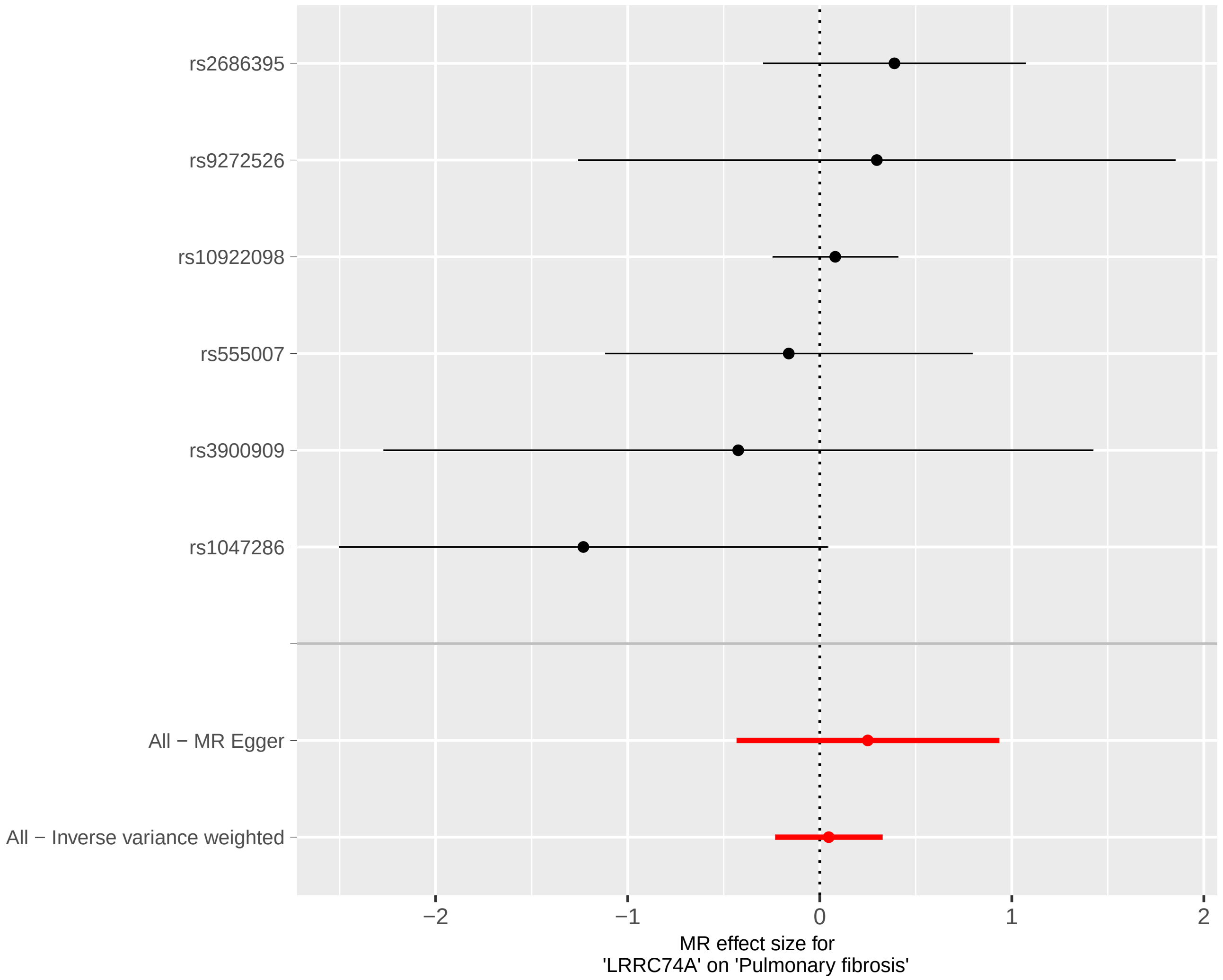

rs2686395
rs9272526
rs10922098
rs555007
rs3900909
rs1047286
All − MR Egger
All − Inverse variance weighted
−2
−1
0
1
2
MR effect size for
'LRRC74A' on 'Pulmonary fibrosis'

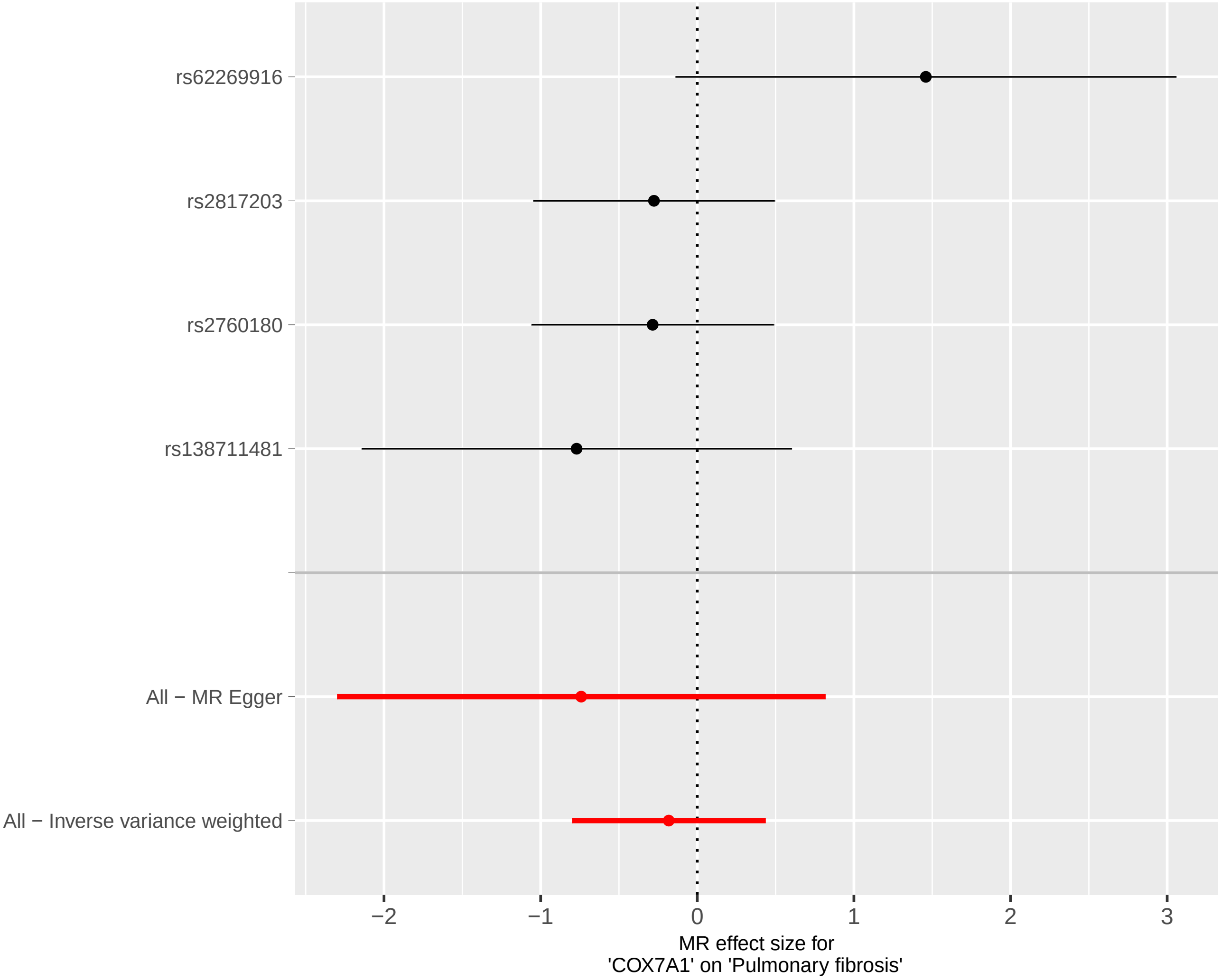

rs62269916
rs2817203
rs2760180
rs138711481
All − MR Egger
All − Inverse variance weighted
−2
−1
0
1
2
3
MR effect size for
'COX7A1' on 'Pulmonary fibrosis'

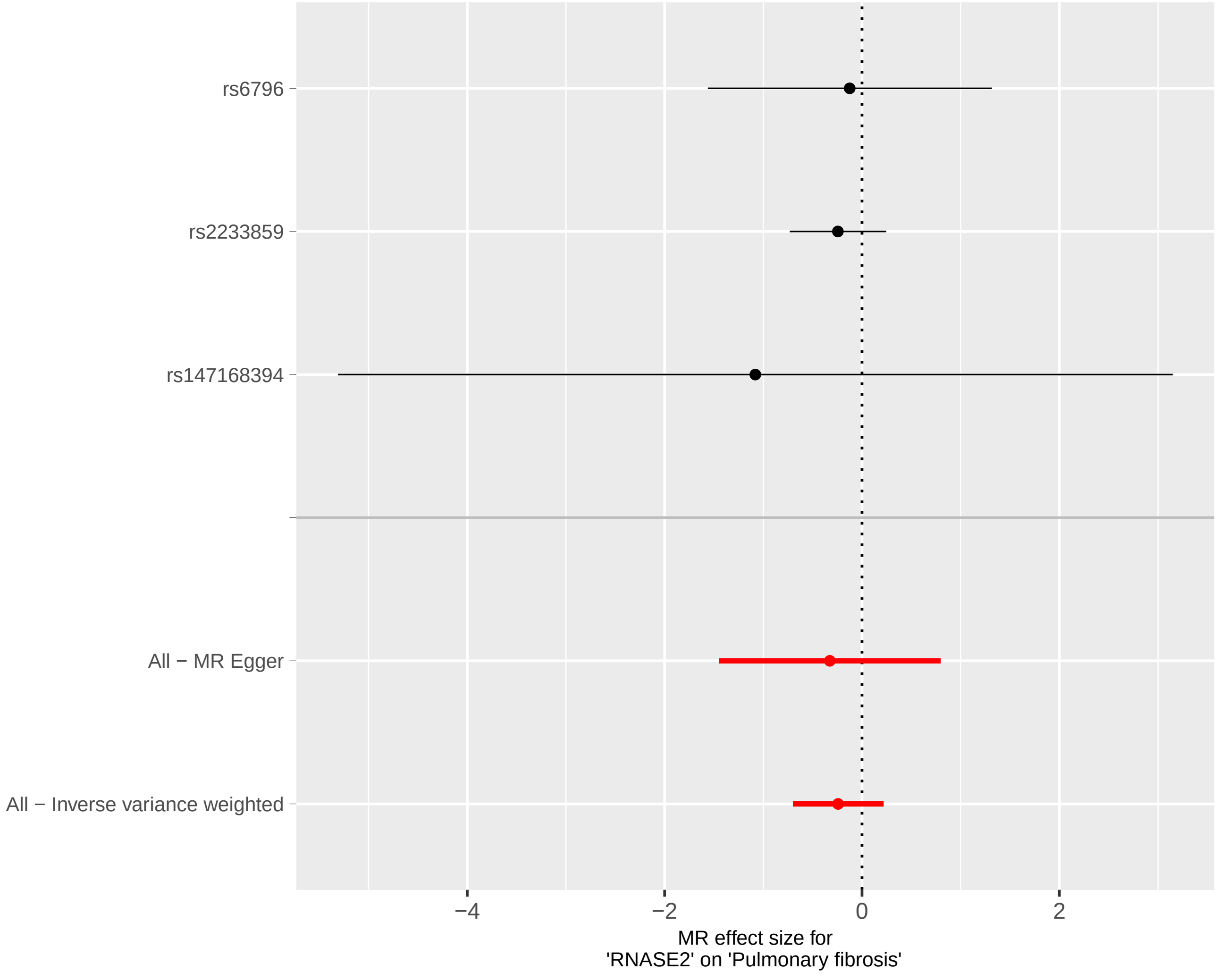

rs6796
rs2233859
rs147168394
All − MR Egger
All − Inverse variance weighted
−4
−2
0
2
MR effect size for
'RNASE2' on 'Pulmonary fibrosis'

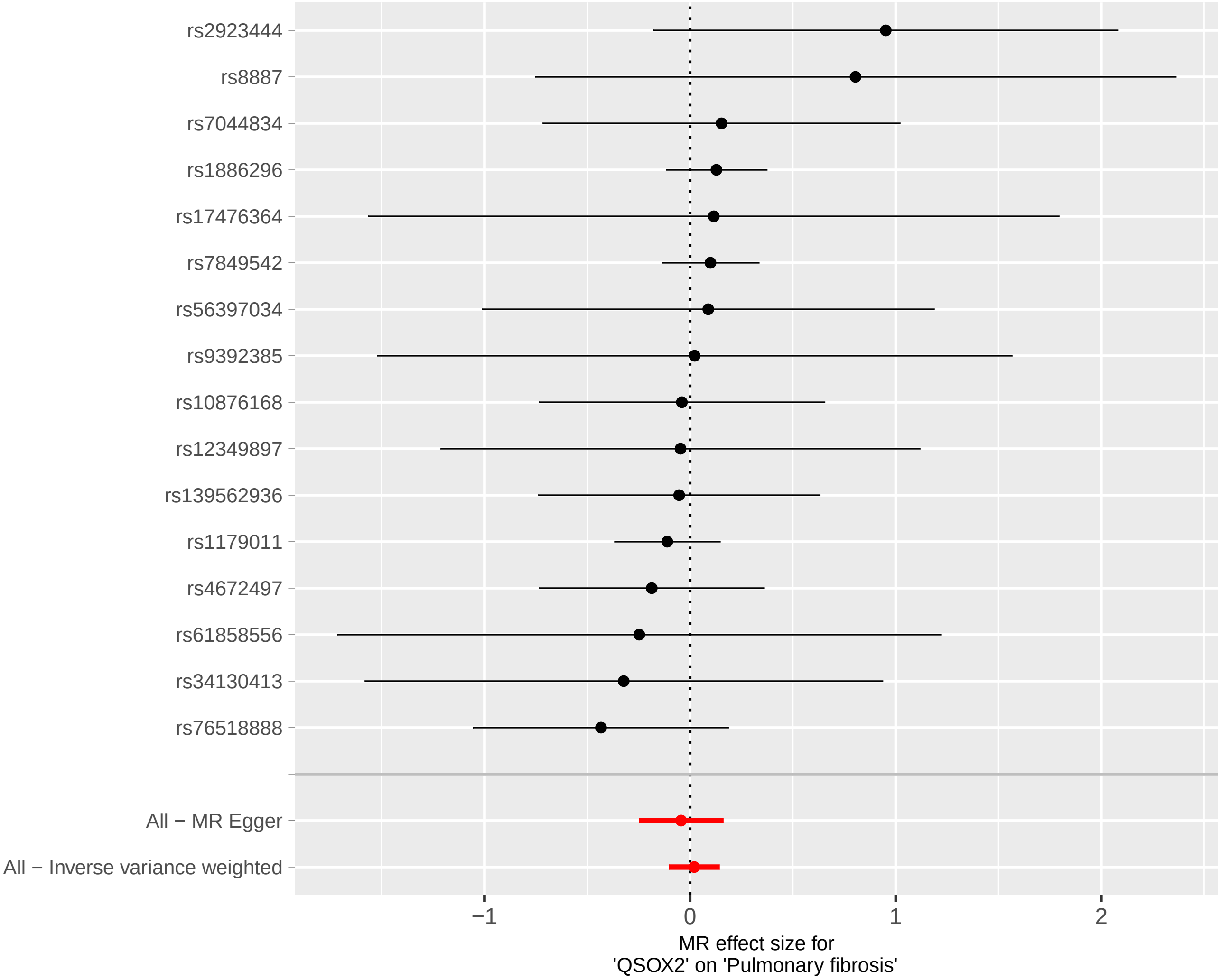
rs2923444
rs8887
rs7044834
rs1886296
rs17476364
rs7849542
rs56397034
rs9392385
rs10876168
rs12349897
rs139562936
rs1179011
rs4672497
rs61858556
rs34130413
rs76518888
All − MR Egger
All − Inverse variance weighted
−1
0
1
2
MR effect size for
'QSOX2' on 'Pulmonary fibrosis'

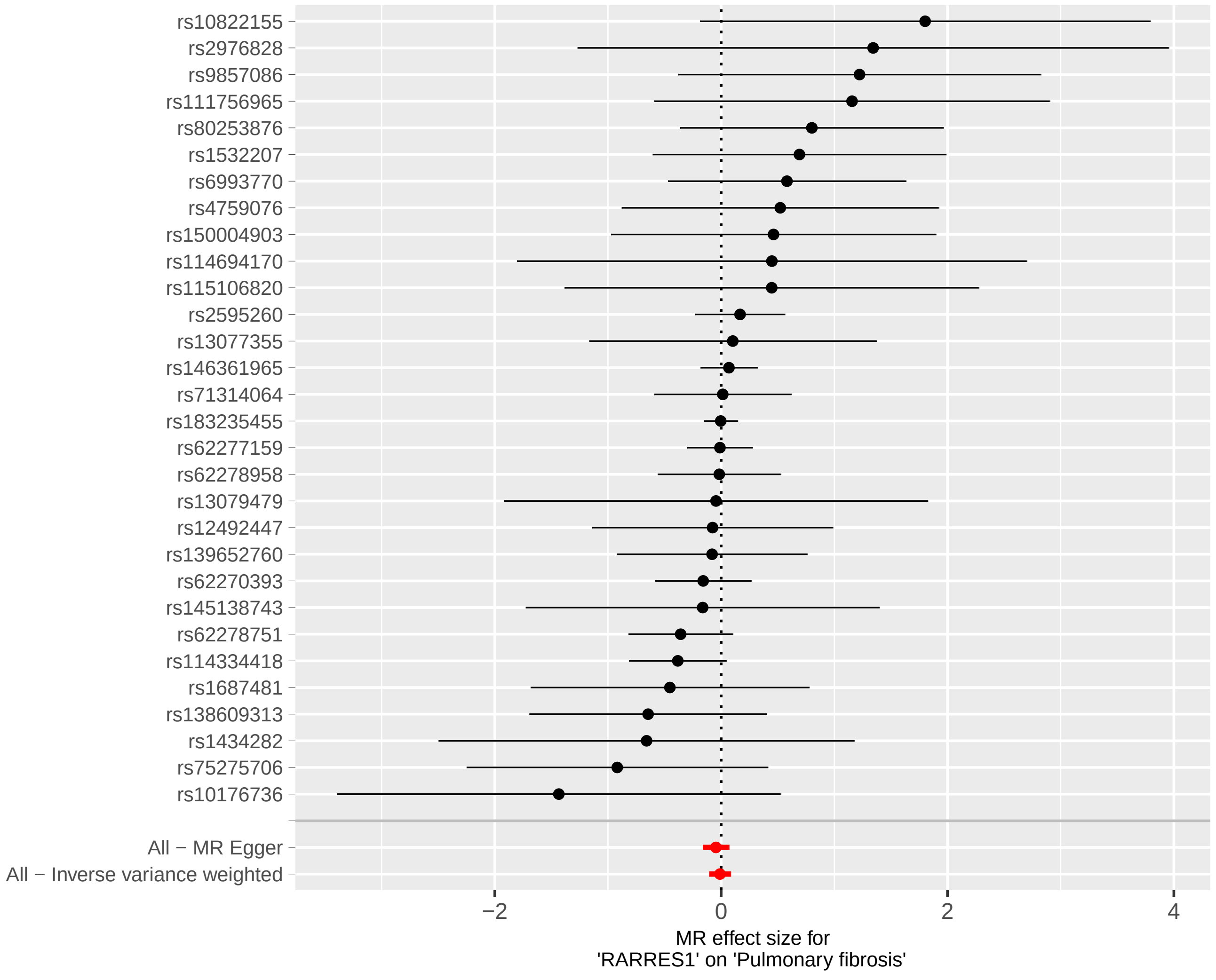

rs10822155
rs2976828
rs9857086
rs111756965
rs80253876
rs1532207
rs6993770
rs4759076
rs150004903
rs114694170
rs115106820
rs2595260
rs13077355
rs146361965
rs71314064
rs183235455
rs62277159
rs62278958
rs13079479
rs12492447
rs139652760
rs62270393
rs145138743
rs62278751
rs114334418
rs1687481
rs138609313
rs1434282
rs75275706
rs10176736
All − MR Egger
All − Inverse variance weighted
−2
0
2
4
MR effect size for
'RARRES1' on 'Pulmonary fibrosis'

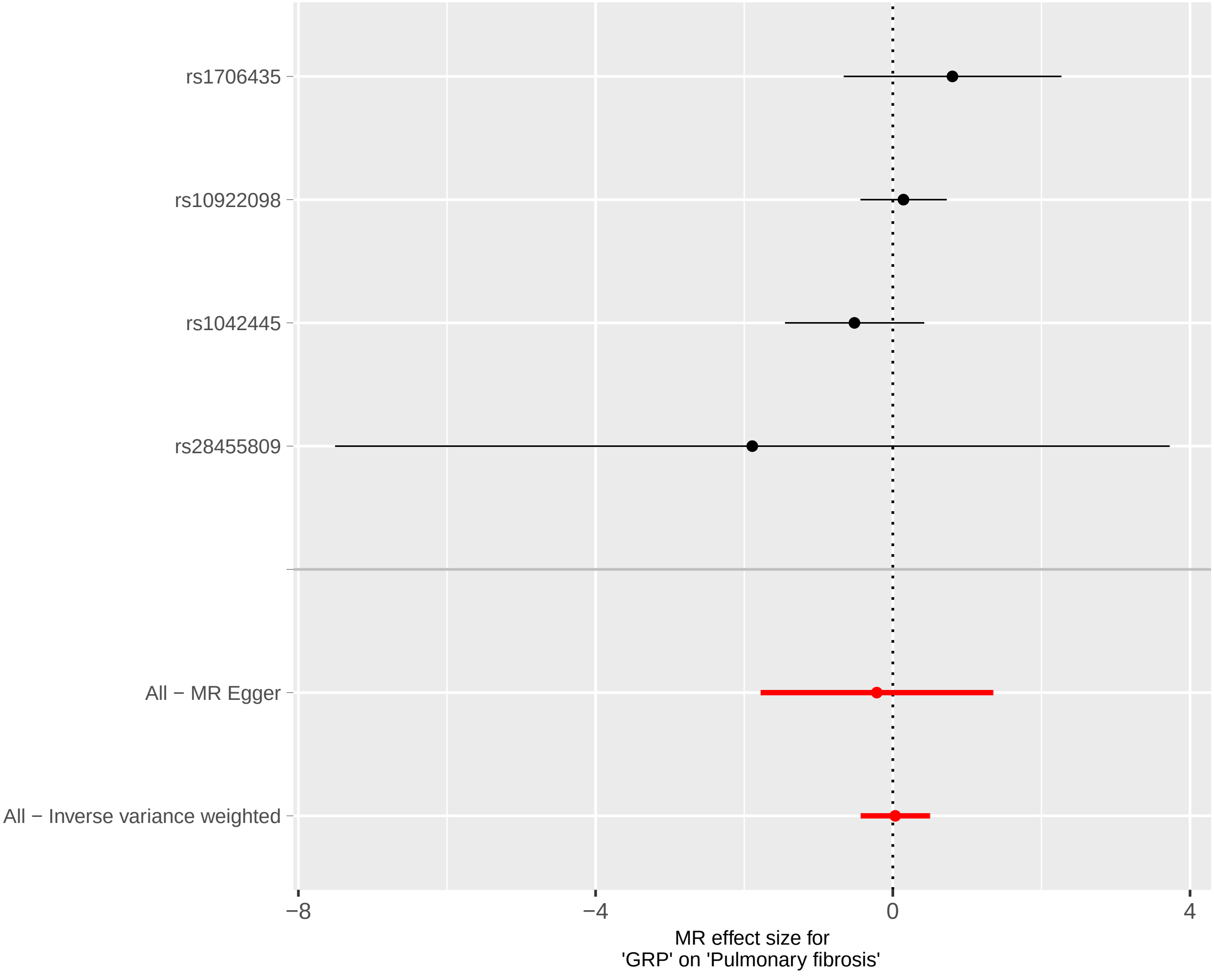
rs1706435
rs10922098
rs1042445
rs28455809
All − MR Egger
All − Inverse variance weighted
−8
−4
0
4
MR effect size for
'GRP' on 'Pulmonary fibrosis'

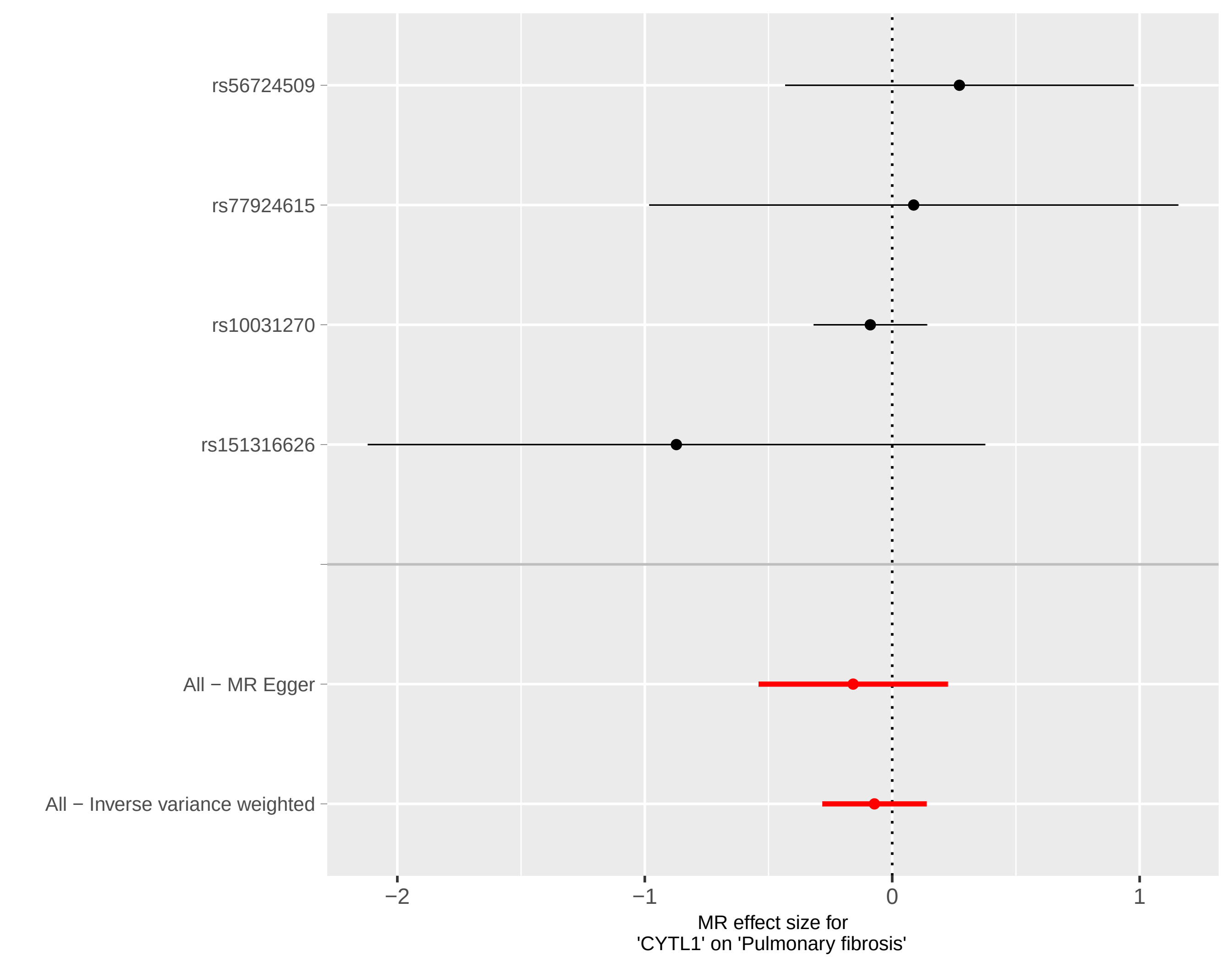

rs56724509
rs77924615
rs10031270
rs151316626
All − MR Egger
All − Inverse variance weighted
−2
−1
0
1
MR effect size for
'CYTL1' on 'Pulmonary fibrosis'

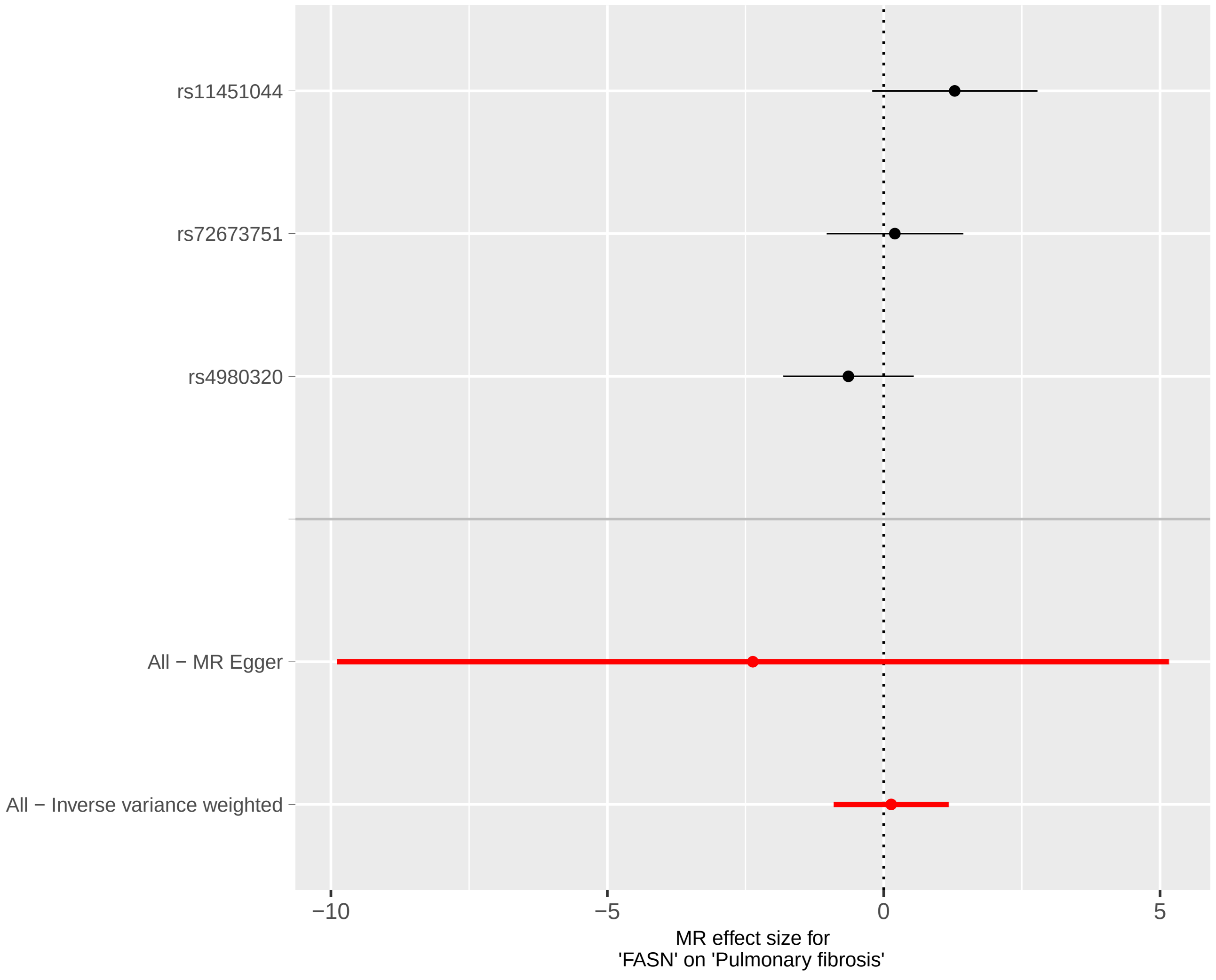

rs11451044
rs72673751
rs4980320
All − MR Egger
All − Inverse variance weighted
−10
−5
0
5
MR effect size for
'FASN' on 'Pulmonary fibrosis'

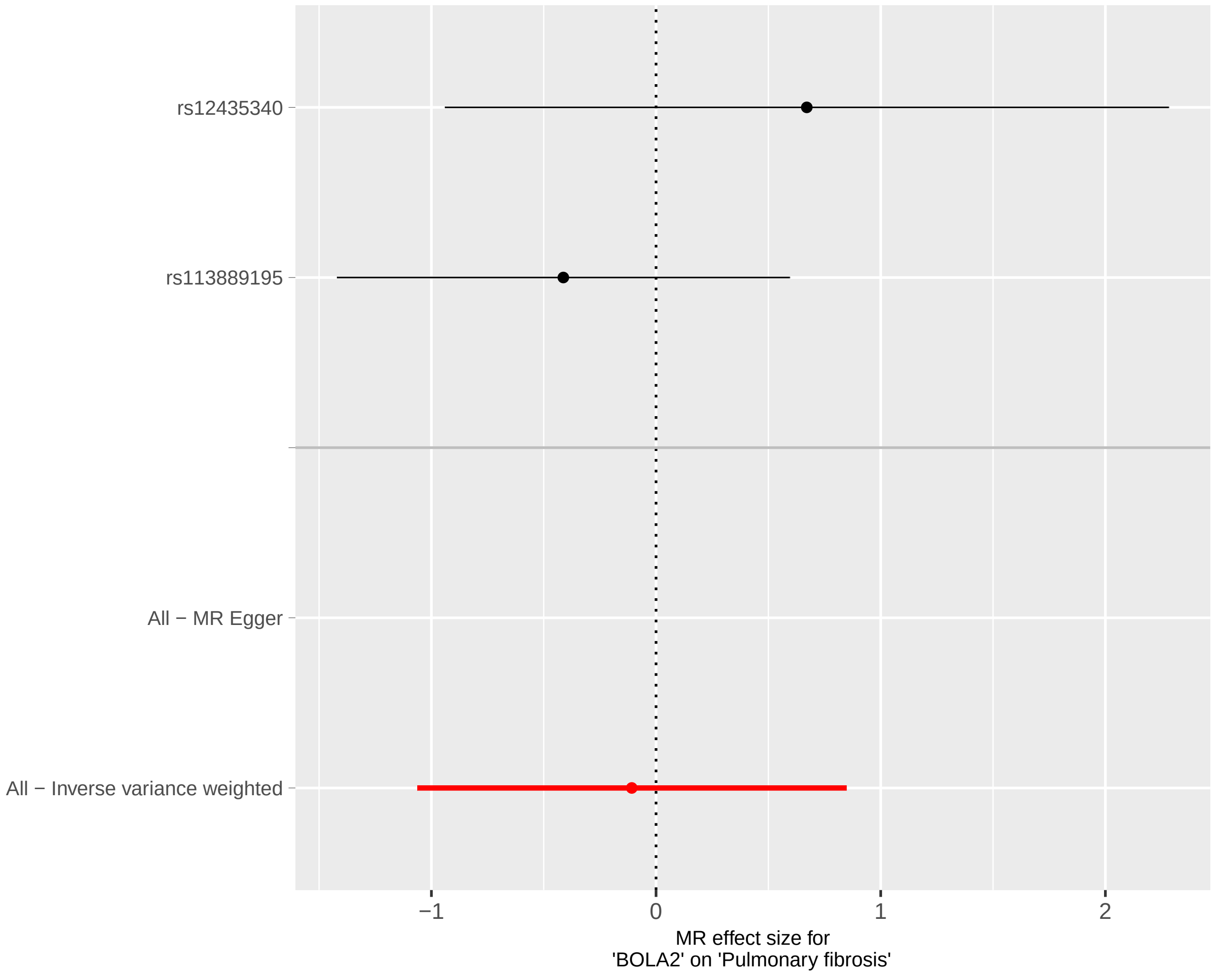

rs12435340
rs113889195
All − MR Egger
All − Inverse variance weighted
−1
0
1
2
MR effect size for
'BOLA2' on 'Pulmonary fibrosis'

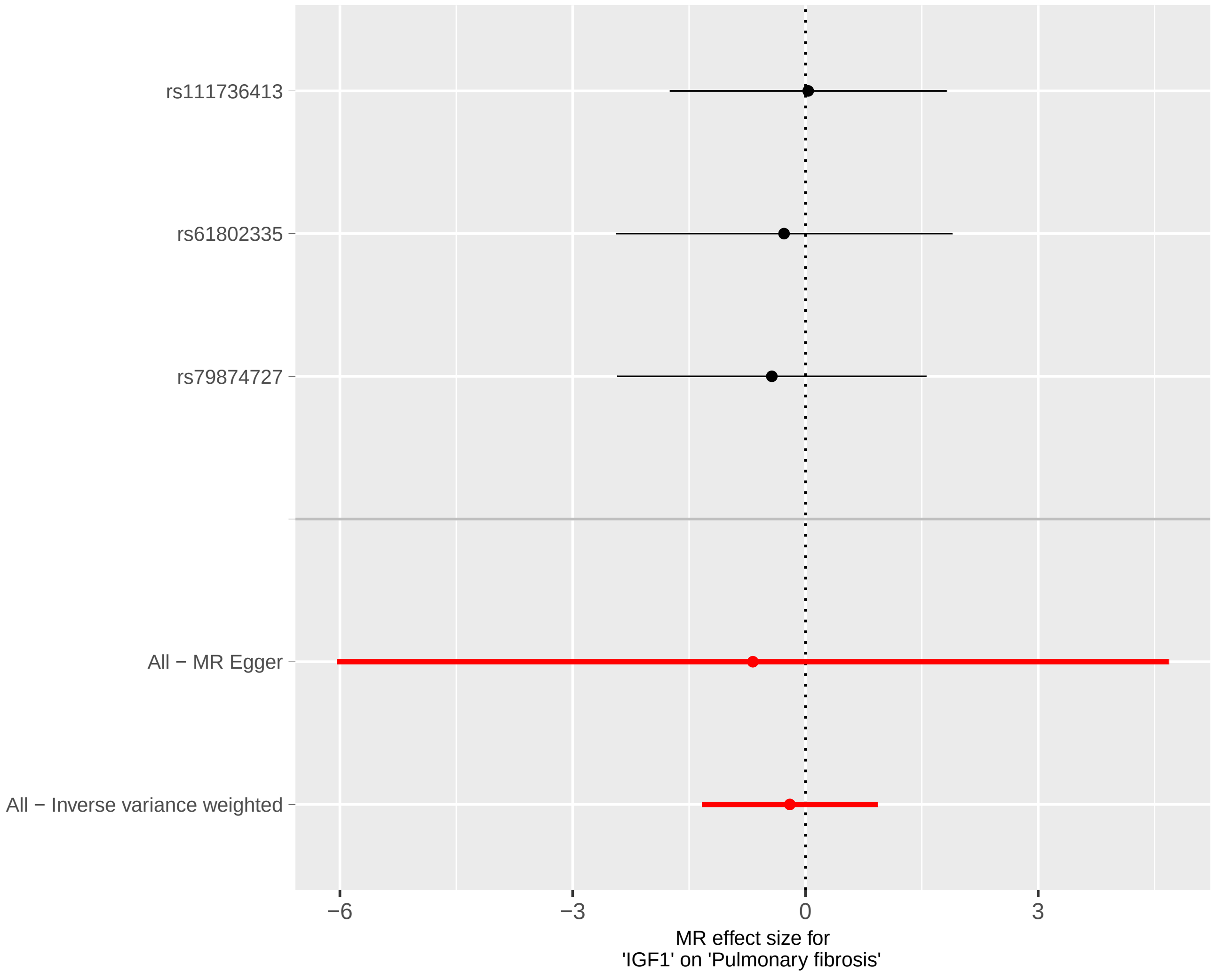

rs111736413
rs61802335
rs79874727
All − MR Egger
All − Inverse variance weighted
−6
−3
0
3
MR effect size for
'IGF1' on 'Pulmonary fibrosis'

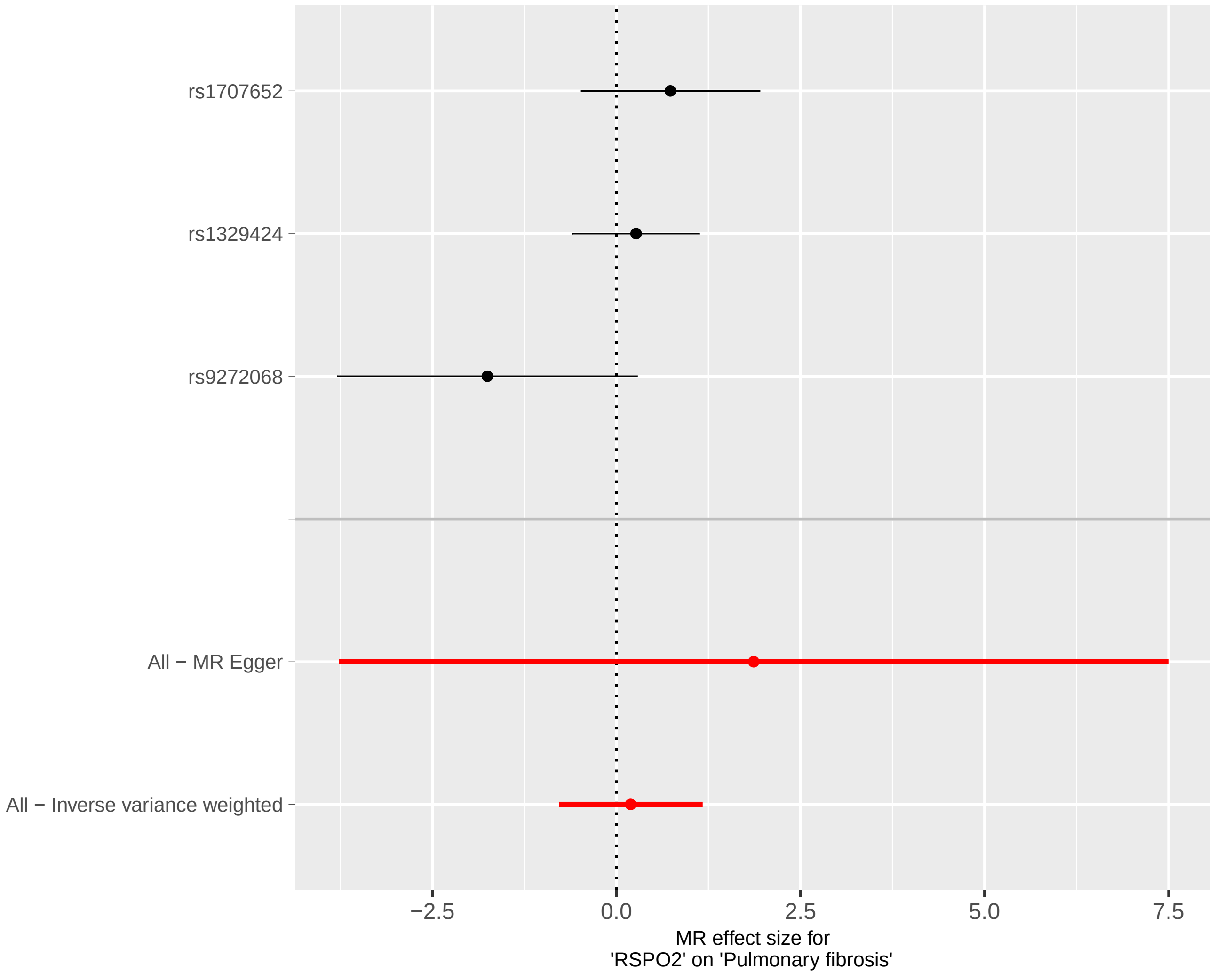

rs1707652
rs1329424
rs9272068
All − MR Egger
All − Inverse variance weighted
−2.5
0.0
2.5
5.0
7.5
MR effect size for
'RSPO2' on 'Pulmonary fibrosis'

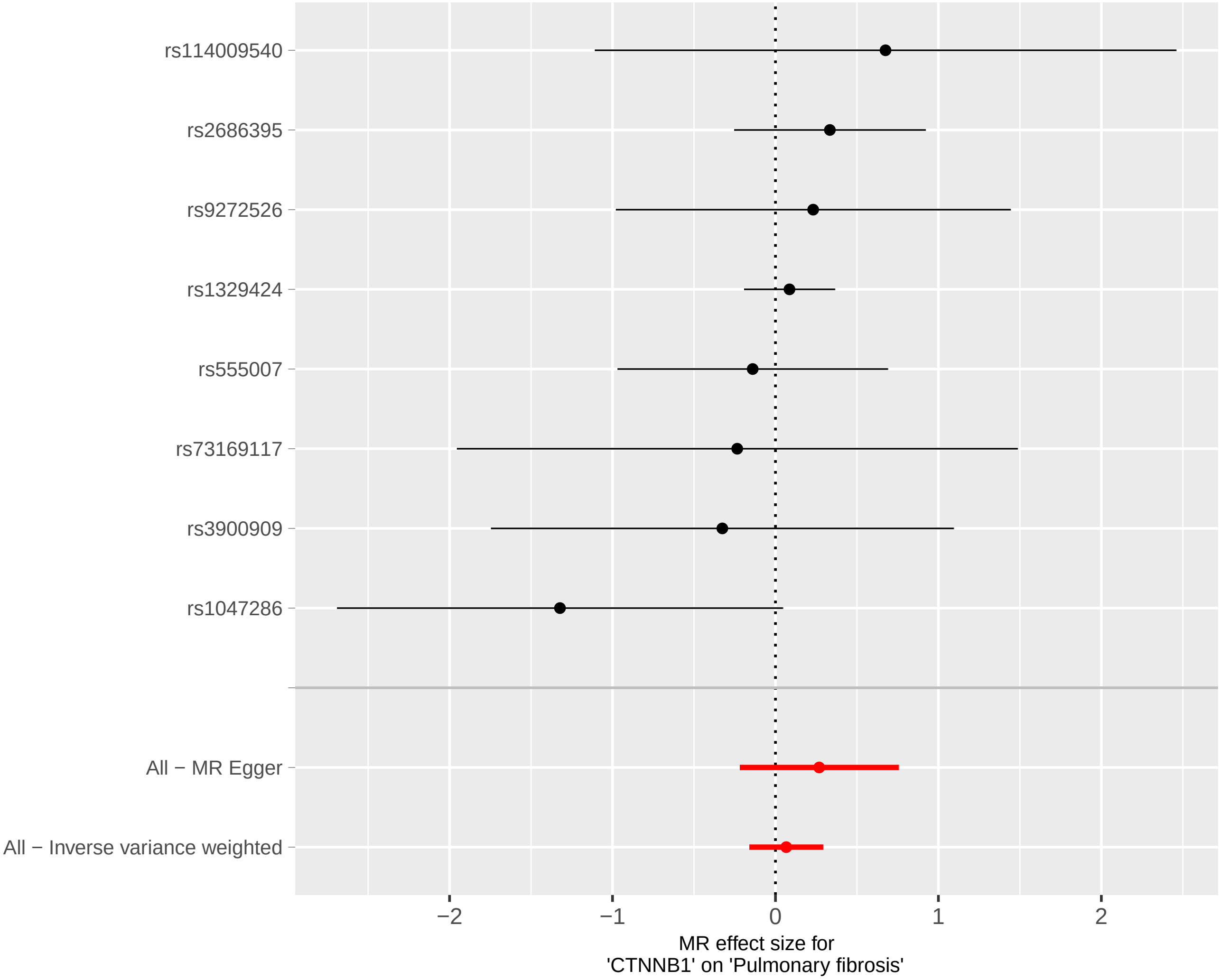
rs114009540
rs2686395
rs9272526
rs1329424
rs555007
rs73169117
rs3900909
rs1047286
All − MR Egger
All − Inverse variance weighted
−2
−1
0
1
2
MR effect size for
'CTNNB1' on 'Pulmonary fibrosis'

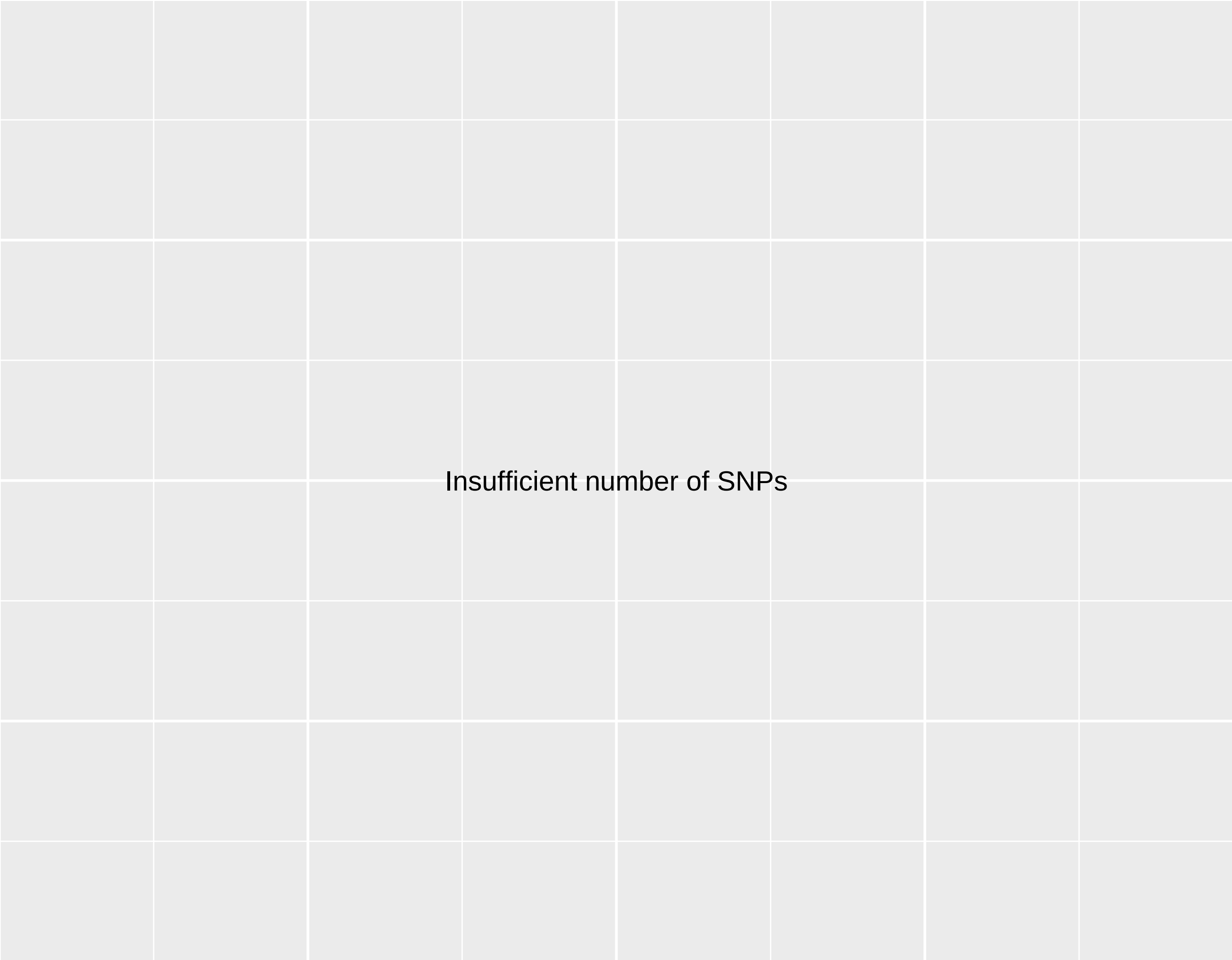
Insufficient number of SNPs

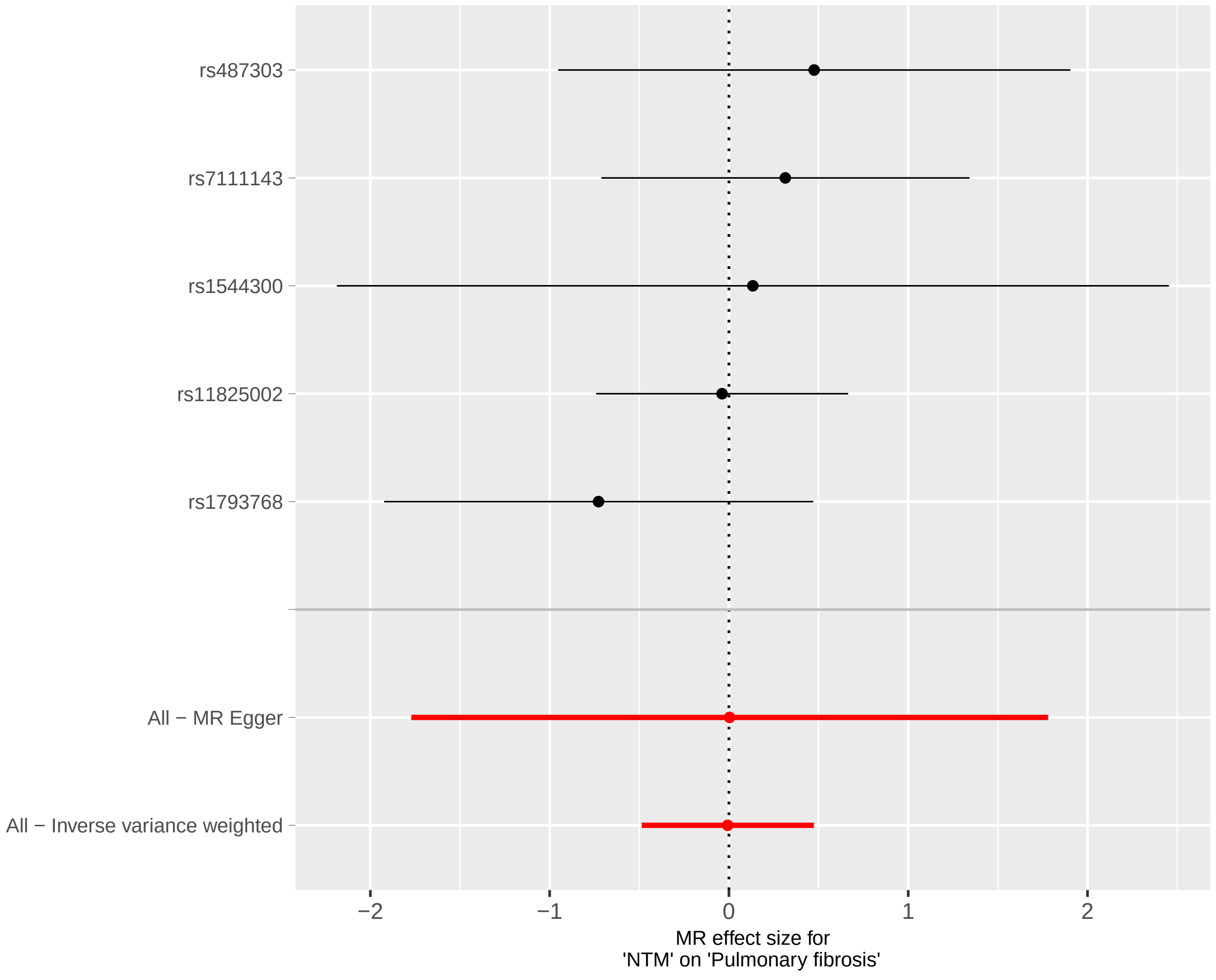
rs487303
rs7111143
rs1544300
rs11825002
rs1793768
All − MR Egger
All − Inverse variance weighted
−2
−1
0
1
2
MR effect size for
'NTM' on 'Pulmonary fibrosis'

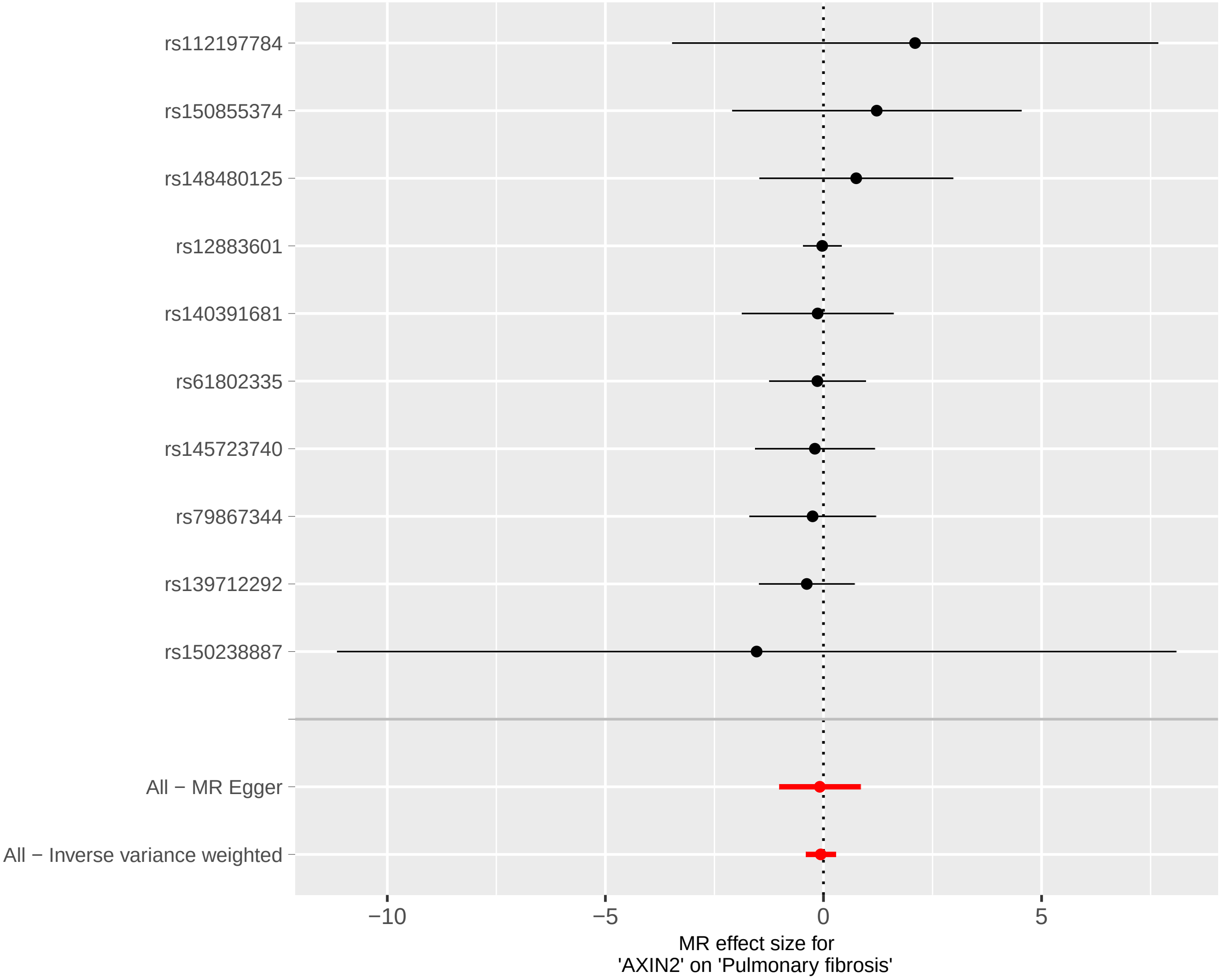
rs112197784
rs150855374
rs148480125
rs12883601
rs140391681
rs61802335
rs145723740
rs79867344
rs139712292
rs150238887
All − MR Egger
All − Inverse variance weighted
−10
−5
0
5
MR effect size for
'AXIN2' on 'Pulmonary fibrosis'

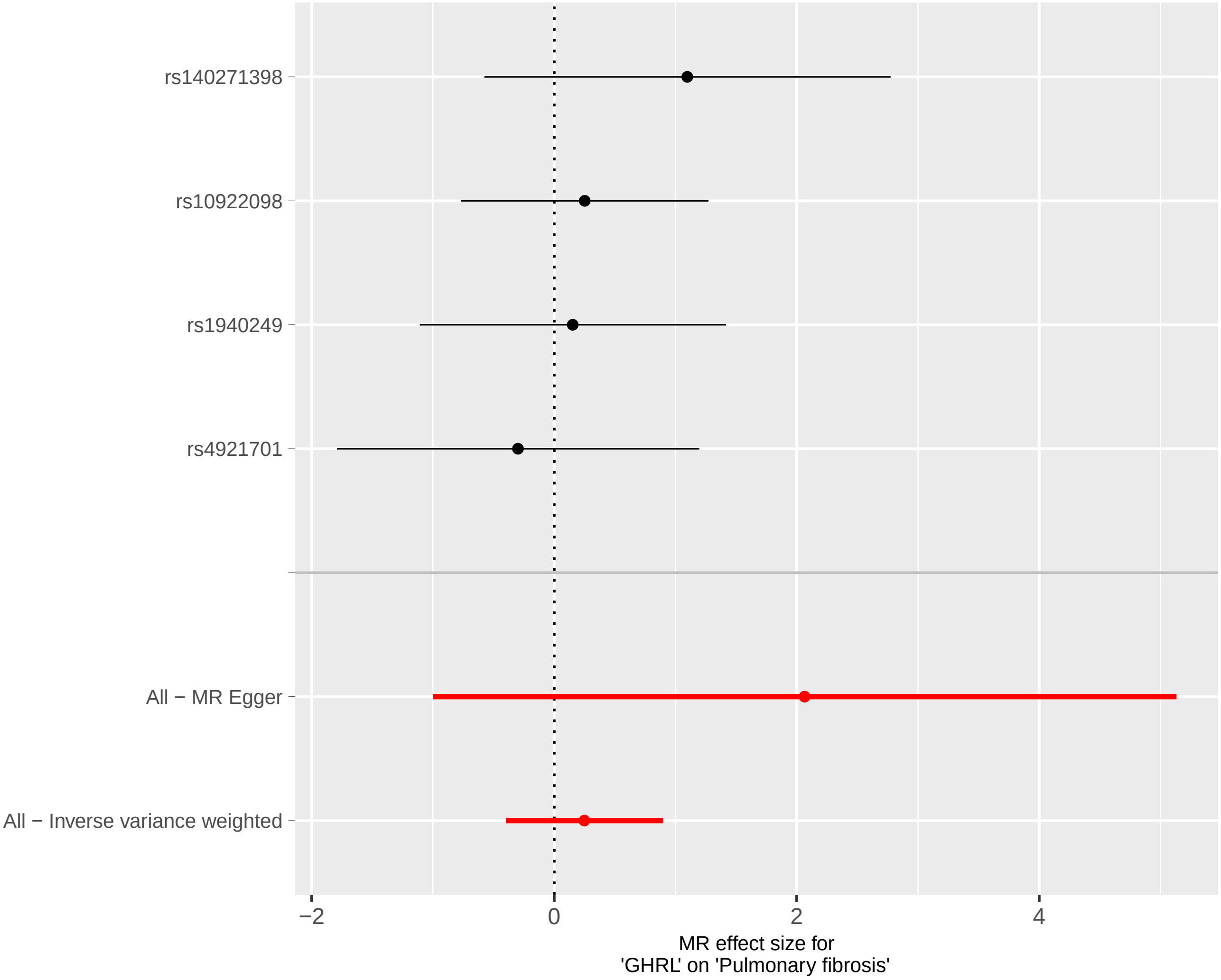

rs140271398
rs10922098
rs1940249
rs4921701
All − MR Egger
All − Inverse variance weighted
−2
0
2
4
MR effect size for
'GHRL' on 'Pulmonary fibrosis'

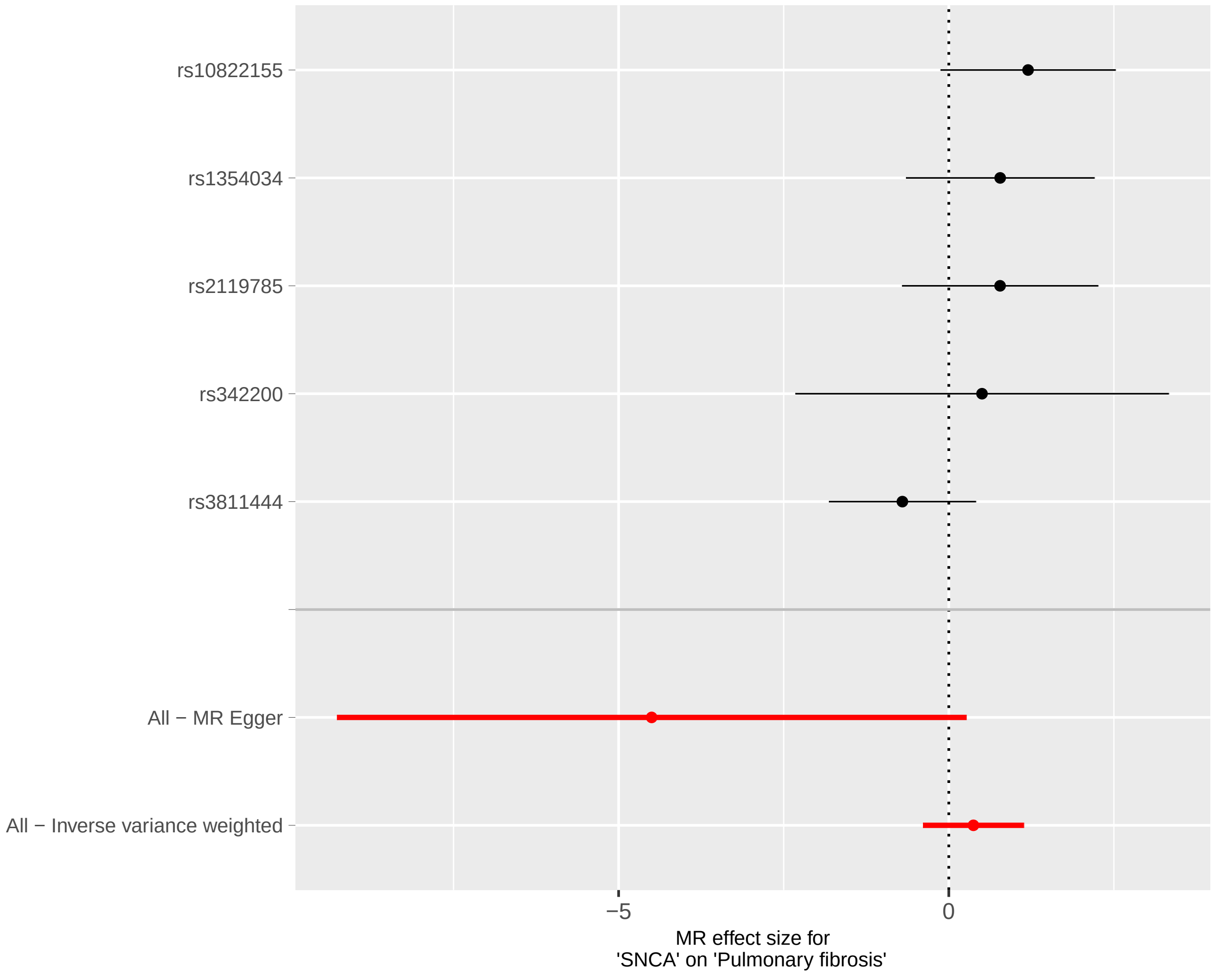
rs10822155
rs1354034
rs2119785
rs342200
rs3811444
All − MR Egger
All − Inverse variance weighted
−5
0
MR effect size for
'SNCA' on 'Pulmonary fibrosis'

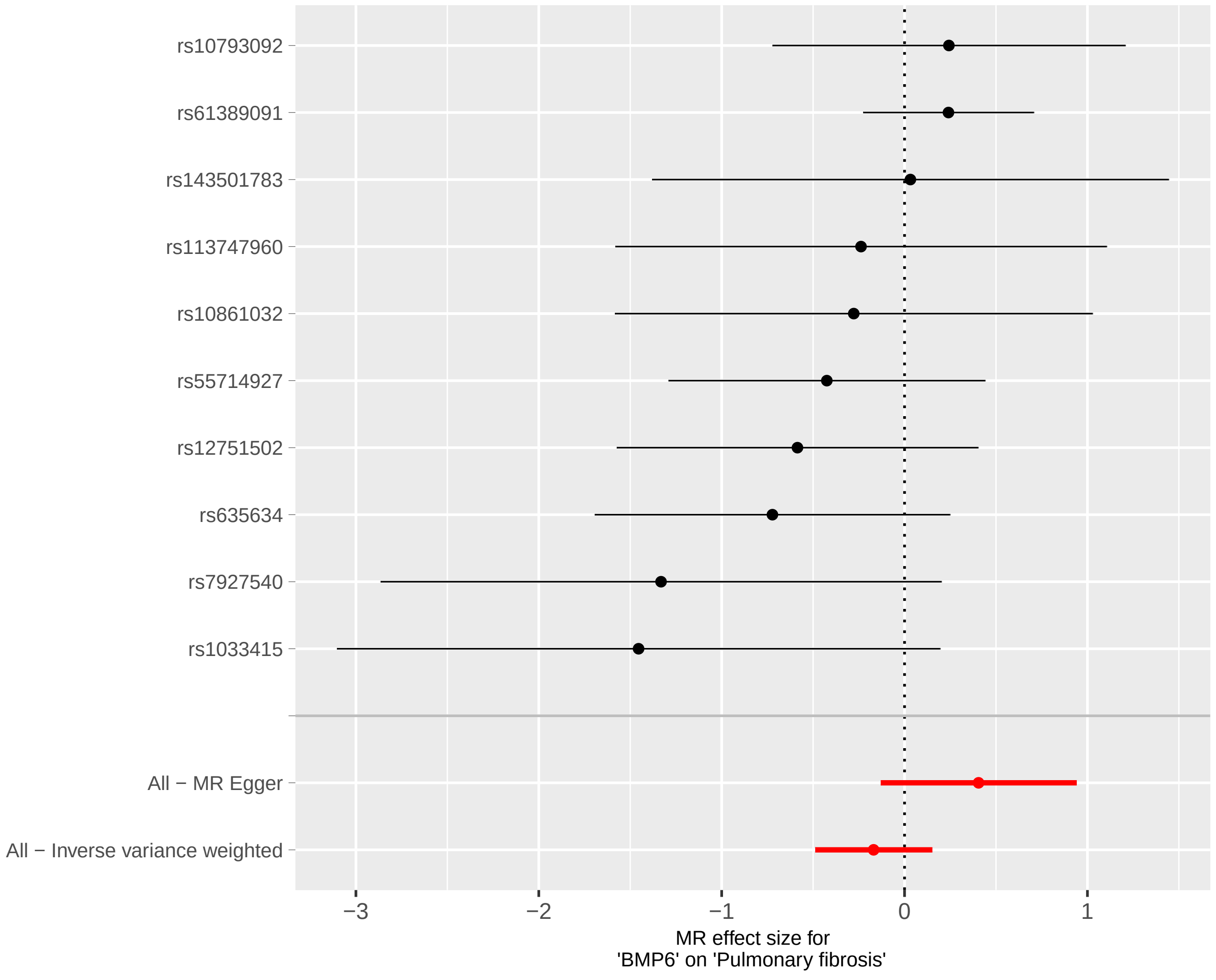

rs10793092
rs61389091
rs143501783
rs113747960
rs10861032
rs55714927
rs12751502
rs635634
rs7927540
rs1033415
All − MR Egger
All − Inverse variance weighted
−3
−2
−1
0
1
MR effect size for
'BMP6' on 'Pulmonary fibrosis'

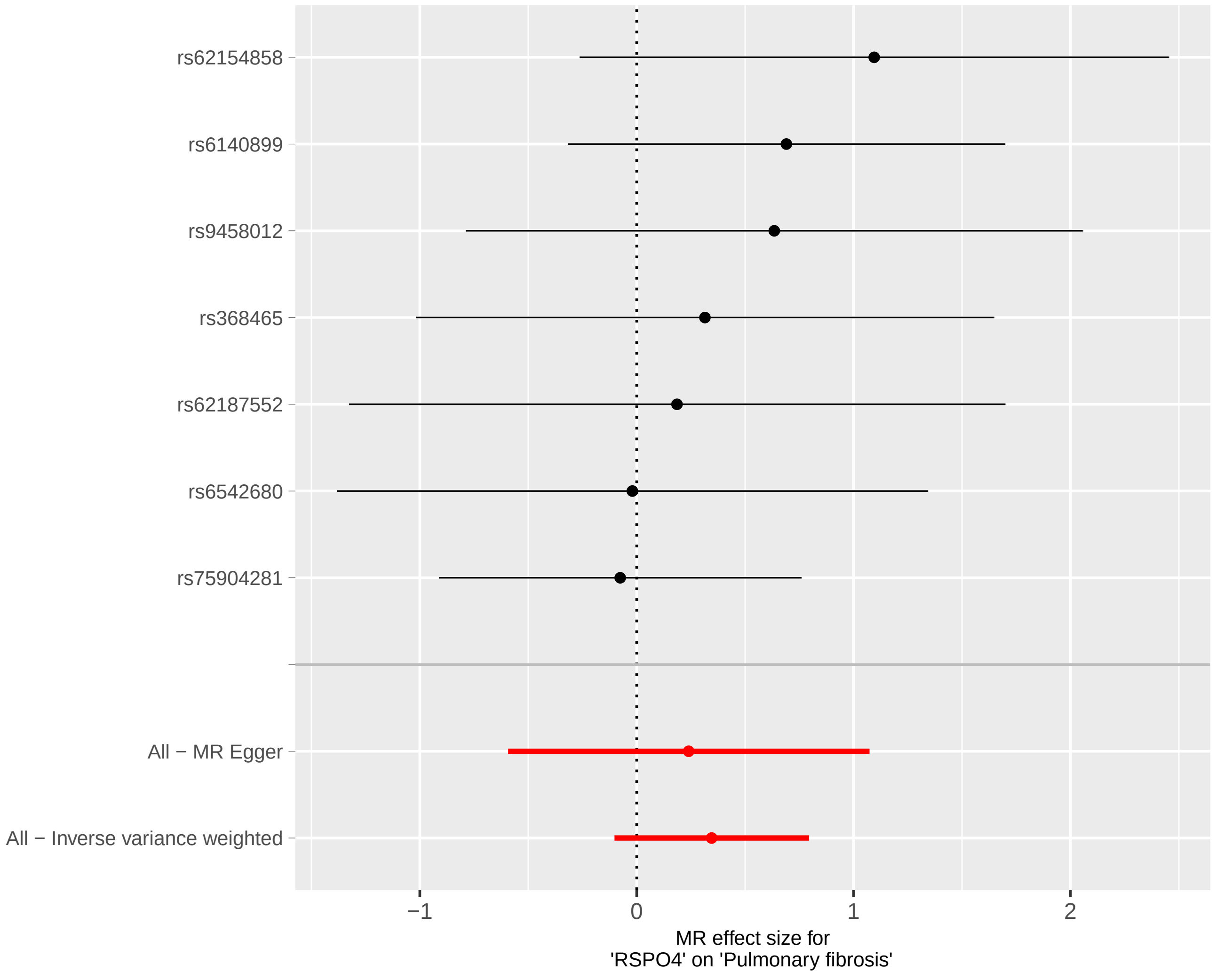
rs62154858
rs6140899
rs9458012
rs368465
rs62187552
rs6542680
rs75904281
All − MR Egger
All − Inverse variance weighted
−1
0
1
2
MR effect size for
'RSPO4' on 'Pulmonary fibrosis'

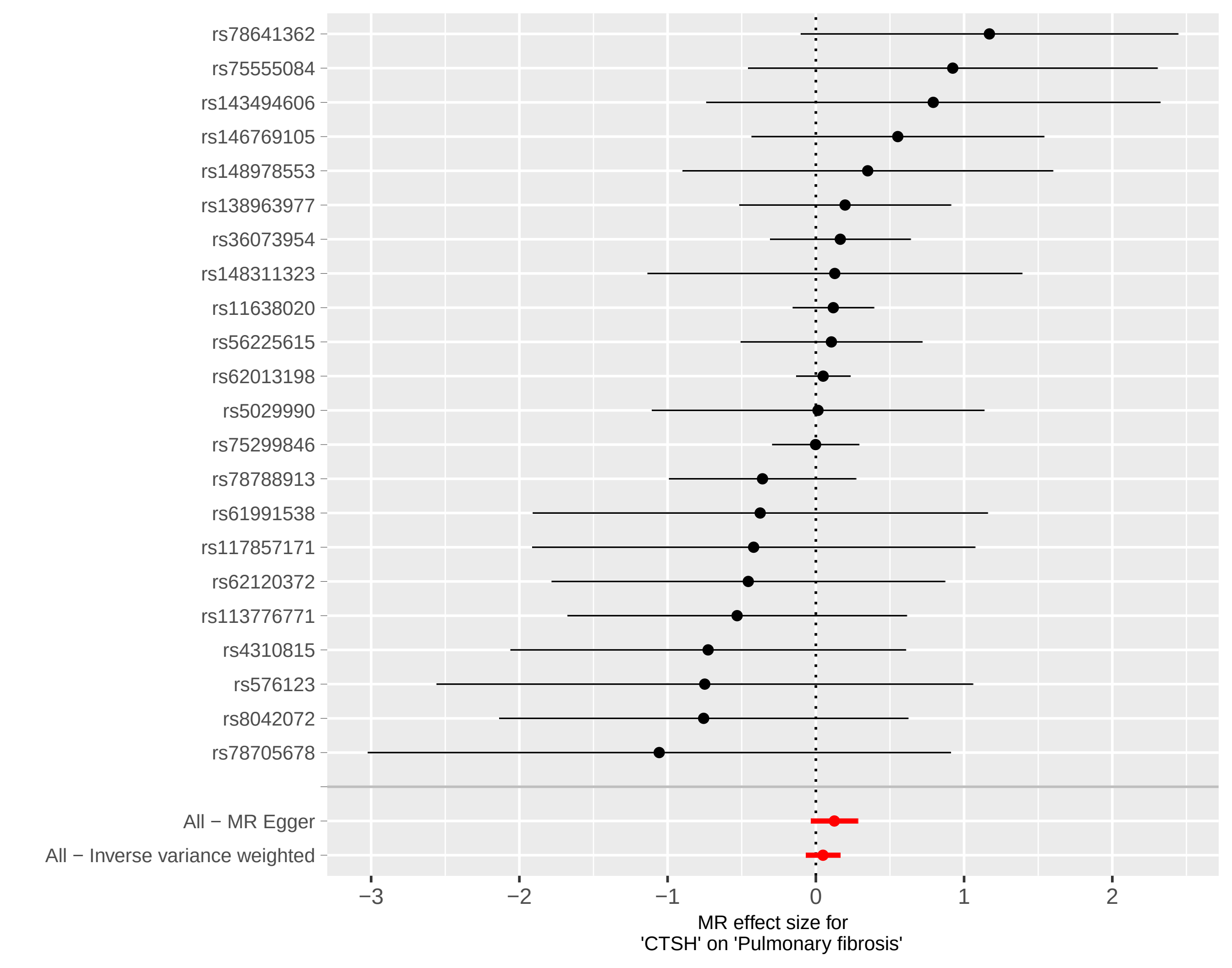
rs78641362
rs75555084
rs143494606
rs146769105
rs148978553
rs138963977
rs36073954
rs148311323
rs11638020
rs56225615
rs62013198
rs5029990
rs75299846
rs78788913
rs61991538
rs117857171
rs62120372
rs113776771
rs4310815
rs576123
rs8042072
rs78705678
All − MR Egger
All − Inverse variance weighted
−3
−2
−1
0
1
2
MR effect size for
'CTSH' on 'Pulmonary fibrosis'

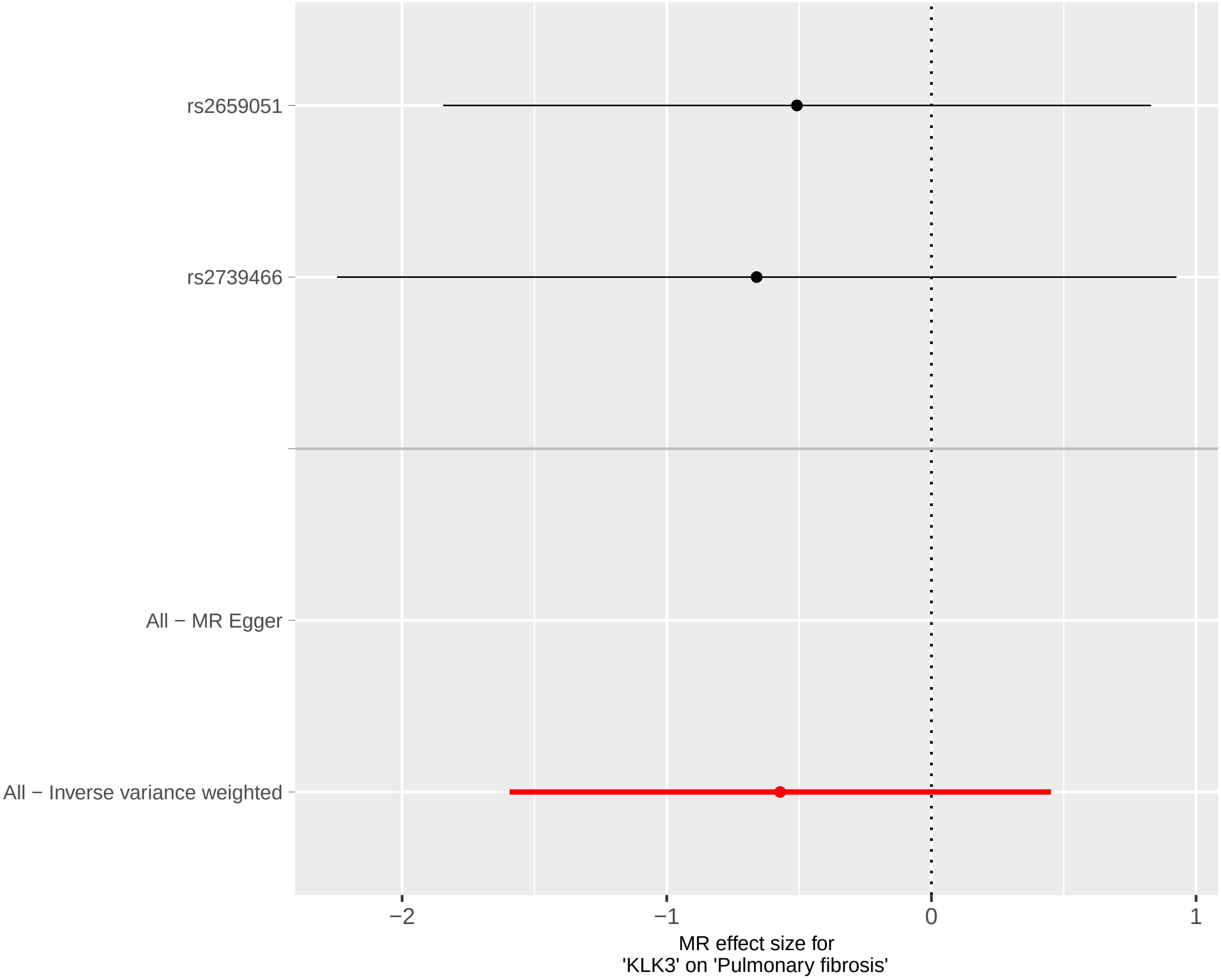

rs2659051
rs2739466
All − MR Egger
All − Inverse variance weighted
−2
−1
0
1
MR effect size for
'KLK3' on 'Pulmonary fibrosis'

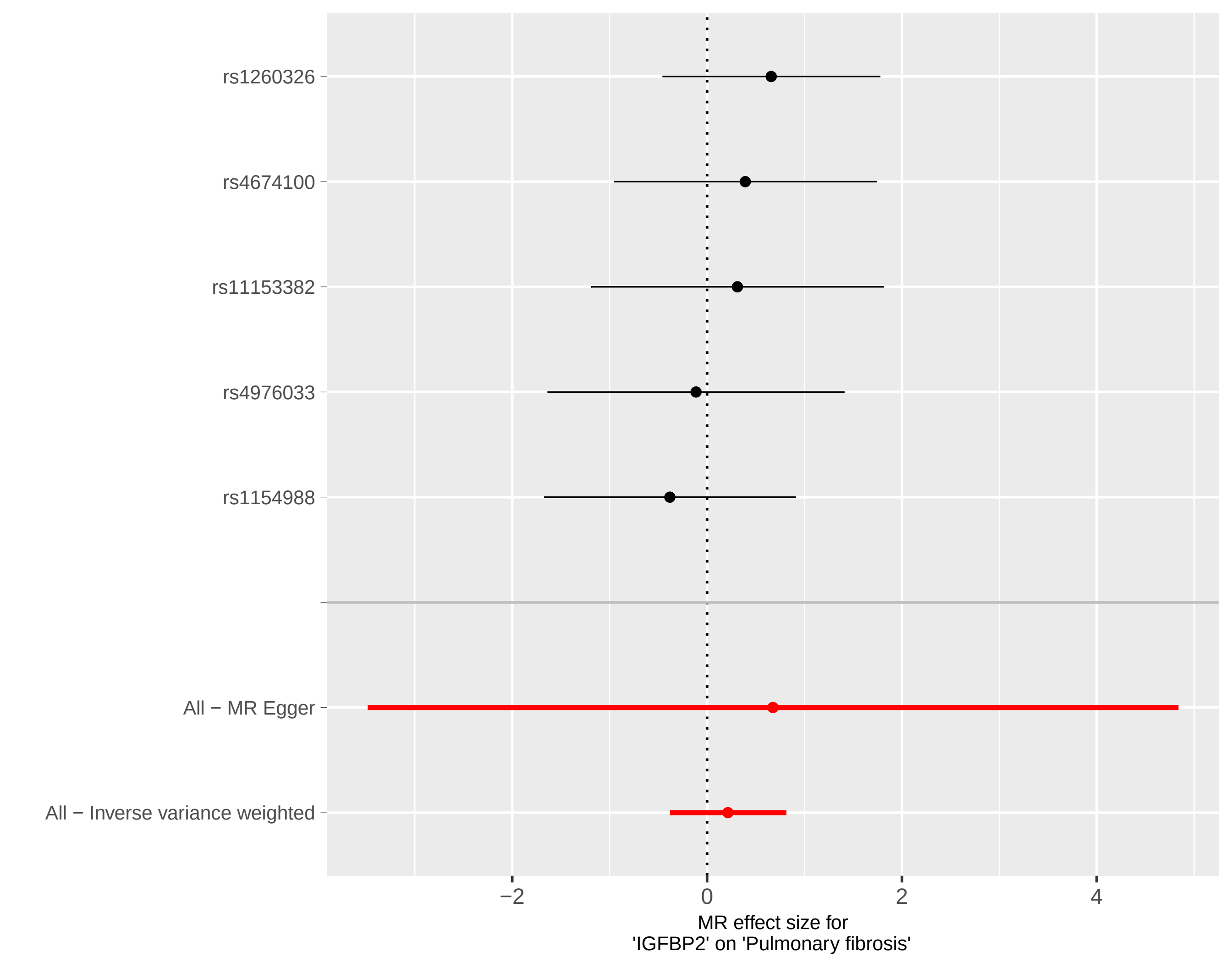
rs1260326
rs4674100
rs11153382
rs4976033
rs1154988
All − MR Egger
All − Inverse variance weighted
−2
0
2
4
MR effect size for
'IGFBP2' on 'Pulmonary fibrosis'

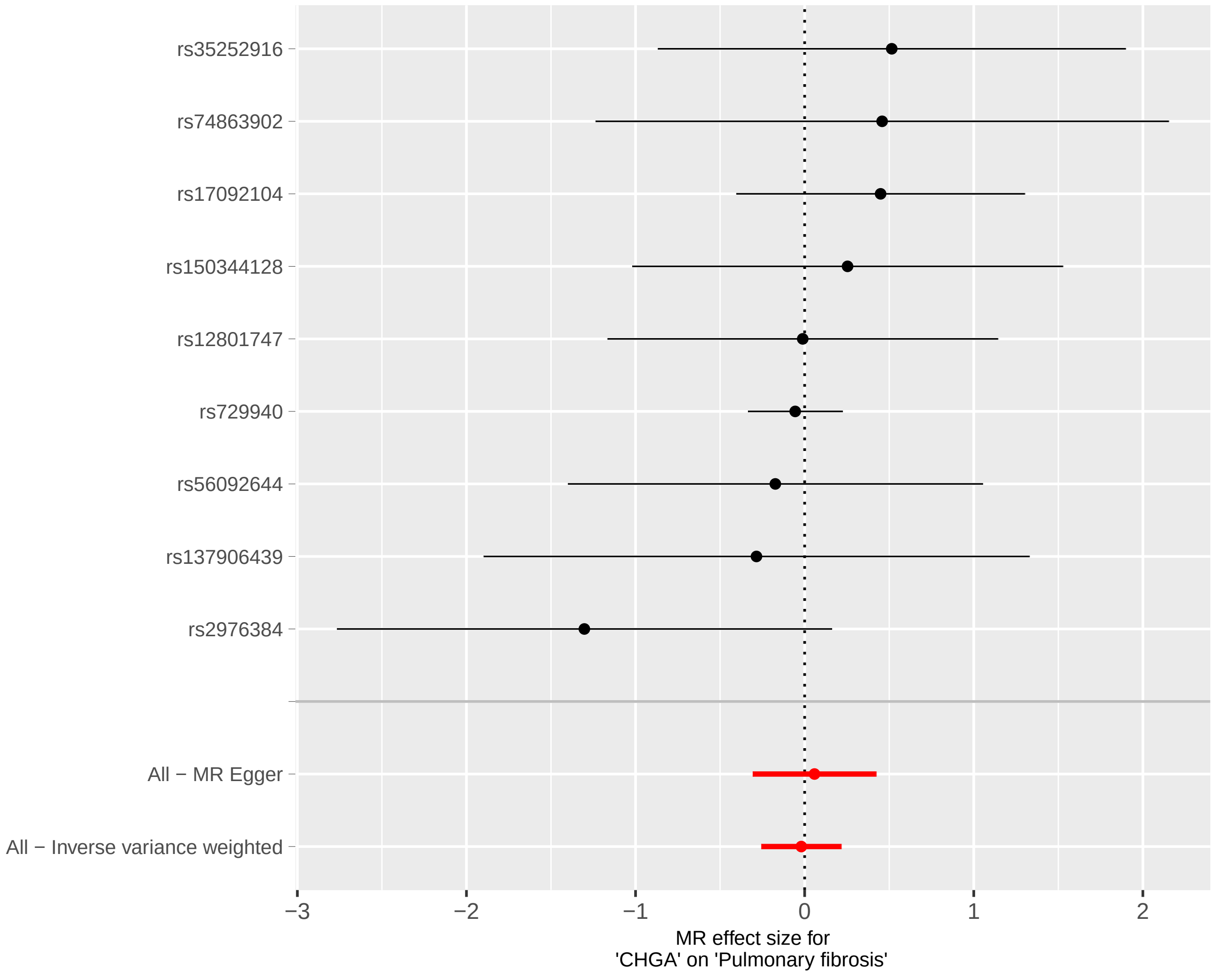

rs35252916
rs74863902
rs17092104
rs150344128
rs12801747
rs729940
rs56092644
rs137906439
rs2976384
All − MR Egger
All − Inverse variance weighted
−3
−2
−1
0
1
2
MR effect size for
'CHGA' on 'Pulmonary fibrosis'

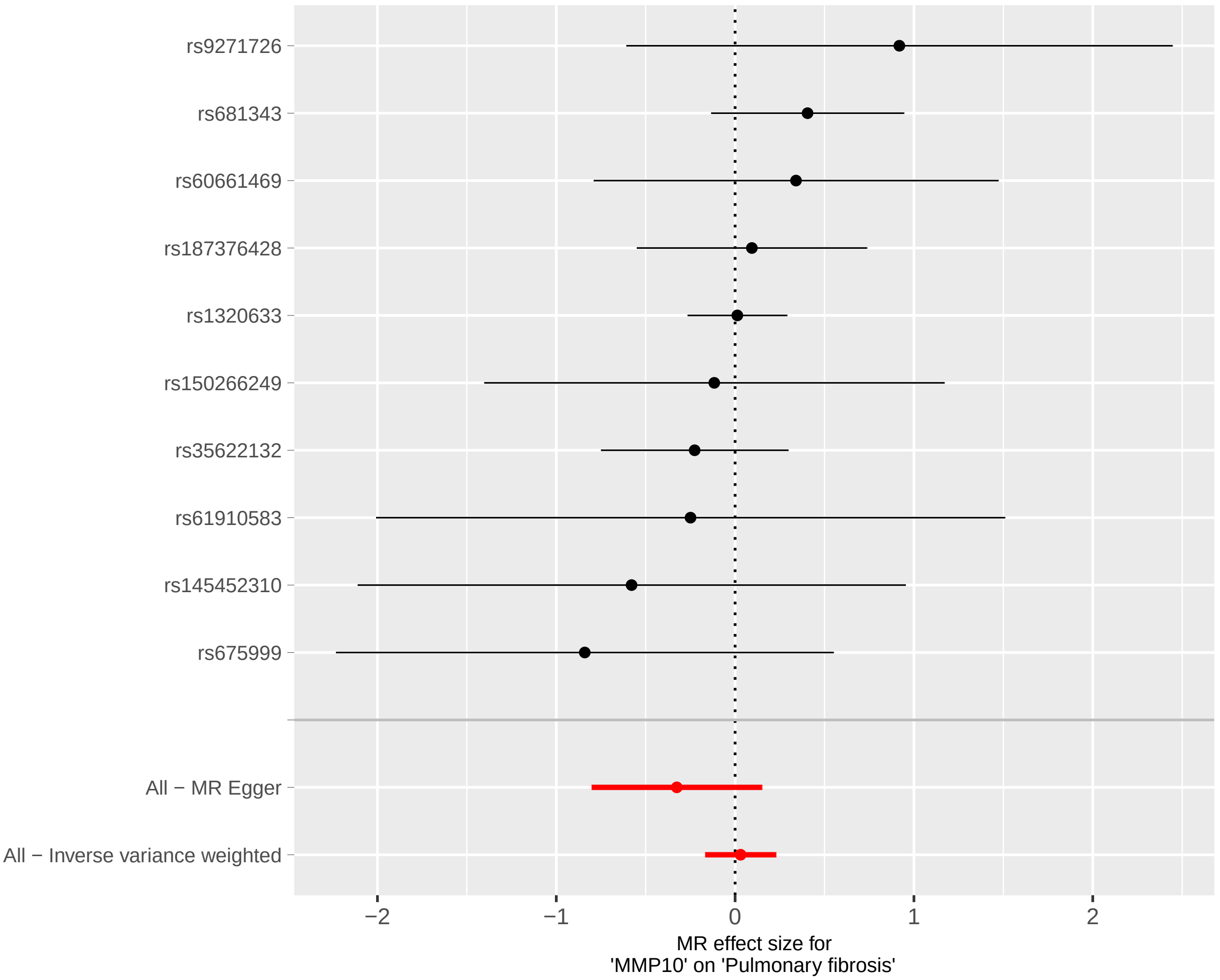

rs9271726
rs681343
rs60661469
rs187376428
rs1320633
rs150266249
rs35622132
rs61910583
rs145452310
rs675999
All − MR Egger
All − Inverse variance weighted
−2
−1
0
1
2
MR effect size for
'MMP10' on 'Pulmonary fibrosis'

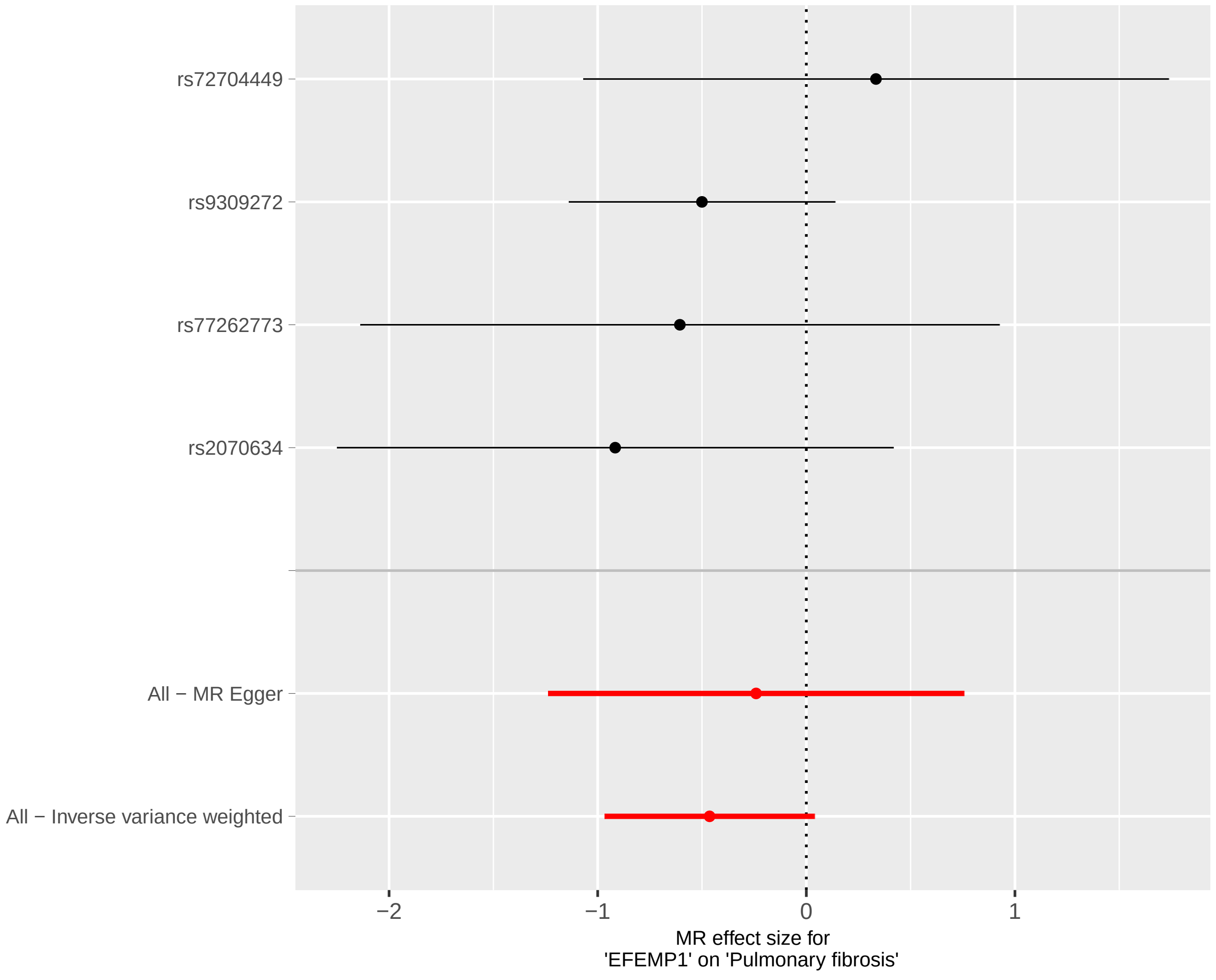

rs72704449
rs9309272
rs77262773
rs2070634
All − MR Egger
All − Inverse variance weighted
−2
−1
0
1
MR effect size for
'EFEMP1' on 'Pulmonary fibrosis'

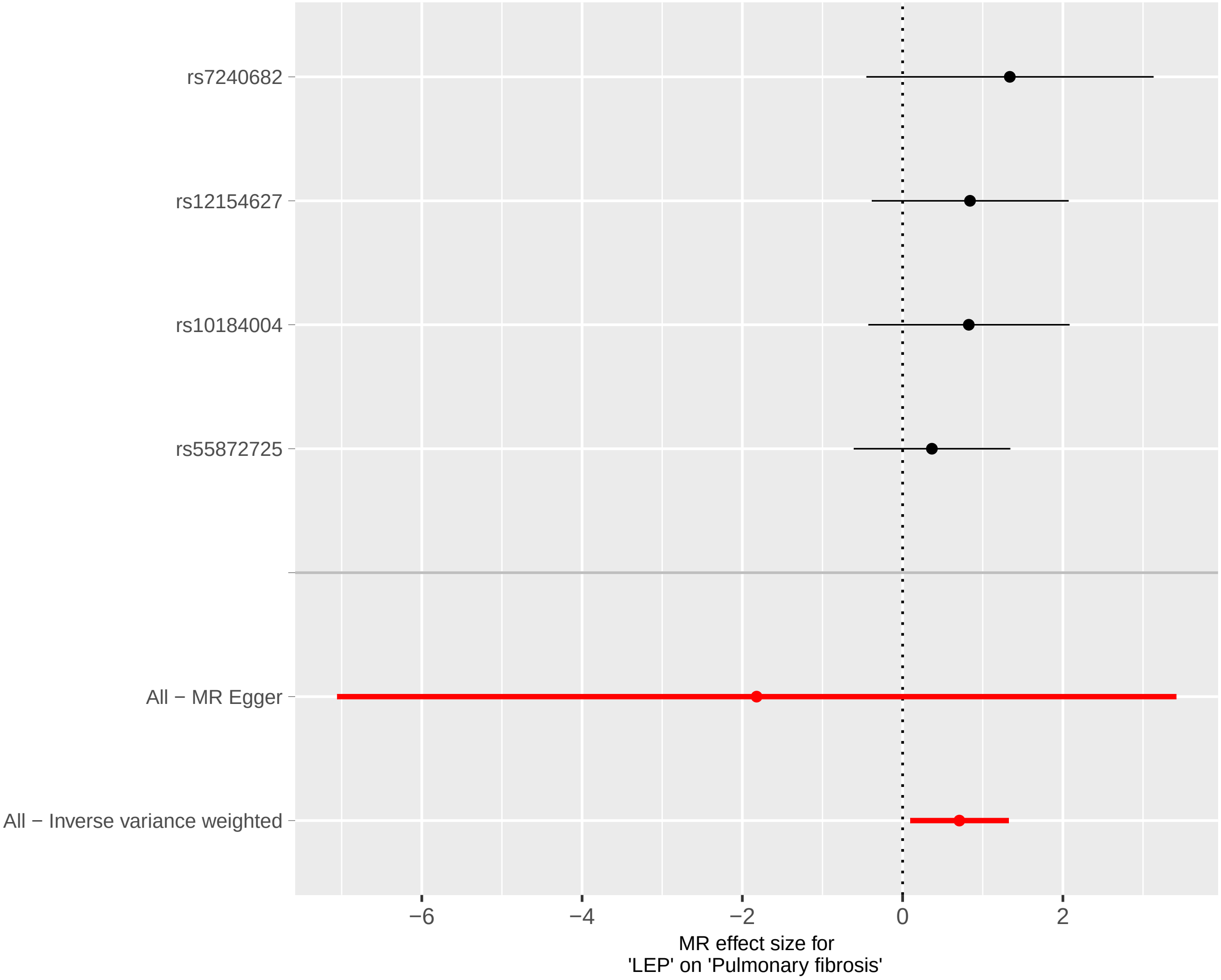
rs7240682
rs12154627
rs10184004
rs55872725
All − MR Egger
All − Inverse variance weighted
−6
−4
−2
0
2
MR effect size for
'LEP' on 'Pulmonary fibrosis'

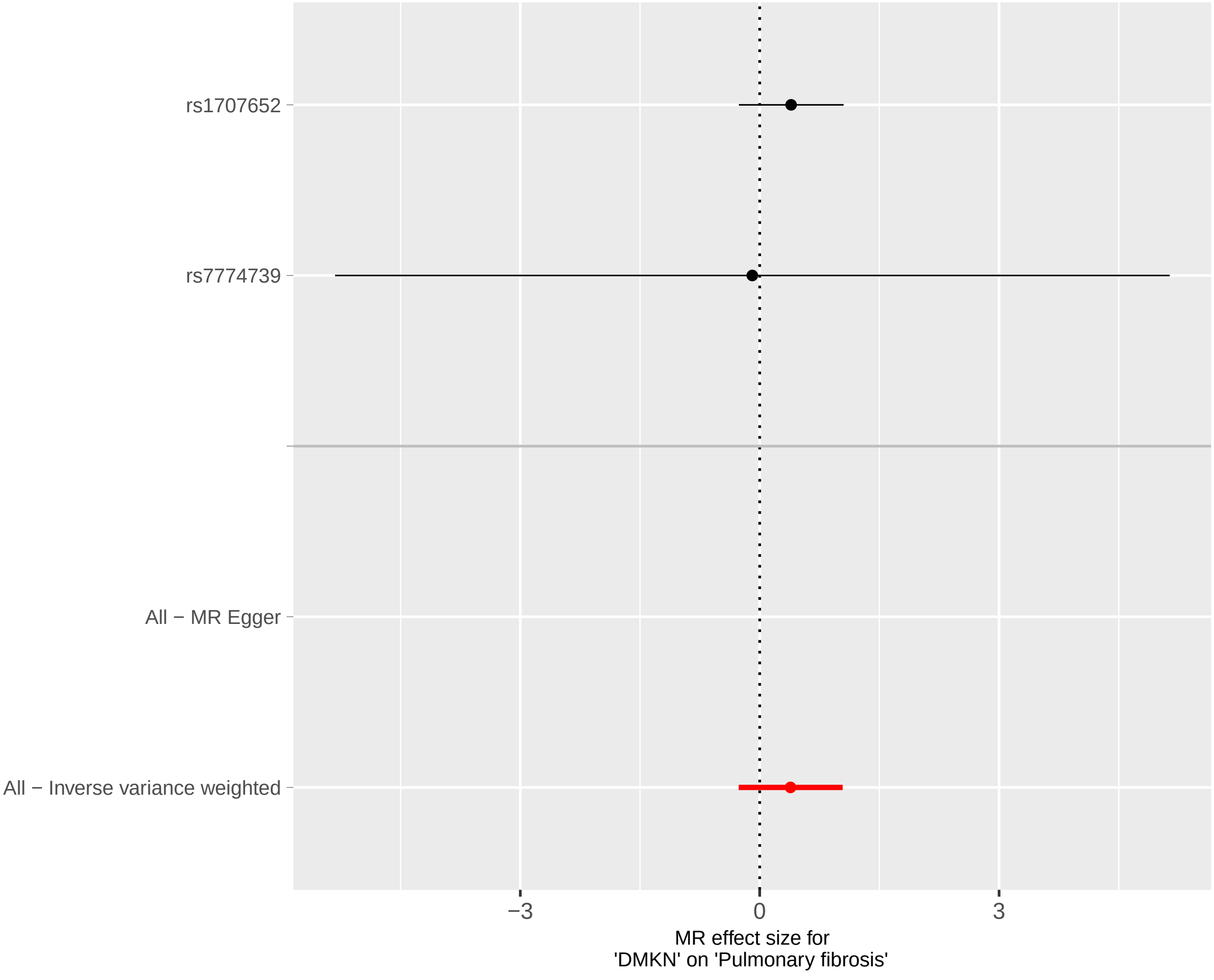

rs1707652
rs7774739
All − MR Egger
All − Inverse variance weighted
−3
0
3
MR effect size for
'DMKN' on 'Pulmonary fibrosis'

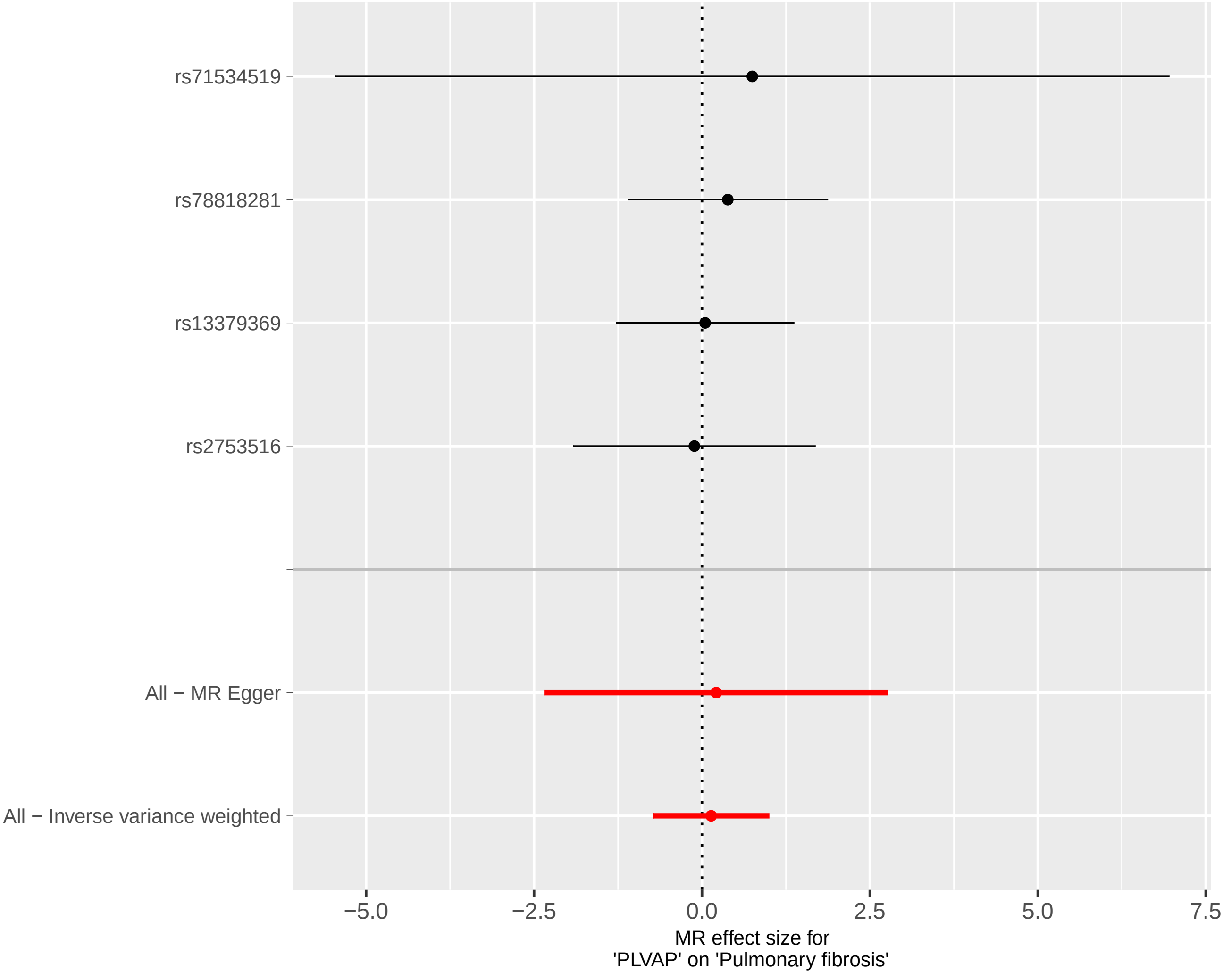

rs71534519
rs78818281
rs13379369
rs2753516
All − MR Egger
All − Inverse variance weighted
−5.0
−2.5
0.0
2.5
5.0
7.5
MR effect size for
'PLVAP' on 'Pulmonary fibrosis'

Insufficient number of SNPs

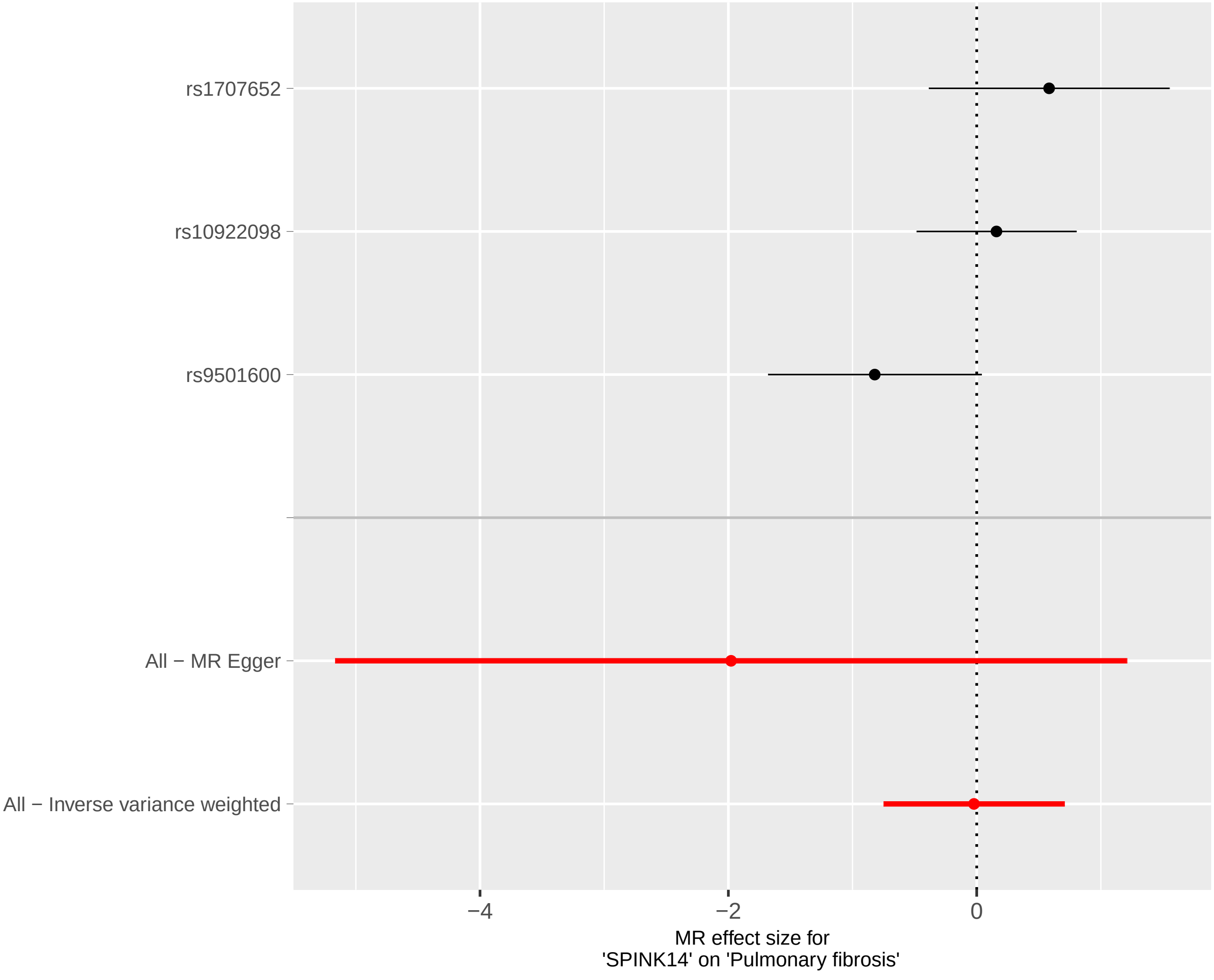

rs1707652
rs10922098
rs9501600
All − MR Egger
All − Inverse variance weighted
−4
−2
0
MR effect size for
'SPINK14' on 'Pulmonary fibrosis'

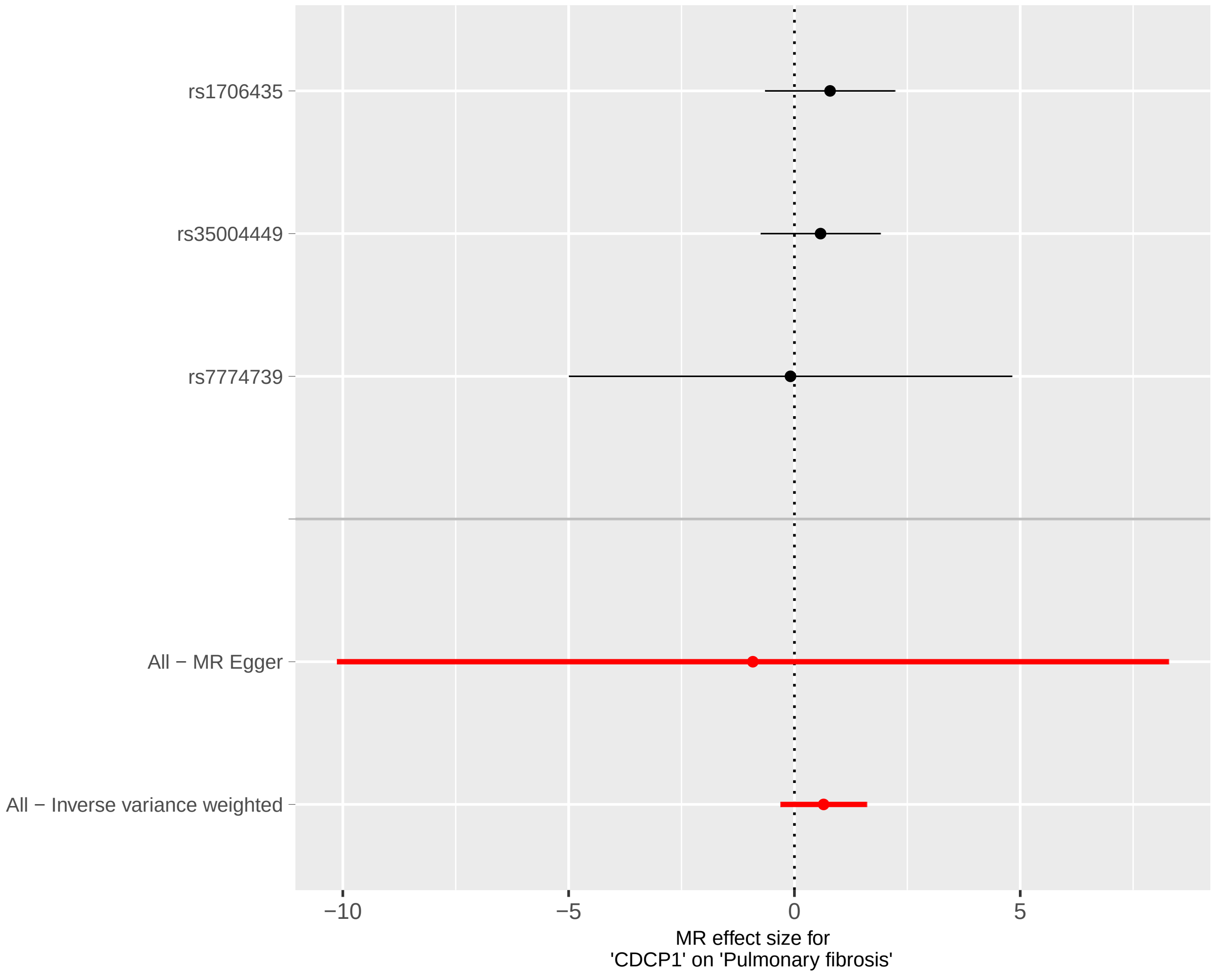

rs1706435
rs35004449
rs7774739
All − MR Egger
All − Inverse variance weighted
−10
−5
0
5
MR effect size for
'CDCP1' on 'Pulmonary fibrosis'

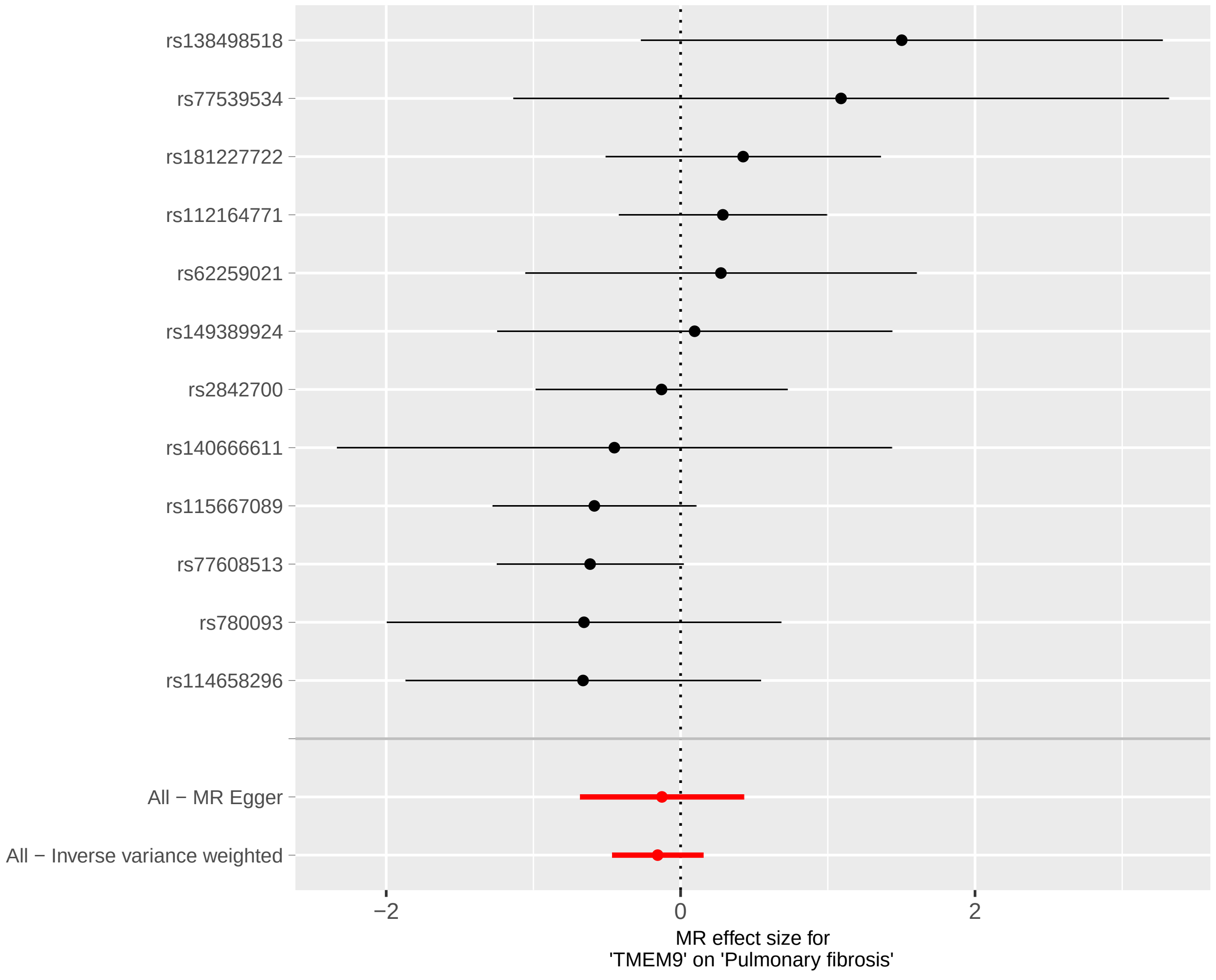

rs138498518
rs77539534
rs181227722
rs112164771
rs62259021
rs149389924
rs2842700
rs140666611
rs115667089
rs77608513
rs780093
rs114658296
All − MR Egger
All − Inverse variance weighted
−2
0
2
MR effect size for
'TMEM9' on 'Pulmonary fibrosis'

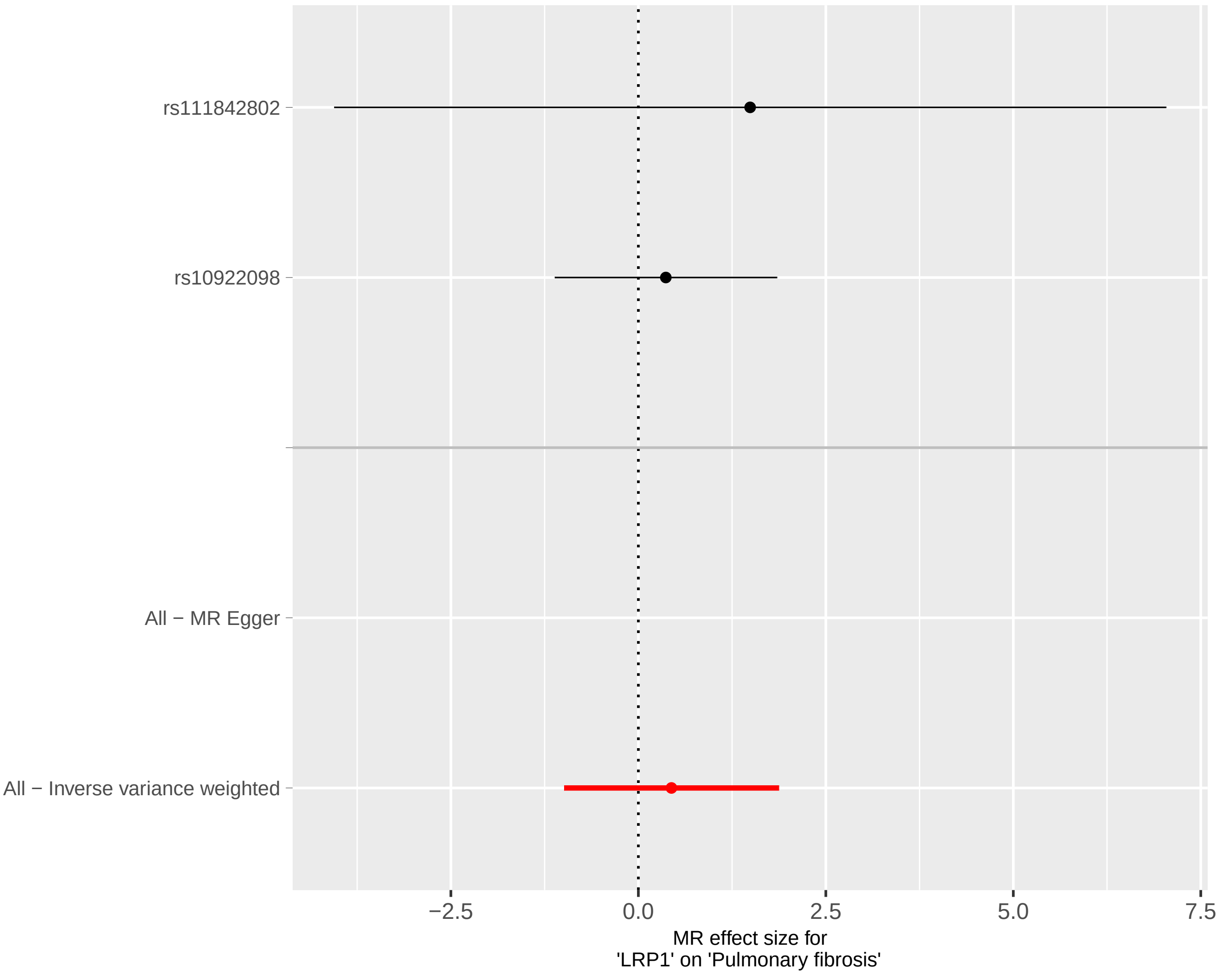
rs111842802
rs10922098
All − MR Egger
All − Inverse variance weighted
−2.5
0.0
2.5
5.0
7.5
MR effect size for
'LRP1' on 'Pulmonary fibrosis'

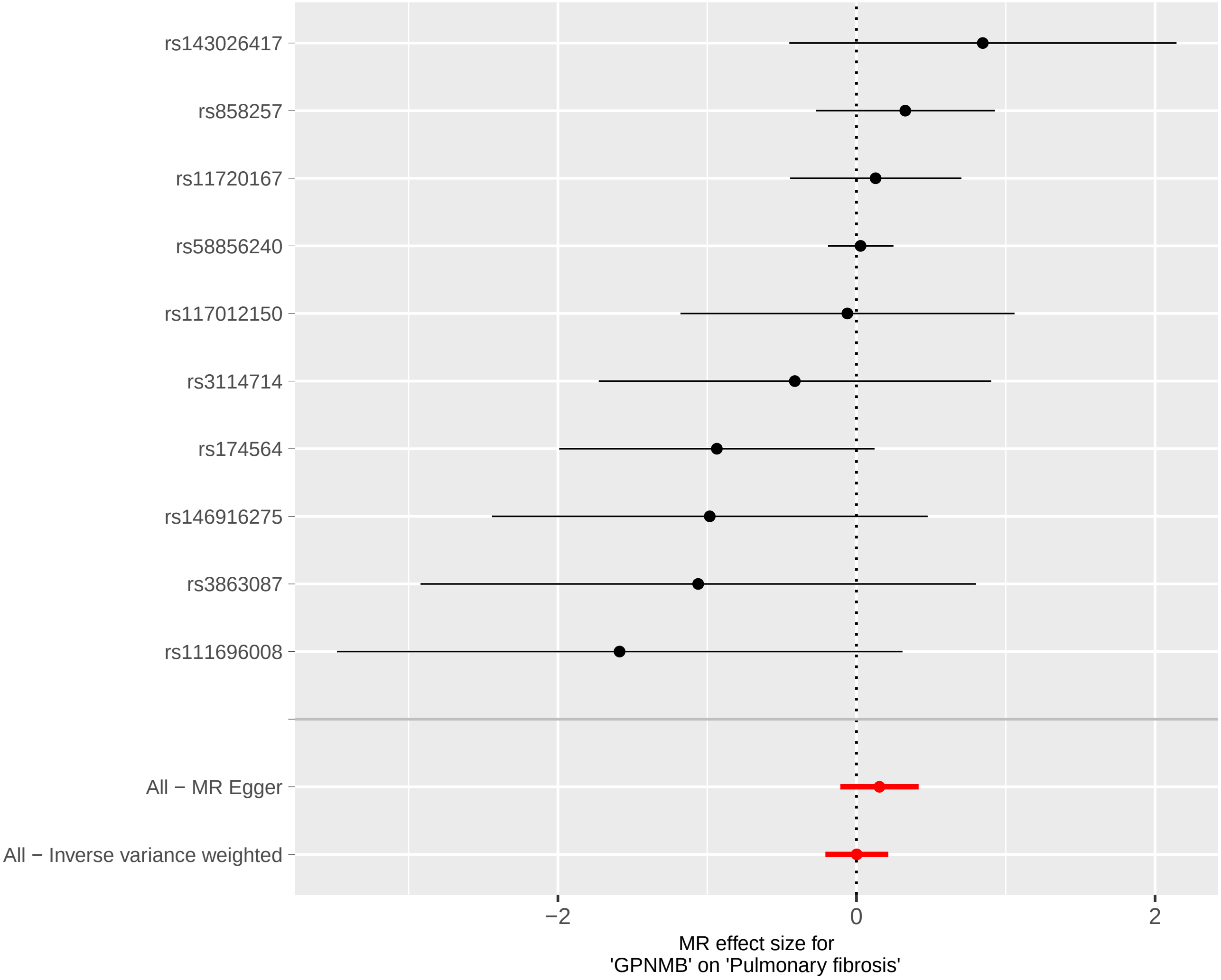
rs143026417
rs858257
rs11720167
rs58856240
rs117012150
rs3114714
rs174564
rs146916275
rs3863087
rs111696008
All − MR Egger
All − Inverse variance weighted
−2
0
2
MR effect size for
'GPNMB' on 'Pulmonary fibrosis'

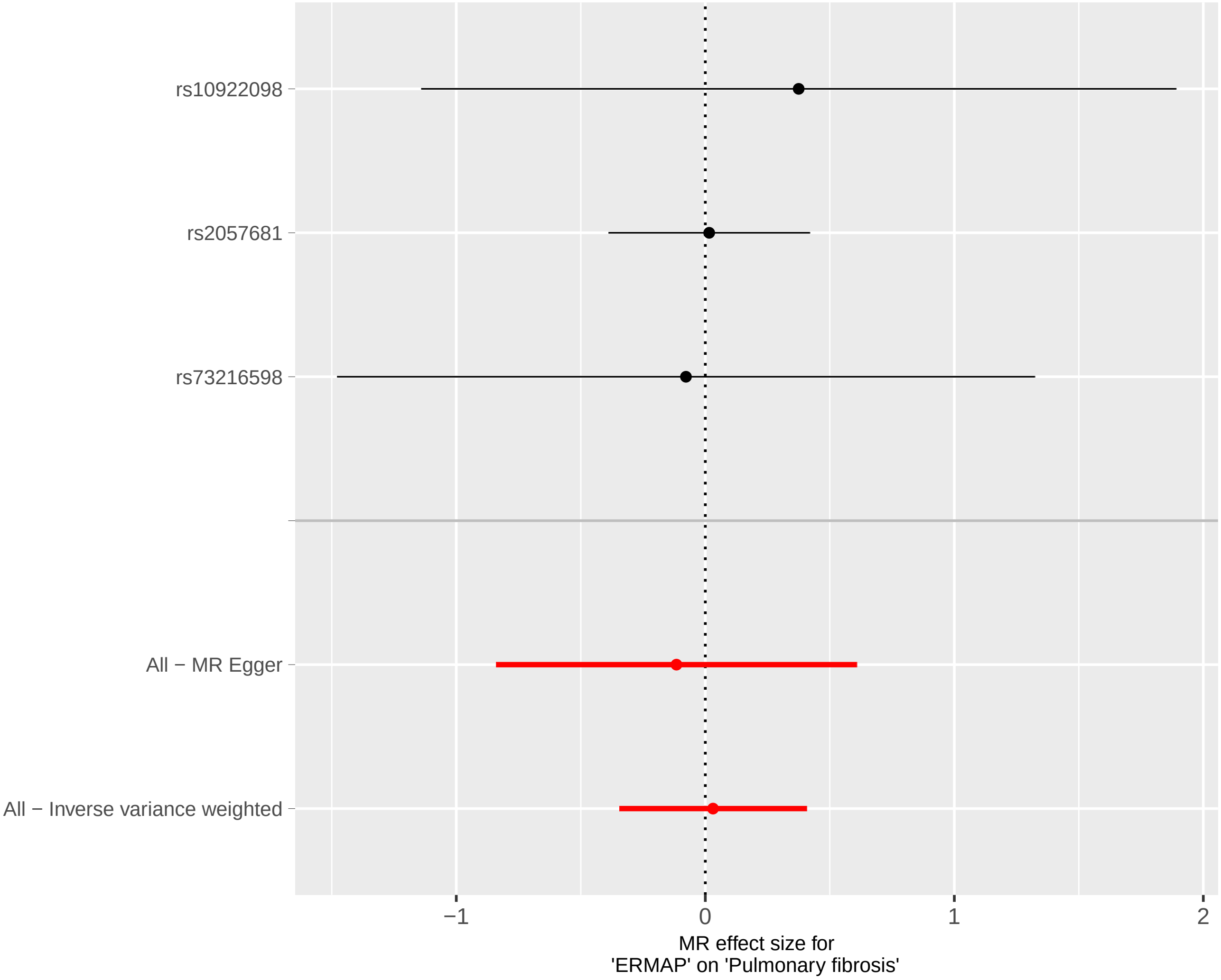

rs10922098
rs2057681
rs73216598
All − MR Egger
All − Inverse variance weighted
−1
0
1
2
MR effect size for
'ERMAP' on 'Pulmonary fibrosis'

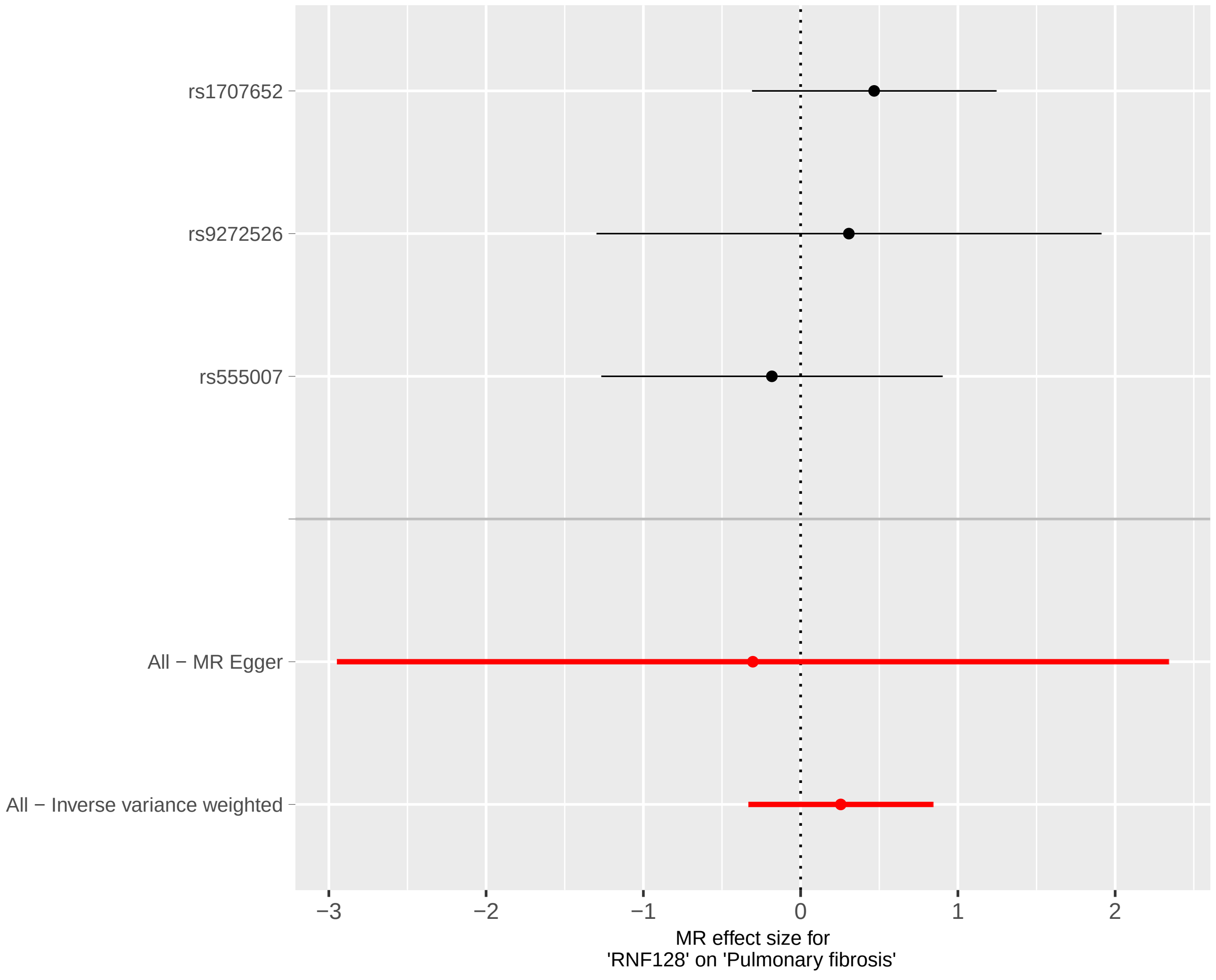

rs1707652
rs9272526
rs555007
All − MR Egger
All − Inverse variance weighted
−3
−2
−1
0
1
2
MR effect size for
'RNF128' on 'Pulmonary fibrosis'

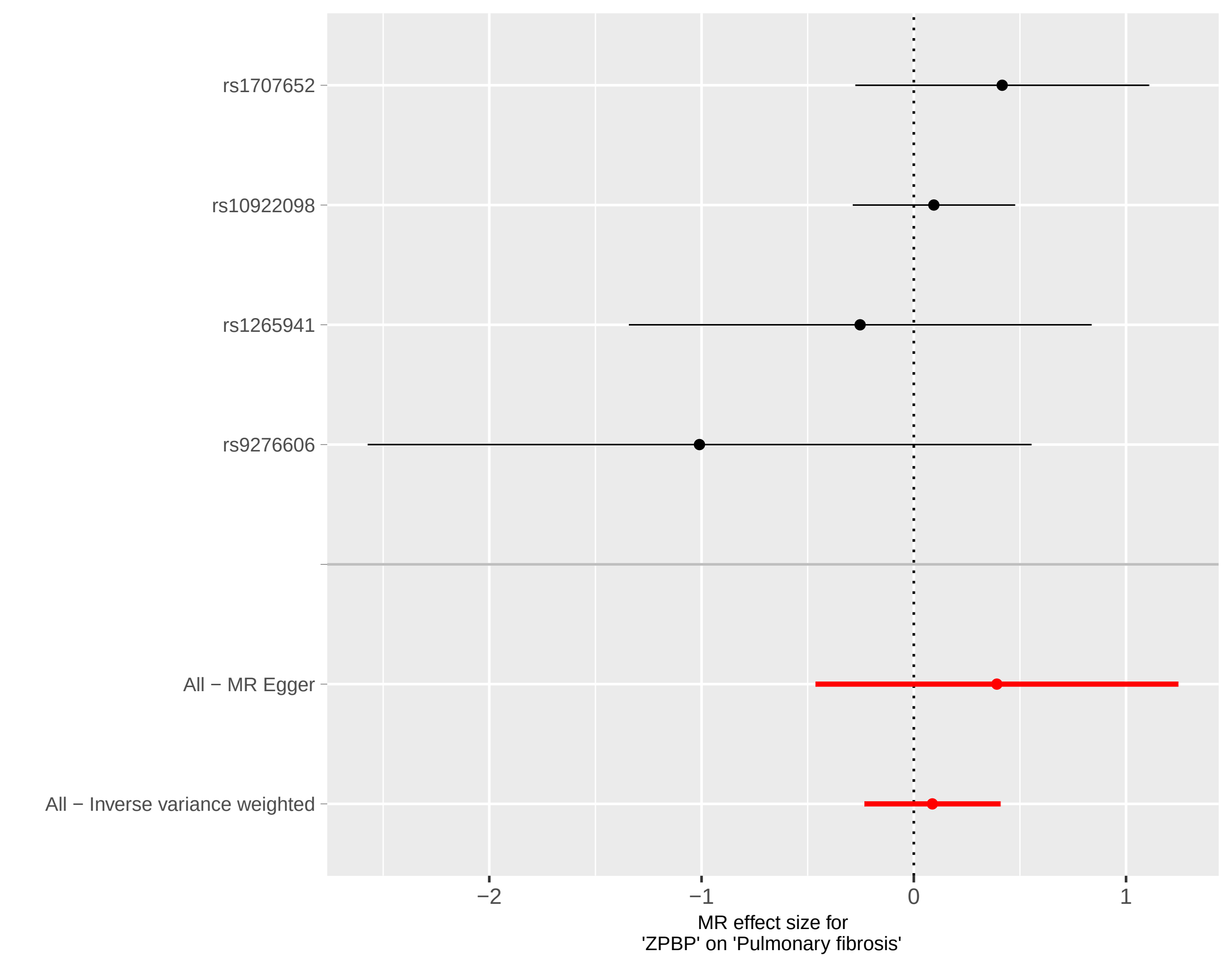
rs1707652
rs10922098
rs1265941
rs9276606
All − MR Egger
All − Inverse variance weighted
−2
−1
0
1
MR effect size for
'ZPBP' on 'Pulmonary fibrosis'

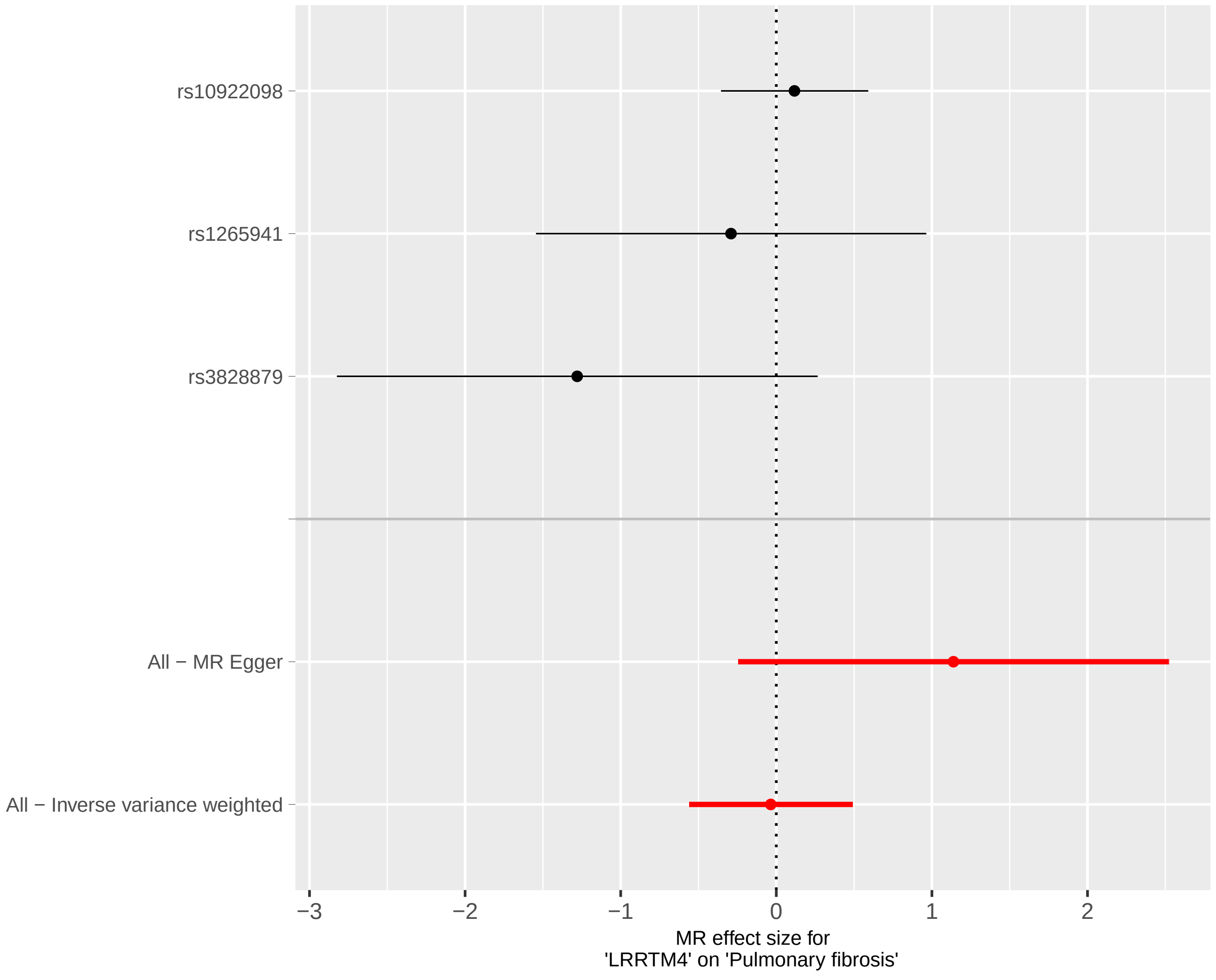

rs10922098
rs1265941
rs3828879
All − MR Egger
All − Inverse variance weighted
−3
−2
−1
0
1
2
MR effect size for
'LRRTM4' on 'Pulmonary fibrosis'

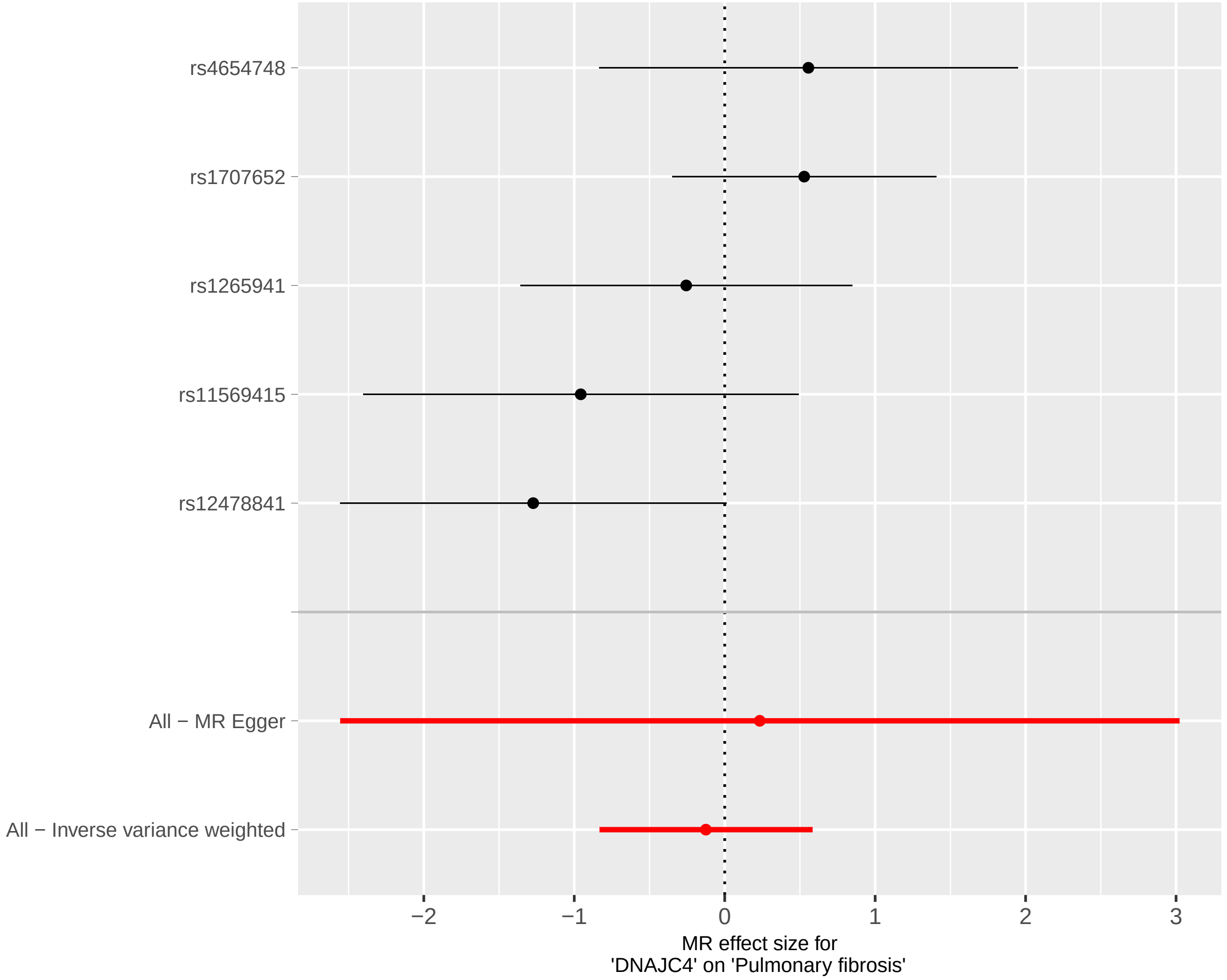

rs4654748
rs1707652
rs1265941
rs11569415
rs12478841
All − MR Egger
All − Inverse variance weighted
−2
−1
0
1
2
3
MR effect size for
'DNAJC4' on 'Pulmonary fibrosis'

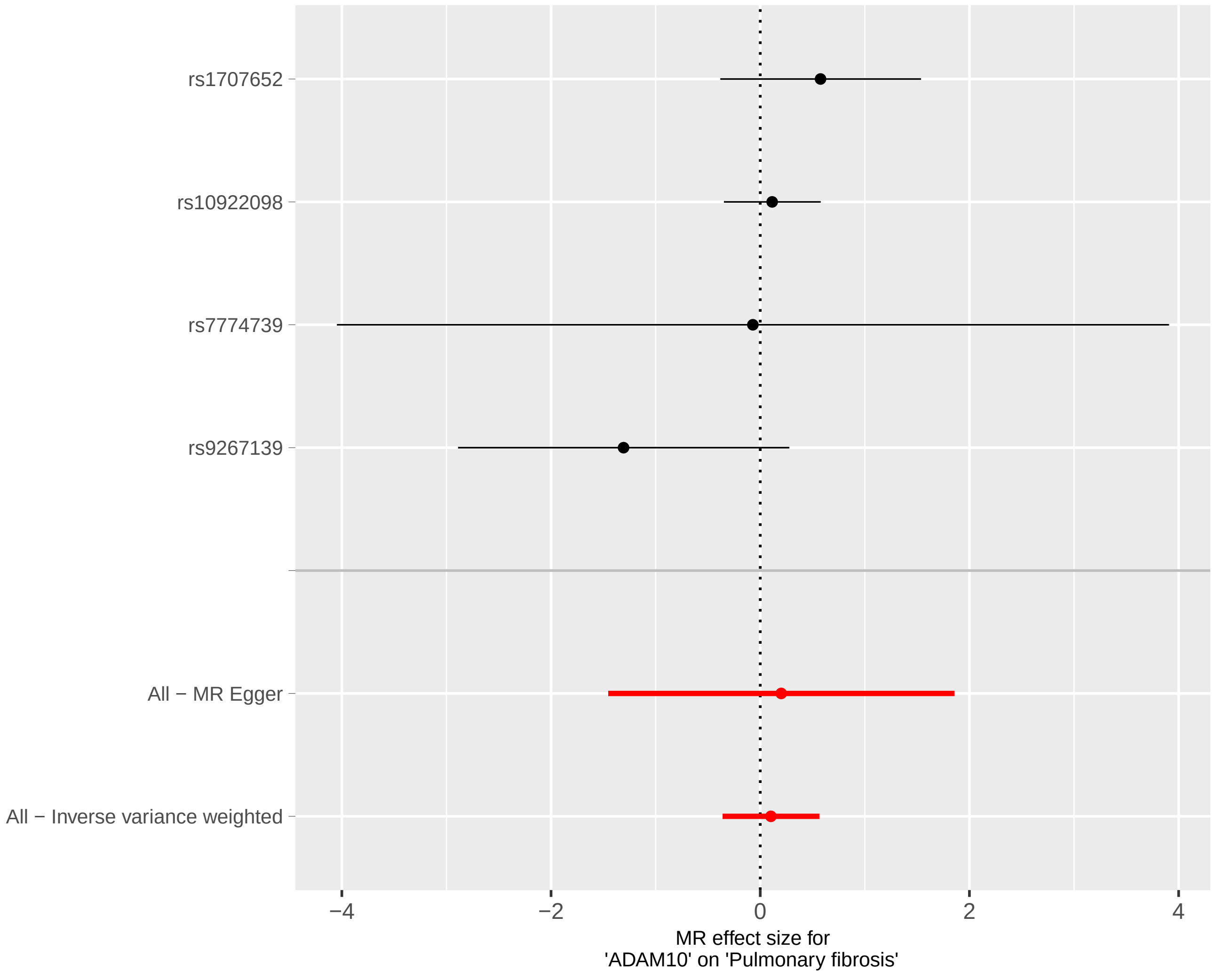

rs1707652
rs10922098
rs7774739
rs9267139
All − MR Egger
All − Inverse variance weighted
−4
−2
0
2
4
MR effect size for
'ADAM10' on 'Pulmonary fibrosis'

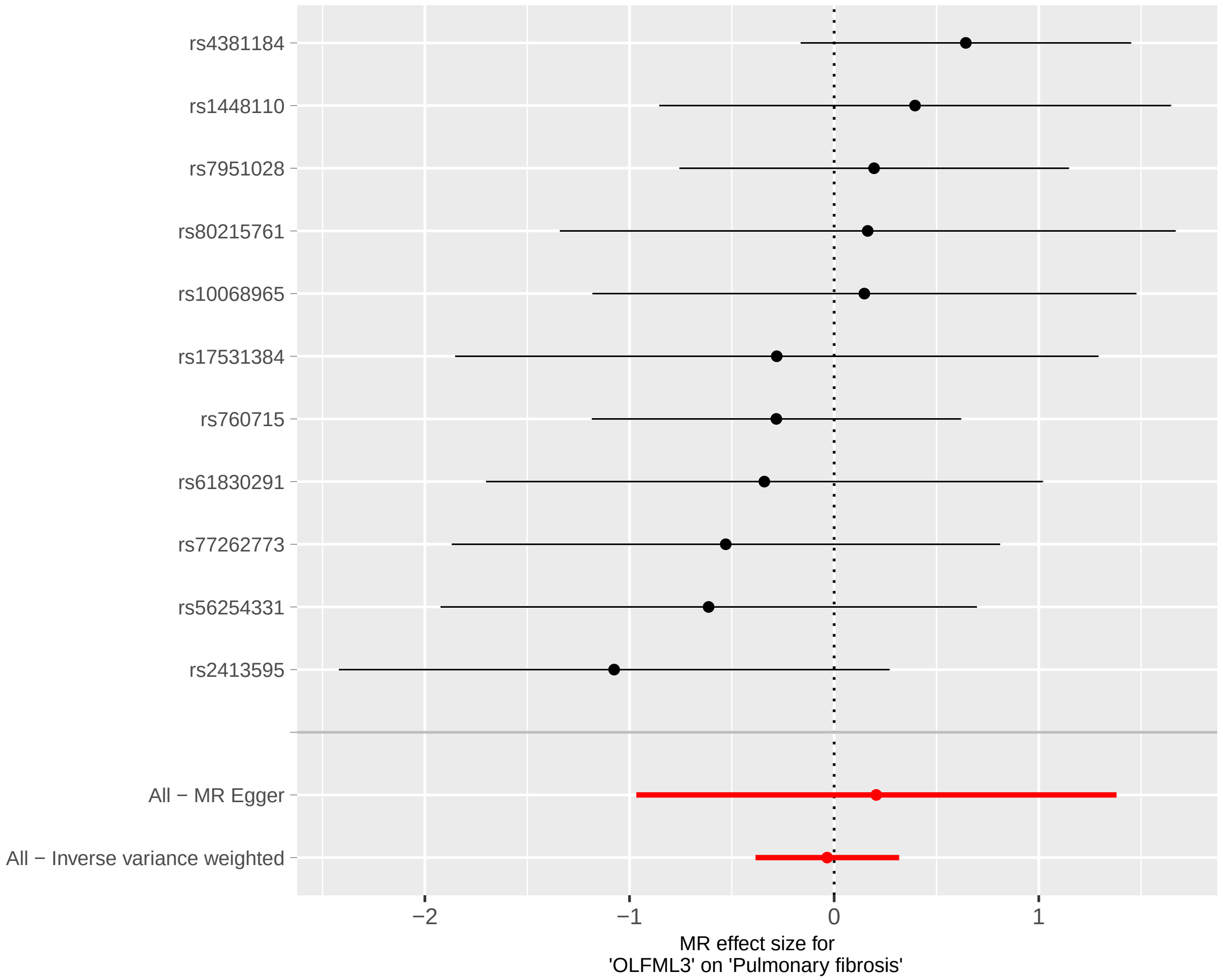
rs4381184
rs1448110
rs7951028
rs80215761
rs10068965
rs17531384
rs760715
rs61830291
rs77262773
rs56254331
rs2413595
All − MR Egger
All − Inverse variance weighted
−2
−1
0
1
MR effect size for
'OLFML3' on 'Pulmonary fibrosis'

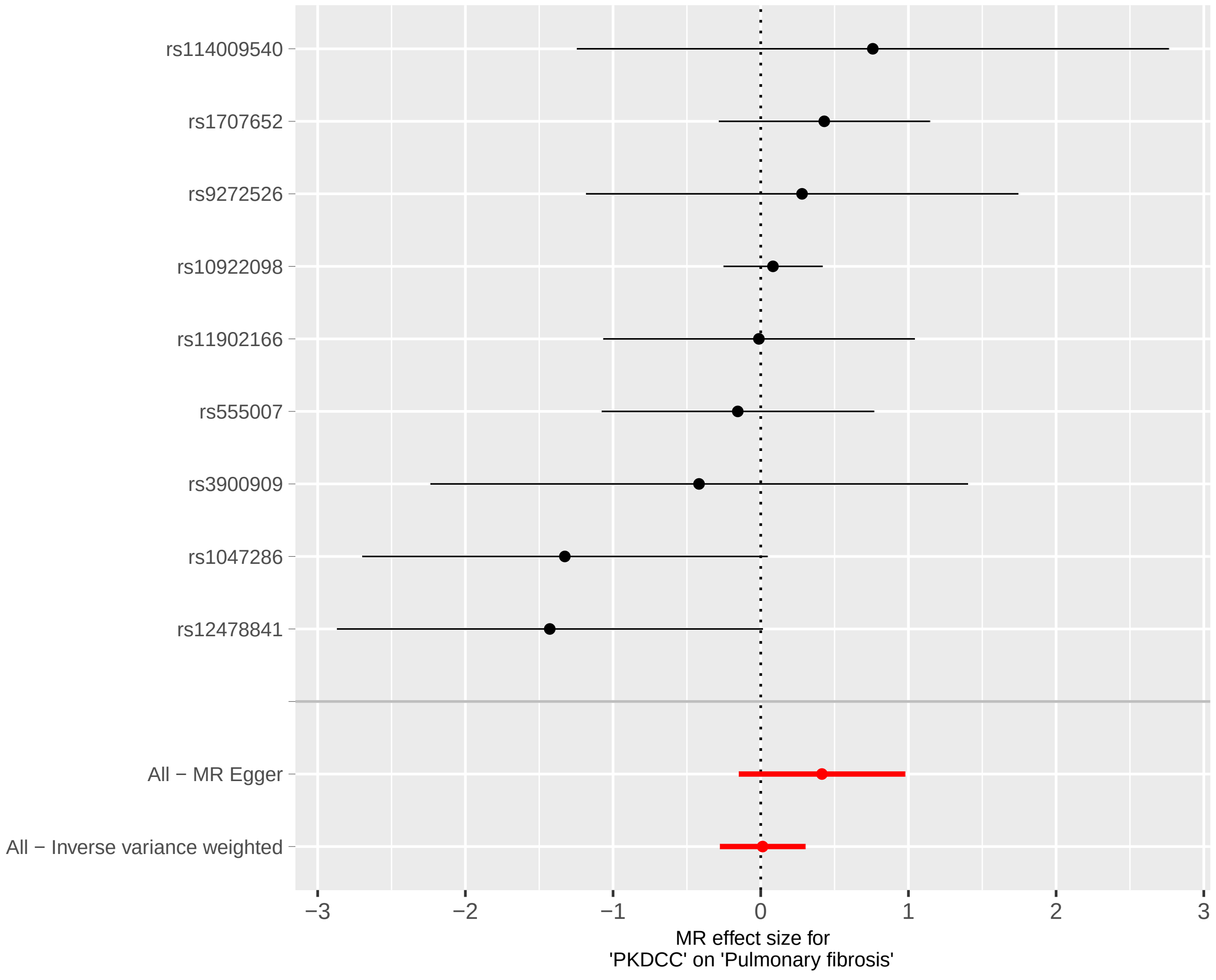

rs114009540
rs1707652
rs9272526
rs10922098
rs11902166
rs555007
rs3900909
rs1047286
rs12478841
All − MR Egger
All − Inverse variance weighted
−3
−2
−1
0
1
2
3
MR effect size for
'PKDCC' on 'Pulmonary fibrosis'

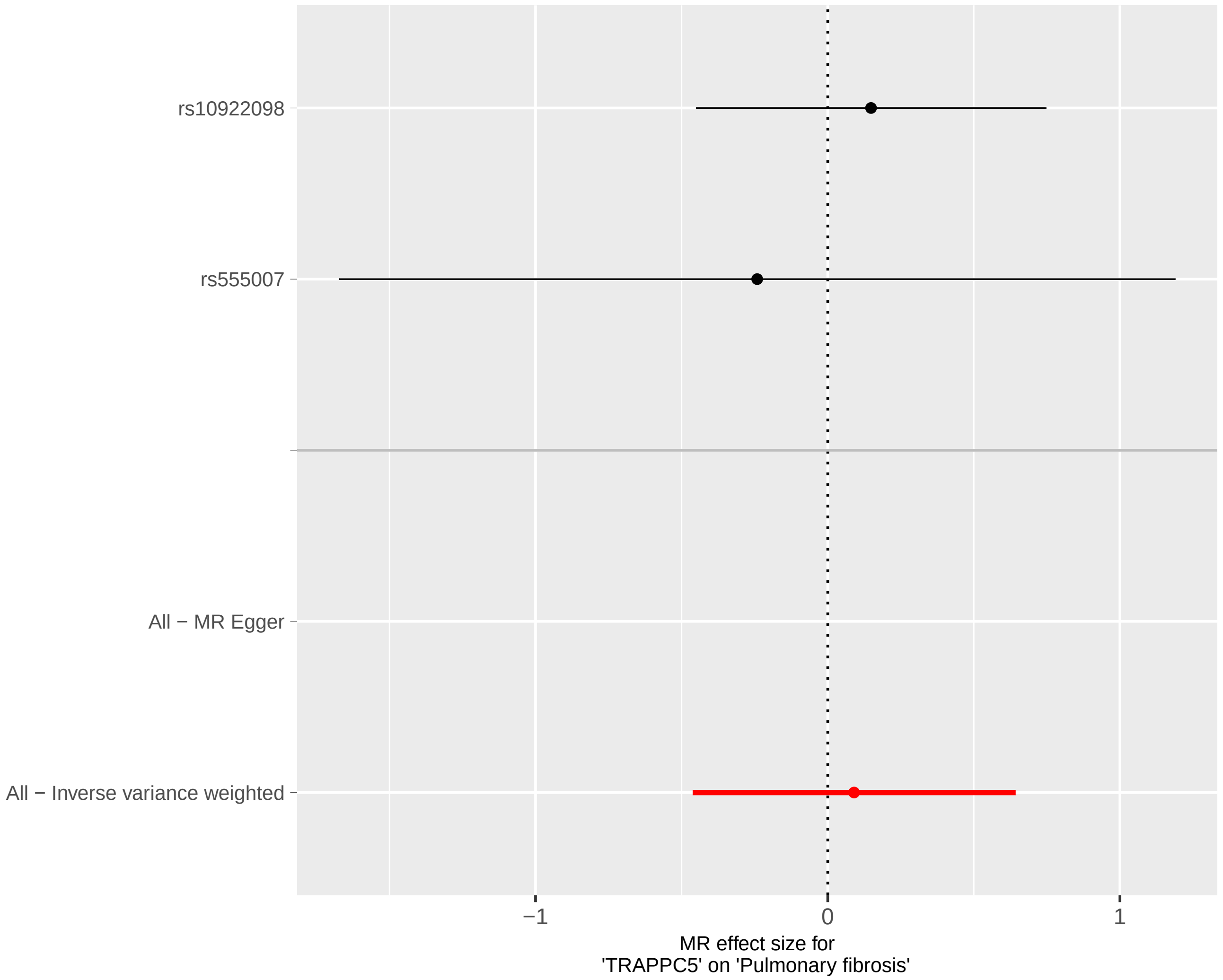

rs10922098
rs555007
All − MR Egger
All − Inverse variance weighted
−1
0
1
MR effect size for
'TRAPPC5' on 'Pulmonary fibrosis'

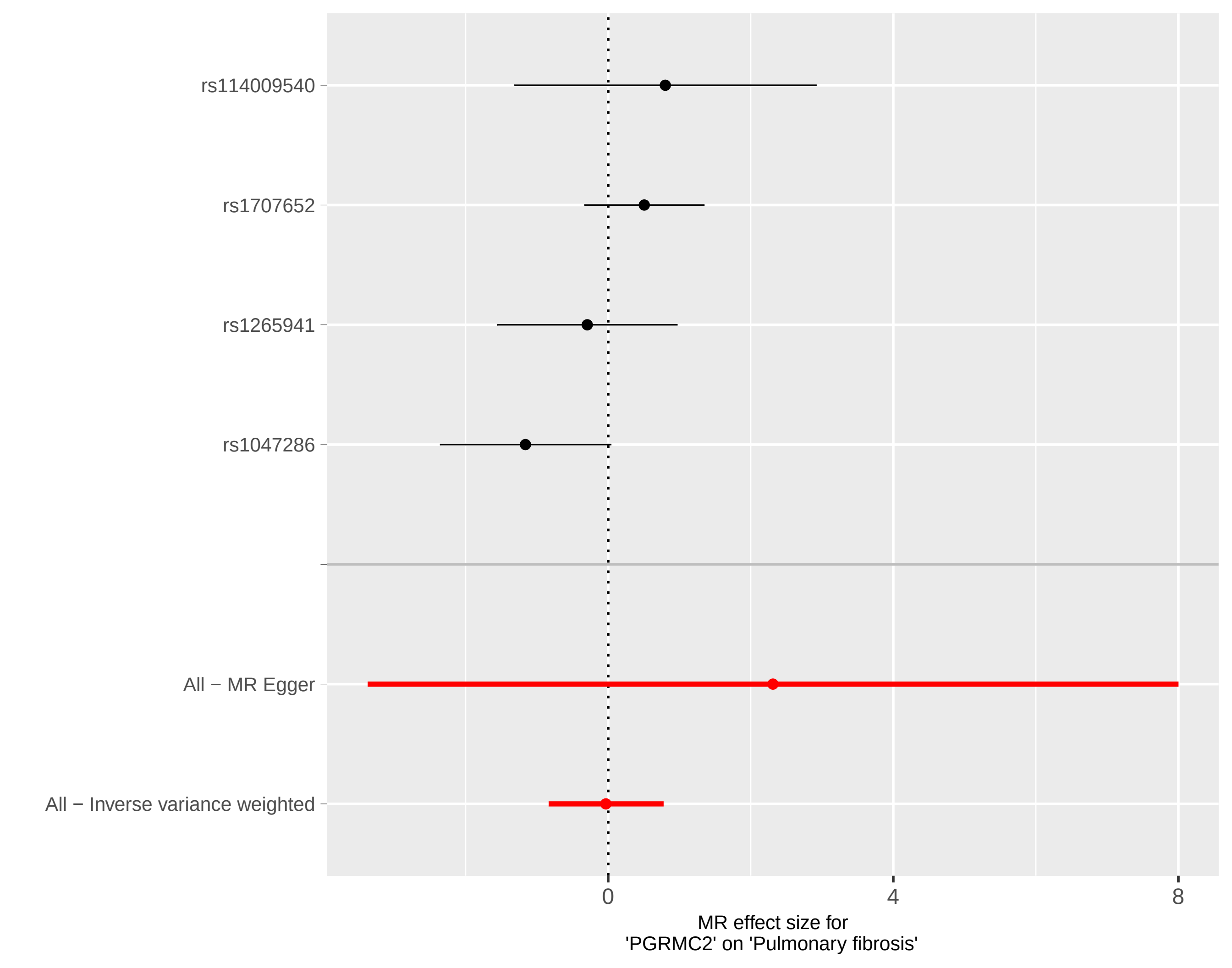

rs114009540
rs1707652
rs1265941
rs1047286
All − MR Egger
All − Inverse variance weighted
0
4
8
MR effect size for
'PGRMC2' on 'Pulmonary fibrosis'

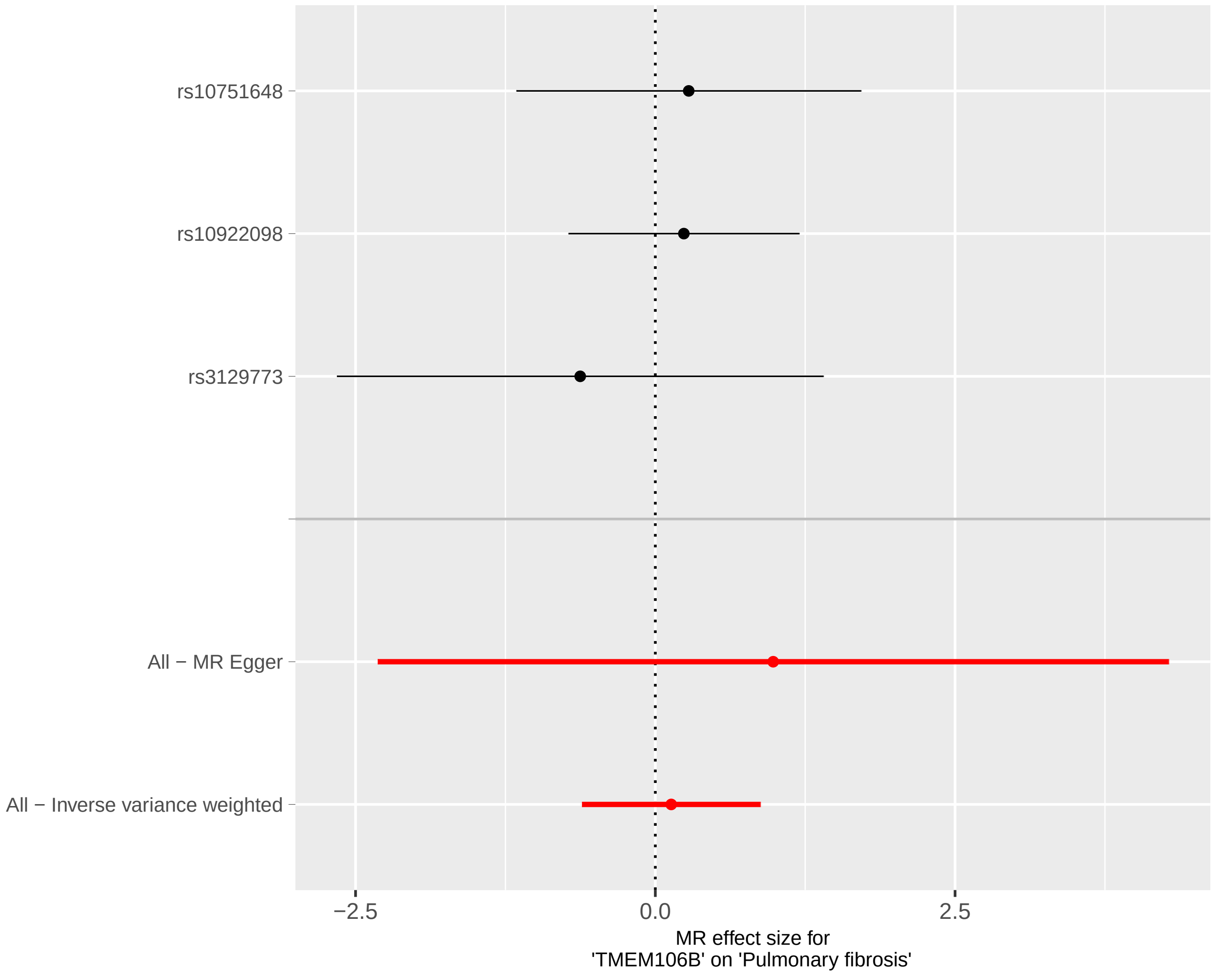
rs10751648
rs10922098
rs3129773
All − MR Egger
All − Inverse variance weighted
−2.5
0.0
2.5
MR effect size for
'TMEM106B' on 'Pulmonary fibrosis'

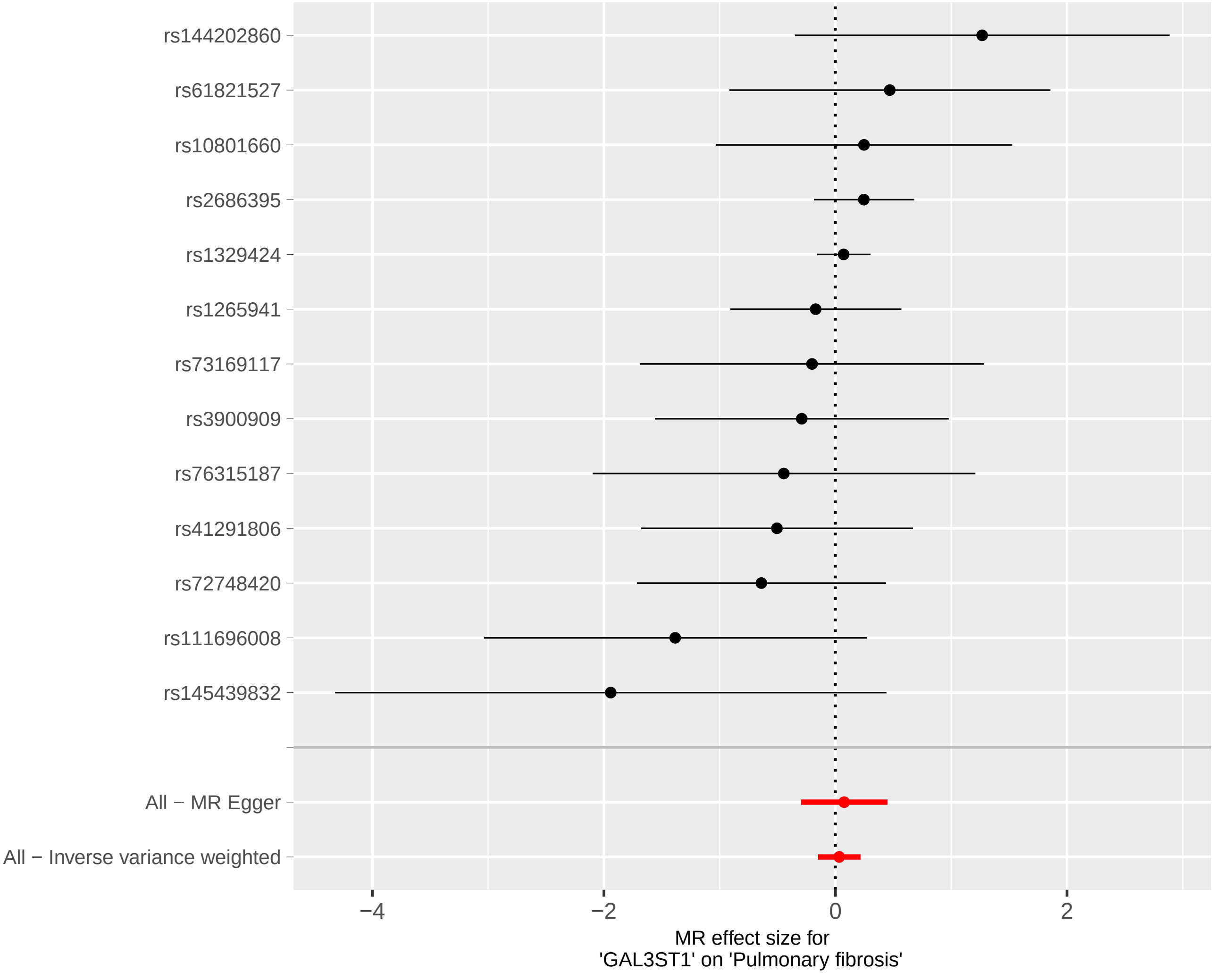
rs144202860
rs61821527
rs10801660
rs2686395
rs1329424
rs1265941
rs73169117
rs3900909
rs76315187
rs41291806
rs72748420
rs111696008
rs145439832
All − MR Egger
All − Inverse variance weighted
−4
−2
0
2
MR effect size for
'GAL3ST1' on 'Pulmonary fibrosis'

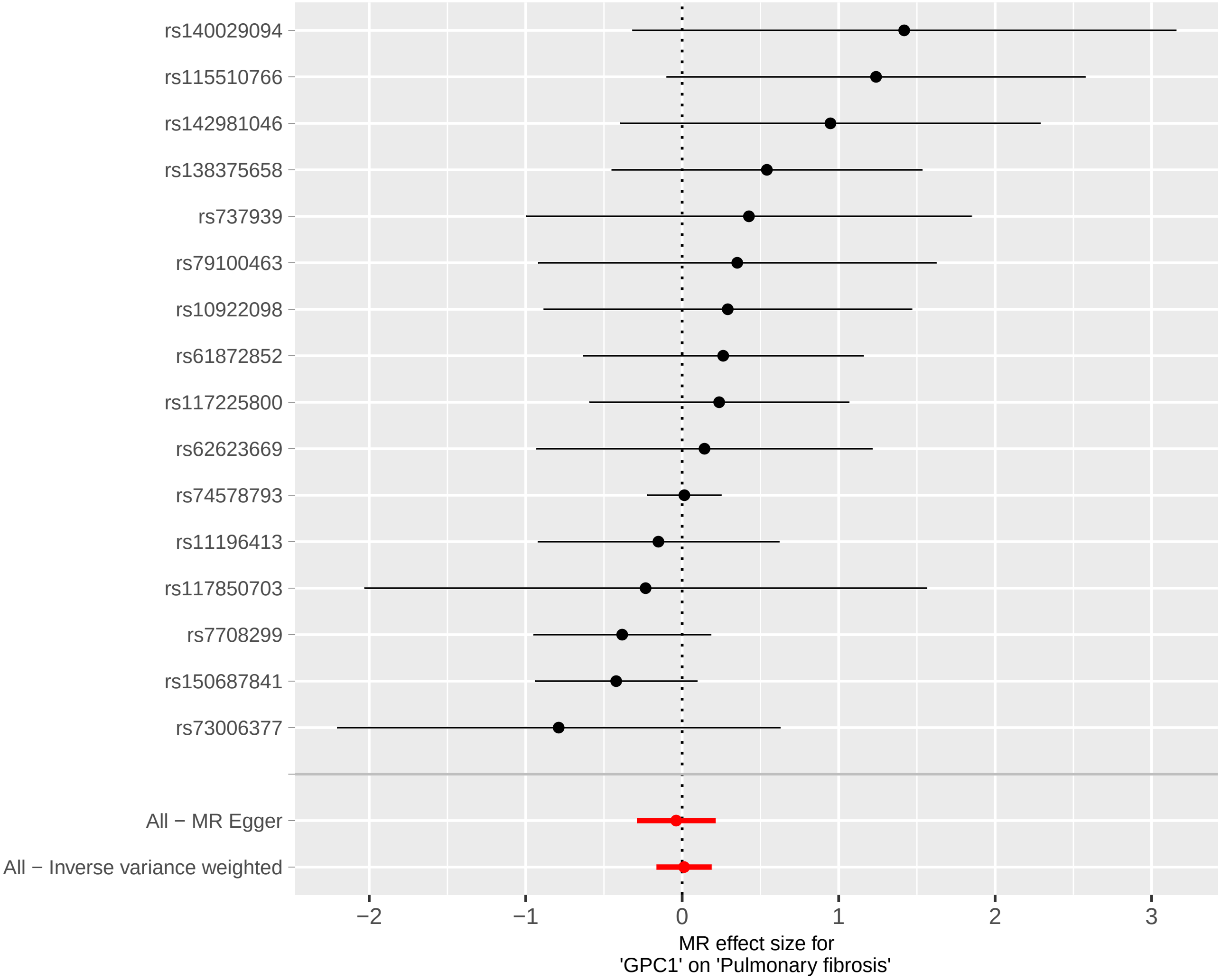
rs140029094
rs115510766
rs142981046
rs138375658
rs737939
rs79100463
rs10922098
rs61872852
rs117225800
rs62623669
rs74578793
rs11196413
rs117850703
rs7708299
rs150687841
rs73006377
All − MR Egger
All − Inverse variance weighted
−2
−1
0
1
2
3
MR effect size for
'GPC1' on 'Pulmonary fibrosis'

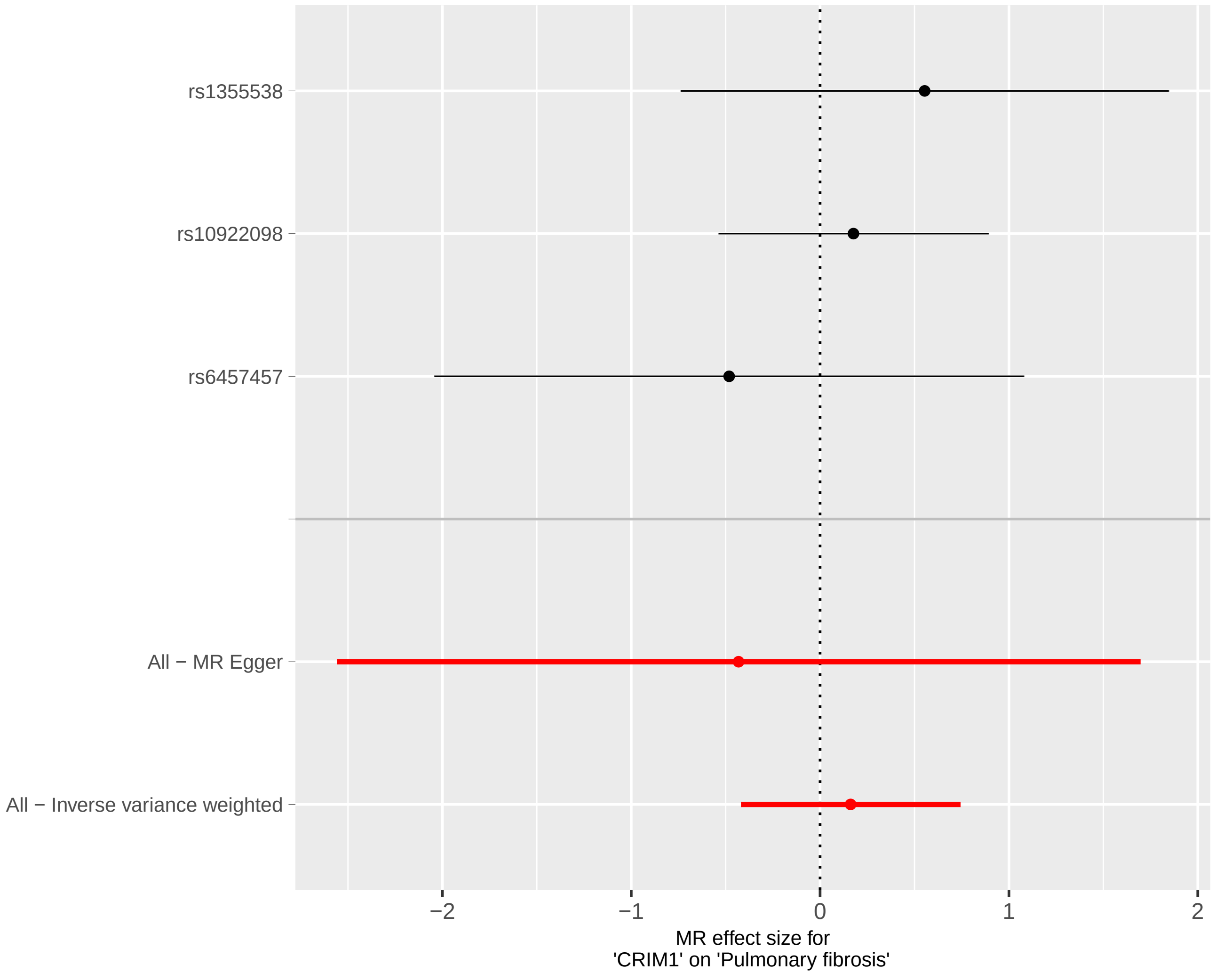

rs1355538
rs10922098
rs6457457
All − MR Egger
All − Inverse variance weighted
−2
−1
0
1
2
MR effect size for
'CRIM1' on 'Pulmonary fibrosis'

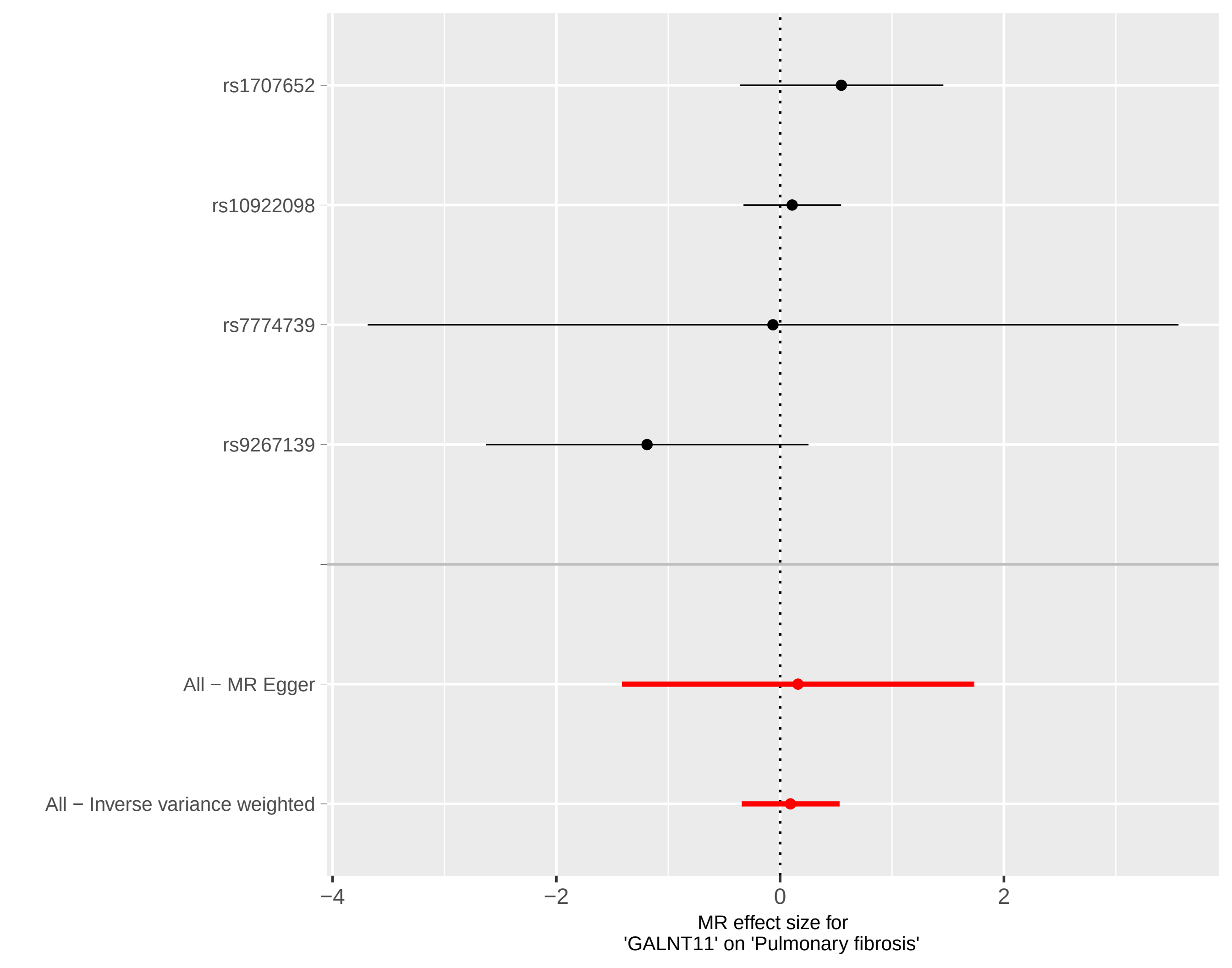
rs1707652
rs10922098
rs7774739
rs9267139
All − MR Egger
All − Inverse variance weighted
−4
−2
0
2
MR effect size for
'GALNT11' on 'Pulmonary fibrosis'

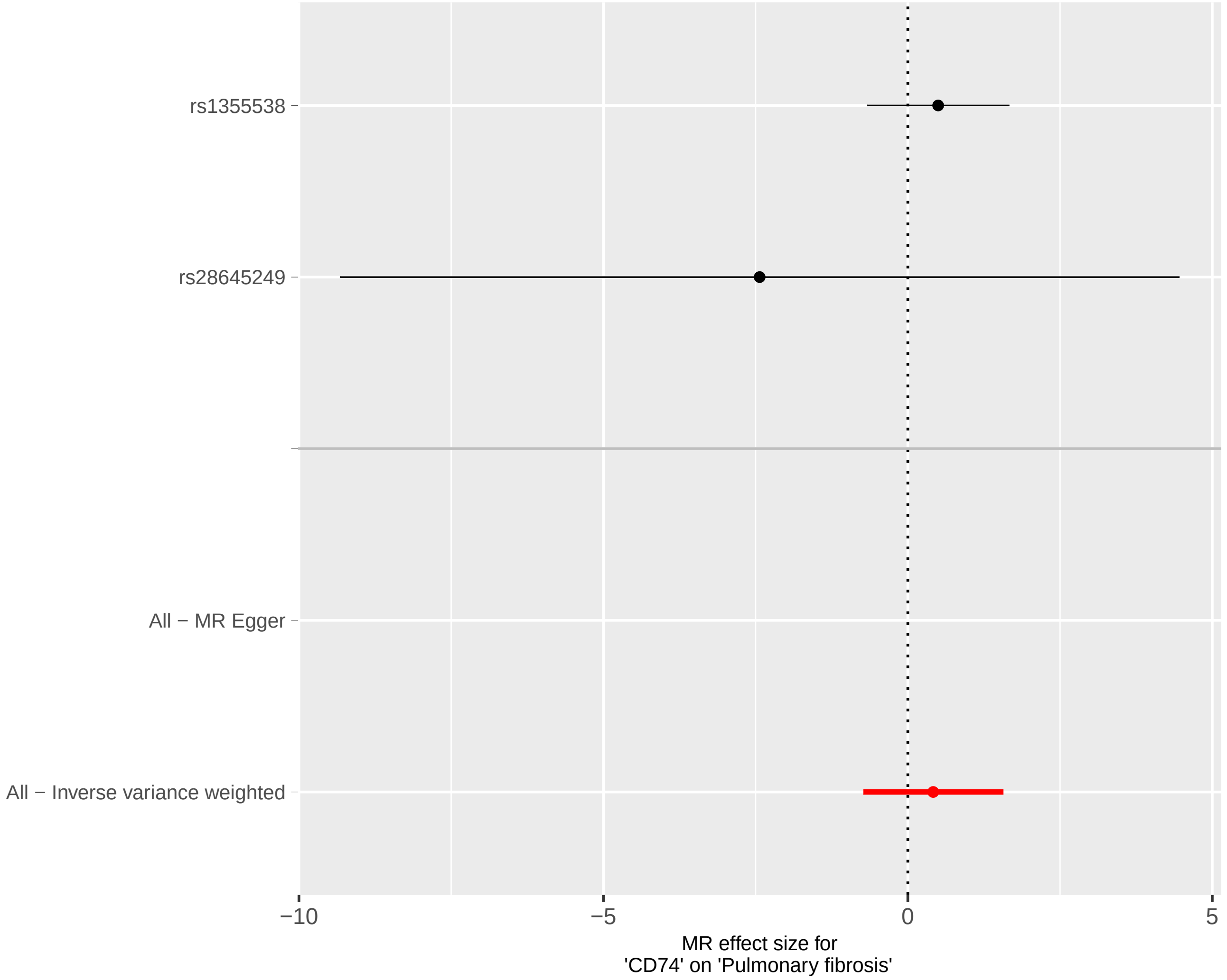
rs1355538
rs28645249
All − MR Egger
All − Inverse variance weighted
−10
−5
0
5
MR effect size for
'CD74' on 'Pulmonary fibrosis'

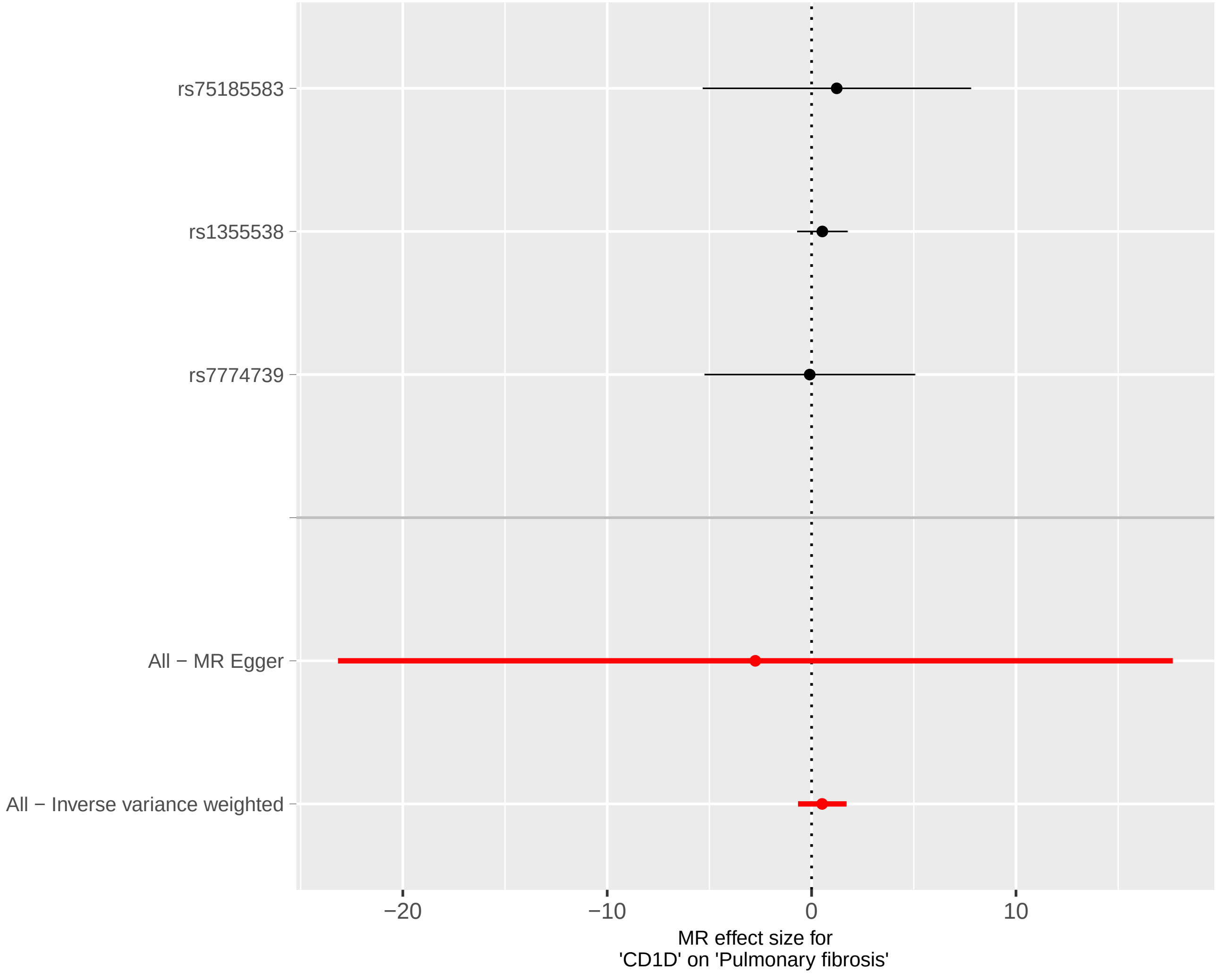

rs75185583
rs1355538
rs7774739
All − MR Egger
All − Inverse variance weighted
−20
−10
0
10
MR effect size for
'CD1D' on 'Pulmonary fibrosis'

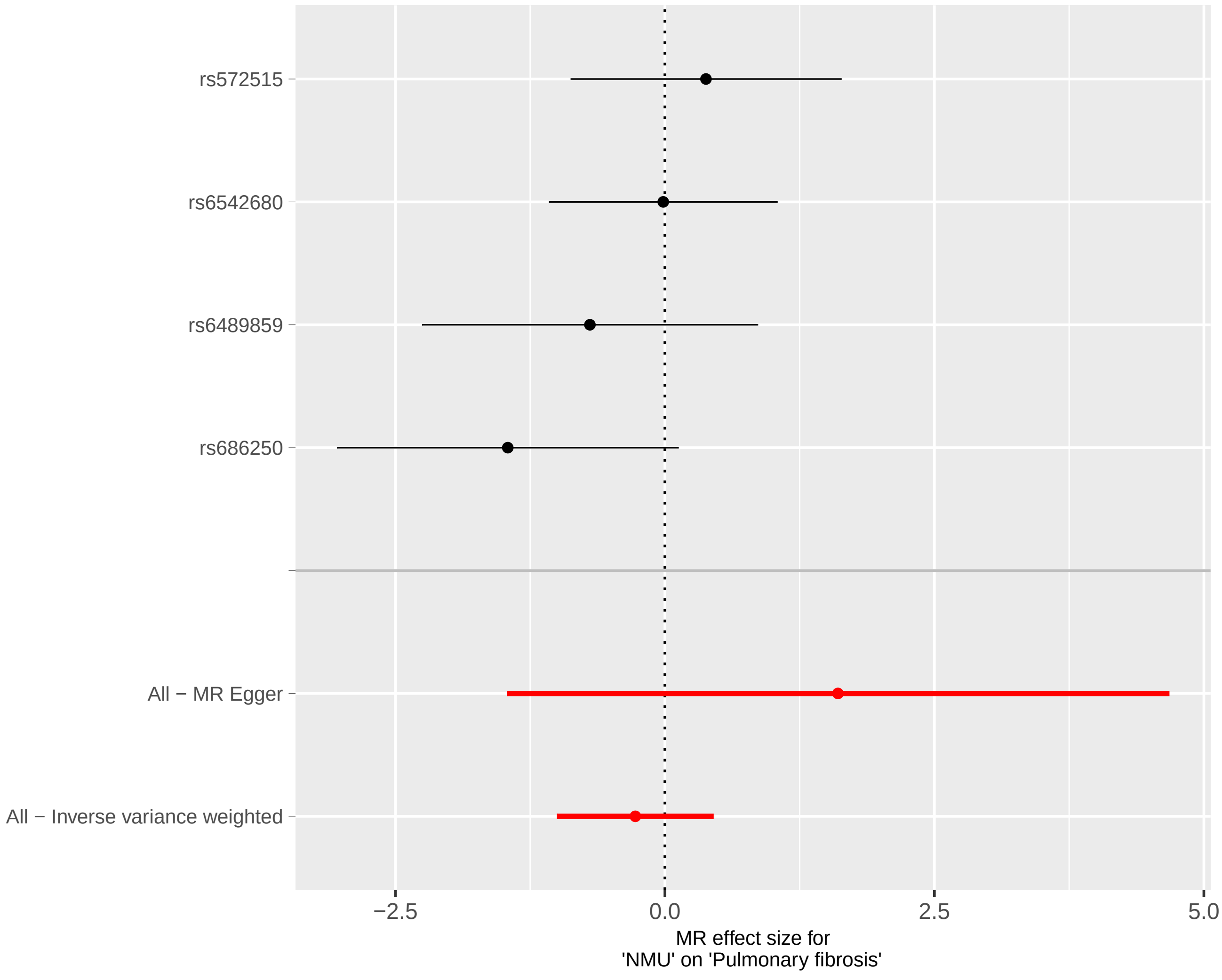

rs572515
rs6542680
rs6489859
rs686250
All − MR Egger
All − Inverse variance weighted
−2.5
0.0
2.5
5.0
MR effect size for
'NMU' on 'Pulmonary fibrosis'

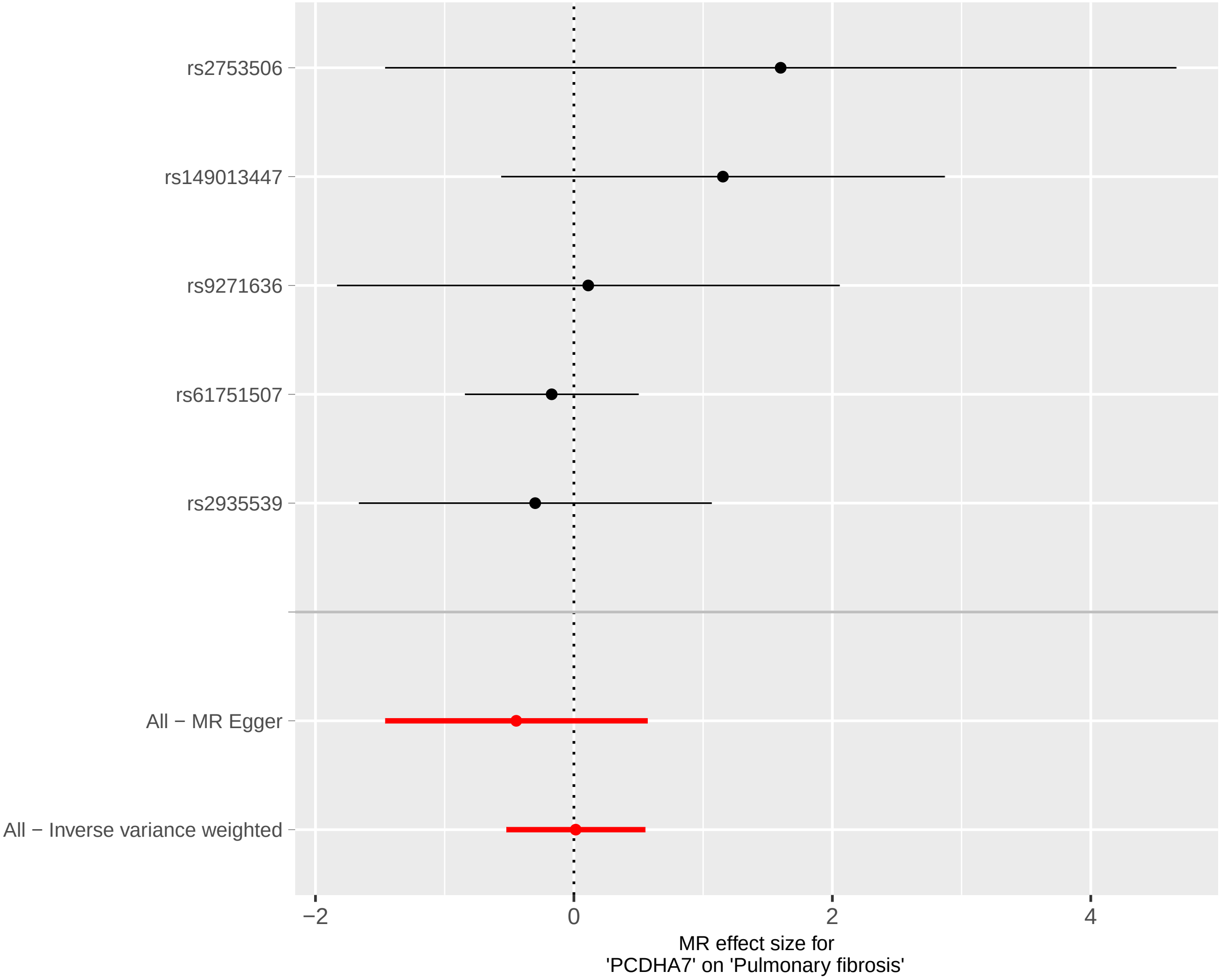
rs2753506
rs149013447
rs9271636
rs61751507
rs2935539
All − MR Egger
All − Inverse variance weighted
−2
0
2
4
MR effect size for
'PCDHA7' on 'Pulmonary fibrosis'

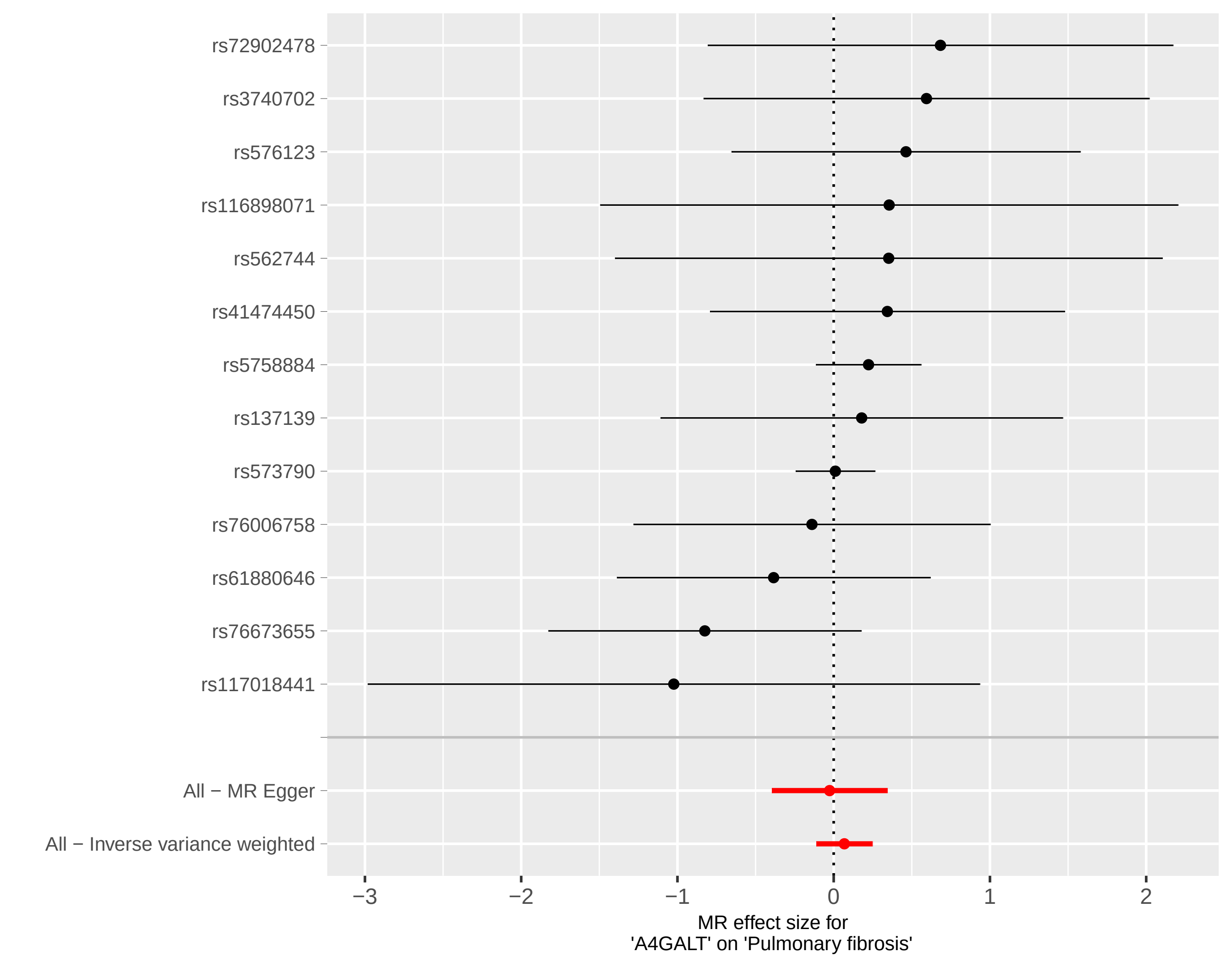

rs72902478
rs3740702
rs576123
rs116898071
rs562744
rs41474450
rs5758884
rs137139
rs573790
rs76006758
rs61880646
rs76673655
rs117018441
All − MR Egger
All − Inverse variance weighted
−3
−2
−1
0
1
2
MR effect size for
'A4GALT' on 'Pulmonary fibrosis'

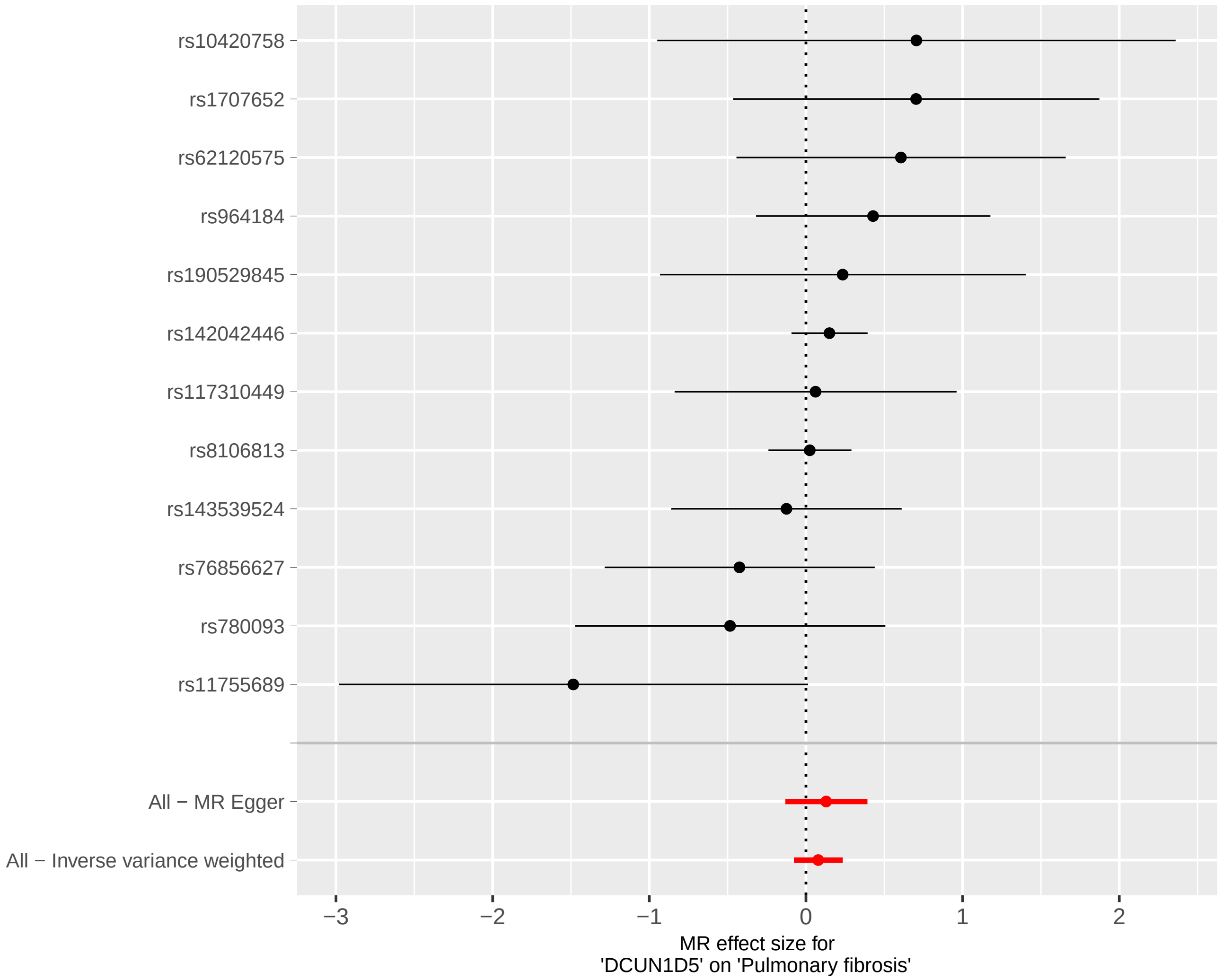
rs10420758
rs1707652
rs62120575
rs964184
rs190529845
rs142042446
rs117310449
rs8106813
rs143539524
rs76856627
rs780093
rs11755689
All − MR Egger
All − Inverse variance weighted
−3
−2
−1
0
1
2
MR effect size for
'DCUN1D5' on 'Pulmonary fibrosis'

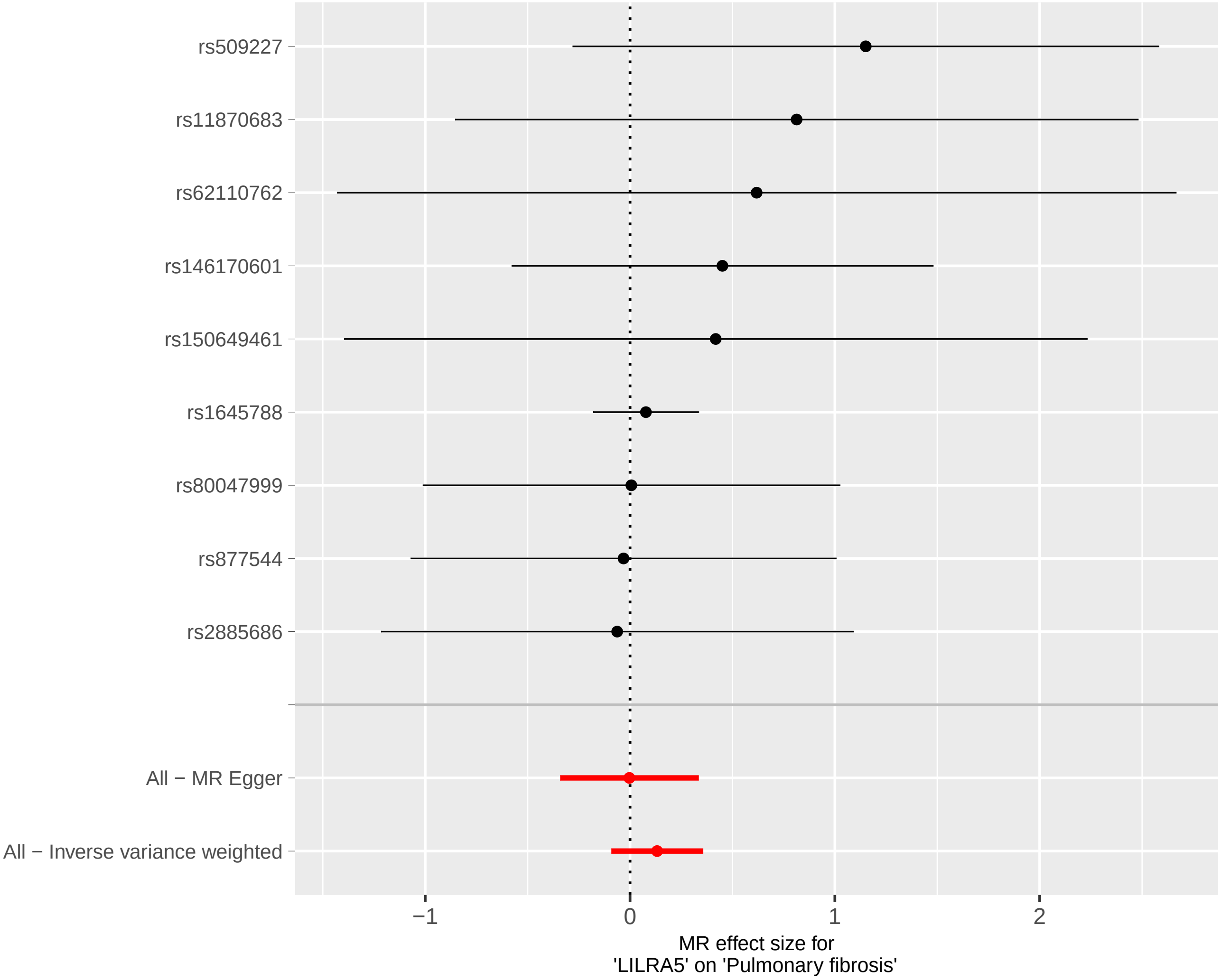

rs509227
rs11870683
rs62110762
rs146170601
rs150649461
rs1645788
rs80047999
rs877544
rs2885686
All − MR Egger
All − Inverse variance weighted
−1
0
1
2
MR effect size for
'LILRA5' on 'Pulmonary fibrosis'

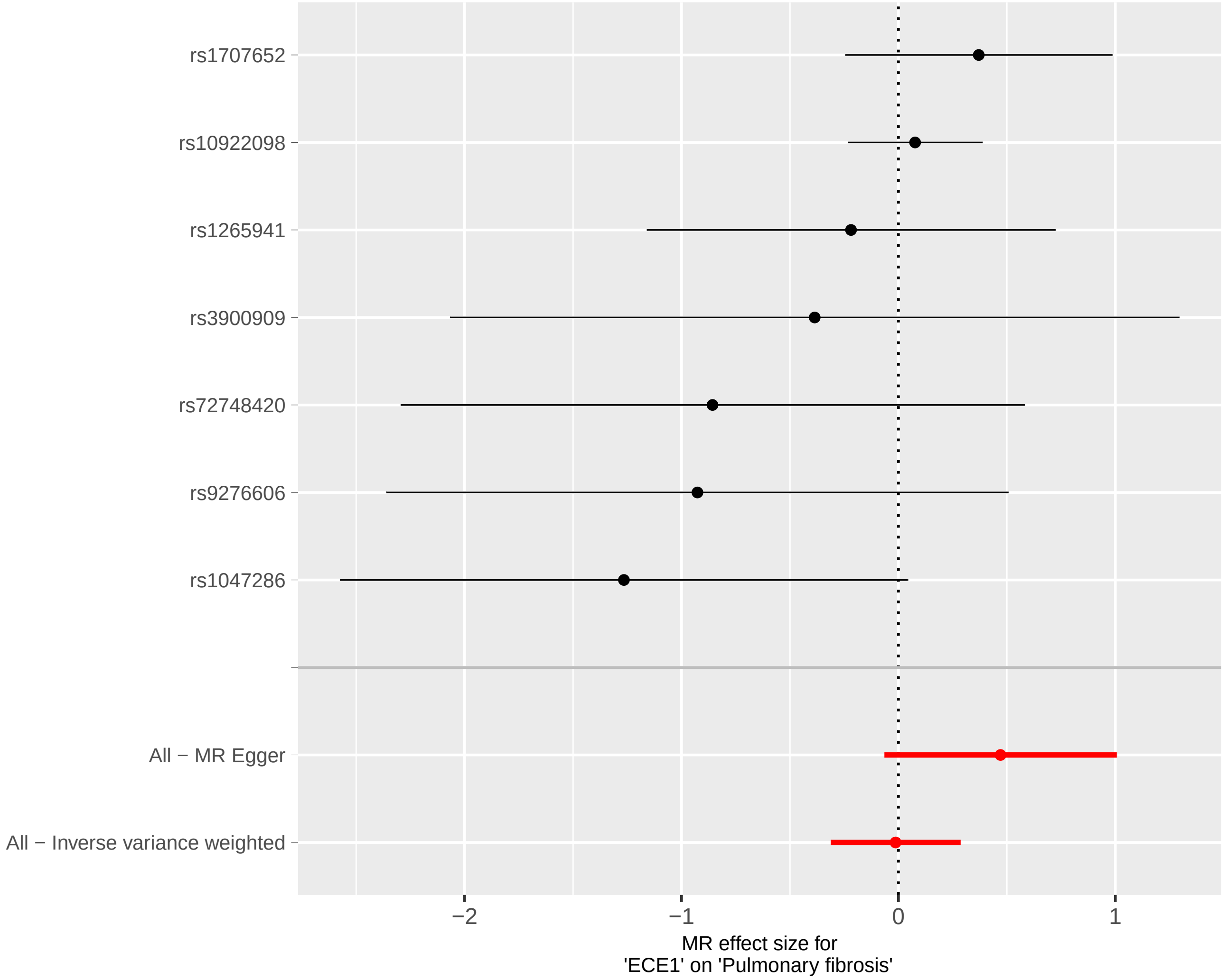
rs1707652
rs10922098
rs1265941
rs3900909
rs72748420
rs9276606
rs1047286
All − MR Egger
All − Inverse variance weighted
−2
−1
0
1
MR effect size for
'ECE1' on 'Pulmonary fibrosis'

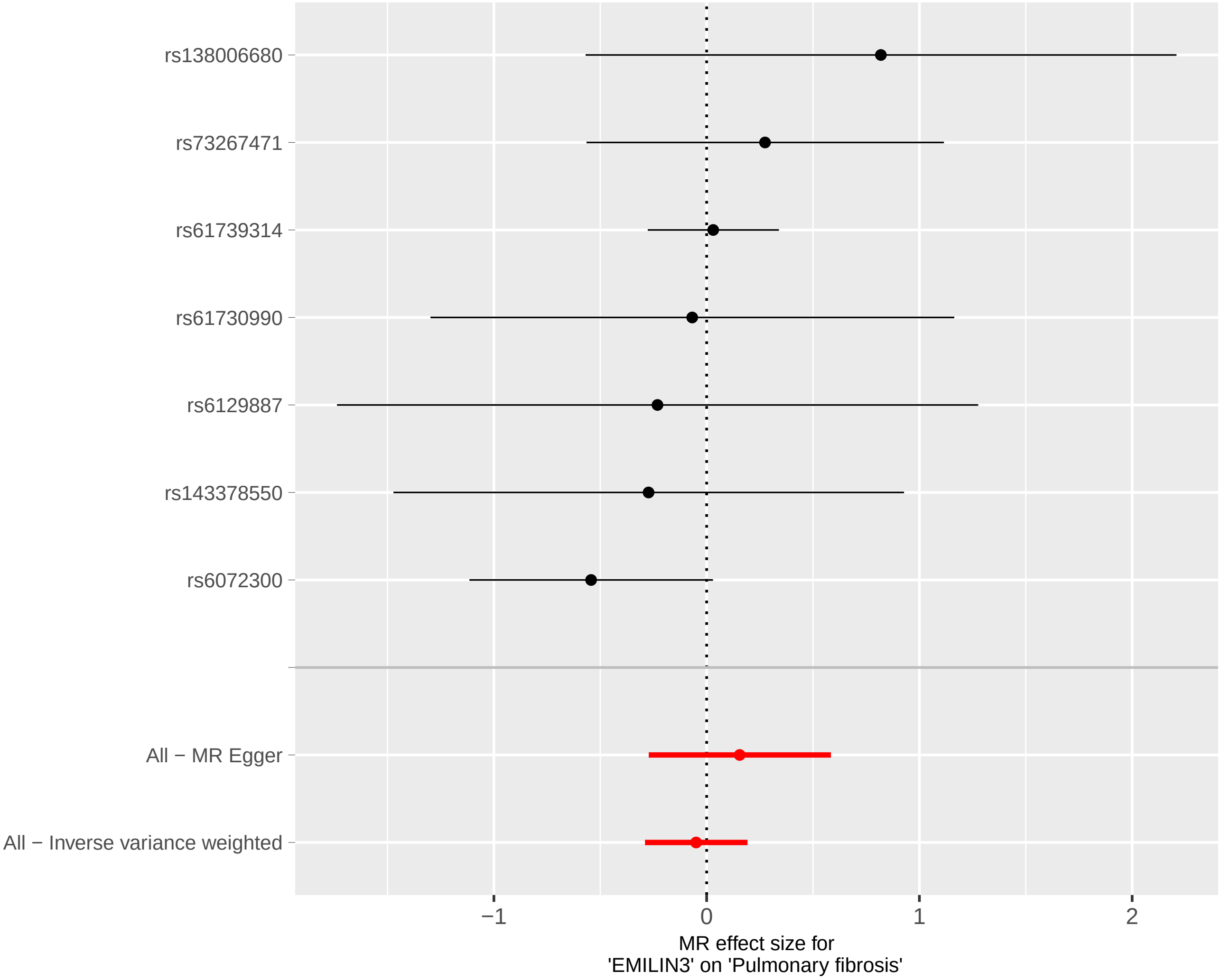

rs138006680
rs73267471
rs61739314
rs61730990
rs6129887
rs143378550
rs6072300
All − MR Egger
All − Inverse variance weighted
−1
0
1
2
MR effect size for
'EMILIN3' on 'Pulmonary fibrosis'

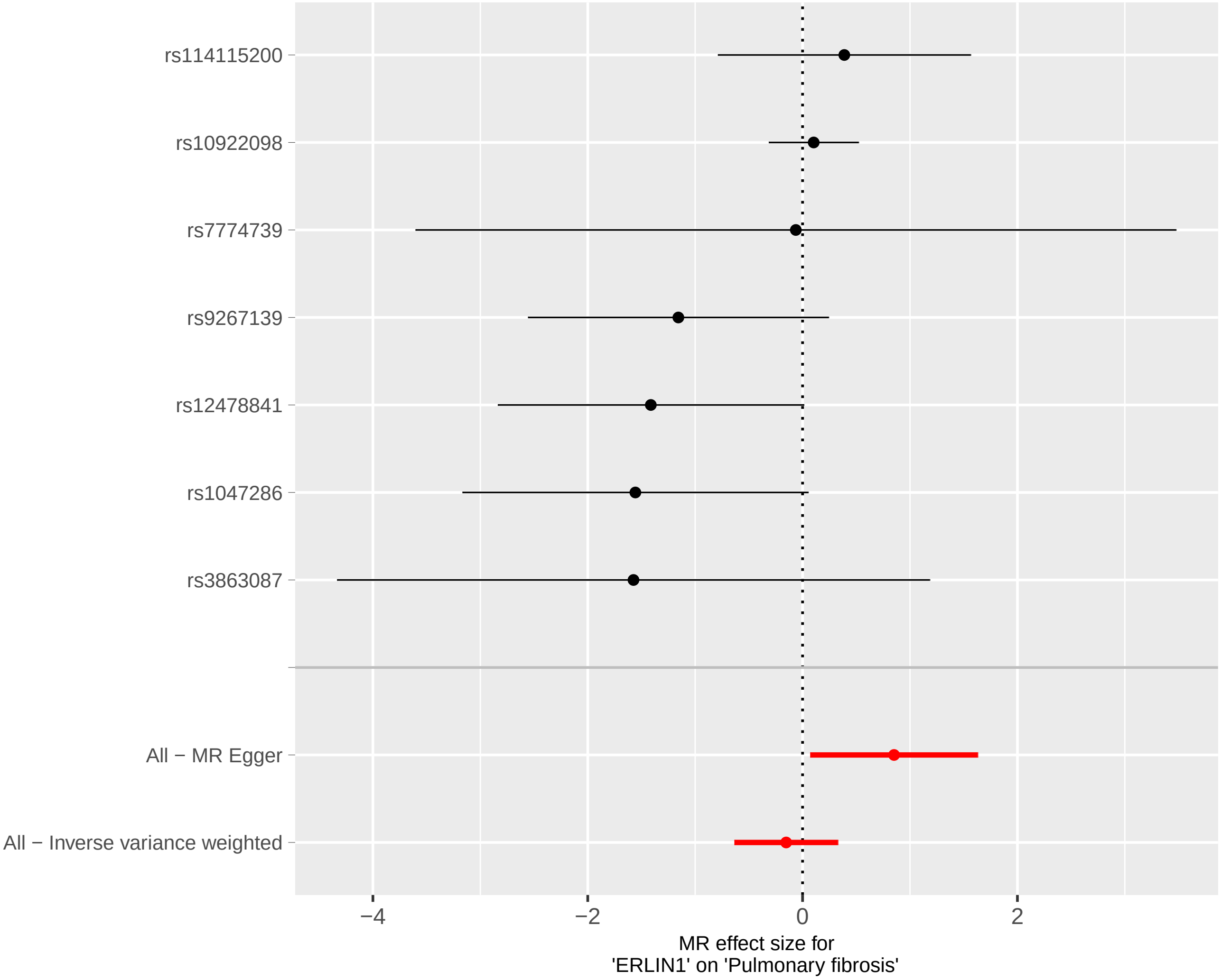
rs114115200
rs10922098
rs7774739
rs9267139
rs12478841
rs1047286
rs3863087
All − MR Egger
All − Inverse variance weighted
−4
−2
0
2
MR effect size for
'ERLIN1' on 'Pulmonary fibrosis'

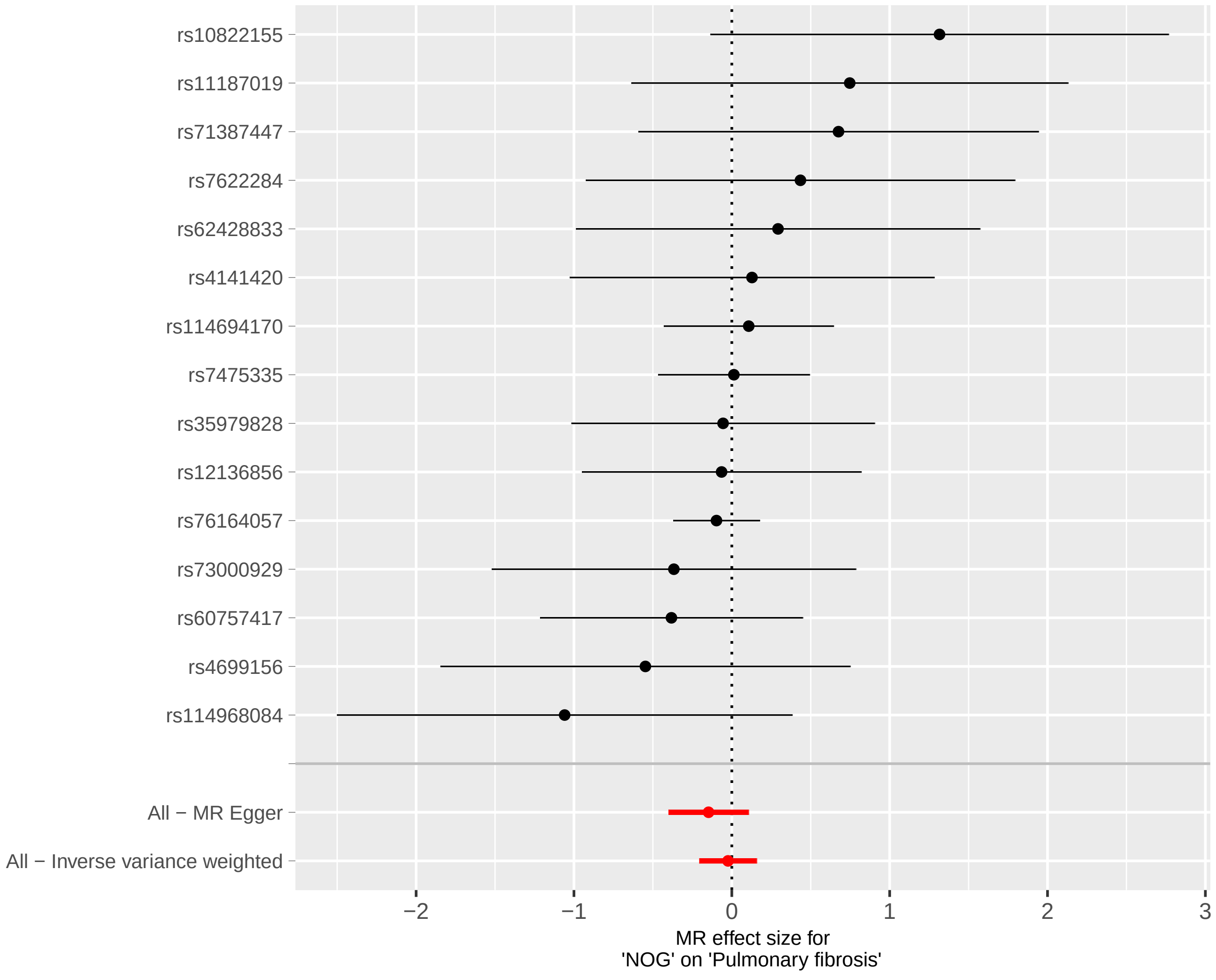

rs10822155
rs11187019
rs71387447
rs7622284
rs62428833
rs4141420
rs114694170
rs7475335
rs35979828
rs12136856
rs76164057
rs73000929
rs60757417
rs4699156
rs114968084
All − MR Egger
All − Inverse variance weighted
−2
−1
0
1
2
3
MR effect size for
'NOG' on 'Pulmonary fibrosis'

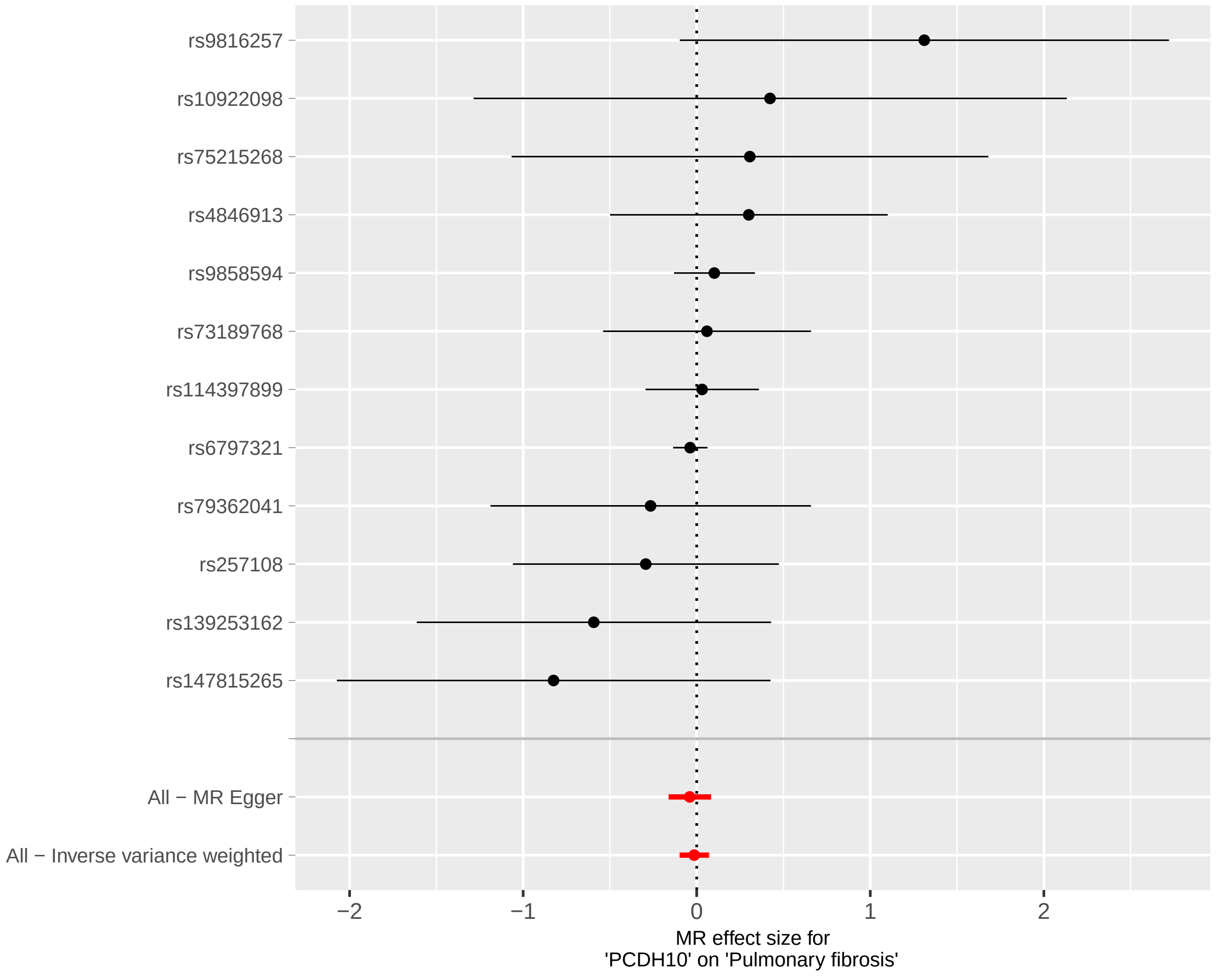

rs9816257
rs10922098
rs75215268
rs4846913
rs9858594
rs73189768
rs114397899
rs6797321
rs79362041
rs257108
rs139253162
rs147815265
All − MR Egger
All − Inverse variance weighted
−2
−1
0
1
2
MR effect size for
'PCDH10' on 'Pulmonary fibrosis'

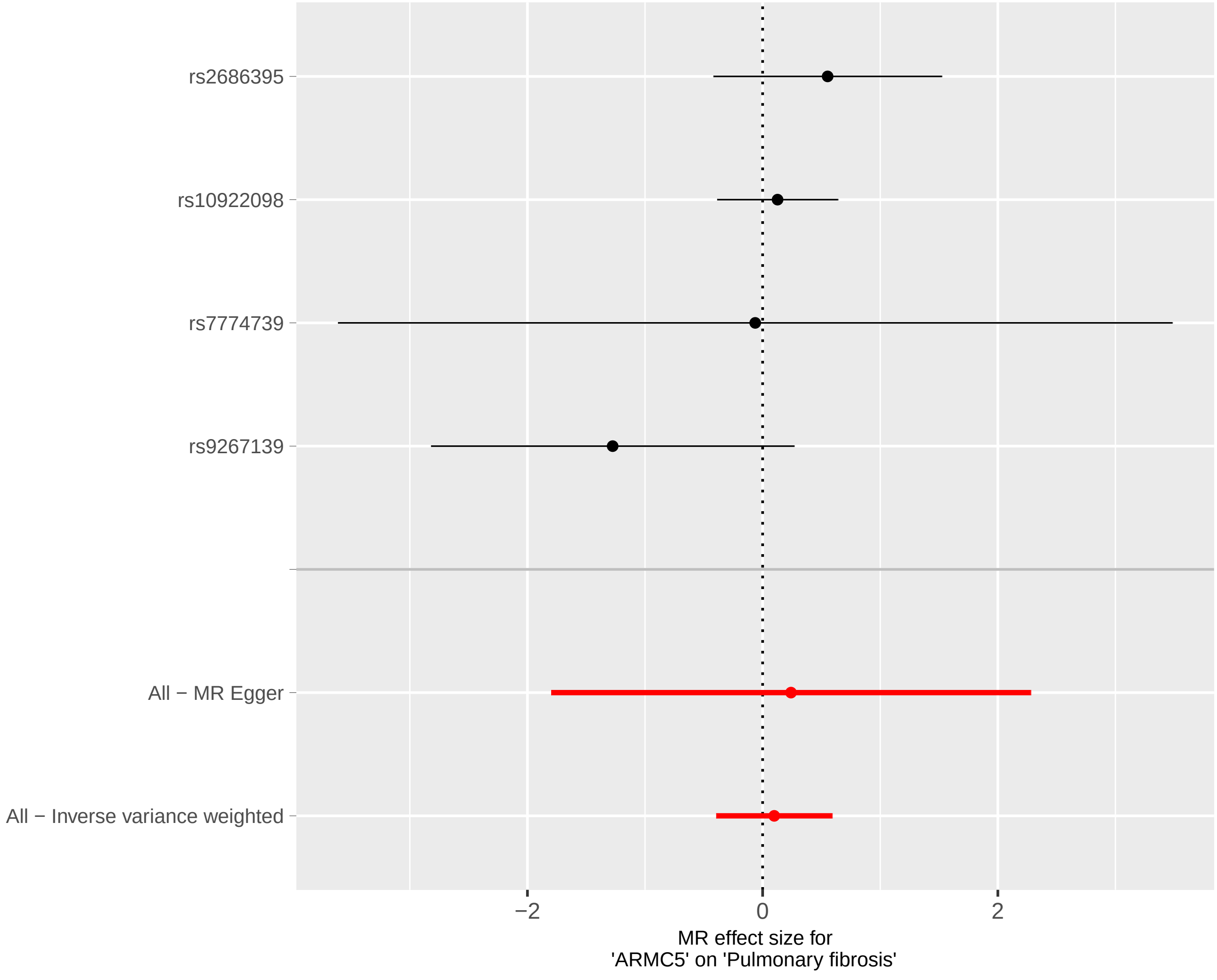
rs2686395
rs10922098
rs7774739
rs9267139
All − MR Egger
All − Inverse variance weighted
−2
0
2
MR effect size for
'ARMC5' on 'Pulmonary fibrosis'

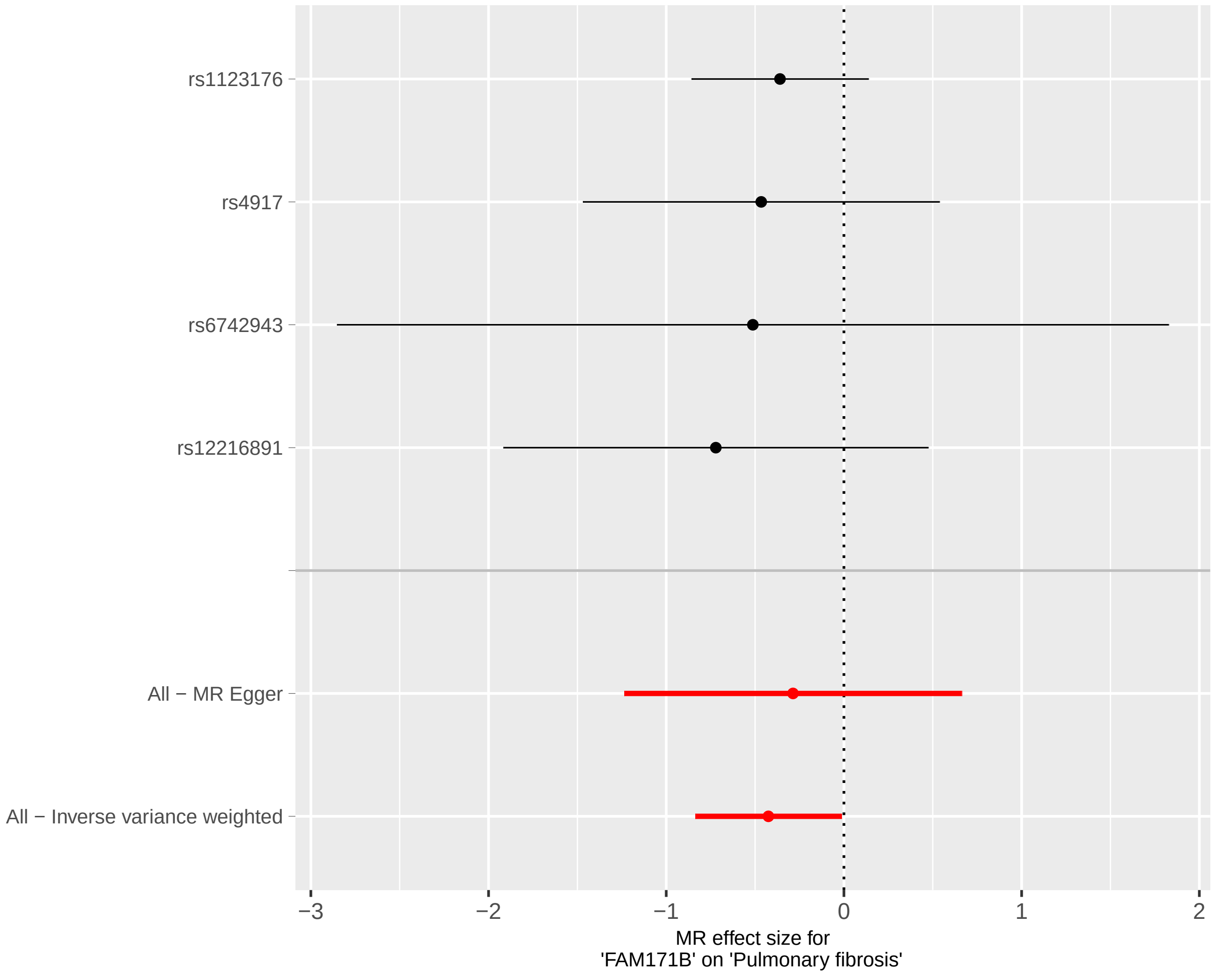
rs1123176
rs4917
rs6742943
rs12216891
All − MR Egger
All − Inverse variance weighted
−3
−2
−1
0
1
2
MR effect size for
'FAM171B' on 'Pulmonary fibrosis'

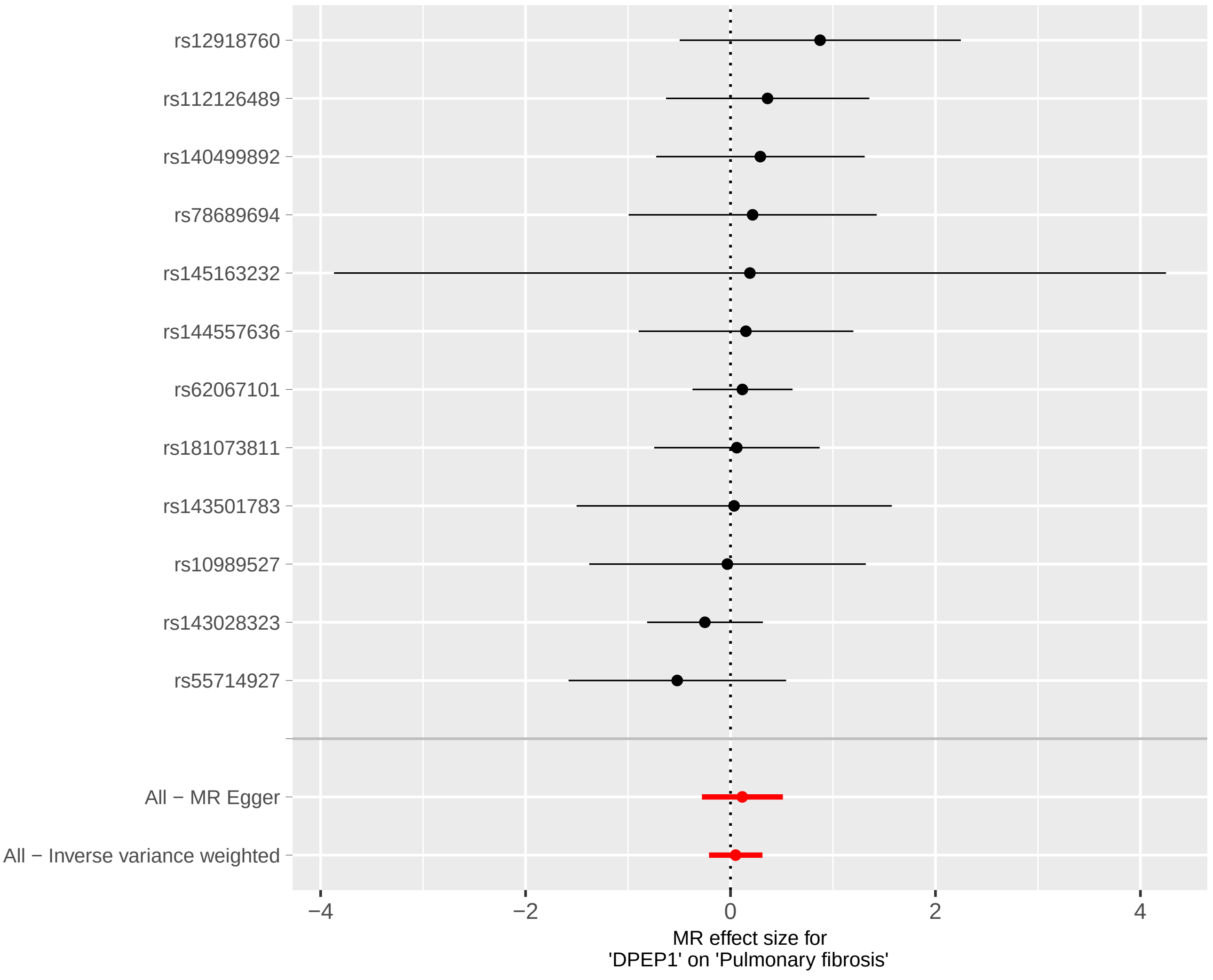
rs12918760
rs112126489
rs140499892
rs78689694
rs145163232
rs144557636
rs62067101
rs181073811
rs143501783
rs10989527
rs143028323
rs55714927
All − MR Egger
All − Inverse variance weighted
−4
−2
0
2
4
MR effect size for
'DPEP1' on 'Pulmonary fibrosis'

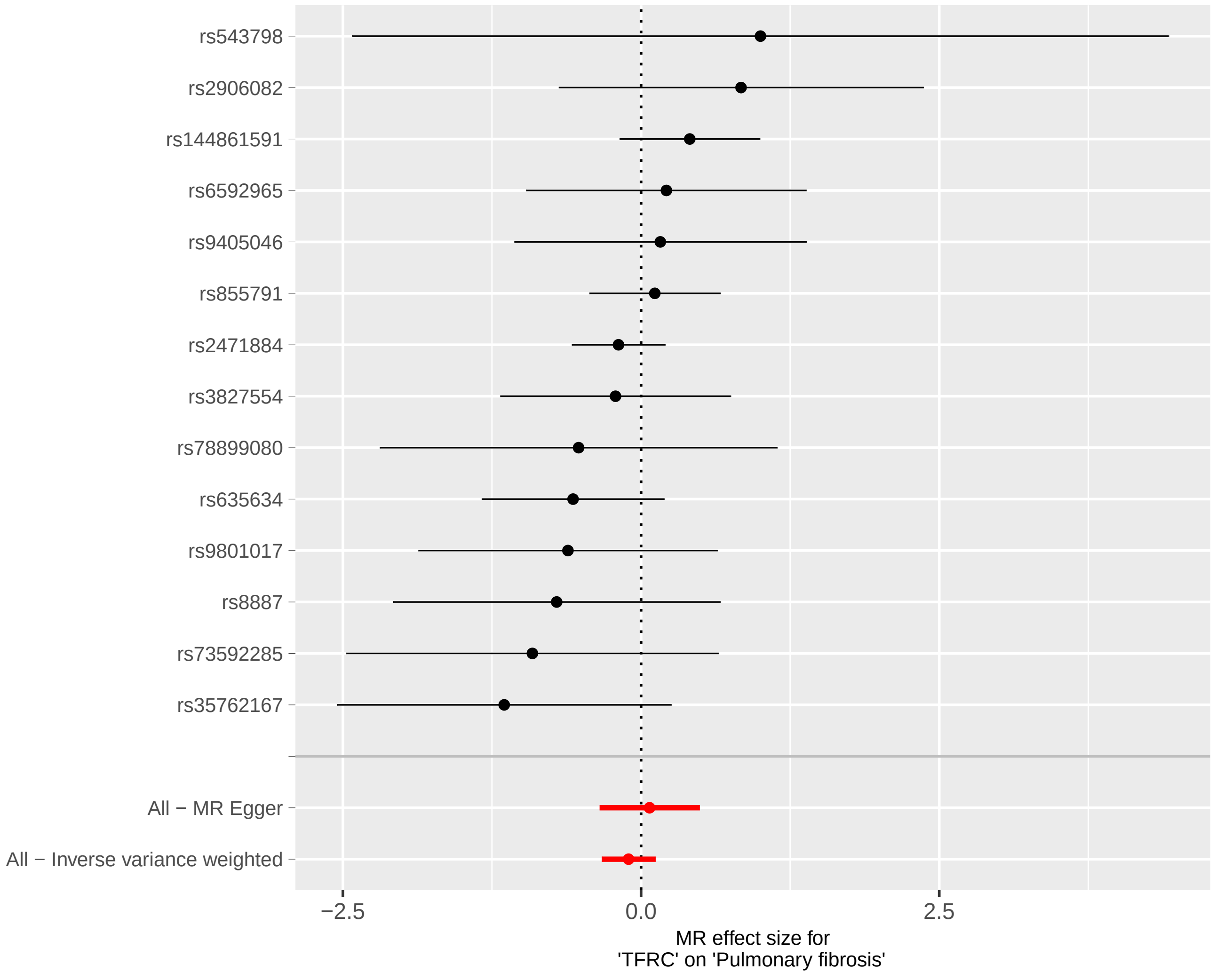
rs543798
rs2906082
rs144861591
rs6592965
rs9405046
rs855791
rs2471884
rs3827554
rs78899080
rs635634
rs9801017
rs8887
rs73592285
rs35762167
All − MR Egger
All − Inverse variance weighted
−2.5
0.0
2.5
MR effect size for
'TFRC' on 'Pulmonary fibrosis'

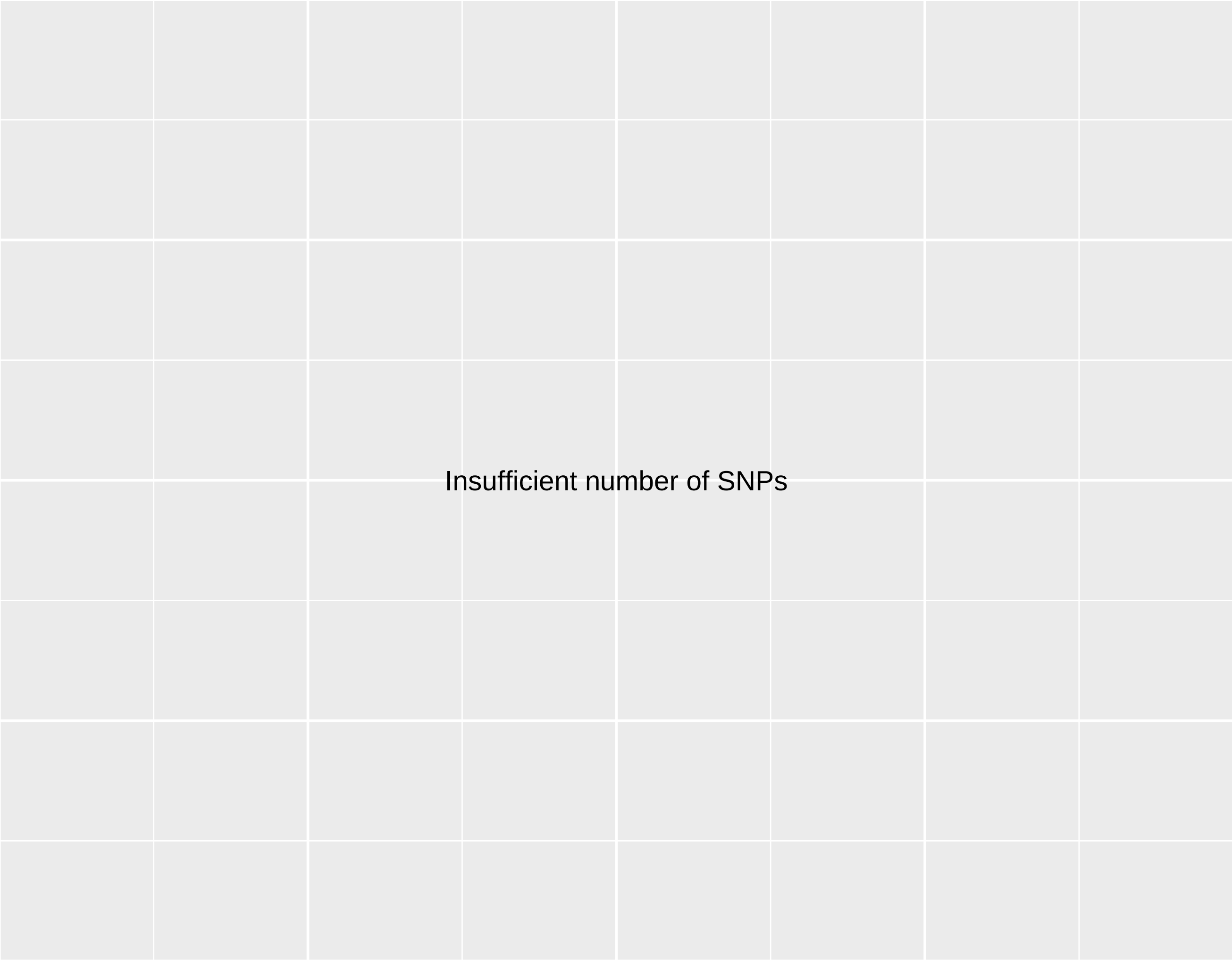
Insufficient number of SNPs

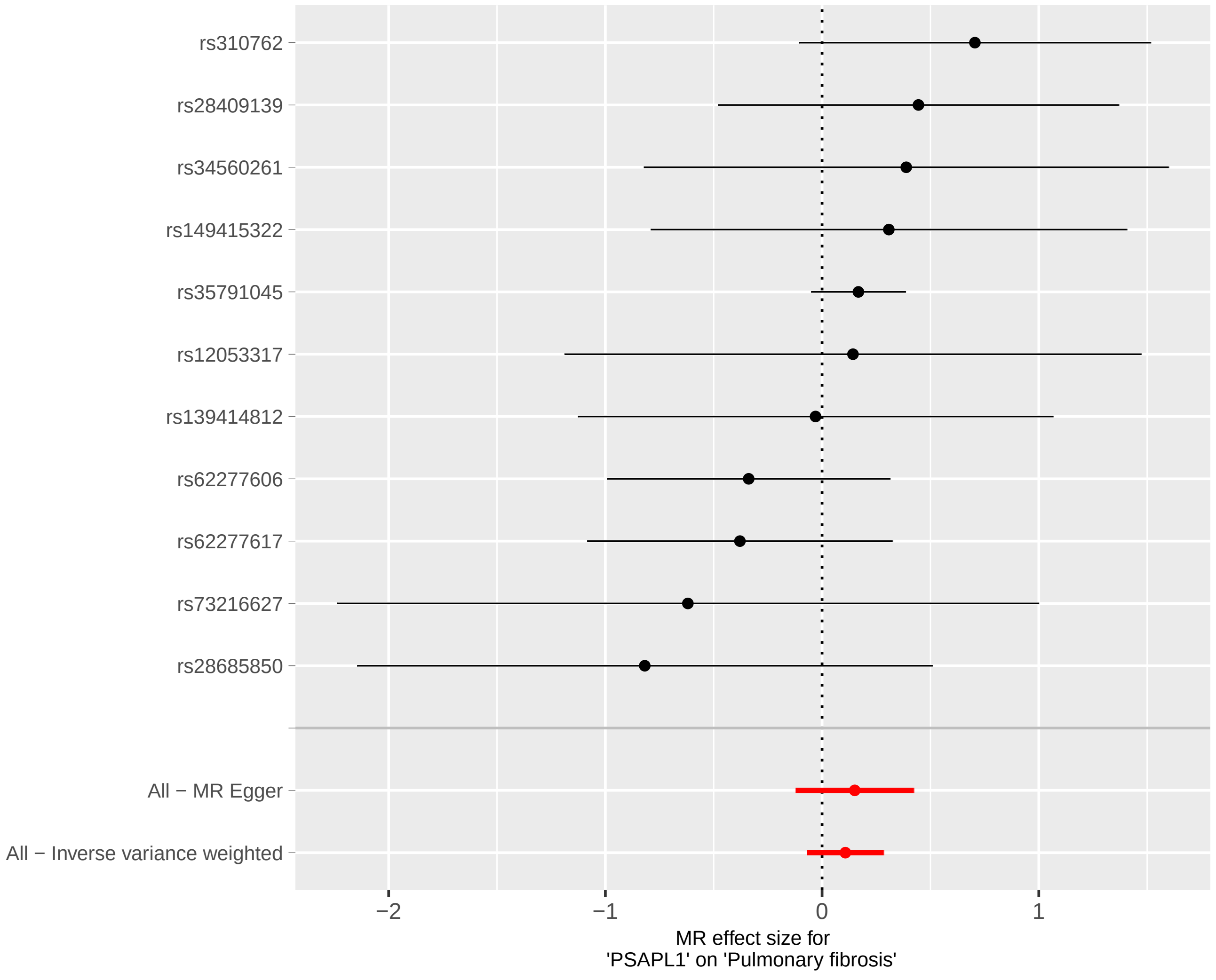

rs310762
rs28409139
rs34560261
rs149415322
rs35791045
rs12053317
rs139414812
rs62277606
rs62277617
rs73216627
rs28685850
All − MR Egger
All − Inverse variance weighted
−2
−1
0
1
MR effect size for
'PSAPL1' on 'Pulmonary fibrosis'

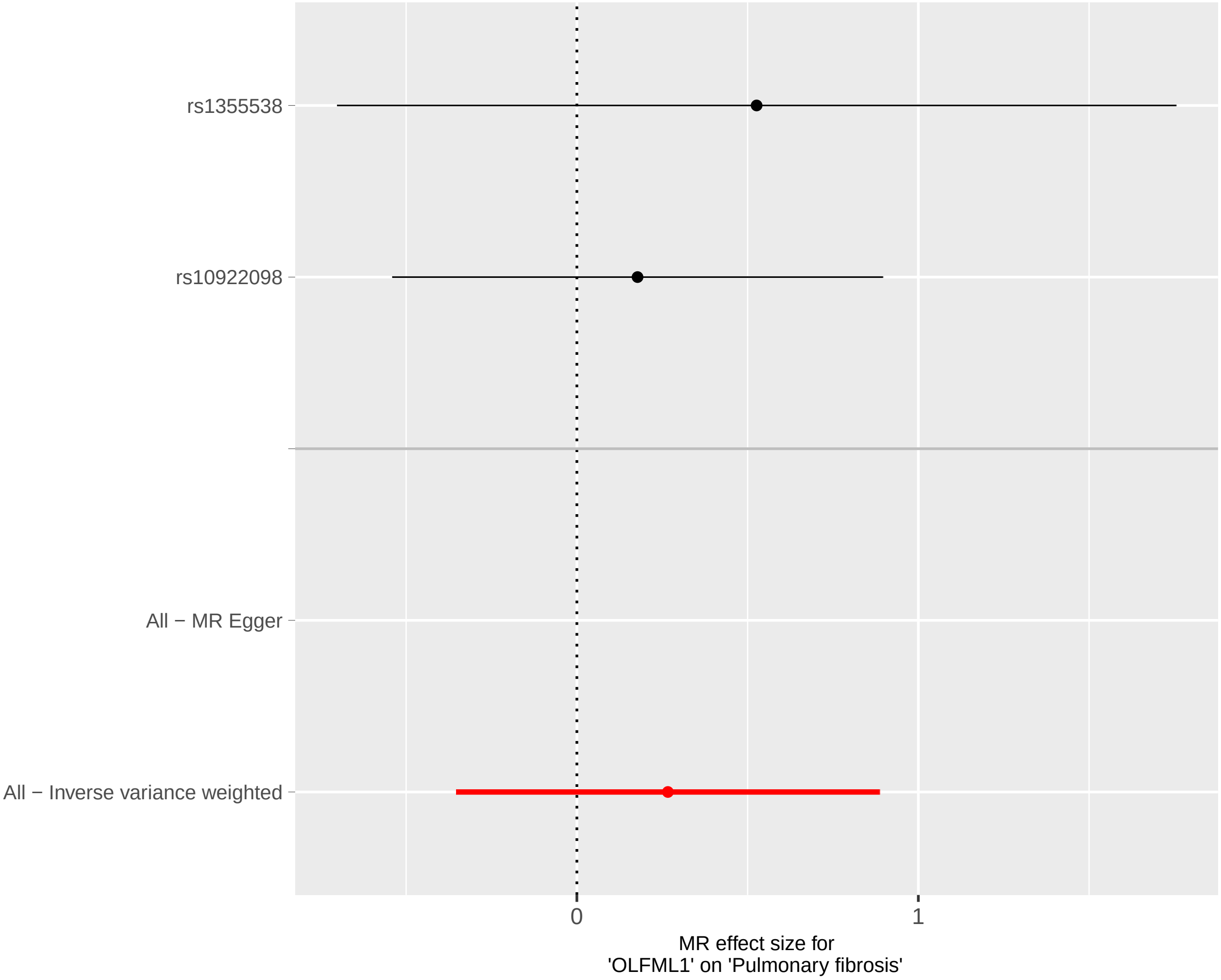
rs1355538
rs10922098
All − MR Egger
All − Inverse variance weighted
0
1
MR effect size for
'OLFML1' on 'Pulmonary fibrosis'

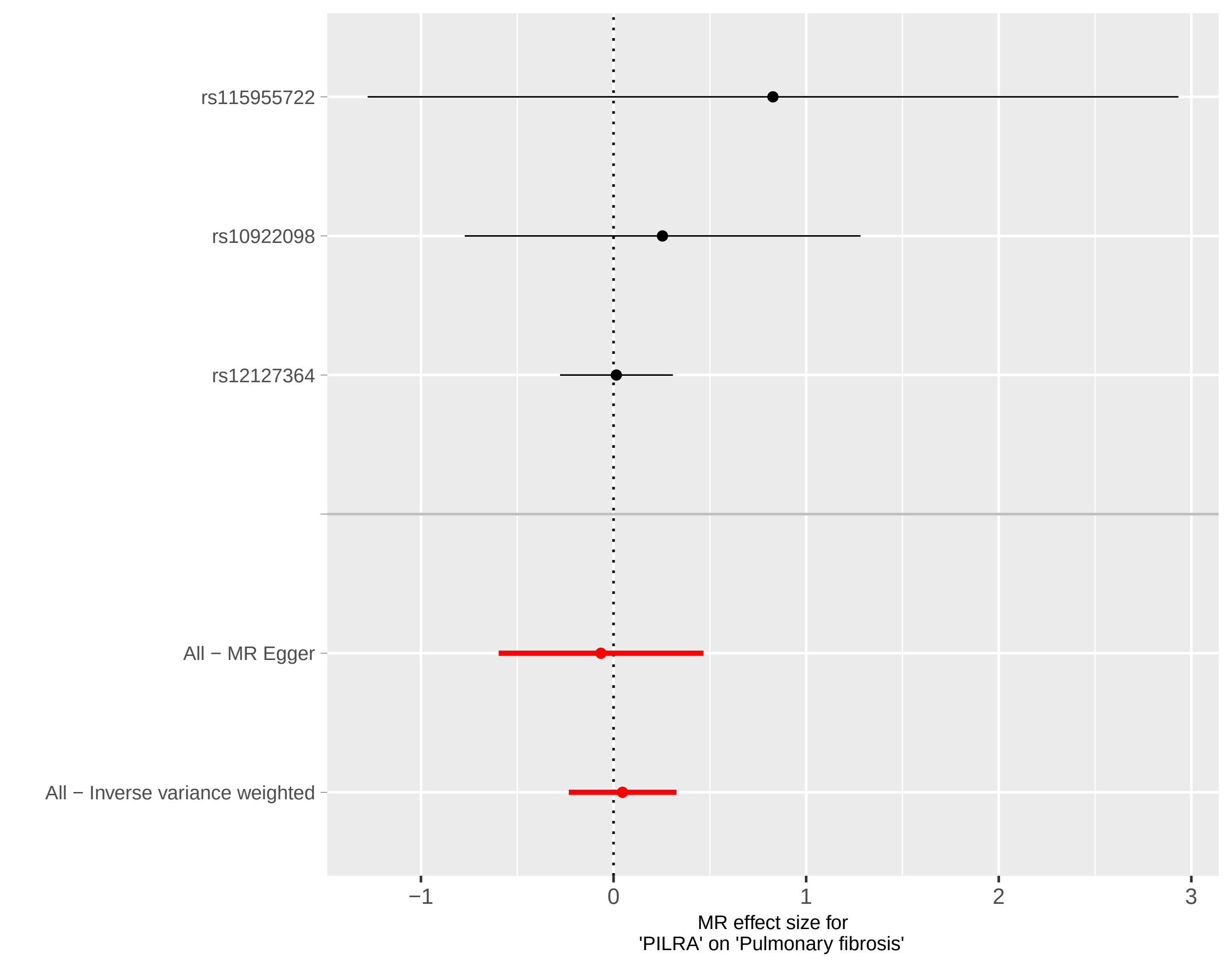
rs115955722
rs10922098
rs12127364
All − MR Egger
All − Inverse variance weighted
−1
0
1
2
3
MR effect size for
'PILRA' on 'Pulmonary fibrosis'

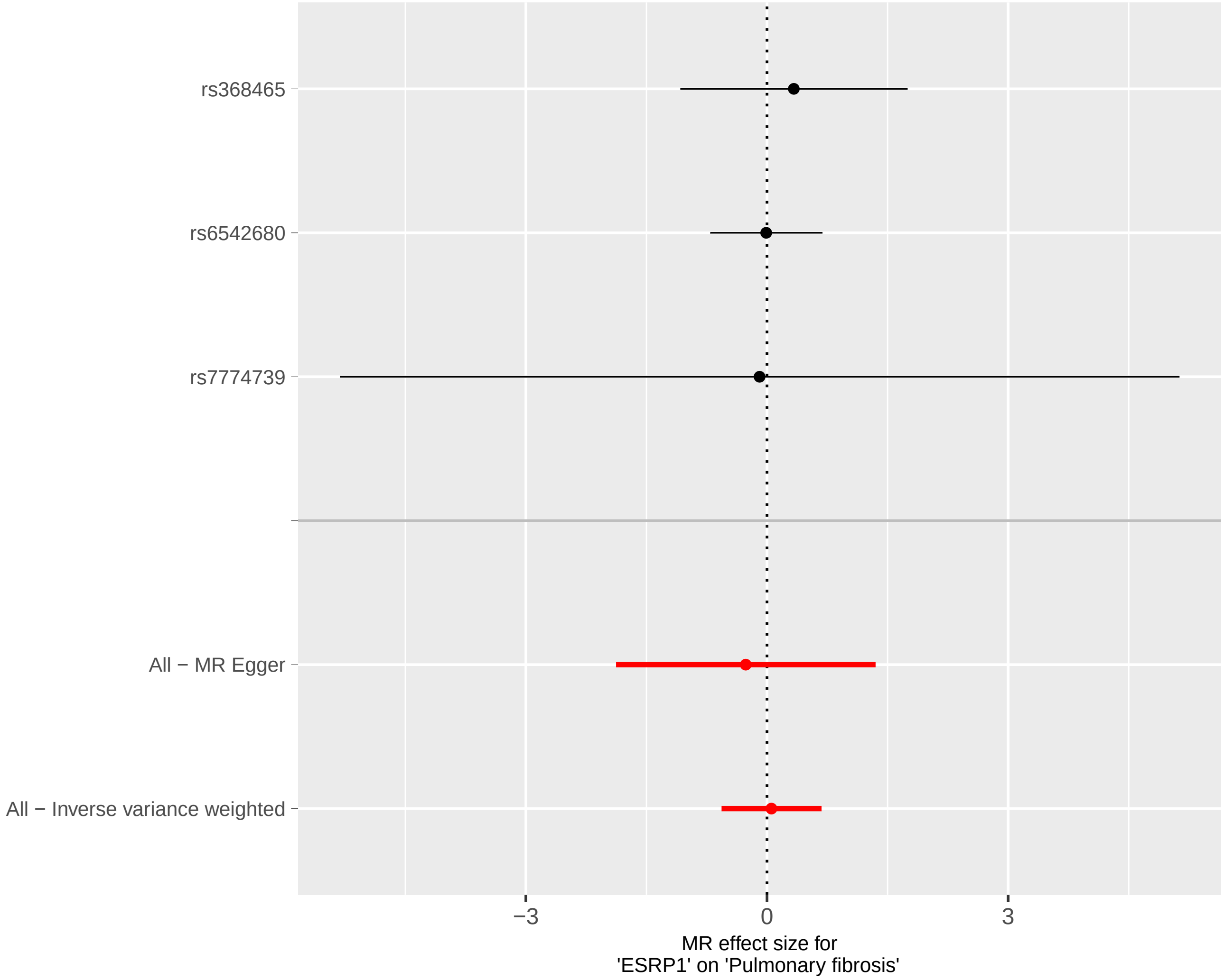
rs368465
rs6542680
rs7774739
All − MR Egger
All − Inverse variance weighted
−3
0
3
MR effect size for
'ESRP1' on 'Pulmonary fibrosis'

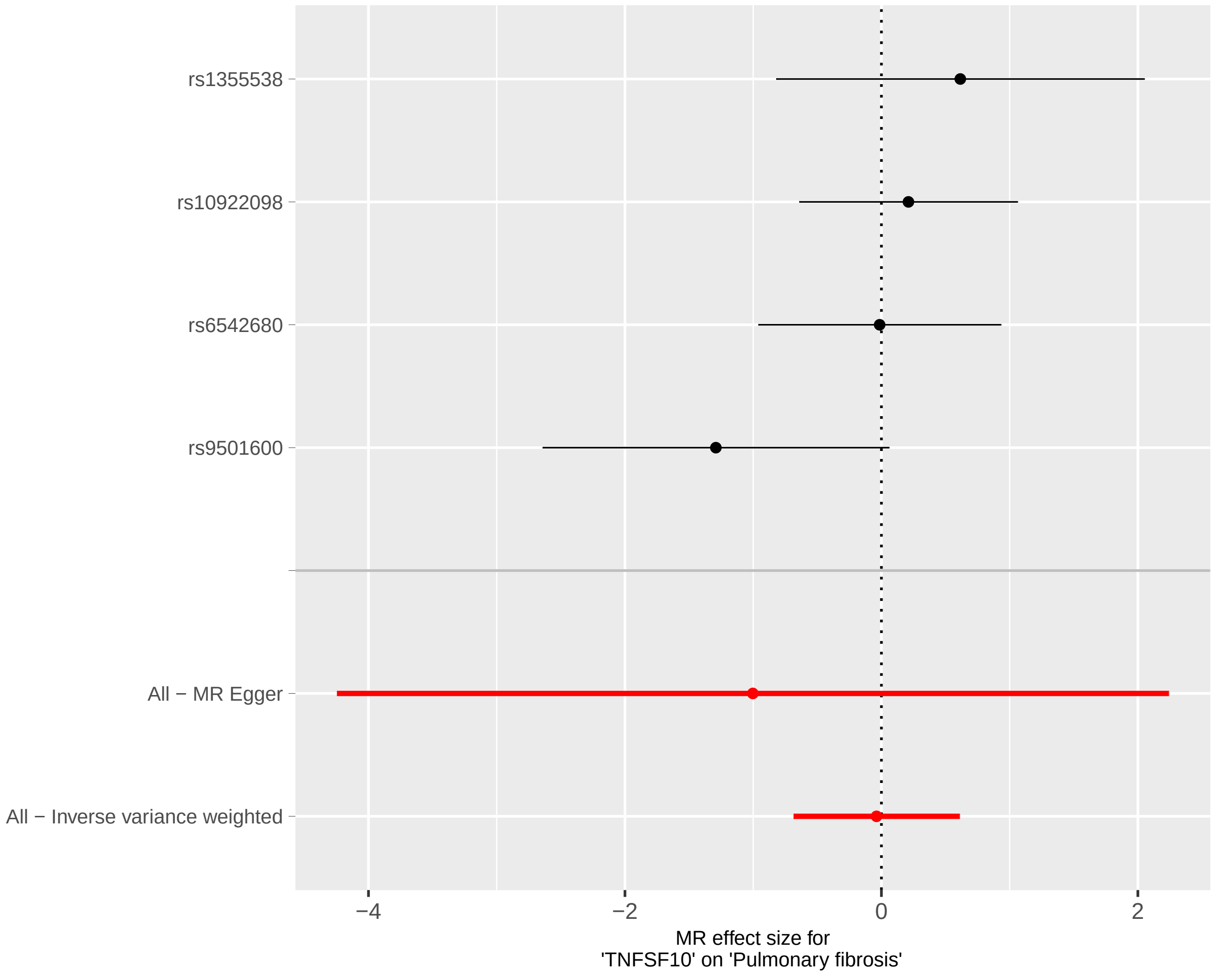

rs1355538
rs10922098
rs6542680
rs9501600
All − MR Egger
All − Inverse variance weighted
−4
−2
0
2
MR effect size for
'TNFSF10' on 'Pulmonary fibrosis'

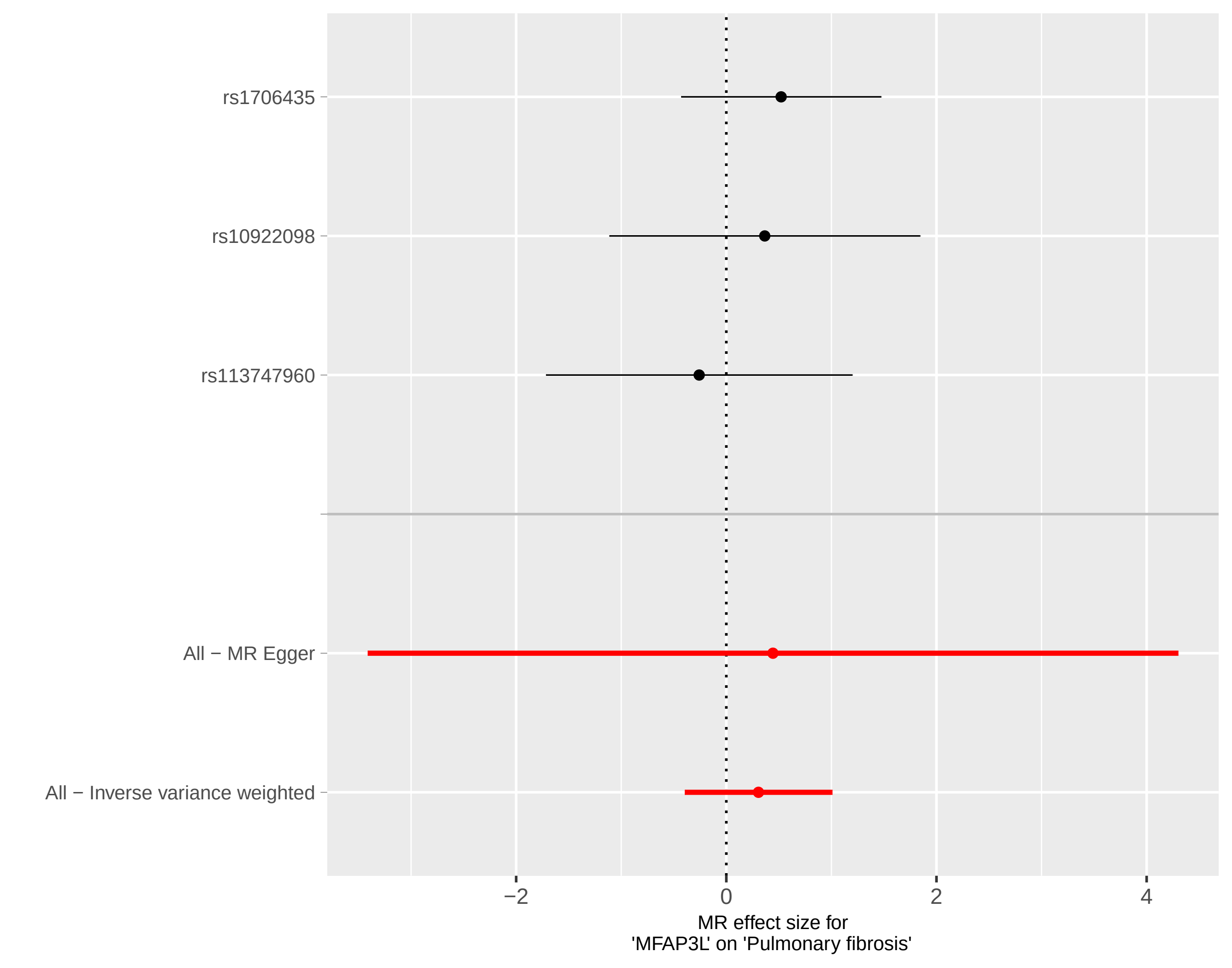
rs1706435
rs10922098
rs113747960
All − MR Egger
All − Inverse variance weighted
−2
0
2
4
MR effect size for
'MFAP3L' on 'Pulmonary fibrosis'

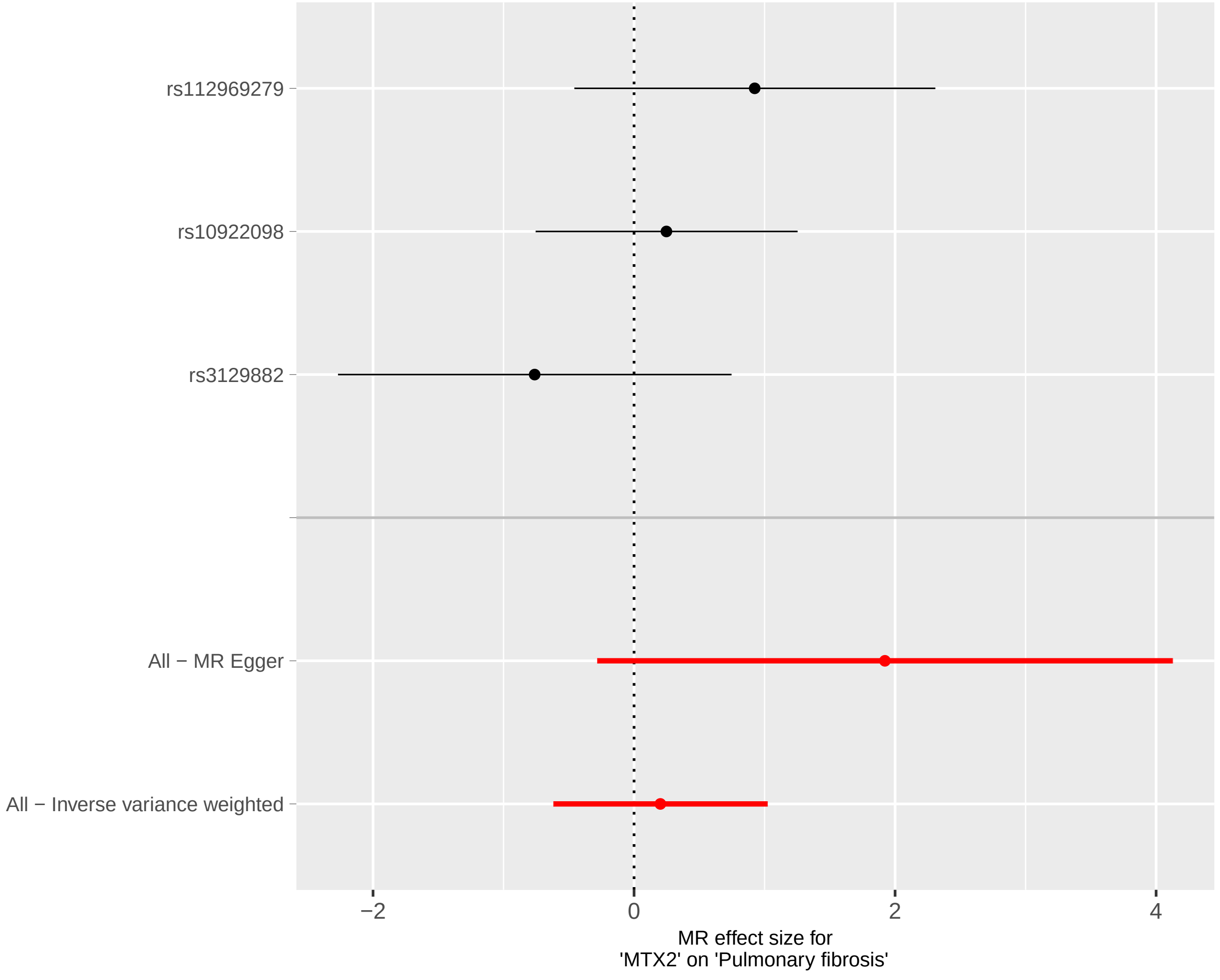

rs112969279
rs10922098
rs3129882
All − MR Egger
All − Inverse variance weighted
−2
0
2
4
MR effect size for
'MTX2' on 'Pulmonary fibrosis'

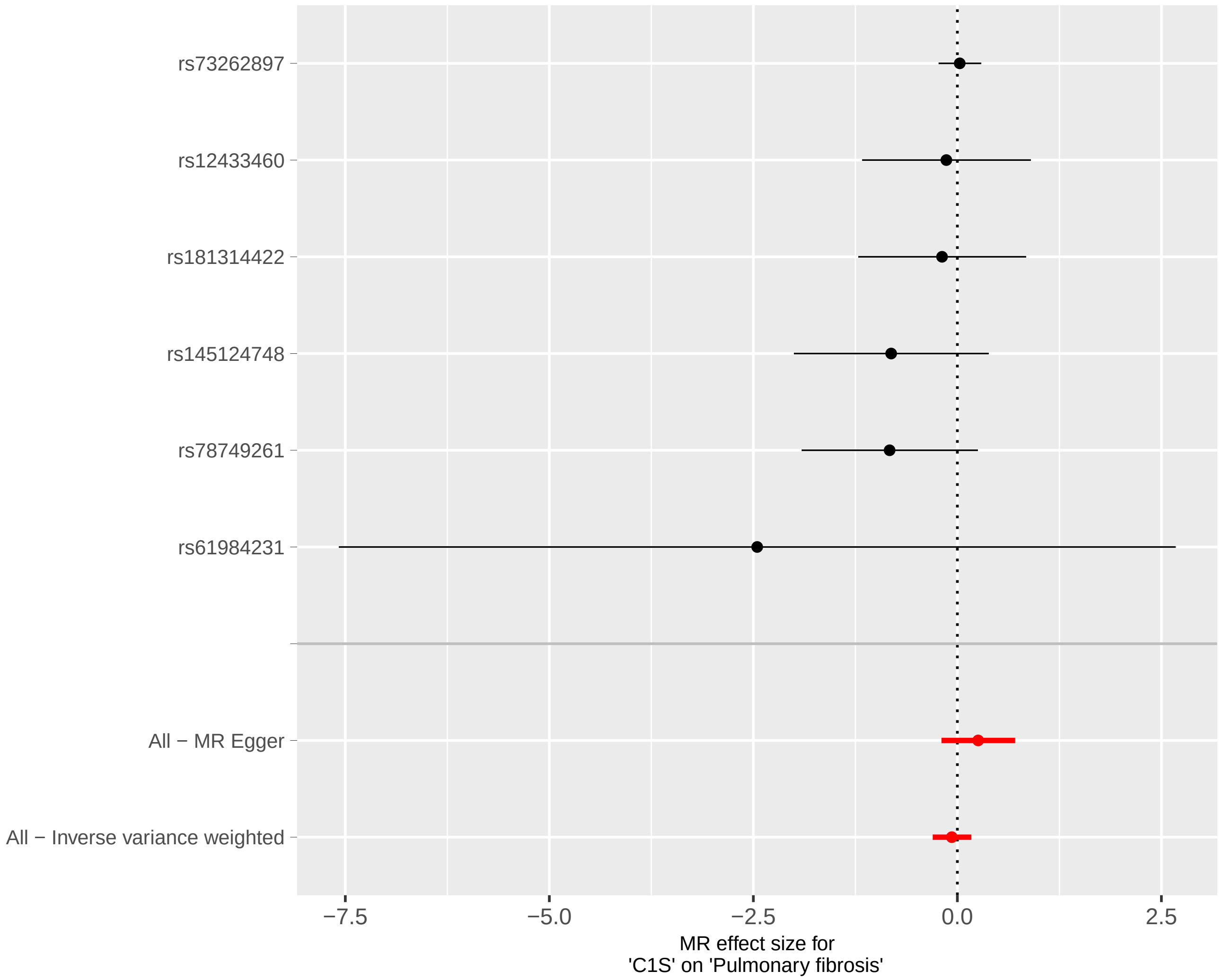

rs73262897
rs12433460
rs181314422
rs145124748
rs78749261
rs61984231
All − MR Egger
All − Inverse variance weighted
−7.5
−5.0
−2.5
0.0
2.5
MR effect size for
'C1S' on 'Pulmonary fibrosis'

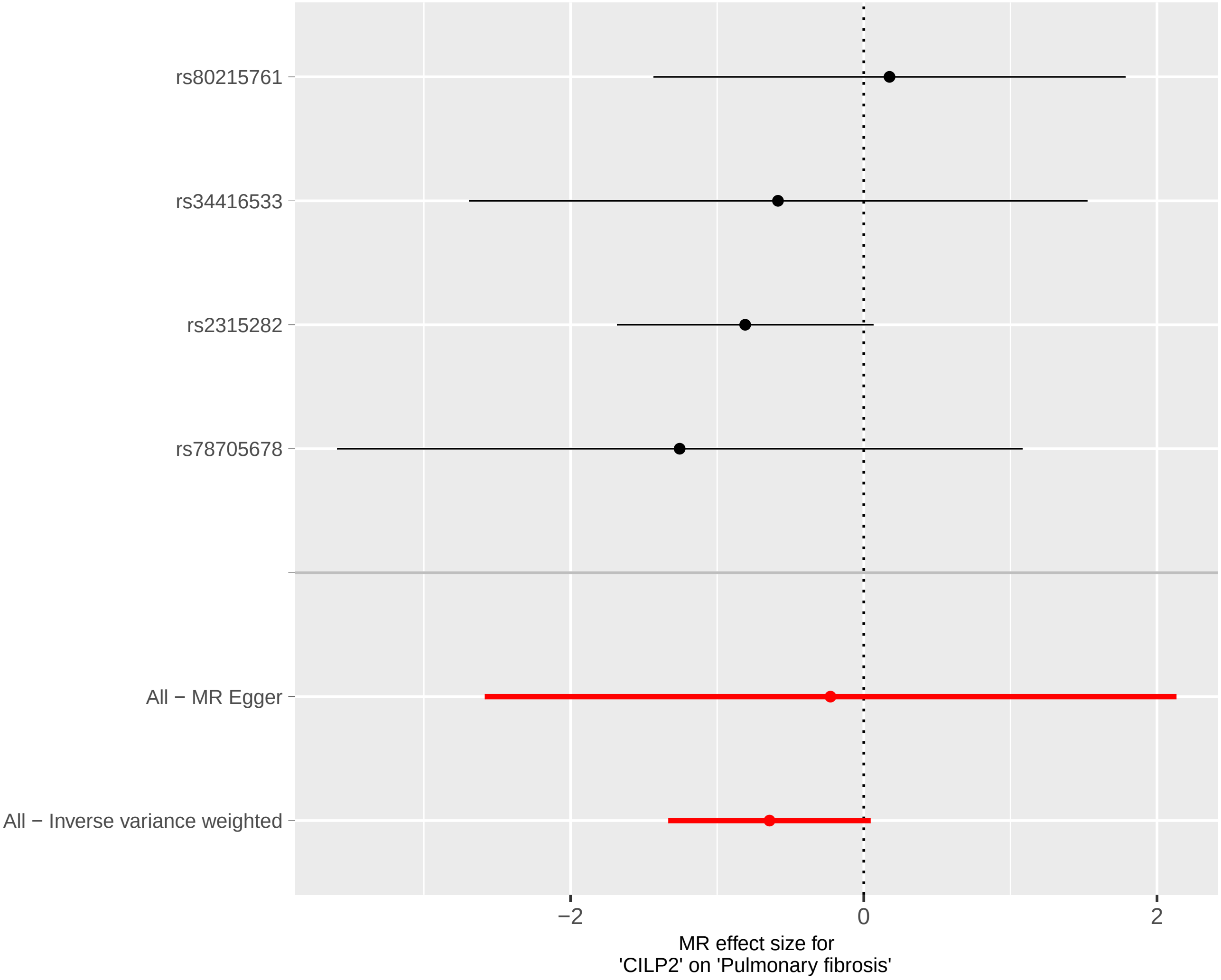

rs80215761
rs34416533
rs2315282
rs78705678
All − MR Egger
All − Inverse variance weighted
−2
0
2
MR effect size for
'CILP2' on 'Pulmonary fibrosis'

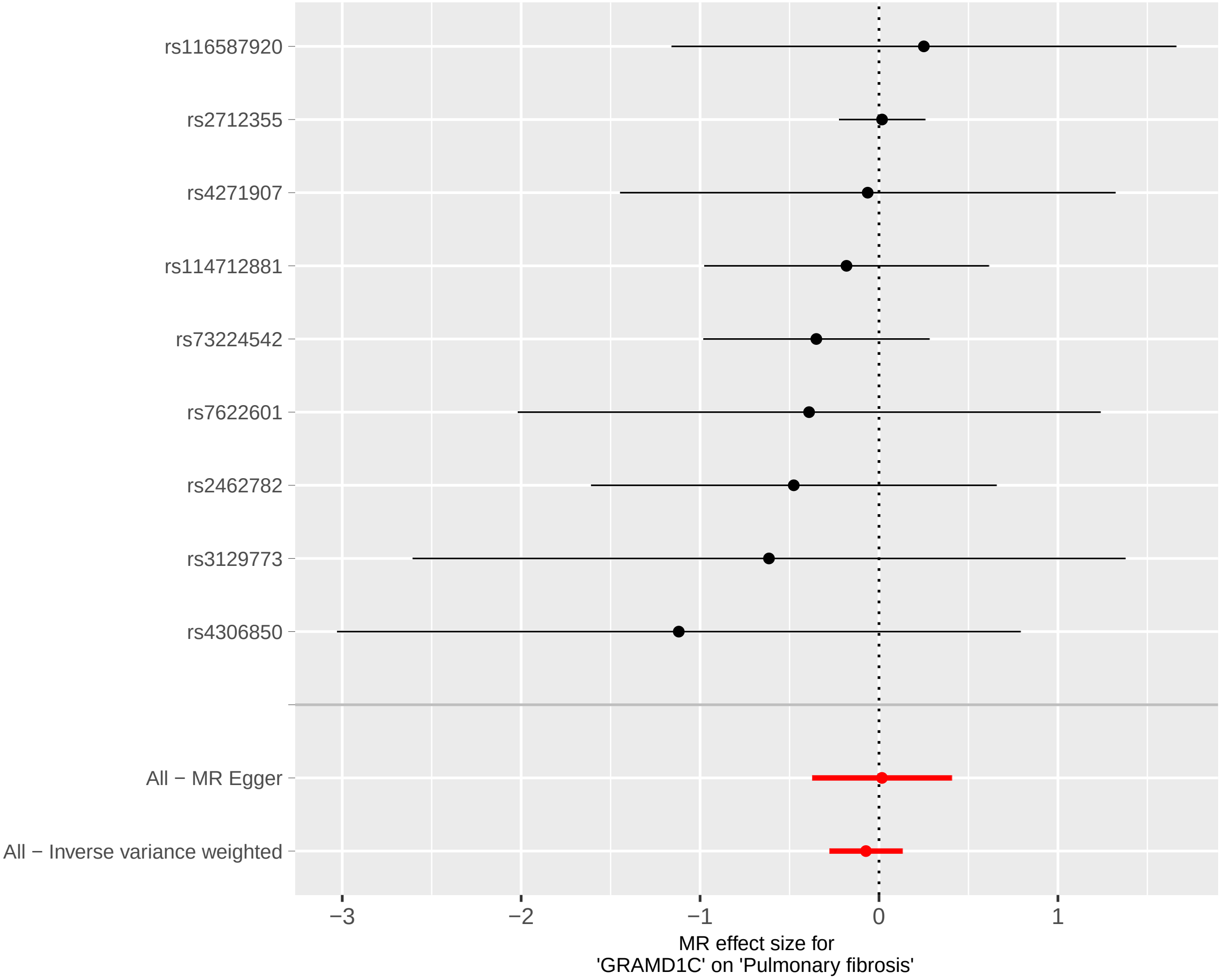

rs116587920
rs2712355
rs4271907
rs114712881
rs73224542
rs7622601
rs2462782
rs3129773
rs4306850
All − MR Egger
All − Inverse variance weighted
−3
−2
−1
0
1
MR effect size for
'GRAMD1C' on 'Pulmonary fibrosis'

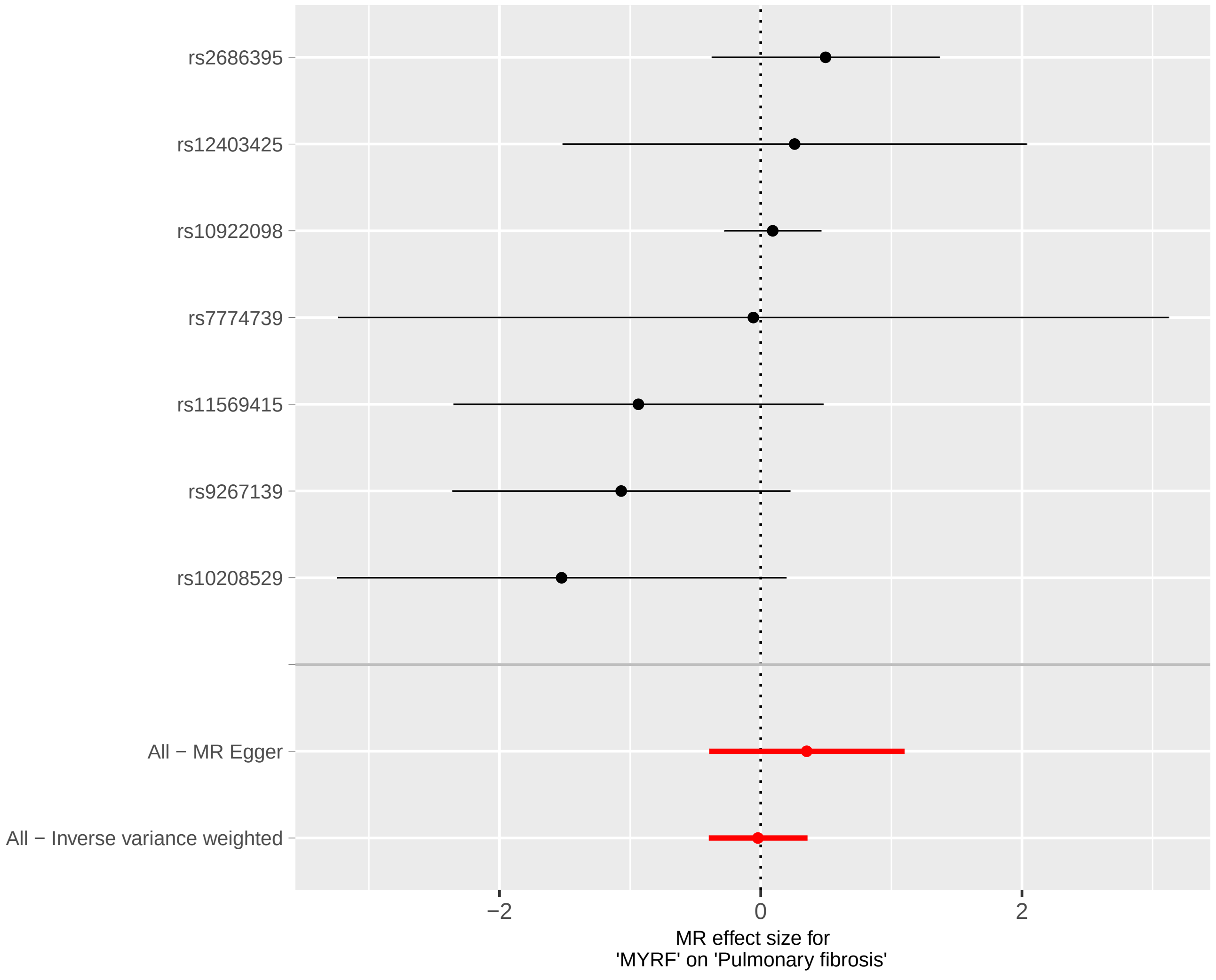

rs2686395
rs12403425
rs10922098
rs7774739
rs11569415
rs9267139
rs10208529
All − MR Egger
All − Inverse variance weighted
−2
0
2
MR effect size for
'MYRF' on 'Pulmonary fibrosis'

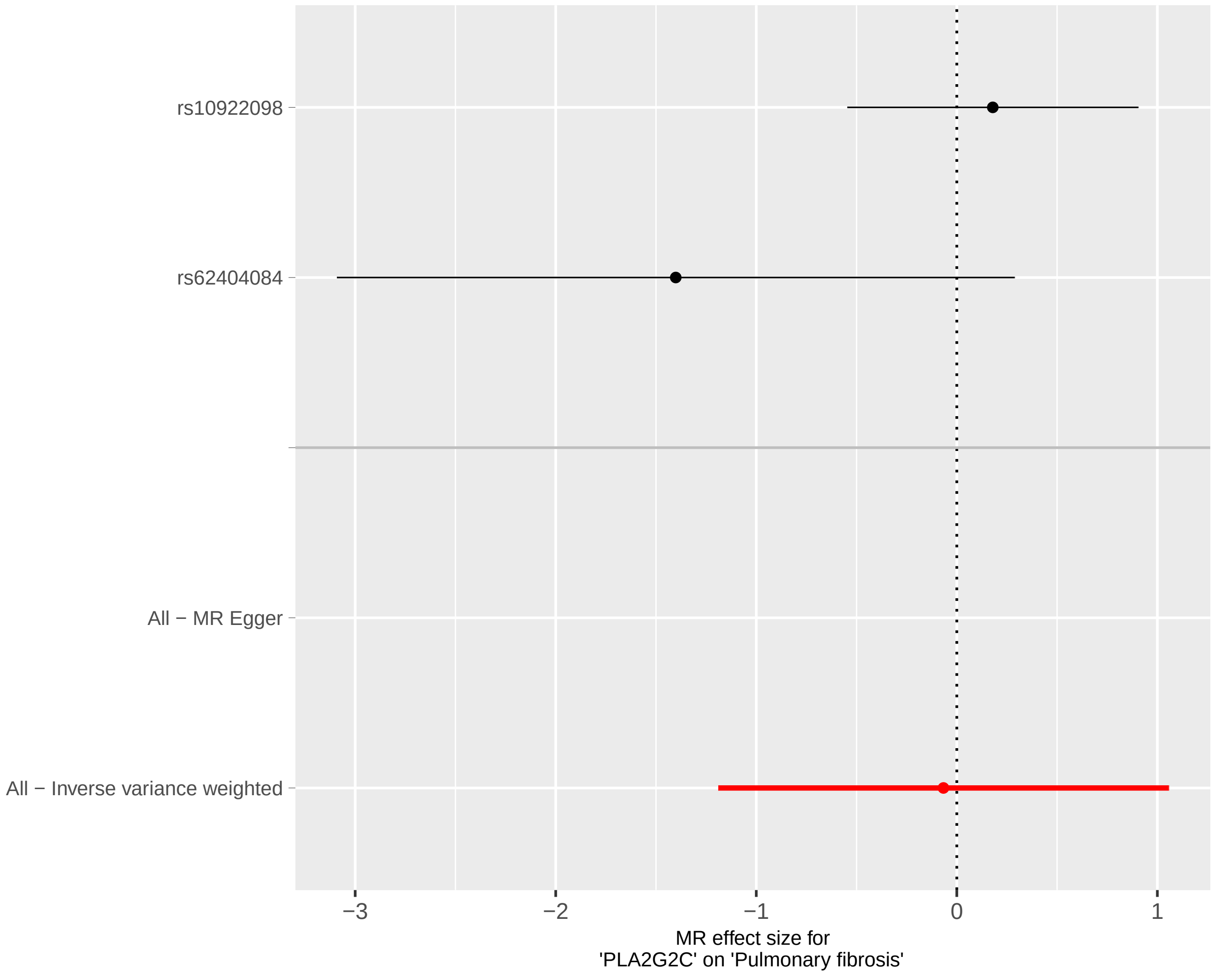
rs10922098
rs62404084
All − MR Egger
All − Inverse variance weighted
−3
−2
−1
0
1
MR effect size for
'PLA2G2C' on 'Pulmonary fibrosis'

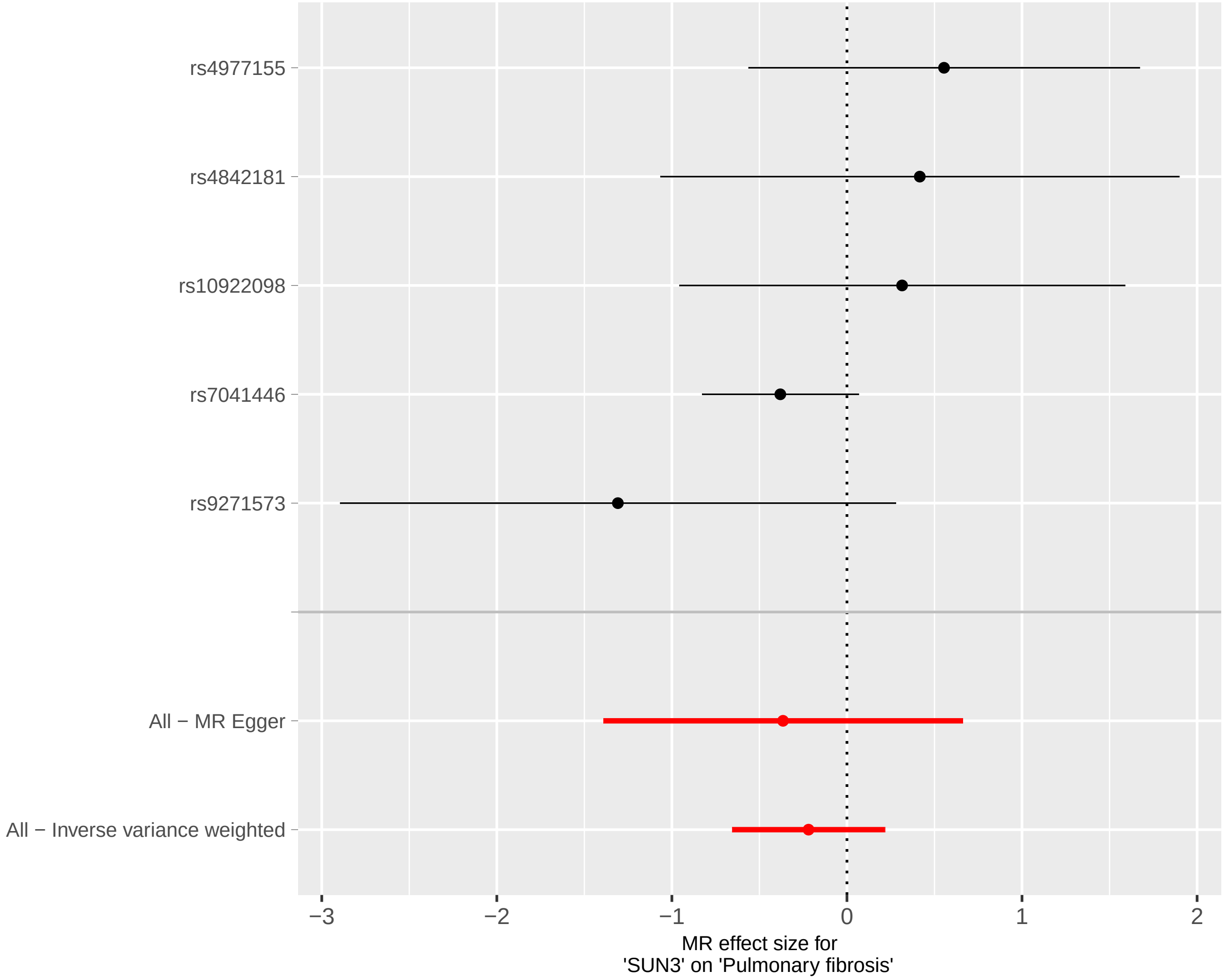
rs4977155
rs4842181
rs10922098
rs7041446
rs9271573
All − MR Egger
All − Inverse variance weighted
−3
−2
−1
0
1
2
MR effect size for
'SUN3' on 'Pulmonary fibrosis'

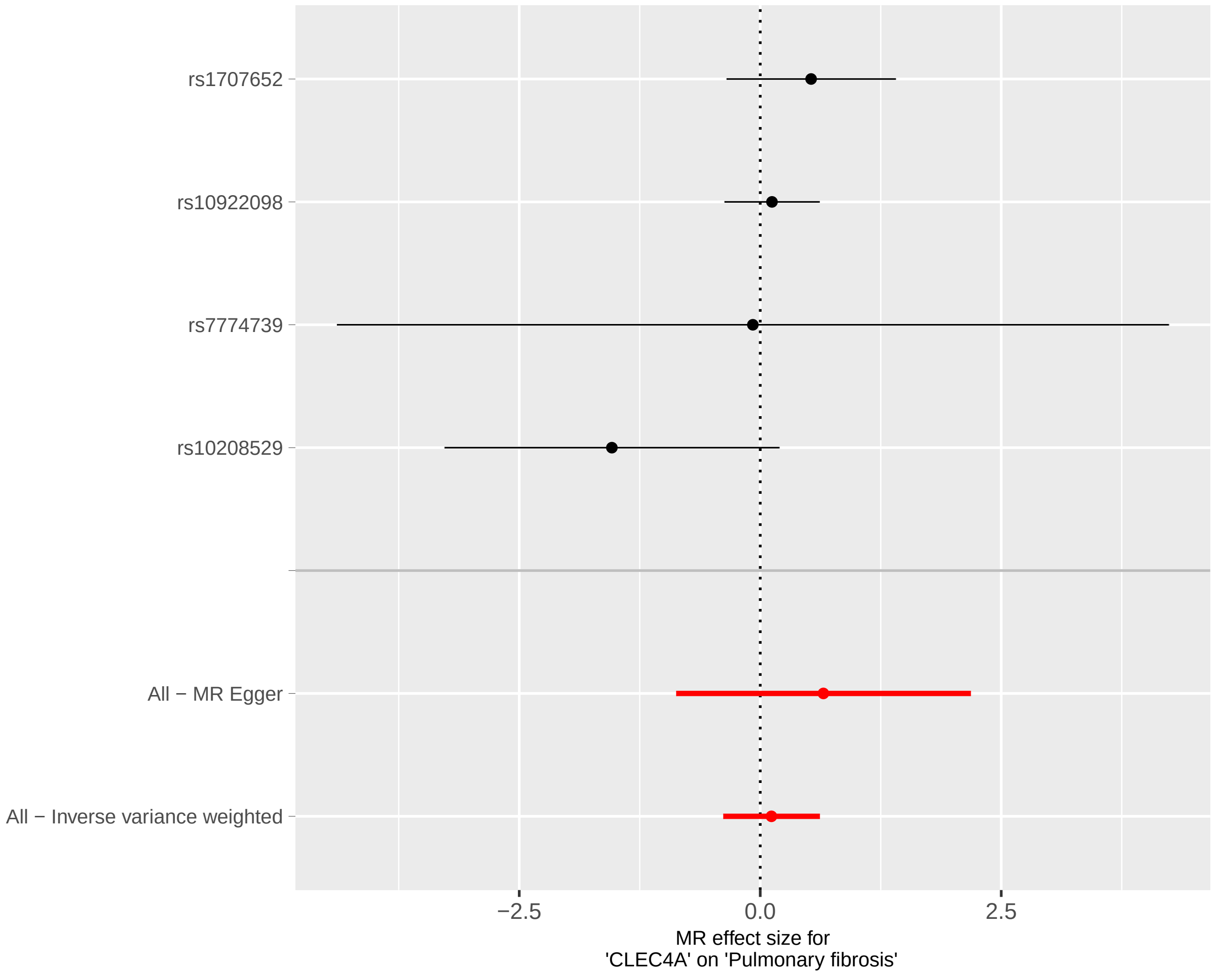

rs1707652
rs10922098
rs7774739
rs10208529
All − MR Egger
All − Inverse variance weighted
−2.5
0.0
2.5
MR effect size for
'CLEC4A' on 'Pulmonary fibrosis'

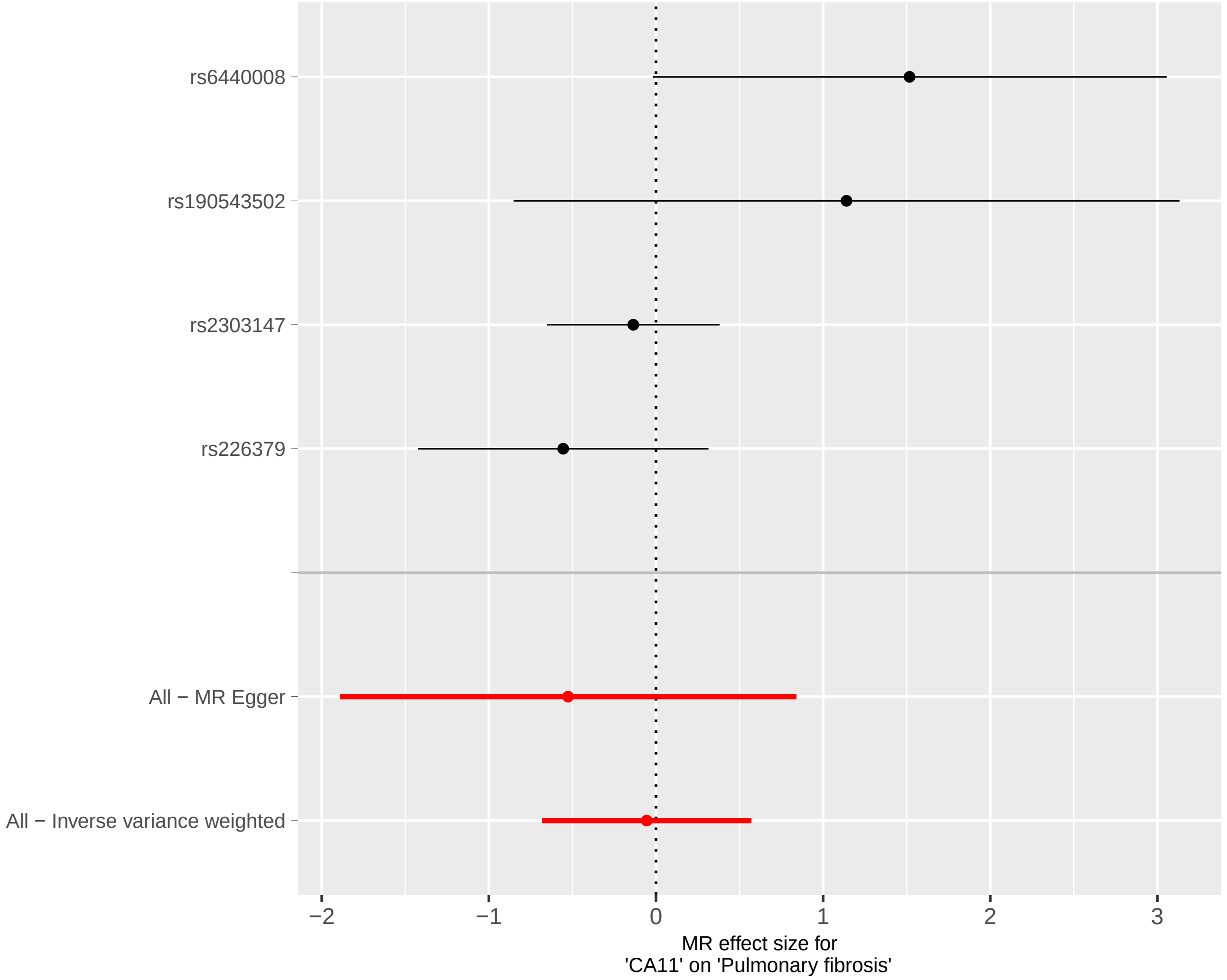

rs6440008
rs190543502
rs2303147
rs226379
All − MR Egger
All − Inverse variance weighted
−2
−1
0
1
2
3
MR effect size for
'CA11' on 'Pulmonary fibrosis'

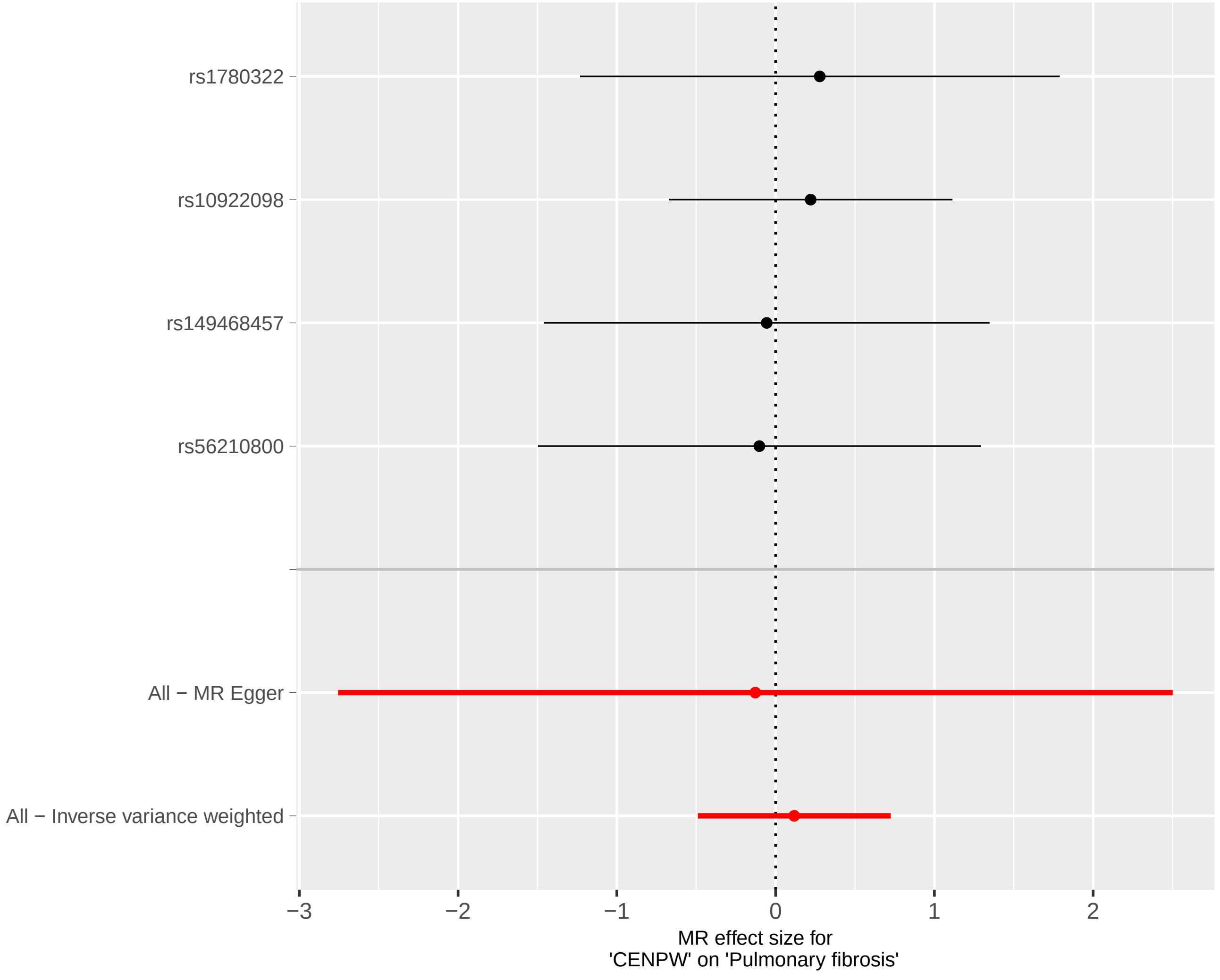

rs1780322
rs10922098
rs149468457
rs56210800
All − MR Egger
All − Inverse variance weighted
−3
−2
−1
0
1
2
MR effect size for
'CENPW' on 'Pulmonary fibrosis'

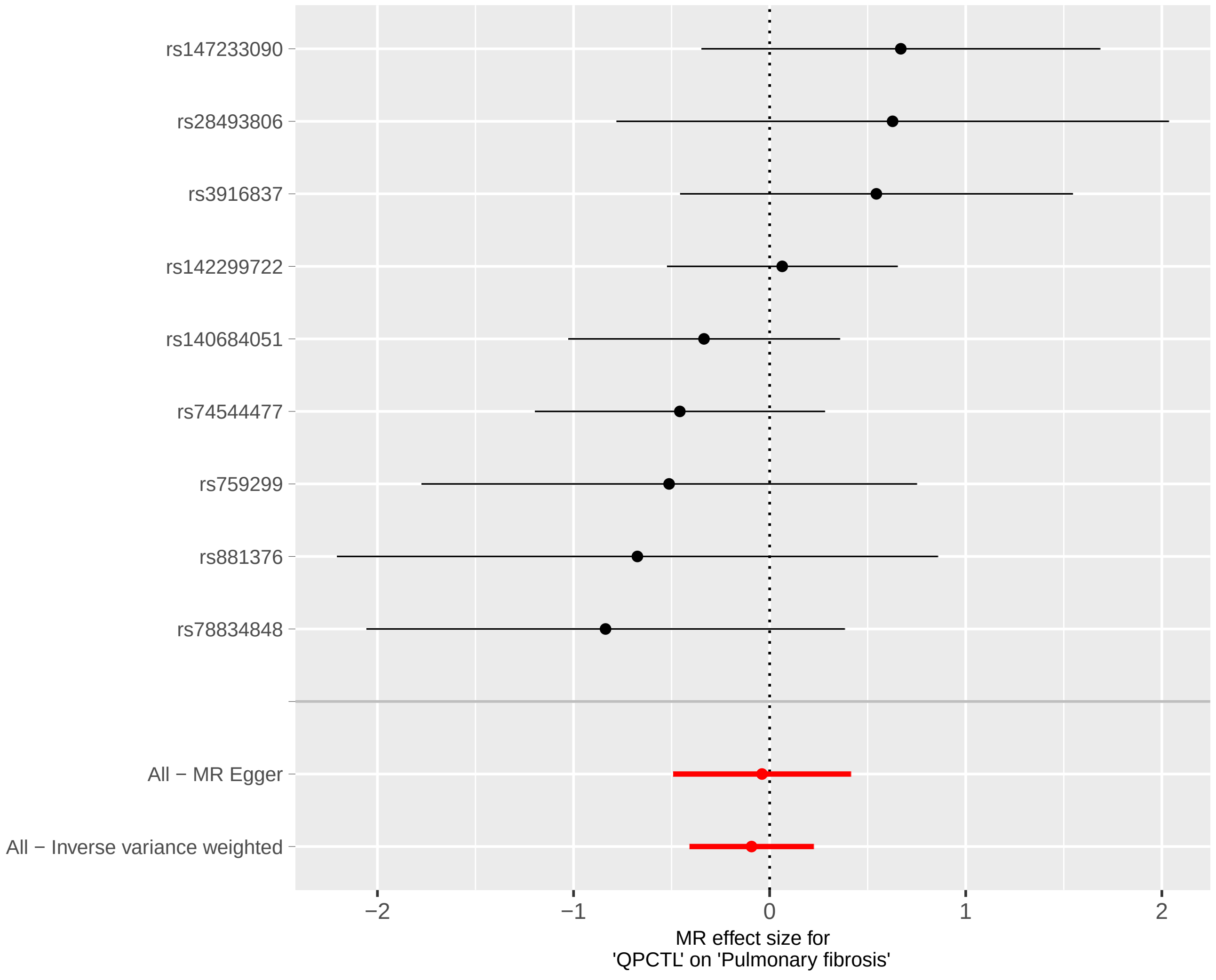

rs147233090
rs28493806
rs3916837
rs142299722
rs140684051
rs74544477
rs759299
rs881376
rs78834848
All − MR Egger
All − Inverse variance weighted
−2
−1
0
1
2
MR effect size for
'QPCTL' on 'Pulmonary fibrosis'

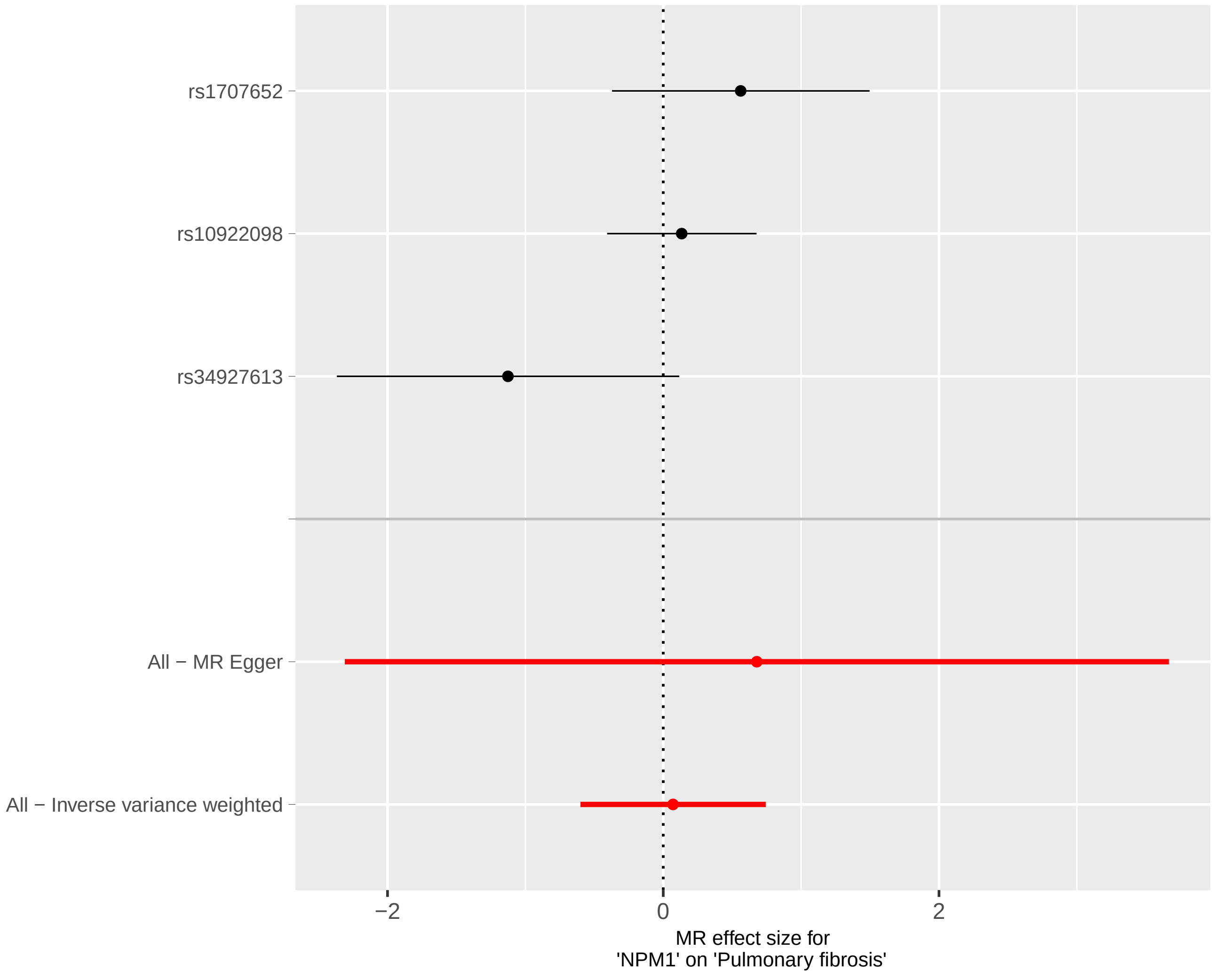
rs1707652
rs10922098
rs34927613
All − MR Egger
All − Inverse variance weighted
−2
0
2
MR effect size for
'NPM1' on 'Pulmonary fibrosis'

Insufficient number of SNPs

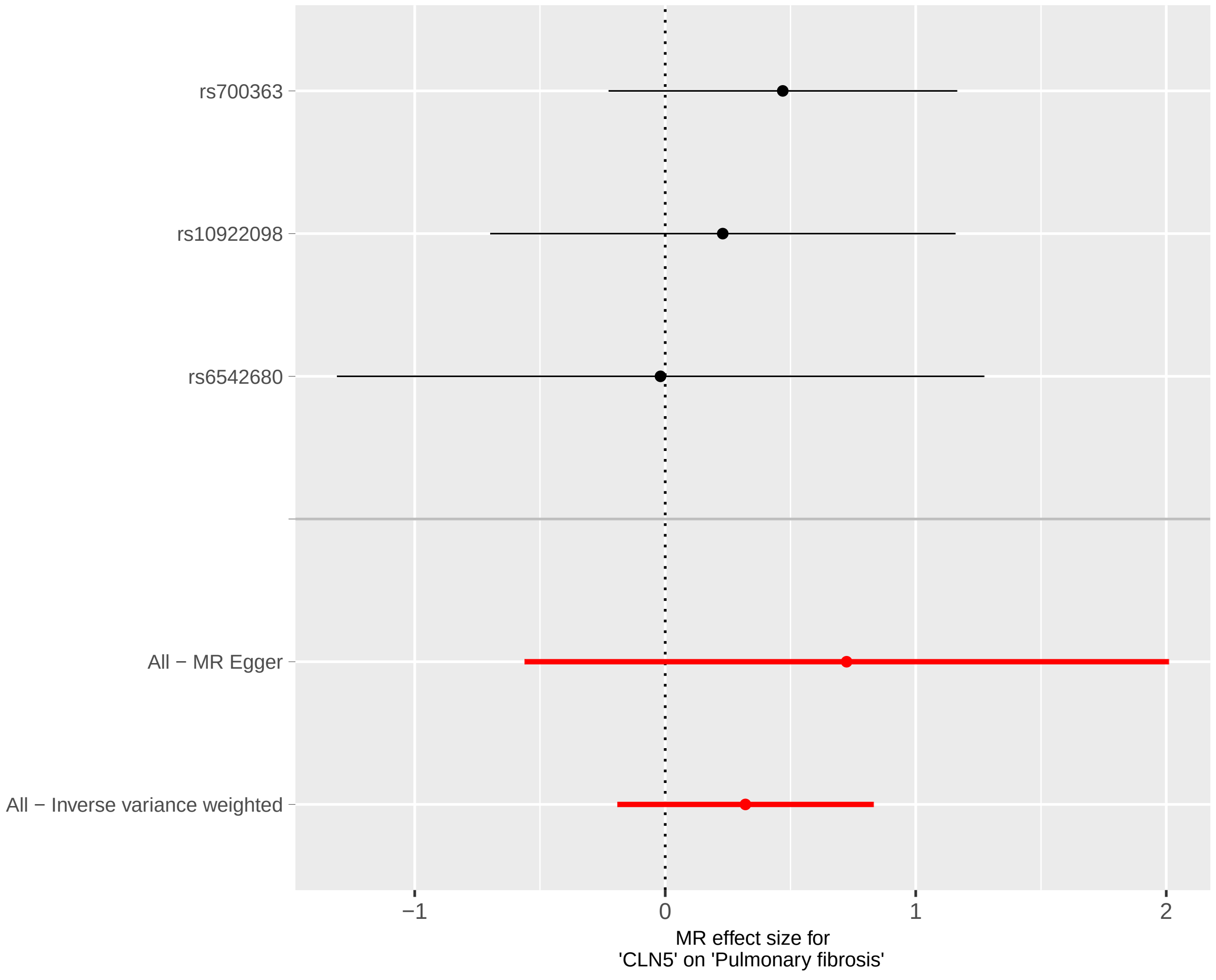
rs700363
rs10922098
rs6542680
All − MR Egger
All − Inverse variance weighted
−1
0
1
2
MR effect size for
'CLN5' on 'Pulmonary fibrosis'

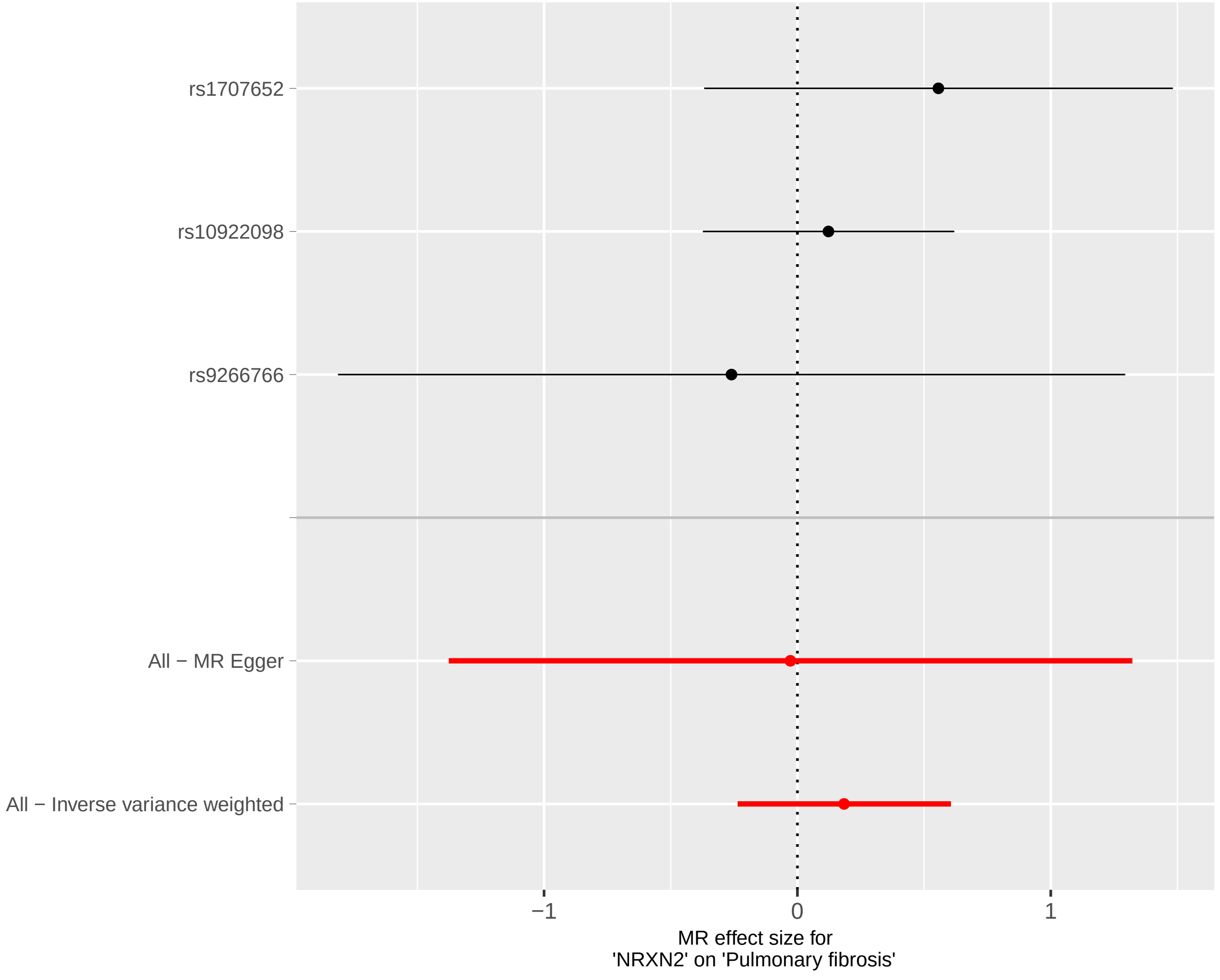

rs1707652
rs10922098
rs9266766
All − MR Egger
All − Inverse variance weighted
−1
0
1
MR effect size for
'NRXN2' on 'Pulmonary fibrosis'

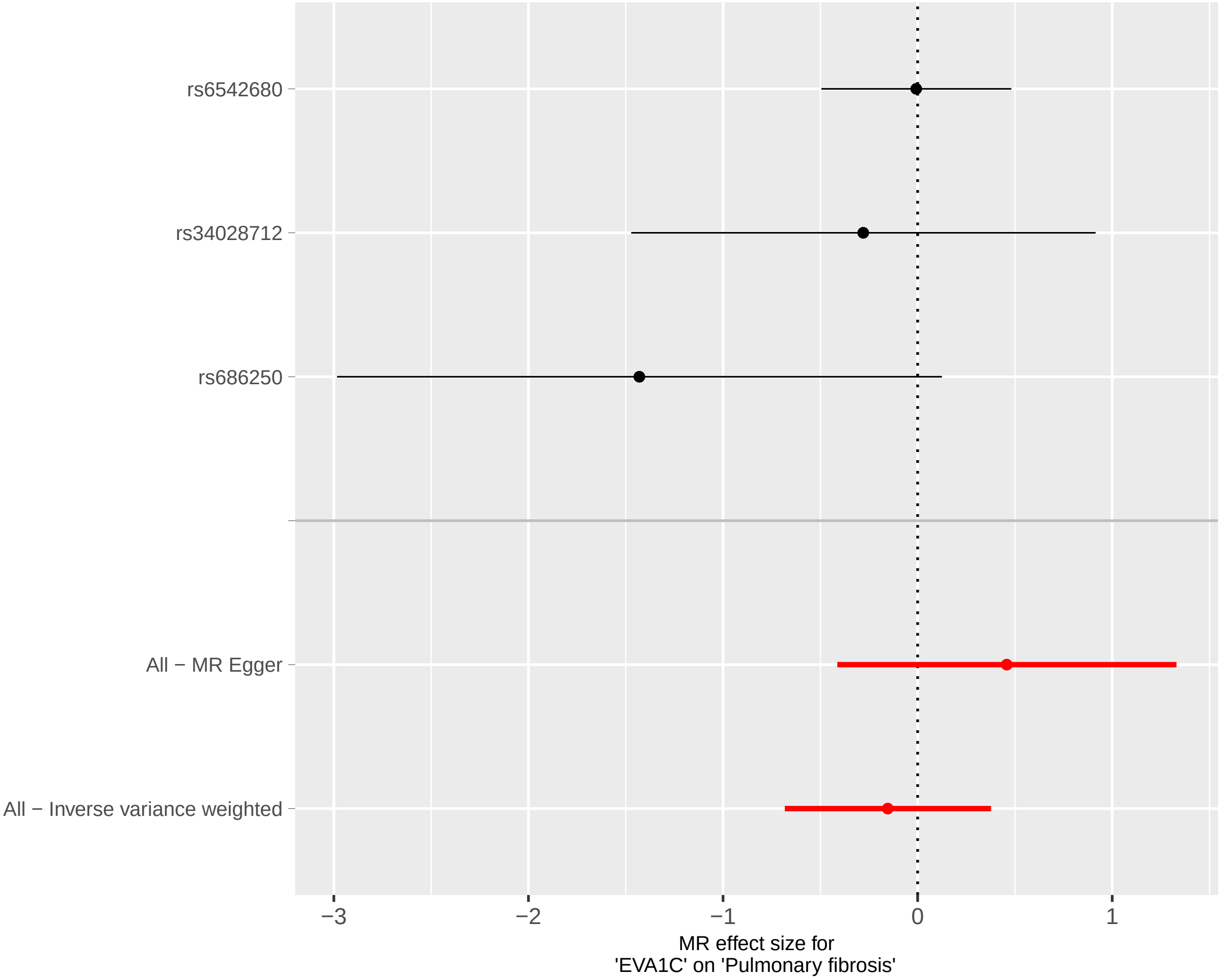
rs6542680
rs34028712
rs686250
All − MR Egger
All − Inverse variance weighted
−3
−2
−1
0
1
MR effect size for
'EVA1C' on 'Pulmonary fibrosis'

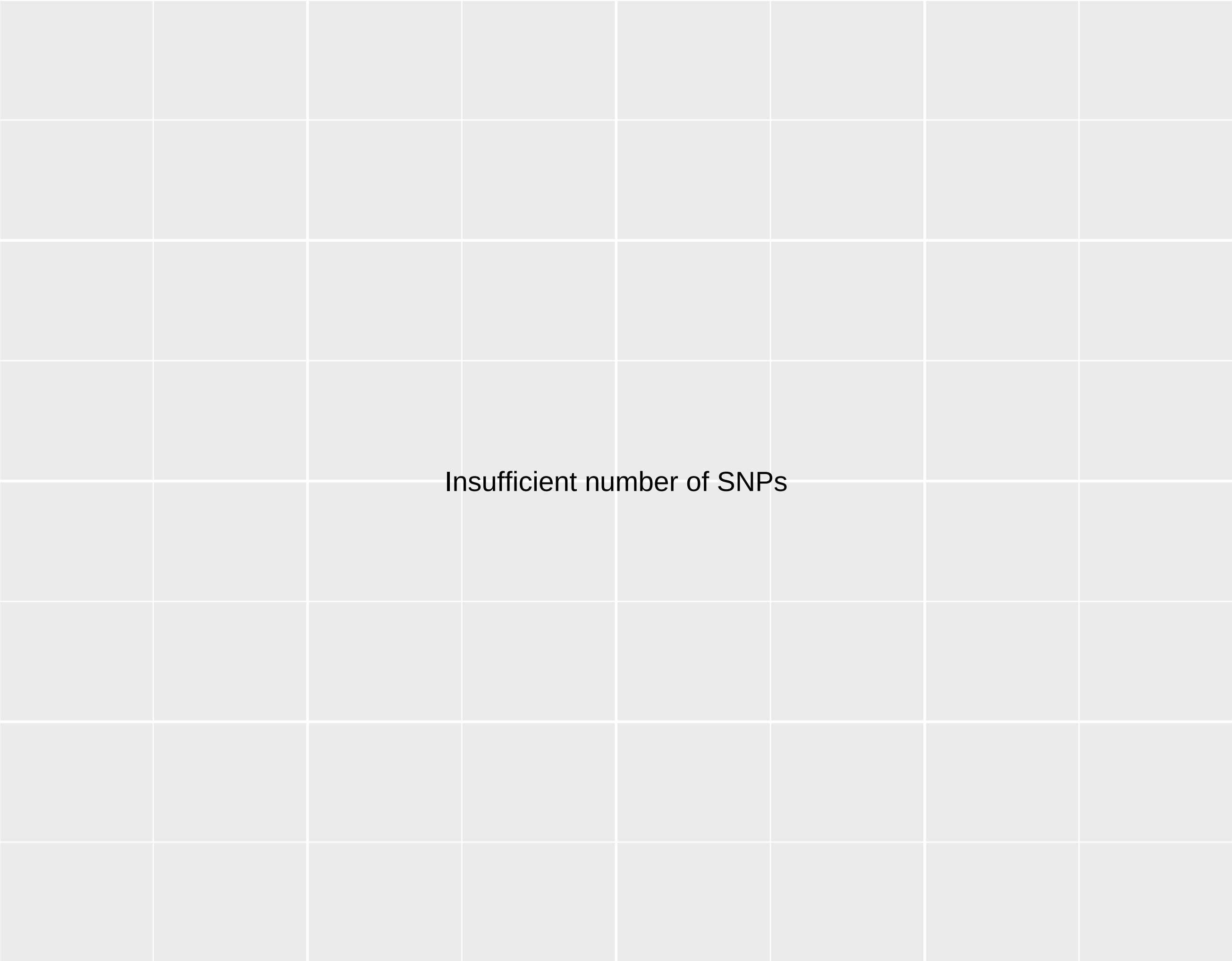
Insufficient number of SNPs

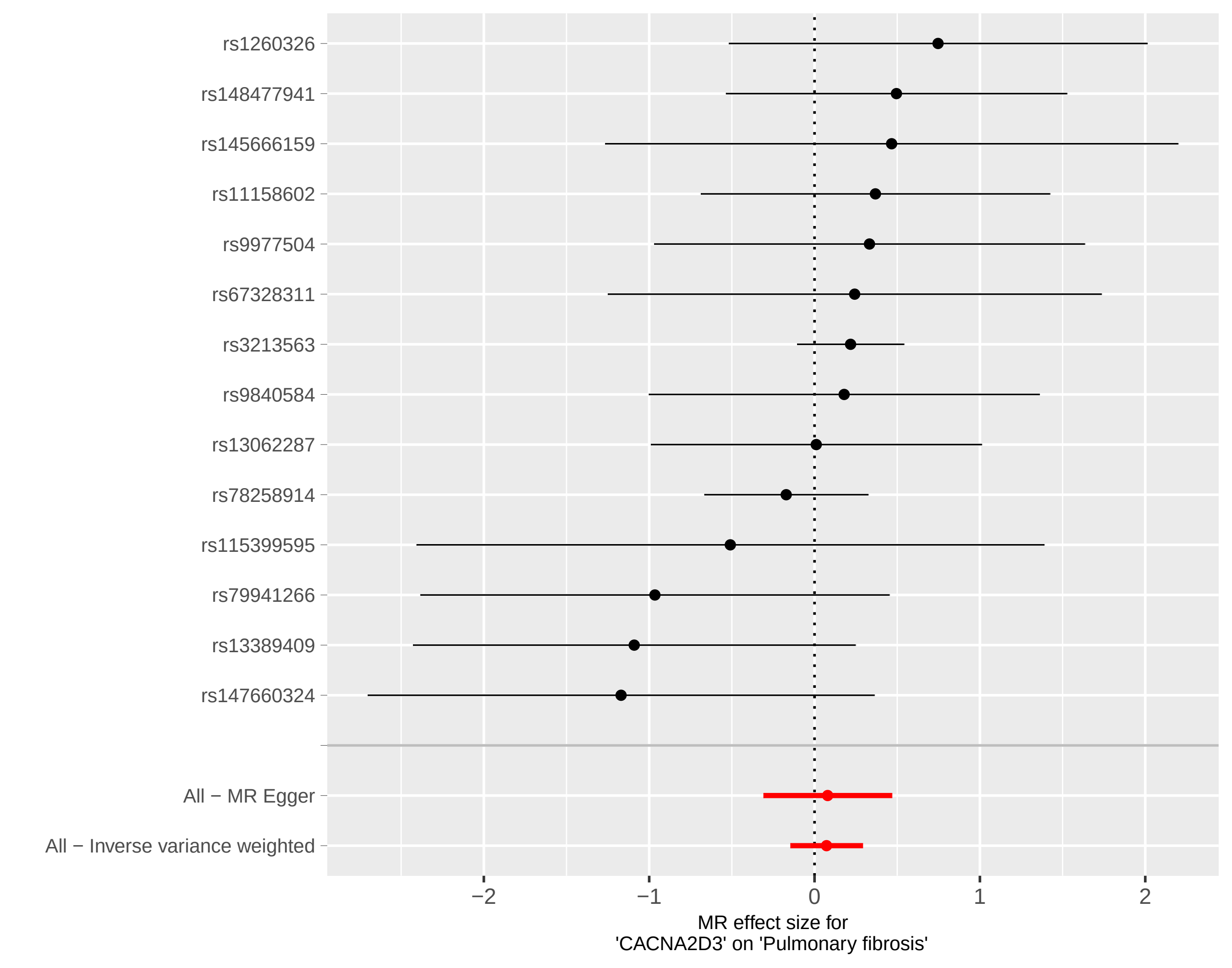

rs1260326
rs148477941
rs145666159
rs11158602
rs9977504
rs67328311
rs3213563
rs9840584
rs13062287
rs78258914
rs115399595
rs79941266
rs13389409
rs147660324
All − MR Egger
All − Inverse variance weighted
−2
−1
0
1
2
MR effect size for
'CACNA2D3' on 'Pulmonary fibrosis'

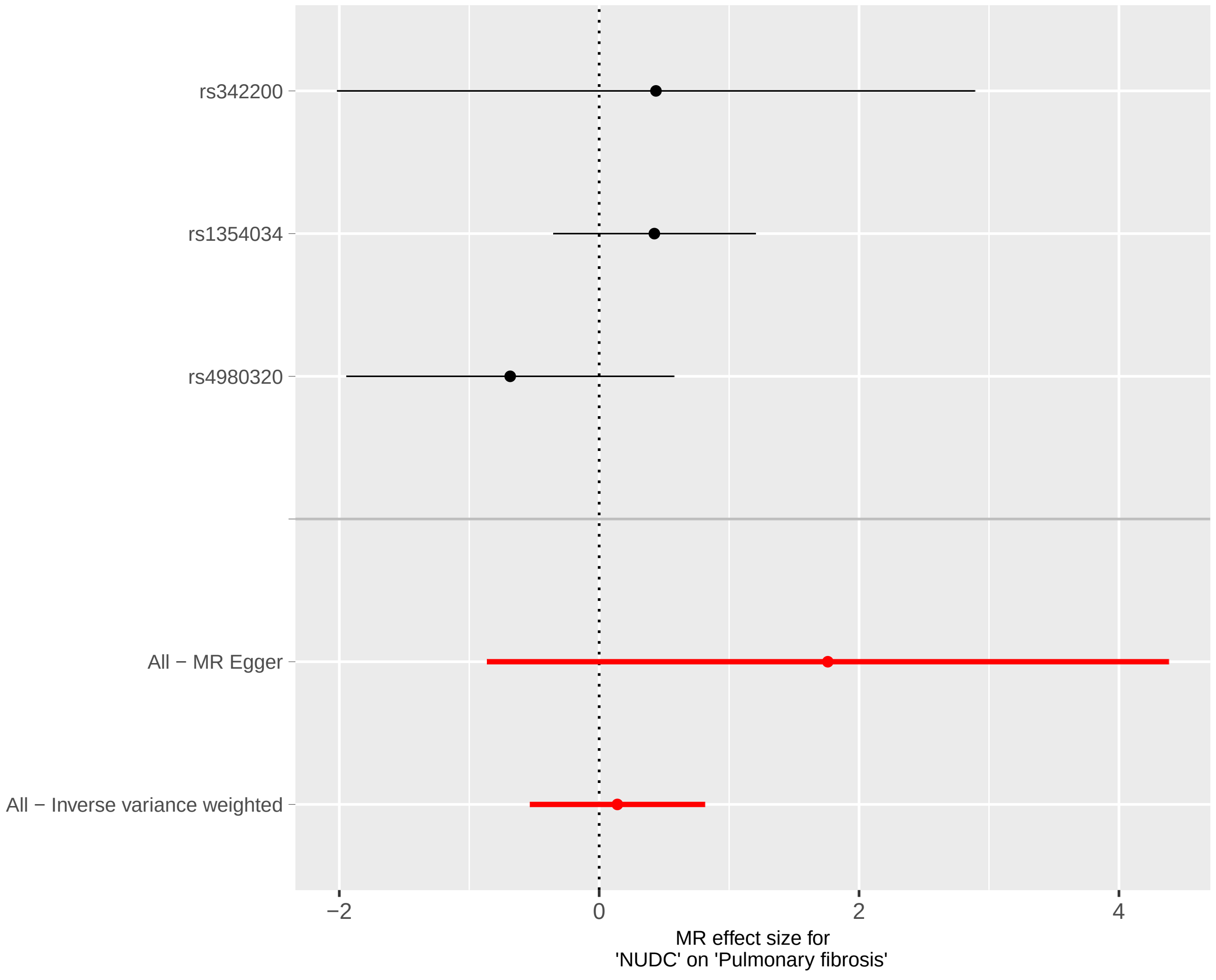

rs342200
rs1354034
rs4980320
All − MR Egger
All − Inverse variance weighted
−2
0
2
4
MR effect size for
'NUDC' on 'Pulmonary fibrosis'

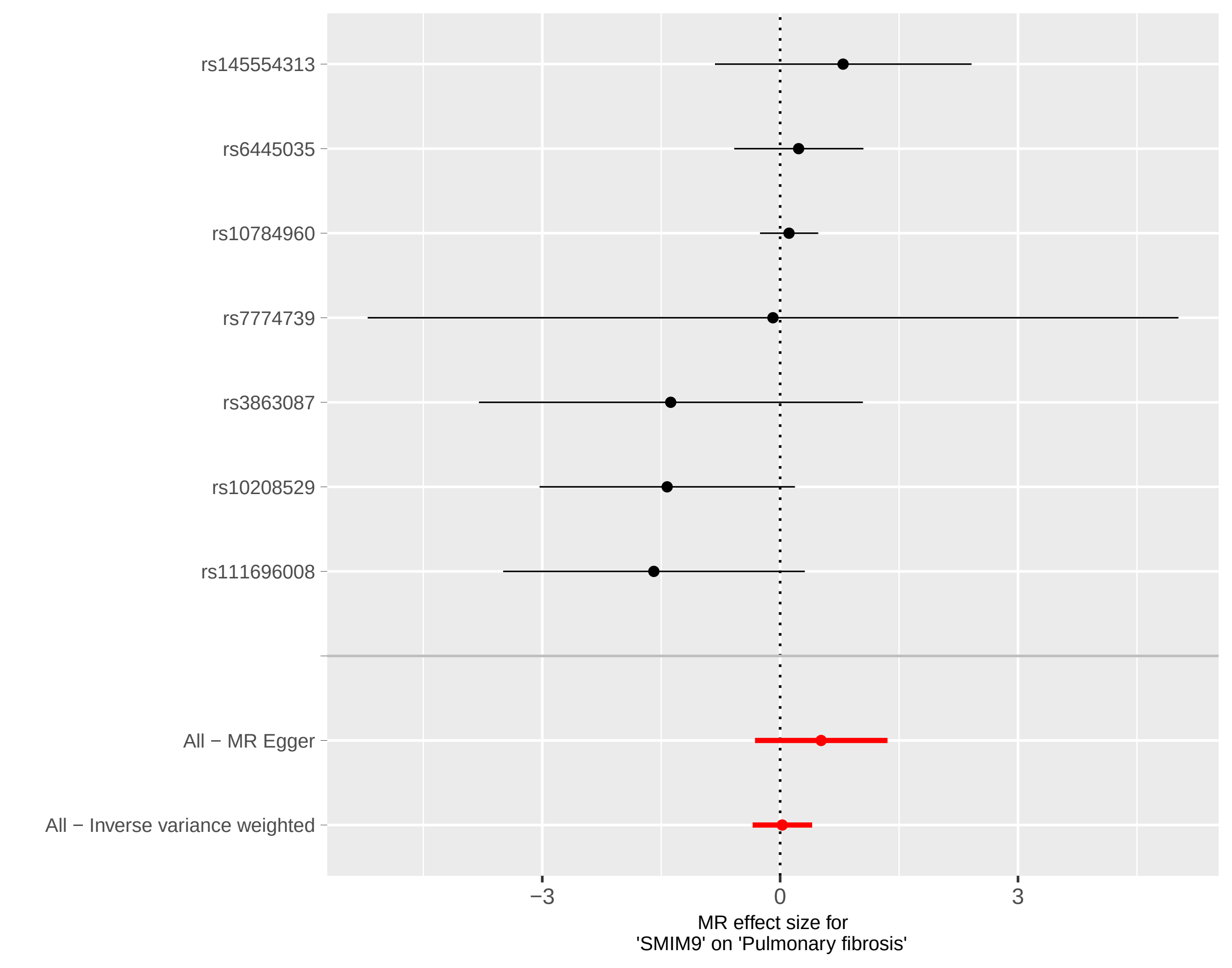
rs145554313
rs6445035
rs10784960
rs7774739
rs3863087
rs10208529
rs111696008
All − MR Egger
All − Inverse variance weighted
−3
0
3
MR effect size for
'SMIM9' on 'Pulmonary fibrosis'

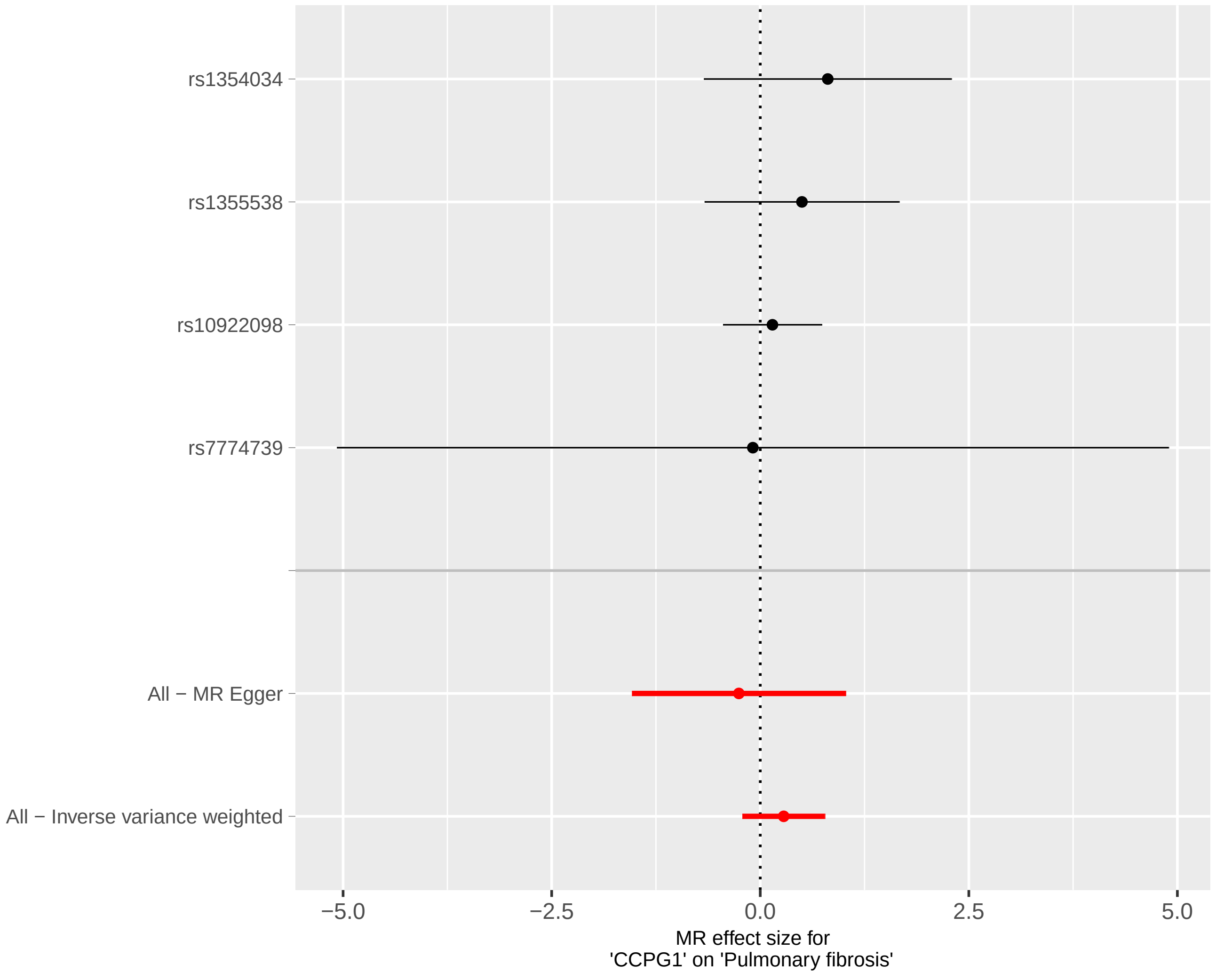
rs1354034
rs1355538
rs10922098
rs7774739
All − MR Egger
All − Inverse variance weighted
−5.0
−2.5
0.0
2.5
5.0
MR effect size for
'CCPG1' on 'Pulmonary fibrosis'

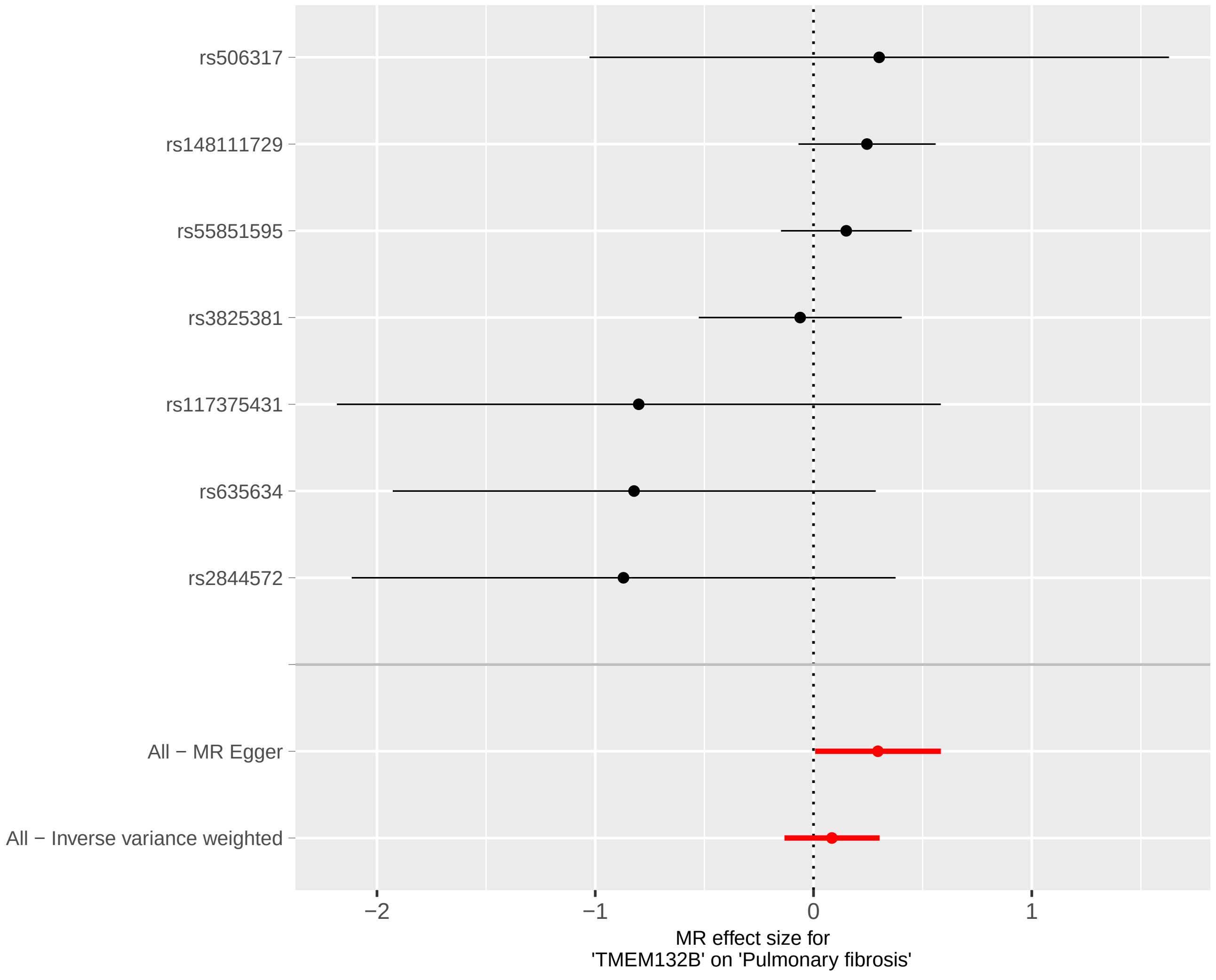

rs506317
rs148111729
rs55851595
rs3825381
rs117375431
rs635634
rs2844572
All − MR Egger
All − Inverse variance weighted
−2
−1
0
1
MR effect size for
'TMEM132B' on 'Pulmonary fibrosis'

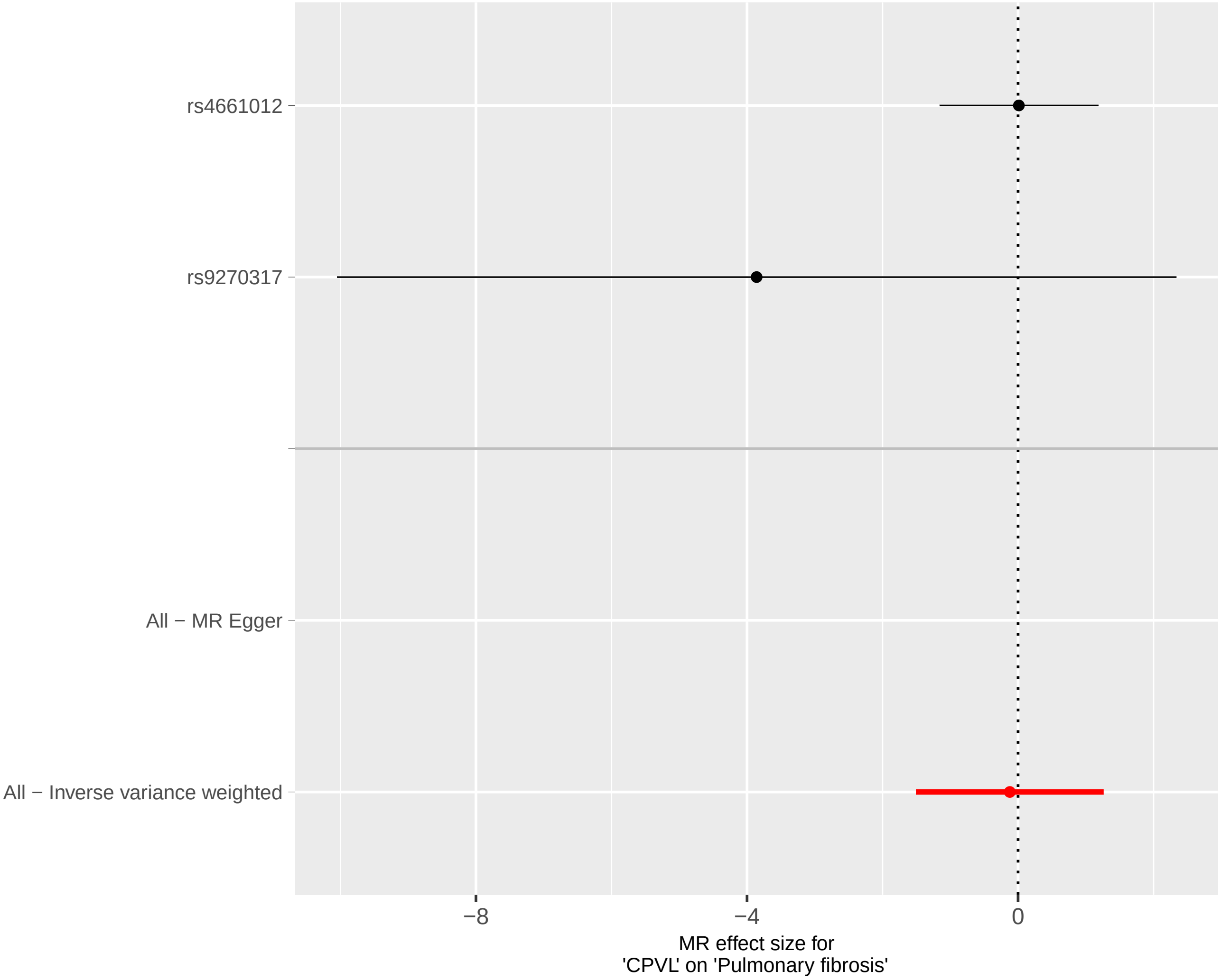
rs4661012
rs9270317
All − MR Egger
All − Inverse variance weighted
−8
−4
0
MR effect size for
'CPVL' on 'Pulmonary fibrosis'

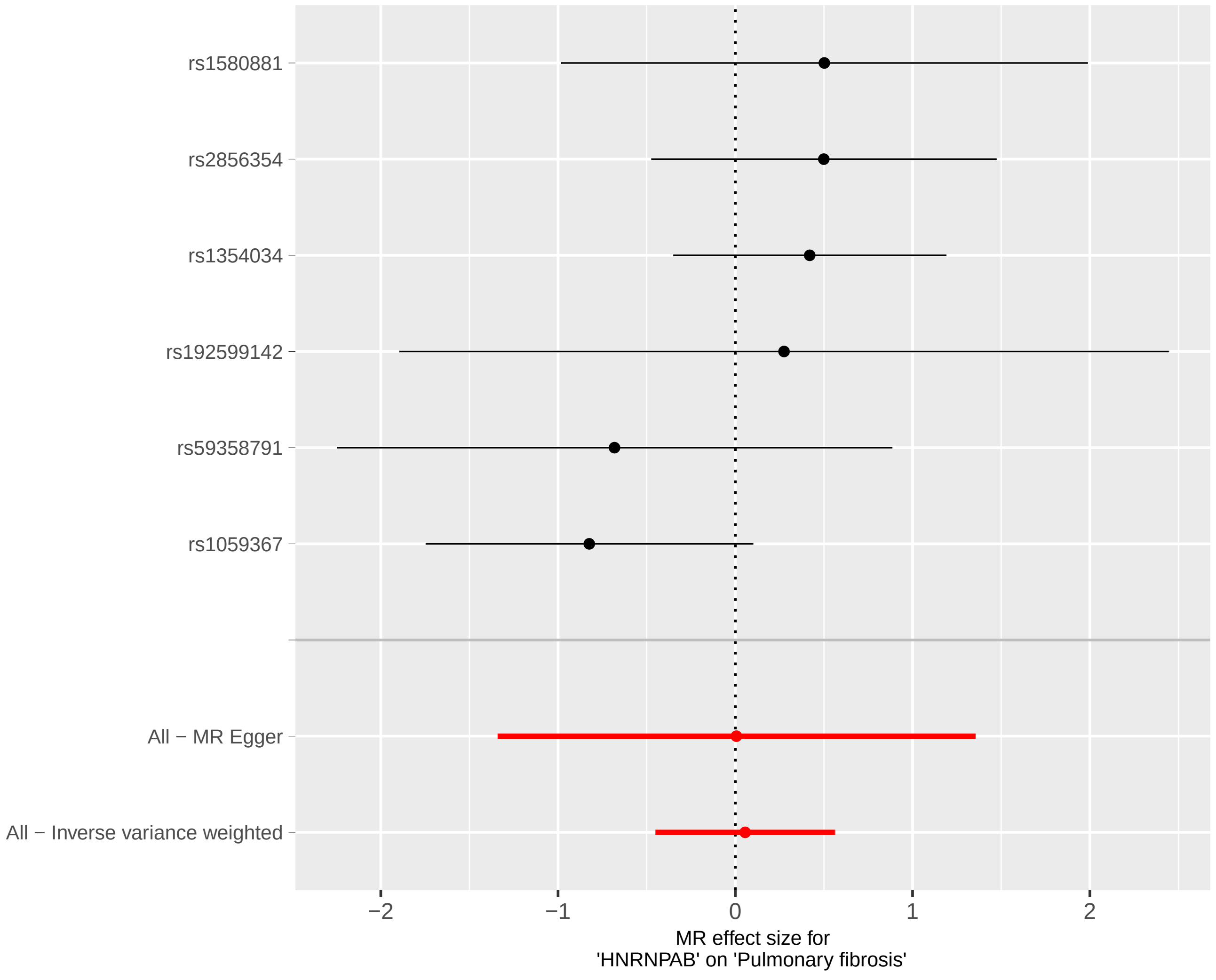
rs1580881
rs2856354
rs1354034
rs192599142
rs59358791
rs1059367
All − MR Egger
All − Inverse variance weighted
−2
−1
0
1
2
MR effect size for
'HNRNPAB' on 'Pulmonary fibrosis'

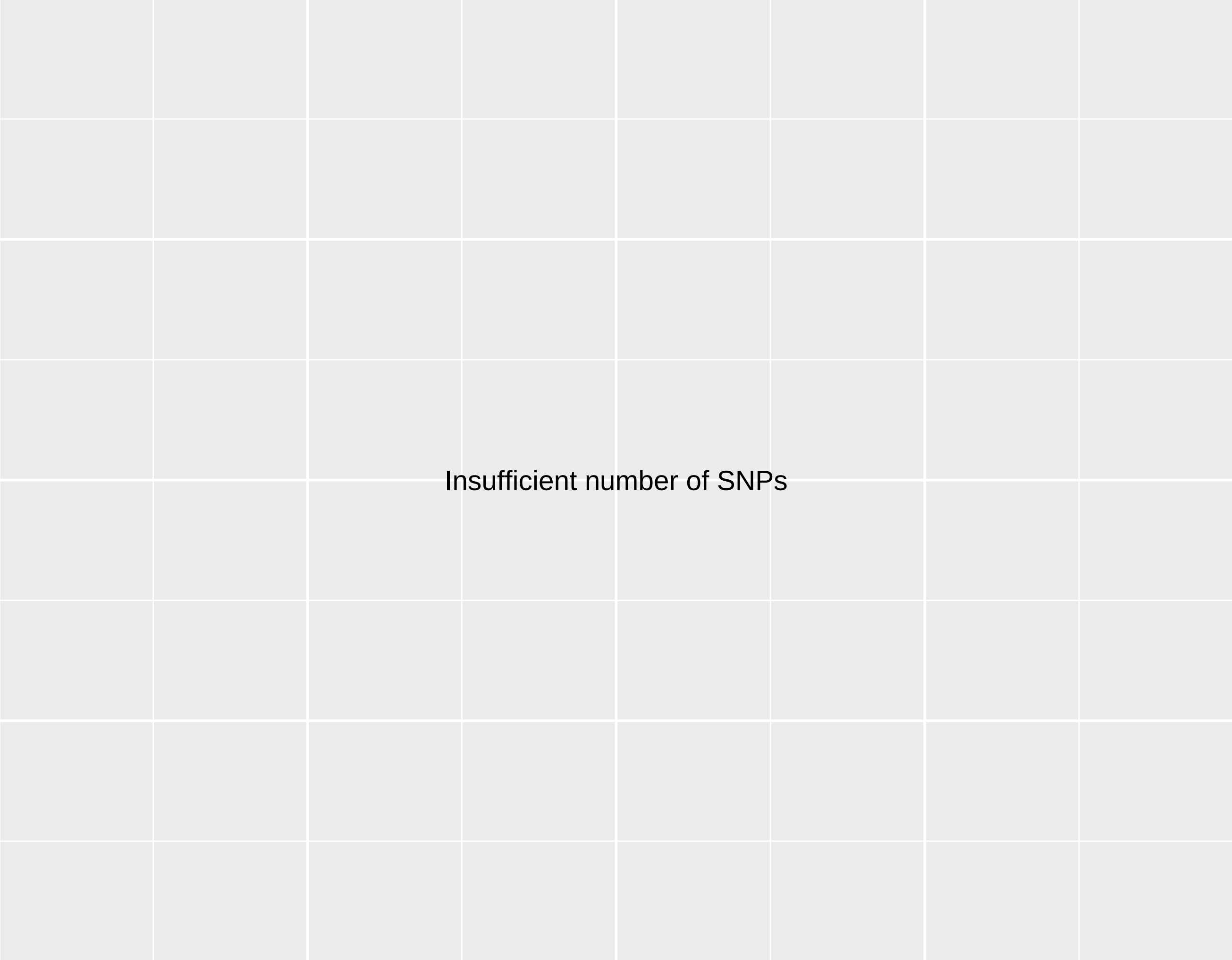
Insufficient number of SNPs

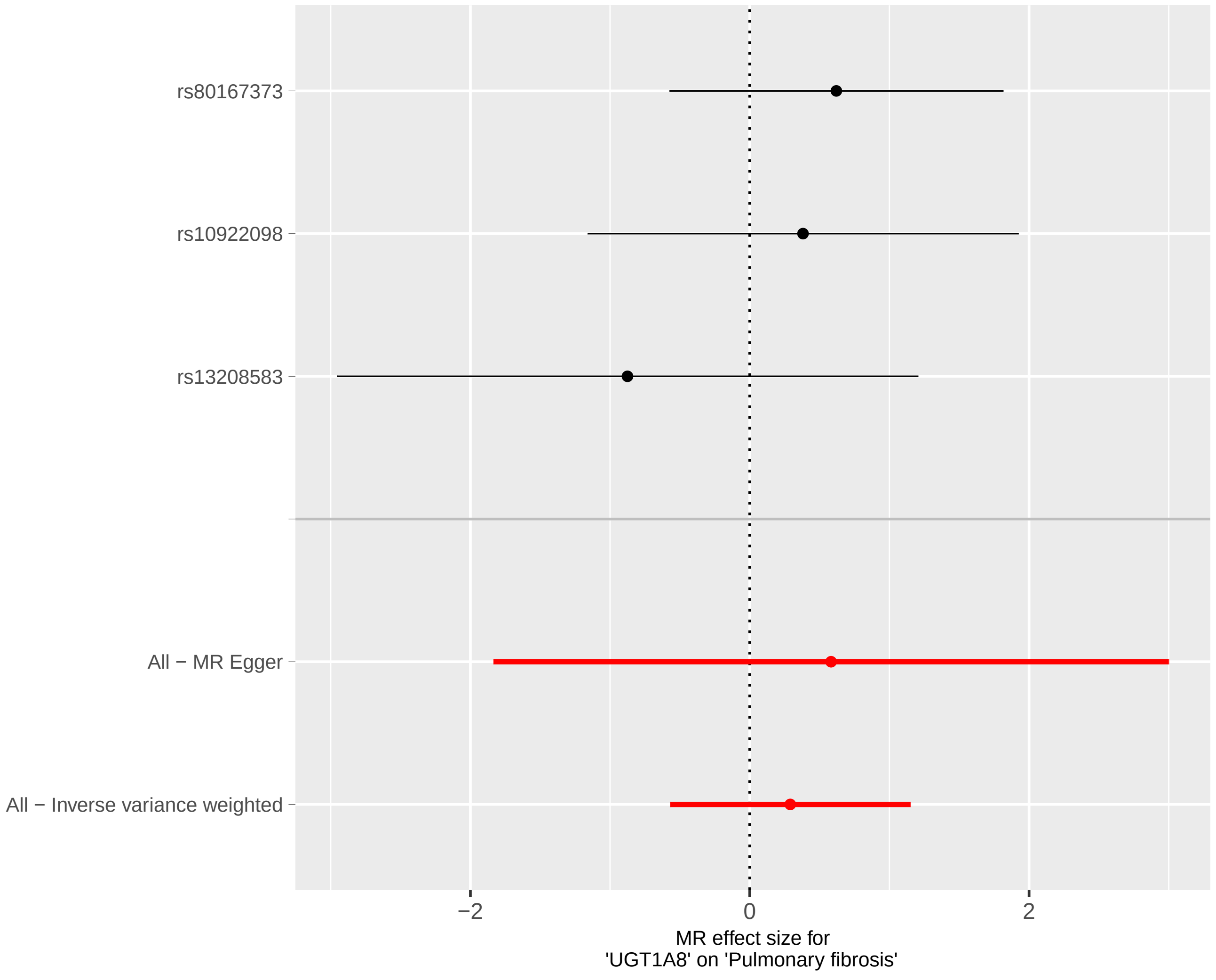
rs80167373
rs10922098
rs13208583
All − MR Egger
All − Inverse variance weighted
−2
0
2
MR effect size for
'UGT1A8' on 'Pulmonary fibrosis'

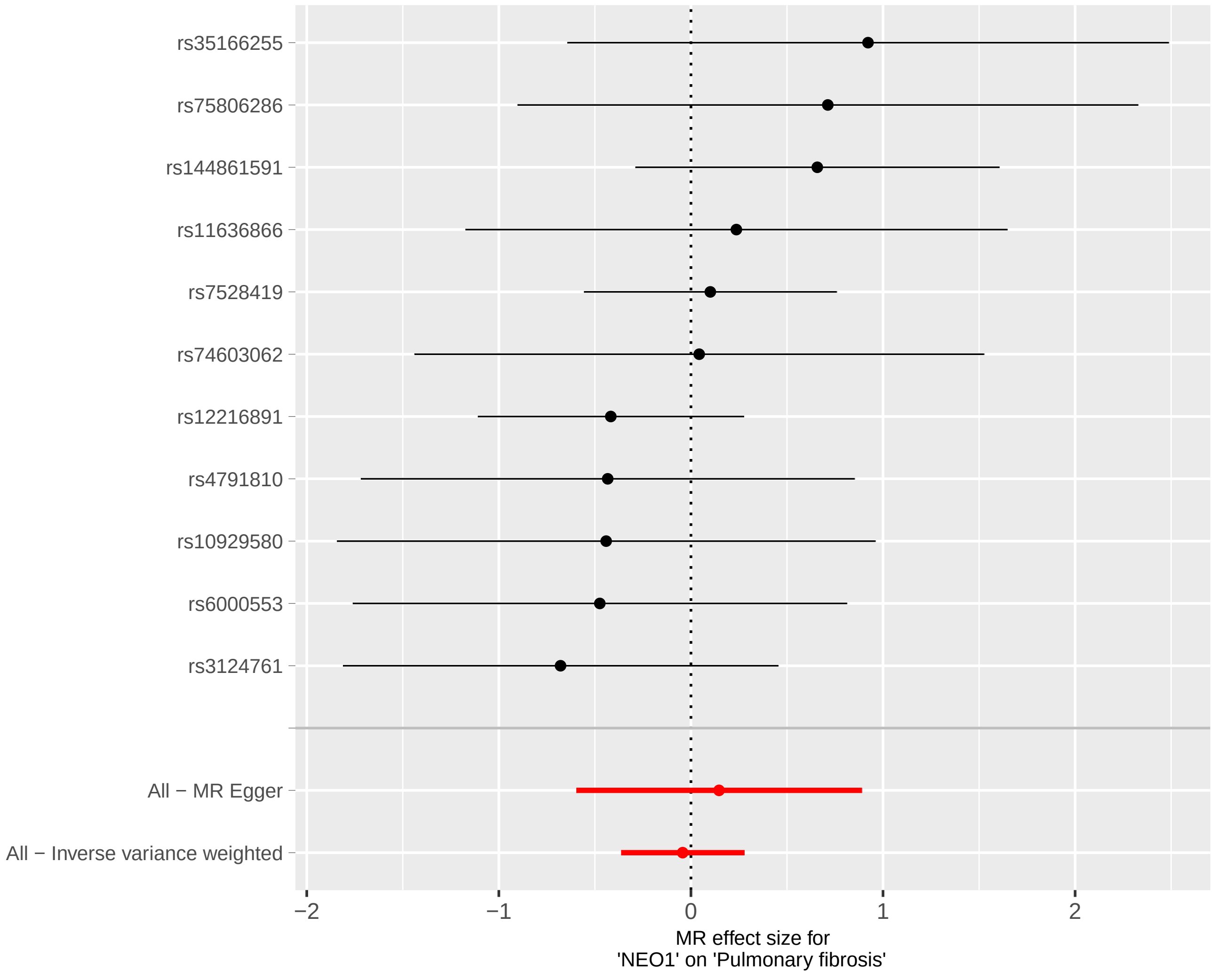
rs35166255
rs75806286
rs144861591
rs11636866
rs7528419
rs74603062
rs12216891
rs4791810
rs10929580
rs6000553
rs3124761
All − MR Egger
All − Inverse variance weighted
−2
−1
0
1
2
MR effect size for
'NEO1' on 'Pulmonary fibrosis'

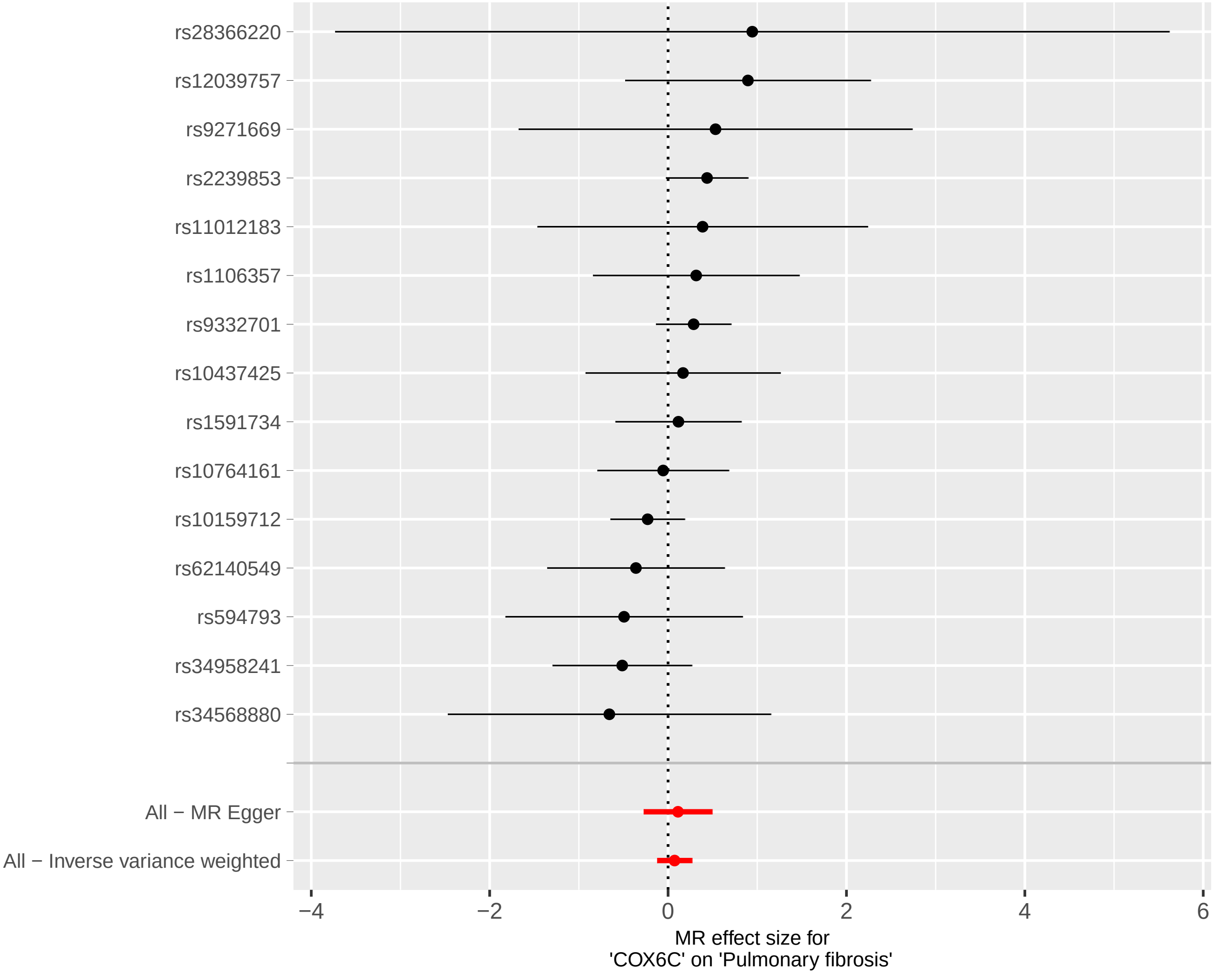

rs28366220
rs12039757
rs9271669
rs2239853
rs11012183
rs1106357
rs9332701
rs10437425
rs1591734
rs10764161
rs10159712
rs62140549
rs594793
rs34958241
rs34568880
All − MR Egger
All − Inverse variance weighted
−4
−2
0
2
4
6
MR effect size for
'COX6C' on 'Pulmonary fibrosis'

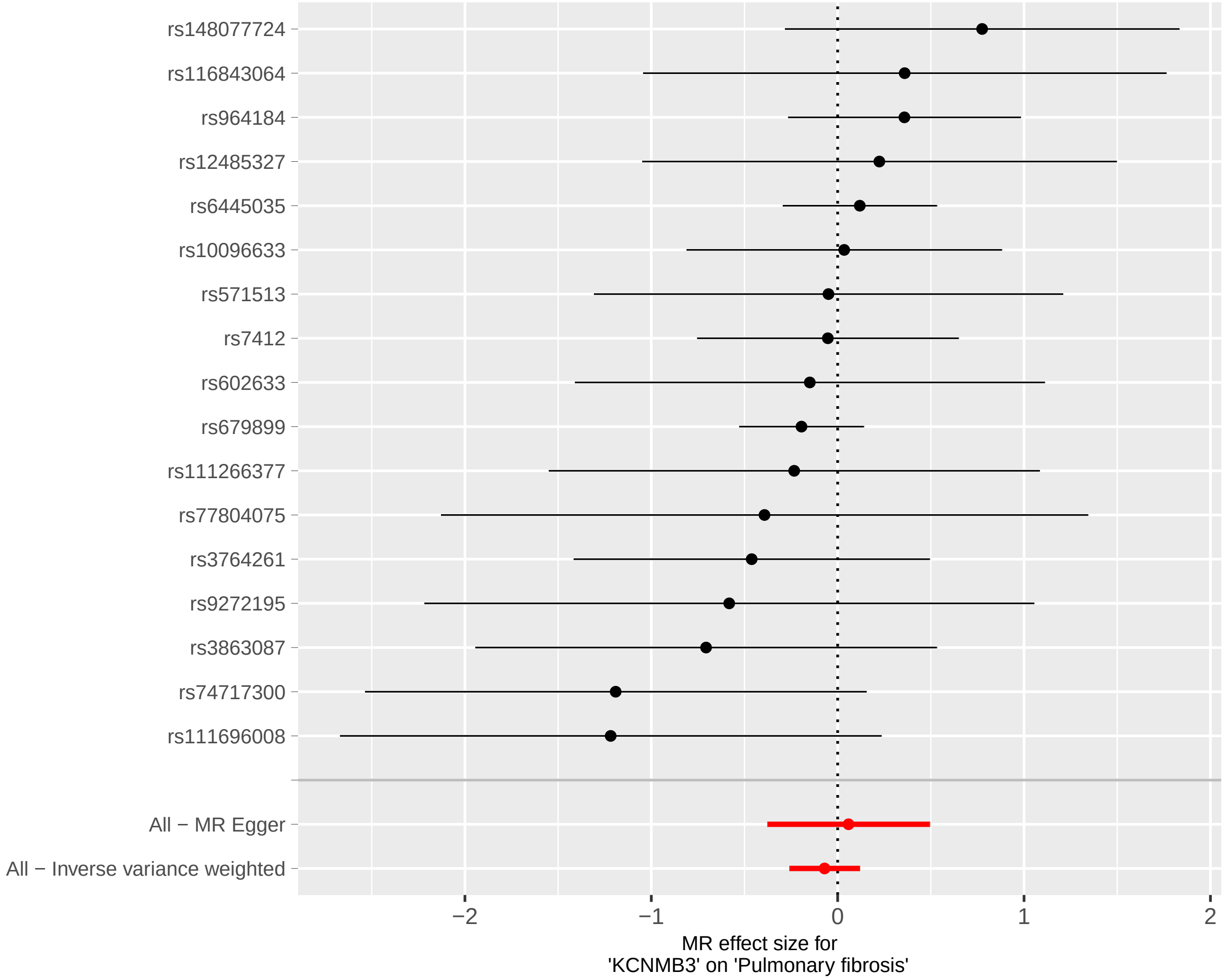
rs148077724
rs116843064
rs964184
rs12485327
rs6445035
rs10096633
rs571513
rs7412
rs602633
rs679899
rs111266377
rs77804075
rs3764261
rs9272195
rs3863087
rs74717300
rs111696008
All − MR Egger
All − Inverse variance weighted
−2
−1
0
1
2
MR effect size for
'KCNMB3' on 'Pulmonary fibrosis'

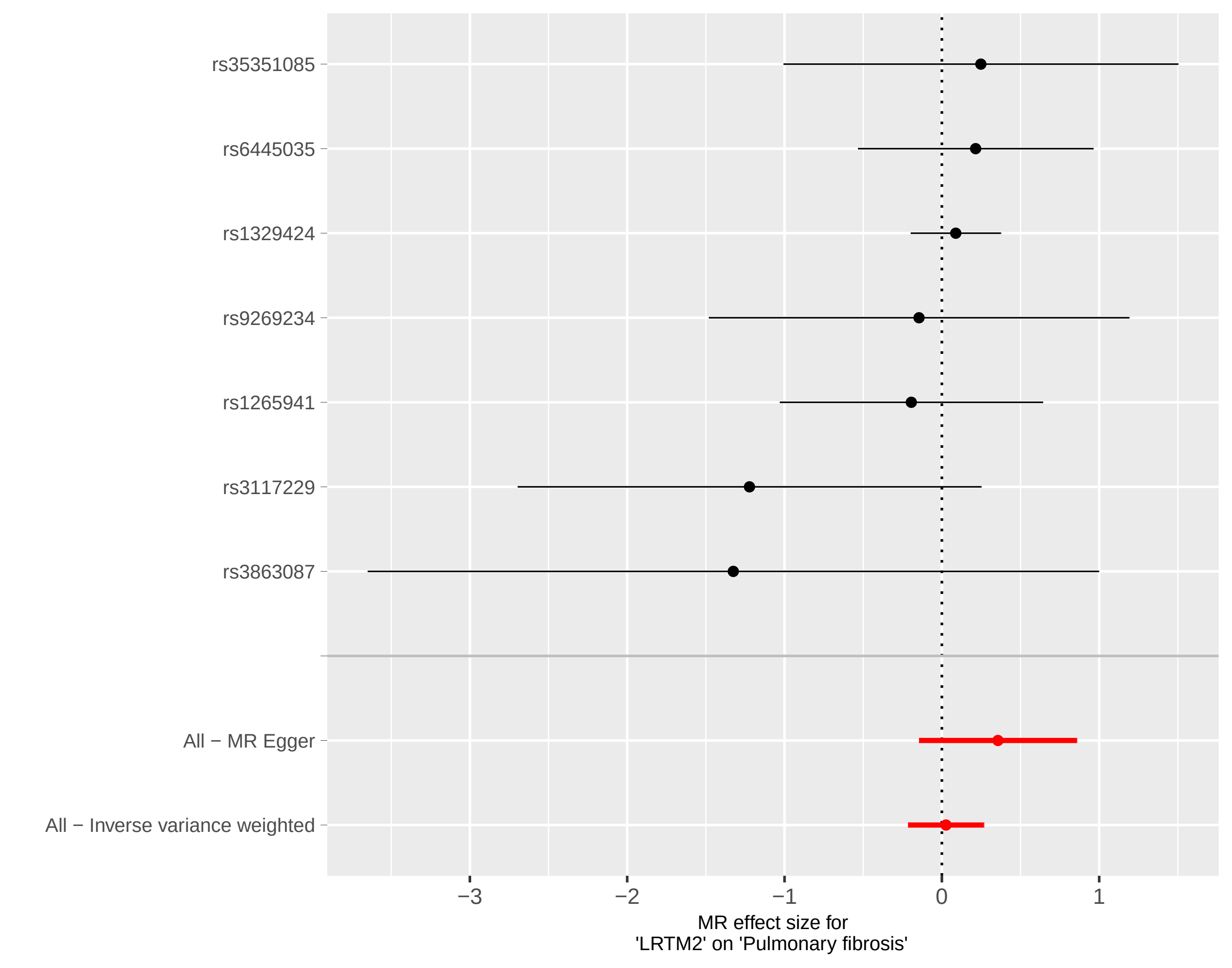
rs35351085
rs6445035
rs1329424
rs9269234
rs1265941
rs3117229
rs3863087
All − MR Egger
All − Inverse variance weighted
−3
−2
−1
0
1
MR effect size for
'LRTM2' on 'Pulmonary fibrosis'

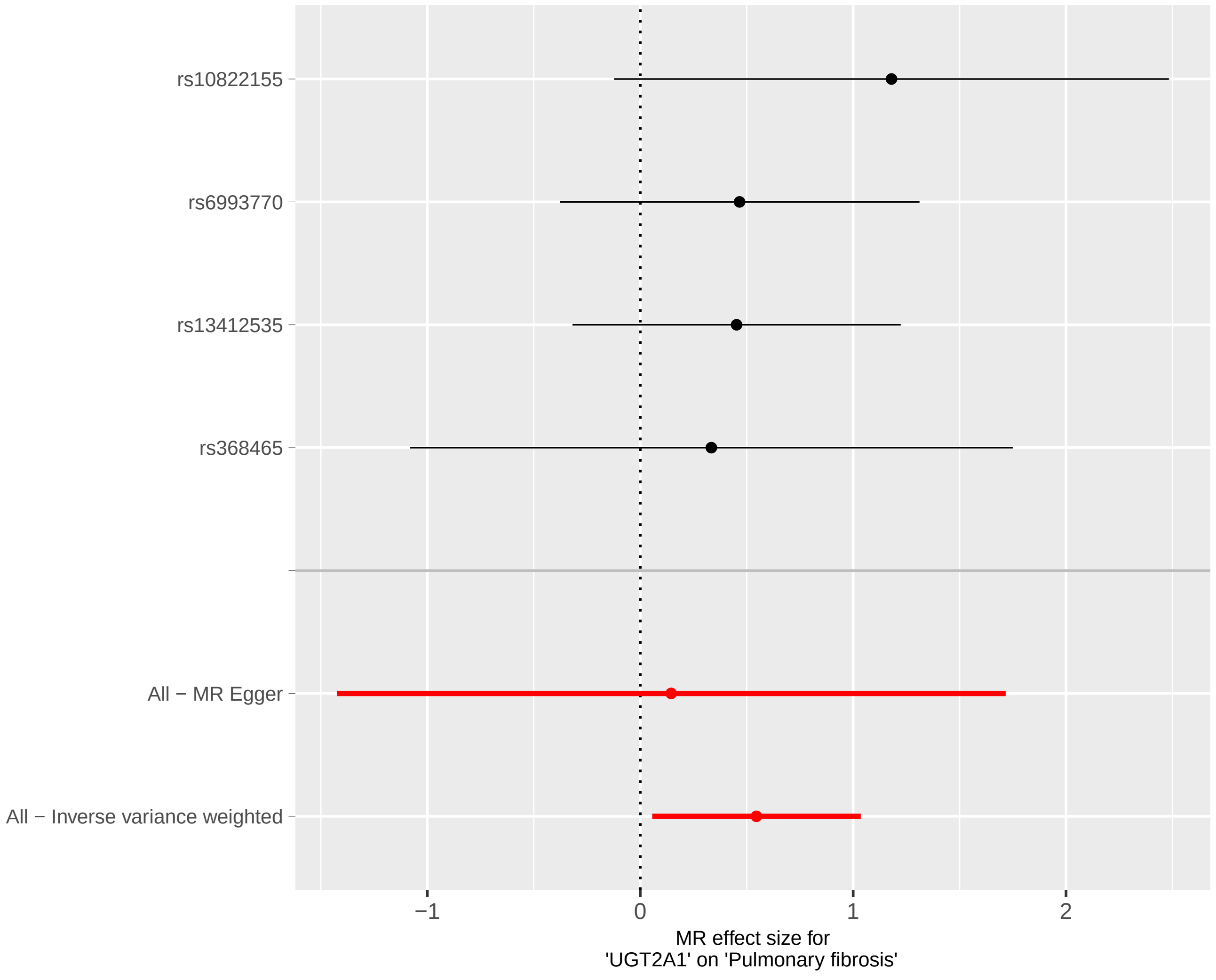

rs10822155
rs6993770
rs13412535
rs368465
All − MR Egger
All − Inverse variance weighted
−1
0
1
2
MR effect size for
'UGT2A1' on 'Pulmonary fibrosis'

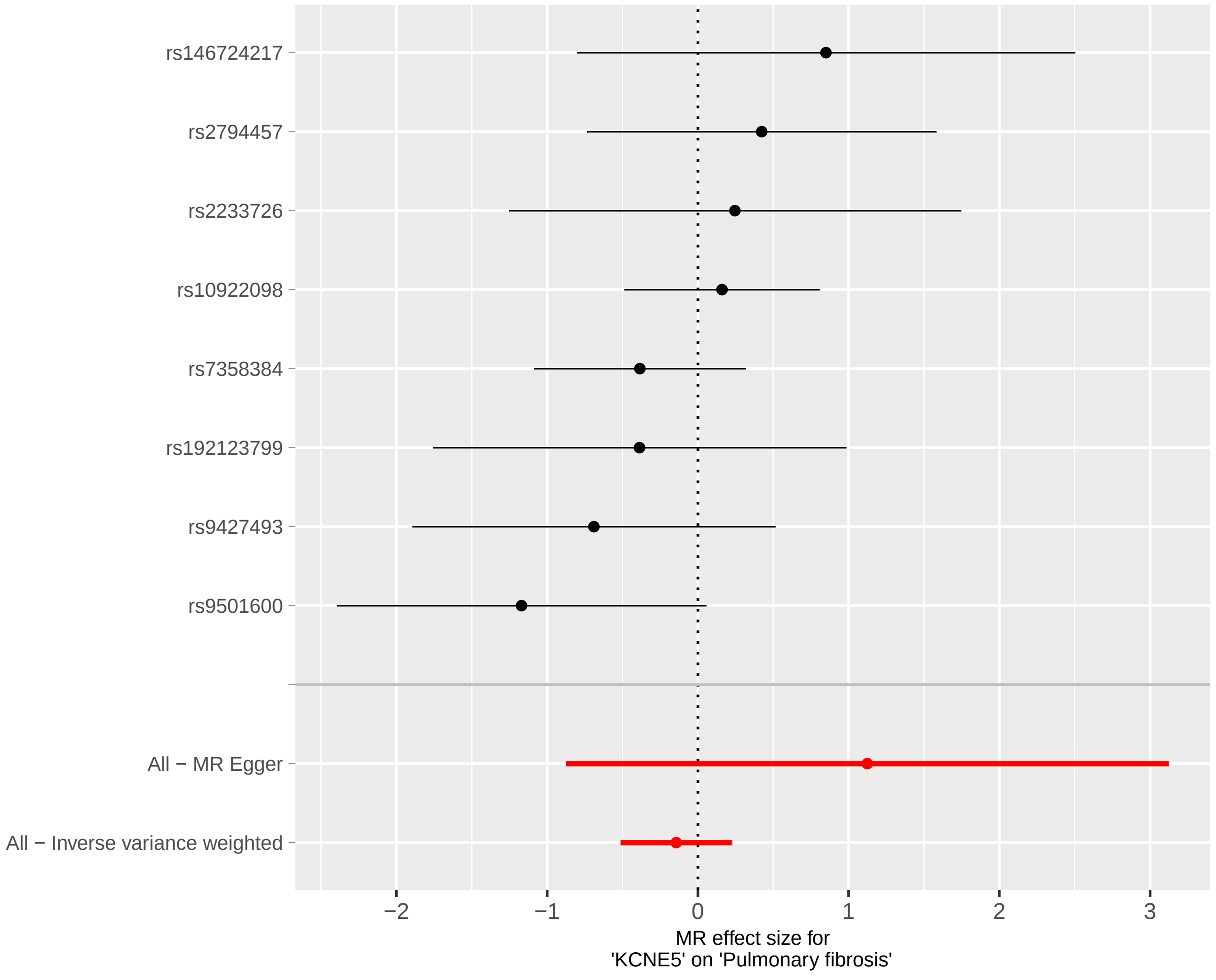
rs146724217
rs2794457
rs2233726
rs10922098
rs7358384
rs192123799
rs9427493
rs9501600
All − MR Egger
All − Inverse variance weighted
−2
−1
0
1
2
3
MR effect size for
'KCNE5' on 'Pulmonary fibrosis'

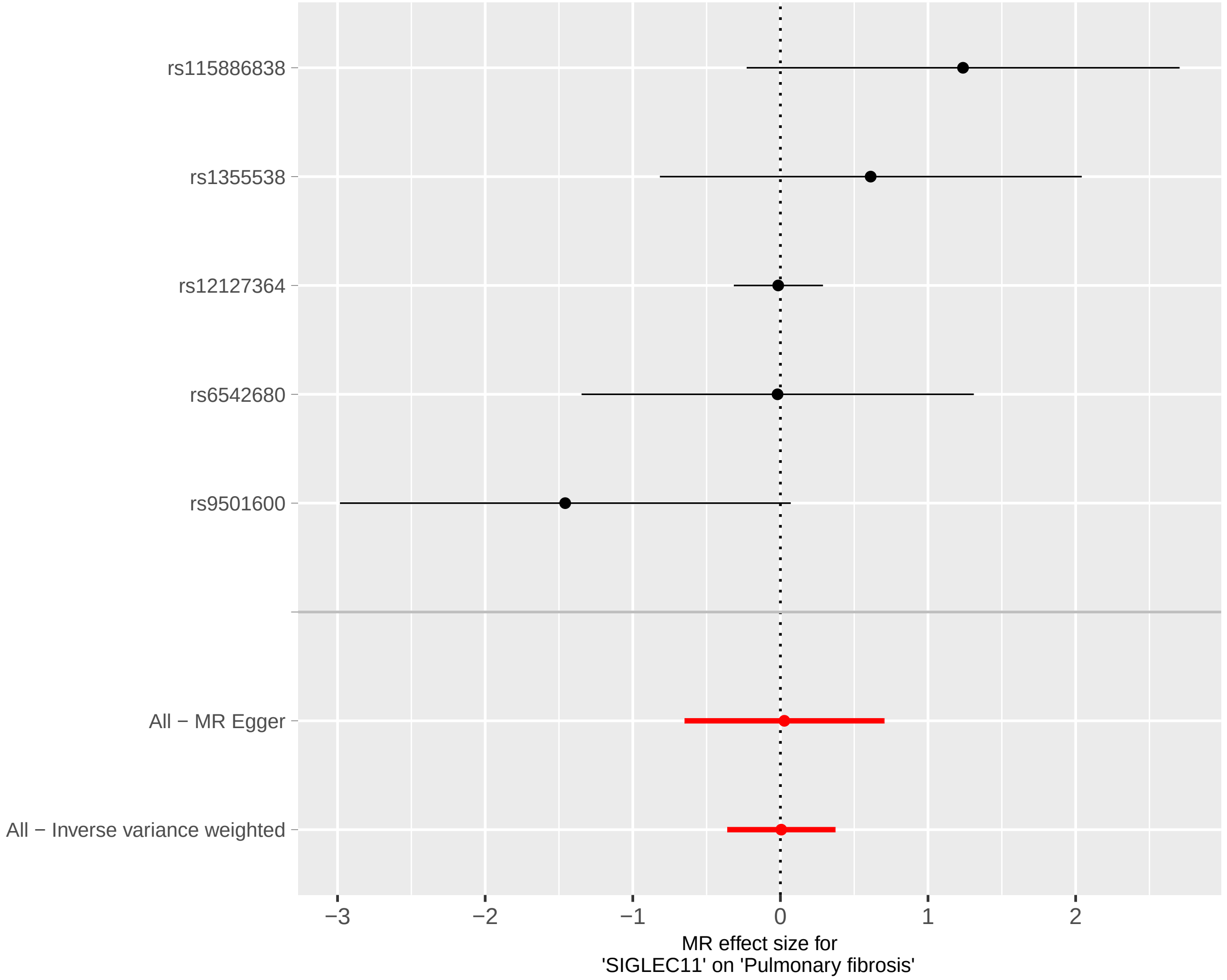
rs115886838
rs1355538
rs12127364
rs6542680
rs9501600
All − MR Egger
All − Inverse variance weighted
−3
−2
−1
0
1
2
MR effect size for
'SIGLEC11' on 'Pulmonary fibrosis'

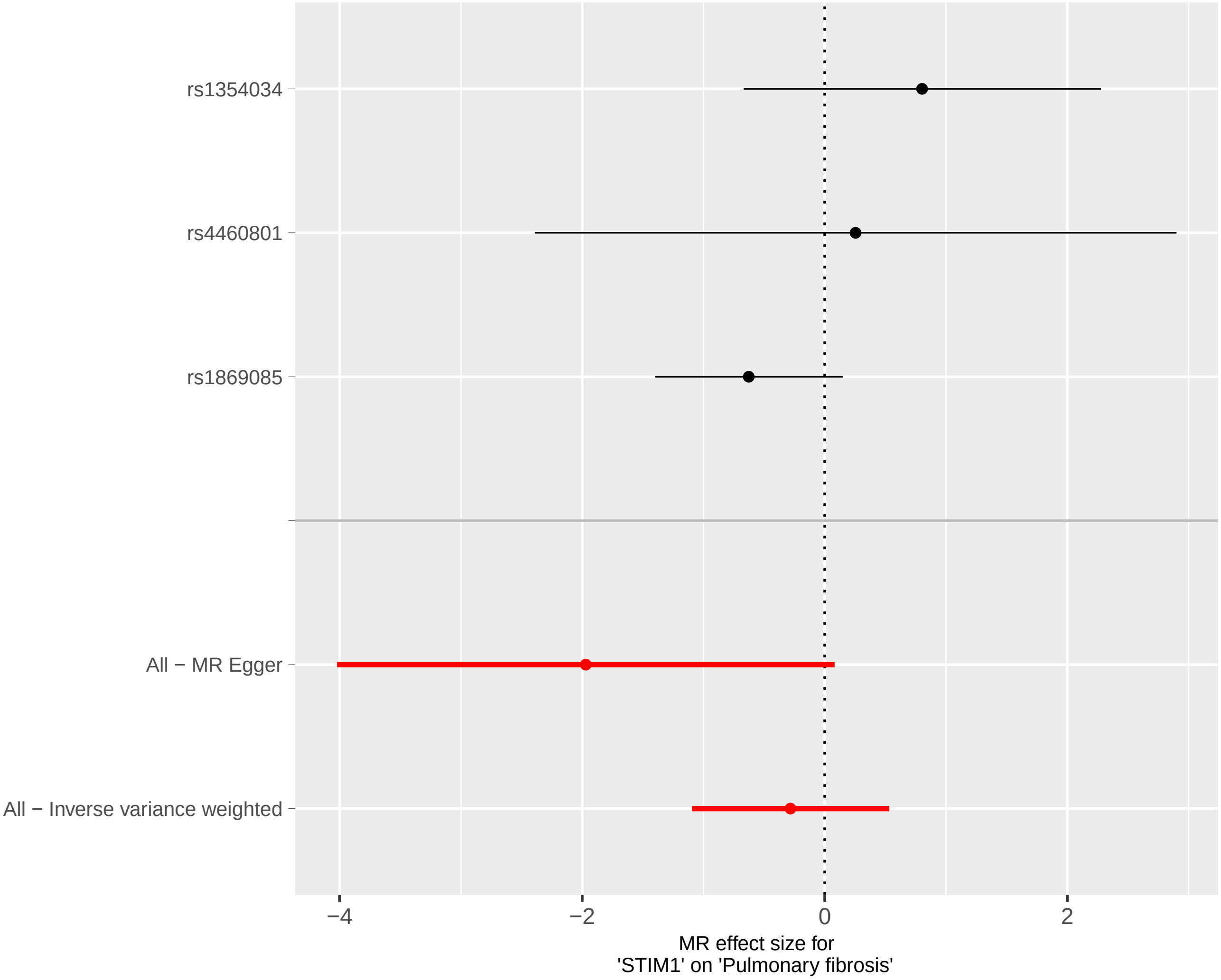
rs1354034
rs4460801
rs1869085
All − MR Egger
All − Inverse variance weighted
−4
−2
0
2
MR effect size for
'STIM1' on 'Pulmonary fibrosis'

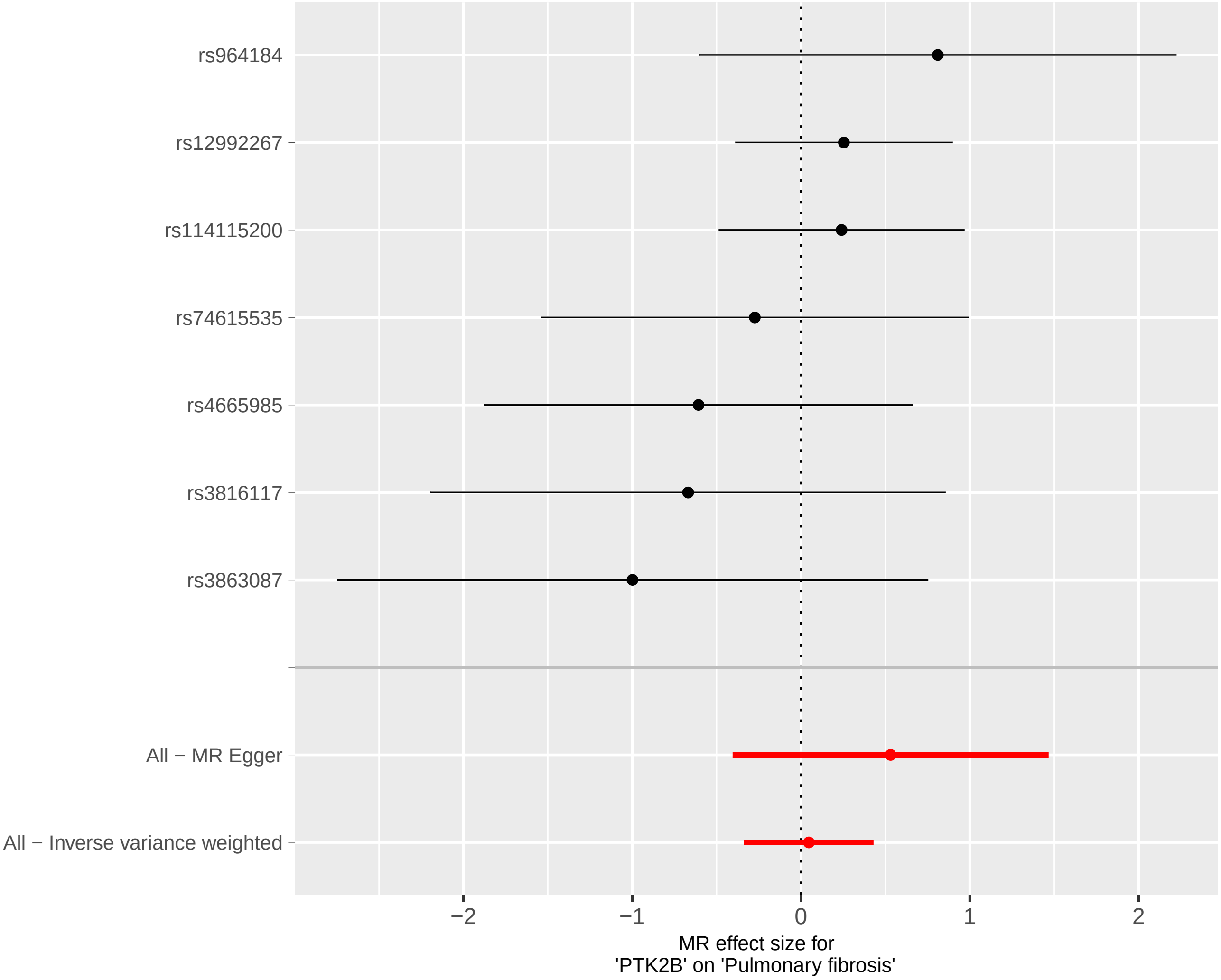

rs964184
rs12992267
rs114115200
rs74615535
rs4665985
rs3816117
rs3863087
All − MR Egger
All − Inverse variance weighted
−2
−1
0
1
2
MR effect size for
'PTK2B' on 'Pulmonary fibrosis'

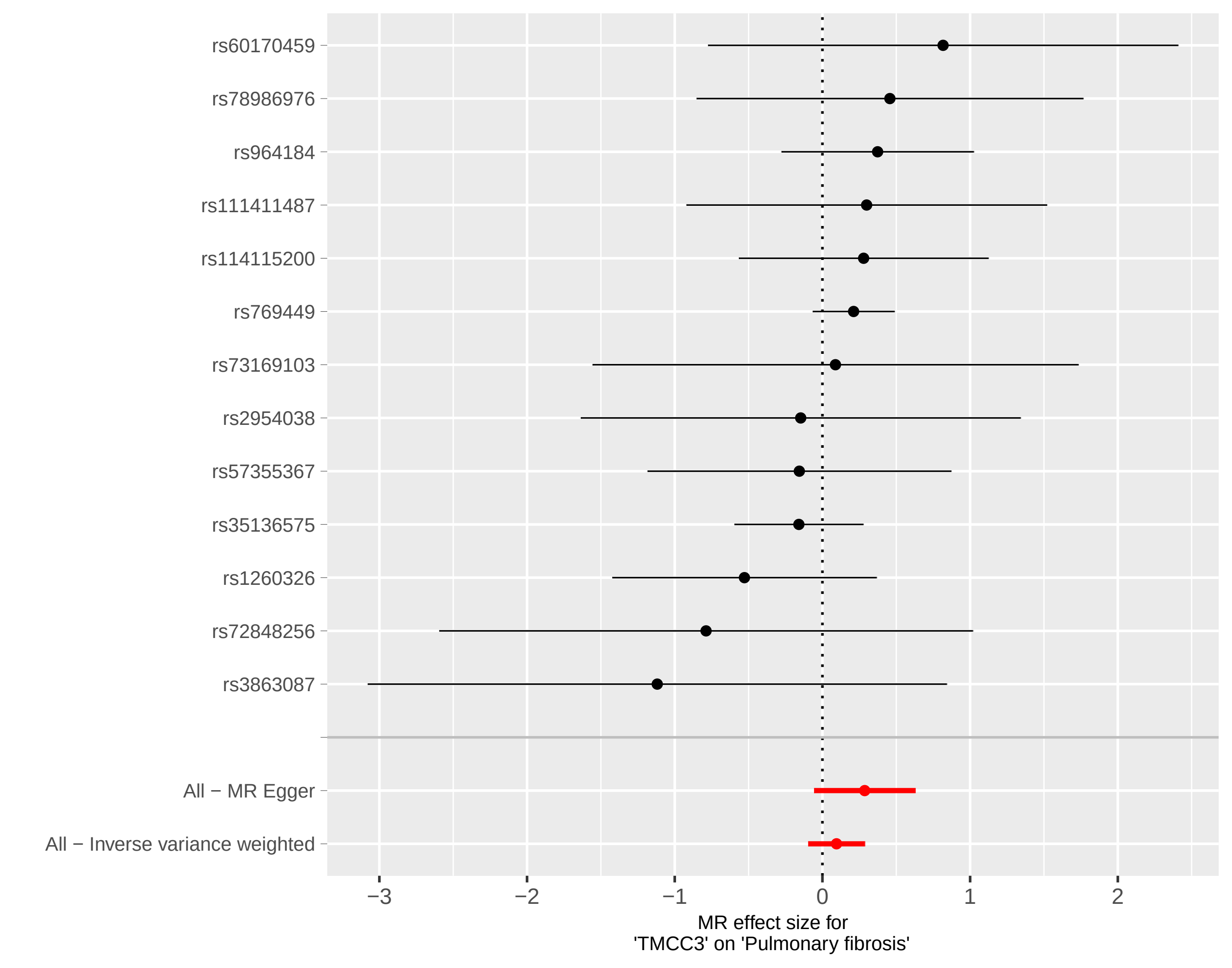
rs60170459
rs78986976
rs964184
rs111411487
rs114115200
rs769449
rs73169103
rs2954038
rs57355367
rs35136575
rs1260326
rs72848256
rs3863087
All − MR Egger
All − Inverse variance weighted
−3
−2
−1
0
1
2
MR effect size for
'TMCC3' on 'Pulmonary fibrosis'

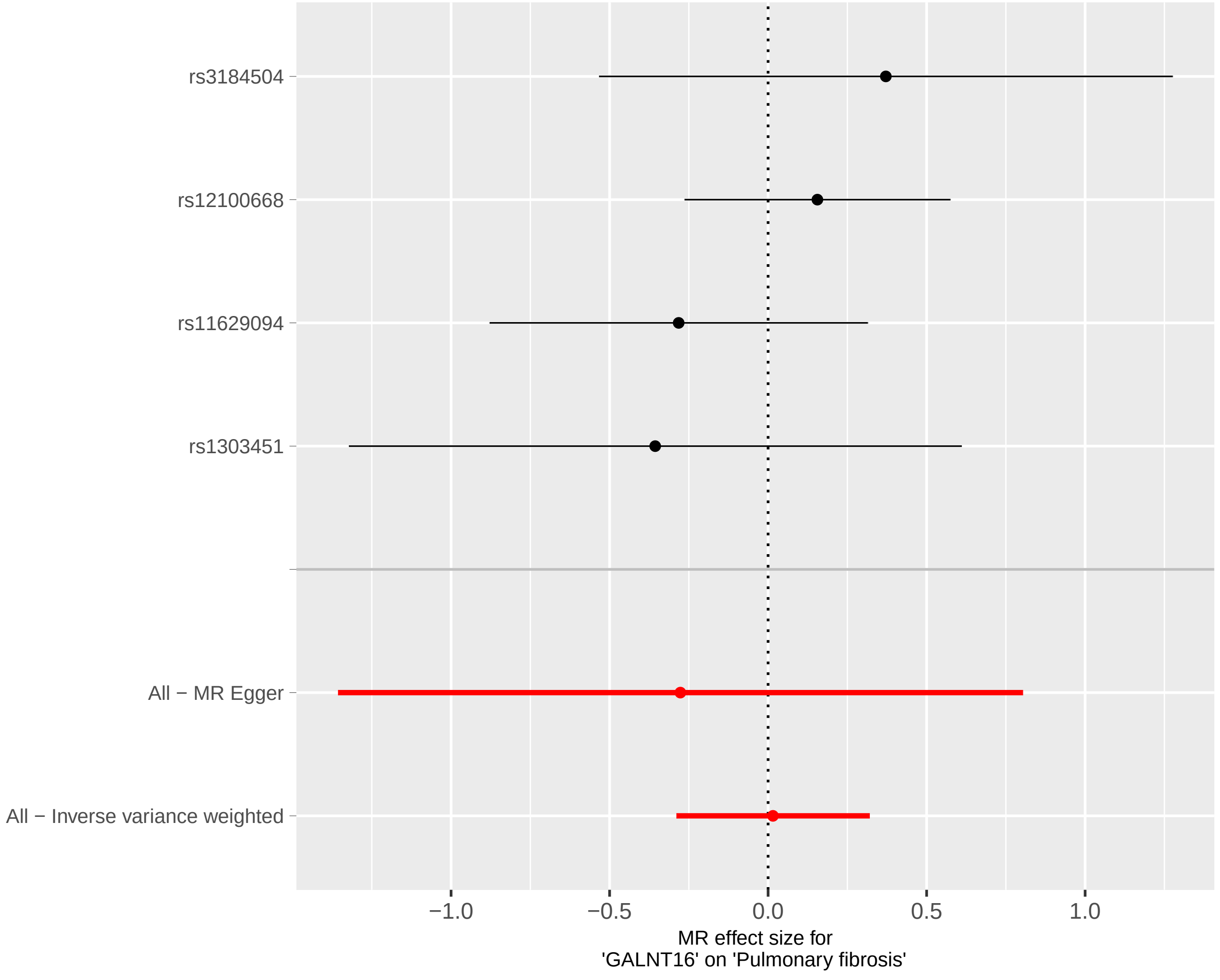

rs3184504
rs12100668
rs11629094
rs1303451
All − MR Egger
All − Inverse variance weighted
−1.0
−0.5
0.0
0.5
1.0
MR effect size for
'GALNT16' on 'Pulmonary fibrosis'

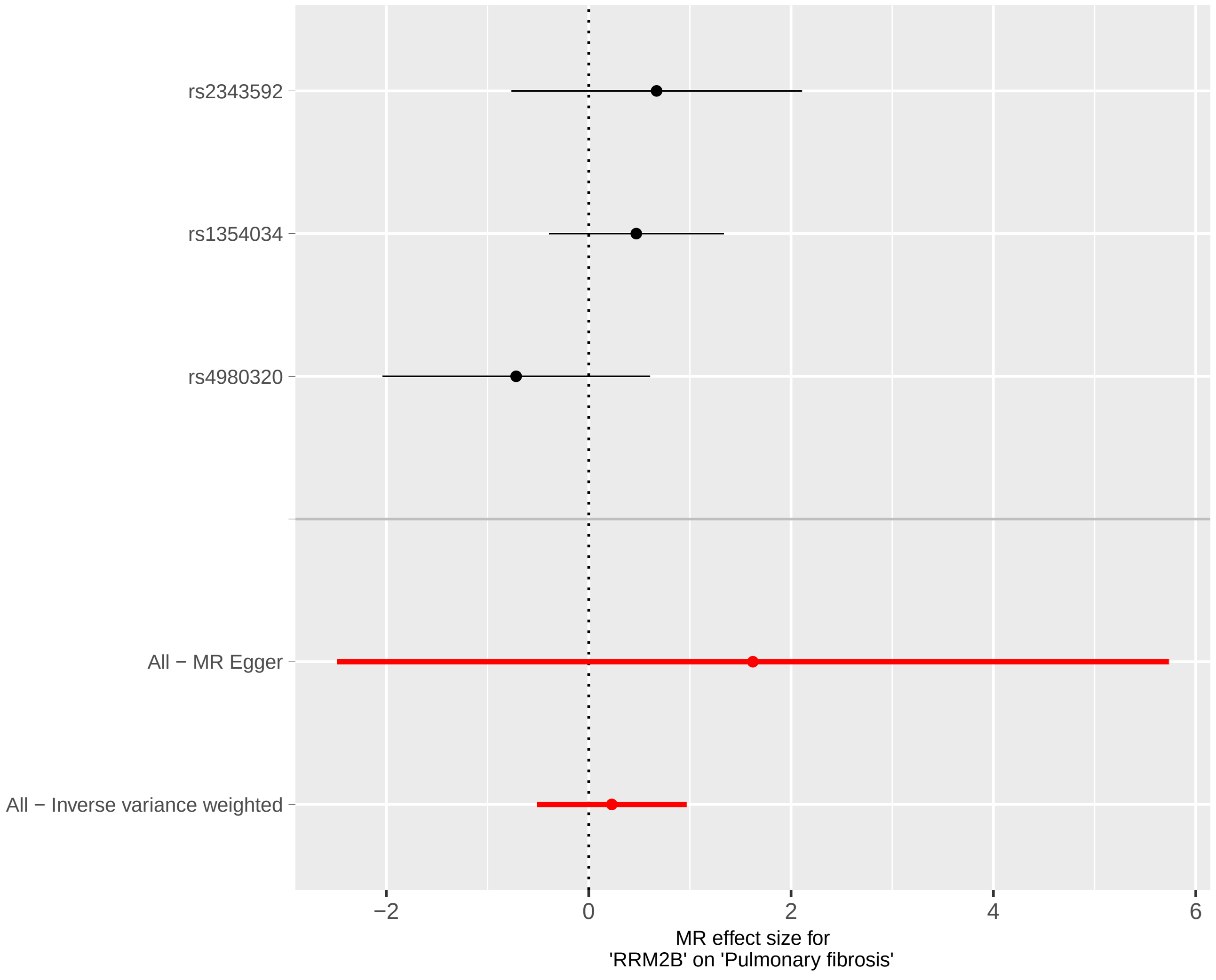

rs2343592
rs1354034
rs4980320
All − MR Egger
All − Inverse variance weighted
−2
0
2
4
6
MR effect size for
'RRM2B' on 'Pulmonary fibrosis'

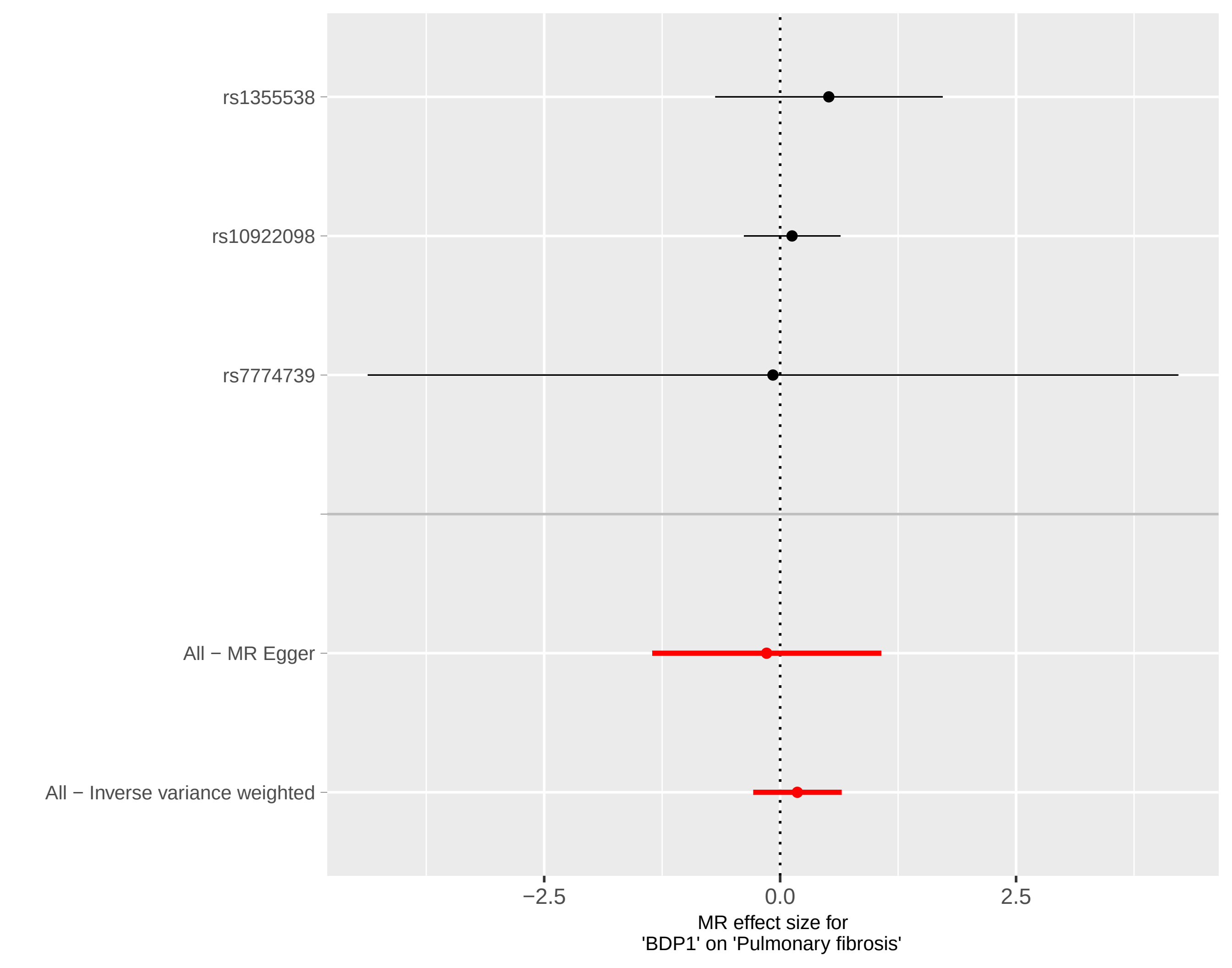
rs1355538
rs10922098
rs7774739
All − MR Egger
All − Inverse variance weighted
−2.5
0.0
2.5
MR effect size for
'BDP1' on 'Pulmonary fibrosis'

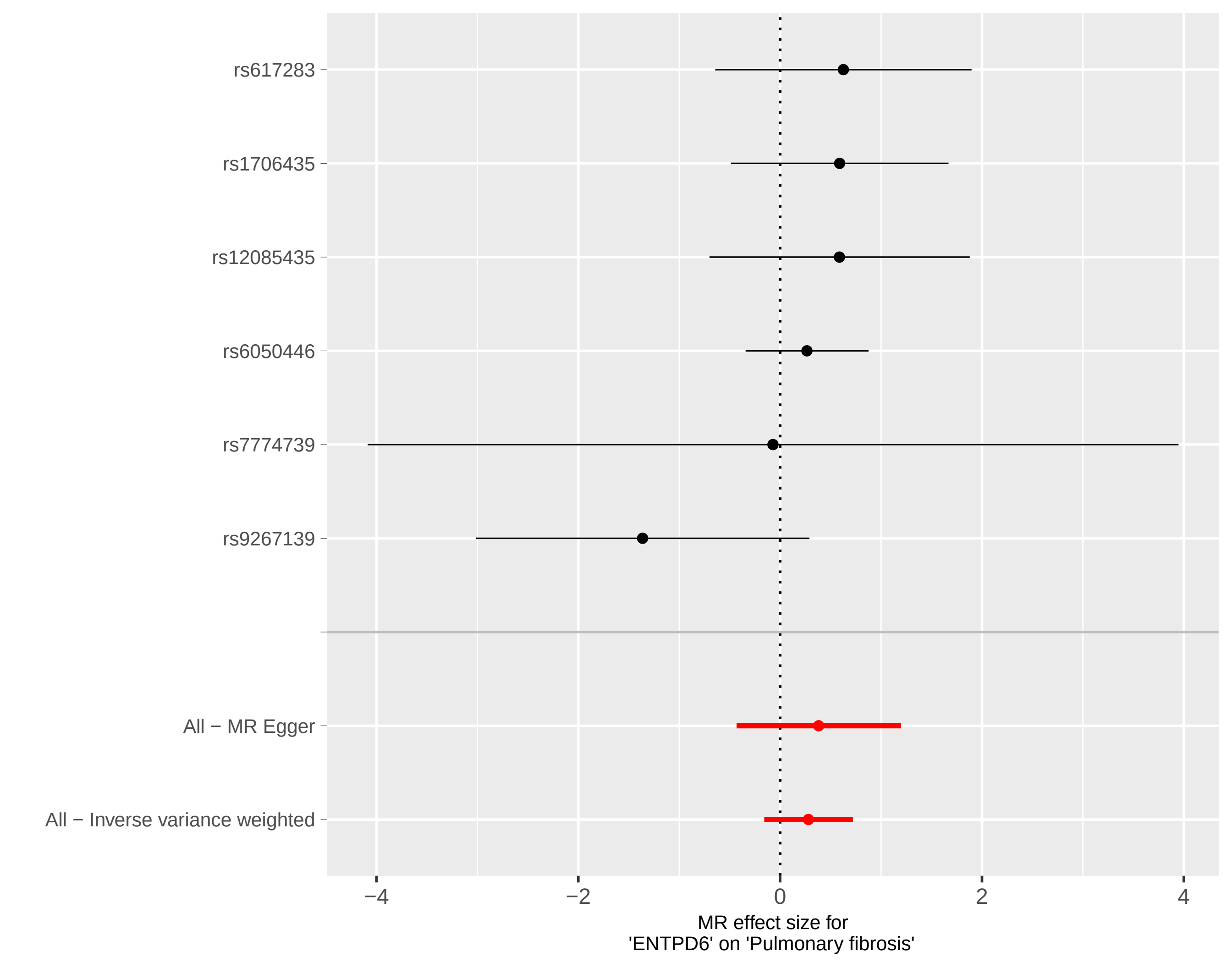
rs617283
rs1706435
rs12085435
rs6050446
rs7774739
rs9267139
All − MR Egger
All − Inverse variance weighted
−4
−2
0
2
4
MR effect size for
'ENTPD6' on 'Pulmonary fibrosis'

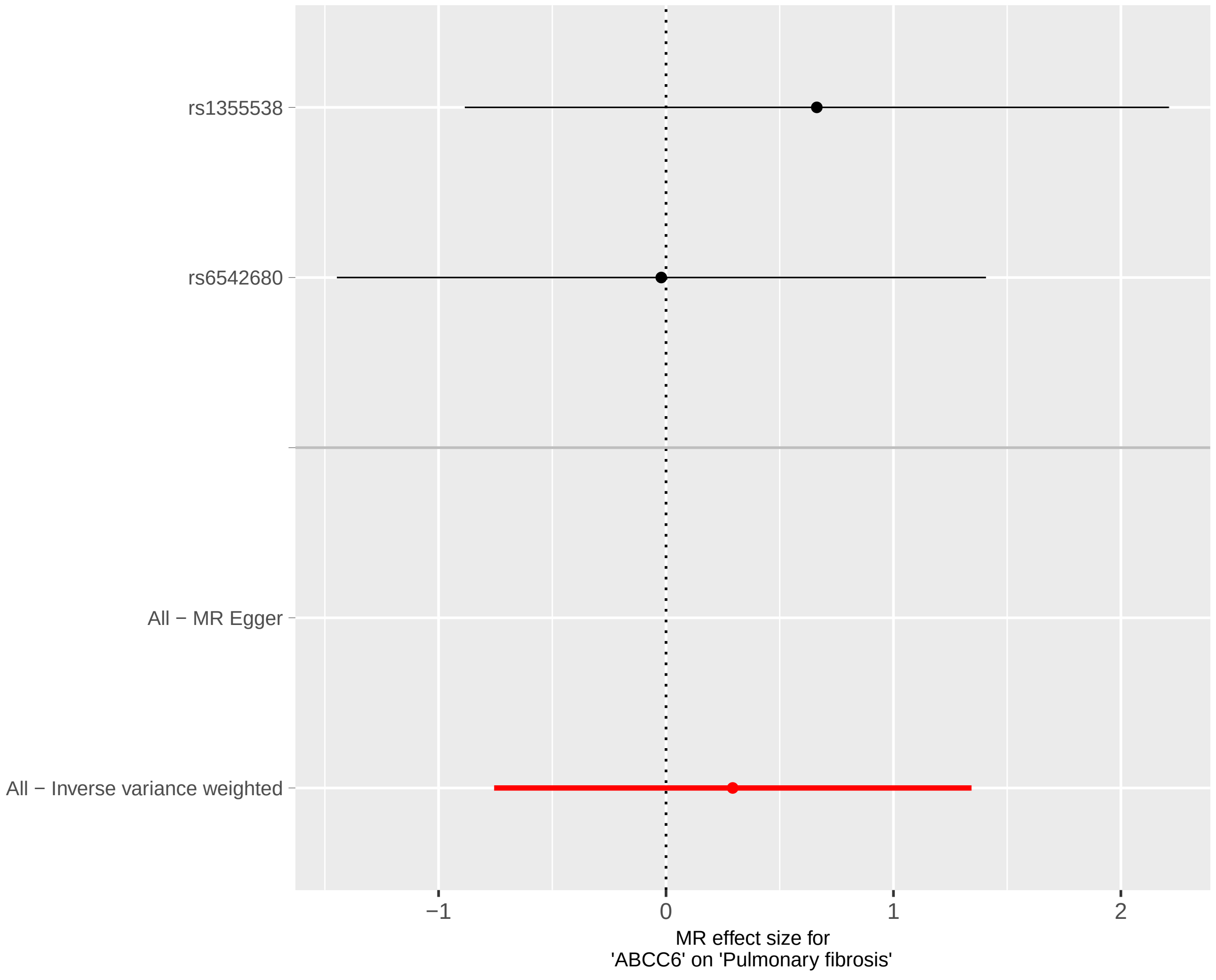

rs1355538
rs6542680
All − MR Egger
All − Inverse variance weighted
−1
0
1
2
MR effect size for
'ABCC6' on 'Pulmonary fibrosis'

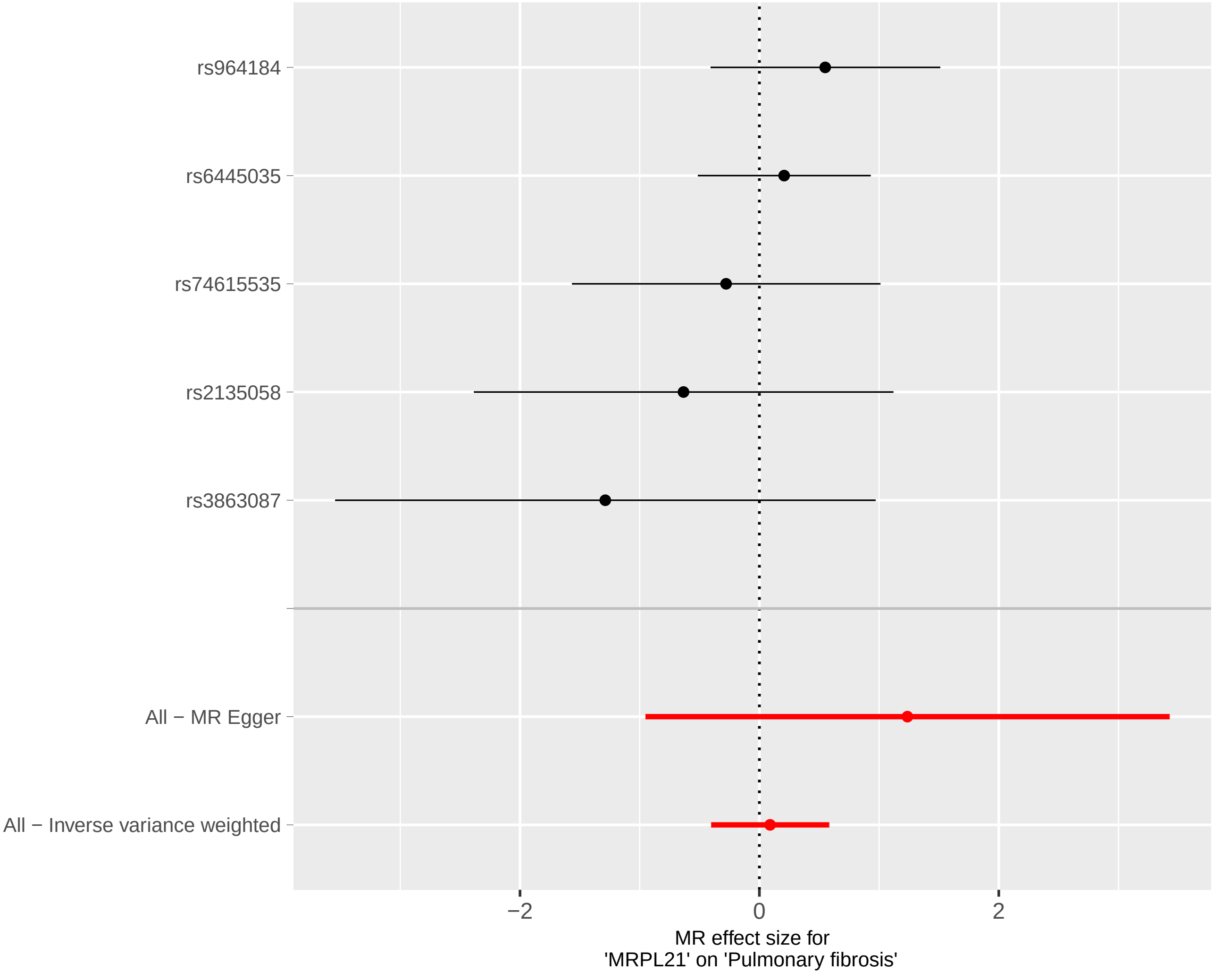
rs964184
rs6445035
rs74615535
rs2135058
rs3863087
All − MR Egger
All − Inverse variance weighted
−2
0
2
MR effect size for
'MRPL21' on 'Pulmonary fibrosis'

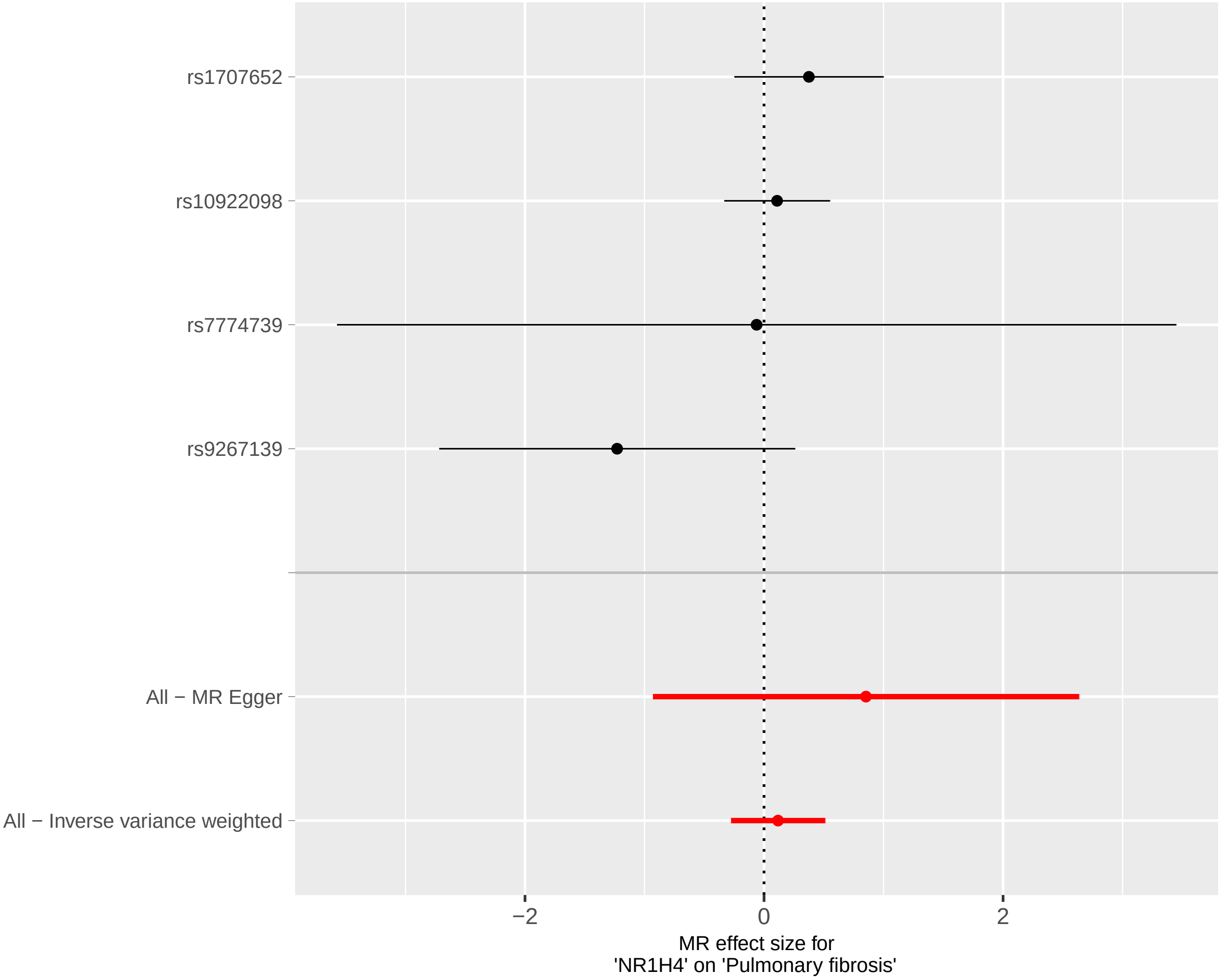
rs1707652
rs10922098
rs7774739
rs9267139
All − MR Egger
All − Inverse variance weighted
−2
0
2
MR effect size for
'NR1H4' on 'Pulmonary fibrosis'

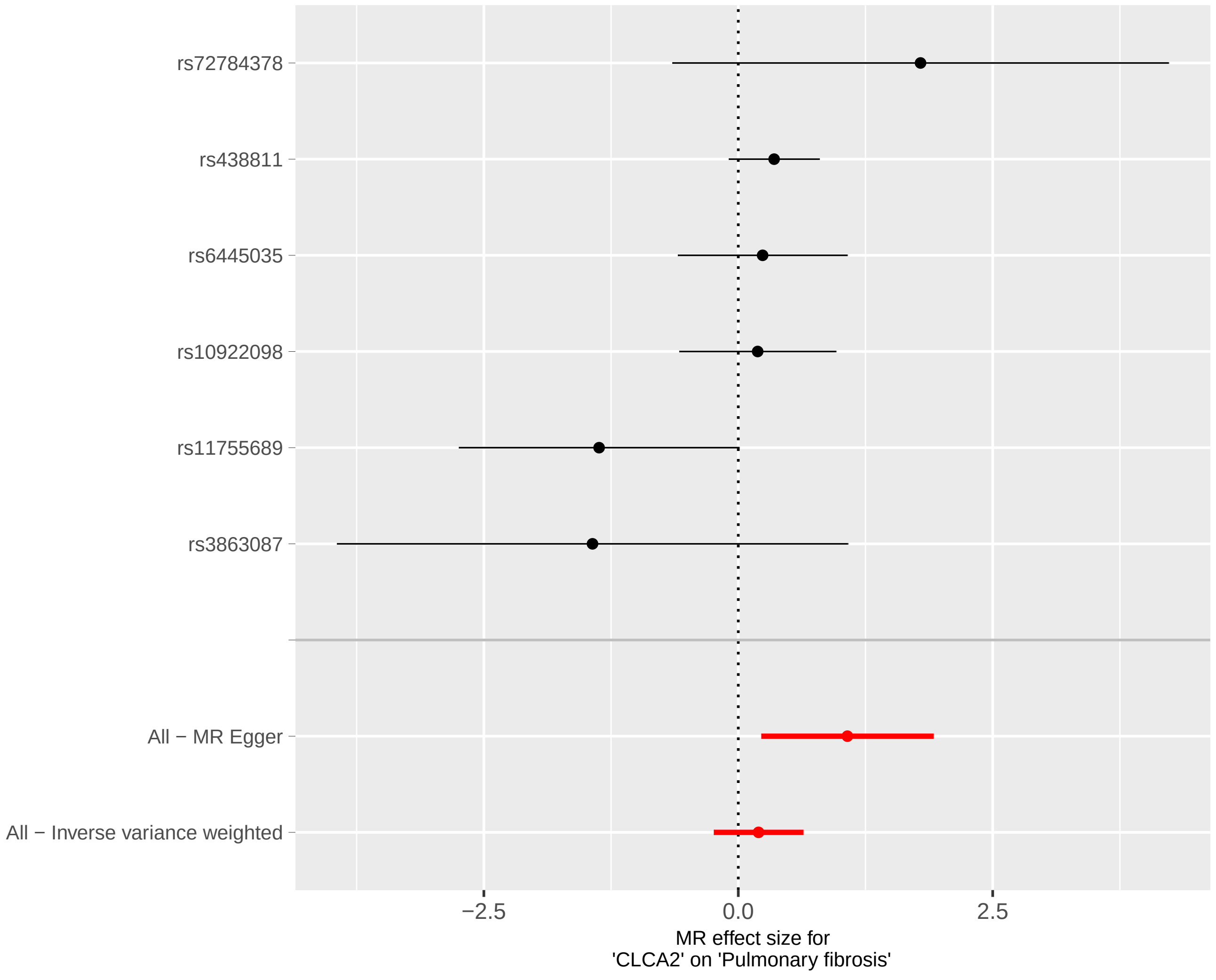
rs72784378
rs438811
rs6445035
rs10922098
rs11755689
rs3863087
All − MR Egger
All − Inverse variance weighted
−2.5
0.0
2.5
MR effect size for
'CLCA2' on 'Pulmonary fibrosis'

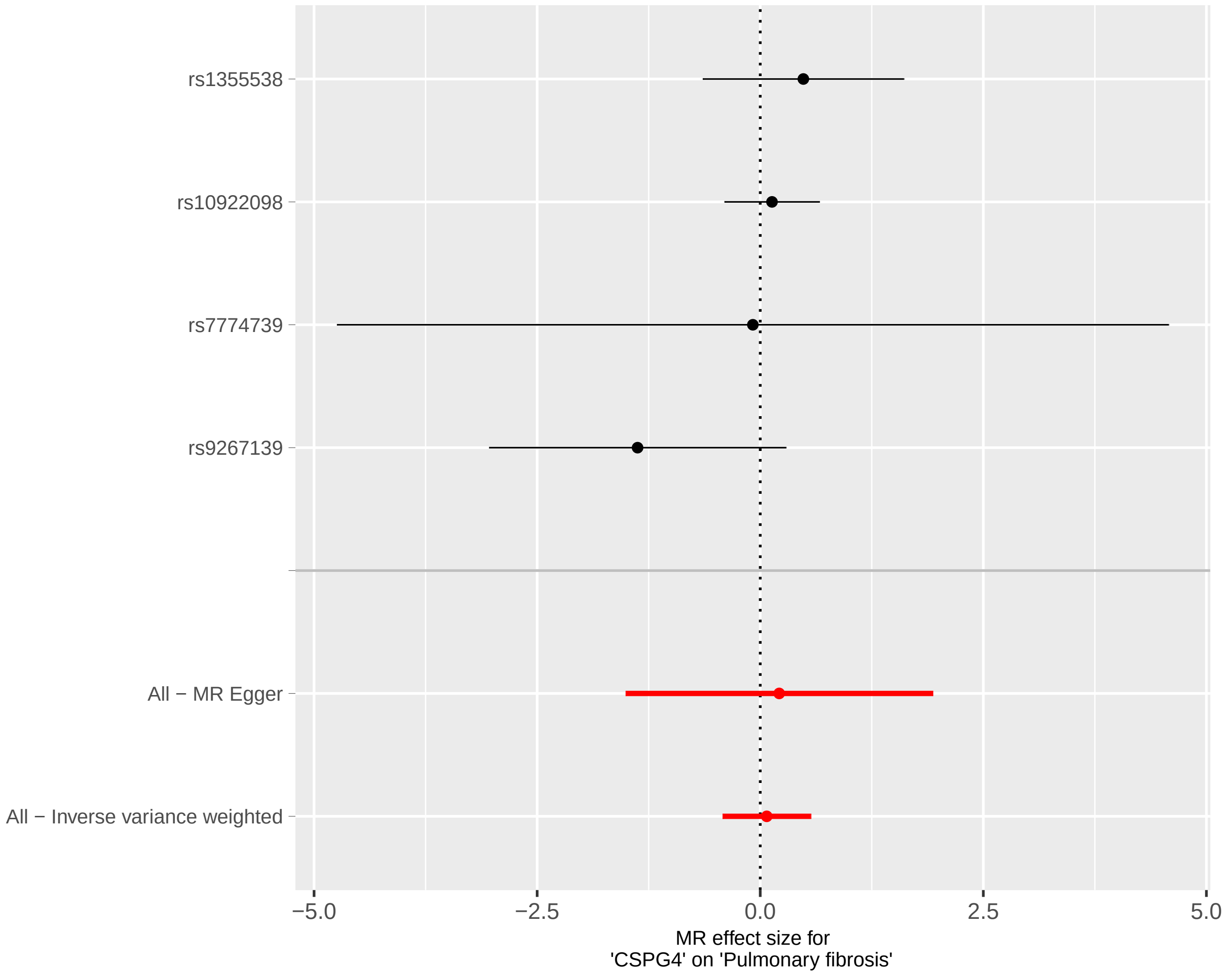
rs1355538
rs10922098
rs7774739
rs9267139
All − MR Egger
All − Inverse variance weighted
−5.0
−2.5
0.0
2.5
5.0
MR effect size for
'CSPG4' on 'Pulmonary fibrosis'

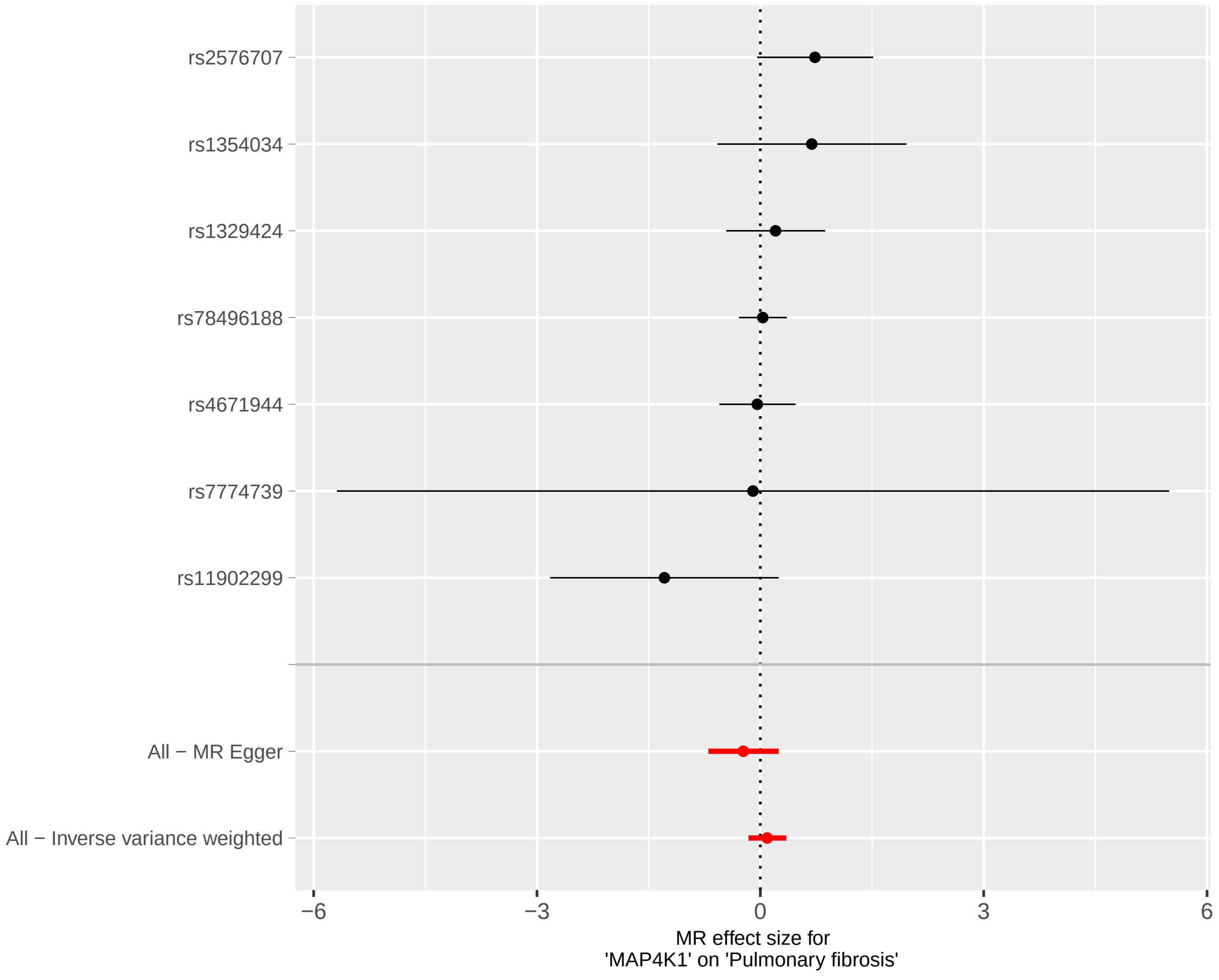
rs2576707
rs1354034
rs1329424
rs78496188
rs4671944
rs7774739
rs11902299
All − MR Egger
All − Inverse variance weighted
−6
−3
0
3
6
MR effect size for
'MAP4K1' on 'Pulmonary fibrosis'

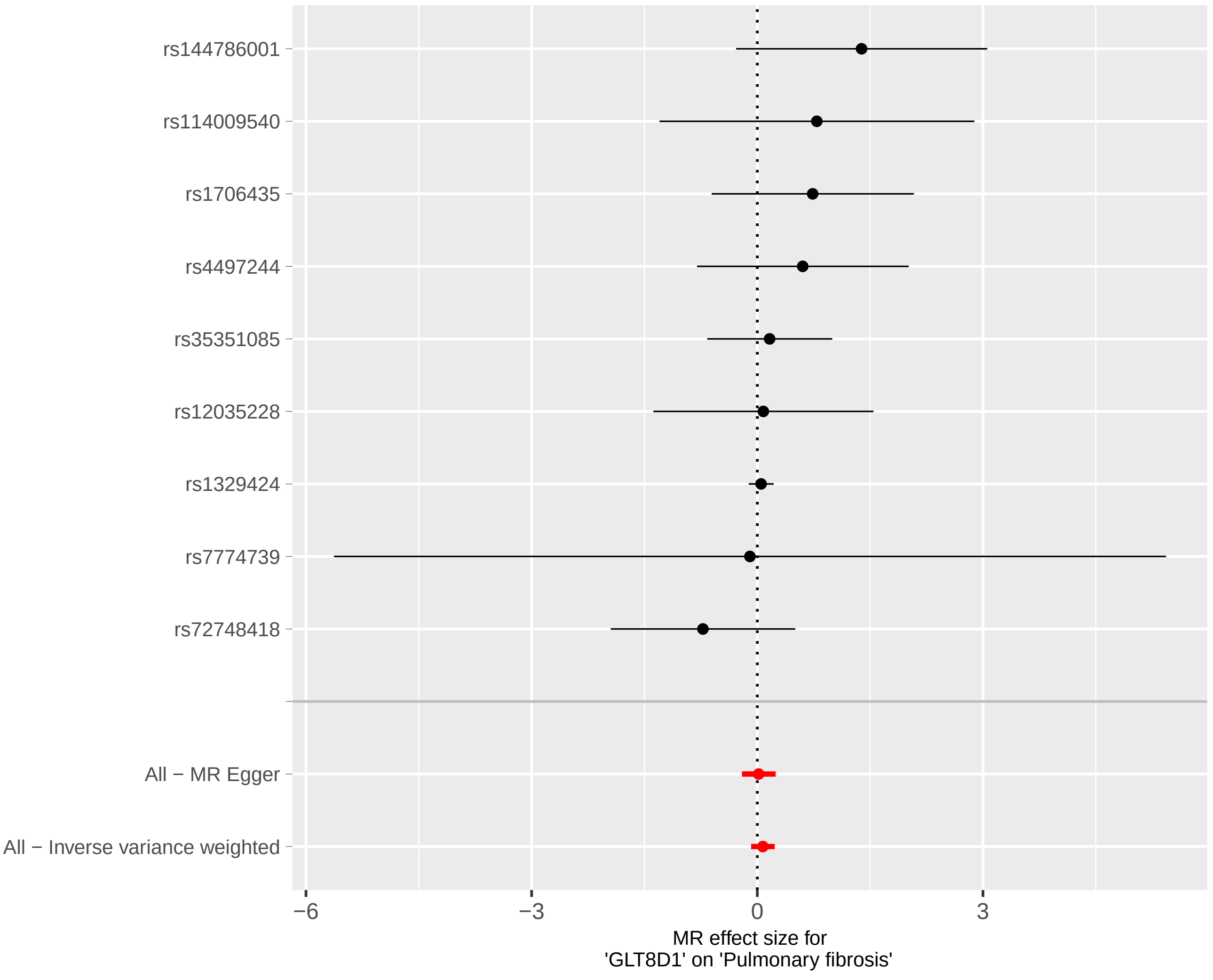
rs144786001
rs114009540
rs1706435
rs4497244
rs35351085
rs12035228
rs1329424
rs7774739
rs72748418
All − MR Egger
All − Inverse variance weighted
−6
−3
0
3
MR effect size for
'GLT8D1' on 'Pulmonary fibrosis'

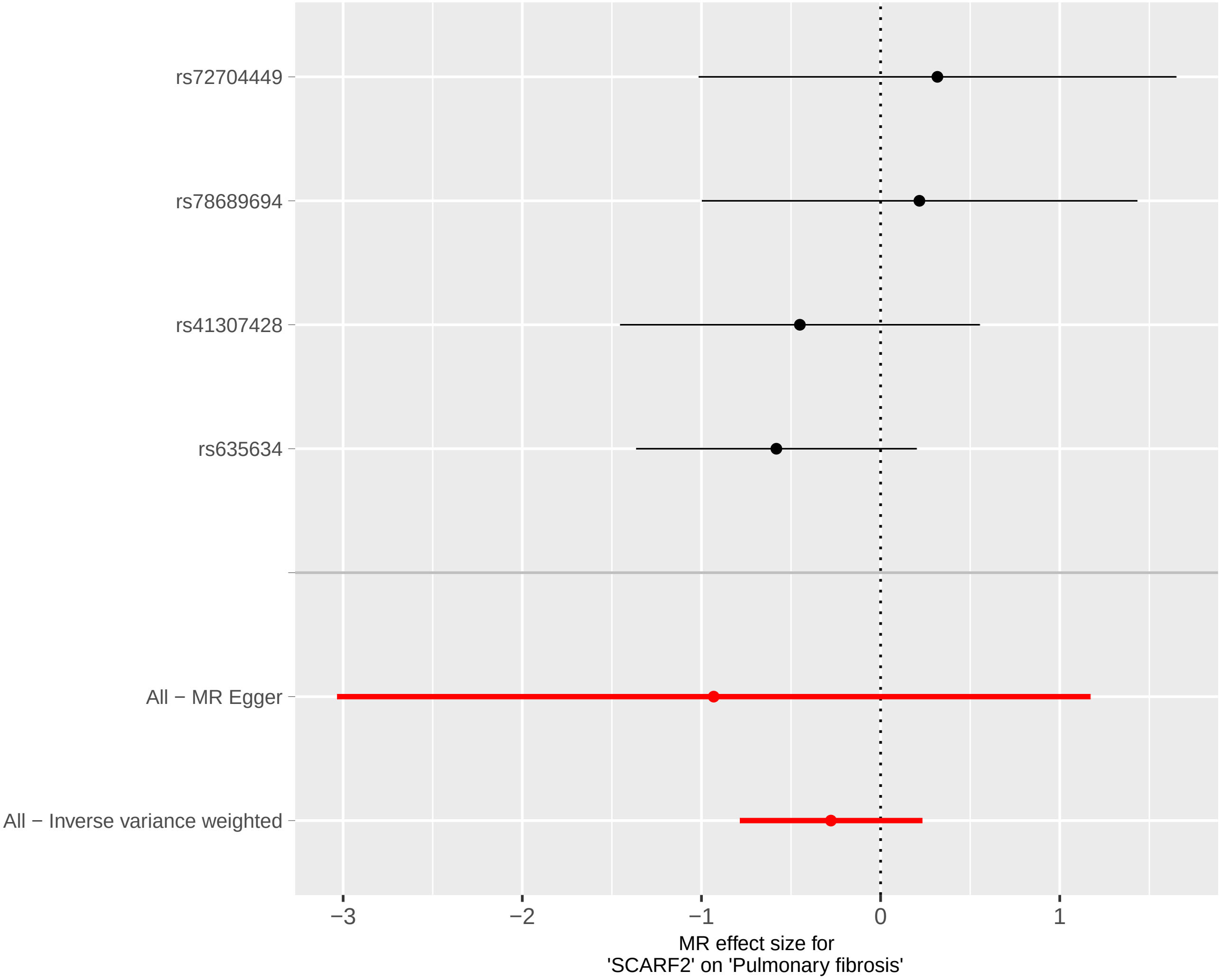
rs72704449
rs78689694
rs41307428
rs635634
All − MR Egger
All − Inverse variance weighted
−3
−2
−1
0
1
MR effect size for
'SCARF2' on 'Pulmonary fibrosis'

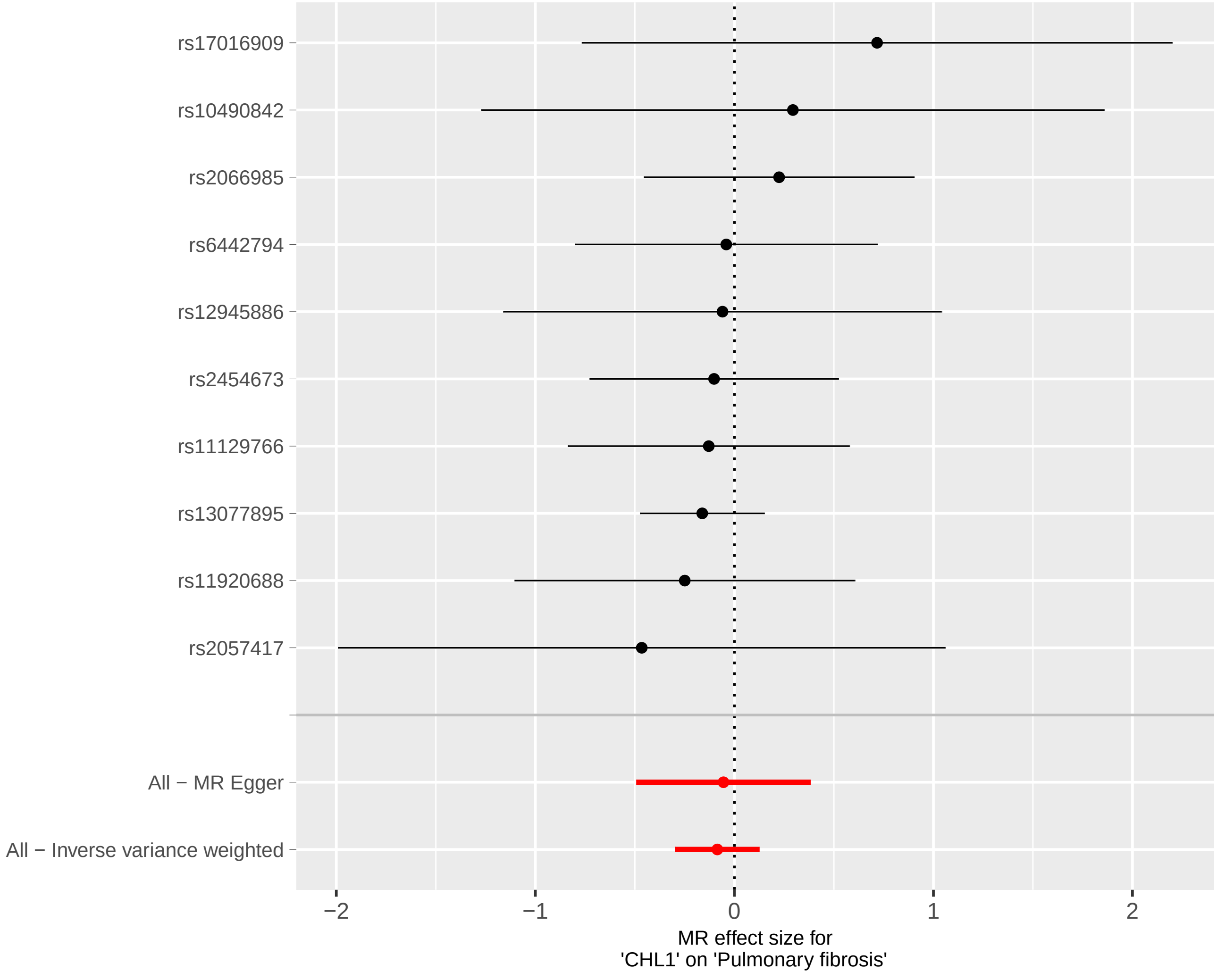
rs17016909
rs10490842
rs2066985
rs6442794
rs12945886
rs2454673
rs11129766
rs13077895
rs11920688
rs2057417
All − MR Egger
All − Inverse variance weighted
−2
−1
0
1
2
MR effect size for
'CHL1' on 'Pulmonary fibrosis'

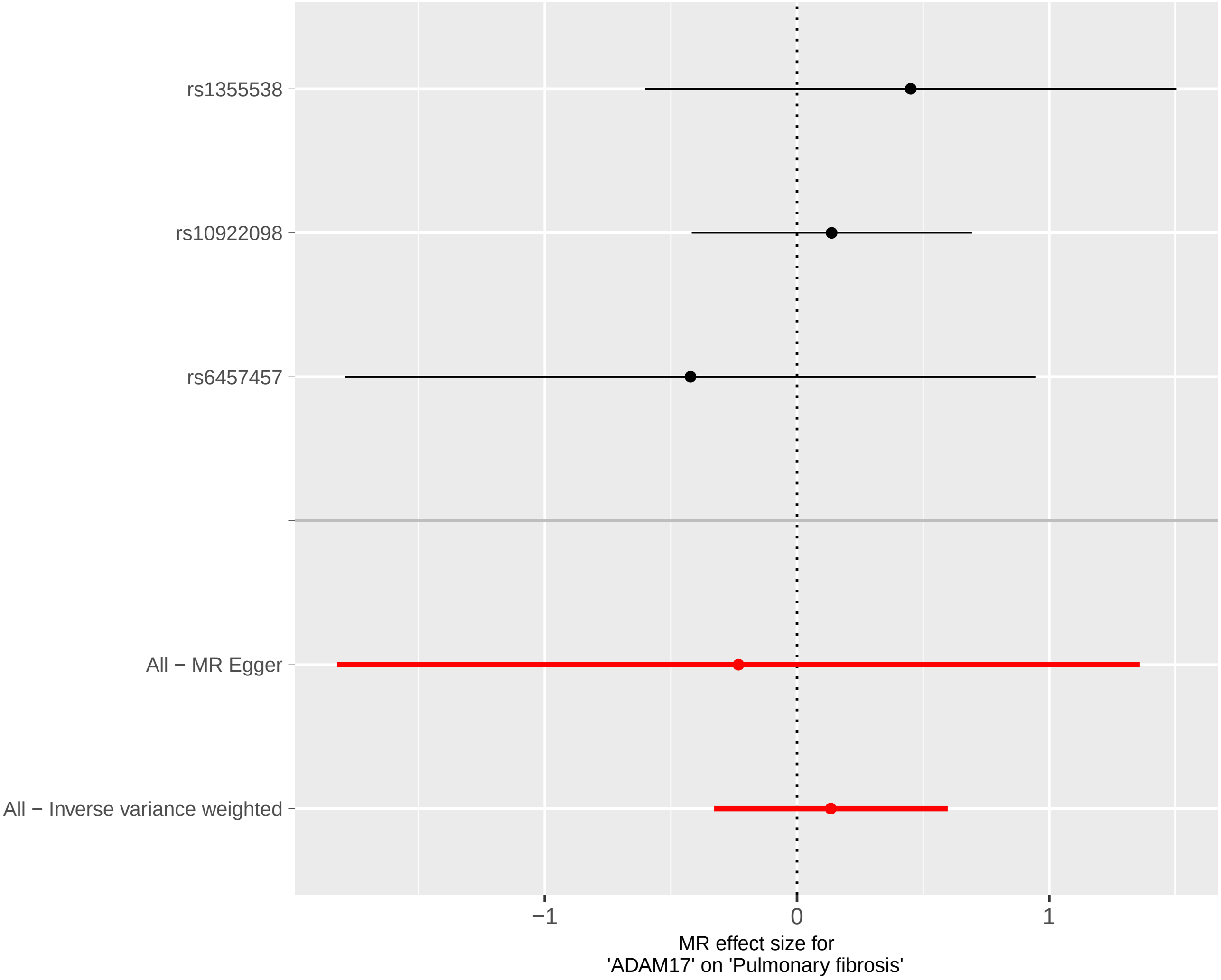

rs1355538
rs10922098
rs6457457
All − MR Egger
All − Inverse variance weighted
−1
0
1
MR effect size for
'ADAM17' on 'Pulmonary fibrosis'

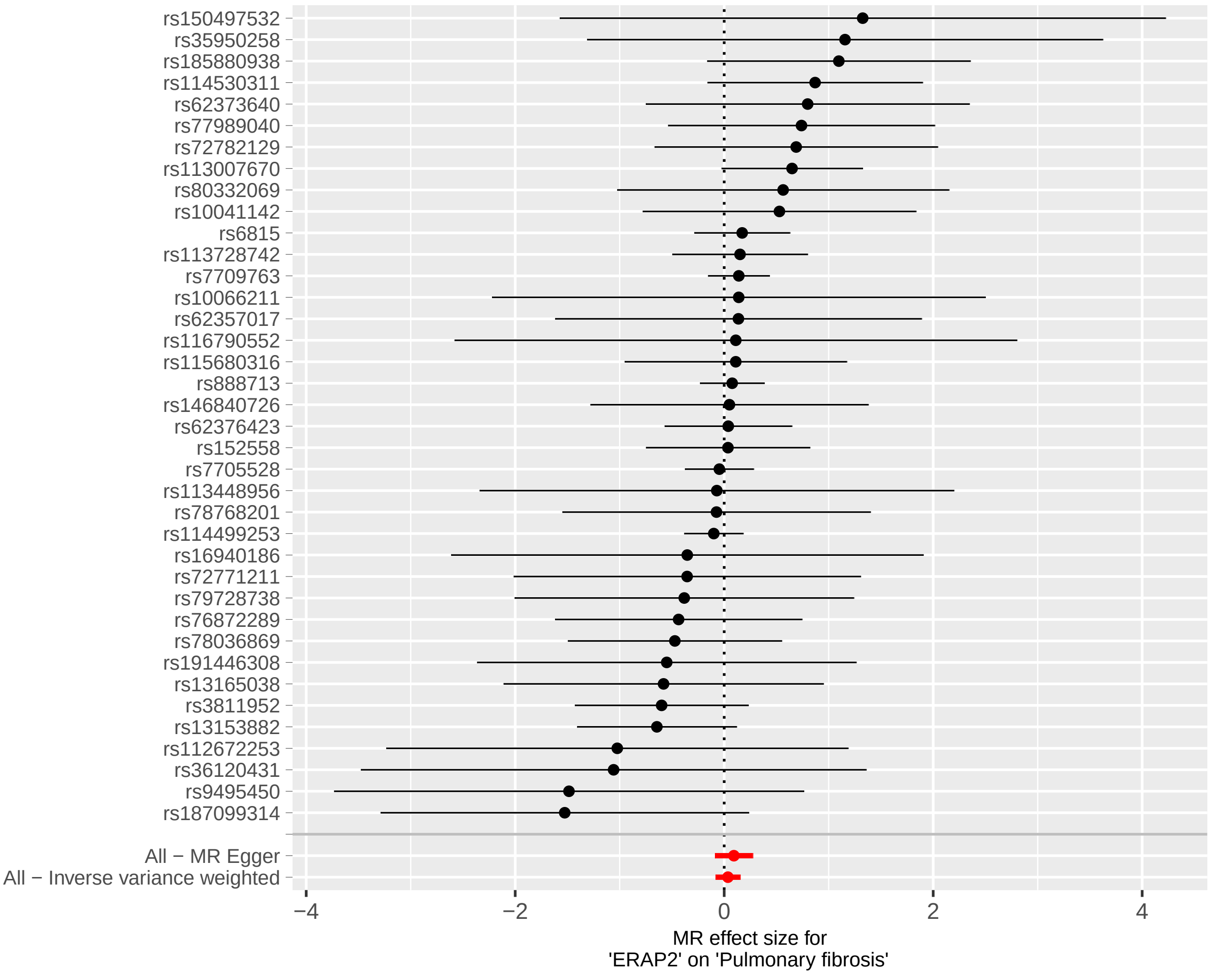
rs150497532
rs35950258
rs185880938
rs114530311
rs62373640
rs77989040
rs72782129
rs113007670
rs80332069
rs10041142
rs6815
rs113728742
rs7709763
rs10066211
rs62357017
rs116790552
rs115680316
rs888713
rs146840726
rs62376423
rs152558
rs7705528
rs113448956
rs78768201
rs114499253
rs16940186
rs72771211
rs79728738
rs76872289
rs78036869
rs191446308
rs13165038
rs3811952
rs13153882
rs112672253
rs36120431
rs9495450
rs187099314
All − MR Egger
All − Inverse variance weighted
−4
−2
0
2
4
MR effect size for
'ERAP2' on 'Pulmonary fibrosis'

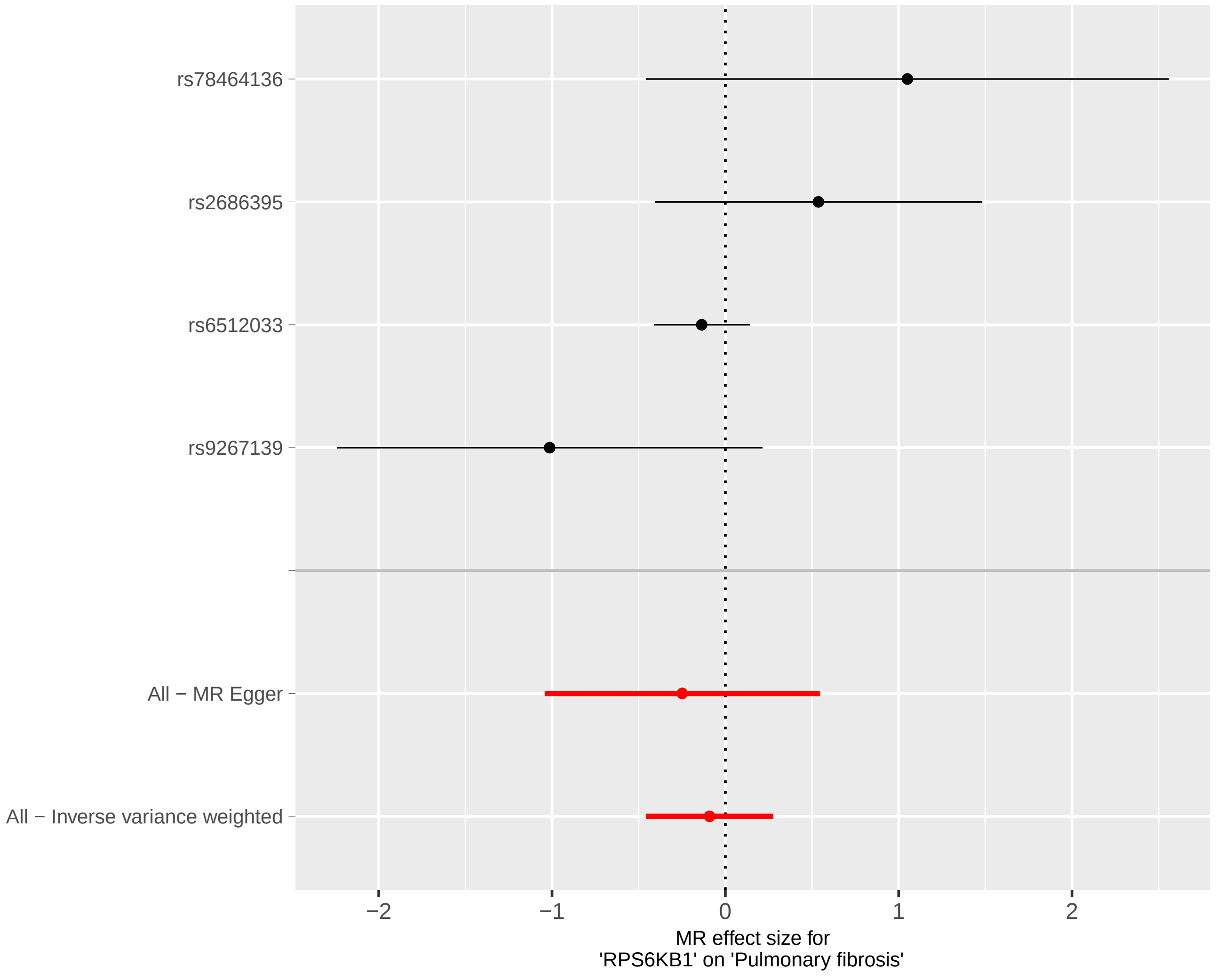
rs78464136
rs2686395
rs6512033
rs9267139
All − MR Egger
All − Inverse variance weighted
−2
−1
0
1
2
MR effect size for
'RPS6KB1' on 'Pulmonary fibrosis'

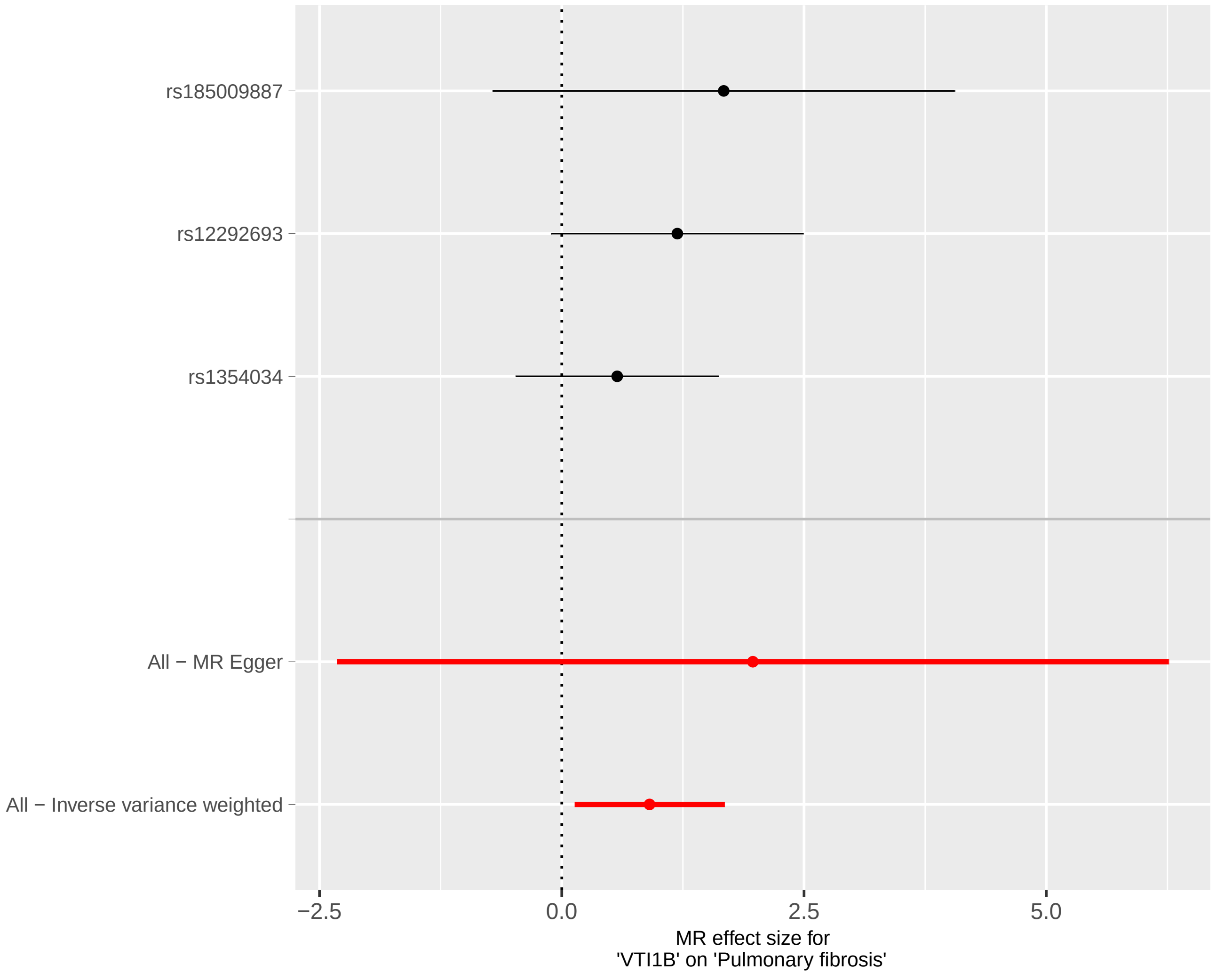

rs185009887
rs12292693
rs1354034
All − MR Egger
All − Inverse variance weighted
−2.5
0.0
2.5
5.0
MR effect size for
'VTI1B' on 'Pulmonary fibrosis'

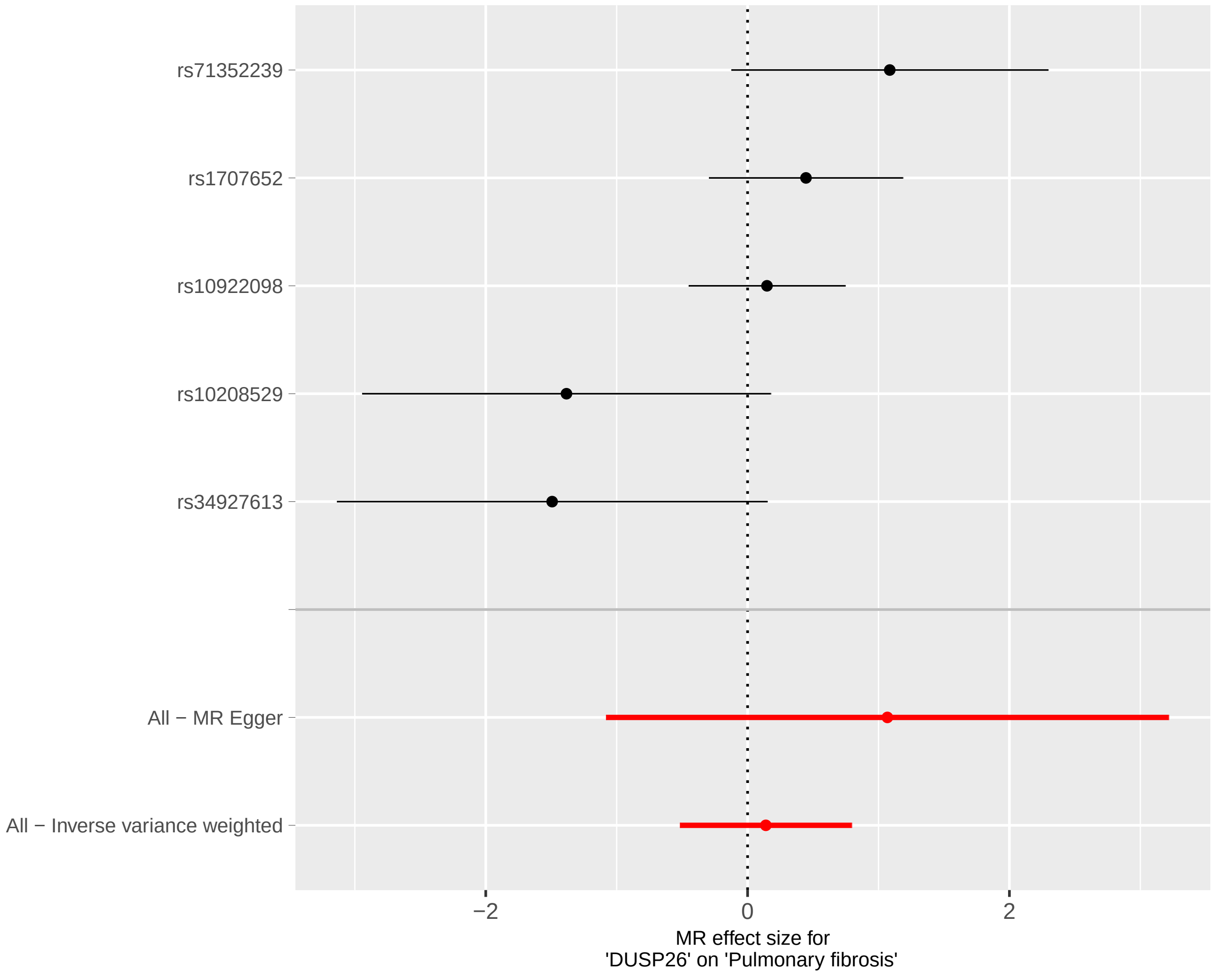

rs71352239
rs1707652
rs10922098
rs10208529
rs34927613
All − MR Egger
All − Inverse variance weighted
−2
0
2
MR effect size for
'DUSP26' on 'Pulmonary fibrosis'

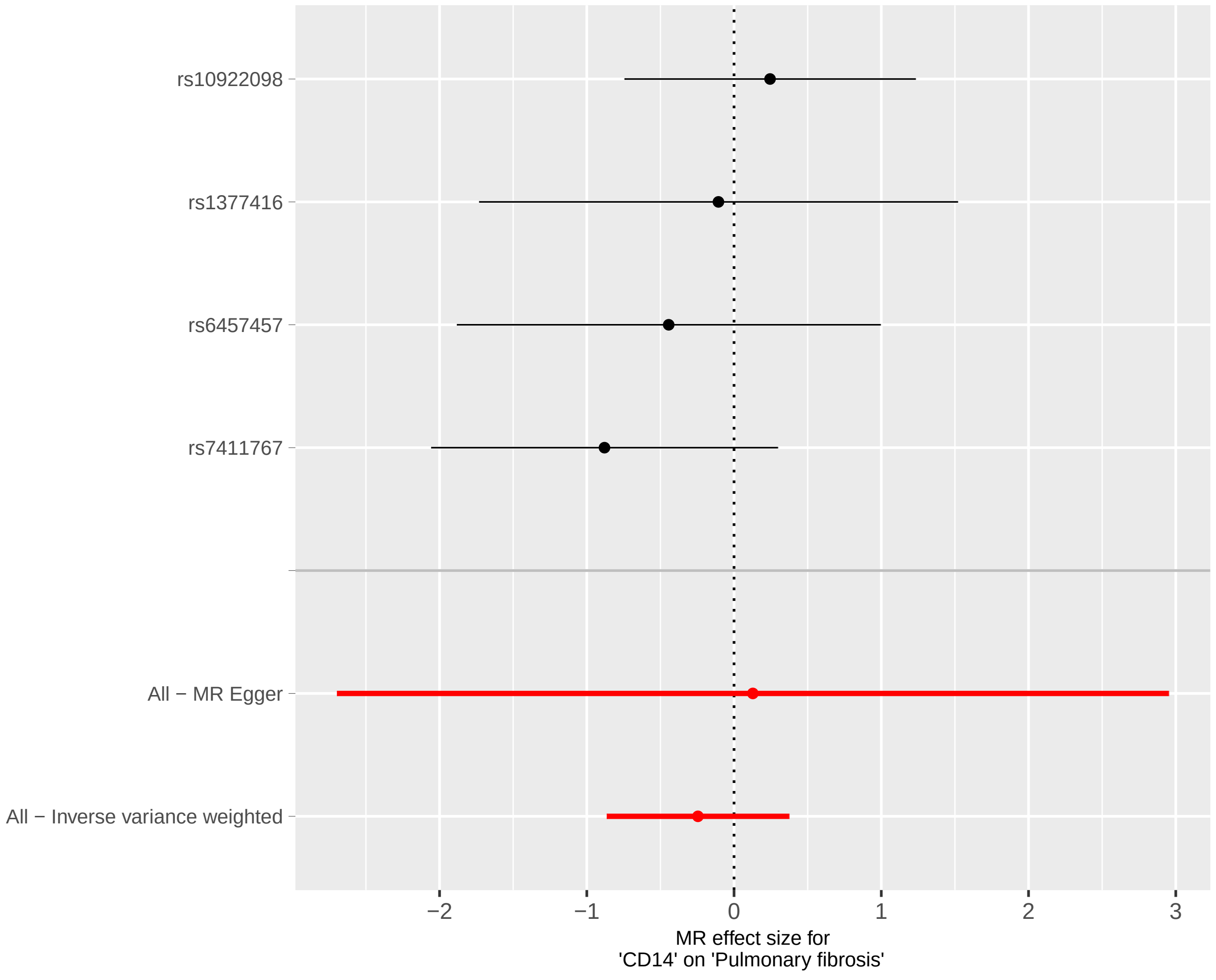

rs10922098
rs1377416
rs6457457
rs7411767
All − MR Egger
All − Inverse variance weighted
−2
−1
0
1
2
3
MR effect size for
'CD14' on 'Pulmonary fibrosis'

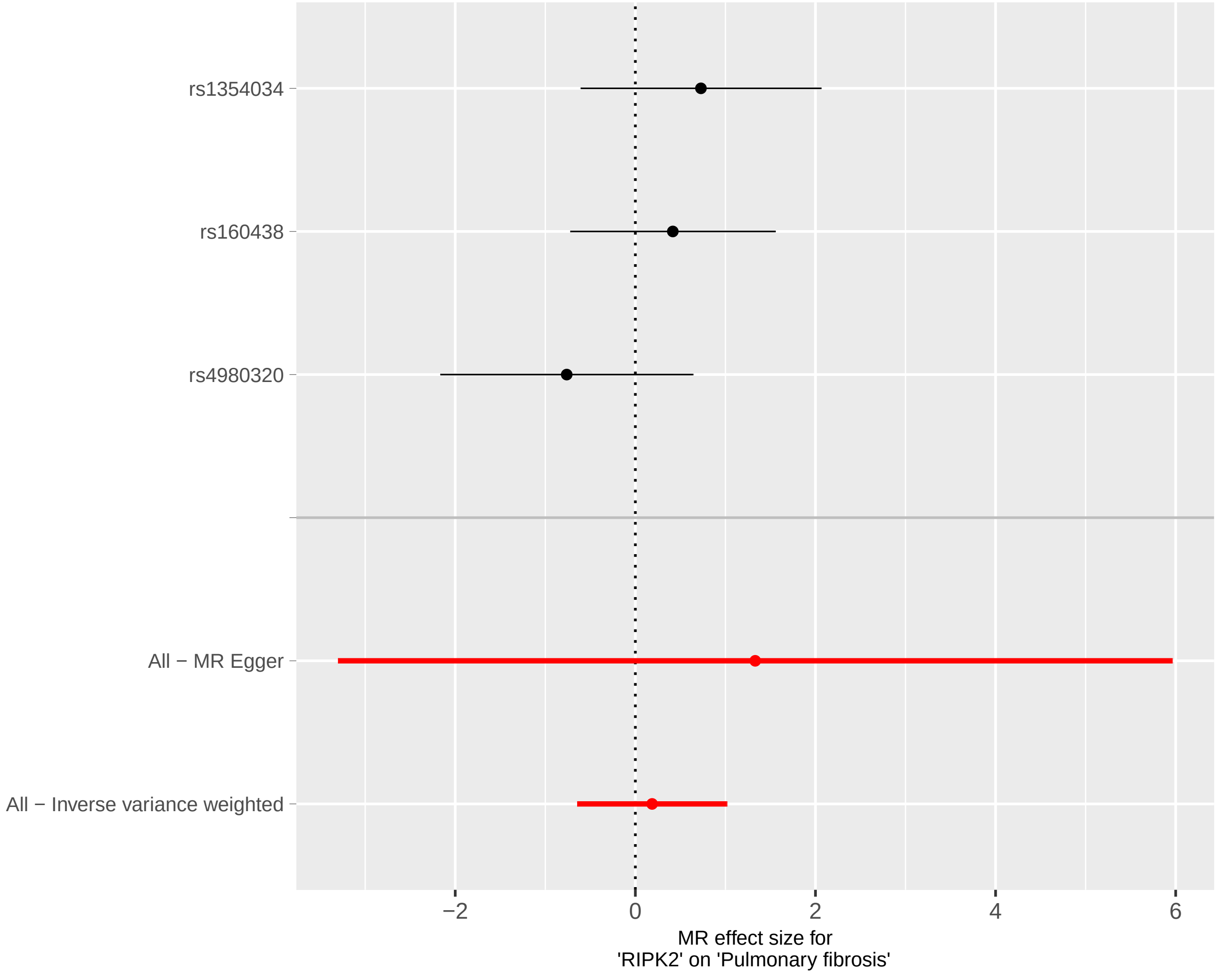
rs1354034
rs160438
rs4980320
All − MR Egger
All − Inverse variance weighted
−2
0
2
4
6
MR effect size for
'RIPK2' on 'Pulmonary fibrosis'

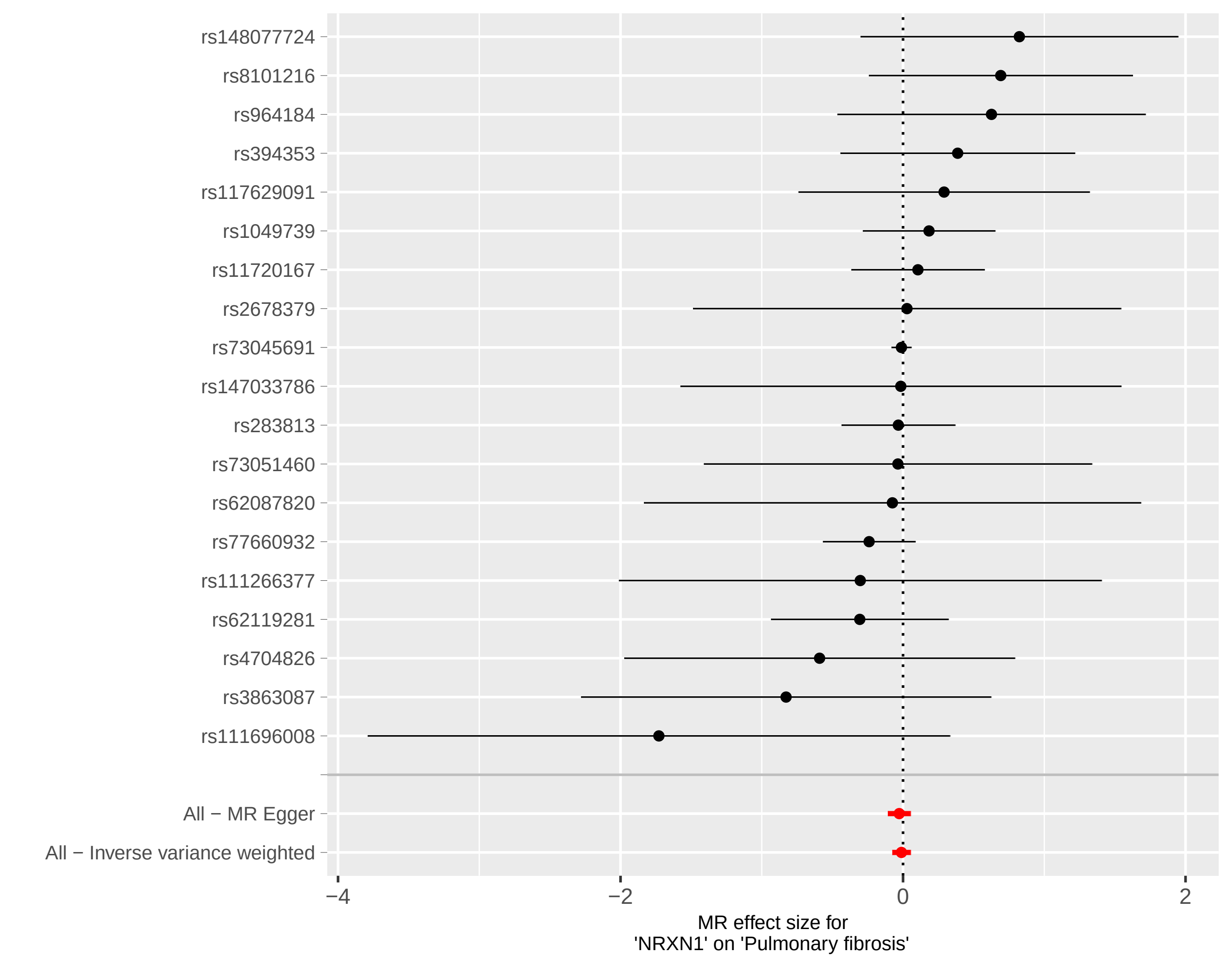
rs148077724
rs8101216
rs964184
rs394353
rs117629091
rs1049739
rs11720167
rs2678379
rs73045691
rs147033786
rs283813
rs73051460
rs62087820
rs77660932
rs111266377
rs62119281
rs4704826
rs3863087
rs111696008
All − MR Egger
All − Inverse variance weighted
−4
−2
0
2
MR effect size for
'NRXN1' on 'Pulmonary fibrosis'

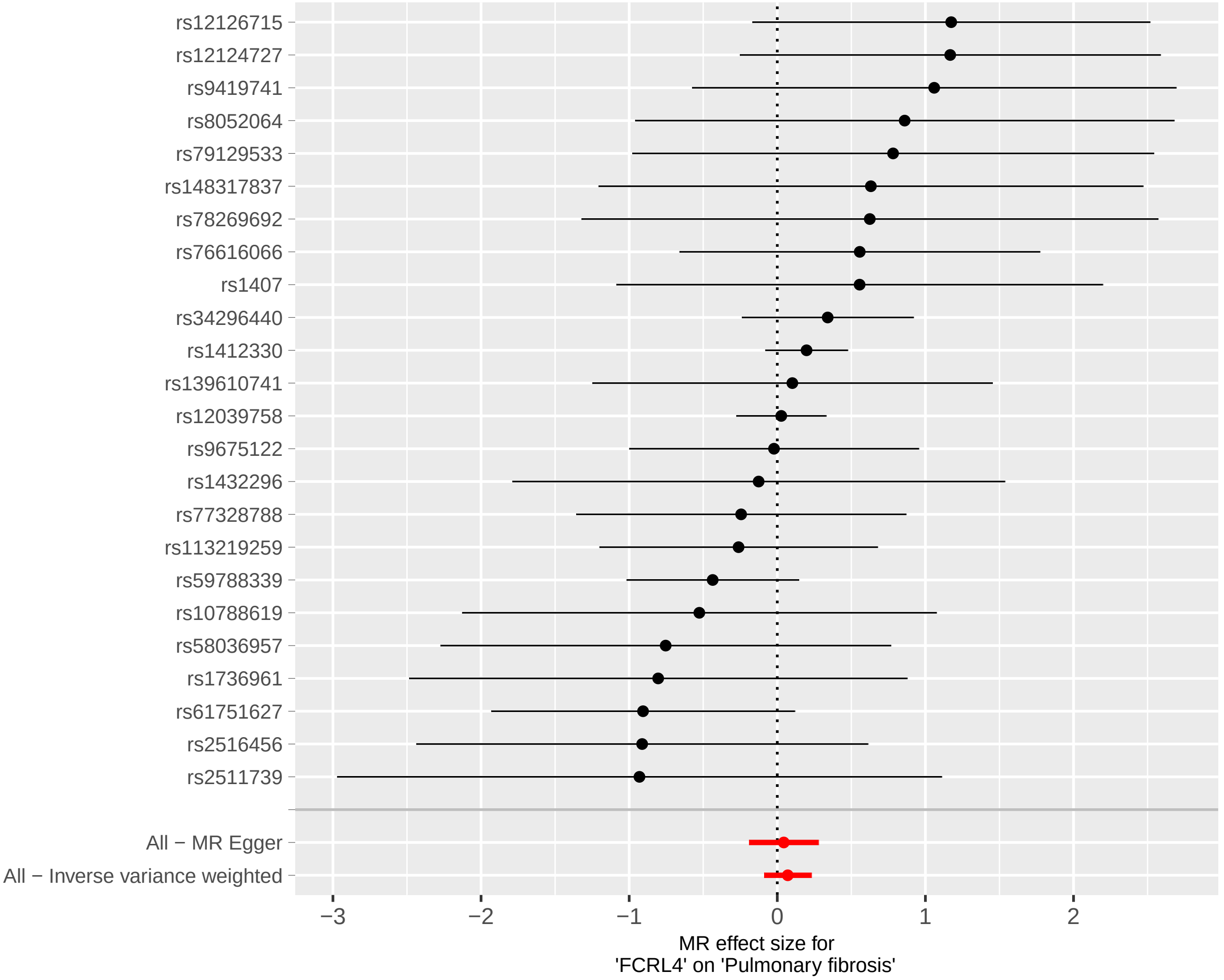

rs12126715
rs12124727
rs9419741
rs8052064
rs79129533
rs148317837
rs78269692
rs76616066
rs1407
rs34296440
rs1412330
rs139610741
rs12039758
rs9675122
rs1432296
rs77328788
rs113219259
rs59788339
rs10788619
rs58036957
rs1736961
rs61751627
rs2516456
rs2511739
All − MR Egger
All − Inverse variance weighted
−3
−2
−1
0
1
2
MR effect size for
'FCRL4' on 'Pulmonary fibrosis'

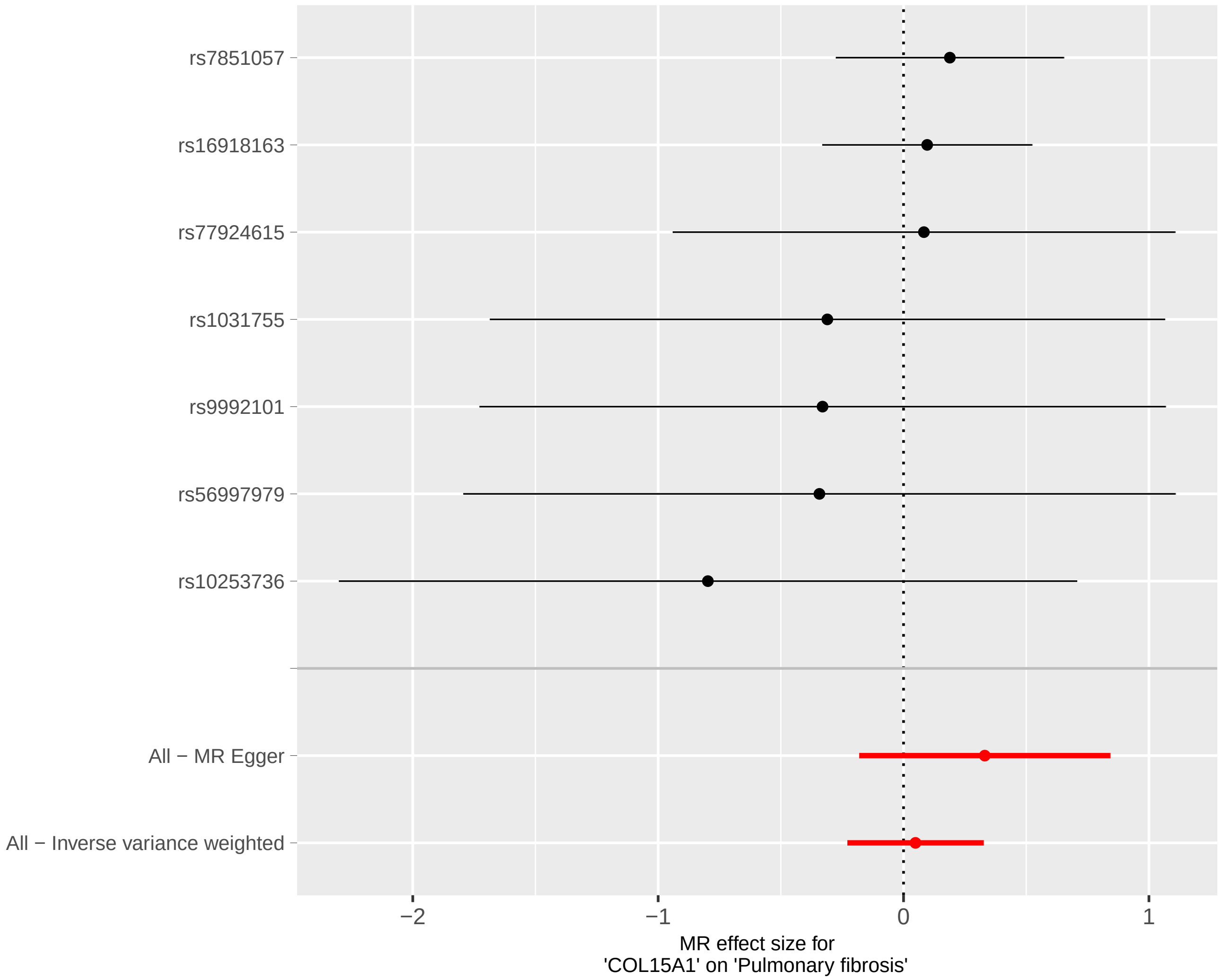
rs7851057
rs16918163
rs77924615
rs1031755
rs9992101
rs56997979
rs10253736
All − MR Egger
All − Inverse variance weighted
−2
−1
0
1
MR effect size for
'COL15A1' on 'Pulmonary fibrosis'

Insufficient number of SNPs

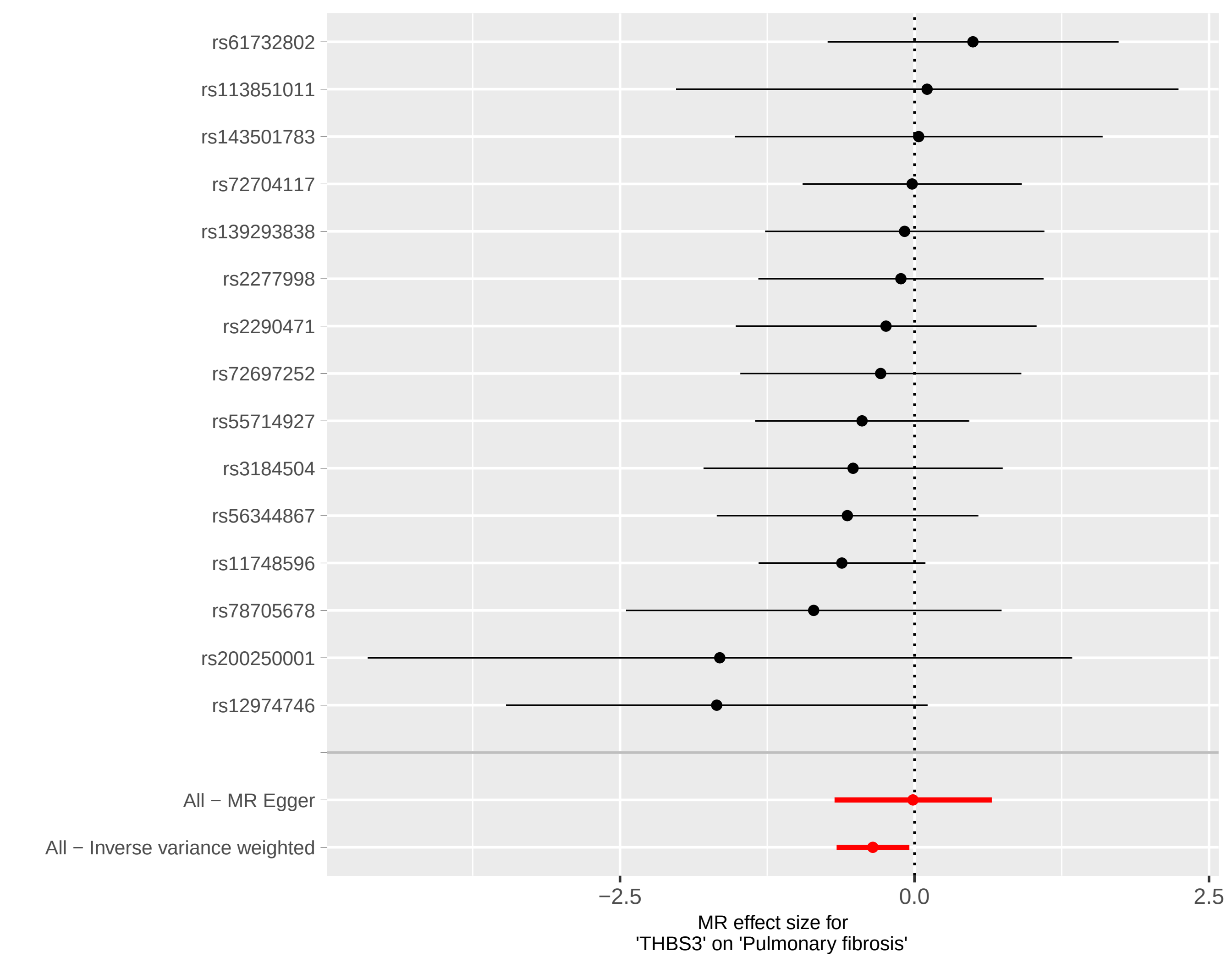

rs61732802
rs113851011
rs143501783
rs72704117
rs139293838
rs2277998
rs2290471
rs72697252
rs55714927
rs3184504
rs56344867
rs11748596
rs78705678
rs200250001
rs12974746
All − MR Egger
All − Inverse variance weighted
−2.5
0.0
2.5
MR effect size for
'THBS3' on 'Pulmonary fibrosis'

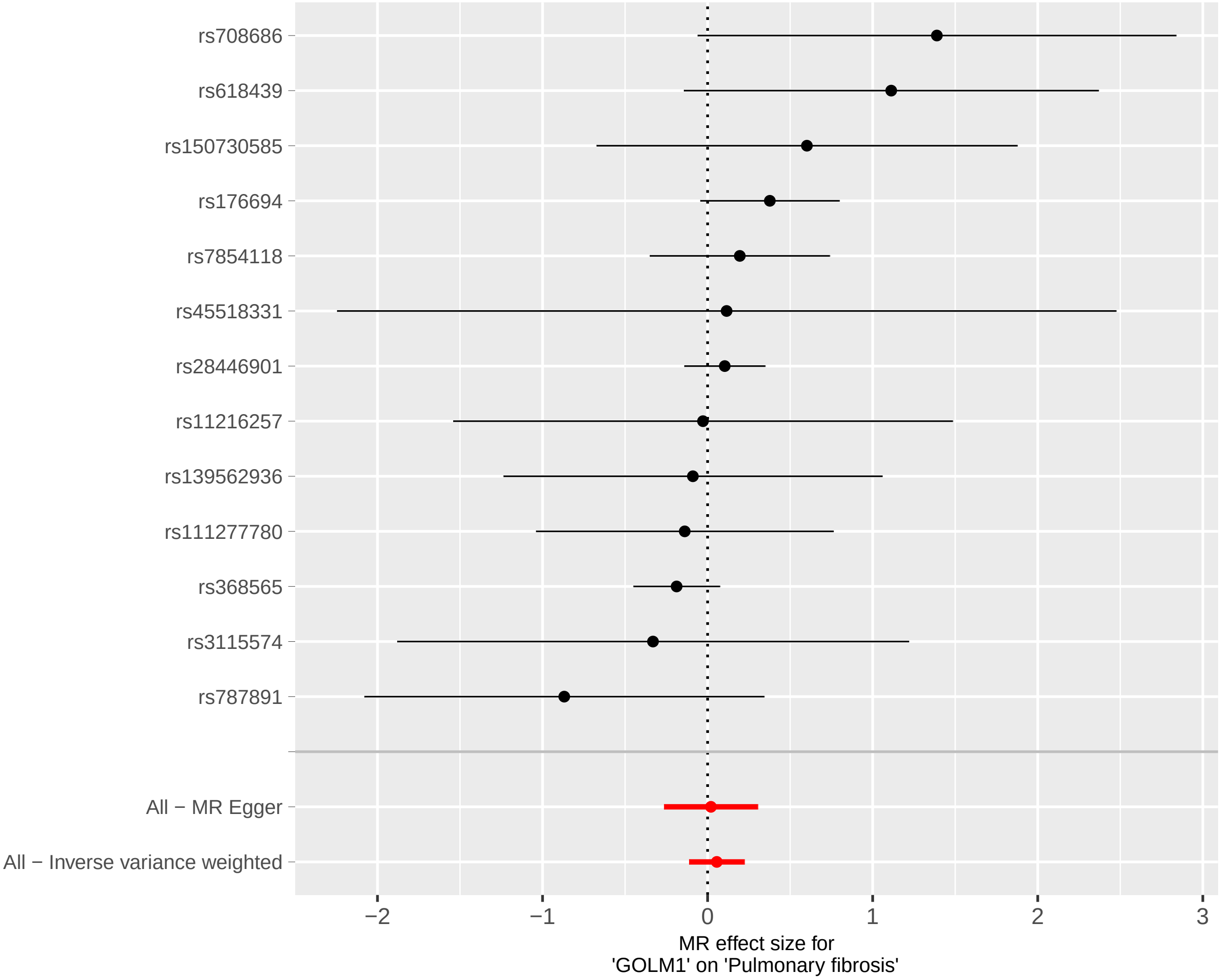
rs708686
rs618439
rs150730585
rs176694
rs7854118
rs45518331
rs28446901
rs11216257
rs139562936
rs111277780
rs368565
rs3115574
rs787891
All − MR Egger
All − Inverse variance weighted
−2
−1
0
1
2
3
MR effect size for
'GOLM1' on 'Pulmonary fibrosis'

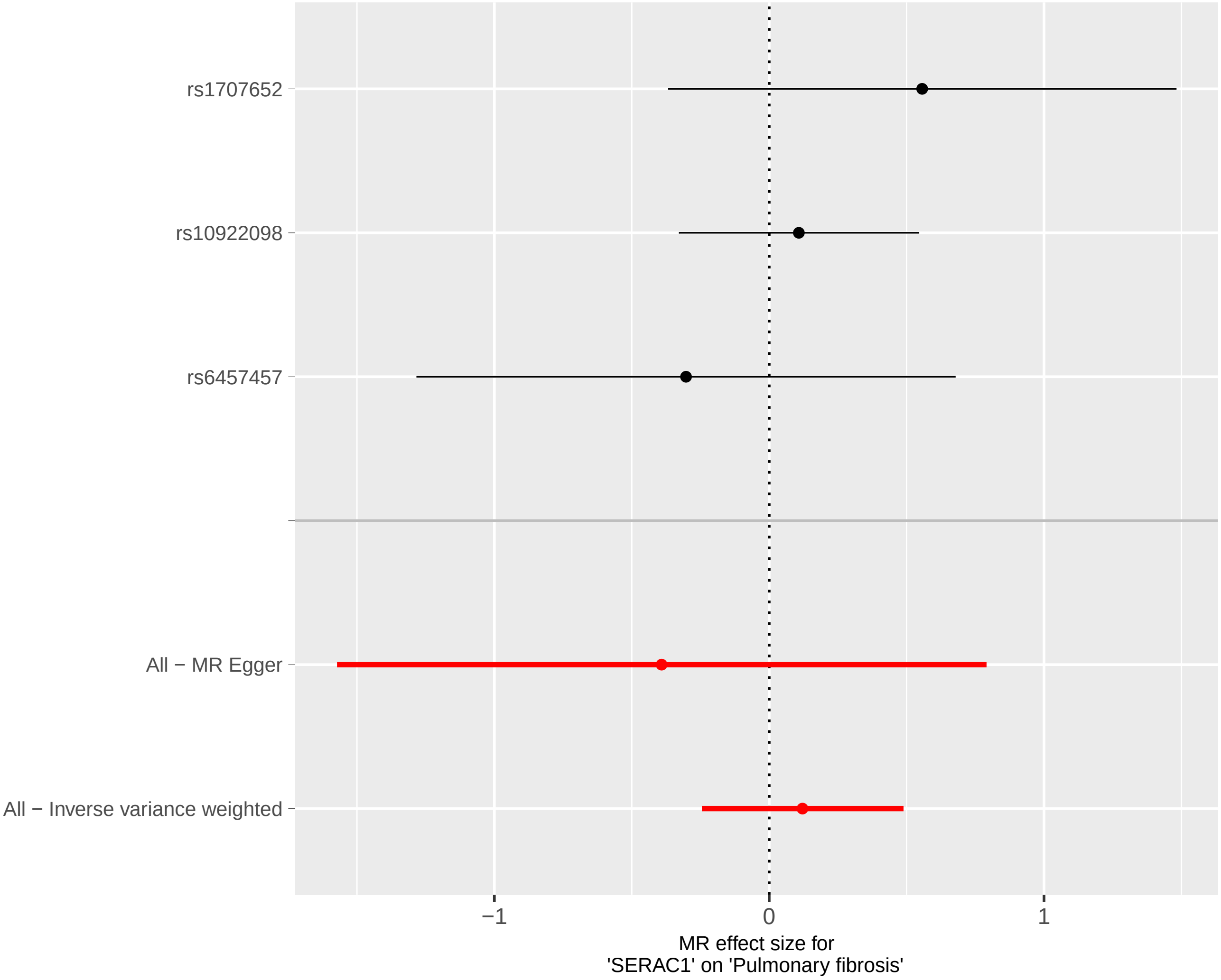

rs1707652
rs10922098
rs6457457
All − MR Egger
All − Inverse variance weighted
−1
0
1
MR effect size for
'SERAC1' on 'Pulmonary fibrosis'

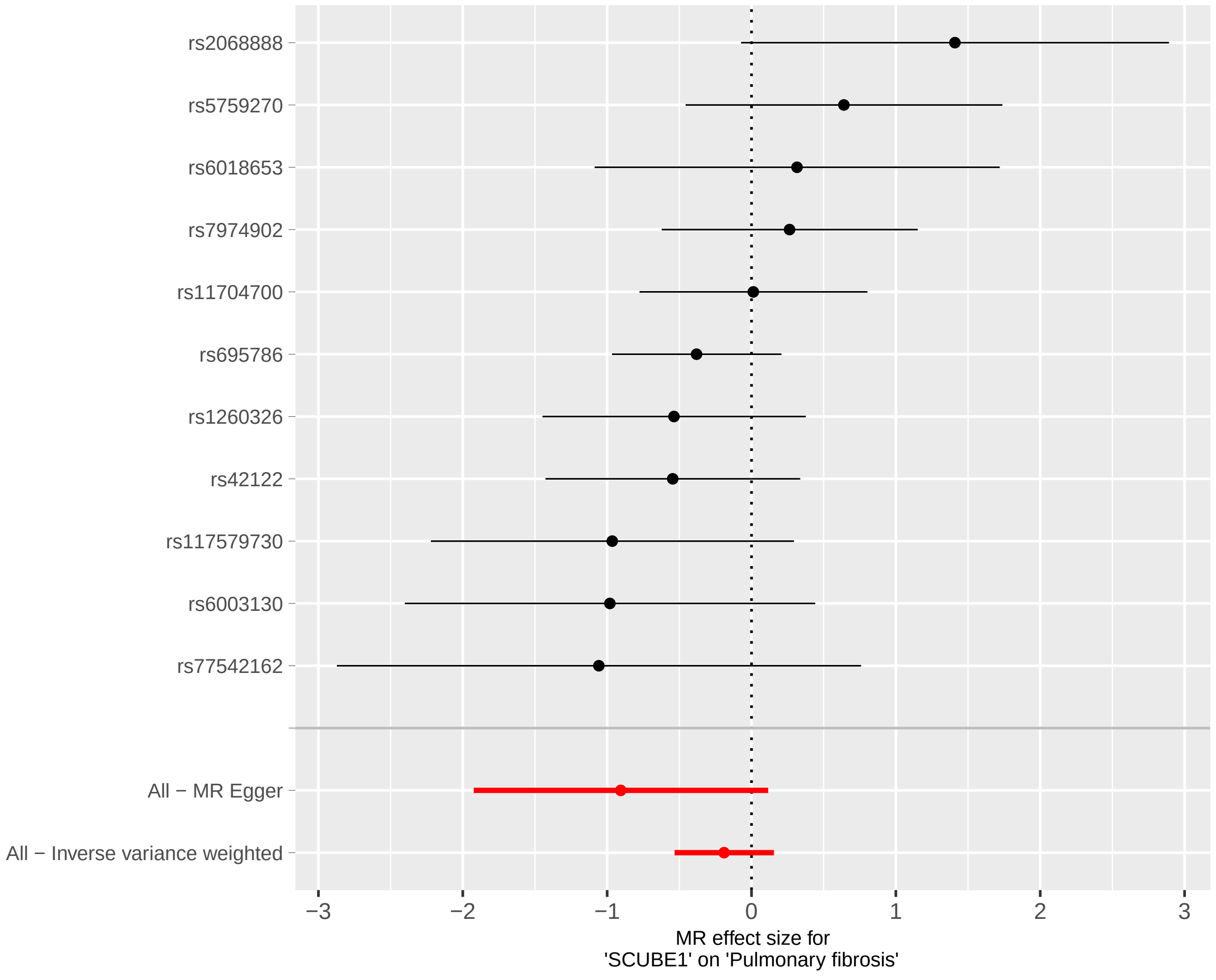
rs2068888
rs5759270
rs6018653
rs7974902
rs11704700
rs695786
rs1260326
rs42122
rs117579730
rs6003130
rs77542162
All − MR Egger
All − Inverse variance weighted
−3
−2
−1
0
1
2
3
MR effect size for
'SCUBE1' on 'Pulmonary fibrosis'

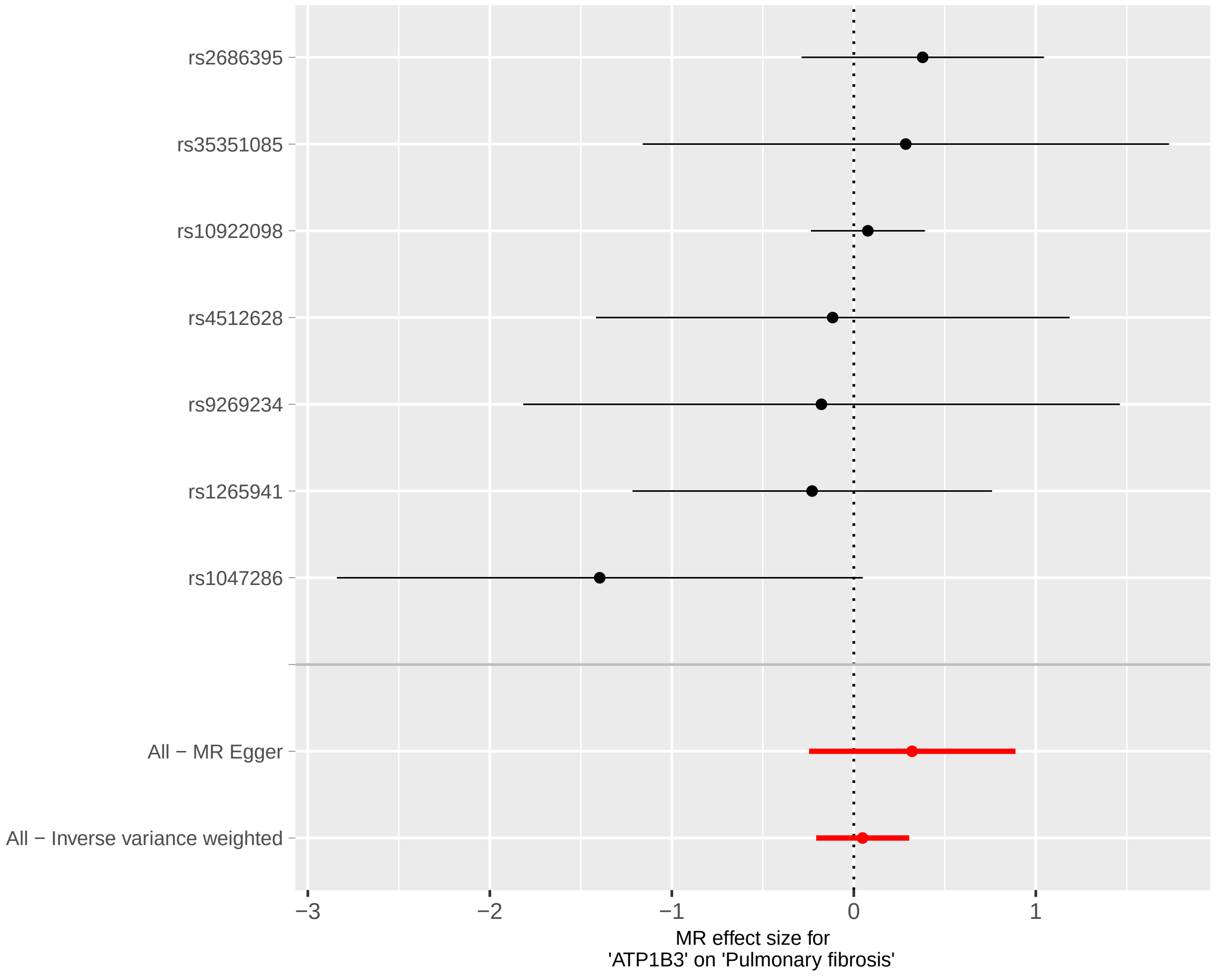
rs2686395
rs35351085
rs10922098
rs4512628
rs9269234
rs1265941
rs1047286
All − MR Egger
All − Inverse variance weighted
−3
−2
−1
0
1
MR effect size for
'ATP1B3' on 'Pulmonary fibrosis'

Insufficient number of SNPs

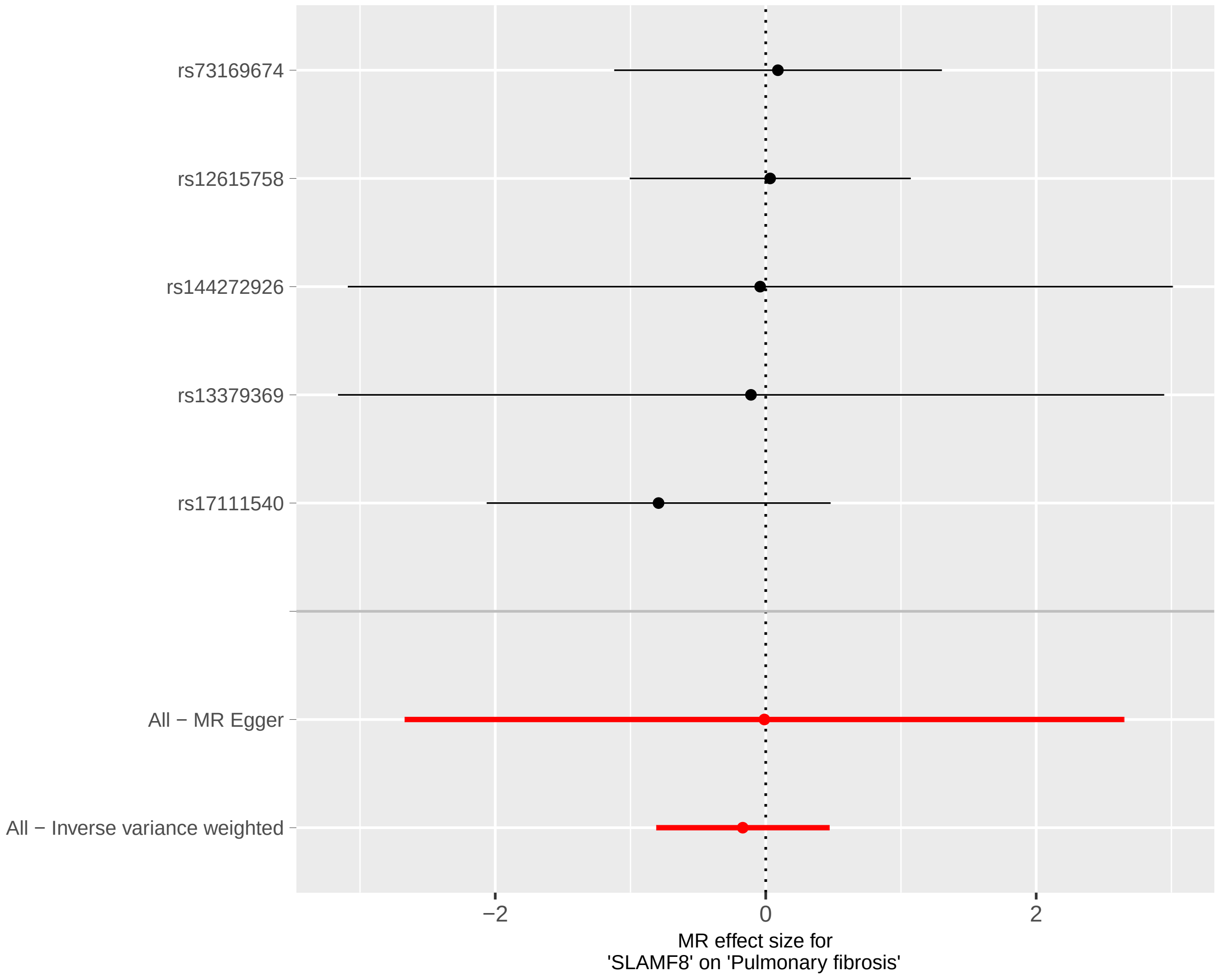

rs73169674
rs12615758
rs144272926
rs13379369
rs17111540
All − MR Egger
All − Inverse variance weighted
−2
0
2
MR effect size for
'SLAMF8' on 'Pulmonary fibrosis'

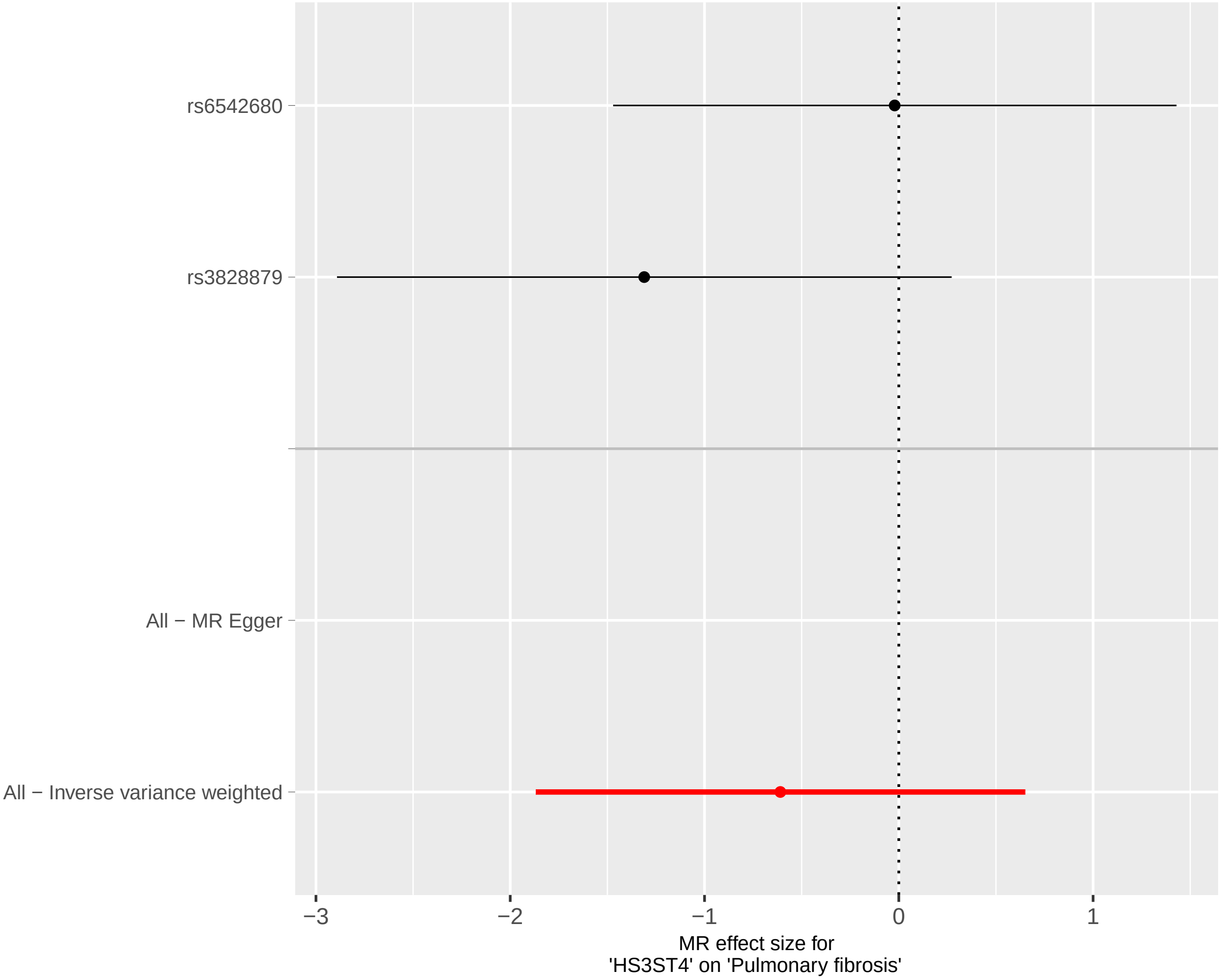
rs6542680
rs3828879
All − MR Egger
All − Inverse variance weighted
−3
−2
−1
0
1
MR effect size for
'HS3ST4' on 'Pulmonary fibrosis'

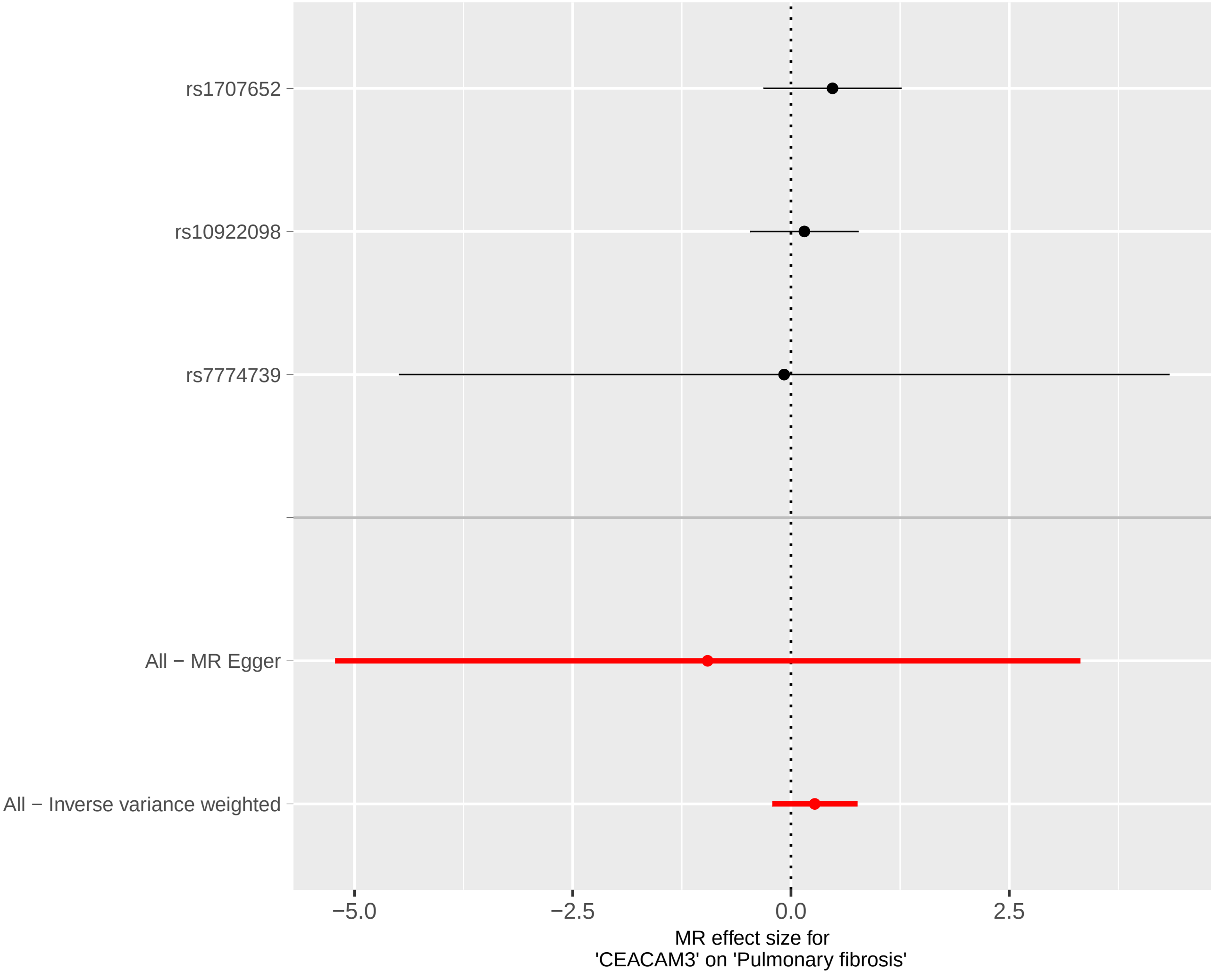
rs1707652
rs10922098
rs7774739
All − MR Egger
All − Inverse variance weighted
−5.0
−2.5
0.0
2.5
MR effect size for
'CEACAM3' on 'Pulmonary fibrosis'

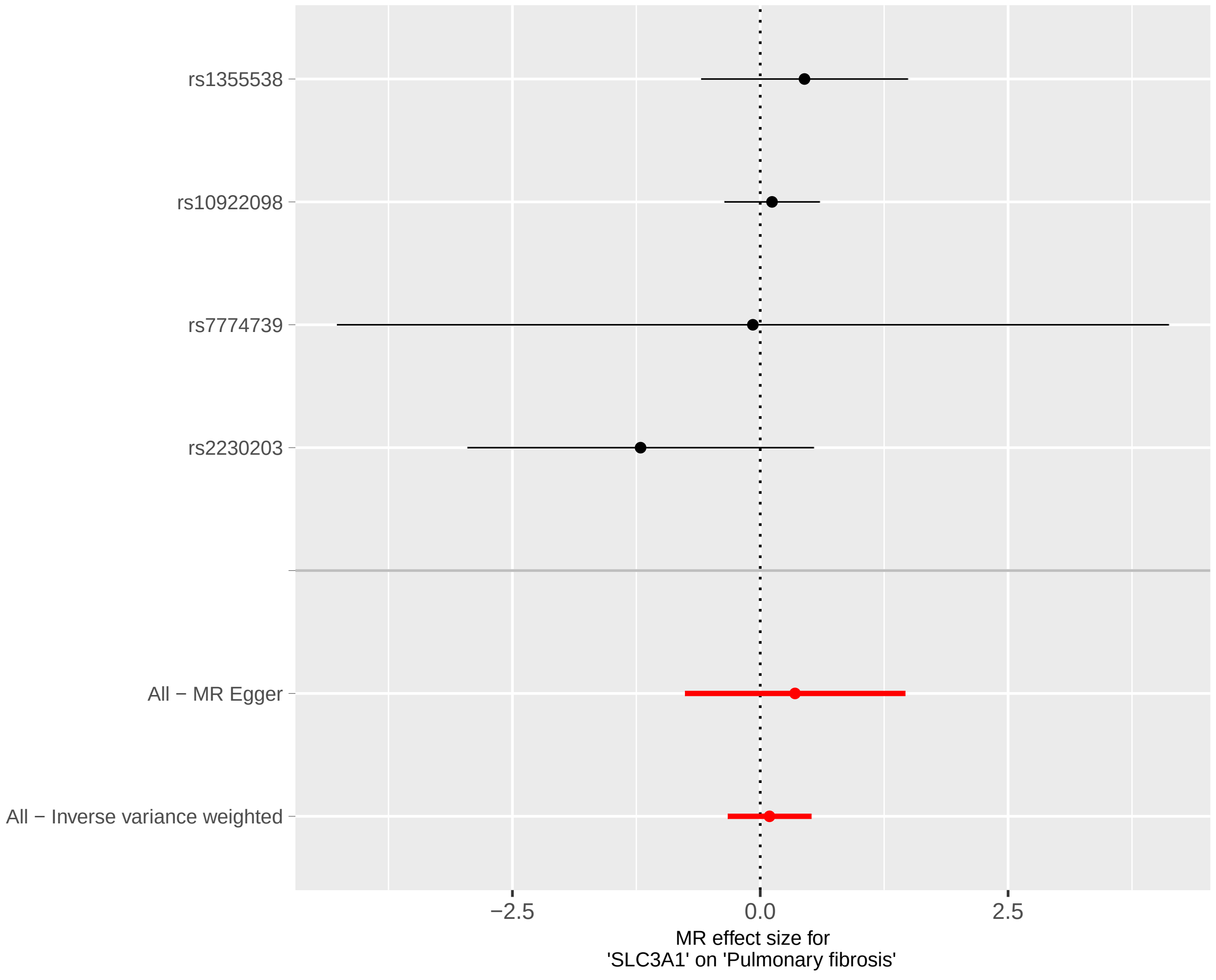
rs1355538
rs10922098
rs7774739
rs2230203
All − MR Egger
All − Inverse variance weighted
−2.5
0.0
2.5
MR effect size for
'SLC3A1' on 'Pulmonary fibrosis'

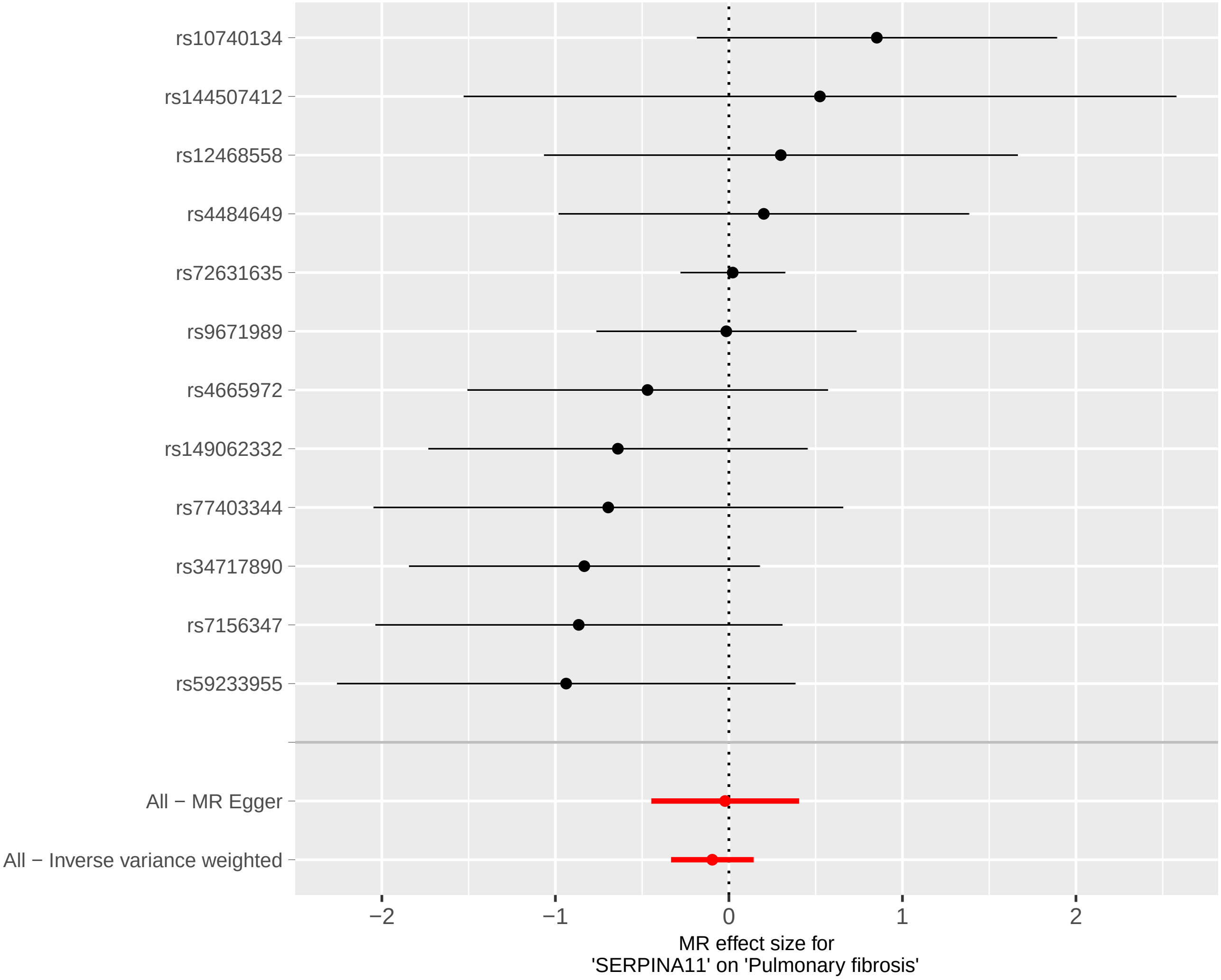

rs10740134
rs144507412
rs12468558
rs4484649
rs72631635
rs9671989
rs4665972
rs149062332
rs77403344
rs34717890
rs7156347
rs59233955
All − MR Egger
All − Inverse variance weighted
−2
−1
0
1
2
MR effect size for
'SERPINA11' on 'Pulmonary fibrosis'

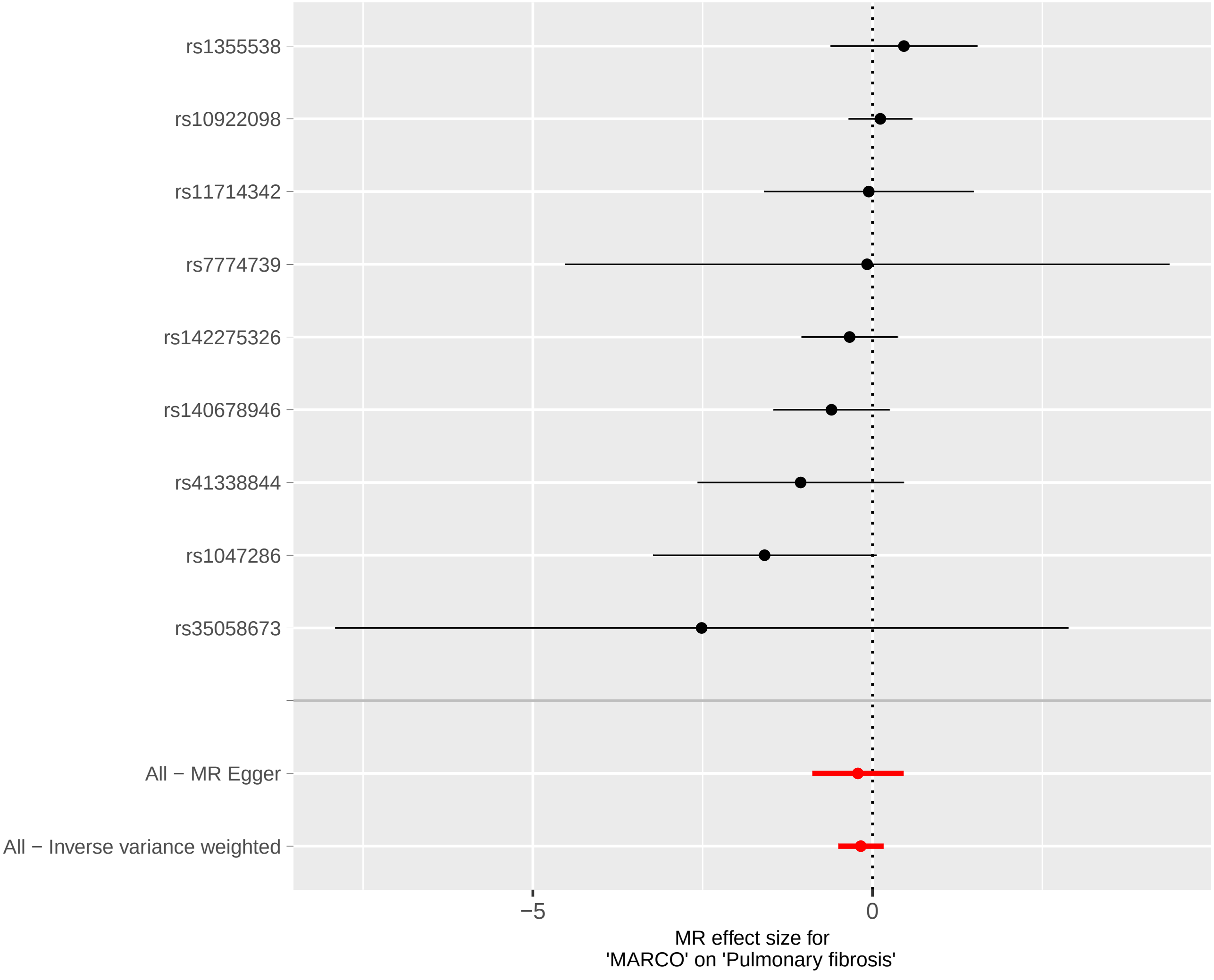
rs1355538
rs10922098
rs11714342
rs7774739
rs142275326
rs140678946
rs41338844
rs1047286
rs35058673
All − MR Egger
All − Inverse variance weighted
−5
0
MR effect size for
'MARCO' on 'Pulmonary fibrosis'

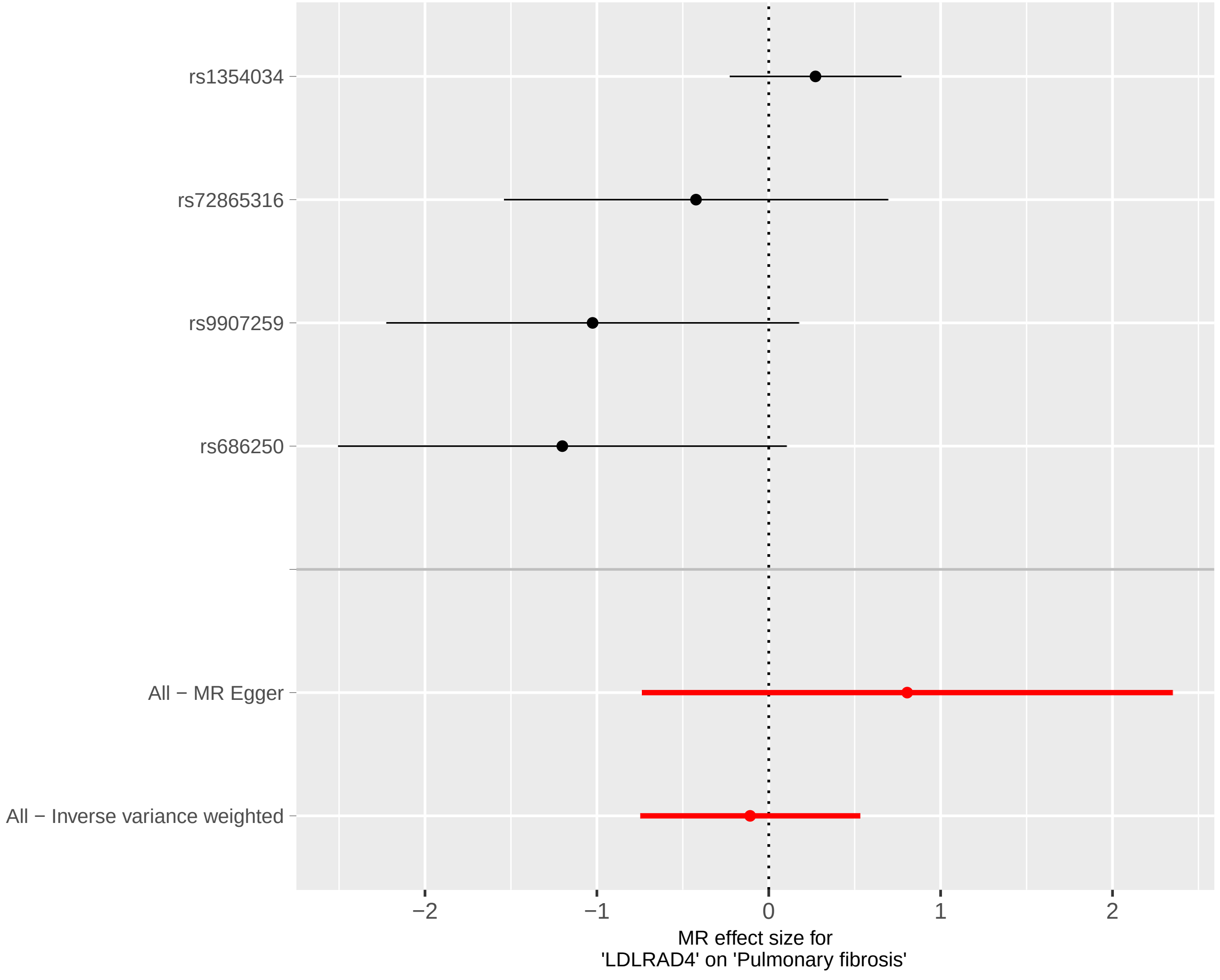

rs1354034
rs72865316
rs9907259
rs686250
All − MR Egger
All − Inverse variance weighted
−2
−1
0
1
2
MR effect size for
'LDLRAD4' on 'Pulmonary fibrosis'

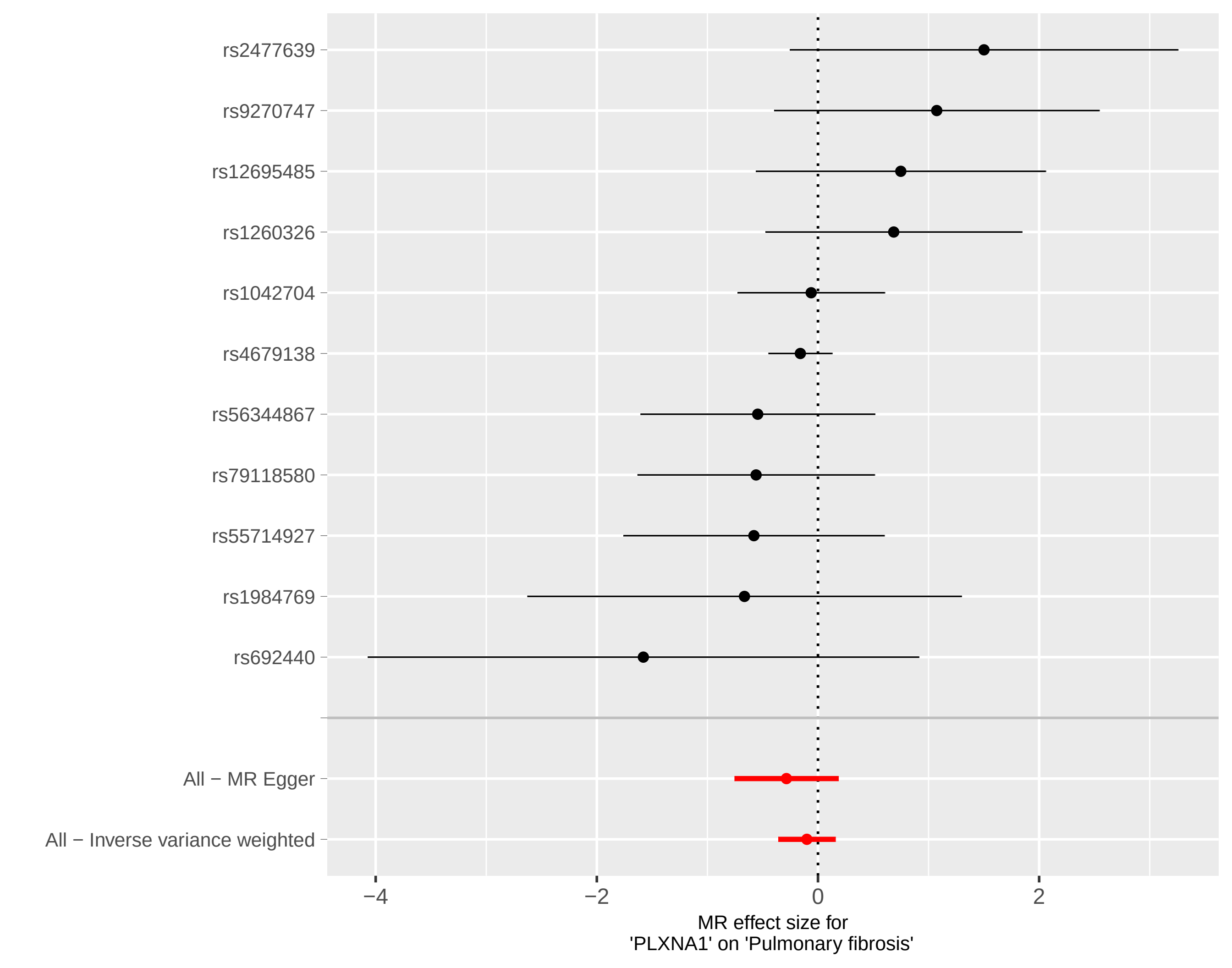

rs2477639
rs9270747
rs12695485
rs1260326
rs1042704
rs4679138
rs56344867
rs79118580
rs55714927
rs1984769
rs692440
All − MR Egger
All − Inverse variance weighted
−4
−2
0
2
MR effect size for
'PLXNA1' on 'Pulmonary fibrosis'

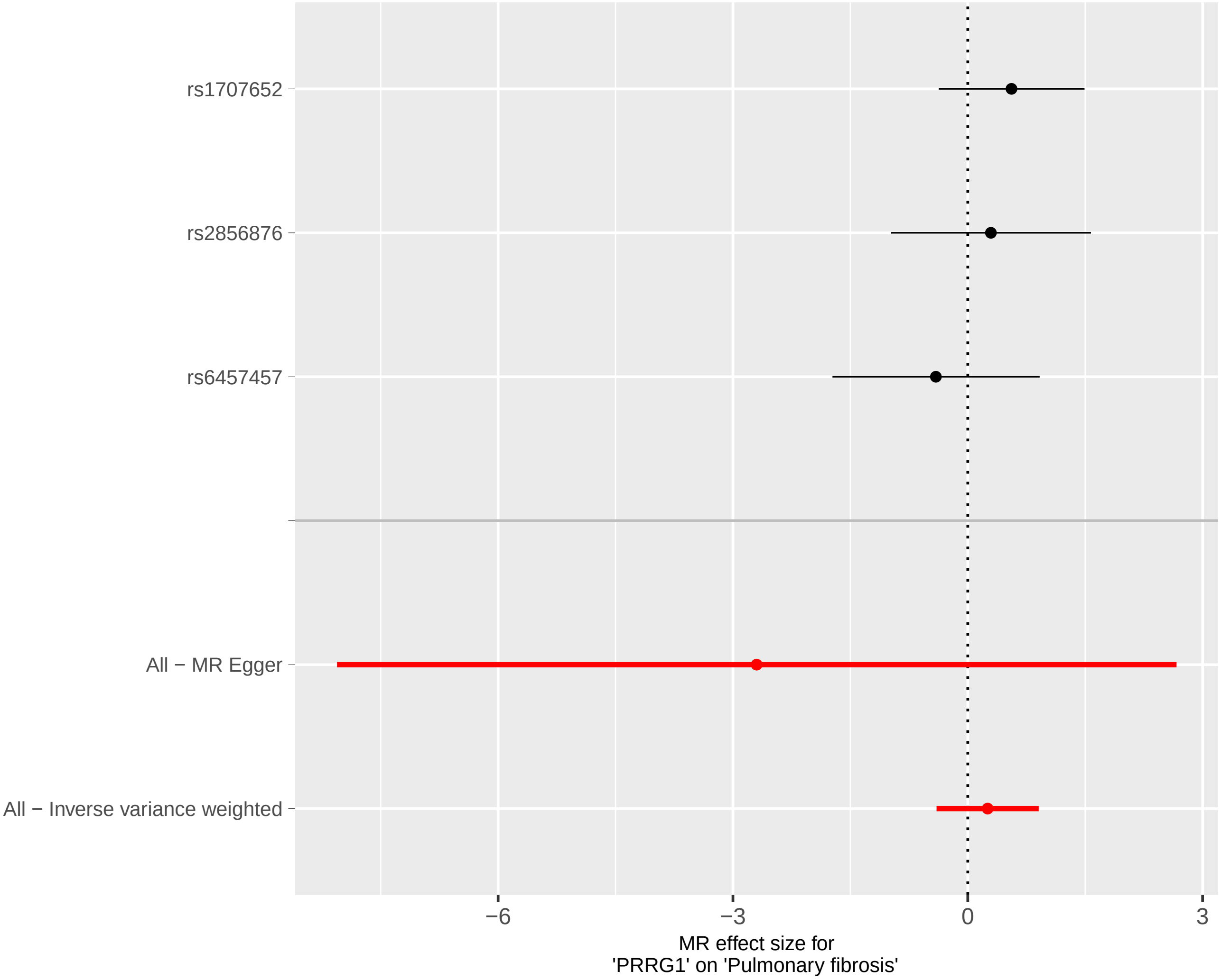

rs1707652
rs2856876
rs6457457
All − MR Egger
All − Inverse variance weighted
−6
−3
0
3
MR effect size for
'PRRG1' on 'Pulmonary fibrosis'

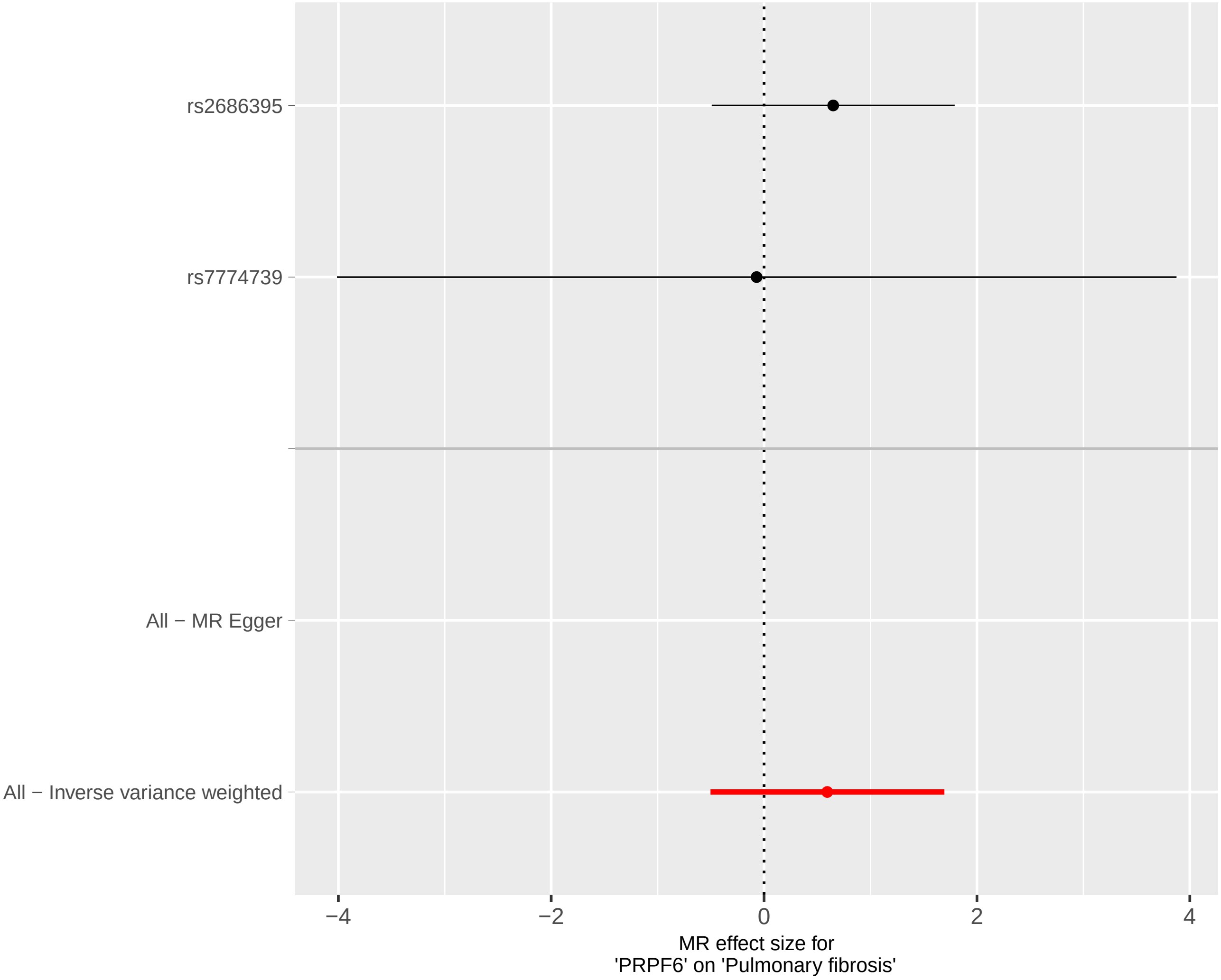
rs2686395
rs7774739
All − MR Egger
All − Inverse variance weighted
−4
−2
0
2
4
MR effect size for
'PRPF6' on 'Pulmonary fibrosis'

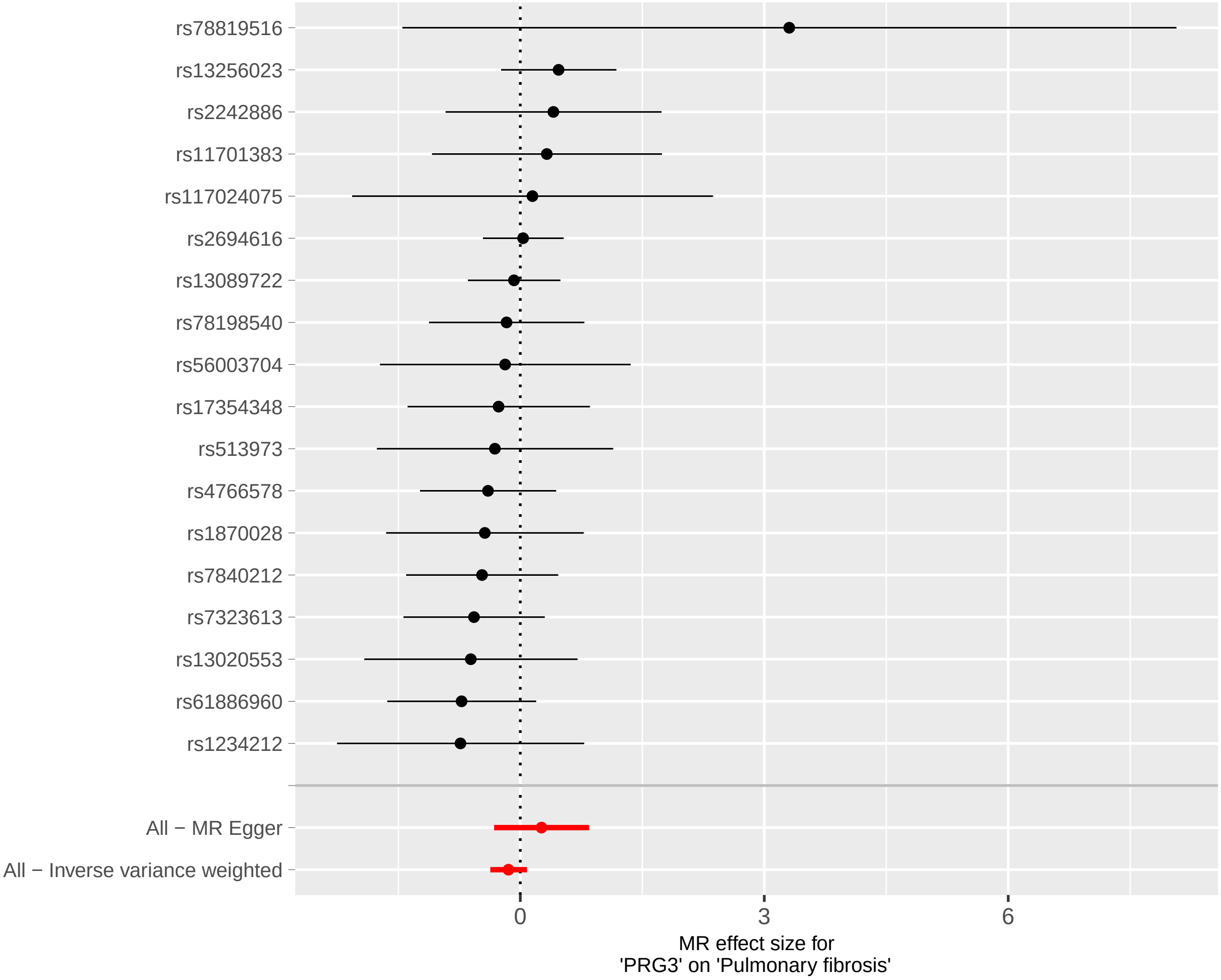
rs78819516
rs13256023
rs2242886
rs11701383
rs117024075
rs2694616
rs13089722
rs78198540
rs56003704
rs17354348
rs513973
rs4766578
rs1870028
rs7840212
rs7323613
rs13020553
rs61886960
rs1234212
All − MR Egger
All − Inverse variance weighted
0
3
6
MR effect size for
'PRG3' on 'Pulmonary fibrosis'

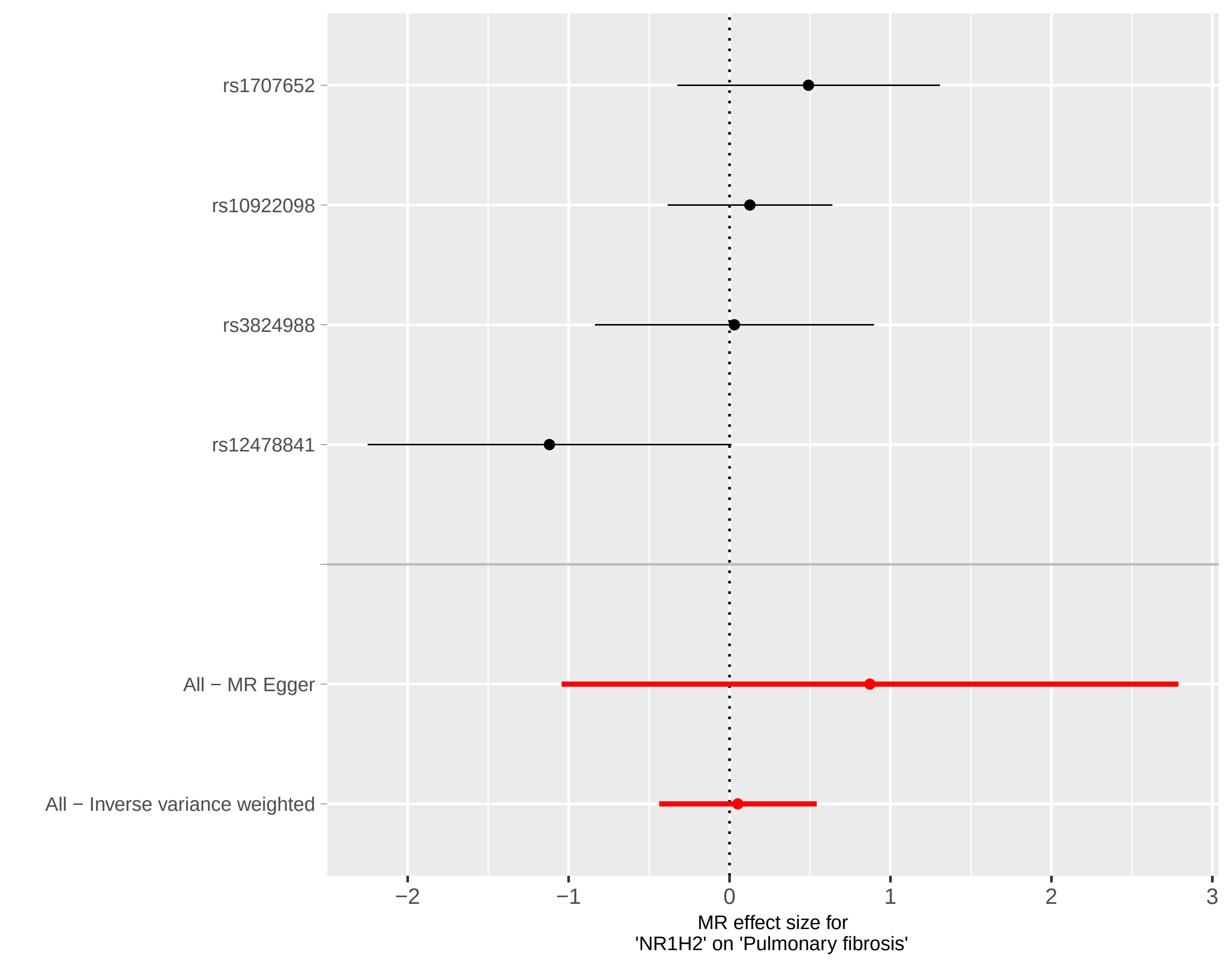
rs1707652
rs10922098
rs3824988
rs12478841
All − MR Egger
All − Inverse variance weighted
−2
−1
0
1
2
3
MR effect size for
'NR1H2' on 'Pulmonary fibrosis'

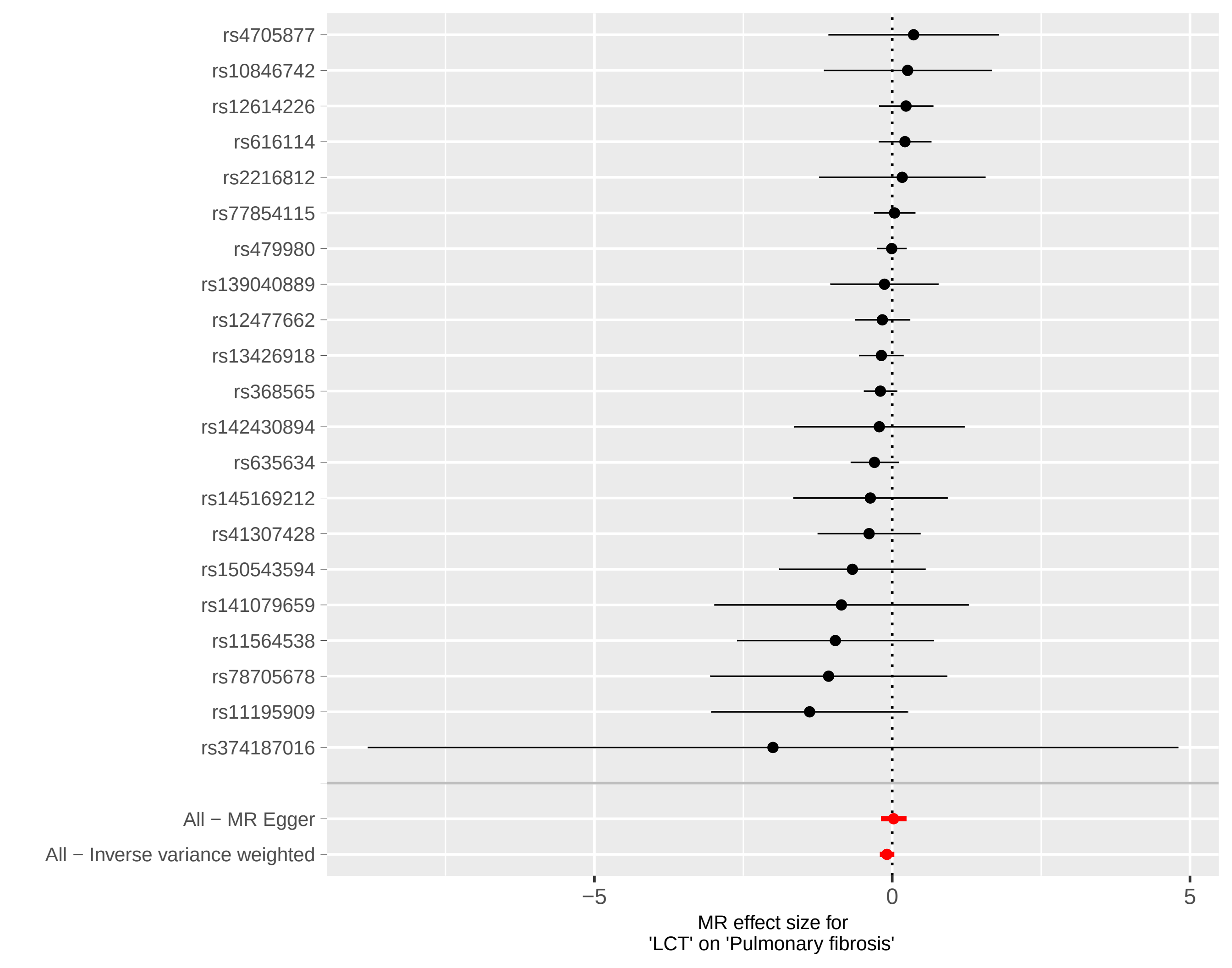
rs4705877
rs10846742
rs12614226
rs616114
rs2216812
rs77854115
rs479980
rs139040889
rs12477662
rs13426918
rs368565
rs142430894
rs635634
rs145169212
rs41307428
rs150543594
rs141079659
rs11564538
rs78705678
rs11195909
rs374187016
All − MR Egger
All − Inverse variance weighted
−5
0
5
MR effect size for
'LCT' on 'Pulmonary fibrosis'

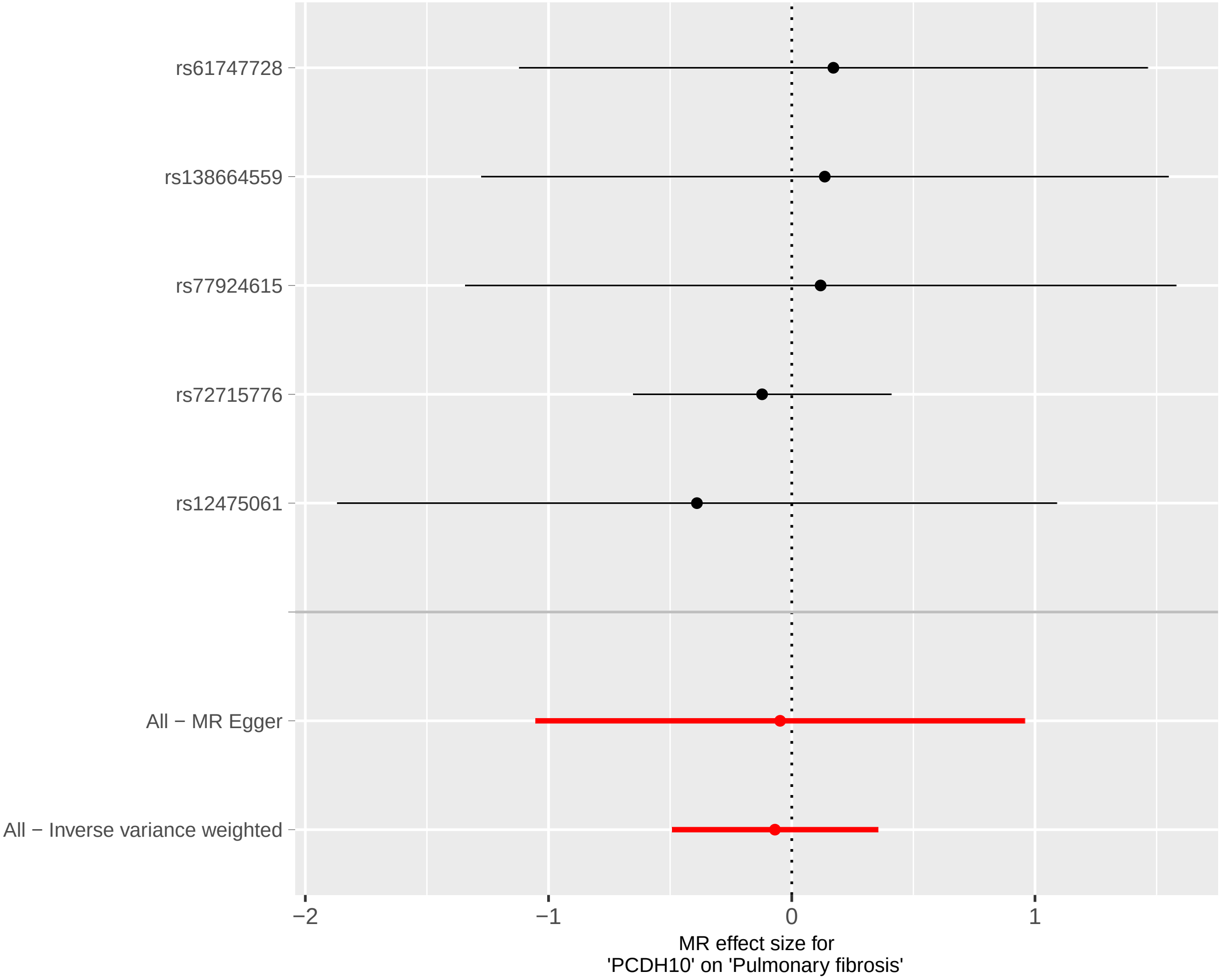

rs61747728
rs138664559
rs77924615
rs72715776
rs12475061
All − MR Egger
All − Inverse variance weighted
−2
−1
0
1
MR effect size for
'PCDH10' on 'Pulmonary fibrosis'

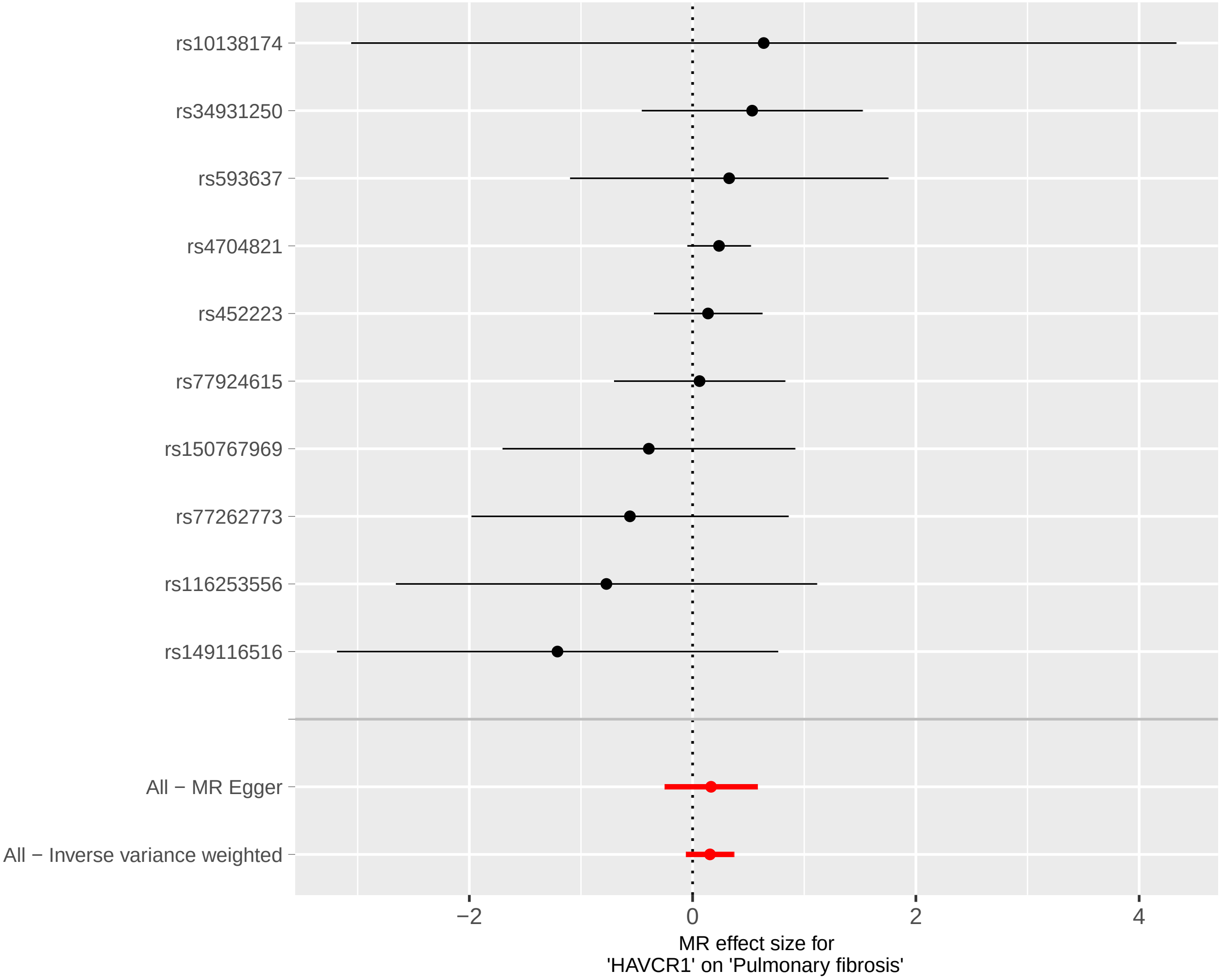

rs10138174
rs34931250
rs593637
rs4704821
rs452223
rs77924615
rs150767969
rs77262773
rs116253556
rs149116516
All − MR Egger
All − Inverse variance weighted
−2
0
2
4
MR effect size for
'HAVCR1' on 'Pulmonary fibrosis'

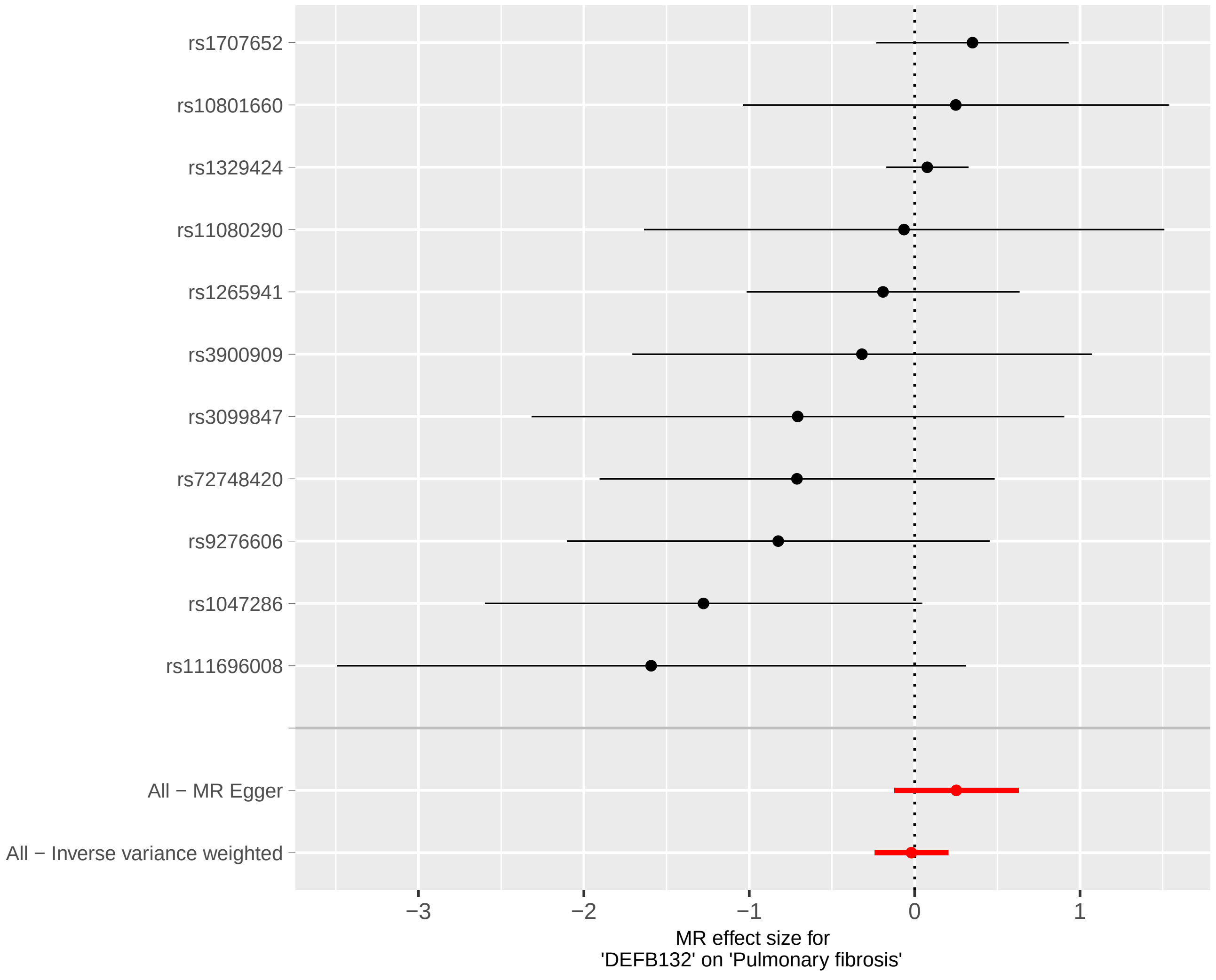

rs1707652
rs10801660
rs1329424
rs11080290
rs1265941
rs3900909
rs3099847
rs72748420
rs9276606
rs1047286
rs111696008
All − MR Egger
All − Inverse variance weighted
−3
−2
−1
0
1
MR effect size for
'DEFB132' on 'Pulmonary fibrosis'

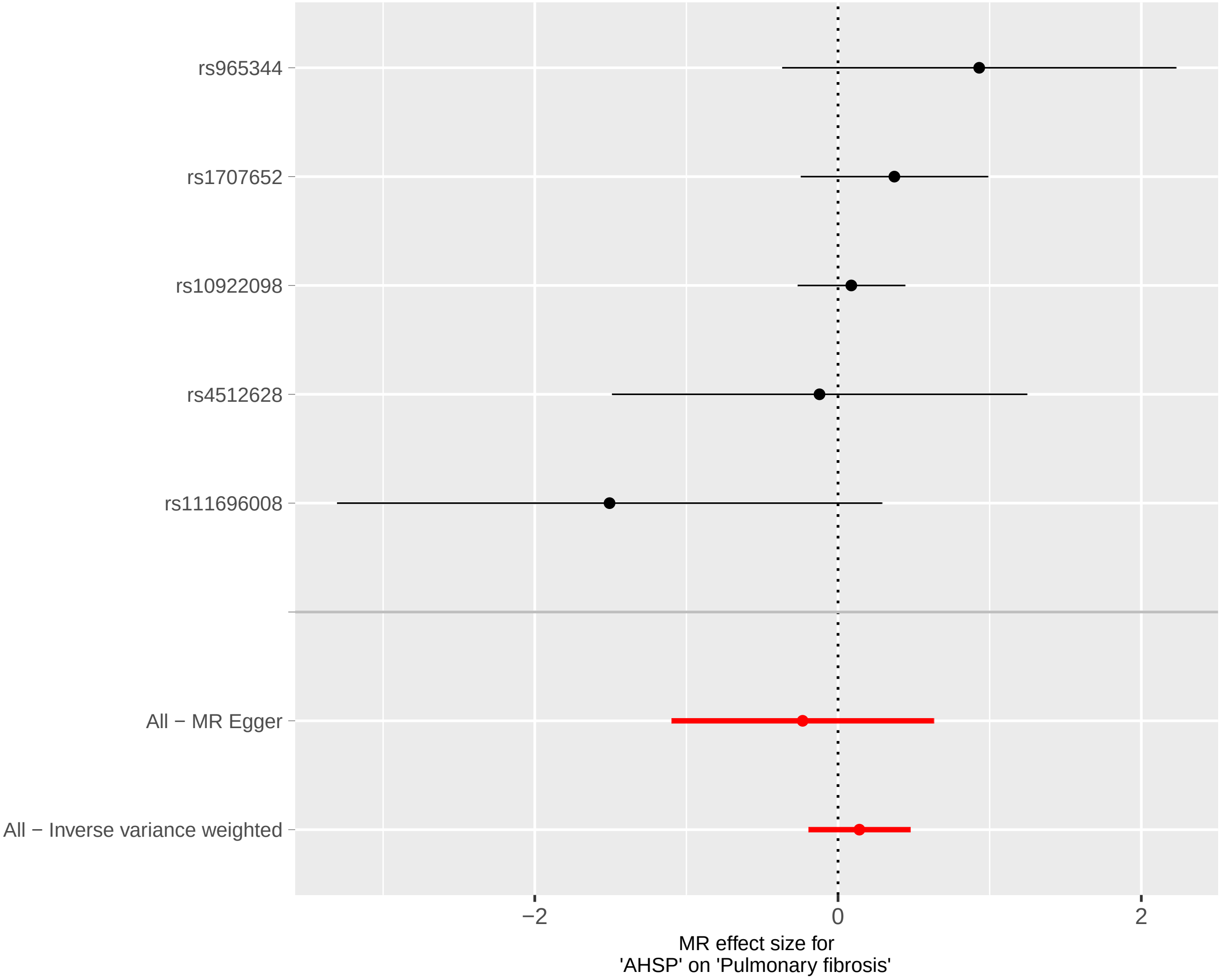

rs965344
rs1707652
rs10922098
rs4512628
rs111696008
All − MR Egger
All − Inverse variance weighted
−2
0
2
MR effect size for
'AHSP' on 'Pulmonary fibrosis'

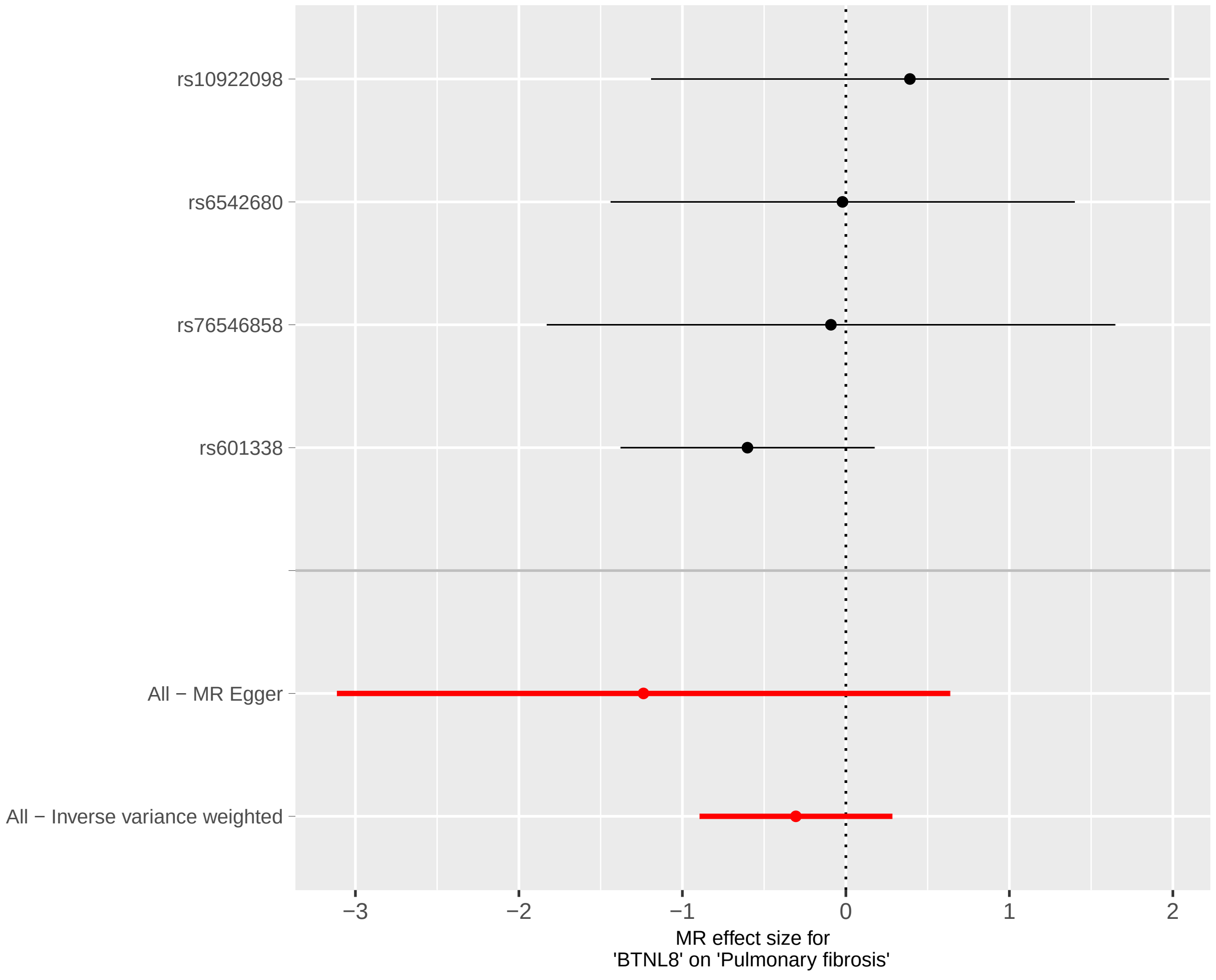

rs10922098
rs6542680
rs76546858
rs601338
All − MR Egger
All − Inverse variance weighted
−3
−2
−1
0
1
2
MR effect size for
'BTNL8' on 'Pulmonary fibrosis'

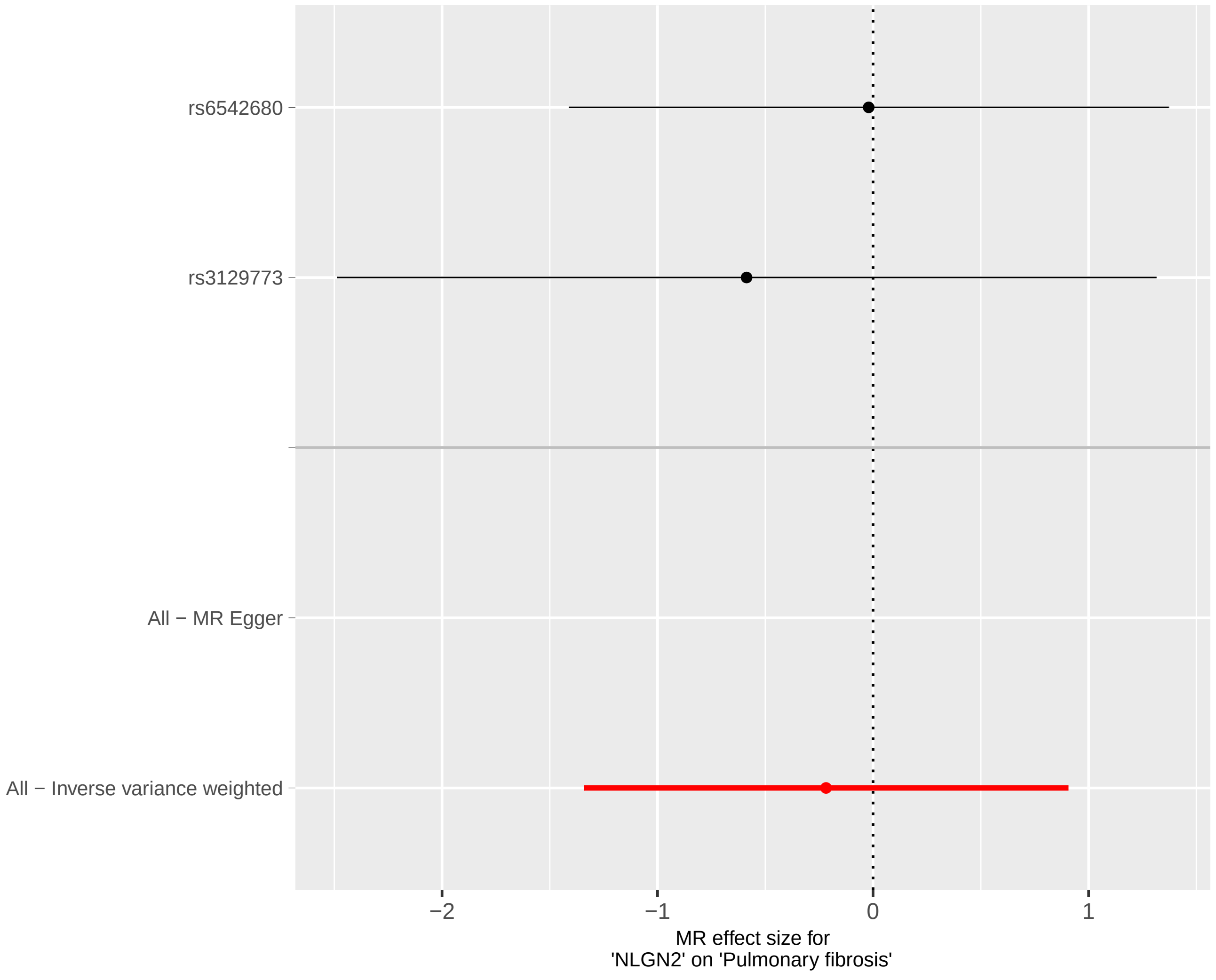
rs6542680
rs3129773
All − MR Egger
All − Inverse variance weighted
−2
−1
0
1
MR effect size for
'NLGN2' on 'Pulmonary fibrosis'

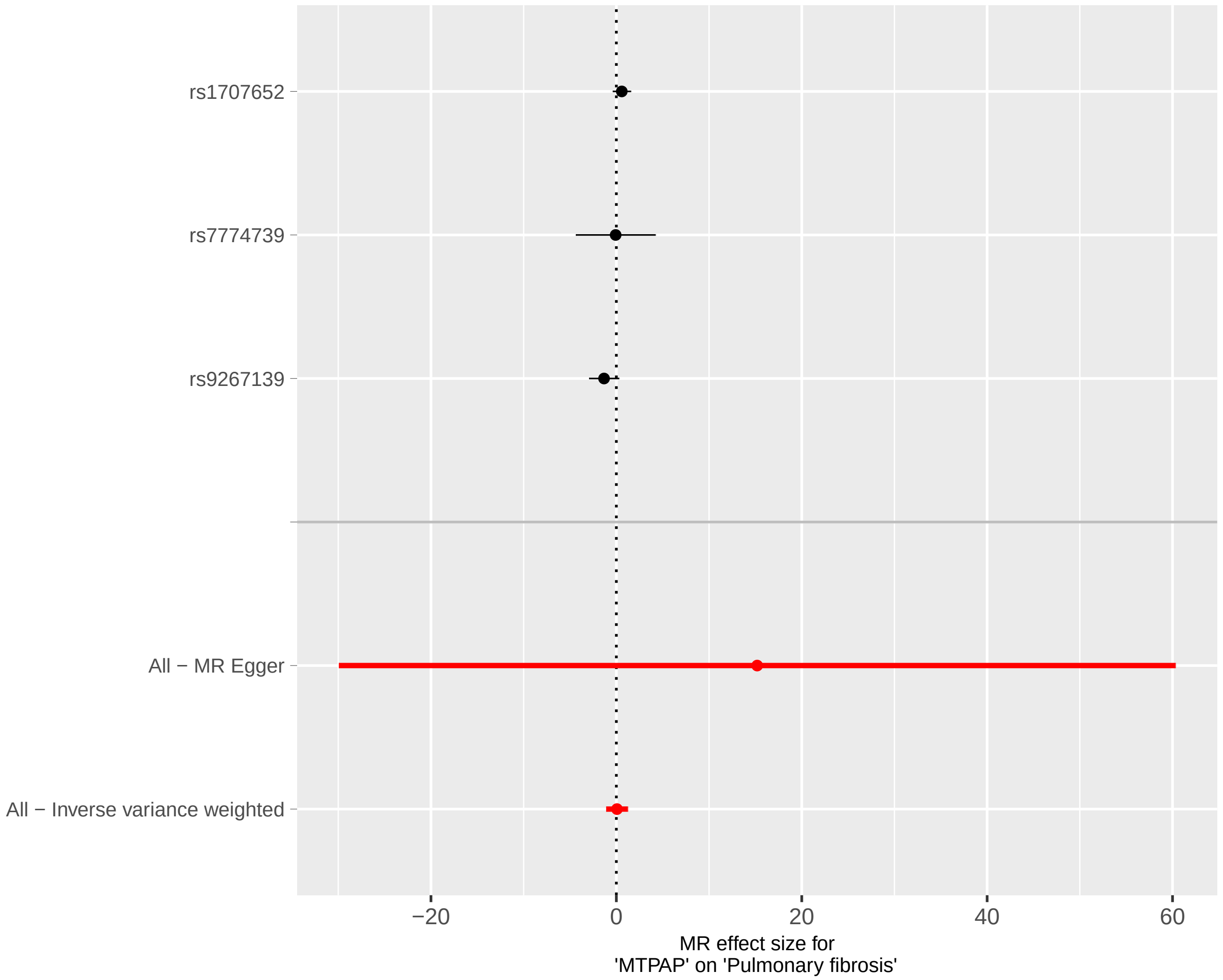

rs1707652
rs7774739
rs9267139
All − MR Egger
All − Inverse variance weighted
−20
0
20
40
60
MR effect size for
'MTPAP' on 'Pulmonary fibrosis'

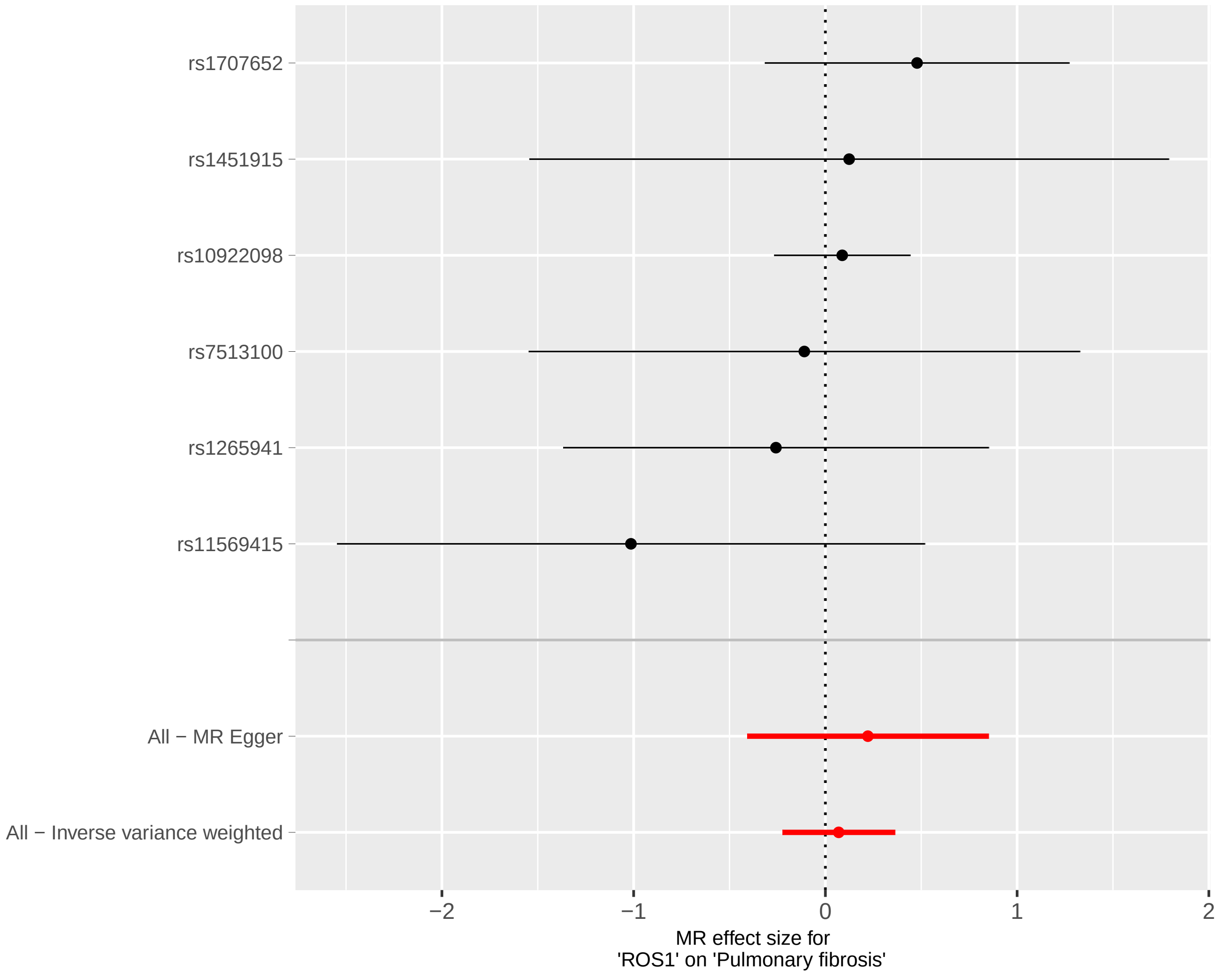
rs1707652
rs1451915
rs10922098
rs7513100
rs1265941
rs11569415
All − MR Egger
All − Inverse variance weighted
−2
−1
0
1
2
MR effect size for
'ROS1' on 'Pulmonary fibrosis'

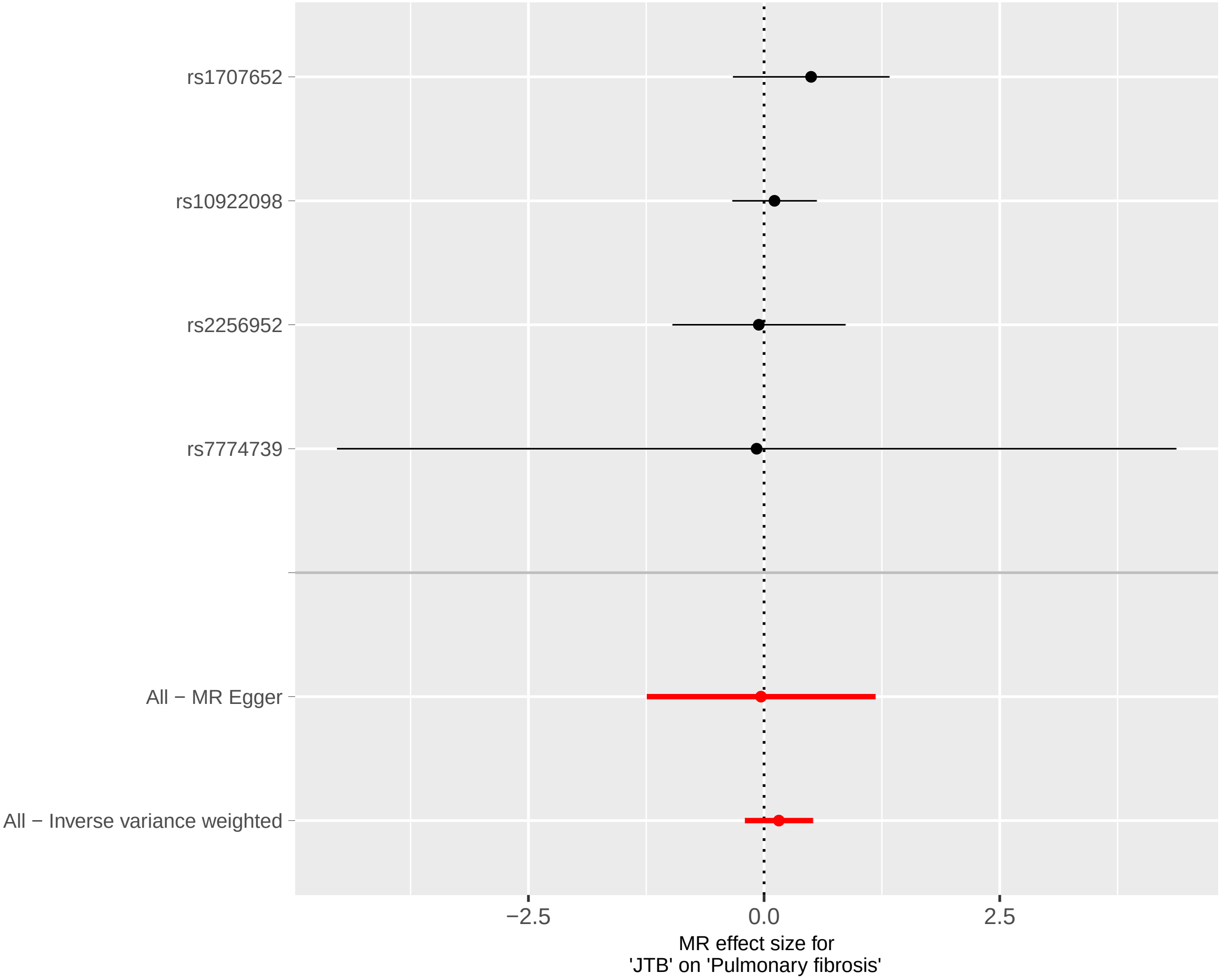
rs1707652
rs10922098
rs2256952
rs7774739
All − MR Egger
All − Inverse variance weighted
−2.5
0.0
2.5
MR effect size for
'JTB' on 'Pulmonary fibrosis'

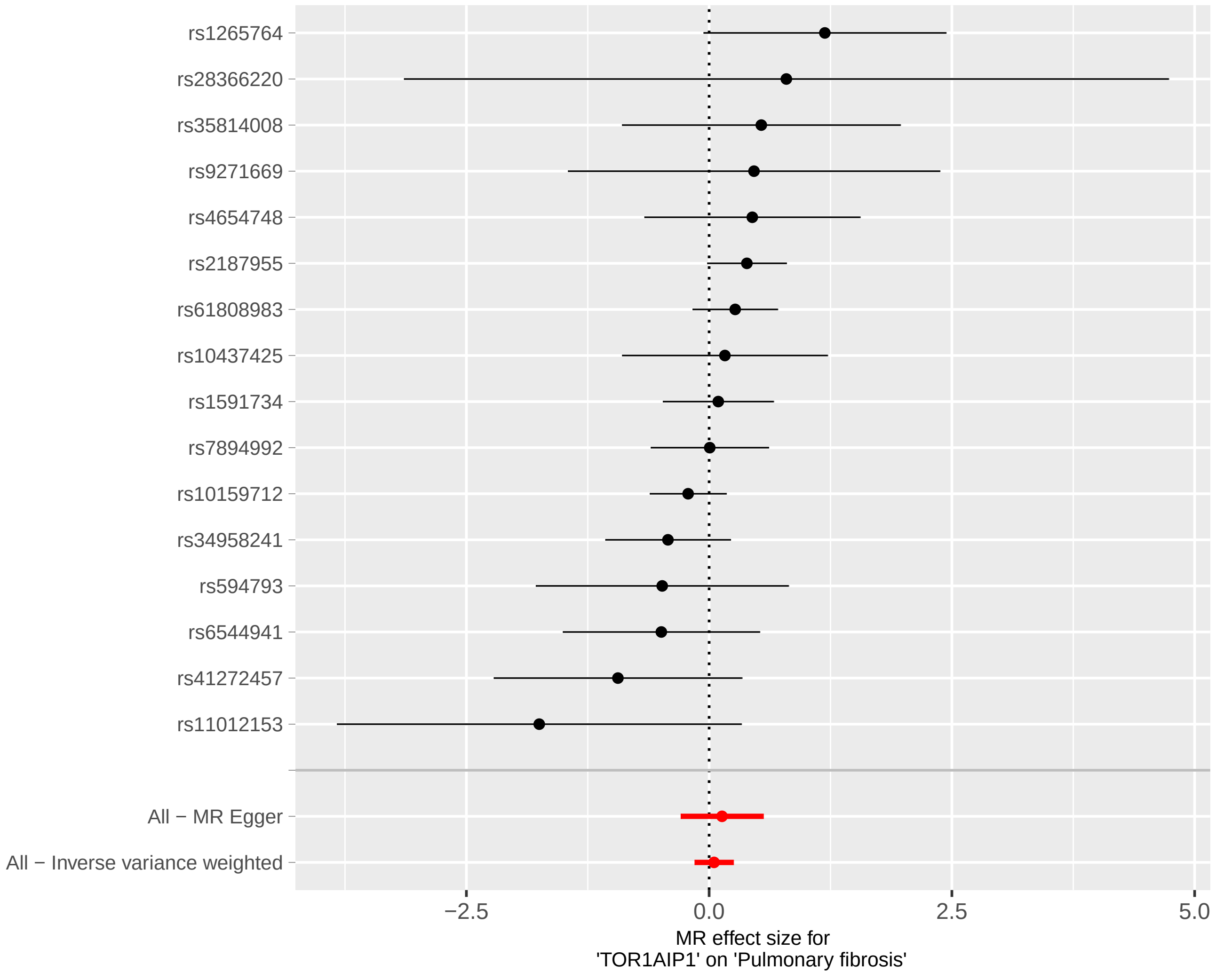
rs1265764
rs28366220
rs35814008
rs9271669
rs4654748
rs2187955
rs61808983
rs10437425
rs1591734
rs7894992
rs10159712
rs34958241
rs594793
rs6544941
rs41272457
rs11012153
All − MR Egger
All − Inverse variance weighted
−2.5
0.0
2.5
5.0
MR effect size for
'TOR1AIP1' on 'Pulmonary fibrosis'

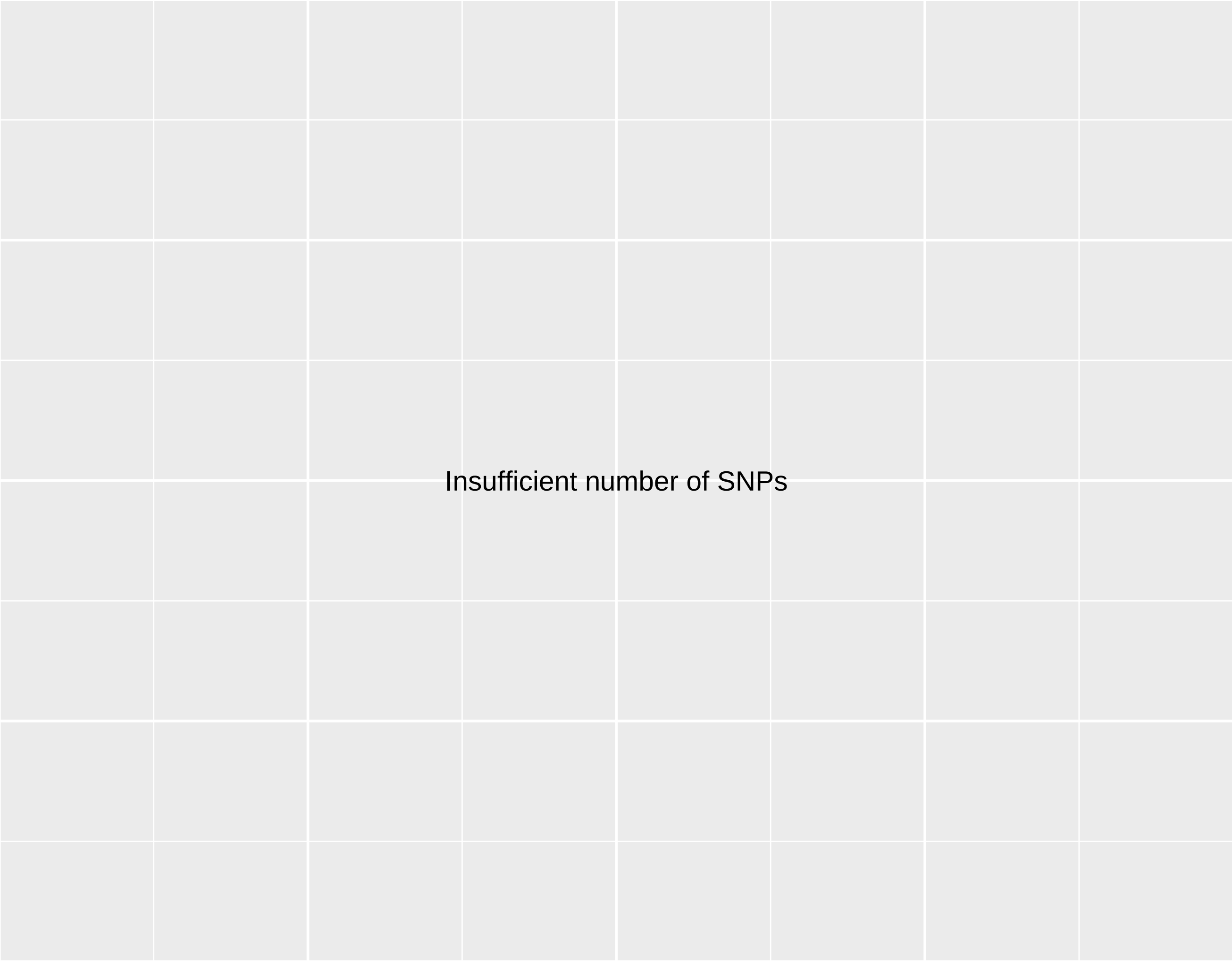
Insufficient number of SNPs

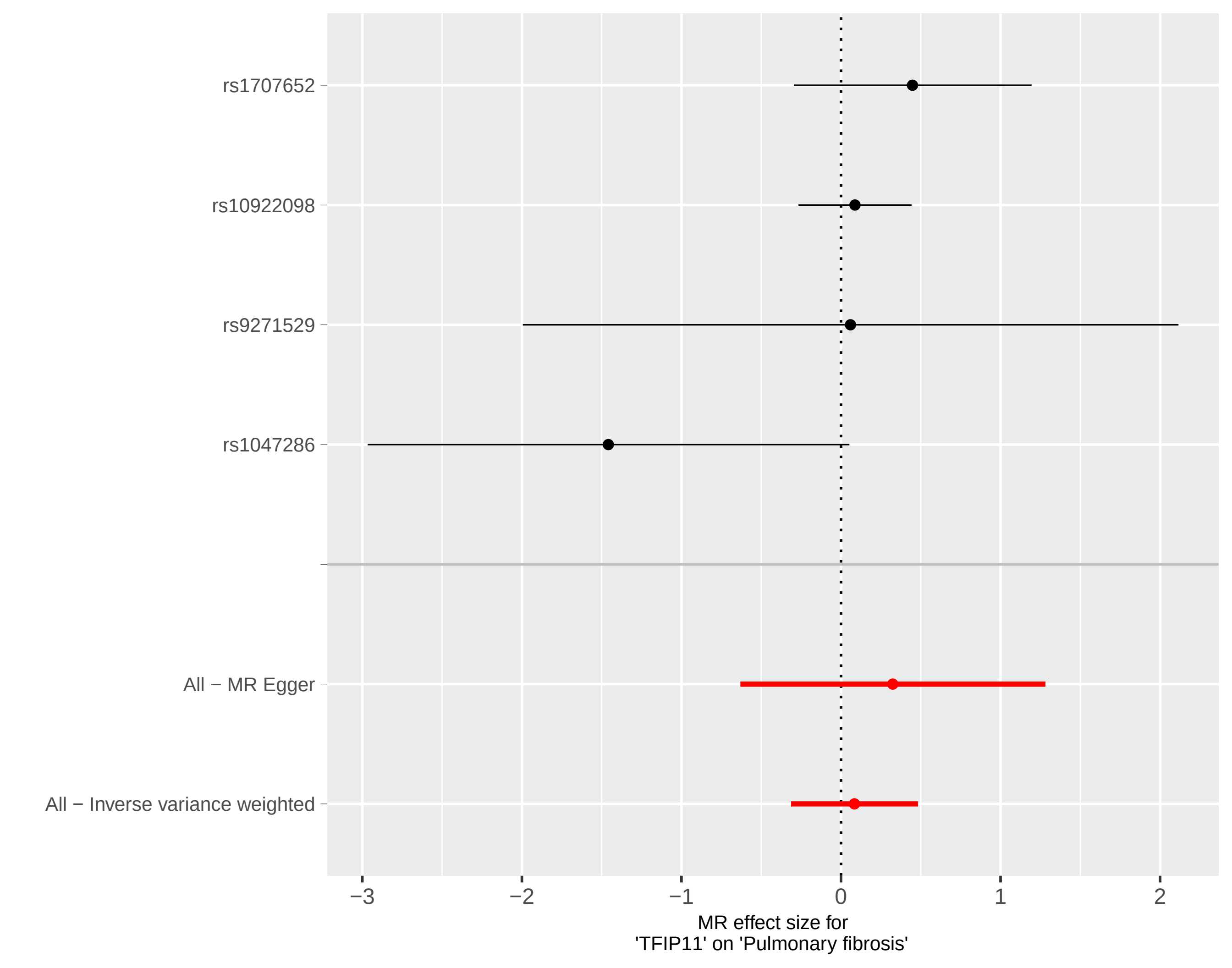

rs1707652
rs10922098
rs9271529
rs1047286
All − MR Egger
All − Inverse variance weighted
−3
−2
−1
0
1
2
MR effect size for
'TFIP11' on 'Pulmonary fibrosis'

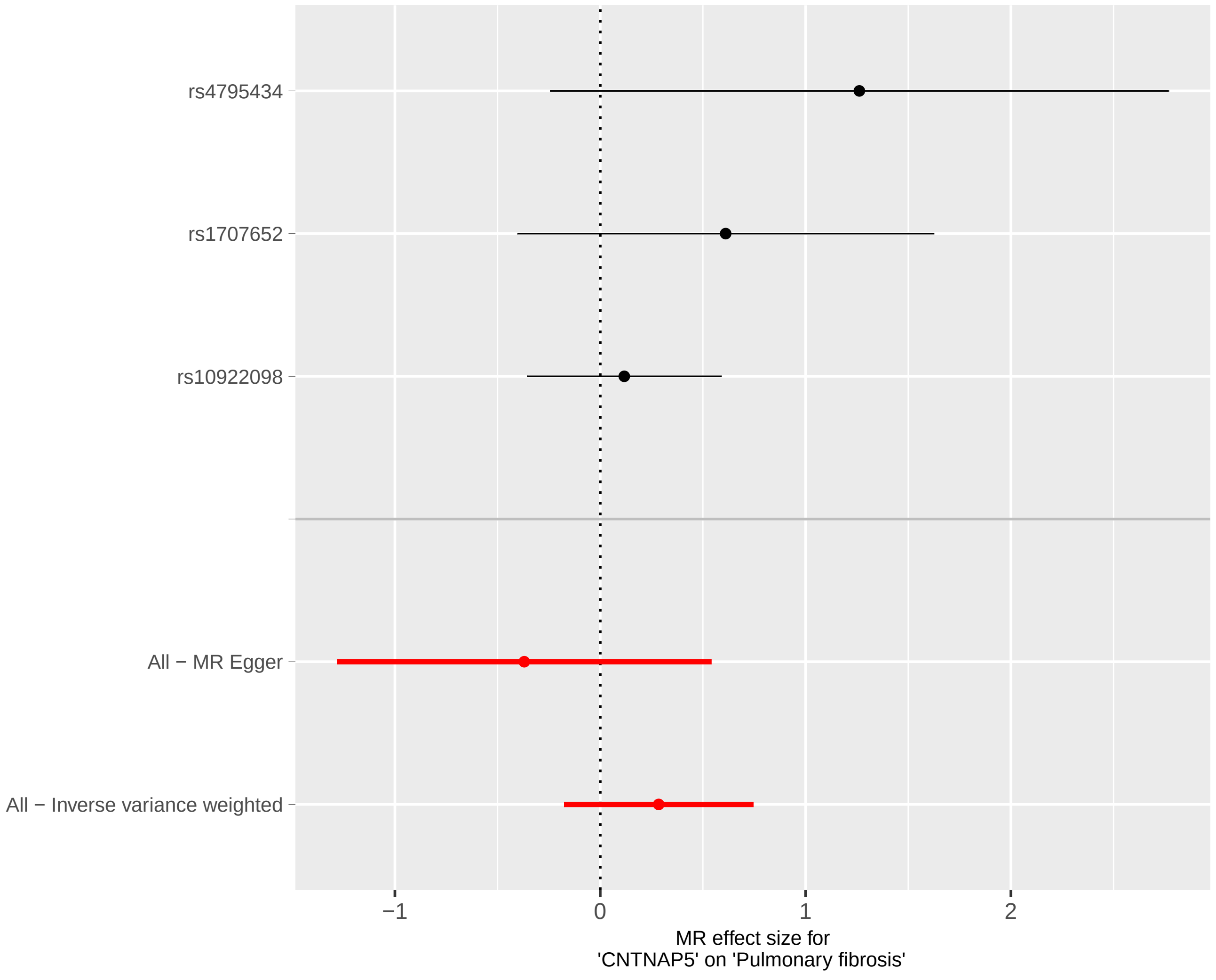

rs4795434
rs1707652
rs10922098
All − MR Egger
All − Inverse variance weighted
−1
0
1
2
MR effect size for
'CNTNAP5' on 'Pulmonary fibrosis'

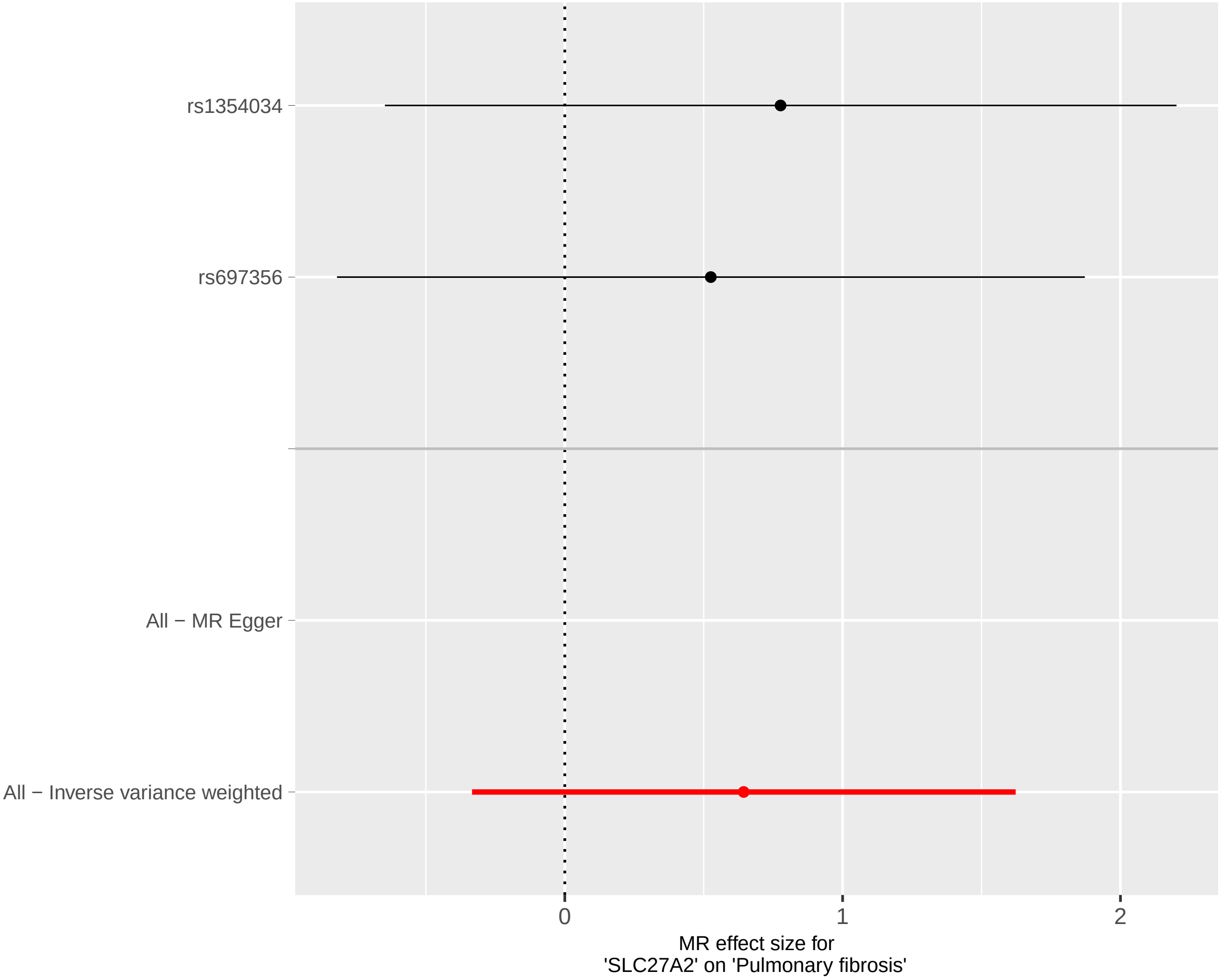

rs1354034
rs697356
All − MR Egger
All − Inverse variance weighted
0
1
2
MR effect size for
'SLC27A2' on 'Pulmonary fibrosis'

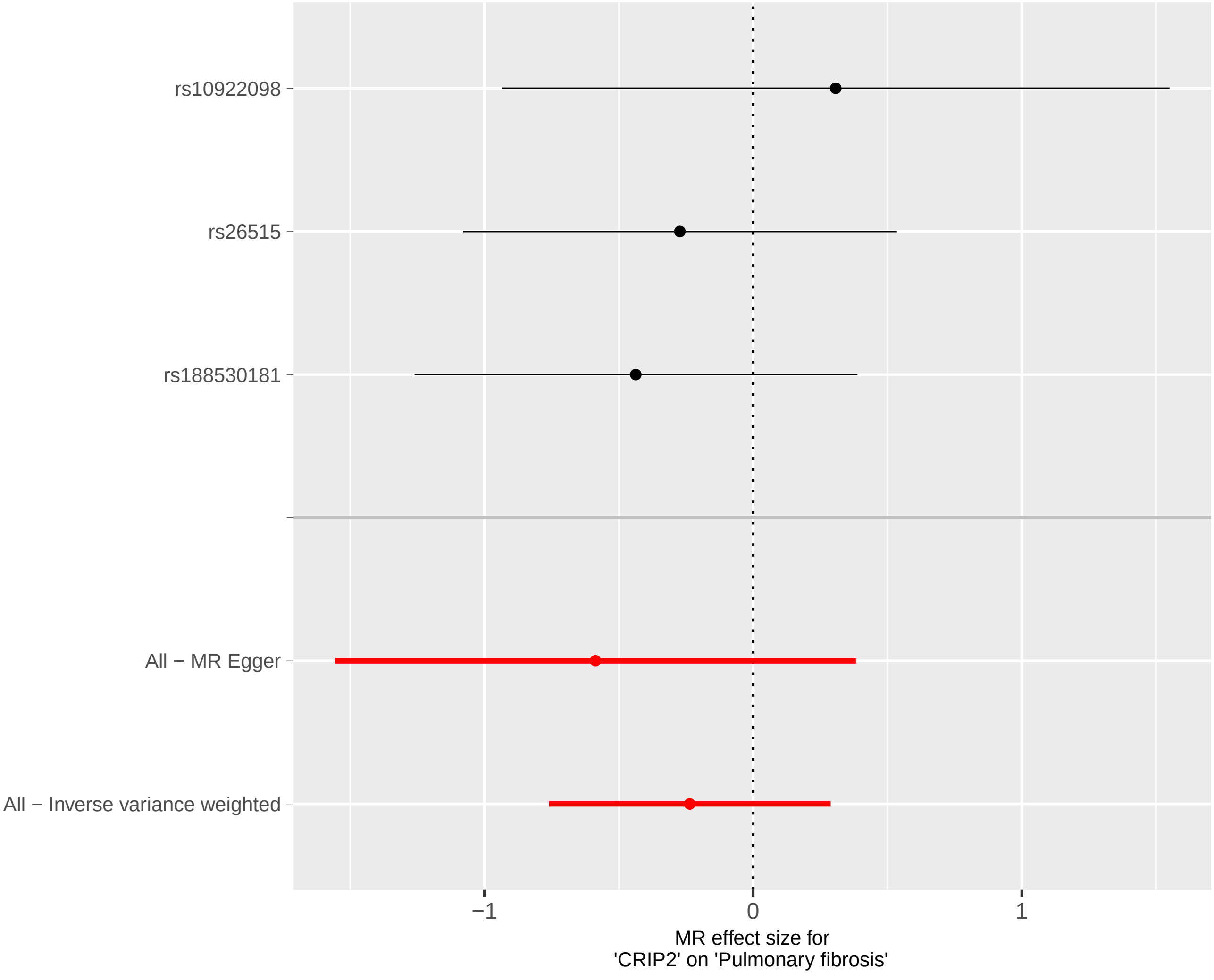

rs10922098
rs26515
rs188530181
All − MR Egger
All − Inverse variance weighted
−1
0
1
MR effect size for
'CRIP2' on 'Pulmonary fibrosis'

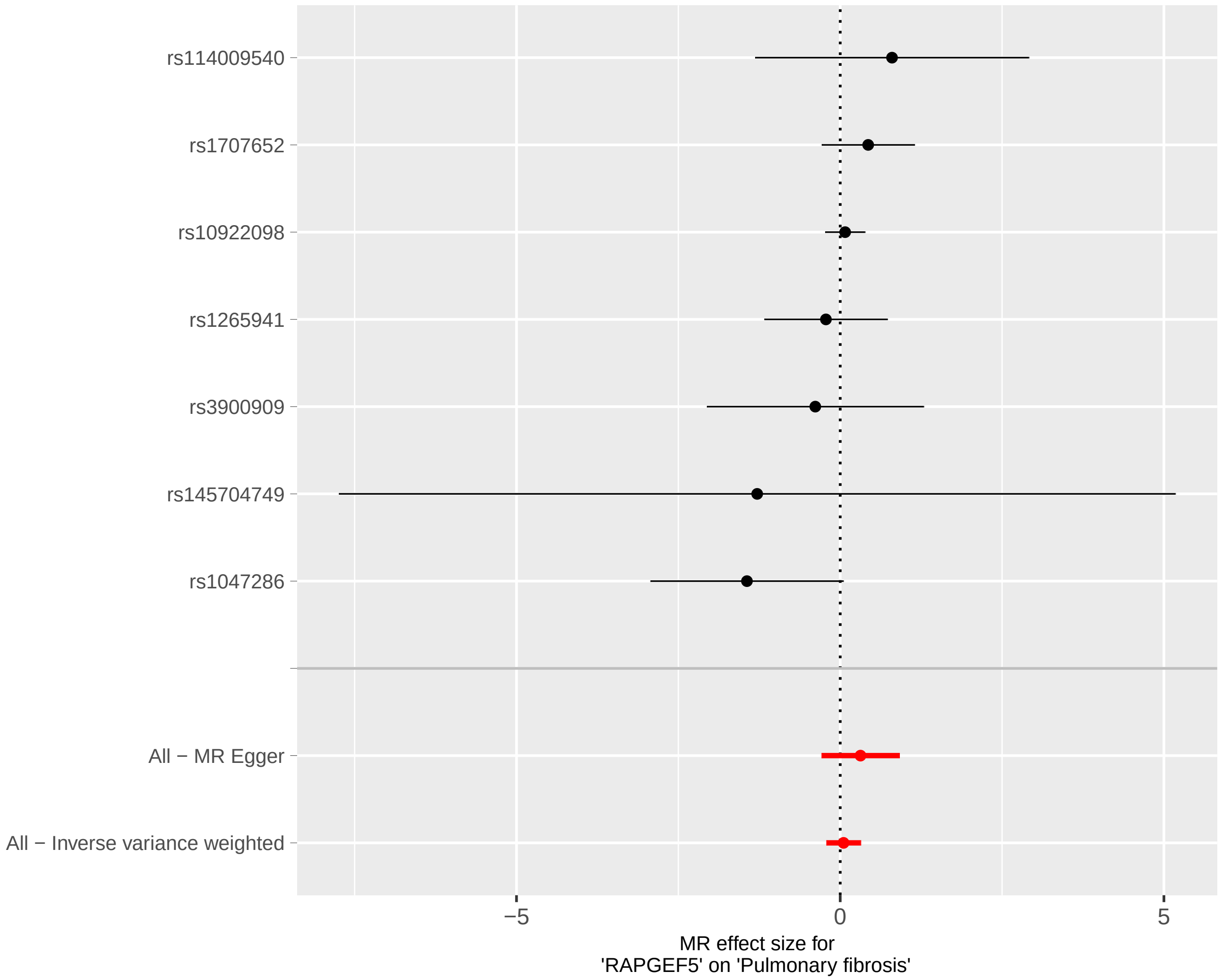
rs114009540
rs1707652
rs10922098
rs1265941
rs3900909
rs145704749
rs1047286
All − MR Egger
All − Inverse variance weighted
−5
0
5
MR effect size for
'RAPGEF5' on 'Pulmonary fibrosis'

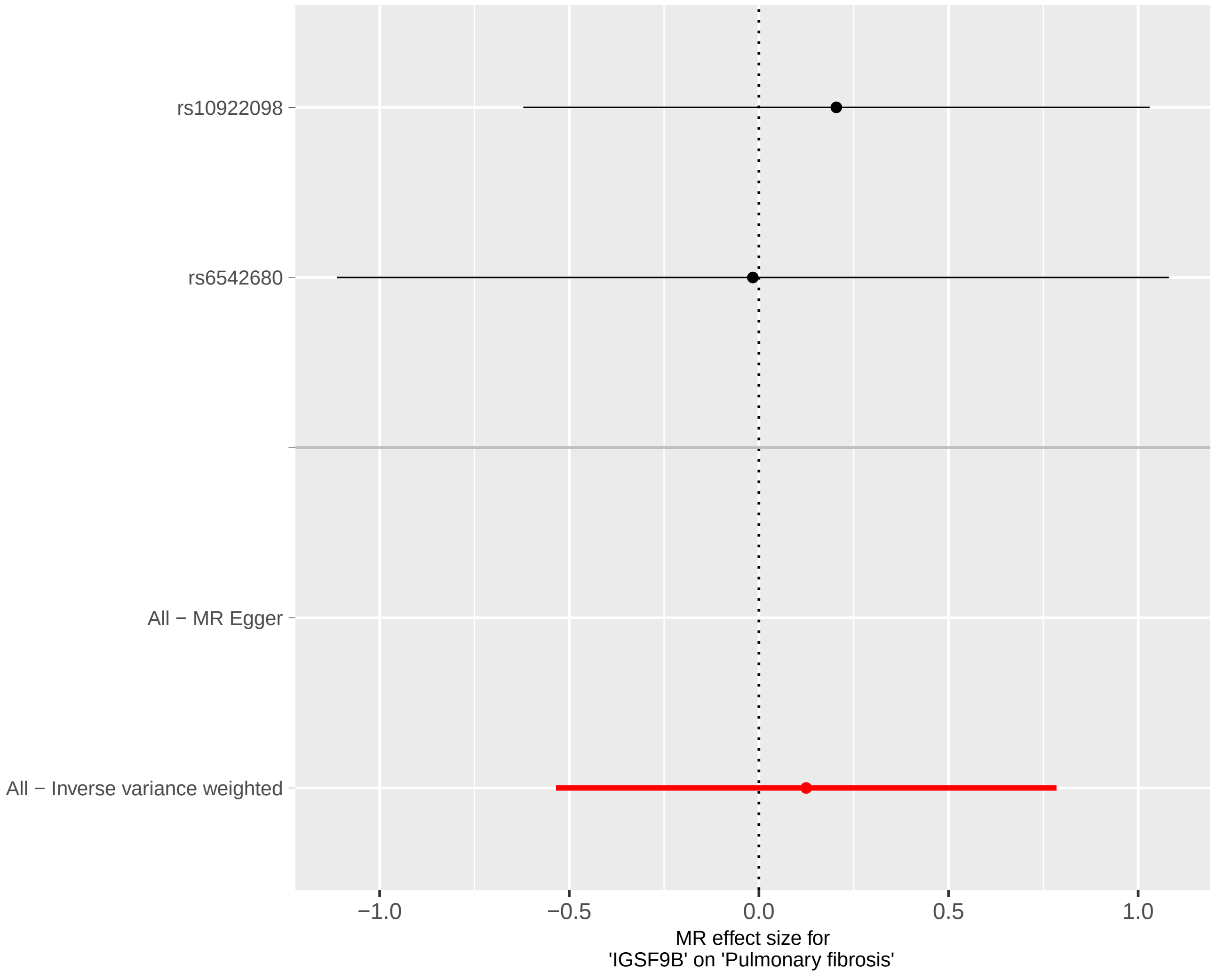

rs10922098
rs6542680
All − MR Egger
All − Inverse variance weighted
−1.0
−0.5
0.0
0.5
1.0
MR effect size for
'IGSF9B' on 'Pulmonary fibrosis'

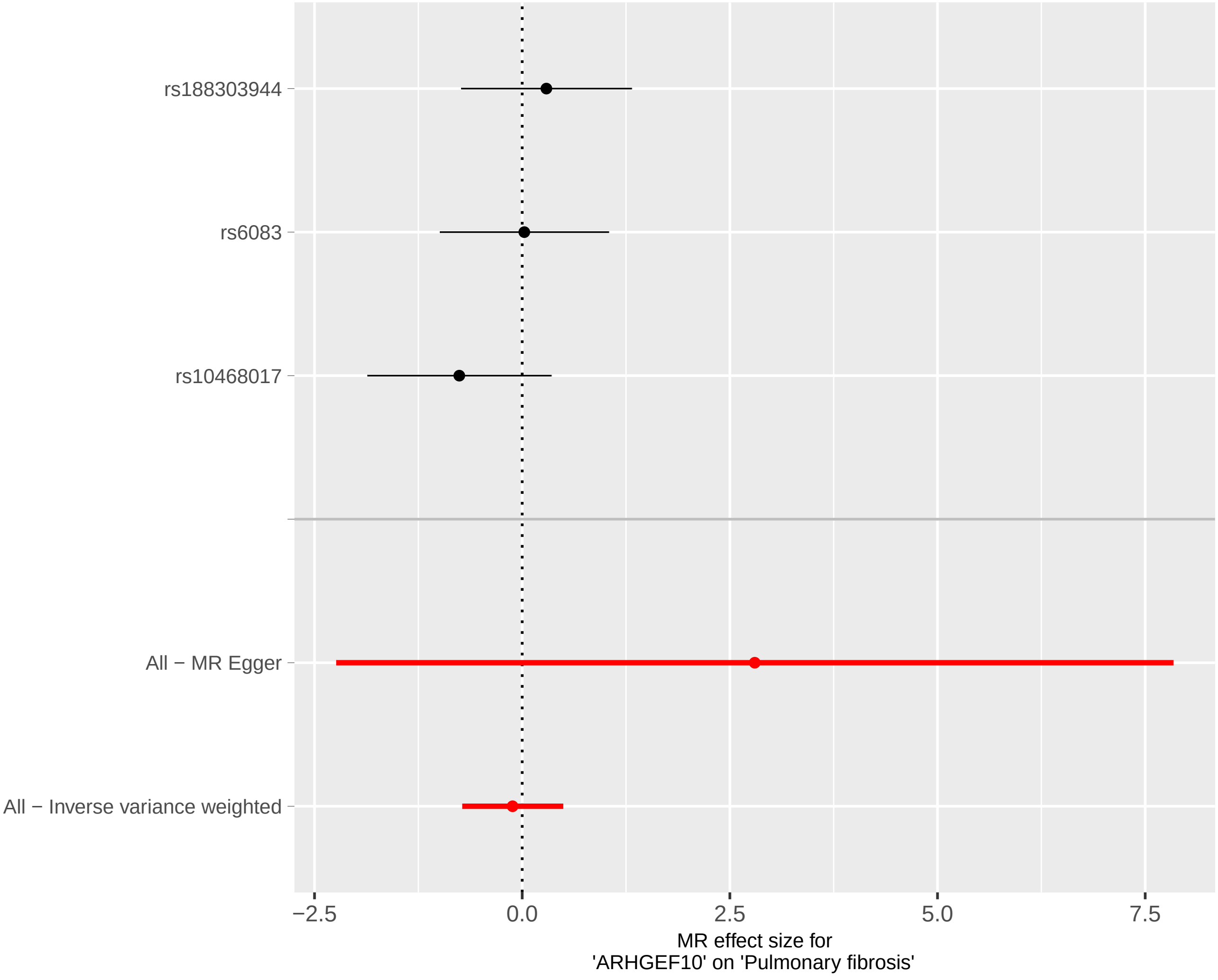
rs188303944
rs6083
rs10468017
All − MR Egger
All − Inverse variance weighted
−2.5
0.0
2.5
5.0
7.5
MR effect size for
'ARHGEF10' on 'Pulmonary fibrosis'

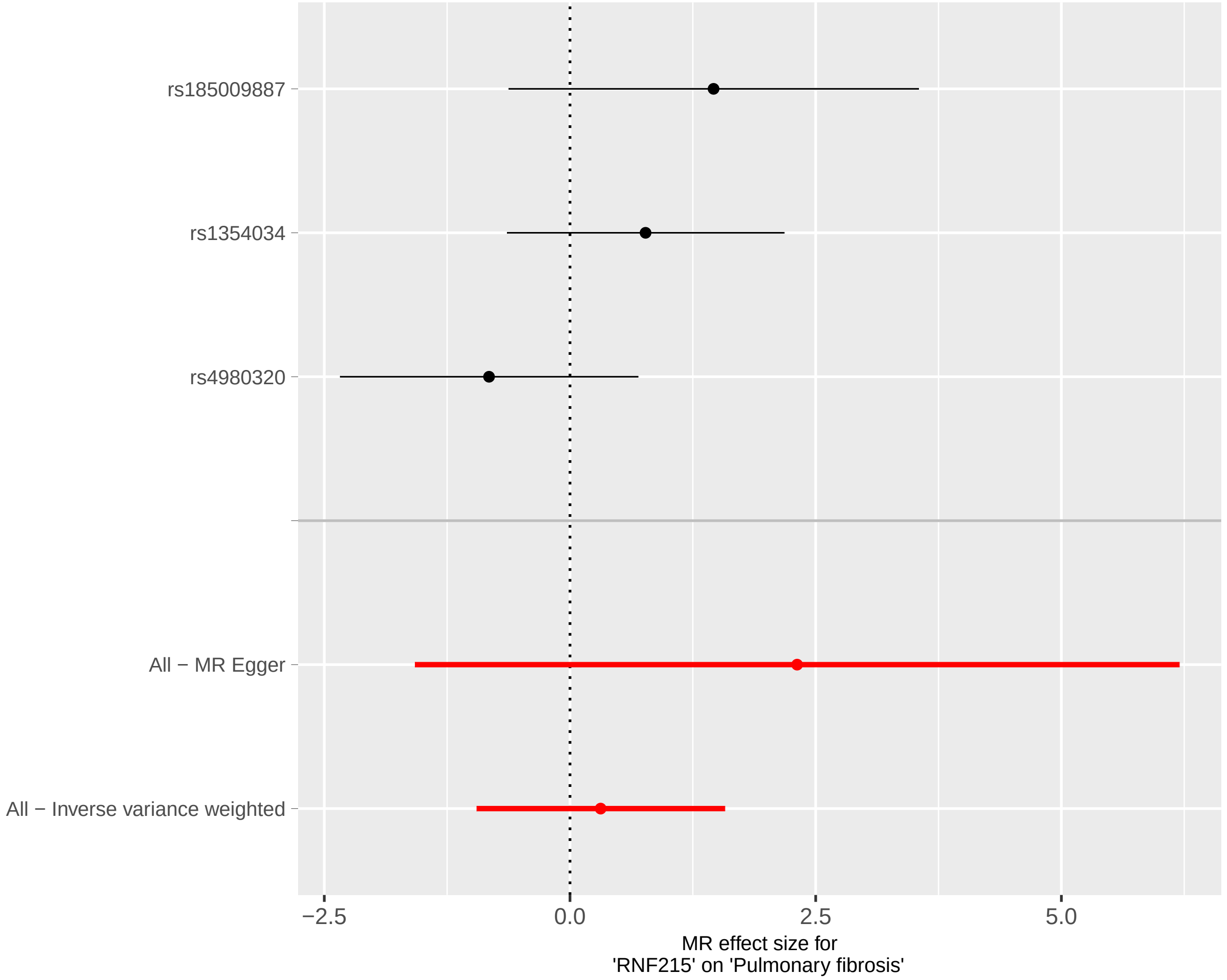
rs185009887
rs1354034
rs4980320
All − MR Egger
All − Inverse variance weighted
−2.5
0.0
2.5
5.0
MR effect size for
'RNF215' on 'Pulmonary fibrosis'

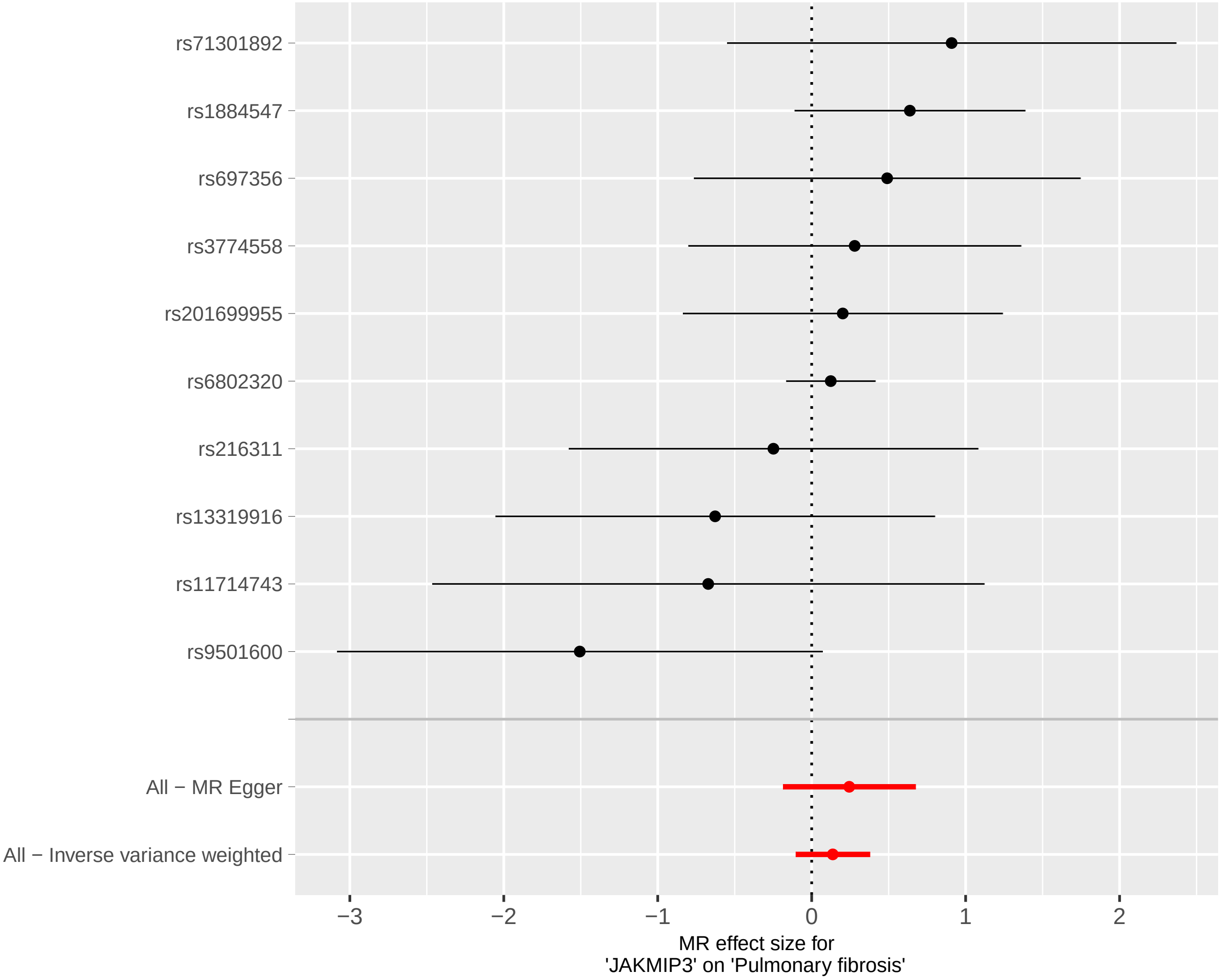

rs71301892
rs1884547
rs697356
rs3774558
rs201699955
rs6802320
rs216311
rs13319916
rs11714743
rs9501600
All − MR Egger
All − Inverse variance weighted
−3
−2
−1
0
1
2
MR effect size for
'JAKMIP3' on 'Pulmonary fibrosis'

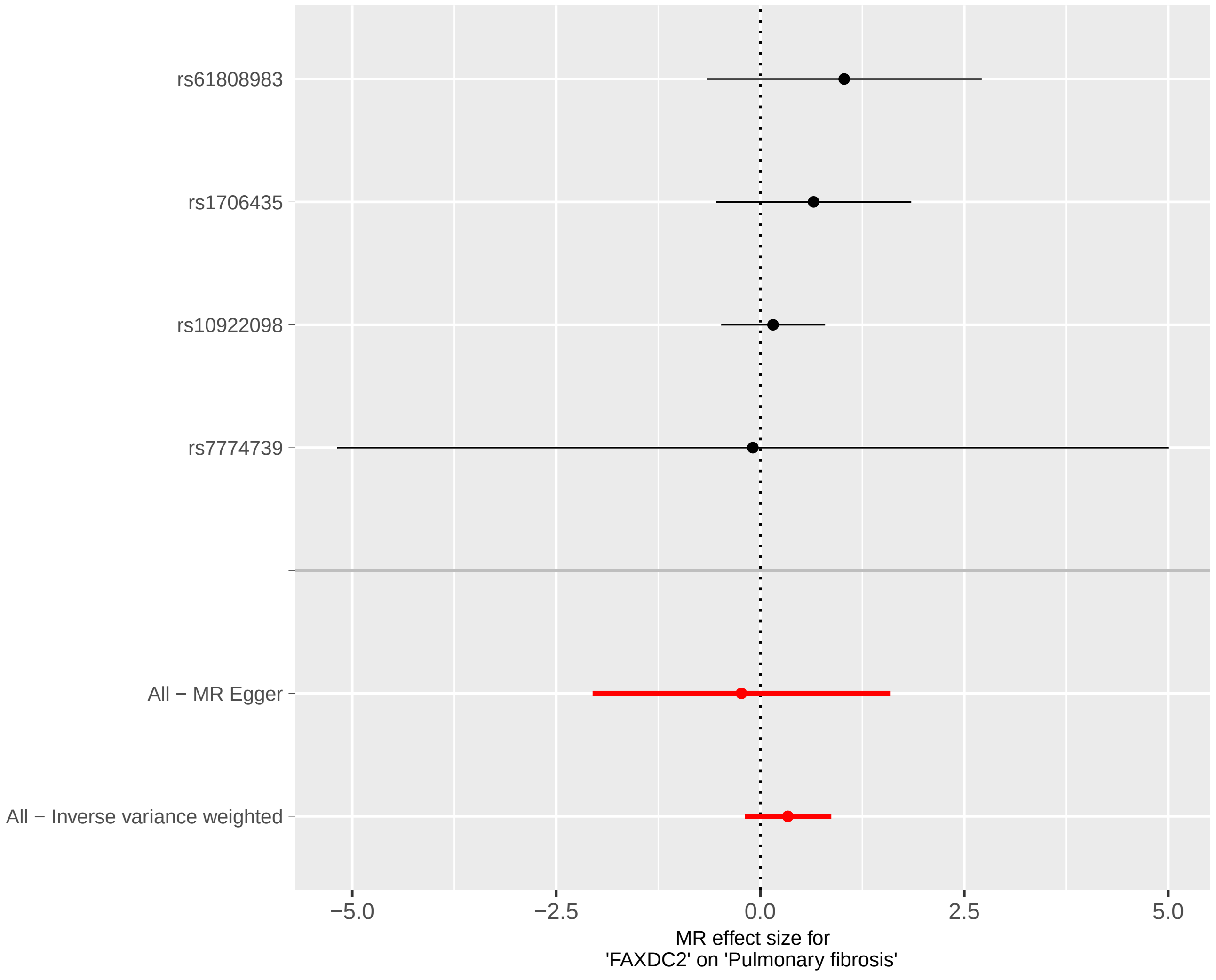
rs61808983
rs1706435
rs10922098
rs7774739
All − MR Egger
All − Inverse variance weighted
−5.0
−2.5
0.0
2.5
5.0
MR effect size for
'FAXDC2' on 'Pulmonary fibrosis'

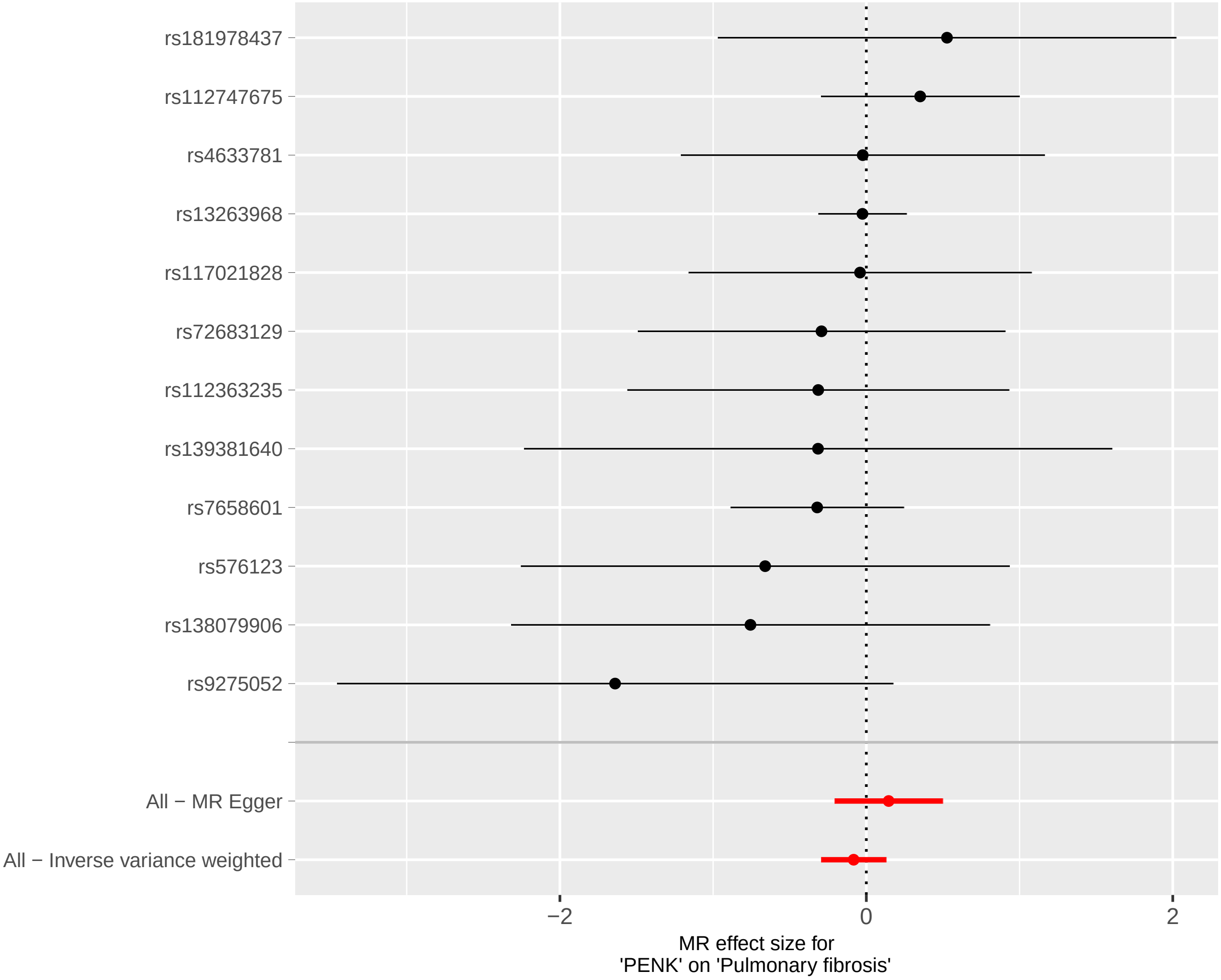

rs181978437
rs112747675
rs4633781
rs13263968
rs117021828
rs72683129
rs112363235
rs139381640
rs7658601
rs576123
rs138079906
rs9275052
All − MR Egger
All − Inverse variance weighted
−2
0
2
MR effect size for
'PENK' on 'Pulmonary fibrosis'

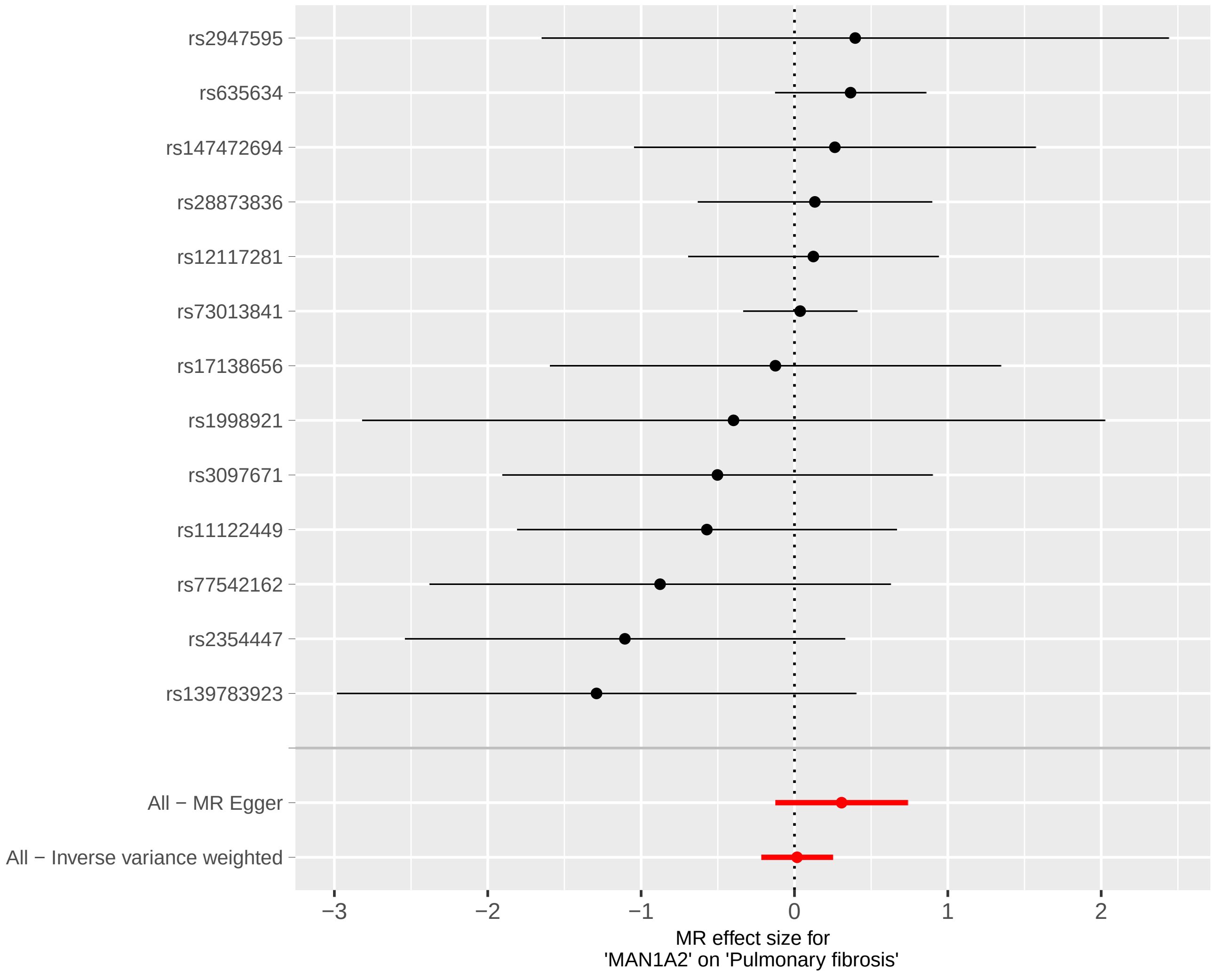
rs2947595
rs635634
rs147472694
rs28873836
rs12117281
rs73013841
rs17138656
rs1998921
rs3097671
rs11122449
rs77542162
rs2354447
rs139783923
All − MR Egger
All − Inverse variance weighted
−3
−2
−1
0
1
2
MR effect size for
'MAN1A2' on 'Pulmonary fibrosis'

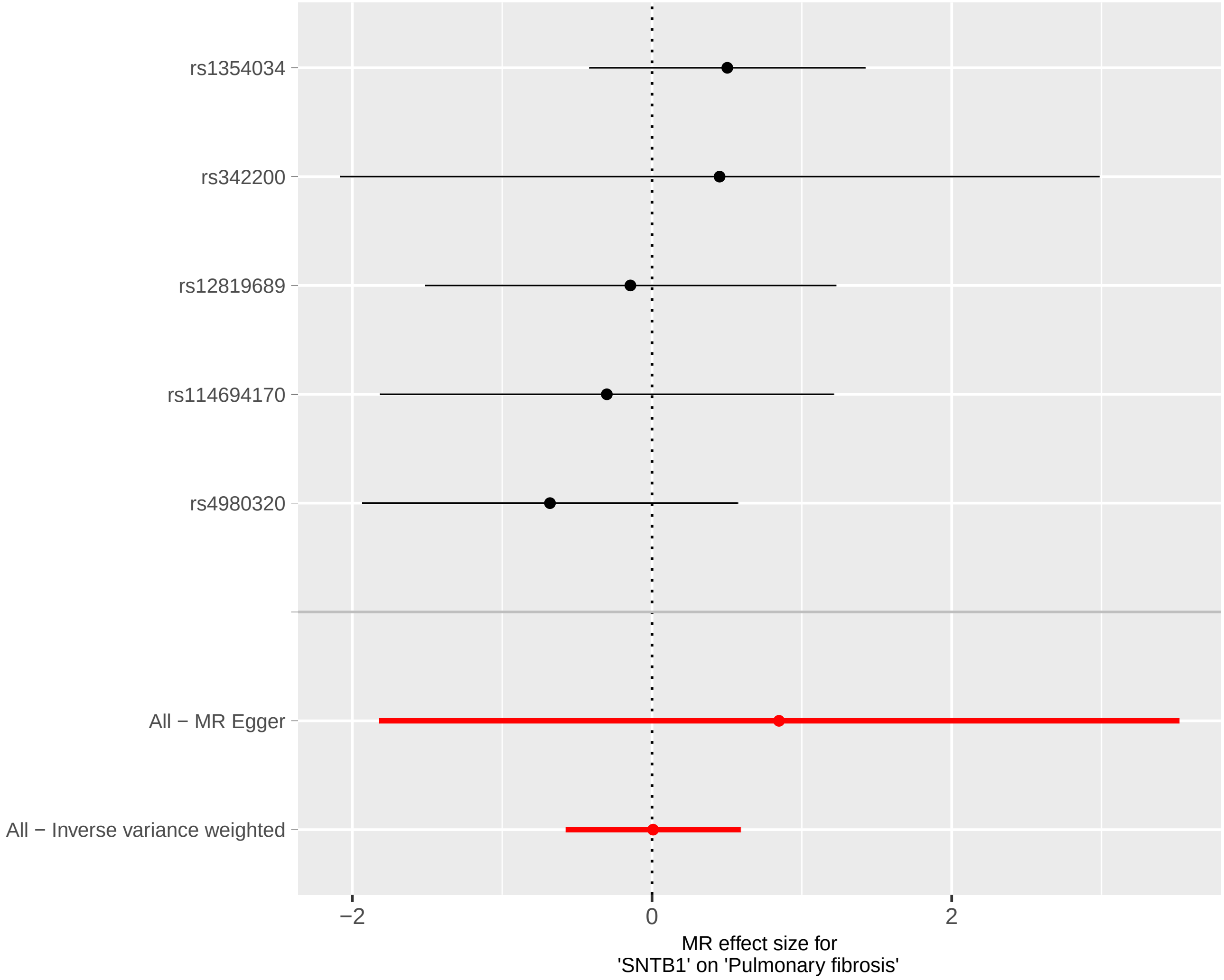
rs1354034
rs342200
rs12819689
rs114694170
rs4980320
All − MR Egger
All − Inverse variance weighted
−2
0
2
MR effect size for
'SNTB1' on 'Pulmonary fibrosis'

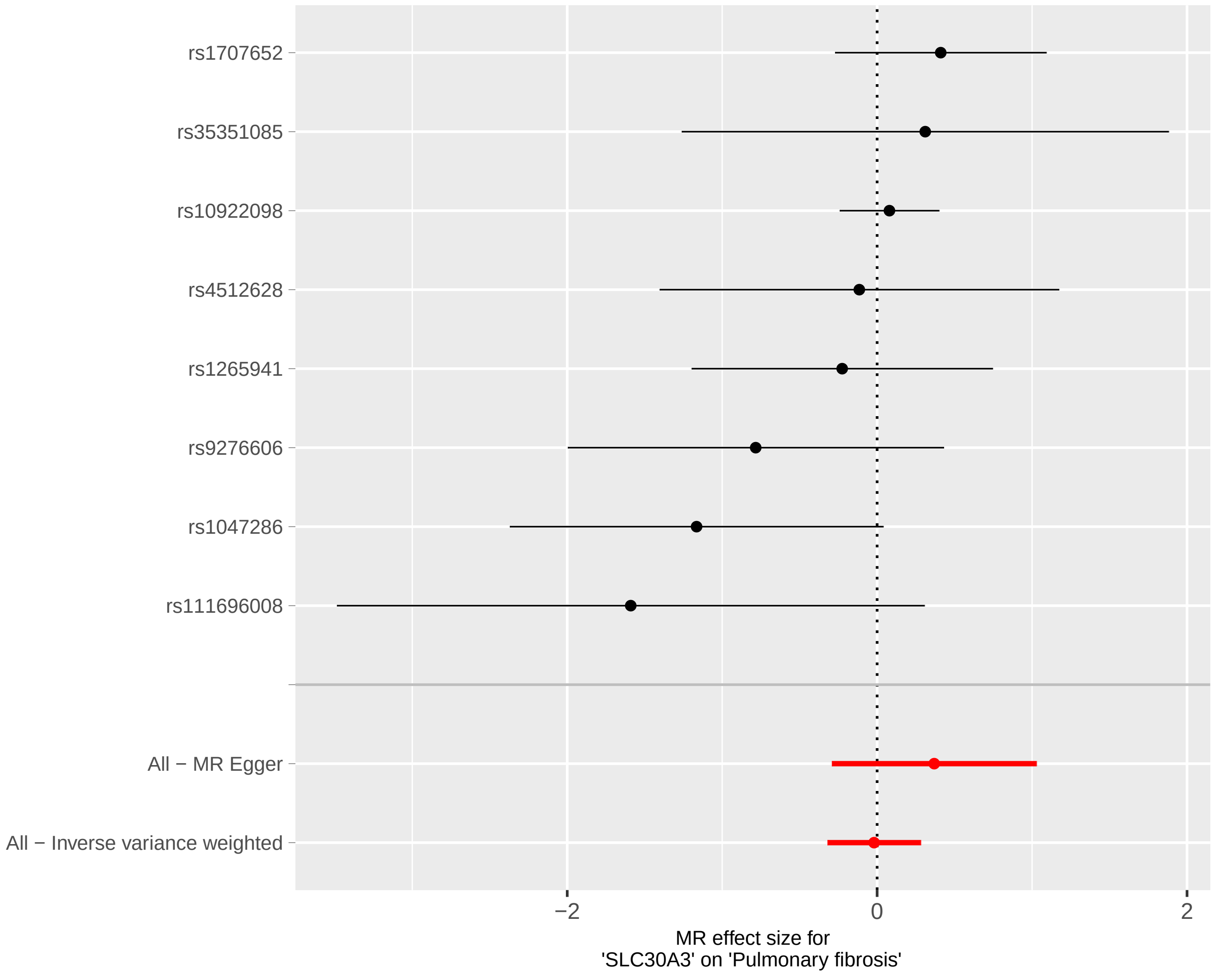
rs1707652
rs35351085
rs10922098
rs4512628
rs1265941
rs9276606
rs1047286
rs111696008
All − MR Egger
All − Inverse variance weighted
−2
0
2
MR effect size for
'SLC30A3' on 'Pulmonary fibrosis'

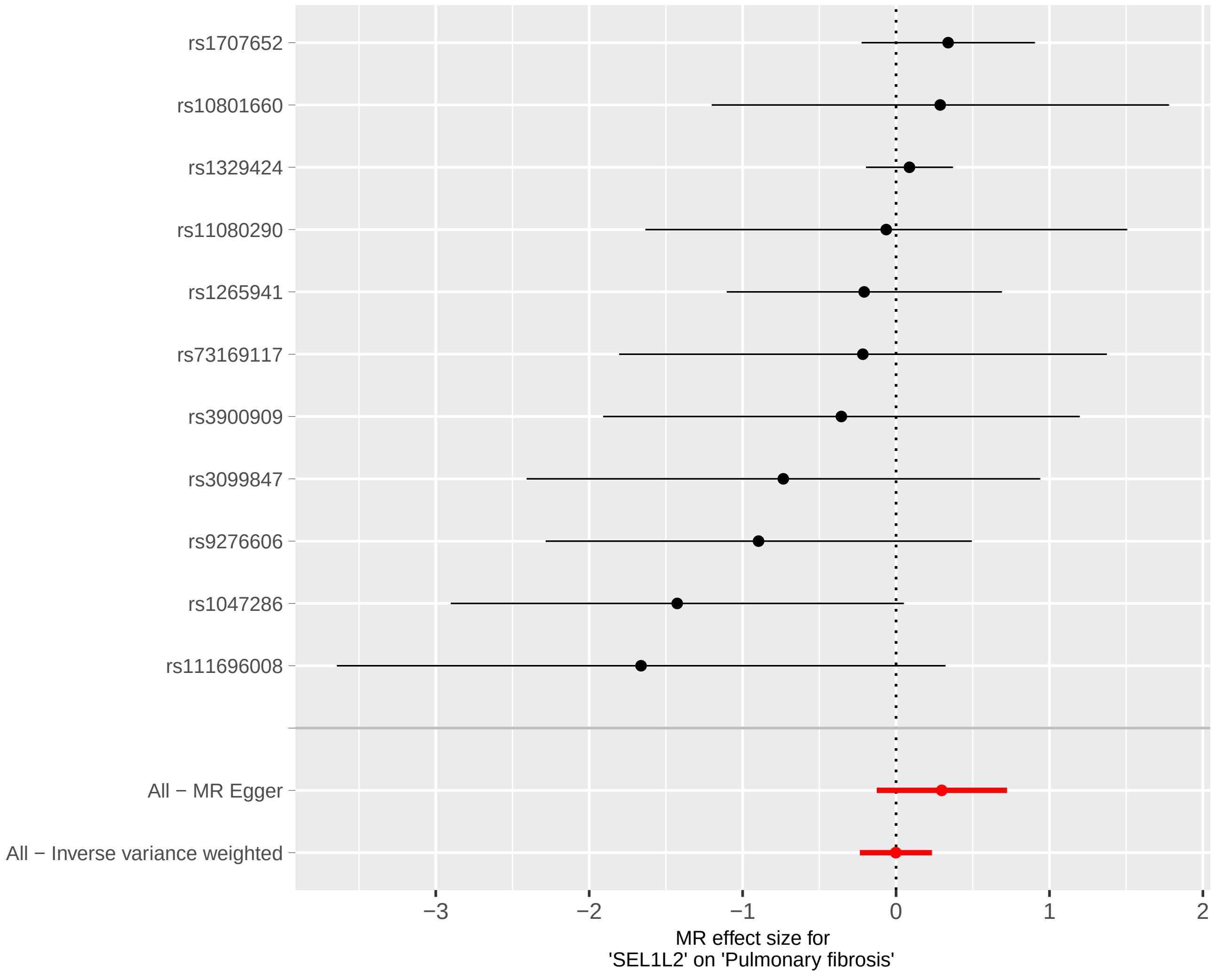

rs1707652
rs10801660
rs1329424
rs11080290
rs1265941
rs73169117
rs3900909
rs3099847
rs9276606
rs1047286
rs111696008
All − MR Egger
All − Inverse variance weighted
−3
−2
−1
0
1
2
MR effect size for
'SEL1L2' on 'Pulmonary fibrosis'

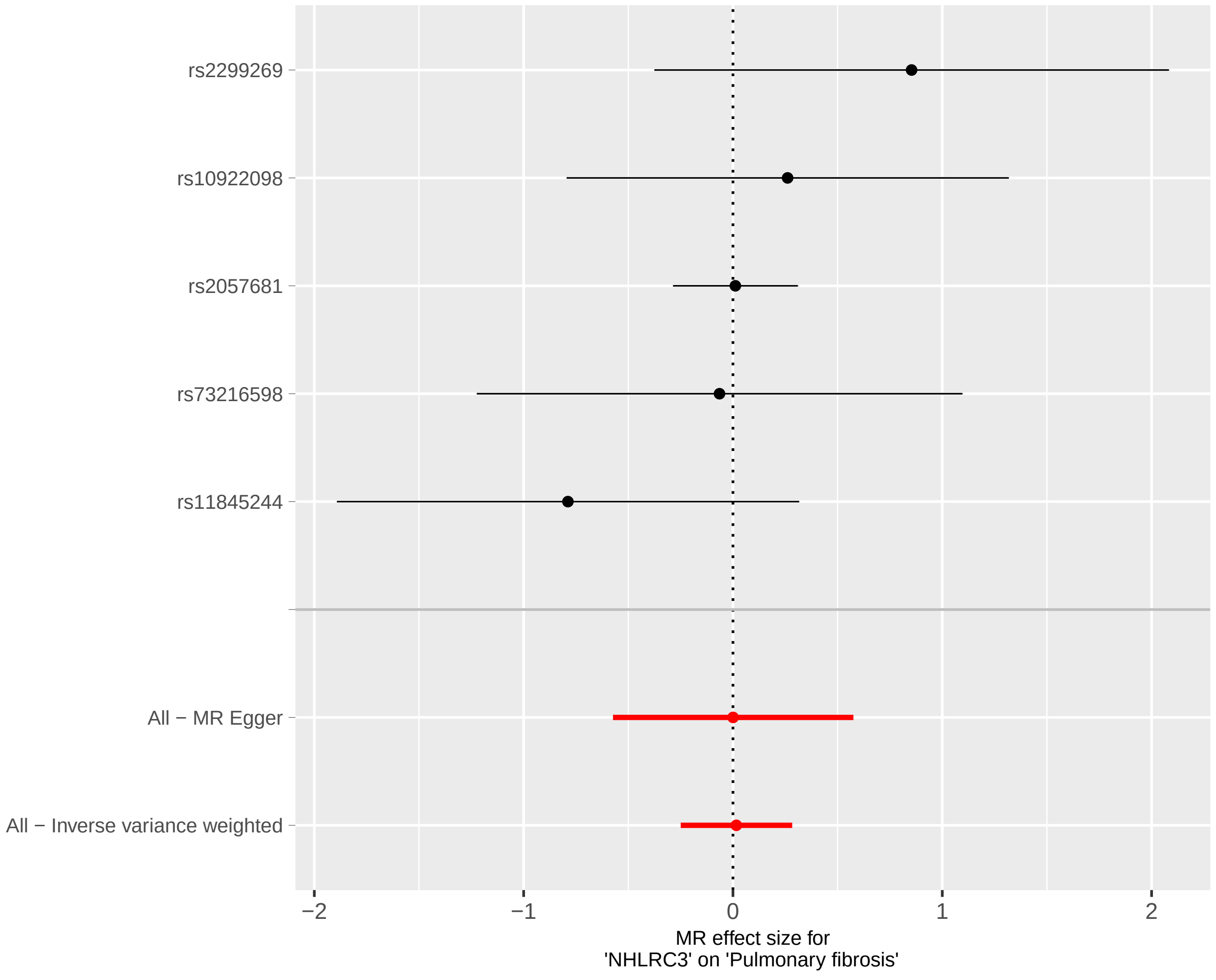
rs2299269
rs10922098
rs2057681
rs73216598
rs11845244
All − MR Egger
All − Inverse variance weighted
−2
−1
0
1
2
MR effect size for
'NHLRC3' on 'Pulmonary fibrosis'

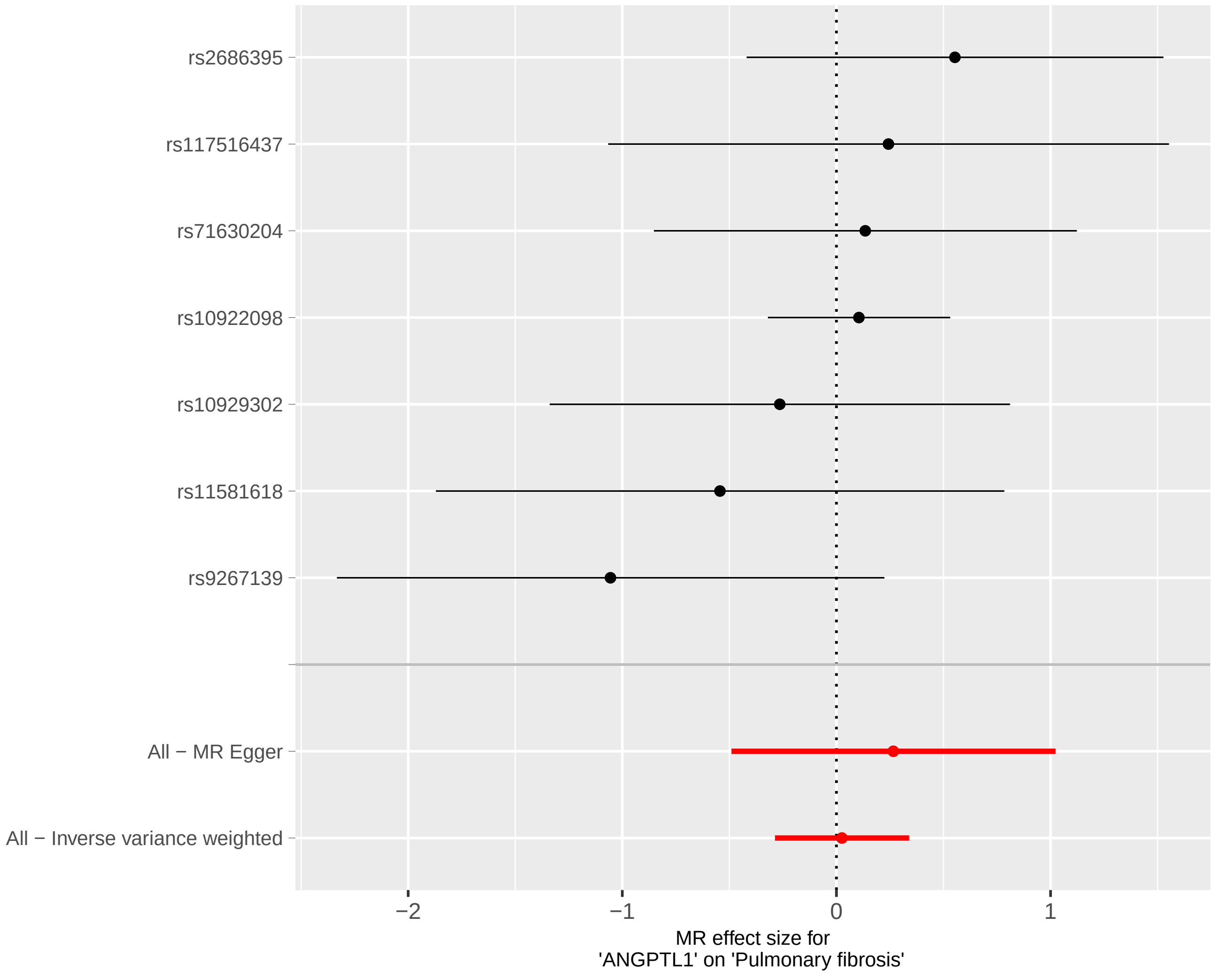
rs2686395
rs117516437
rs71630204
rs10922098
rs10929302
rs11581618
rs9267139
All − MR Egger
All − Inverse variance weighted
−2
−1
0
1
MR effect size for
'ANGPTL1' on 'Pulmonary fibrosis'

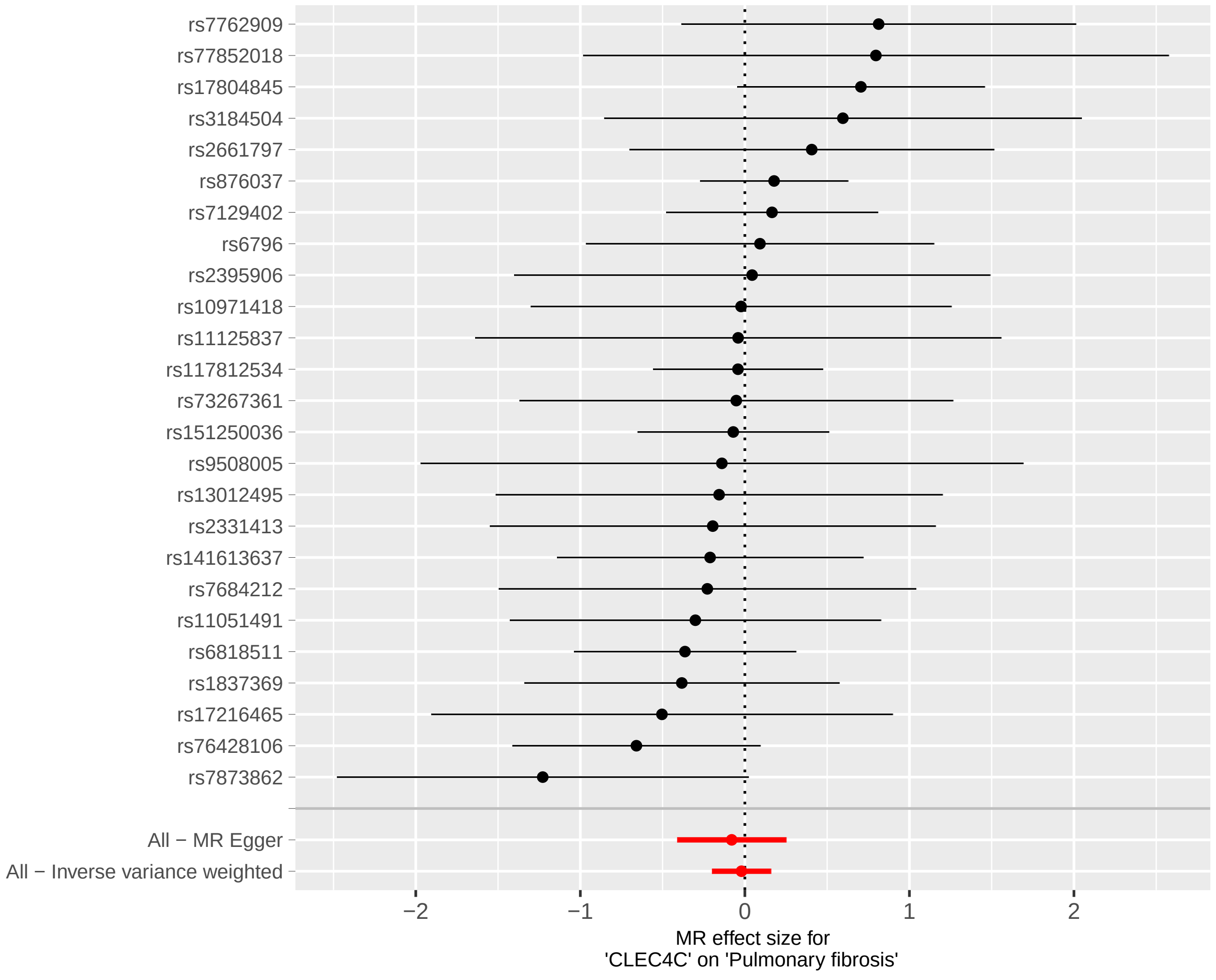

rs7762909
rs77852018
rs17804845
rs3184504
rs2661797
rs876037
rs7129402
rs6796
rs2395906
rs10971418
rs11125837
rs117812534
rs73267361
rs151250036
rs9508005
rs13012495
rs2331413
rs141613637
rs7684212
rs11051491
rs6818511
rs1837369
rs17216465
rs76428106
rs7873862
All − MR Egger
All − Inverse variance weighted
−2
−1
0
1
2
MR effect size for
'CLEC4C' on 'Pulmonary fibrosis'

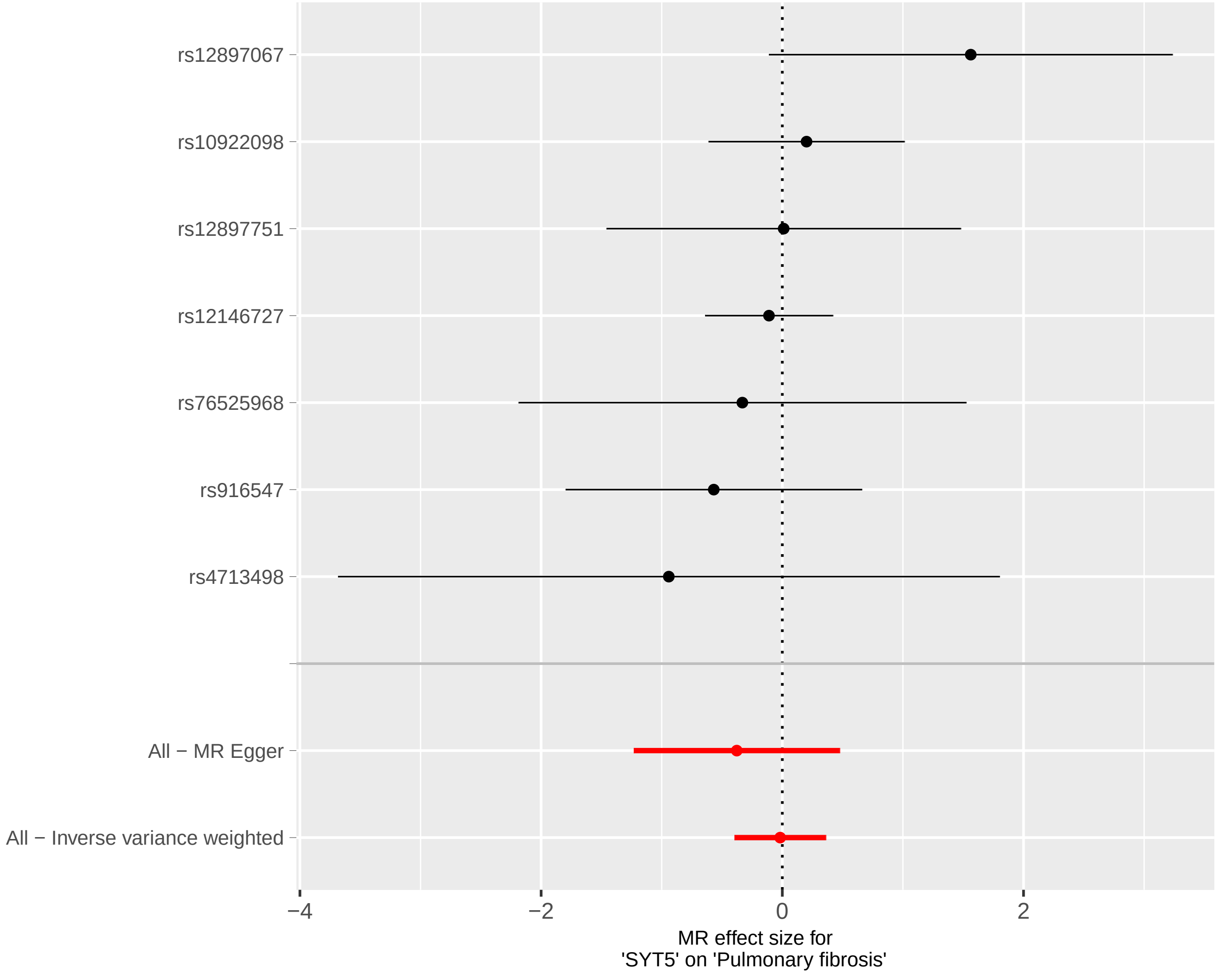
rs12897067
rs10922098
rs12897751
rs12146727
rs76525968
rs916547
rs4713498
All − MR Egger
All − Inverse variance weighted
−4
−2
0
2
MR effect size for
'SYT5' on 'Pulmonary fibrosis'

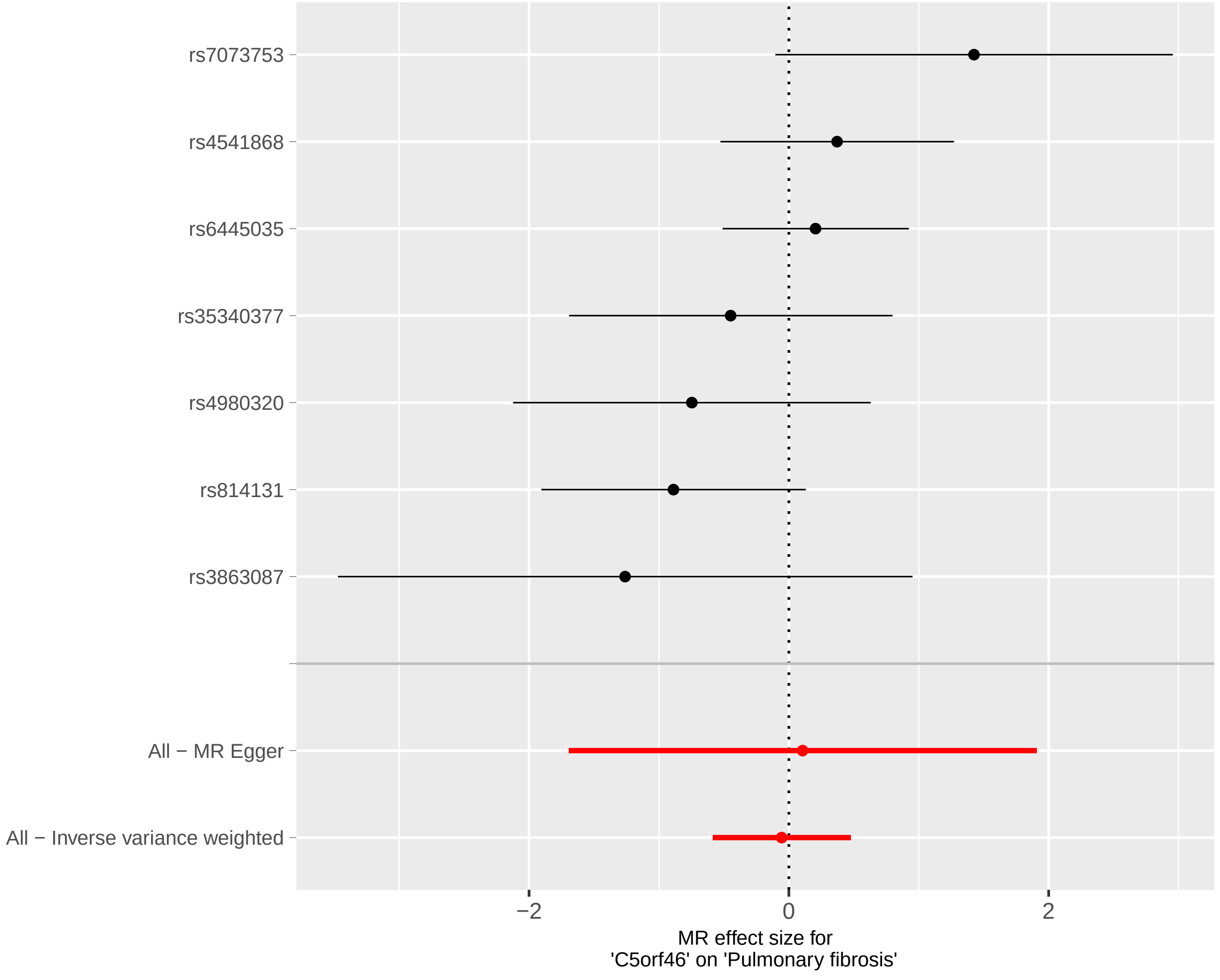
rs7073753
rs4541868
rs6445035
rs35340377
rs4980320
rs814131
rs3863087
All − MR Egger
All − Inverse variance weighted
−2
0
2
MR effect size for
'C5orf46' on 'Pulmonary fibrosis'

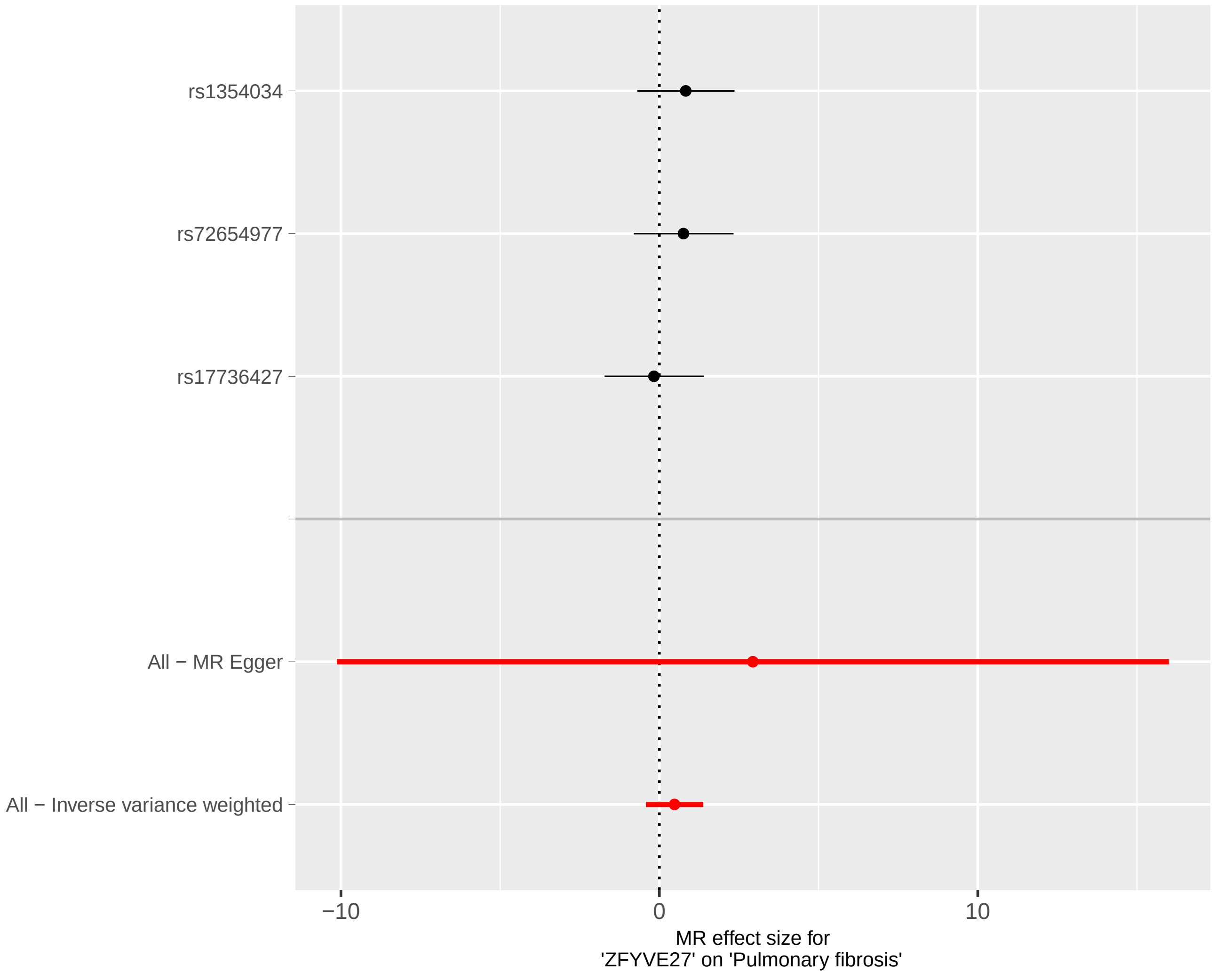
rs1354034
rs72654977
rs17736427
All − MR Egger
All − Inverse variance weighted
−10
0
10
MR effect size for
'ZFYVE27' on 'Pulmonary fibrosis'

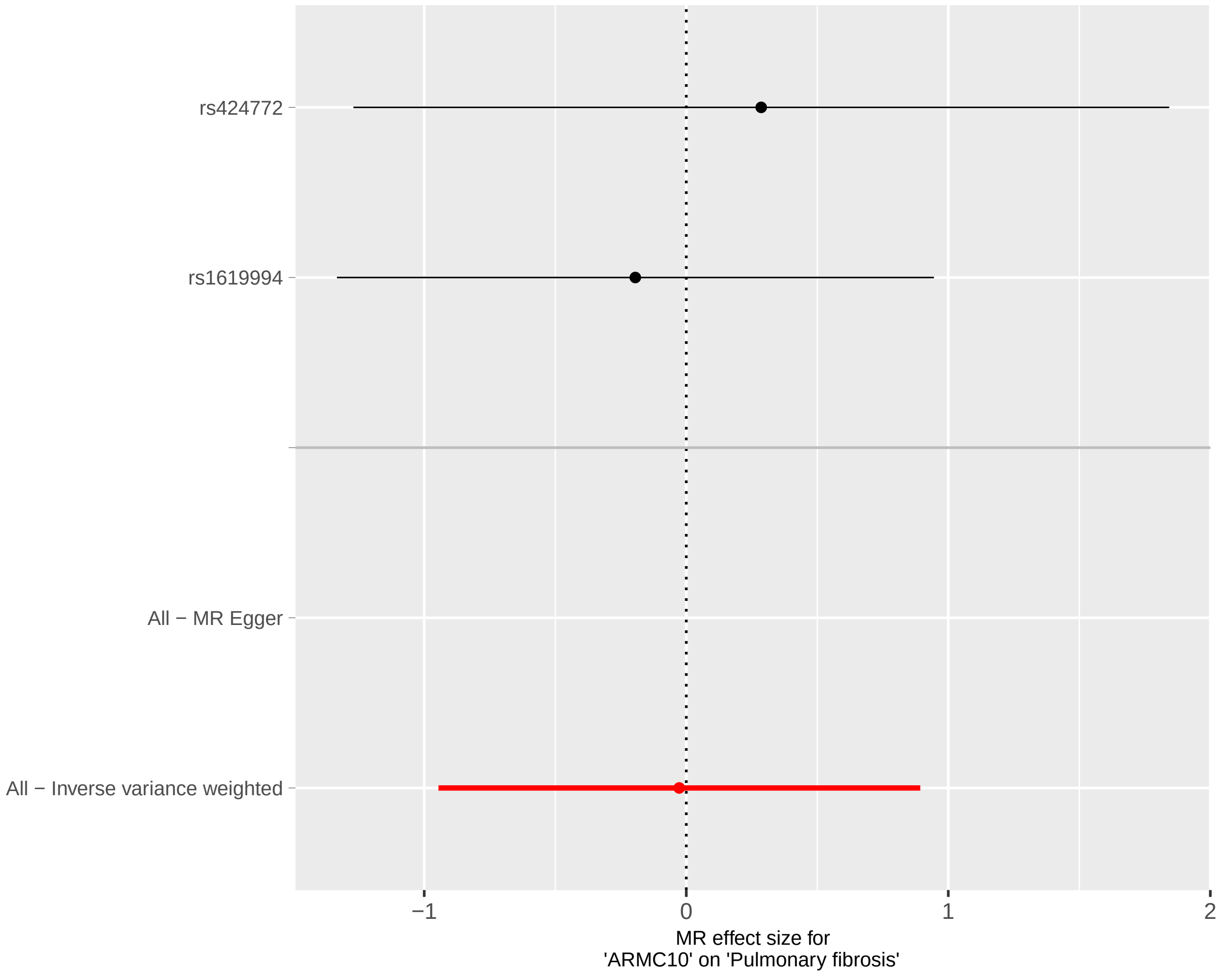
rs424772
rs1619994
All − MR Egger
All − Inverse variance weighted
−1
0
1
2
MR effect size for
'ARMC10' on 'Pulmonary fibrosis'

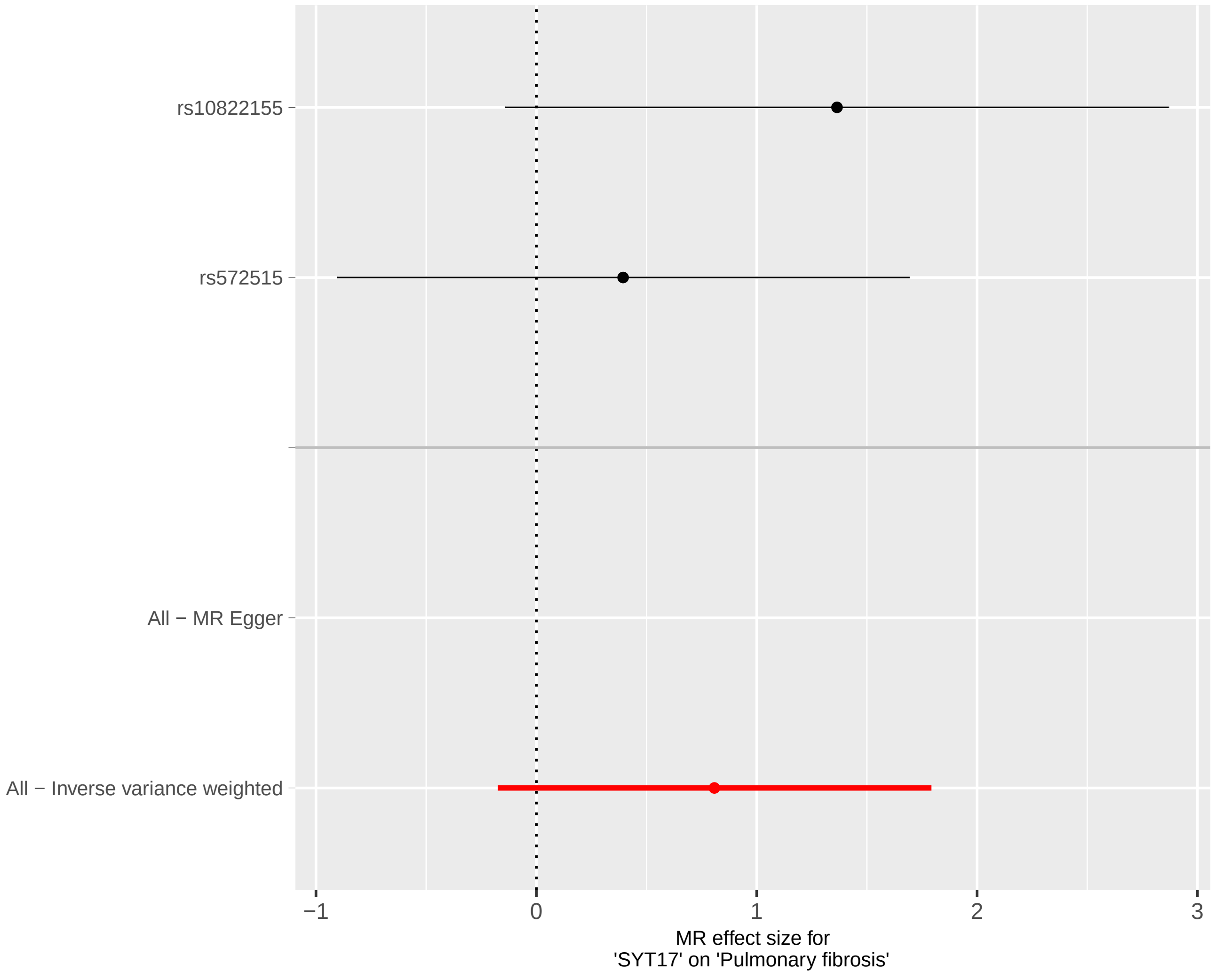

rs10822155
rs572515
All − MR Egger
All − Inverse variance weighted
−1
0
1
2
3
MR effect size for
'SYT17' on 'Pulmonary fibrosis'

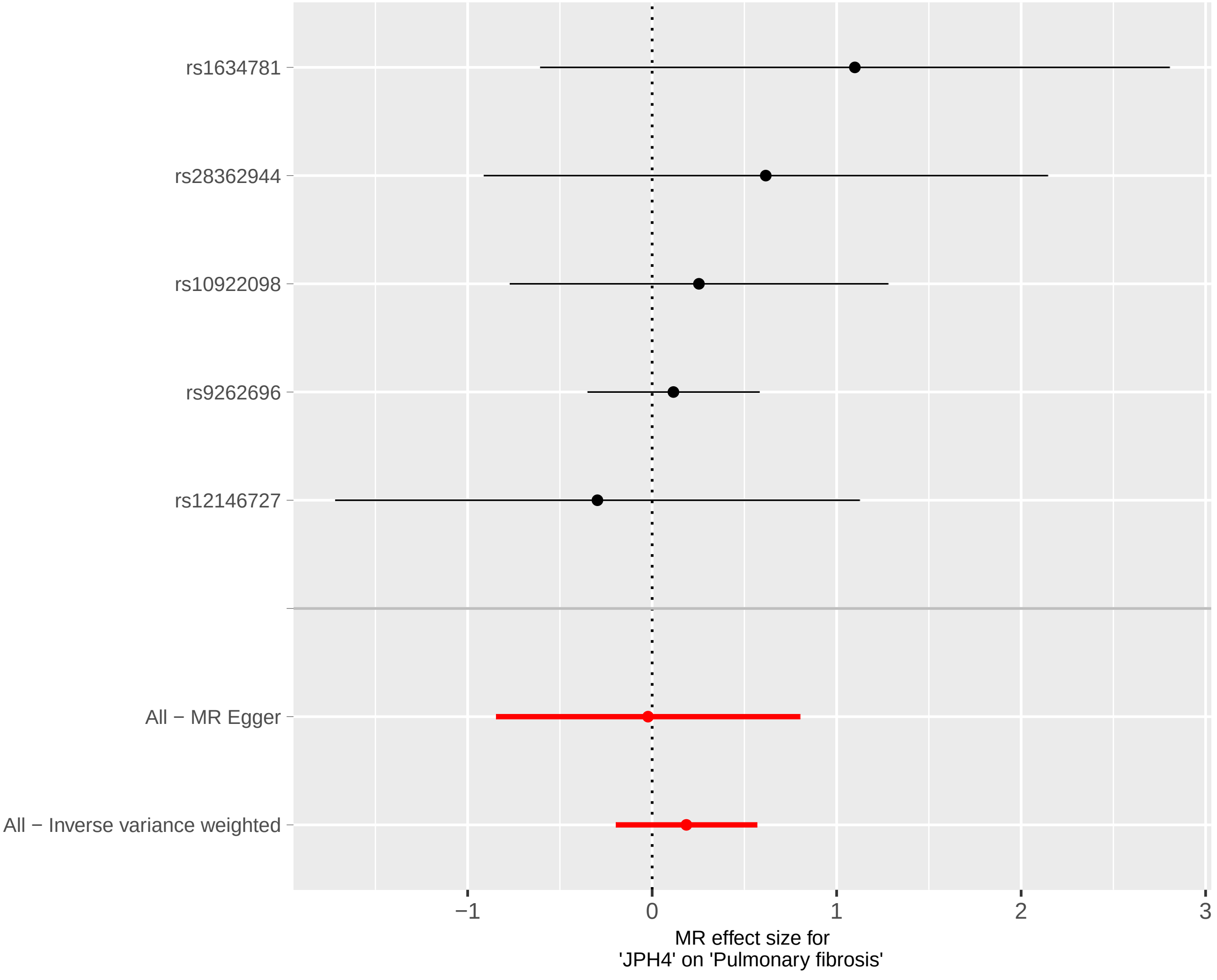

rs1634781
rs28362944
rs10922098
rs9262696
rs12146727
All − MR Egger
All − Inverse variance weighted
−1
0
1
2
3
MR effect size for
'JPH4' on 'Pulmonary fibrosis'

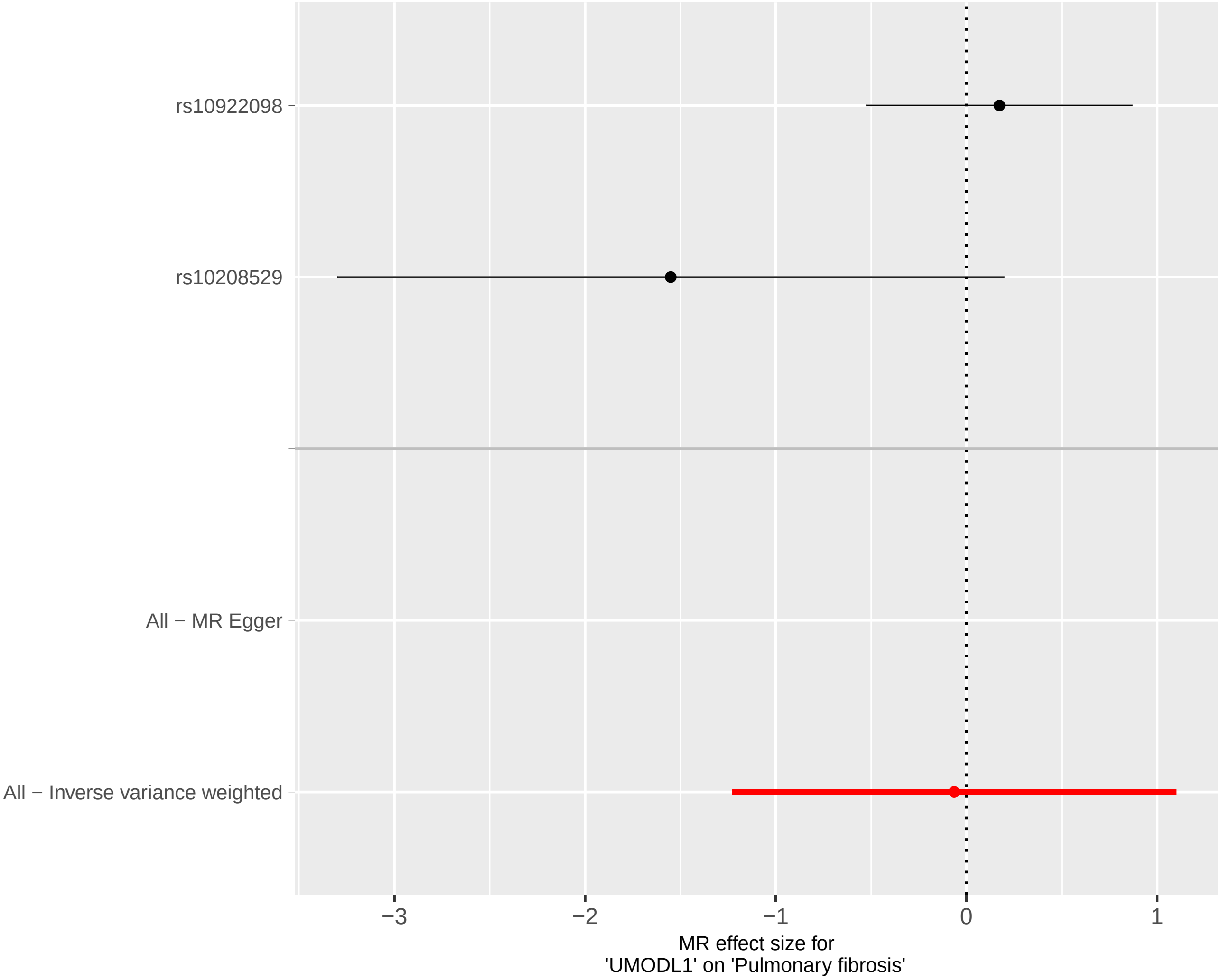
rs10922098
rs10208529
All − MR Egger
All − Inverse variance weighted
−3
−2
−1
0
1
MR effect size for
'UMODL1' on 'Pulmonary fibrosis'

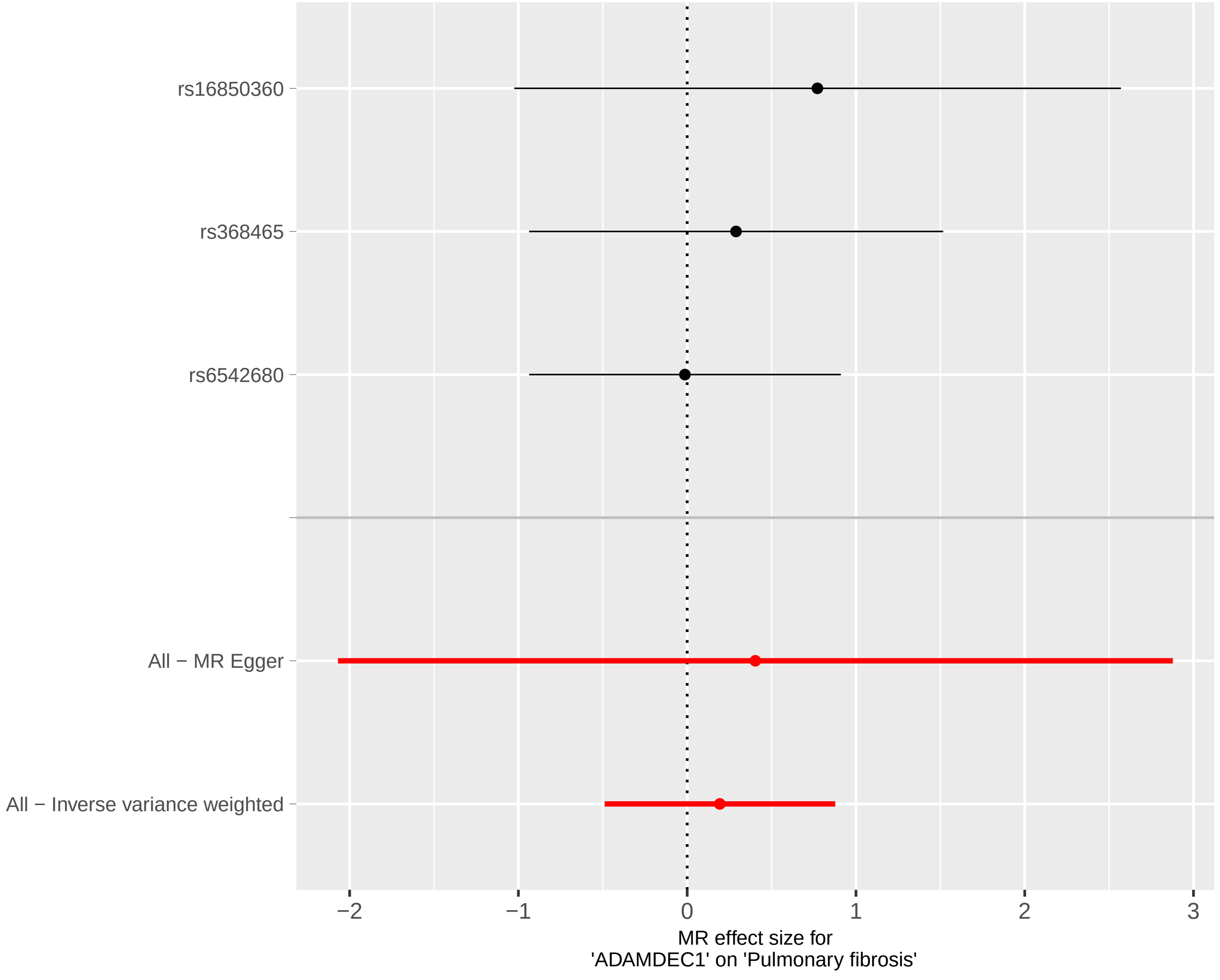

rs16850360
rs368465
rs6542680
All − MR Egger
All − Inverse variance weighted
−2
−1
0
1
2
3
MR effect size for
'ADAMDEC1' on 'Pulmonary fibrosis'

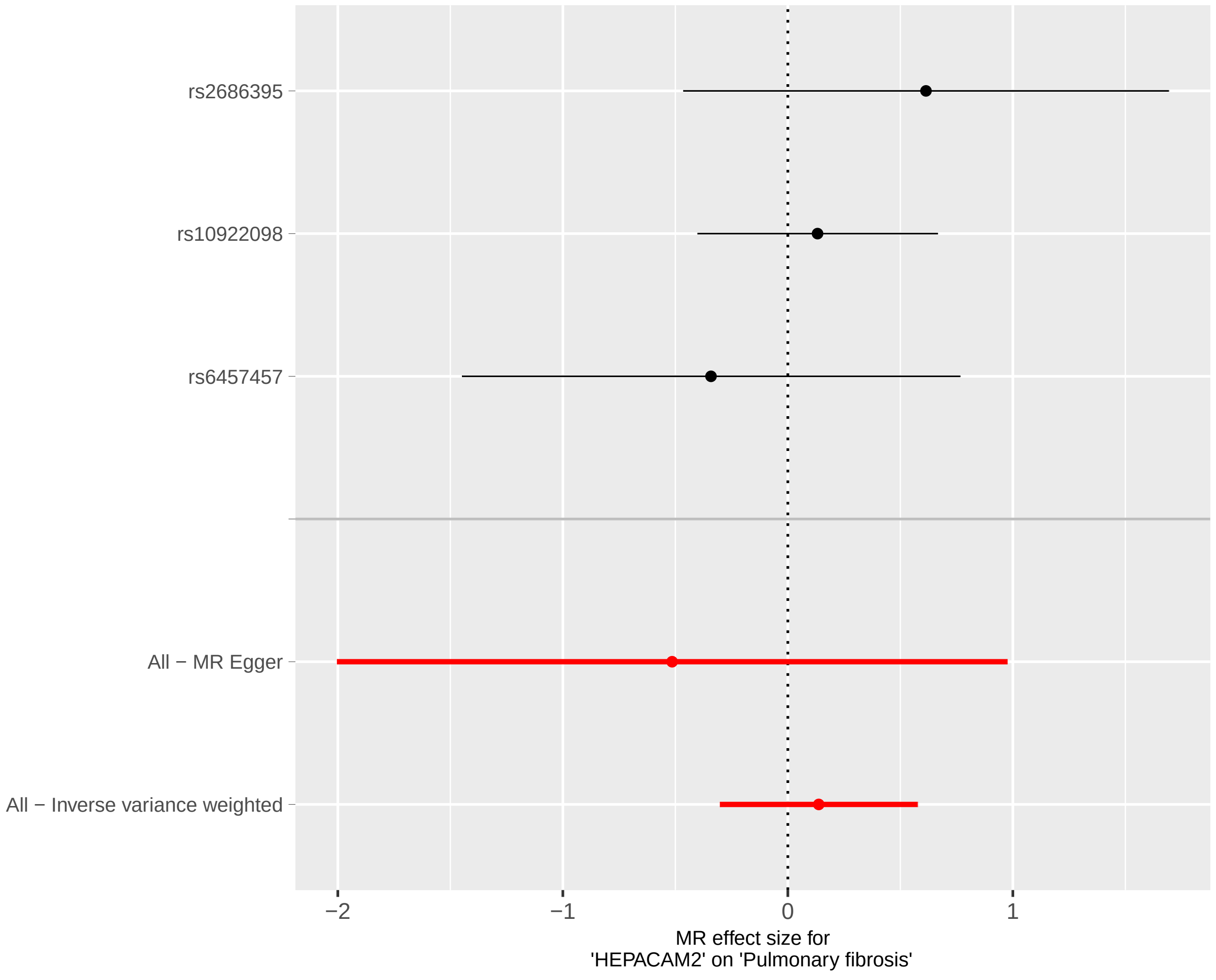

rs2686395
rs10922098
rs6457457
All − MR Egger
All − Inverse variance weighted
−2
−1
0
1
MR effect size for
'HEPACAM2' on 'Pulmonary fibrosis'

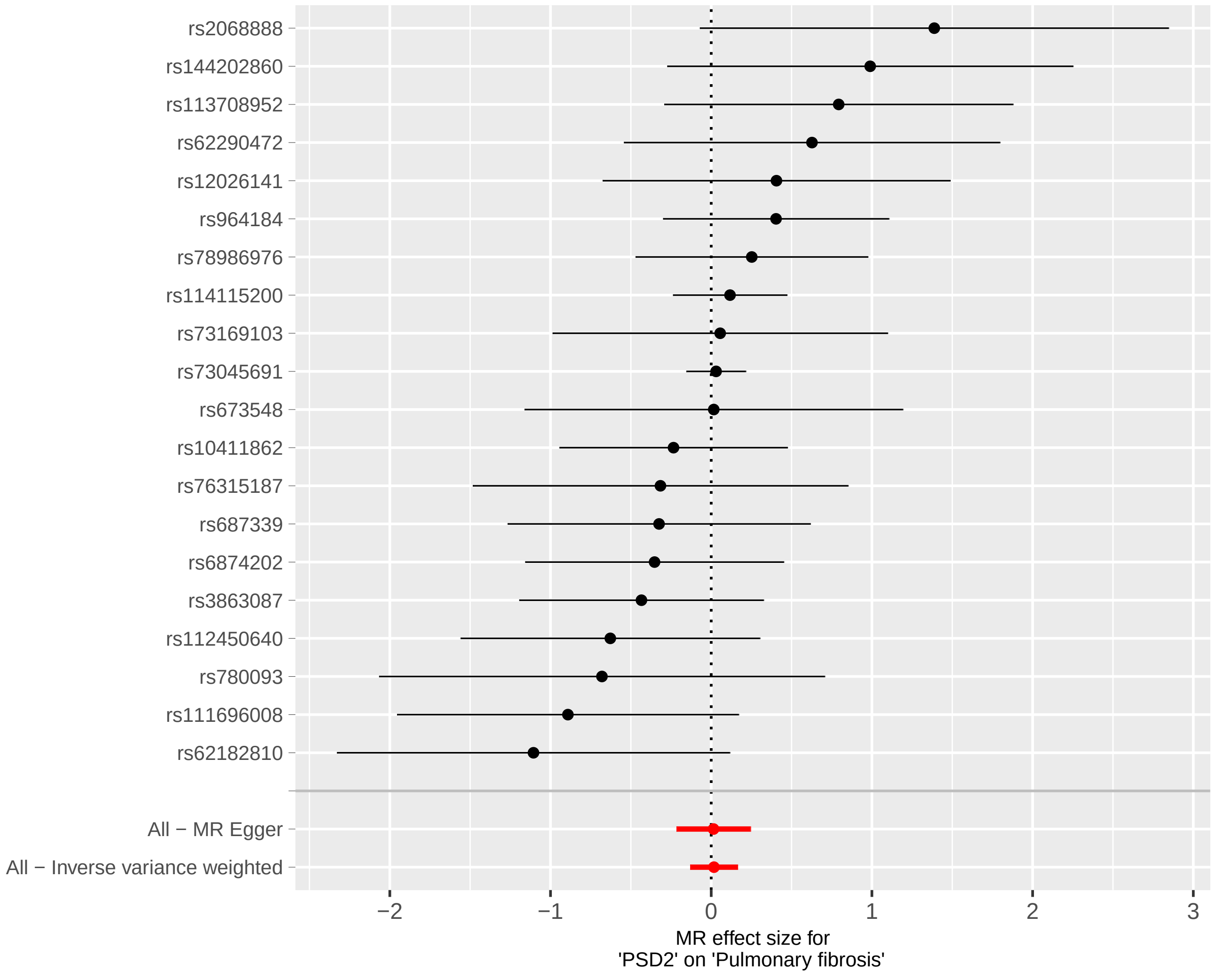

rs2068888
rs144202860
rs113708952
rs62290472
rs12026141
rs964184
rs78986976
rs114115200
rs73169103
rs73045691
rs673548
rs10411862
rs76315187
rs687339
rs6874202
rs3863087
rs112450640
rs780093
rs111696008
rs62182810
All − MR Egger
All − Inverse variance weighted
−2
−1
0
1
2
3
MR effect size for
'PSD2' on 'Pulmonary fibrosis'

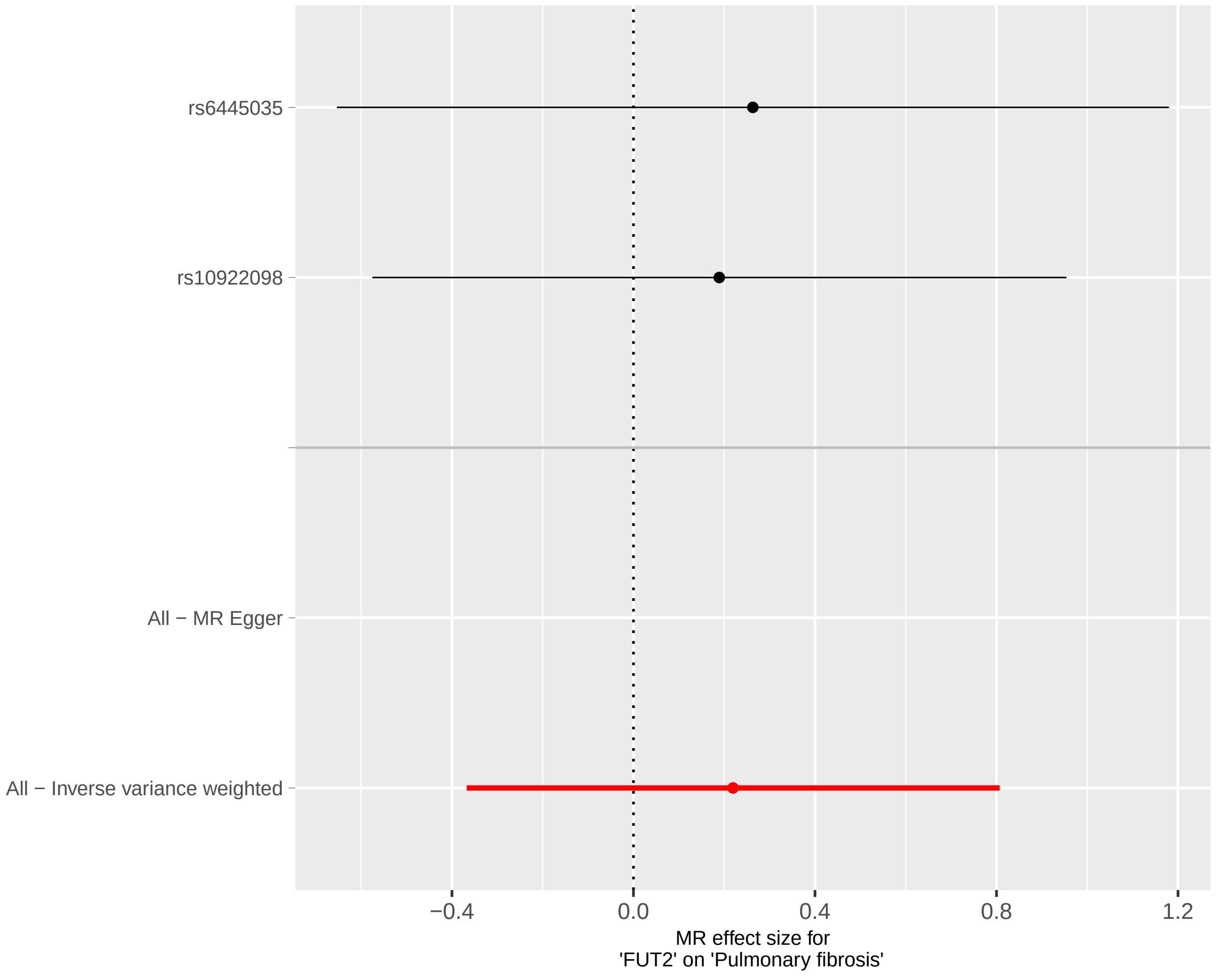
rs6445035
rs10922098
All − MR Egger
All − Inverse variance weighted
−0.4
0.0
0.4
0.8
1.2
MR effect size for
'FUT2' on 'Pulmonary fibrosis'

Insufficient number of SNPs

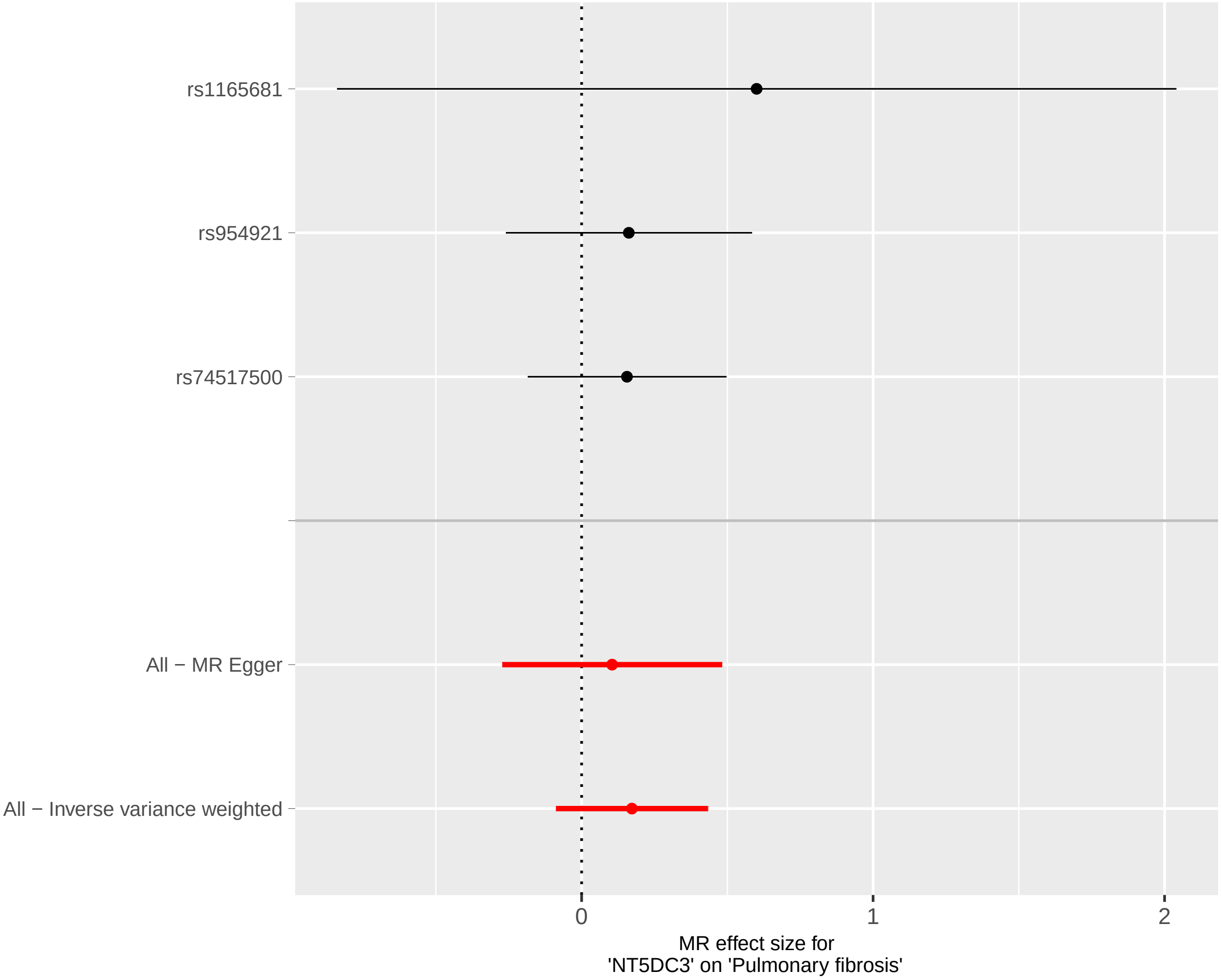
rs1165681
rs954921
rs74517500
All − MR Egger
All − Inverse variance weighted
0
1
2
MR effect size for
'NT5DC3' on 'Pulmonary fibrosis'

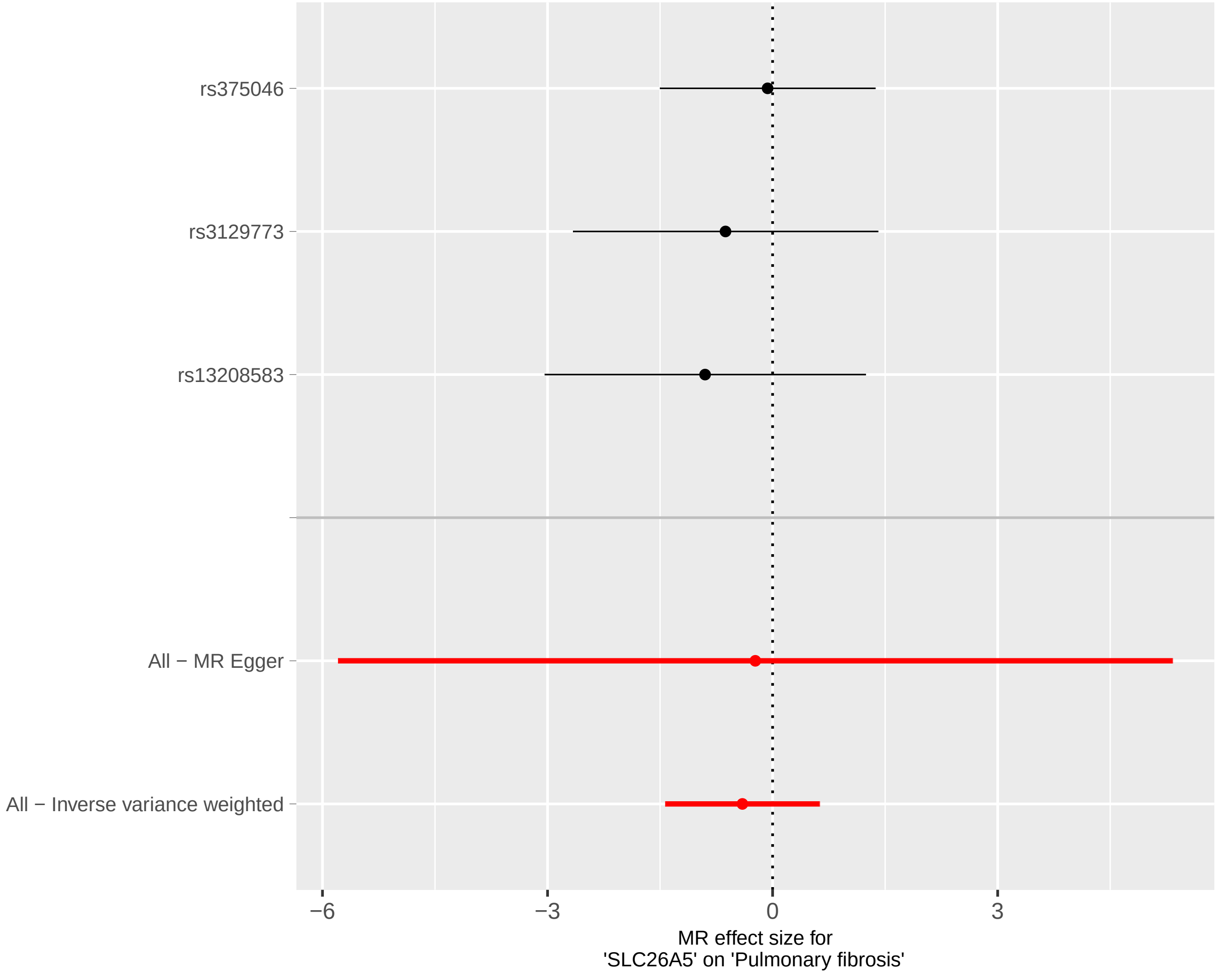
rs375046
rs3129773
rs13208583
All − MR Egger
All − Inverse variance weighted
−6
−3
0
3
MR effect size for
'SLC26A5' on 'Pulmonary fibrosis'

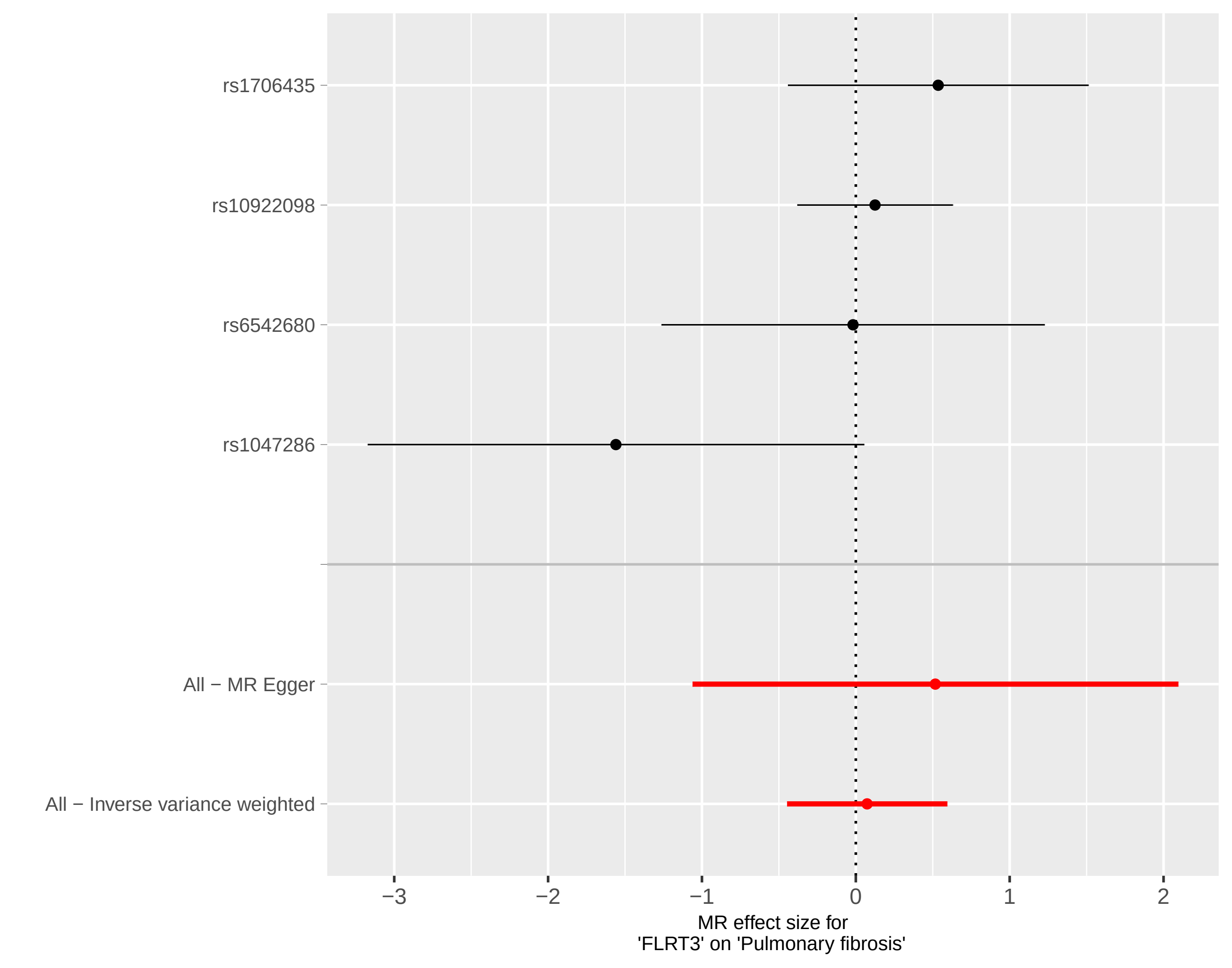
rs1706435
rs10922098
rs6542680
rs1047286
All − MR Egger
All − Inverse variance weighted
−3
−2
−1
0
1
2
MR effect size for
'FLRT3' on 'Pulmonary fibrosis'

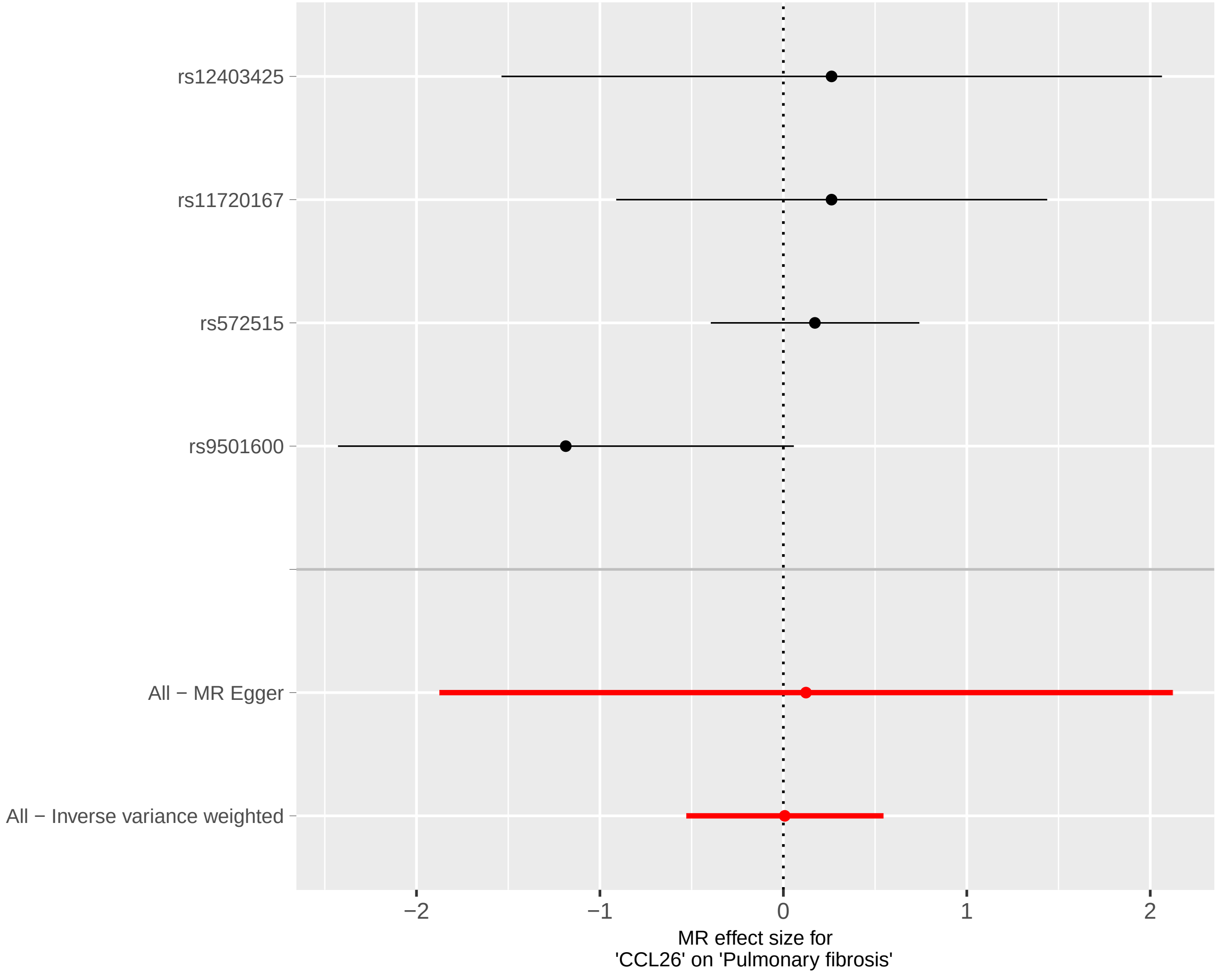
rs12403425
rs11720167
rs572515
rs9501600
All − MR Egger
All − Inverse variance weighted
−2
−1
0
1
2
MR effect size for
'CCL26' on 'Pulmonary fibrosis'

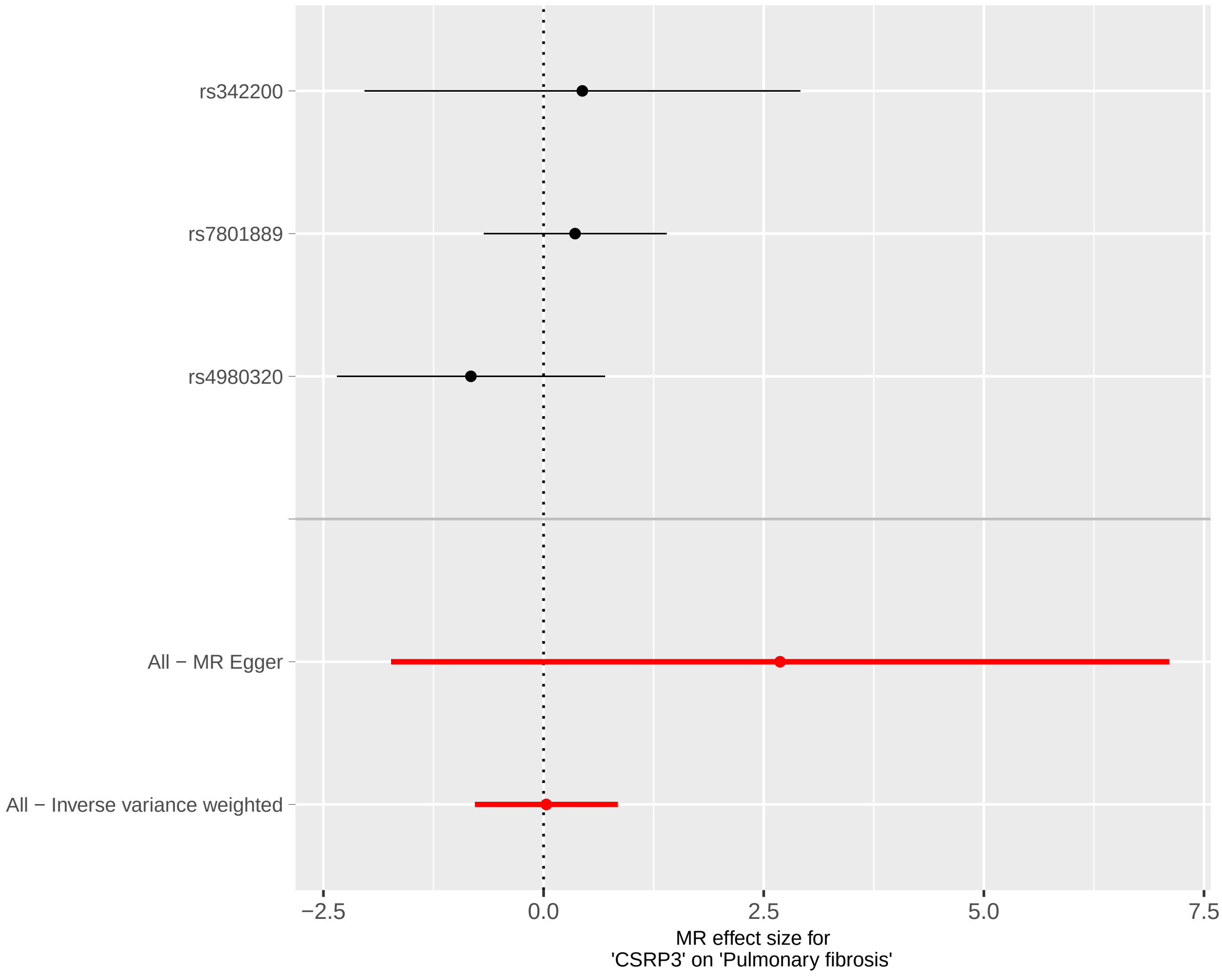
rs342200
rs7801889
rs4980320
All − MR Egger
All − Inverse variance weighted
−2.5
0.0
2.5
5.0
7.5
MR effect size for
'CSRP3' on 'Pulmonary fibrosis'

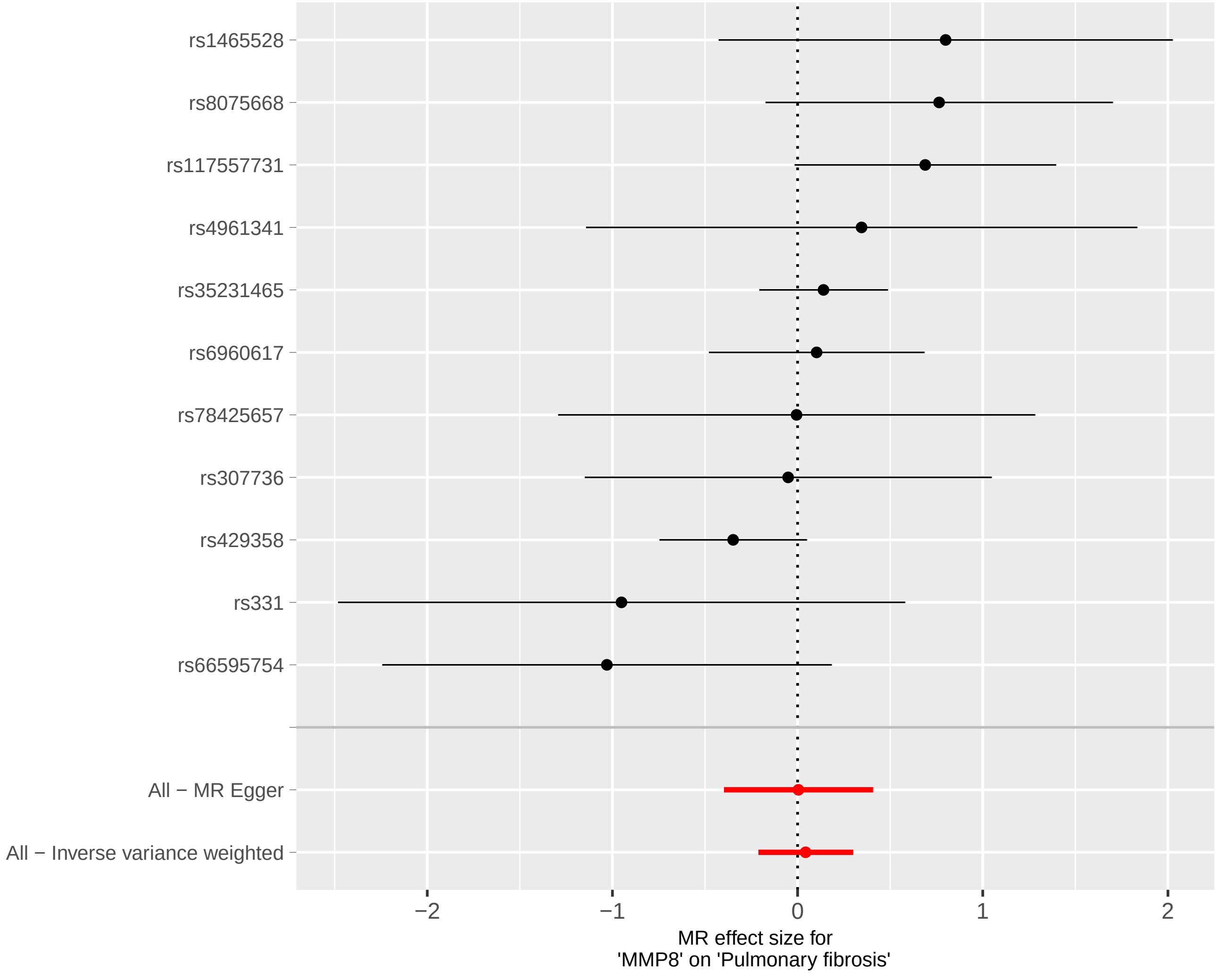

rs1465528
rs8075668
rs117557731
rs4961341
rs35231465
rs6960617
rs78425657
rs307736
rs429358
rs331
rs66595754
All − MR Egger
All − Inverse variance weighted
−2
−1
0
1
2
MR effect size for
'MMP8' on 'Pulmonary fibrosis'

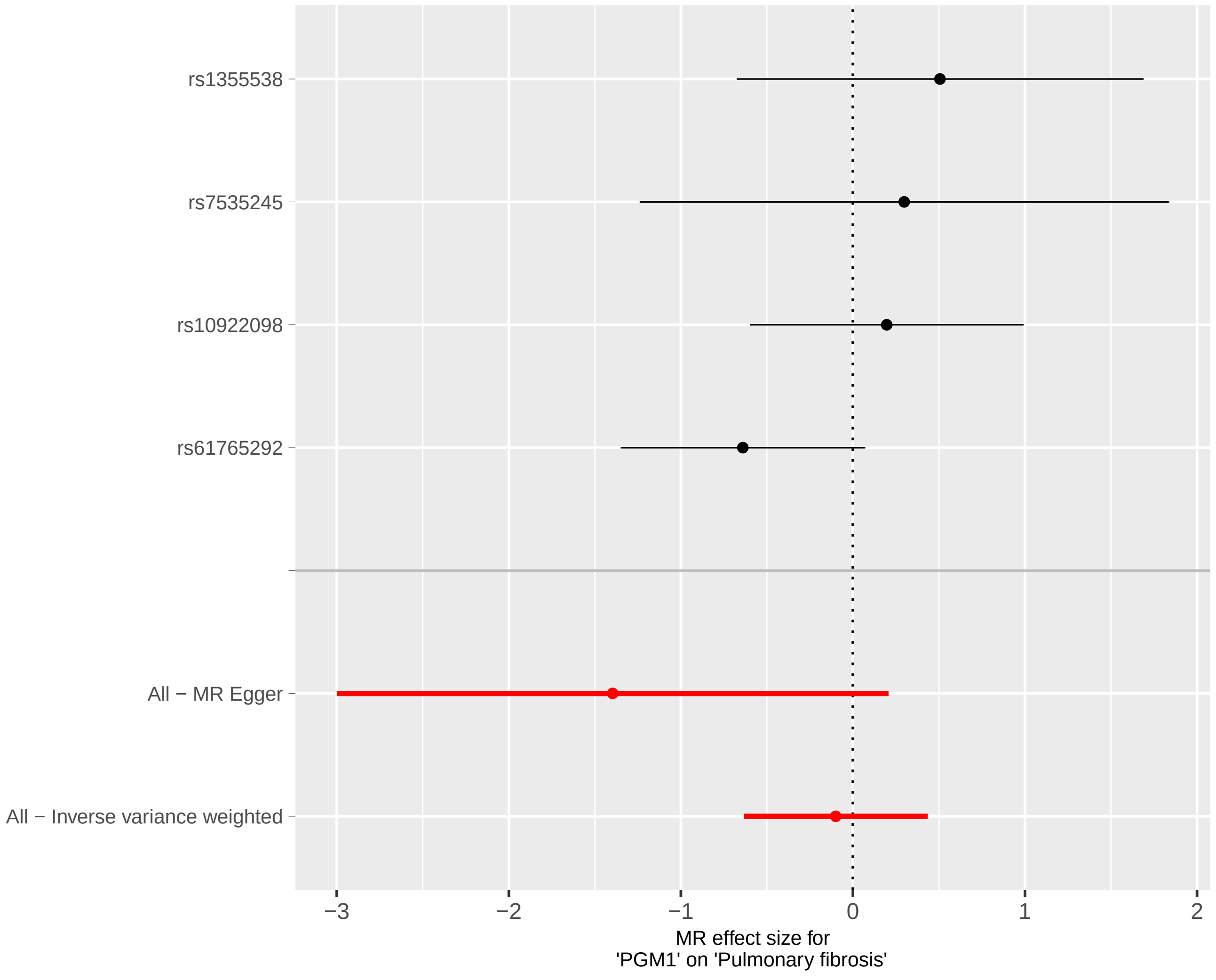

rs1355538
rs7535245
rs10922098
rs61765292
All − MR Egger
All − Inverse variance weighted
−3
−2
−1
0
1
2
MR effect size for
'PGM1' on 'Pulmonary fibrosis'

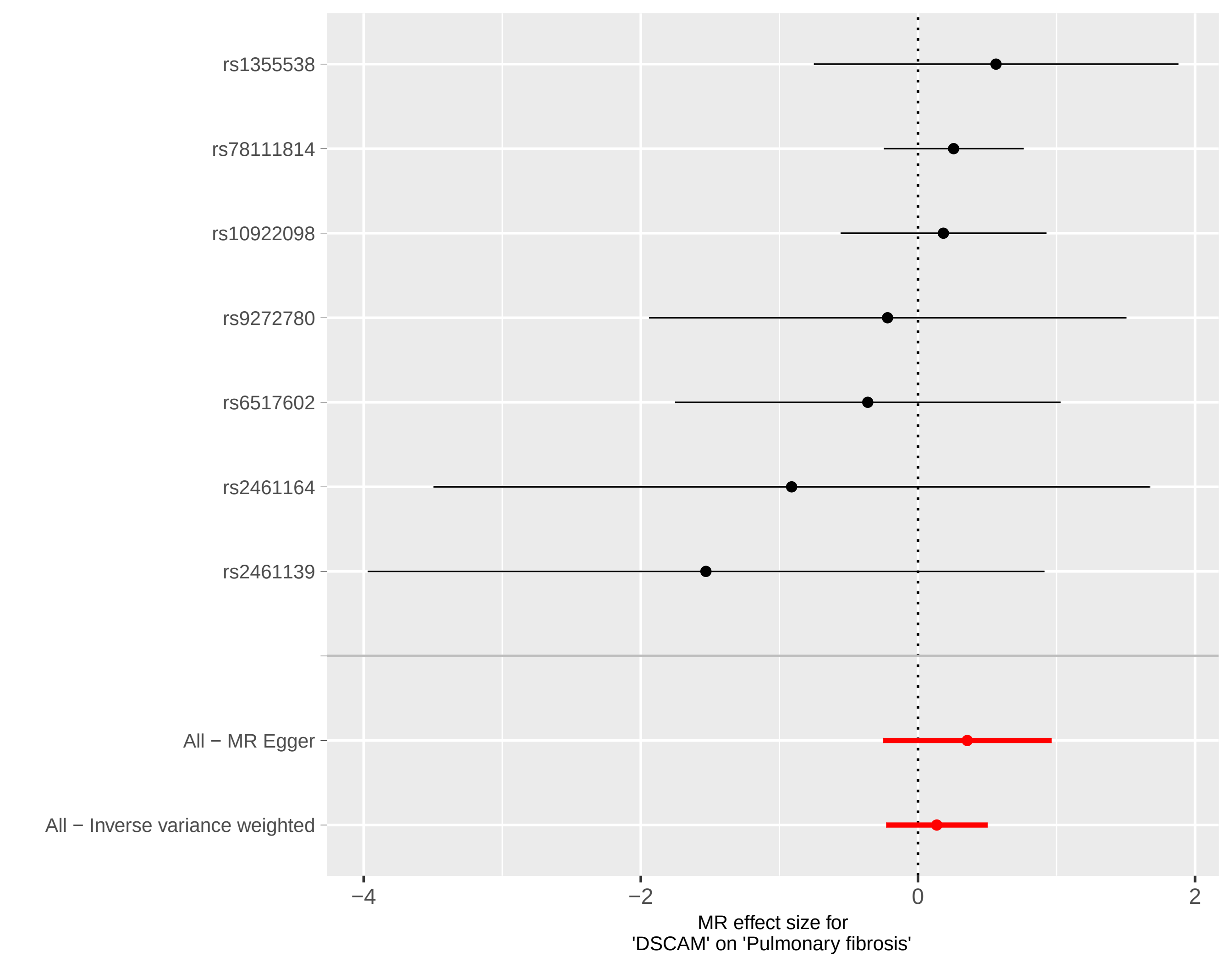
rs1355538
rs78111814
rs10922098
rs9272780
rs6517602
rs2461164
rs2461139
All − MR Egger
All − Inverse variance weighted
−4
−2
0
2
MR effect size for
'DSCAM' on 'Pulmonary fibrosis'

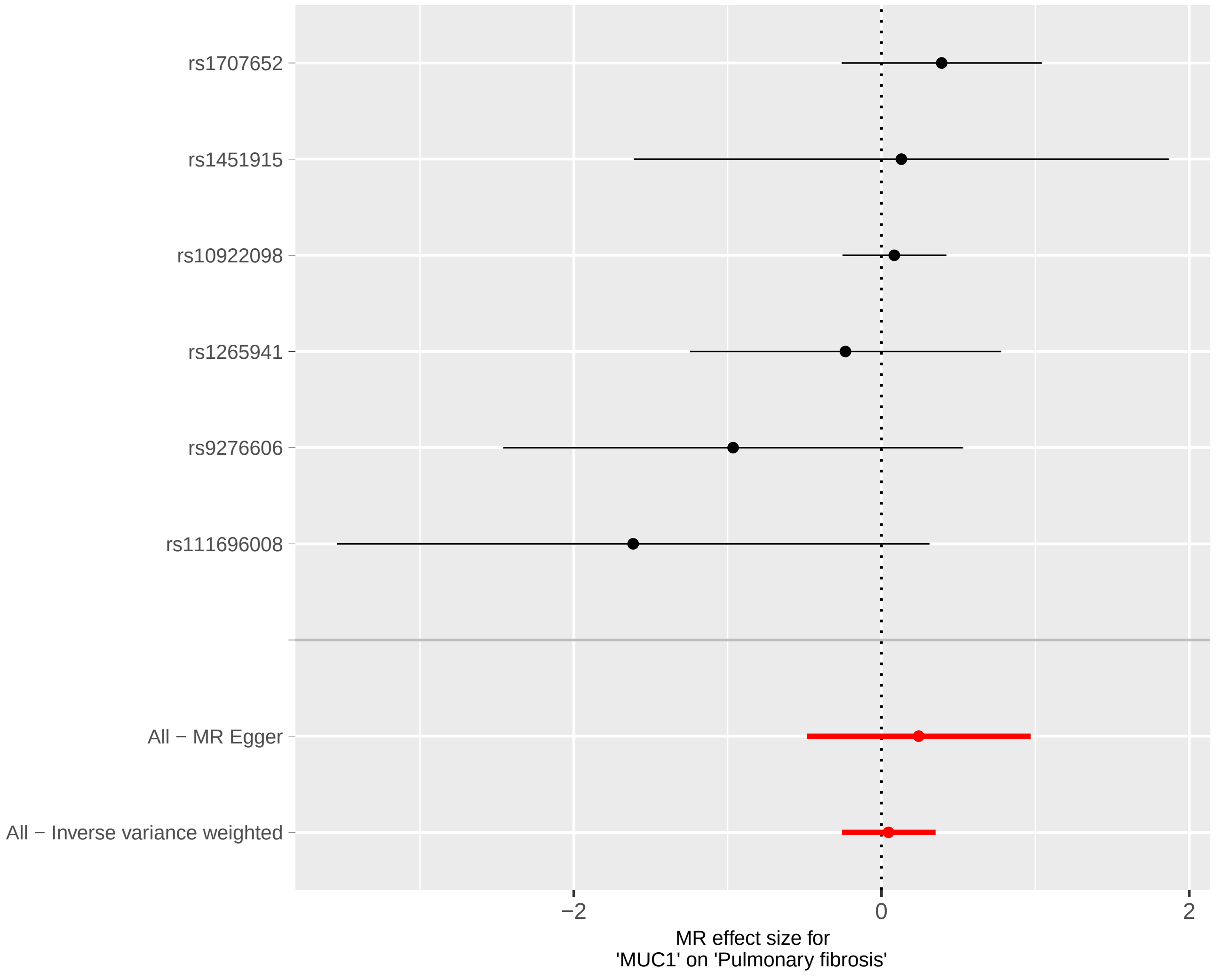
rs1707652
rs1451915
rs10922098
rs1265941
rs9276606
rs111696008
All − MR Egger
All − Inverse variance weighted
−2
0
2
MR effect size for
'MUC1' on 'Pulmonary fibrosis'

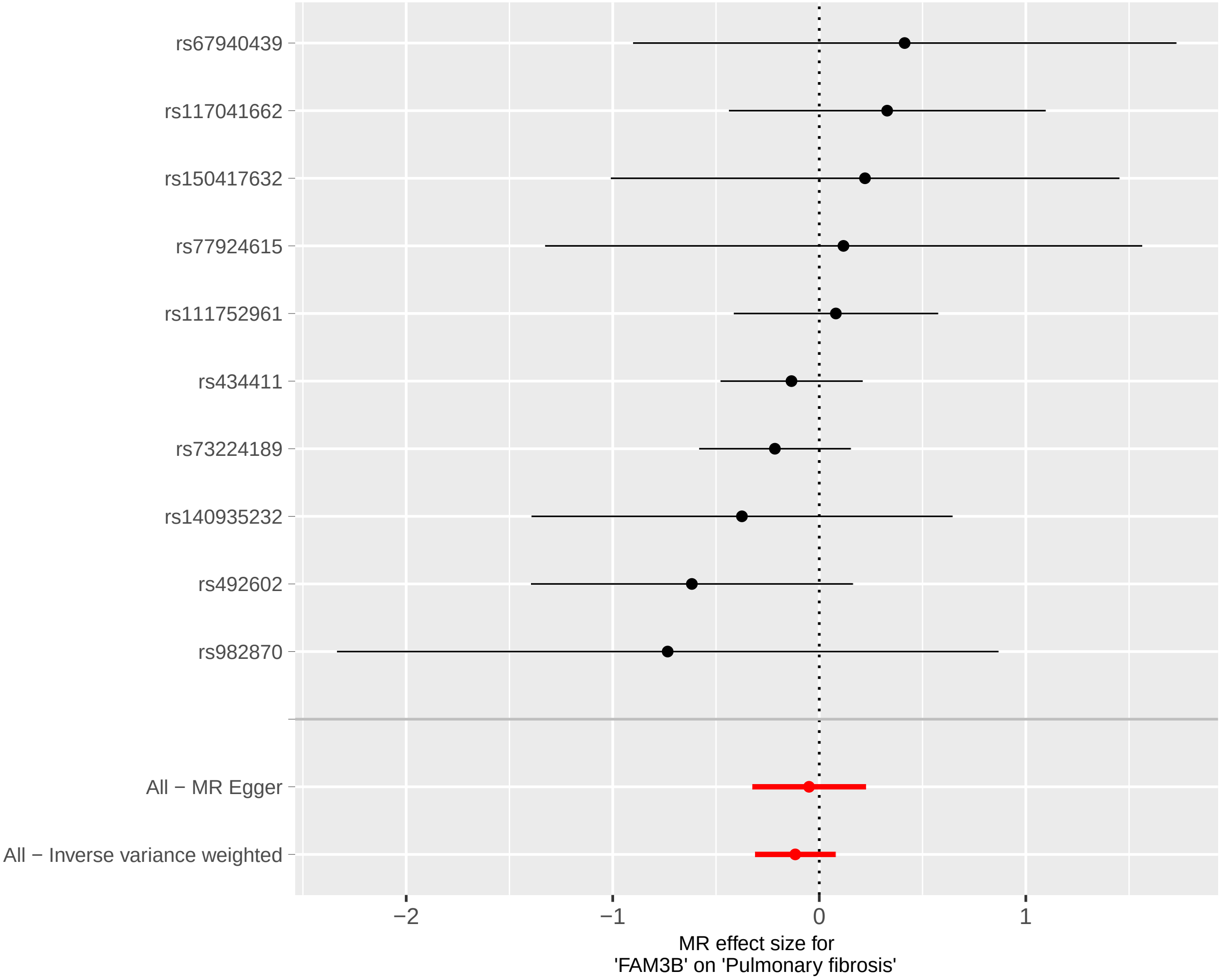
rs67940439
rs117041662
rs150417632
rs77924615
rs111752961
rs434411
rs73224189
rs140935232
rs492602
rs982870
All − MR Egger
All − Inverse variance weighted
−2
−1
0
1
MR effect size for
'FAM3B' on 'Pulmonary fibrosis'

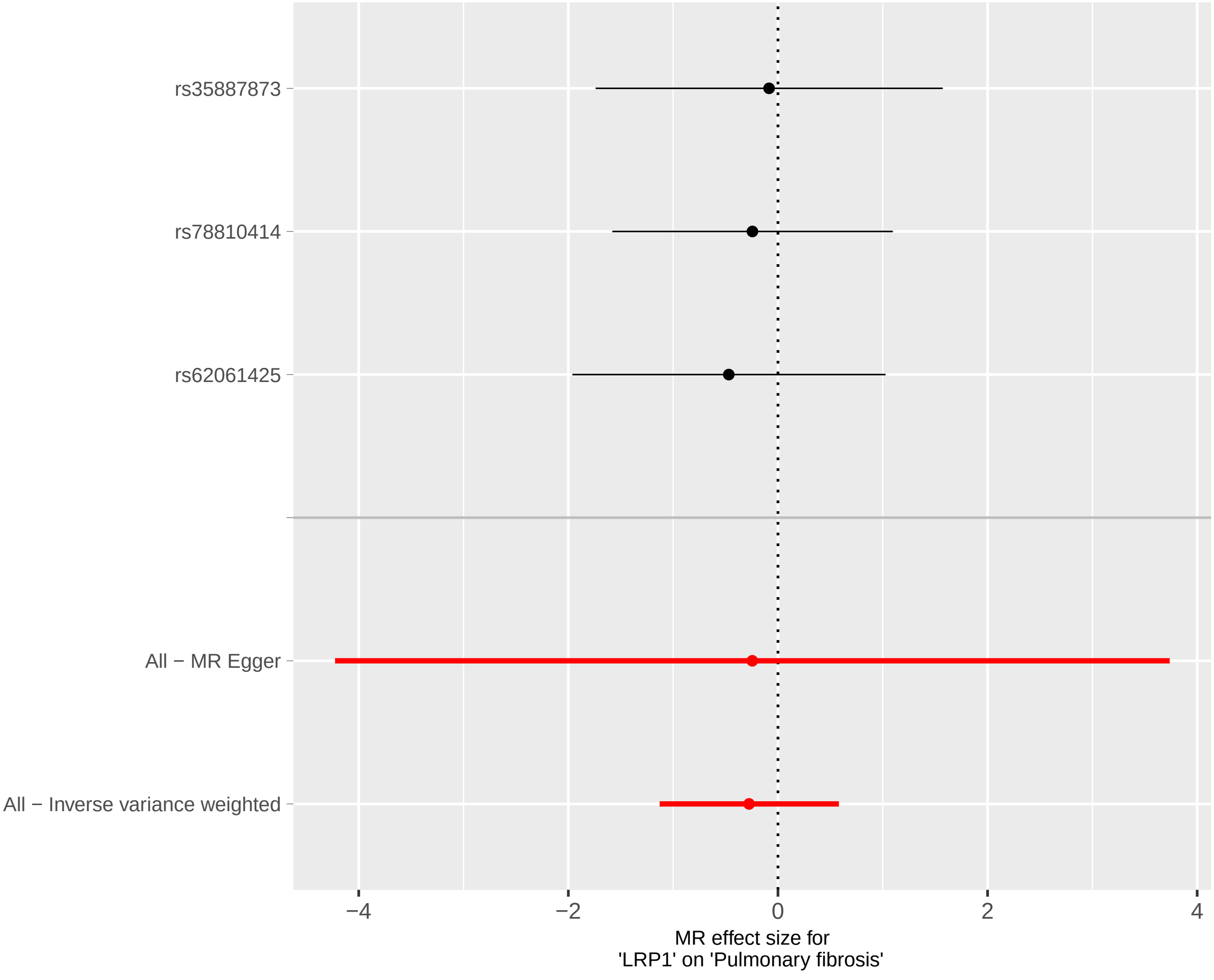

rs35887873
rs78810414
rs62061425
All − MR Egger
All − Inverse variance weighted
−4
−2
0
2
4
MR effect size for
'LRP1' on 'Pulmonary fibrosis'

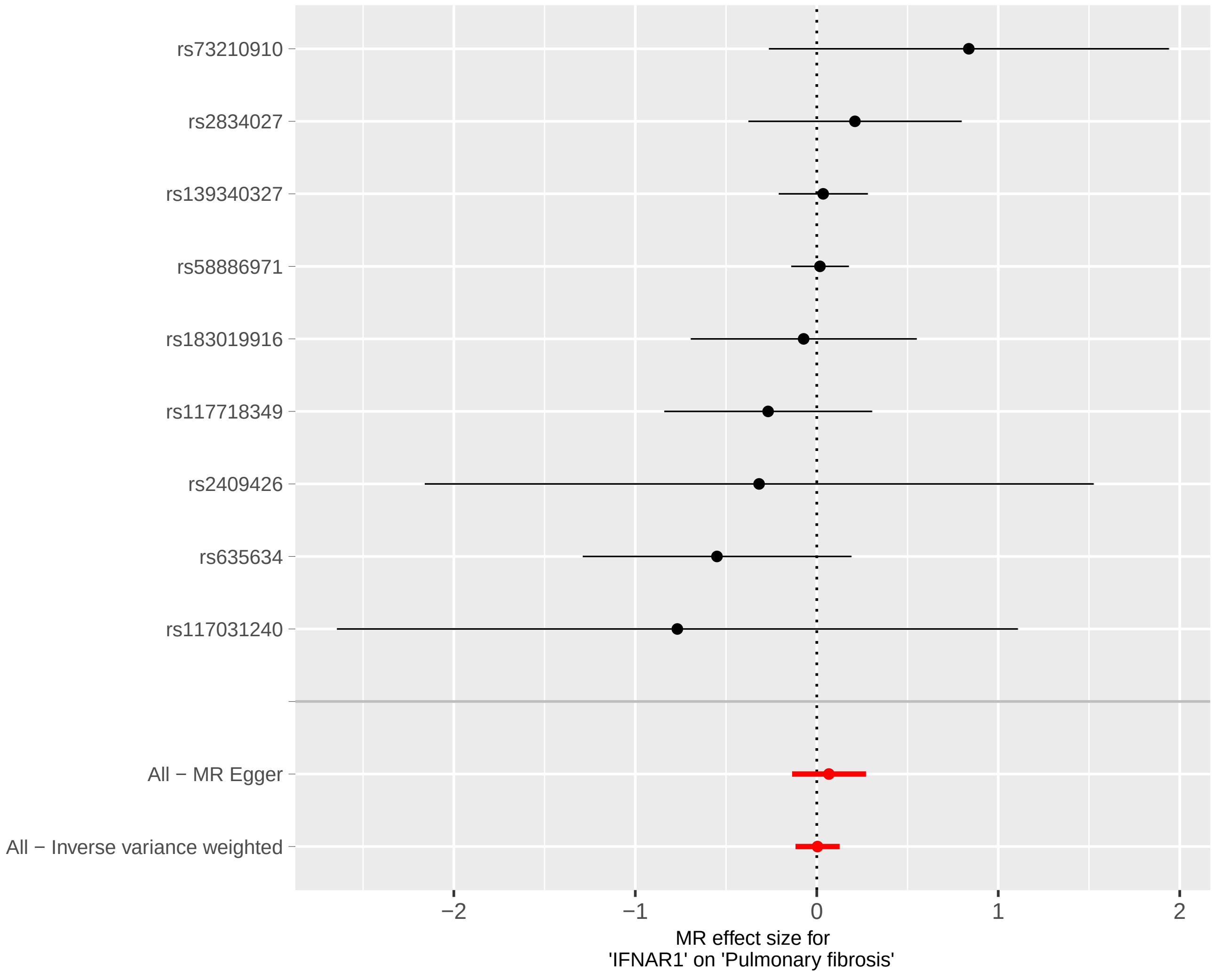
rs73210910
rs2834027
rs139340327
rs58886971
rs183019916
rs117718349
rs2409426
rs635634
rs117031240
All − MR Egger
All − Inverse variance weighted
−2
−1
0
1
2
MR effect size for
'IFNAR1' on 'Pulmonary fibrosis'

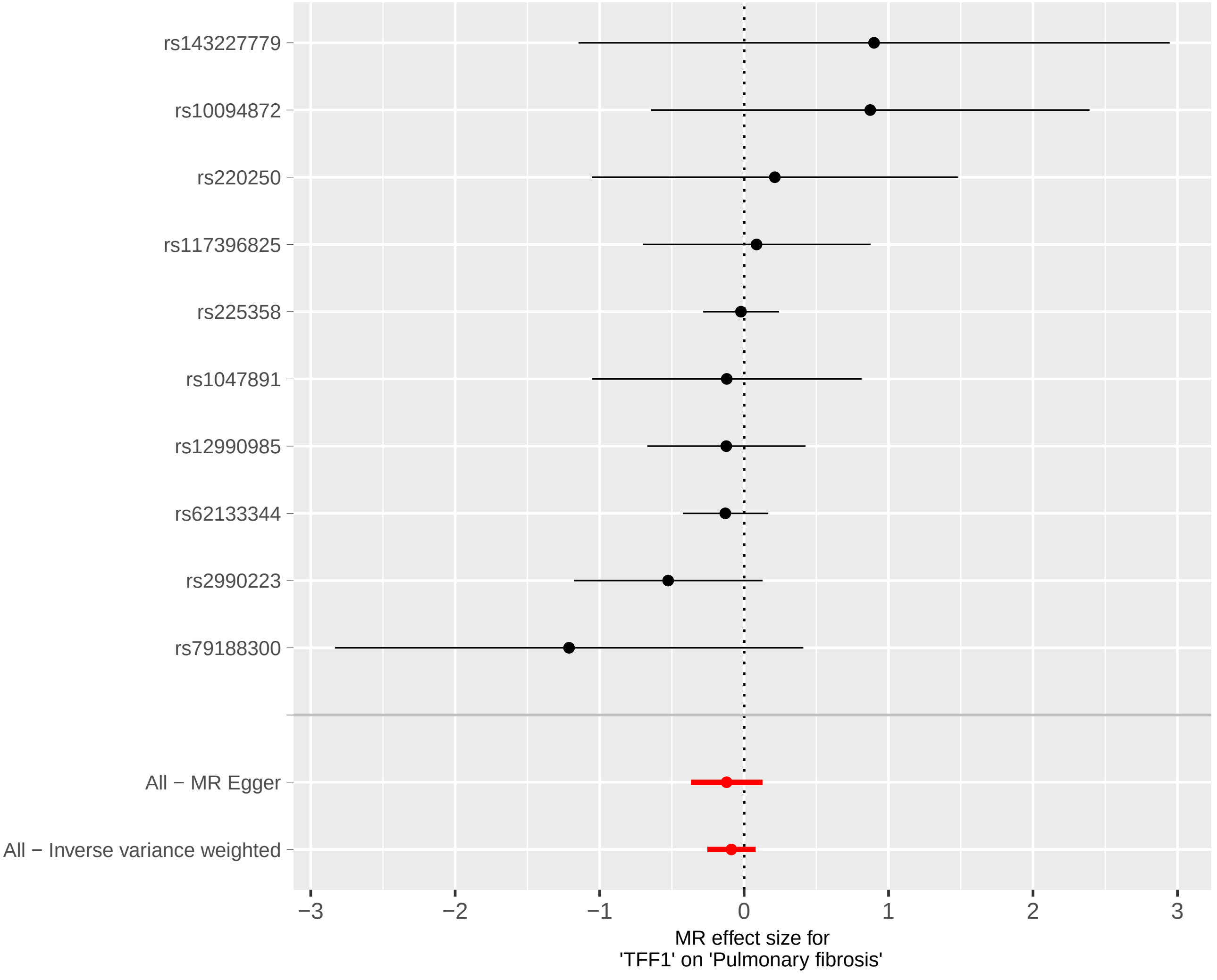
rs143227779
rs10094872
rs220250
rs117396825
rs225358
rs1047891
rs12990985
rs62133344
rs2990223
rs79188300
All − MR Egger
All − Inverse variance weighted
−3
−2
−1
0
1
2
3
MR effect size for
'TFF1' on 'Pulmonary fibrosis'

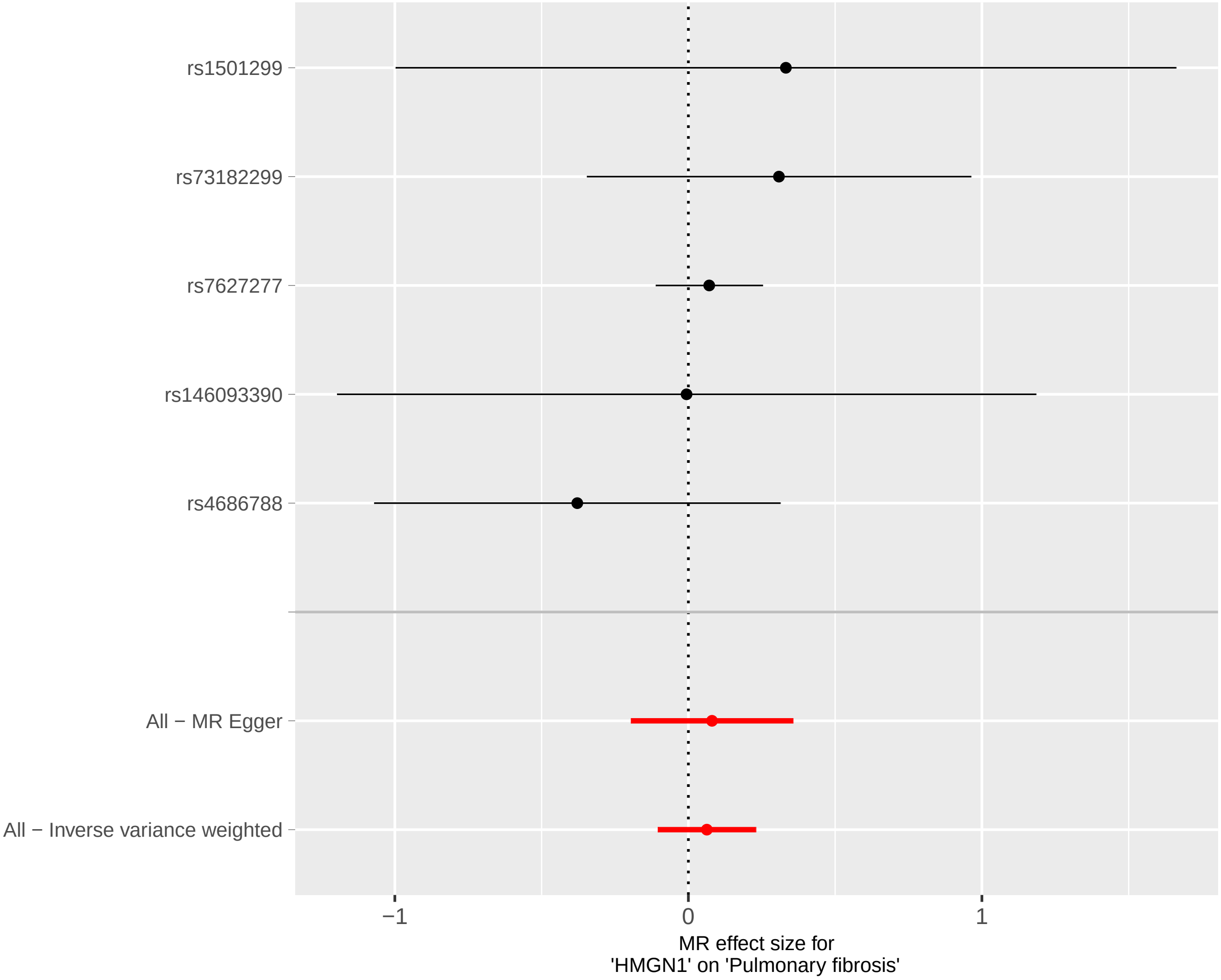

rs1501299
rs73182299
rs7627277
rs146093390
rs4686788
All − MR Egger
All − Inverse variance weighted
−1
0
1
MR effect size for
'HMGN1' on 'Pulmonary fibrosis'

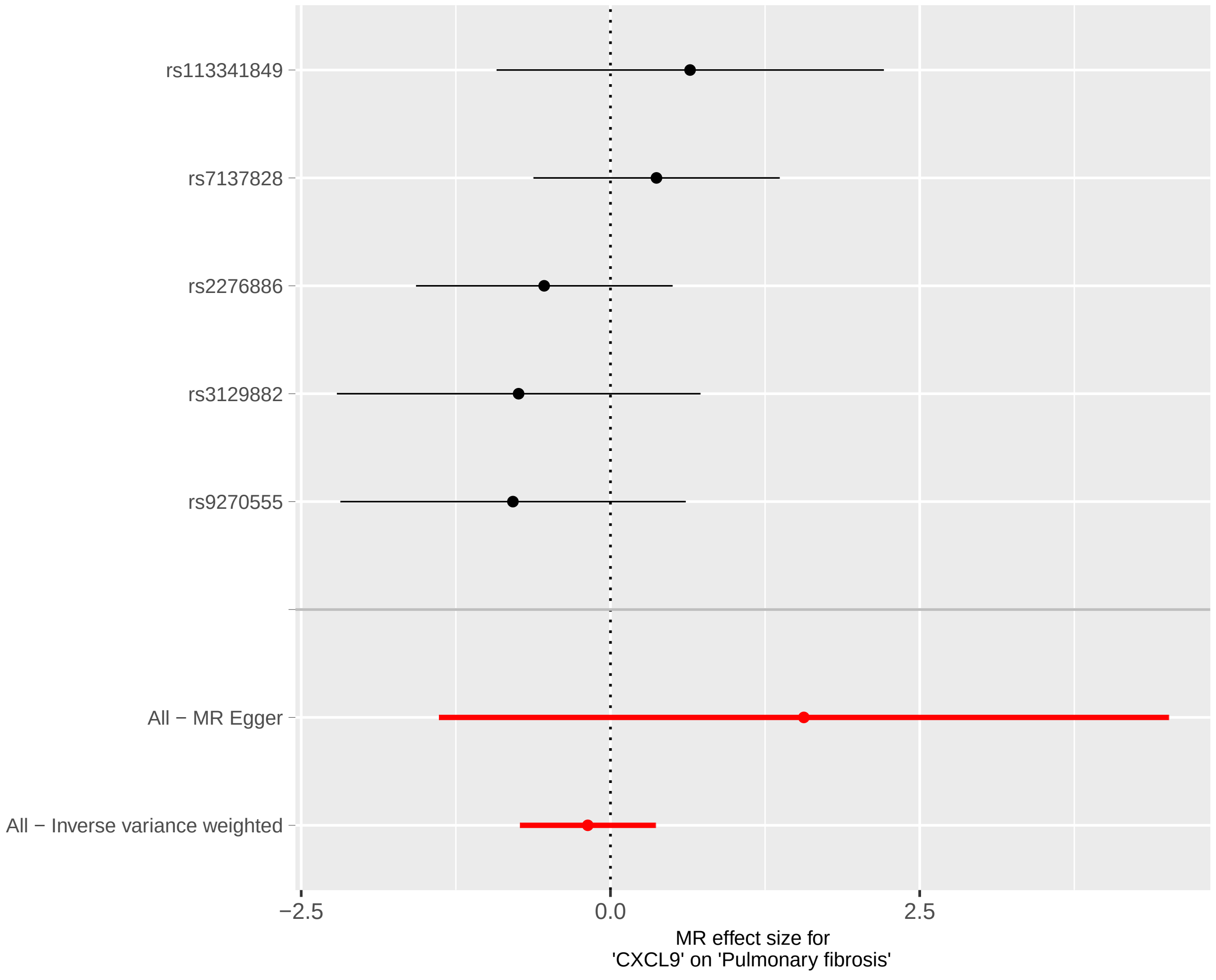

rs113341849
rs7137828
rs2276886
rs3129882
rs9270555
All − MR Egger
All − Inverse variance weighted
−2.5
0.0
2.5
MR effect size for
'CXCL9' on 'Pulmonary fibrosis'

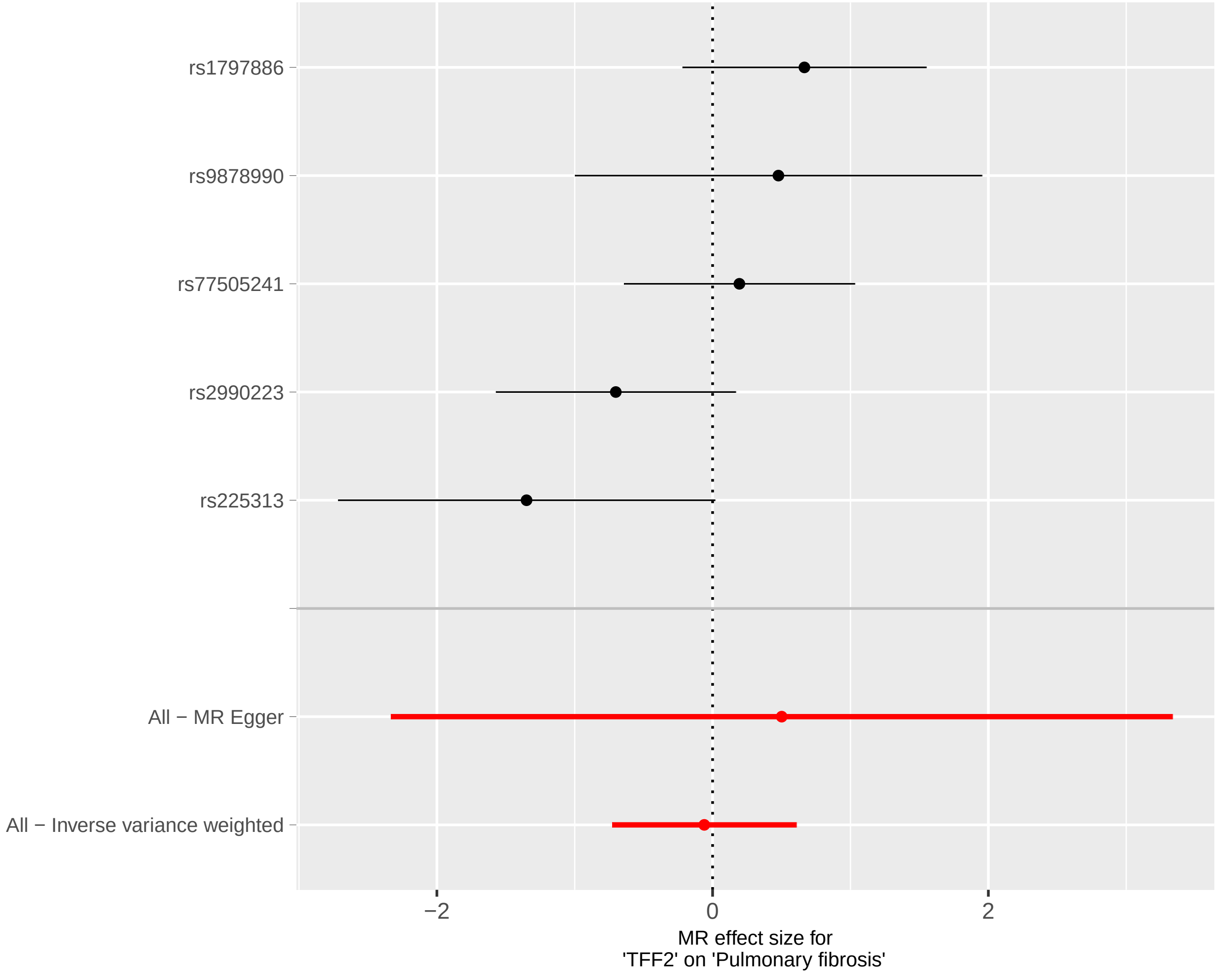
rs1797886
rs9878990
rs77505241
rs2990223
rs225313
All − MR Egger
All − Inverse variance weighted
−2
0
2
MR effect size for
'TFF2' on 'Pulmonary fibrosis'

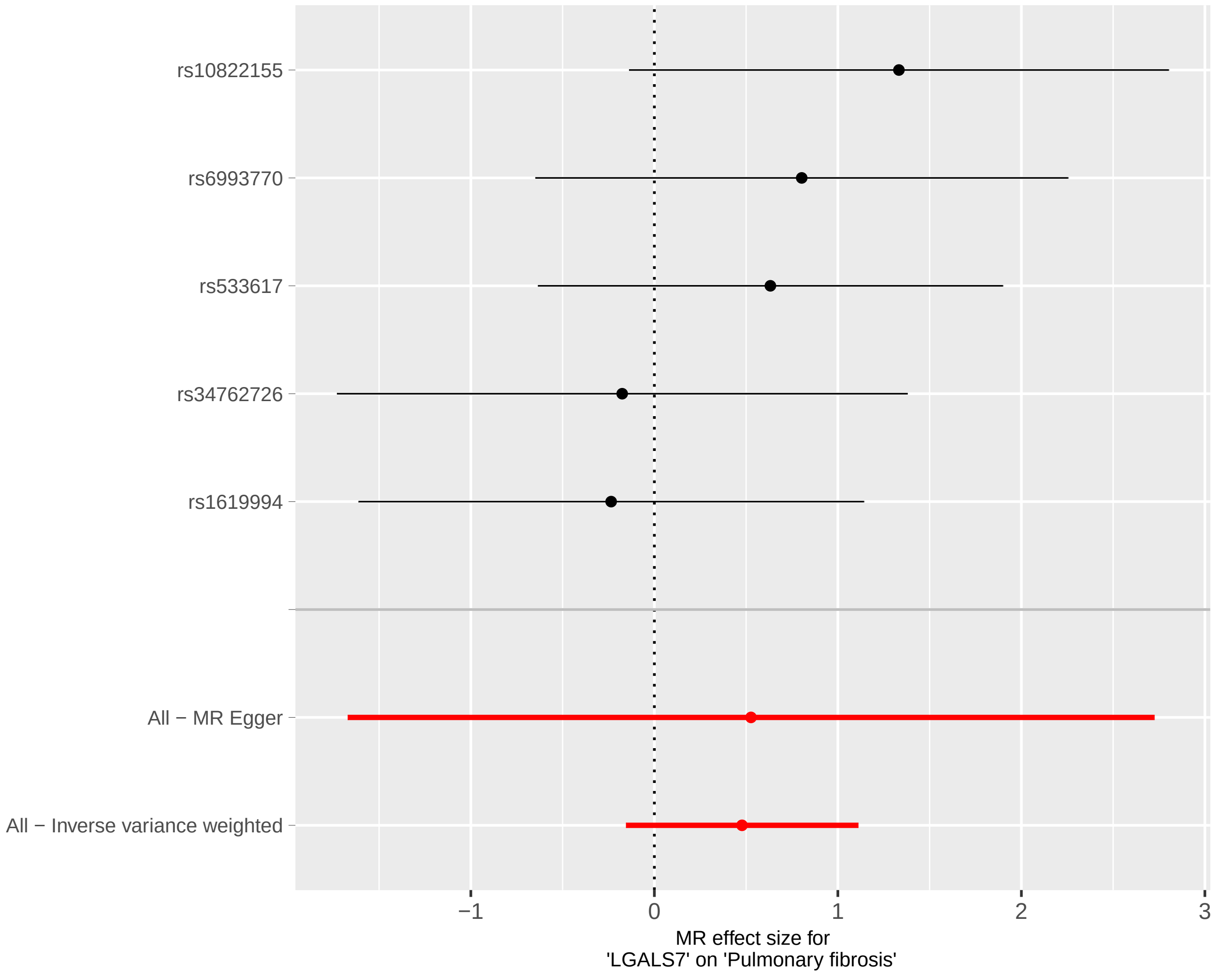
rs10822155
rs6993770
rs533617
rs34762726
rs1619994
All − MR Egger
All − Inverse variance weighted
−1
0
1
2
3
MR effect size for
'LGALS7' on 'Pulmonary fibrosis'

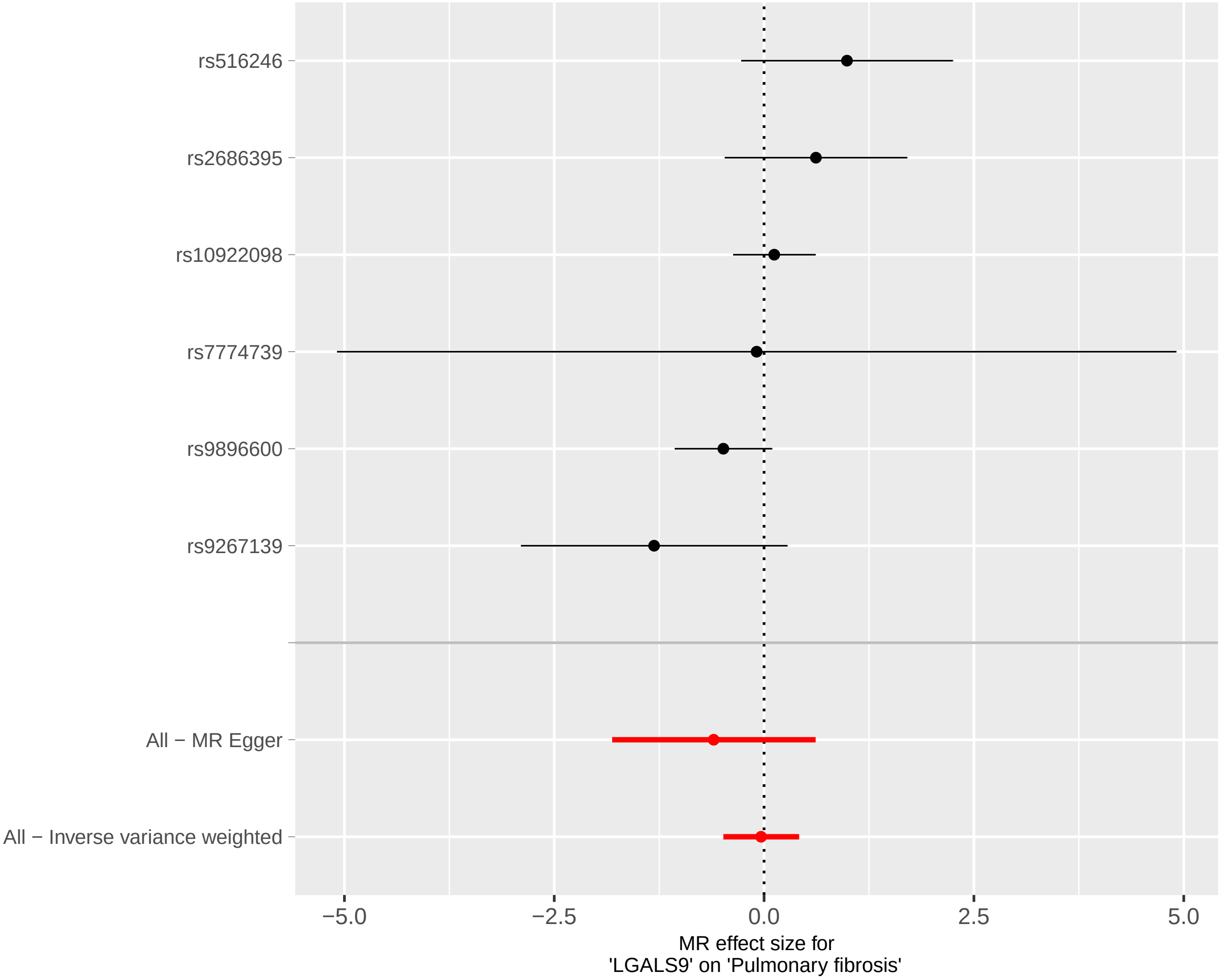
rs516246
rs2686395
rs10922098
rs7774739
rs9896600
rs9267139
All − MR Egger
All − Inverse variance weighted
−5.0
−2.5
0.0
2.5
5.0
MR effect size for
'LGALS9' on 'Pulmonary fibrosis'

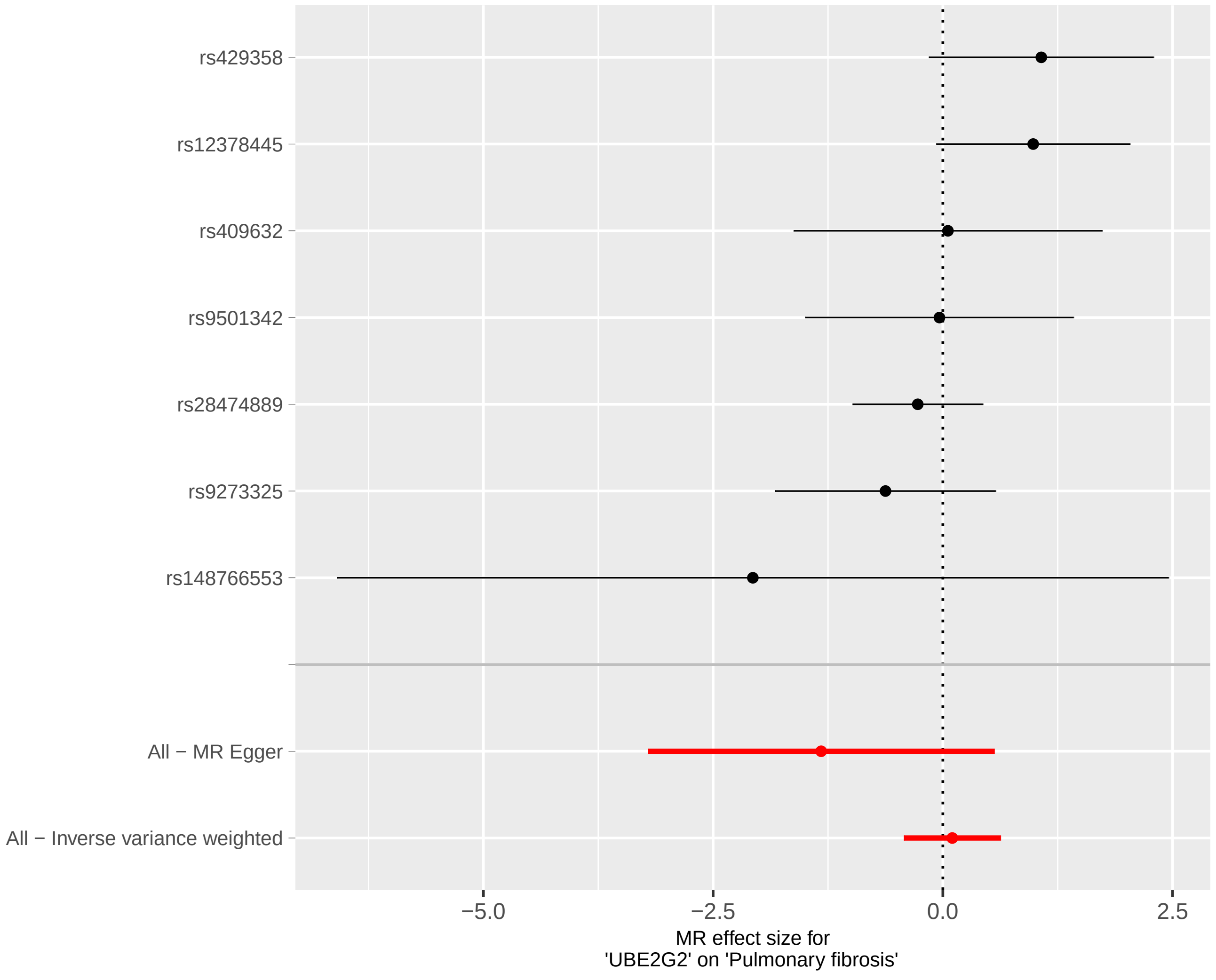

rs429358
rs12378445
rs409632
rs9501342
rs28474889
rs9273325
rs148766553
All − MR Egger
All − Inverse variance weighted
−5.0
−2.5
0.0
2.5
MR effect size for
'UBE2G2' on 'Pulmonary fibrosis'

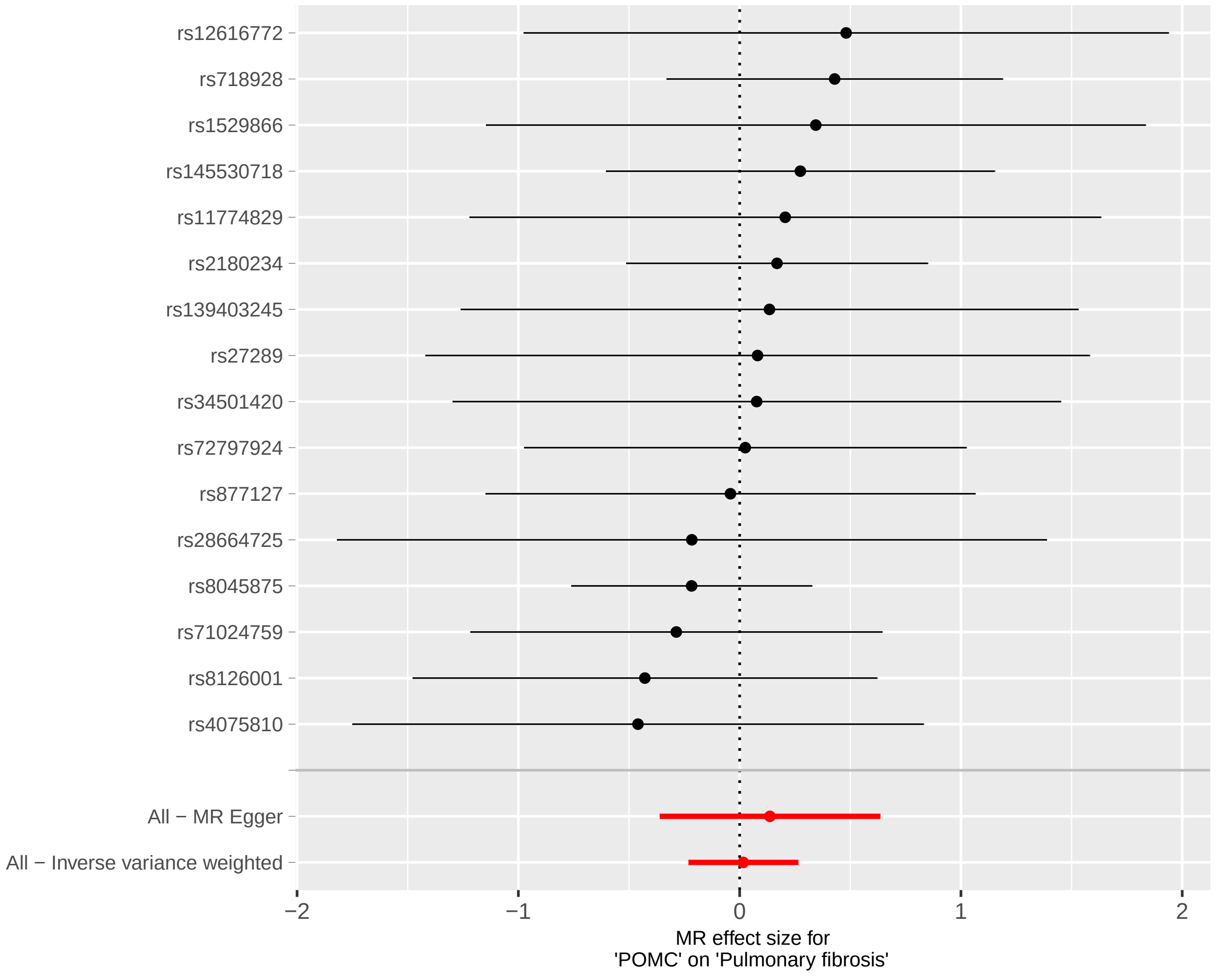

rs12616772
rs718928
rs1529866
rs145530718
rs11774829
rs2180234
rs139403245
rs27289
rs34501420
rs72797924
rs877127
rs28664725
rs8045875
rs71024759
rs8126001
rs4075810
All − MR Egger
All − Inverse variance weighted
−2
−1
0
1
2
MR effect size for
'POMC' on 'Pulmonary fibrosis'

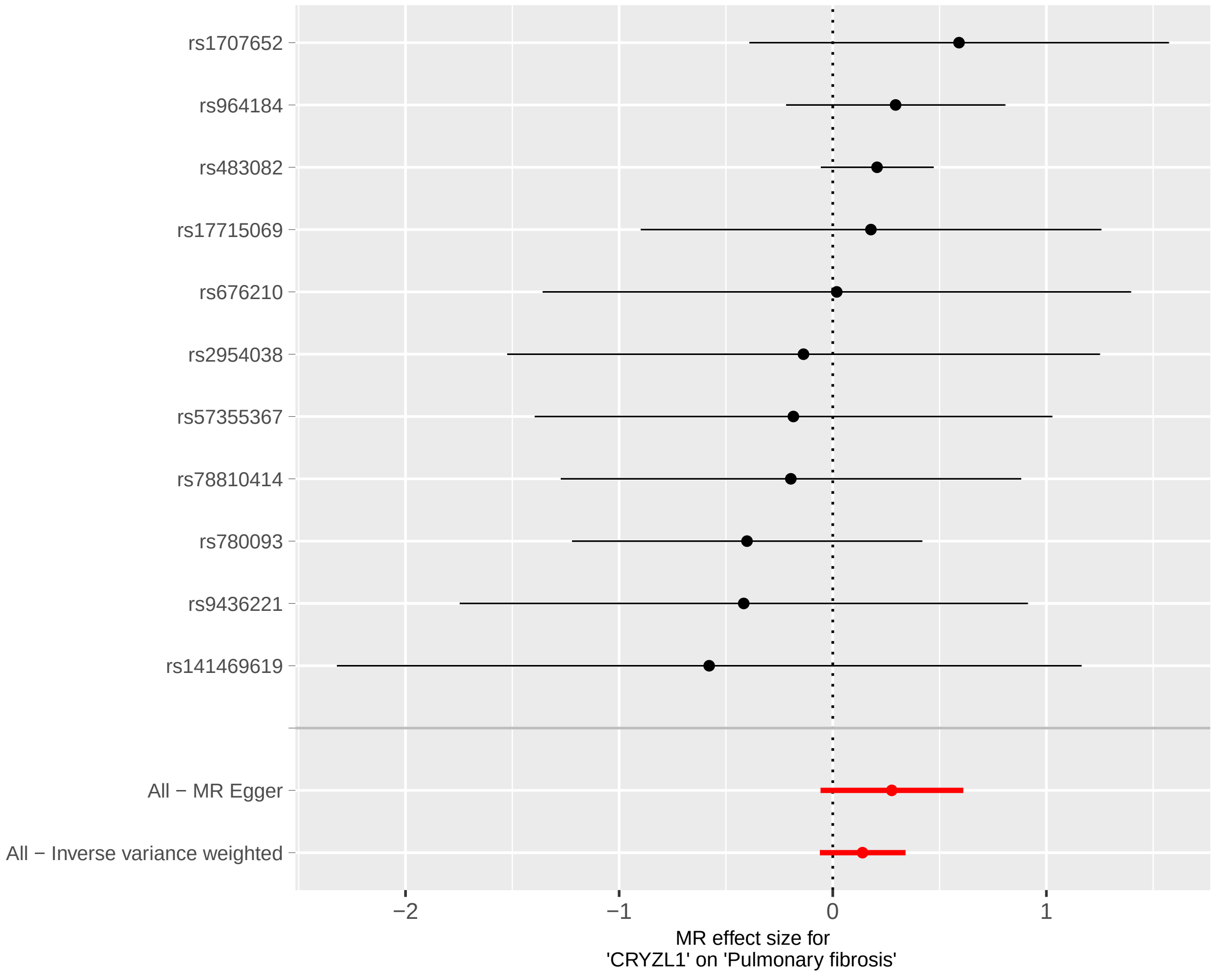
rs1707652
rs964184
rs483082
rs17715069
rs676210
rs2954038
rs57355367
rs78810414
rs780093
rs9436221
rs141469619
All − MR Egger
All − Inverse variance weighted
−2
−1
0
1
MR effect size for
'CRYZL1' on 'Pulmonary fibrosis'

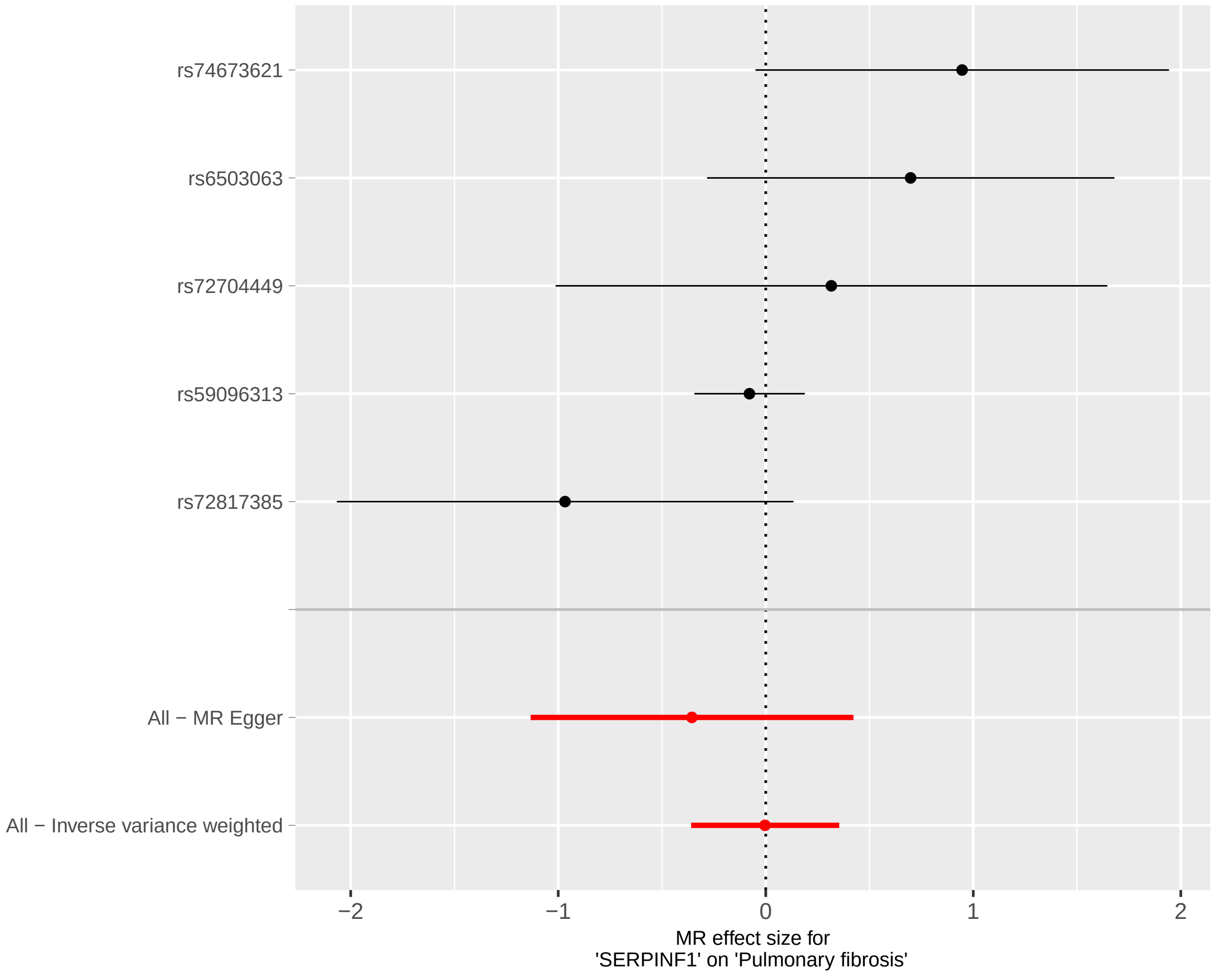
rs74673621
rs6503063
rs72704449
rs59096313
rs72817385
All − MR Egger
All − Inverse variance weighted
−2
−1
0
1
2
MR effect size for
'SERPINF1' on 'Pulmonary fibrosis'

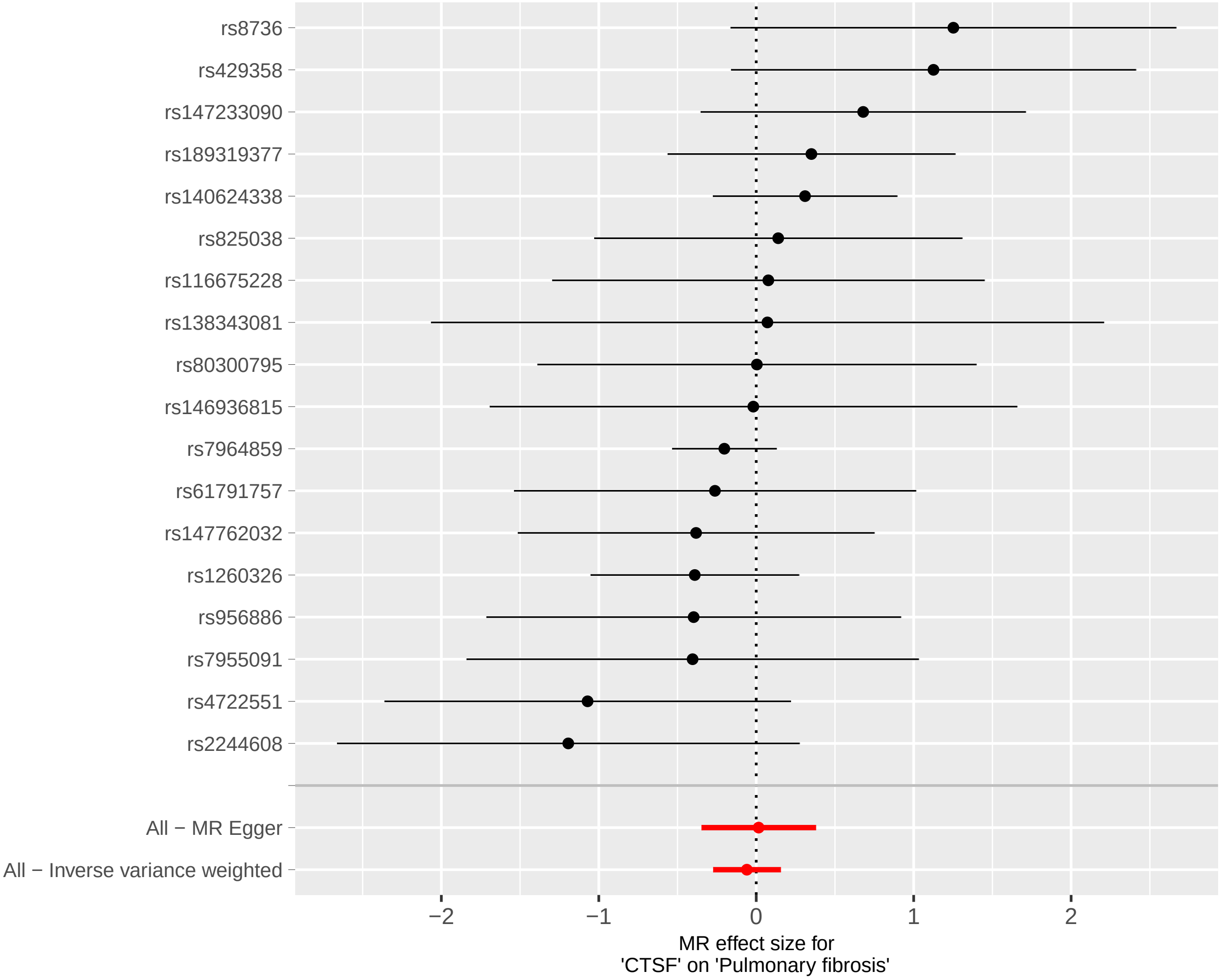

rs8736
rs429358
rs147233090
rs189319377
rs140624338
rs825038
rs116675228
rs138343081
rs80300795
rs146936815
rs7964859
rs61791757
rs147762032
rs1260326
rs956886
rs7955091
rs4722551
rs2244608
All − MR Egger
All − Inverse variance weighted
−2
−1
0
1
2
MR effect size for
'CTSF' on 'Pulmonary fibrosis'

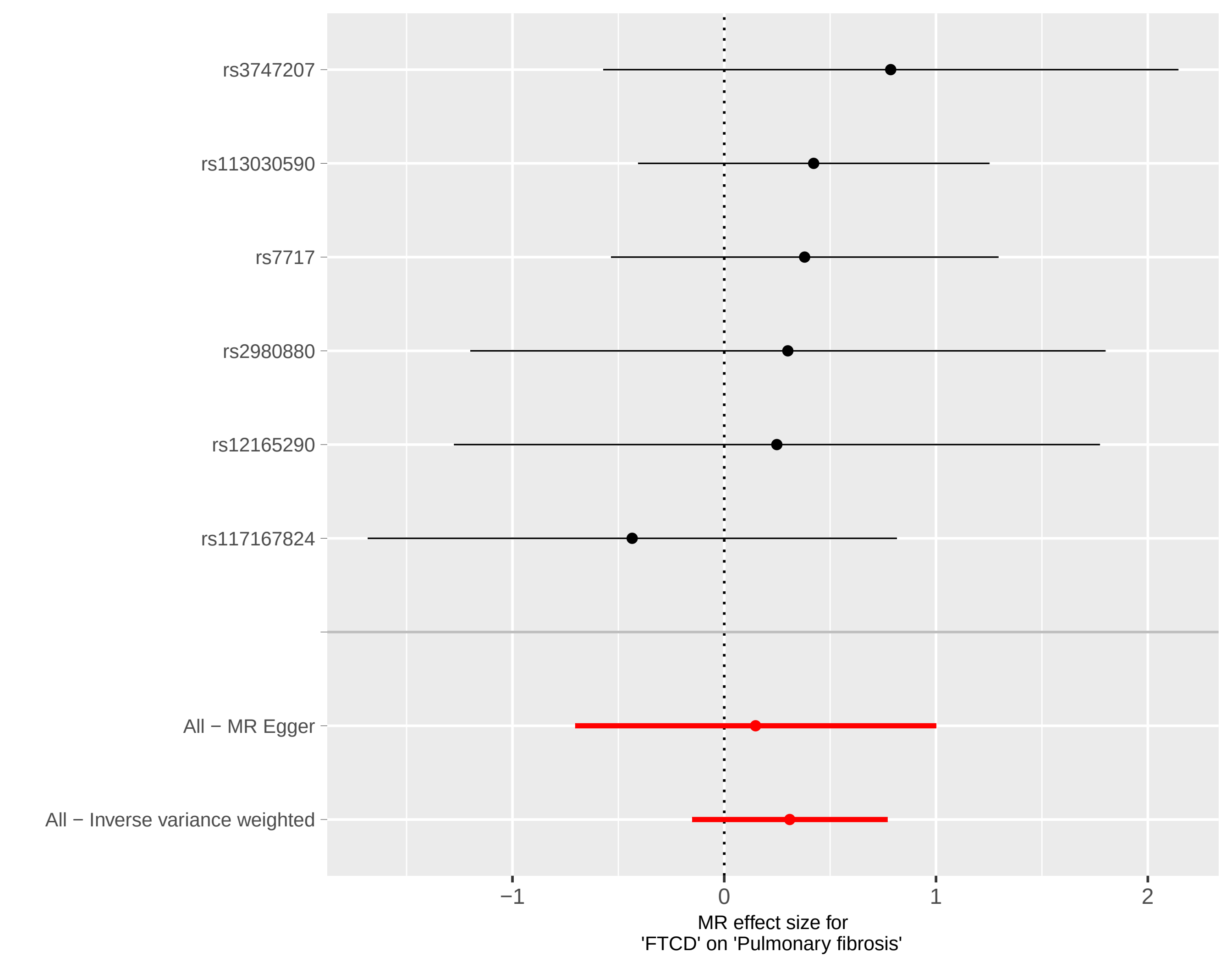

rs3747207
rs113030590
rs7717
rs2980880
rs12165290
rs117167824
All − MR Egger
All − Inverse variance weighted
−1
0
1
2
MR effect size for
'FTCD' on 'Pulmonary fibrosis'

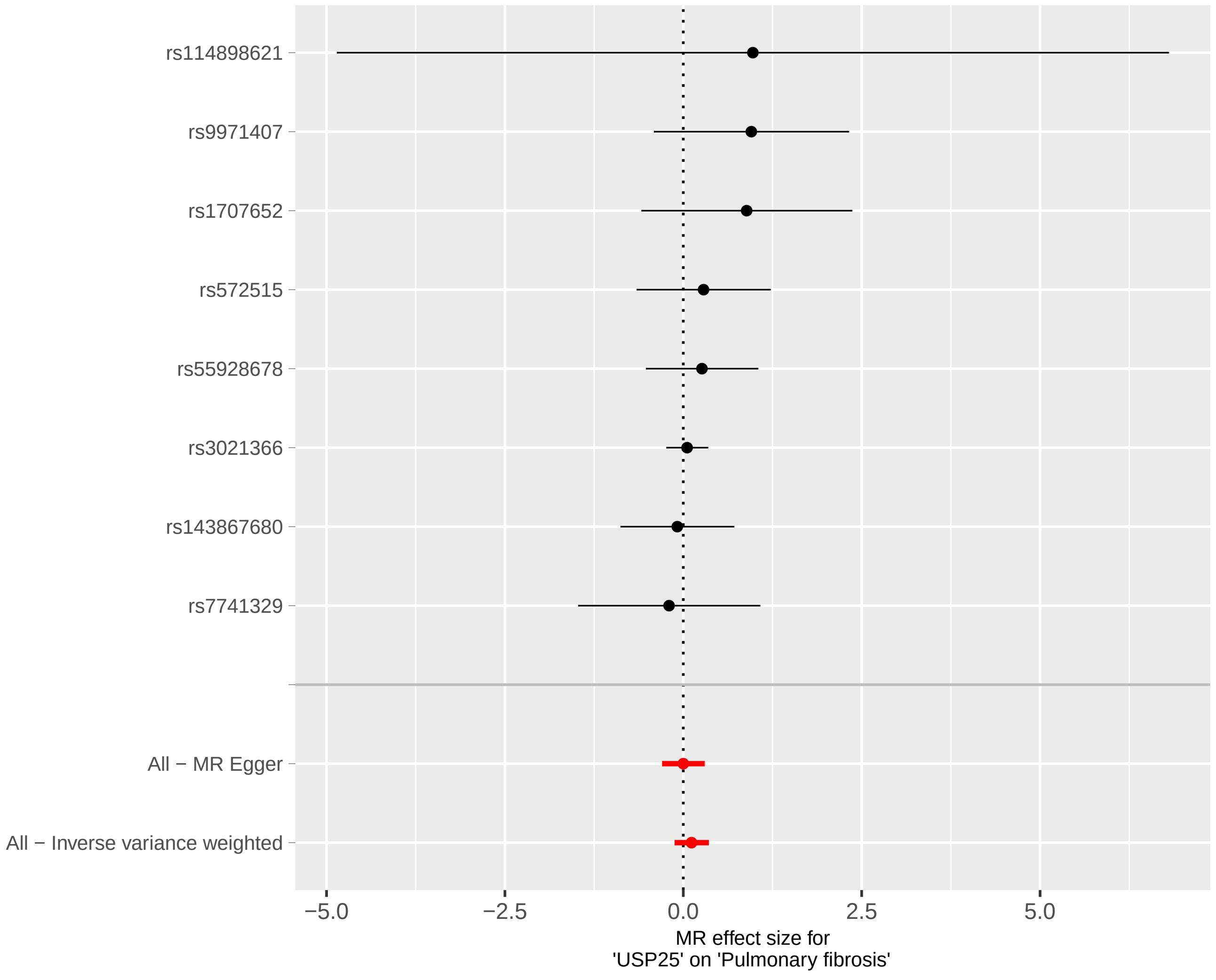
rs114898621
rs9971407
rs1707652
rs572515
rs55928678
rs3021366
rs143867680
rs7741329
All − MR Egger
All − Inverse variance weighted
−5.0
−2.5
0.0
2.5
5.0
MR effect size for
'USP25' on 'Pulmonary fibrosis'

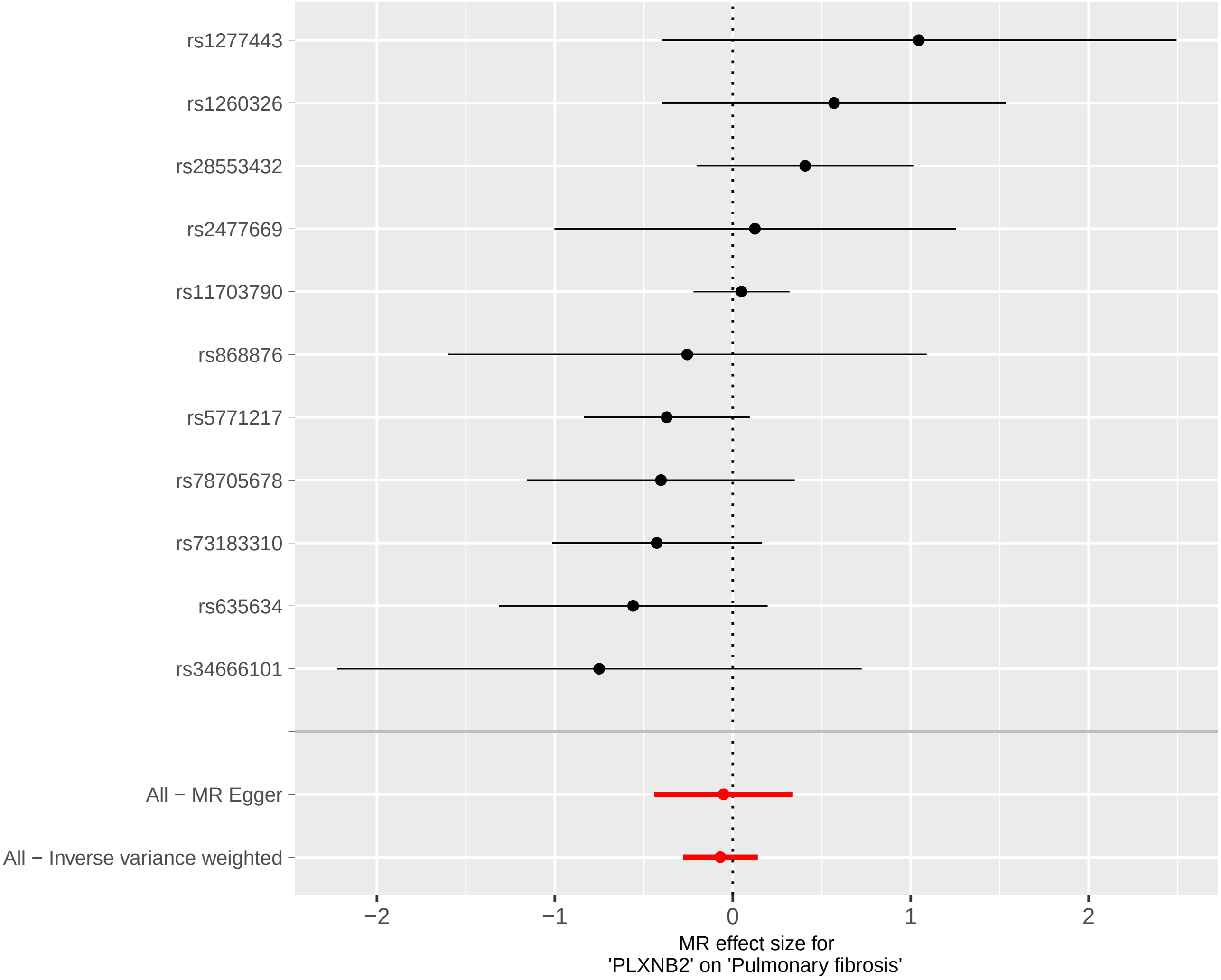

rs1277443
rs1260326
rs28553432
rs2477669
rs11703790
rs868876
rs5771217
rs78705678
rs73183310
rs635634
rs34666101
All − MR Egger
All − Inverse variance weighted
−2
−1
0
1
2
MR effect size for
'PLXNB2' on 'Pulmonary fibrosis'

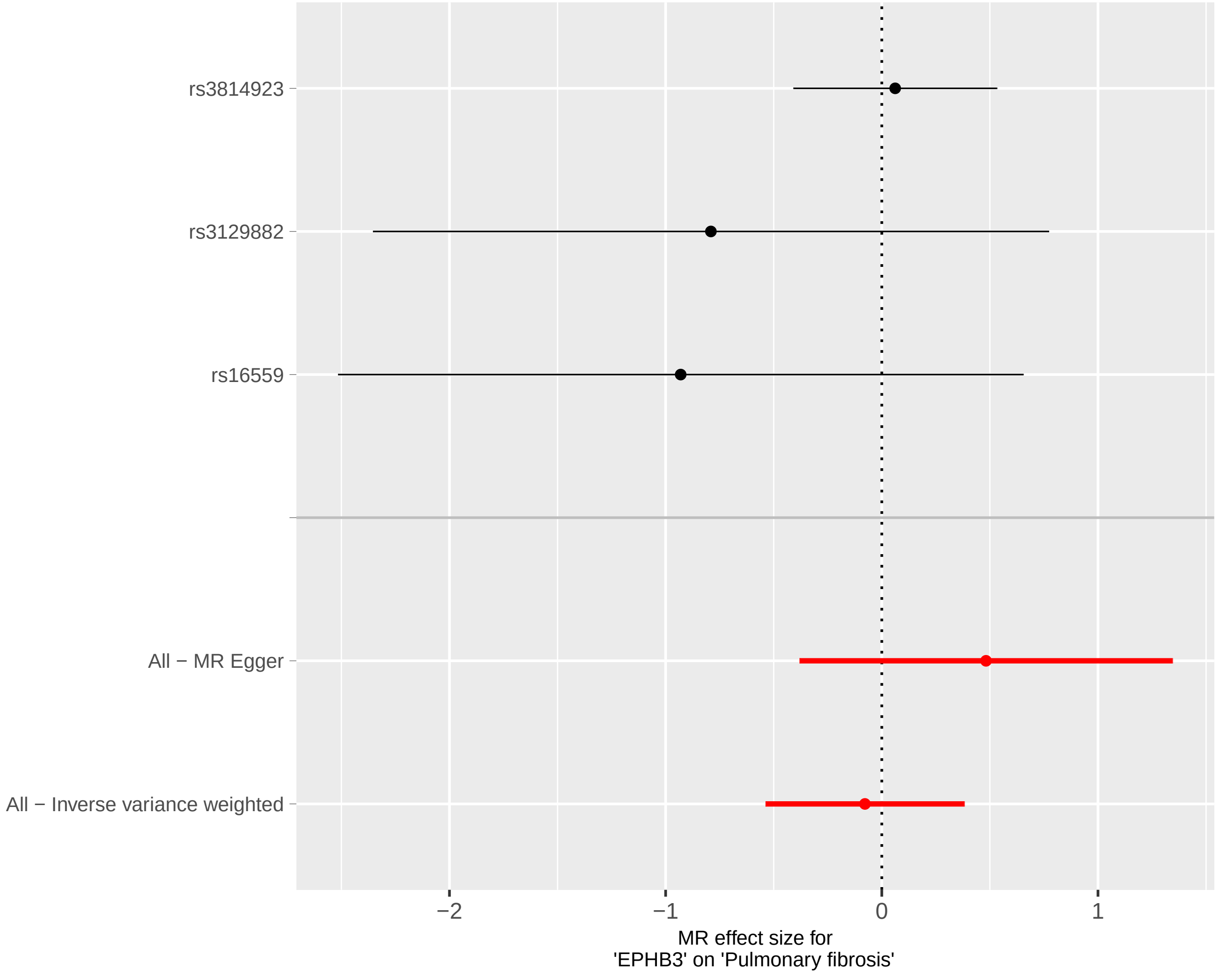
rs3814923
rs3129882
rs16559
All − MR Egger
All − Inverse variance weighted
−2
−1
0
1
MR effect size for
'EPHB3' on 'Pulmonary fibrosis'

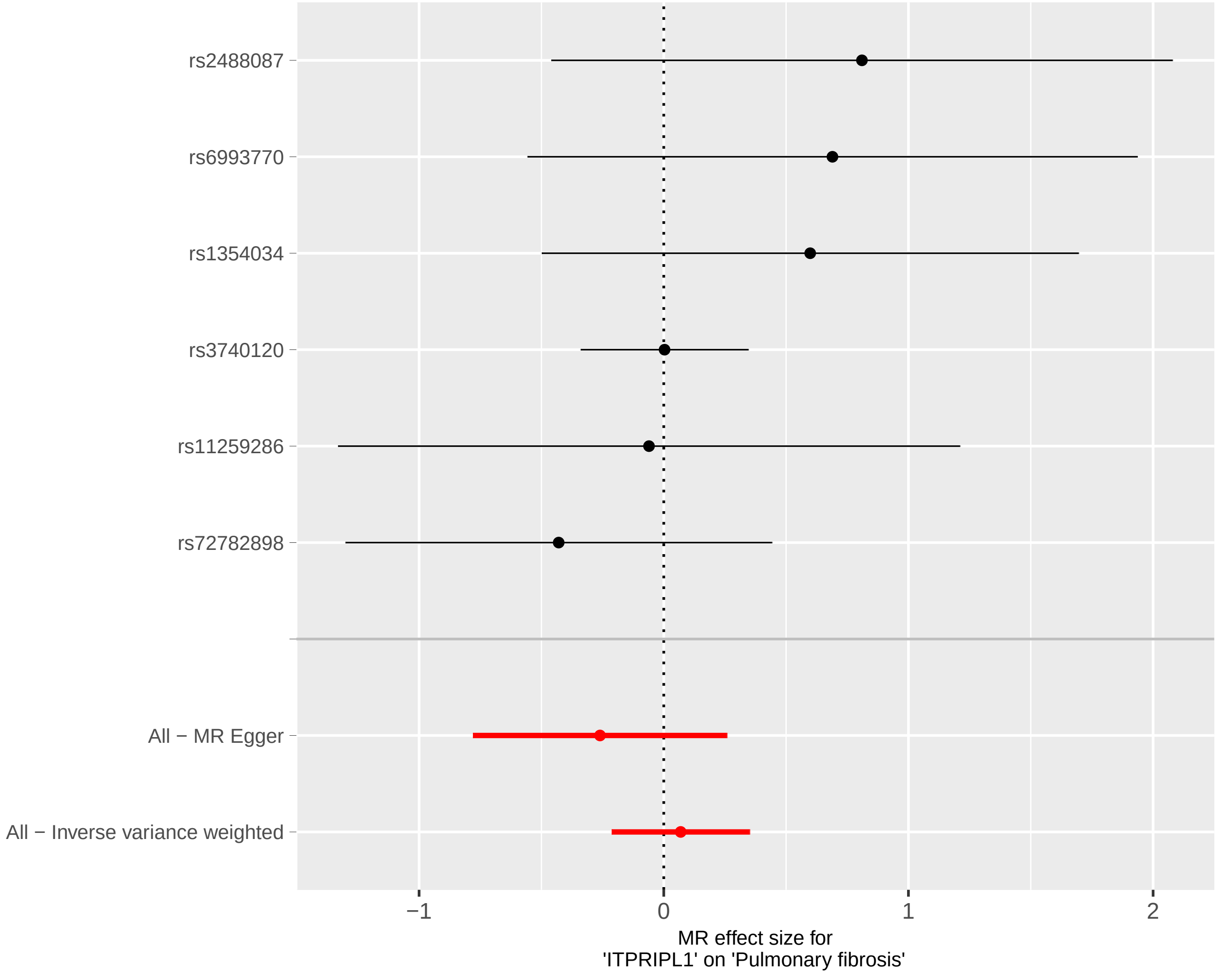

rs2488087
rs6993770
rs1354034
rs3740120
rs11259286
rs72782898
All − MR Egger
All − Inverse variance weighted
−1
0
1
2
MR effect size for
'ITPRIPL1' on 'Pulmonary fibrosis'

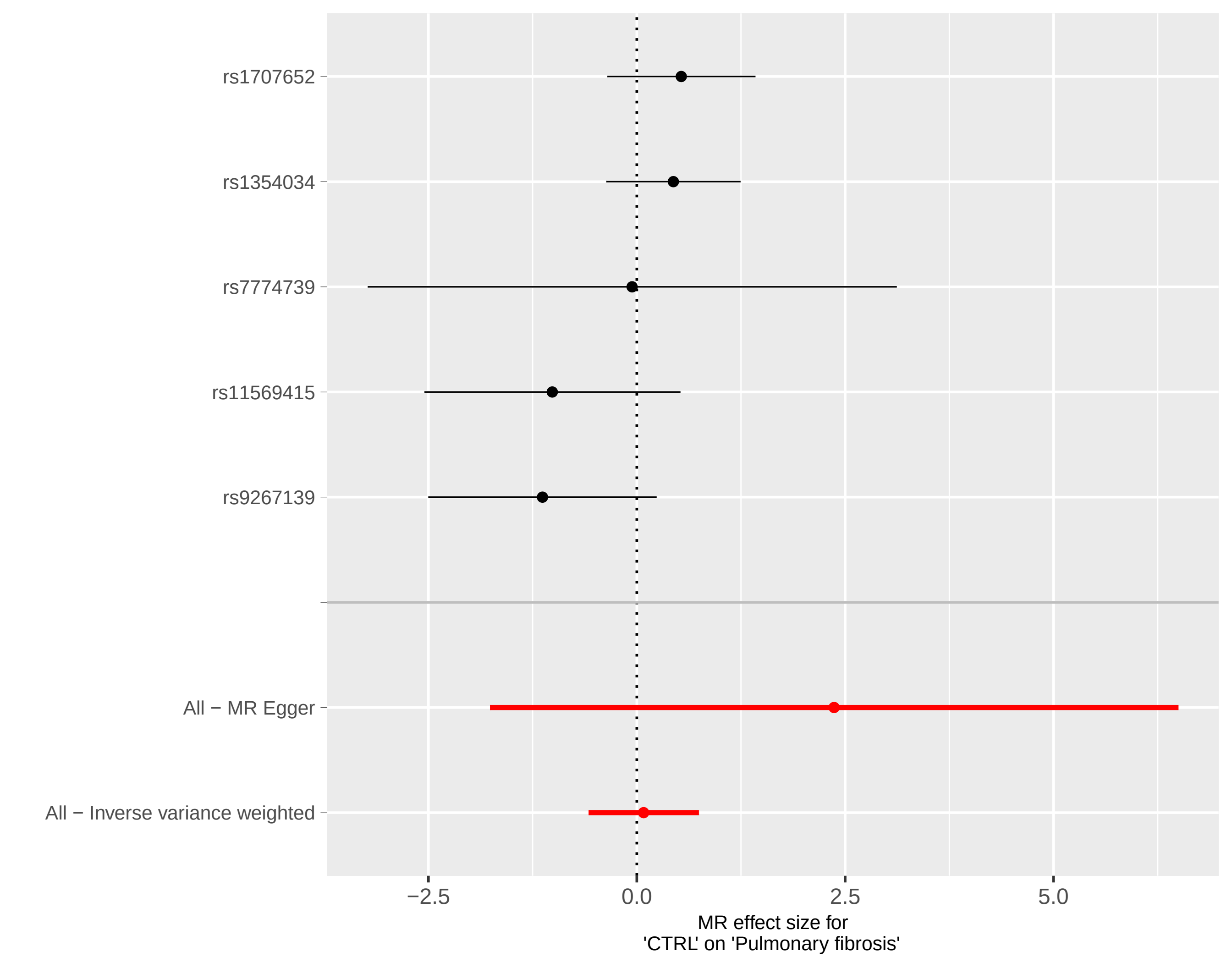
rs1707652
rs1354034
rs7774739
rs11569415
rs9267139
All − MR Egger
All − Inverse variance weighted
−2.5
0.0
2.5
5.0
MR effect size for
'CTRL' on 'Pulmonary fibrosis'

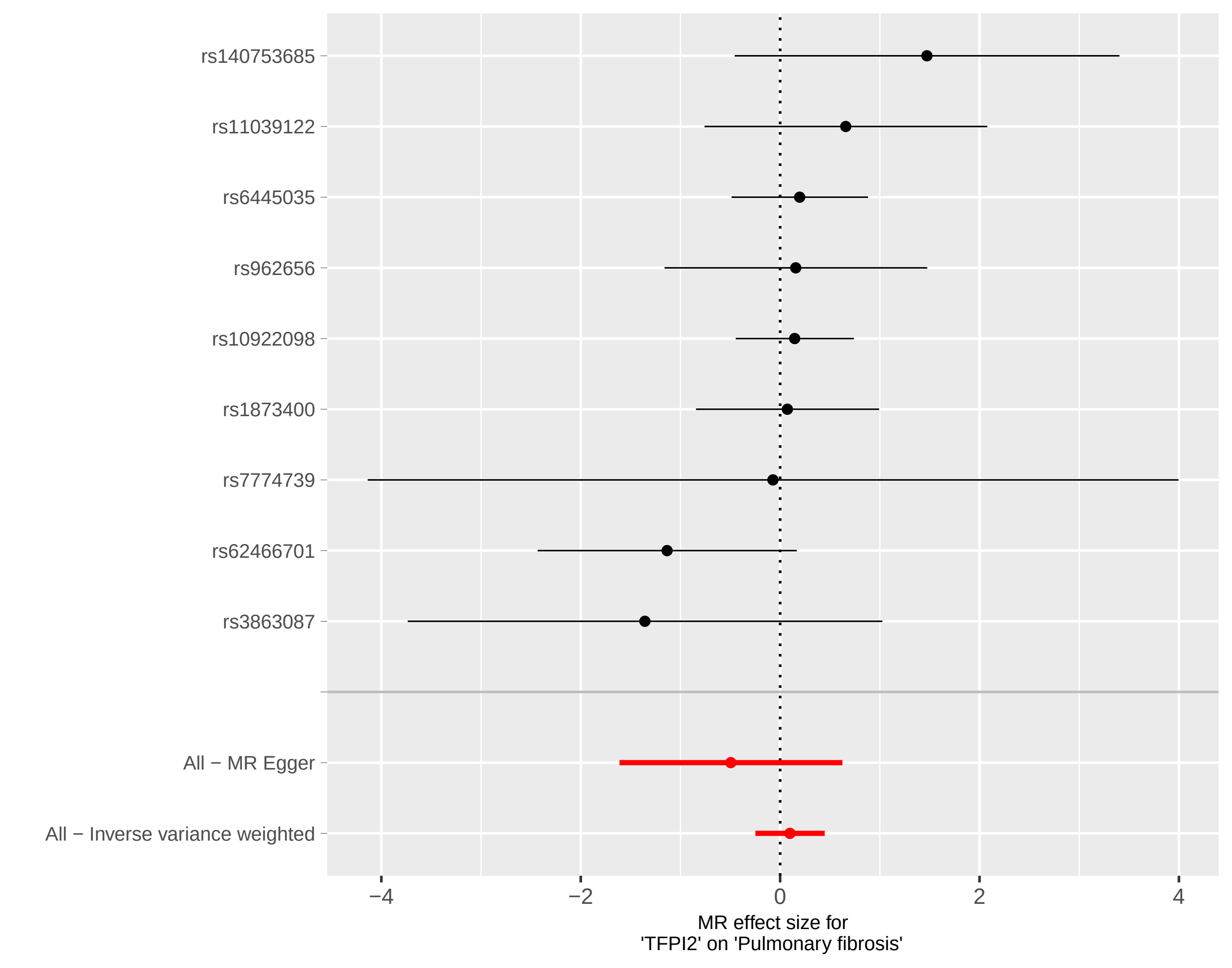
rs140753685
rs11039122
rs6445035
rs962656
rs10922098
rs1873400
rs7774739
rs62466701
rs3863087
All − MR Egger
All − Inverse variance weighted
−4
−2
0
2
4
MR effect size for
'TFPI2' on 'Pulmonary fibrosis'

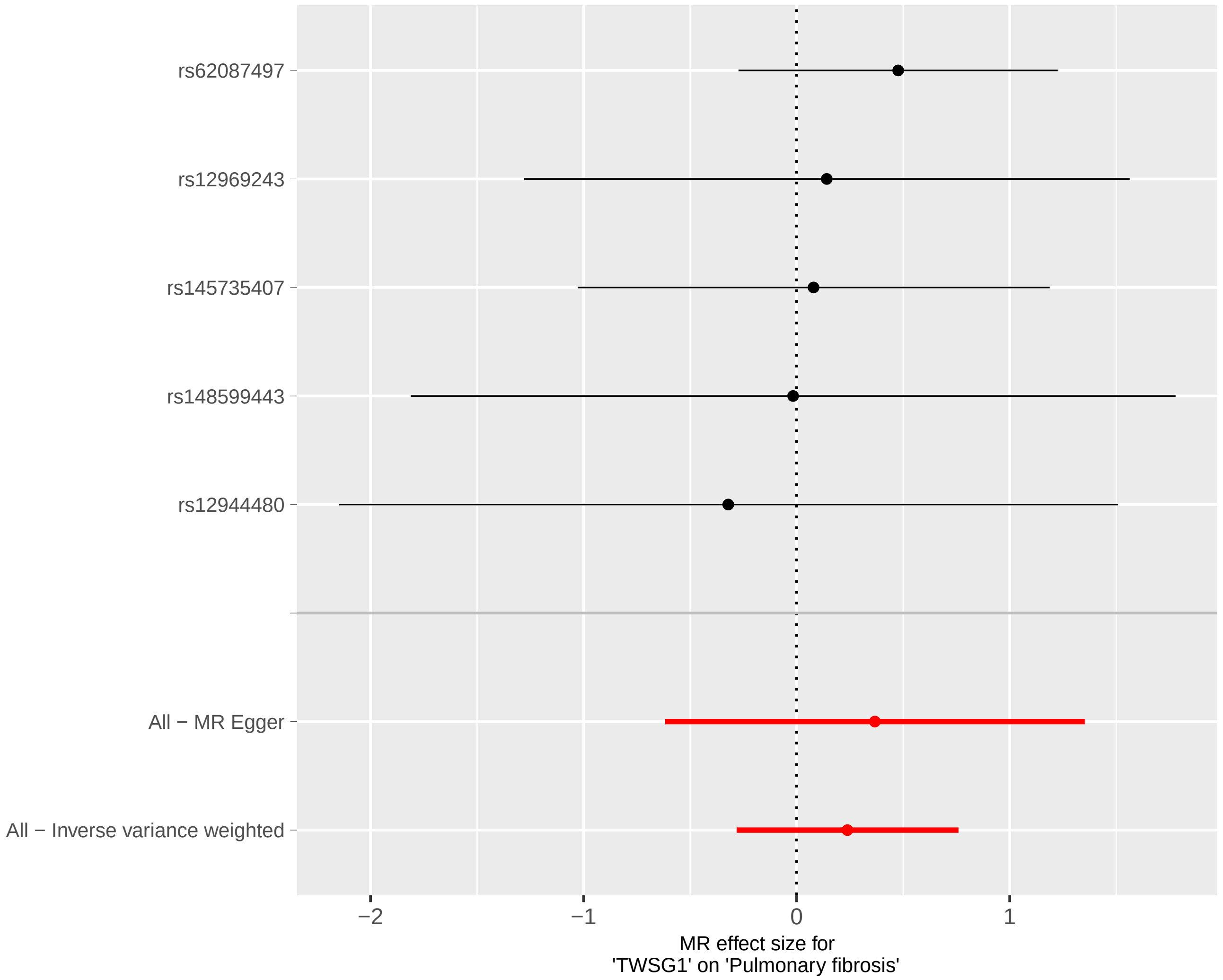

rs62087497
rs12969243
rs145735407
rs148599443
rs12944480
All − MR Egger
All − Inverse variance weighted
−2
−1
0
1
MR effect size for
'TWSG1' on 'Pulmonary fibrosis'

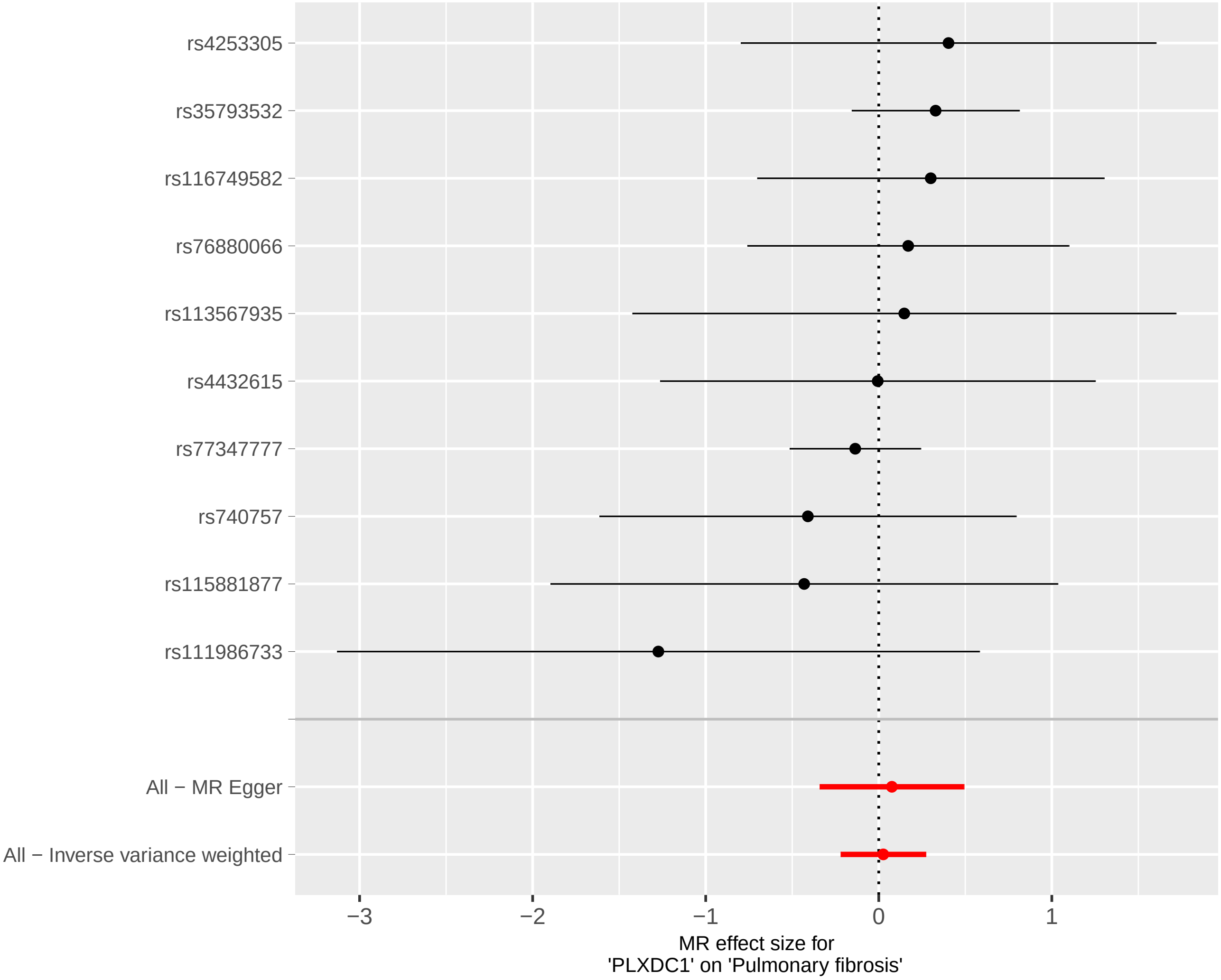
rs4253305
rs35793532
rs116749582
rs76880066
rs113567935
rs4432615
rs77347777
rs740757
rs115881877
rs111986733
All − MR Egger
All − Inverse variance weighted
−3
−2
−1
0
1
MR effect size for
'PLXDC1' on 'Pulmonary fibrosis'

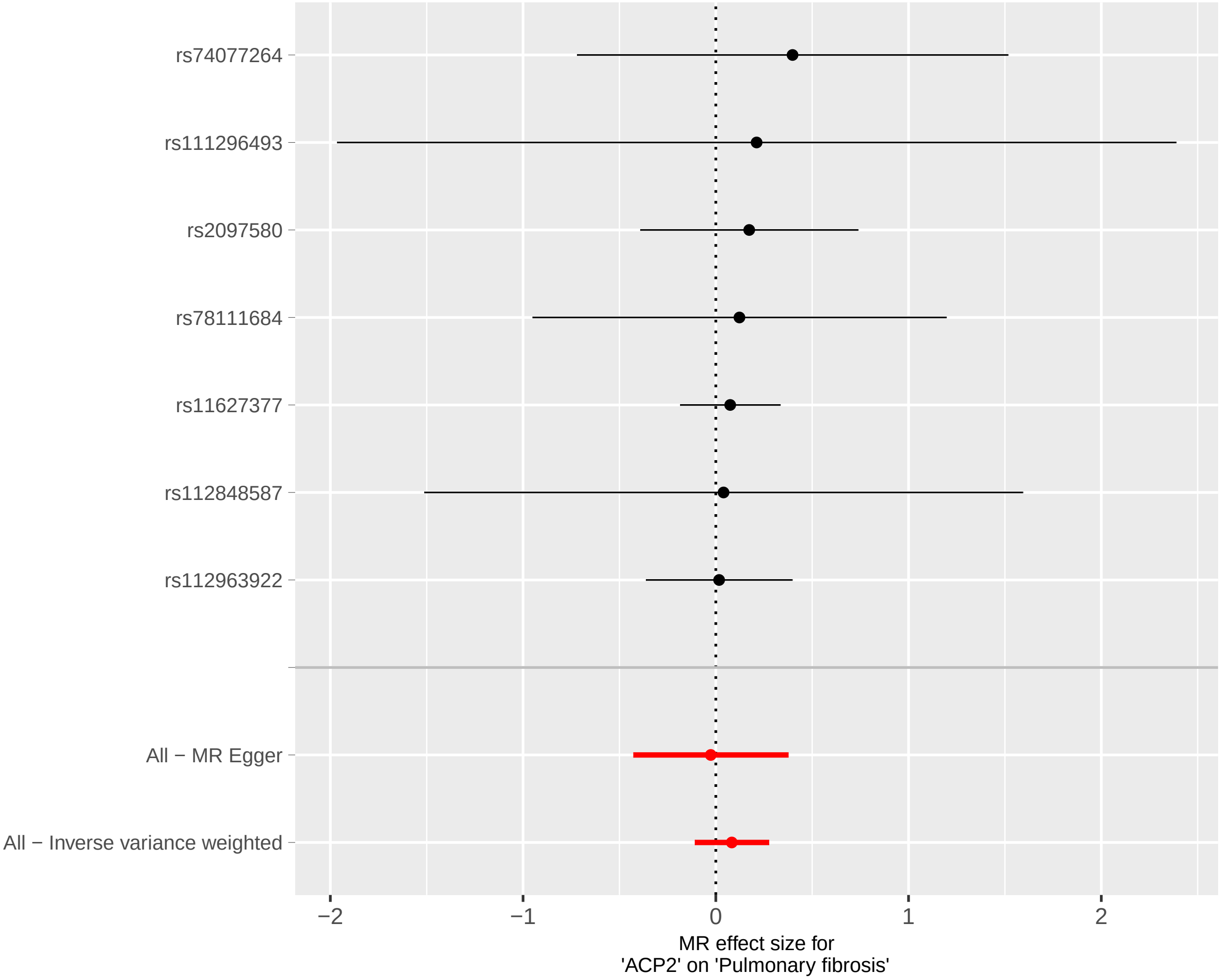
rs74077264
rs111296493
rs2097580
rs78111684
rs11627377
rs112848587
rs112963922
All − MR Egger
All − Inverse variance weighted
−2
−1
0
1
2
MR effect size for
'ACP2' on 'Pulmonary fibrosis'

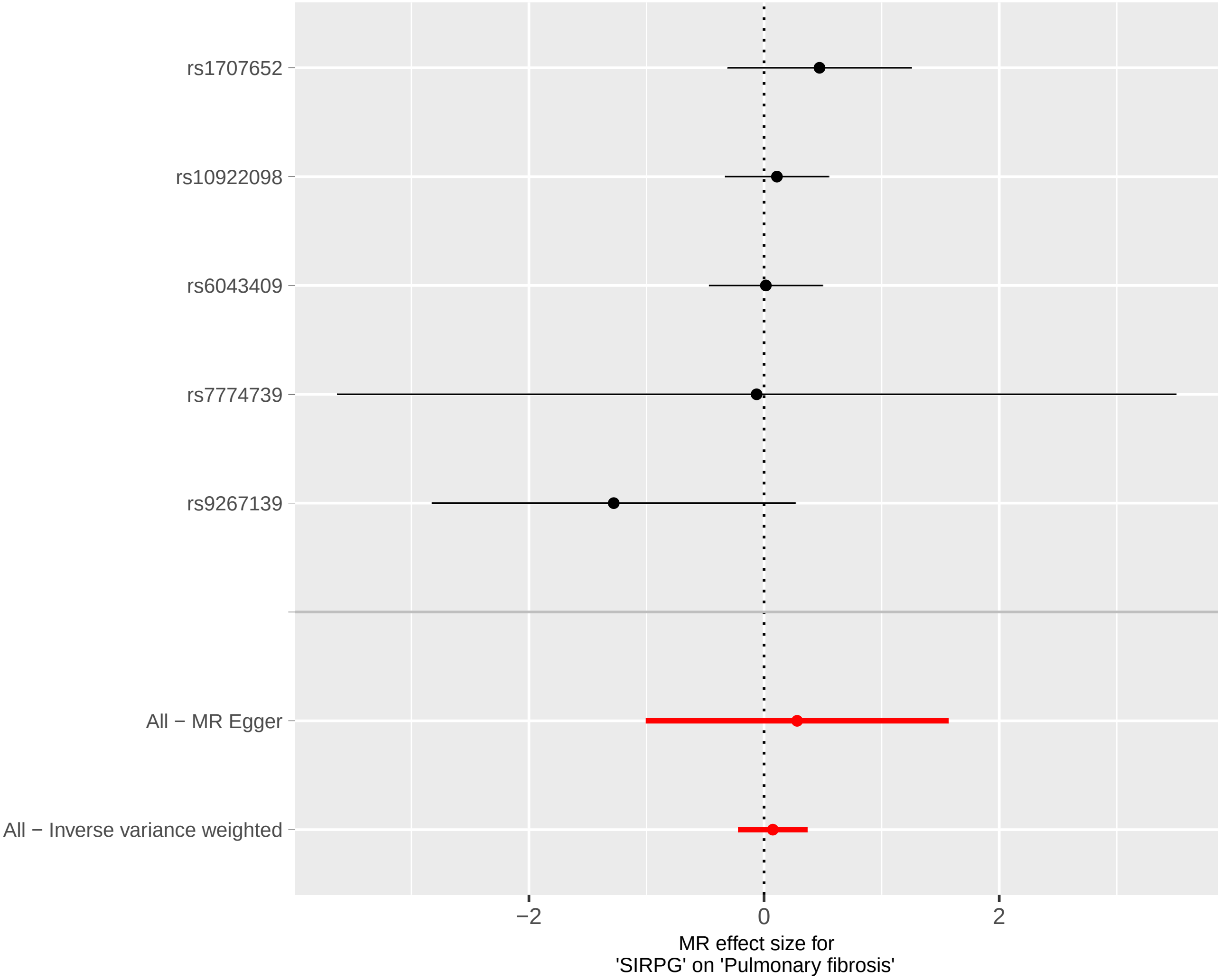
rs1707652
rs10922098
rs6043409
rs7774739
rs9267139
All − MR Egger
All − Inverse variance weighted
−2
0
2
MR effect size for
'SIRPG' on 'Pulmonary fibrosis'

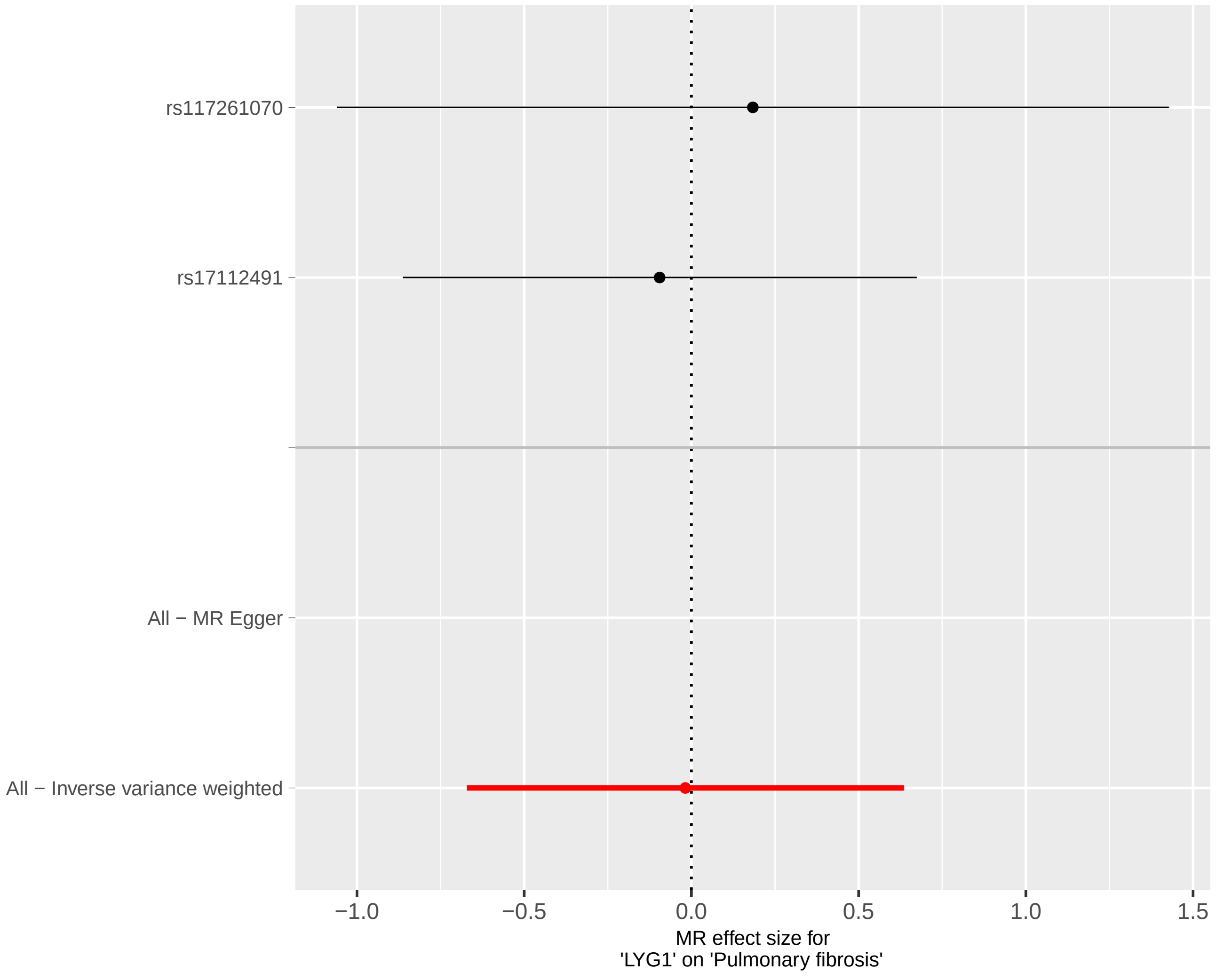

rs117261070
rs17112491
All − MR Egger
All − Inverse variance weighted
−1.0
−0.5
0.0
0.5
1.0
1.5
MR effect size for
'LYG1' on 'Pulmonary fibrosis'

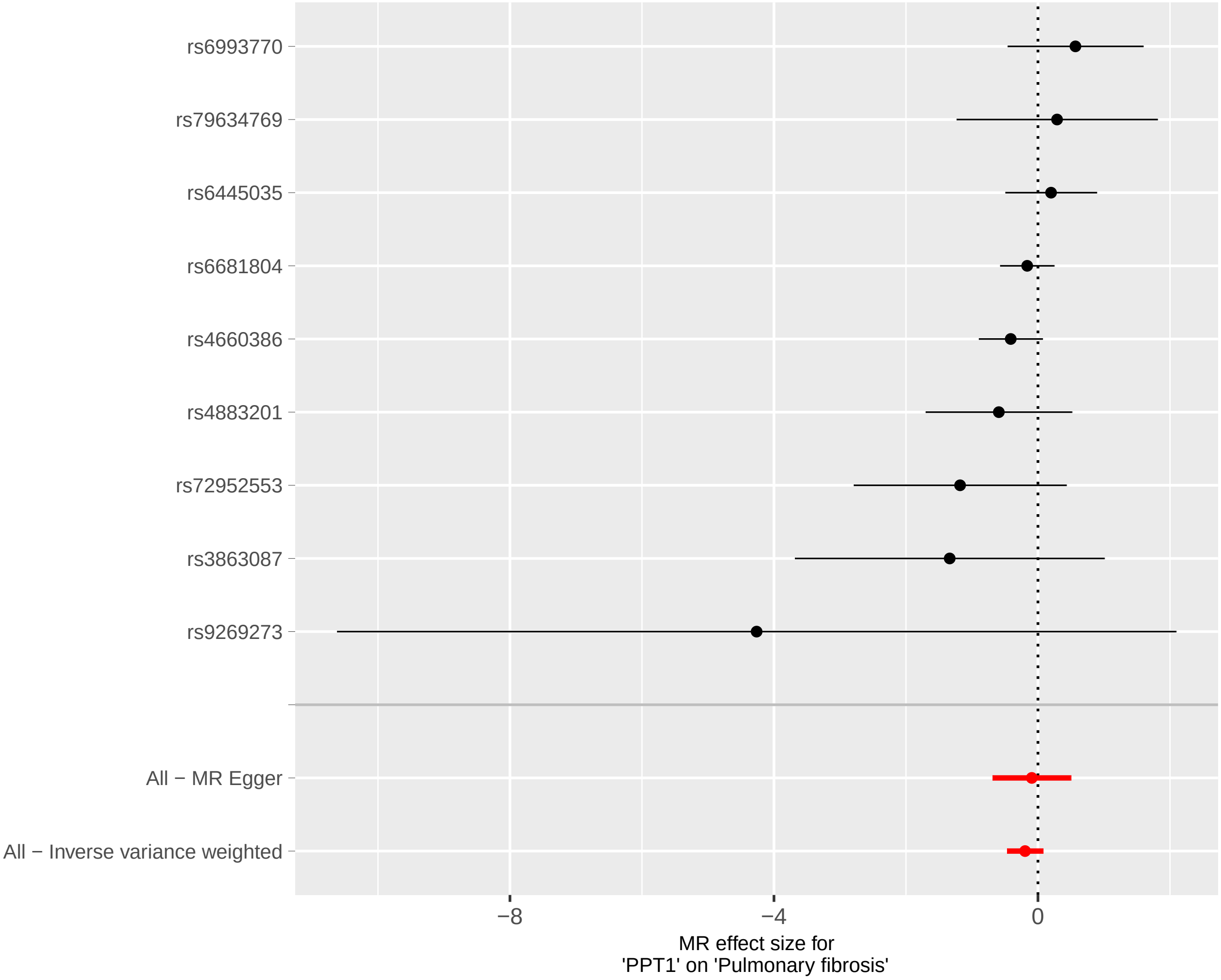
rs6993770
rs79634769
rs6445035
rs6681804
rs4660386
rs4883201
rs72952553
rs3863087
rs9269273
All − MR Egger
All − Inverse variance weighted
−8
−4
0
MR effect size for
'PPT1' on 'Pulmonary fibrosis'

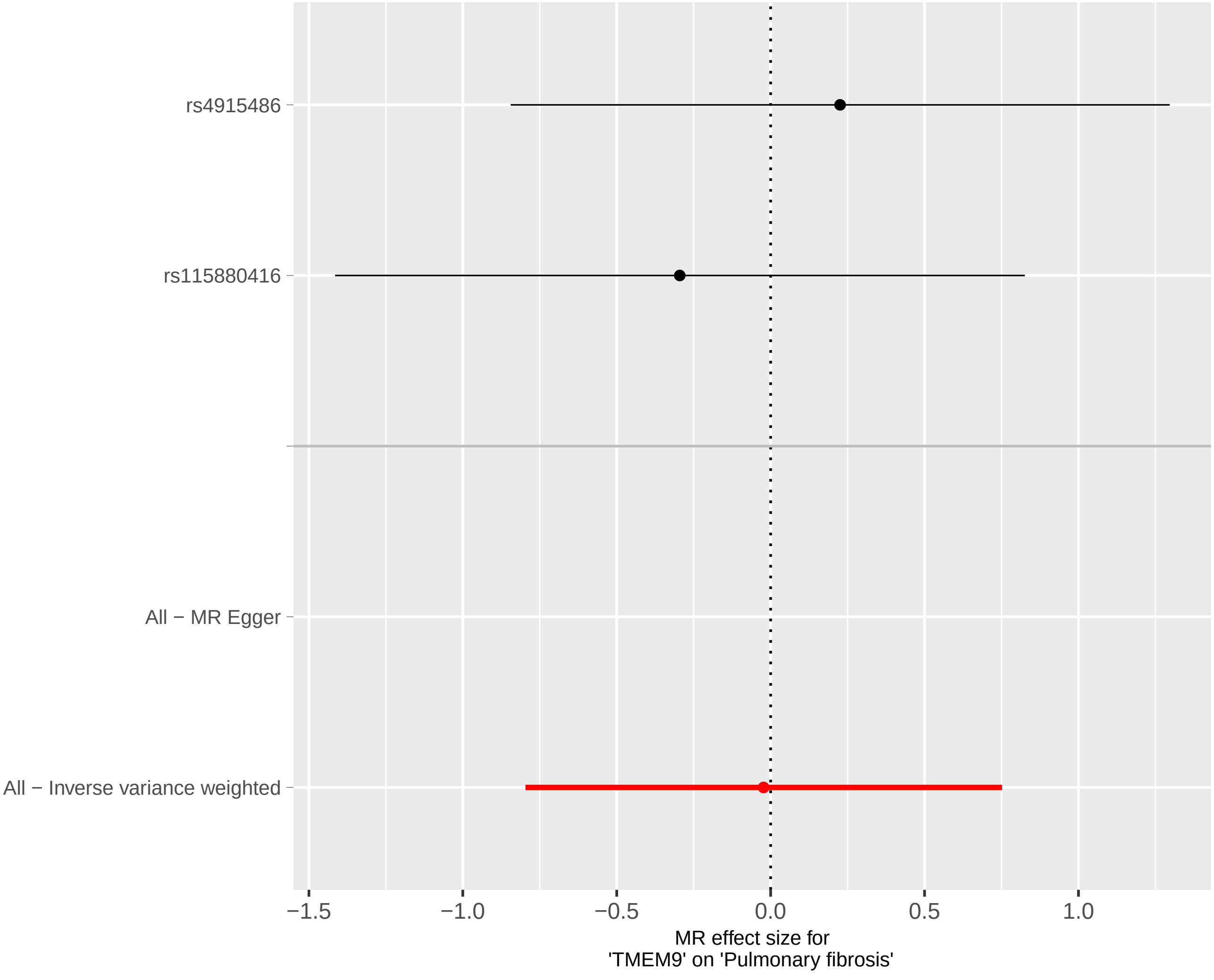

rs4915486
rs115880416
All − MR Egger
All − Inverse variance weighted
−1.5
−1.0
−0.5
0.0
0.5
1.0
MR effect size for
'TMEM9' on 'Pulmonary fibrosis'

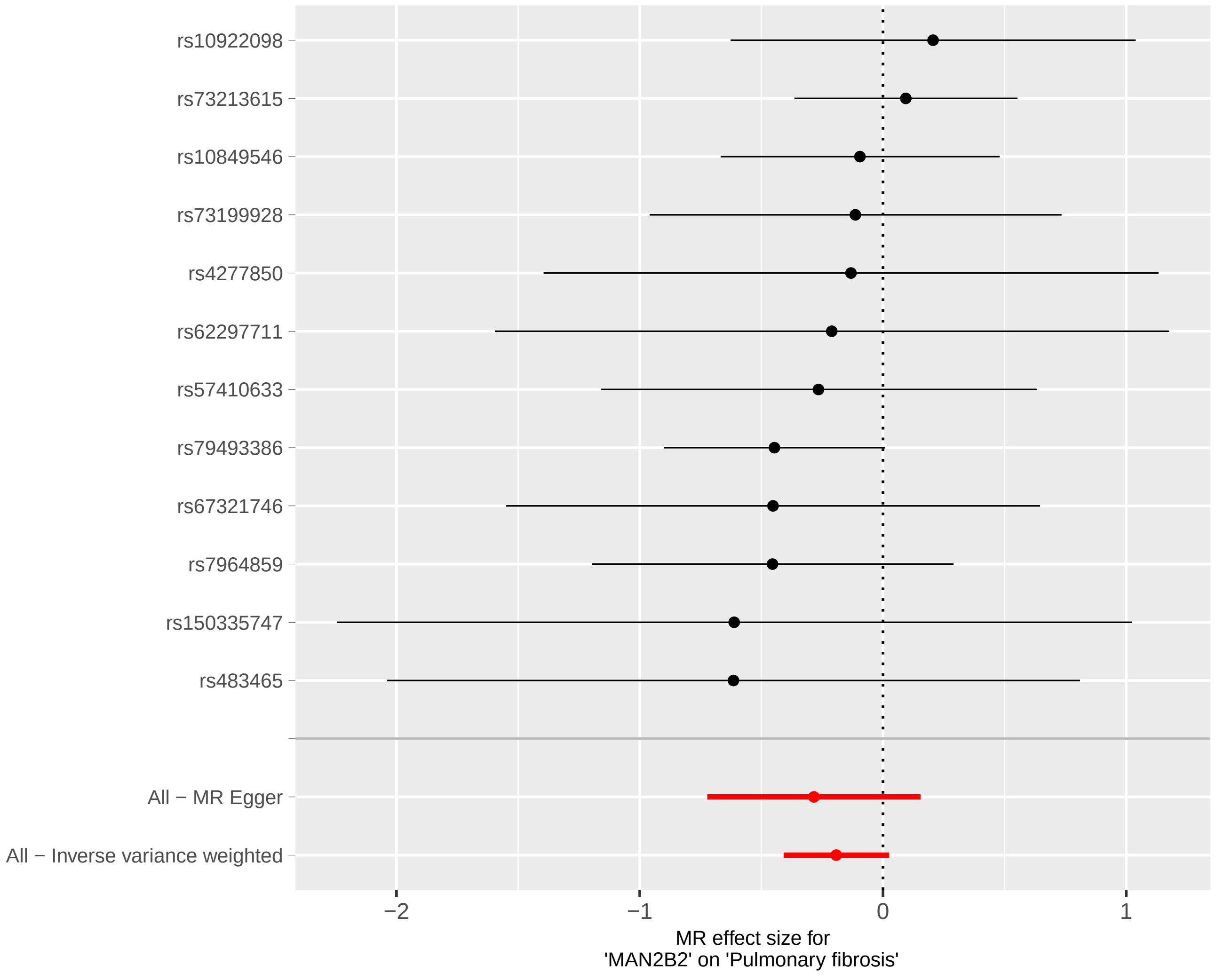

rs10922098
rs73213615
rs10849546
rs73199928
rs4277850
rs62297711
rs57410633
rs79493386
rs67321746
rs7964859
rs150335747
rs483465
All − MR Egger
All − Inverse variance weighted
−2
−1
0
1
MR effect size for
'MAN2B2' on 'Pulmonary fibrosis'

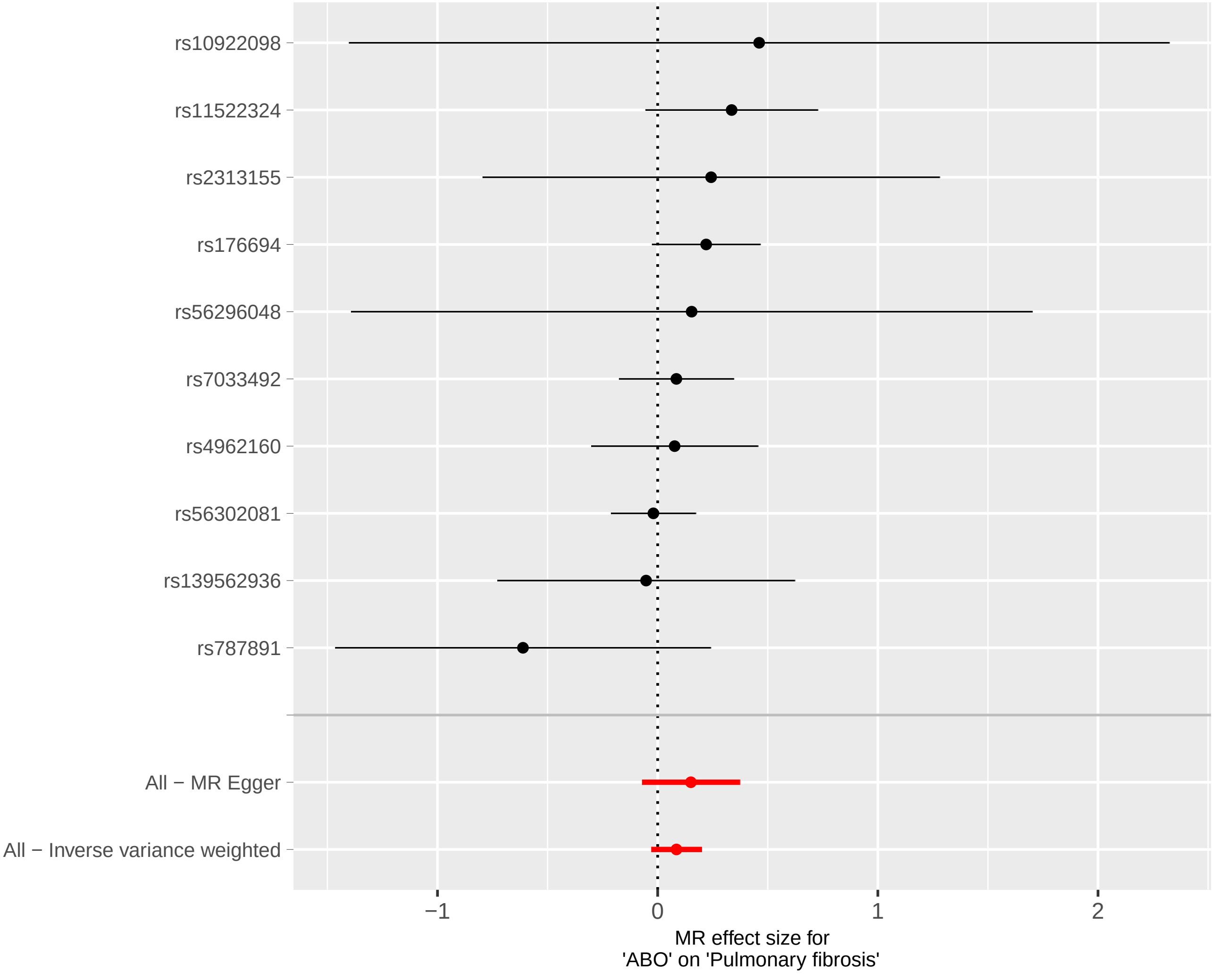
rs10922098
rs11522324
rs2313155
rs176694
rs56296048
rs7033492
rs4962160
rs56302081
rs139562936
rs787891
All − MR Egger
All − Inverse variance weighted
−1
0
1
2
MR effect size for
'ABO' on 'Pulmonary fibrosis'

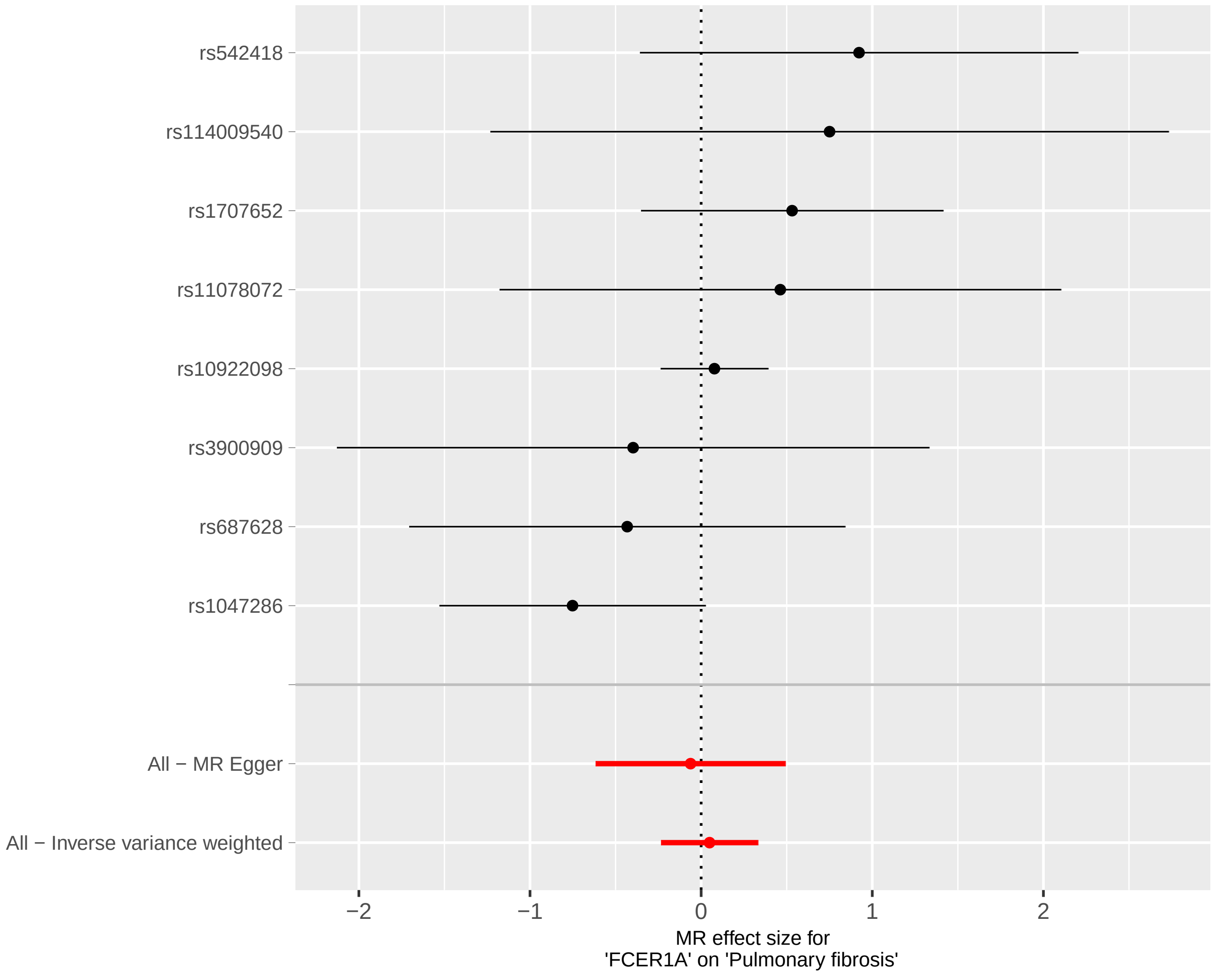

rs542418
rs114009540
rs1707652
rs11078072
rs10922098
rs3900909
rs687628
rs1047286
All − MR Egger
All − Inverse variance weighted
−2
−1
0
1
2
MR effect size for
'FCER1A' on 'Pulmonary fibrosis'

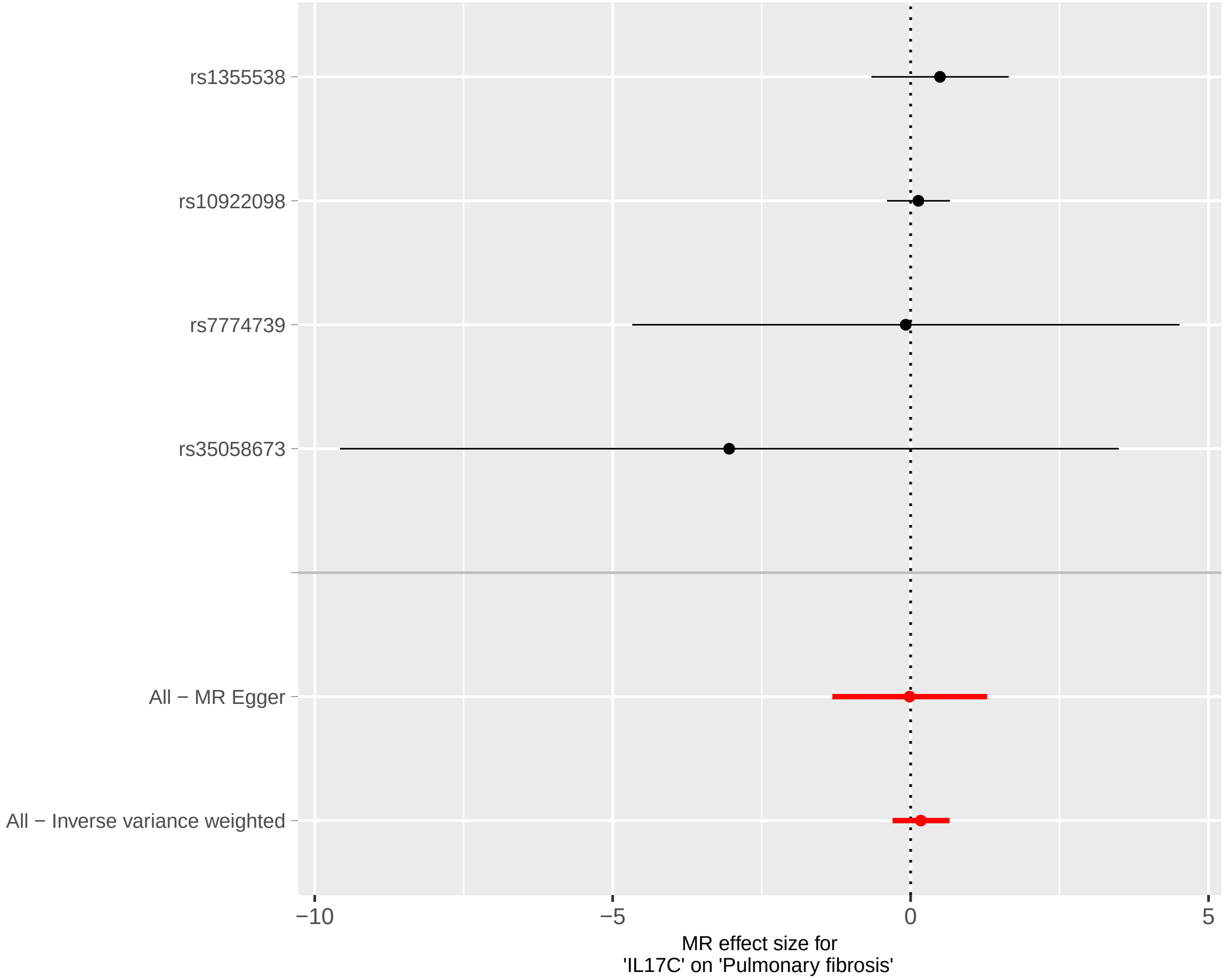

rs1355538
rs10922098
rs7774739
rs35058673
All − MR Egger
All − Inverse variance weighted
−10
−5
0
5
MR effect size for
'IL17C' on 'Pulmonary fibrosis'

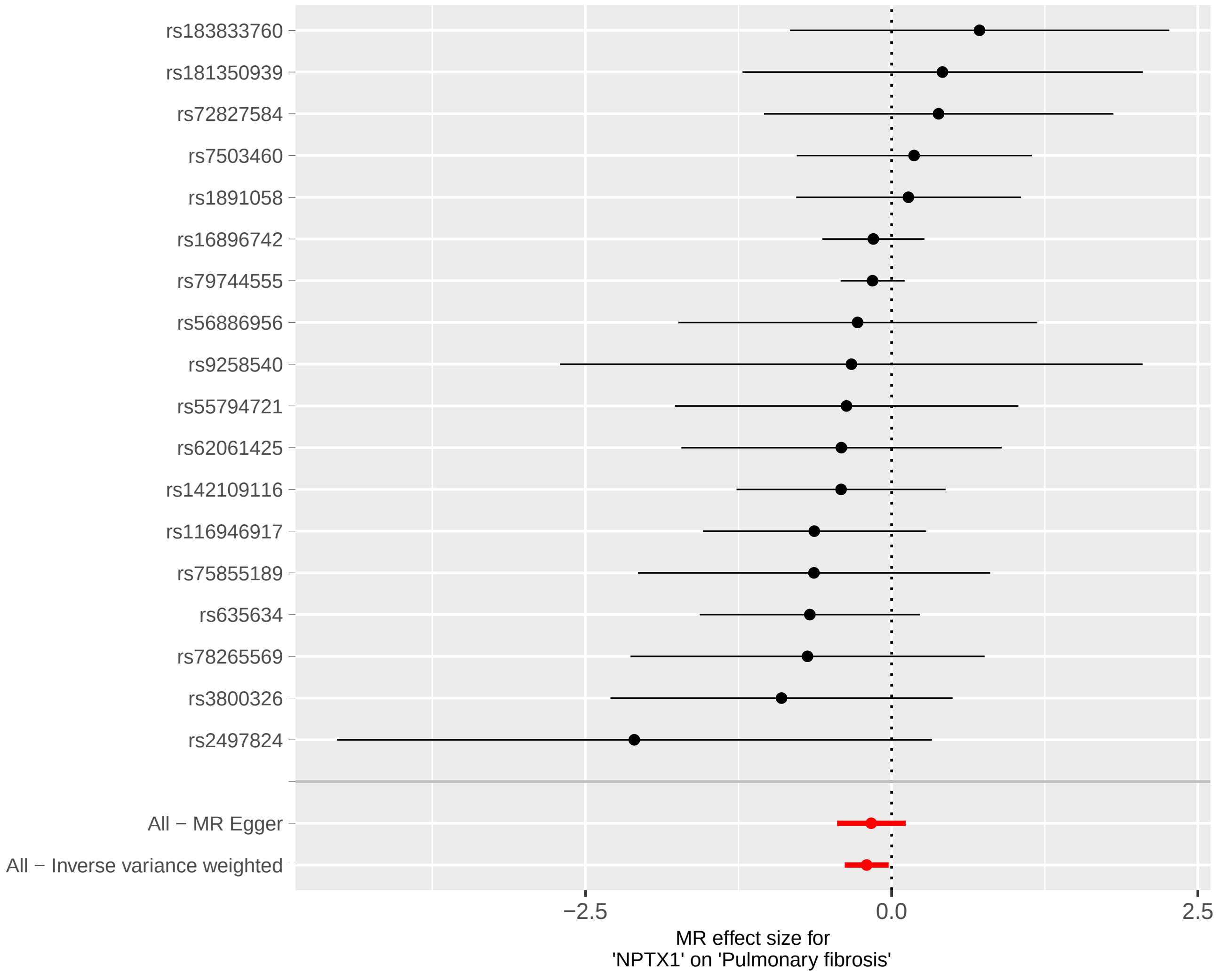

rs183833760
rs181350939
rs72827584
rs7503460
rs1891058
rs16896742
rs79744555
rs56886956
rs9258540
rs55794721
rs62061425
rs142109116
rs116946917
rs75855189
rs635634
rs78265569
rs3800326
rs2497824
All − MR Egger
All − Inverse variance weighted
−2.5
0.0
2.5
MR effect size for
'NPTX1' on 'Pulmonary fibrosis'

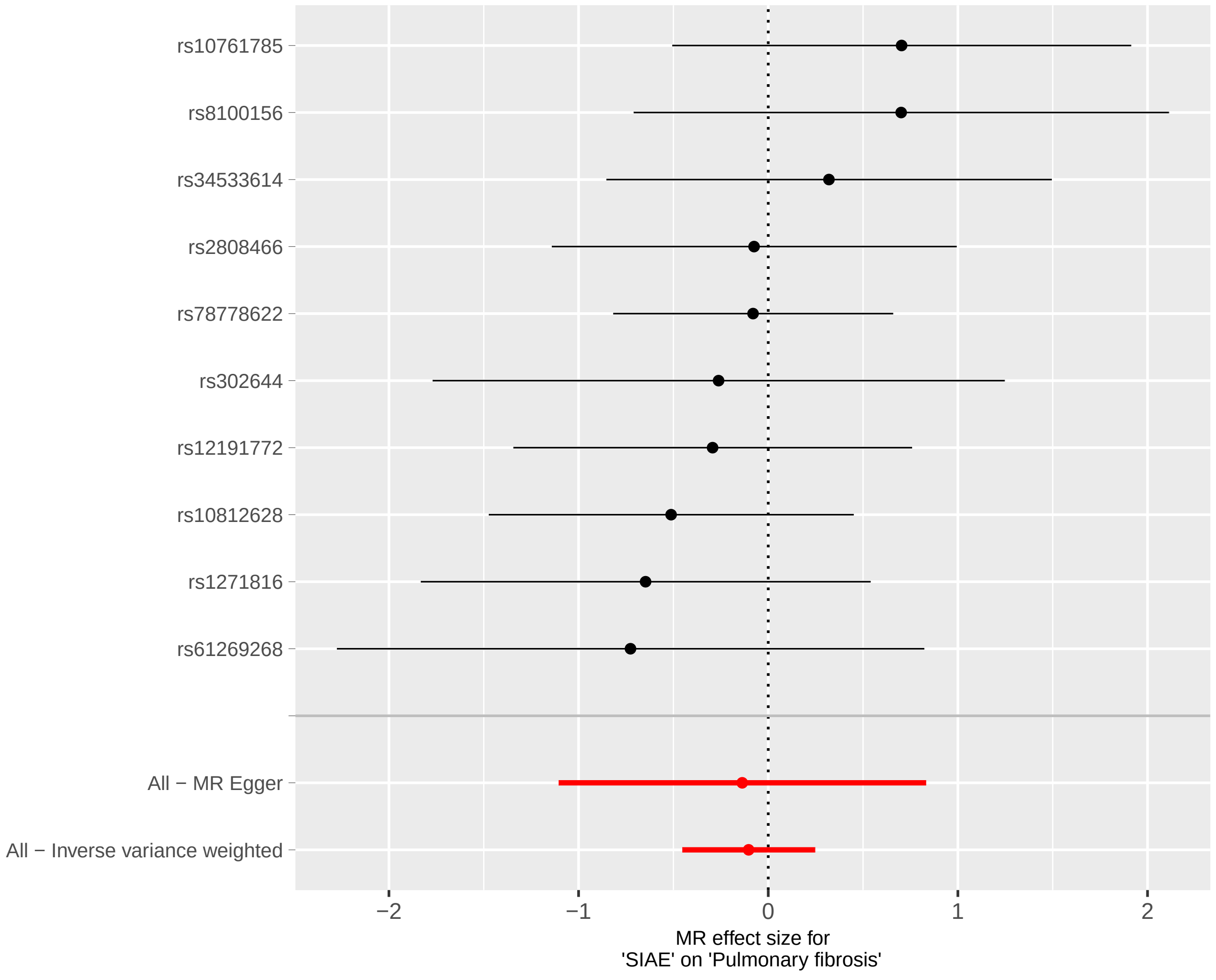
rs10761785
rs8100156
rs34533614
rs2808466
rs78778622
rs302644
rs12191772
rs10812628
rs1271816
rs61269268
All − MR Egger
All − Inverse variance weighted
−2
−1
0
1
2
MR effect size for
'SIAE' on 'Pulmonary fibrosis'

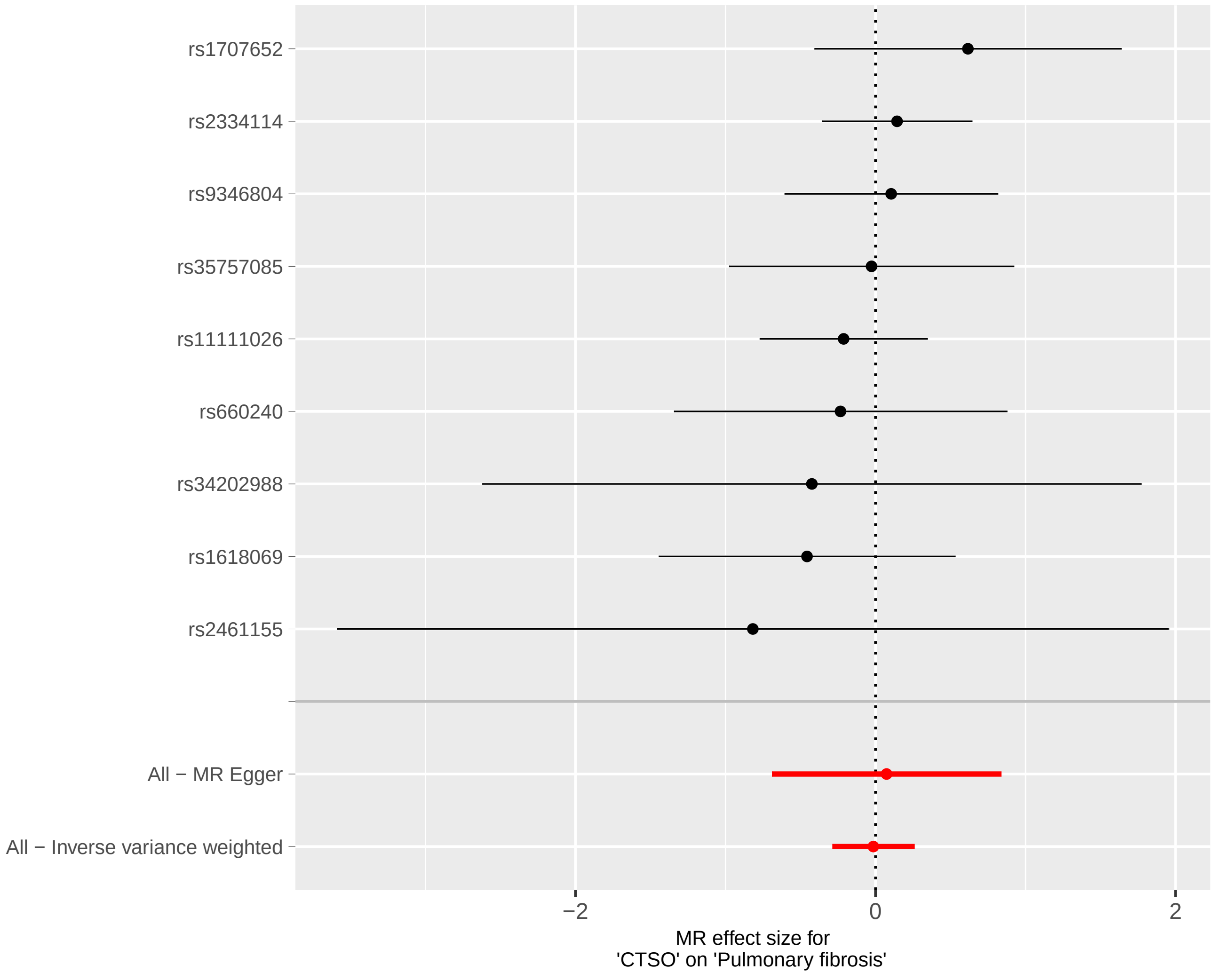

rs1707652
rs2334114
rs9346804
rs35757085
rs11111026
rs660240
rs34202988
rs1618069
rs2461155
All − MR Egger
All − Inverse variance weighted
−2
0
2
MR effect size for
'CTSO' on 'Pulmonary fibrosis'

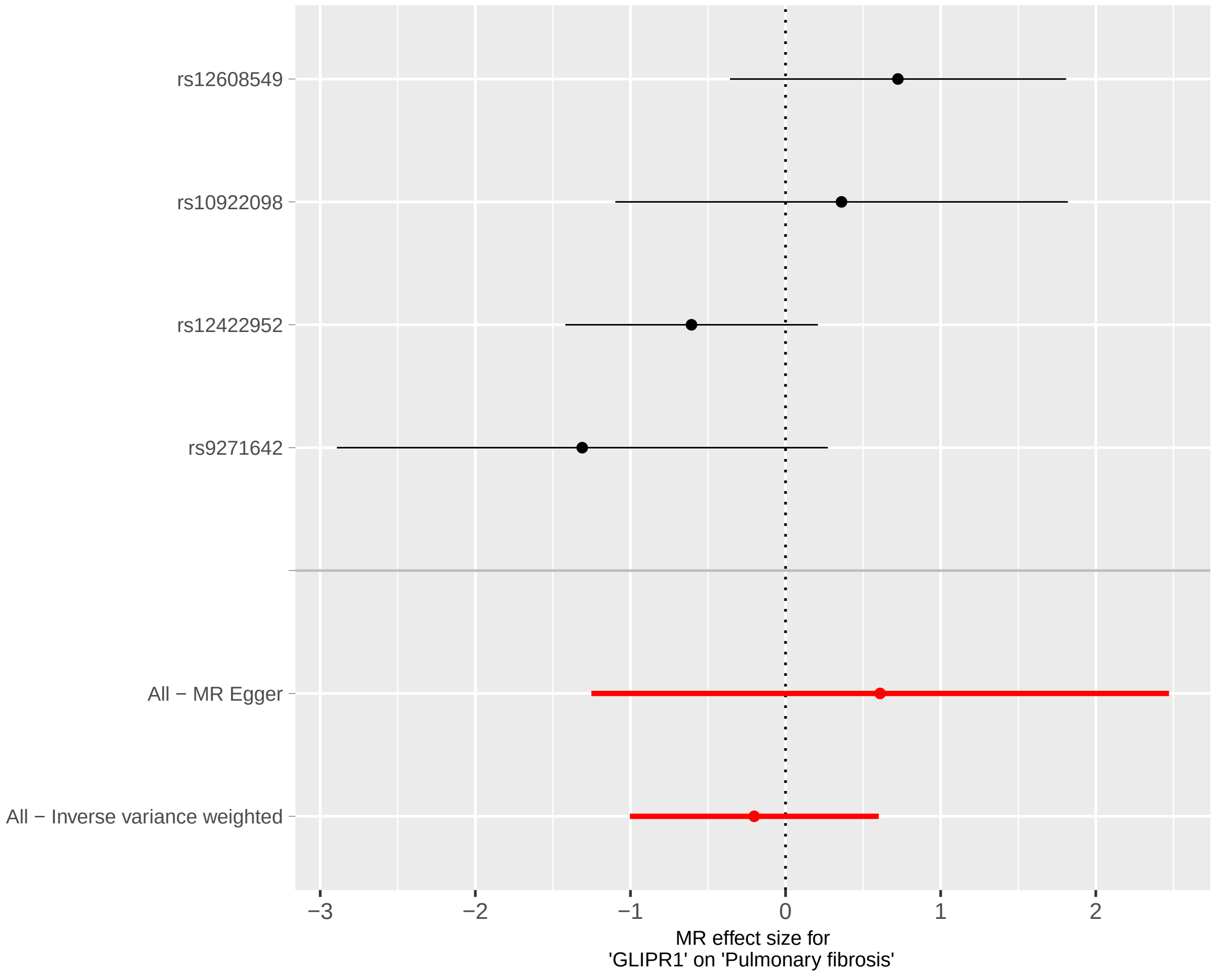

rs12608549
rs10922098
rs12422952
rs9271642
All − MR Egger
All − Inverse variance weighted
−3
−2
−1
0
1
2
MR effect size for
'GLIPR1' on 'Pulmonary fibrosis'

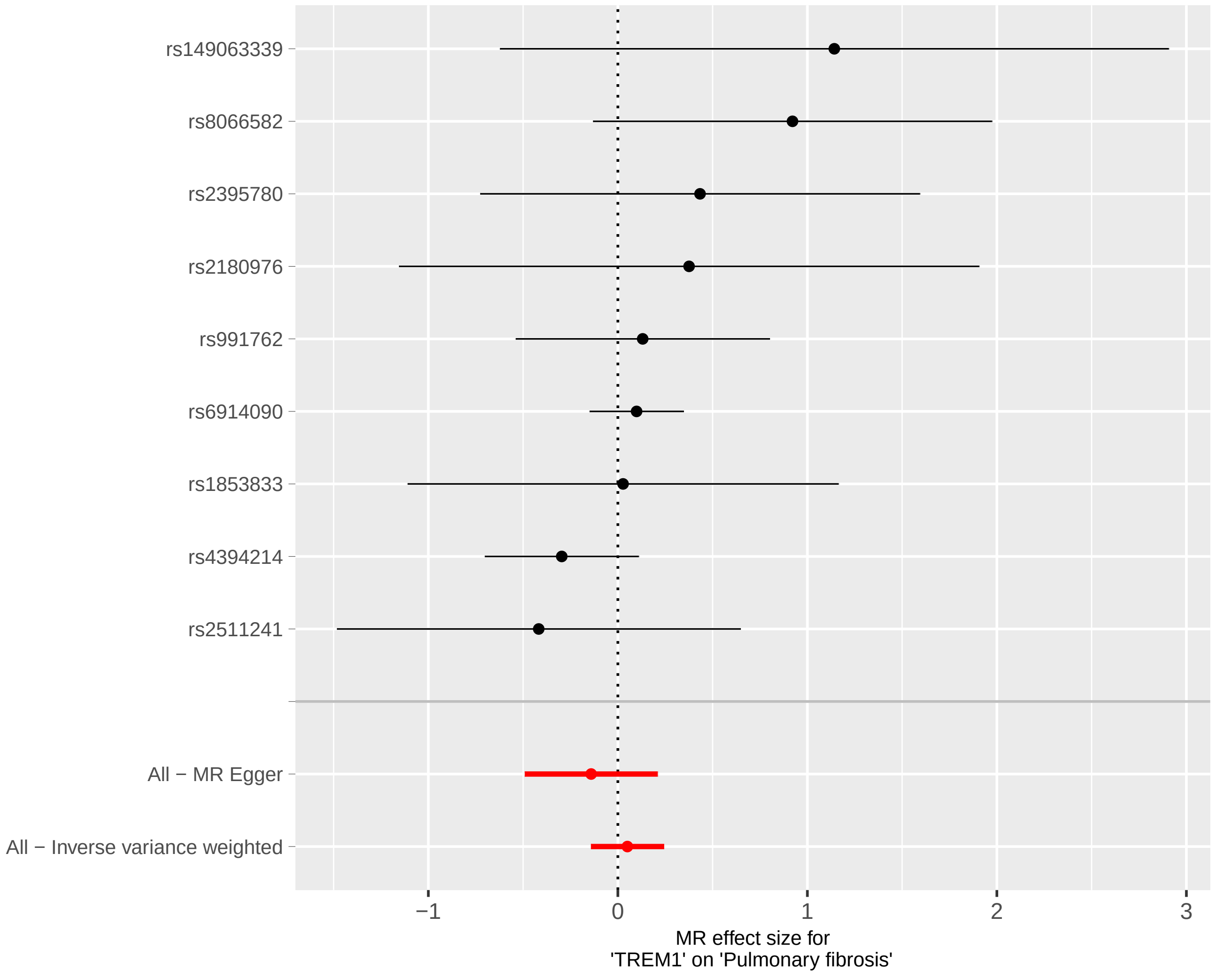
rs149063339
rs8066582
rs2395780
rs2180976
rs991762
rs6914090
rs1853833
rs4394214
rs2511241
All − MR Egger
All − Inverse variance weighted
−1
0
1
2
3
MR effect size for
'TREM1' on 'Pulmonary fibrosis'

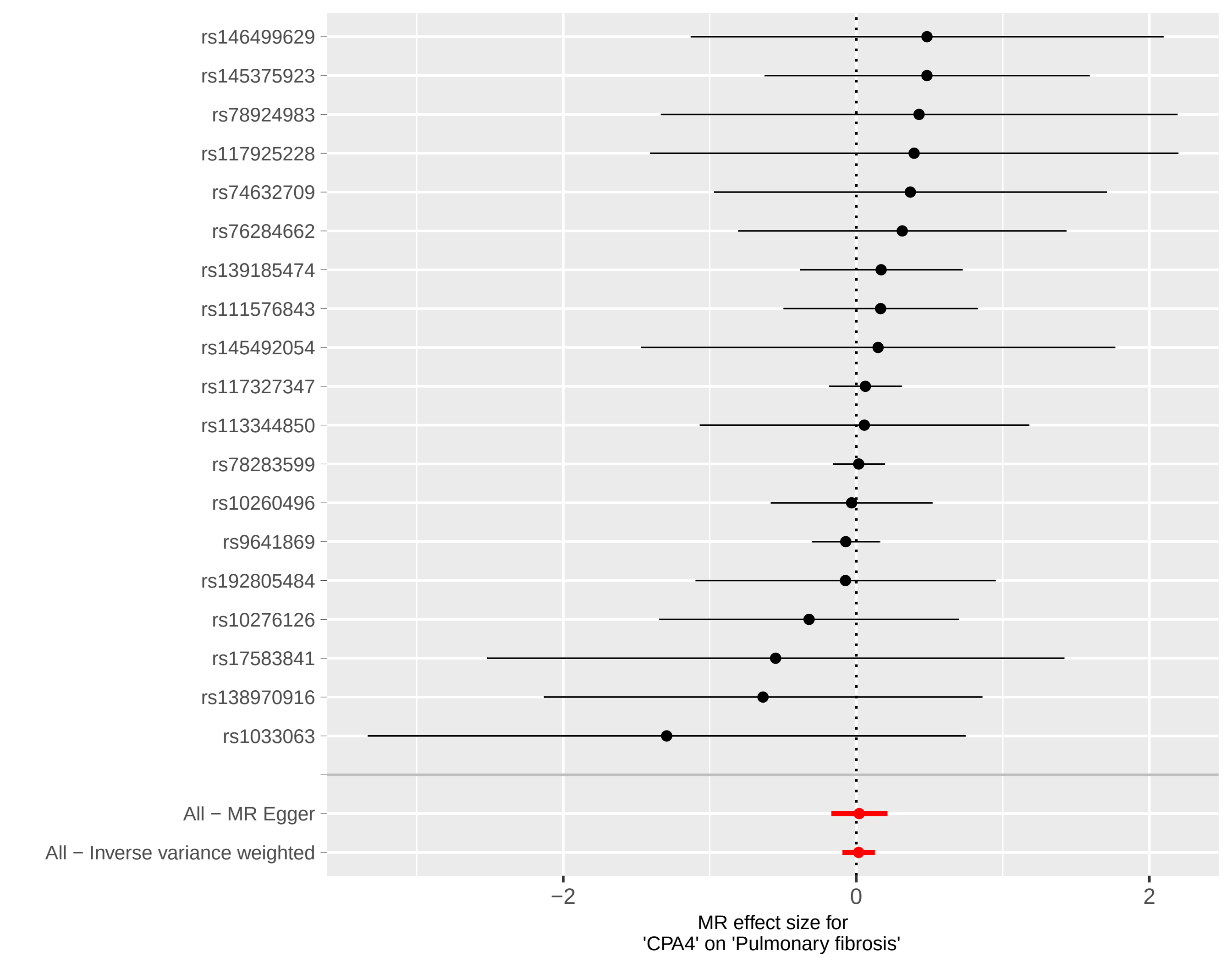
rs146499629
rs145375923
rs78924983
rs117925228
rs74632709
rs76284662
rs139185474
rs111576843
rs145492054
rs117327347
rs113344850
rs78283599
rs10260496
rs9641869
rs192805484
rs10276126
rs17583841
rs138970916
rs1033063
All − MR Egger
All − Inverse variance weighted
−2
0
2
MR effect size for
'CPA4' on 'Pulmonary fibrosis'

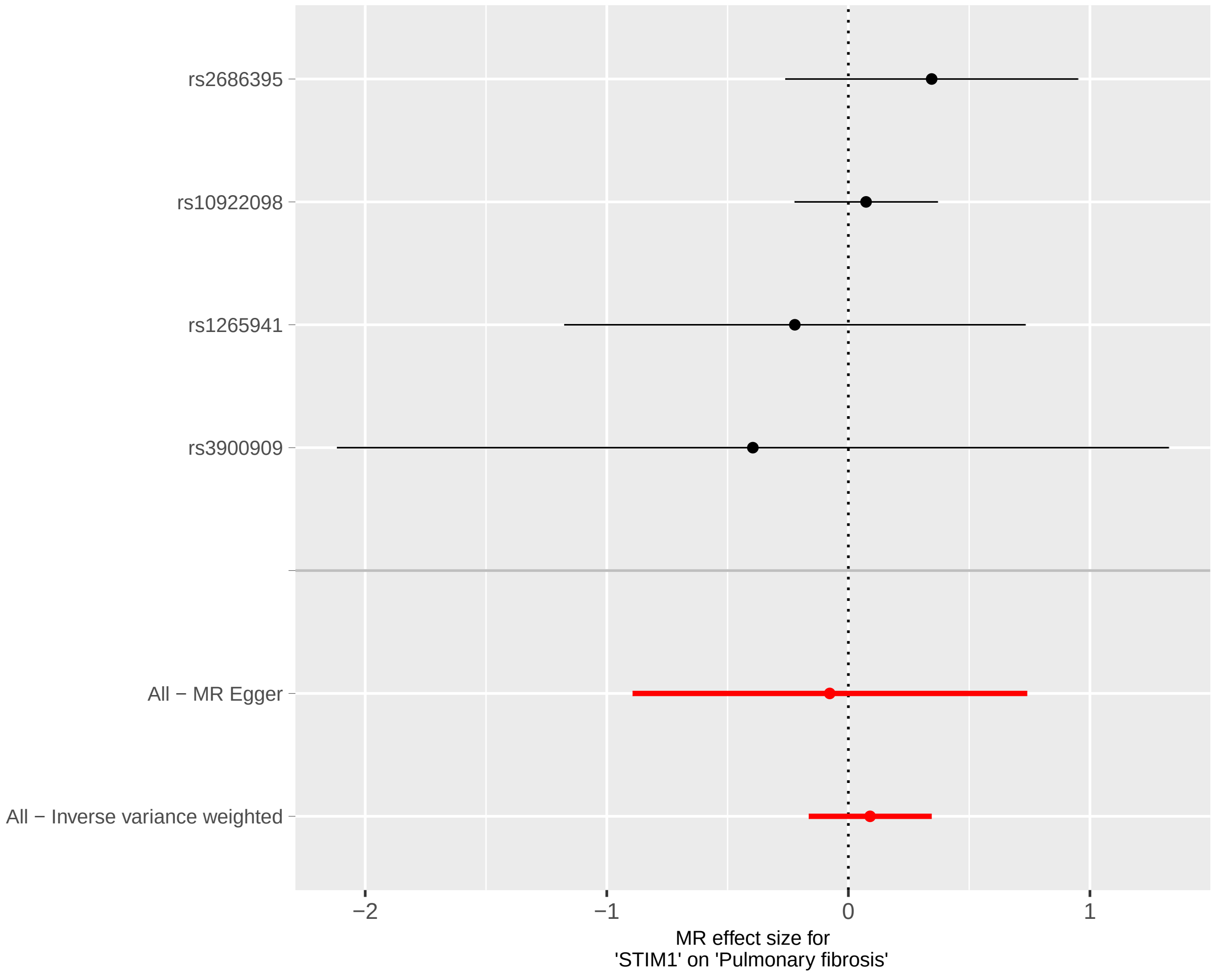
rs2686395
rs10922098
rs1265941
rs3900909
All − MR Egger
All − Inverse variance weighted
−2
−1
0
1
MR effect size for
'STIM1' on 'Pulmonary fibrosis'

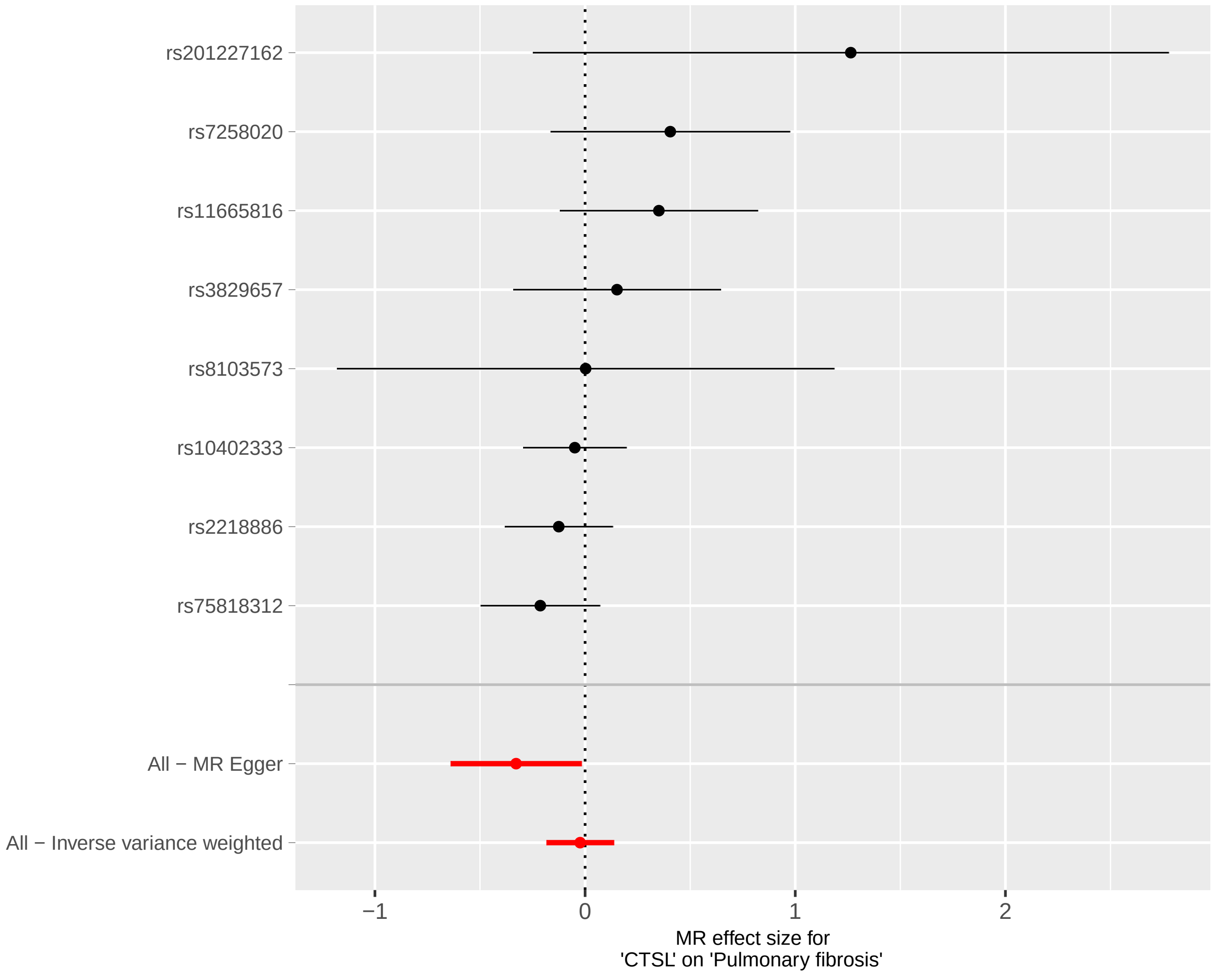
rs201227162
rs7258020
rs11665816
rs3829657
rs8103573
rs10402333
rs2218886
rs75818312
All − MR Egger
All − Inverse variance weighted
−1
0
1
2
MR effect size for
'CTSL' on 'Pulmonary fibrosis'

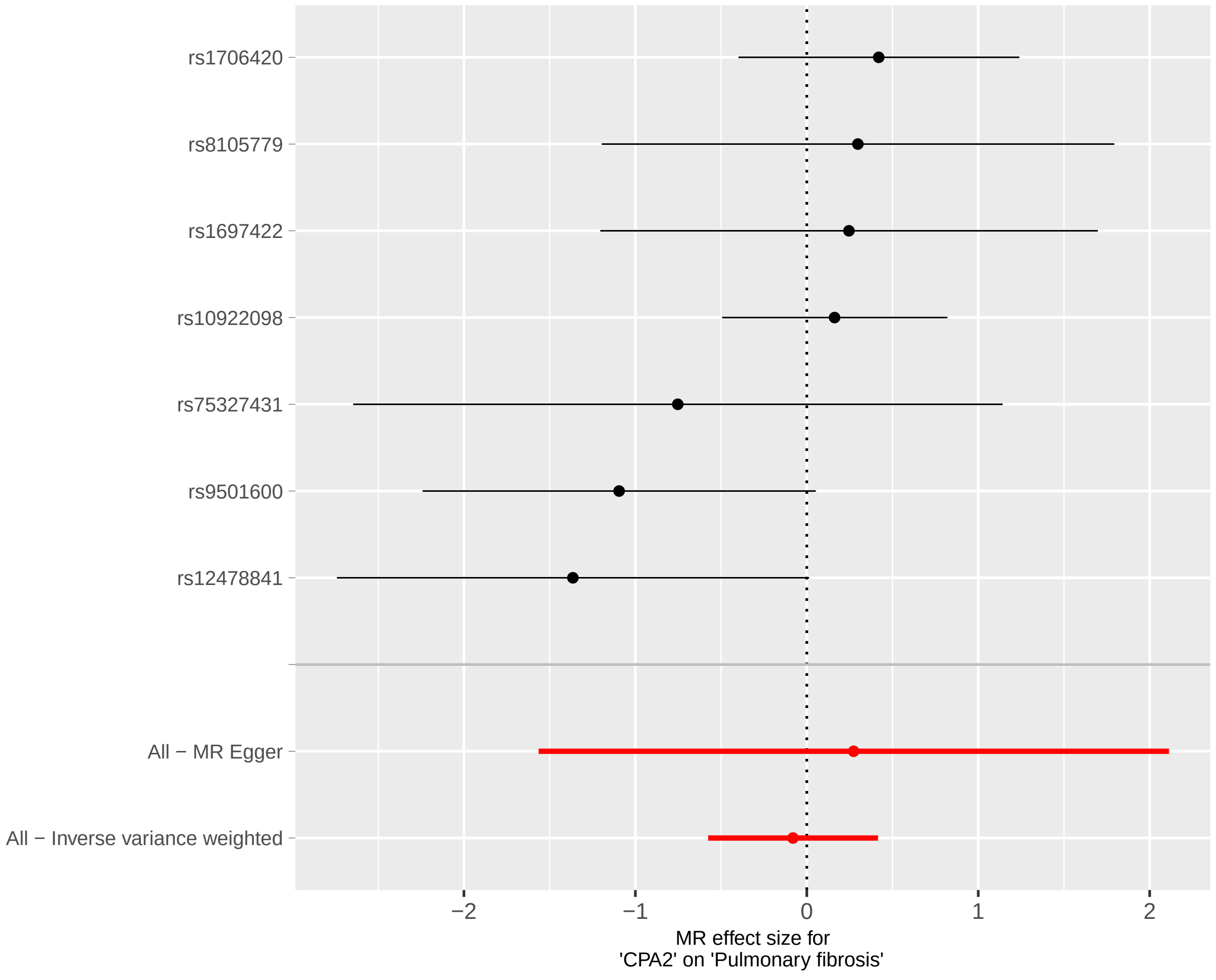

rs1706420
rs8105779
rs1697422
rs10922098
rs75327431
rs9501600
rs12478841
All − MR Egger
All − Inverse variance weighted
−2
−1
0
1
2
MR effect size for
'CPA2' on 'Pulmonary fibrosis'

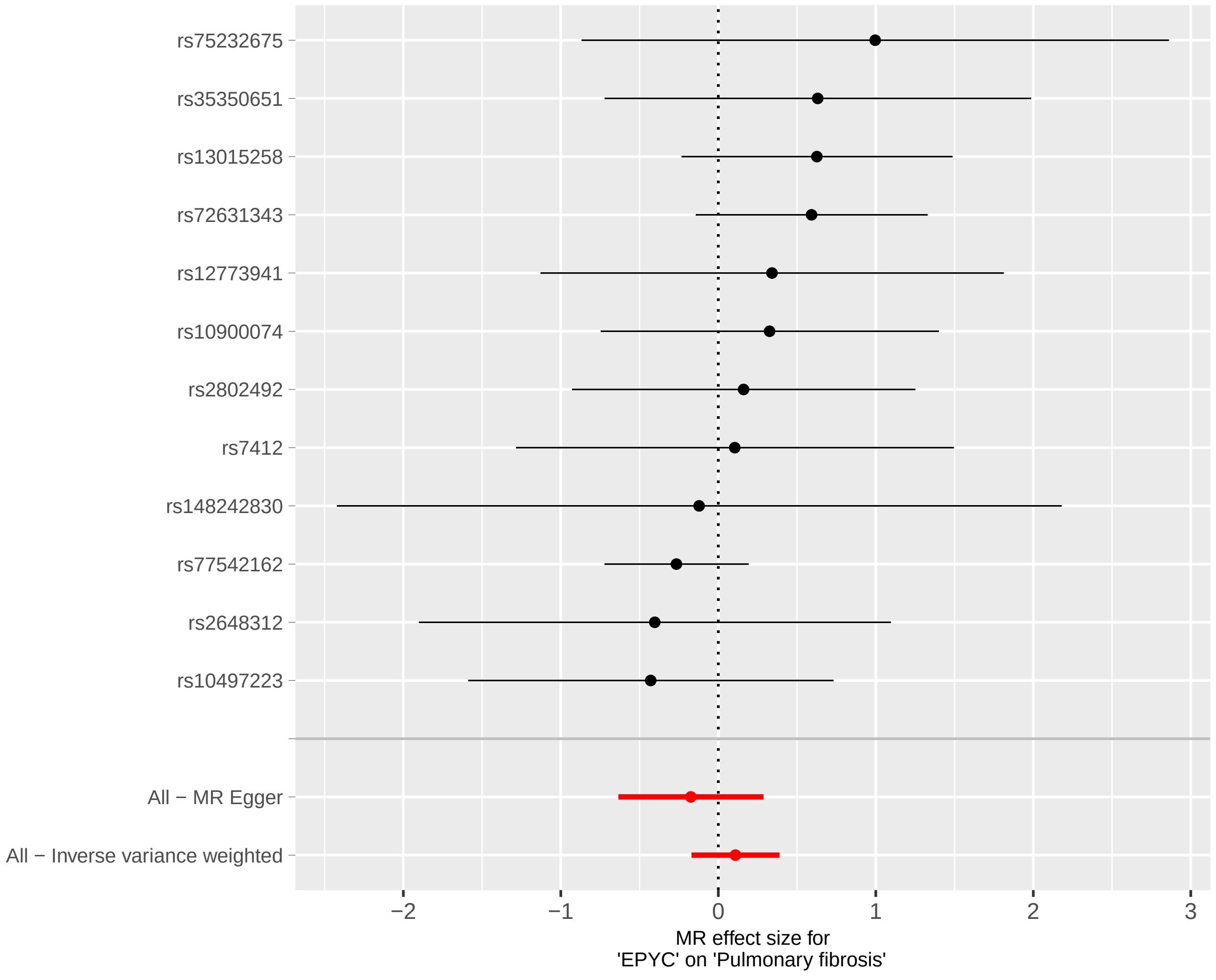

rs75232675
rs35350651
rs13015258
rs72631343
rs12773941
rs10900074
rs2802492
rs7412
rs148242830
rs77542162
rs2648312
rs10497223
All − MR Egger
All − Inverse variance weighted
−2
−1
0
1
2
3
MR effect size for
'EPYC' on 'Pulmonary fibrosis'

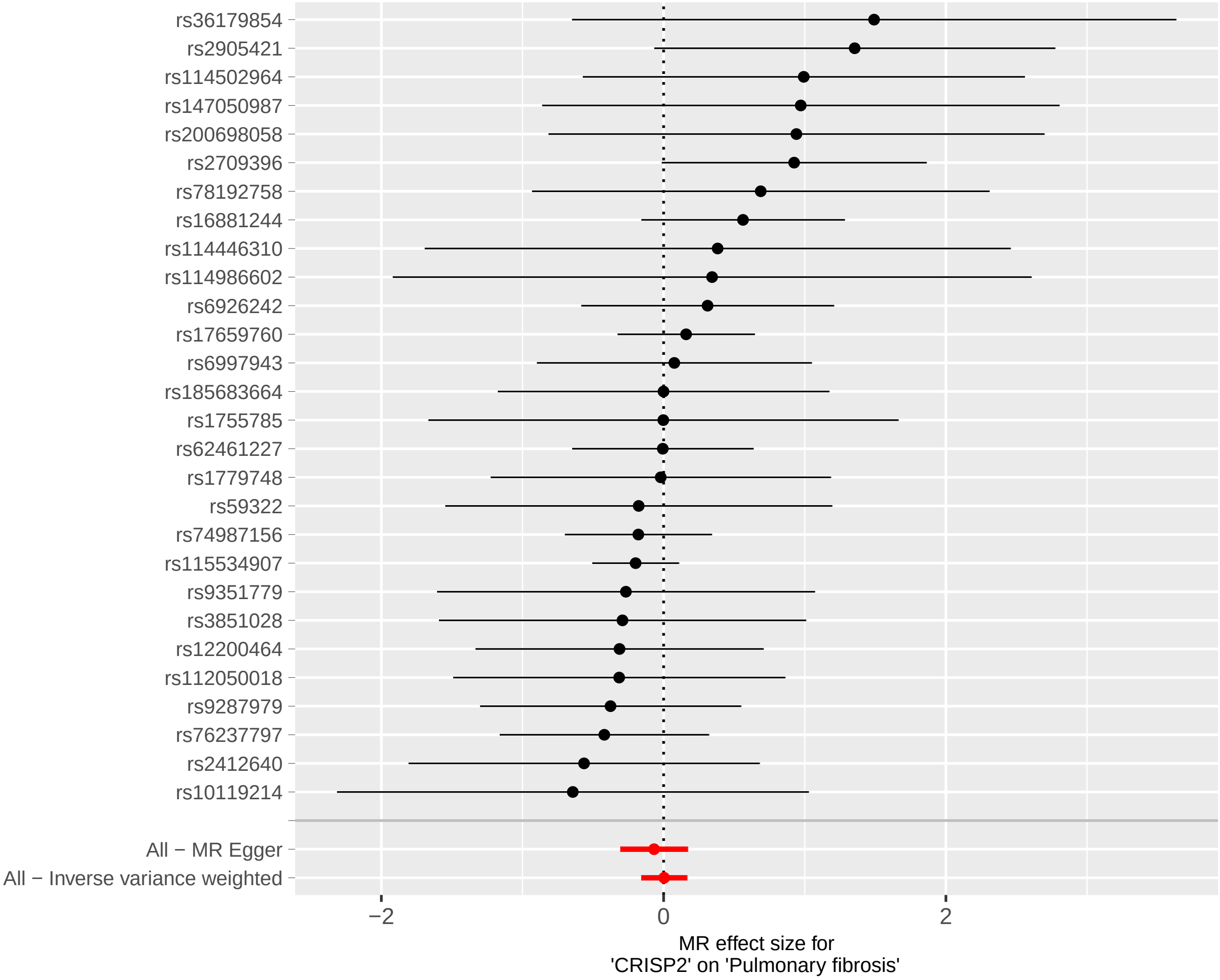
rs36179854
rs2905421
rs114502964
rs147050987
rs200698058
rs2709396
rs78192758
rs16881244
rs114446310
rs114986602
rs6926242
rs17659760
rs6997943
rs185683664
rs1755785
rs62461227
rs1779748
rs59322
rs74987156
rs115534907
rs9351779
rs3851028
rs12200464
rs112050018
rs9287979
rs76237797
rs2412640
rs10119214
All − MR Egger
All − Inverse variance weighted
−2
0
2
MR effect size for
'CRISP2' on 'Pulmonary fibrosis'

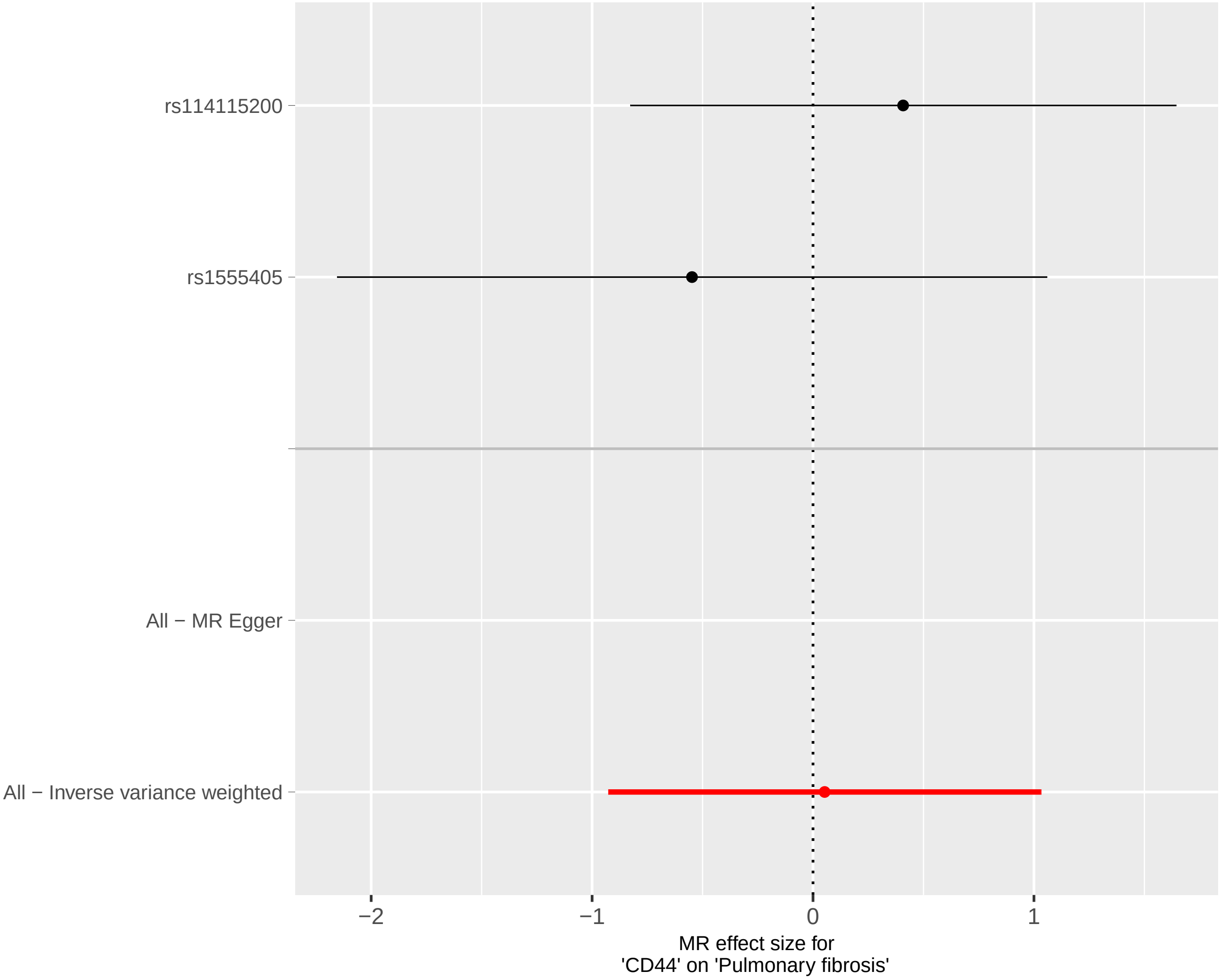
rs114115200
rs1555405
All − MR Egger
All − Inverse variance weighted
−2
−1
0
1
MR effect size for
'CD44' on 'Pulmonary fibrosis'

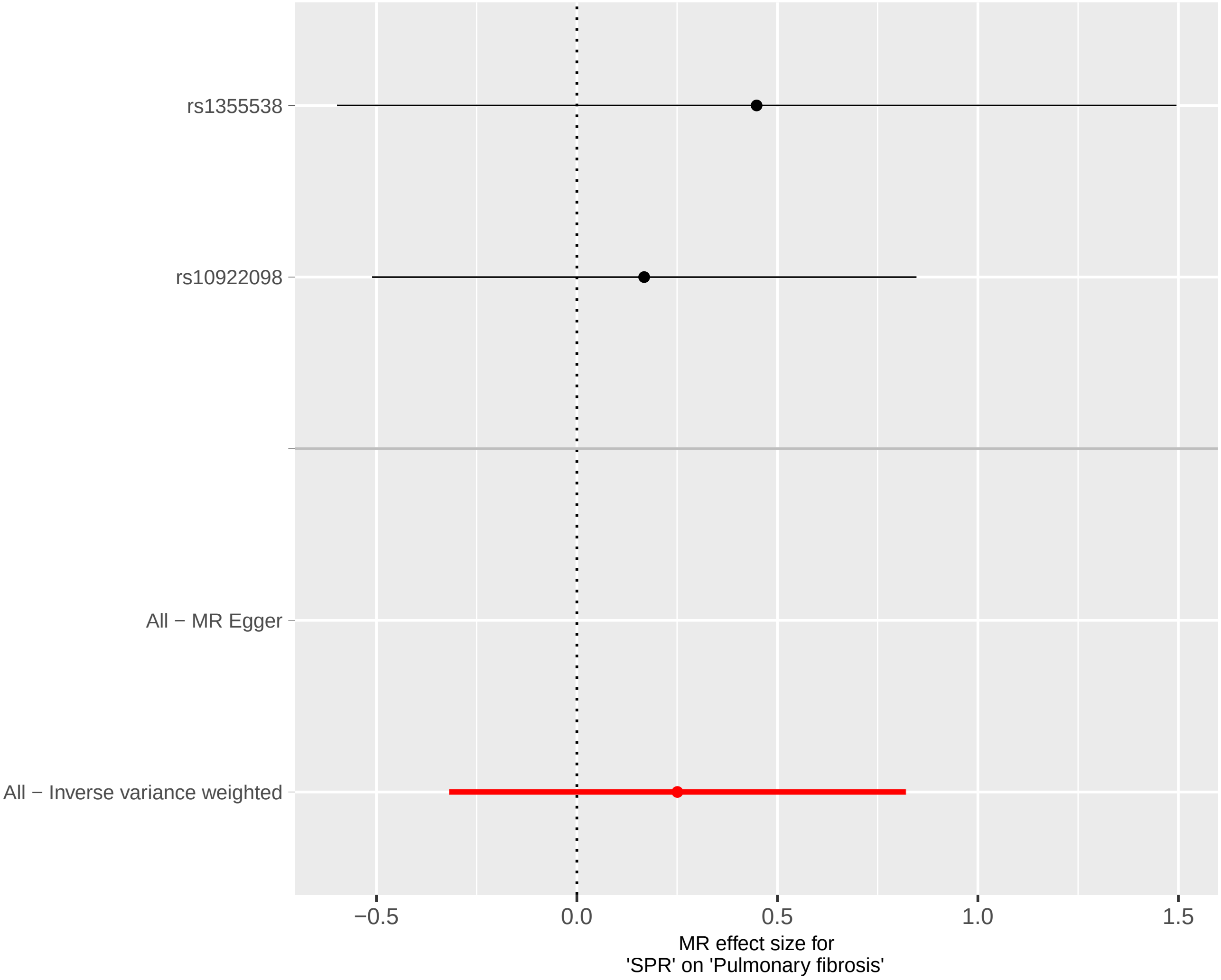
rs1355538
rs10922098
All − MR Egger
All − Inverse variance weighted
−0.5
0.0
0.5
1.0
1.5
MR effect size for
'SPR' on 'Pulmonary fibrosis'

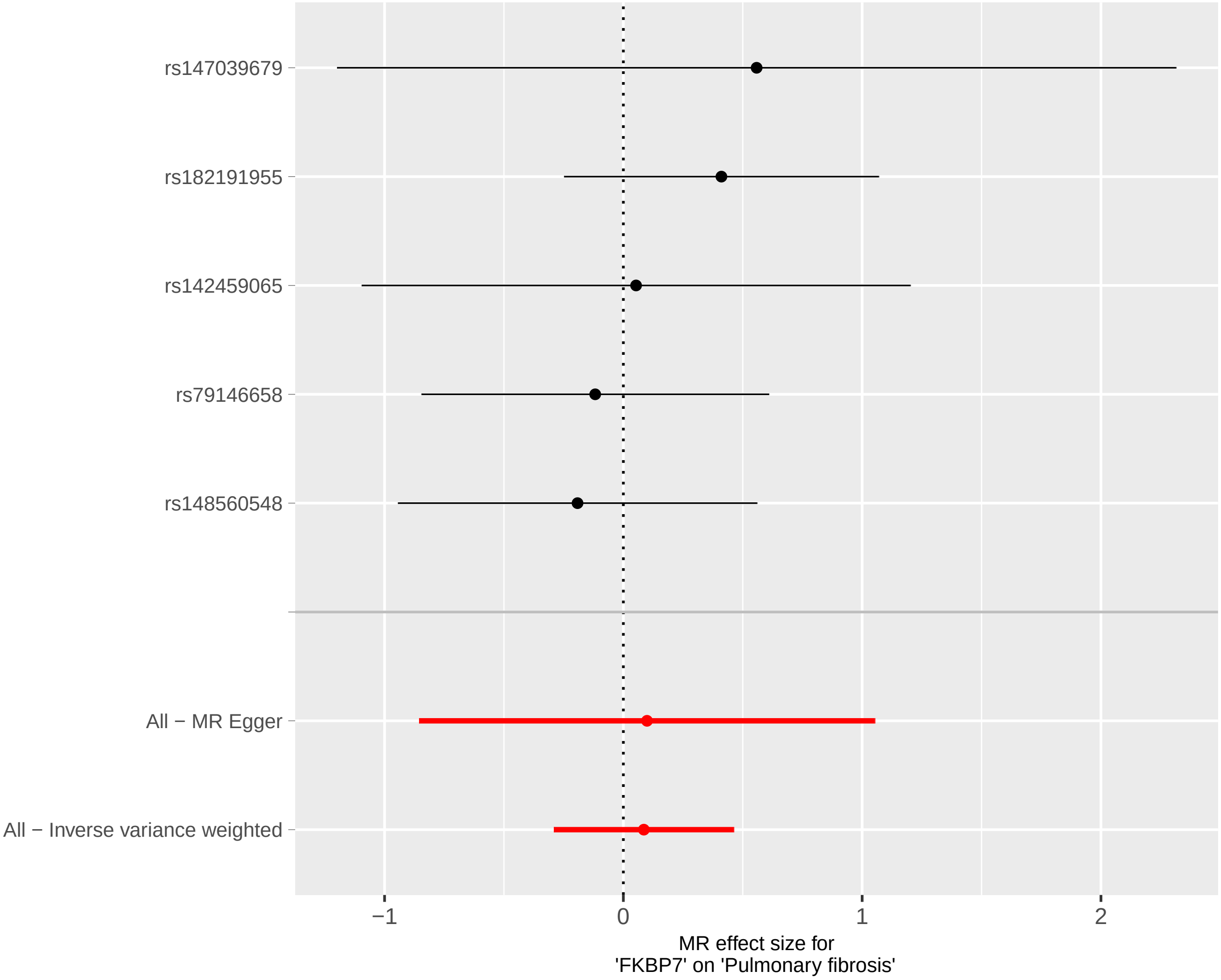
rs147039679
rs182191955
rs142459065
rs79146658
rs148560548
All − MR Egger
All − Inverse variance weighted
−1
0
1
2
MR effect size for
'FKBP7' on 'Pulmonary fibrosis'

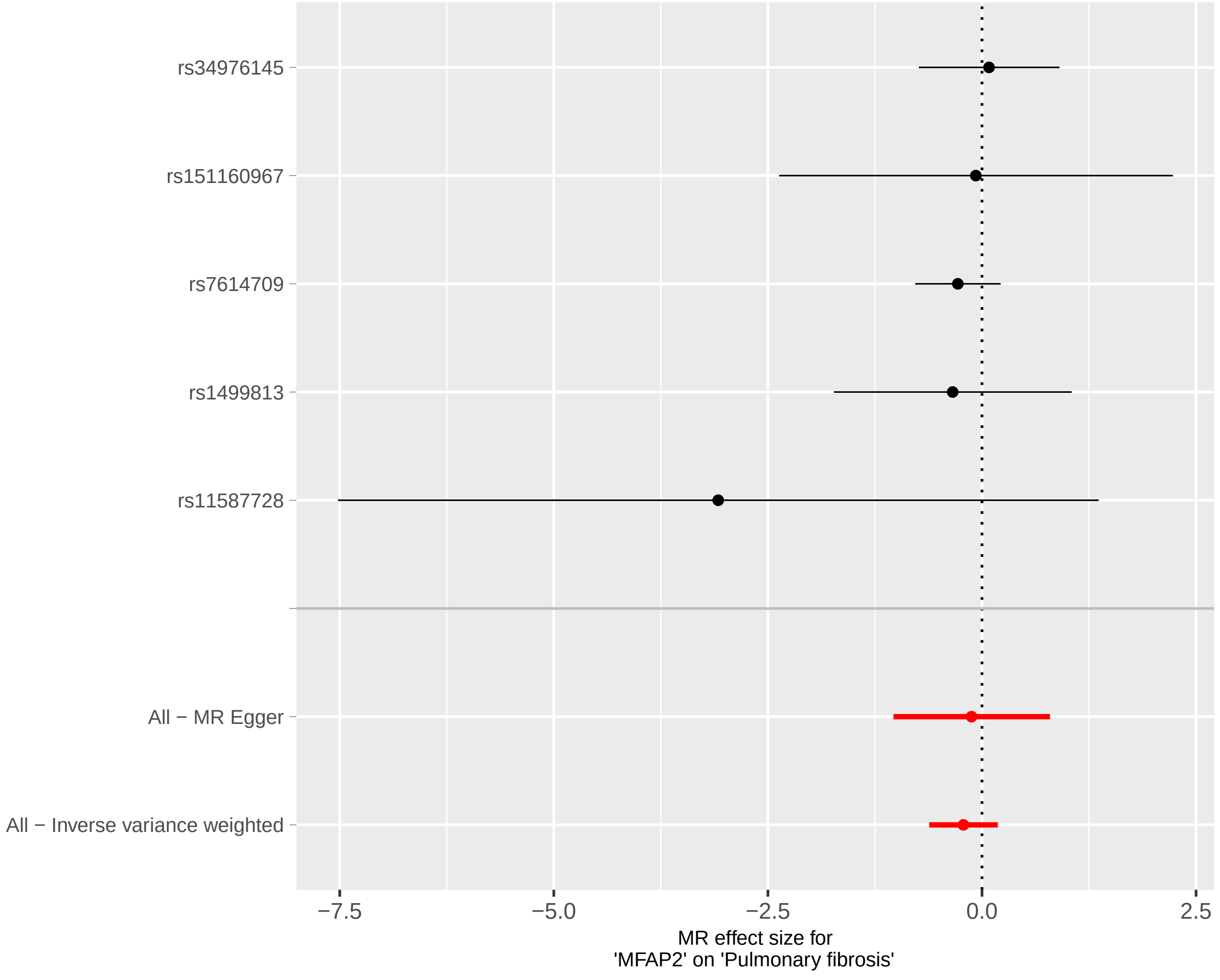

rs34976145
rs151160967
rs7614709
rs1499813
rs11587728
All − MR Egger
All − Inverse variance weighted
−7.5
−5.0
−2.5
0.0
2.5
MR effect size for
'MFAP2' on 'Pulmonary fibrosis'

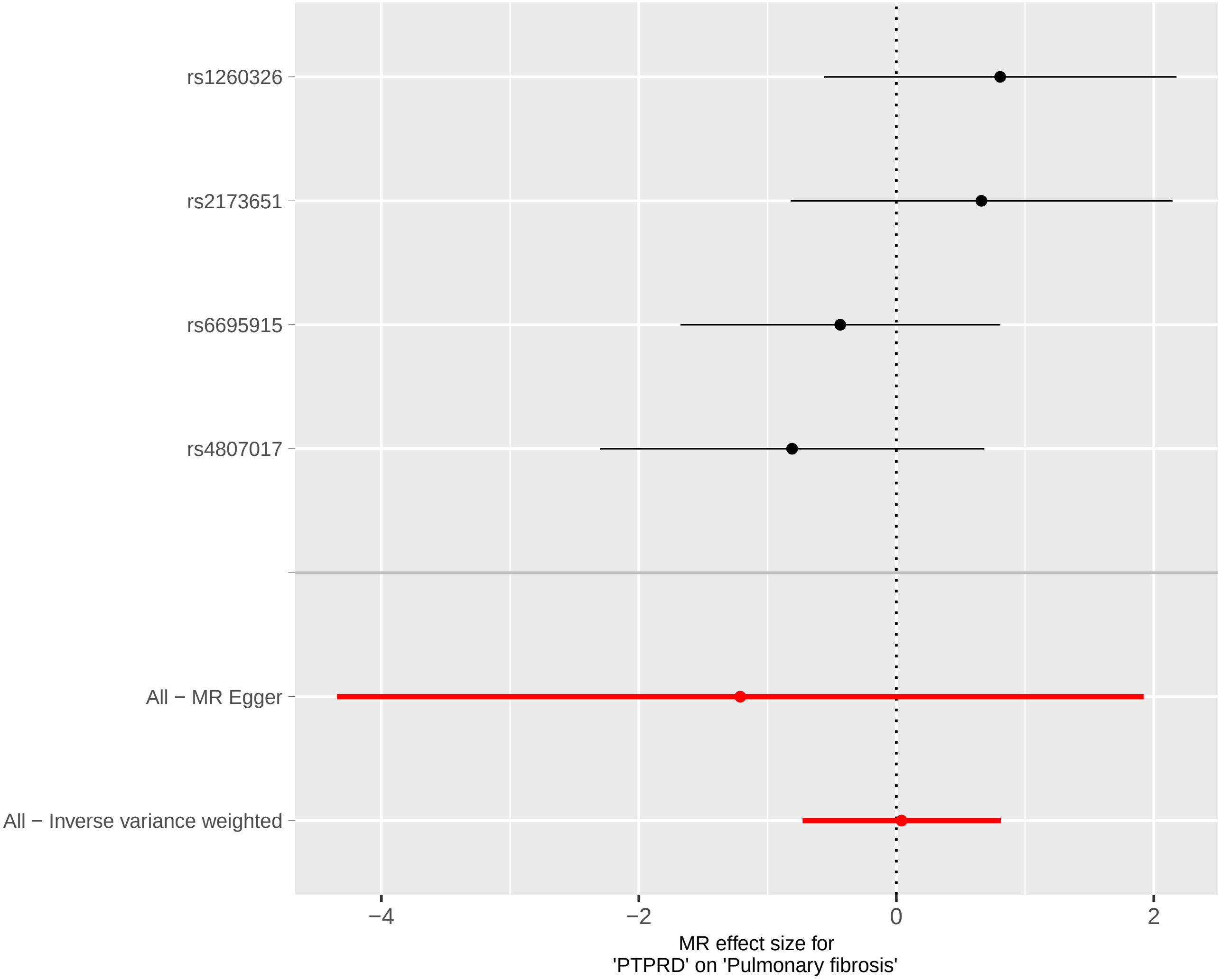

rs1260326
rs2173651
rs6695915
rs4807017
All − MR Egger
All − Inverse variance weighted
−4
−2
0
2
MR effect size for
'PTPRD' on 'Pulmonary fibrosis'

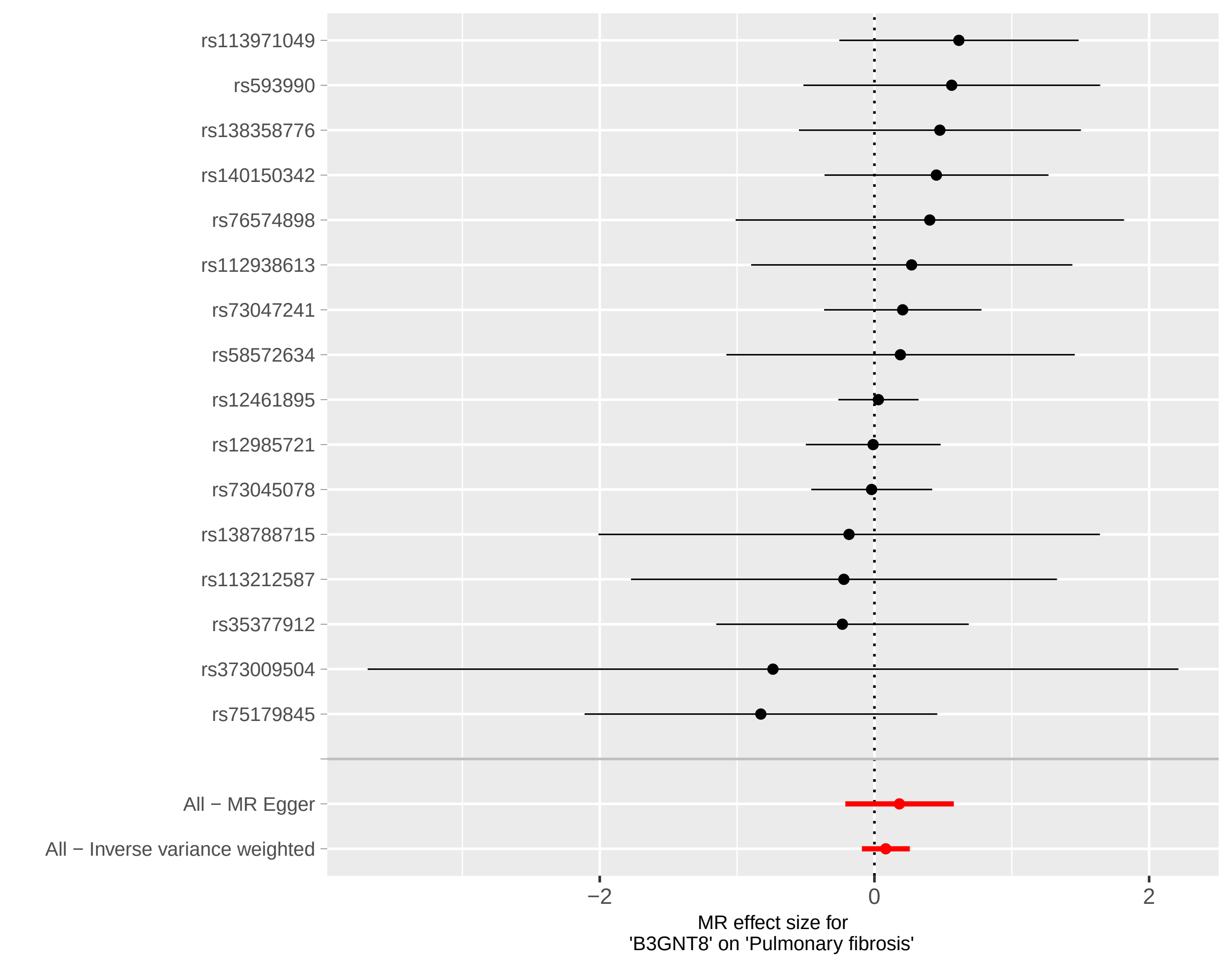

rs113971049
rs593990
rs138358776
rs140150342
rs76574898
rs112938613
rs73047241
rs58572634
rs12461895
rs12985721
rs73045078
rs138788715
rs113212587
rs35377912
rs373009504
rs75179845
All − MR Egger
All − Inverse variance weighted
−2
0
2
MR effect size for
'B3GNT8' on 'Pulmonary fibrosis'

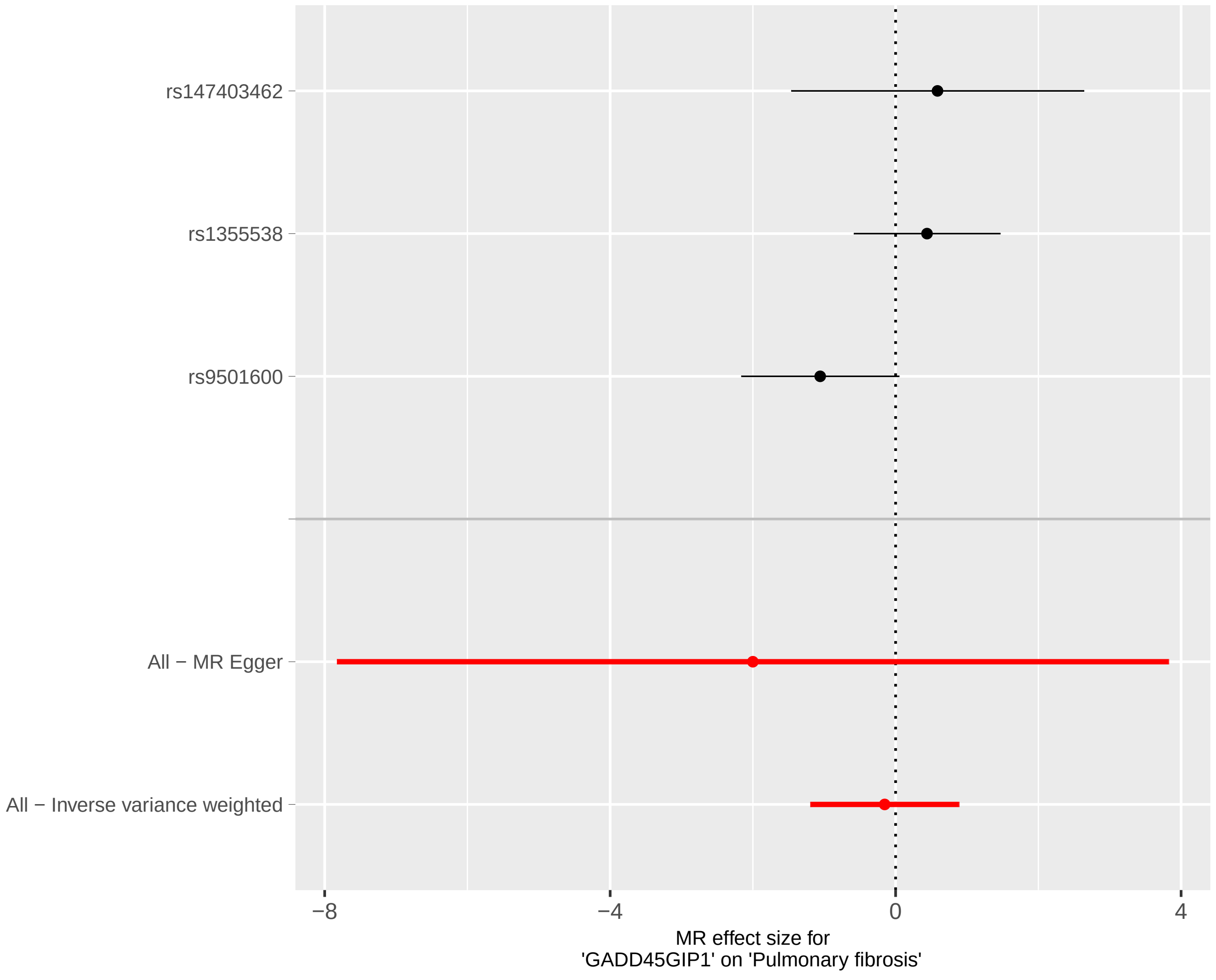

rs147403462
rs1355538
rs9501600
All − MR Egger
All − Inverse variance weighted
−8
−4
0
4
MR effect size for
'GADD45GIP1' on 'Pulmonary fibrosis'

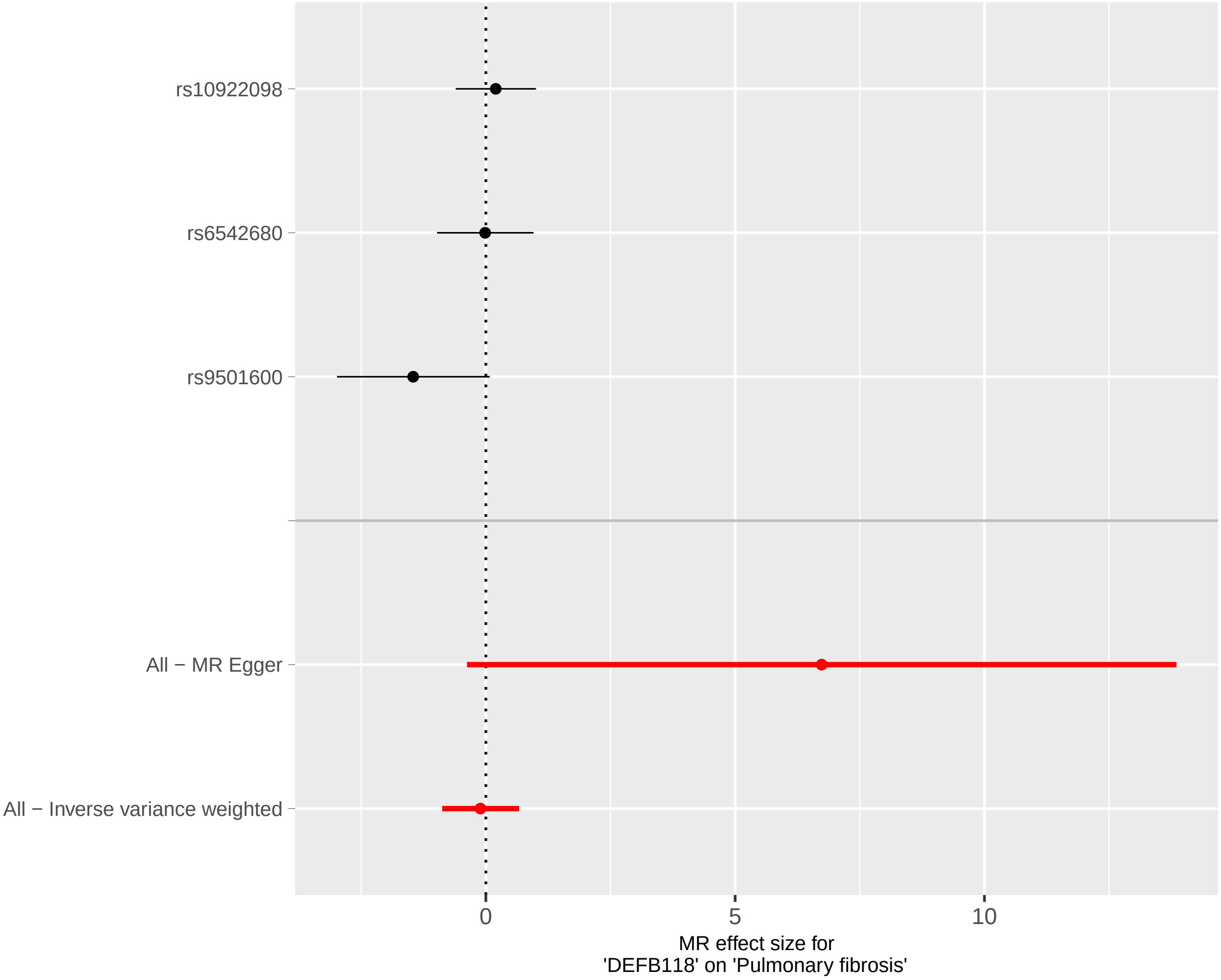

rs10922098
rs6542680
rs9501600
All − MR Egger
All − Inverse variance weighted
0
5
10
MR effect size for
'DEFB118' on 'Pulmonary fibrosis'

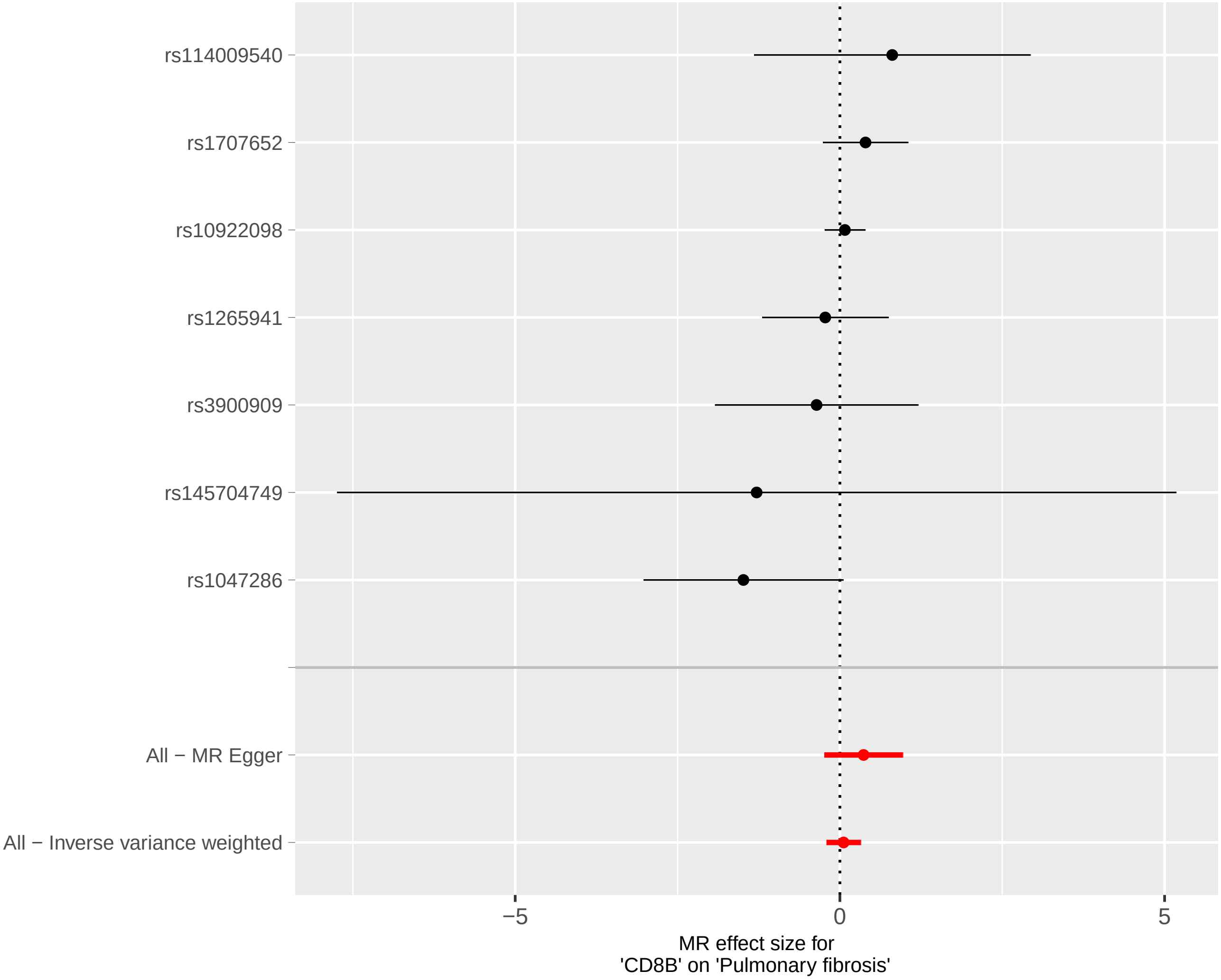

rs114009540
rs1707652
rs10922098
rs1265941
rs3900909
rs145704749
rs1047286
All − MR Egger
All − Inverse variance weighted
−5
0
5
MR effect size for
'CD8B' on 'Pulmonary fibrosis'

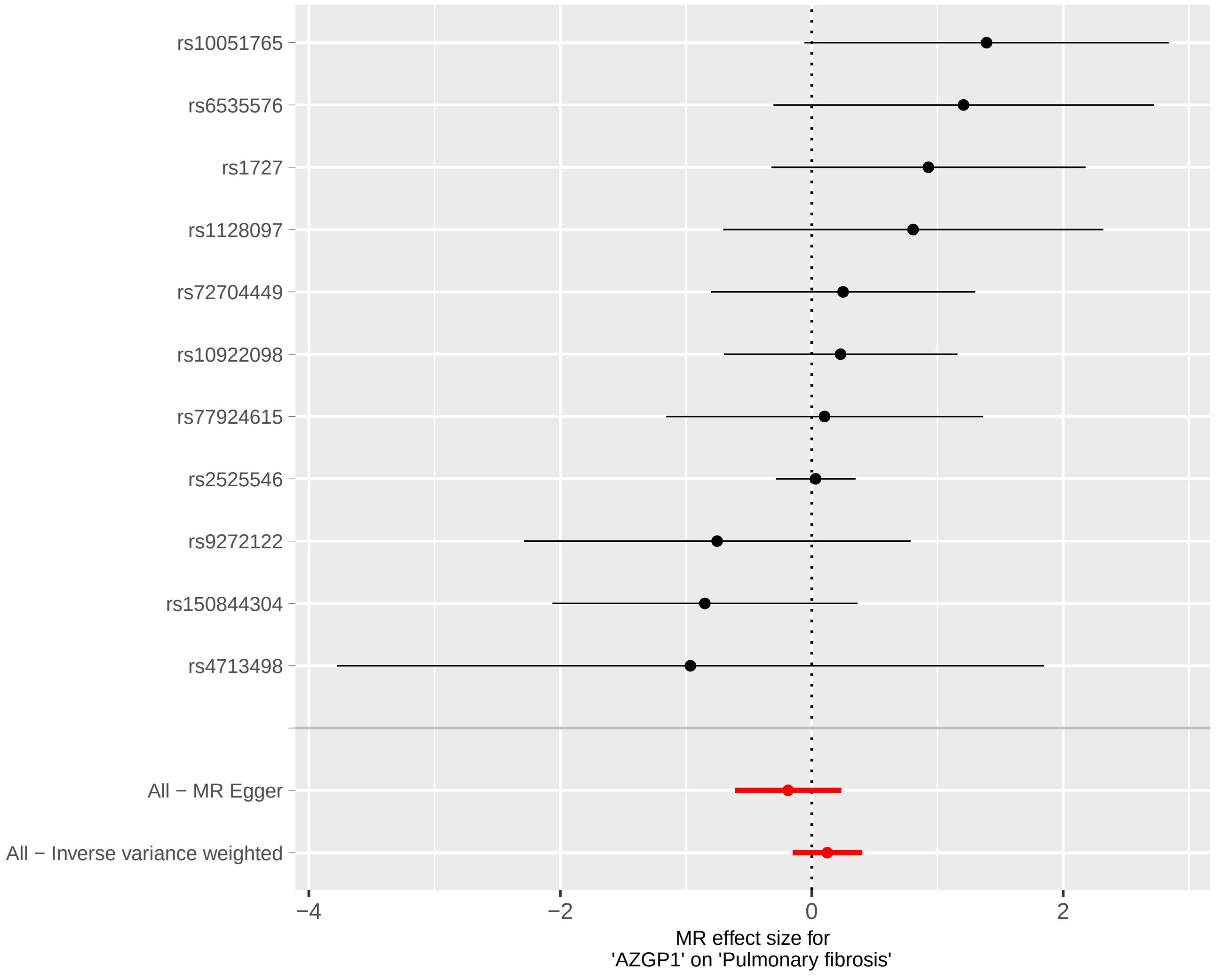

rs10051765
rs6535576
rs1727
rs1128097
rs72704449
rs10922098
rs77924615
rs2525546
rs9272122
rs150844304
rs4713498
All − MR Egger
All − Inverse variance weighted
−4
−2
0
2
MR effect size for
'AZGP1' on 'Pulmonary fibrosis'

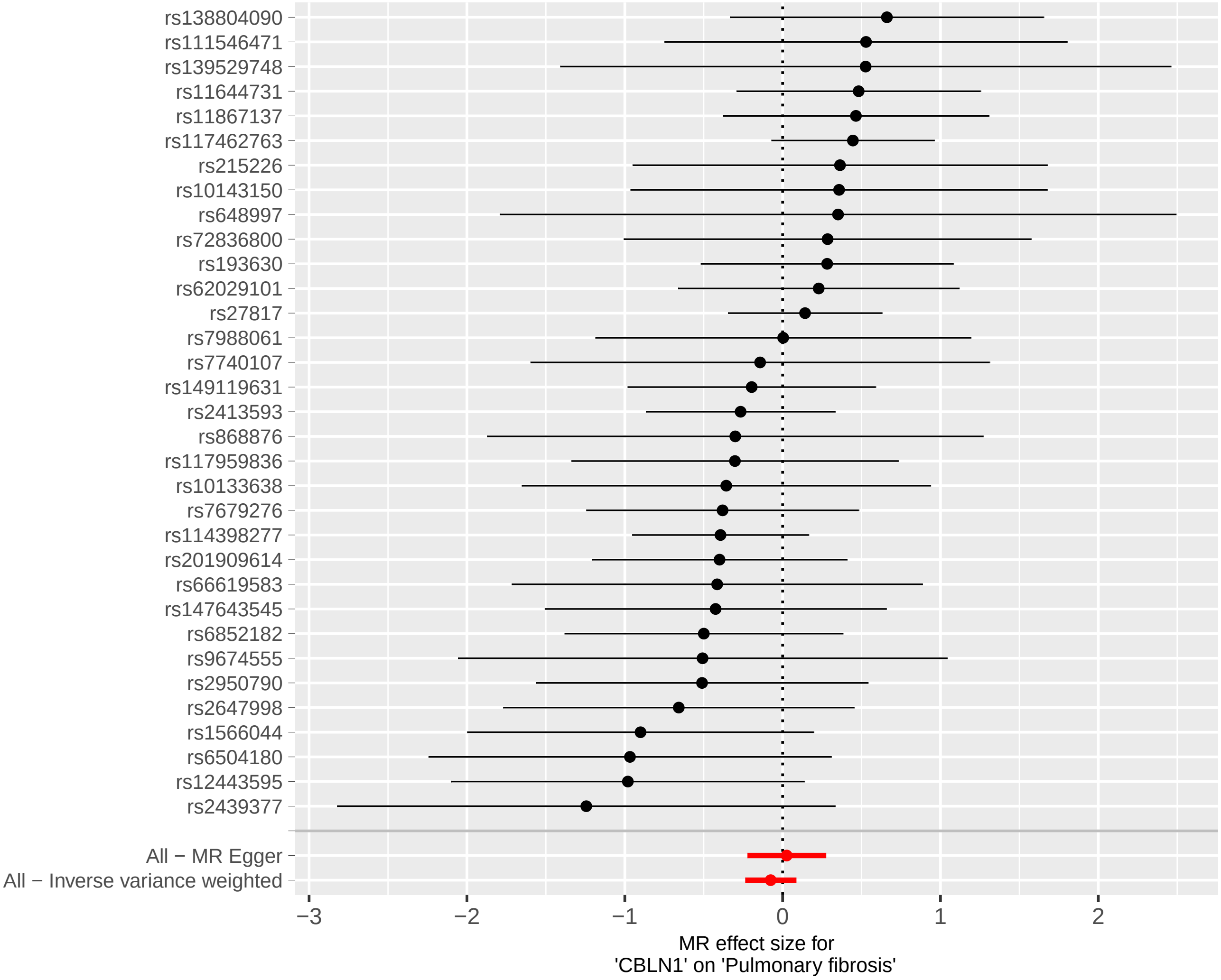
rs138804090
rs111546471
rs139529748
rs11644731
rs11867137
rs117462763
rs215226
rs10143150
rs648997
rs72836800
rs193630
rs62029101
rs27817
rs7988061
rs7740107
rs149119631
rs2413593
rs868876
rs117959836
rs10133638
rs7679276
rs114398277
rs201909614
rs66619583
rs147643545
rs6852182
rs9674555
rs2950790
rs2647998
rs1566044
rs6504180
rs12443595
rs2439377
All − MR Egger
All − Inverse variance weighted
−3
−2
−1
0
1
2
MR effect size for
'CBLN1' on 'Pulmonary fibrosis'

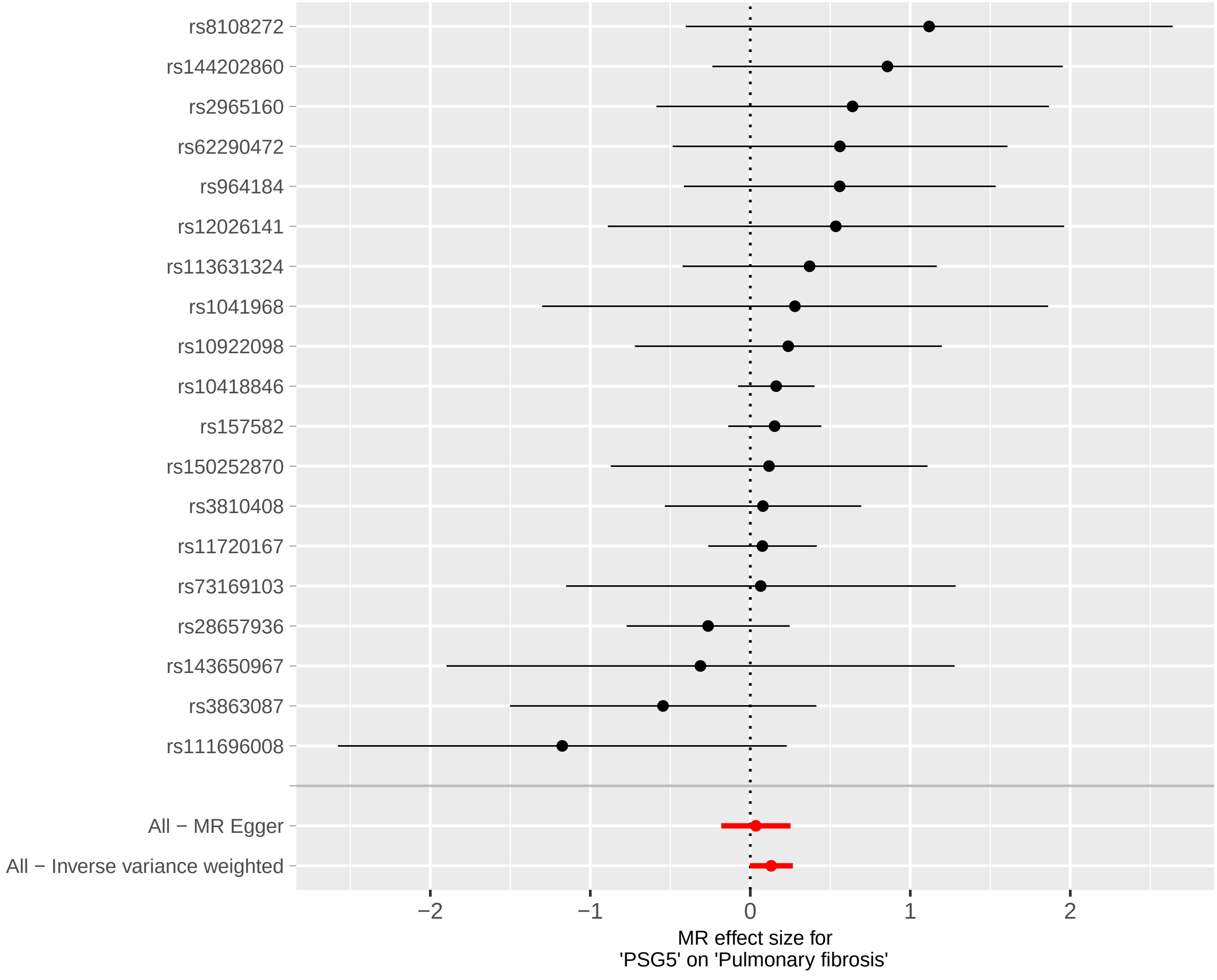
rs8108272
rs144202860
rs2965160
rs62290472
rs964184
rs12026141
rs113631324
rs1041968
rs10922098
rs10418846
rs157582
rs150252870
rs3810408
rs11720167
rs73169103
rs28657936
rs143650967
rs3863087
rs111696008
All − MR Egger
All − Inverse variance weighted
−2
−1
0
1
2
MR effect size for
'PSG5' on 'Pulmonary fibrosis'

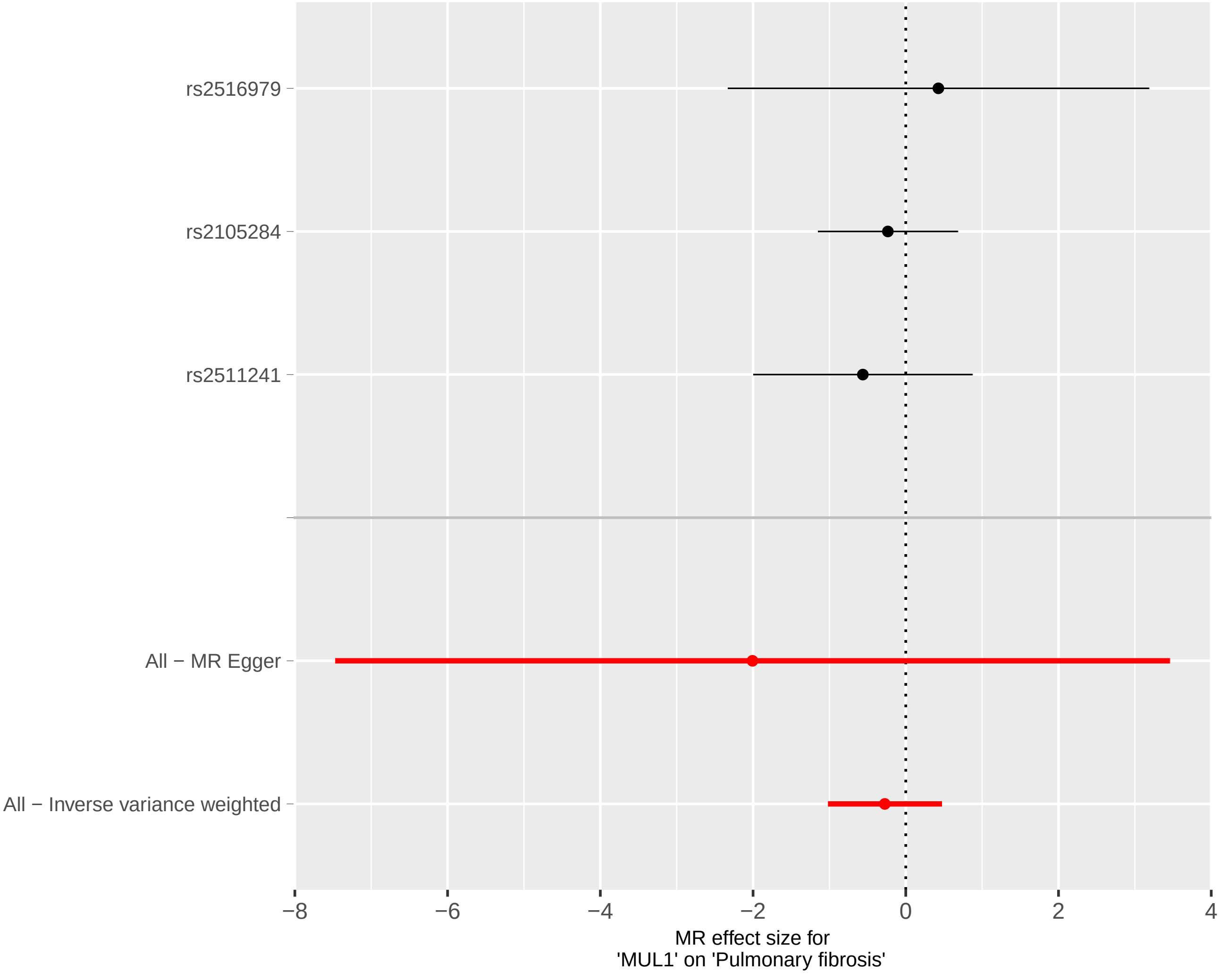

rs2516979
rs2105284
rs2511241
All − MR Egger
All − Inverse variance weighted
−8
−6
−4
−2
0
2
4
MR effect size for
'MUL1' on 'Pulmonary fibrosis'

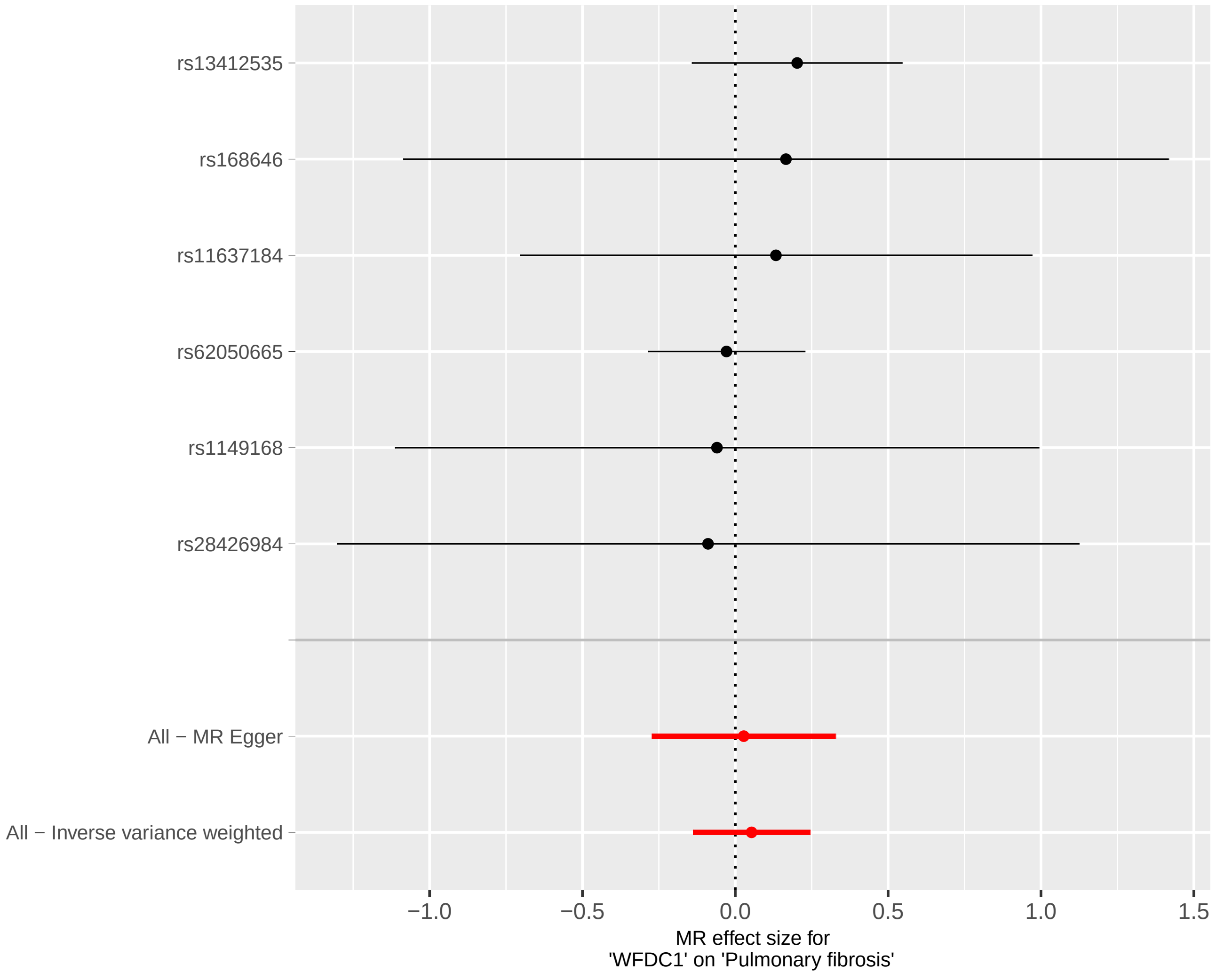
rs13412535
rs168646
rs11637184
rs62050665
rs1149168
rs28426984
All − MR Egger
All − Inverse variance weighted
−1.0
−0.5
0.0
0.5
1.0
1.5
MR effect size for
'WFDC1' on 'Pulmonary fibrosis'

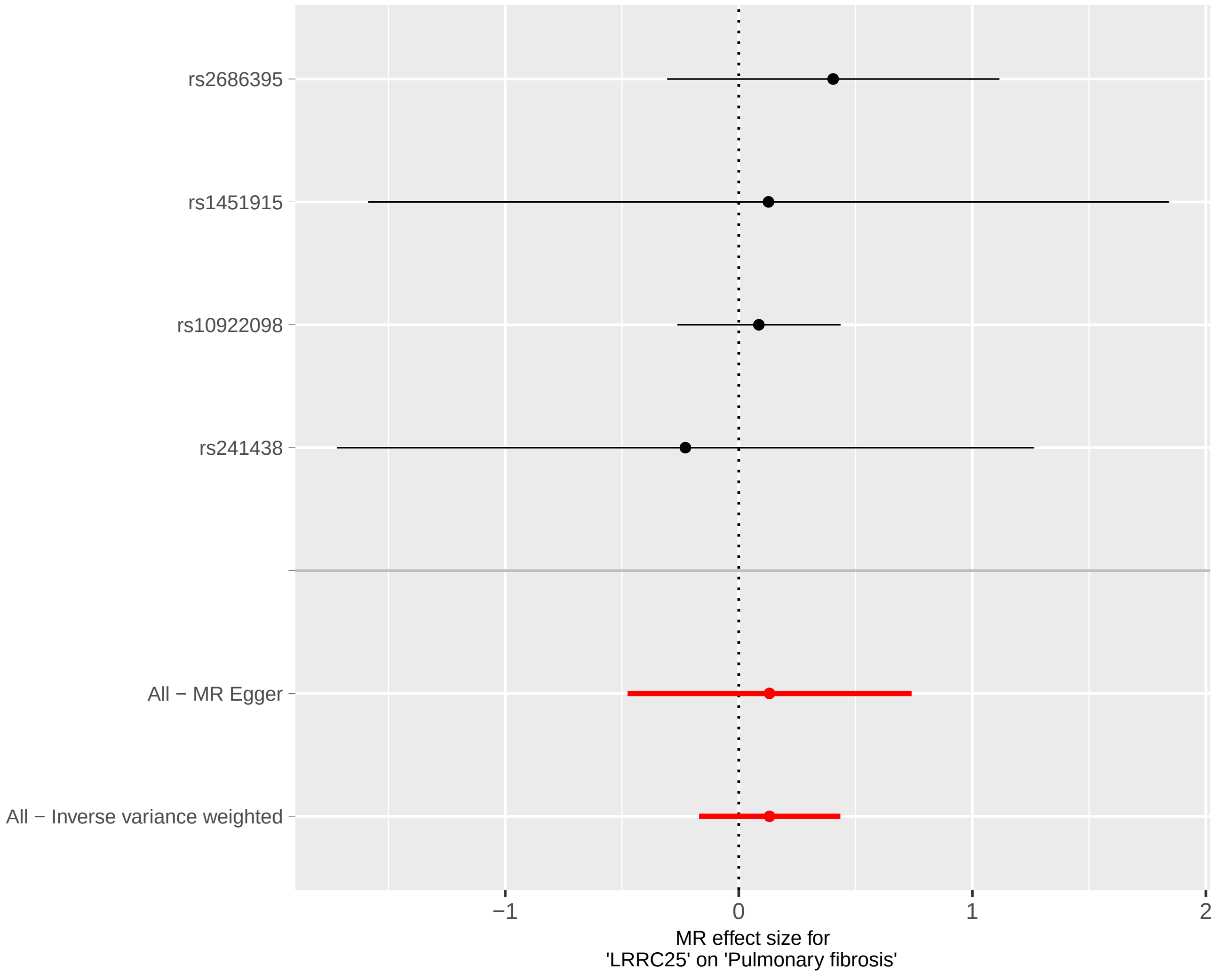
rs2686395
rs1451915
rs10922098
rs241438
All − MR Egger
All − Inverse variance weighted
−1
0
1
2
MR effect size for
'LRRC25' on 'Pulmonary fibrosis'

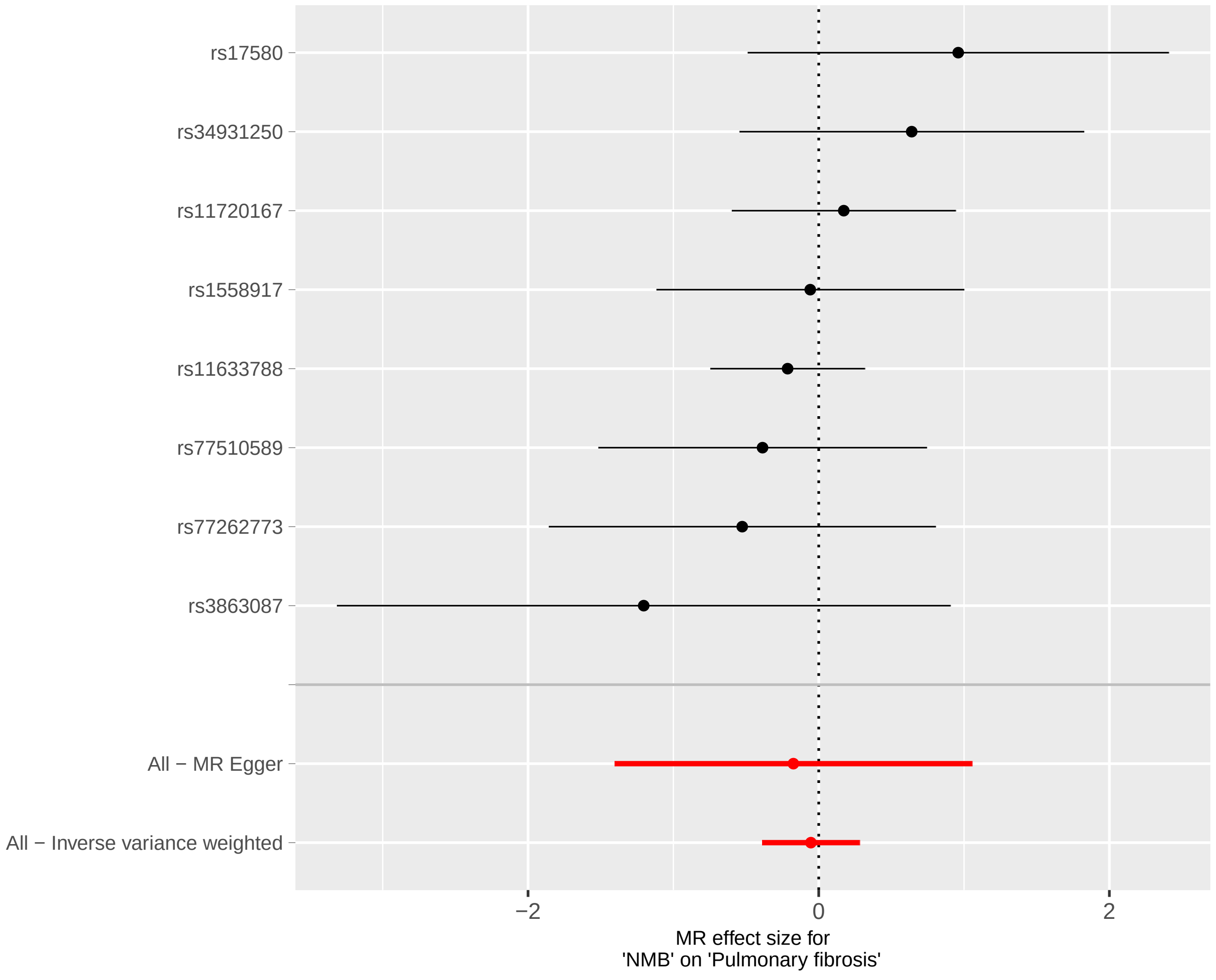

rs17580
rs34931250
rs11720167
rs1558917
rs11633788
rs77510589
rs77262773
rs3863087
All − MR Egger
All − Inverse variance weighted
−2
0
2
MR effect size for
'NMB' on 'Pulmonary fibrosis'

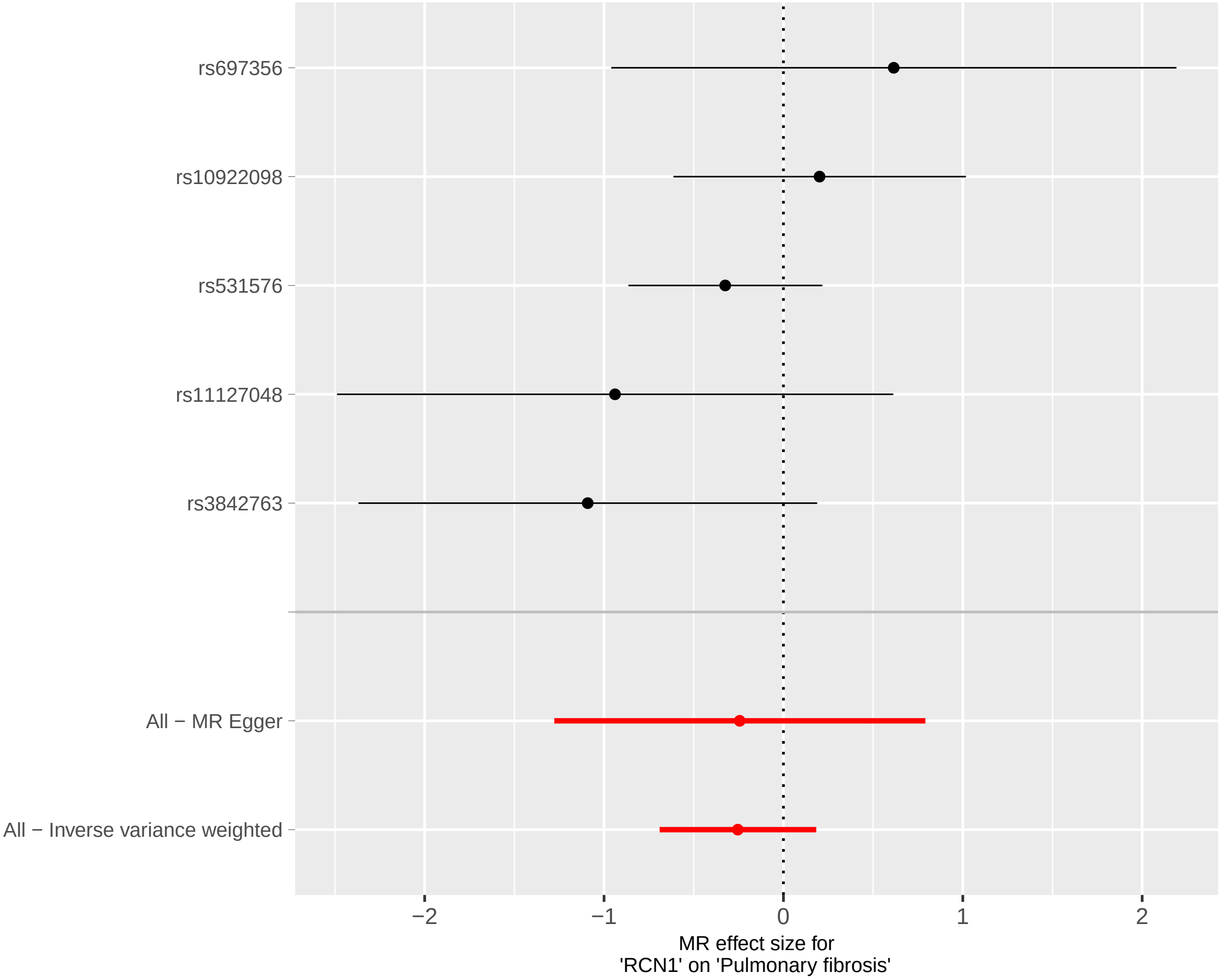

rs697356
rs10922098
rs531576
rs11127048
rs3842763
All − MR Egger
All − Inverse variance weighted
−2
−1
0
1
2
MR effect size for
'RCN1' on 'Pulmonary fibrosis'

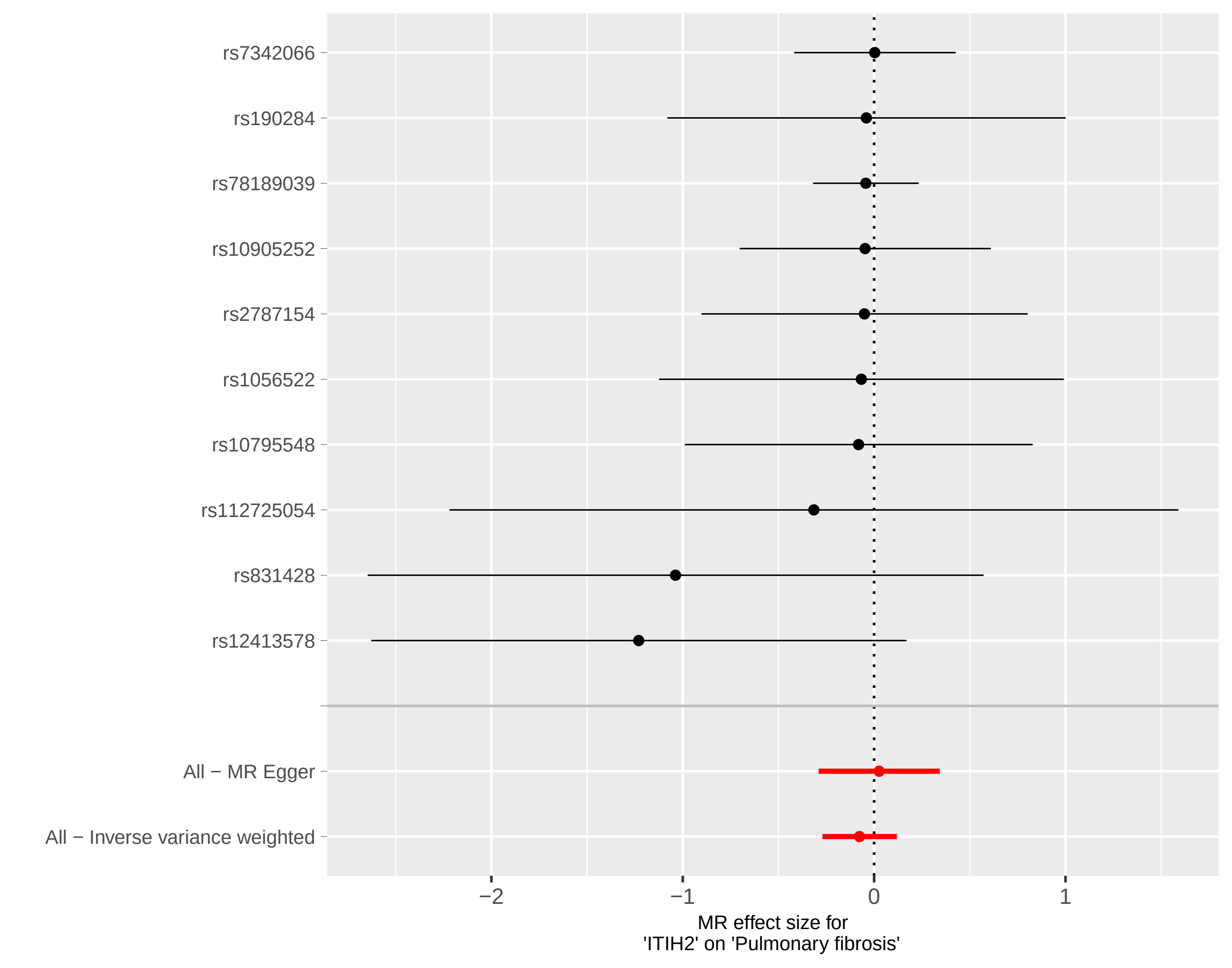
rs7342066
rs190284
rs78189039
rs10905252
rs2787154
rs1056522
rs10795548
rs112725054
rs831428
rs12413578
All − MR Egger
All − Inverse variance weighted
−2
−1
0
1
MR effect size for
'ITIH2' on 'Pulmonary fibrosis'

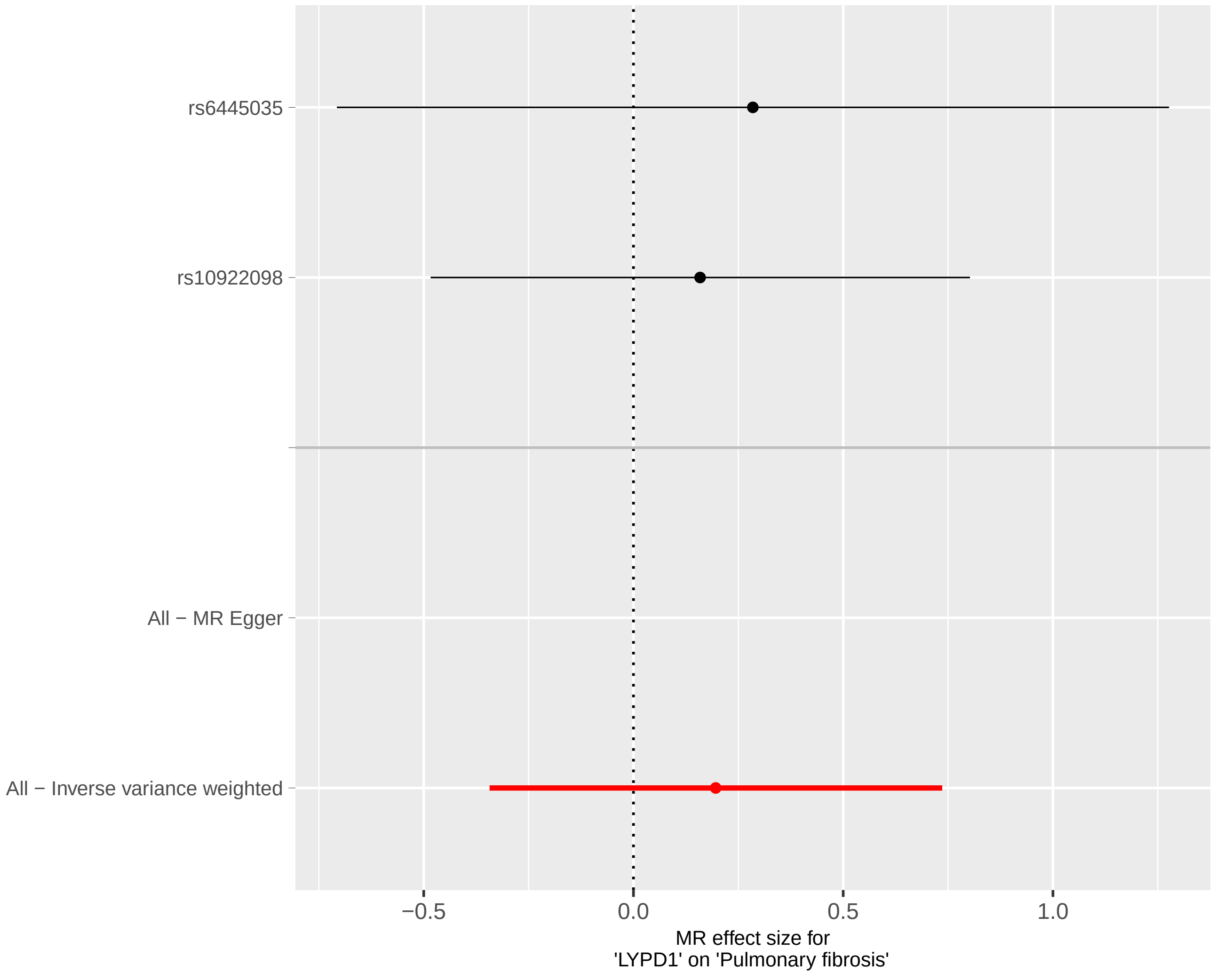

rs6445035
rs10922098
All − MR Egger
All − Inverse variance weighted
−0.5
0.0
0.5
1.0
MR effect size for
'LYPD1' on 'Pulmonary fibrosis'

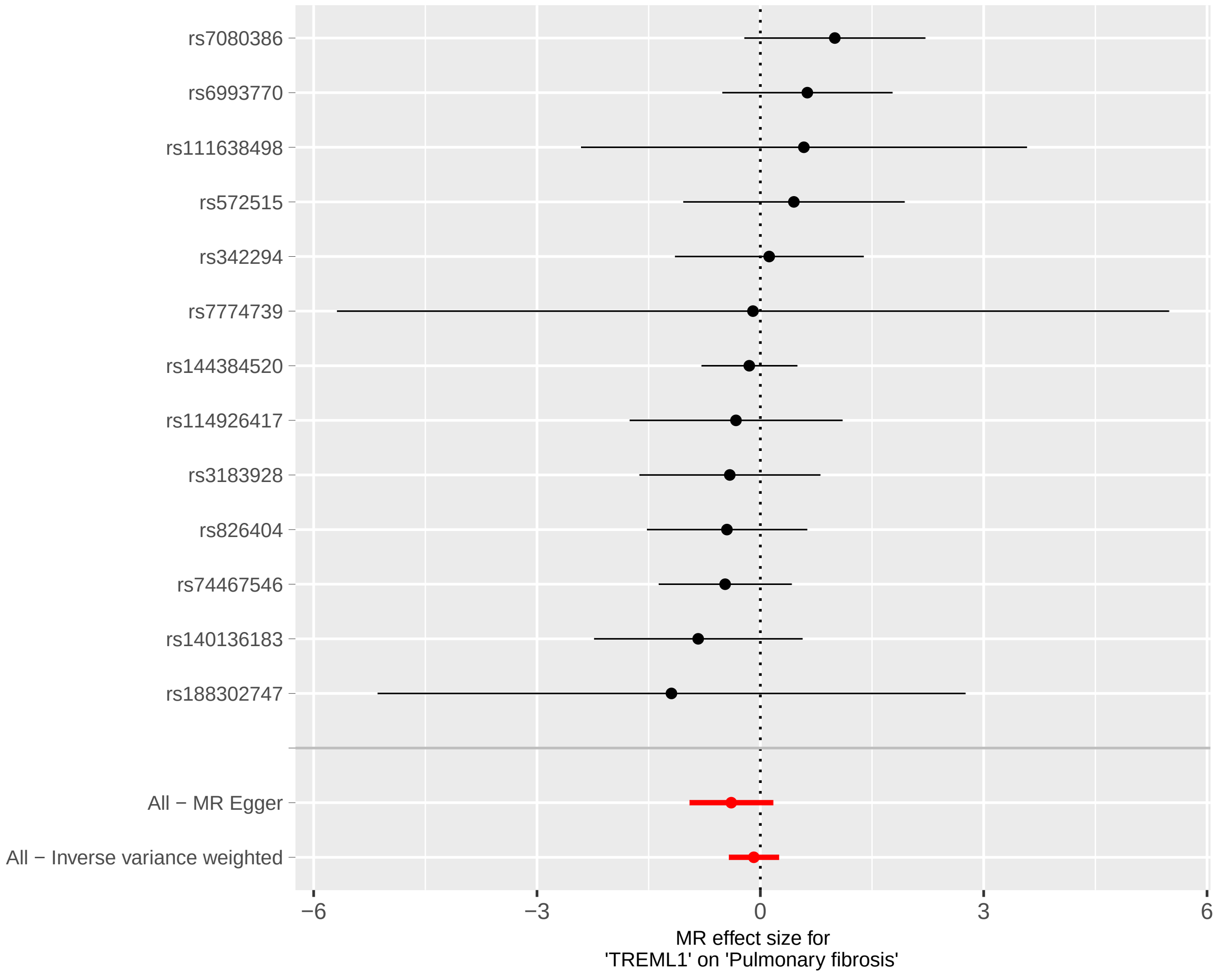
rs7080386
rs6993770
rs111638498
rs572515
rs342294
rs7774739
rs144384520
rs114926417
rs3183928
rs826404
rs74467546
rs140136183
rs188302747
All − MR Egger
All − Inverse variance weighted
−6
−3
0
3
6
MR effect size for
'TREML1' on 'Pulmonary fibrosis'

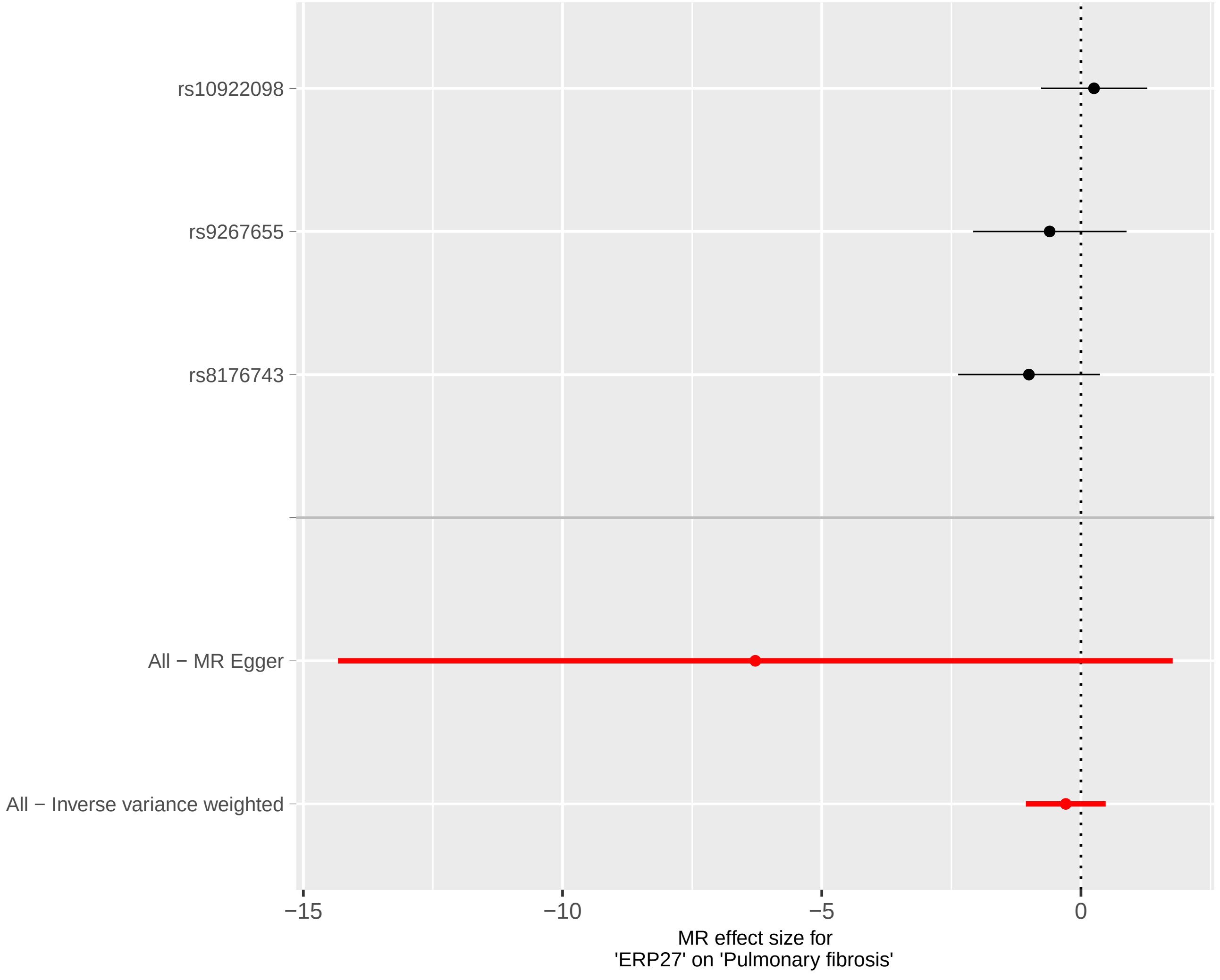
rs10922098
rs9267655
rs8176743
All − MR Egger
All − Inverse variance weighted
−15
−10
−5
0
MR effect size for
'ERP27' on 'Pulmonary fibrosis'

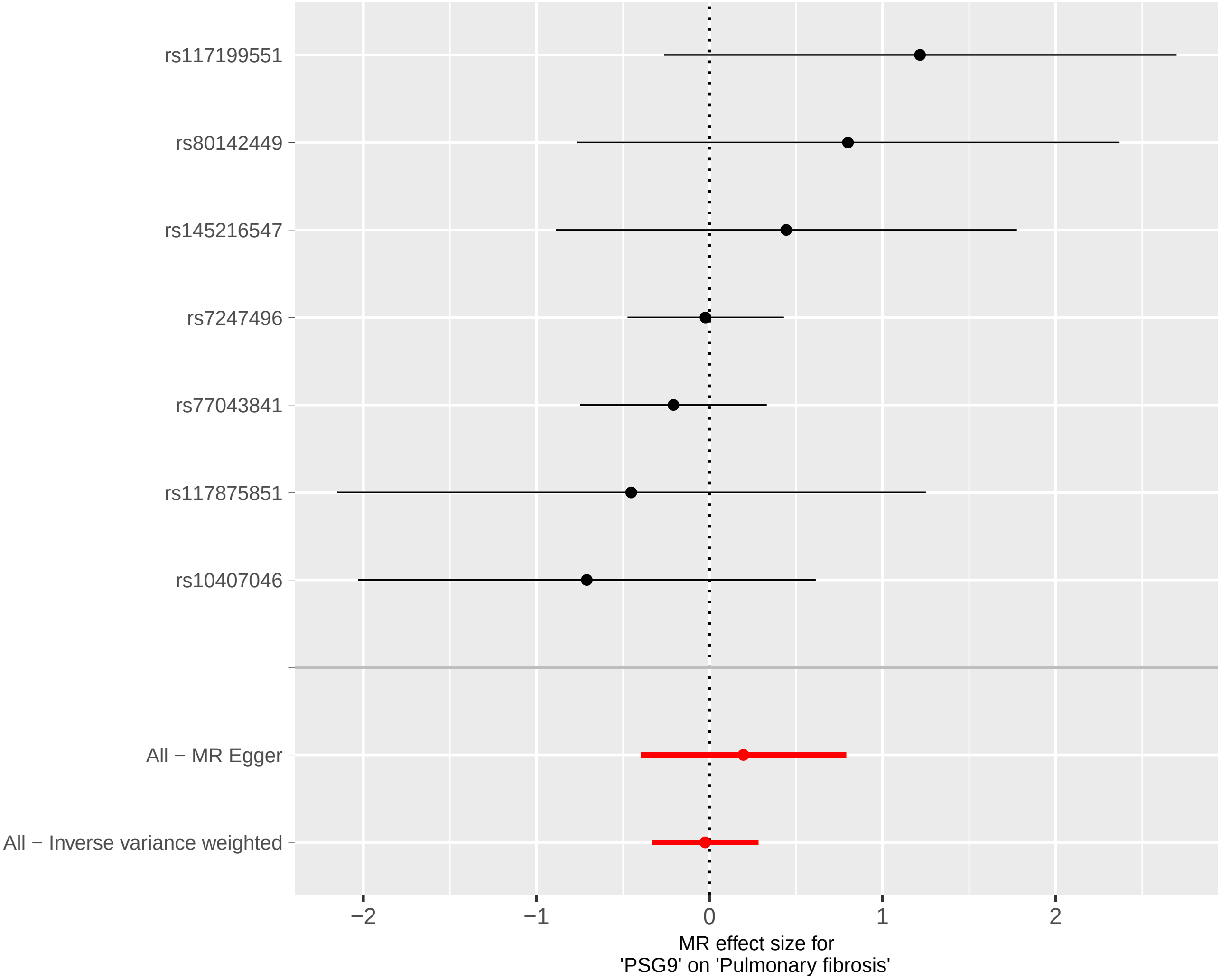
rs117199551
rs80142449
rs145216547
rs7247496
rs77043841
rs117875851
rs10407046
All − MR Egger
All − Inverse variance weighted
−2
−1
0
1
2
MR effect size for
'PSG9' on 'Pulmonary fibrosis'

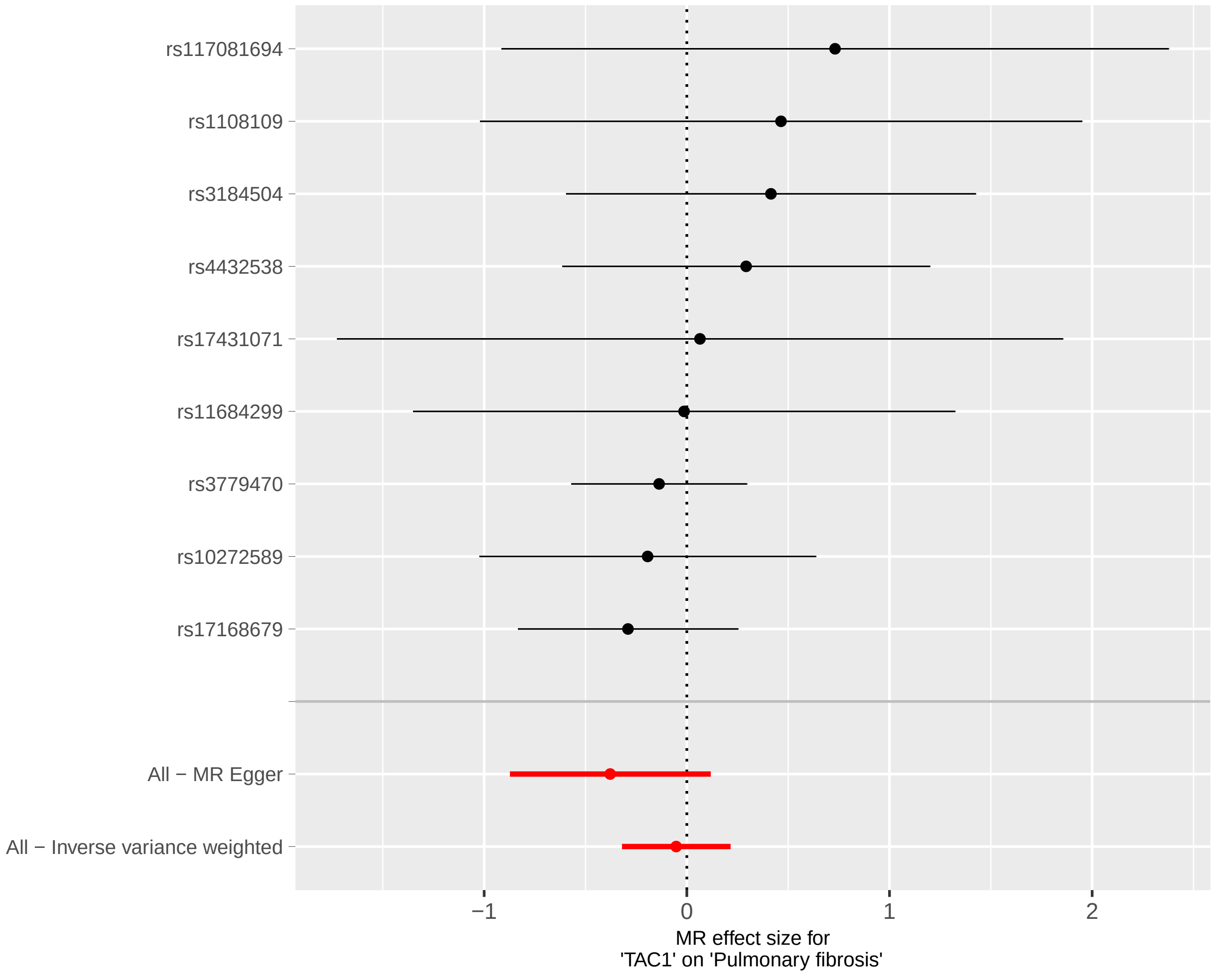
rs117081694
rs1108109
rs3184504
rs4432538
rs17431071
rs11684299
rs3779470
rs10272589
rs17168679
All − MR Egger
All − Inverse variance weighted
−1
0
1
2
MR effect size for
'TAC1' on 'Pulmonary fibrosis'

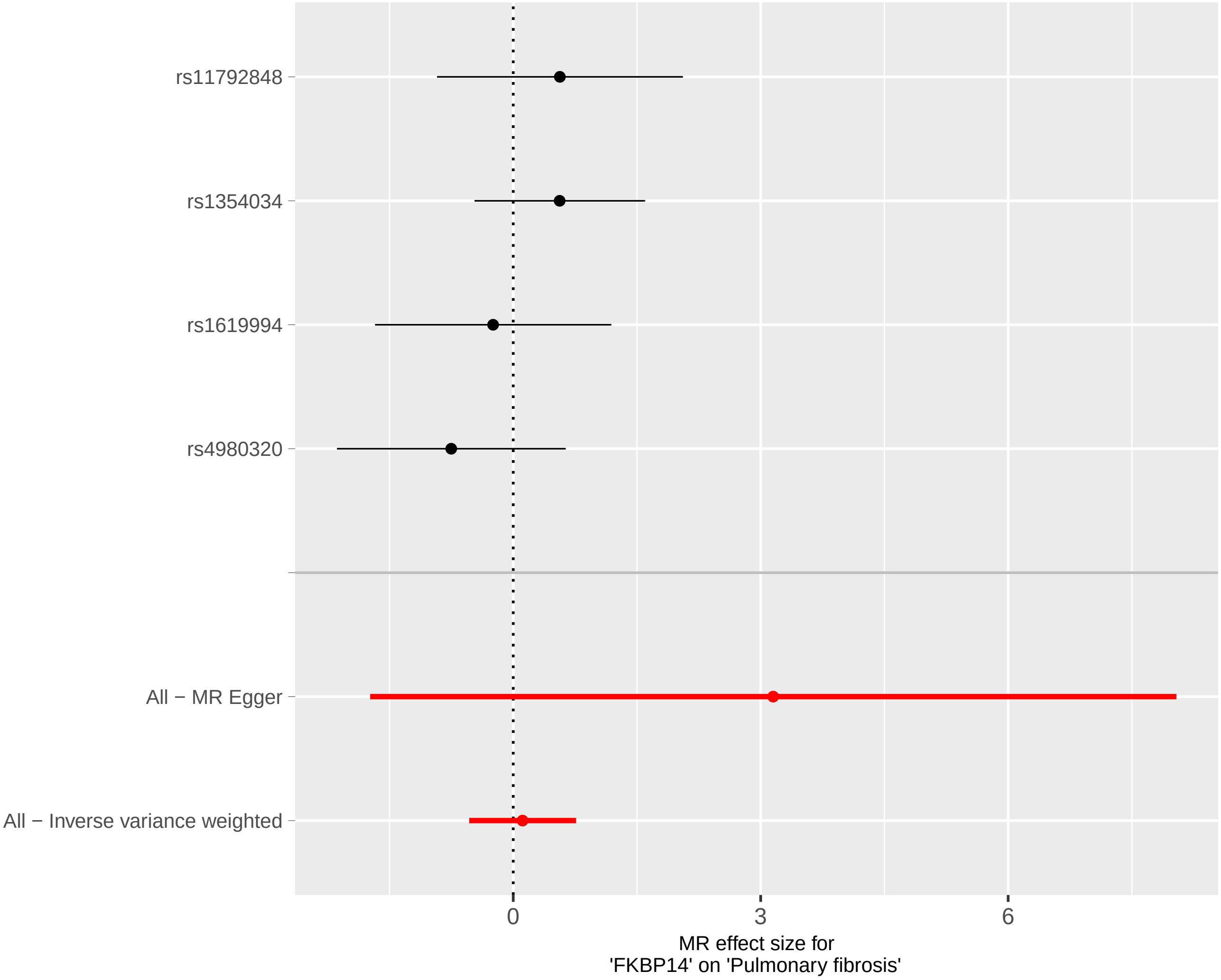
rs11792848
rs1354034
rs1619994
rs4980320
All − MR Egger
All − Inverse variance weighted
0
3
6
MR effect size for
'FKBP14' on 'Pulmonary fibrosis'

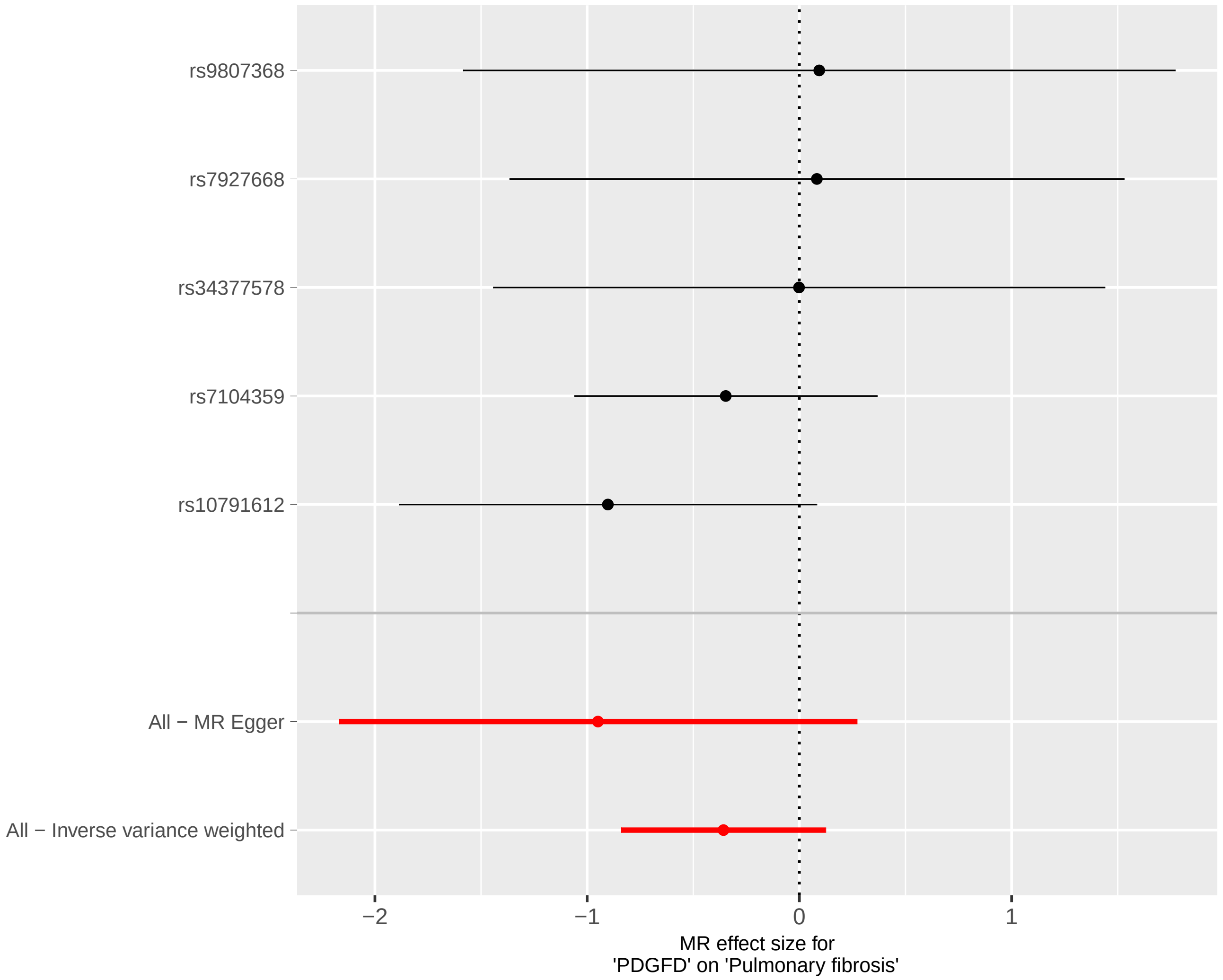

rs9807368
rs7927668
rs34377578
rs7104359
rs10791612
All − MR Egger
All − Inverse variance weighted
−2
−1
0
1
MR effect size for
'PDGFD' on 'Pulmonary fibrosis'

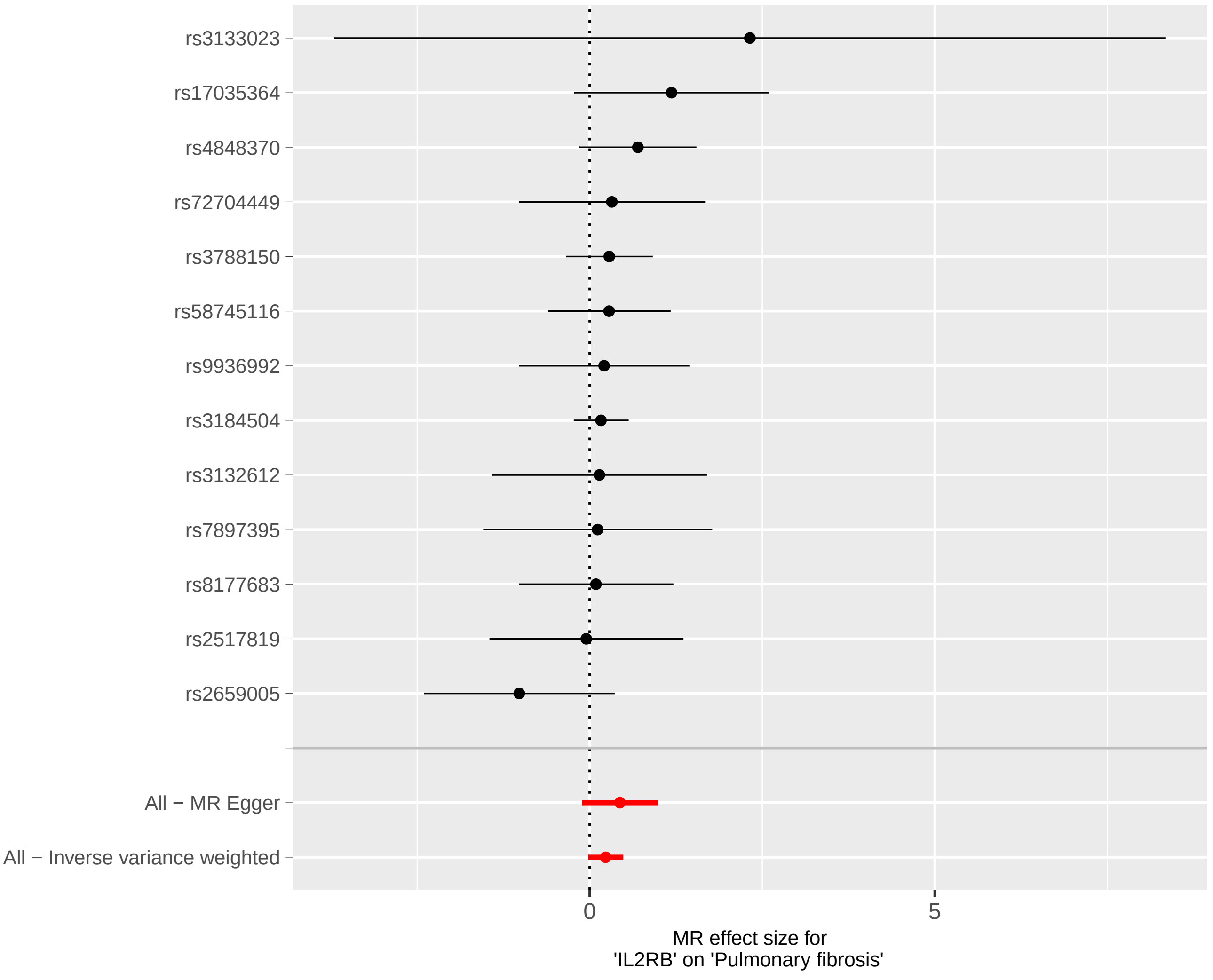
rs3133023
rs17035364
rs4848370
rs72704449
rs3788150
rs58745116
rs9936992
rs3184504
rs3132612
rs7897395
rs8177683
rs2517819
rs2659005
All − MR Egger
All − Inverse variance weighted
0
5
MR effect size for
'IL2RB' on 'Pulmonary fibrosis'

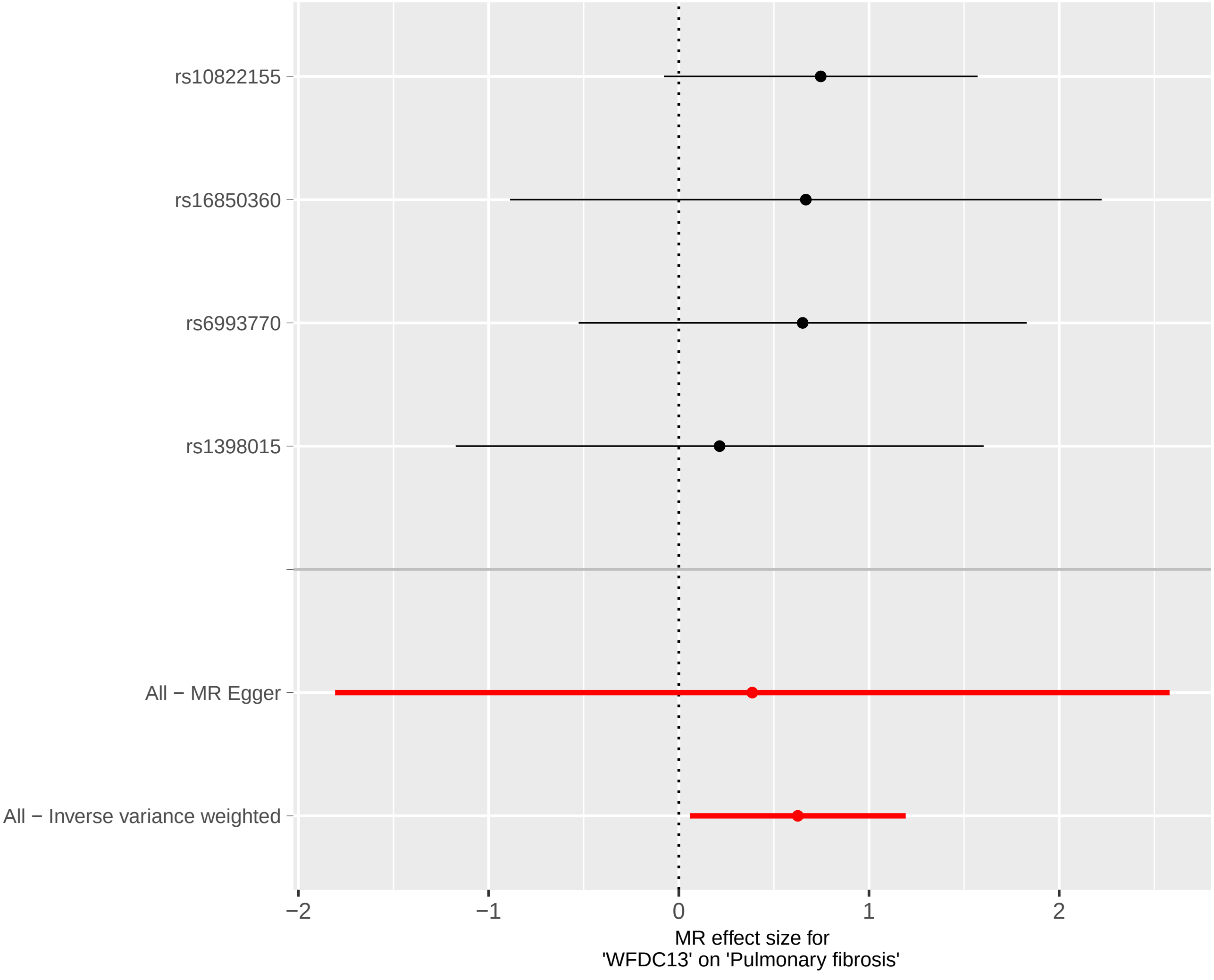

rs10822155
rs16850360
rs6993770
rs1398015
All − MR Egger
All − Inverse variance weighted
−2
−1
0
1
2
MR effect size for
'WFDC13' on 'Pulmonary fibrosis'

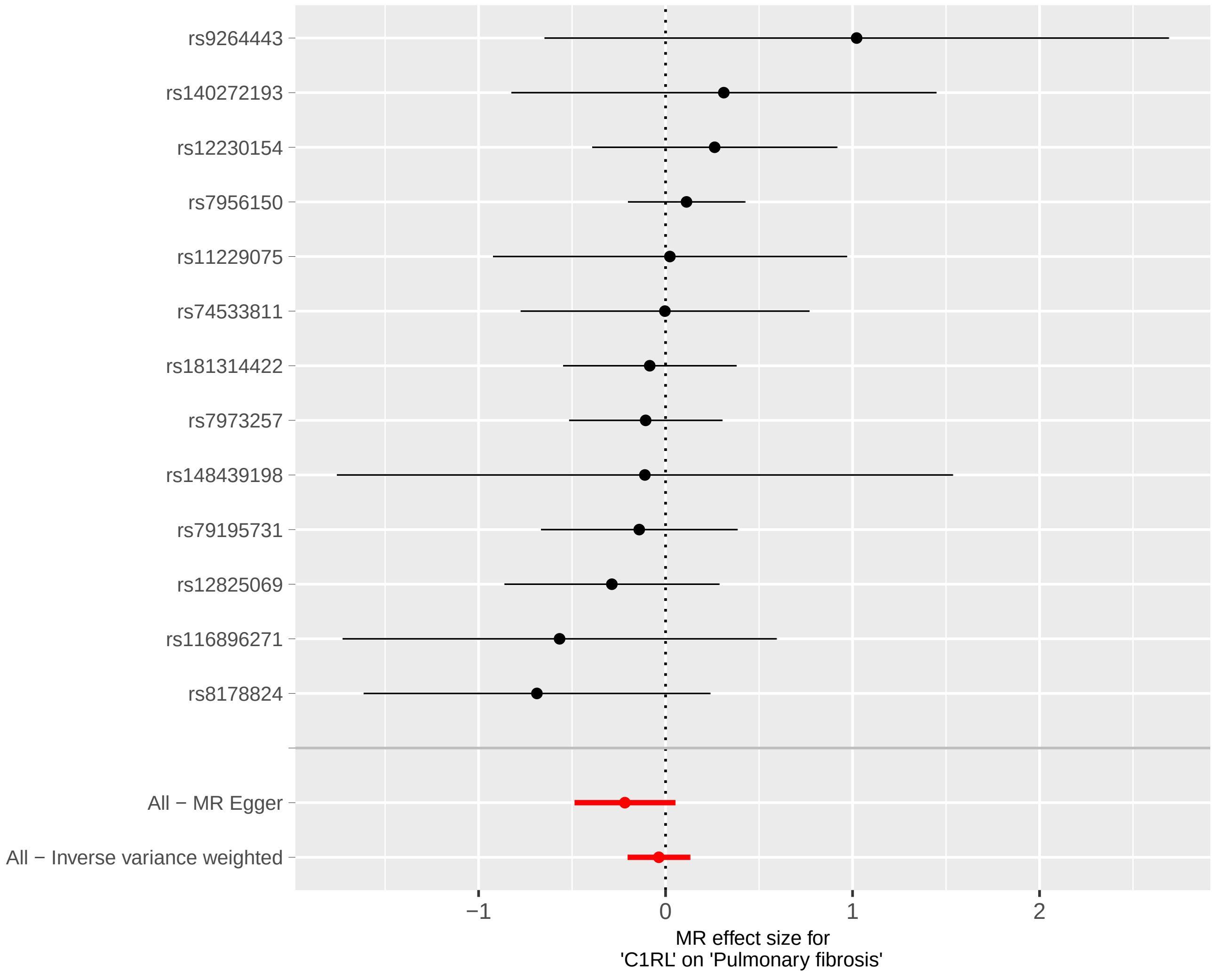
rs9264443
rs140272193
rs12230154
rs7956150
rs11229075
rs74533811
rs181314422
rs7973257
rs148439198
rs79195731
rs12825069
rs116896271
rs8178824
All − MR Egger
All − Inverse variance weighted
−1
0
1
2
MR effect size for
'C1RL' on 'Pulmonary fibrosis'

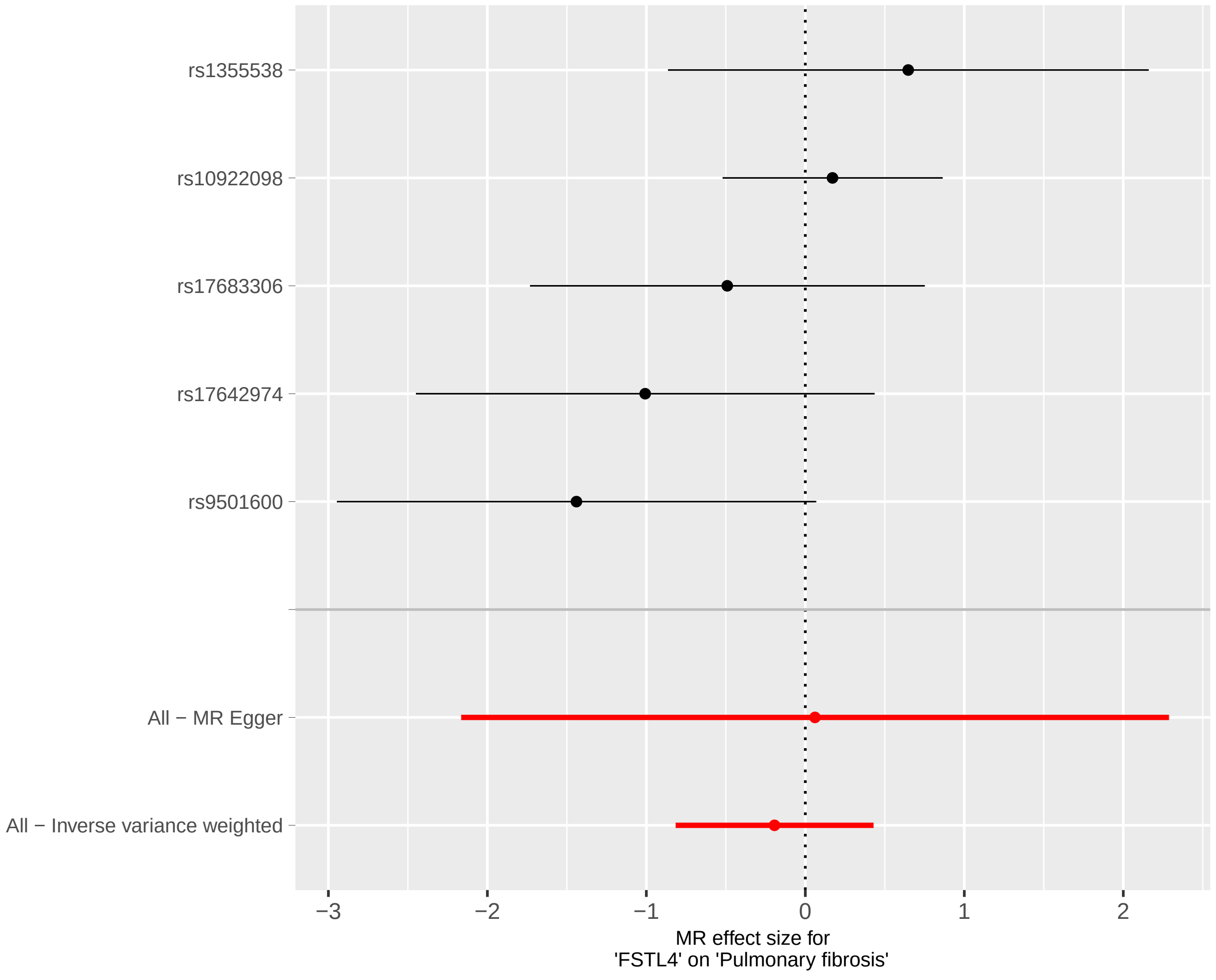
rs1355538
rs10922098
rs17683306
rs17642974
rs9501600
All − MR Egger
All − Inverse variance weighted
−3
−2
−1
0
1
2
MR effect size for
'FSTL4' on 'Pulmonary fibrosis'

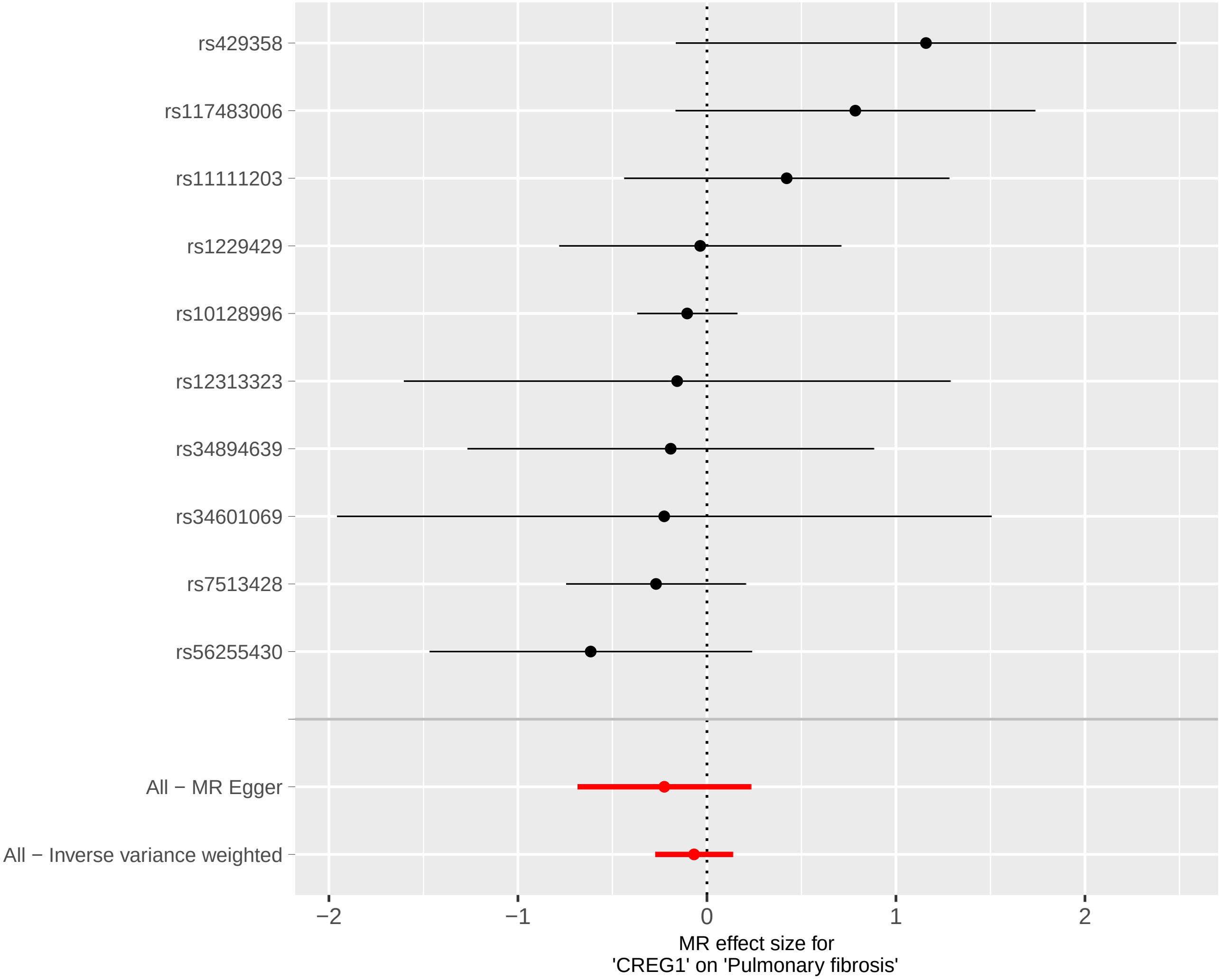

rs429358
rs117483006
rs11111203
rs1229429
rs10128996
rs12313323
rs34894639
rs34601069
rs7513428
rs56255430
All − MR Egger
All − Inverse variance weighted
−2
−1
0
1
2
MR effect size for
'CREG1' on 'Pulmonary fibrosis'

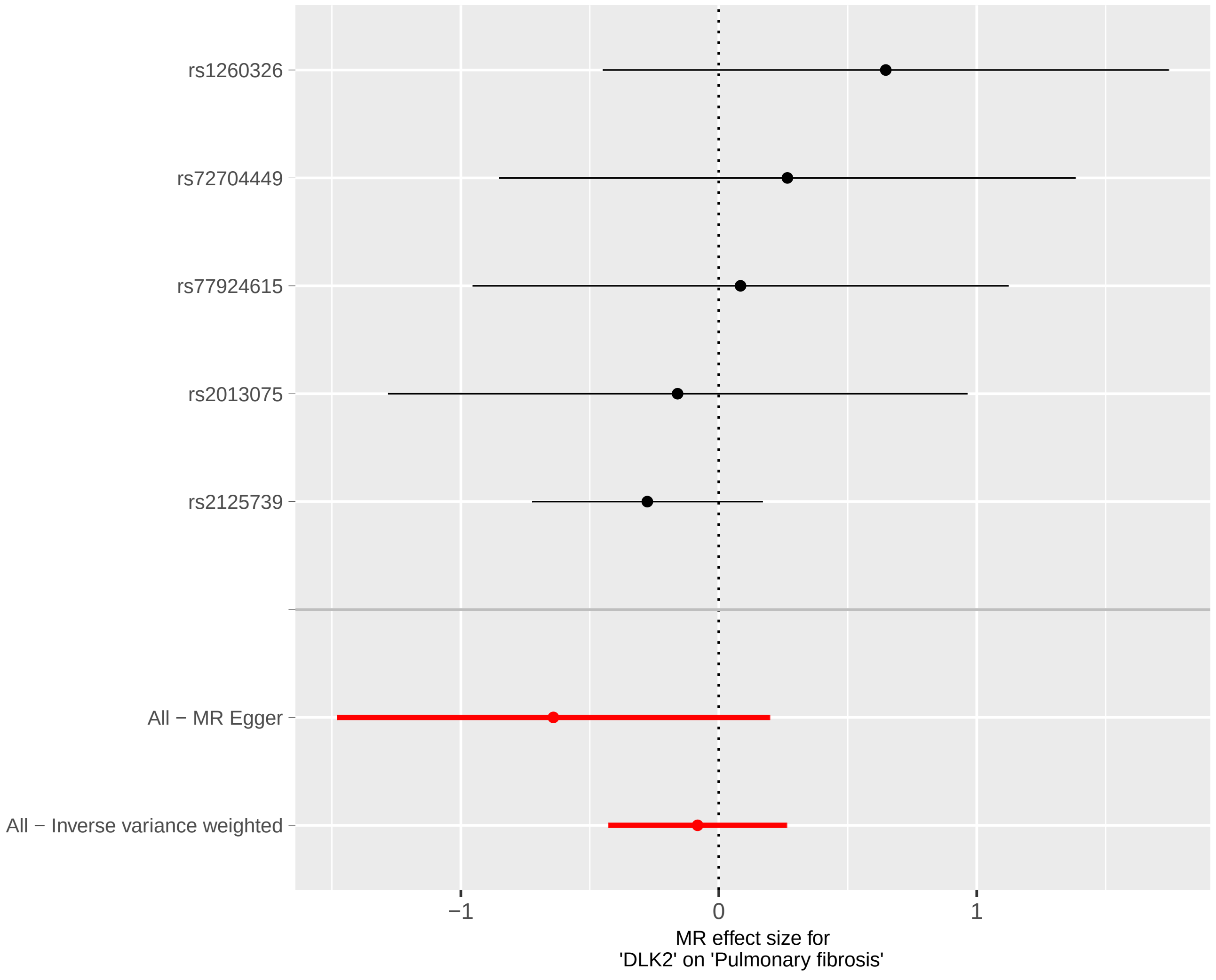
rs1260326
rs72704449
rs77924615
rs2013075
rs2125739
All − MR Egger
All − Inverse variance weighted
−1
0
1
MR effect size for
'DLK2' on 'Pulmonary fibrosis'

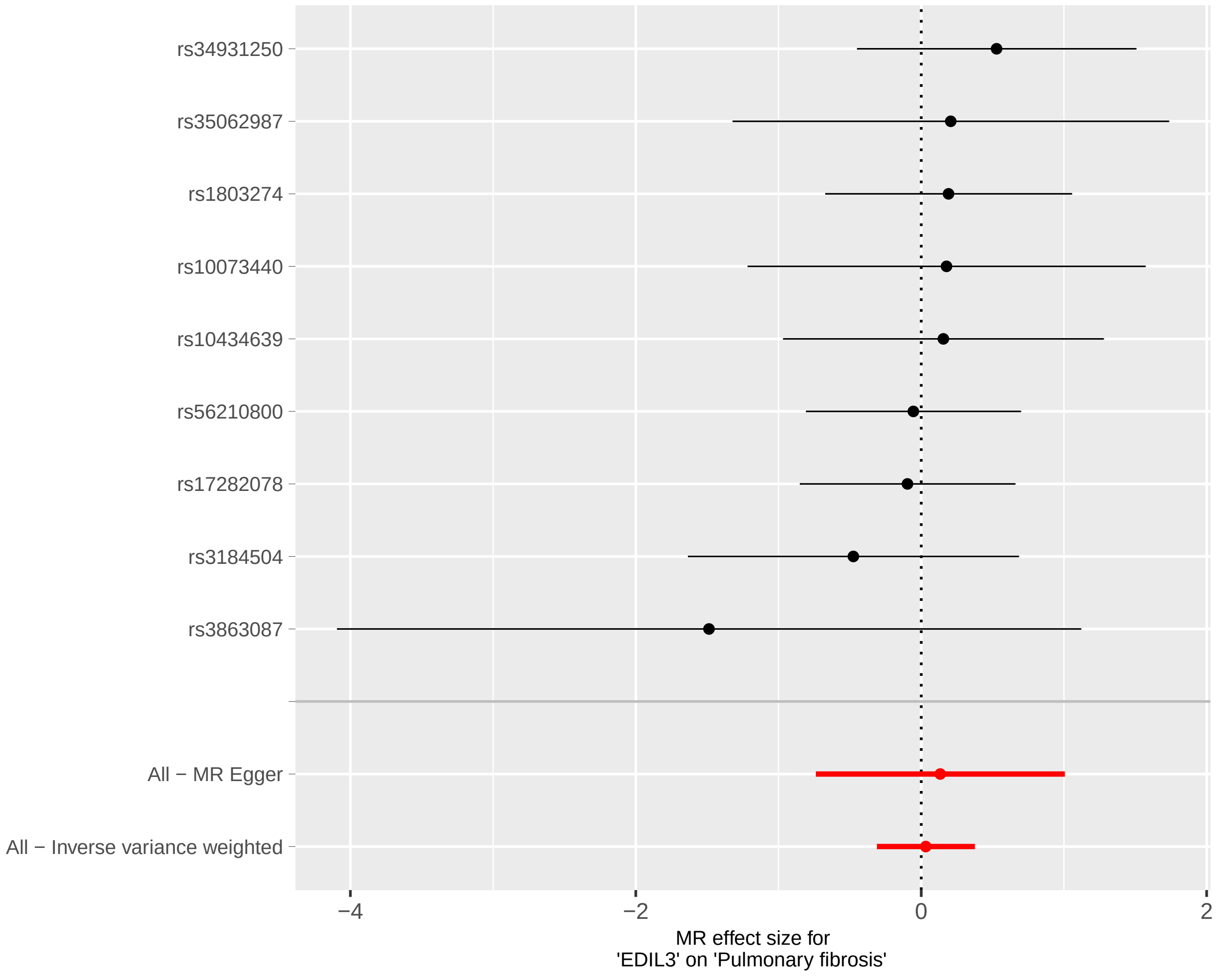

rs34931250
rs35062987
rs1803274
rs10073440
rs10434639
rs56210800
rs17282078
rs3184504
rs3863087
All − MR Egger
All − Inverse variance weighted
−4
−2
0
2
MR effect size for
'EDIL3' on 'Pulmonary fibrosis'

Insufficient number of SNPs

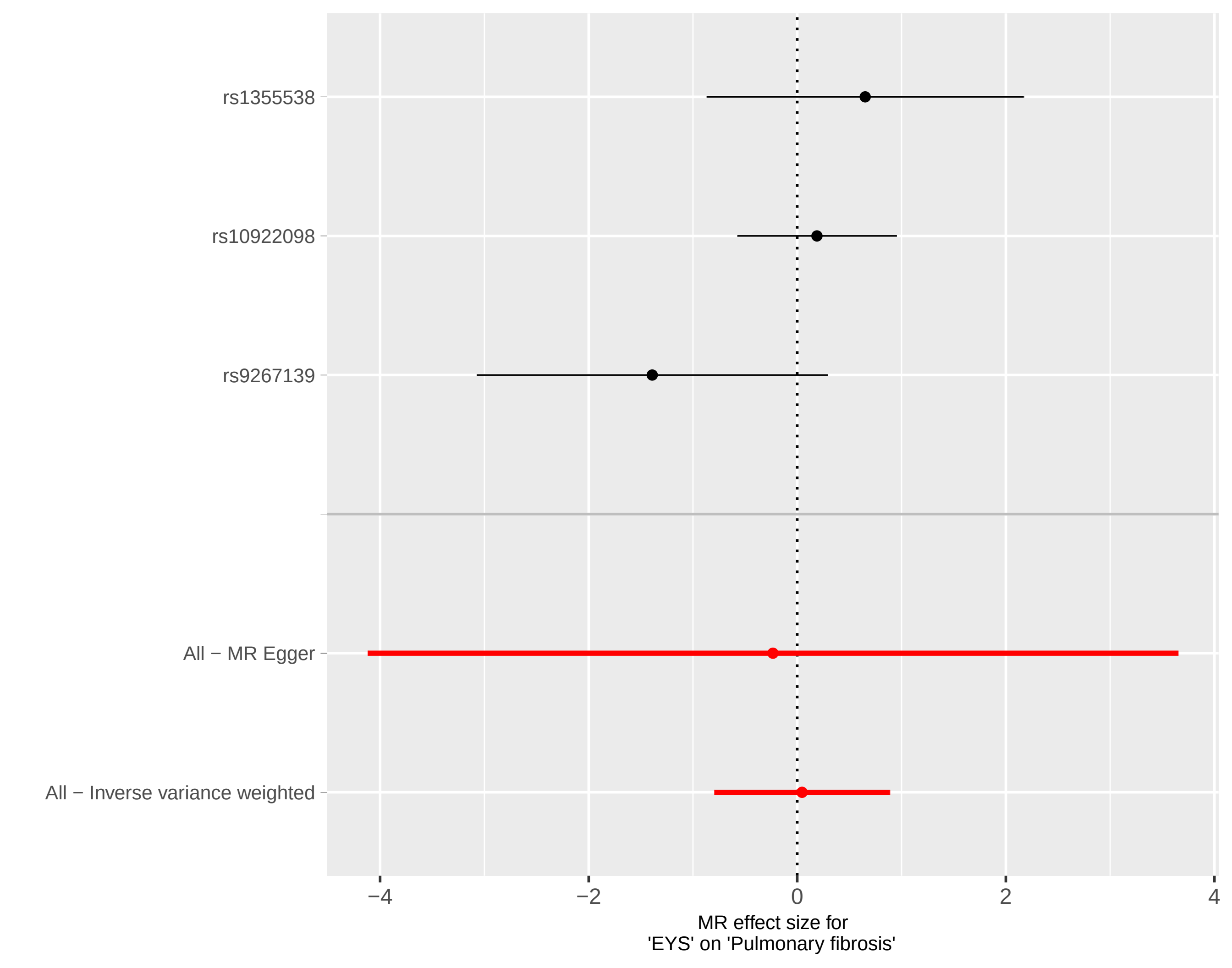
rs1355538
rs10922098
rs9267139
All − MR Egger
All − Inverse variance weighted
−4
−2
0
2
4
MR effect size for
'EYS' on 'Pulmonary fibrosis'

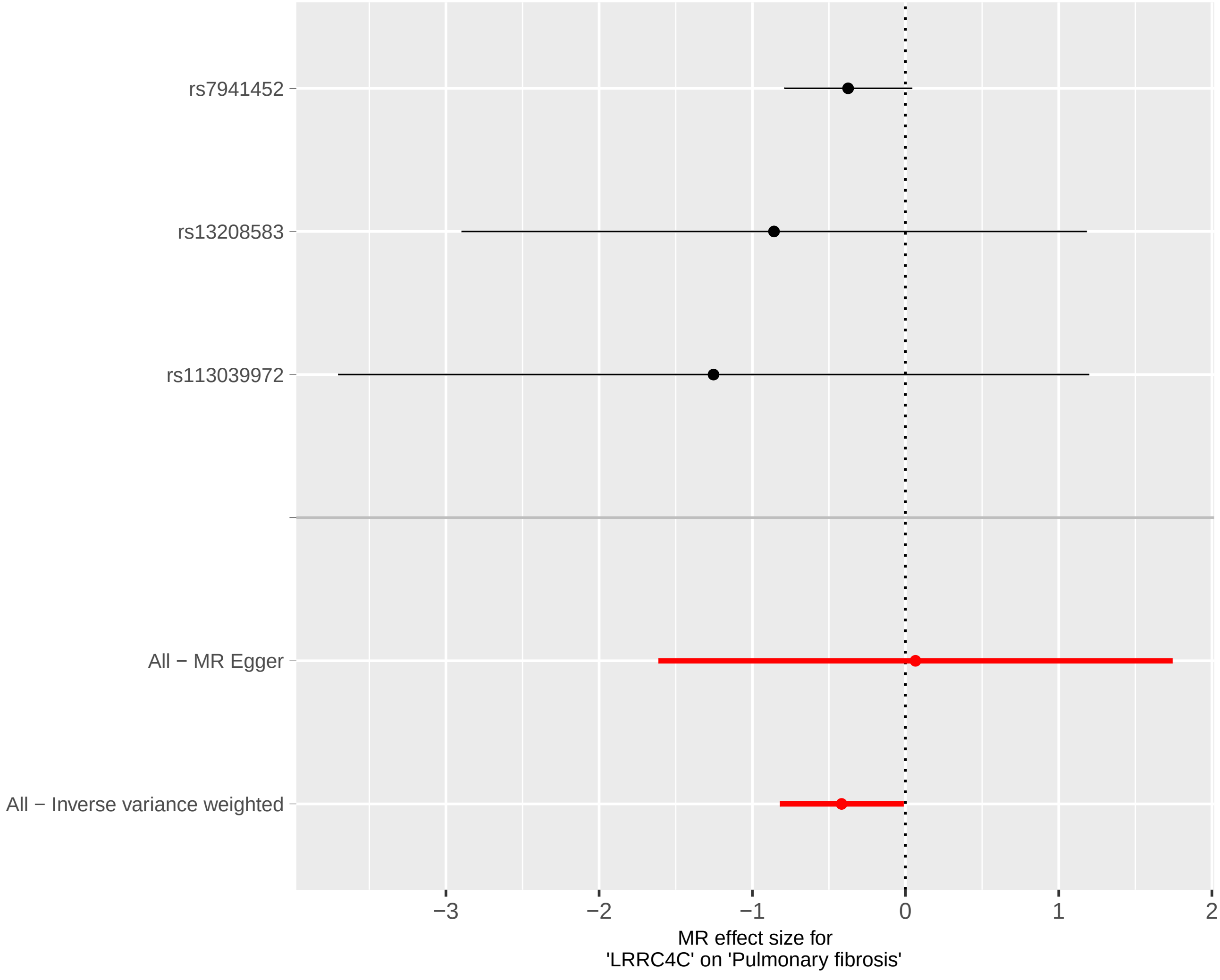
rs7941452
rs13208583
rs113039972
All − MR Egger
All − Inverse variance weighted
−3
−2
−1
0
1
2
MR effect size for
'LRRC4C' on 'Pulmonary fibrosis'

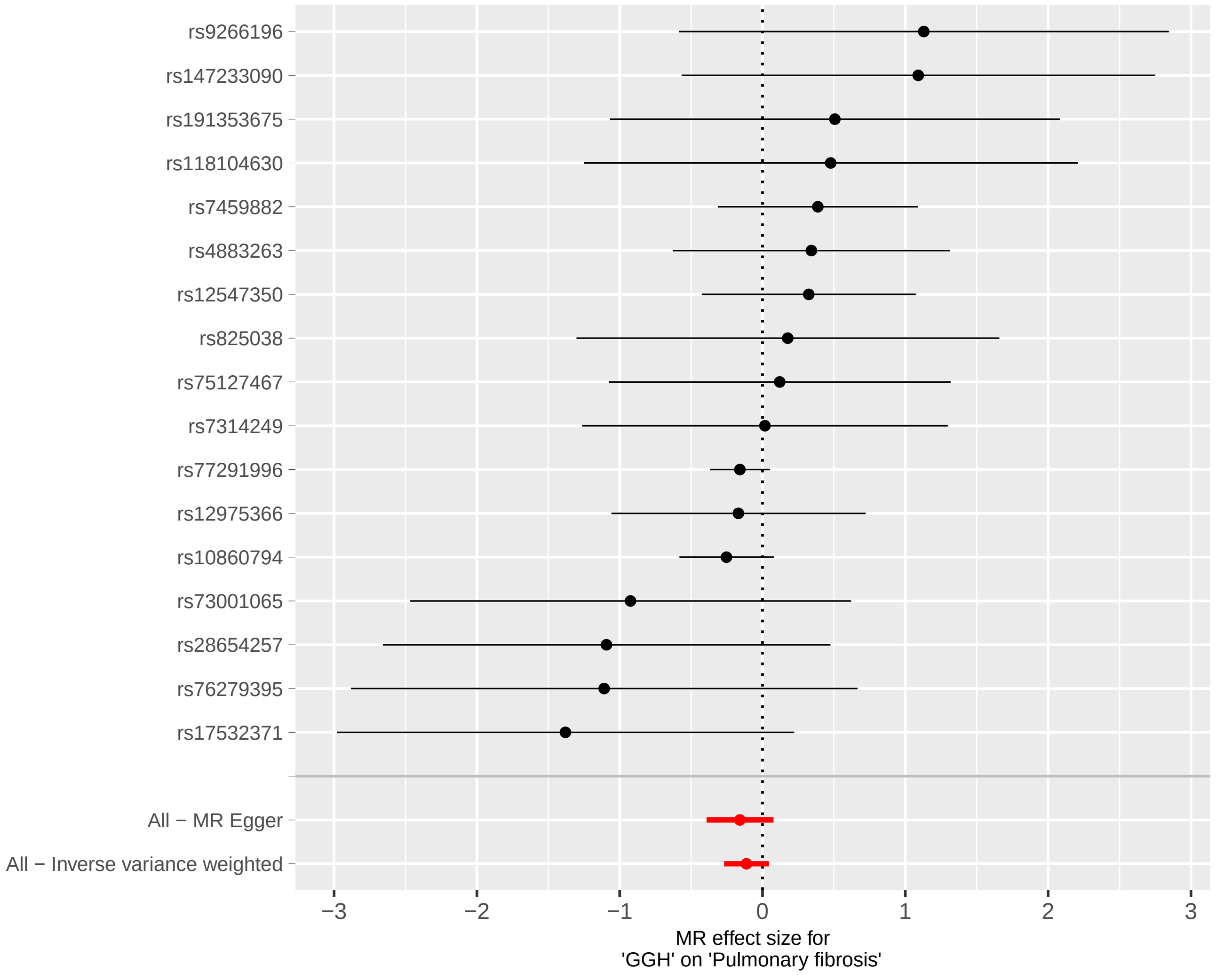
rs9266196
rs147233090
rs191353675
rs118104630
rs7459882
rs4883263
rs12547350
rs825038
rs75127467
rs7314249
rs77291996
rs12975366
rs10860794
rs73001065
rs28654257
rs76279395
rs17532371
All − MR Egger
All − Inverse variance weighted
−3
−2
−1
0
1
2
3
MR effect size for
'GGH' on 'Pulmonary fibrosis'

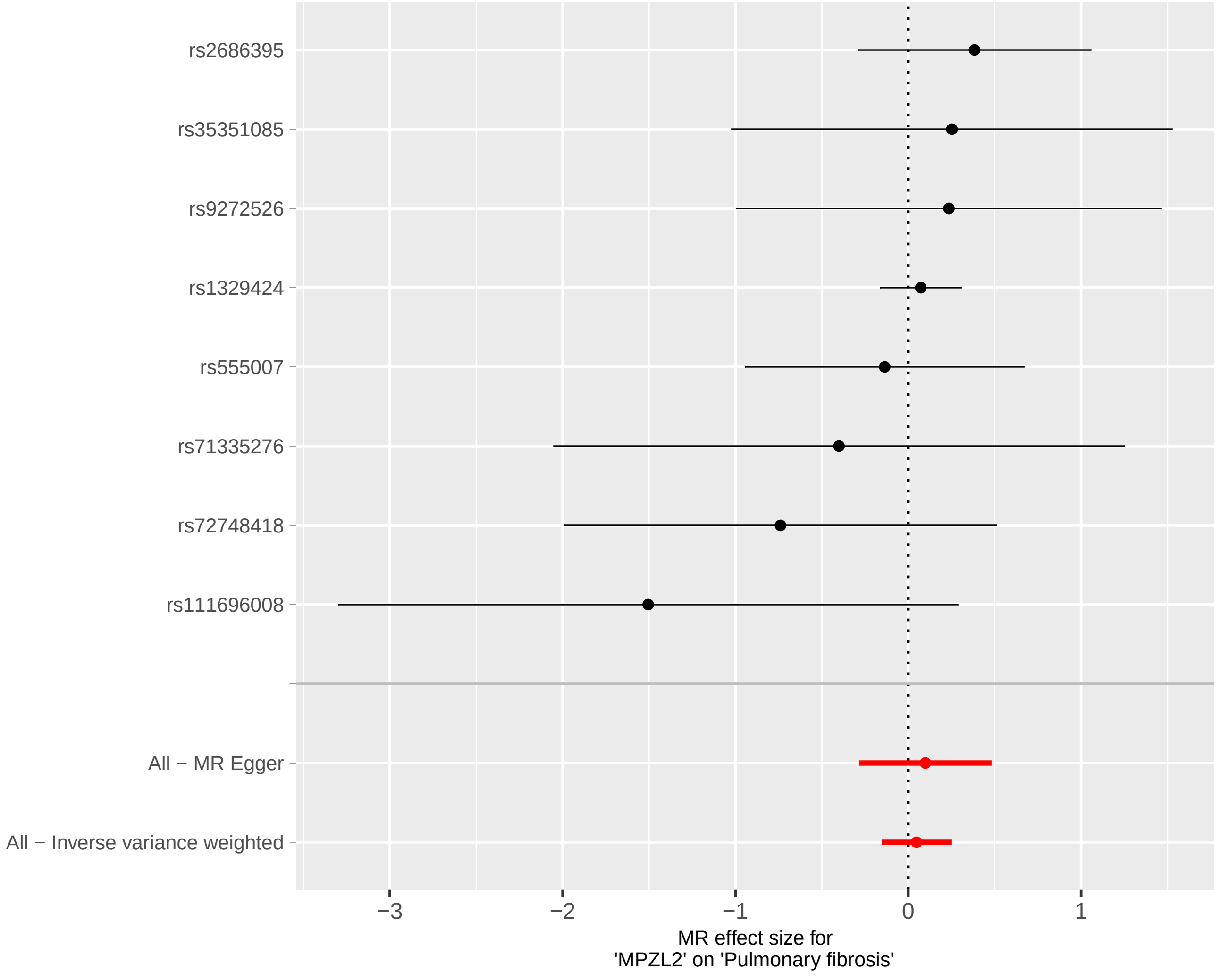

rs2686395
rs35351085
rs9272526
rs1329424
rs555007
rs71335276
rs72748418
rs111696008
All − MR Egger
All − Inverse variance weighted
−3
−2
−1
0
1
MR effect size for
'MPZL2' on 'Pulmonary fibrosis'

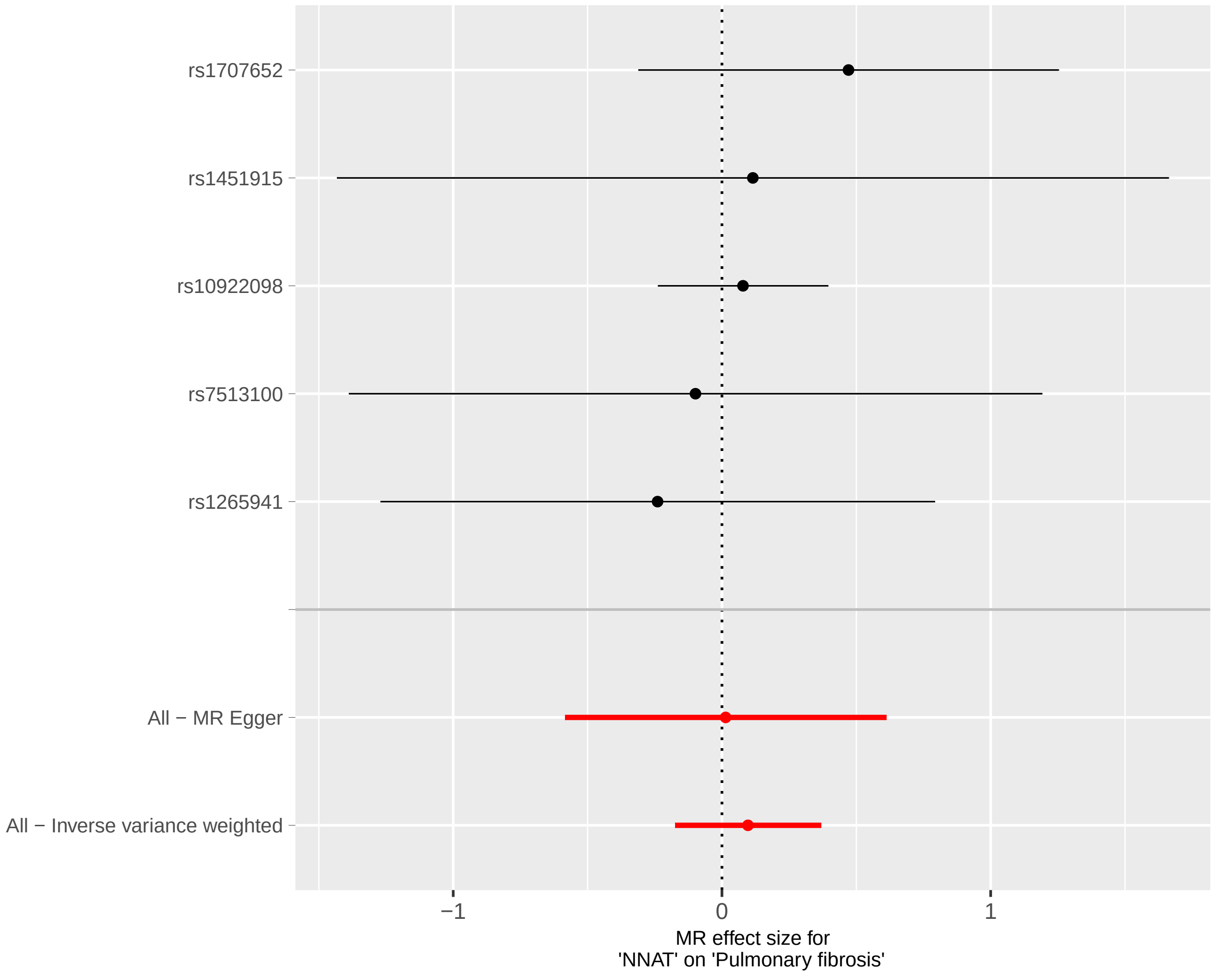

rs1707652
rs1451915
rs10922098
rs7513100
rs1265941
All − MR Egger
All − Inverse variance weighted
−1
0
1
MR effect size for
'NNAT' on 'Pulmonary fibrosis'

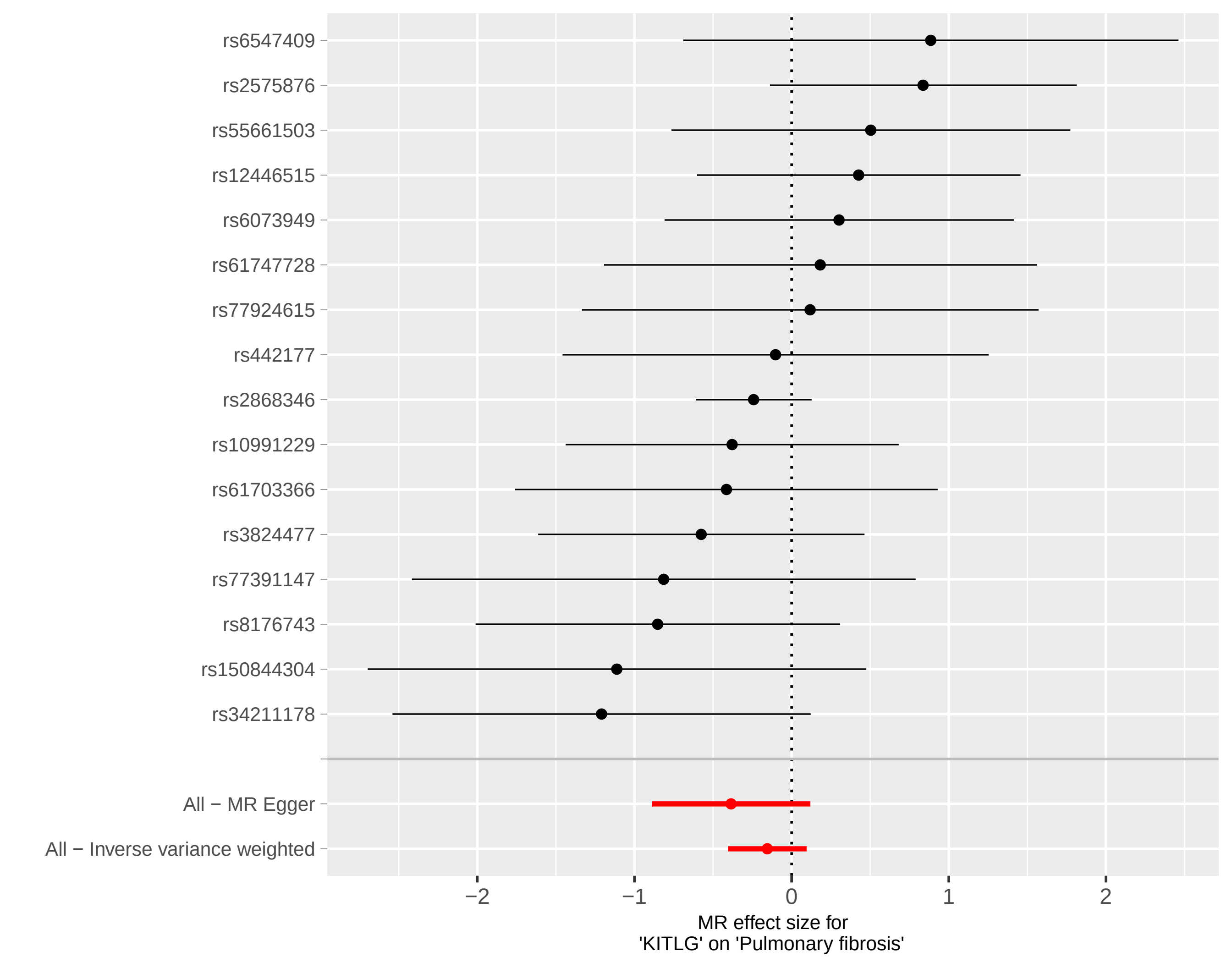
rs6547409
rs2575876
rs55661503
rs12446515
rs6073949
rs61747728
rs77924615
rs442177
rs2868346
rs10991229
rs61703366
rs3824477
rs77391147
rs8176743
rs150844304
rs34211178
All − MR Egger
All − Inverse variance weighted
−2
−1
0
1
2
MR effect size for
'KITLG' on 'Pulmonary fibrosis'

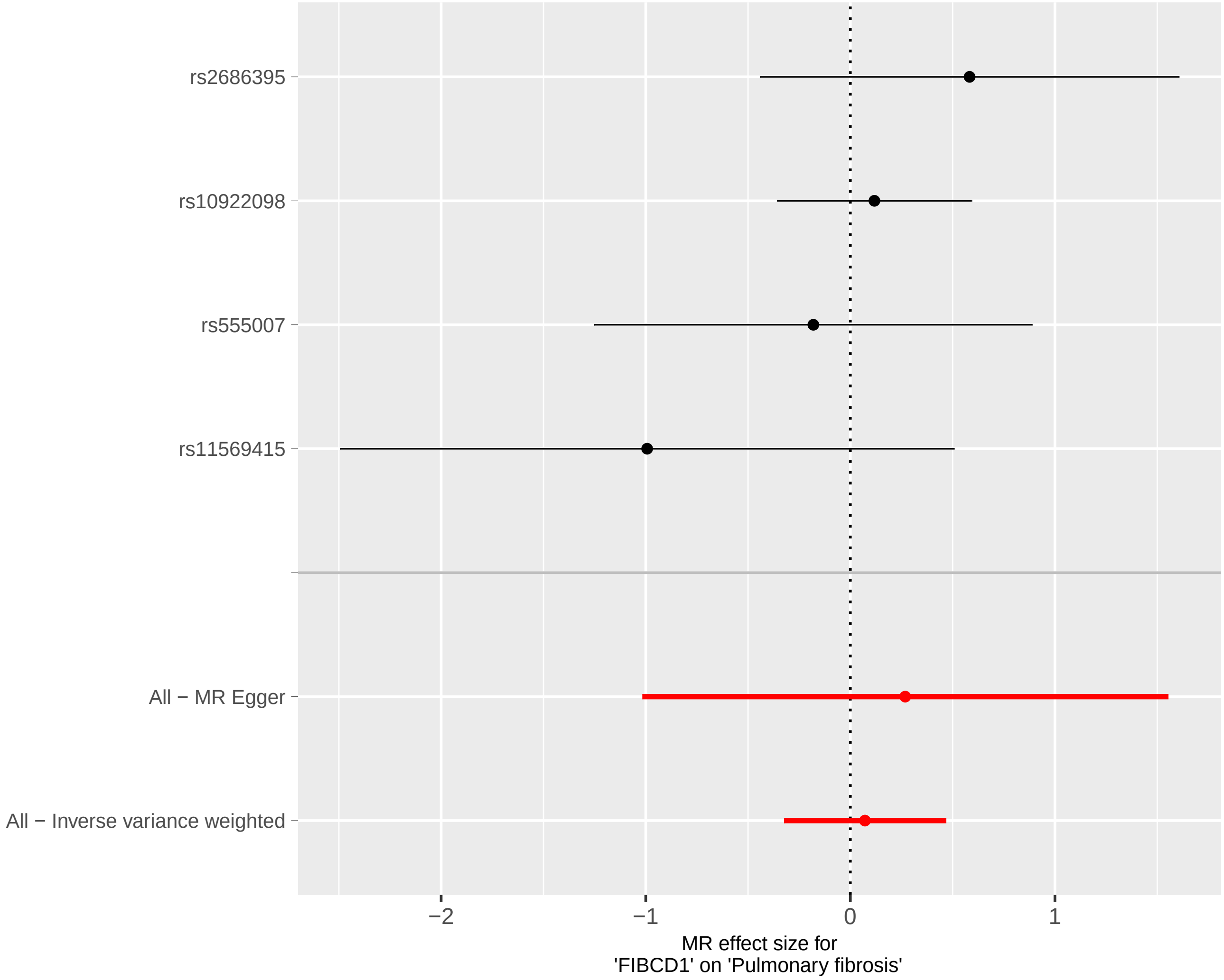
rs2686395
rs10922098
rs555007
rs11569415
All − MR Egger
All − Inverse variance weighted
−2
−1
0
1
MR effect size for
'FIBCD1' on 'Pulmonary fibrosis'

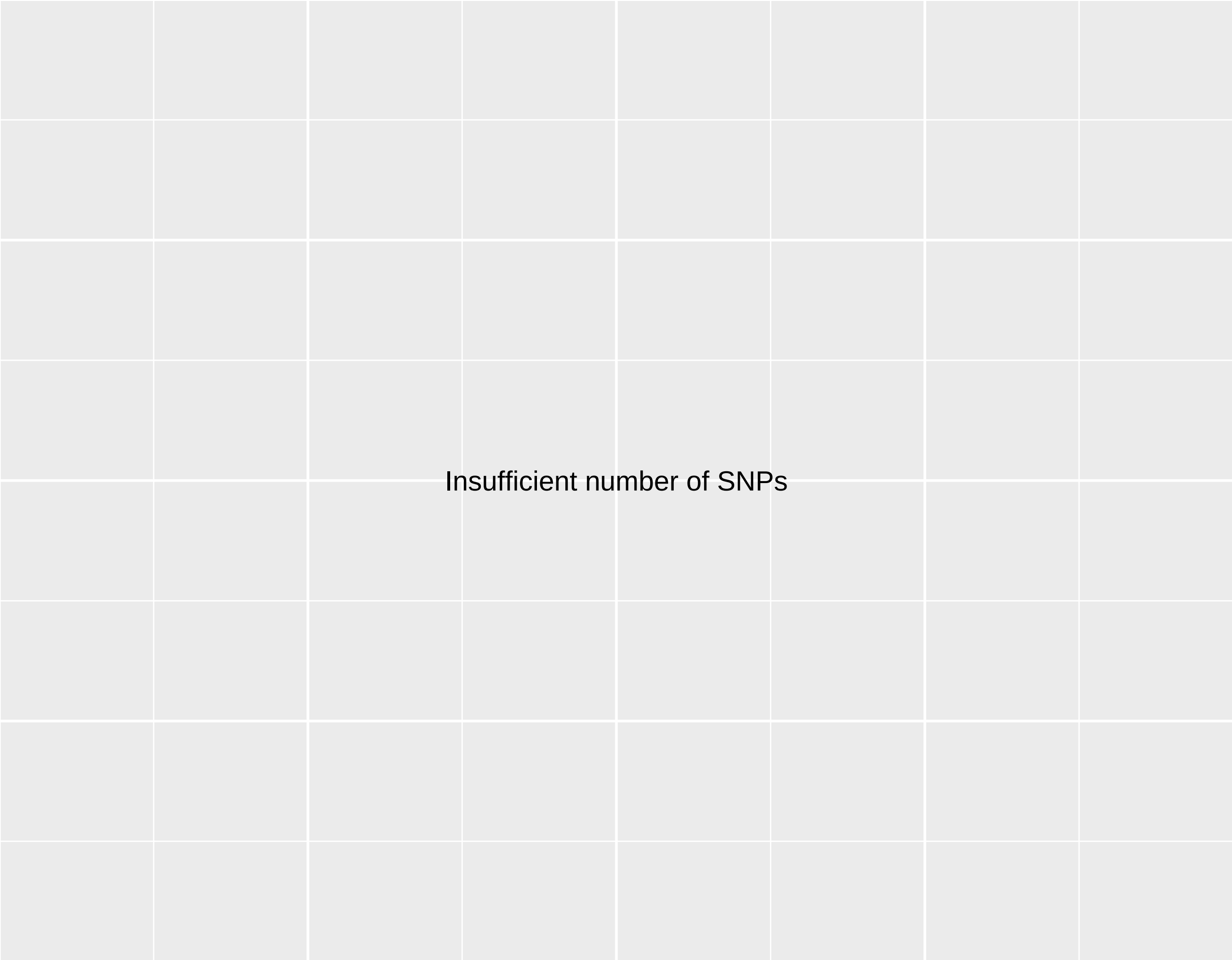
Insufficient number of SNPs

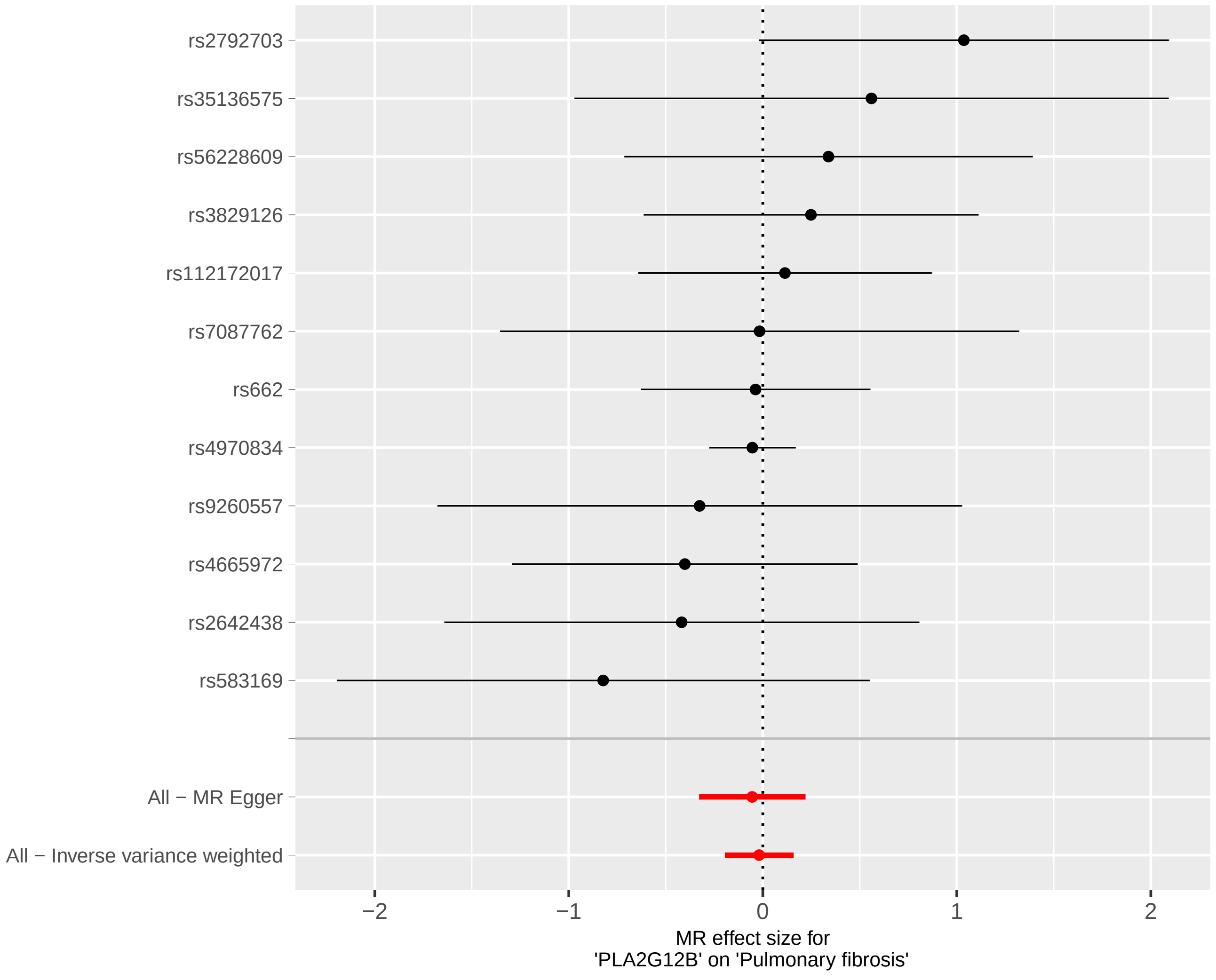

rs2792703
rs35136575
rs56228609
rs3829126
rs112172017
rs7087762
rs662
rs4970834
rs9260557
rs4665972
rs2642438
rs583169
All − MR Egger
All − Inverse variance weighted
−2
−1
0
1
2
MR effect size for
'PLA2G12B' on 'Pulmonary fibrosis'

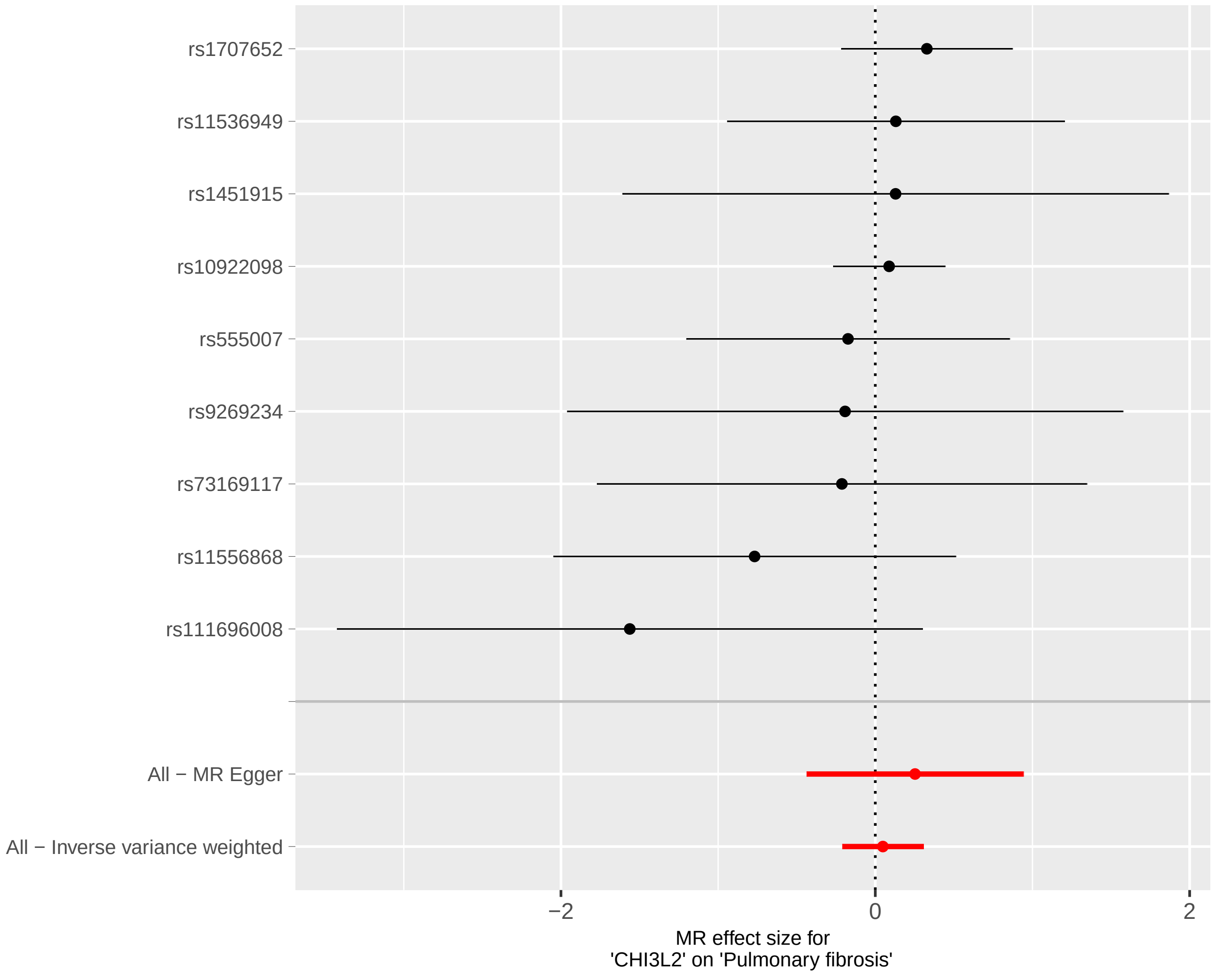
rs1707652
rs11536949
rs1451915
rs10922098
rs555007
rs9269234
rs73169117
rs11556868
rs111696008
All − MR Egger
All − Inverse variance weighted
−2
0
2
MR effect size for
'CHI3L2' on 'Pulmonary fibrosis'

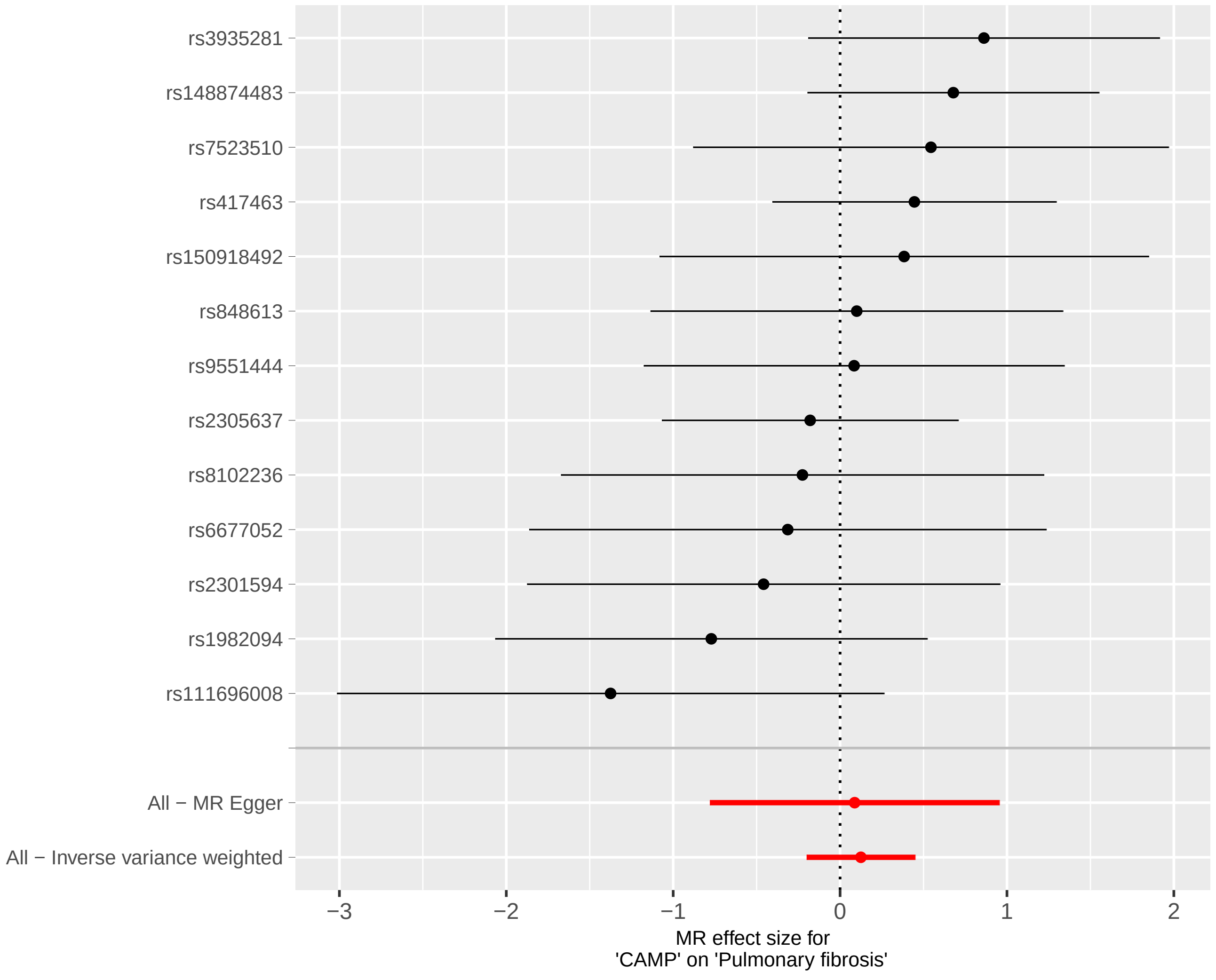
rs3935281
rs148874483
rs7523510
rs417463
rs150918492
rs848613
rs9551444
rs2305637
rs8102236
rs6677052
rs2301594
rs1982094
rs111696008
All − MR Egger
All − Inverse variance weighted
−3
−2
−1
0
1
2
MR effect size for
'CAMP' on 'Pulmonary fibrosis'

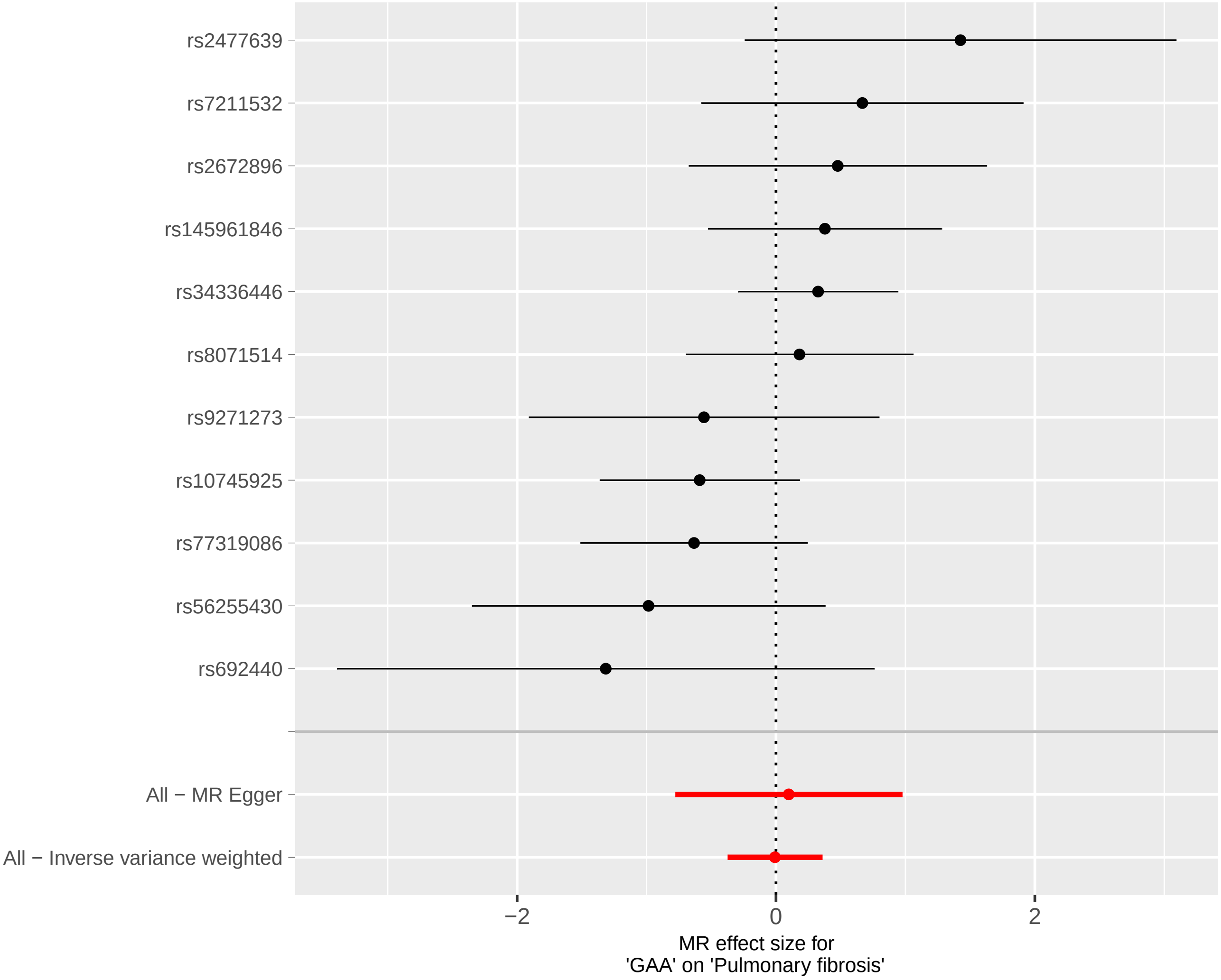
rs2477639
rs7211532
rs2672896
rs145961846
rs34336446
rs8071514
rs9271273
rs10745925
rs77319086
rs56255430
rs692440
All − MR Egger
All − Inverse variance weighted
−2
0
2
MR effect size for
'GAA' on 'Pulmonary fibrosis'

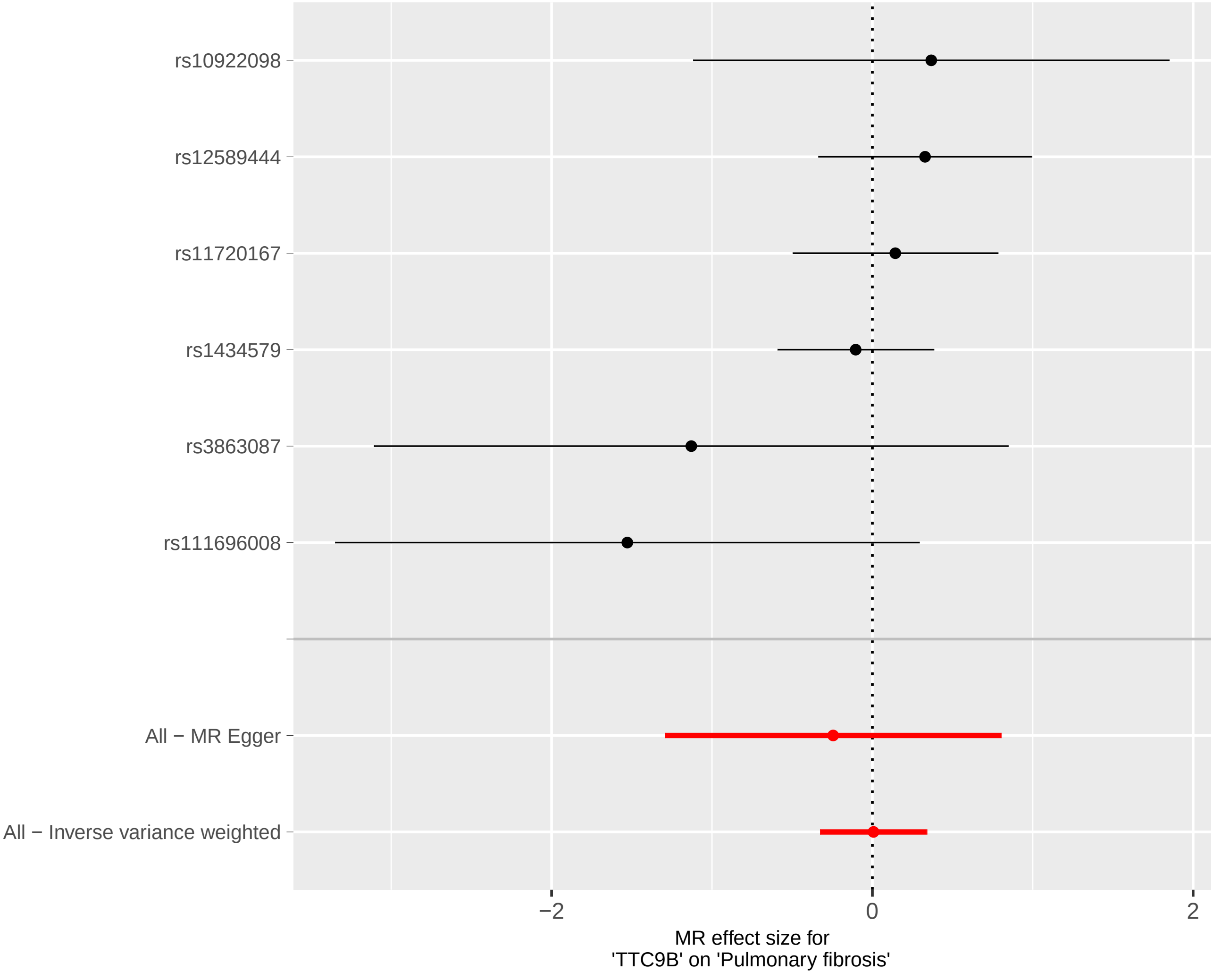

rs10922098
rs12589444
rs11720167
rs1434579
rs3863087
rs111696008
All − MR Egger
All − Inverse variance weighted
−2
0
2
MR effect size for
'TTC9B' on 'Pulmonary fibrosis'

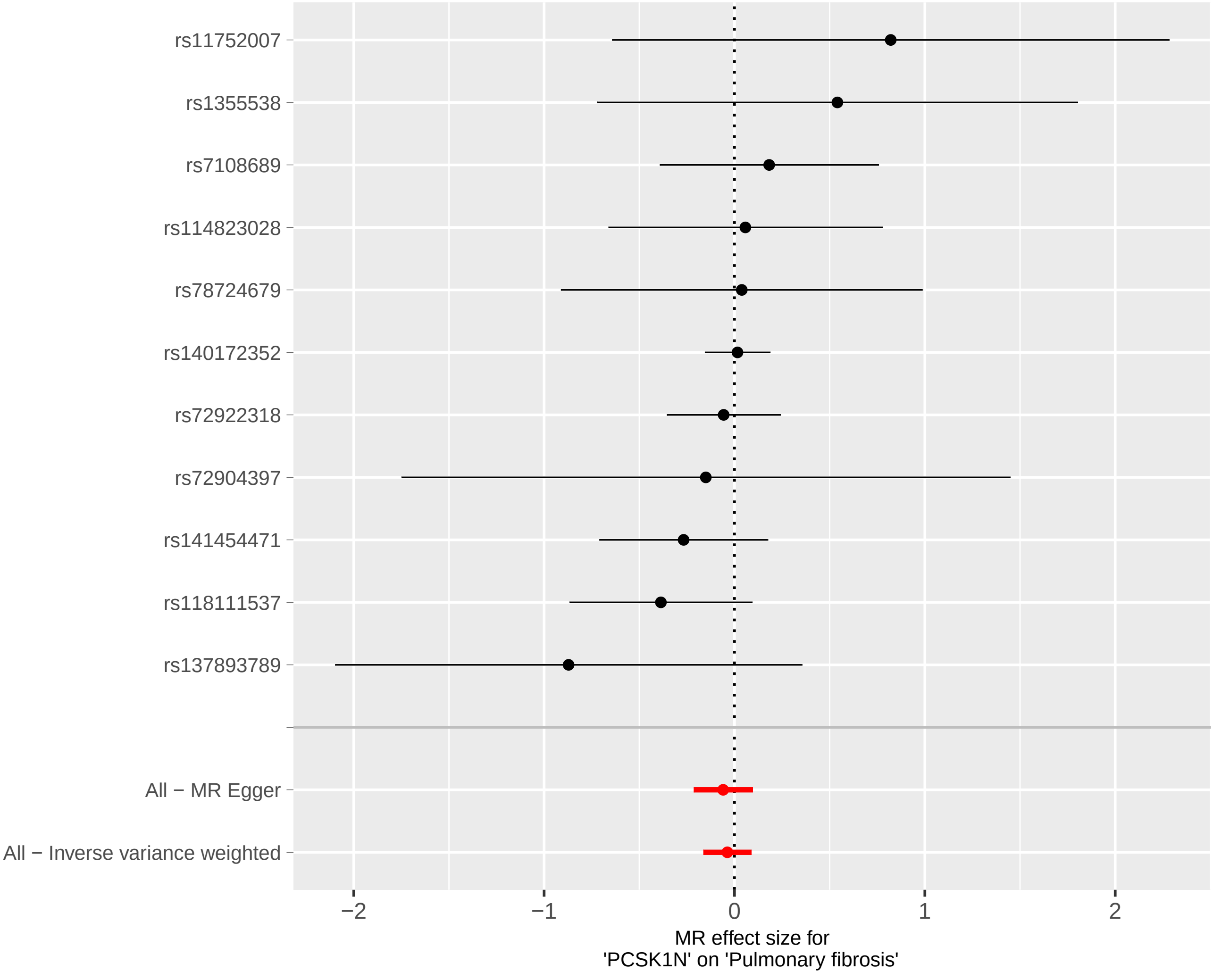

rs11752007
rs1355538
rs7108689
rs114823028
rs78724679
rs140172352
rs72922318
rs72904397
rs141454471
rs118111537
rs137893789
All − MR Egger
All − Inverse variance weighted
−2
−1
0
1
2
MR effect size for
'PCSK1N' on 'Pulmonary fibrosis'

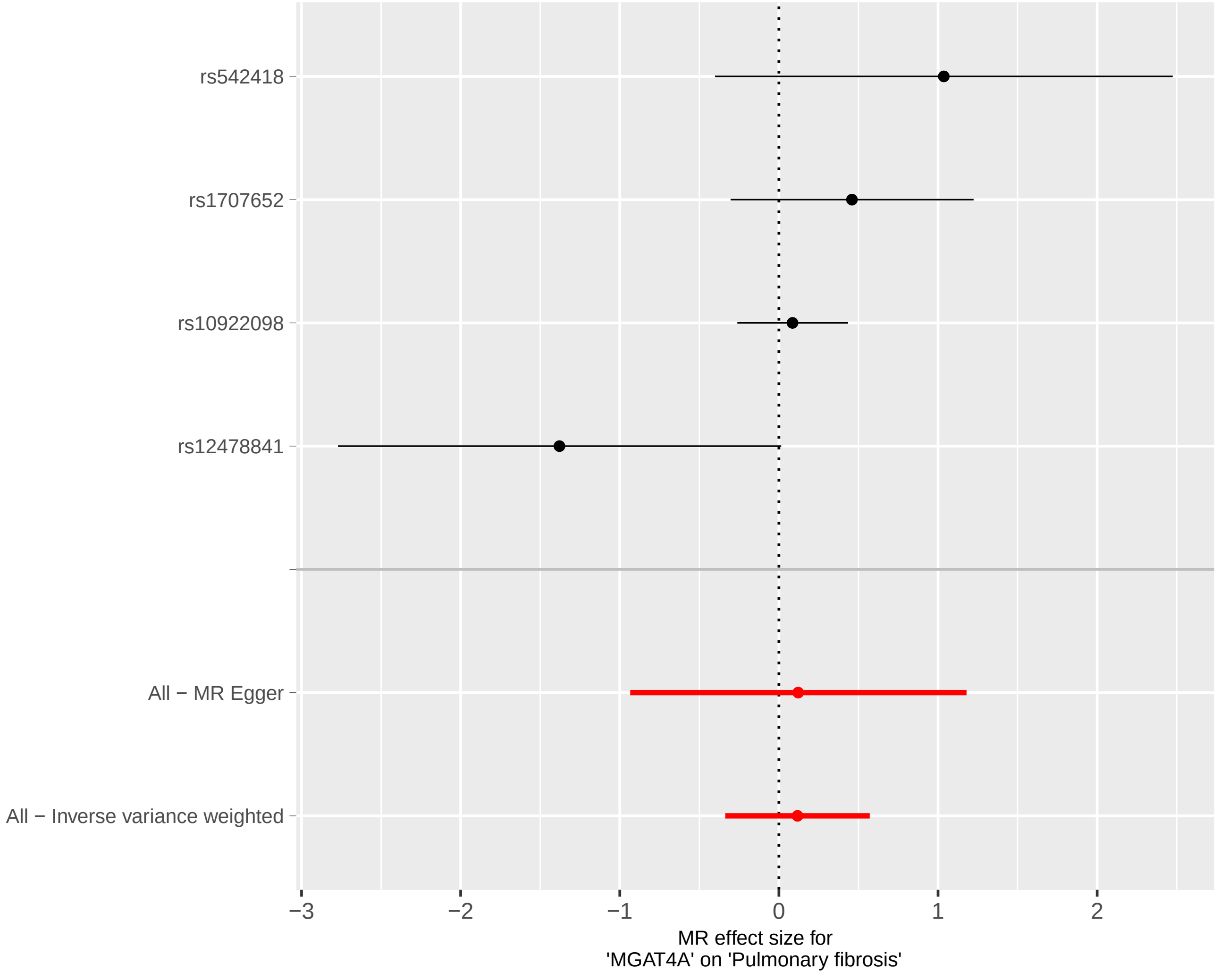

rs542418
rs1707652
rs10922098
rs12478841
All − MR Egger
All − Inverse variance weighted
−3
−2
−1
0
1
2
MR effect size for
'MGAT4A' on 'Pulmonary fibrosis'

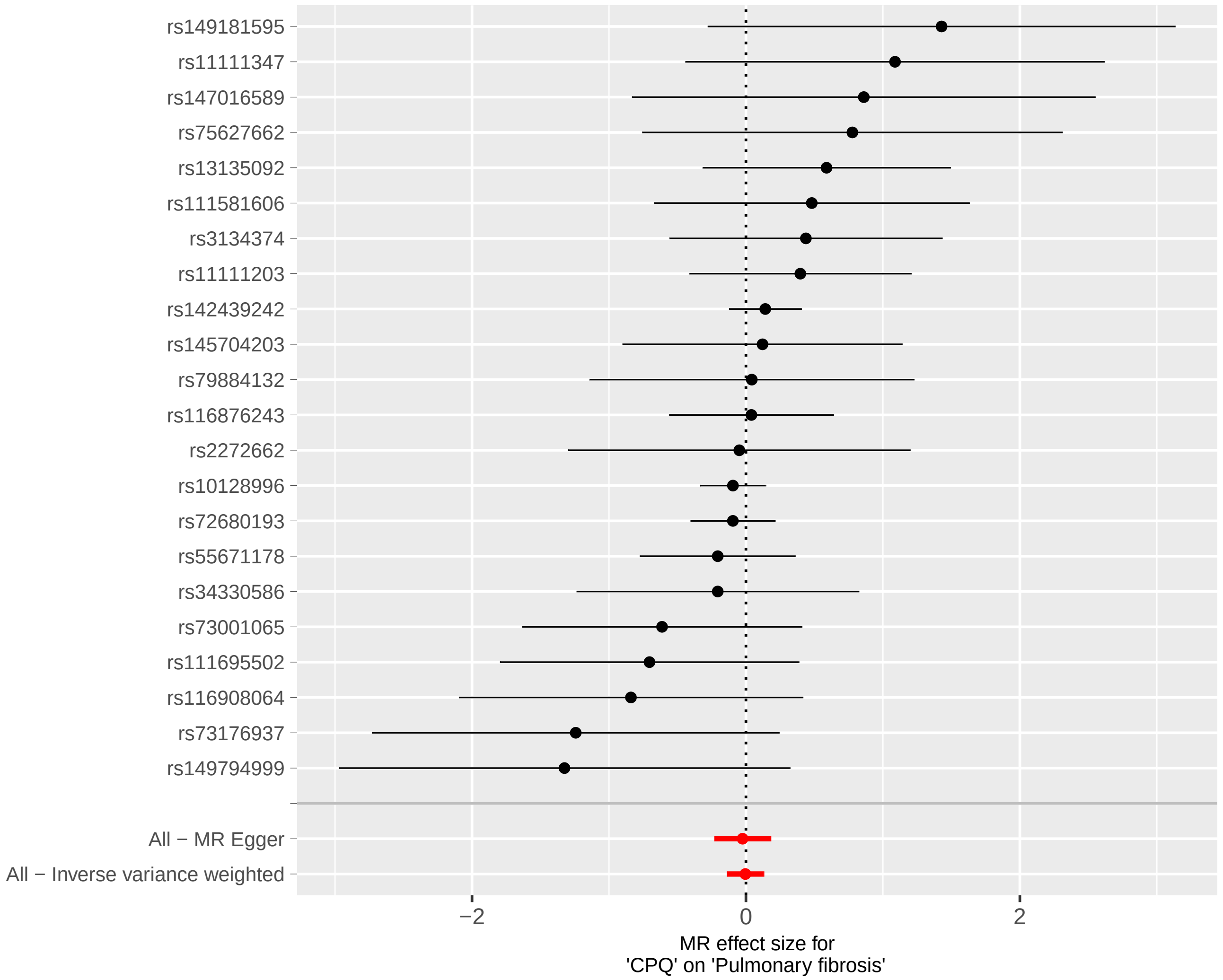

rs149181595
rs11111347
rs147016589
rs75627662
rs13135092
rs111581606
rs3134374
rs11111203
rs142439242
rs145704203
rs79884132
rs116876243
rs2272662
rs10128996
rs72680193
rs55671178
rs34330586
rs73001065
rs111695502
rs116908064
rs73176937
rs149794999
All − MR Egger
All − Inverse variance weighted
−2
0
2
MR effect size for
'CPQ' on 'Pulmonary fibrosis'

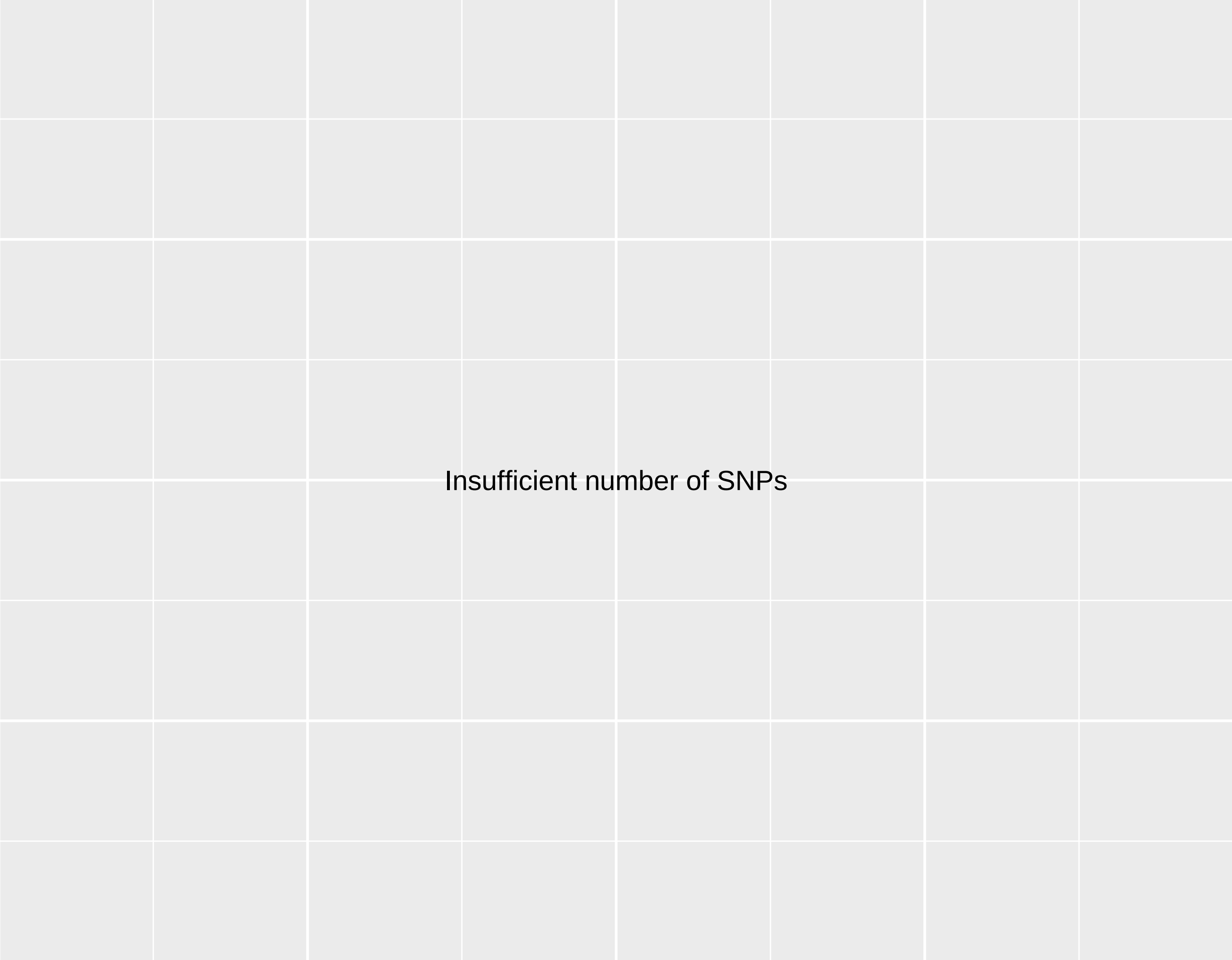
Insufficient number of SNPs

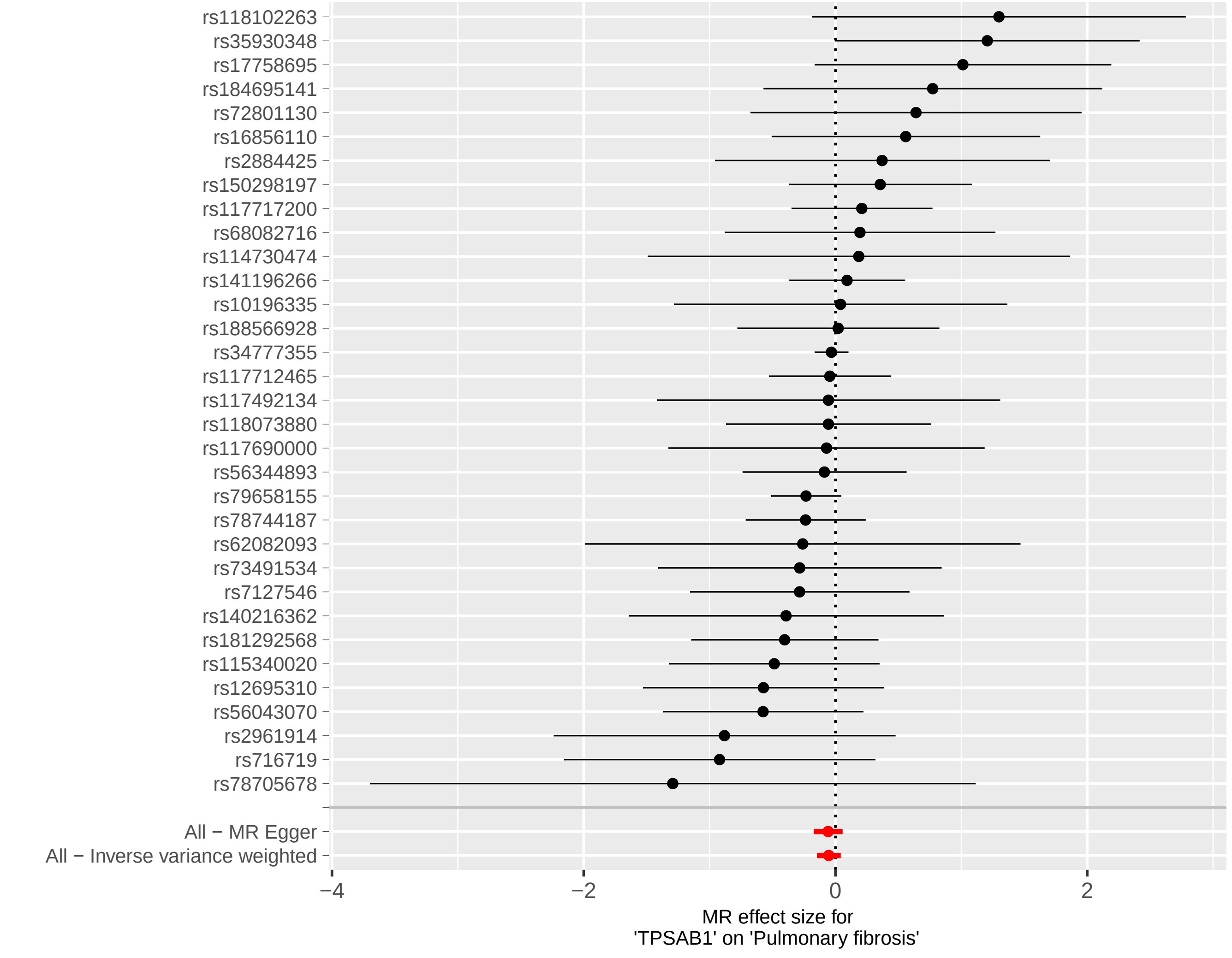
rs118102263
rs35930348
rs17758695
rs184695141
rs72801130
rs16856110
rs2884425
rs150298197
rs117717200
rs68082716
rs114730474
rs141196266
rs10196335
rs188566928
rs34777355
rs117712465
rs117492134
rs118073880
rs117690000
rs56344893
rs79658155
rs78744187
rs62082093
rs73491534
rs7127546
rs140216362
rs181292568
rs115340020
rs12695310
rs56043070
rs2961914
rs716719
rs78705678
All − MR Egger
All − Inverse variance weighted
−4
−2
0
2
MR effect size for
'TPSAB1' on 'Pulmonary fibrosis'

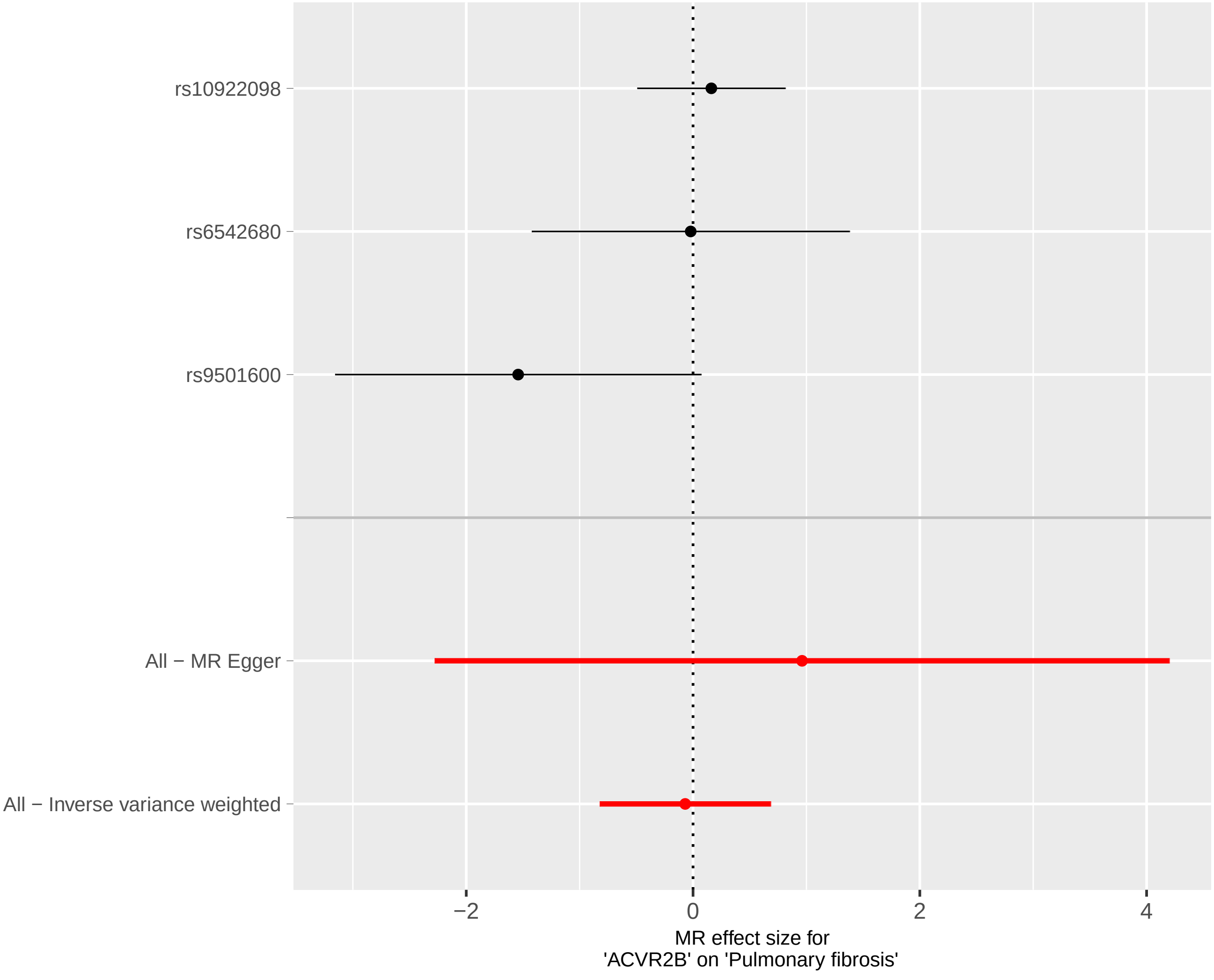
rs10922098
rs6542680
rs9501600
All − MR Egger
All − Inverse variance weighted
−2
0
2
4
MR effect size for
'ACVR2B' on 'Pulmonary fibrosis'

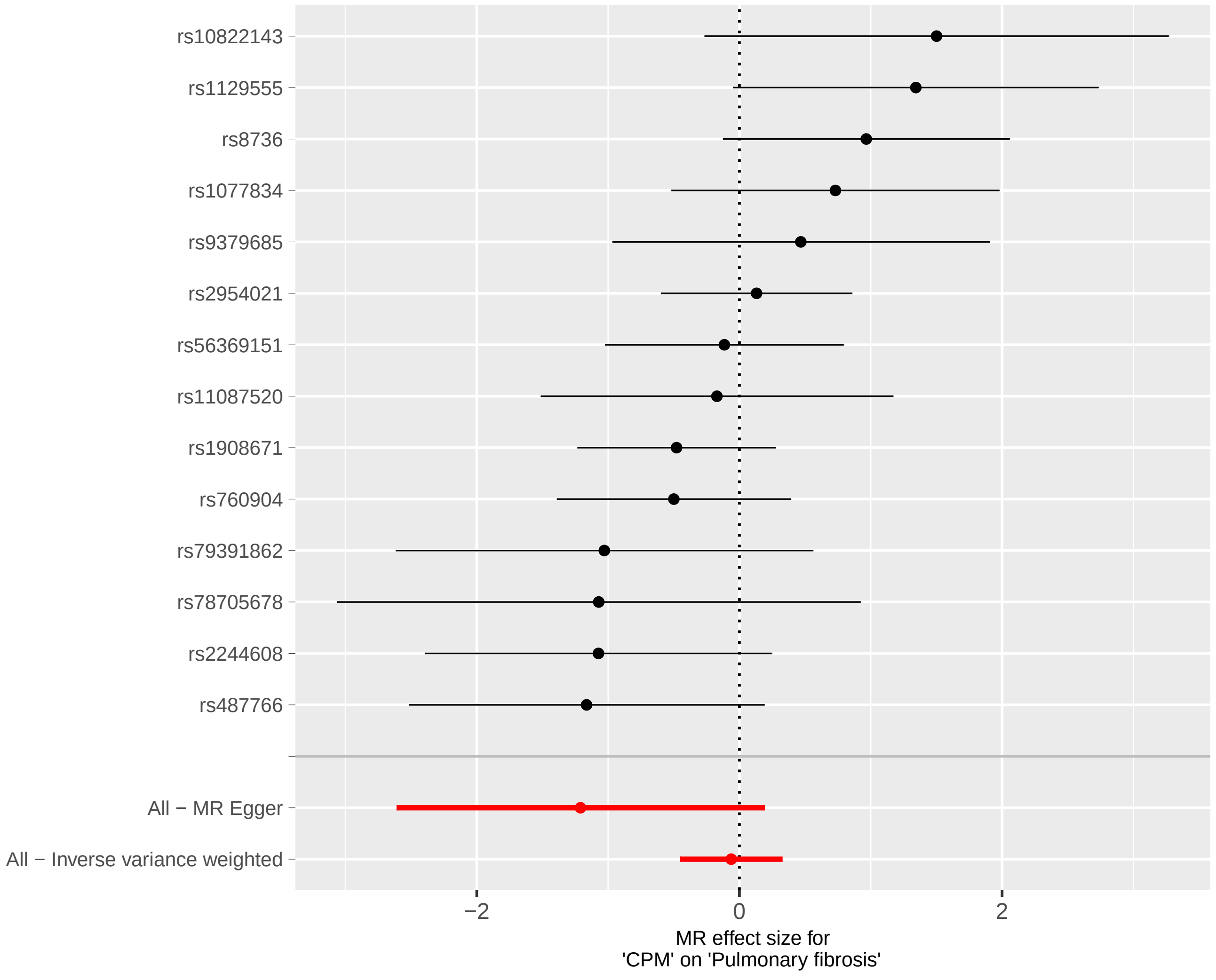
rs10822143
rs1129555
rs8736
rs1077834
rs9379685
rs2954021
rs56369151
rs11087520
rs1908671
rs760904
rs79391862
rs78705678
rs2244608
rs487766
All − MR Egger
All − Inverse variance weighted
−2
0
2
MR effect size for
'CPM' on 'Pulmonary fibrosis'

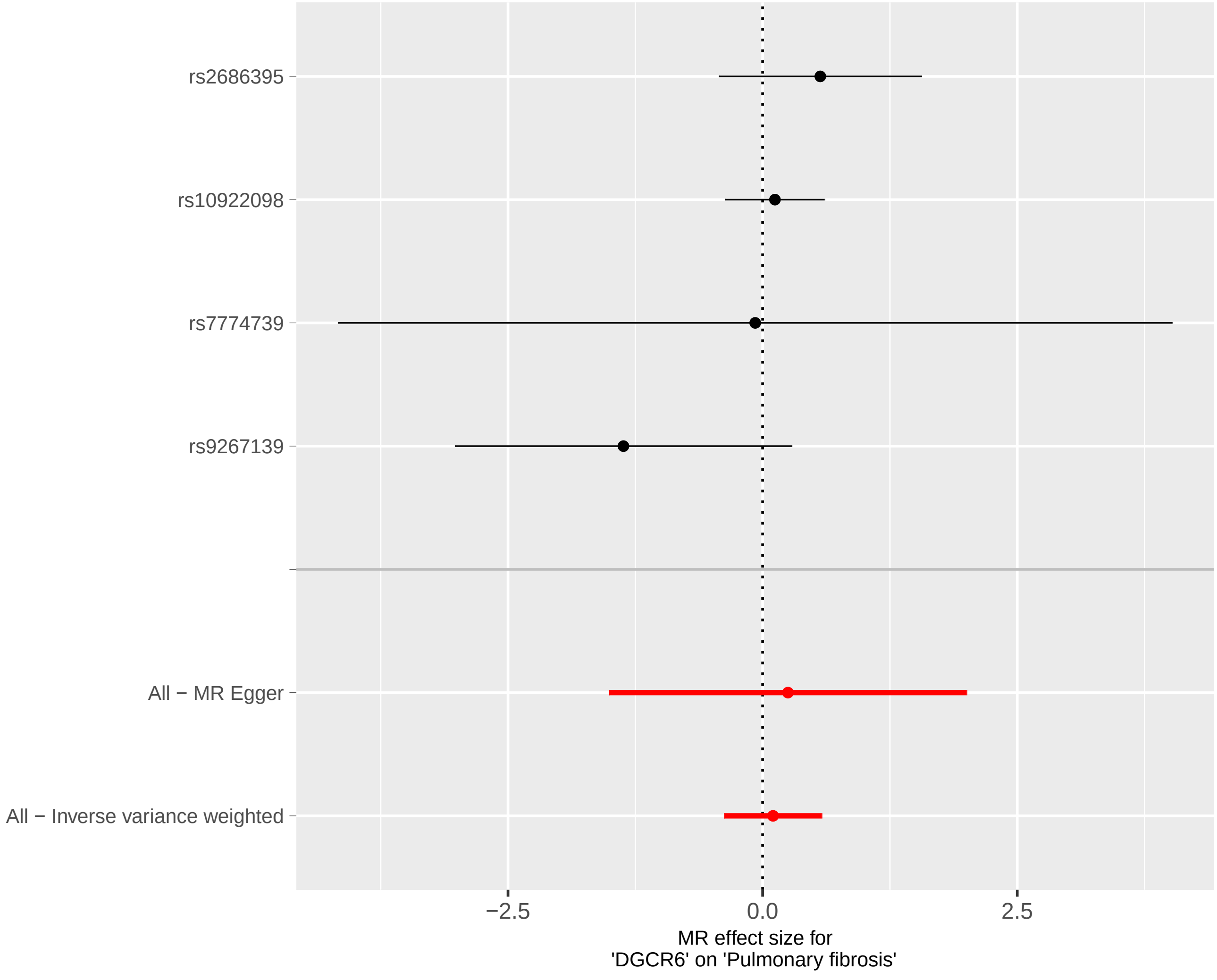
rs2686395
rs10922098
rs7774739
rs9267139
All − MR Egger
All − Inverse variance weighted
−2.5
0.0
2.5
MR effect size for
'DGCR6' on 'Pulmonary fibrosis'

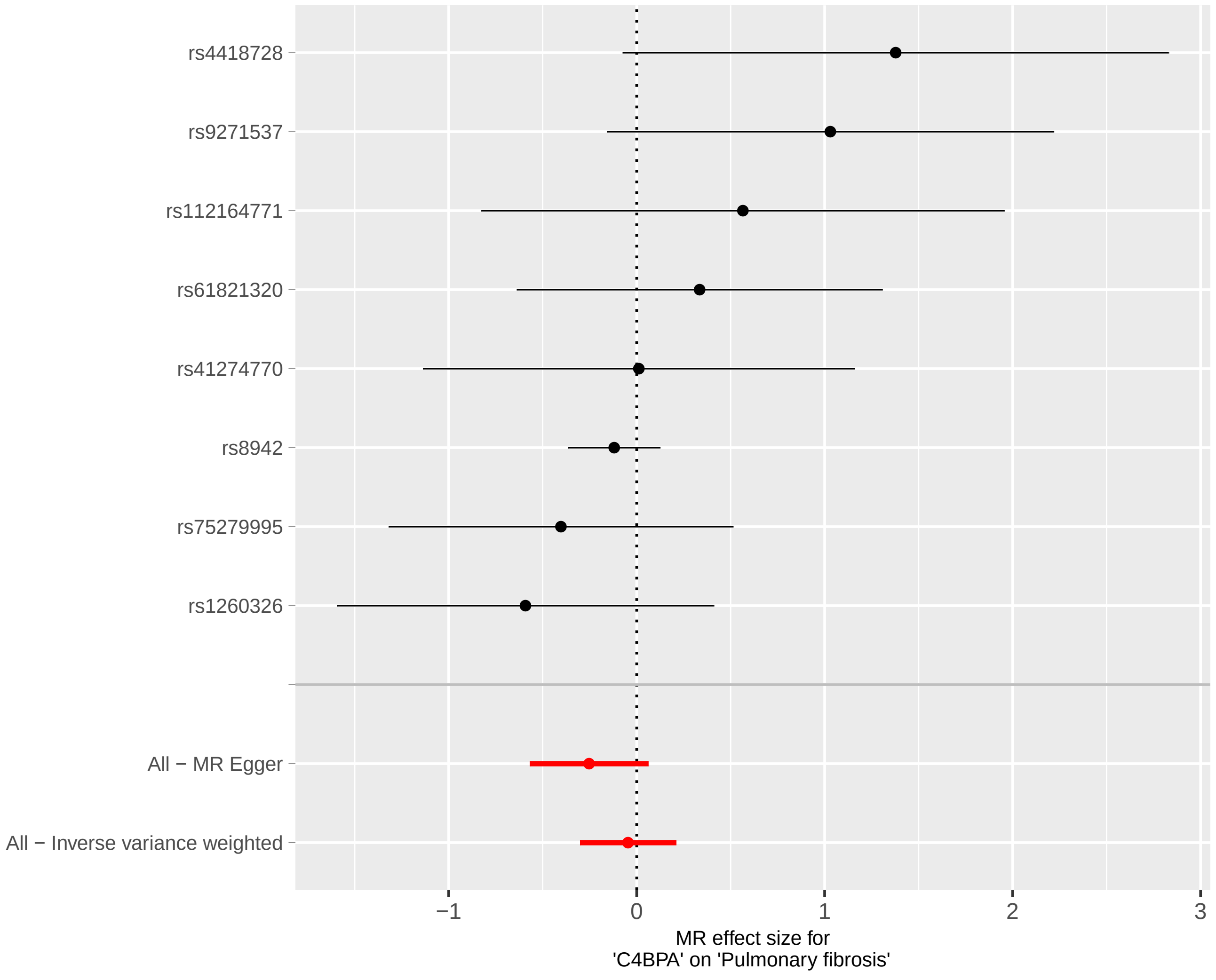

rs4418728
rs9271537
rs112164771
rs61821320
rs41274770
rs8942
rs75279995
rs1260326
All − MR Egger
All − Inverse variance weighted
−1
0
1
2
3
MR effect size for
'C4BPA' on 'Pulmonary fibrosis'

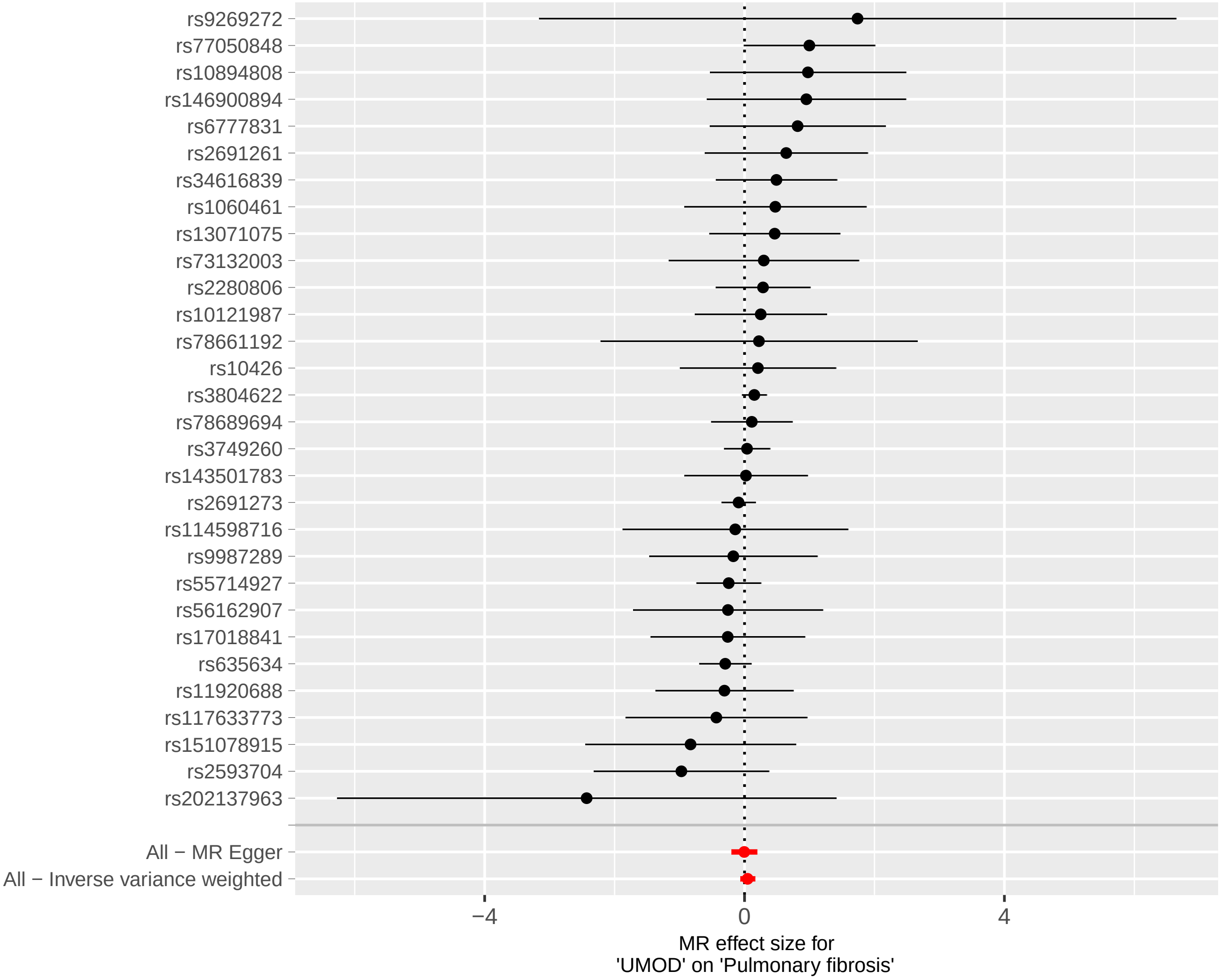
rs9269272
rs77050848
rs10894808
rs146900894
rs6777831
rs2691261
rs34616839
rs1060461
rs13071075
rs73132003
rs2280806
rs10121987
rs78661192
rs10426
rs3804622
rs78689694
rs3749260
rs143501783
rs2691273
rs114598716
rs9987289
rs55714927
rs56162907
rs17018841
rs635634
rs11920688
rs117633773
rs151078915
rs2593704
rs202137963
All − MR Egger
All − Inverse variance weighted
−4
0
4
MR effect size for
'UMOD' on 'Pulmonary fibrosis'

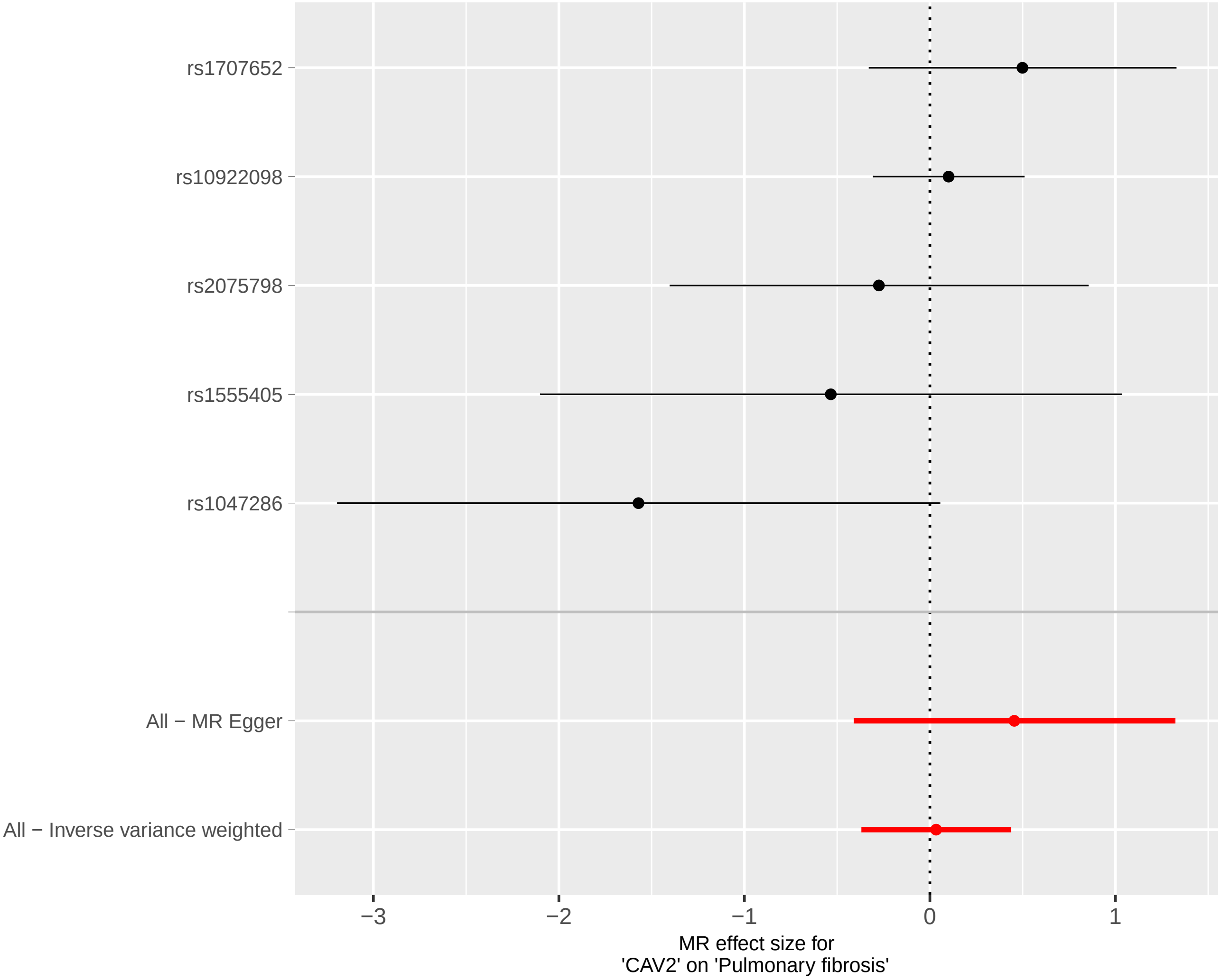
rs1707652
rs10922098
rs2075798
rs1555405
rs1047286
All − MR Egger
All − Inverse variance weighted
−3
−2
−1
0
1
MR effect size for
'CAV2' on 'Pulmonary fibrosis'

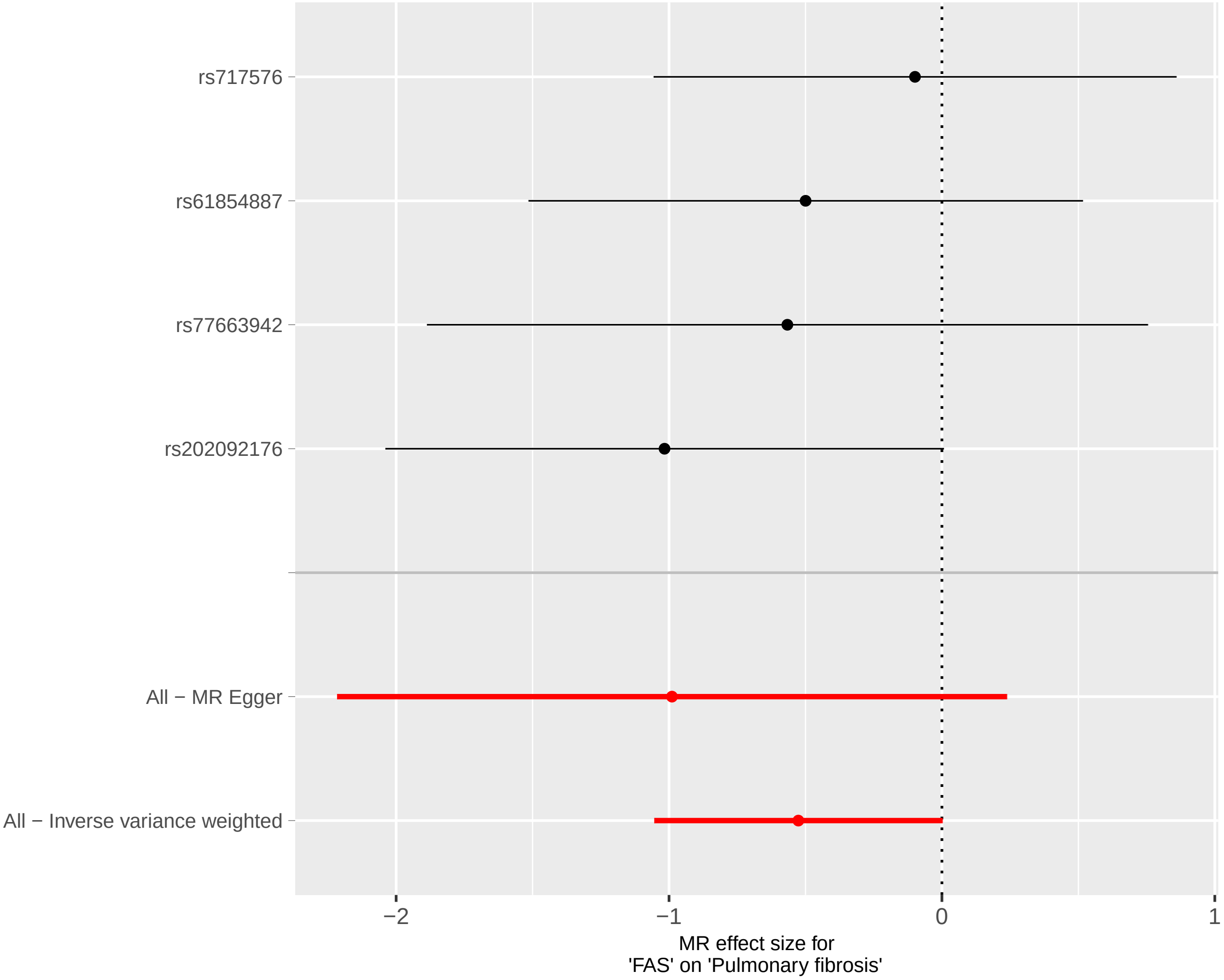

rs717576
rs61854887
rs77663942
rs202092176
All − MR Egger
All − Inverse variance weighted
−2
−1
0
1
MR effect size for
'FAS' on 'Pulmonary fibrosis'

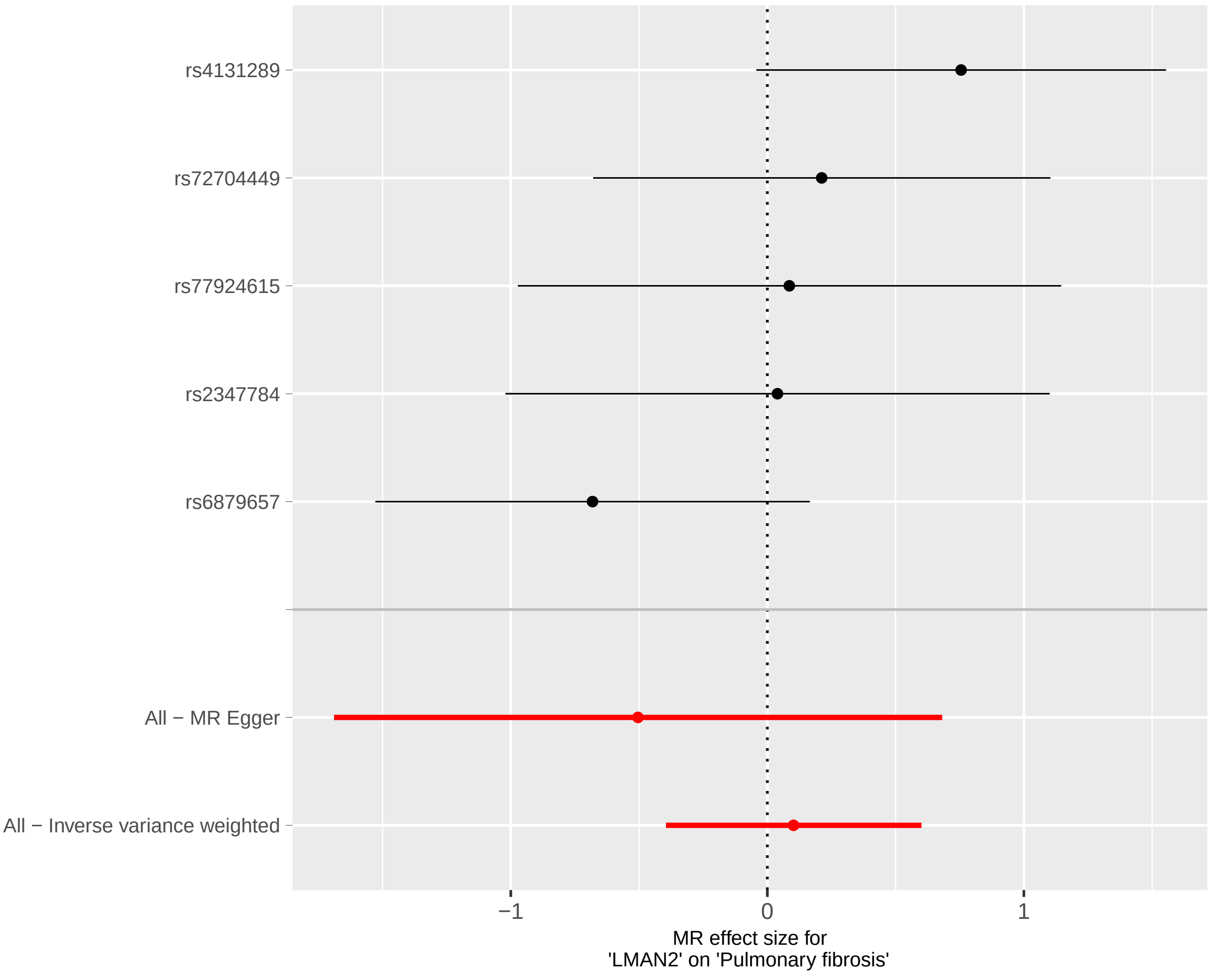

rs4131289
rs72704449
rs77924615
rs2347784
rs6879657
All − MR Egger
All − Inverse variance weighted
−1
0
1
MR effect size for
'LMAN2' on 'Pulmonary fibrosis'

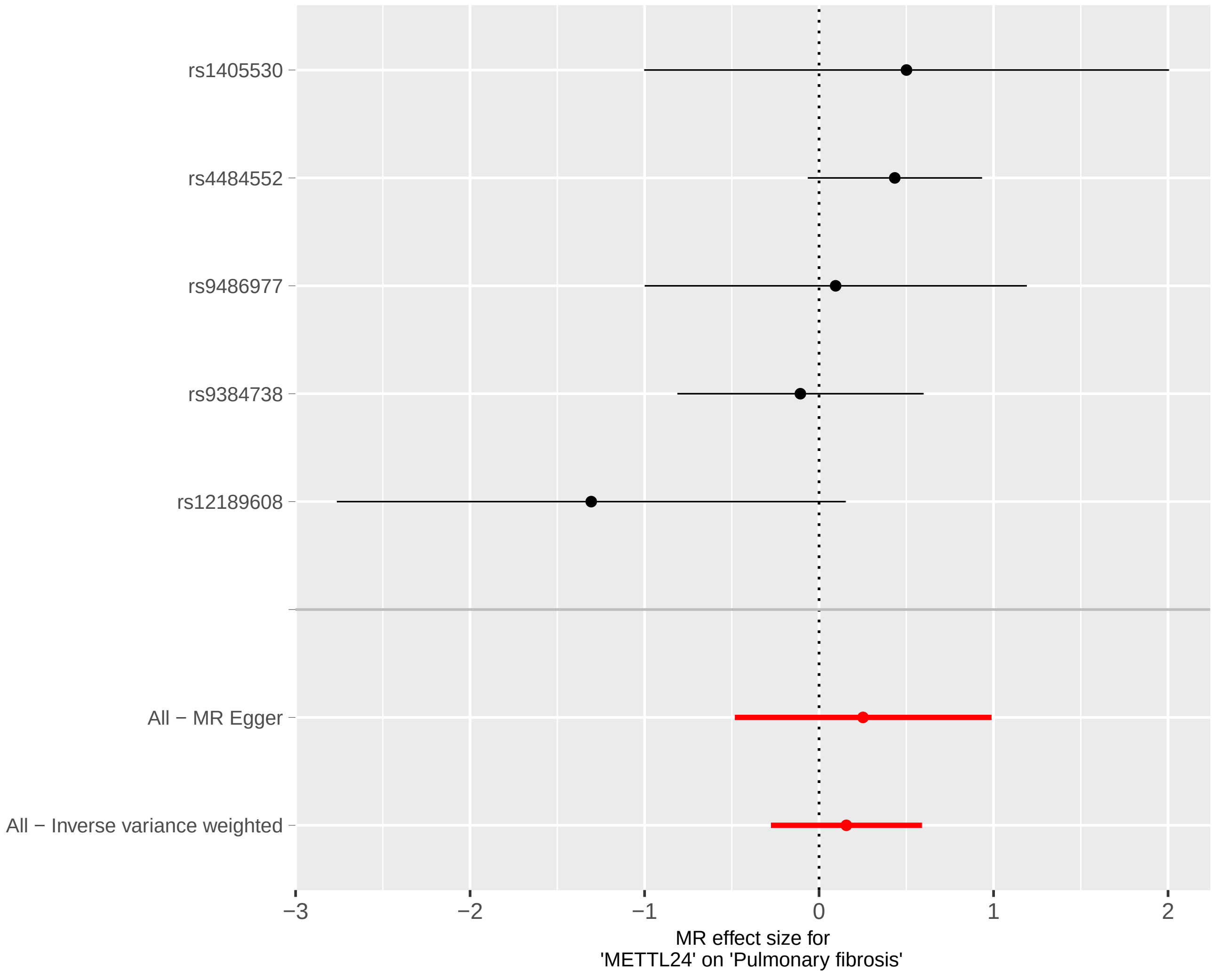
rs1405530
rs4484552
rs9486977
rs9384738
rs12189608
All − MR Egger
All − Inverse variance weighted
−3
−2
−1
0
1
2
MR effect size for
'METTL24' on 'Pulmonary fibrosis'

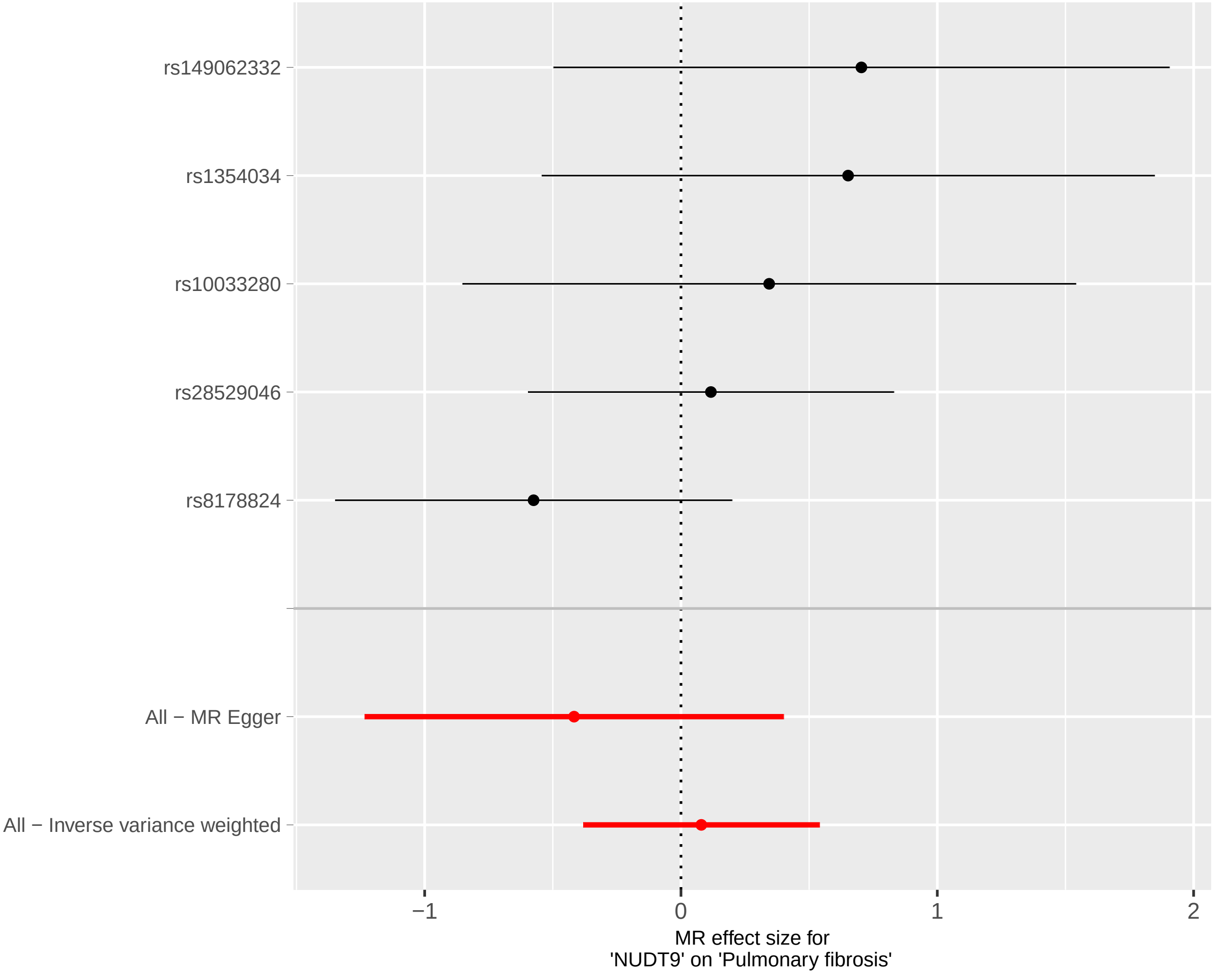
rs149062332
rs1354034
rs10033280
rs28529046
rs8178824
All − MR Egger
All − Inverse variance weighted
−1
0
1
2
MR effect size for
'NUDT9' on 'Pulmonary fibrosis'

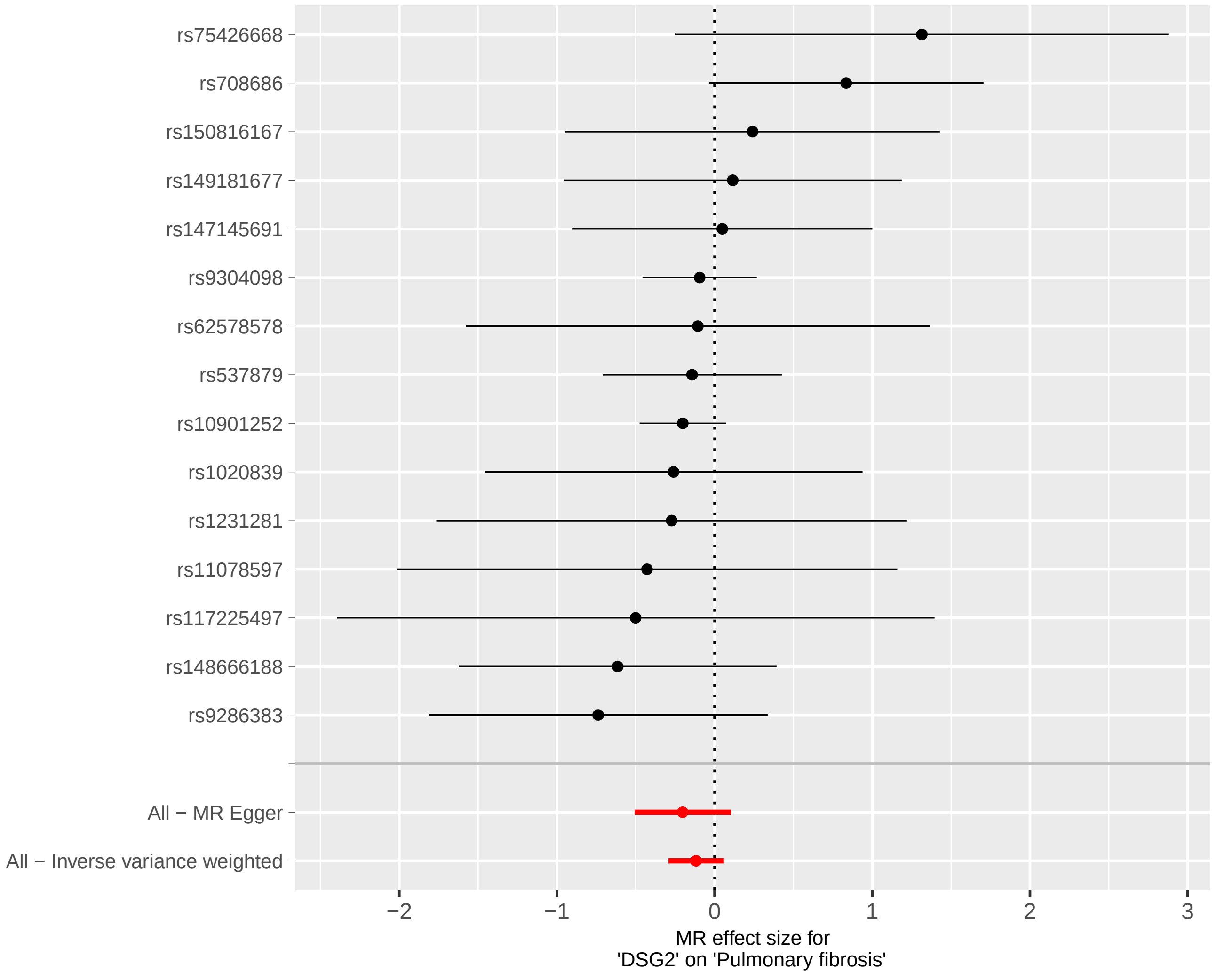
rs75426668
rs708686
rs150816167
rs149181677
rs147145691
rs9304098
rs62578578
rs537879
rs10901252
rs1020839
rs1231281
rs11078597
rs117225497
rs148666188
rs9286383
All − MR Egger
All − Inverse variance weighted
−2
−1
0
1
2
3
MR effect size for
'DSG2' on 'Pulmonary fibrosis'

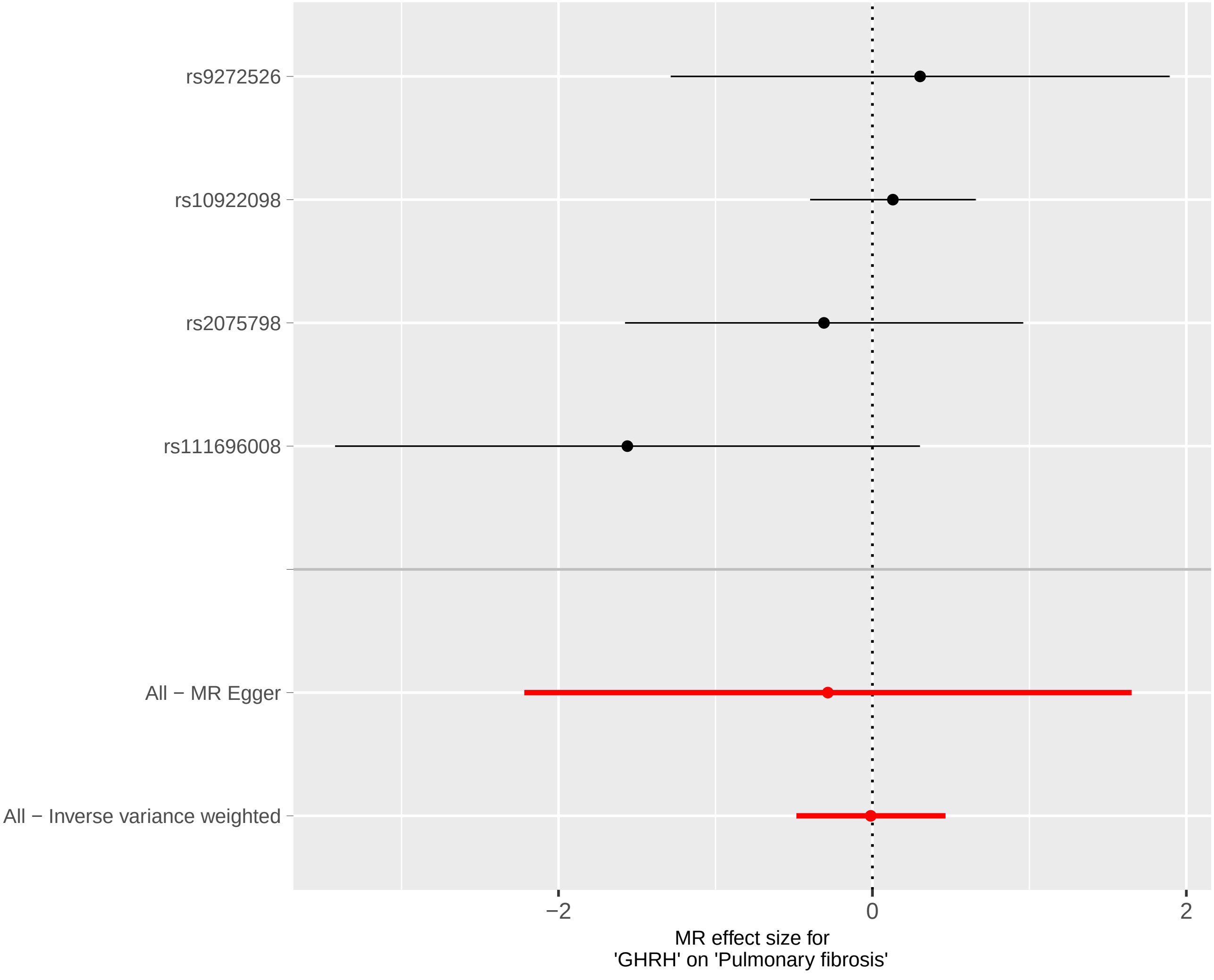
rs9272526
rs10922098
rs2075798
rs11696008
All − MR Egger
All − Inverse variance weighted
−2
0
2
MR effect size for
'GHRH' on 'Pulmonary fibrosis'

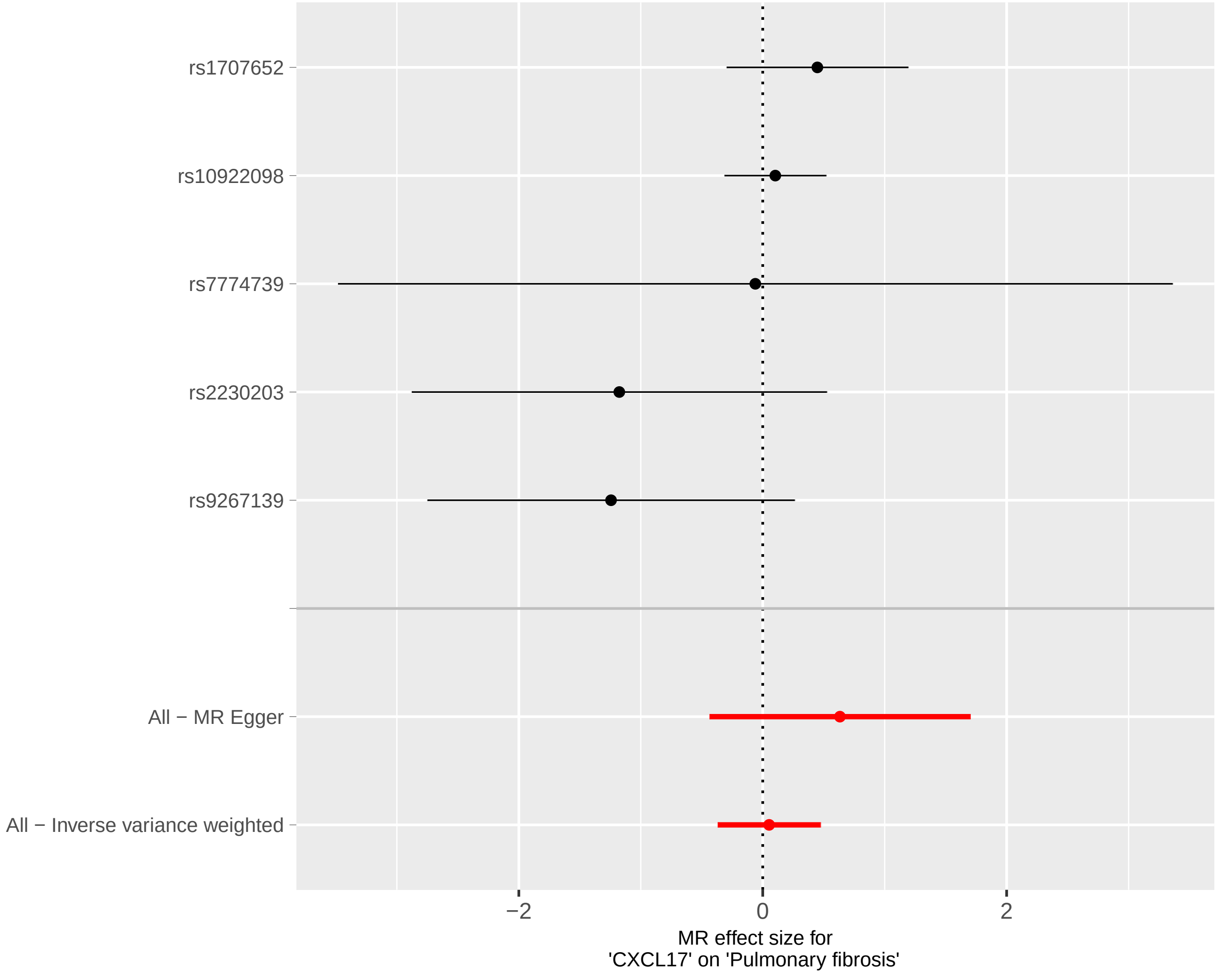
rs1707652
rs10922098
rs7774739
rs2230203
rs9267139
All − MR Egger
All − Inverse variance weighted
−2
0
2
MR effect size for
'CXCL17' on 'Pulmonary fibrosis'

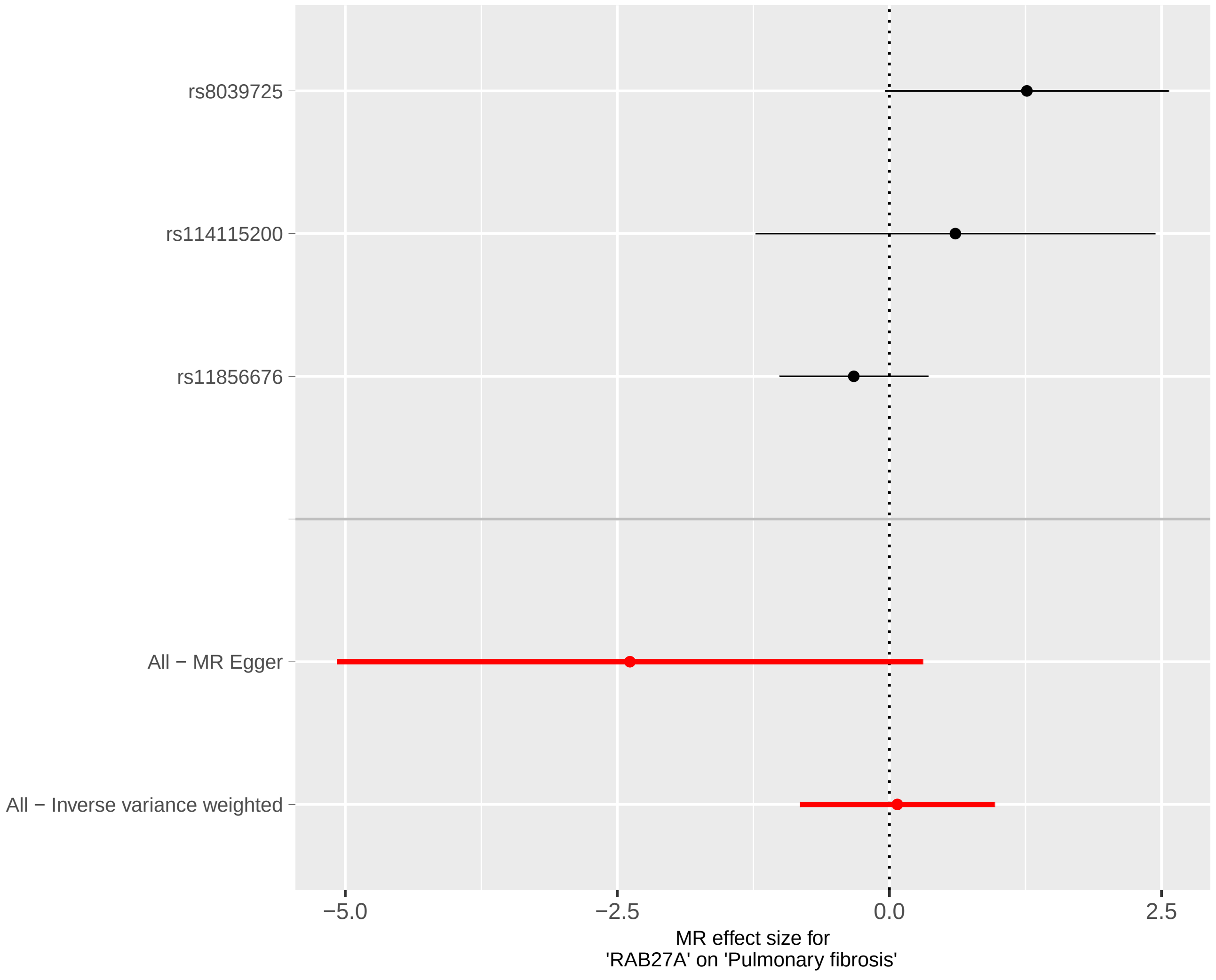

rs8039725
rs114115200
rs11856676
All − MR Egger
All − Inverse variance weighted
−5.0
−2.5
0.0
2.5
MR effect size for
'RAB27A' on 'Pulmonary fibrosis'

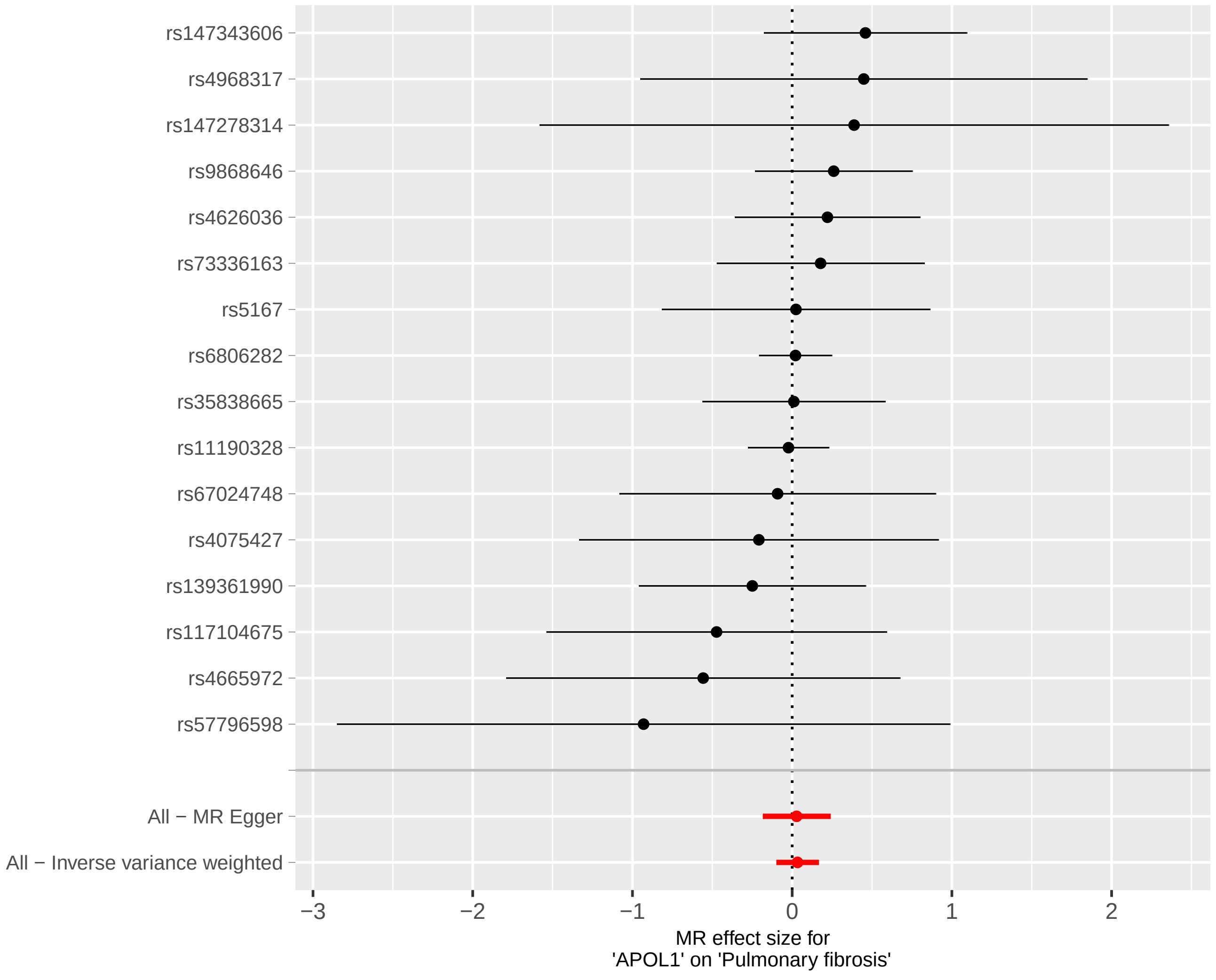
rs147343606
rs4968317
rs147278314
rs9868646
rs4626036
rs73336163
rs5167
rs6806282
rs35838665
rs11190328
rs67024748
rs4075427
rs139361990
rs117104675
rs4665972
rs57796598
All − MR Egger
All − Inverse variance weighted
−3
−2
−1
0
1
2
MR effect size for
'APOL1' on 'Pulmonary fibrosis'

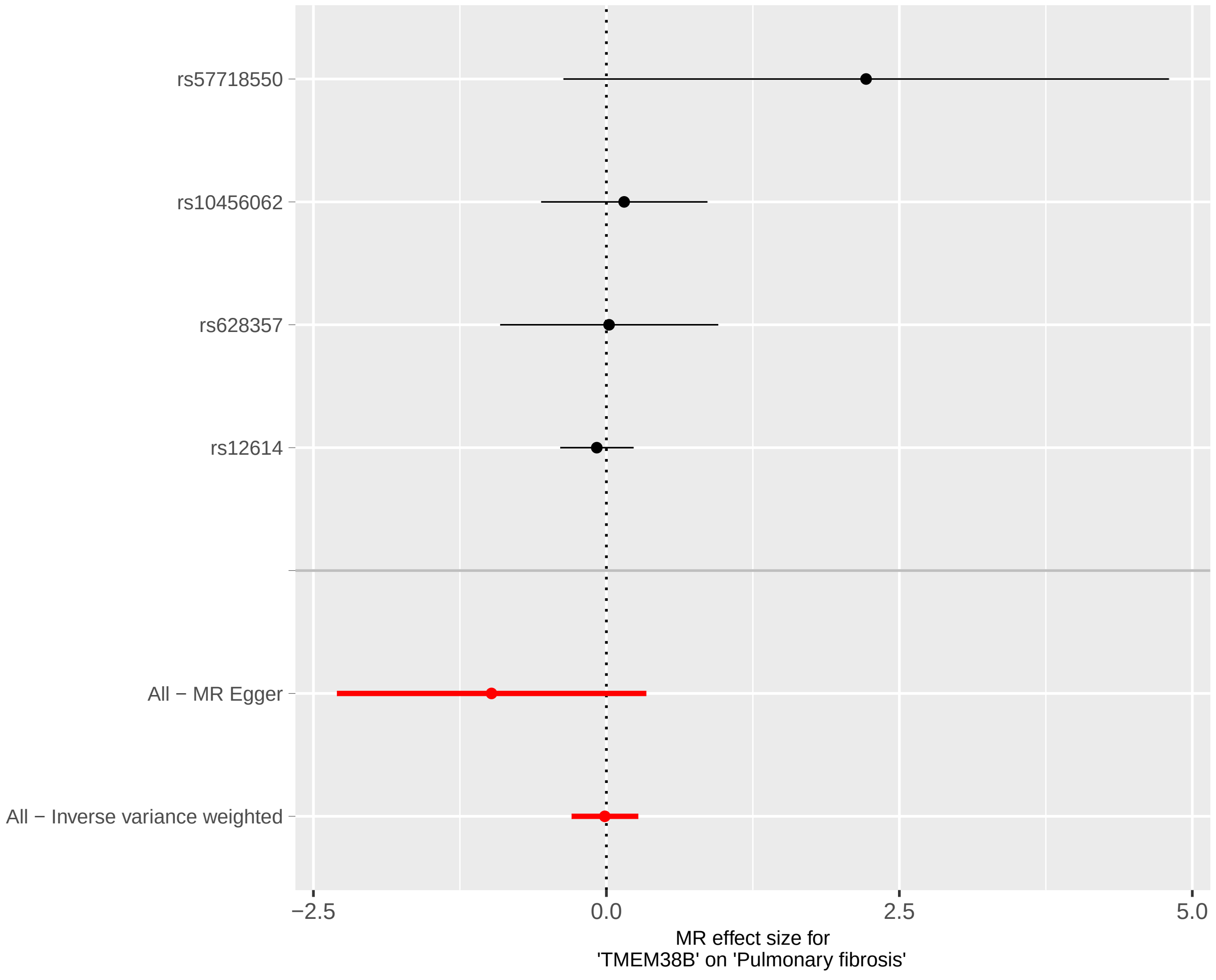
rs57718550
rs10456062
rs628357
rs12614
All − MR Egger
All − Inverse variance weighted
−2.5
0.0
2.5
5.0
MR effect size for
'TMEM38B' on 'Pulmonary fibrosis'

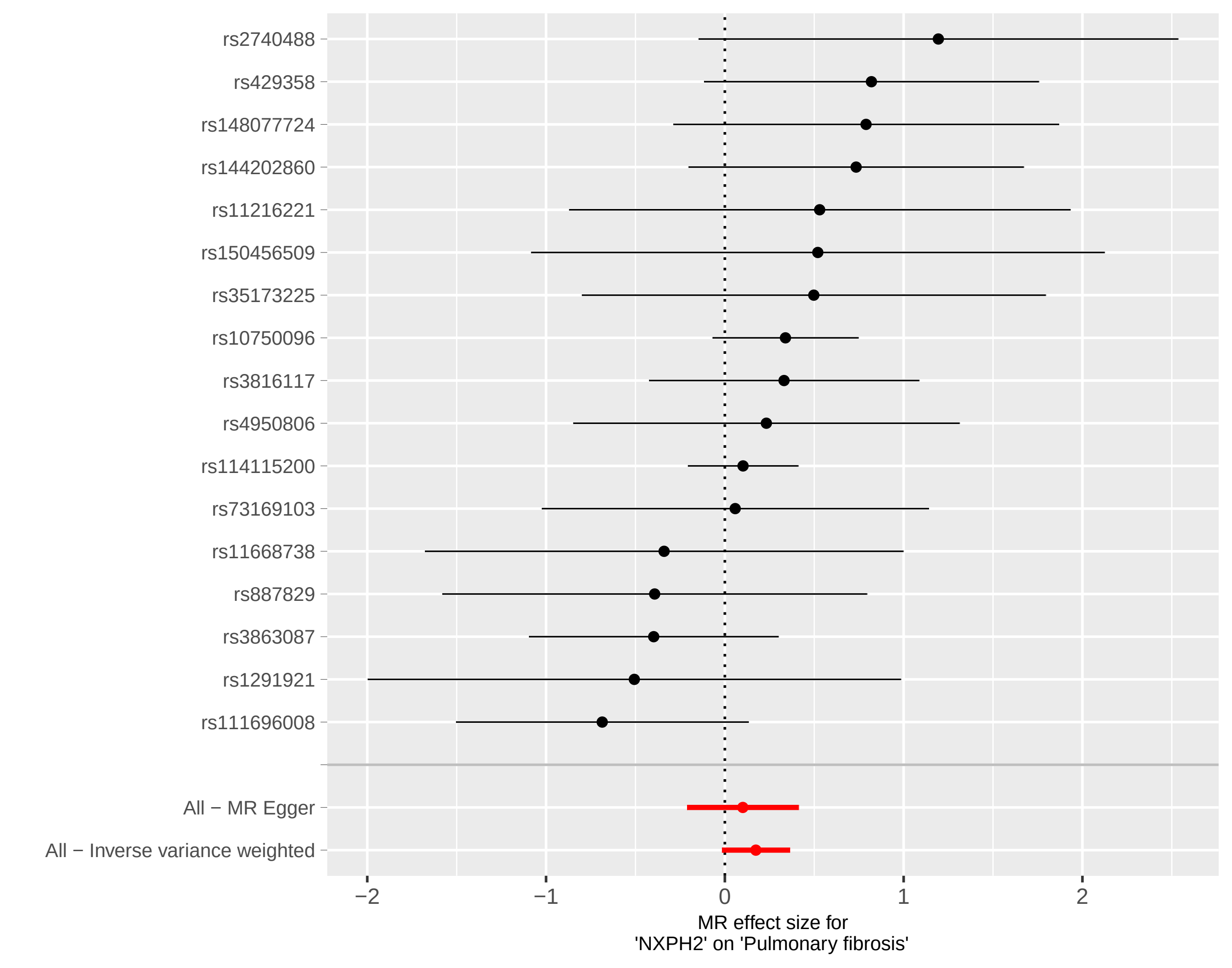
rs2740488
rs429358
rs148077724
rs144202860
rs11216221
rs150456509
rs35173225
rs10750096
rs3816117
rs4950806
rs114115200
rs73169103
rs11668738
rs887829
rs3863087
rs1291921
rs111696008
All − MR Egger
All − Inverse variance weighted
−2
−1
0
1
2
MR effect size for
'NXPH2' on 'Pulmonary fibrosis'

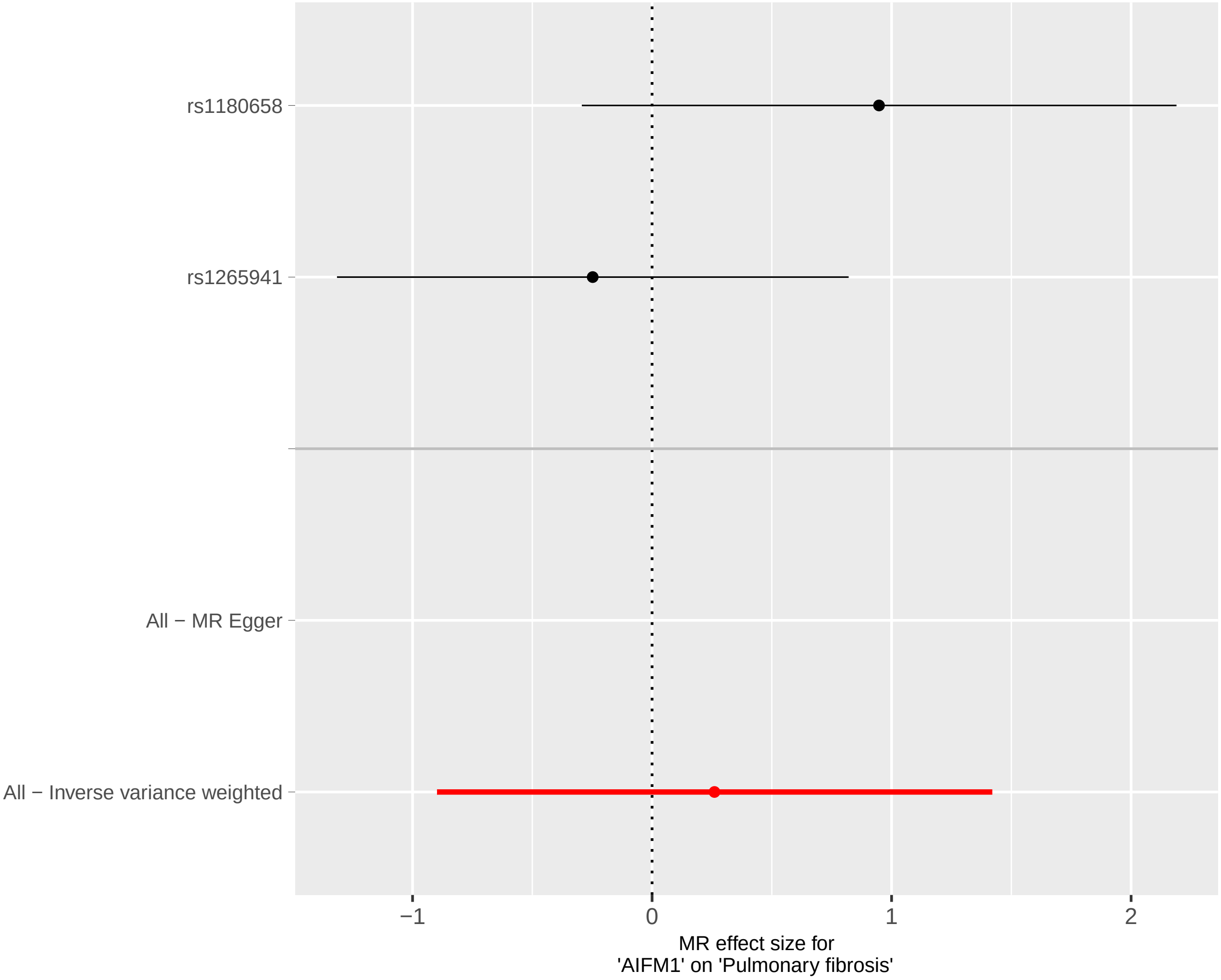
rs1180658
rs1265941
All − MR Egger
All − Inverse variance weighted
−1
0
1
2
MR effect size for
'AIFM1' on 'Pulmonary fibrosis'

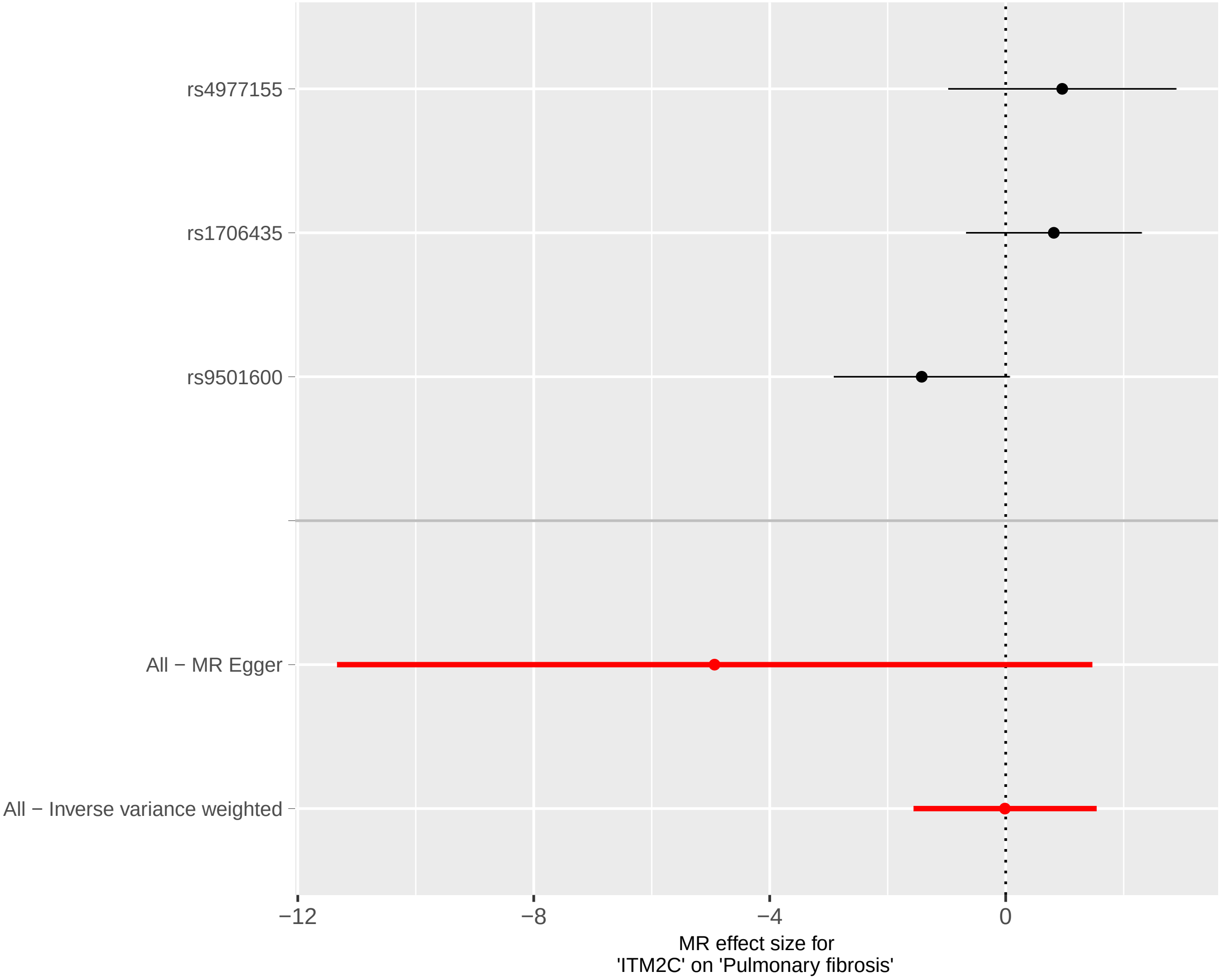
rs4977155
rs1706435
rs9501600
All − MR Egger
All − Inverse variance weighted
−12
−8
−4
0
MR effect size for
'ITM2C' on 'Pulmonary fibrosis'

Insufficient number of SNPs

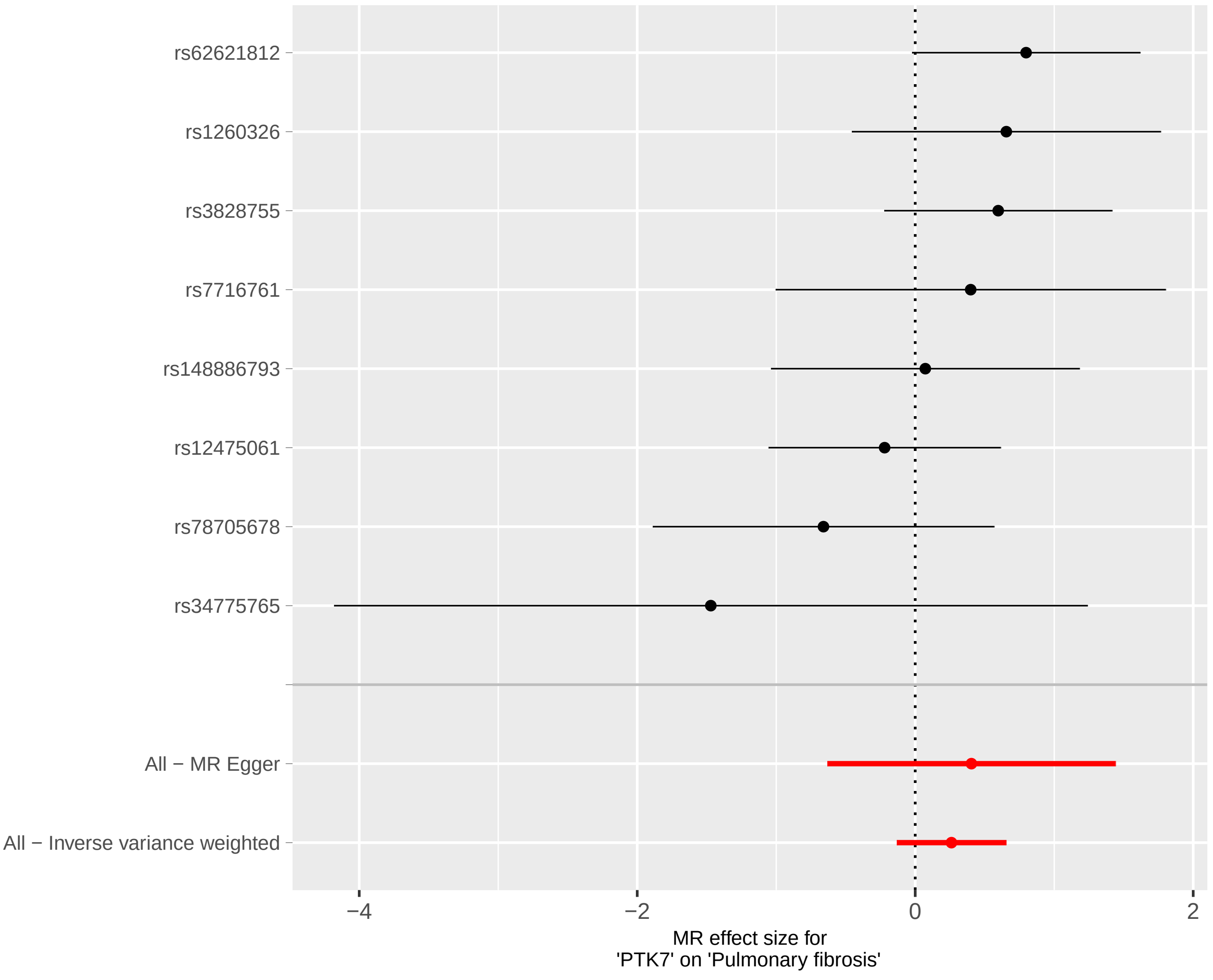

rs62621812
rs1260326
rs3828755
rs7716761
rs148886793
rs12475061
rs78705678
rs34775765
All − MR Egger
All − Inverse variance weighted
−4
−2
0
2
MR effect size for
'PTK7' on 'Pulmonary fibrosis'

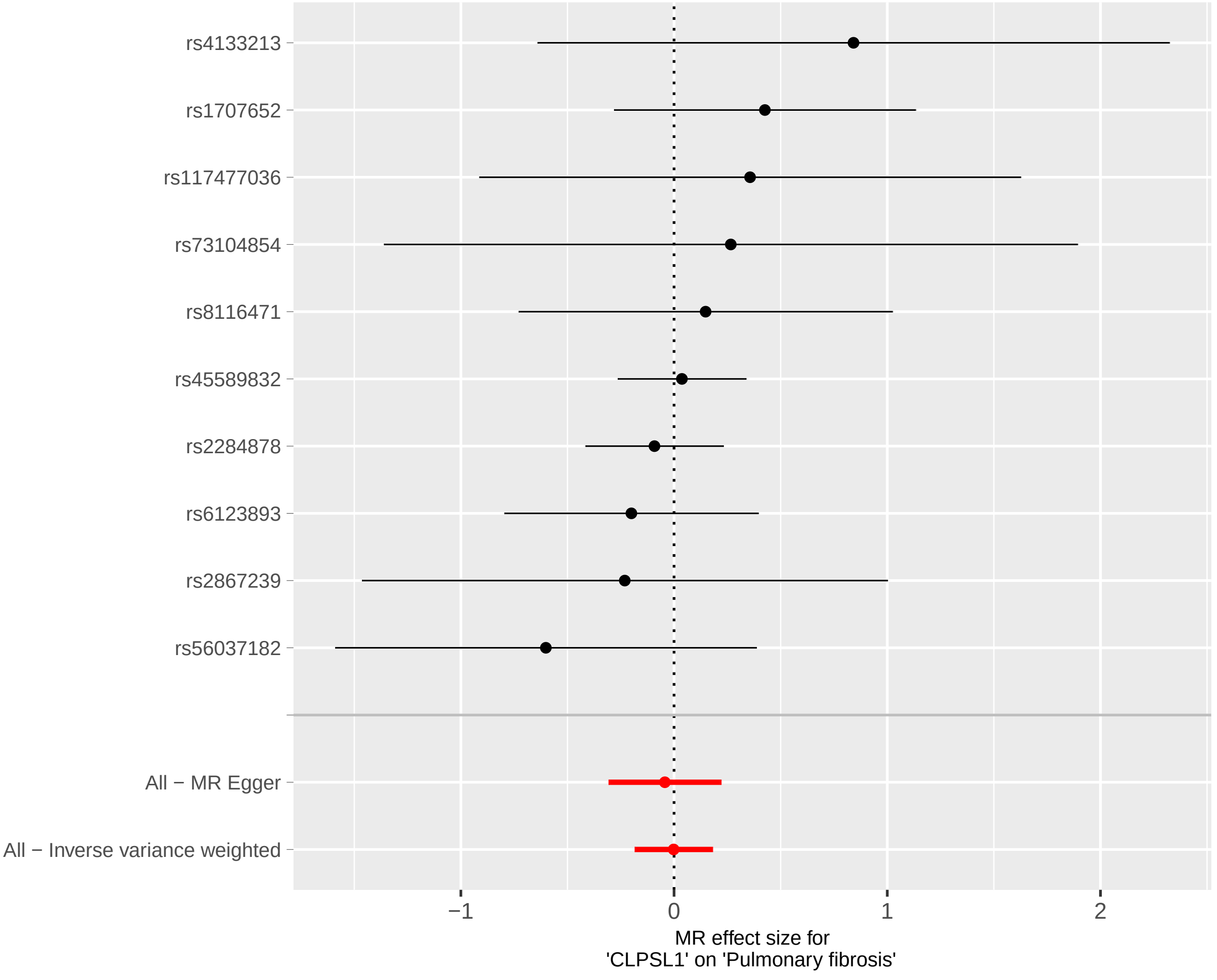

rs4133213
rs1707652
rs117477036
rs73104854
rs8116471
rs45589832
rs2284878
rs6123893
rs2867239
rs56037182
All − MR Egger
All − Inverse variance weighted
−1
0
1
2
MR effect size for
'CLPSL1' on 'Pulmonary fibrosis'

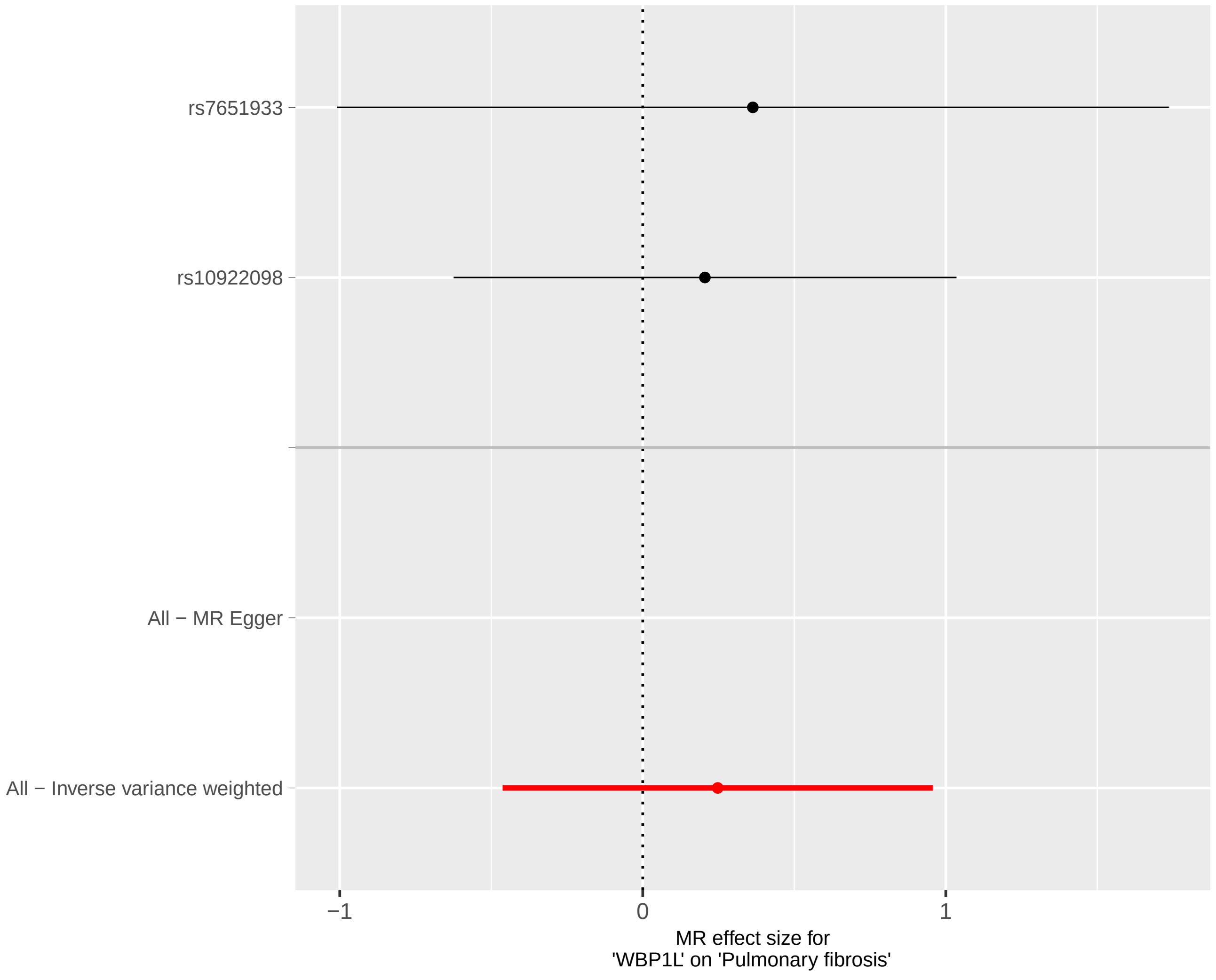
rs7651933
rs10922098
All − MR Egger
All − Inverse variance weighted
−1
0
1
MR effect size for
'WBP1L' on 'Pulmonary fibrosis'

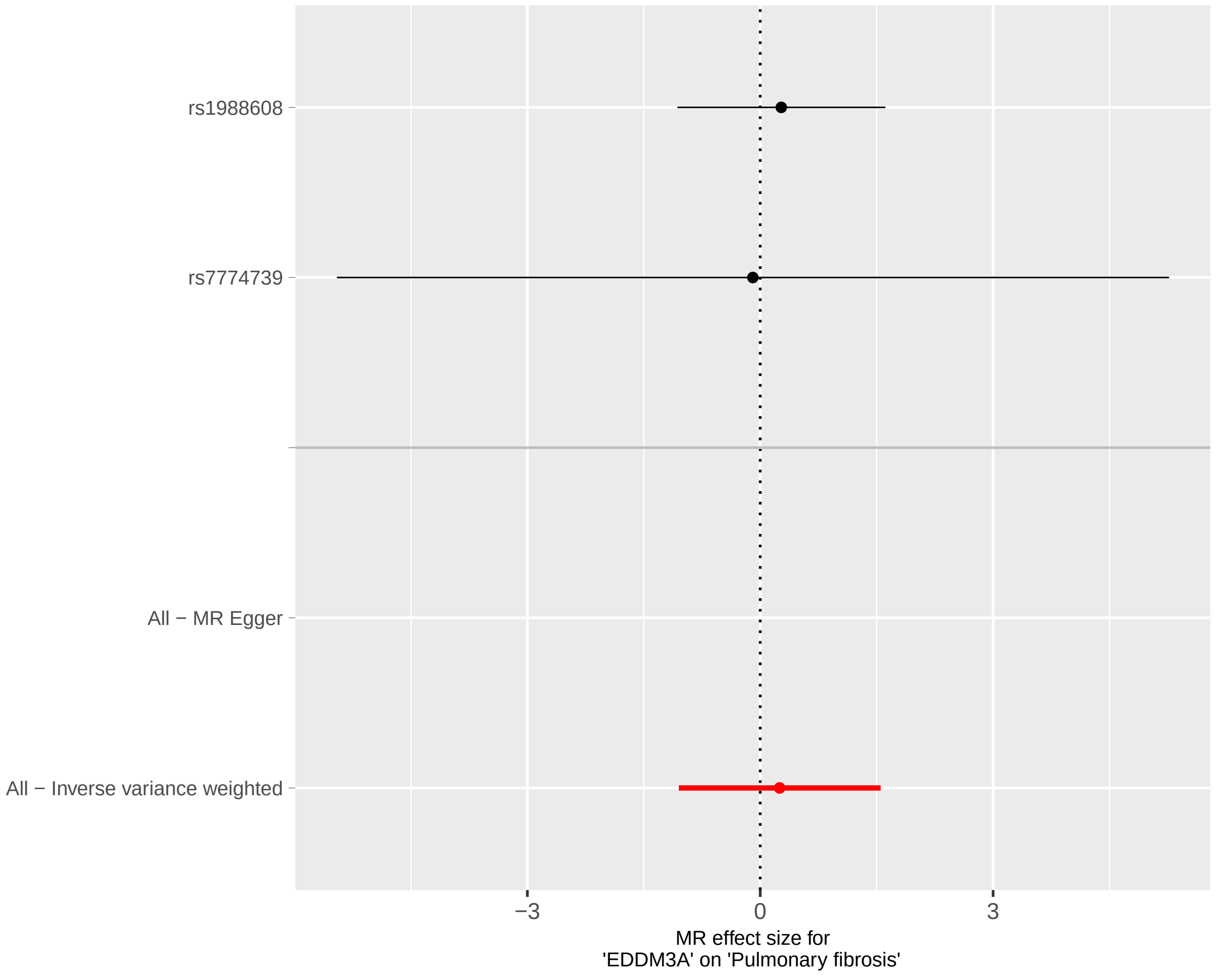

rs1988608
rs7774739
All − MR Egger
All − Inverse variance weighted
−3
0
3
MR effect size for
'EDDM3A' on 'Pulmonary fibrosis'

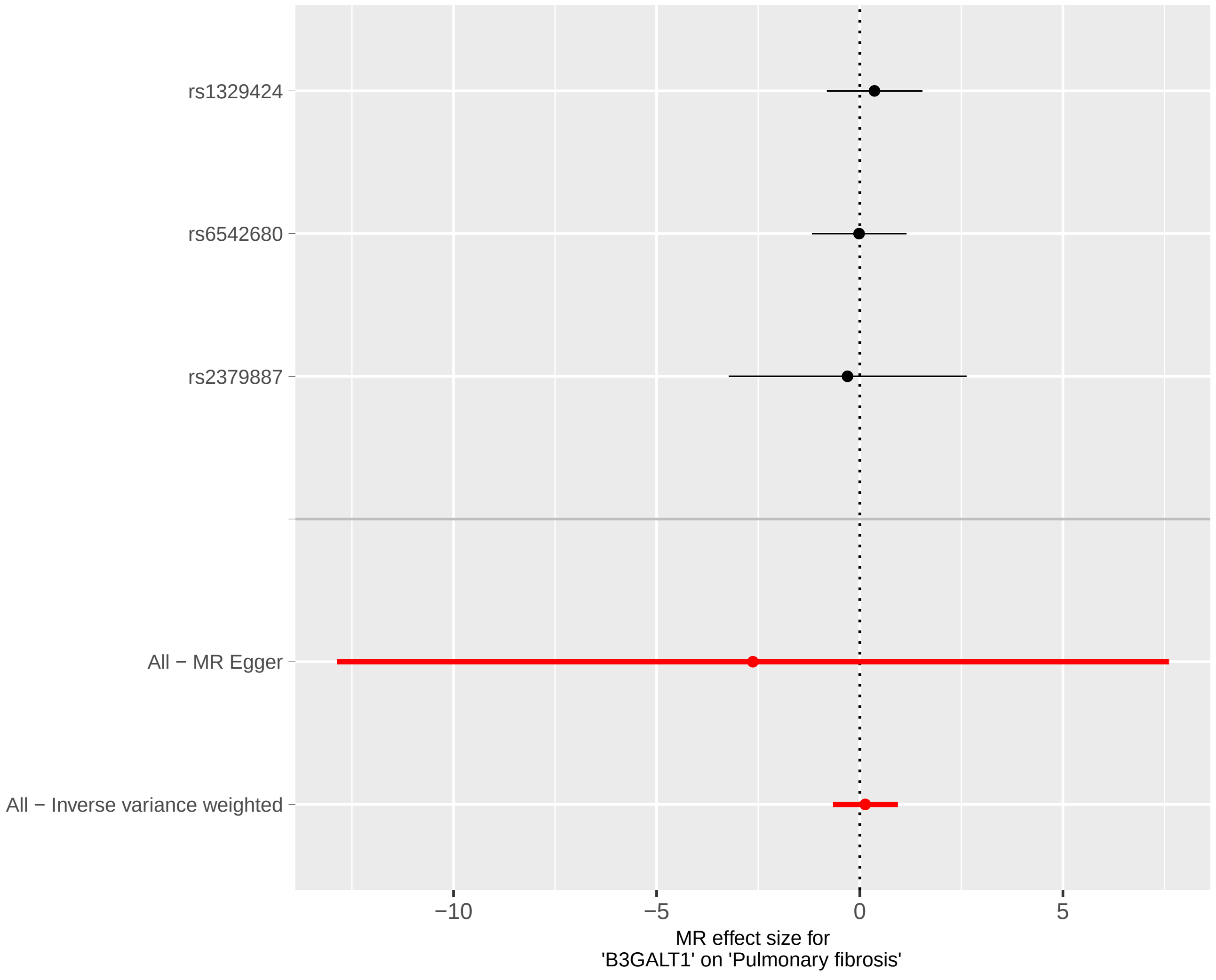
rs1329424
rs6542680
rs2379887
All − MR Egger
All − Inverse variance weighted
−10
−5
0
5
MR effect size for
'B3GALT1' on 'Pulmonary fibrosis'

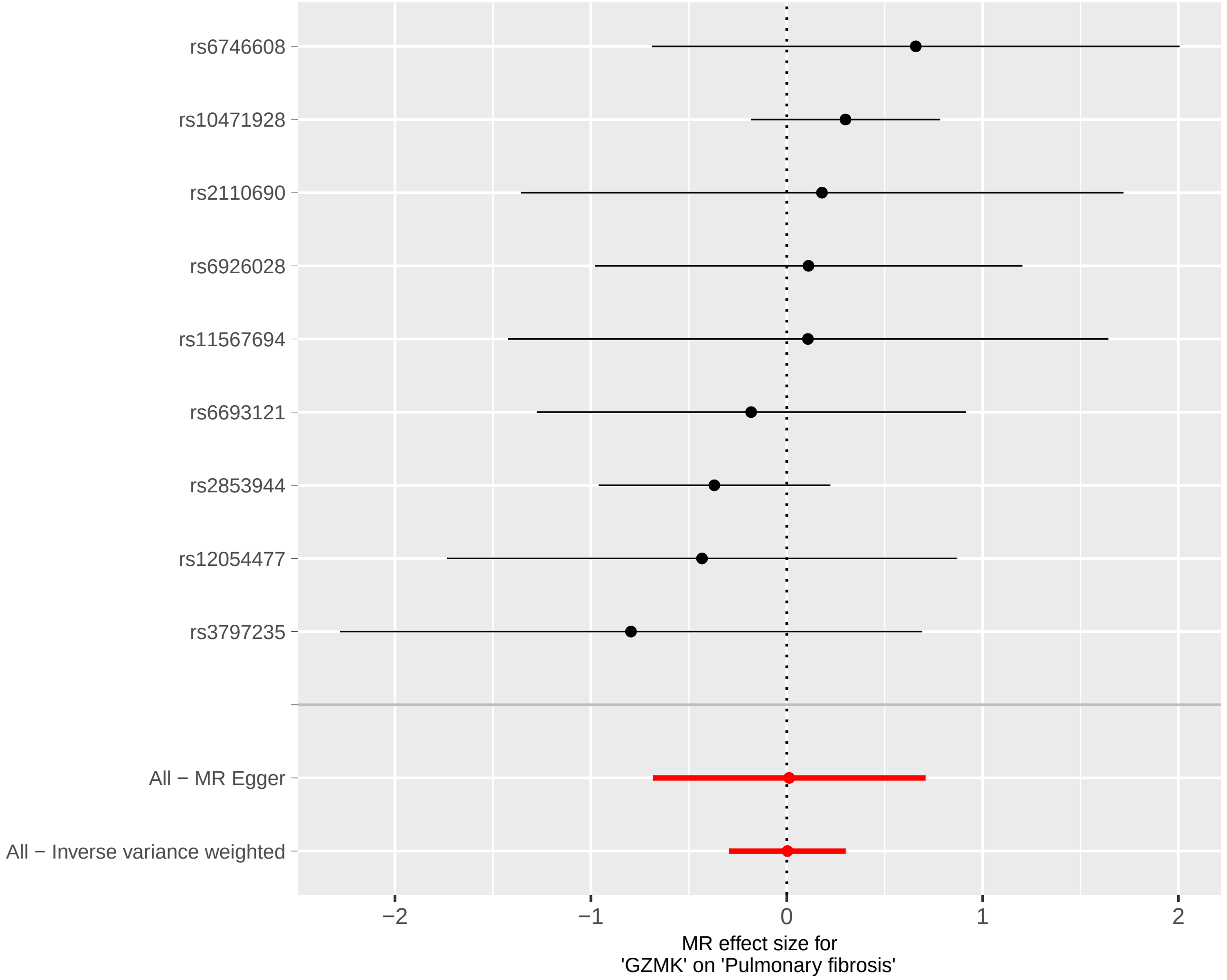
rs6746608
rs10471928
rs2110690
rs6926028
rs11567694
rs6693121
rs2853944
rs12054477
rs3797235
All − MR Egger
All − Inverse variance weighted
−2
−1
0
1
2
MR effect size for
'GZMK' on 'Pulmonary fibrosis'

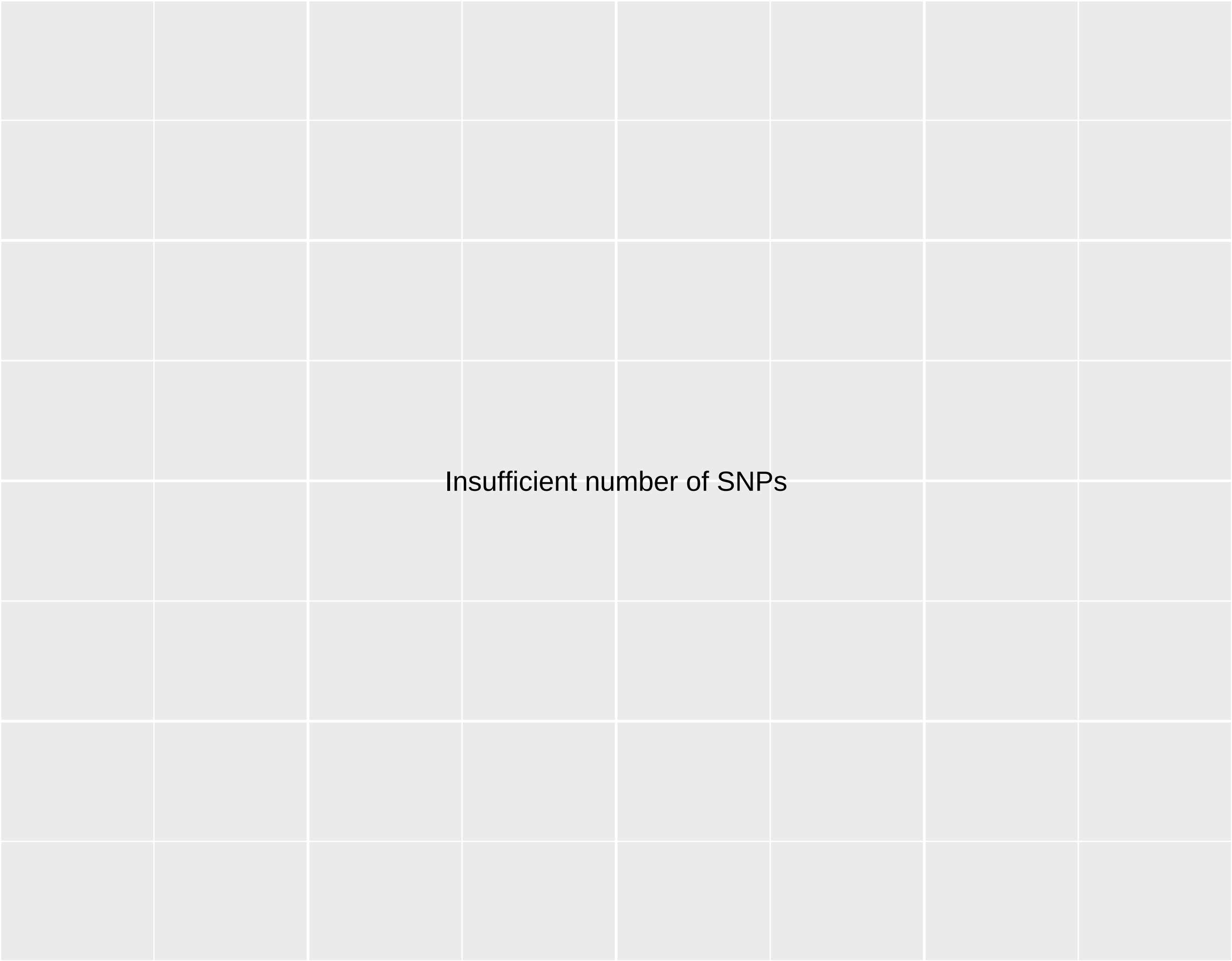
Insufficient number of SNPs

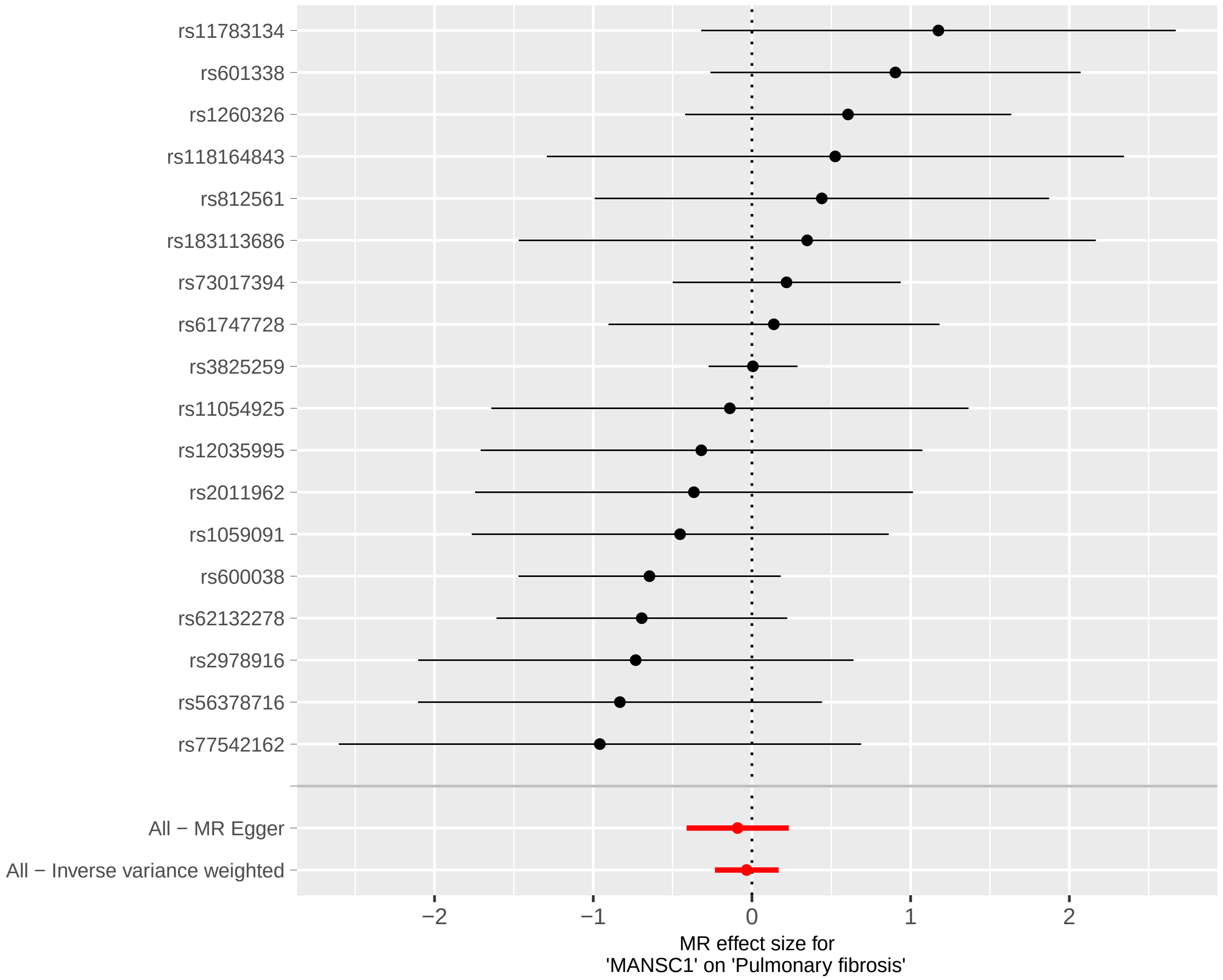

rs11783134
rs601338
rs1260326
rs118164843
rs812561
rs183113686
rs73017394
rs61747728
rs3825259
rs11054925
rs12035995
rs2011962
rs1059091
rs600038
rs62132278
rs2978916
rs56378716
rs77542162
All − MR Egger
All − Inverse variance weighted
−2
−1
0
1
2
MR effect size for
'MANSC1' on 'Pulmonary fibrosis'

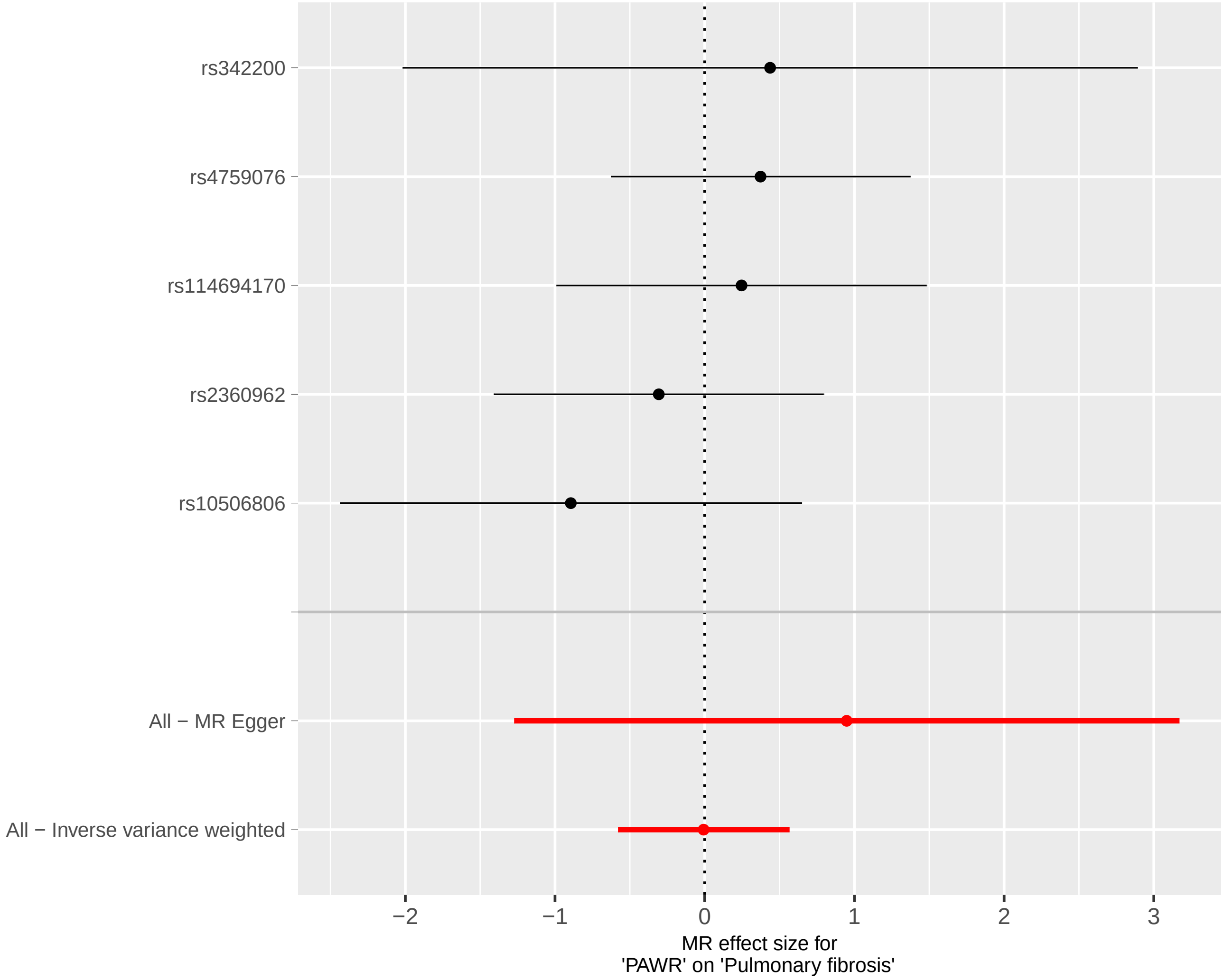
rs342200
rs4759076
rs114694170
rs2360962
rs10506806
All − MR Egger
All − Inverse variance weighted
−2
−1
0
1
2
3
MR effect size for
'PAWR' on 'Pulmonary fibrosis'

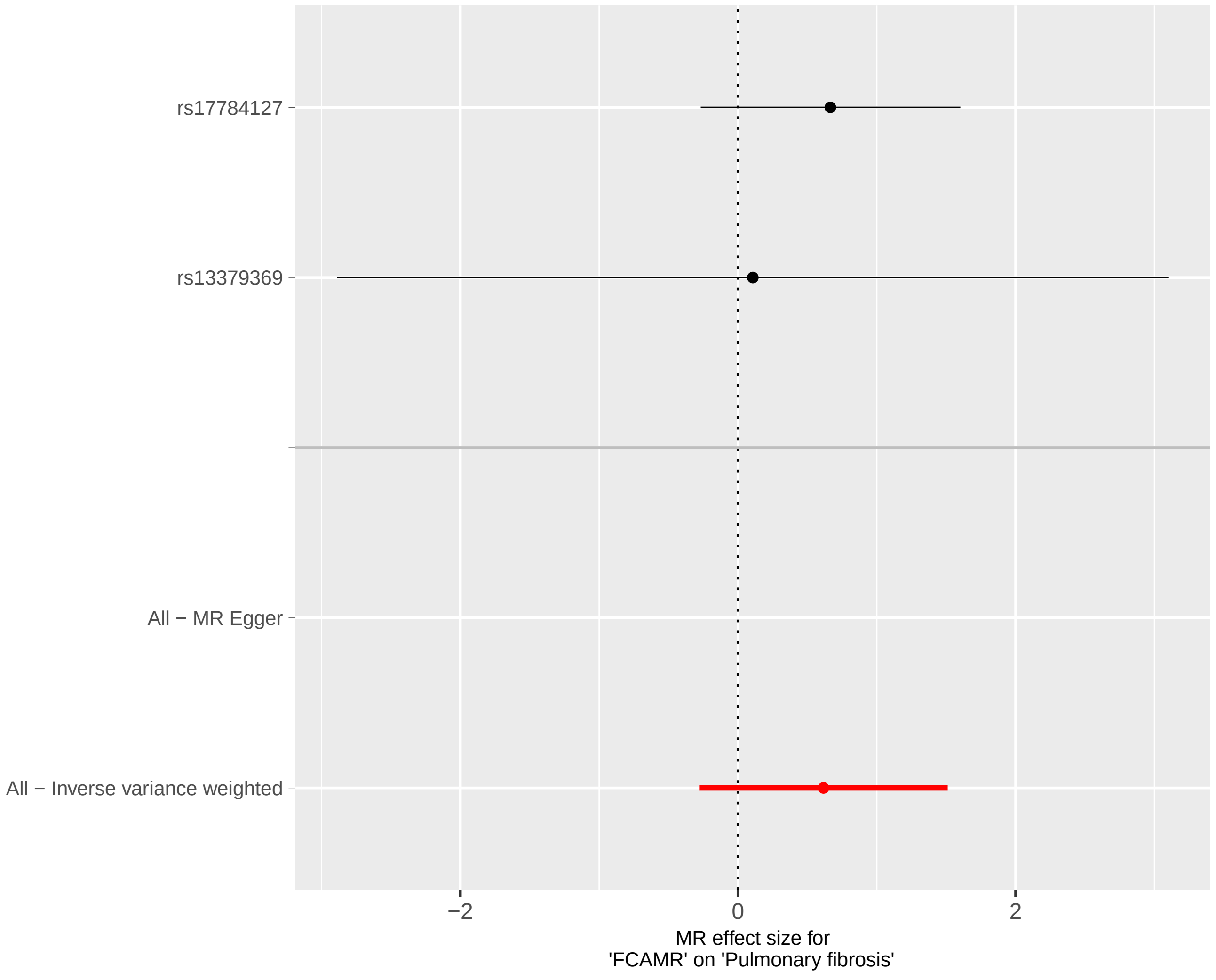

rs17784127
rs13379369
All − MR Egger
All − Inverse variance weighted
−2
0
2
MR effect size for
'FCAMR' on 'Pulmonary fibrosis'

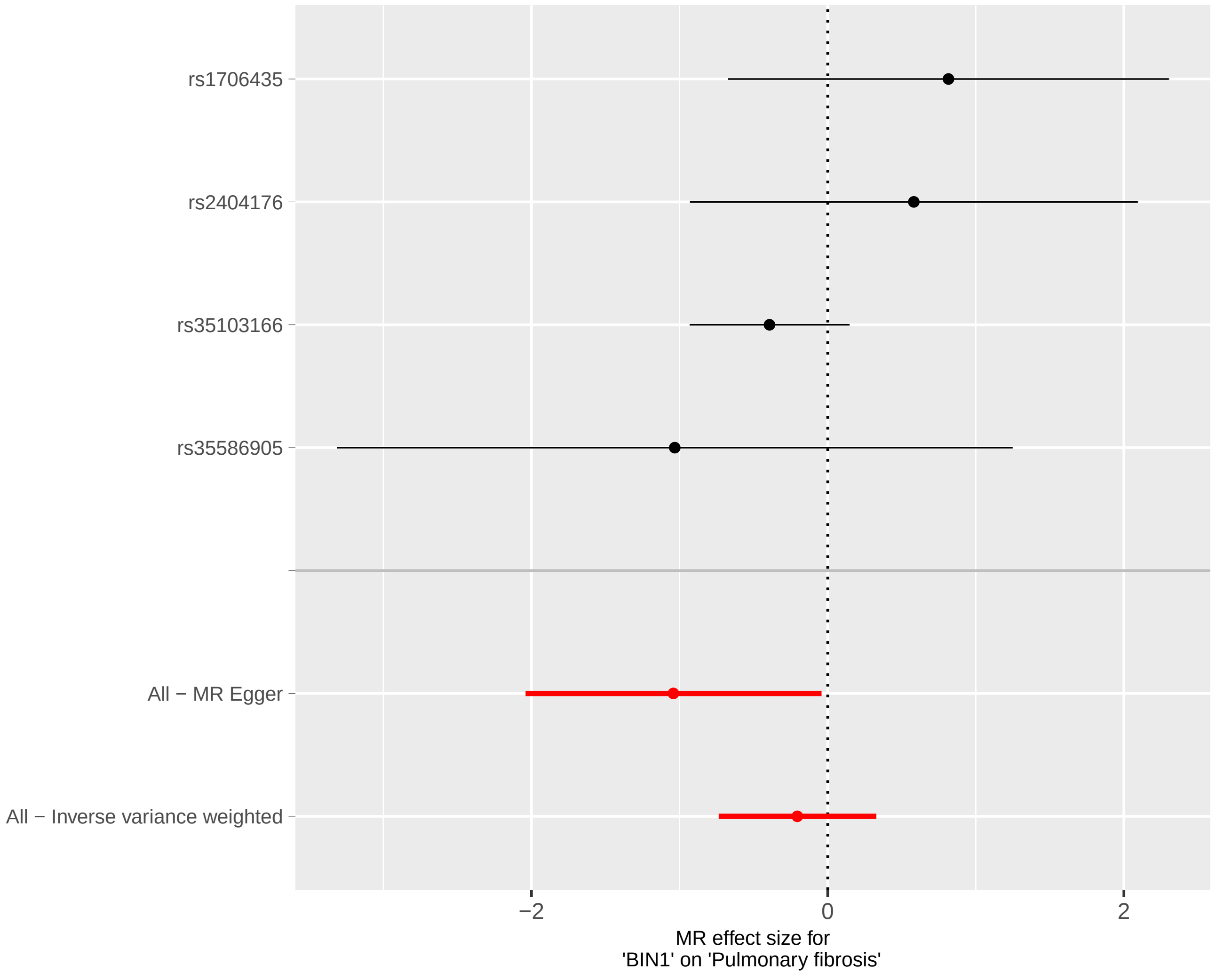
rs1706435
rs2404176
rs35103166
rs35586905
All − MR Egger
All − Inverse variance weighted
−2
0
2
MR effect size for
'BIN1' on 'Pulmonary fibrosis'

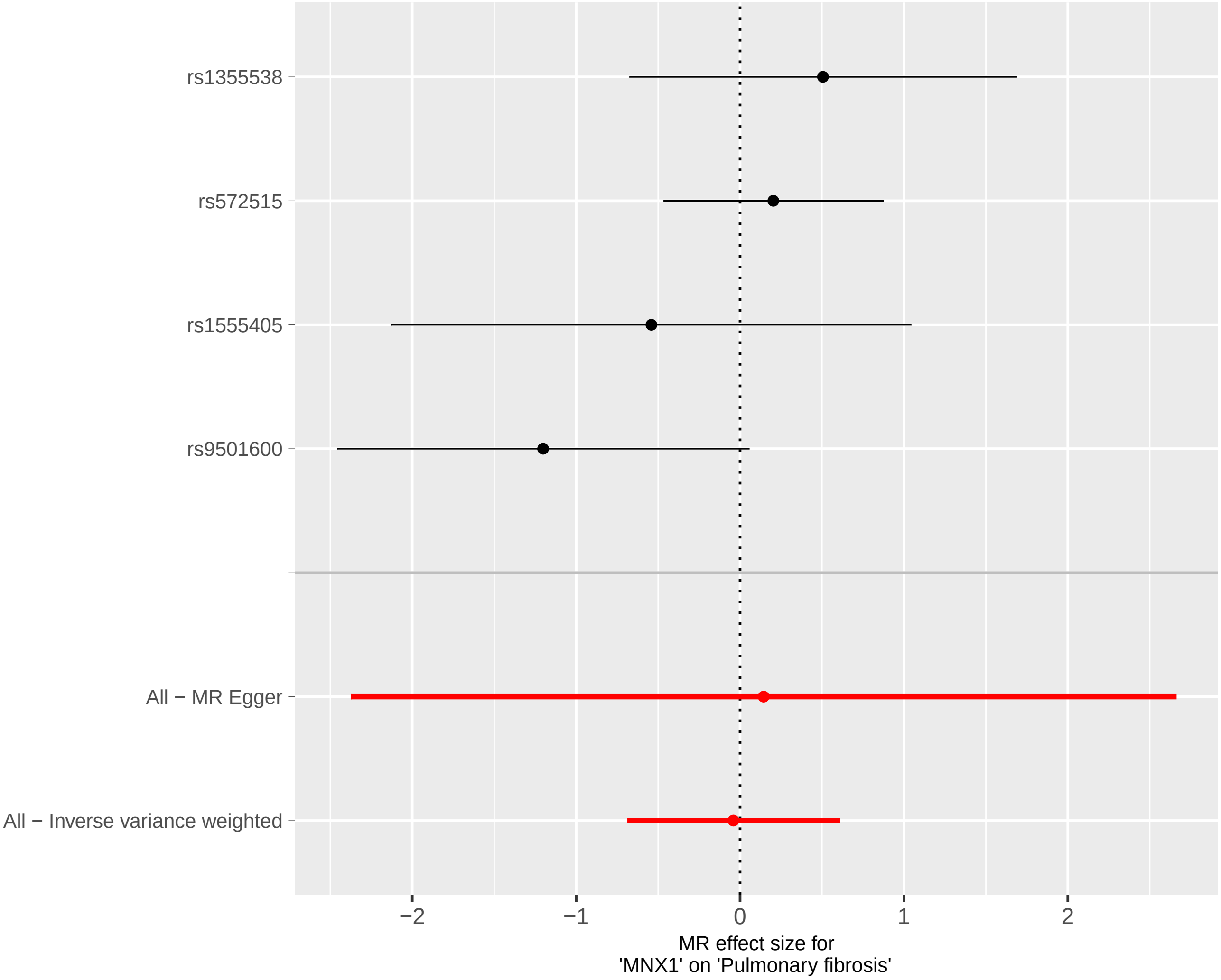

rs1355538
rs572515
rs1555405
rs9501600
All − MR Egger
All − Inverse variance weighted
−2
−1
0
1
2
MR effect size for
'MNX1' on 'Pulmonary fibrosis'

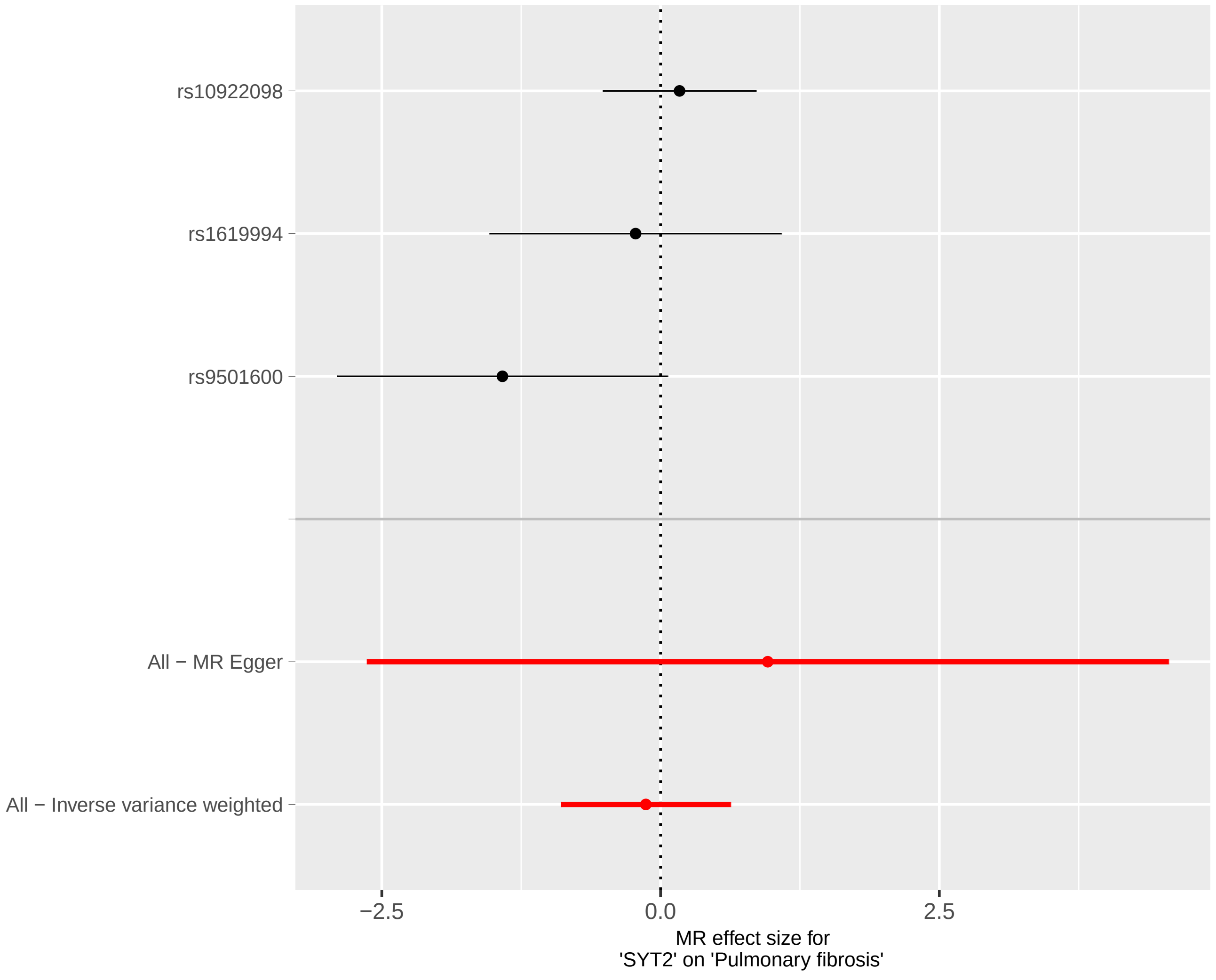

rs10922098
rs1619994
rs9501600
All − MR Egger
All − Inverse variance weighted
−2.5
0.0
2.5
MR effect size for
'SYT2' on 'Pulmonary fibrosis'

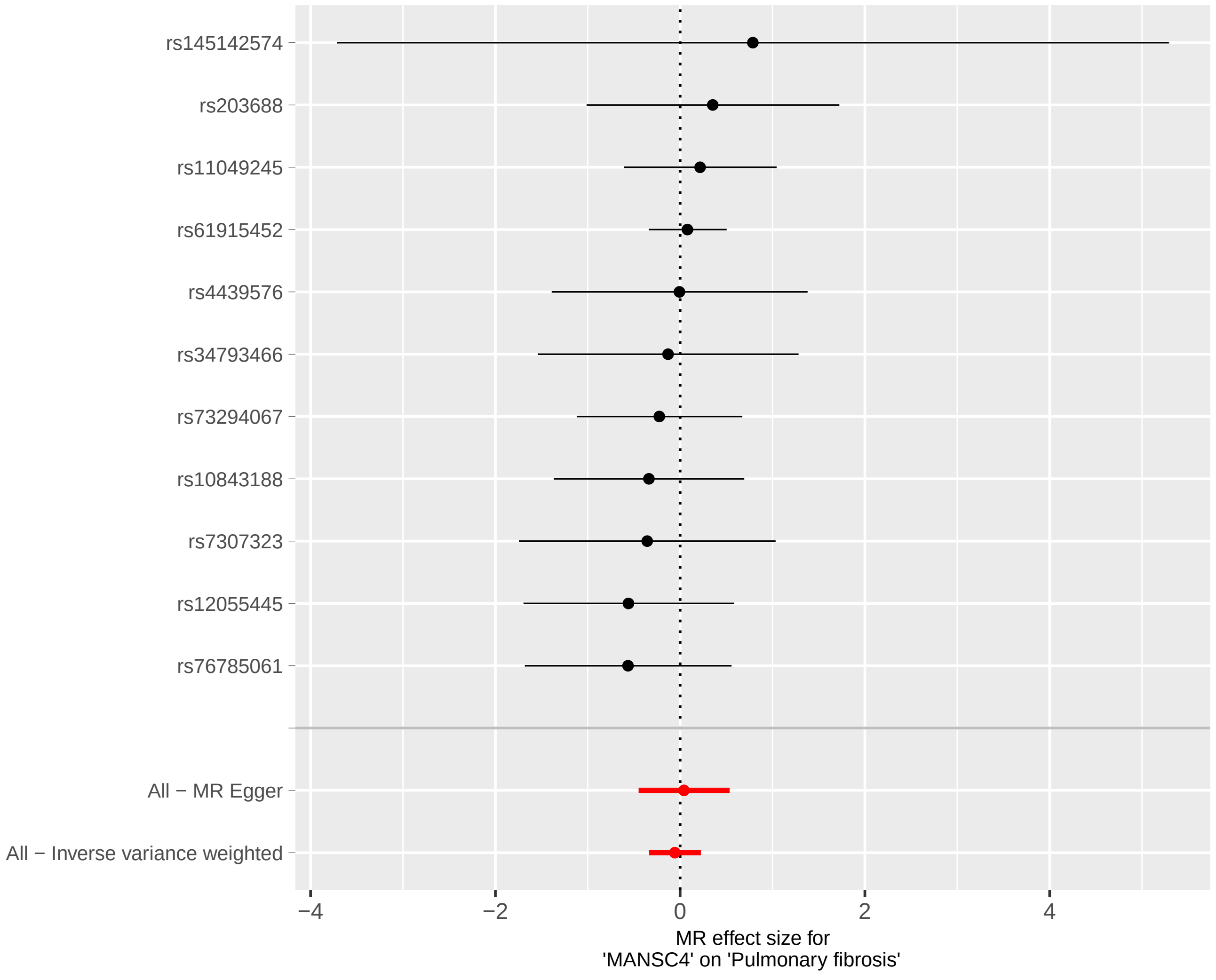

rs145142574
rs203688
rs11049245
rs61915452
rs4439576
rs34793466
rs73294067
rs10843188
rs7307323
rs12055445
rs76785061
All − MR Egger
All − Inverse variance weighted
−4
−2
0
2
4
MR effect size for
'MANSC4' on 'Pulmonary fibrosis'

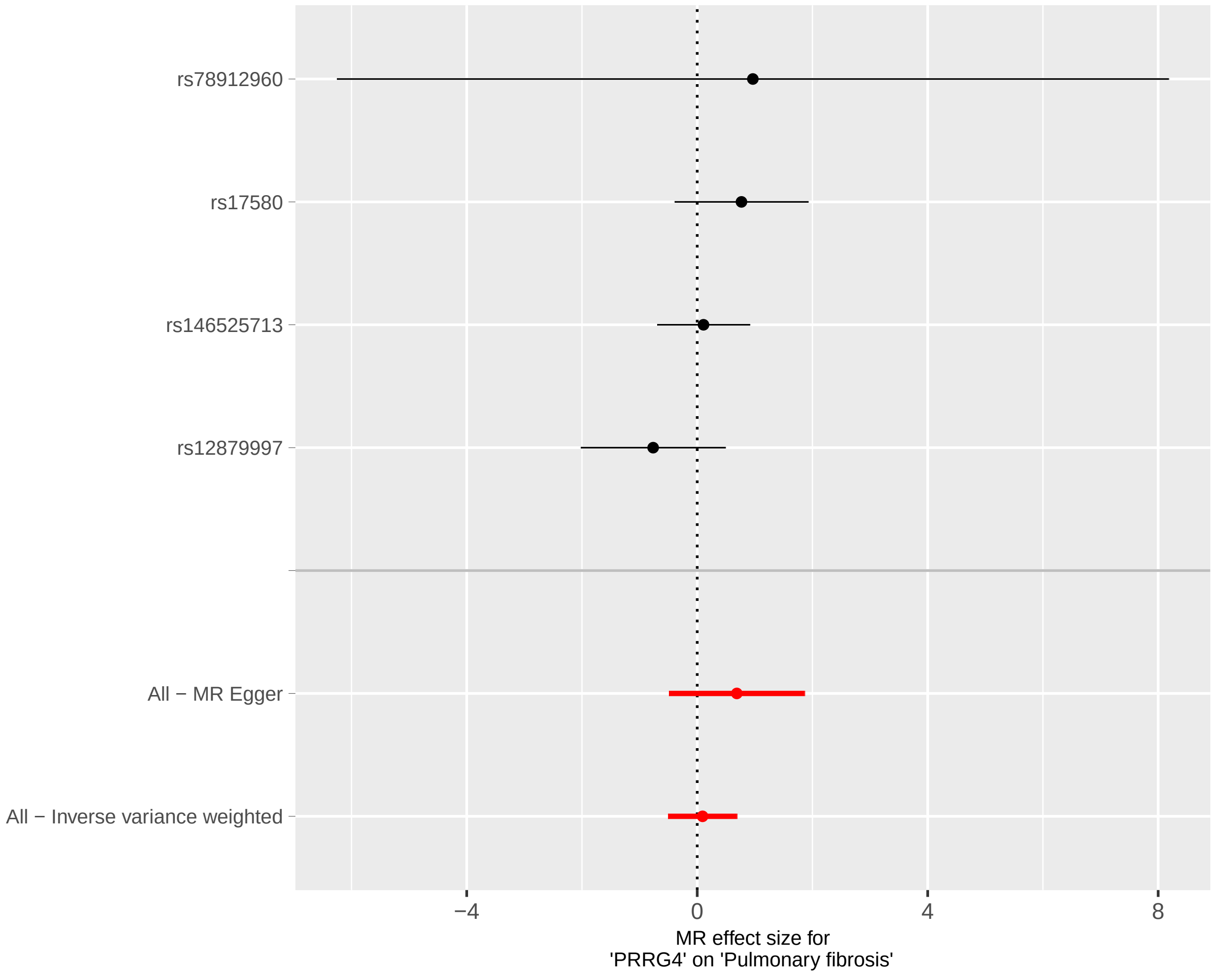

rs78912960
rs17580
rs146525713
rs12879997
All − MR Egger
All − Inverse variance weighted
−4
0
4
8
MR effect size for
'PRRG4' on 'Pulmonary fibrosis'

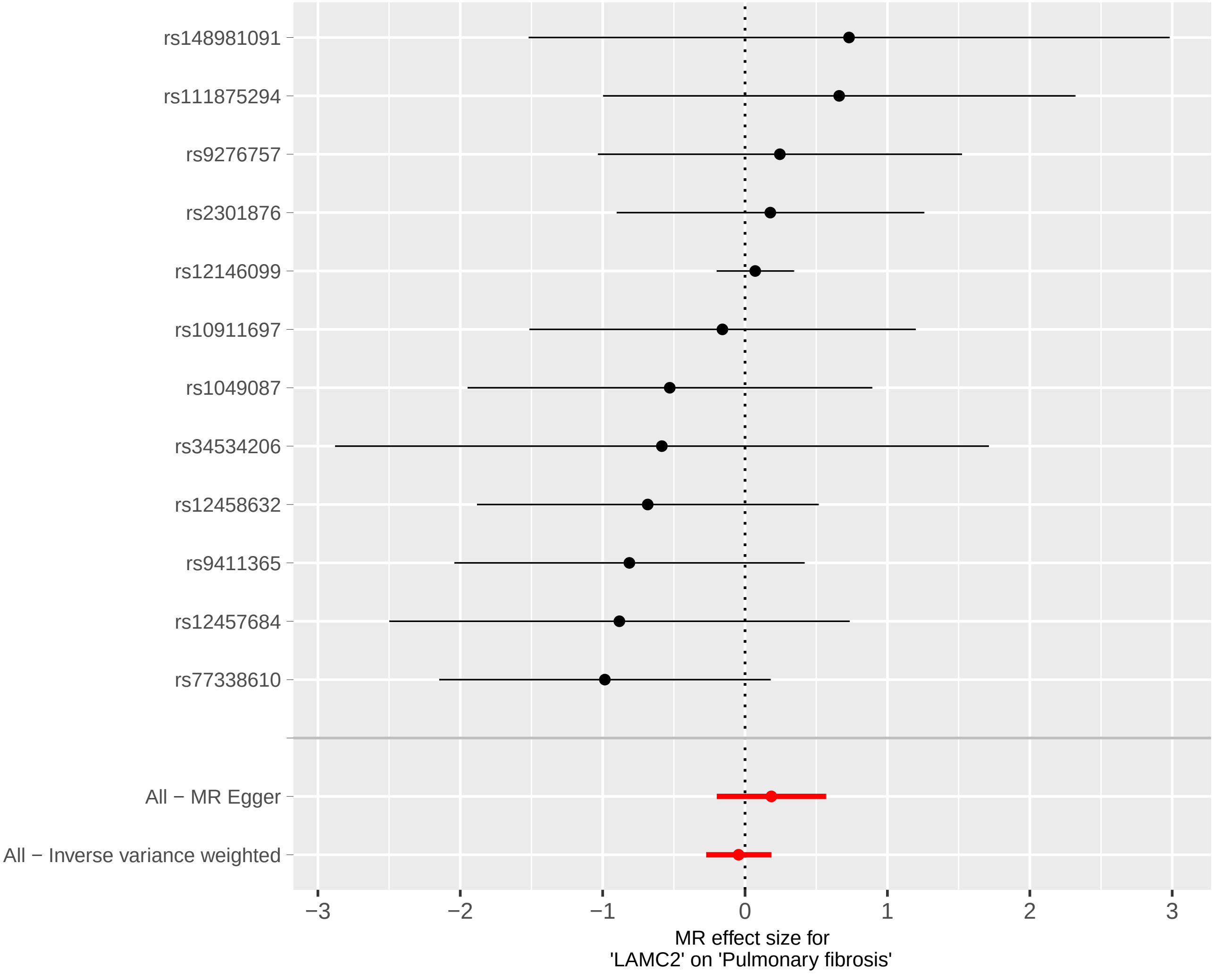
rs148981091
rs111875294
rs9276757
rs2301876
rs12146099
rs10911697
rs1049087
rs34534206
rs12458632
rs9411365
rs12457684
rs77338610
All − MR Egger
All − Inverse variance weighted
−3
−2
−1
0
1
2
3
MR effect size for
'LAMC2' on 'Pulmonary fibrosis'

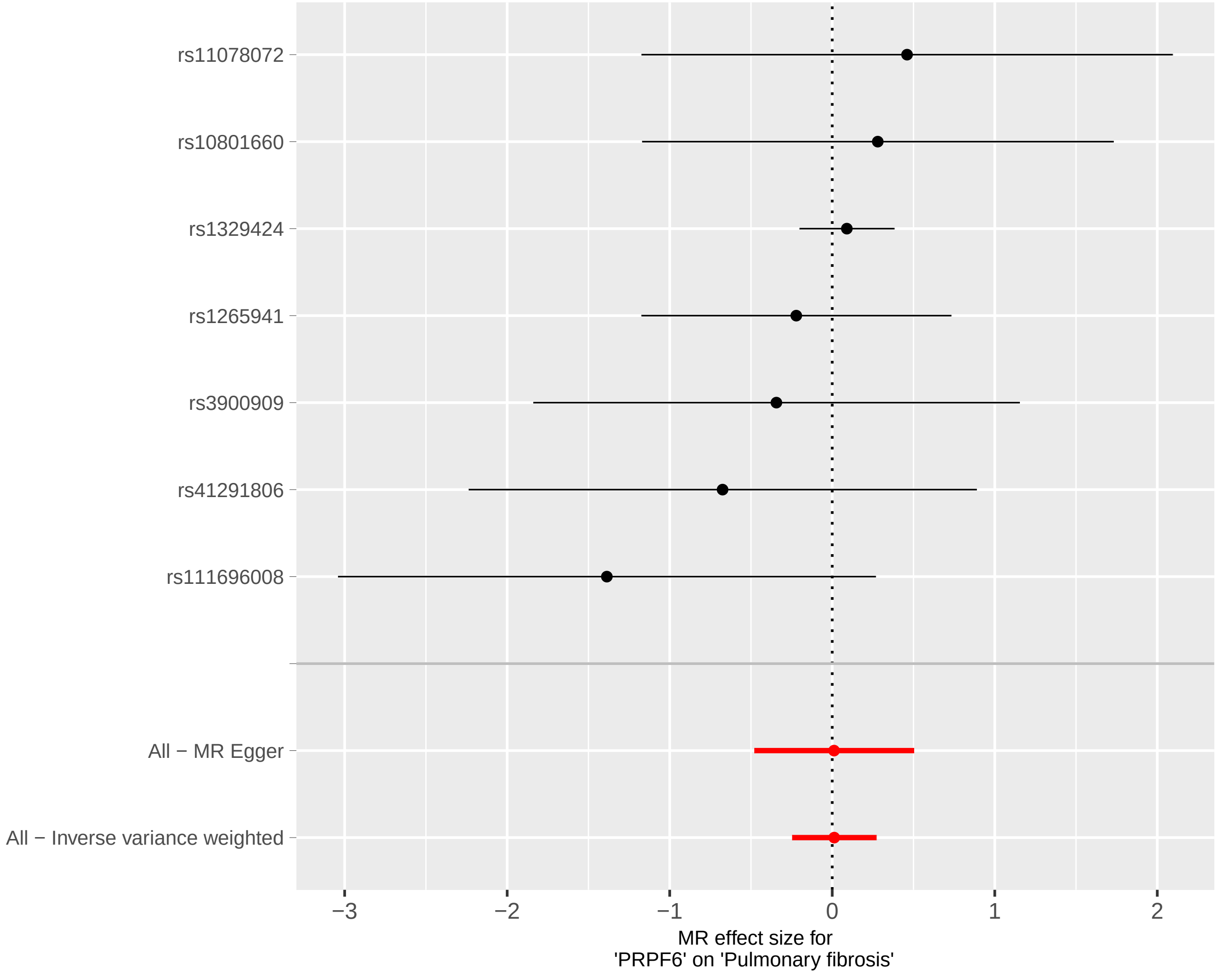
rs11078072
rs10801660
rs1329424
rs1265941
rs3900909
rs41291806
rs111696008
All − MR Egger
All − Inverse variance weighted
−3
−2
−1
0
1
2
MR effect size for
'PRPF6' on 'Pulmonary fibrosis'

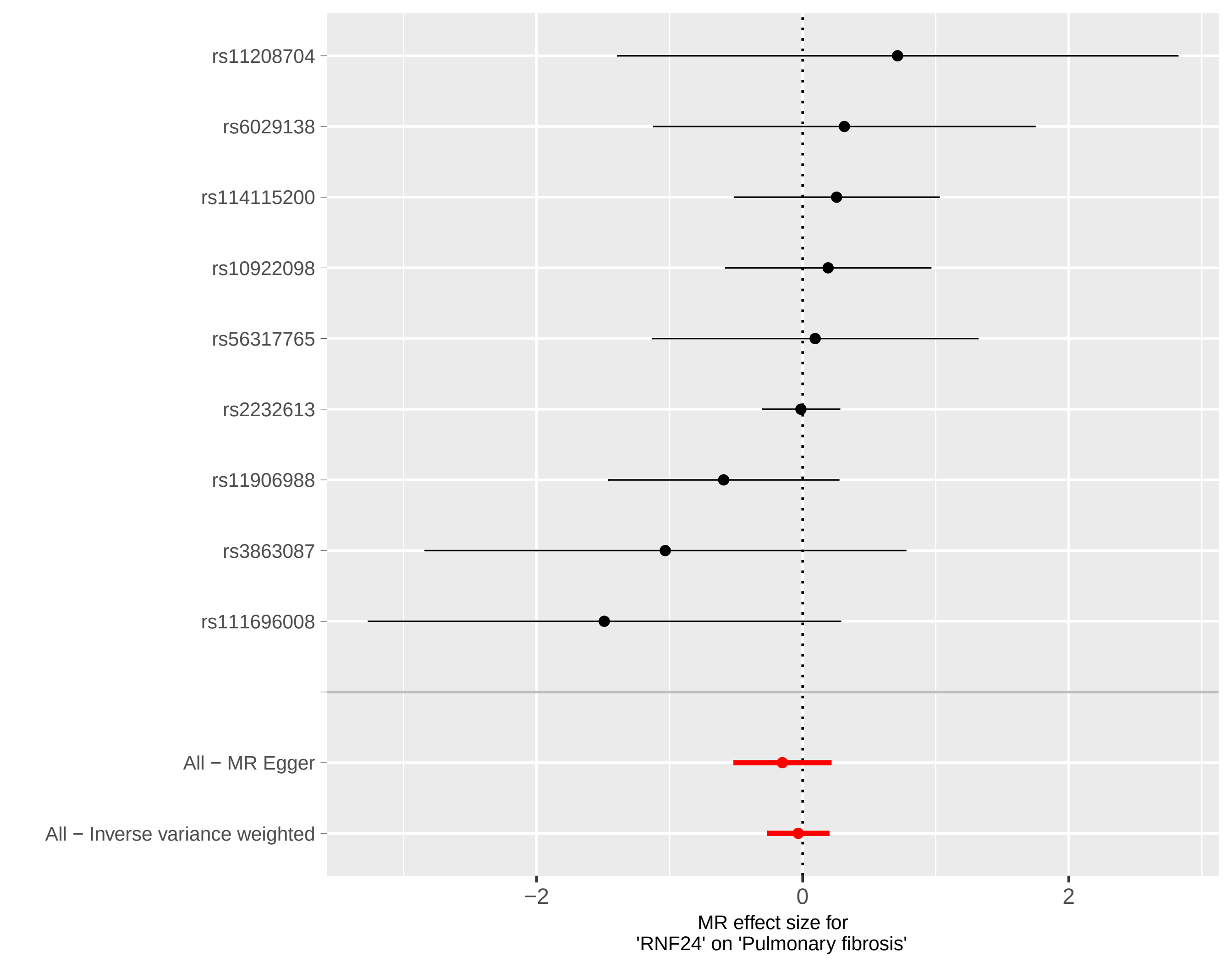

rs11208704
rs6029138
rs114115200
rs10922098
rs56317765
rs2232613
rs11906988
rs3863087
rs111696008
All − MR Egger
All − Inverse variance weighted
−2
0
2
MR effect size for
'RNF24' on 'Pulmonary fibrosis'

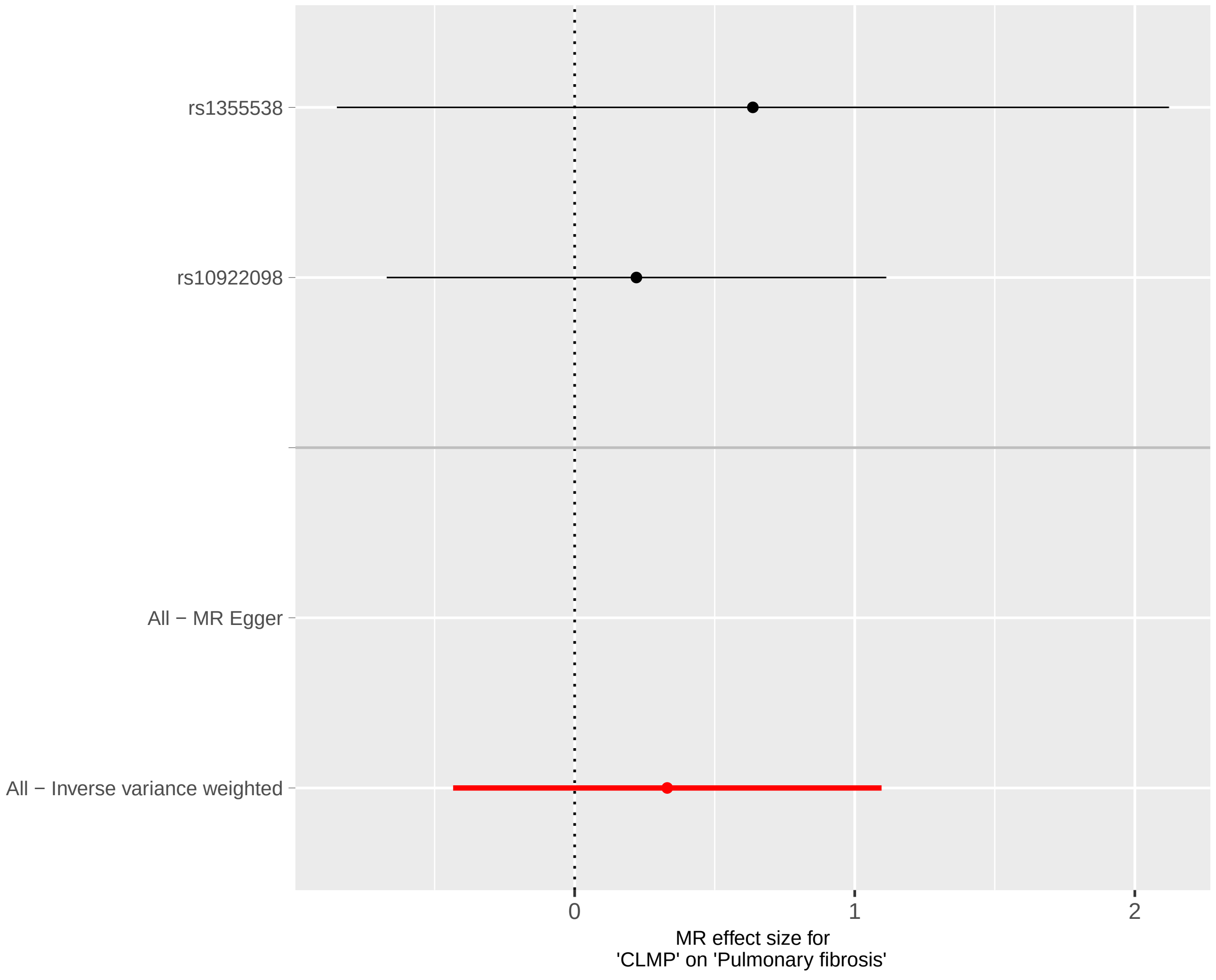

rs1355538
rs10922098
All − MR Egger
All − Inverse variance weighted
0
1
2
MR effect size for
'CLMP' on 'Pulmonary fibrosis'

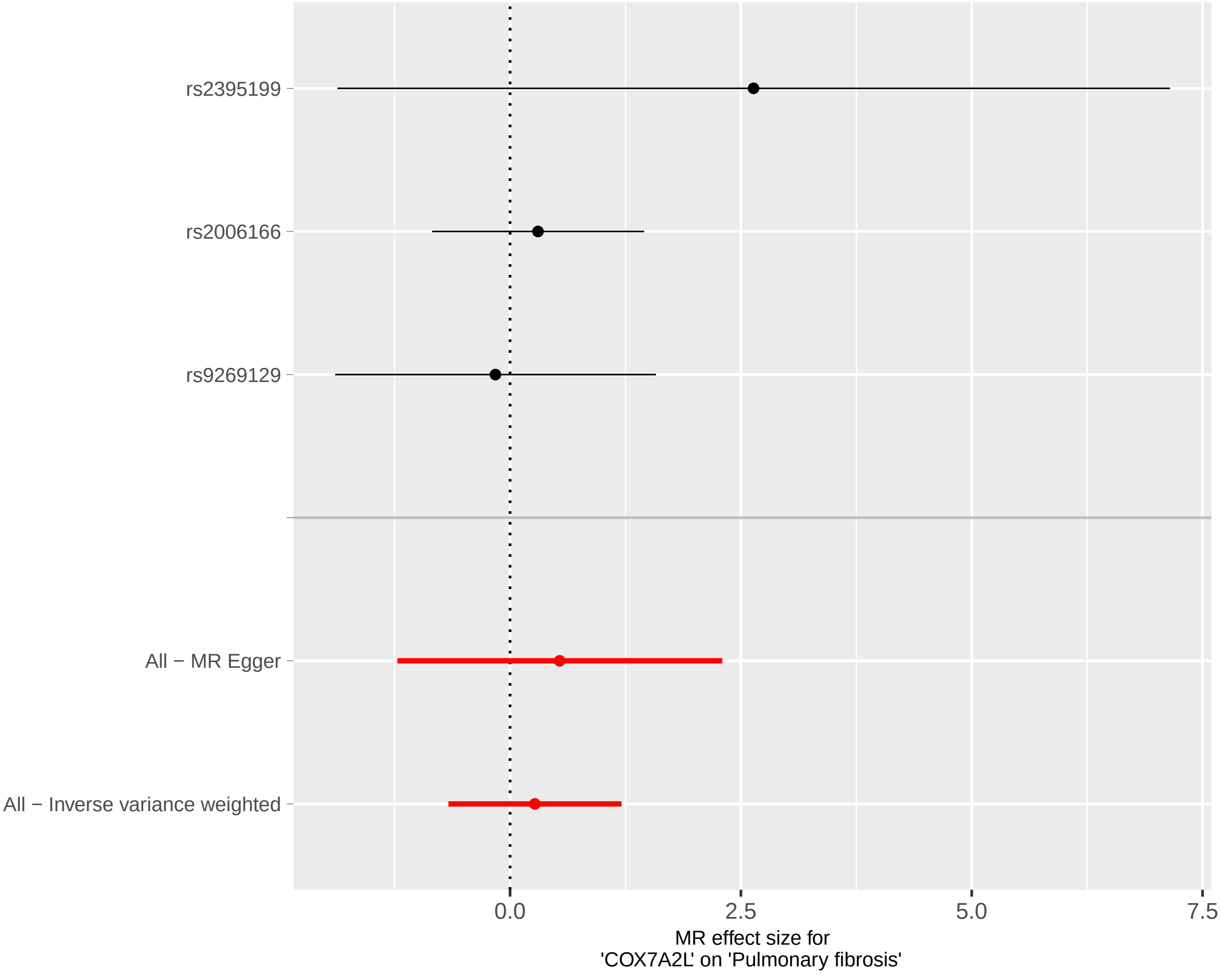
rs2395199
rs2006166
rs9269129
All − MR Egger
All − Inverse variance weighted
0.0
2.5
5.0
7.5
MR effect size for
'COX7A2L' on 'Pulmonary fibrosis'

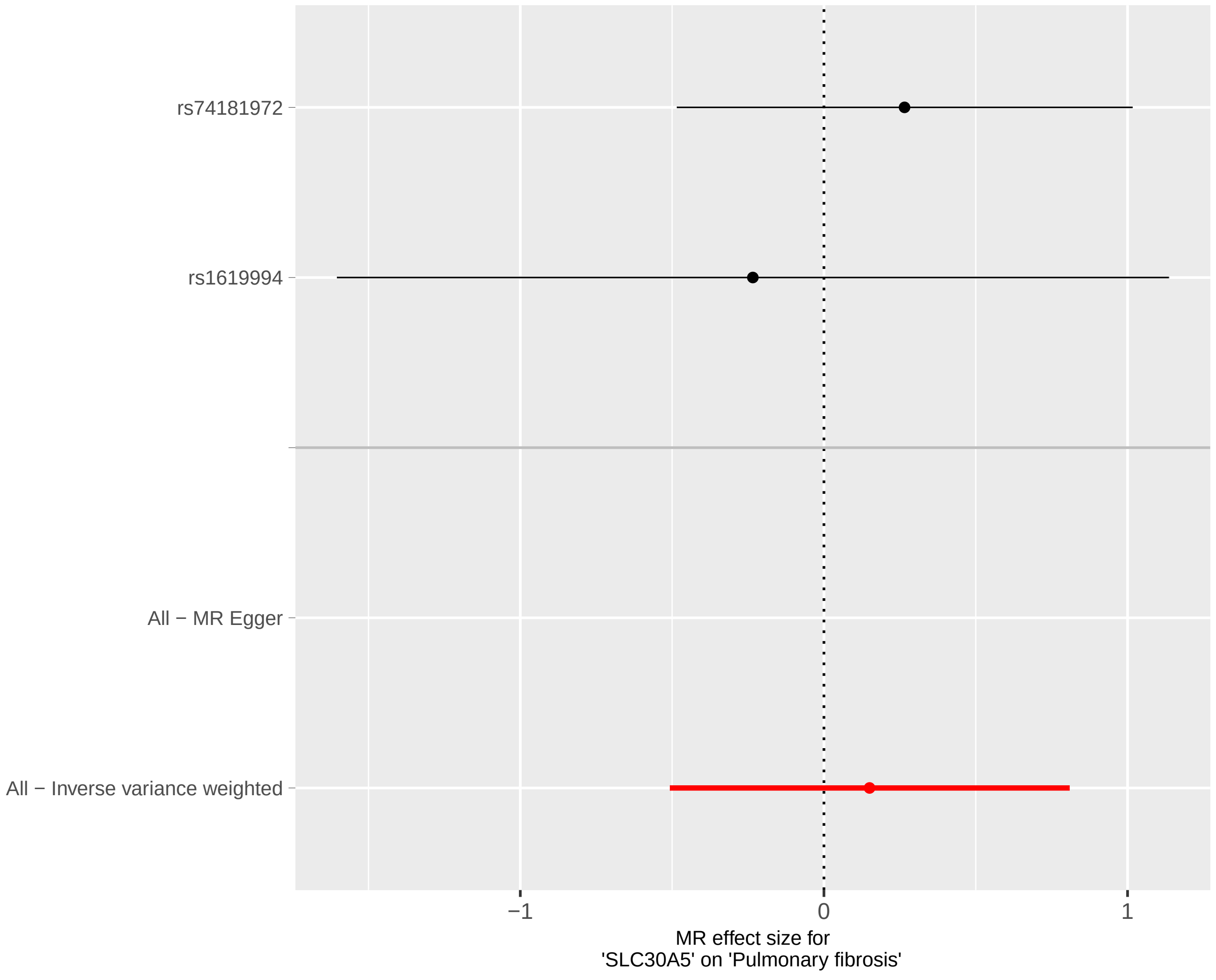
rs74181972
rs1619994
All − MR Egger
All − Inverse variance weighted
−1
0
1
MR effect size for
'SLC30A5' on 'Pulmonary fibrosis'

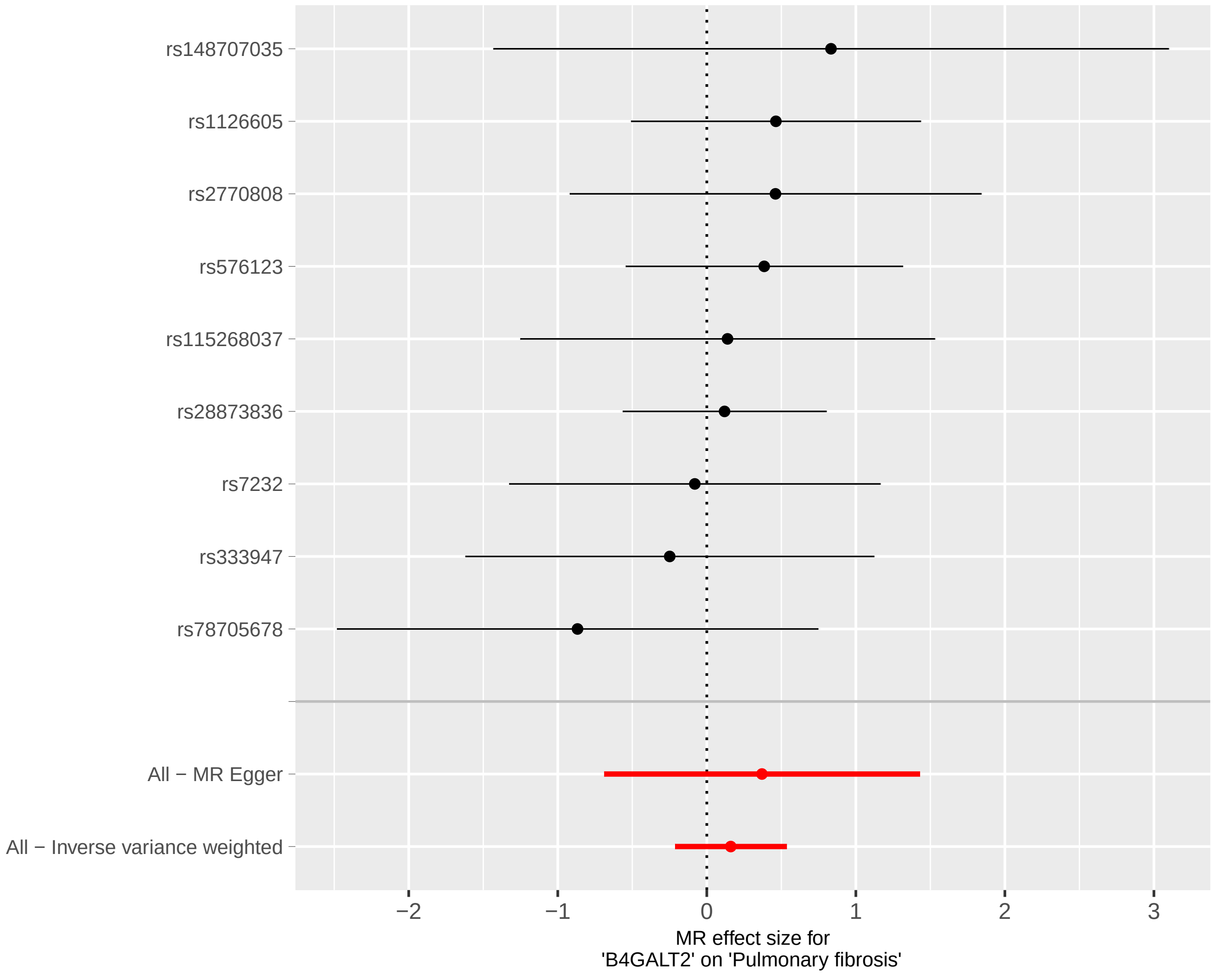

rs148707035
rs1126605
rs2770808
rs576123
rs115268037
rs28873836
rs7232
rs333947
rs78705678
All − MR Egger
All − Inverse variance weighted
−2
−1
0
1
2
3
MR effect size for
'B4GALT2' on 'Pulmonary fibrosis'

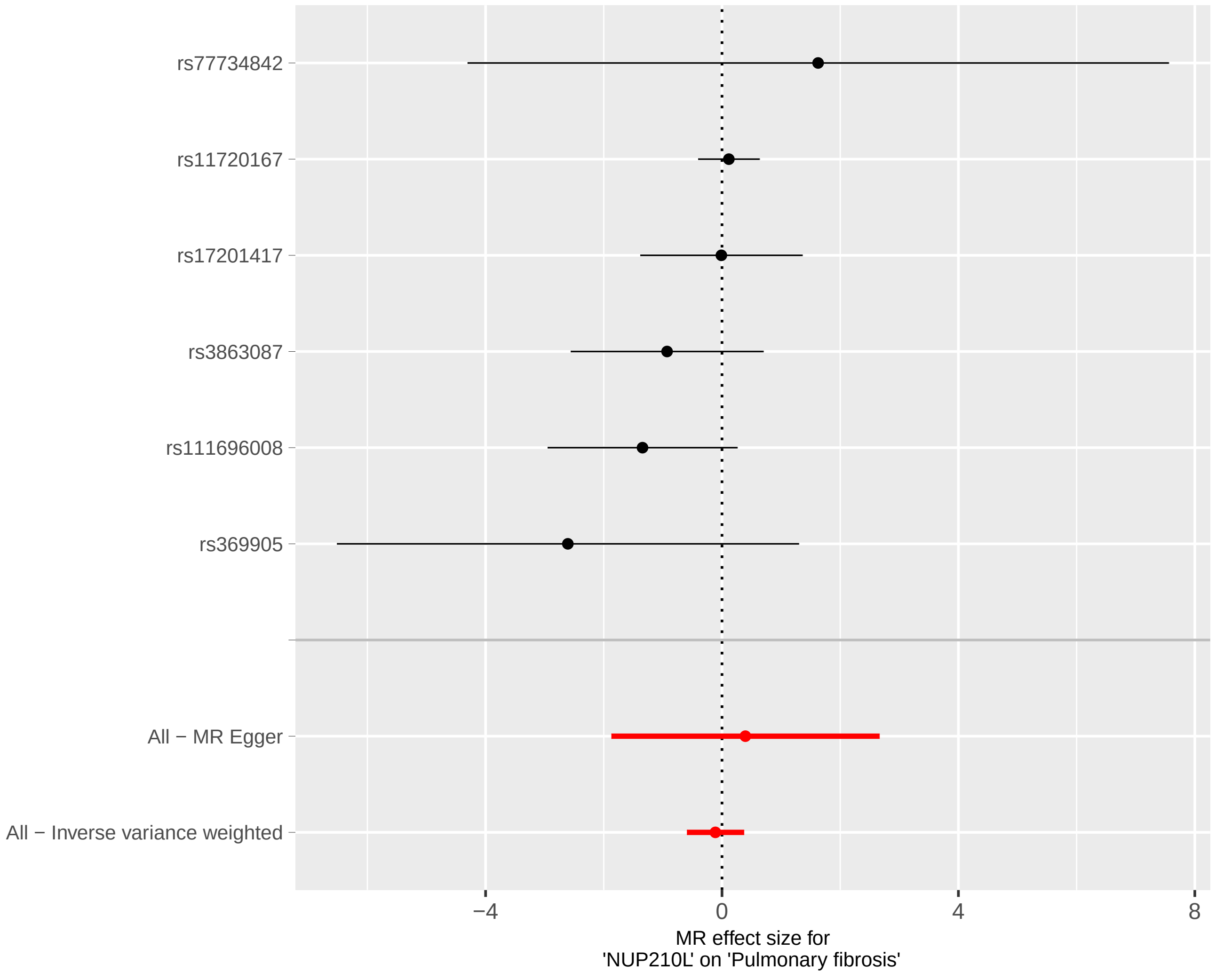
rs77734842
rs11720167
rs17201417
rs3863087
rs111696008
rs369905
All − MR Egger
All − Inverse variance weighted
−4
0
4
8
MR effect size for
'NUP210L' on 'Pulmonary fibrosis'

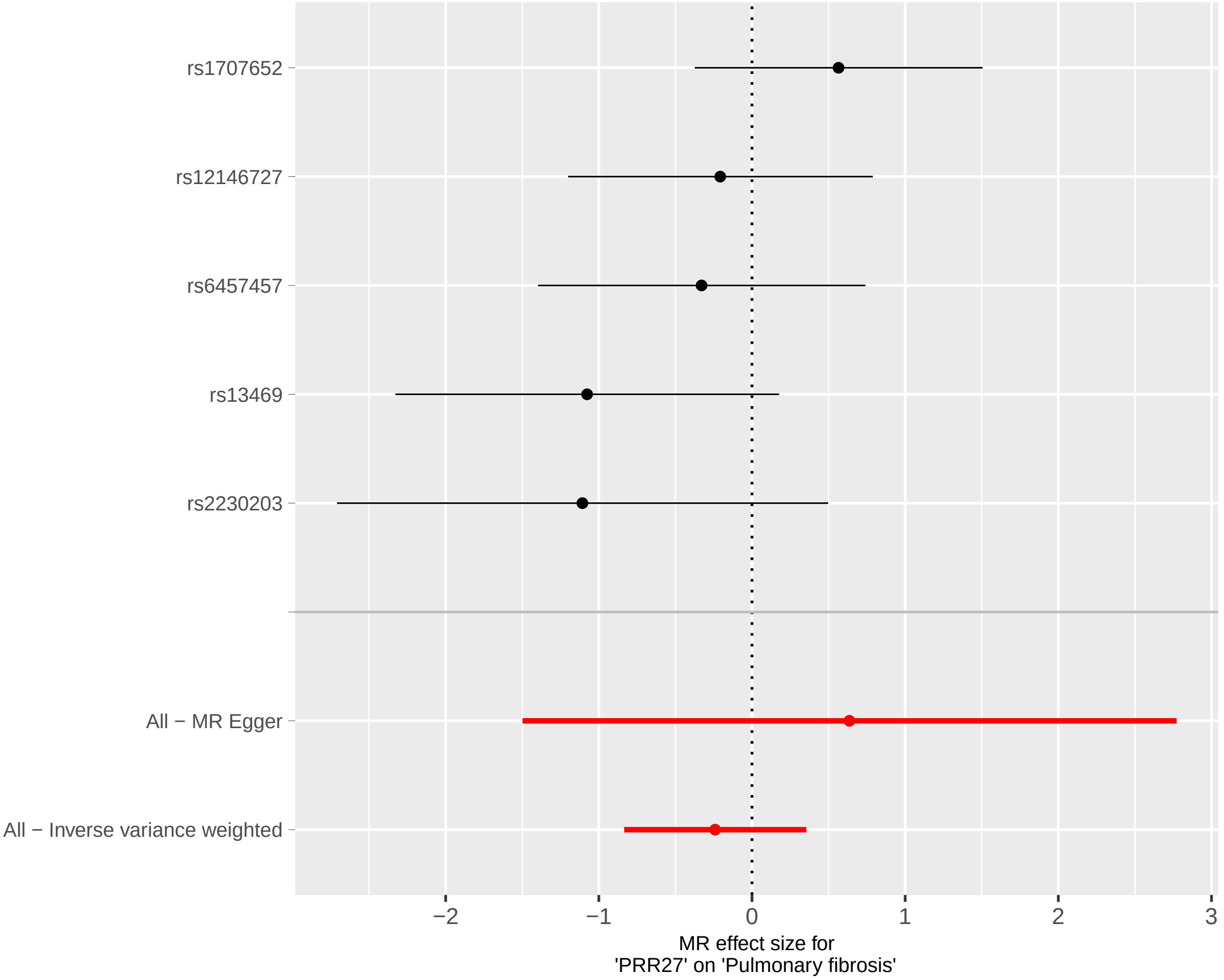
rs1707652
rs12146727
rs6457457
rs13469
rs2230203
All − MR Egger
All − Inverse variance weighted
−2
−1
0
1
2
3
MR effect size for
'PRR27' on 'Pulmonary fibrosis'

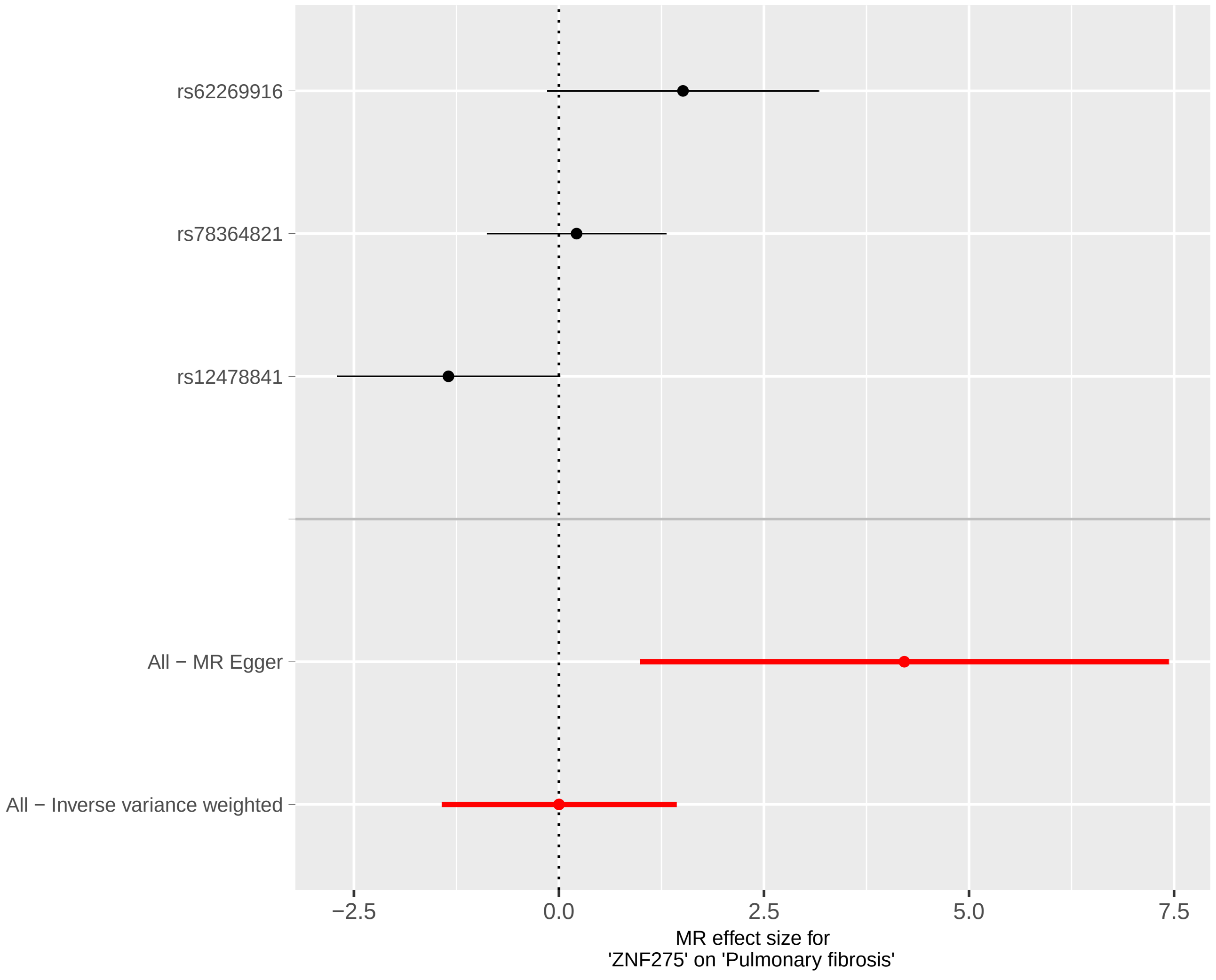

rs62269916
rs78364821
rs12478841
All − MR Egger
All − Inverse variance weighted
−2.5
0.0
2.5
5.0
7.5
MR effect size for
'ZNF275' on 'Pulmonary fibrosis'

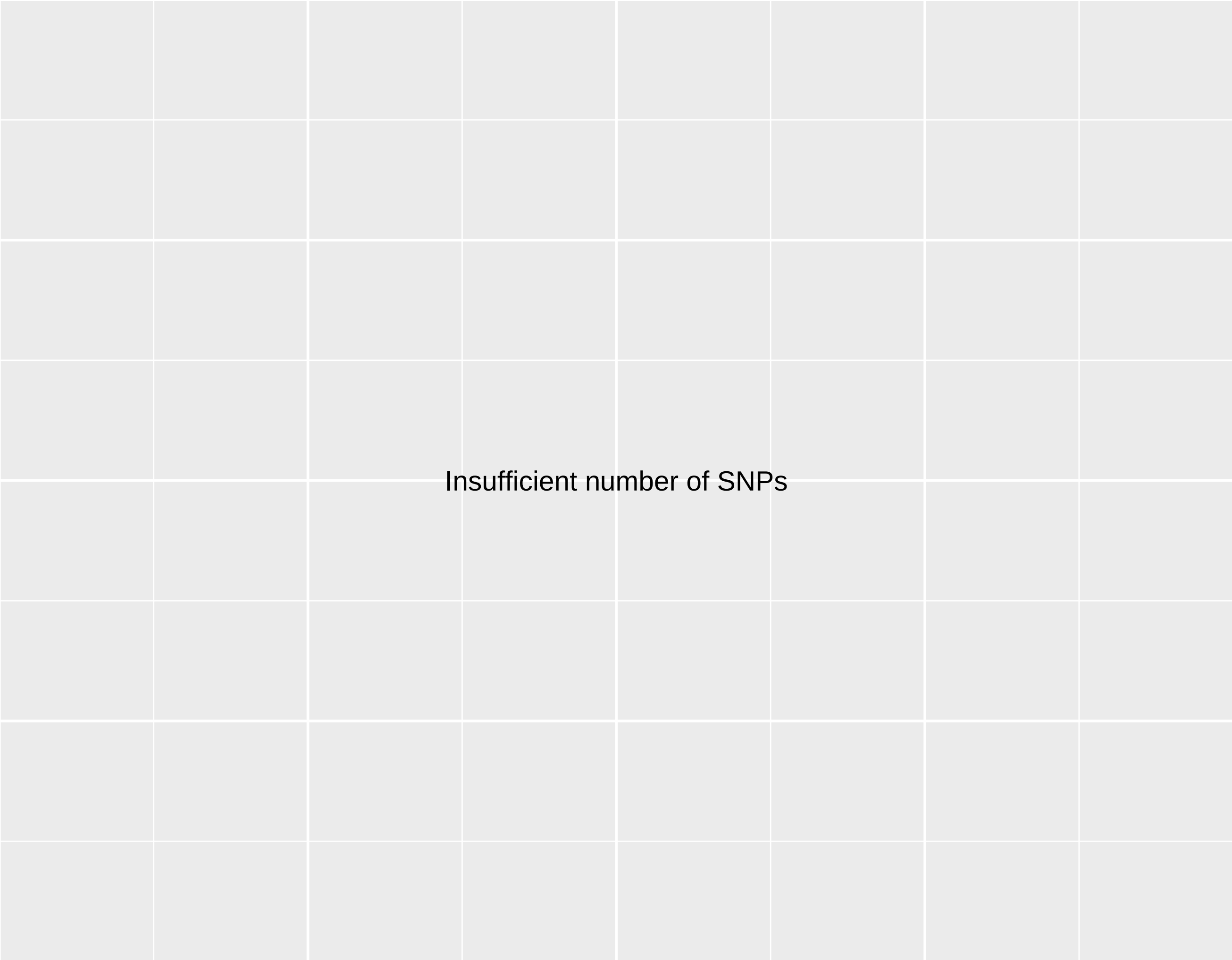
Insufficient number of SNPs

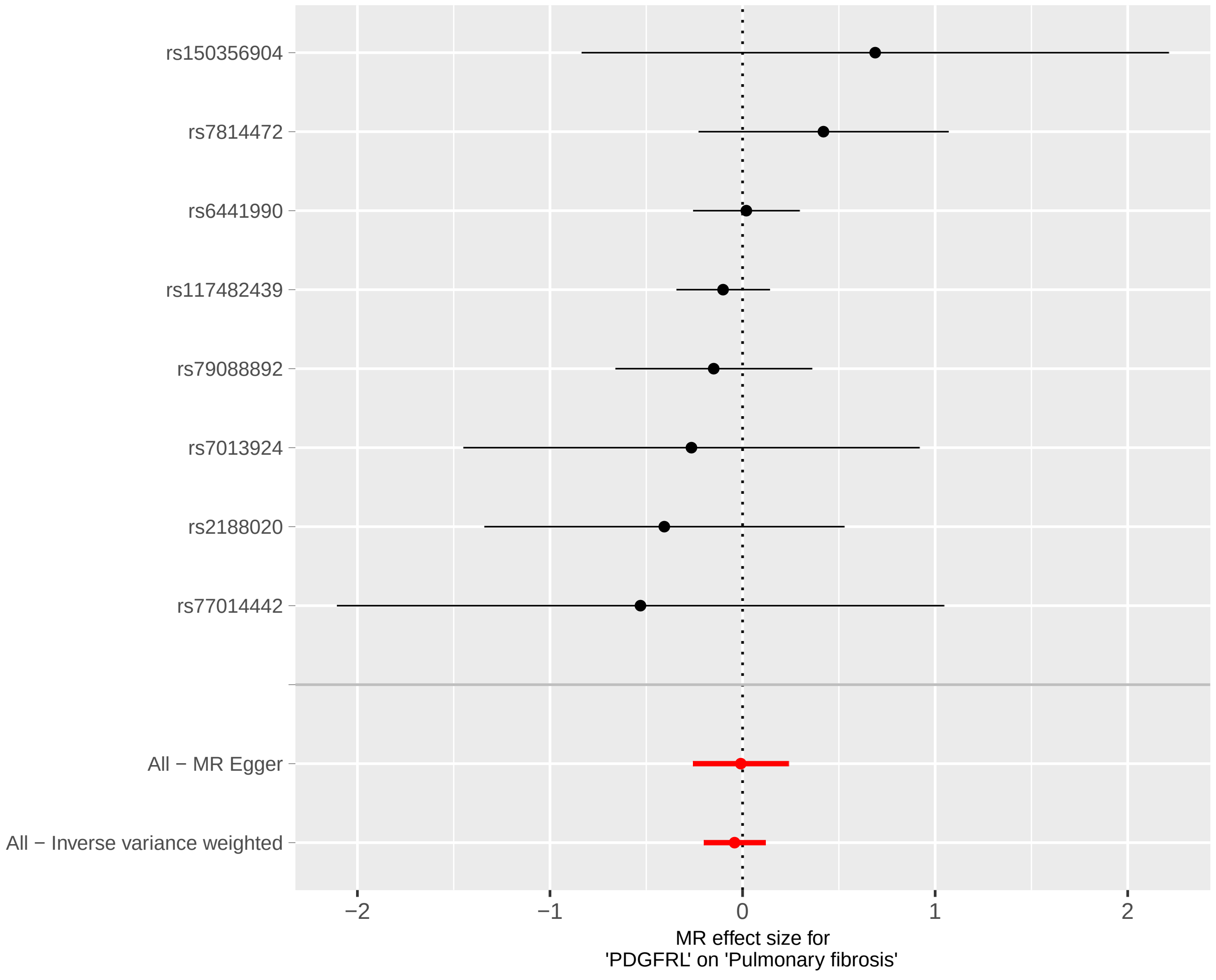

rs150356904
rs7814472
rs6441990
rs117482439
rs79088892
rs7013924
rs2188020
rs77014442
All − MR Egger
All − Inverse variance weighted
−2
−1
0
1
2
MR effect size for
'PDGFRL' on 'Pulmonary fibrosis'

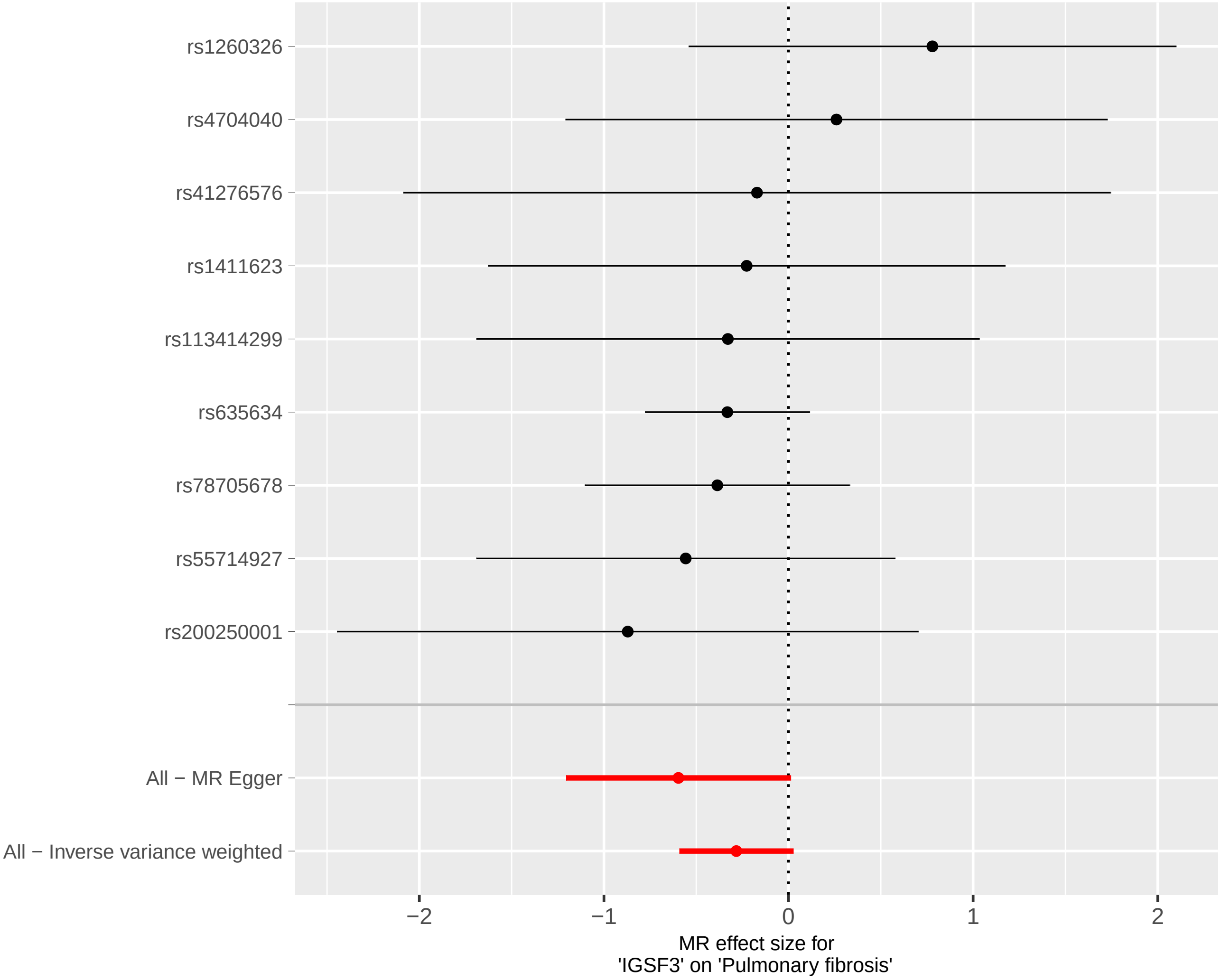

rs1260326
rs4704040
rs41276576
rs1411623
rs113414299
rs635634
rs78705678
rs55714927
rs200250001
All − MR Egger
All − Inverse variance weighted
−2
−1
0
1
2
MR effect size for
'IGSF3' on 'Pulmonary fibrosis'

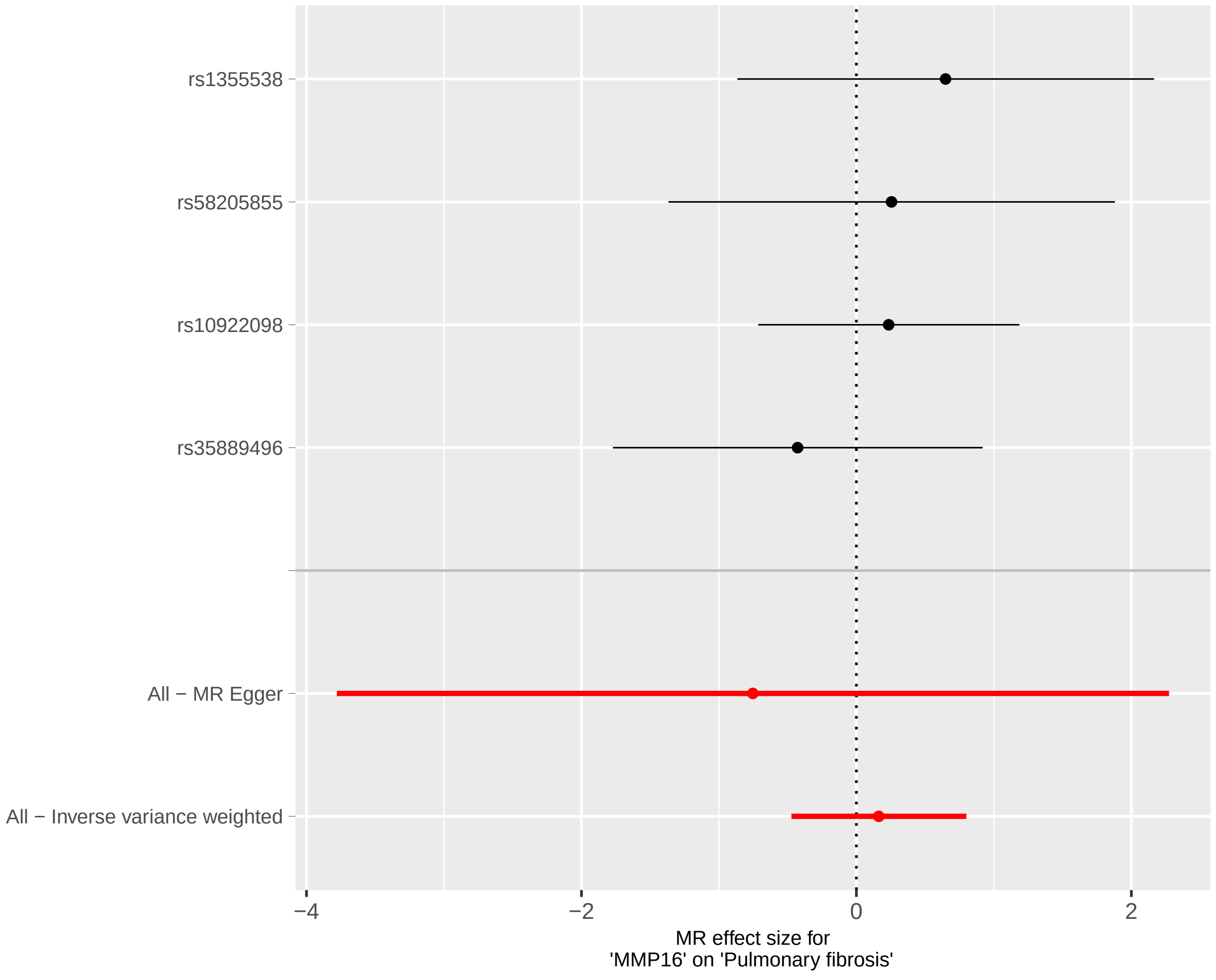

rs1355538
rs58205855
rs10922098
rs35889496
All − MR Egger
All − Inverse variance weighted
−4
−2
0
2
MR effect size for
'MMP16' on 'Pulmonary fibrosis'

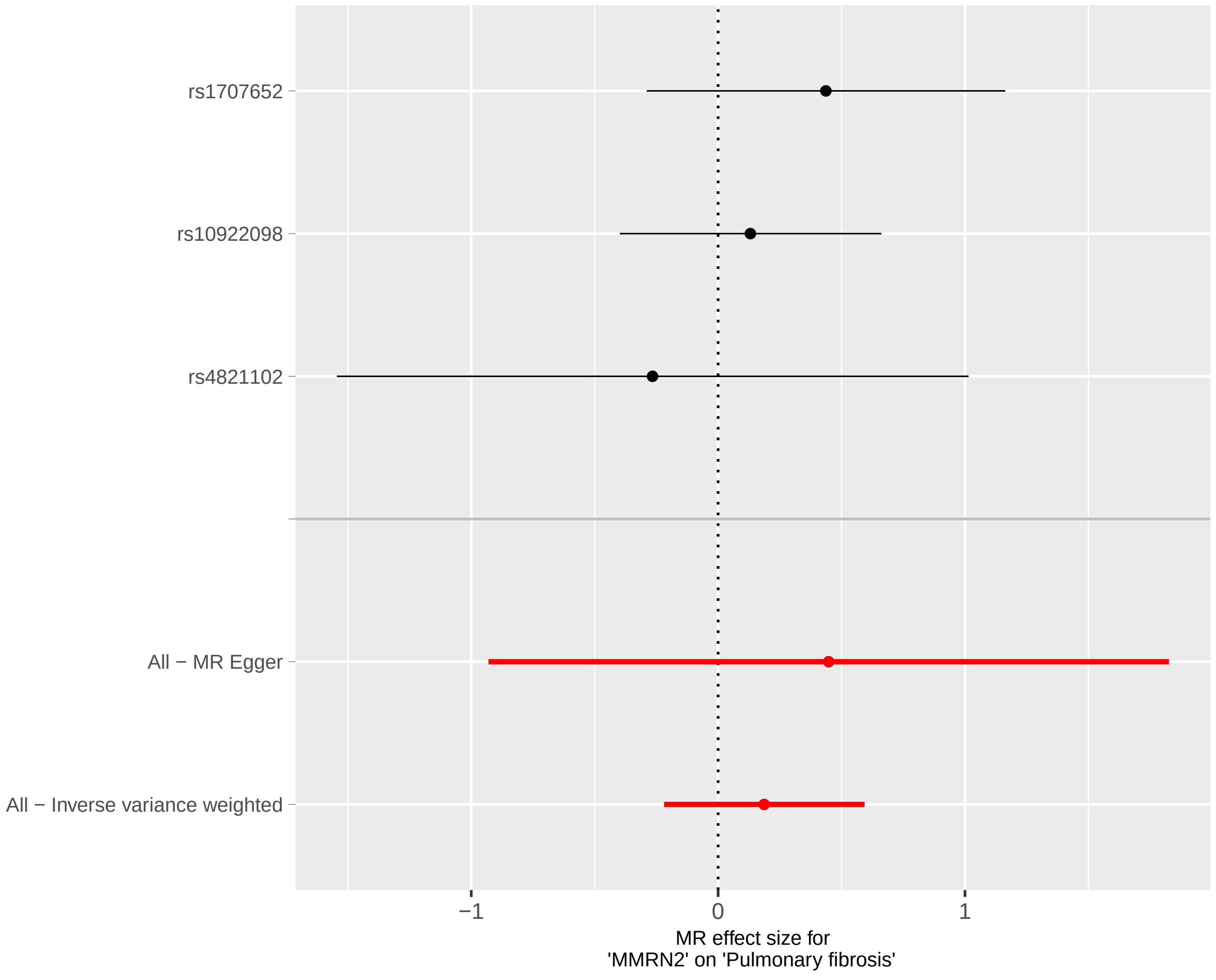
rs1707652
rs10922098
rs4821102
All − MR Egger
All − Inverse variance weighted
−1
0
1
MR effect size for
'MMRN2' on 'Pulmonary fibrosis'

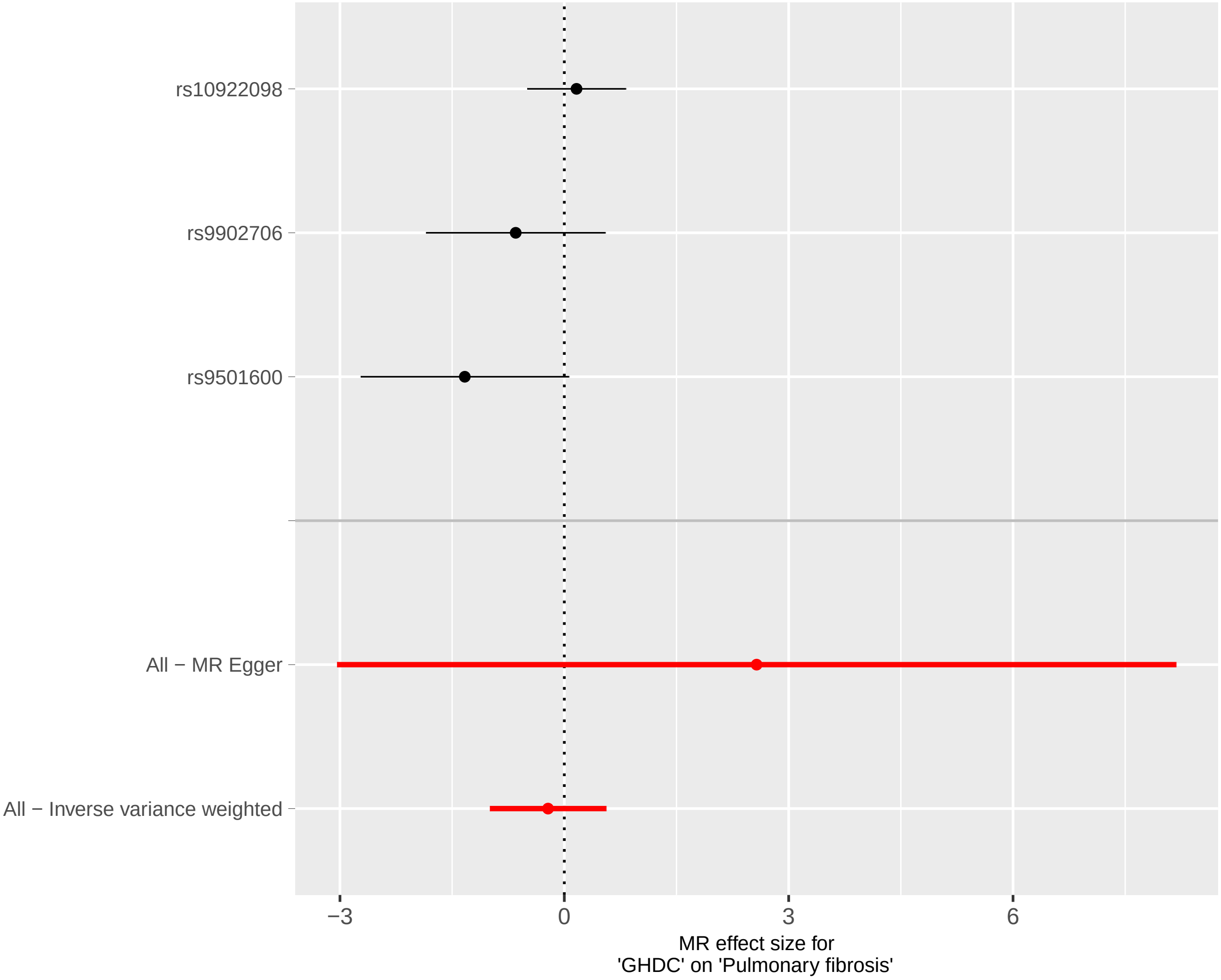
rs10922098
rs9902706
rs9501600
All − MR Egger
All − Inverse variance weighted
−3
0
3
6
MR effect size for
'GHDC' on 'Pulmonary fibrosis'

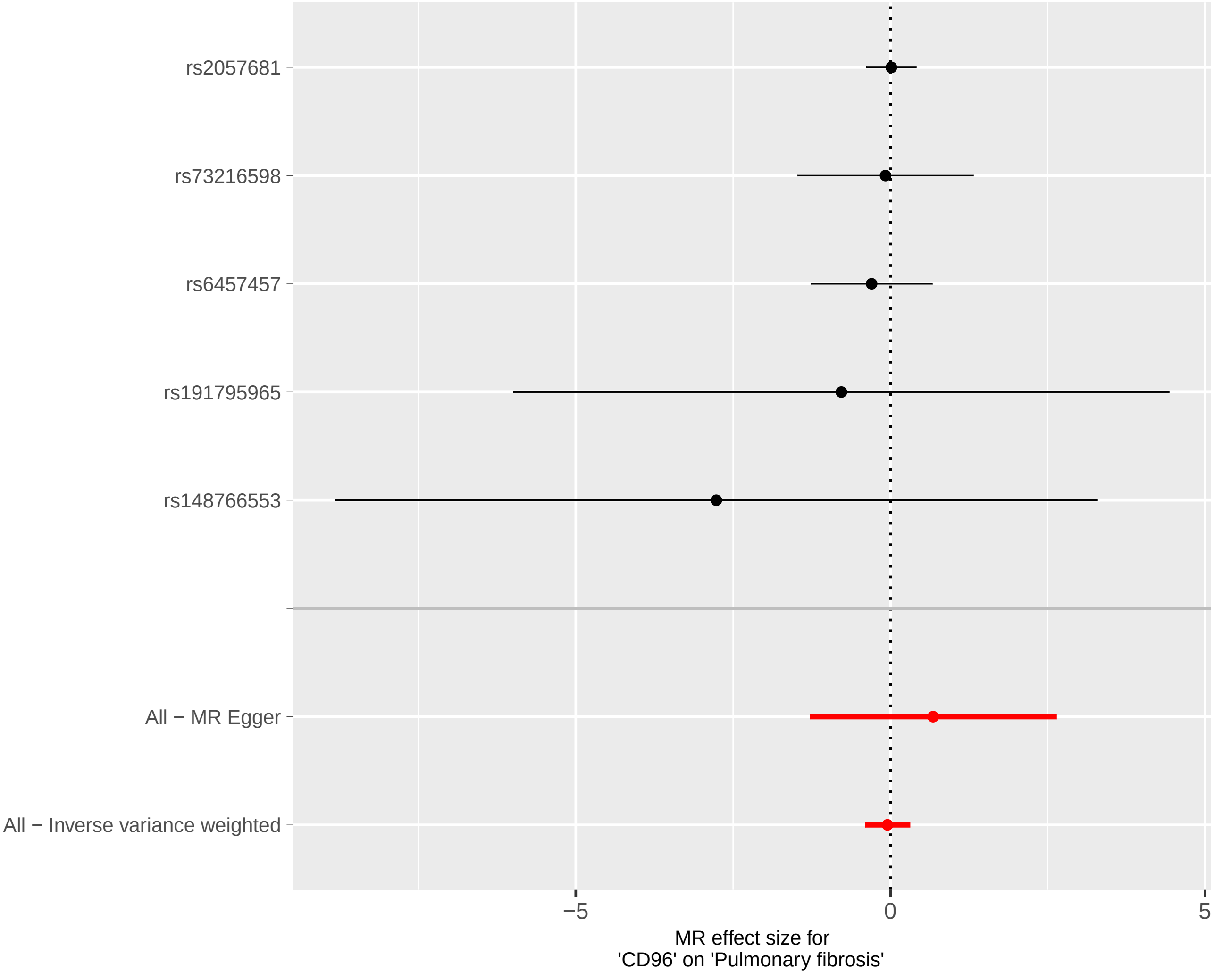
rs2057681
rs73216598
rs6457457
rs191795965
rs148766553
All − MR Egger
All − Inverse variance weighted
−5
0
5
MR effect size for
'CD96' on 'Pulmonary fibrosis'

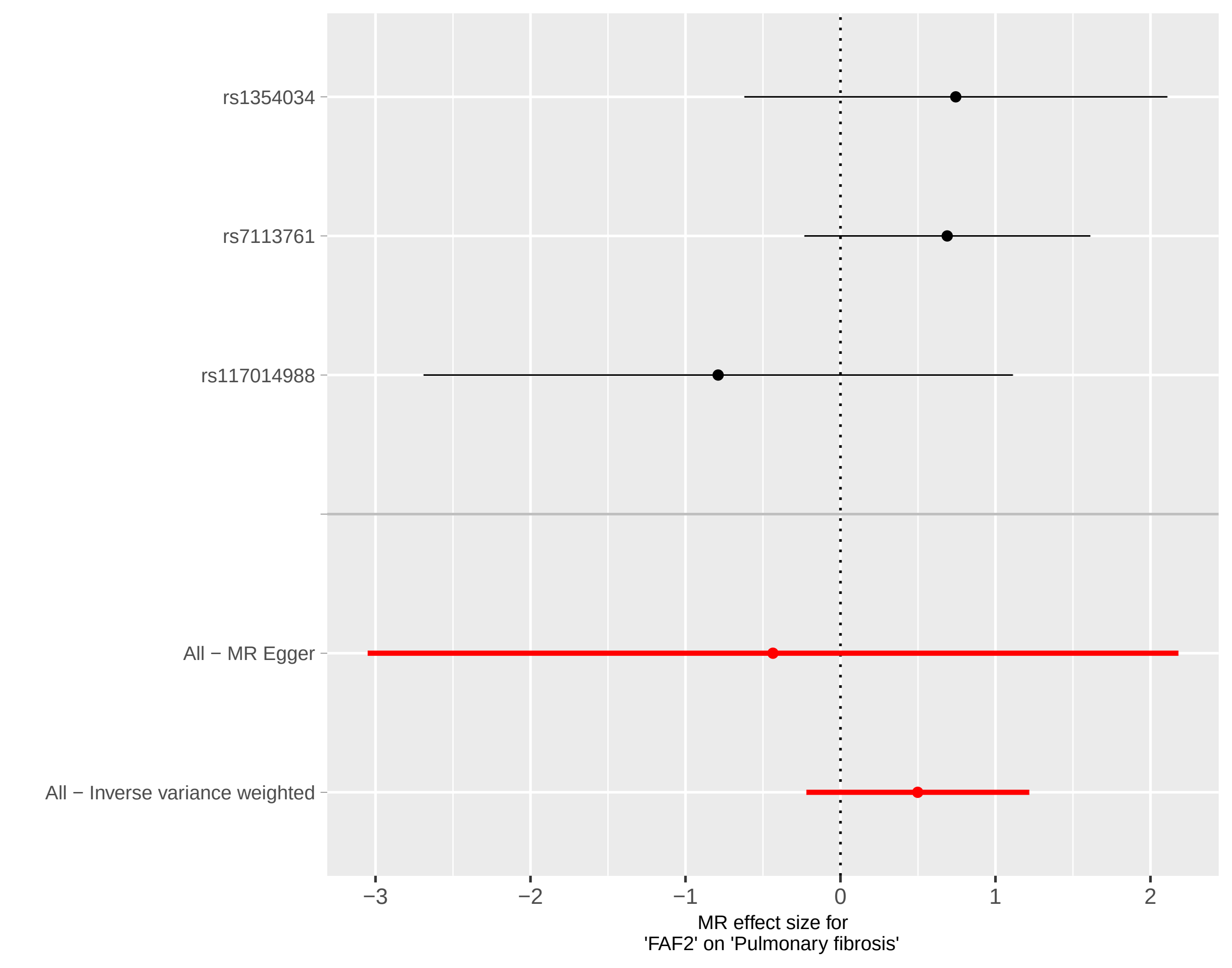
rs1354034
rs7113761
rs117014988
All − MR Egger
All − Inverse variance weighted
−3
−2
−1
0
1
2
MR effect size for
'FAF2' on 'Pulmonary fibrosis'

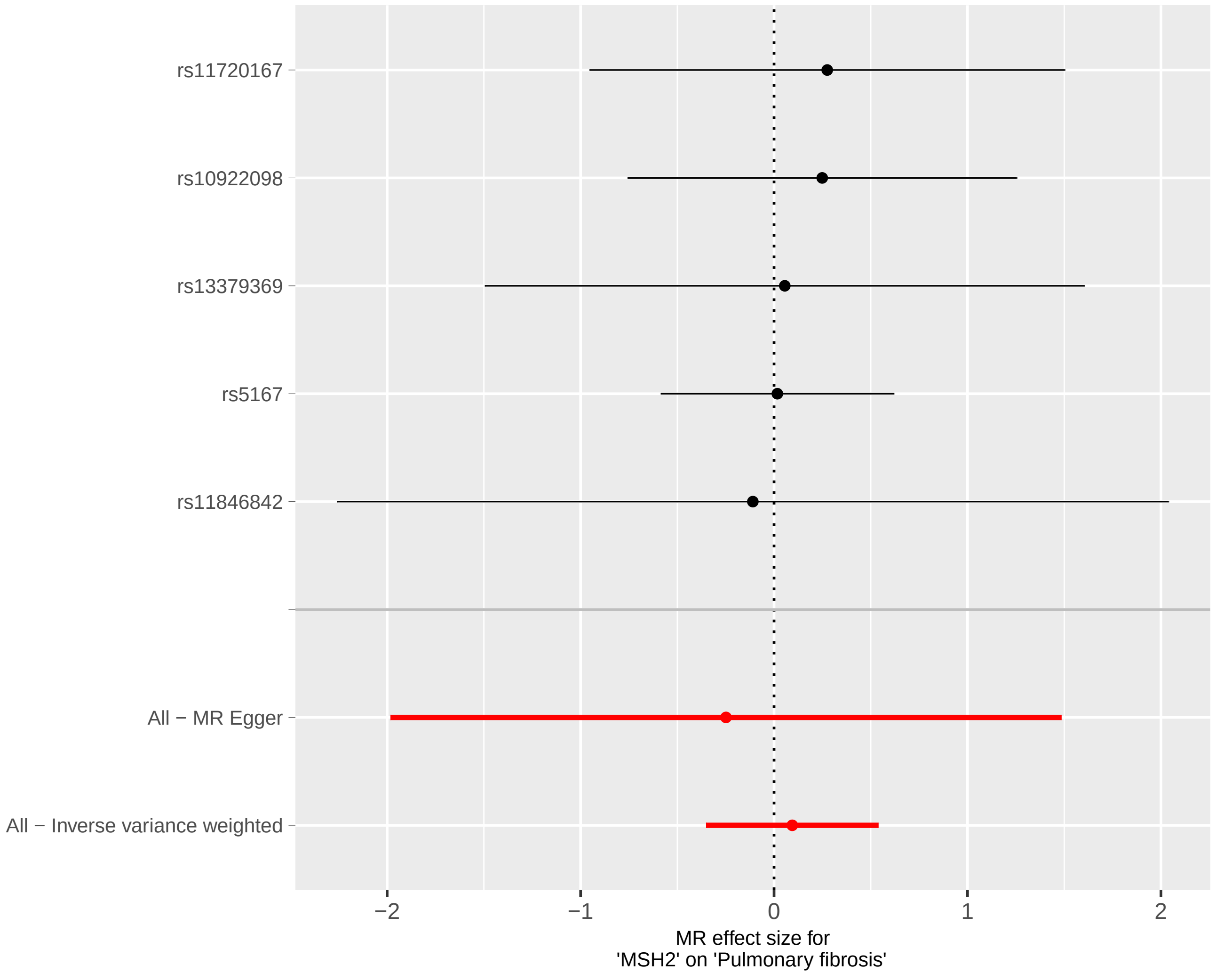

rs11720167
rs10922098
rs13379369
rs5167
rs11846842
All − MR Egger
All − Inverse variance weighted
−2
−1
0
1
2
MR effect size for
'MSH2' on 'Pulmonary fibrosis'

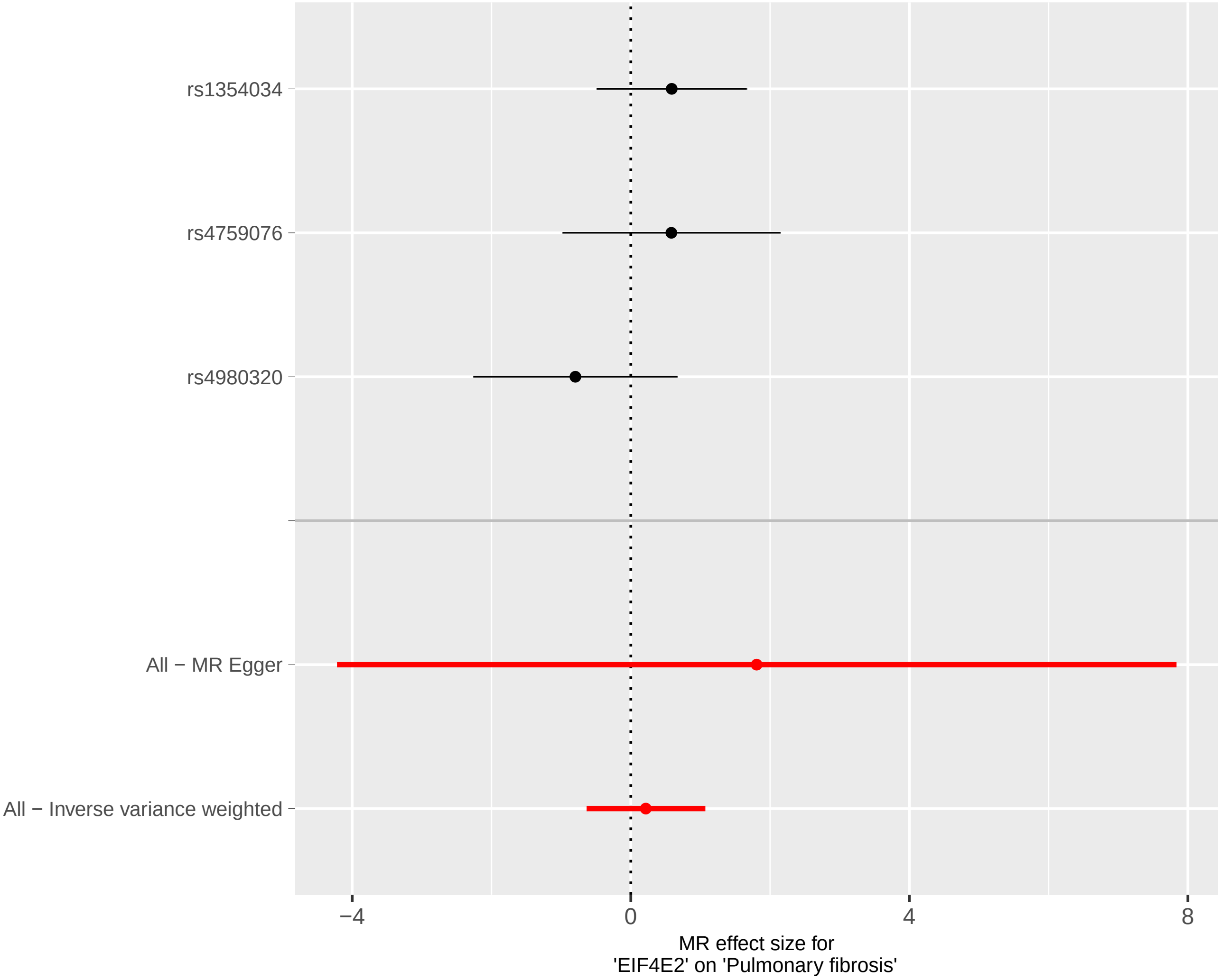

rs1354034
rs4759076
rs4980320
All − MR Egger
All − Inverse variance weighted
−4
0
4
8
MR effect size for
'EIF4E2' on 'Pulmonary fibrosis'

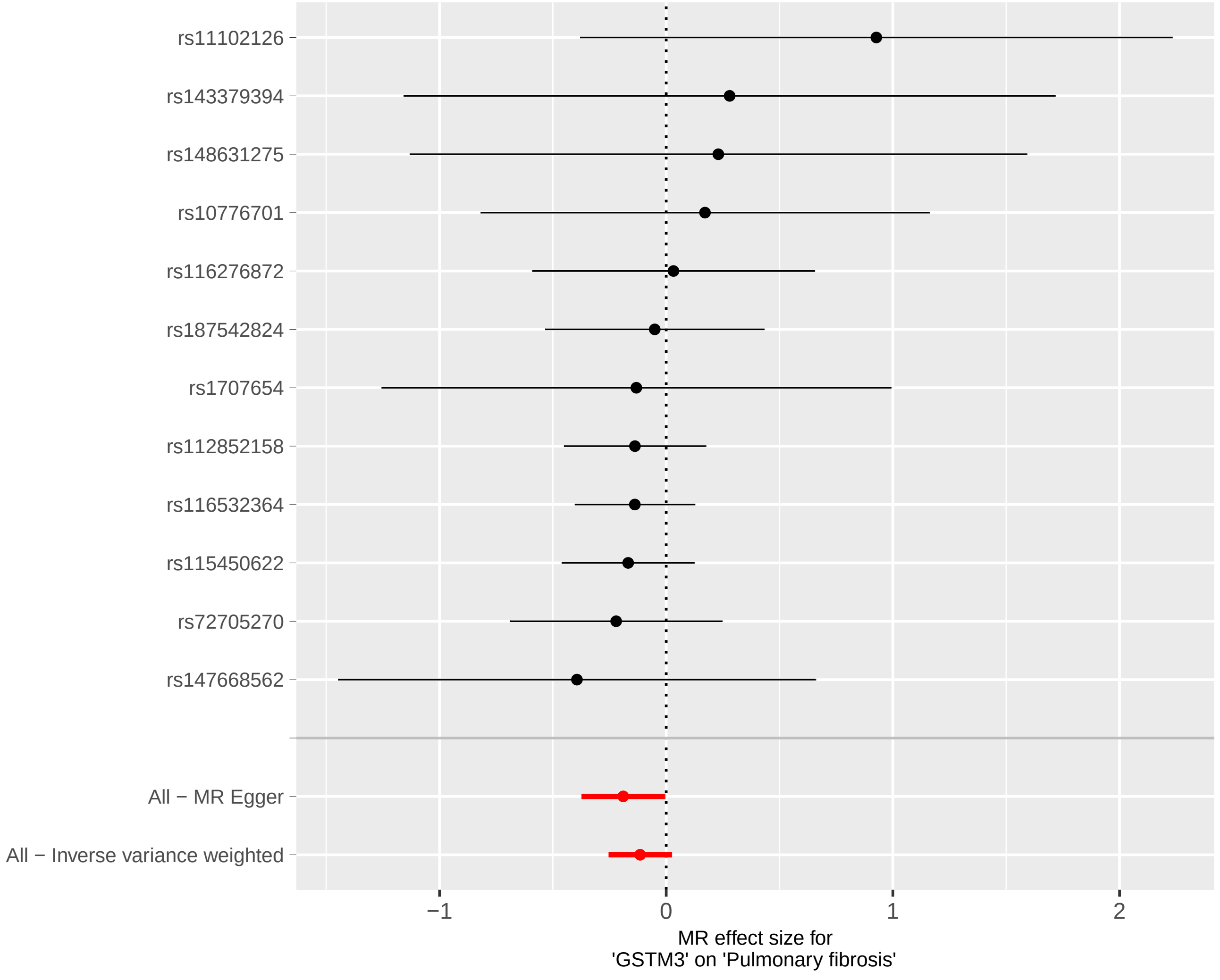
rs11102126
rs143379394
rs148631275
rs10776701
rs116276872
rs187542824
rs1707654
rs112852158
rs116532364
rs115450622
rs72705270
rs147668562
All − MR Egger
All − Inverse variance weighted
−1
0
1
2
MR effect size for
'GSTM3' on 'Pulmonary fibrosis'

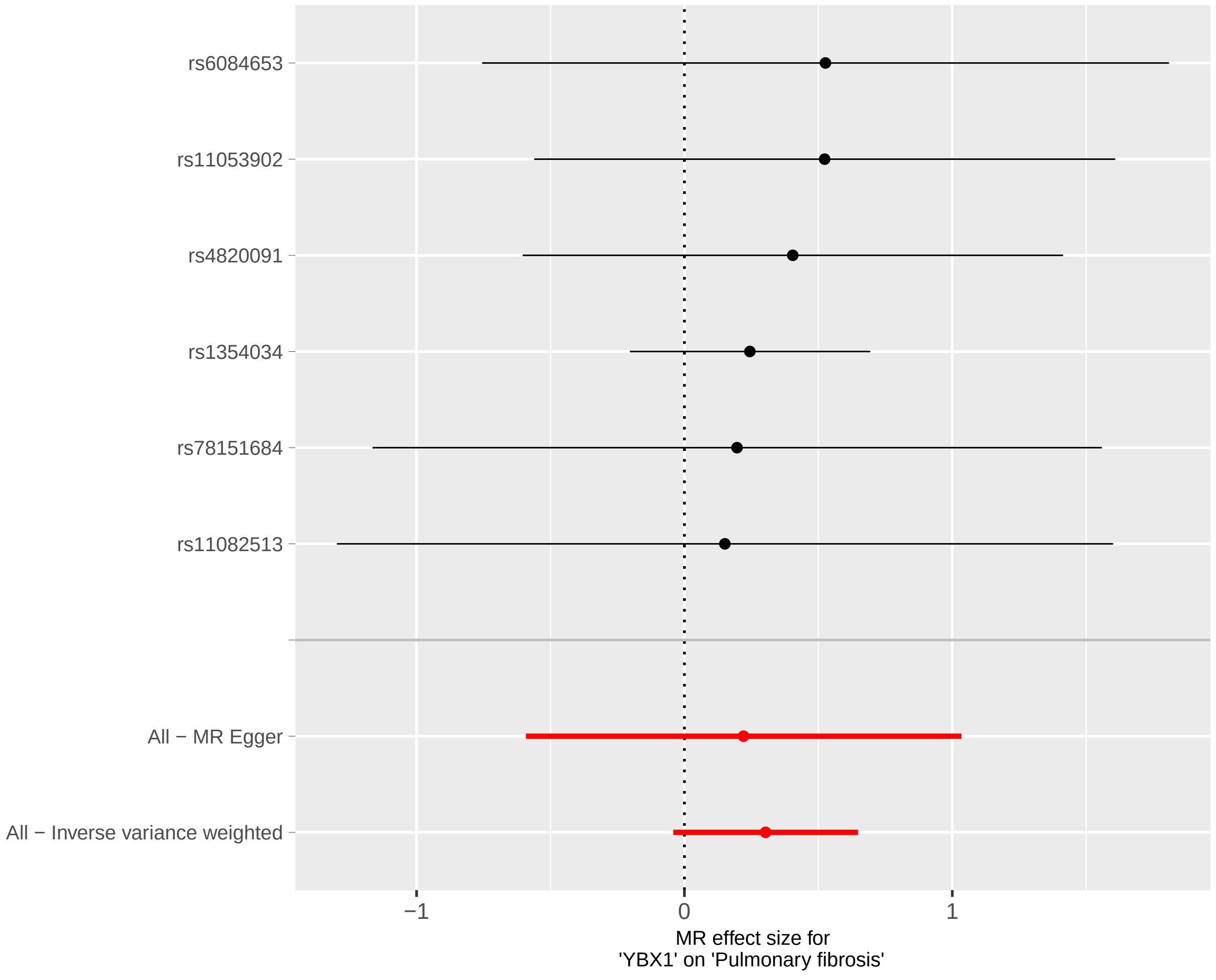

rs6084653
rs11053902
rs4820091
rs1354034
rs78151684
rs11082513
All − MR Egger
All − Inverse variance weighted
−1
0
1
MR effect size for
'YBX1' on 'Pulmonary fibrosis'

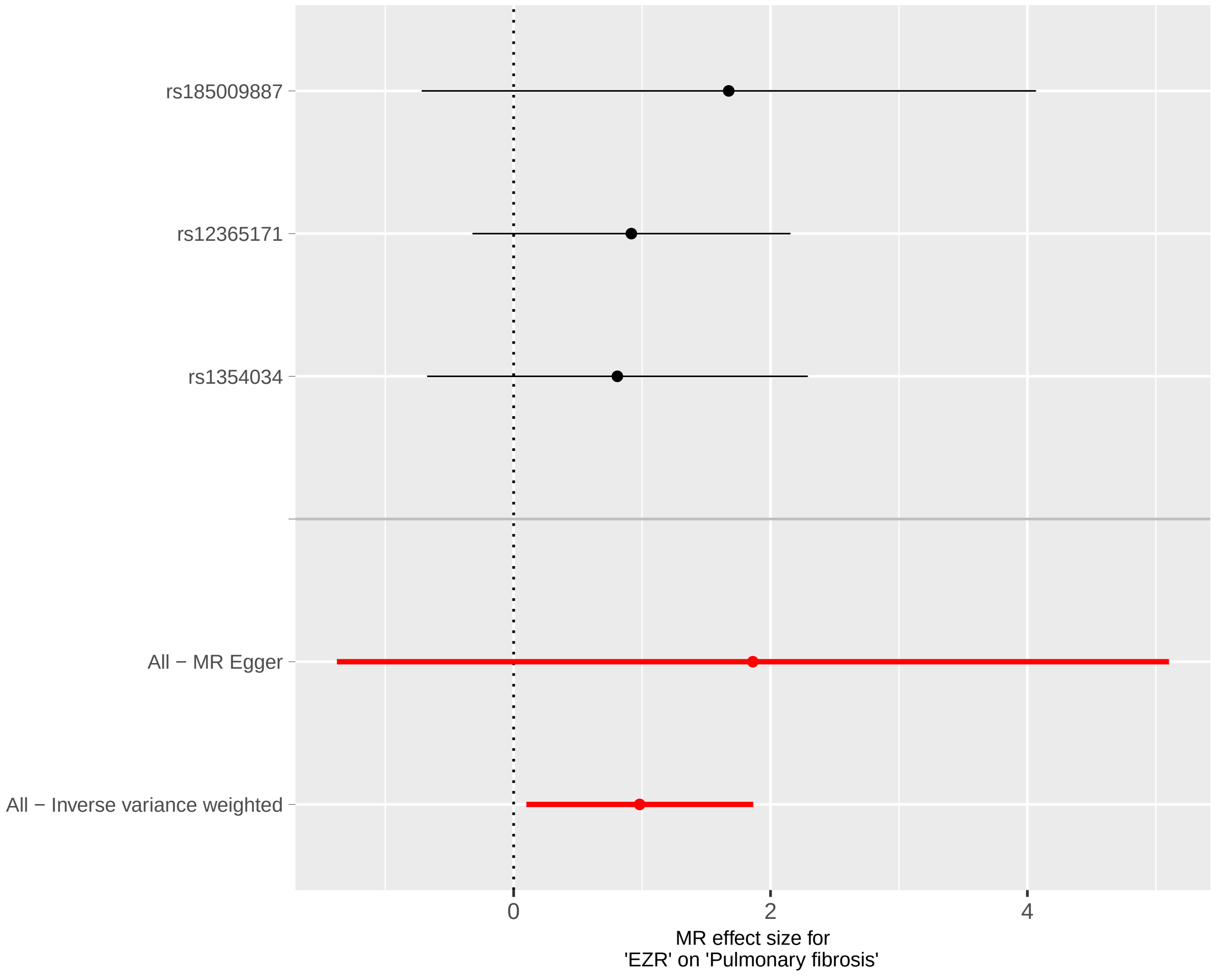

rs185009887
rs12365171
rs1354034
All − MR Egger
All − Inverse variance weighted
0
2
4
MR effect size for
'EZR' on 'Pulmonary fibrosis'

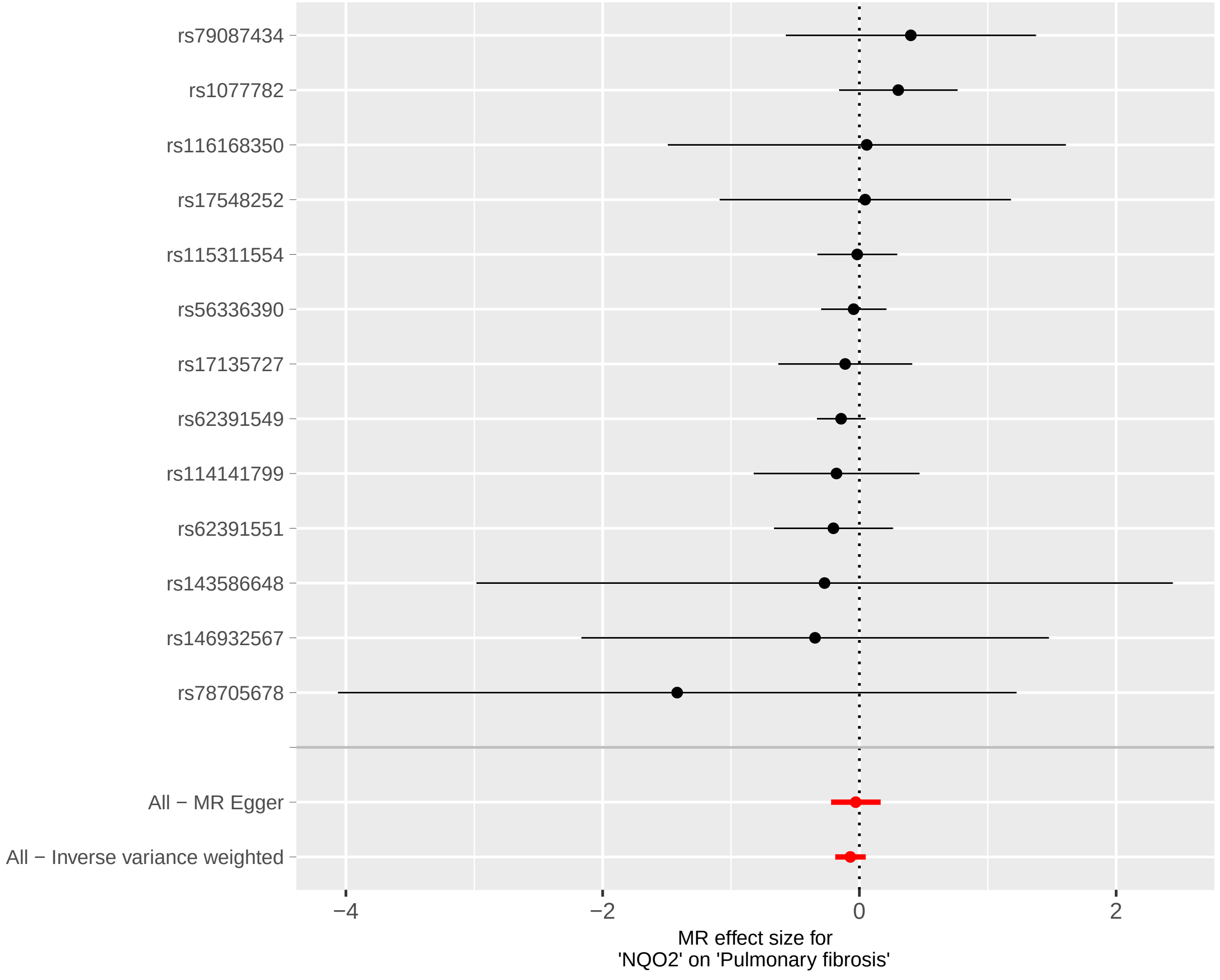
rs79087434
rs1077782
rs116168350
rs17548252
rs115311554
rs56336390
rs17135727
rs62391549
rs114141799
rs62391551
rs143586648
rs146932567
rs78705678
All − MR Egger
All − Inverse variance weighted
−4
−2
0
2
MR effect size for
'NQO2' on 'Pulmonary fibrosis'

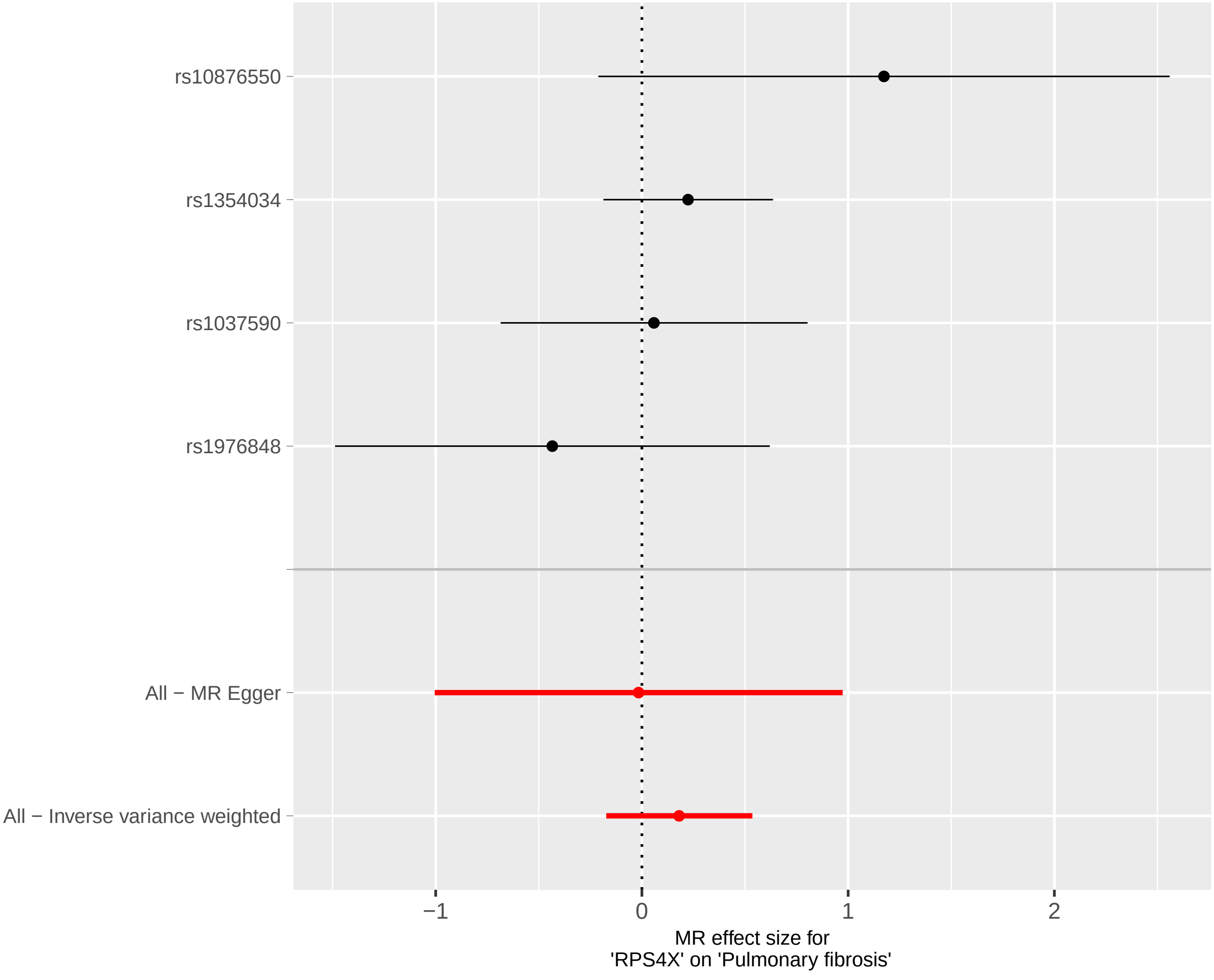

rs10876550
rs1354034
rs1037590
rs1976848
All − MR Egger
All − Inverse variance weighted
−1
0
1
2
MR effect size for
'RPS4X' on 'Pulmonary fibrosis'

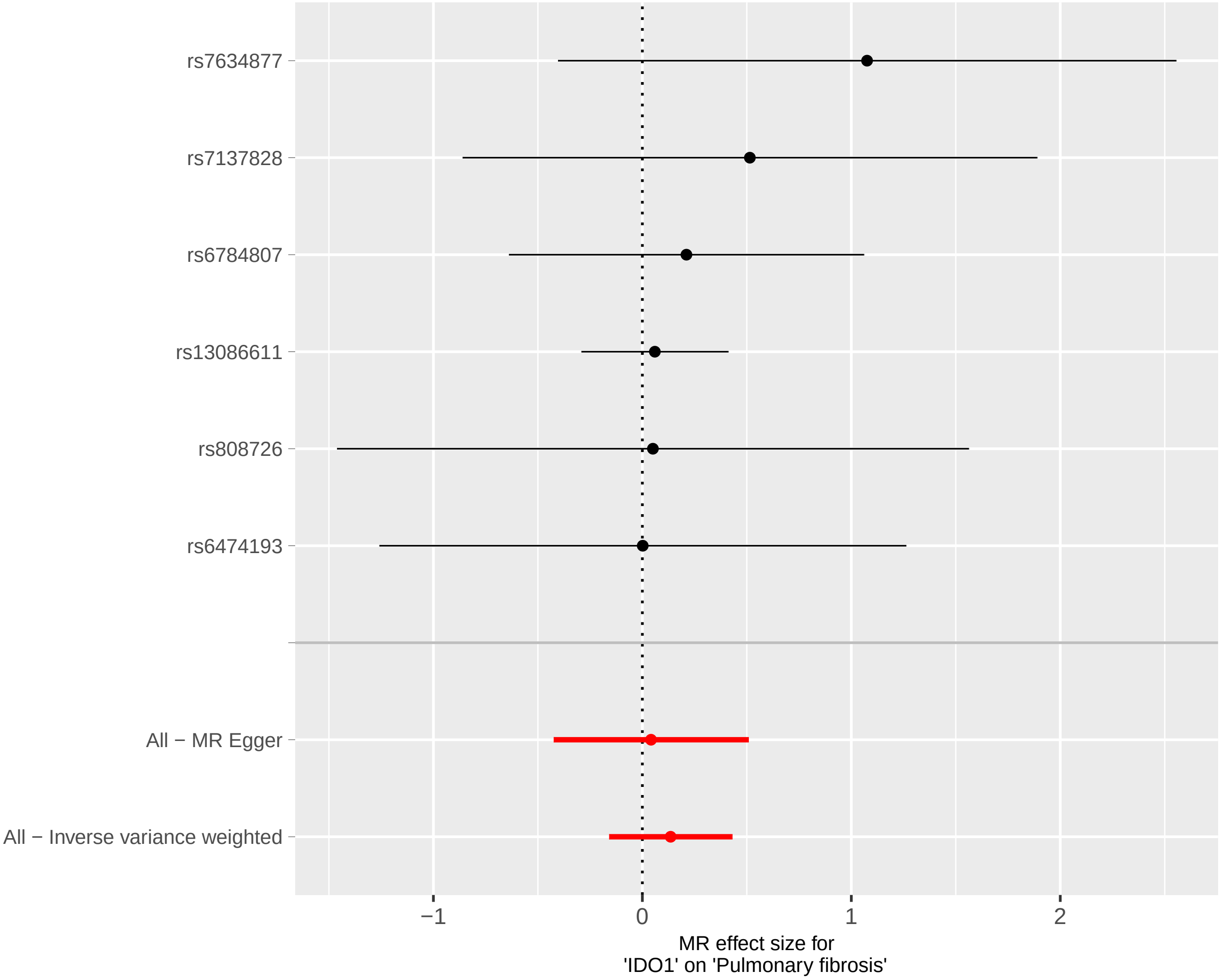

rs7634877
rs7137828
rs6784807
rs13086611
rs808726
rs6474193
All − MR Egger
All − Inverse variance weighted
−1
0
1
2
MR effect size for
'IDO1' on 'Pulmonary fibrosis'

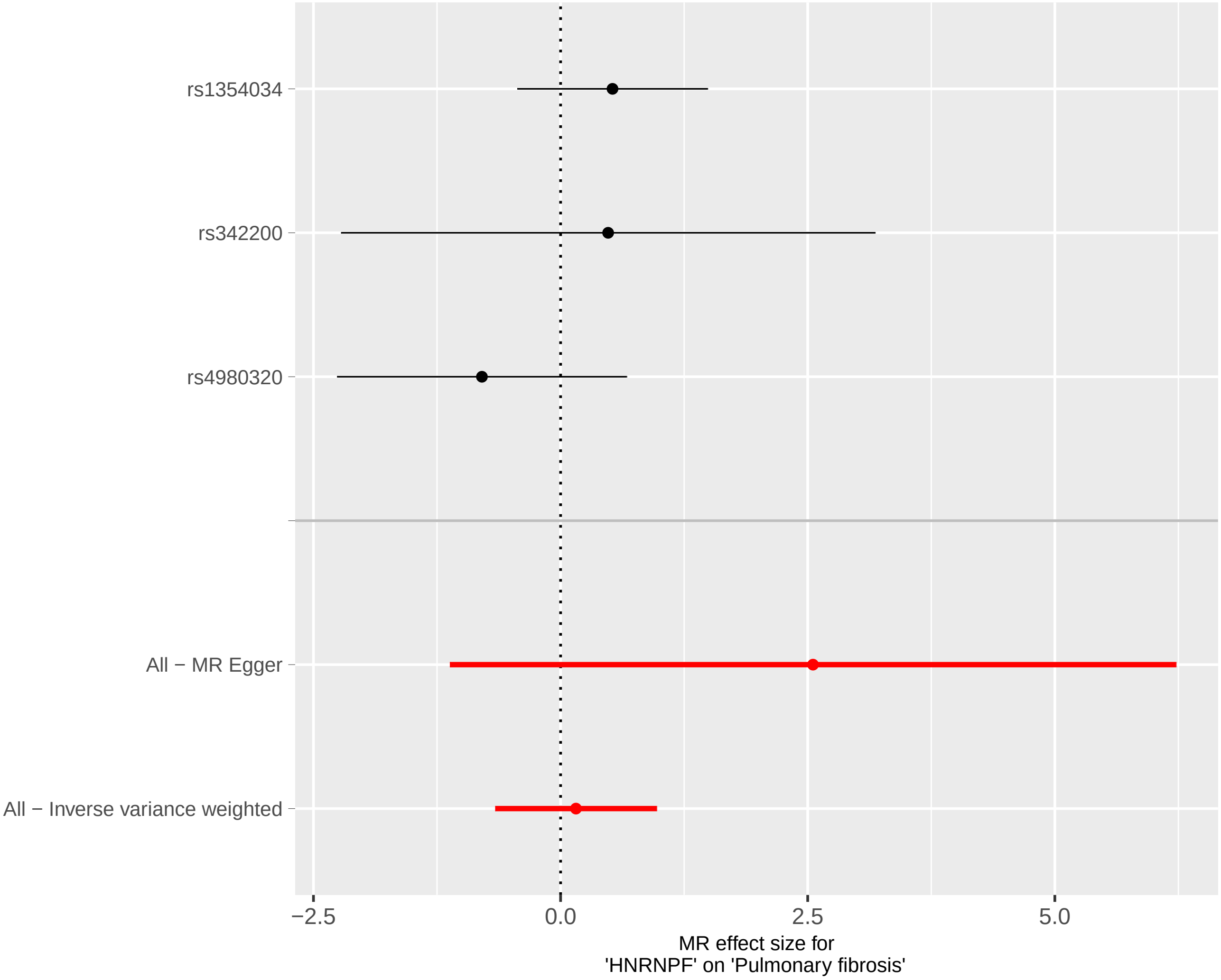
rs1354034
rs342200
rs4980320
All − MR Egger
All − Inverse variance weighted
−2.5
0.0
2.5
5.0
MR effect size for
'HNRNPF' on 'Pulmonary fibrosis'

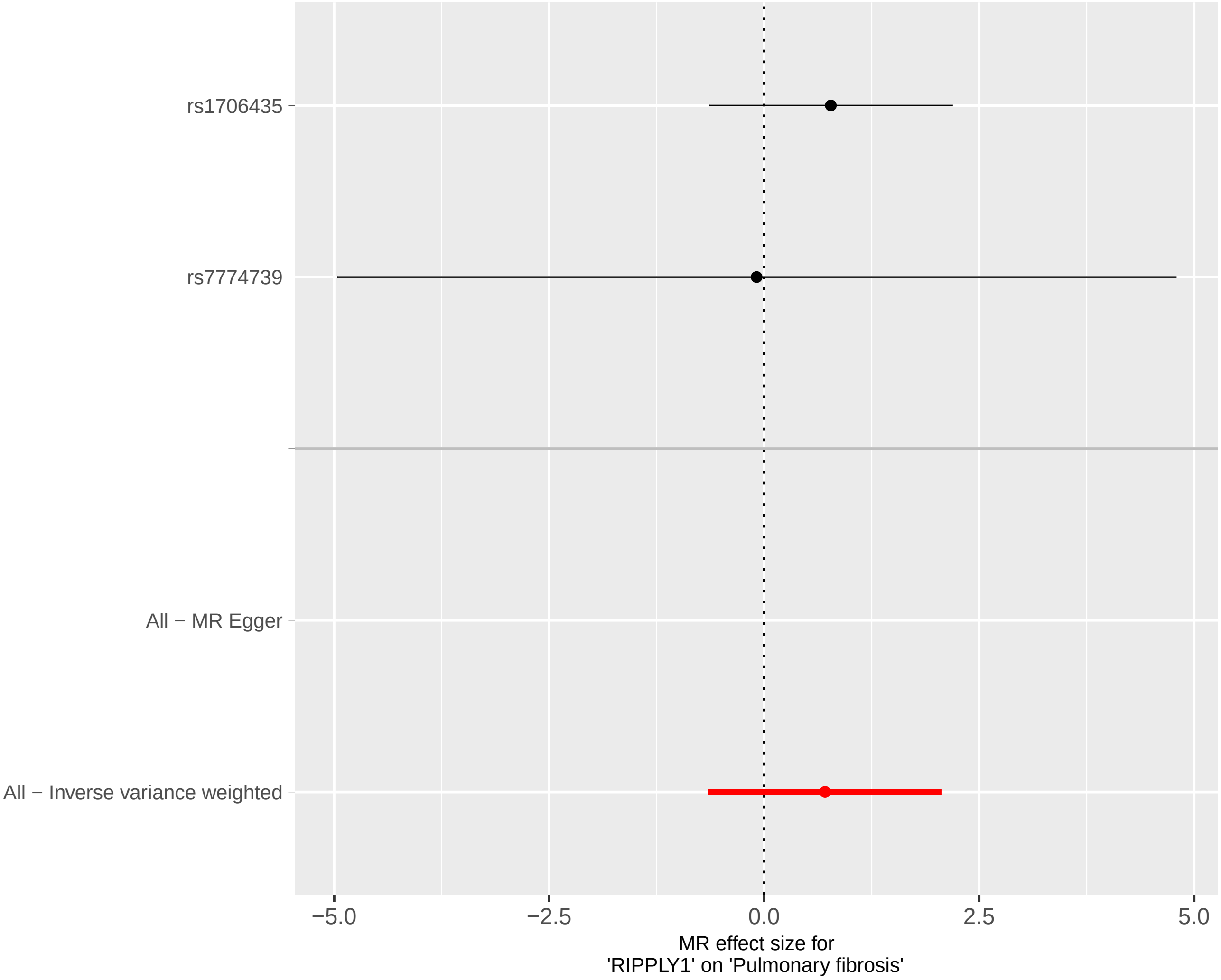

rs1706435
rs7774739
All − MR Egger
All − Inverse variance weighted
−5.0
−2.5
0.0
2.5
5.0
MR effect size for
'RIPPLY1' on 'Pulmonary fibrosis'

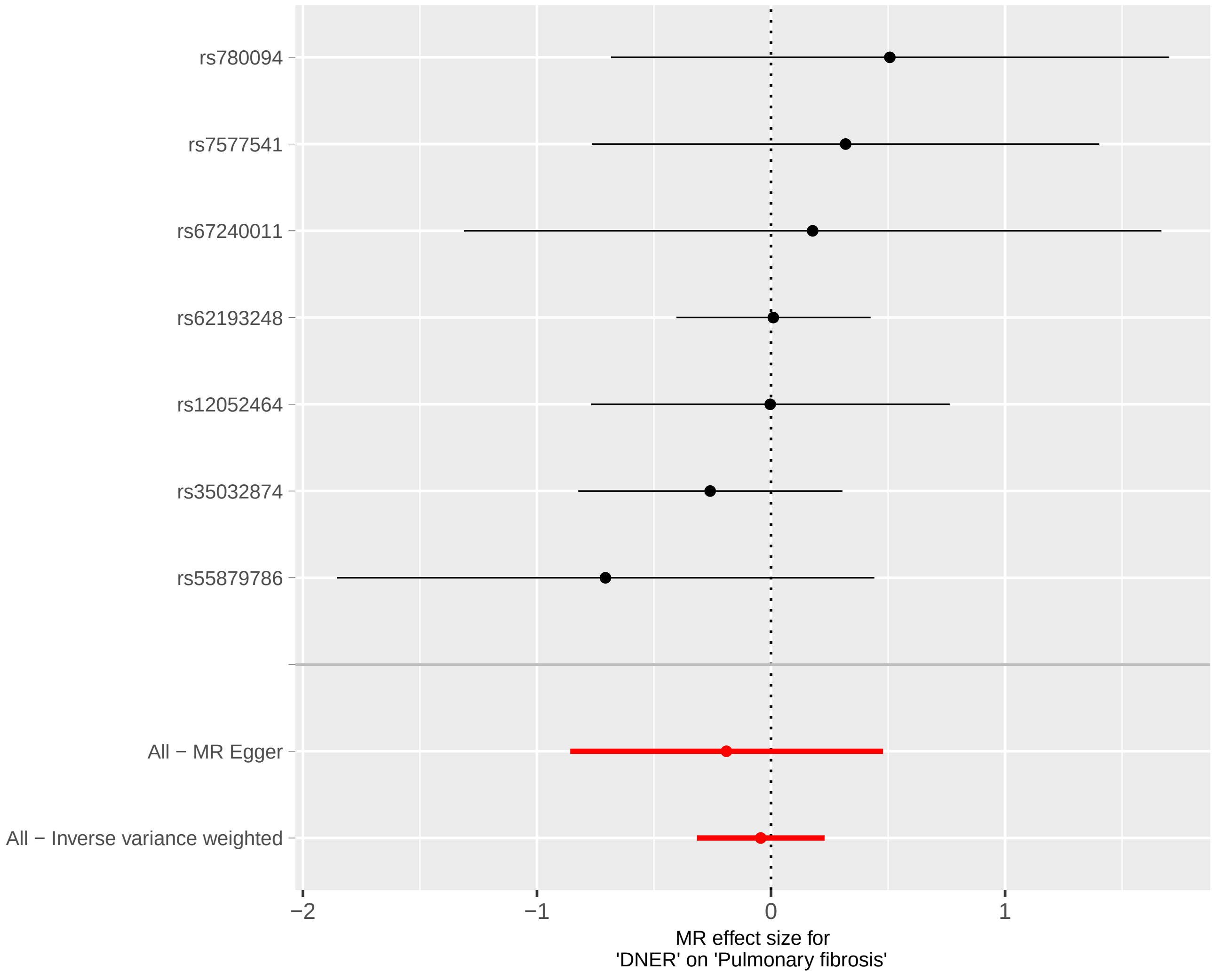
rs780094
rs7577541
rs67240011
rs62193248
rs12052464
rs35032874
rs55879786
All − MR Egger
All − Inverse variance weighted
−2
−1
0
1
MR effect size for
'DNER' on 'Pulmonary fibrosis'

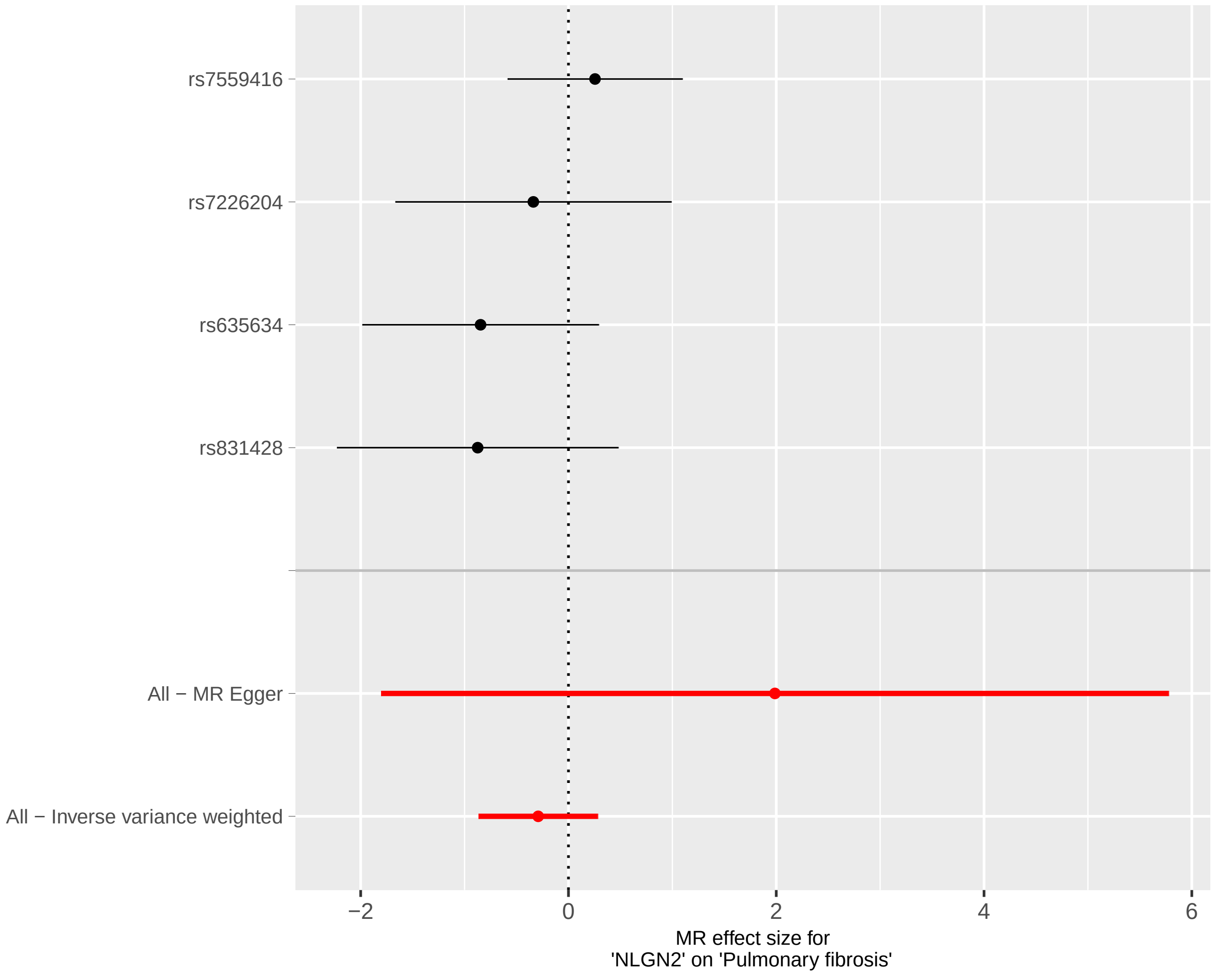

rs7559416
rs7226204
rs635634
rs831428
All − MR Egger
All − Inverse variance weighted
−2
0
2
4
6
MR effect size for
'NLGN2' on 'Pulmonary fibrosis'

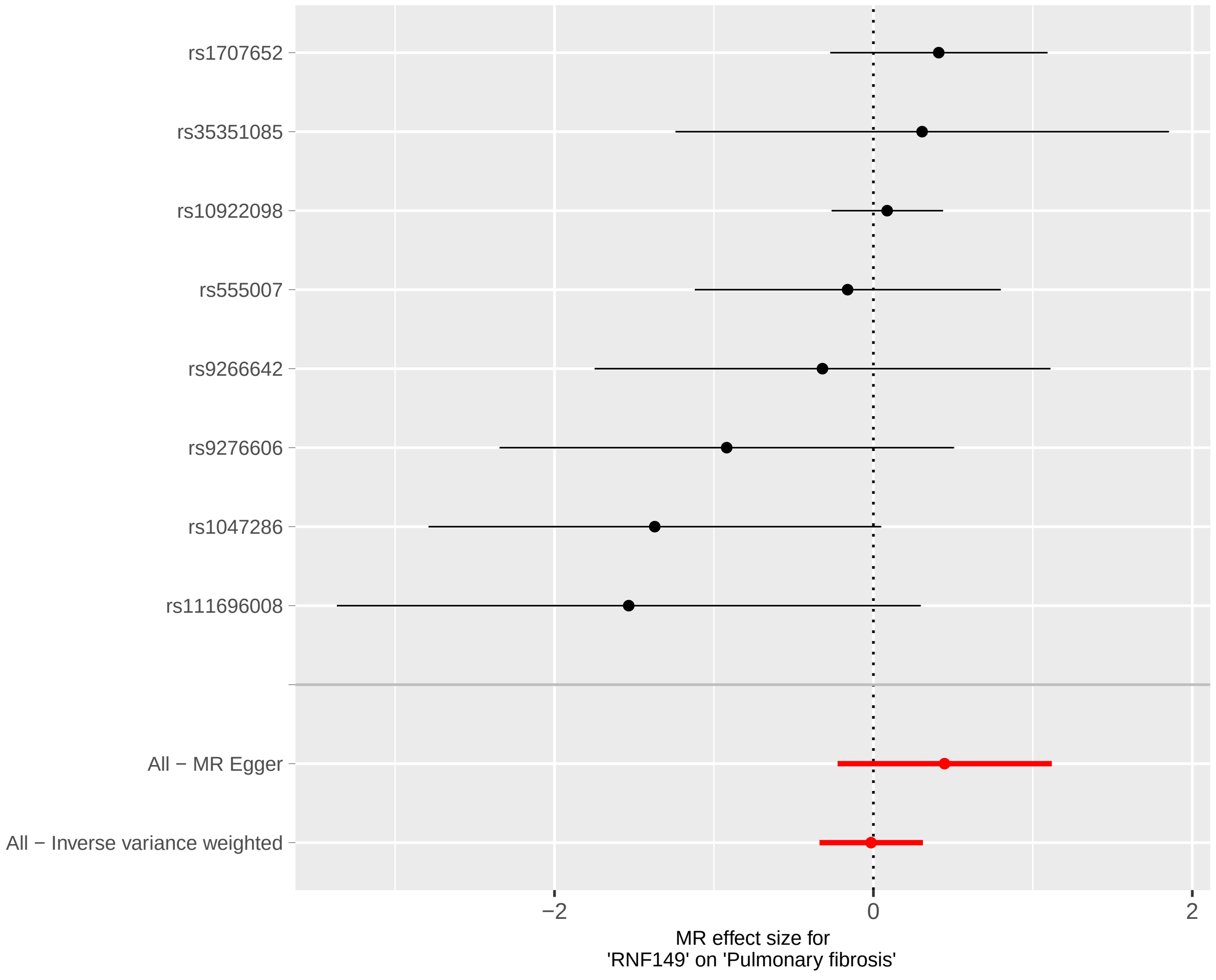
rs1707652
rs35351085
rs10922098
rs555007
rs9266642
rs9276606
rs1047286
rs111696008
All − MR Egger
All − Inverse variance weighted
−2
0
2
MR effect size for
'RNF149' on 'Pulmonary fibrosis'

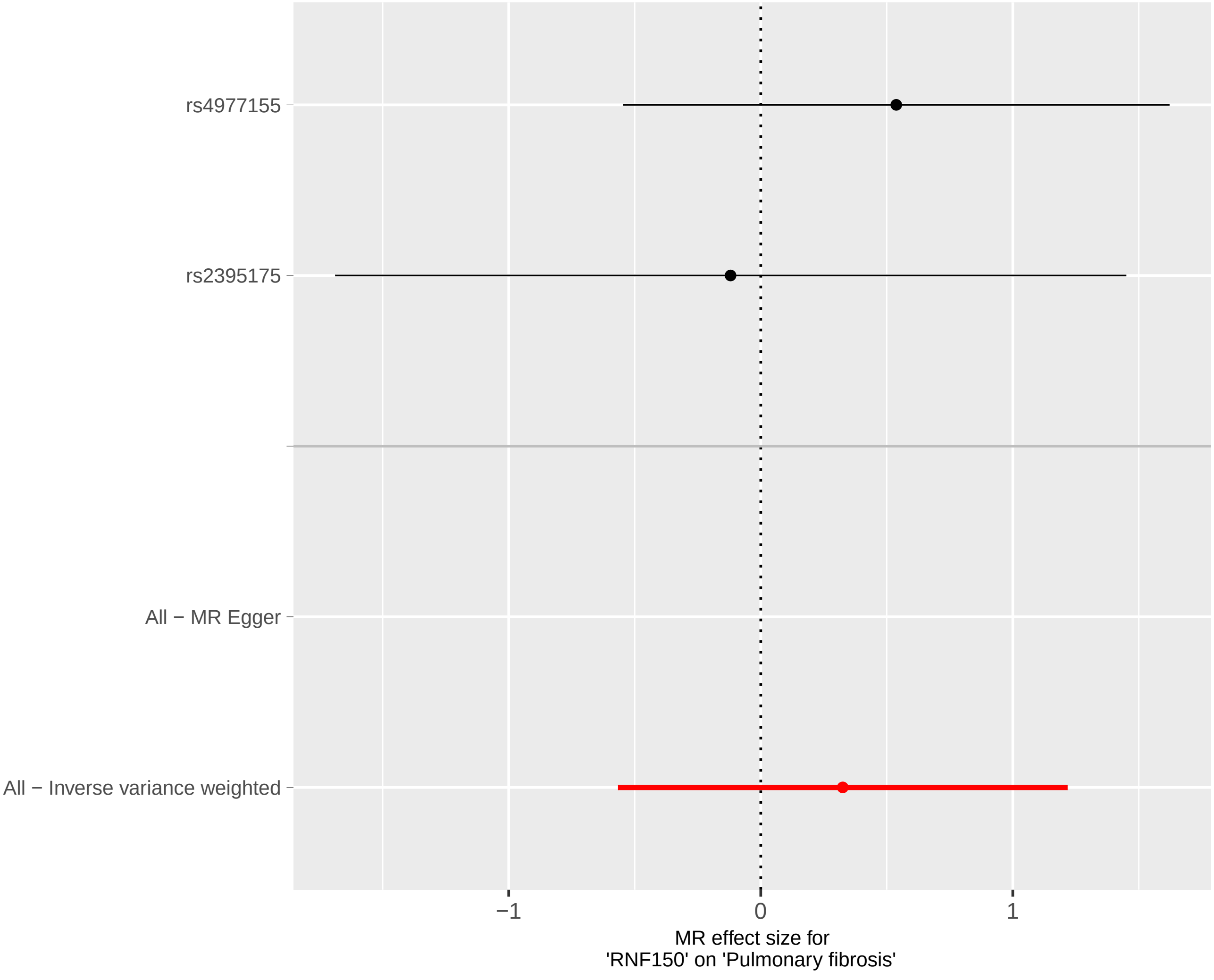

rs4977155
rs2395175
All − MR Egger
All − Inverse variance weighted
−1
0
1
MR effect size for
'RNF150' on 'Pulmonary fibrosis'

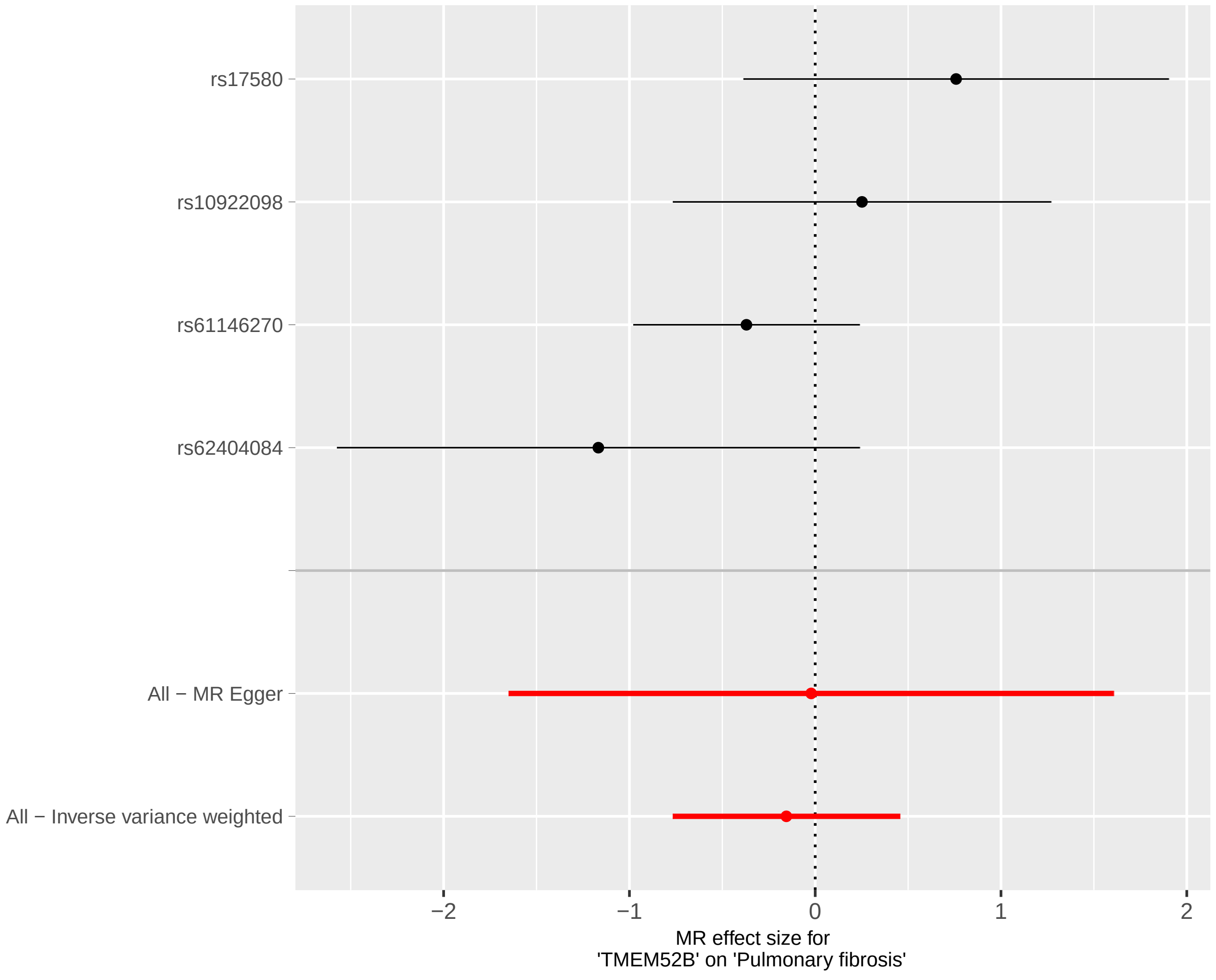
rs17580
rs10922098
rs61146270
rs62404084
All − MR Egger
All − Inverse variance weighted
−2
−1
0
1
2
MR effect size for
'TMEM52B' on 'Pulmonary fibrosis'

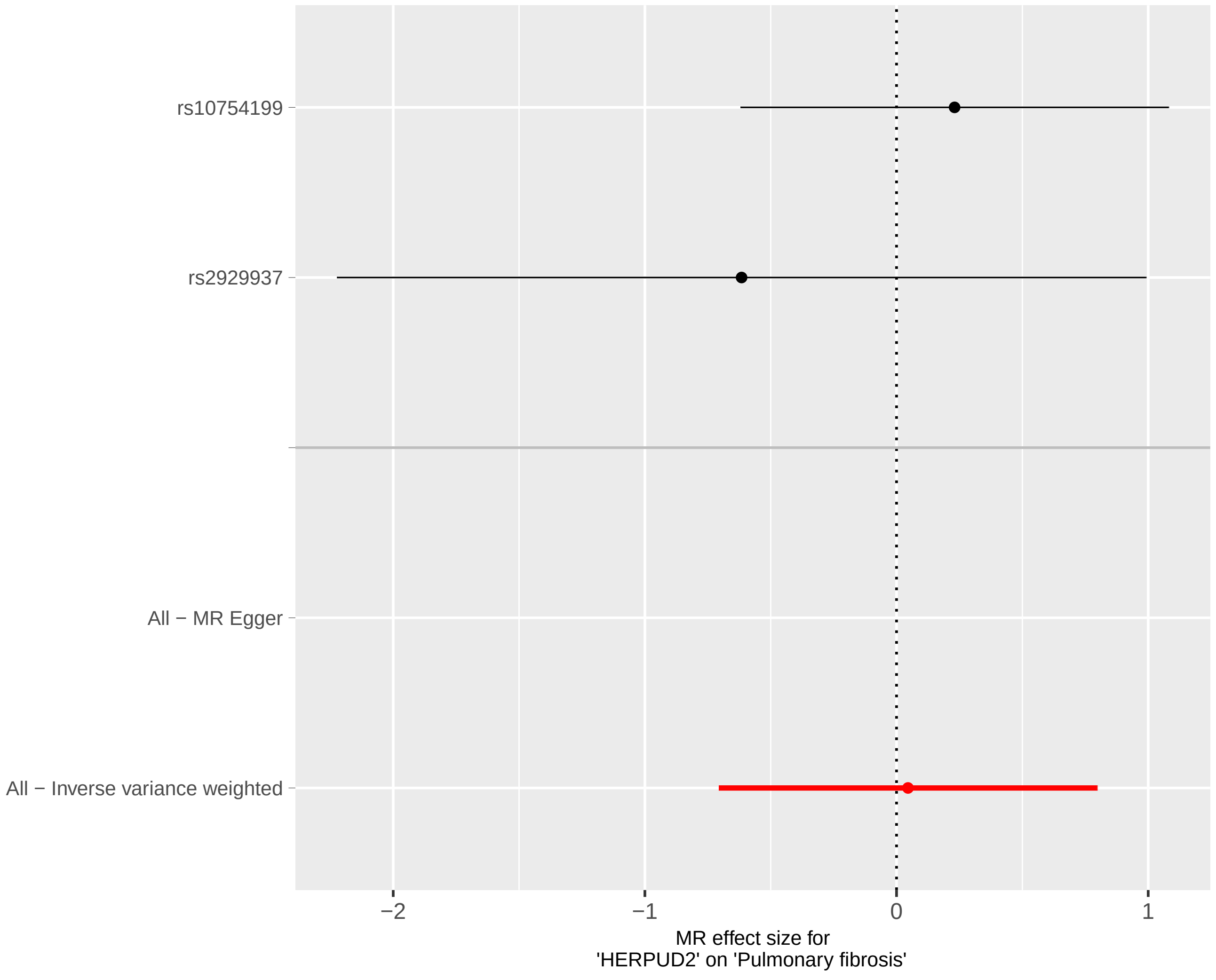

rs10754199
rs2929937
All − MR Egger
All − Inverse variance weighted
−2
−1
0
1
MR effect size for
'HERPUD2' on 'Pulmonary fibrosis'

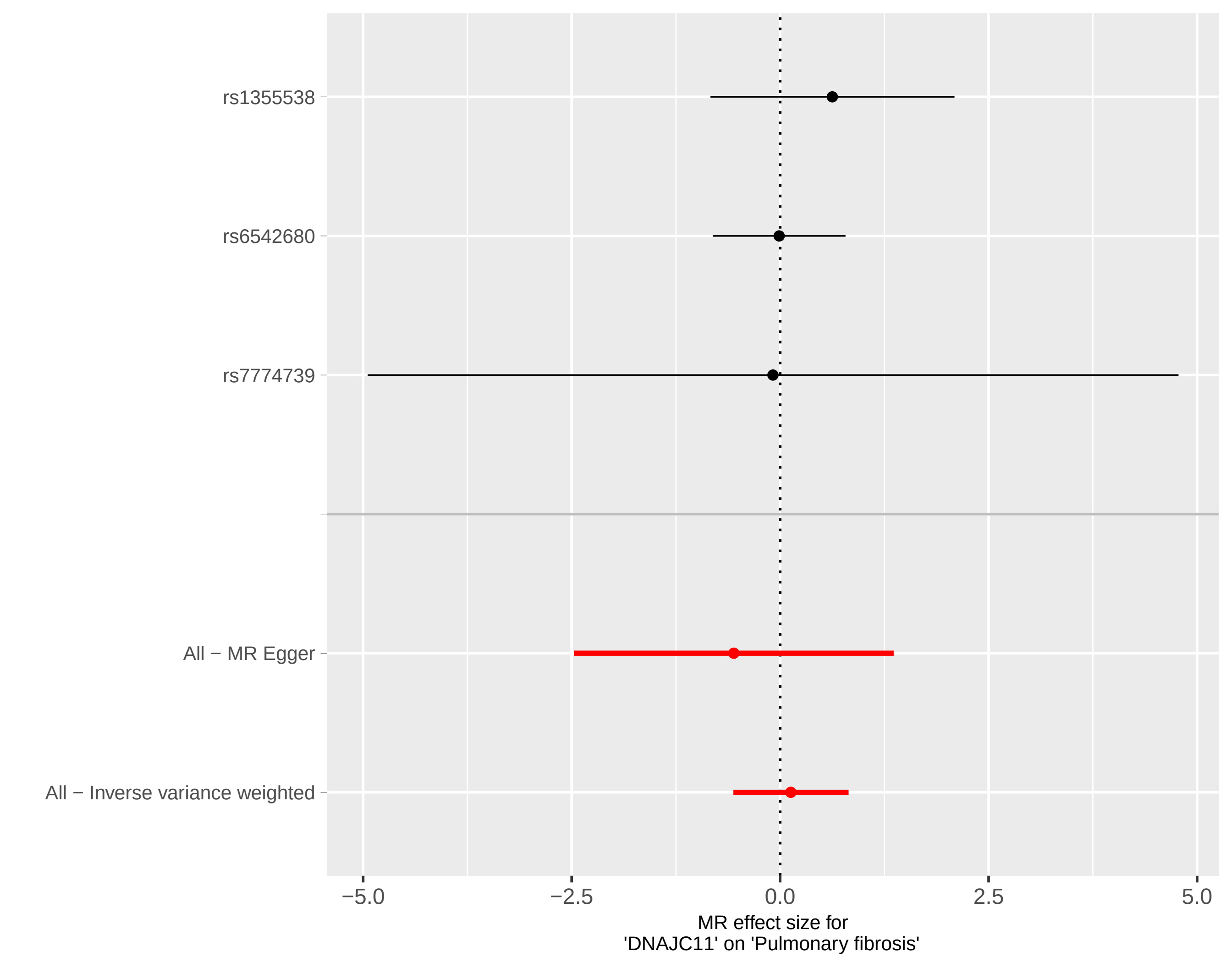

rs1355538
rs6542680
rs7774739
All − MR Egger
All − Inverse variance weighted
−5.0
−2.5
0.0
2.5
5.0
MR effect size for
'DNAJC11' on 'Pulmonary fibrosis'

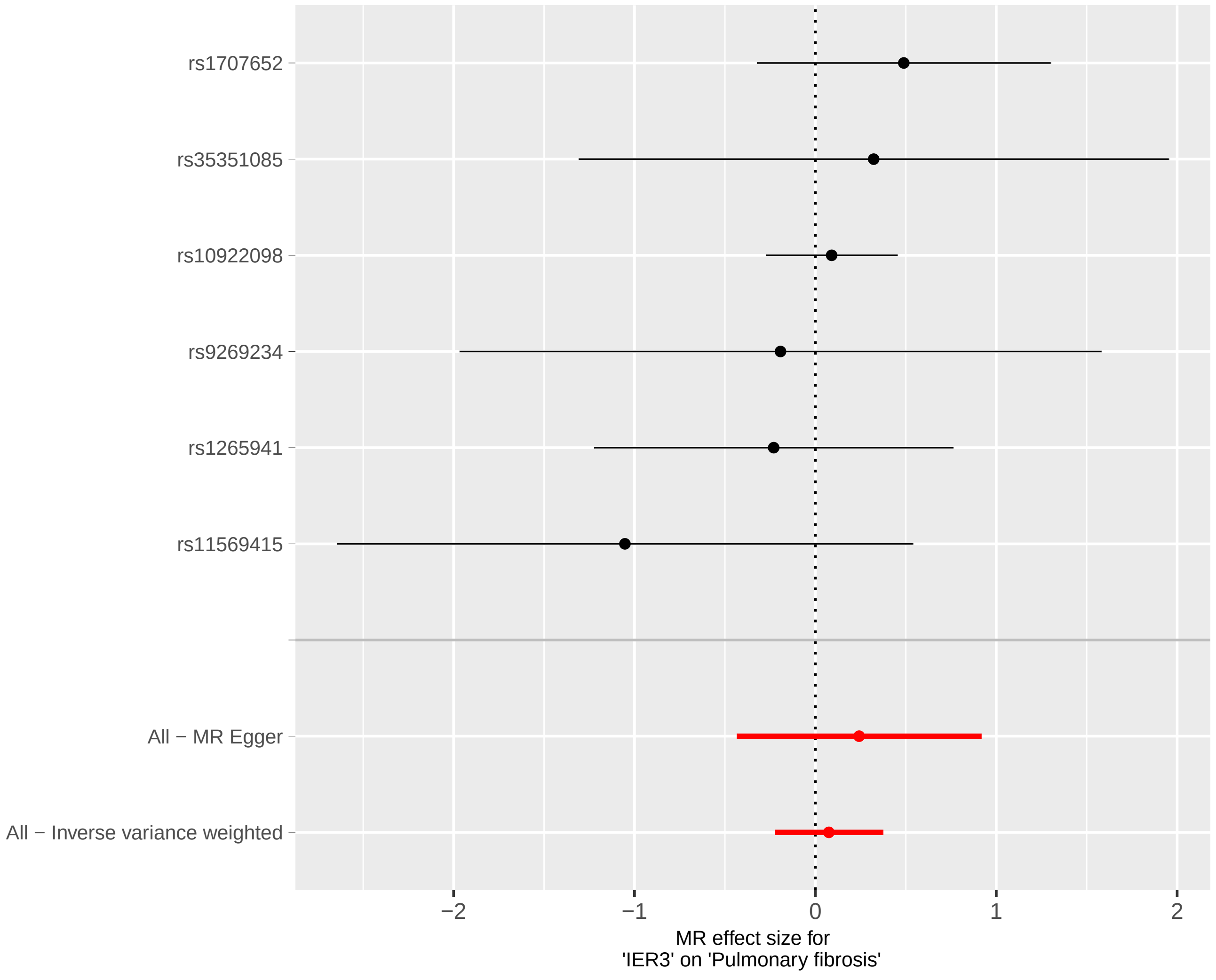

rs1707652
rs35351085
rs10922098
rs9269234
rs1265941
rs11569415
All − MR Egger
All − Inverse variance weighted
−2
−1
0
1
2
MR effect size for
'IER3' on 'Pulmonary fibrosis'

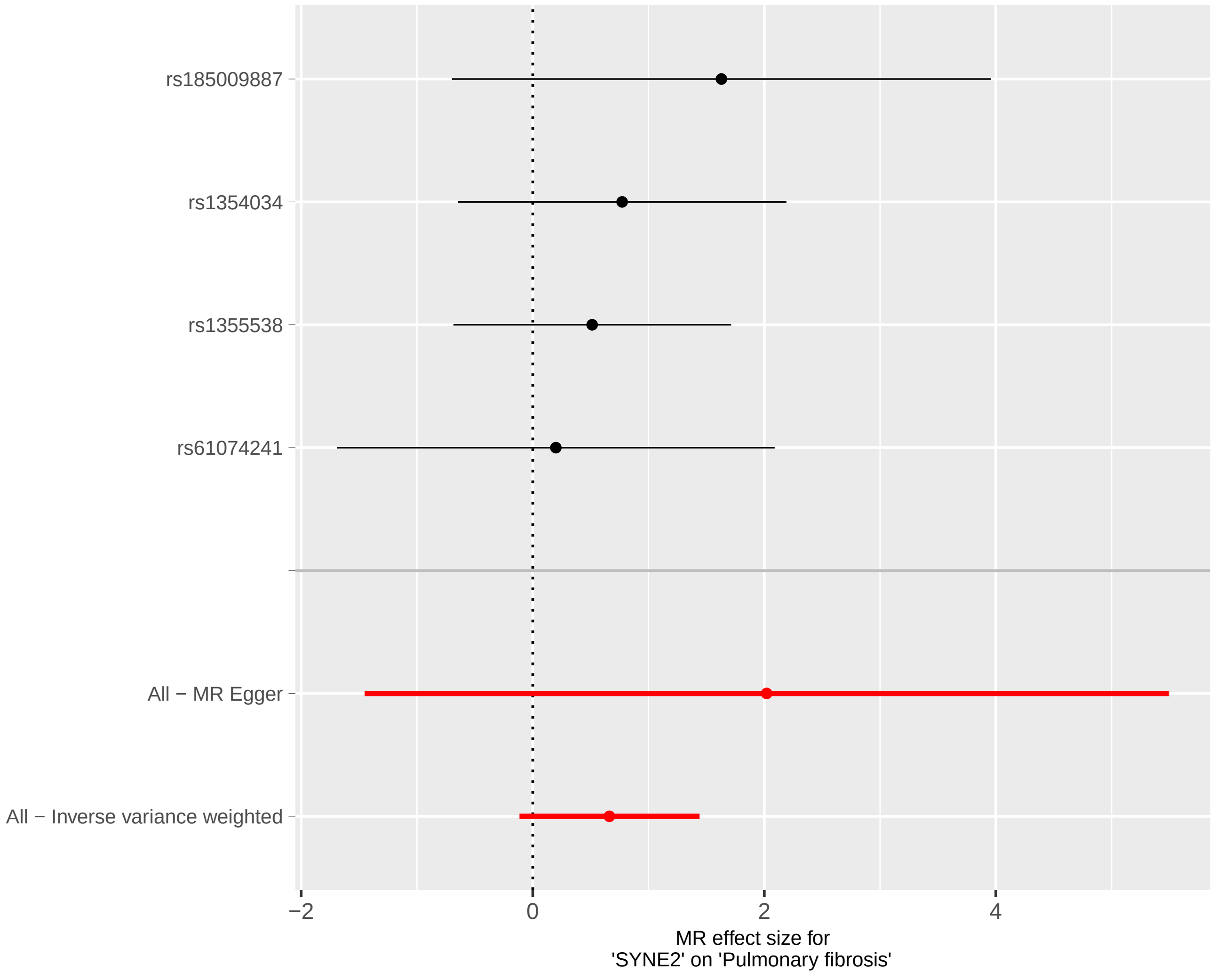

rs185009887
rs1354034
rs1355538
rs61074241
All − MR Egger
All − Inverse variance weighted
−2
0
2
4
MR effect size for
'SYNE2' on 'Pulmonary fibrosis'

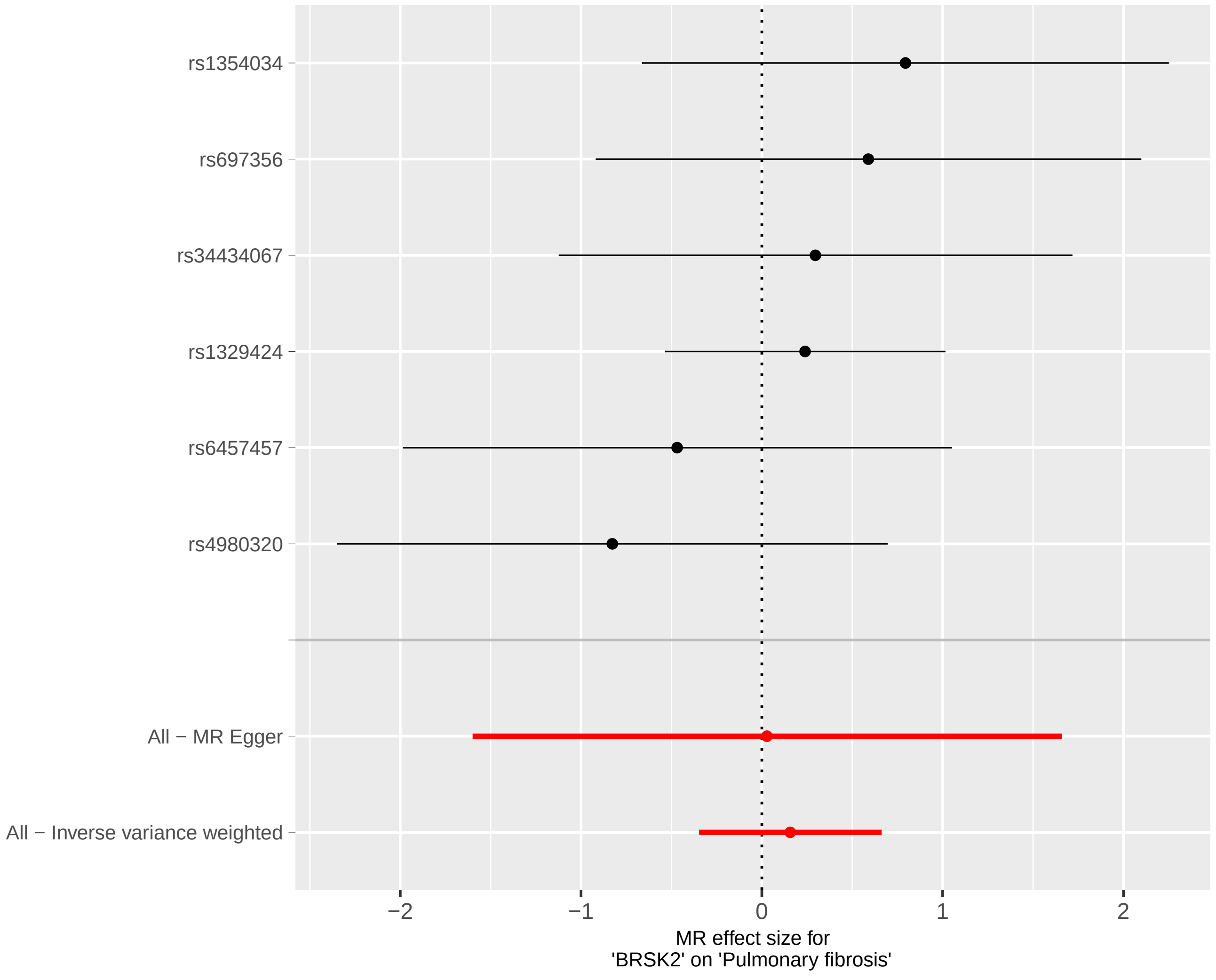

rs1354034
rs697356
rs34434067
rs1329424
rs6457457
rs4980320
All − MR Egger
All − Inverse variance weighted
−2
−1
0
1
2
MR effect size for
'BRSK2' on 'Pulmonary fibrosis'

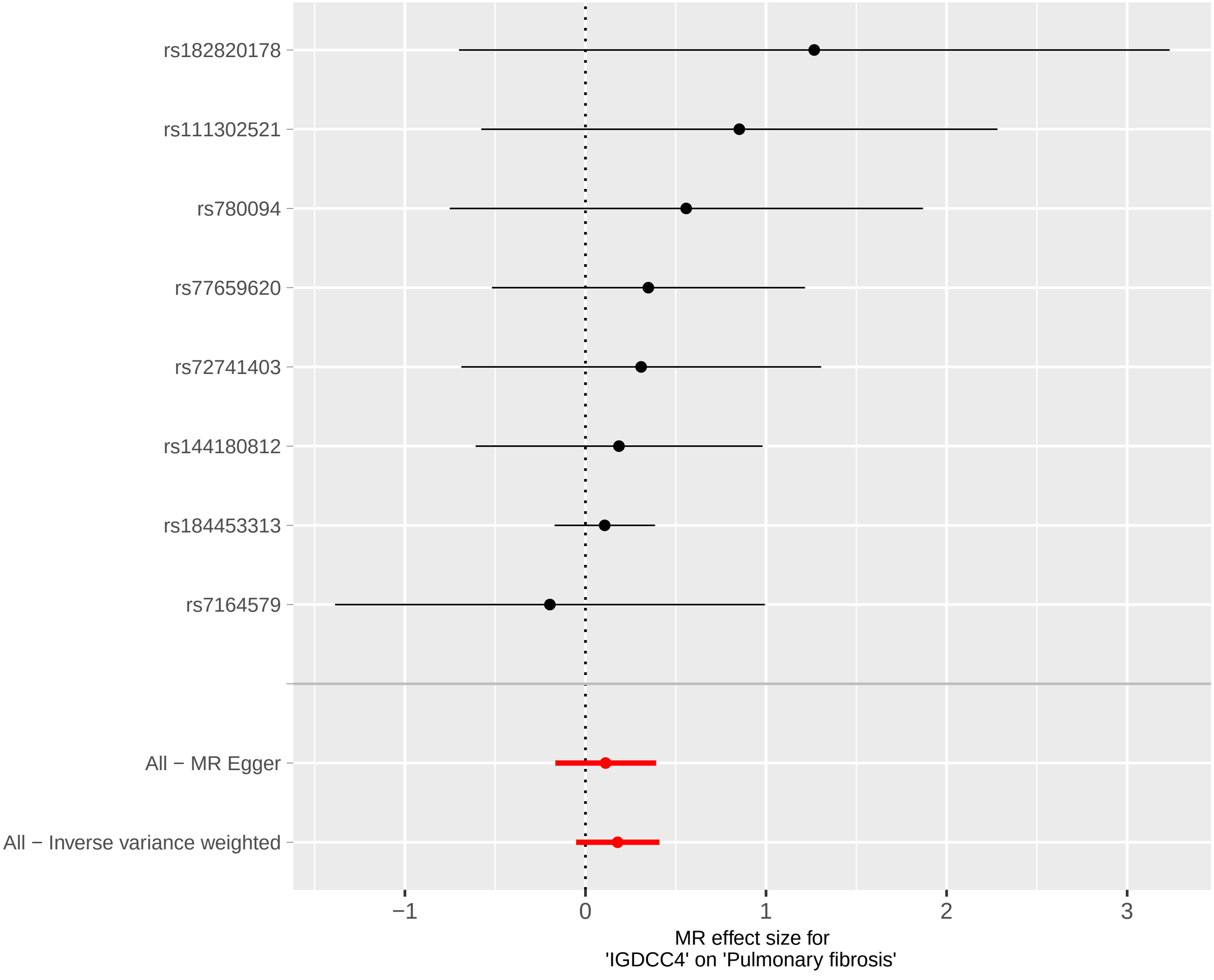

rs182820178
rs111302521
rs780094
rs77659620
rs72741403
rs144180812
rs184453313
rs7164579
All − MR Egger
All − Inverse variance weighted
−1
0
1
2
3
MR effect size for
'IGDCC4' on 'Pulmonary fibrosis'

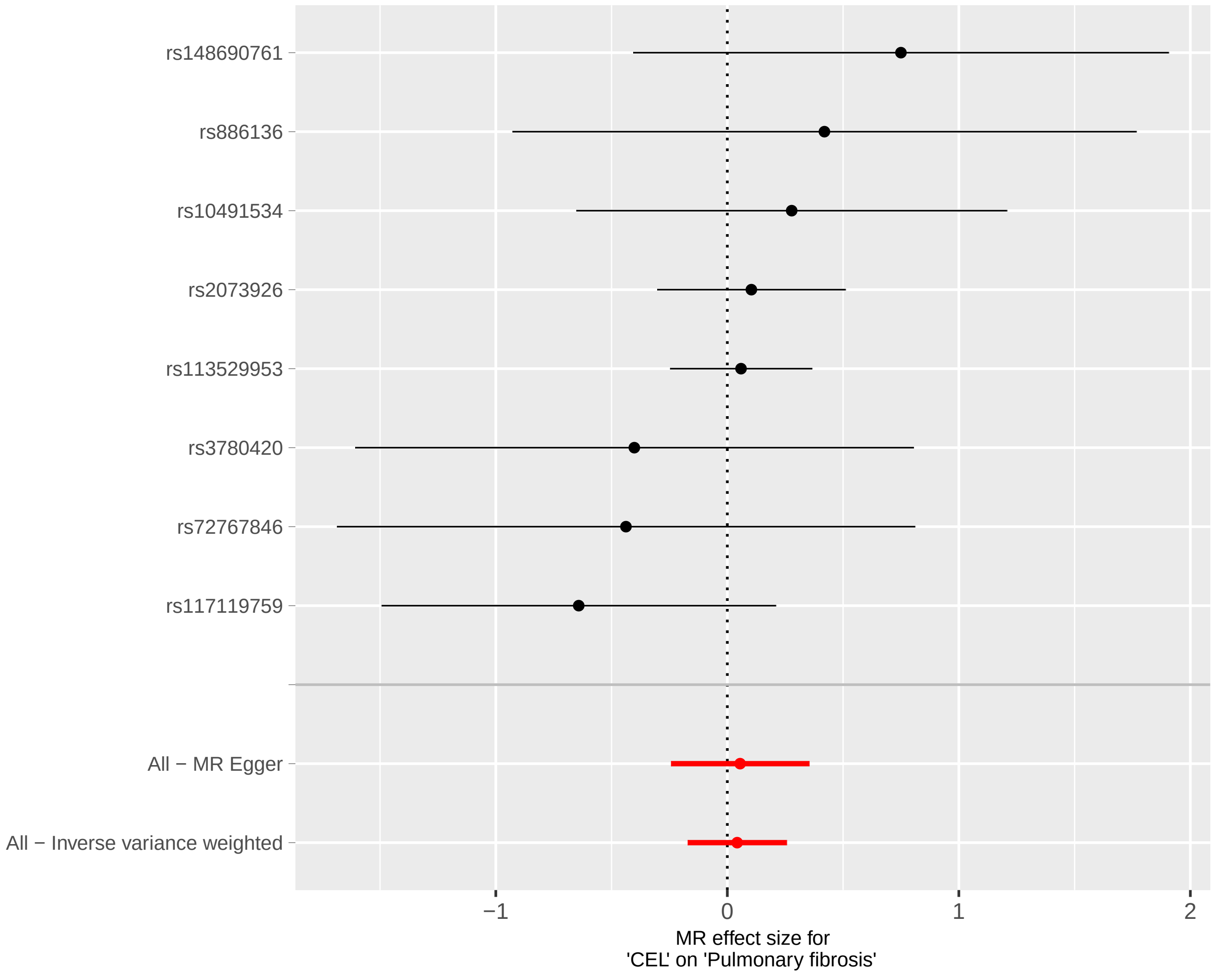
rs148690761
rs886136
rs10491534
rs2073926
rs113529953
rs3780420
rs72767846
rs117119759
All − MR Egger
All − Inverse variance weighted
−1
0
1
2
MR effect size for
'CEL' on 'Pulmonary fibrosis'

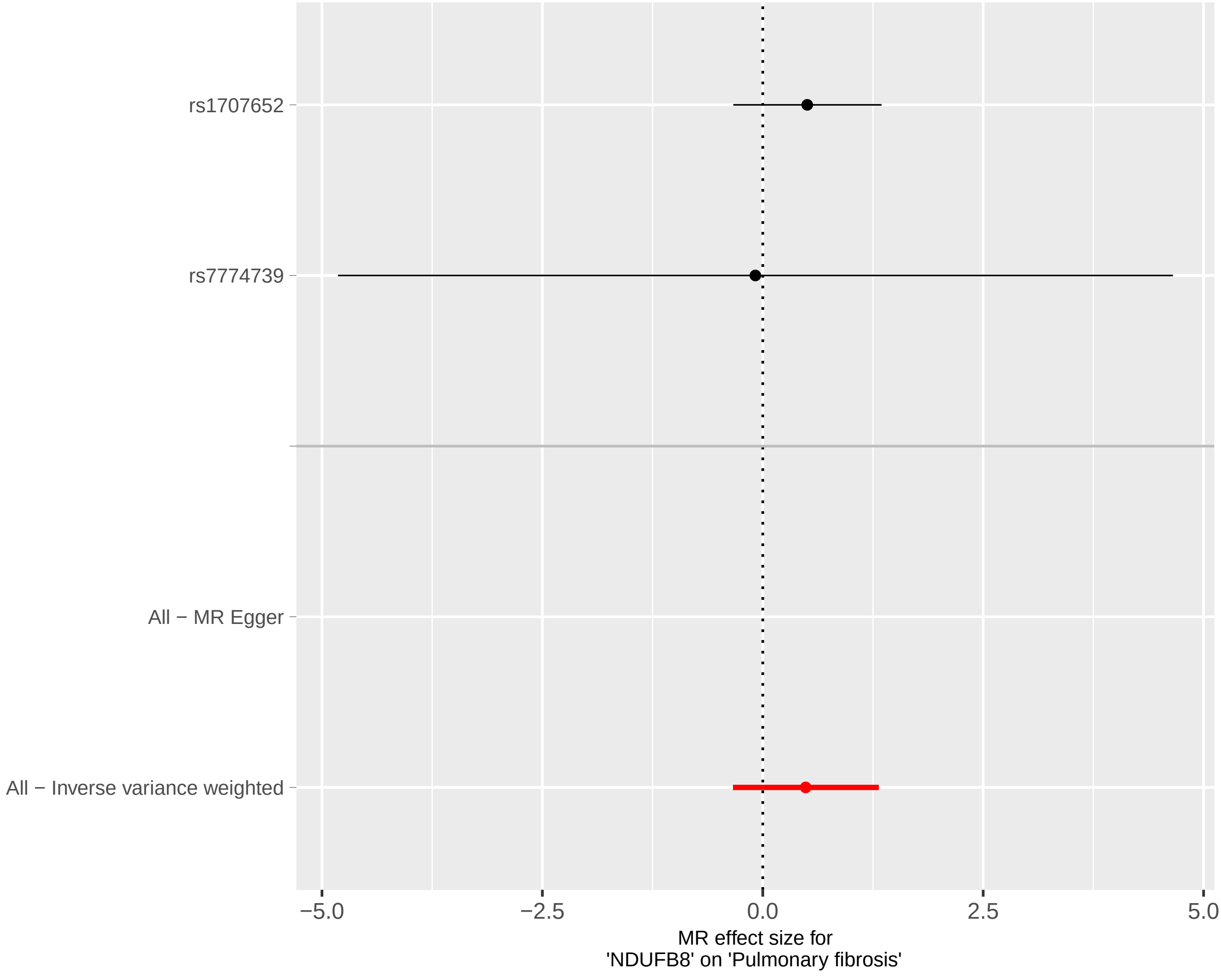
rs1707652
rs7774739
All − MR Egger
All − Inverse variance weighted
−5.0
−2.5
0.0
2.5
5.0
MR effect size for
'NDUFB8' on 'Pulmonary fibrosis'

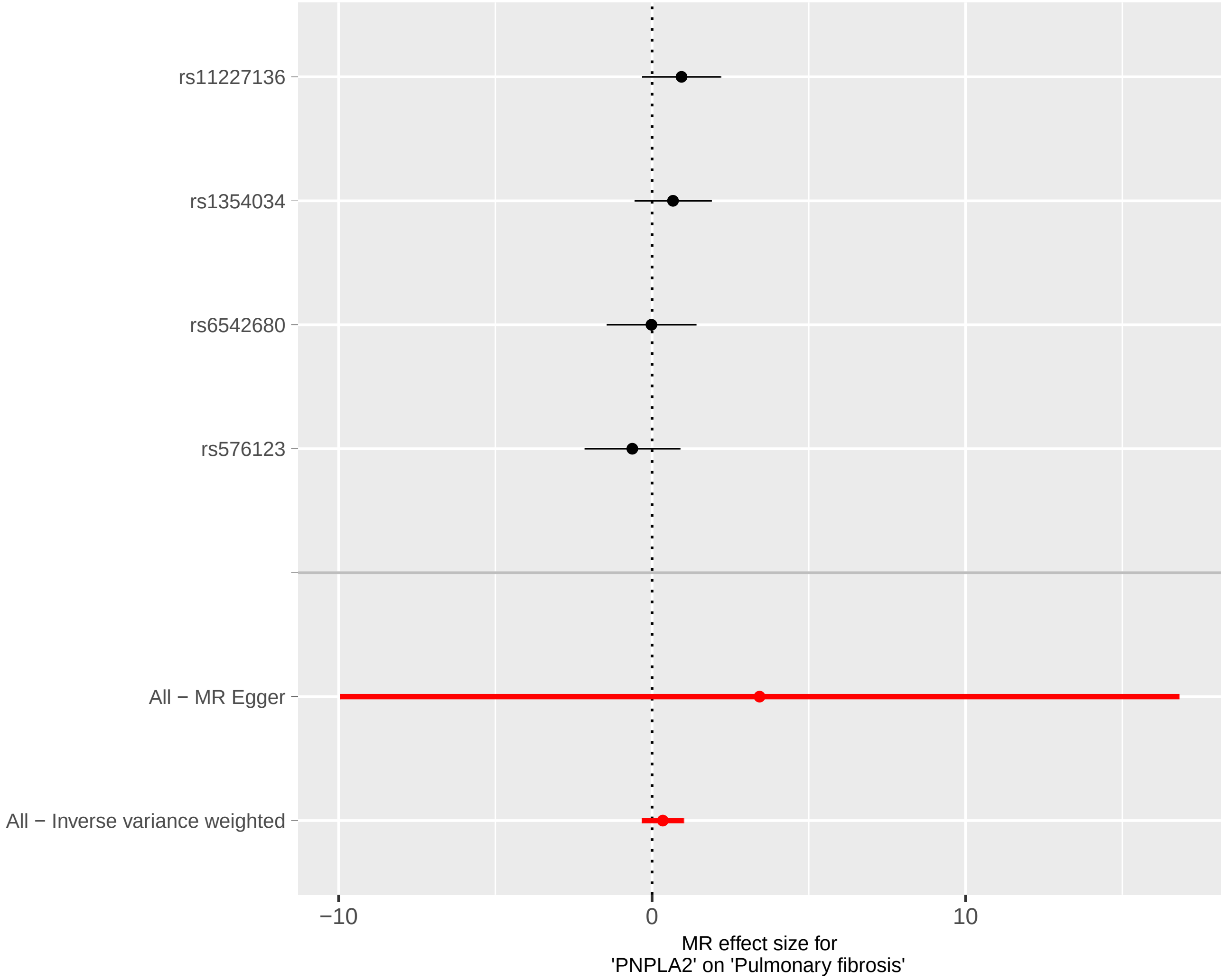
rs11227136
rs1354034
rs6542680
rs576123
All − MR Egger
All − Inverse variance weighted
−10
0
10
MR effect size for
'PNPLA2' on 'Pulmonary fibrosis'

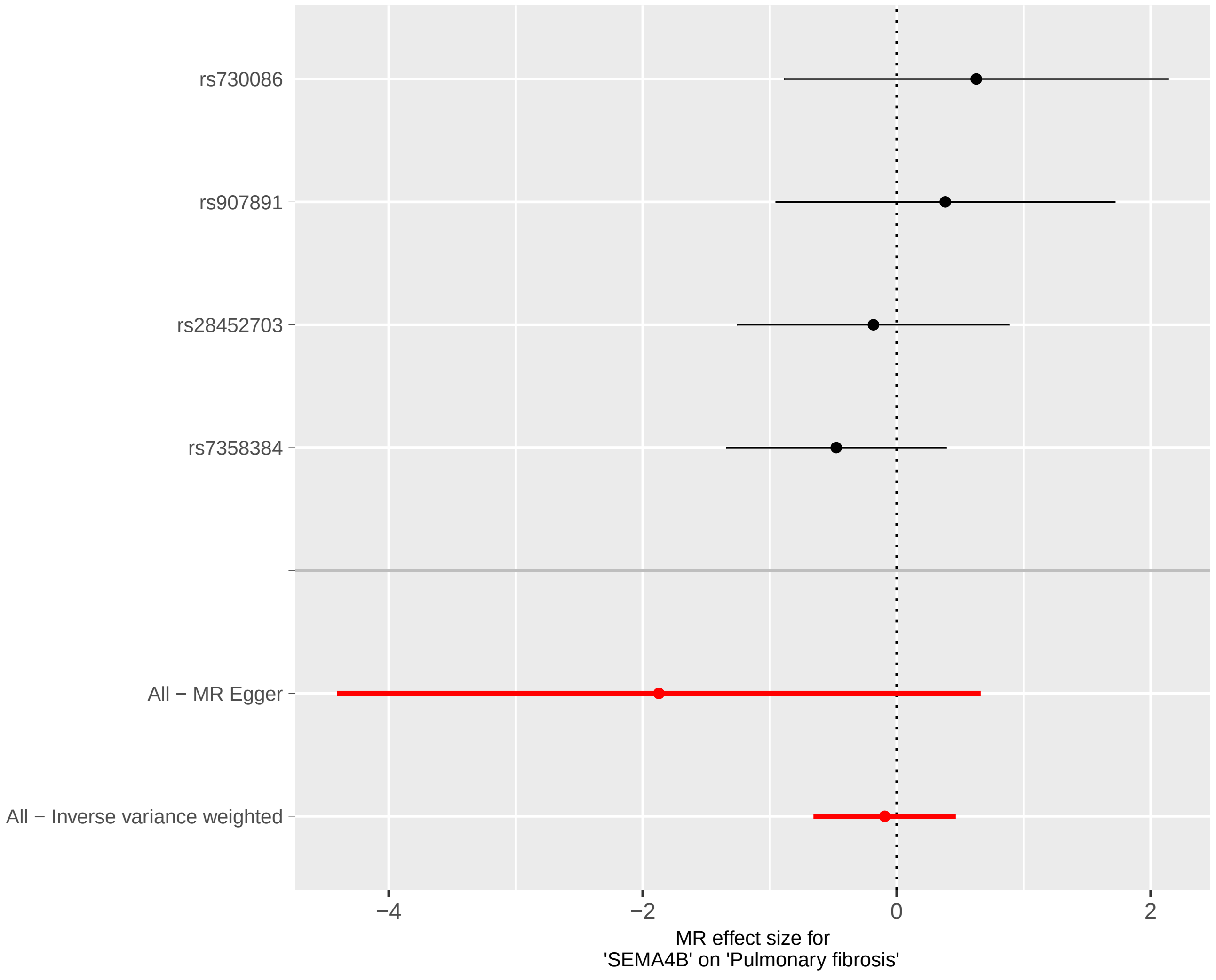
rs730086
rs907891
rs28452703
rs7358384
All − MR Egger
All − Inverse variance weighted
−4
−2
0
2
MR effect size for
'SEMA4B' on 'Pulmonary fibrosis'

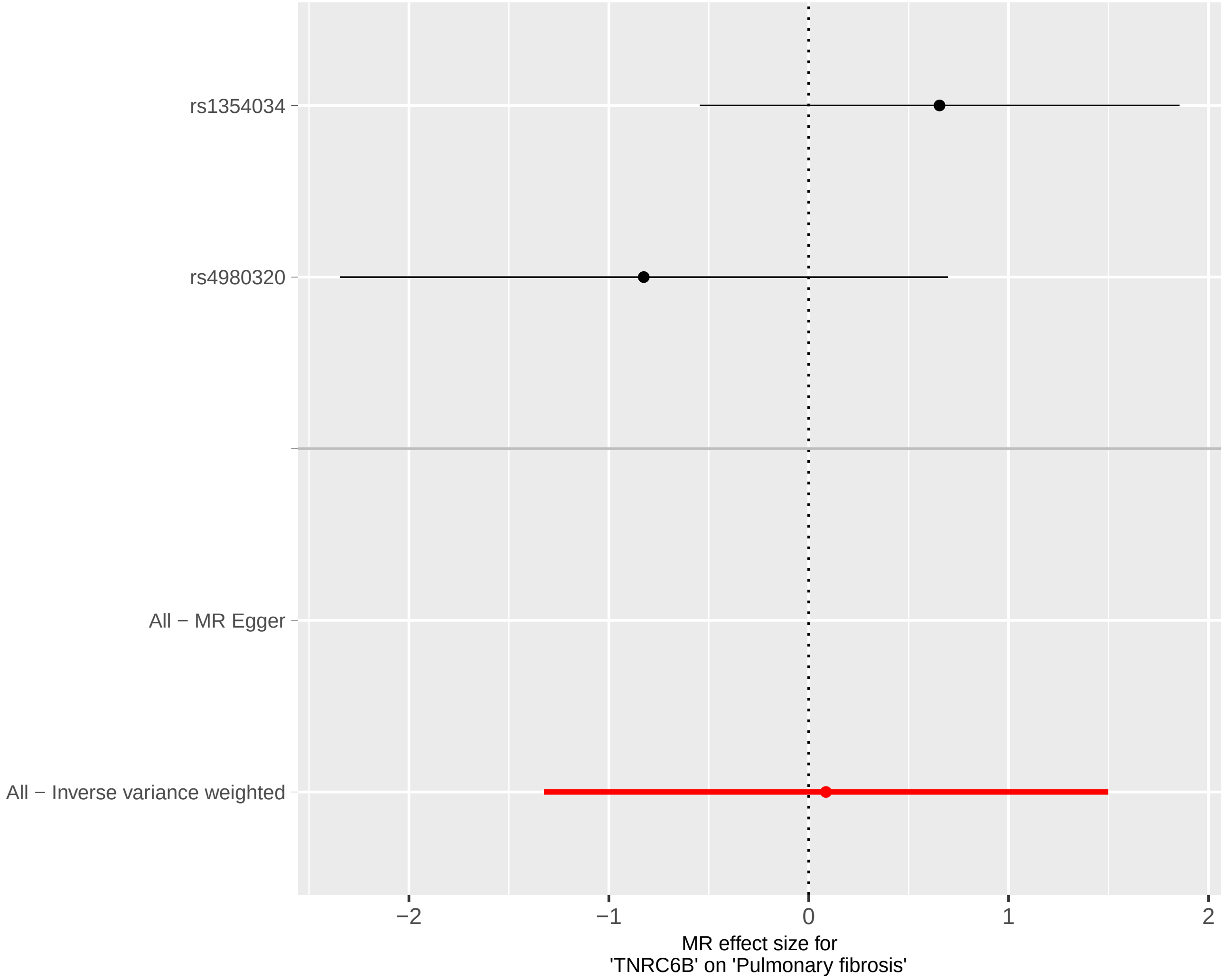
rs1354034
rs4980320
All − MR Egger
All − Inverse variance weighted
−2
−1
0
1
2
MR effect size for
'TNRC6B' on 'Pulmonary fibrosis'

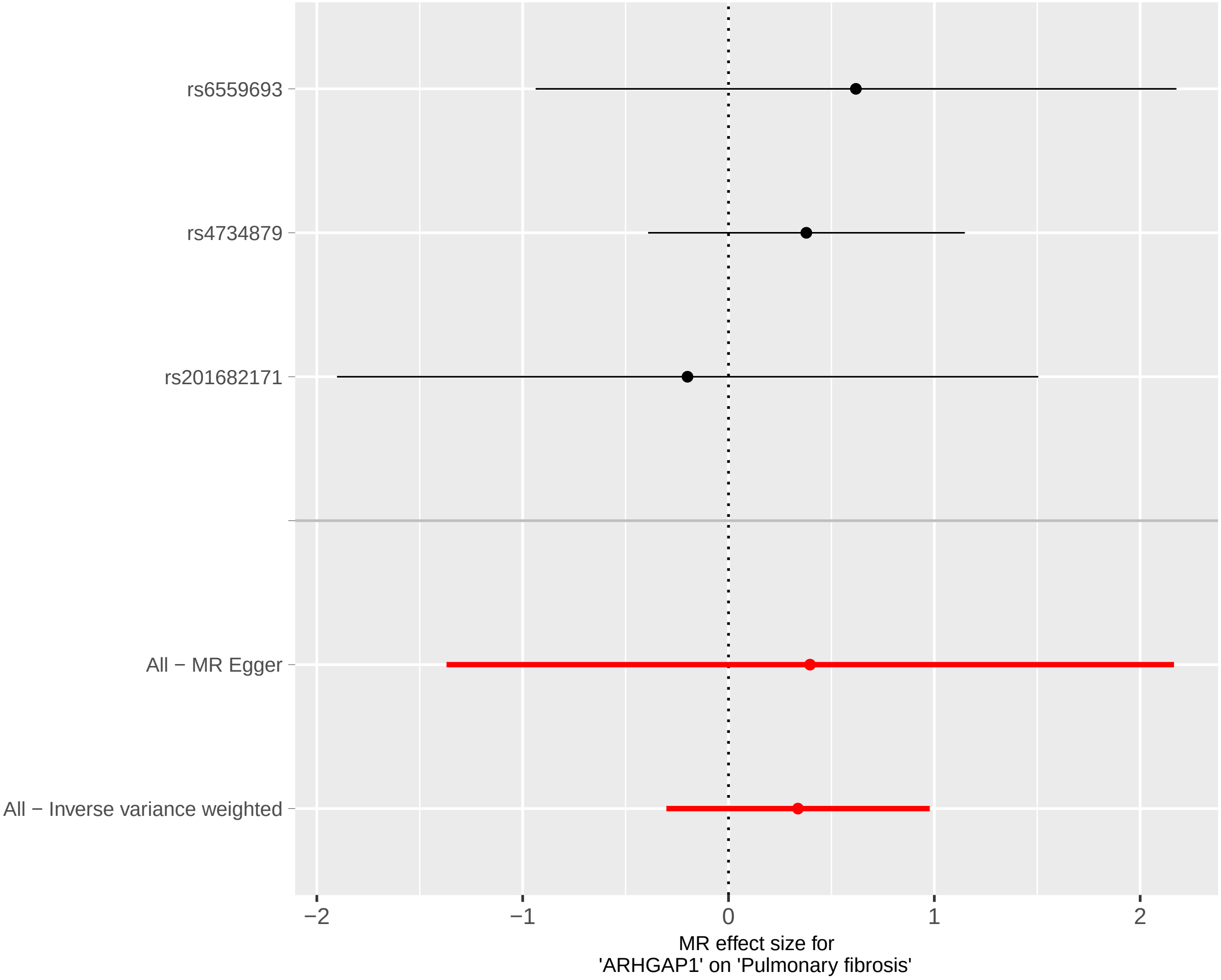
rs6559693
rs4734879
rs201682171
All − MR Egger
All − Inverse variance weighted
−2
−1
0
1
2
MR effect size for
'ARHGAP1' on 'Pulmonary fibrosis'

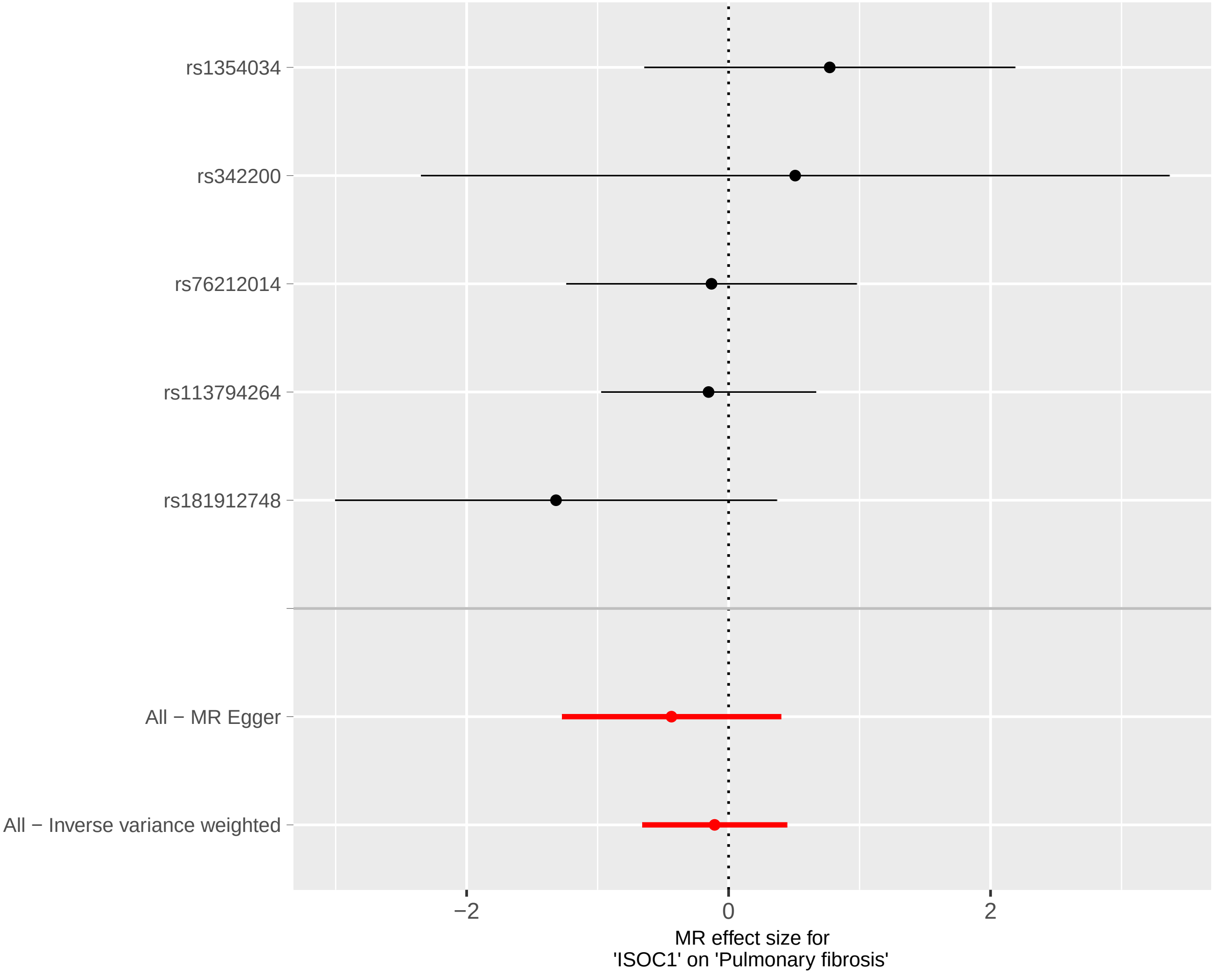

rs1354034
rs342200
rs76212014
rs113794264
rs181912748
All − MR Egger
All − Inverse variance weighted
−2
0
2
MR effect size for
'ISOC1' on 'Pulmonary fibrosis'

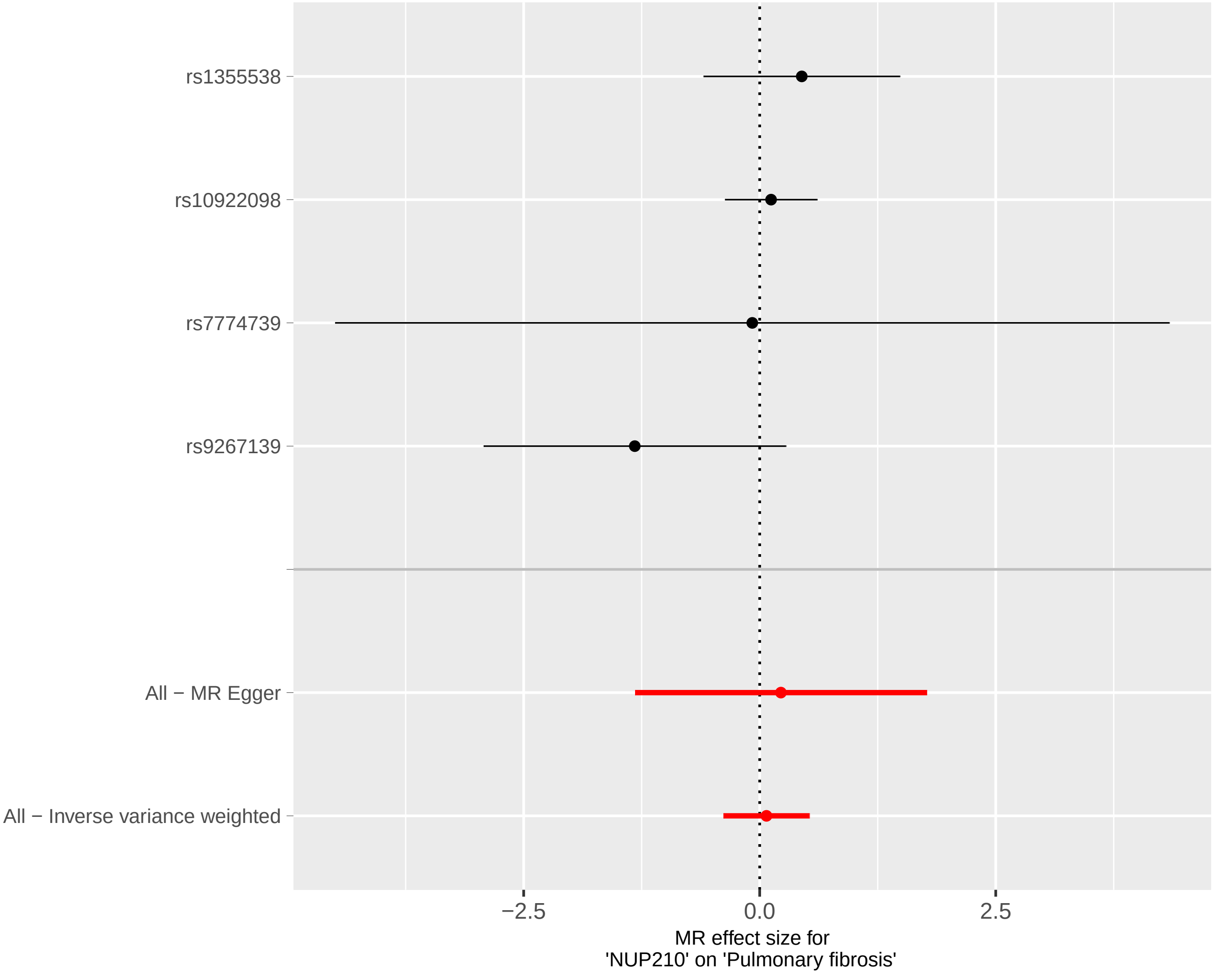
rs1355538
rs10922098
rs7774739
rs9267139
All − MR Egger
All − Inverse variance weighted
−2.5
0.0
2.5
MR effect size for
'NUP210' on 'Pulmonary fibrosis'

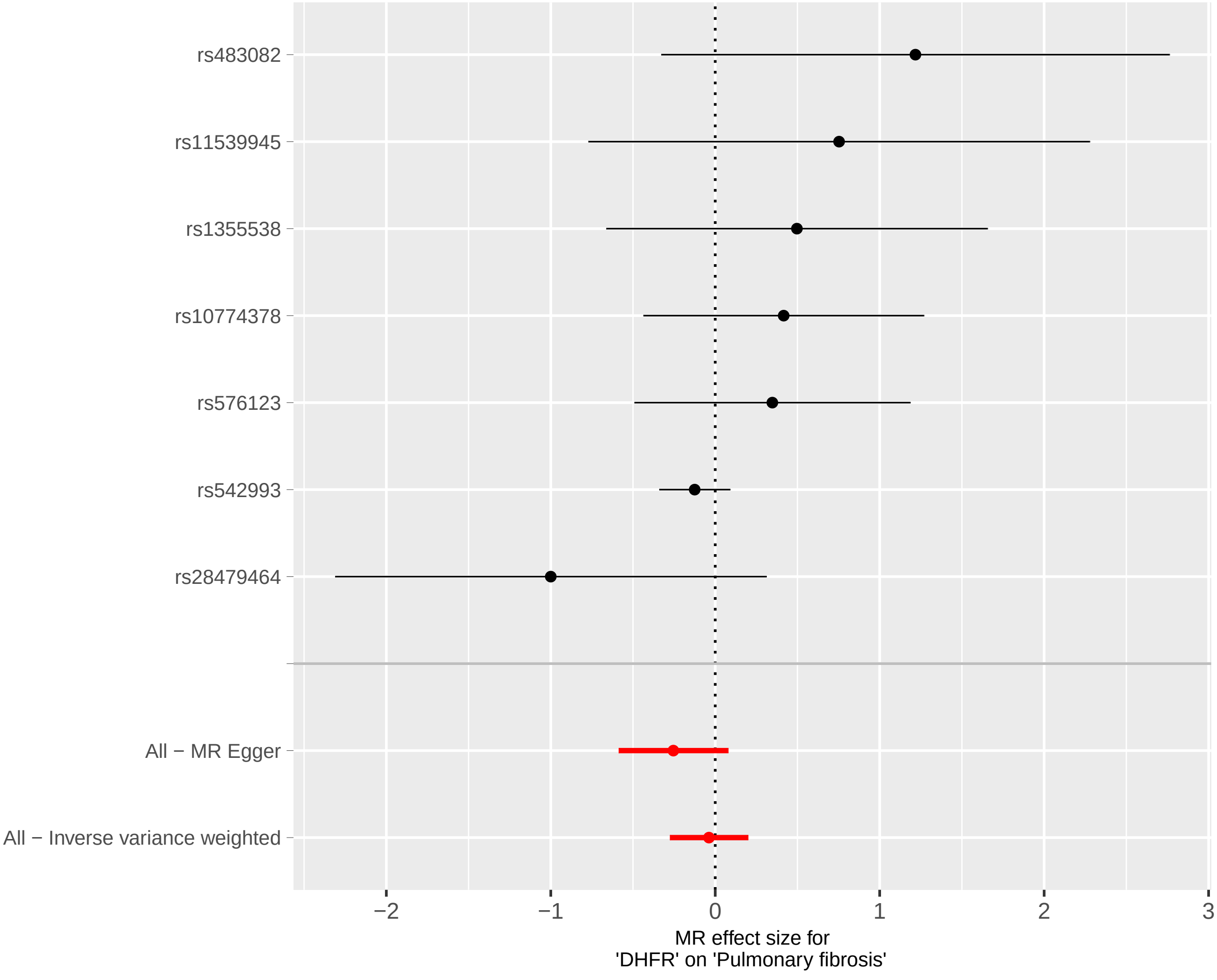
rs483082
rs11539945
rs1355538
rs10774378
rs576123
rs542993
rs28479464
All − MR Egger
All − Inverse variance weighted
−2
−1
0
1
2
3
MR effect size for
'DHFR' on 'Pulmonary fibrosis'

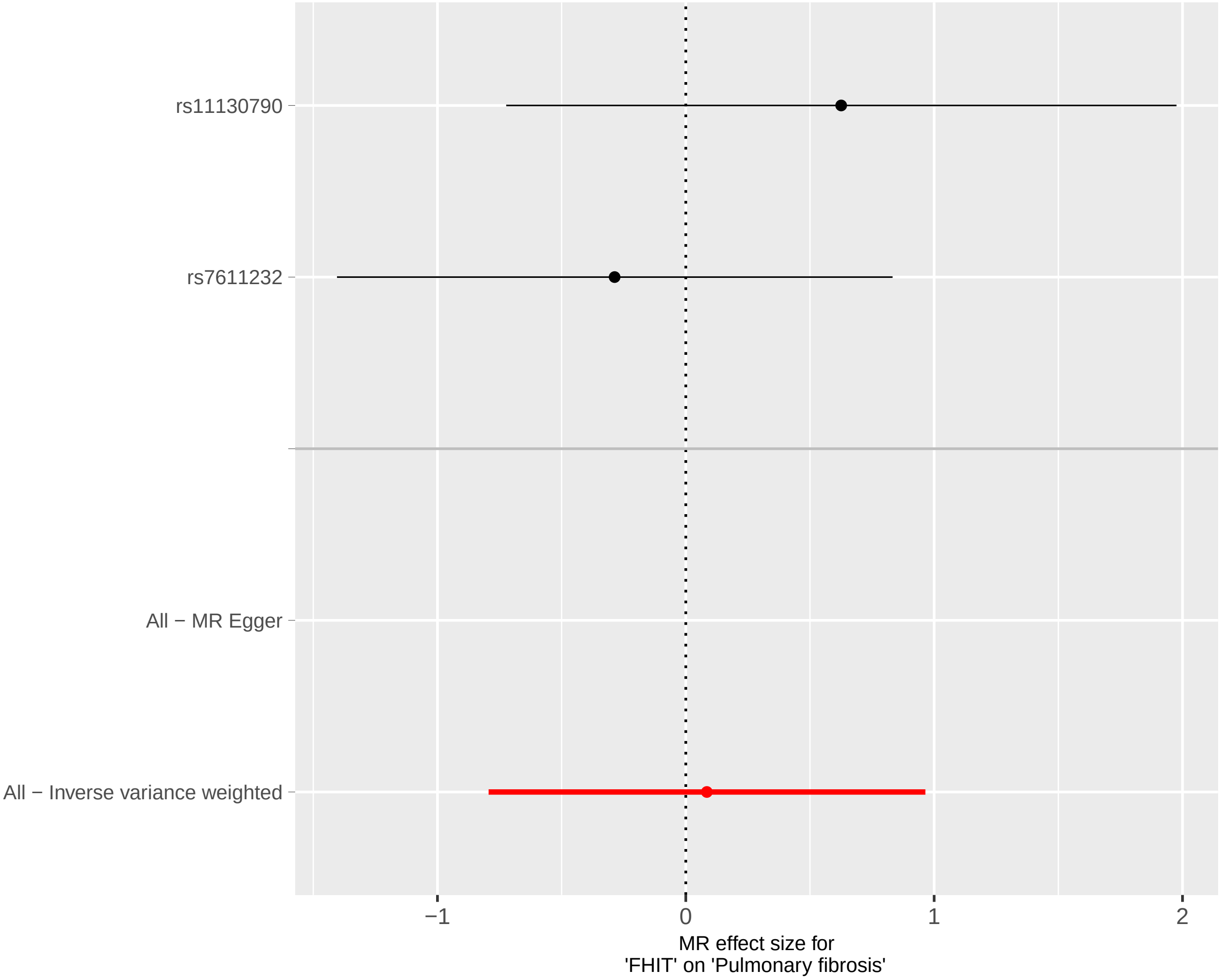
rs11130790
rs7611232
All − MR Egger
All − Inverse variance weighted
−1
0
1
2
MR effect size for
'FHIT' on 'Pulmonary fibrosis'

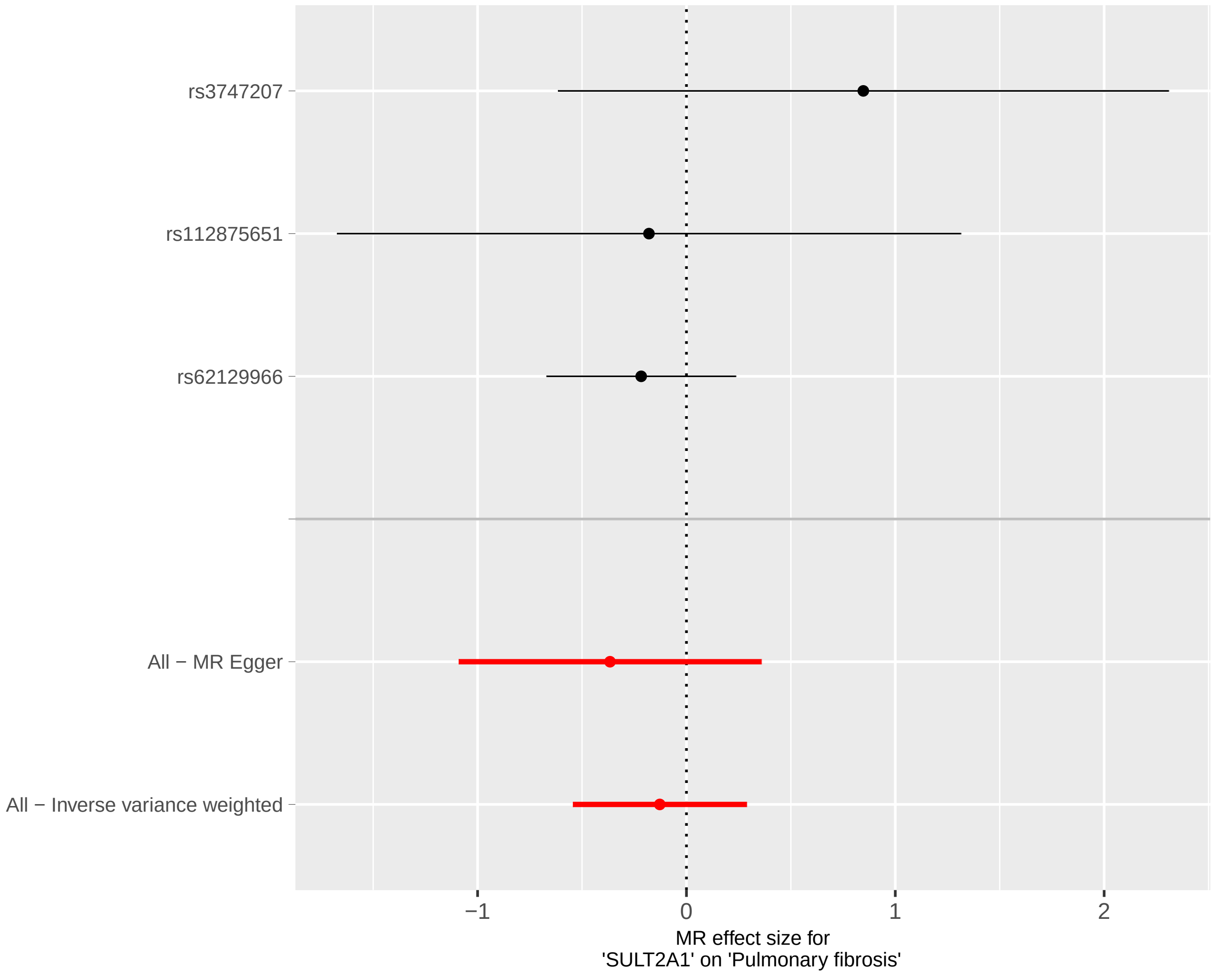
rs3747207
rs112875651
rs62129966
All − MR Egger
All − Inverse variance weighted
−1
0
1
2
MR effect size for
'SULT2A1' on 'Pulmonary fibrosis'

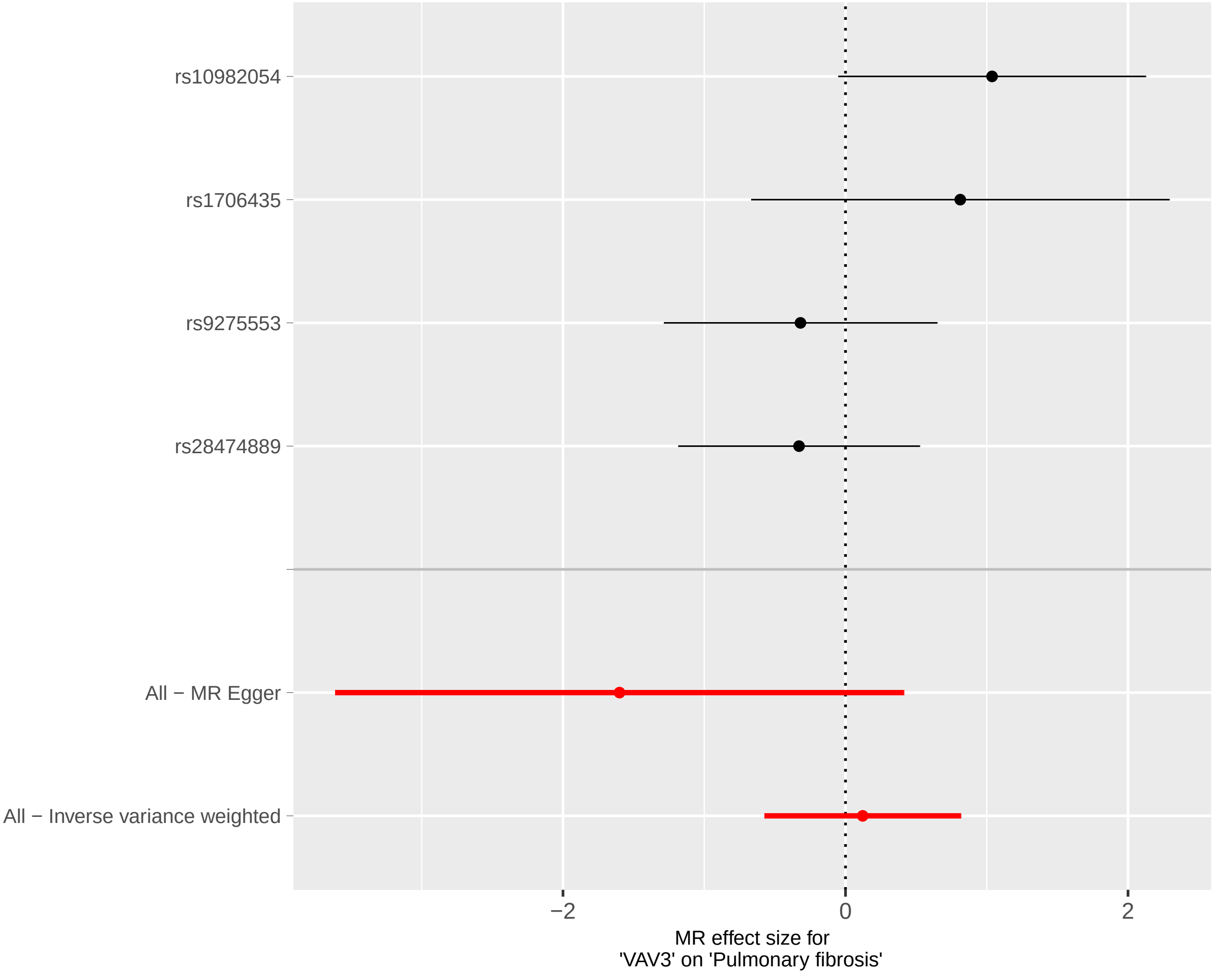
rs10982054
rs1706435
rs9275553
rs28474889
All − MR Egger
All − Inverse variance weighted
−2
0
2
MR effect size for
'VAV3' on 'Pulmonary fibrosis'

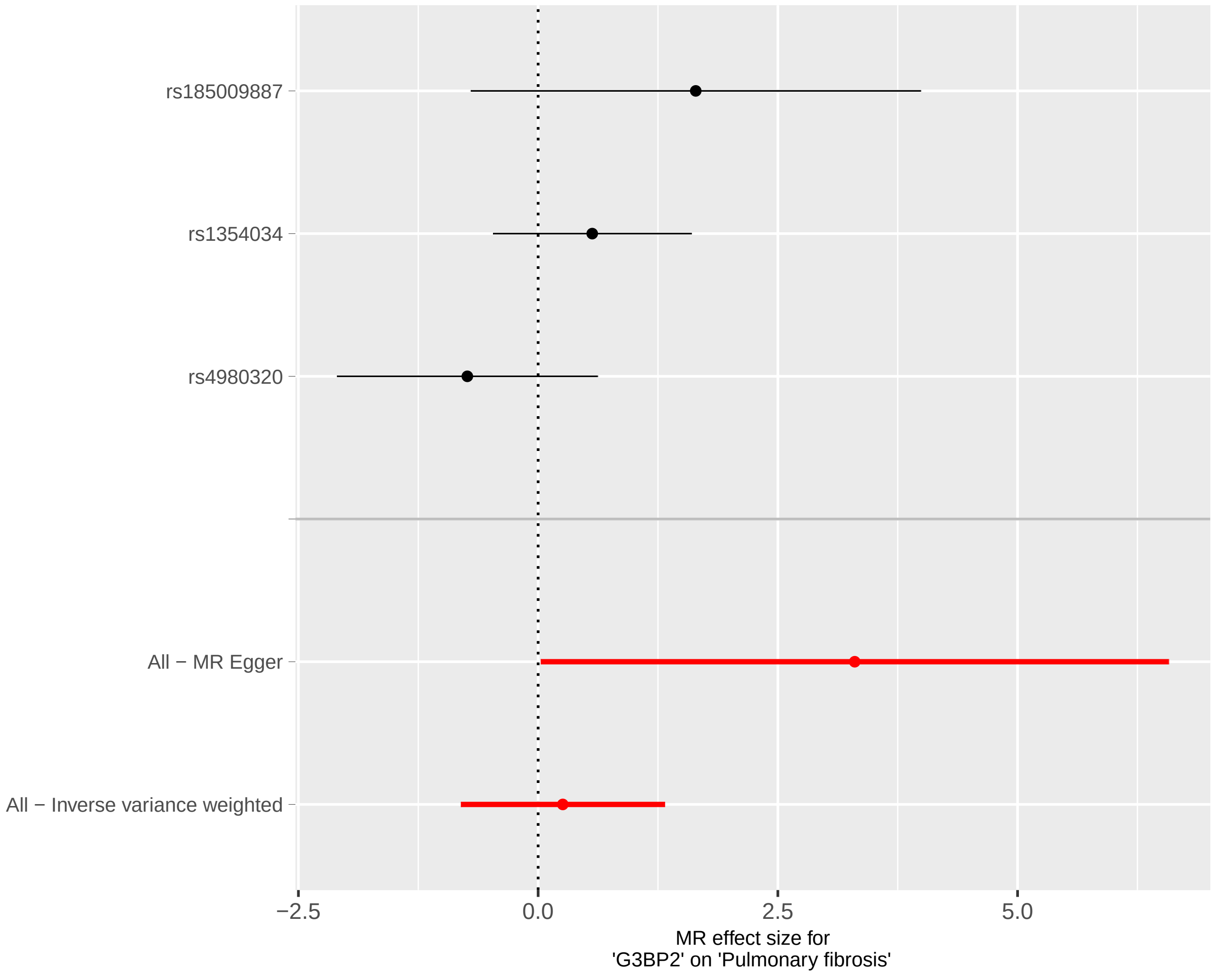
rs185009887
rs1354034
rs4980320
All − MR Egger
All − Inverse variance weighted
−2.5
0.0
2.5
5.0
MR effect size for
'G3BP2' on 'Pulmonary fibrosis'

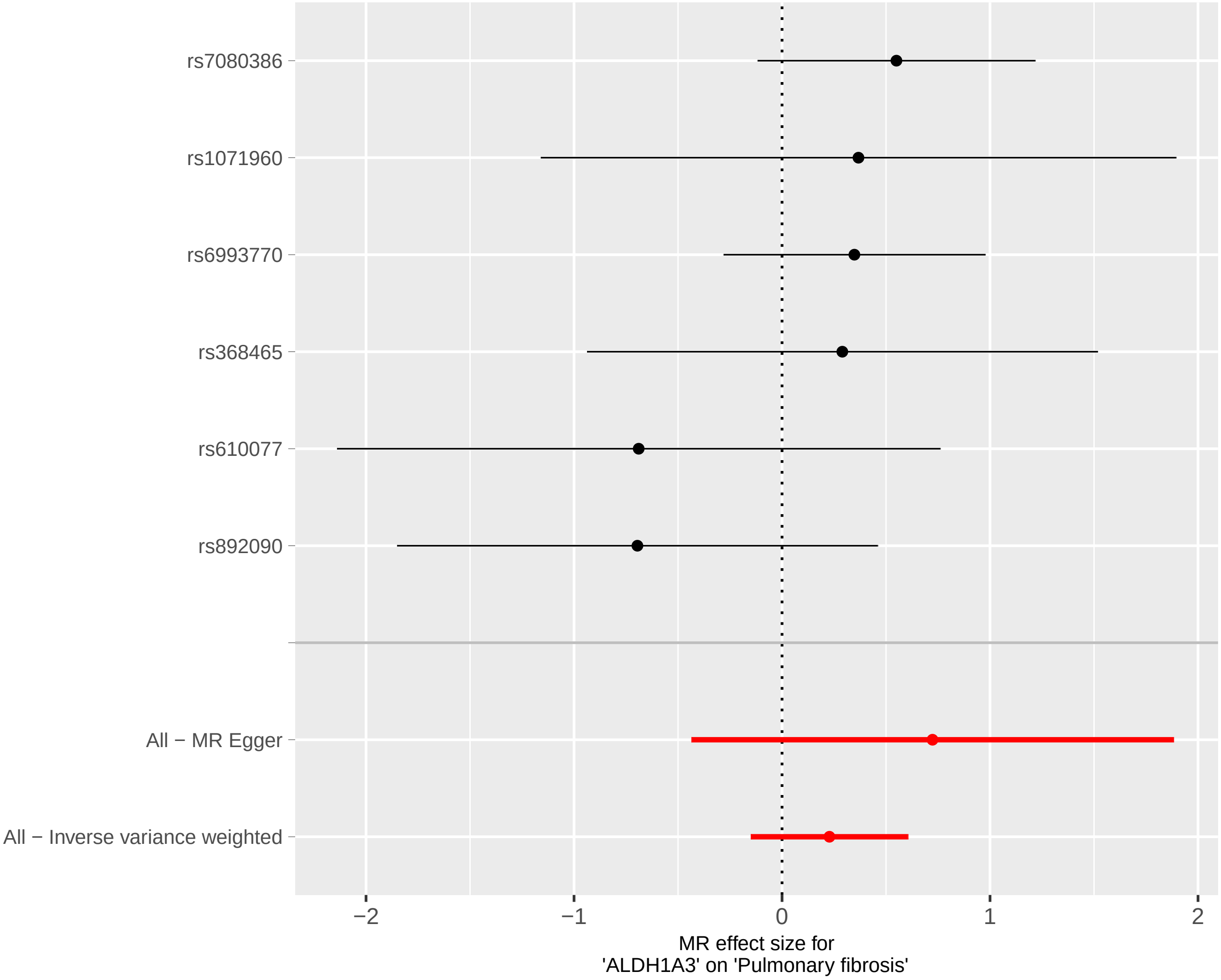

rs7080386
rs1071960
rs6993770
rs368465
rs610077
rs892090
All − MR Egger
All − Inverse variance weighted
−2
−1
0
1
2
MR effect size for
'ALDH1A3' on 'Pulmonary fibrosis'

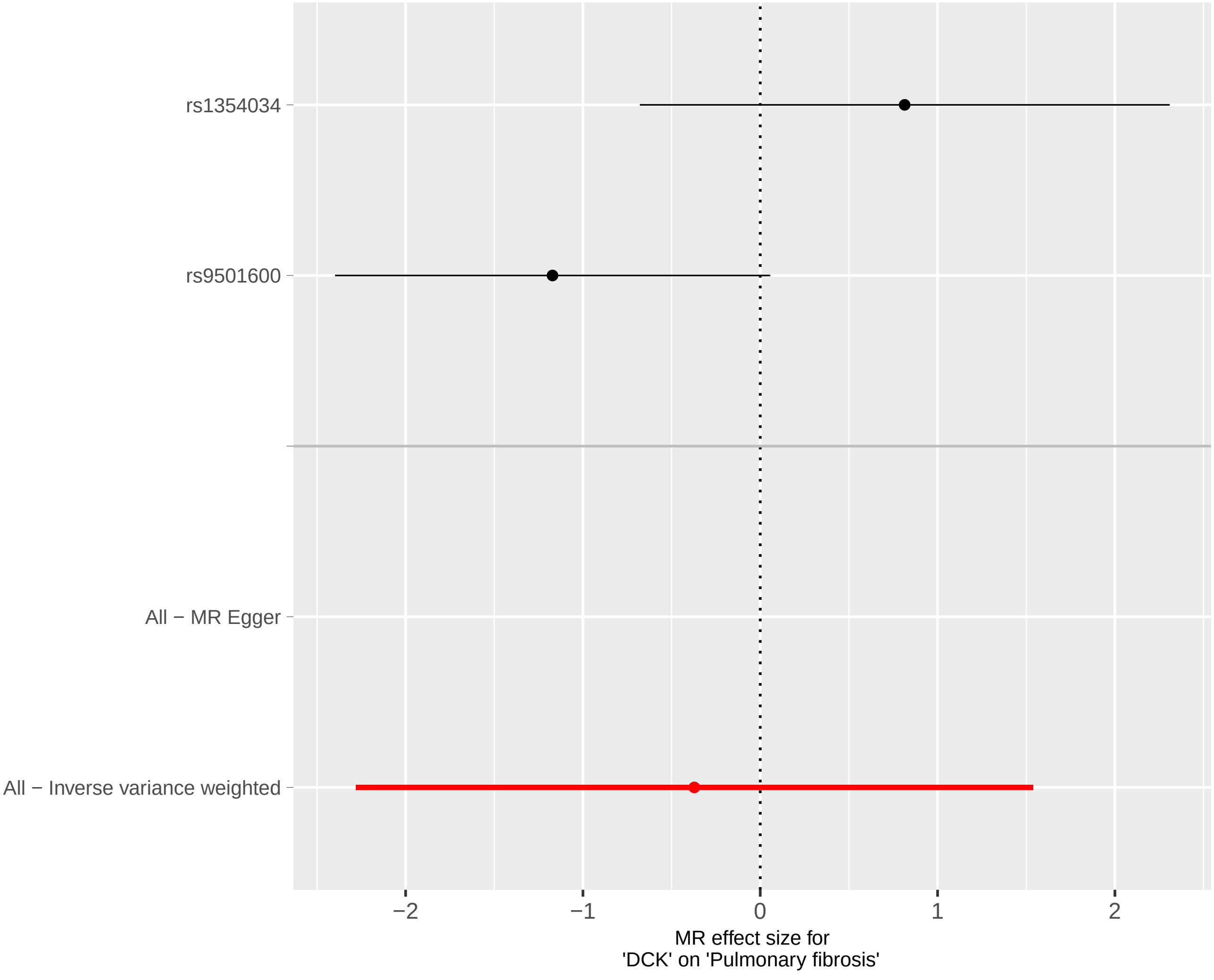
rs1354034
rs9501600
All − MR Egger
All − Inverse variance weighted
−2
−1
0
1
2
MR effect size for
'DCK' on 'Pulmonary fibrosis'

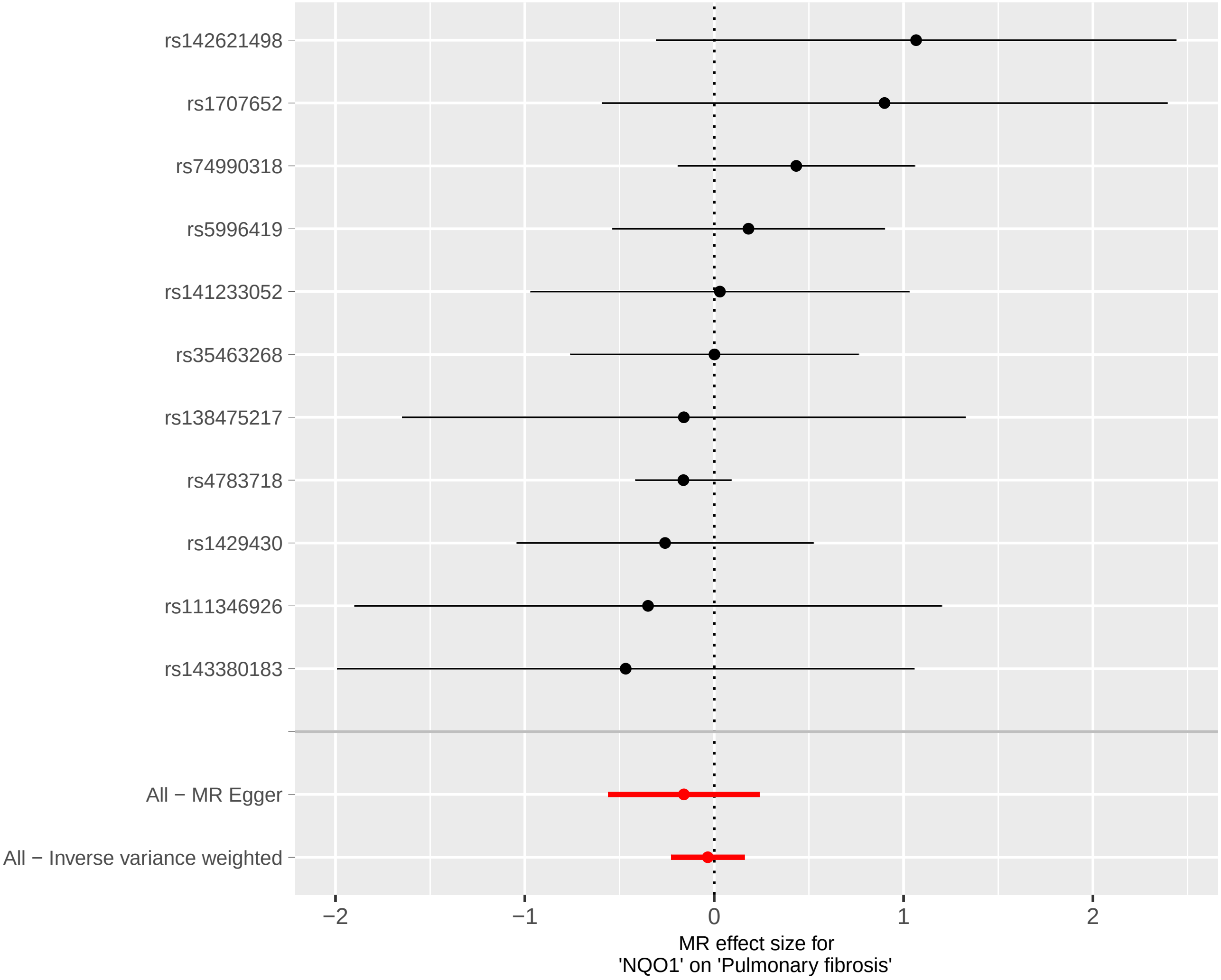
rs142621498
rs1707652
rs74990318
rs5996419
rs141233052
rs35463268
rs138475217
rs4783718
rs1429430
rs111346926
rs143380183
All − MR Egger
All − Inverse variance weighted
−2
−1
0
1
2
MR effect size for
'NQO1' on 'Pulmonary fibrosis'

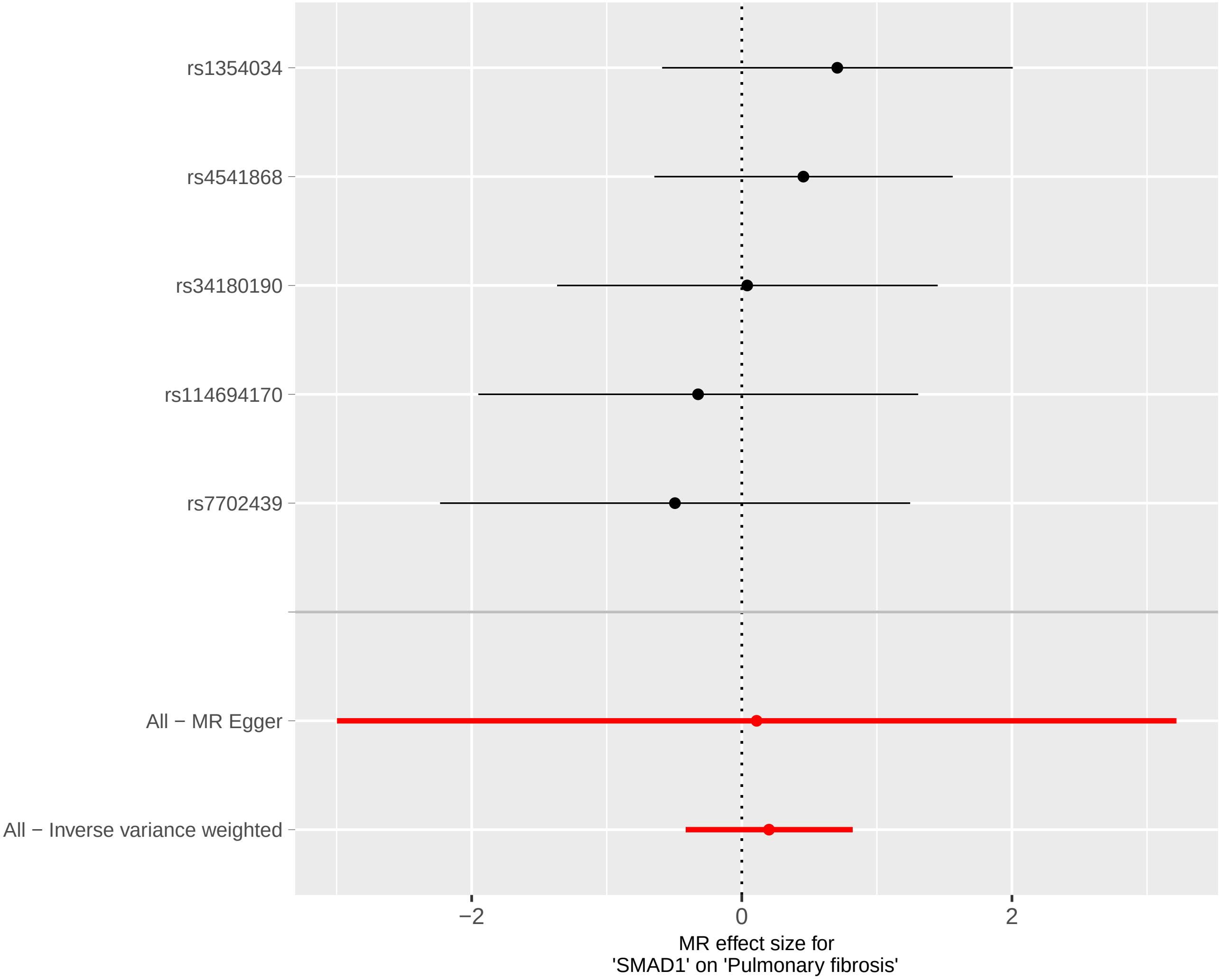
rs1354034
rs4541868
rs34180190
rs114694170
rs7702439
All − MR Egger
All − Inverse variance weighted
−2
0
2
MR effect size for
'SMAD1' on 'Pulmonary fibrosis'

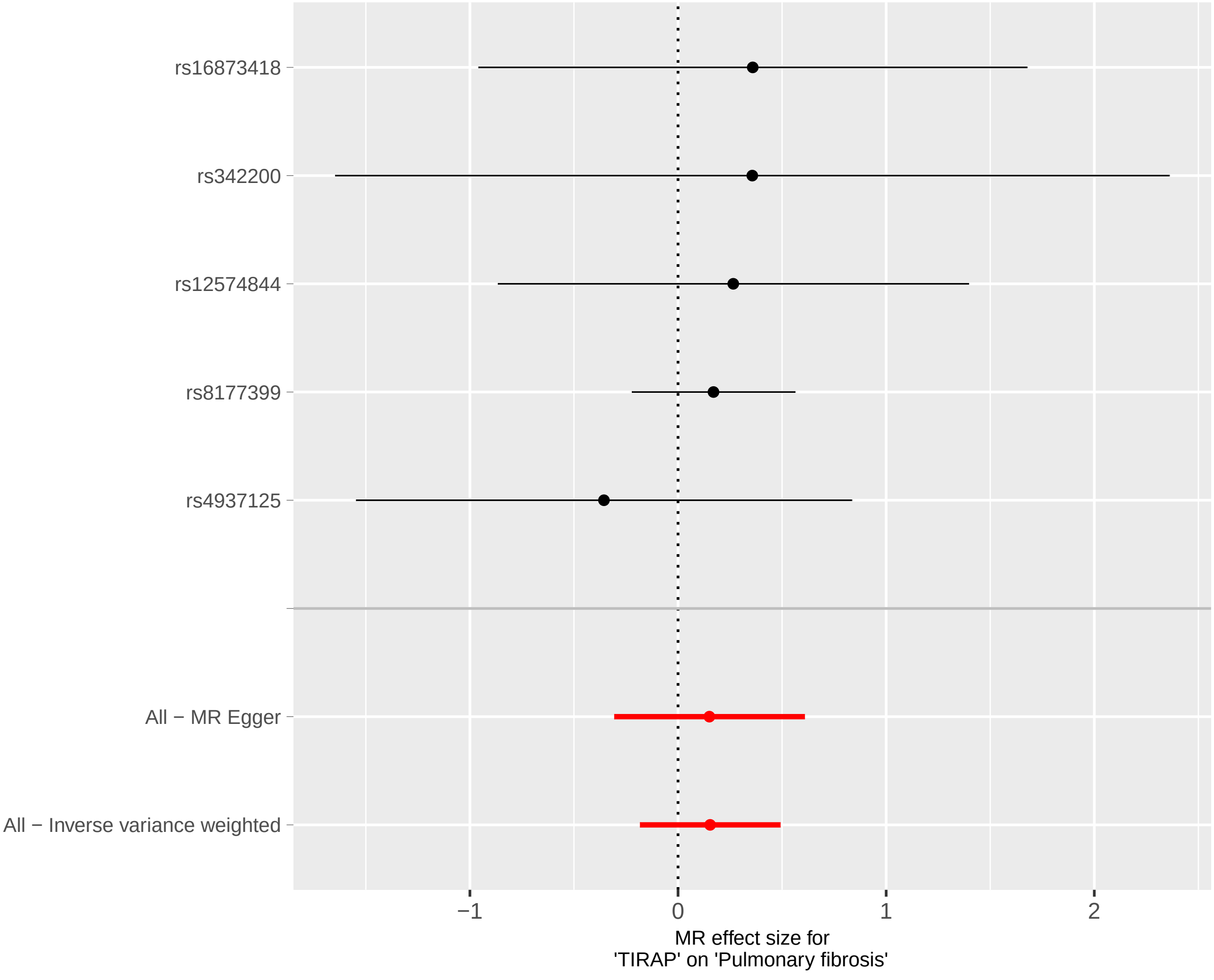
rs16873418
rs342200
rs12574844
rs8177399
rs4937125
All − MR Egger
All − Inverse variance weighted
−1
0
1
2
MR effect size for
'TIRAP' on 'Pulmonary fibrosis'

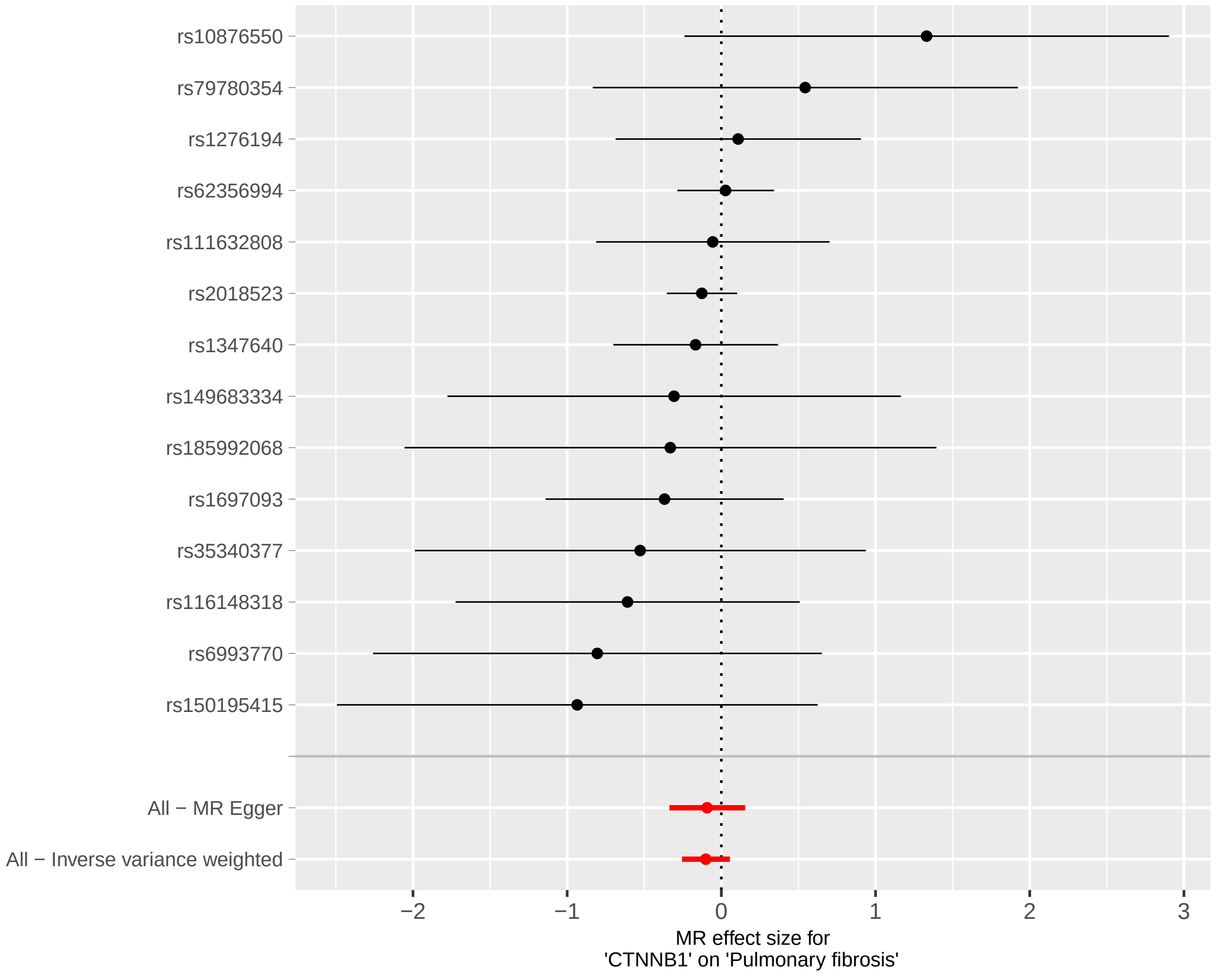
rs10876550
rs79780354
rs1276194
rs62356994
rs111632808
rs2018523
rs1347640
rs149683334
rs185992068
rs1697093
rs35340377
rs116148318
rs6993770
rs150195415
All − MR Egger
All − Inverse variance weighted
−2
−1
0
1
2
3
MR effect size for
'CTNNB1' on 'Pulmonary fibrosis'

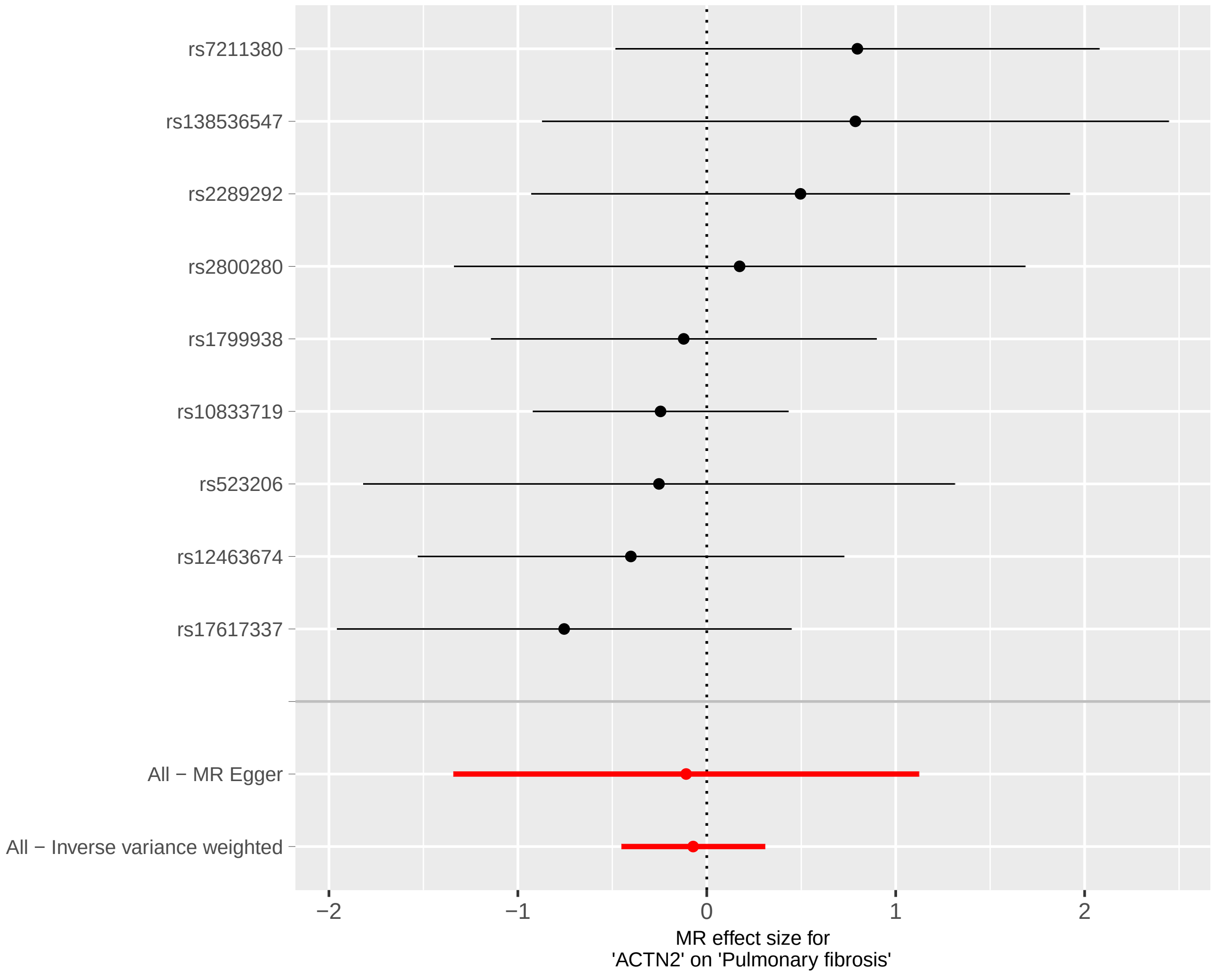

rs7211380
rs138536547
rs2289292
rs2800280
rs1799938
rs10833719
rs523206
rs12463674
rs17617337
All − MR Egger
All − Inverse variance weighted
−2
−1
0
1
2
MR effect size for
'ACTN2' on 'Pulmonary fibrosis'

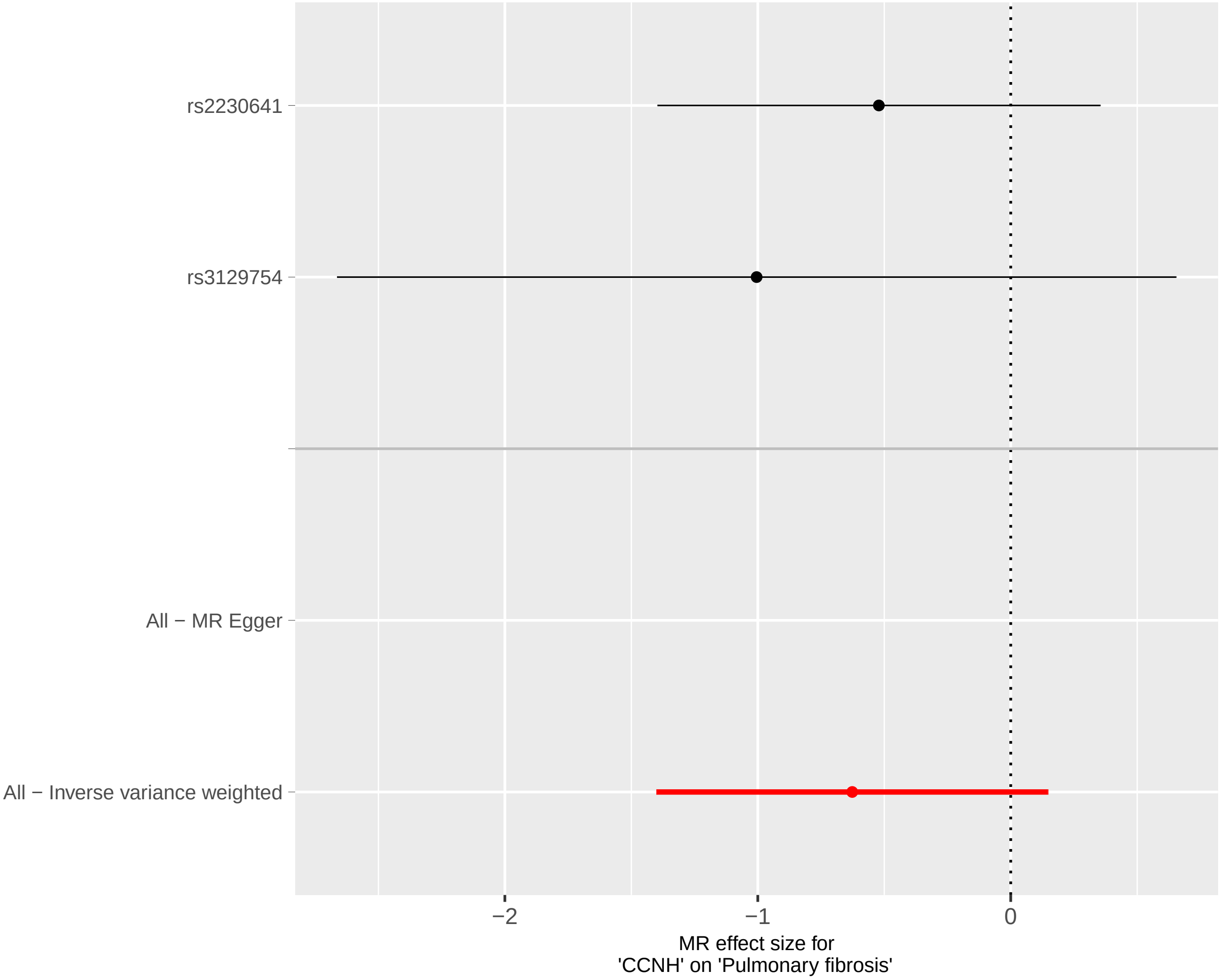

rs2230641
rs3129754
All − MR Egger
All − Inverse variance weighted
−2
−1
0
MR effect size for
'CCNH' on 'Pulmonary fibrosis'

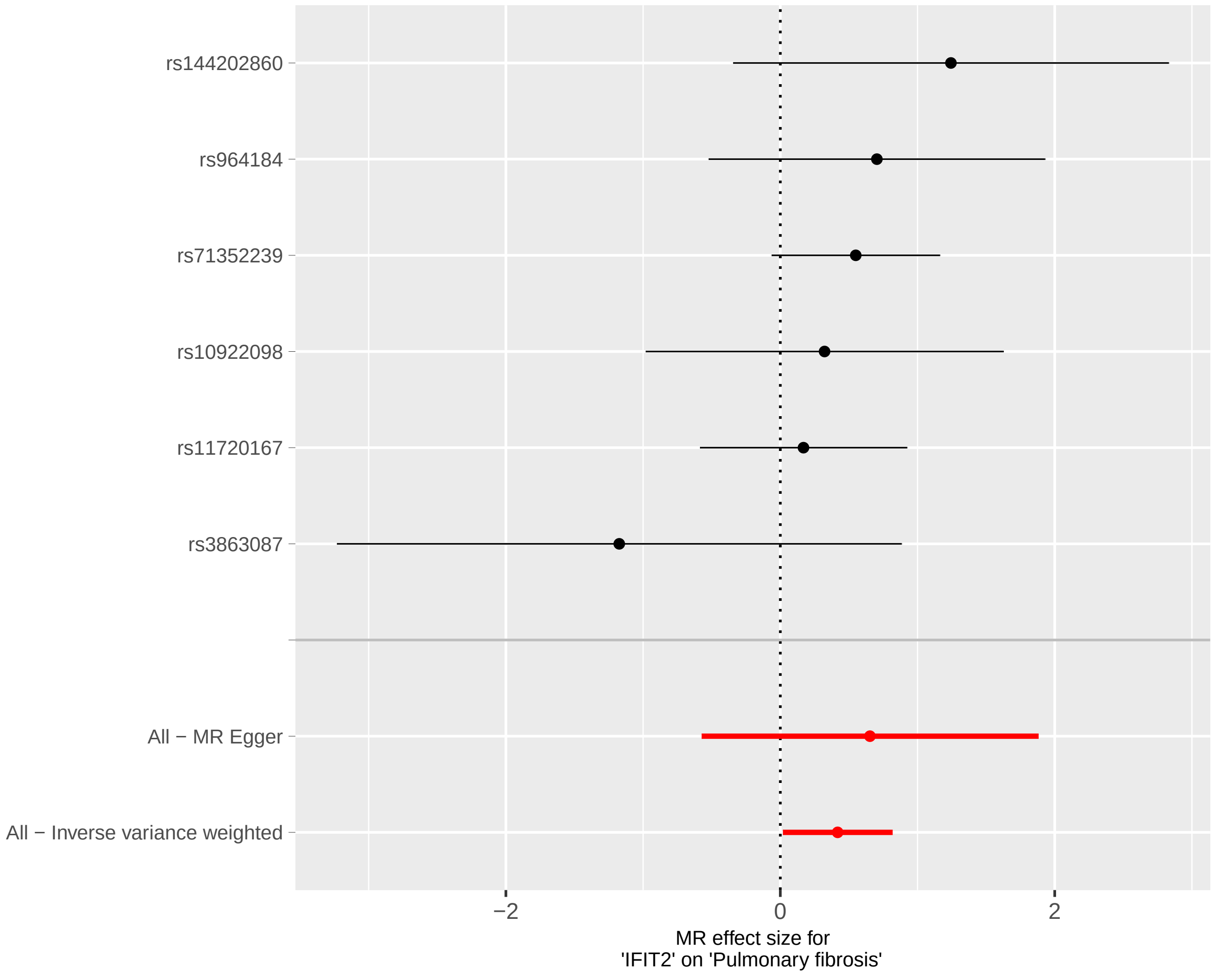

rs144202860
rs964184
rs71352239
rs10922098
rs11720167
rs3863087
All − MR Egger
All − Inverse variance weighted
−2
0
2
MR effect size for
'IFIT2' on 'Pulmonary fibrosis'

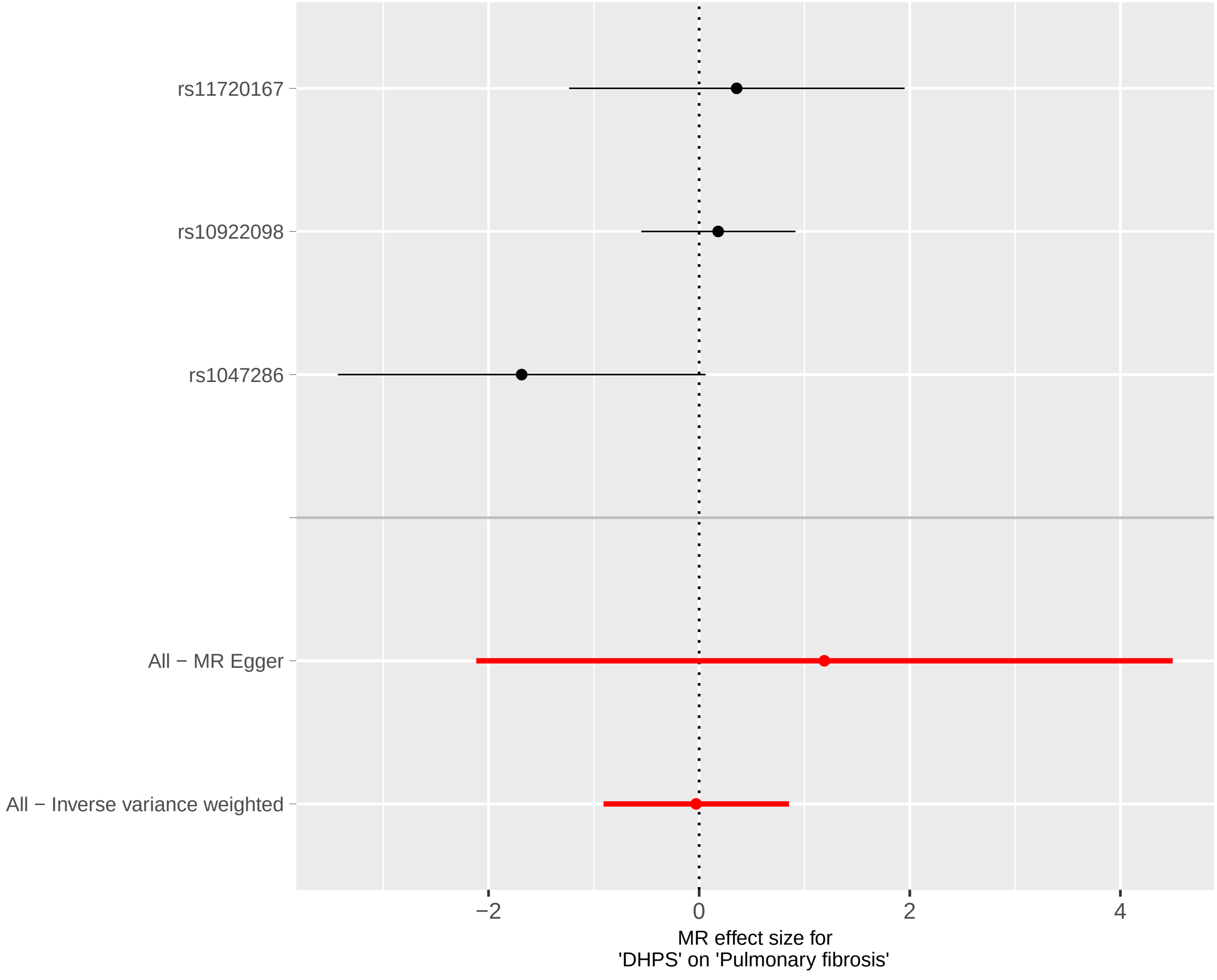
rs11720167
rs10922098
rs1047286
All − MR Egger
All − Inverse variance weighted
−2
0
2
4
MR effect size for
'DHPS' on 'Pulmonary fibrosis'

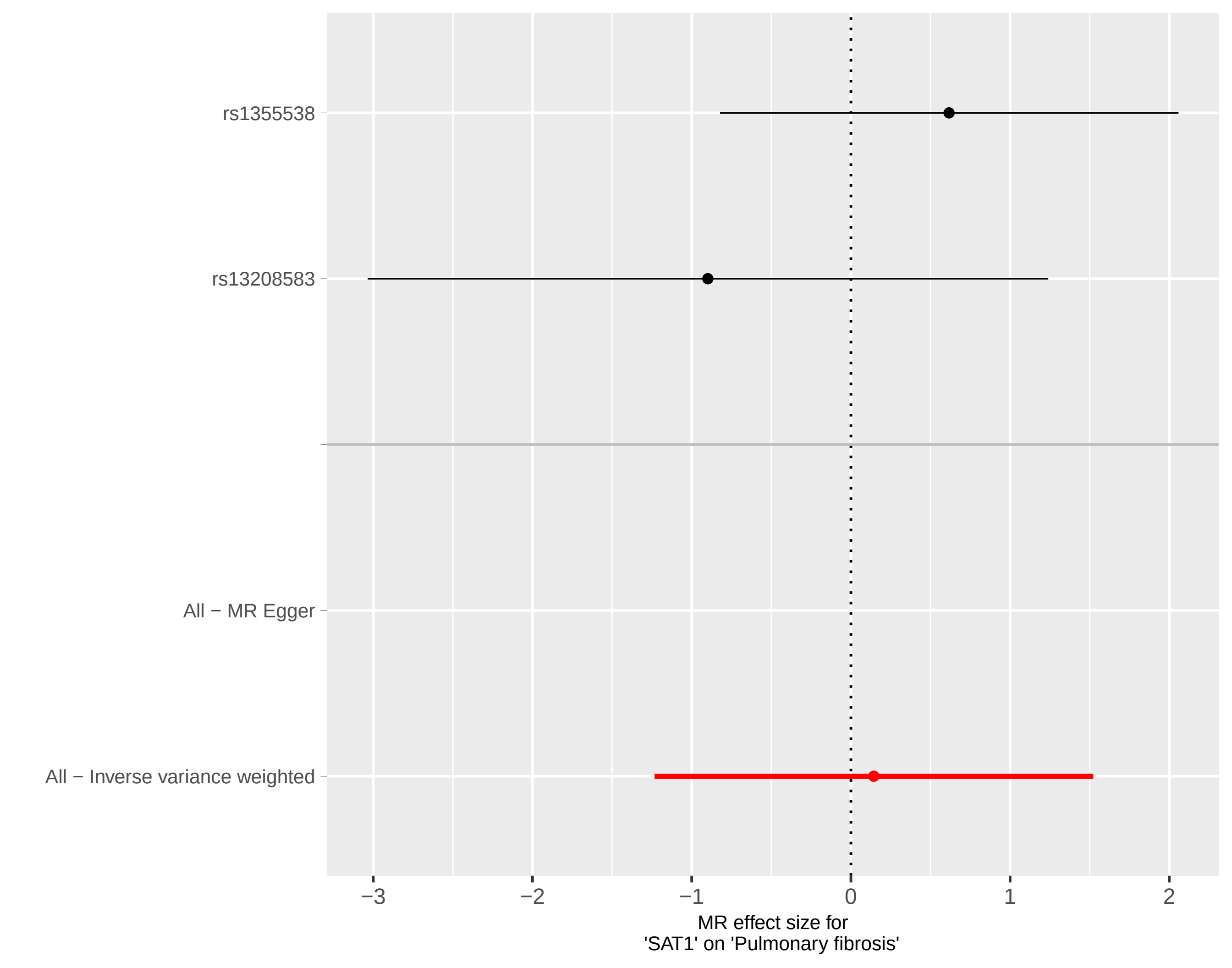

rs1355538
rs13208583
All − MR Egger
All − Inverse variance weighted
−3
−2
−1
0
1
2
MR effect size for
'SAT1' on 'Pulmonary fibrosis'

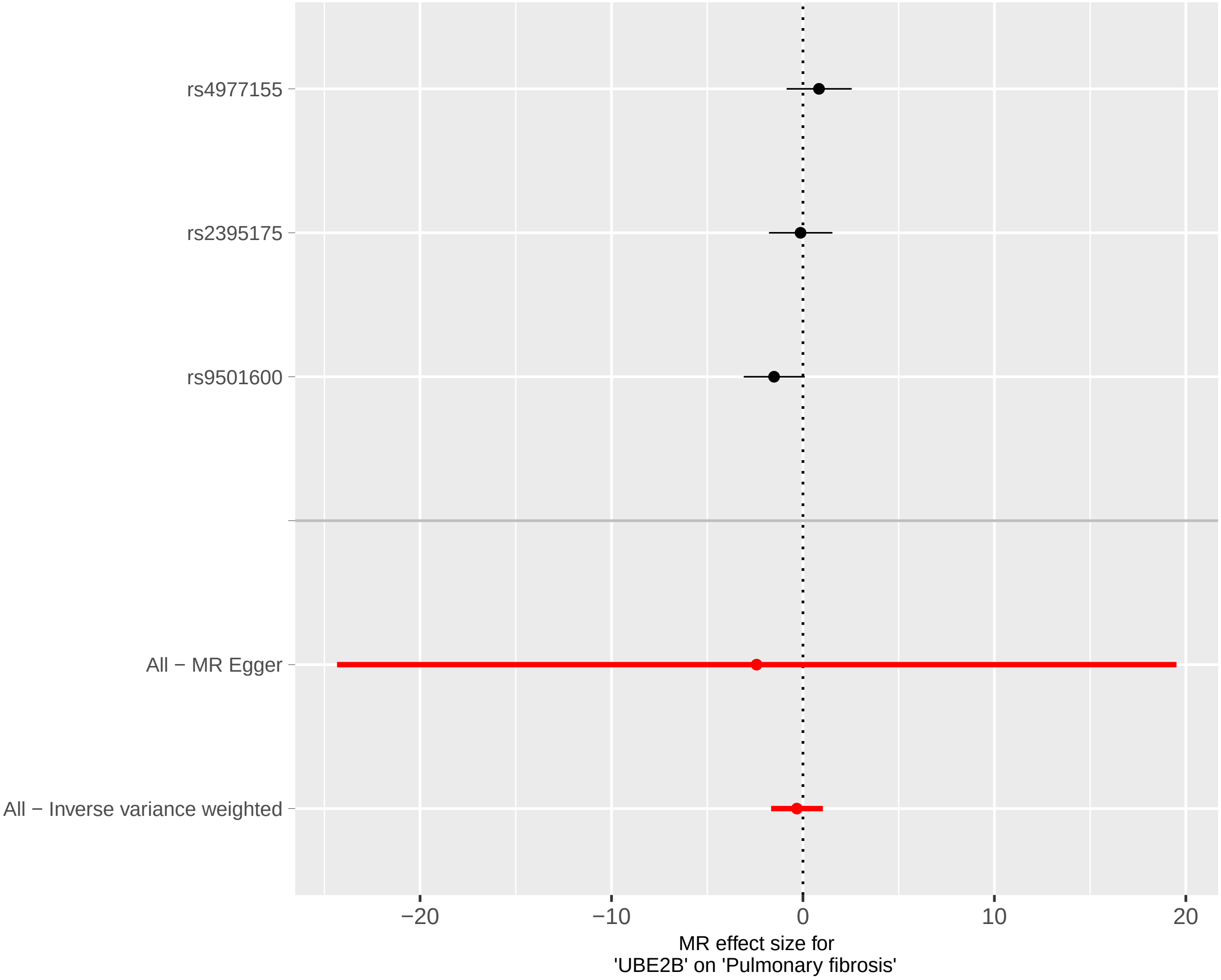

rs4977155
rs2395175
rs9501600
All − MR Egger
All − Inverse variance weighted
−20
−10
0
10
20
MR effect size for
'UBE2B' on 'Pulmonary fibrosis'

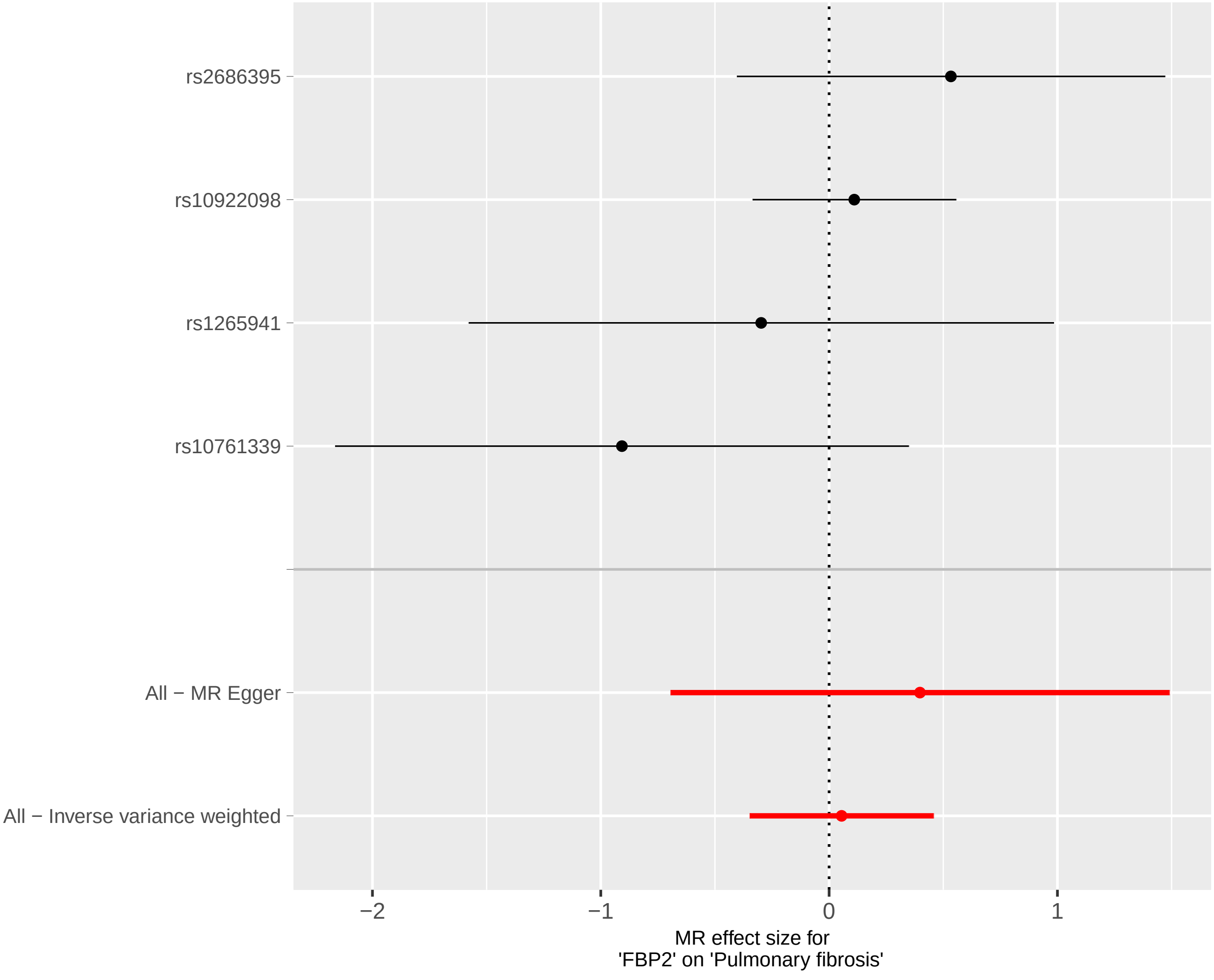

rs2686395
rs10922098
rs1265941
rs10761339
All − MR Egger
All − Inverse variance weighted
−2
−1
0
1
MR effect size for
'FBP2' on 'Pulmonary fibrosis'

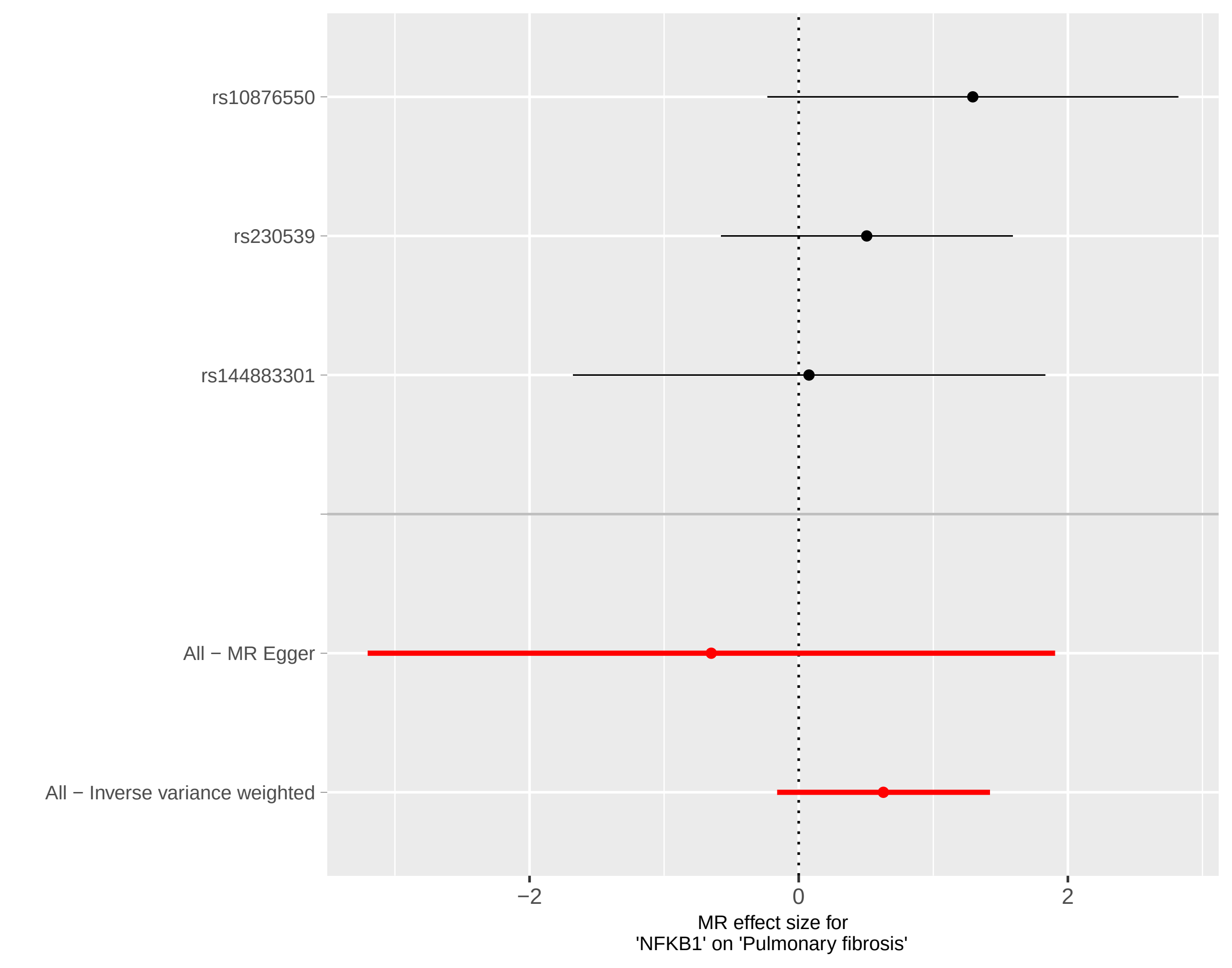

rs10876550
rs230539
rs144883301
All − MR Egger
All − Inverse variance weighted
−2
0
2
MR effect size for
'NFKB1' on 'Pulmonary fibrosis'

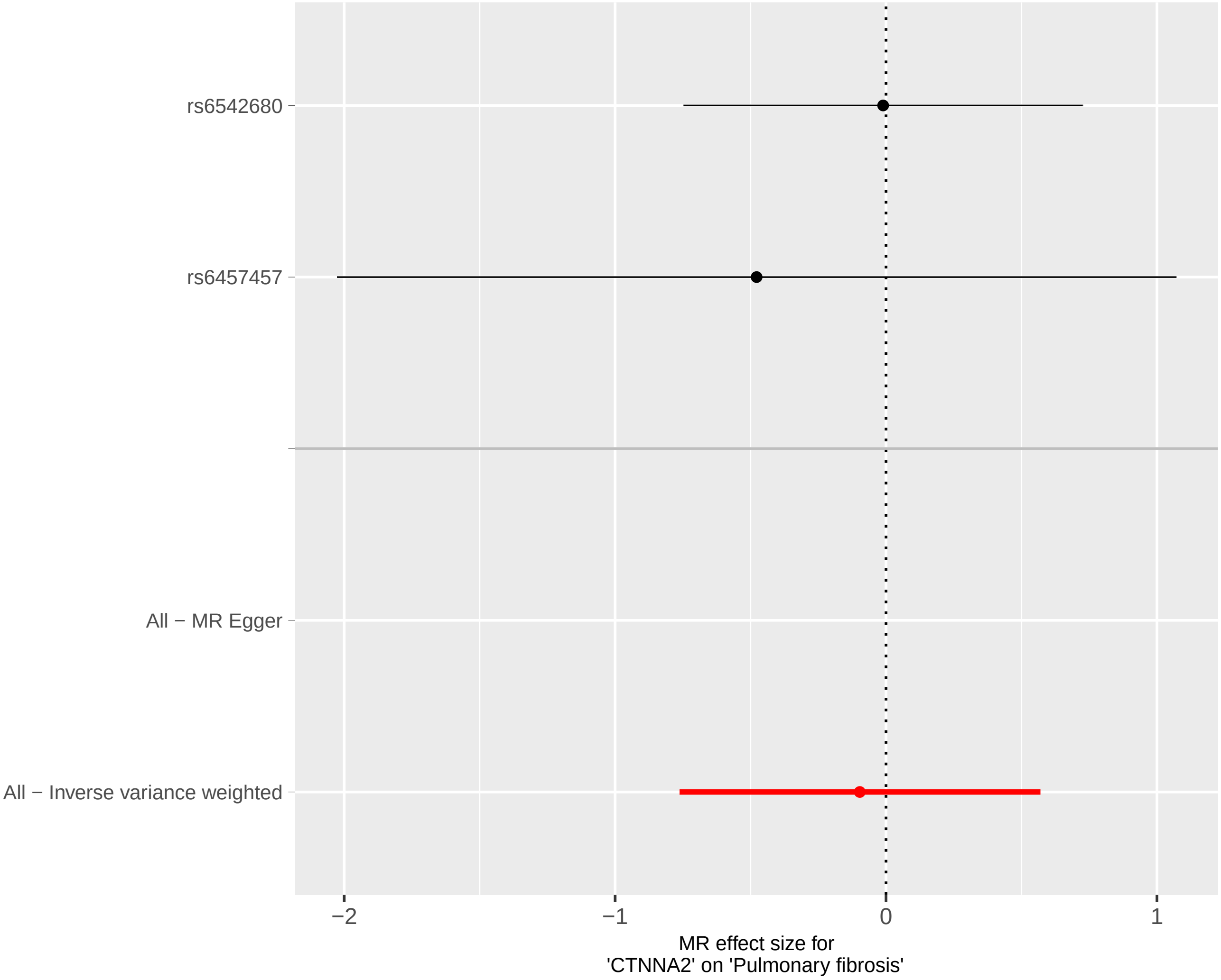

rs6542680
rs6457457
All − MR Egger
All − Inverse variance weighted
−2
−1
0
1
MR effect size for
'CTNNA2' on 'Pulmonary fibrosis'

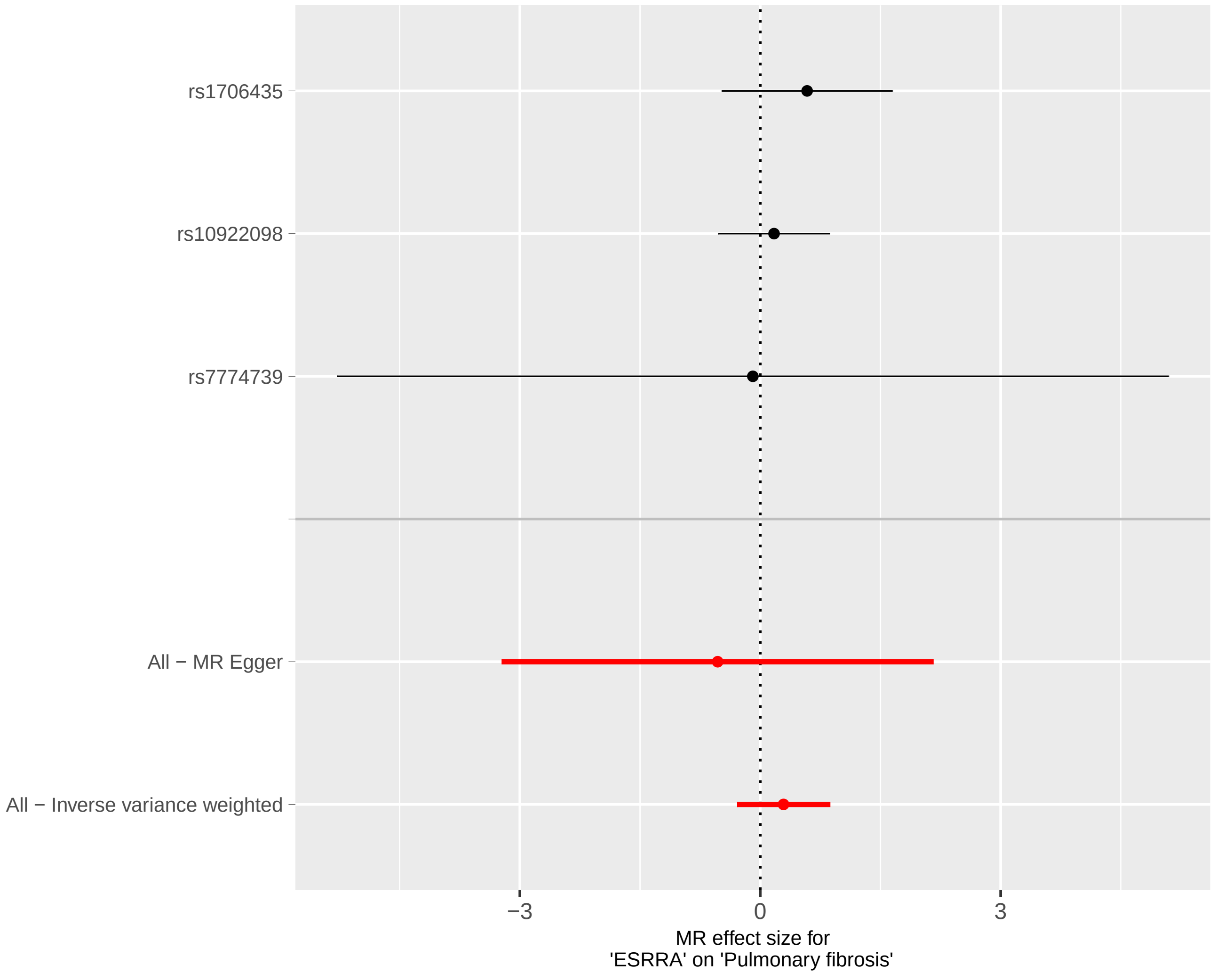
rs1706435
rs10922098
rs7774739
All − MR Egger
All − Inverse variance weighted
−3
0
3
MR effect size for
'ESRRA' on 'Pulmonary fibrosis'

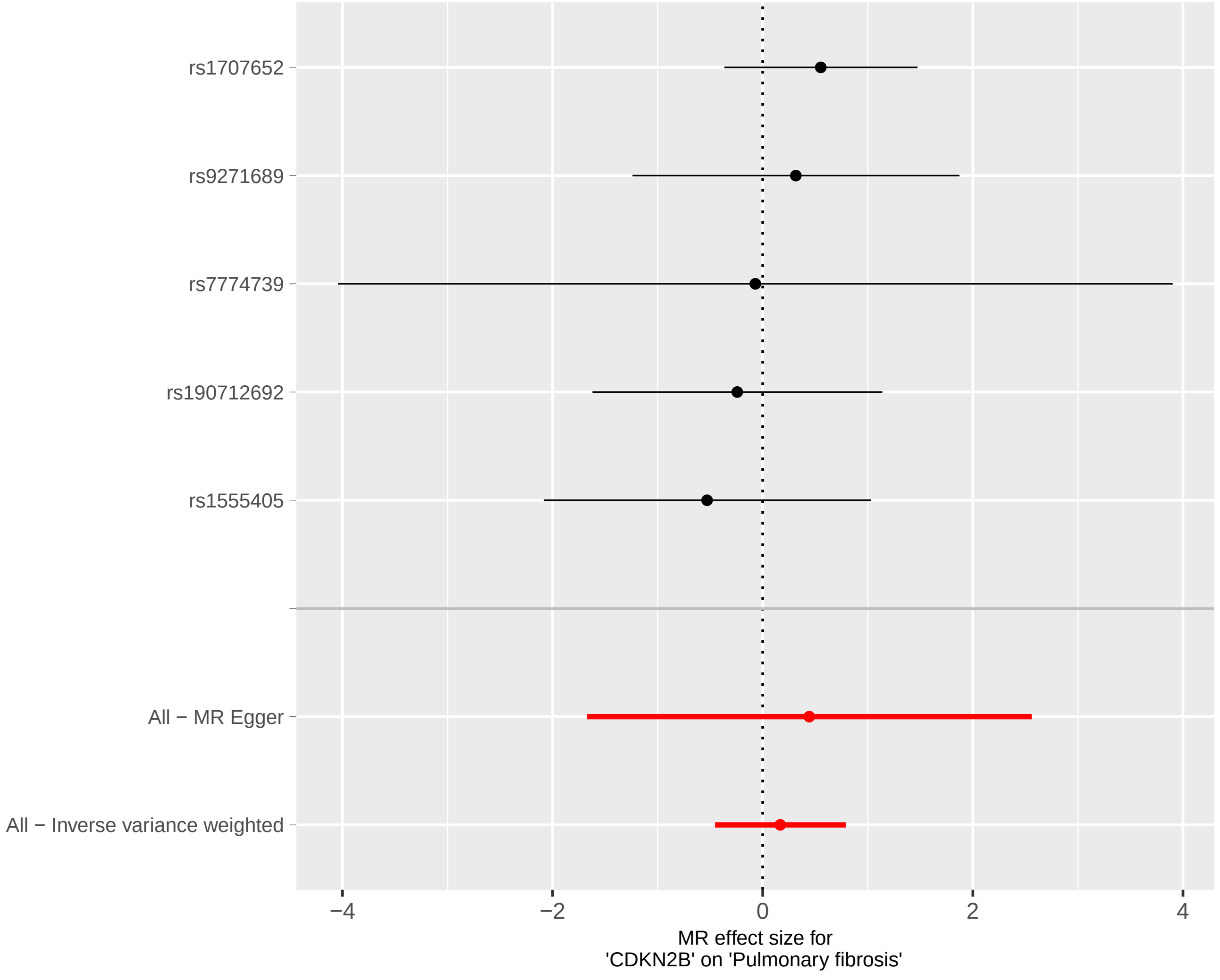

rs1707652
rs9271689
rs7774739
rs190712692
rs1555405
All − MR Egger
All − Inverse variance weighted
−4
−2
0
2
4
MR effect size for
'CDKN2B' on 'Pulmonary fibrosis'

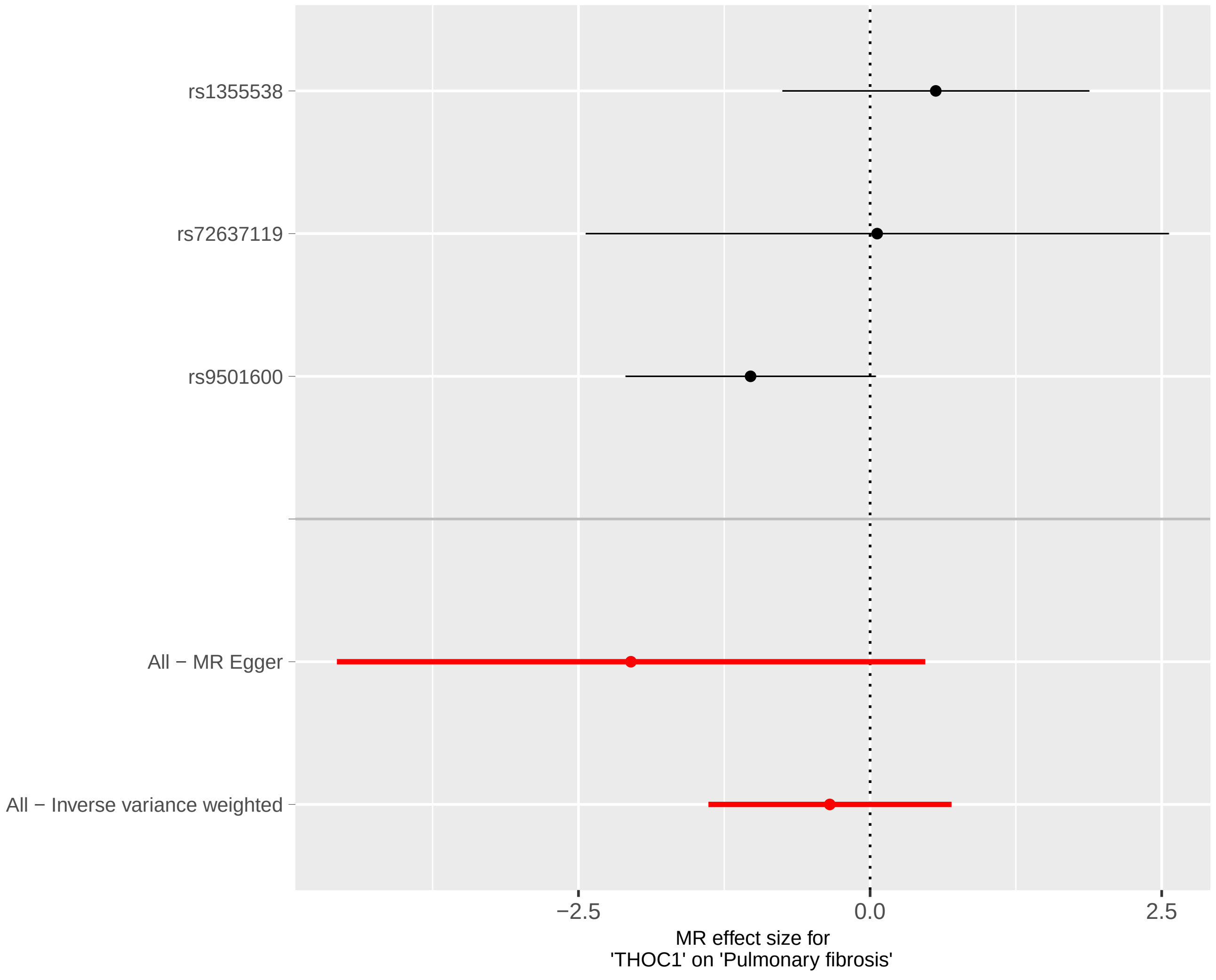
rs1355538
rs72637119
rs9501600
All − MR Egger
All − Inverse variance weighted
−2.5
0.0
2.5
MR effect size for
'THOC1' on 'Pulmonary fibrosis'

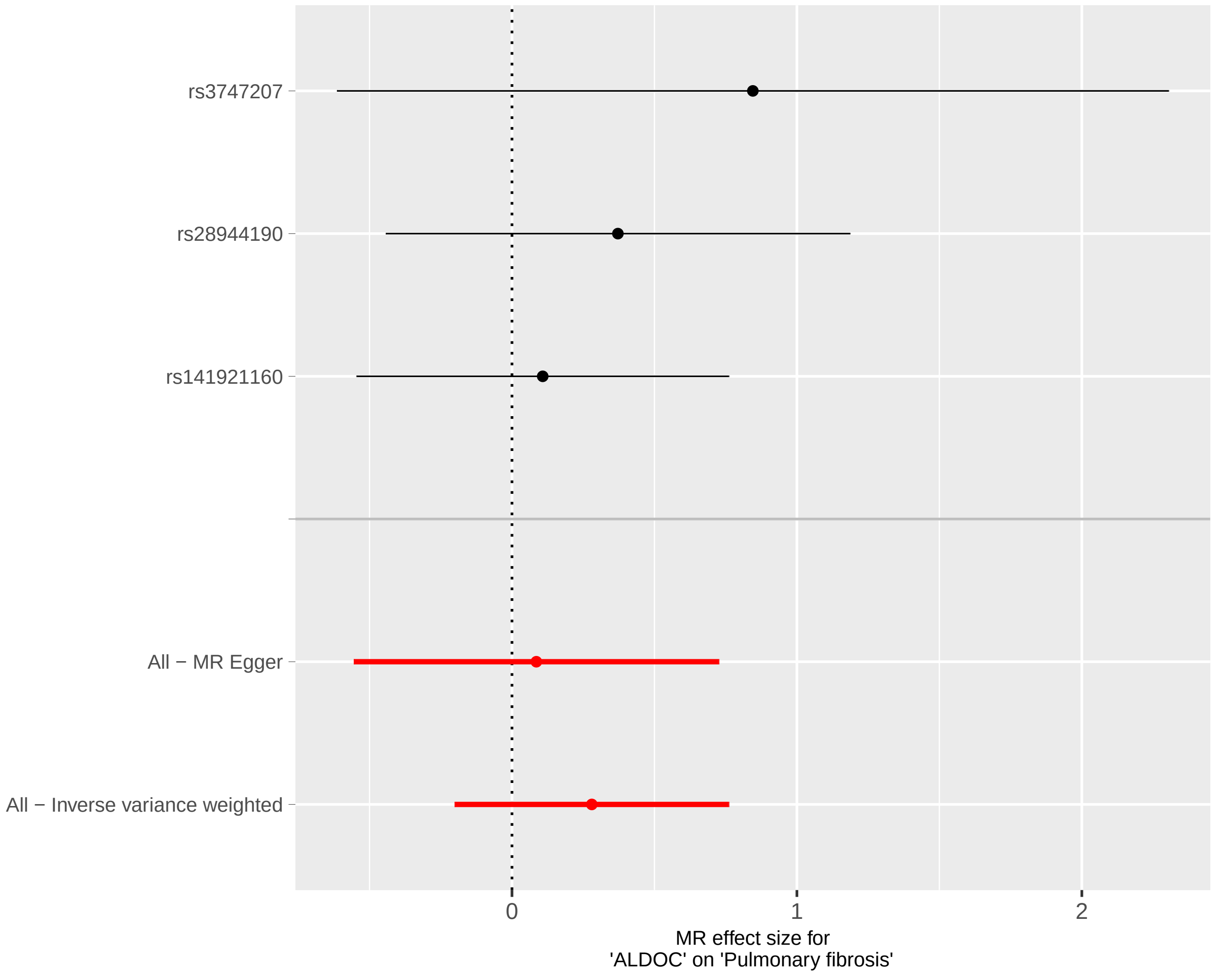

rs3747207
rs28944190
rs141921160
All − MR Egger
All − Inverse variance weighted
0
1
2
MR effect size for
'ALDOC' on 'Pulmonary fibrosis'

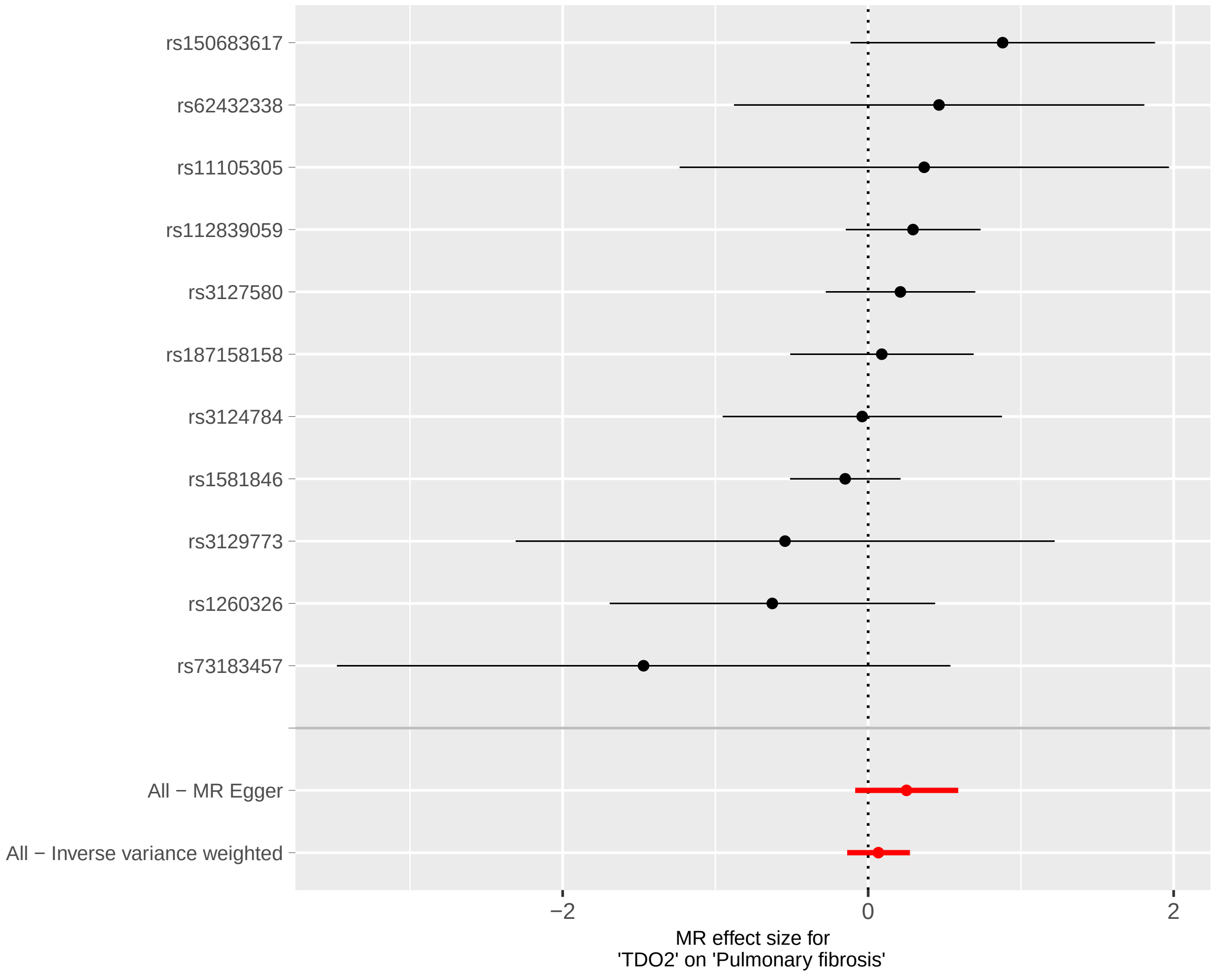
rs150683617
rs62432338
rs11105305
rs112839059
rs3127580
rs187158158
rs3124784
rs1581846
rs3129773
rs1260326
rs73183457
All − MR Egger
All − Inverse variance weighted
−2
0
2
MR effect size for
'TDO2' on 'Pulmonary fibrosis'

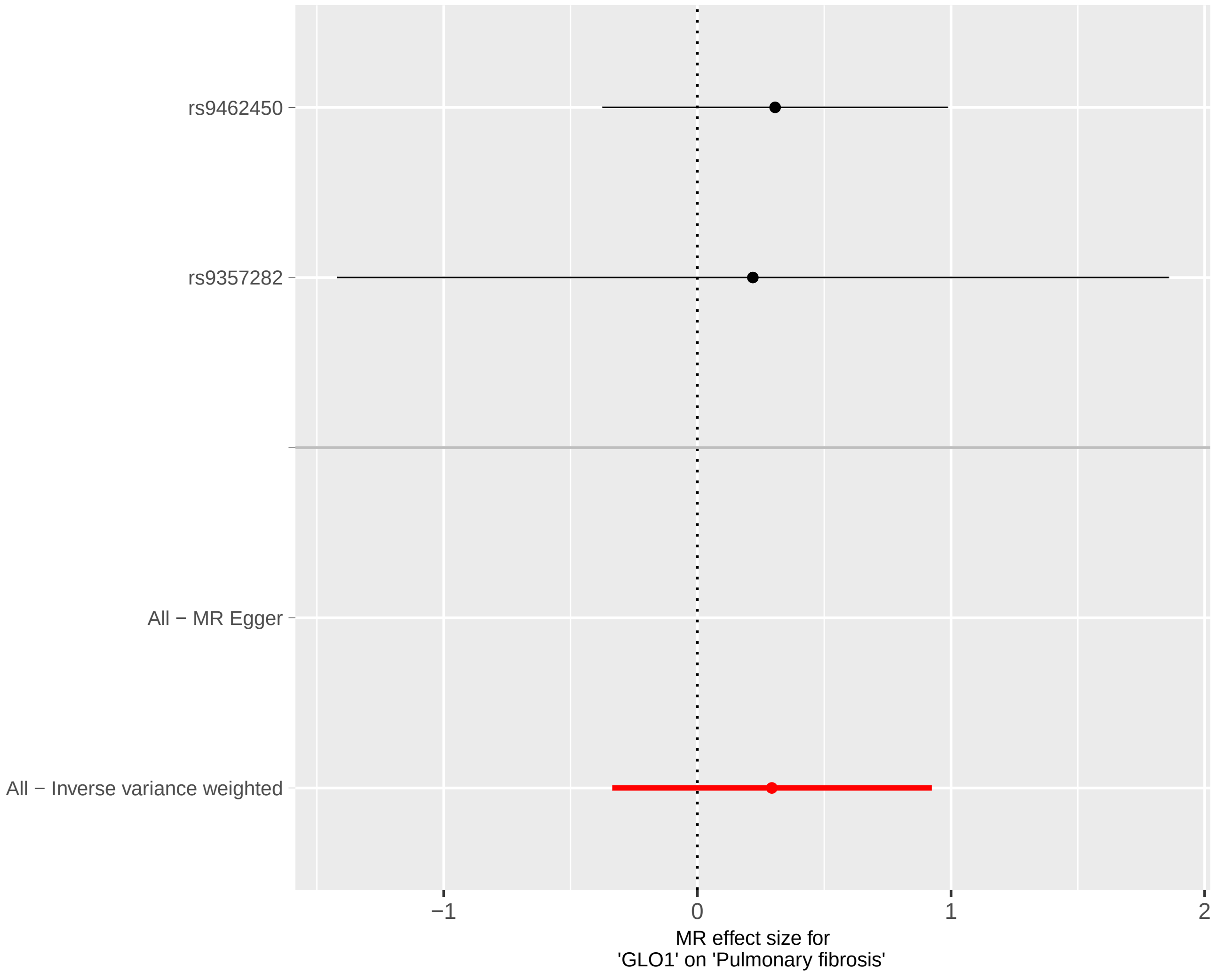
rs9462450
rs9357282
All − MR Egger
All − Inverse variance weighted
−1
0
1
2
MR effect size for
'GLO1' on 'Pulmonary fibrosis'

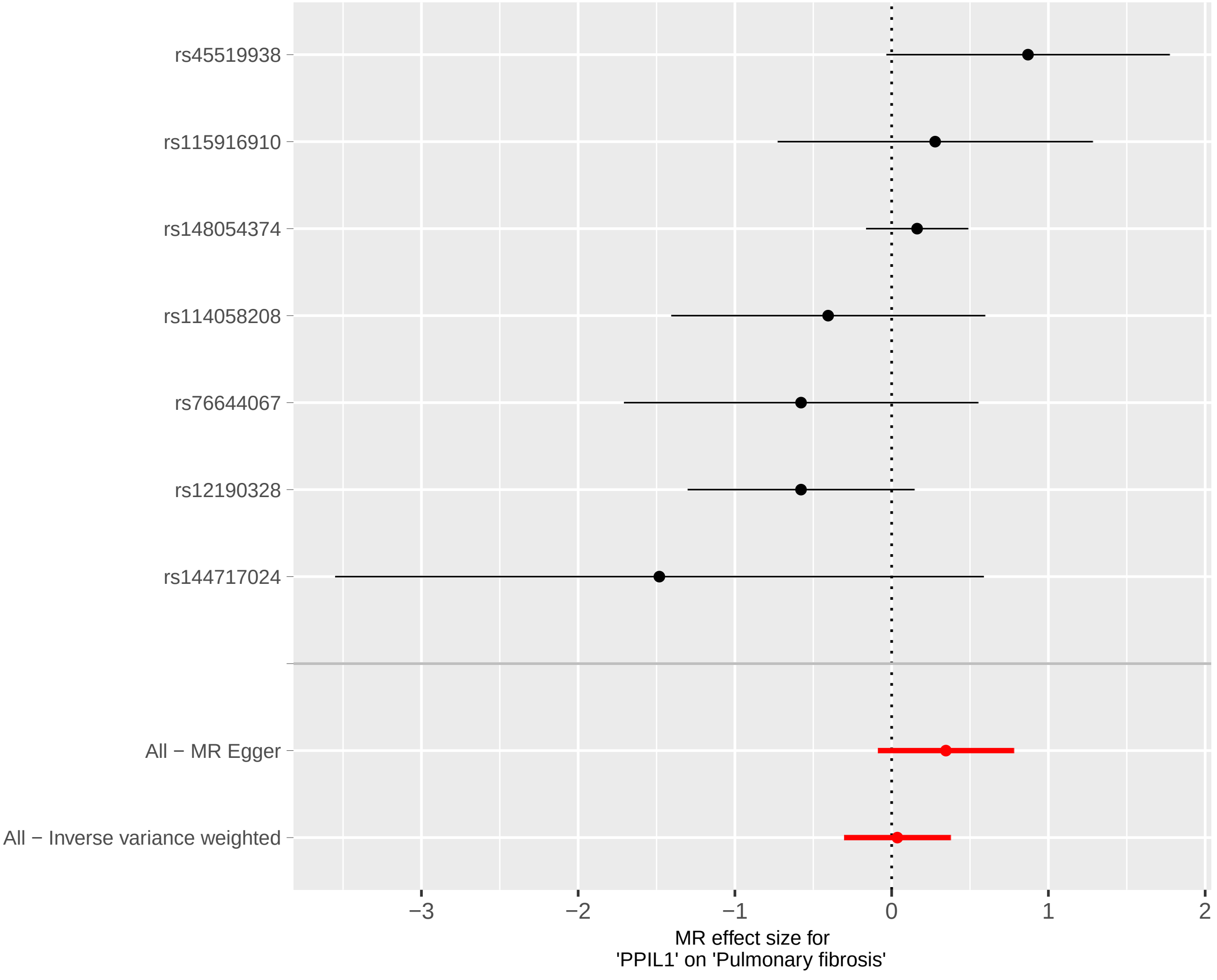
rs45519938
rs115916910
rs148054374
rs114058208
rs76644067
rs12190328
rs144717024
All − MR Egger
All − Inverse variance weighted
−3
−2
−1
0
1
2
MR effect size for
'PPIL1' on 'Pulmonary fibrosis'

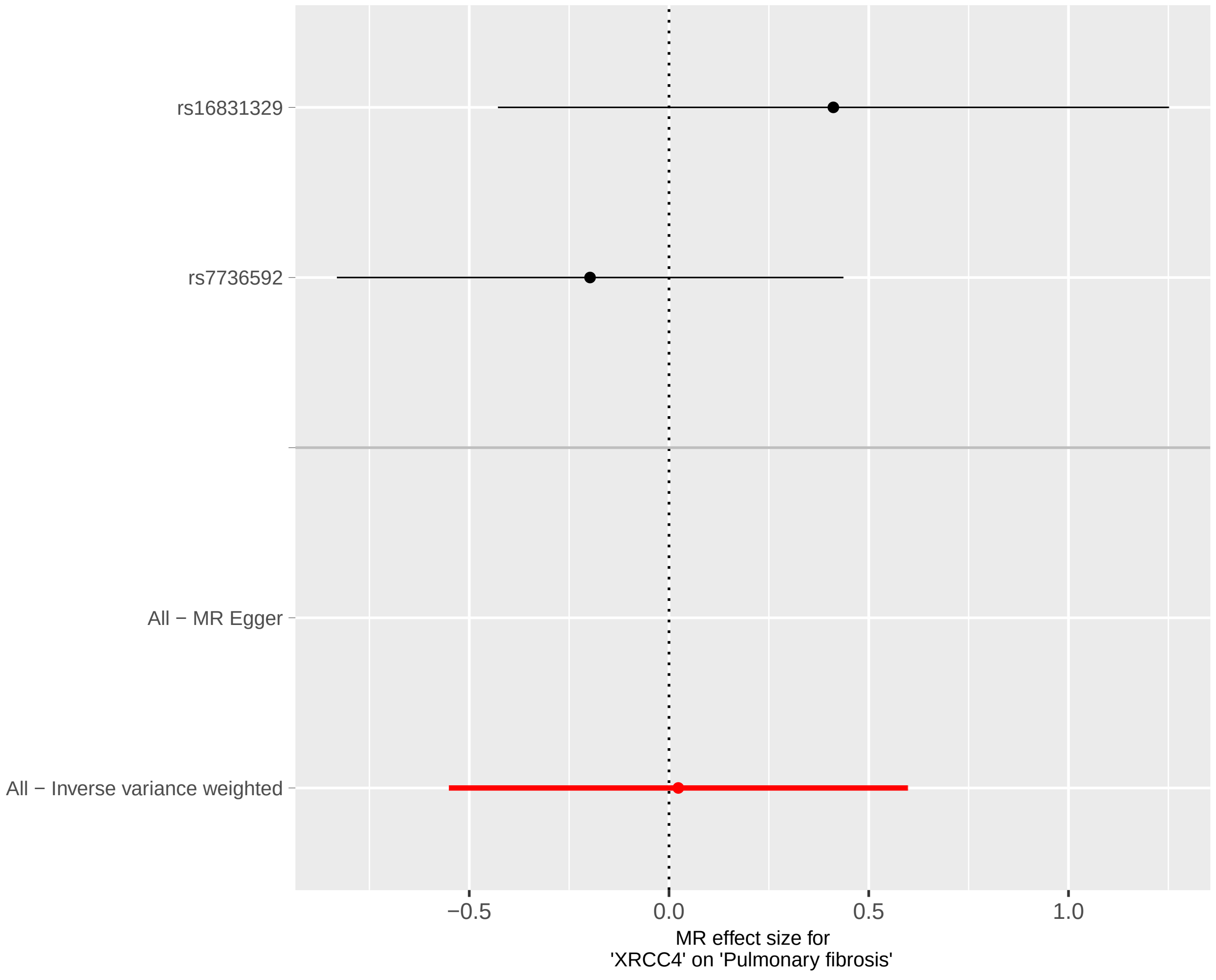
rs16831329
rs7736592
All − MR Egger
All − Inverse variance weighted
−0.5
0.0
0.5
1.0
MR effect size for
'XRCC4' on 'Pulmonary fibrosis'

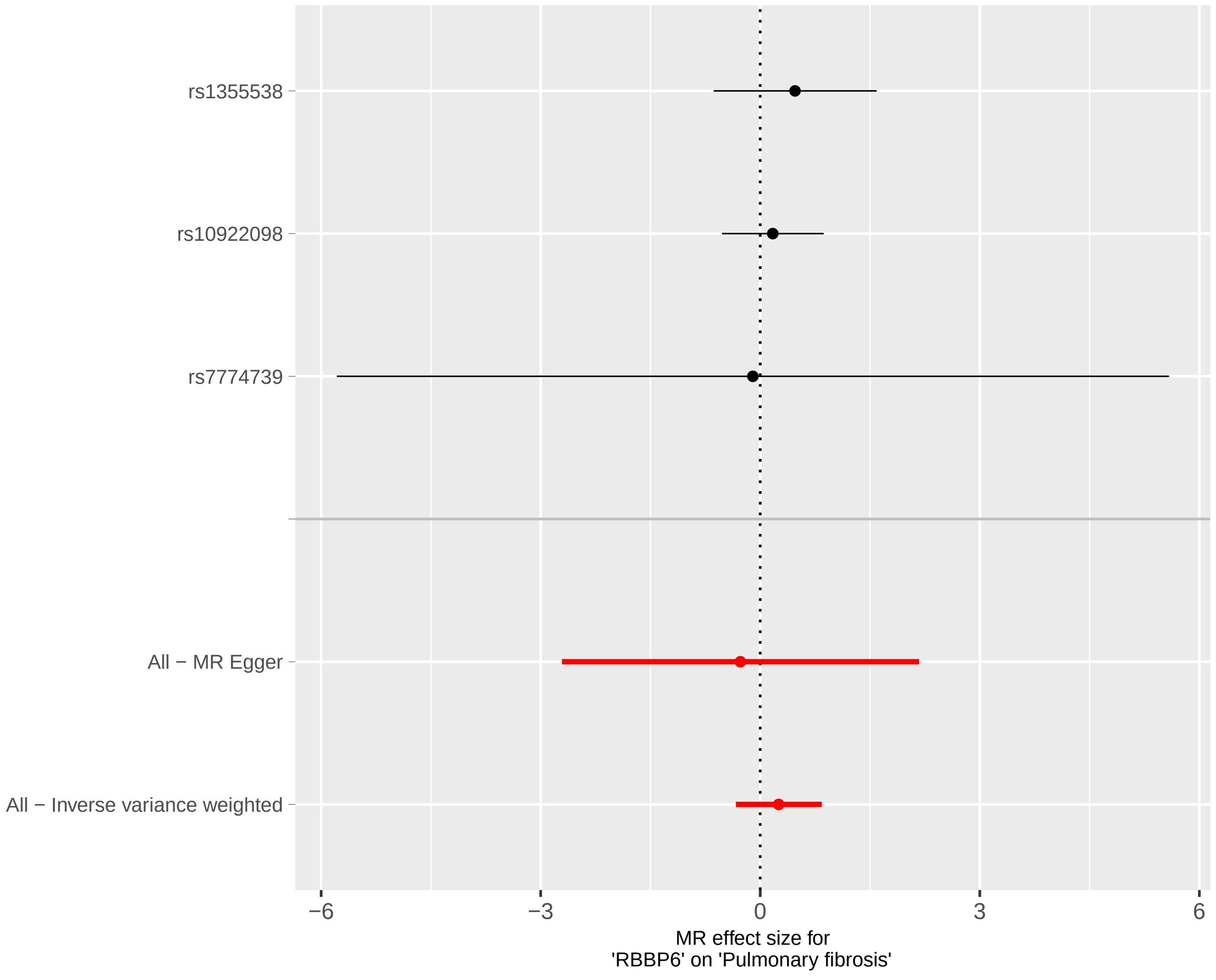

rs1355538
rs10922098
rs7774739
All − MR Egger
All − Inverse variance weighted
−6
−3
0
3
6
MR effect size for
'RBBP6' on 'Pulmonary fibrosis'

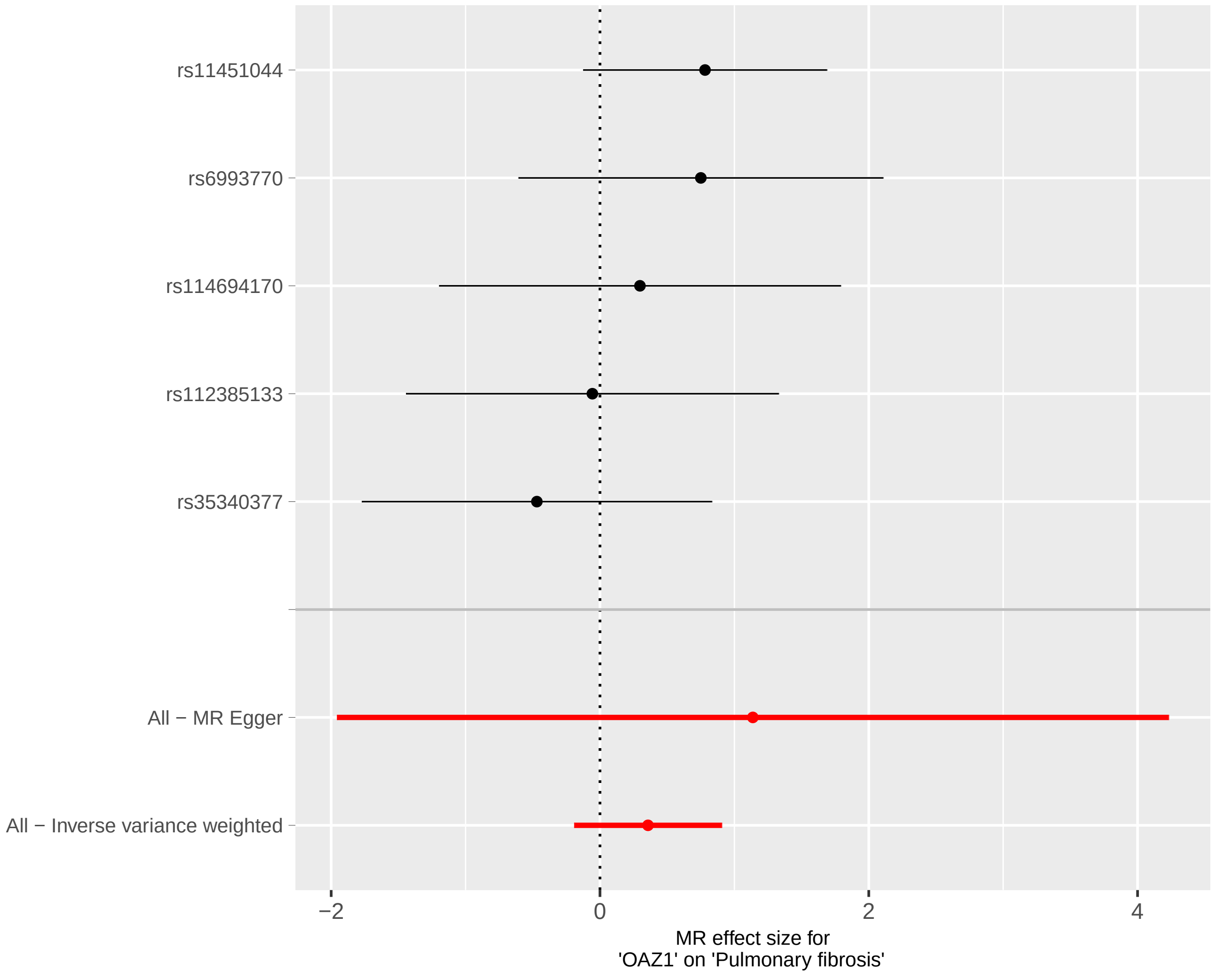

rs11451044
rs6993770
rs114694170
rs112385133
rs35340377
All − MR Egger
All − Inverse variance weighted
−2
0
2
4
MR effect size for
'OAZ1' on 'Pulmonary fibrosis'

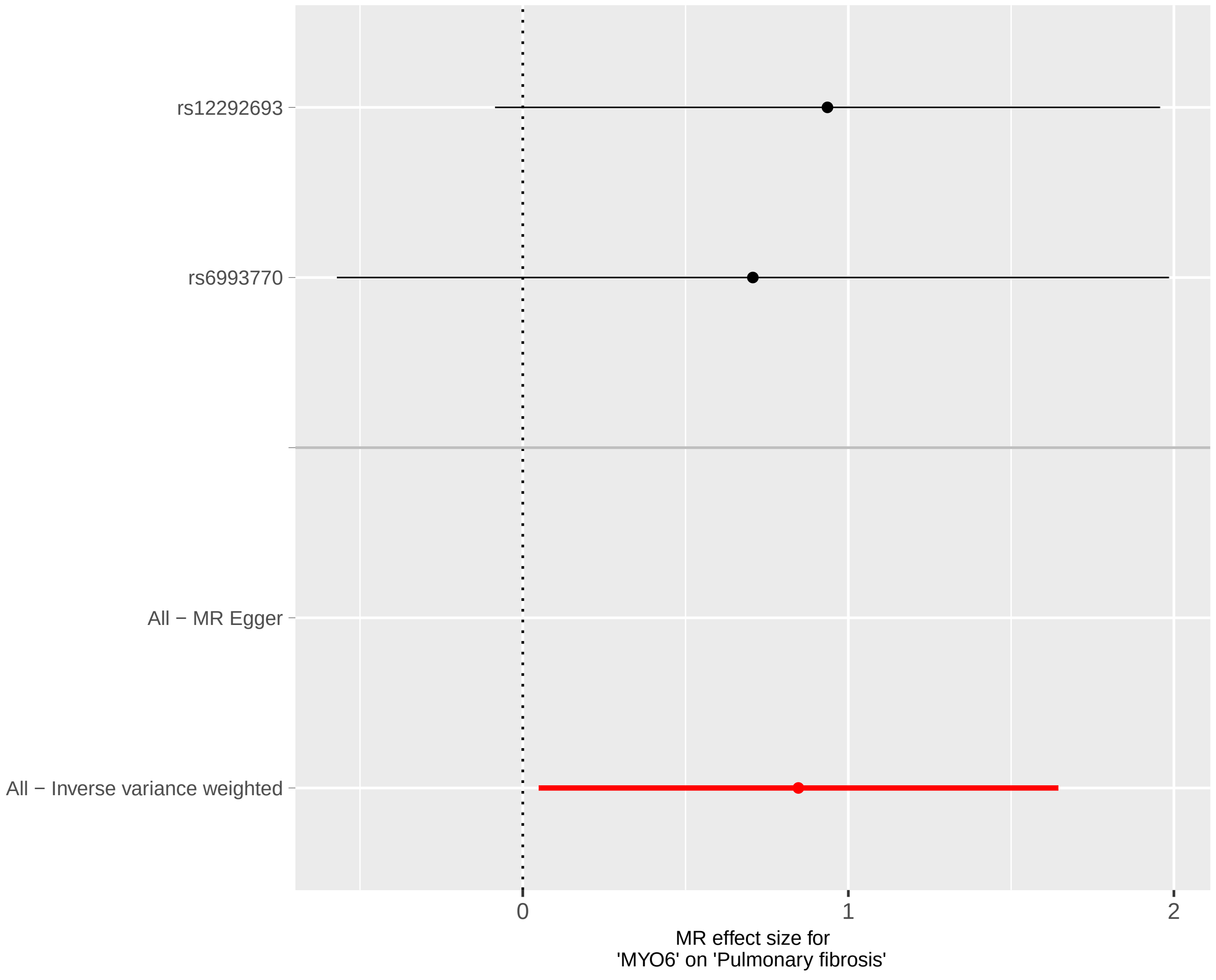

rs12292693
rs6993770
All − MR Egger
All − Inverse variance weighted
0
1
2
MR effect size for
'MYO6' on 'Pulmonary fibrosis'

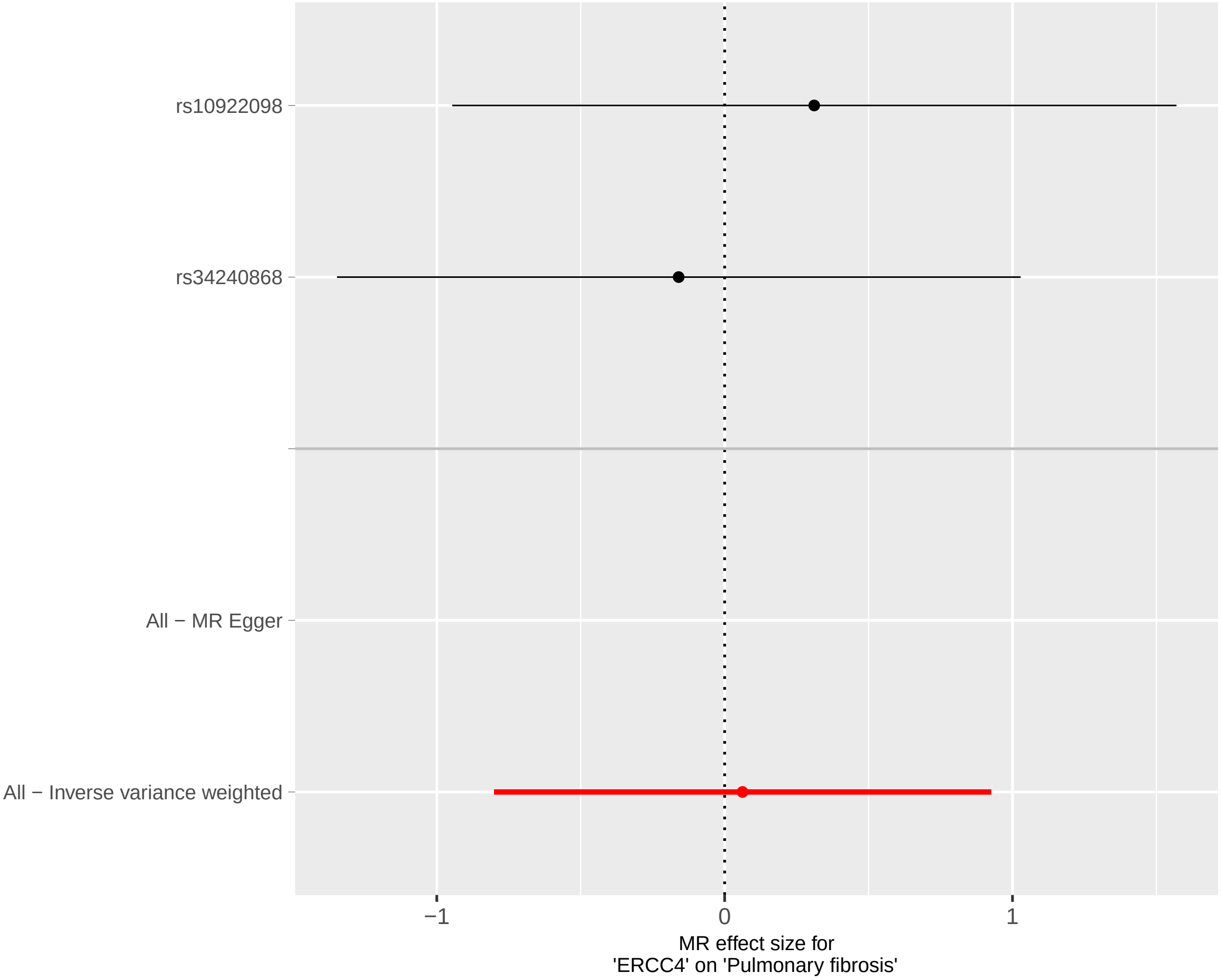

rs10922098
rs34240868
All − MR Egger
All − Inverse variance weighted
−1
0
1
MR effect size for
'ERCC4' on 'Pulmonary fibrosis'

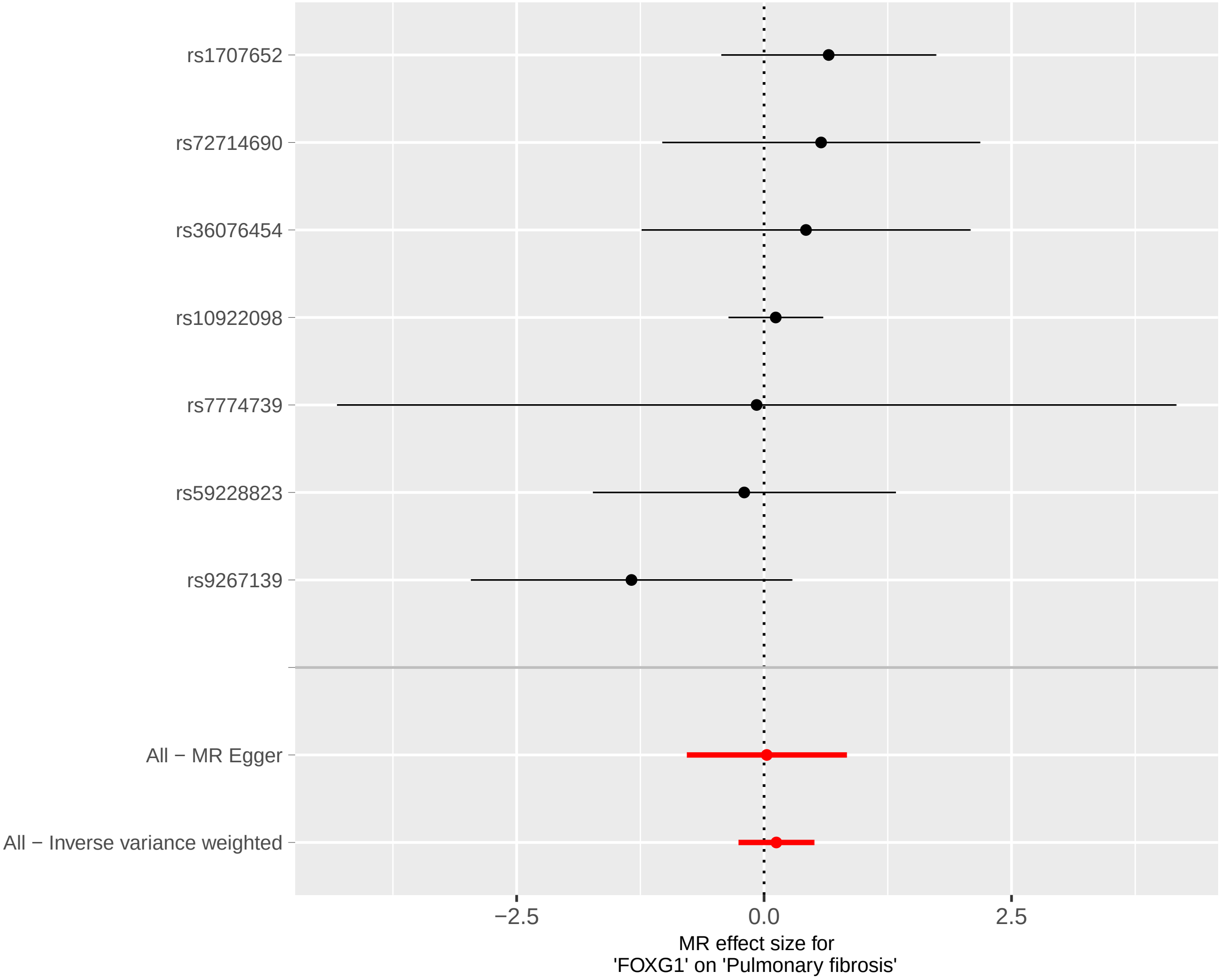
rs1707652
rs72714690
rs36076454
rs10922098
rs7774739
rs59228823
rs9267139
All − MR Egger
All − Inverse variance weighted
−2.5
0.0
2.5
MR effect size for
'FOXG1' on 'Pulmonary fibrosis'

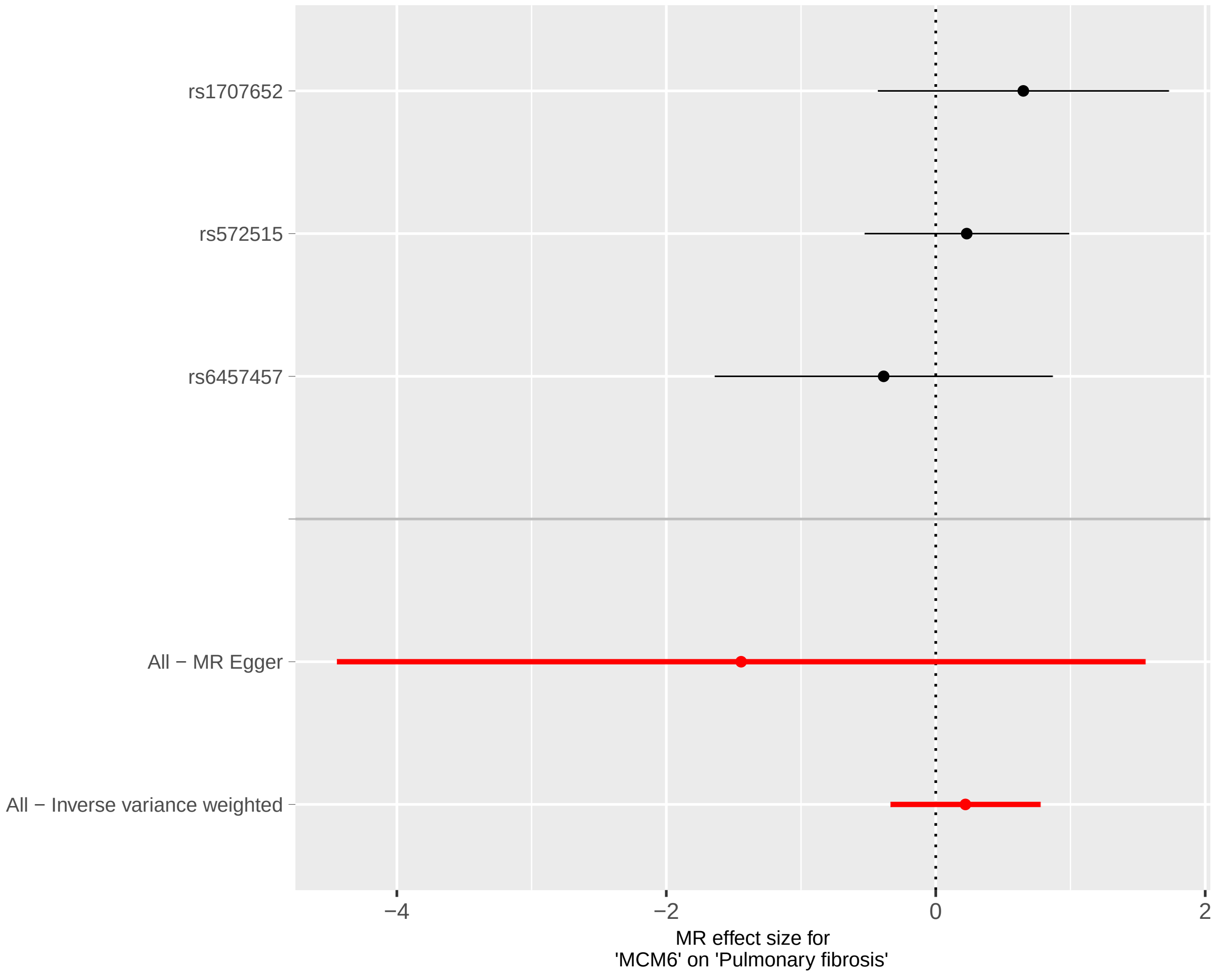
rs1707652
rs572515
rs6457457
All − MR Egger
All − Inverse variance weighted
−4
−2
0
2
MR effect size for
'MCM6' on 'Pulmonary fibrosis'

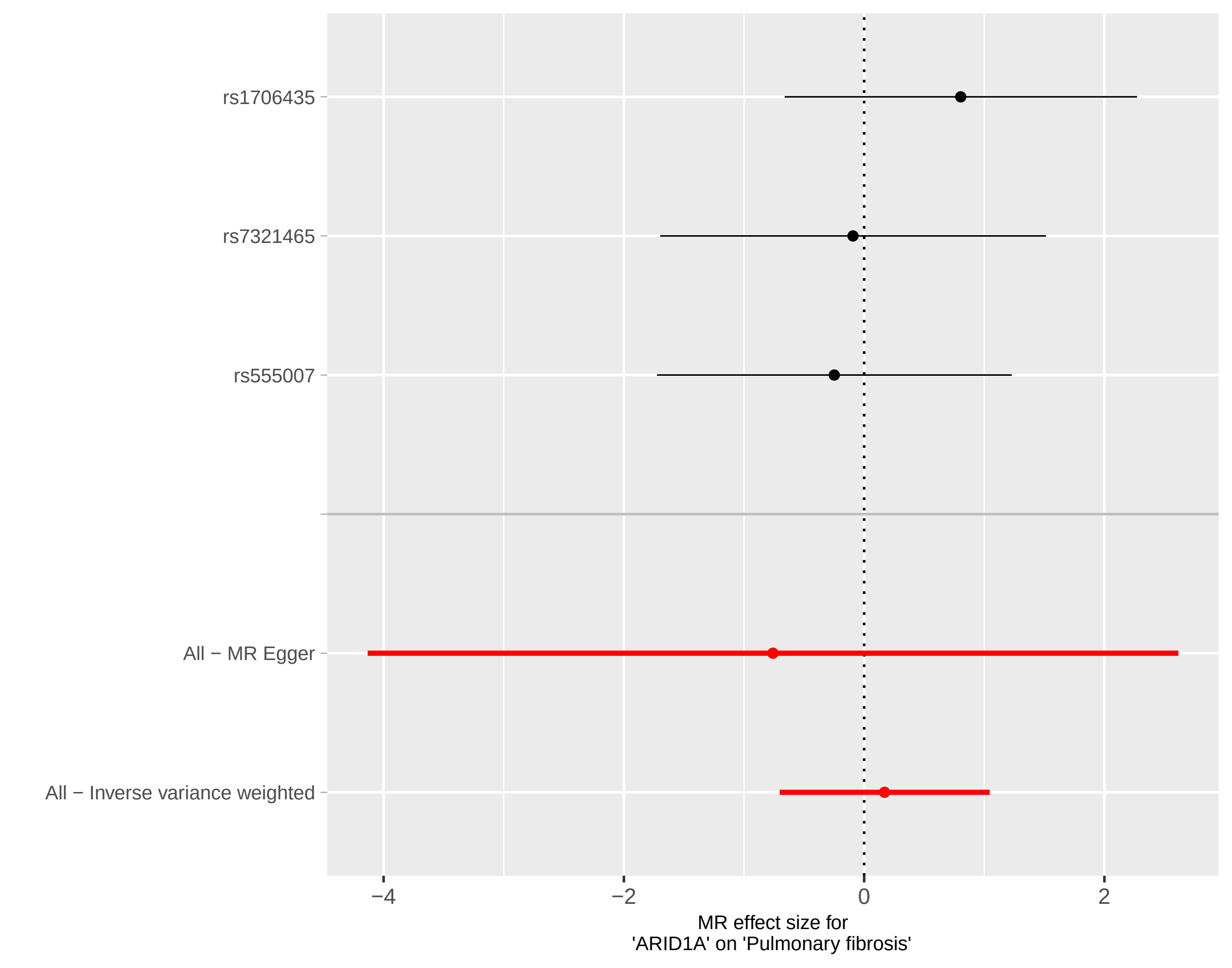
rs1706435
rs7321465
rs555007
All − MR Egger
All − Inverse variance weighted
−4
−2
0
2
MR effect size for
'ARID1A' on 'Pulmonary fibrosis'

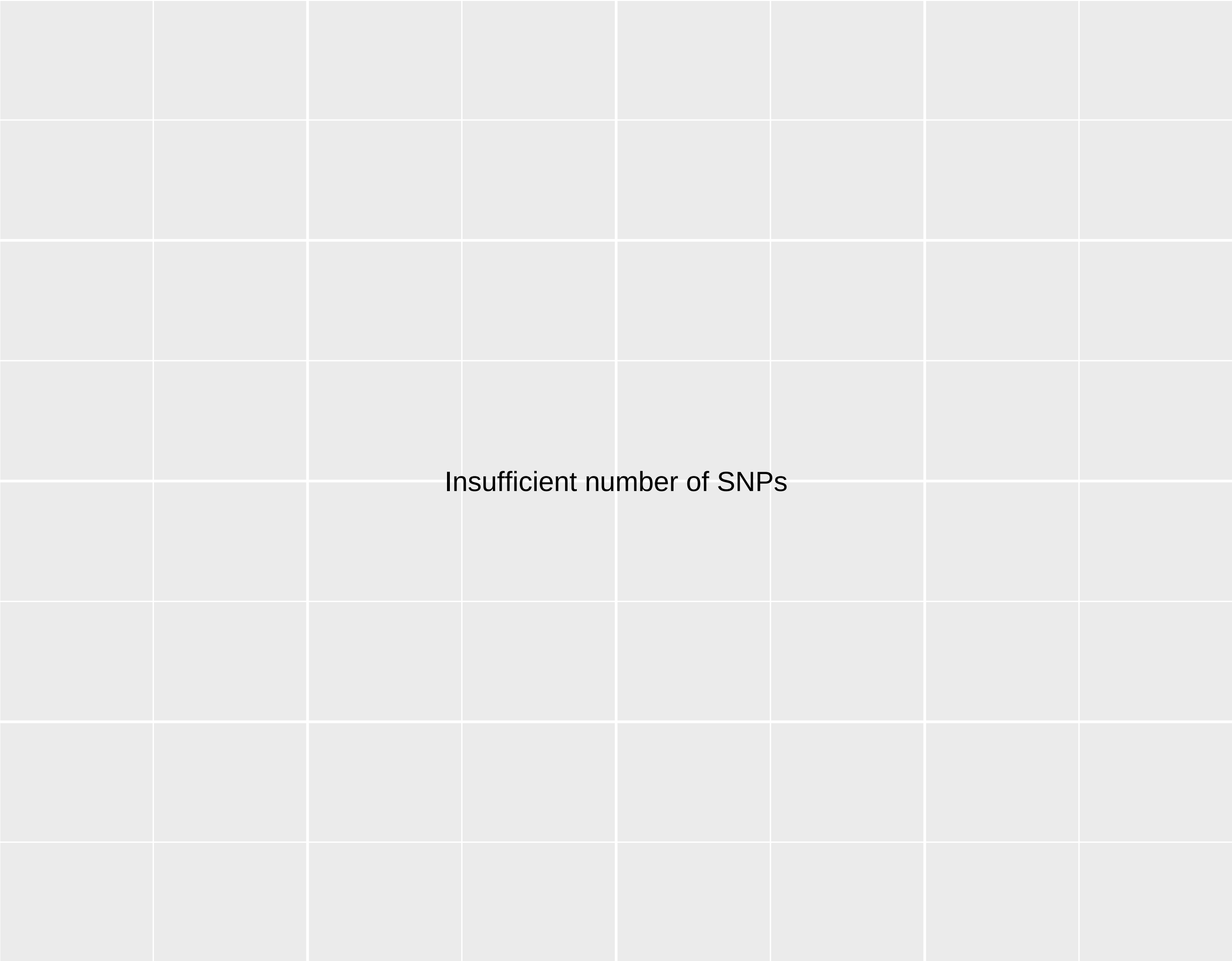
Insufficient number of SNPs

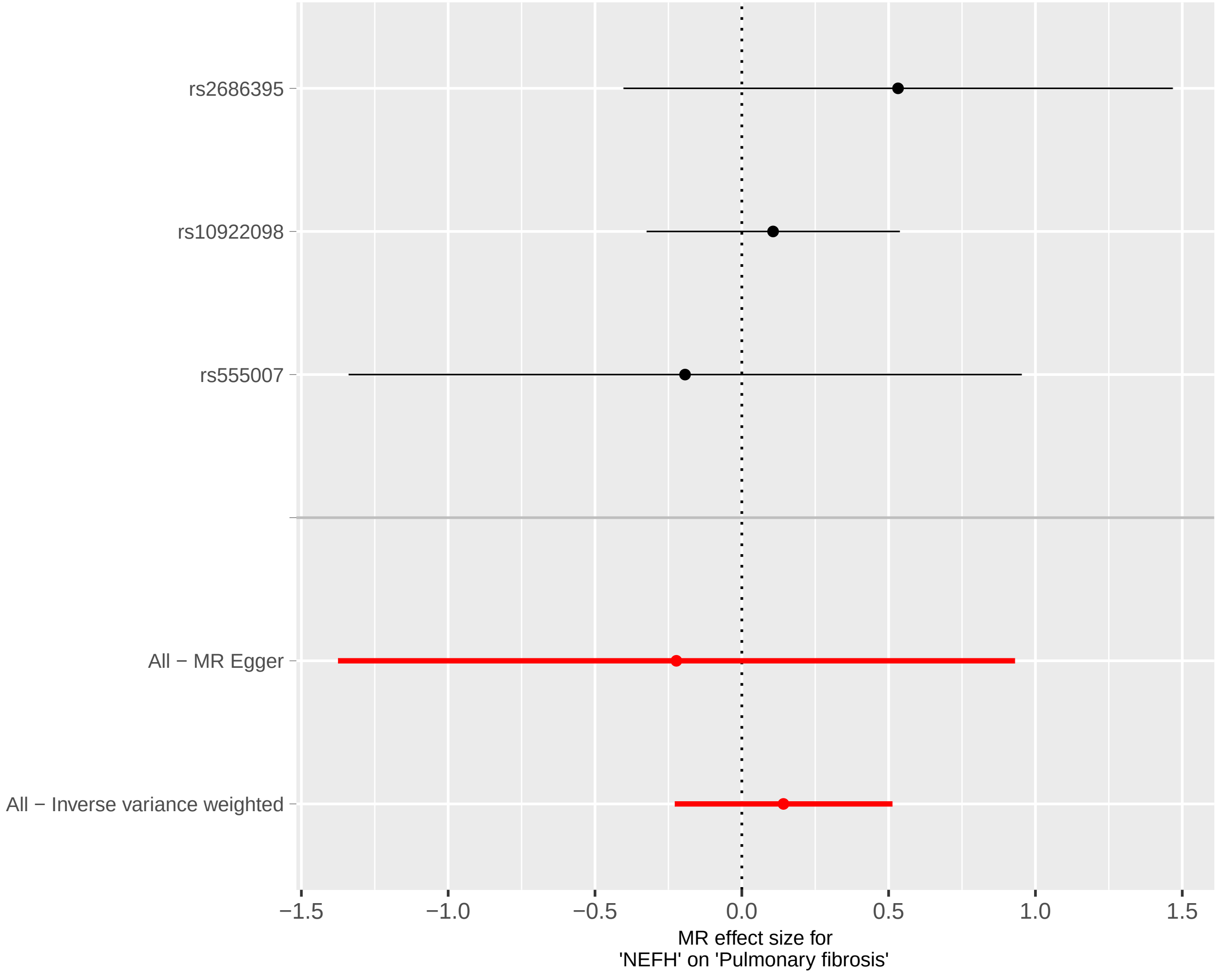

rs2686395
rs10922098
rs555007
All − MR Egger
All − Inverse variance weighted
−1.5
−1.0
−0.5
0.0
0.5
1.0
1.5
MR effect size for
'NEFH' on 'Pulmonary fibrosis'

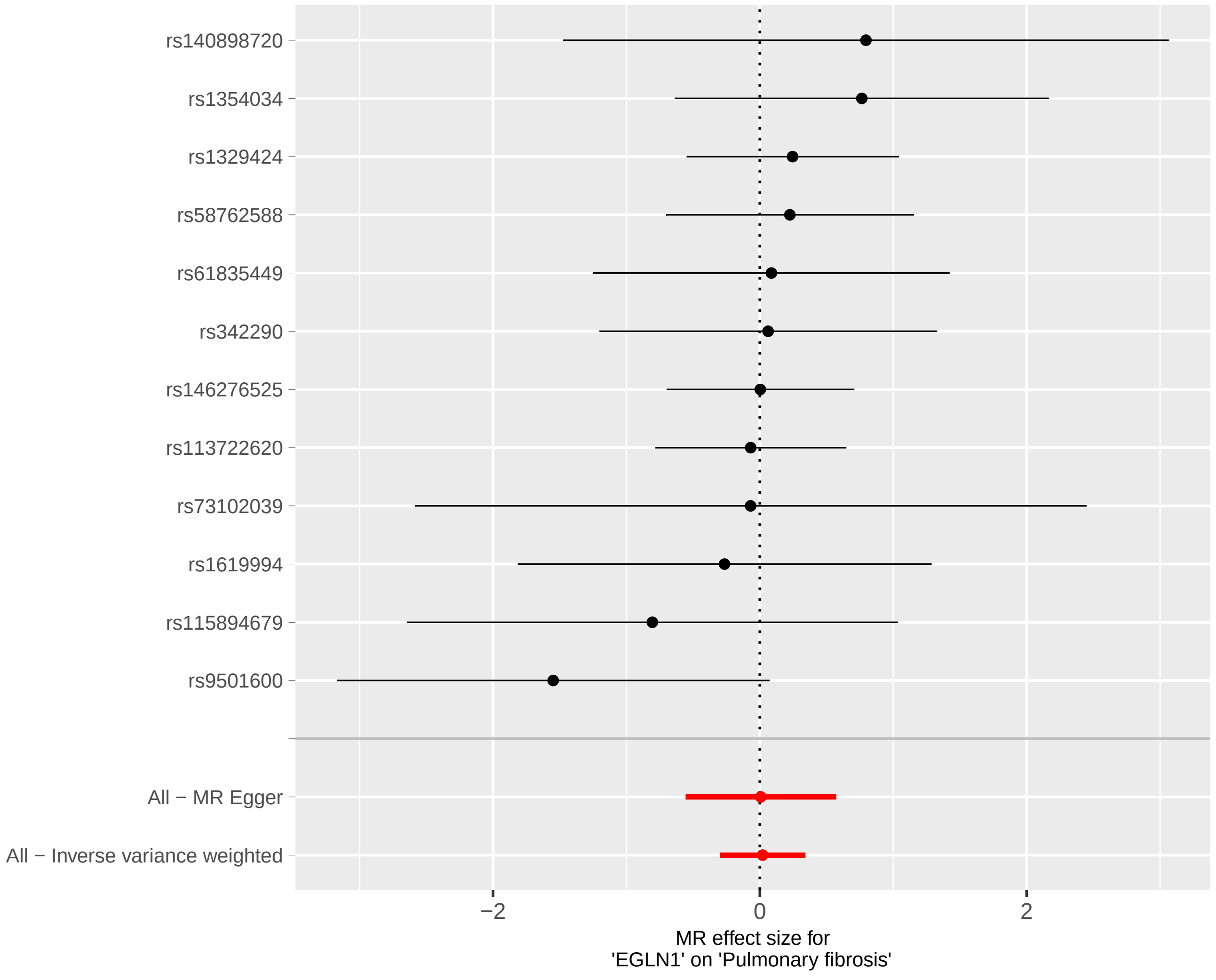
rs140898720
rs1354034
rs1329424
rs58762588
rs61835449
rs342290
rs146276525
rs113722620
rs73102039
rs1619994
rs115894679
rs9501600
All − MR Egger
All − Inverse variance weighted
−2
0
2
MR effect size for
'EGLN1' on 'Pulmonary fibrosis'

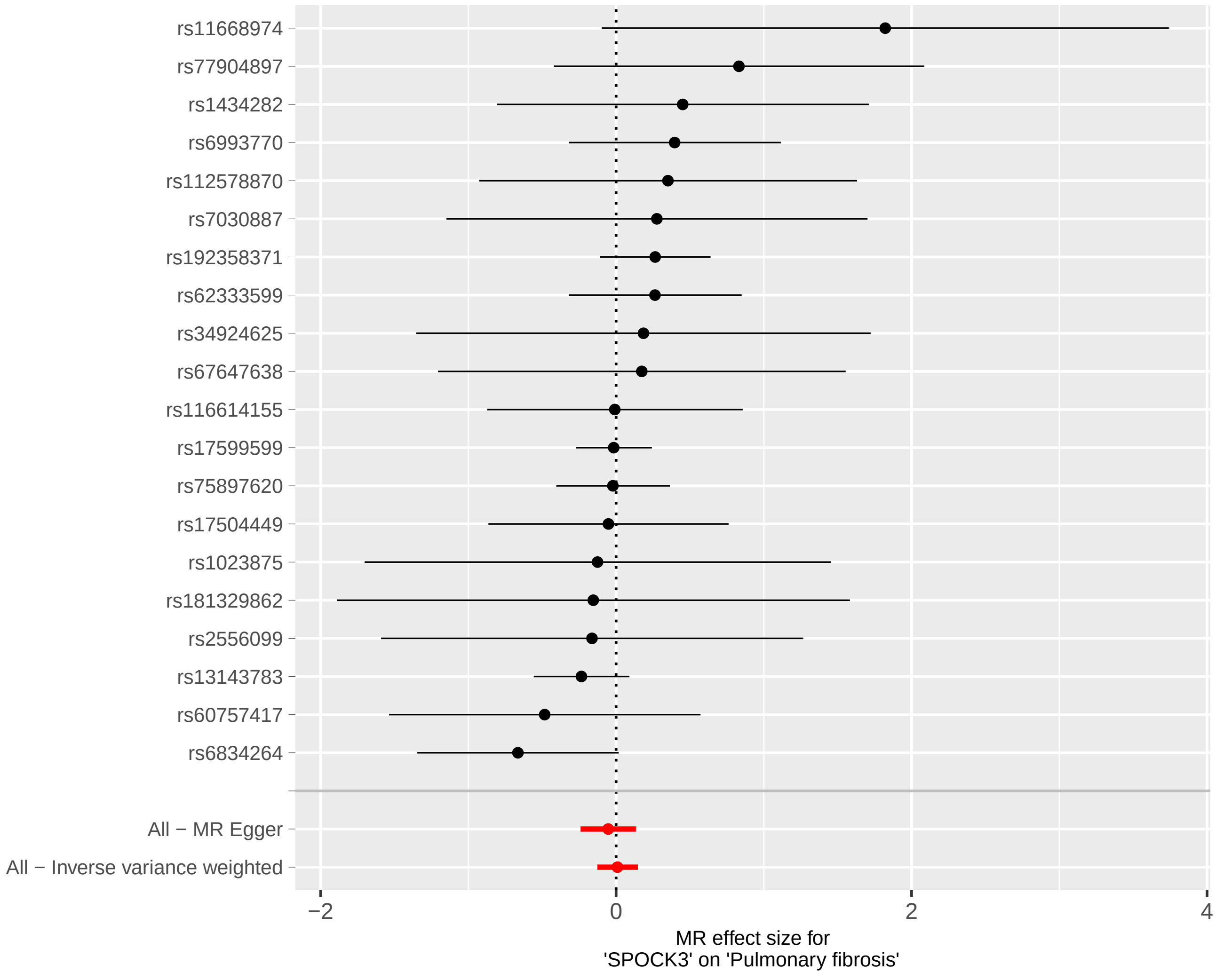

rs11668974
rs77904897
rs1434282
rs6993770
rs112578870
rs7030887
rs192358371
rs62333599
rs34924625
rs67647638
rs116614155
rs17599599
rs75897620
rs17504449
rs1023875
rs181329862
rs2556099
rs13143783
rs60757417
rs6834264
All − MR Egger
All − Inverse variance weighted
−2
0
2
4
MR effect size for
'SPOCK3' on 'Pulmonary fibrosis'

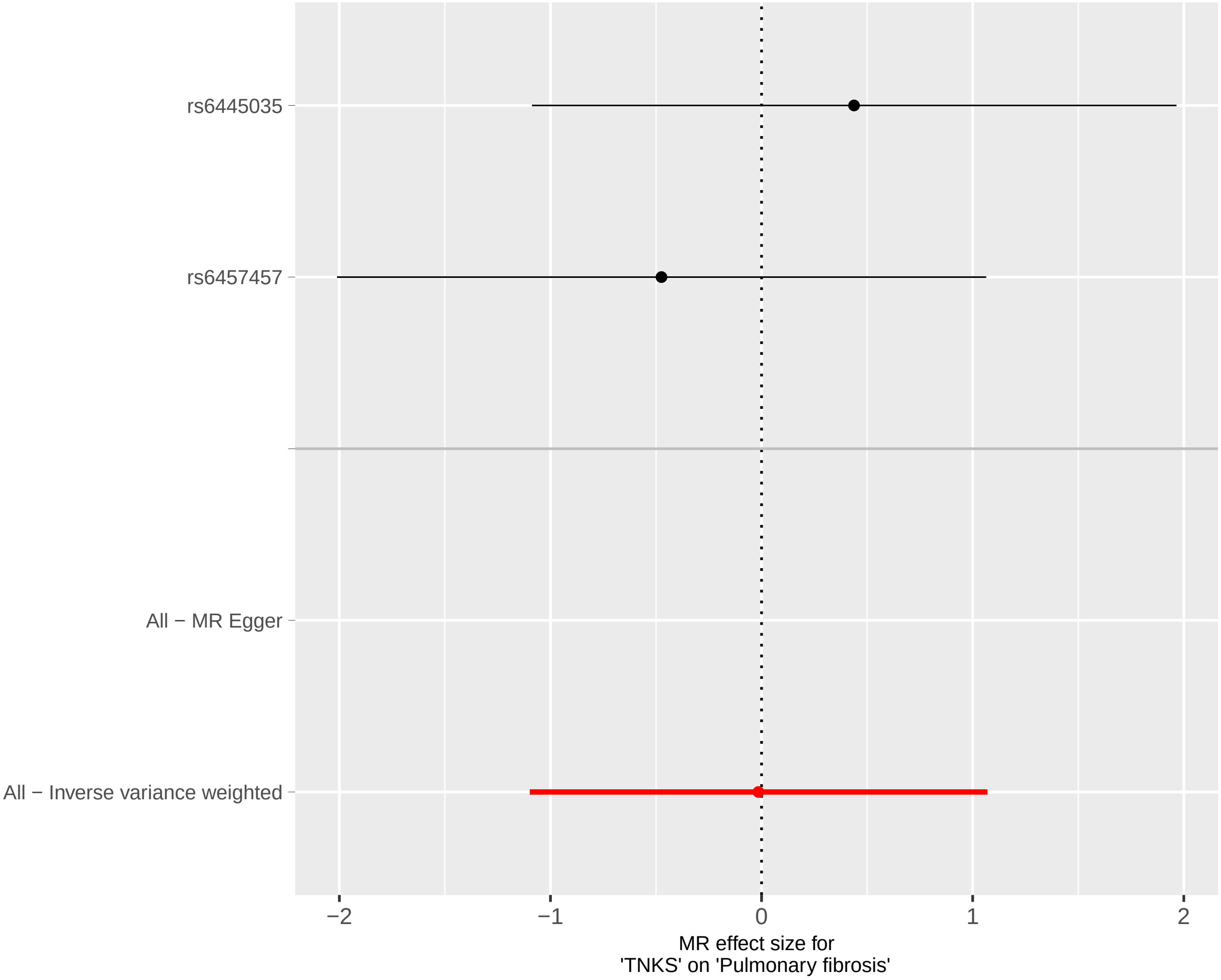
rs6445035
rs6457457
All − MR Egger
All − Inverse variance weighted
−2
−1
0
1
2
MR effect size for
'TNKS' on 'Pulmonary fibrosis'

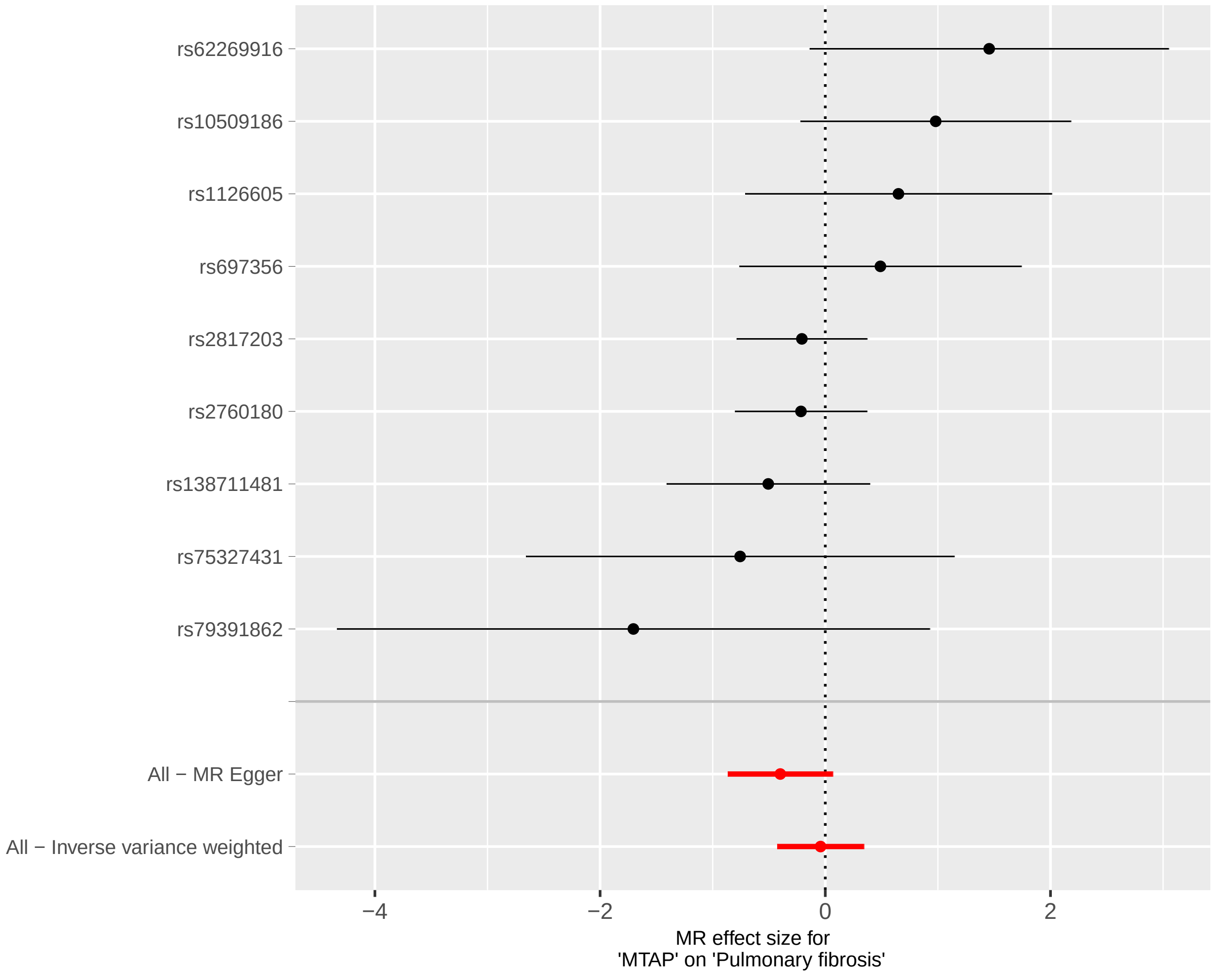

rs62269916
rs10509186
rs1126605
rs697356
rs2817203
rs2760180
rs138711481
rs75327431
rs79391862
All − MR Egger
All − Inverse variance weighted
−4
−2
0
2
MR effect size for
'MTAP' on 'Pulmonary fibrosis'

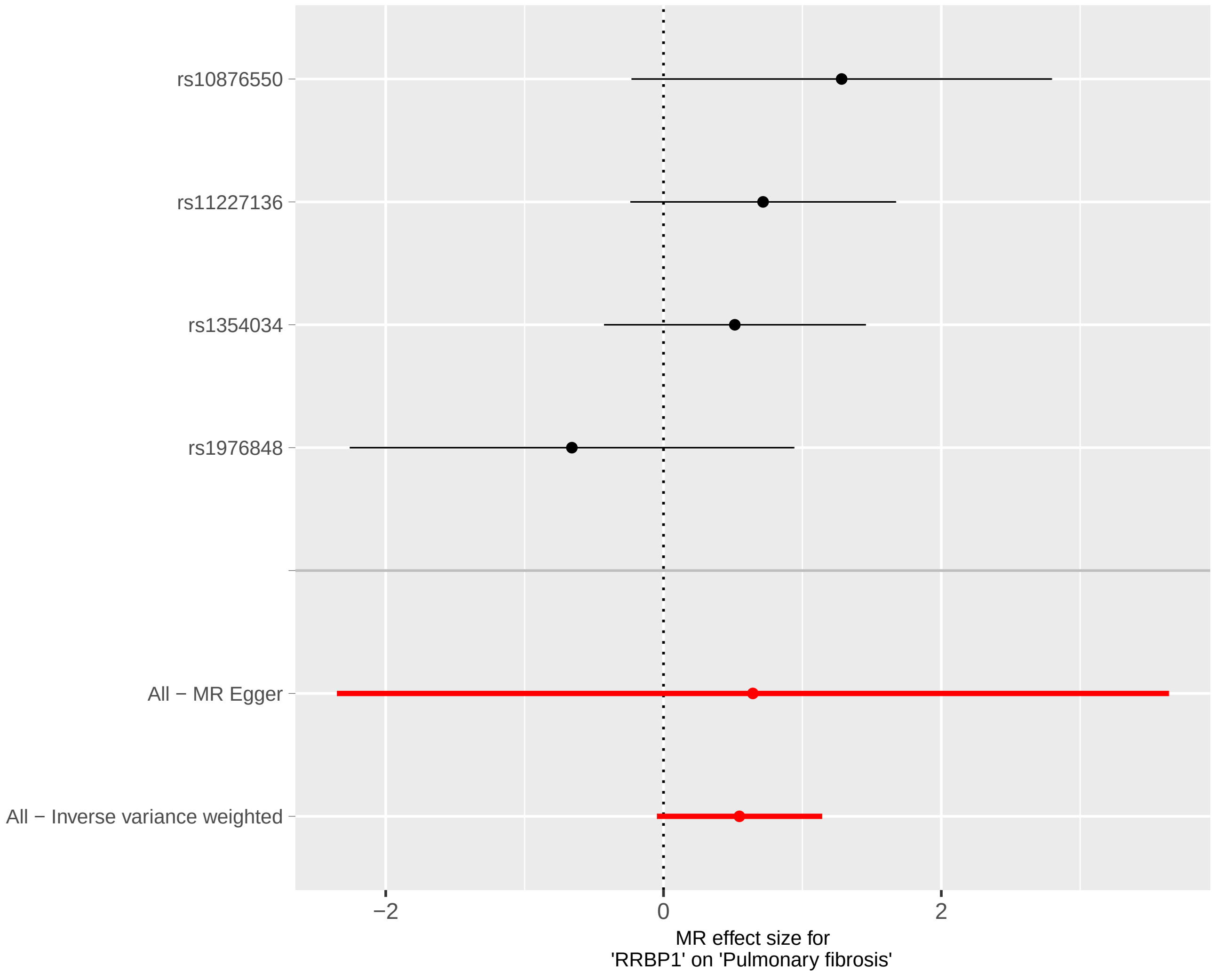
rs10876550
rs11227136
rs1354034
rs1976848
All − MR Egger
All − Inverse variance weighted
−2
0
2
MR effect size for
'RRBP1' on 'Pulmonary fibrosis'

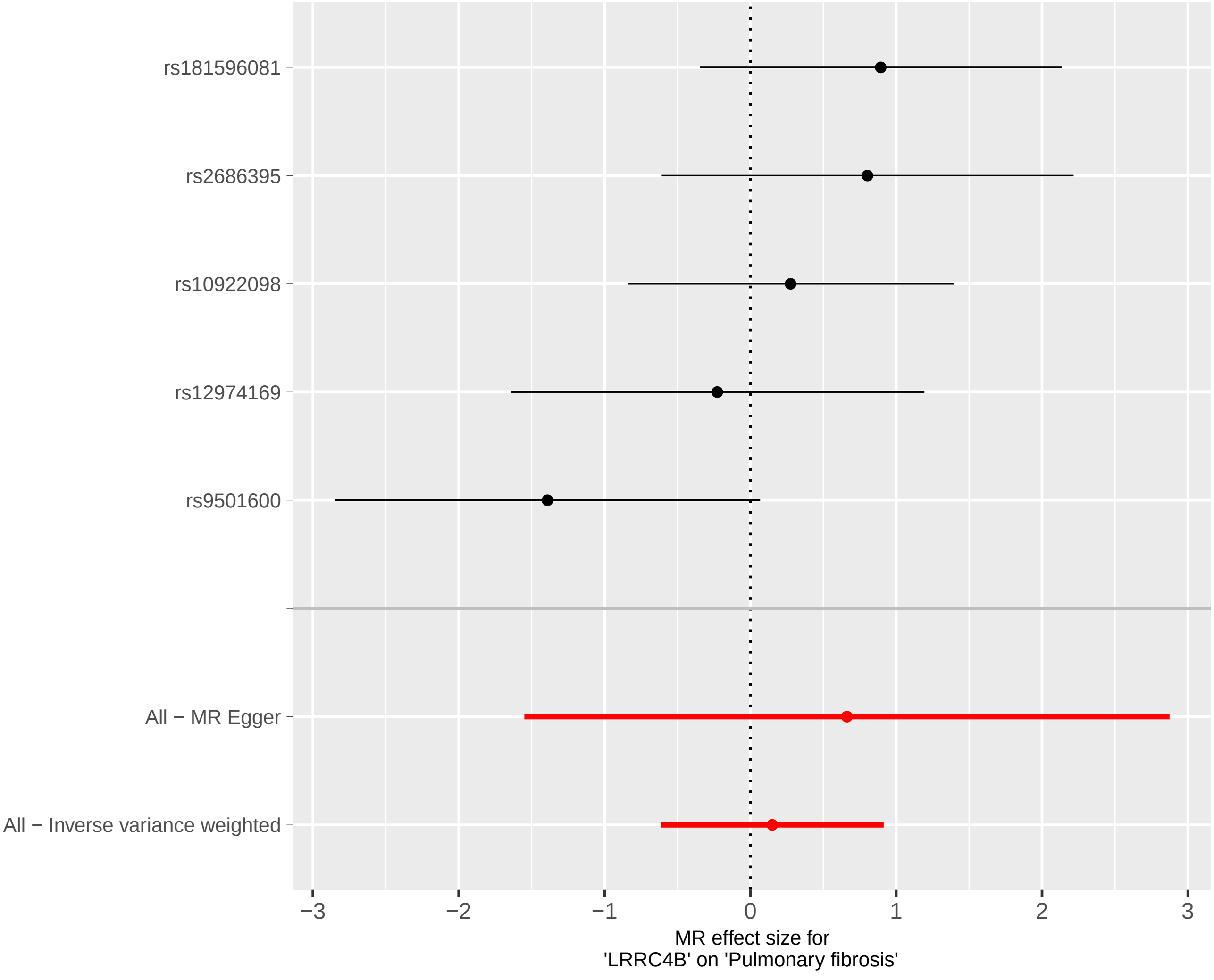

rs181596081
rs2686395
rs10922098
rs12974169
rs9501600
All − MR Egger
All − Inverse variance weighted
−3
−2
−1
0
1
2
3
MR effect size for
'LRRC4B' on 'Pulmonary fibrosis'

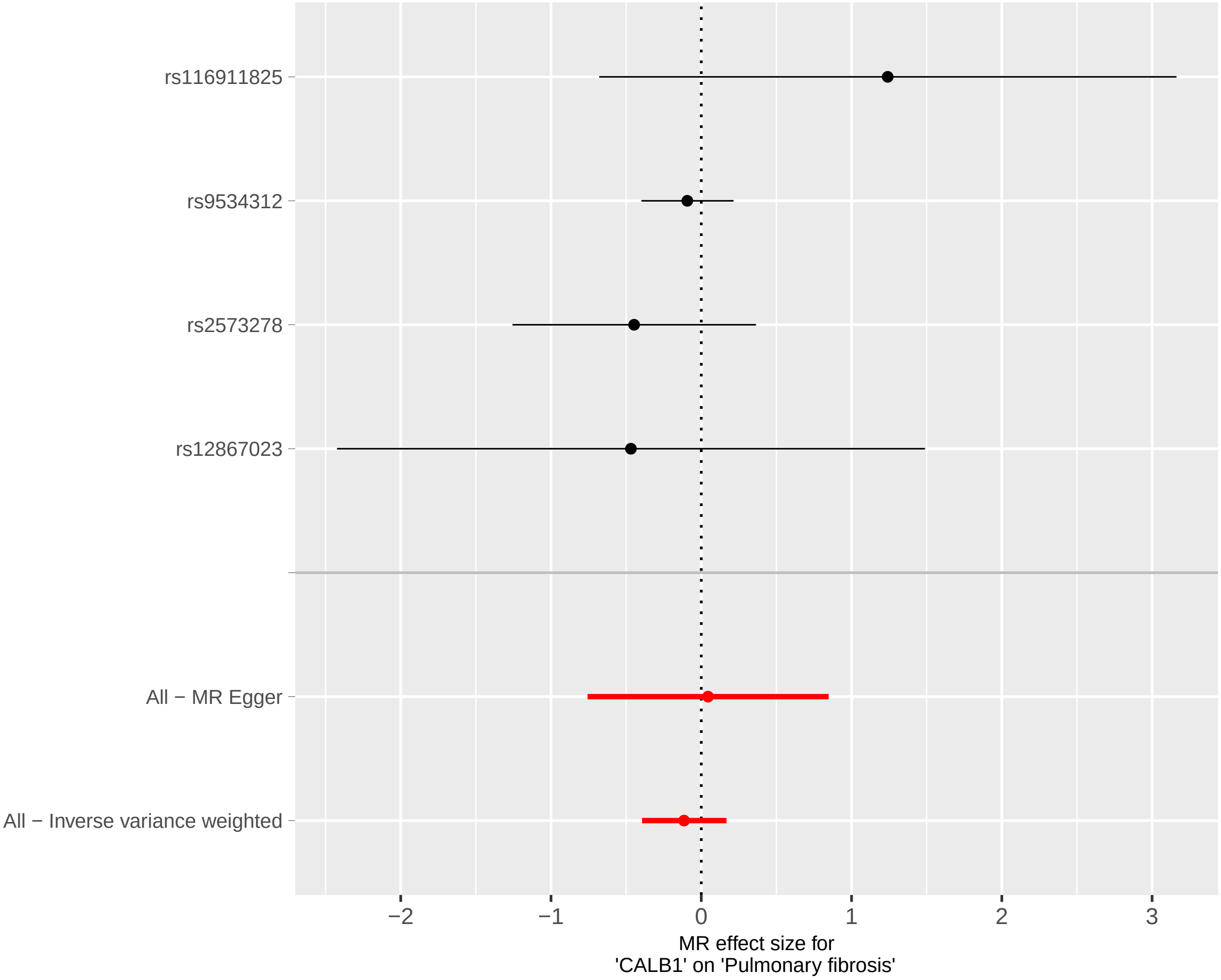
rs116911825
rs9534312
rs2573278
rs12867023
All − MR Egger
All − Inverse variance weighted
−2
−1
0
1
2
3
MR effect size for
'CALB1' on 'Pulmonary fibrosis'

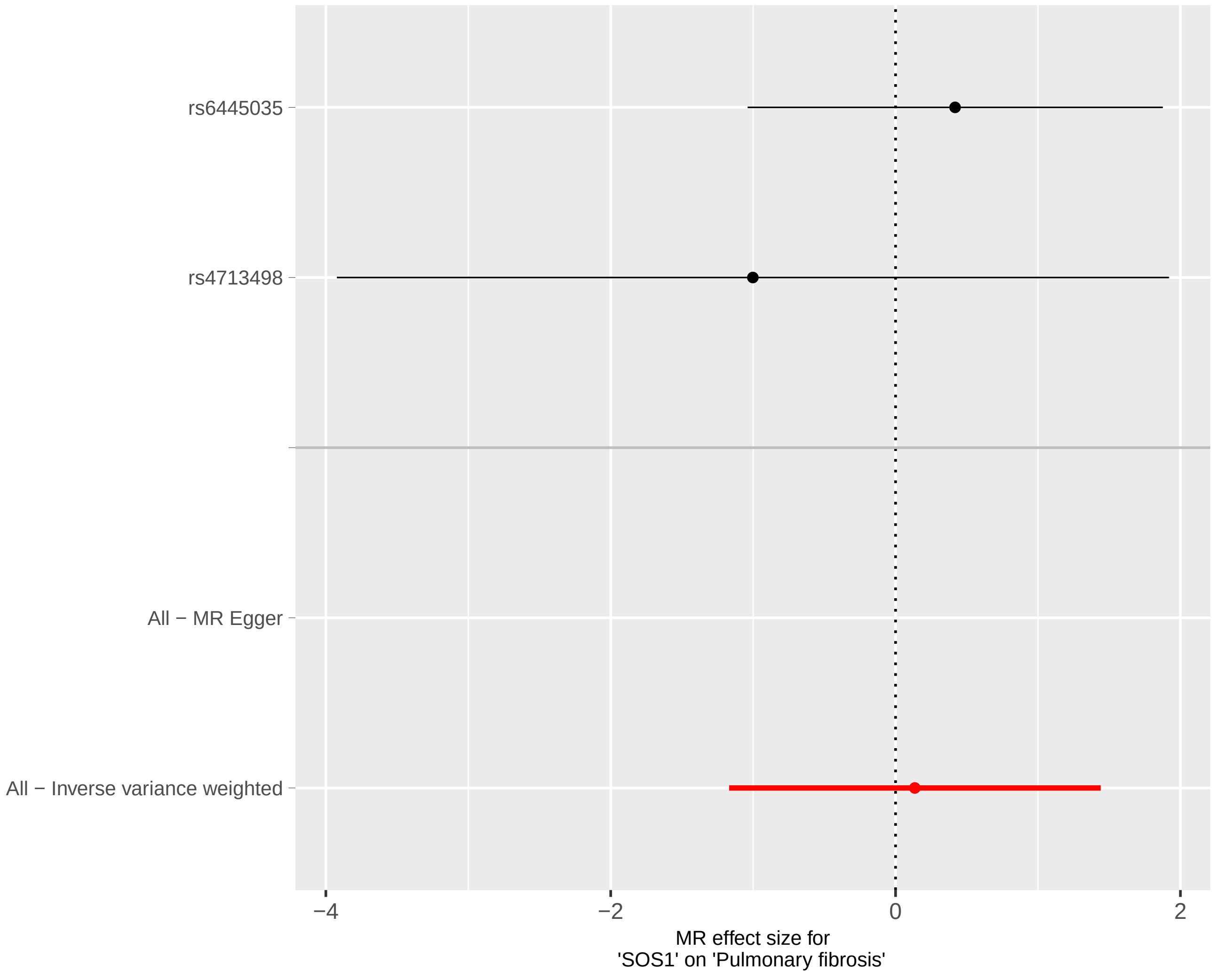

rs6445035
rs4713498
All − MR Egger
All − Inverse variance weighted
−4
−2
0
2
MR effect size for
'SOS1' on 'Pulmonary fibrosis'

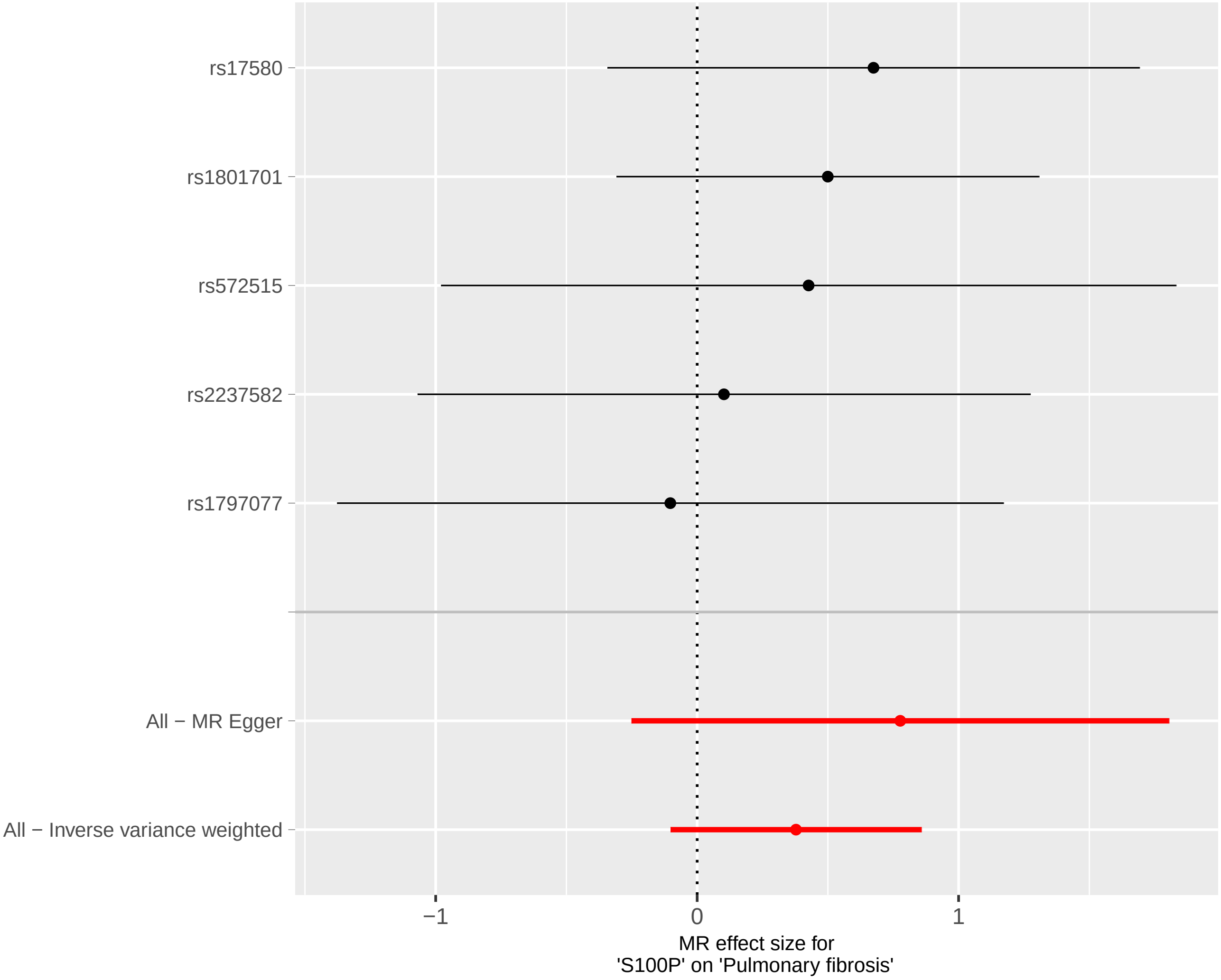
rs17580
rs1801701
rs572515
rs2237582
rs1797077
All − MR Egger
All − Inverse variance weighted
−1
0
1
MR effect size for
'S100P' on 'Pulmonary fibrosis'

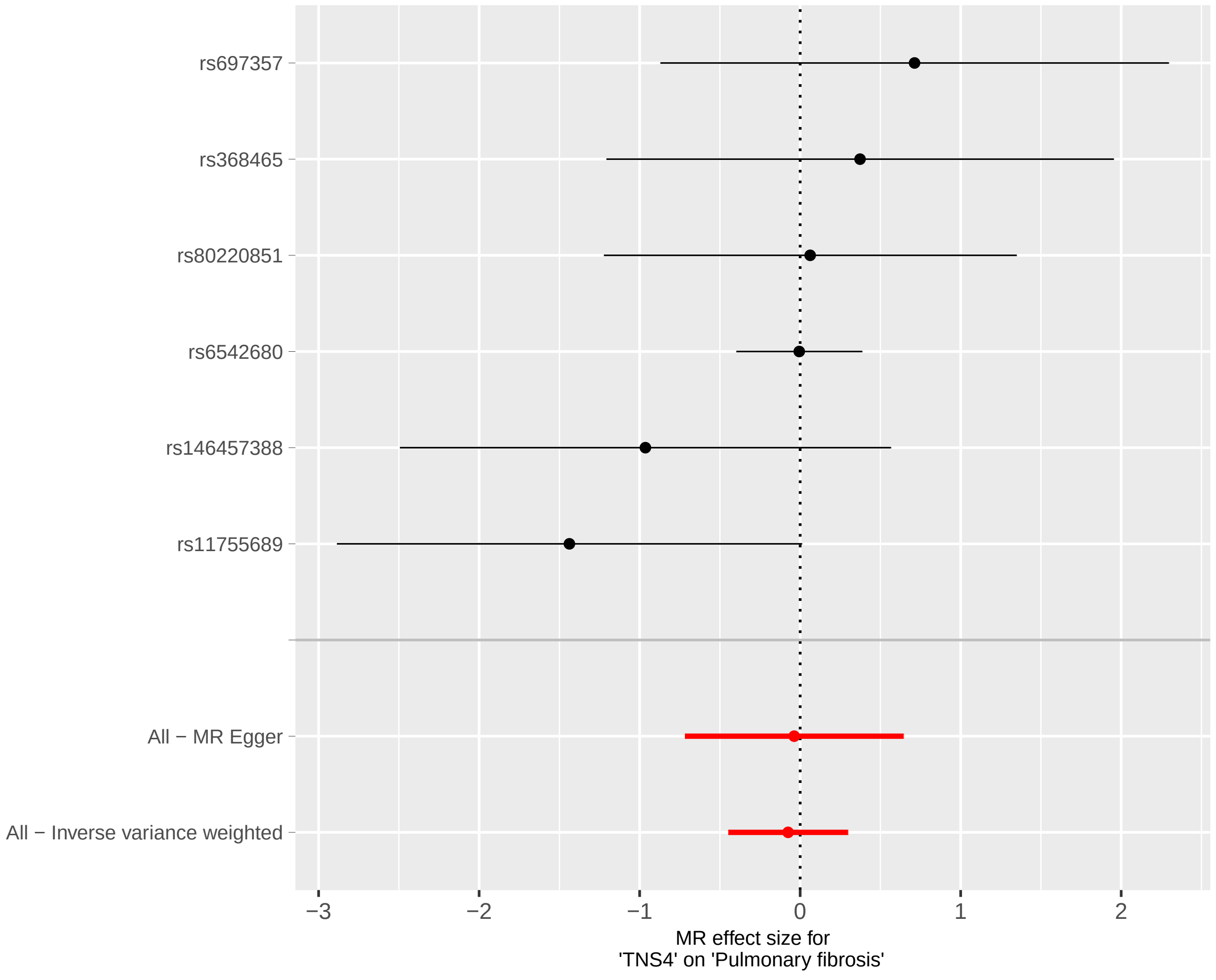
rs697357
rs368465
rs80220851
rs6542680
rs146457388
rs11755689
All − MR Egger
All − Inverse variance weighted
−3
−2
−1
0
1
2
MR effect size for
'TNS4' on 'Pulmonary fibrosis'

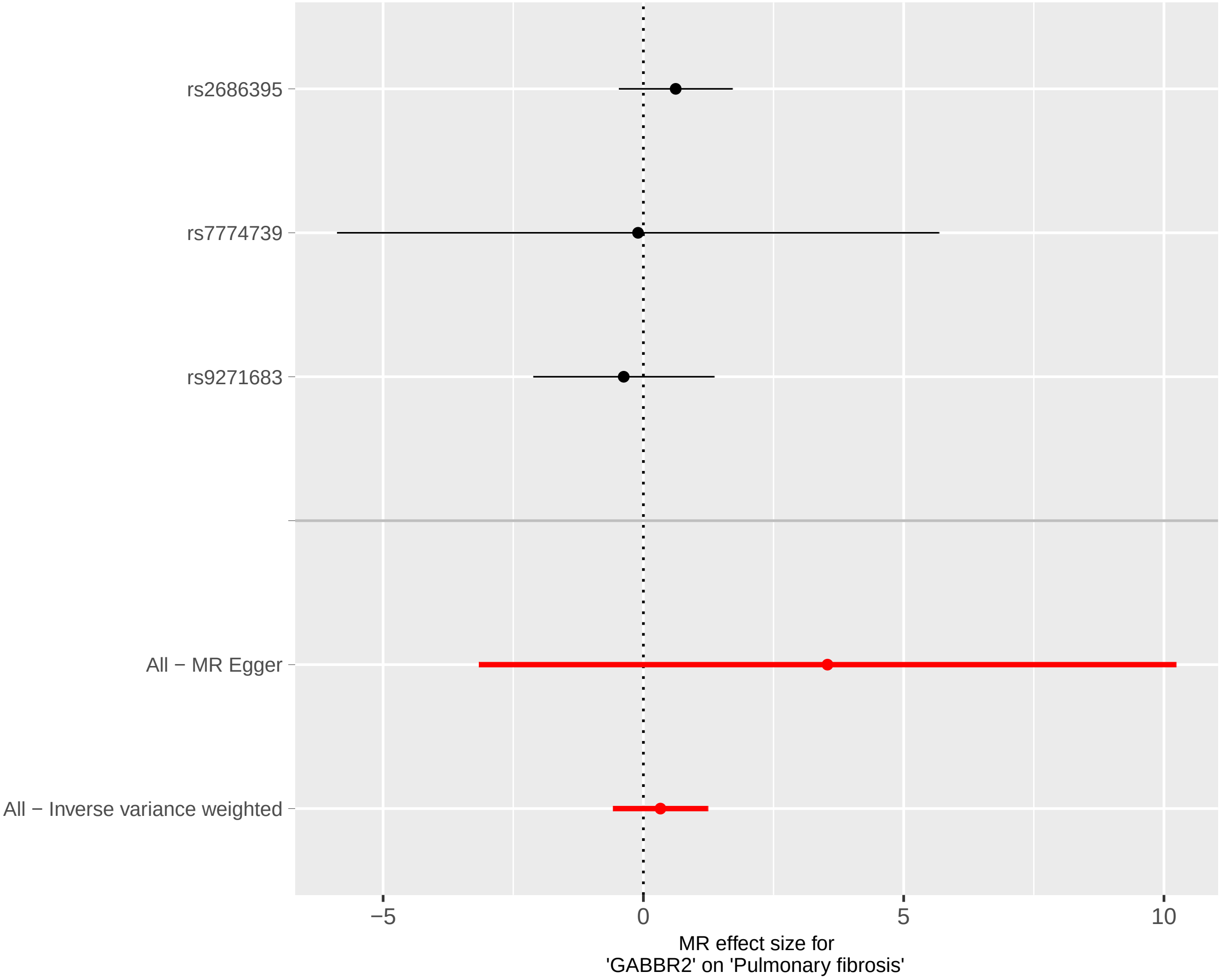

rs2686395
rs7774739
rs9271683
All − MR Egger
All − Inverse variance weighted
−5
0
5
10
MR effect size for
'GABBR2' on 'Pulmonary fibrosis'

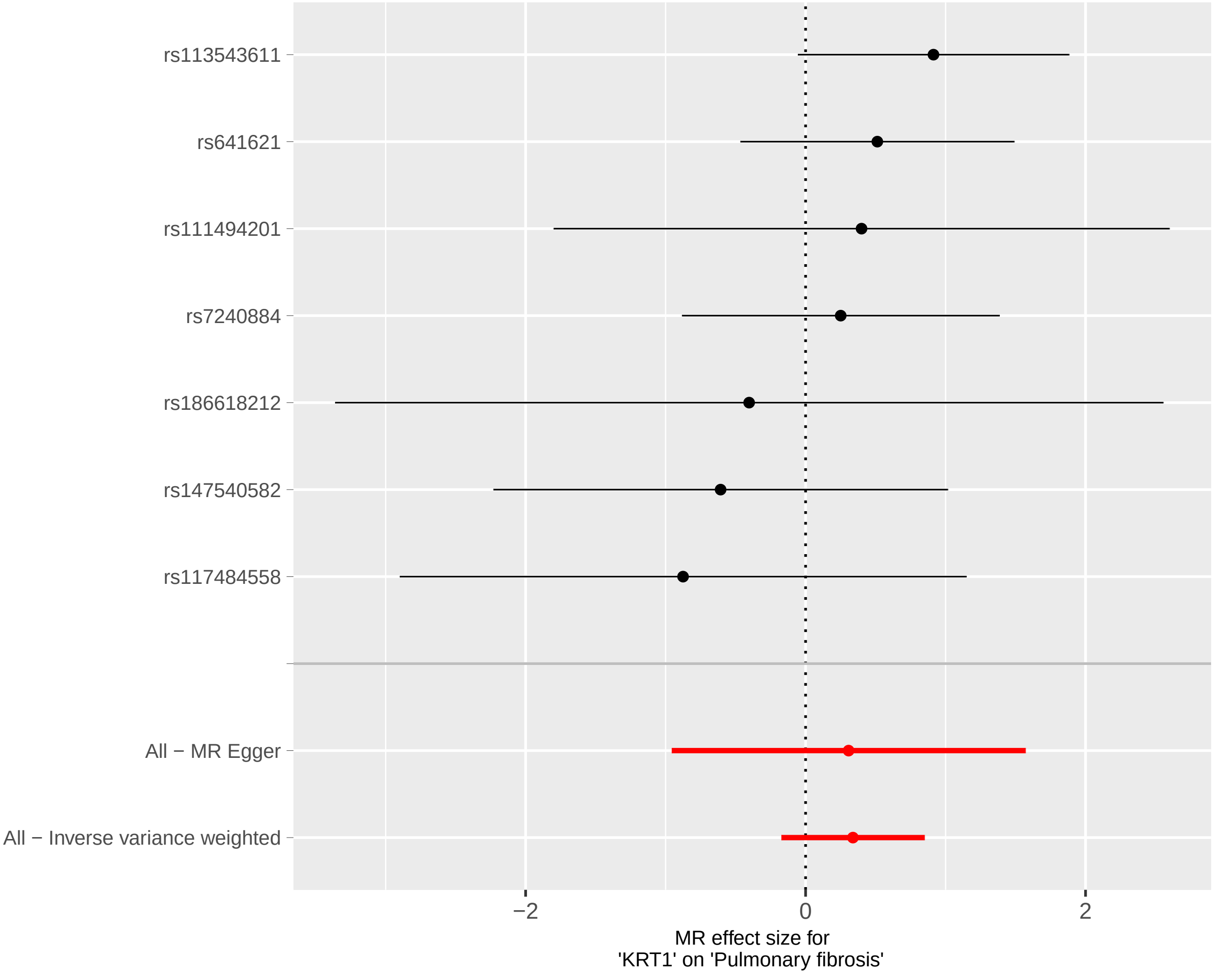
rs113543611
rs641621
rs111494201
rs7240884
rs186618212
rs147540582
rs117484558
All − MR Egger
All − Inverse variance weighted
−2
0
2
MR effect size for
'KRT1' on 'Pulmonary fibrosis'

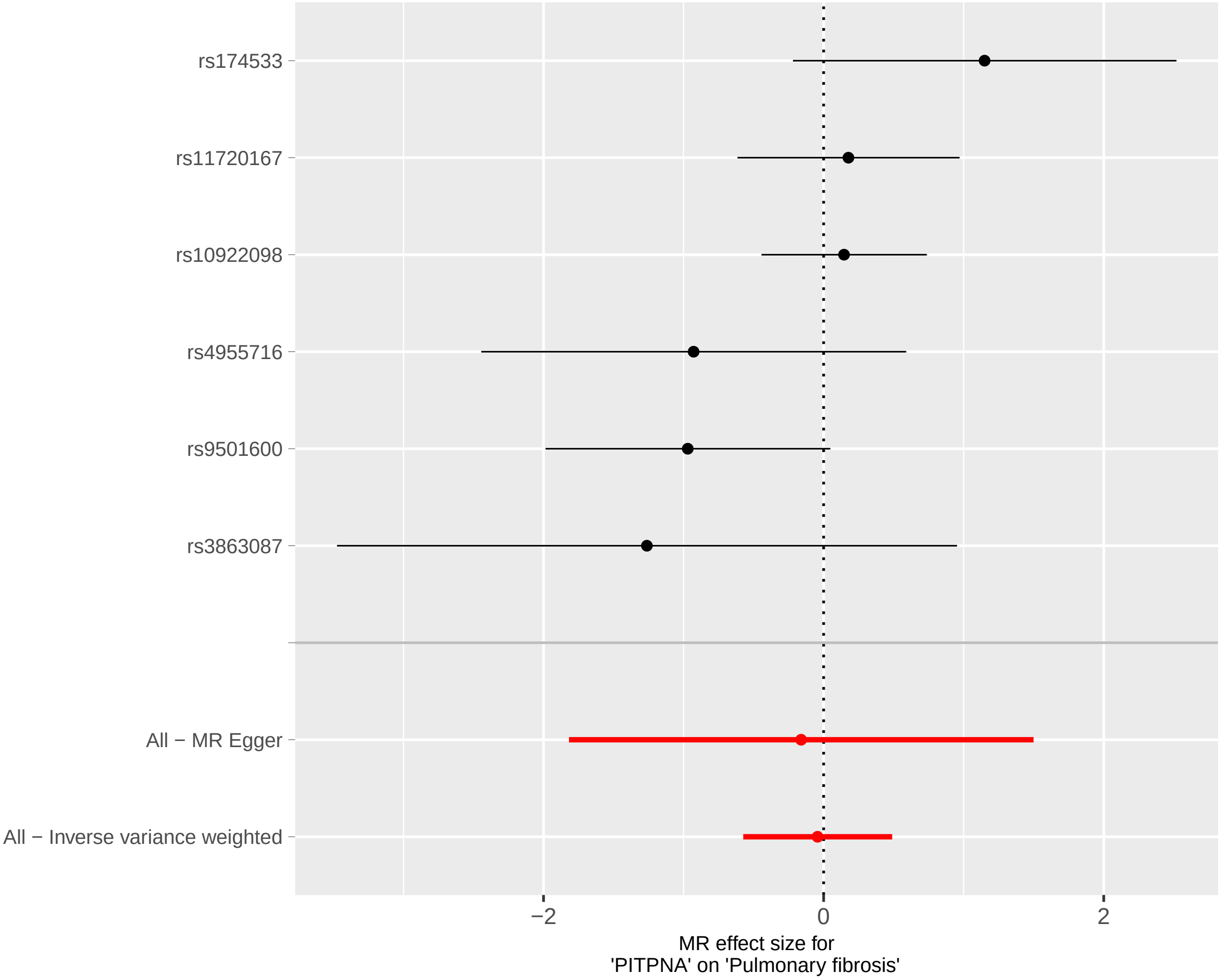
rs174533
rs11720167
rs10922098
rs4955716
rs9501600
rs3863087
All − MR Egger
All − Inverse variance weighted
−2
0
2
MR effect size for
'PITPNA' on 'Pulmonary fibrosis'

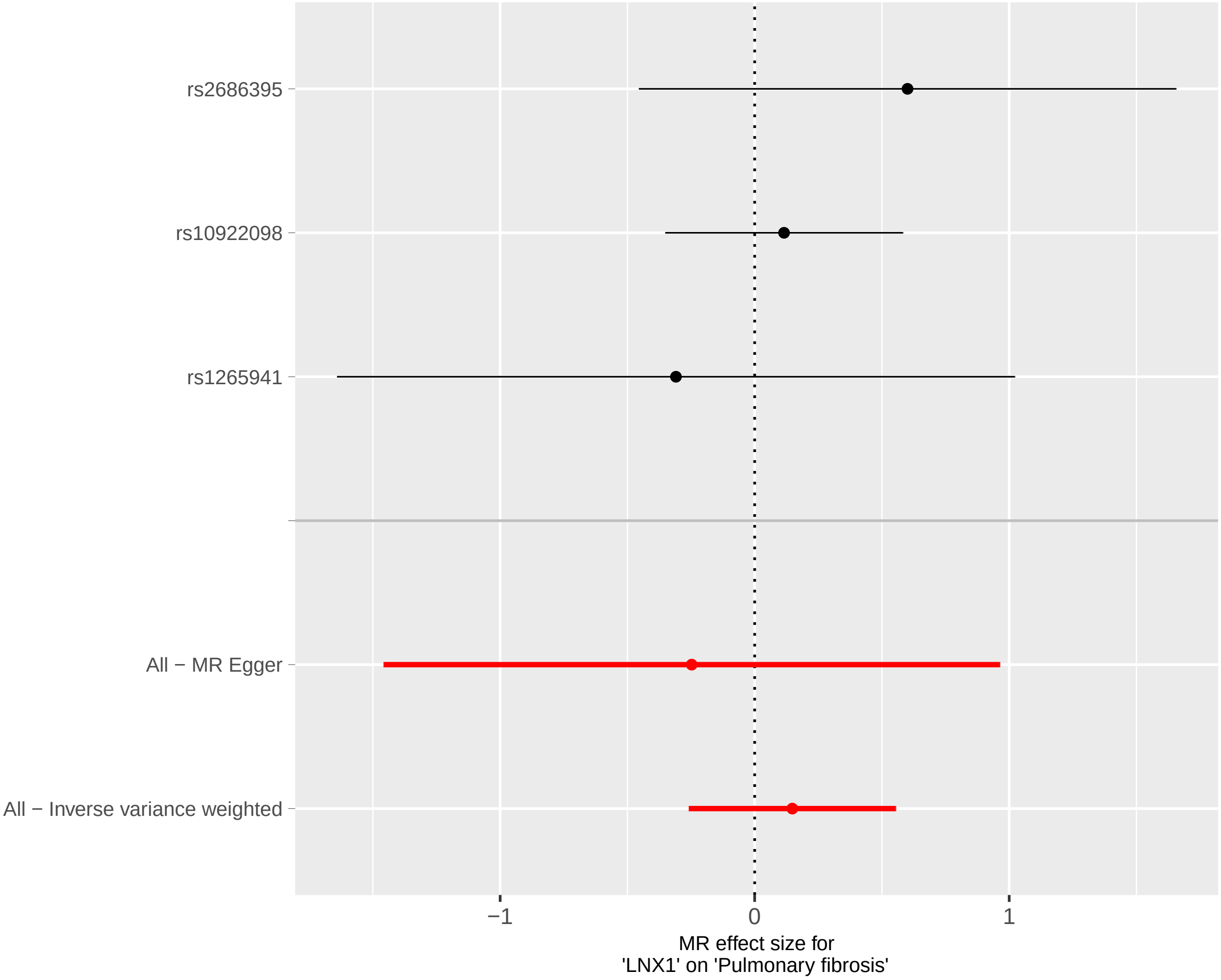

rs2686395
rs10922098
rs1265941
All − MR Egger
All − Inverse variance weighted
−1
0
1
MR effect size for
'LNX1' on 'Pulmonary fibrosis'

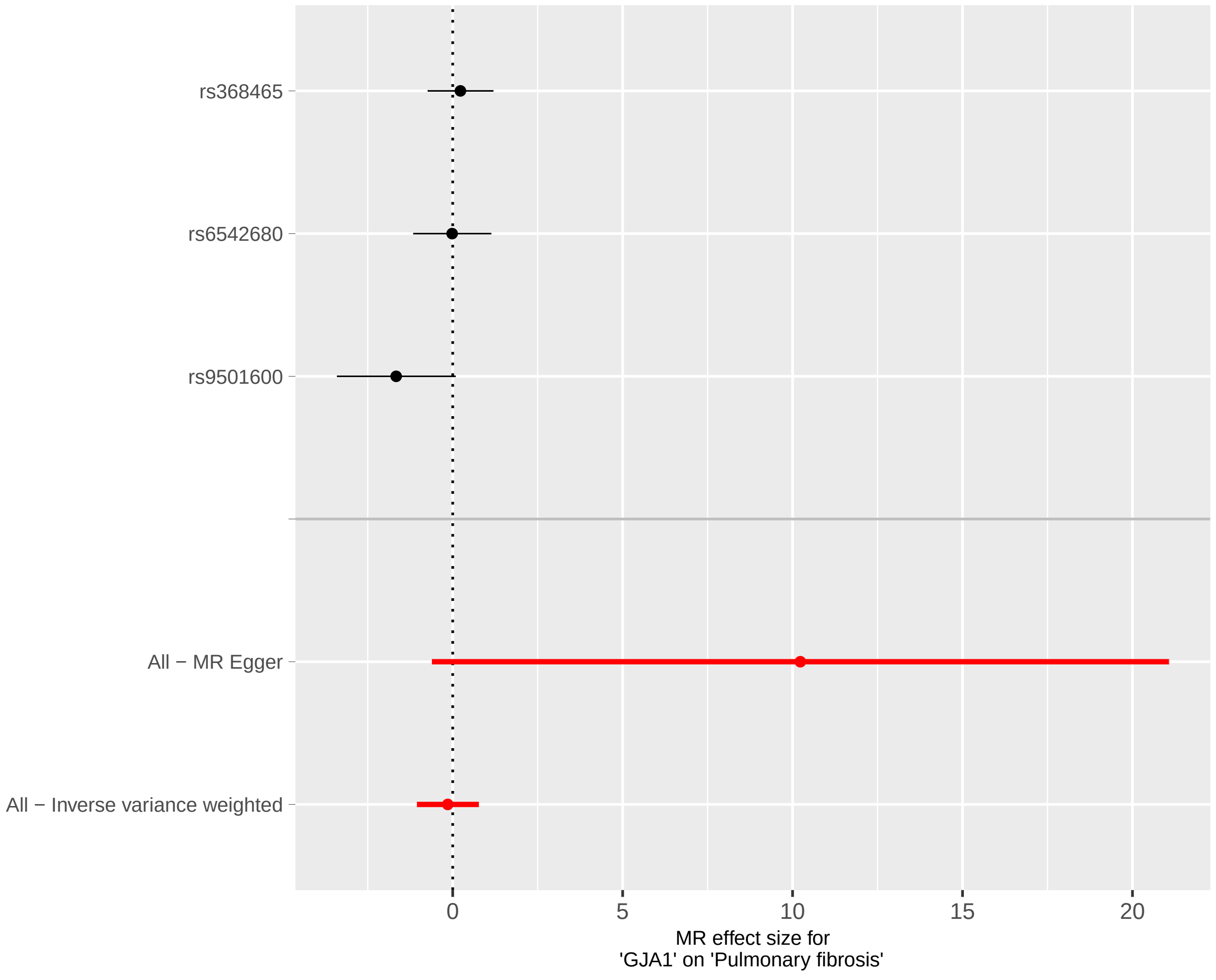

rs368465
rs6542680
rs9501600
All − MR Egger
All − Inverse variance weighted
0
5
10
15
20
MR effect size for
'GJA1' on 'Pulmonary fibrosis'

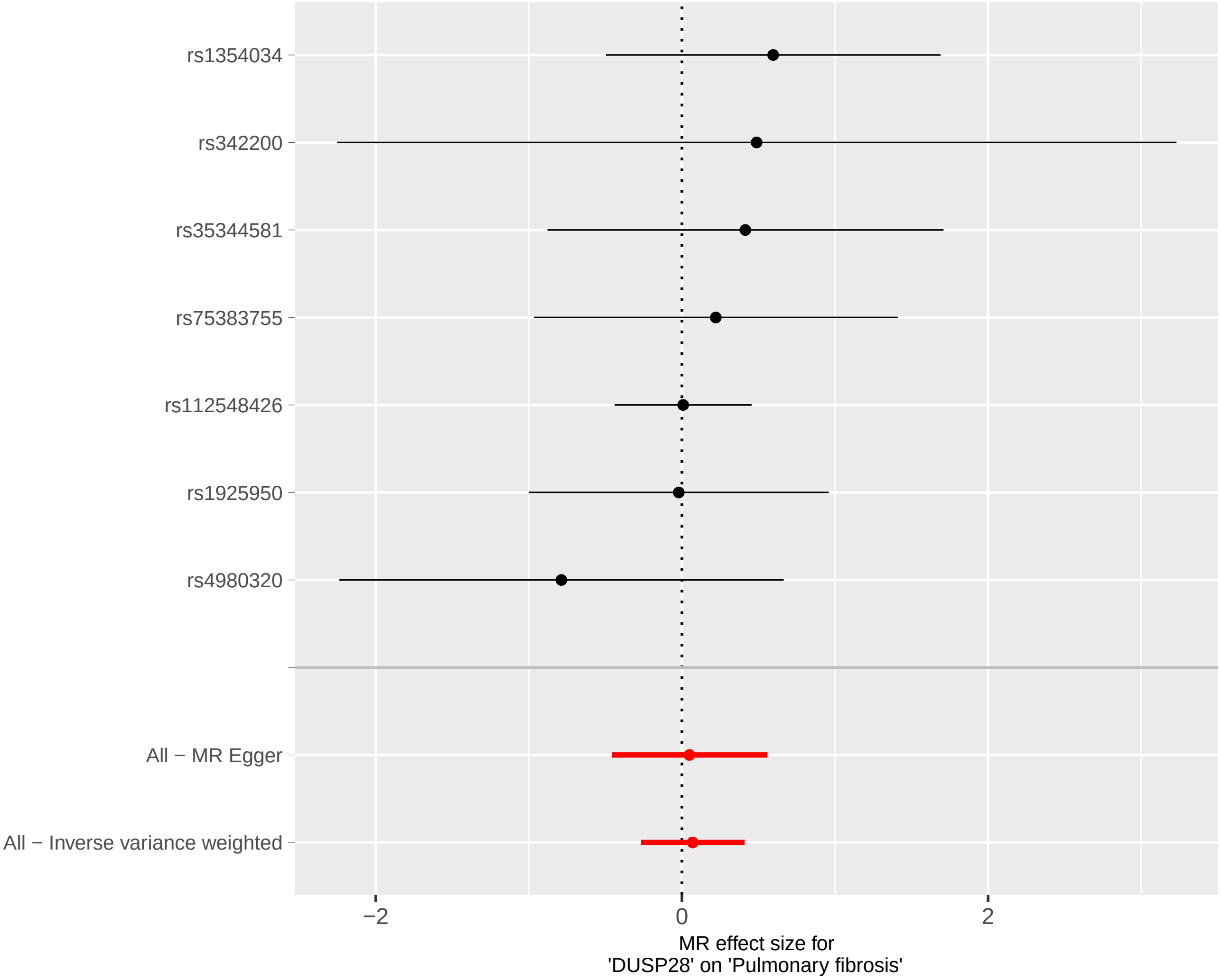
rs1354034
rs342200
rs35344581
rs75383755
rs112548426
rs1925950
rs4980320
All − MR Egger
All − Inverse variance weighted
−2
0
2
MR effect size for
'DUSP28' on 'Pulmonary fibrosis'

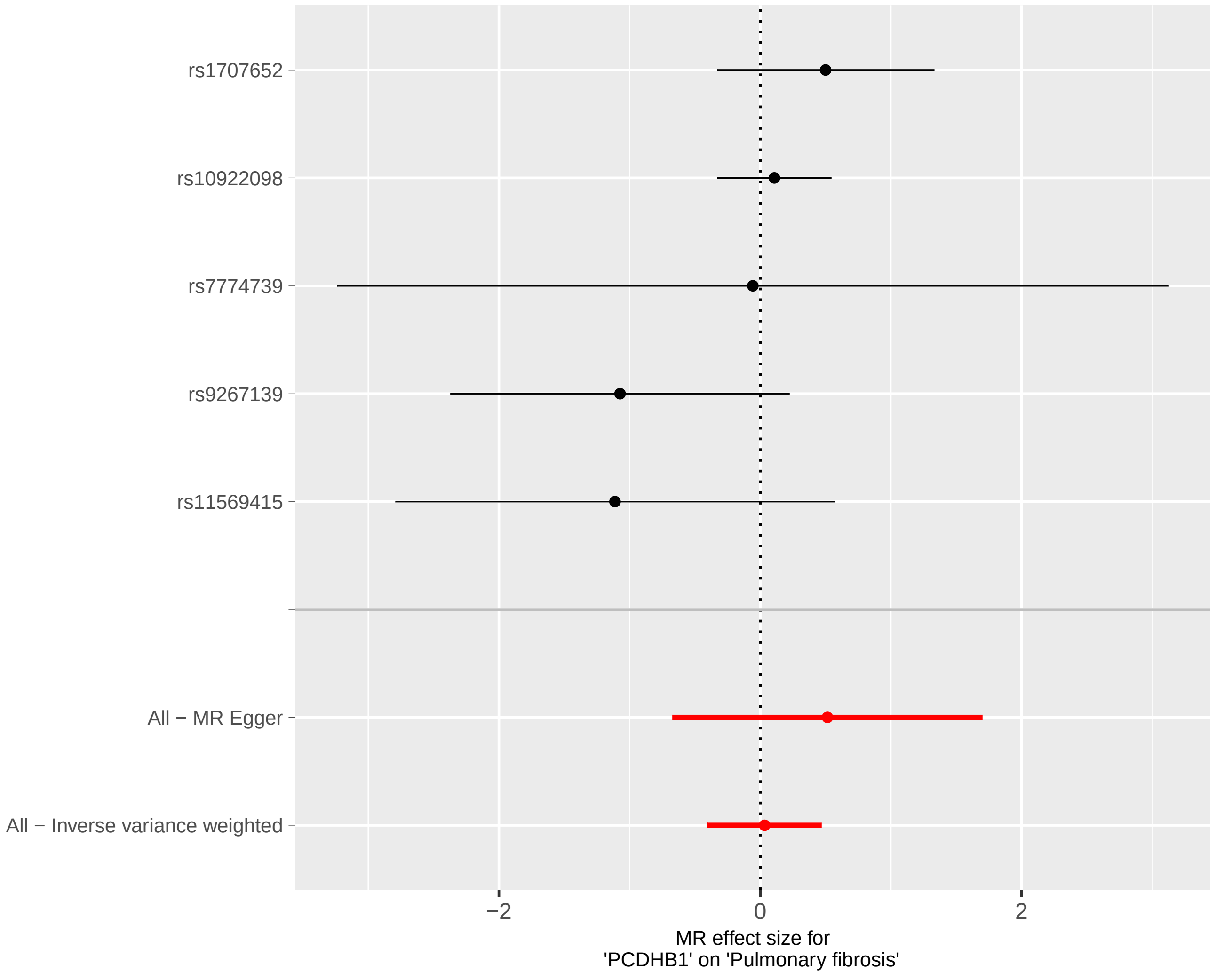

rs1707652
rs10922098
rs7774739
rs9267139
rs11569415
All − MR Egger
All − Inverse variance weighted
−2
0
2
MR effect size for
'PCDHB1' on 'Pulmonary fibrosis'

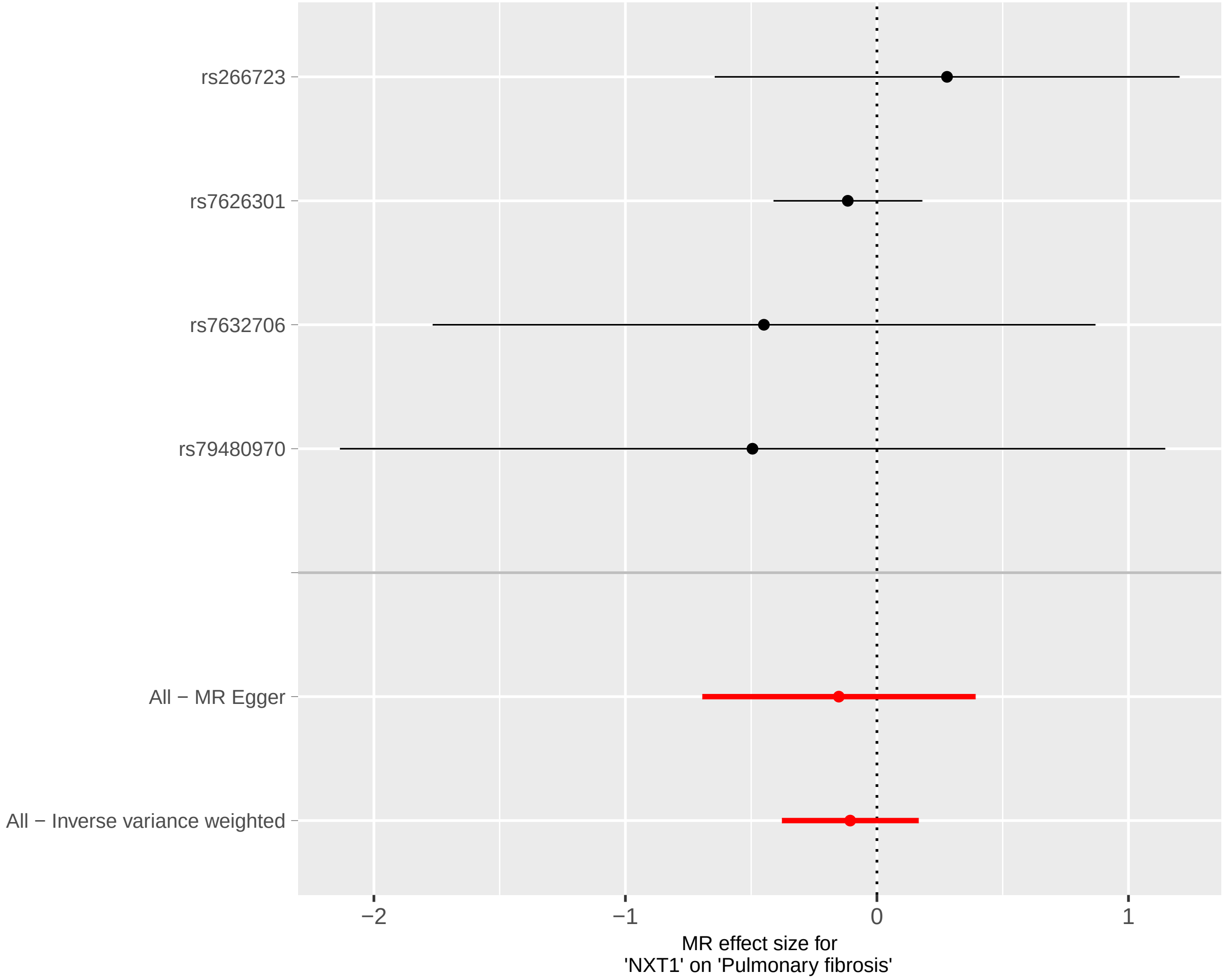
rs266723
rs7626301
rs7632706
rs79480970
All − MR Egger
All − Inverse variance weighted
−2
−1
0
1
MR effect size for
'NXT1' on 'Pulmonary fibrosis'

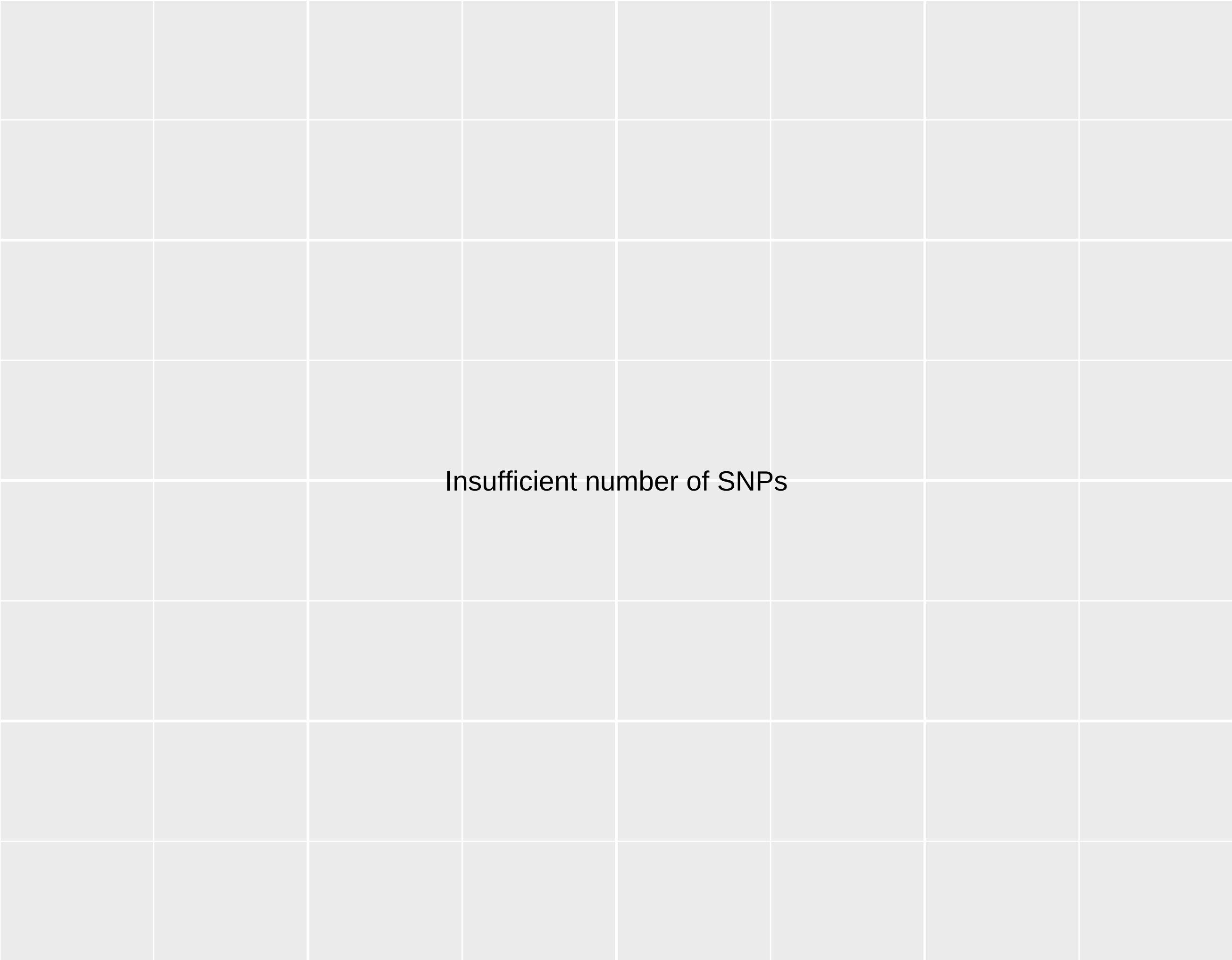
Insufficient number of SNPs

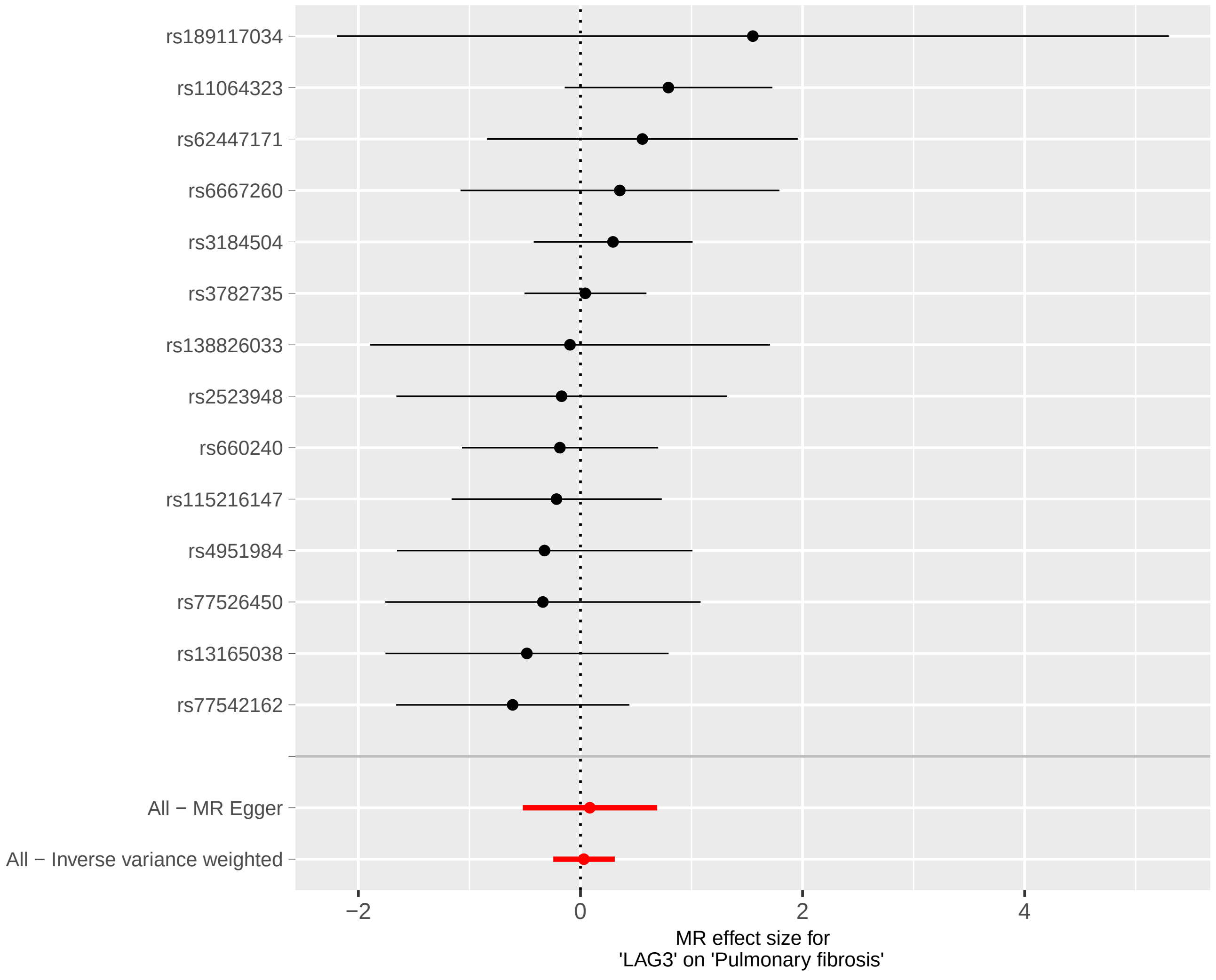

rs189117034
rs11064323
rs62447171
rs6667260
rs3184504
rs3782735
rs138826033
rs2523948
rs660240
rs115216147
rs4951984
rs77526450
rs13165038
rs77542162
All − MR Egger
All − Inverse variance weighted
−2
0
2
4
MR effect size for
'LAG3' on 'Pulmonary fibrosis'

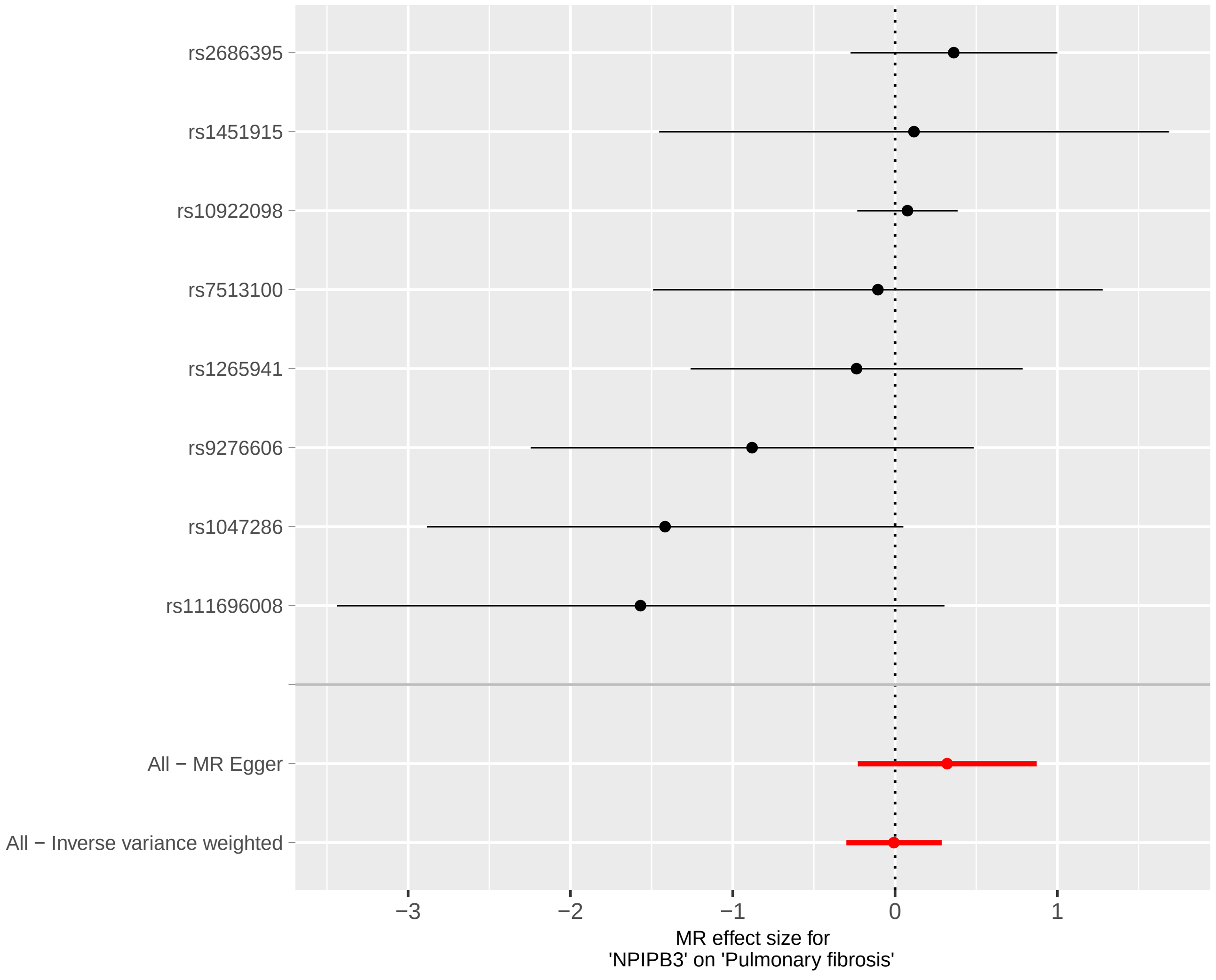

rs2686395
rs1451915
rs10922098
rs7513100
rs1265941
rs9276606
rs1047286
rs111696008
All − MR Egger
All − Inverse variance weighted
−3
−2
−1
0
1
MR effect size for
'NPIPB3' on 'Pulmonary fibrosis'

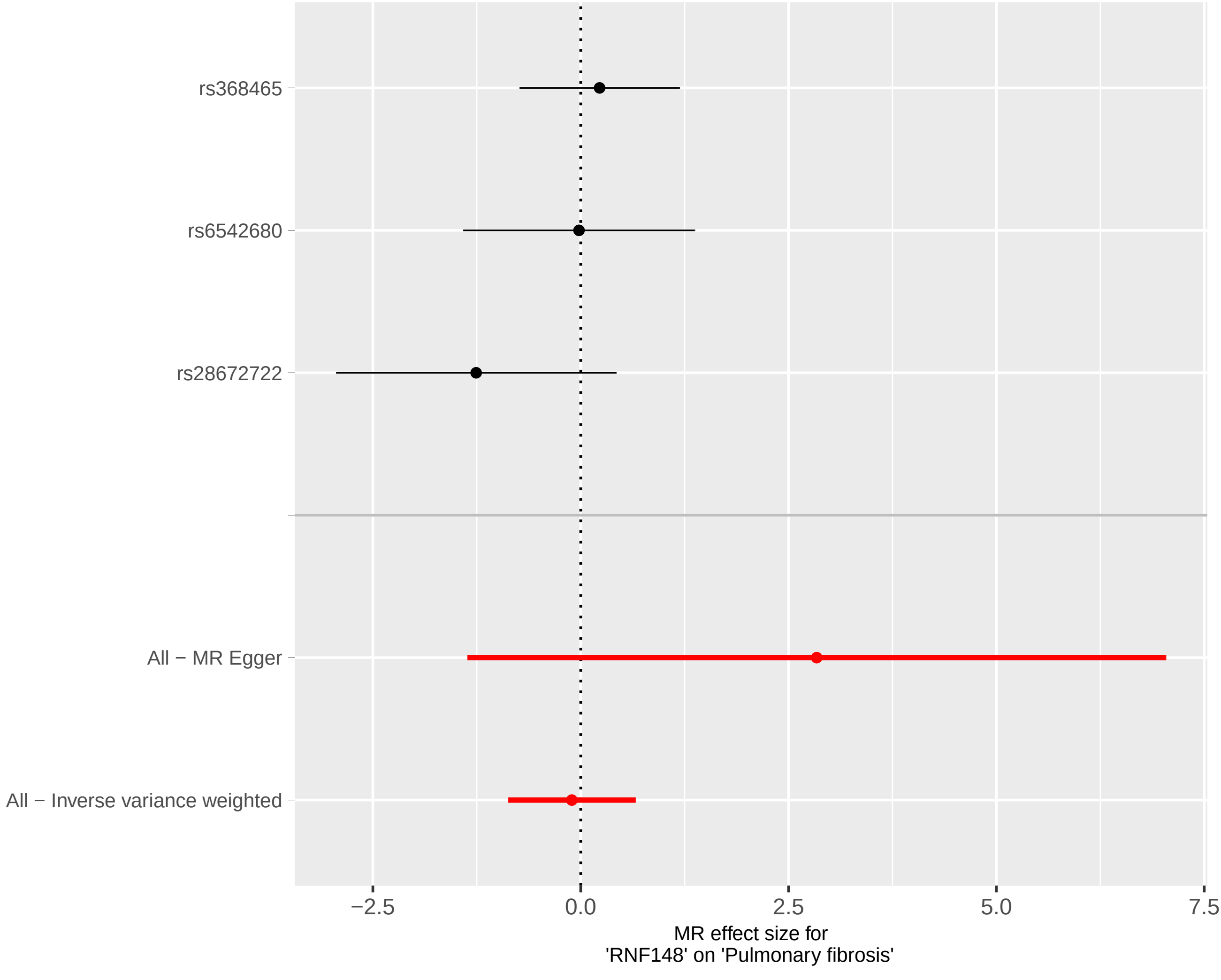
rs368465
rs6542680
rs28672722
All − MR Egger
All − Inverse variance weighted
−2.5
0.0
2.5
5.0
7.5
MR effect size for
'RNF148' on 'Pulmonary fibrosis'

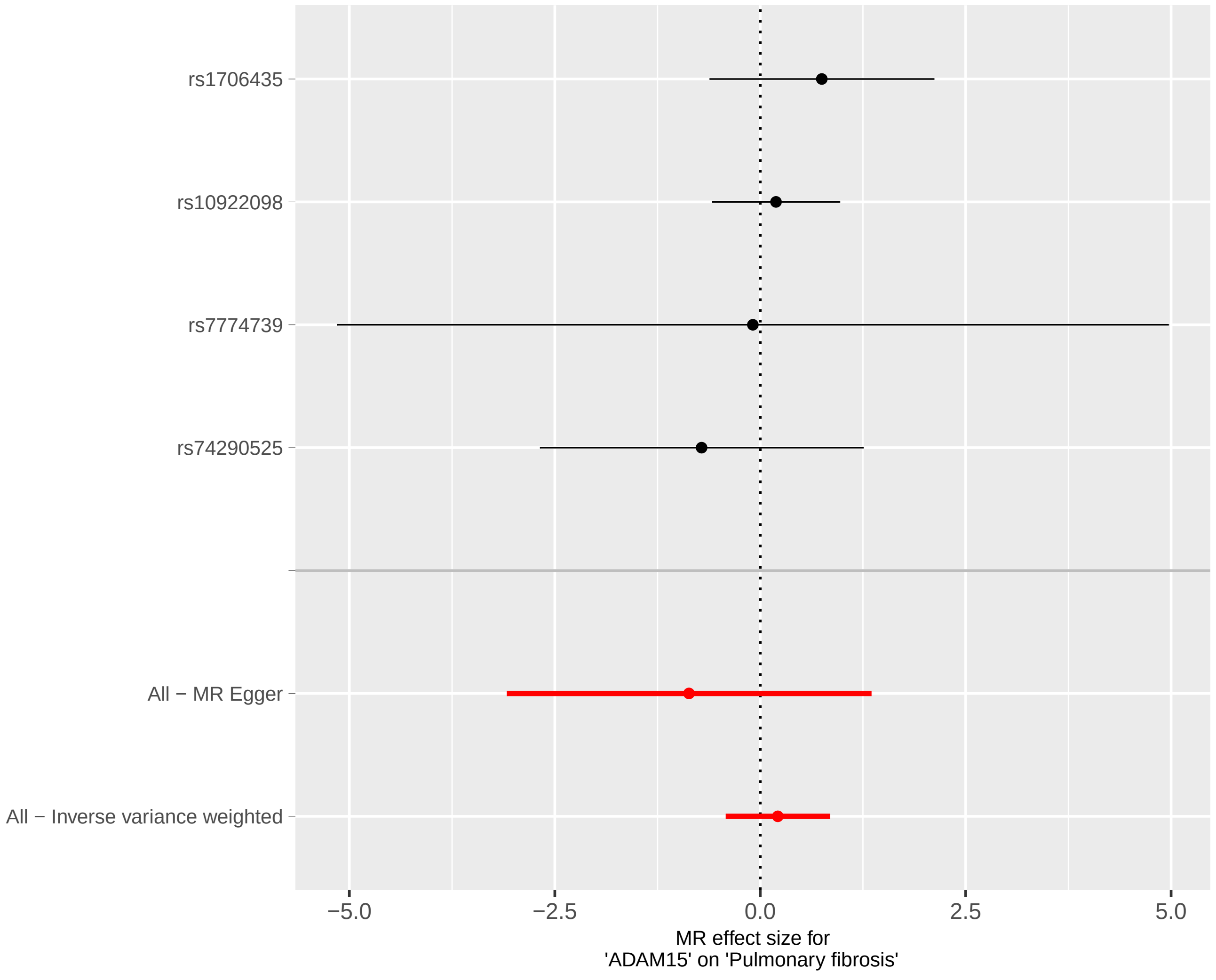
rs1706435
rs10922098
rs7774739
rs74290525
All − MR Egger
All − Inverse variance weighted
−5.0
−2.5
0.0
2.5
5.0
MR effect size for
'ADAM15' on 'Pulmonary fibrosis'

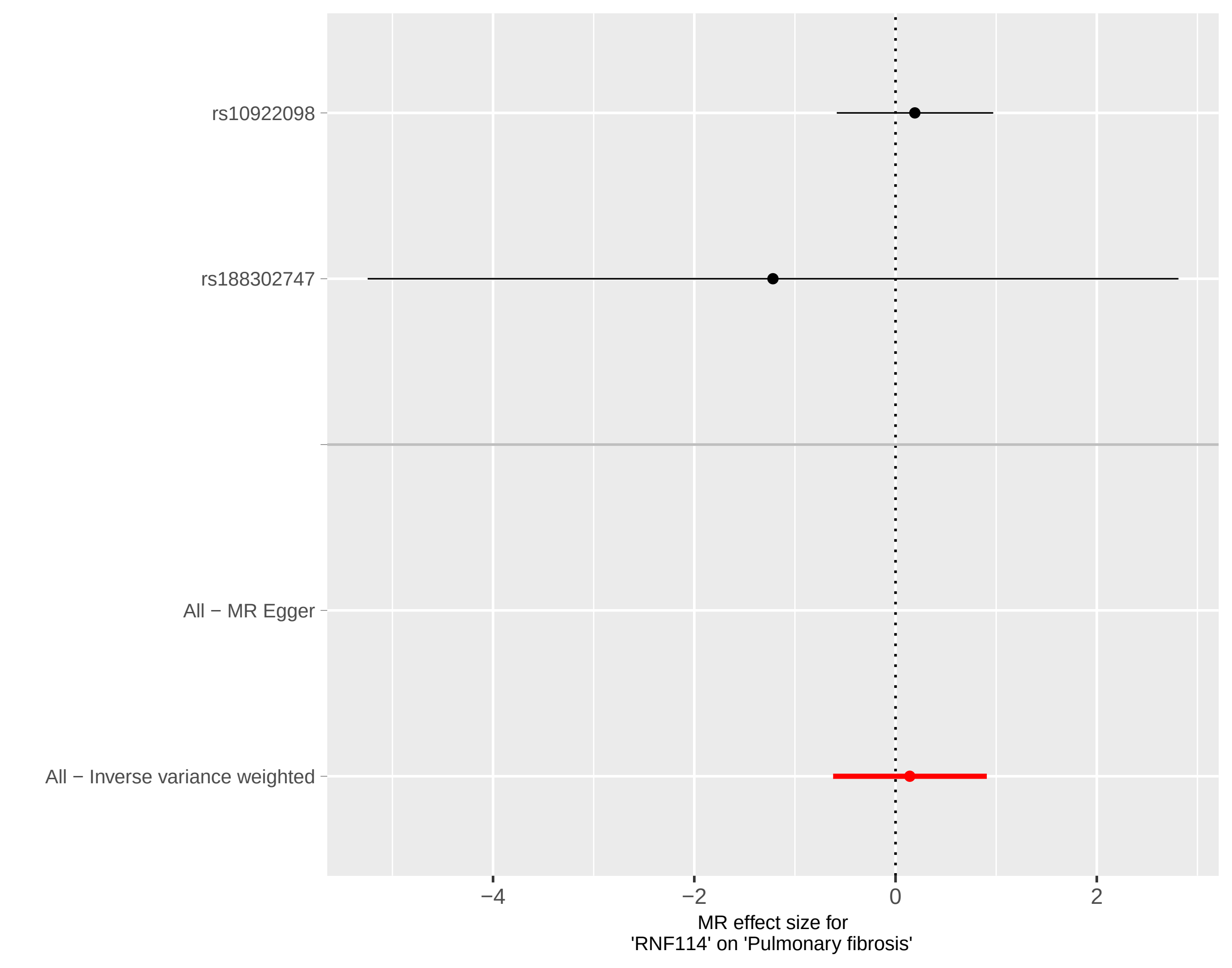

rs10922098
rs188302747
All − MR Egger
All − Inverse variance weighted
−4
−2
0
2
MR effect size for
'RNF114' on 'Pulmonary fibrosis'

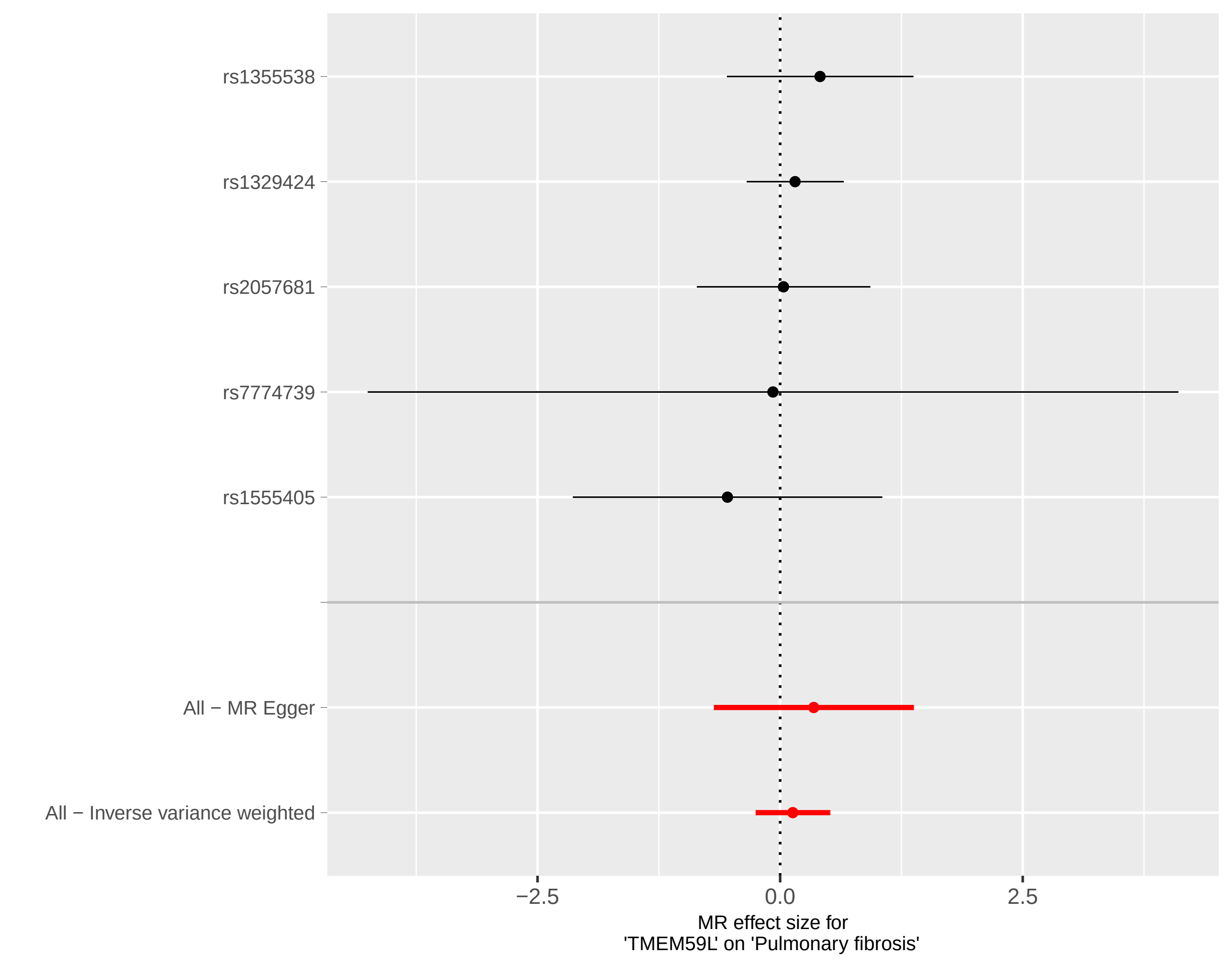
rs1355538
rs1329424
rs2057681
rs7774739
rs1555405
All − MR Egger
All − Inverse variance weighted
−2.5
0.0
2.5
MR effect size for
'TMEM59L' on 'Pulmonary fibrosis'

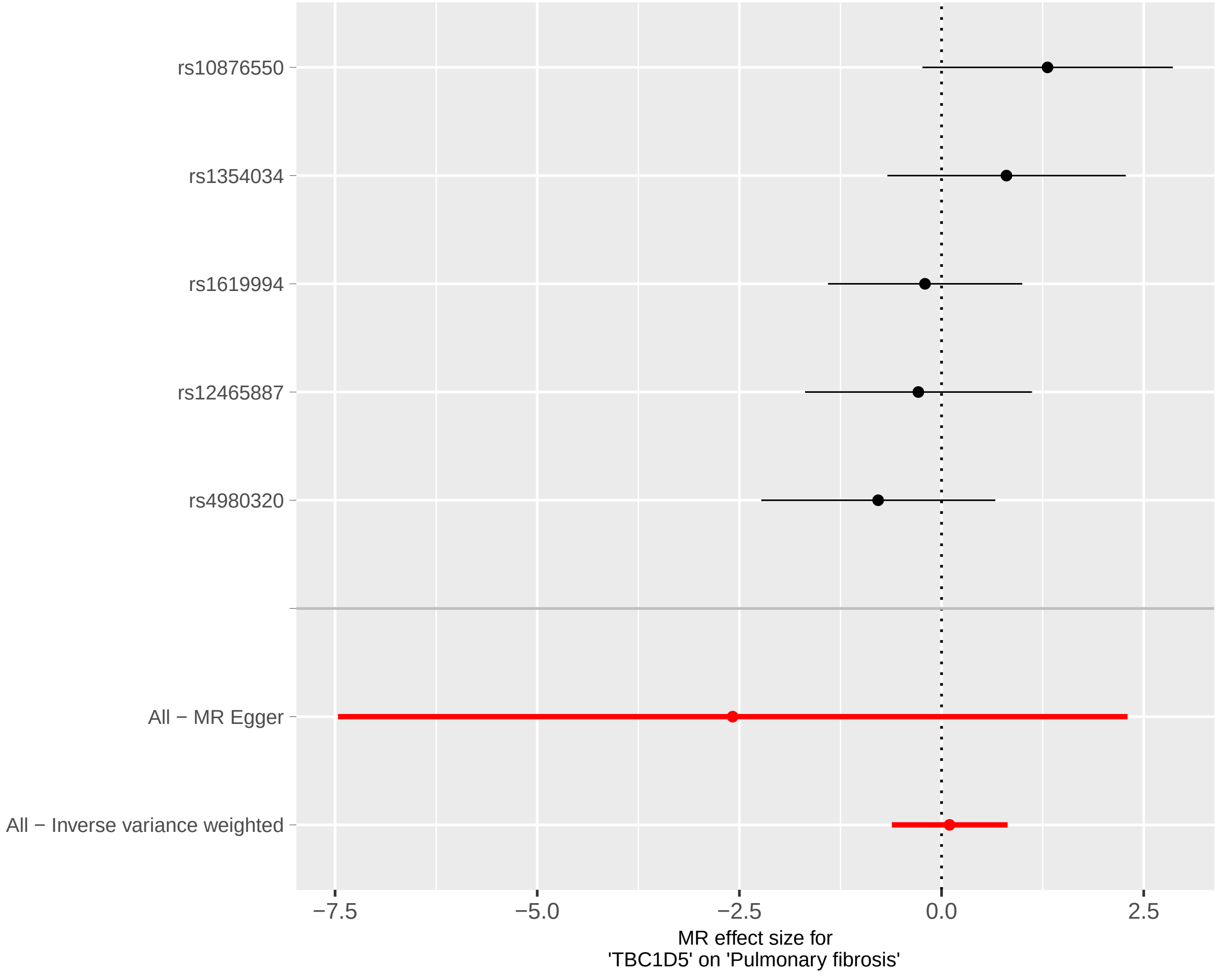
rs10876550
rs1354034
rs1619994
rs12465887
rs4980320
All − MR Egger
All − Inverse variance weighted
−7.5
−5.0
−2.5
0.0
2.5
MR effect size for
'TBC1D5' on 'Pulmonary fibrosis'

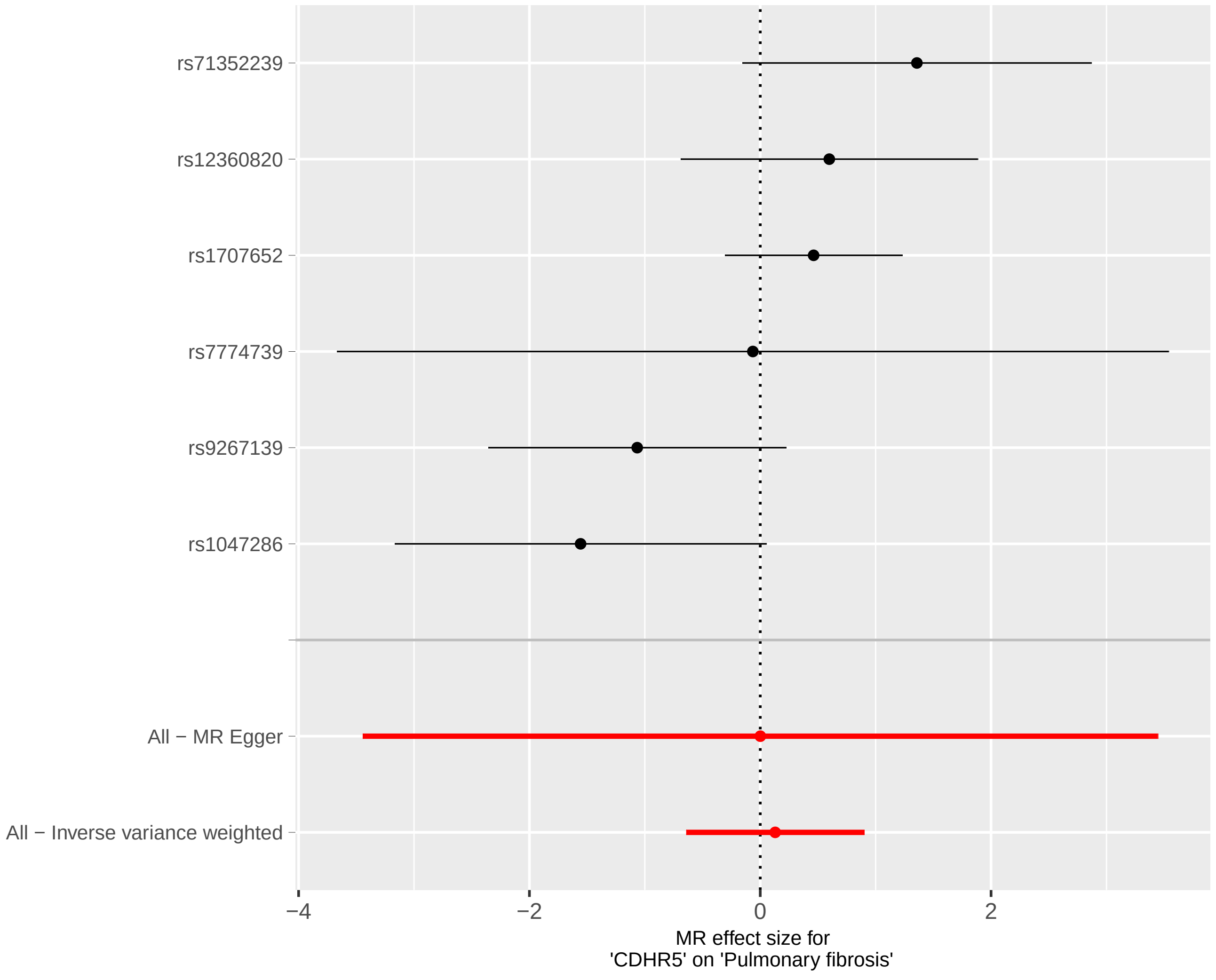

rs71352239
rs12360820
rs1707652
rs7774739
rs9267139
rs1047286
All − MR Egger
All − Inverse variance weighted
−4
−2
0
2
MR effect size for
'CDHR5' on 'Pulmonary fibrosis'

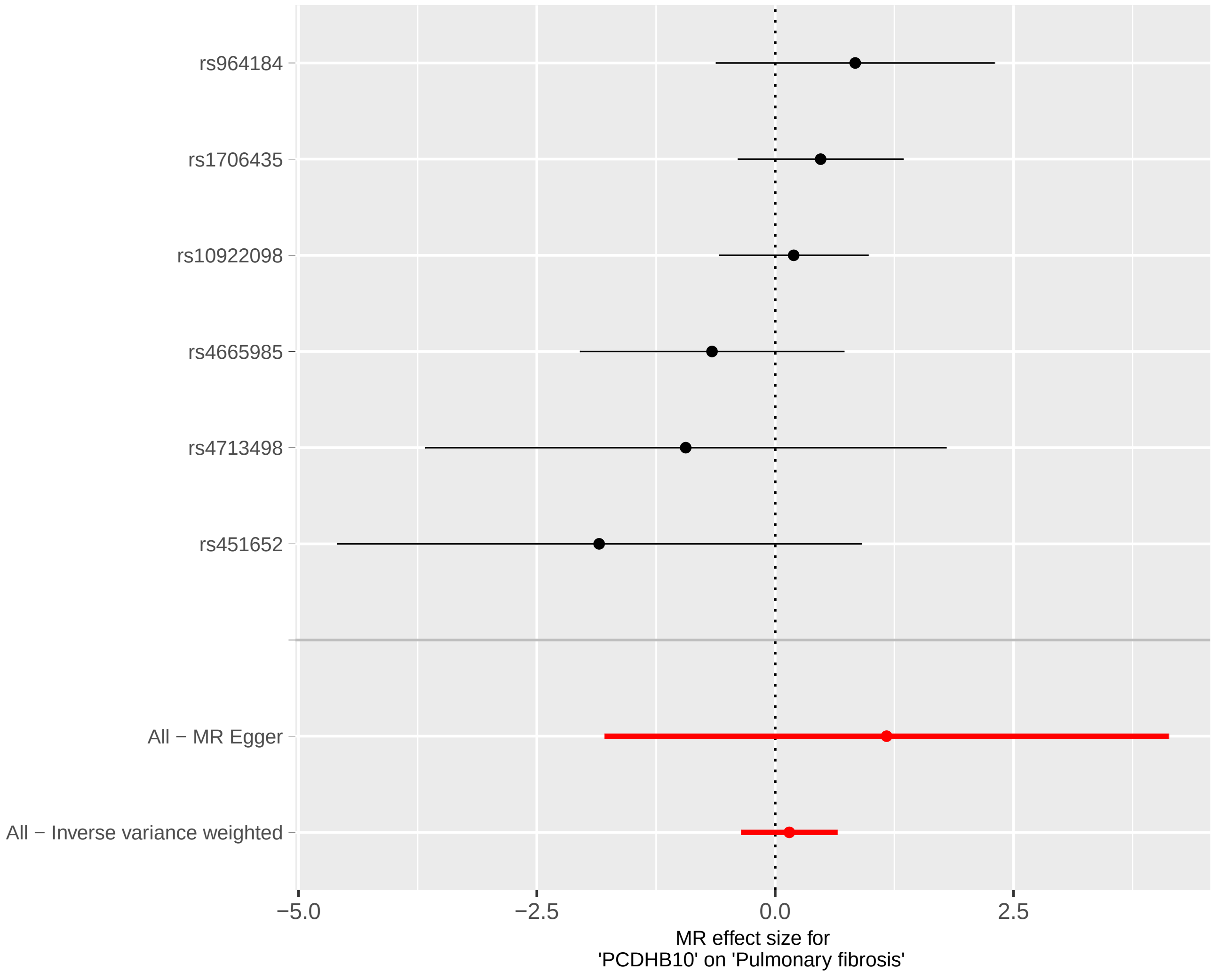

rs964184
rs1706435
rs10922098
rs4665985
rs4713498
rs451652
All − MR Egger
All − Inverse variance weighted
−5.0
−2.5
0.0
2.5
MR effect size for
'PCDHB10' on 'Pulmonary fibrosis'

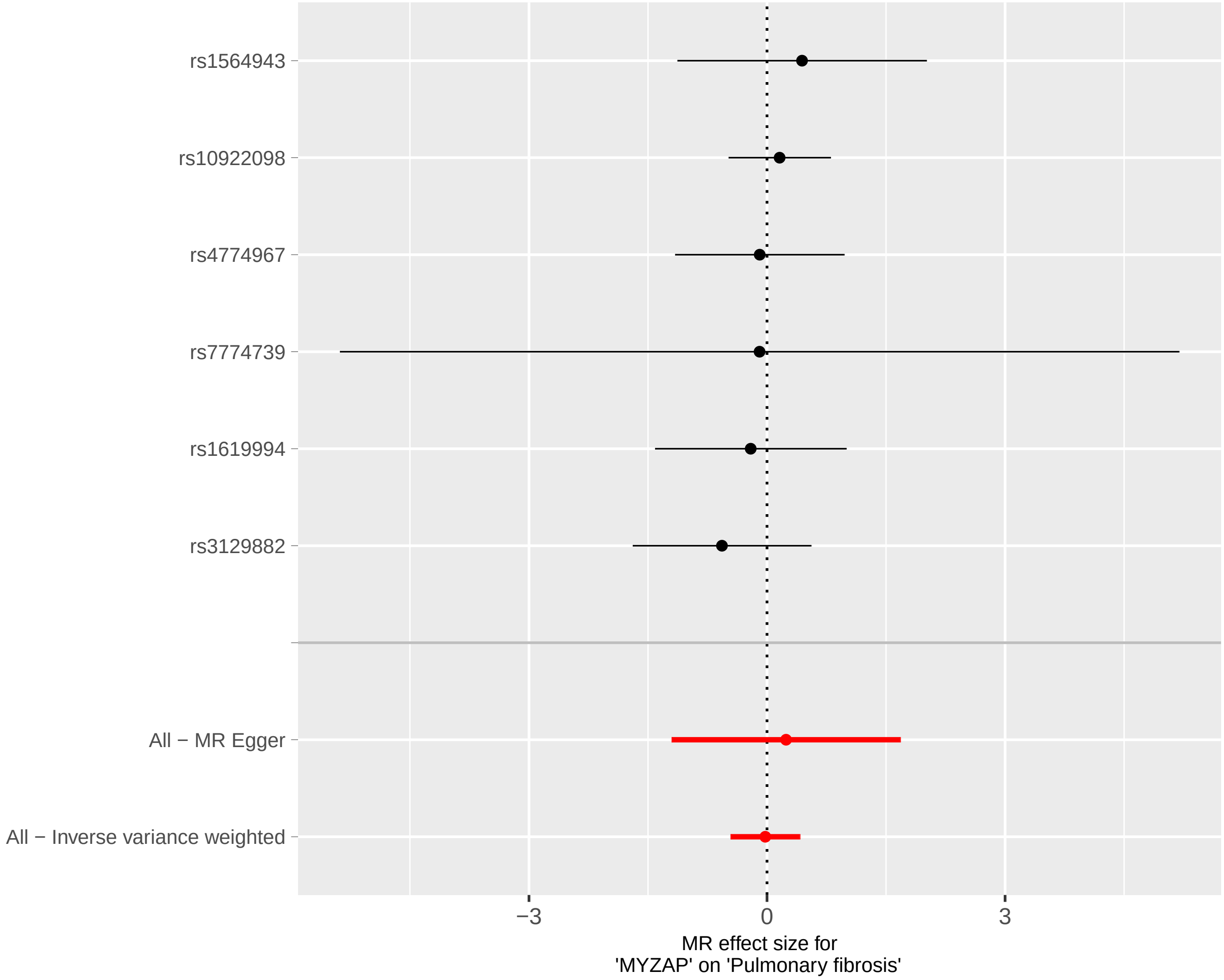
rs1564943
rs10922098
rs4774967
rs7774739
rs1619994
rs3129882
All − MR Egger
All − Inverse variance weighted
−3
0
3
MR effect size for
'MYZAP' on 'Pulmonary fibrosis'

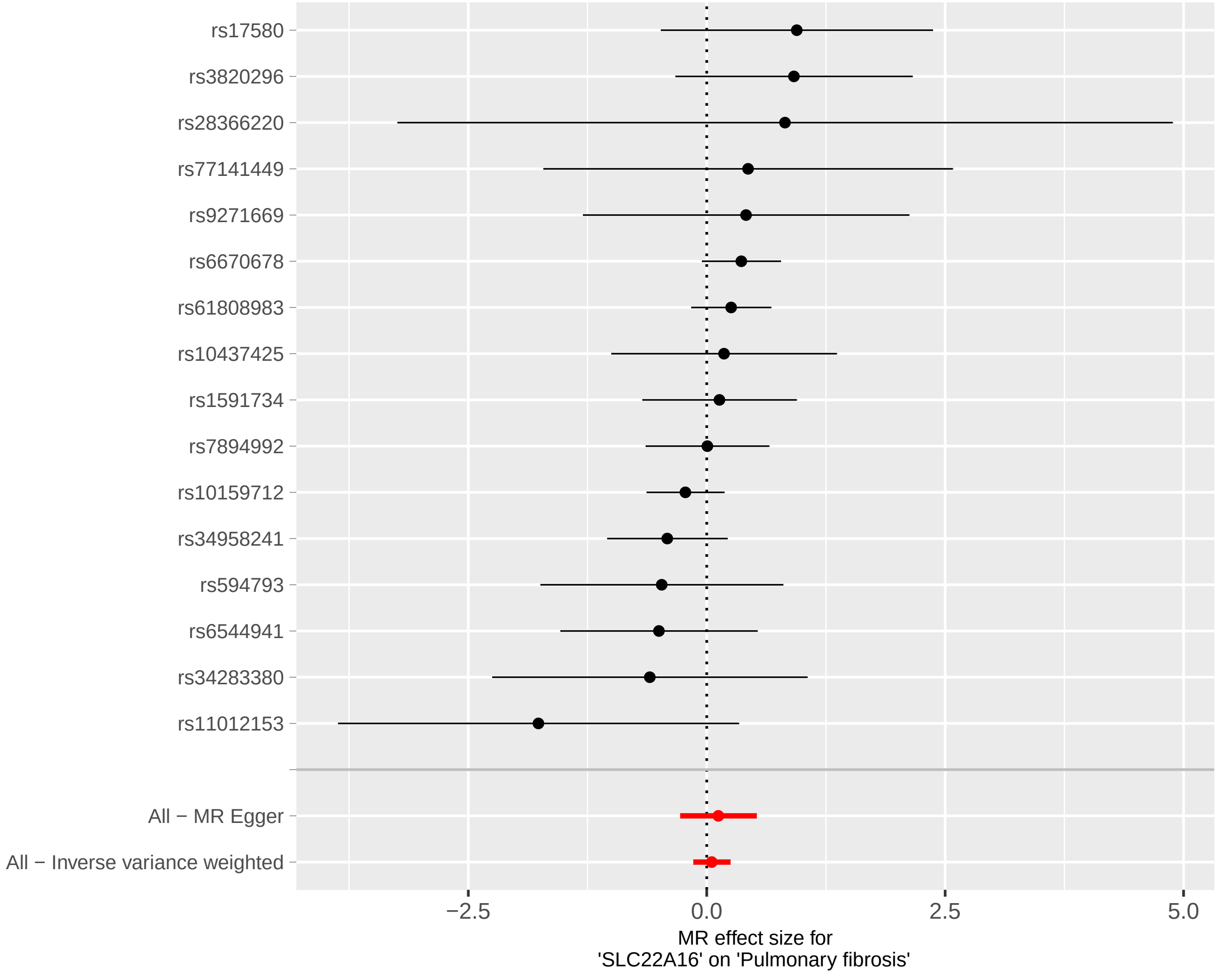
rs17580
rs3820296
rs28366220
rs77141449
rs9271669
rs6670678
rs61808983
rs10437425
rs1591734
rs7894992
rs10159712
rs34958241
rs594793
rs6544941
rs34283380
rs11012153
All − MR Egger
All − Inverse variance weighted
−2.5
0.0
2.5
5.0
MR effect size for
'SLC22A16' on 'Pulmonary fibrosis'

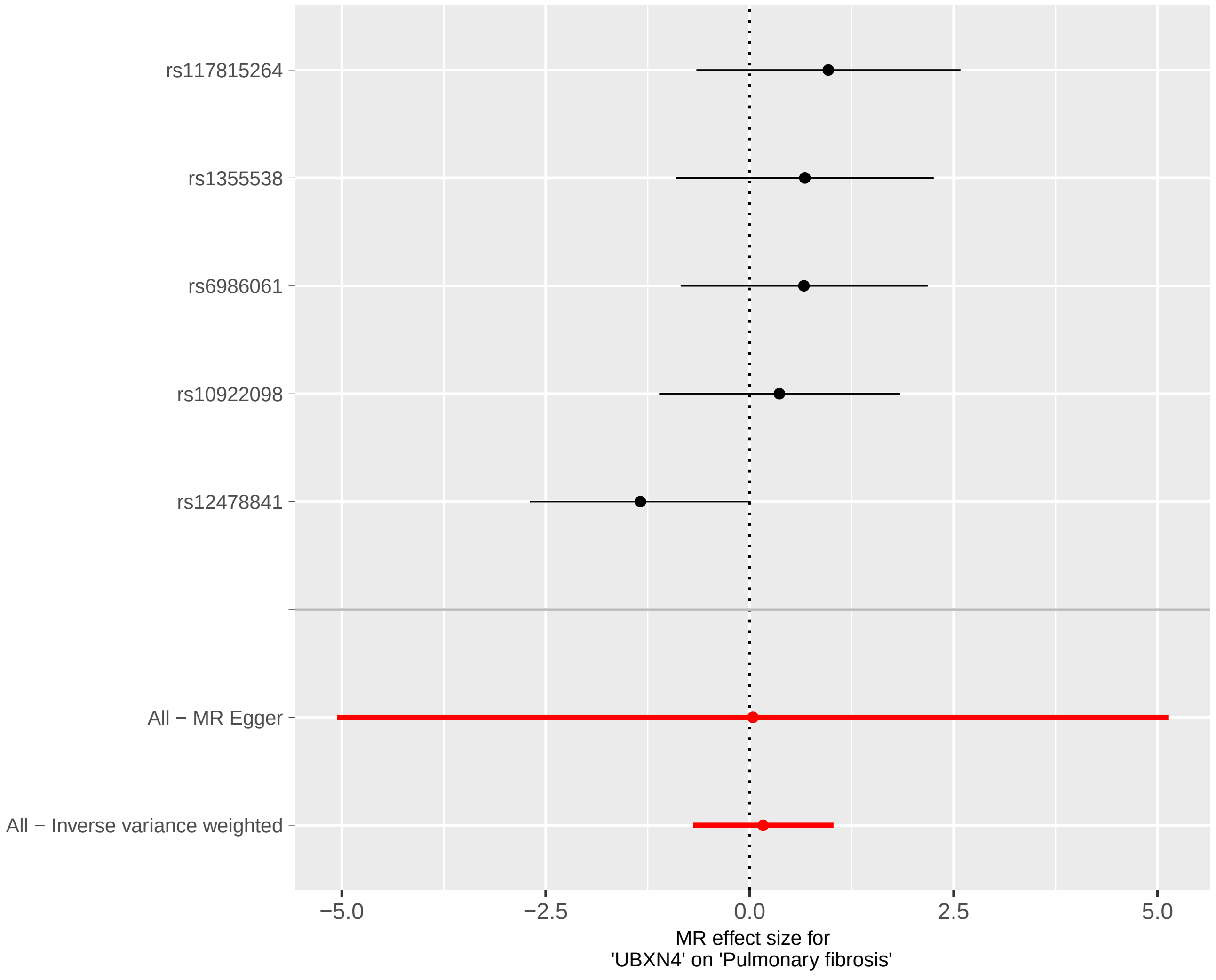

rs117815264
rs1355538
rs6986061
rs10922098
rs12478841
All − MR Egger
All − Inverse variance weighted
−5.0
−2.5
0.0
2.5
5.0
MR effect size for
'UBXN4' on 'Pulmonary fibrosis'

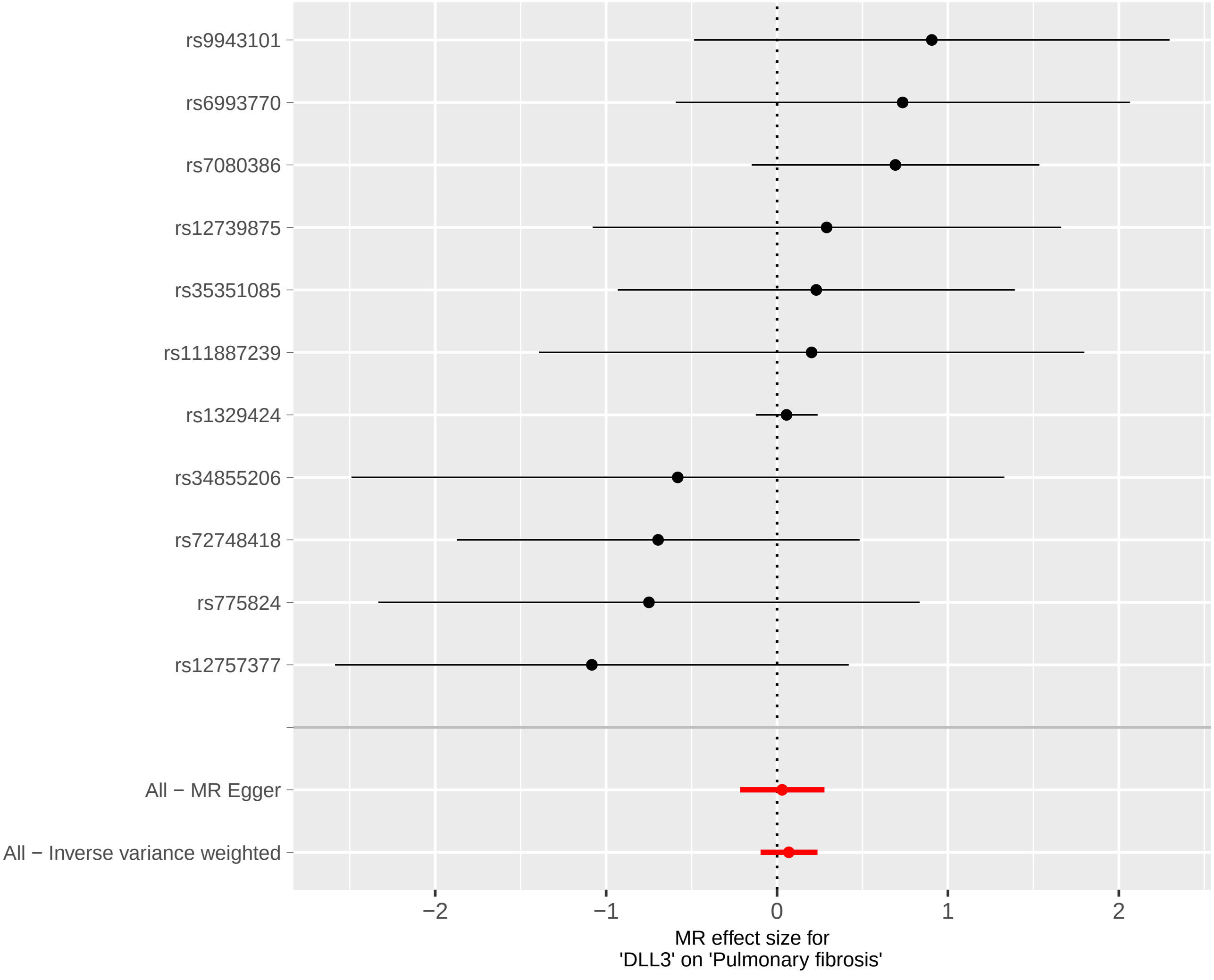

rs9943101
rs6993770
rs7080386
rs12739875
rs35351085
rs111887239
rs1329424
rs34855206
rs72748418
rs775824
rs12757377
All − MR Egger
All − Inverse variance weighted
−2
−1
0
1
2
MR effect size for
'DLL3' on 'Pulmonary fibrosis'

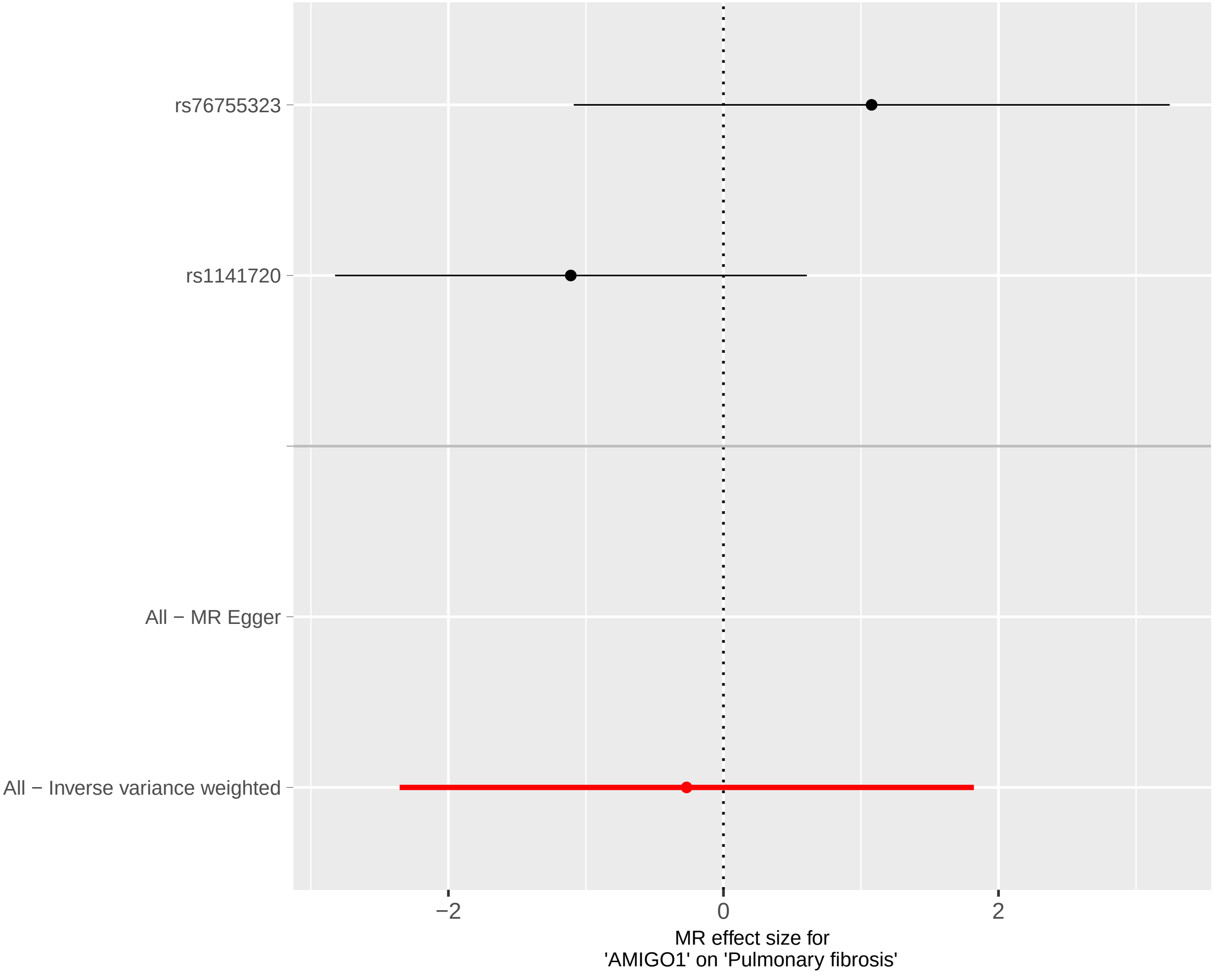
rs76755323
rs1141720
All − MR Egger
All − Inverse variance weighted
−2
0
2
MR effect size for
'AMIGO1' on 'Pulmonary fibrosis'

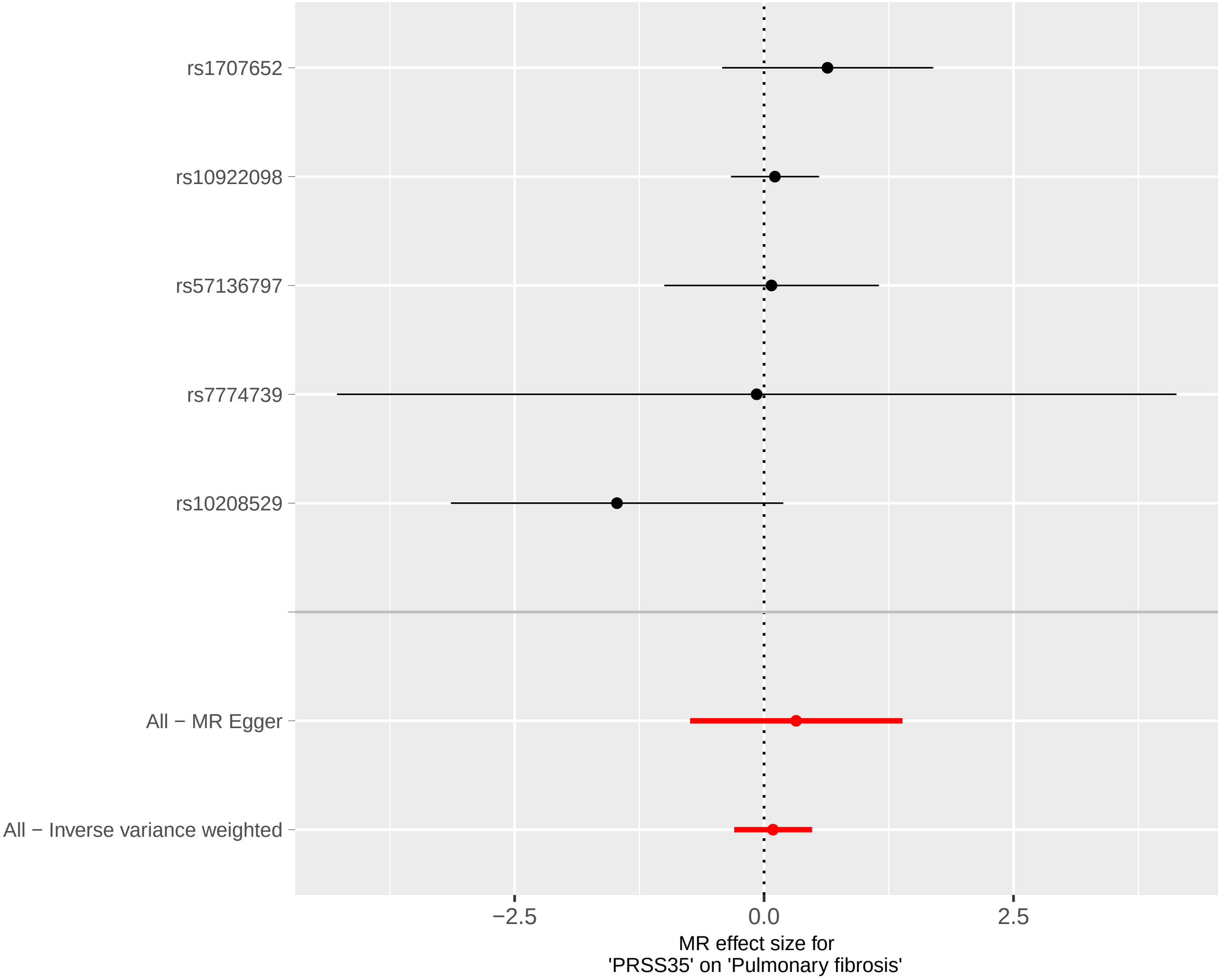

rs1707652
rs10922098
rs57136797
rs7774739
rs10208529
All − MR Egger
All − Inverse variance weighted
−2.5
0.0
2.5
MR effect size for
'PRSS35' on 'Pulmonary fibrosis'

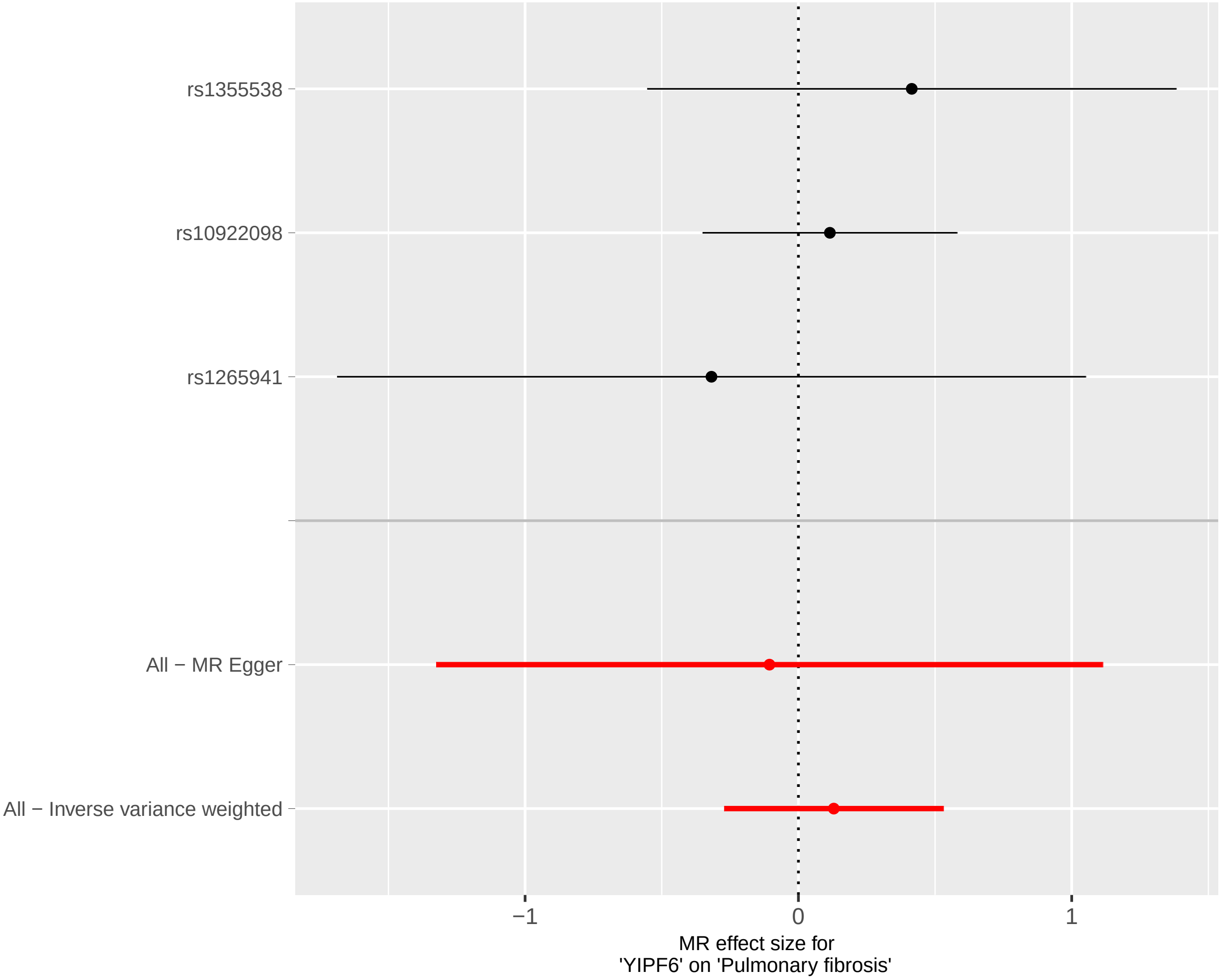

rs1355538
rs10922098
rs1265941
All − MR Egger
All − Inverse variance weighted
−1
0
1
MR effect size for
'YIPF6' on 'Pulmonary fibrosis'

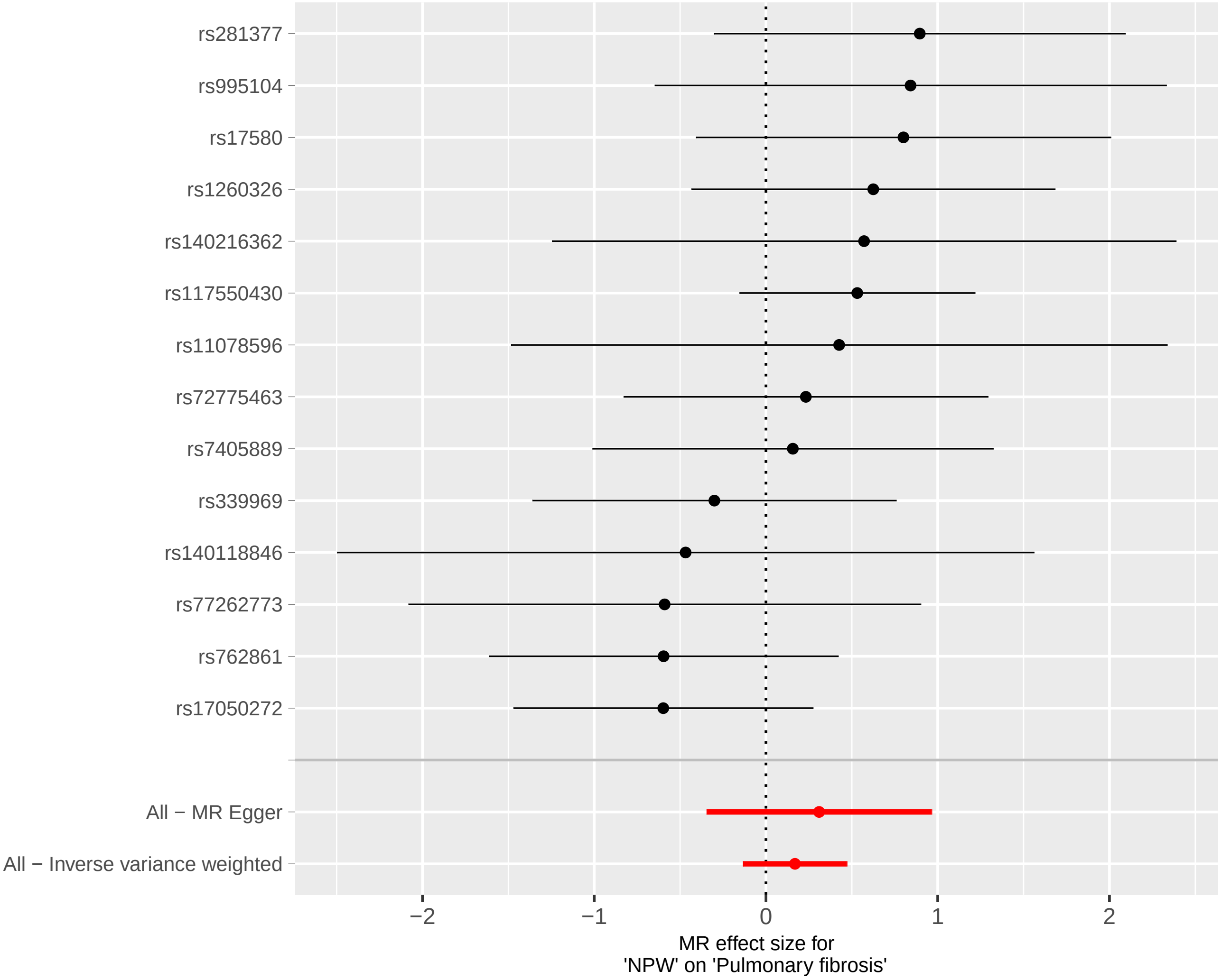
rs281377
rs995104
rs17580
rs1260326
rs140216362
rs117550430
rs11078596
rs72775463
rs7405889
rs339969
rs140118846
rs77262773
rs762861
rs17050272
All − MR Egger
All − Inverse variance weighted
−2
−1
0
1
2
MR effect size for
'NPW' on 'Pulmonary fibrosis'

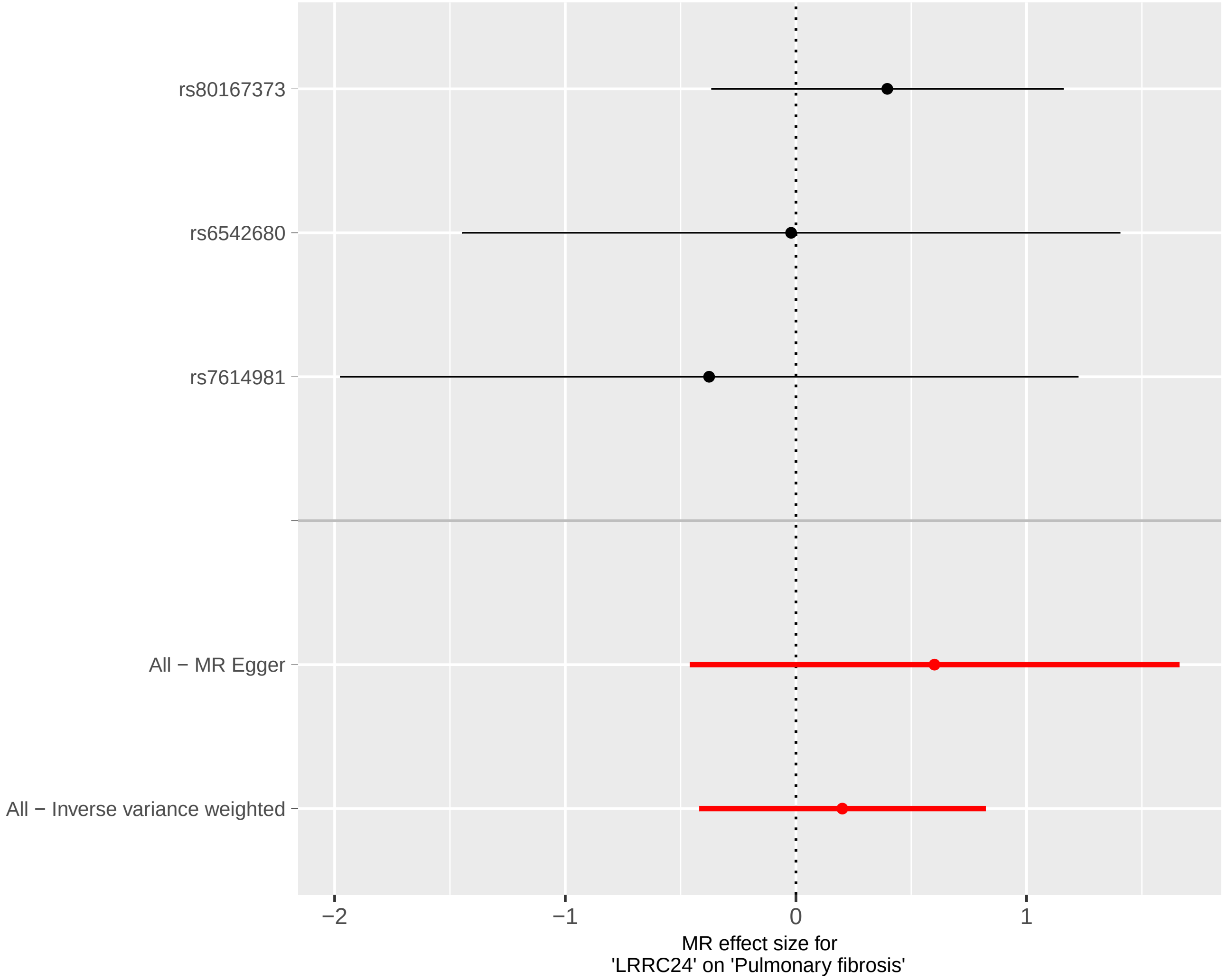
rs80167373
rs6542680
rs7614981
All − MR Egger
All − Inverse variance weighted
−2
−1
0
1
MR effect size for
'LRRC24' on 'Pulmonary fibrosis'

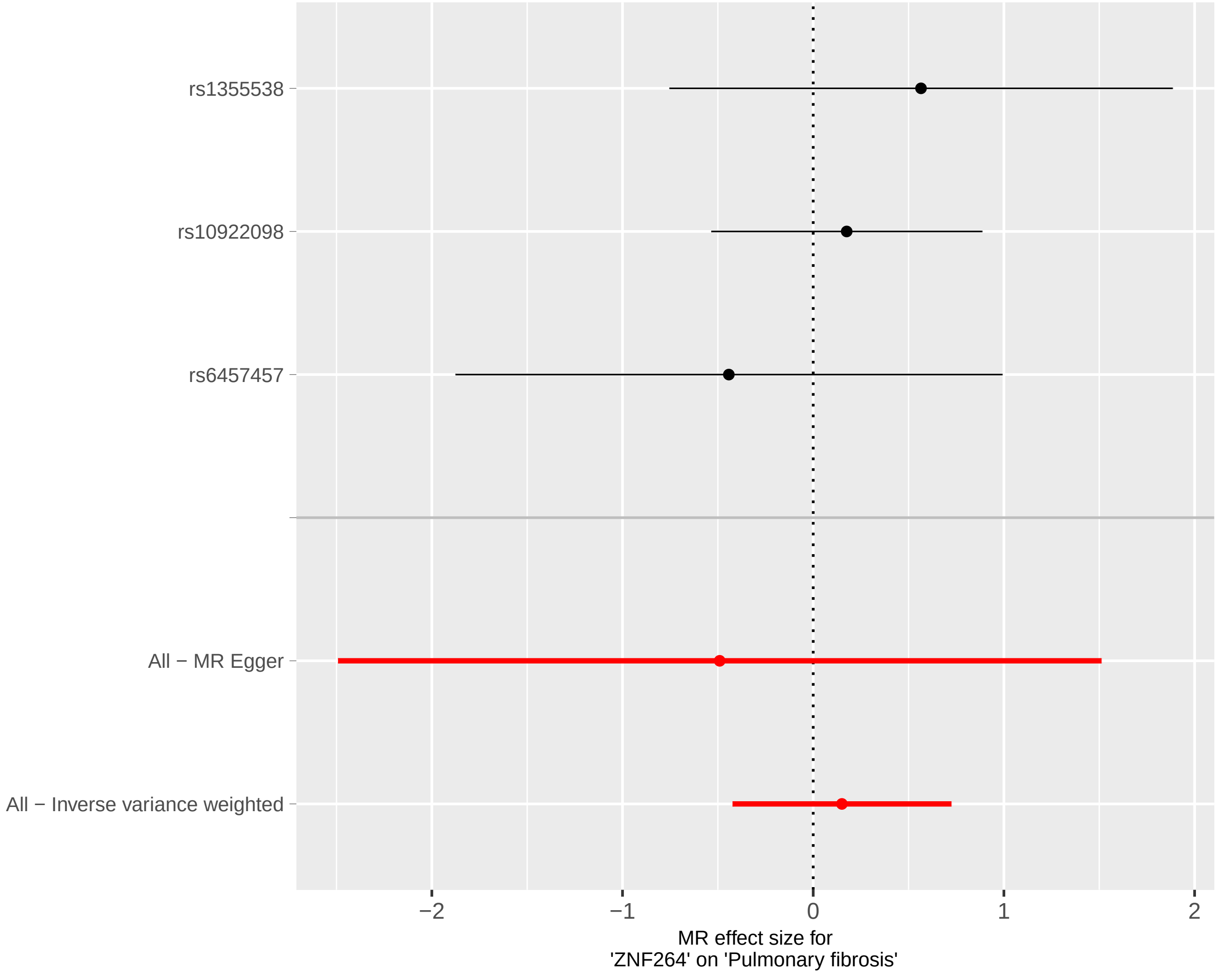
rs1355538
rs10922098
rs6457457
All − MR Egger
All − Inverse variance weighted
−2
−1
0
1
2
MR effect size for
'ZNF264' on 'Pulmonary fibrosis'

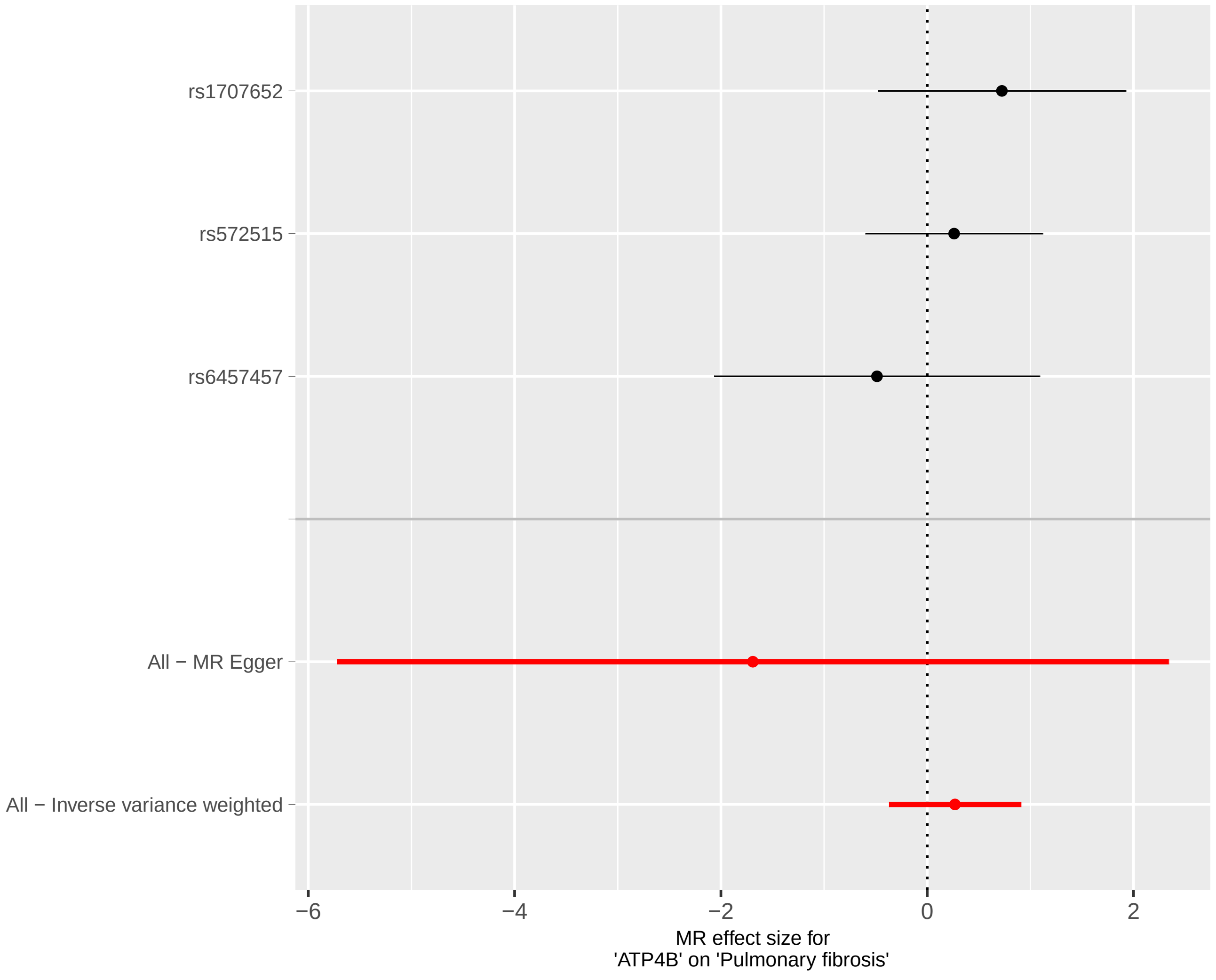
rs1707652
rs572515
rs6457457
All − MR Egger
All − Inverse variance weighted
−6
−4
−2
0
2
MR effect size for
'ATP4B' on 'Pulmonary fibrosis'

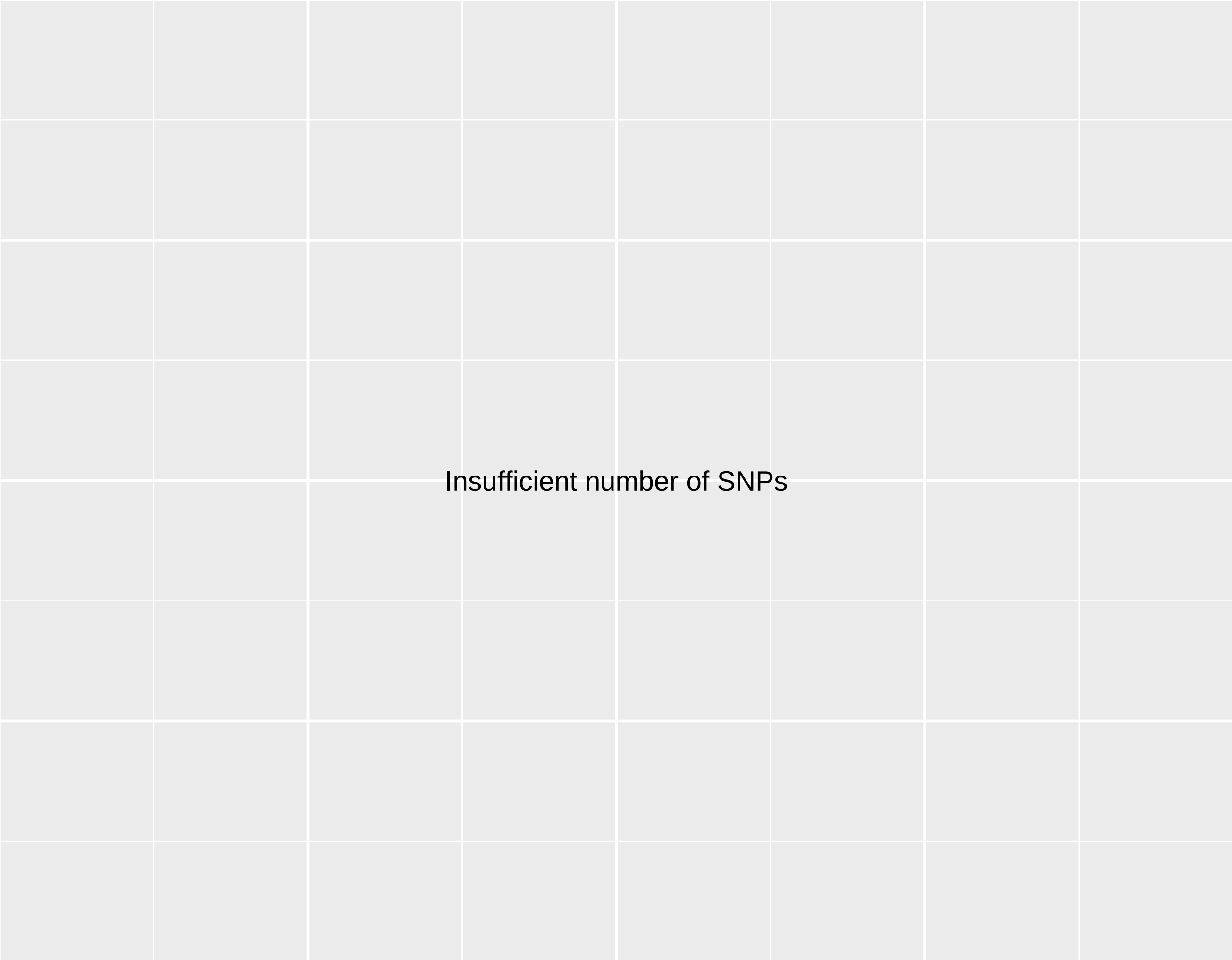
Insufficient number of SNPs
