# Supplementary material for: Assessing the Causal Relationship Between Plasma Proteins and Pulmonary Fibrosis: A Systematic Analysis Based on Mendelian Randomization
Source: Biology (Basel). 2025 Feb 14;14(2):200. doi: 10.3390/biology14020200 (PMC11852313; doi:10.3390/biology14020200)
Supplement: Supplementary file 1 [file Figure S2.pdf › Supplementary Material S1/Figure S2.pdf]

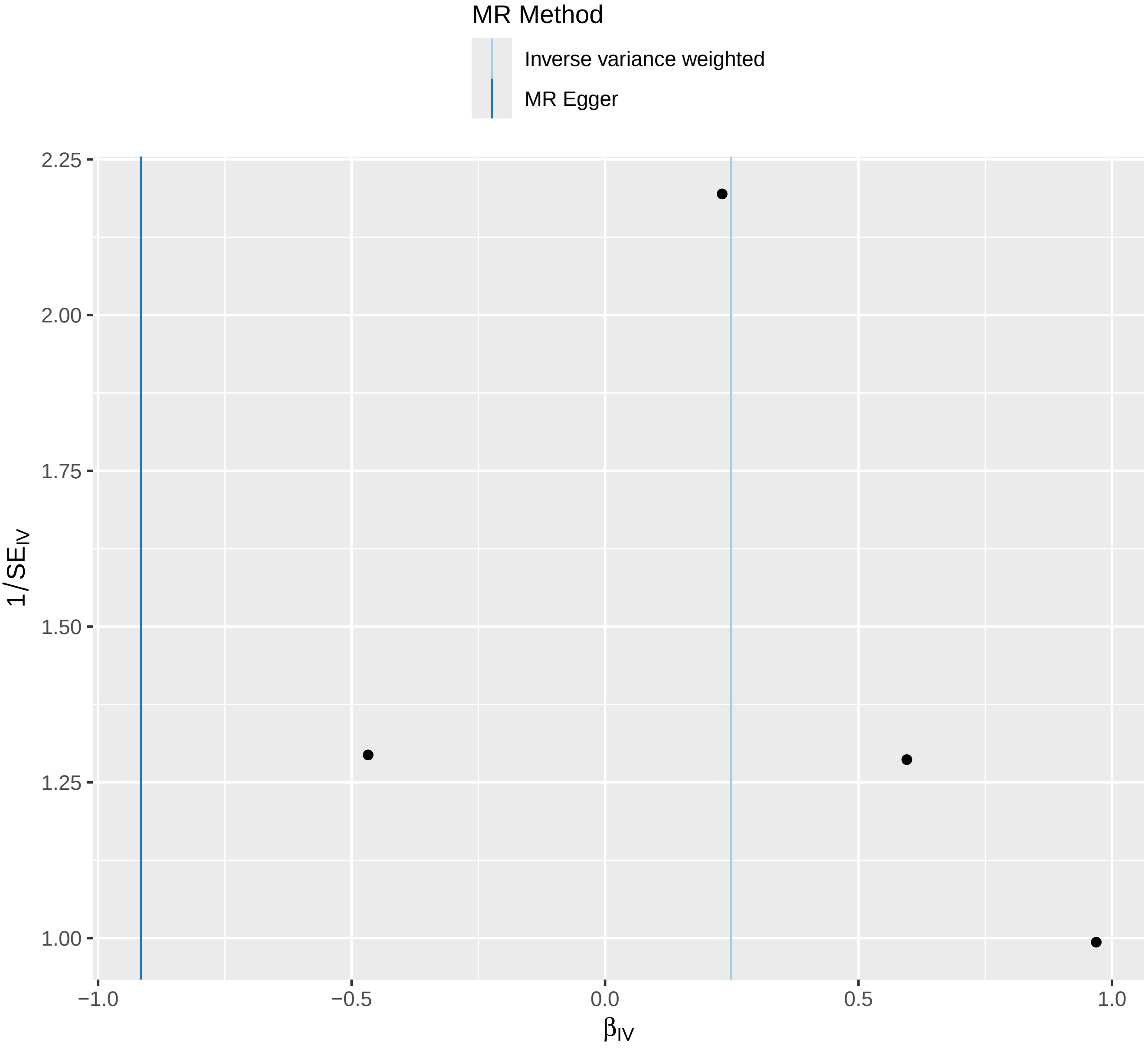

MR Method
Inverse variance weighted
MR Egger
2.25
2.00
1.75
1.50
1.25
1.00
1/SE_IV
−1.0
−0.5
0.0
0.5
1.0
β_IV

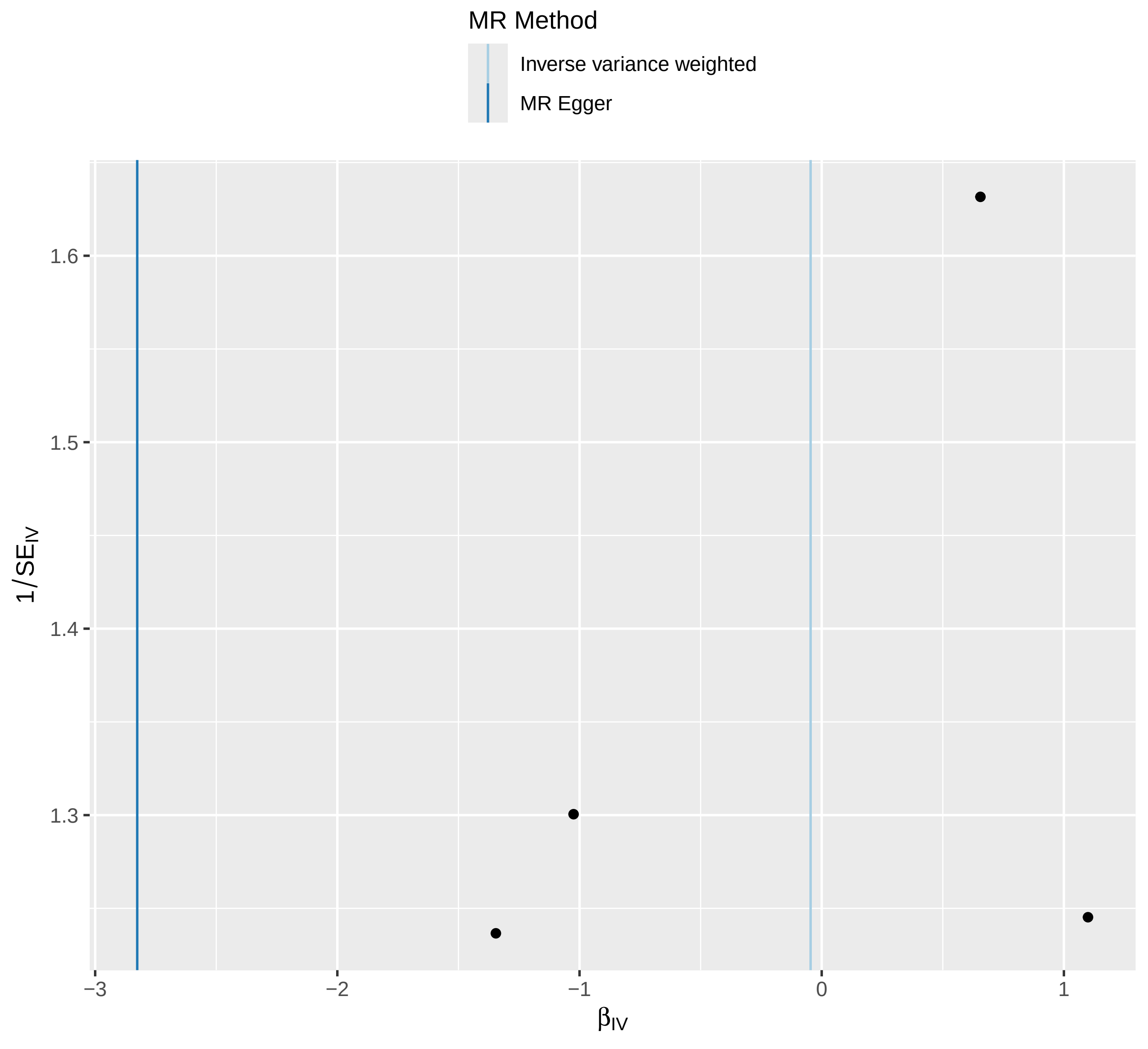
MR Method
Inverse variance weighted
MR Egger
1.6
1.5
1.4
1.3
1/SE_IV
−3
−2
−1
0
1
β_IV

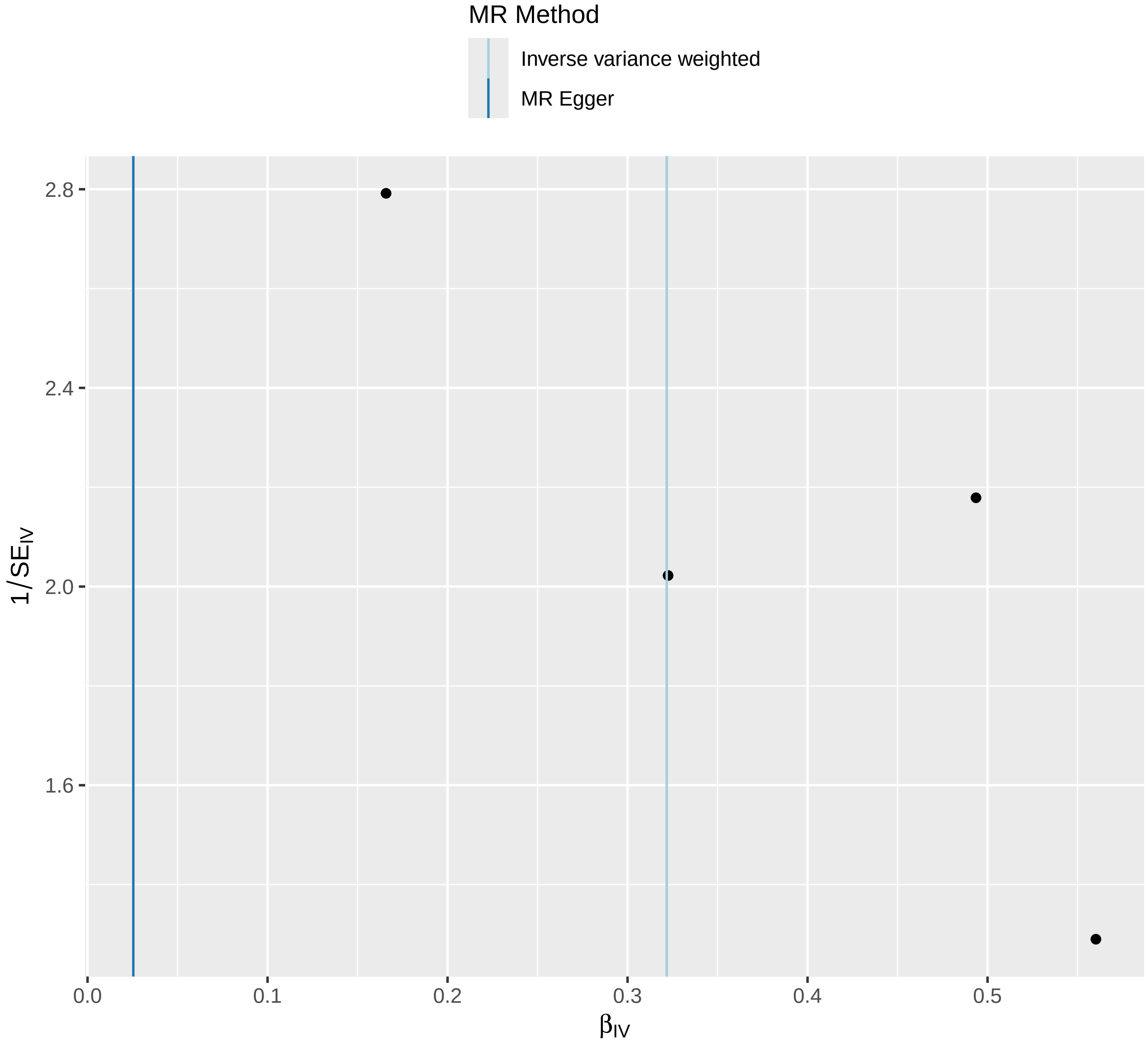
MR Method
Inverse variance weighted
MR Egger
2.8
2.4
2.0
1.6
1/SE_IV
0.0
0.1
0.2
0.3
0.4
0.5
β_IV

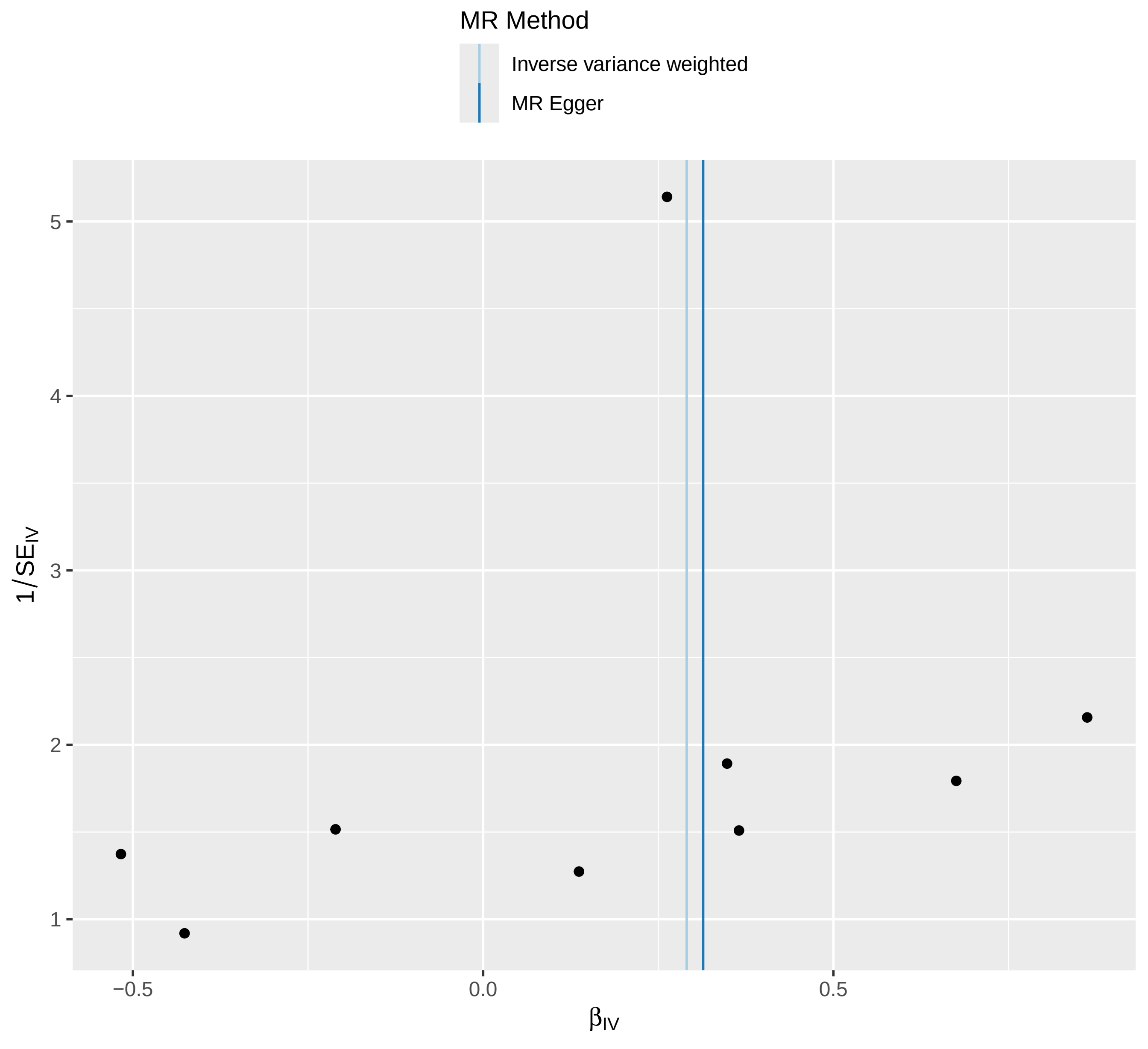
MR Method
Inverse variance weighted
MR Egger
1/SE_IV
5
4
3
2
1
−0.5
0.0
0.5
β_IV

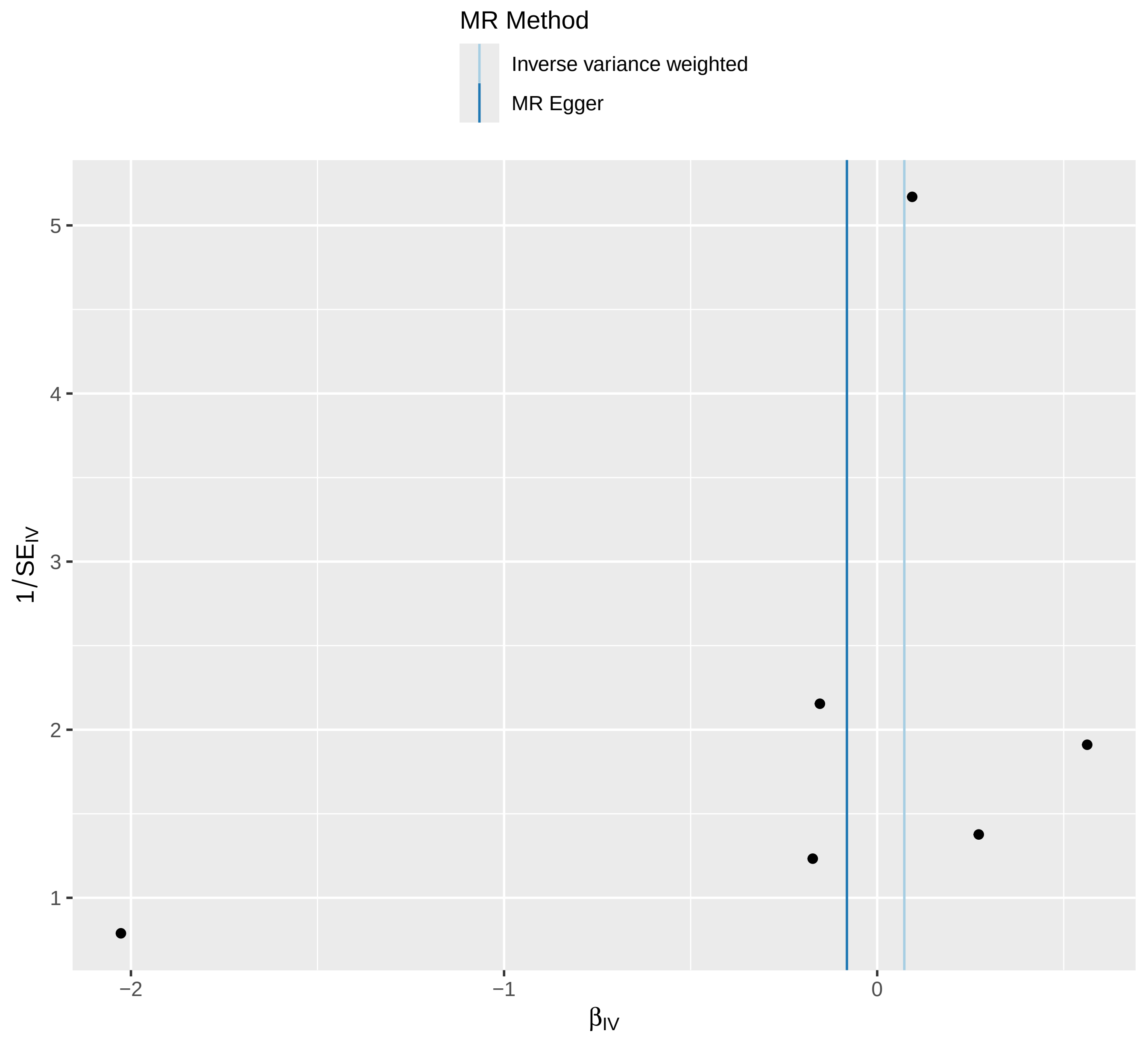

MR Method
Inverse variance weighted
MR Egger
5
4
3
2
1
1/SE_IV
−2
−1
0
β_IV

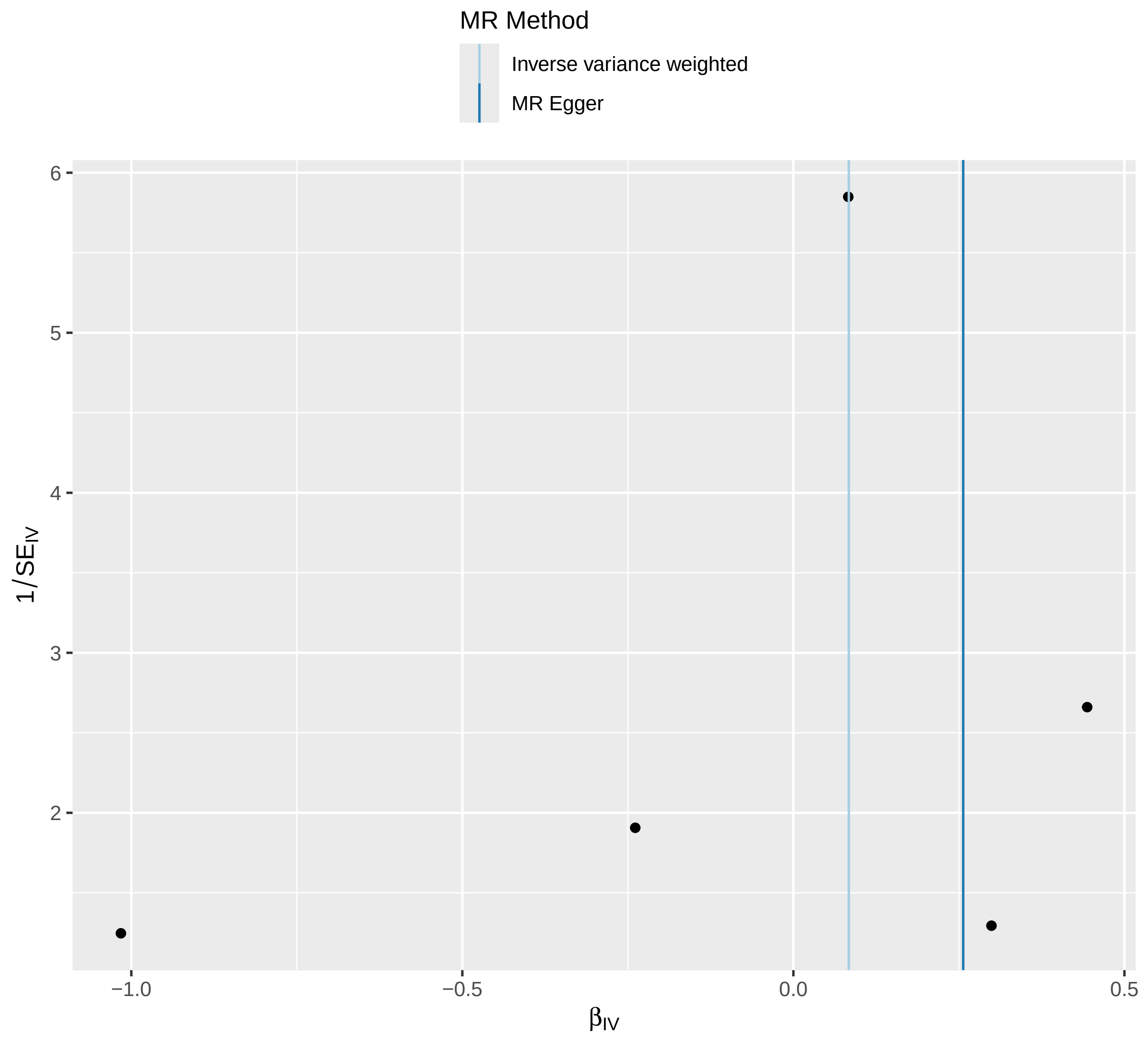
MR Method
Inverse variance weighted
MR Egger
6
5
4
3
2
1/SE_IV
−1.0
−0.5
0.0
0.5
β_IV

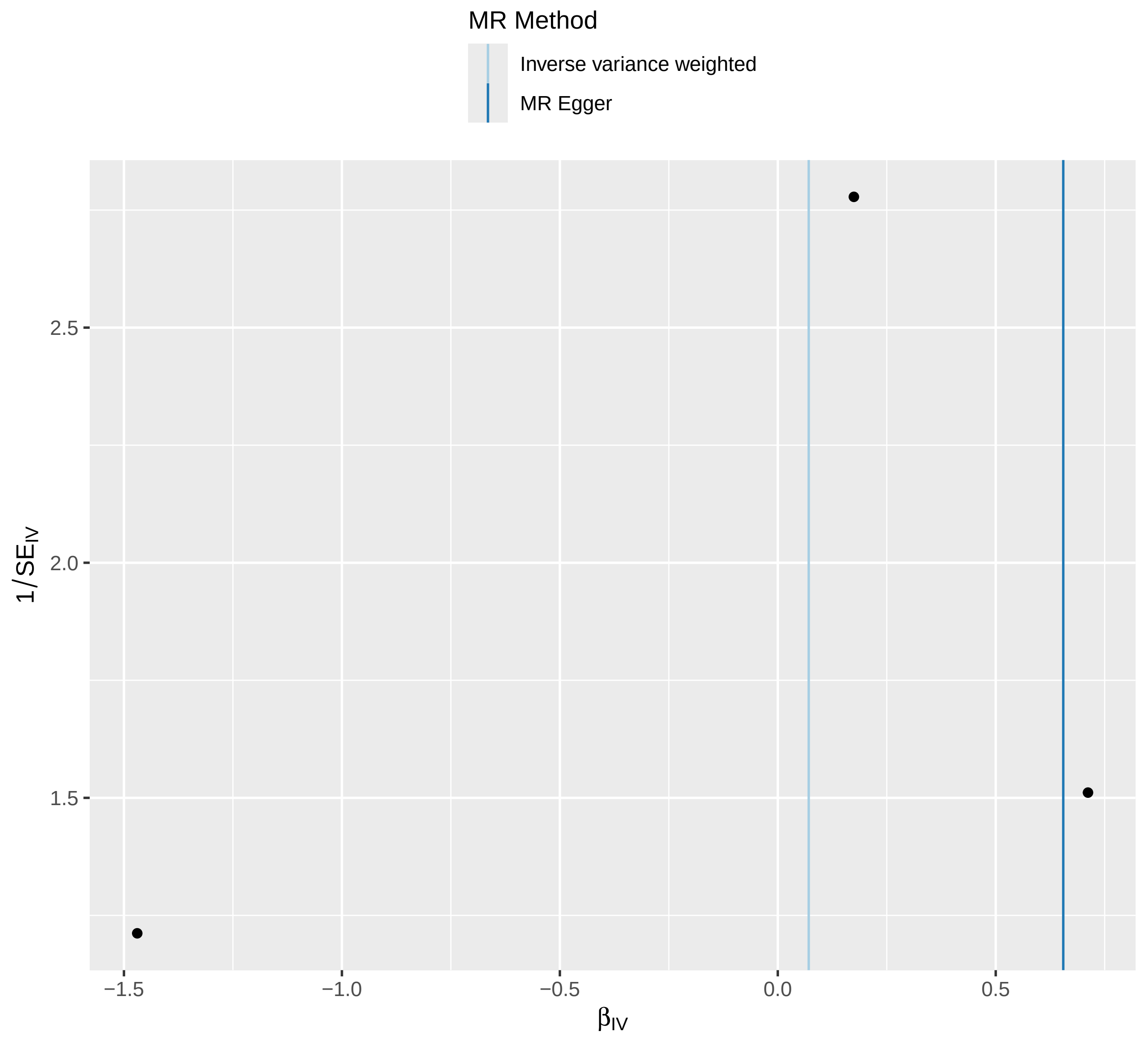
MR Method
Inverse variance weighted
MR Egger
2.5
2.0
1.5
1/SE_IV
−1.5
−1.0
−0.5
0.0
0.5
β_IV

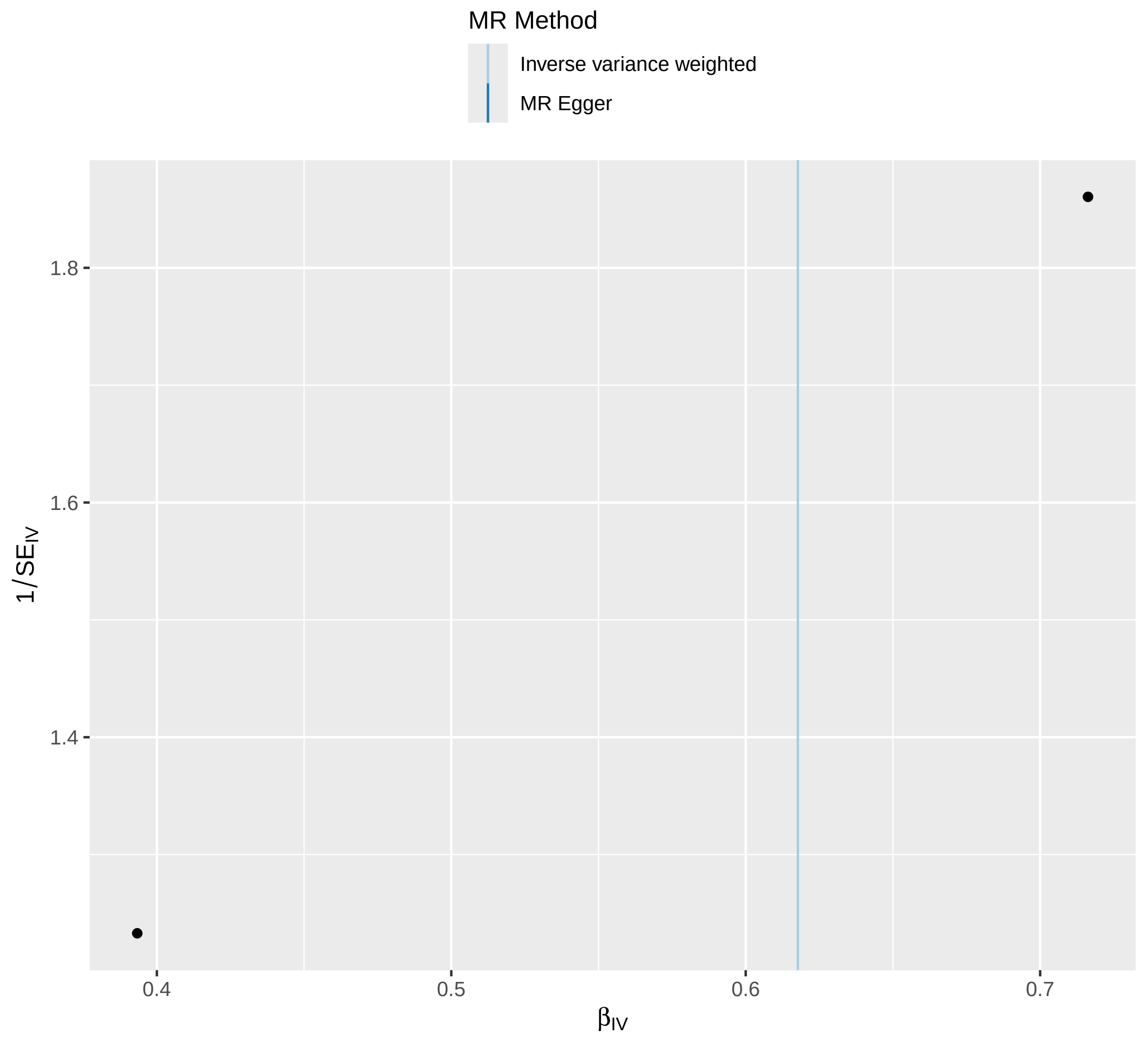
MR Method
Inverse variance weighted
MR Egger
1.8
1.6
1.4
0.4
0.5
0.6
0.7
$1/SE_{IV}$
β_{IV}

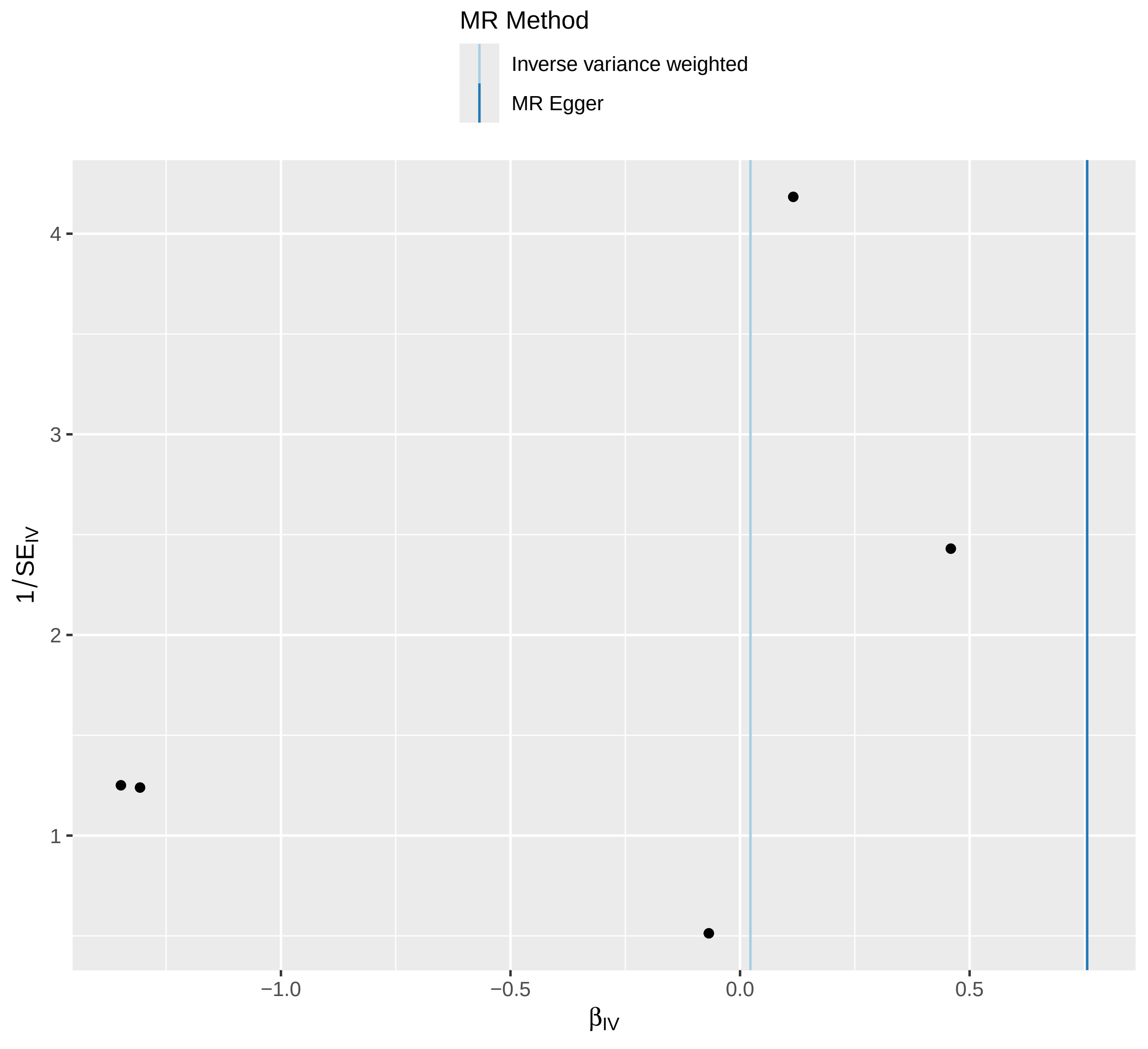

MR Method
Inverse variance weighted
MR Egger
4
3
2
1
$1/SE_{IV}$
−1.0
−0.5
0.0
0.5
β_{IV}

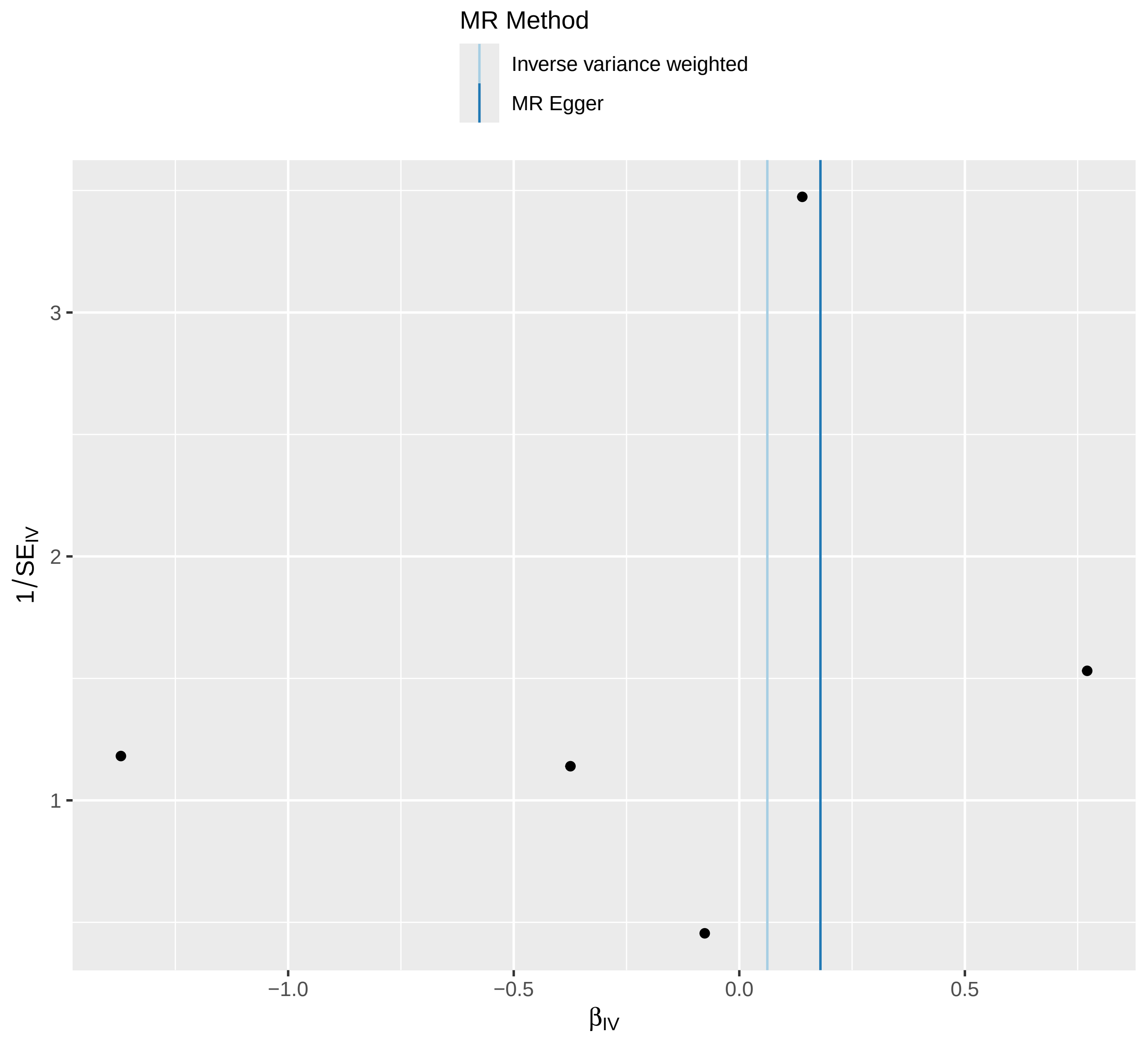
MR Method
Inverse variance weighted
MR Egger
3
2
1
1/SE_IV
−1.0
−0.5
0.0
0.5
β_IV

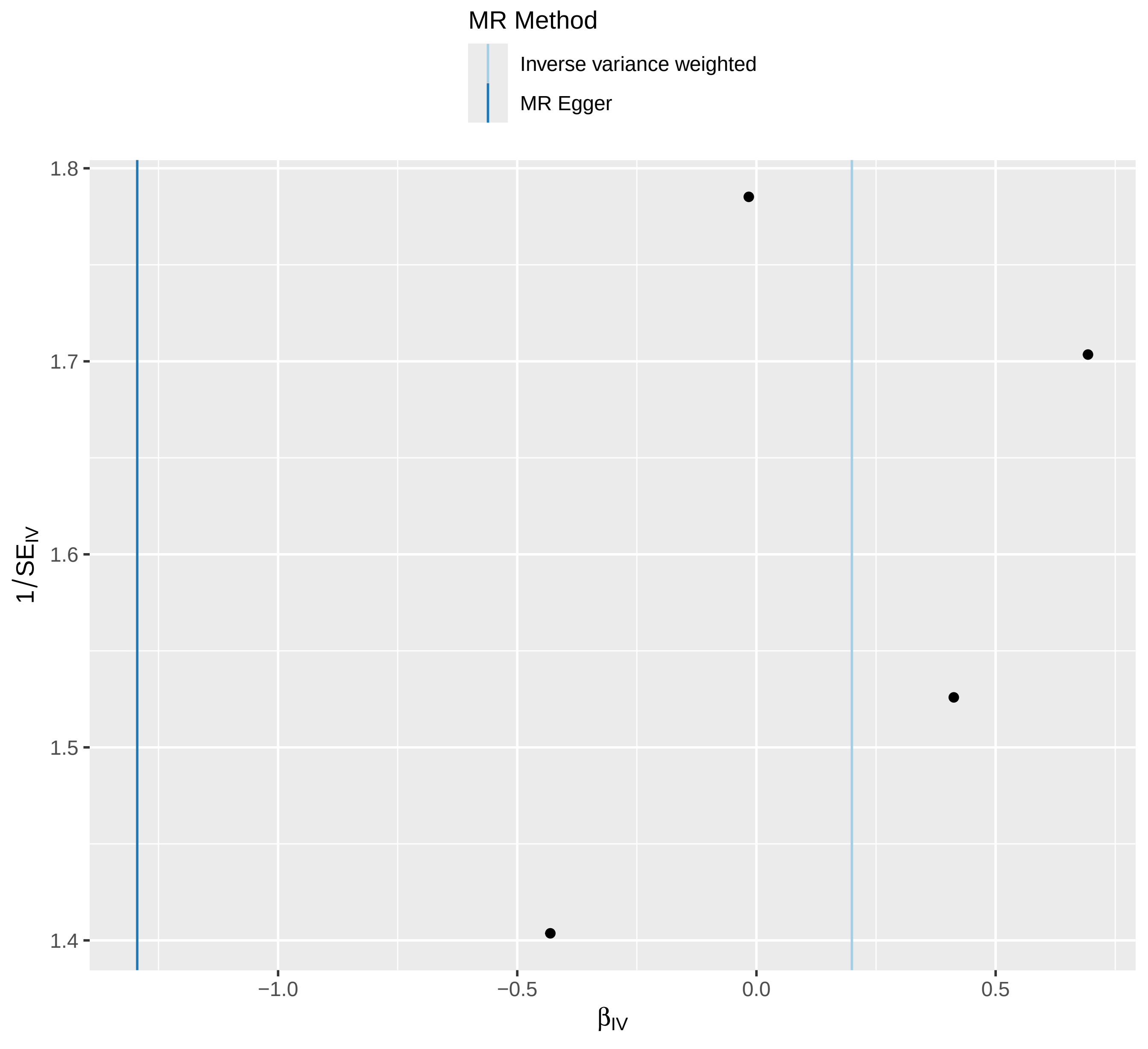

MR Method
Inverse variance weighted
MR Egger
1.8
1.7
1.6
1.5
1.4
1/SE_IV
−1.0
−0.5
0.0
0.5
β_IV

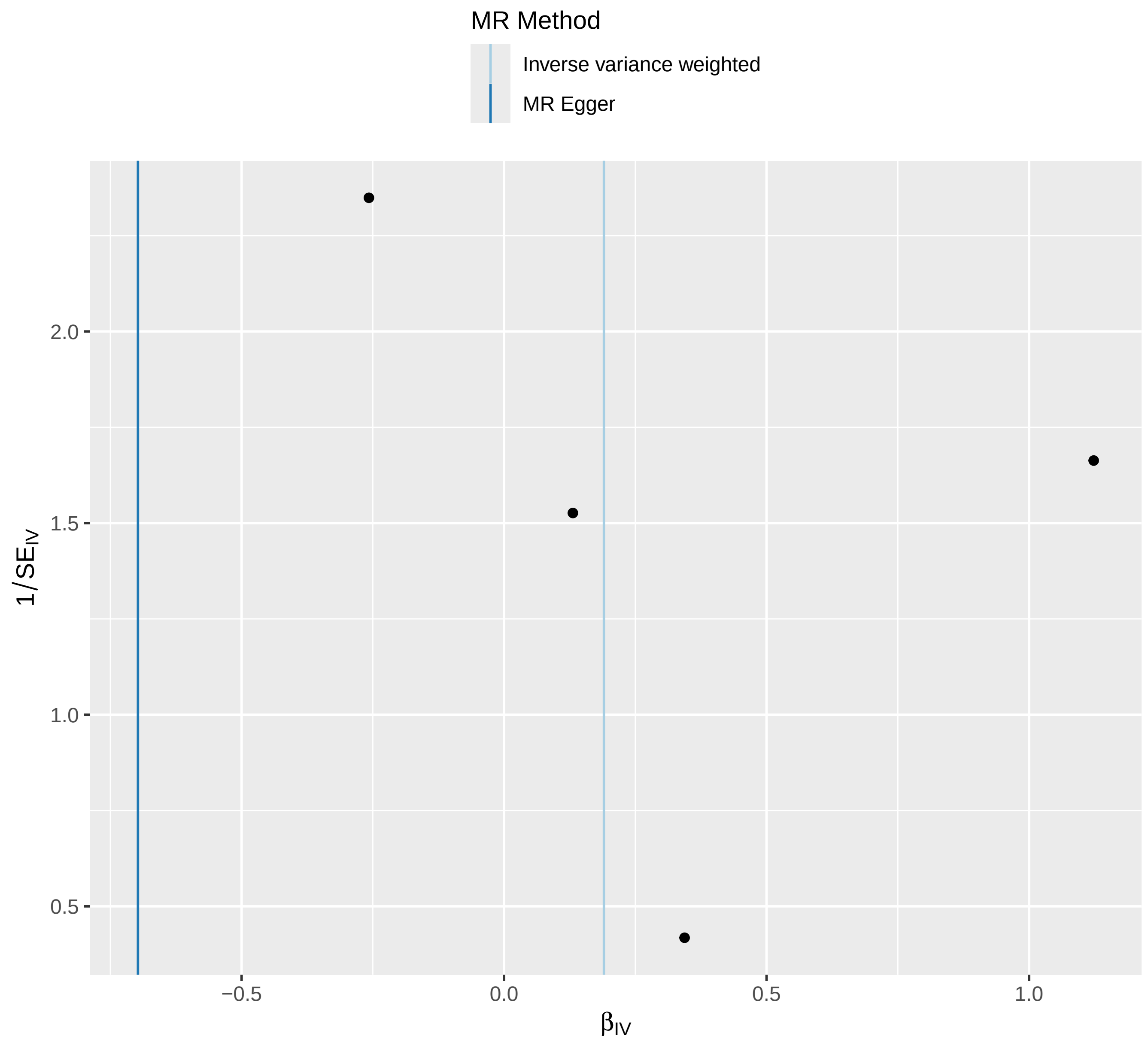
MR Method
Inverse variance weighted
MR Egger
2.0
1.5
1.0
0.5
1/SE_IV
−0.5
0.0
0.5
1.0
β_IV

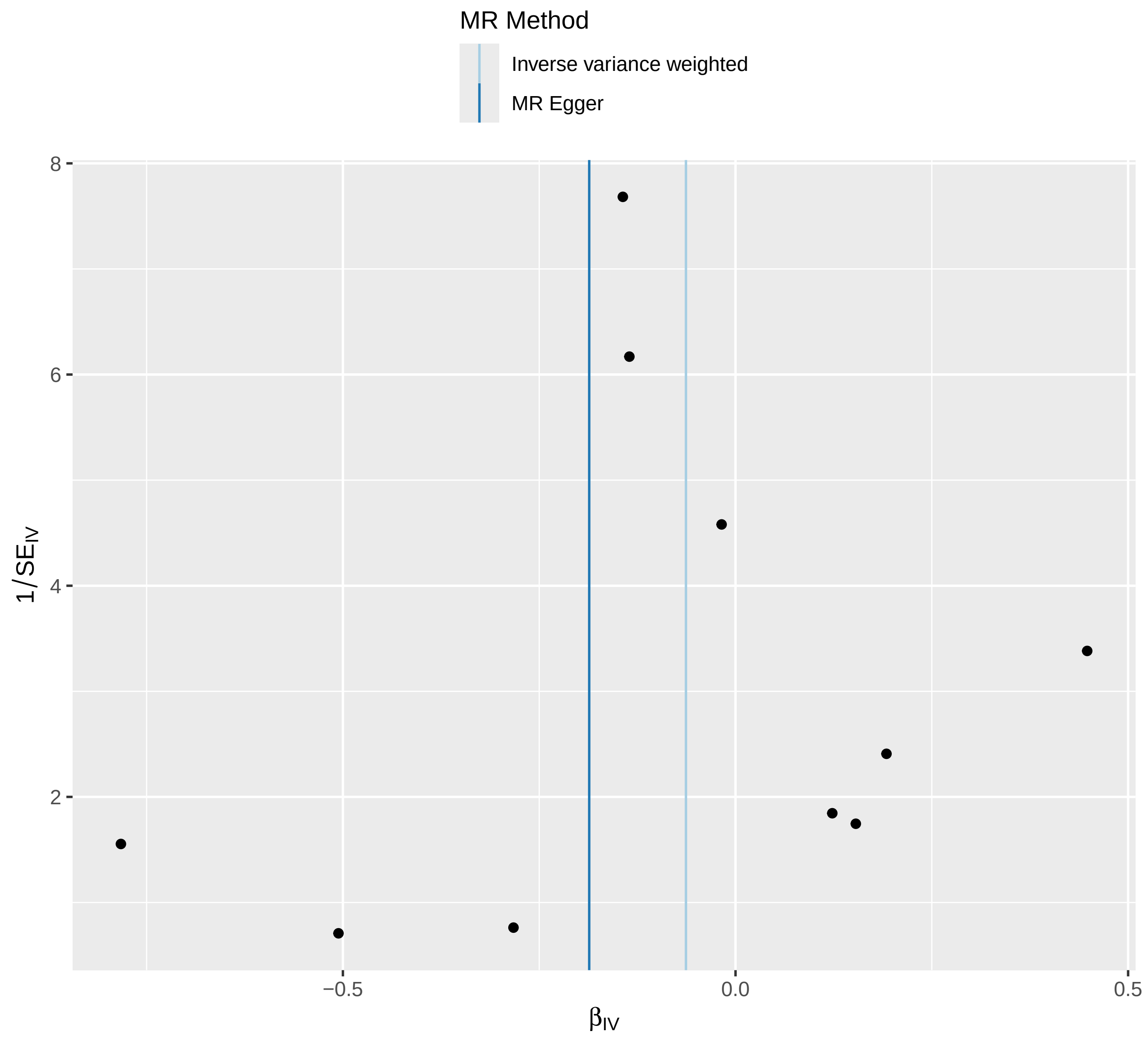
MR Method
Inverse variance weighted
MR Egger
8
6
4
2
1/SE_IV
−0.5
0.0
0.5
β_IV

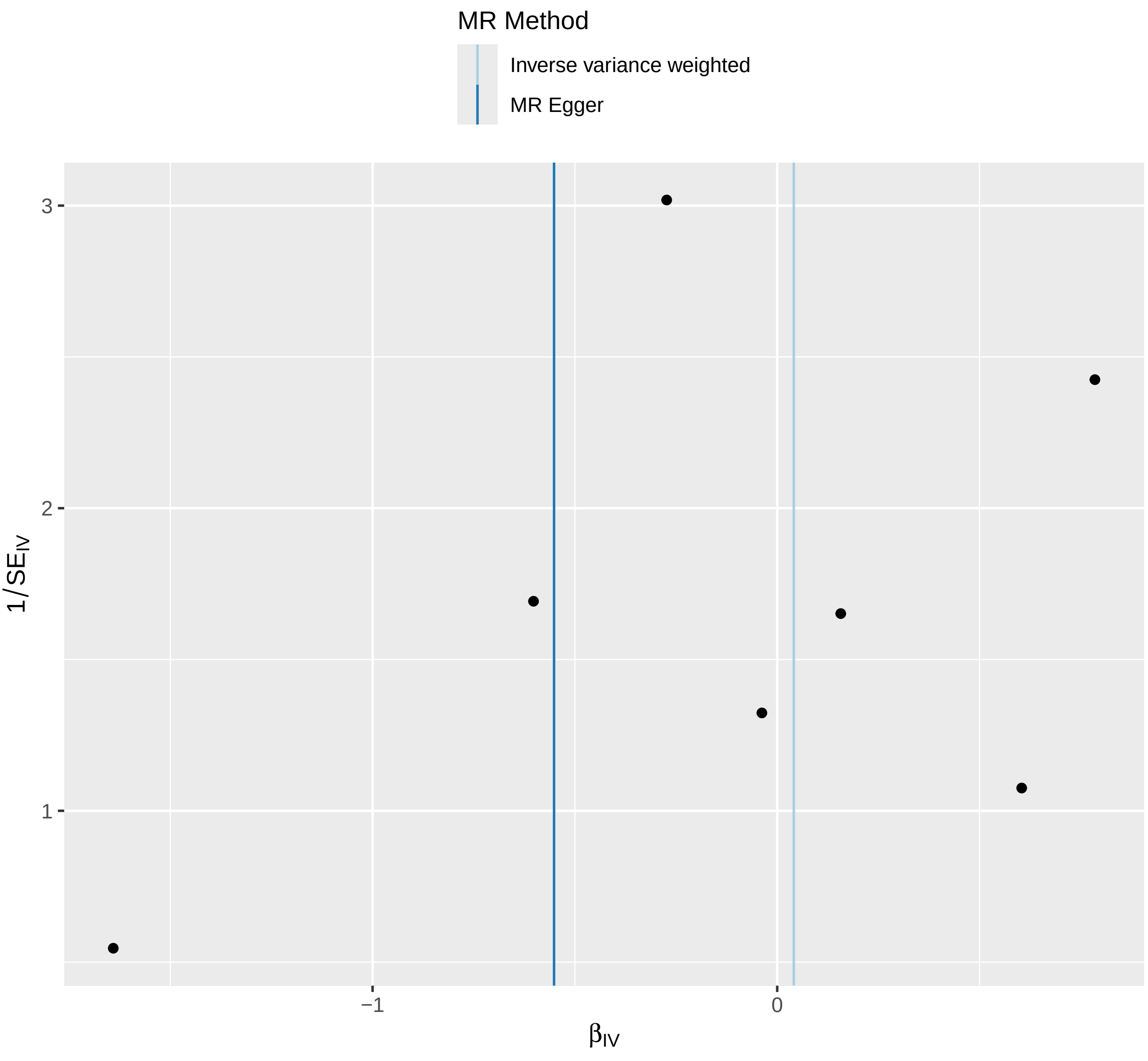

MR Method
Inverse variance weighted
MR Egger
3
2
1
1/SE_IV
−1
0
β_IV

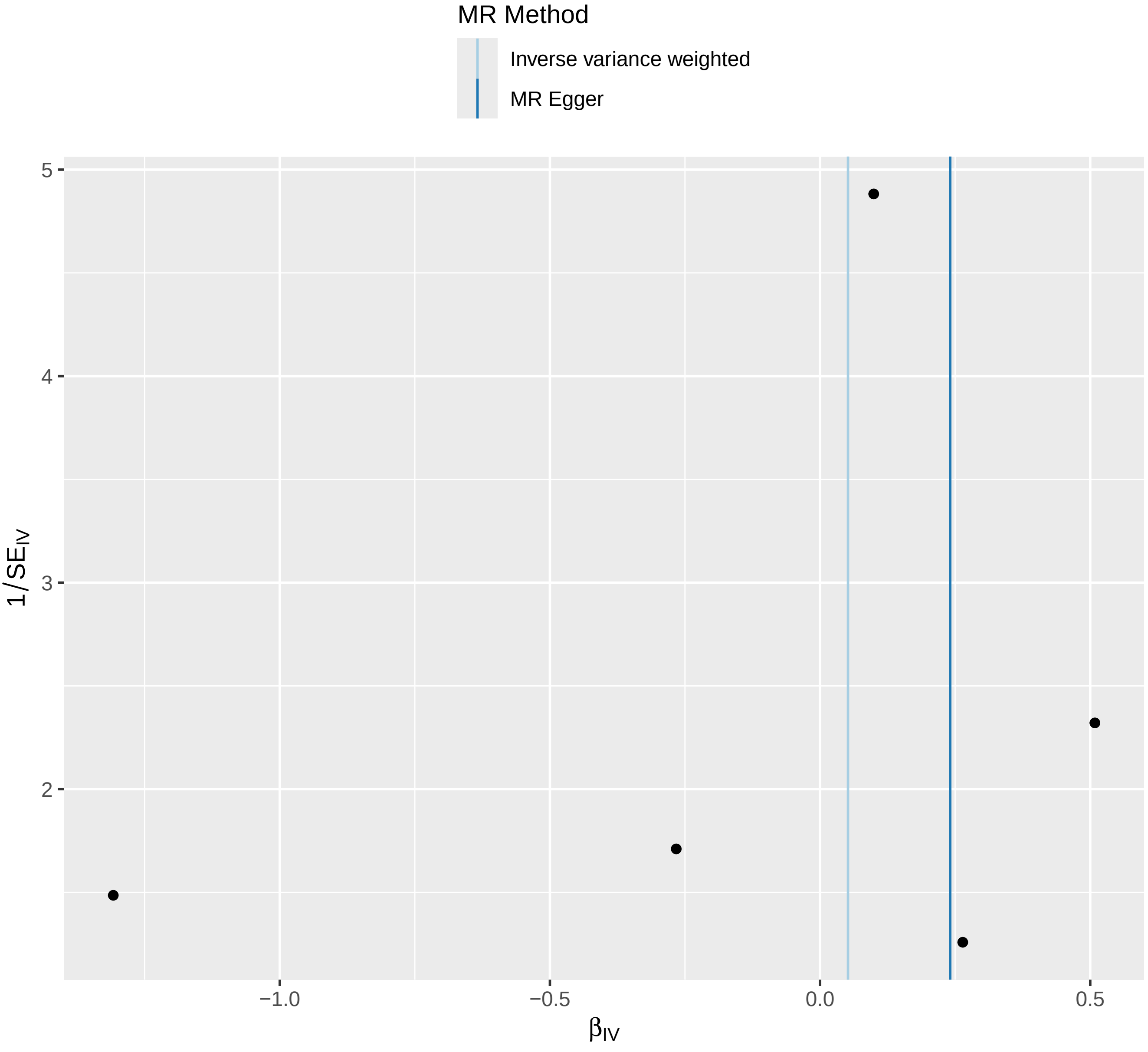

MR Method
Inverse variance weighted
MR Egger
5
4
3
2
1/SE$_{IV}$
−1.0
−0.5
0.0
0.5
β_{IV}

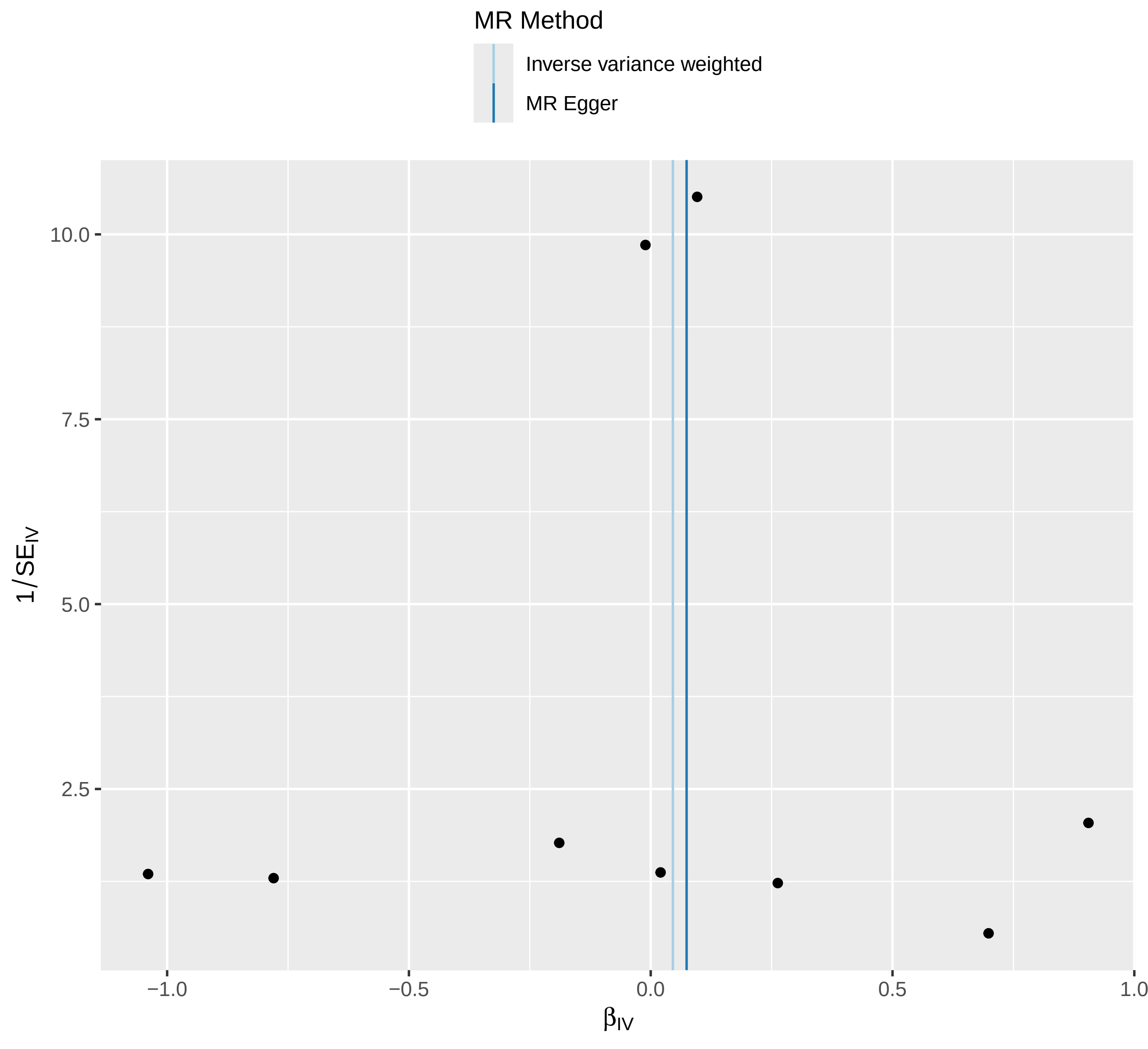

MR Method
Inverse variance weighted
MR Egger
10.0
7.5
5.0
2.5
1/SE_IV
−1.0
−0.5
0.0
0.5
1.0
β_IV

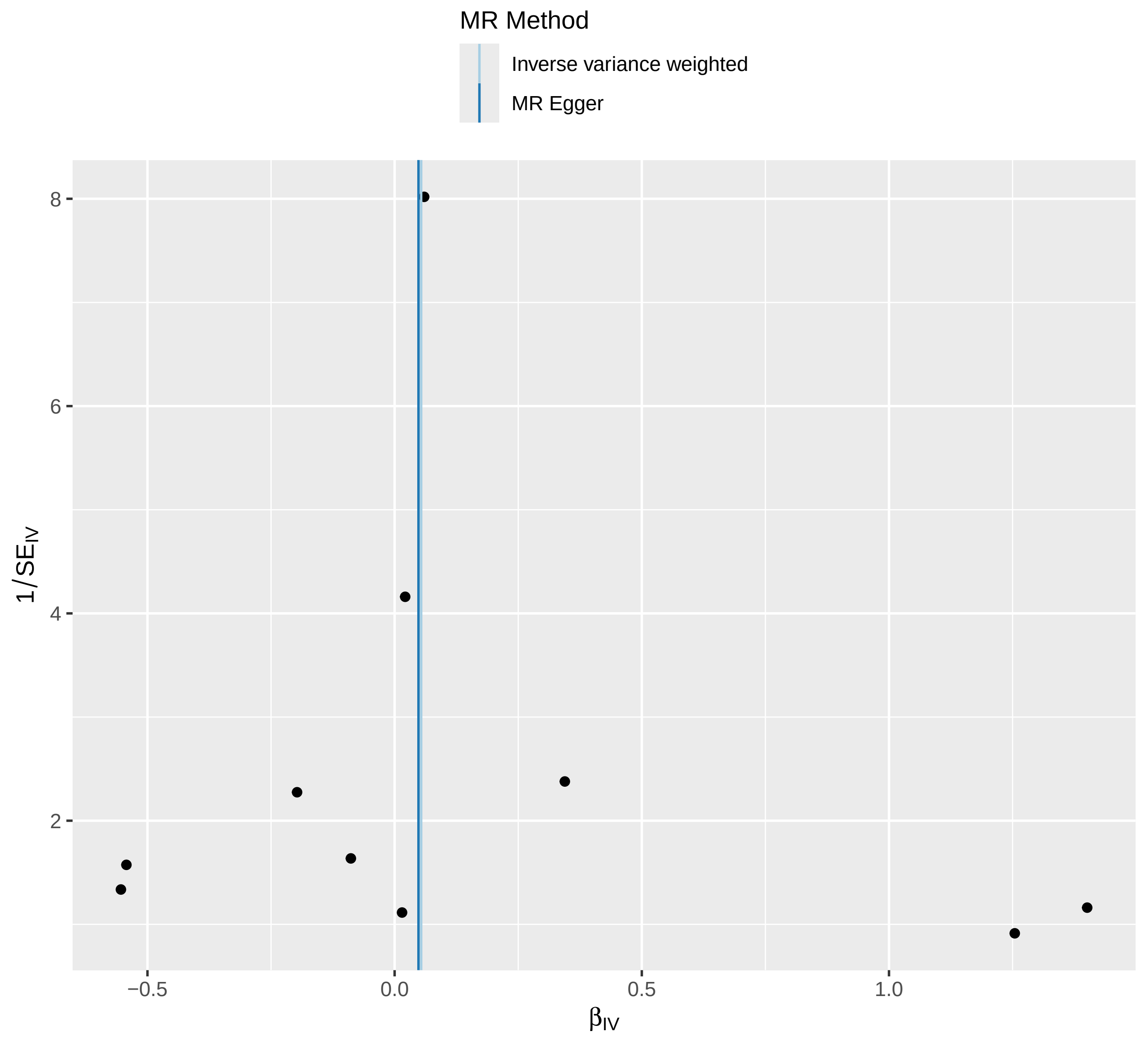

MR Method
Inverse variance weighted
MR Egger
1/SE_IV
8
6
4
2
−0.5
0.0
0.5
1.0
β_IV

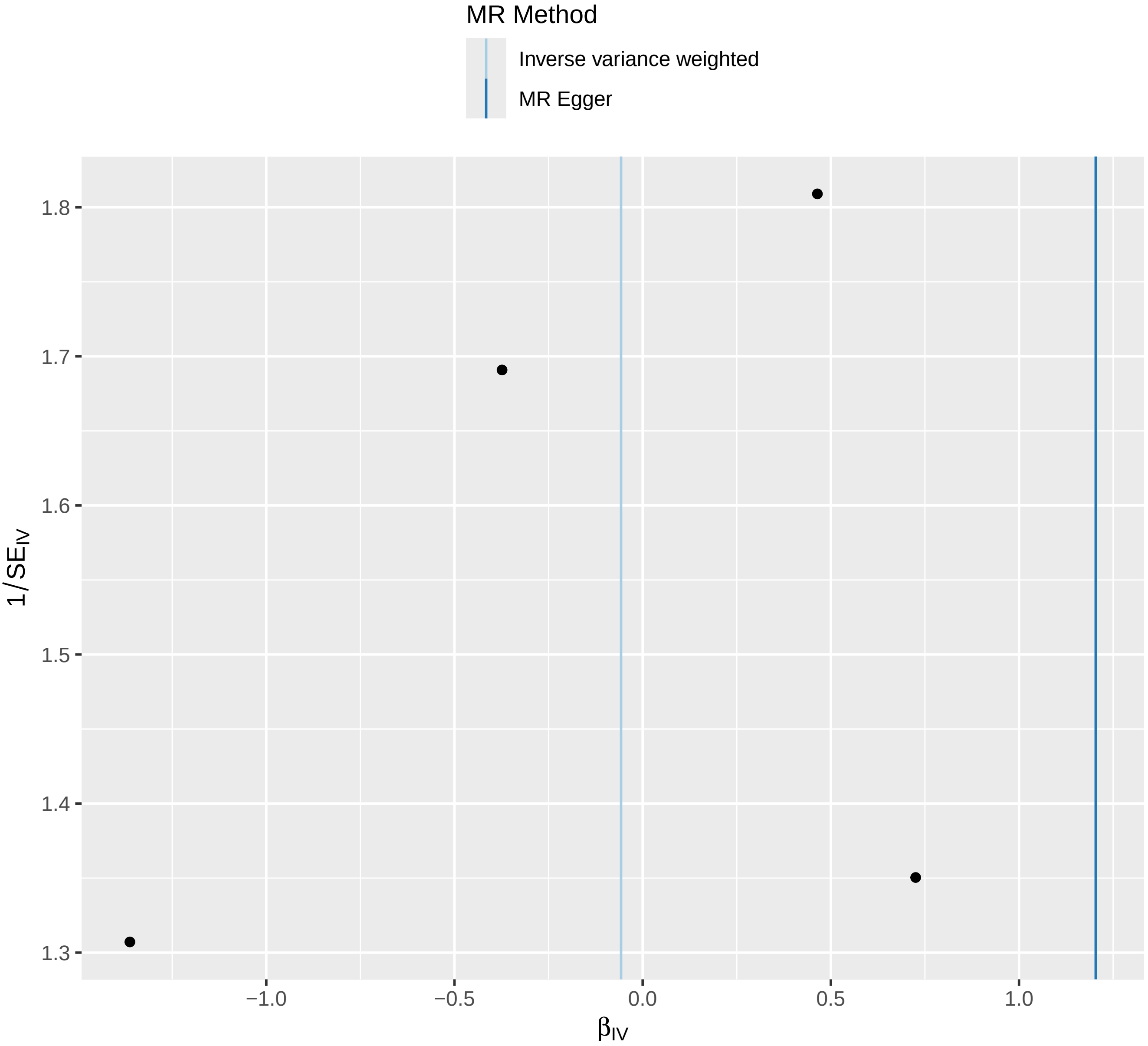
MR Method
Inverse variance weighted
MR Egger
1/SE$_{IV}$
1.8
1.7
1.6
1.5
1.4
1.3
−1.0
−0.5
0.0
0.5
1.0
β$_{IV}$

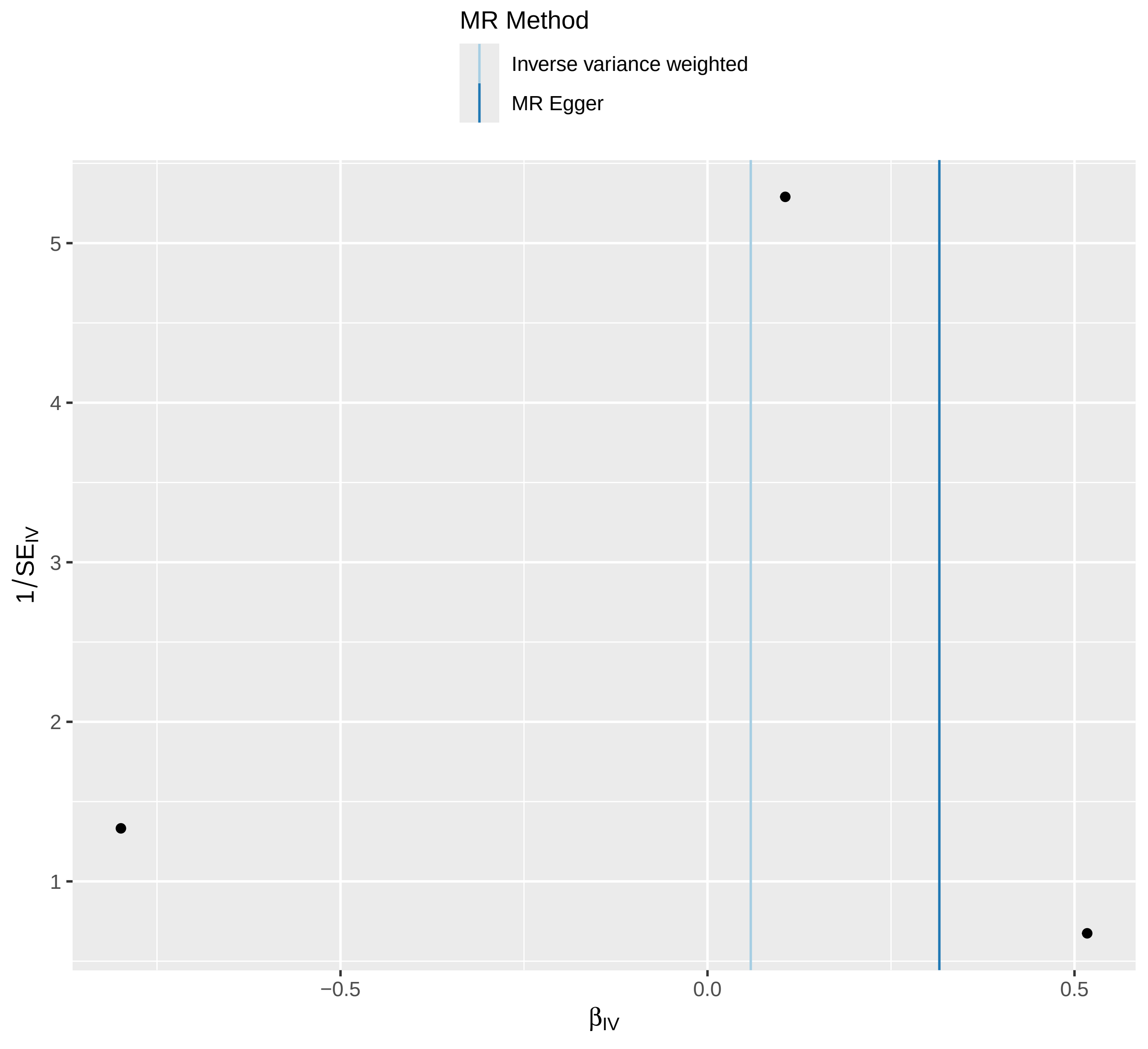
MR Method
Inverse variance weighted
MR Egger
1/SE_IV
5
4
3
2
1
−0.5
0.0
0.5
β_IV

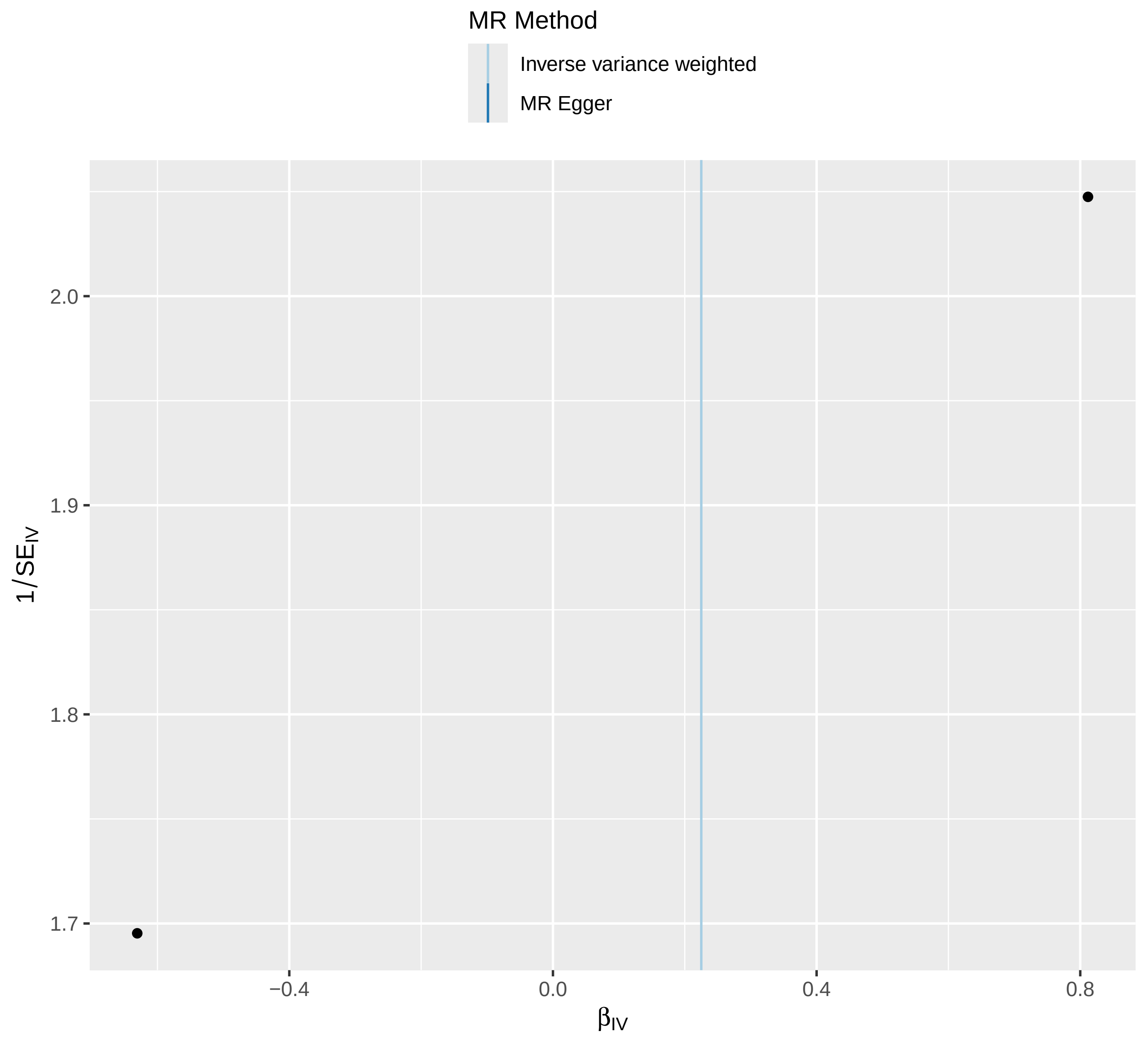
MR Method
Inverse variance weighted
MR Egger
2.0
1.9
1.8
1.7
1/SE$_{IV}$
−0.4
0.0
0.4
0.8
β$_{IV}$

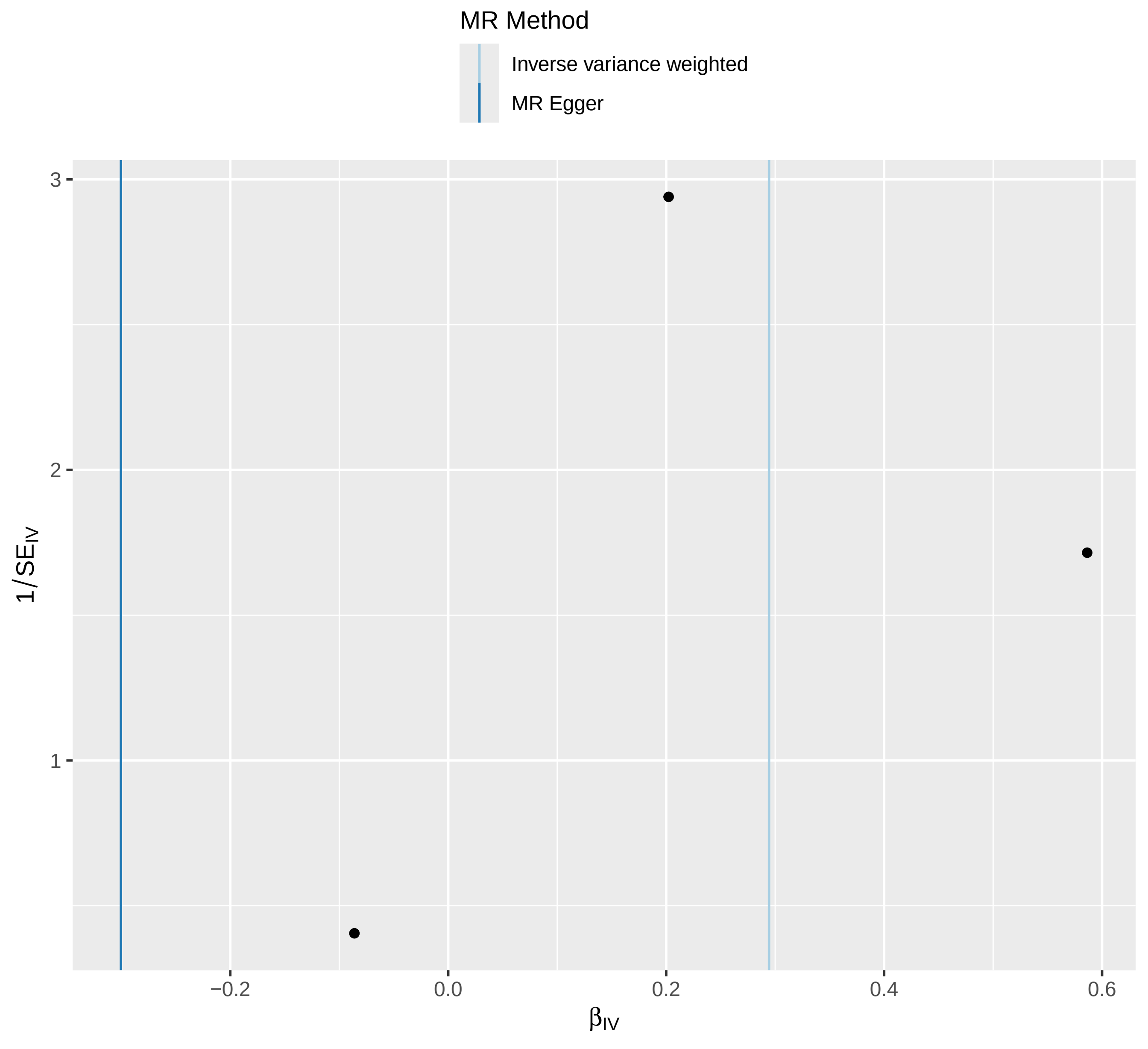

MR Method
Inverse variance weighted
MR Egger
3
2
1
1/SE_IV
−0.2
0.0
0.2
0.4
0.6
β_IV

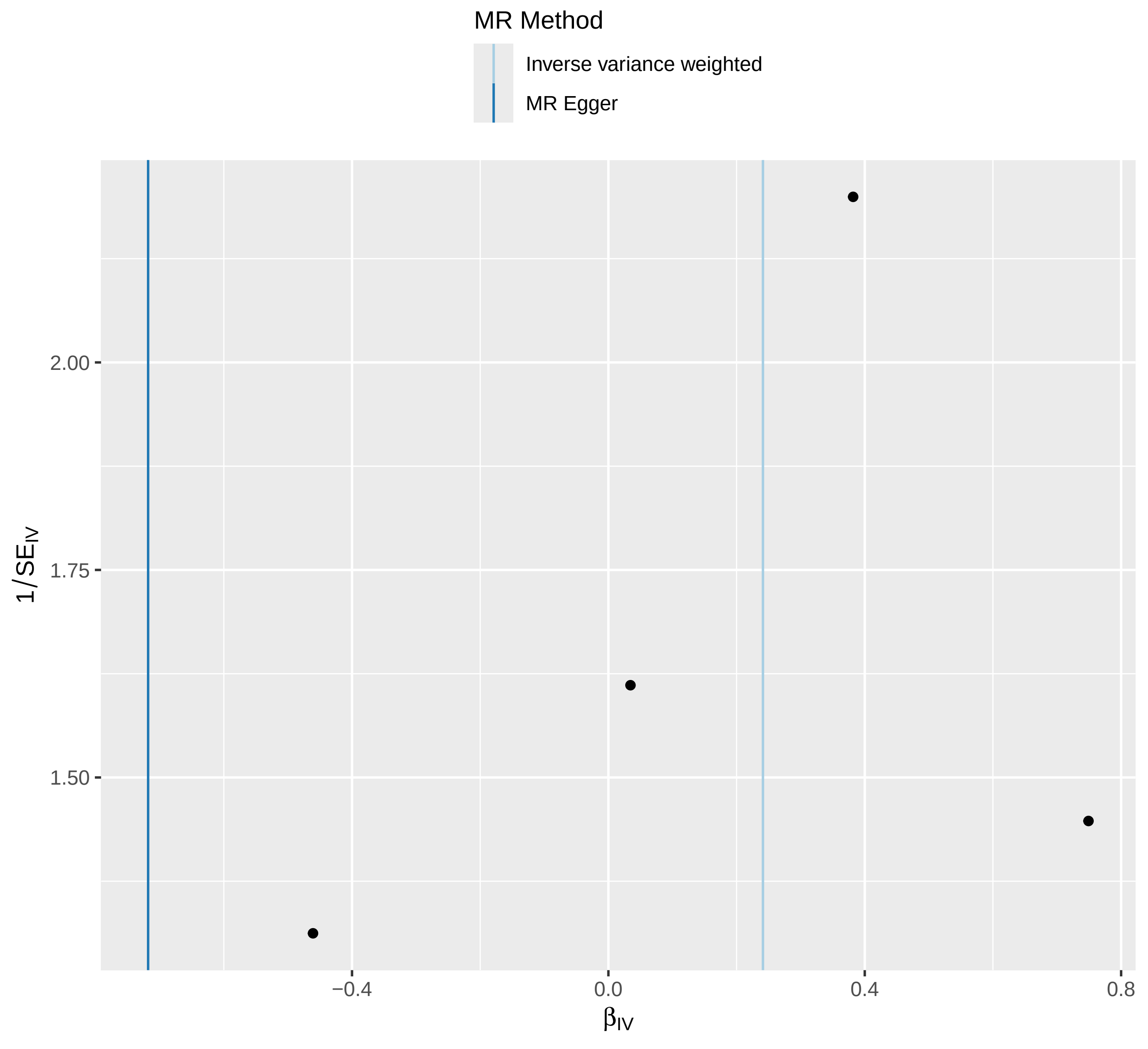
MR Method
Inverse variance weighted
MR Egger
2.00
1.75
1.50
1/SE_{IV}
−0.4
0.0
0.4
0.8
β_{IV}

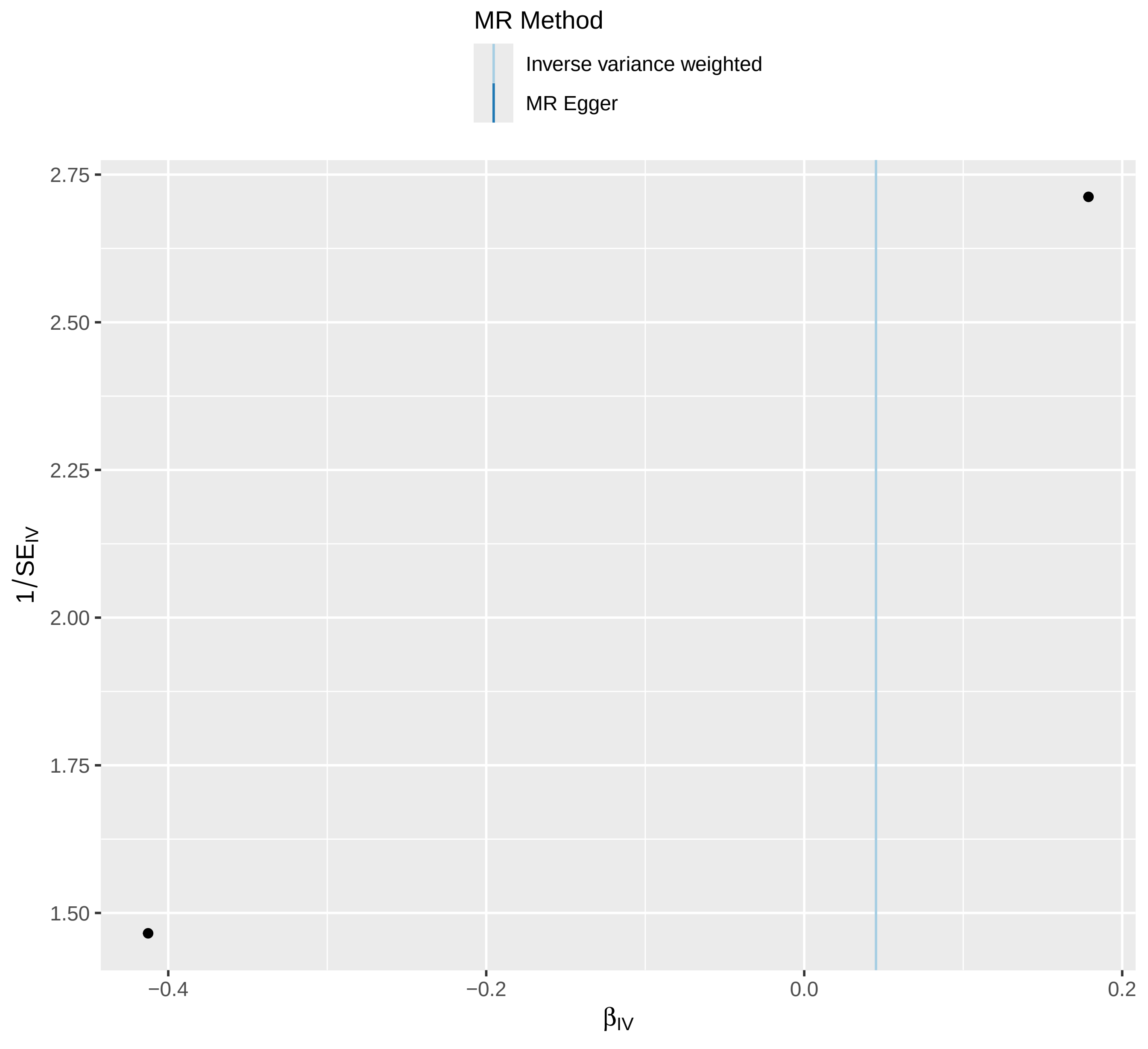

MR Method
Inverse variance weighted
MR Egger
2.75
2.50
2.25
2.00
1.75
1.50
1/SE_IV
−0.4
−0.2
0.0
0.2
β_IV

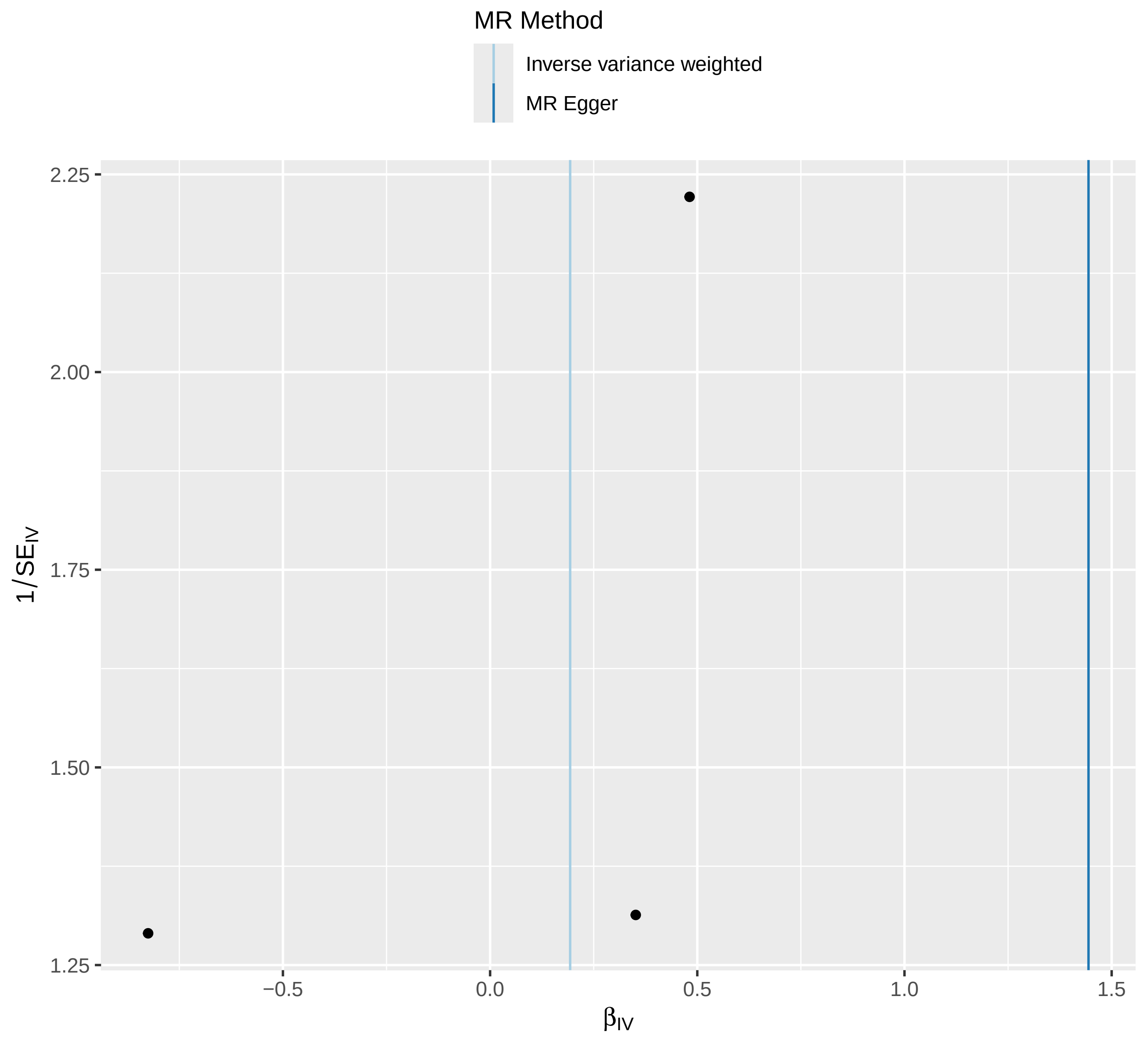
MR Method
Inverse variance weighted
MR Egger
2.25
2.00
1.75
1.50
1.25
−0.5
0.0
0.5
1.0
1.5
$1/SE_{IV}$
β_{IV}

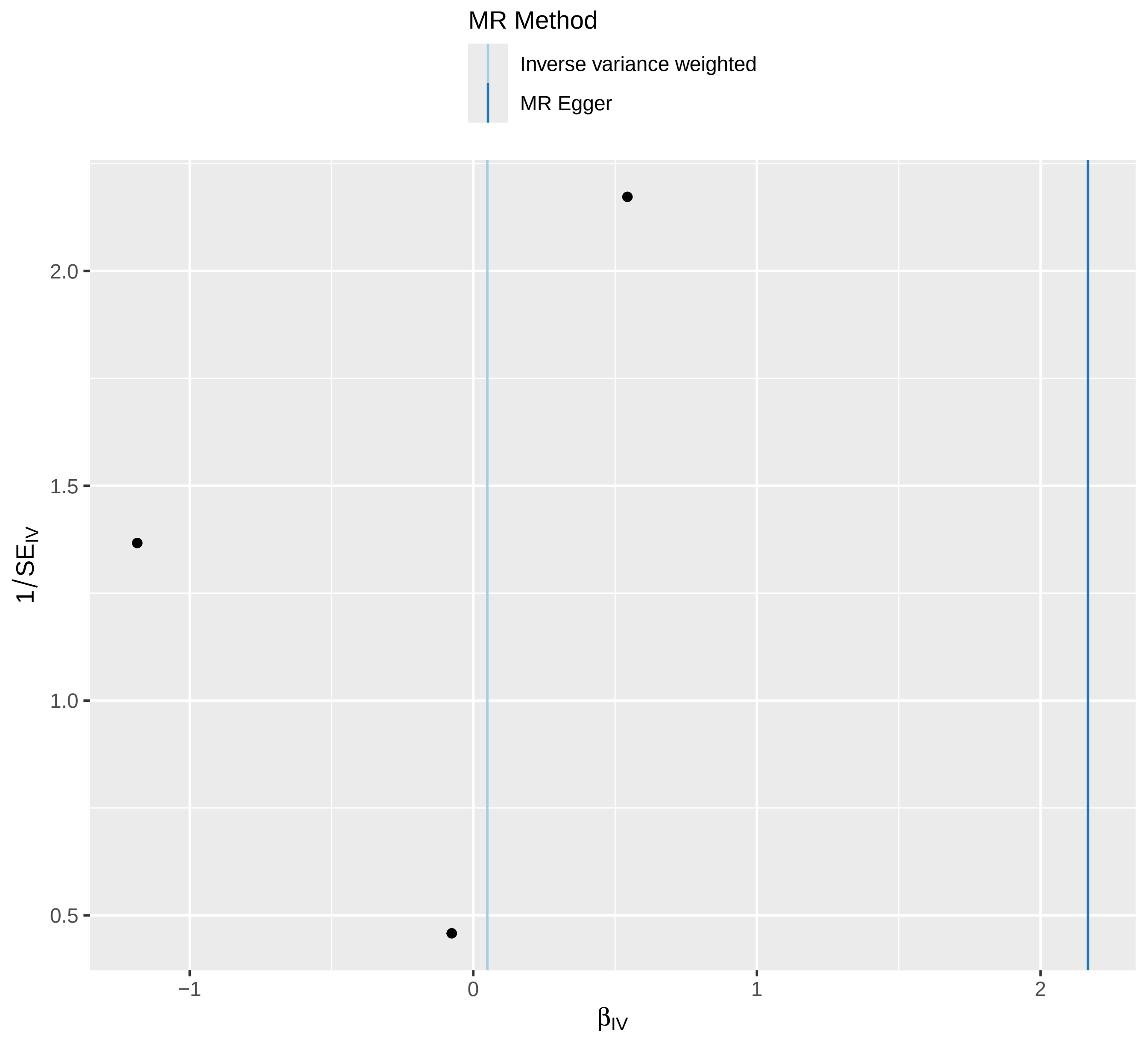
MR Method
Inverse variance weighted
MR Egger
2.0
1.5
1.0
0.5
1/SE_IV
−1
0
1
2
β_IV

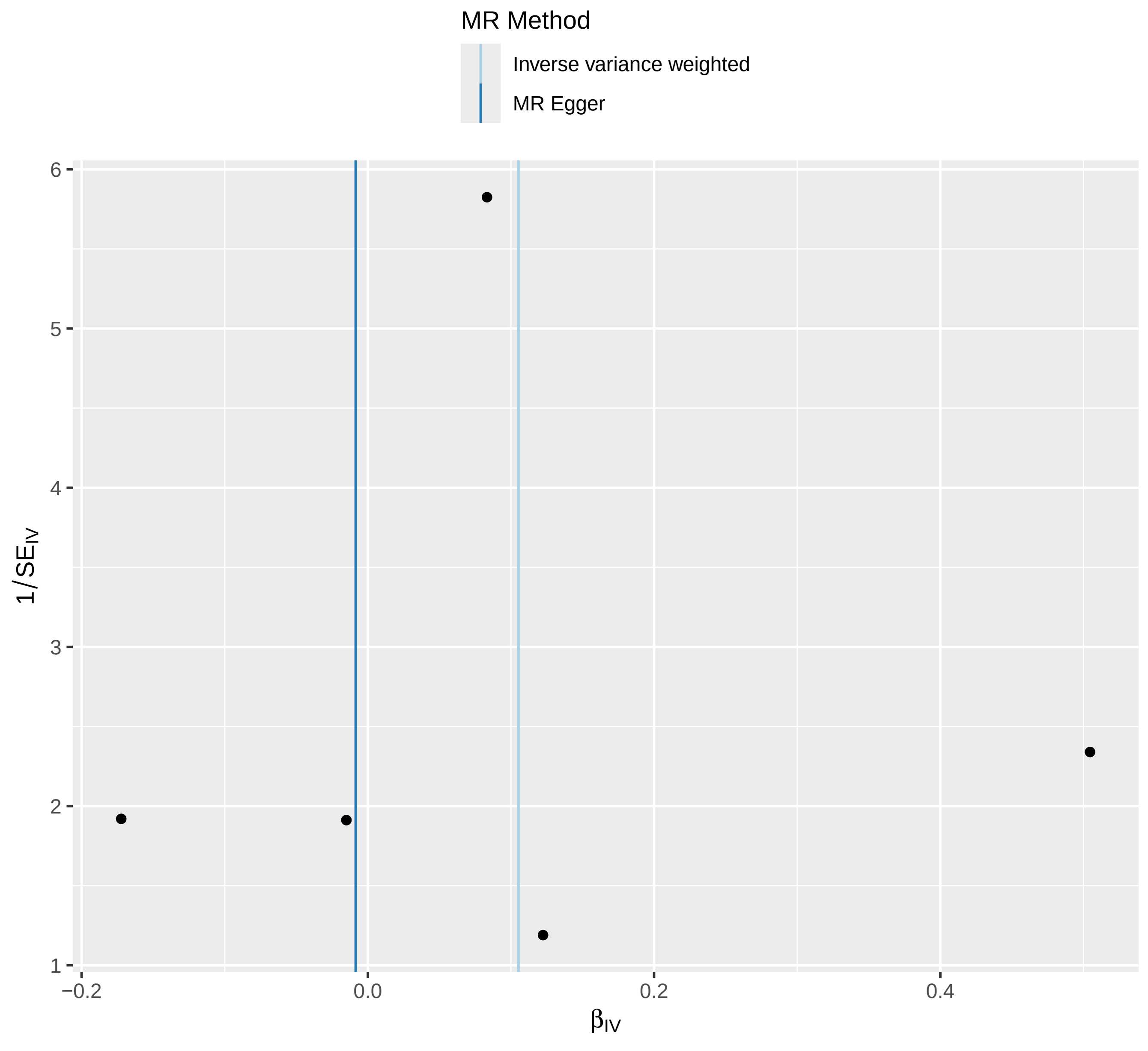
MR Method
Inverse variance weighted
MR Egger
1/SE_IV
β_IV
6
5
4
3
2
1
−0.2
0.0
0.2
0.4

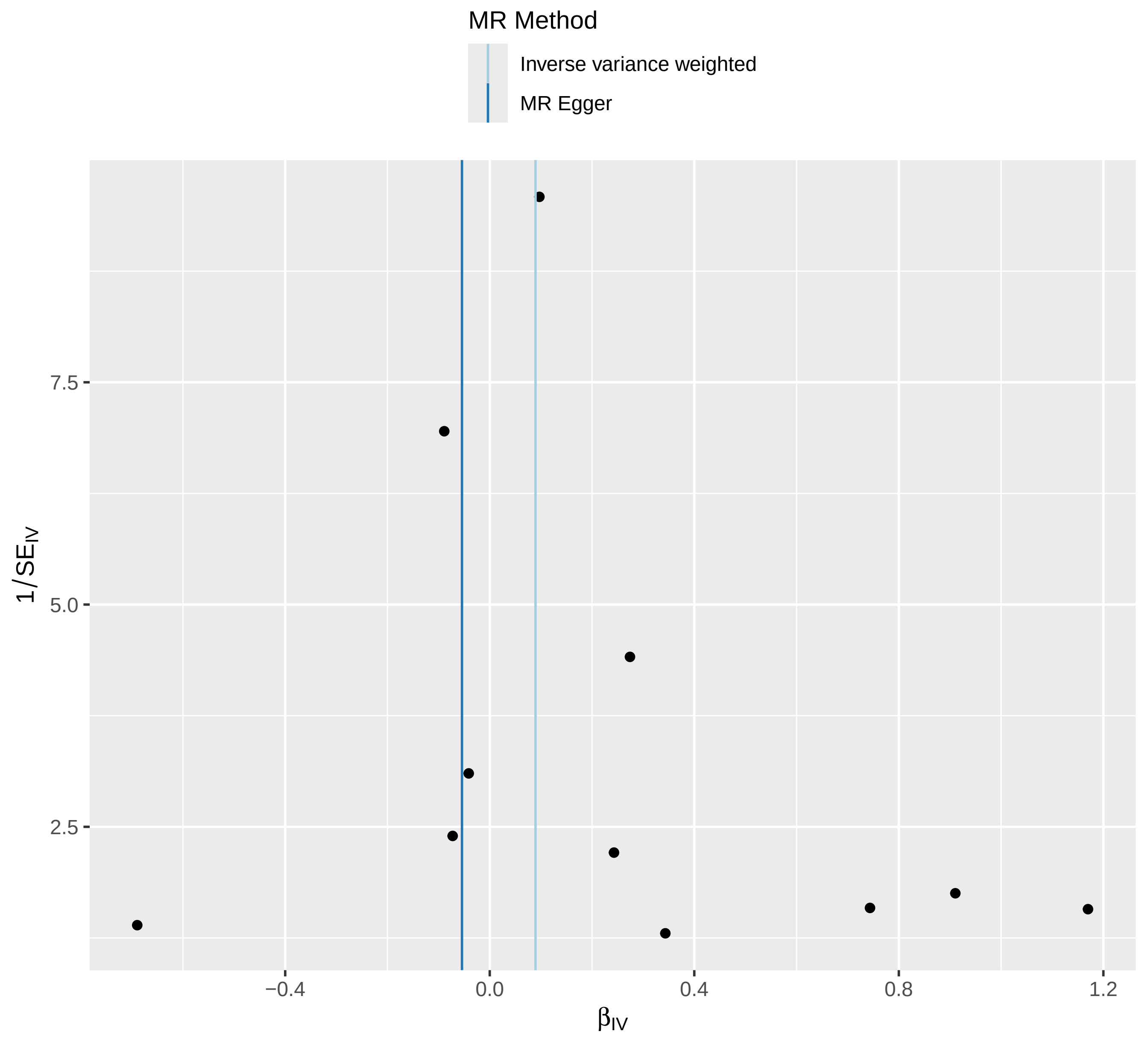

MR Method
Inverse variance weighted
MR Egger
7.5
5.0
2.5
1/SE_IV
−0.4
0.0
0.4
0.8
1.2
β_IV

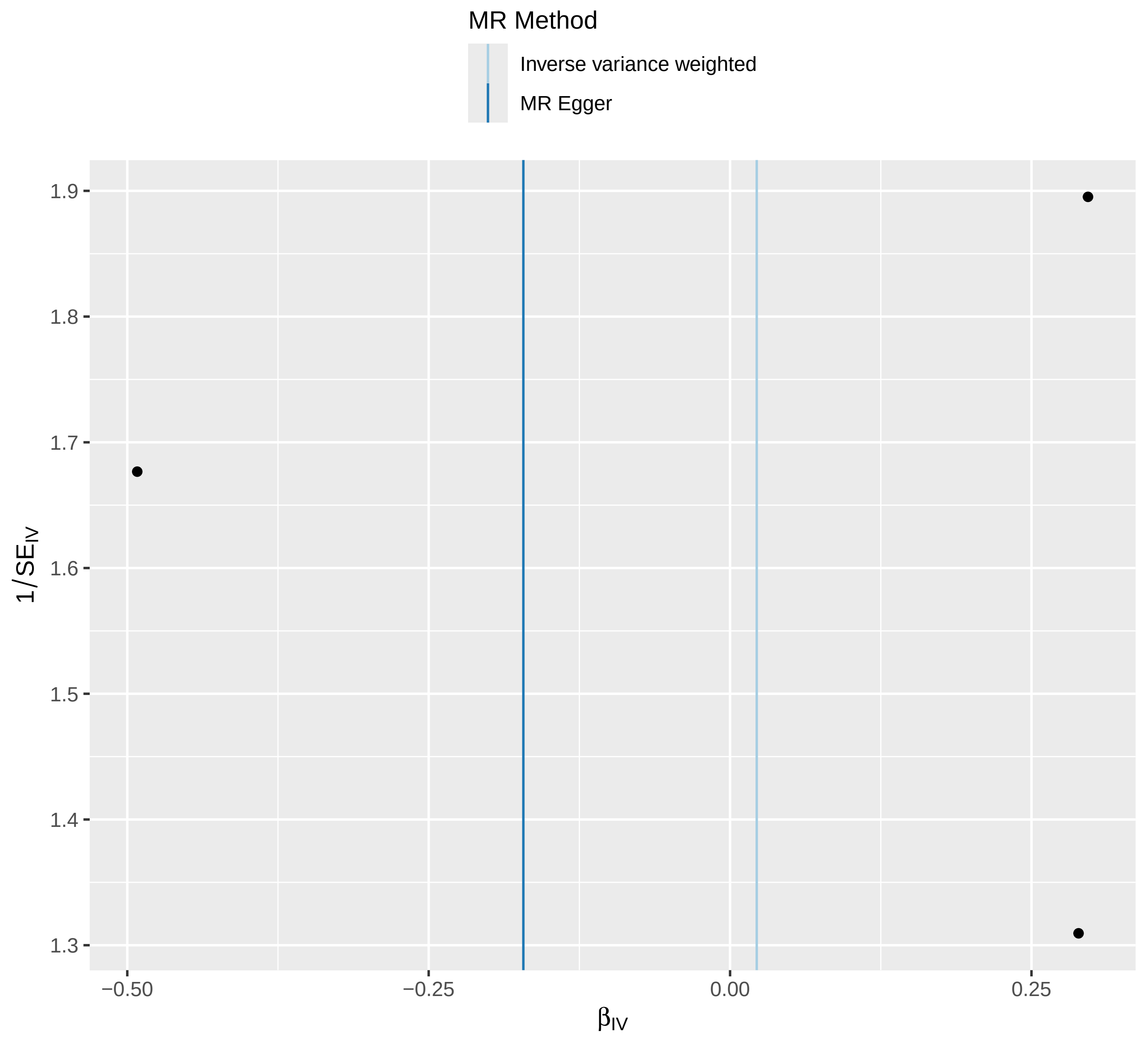
MR Method
Inverse variance weighted
MR Egger
1.9
1.8
1.7
1.6
1.5
1.4
1.3
1/SE_IV
−0.50
−0.25
0.00
0.25
β_IV

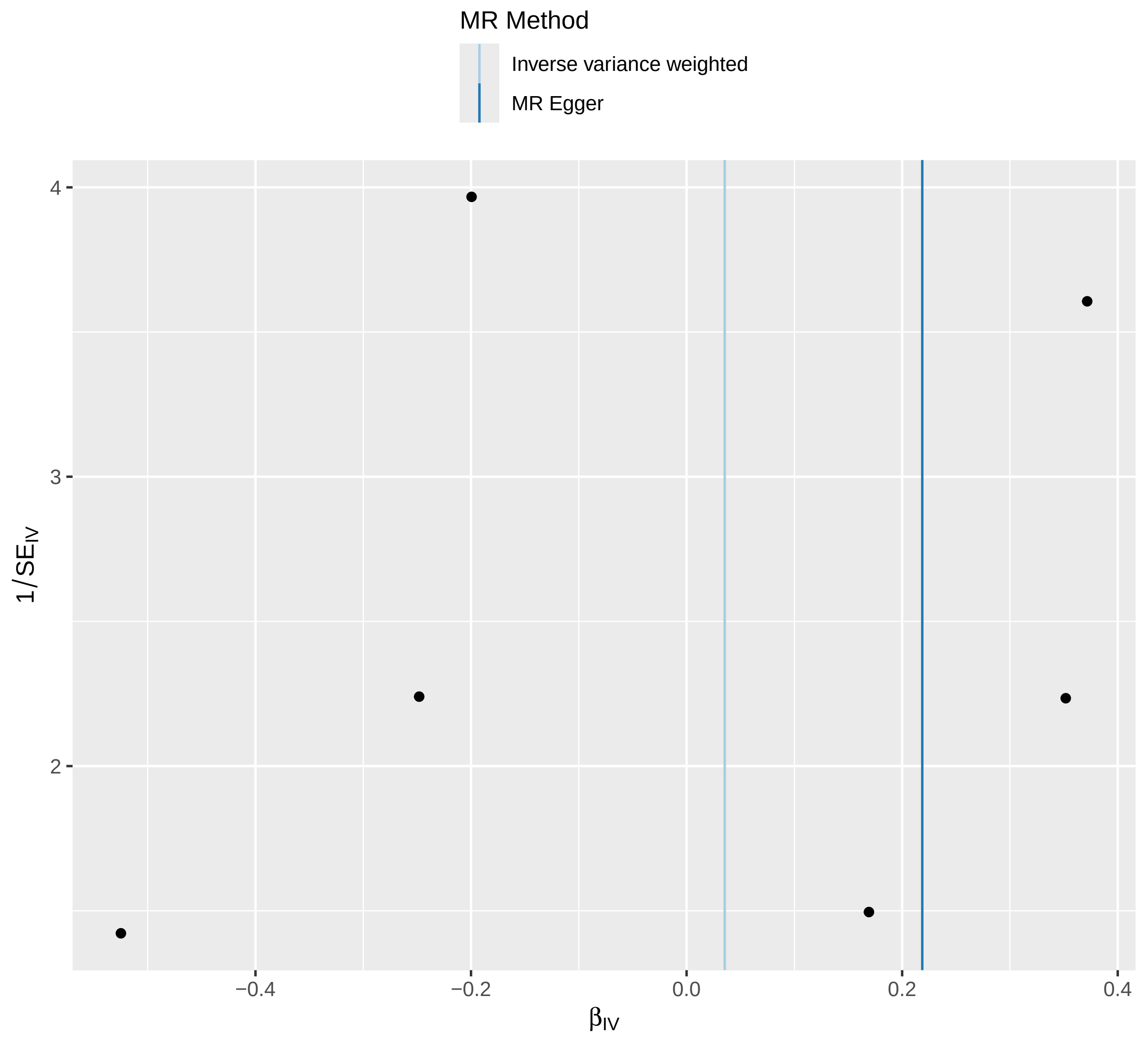

MR Method
Inverse variance weighted
MR Egger
4
3
2
1/SE_IV
−0.4
−0.2
0.0
0.2
0.4
β_IV

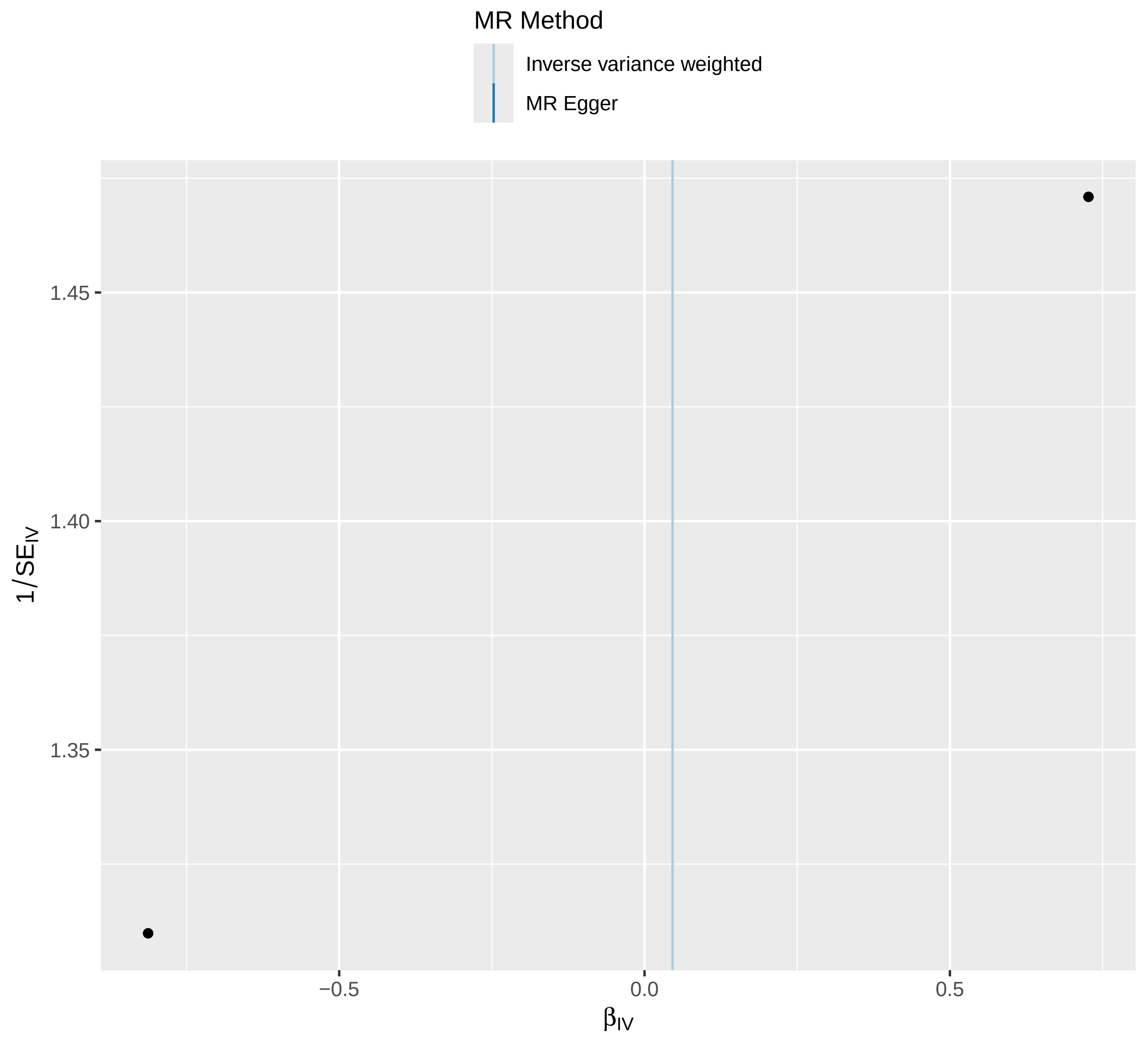
MR Method
Inverse variance weighted
MR Egger
1.45
1.40
1.35
1/SE_IV
−0.5
0.0
0.5
β_IV

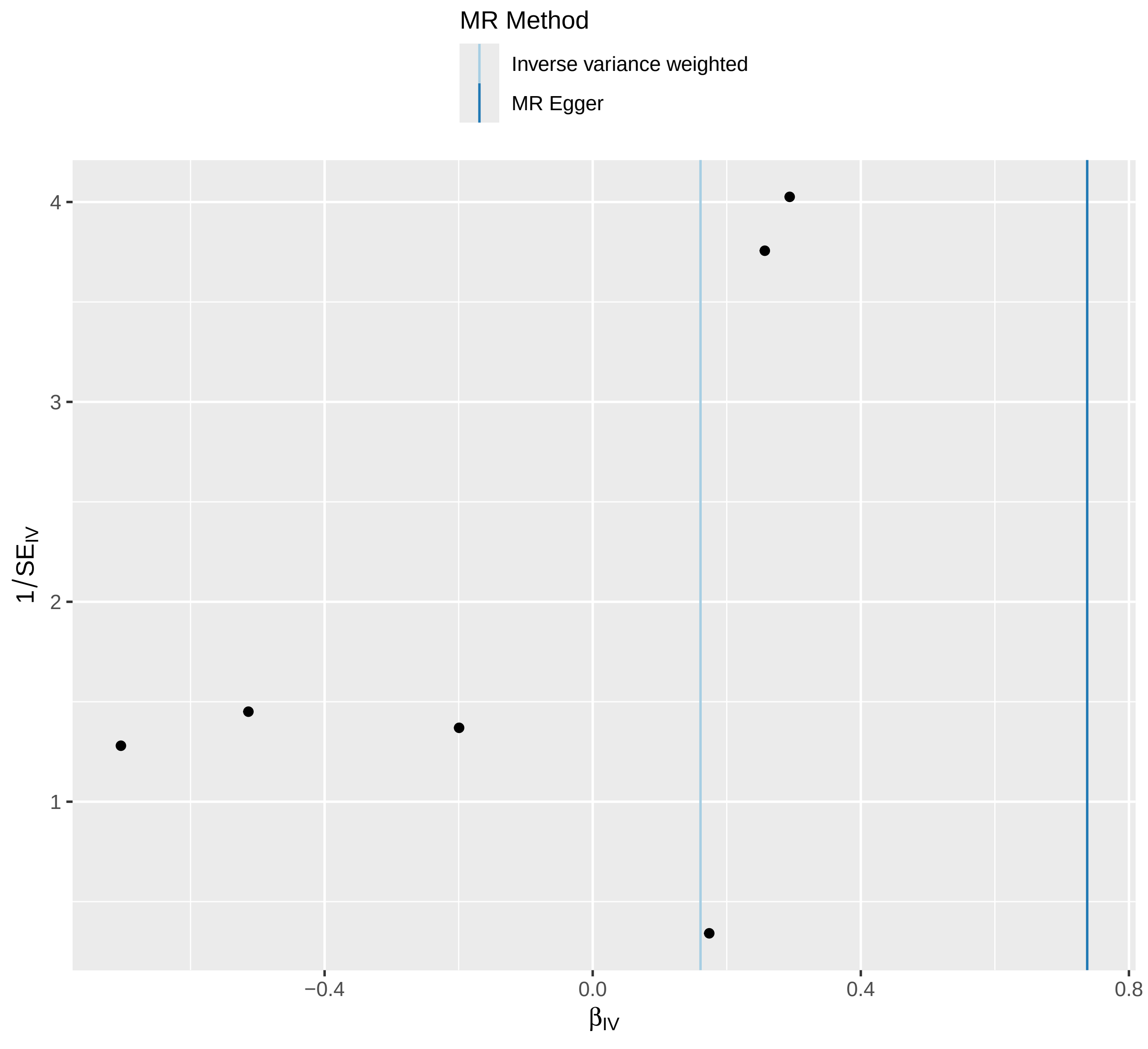
MR Method
Inverse variance weighted
MR Egger
4
3
2
1
−0.4
0.0
0.4
0.8
$1/SE_{IV}$
β_{IV}

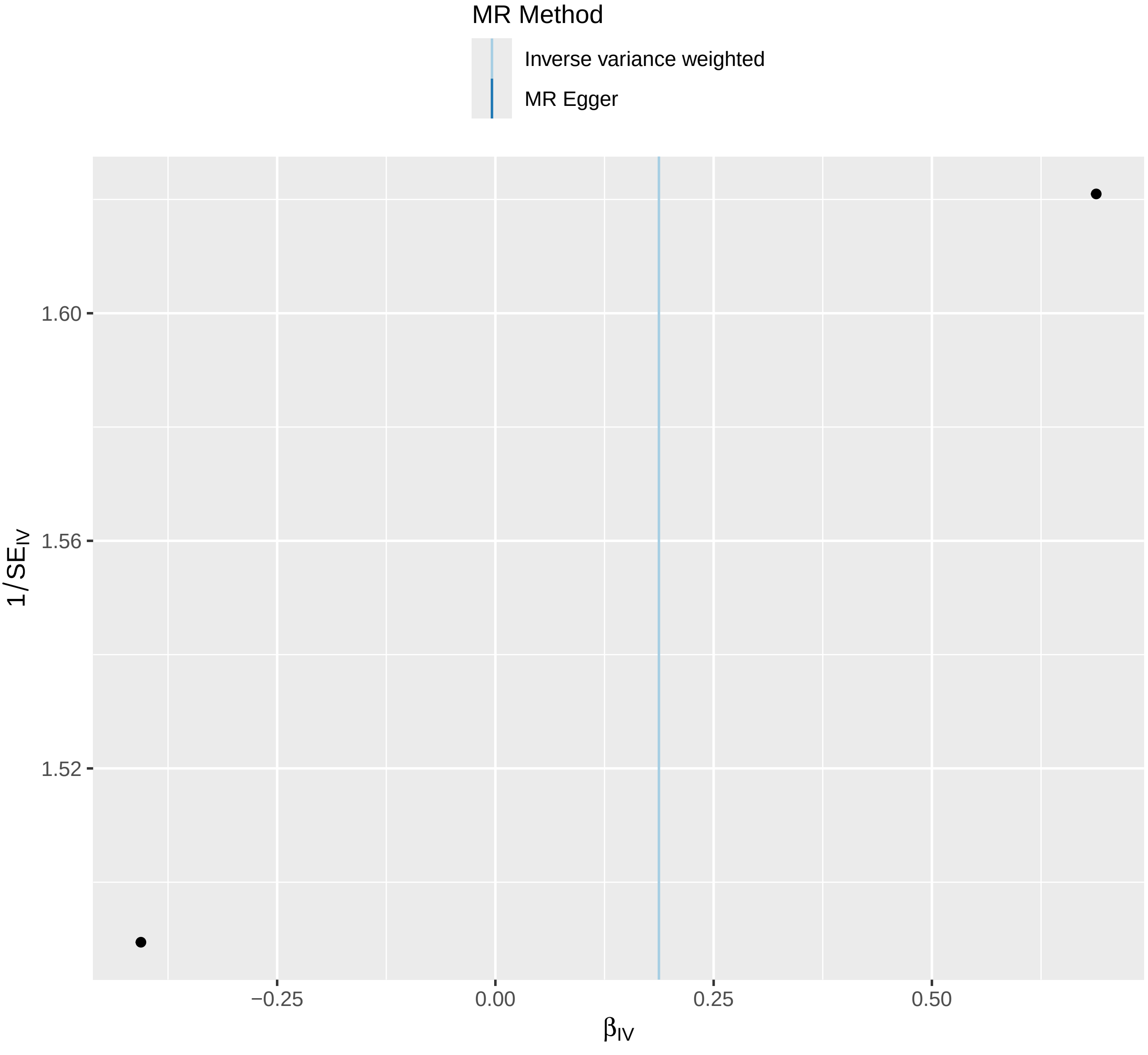

MR Method
Inverse variance weighted
MR Egger
1.60
1.56
1.52
−0.25
0.00
0.25
0.50
β_{IV}
$1/SE_{IV}$

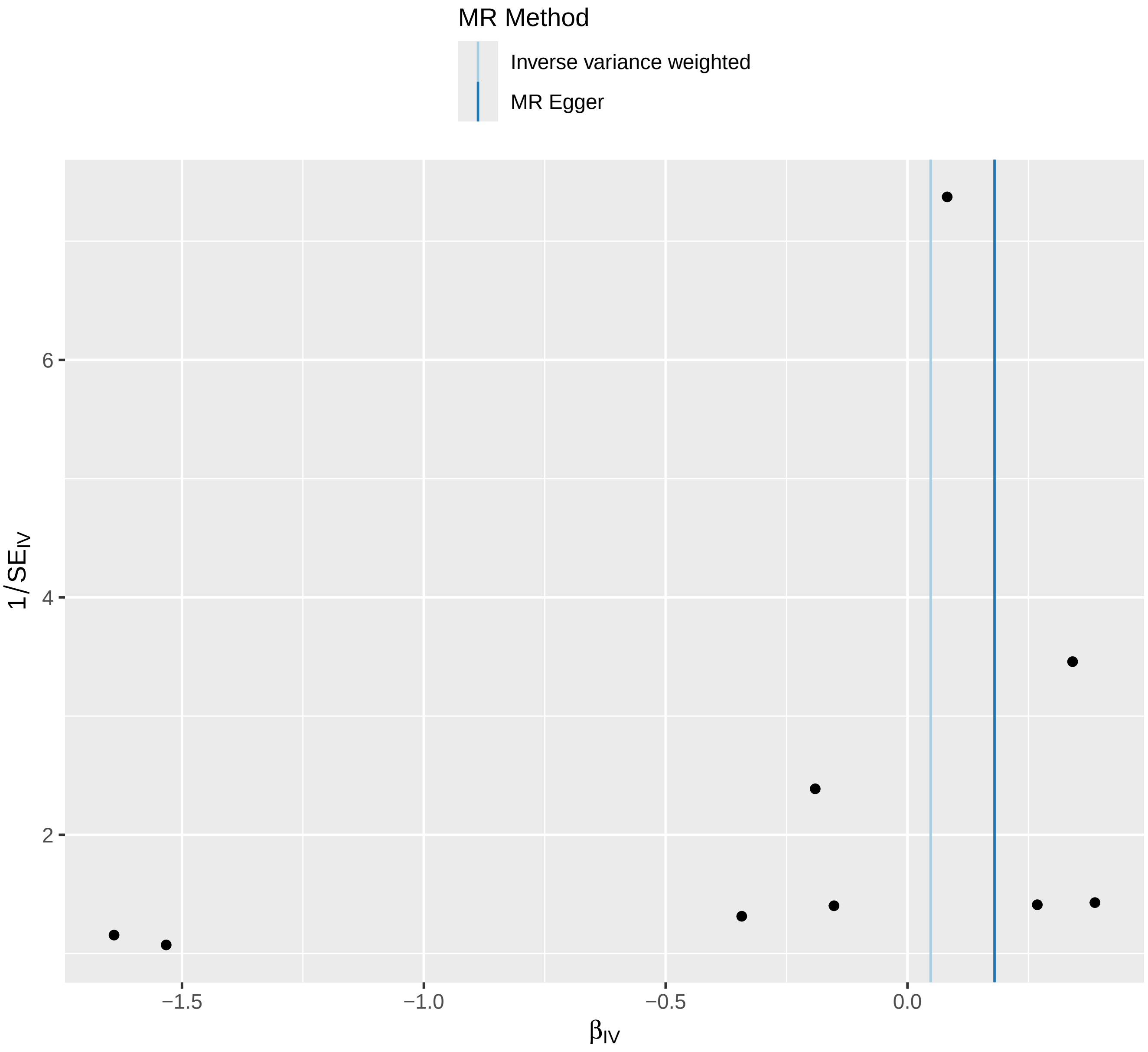

MR Method
Inverse variance weighted
MR Egger
1/SE_IV
6
4
2
−1.5
−1.0
−0.5
0.0
β_IV

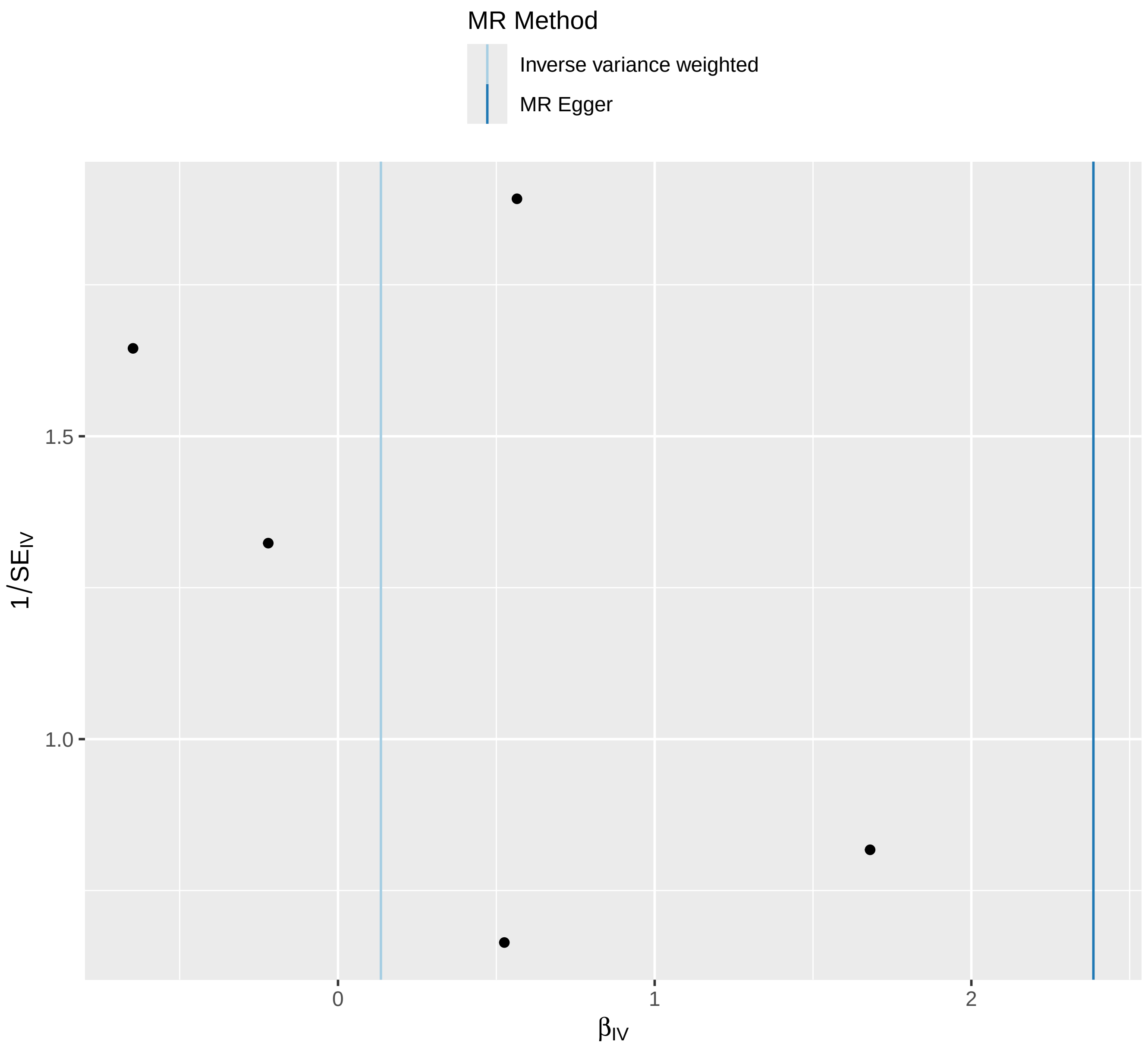

MR Method
Inverse variance weighted
MR Egger
1.5
1.0
0
1
2
1/SE_IV
β_IV

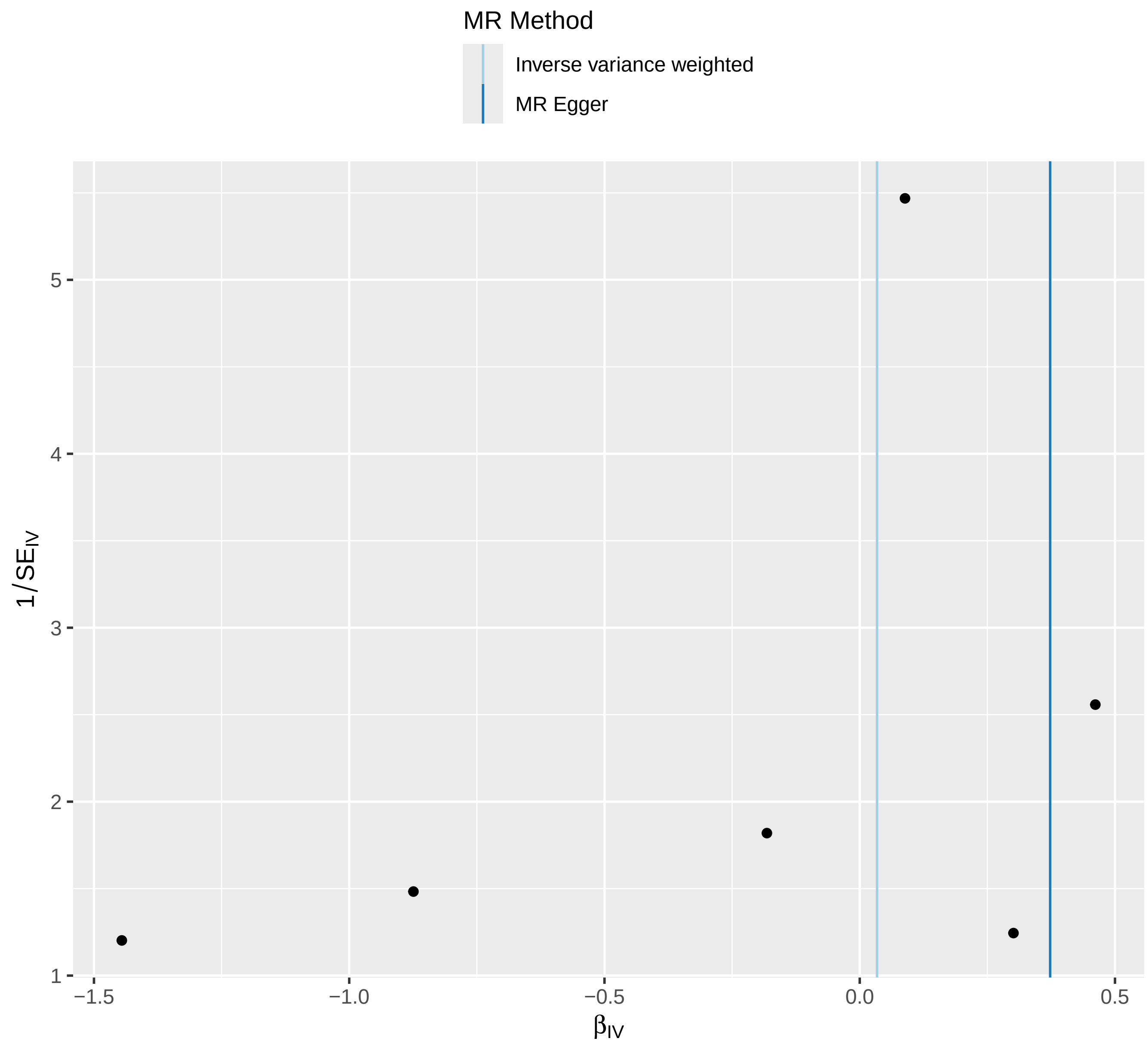

MR Method
Inverse variance weighted
MR Egger
5
4
3
2
1
1/SE_IV
−1.5
−1.0
−0.5
0.0
0.5
β_IV

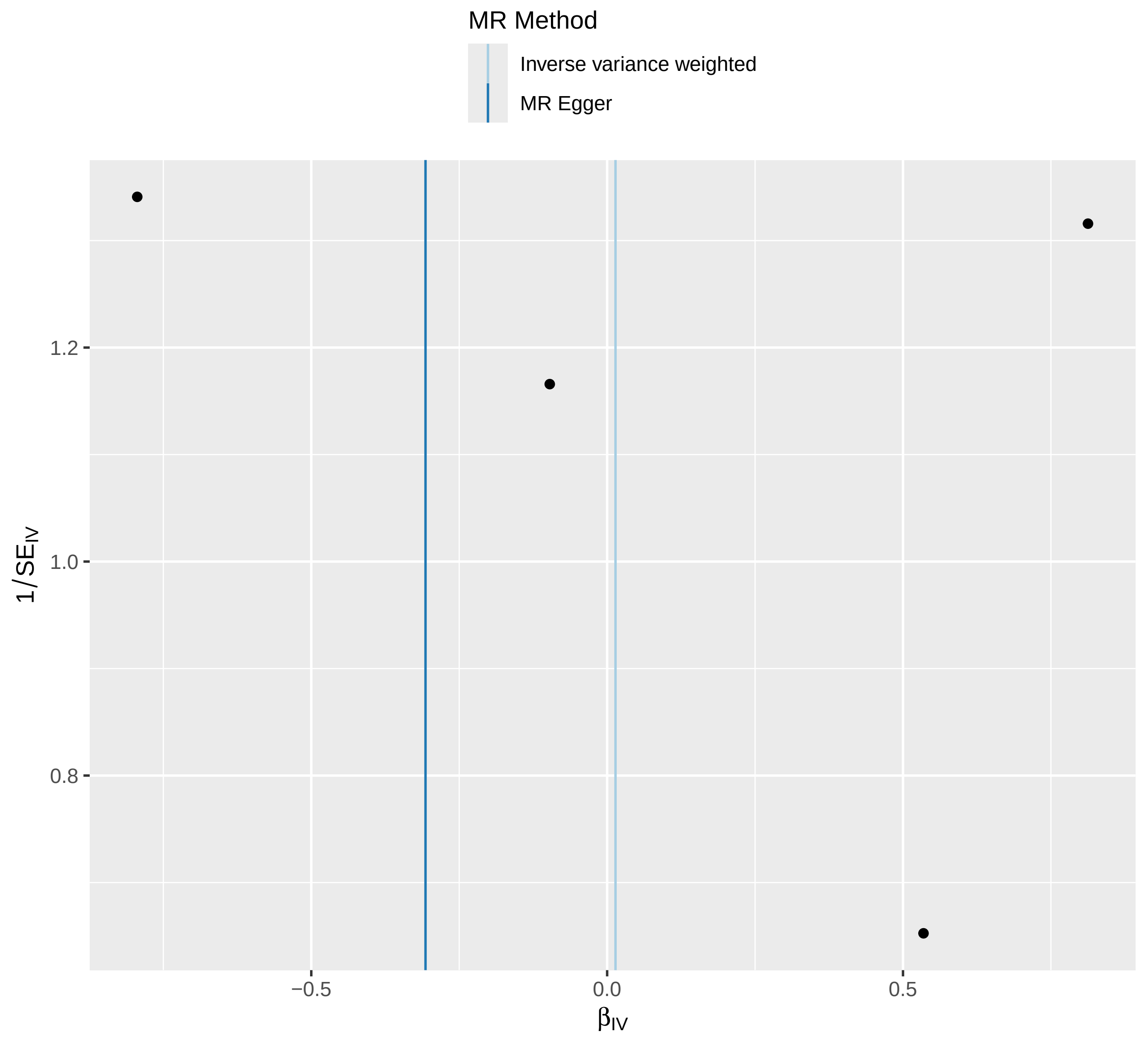
MR Method
Inverse variance weighted
MR Egger
1.2
1.0
0.8
1/SE_IV
−0.5
0.0
0.5
β_IV

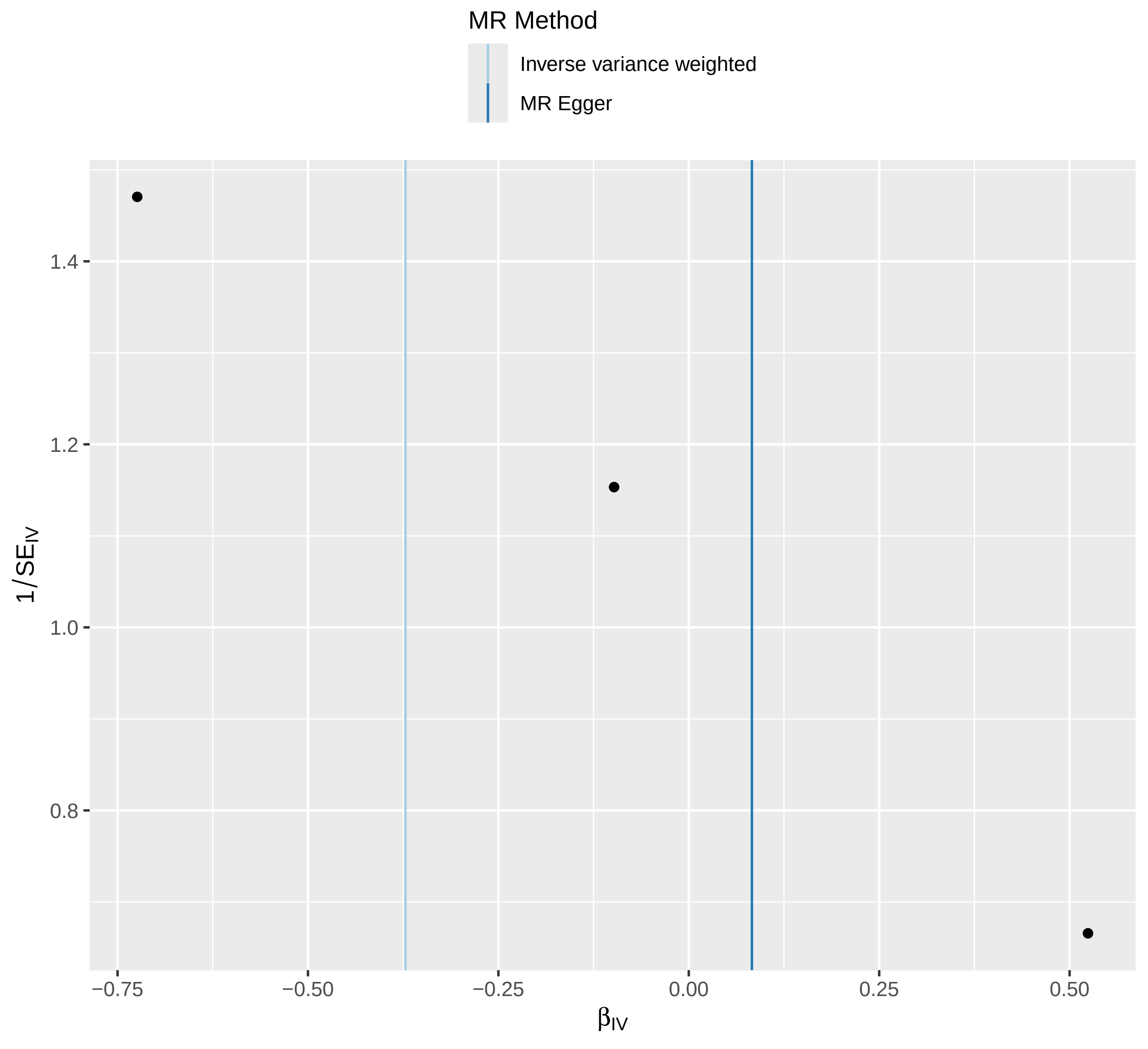
MR Method
Inverse variance weighted
MR Egger
1/SE_IV
1.4
1.2
1.0
0.8
−0.75
−0.50
−0.25
0.00
0.25
0.50
β_IV

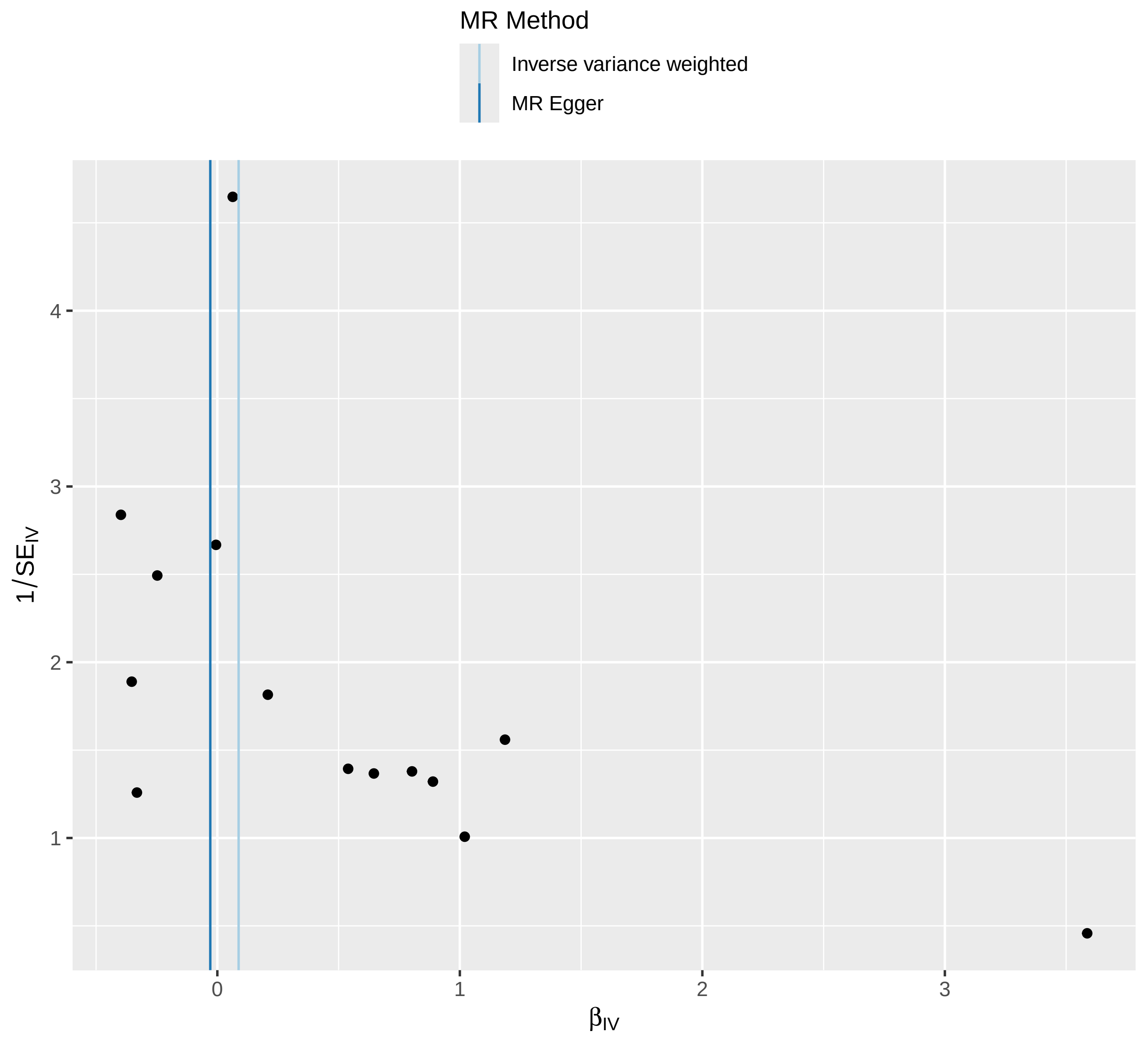
MR Method
Inverse variance weighted
MR Egger
4
3
2
1
0
1
2
3
$1/SE_{IV}$
β_{IV}

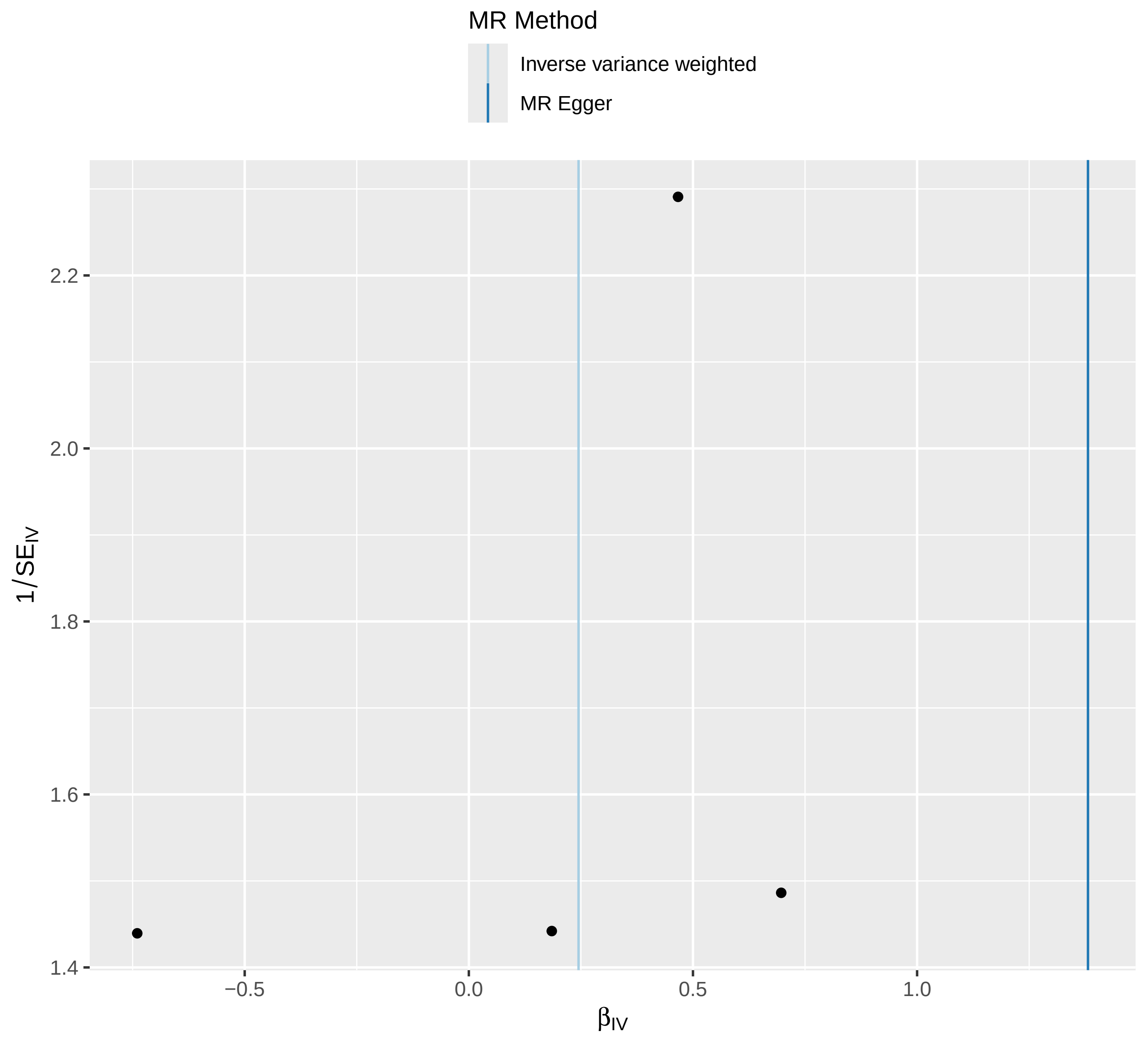

MR Method
Inverse variance weighted
MR Egger
2.2
2.0
1.8
1.6
1.4
1/SE_IV
−0.5
0.0
0.5
1.0
β_IV

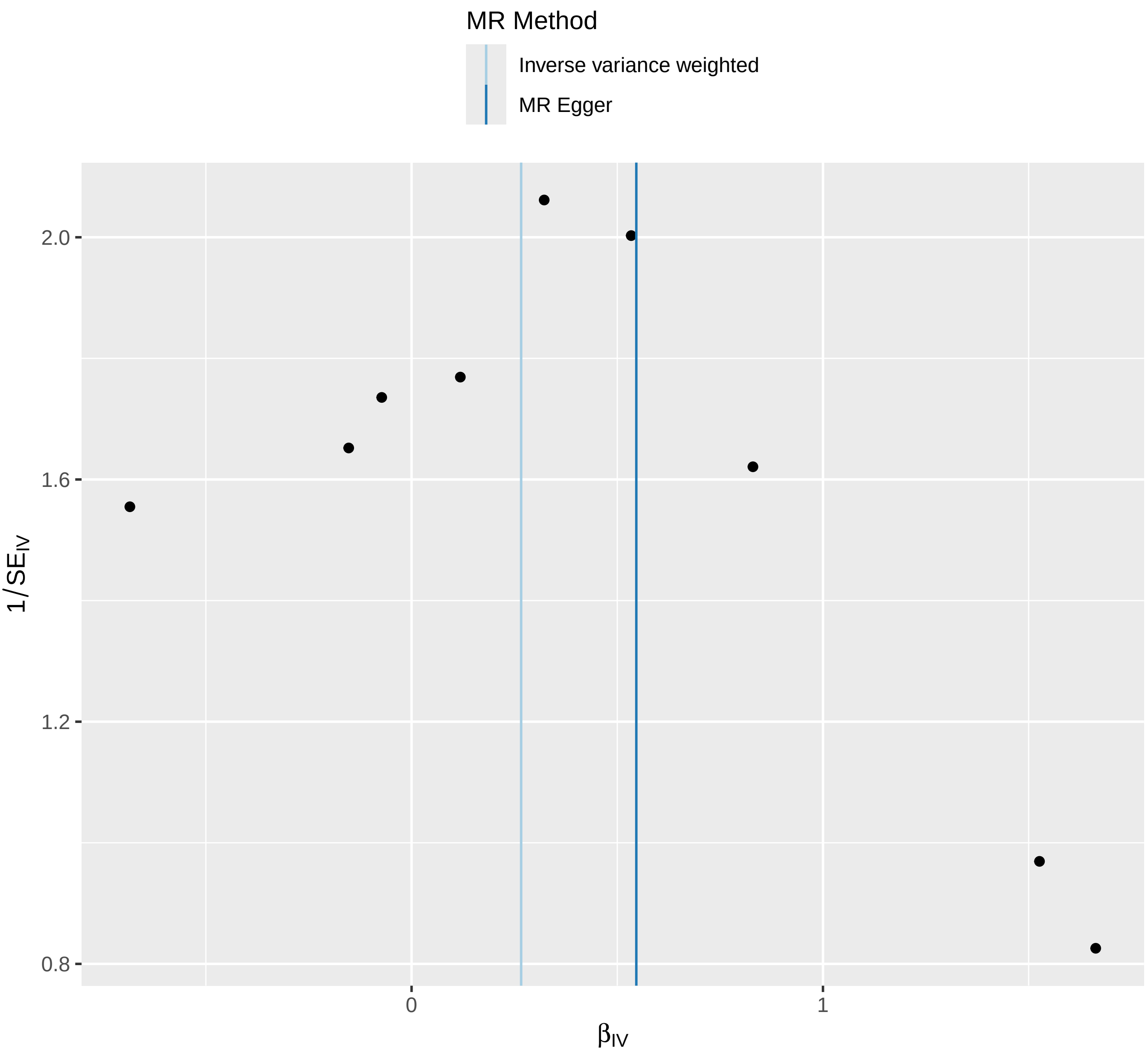
MR Method
Inverse variance weighted
MR Egger
2.0
1.6
1.2
0.8
1/SE$_{IV}$
0
1
β$_{IV}$

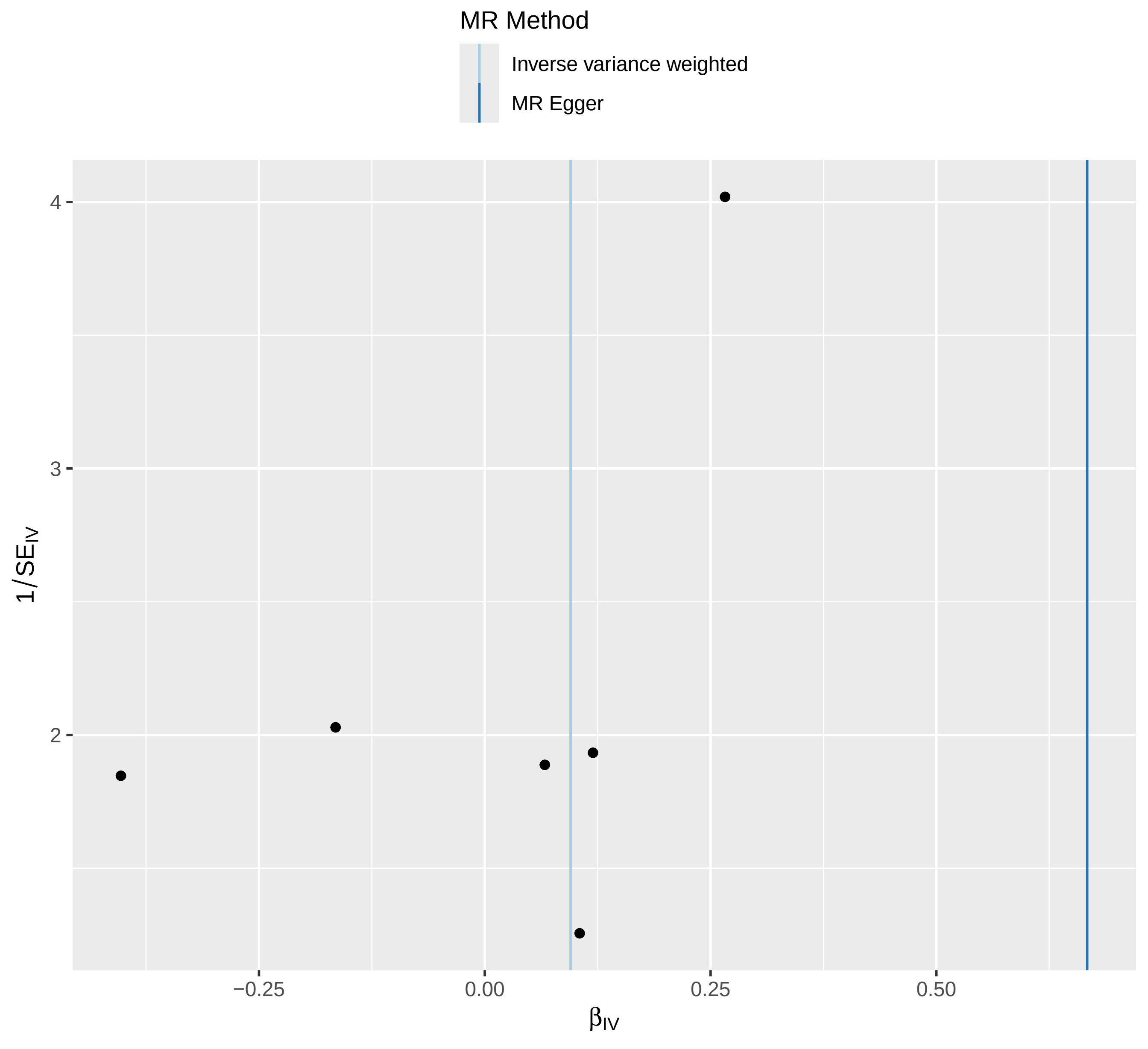

MR Method
Inverse variance weighted
MR Egger
4
3
2
1/SE_IV
−0.25
0.00
0.25
0.50
β_IV

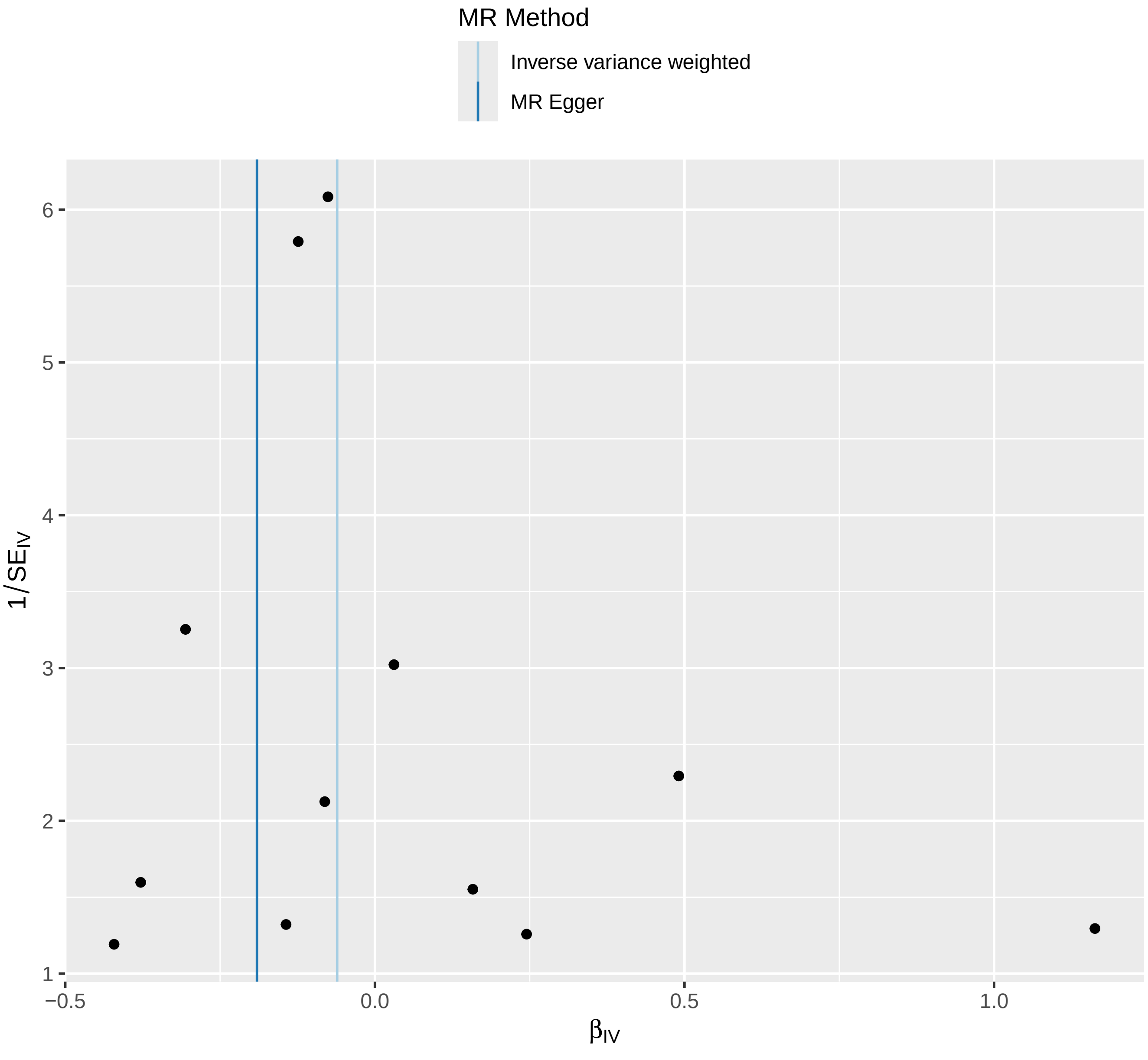

MR Method
Inverse variance weighted
MR Egger
6
5
4
3
2
1
1/SE$_{IV}$
−0.5
0.0
0.5
1.0
β$_{IV}$

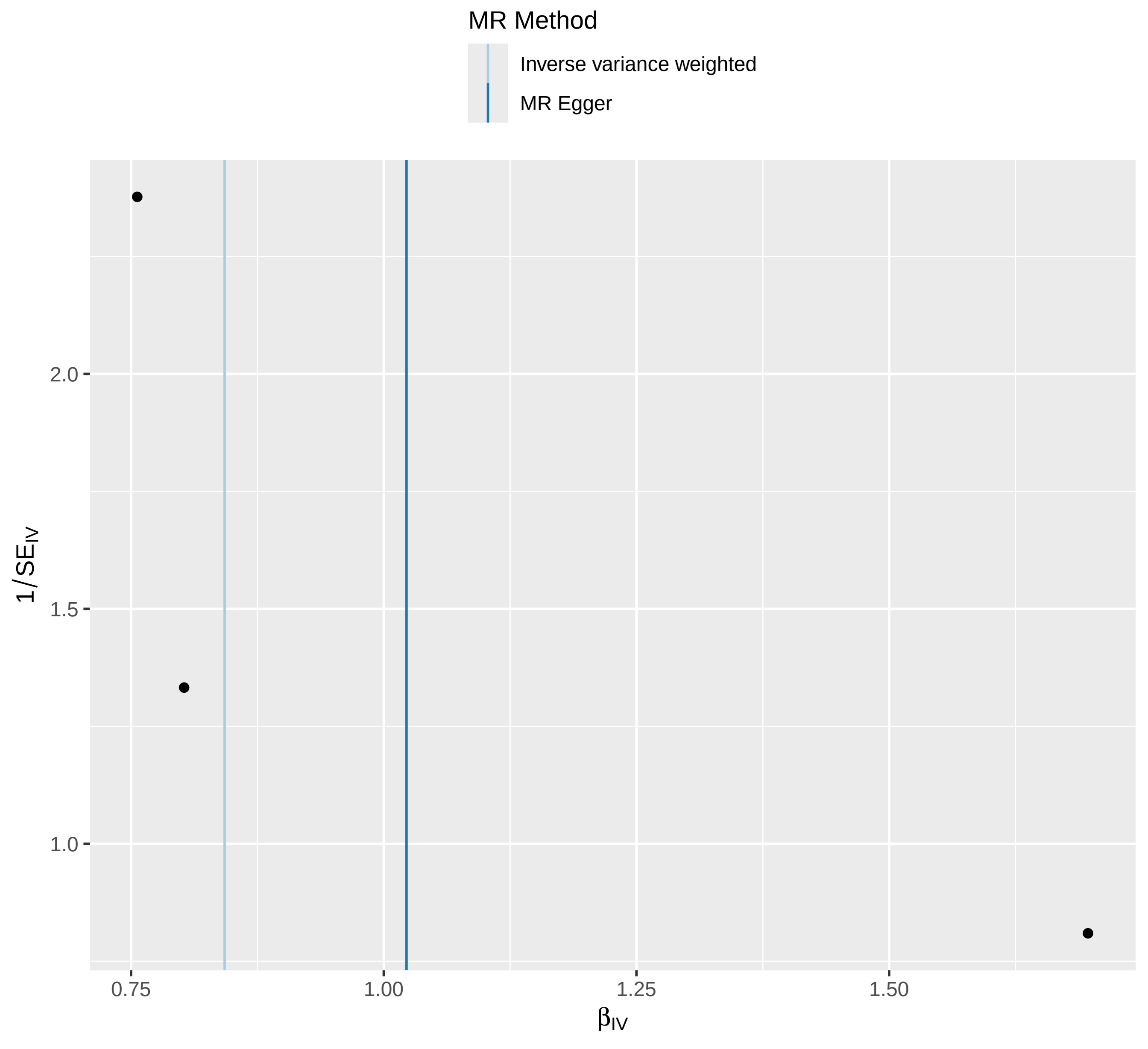
MR Method
Inverse variance weighted
MR Egger
2.0
1.5
1.0
0.75
1.00
1.25
1.50
$1/SE_{IV}$
β_{IV}

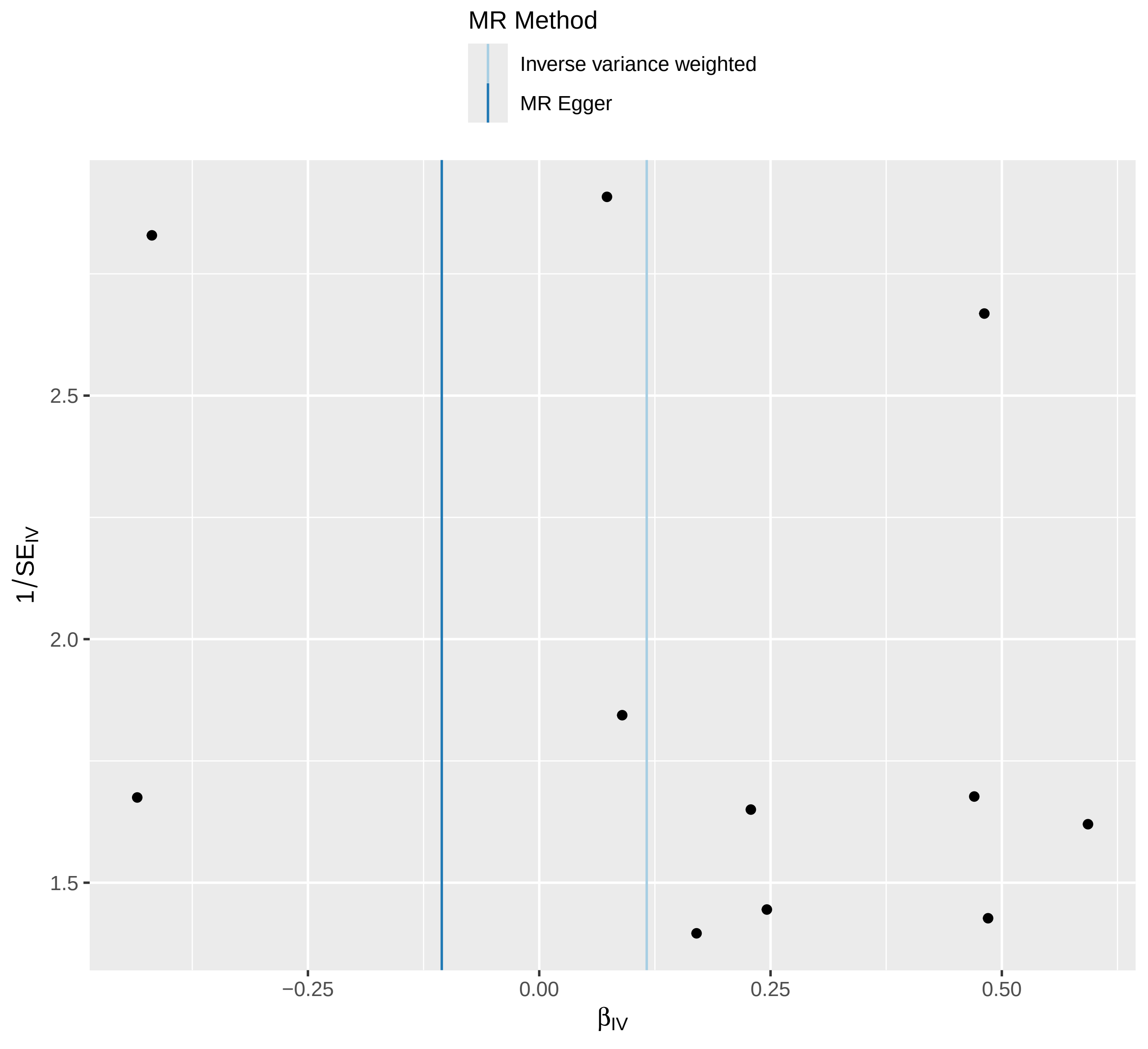

MR Method
Inverse variance weighted
MR Egger
2.5
2.0
1.5
1/SE$_{IV}$
−0.25
0.00
0.25
0.50
β$_{IV}$

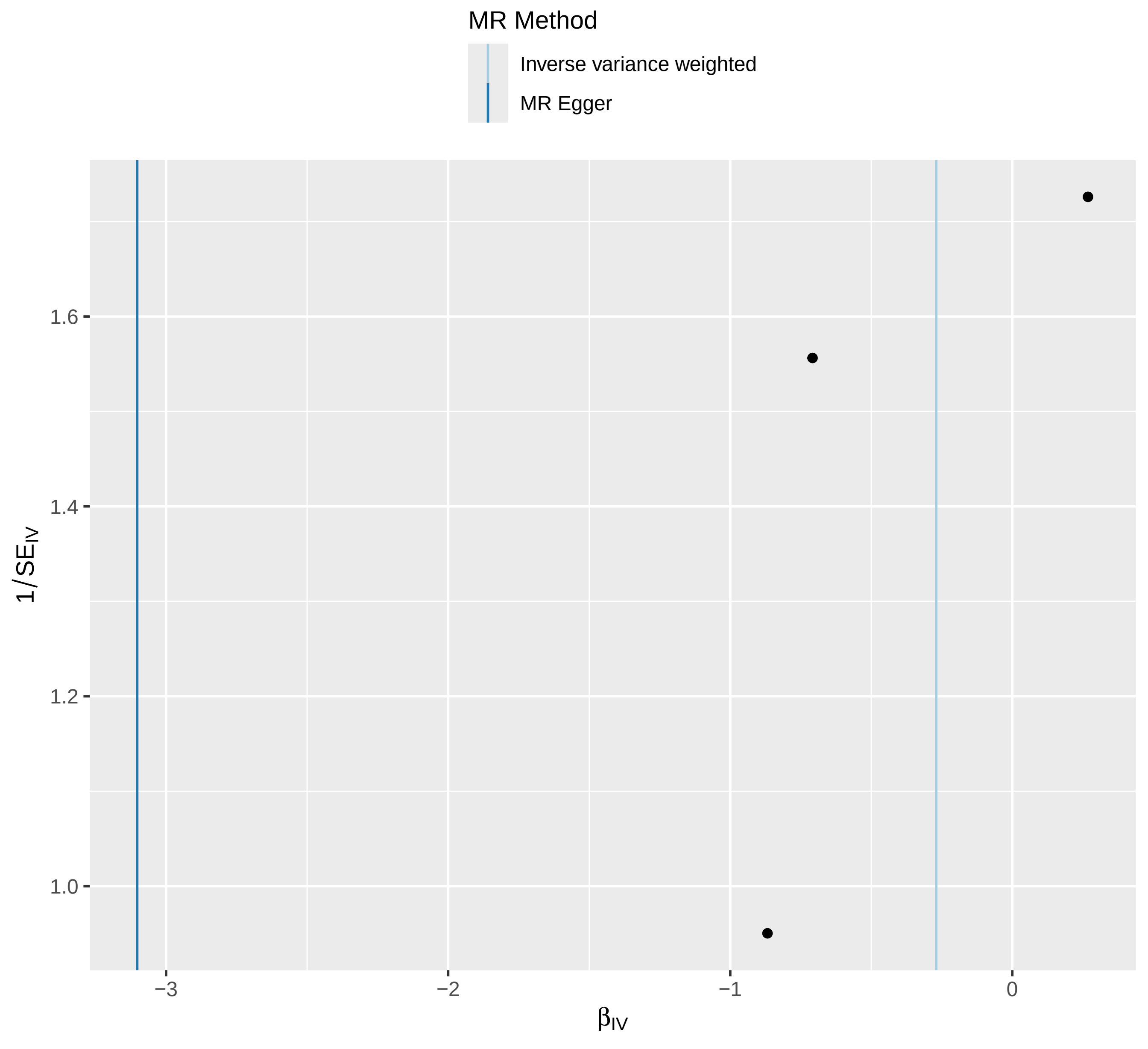
MR Method
Inverse variance weighted
MR Egger
1.6
1.4
1.2
1.0
1/SE_IV
−3
−2
−1
0
β_IV

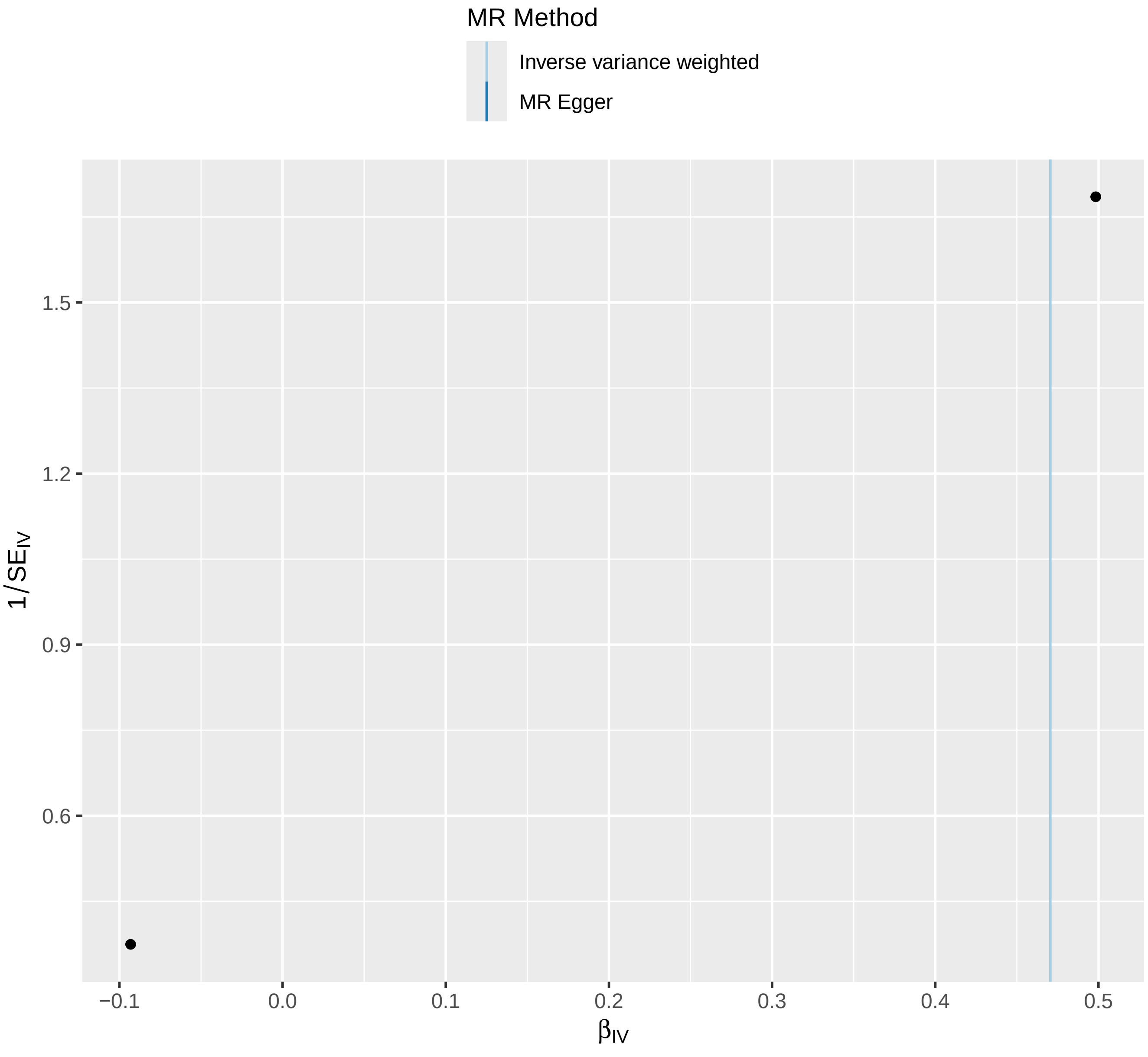
MR Method
Inverse variance weighted
MR Egger
1.5
1.2
0.9
0.6
$1/SE_{IV}$
−0.1
0.0
0.1
0.2
0.3
0.4
0.5
β_{IV}

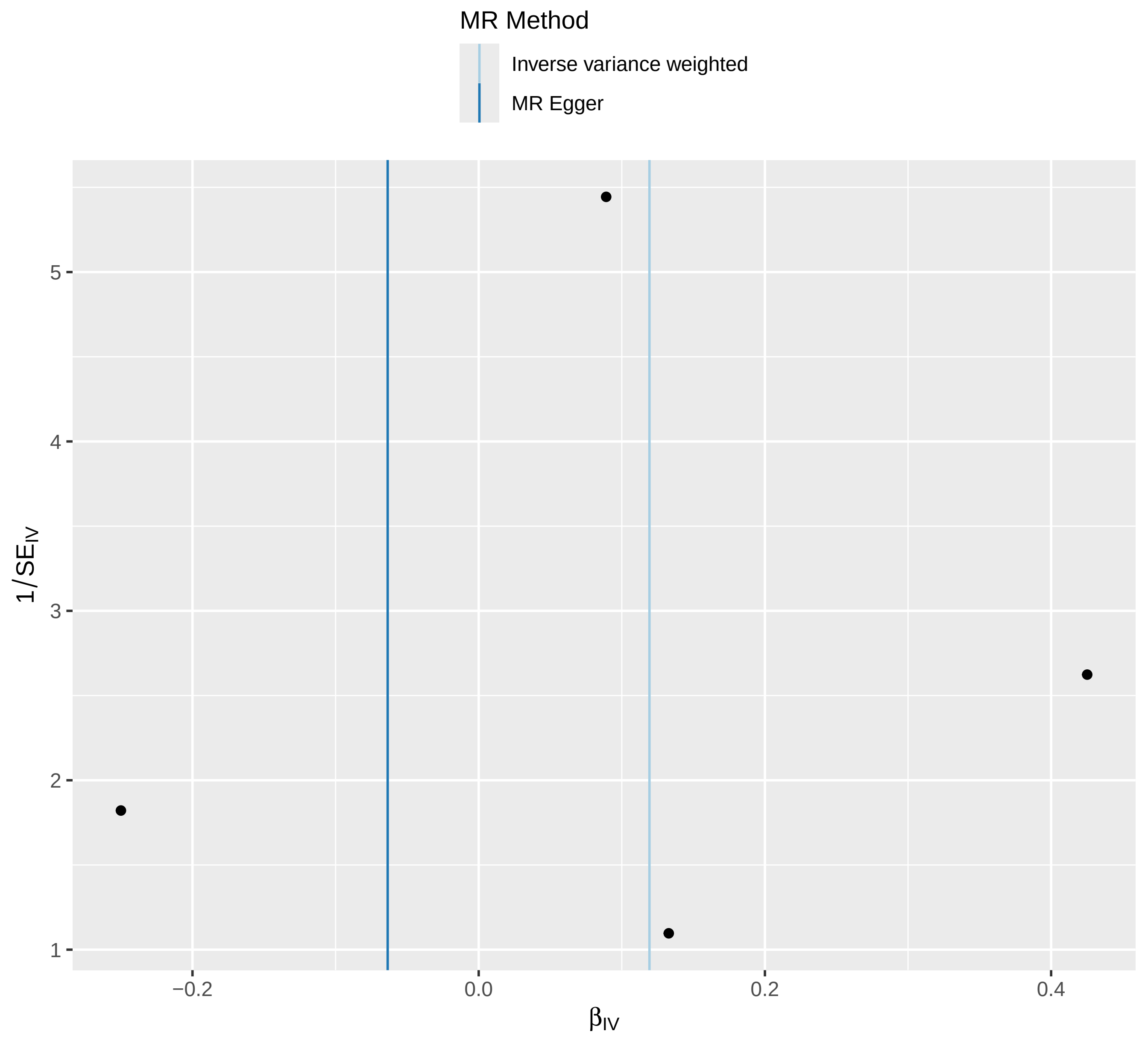

MR Method
Inverse variance weighted
MR Egger
5
4
3
2
1
1/SE_IV
−0.2
0.0
0.2
0.4
β_IV

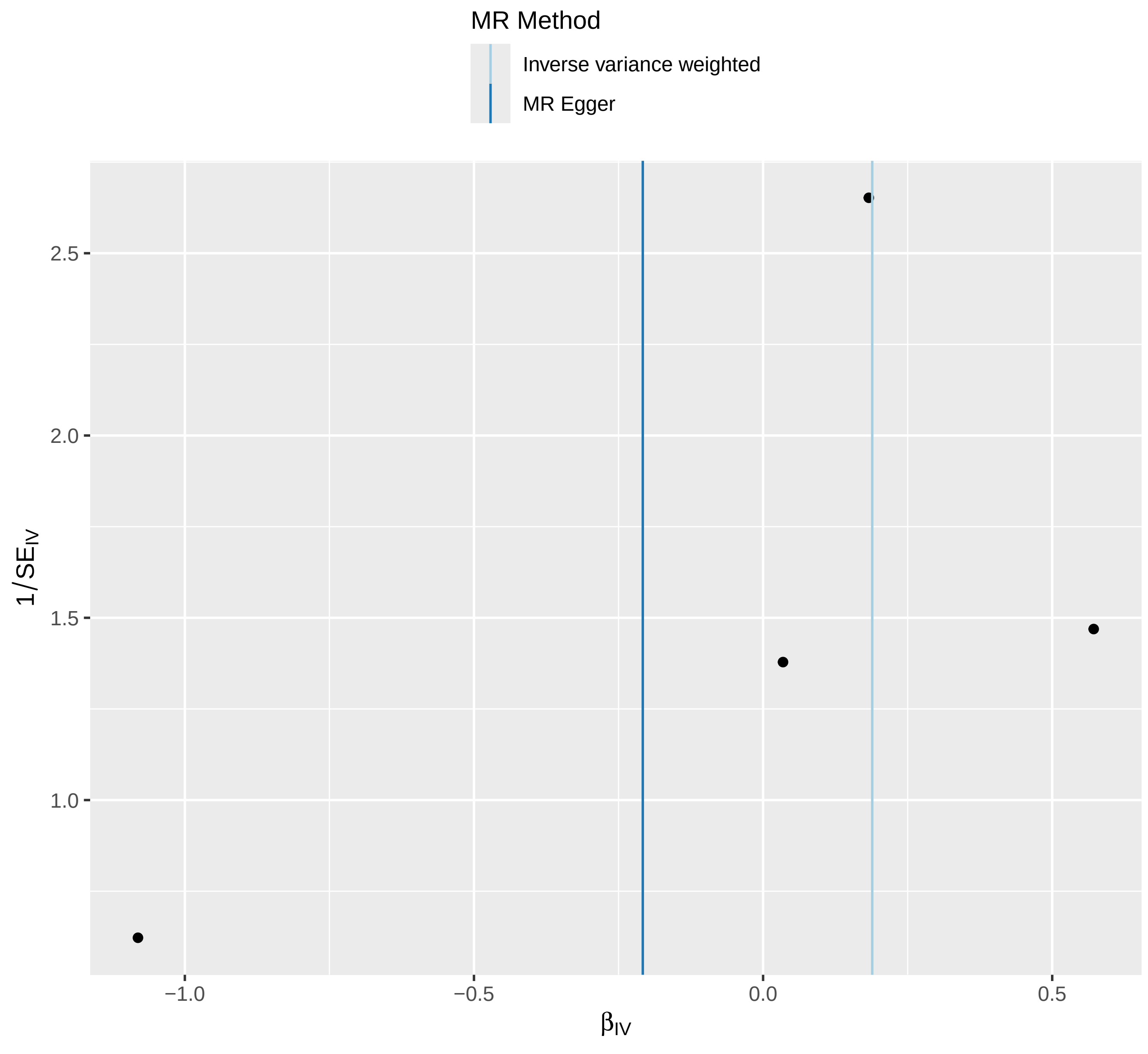

MR Method
Inverse variance weighted
MR Egger
2.5
2.0
1.5
1.0
1/SE$_{IV}$
−1.0
−0.5
0.0
0.5
β_{IV}

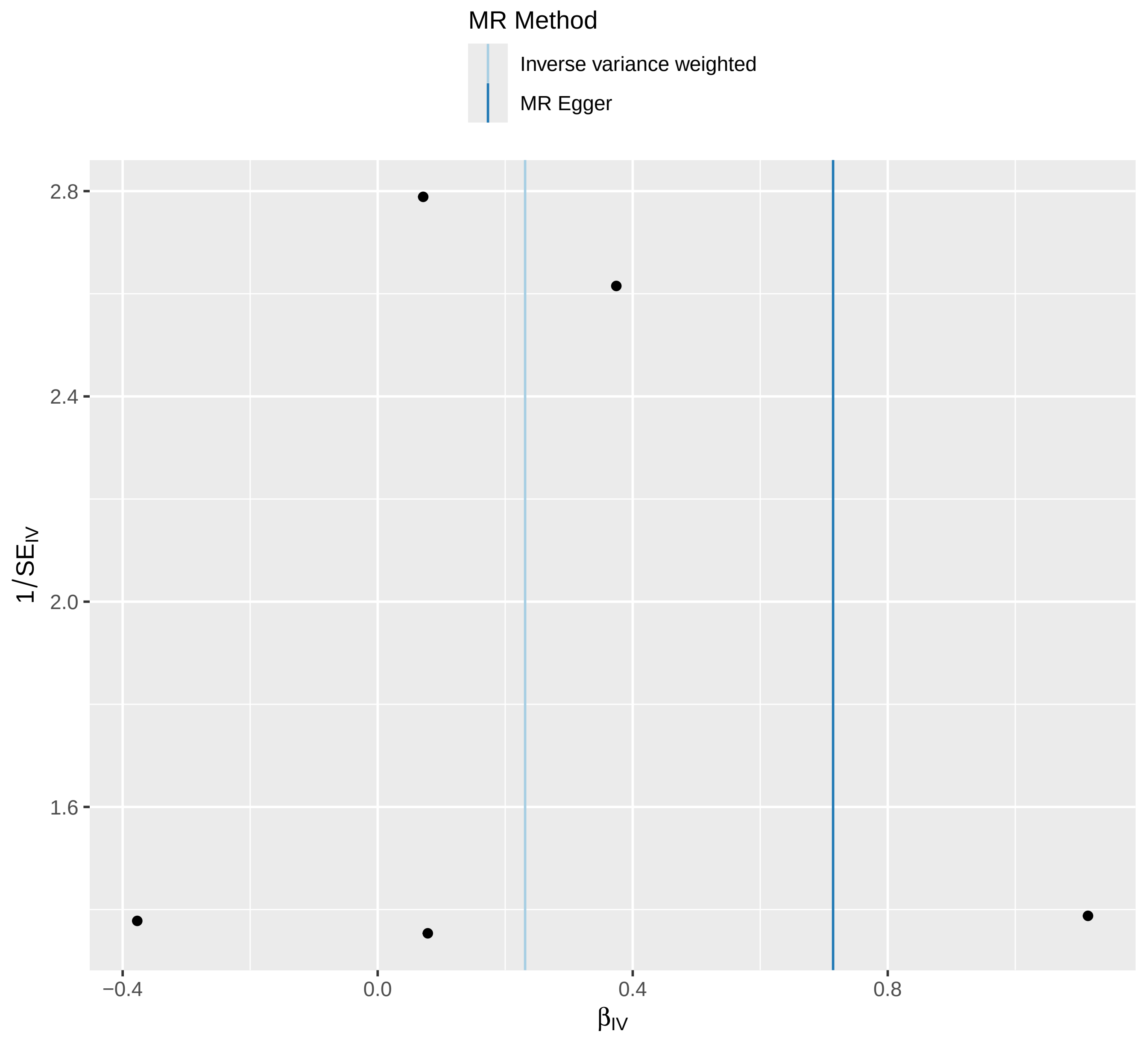

MR Method
Inverse variance weighted
MR Egger
2.8
2.4
2.0
1.6
$1/SE_{IV}$
−0.4
0.0
0.4
0.8
β_{IV}

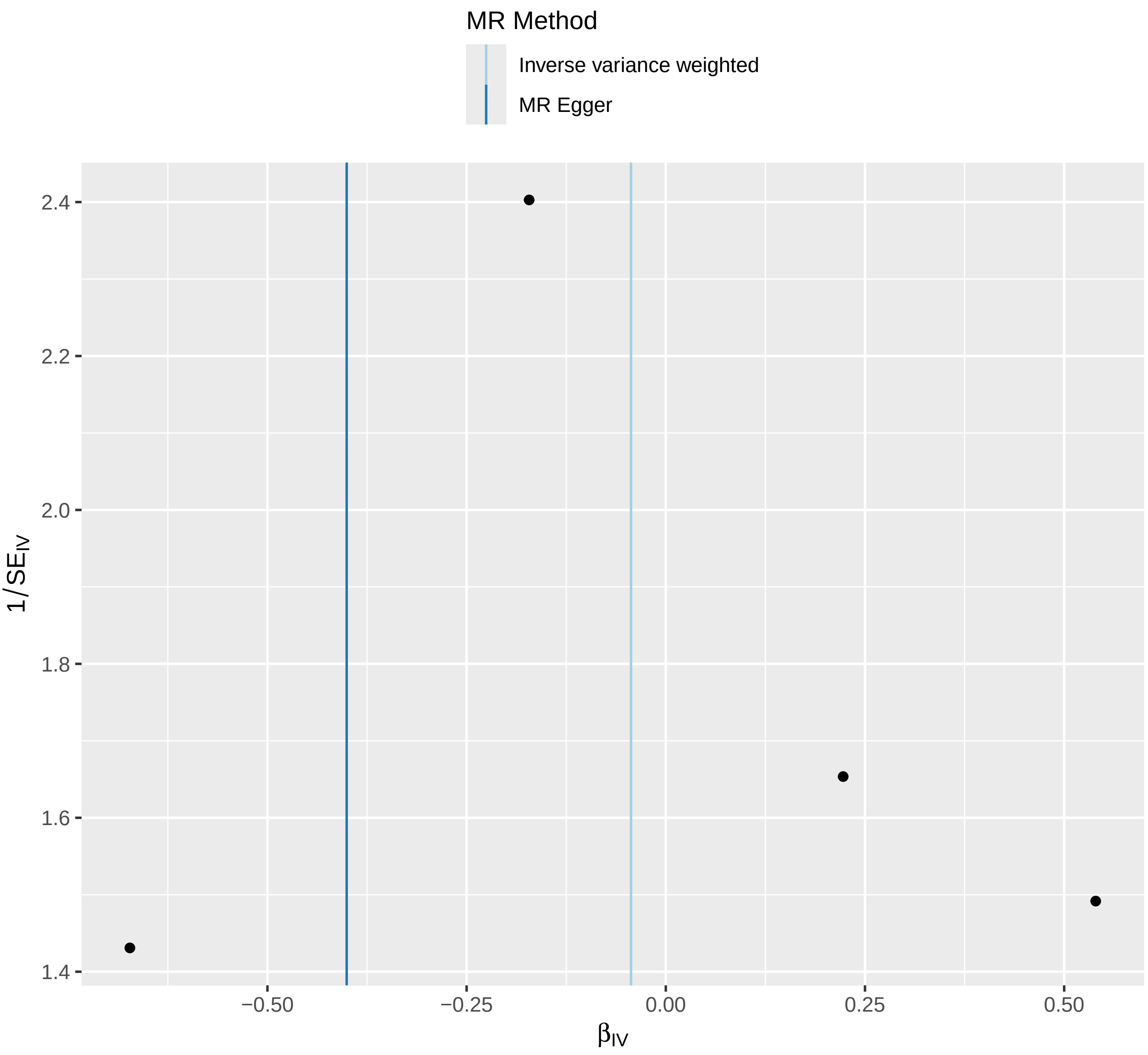
MR Method
Inverse variance weighted
MR Egger
1/SE$_{IV}$
2.4
2.2
2.0
1.8
1.6
1.4
−0.50
−0.25
0.00
0.25
0.50
β$_{IV}$

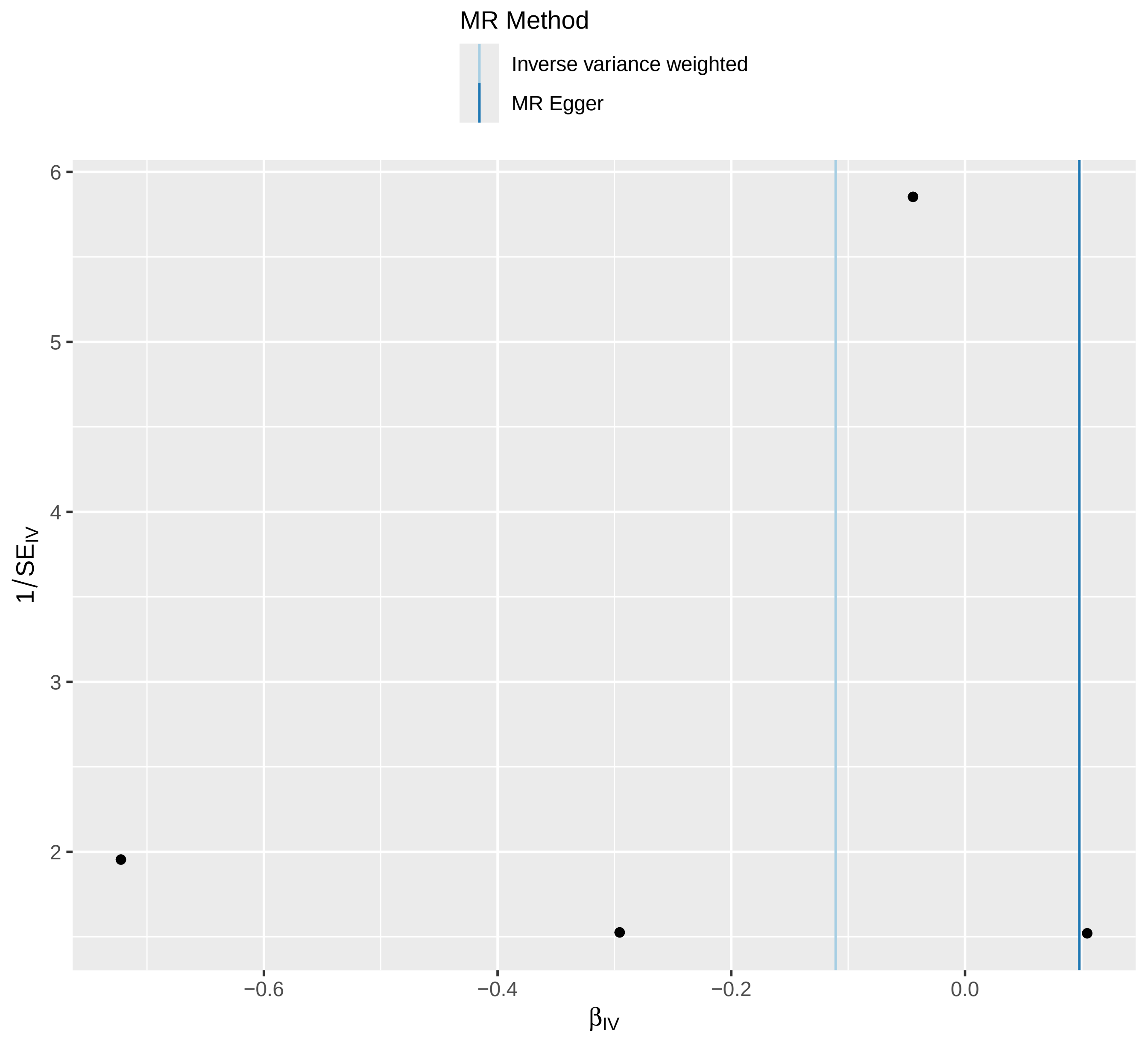

MR Method
Inverse variance weighted
MR Egger
6
5
4
3
2
1/SE_IV
−0.6
−0.4
−0.2
0.0
β_IV

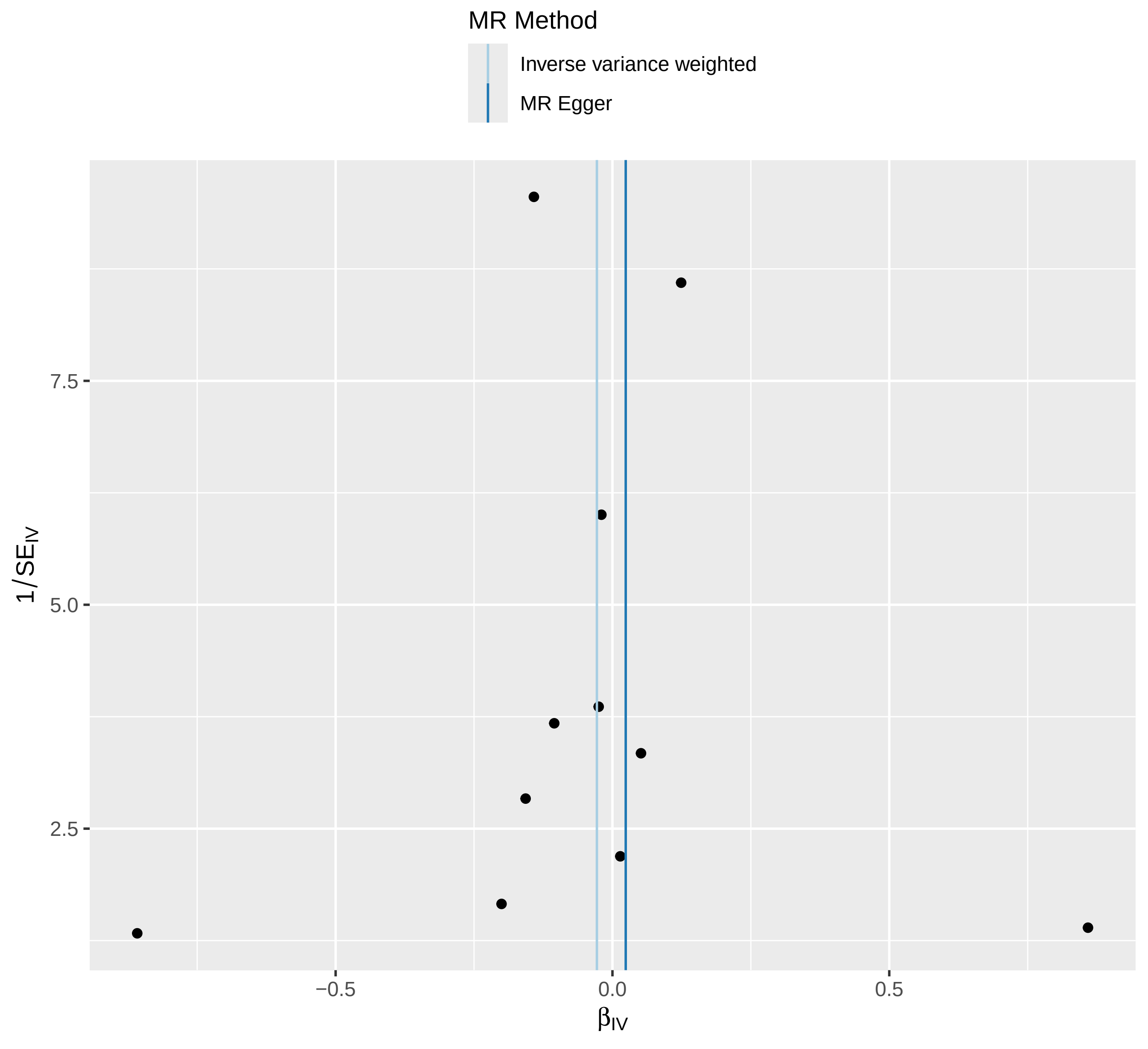

MR Method
Inverse variance weighted
MR Egger
7.5
5.0
2.5
1/SE_IV
−0.5
0.0
0.5
β_IV

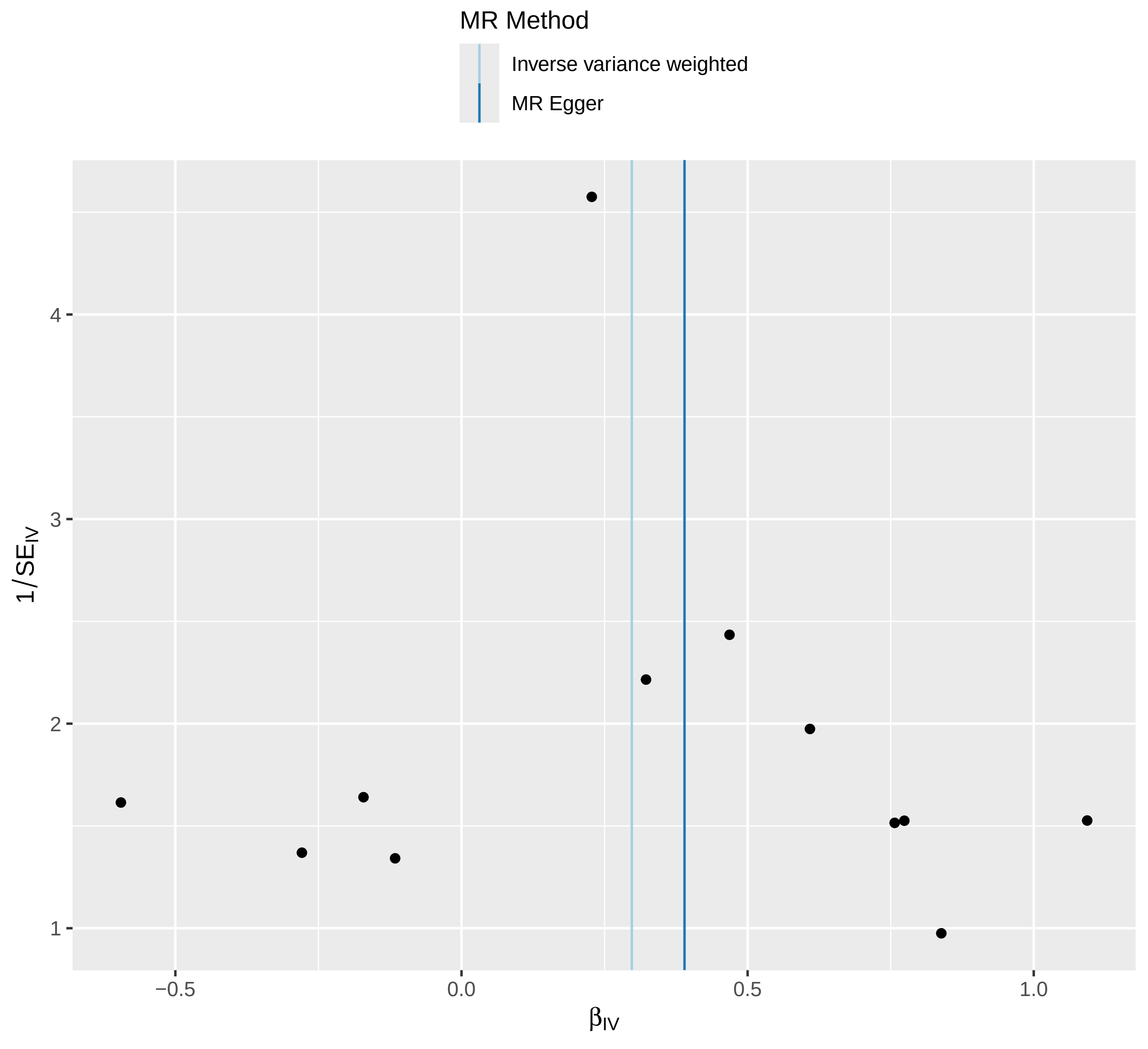

MR Method
Inverse variance weighted
MR Egger
4
3
2
1
1/SE_IV
−0.5
0.0
0.5
1.0
β_IV

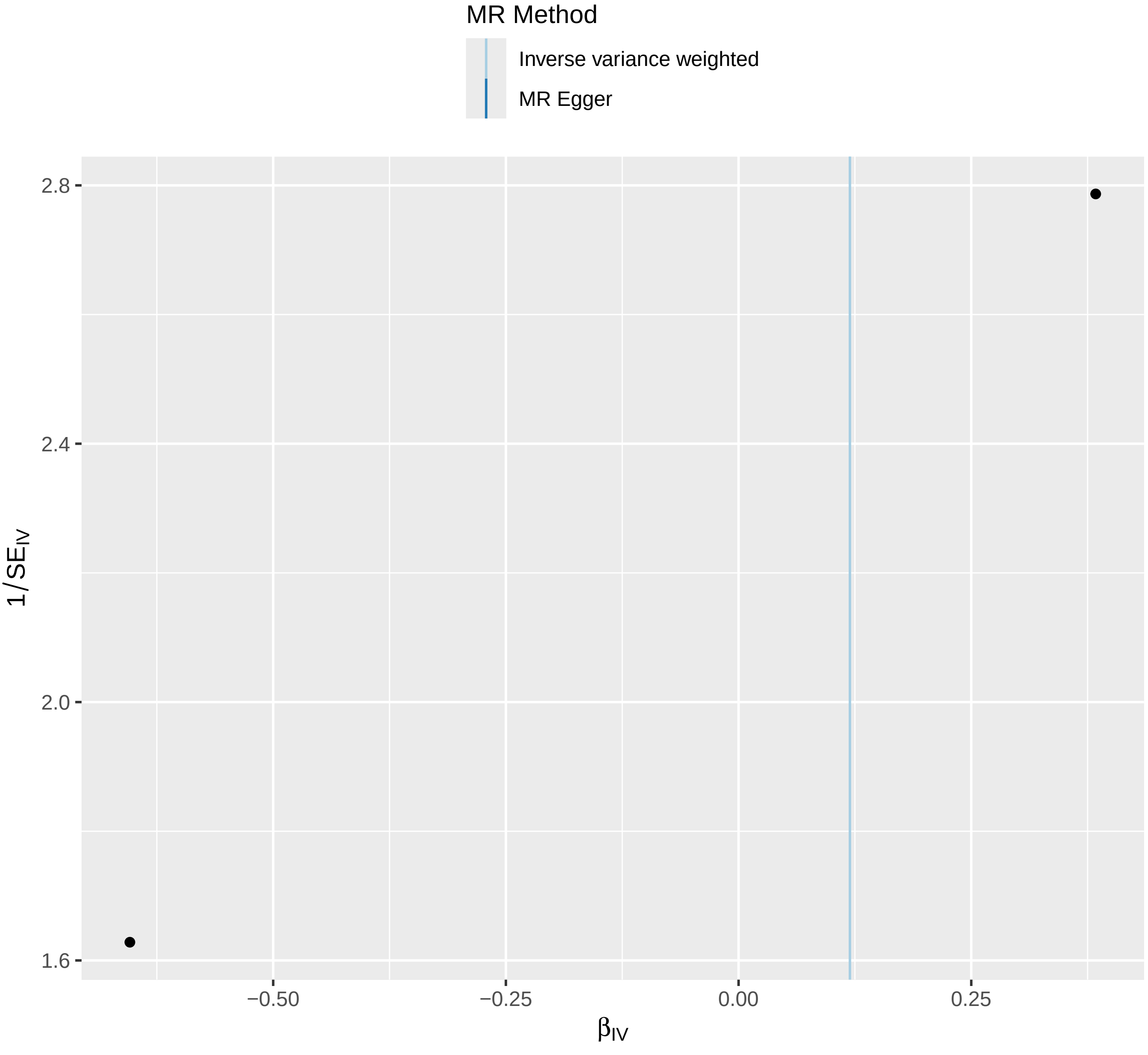

MR Method
Inverse variance weighted
MR Egger
2.8
2.4
2.0
1.6
1/SE_IV
−0.50
−0.25
0.00
0.25
β_IV

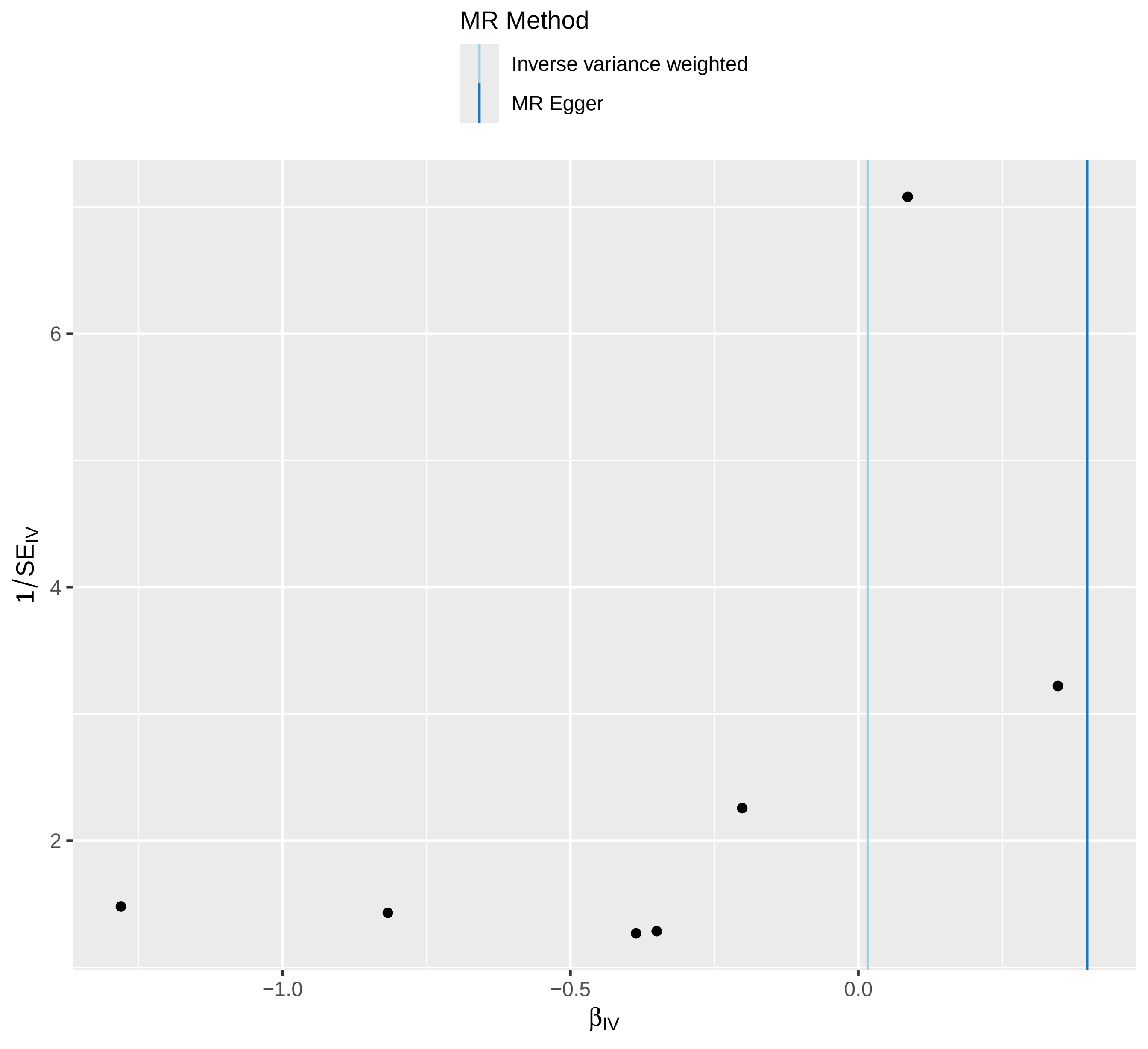

MR Method
Inverse variance weighted
MR Egger
6
4
2
1/SE_IV
−1.0
−0.5
0.0
β_IV

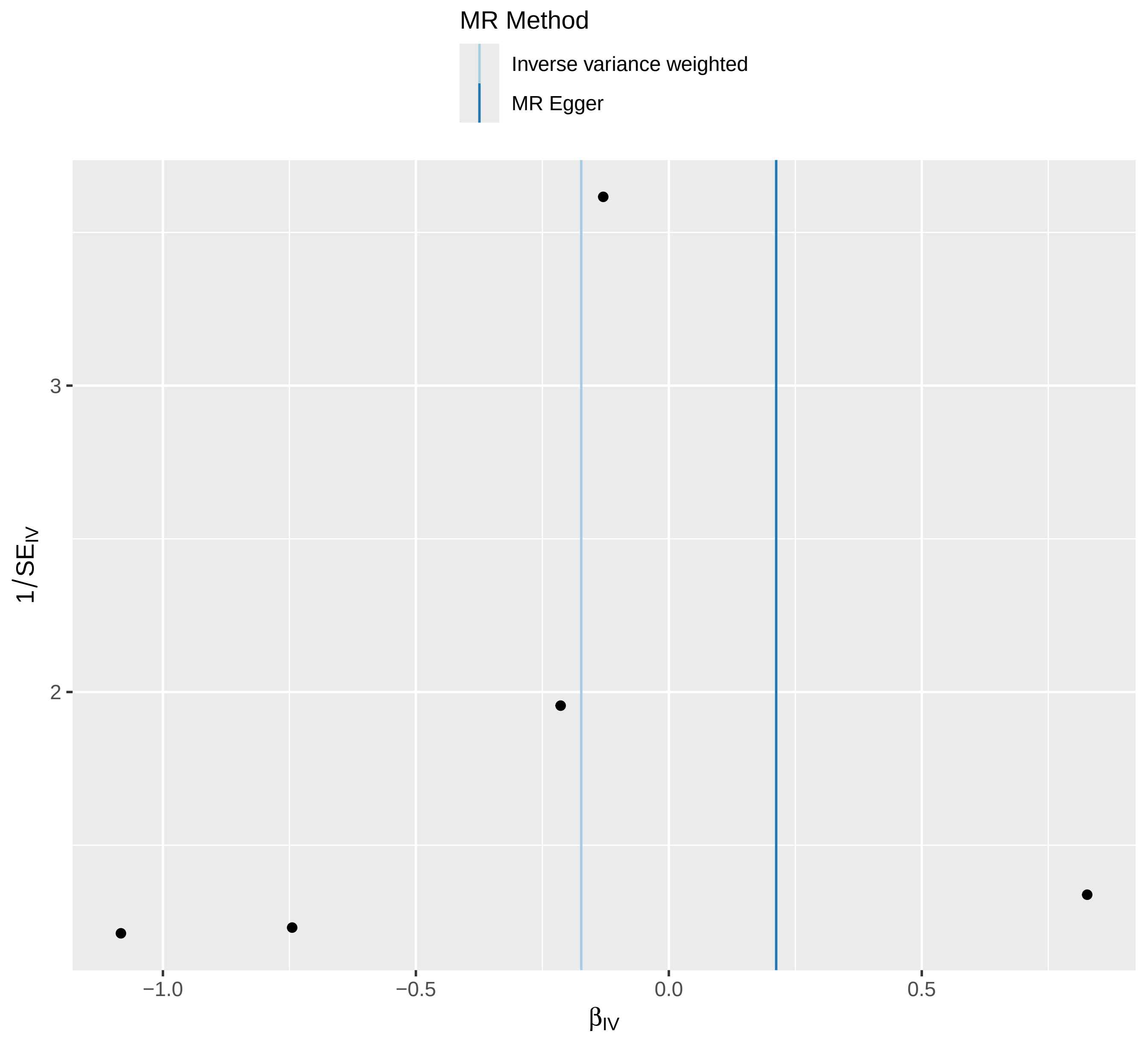
MR Method
Inverse variance weighted
MR Egger
3
2
1/SE_IV
−1.0
−0.5
0.0
0.5
β_IV

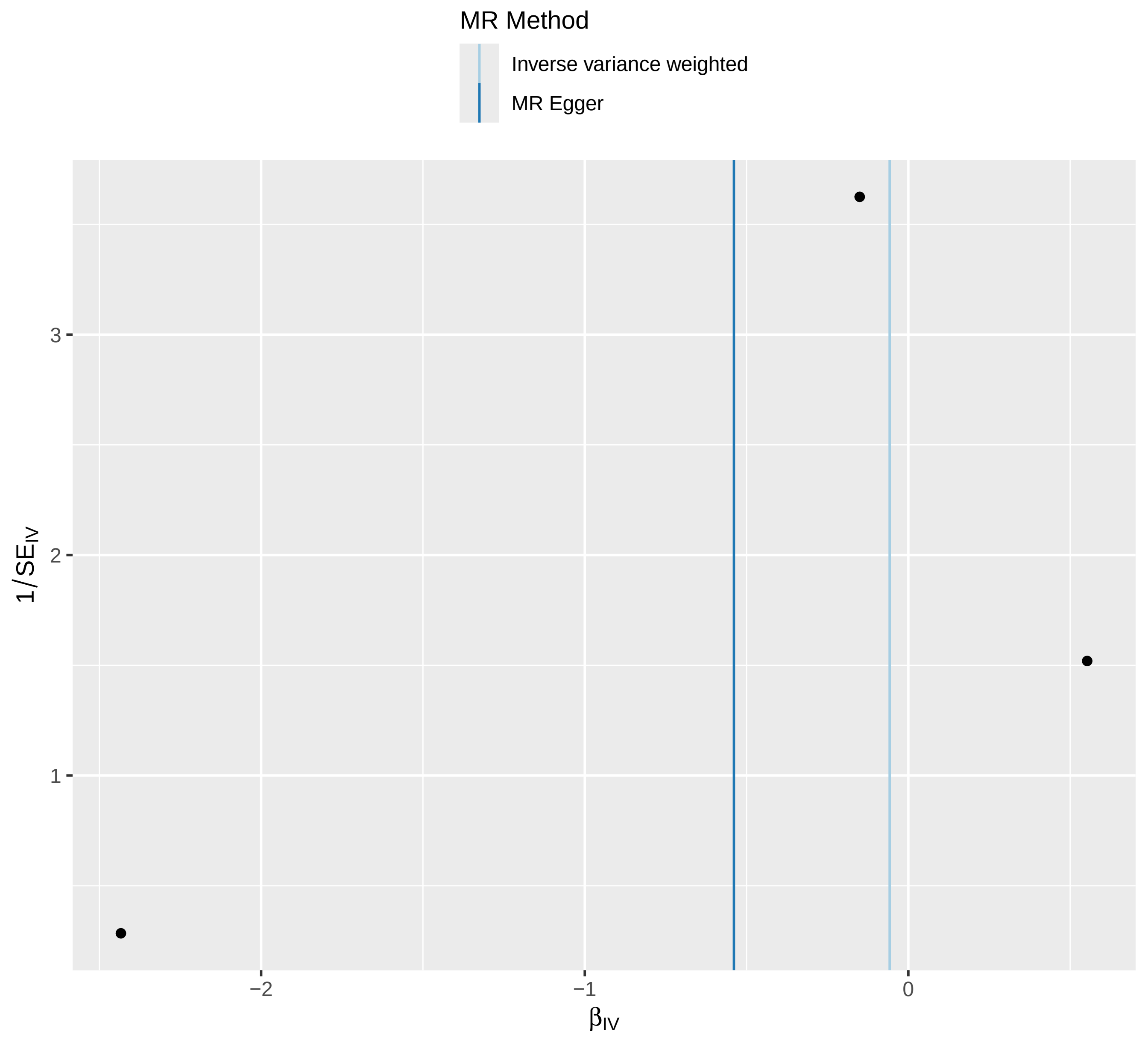
MR Method
Inverse variance weighted
MR Egger
1/SE_IV
3
2
1
−2
−1
0
β_IV

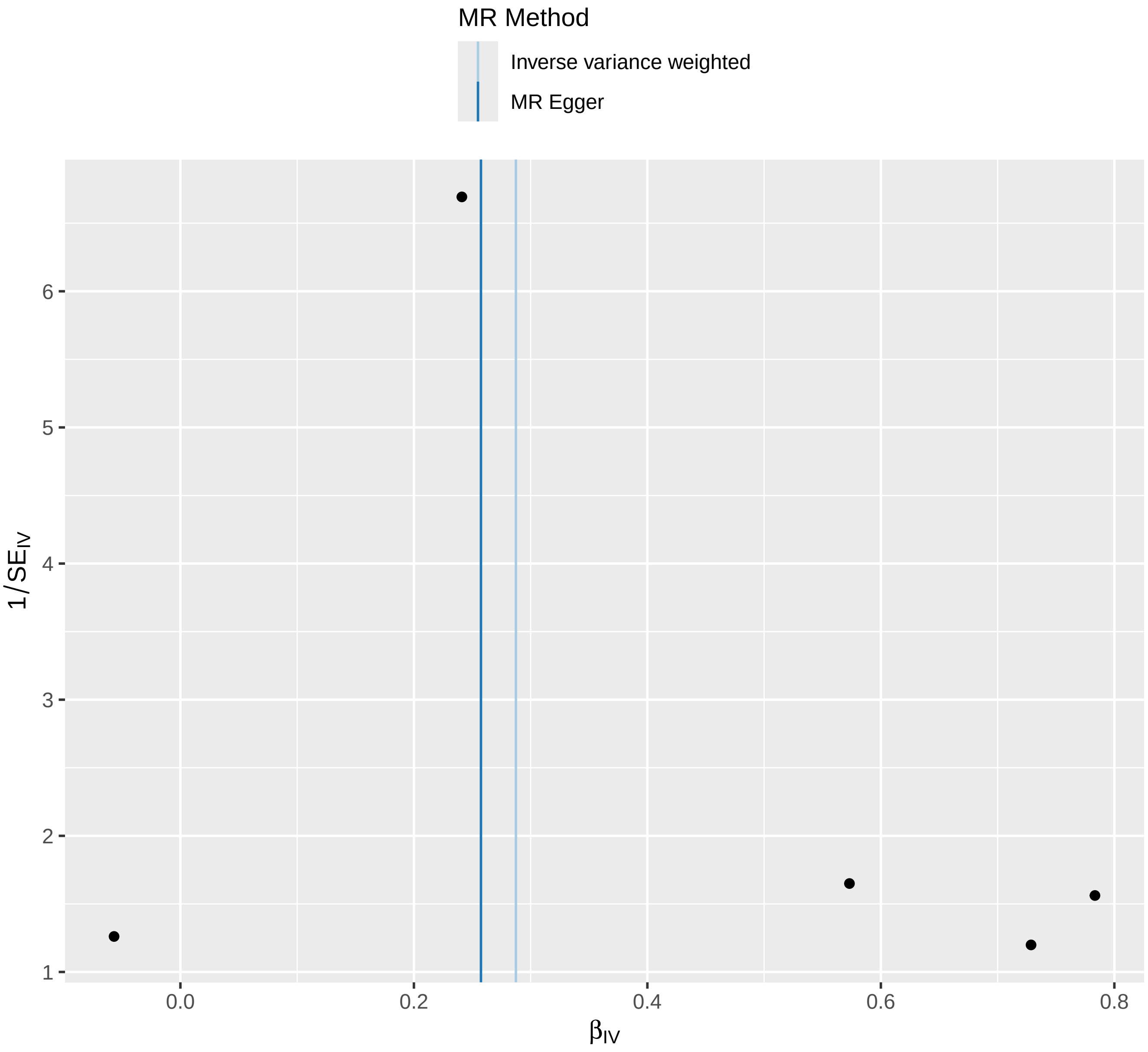
MR Method
Inverse variance weighted
MR Egger
1/SE_IV
6
5
4
3
2
1
0.0
0.2
0.4
0.6
0.8
β_IV

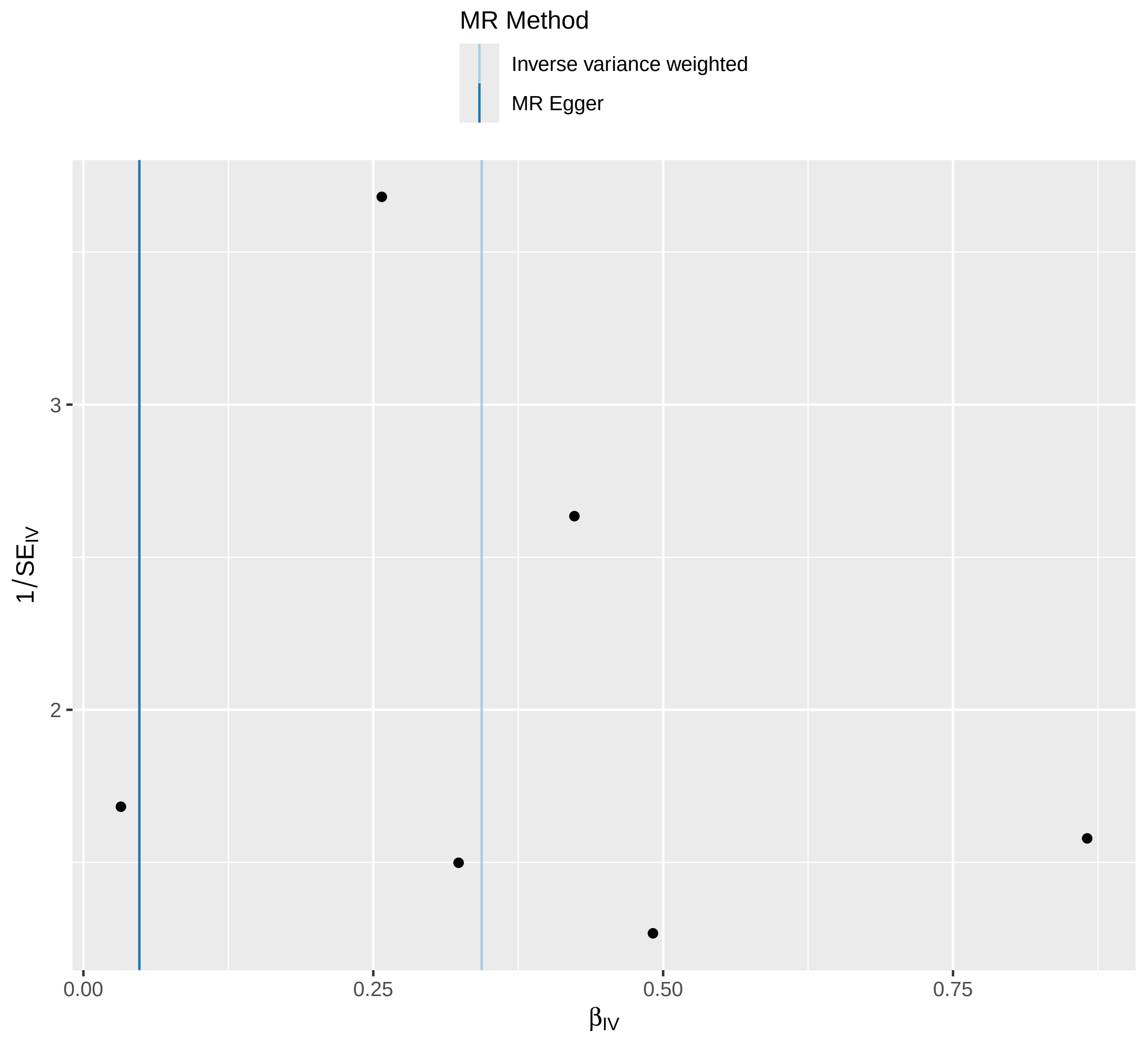

MR Method
Inverse variance weighted
MR Egger
1/SE_IV
3
2
0.00
0.25
0.50
0.75
β_IV

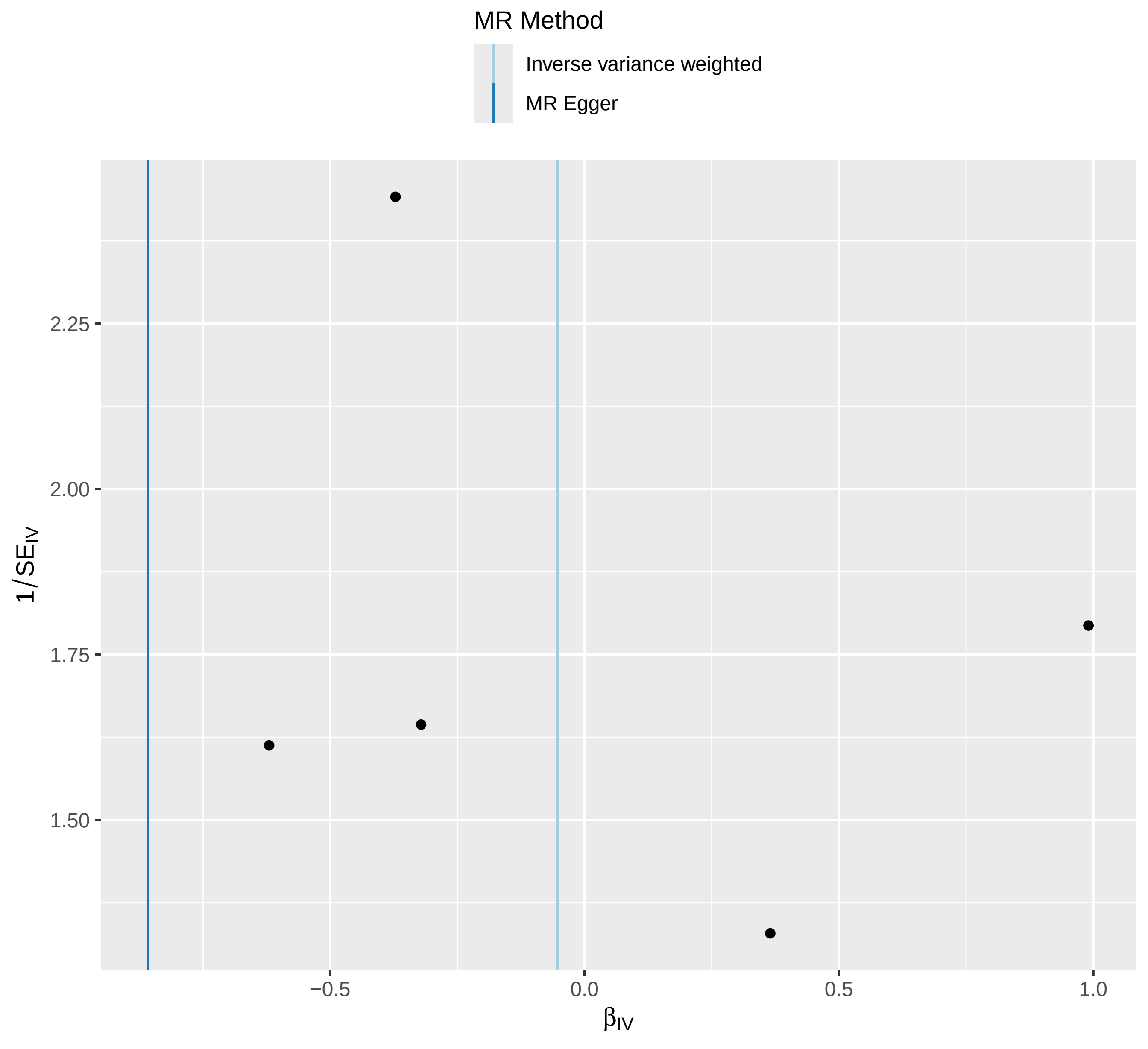

MR Method
Inverse variance weighted
MR Egger
2.25
2.00
1.75
1.50
1/SE_IV
−0.5
0.0
0.5
1.0
β_IV

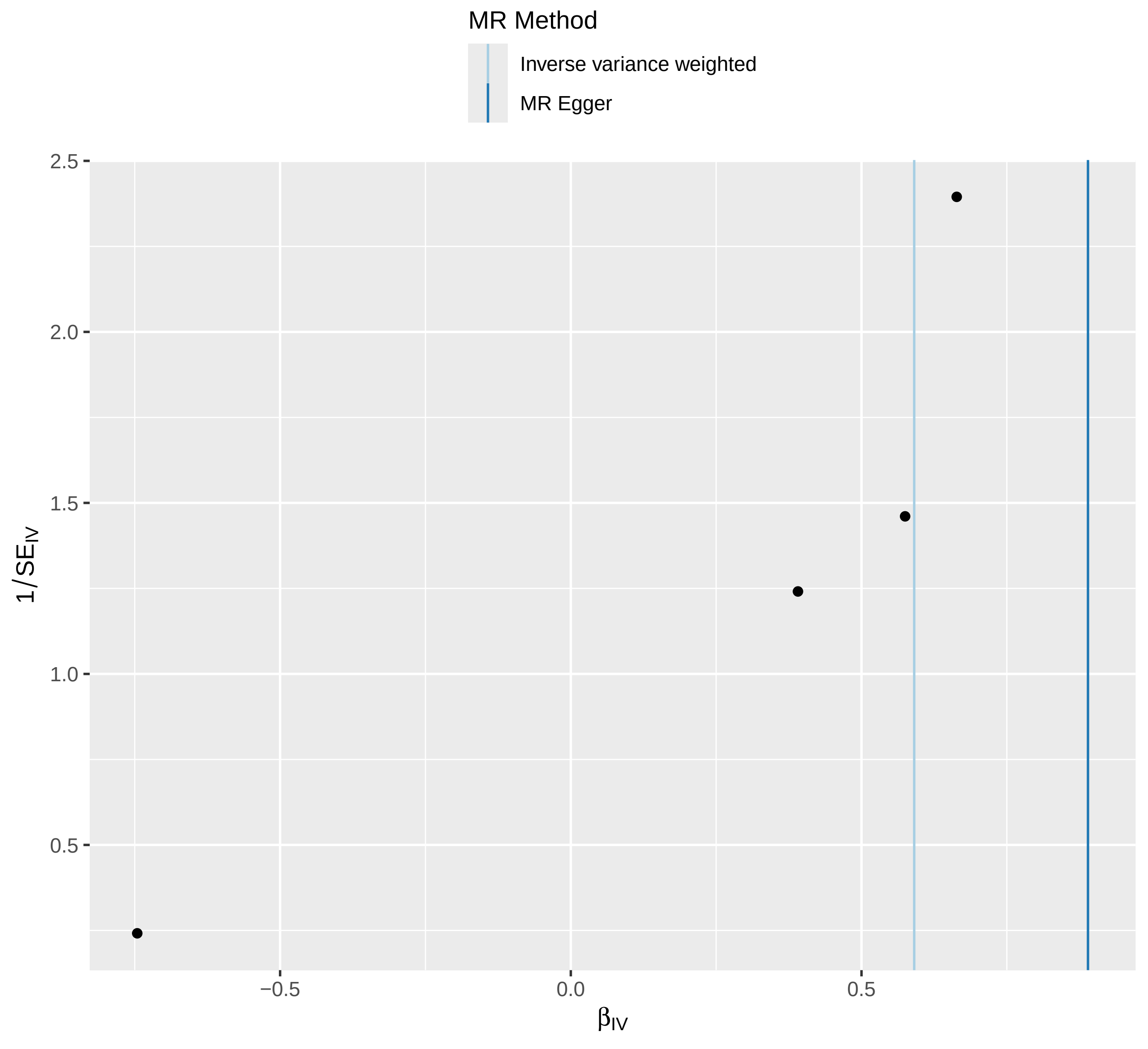

MR Method
Inverse variance weighted
MR Egger
2.5
2.0
1.5
1.0
0.5
1/SE_IV
−0.5
0.0
0.5
β_IV

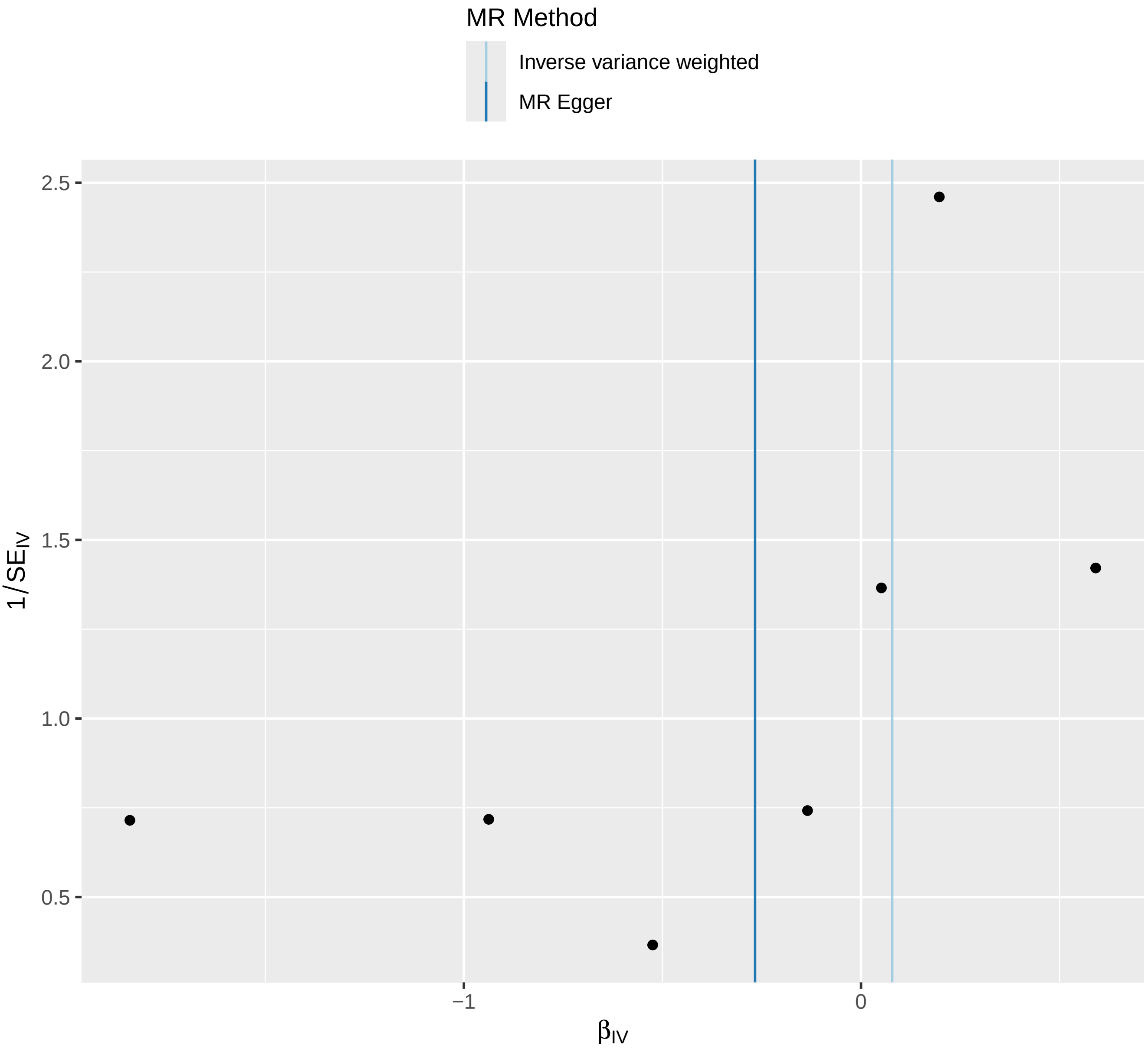

MR Method
Inverse variance weighted
MR Egger
2.5
2.0
1.5
1.0
0.5
$1/SE_{IV}$
−1
0
β_{IV}

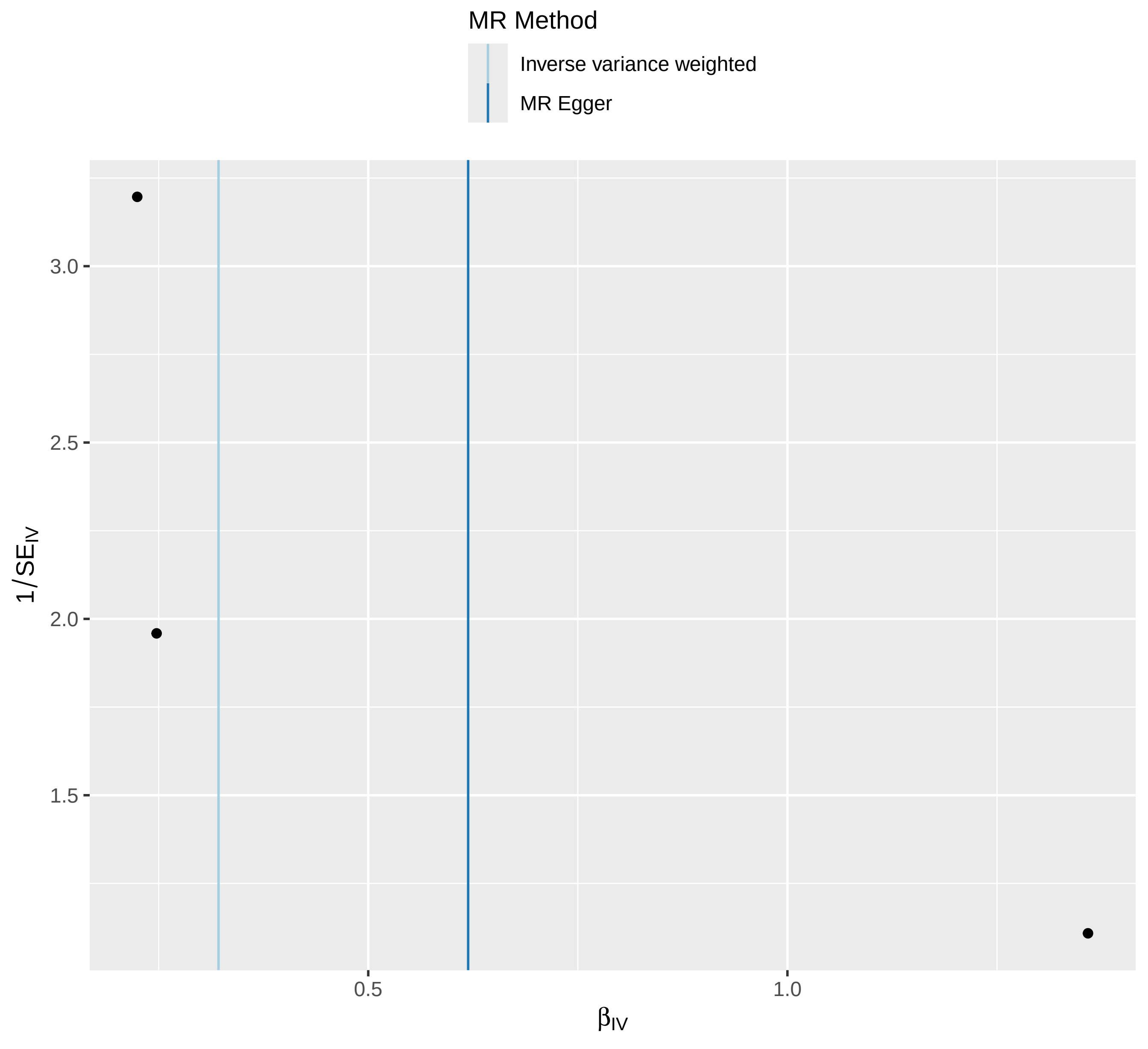
MR Method
Inverse variance weighted
MR Egger
3.0
2.5
2.0
1.5
1/SE_IV
0.5
1.0
β_IV

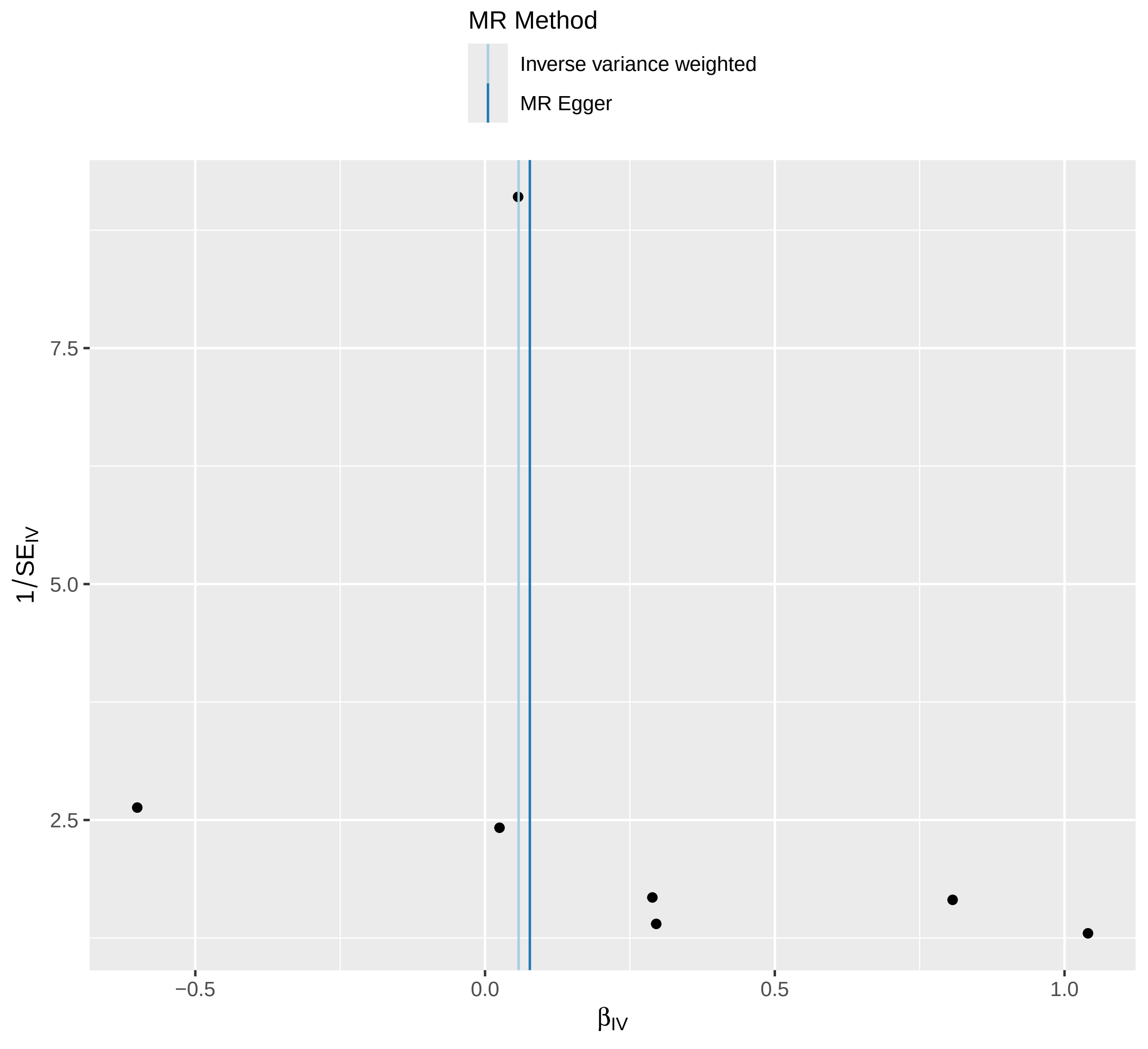
MR Method
Inverse variance weighted
MR Egger
7.5
5.0
2.5
$1/SE_{IV}$
−0.5
0.0
0.5
1.0
β_{IV}

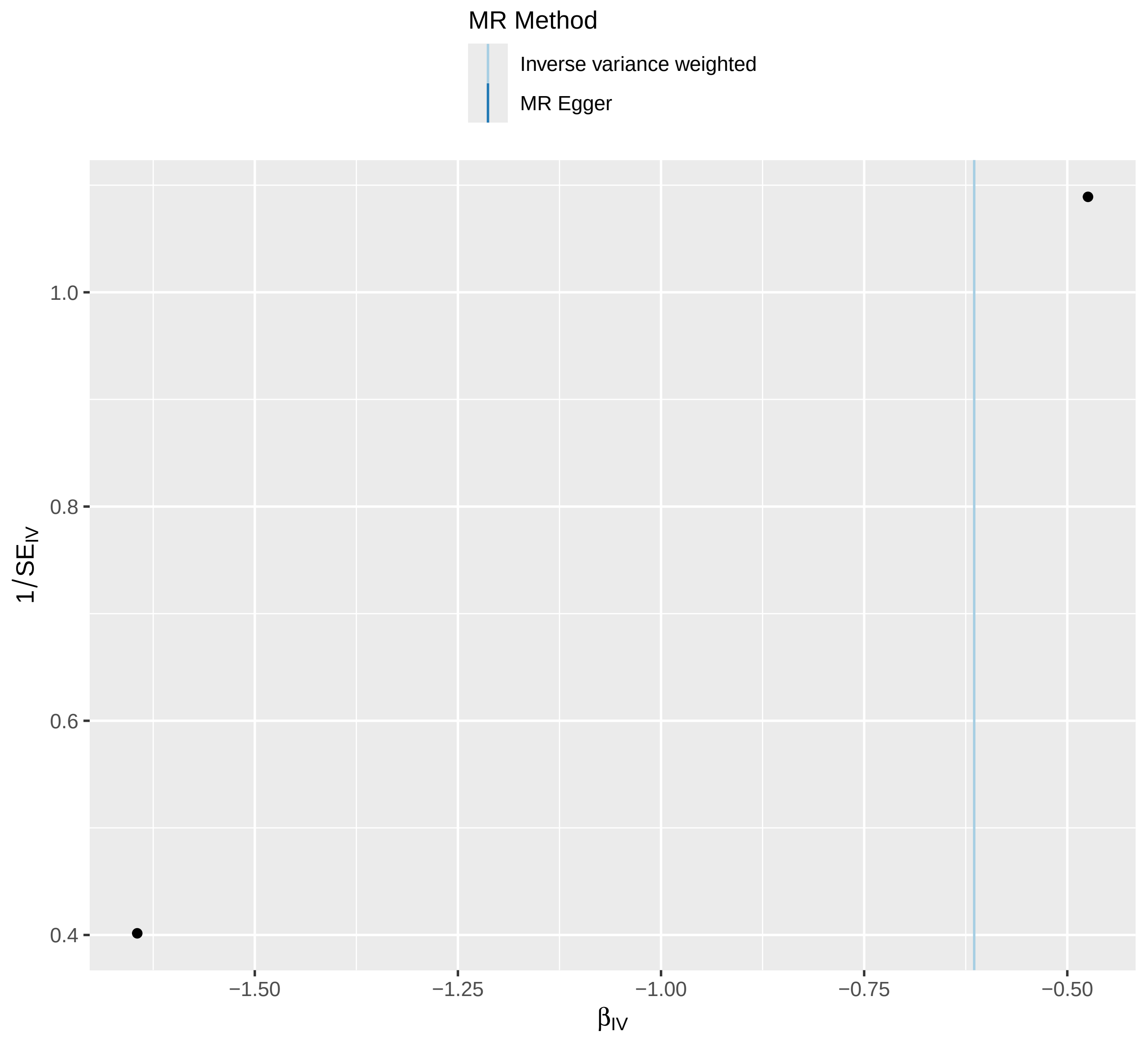

MR Method
Inverse variance weighted
MR Egger
1.0
0.8
0.6
0.4
$1/SE_{IV}$
−1.50
−1.25
−1.00
−0.75
−0.50
β_{IV}

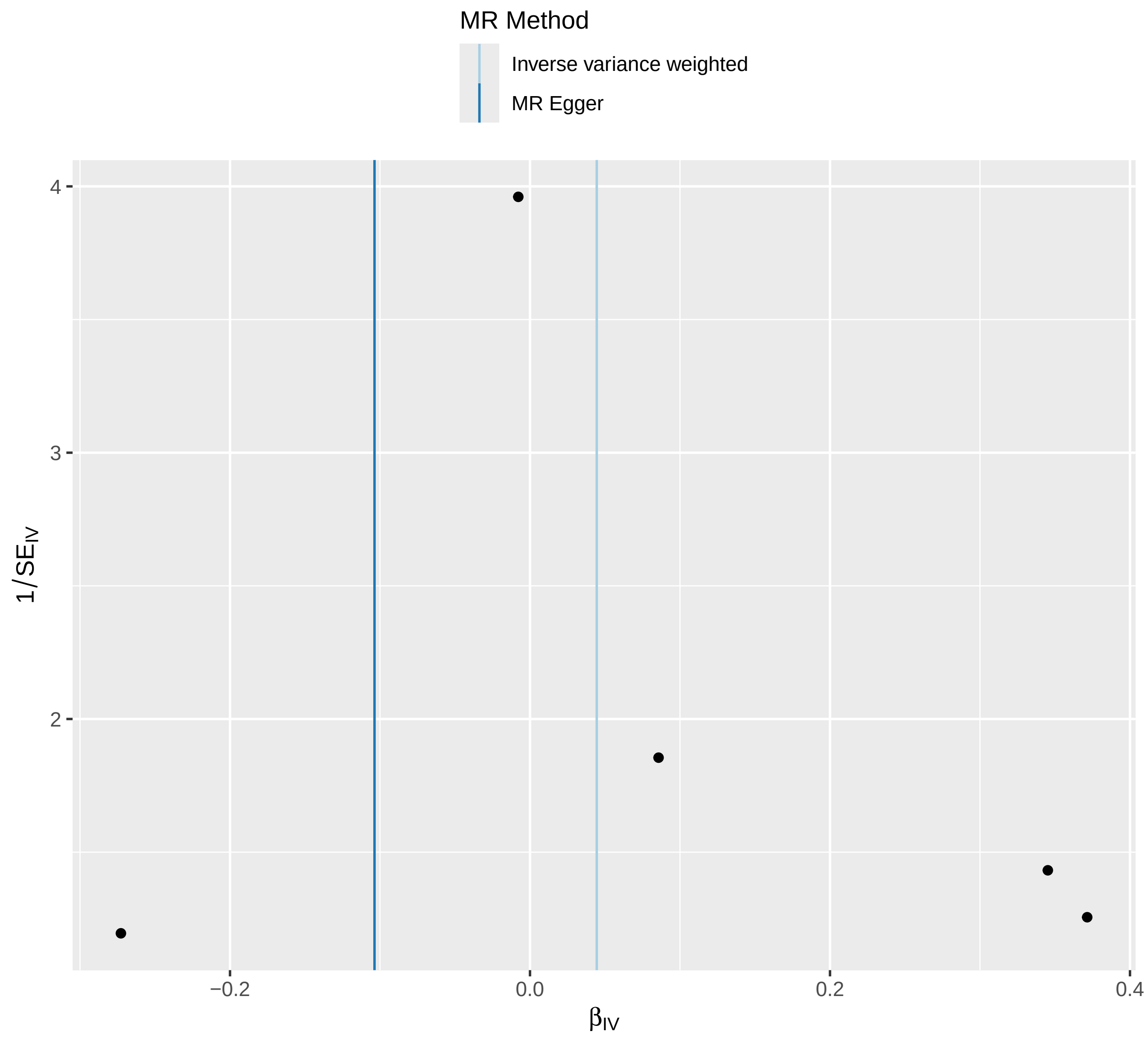
MR Method
Inverse variance weighted
MR Egger
4
3
2
1/SE_IV
−0.2
0.0
0.2
0.4
β_IV

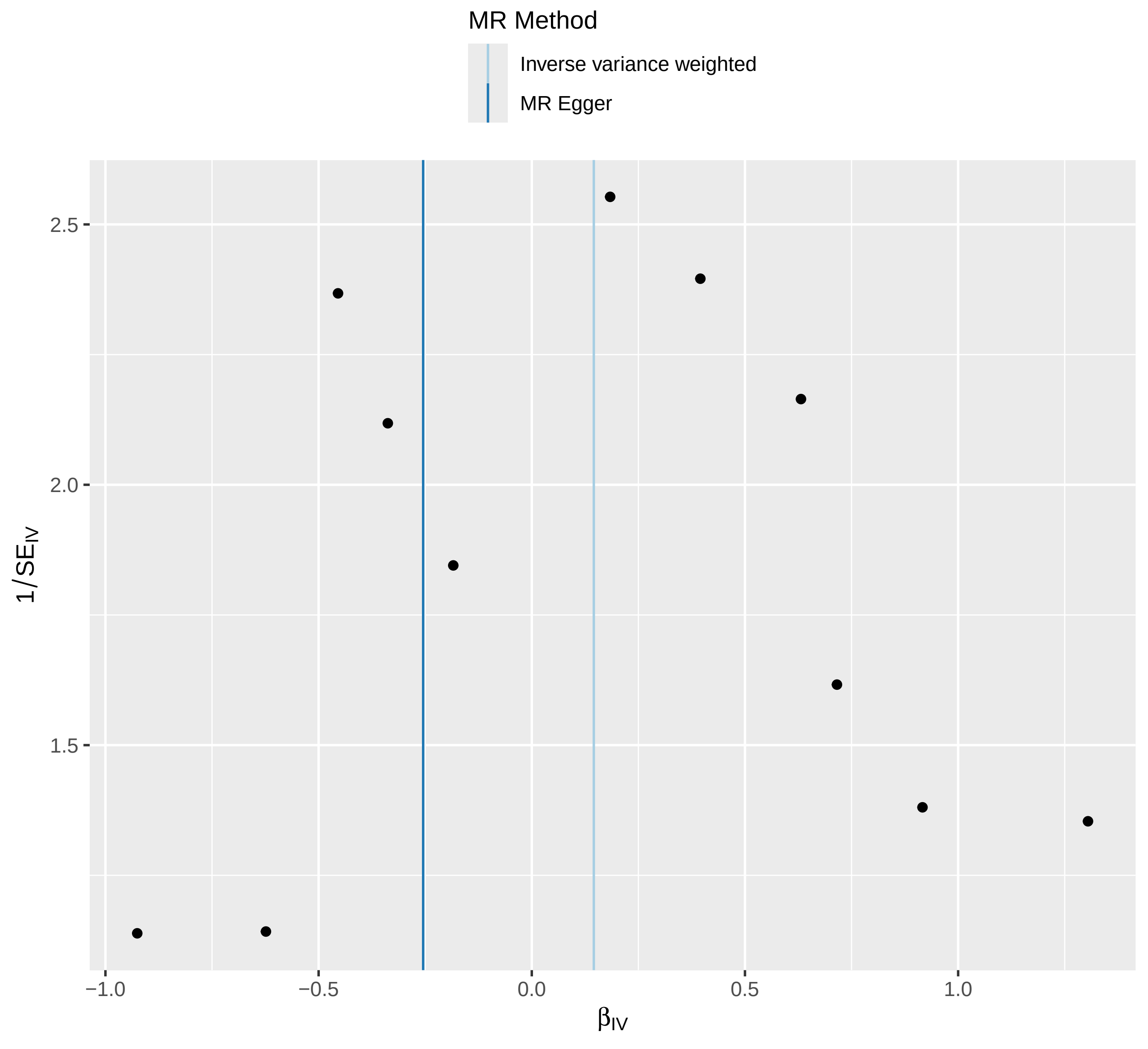

MR Method
Inverse variance weighted
MR Egger
2.5
2.0
1.5
1/SE_IV
−1.0
−0.5
0.0
0.5
1.0
β_IV

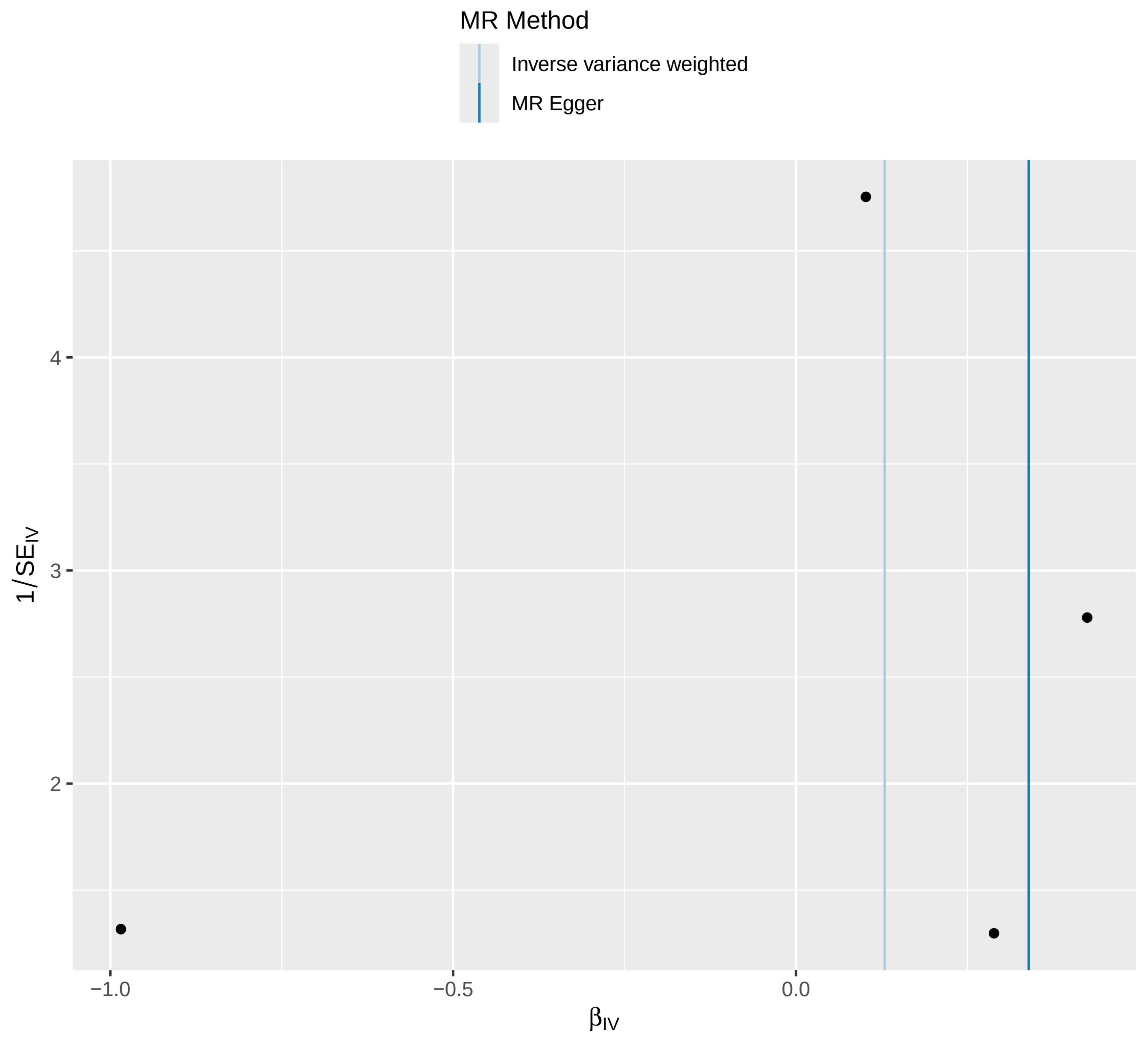

MR Method
Inverse variance weighted
MR Egger
4
3
2
1/SE$_{IV}$
−1.0
−0.5
0.0
β$_{IV}$

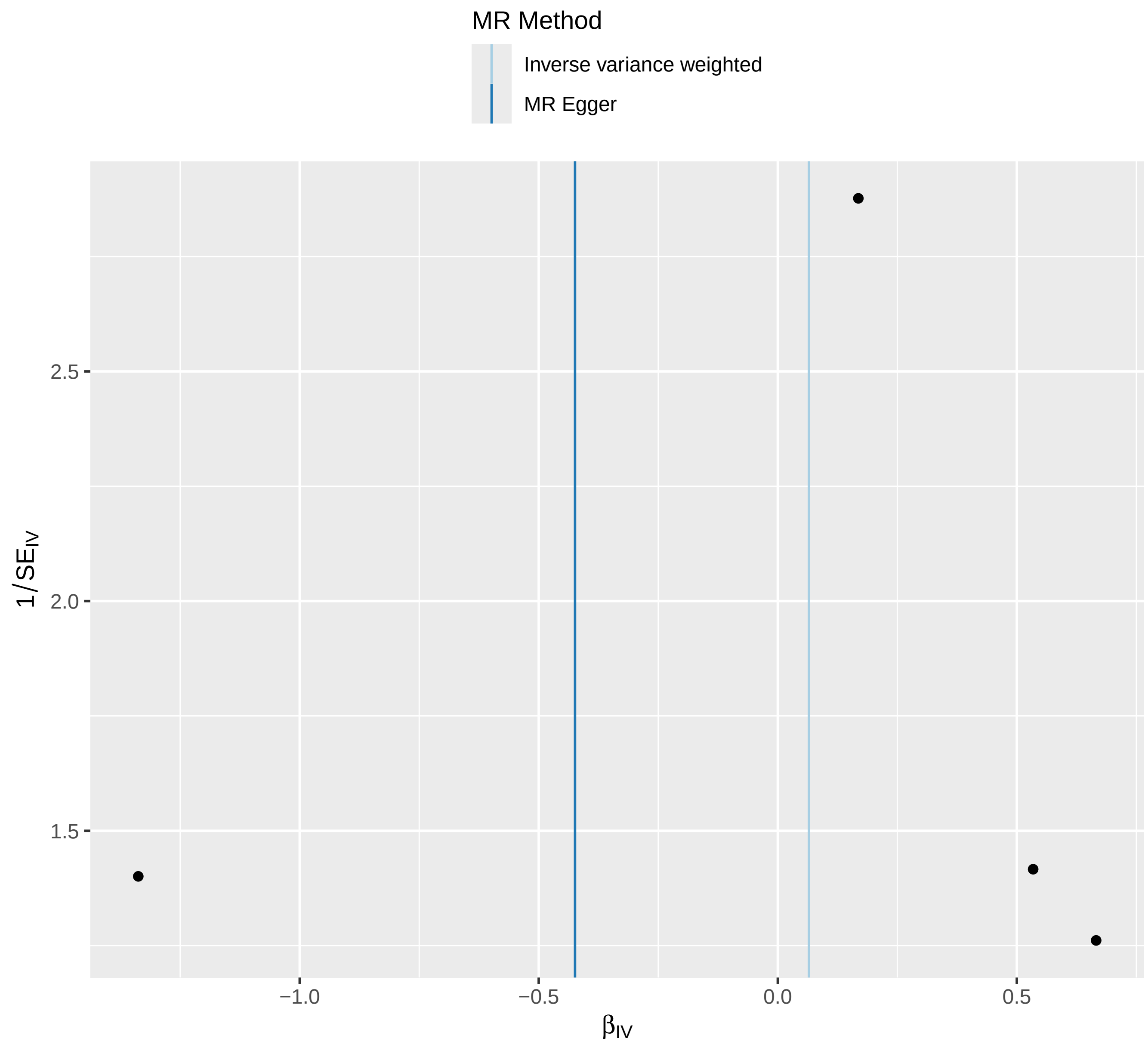

MR Method
Inverse variance weighted
MR Egger
2.5
2.0
1.5
1/SE_IV
−1.0
−0.5
0.0
0.5
β_IV

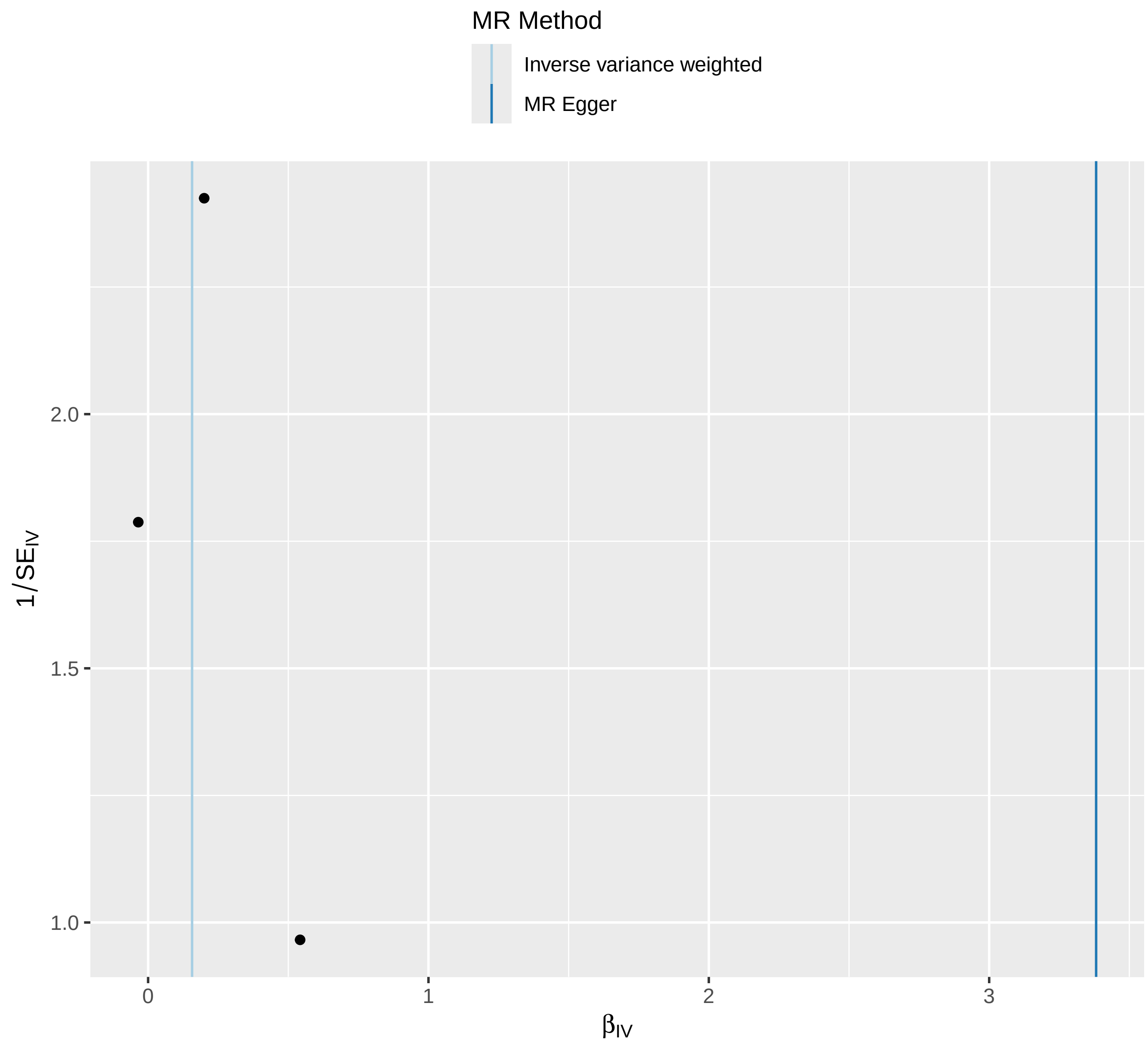
MR Method
Inverse variance weighted
MR Egger
2.0
1.5
1.0
$1/SE_{IV}$
0
1
2
3
β_{IV}

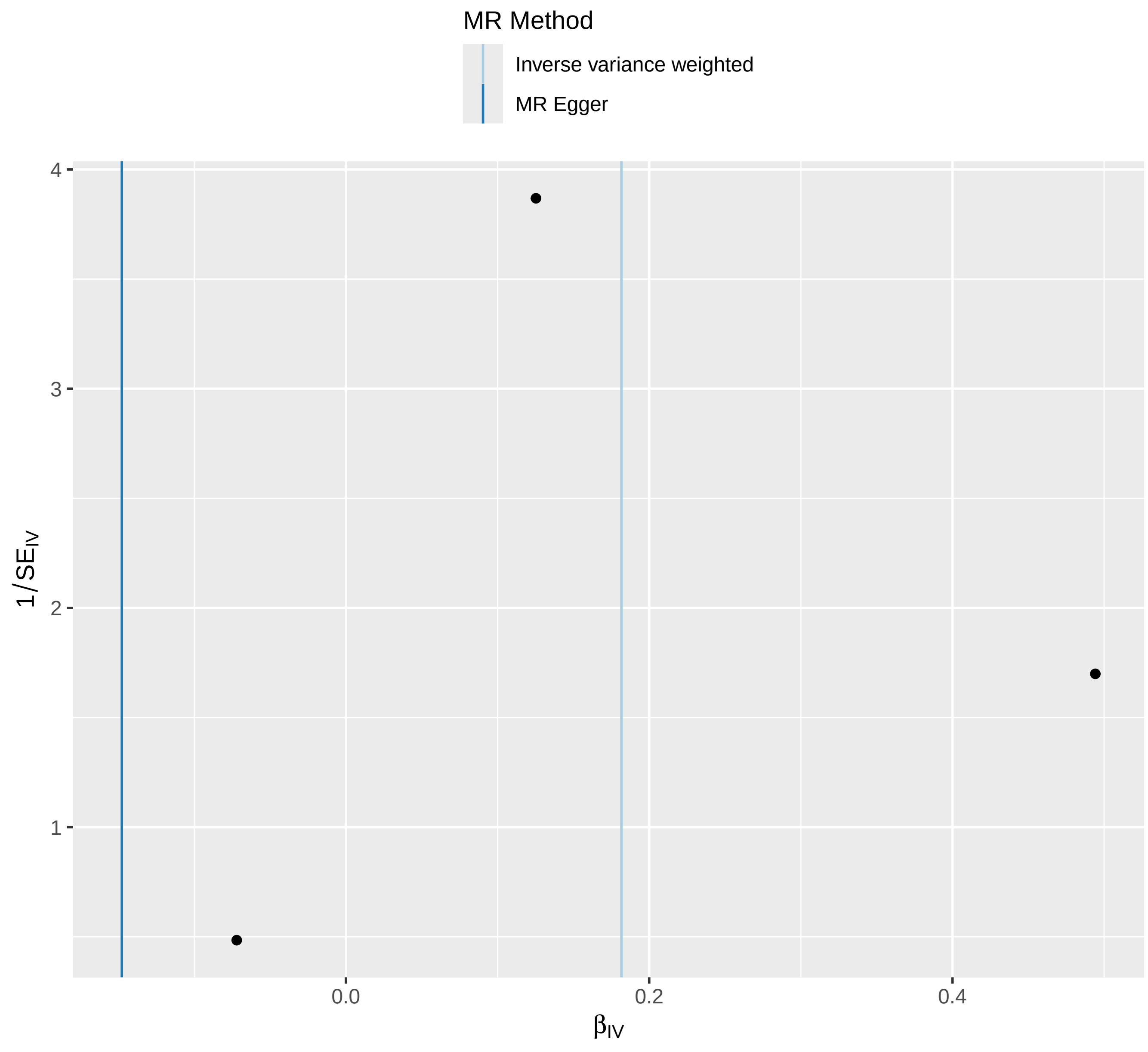
MR Method
Inverse variance weighted
MR Egger
4
3
2
1
1/SE_IV
0.0
0.2
0.4
β_IV

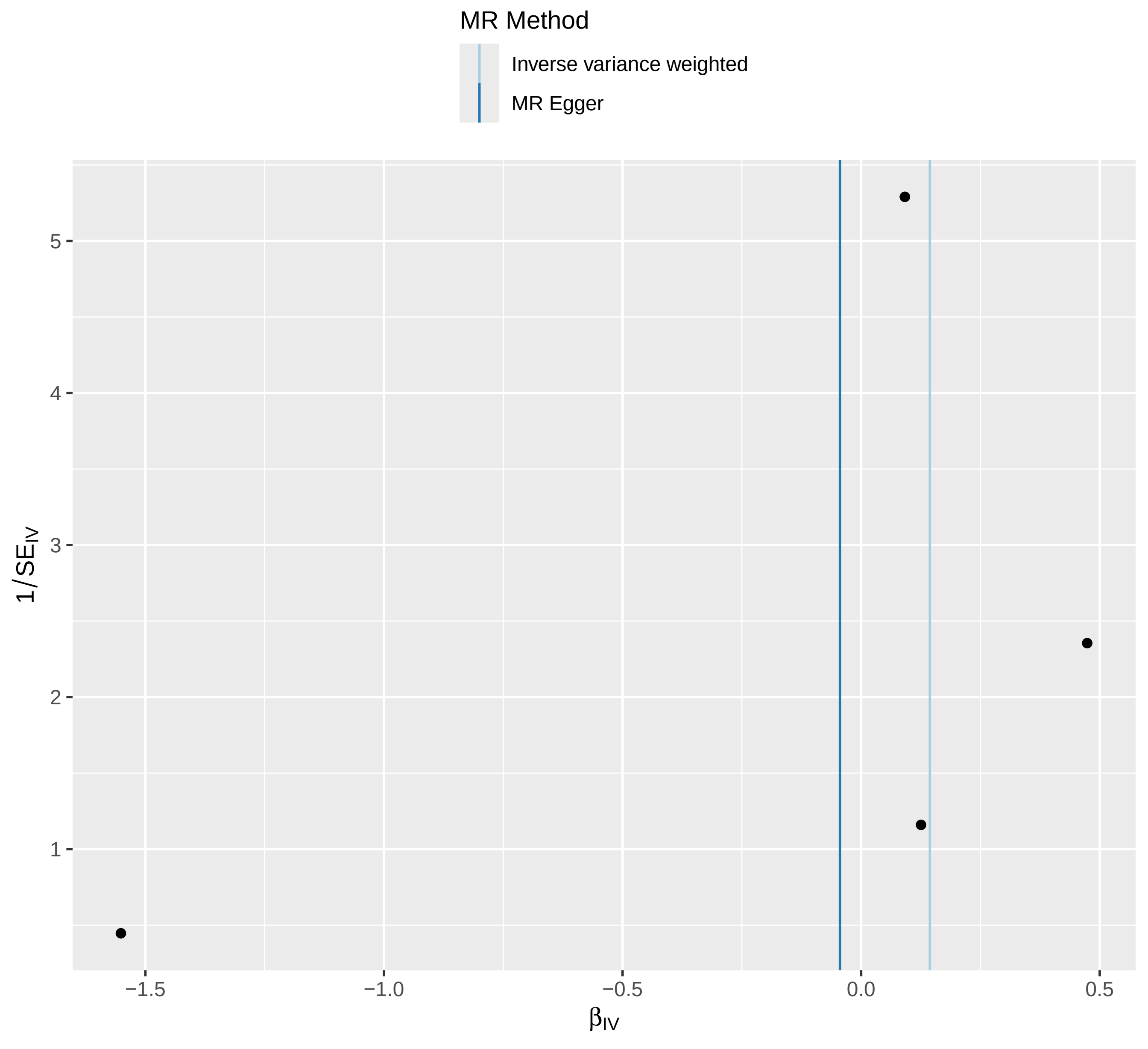

MR Method
Inverse variance weighted
MR Egger
5
4
3
2
1
1/SE_IV
−1.5
−1.0
−0.5
0.0
0.5
β_IV

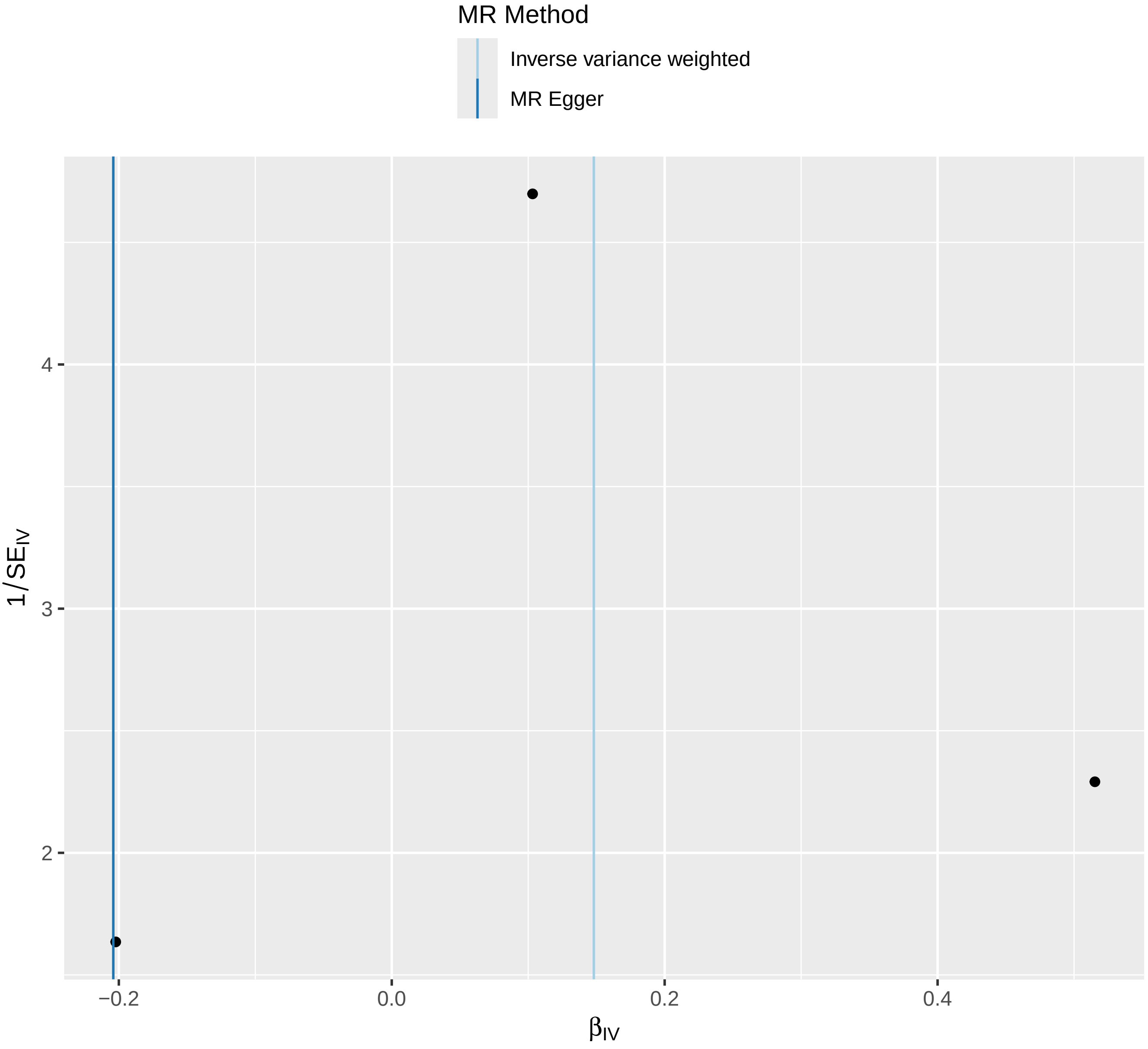
MR Method
Inverse variance weighted
MR Egger
4
3
2
1/SE$_{IV}$
−0.2
0.0
0.2
0.4
β$_{IV}$

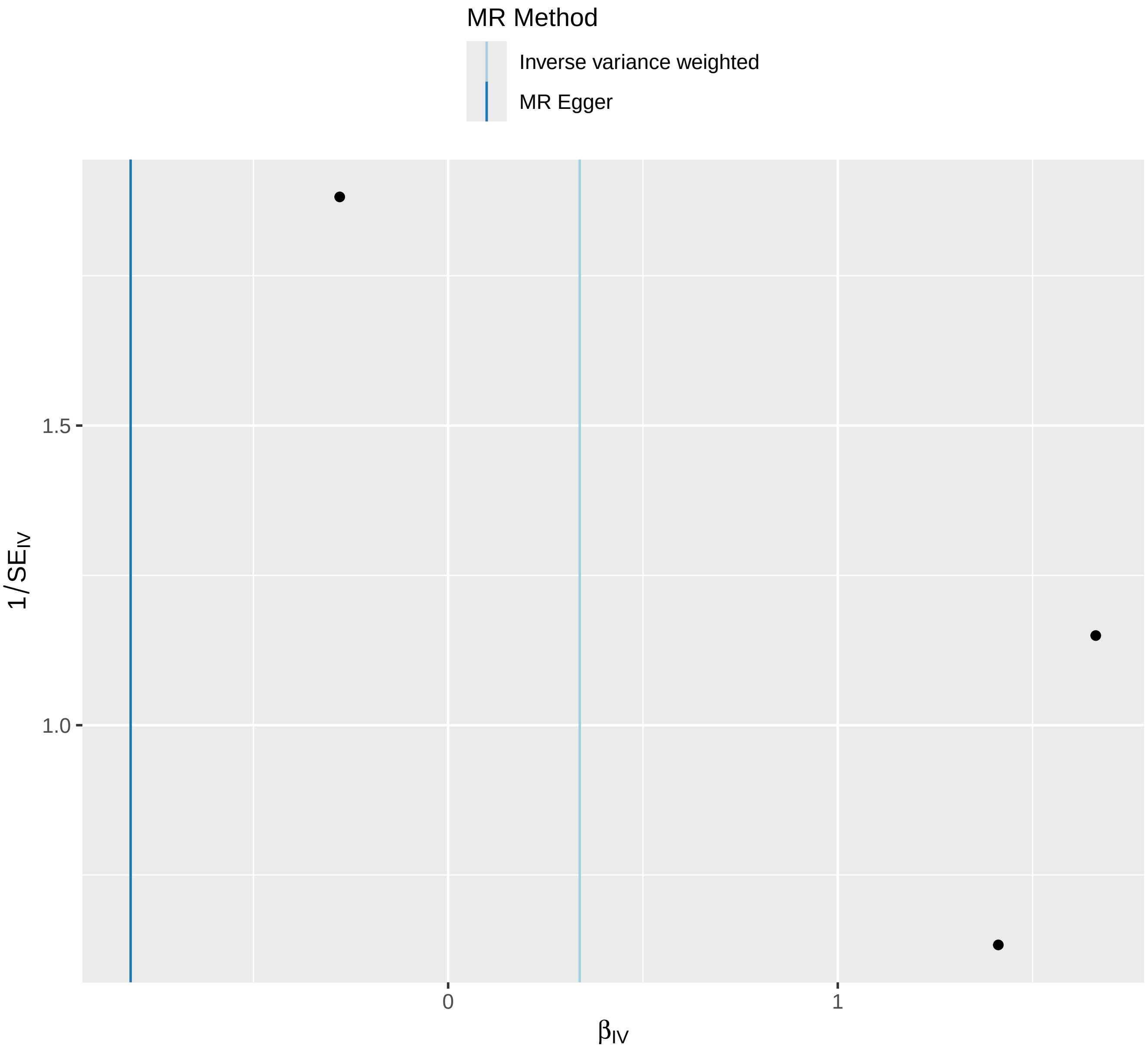
MR Method
Inverse variance weighted
MR Egger
1.5
1.0
0
1
$1/SE_{IV}$
β_{IV}

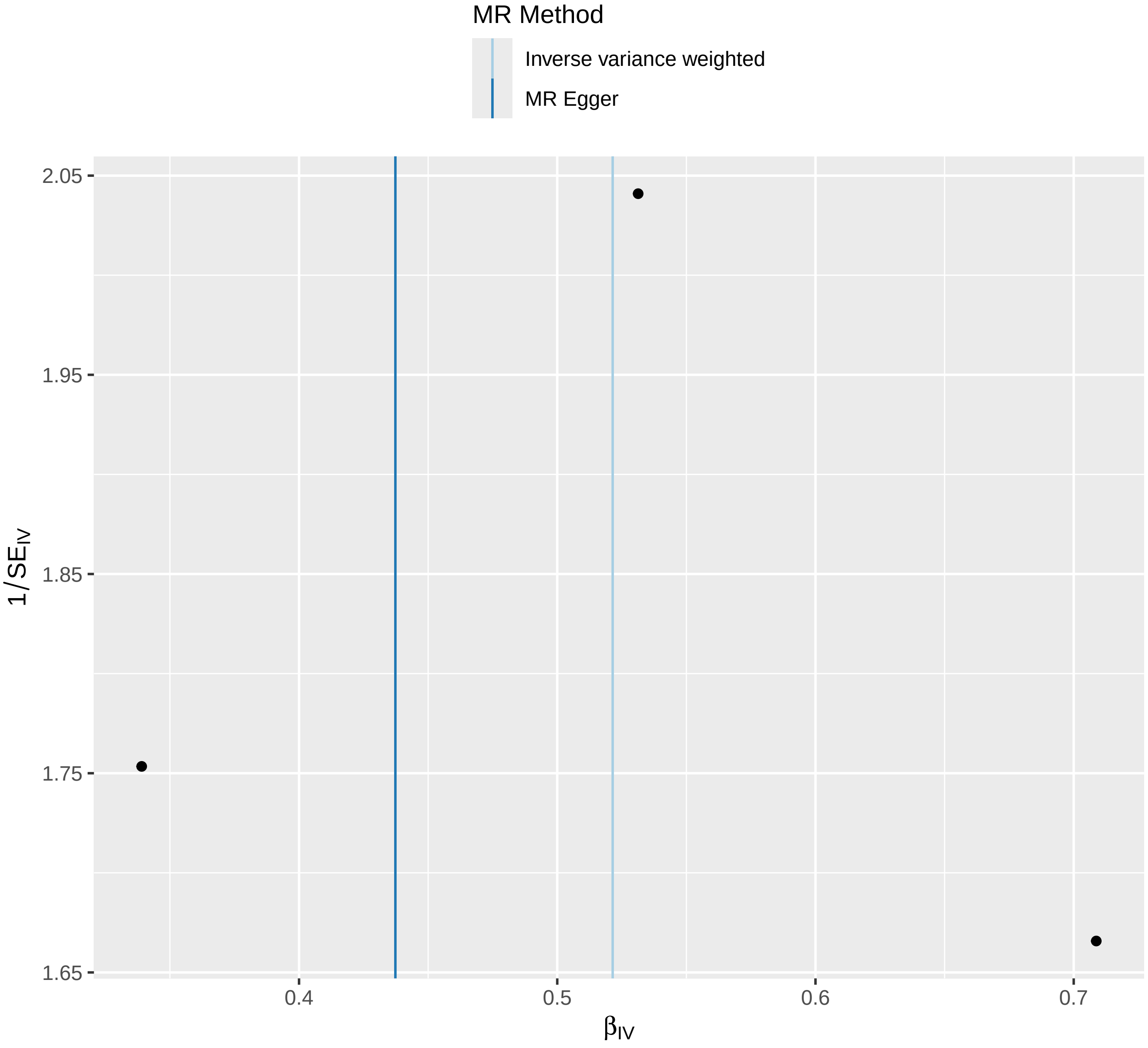

MR Method
Inverse variance weighted
MR Egger
2.05
1.95
1.85
1.75
1.65
$1/SE_{IV}$
0.4
0.5
0.6
0.7
β_{IV}

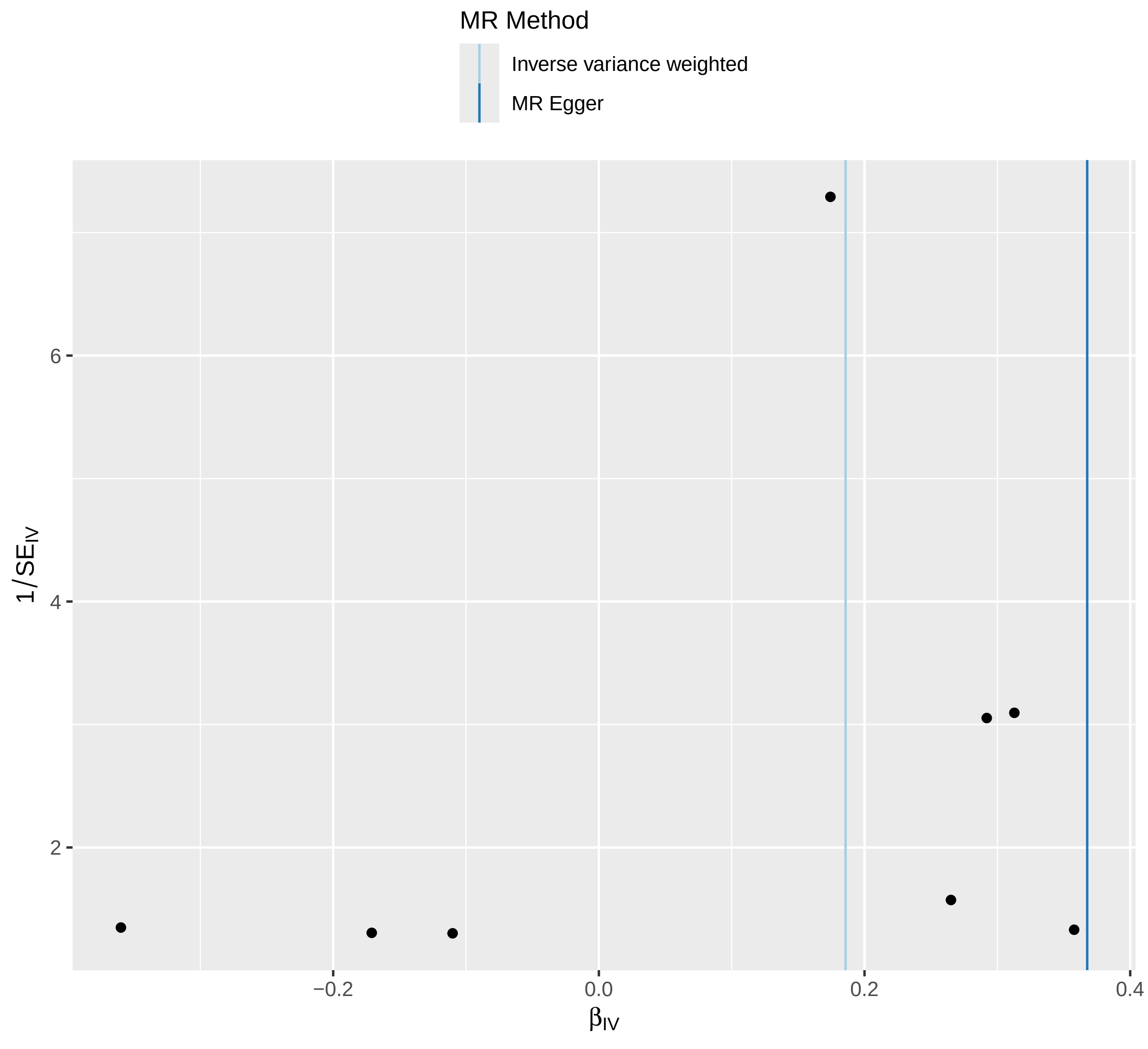

MR Method
Inverse variance weighted
MR Egger
1/SE_IV
β_IV
6
4
2
−0.2
0.0
0.2
0.4

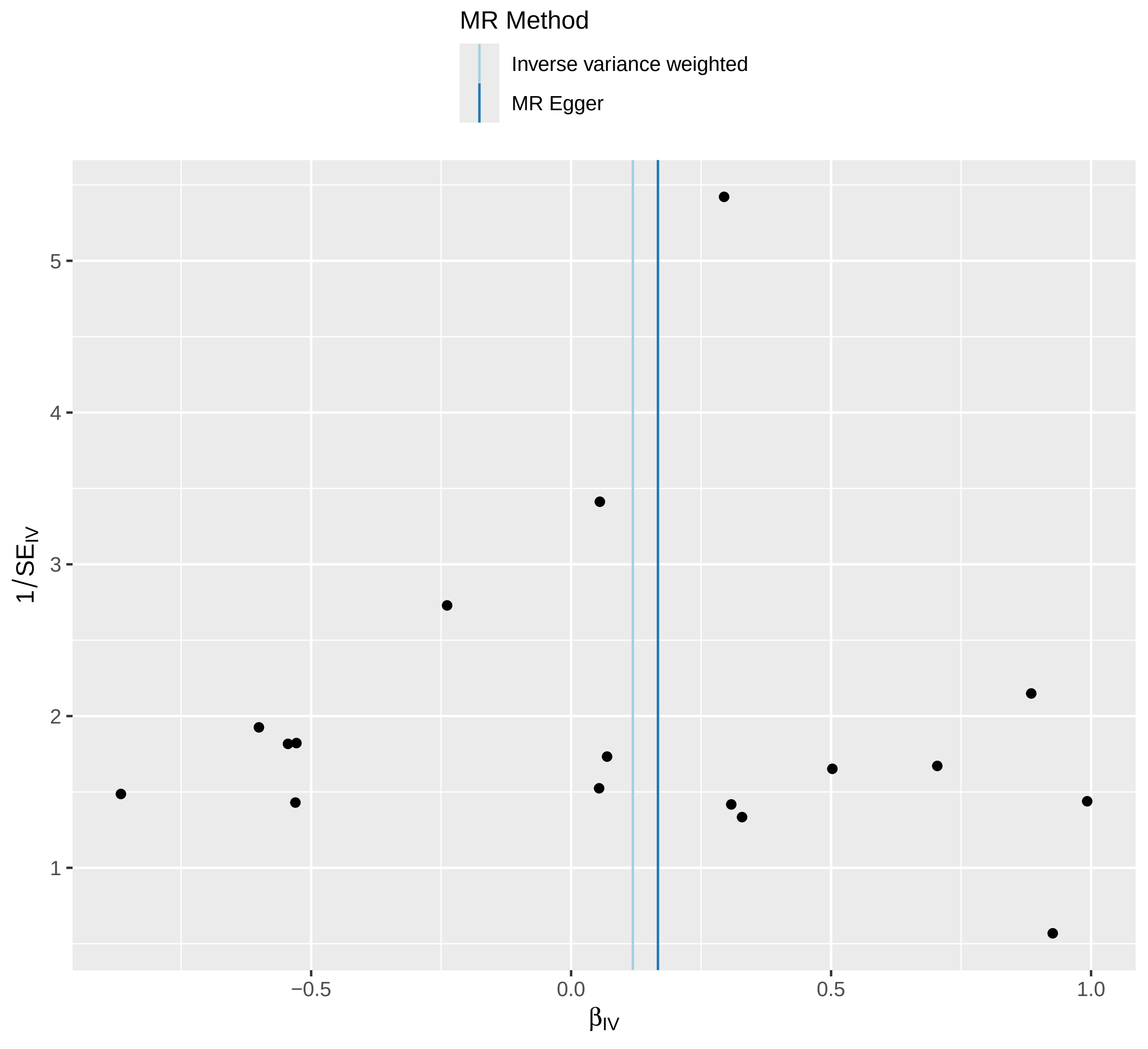
MR Method
Inverse variance weighted
MR Egger
5
4
3
2
1
$1/SE_{IV}$
−0.5
0.0
0.5
1.0
β_{IV}

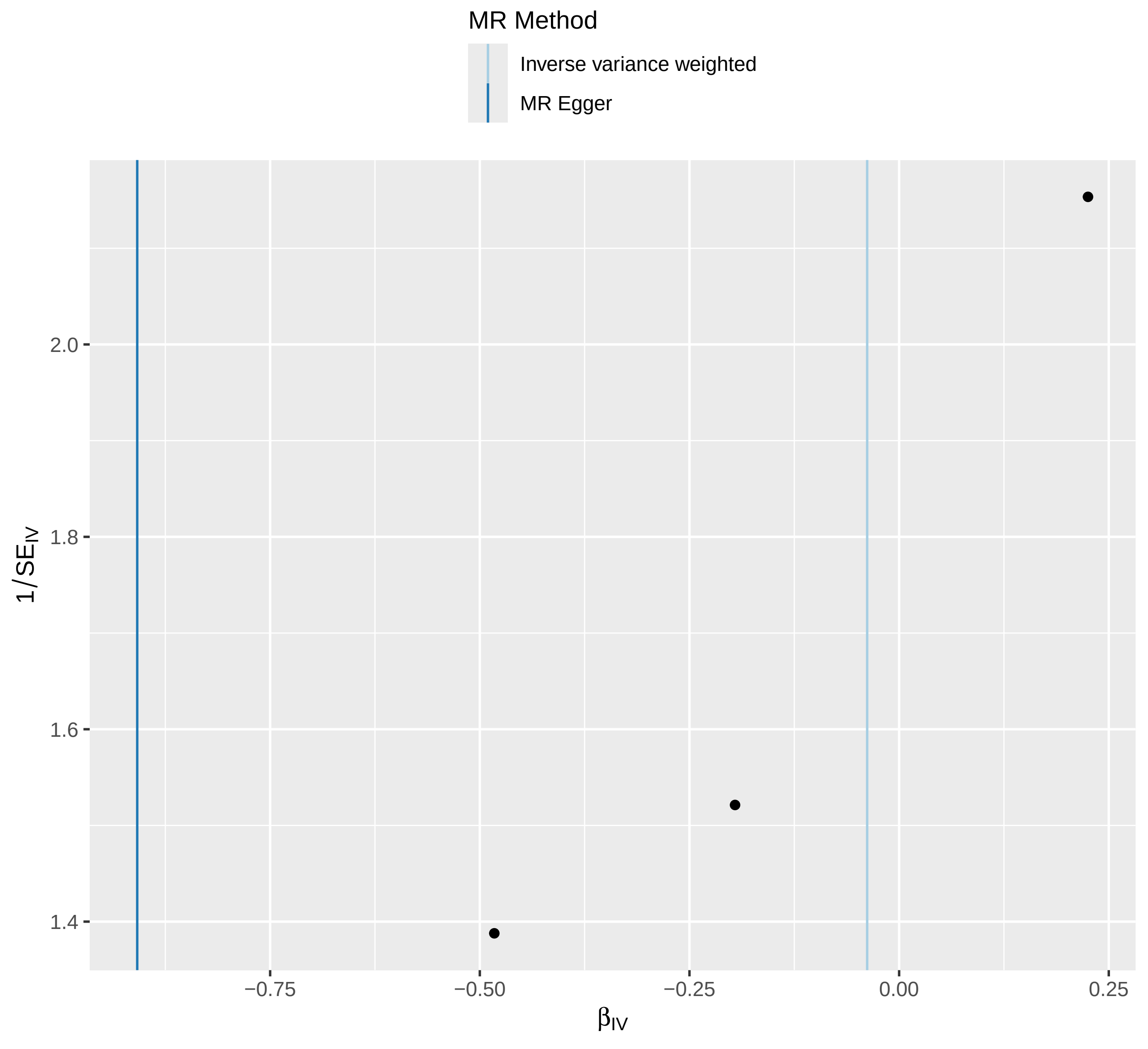

MR Method
Inverse variance weighted
MR Egger
2.0
1.8
1.6
1.4
1/SE_IV
−0.75
−0.50
−0.25
0.00
0.25
β_IV

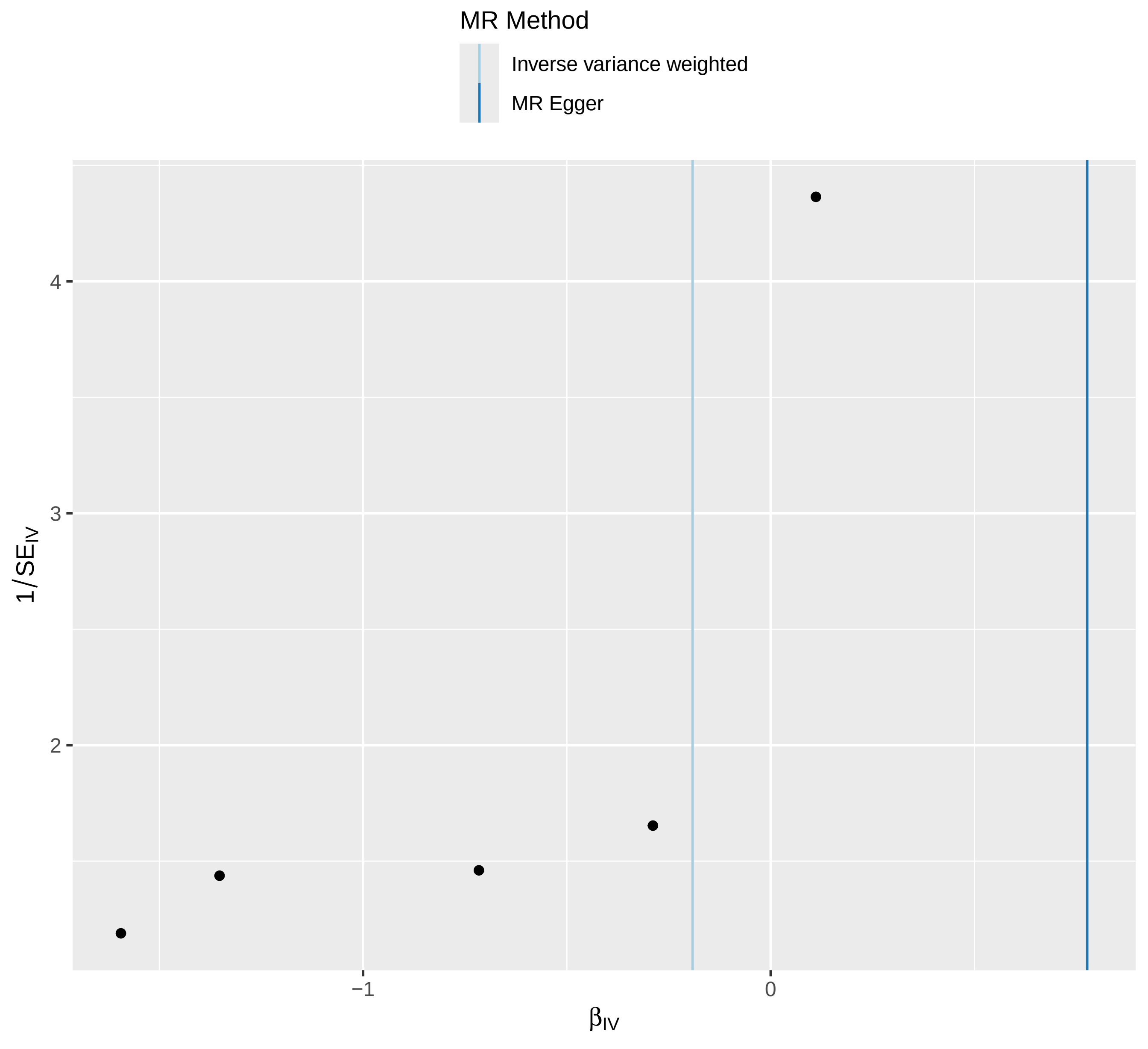
MR Method
Inverse variance weighted
MR Egger
4
3
2
1/SE_IV
−1
0
β_IV

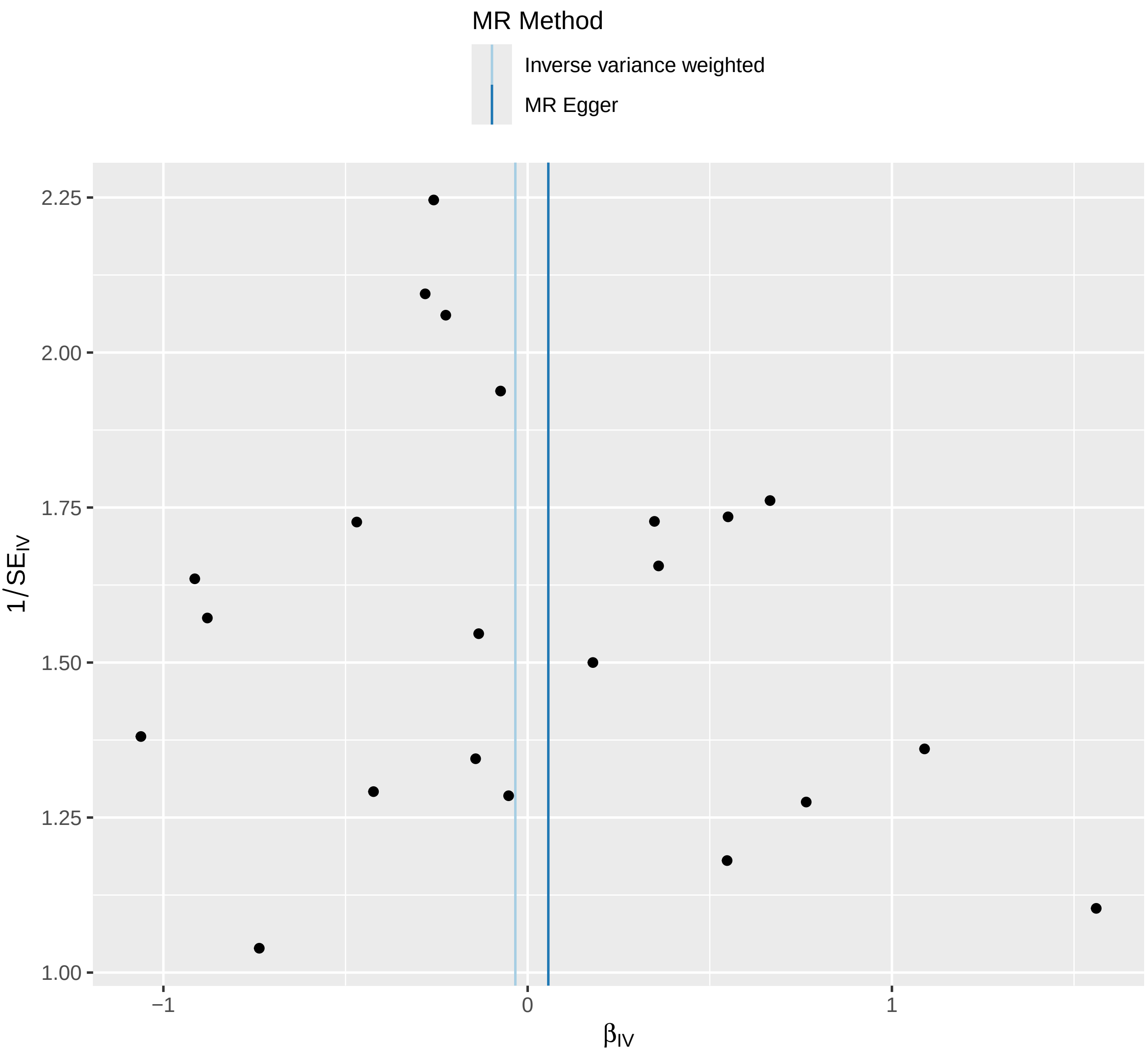
MR Method
Inverse variance weighted
MR Egger
2.25
2.00
1.75
1.50
1.25
1.00
1/SE_IV
−1
0
1
β_IV

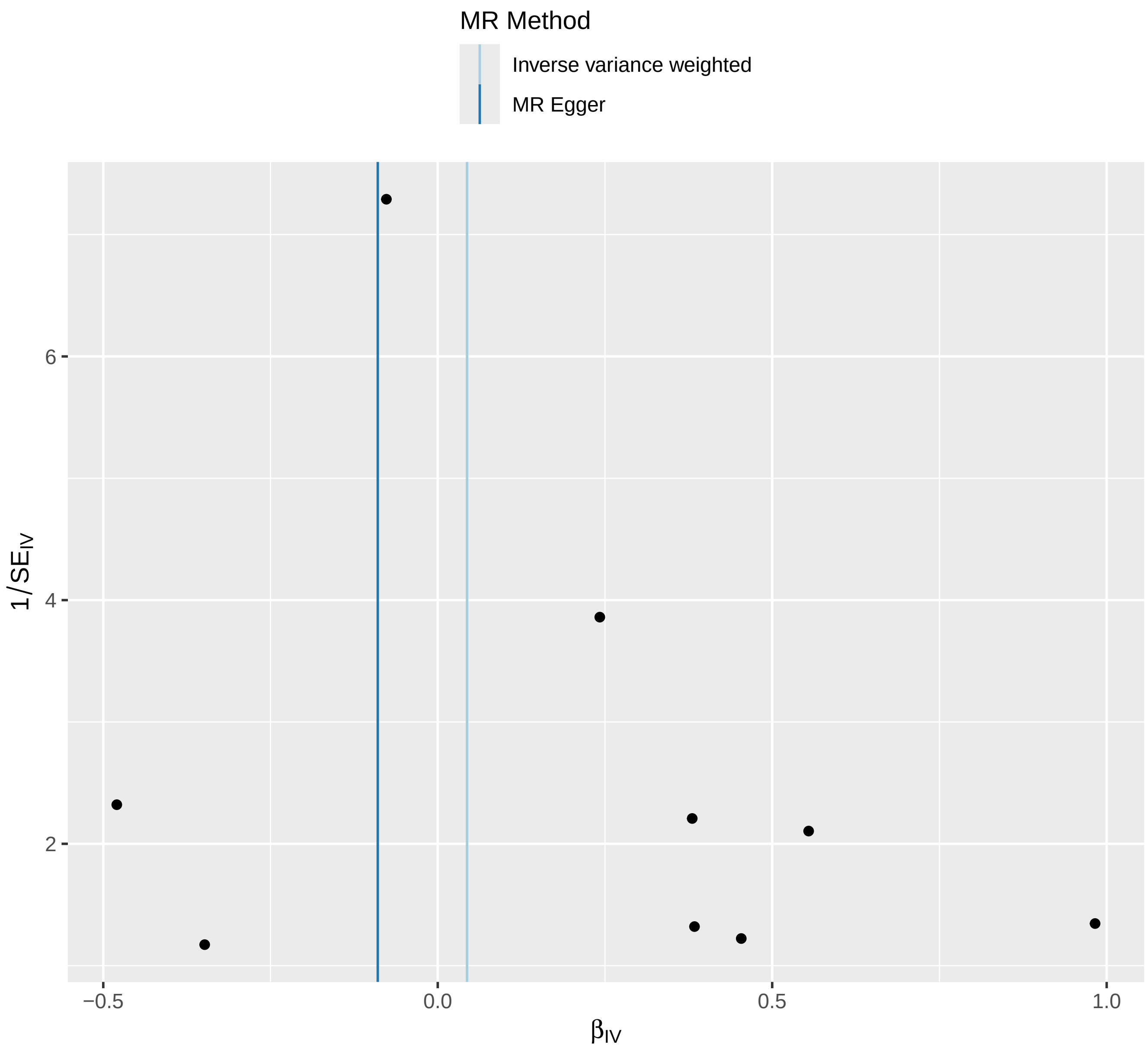
MR Method
Inverse variance weighted
MR Egger
6
4
2
1/SE$_{IV}$
−0.5
0.0
0.5
1.0
β$_{IV}$

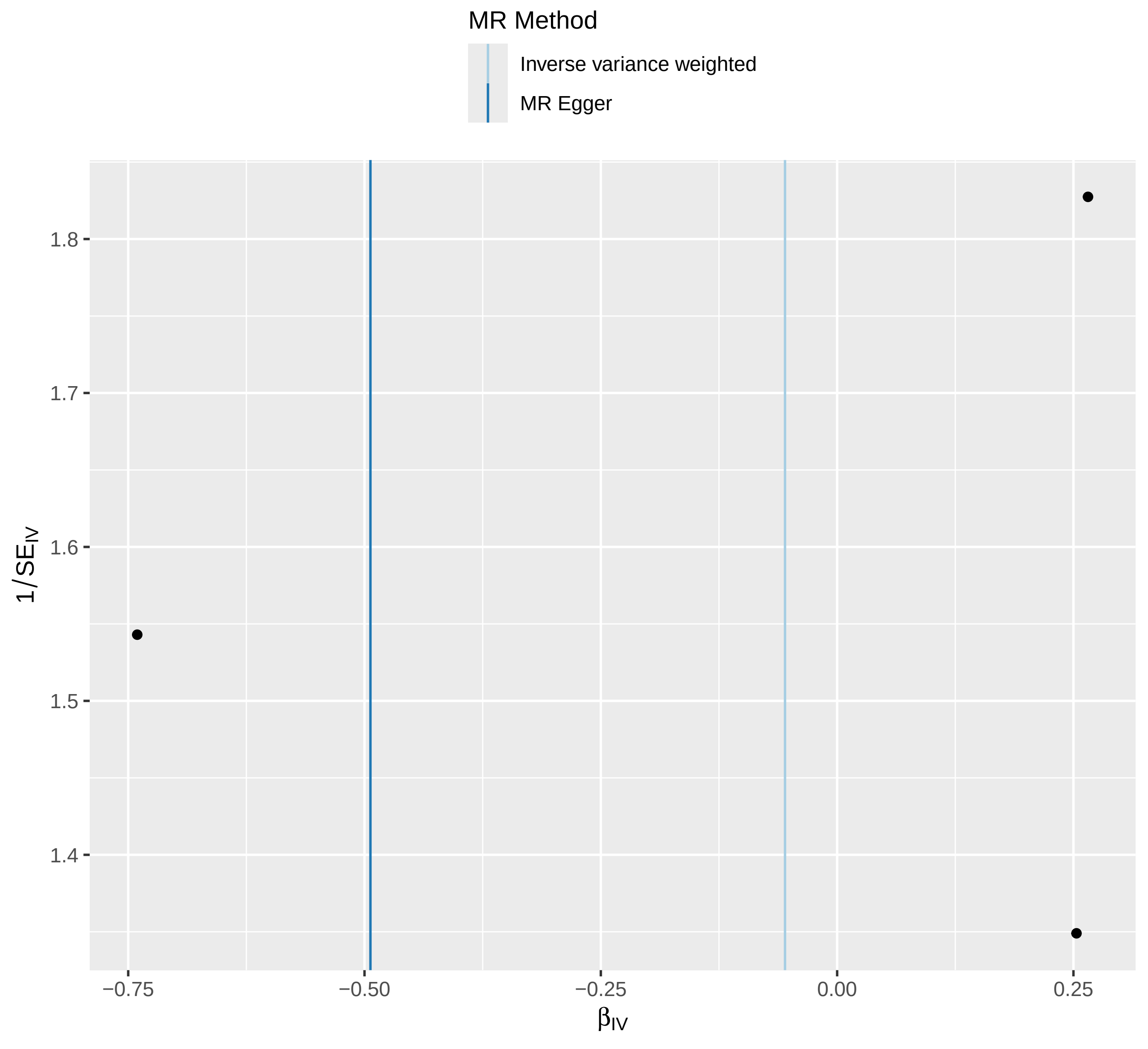
MR Method
Inverse variance weighted
MR Egger
1.8
1.7
1.6
1.5
1.4
1/SE_IV
−0.75
−0.50
−0.25
0.00
0.25
β_IV

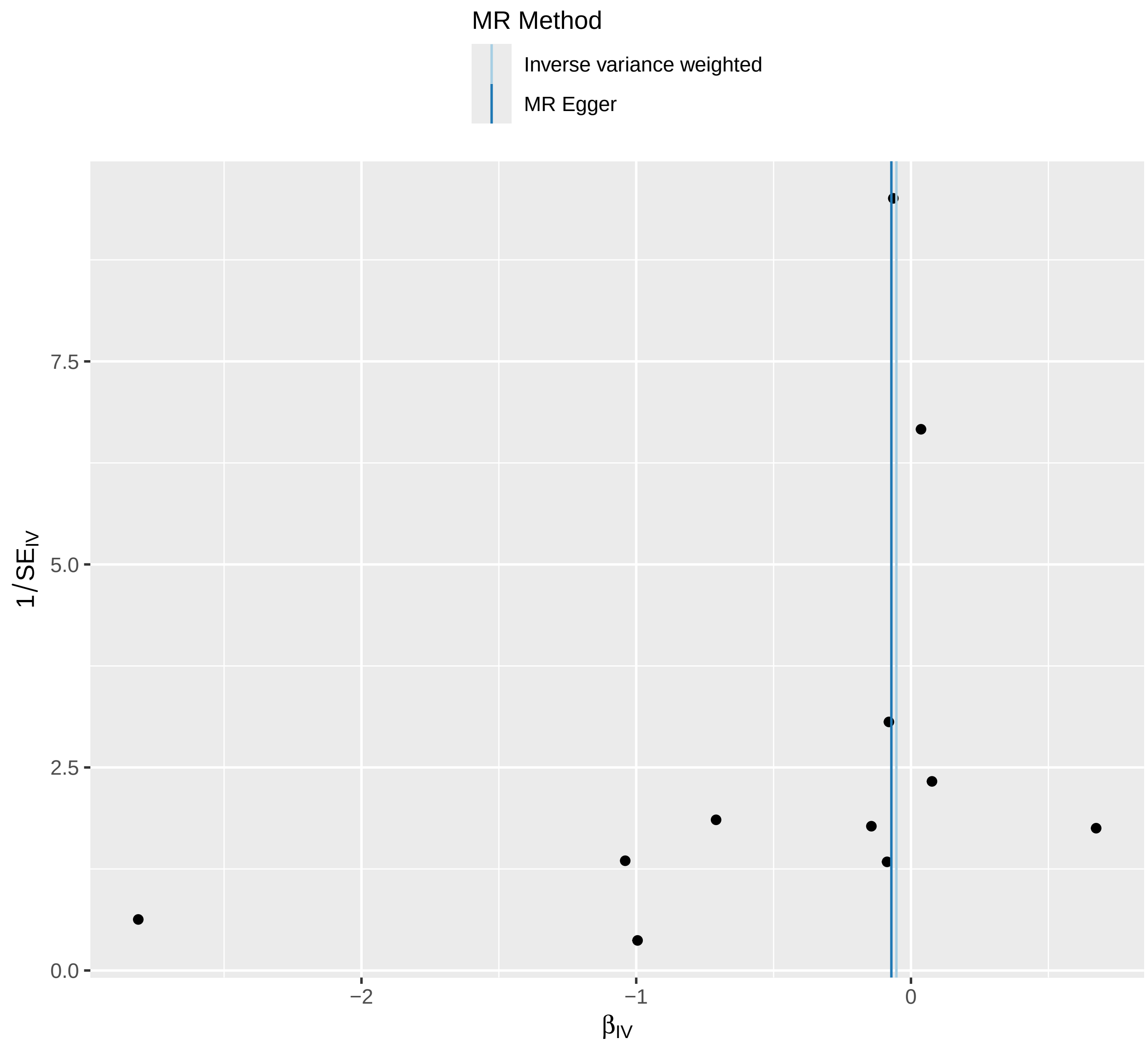
MR Method
Inverse variance weighted
MR Egger
7.5
5.0
2.5
0.0
1/SE_IV
−2
−1
0
β_IV

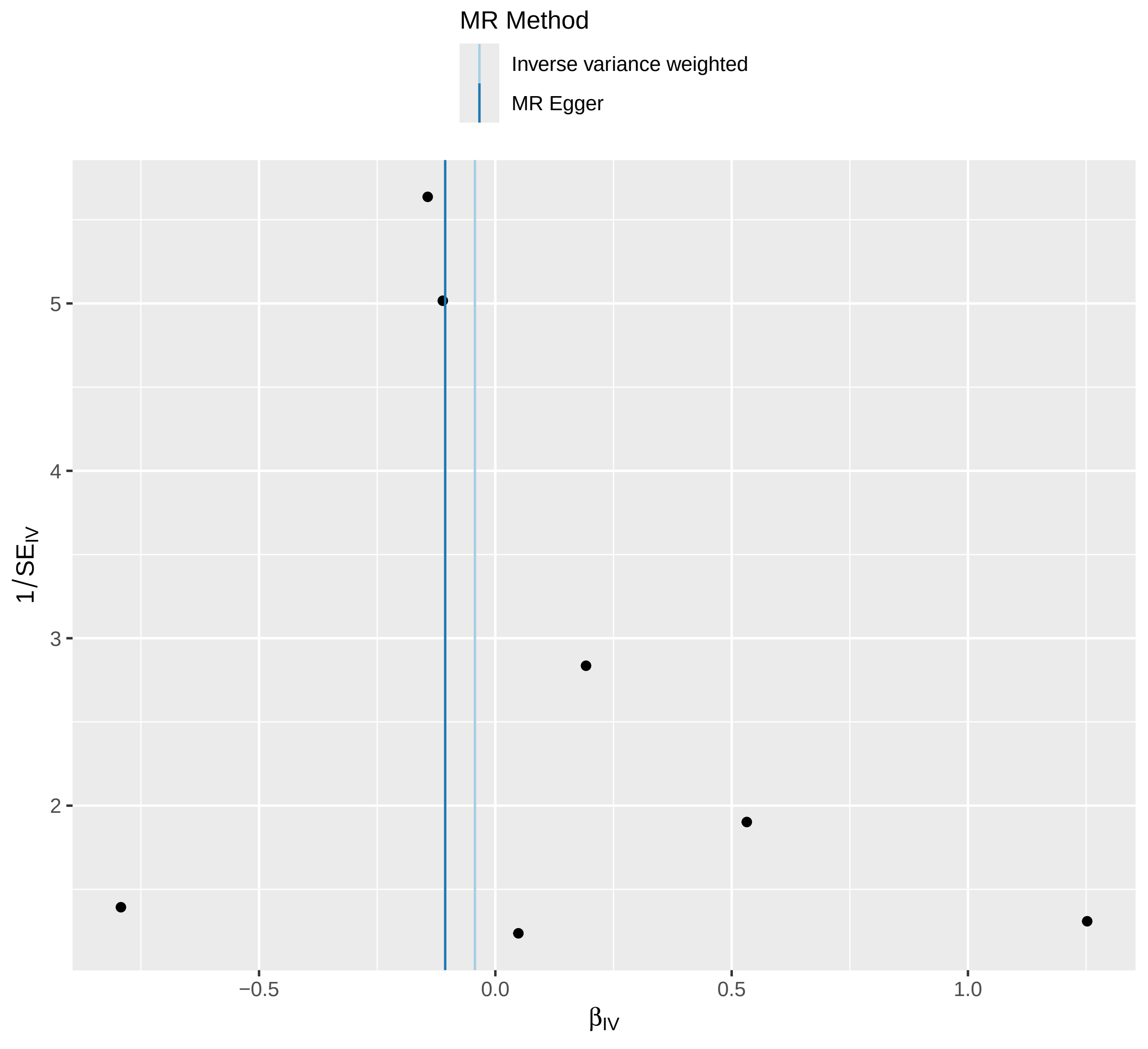

MR Method
Inverse variance weighted
MR Egger
5
4
3
2
1/SE_IV
−0.5
0.0
0.5
1.0
β_IV

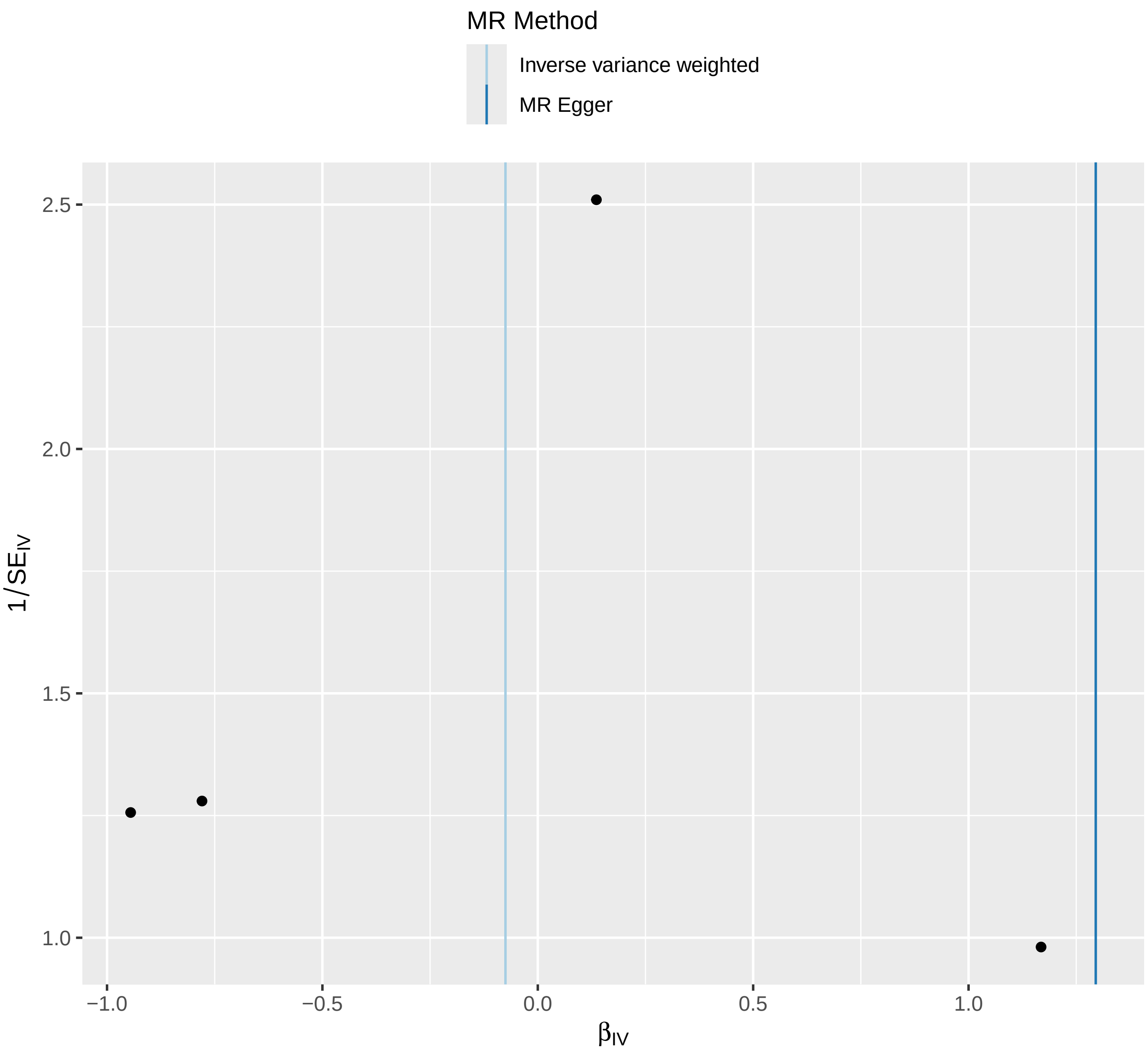
MR Method
Inverse variance weighted
MR Egger
2.5
2.0
1.5
1.0
1/SE_IV
−1.0
−0.5
0.0
0.5
1.0
β_IV

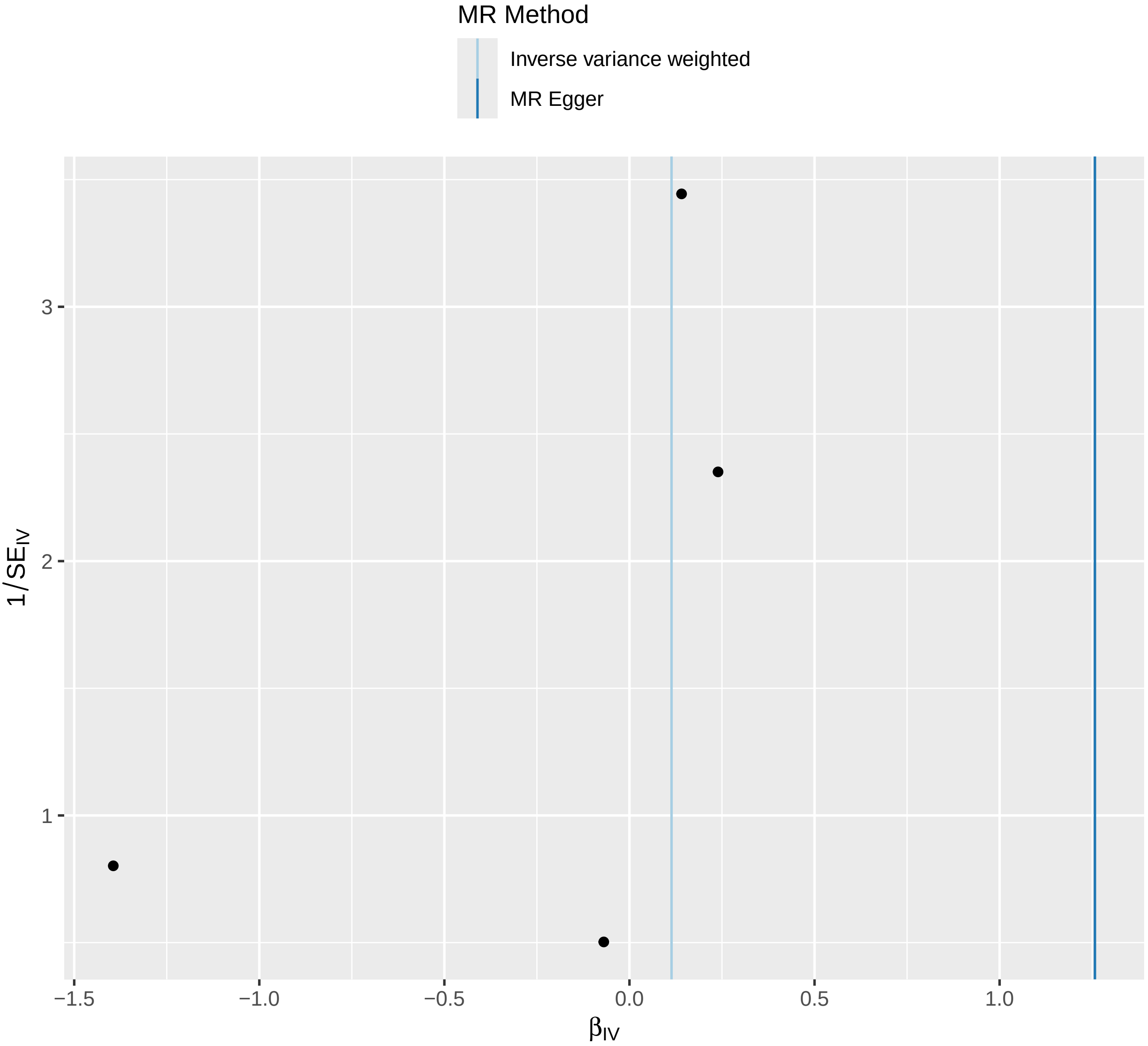

MR Method
Inverse variance weighted
MR Egger
1/SE_IV
3
2
1
−1.5
−1.0
−0.5
0.0
0.5
1.0
β_IV

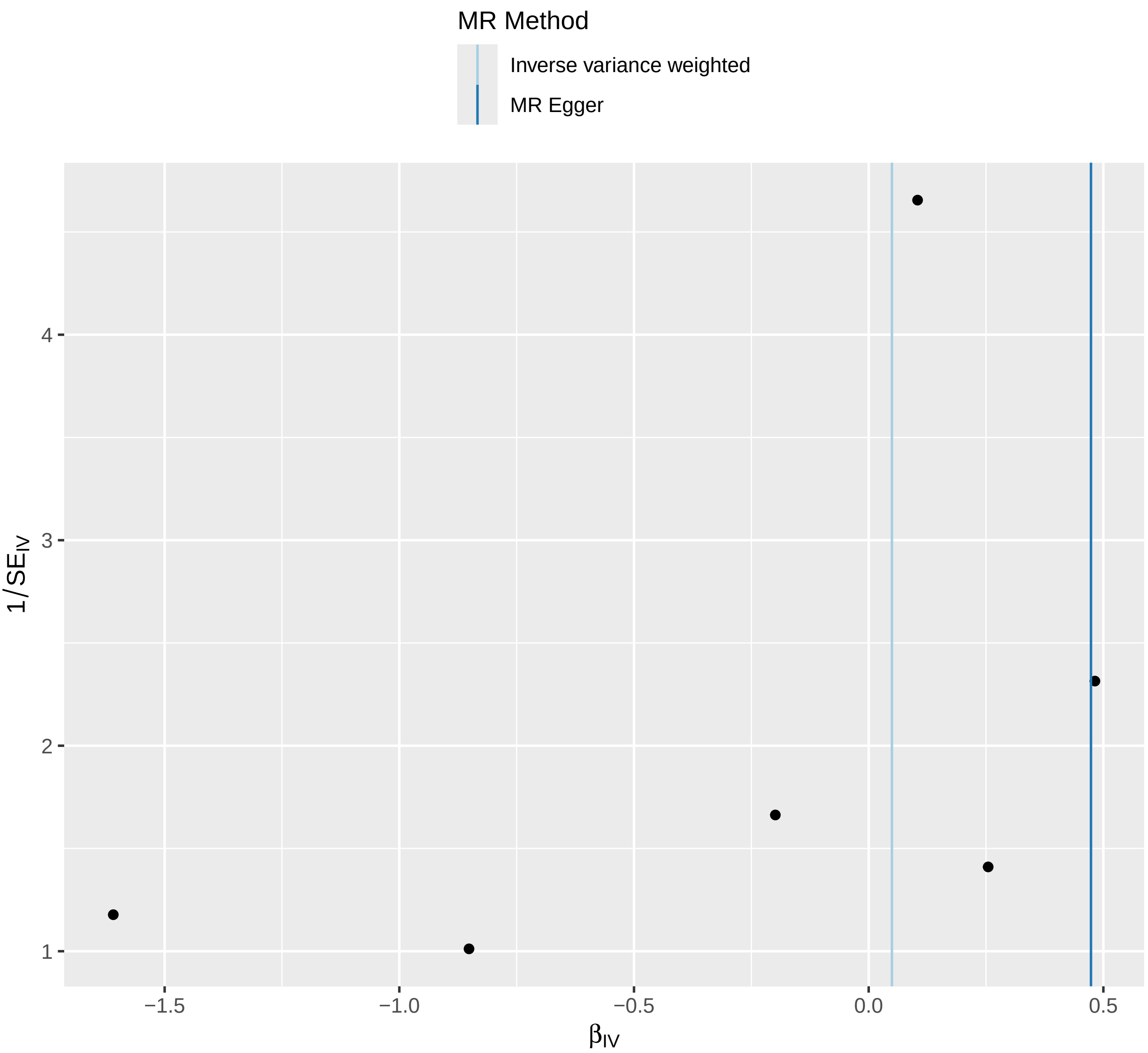
MR Method
Inverse variance weighted
MR Egger
1/SE_IV
β_IV
4
3
2
1
−1.5
−1.0
−0.5
0.0
0.5

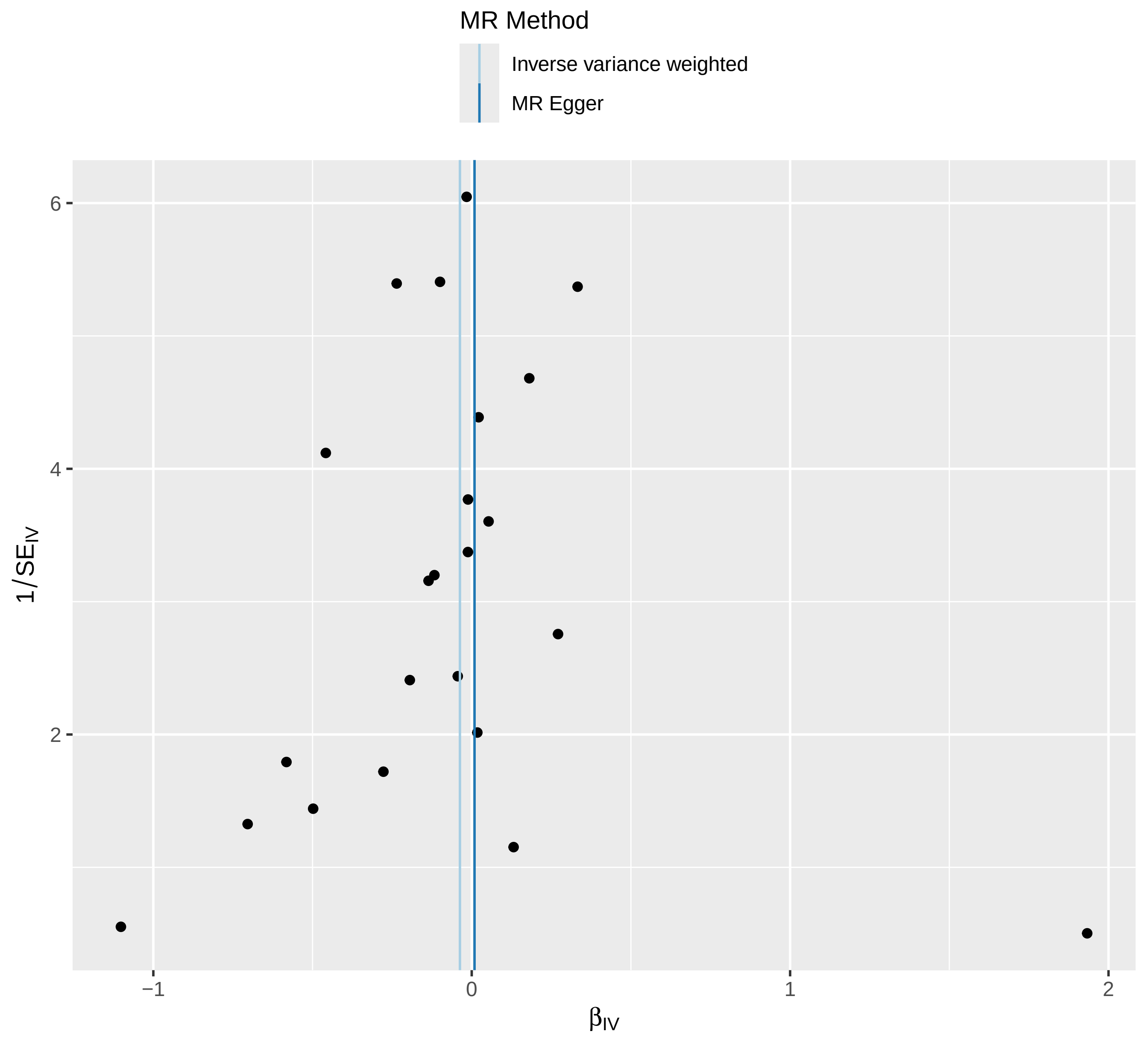
MR Method
Inverse variance weighted
MR Egger
1/SE_IV
6
4
2
−1
0
1
2
β_IV

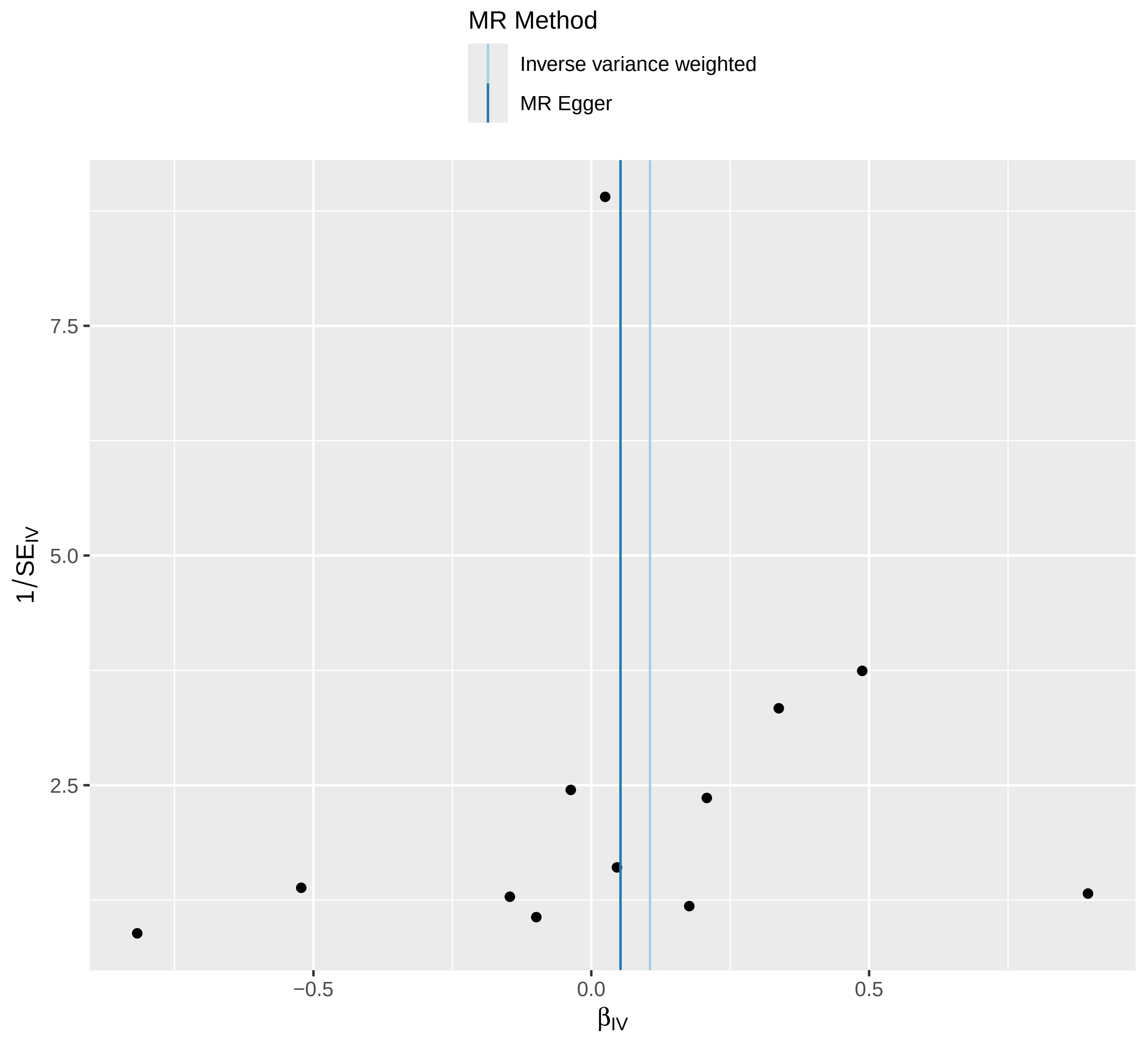

MR Method
Inverse variance weighted
MR Egger
7.5
5.0
2.5
1/SE_IV
−0.5
0.0
0.5
β_IV

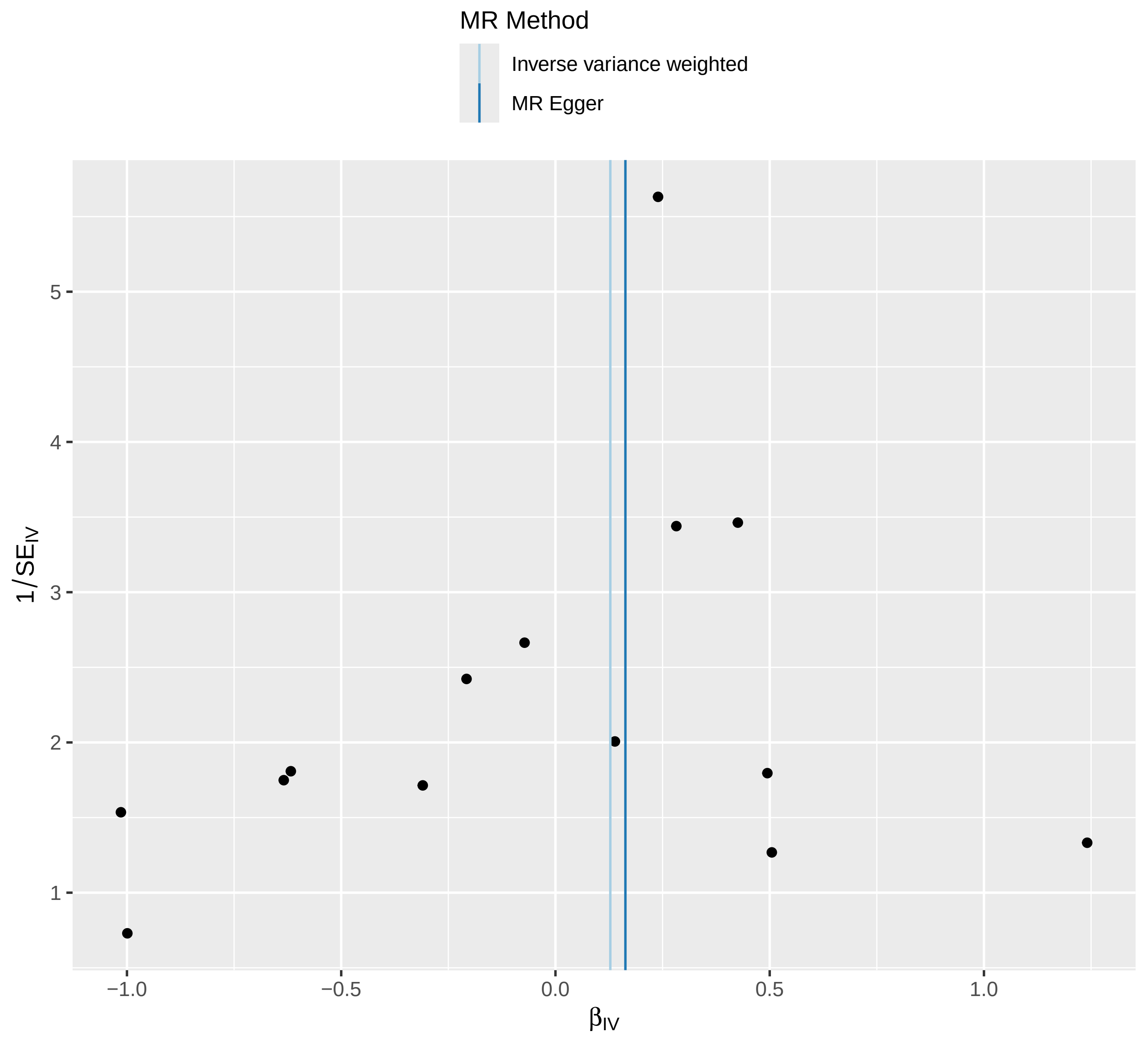

MR Method
Inverse variance weighted
MR Egger
1/SE_IV
5
4
3
2
1
−1.0
−0.5
0.0
0.5
1.0
β_IV

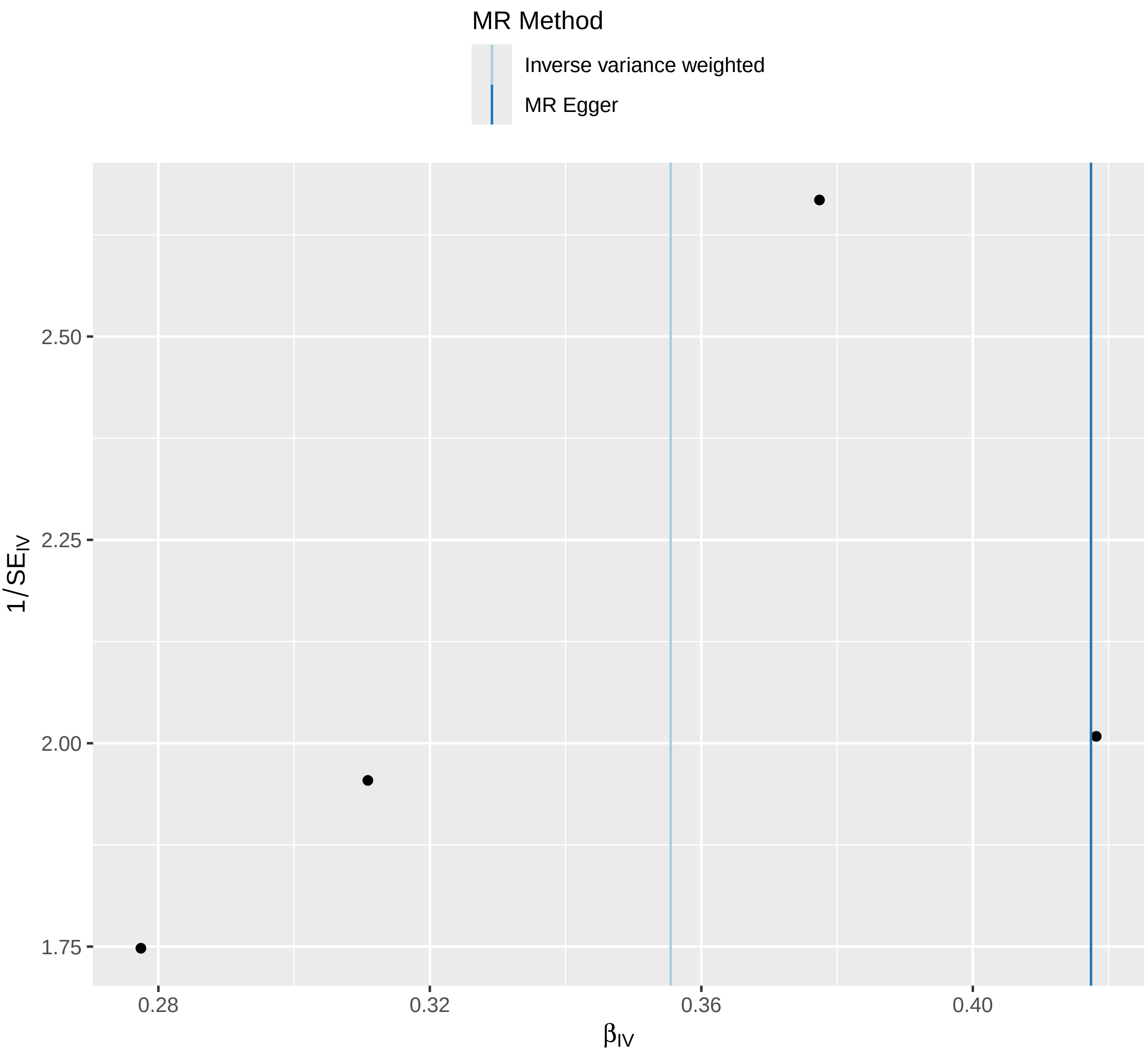

MR Method
Inverse variance weighted
MR Egger
2.50
2.25
2.00
1.75
$1/SE_{IV}$
0.28
0.32
0.36
0.40
β_{IV}

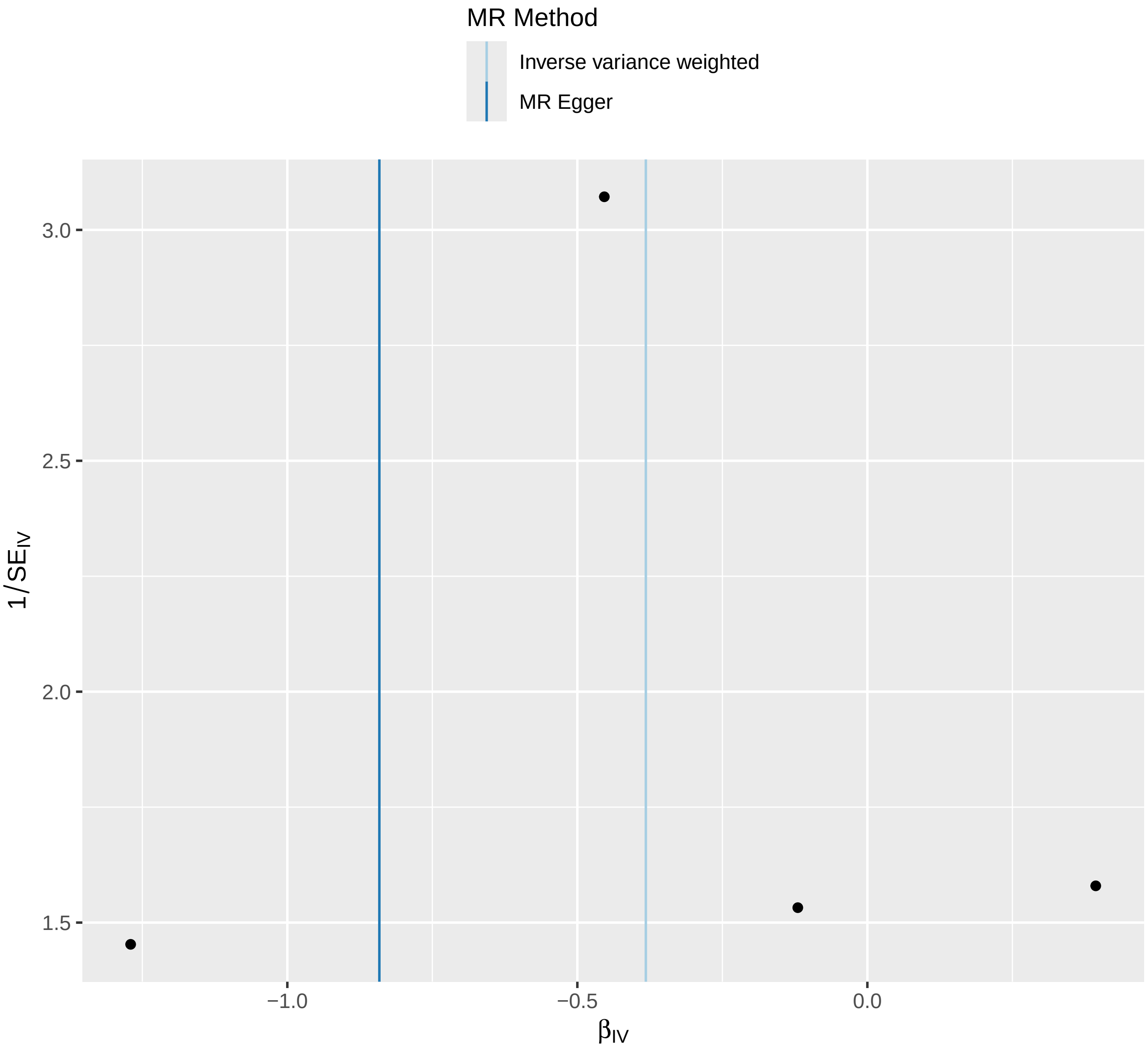
MR Method
Inverse variance weighted
MR Egger
3.0
2.5
2.0
1.5
1/SE_IV
−1.0
−0.5
0.0
β_IV

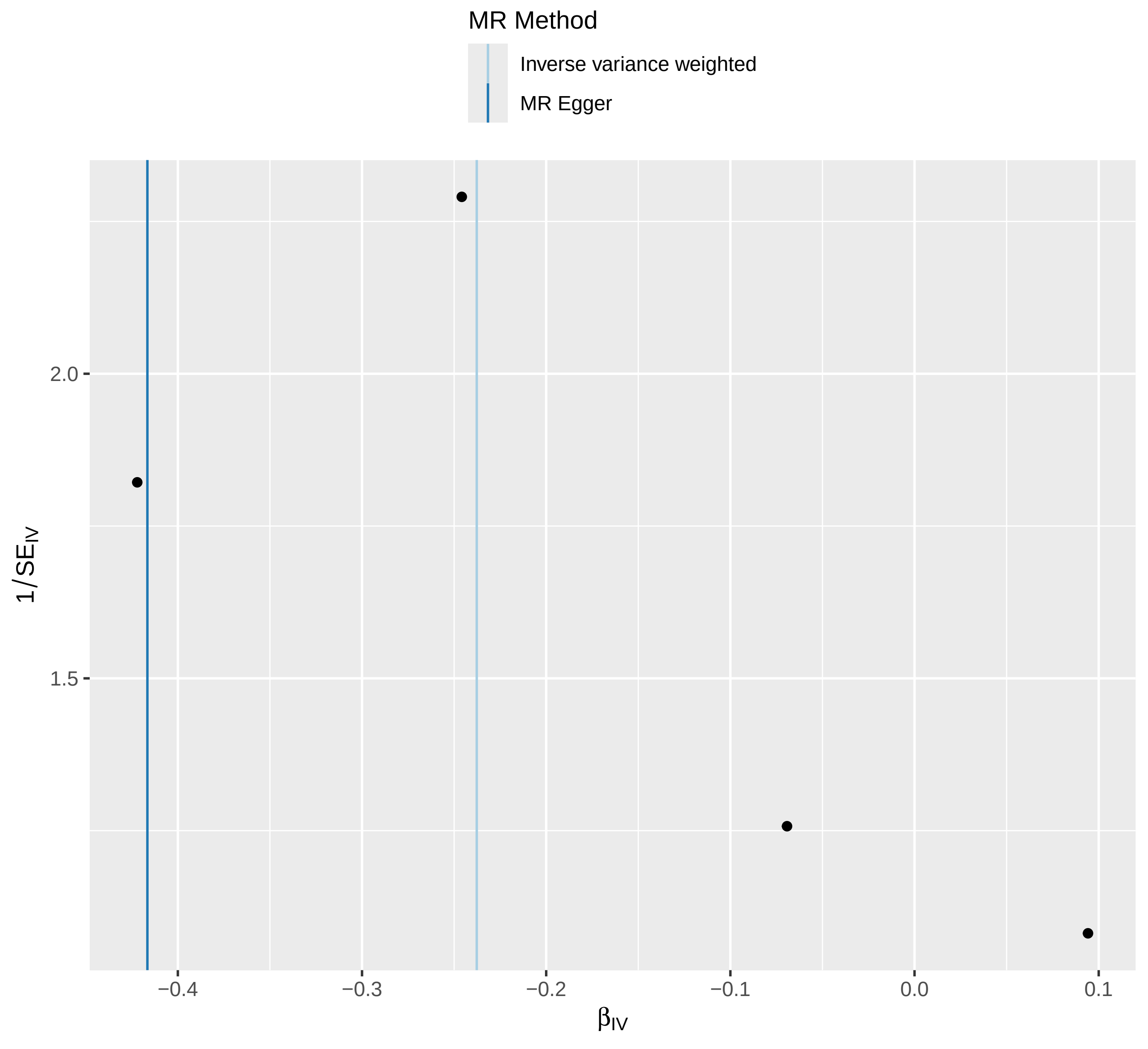

MR Method
Inverse variance weighted
MR Egger
2.0
1.5
1/SE$_{IV}$
−0.4
−0.3
−0.2
−0.1
0.0
0.1
β$_{IV}$

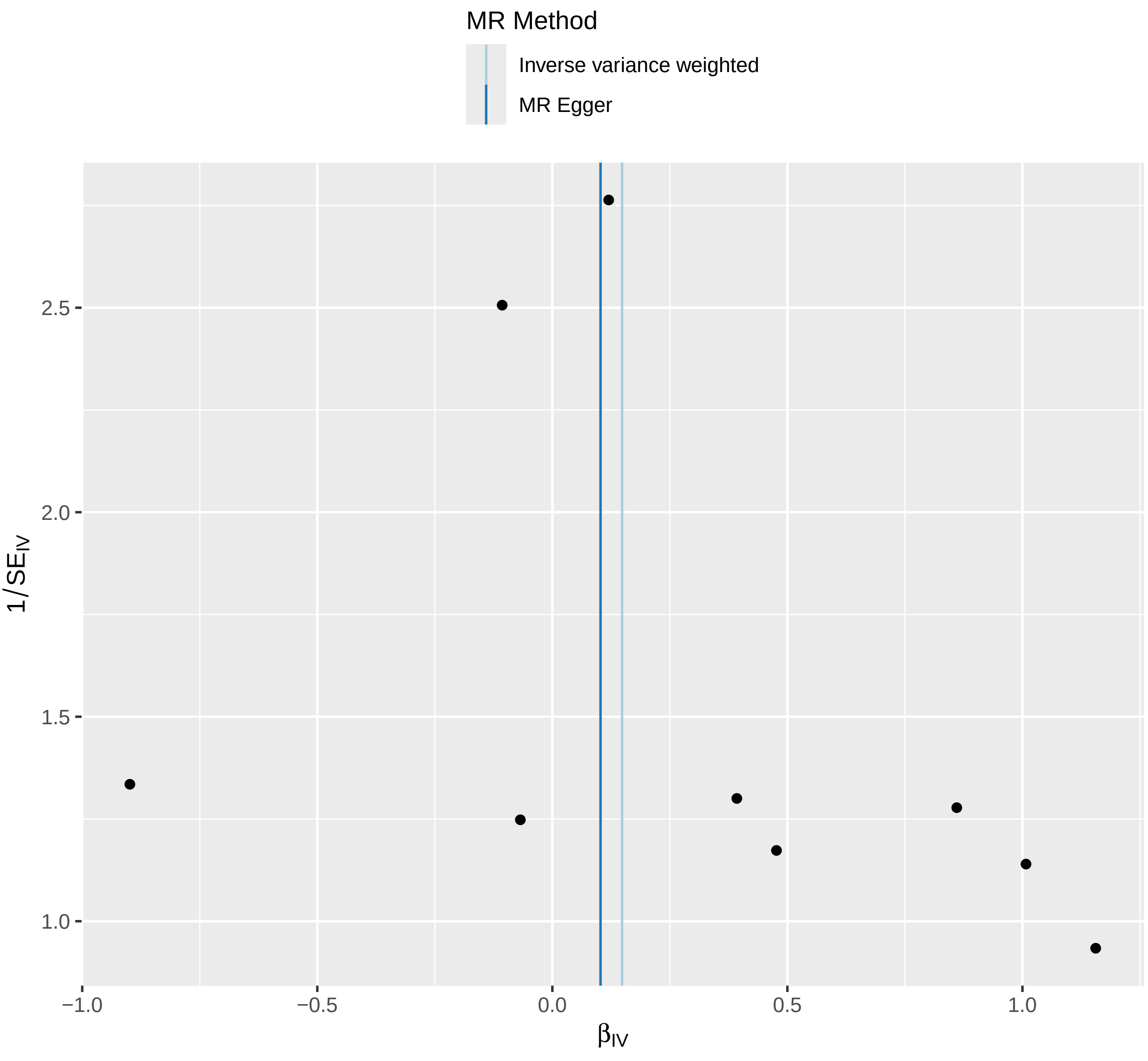

MR Method
Inverse variance weighted
MR Egger
1/SE_IV
2.5
2.0
1.5
1.0
−1.0
−0.5
0.0
0.5
1.0
β_IV

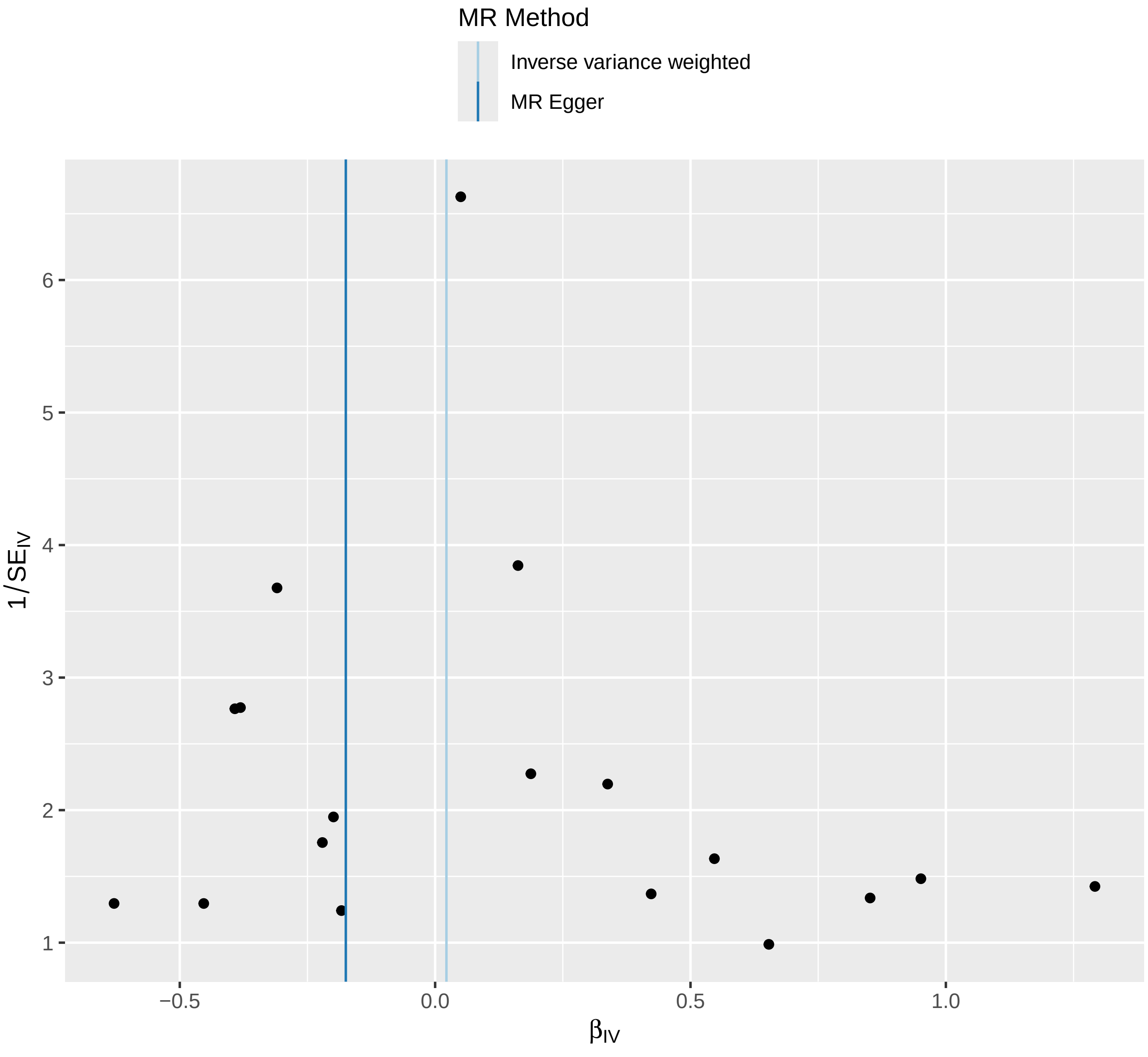

MR Method
Inverse variance weighted
MR Egger
1
2
3
4
5
6
1/SE_IV
−0.5
0.0
0.5
1.0
β_IV

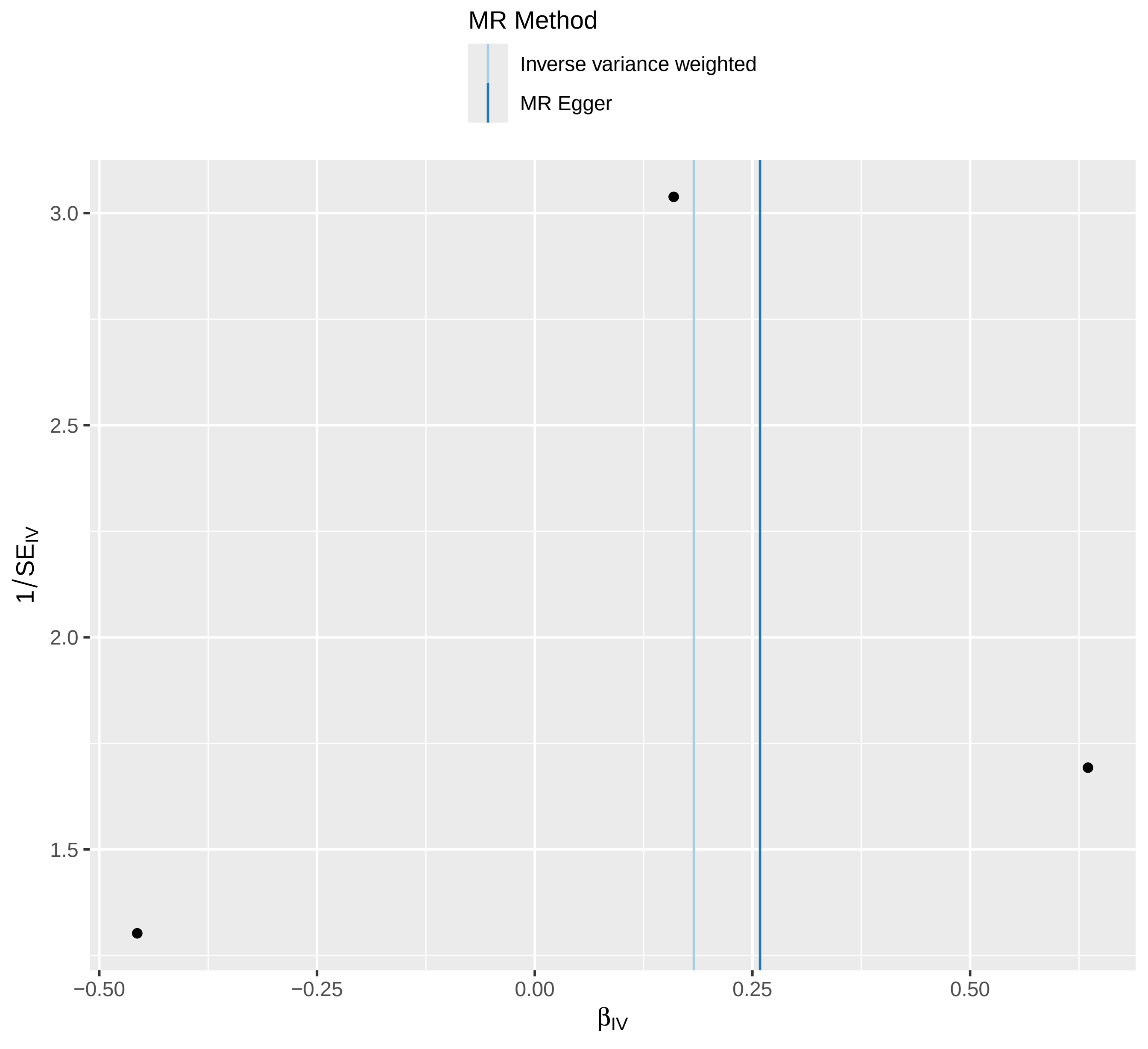

MR Method
Inverse variance weighted
MR Egger
3.0
2.5
2.0
1.5
1/SE_IV
−0.50
−0.25
0.00
0.25
0.50
β_IV

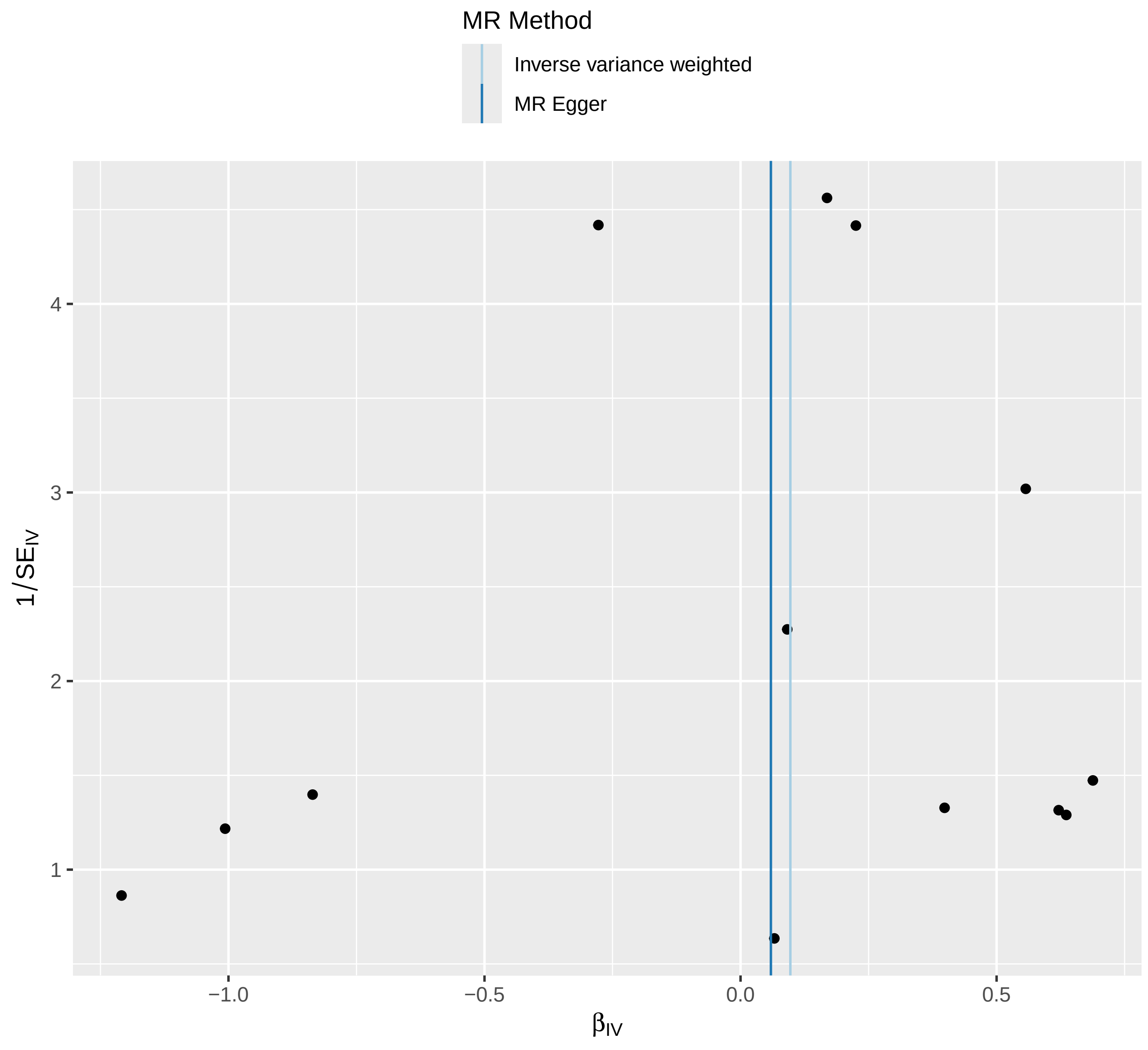

MR Method
Inverse variance weighted
MR Egger
4
3
2
1
1/SE_IV
−1.0
−0.5
0.0
0.5
β_IV

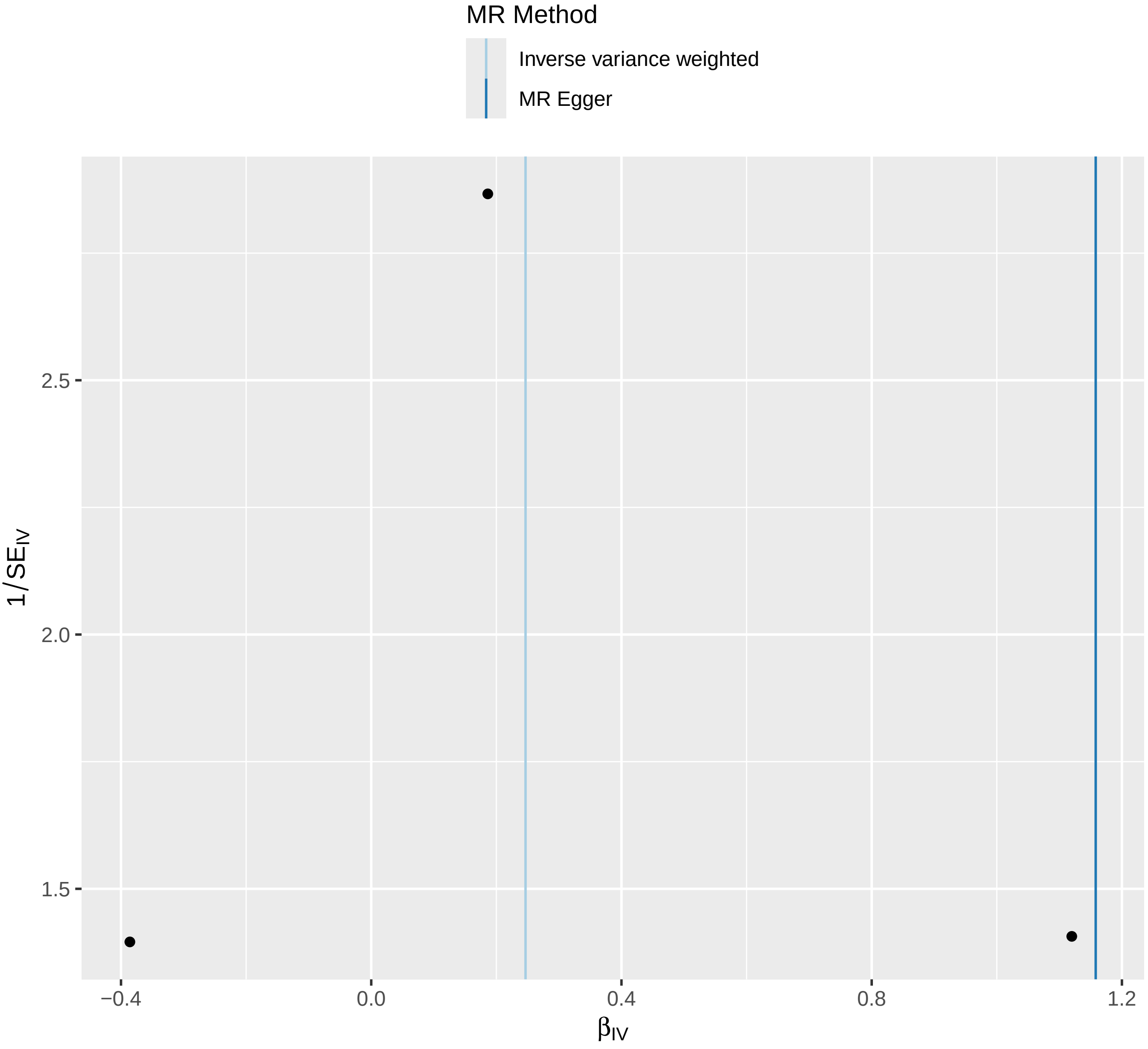

MR Method
Inverse variance weighted
MR Egger
2.5
2.0
1.5
1/SE_IV
−0.4
0.0
0.4
0.8
1.2
β_IV

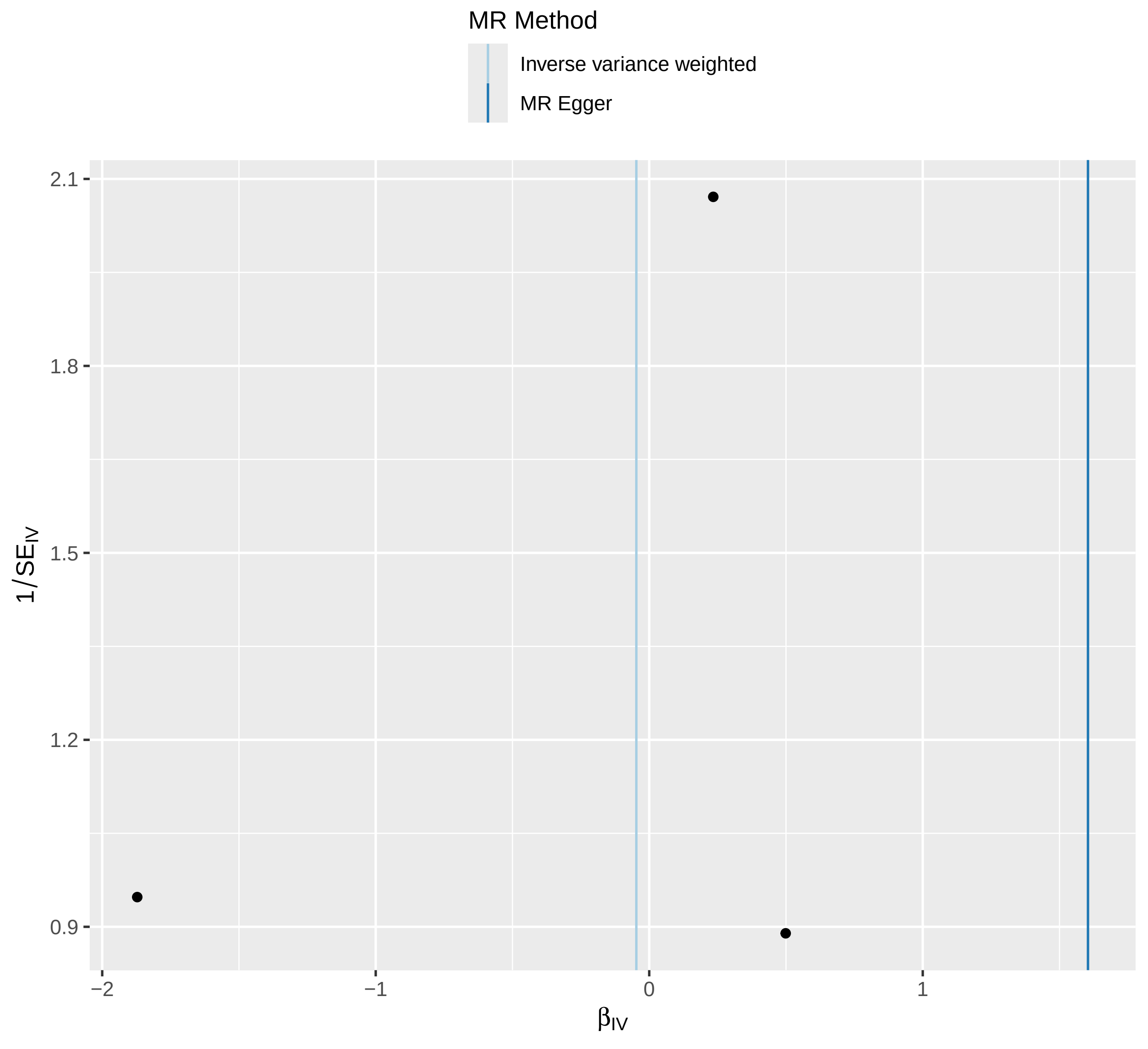

MR Method
Inverse variance weighted
MR Egger
2.1
1.8
1.5
1.2
0.9
1/SE$_{IV}$
−2
−1
0
1
β$_{IV}$

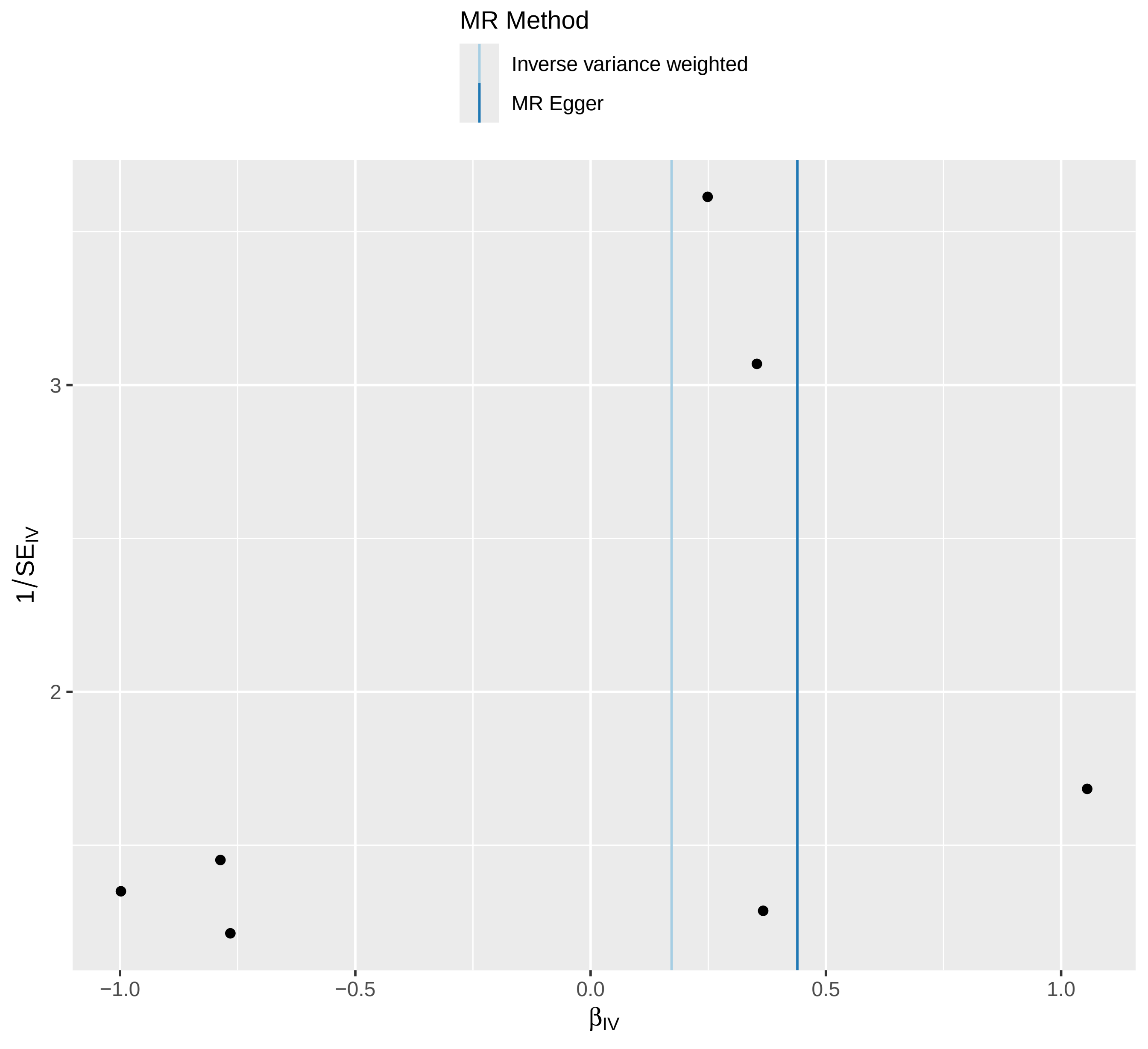
MR Method
Inverse variance weighted
MR Egger
1/SE_IV
3
2
−1.0
−0.5
0.0
0.5
1.0
β_IV

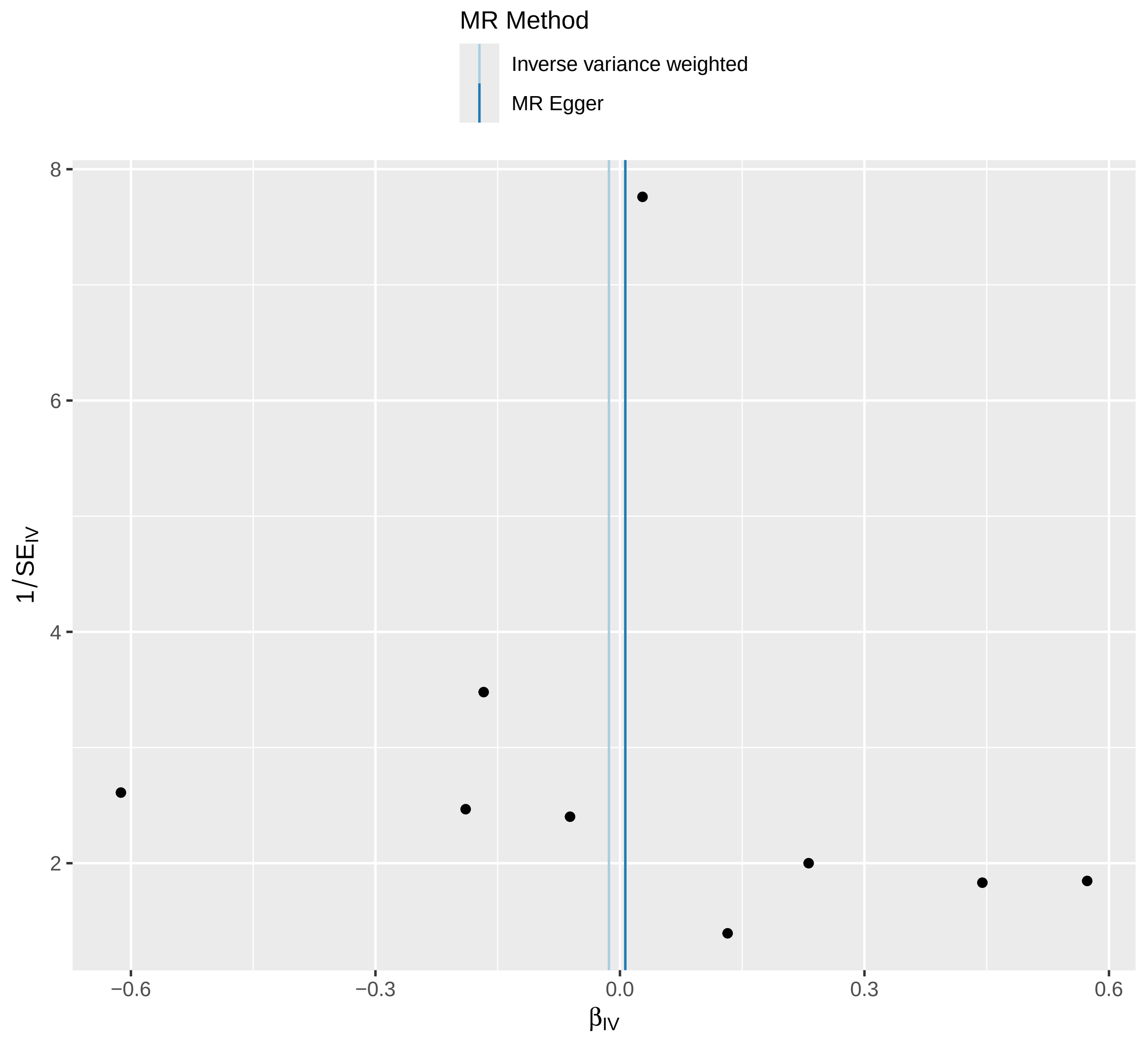

MR Method
Inverse variance weighted
MR Egger
8
6
4
2
1/SE$_{IV}$
−0.6
−0.3
0.0
0.3
0.6
β$_{IV}$

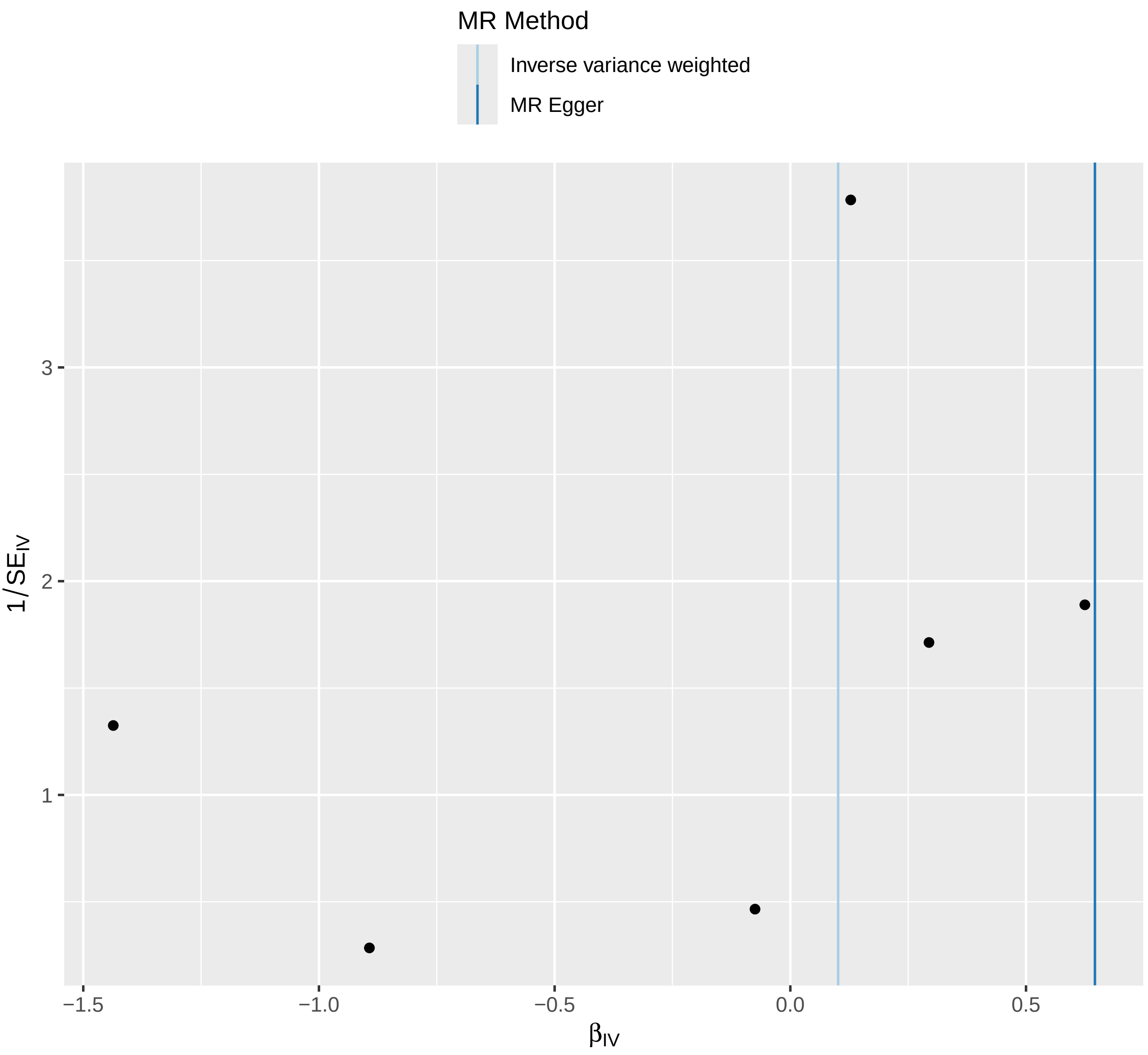

MR Method
Inverse variance weighted
MR Egger
3
2
1
1/SE_IV
−1.5
−1.0
−0.5
0.0
0.5
β_IV

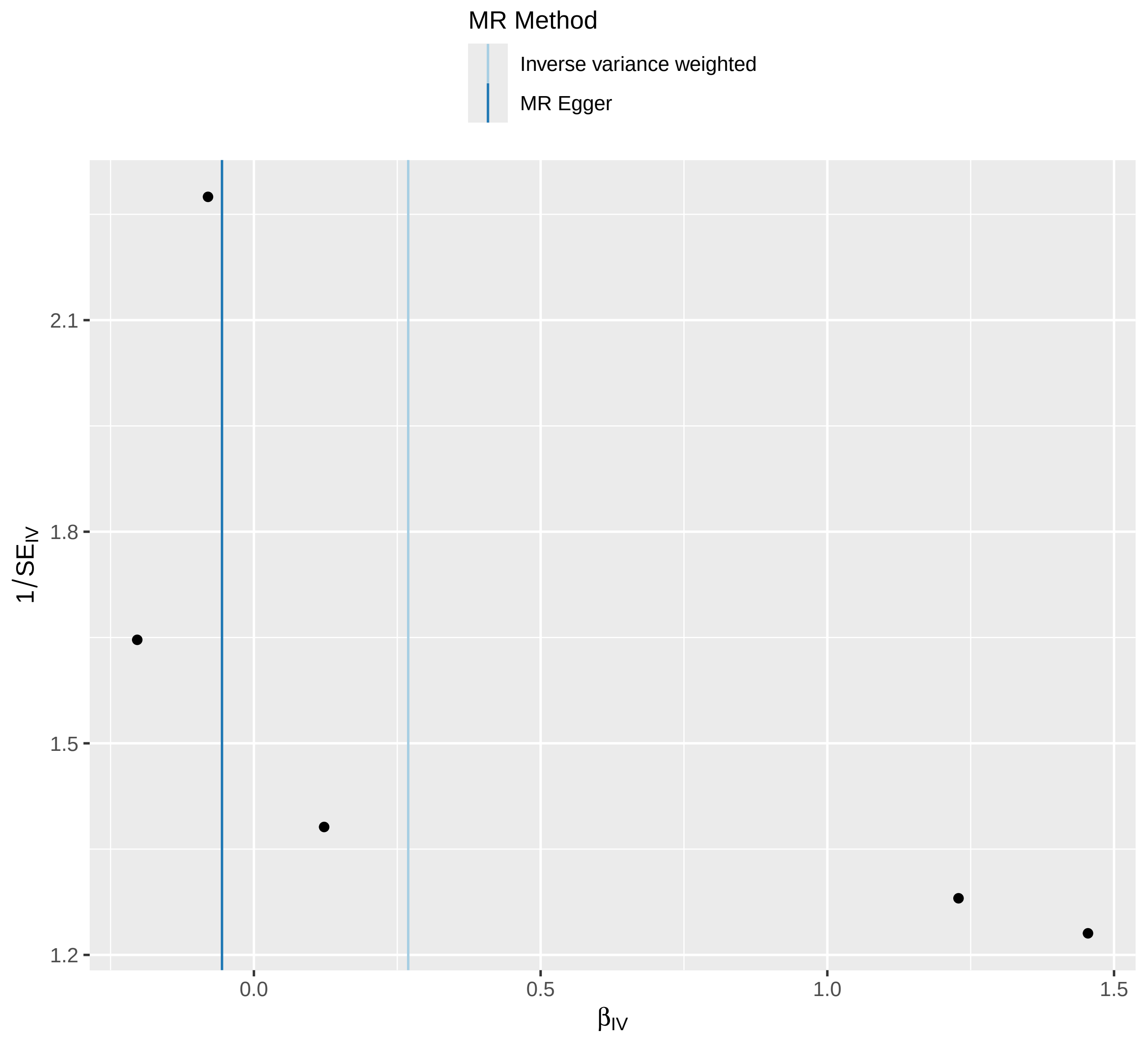

MR Method
Inverse variance weighted
MR Egger
2.1
1.8
1.5
1.2
1/SE_IV
0.0
0.5
1.0
1.5
β_IV

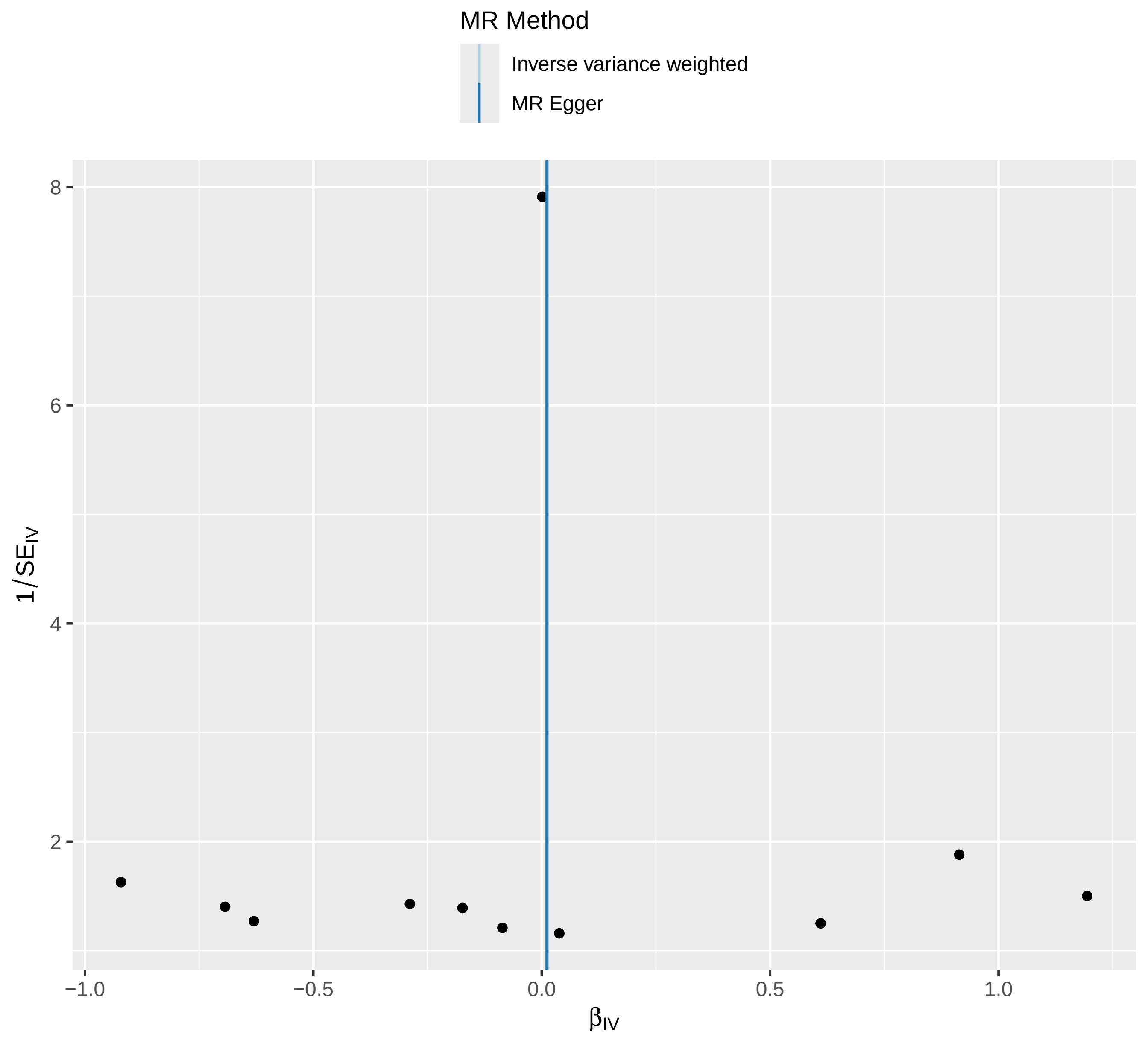
MR Method
Inverse variance weighted
MR Egger
8
6
4
2
1/SE_IV
−1.0
−0.5
0.0
0.5
1.0
β_IV

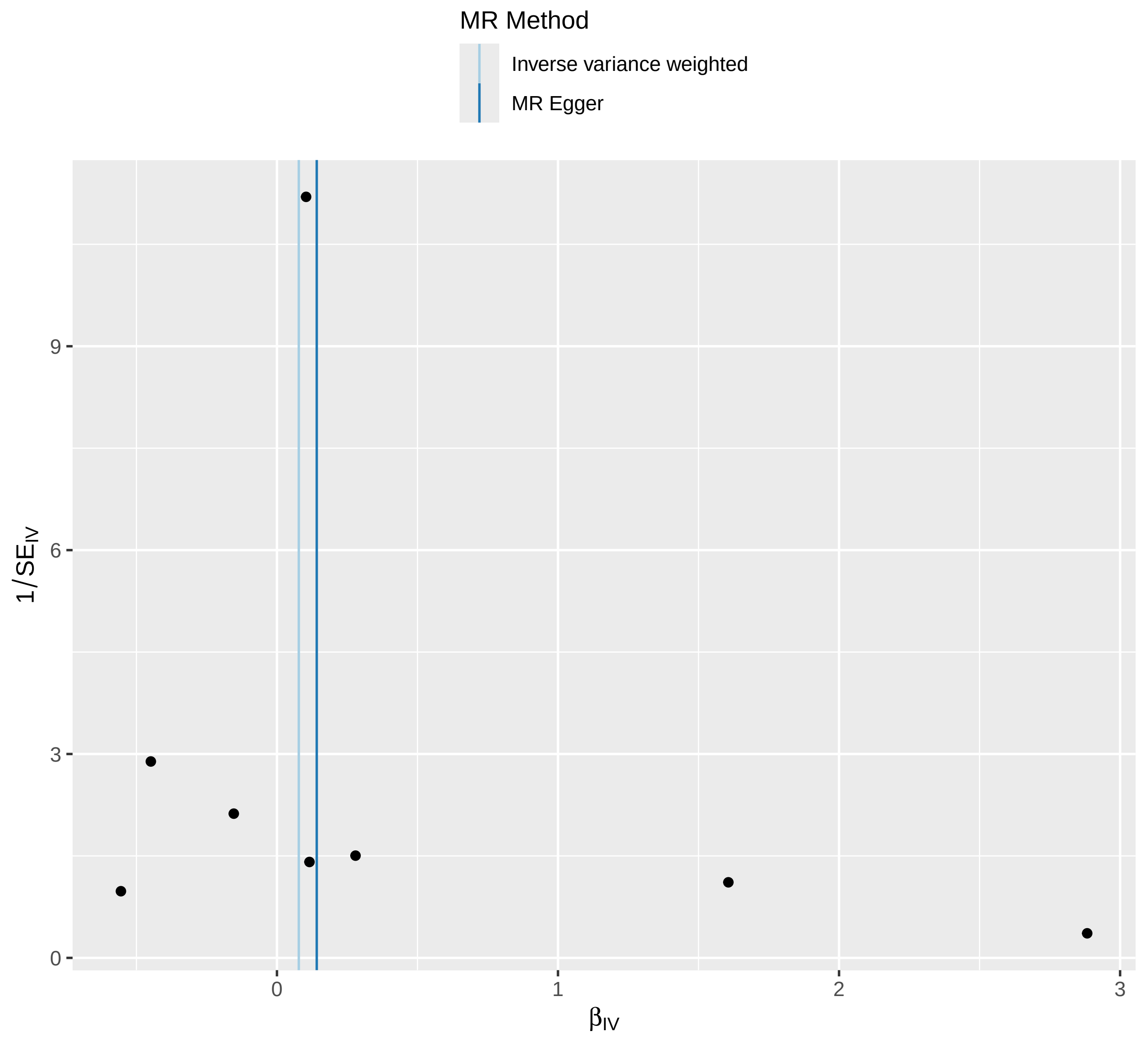
MR Method
Inverse variance weighted
MR Egger
1/SE_IV
9
6
3
0
0
1
2
3
β_IV

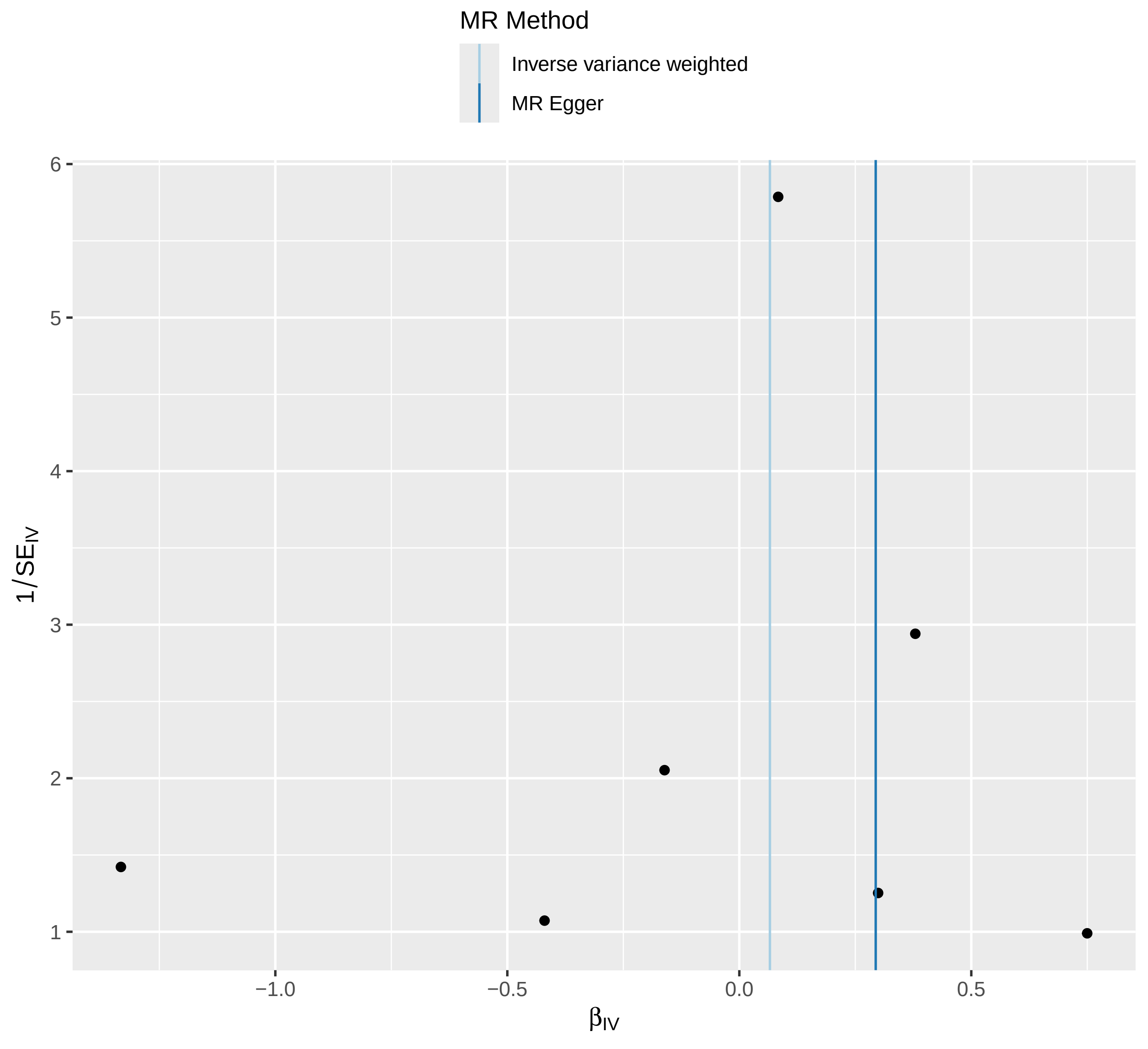

MR Method
Inverse variance weighted
MR Egger
6
5
4
3
2
1
1/SE$_{IV}$
−1.0
−0.5
0.0
0.5
β$_{IV}$

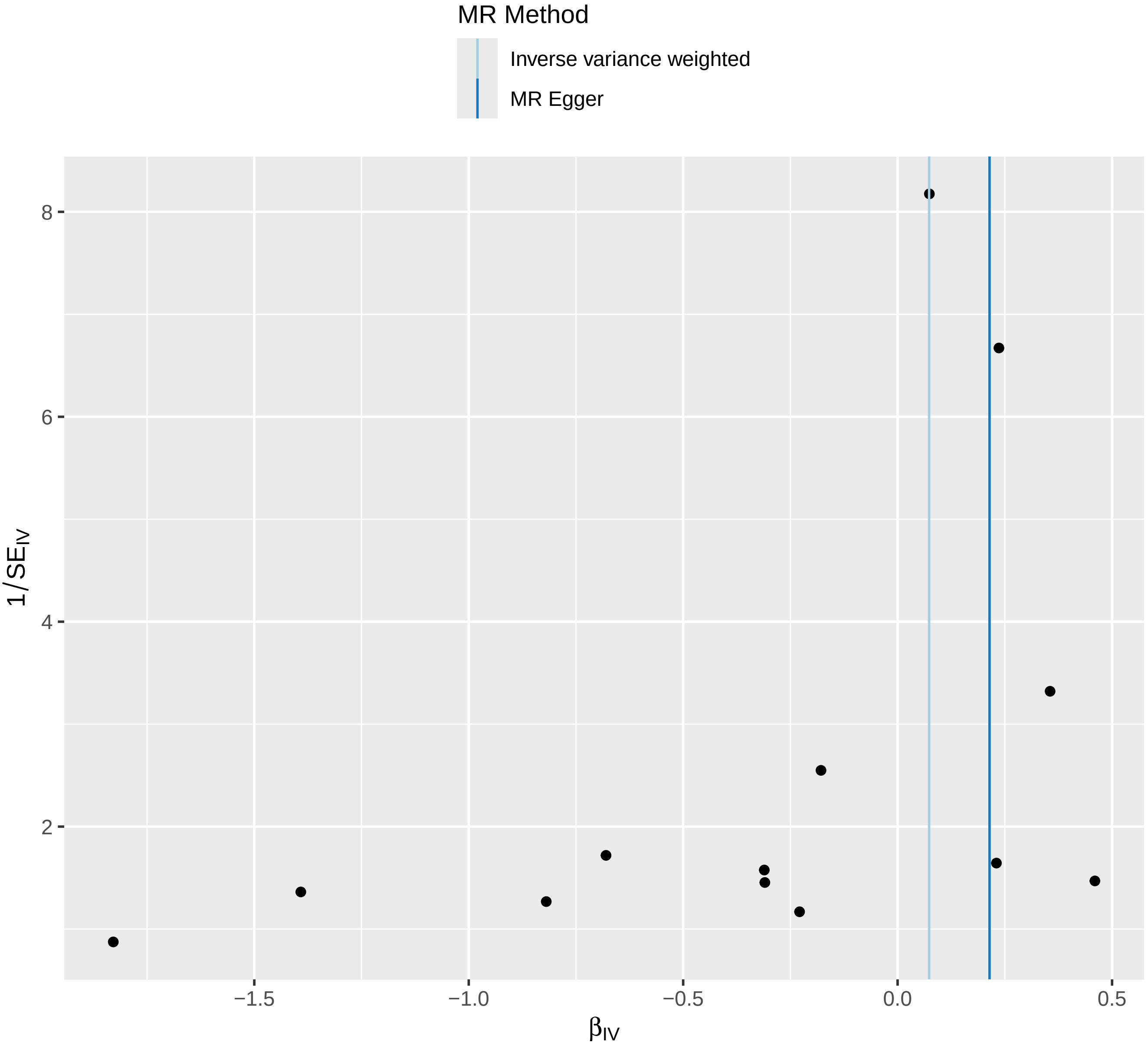

MR Method
Inverse variance weighted
MR Egger
8
6
4
2
1/SE$_{IV}$
−1.5
−1.0
−0.5
0.0
0.5
β$_{IV}$

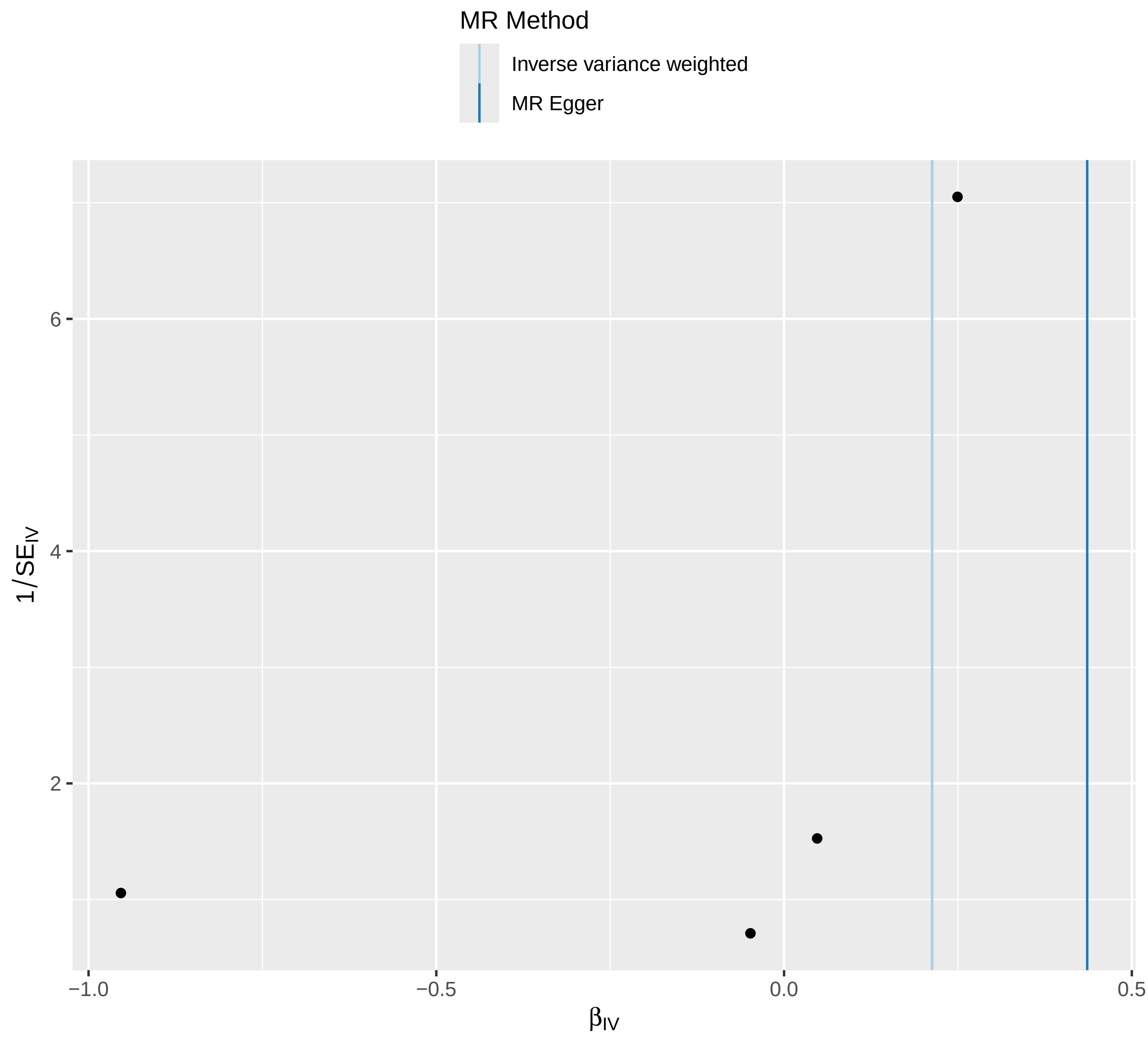

MR Method
Inverse variance weighted
MR Egger
6
4
2
1/SE_IV
−1.0
−0.5
0.0
0.5
β_IV

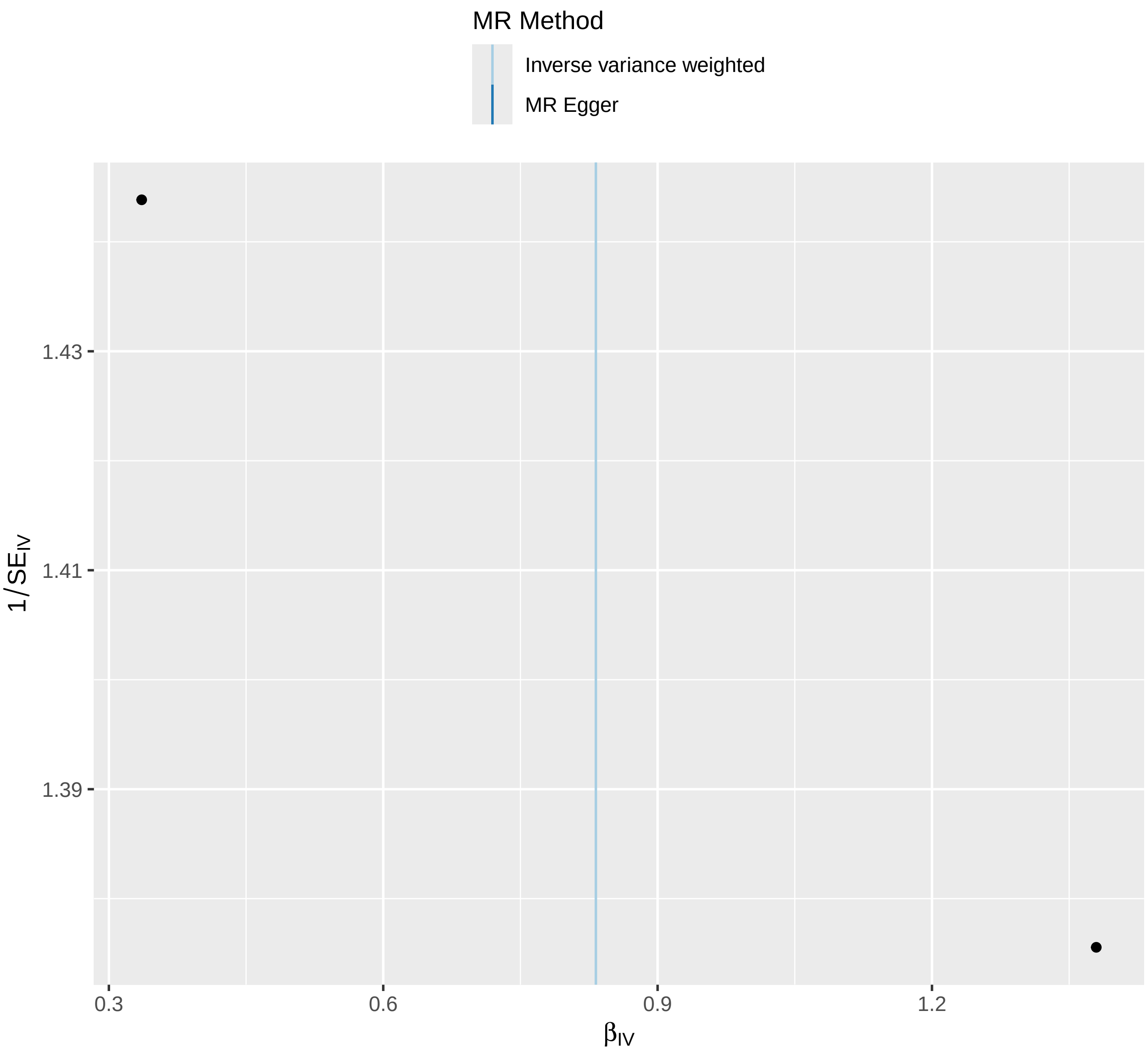
MR Method
Inverse variance weighted
MR Egger
1.43
1.41
1.39
0.3
0.6
0.9
1.2
β_{IV}
$1/SE_{IV}$

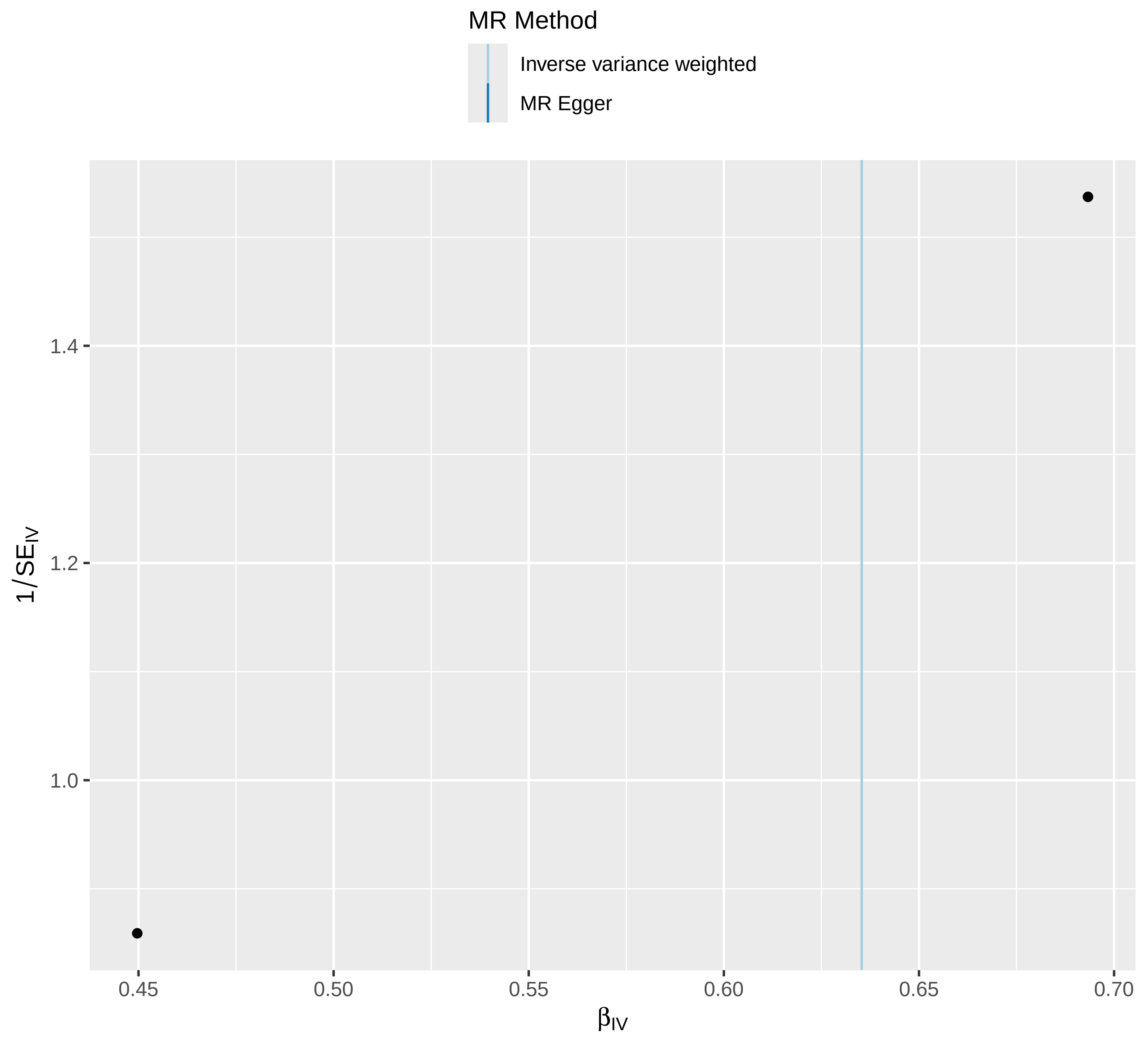
MR Method
Inverse variance weighted
MR Egger
1.4
1.2
1.0
$1/SE_{IV}$
0.45
0.50
0.55
0.60
0.65
0.70
β_{IV}

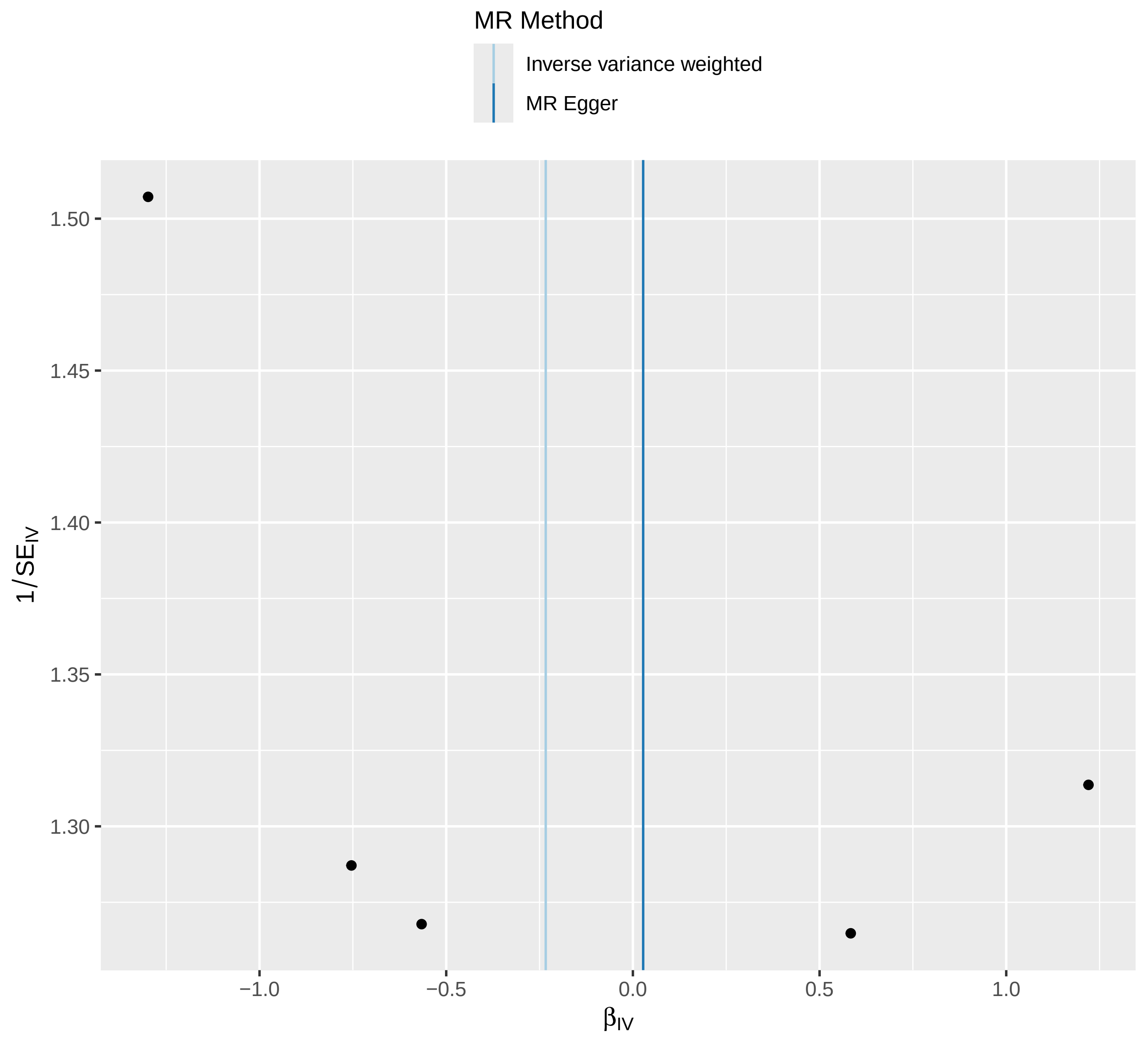

MR Method
Inverse variance weighted
MR Egger
1.50
1.45
1.40
1.35
1.30
1/SE_IV
−1.0
−0.5
0.0
0.5
1.0
β_IV

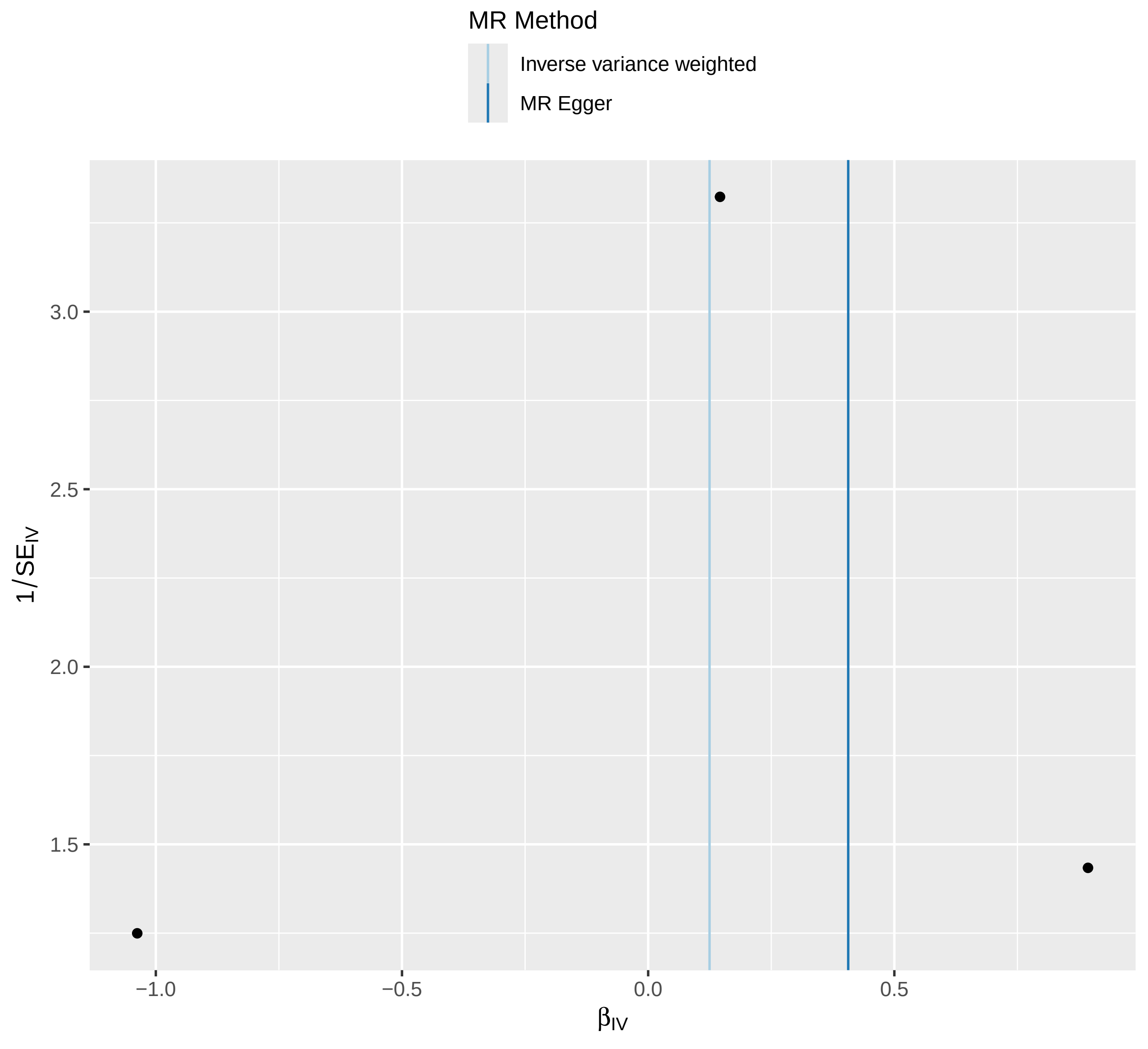

MR Method
Inverse variance weighted
MR Egger
3.0
2.5
2.0
1.5
1/SE_IV
−1.0
−0.5
0.0
0.5
β_IV

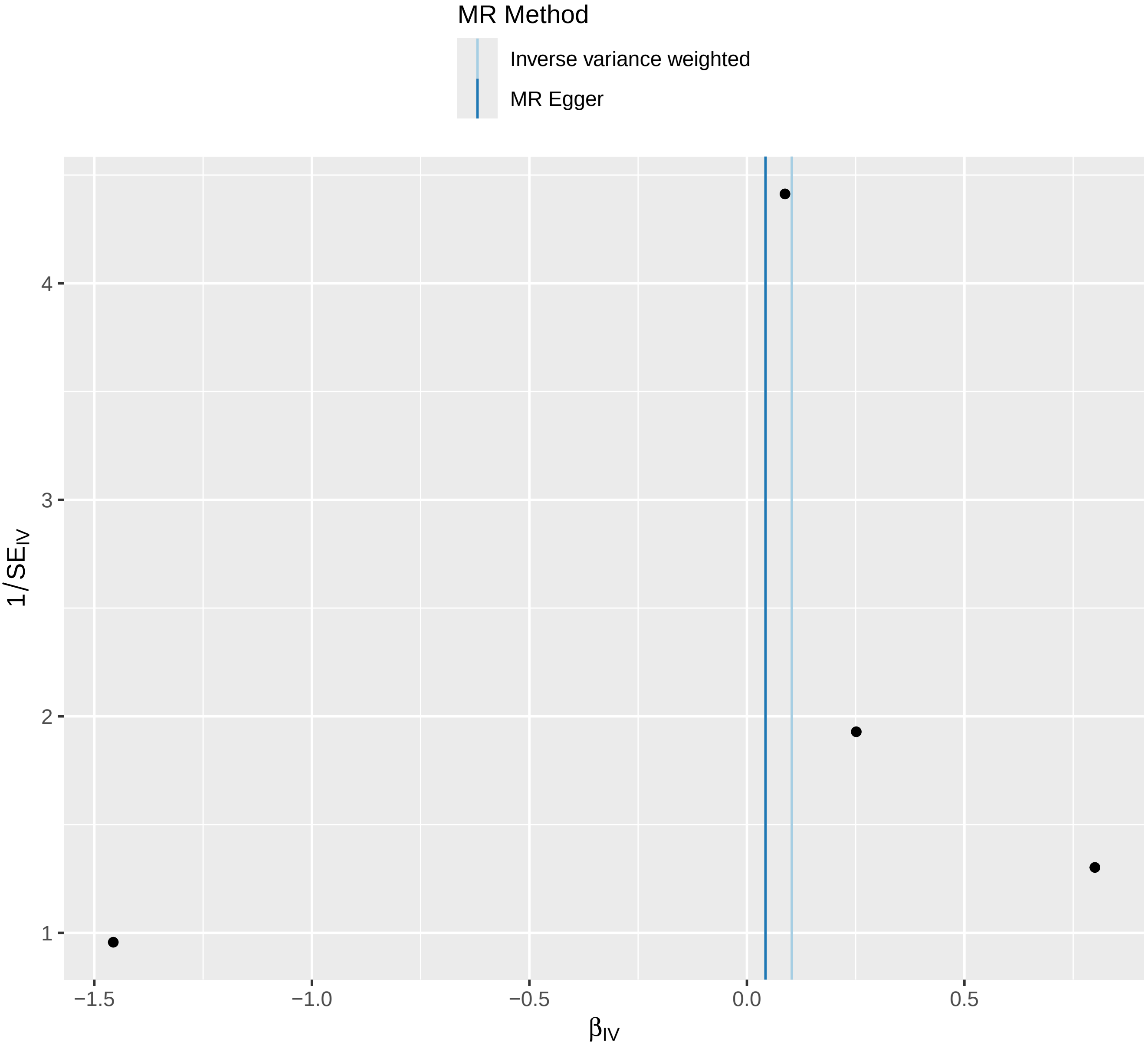

MR Method
Inverse variance weighted
MR Egger
4
3
2
1
1/SE$_{IV}$
−1.5
−1.0
−0.5
0.0
0.5
β$_{IV}$

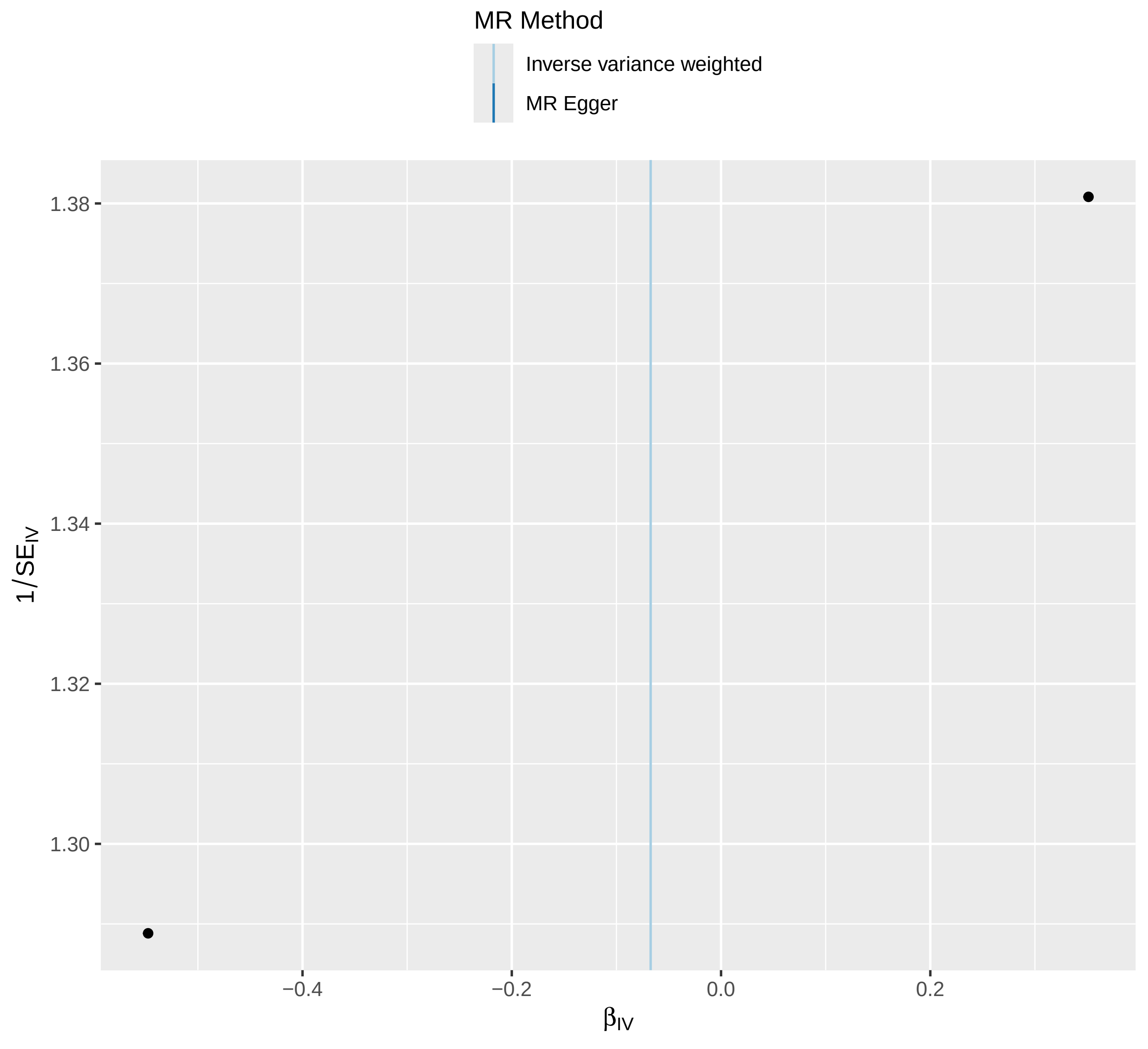
MR Method
Inverse variance weighted
MR Egger
1.38
1.36
1.34
1.32
1.30
1/SE_IV
−0.4
−0.2
0.0
0.2
β_IV

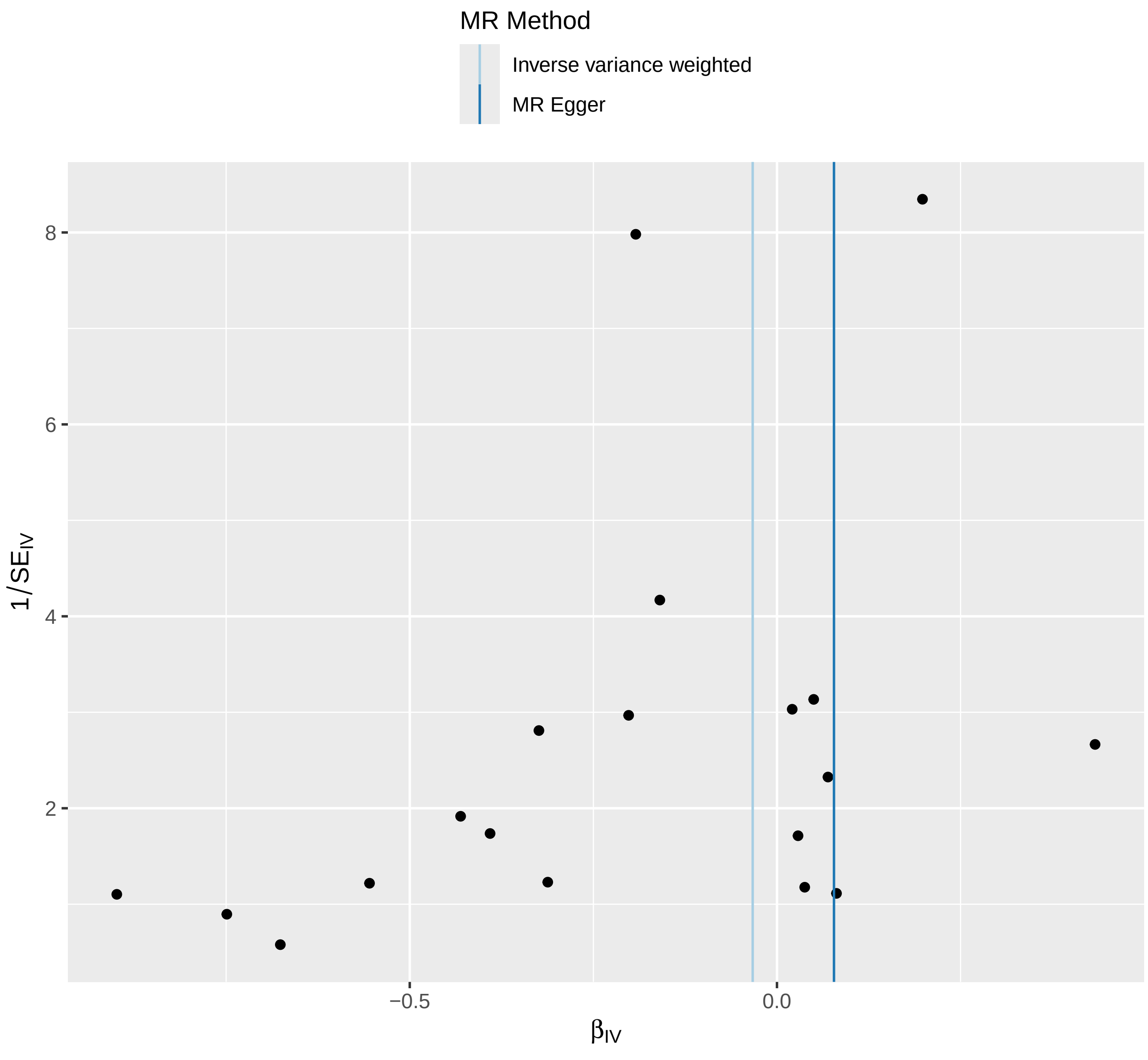

MR Method
Inverse variance weighted
MR Egger
8
6
4
2
1/SE_IV
−0.5
0.0
β_IV

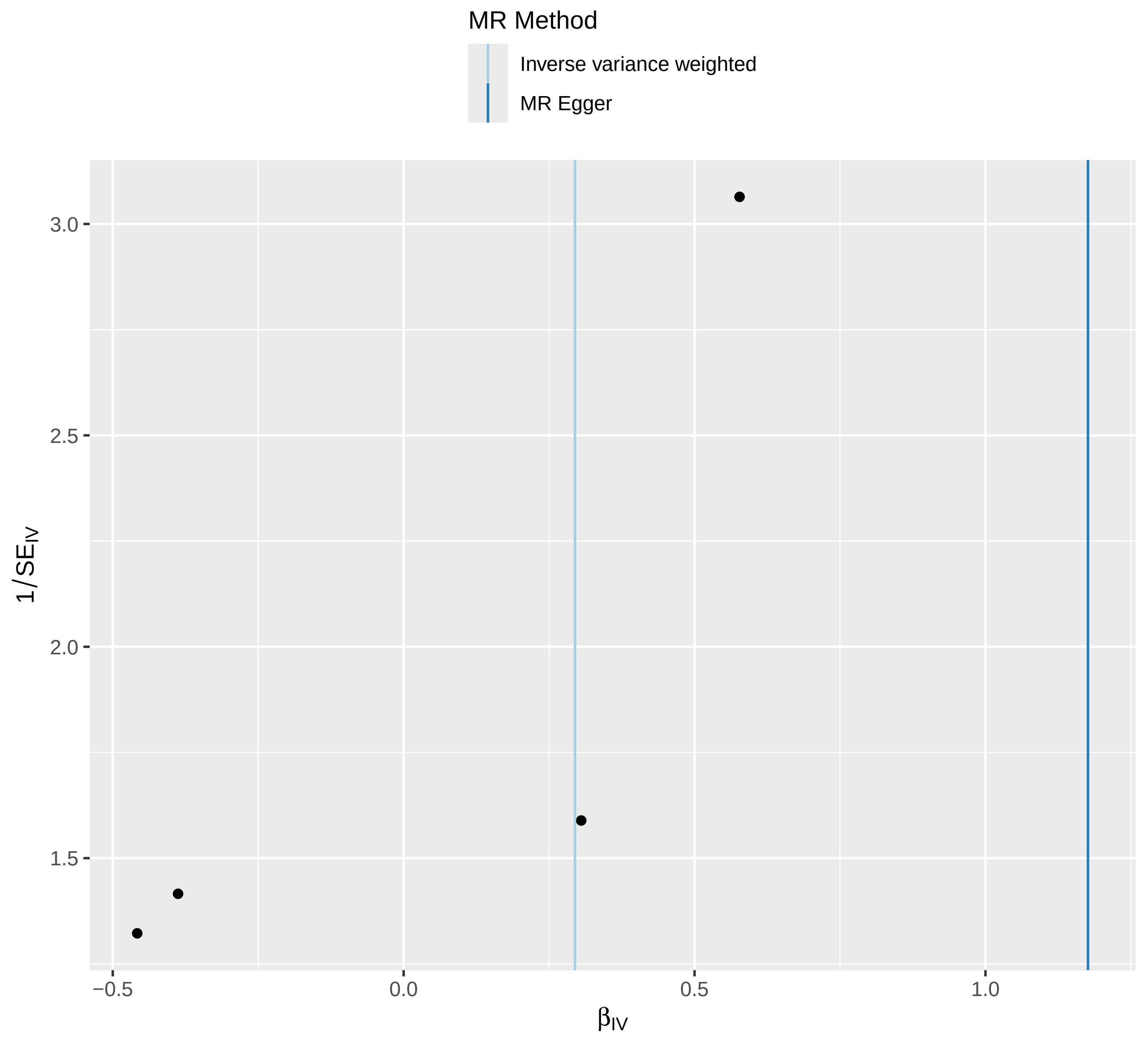

MR Method
Inverse variance weighted
MR Egger
3.0
2.5
2.0
1.5
1/SE_IV
−0.5
0.0
0.5
1.0
β_IV

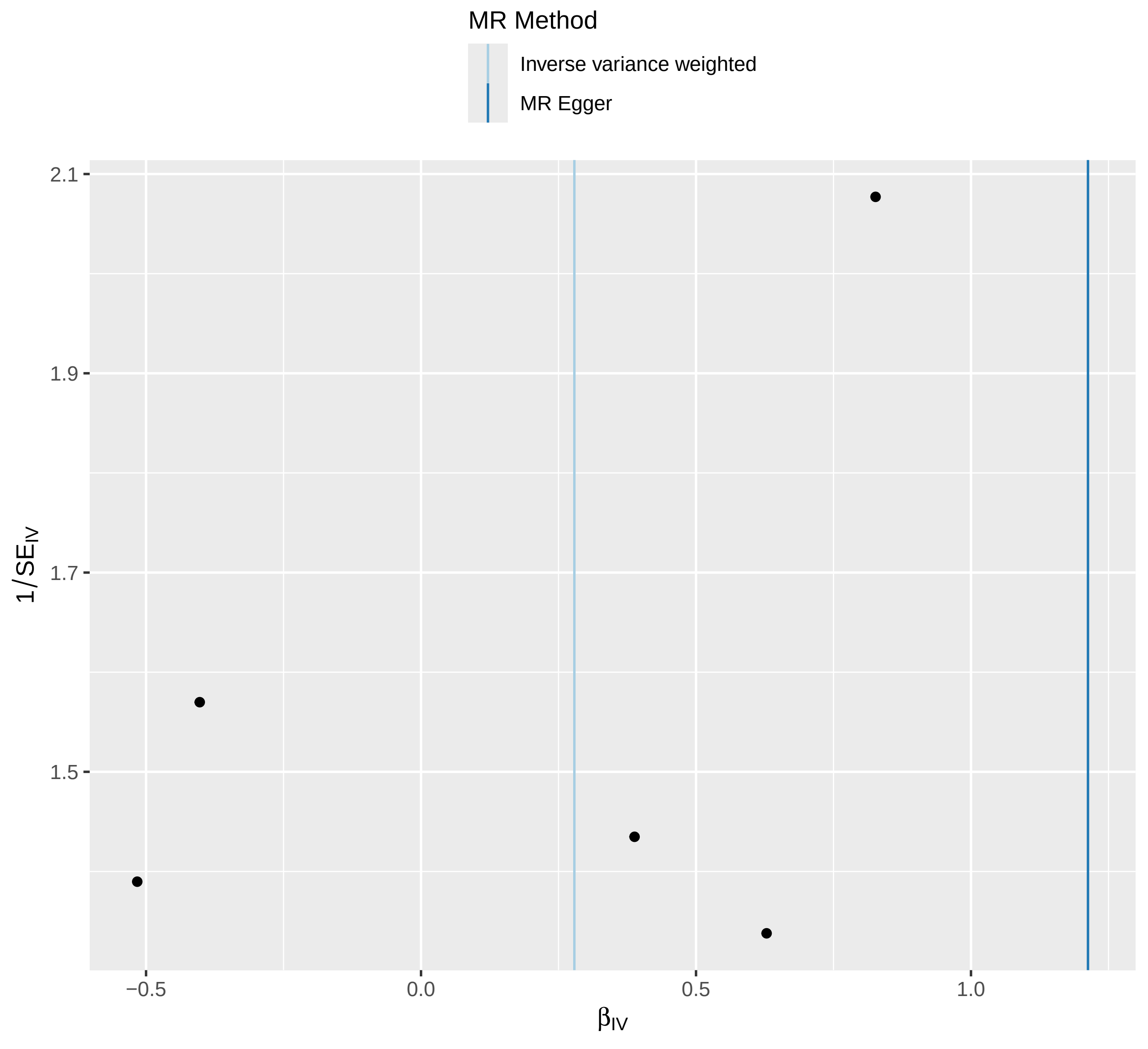

MR Method
Inverse variance weighted
MR Egger
2.1
1.9
1.7
1.5
1/SE$_{IV}$
−0.5
0.0
0.5
1.0
β$_{IV}$

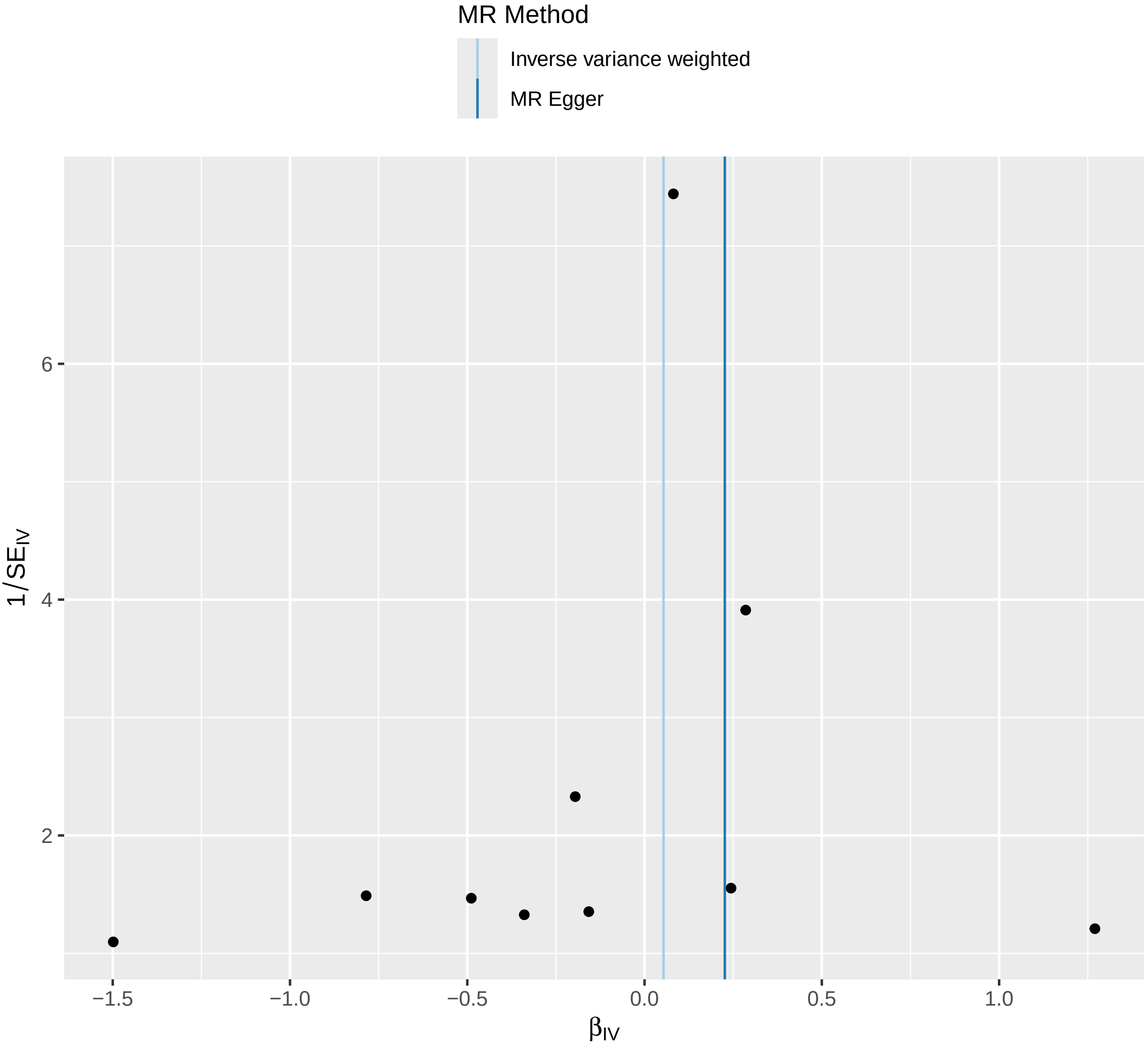

MR Method
Inverse variance weighted
MR Egger
6
4
2
1/SE_IV
−1.5
−1.0
−0.5
0.0
0.5
1.0
β_IV

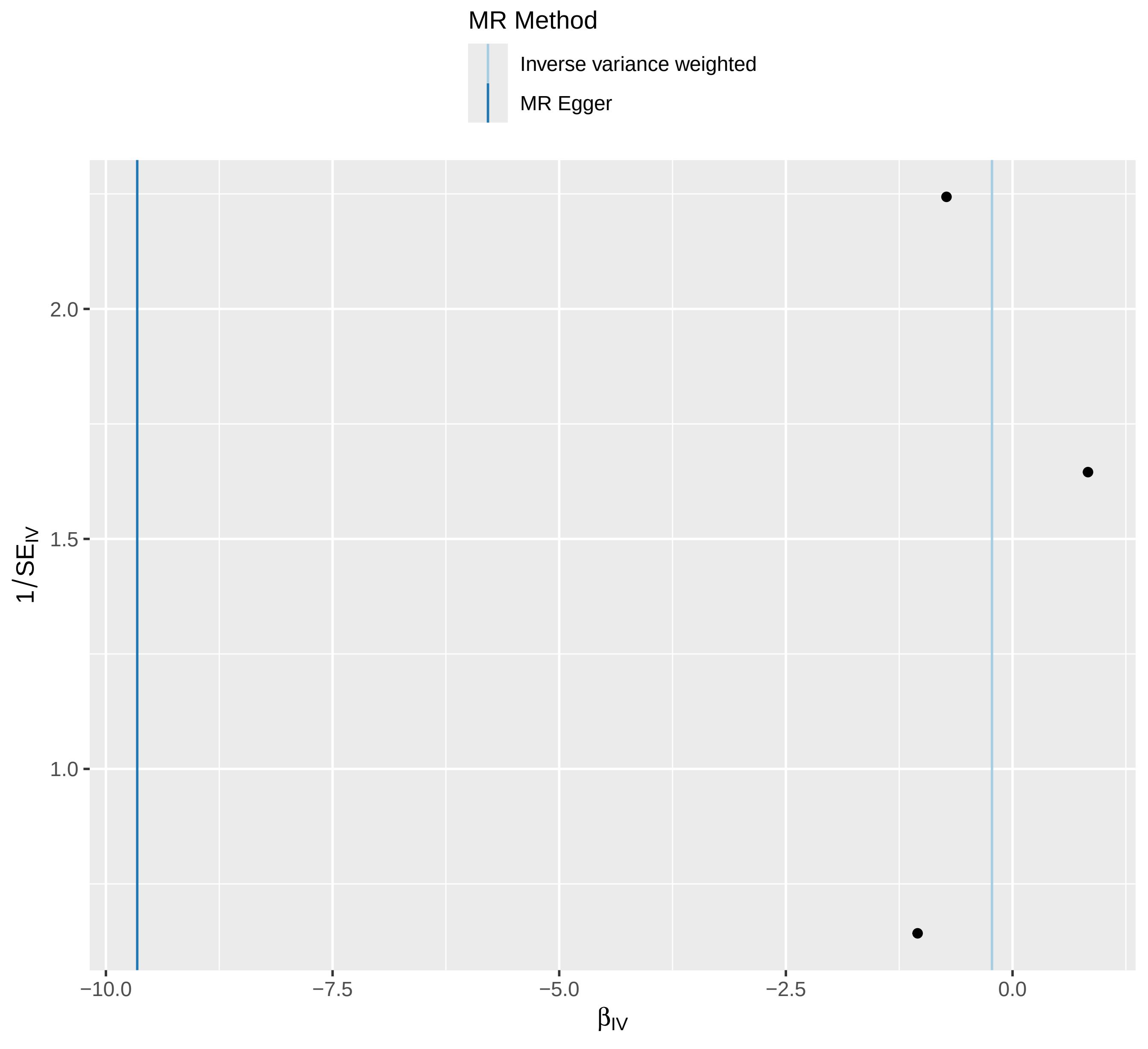
MR Method
Inverse variance weighted
MR Egger
2.0
1.5
1.0
1/SE_IV
−10.0
−7.5
−5.0
−2.5
0.0
β_IV

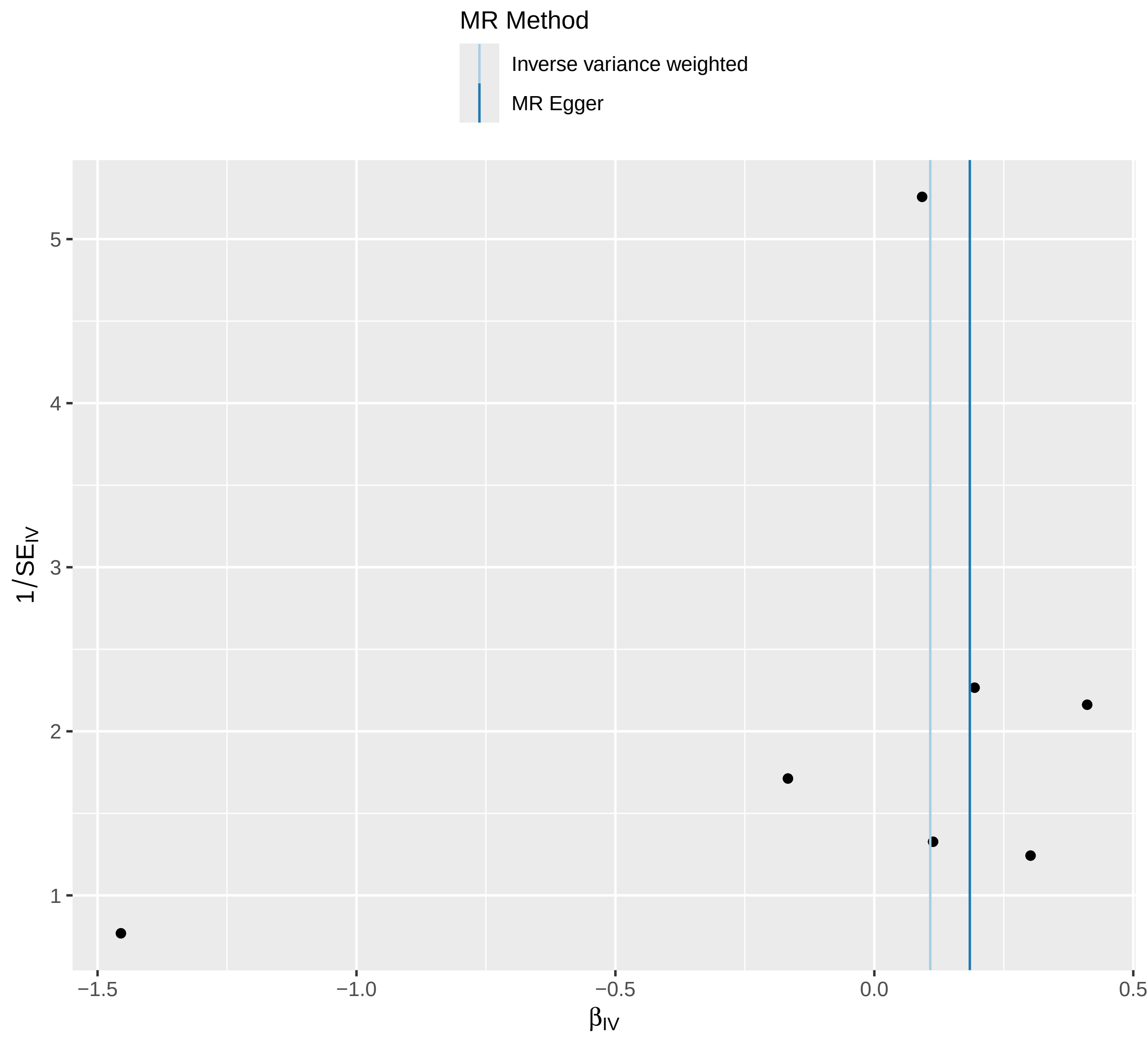
MR Method
Inverse variance weighted
MR Egger
1/SE_IV
β_IV
5
4
3
2
1
−1.5
−1.0
−0.5
0.0
0.5

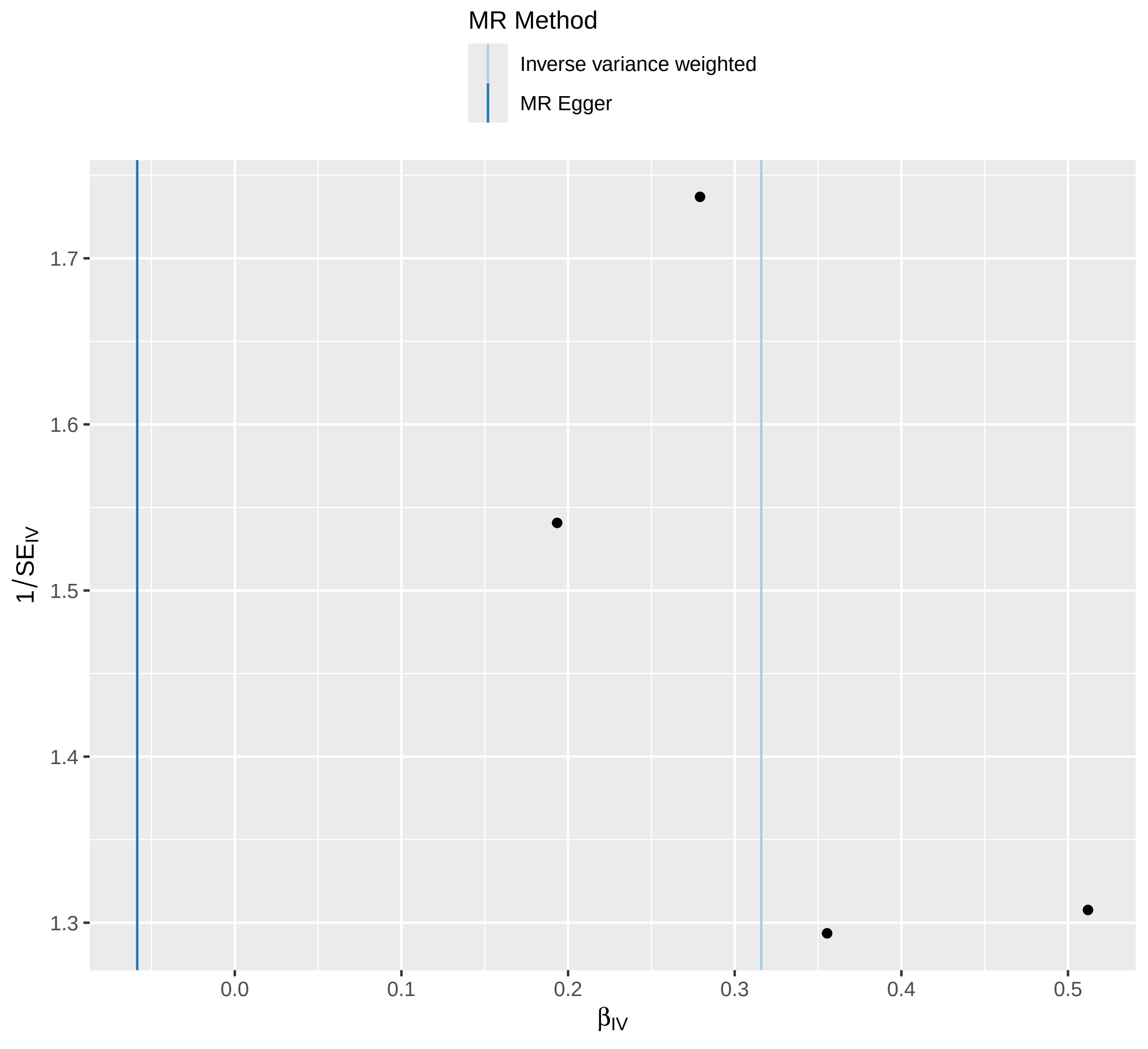

MR Method
Inverse variance weighted
MR Egger
1.7
1.6
1.5
1.4
1.3
$1/SE_{IV}$
0.0
0.1
0.2
0.3
0.4
0.5
β_{IV}

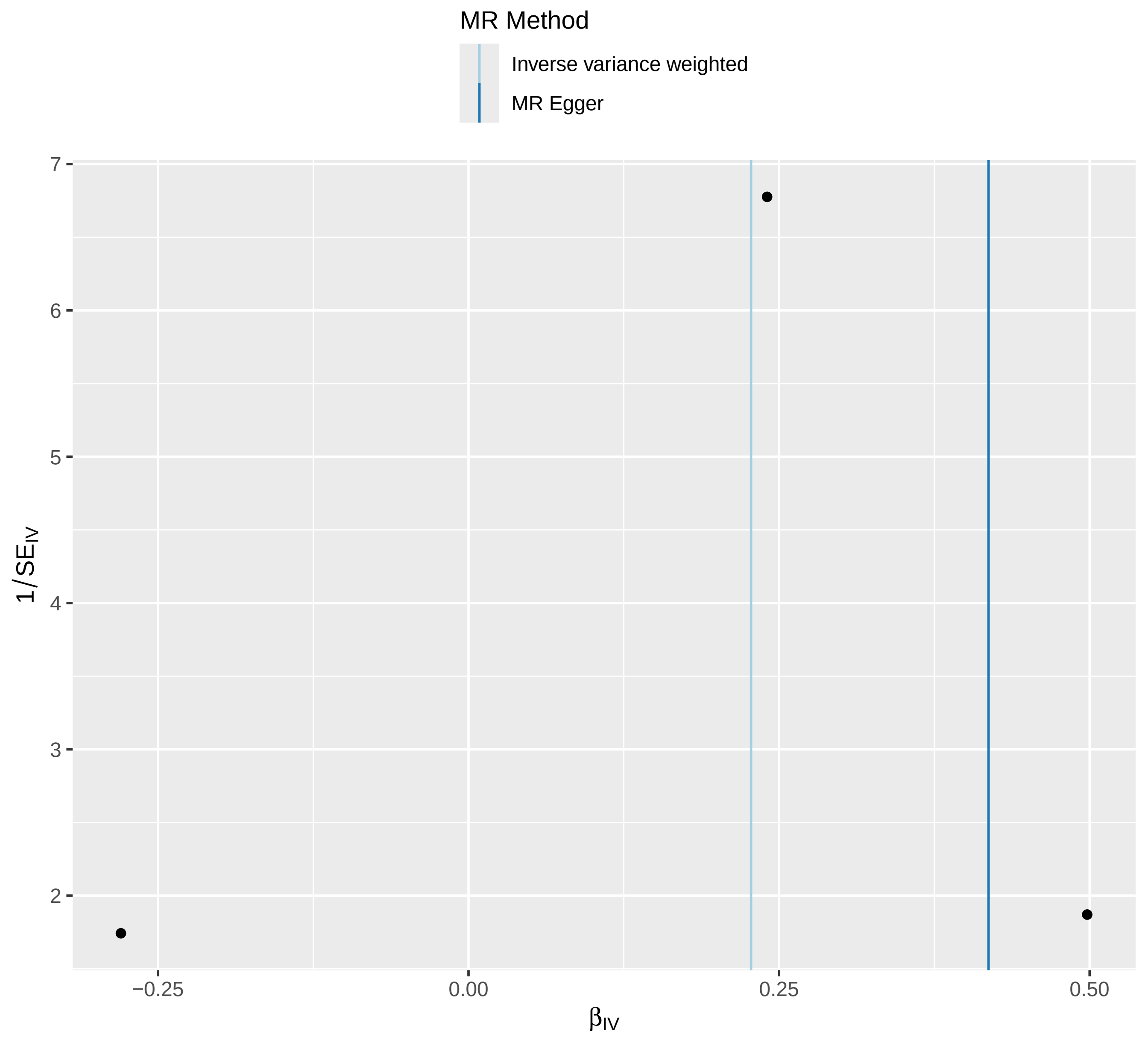
MR Method
Inverse variance weighted
MR Egger
7
6
5
4
3
2
1/SE_IV
−0.25
0.00
0.25
0.50
β_IV

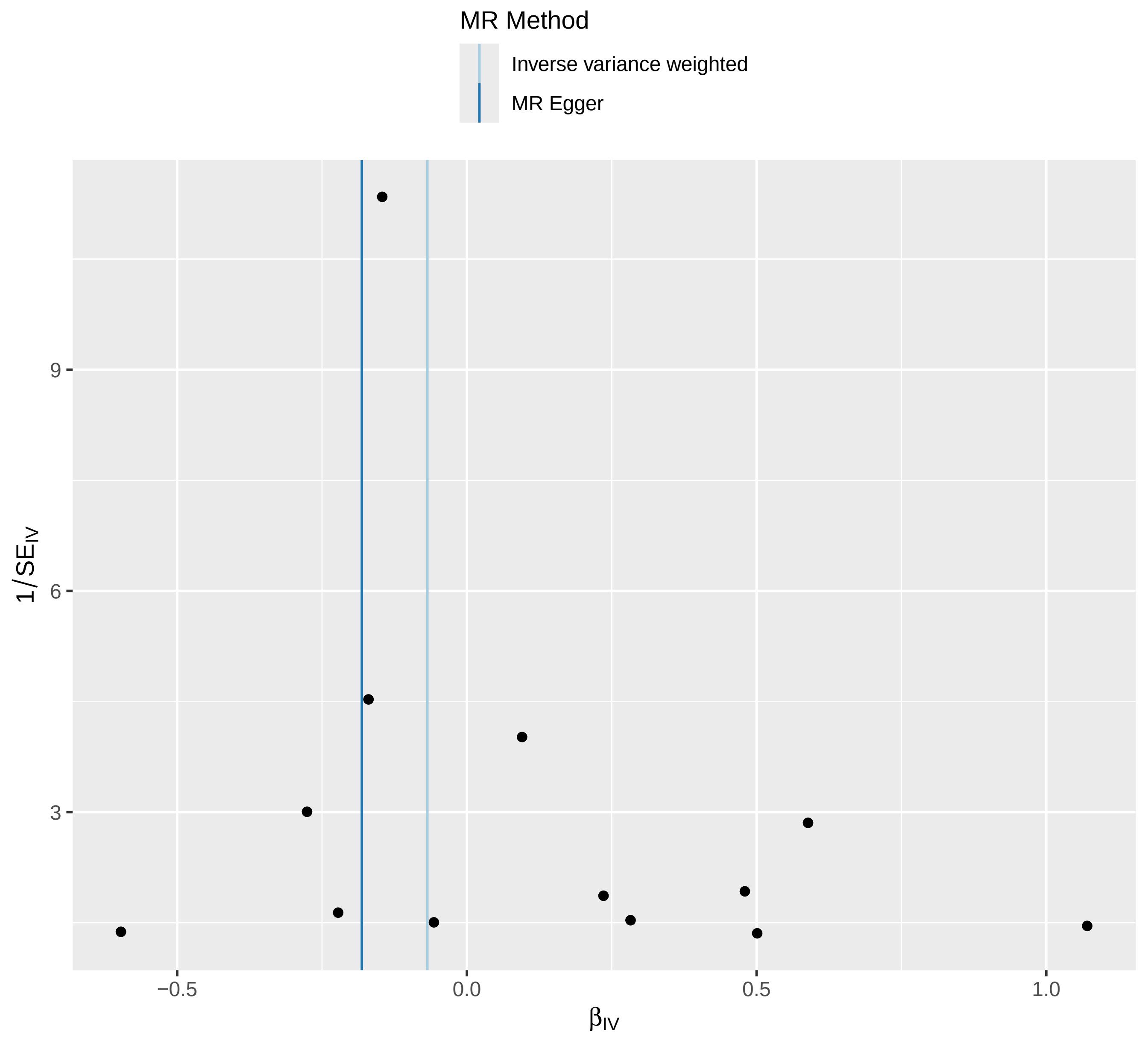
MR Method
Inverse variance weighted
MR Egger
1/SE_IV
9
6
3
−0.5
0.0
0.5
1.0
β_IV

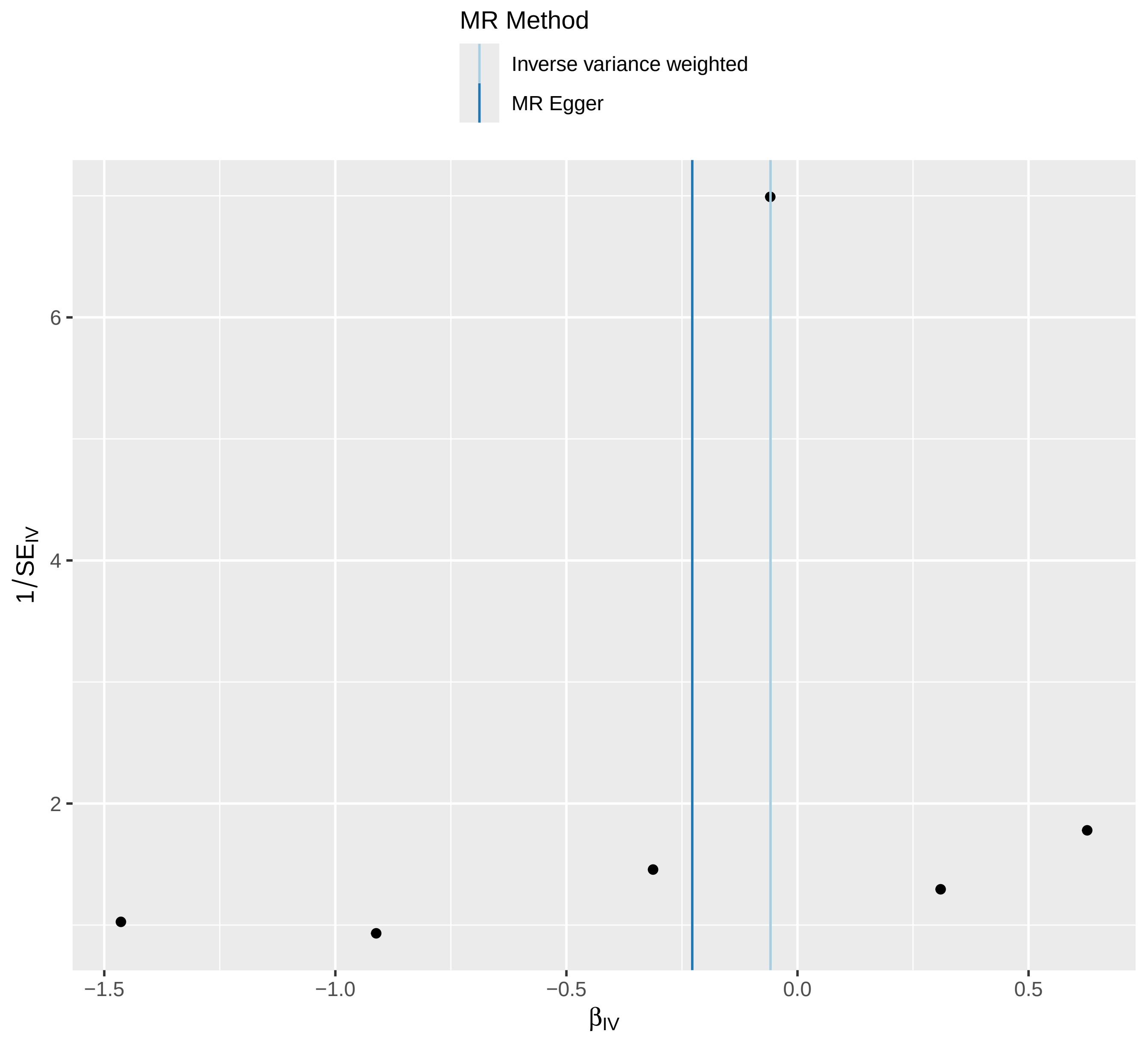

MR Method
Inverse variance weighted
MR Egger
6
4
2
1/SE$_{IV}$
−1.5
−1.0
−0.5
0.0
0.5
β$_{IV}$

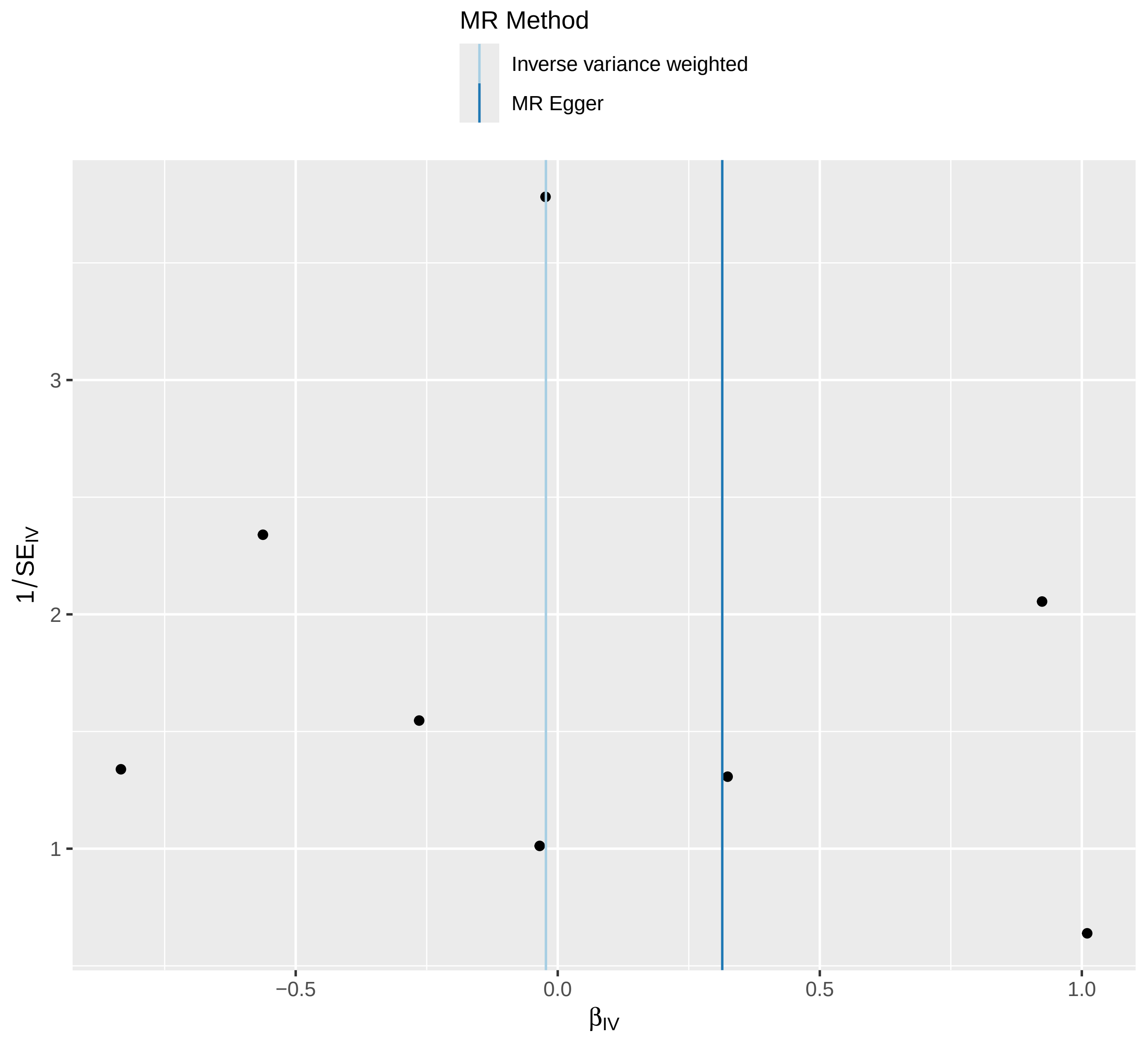

MR Method
Inverse variance weighted
MR Egger
3
2
1
1/SE$_{IV}$
−0.5
0.0
0.5
1.0
β_{IV}

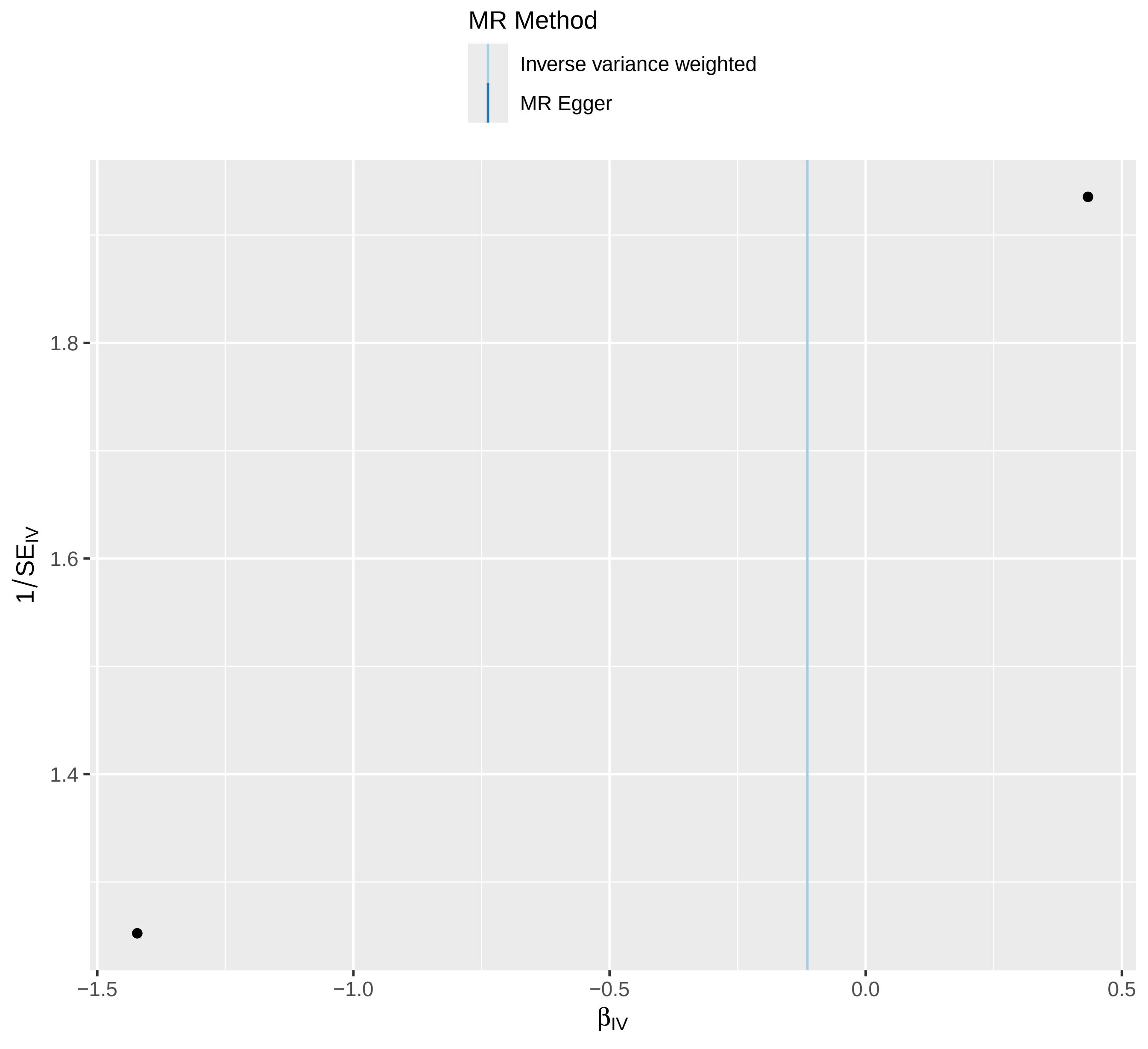

MR Method
Inverse variance weighted
MR Egger
1.8
1.6
1.4
1/SE_IV
−1.5
−1.0
−0.5
0.0
0.5
β_IV

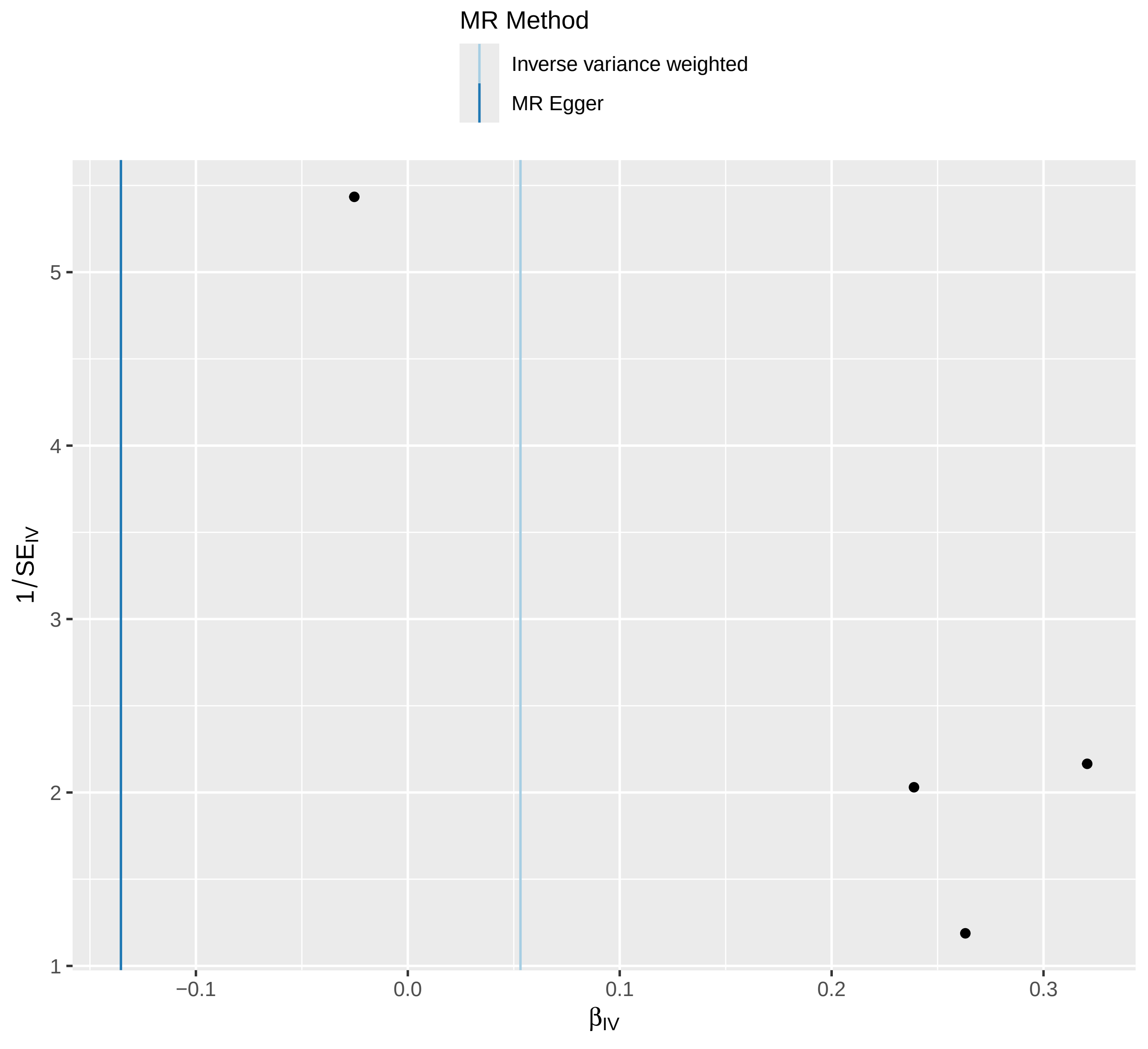
MR Method
Inverse variance weighted
MR Egger
5
4
3
2
1
1/SE_IV
−0.1
0.0
0.1
0.2
0.3
β_IV

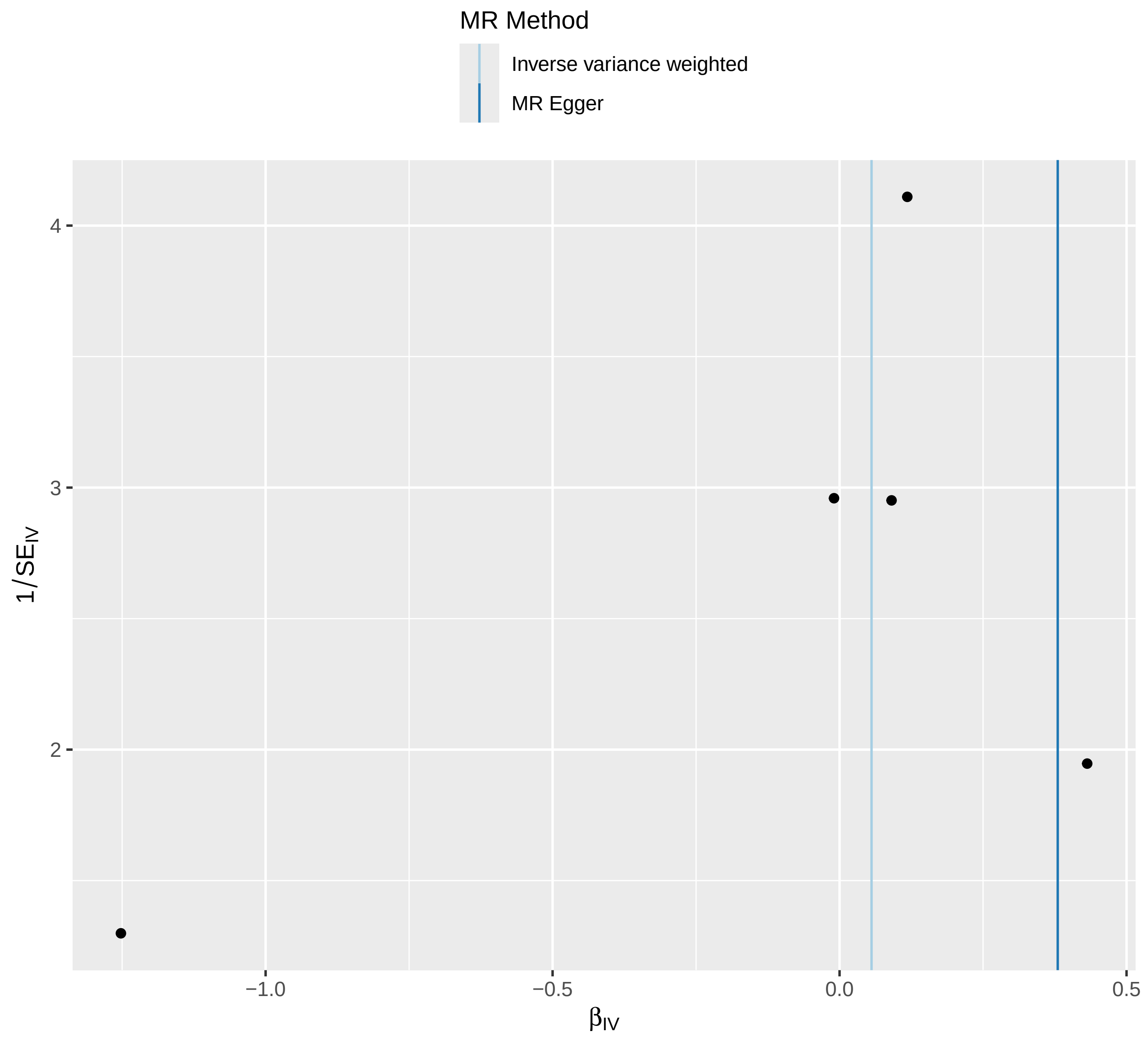
MR Method
Inverse variance weighted
MR Egger
4
3
2
1/SE_IV
−1.0
−0.5
0.0
0.5
β_IV

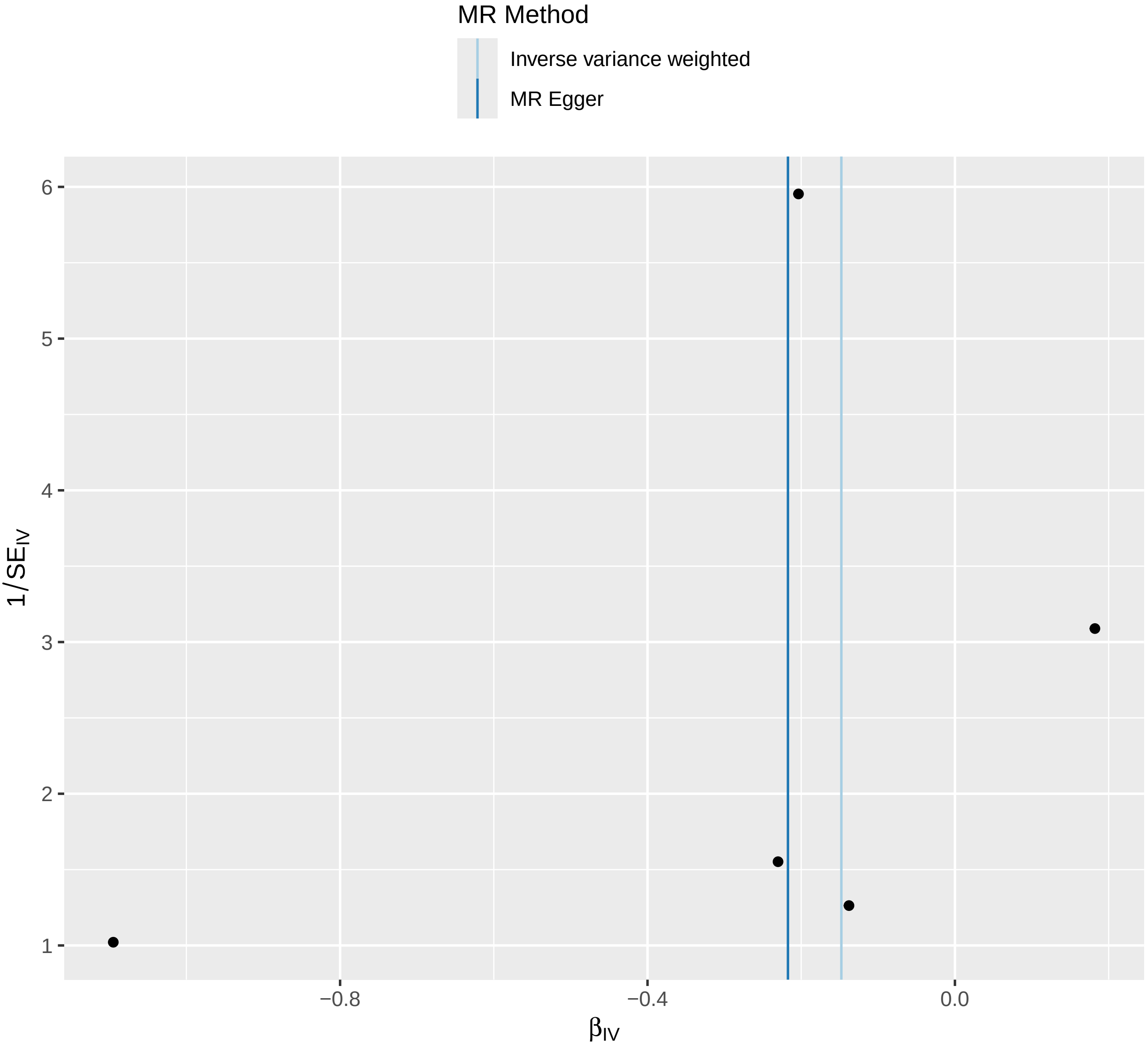
MR Method
Inverse variance weighted
MR Egger
6
5
4
3
2
1
1/SE_IV
−0.8
−0.4
0.0
β_IV

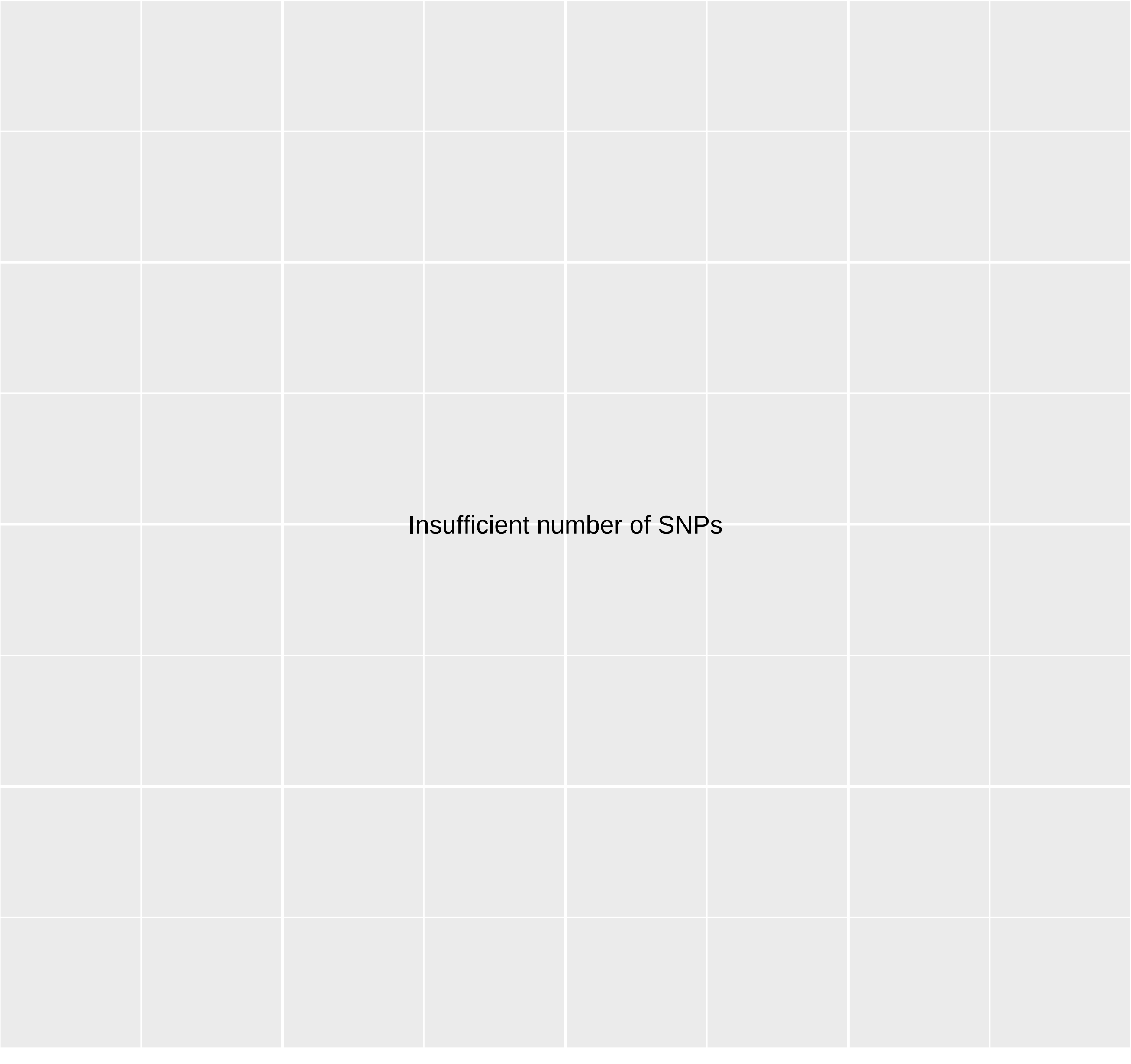
Insufficient number of SNPs

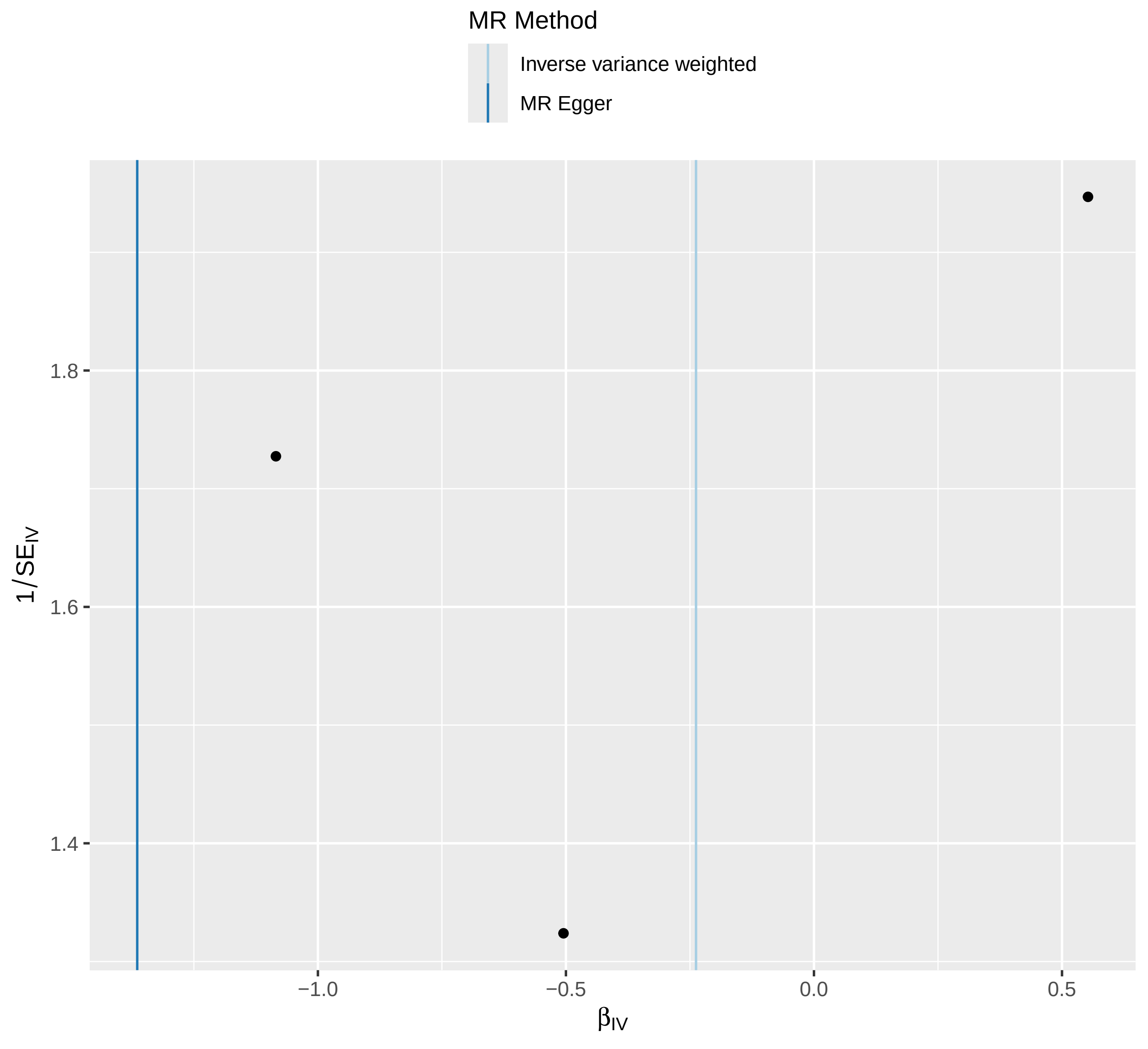

MR Method
Inverse variance weighted
MR Egger
1.8
1.6
1.4
1/SE_IV
−1.0
−0.5
0.0
0.5
β_IV

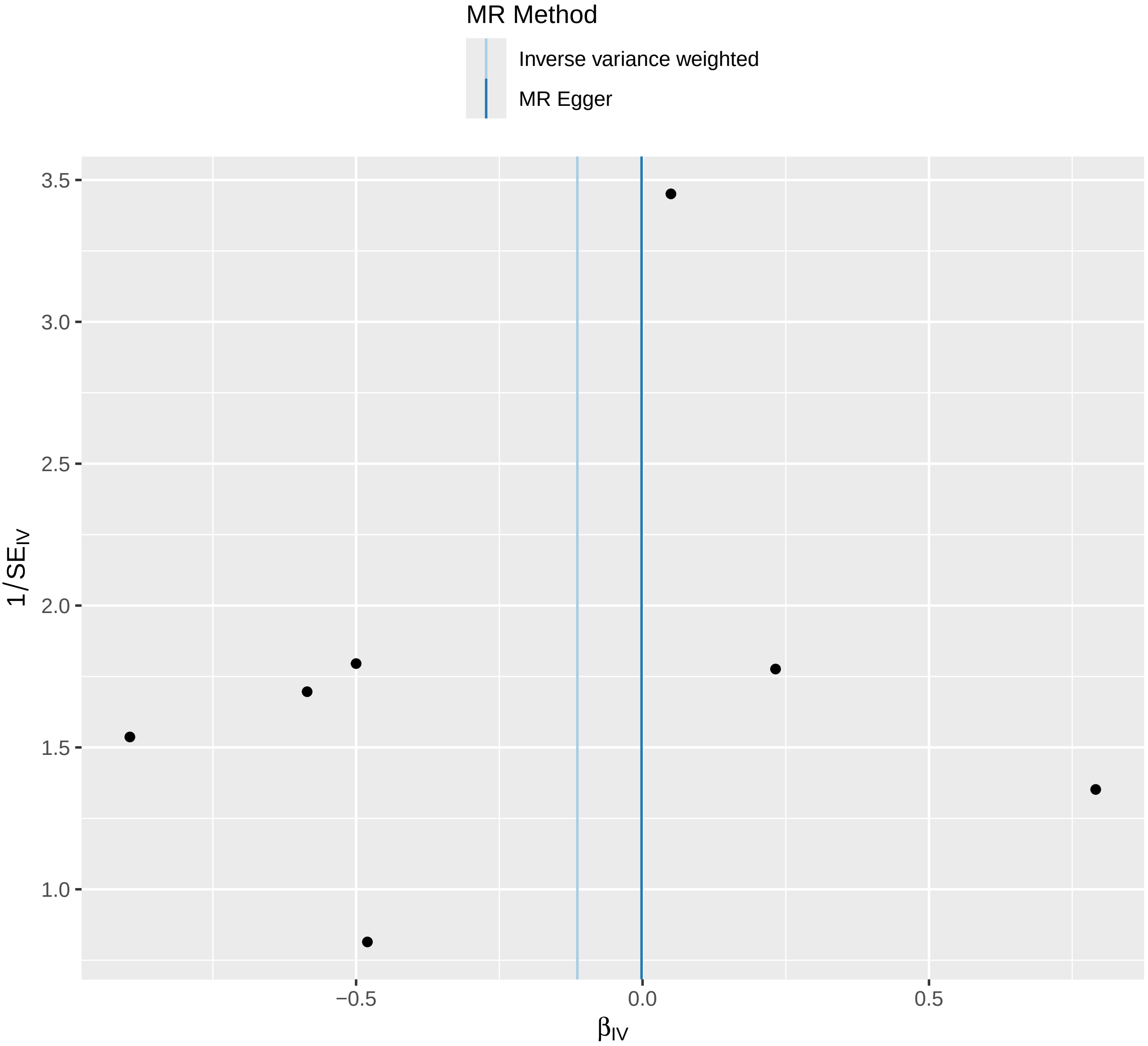
MR Method
Inverse variance weighted
MR Egger
3.5
3.0
2.5
2.0
1.5
1.0
1/SE_IV
−0.5
0.0
0.5
β_IV

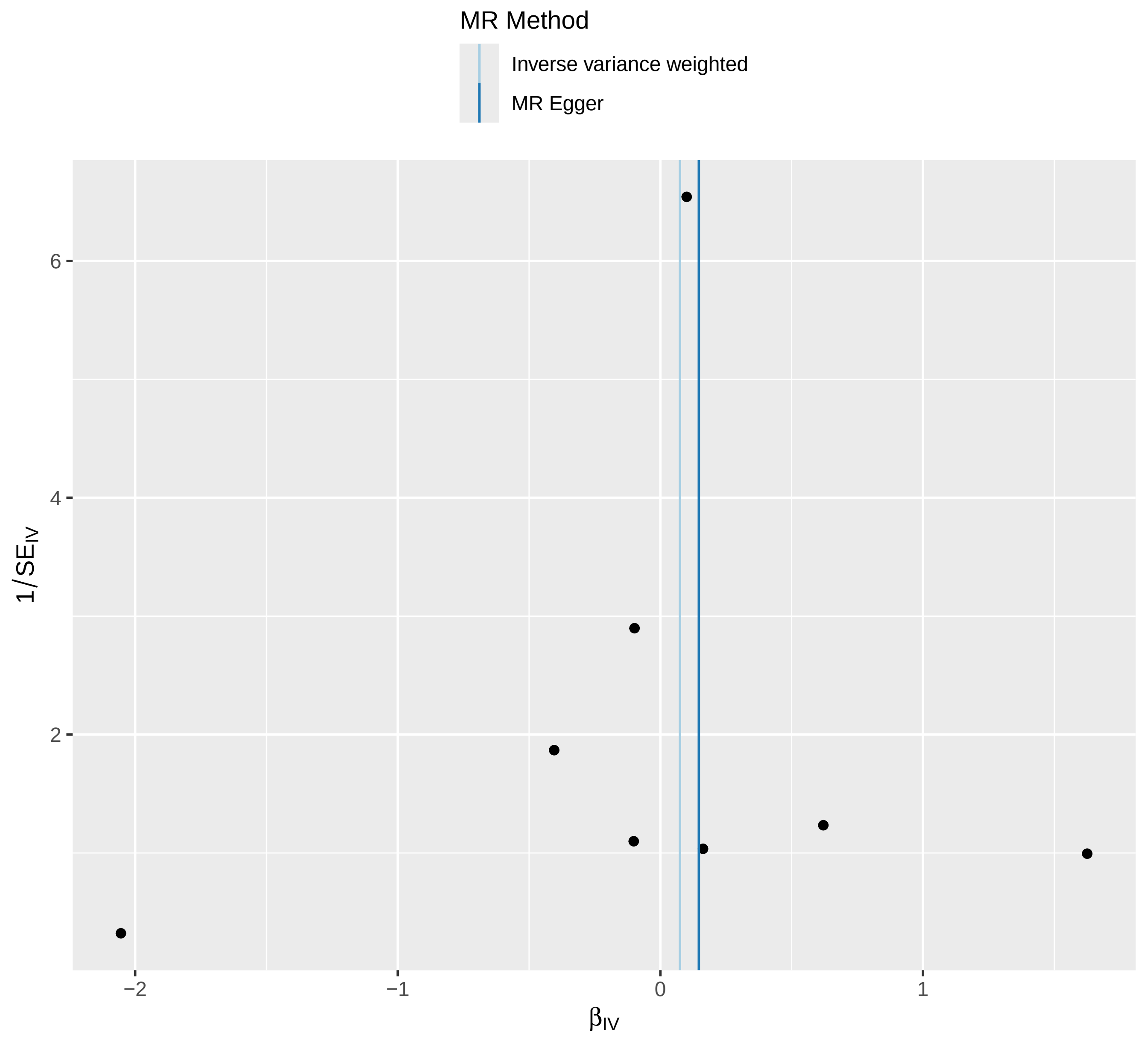
MR Method
Inverse variance weighted
MR Egger
6
4
2
1/SE_IV
−2
−1
0
1
β_IV

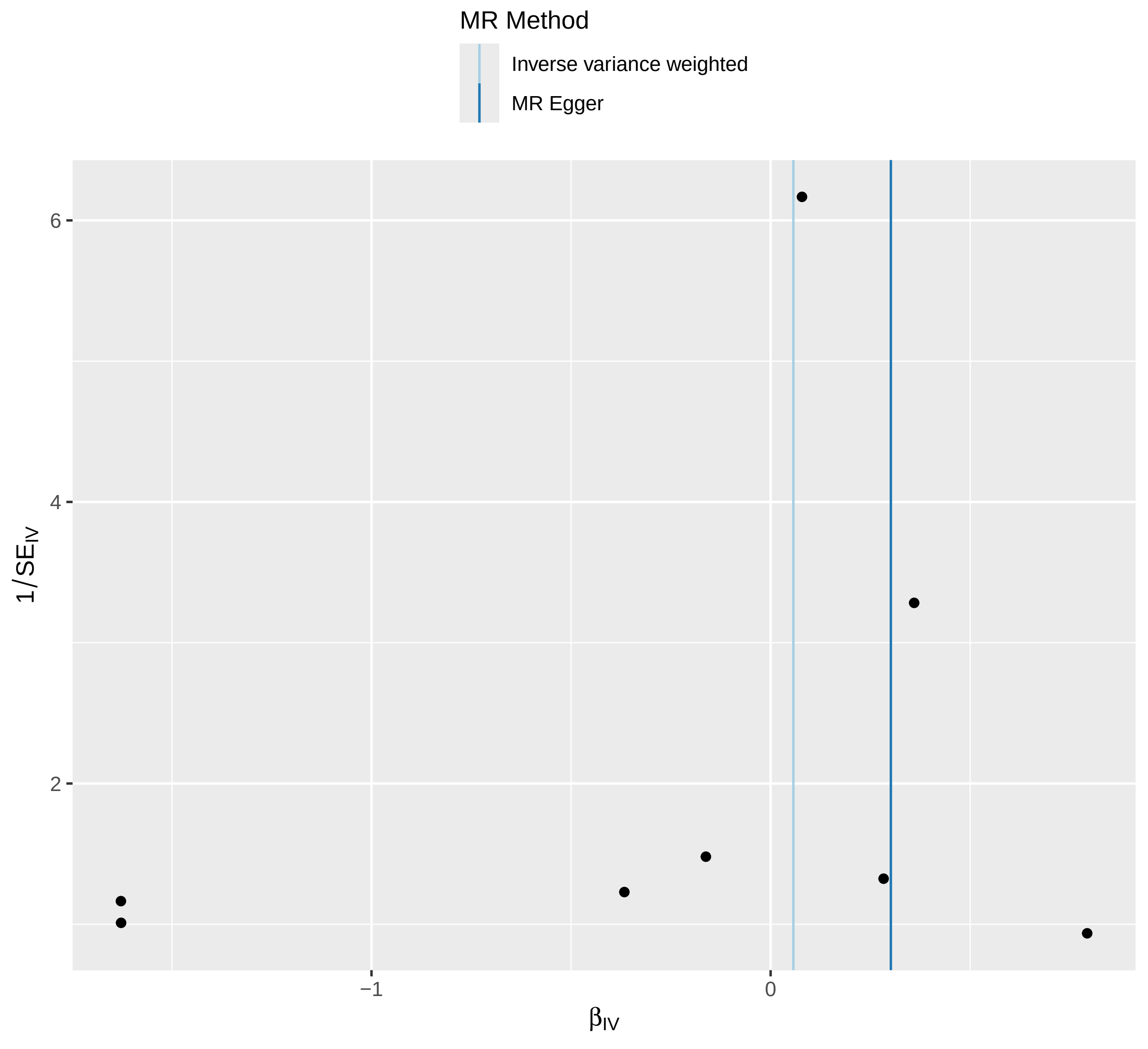
MR Method
Inverse variance weighted
MR Egger
6
4
2
1/SE$_{IV}$
−1
0
β$_{IV}$

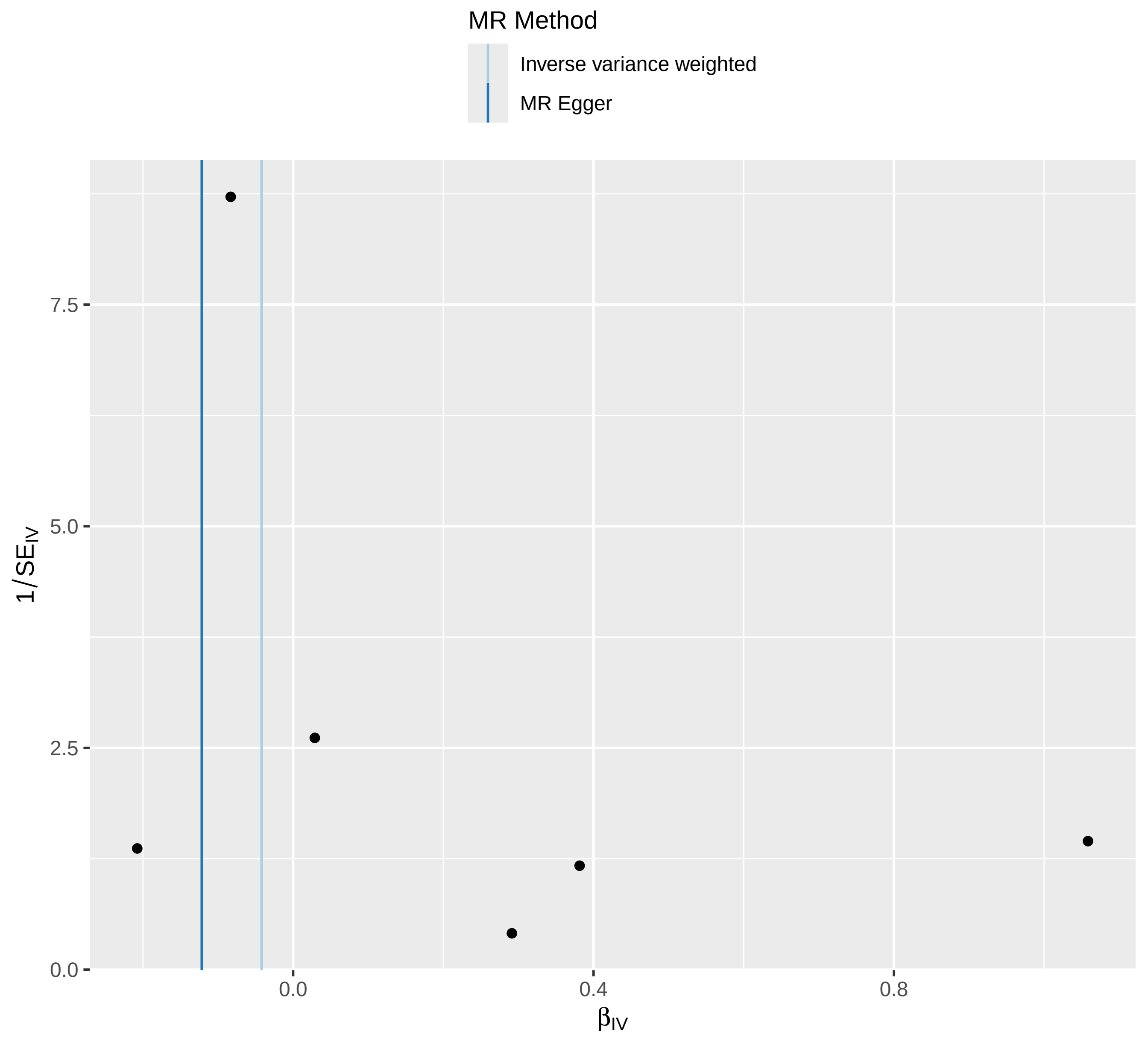

MR Method
Inverse variance weighted
MR Egger
7.5
5.0
2.5
0.0
1/SE_IV
0.0
0.4
0.8
β_IV

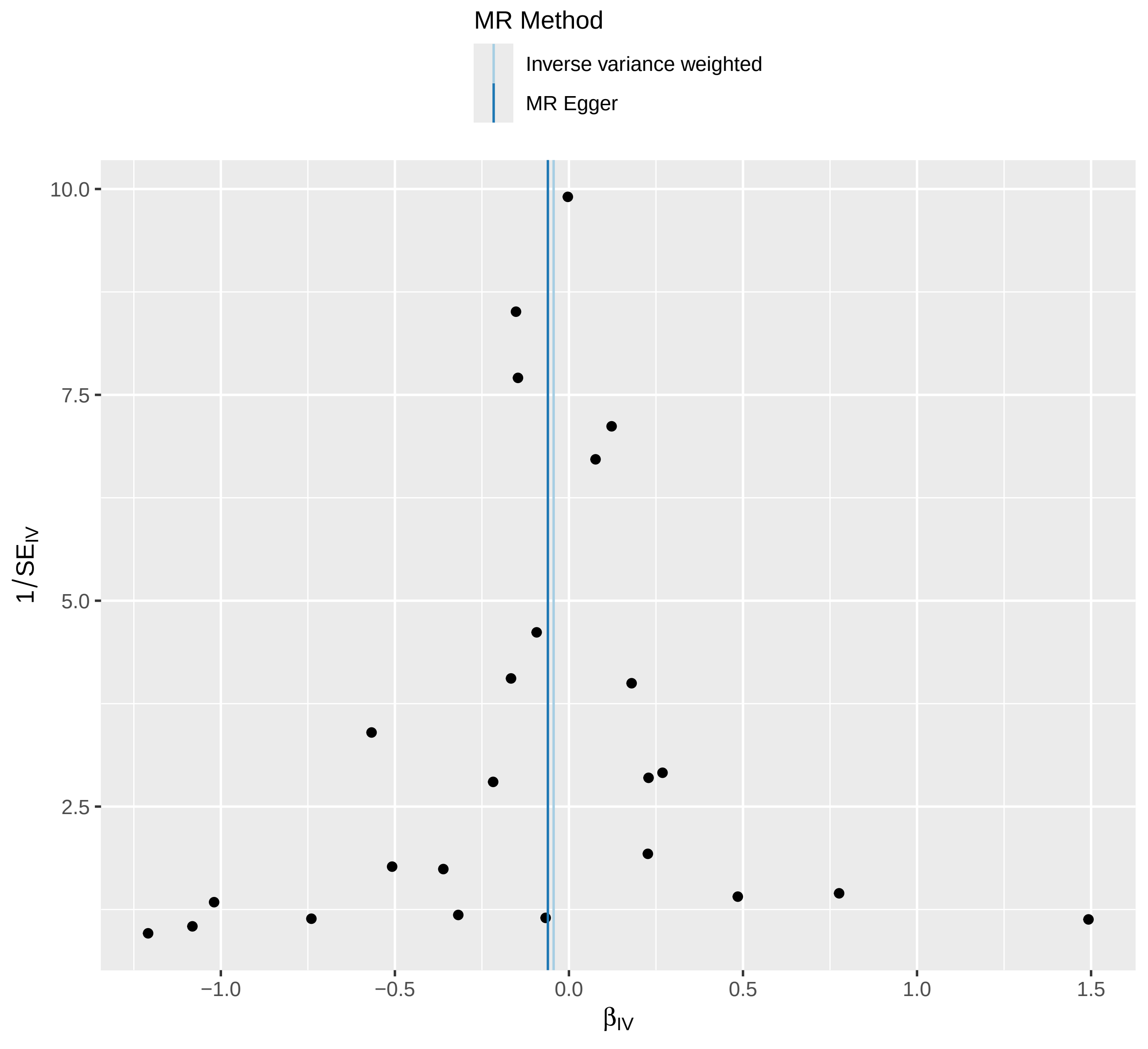
MR Method
Inverse variance weighted
MR Egger
10.0
7.5
5.0
2.5
$1/SE_{IV}$
−1.0
−0.5
0.0
0.5
1.0
1.5
β_{IV}

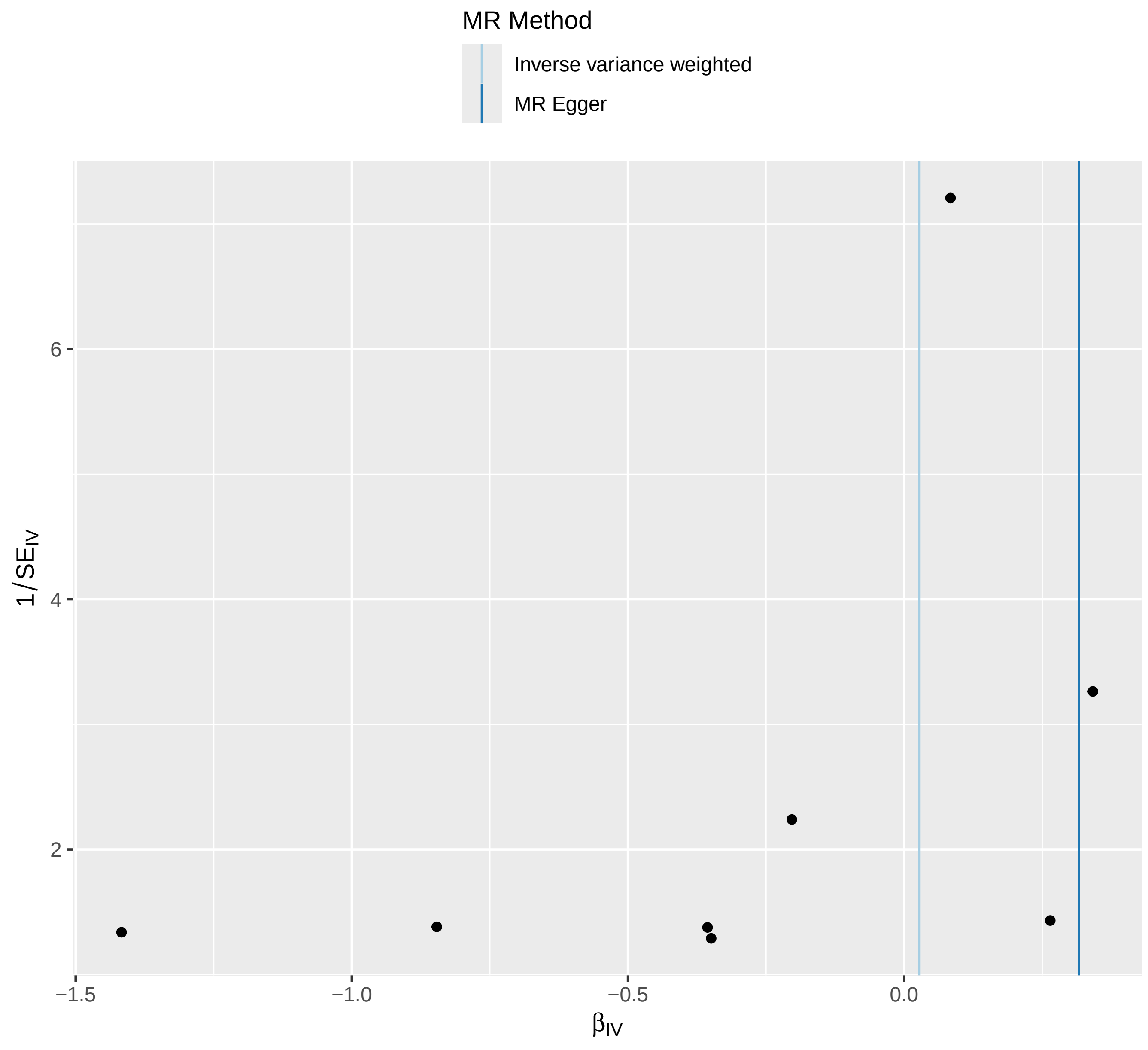

MR Method
Inverse variance weighted
MR Egger
6
4
2
1/SE_IV
−1.5
−1.0
−0.5
0.0
β_IV

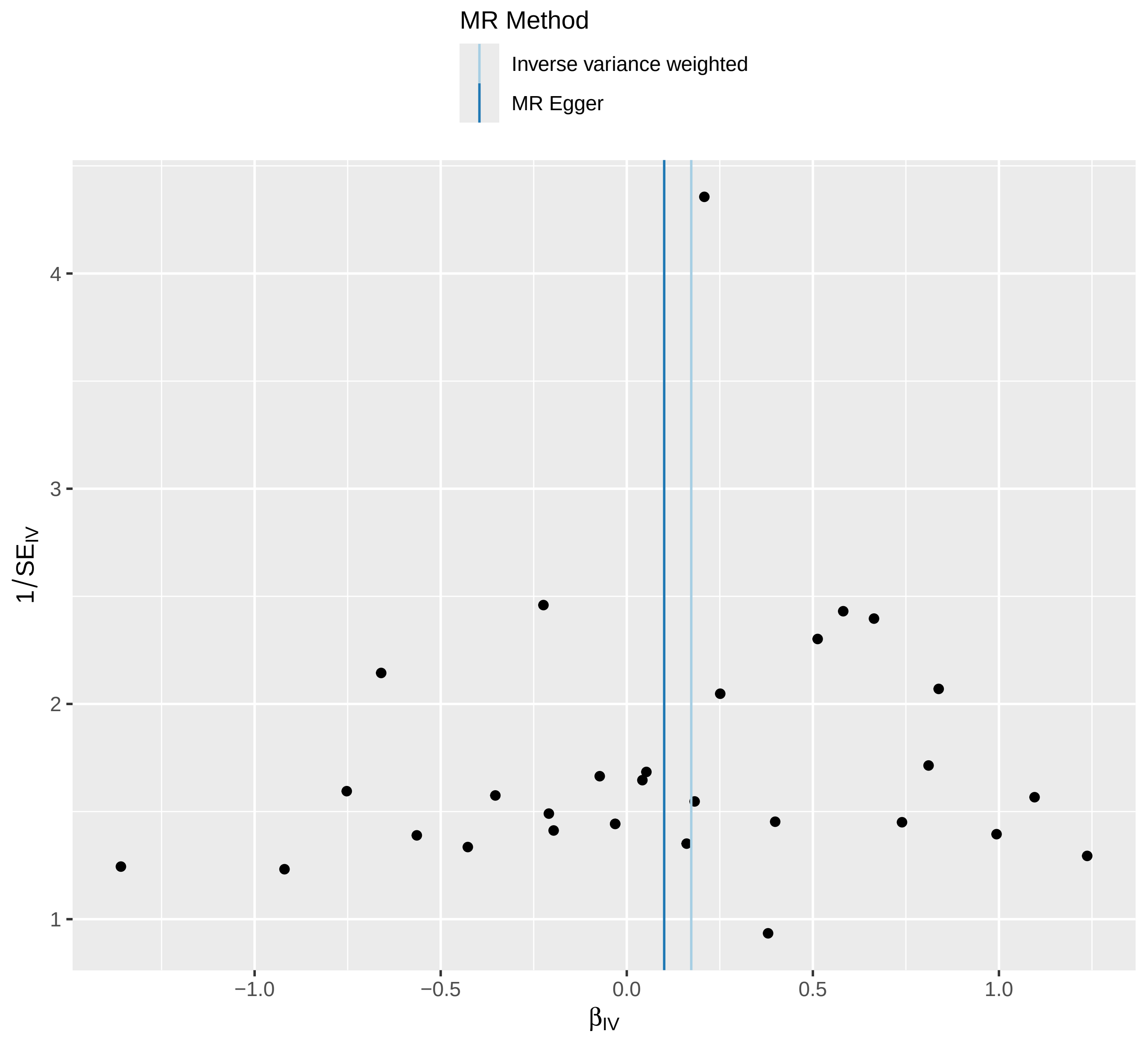
MR Method
Inverse variance weighted
MR Egger
1/SE_IV
4
3
2
1
−1.0
−0.5
0.0
0.5
1.0
β_IV

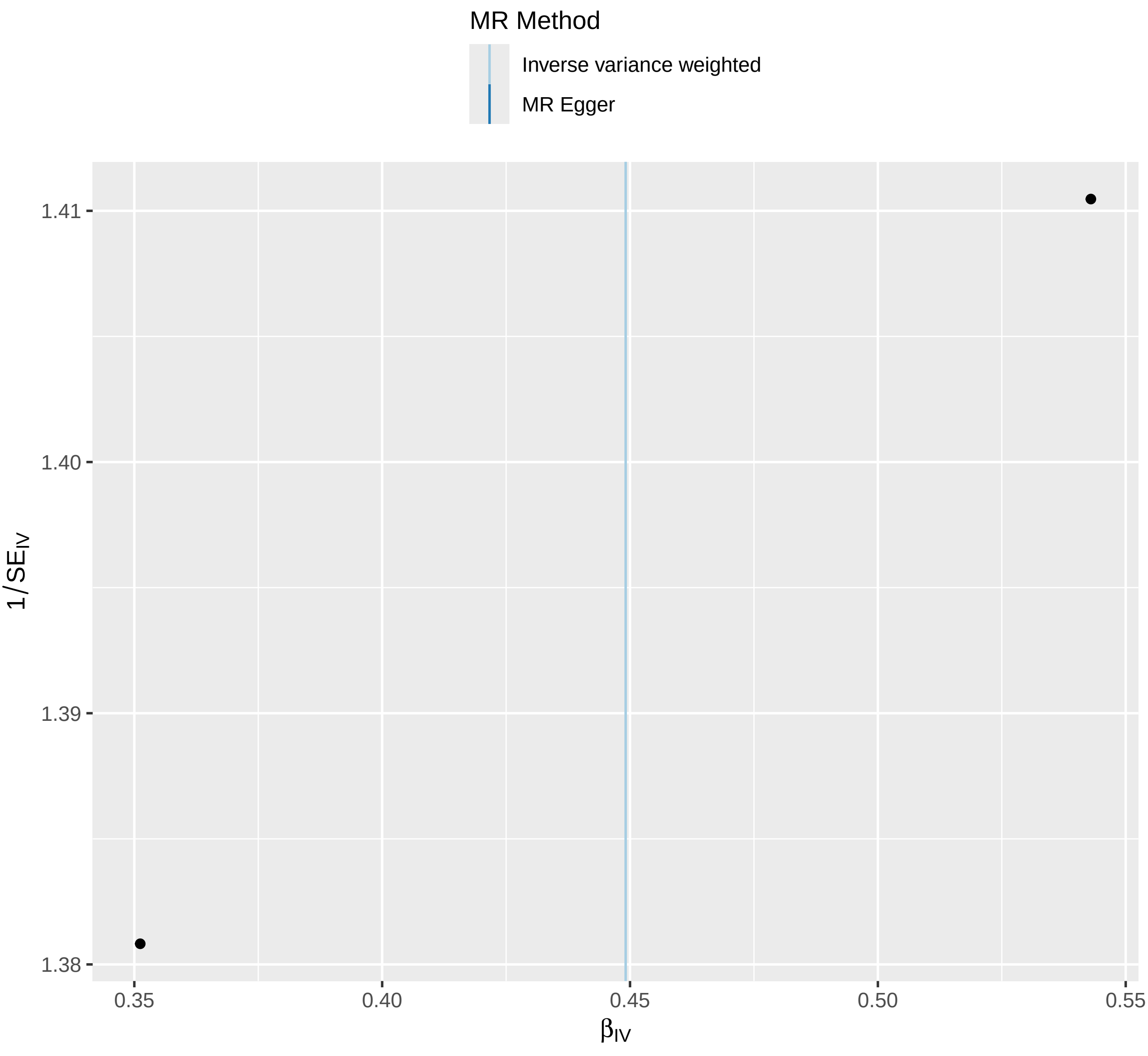
MR Method
Inverse variance weighted
MR Egger
1.41
1.40
1.39
1.38
1/SE_IV
0.35
0.40
0.45
0.50
0.55
β_IV

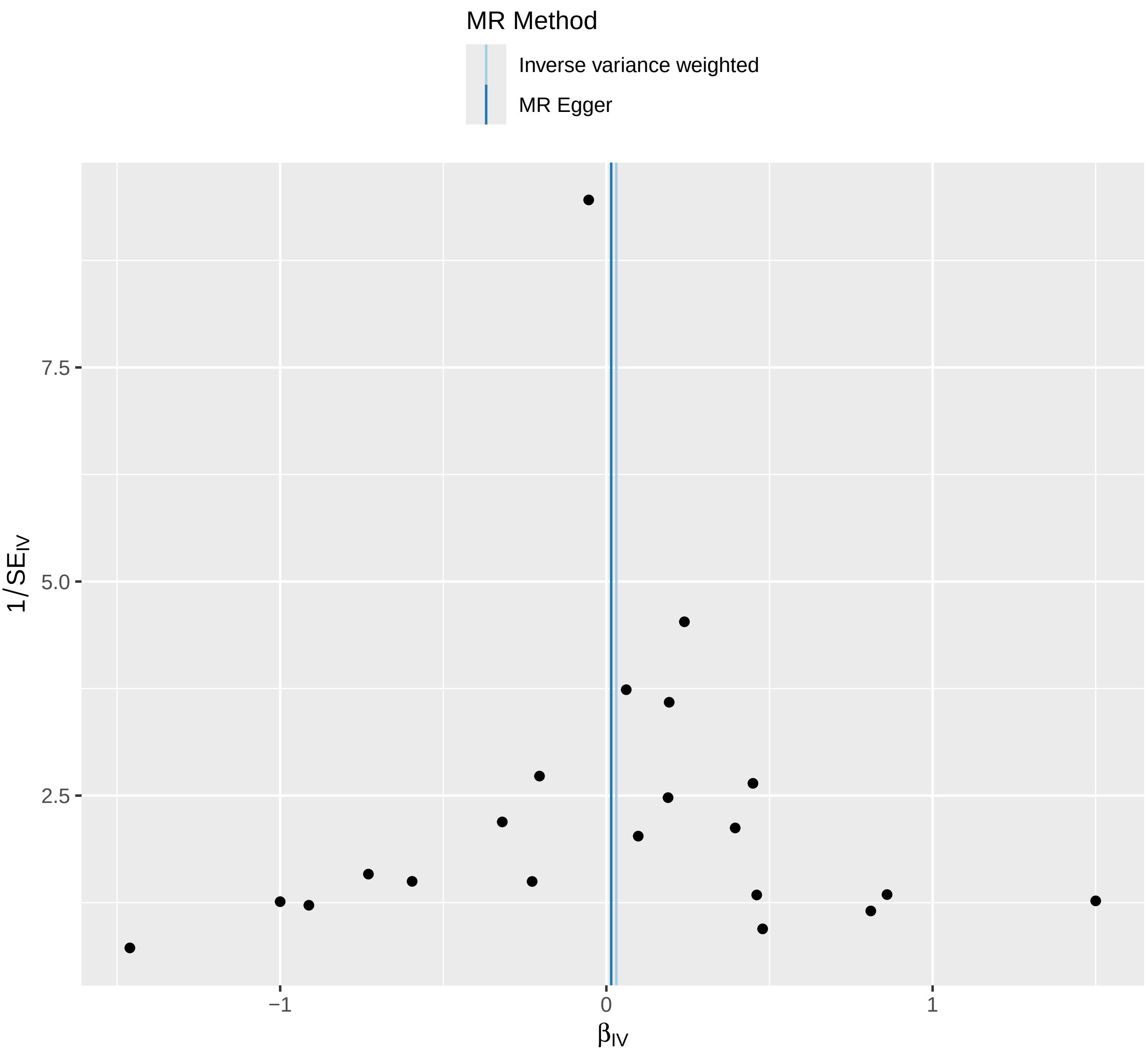
MR Method
Inverse variance weighted
MR Egger
7.5
5.0
2.5
$1/SE_{IV}$
−1
0
1
β_{IV}

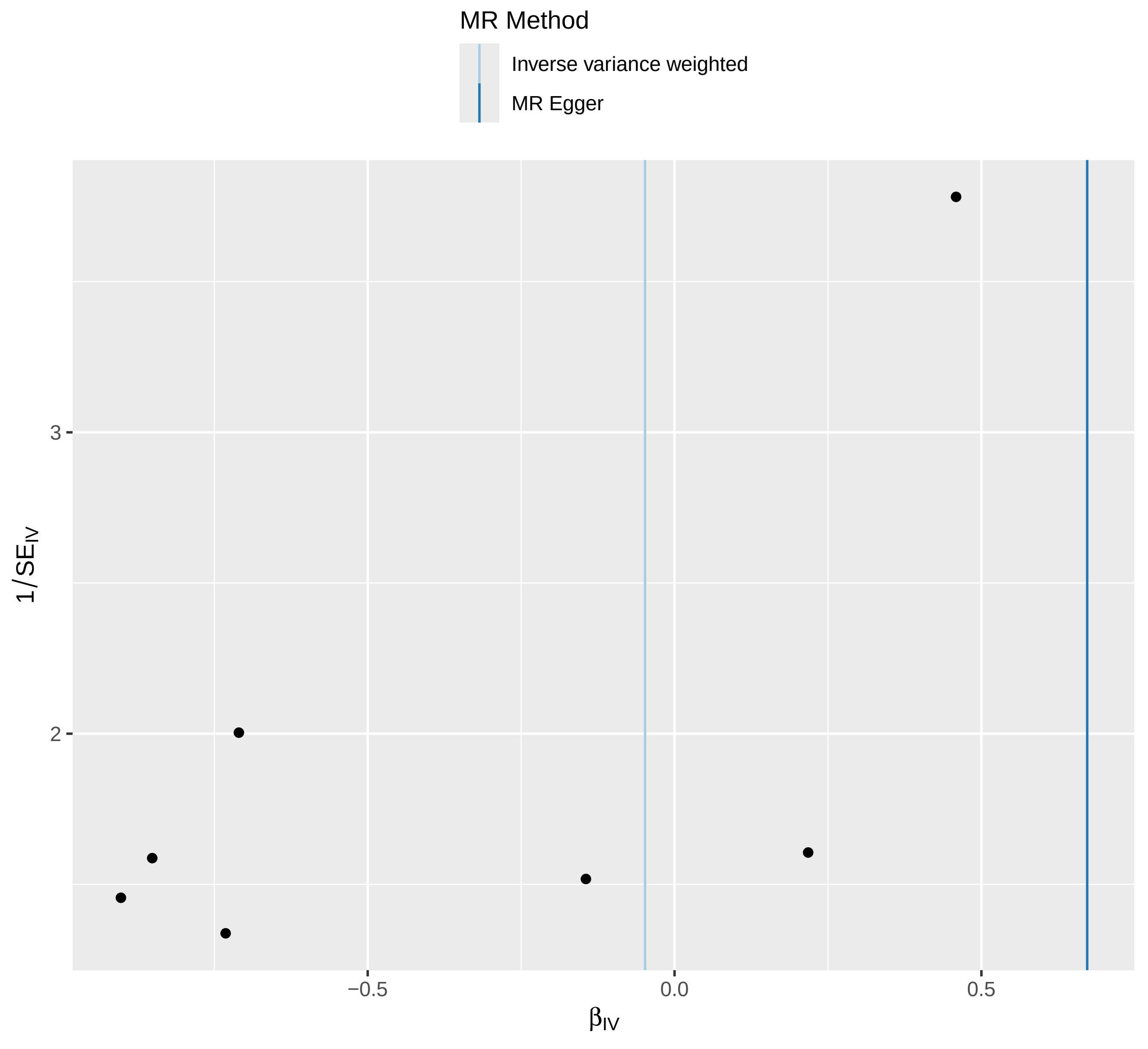

MR Method
Inverse variance weighted
MR Egger
3
2
$1/SE_{IV}$
−0.5
0.0
0.5
β_{IV}

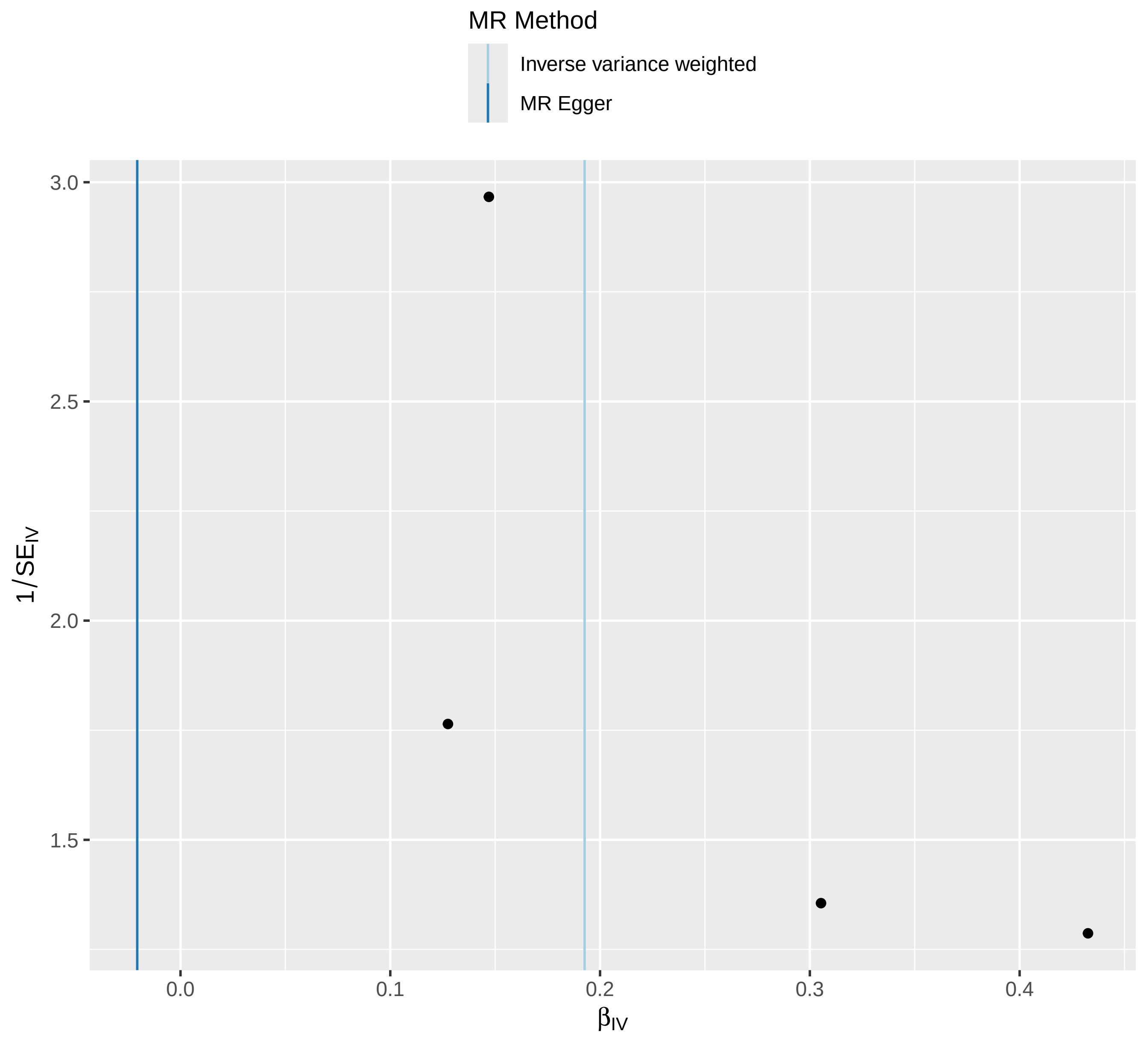
MR Method
Inverse variance weighted
MR Egger
3.0
2.5
2.0
1.5
1/SE_IV
0.0
0.1
0.2
0.3
0.4
β_IV

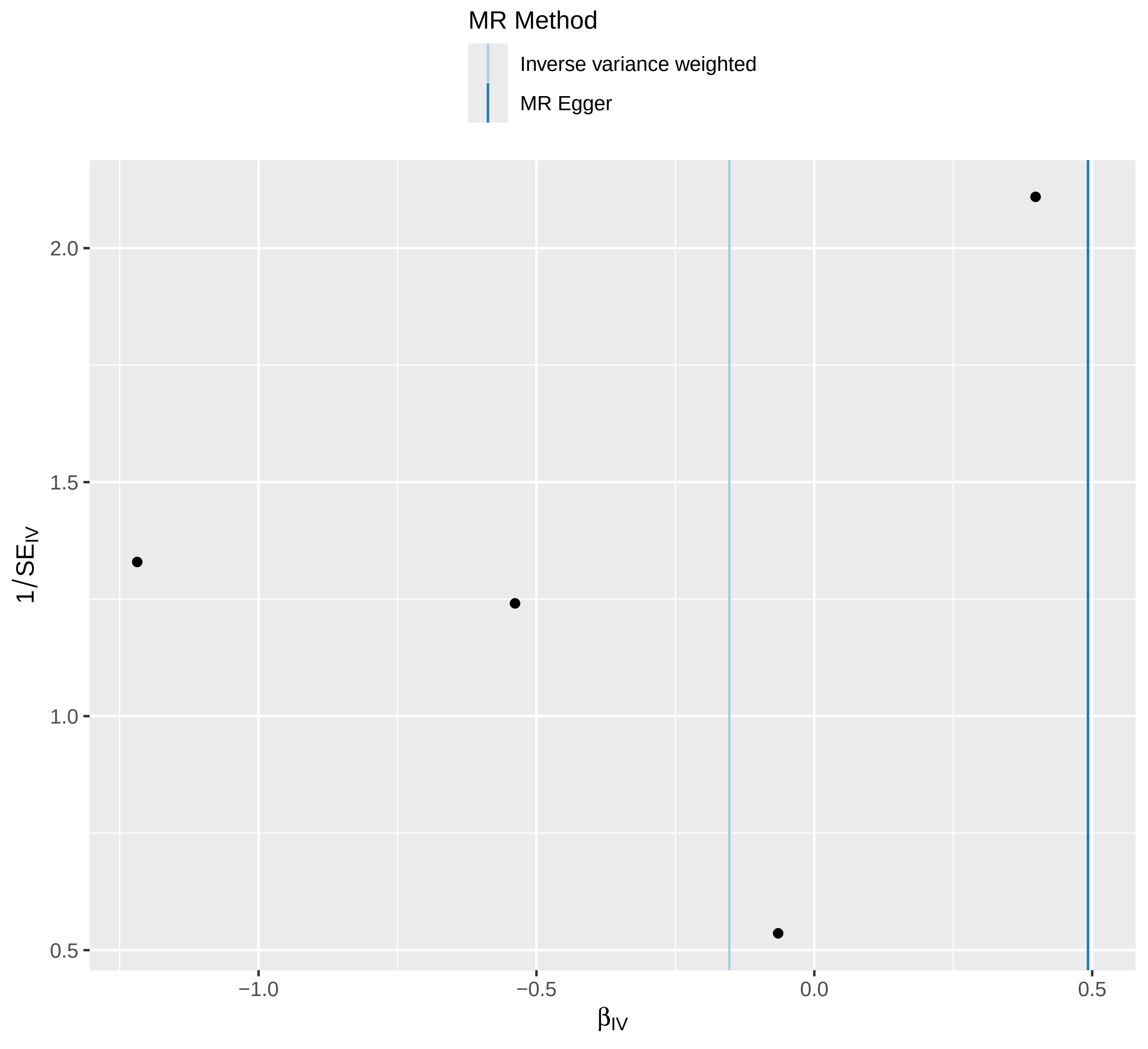
MR Method
Inverse variance weighted
MR Egger
2.0
1.5
1.0
0.5
1/SE_IV
−1.0
−0.5
0.0
0.5
β_IV

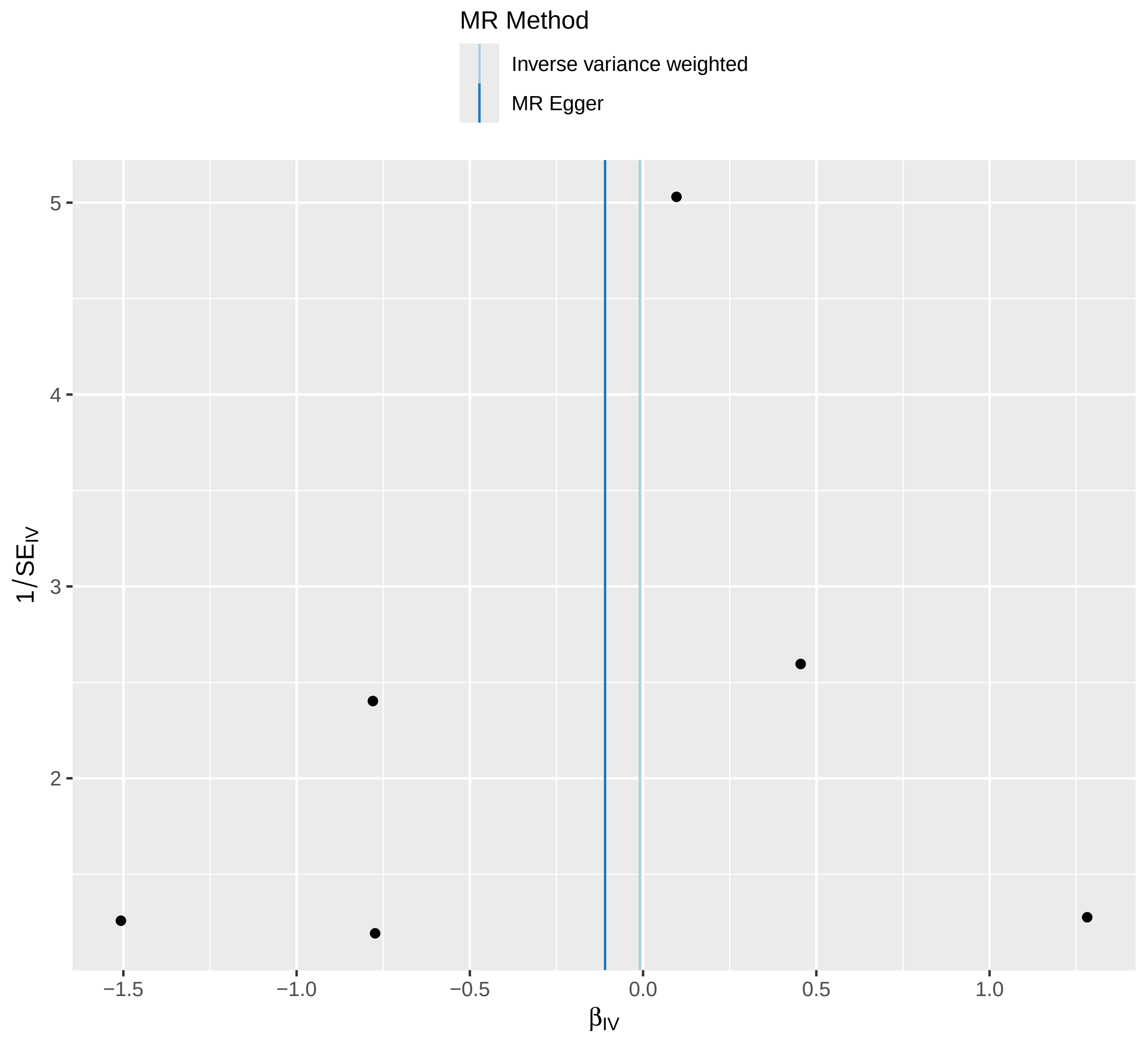
MR Method
Inverse variance weighted
MR Egger
5
4
3
2
1/SE_IV
−1.5
−1.0
−0.5
0.0
0.5
1.0
β_IV

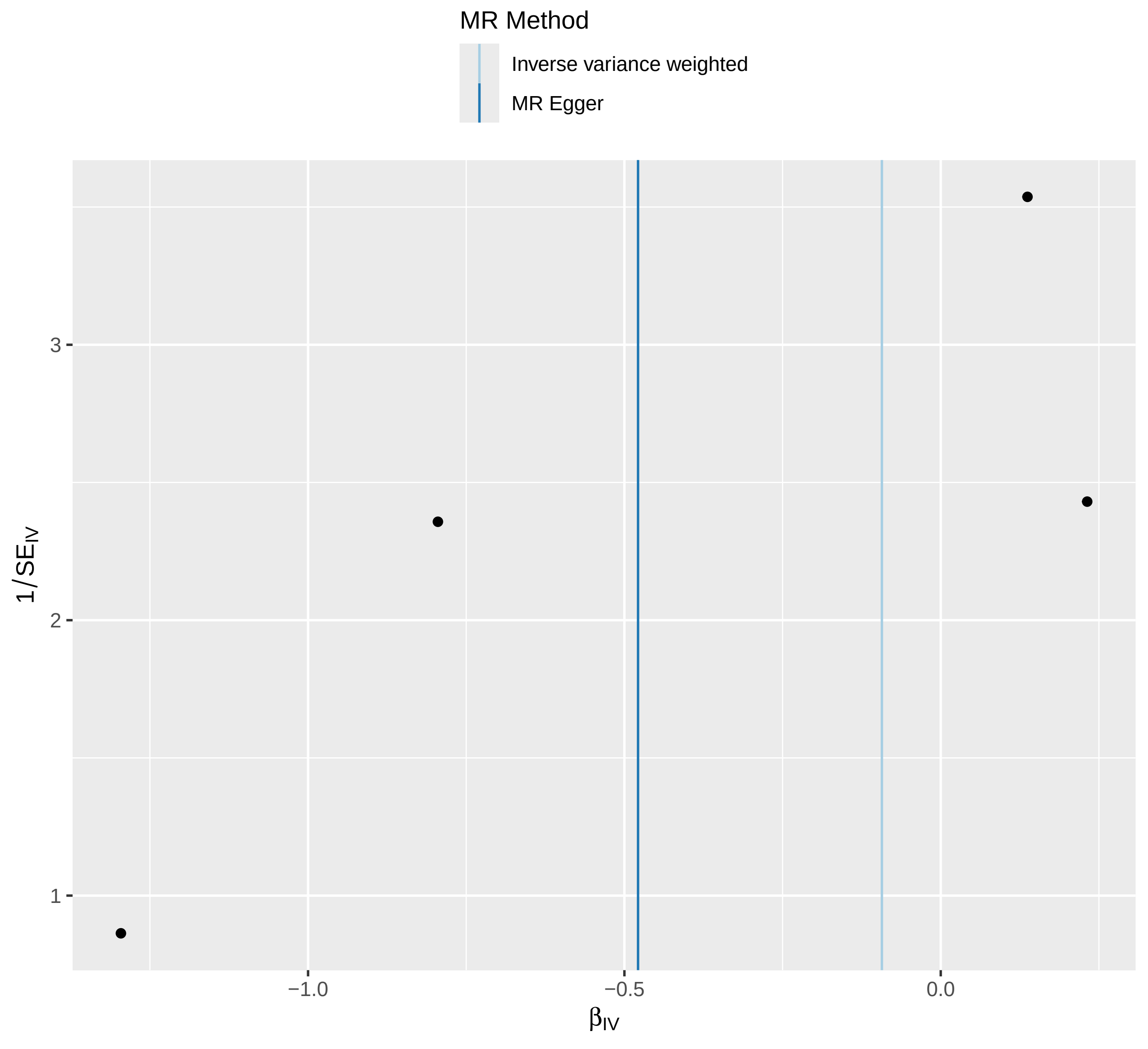

MR Method
Inverse variance weighted
MR Egger
3
2
1
1/SE_IV
−1.0
−0.5
0.0
β_IV

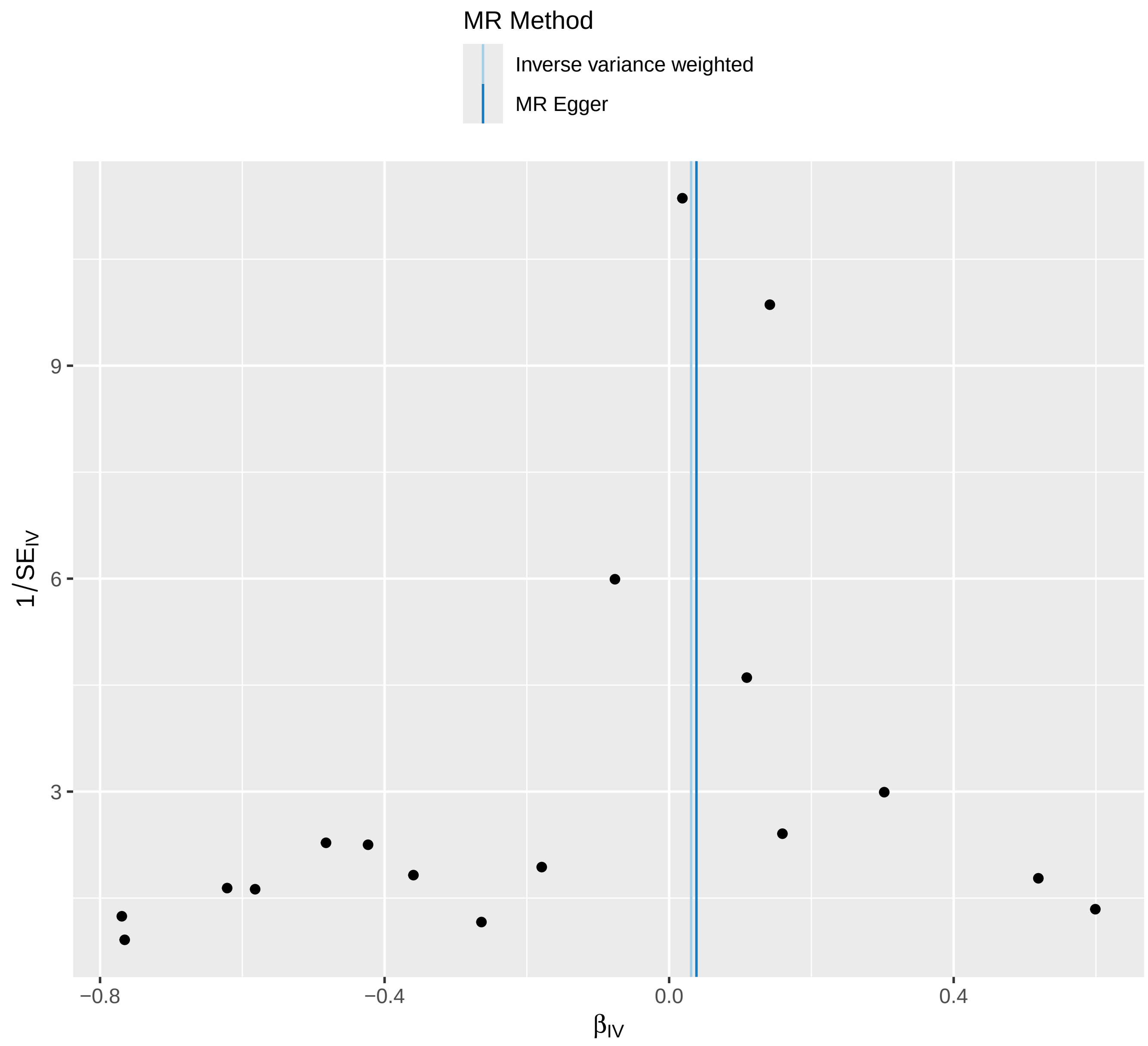
MR Method
Inverse variance weighted
MR Egger
9
6
3
1/SE$_{IV}$
−0.8
−0.4
0.0
0.4
β$_{IV}$

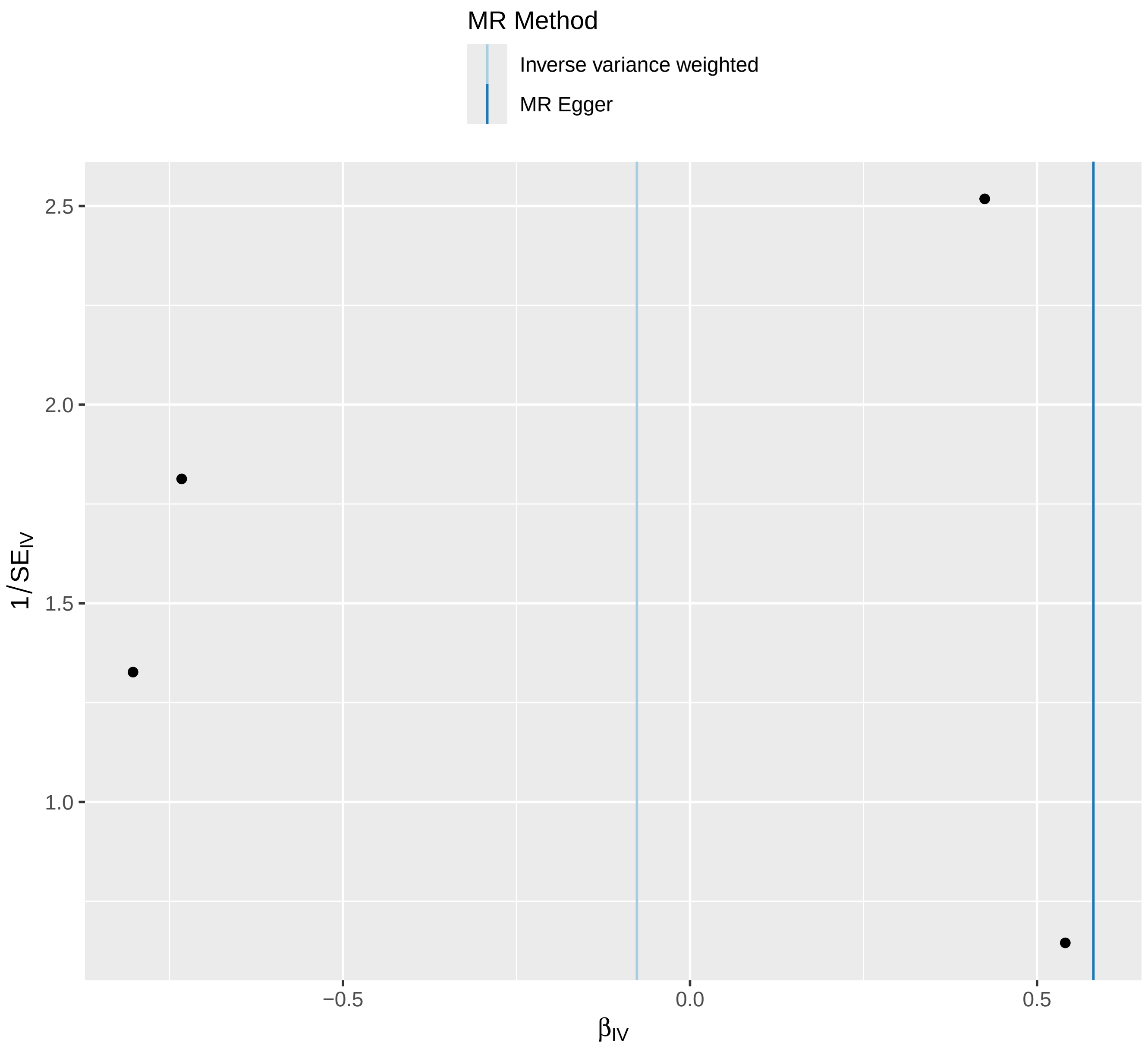
MR Method
Inverse variance weighted
MR Egger
2.5
2.0
1.5
1.0
1/SE_IV
−0.5
0.0
0.5
β_IV

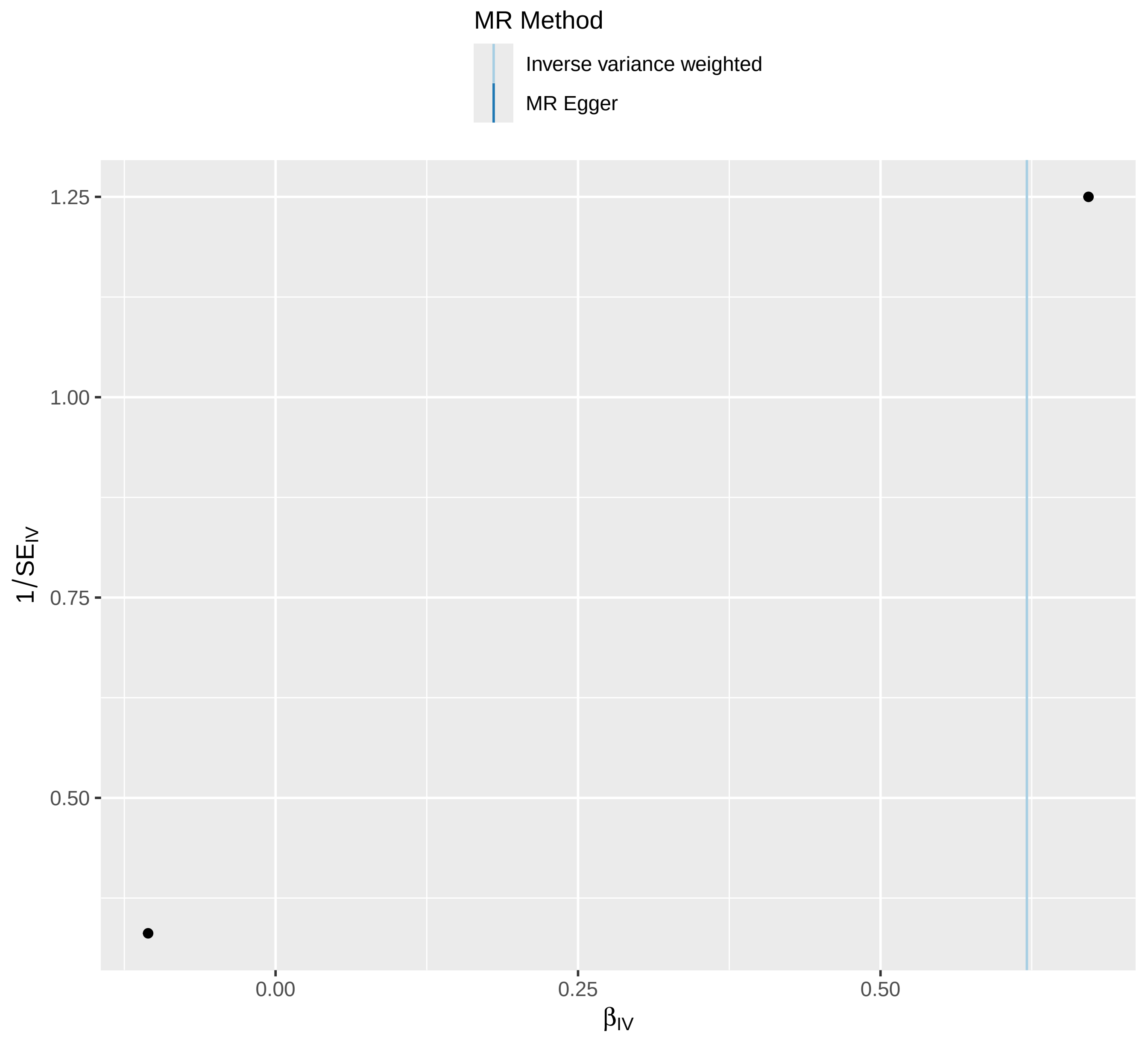
MR Method
Inverse variance weighted
MR Egger
1.25
1.00
0.75
0.50
0.00
0.25
0.50
1/SE_IV
β_IV

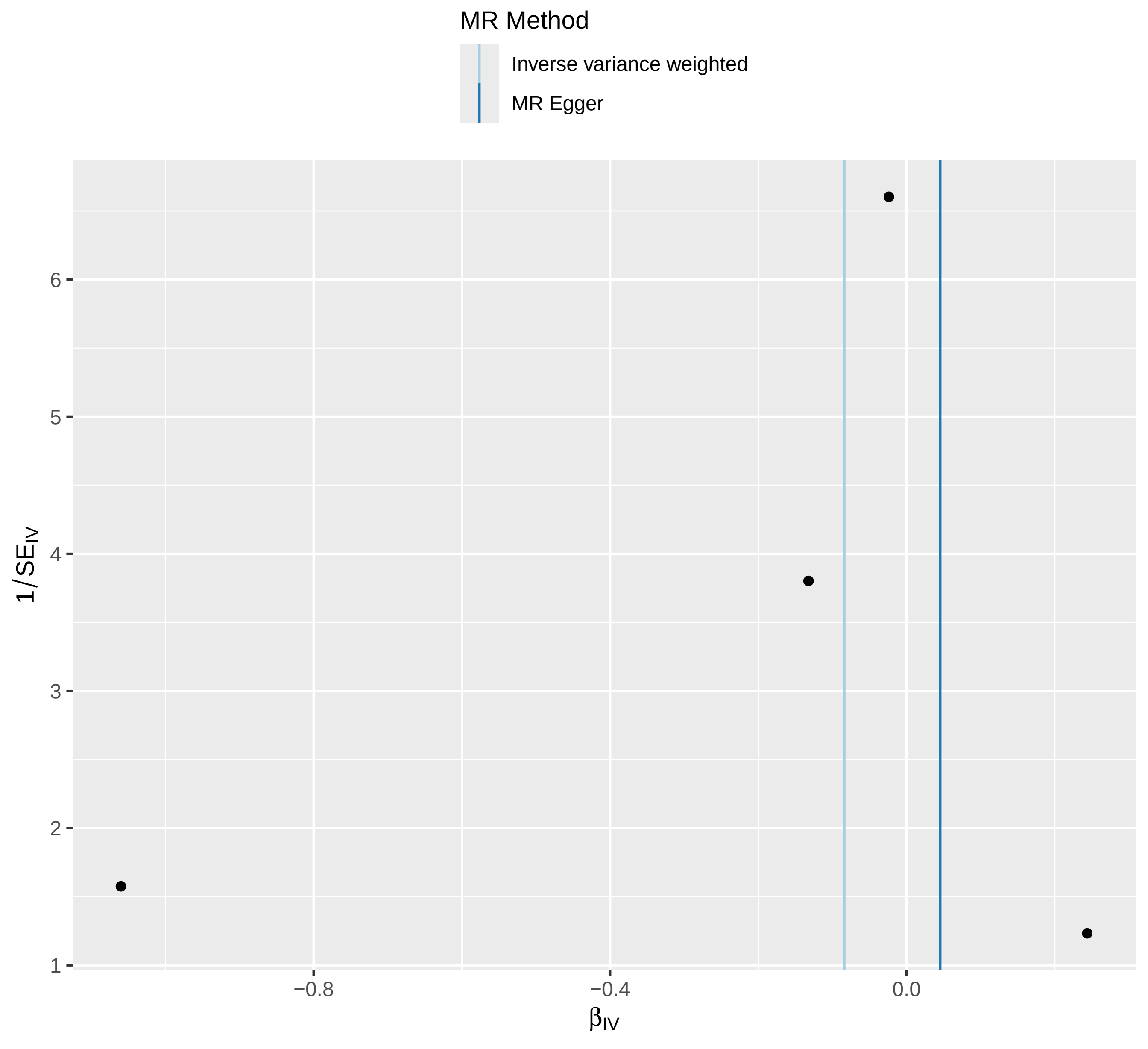

MR Method
Inverse variance weighted
MR Egger
6
5
4
3
2
1
1/SE$_{IV}$
−0.8
−0.4
0.0
β$_{IV}$

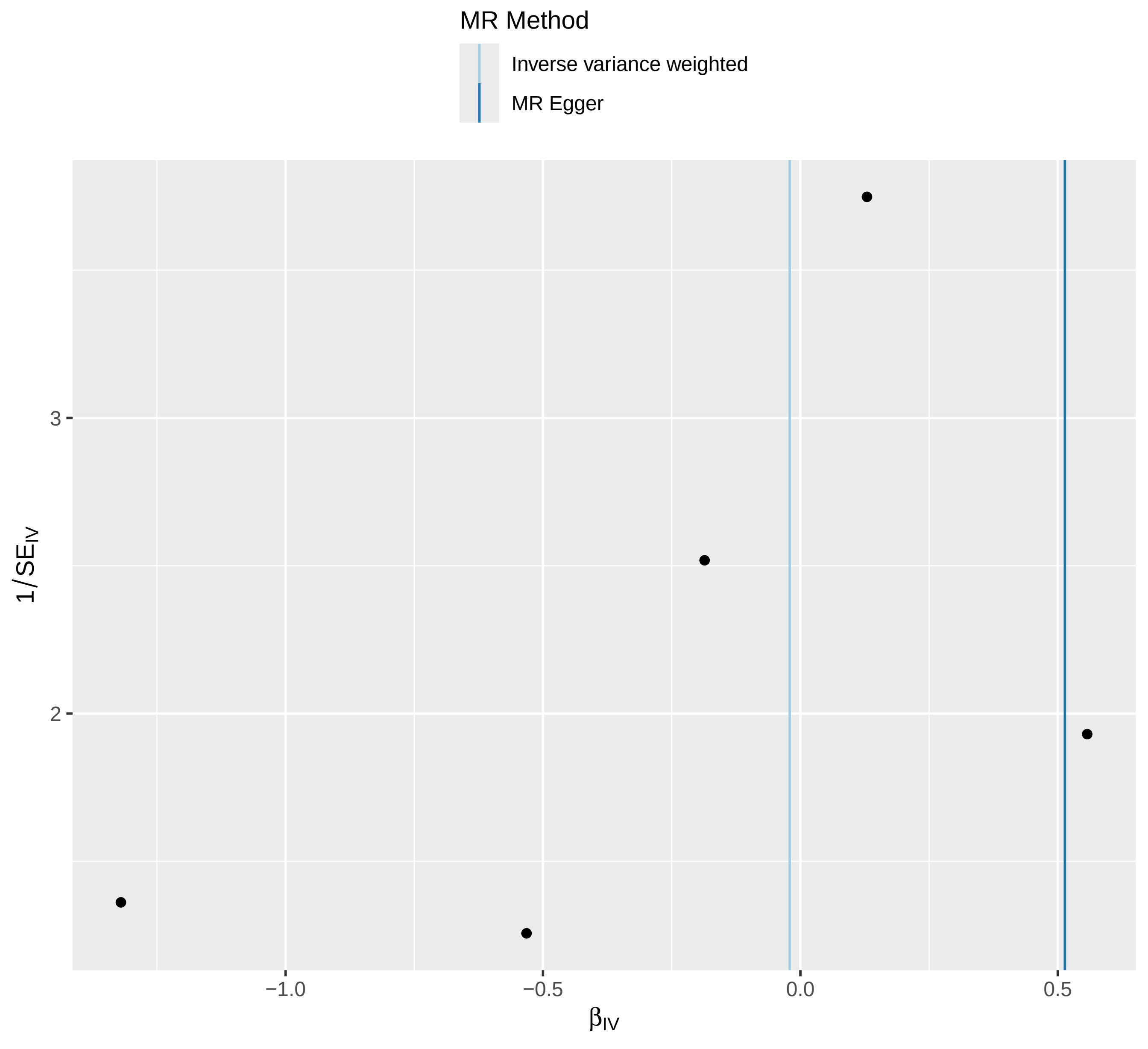

MR Method
Inverse variance weighted
MR Egger
3
2
1/SE_IV
−1.0
−0.5
0.0
0.5
β_IV

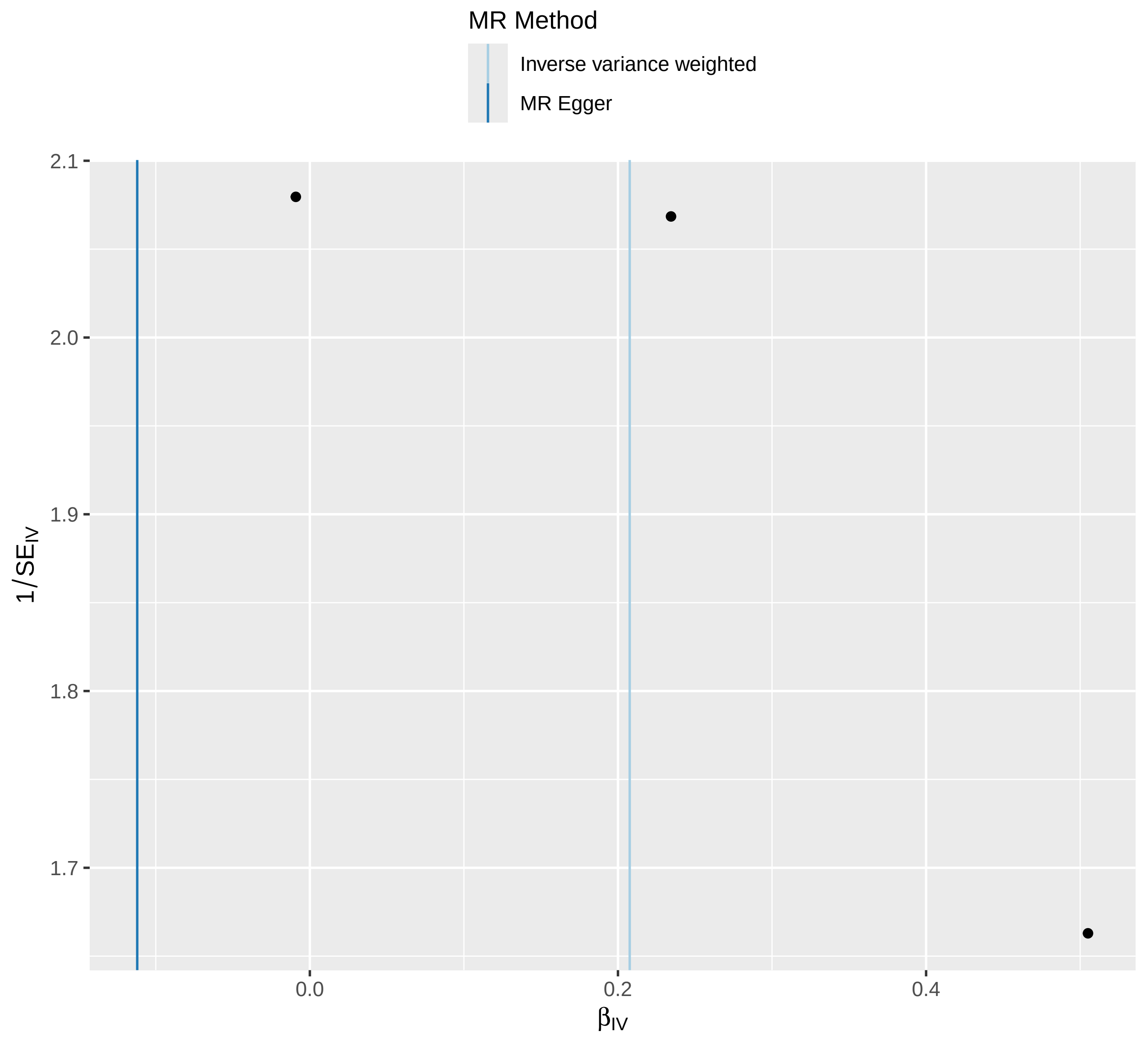

MR Method
Inverse variance weighted
MR Egger
2.1
2.0
1.9
1.8
1.7
1/SE_IV
0.0
0.2
0.4
β_IV

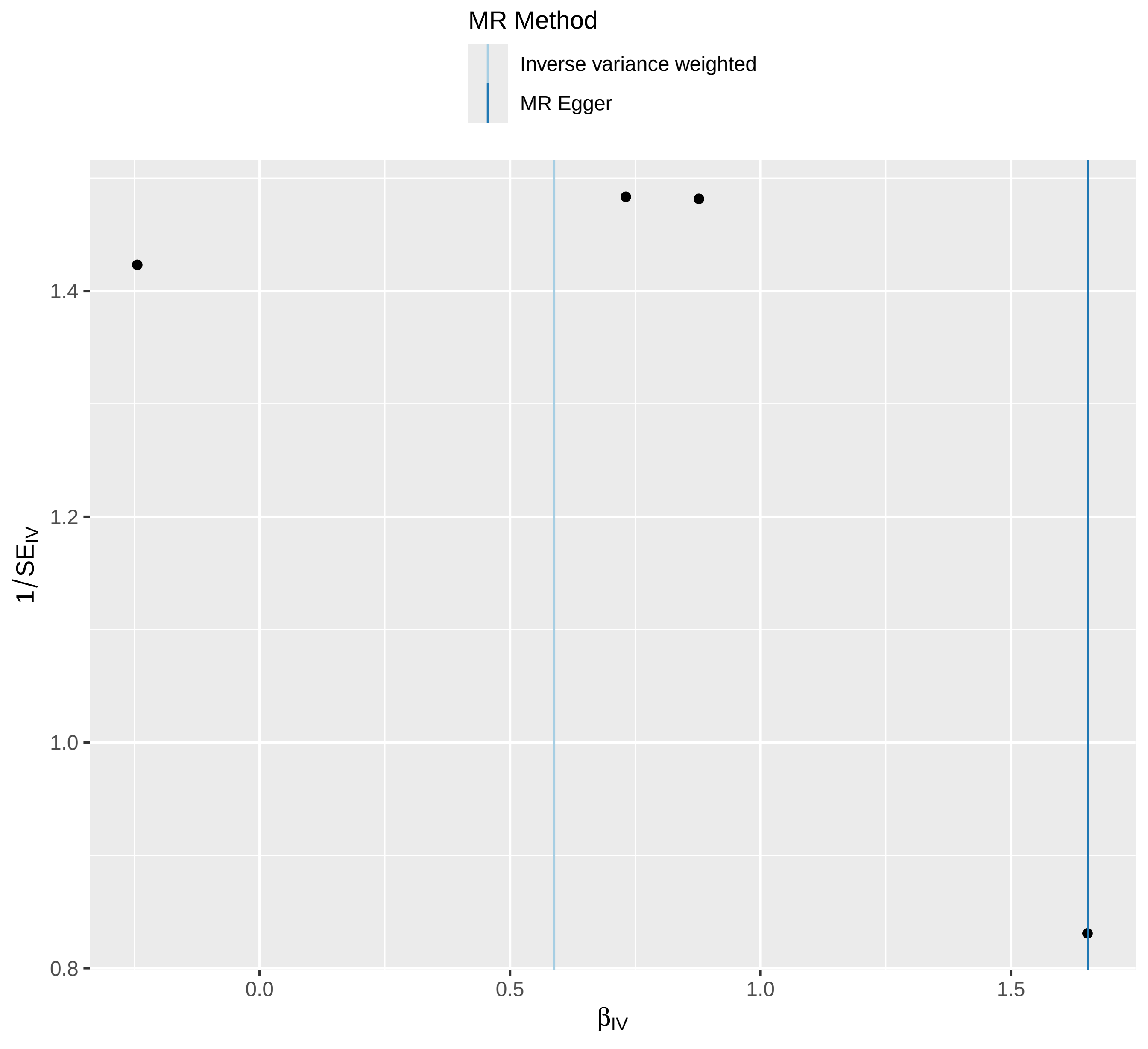
MR Method
Inverse variance weighted
MR Egger
1.4
1.2
1.0
0.8
1/SE_IV
0.0
0.5
1.0
1.5
β_IV

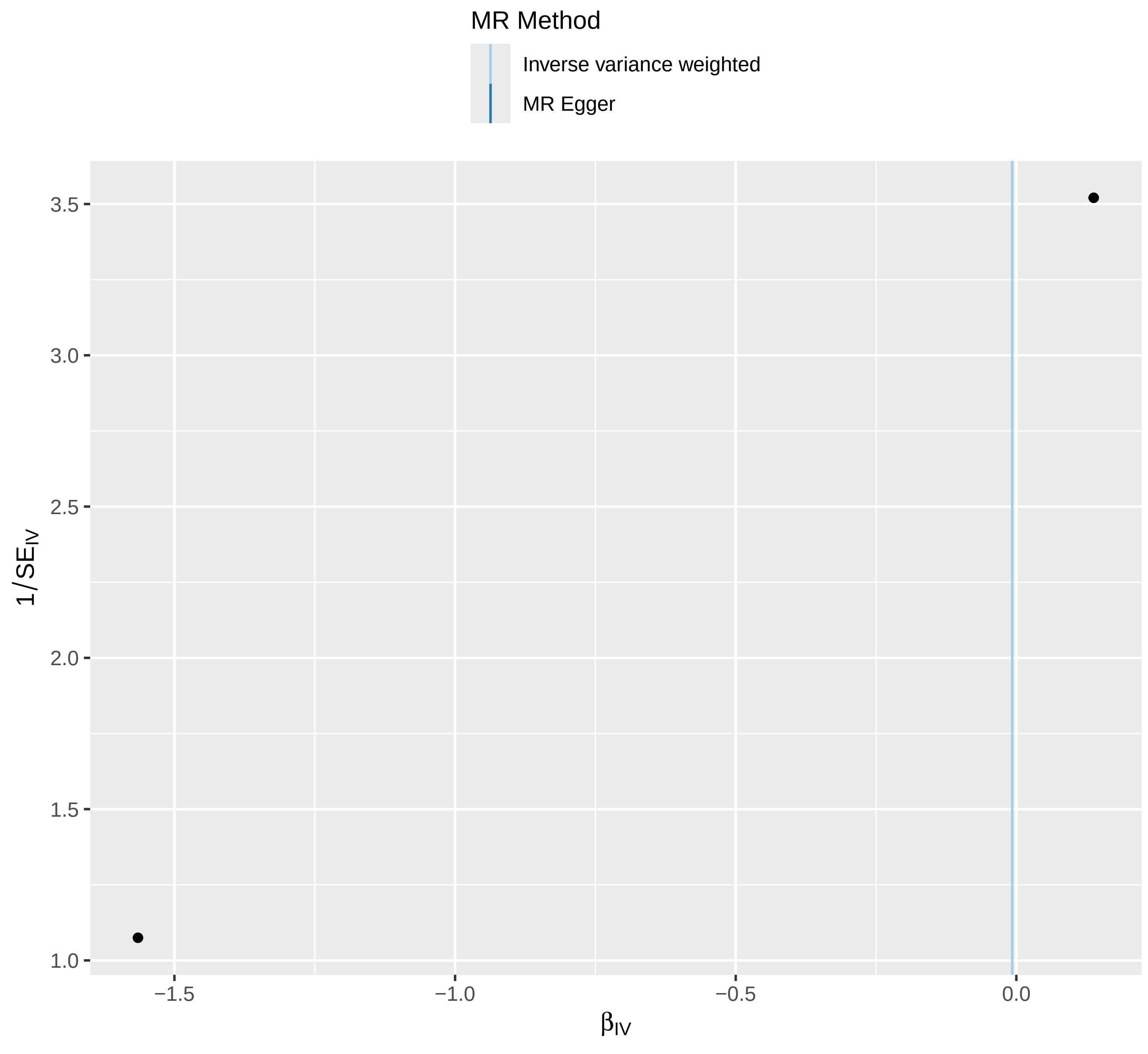
MR Method
Inverse variance weighted
MR Egger
3.5
3.0
2.5
2.0
1.5
1.0
1/SE_IV
−1.5
−1.0
−0.5
0.0
β_IV

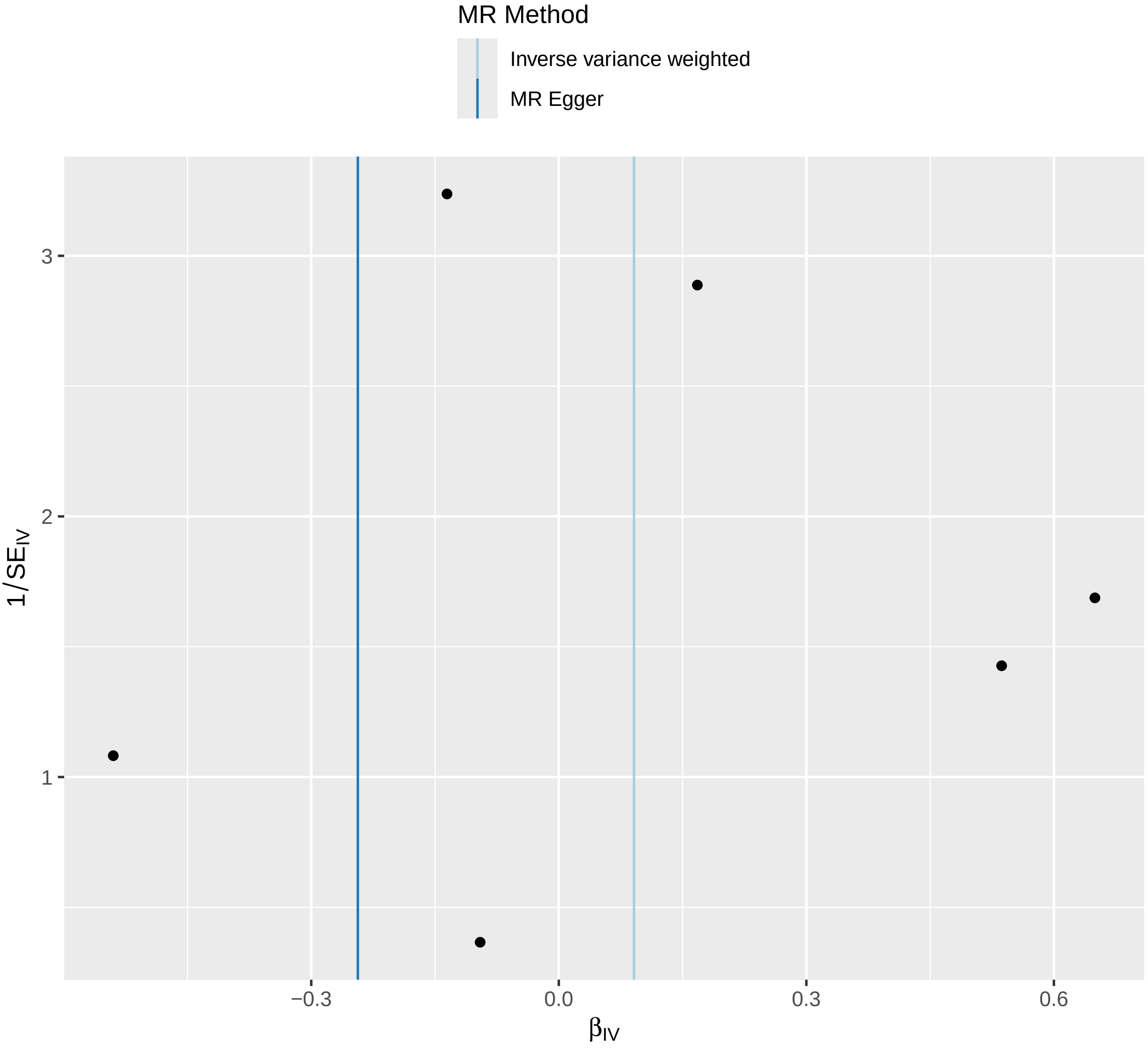
MR Method
Inverse variance weighted
MR Egger
3
2
1
1/SE_IV
−0.3
0.0
0.3
0.6
β_IV

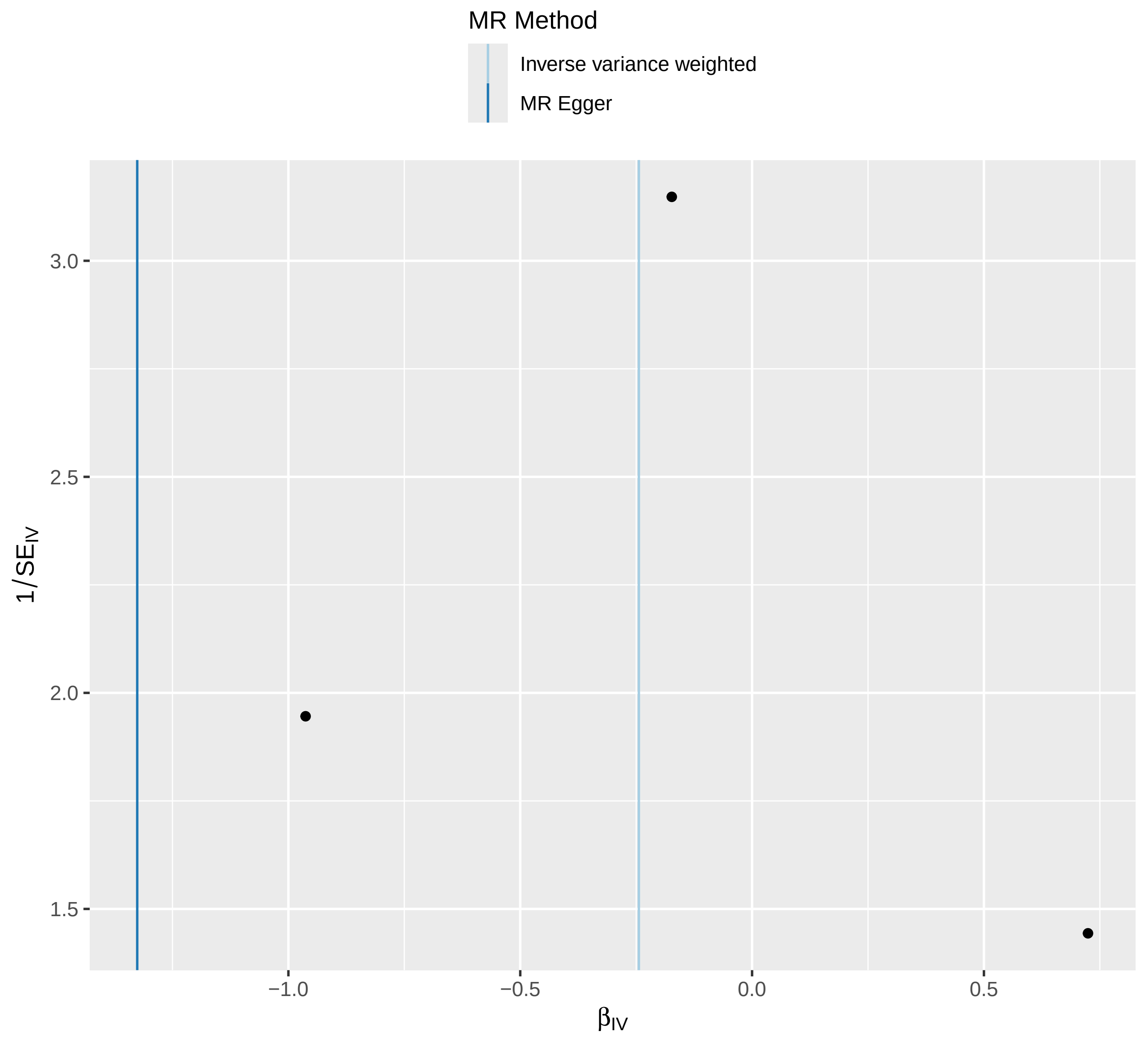
MR Method
Inverse variance weighted
MR Egger
3.0
2.5
2.0
1.5
$1/SE_{IV}$
−1.0
−0.5
0.0
0.5
β_{IV}

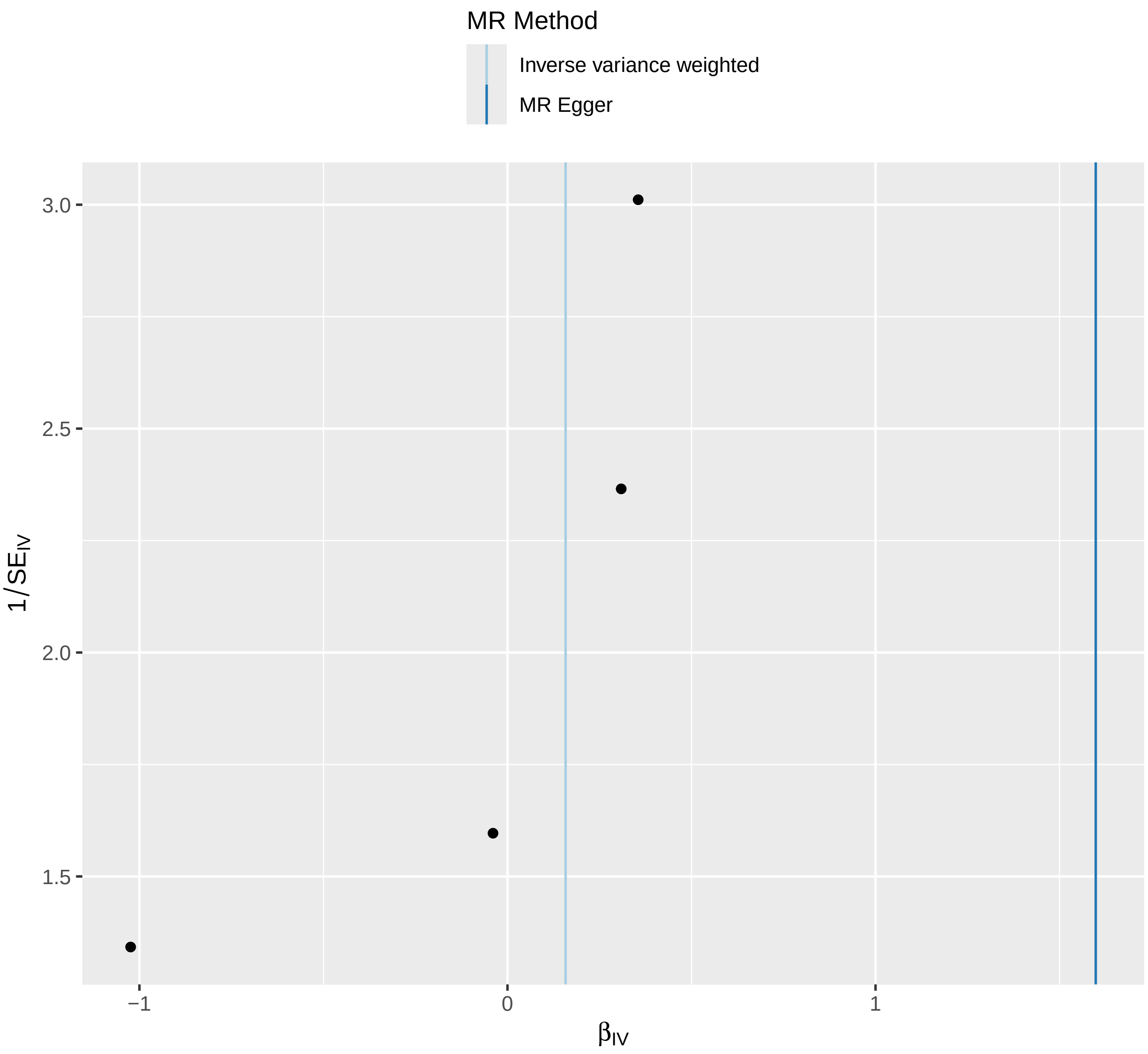

MR Method
Inverse variance weighted
MR Egger
3.0
2.5
2.0
1.5
1/SE_IV
−1
0
1
β_IV

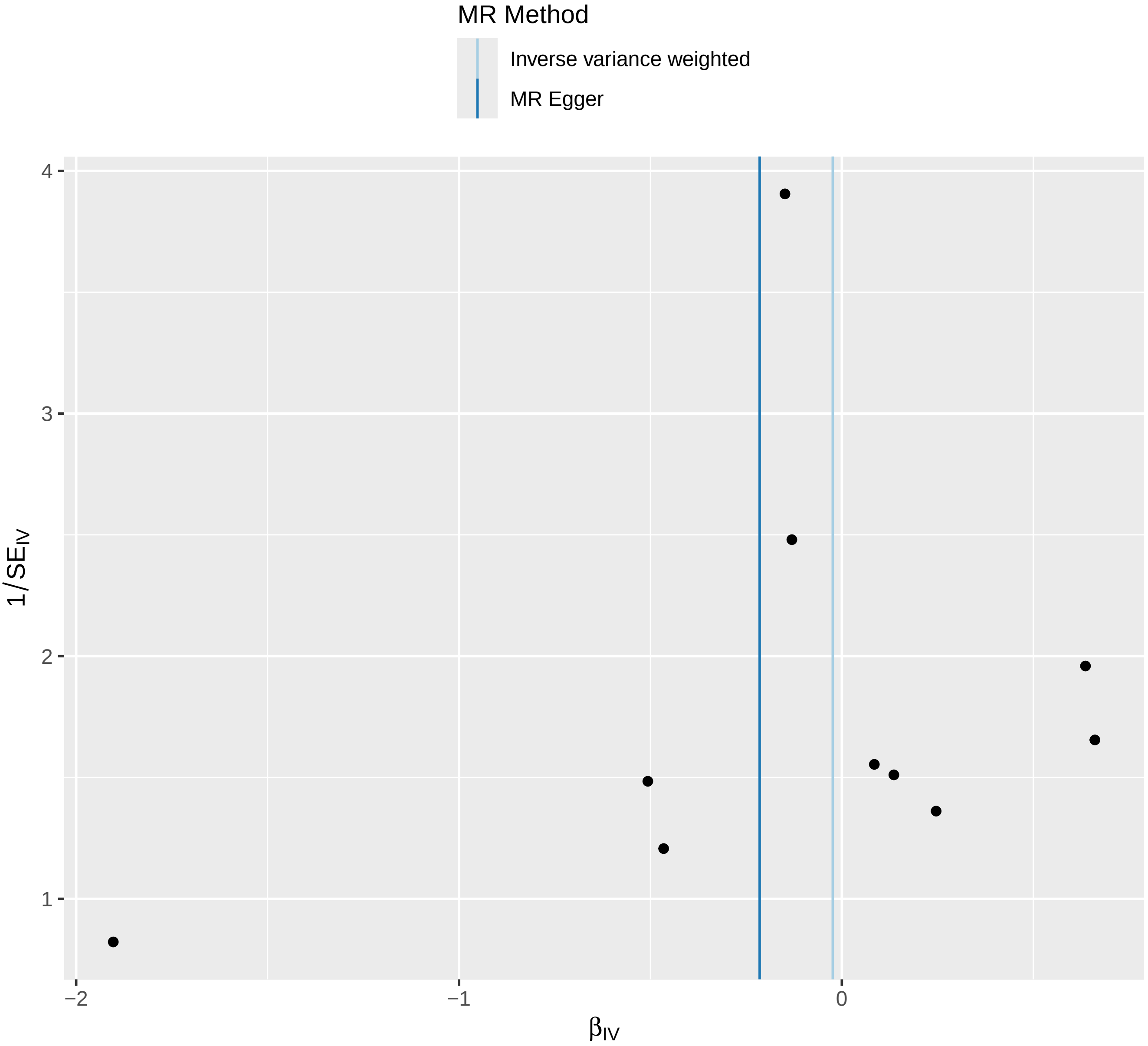
MR Method
Inverse variance weighted
MR Egger
4
3
2
1
−2
−1
0
$1/SE_{IV}$
β_{IV}

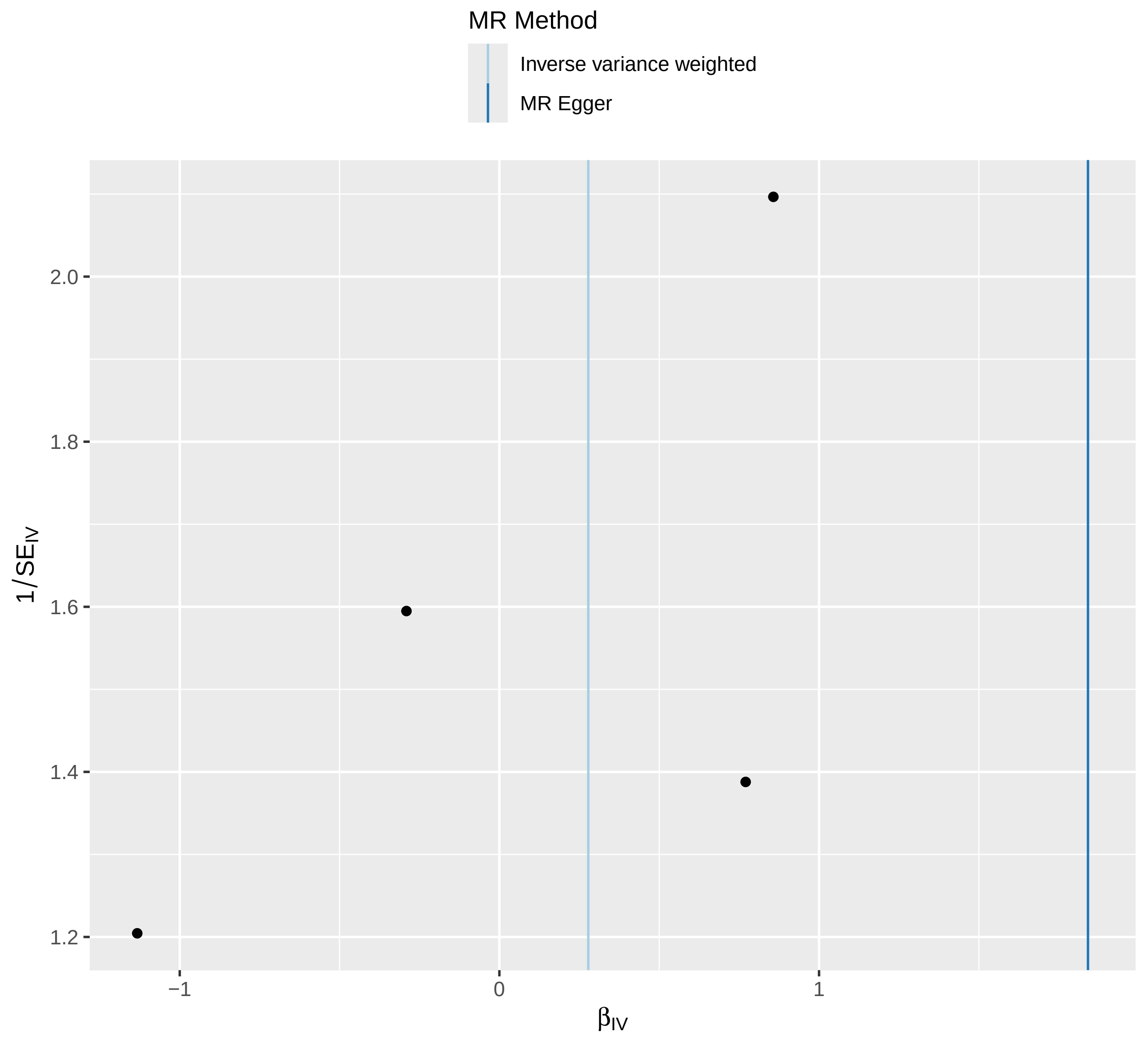
MR Method
Inverse variance weighted
MR Egger
2.0
1.8
1.6
1.4
1.2
$1/SE_{IV}$
−1
0
1
β_{IV}

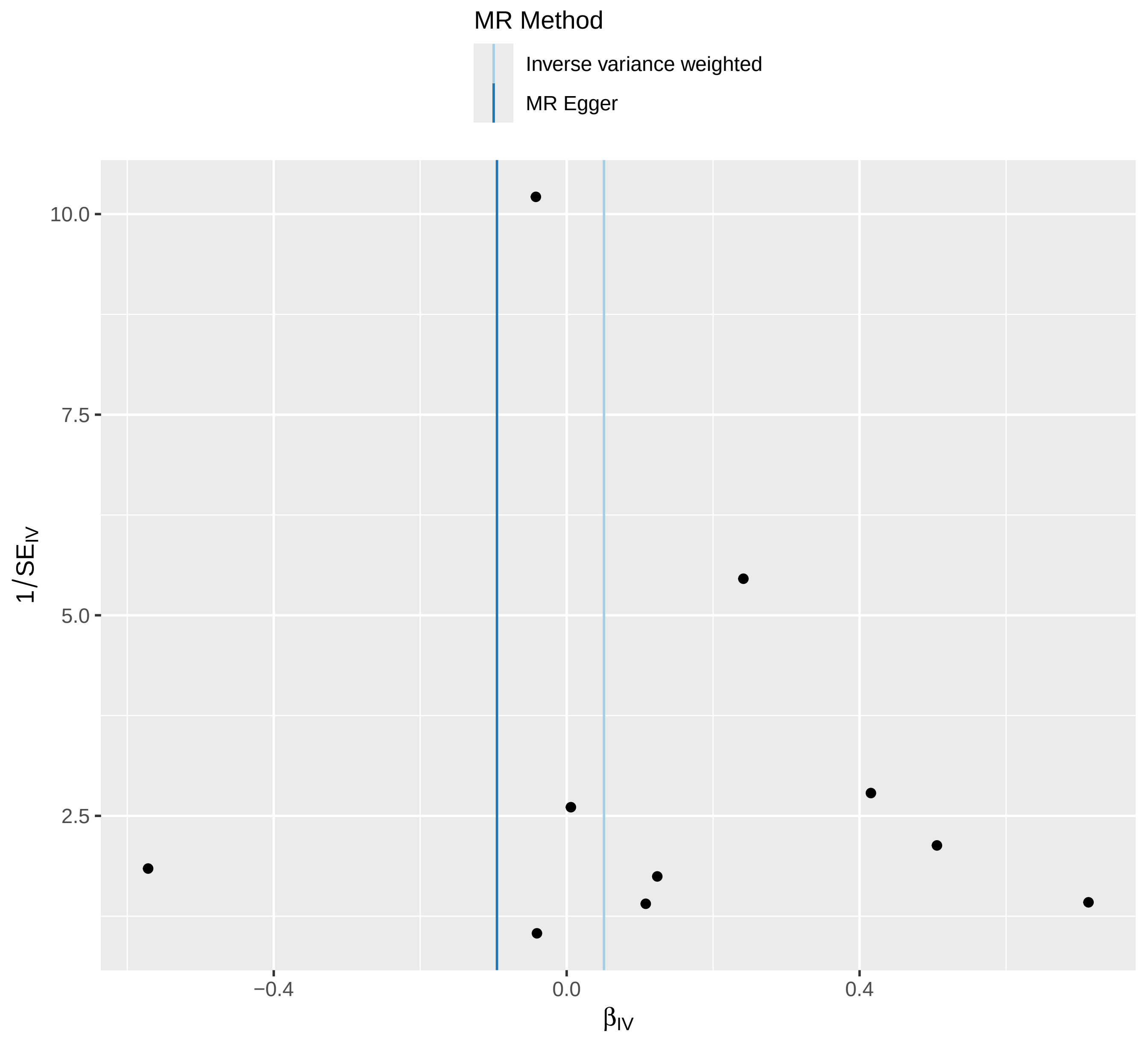
MR Method
Inverse variance weighted
MR Egger
10.0
7.5
5.0
2.5
1/SE_IV
−0.4
0.0
0.4
β_IV

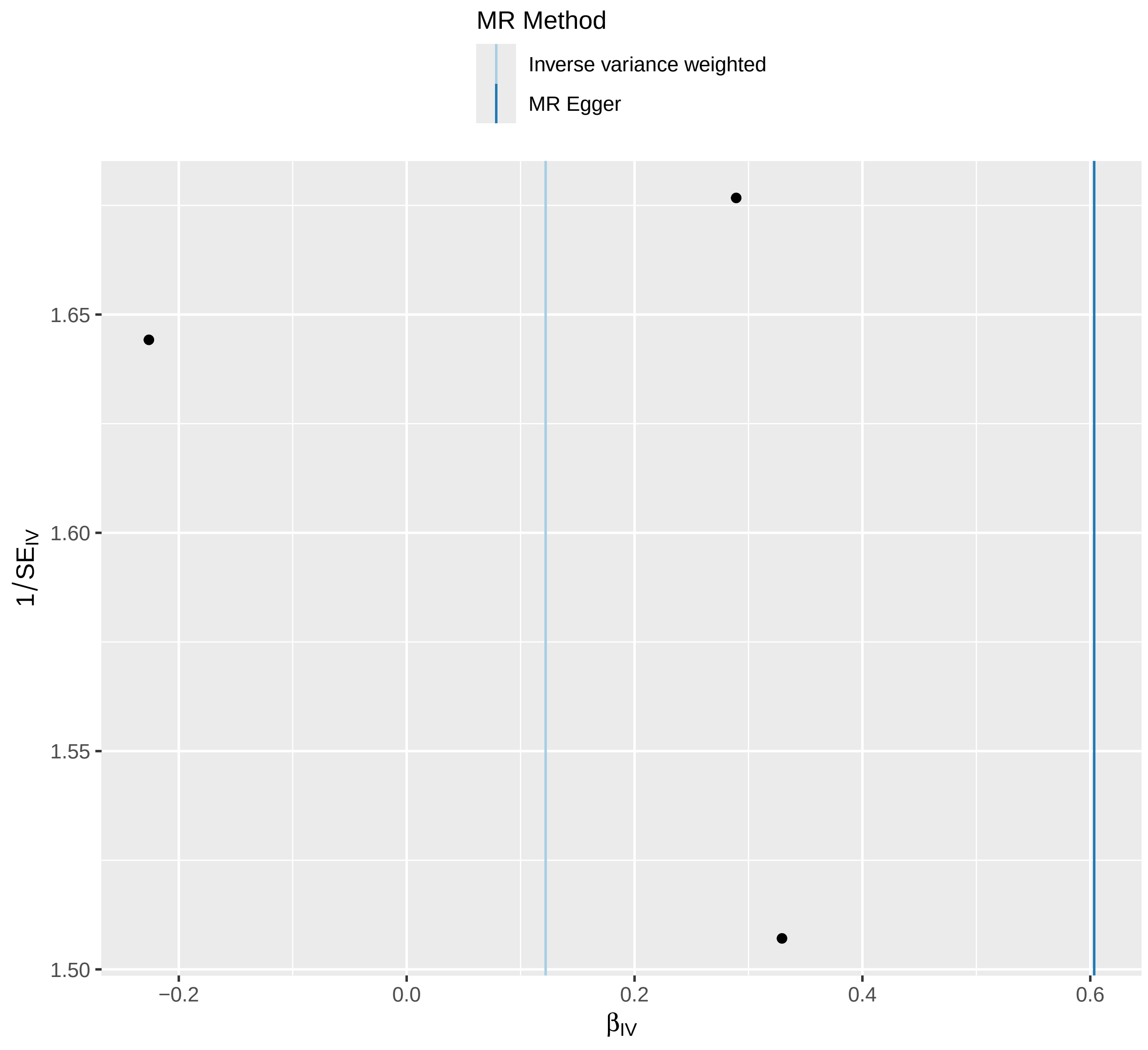

MR Method
Inverse variance weighted
MR Egger
1.65
1.60
1.55
1.50
$1/SE_{IV}$
−0.2
0.0
0.2
0.4
0.6
β_{IV}

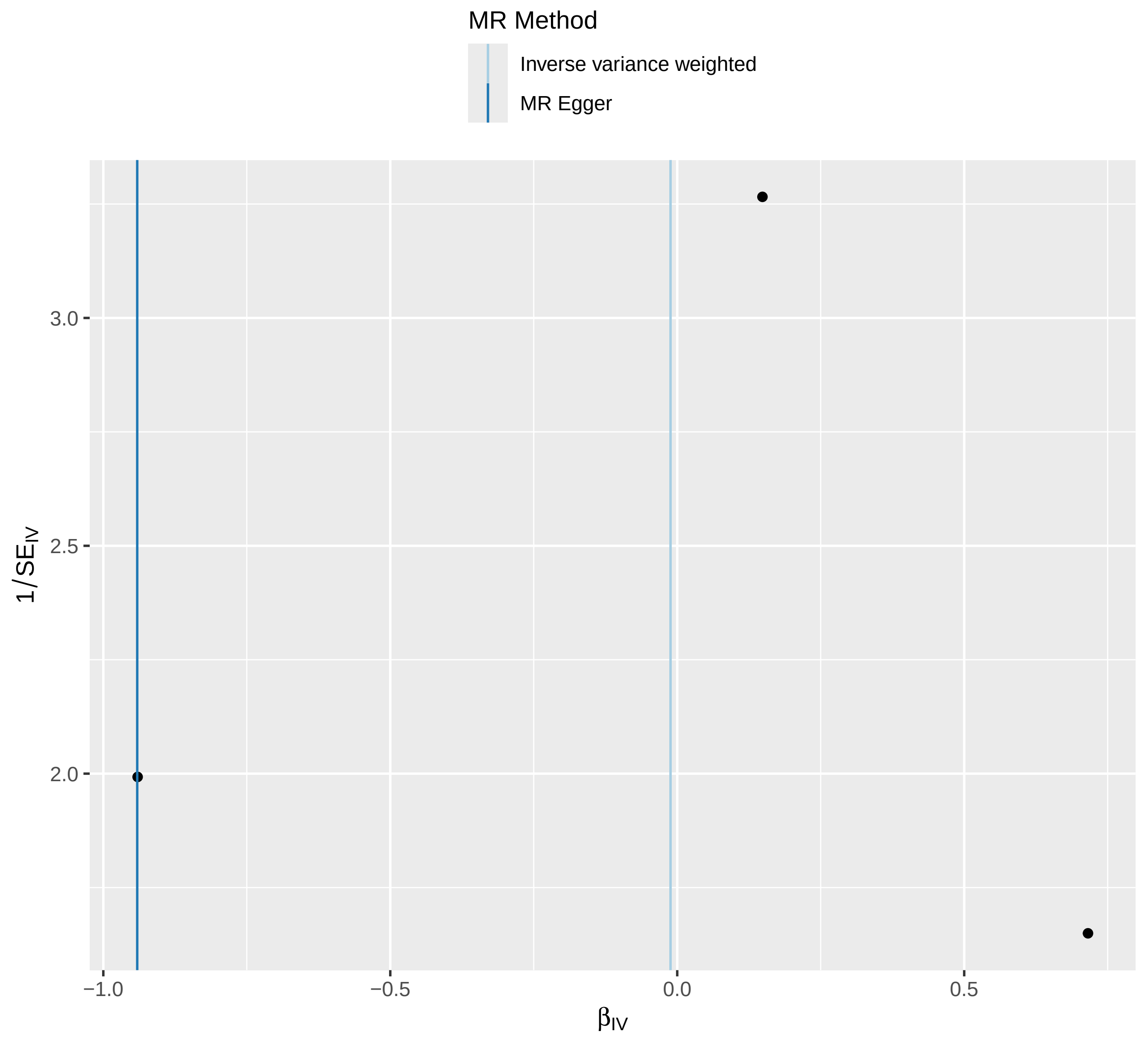
MR Method
Inverse variance weighted
MR Egger
3.0
2.5
2.0
1/SE$_{IV}$
−1.0
−0.5
0.0
0.5
β$_{IV}$

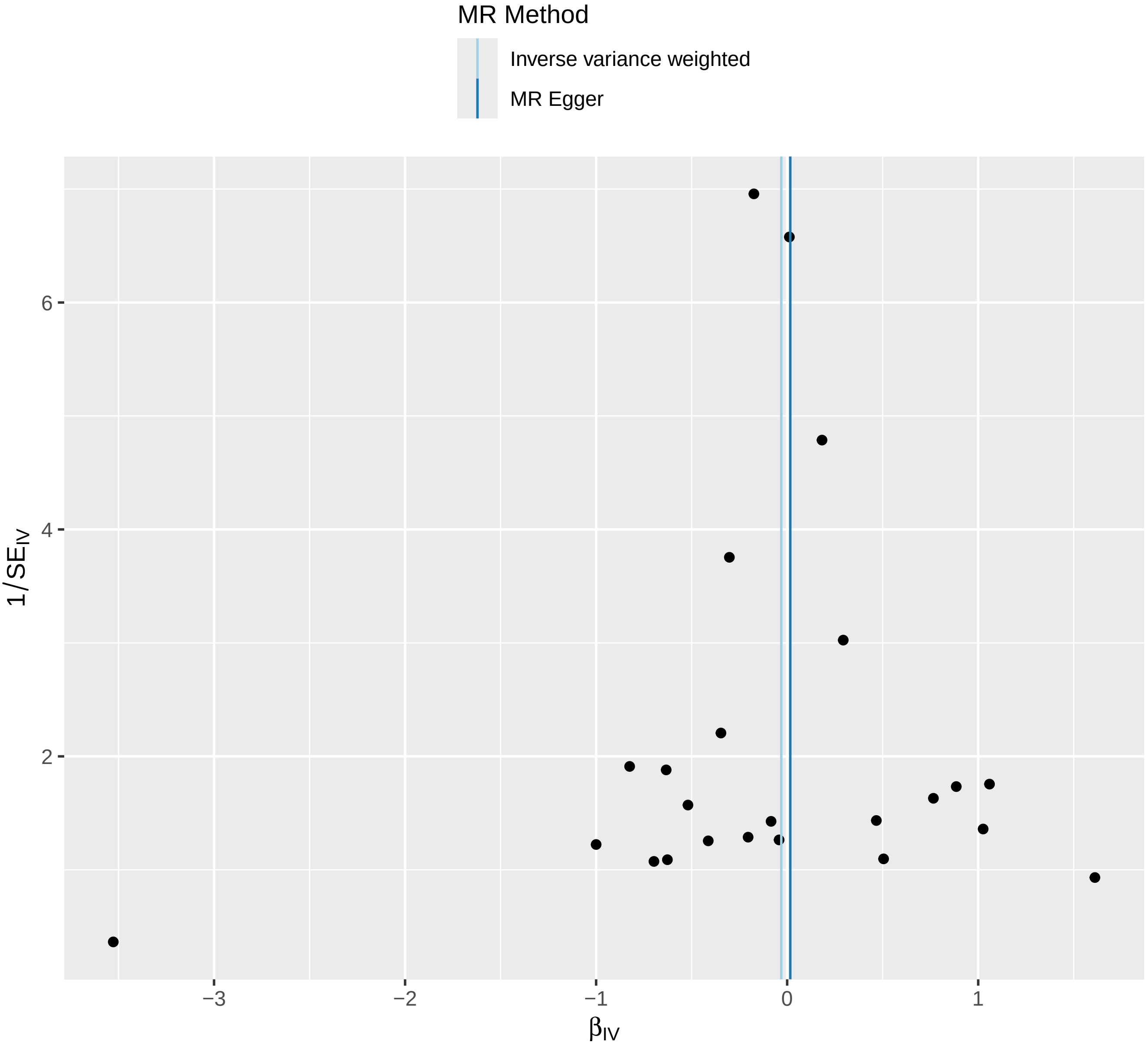

MR Method
Inverse variance weighted
MR Egger
6
4
2
1/SE_IV
−3
−2
−1
0
1
β_IV

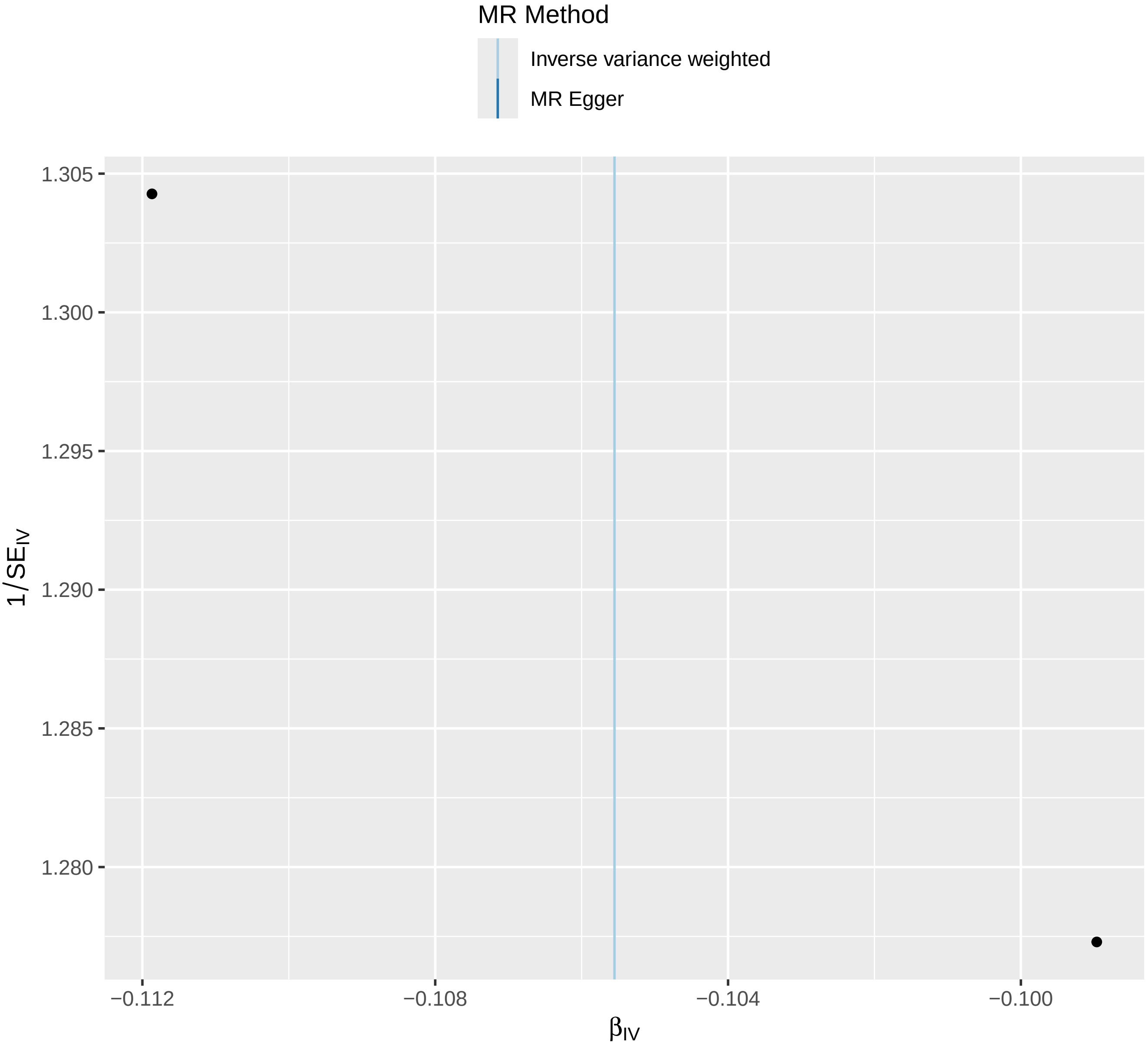

MR Method
Inverse variance weighted
MR Egger
1.305
1.300
1.295
1.290
1.285
1.280
1/SE_IV
−0.112
−0.108
−0.104
−0.100
β_IV

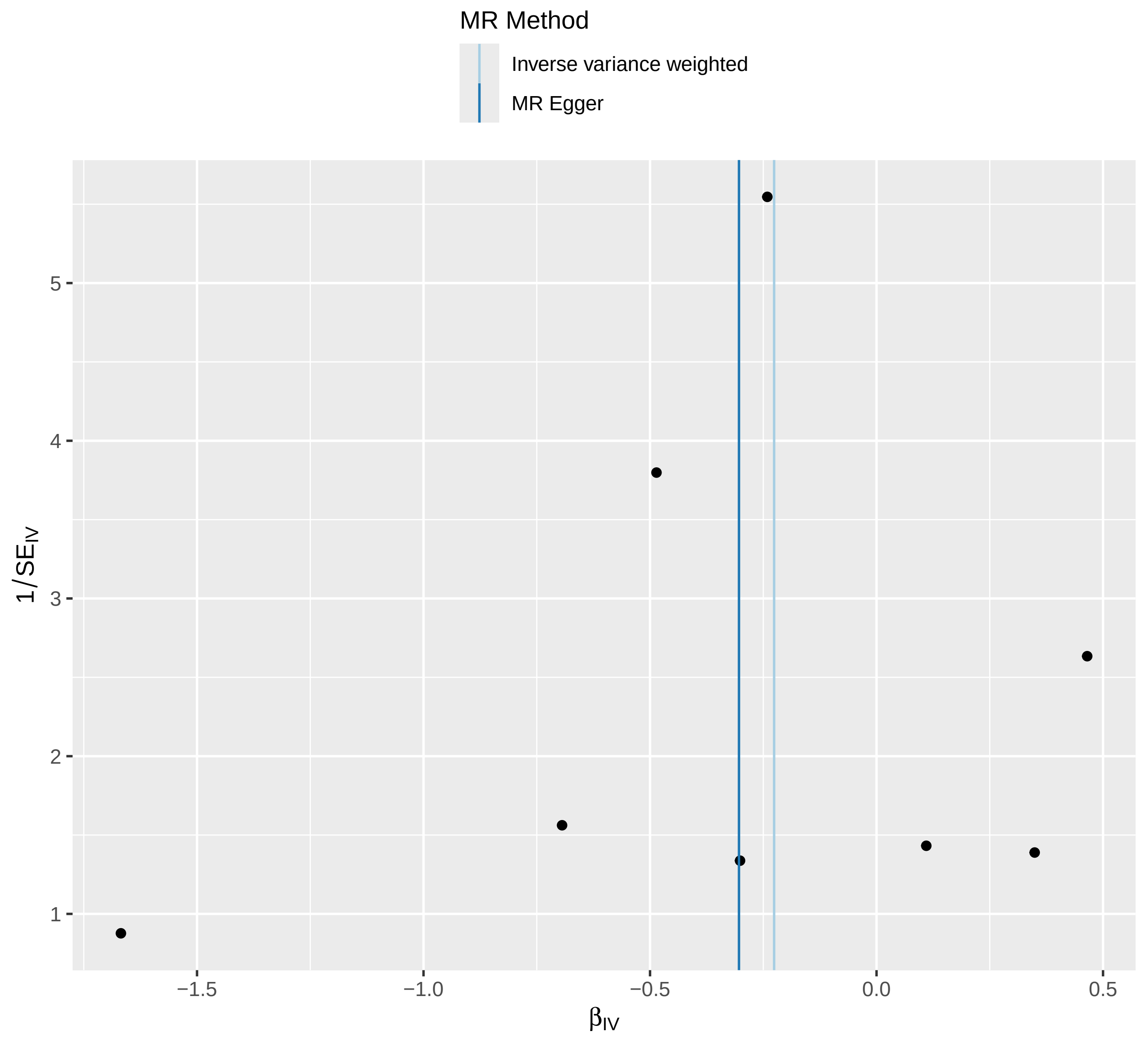
MR Method
Inverse variance weighted
MR Egger
1/SE_IV
β_IV
5
4
3
2
1
−1.5
−1.0
−0.5
0.0
0.5

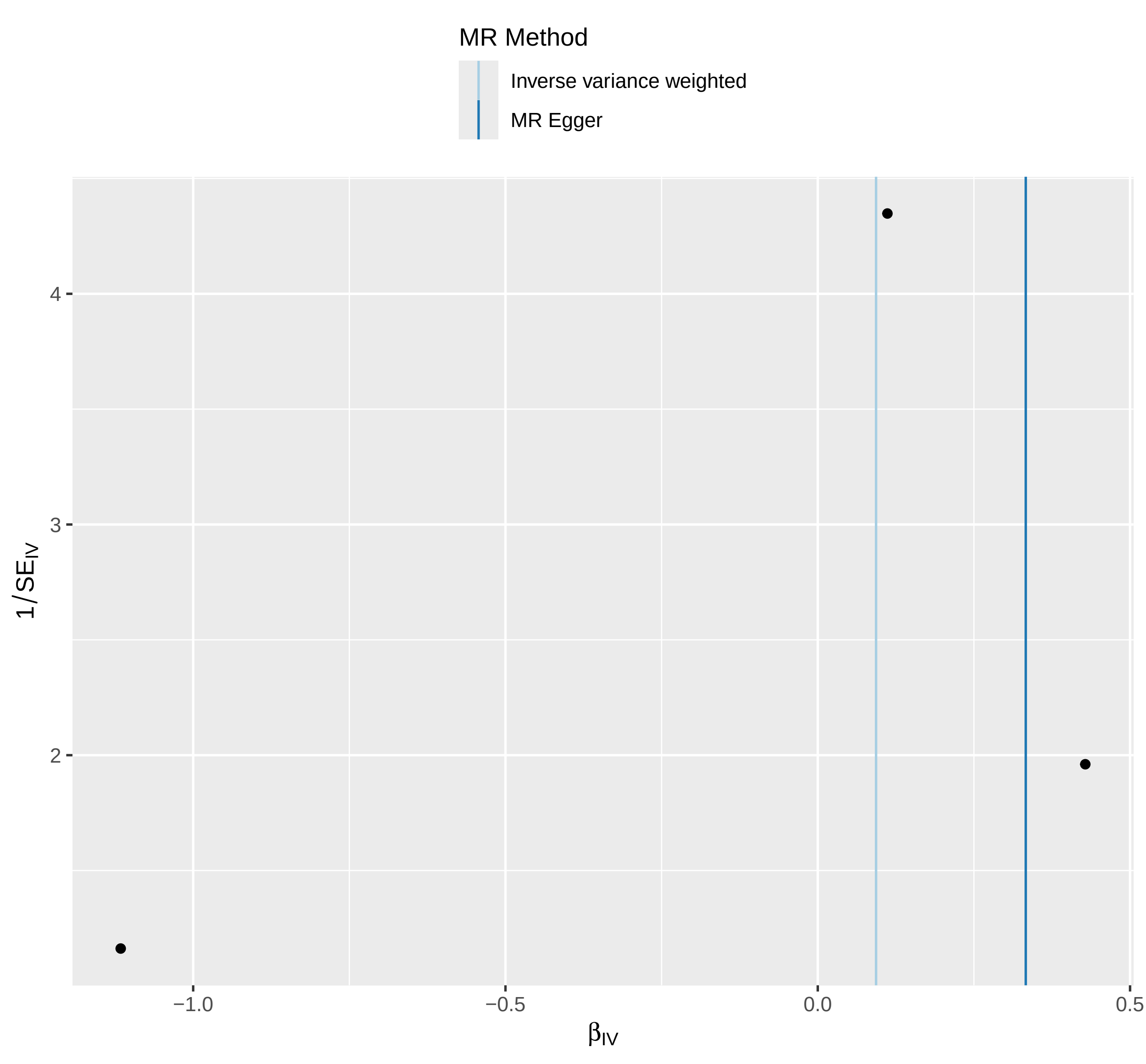
MR Method
Inverse variance weighted
MR Egger
4
3
2
1/SE_IV
−1.0
−0.5
0.0
0.5
β_IV

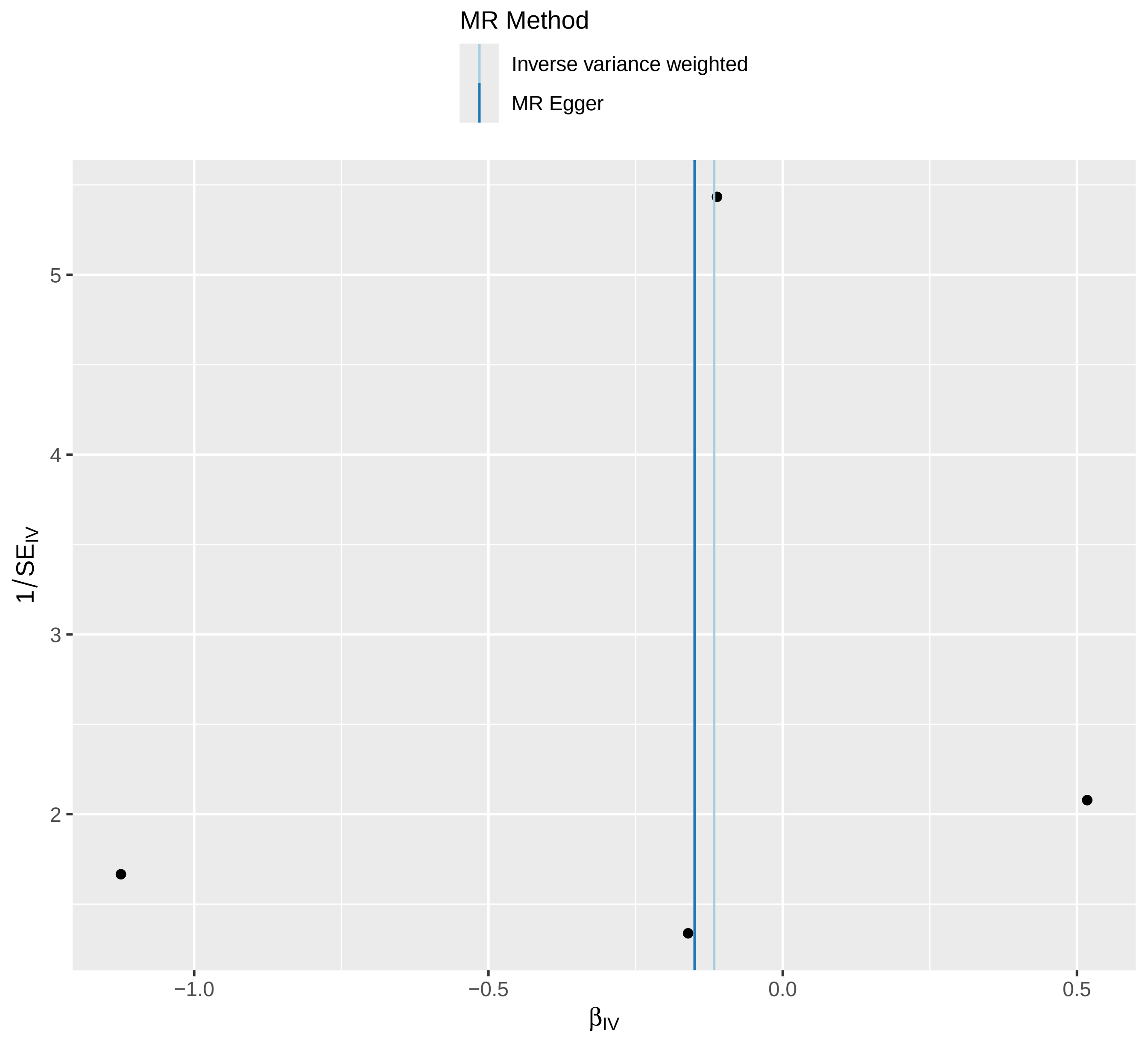
MR Method
Inverse variance weighted
MR Egger
5
4
3
2
1/SE_IV
−1.0
−0.5
0.0
0.5
β_IV

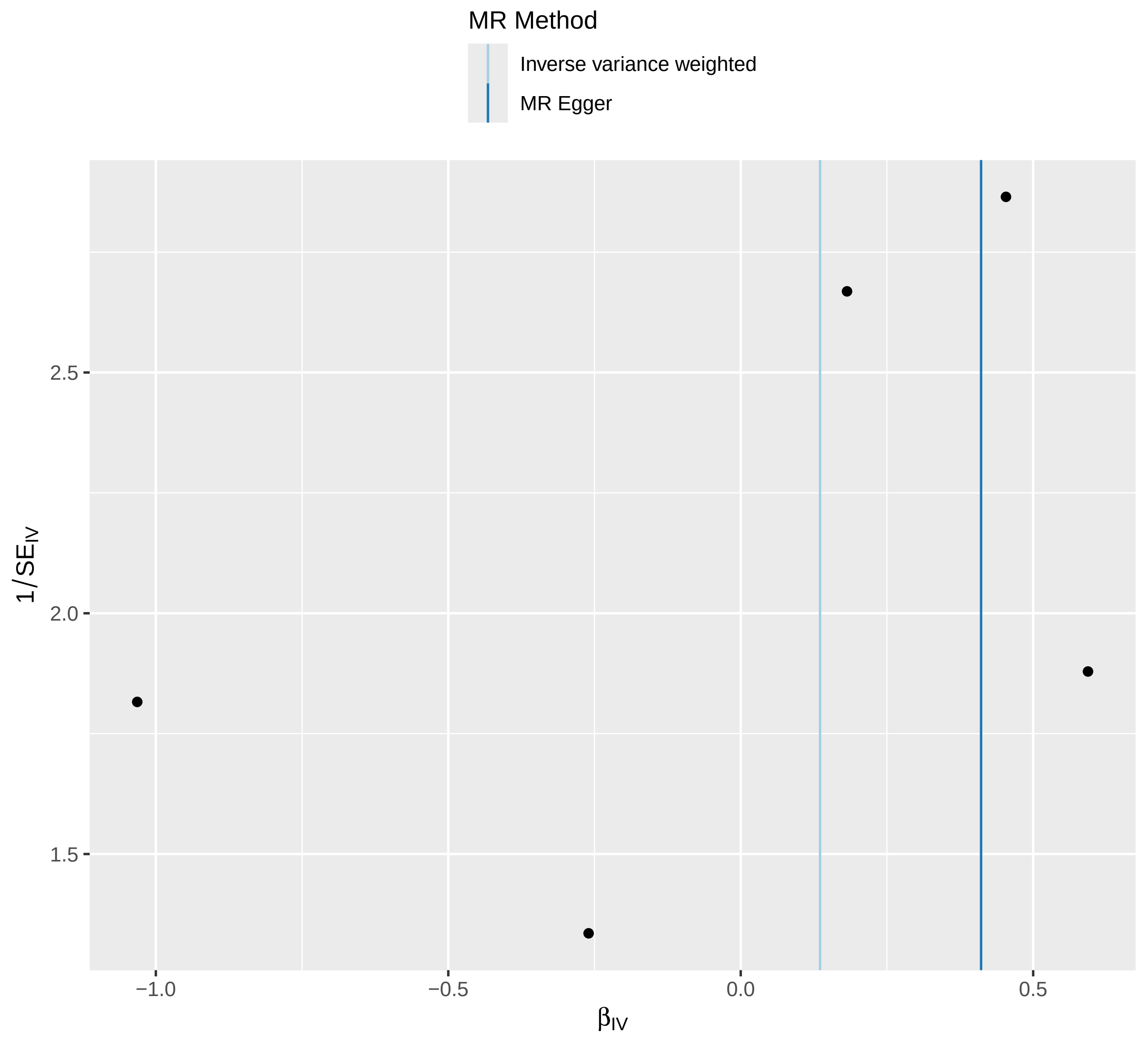
MR Method
Inverse variance weighted
MR Egger
2.5
2.0
1.5
1/SE$_{IV}$
−1.0
−0.5
0.0
0.5
β$_{IV}$

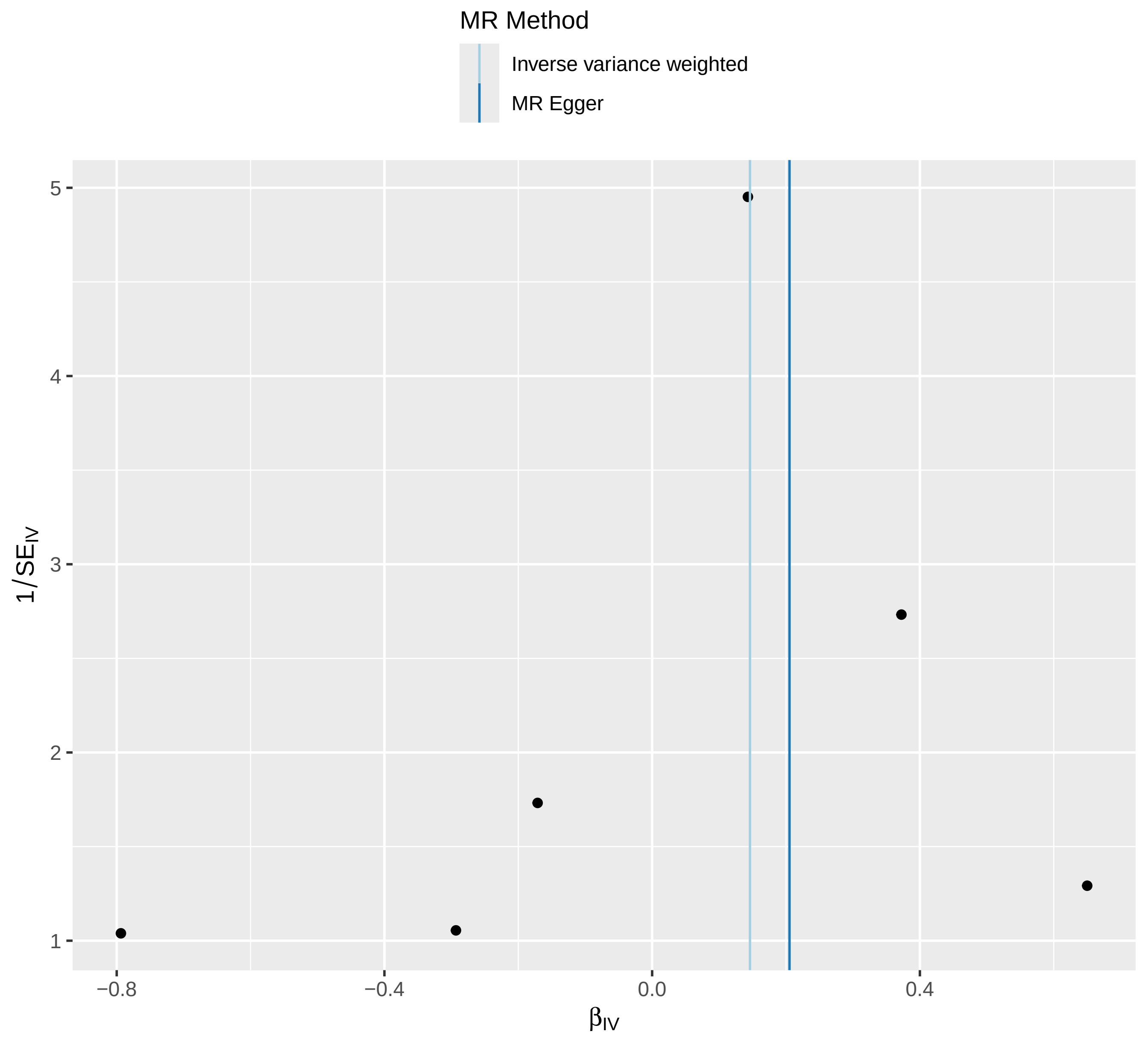
MR Method
Inverse variance weighted
MR Egger
5
4
3
2
1
1/SE_IV
−0.8
−0.4
0.0
0.4
β_IV

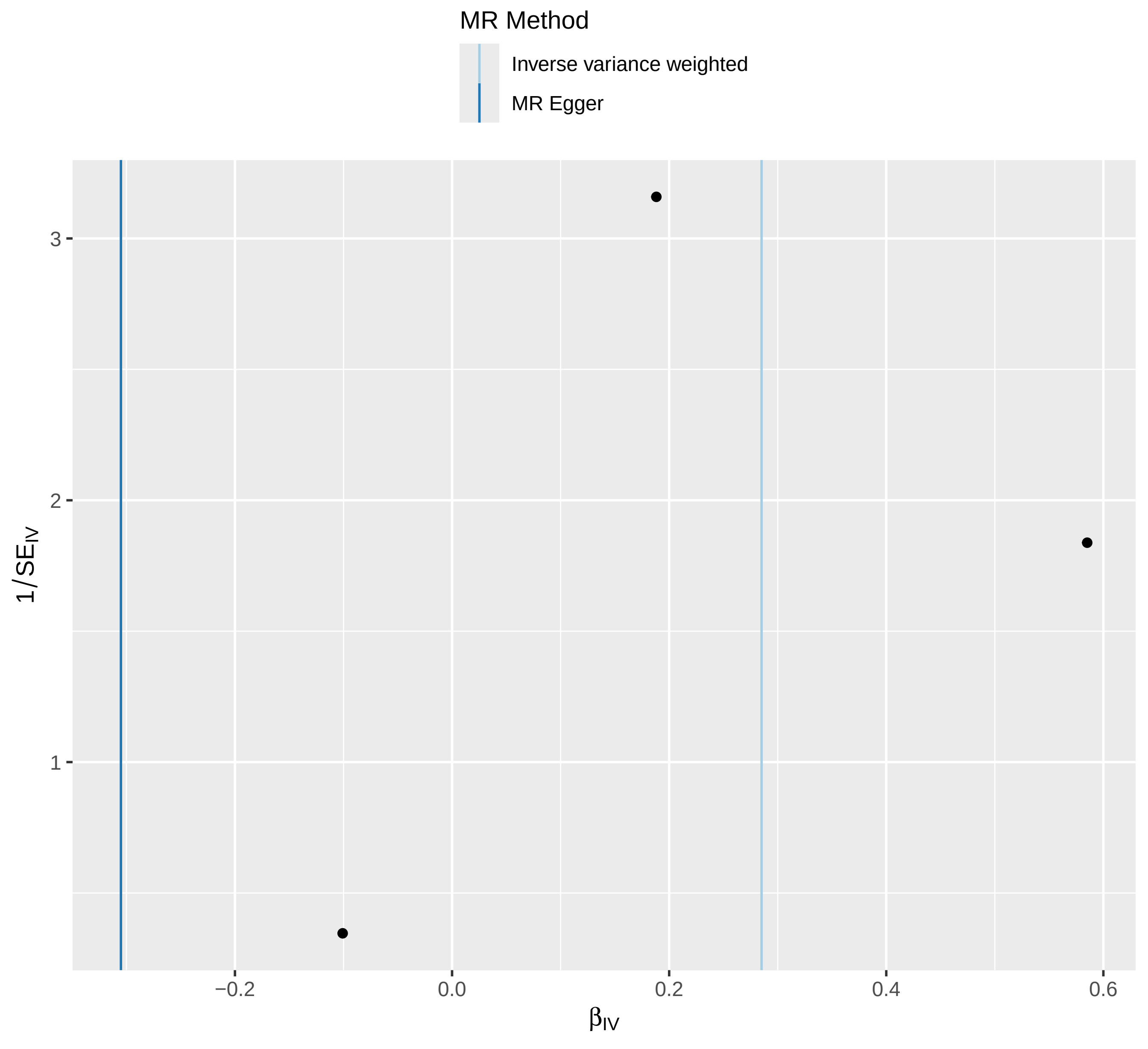
MR Method
Inverse variance weighted
MR Egger
1/SE_IV
3
2
1
−0.2
0.0
0.2
0.4
0.6
β_IV

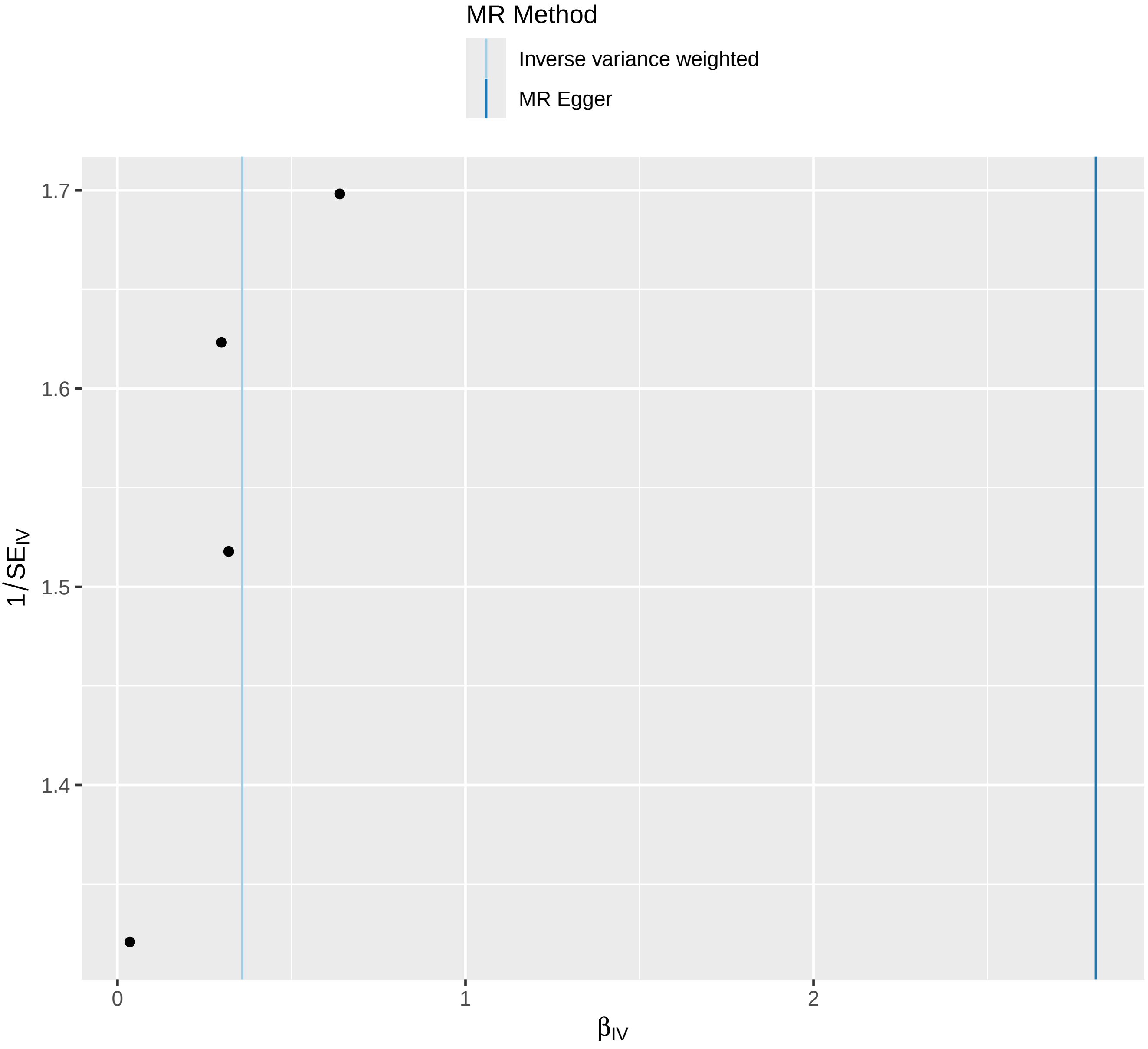

MR Method
Inverse variance weighted
MR Egger
1.7
1.6
1.5
1.4
1/SE_IV
0
1
2
β_IV

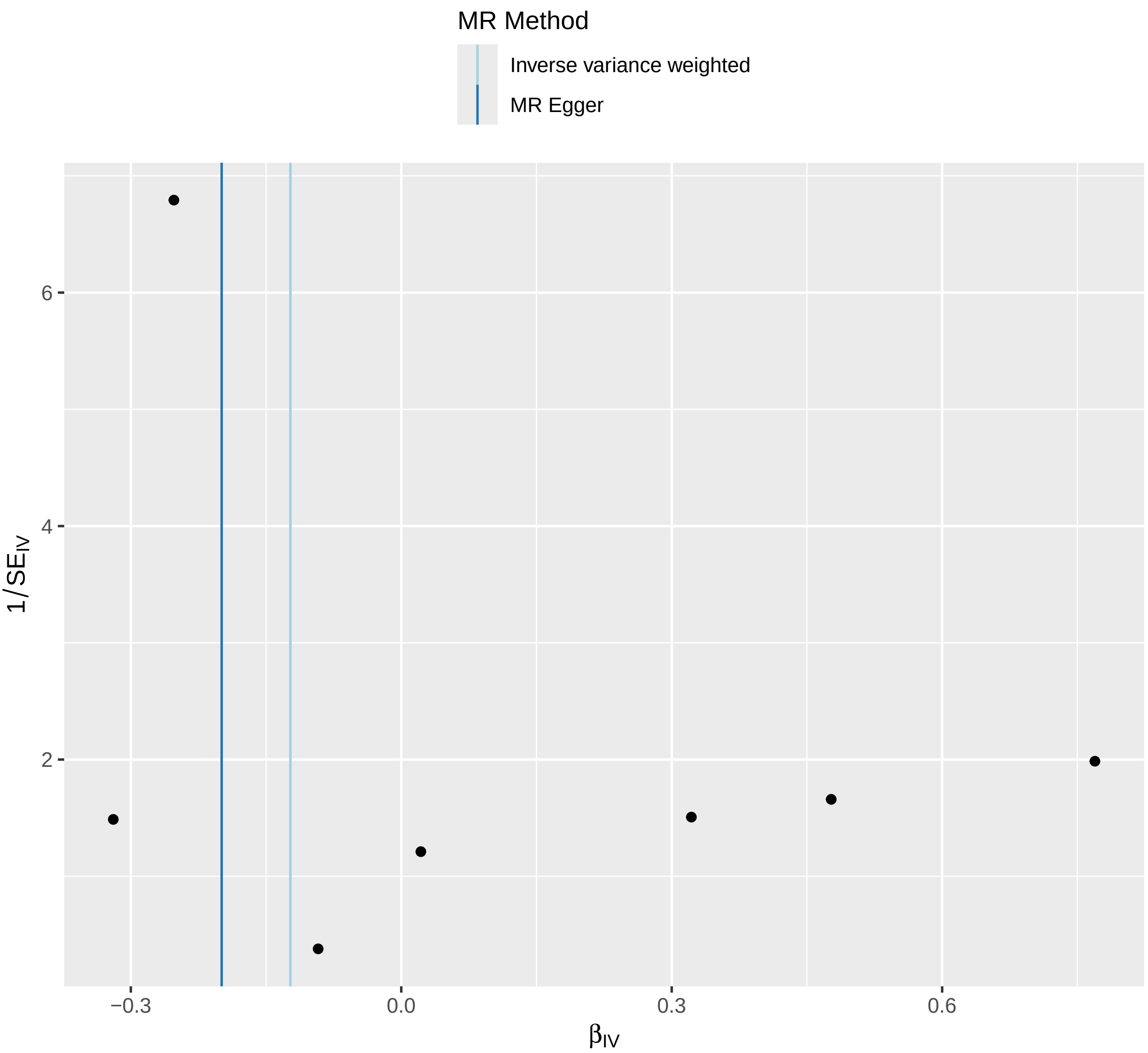
MR Method
Inverse variance weighted
MR Egger
6
4
2
1/SE$_{IV}$
−0.3
0.0
0.3
0.6
β$_{IV}$

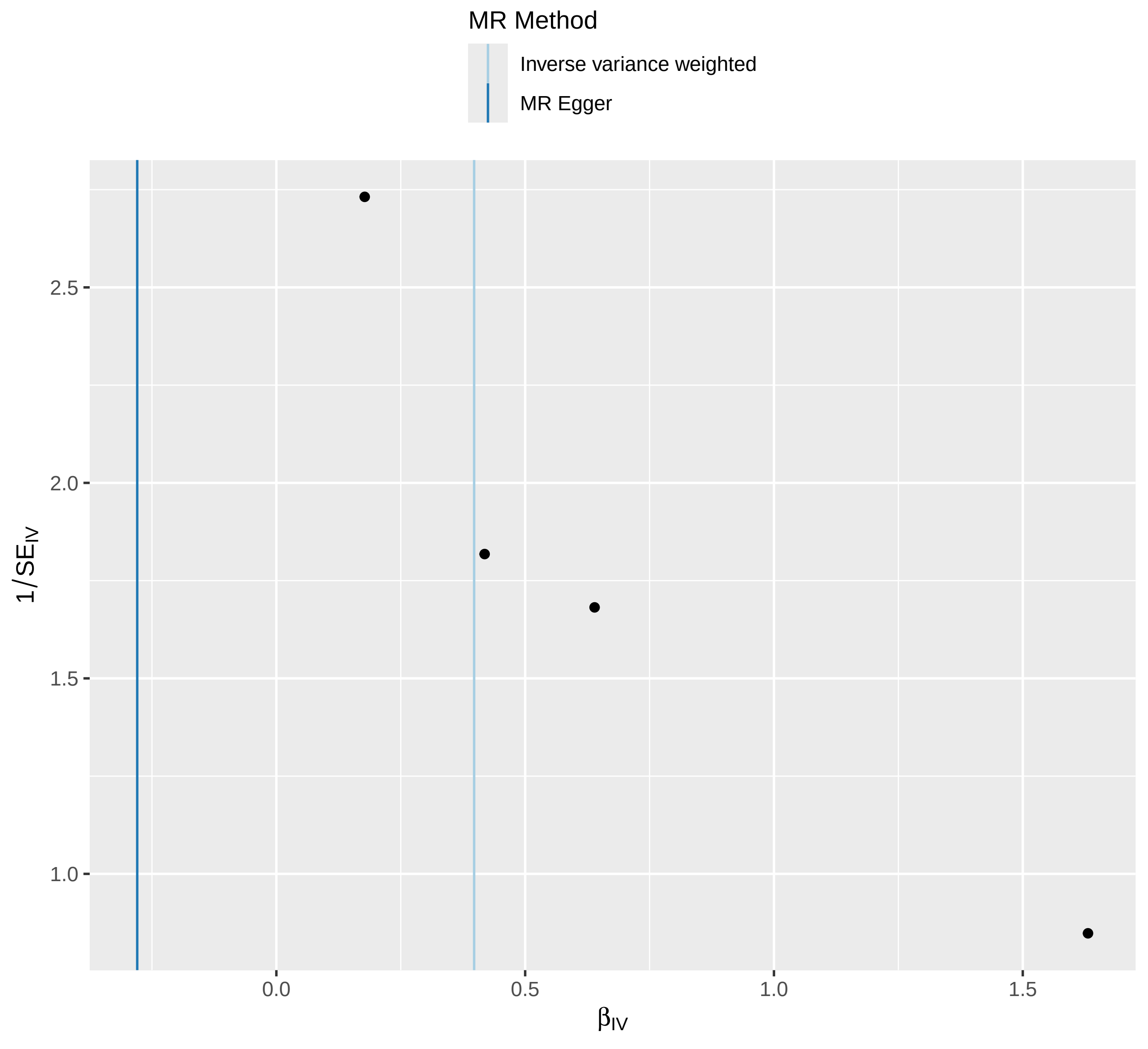

MR Method
Inverse variance weighted
MR Egger
2.5
2.0
1.5
1.0
0.0
0.5
1.0
1.5
$1/SE_{IV}$
β_{IV}

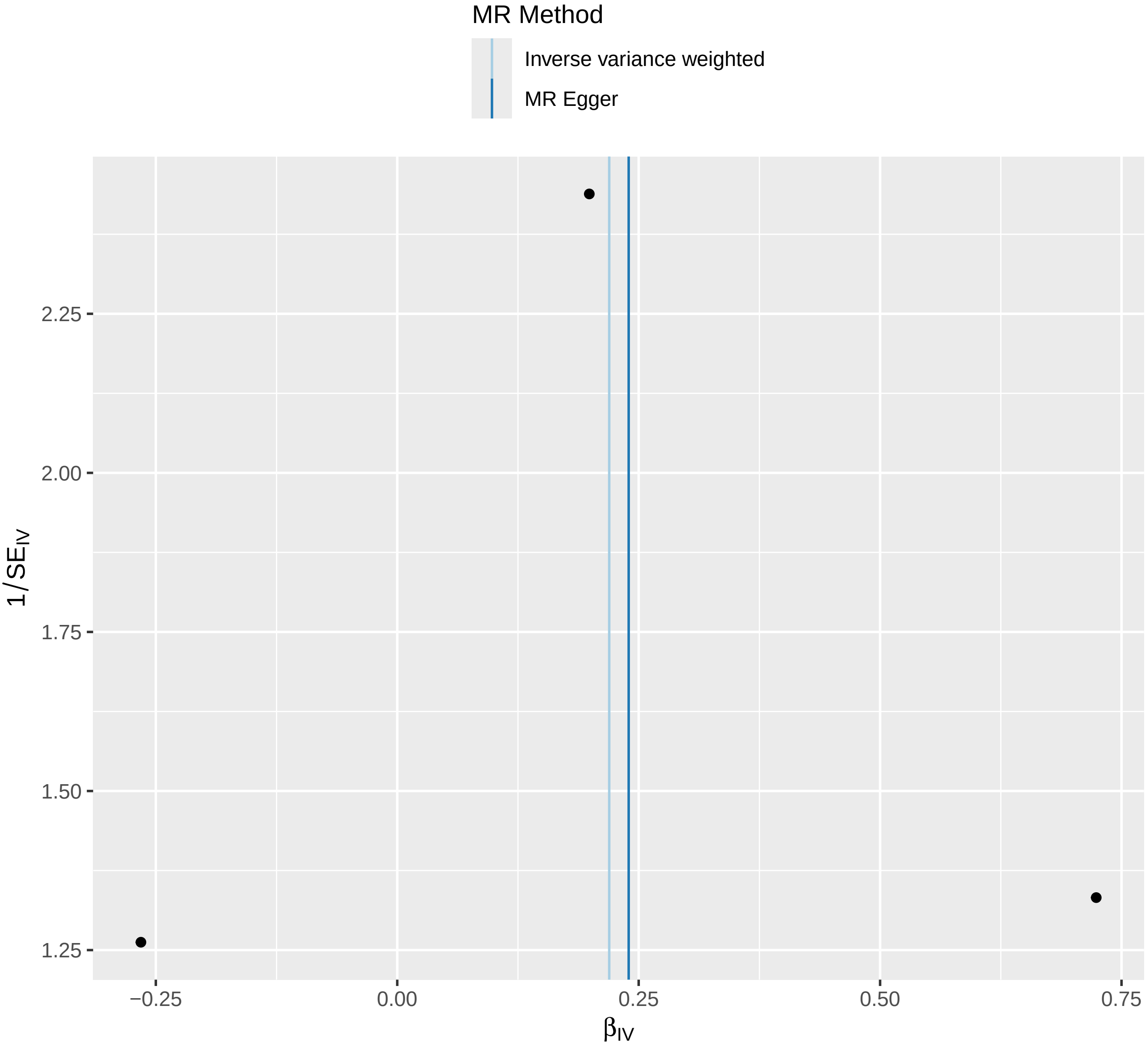

MR Method
Inverse variance weighted
MR Egger
2.25
2.00
1.75
1.50
1.25
1/SE_IV
−0.25
0.00
0.25
0.50
0.75
β_IV

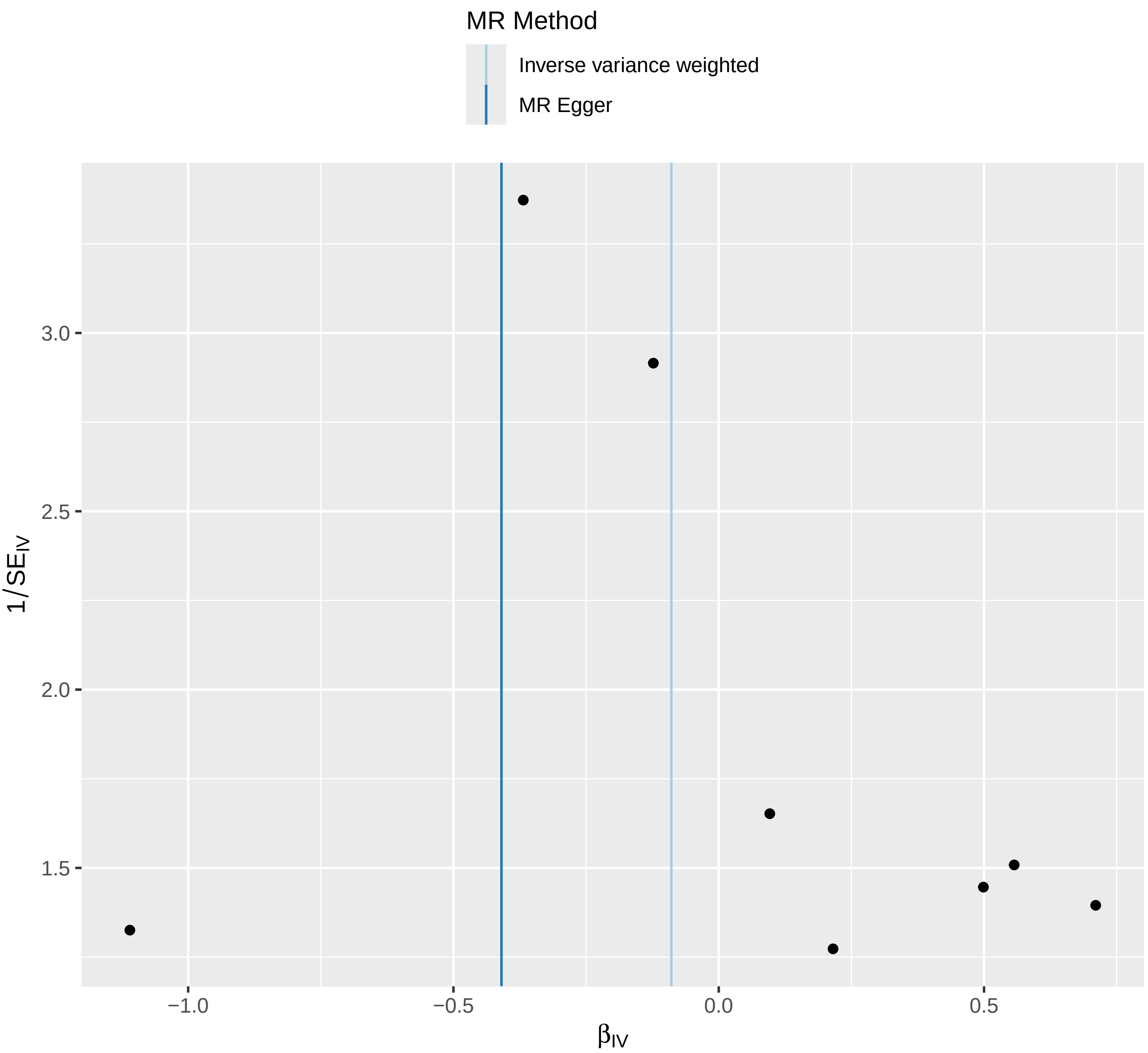
MR Method
Inverse variance weighted
MR Egger
3.0
2.5
2.0
1.5
1/SE_IV
−1.0
−0.5
0.0
0.5
β_IV

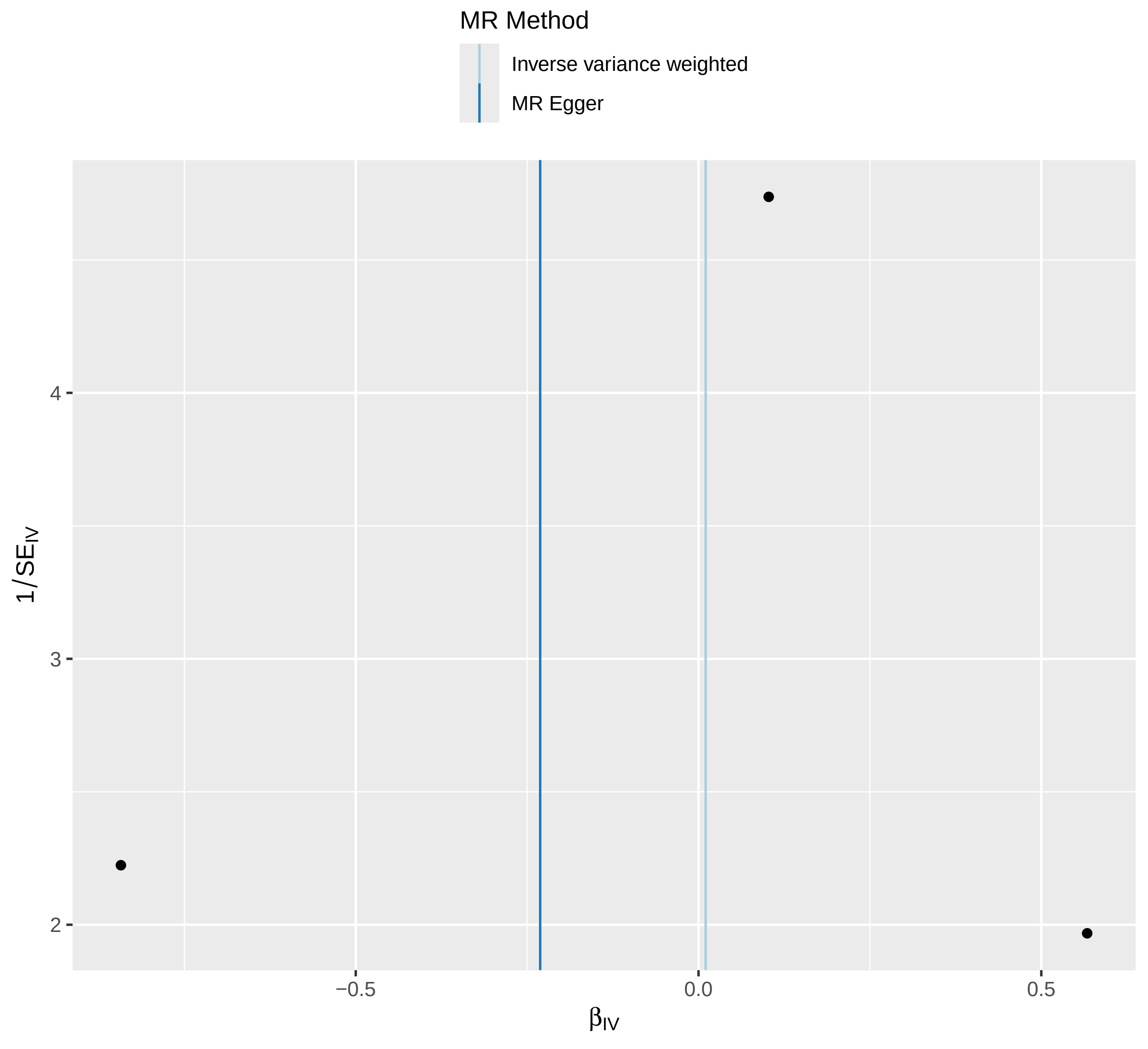

MR Method
Inverse variance weighted
MR Egger
4
3
2
1/SE_IV
−0.5
0.0
0.5
β_IV

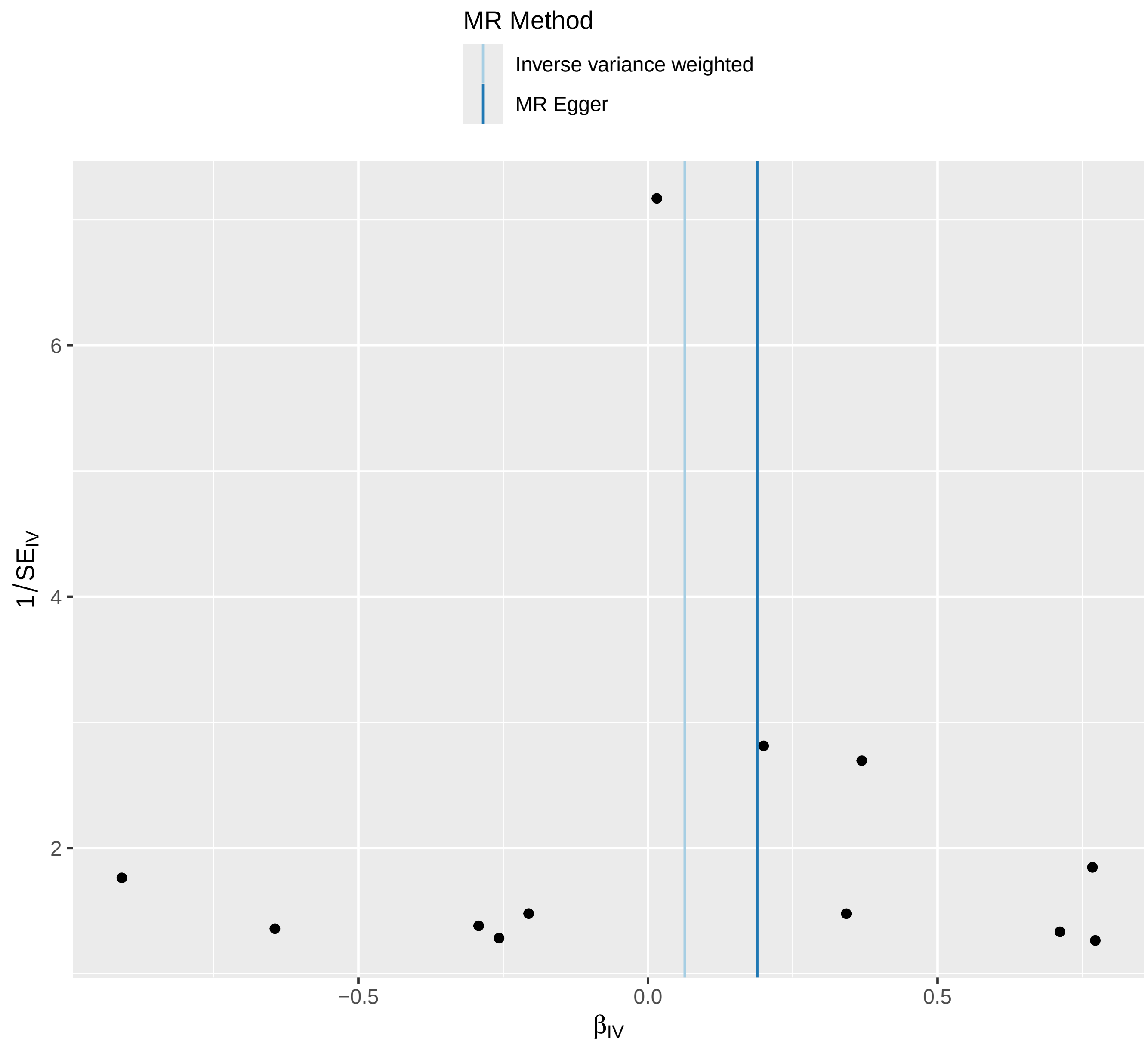
MR Method
Inverse variance weighted
MR Egger
6
4
2
−0.5
0.0
0.5
1/SE_IV
β_IV

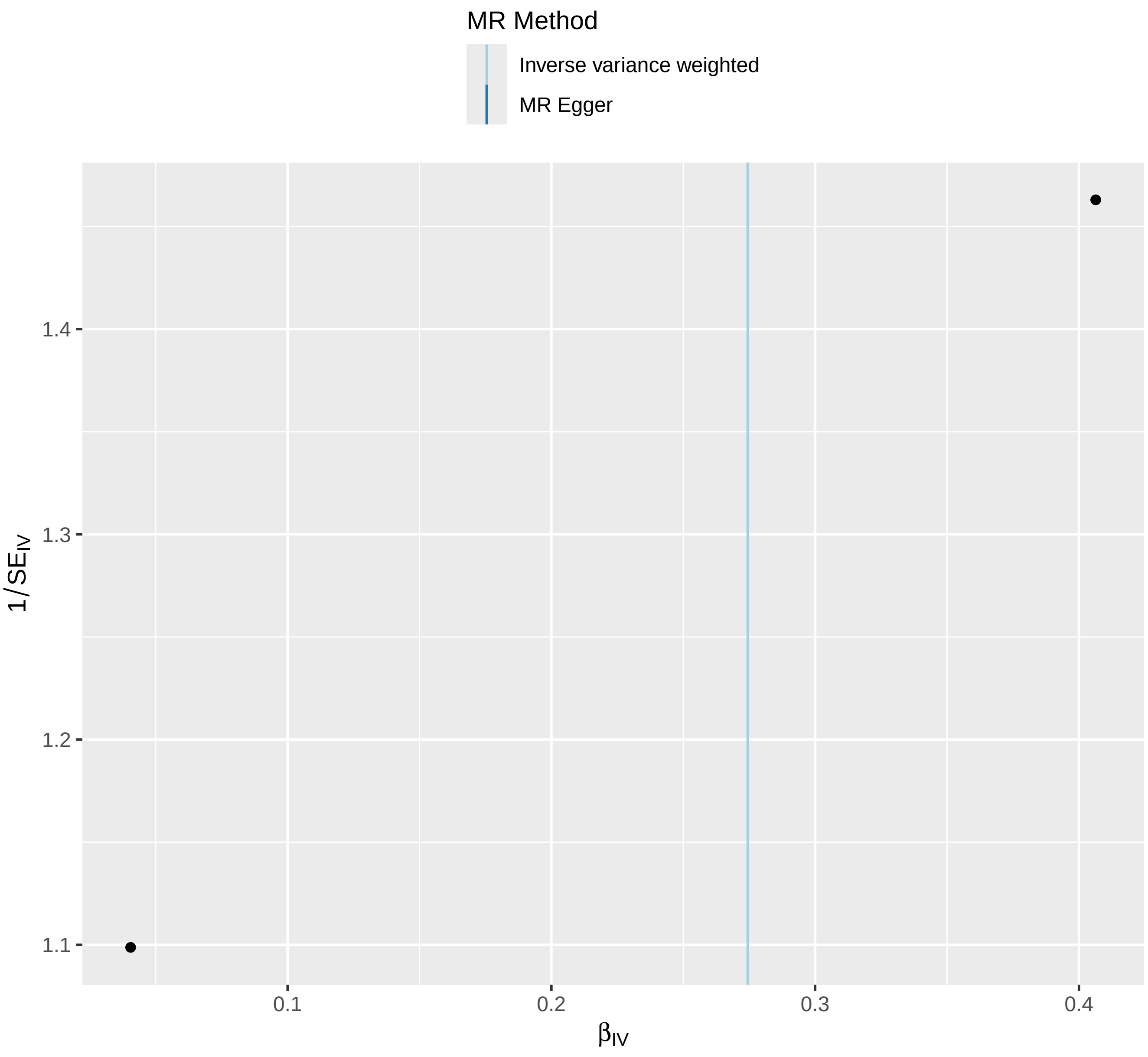

MR Method
Inverse variance weighted
MR Egger
1.4
1.3
1.2
1.1
1/SE_IV
0.1
0.2
0.3
0.4
β_IV

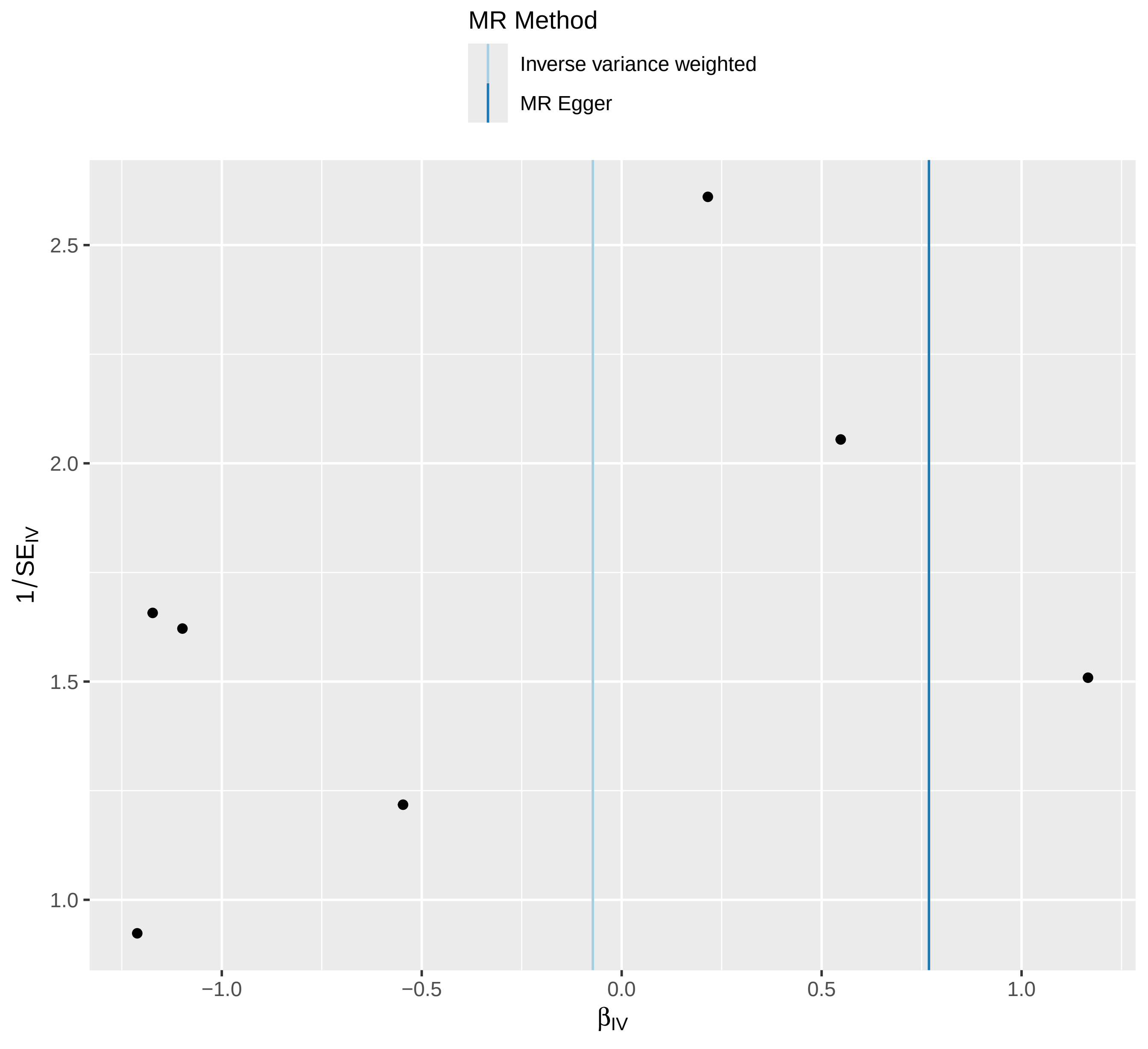
MR Method
Inverse variance weighted
MR Egger
2.5
2.0
1.5
1.0
$1/SE_{IV}$
−1.0
−0.5
0.0
0.5
1.0
β_{IV}

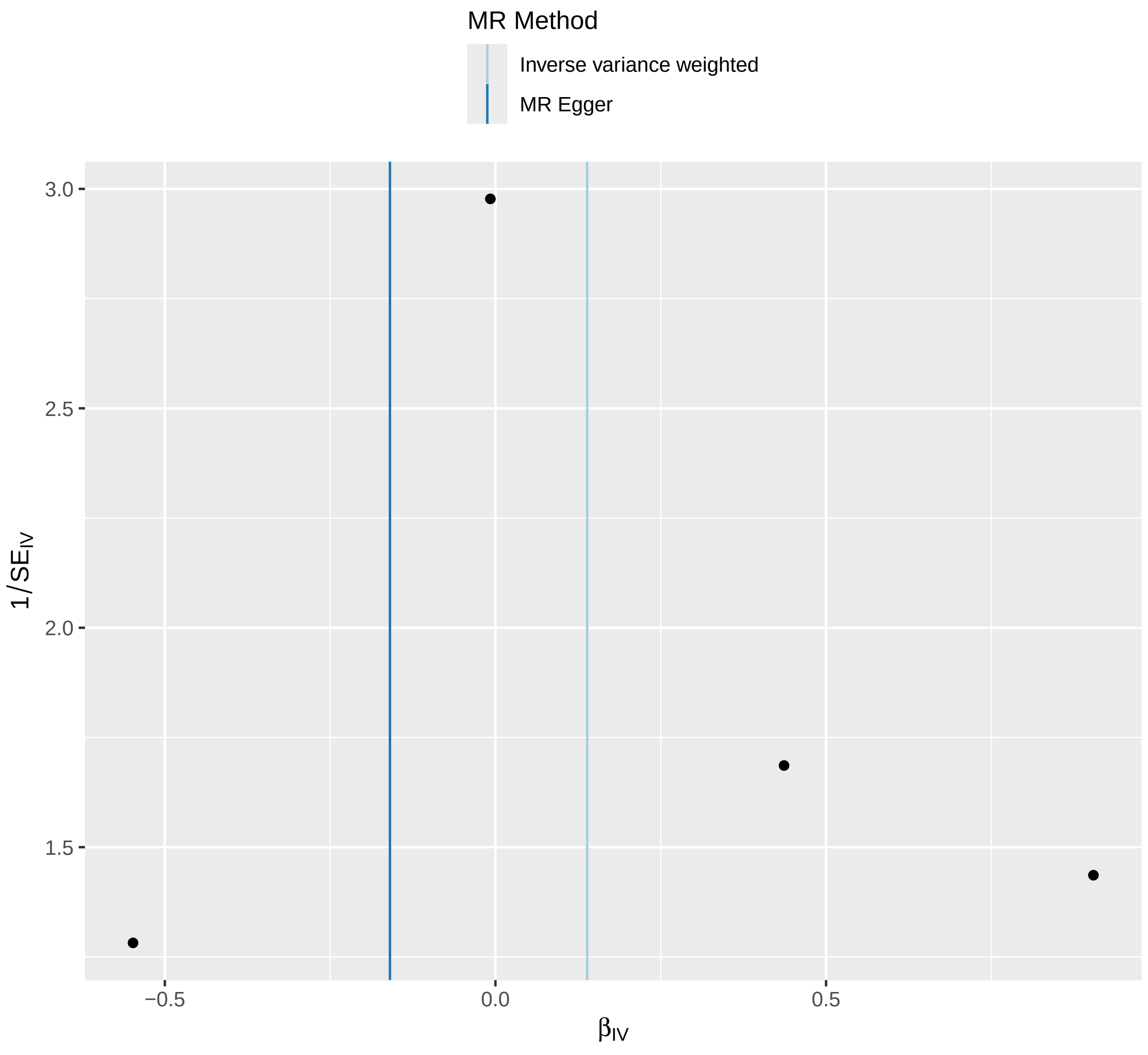
MR Method
Inverse variance weighted
MR Egger
3.0
2.5
2.0
1.5
1/SE_IV
−0.5
0.0
0.5
β_IV

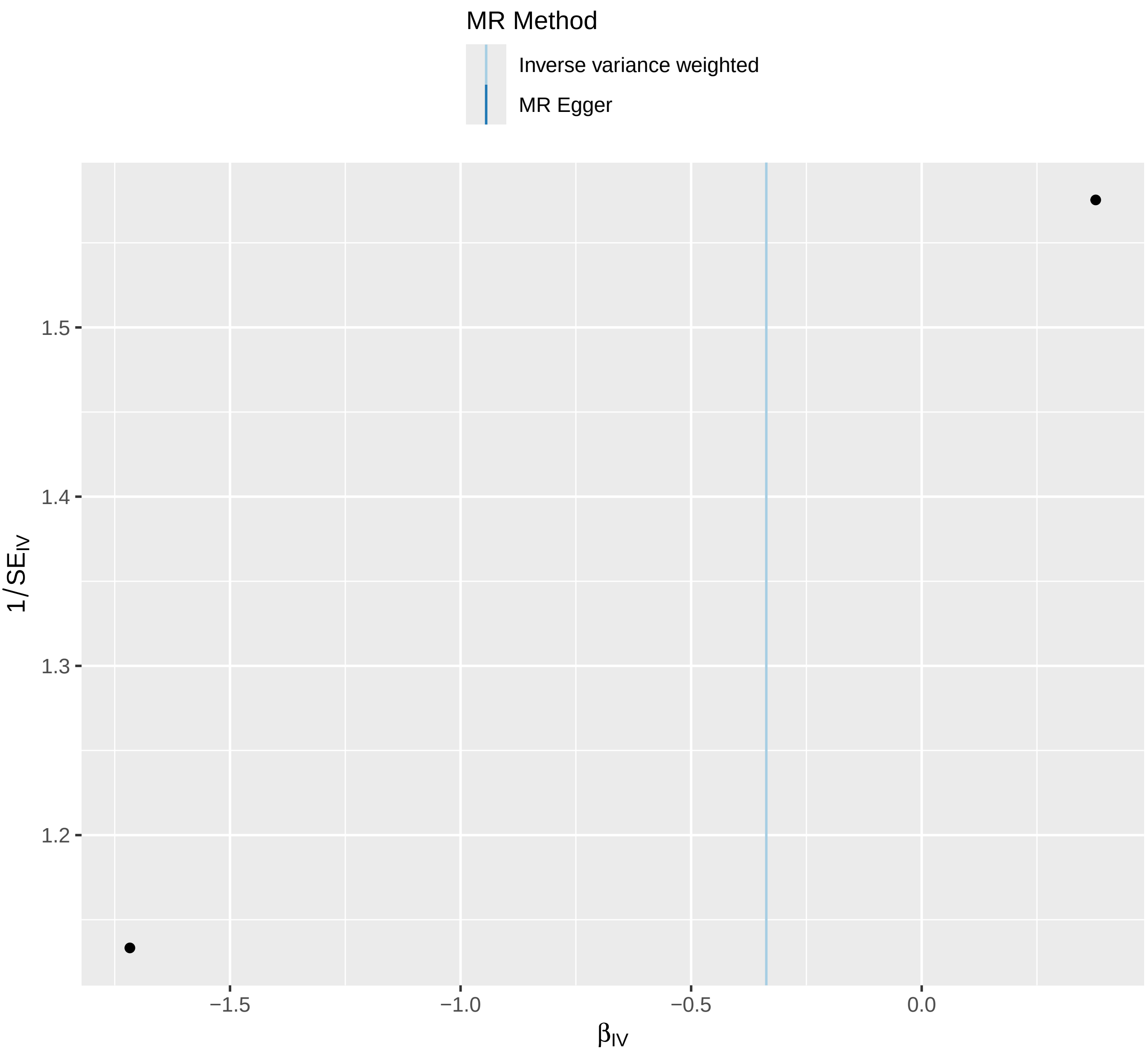
MR Method
Inverse variance weighted
MR Egger
1/SE_IV
1.5
1.4
1.3
1.2
−1.5
−1.0
−0.5
0.0
β_IV

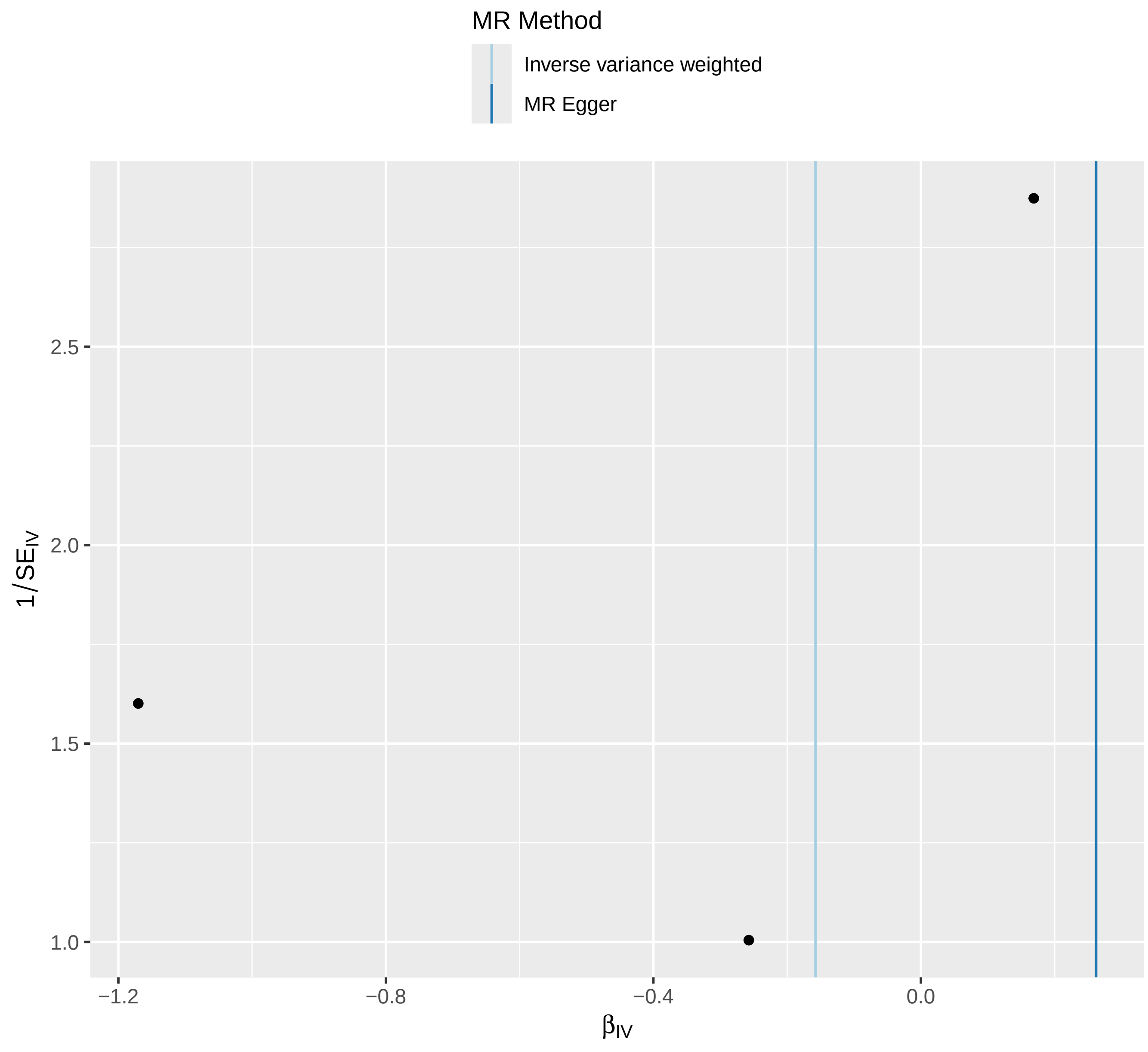

MR Method
Inverse variance weighted
MR Egger
1/SE_IV
2.5
2.0
1.5
1.0
−1.2
−0.8
−0.4
0.0
β_IV

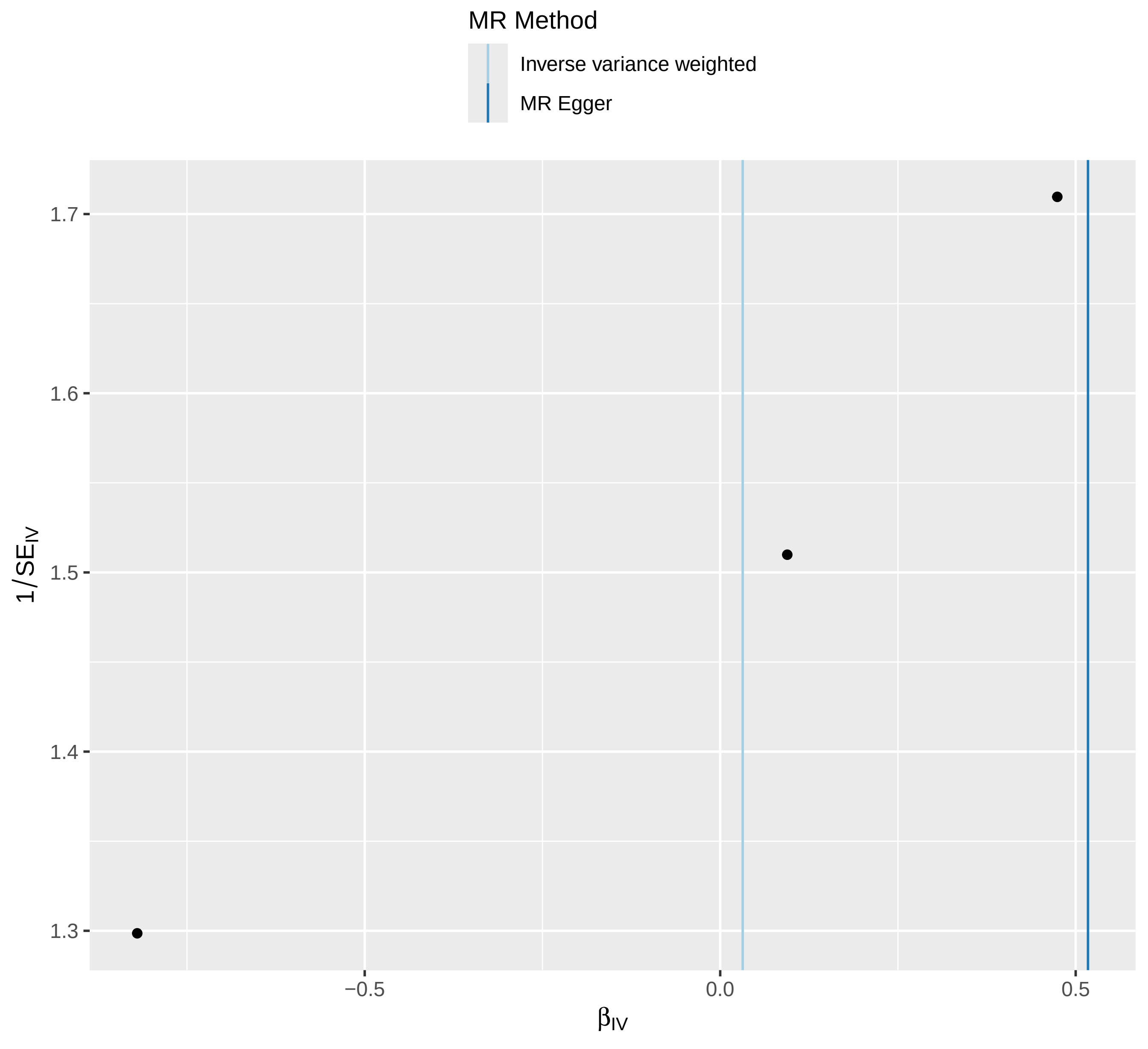
MR Method
Inverse variance weighted
MR Egger
1.7
1.6
1.5
1.4
1.3
1/SE_IV
−0.5
0.0
0.5
β_IV

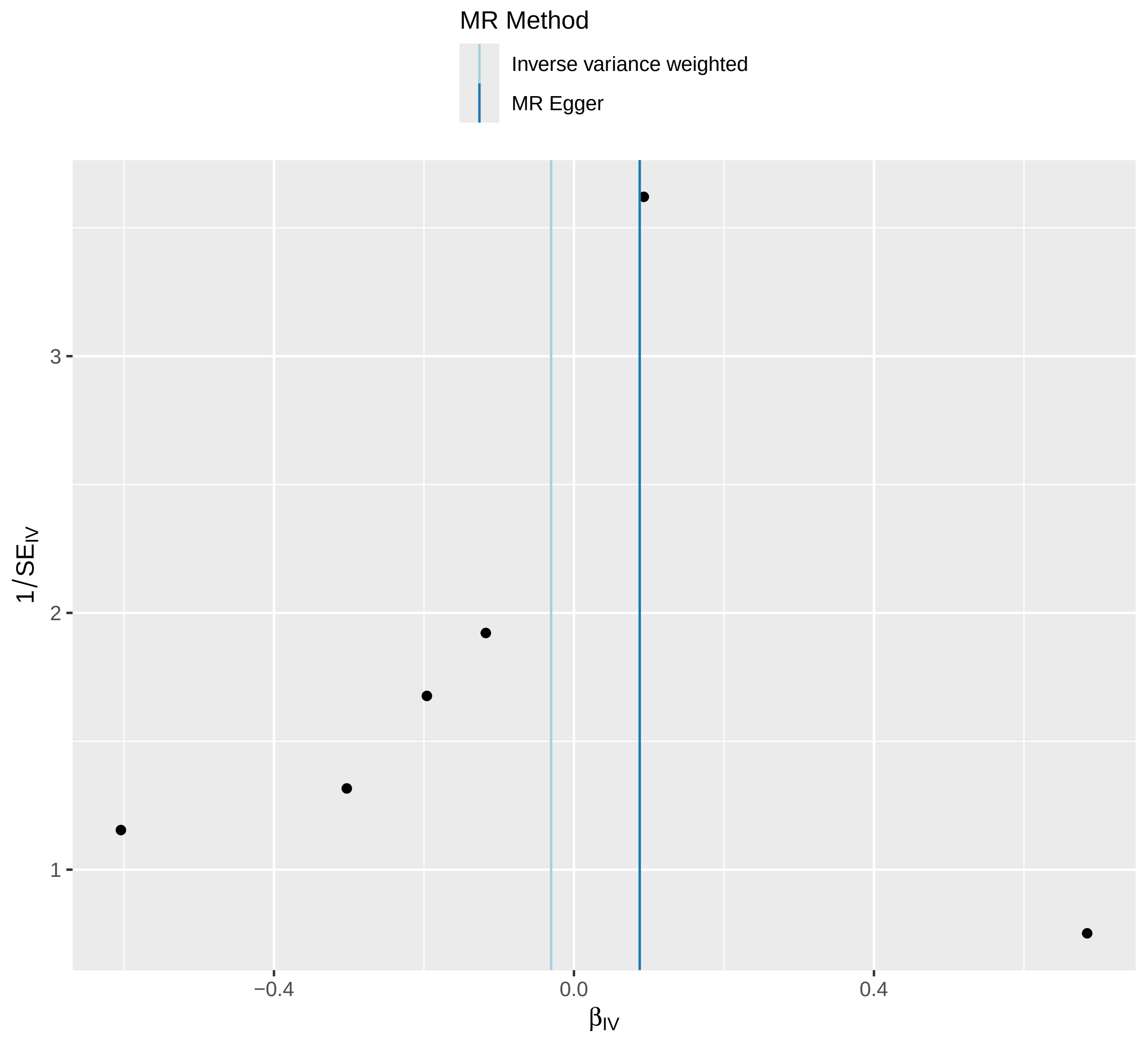

MR Method
Inverse variance weighted
MR Egger
1/SE_IV
3
2
1
−0.4
0.0
0.4
β_IV

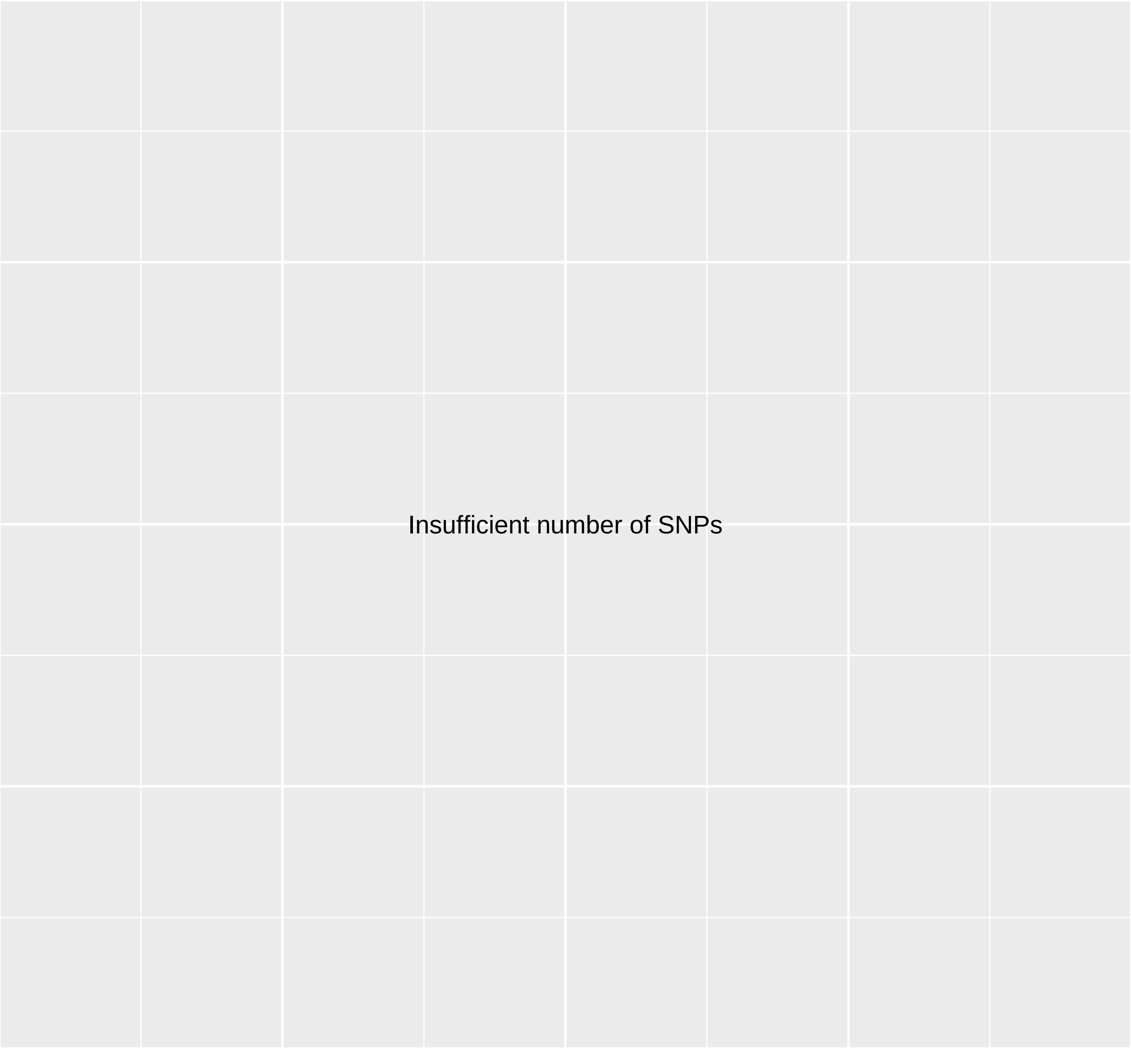
Insufficient number of SNPs

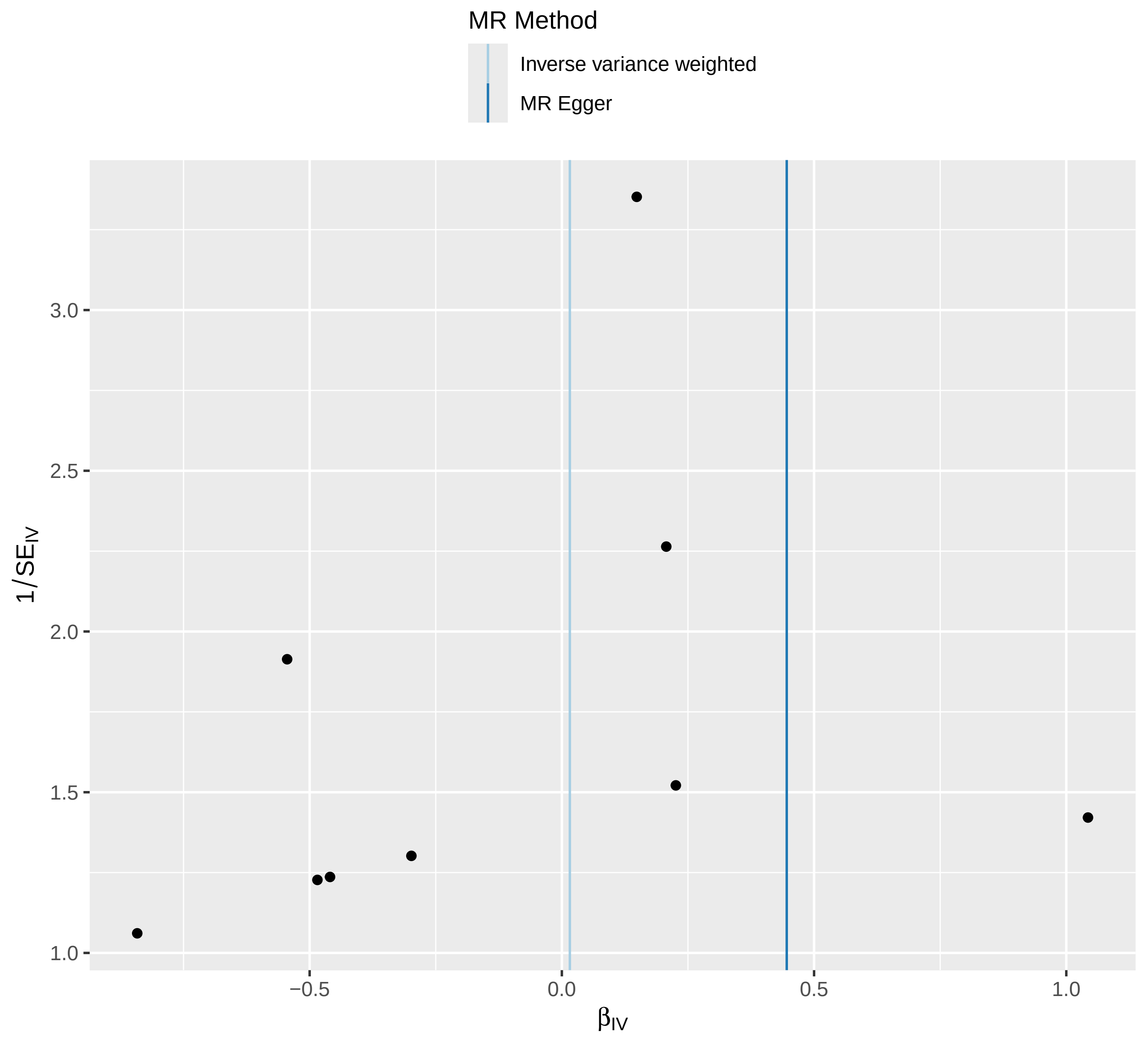
MR Method
Inverse variance weighted
MR Egger
1/SE_IV
3.0
2.5
2.0
1.5
1.0
−0.5
0.0
0.5
1.0
β_IV

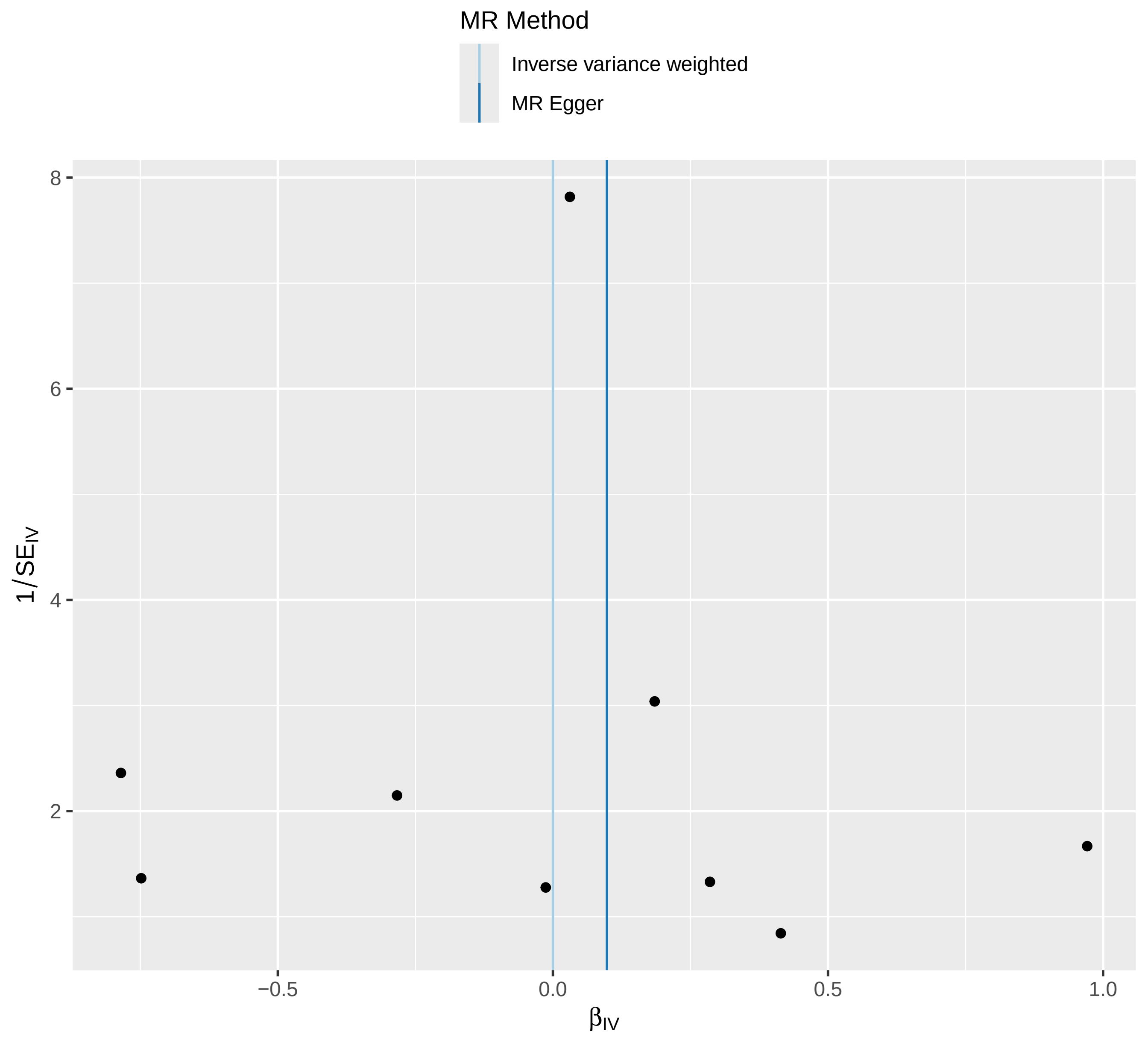
MR Method
Inverse variance weighted
MR Egger
8
6
4
2
1/SE$_{IV}$
−0.5
0.0
0.5
1.0
β$_{IV}$

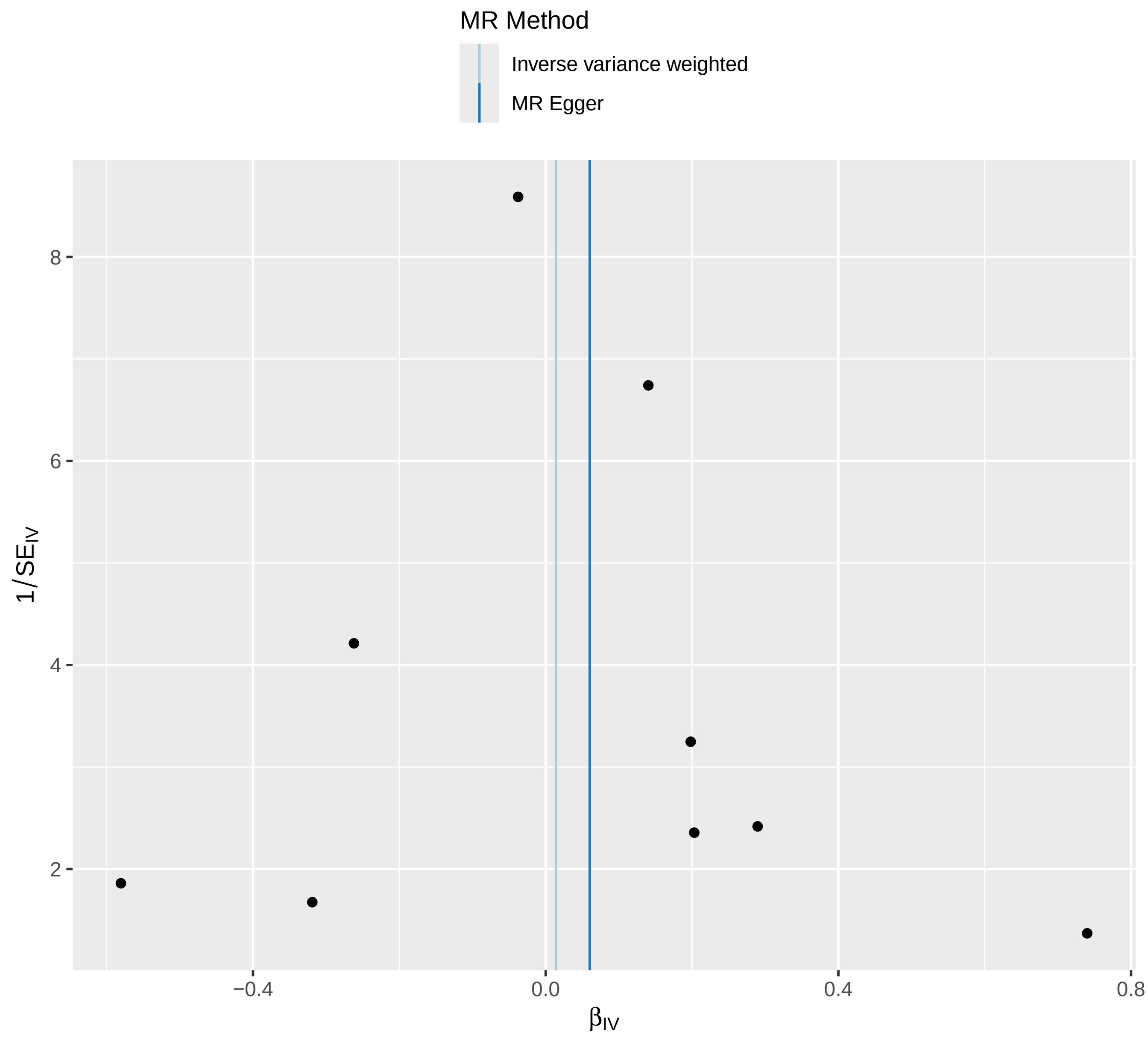

MR Method
Inverse variance weighted
MR Egger
8
6
4
2
−0.4
0.0
0.4
0.8
$1/SE_{IV}$
β_{IV}

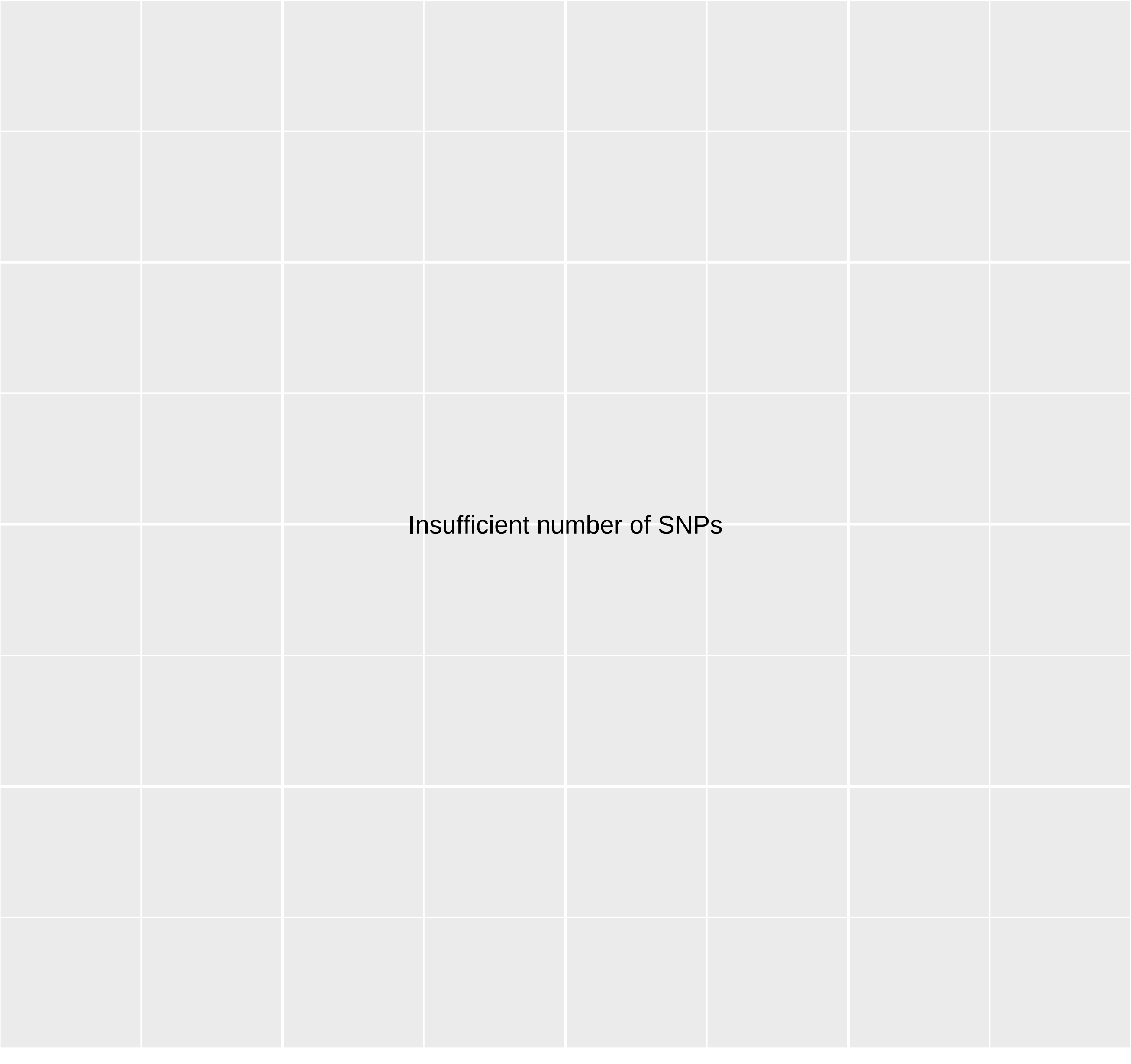
Insufficient number of SNPs

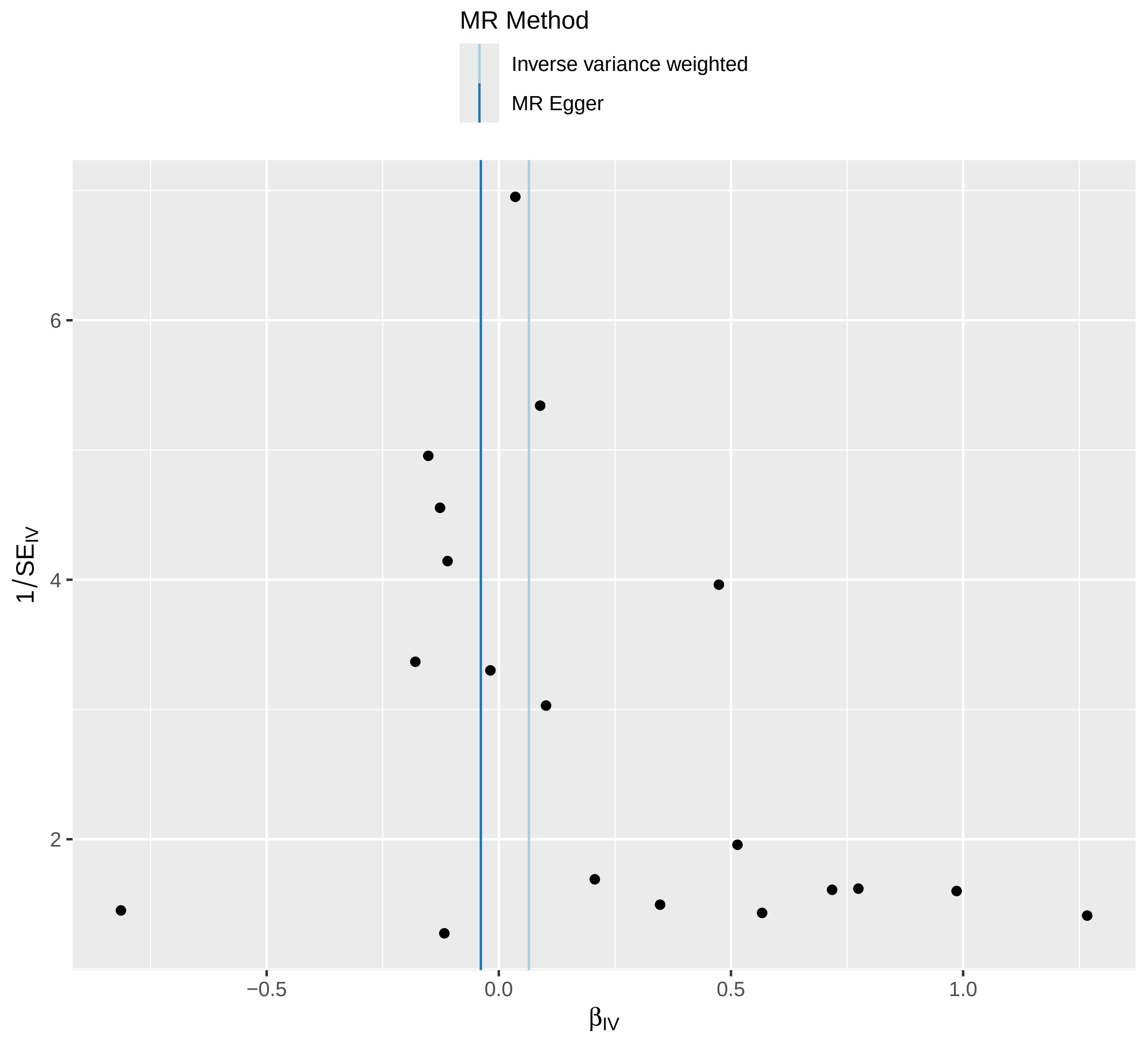
MR Method
Inverse variance weighted
MR Egger
6
4
2
1/SE_IV
−0.5
0.0
0.5
1.0
β_IV

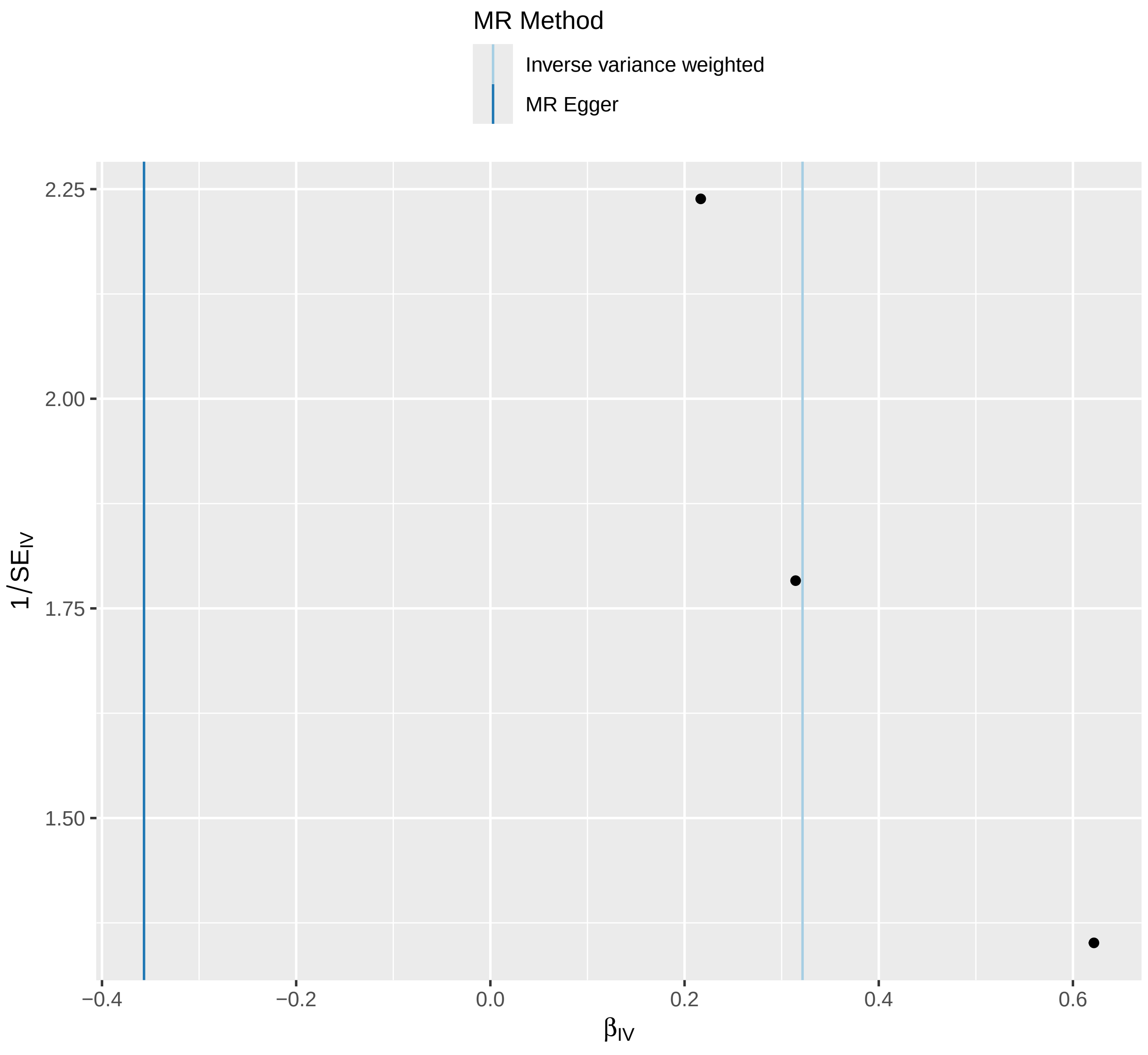

MR Method
Inverse variance weighted
MR Egger
2.25
2.00
1.75
1.50
1/SE$_{IV}$
−0.4
−0.2
0.0
0.2
0.4
0.6
β$_{IV}$

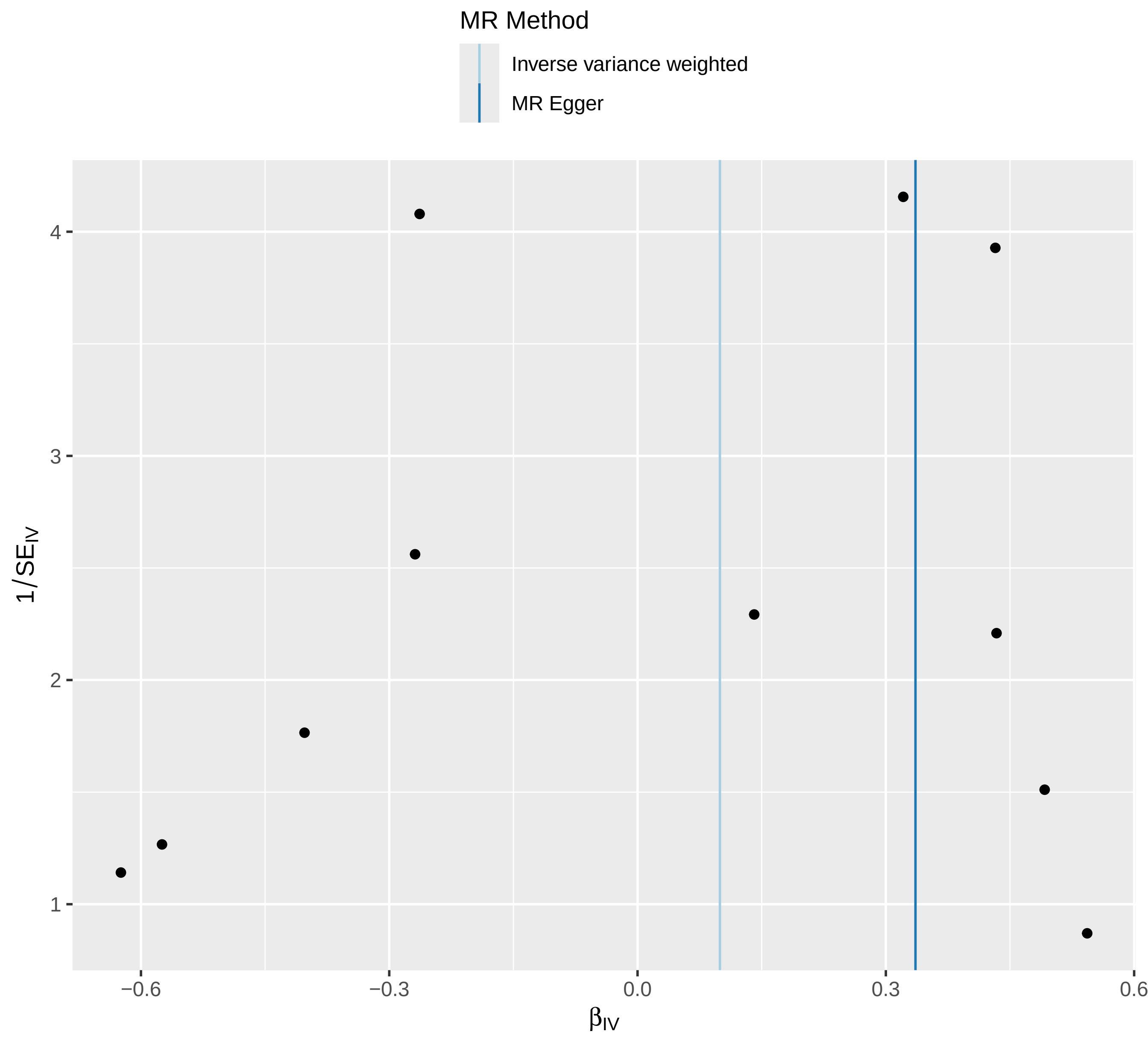

MR Method
Inverse variance weighted
MR Egger
4
3
2
1
1/SE$_{IV}$
−0.6
−0.3
0.0
0.3
0.6
β$_{IV}$

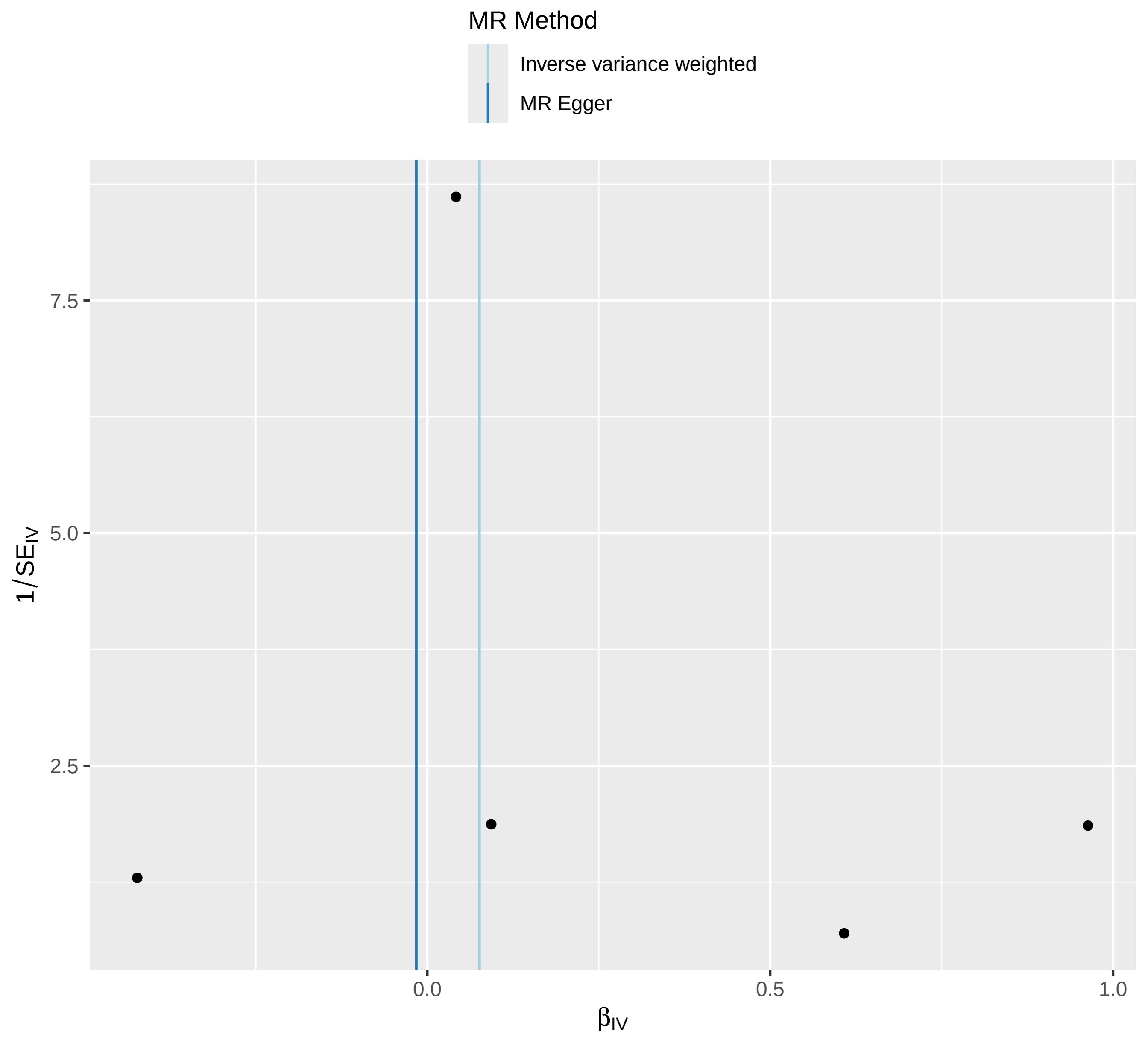

MR Method
Inverse variance weighted
MR Egger
7.5
5.0
2.5
$1/SE_{IV}$
0.0
0.5
1.0
β_{IV}

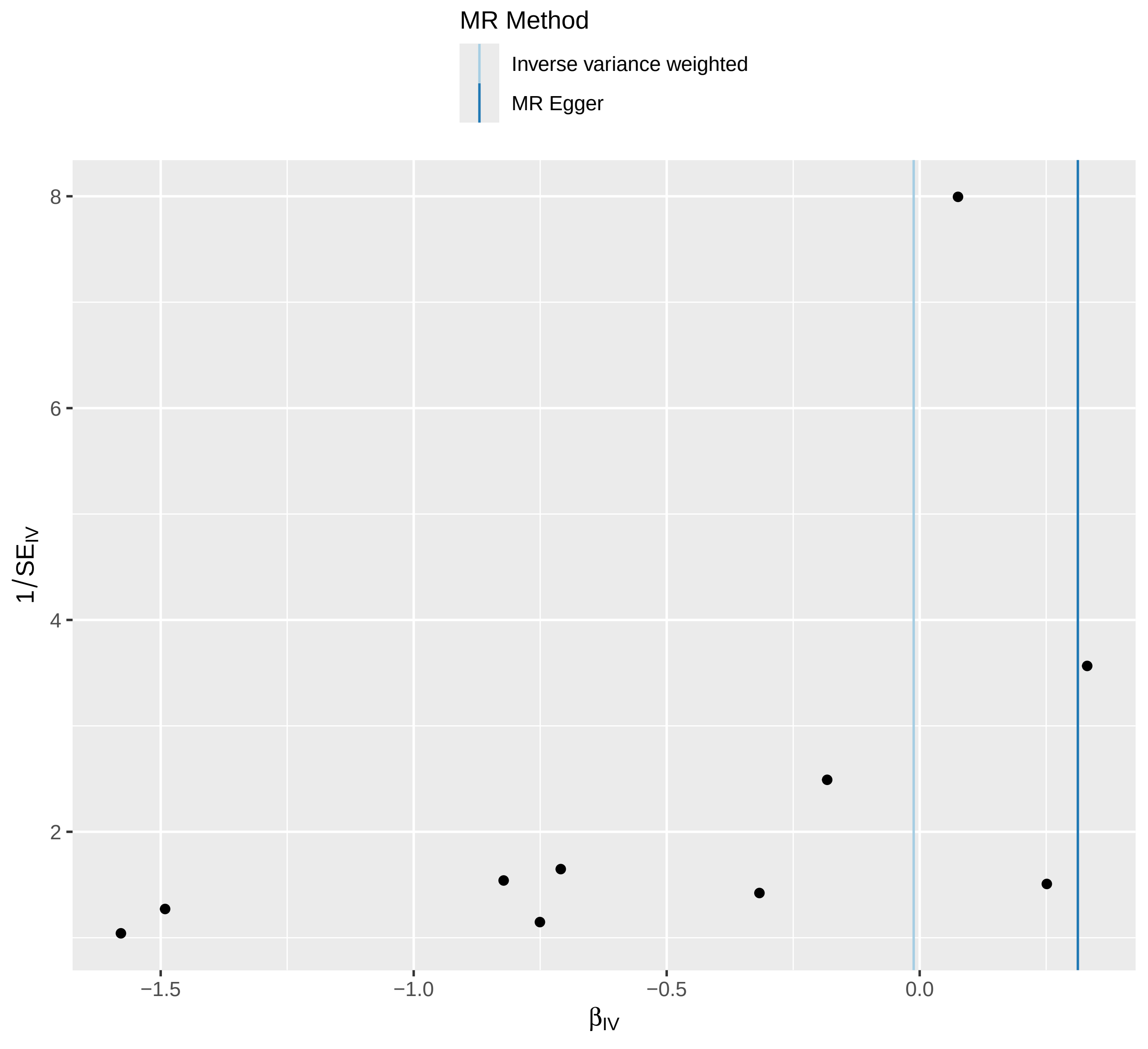
MR Method
Inverse variance weighted
MR Egger
8
6
4
2
1/SE_IV
−1.5
−1.0
−0.5
0.0
β_IV

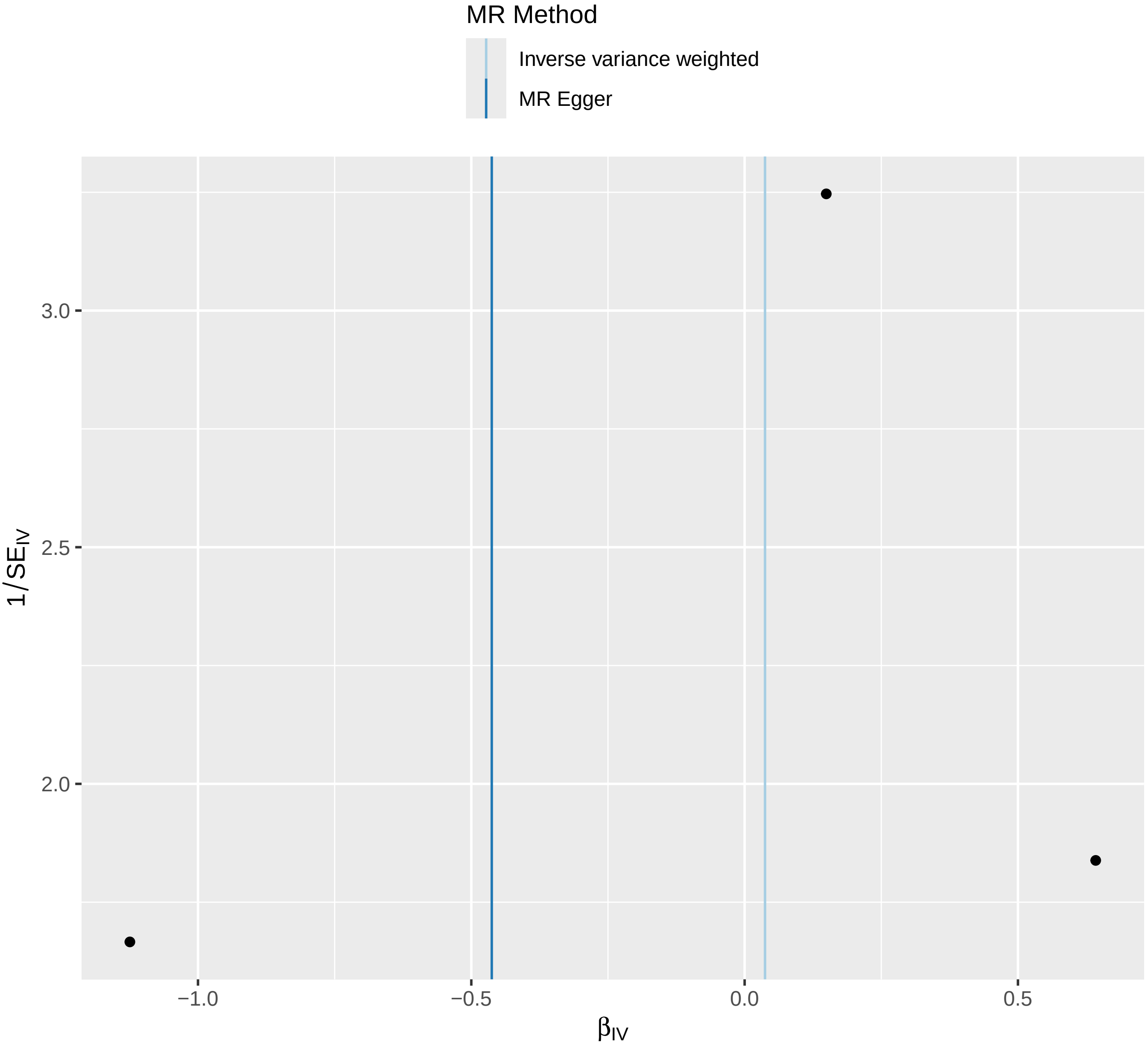
MR Method
Inverse variance weighted
MR Egger
3.0
2.5
2.0
1/SE_IV
−1.0
−0.5
0.0
0.5
β_IV

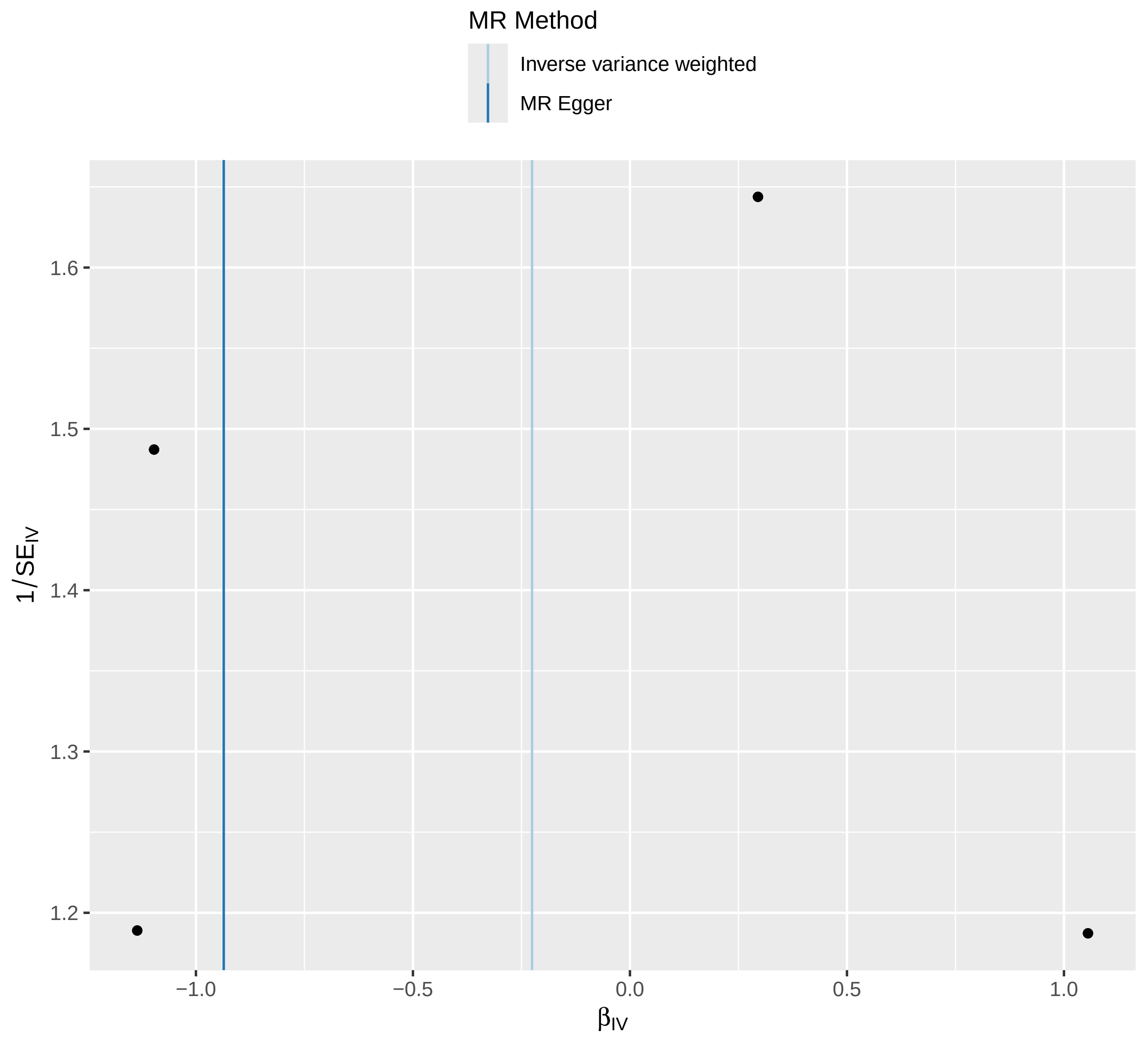
MR Method
Inverse variance weighted
MR Egger
1.6
1.5
1.4
1.3
1.2
1/SE_IV
−1.0
−0.5
0.0
0.5
1.0
β_IV

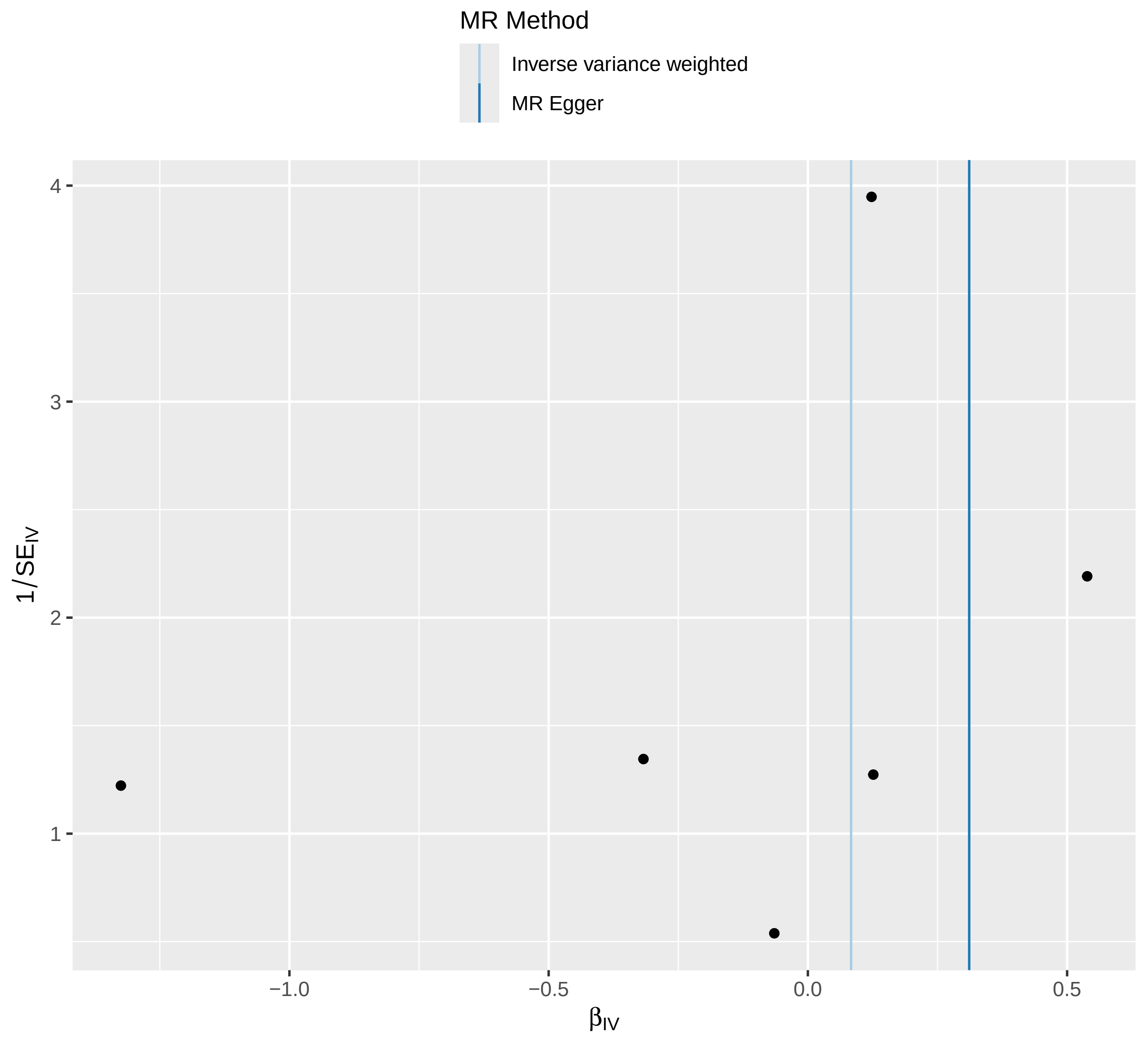
MR Method
Inverse variance weighted
MR Egger
4
3
2
1
1/SE$_{IV}$
−1.0
−0.5
0.0
0.5
β$_{IV}$

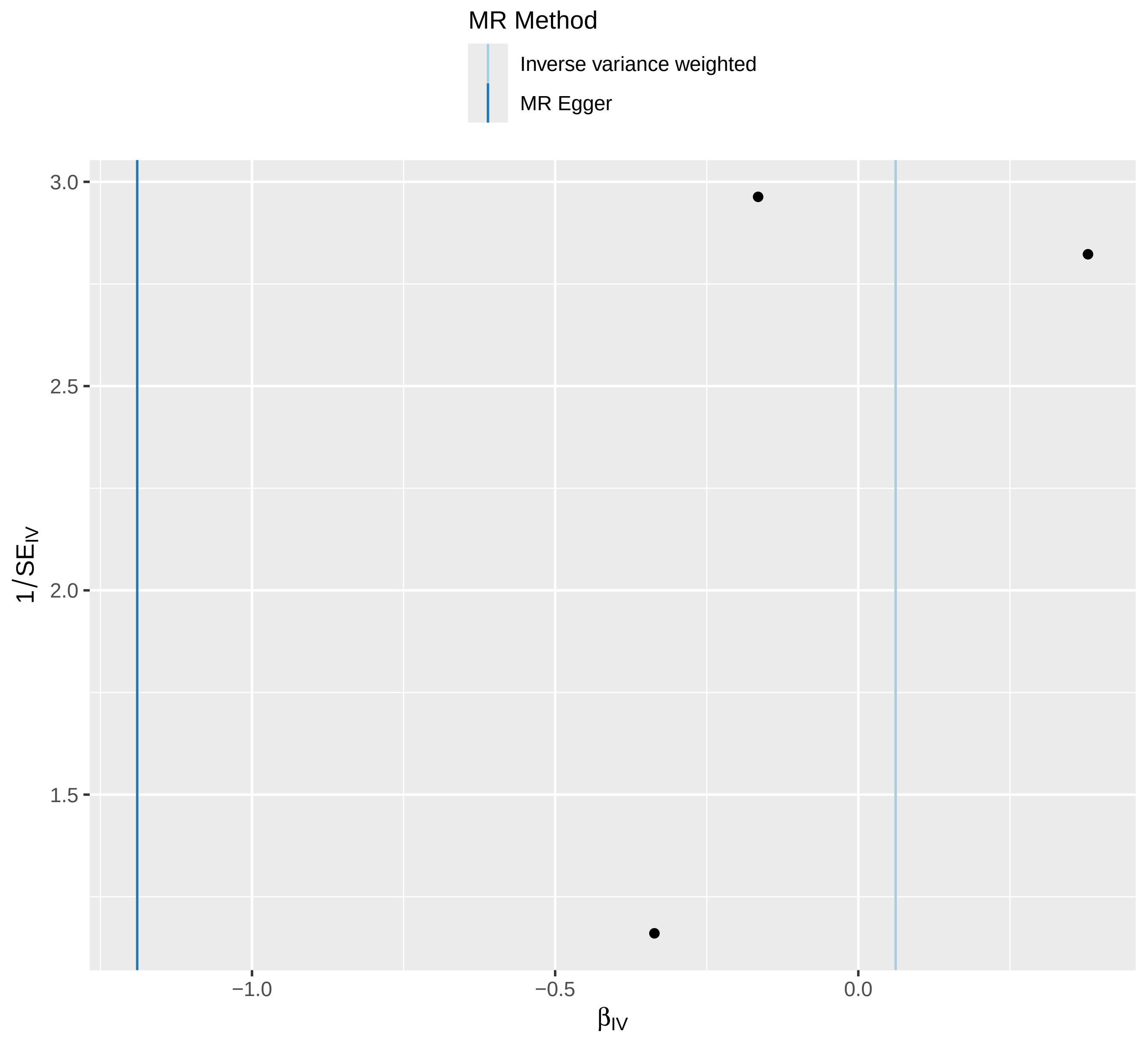
MR Method
Inverse variance weighted
MR Egger
3.0
2.5
2.0
1.5
1/SE_IV
−1.0
−0.5
0.0
β_IV

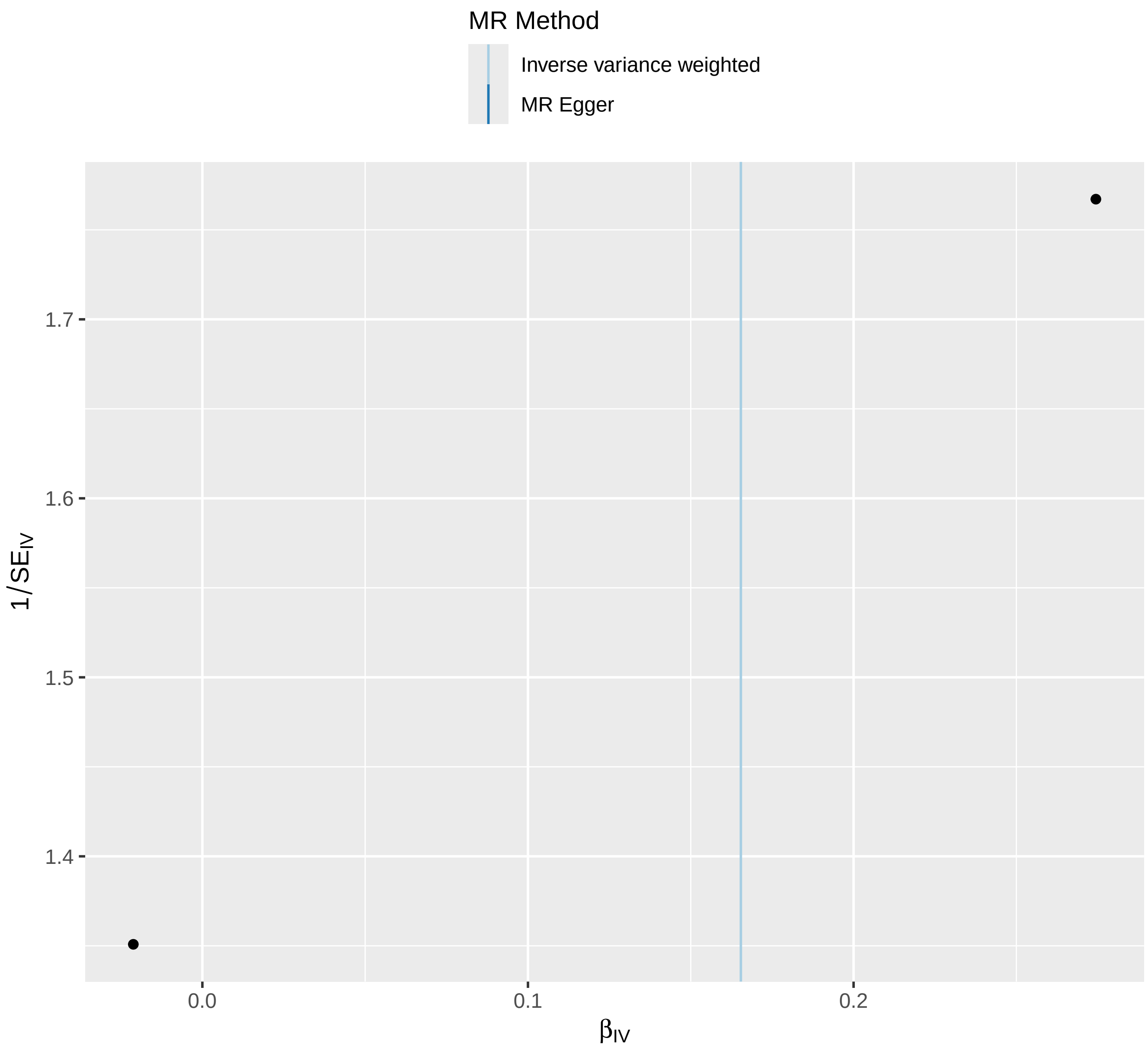

MR Method
Inverse variance weighted
MR Egger
1.7
1.6
1.5
1.4
0.0
0.1
0.2
$1/SE_{IV}$
β_{IV}

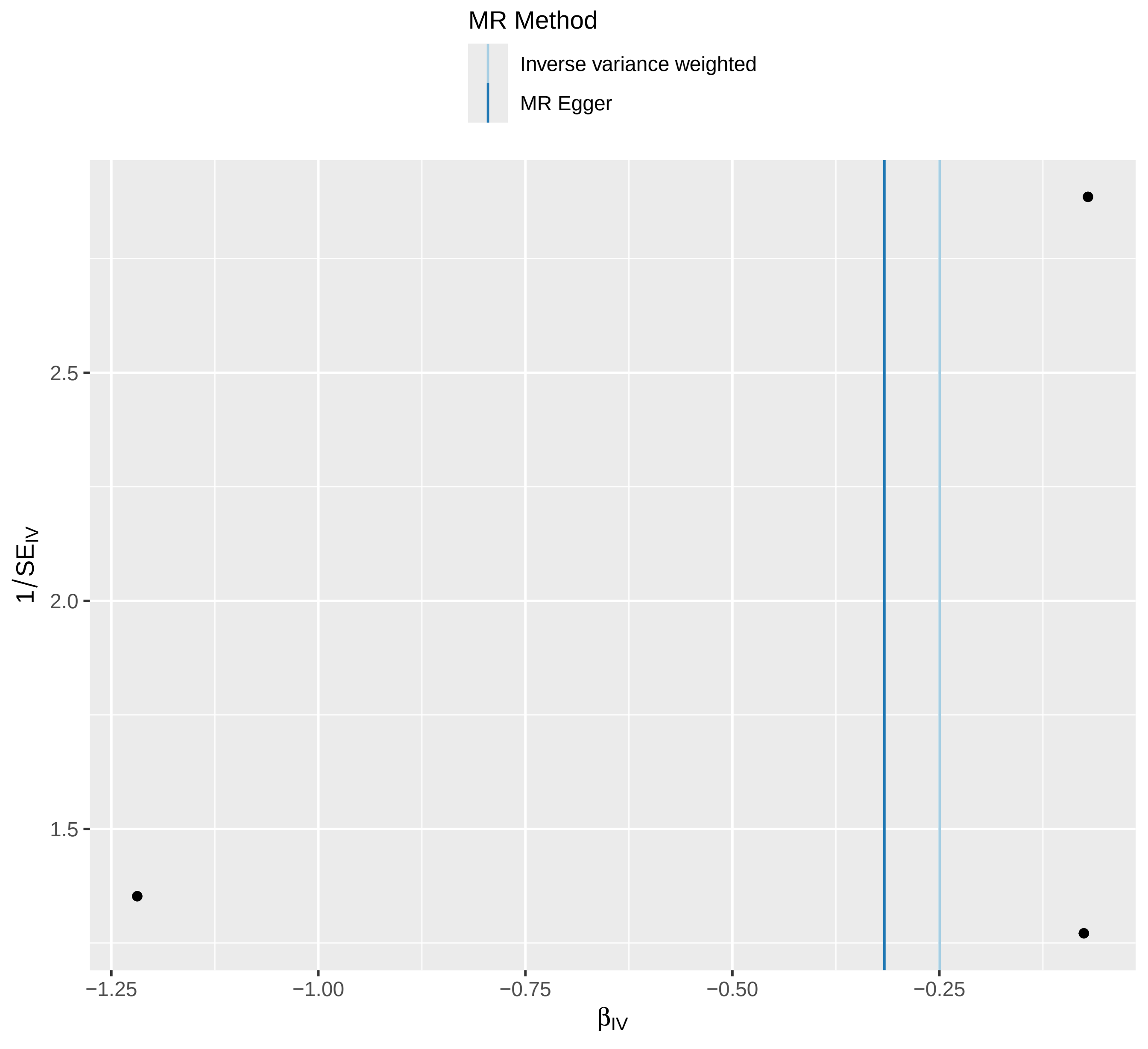

MR Method
Inverse variance weighted
MR Egger
2.5
2.0
1.5
1/SE_IV
−1.25
−1.00
−0.75
−0.50
−0.25
β_IV

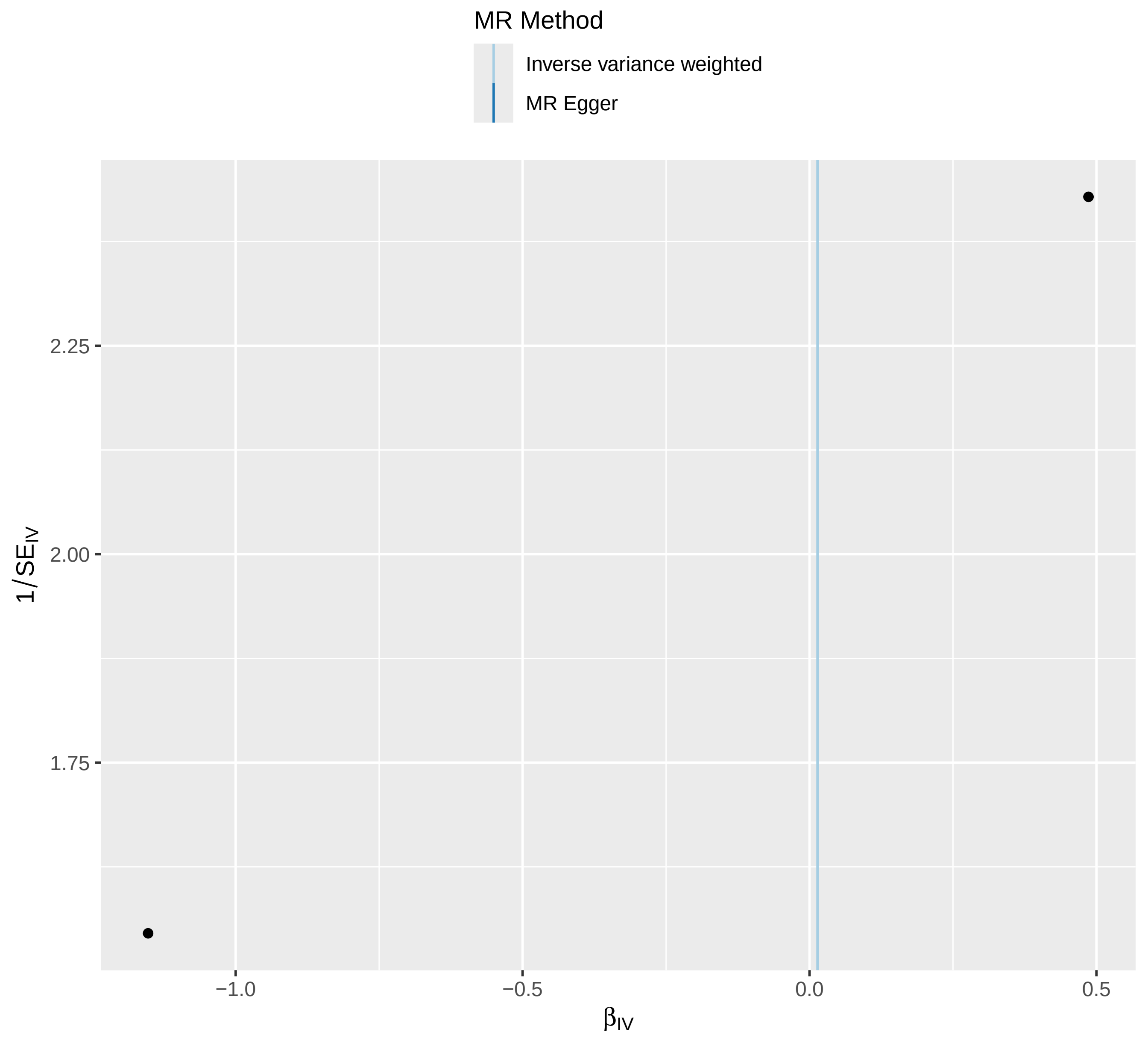
MR Method
Inverse variance weighted
MR Egger
1/SE_IV
2.25
2.00
1.75
−1.0
−0.5
0.0
0.5
β_IV

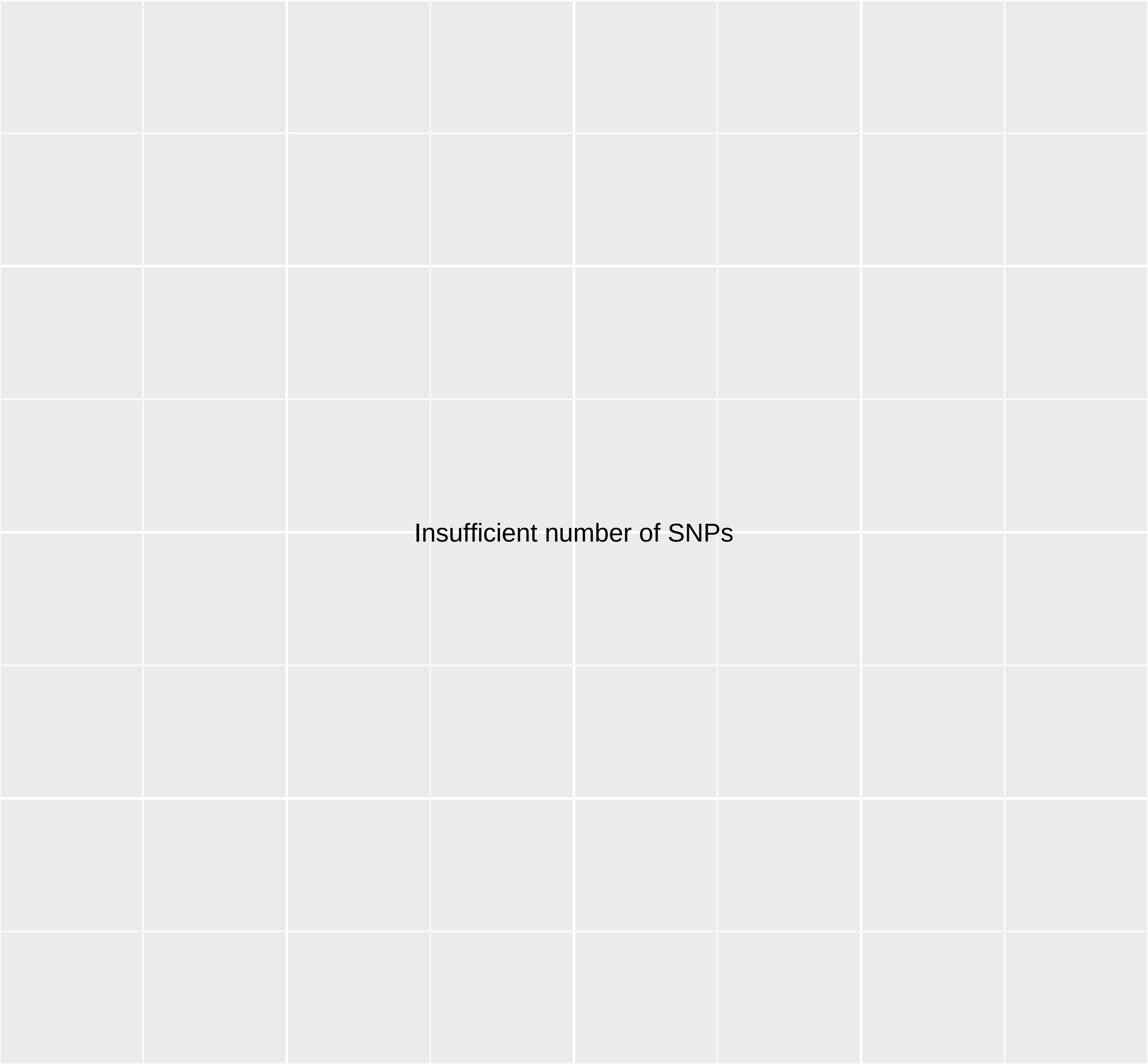
Insufficient number of SNPs

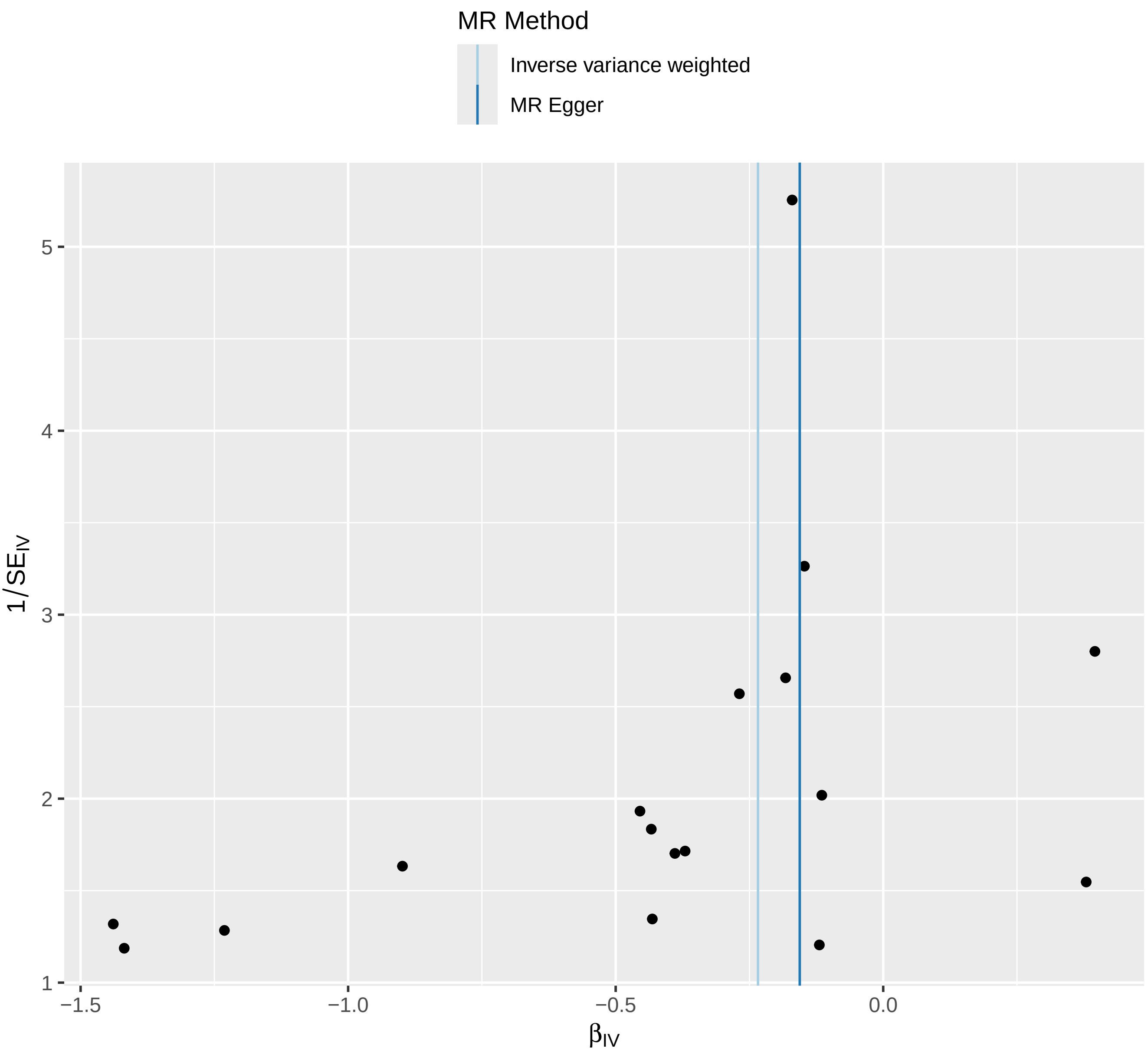
MR Method
Inverse variance weighted
MR Egger
1/SE_IV
5
4
3
2
1
−1.5
−1.0
−0.5
0.0
β_IV

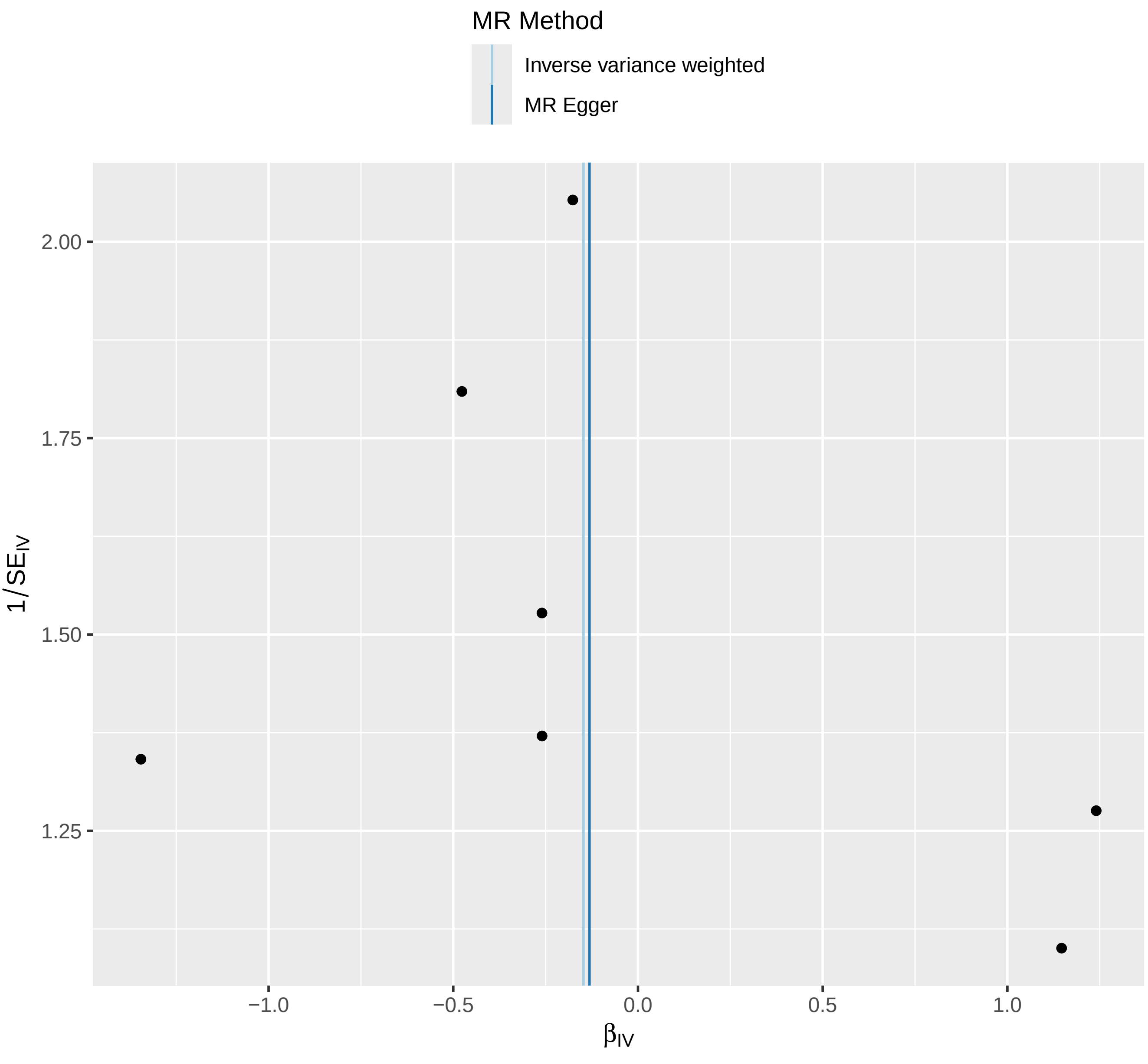

MR Method
Inverse variance weighted
MR Egger
2.00
1.75
1.50
1.25
1/SE_IV
−1.0
−0.5
0.0
0.5
1.0
β_IV

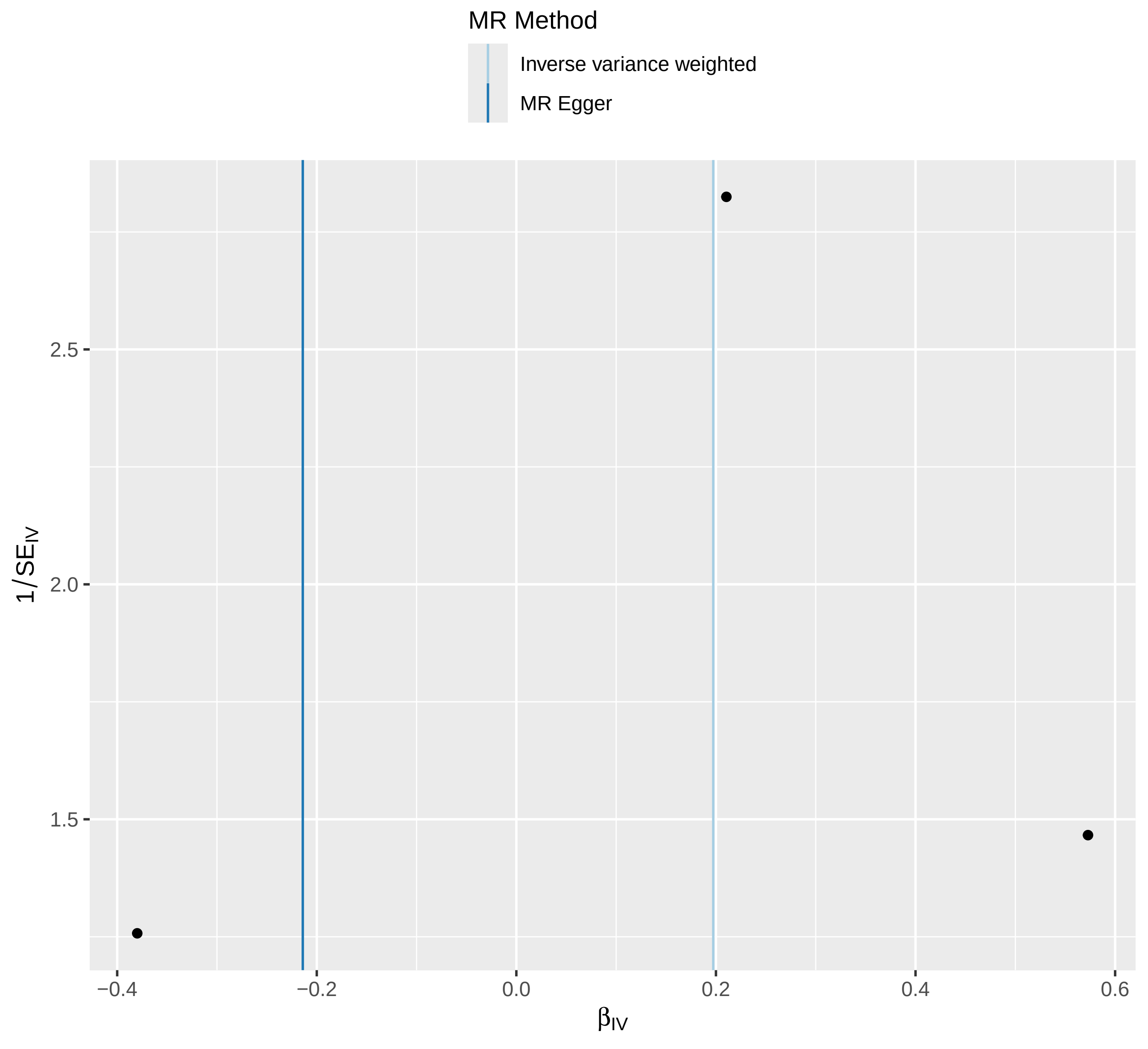

MR Method
Inverse variance weighted
MR Egger
1/SE_IV
2.5
2.0
1.5
−0.4
−0.2
0.0
0.2
0.4
0.6
β_IV

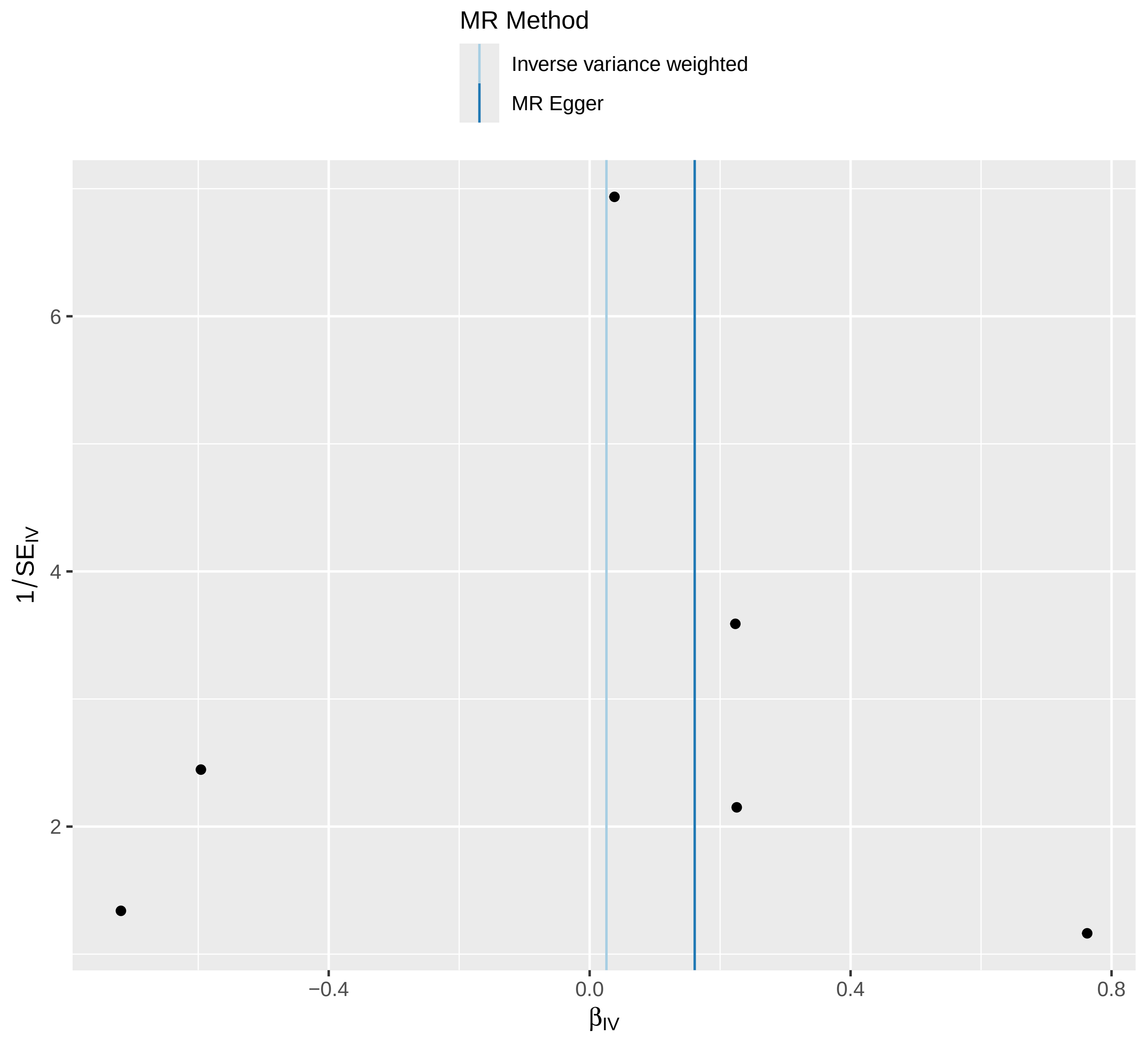
MR Method
Inverse variance weighted
MR Egger
6
4
2
$1/SE_{IV}$
−0.4
0.0
0.4
0.8
β_{IV}

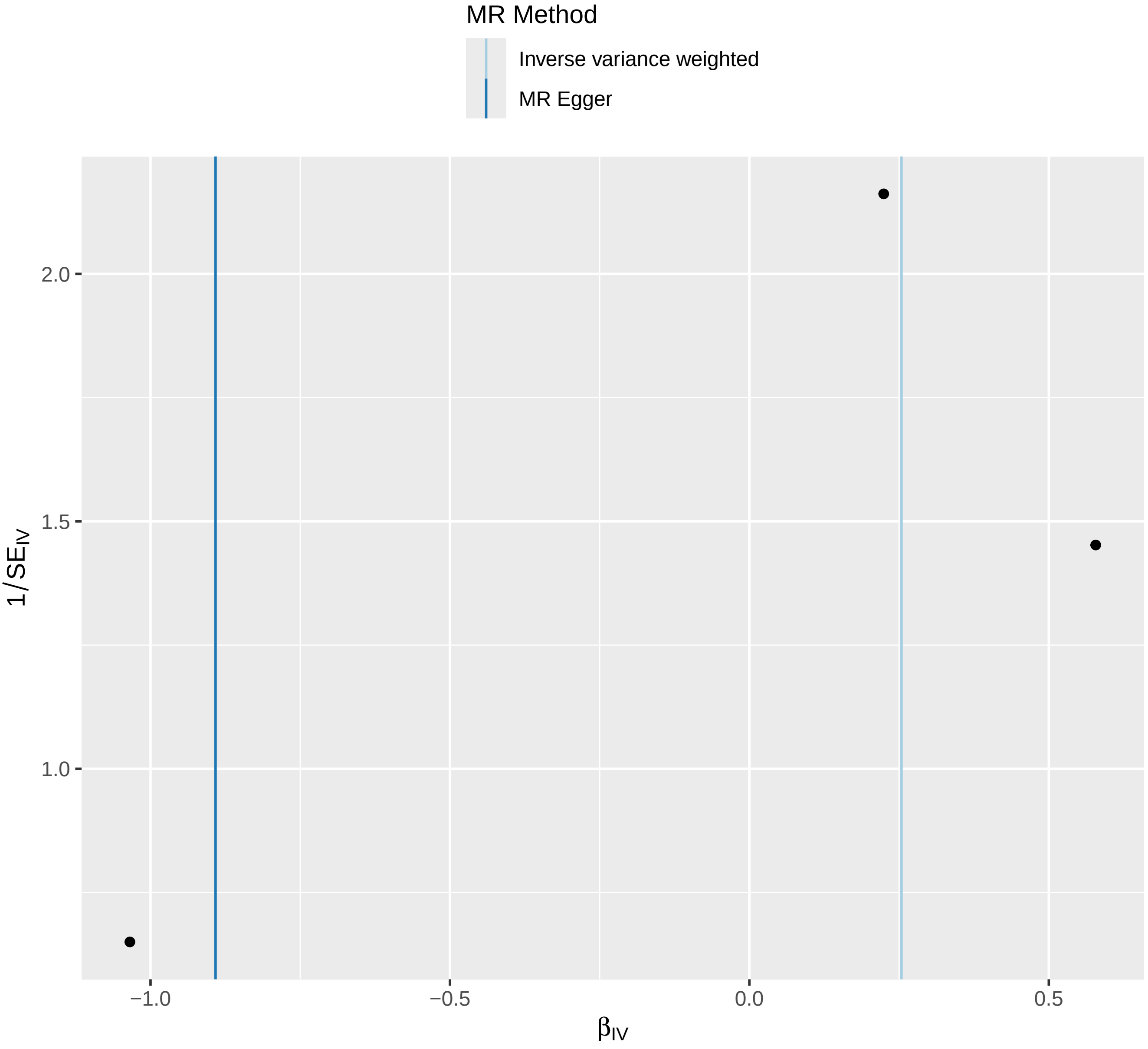
MR Method
Inverse variance weighted
MR Egger
2.0
1.5
1.0
1/SE_IV
−1.0
−0.5
0.0
0.5
β_IV

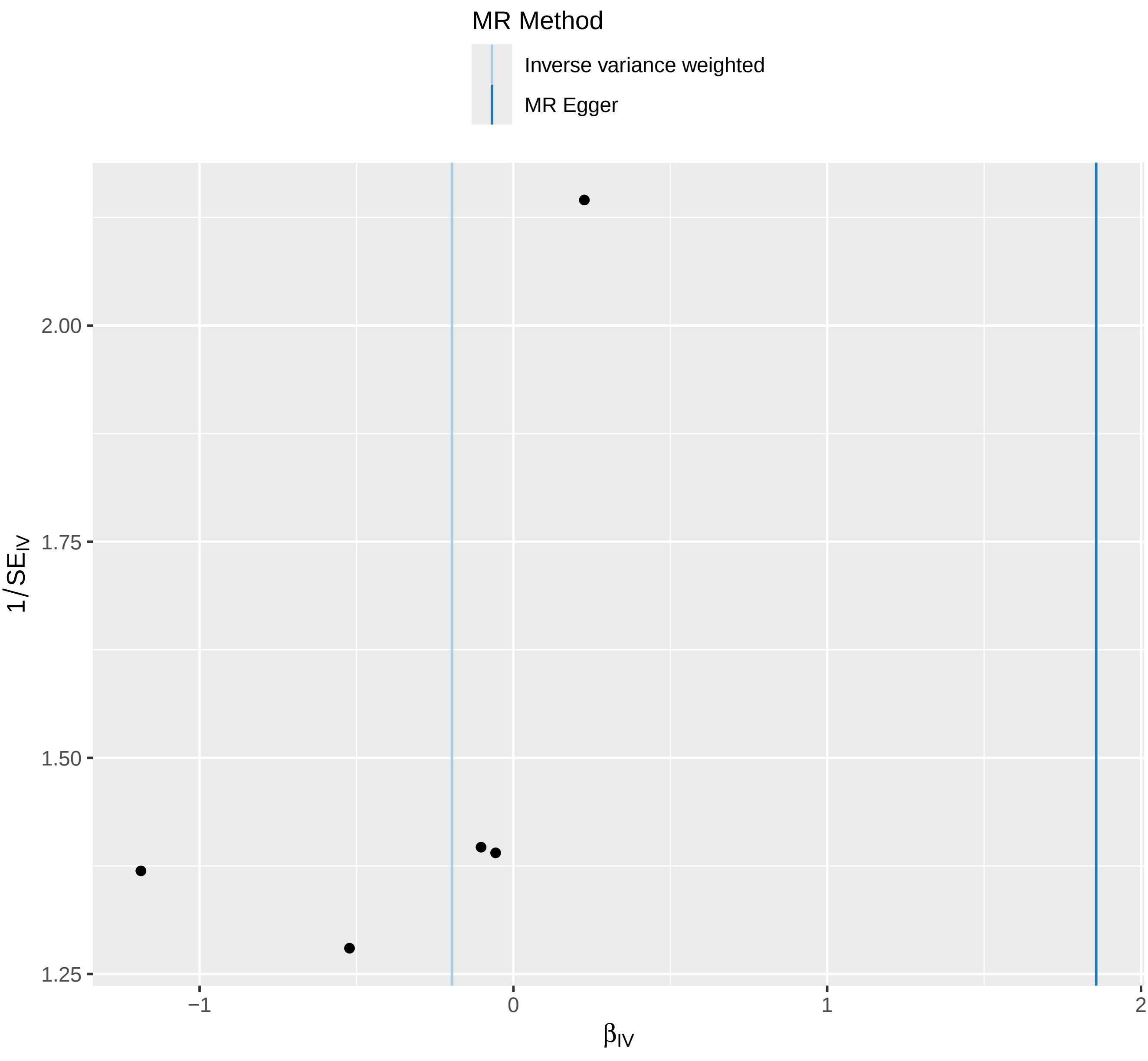

MR Method
Inverse variance weighted
MR Egger
2.00
1.75
1.50
1.25
1/SE_IV
−1
0
1
2
β_IV

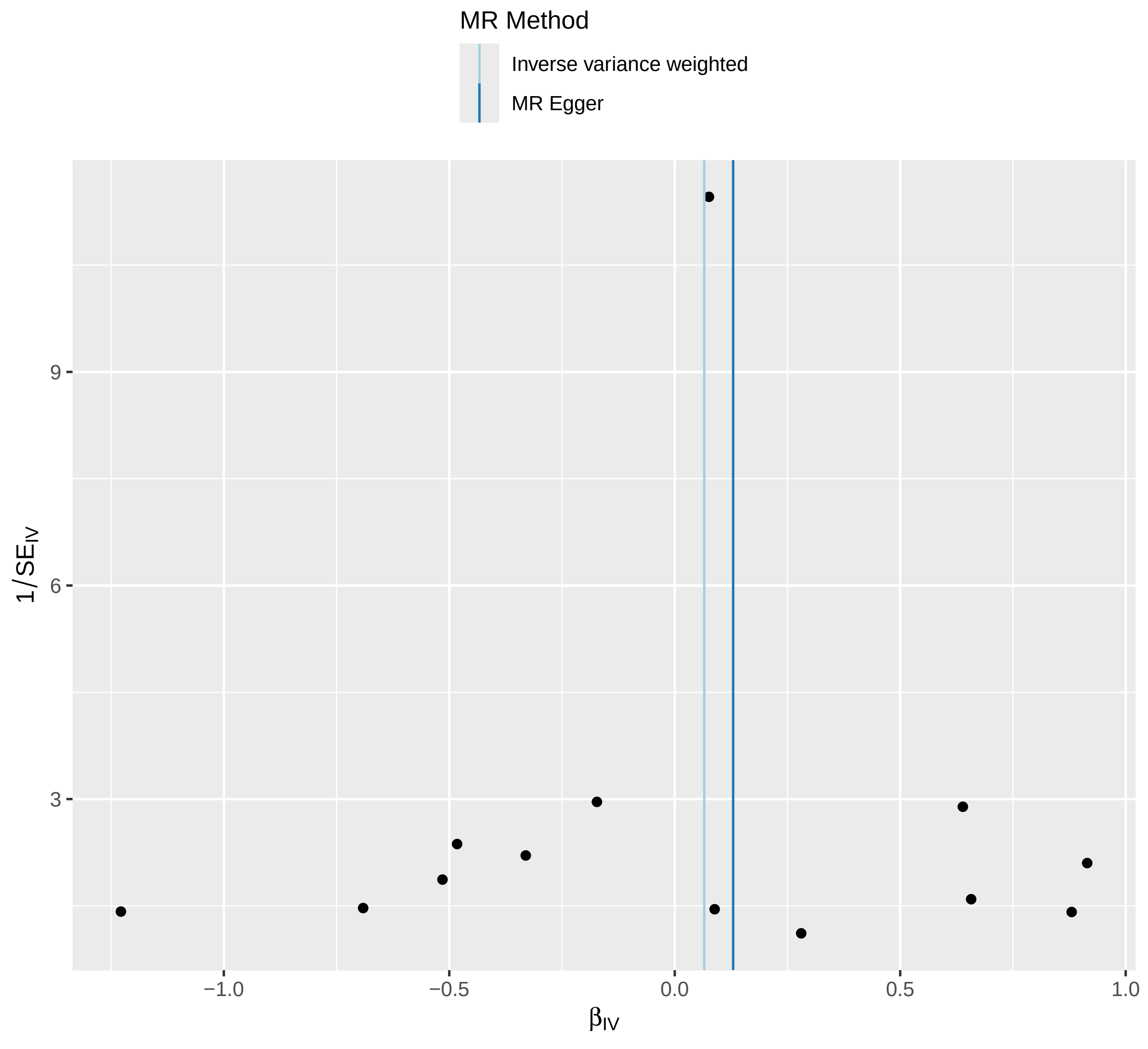
MR Method
Inverse variance weighted
MR Egger
9
6
3
1/SE$_{IV}$
−1.0
−0.5
0.0
0.5
1.0
β$_{IV}$

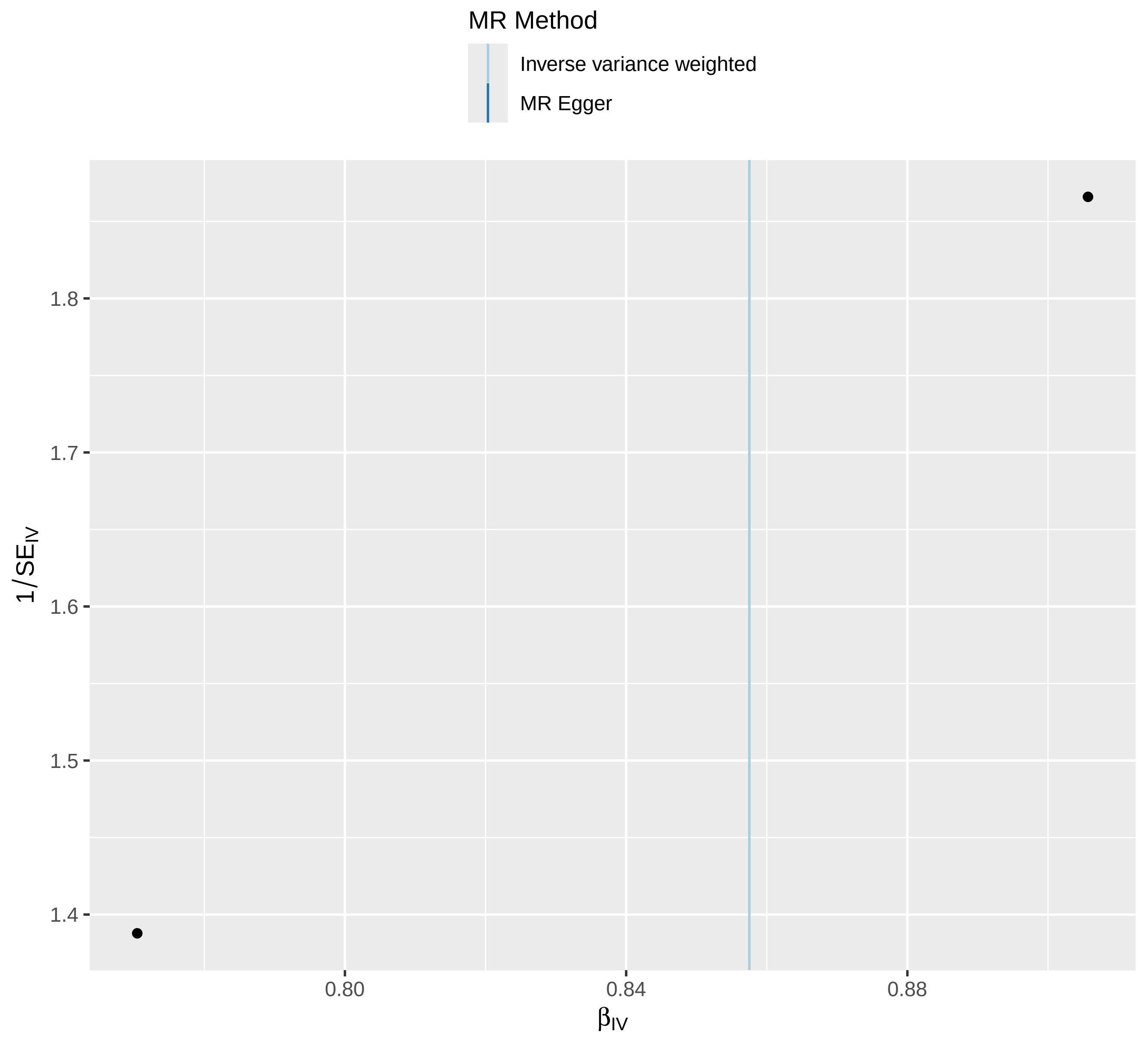
MR Method
Inverse variance weighted
MR Egger
1.8
1.7
1.6
1.5
1.4
$1/SE_{IV}$
0.80
0.84
0.88
β_{IV}

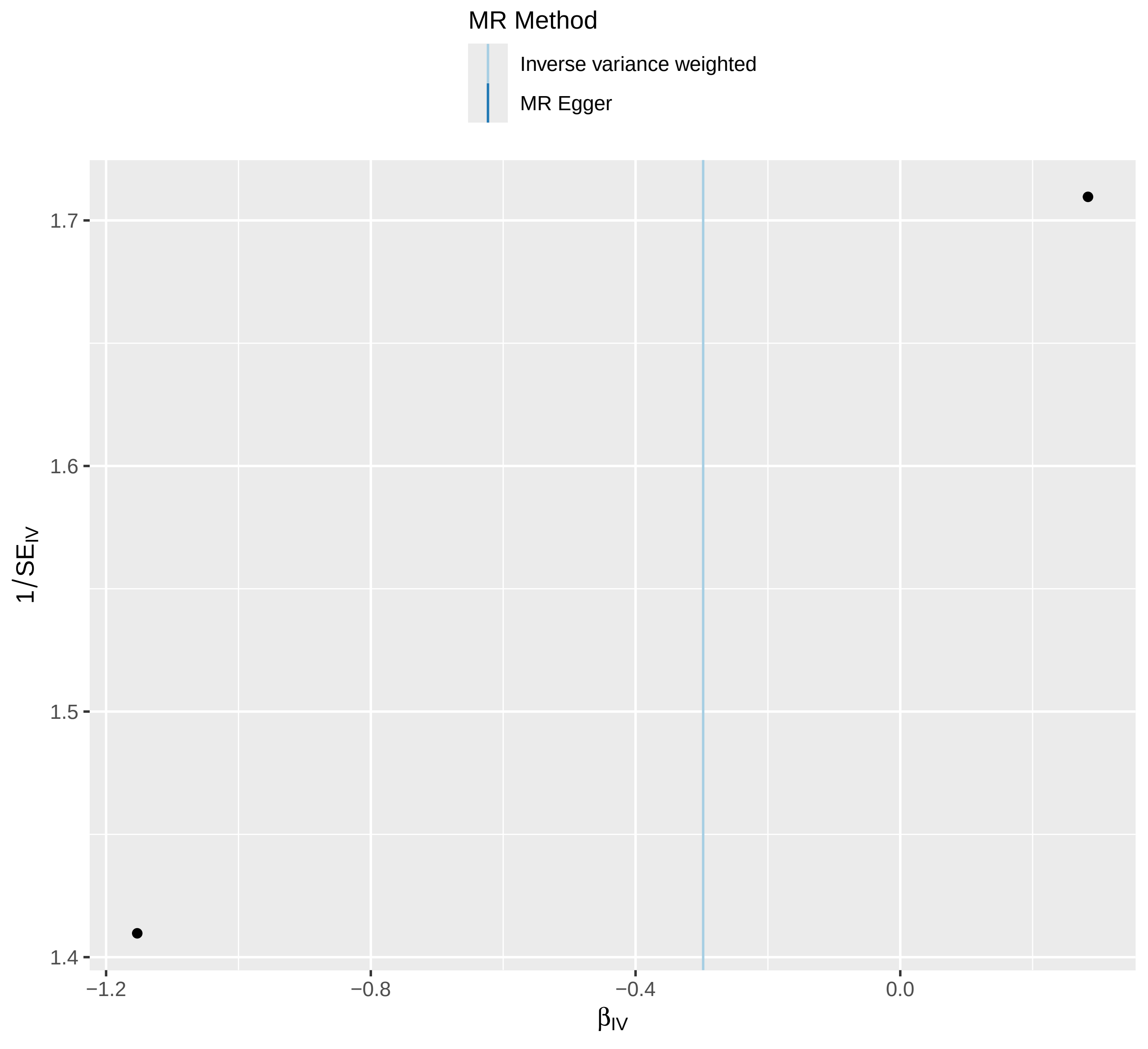

MR Method
Inverse variance weighted
MR Egger
1.7
1.6
1.5
1.4
1/SE_IV
−1.2
−0.8
−0.4
0.0
β_IV

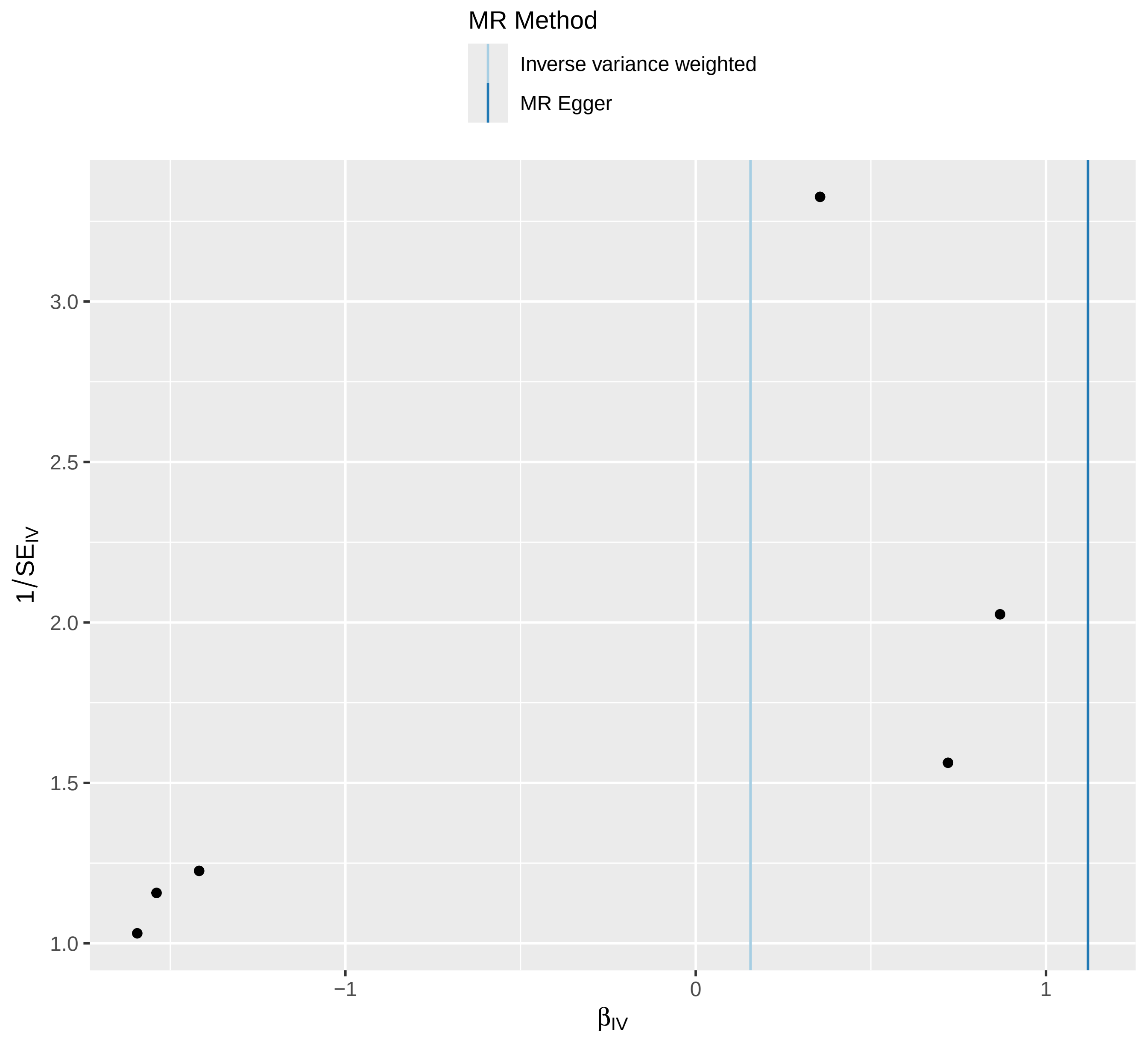
MR Method
Inverse variance weighted
MR Egger
3.0
2.5
2.0
1.5
1.0
1/SE_IV
−1
0
1
β_IV

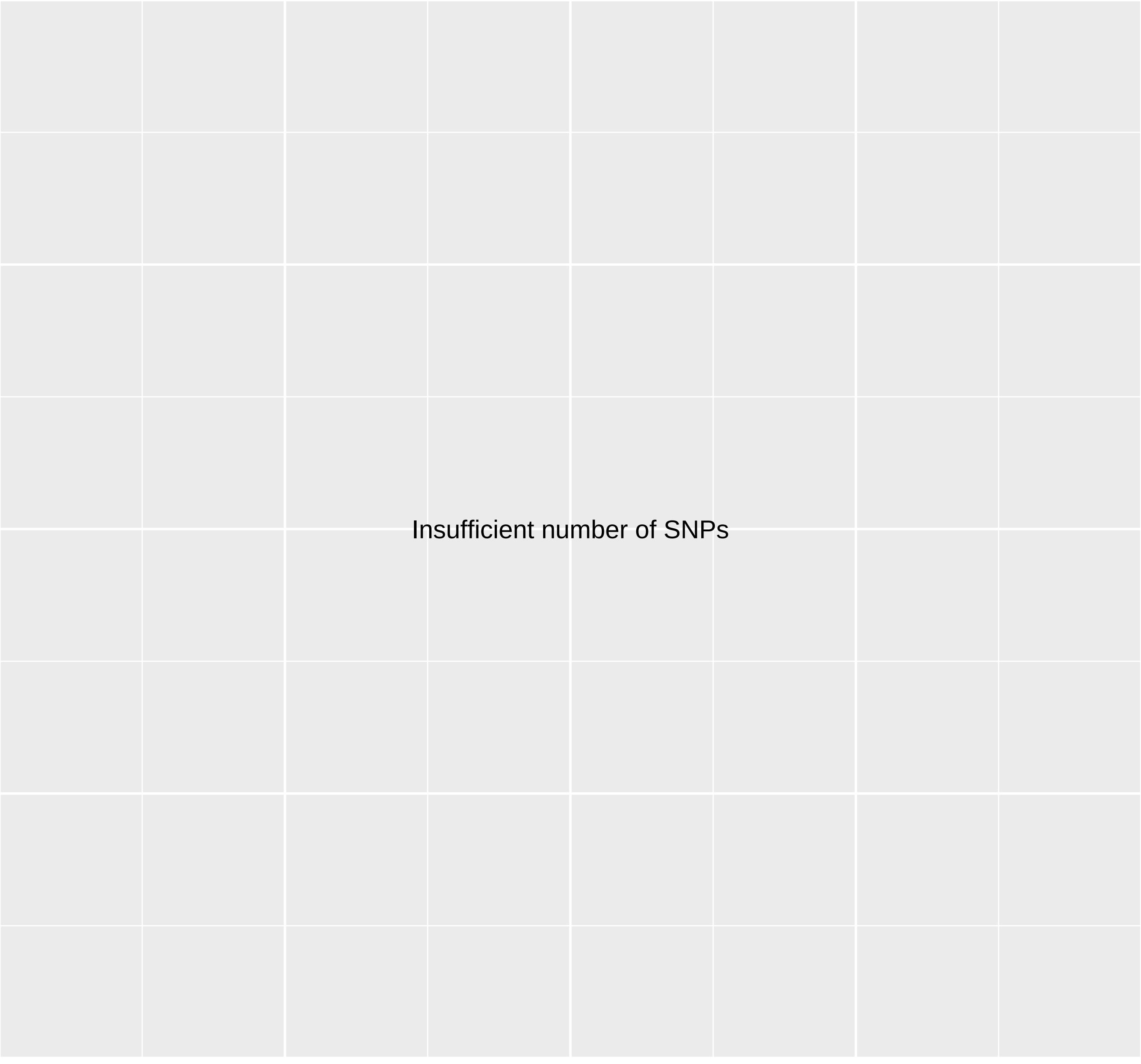
Insufficient number of SNPs

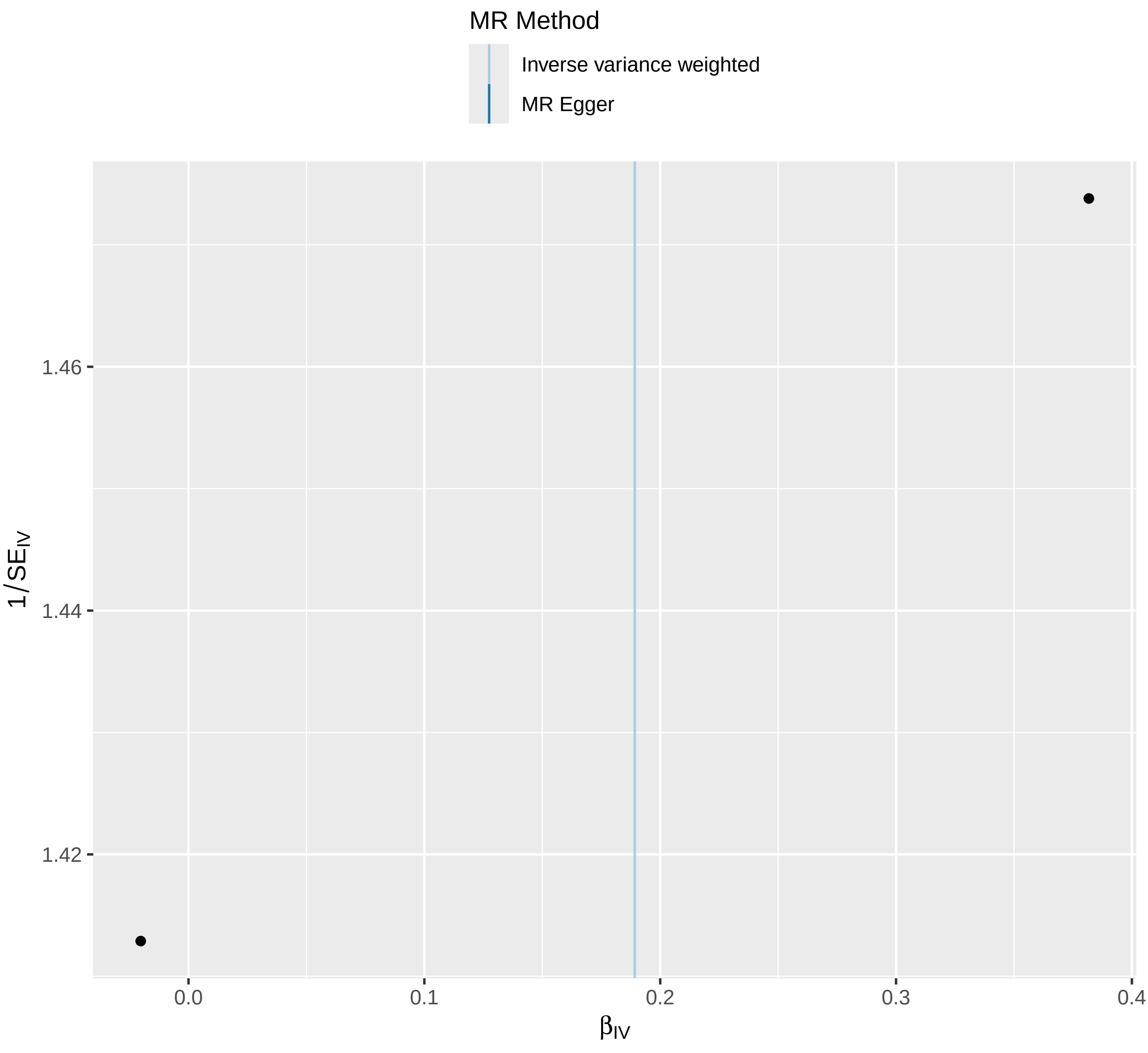
MR Method
Inverse variance weighted
MR Egger
1.46
1.44
1.42
1/SE_IV
0.0
0.1
0.2
0.3
0.4
β_IV

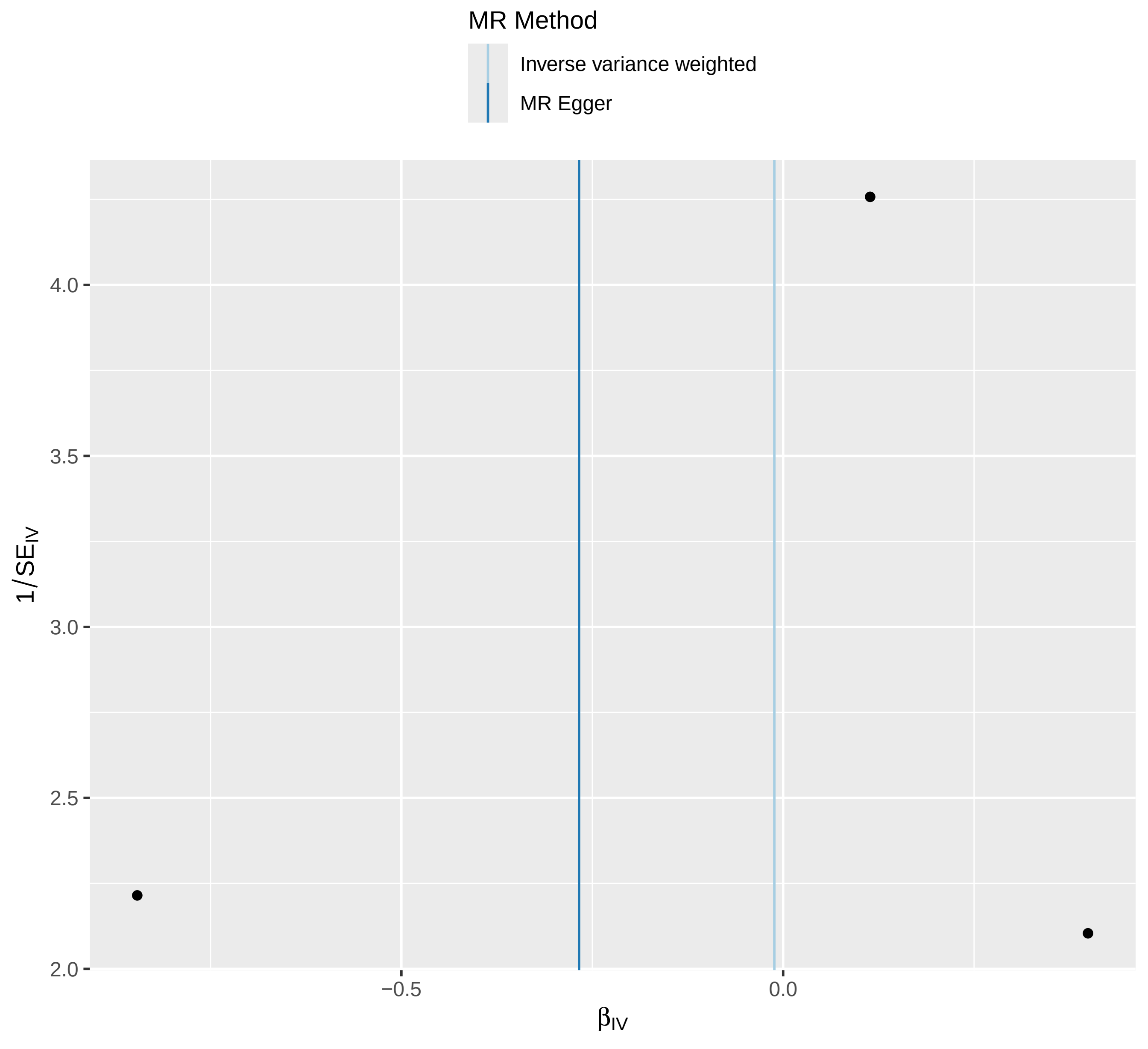

MR Method
Inverse variance weighted
MR Egger
1/SE$_{IV}$
4.0
3.5
3.0
2.5
2.0
−0.5
0.0
β$_{IV}$

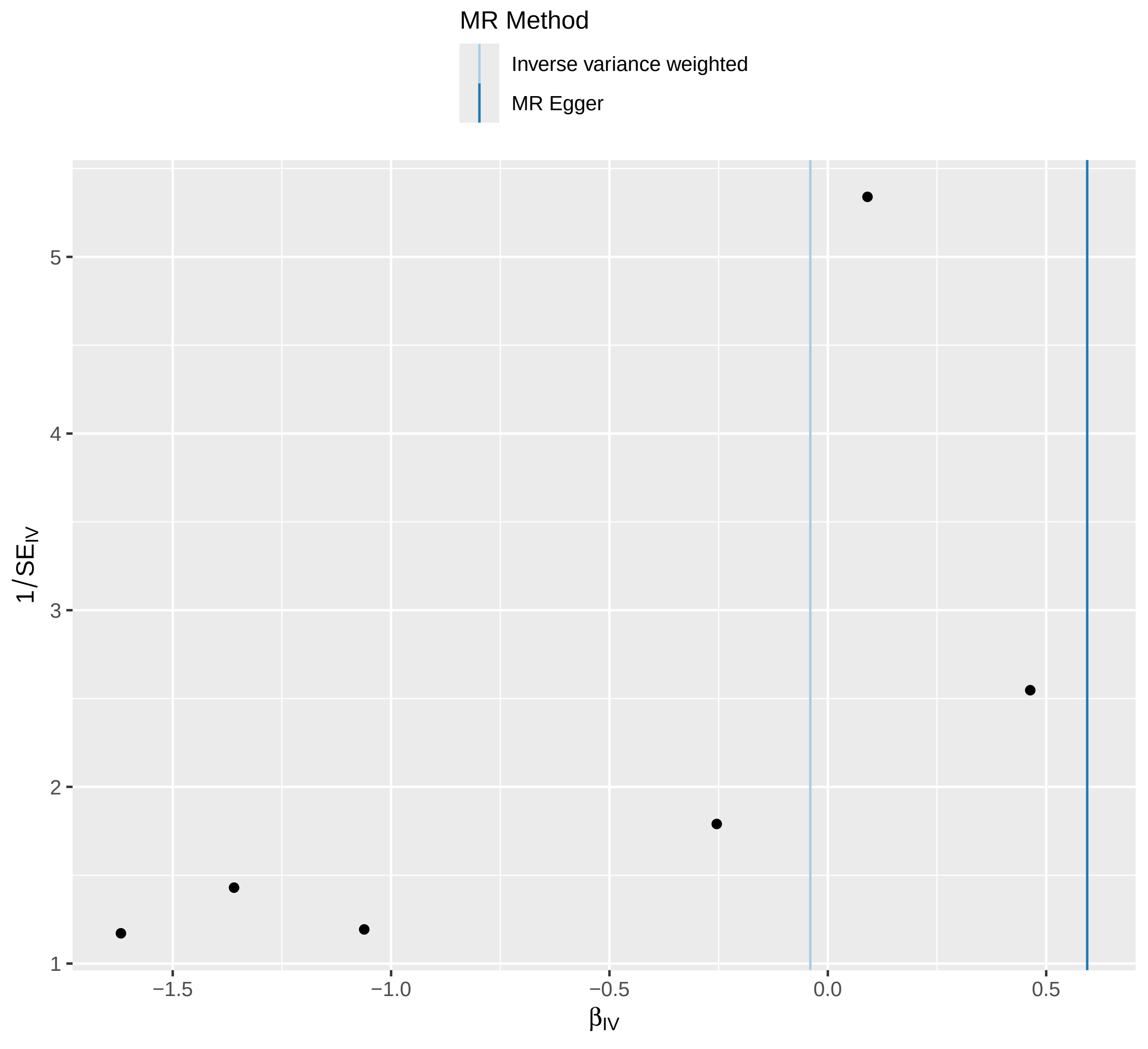

MR Method
Inverse variance weighted
MR Egger
1/SE_IV
5
4
3
2
1
−1.5
−1.0
−0.5
0.0
0.5
β_IV

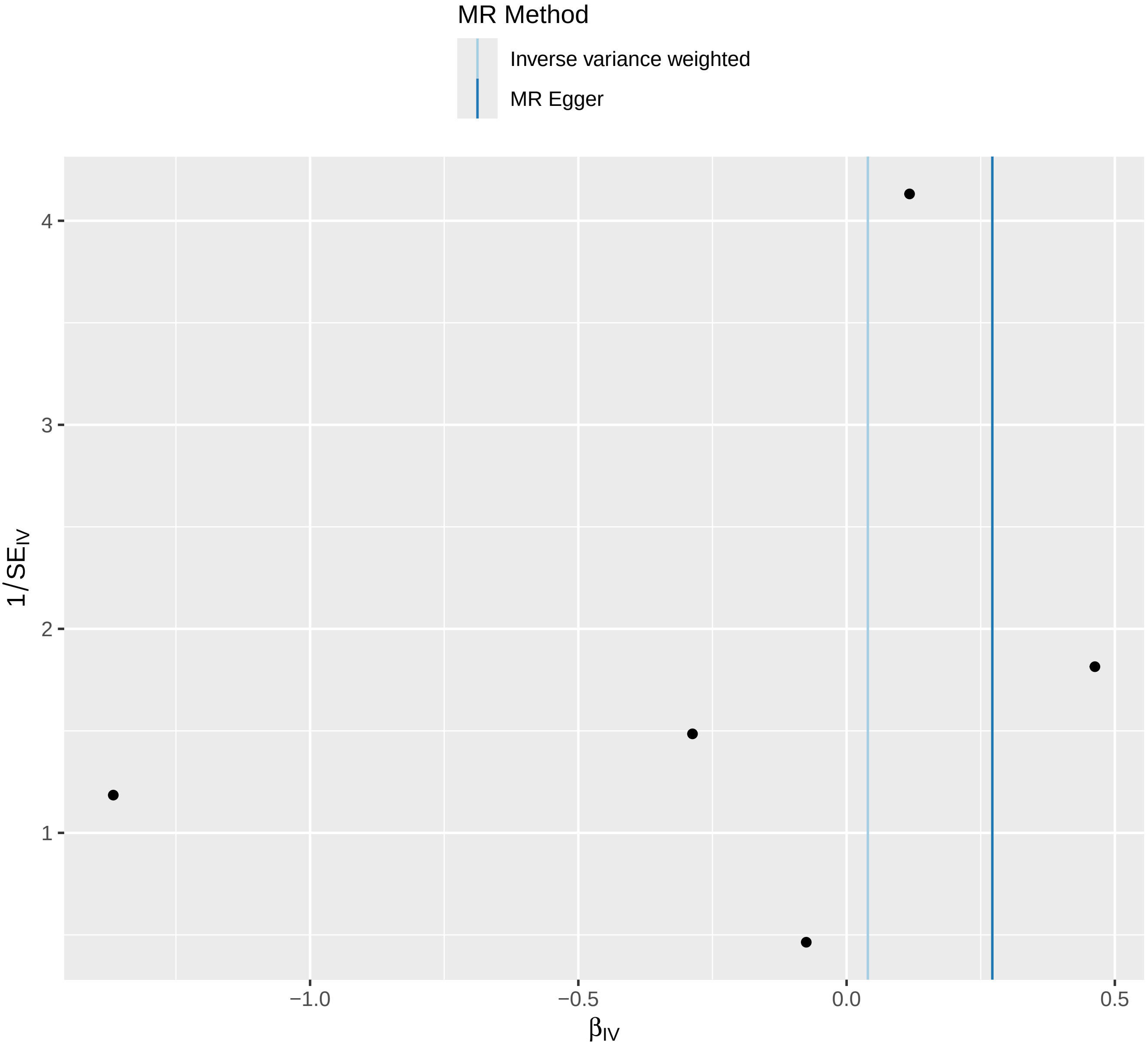
MR Method
Inverse variance weighted
MR Egger
1/SE_IV
4
3
2
1
−1.0
−0.5
0.0
0.5
β_IV

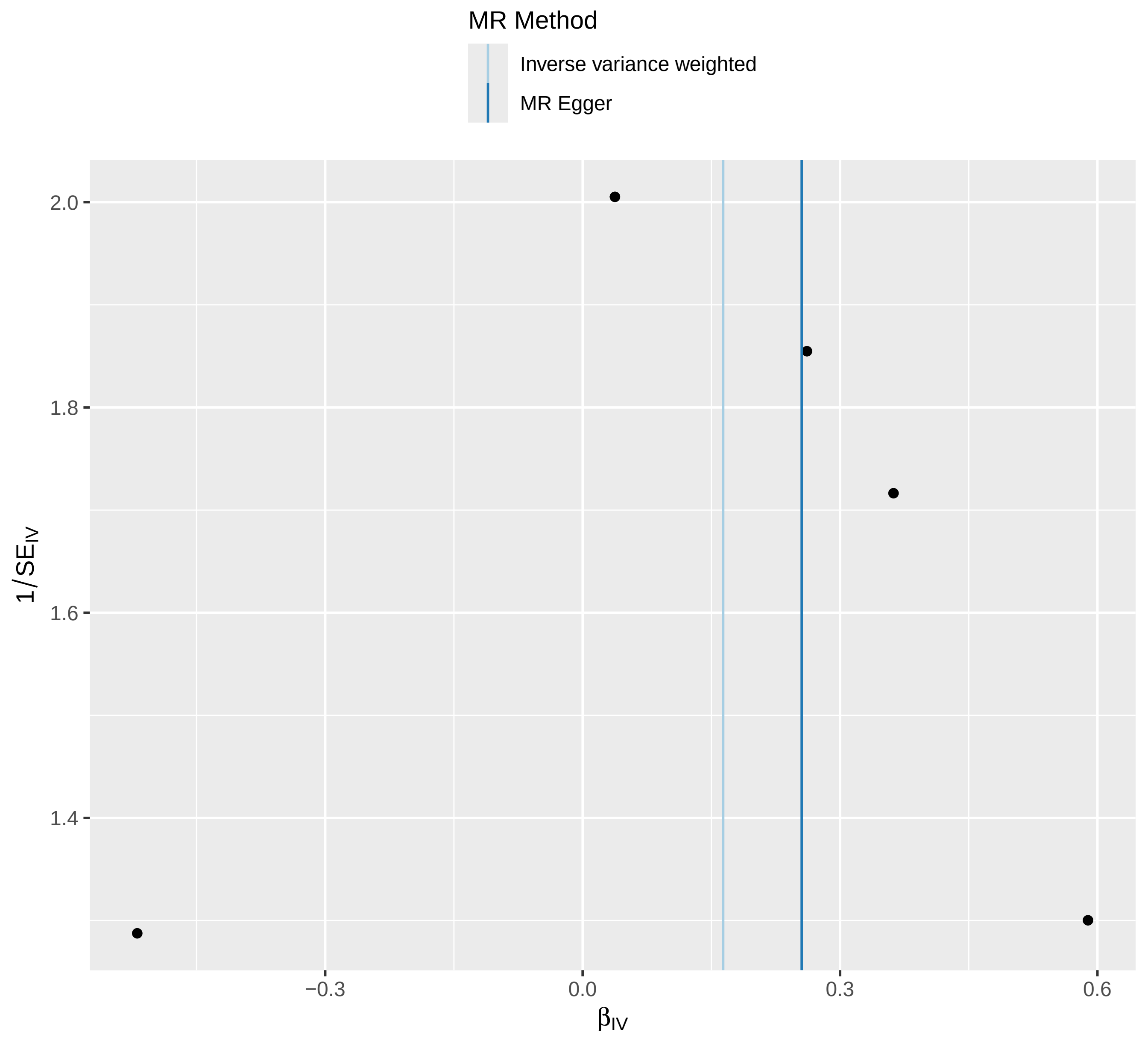
MR Method
Inverse variance weighted
MR Egger
2.0
1.8
1.6
1.4
1/SE_IV
−0.3
0.0
0.3
0.6
β_IV

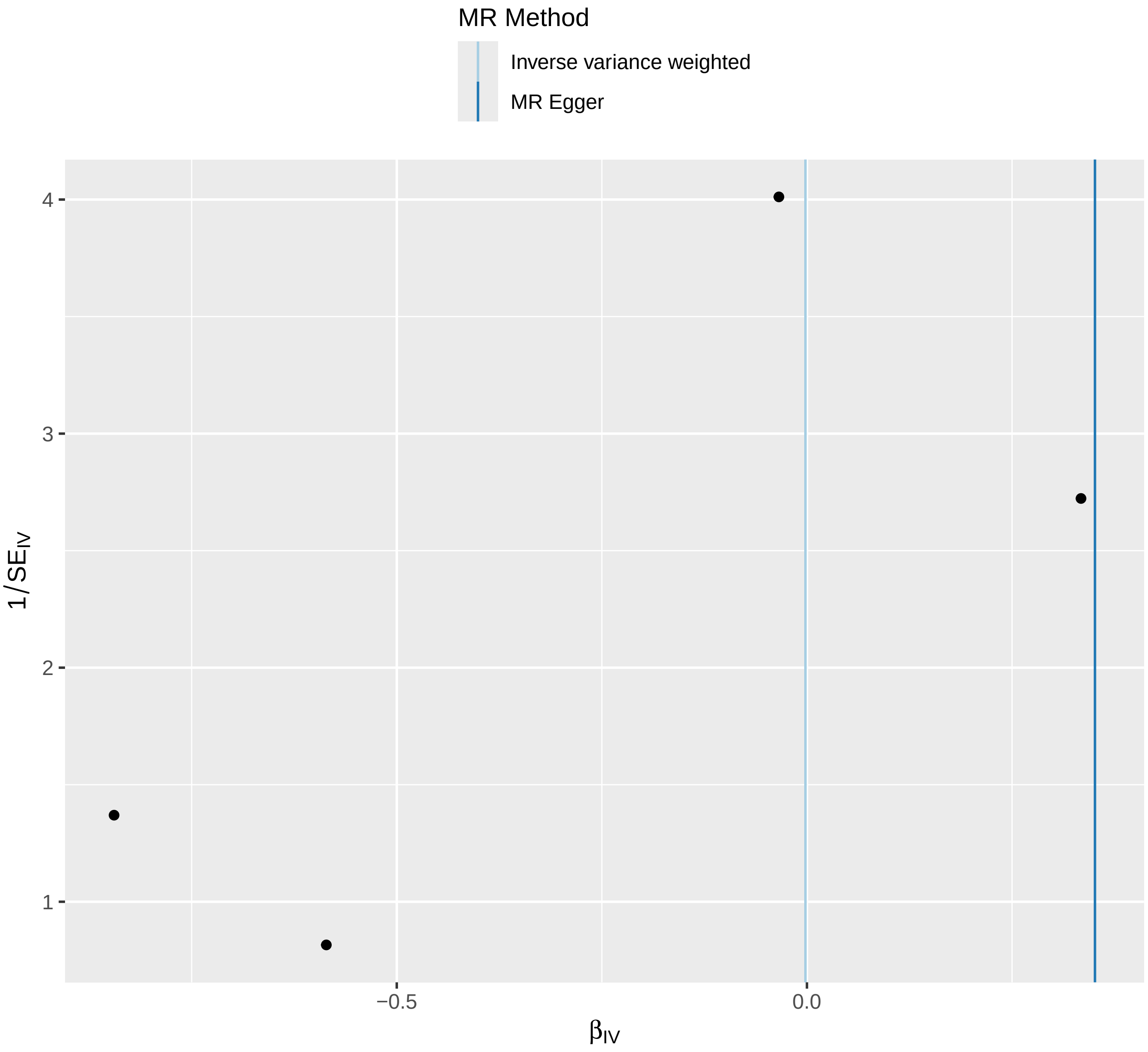
MR Method
Inverse variance weighted
MR Egger
4
3
2
1
1/SE$_{IV}$
−0.5
0.0
β_{IV}

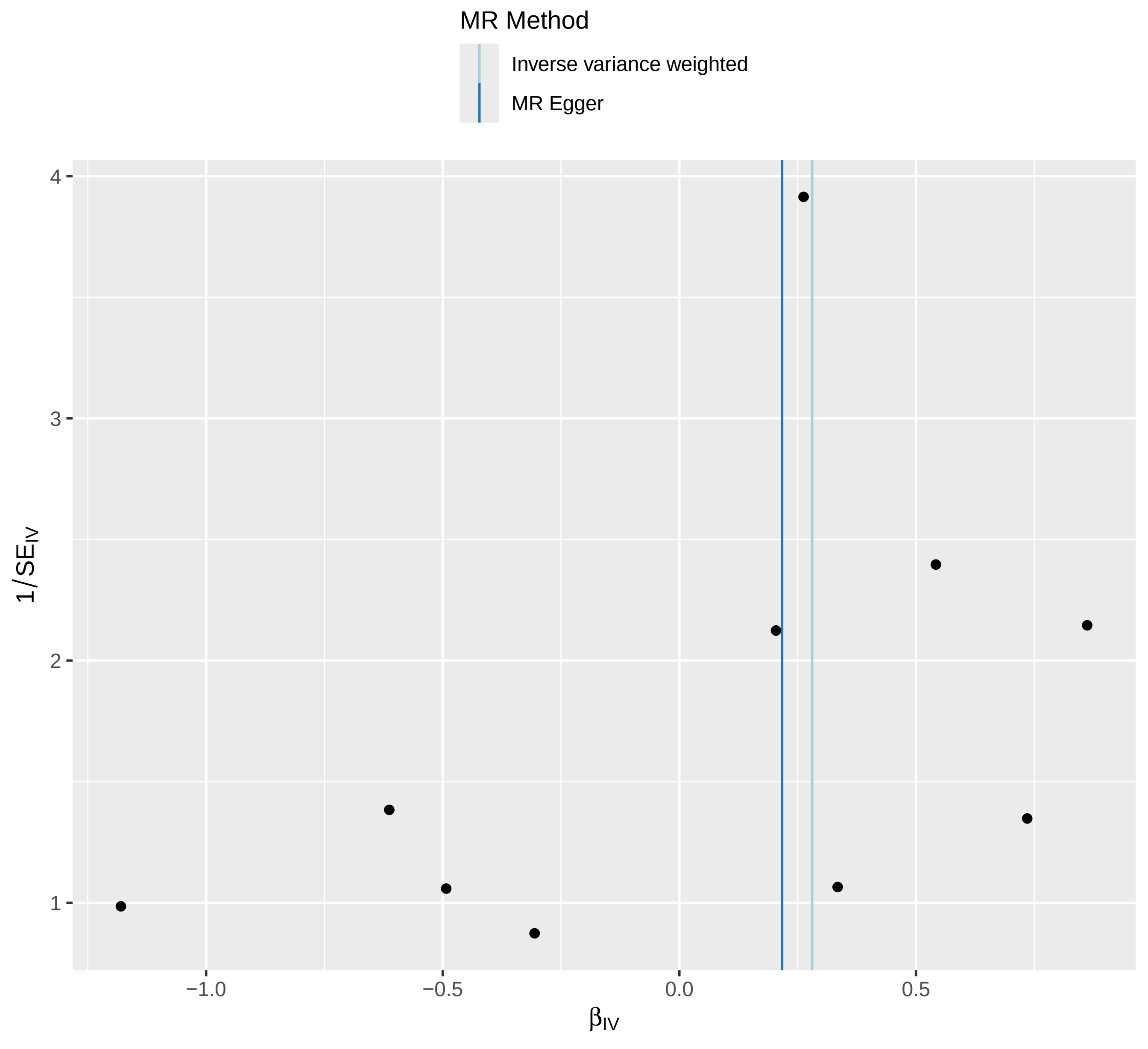

MR Method
Inverse variance weighted
MR Egger
4
3
2
1
1/SE$_{IV}$
−1.0
−0.5
0.0
0.5
β$_{IV}$

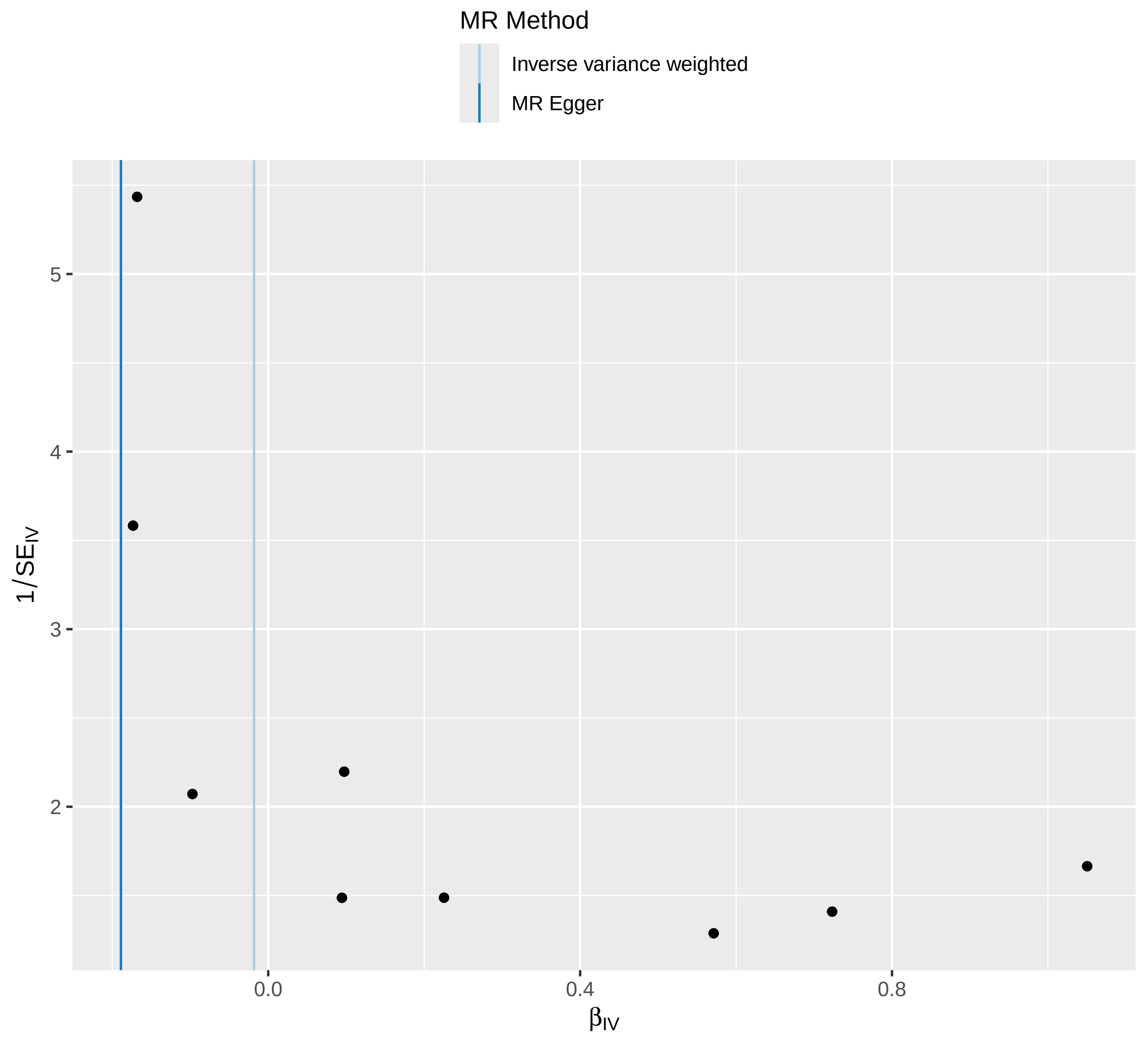
MR Method
Inverse variance weighted
MR Egger
5
4
3
2
1/SE_IV
0.0
0.4
0.8
β_IV

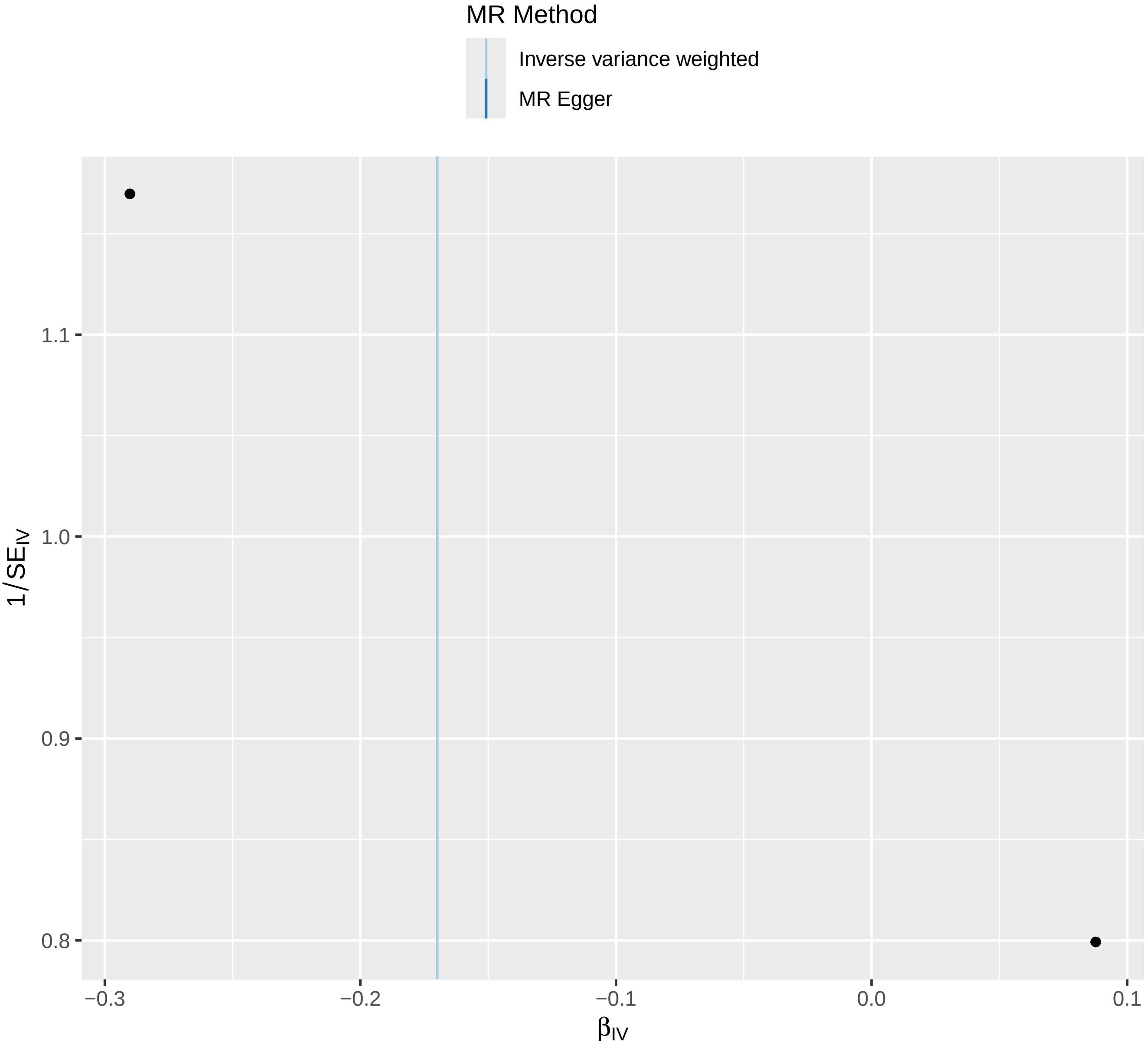
MR Method
Inverse variance weighted
MR Egger
1.1
1.0
0.9
0.8
$1/SE_{IV}$
−0.3
−0.2
−0.1
0.0
0.1
β_{IV}

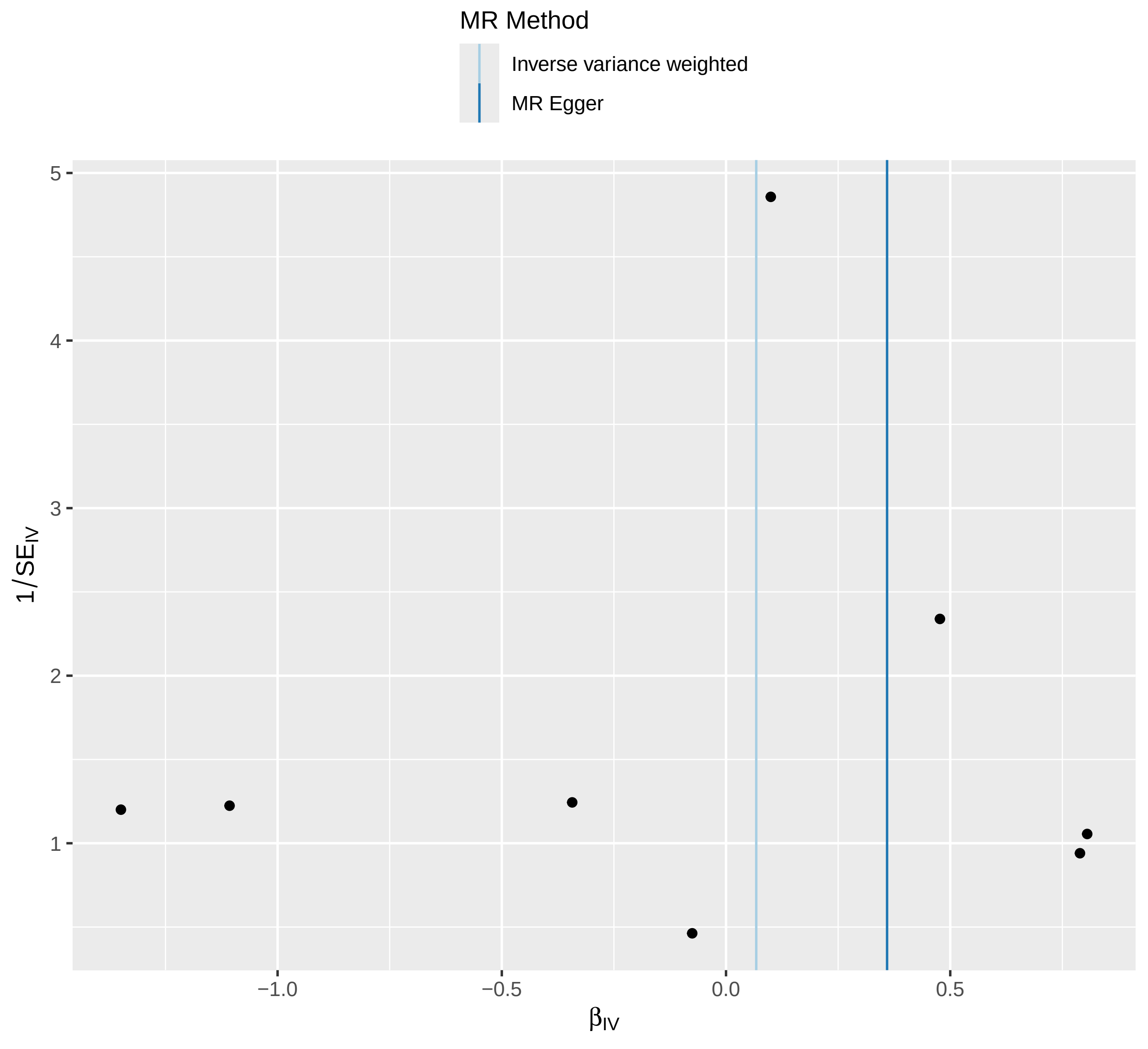
MR Method
Inverse variance weighted
MR Egger
5
4
3
2
1
1/SE_IV
−1.0
−0.5
0.0
0.5
β_IV

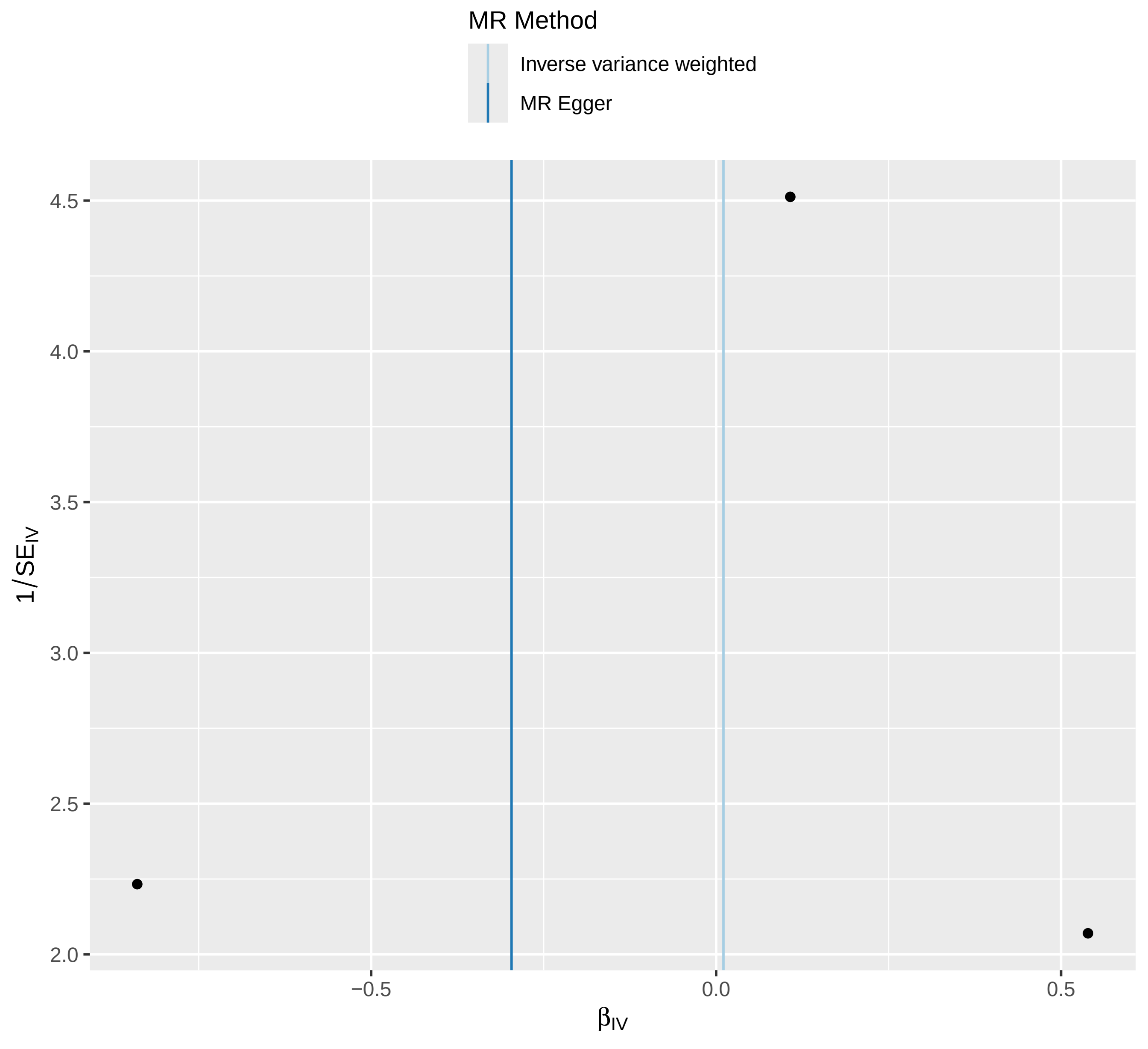
MR Method
Inverse variance weighted
MR Egger
4.5
4.0
3.5
3.0
2.5
2.0
1/SE$_{IV}$
−0.5
0.0
0.5
β$_{IV}$

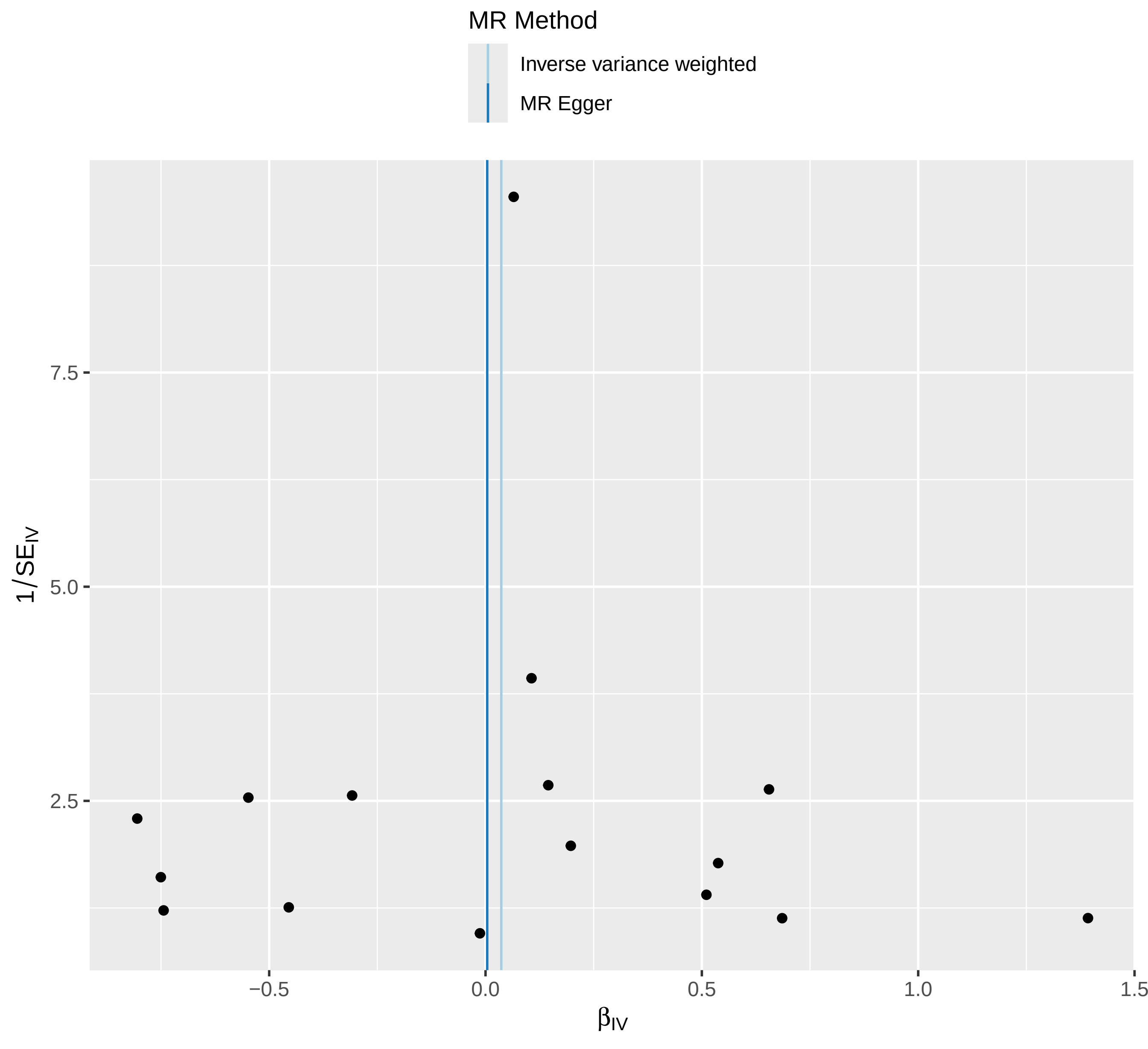

MR Method
Inverse variance weighted
MR Egger
7.5
5.0
2.5
1/SE_IV
−0.5
0.0
0.5
1.0
1.5
β_IV

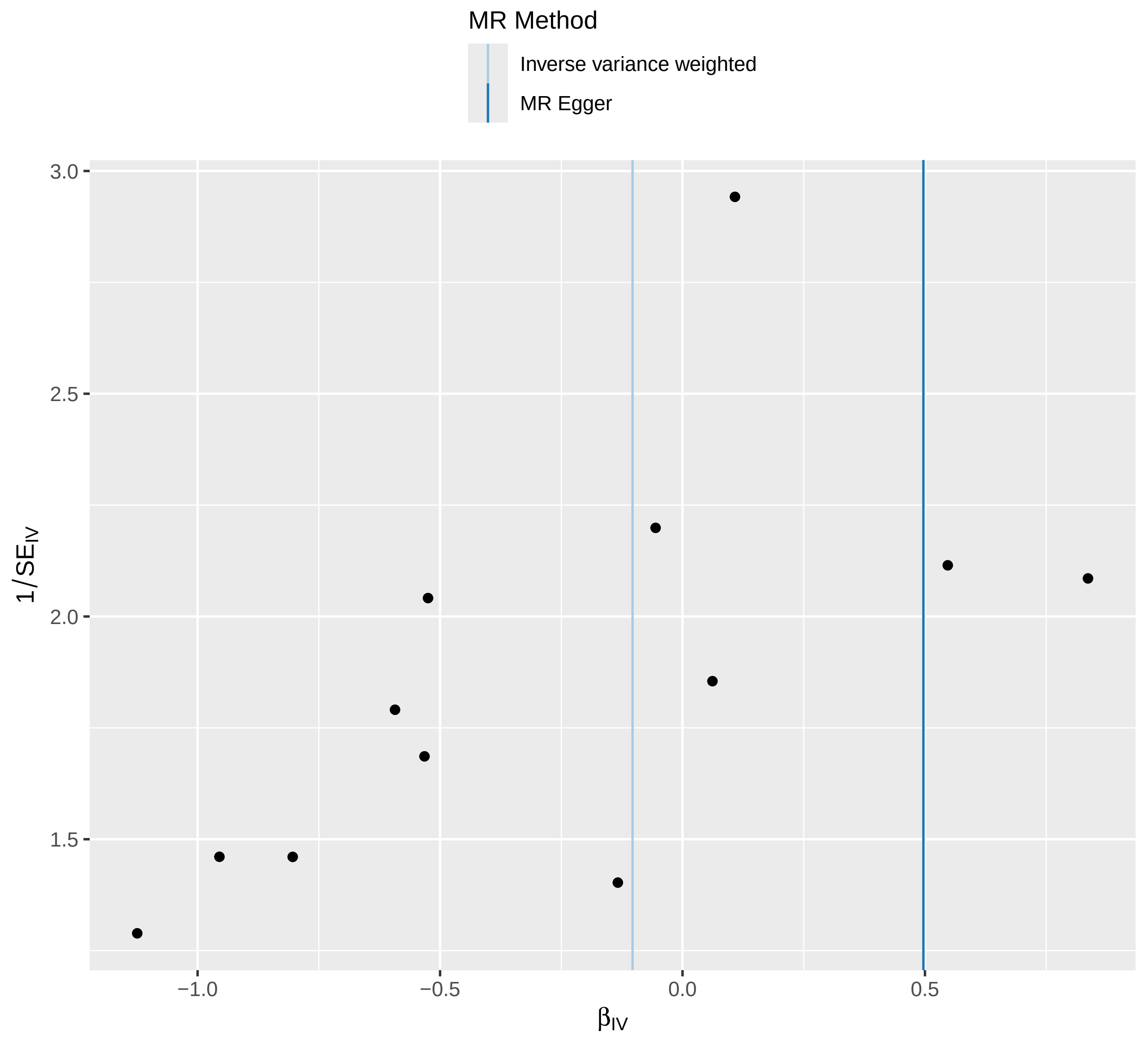
MR Method
Inverse variance weighted
MR Egger
3.0
2.5
2.0
1.5
1/SE$_{IV}$
−1.0
−0.5
0.0
0.5
β$_{IV}$

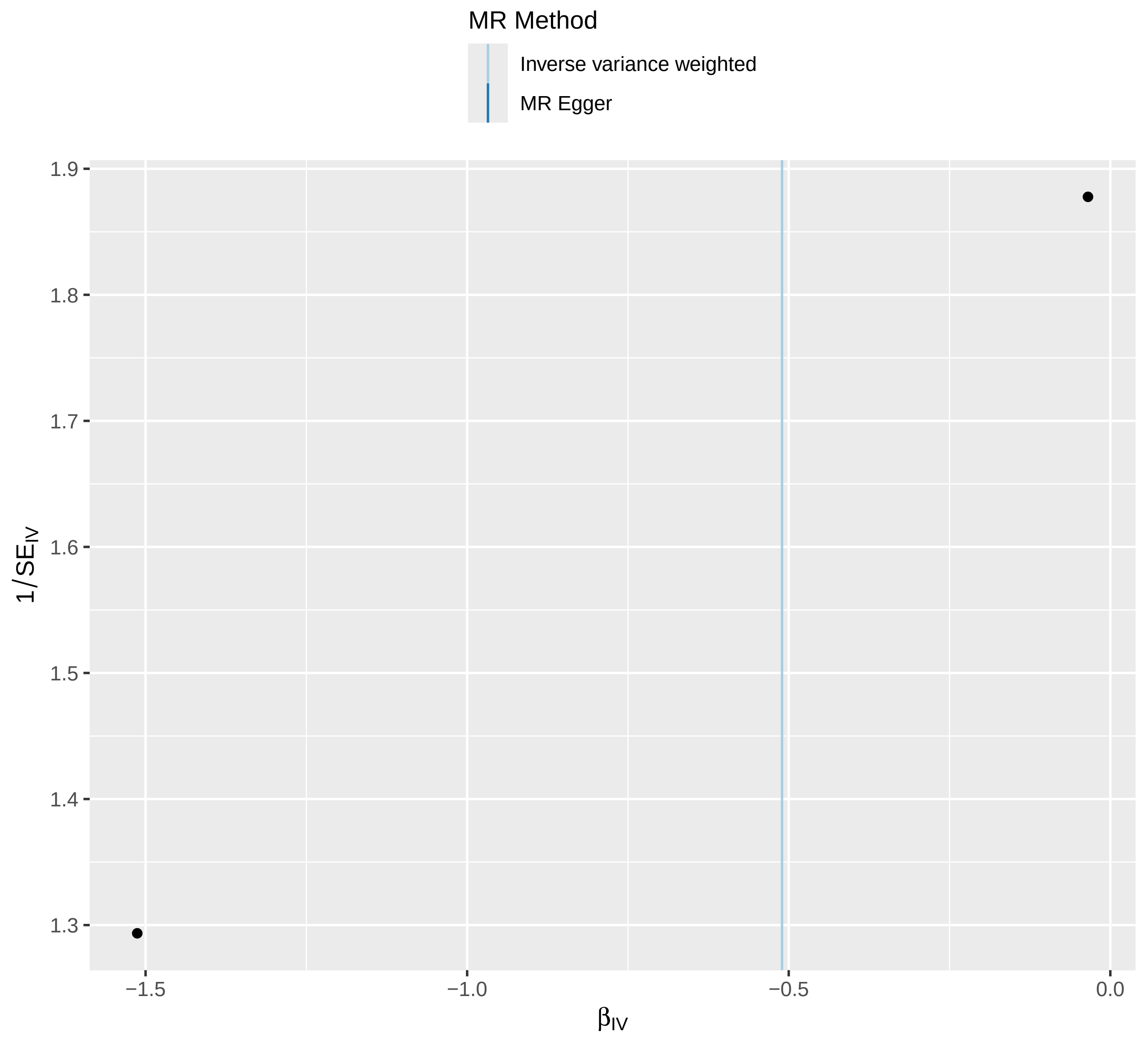
MR Method
Inverse variance weighted
MR Egger
1.9
1.8
1.7
1.6
1.5
1.4
1.3
1/SE$_{IV}$
−1.5
−1.0
−0.5
0.0
β$_{IV}$

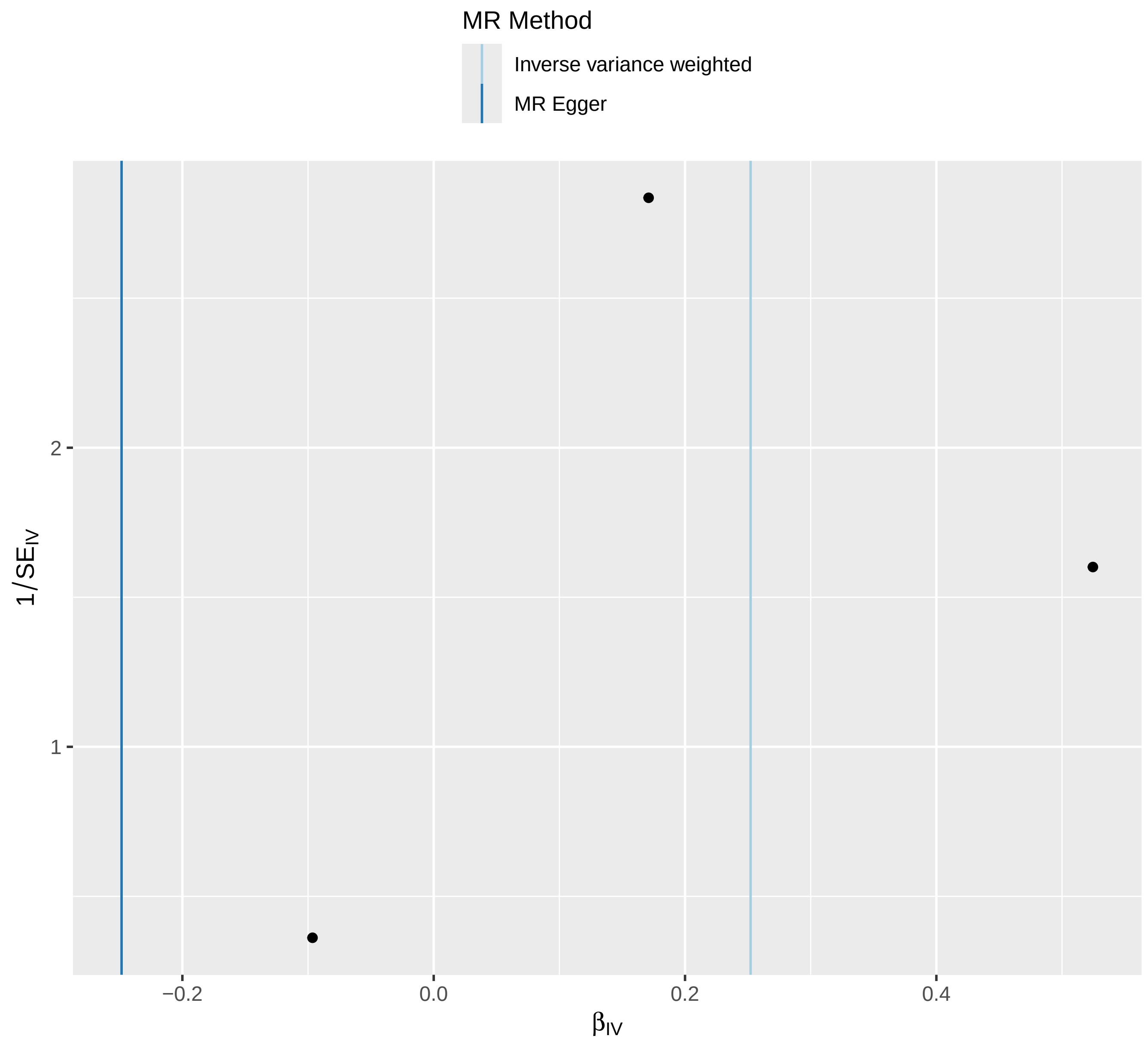
MR Method
Inverse variance weighted
MR Egger
1/SE_IV
2
1
−0.2
0.0
0.2
0.4
β_IV

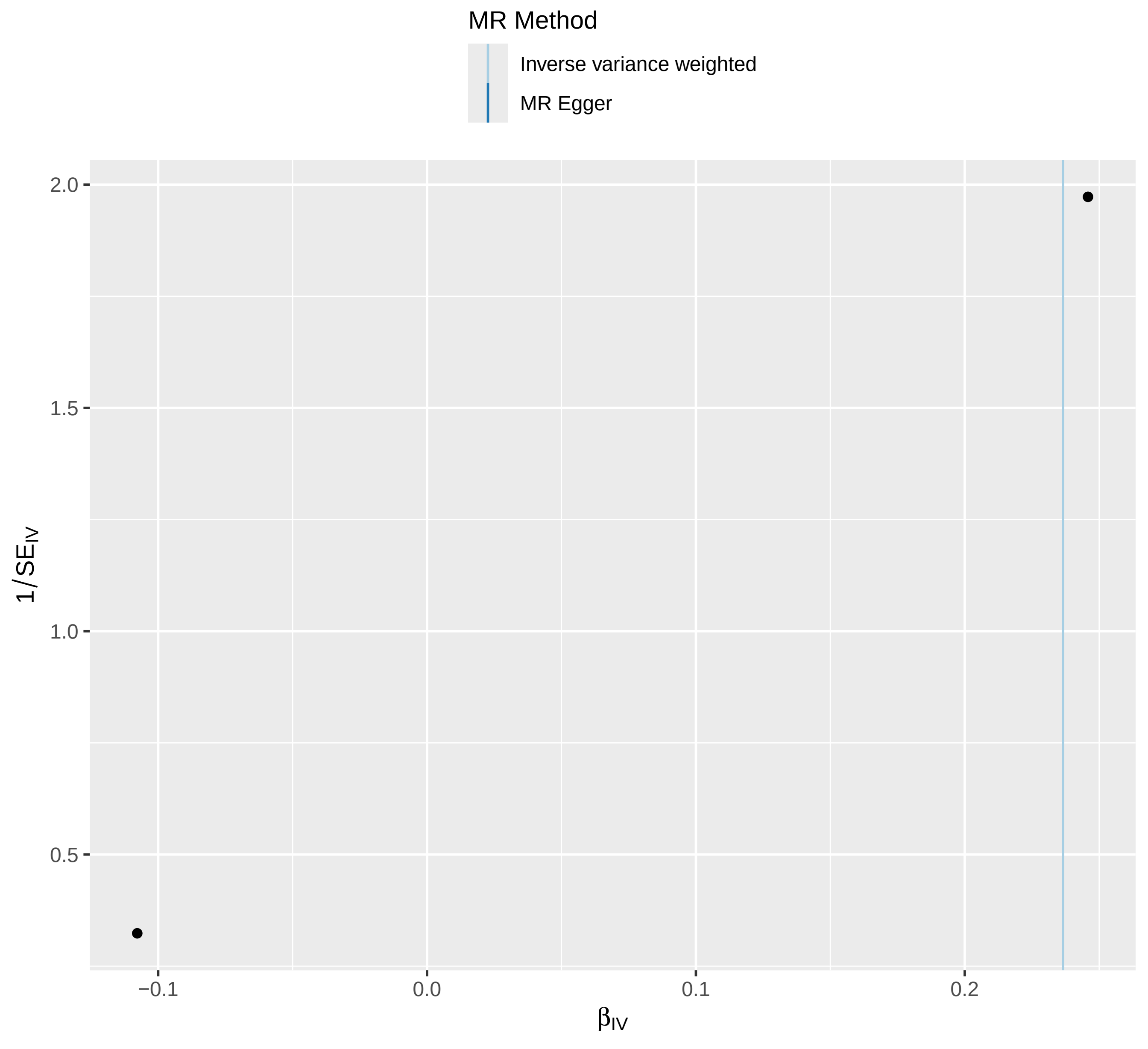
MR Method
Inverse variance weighted
MR Egger
2.0
1.5
1.0
0.5
$1/SE_{IV}$
−0.1
0.0
0.1
0.2
β_{IV}

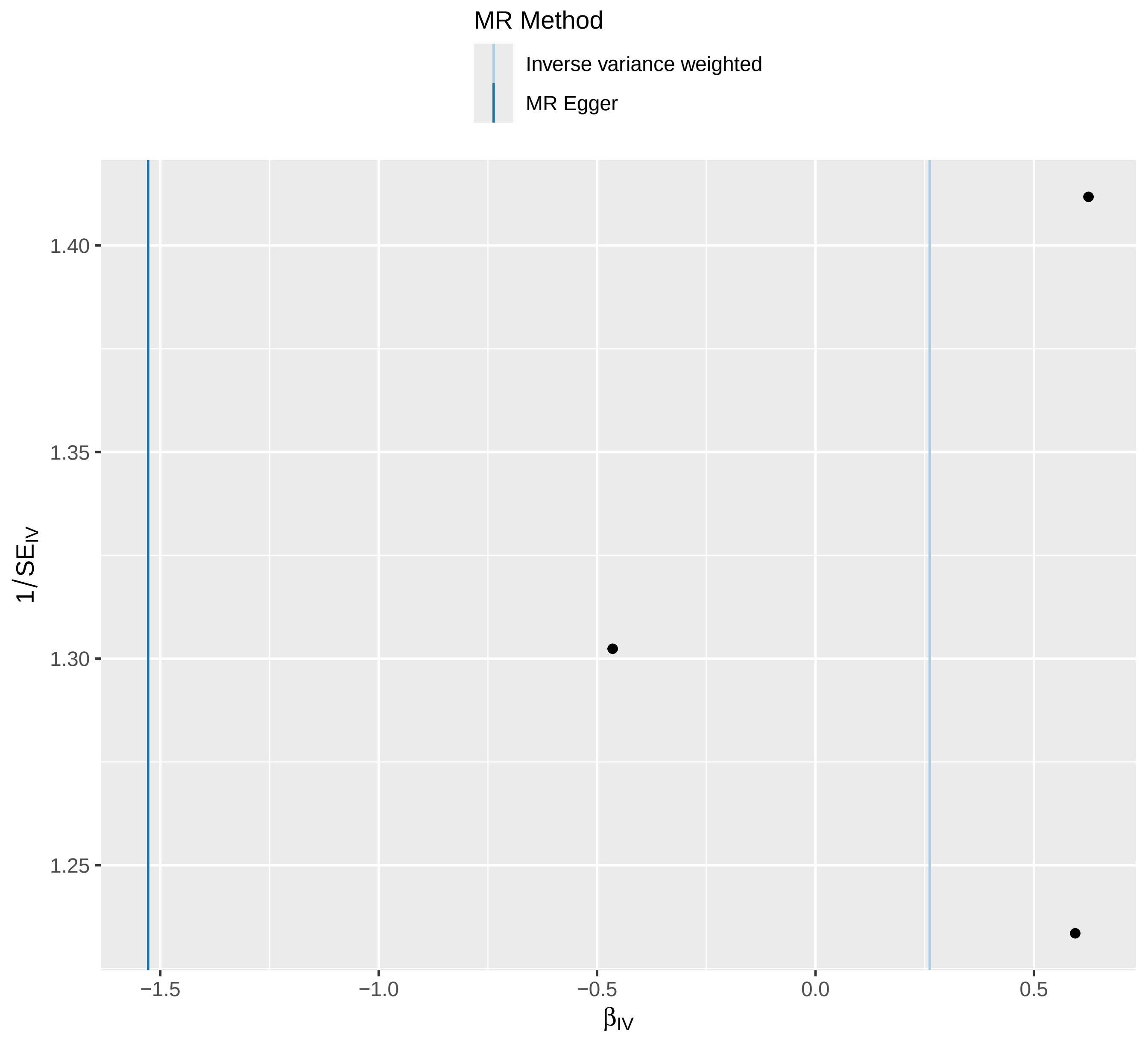
MR Method
Inverse variance weighted
MR Egger
1.40
1.35
1.30
1.25
$1/SE_{IV}$
−1.5
−1.0
−0.5
0.0
0.5
β_{IV}

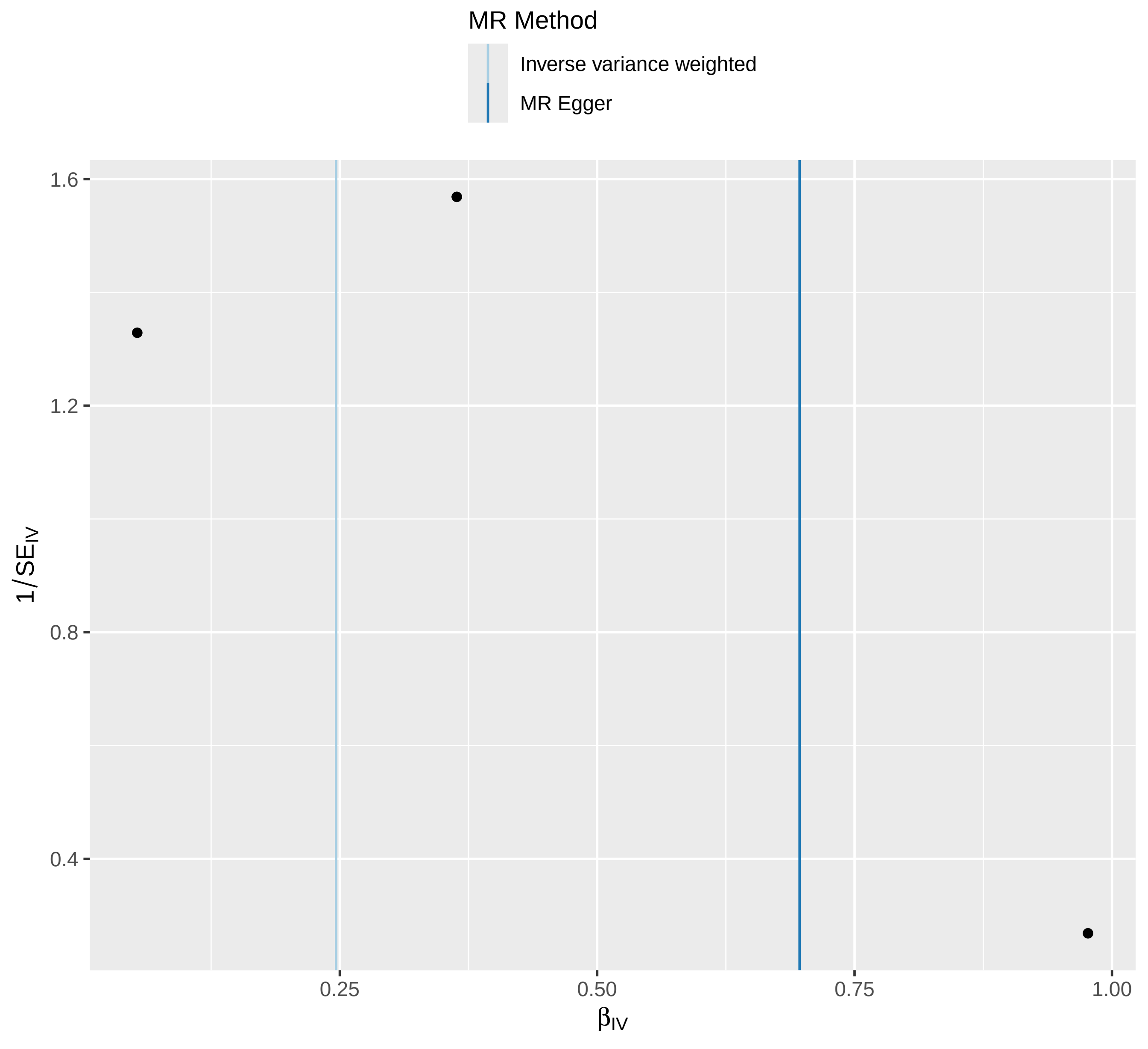

MR Method
Inverse variance weighted
MR Egger
1.6
1.2
0.8
0.4
1/SE_IV
0.25
0.50
0.75
1.00
β_IV

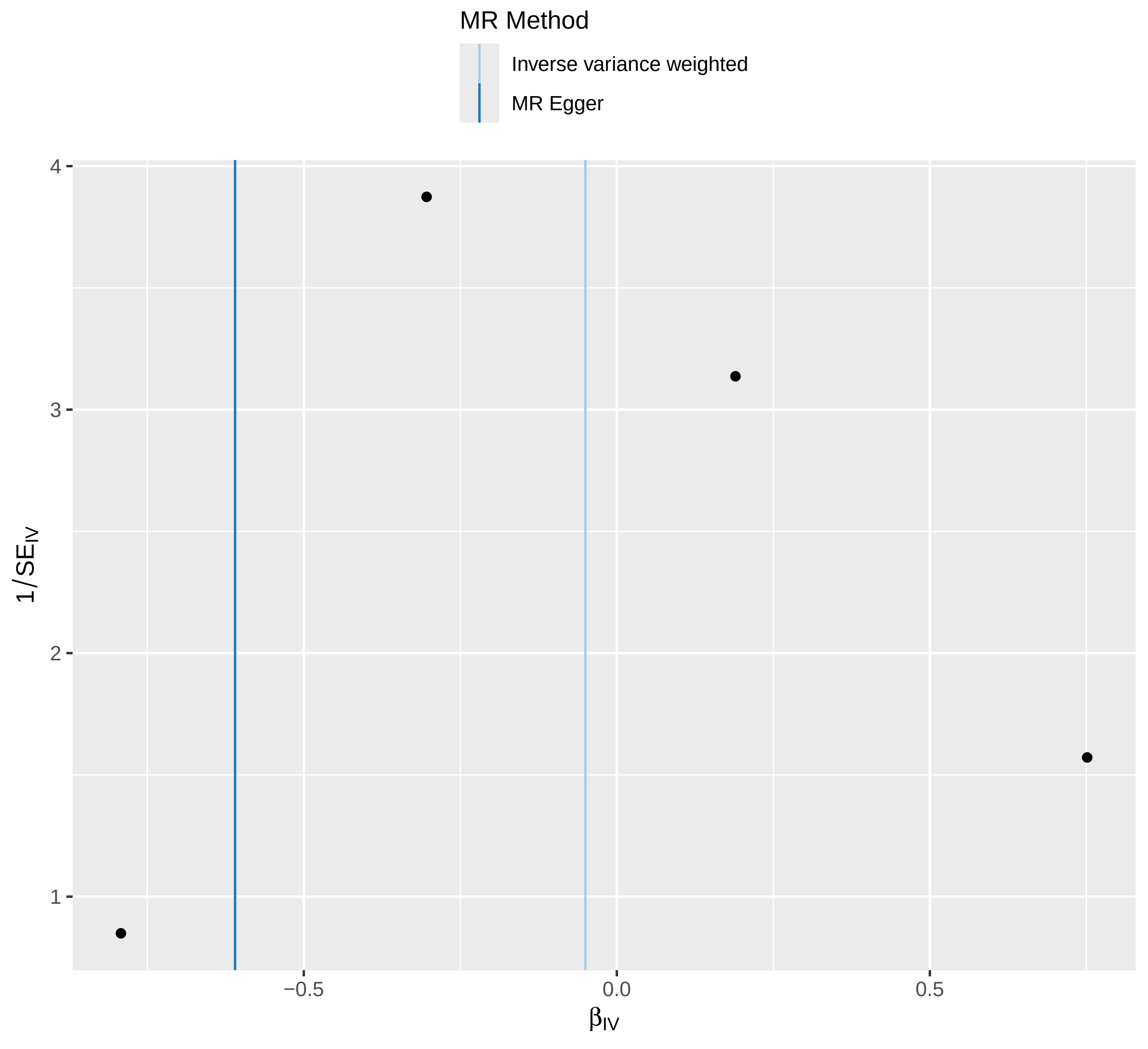
MR Method
Inverse variance weighted
MR Egger
4
3
2
1
1/SE_IV
−0.5
0.0
0.5
β_IV

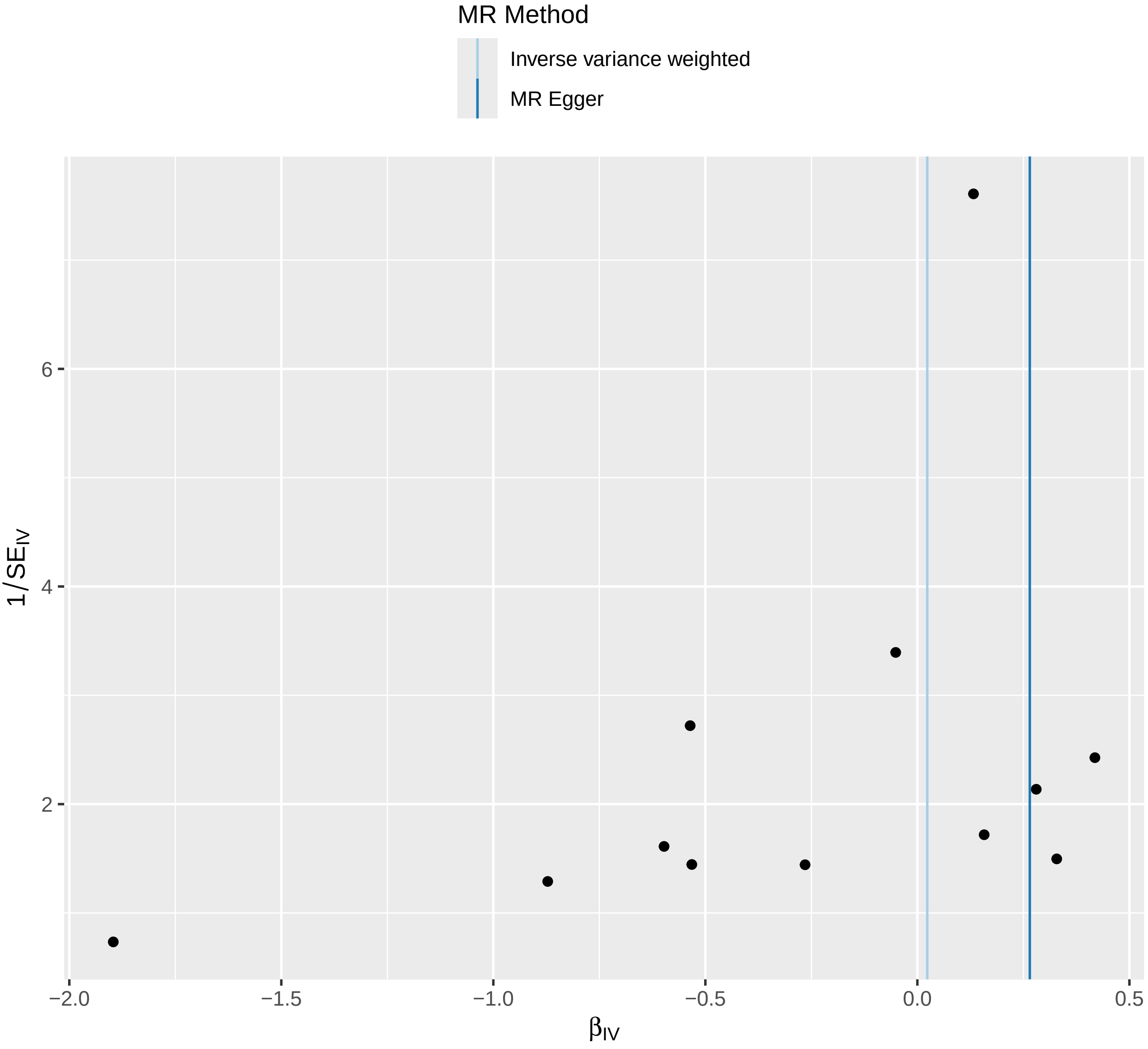
MR Method
Inverse variance weighted
MR Egger
1/SE_IV
2
4
6
−2.0
−1.5
−1.0
−0.5
0.0
0.5
β_IV

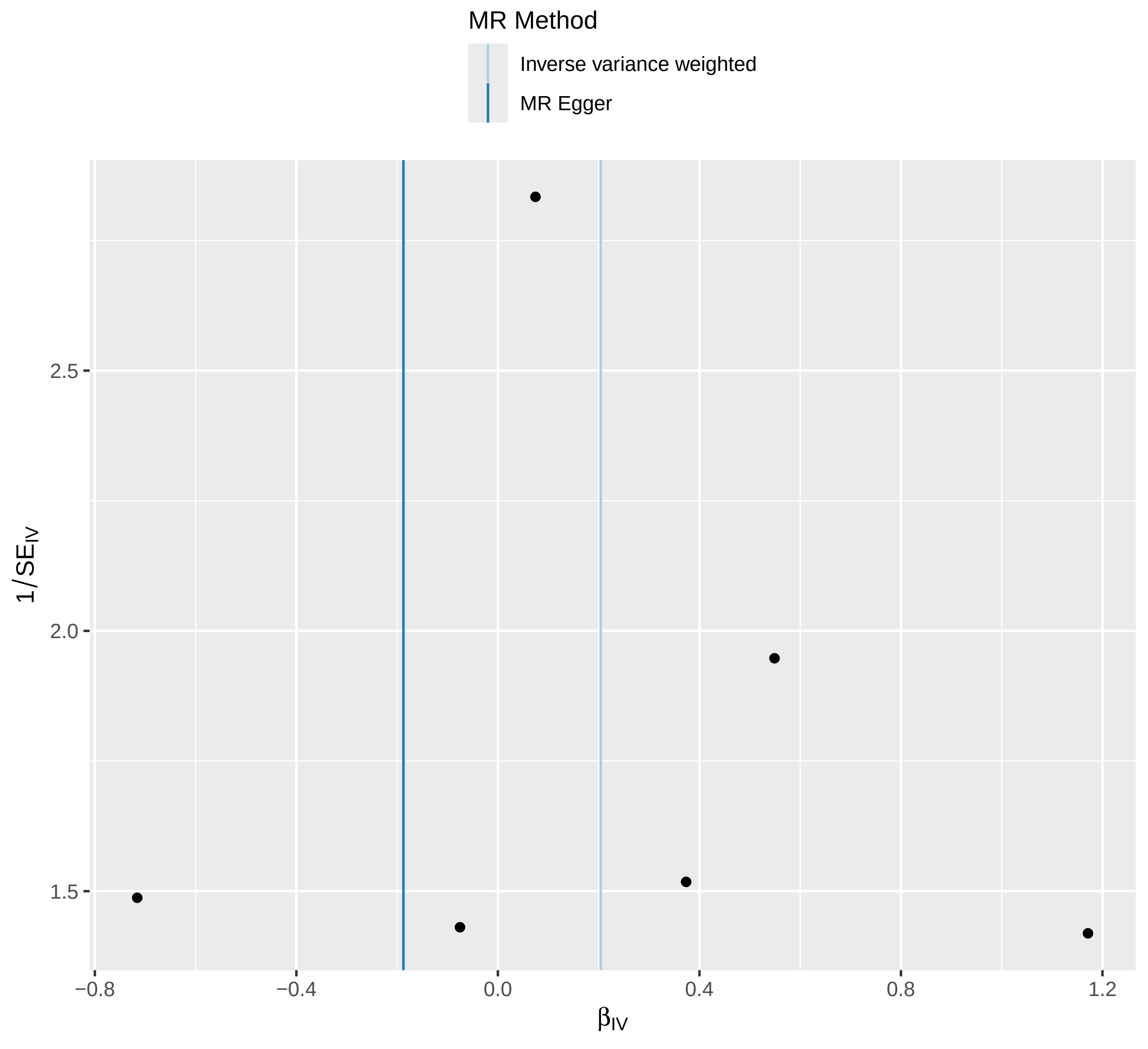
MR Method
Inverse variance weighted
MR Egger
2.5
2.0
1.5
1/SE$_{IV}$
−0.8
−0.4
0.0
0.4
0.8
1.2
β$_{IV}$

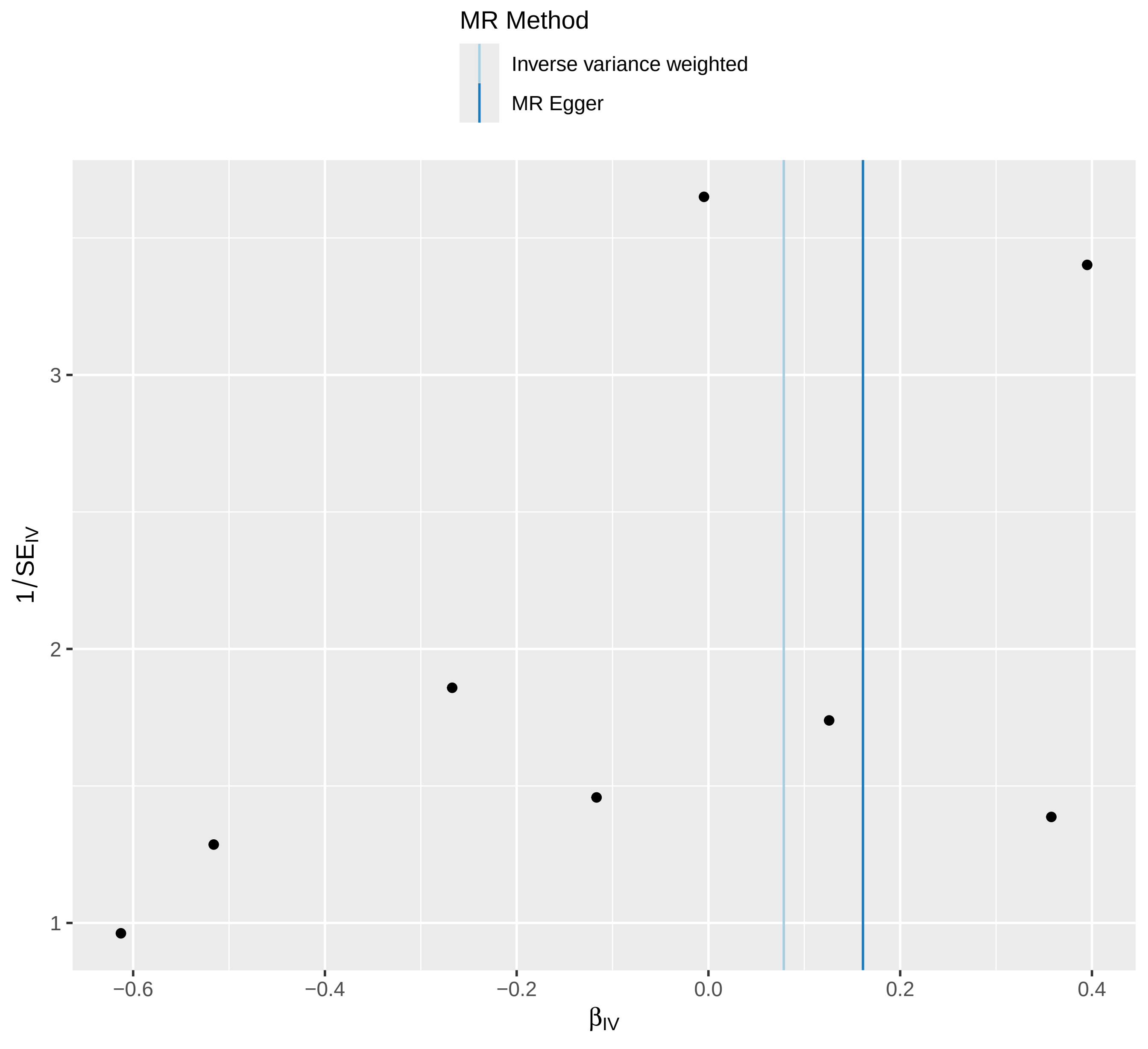
MR Method
Inverse variance weighted
MR Egger
1/SE$_{IV}$
3
2
1
−0.6
−0.4
−0.2
0.0
0.2
0.4
β$_{IV}$

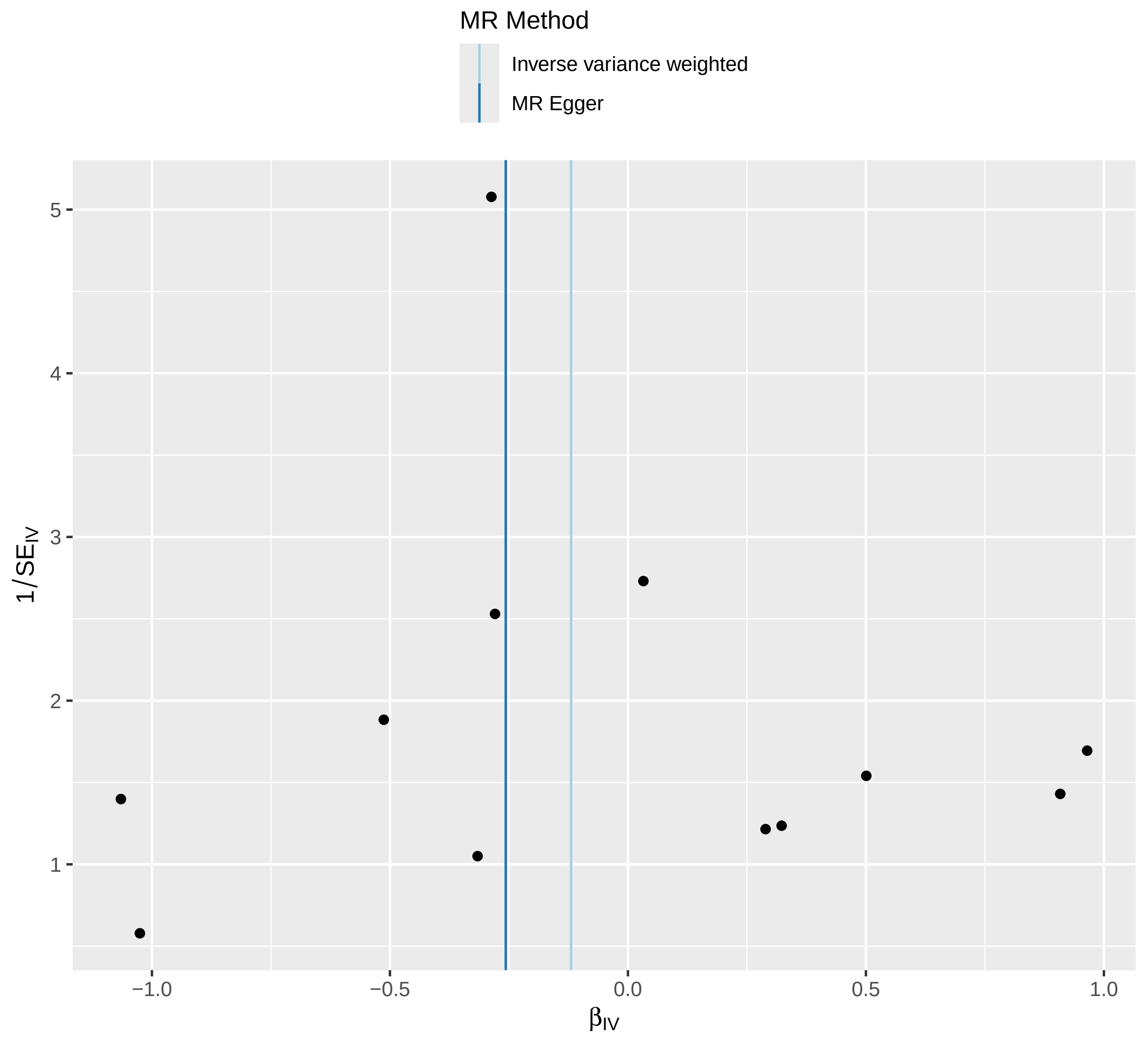

MR Method
Inverse variance weighted
MR Egger
5
4
3
2
1
1/SE$_{IV}$
−1.0
−0.5
0.0
0.5
1.0
β$_{IV}$

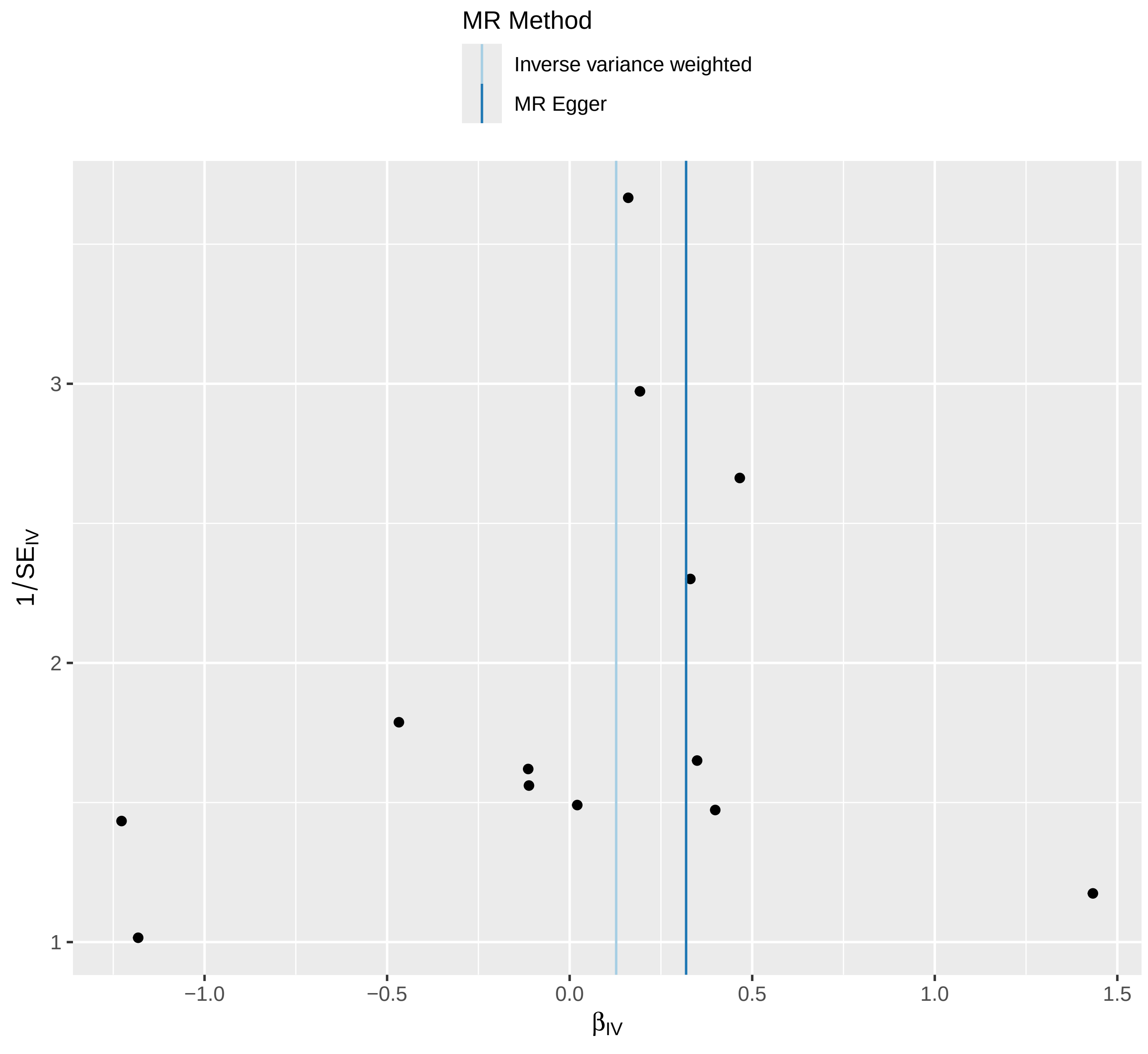

MR Method
Inverse variance weighted
MR Egger
1/SE_IV
3
2
1
−1.0
−0.5
0.0
0.5
1.0
1.5
β_IV

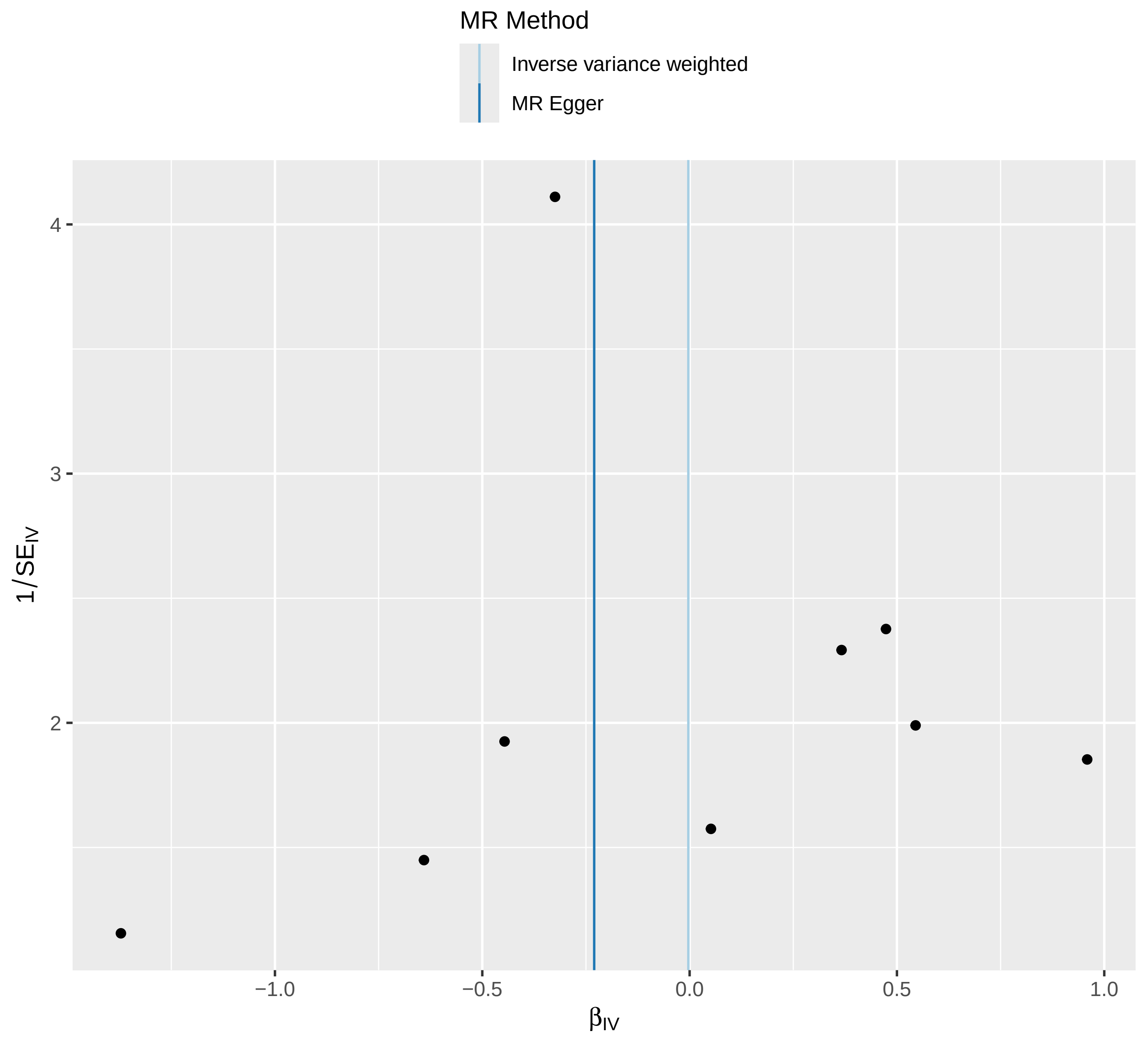
MR Method
Inverse variance weighted
MR Egger
1/SE_IV
β_IV
4
3
2
−1.0
−0.5
0.0
0.5
1.0

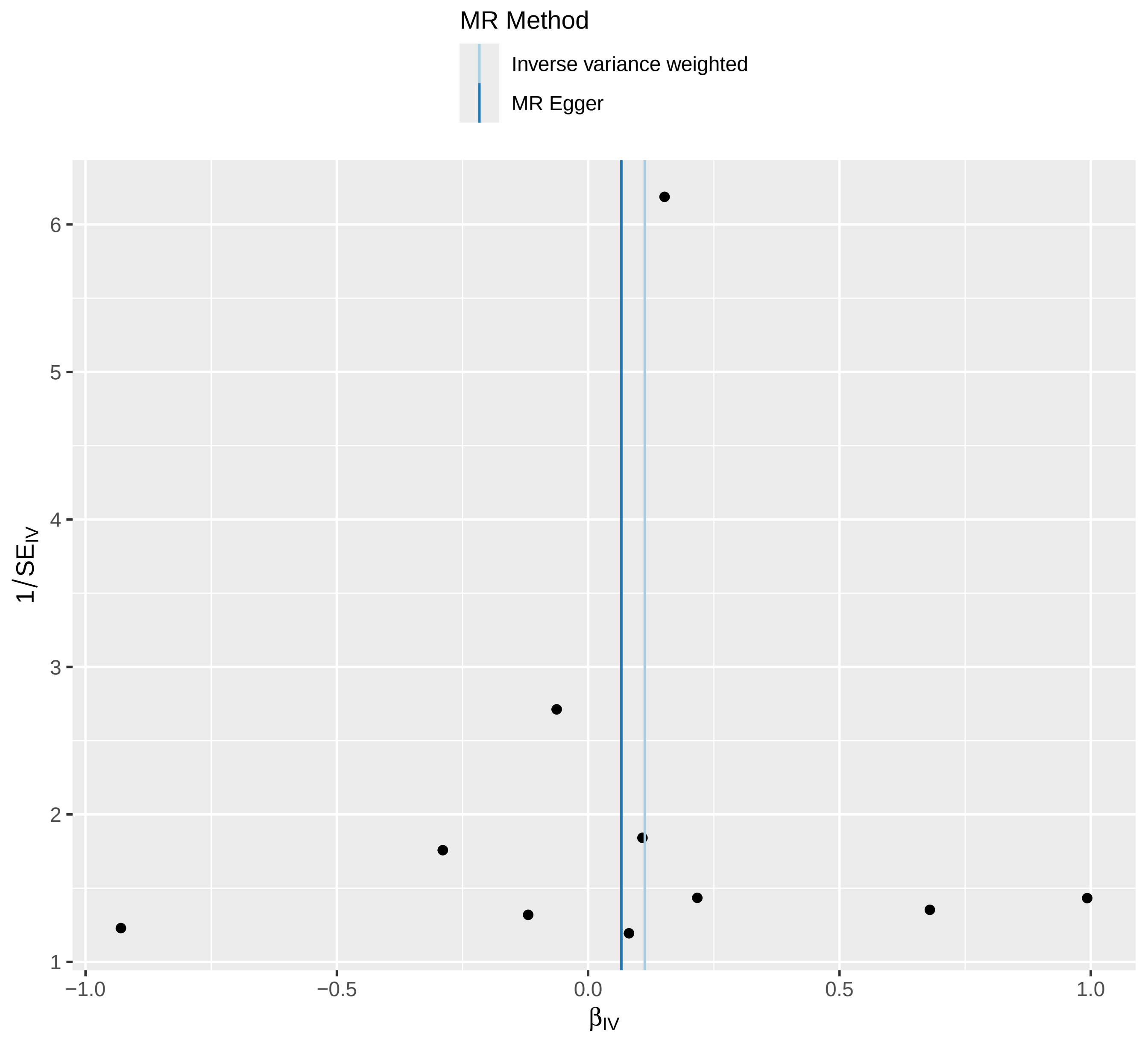
MR Method
Inverse variance weighted
MR Egger
6
5
4
3
2
1
1/SE_IV
−1.0
−0.5
0.0
0.5
1.0
β_IV

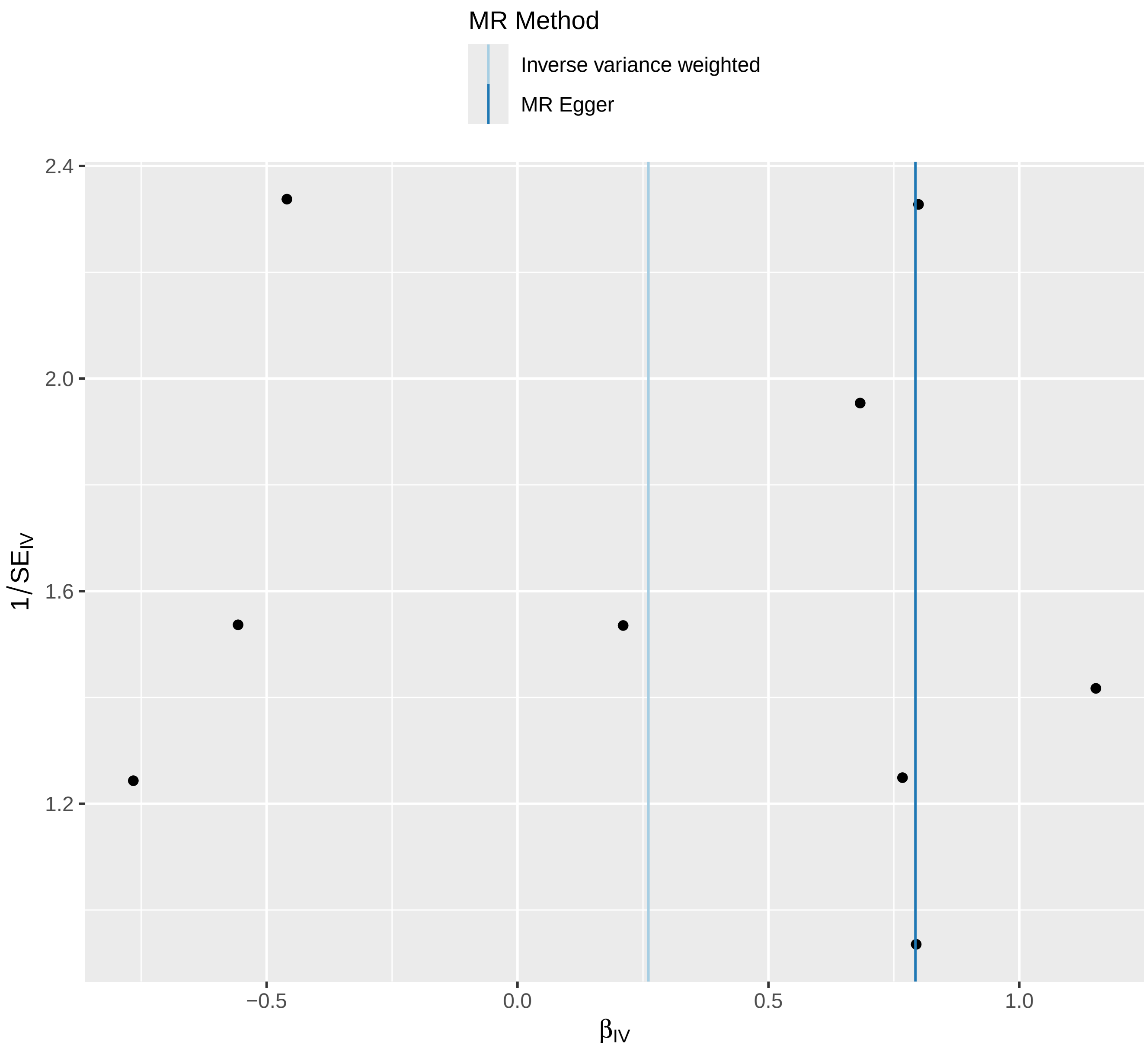
MR Method
Inverse variance weighted
MR Egger
2.4
2.0
1.6
1.2
1/SE_IV
−0.5
0.0
0.5
1.0
β_IV

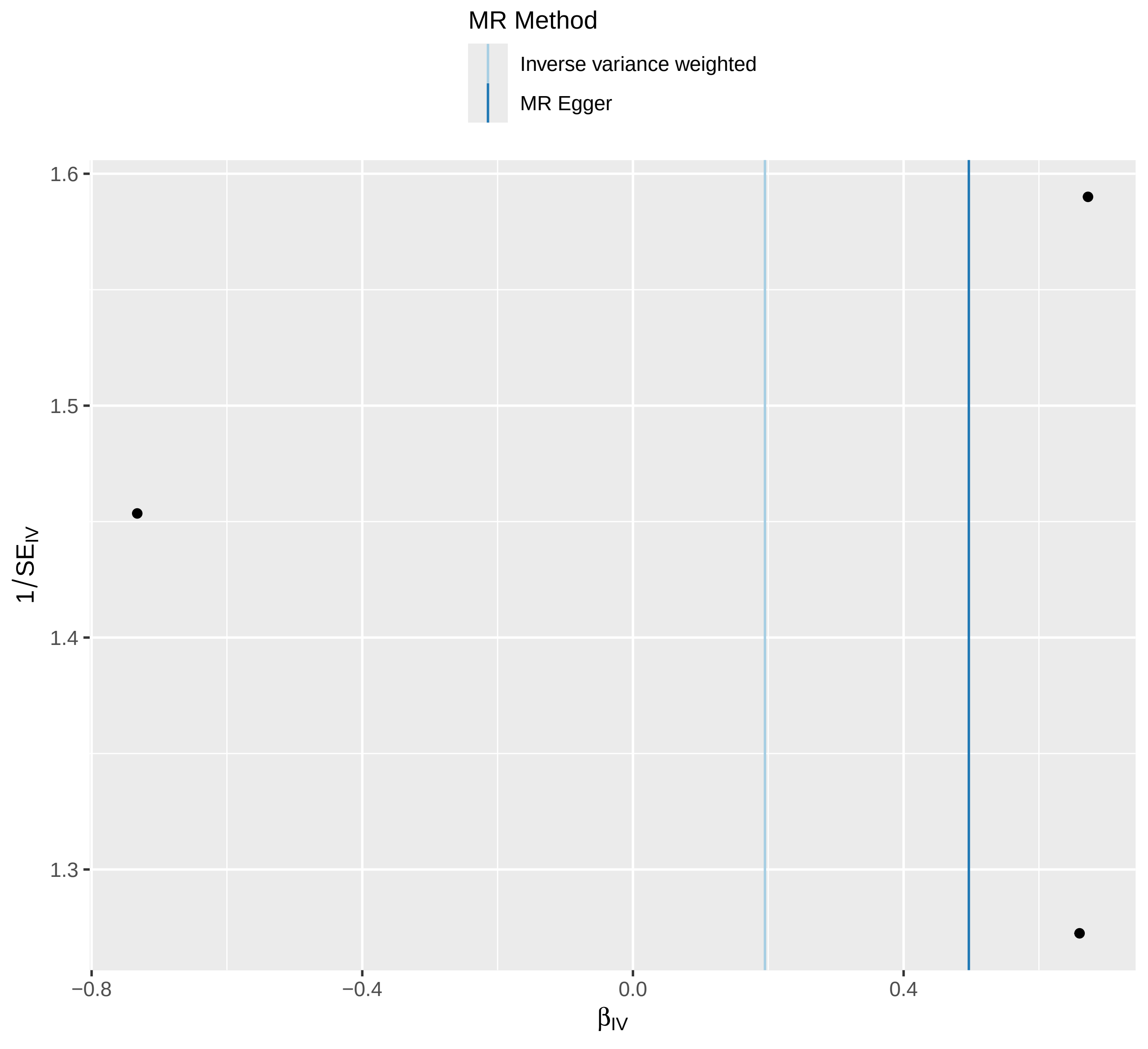
MR Method
Inverse variance weighted
MR Egger
1.6
1.5
1.4
1.3
1/SE_IV
−0.8
−0.4
0.0
0.4
β_IV

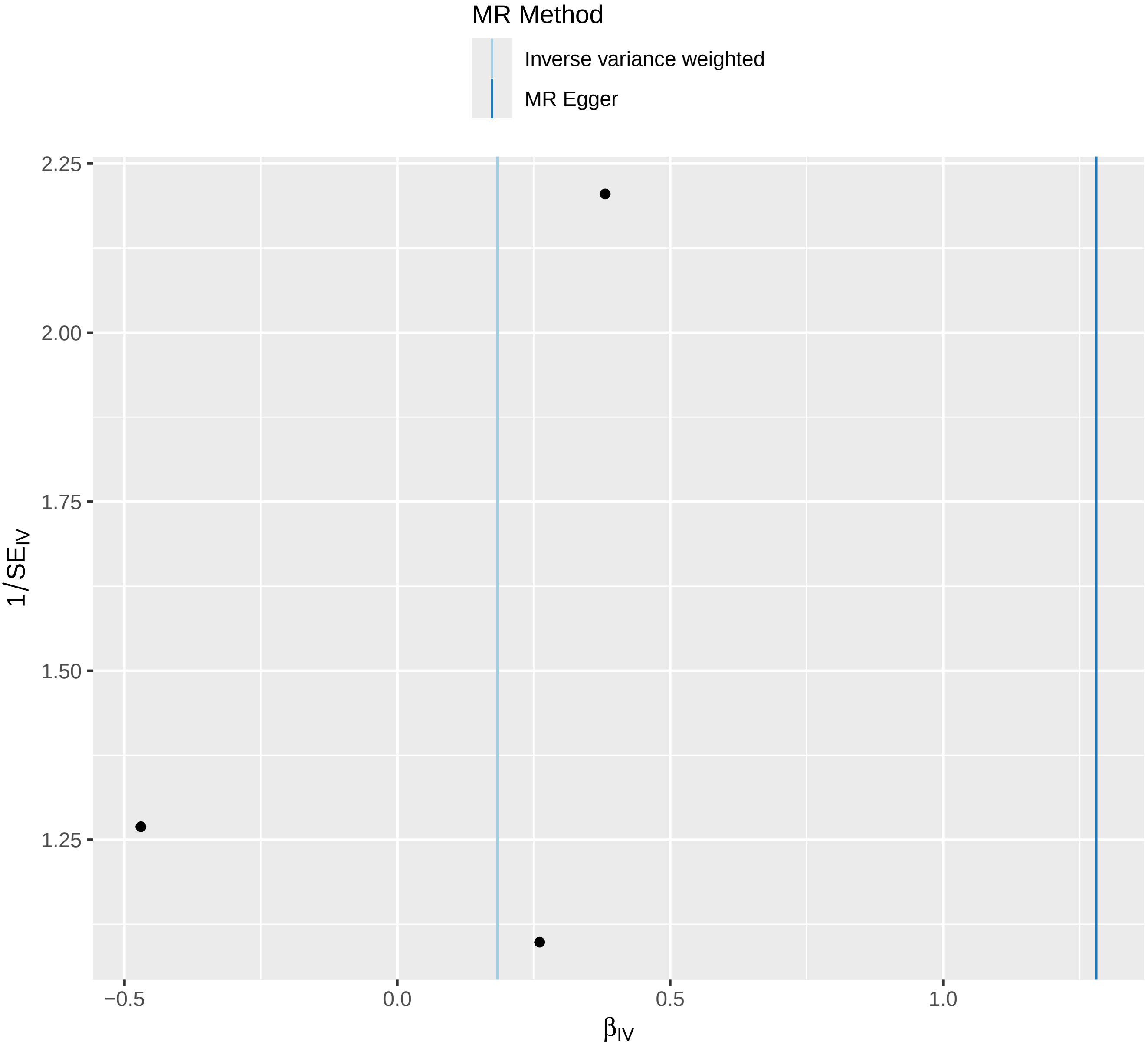

MR Method
Inverse variance weighted
MR Egger
2.25
2.00
1.75
1.50
1.25
1/SE$_{IV}$
−0.5
0.0
0.5
1.0
β_{IV}

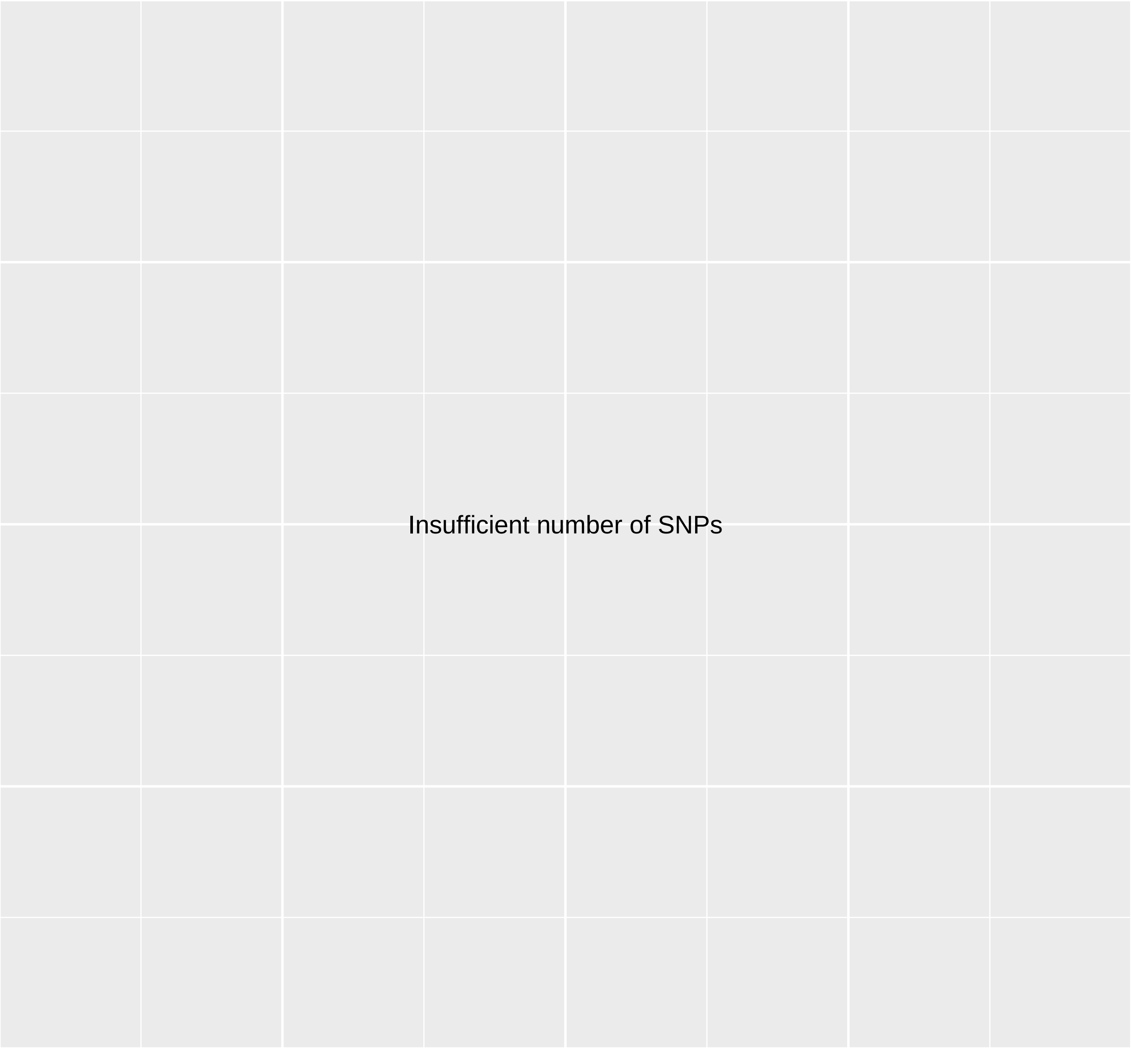
Insufficient number of SNPs

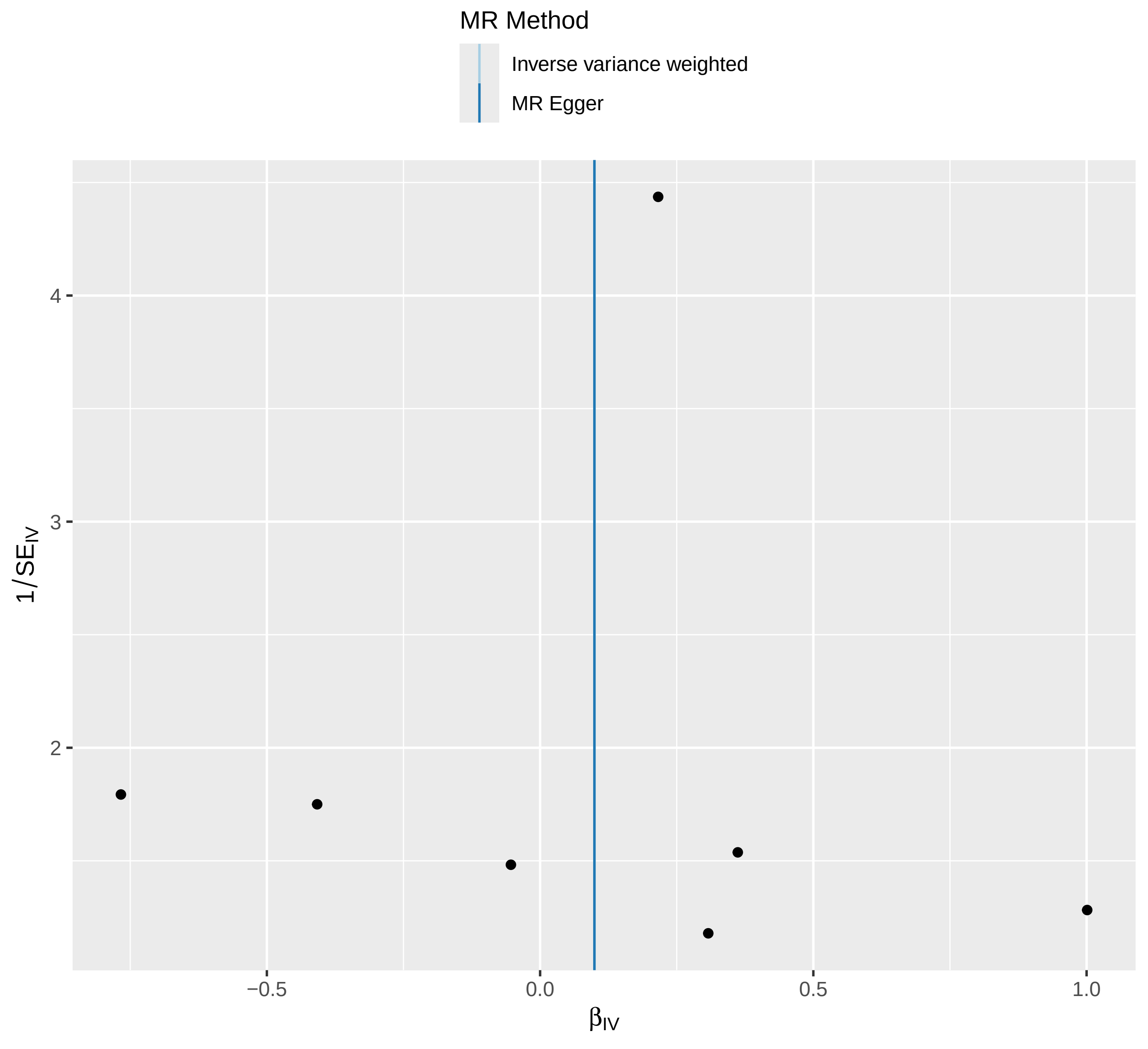
MR Method
Inverse variance weighted
MR Egger
4
3
2
1/SE$_{IV}$
−0.5
0.0
0.5
1.0
β_{IV}

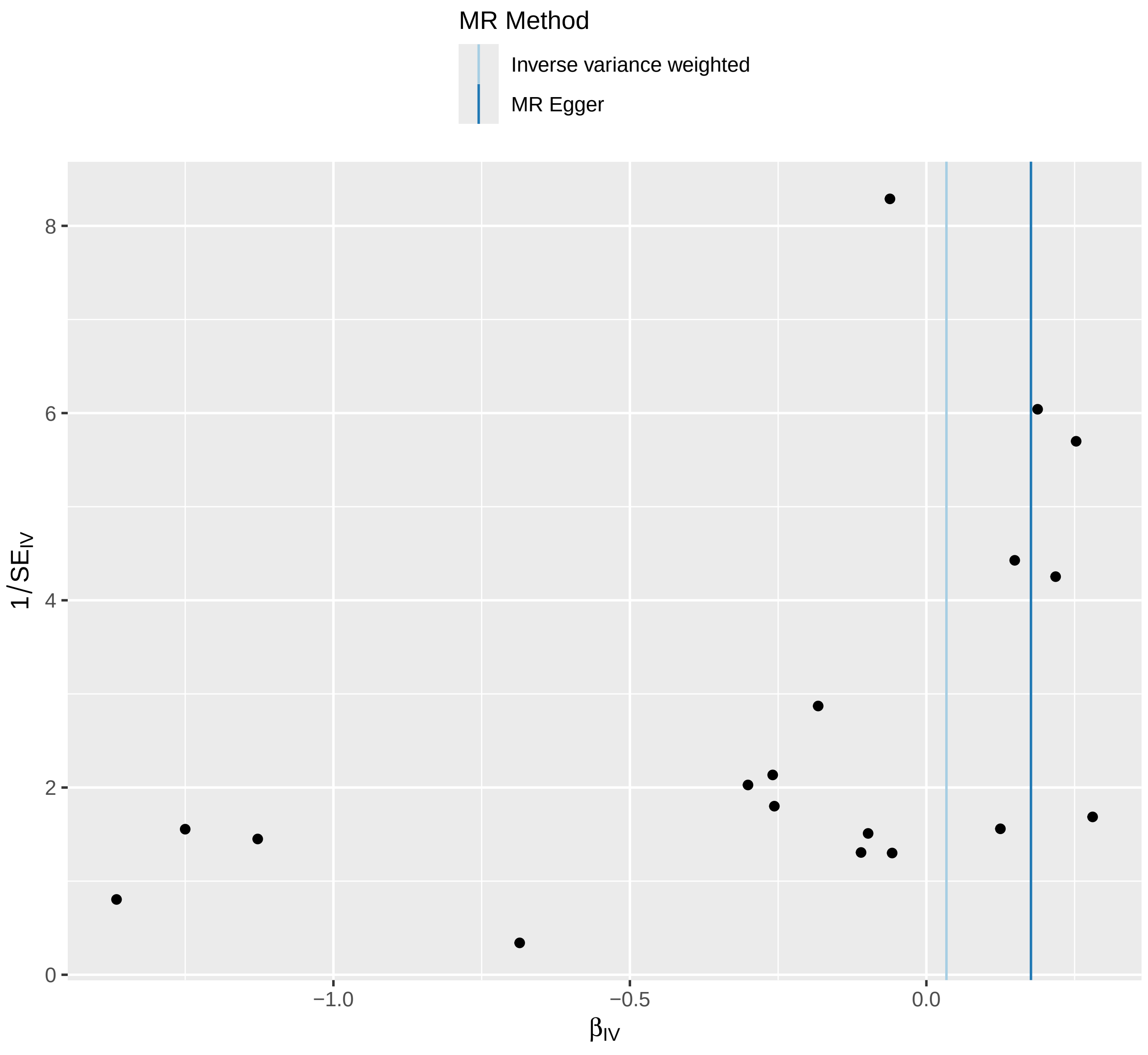
MR Method
Inverse variance weighted
MR Egger
1/SE_IV
β_IV
8
6
4
2
0
−1.0
−0.5
0.0

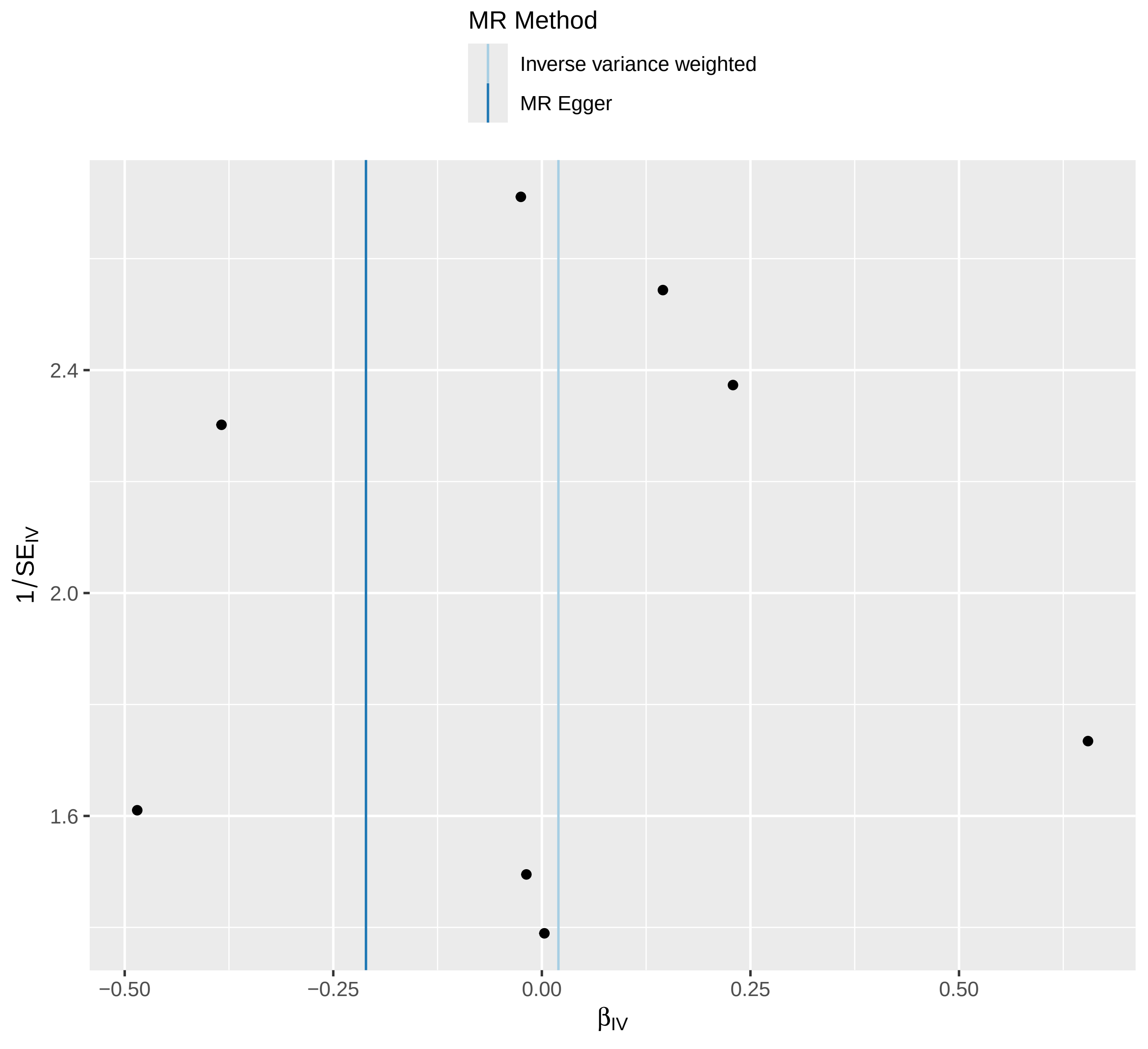
MR Method
Inverse variance weighted
MR Egger
2.4
2.0
1.6
1/SE_IV
−0.50
−0.25
0.00
0.25
0.50
β_IV

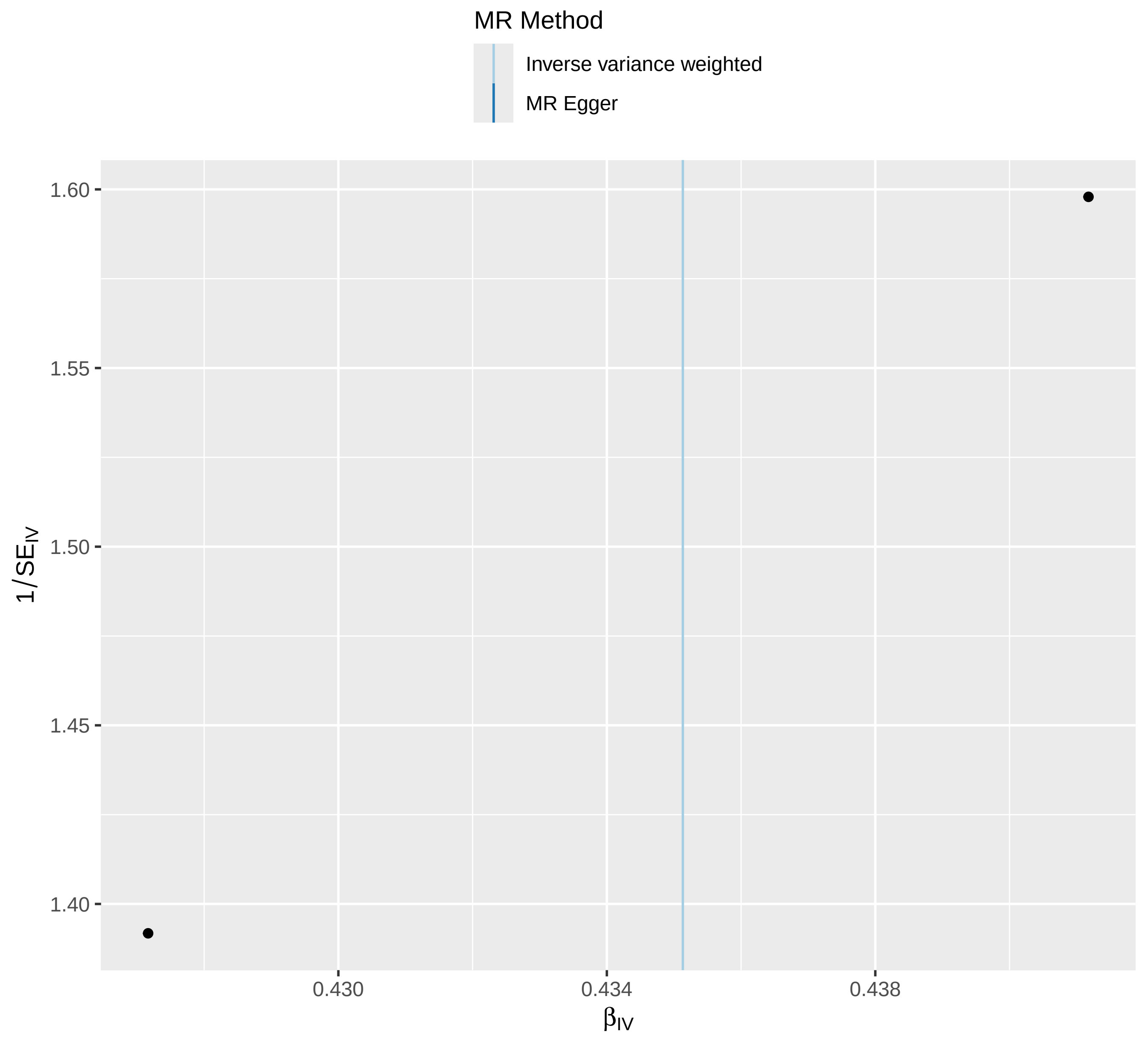

MR Method
Inverse variance weighted
MR Egger
1.60
1.55
1.50
1.45
1.40
1/SE$_{IV}$
0.430
0.434
0.438
β$_{IV}$

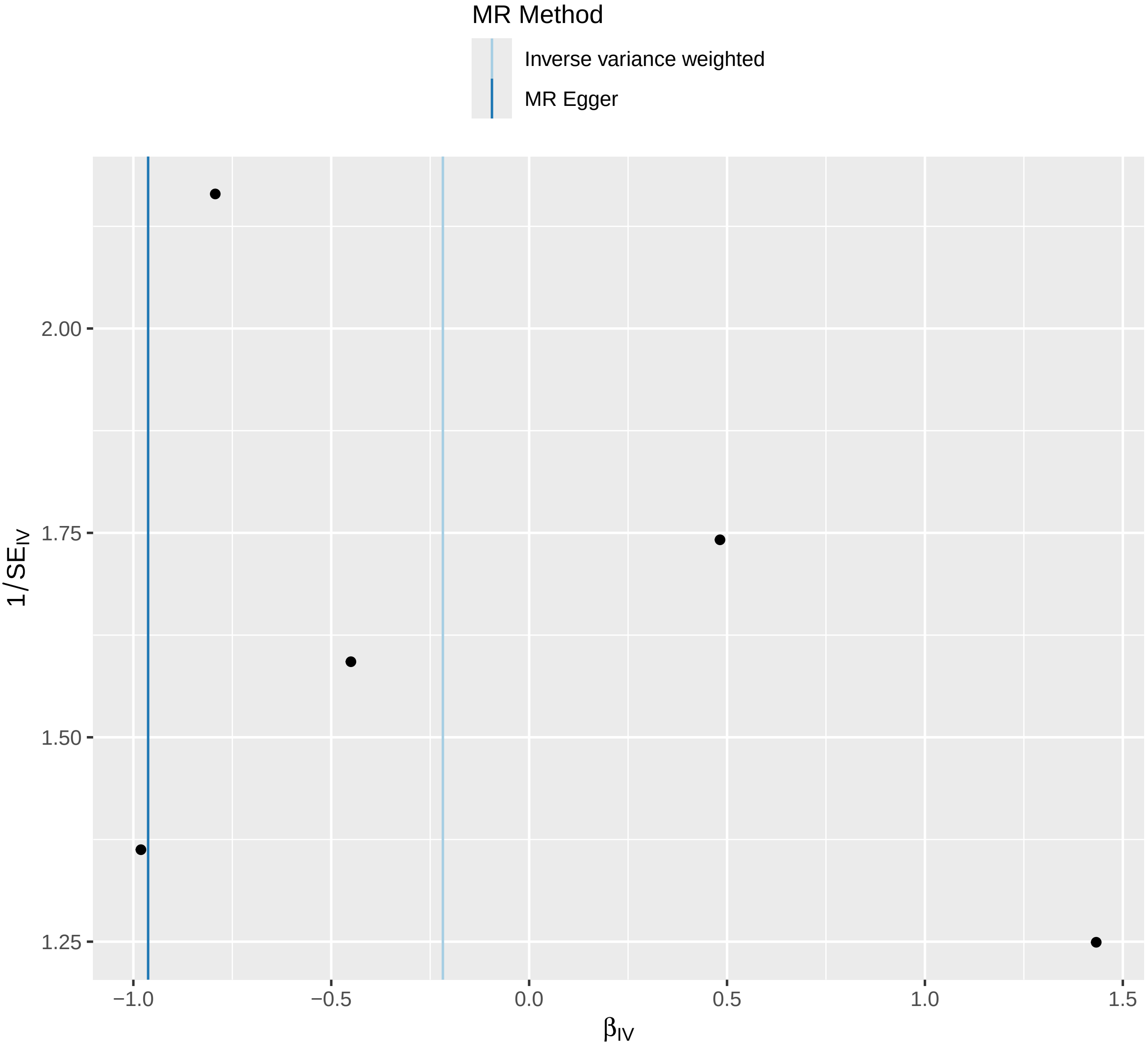
MR Method
Inverse variance weighted
MR Egger
2.00
1.75
1.50
1.25
$1/SE_{IV}$
−1.0
−0.5
0.0
0.5
1.0
1.5
β_{IV}

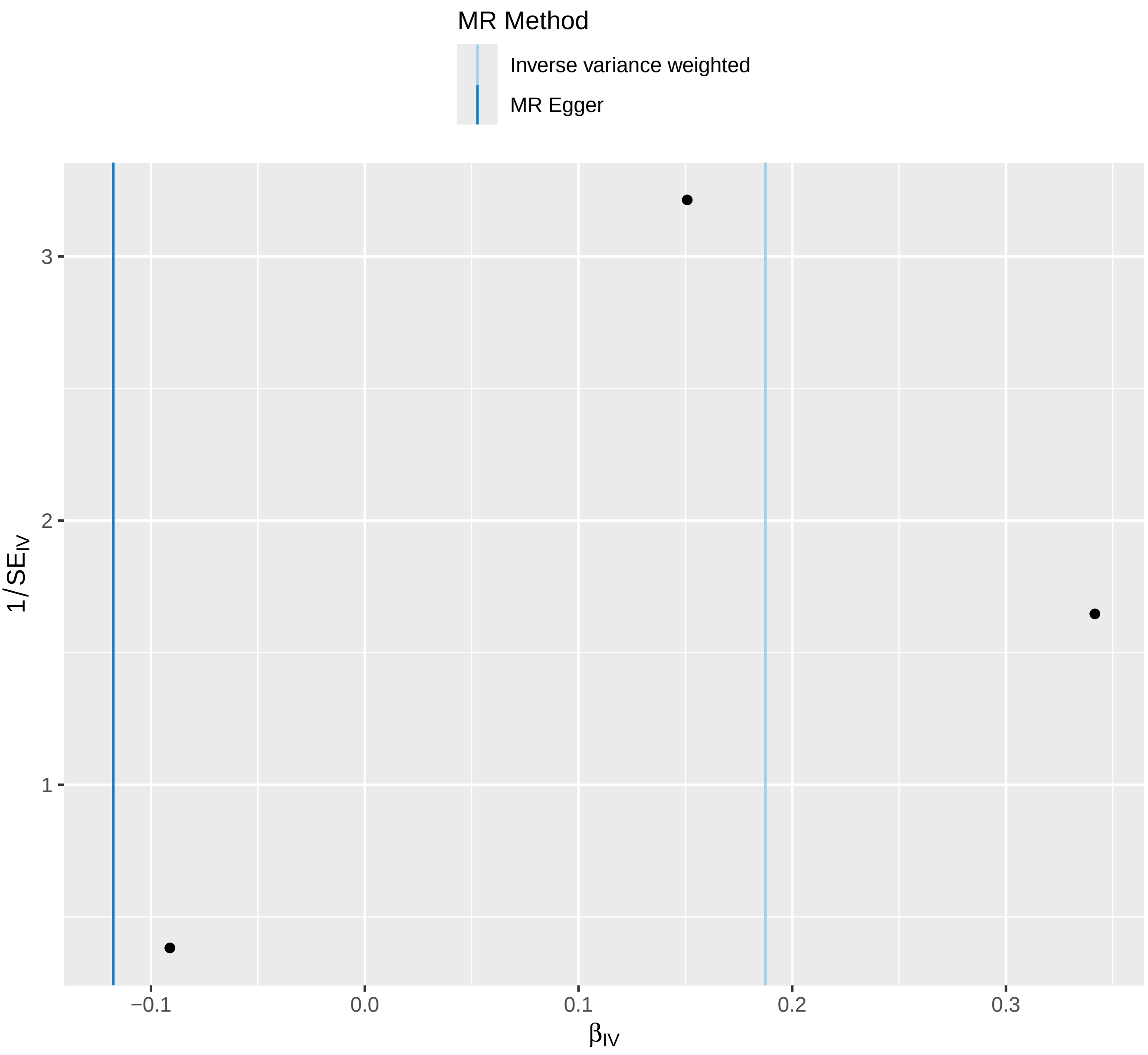
MR Method
Inverse variance weighted
MR Egger
3
2
1
1/SE_IV
−0.1
0.0
0.1
0.2
0.3
β_IV

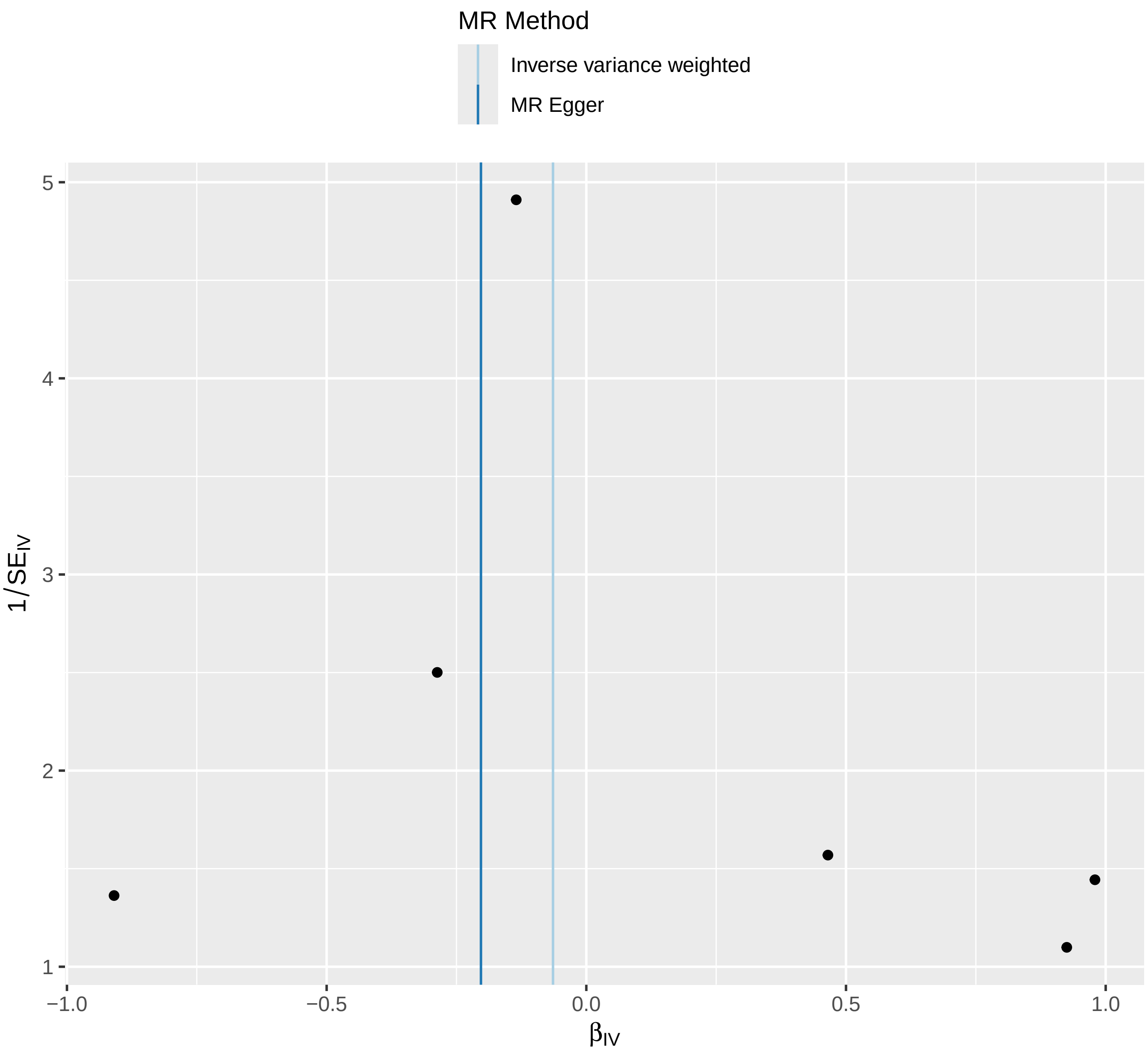

MR Method
Inverse variance weighted
MR Egger
1/SE_IV
5
4
3
2
1
−1.0
−0.5
0.0
0.5
1.0
β_IV

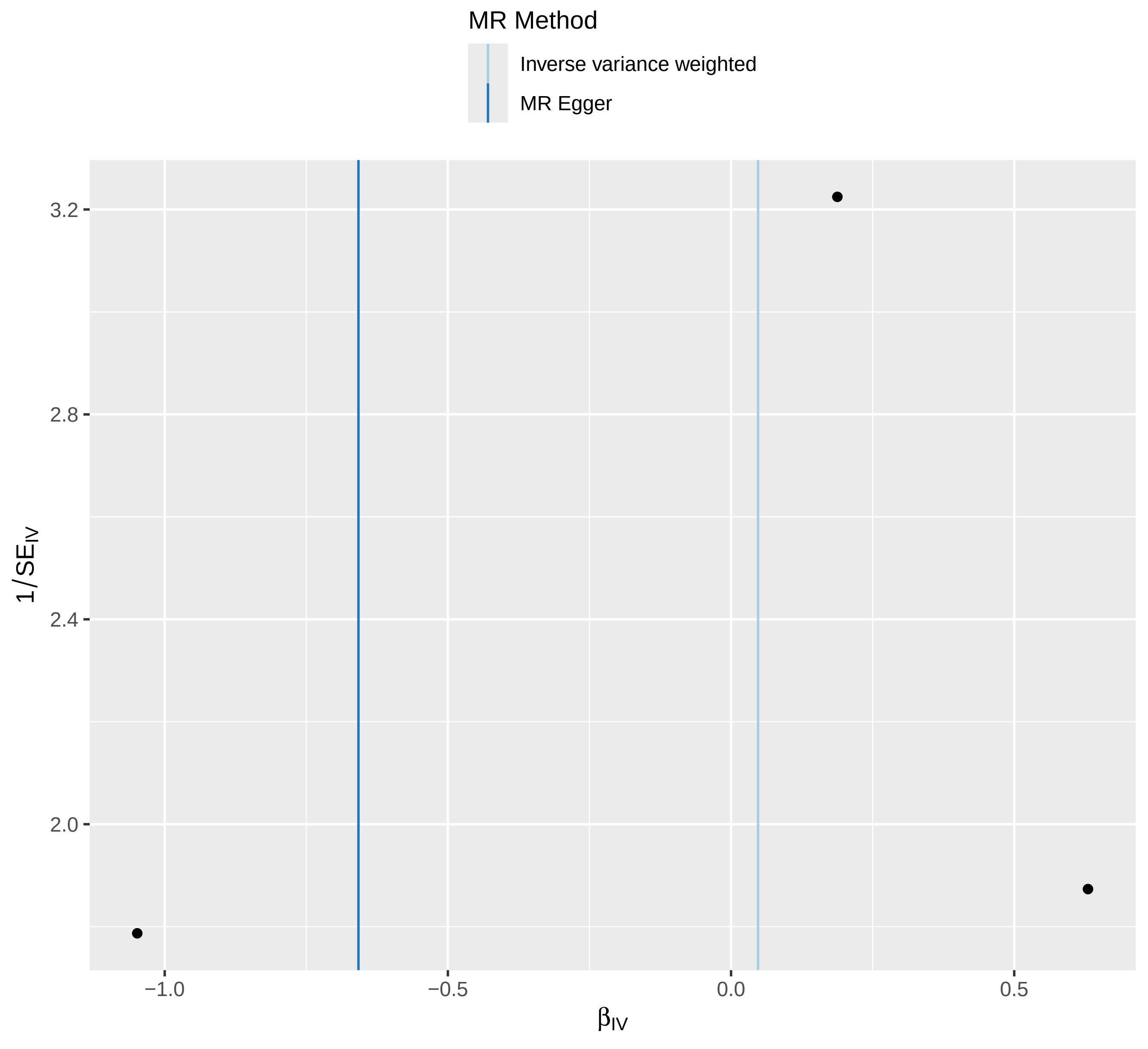
MR Method
Inverse variance weighted
MR Egger
1/SE_IV
β_IV
3.2
2.8
2.4
2.0
−1.0
−0.5
0.0
0.5

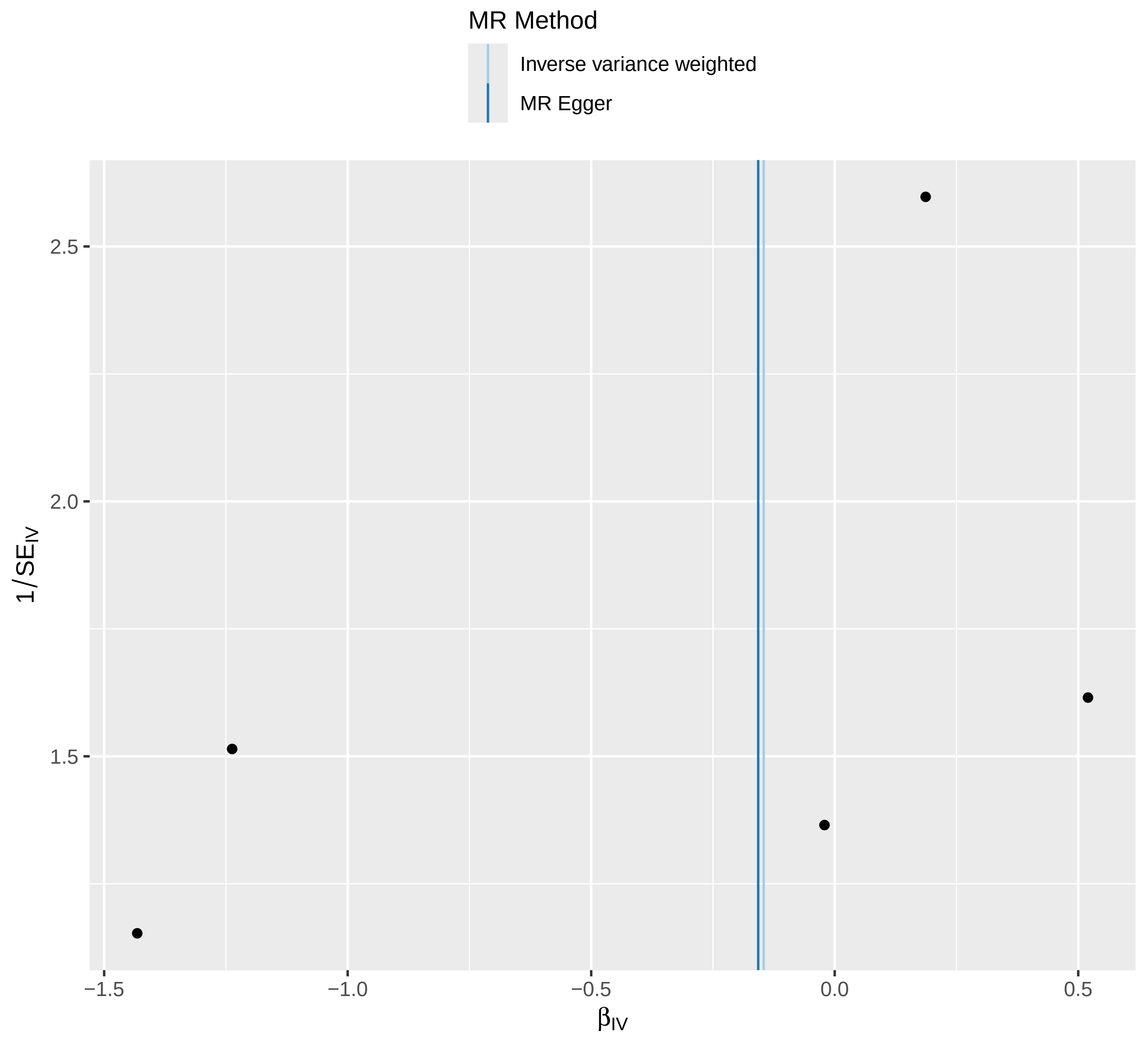
MR Method
Inverse variance weighted
MR Egger
2.5
2.0
1.5
1/SE_IV
−1.5
−1.0
−0.5
0.0
0.5
β_IV

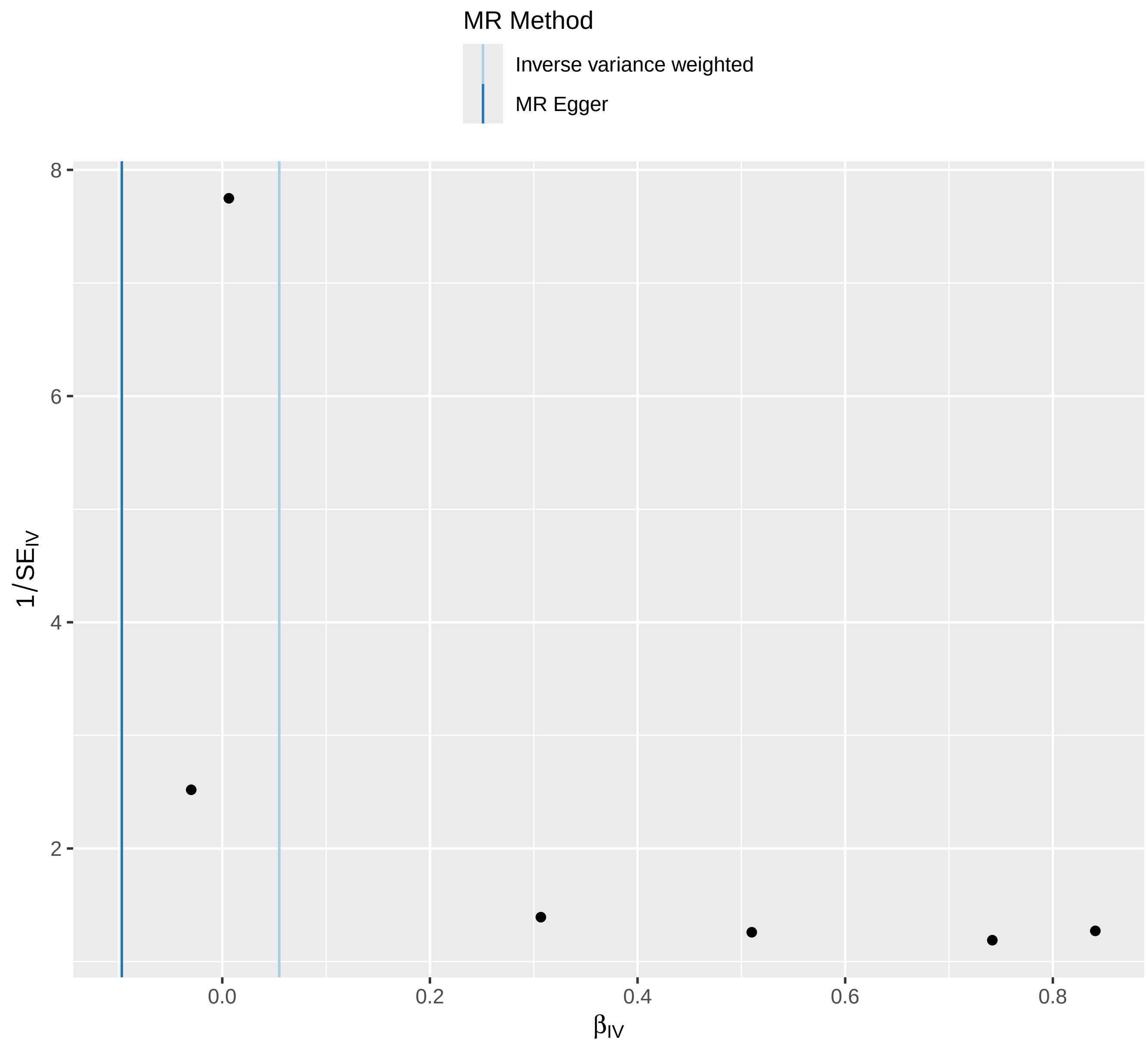
MR Method
Inverse variance weighted
MR Egger
1/SE_IV
8
6
4
2
0.0
0.2
0.4
0.6
0.8
β_IV

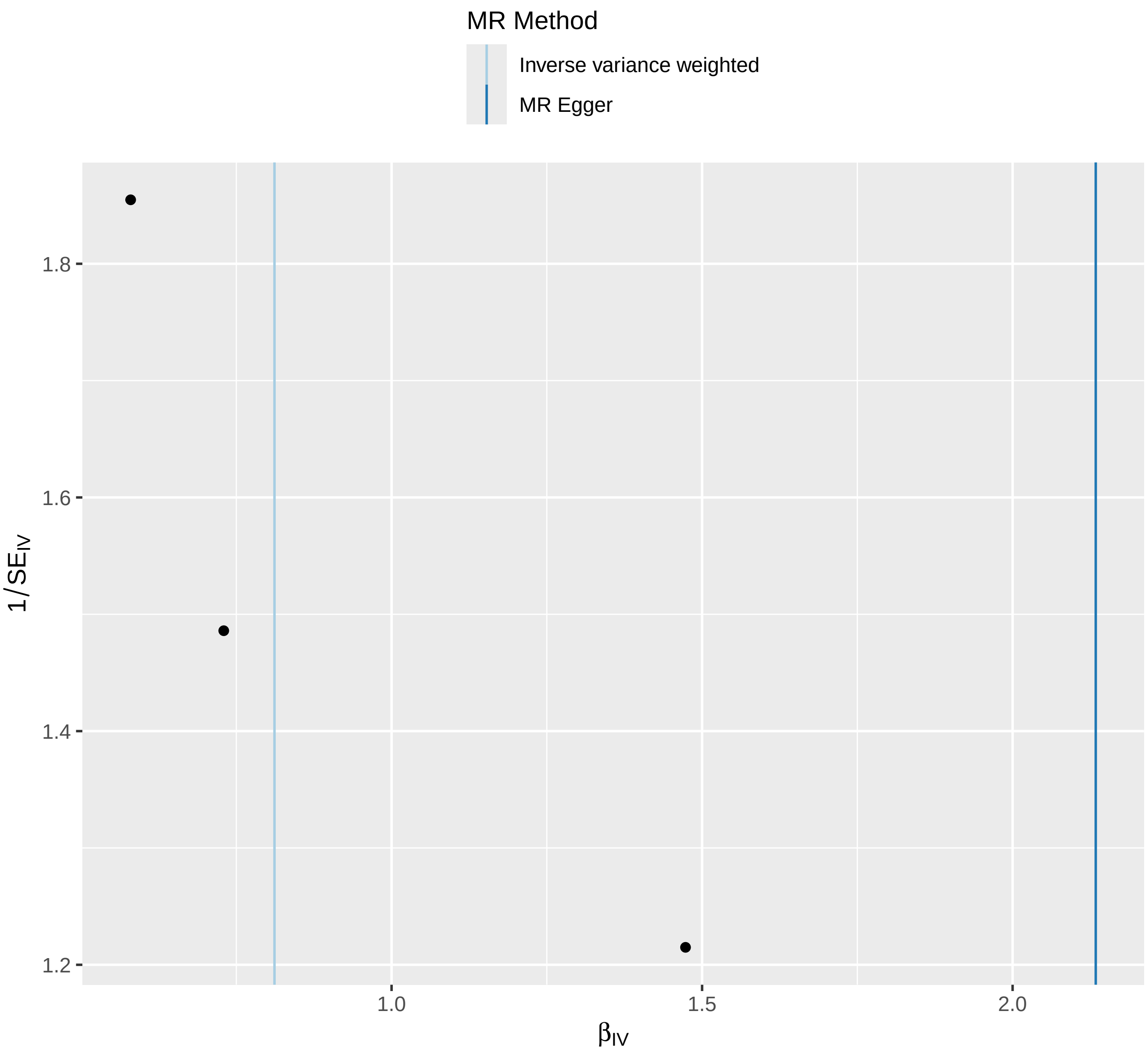
MR Method
Inverse variance weighted
MR Egger
1.8
1.6
1.4
1.2
1/SE$_{IV}$
1.0
1.5
2.0
β$_{IV}$

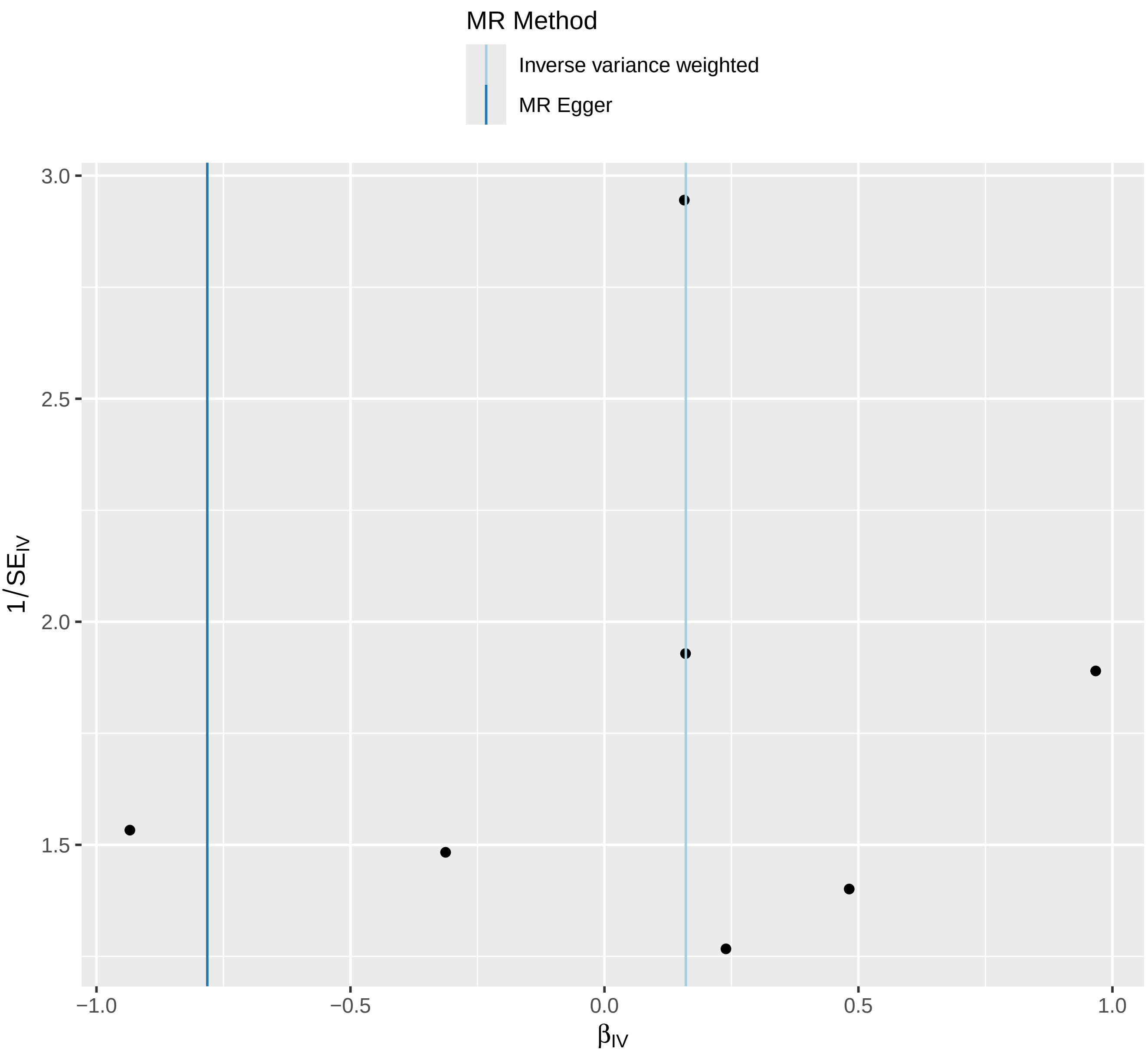

MR Method
Inverse variance weighted
MR Egger
3.0
2.5
2.0
1.5
1/SE$_{IV}$
−1.0
−0.5
0.0
0.5
1.0
β$_{IV}$

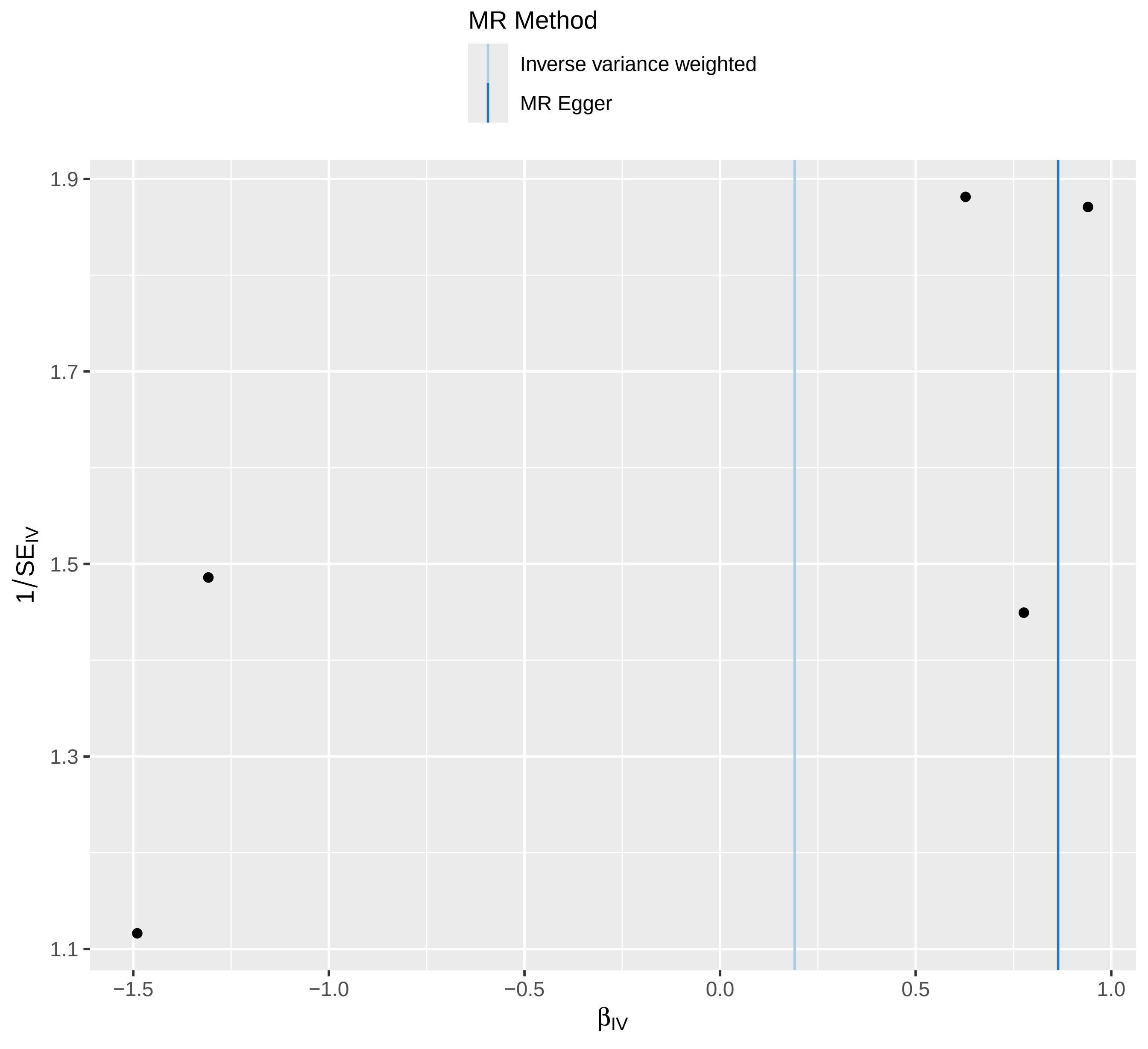
MR Method
Inverse variance weighted
MR Egger
1/SE_IV
1.9
1.7
1.5
1.3
1.1
−1.5
−1.0
−0.5
0.0
0.5
1.0
β_IV

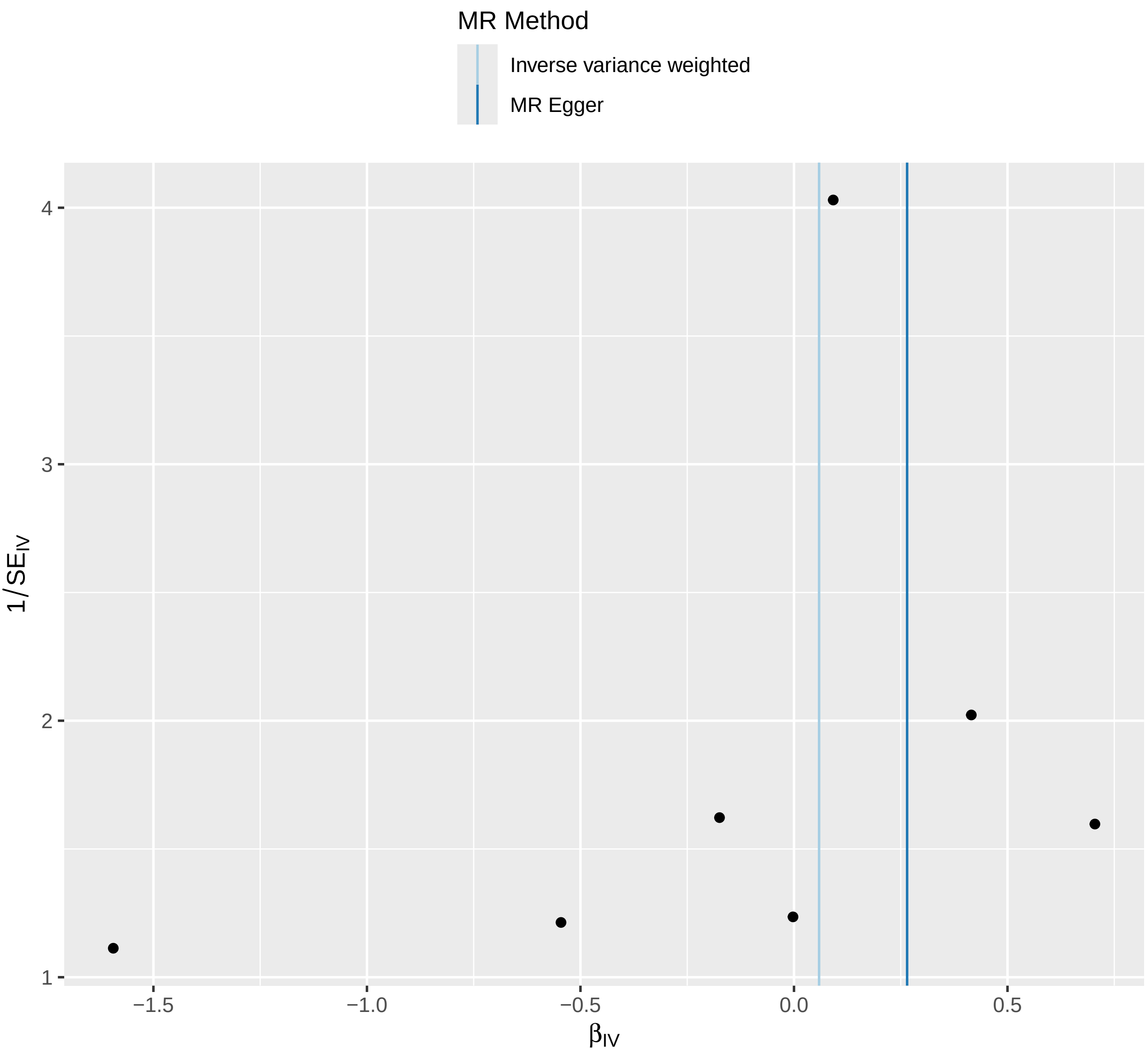

MR Method
Inverse variance weighted
MR Egger
1/SE$_{IV}$
4
3
2
1
−1.5
−1.0
−0.5
0.0
0.5
β$_{IV}$

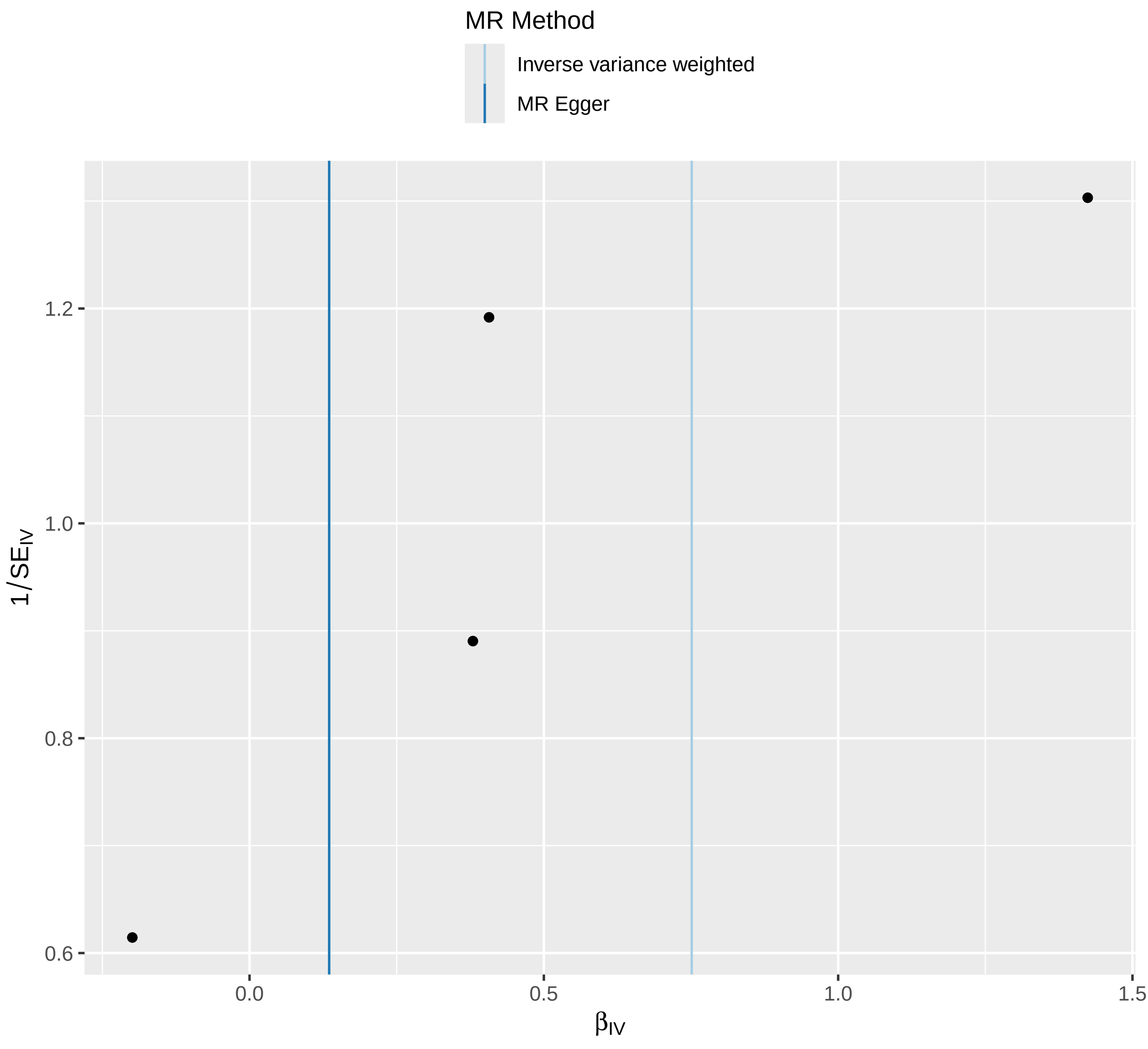

MR Method
Inverse variance weighted
MR Egger
1.2
1.0
0.8
0.6
1/SE_IV
0.0
0.5
1.0
1.5
β_IV

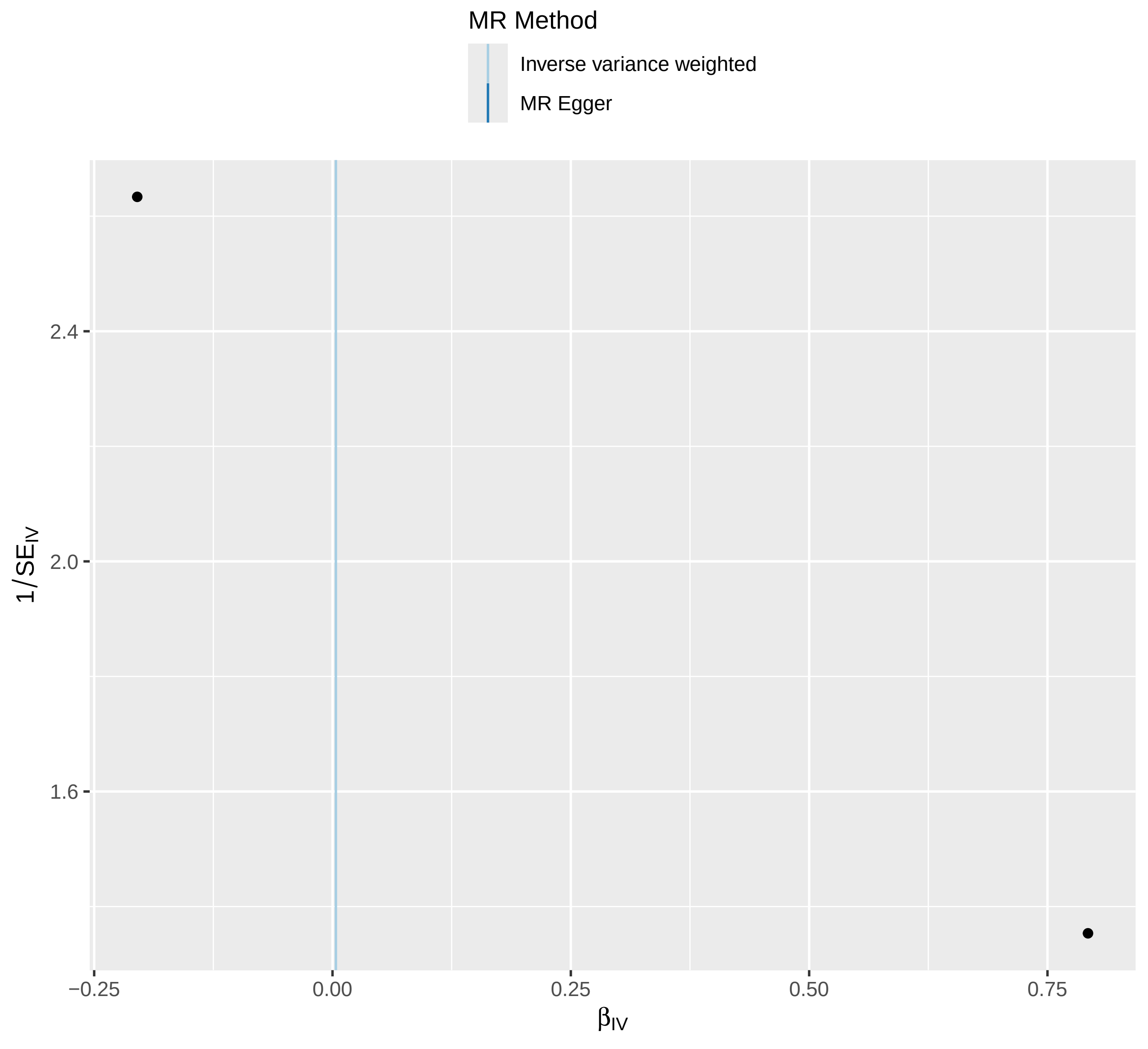

MR Method
Inverse variance weighted
MR Egger
2.4
2.0
1.6
1/SE_IV
−0.25
0.00
0.25
0.50
0.75
β_IV

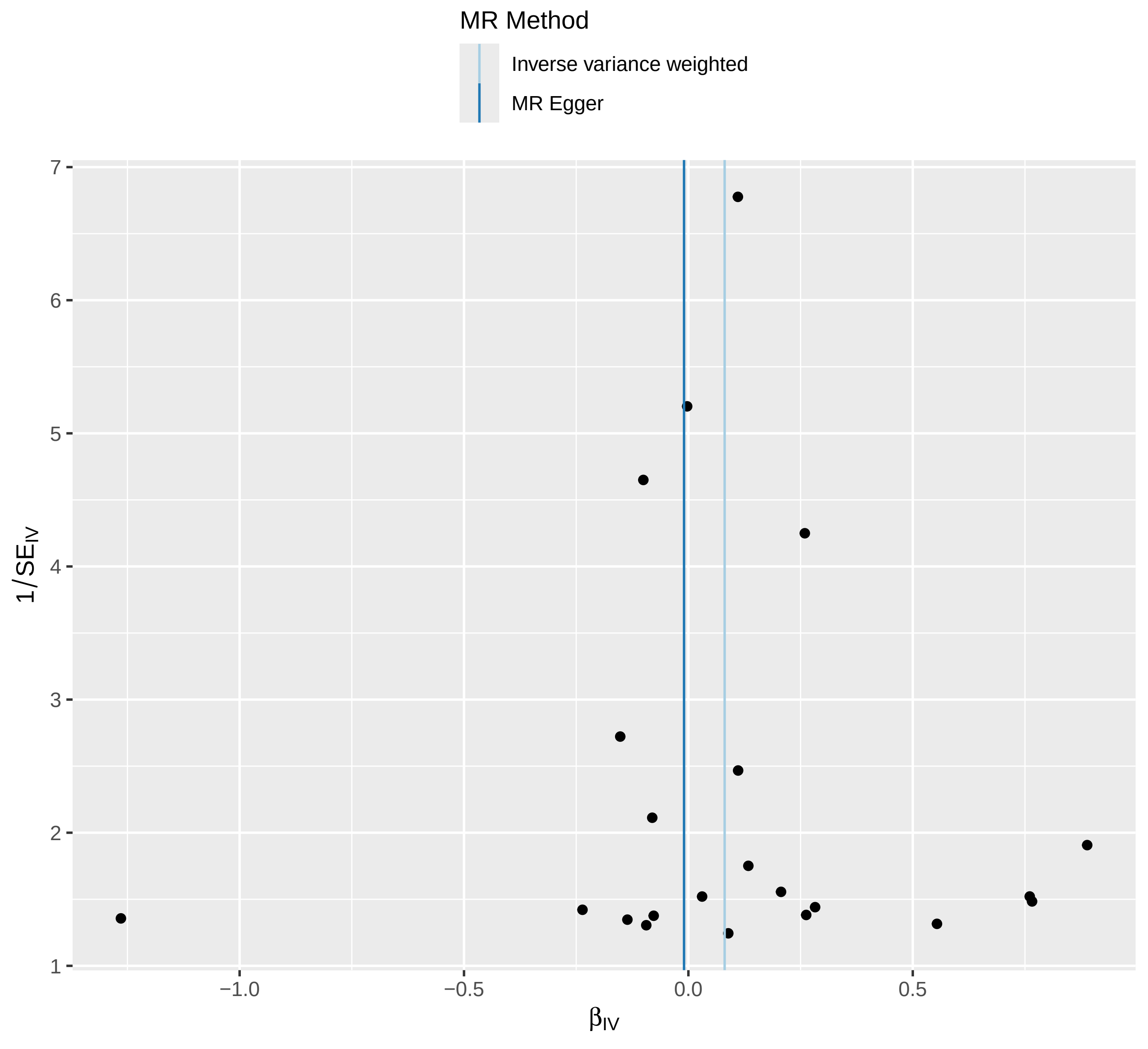

MR Method
Inverse variance weighted
MR Egger
7
6
5
4
3
2
1
1/SE$_{IV}$
−1.0
−0.5
0.0
0.5
β$_{IV}$

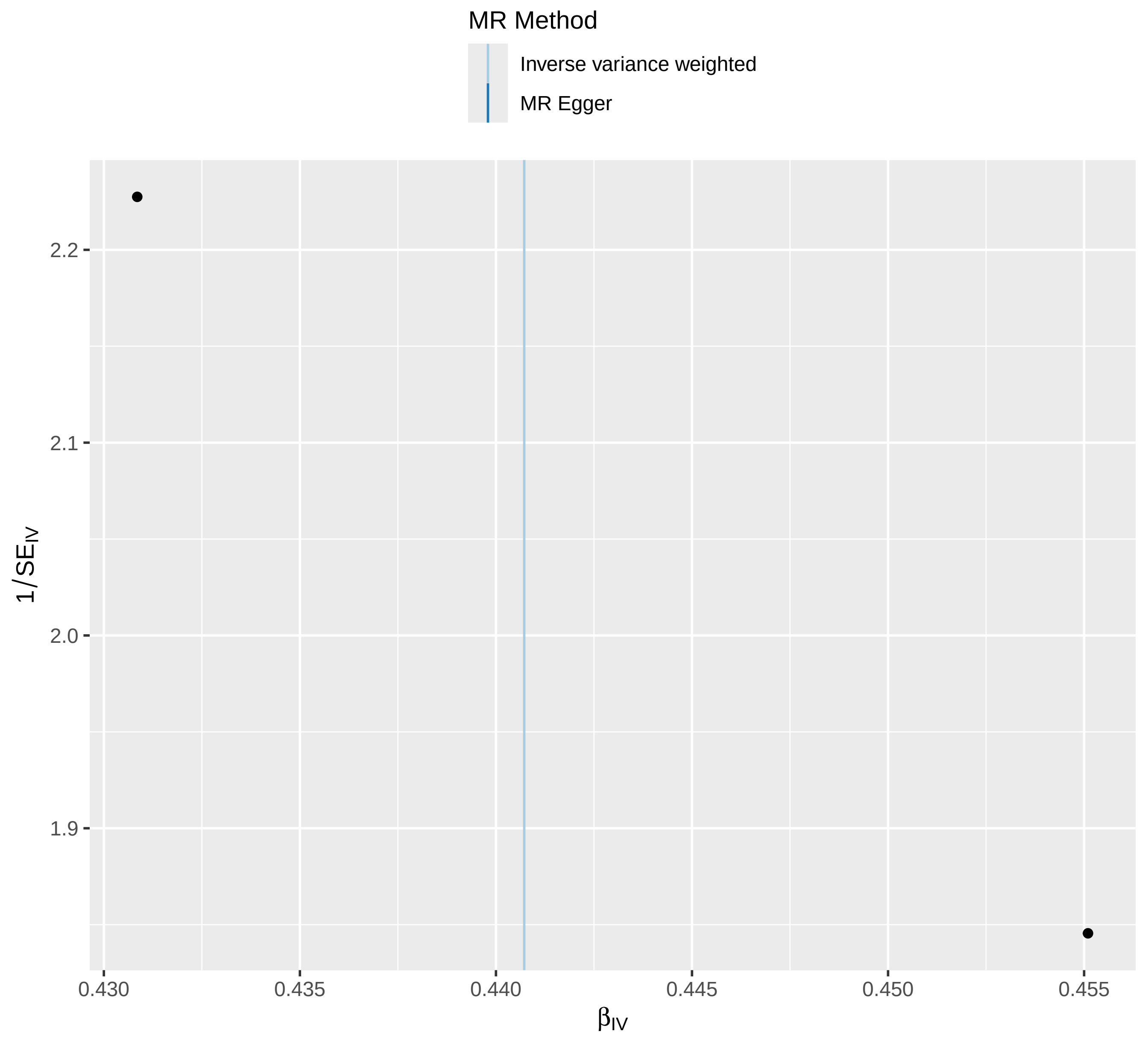
MR Method
Inverse variance weighted
MR Egger
2.2
2.1
2.0
1.9
$1/SE_{IV}$
0.430
0.435
0.440
0.445
0.450
0.455
β_{IV}

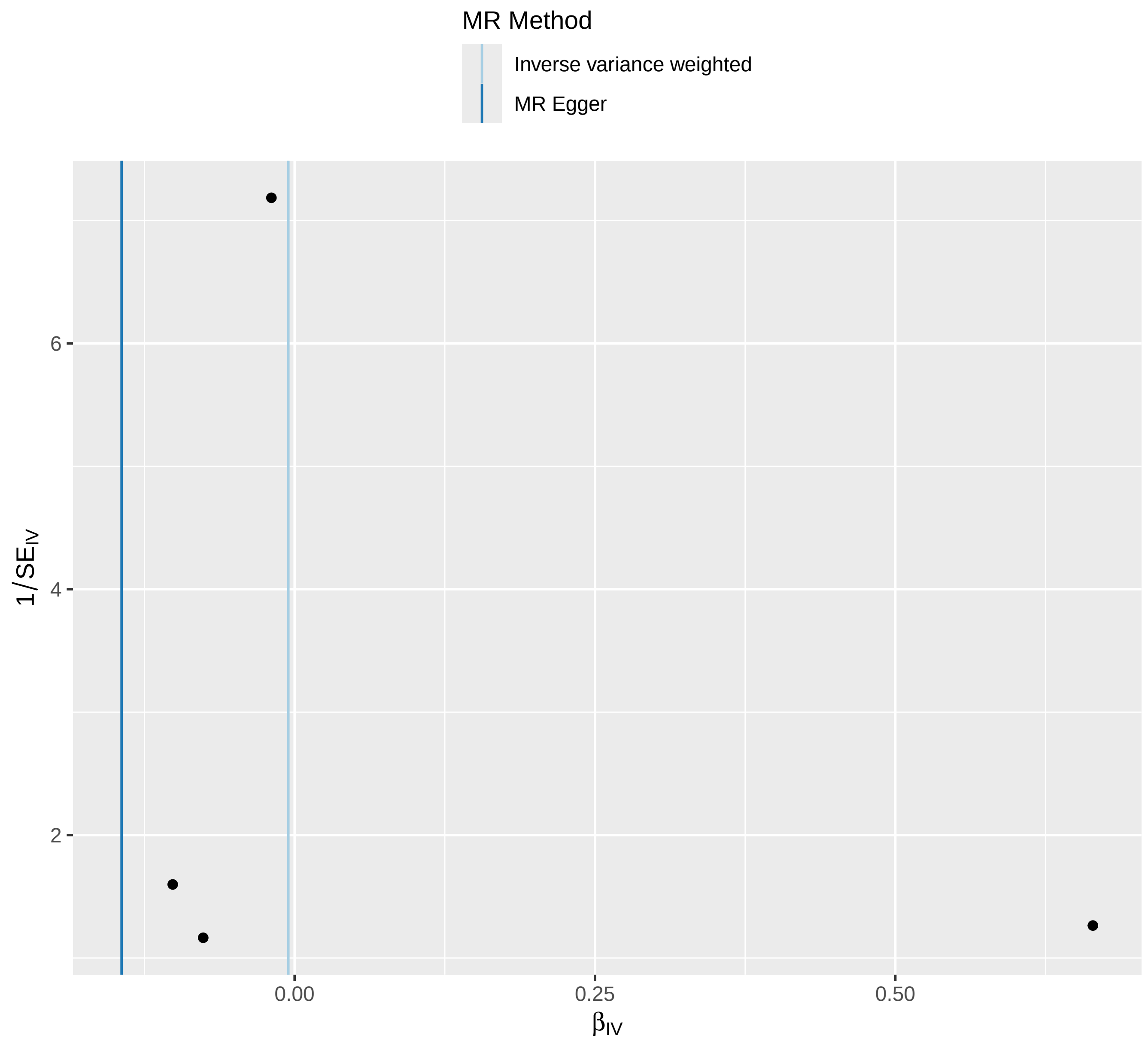
MR Method
Inverse variance weighted
MR Egger
6
4
2
$1/SE_{IV}$
0.00
0.25
0.50
β_{IV}

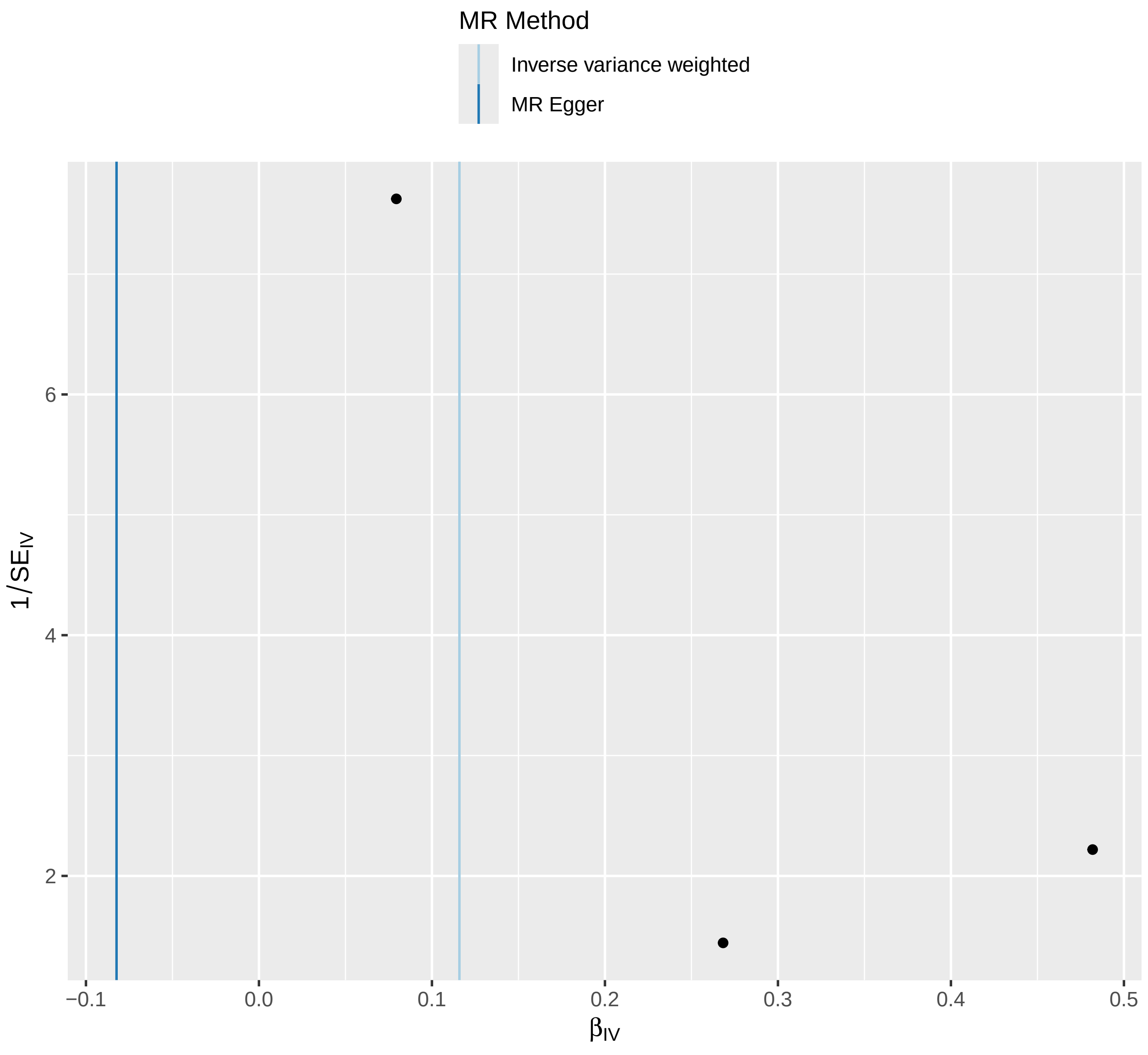
MR Method
Inverse variance weighted
MR Egger
6
4
2
1/SE_IV
−0.1
0.0
0.1
0.2
0.3
0.4
0.5
β_IV

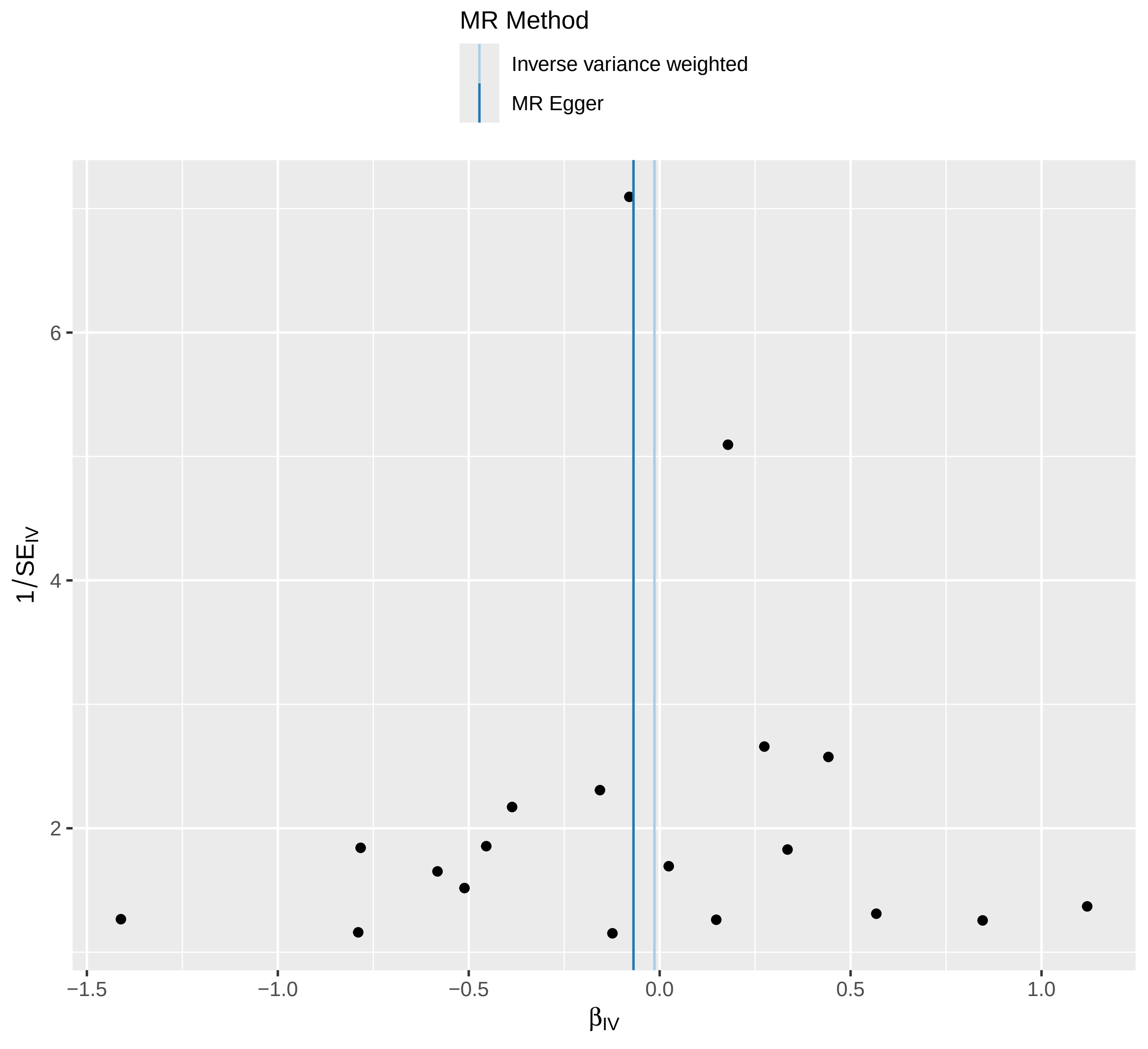
MR Method
Inverse variance weighted
MR Egger
6
4
2
$1/SE_{IV}$
−1.5
−1.0
−0.5
0.0
0.5
1.0
β_{IV}

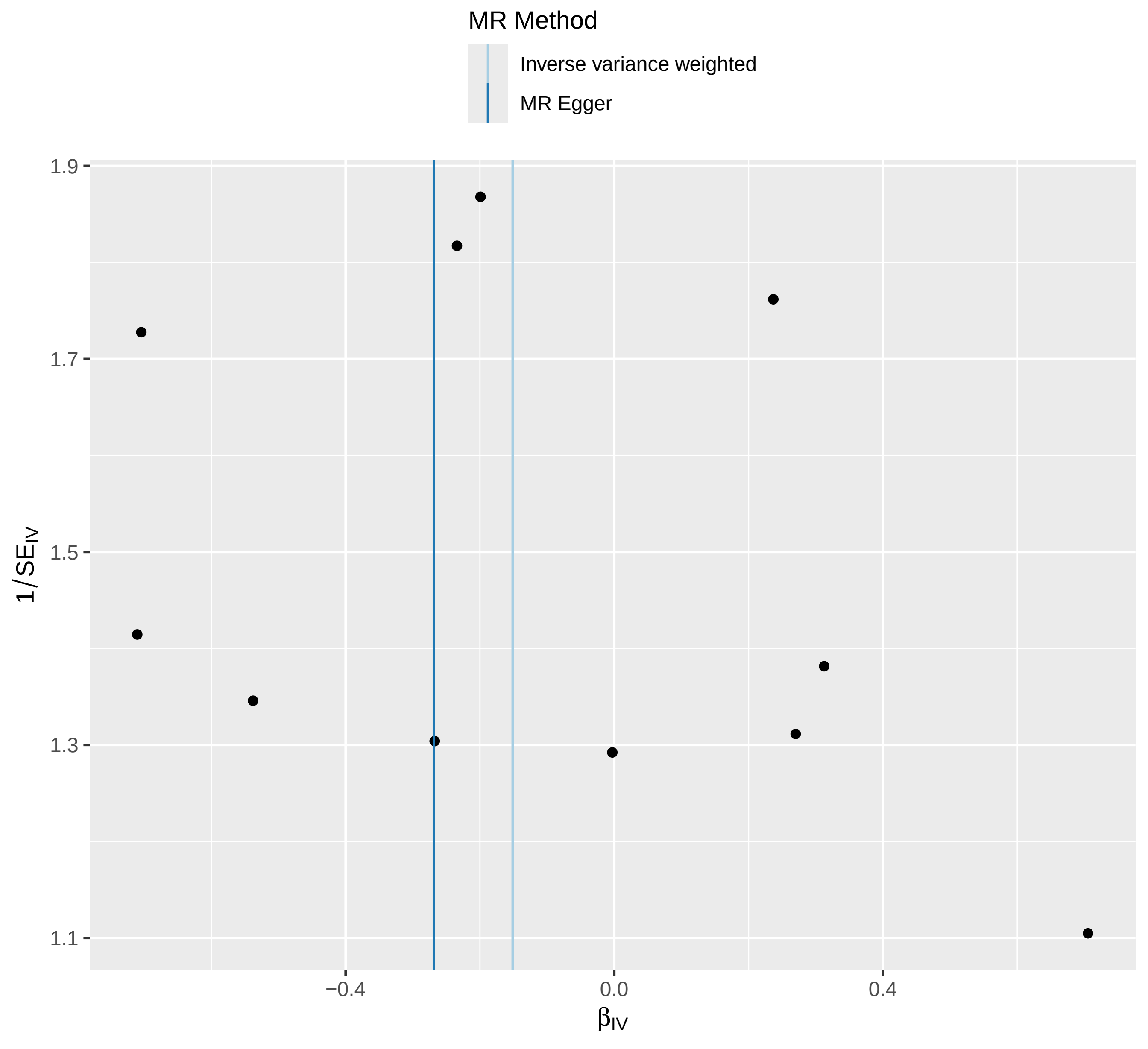
MR Method
Inverse variance weighted
MR Egger
1.9
1.7
1.5
1.3
1.1
$1/SE_{IV}$
−0.4
0.0
0.4
β_{IV}

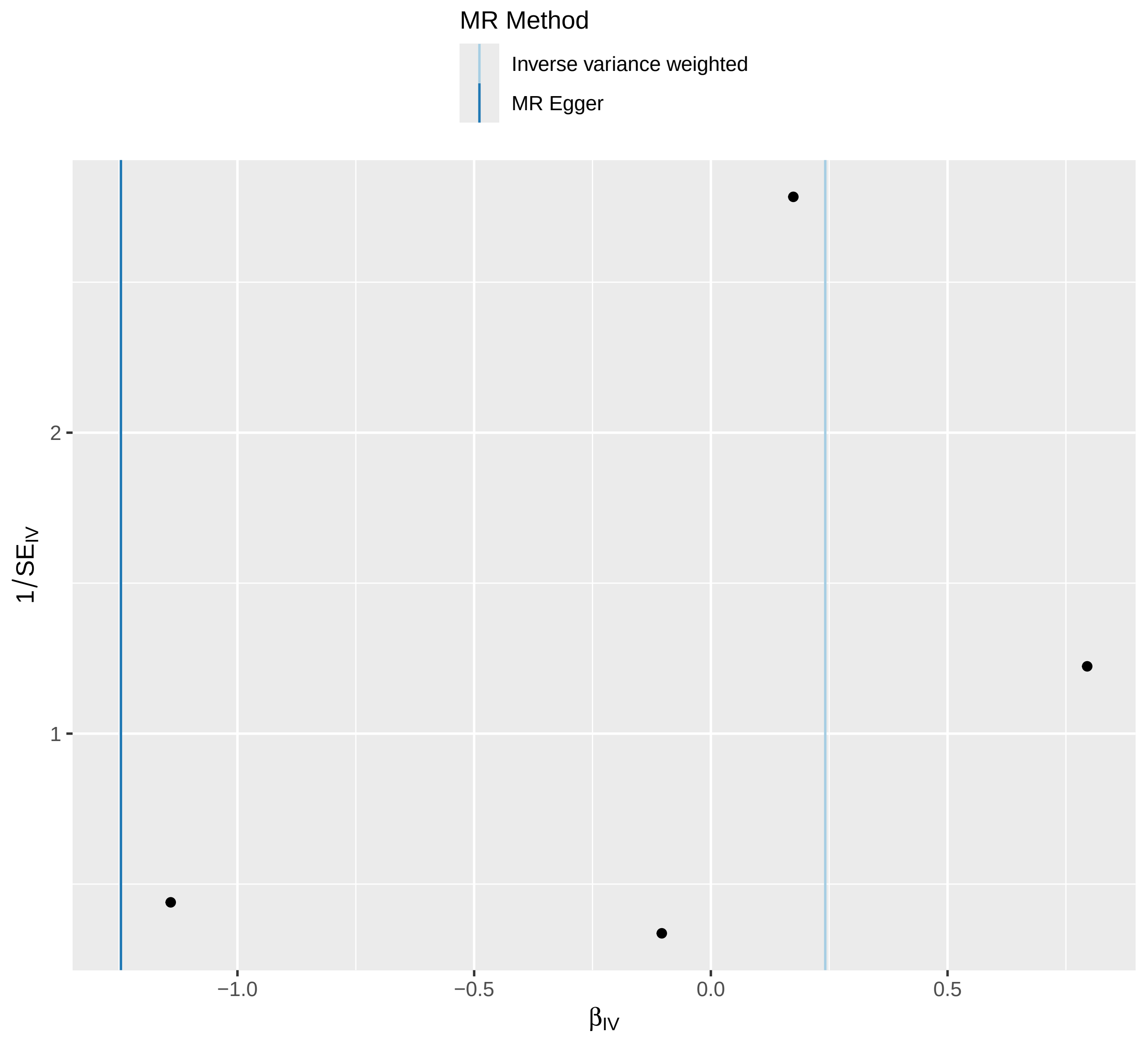
MR Method
Inverse variance weighted
MR Egger
2
1
1/SE_IV
−1.0
−0.5
0.0
0.5
β_IV

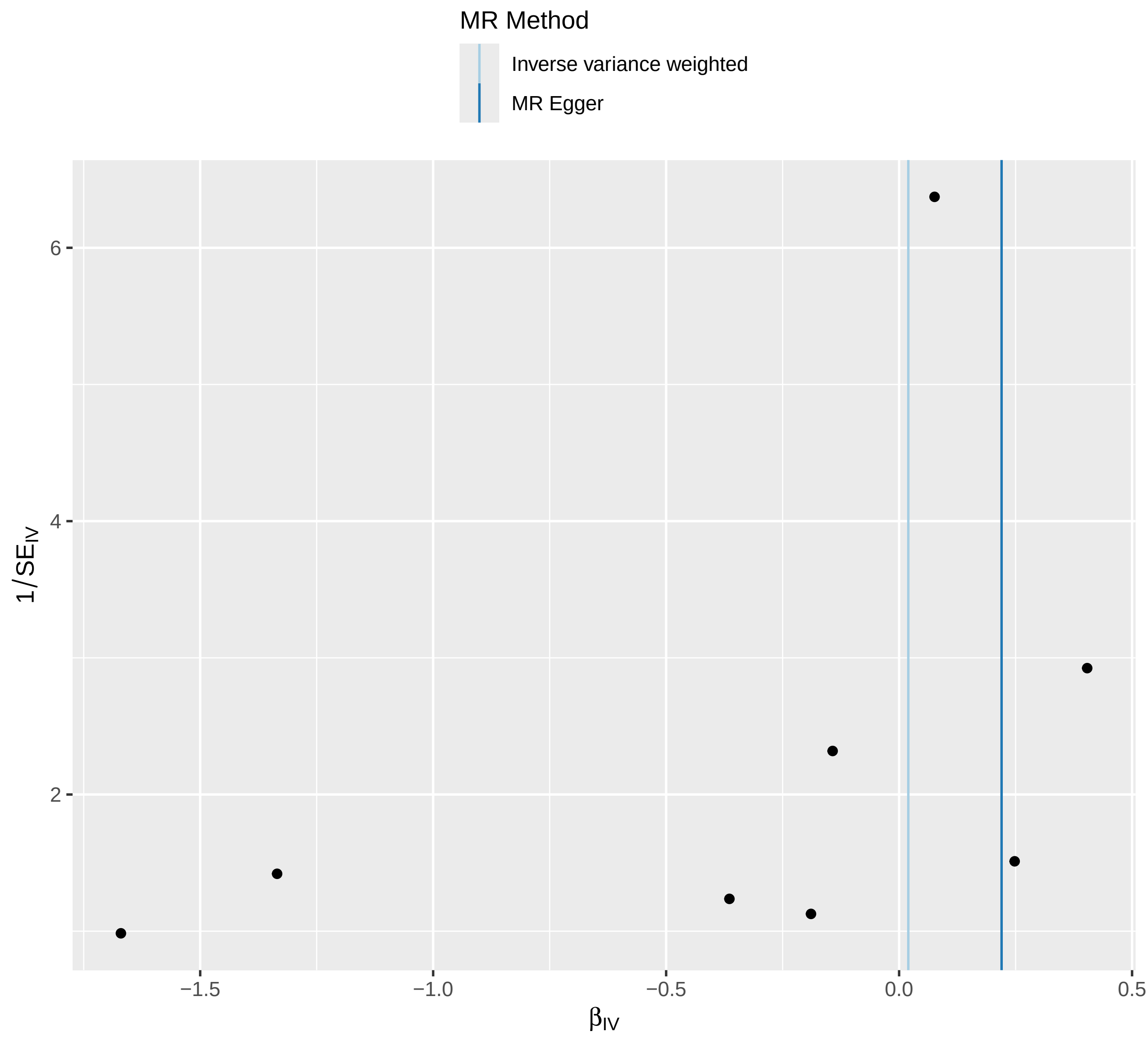
MR Method
Inverse variance weighted
MR Egger
6
4
2
$1/SE_{IV}$
−1.5
−1.0
−0.5
0.0
0.5
β_{IV}

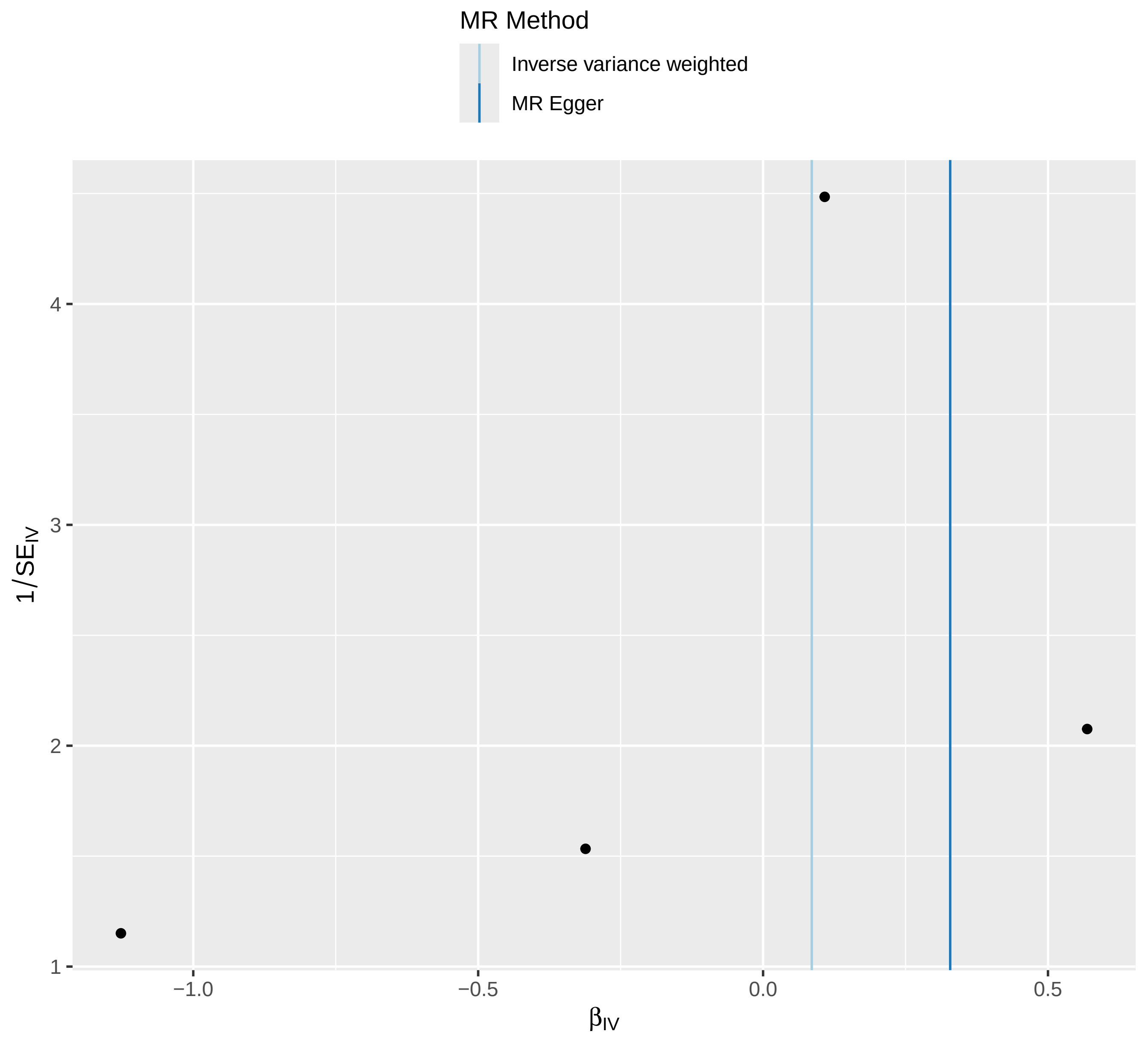
MR Method
Inverse variance weighted
MR Egger
4
3
2
1
1/SE$_{IV}$
−1.0
−0.5
0.0
0.5
β$_{IV}$

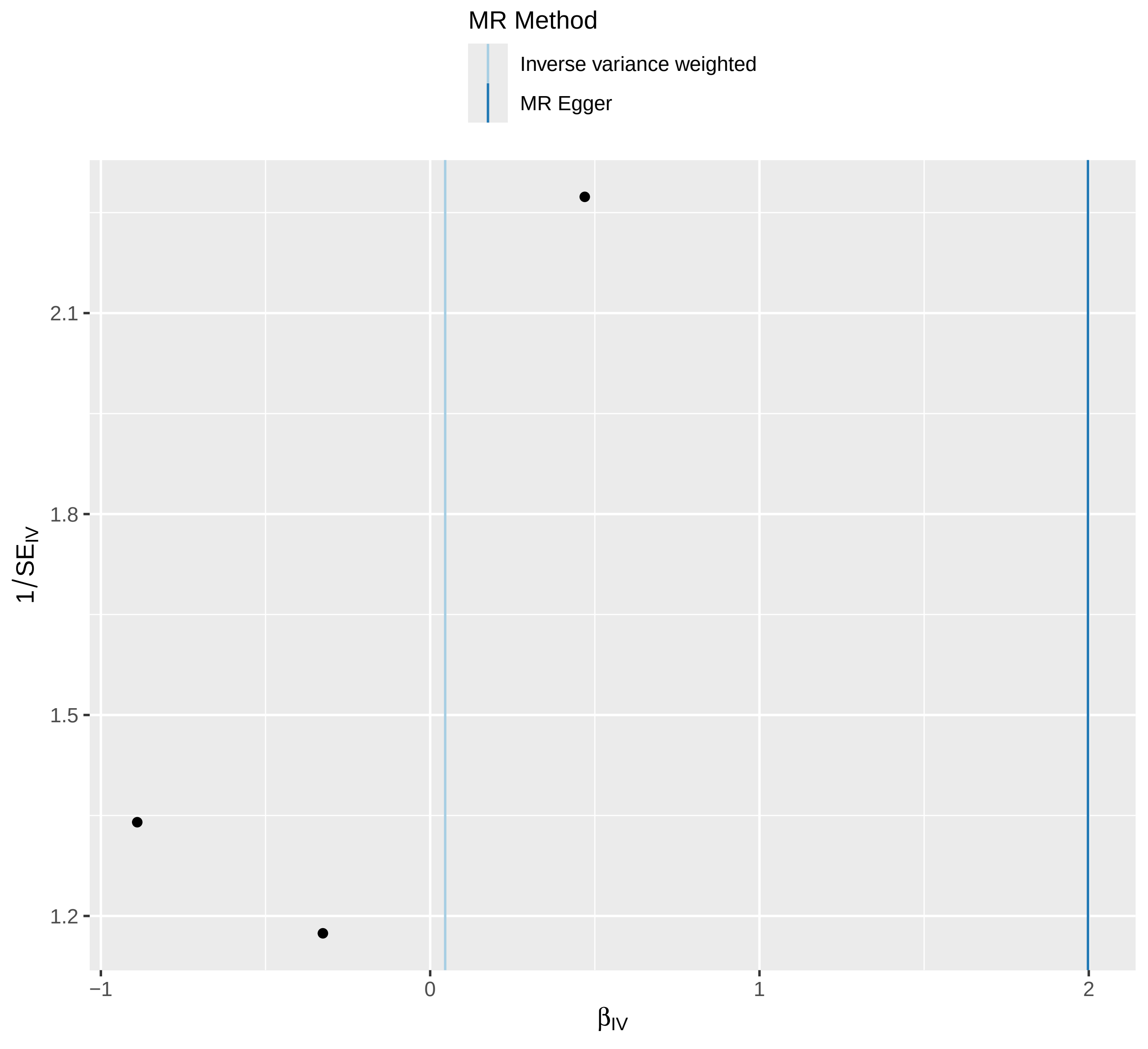
MR Method
Inverse variance weighted
MR Egger
2.1
1.8
1.5
1.2
1/SE_IV
−1
0
1
2
β_IV

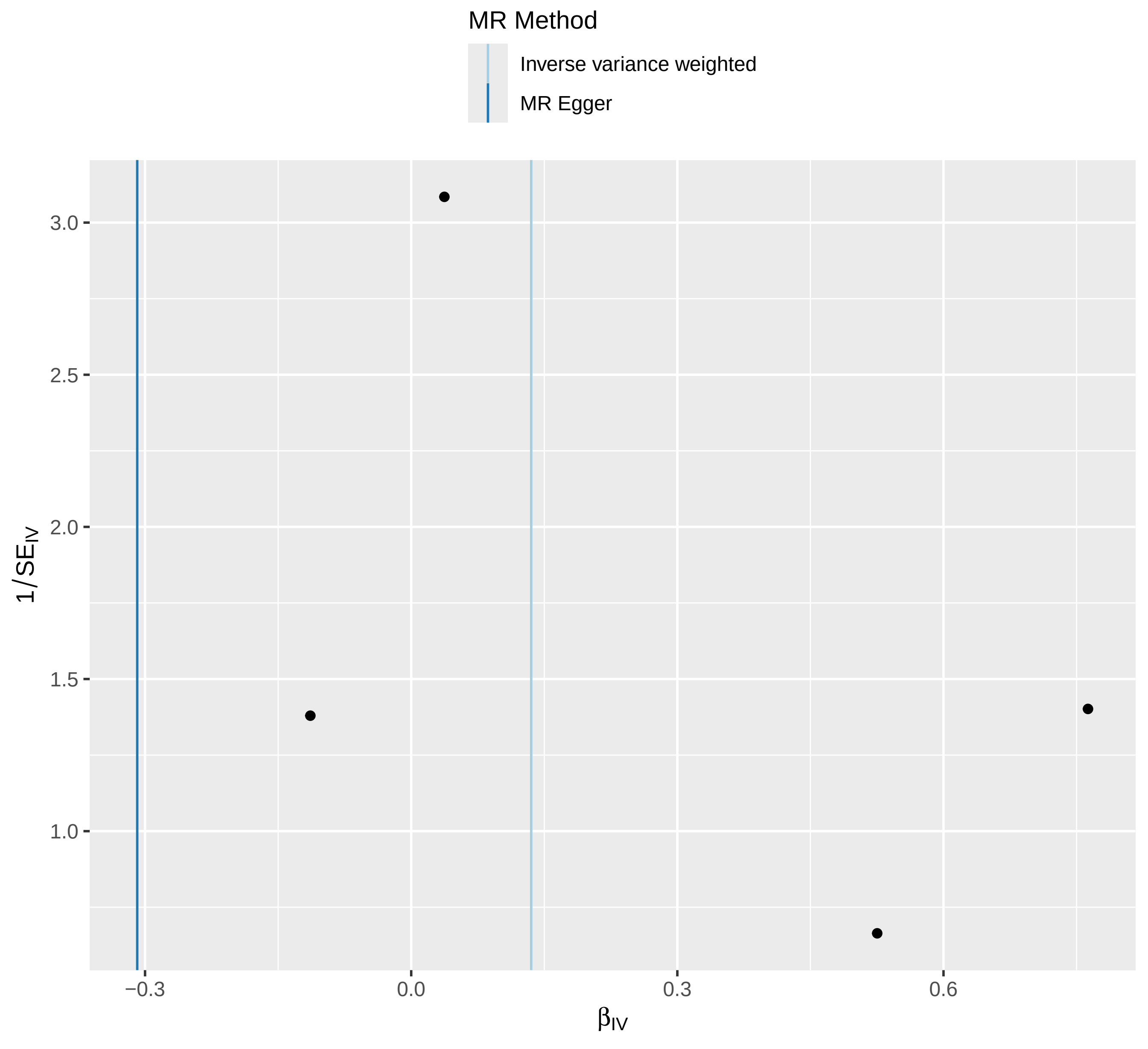

MR Method
Inverse variance weighted
MR Egger
3.0
2.5
2.0
1.5
1.0
1/SE_IV
−0.3
0.0
0.3
0.6
β_IV

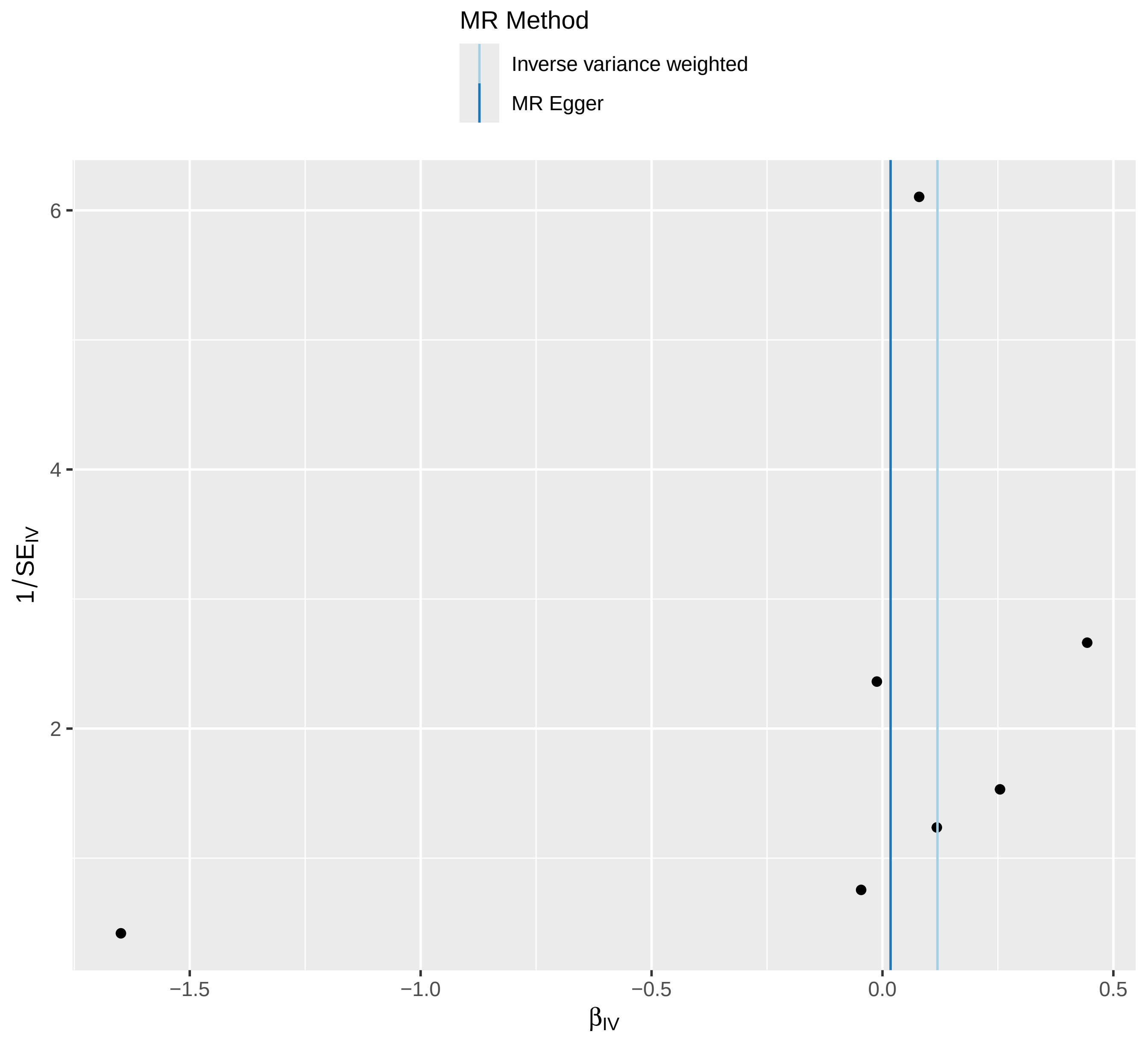

MR Method
Inverse variance weighted
MR Egger
6
4
2
1/SE_IV
−1.5
−1.0
−0.5
0.0
0.5
β_IV

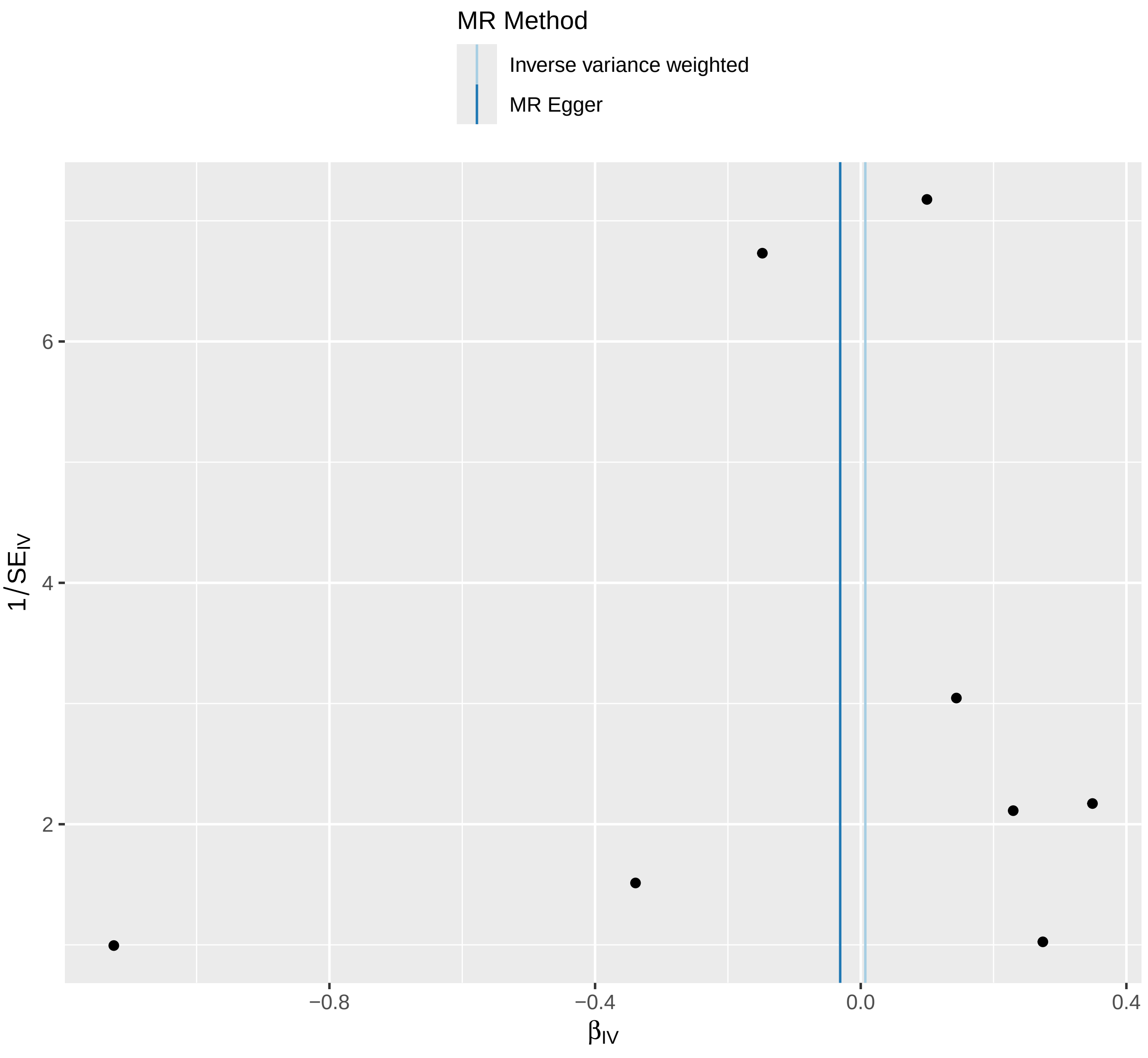

MR Method
Inverse variance weighted
MR Egger
6
4
2
1/SE_IV
−0.8
−0.4
0.0
0.4
β_IV

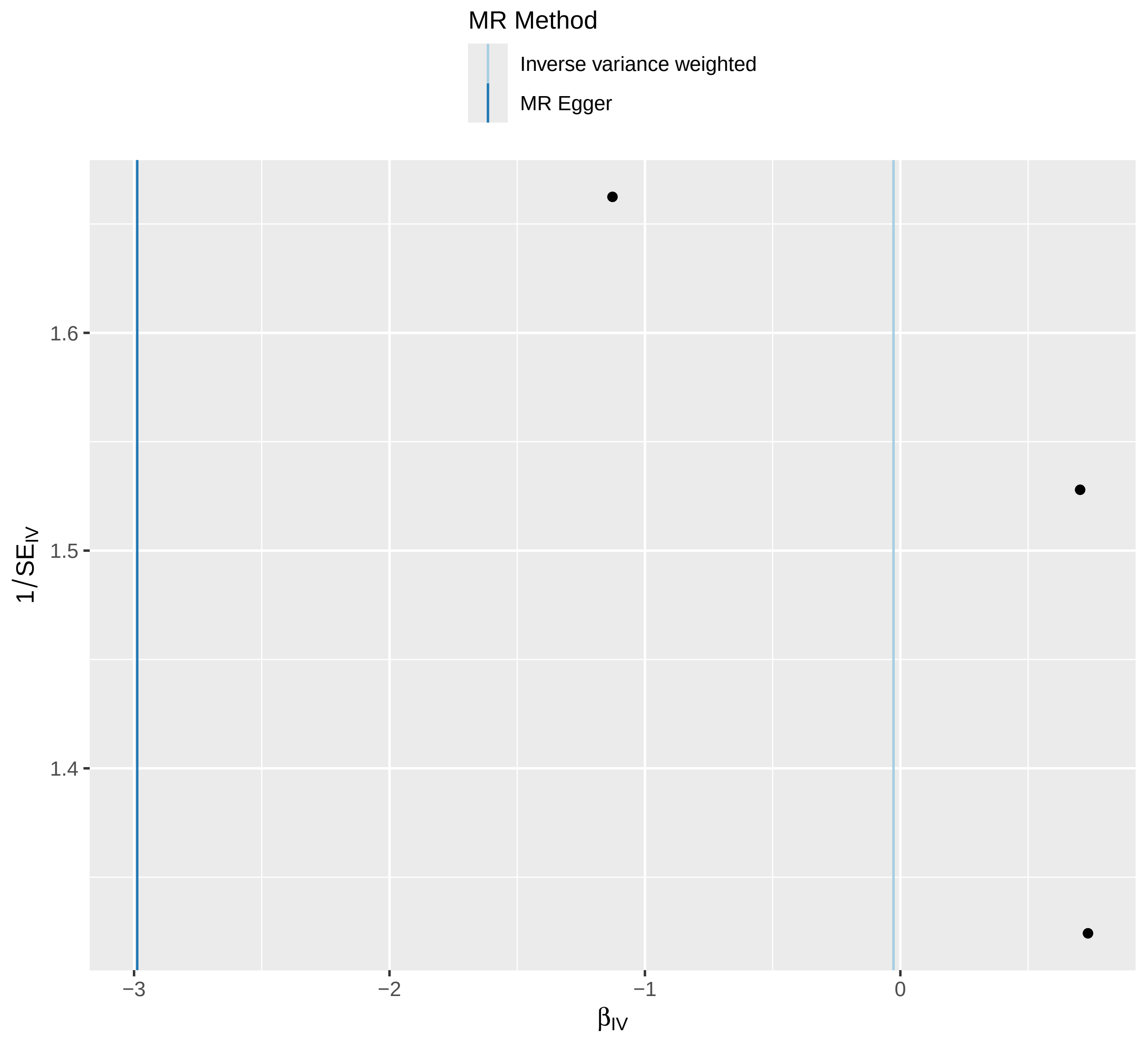

MR Method
Inverse variance weighted
MR Egger
1.6
1.5
1.4
1/SE_IV
−3
−2
−1
0
β_IV

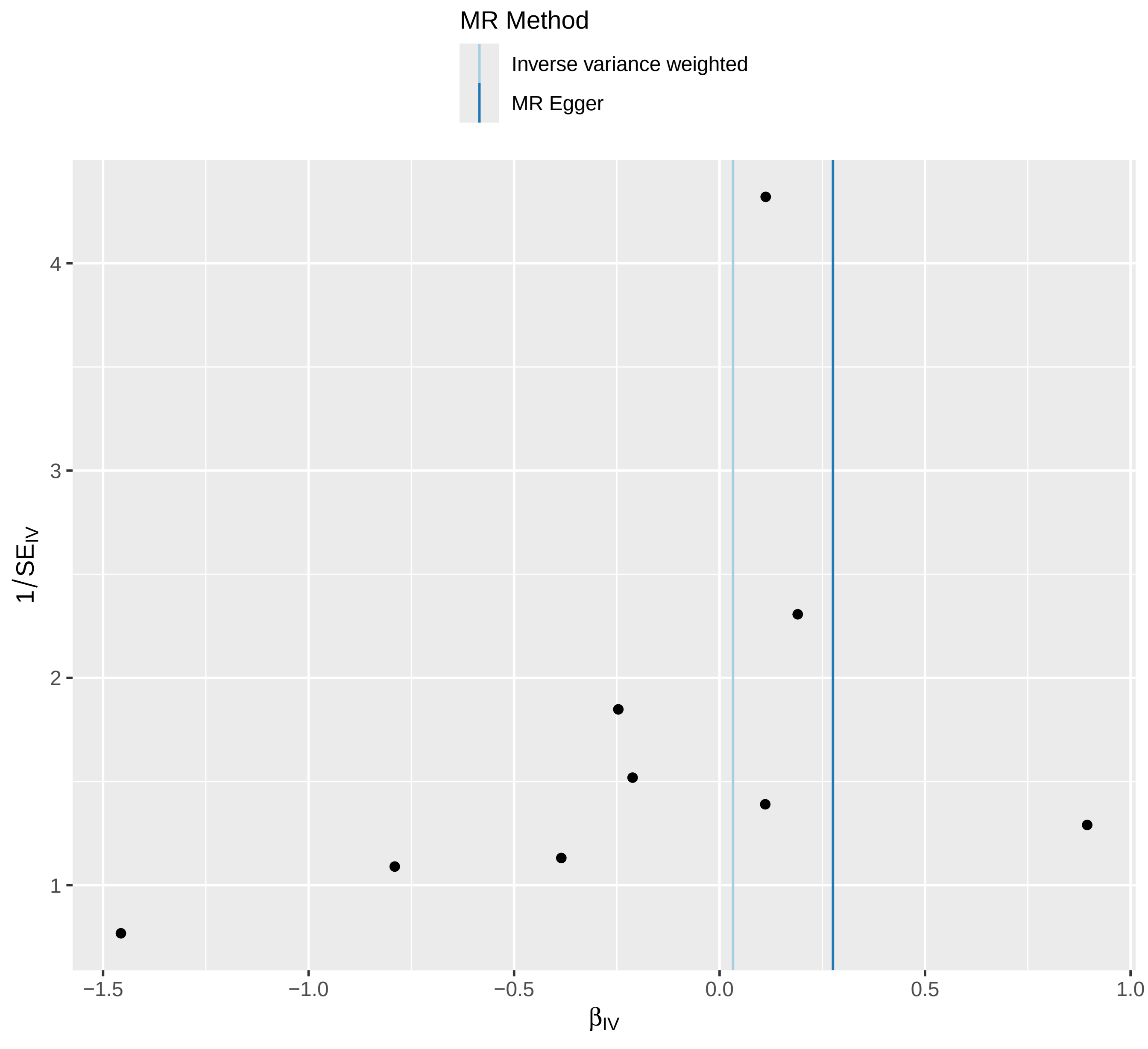
MR Method
Inverse variance weighted
MR Egger
1/SE_IV
β_IV
4
3
2
1
−1.5
−1.0
−0.5
0.0
0.5
1.0

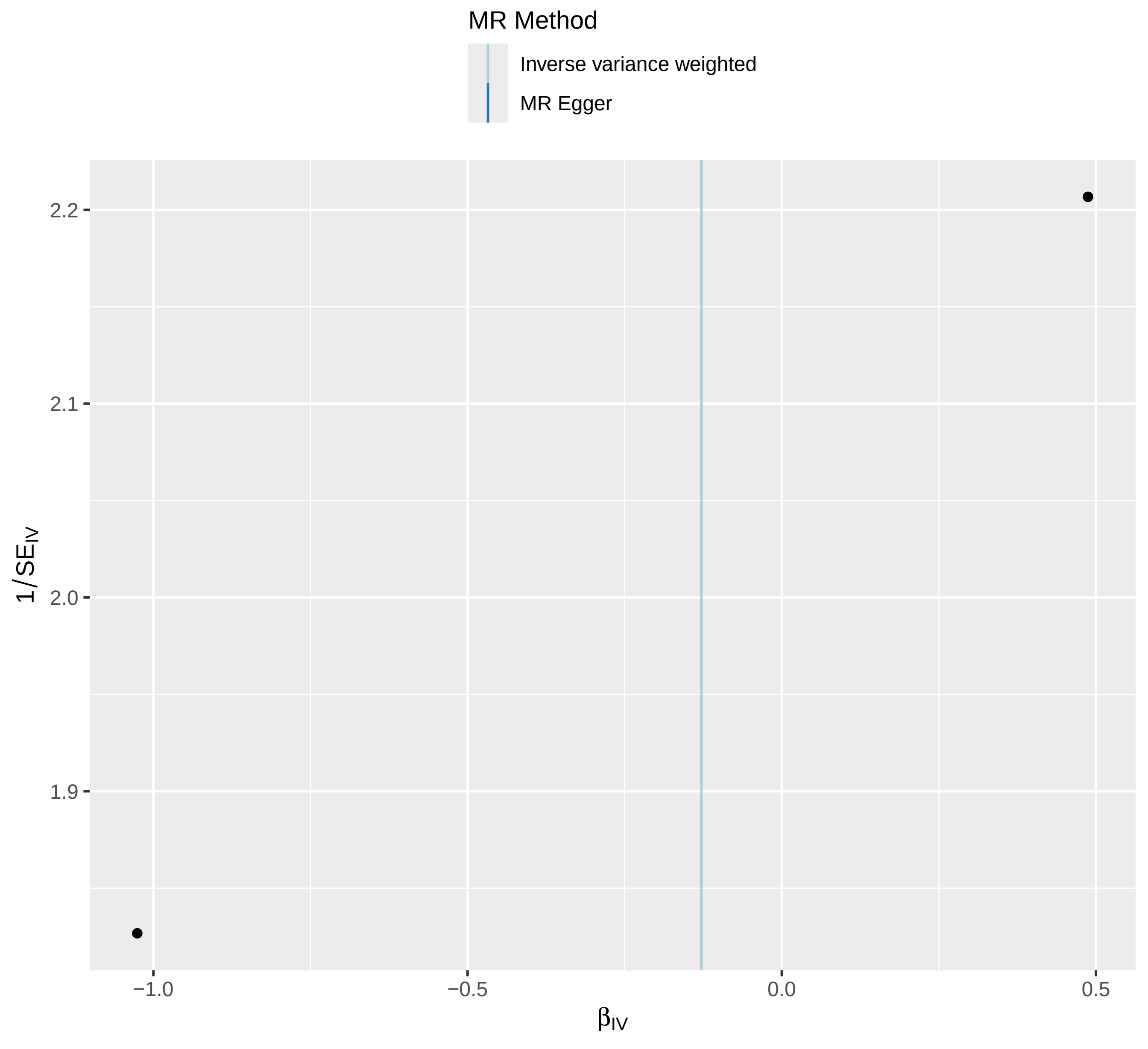
MR Method
Inverse variance weighted
MR Egger
2.2
2.1
2.0
1.9
$1/SE_{IV}$
−1.0
−0.5
0.0
0.5
β_{IV}

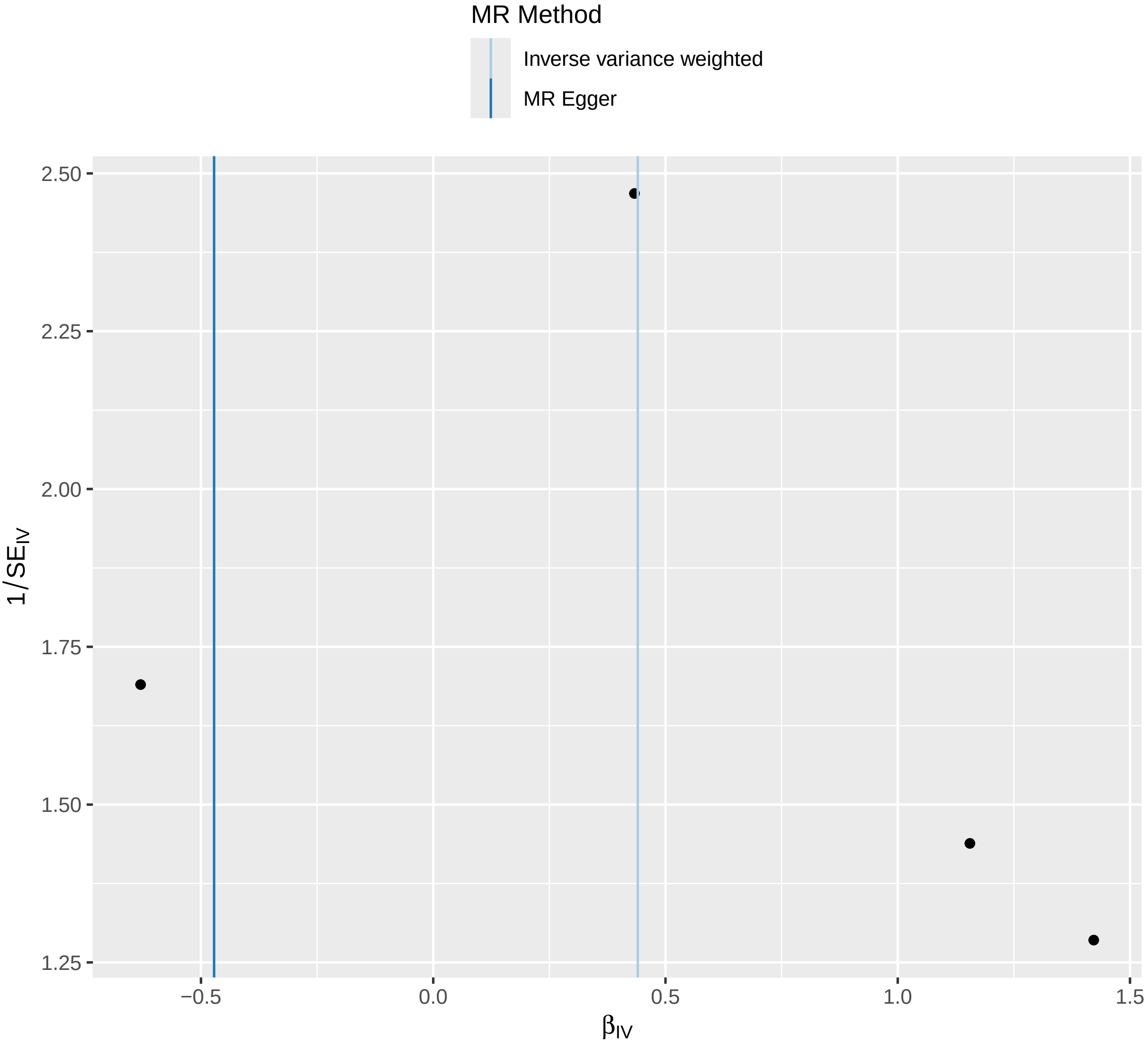

MR Method
Inverse variance weighted
MR Egger
2.50
2.25
2.00
1.75
1.50
1.25
1/SE_IV
−0.5
0.0
0.5
1.0
1.5
β_IV

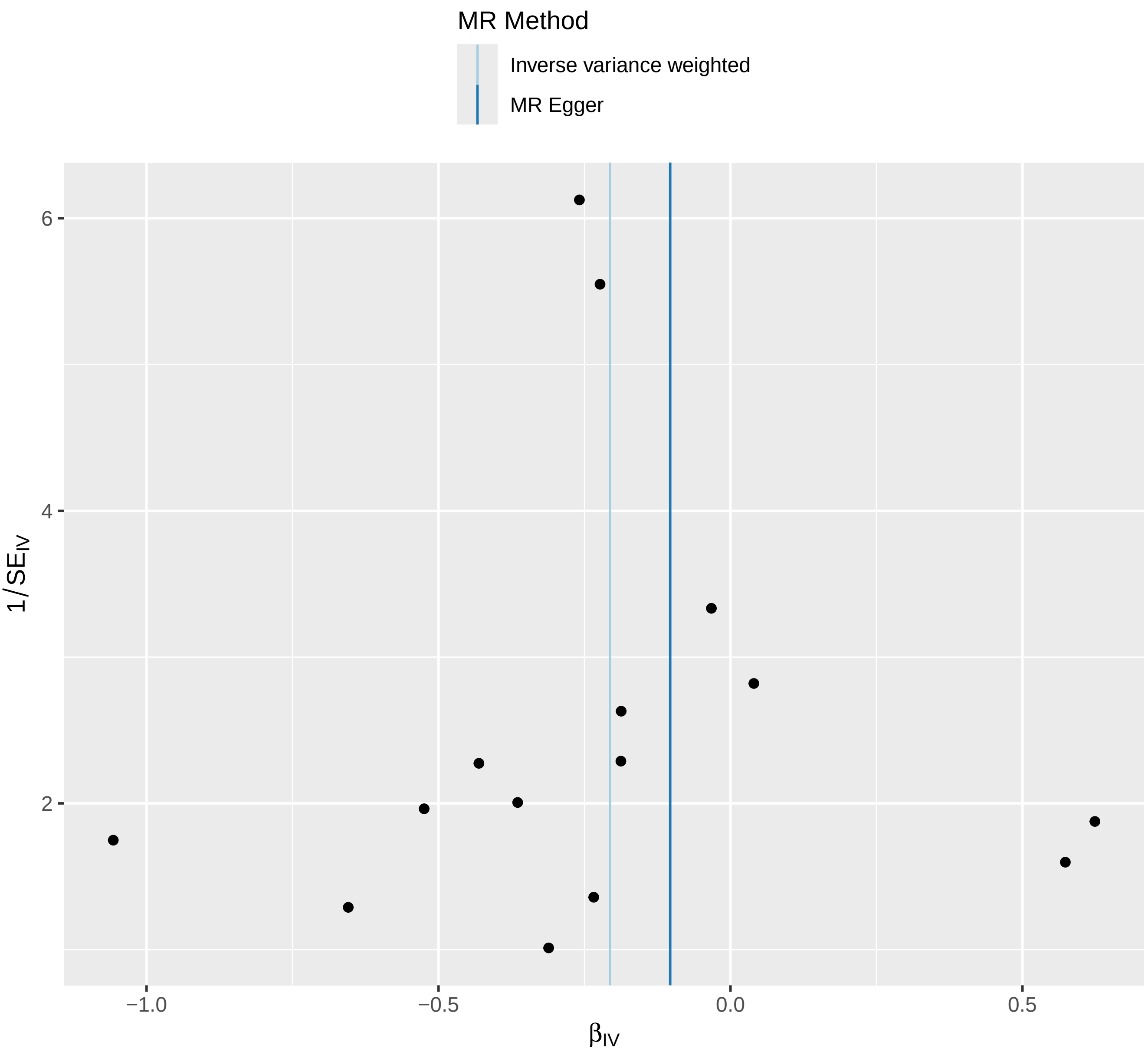
MR Method
Inverse variance weighted
MR Egger
6
4
2
1/SE_IV
−1.0
−0.5
0.0
0.5
β_IV

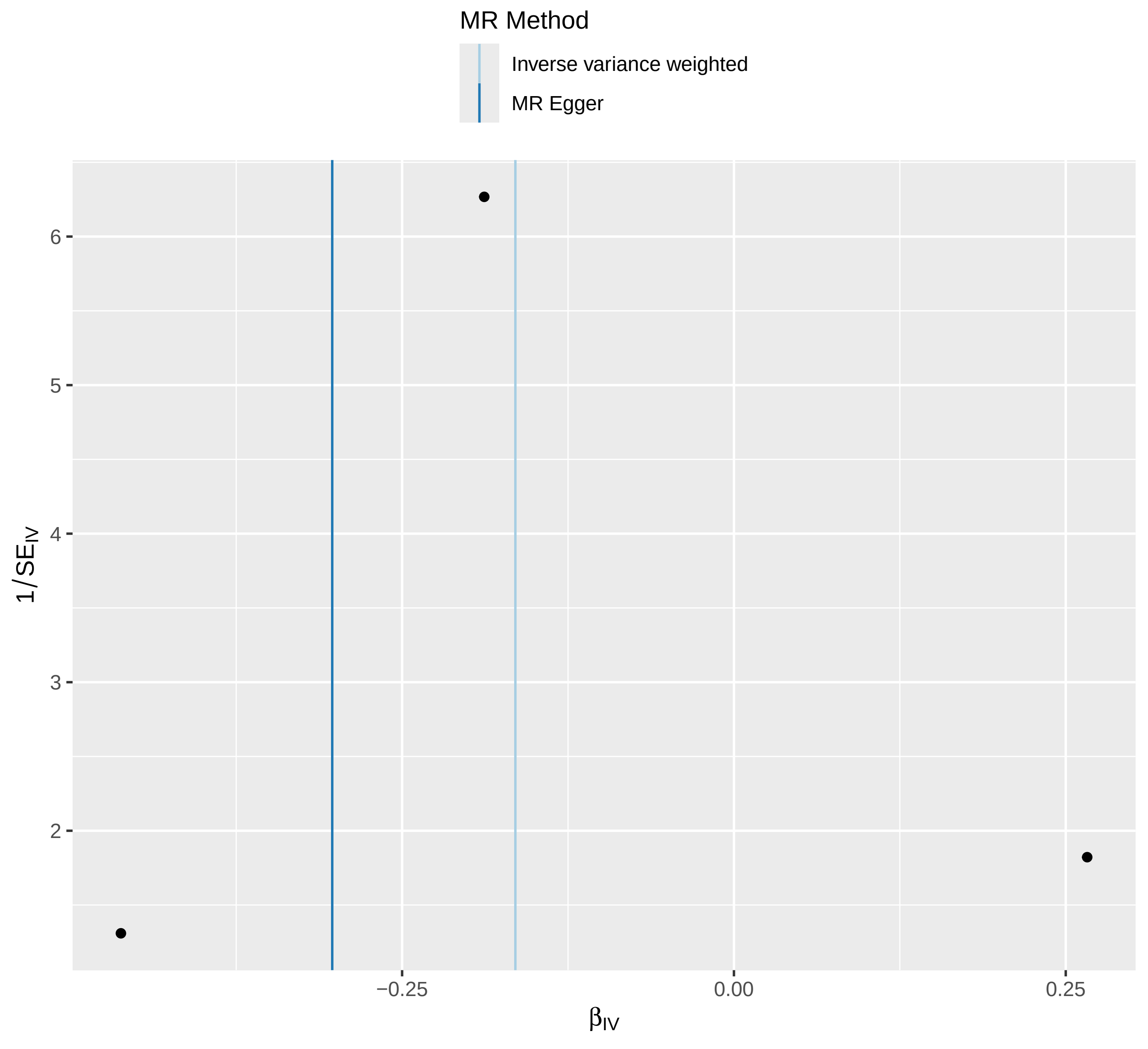
MR Method
Inverse variance weighted
MR Egger
6
5
4
3
2
1/SE$_{IV}$
−0.25
0.00
0.25
β$_{IV}$

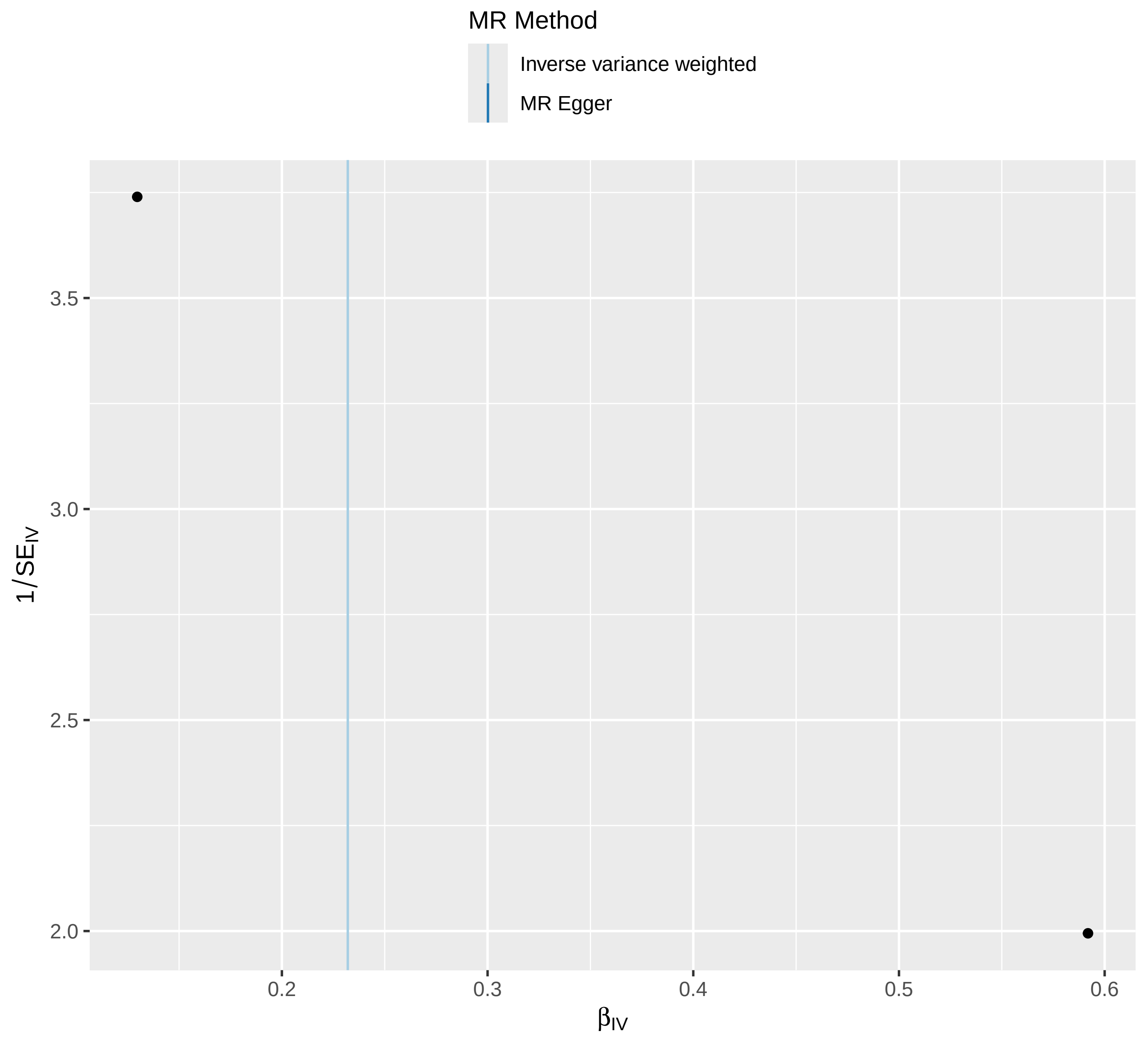

MR Method
Inverse variance weighted
MR Egger
3.5
3.0
2.5
2.0
$1/SE_{IV}$
0.2
0.3
0.4
0.5
0.6
β_{IV}

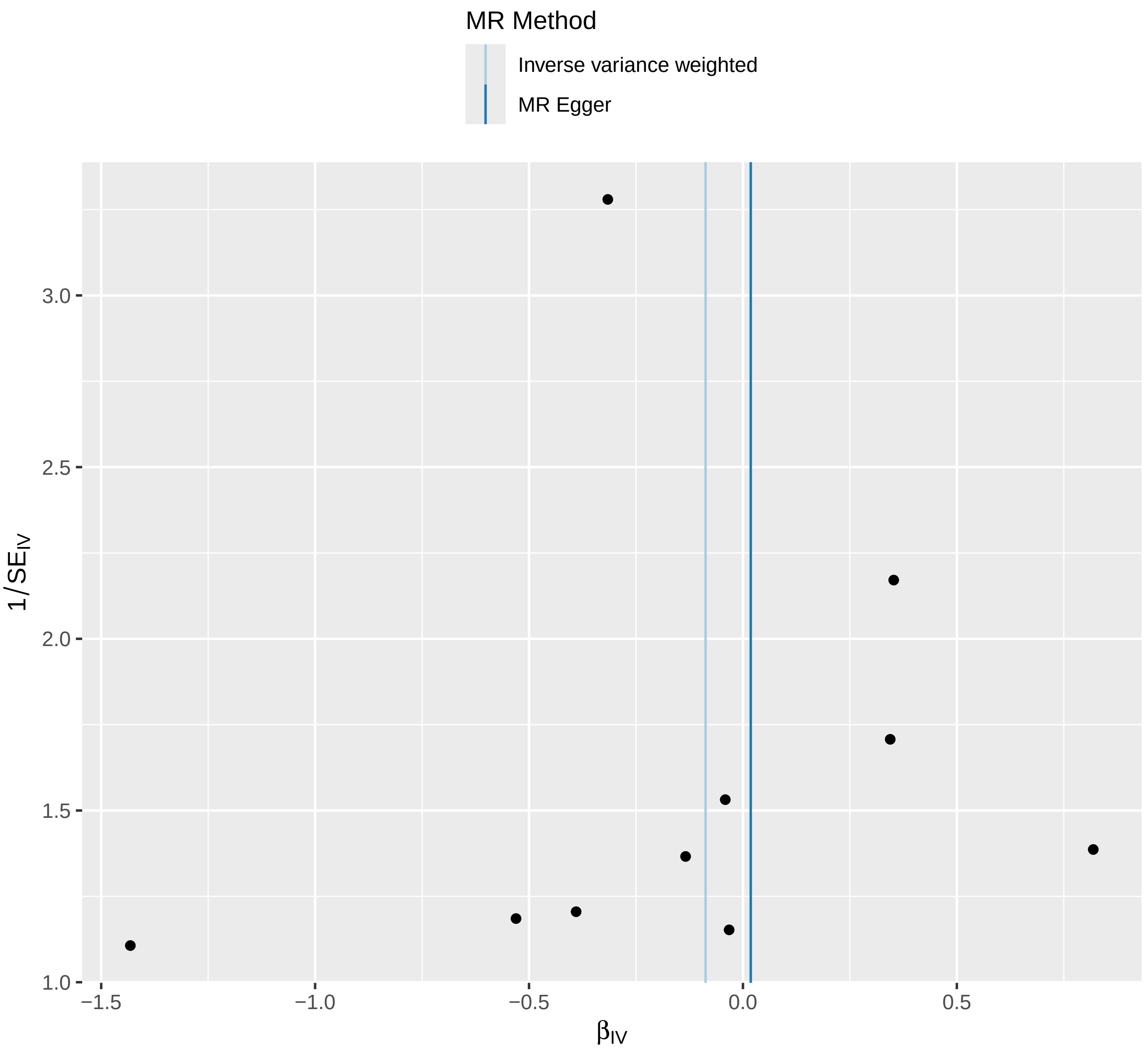

MR Method
Inverse variance weighted
MR Egger
1/SE_IV
3.0
2.5
2.0
1.5
1.0
−1.5
−1.0
−0.5
0.0
0.5
β_IV

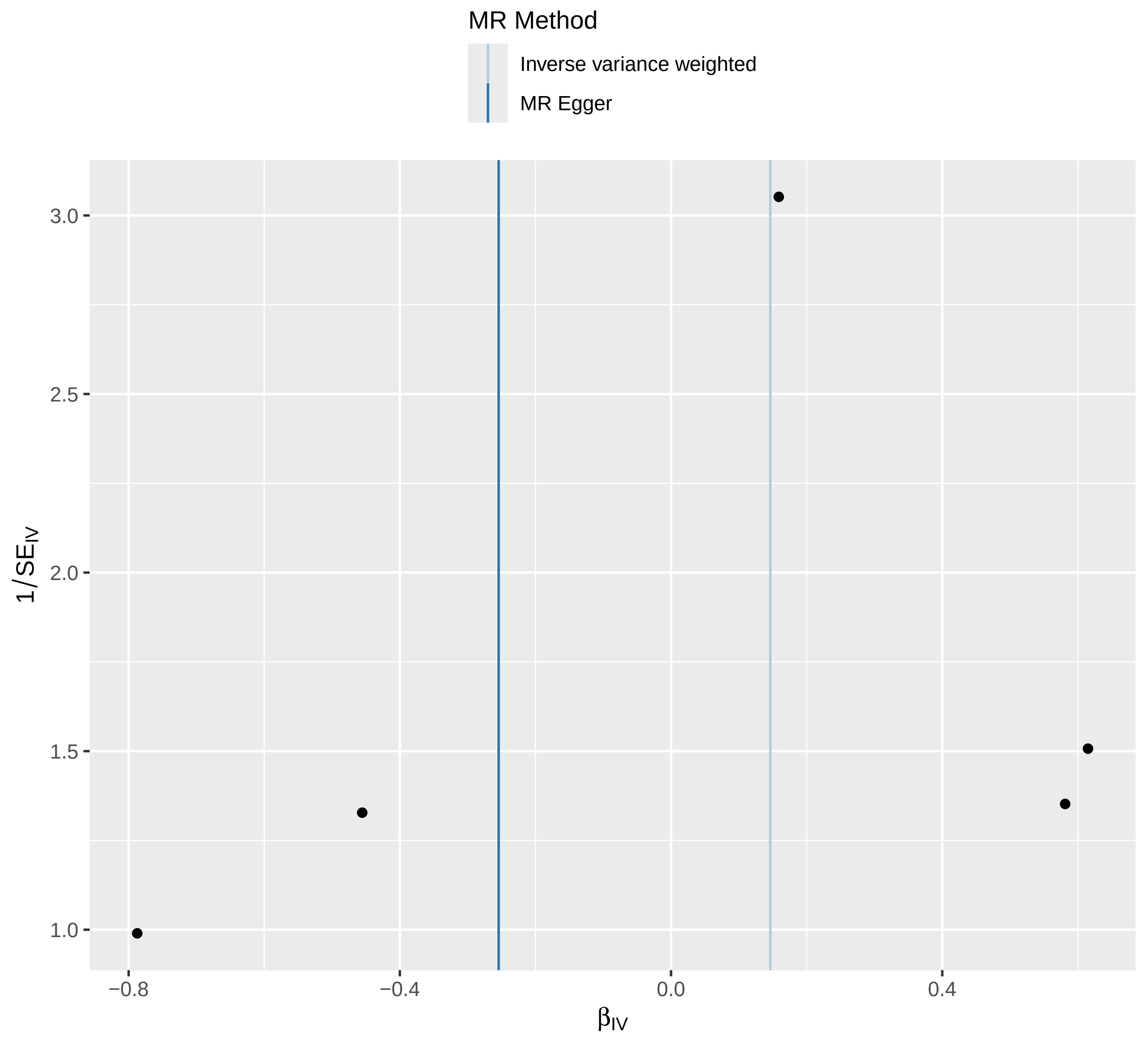
MR Method
Inverse variance weighted
MR Egger
3.0
2.5
2.0
1.5
1.0
$1/SE_{IV}$
−0.8
−0.4
0.0
0.4
β_{IV}

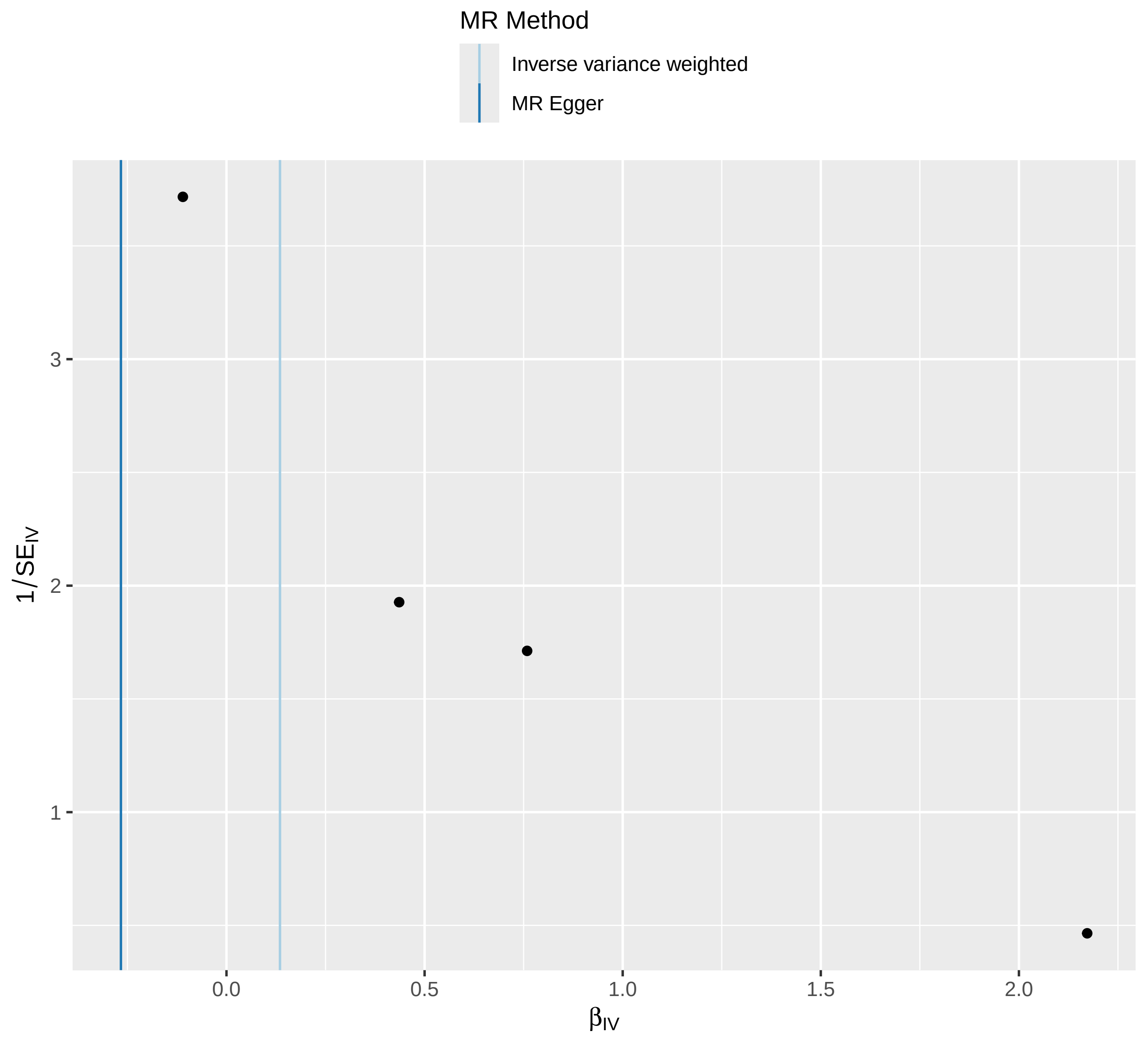
MR Method
Inverse variance weighted
MR Egger
1/SE_IV
3
2
1
0.0
0.5
1.0
1.5
2.0
β_IV

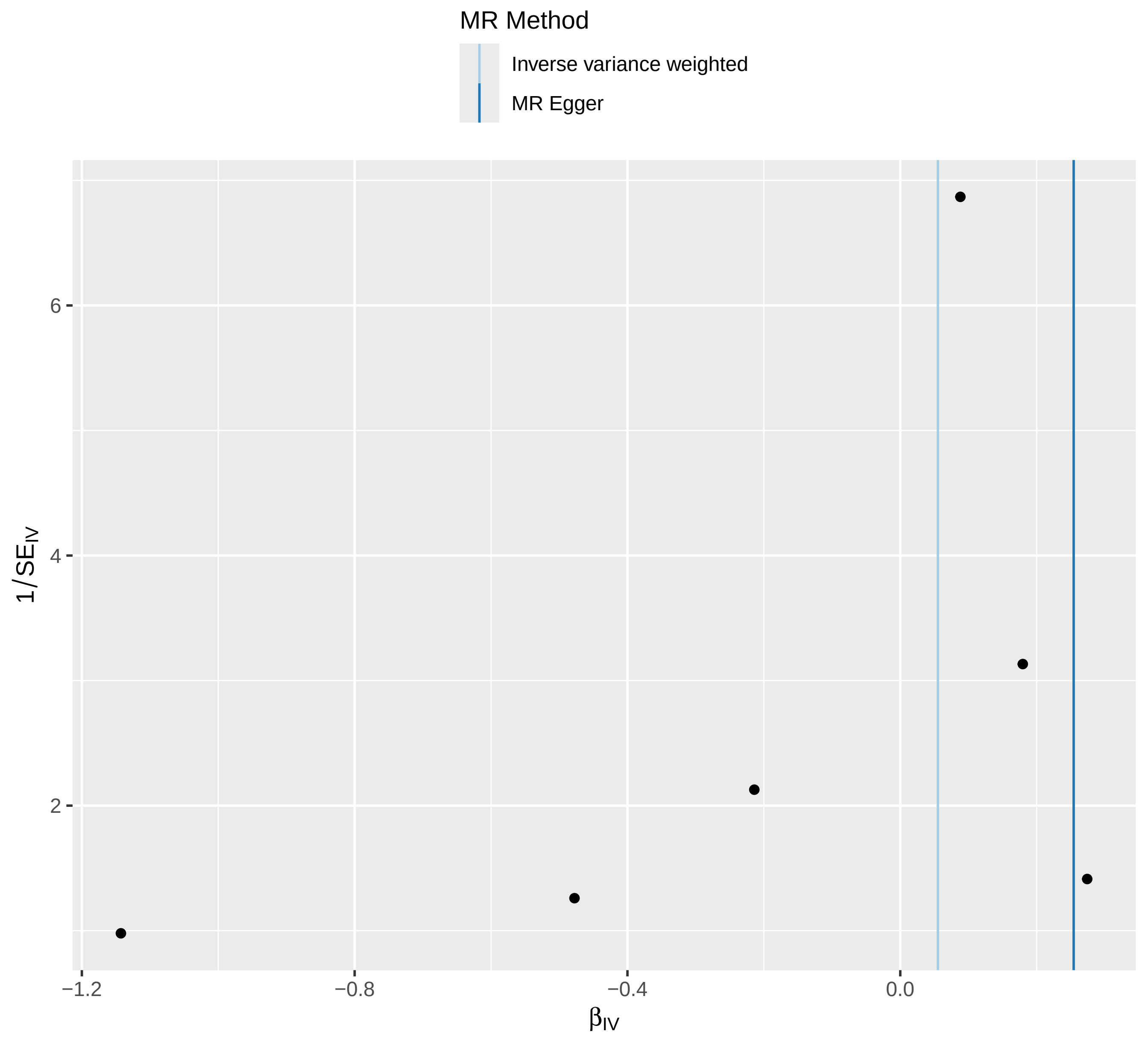

MR Method
Inverse variance weighted
MR Egger
1/SE$_{IV}$
6
4
2
−1.2
−0.8
−0.4
0.0
β$_{IV}$

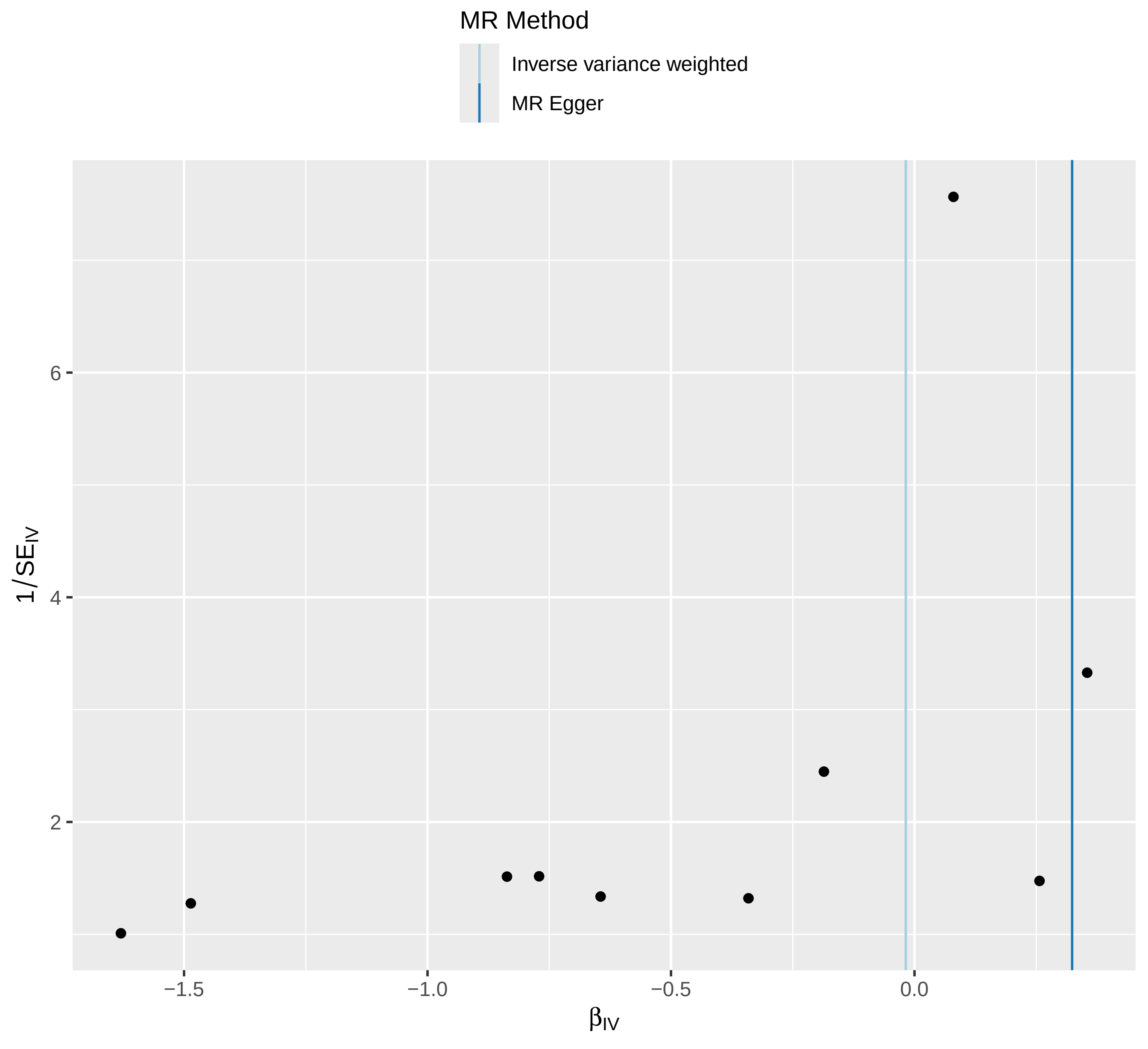

MR Method
Inverse variance weighted
MR Egger
6
4
2
1/SE_IV
−1.5
−1.0
−0.5
0.0
β_IV

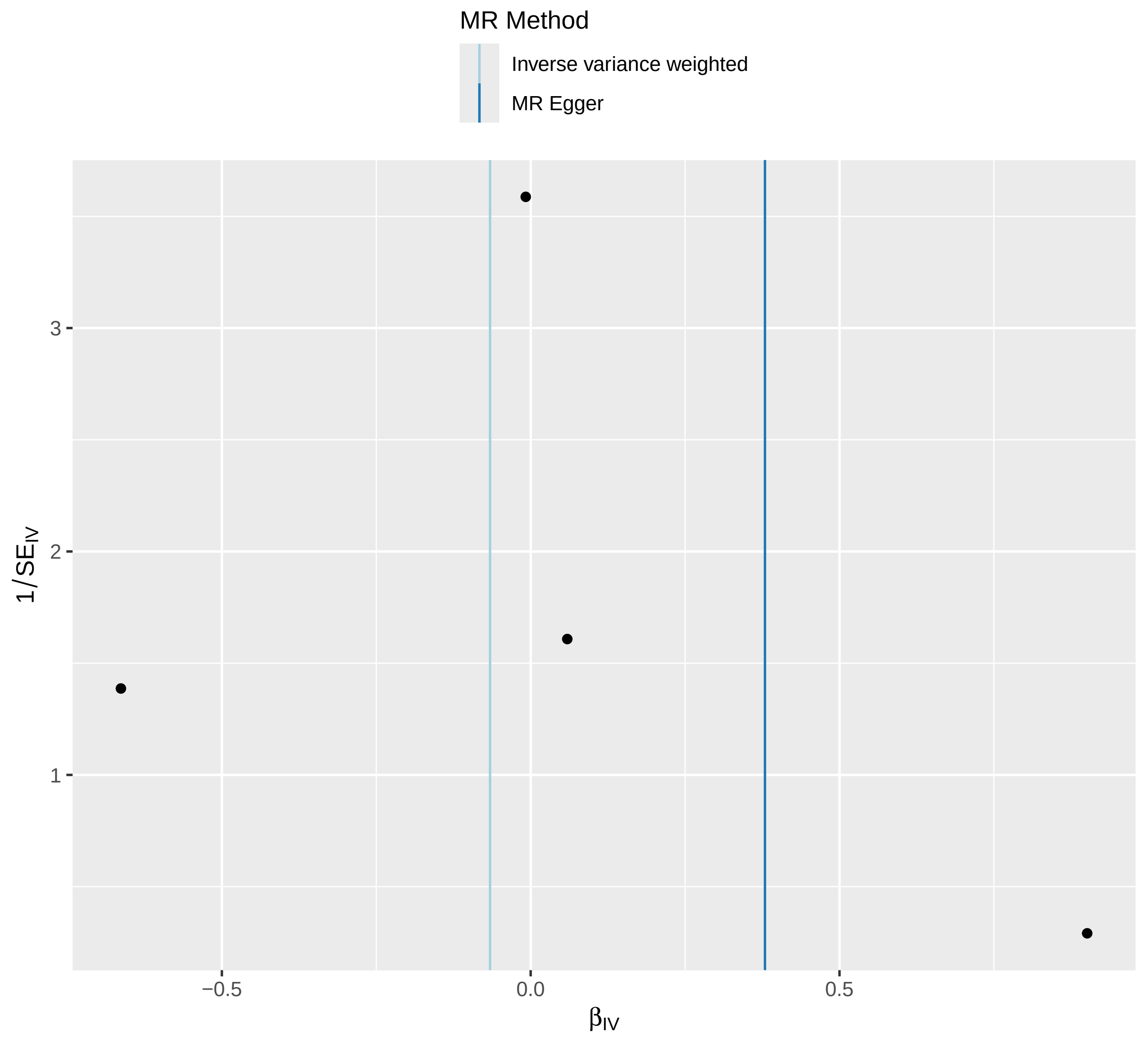
MR Method
Inverse variance weighted
MR Egger
3
2
1
$1/SE_{IV}$
−0.5
0.0
0.5
β_{IV}

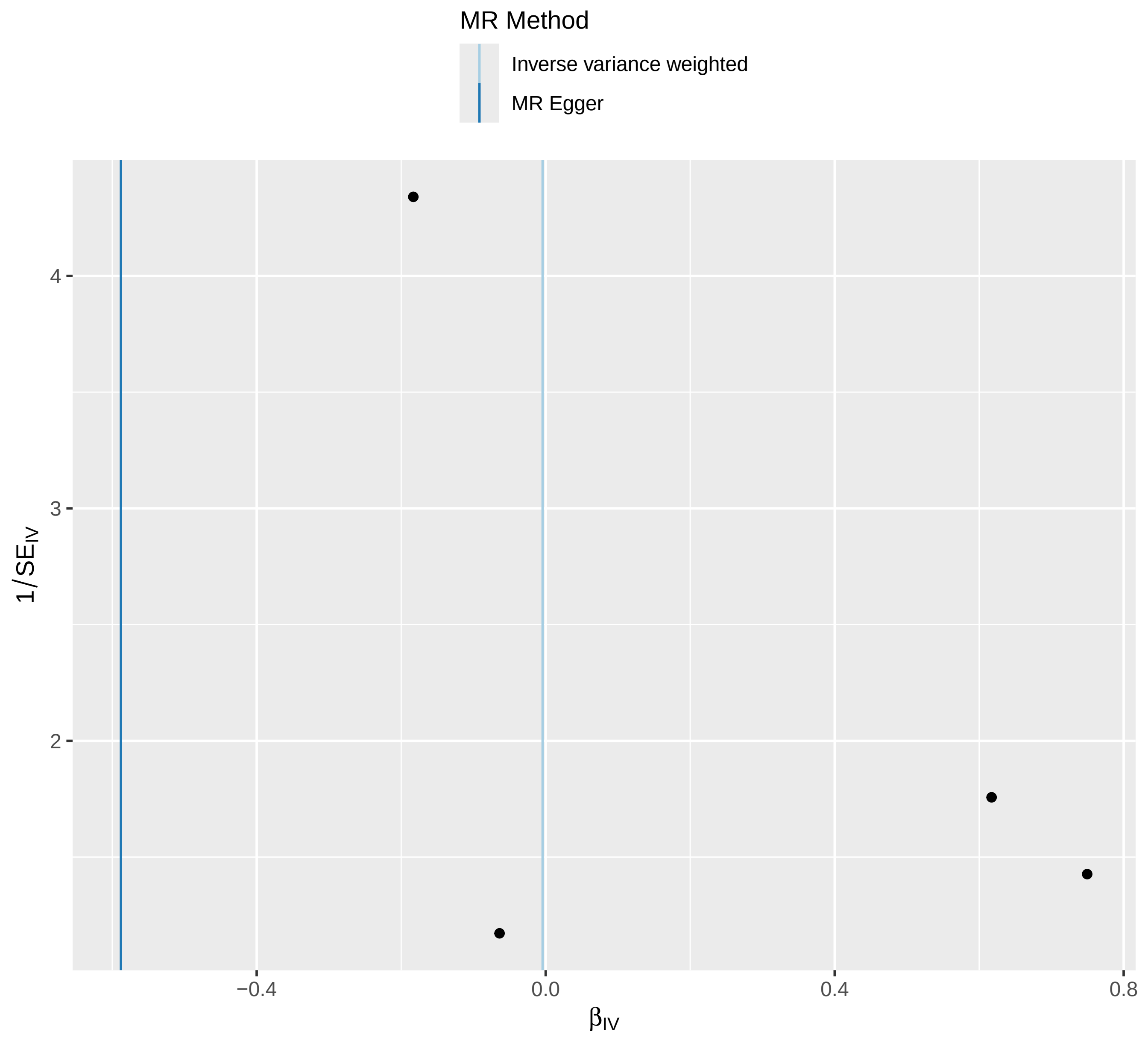

MR Method
Inverse variance weighted
MR Egger
4
3
2
1/SE_IV
−0.4
0.0
0.4
0.8
β_IV

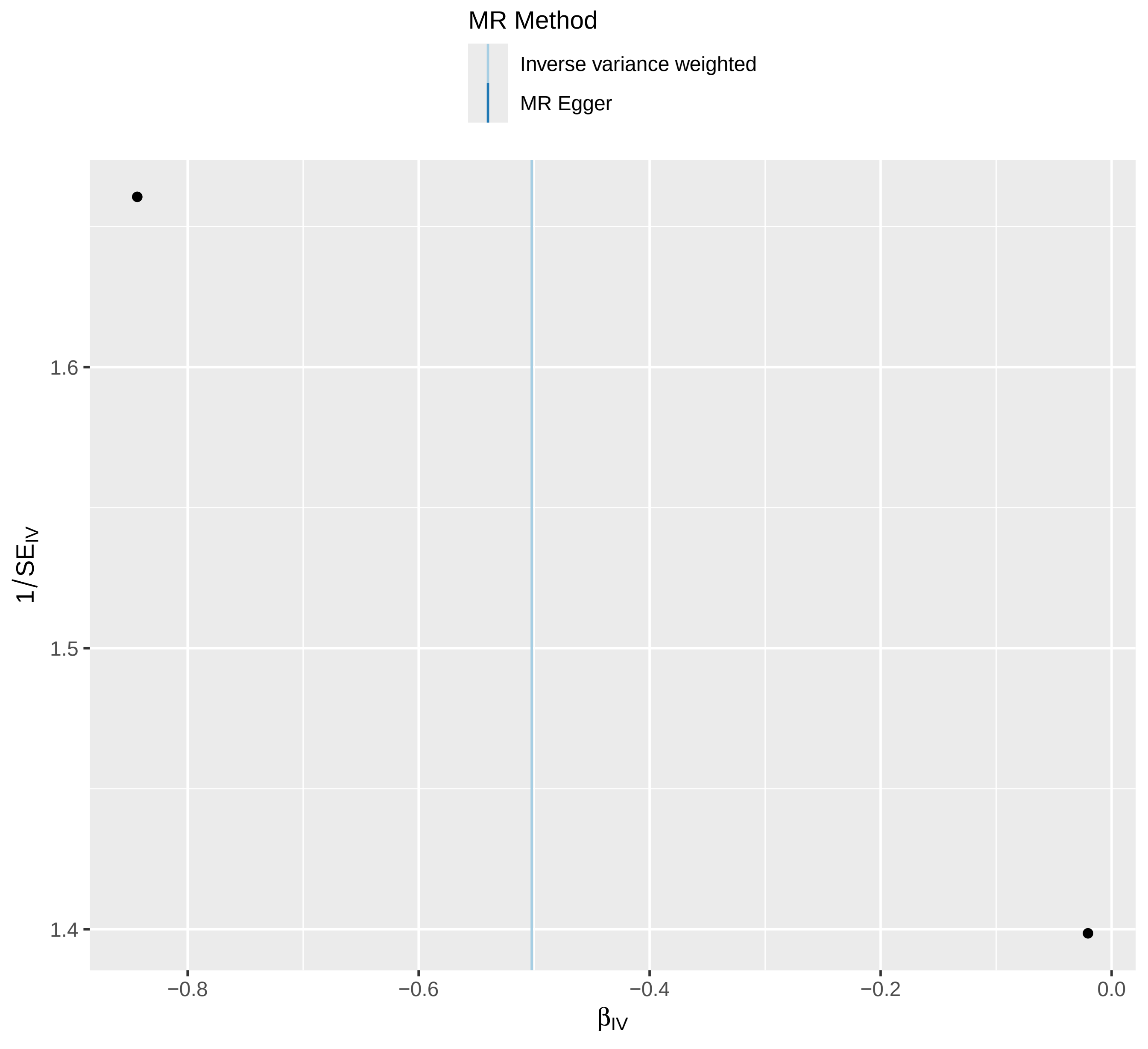
MR Method
Inverse variance weighted
MR Egger
1.6
1.5
1.4
1/SE_IV
−0.8
−0.6
−0.4
−0.2
0.0
β_IV

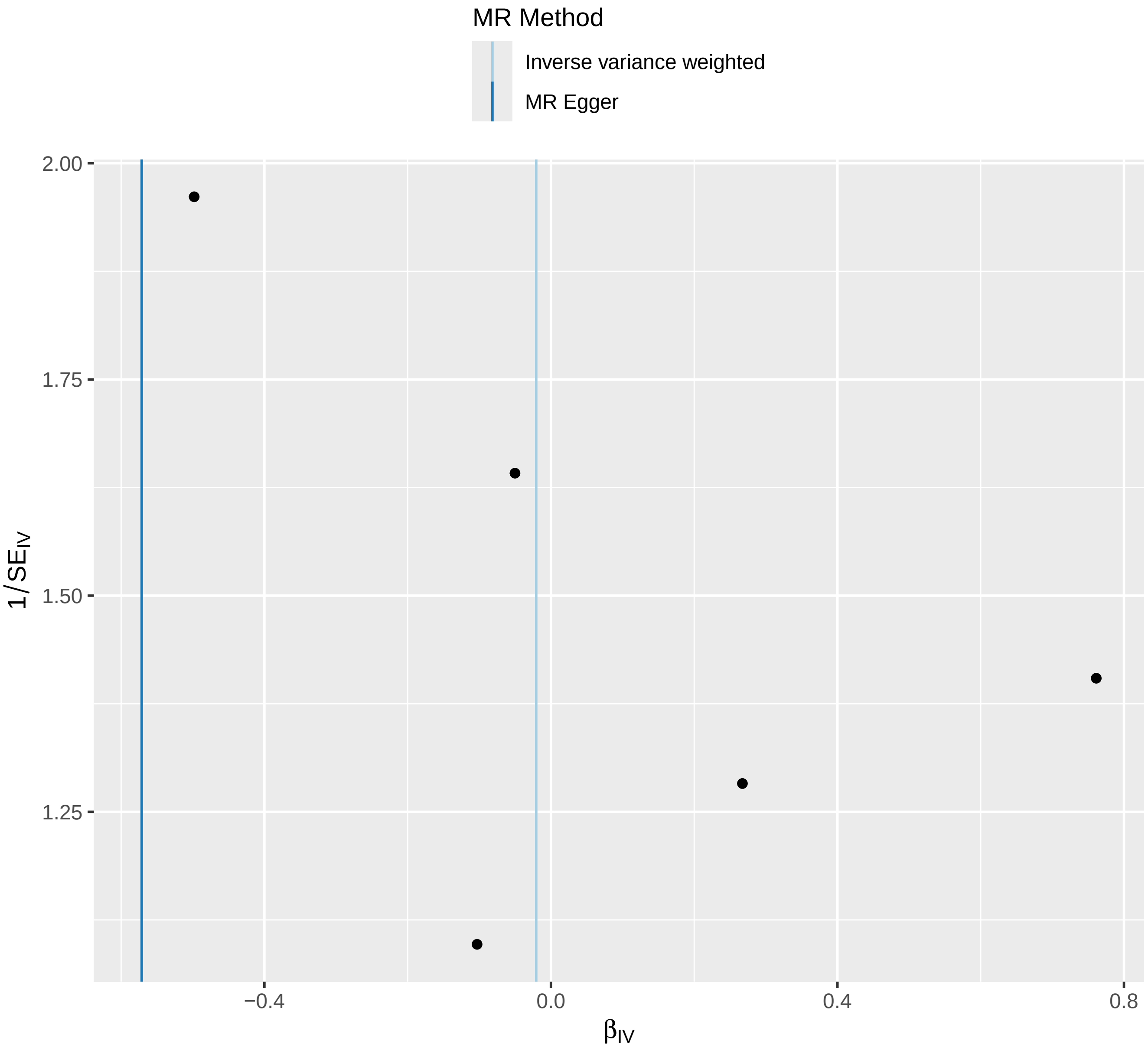
MR Method
Inverse variance weighted
MR Egger
2.00
1.75
1.50
1.25
−0.4
0.0
0.4
0.8
$1/SE_{IV}$
β_{IV}

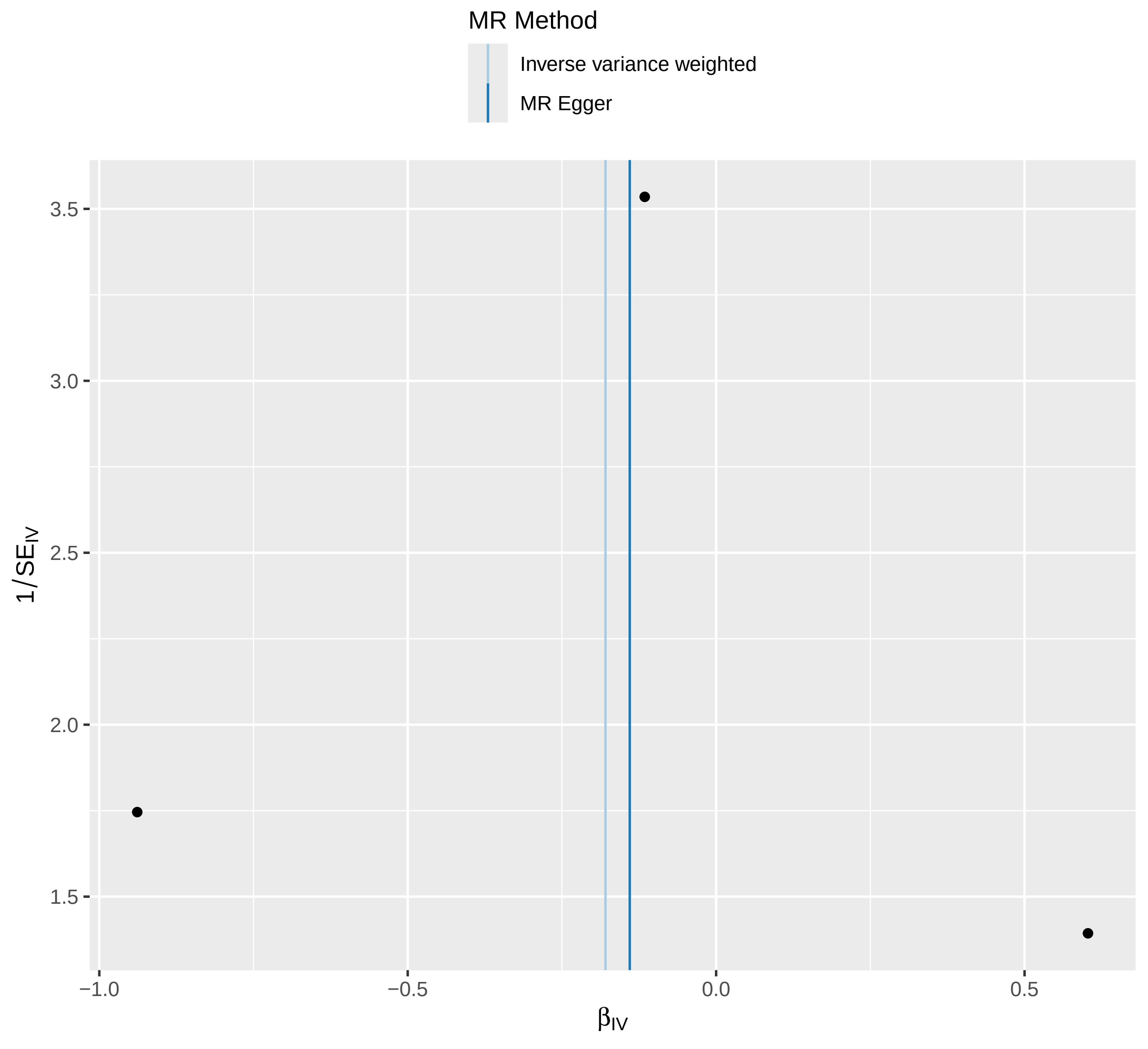

MR Method
Inverse variance weighted
MR Egger
3.5
3.0
2.5
2.0
1.5
$1/SE_{IV}$
−1.0
−0.5
0.0
0.5
β_{IV}

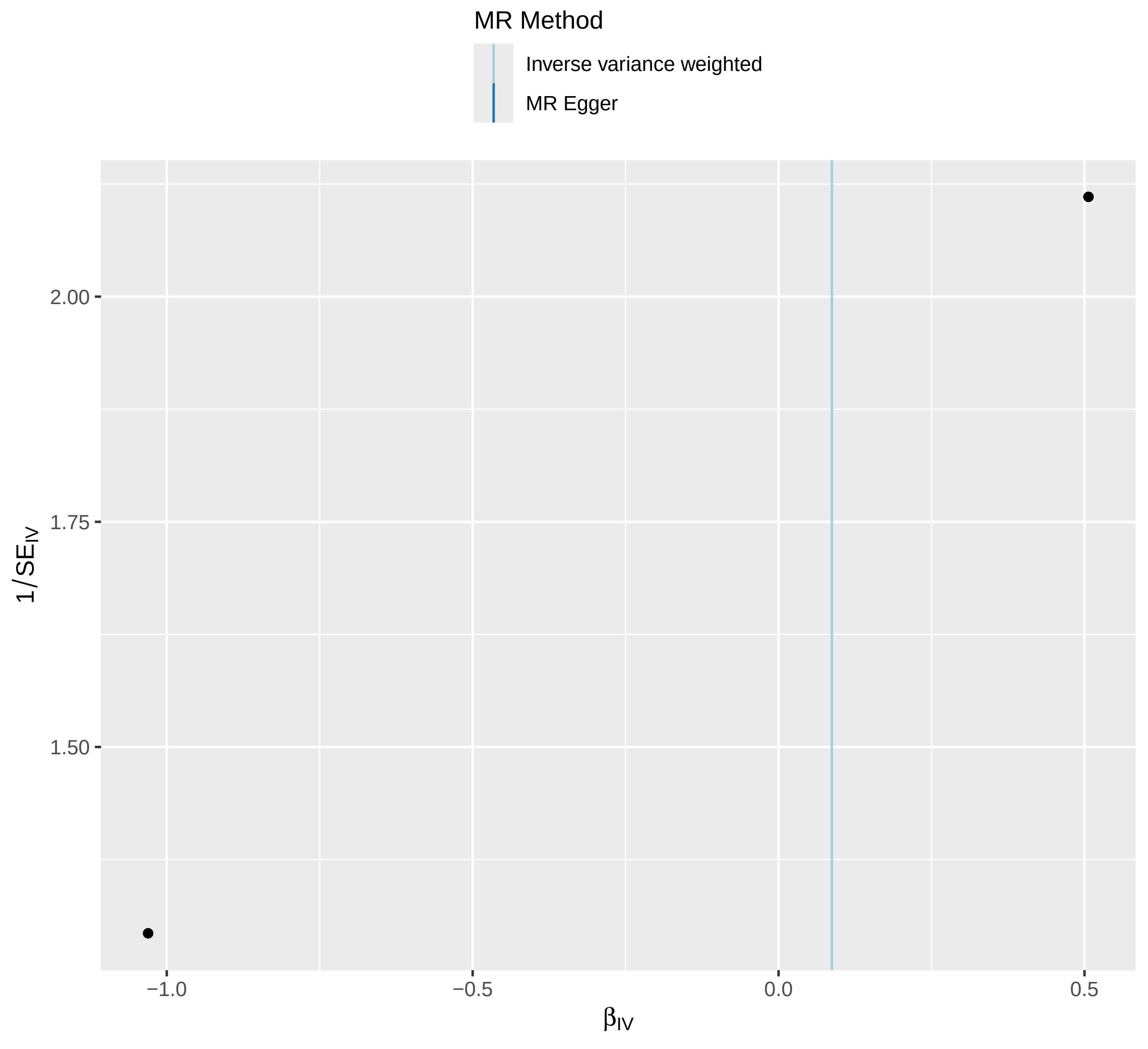

MR Method
Inverse variance weighted
MR Egger
2.00
1.75
1.50
1/SE_IV
−1.0
−0.5
0.0
0.5
β_IV

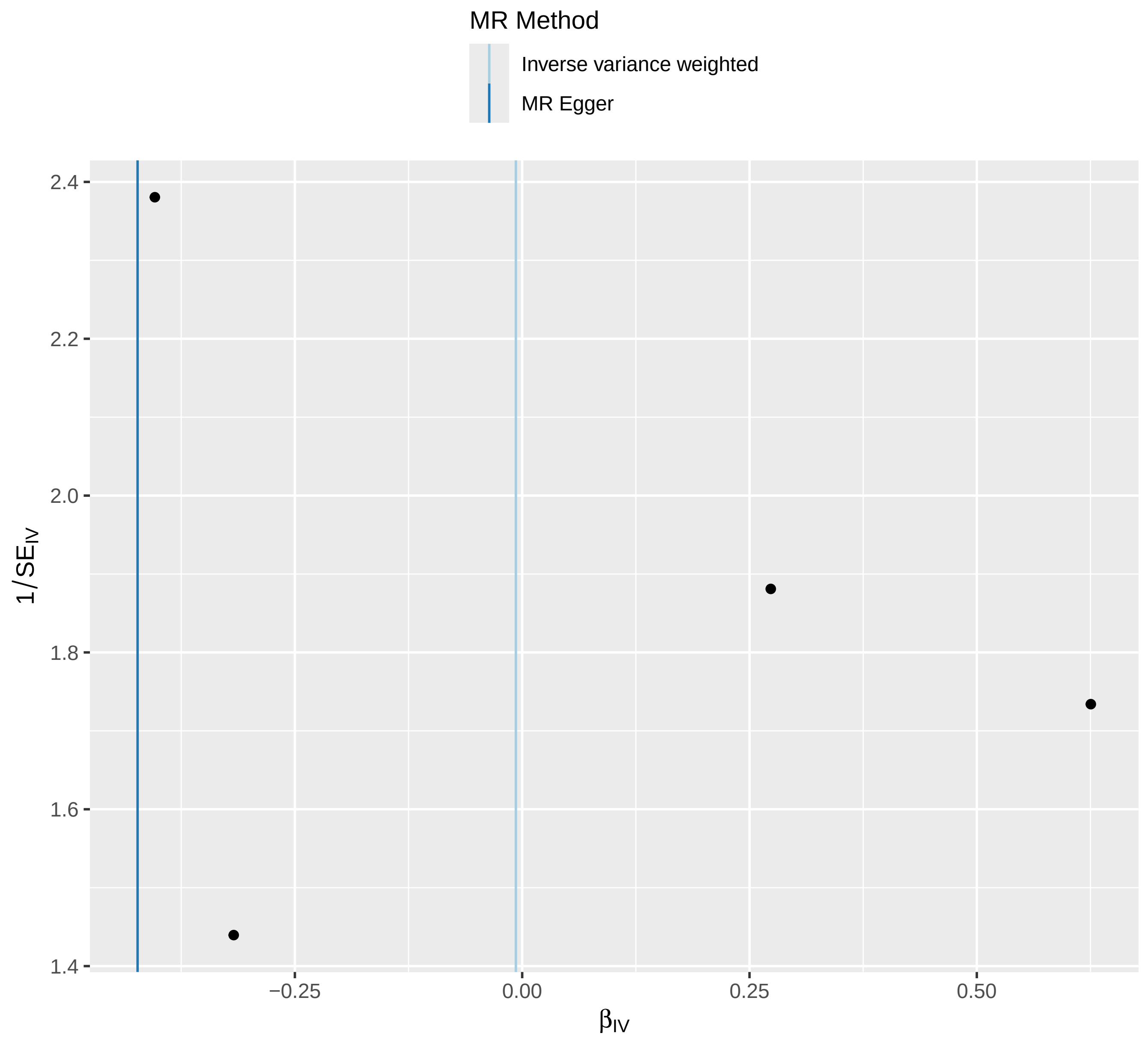
MR Method
Inverse variance weighted
MR Egger
2.4
2.2
2.0
1.8
1.6
1.4
1/SE_IV
−0.25
0.00
0.25
0.50
β_IV

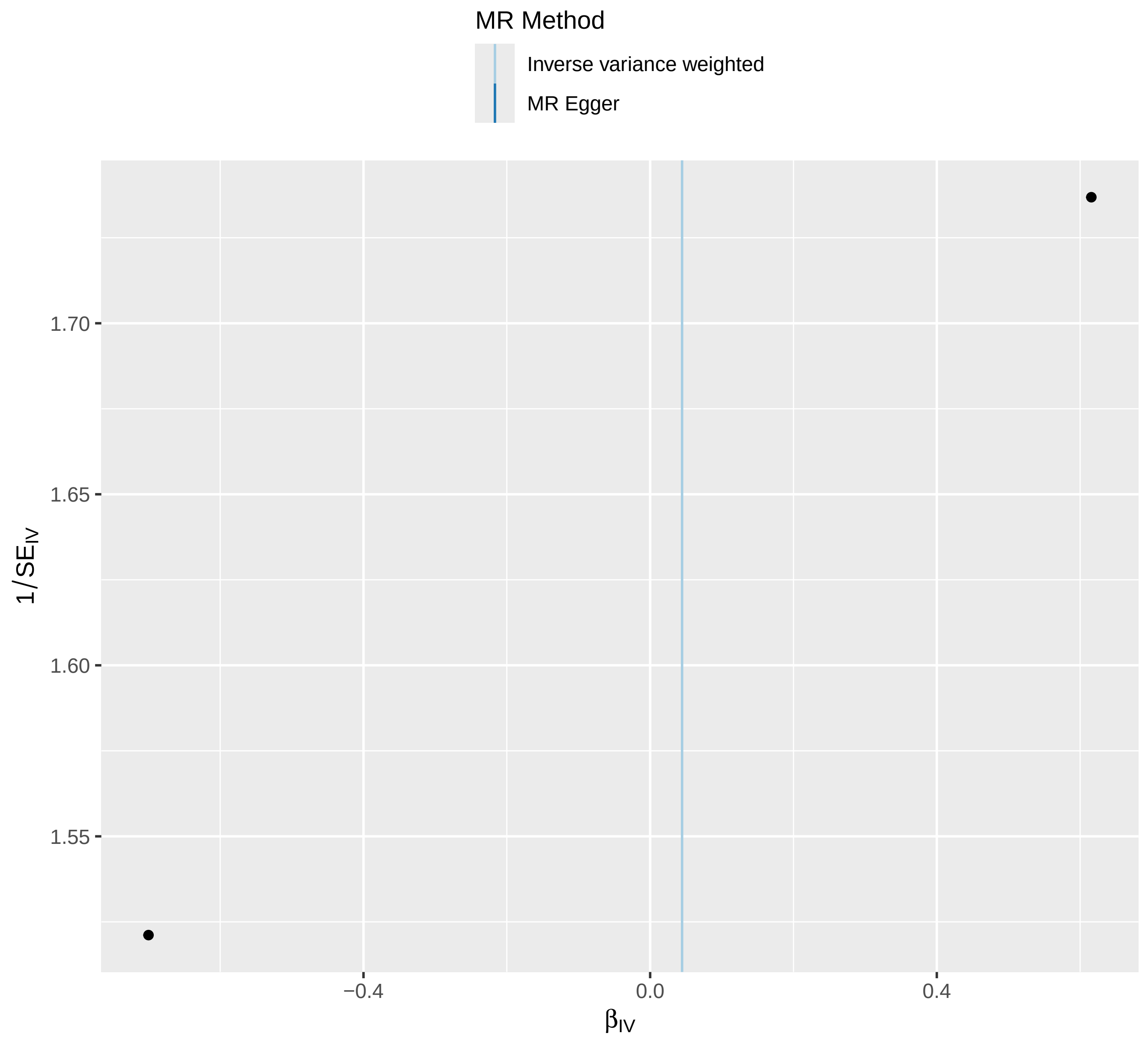
MR Method
Inverse variance weighted
MR Egger
1.70
1.65
1.60
1.55
1/SE_IV
−0.4
0.0
0.4
β_IV

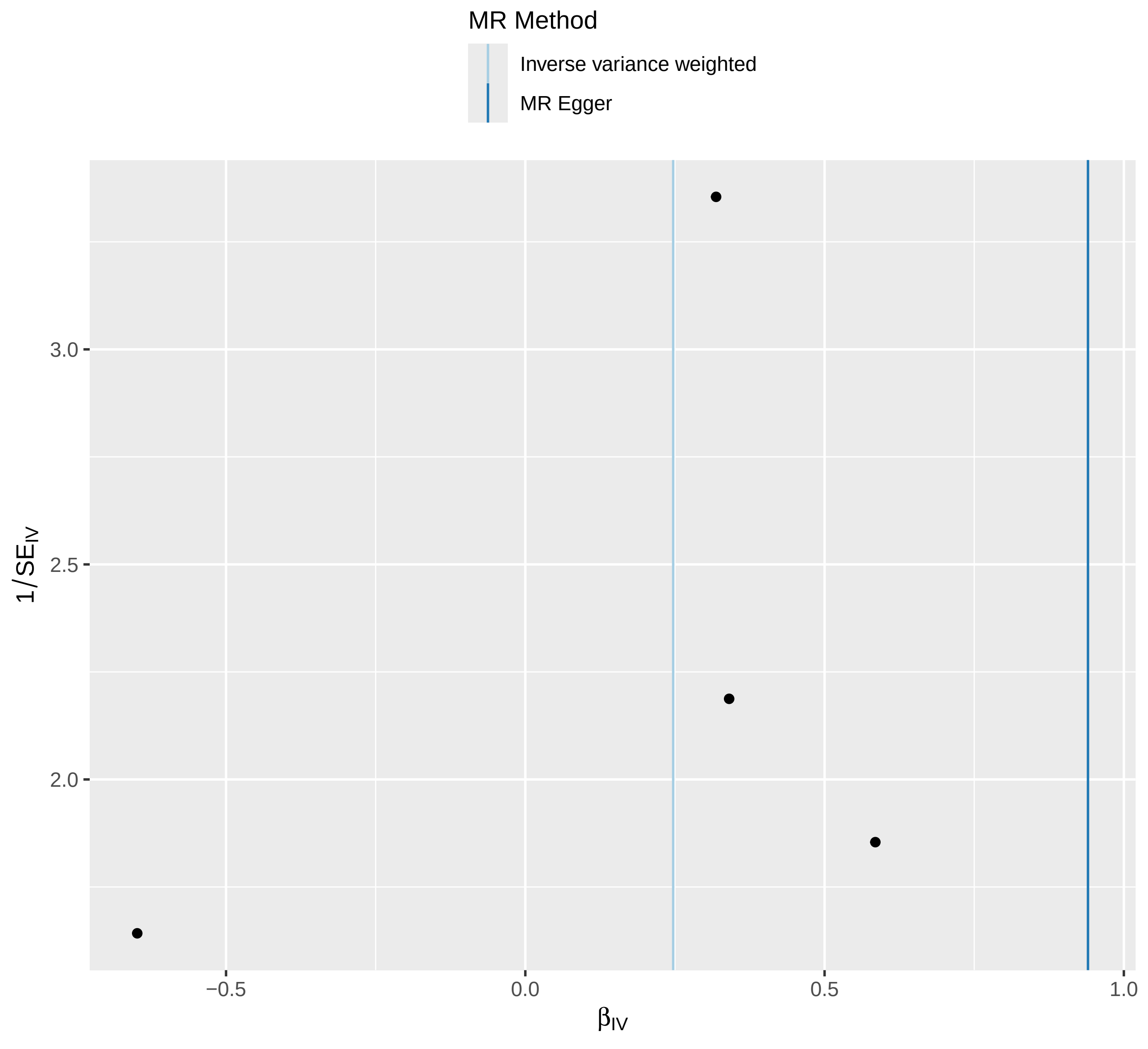
MR Method
Inverse variance weighted
MR Egger
3.0
2.5
2.0
1/SE_IV
−0.5
0.0
0.5
1.0
β_IV

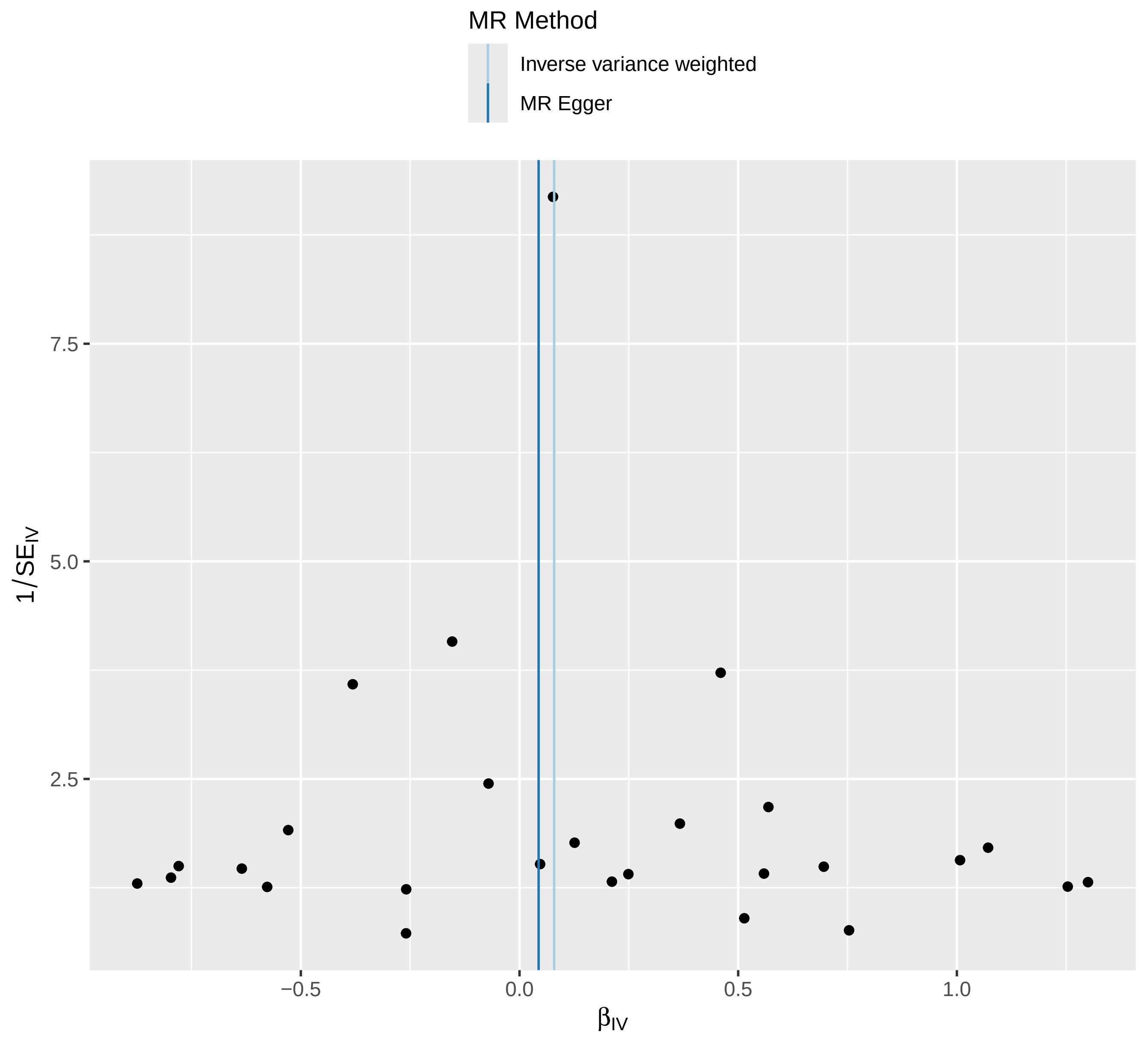

MR Method
Inverse variance weighted
MR Egger
7.5
5.0
2.5
1/SE_IV
−0.5
0.0
0.5
1.0
β_IV

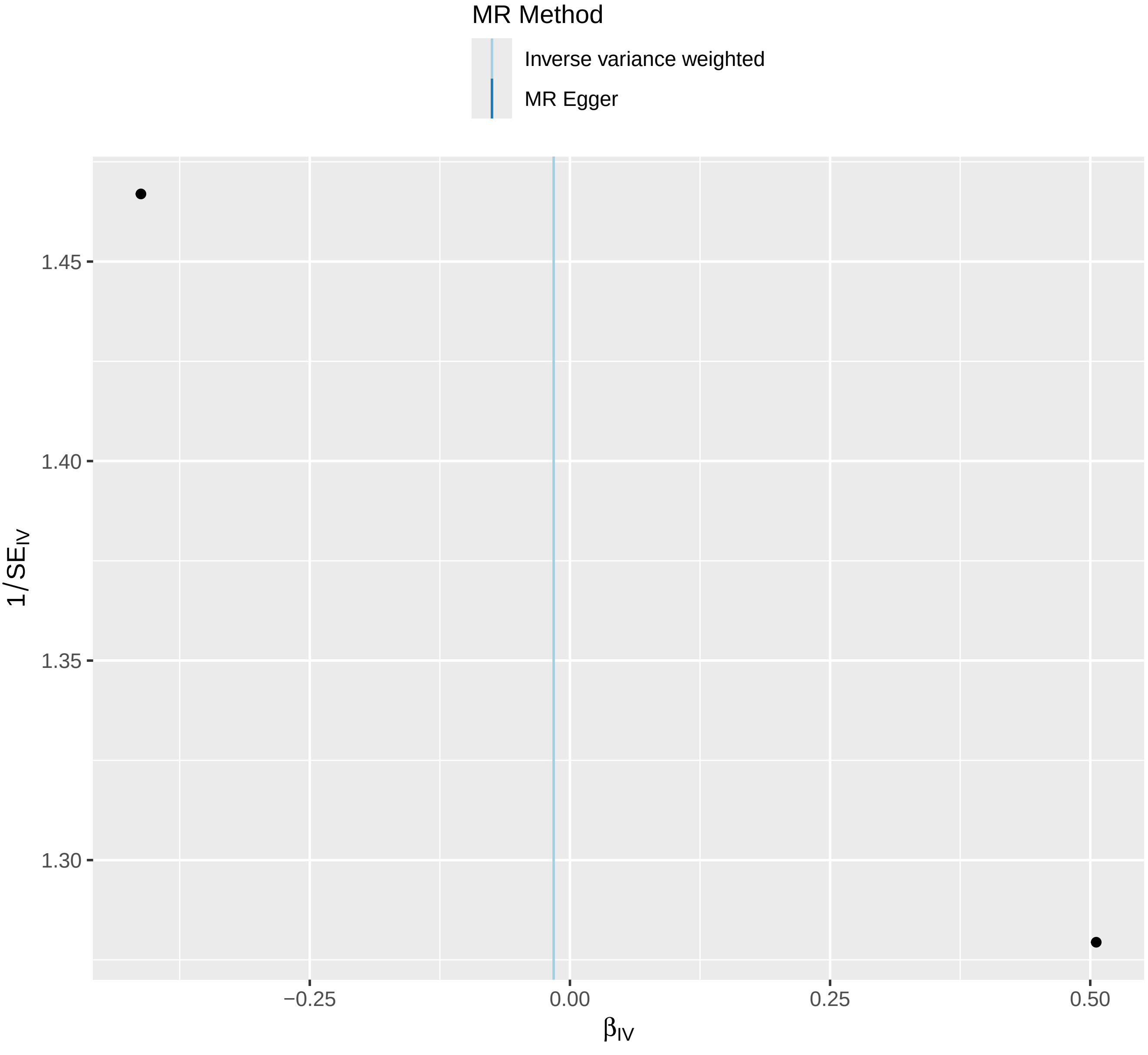

MR Method
Inverse variance weighted
MR Egger
1.45
1.40
1.35
1.30
1/SE$_{IV}$
−0.25
0.00
0.25
0.50
β$_{IV}$

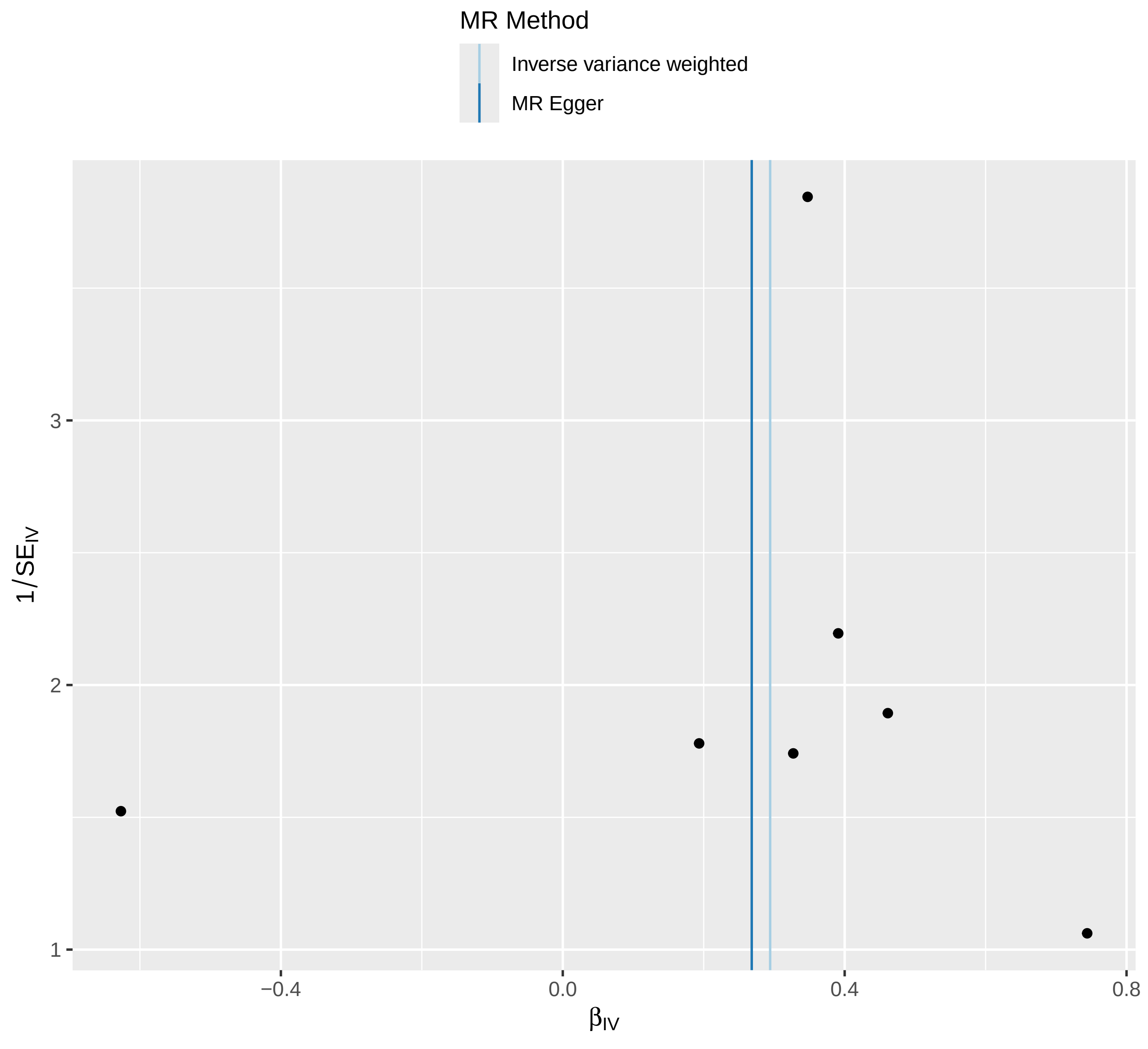

MR Method
Inverse variance weighted
MR Egger
3
2
1
1/SE_IV
−0.4
0.0
0.4
0.8
β_IV

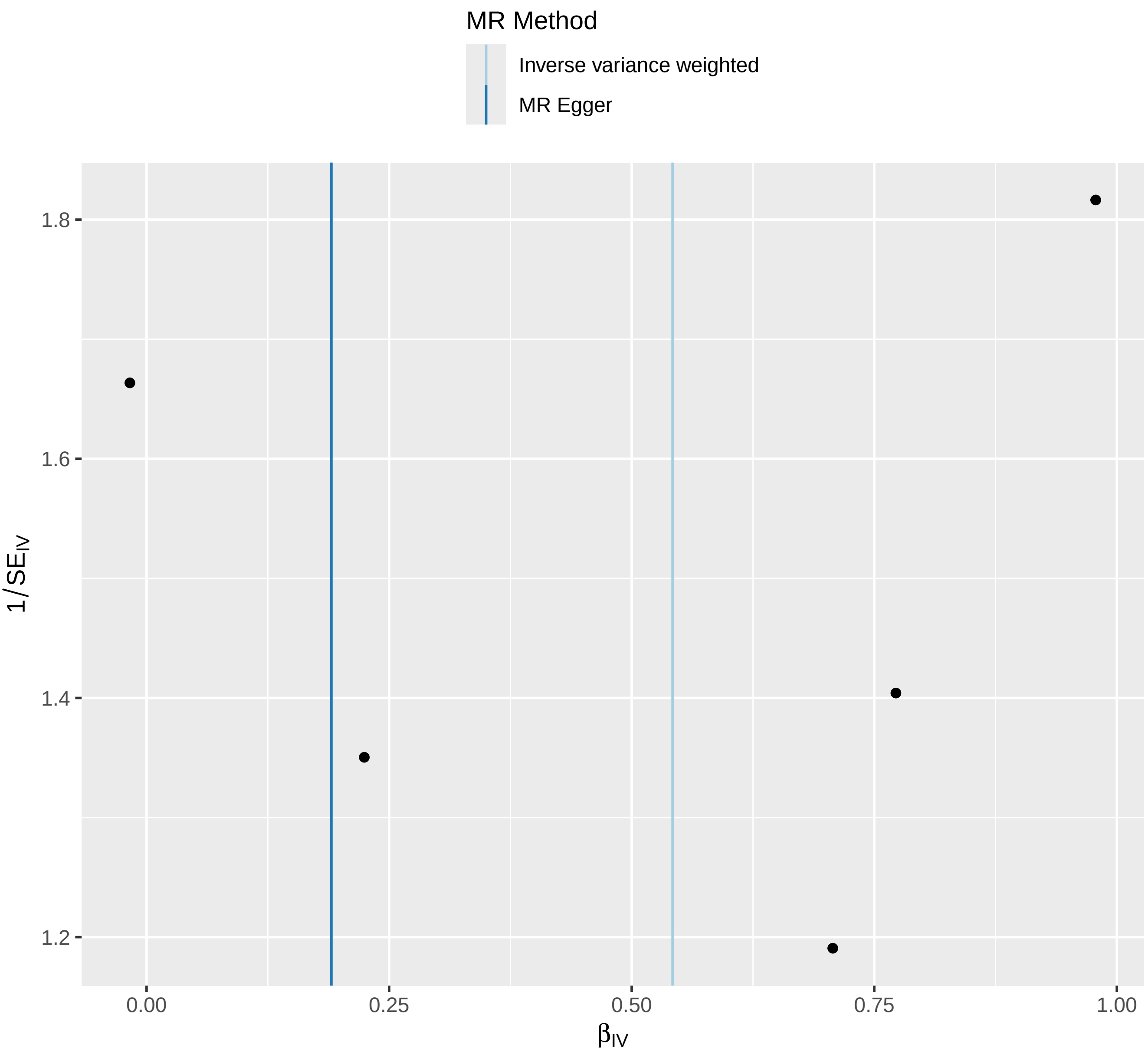

MR Method
Inverse variance weighted
MR Egger
1.8
1.6
1.4
1.2
0.00
0.25
0.50
0.75
1.00
1/SE_IV
β_IV

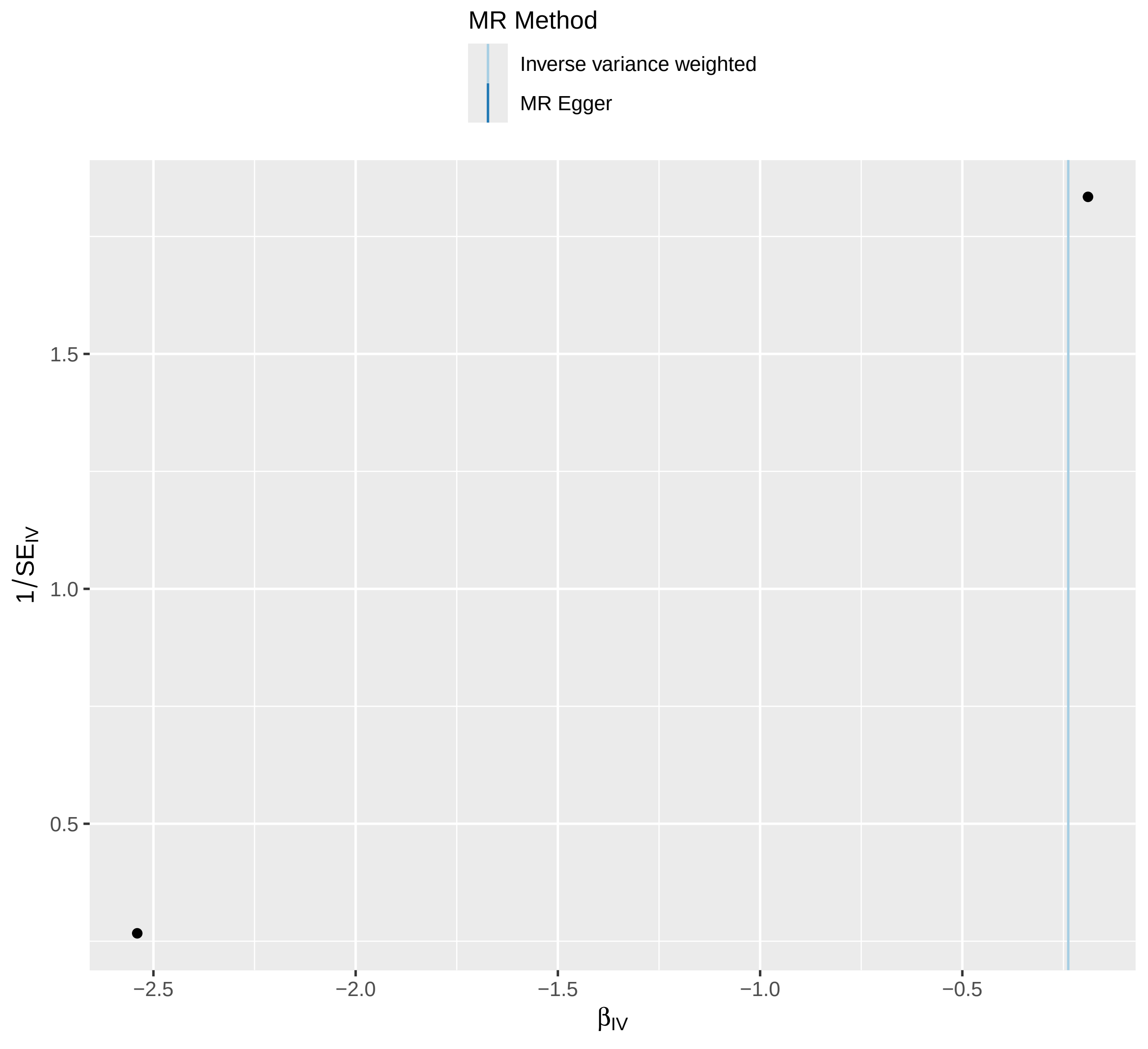

MR Method
Inverse variance weighted
MR Egger
1.5
1.0
0.5
1/SE_IV
−2.5
−2.0
−1.5
−1.0
−0.5
β_IV

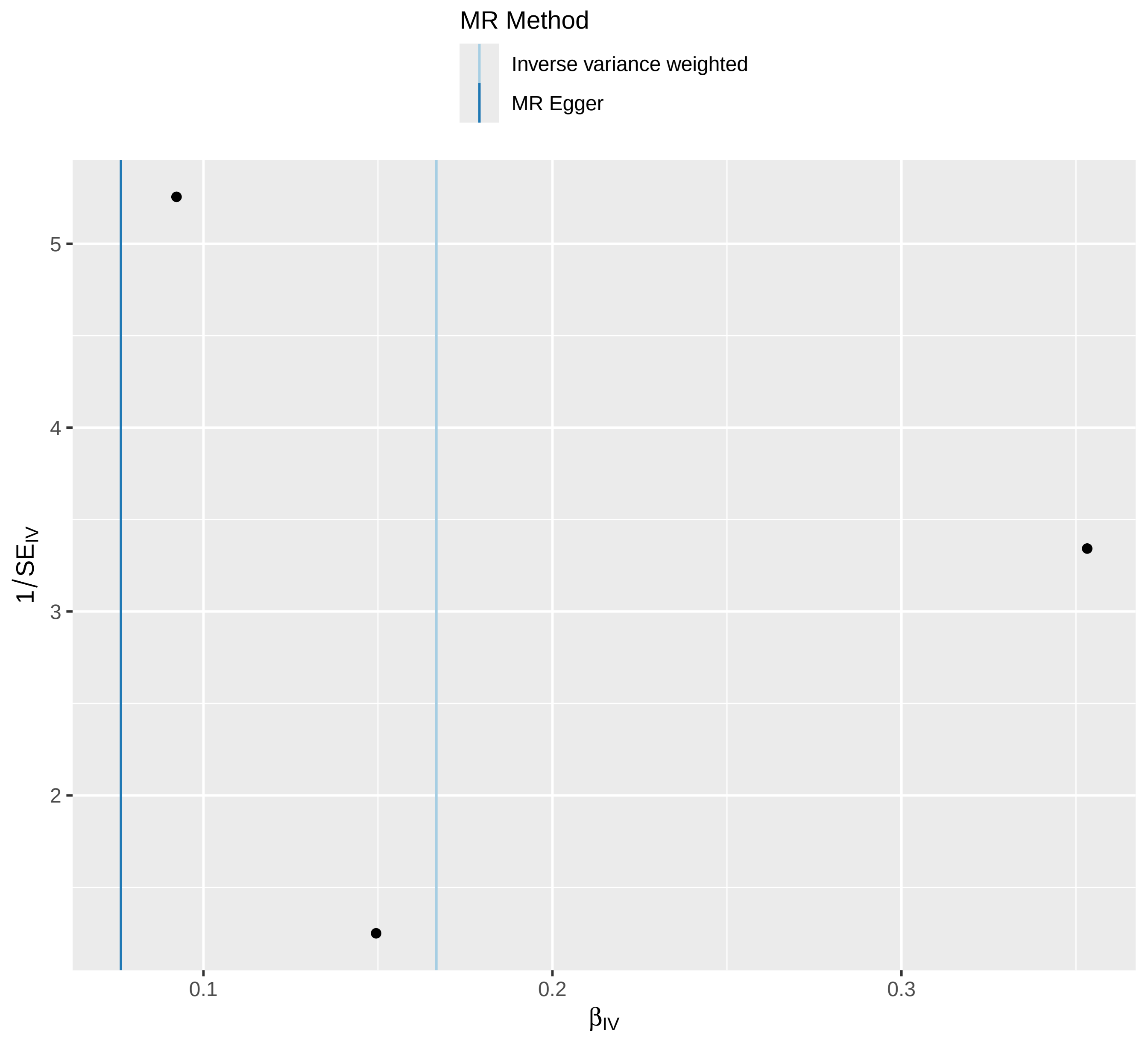
MR Method
Inverse variance weighted
MR Egger
5
4
3
2
$1/SE_{IV}$
0.1
0.2
0.3
β_{IV}

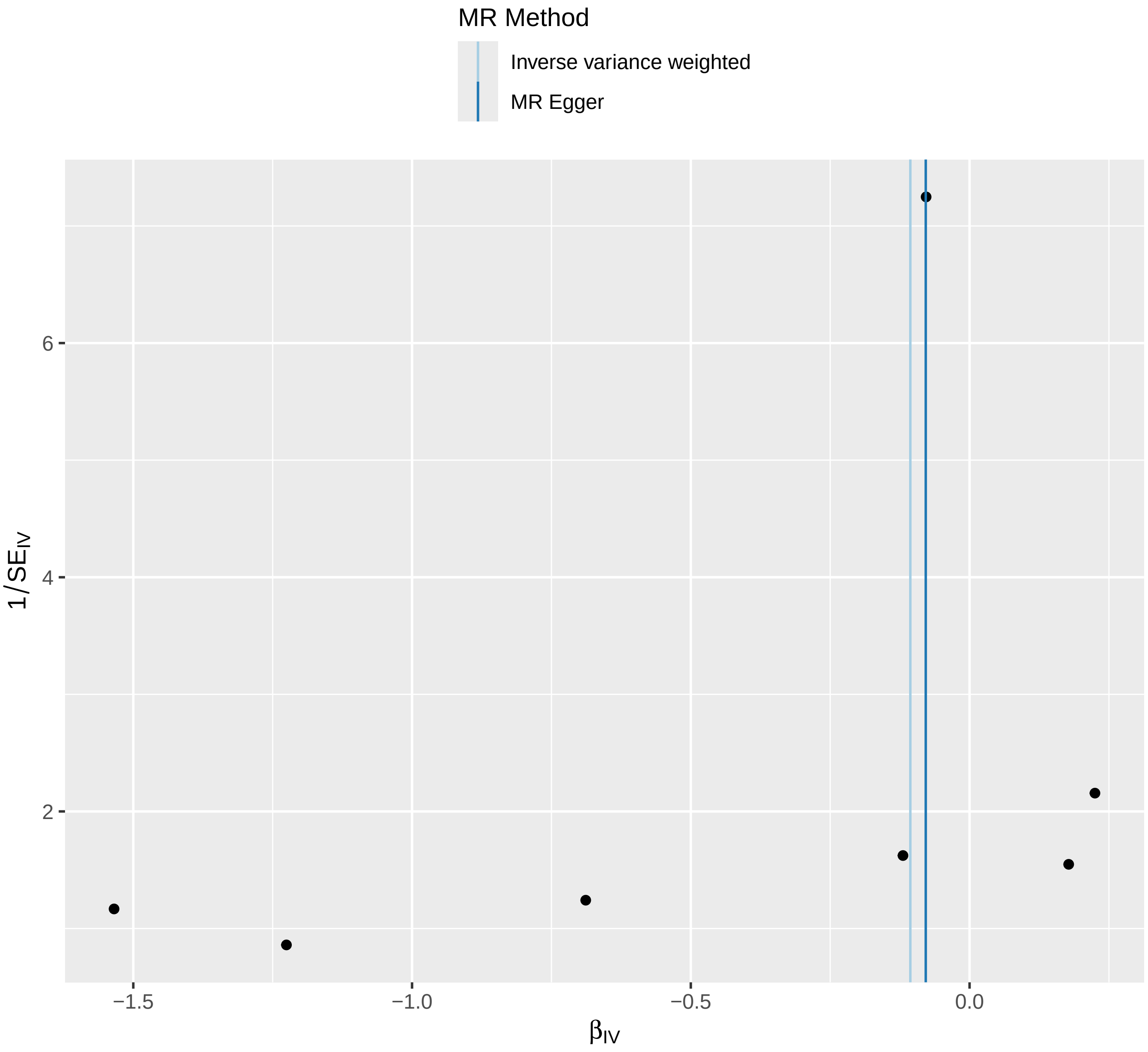

MR Method
Inverse variance weighted
MR Egger
6
4
2
1/SE_IV
−1.5
−1.0
−0.5
0.0
β_IV

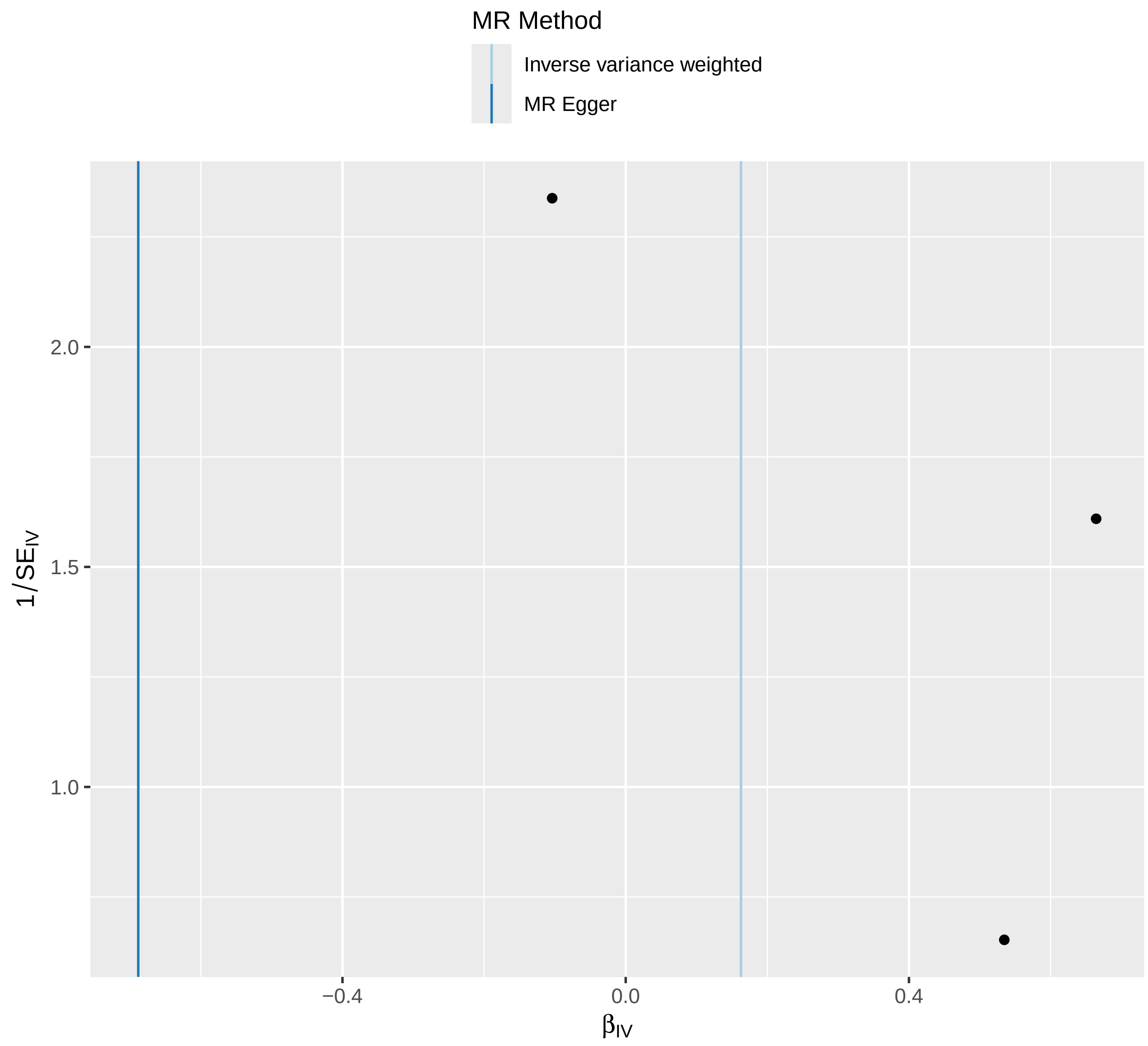
MR Method
Inverse variance weighted
MR Egger
2.0
1.5
1.0
−0.4
0.0
0.4
$1/SE_{IV}$
β_{IV}

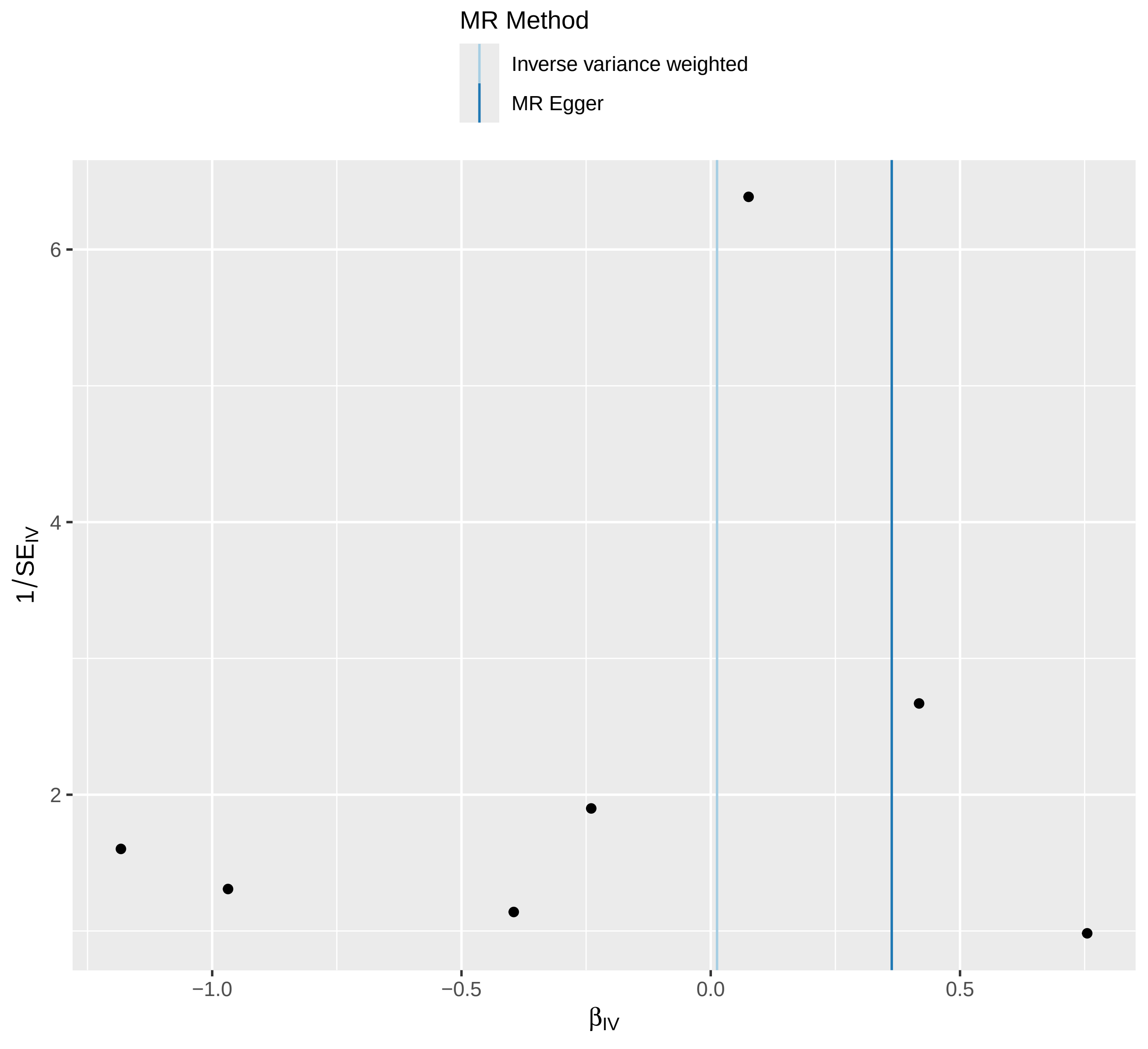

MR Method
Inverse variance weighted
MR Egger
6
4
2
1/SE$_{IV}$
−1.0
−0.5
0.0
0.5
β$_{IV}$

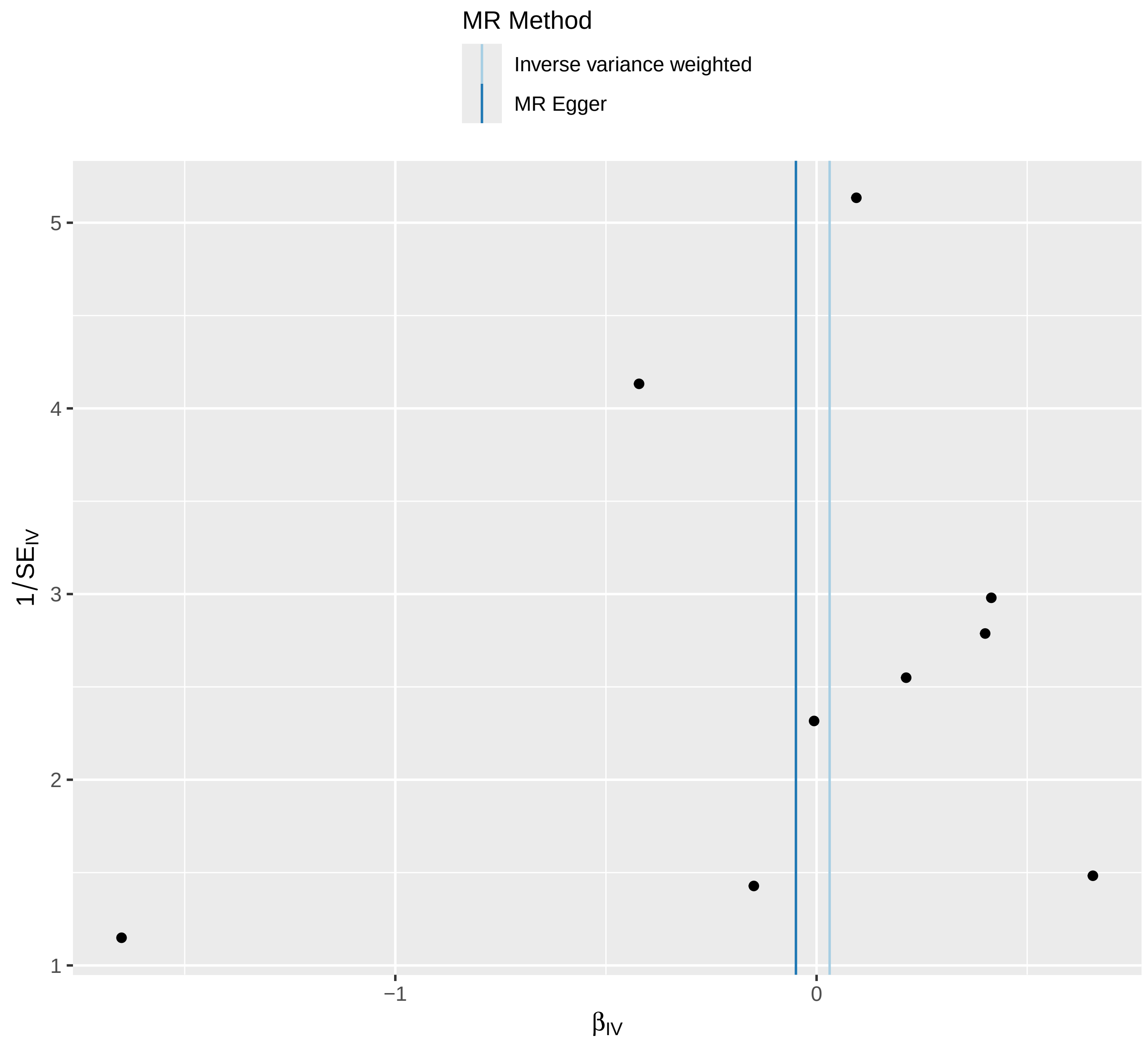

MR Method
Inverse variance weighted
MR Egger
5
4
3
2
1
1/SE_IV
−1
0
β_IV

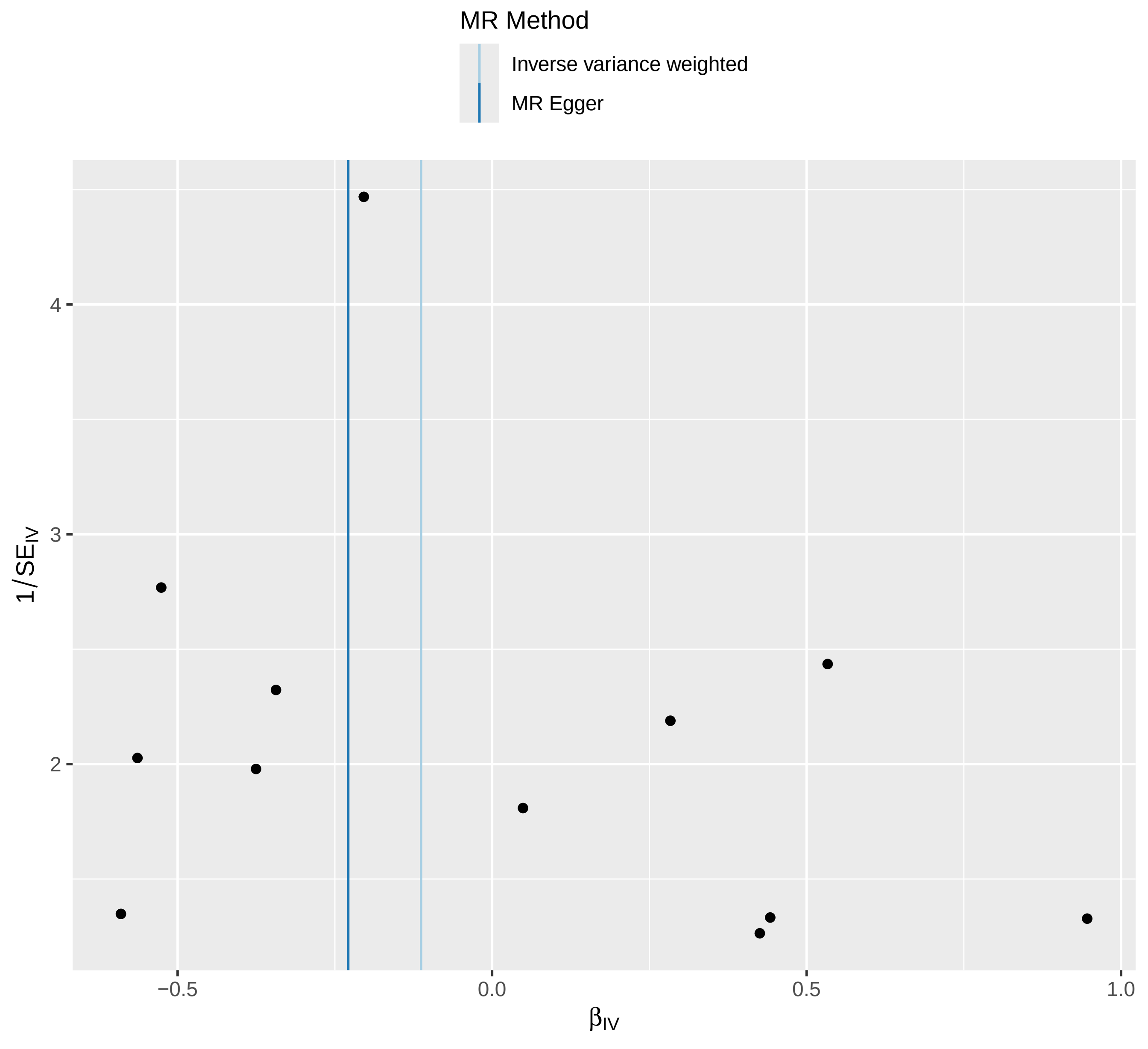
MR Method
Inverse variance weighted
MR Egger
$1/SE_{IV}$
4
3
2
−0.5
0.0
0.5
1.0
β_{IV}

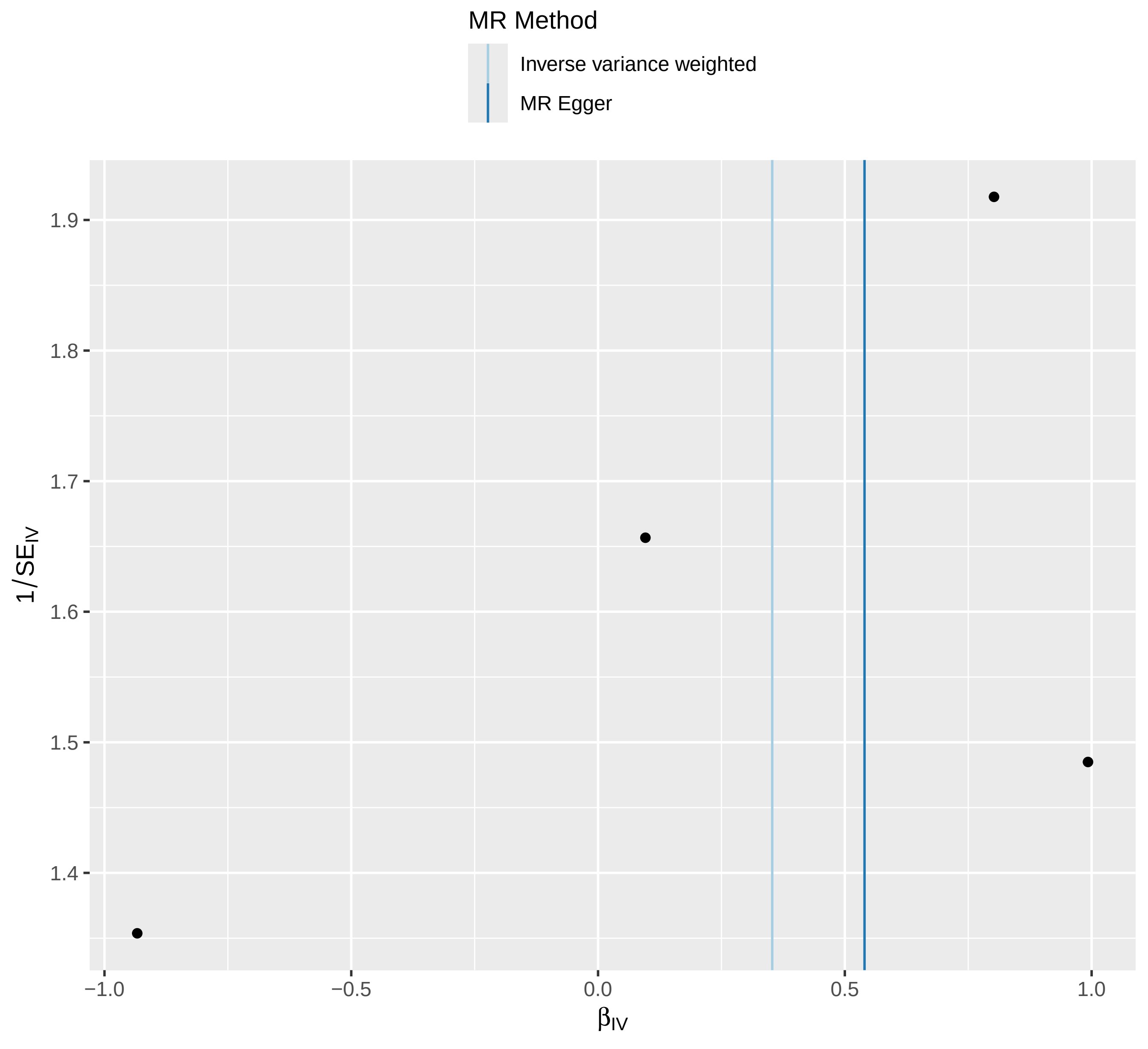

MR Method
Inverse variance weighted
MR Egger
1.9
1.8
1.7
1.6
1.5
1.4
$1/SE_{IV}$
−1.0
−0.5
0.0
0.5
1.0
β_{IV}

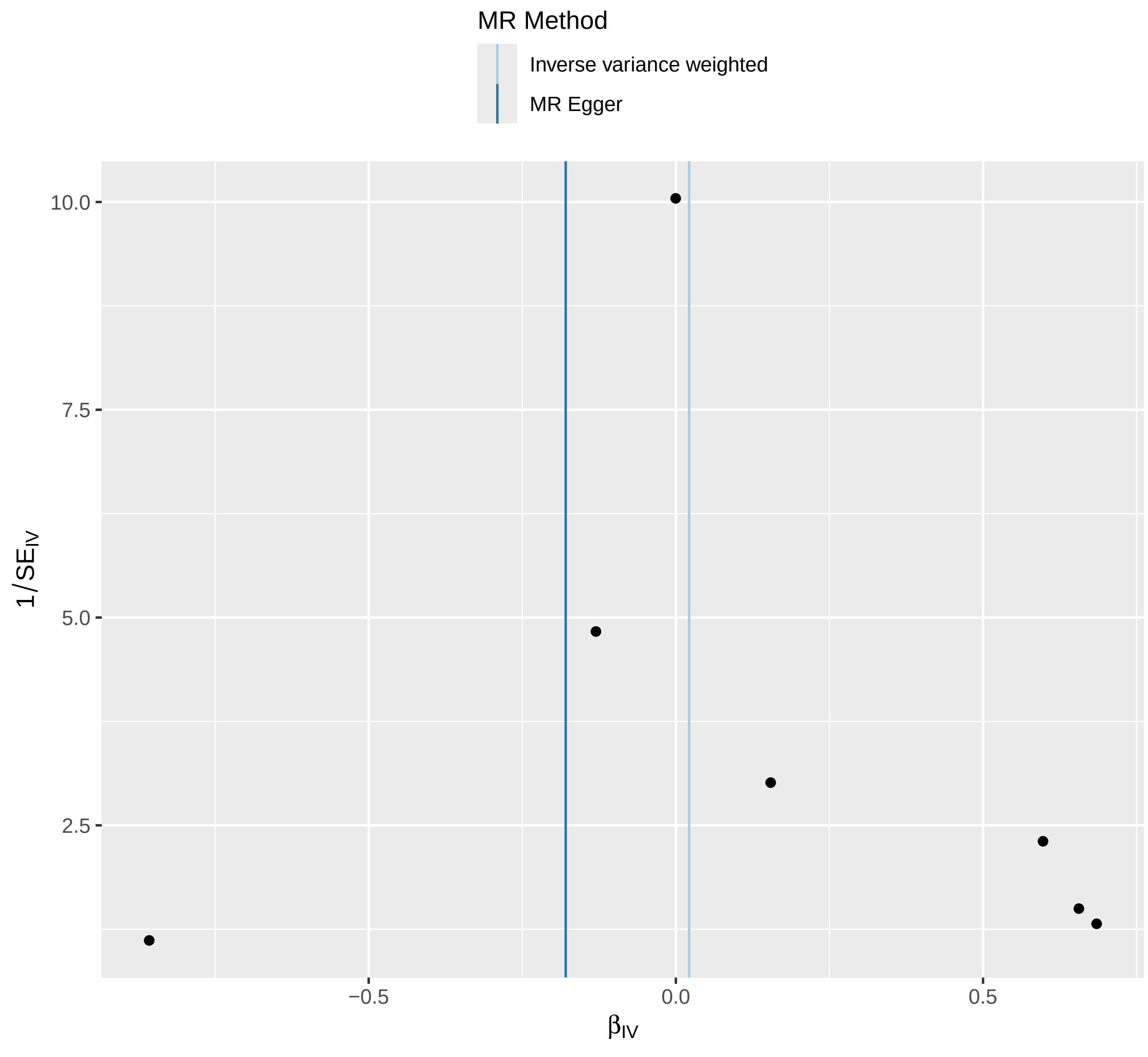
MR Method
Inverse variance weighted
MR Egger
10.0
7.5
5.0
2.5
1/SE_IV
−0.5
0.0
0.5
β_IV

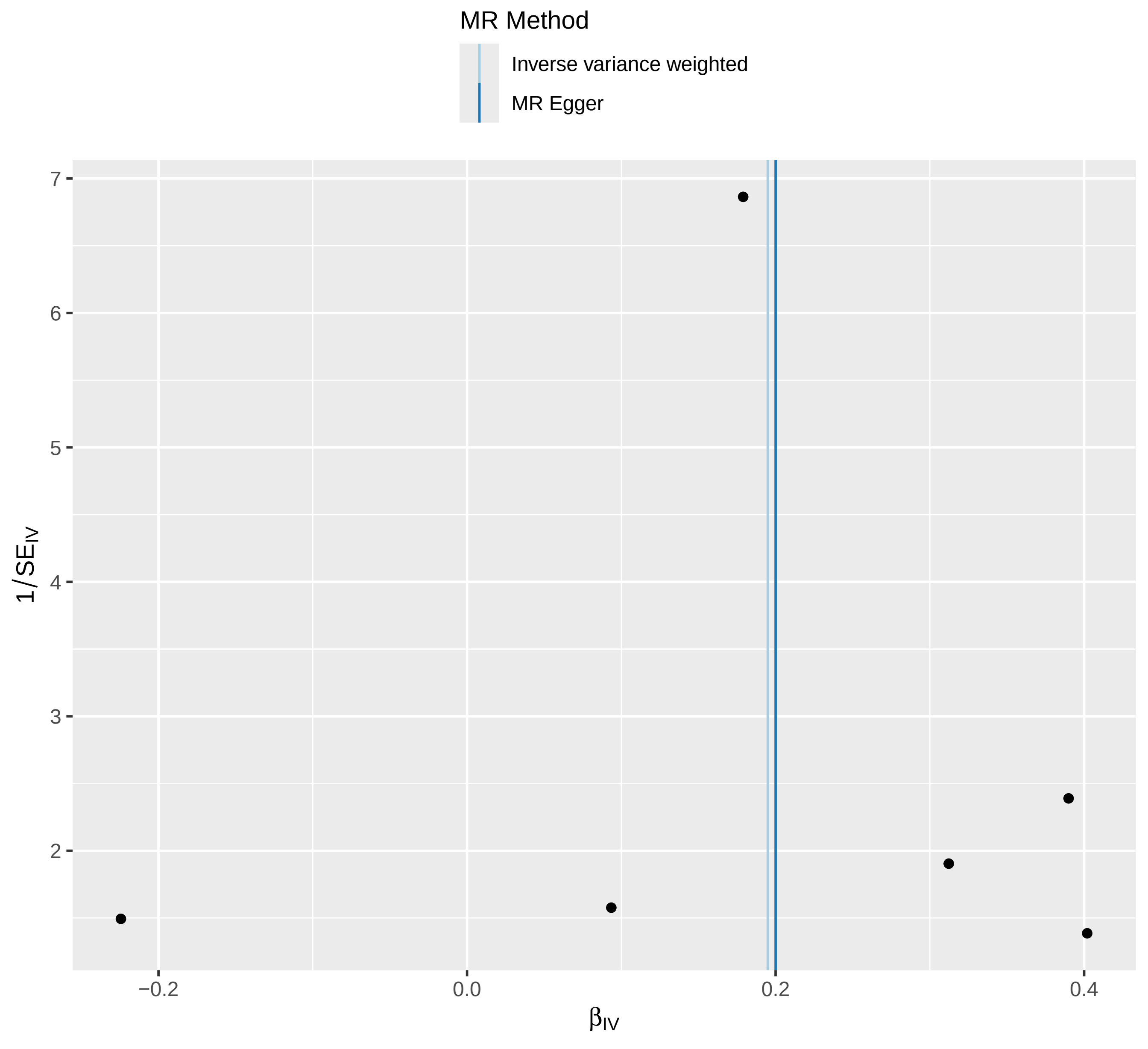
MR Method
Inverse variance weighted
MR Egger
1/SE$_{IV}$
β_{IV}
7
6
5
4
3
2
−0.2
0.0
0.2
0.4

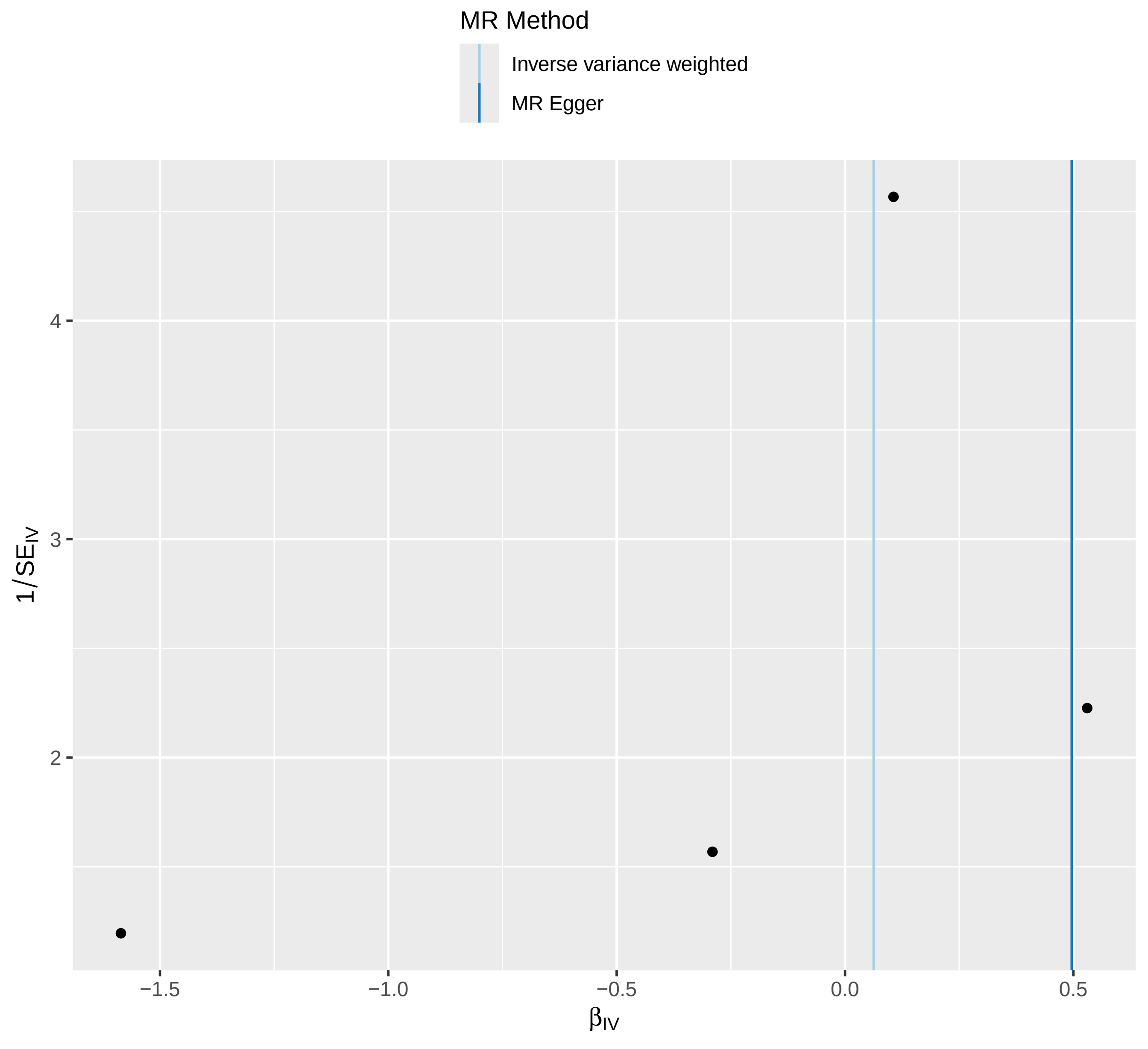
MR Method
Inverse variance weighted
MR Egger
1/SE_IV
4
3
2
−1.5
−1.0
−0.5
0.0
0.5
β_IV

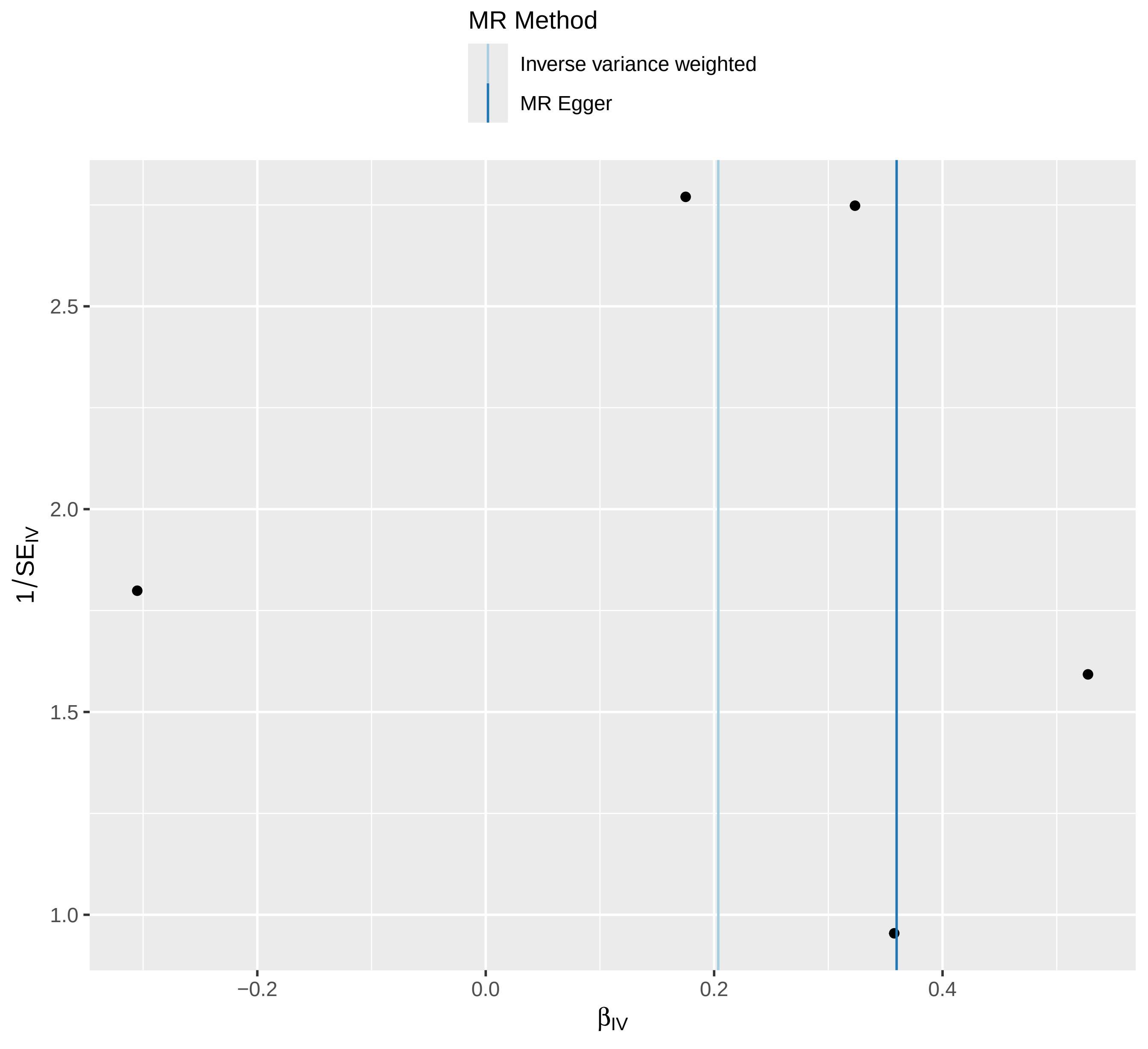
MR Method
Inverse variance weighted
MR Egger
2.5
2.0
1.5
1.0
1/SE$_{IV}$
−0.2
0.0
0.2
0.4
β$_{IV}$

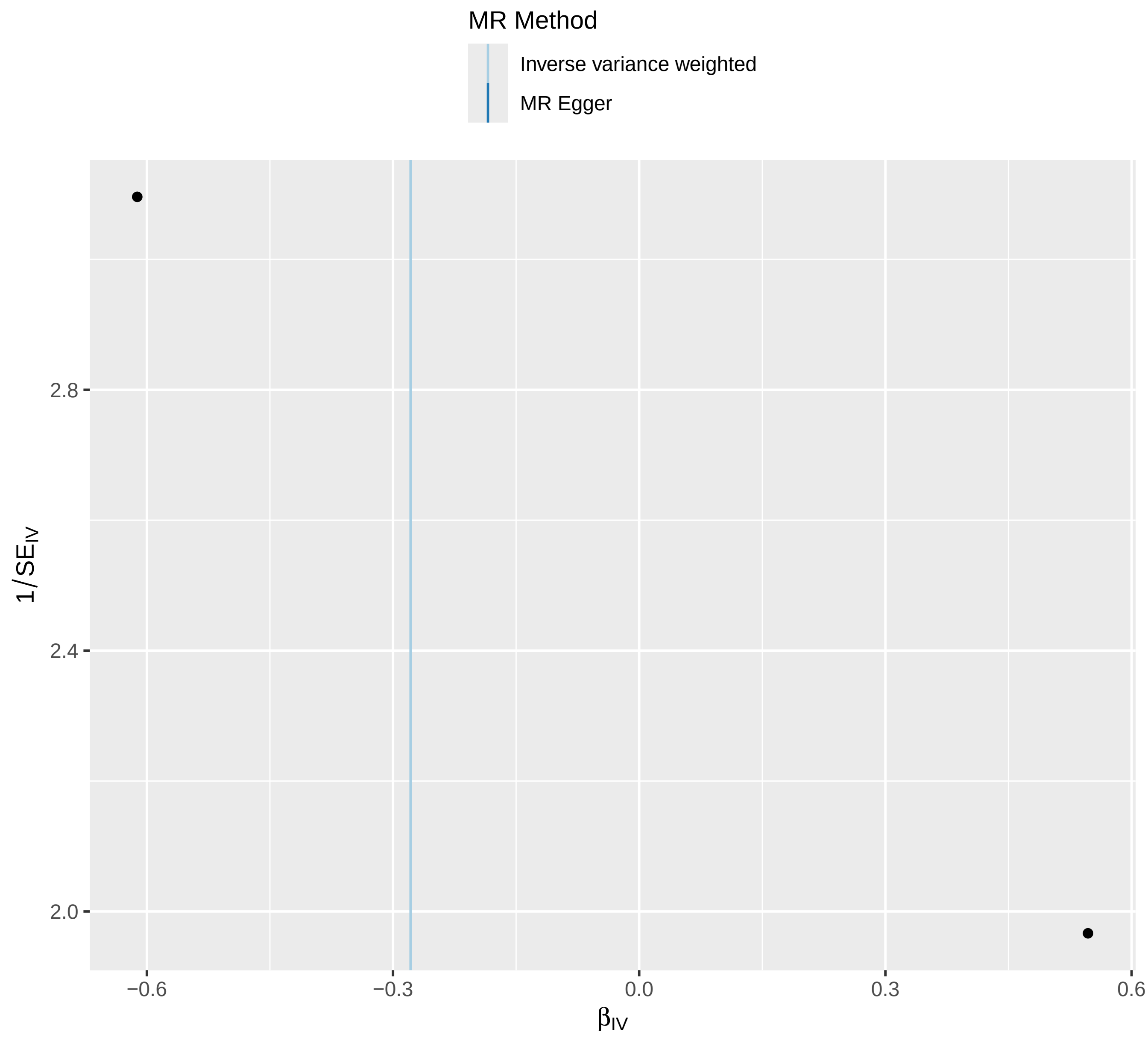

MR Method
Inverse variance weighted
MR Egger
2.8
2.4
2.0
1/SE_{IV}
−0.6
−0.3
0.0
0.3
0.6
β_{IV}

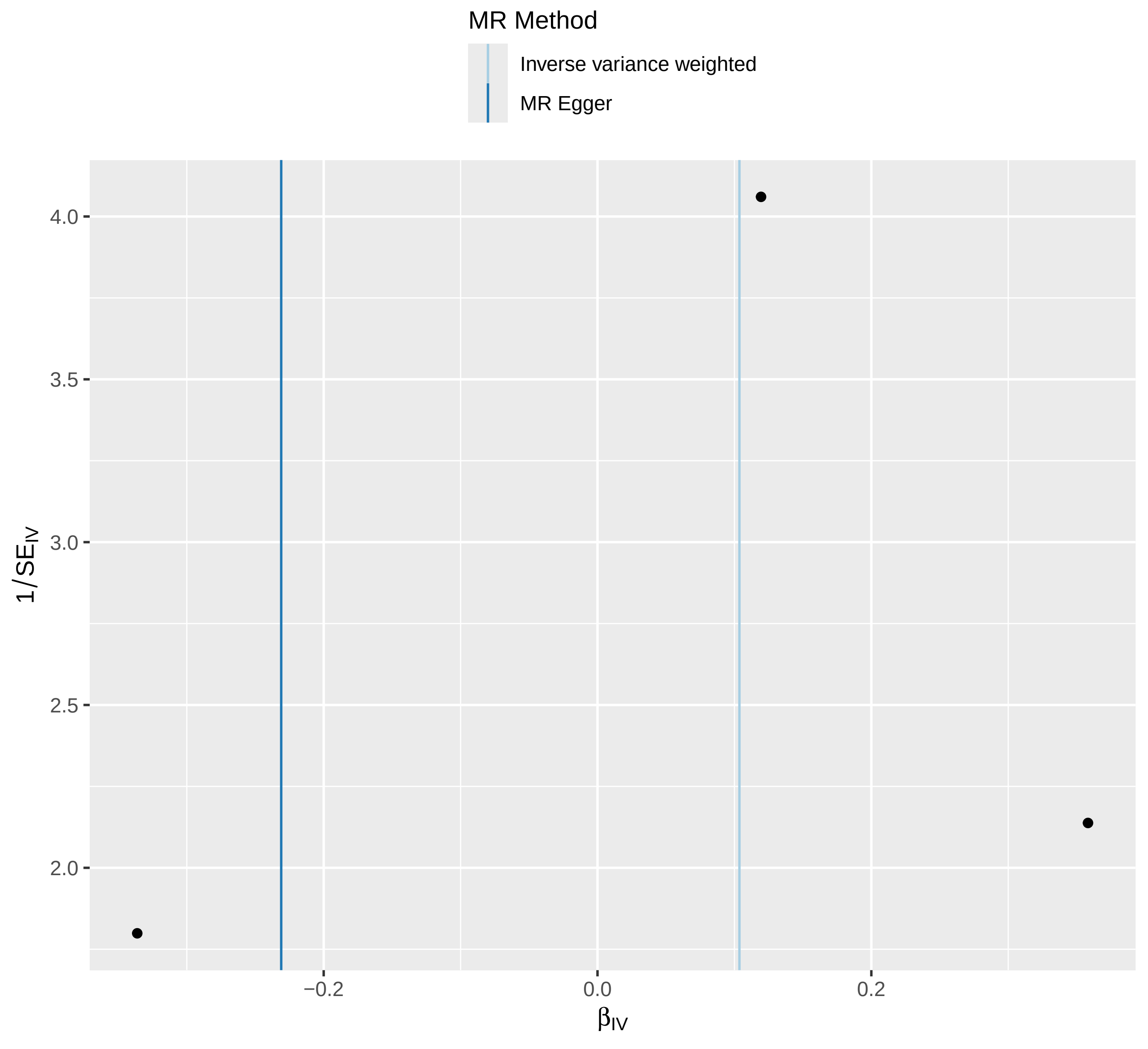
MR Method
Inverse variance weighted
MR Egger
4.0
3.5
3.0
2.5
2.0
1/SE_IV
−0.2
0.0
0.2
β_IV

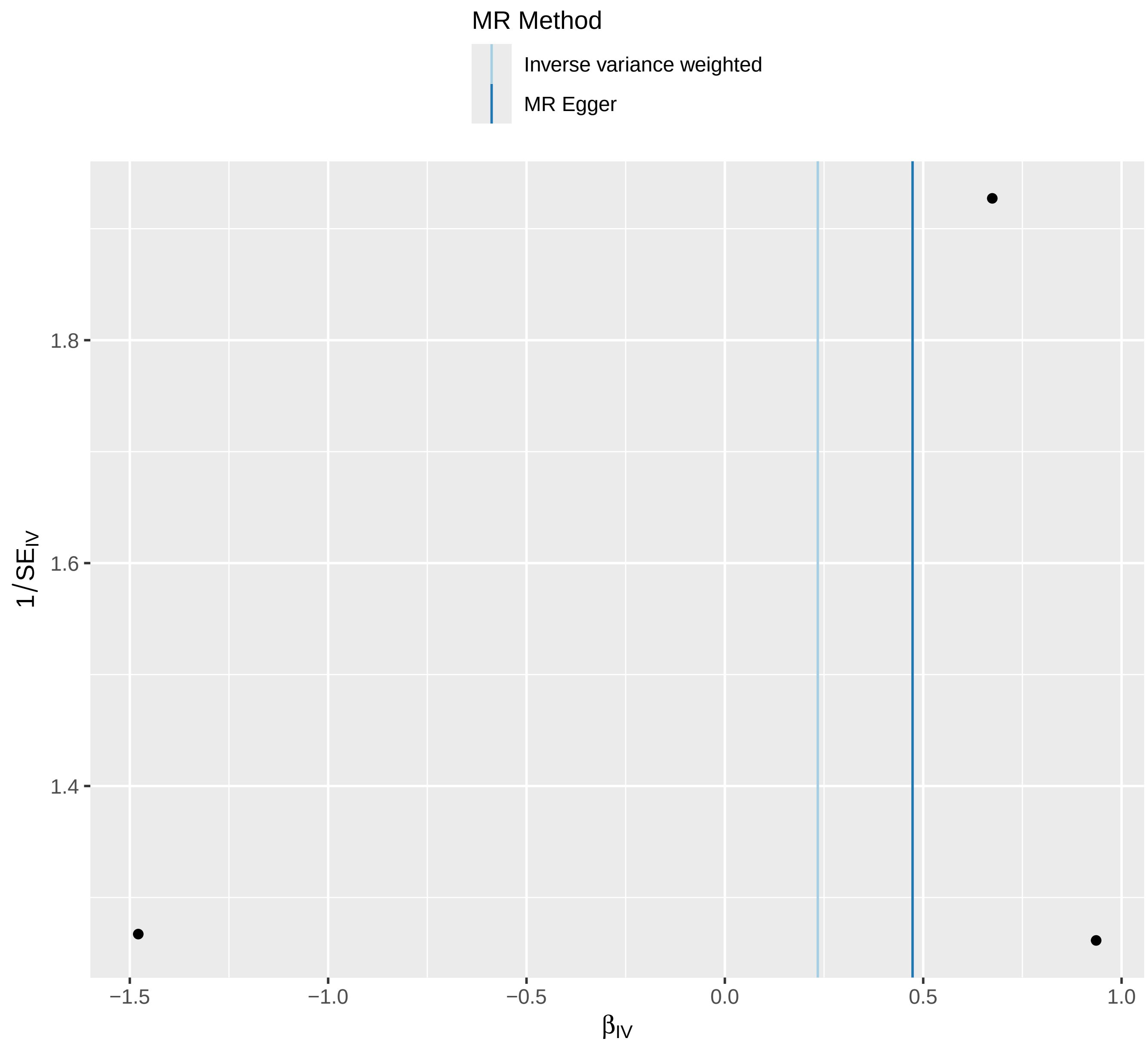

MR Method
Inverse variance weighted
MR Egger
1.8
1.6
1.4
1/SE_IV
−1.5
−1.0
−0.5
0.0
0.5
1.0
β_IV

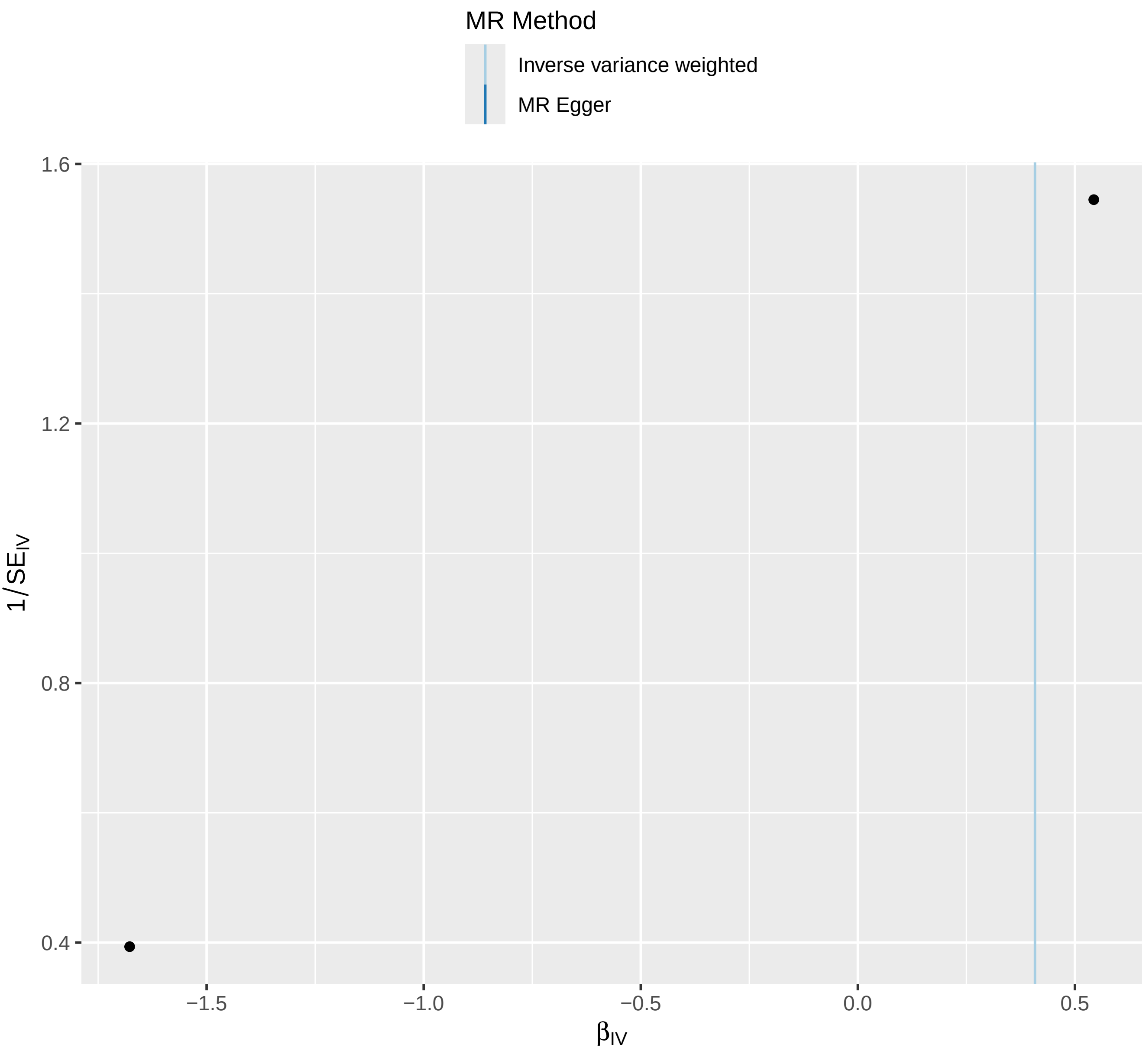

MR Method
Inverse variance weighted
MR Egger
1.6
1.2
0.8
0.4
1/SE_IV
−1.5
−1.0
−0.5
0.0
0.5
β_IV

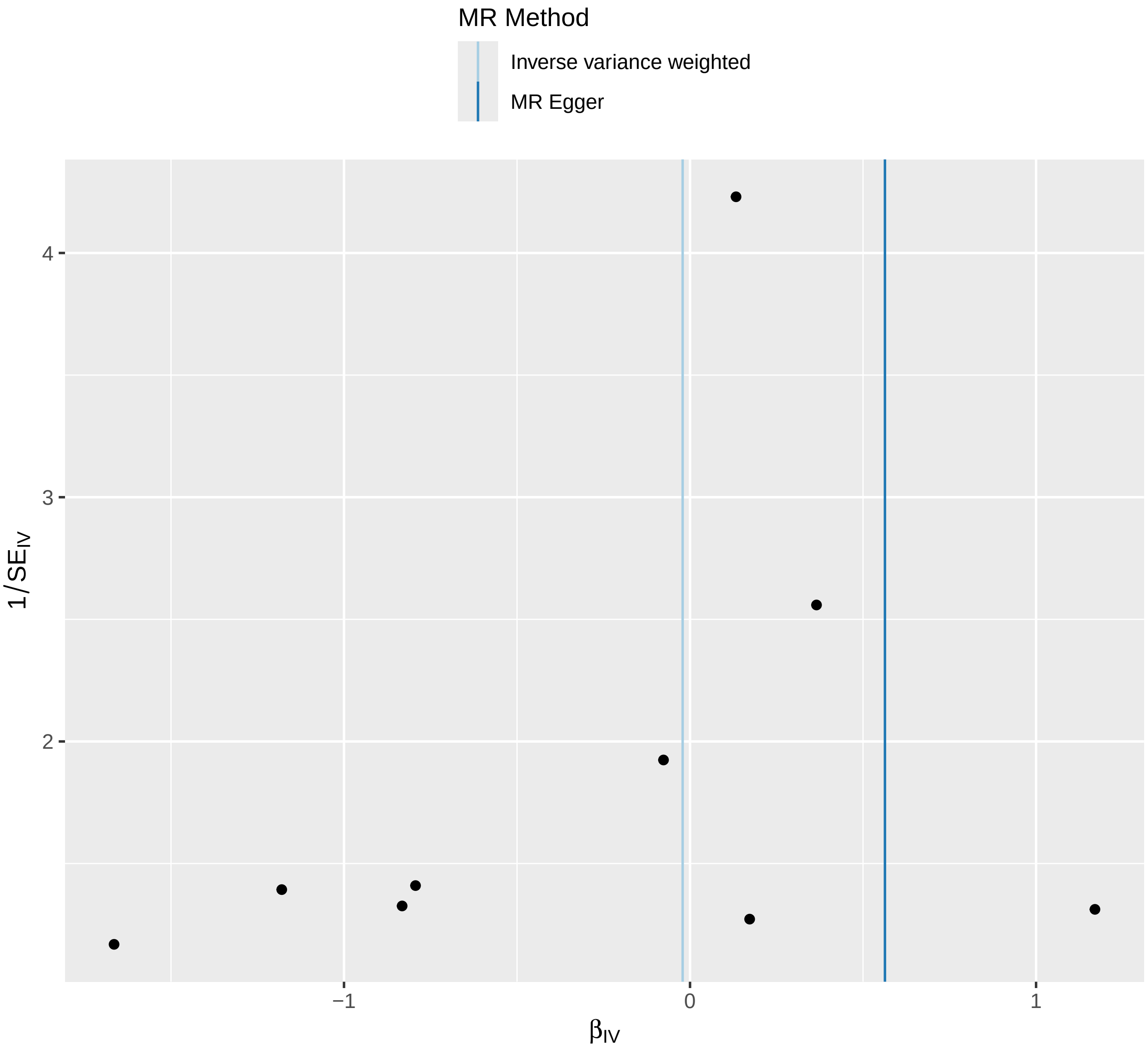

MR Method
Inverse variance weighted
MR Egger
4
3
2
1/SE_IV
−1
0
1
β_IV

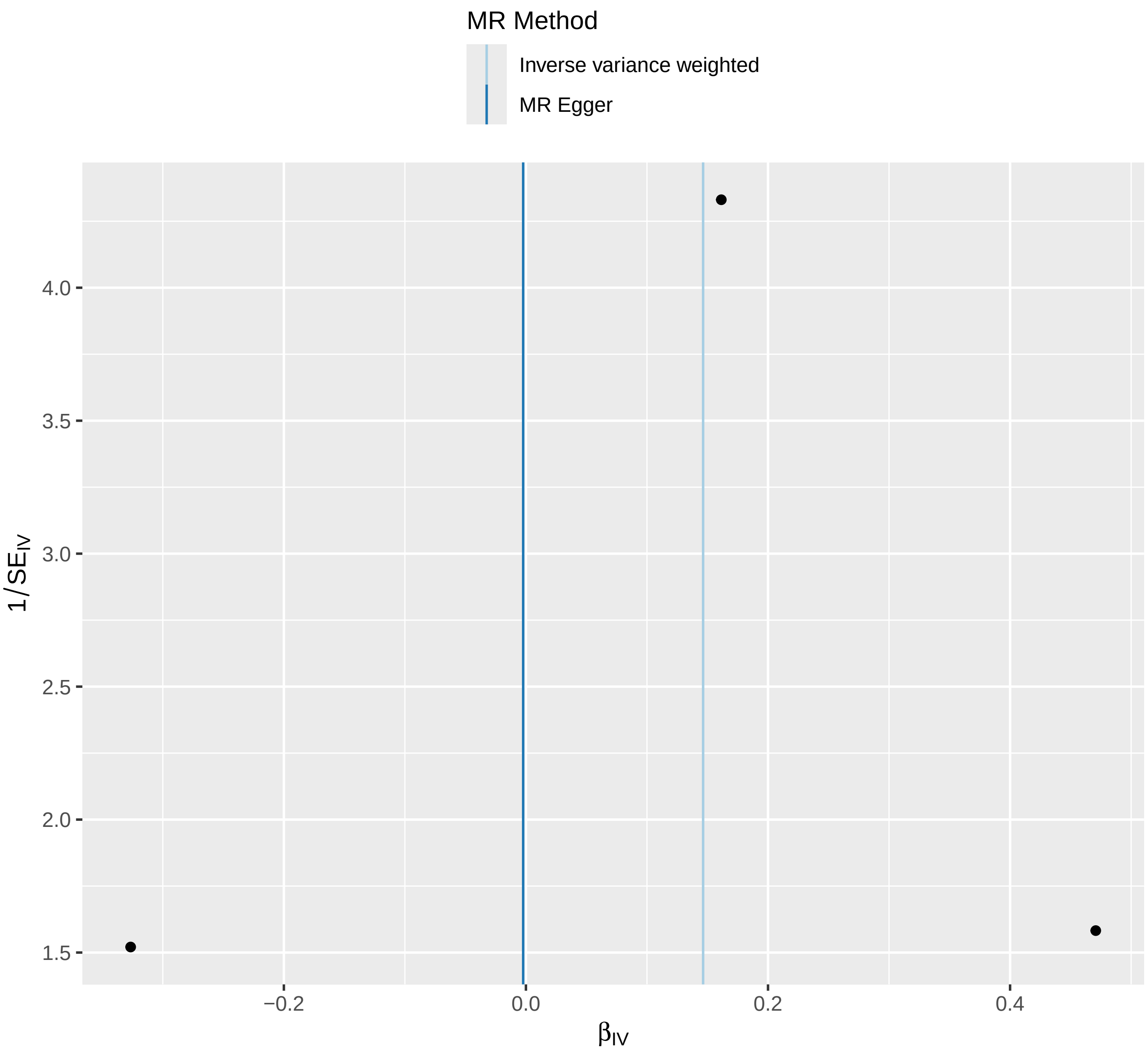

MR Method
Inverse variance weighted
MR Egger
4.0
3.5
3.0
2.5
2.0
1.5
1/SE_IV
−0.2
0.0
0.2
0.4
β_IV

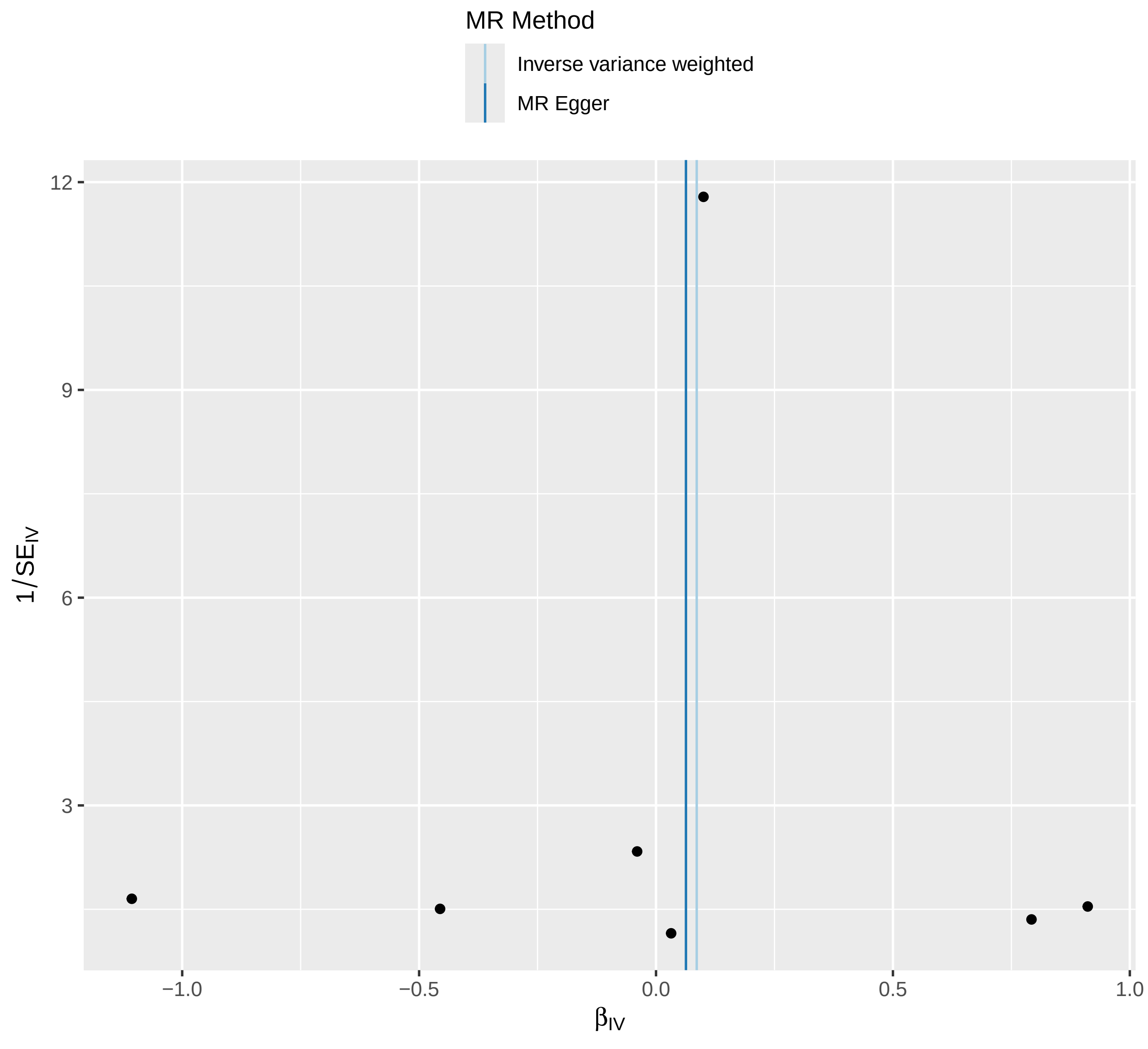

MR Method
Inverse variance weighted
MR Egger
12
9
6
3
1/SE$_{IV}$
−1.0
−0.5
0.0
0.5
1.0
β$_{IV}$

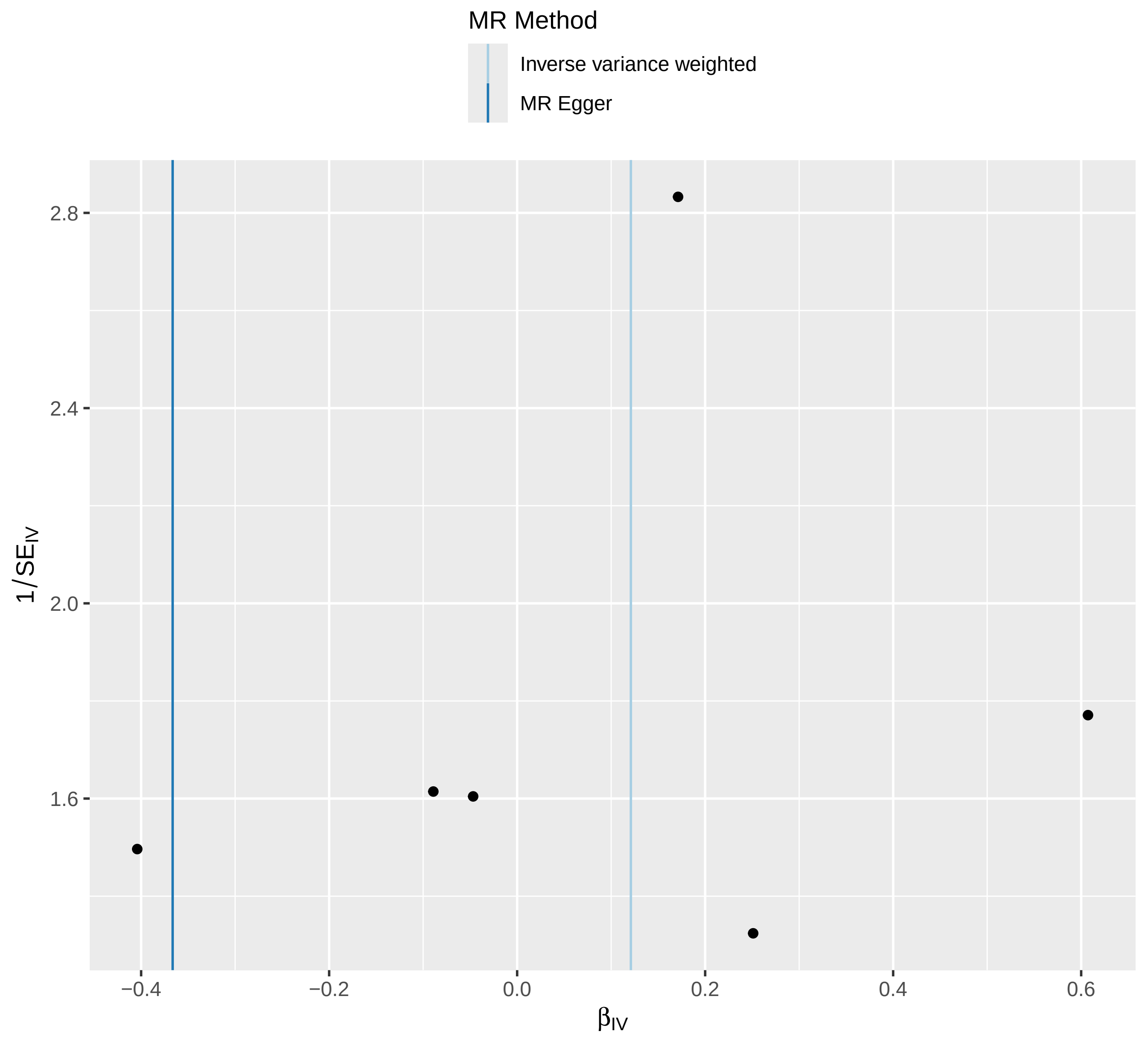
MR Method
Inverse variance weighted
MR Egger
2.8
2.4
2.0
1.6
1/SE_IV
−0.4
−0.2
0.0
0.2
0.4
0.6
β_IV

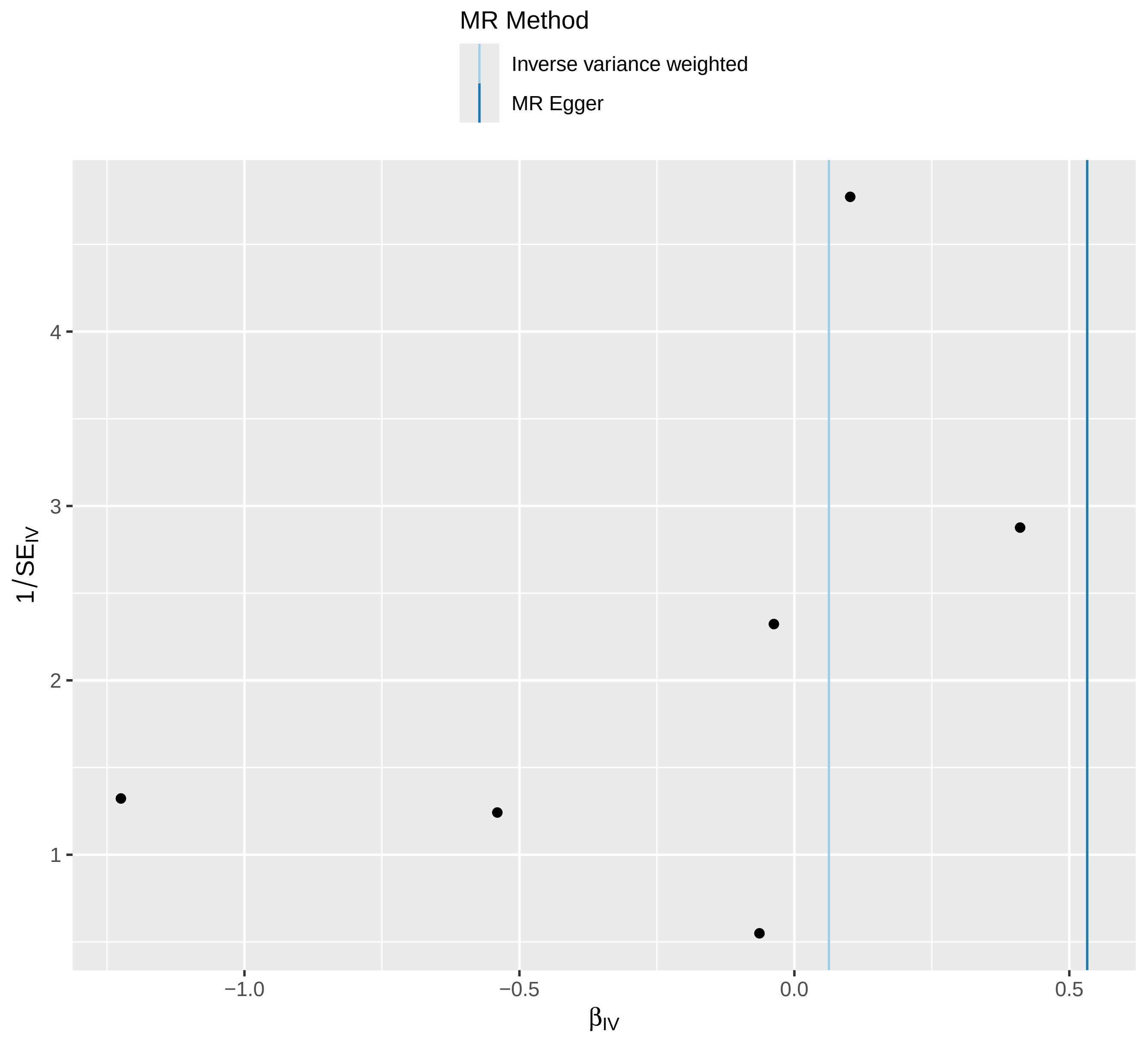

MR Method
Inverse variance weighted
MR Egger
4
3
2
1
1/SE_IV
−1.0
−0.5
0.0
0.5
β_IV

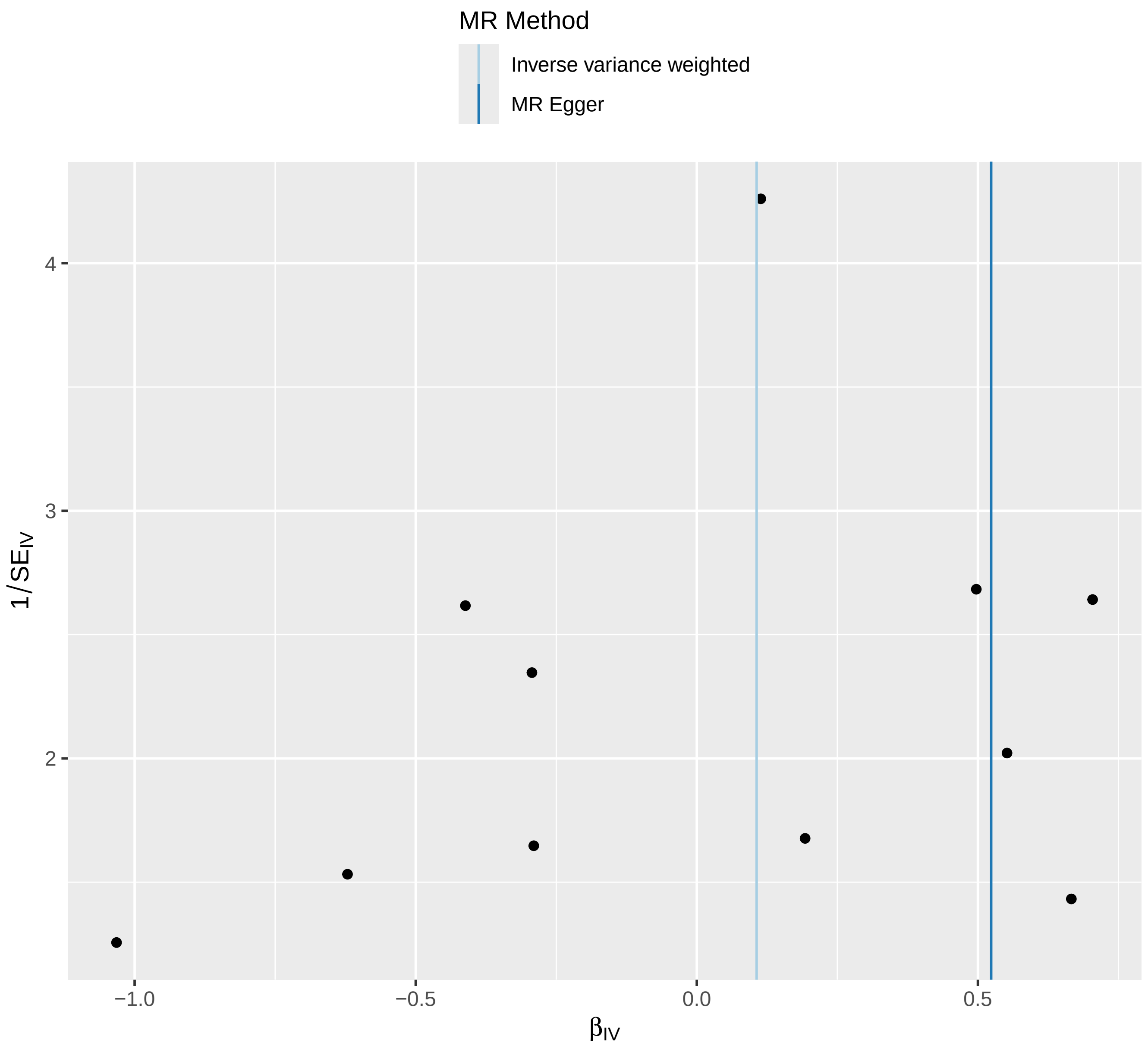
MR Method
Inverse variance weighted
MR Egger
4
3
2
1/SE$_{IV}$
−1.0
−0.5
0.0
0.5
β$_{IV}$

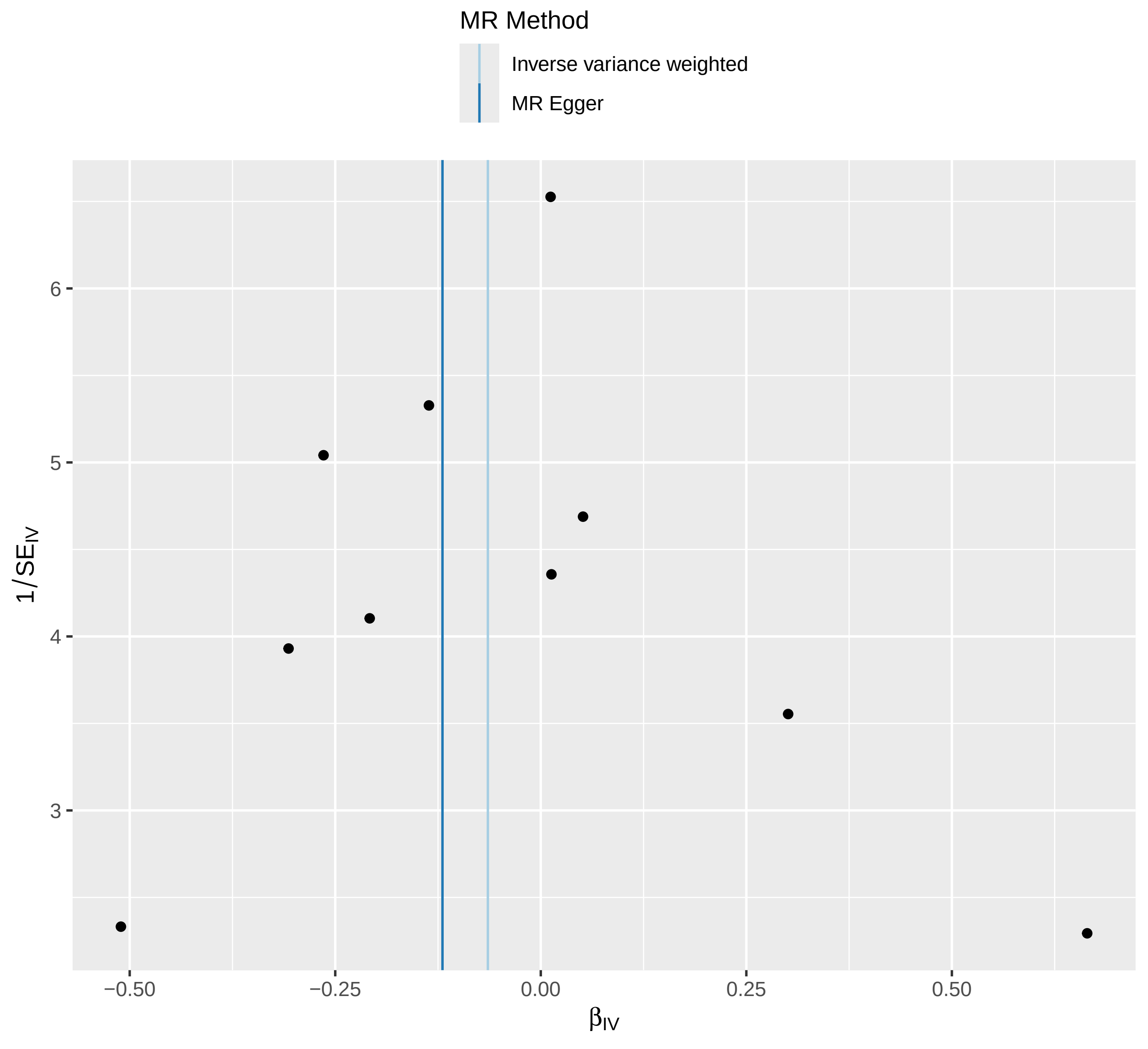

MR Method
Inverse variance weighted
MR Egger
6
5
4
3
1/SE$_{IV}$
−0.50
−0.25
0.00
0.25
0.50
β$_{IV}$

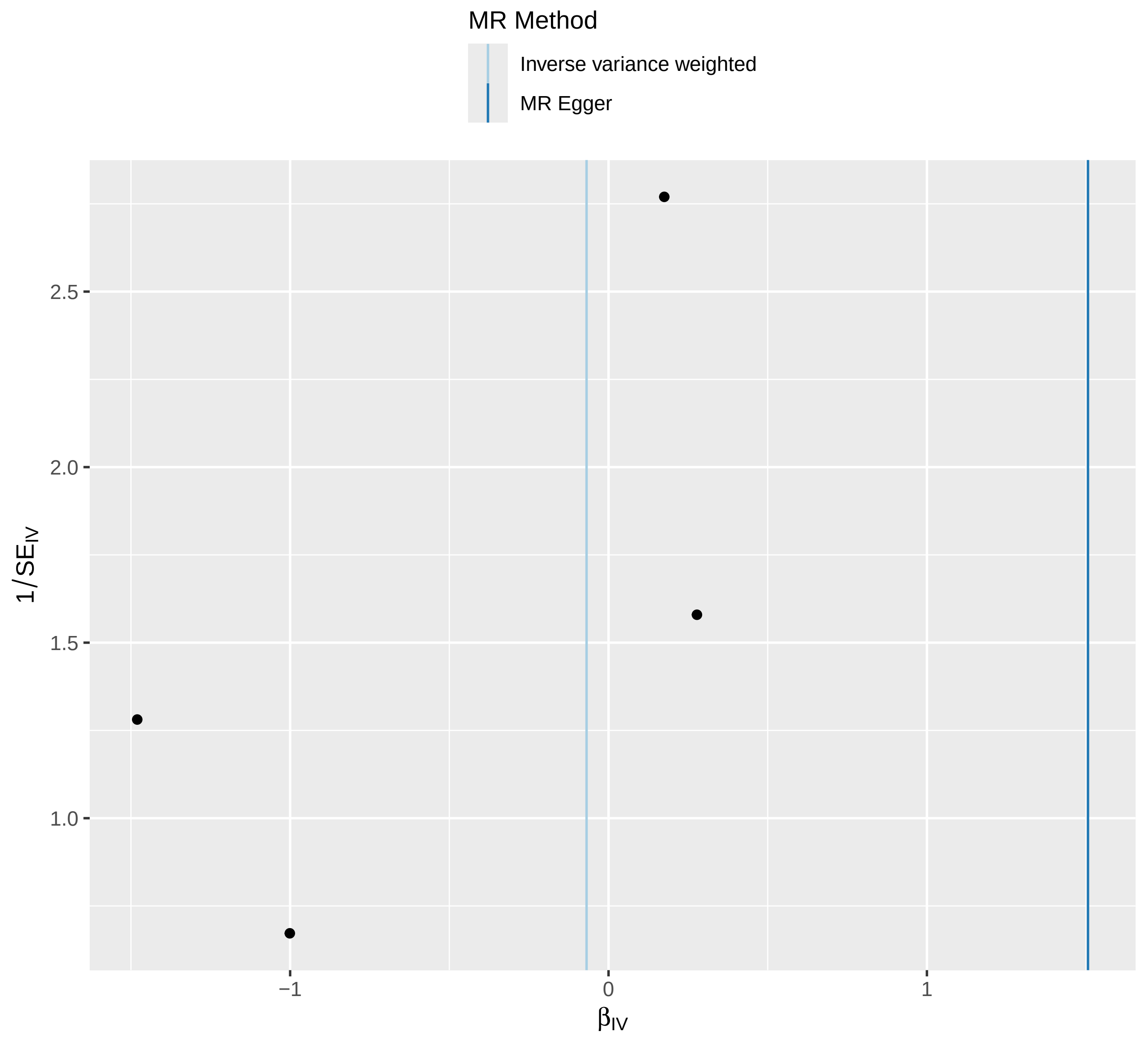

MR Method
Inverse variance weighted
MR Egger
2.5
2.0
1.5
1.0
1/SE_IV
−1
0
1
β_IV

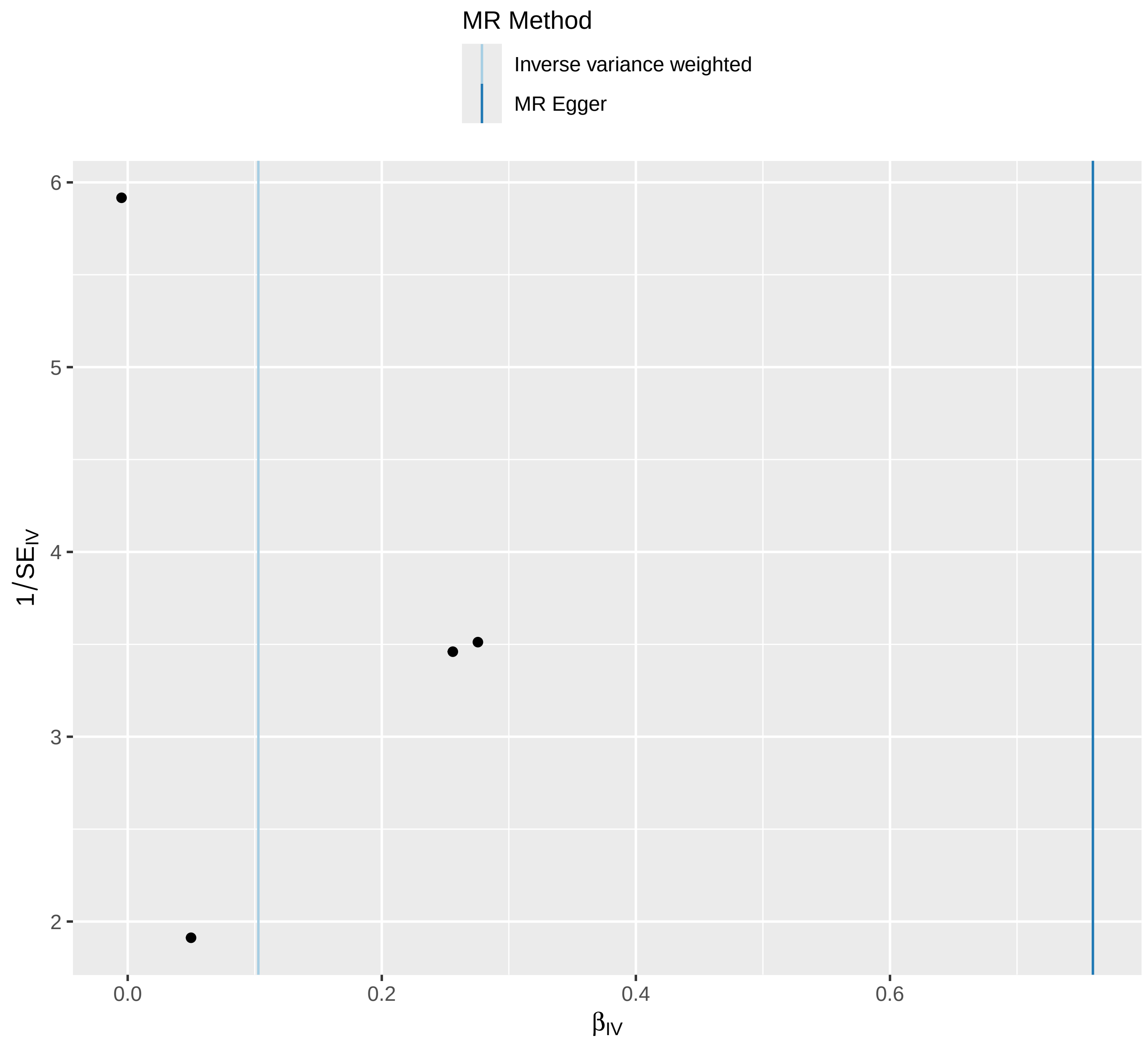
MR Method
Inverse variance weighted
MR Egger
6
5
4
3
2
0.0
0.2
0.4
0.6
$1/SE_{IV}$
β_{IV}

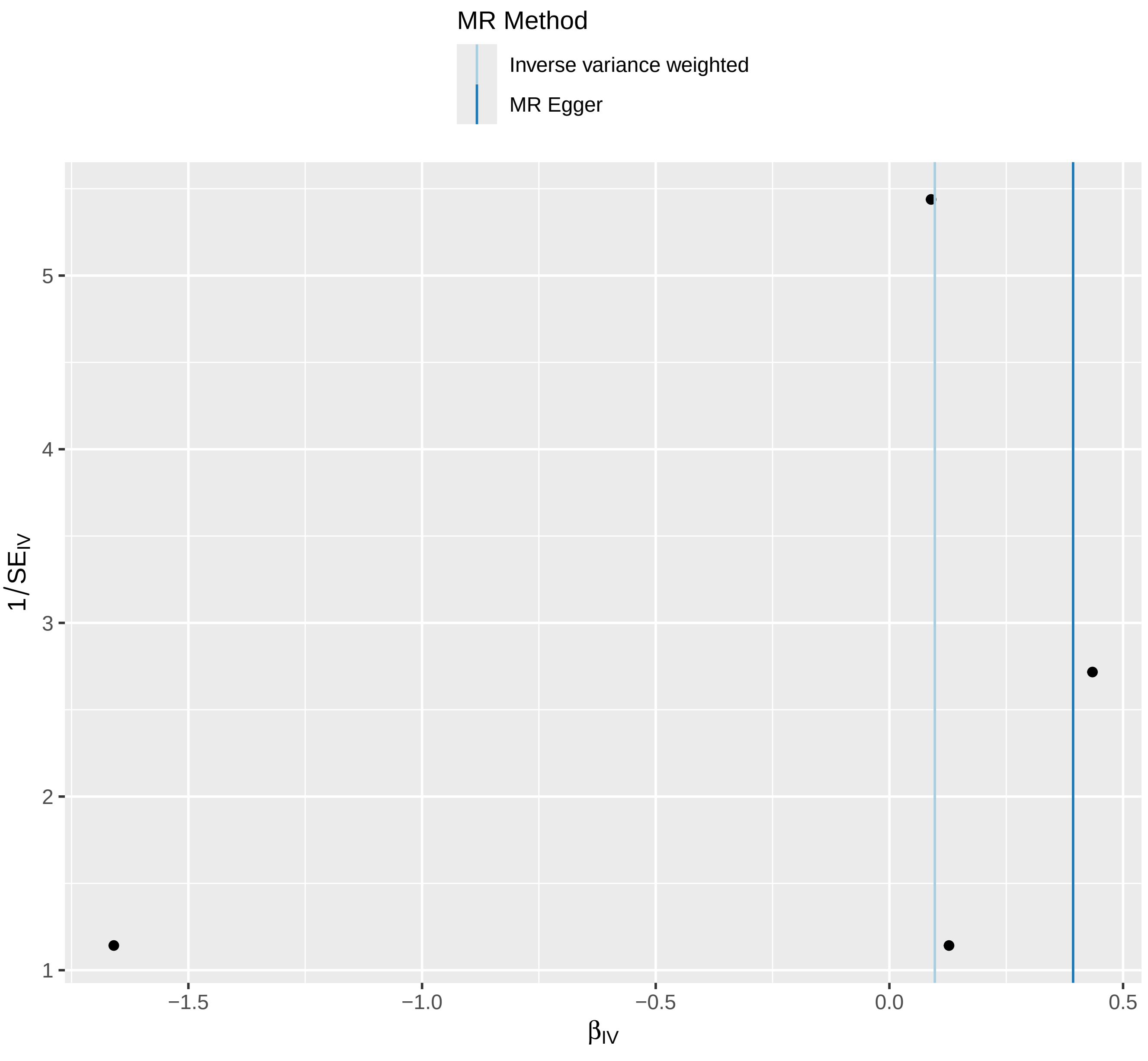
MR Method
Inverse variance weighted
MR Egger
1/SE_IV
5
4
3
2
1
−1.5
−1.0
−0.5
0.0
0.5
β_IV

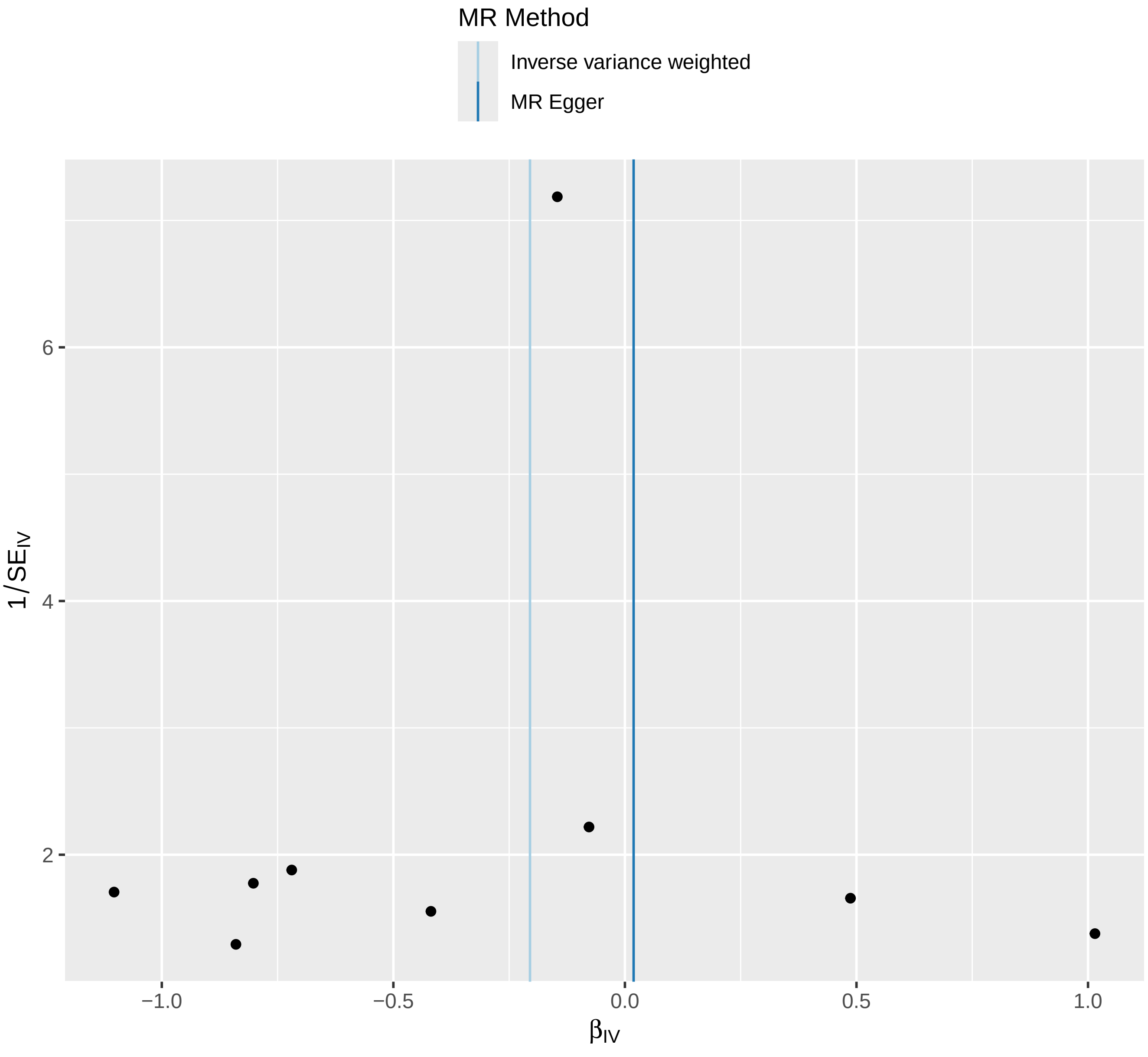

MR Method
Inverse variance weighted
MR Egger
6
4
2
1/SE_IV
−1.0
−0.5
0.0
0.5
1.0
β_IV

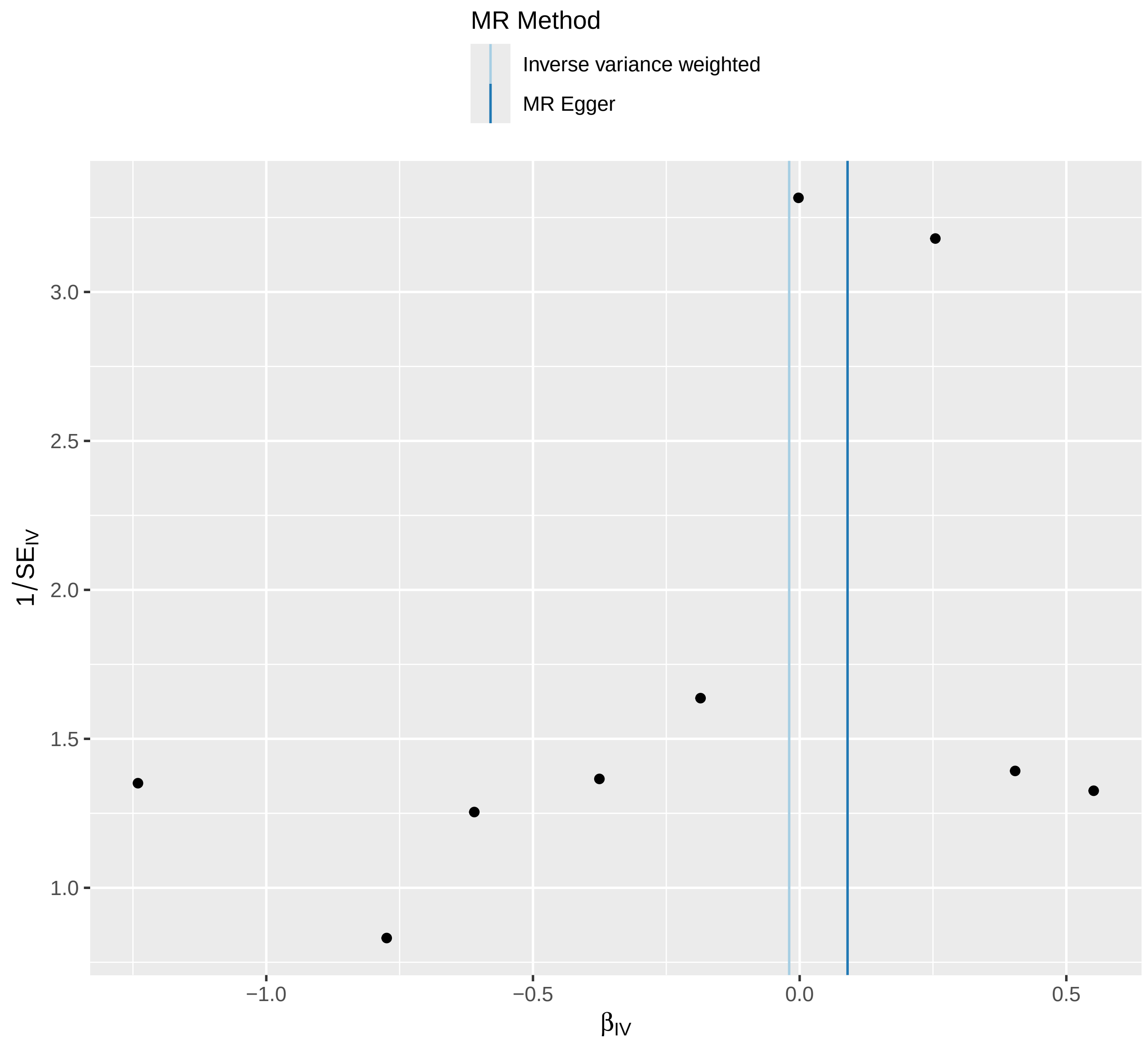

MR Method
Inverse variance weighted
MR Egger
3.0
2.5
2.0
1.5
1.0
1/SE_IV
−1.0
−0.5
0.0
0.5
β_IV

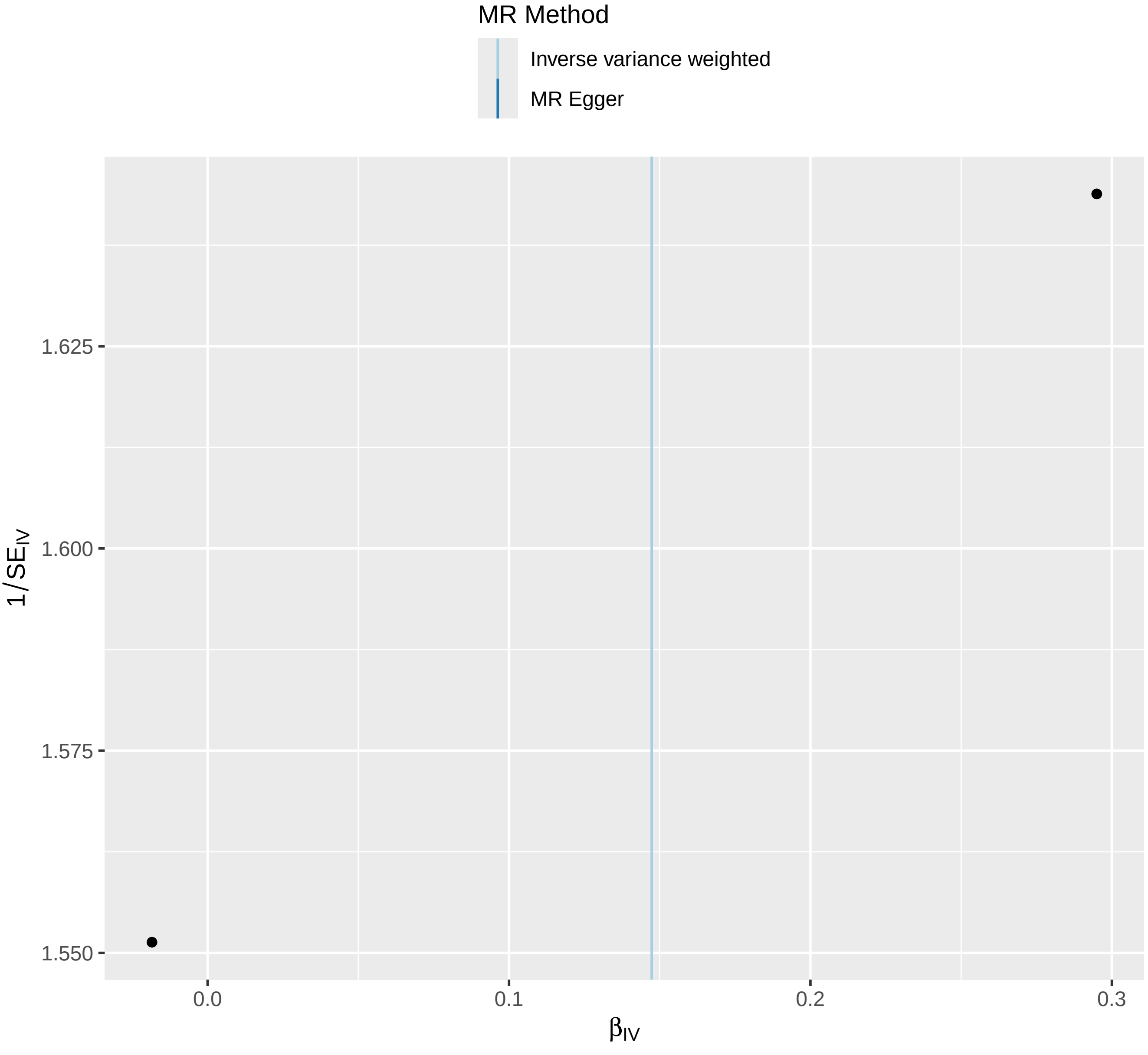

MR Method
Inverse variance weighted
MR Egger
1.625
1.600
1.575
1.550
$1/SE_{IV}$
0.0
0.1
0.2
0.3
β_{IV}

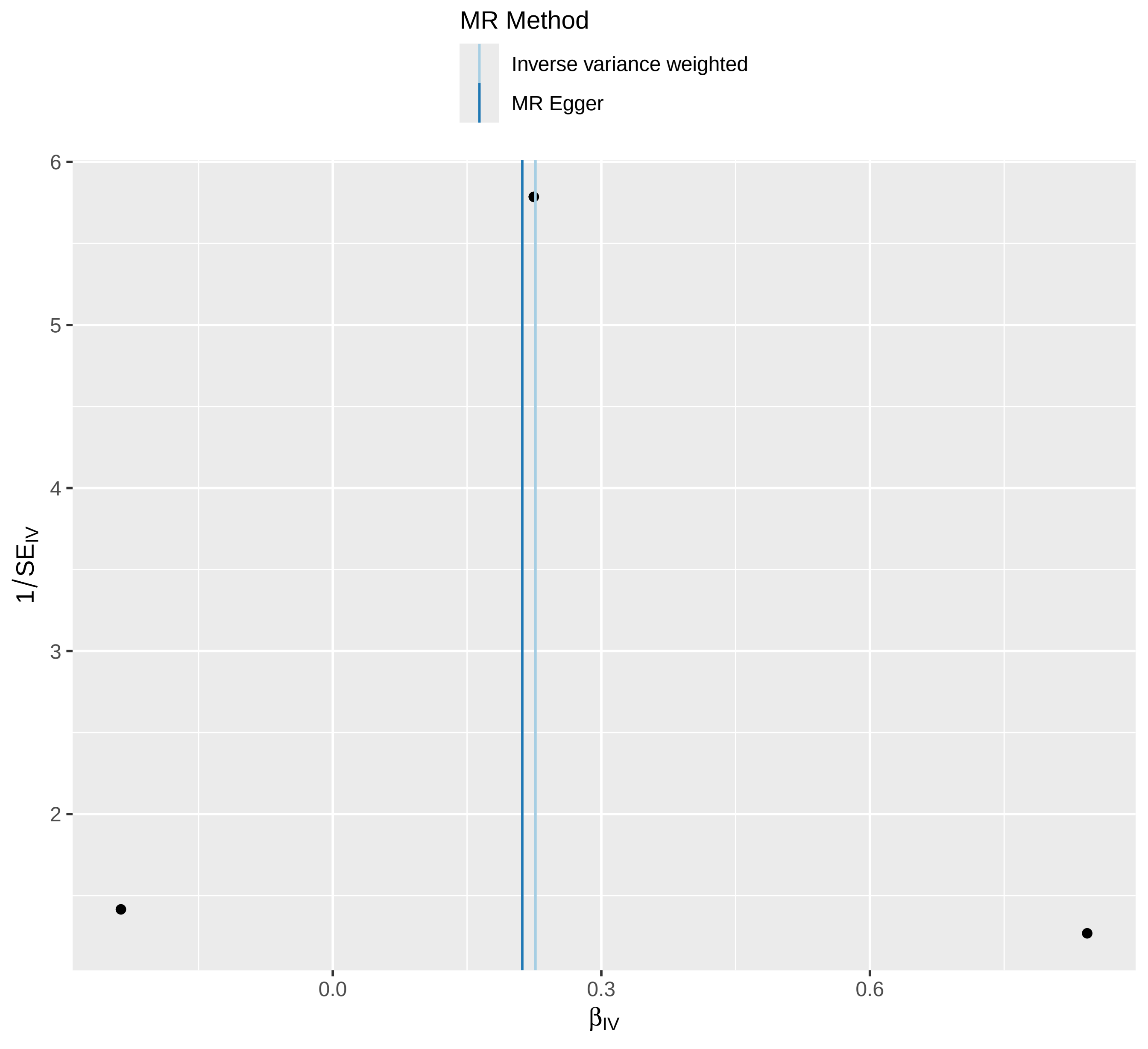

MR Method
Inverse variance weighted
MR Egger
6
5
4
3
2
1/SE_IV
0.0
0.3
0.6
β_IV

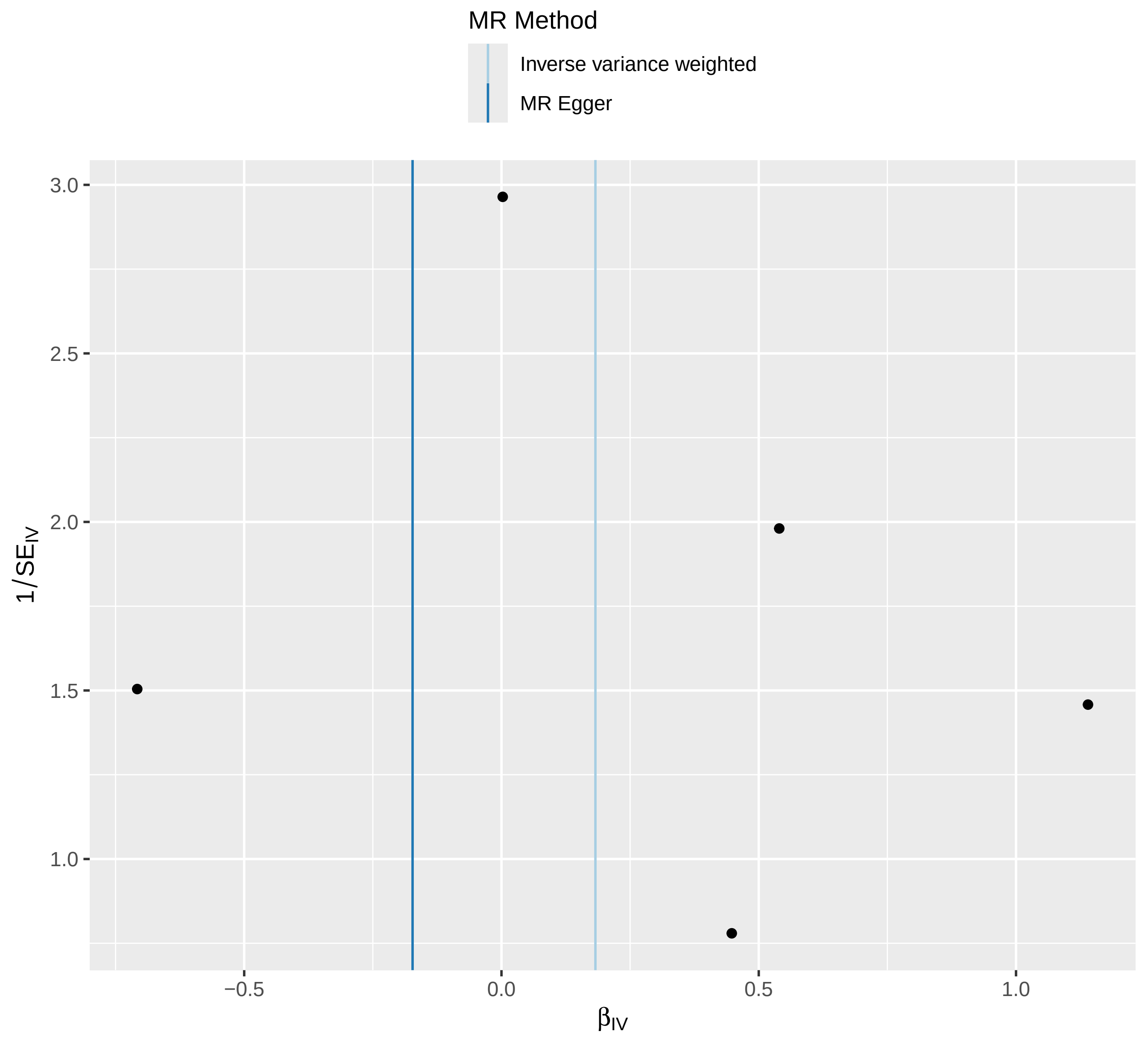
MR Method
Inverse variance weighted
MR Egger
3.0
2.5
2.0
1.5
1.0
1/SE_IV
−0.5
0.0
0.5
1.0
β_IV

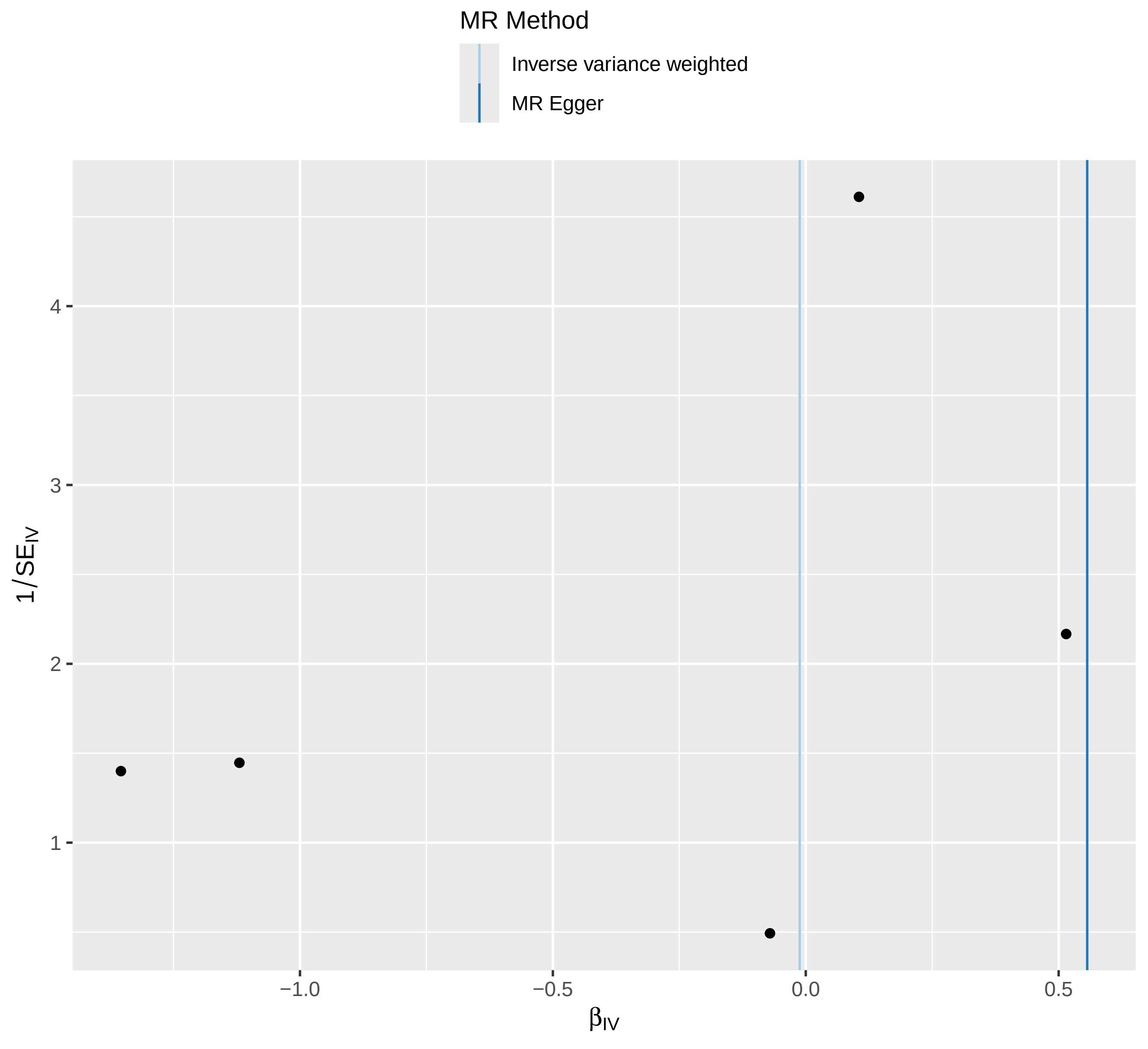
MR Method
Inverse variance weighted
MR Egger
4
3
2
1
1/SE$_{IV}$
−1.0
−0.5
0.0
0.5
β$_{IV}$

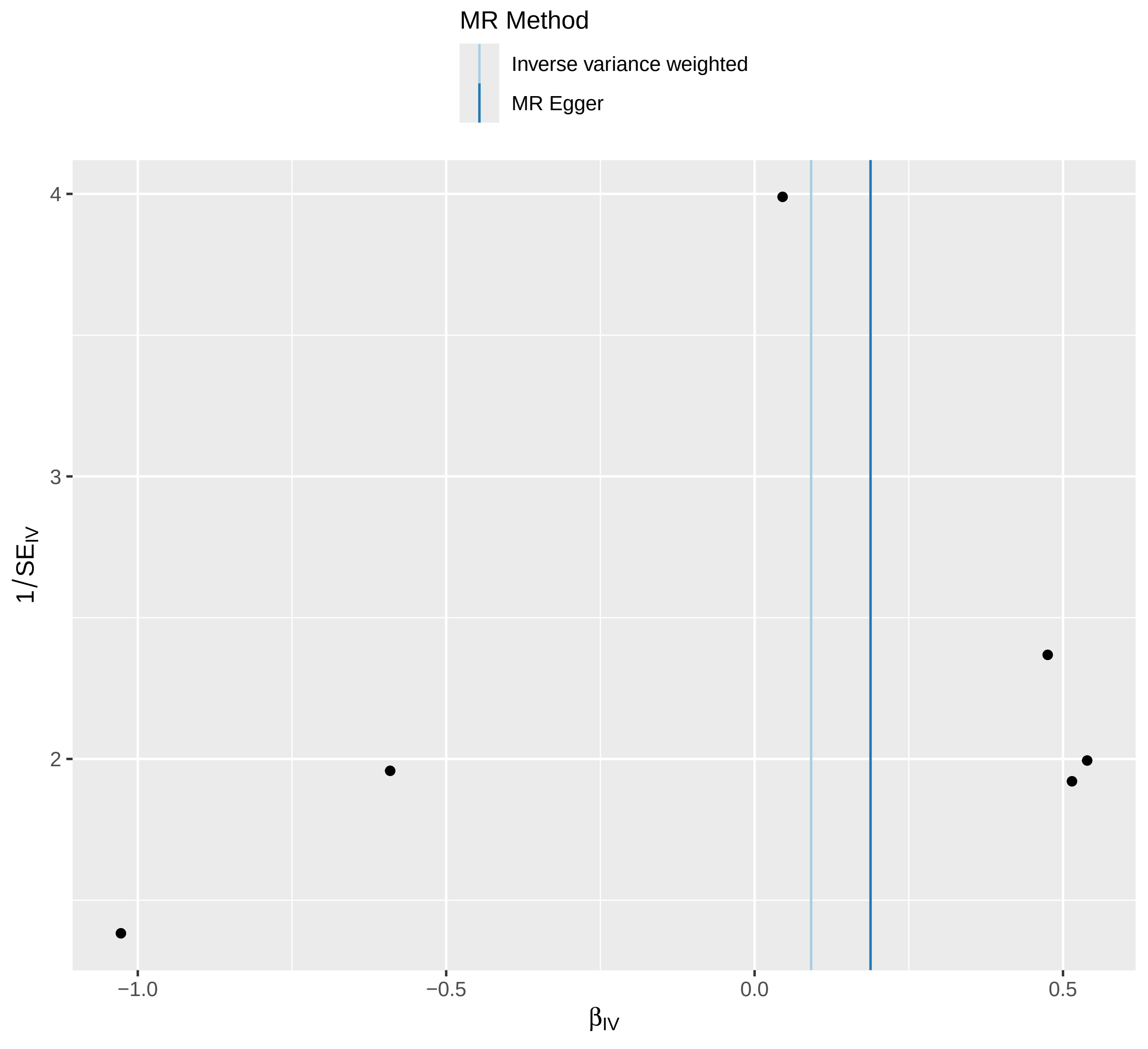

MR Method
Inverse variance weighted
MR Egger
4
3
2
1/SE_IV
−1.0
−0.5
0.0
0.5
β_IV

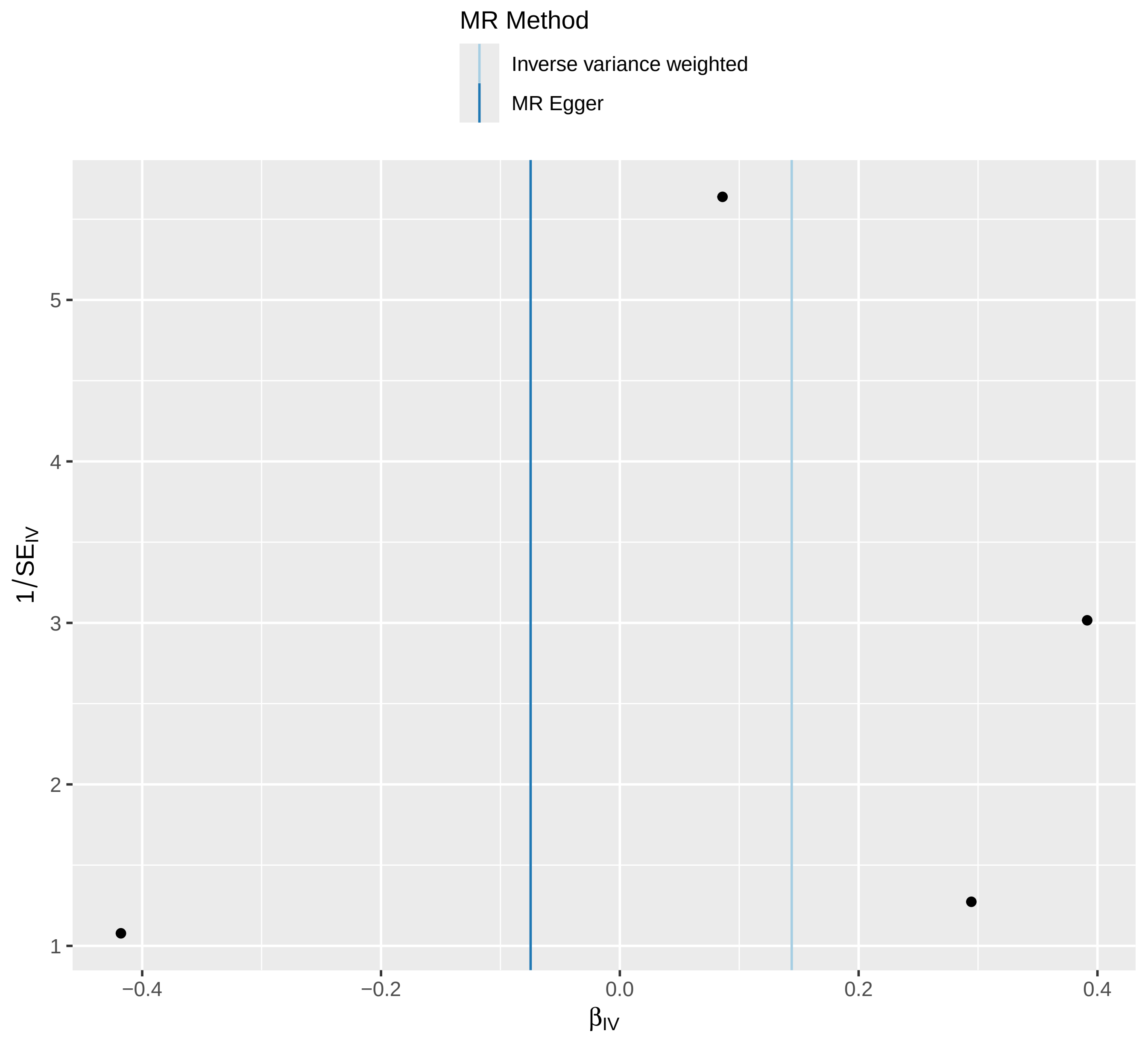
MR Method
Inverse variance weighted
MR Egger
$1/SE_{IV}$
5
4
3
2
1
−0.4
−0.2
0.0
0.2
0.4
β_{IV}

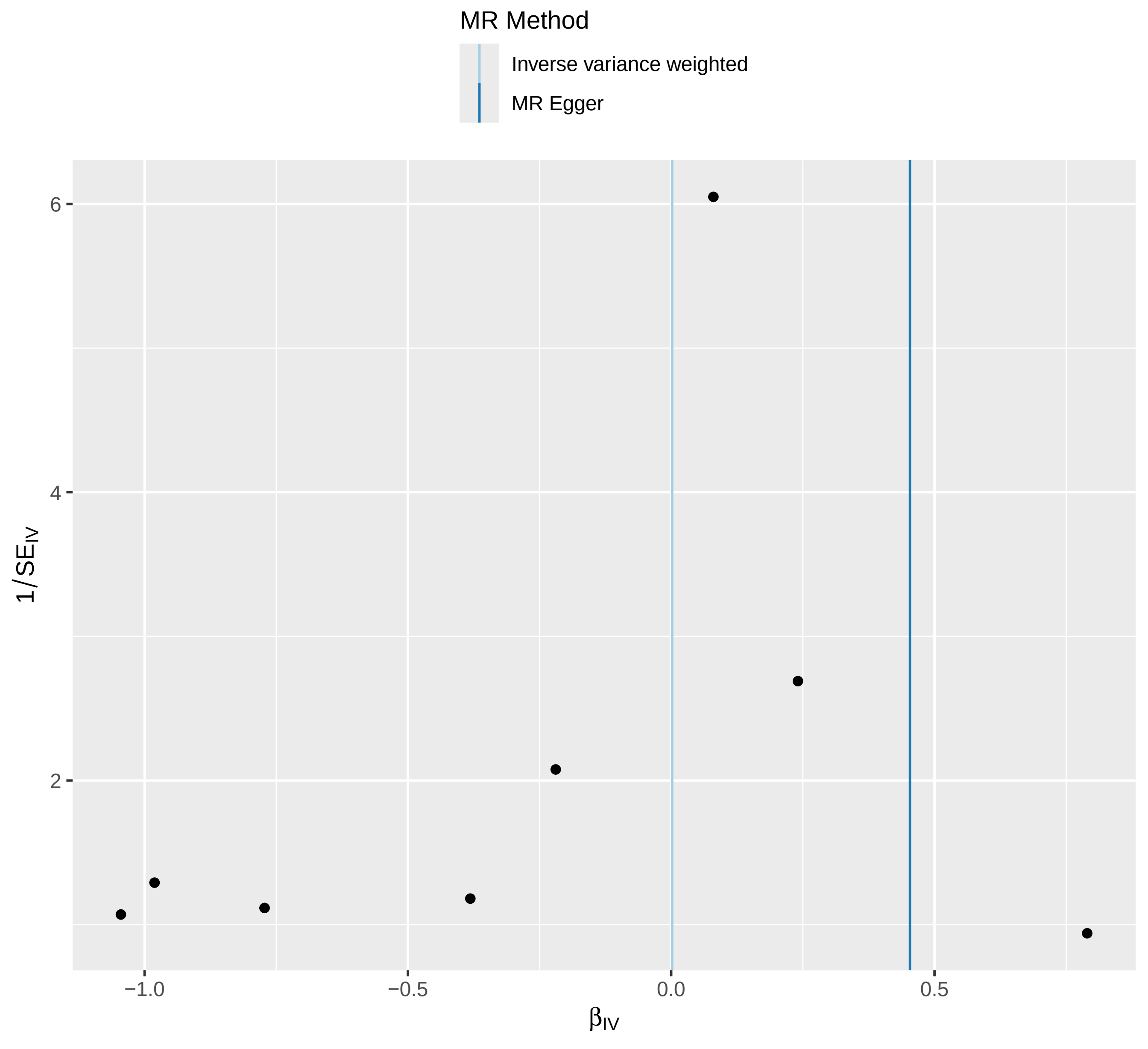

MR Method
Inverse variance weighted
MR Egger
6
4
2
1/SE$_{IV}$
−1.0
−0.5
0.0
0.5
β$_{IV}$

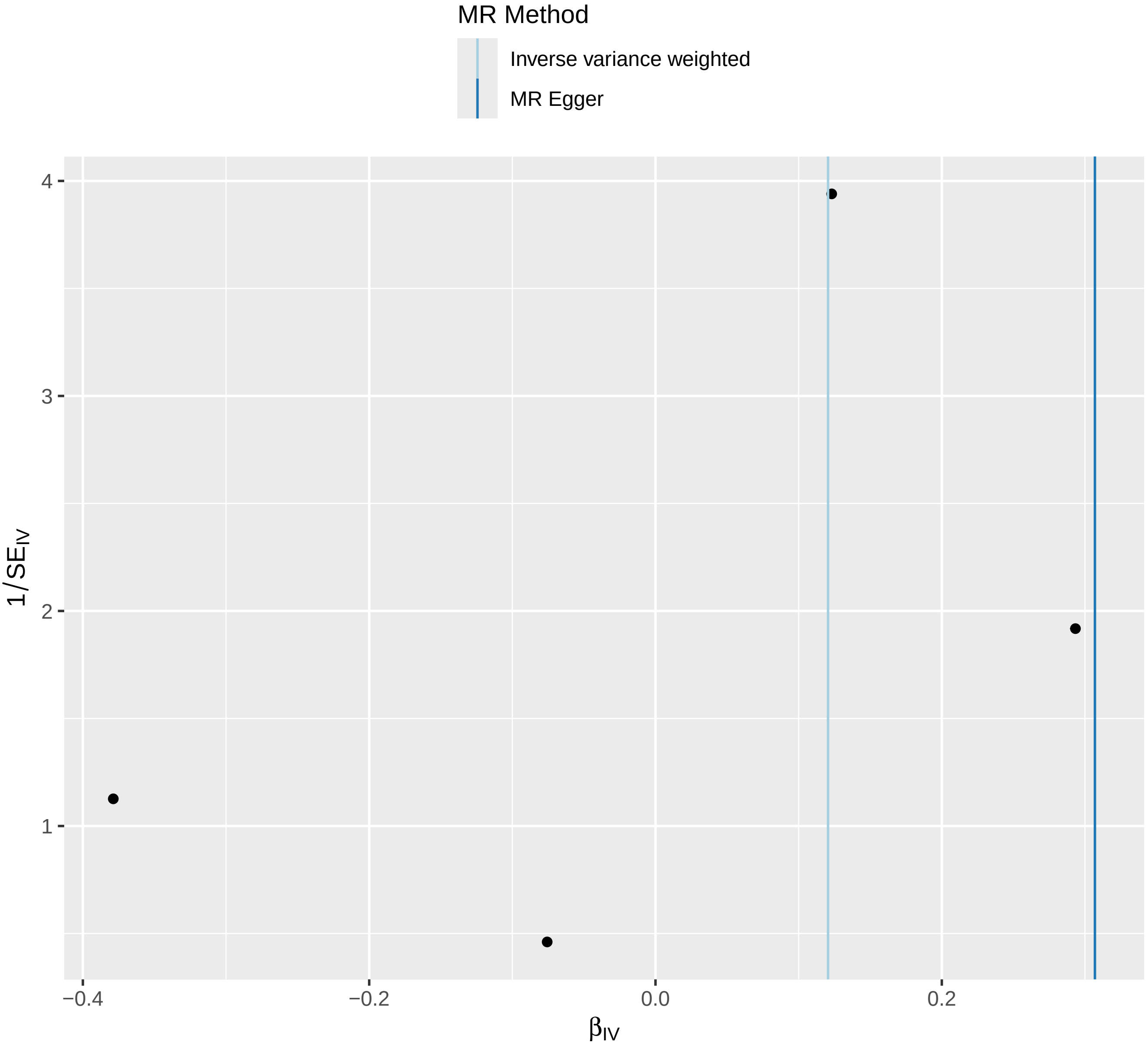

MR Method
Inverse variance weighted
MR Egger
4
3
2
1
$1/SE_{IV}$
−0.4
−0.2
0.0
0.2
β_{IV}

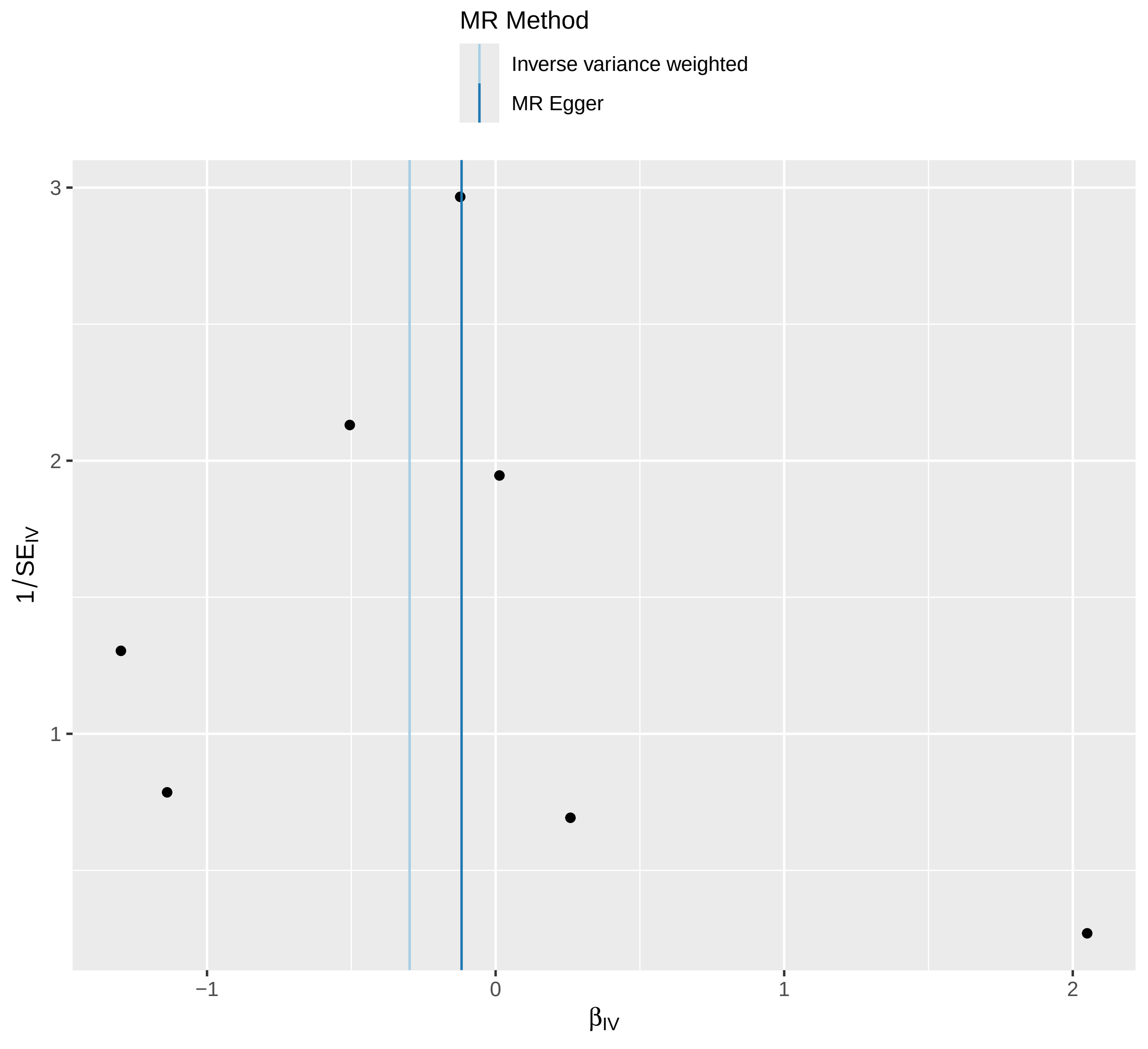

MR Method
Inverse variance weighted
MR Egger
3
2
1
1/SE_IV
−1
0
1
2
β_IV

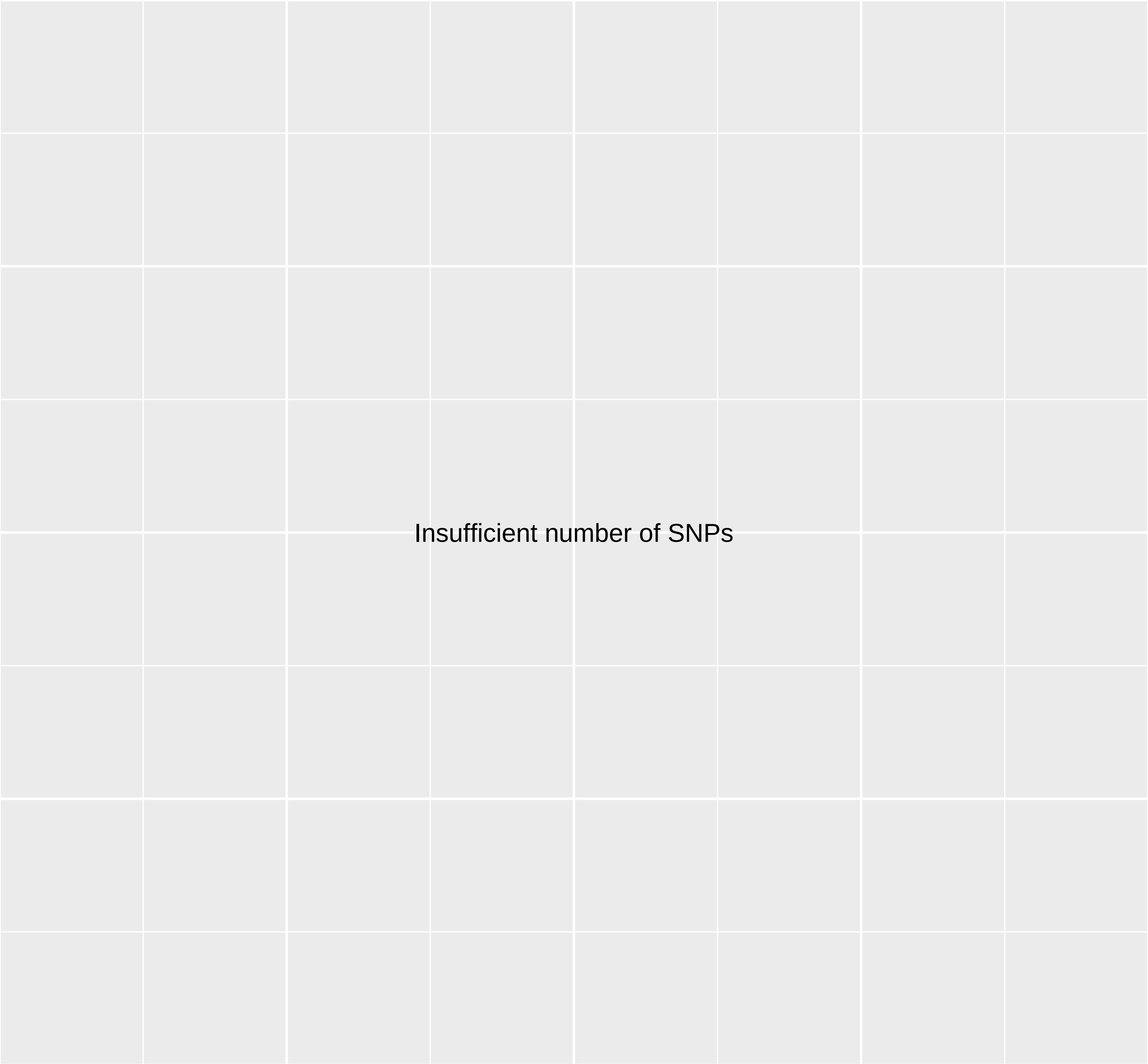
Insufficient number of SNPs

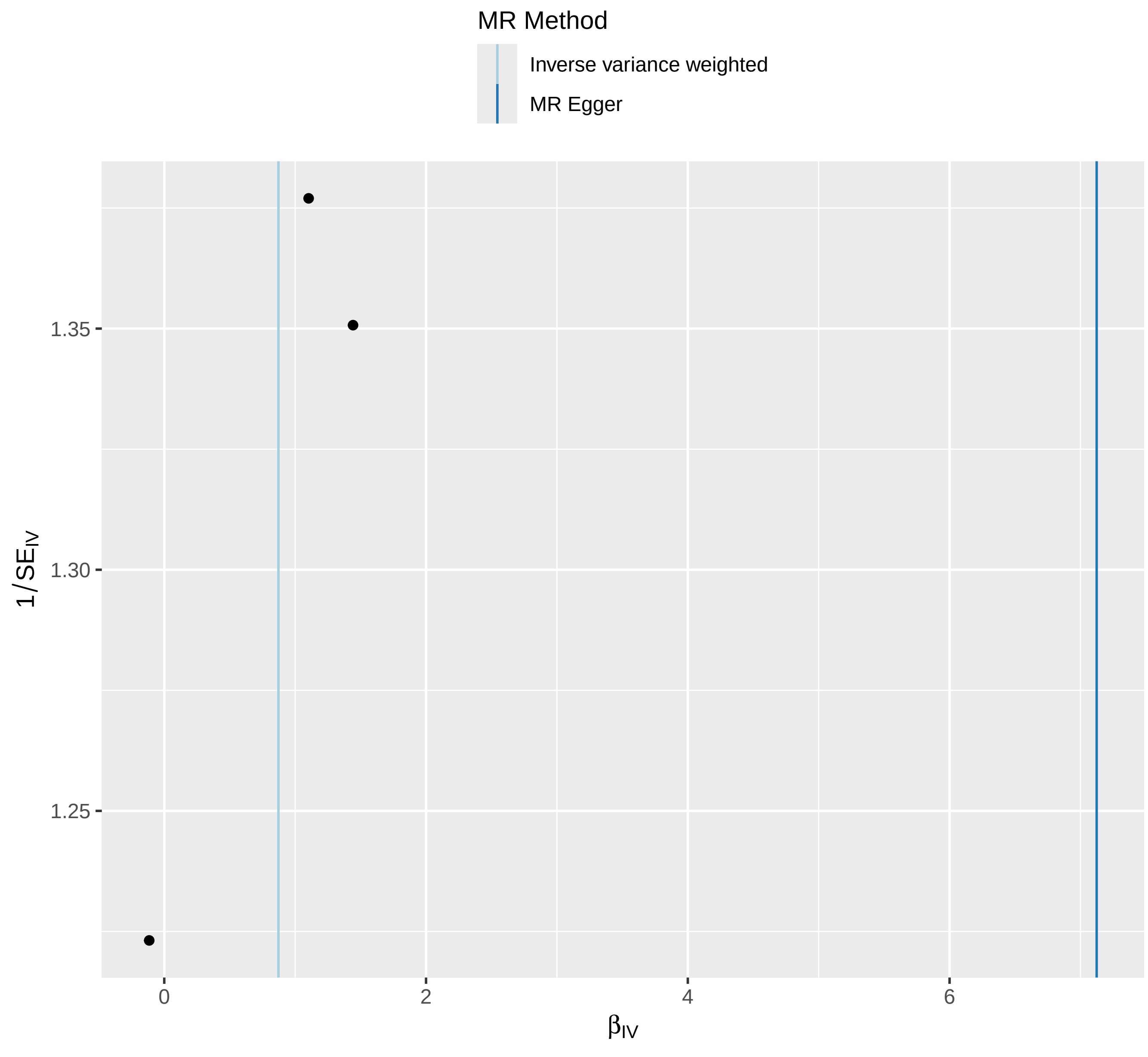
MR Method
Inverse variance weighted
MR Egger
1.35
1.30
1.25
0
2
4
6
β_{IV}
$1/SE_{IV}$

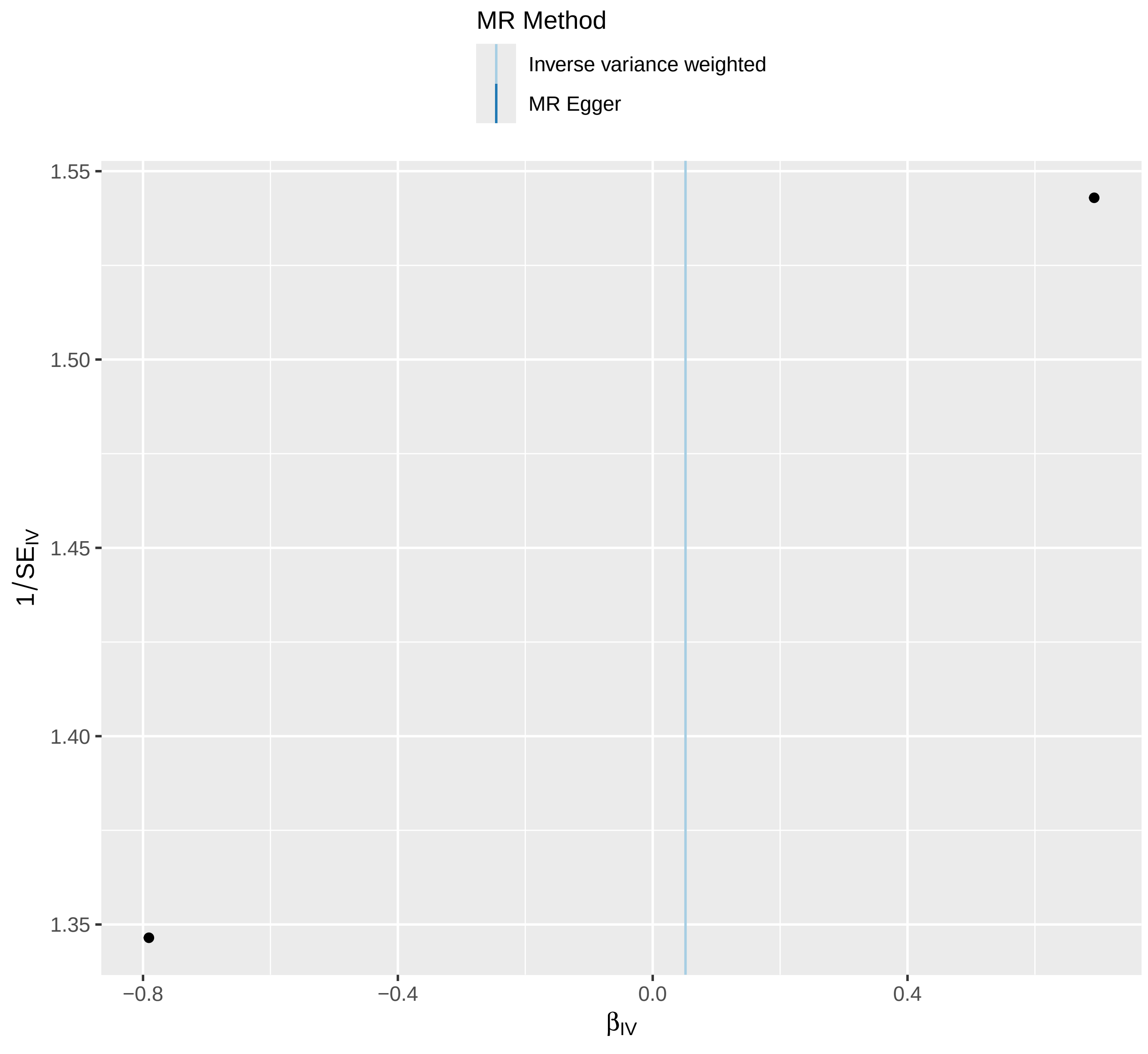

MR Method
Inverse variance weighted
MR Egger
1.55
1.50
1.45
1.40
1.35
1/SE_IV
−0.8
−0.4
0.0
0.4
β_IV

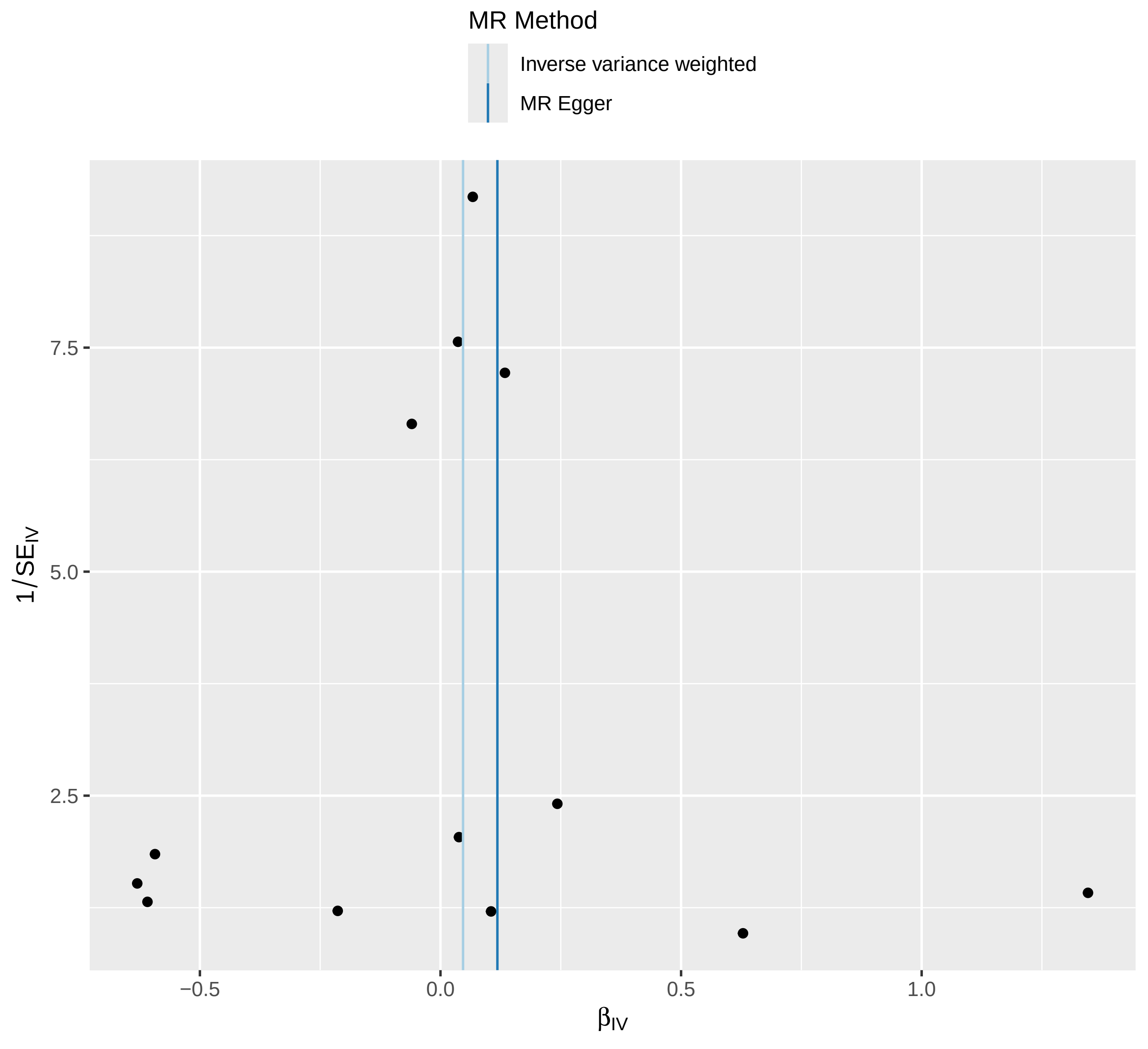
MR Method
Inverse variance weighted
MR Egger
7.5
5.0
2.5
1/SE$_{IV}$
−0.5
0.0
0.5
1.0
β$_{IV}$

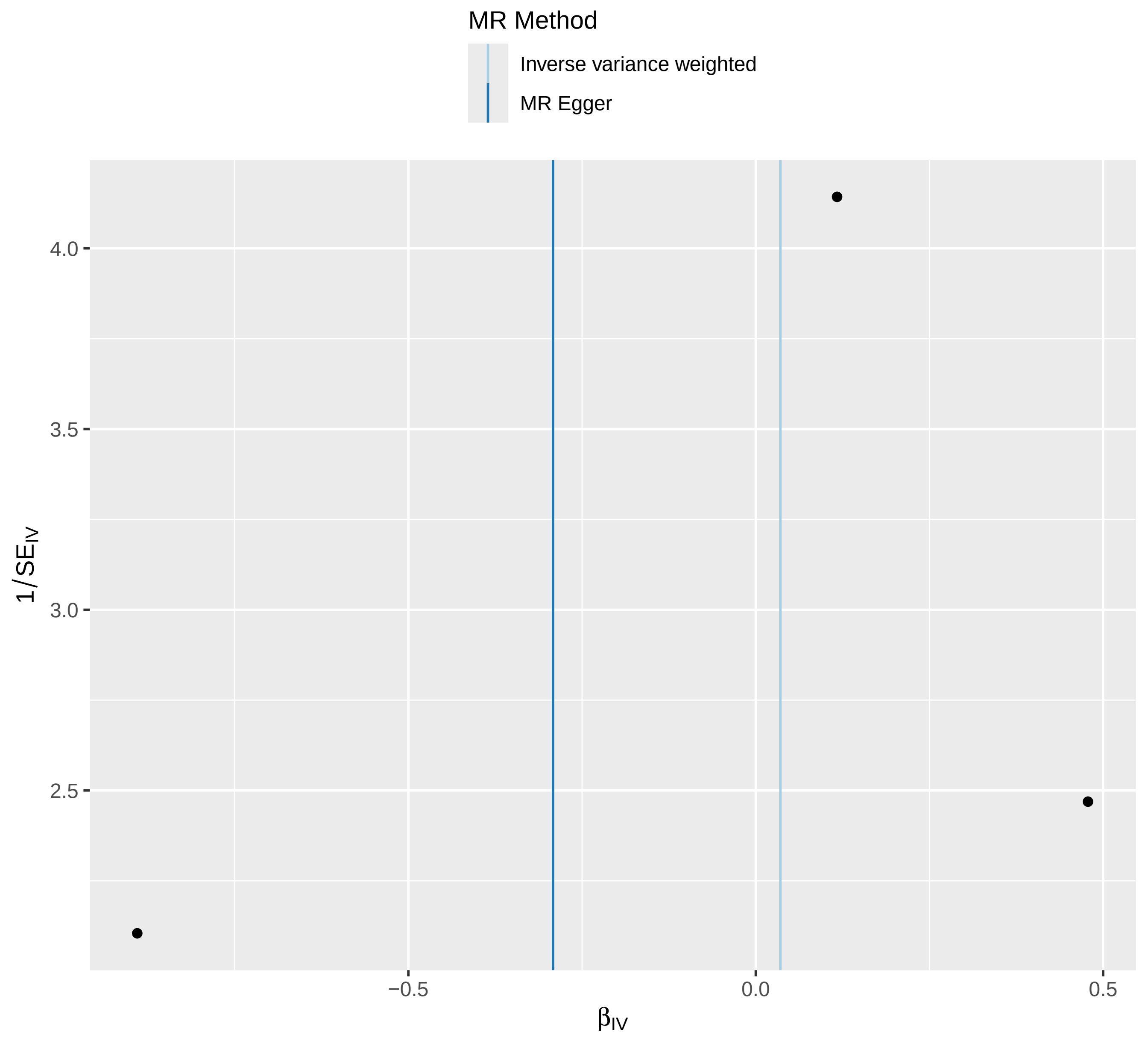
MR Method
Inverse variance weighted
MR Egger
4.0
3.5
3.0
2.5
1/SE$_{IV}$
−0.5
0.0
0.5
β$_{IV}$

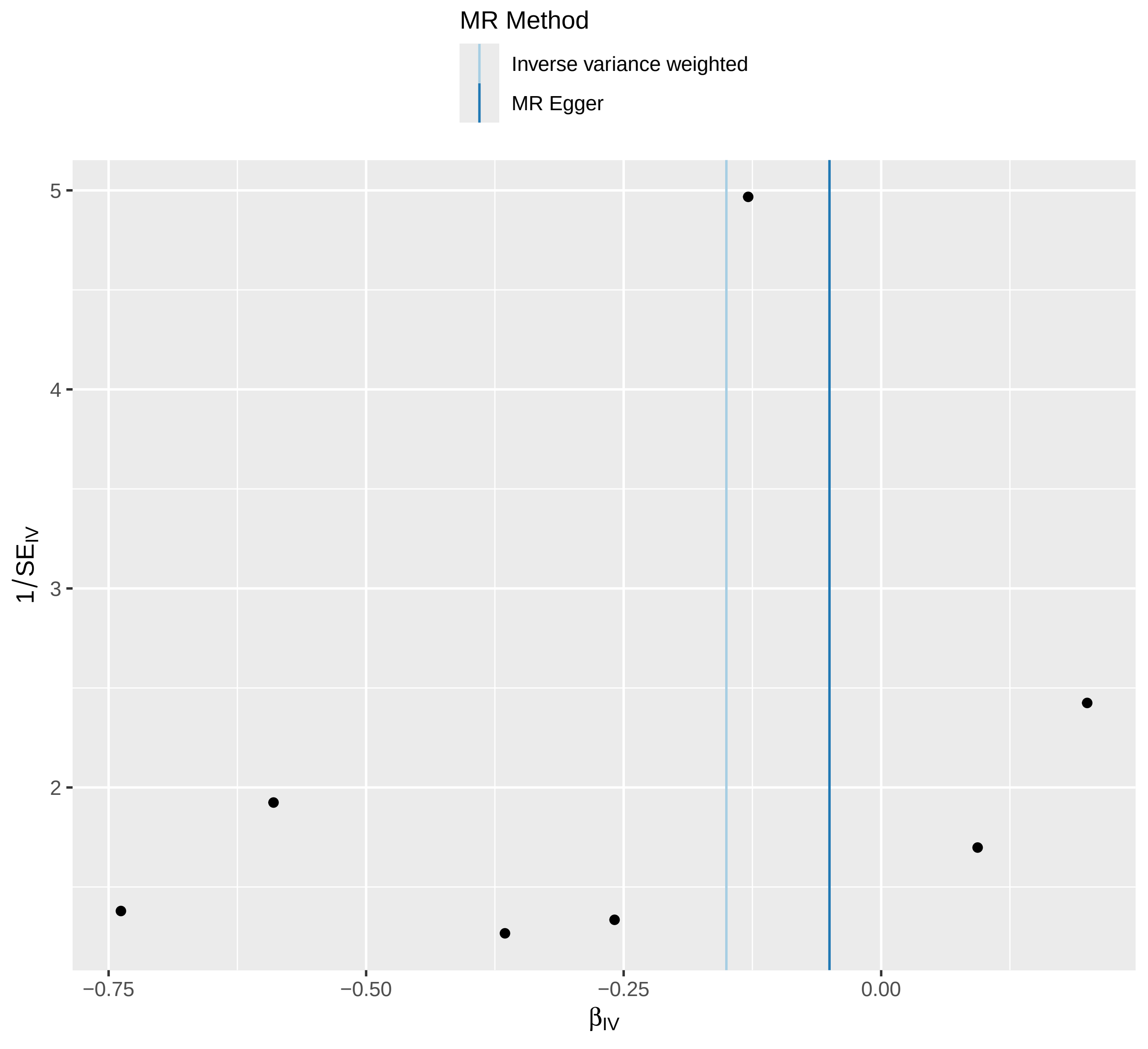
MR Method
Inverse variance weighted
MR Egger
5
4
3
2
1/SE_IV
−0.75
−0.50
−0.25
0.00
β_IV

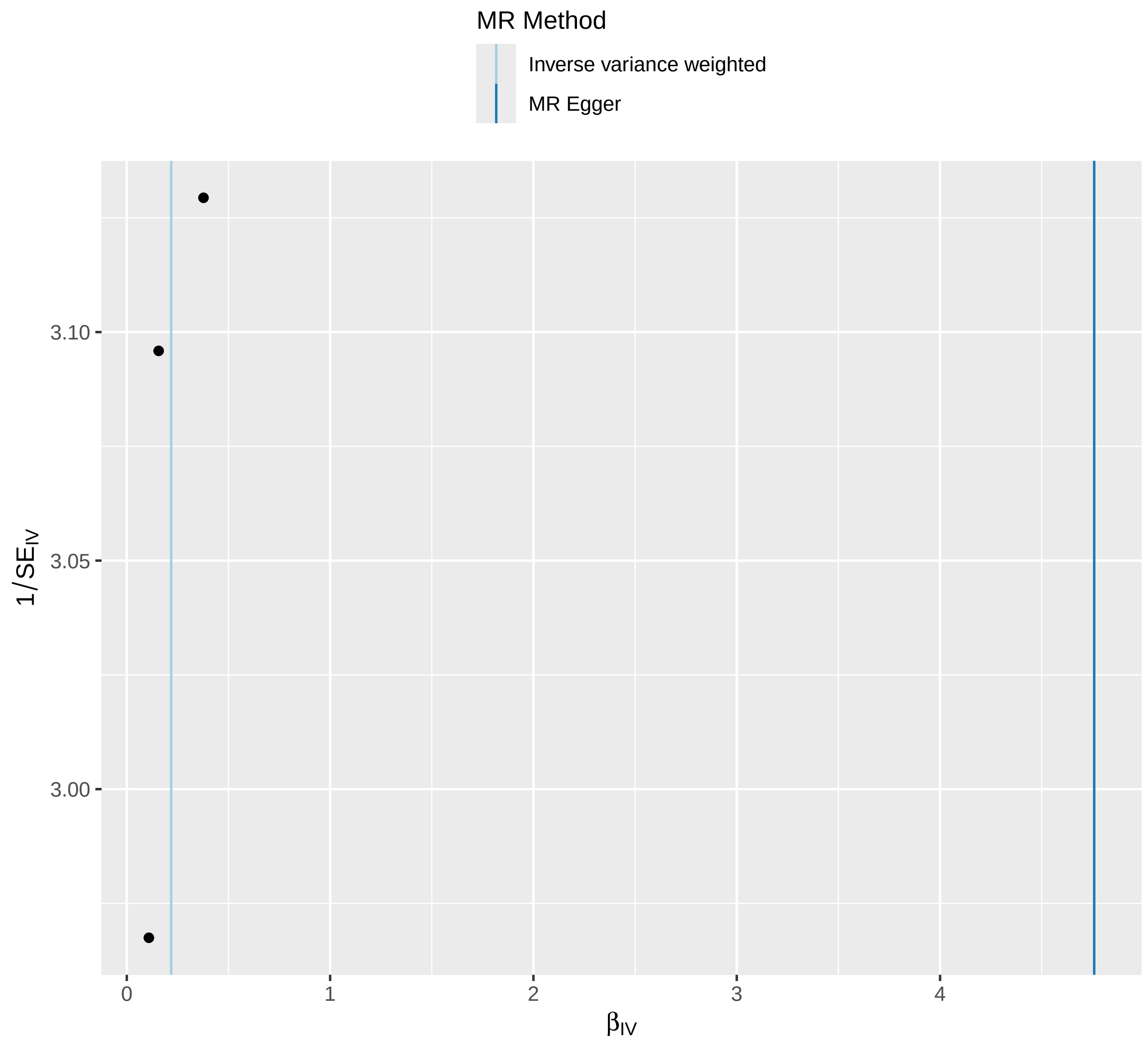

MR Method
Inverse variance weighted
MR Egger
3.10
3.05
3.00
$1/SE_{IV}$
0
1
2
3
4
β_{IV}

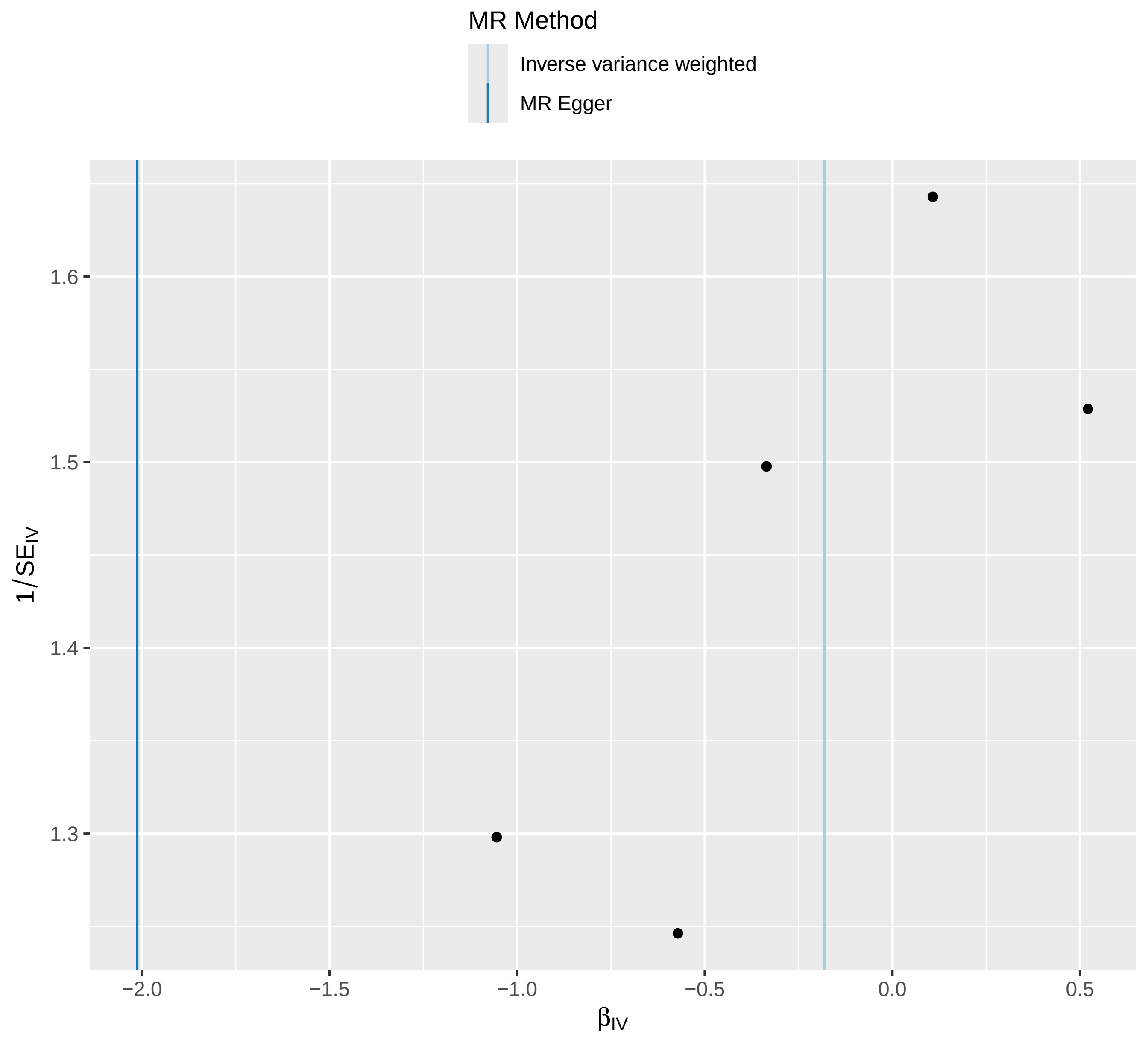
MR Method
Inverse variance weighted
MR Egger
1/SE$_{IV}$
1.3
1.4
1.5
1.6
−2.0
−1.5
−1.0
−0.5
0.0
0.5
β$_{IV}$

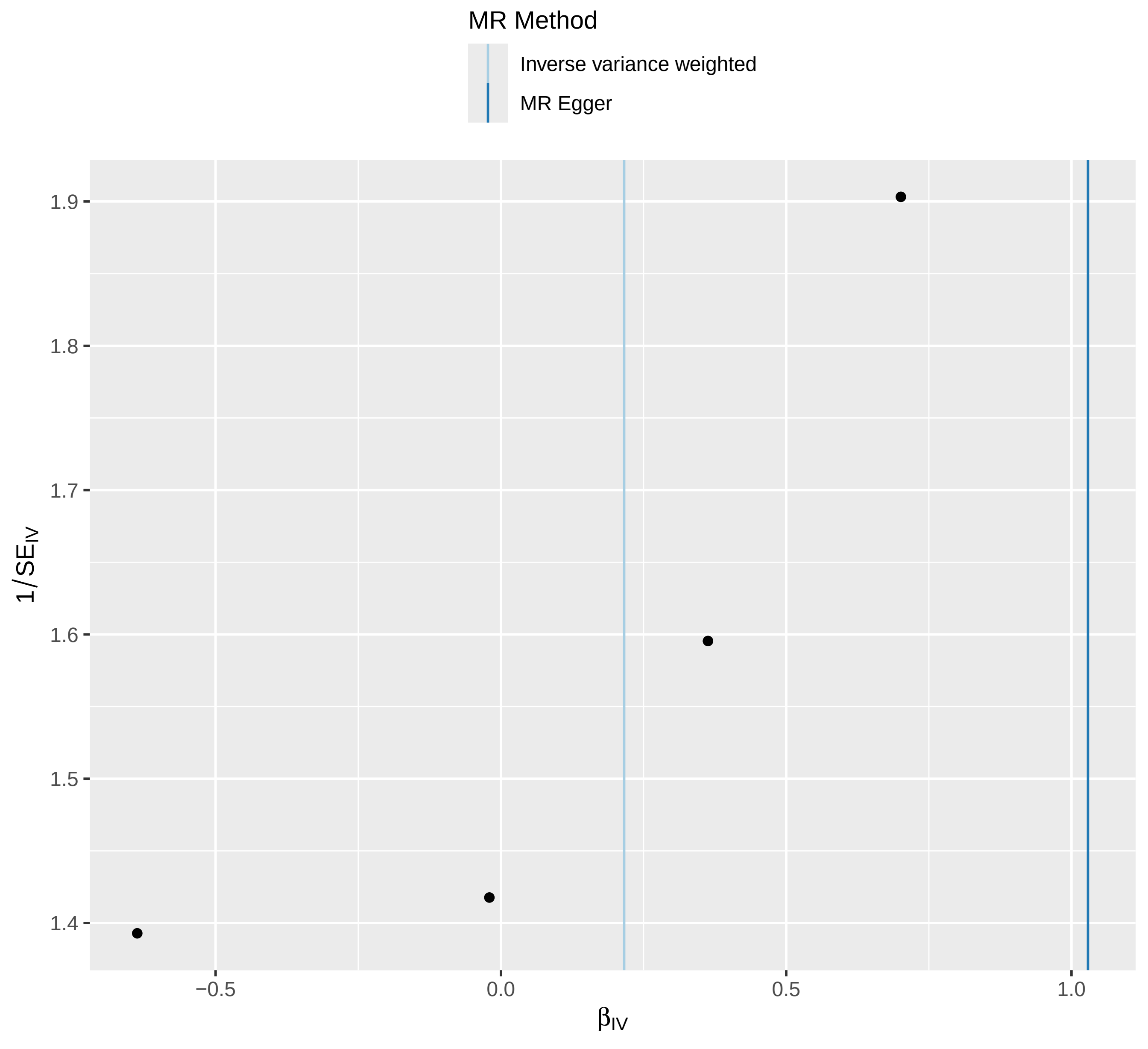

MR Method
Inverse variance weighted
MR Egger
1.9
1.8
1.7
1.6
1.5
1.4
1/SE_IV
−0.5
0.0
0.5
1.0
β_IV

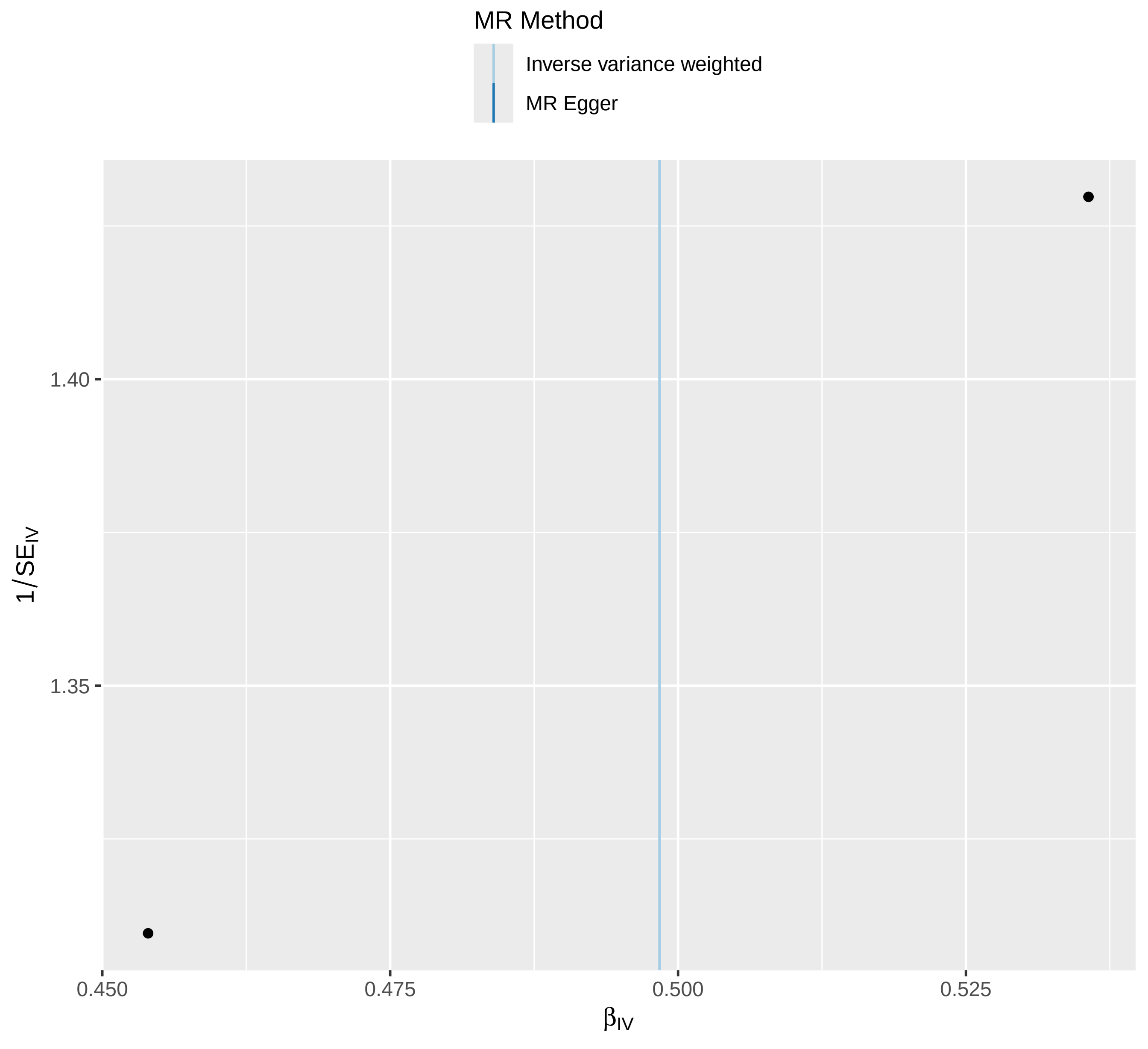
MR Method
Inverse variance weighted
MR Egger
1.40
1.35
1/SE_IV
0.450
0.475
0.500
0.525
β_IV

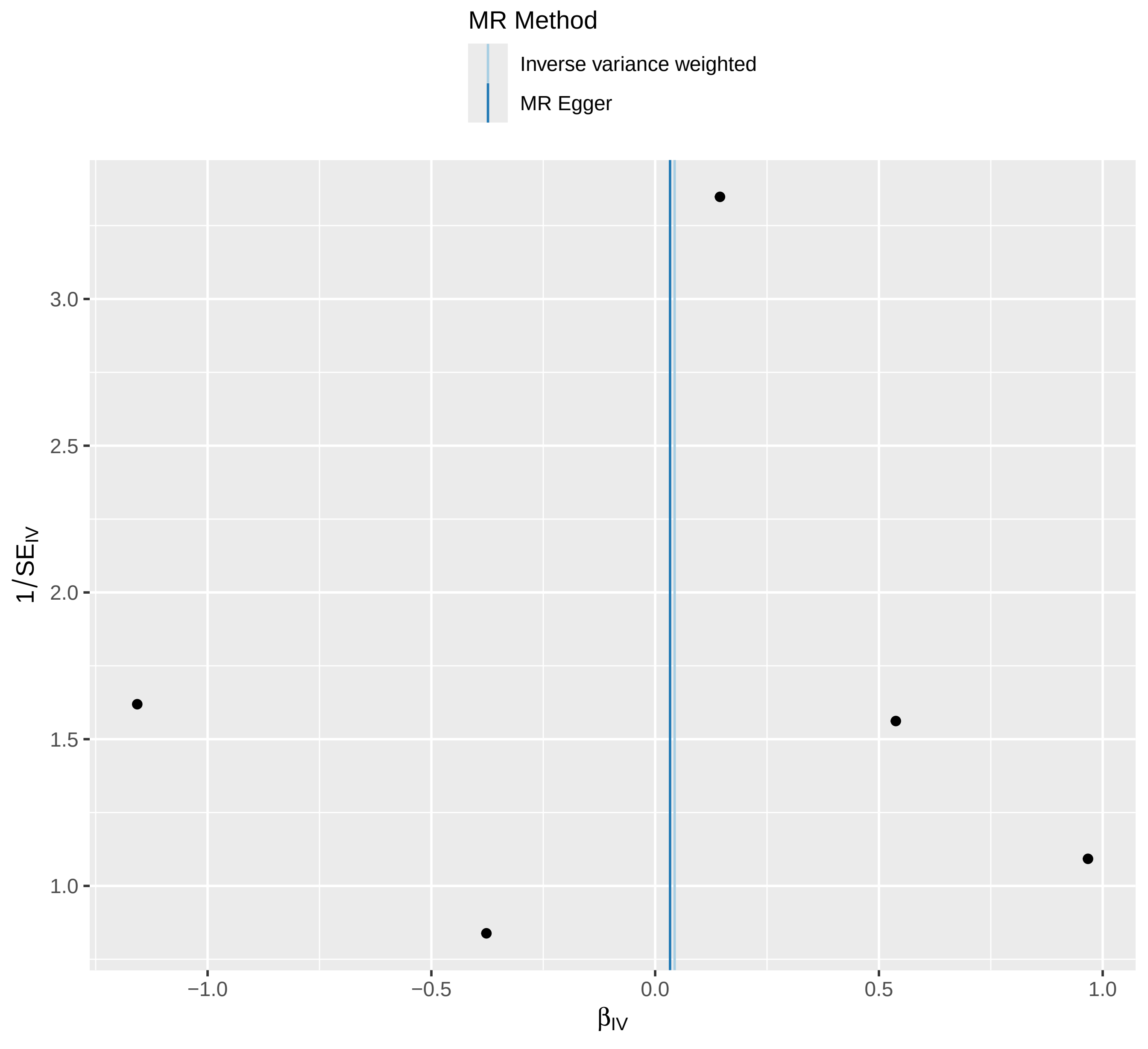

MR Method
Inverse variance weighted
MR Egger
$1/SE_{IV}$
3.0
2.5
2.0
1.5
1.0
−1.0
−0.5
0.0
0.5
1.0
β_{IV}

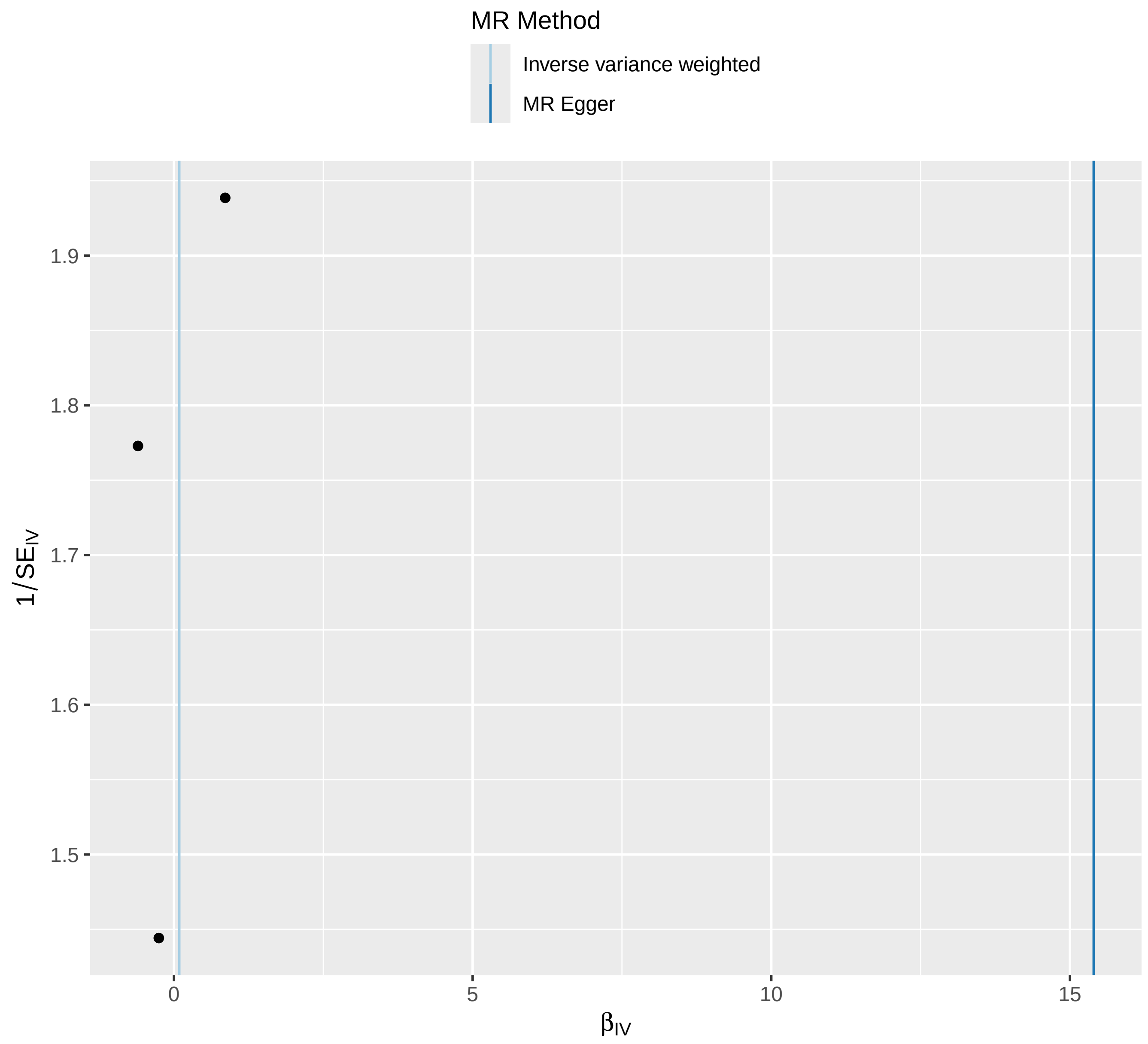
MR Method
Inverse variance weighted
MR Egger
1.9
1.8
1.7
1.6
1.5
1/SE_IV
0
5
10
15
β_IV

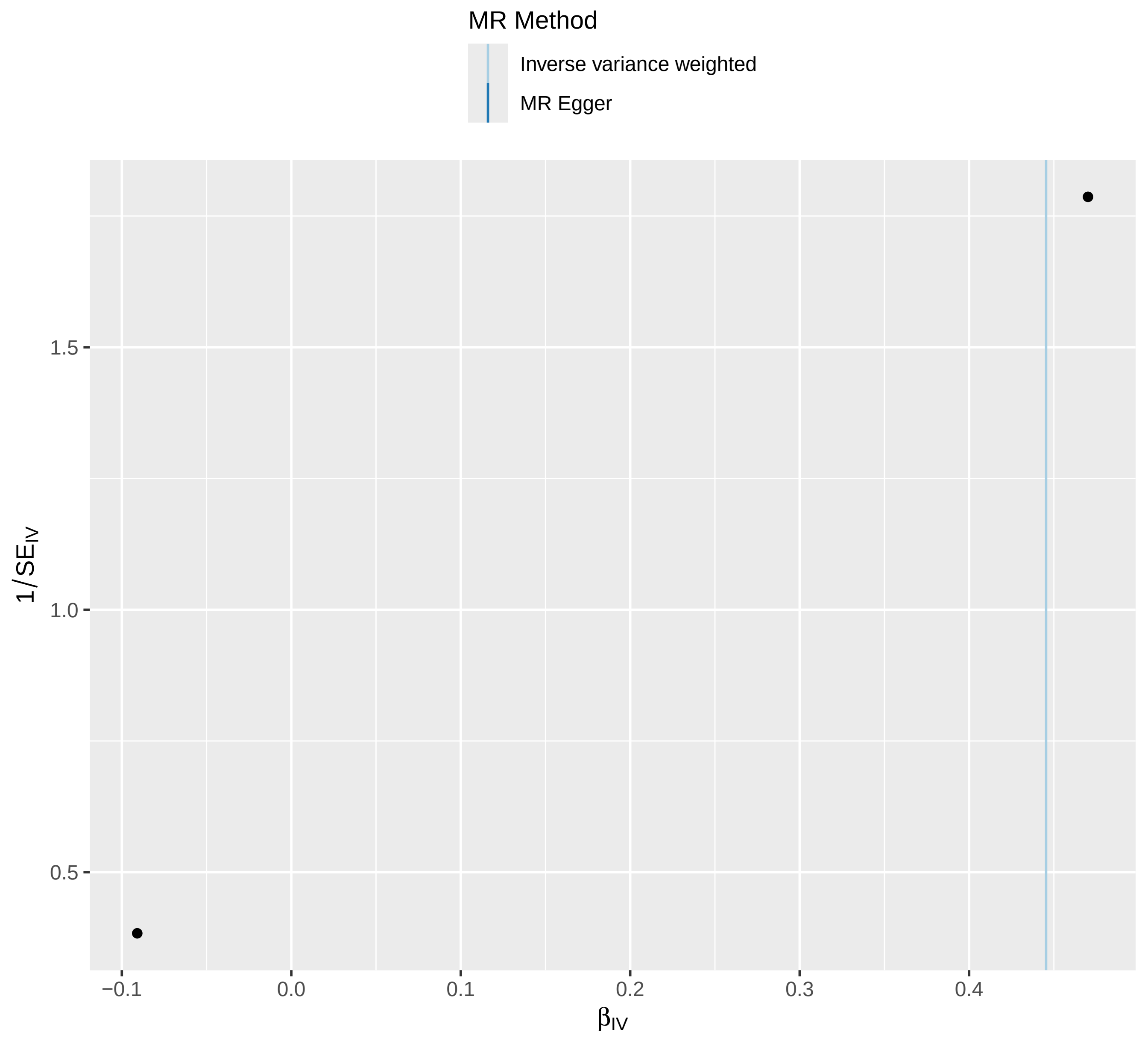

MR Method
Inverse variance weighted
MR Egger
1/SE_IV
1.5
1.0
0.5
−0.1
0.0
0.1
0.2
0.3
0.4
β_IV

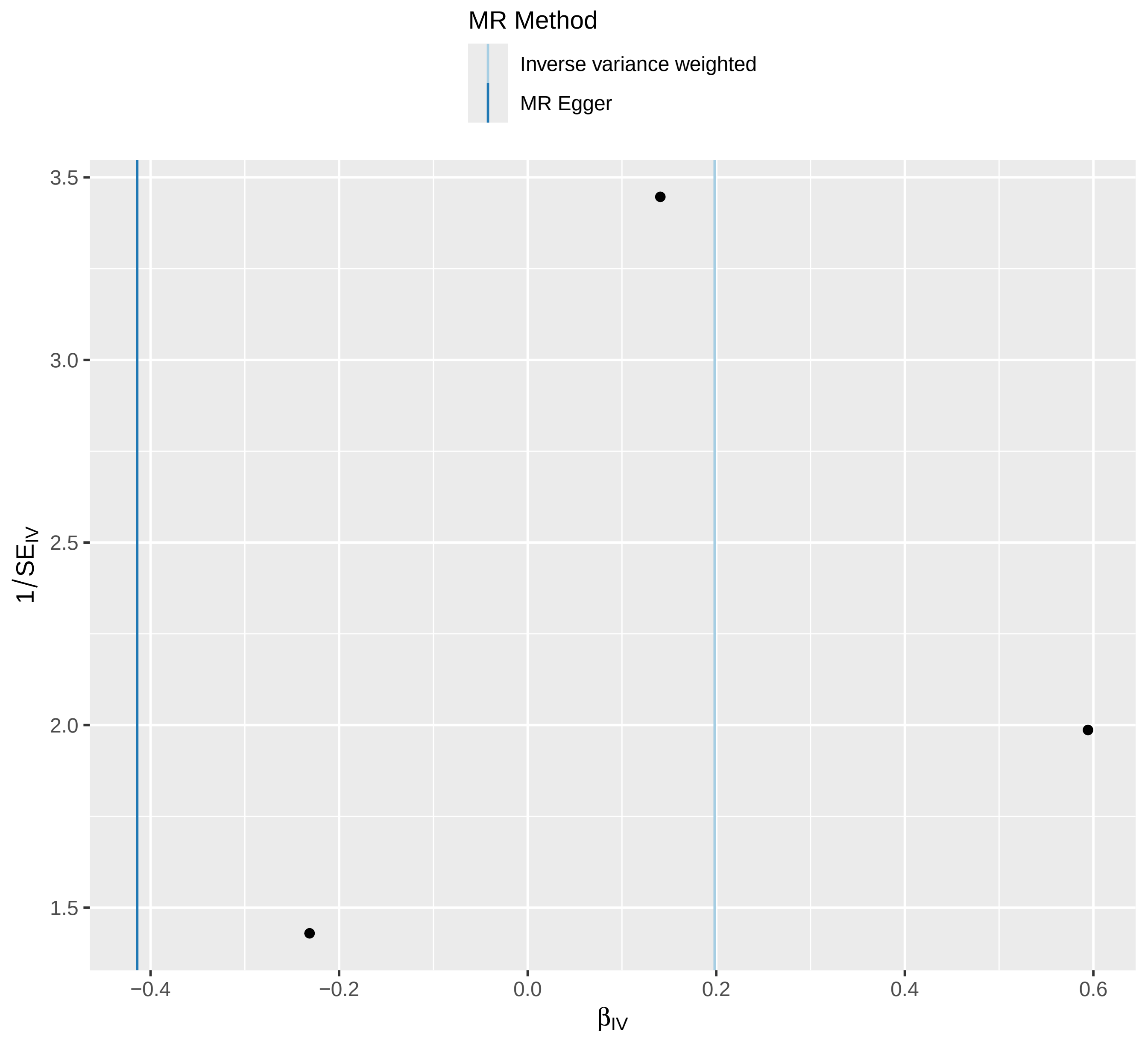
MR Method
Inverse variance weighted
MR Egger
3.5
3.0
2.5
2.0
1.5
1/SE_IV
−0.4
−0.2
0.0
0.2
0.4
0.6
β_IV

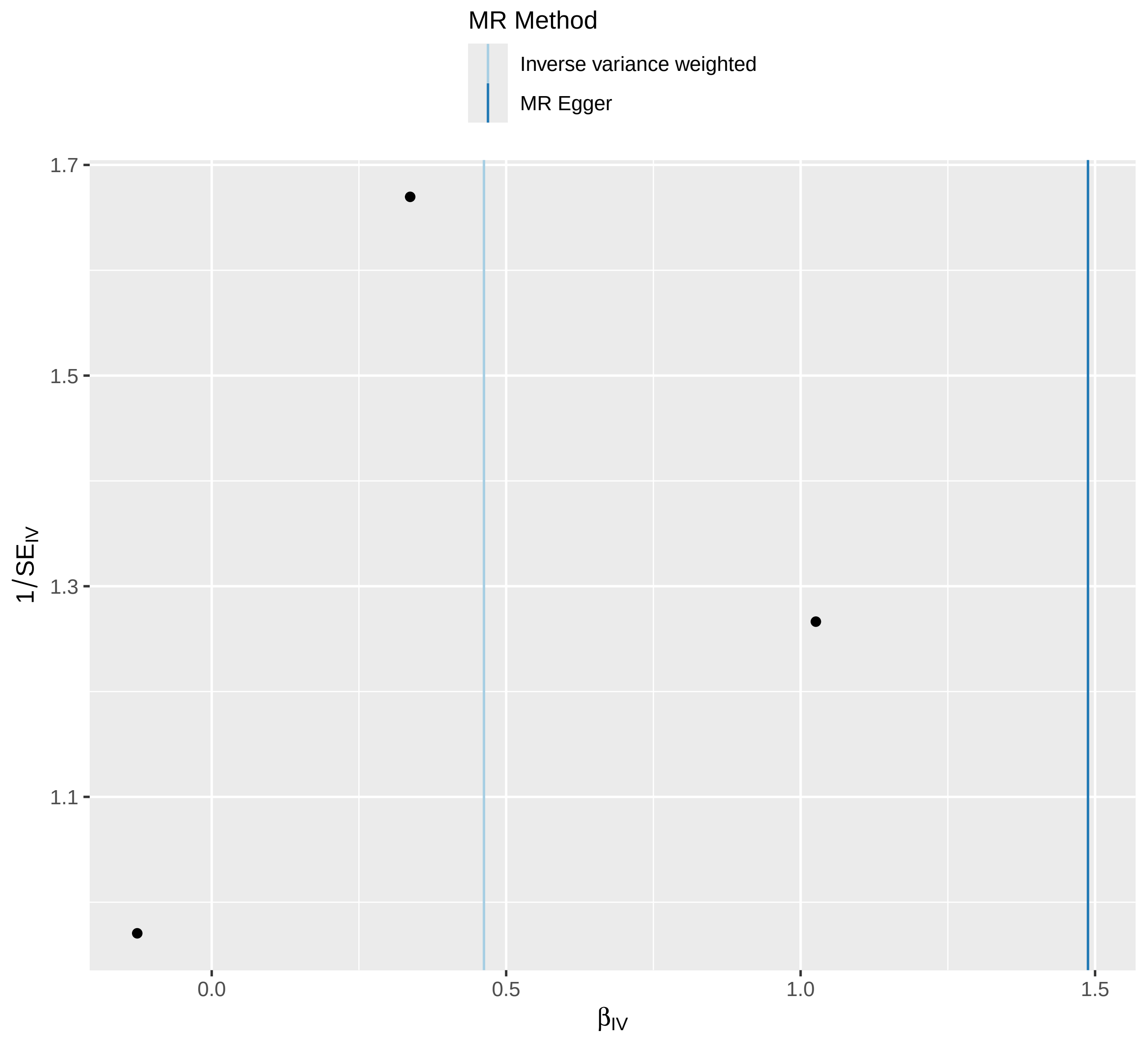
MR Method
Inverse variance weighted
MR Egger
1.7
1.5
1.3
1.1
0.0
0.5
1.0
1.5
$1/SE_{IV}$
β_{IV}

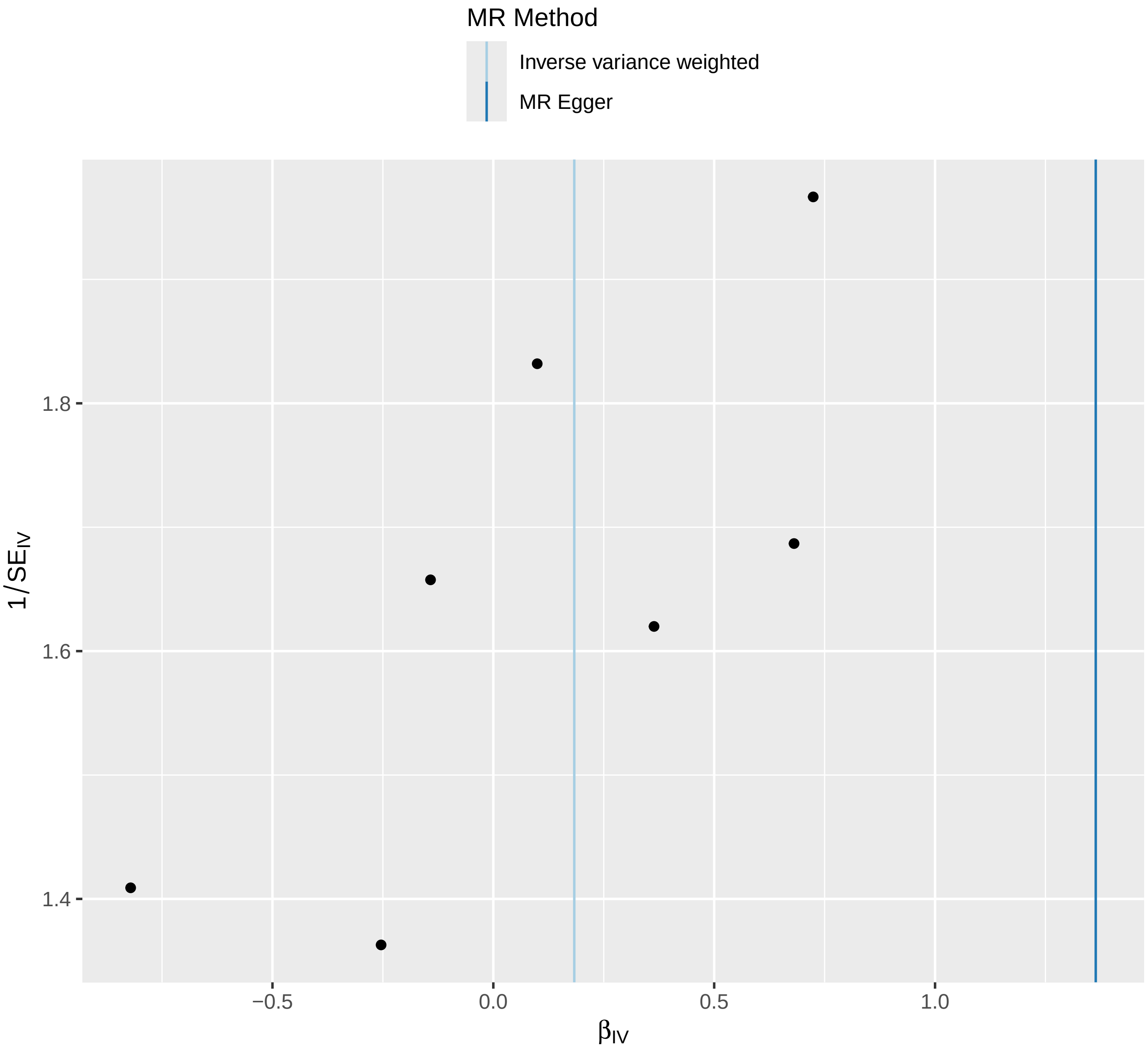
MR Method
Inverse variance weighted
MR Egger
1.8
1.6
1.4
−0.5
0.0
0.5
1.0
1/SE_IV
β_IV

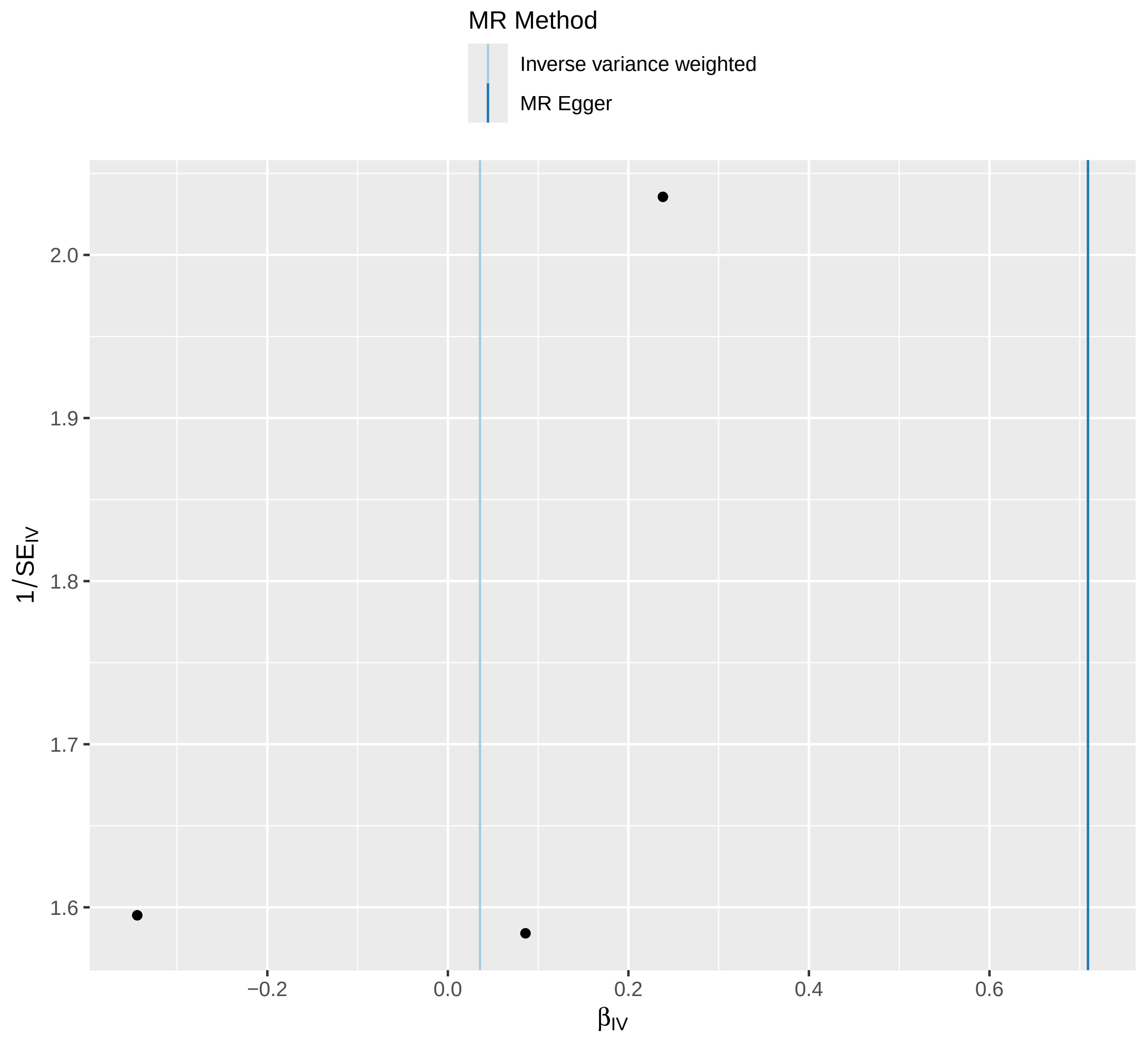
MR Method
Inverse variance weighted
MR Egger
2.0
1.9
1.8
1.7
1.6
1/SE_IV
−0.2
0.0
0.2
0.4
0.6
β_IV

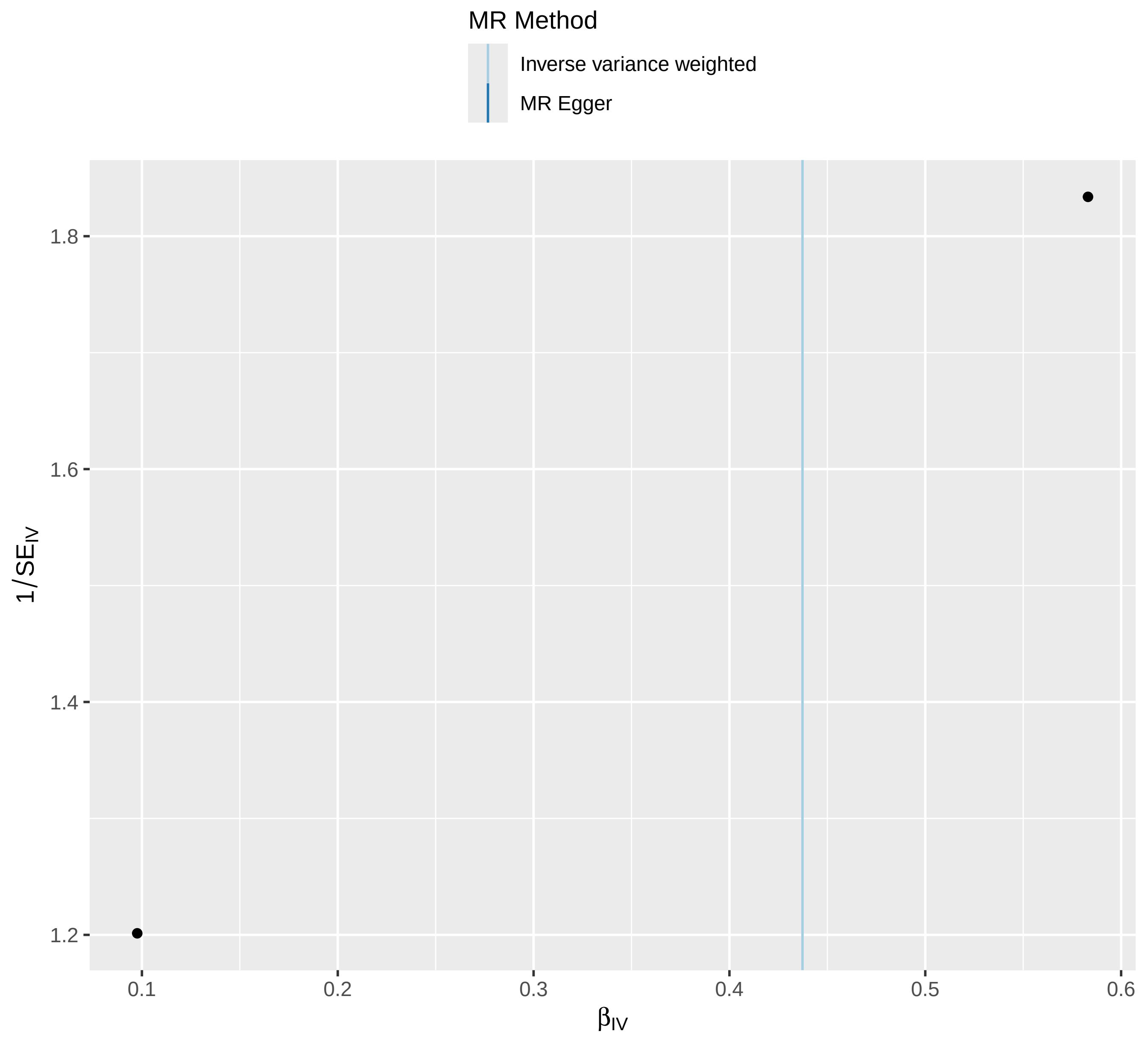

MR Method
Inverse variance weighted
MR Egger
1.8
1.6
1.4
1.2
0.1
0.2
0.3
0.4
0.5
0.6
$1/SE_{IV}$
β_{IV}

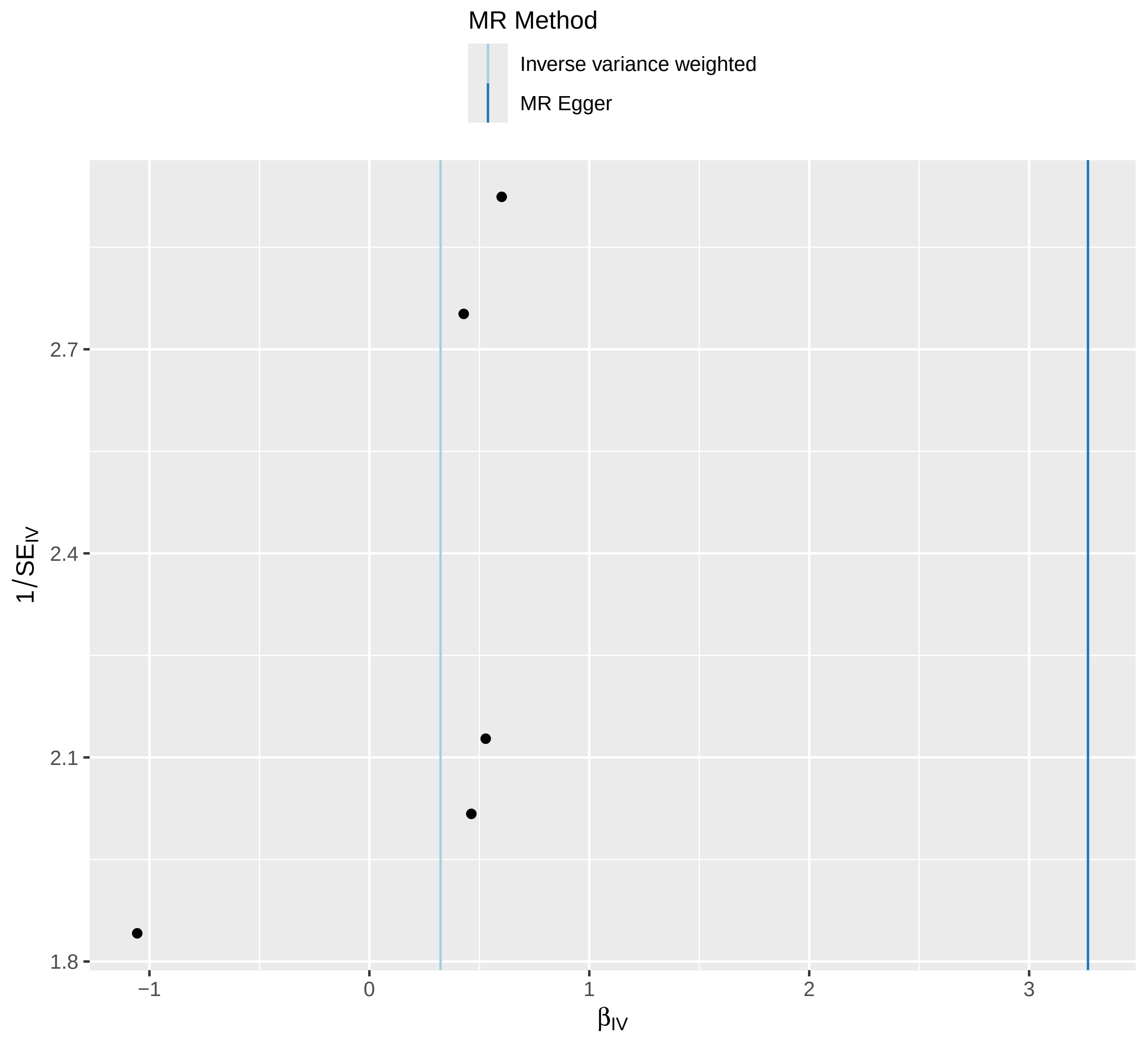
MR Method
Inverse variance weighted
MR Egger
2.7
2.4
2.1
1.8
1/SE$_{IV}$
−1
0
1
2
3
β$_{IV}$

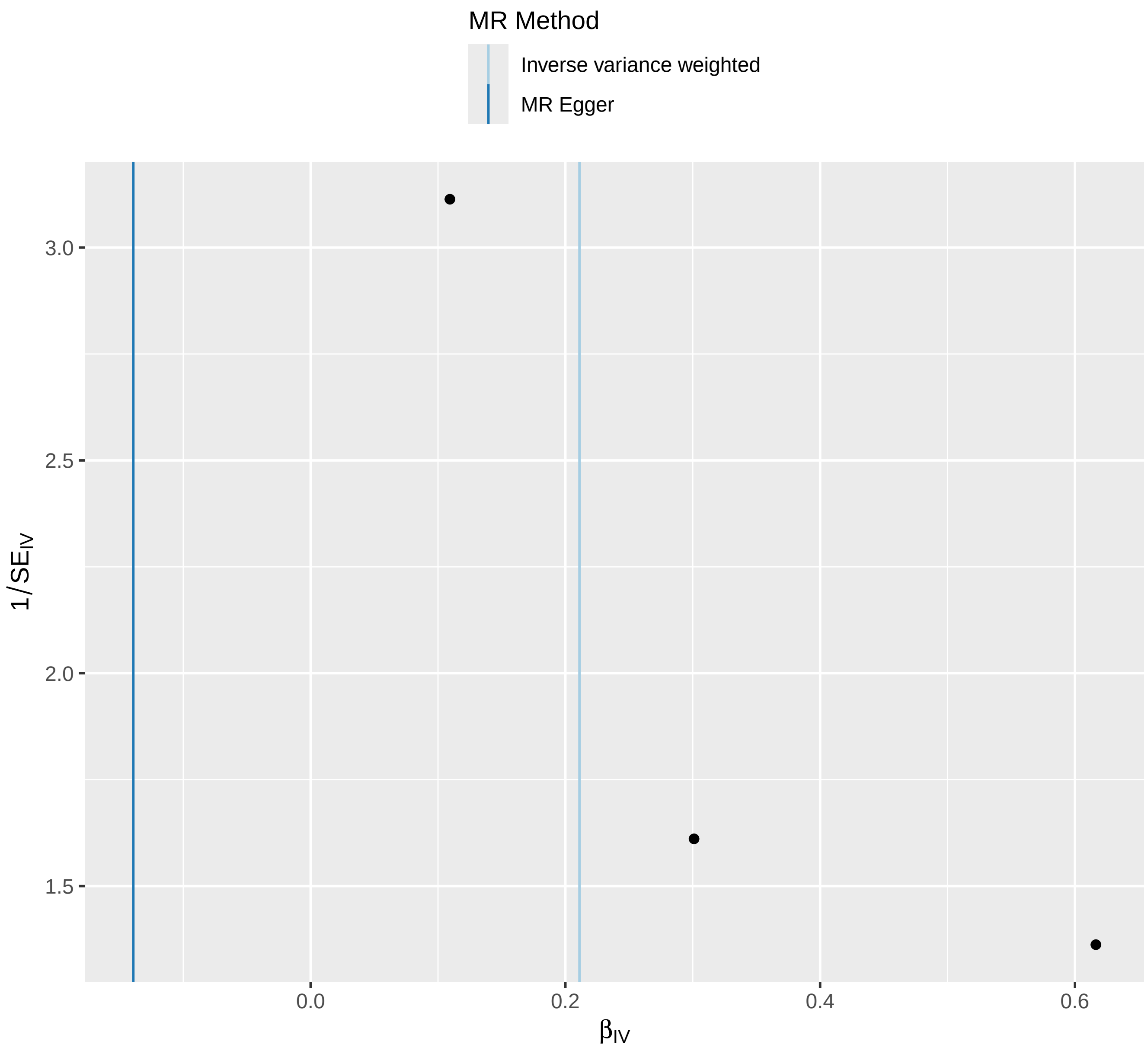
MR Method
Inverse variance weighted
MR Egger
1/SE_IV
3.0
2.5
2.0
1.5
0.0
0.2
0.4
0.6
β_IV

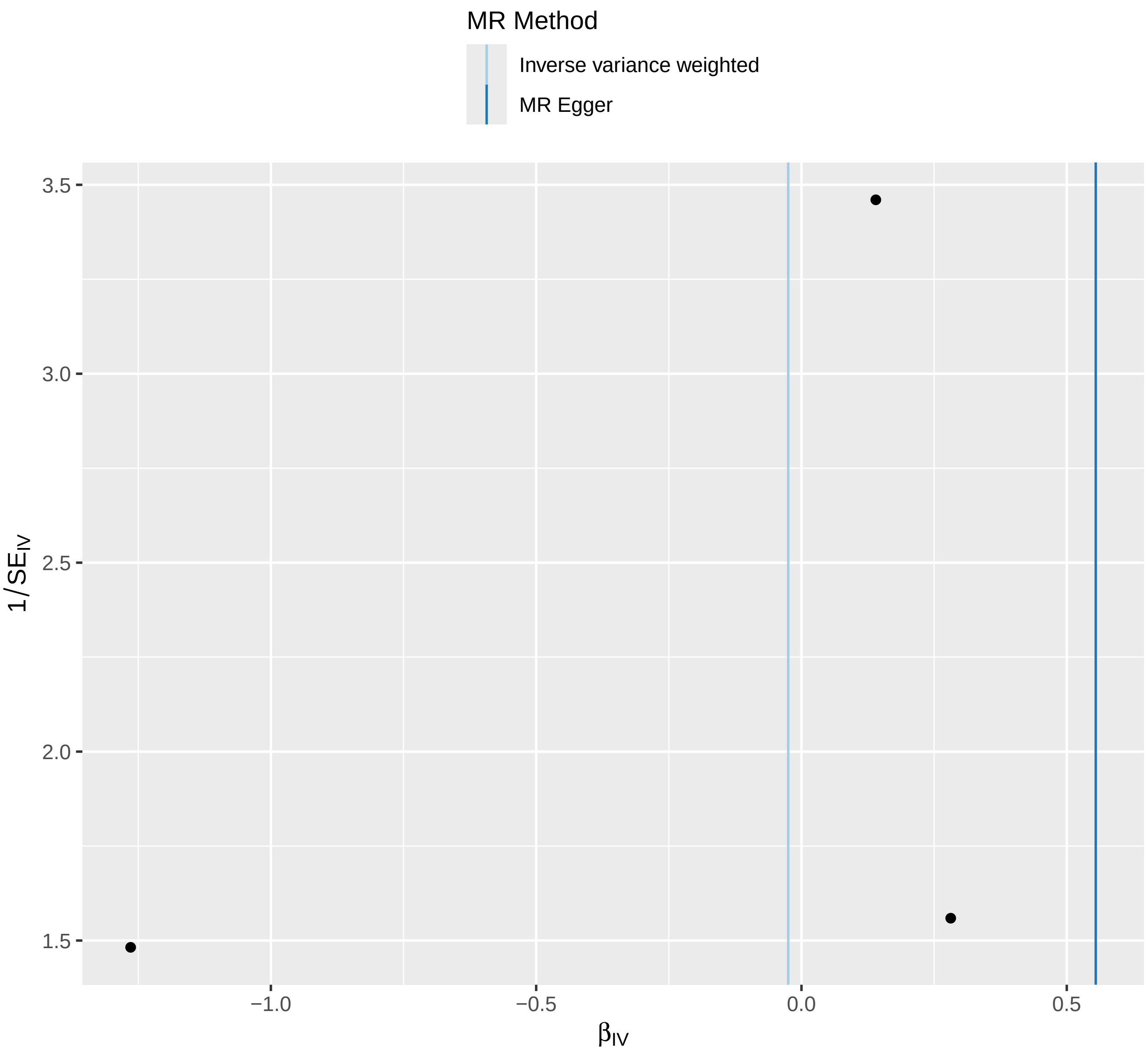

MR Method
Inverse variance weighted
MR Egger
1/SE_IV
3.5
3.0
2.5
2.0
1.5
−1.0
−0.5
0.0
0.5
β_IV

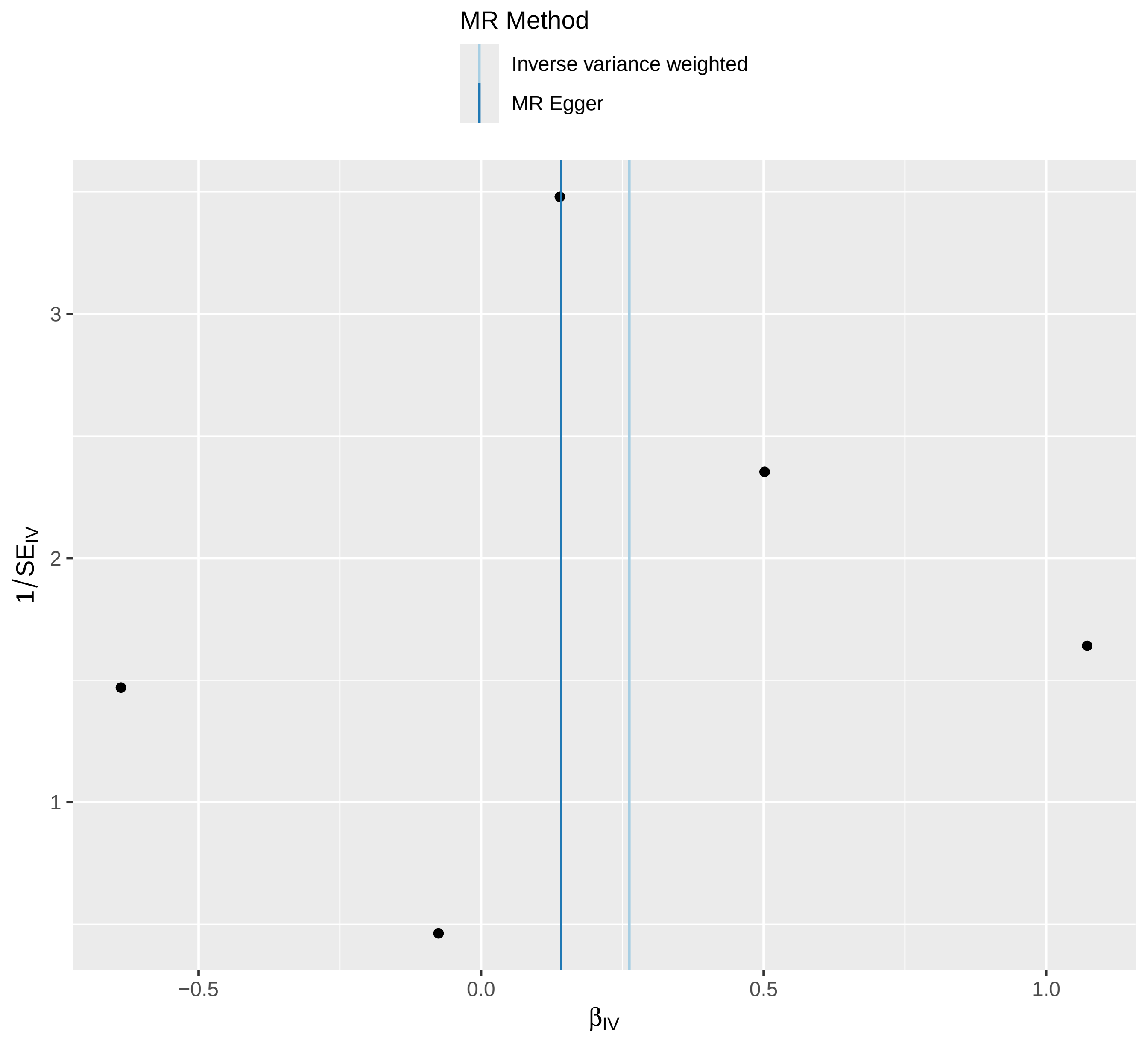
MR Method
Inverse variance weighted
MR Egger
1/SE_IV
3
2
1
−0.5
0.0
0.5
1.0
β_IV

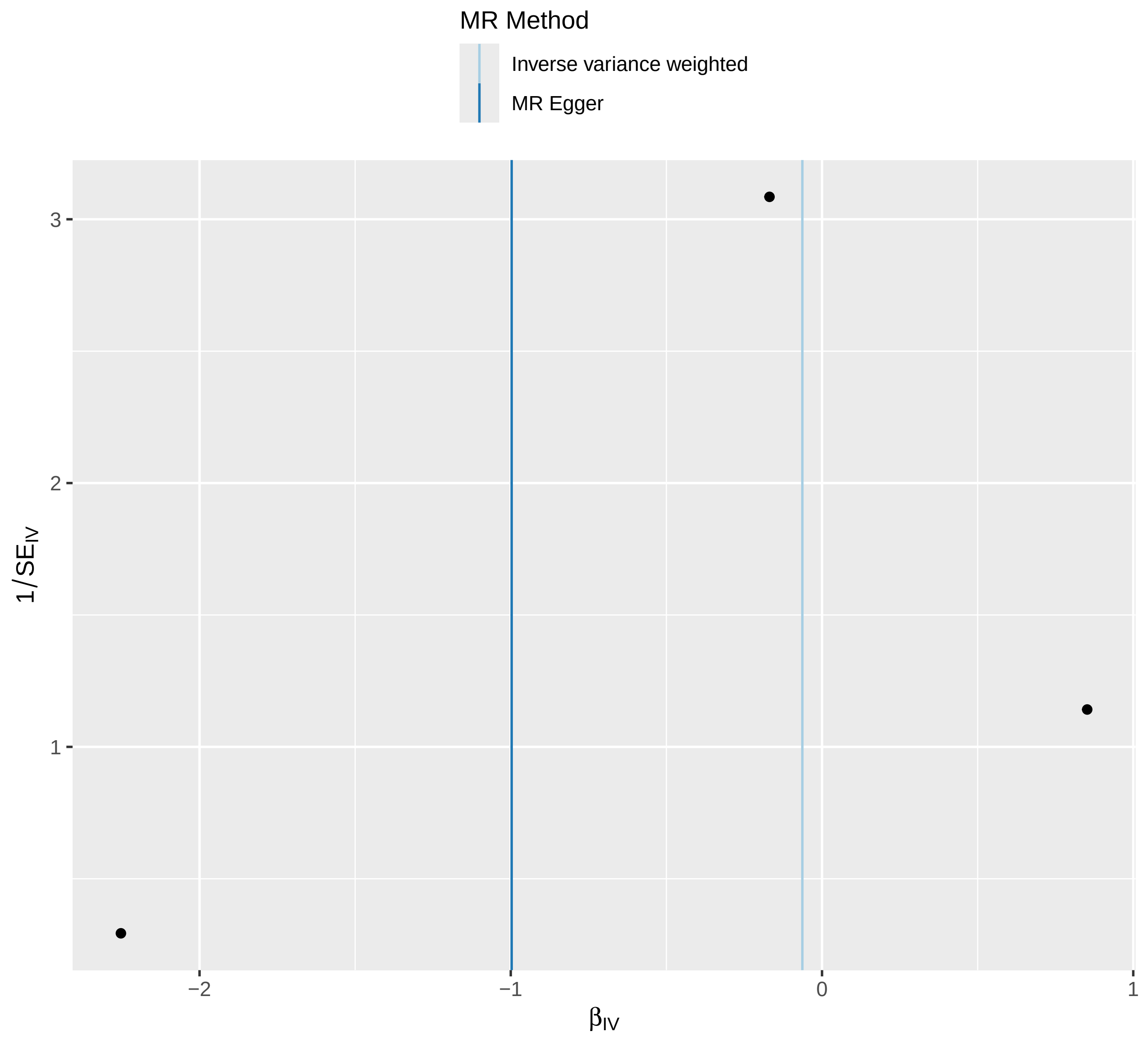

MR Method
Inverse variance weighted
MR Egger
3
2
1
1/SE_IV
−2
−1
0
1
β_IV

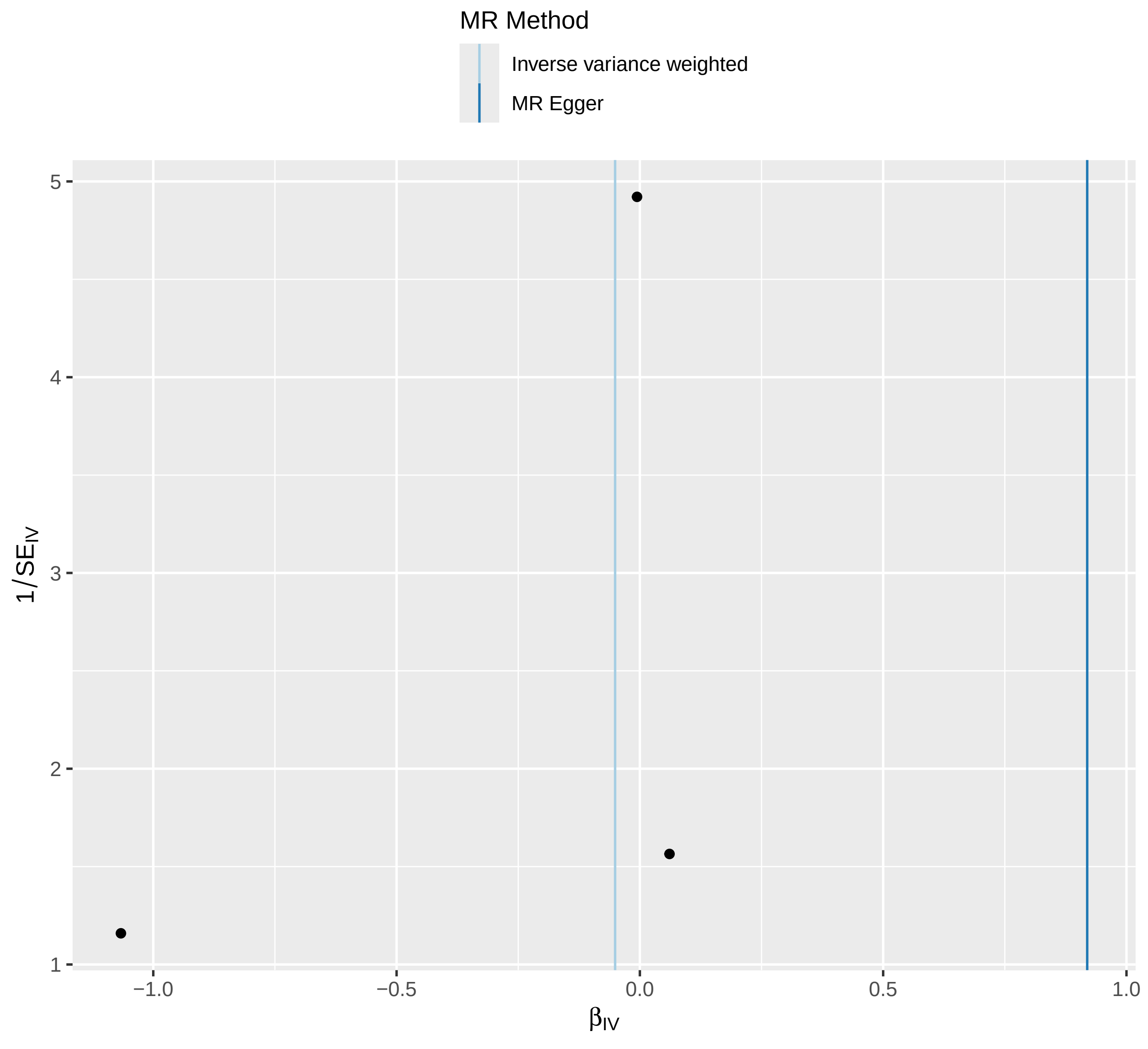

MR Method
Inverse variance weighted
MR Egger
5
4
3
2
1
1/SE_IV
−1.0
−0.5
0.0
0.5
1.0
β_IV

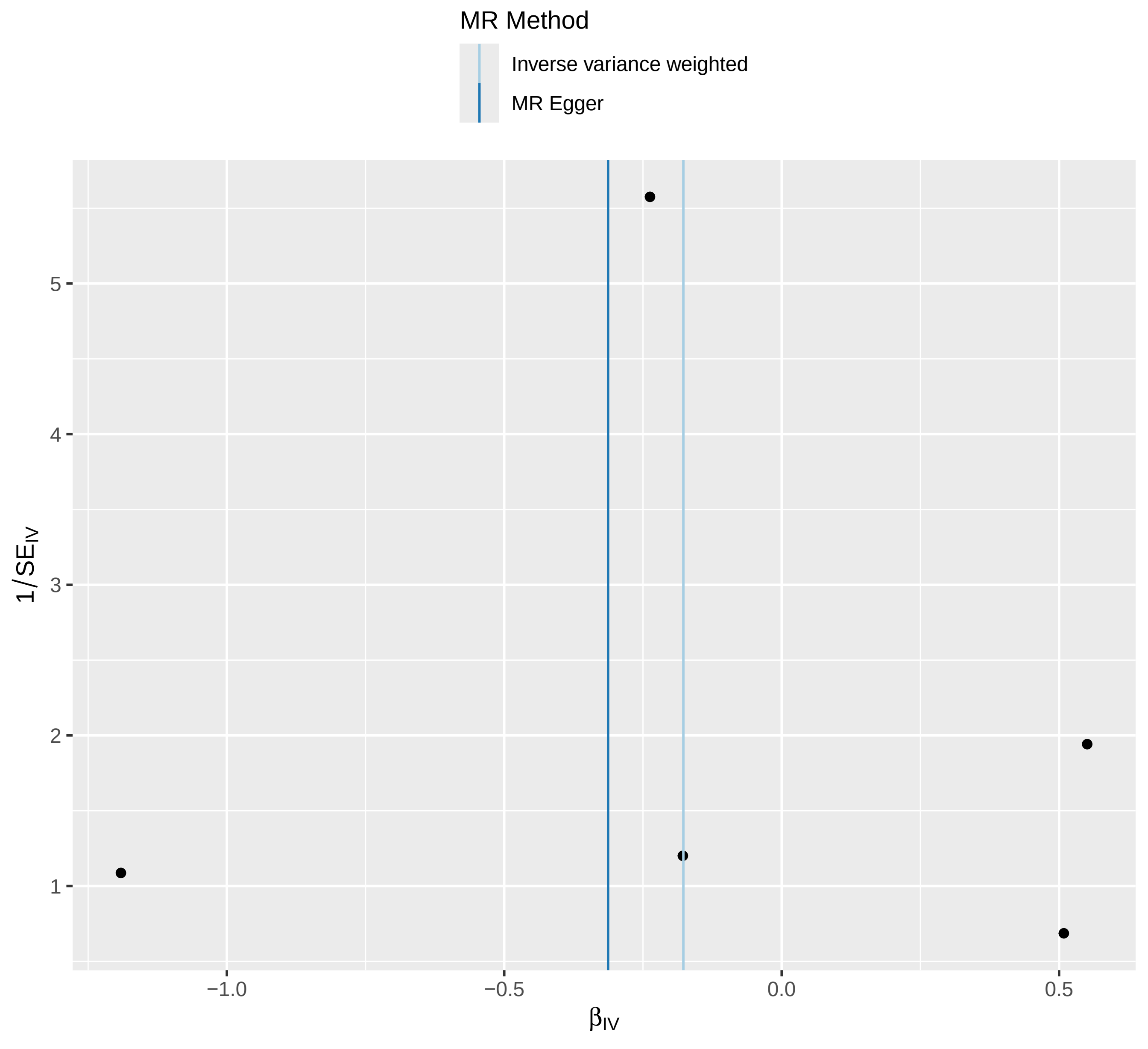
MR Method
Inverse variance weighted
MR Egger
1/SE_IV
5
4
3
2
1
−1.0
−0.5
0.0
0.5
β_IV

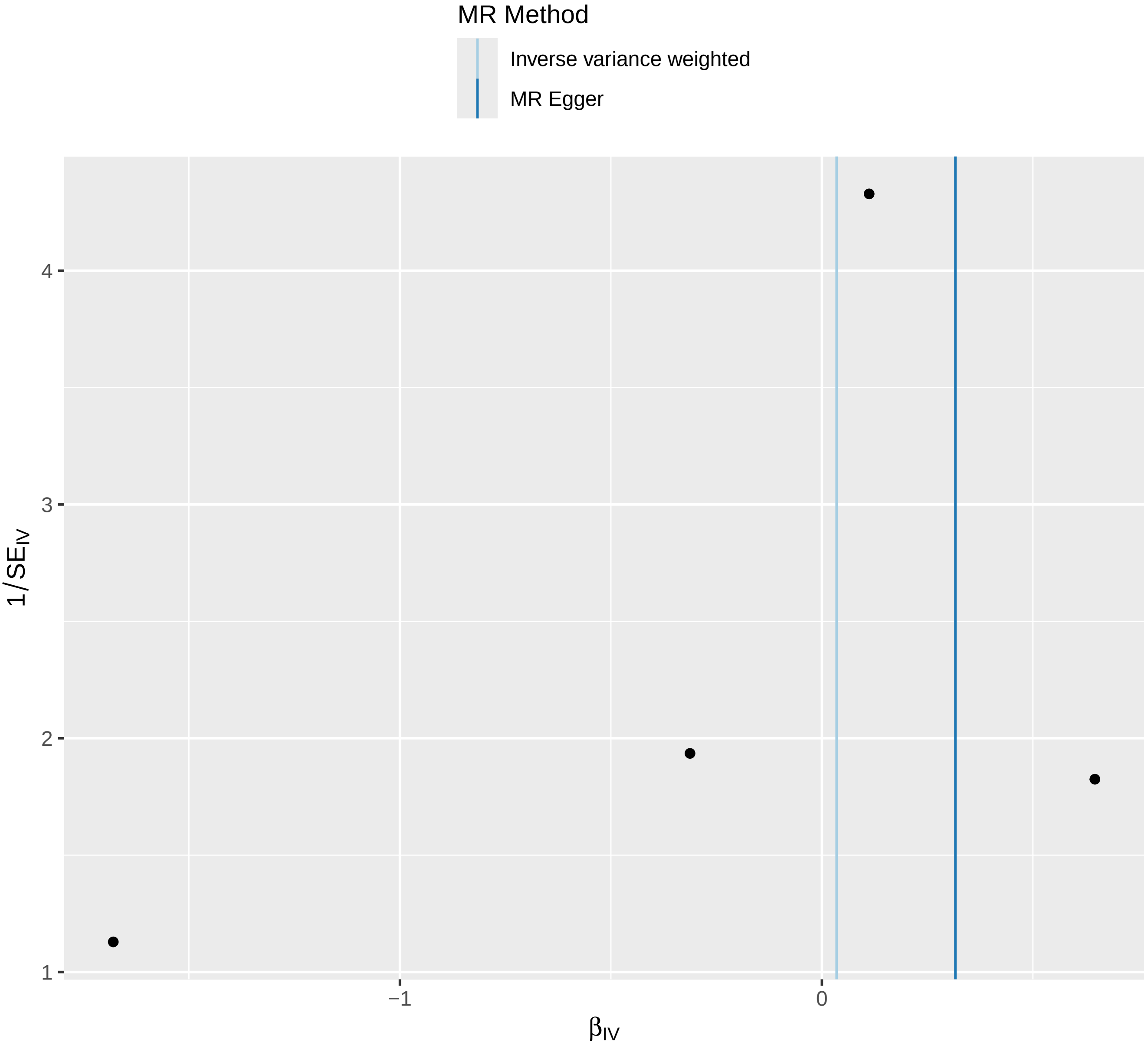

MR Method
Inverse variance weighted
MR Egger
4
3
2
1
−1
0
$1/SE_{IV}$
β_{IV}

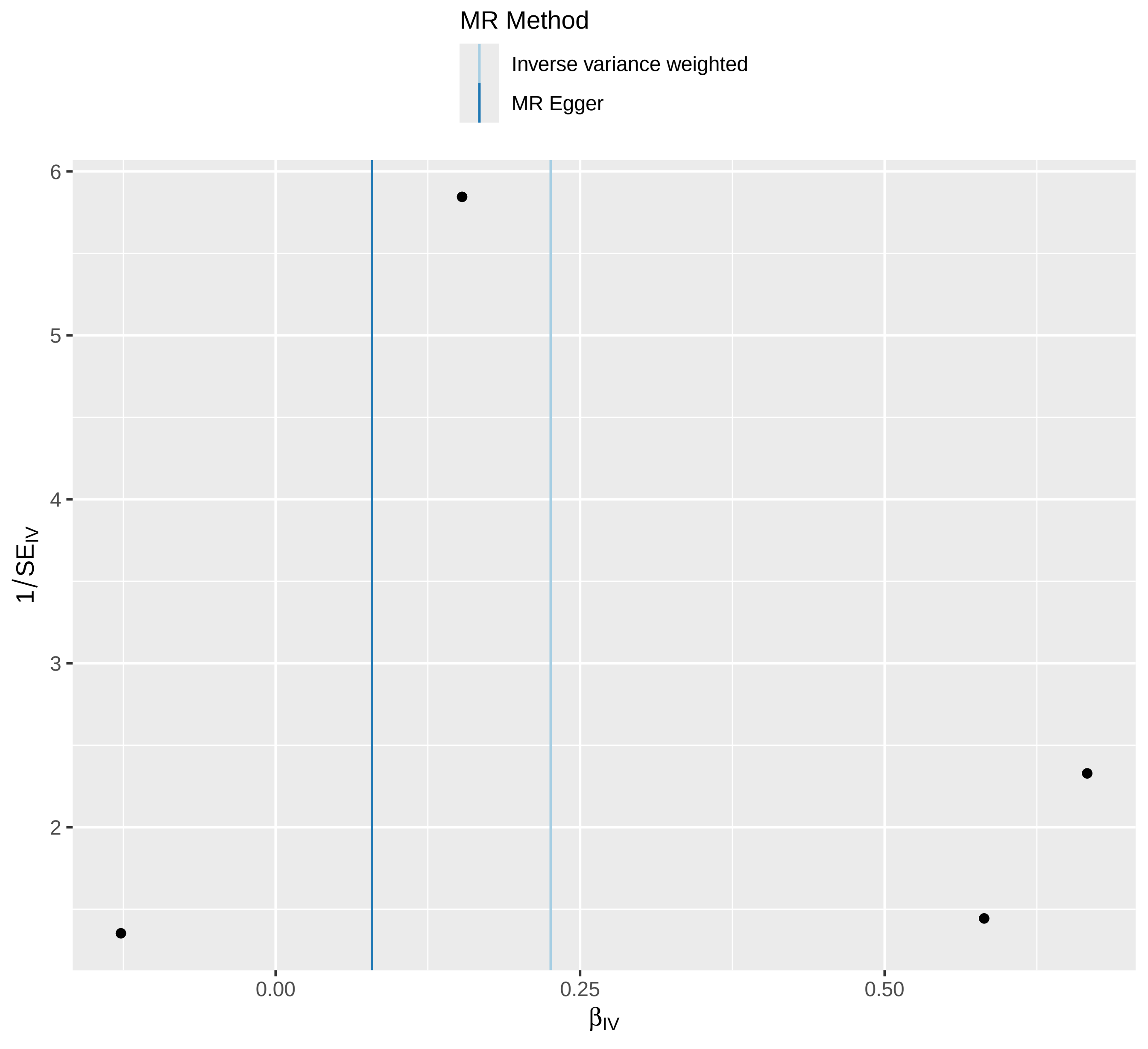

MR Method
Inverse variance weighted
MR Egger
6
5
4
3
2
1/SE$_{IV}$
0.00
0.25
0.50
β_{IV}

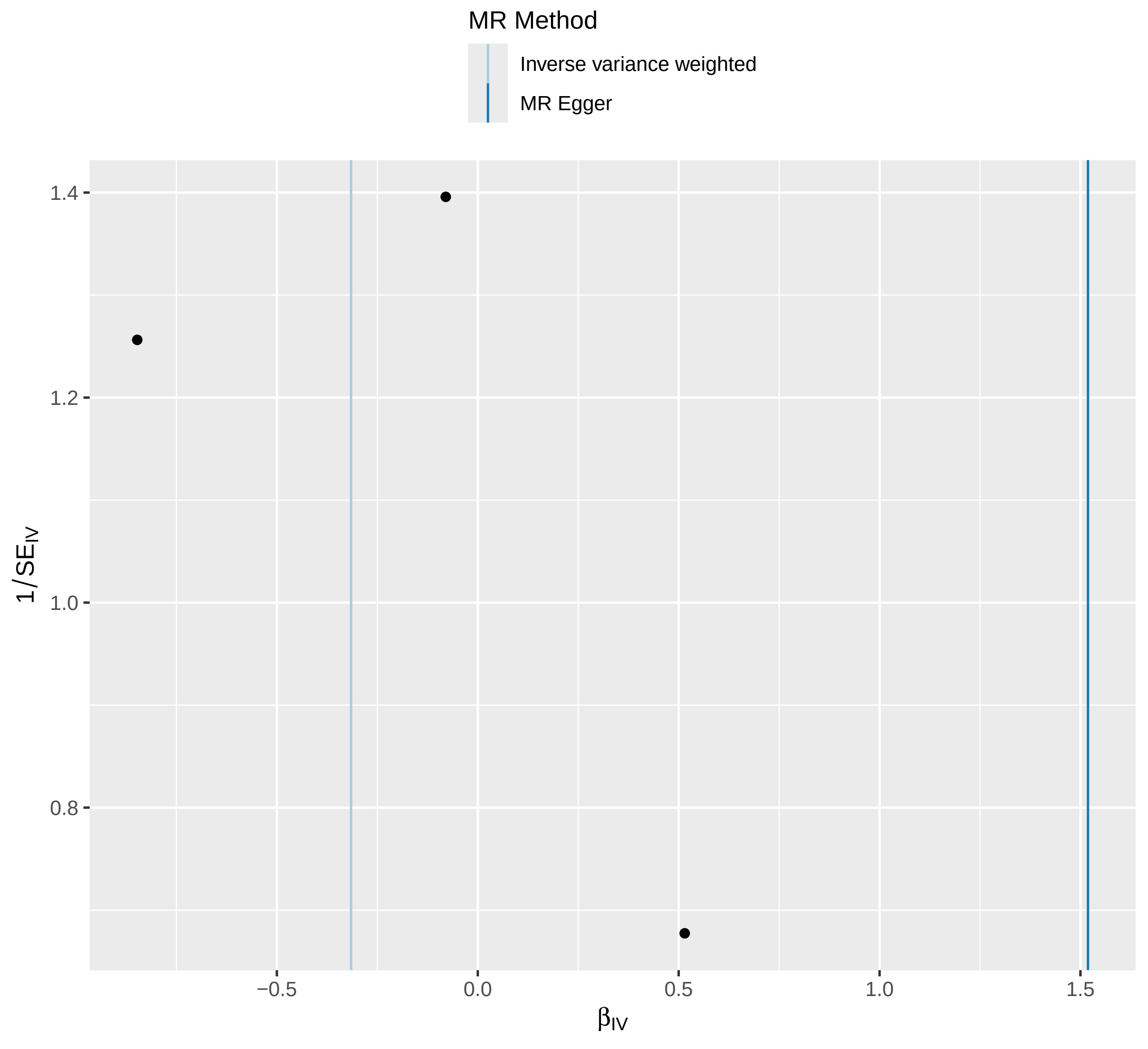

MR Method
Inverse variance weighted
MR Egger
1.4
1.2
1.0
0.8
1/SE_IV
−0.5
0.0
0.5
1.0
1.5
β_IV

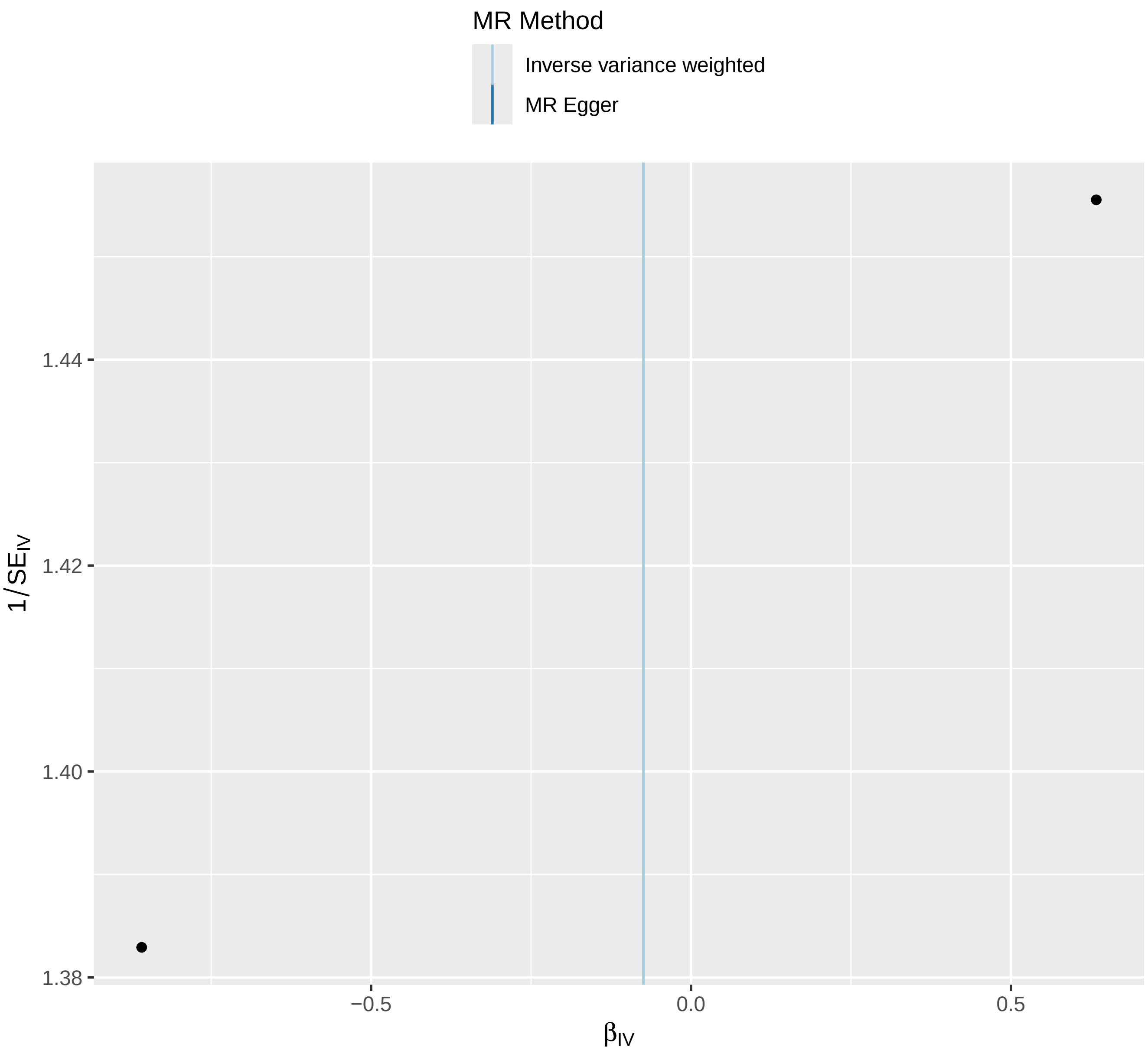
MR Method
Inverse variance weighted
MR Egger
1.44
1.42
1.40
1.38
1/SE$_{IV}$
−0.5
0.0
0.5
β$_{IV}$

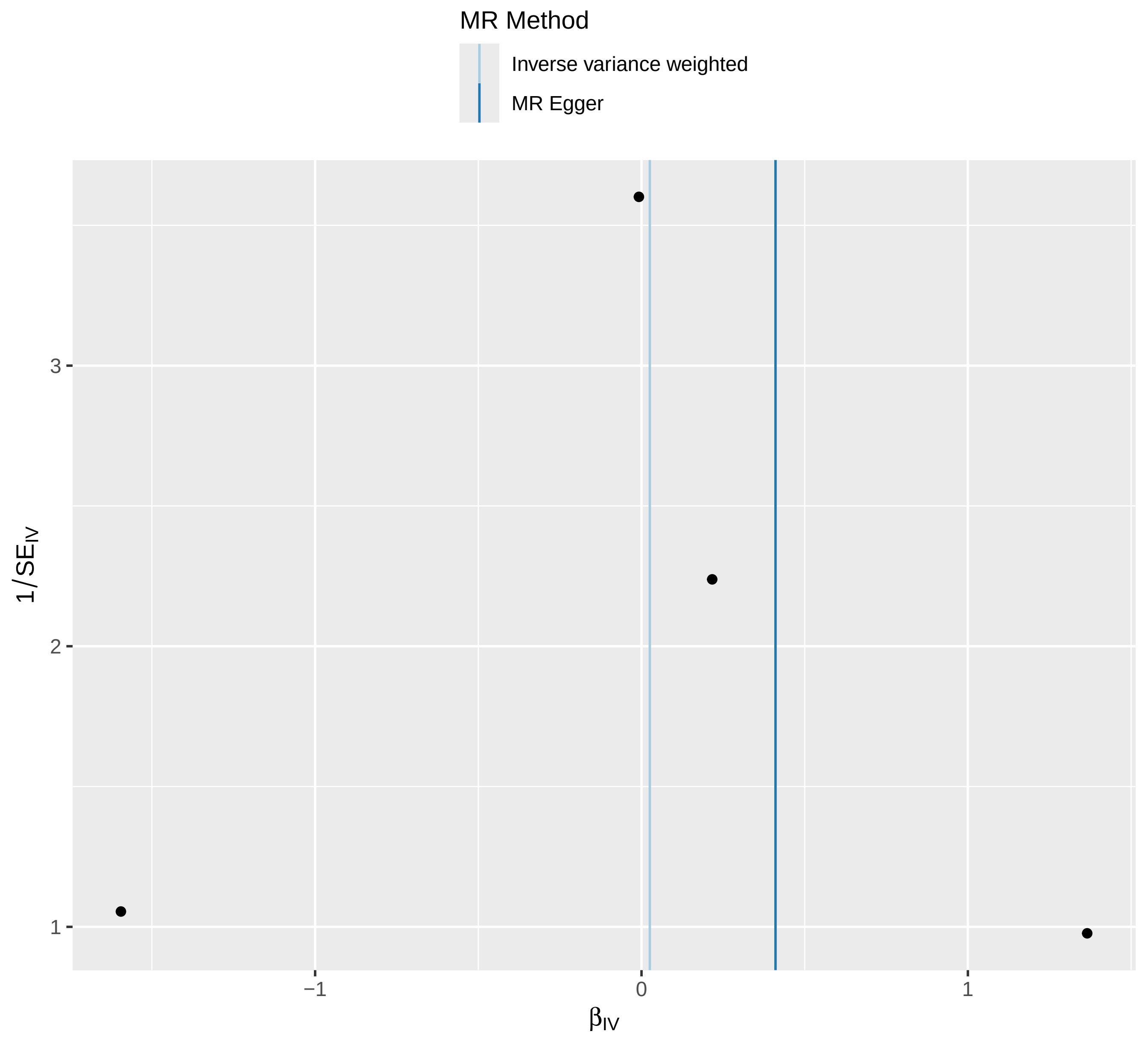
MR Method
Inverse variance weighted
MR Egger
3
2
1
1/SE_IV
−1
0
1
β_IV

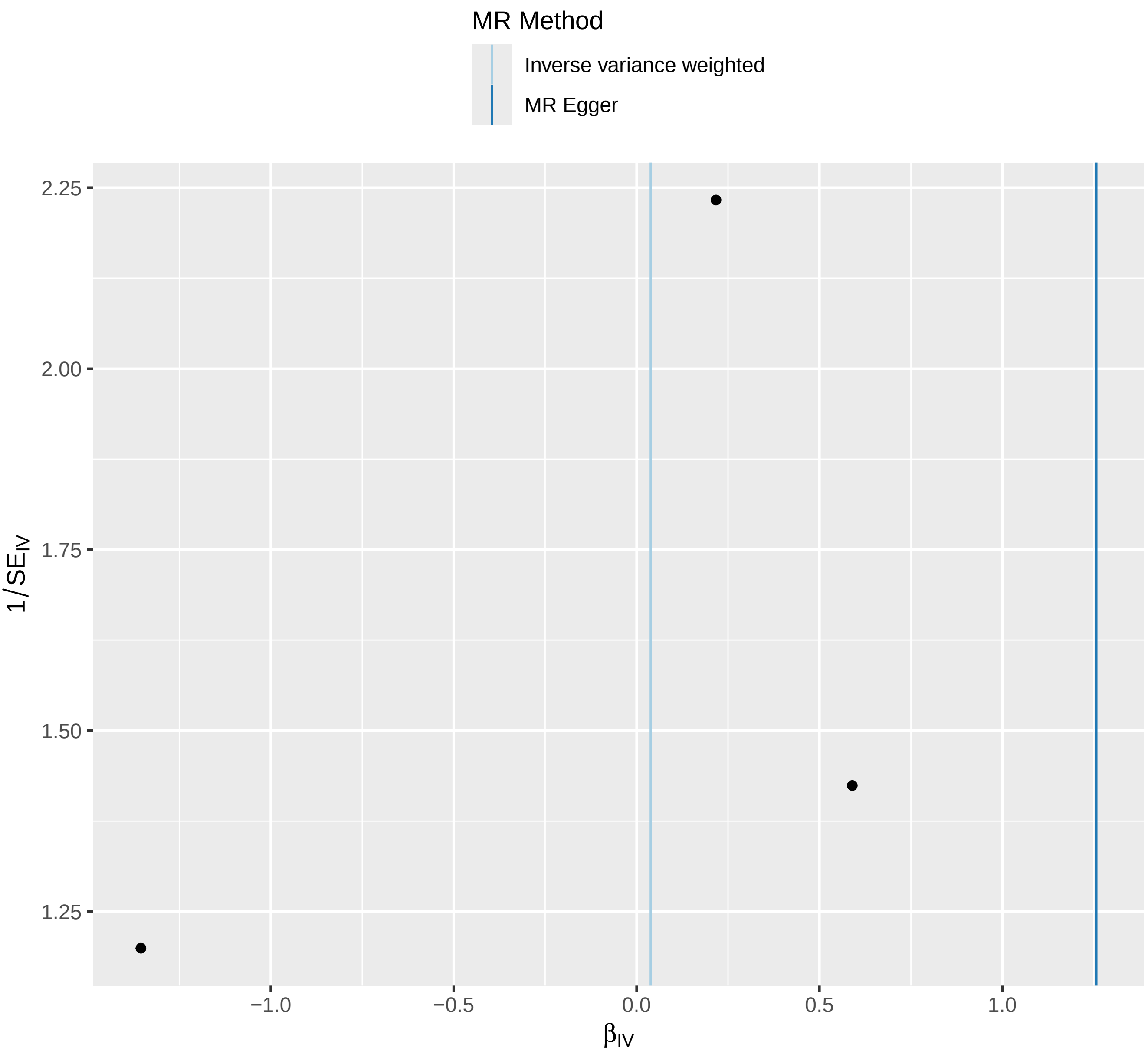
MR Method
Inverse variance weighted
MR Egger
2.25
2.00
1.75
1.50
1.25
$1/SE_{IV}$
−1.0
−0.5
0.0
0.5
1.0
β_{IV}

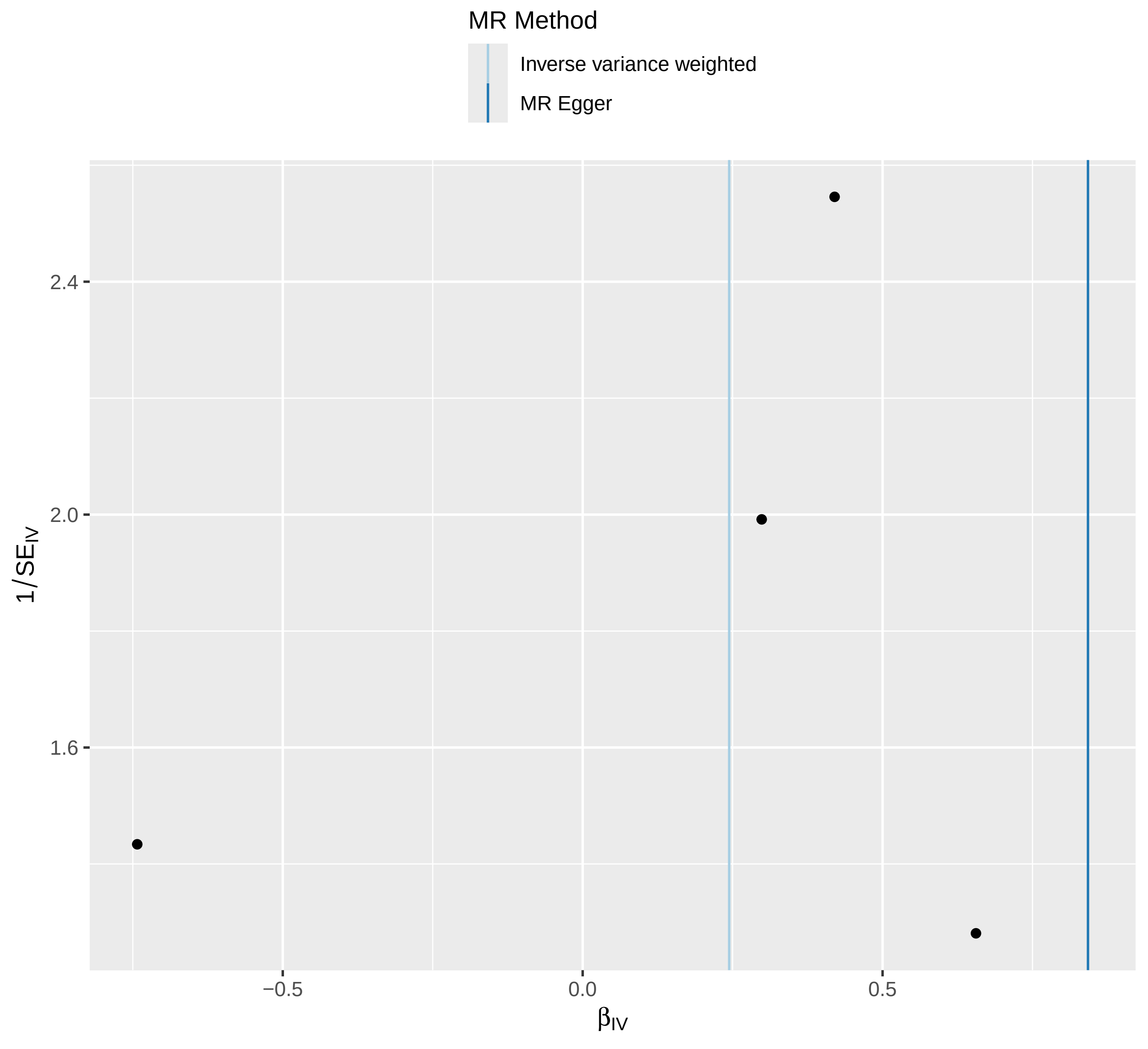

MR Method
Inverse variance weighted
MR Egger
2.4
2.0
1.6
1/SE$_{IV}$
−0.5
0.0
0.5
β$_{IV}$

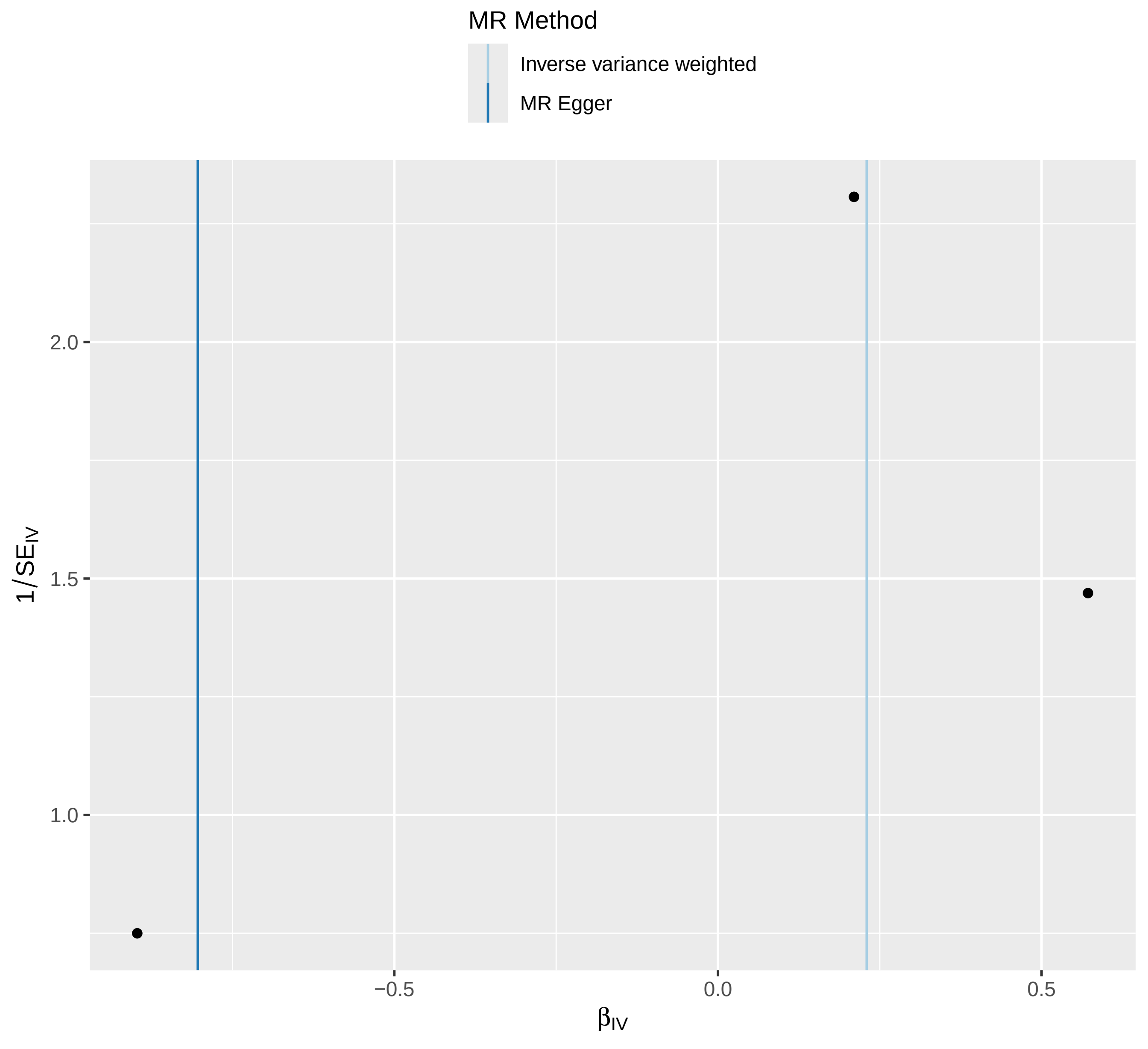
MR Method
Inverse variance weighted
MR Egger
1/SE_{IV}
2.0
1.5
1.0
−0.5
0.0
0.5
β_{IV}

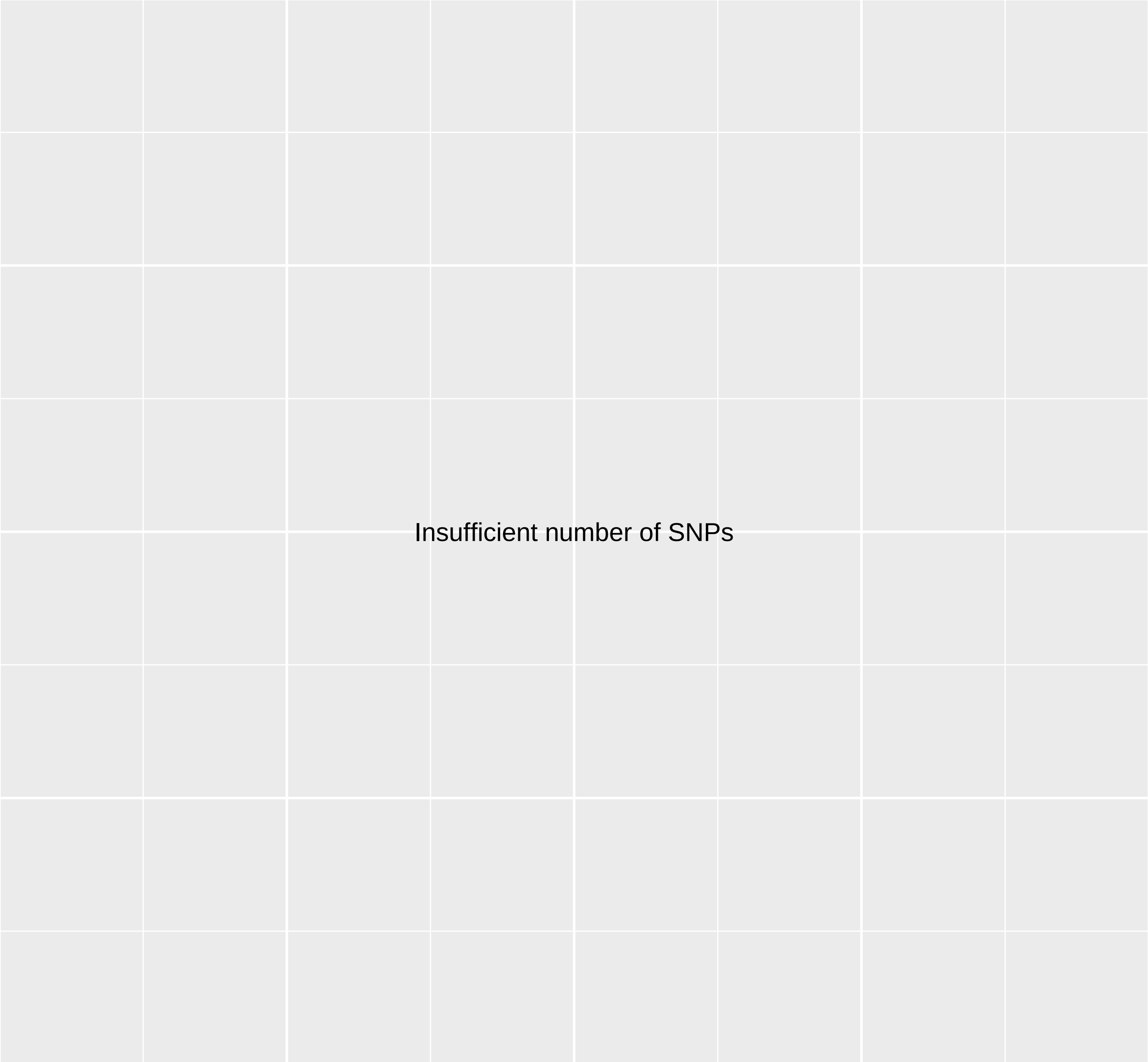
Insufficient number of SNPs

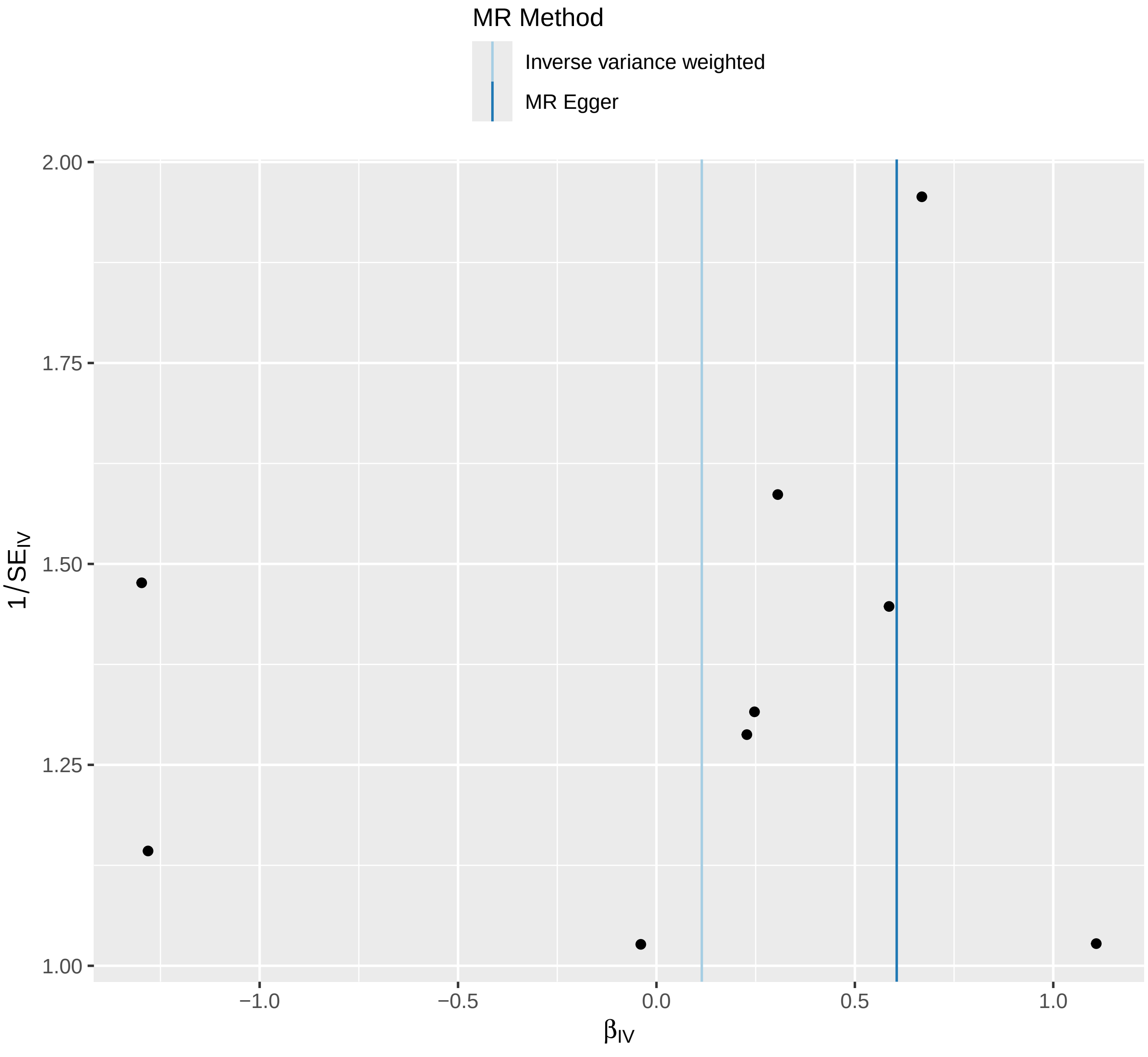

MR Method
Inverse variance weighted
MR Egger
2.00
1.75
1.50
1.25
1.00
$1/SE_{IV}$
−1.0
−0.5
0.0
0.5
1.0
β_{IV}

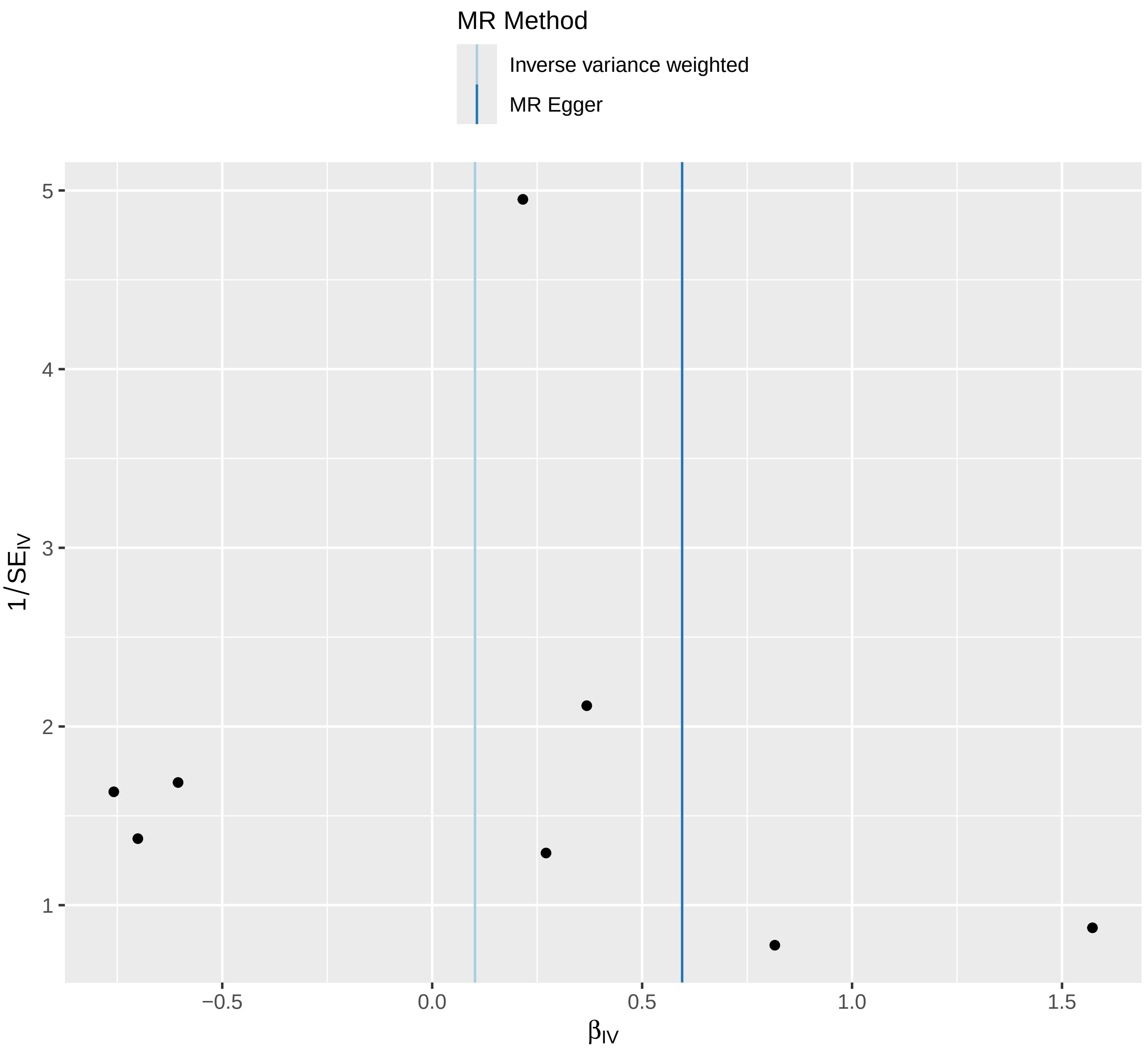

MR Method
Inverse variance weighted
MR Egger
5
4
3
2
1
1/SE_IV
−0.5
0.0
0.5
1.0
1.5
β_IV

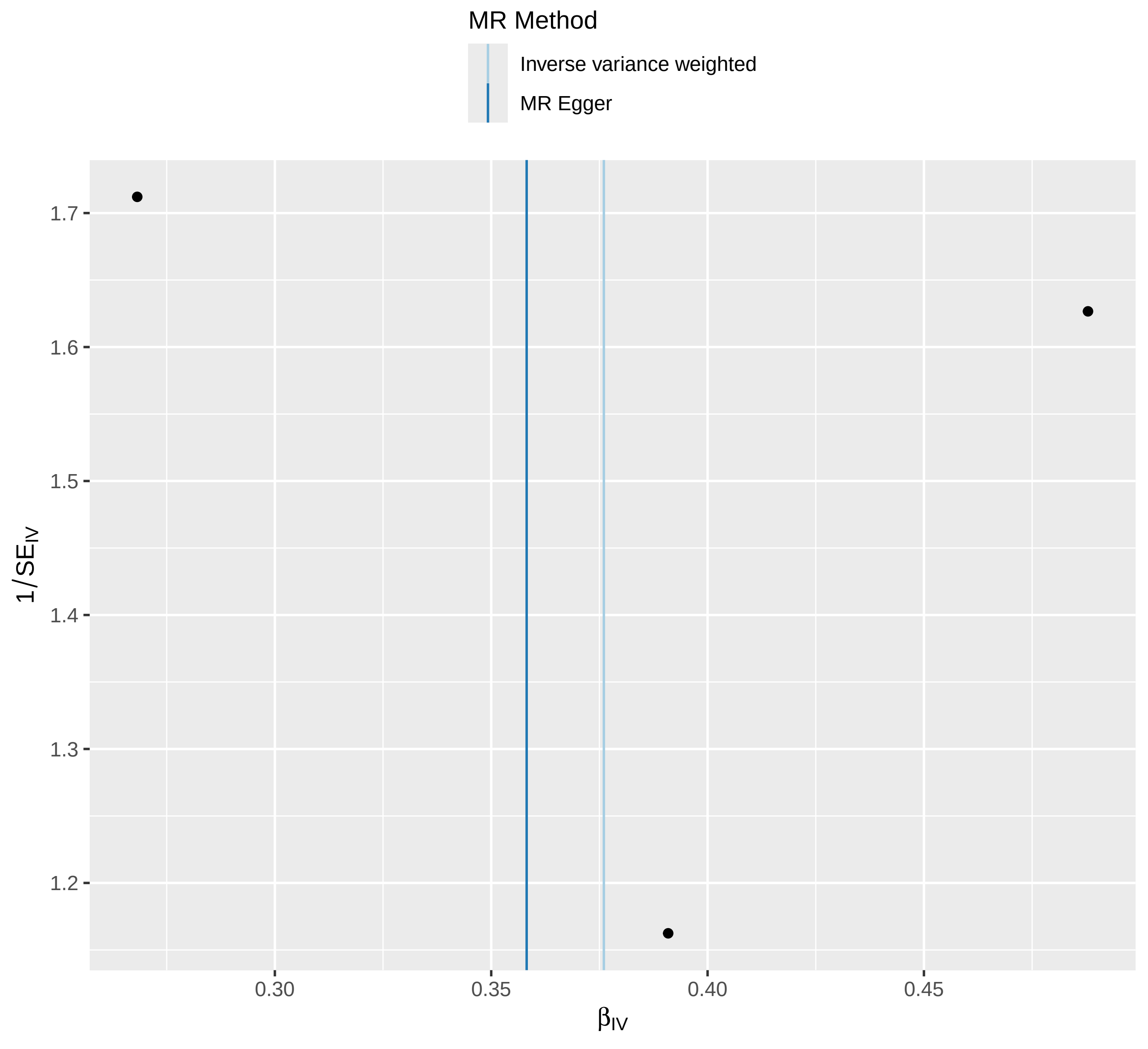
MR Method
Inverse variance weighted
MR Egger
1.7
1.6
1.5
1.4
1.3
1.2
1/SE_IV
0.30
0.35
0.40
0.45
β_IV

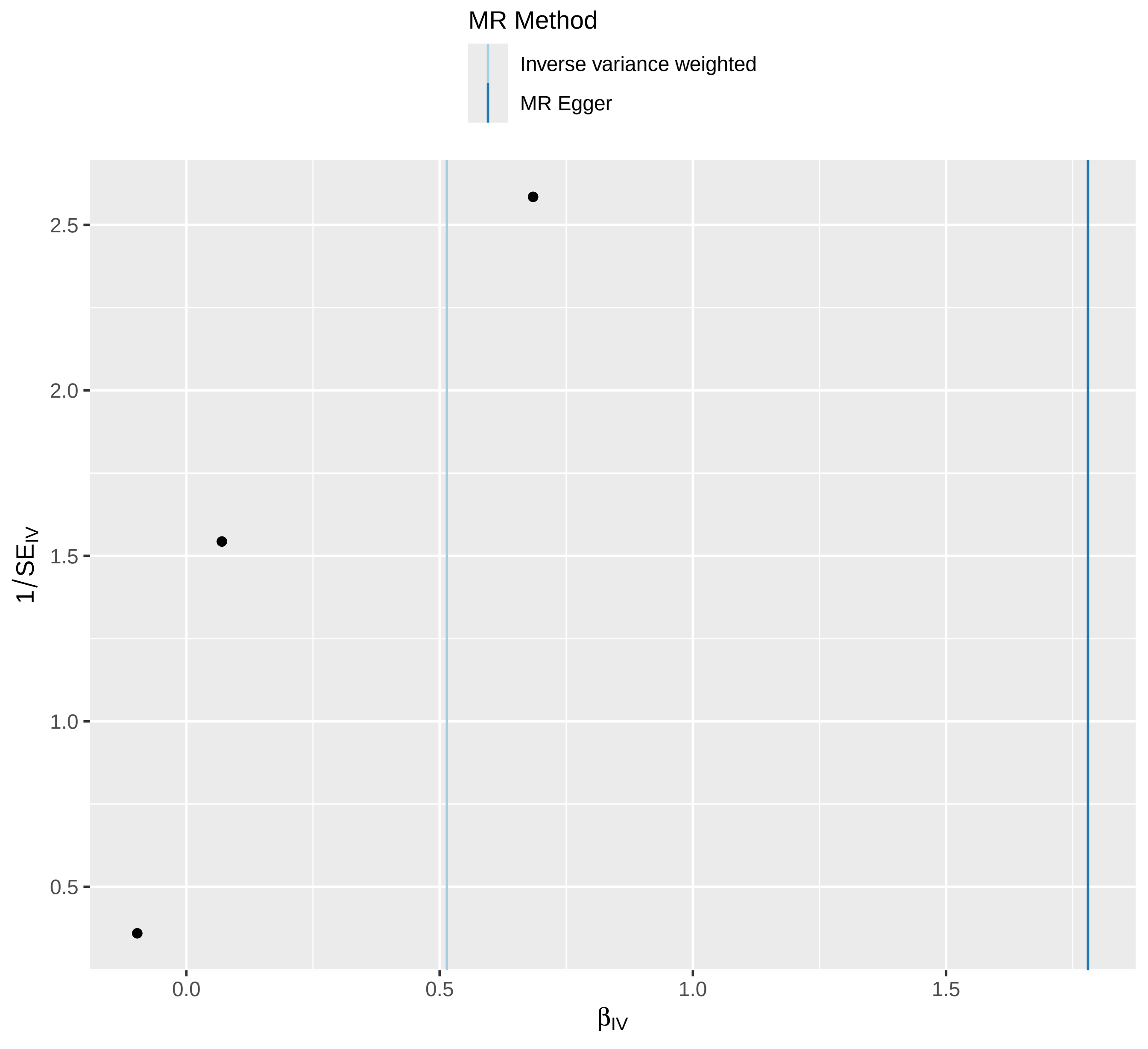

MR Method
Inverse variance weighted
MR Egger
2.5
2.0
1.5
1.0
0.5
$1/SE_{IV}$
0.0
0.5
1.0
1.5
β_{IV}

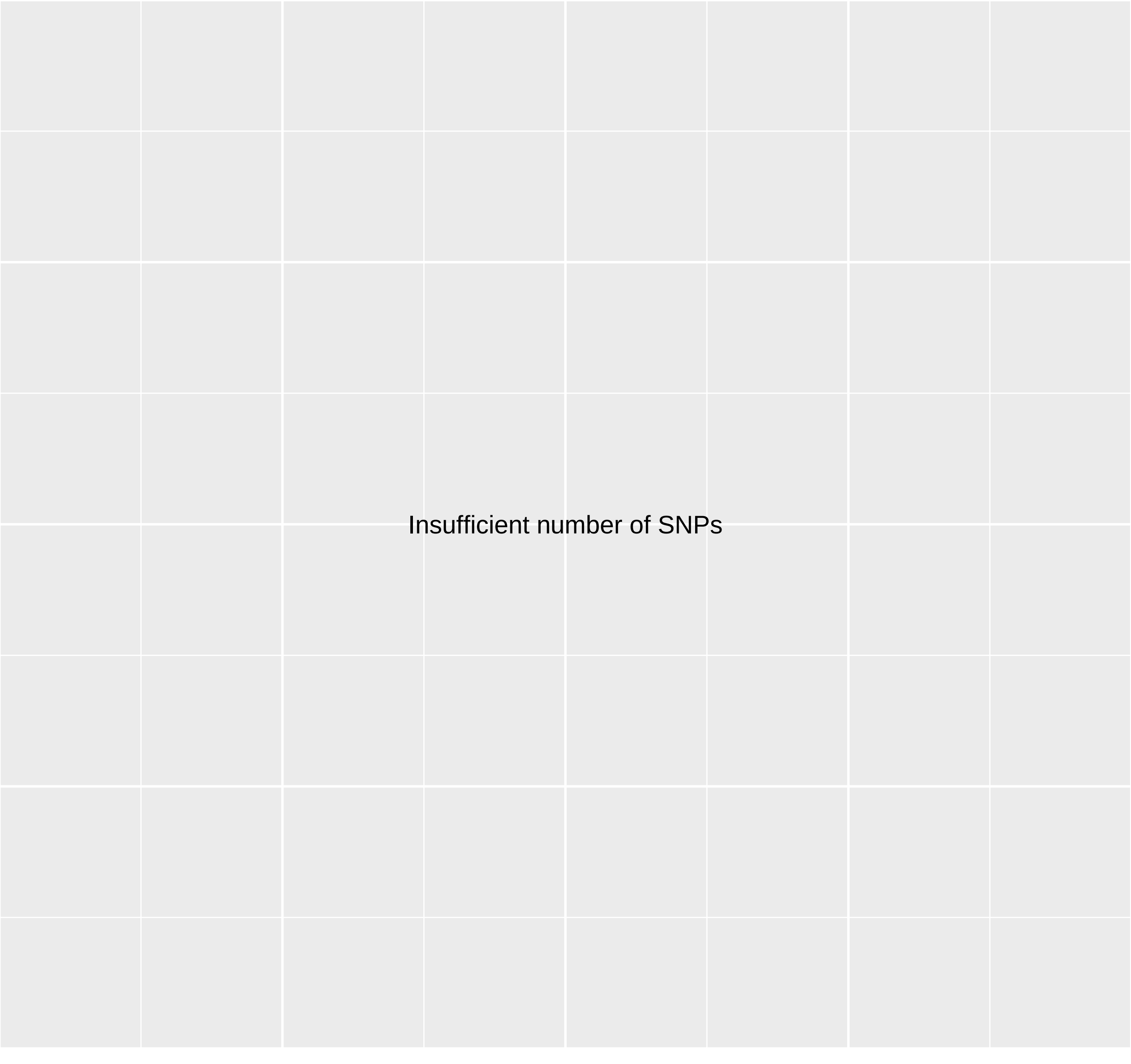
Insufficient number of SNPs

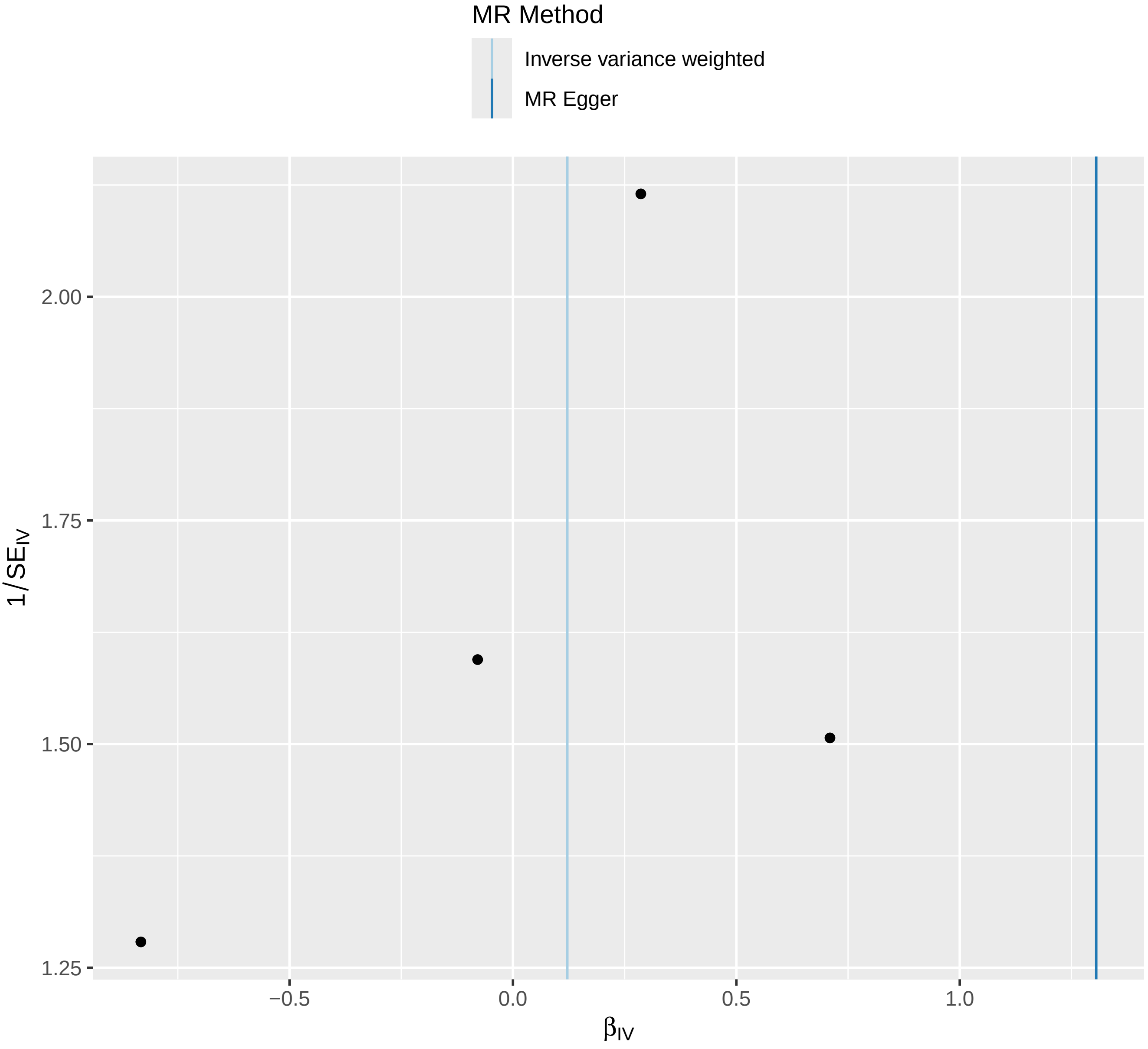

MR Method
Inverse variance weighted
MR Egger
2.00
1.75
1.50
1.25
1/SE$_{IV}$
−0.5
0.0
0.5
1.0
β$_{IV}$

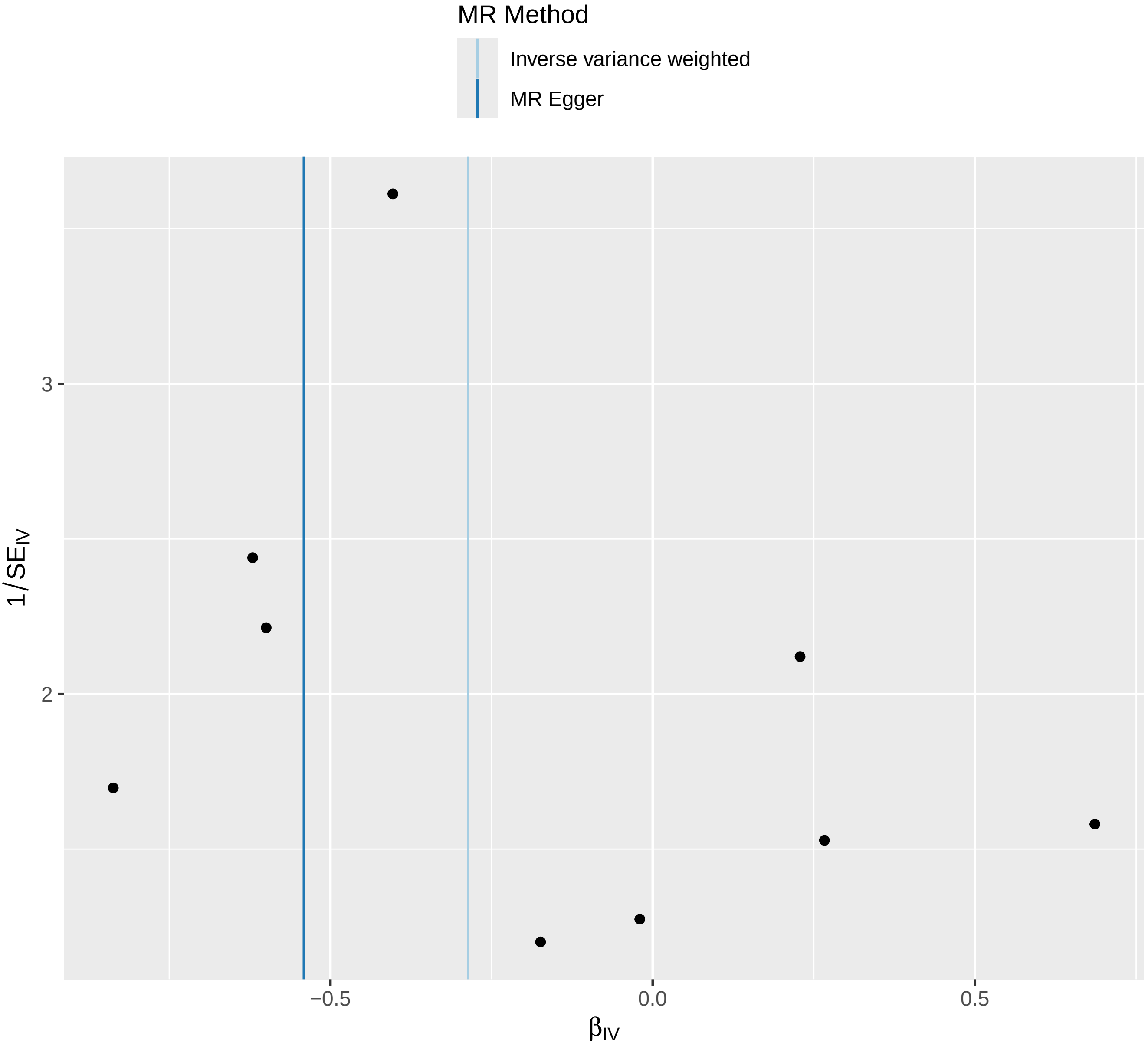

MR Method
Inverse variance weighted
MR Egger
3
2
1/SE_IV
−0.5
0.0
0.5
β_IV

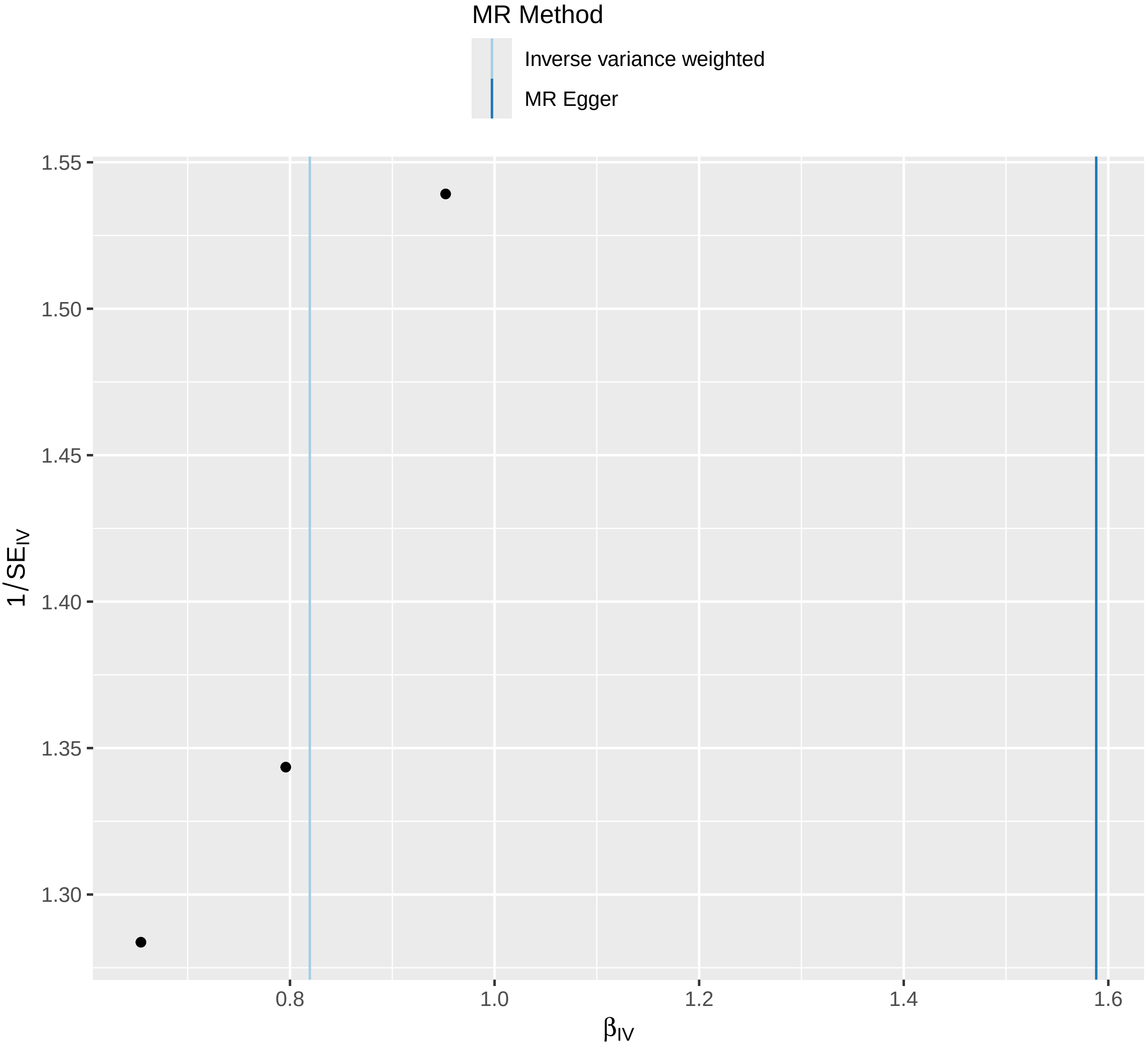

MR Method
Inverse variance weighted
MR Egger
1.55
1.50
1.45
1.40
1.35
1.30
1/SE_IV
0.8
1.0
1.2
1.4
1.6
β_IV

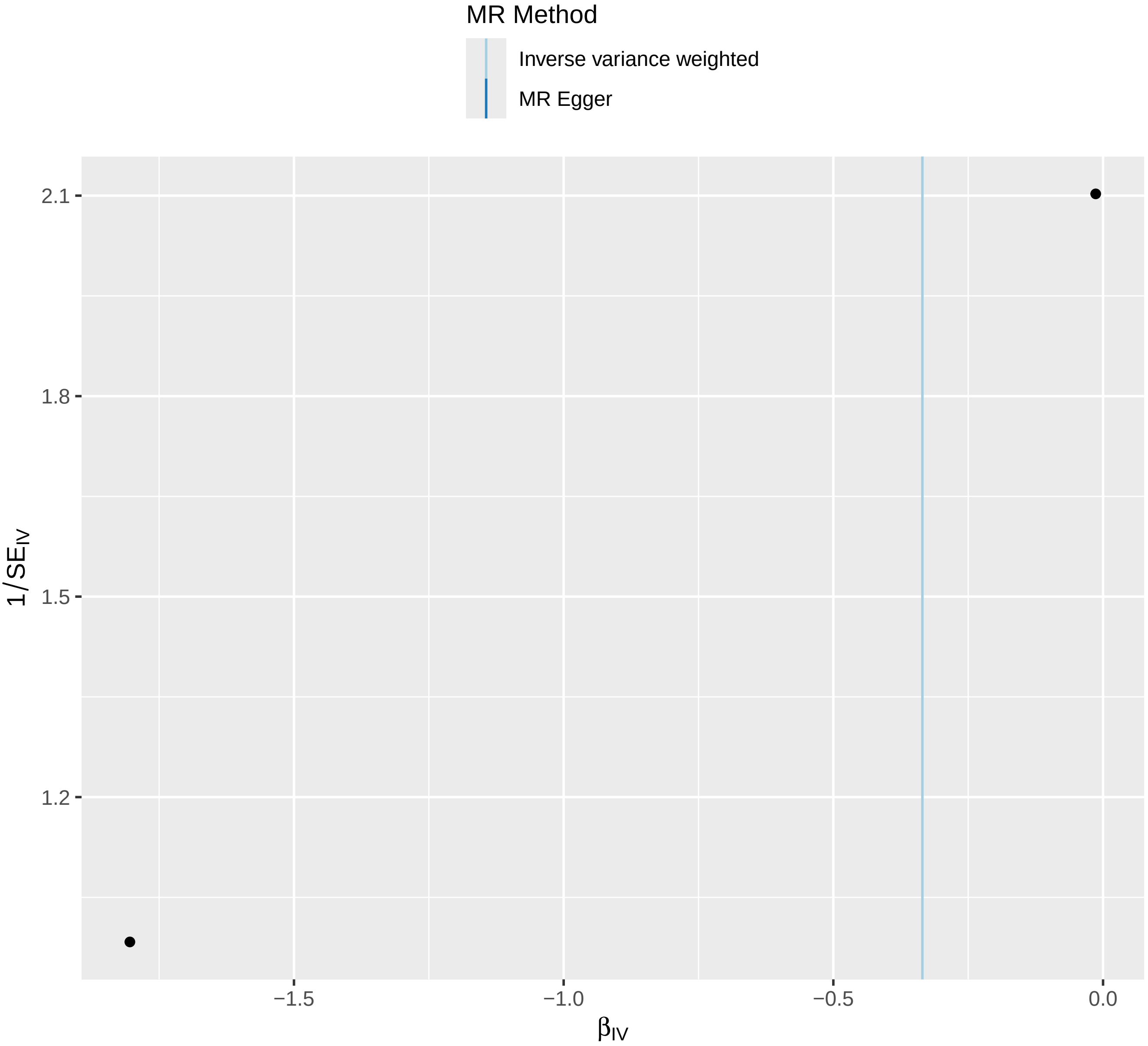

MR Method
Inverse variance weighted
MR Egger
2.1
1.8
1.5
1.2
1/SE_IV
−1.5
−1.0
−0.5
0.0
β_IV

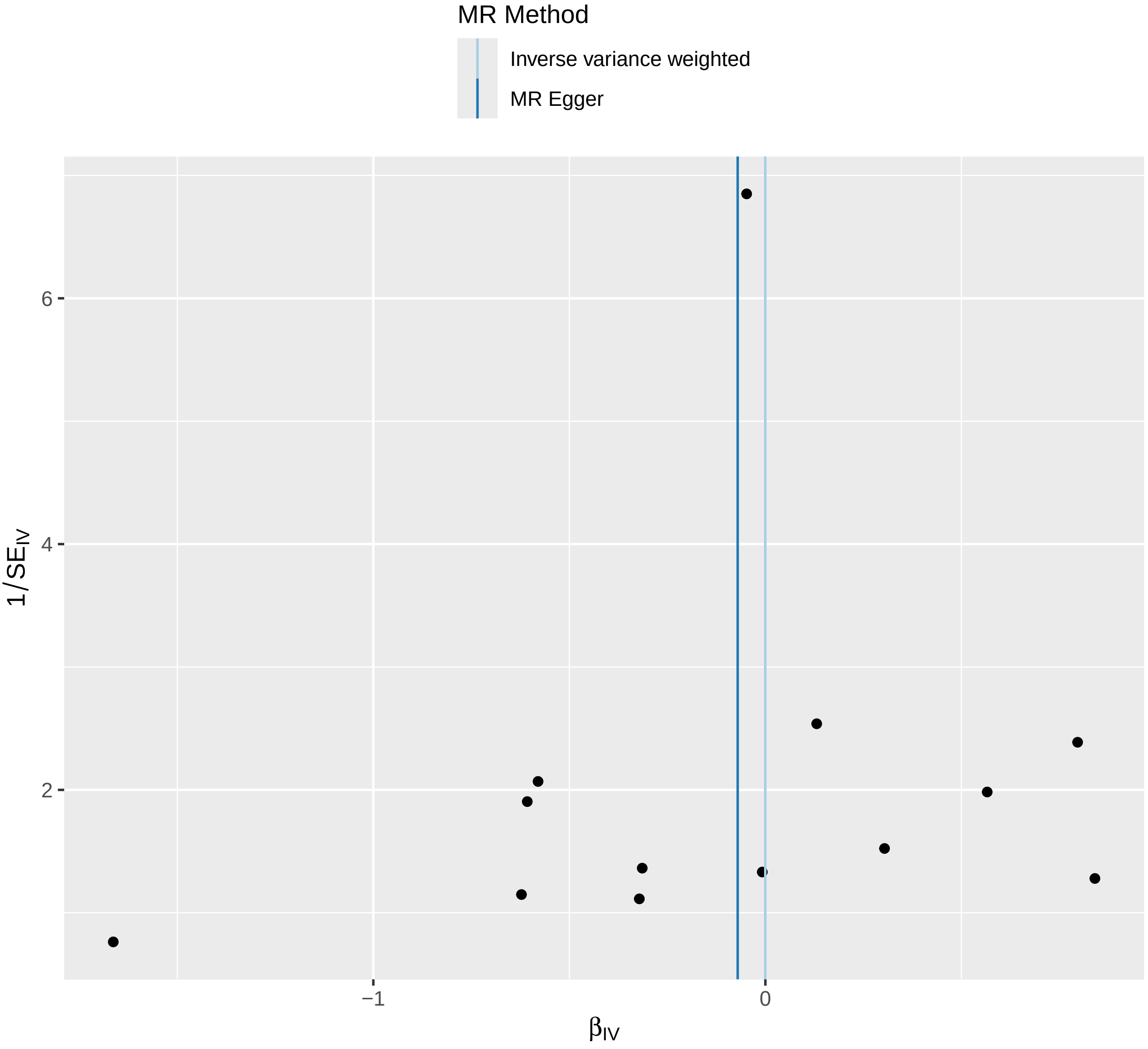
MR Method
Inverse variance weighted
MR Egger
6
4
2
1/SE$_{IV}$
−1
0
β$_{IV}$

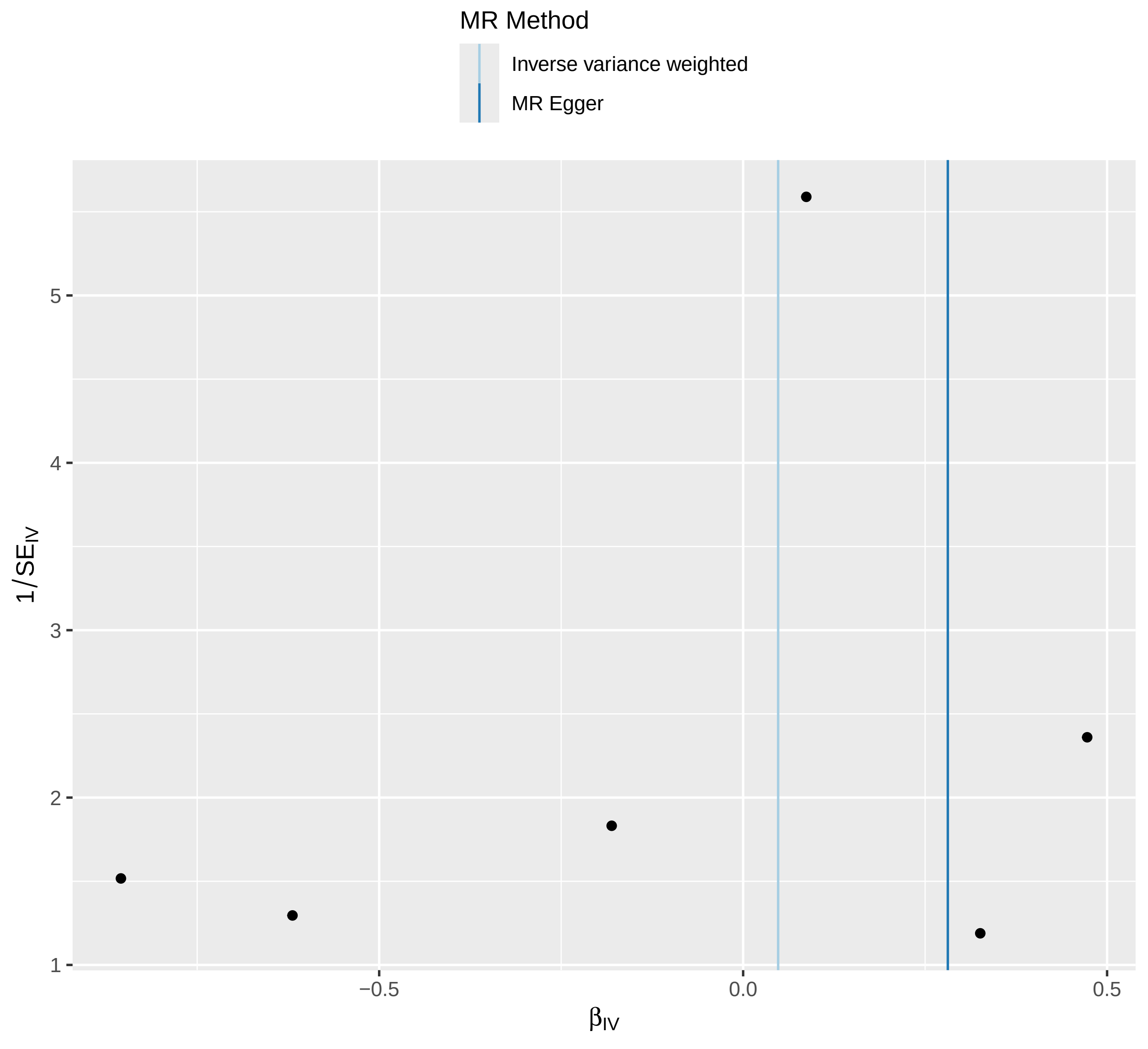

MR Method
Inverse variance weighted
MR Egger
5
4
3
2
1
1/SE$_{IV}$
−0.5
0.0
0.5
β$_{IV}$

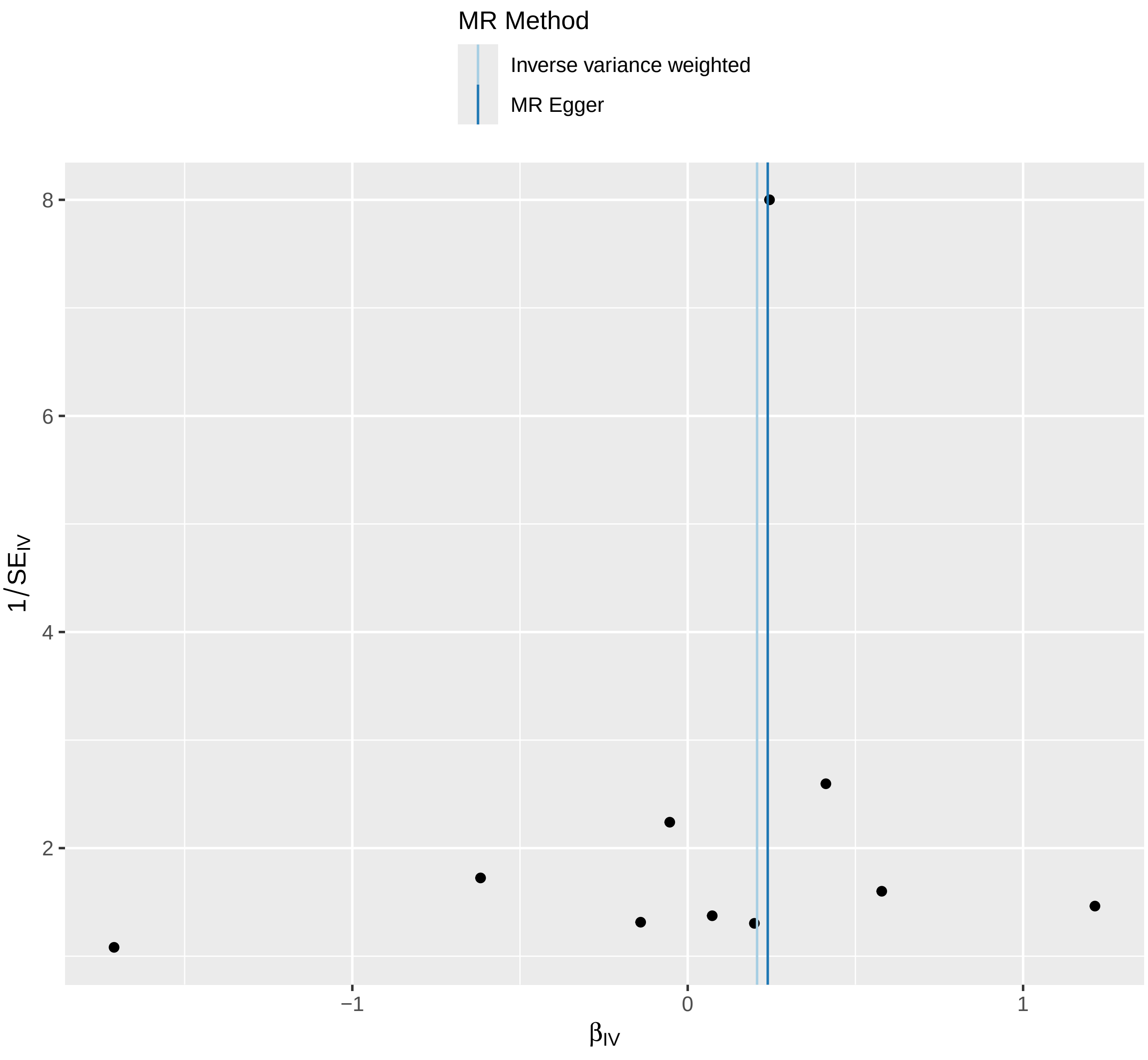

MR Method
Inverse variance weighted
MR Egger
8
6
4
2
1/SE_IV
−1
0
1
β_IV

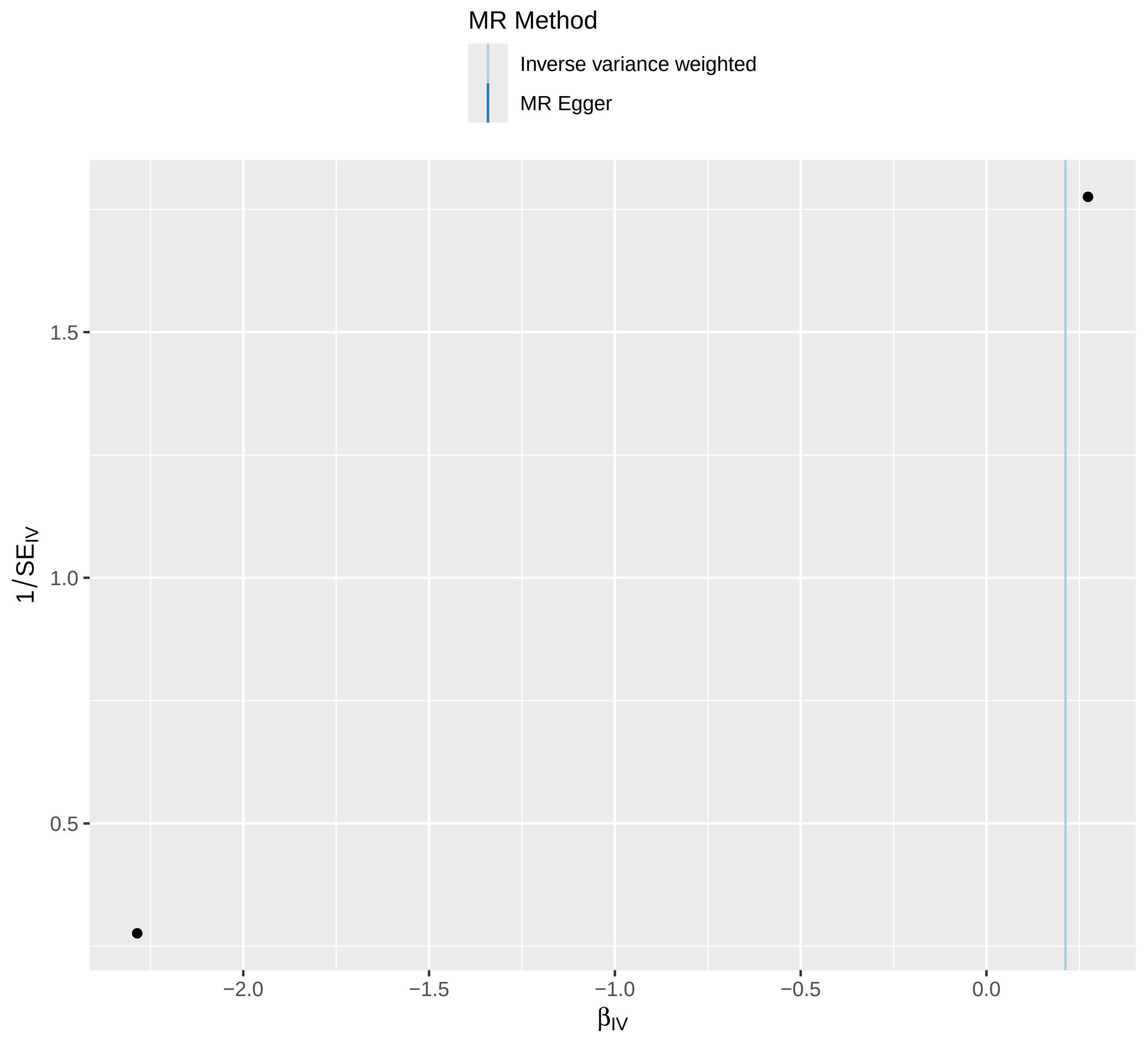
MR Method
Inverse variance weighted
MR Egger
1/SE_IV
1.5
1.0
0.5
−2.0
−1.5
−1.0
−0.5
0.0
β_IV

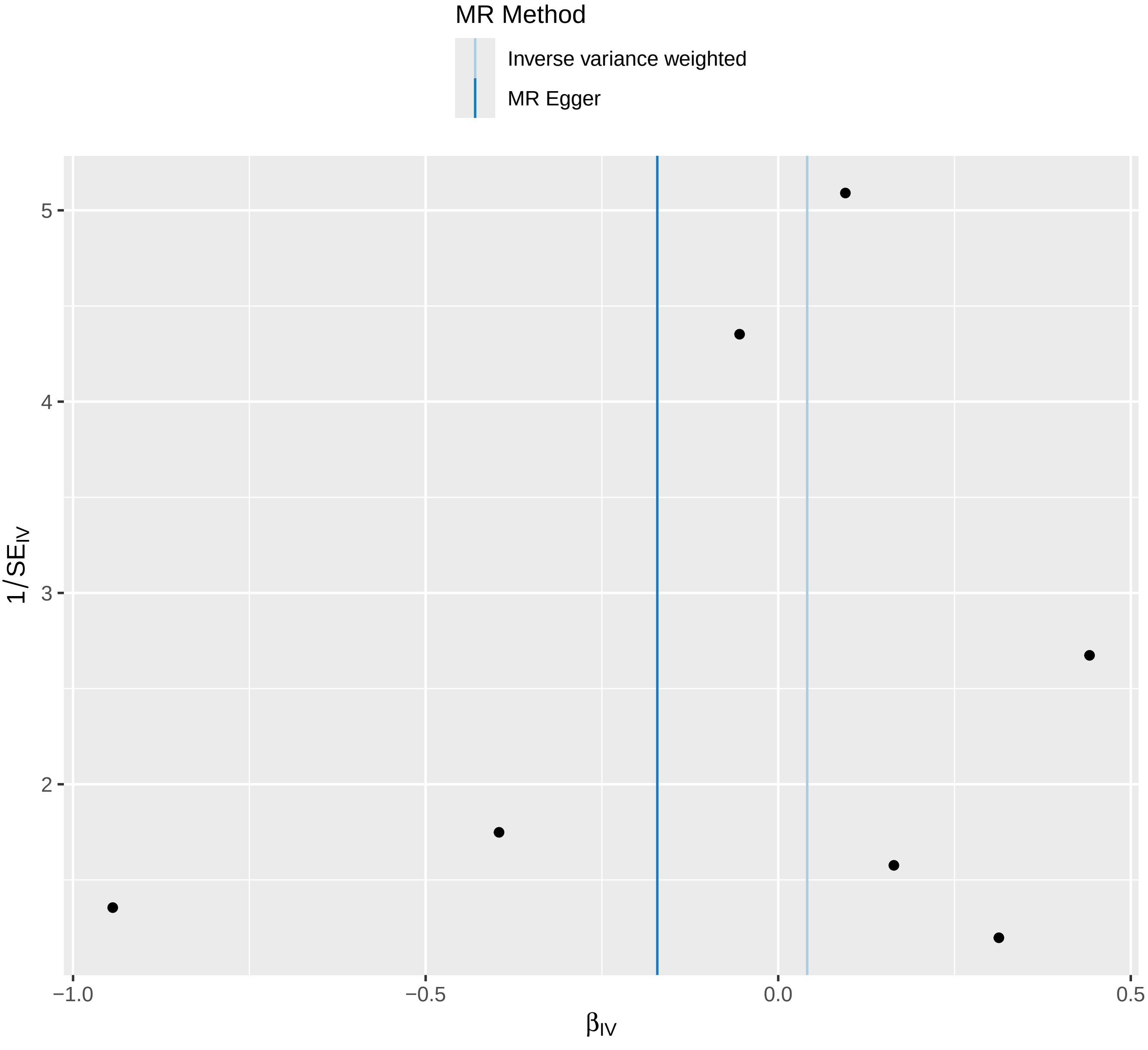

MR Method
Inverse variance weighted
MR Egger
5
4
3
2
1/SE_IV
−1.0
−0.5
0.0
0.5
β_IV

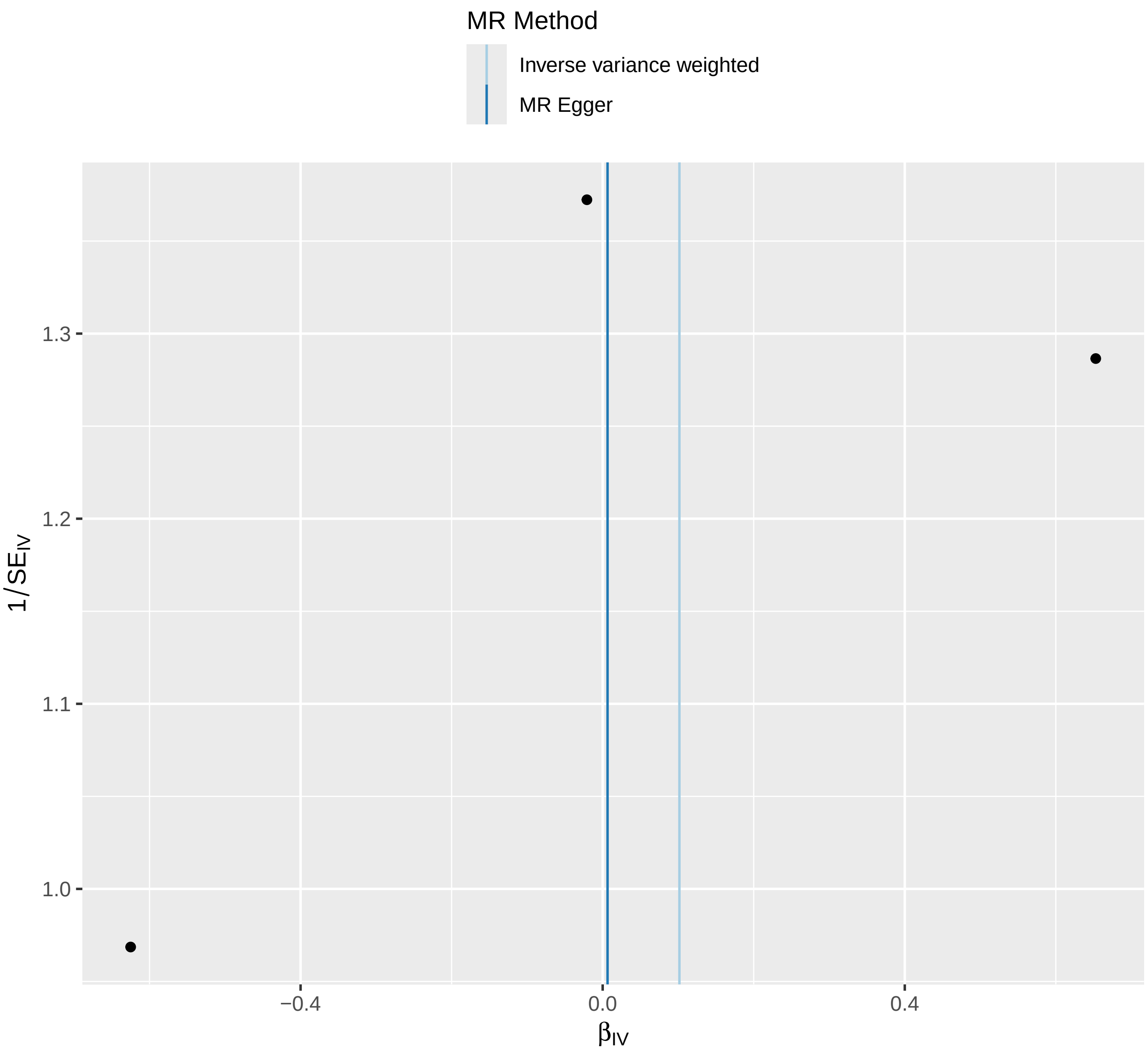
MR Method
Inverse variance weighted
MR Egger
1.3
1.2
1.1
1.0
1/SE$_{IV}$
−0.4
0.0
0.4
β$_{IV}$

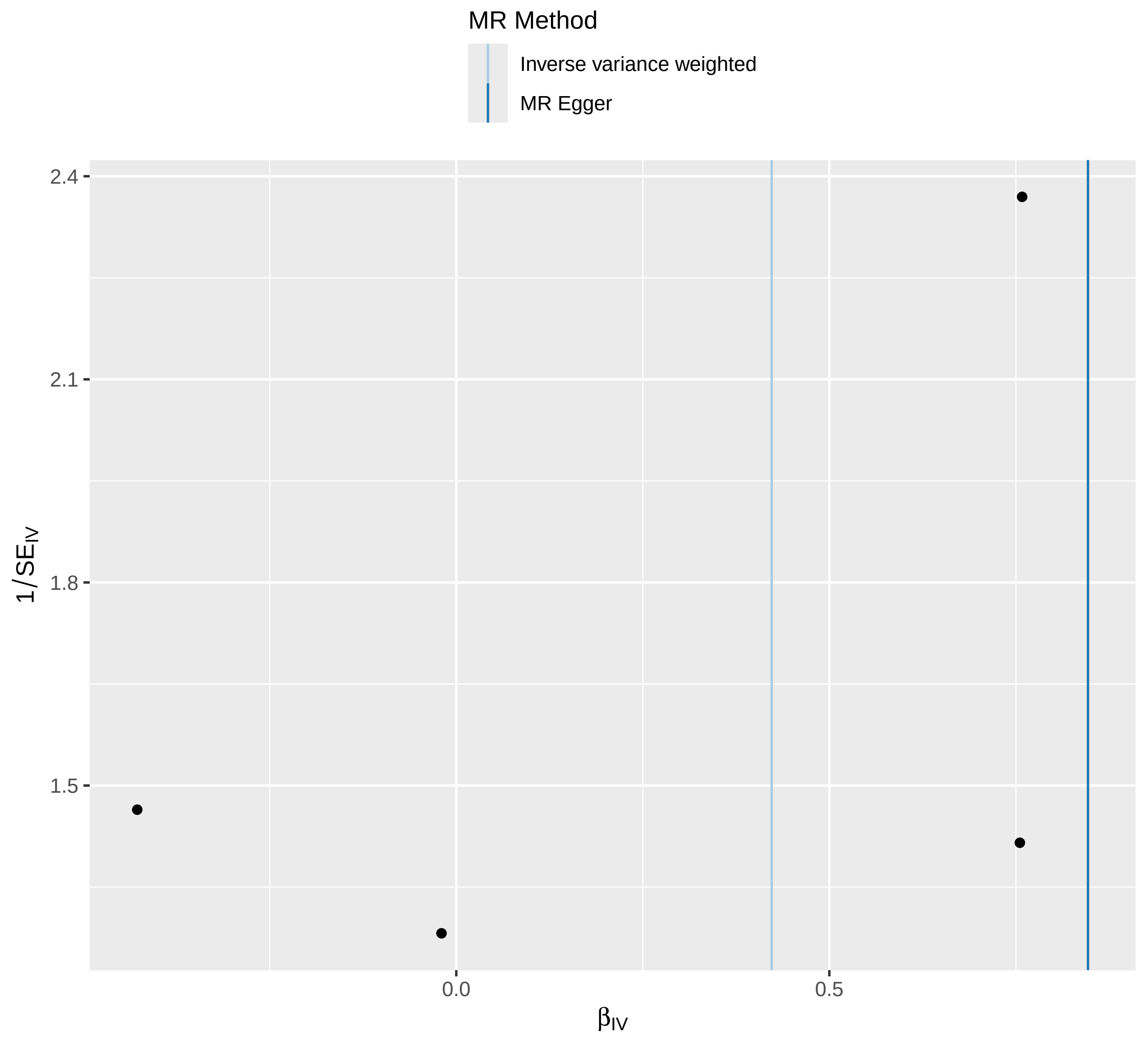
MR Method
Inverse variance weighted
MR Egger
2.4
2.1
1.8
1.5
1/SE_IV
0.0
0.5
β_IV

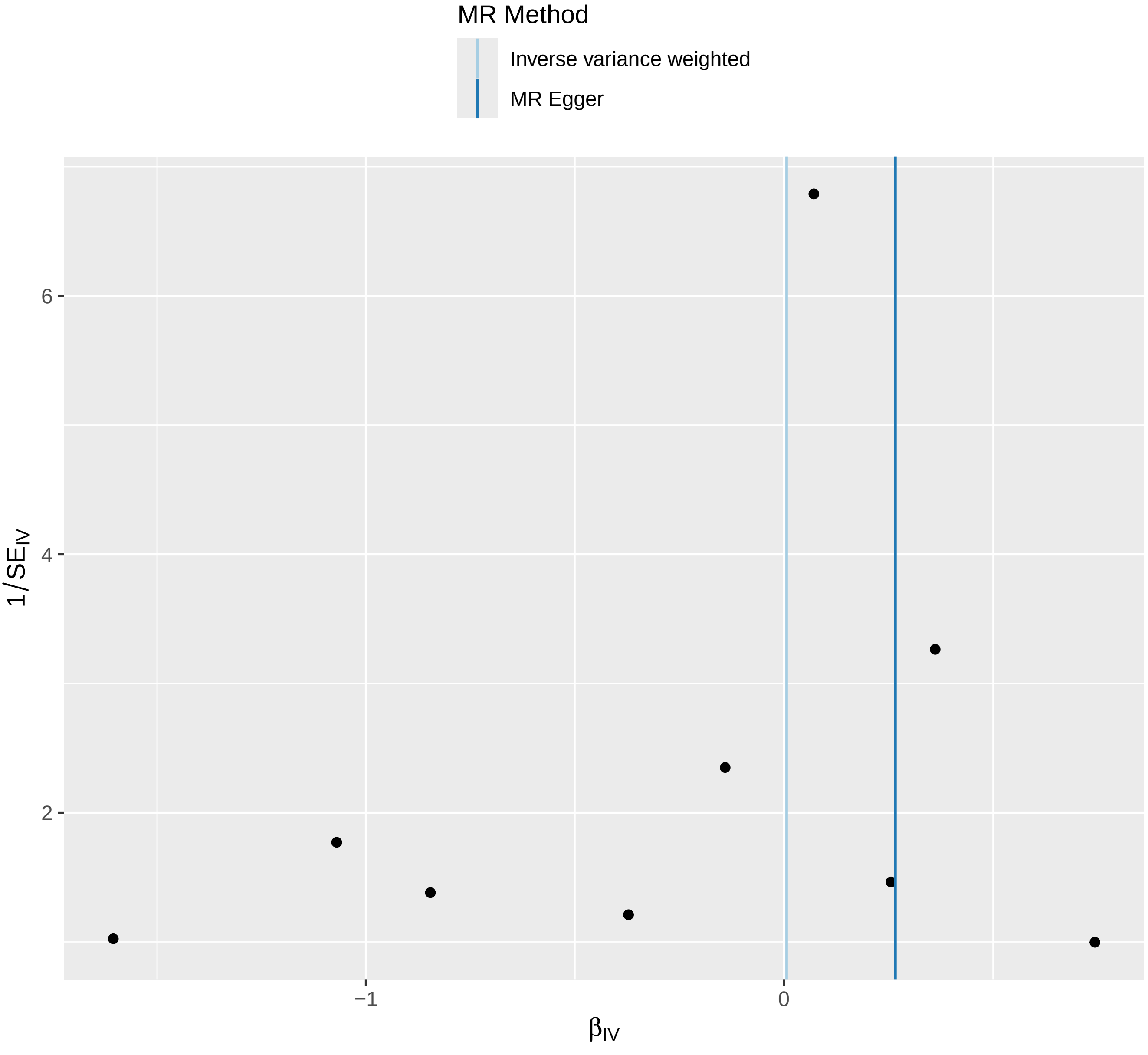

MR Method
Inverse variance weighted
MR Egger
6
4
2
1/SE$_{IV}$
−1
0
β$_{IV}$

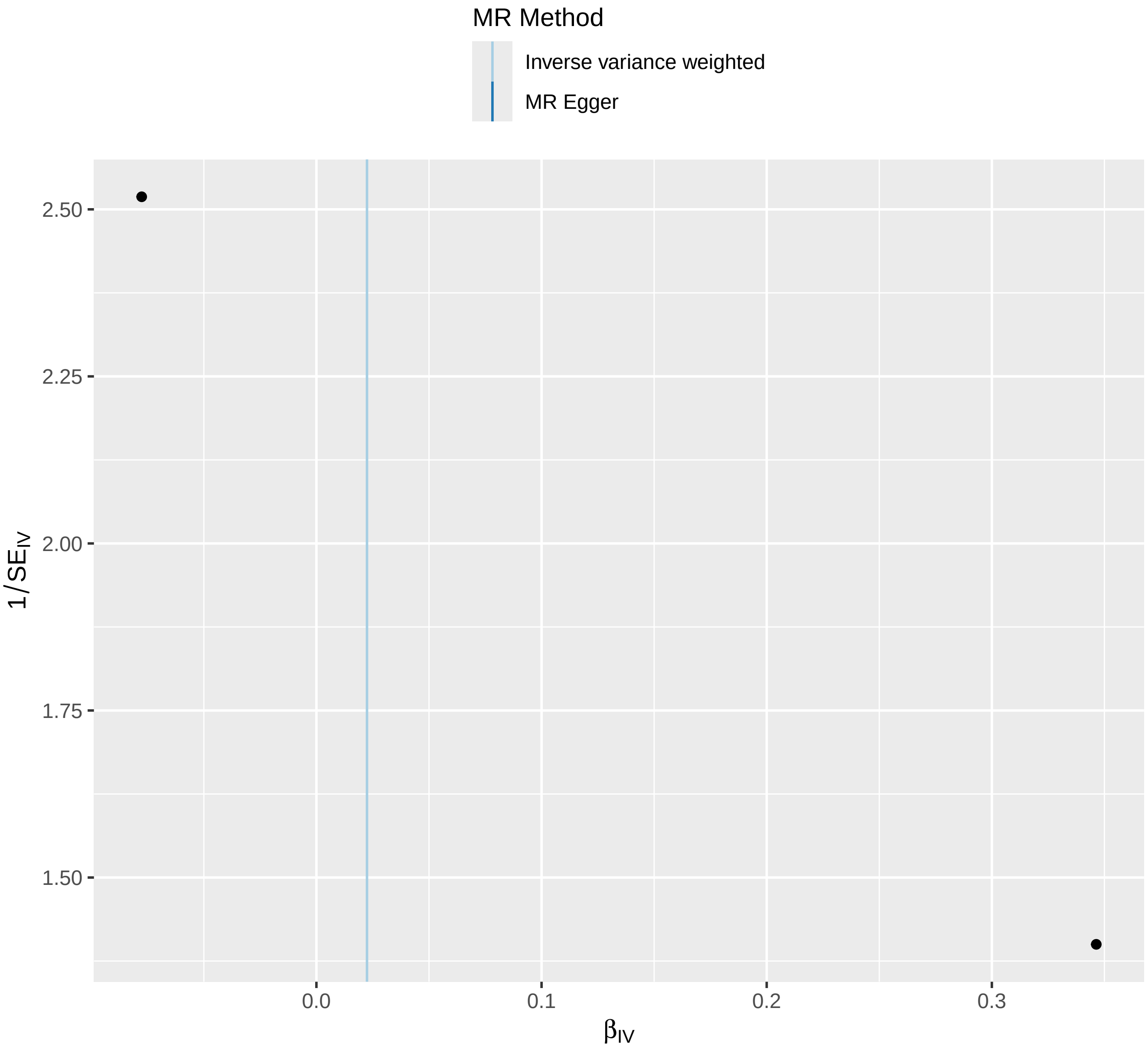
MR Method
Inverse variance weighted
MR Egger
2.50
2.25
2.00
1.75
1.50
$1/SE_{IV}$
0.0
0.1
0.2
0.3
β_{IV}

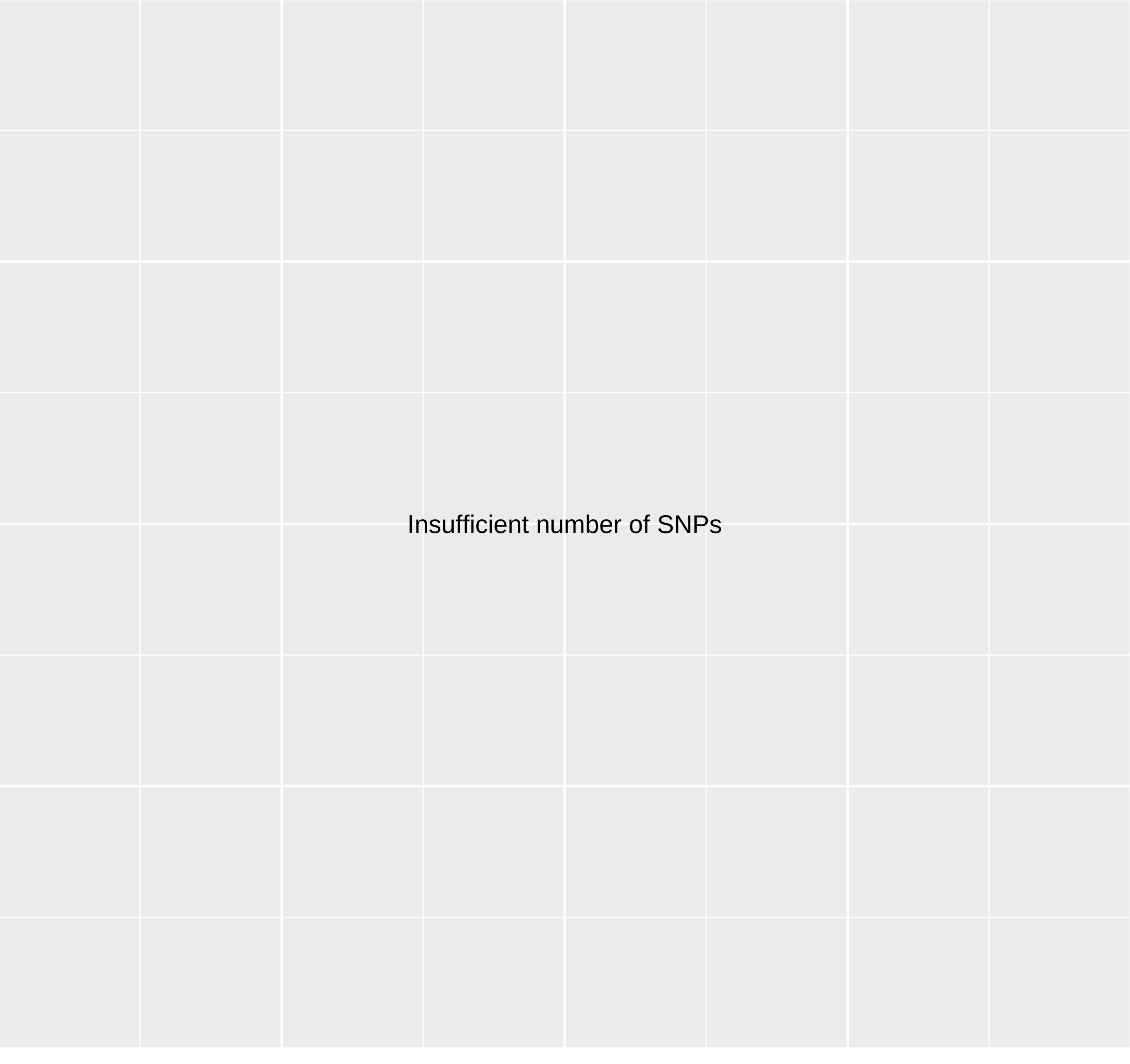
Insufficient number of SNPs

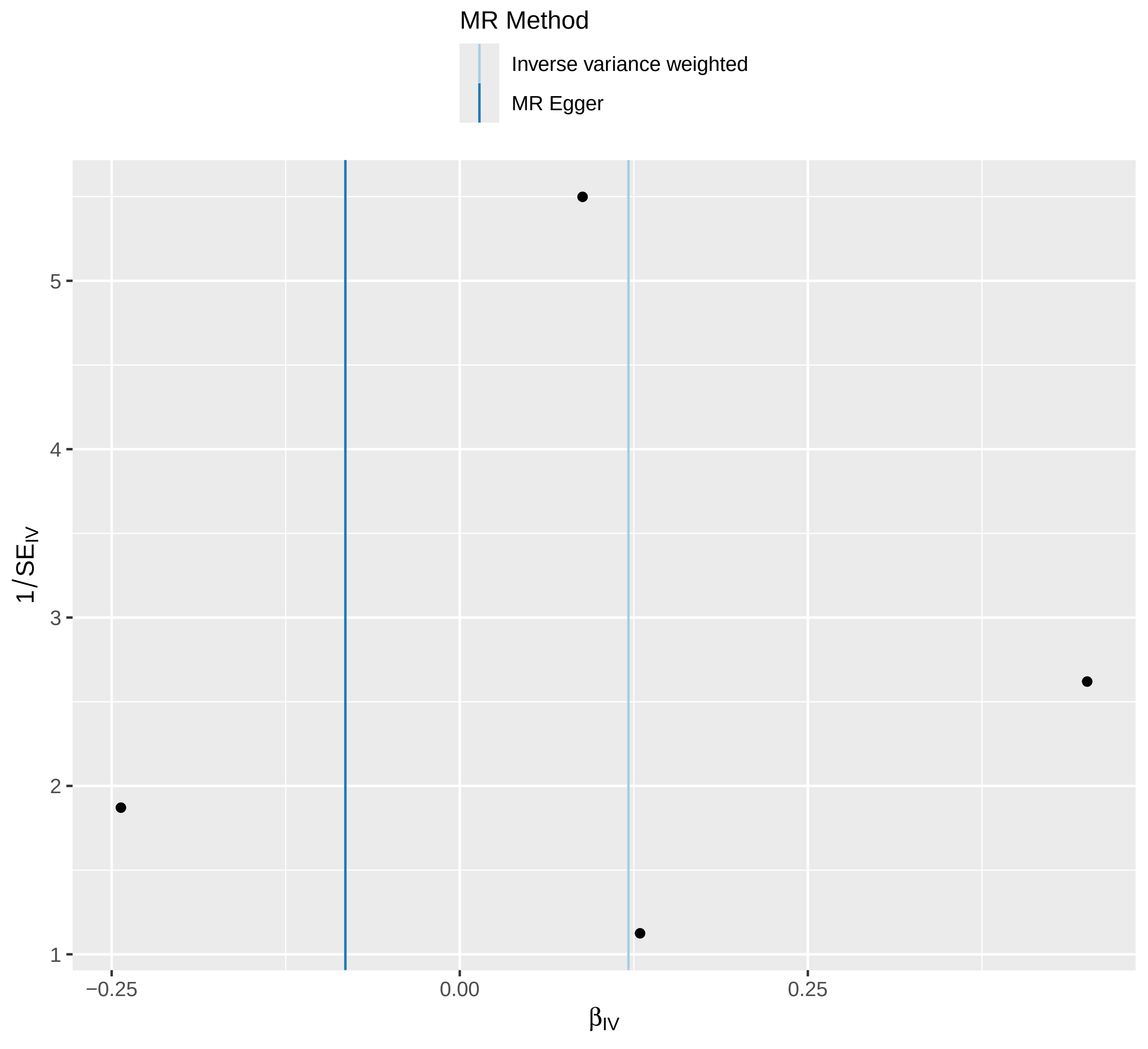

MR Method
Inverse variance weighted
MR Egger
1/SE_IV
5
4
3
2
1
−0.25
0.00
0.25
β_IV

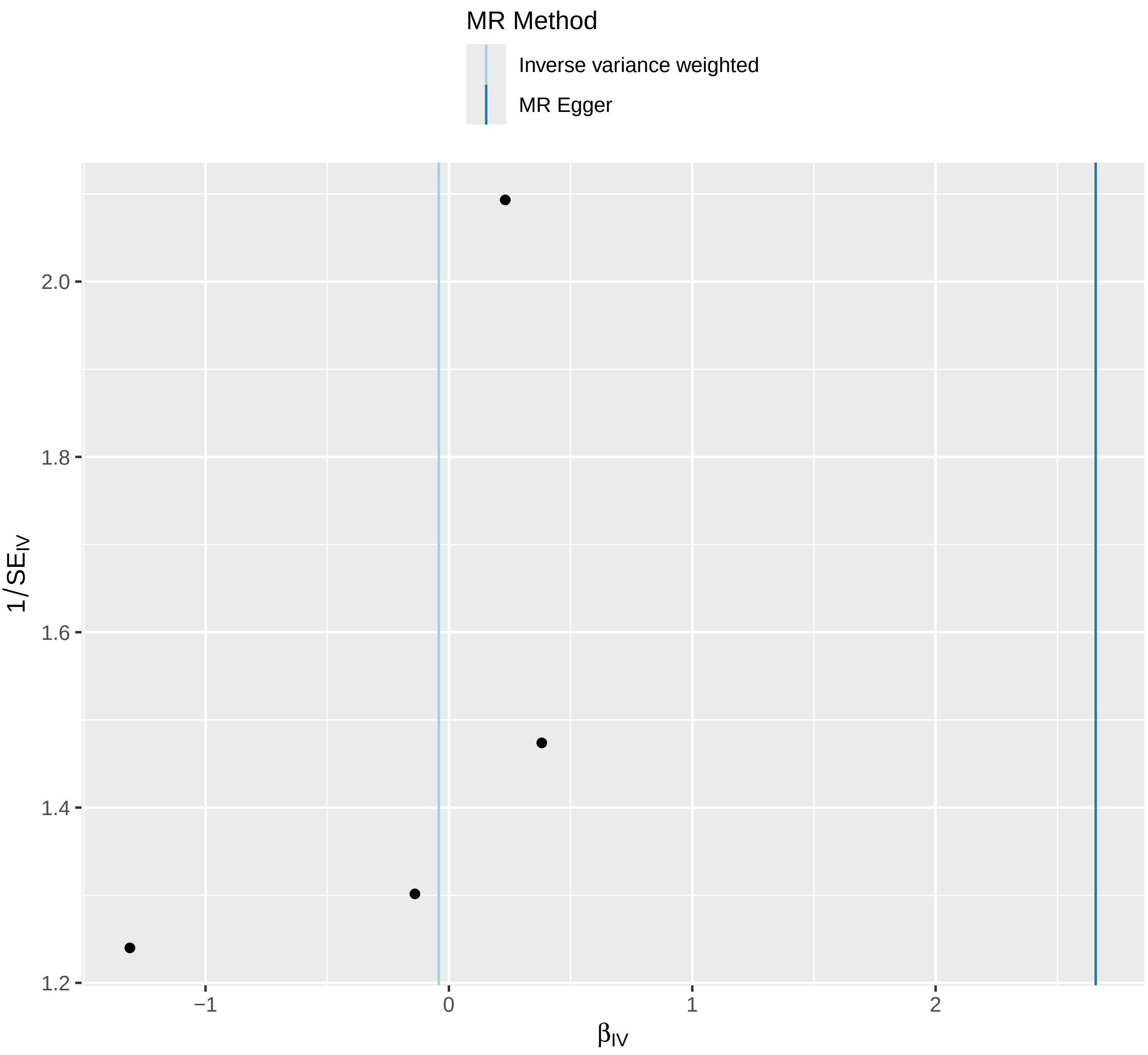
MR Method
Inverse variance weighted
MR Egger
2.0
1.8
1.6
1.4
1.2
1/SE_IV
−1
0
1
2
β_IV

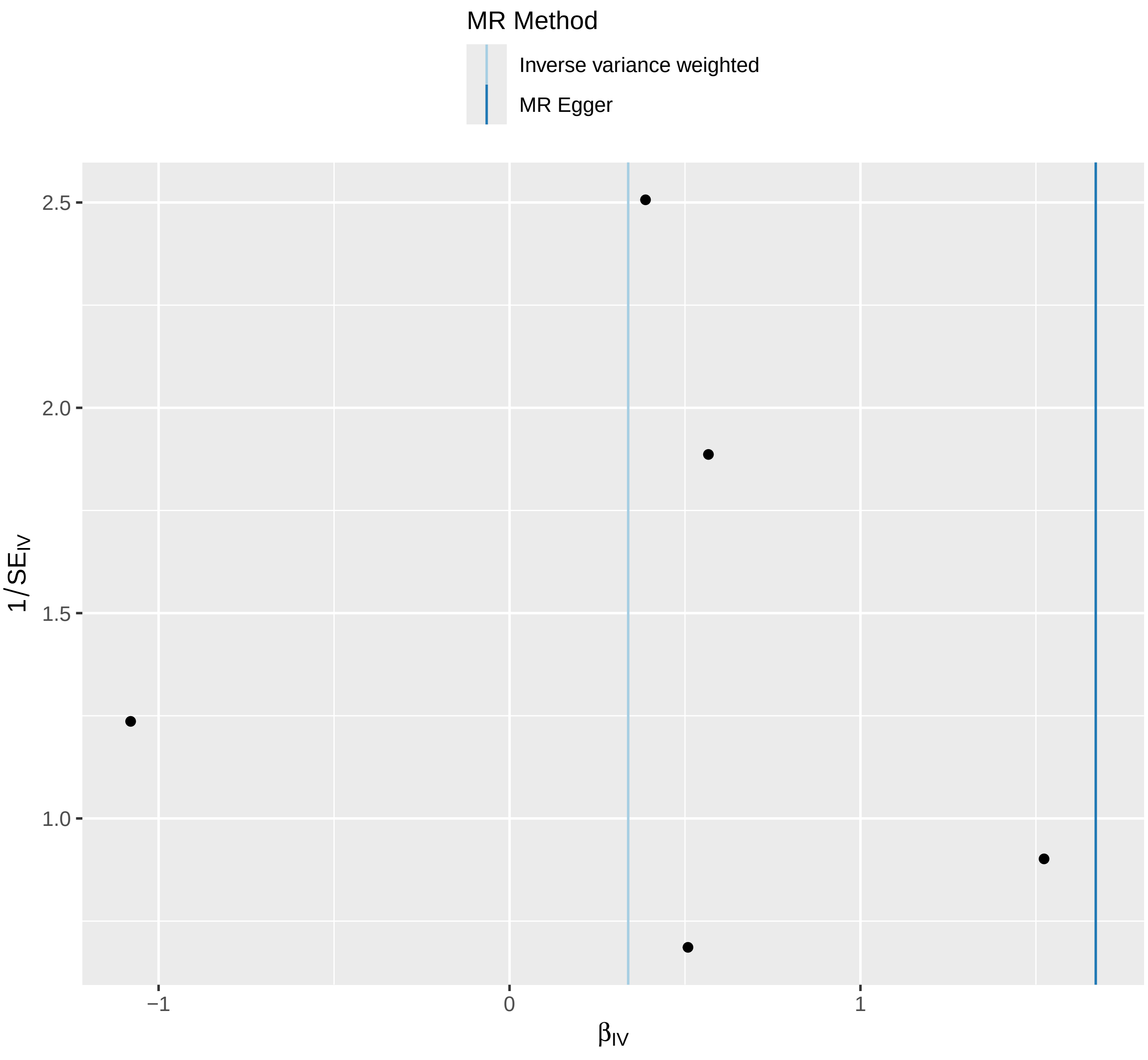
MR Method
Inverse variance weighted
MR Egger
2.5
2.0
1.5
1.0
$1/SE_{IV}$
−1
0
1
β_{IV}

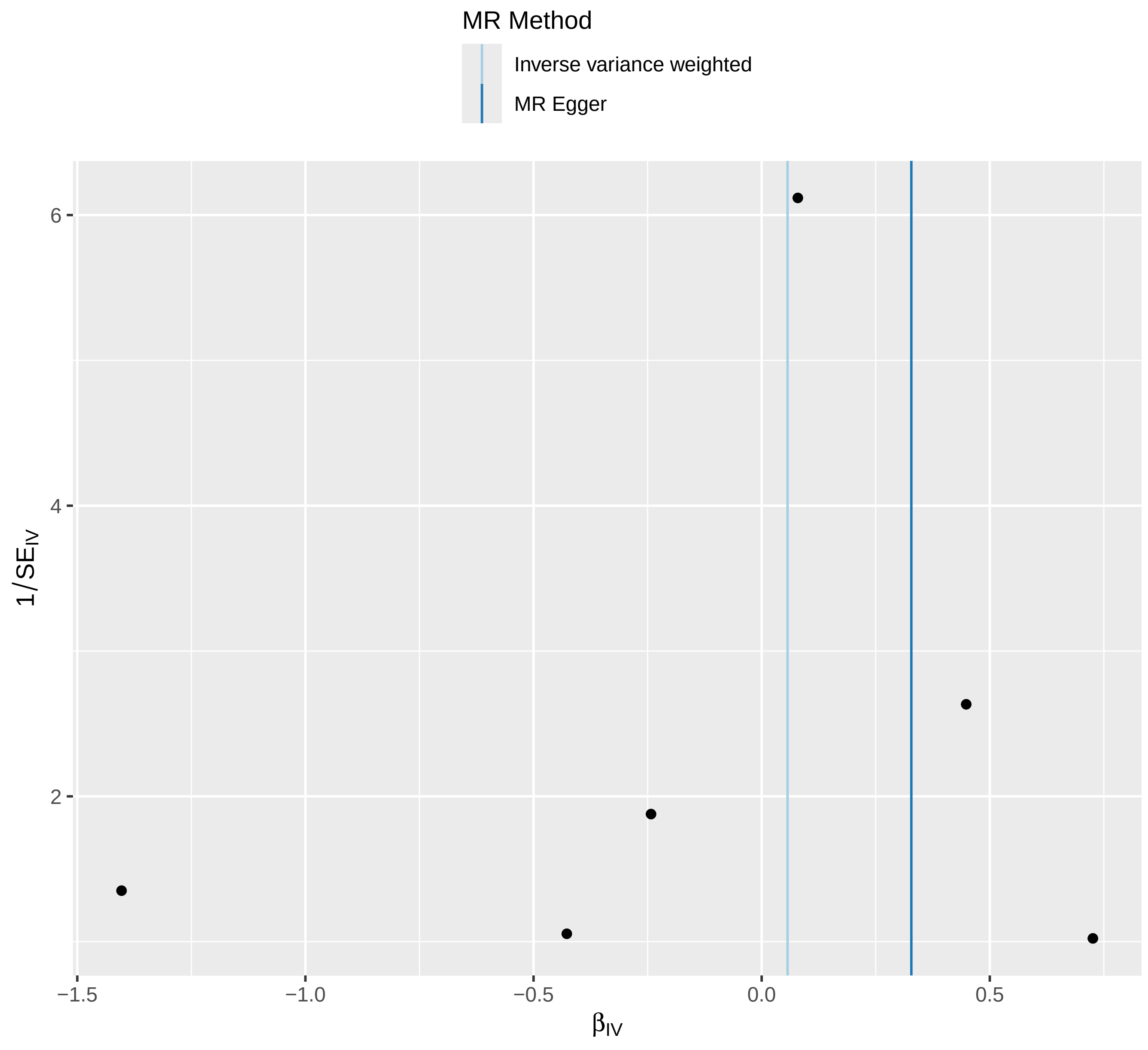

MR Method
Inverse variance weighted
MR Egger
6
4
2
1/SE_IV
−1.5
−1.0
−0.5
0.0
0.5
β_IV

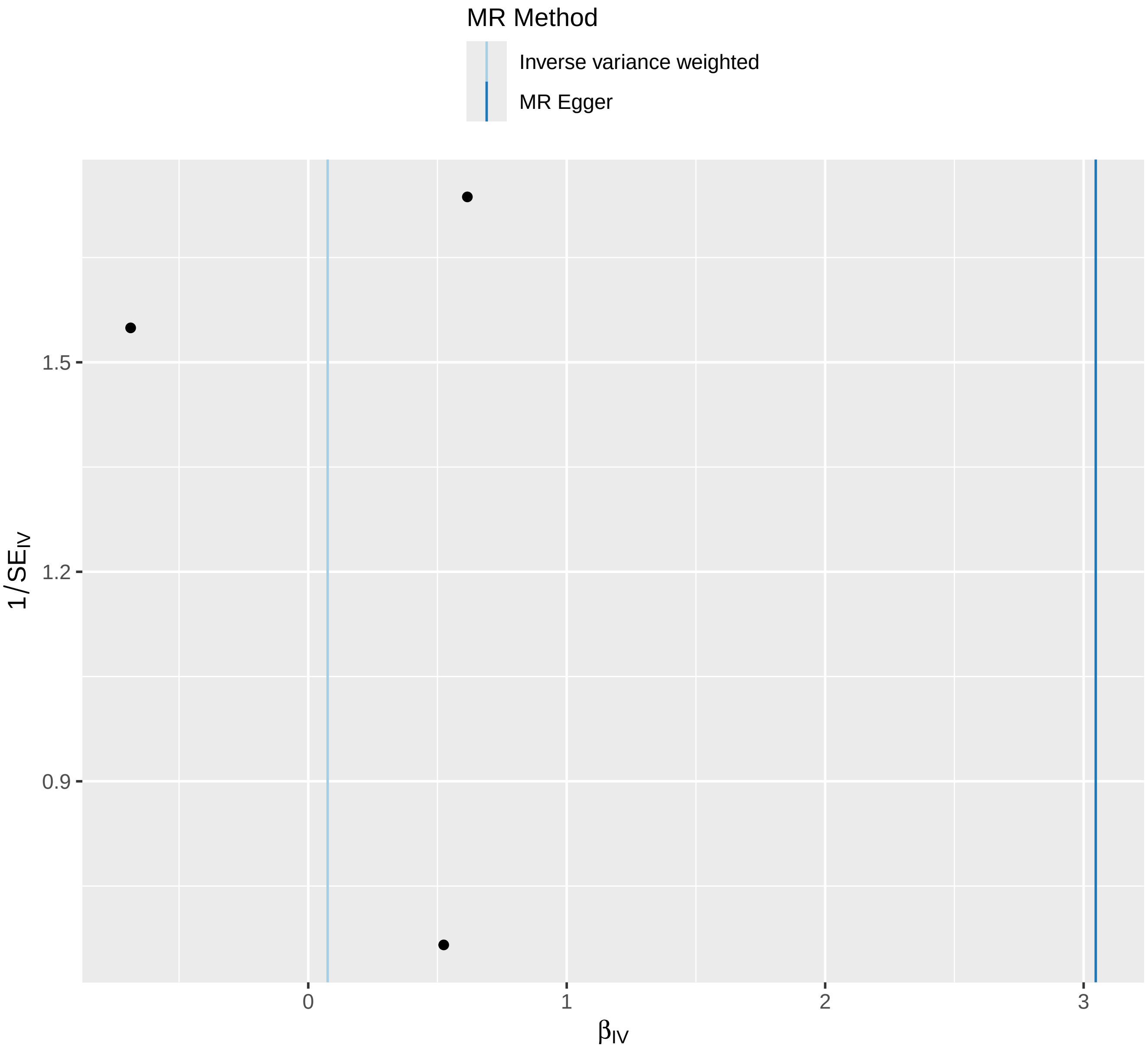
MR Method
Inverse variance weighted
MR Egger
1.5
1.2
0.9
0
1
2
3
$1/SE_{IV}$
β_{IV}

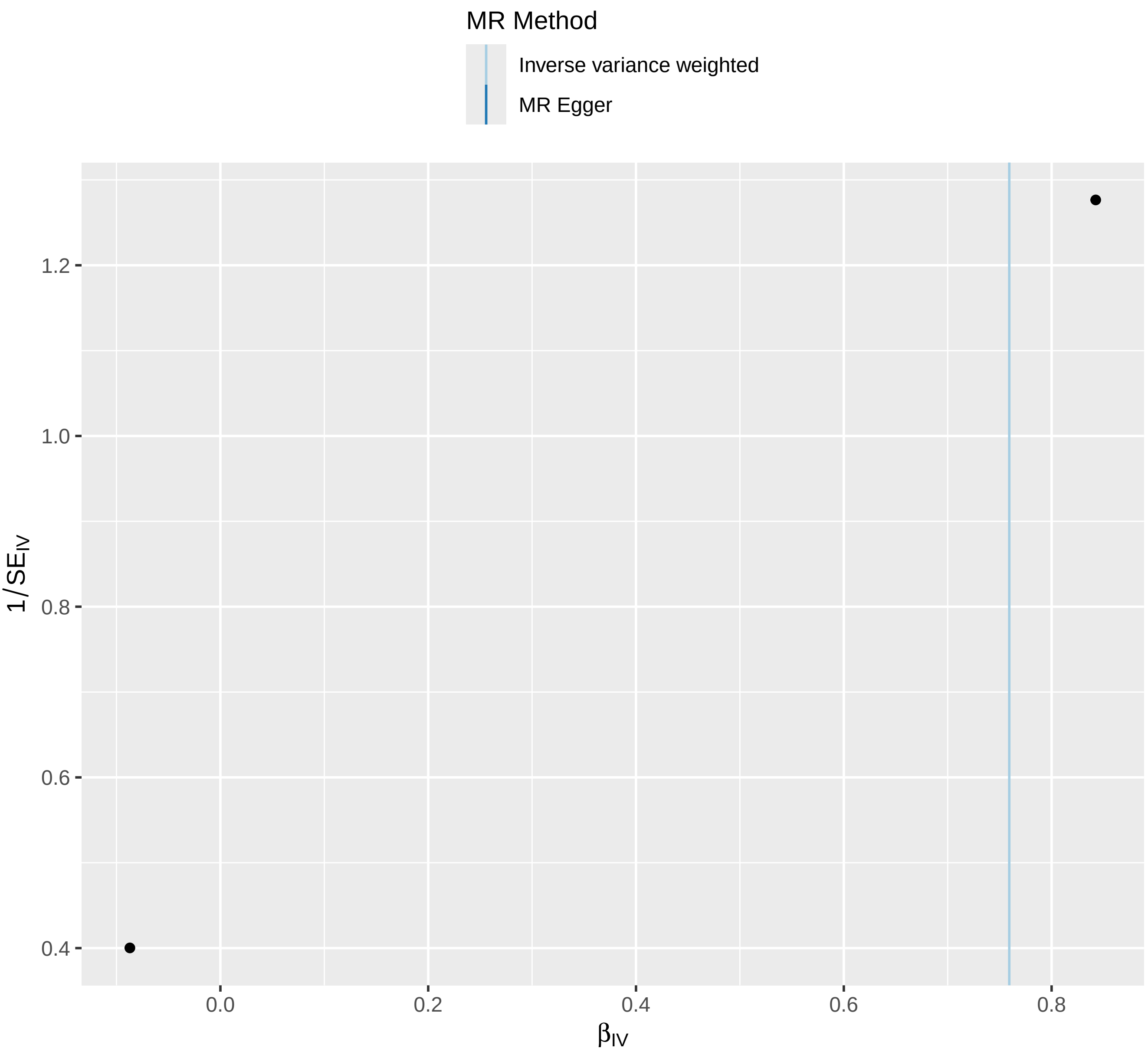

MR Method
Inverse variance weighted
MR Egger
1.2
1.0
0.8
0.6
0.4
1/SE_IV
0.0
0.2
0.4
0.6
0.8
β_IV

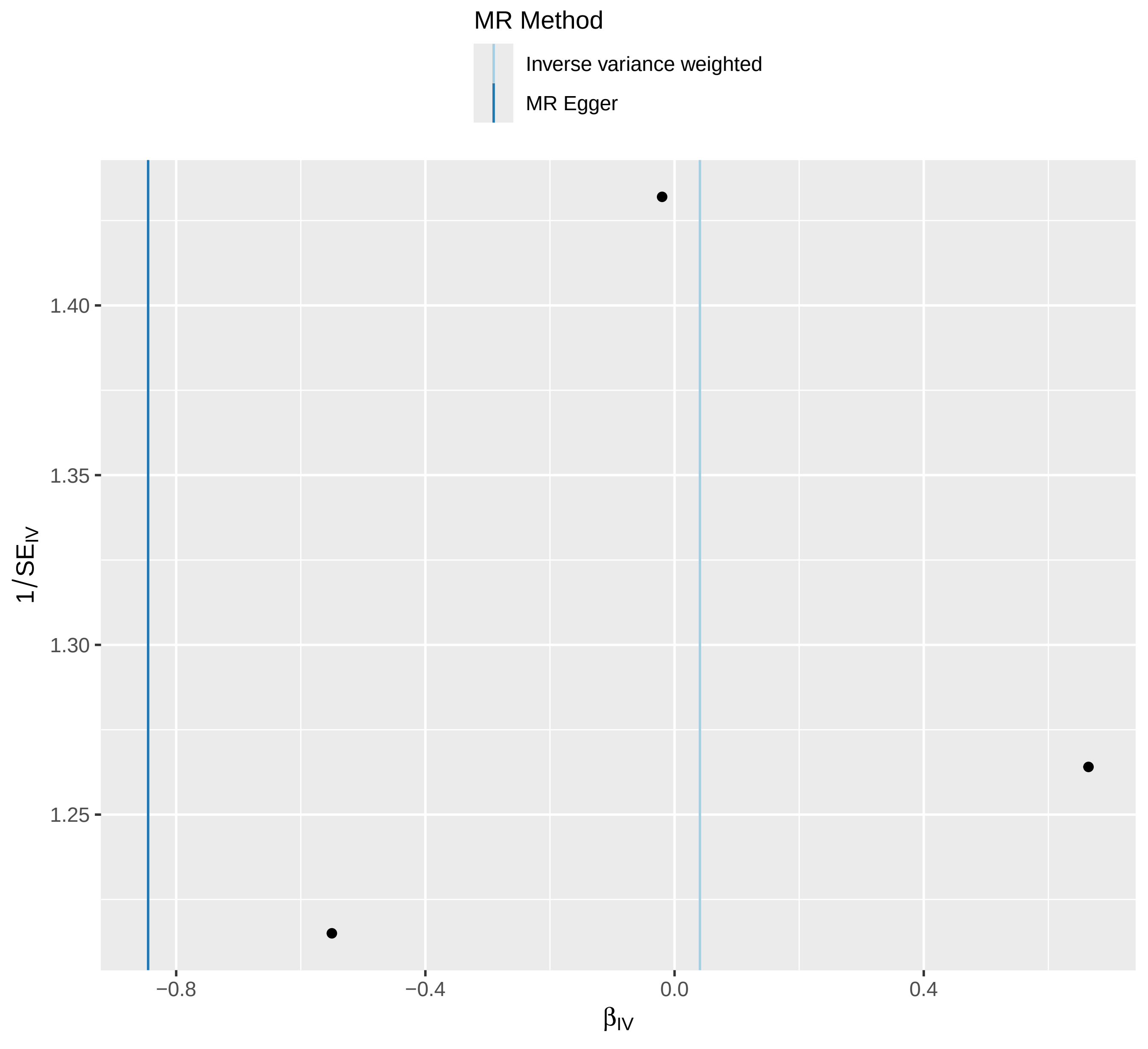
MR Method
Inverse variance weighted
MR Egger
1.40
1.35
1.30
1.25
1/SE_IV
−0.8
−0.4
0.0
0.4
β_IV

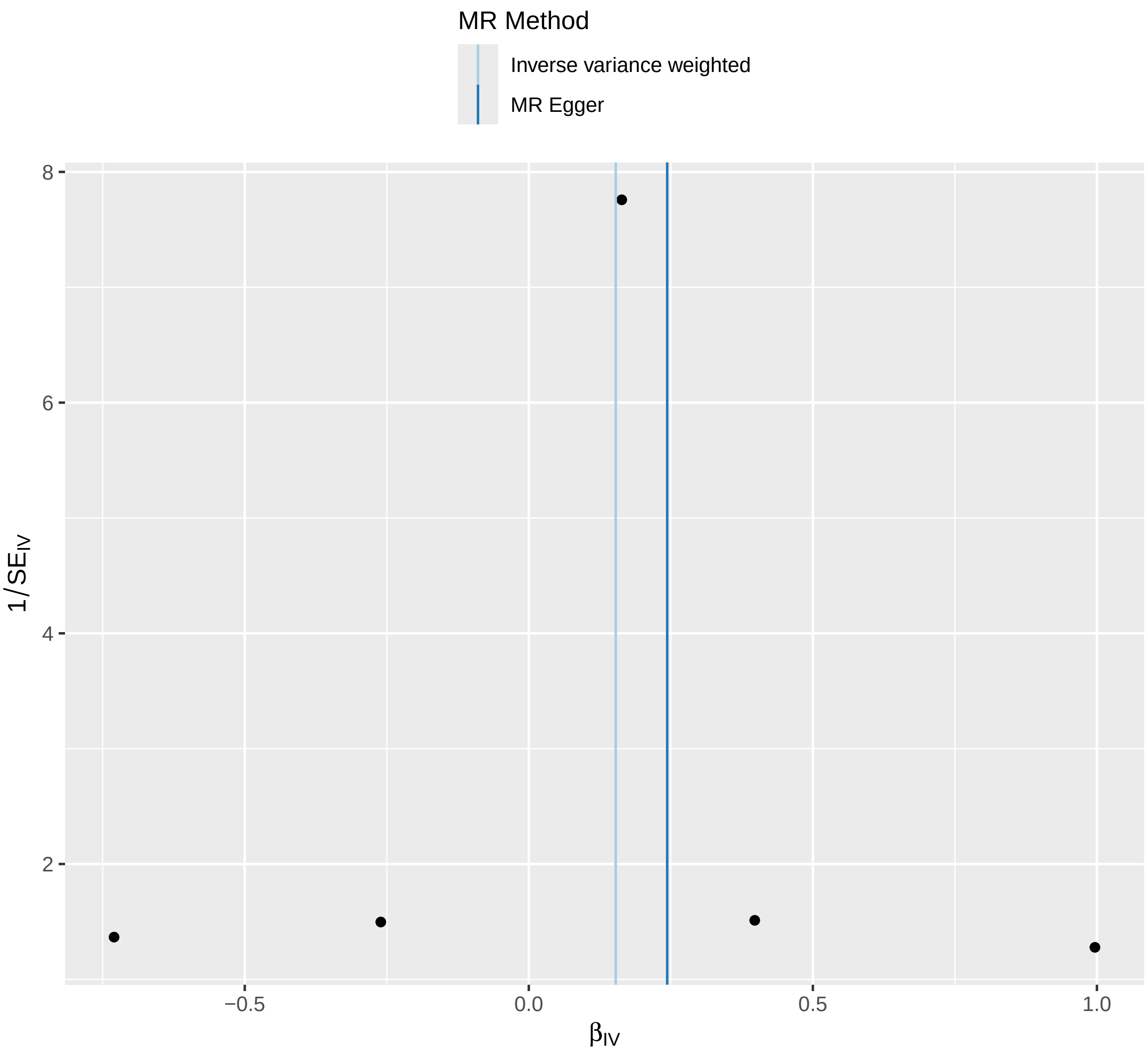

MR Method
Inverse variance weighted
MR Egger
8
6
4
2
1/SE_IV
−0.5
0.0
0.5
1.0
β_IV

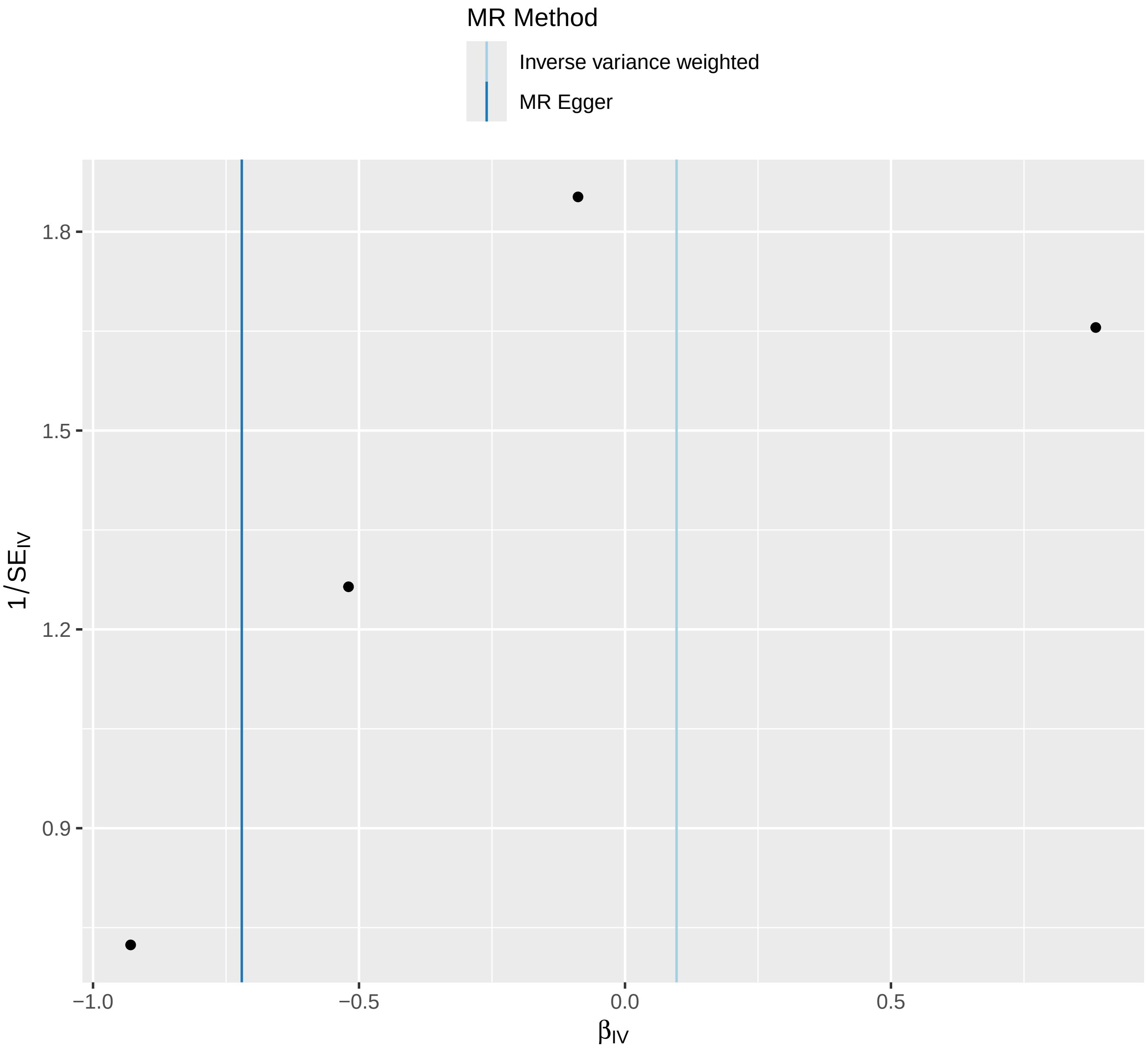
MR Method
Inverse variance weighted
MR Egger
1.8
1.5
1.2
0.9
1/SE$_{IV}$
−1.0
−0.5
0.0
0.5
β$_{IV}$

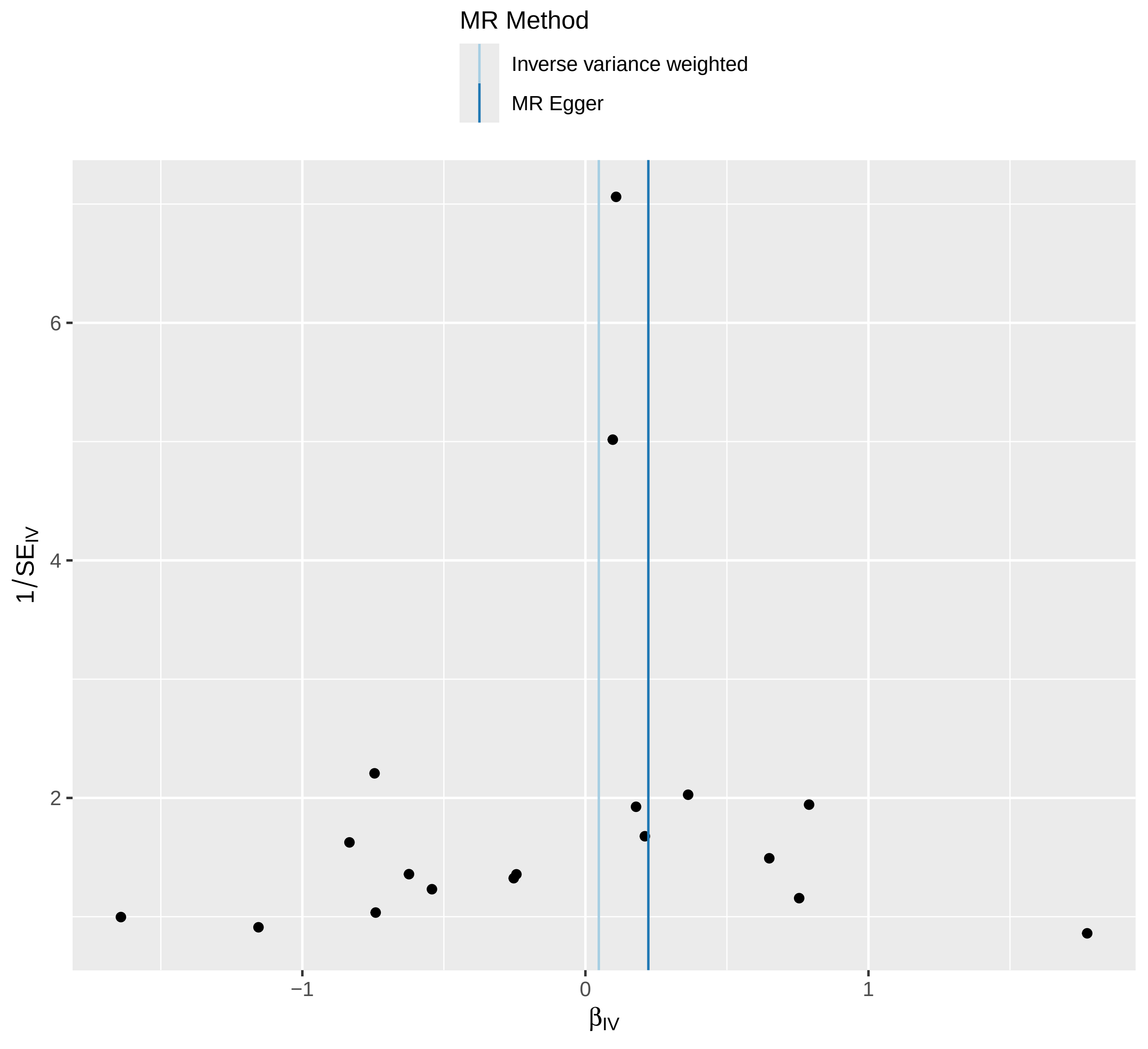

MR Method
Inverse variance weighted
MR Egger
6
4
2
1/SE_IV
−1
0
1
β_IV

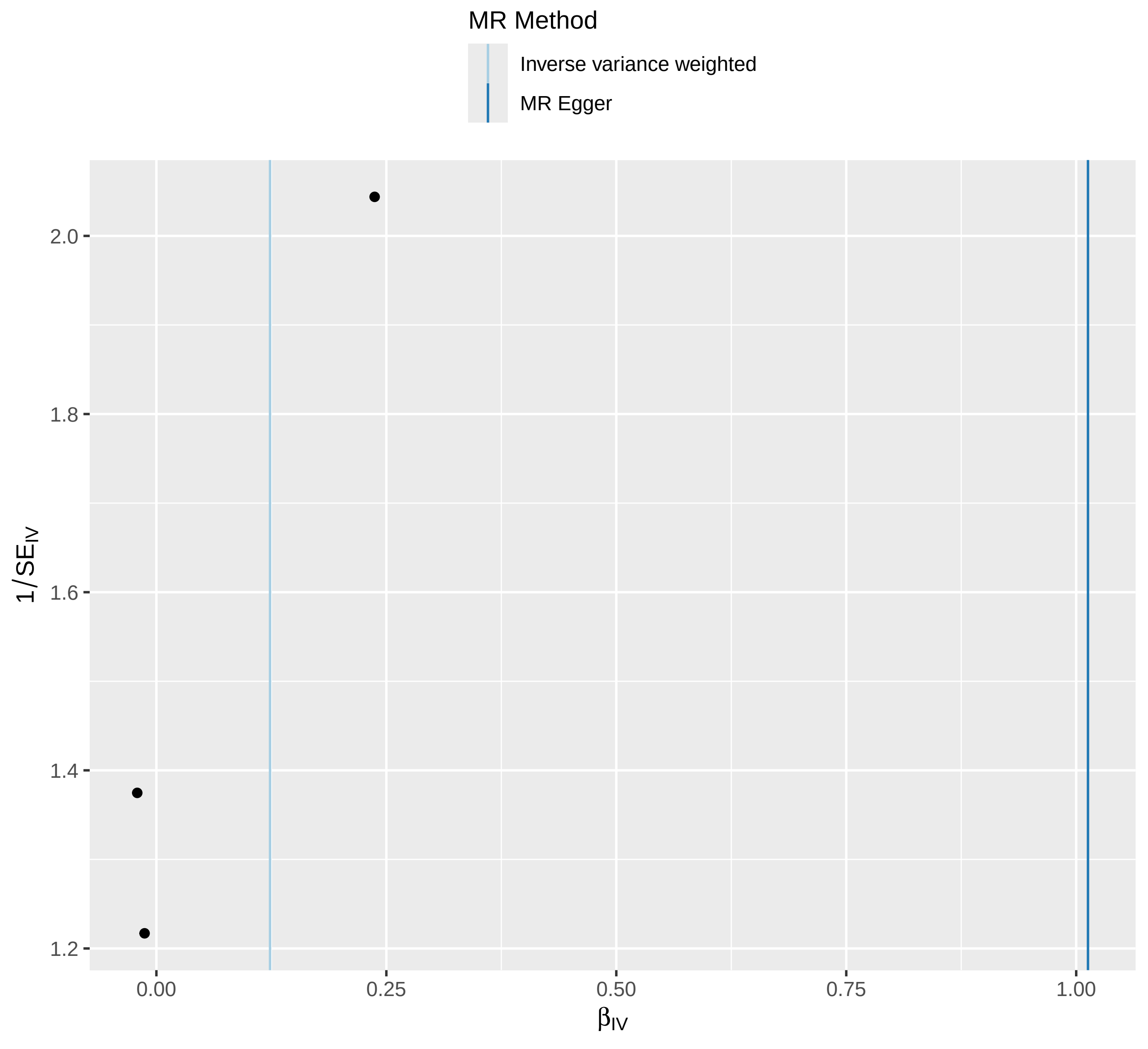

MR Method
Inverse variance weighted
MR Egger
2.0
1.8
1.6
1.4
1.2
1/SE_IV
0.00
0.25
0.50
0.75
1.00
β_IV

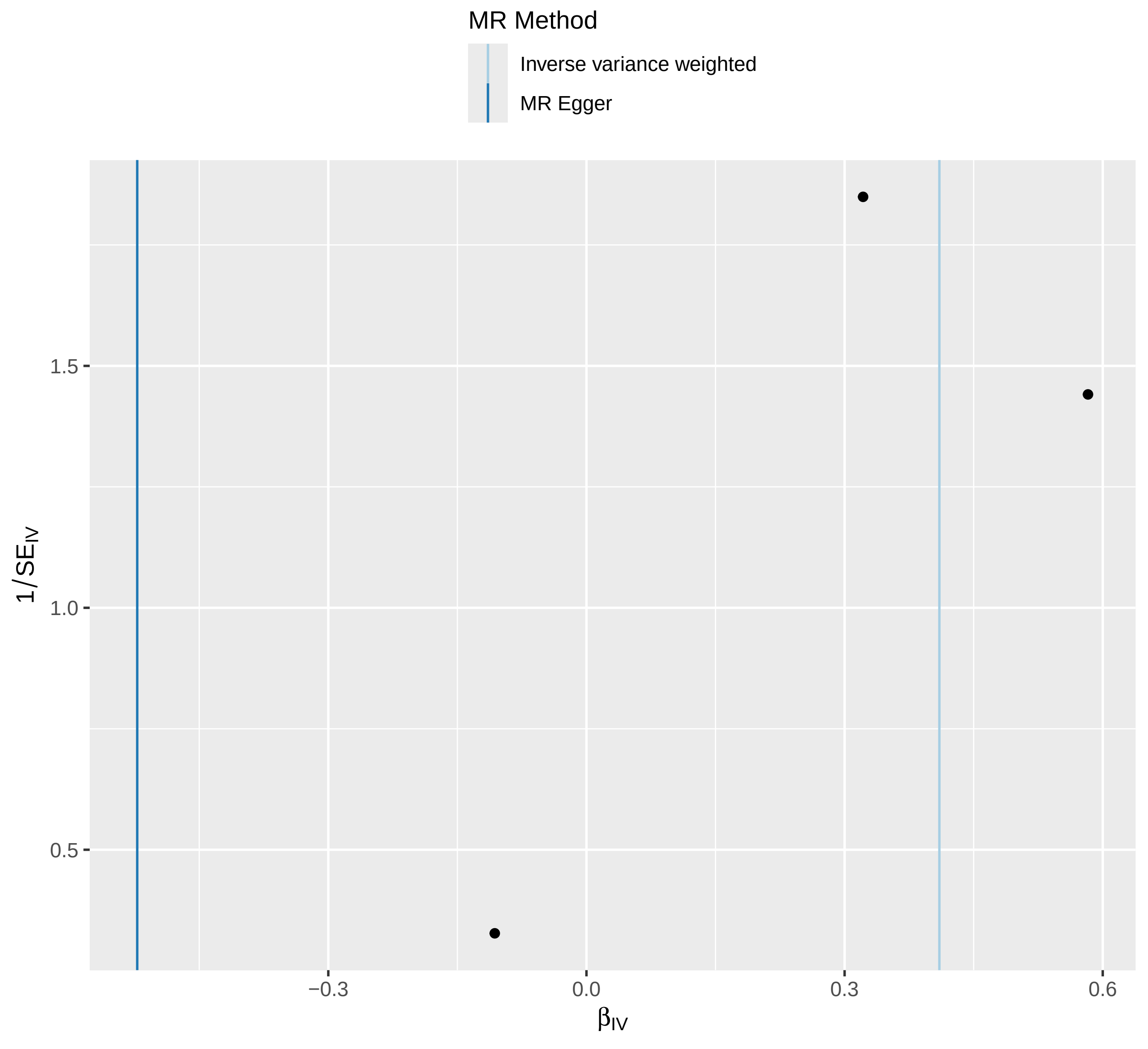

MR Method
Inverse variance weighted
MR Egger
1.5
1.0
0.5
$1/SE_{IV}$
−0.3
0.0
0.3
0.6
β_{IV}

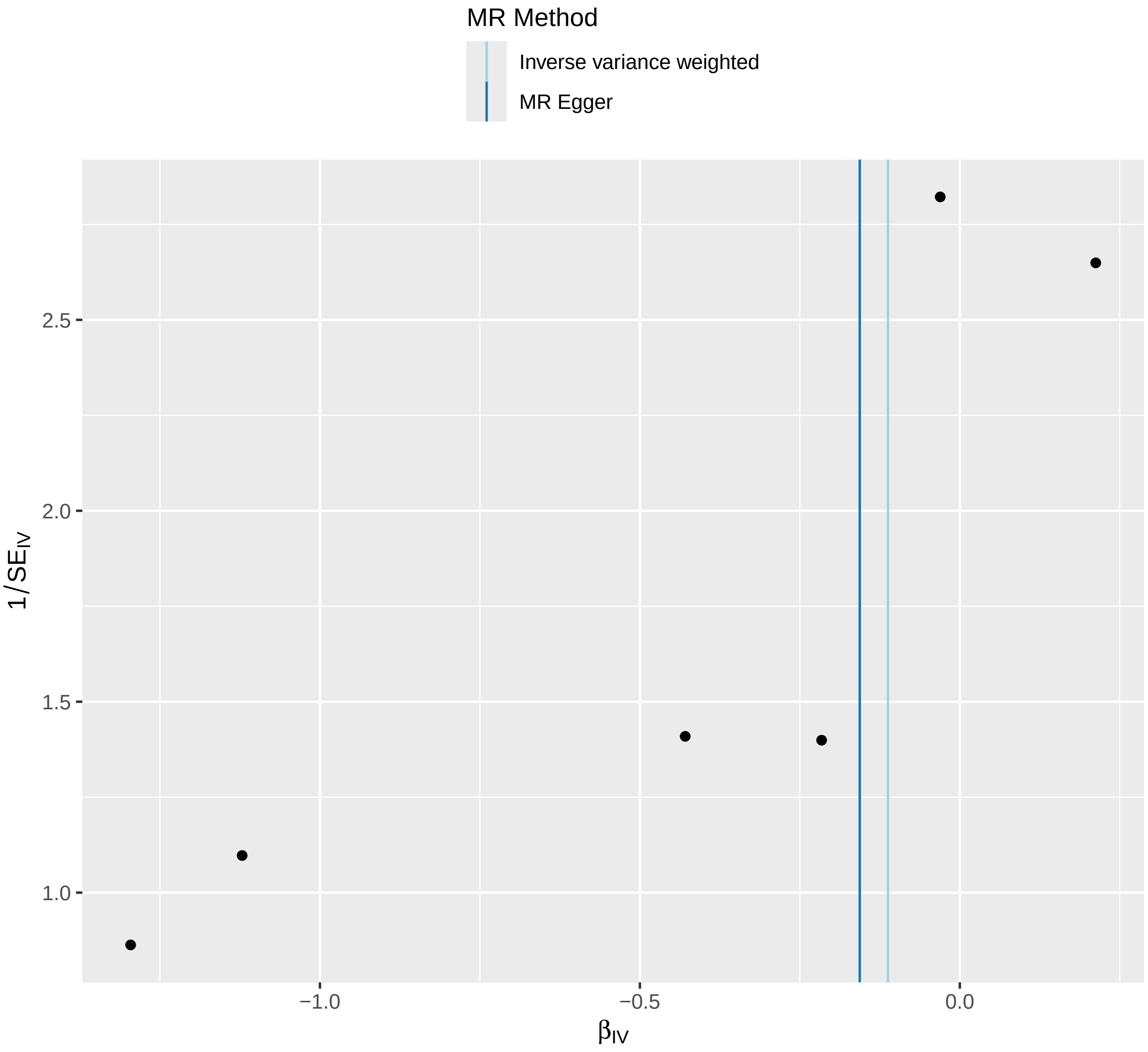
MR Method
Inverse variance weighted
MR Egger
2.5
2.0
1.5
1.0
−1.0
−0.5
0.0
$1/SE_{IV}$
β_{IV}

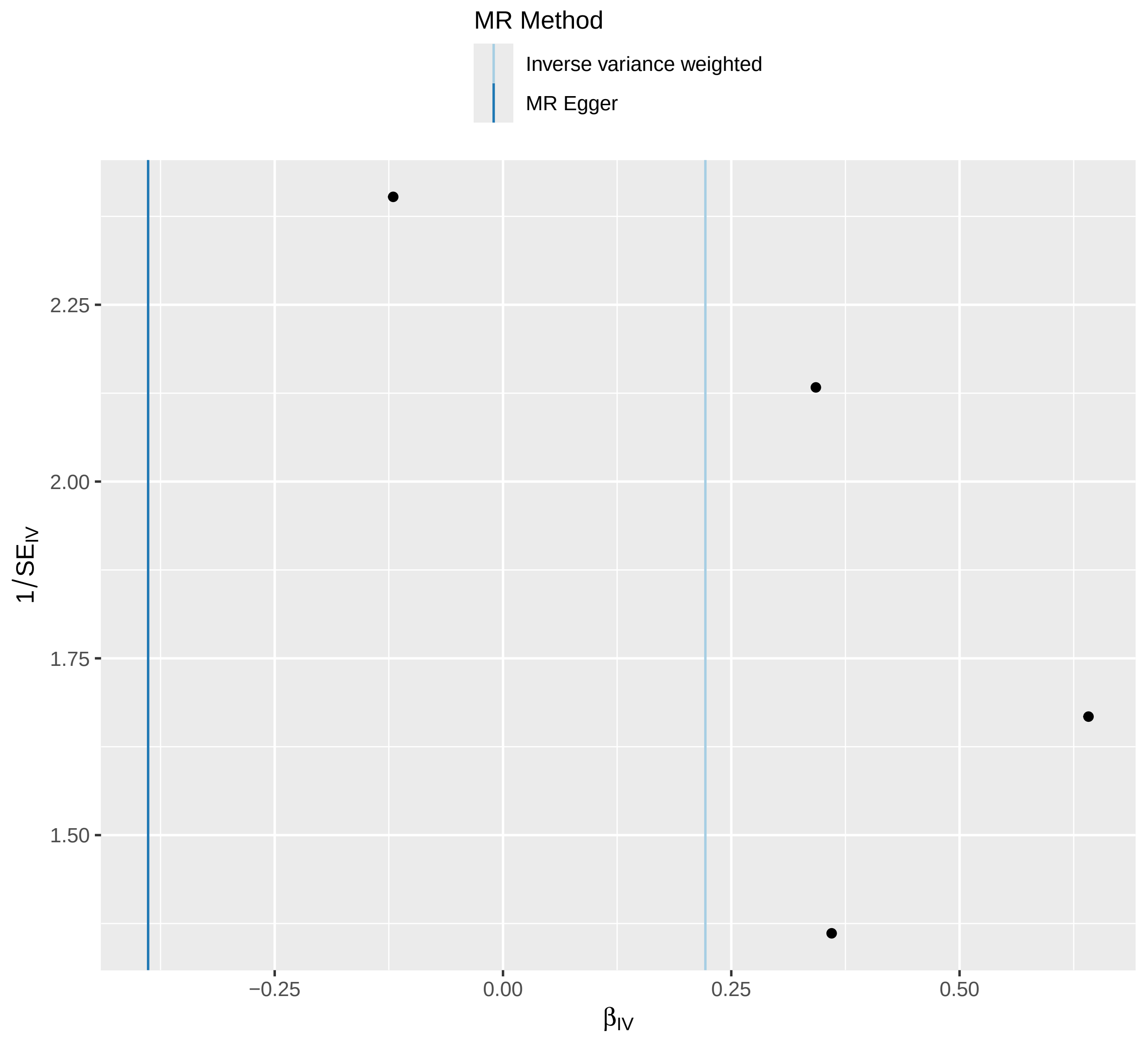
MR Method
Inverse variance weighted
MR Egger
2.25
2.00
1.75
1.50
1/SE$_{IV}$
−0.25
0.00
0.25
0.50
β$_{IV}$

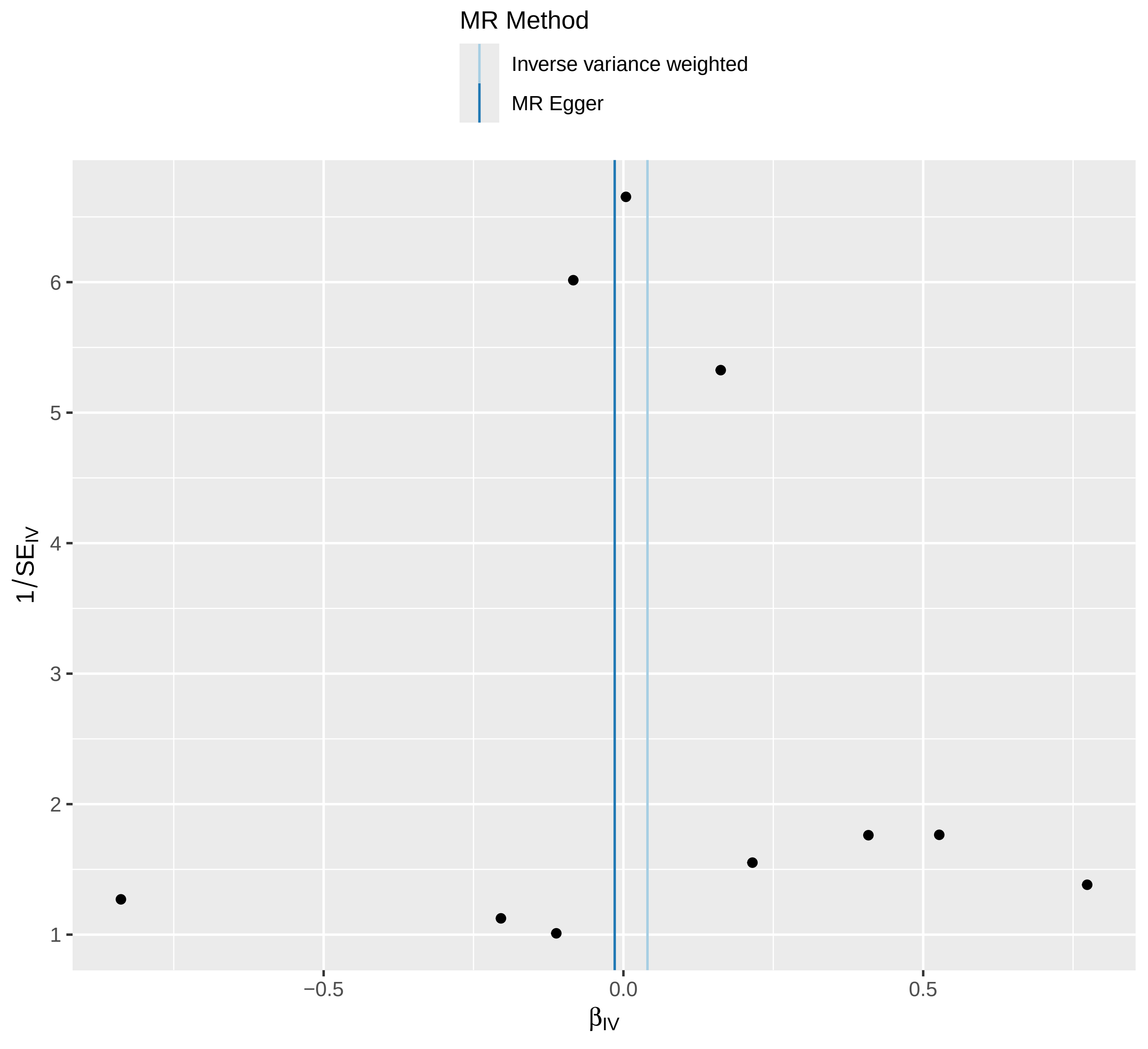
MR Method
Inverse variance weighted
MR Egger
1/SE$_{IV}$
6
5
4
3
2
1
−0.5
0.0
0.5
β$_{IV}$

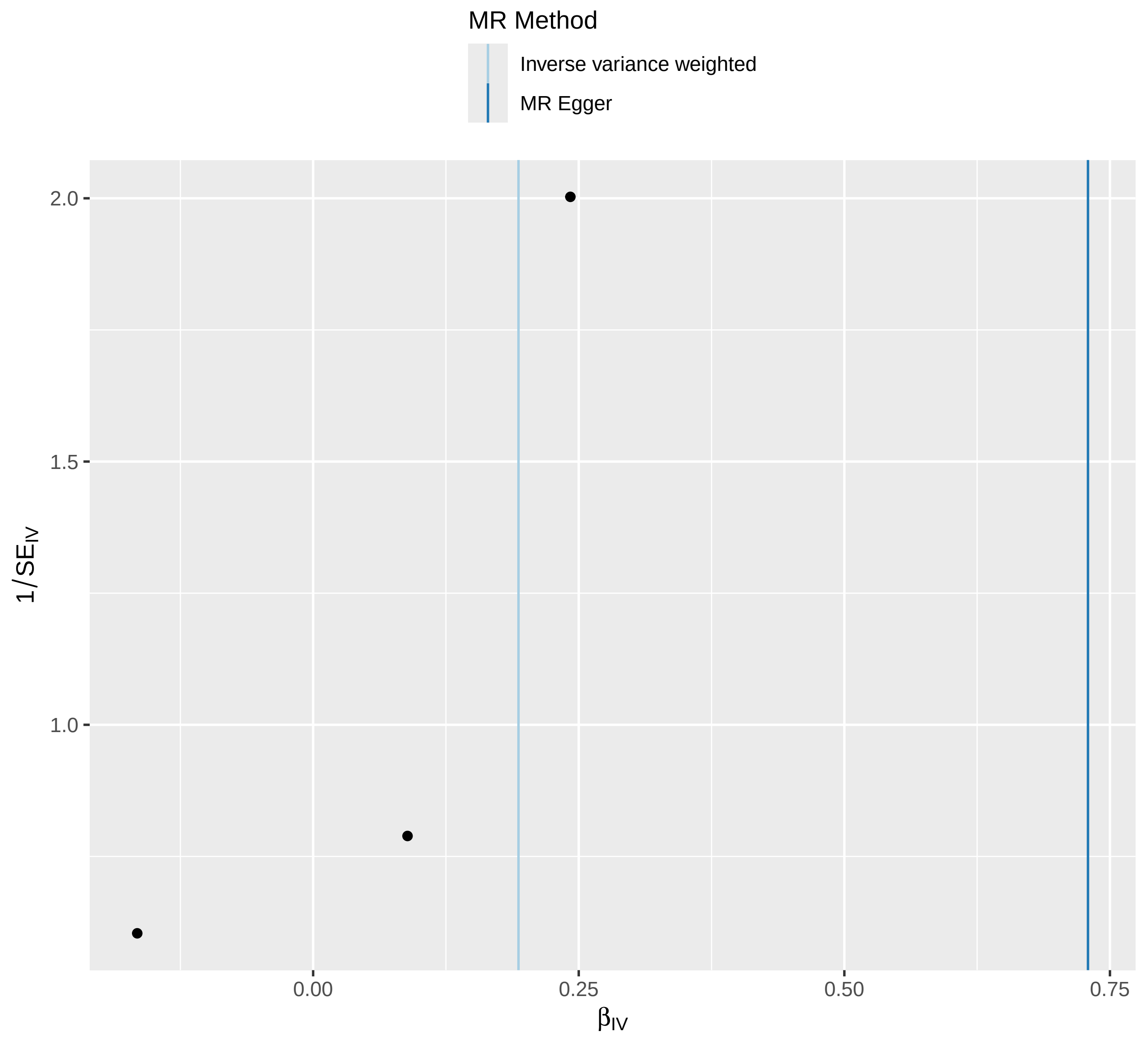
MR Method
Inverse variance weighted
MR Egger
2.0
1.5
1.0
0.00
0.25
0.50
0.75
β_{IV}
$1/SE_{IV}$

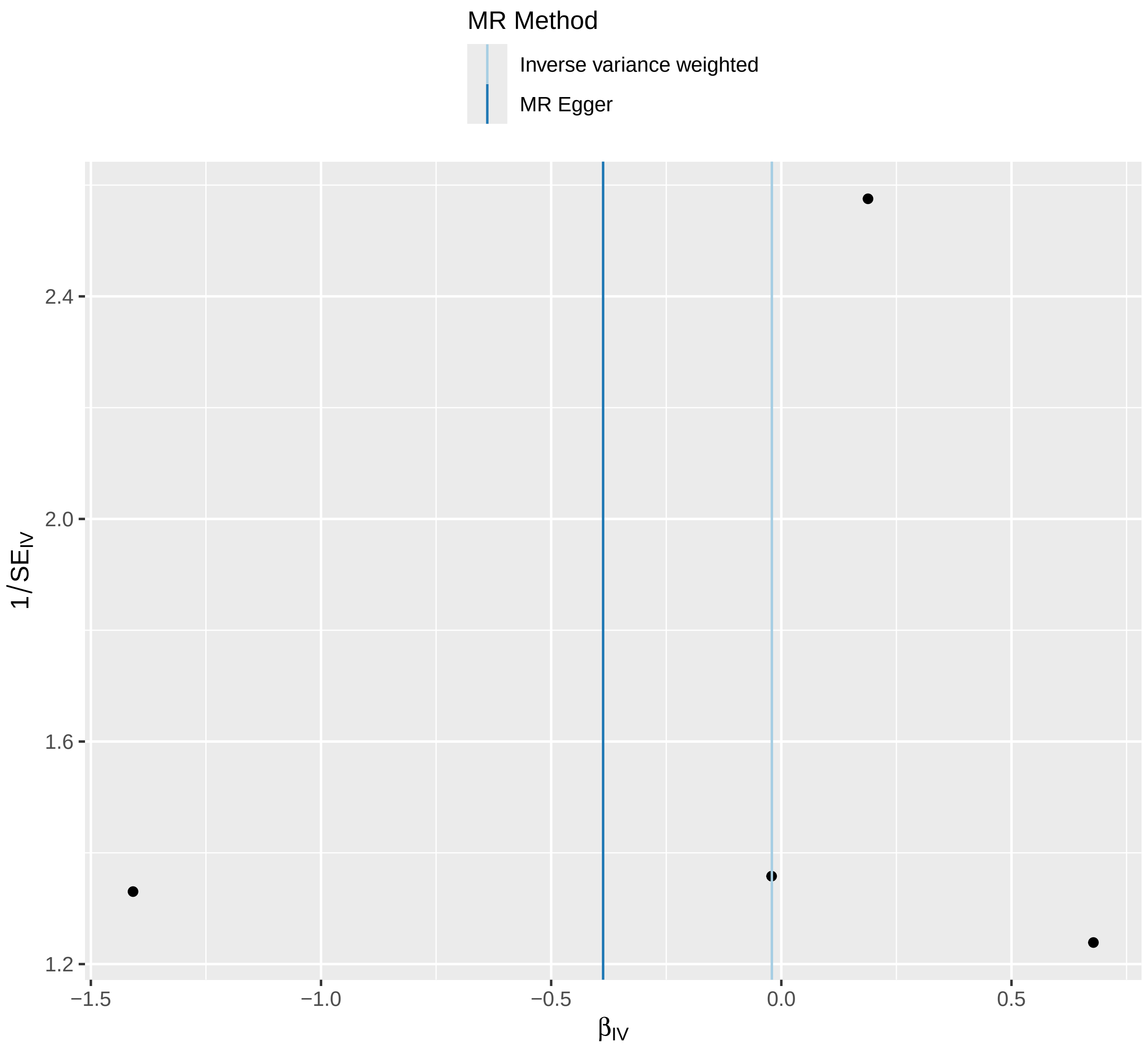
MR Method
Inverse variance weighted
MR Egger
2.4
2.0
1.6
1.2
1/SE_IV
−1.5
−1.0
−0.5
0.0
0.5
β_IV

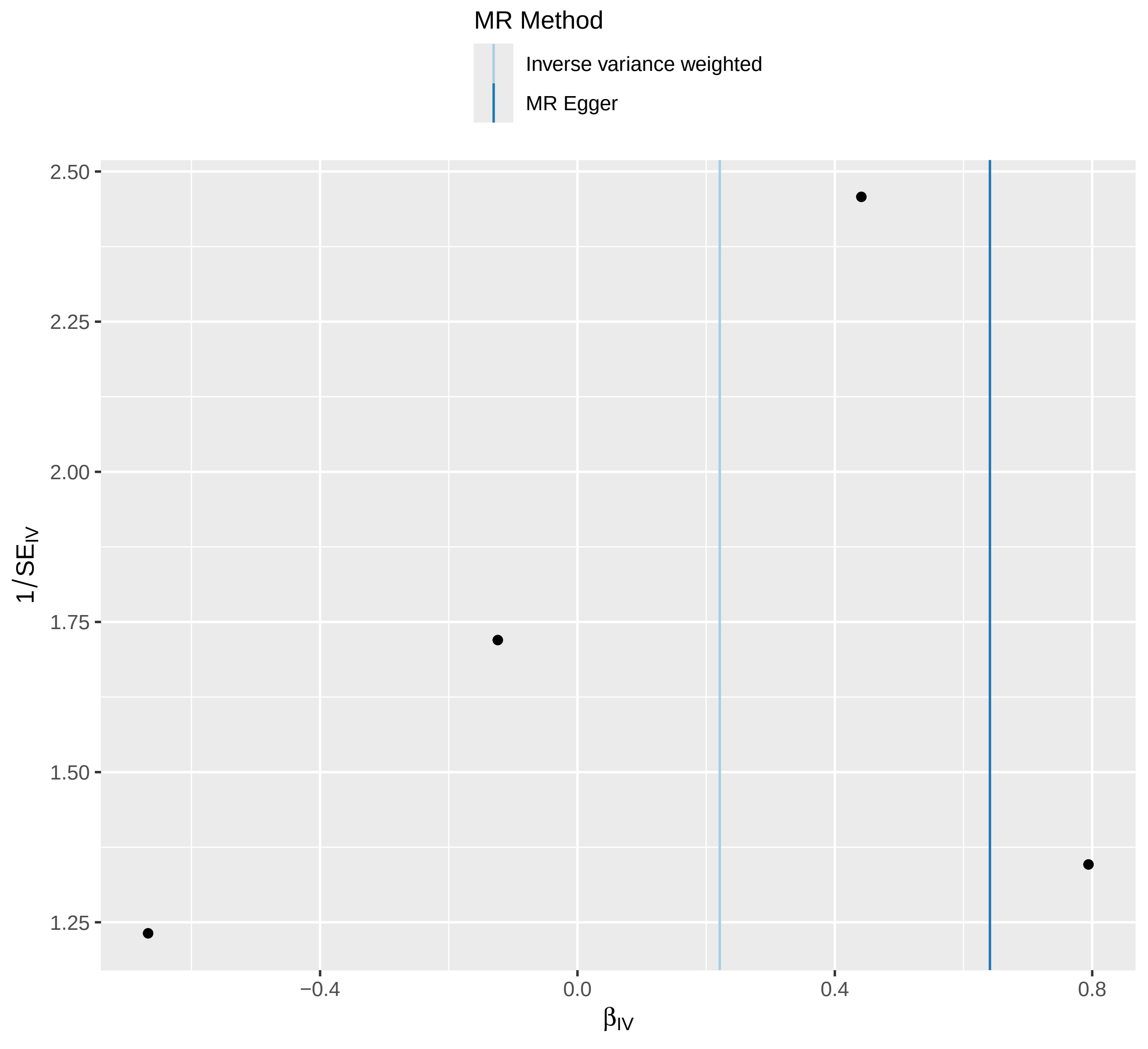
MR Method
Inverse variance weighted
MR Egger
2.50
2.25
2.00
1.75
1.50
1.25
1/SE_IV
−0.4
0.0
0.4
0.8
β_IV

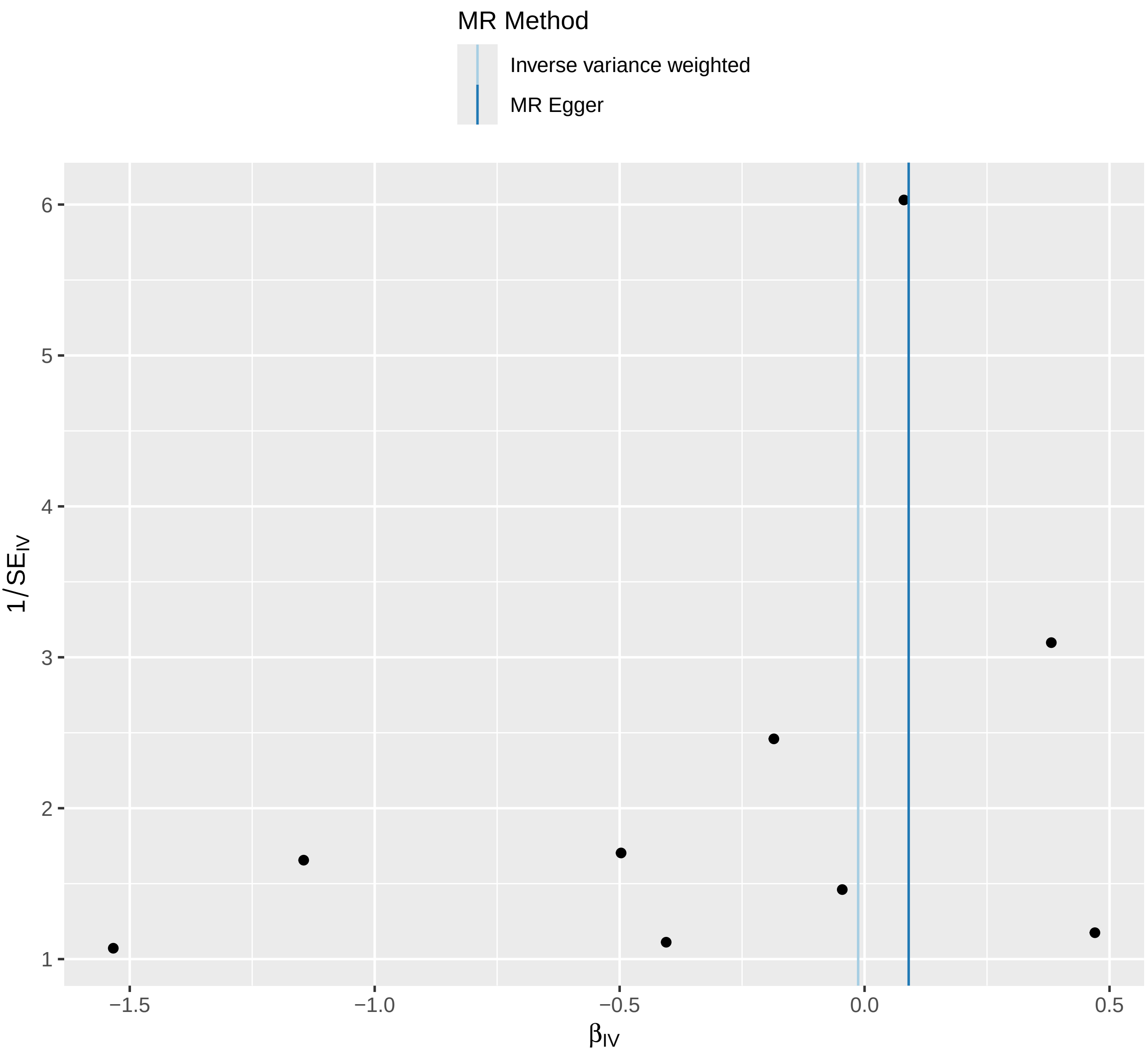

MR Method
Inverse variance weighted
MR Egger
6
5
4
3
2
1
1/SE_IV
−1.5
−1.0
−0.5
0.0
0.5
β_IV

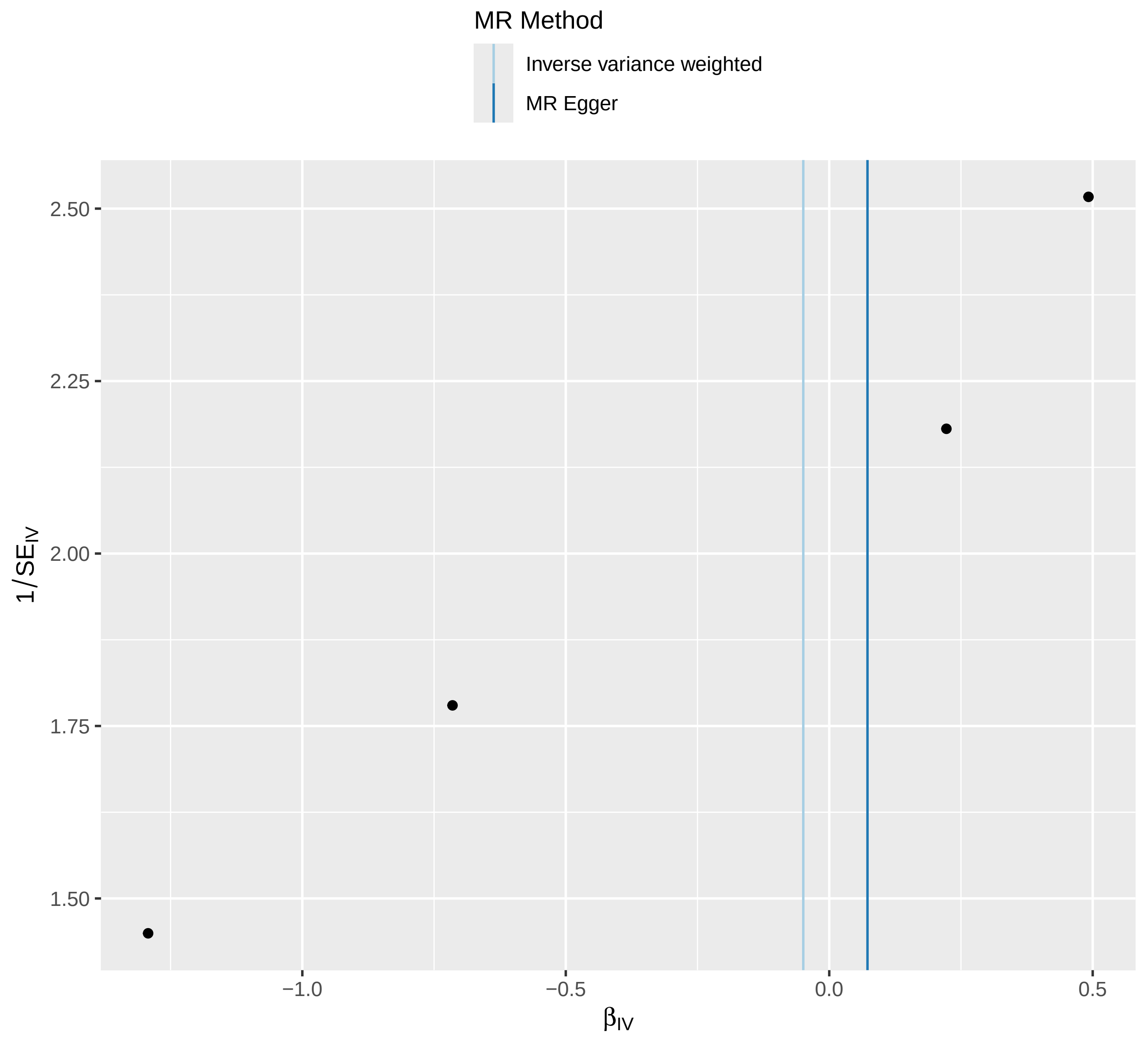

MR Method
Inverse variance weighted
MR Egger
2.50
2.25
2.00
1.75
1.50
1/SE$_{IV}$
−1.0
−0.5
0.0
0.5
β_{IV}

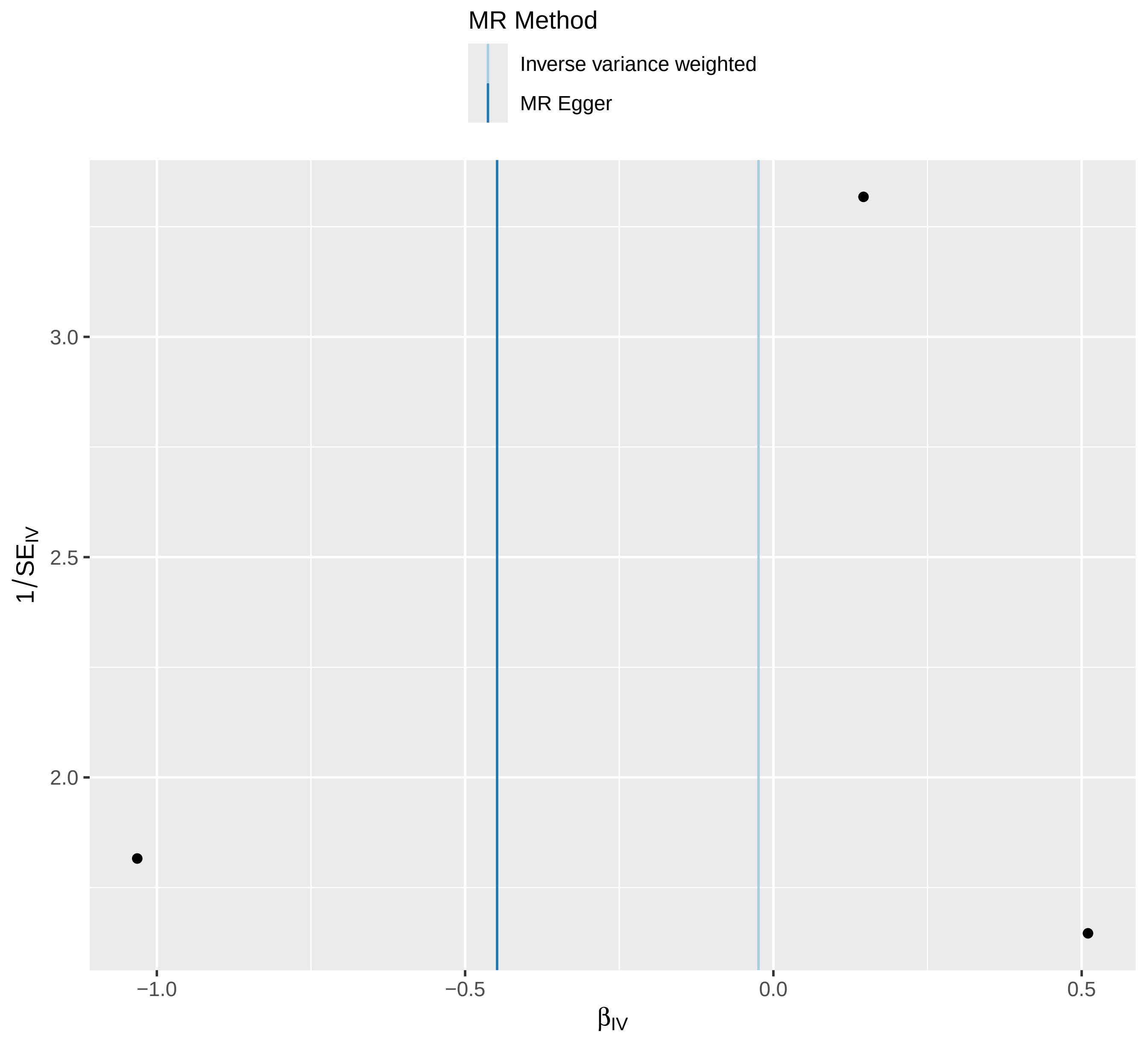
MR Method
Inverse variance weighted
MR Egger
3.0
2.5
2.0
1/SE_IV
−1.0
−0.5
0.0
0.5
β_IV

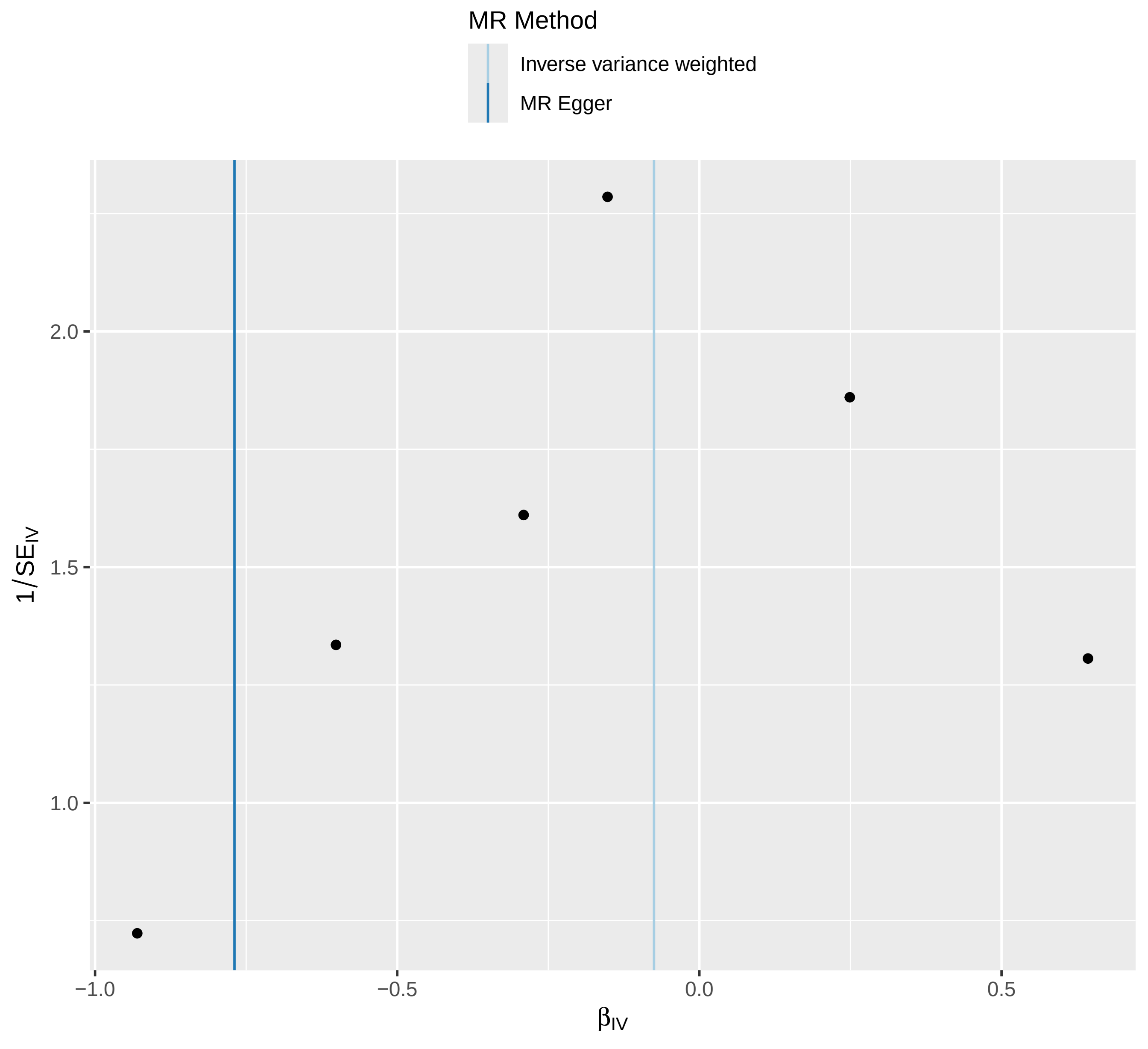
MR Method
Inverse variance weighted
MR Egger
2.0
1.5
1.0
−1.0
−0.5
0.0
0.5
β_{IV}
$1/SE_{IV}$

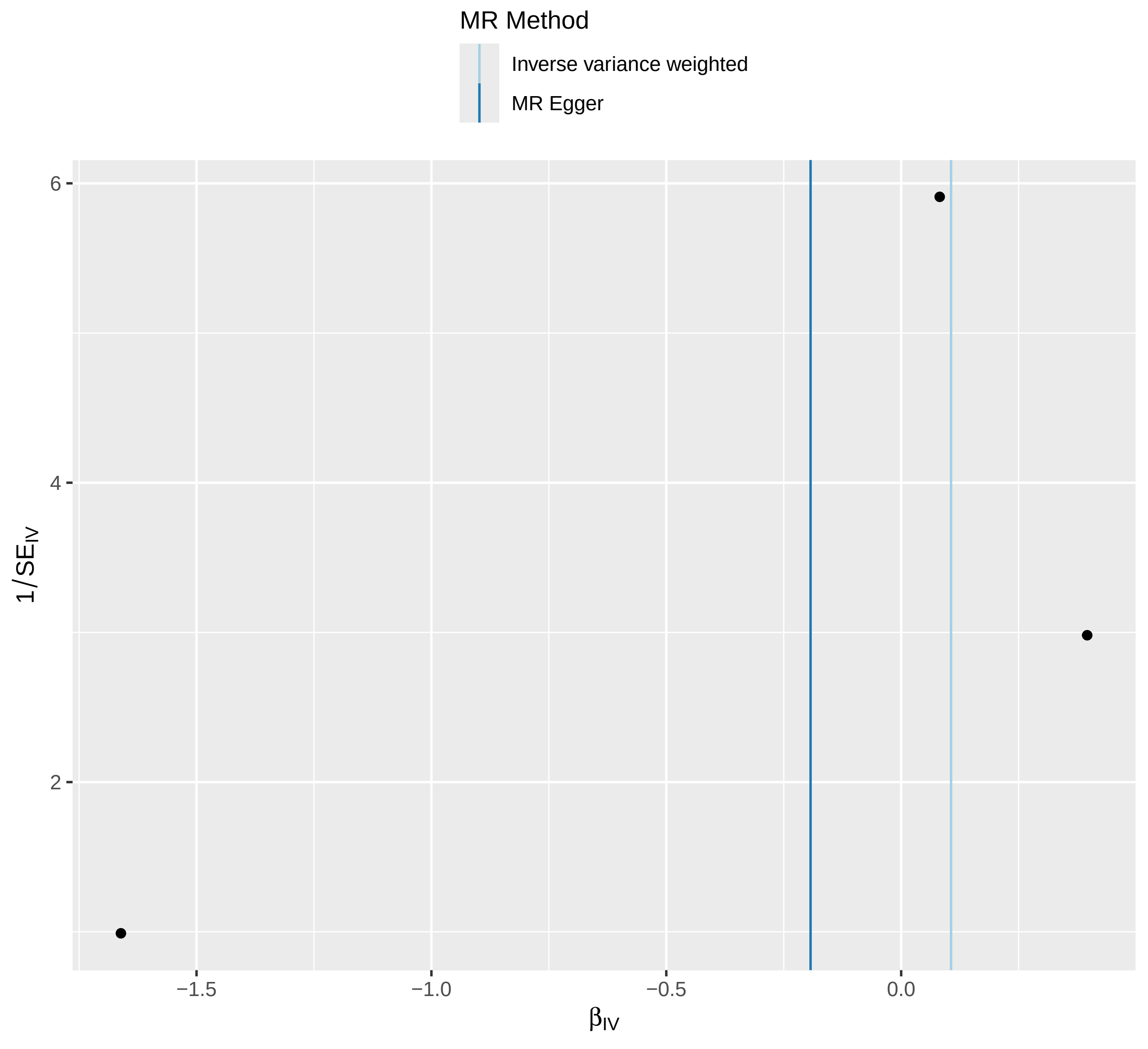

MR Method
Inverse variance weighted
MR Egger
6
4
2
1/SE_IV
−1.5
−1.0
−0.5
0.0
β_IV

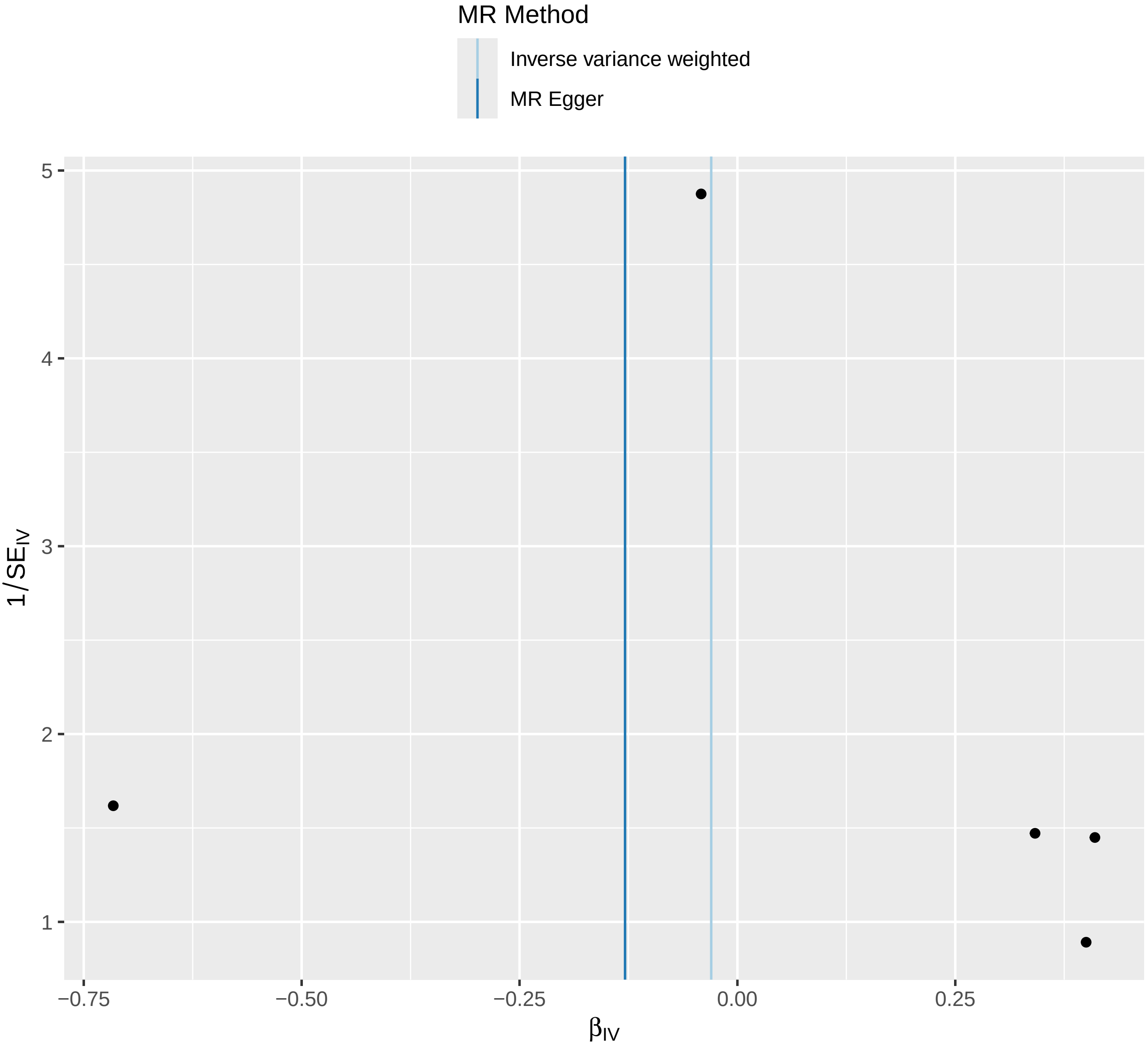

MR Method
Inverse variance weighted
MR Egger
1/SE_{IV}
5
4
3
2
1
−0.75
−0.50
−0.25
0.00
0.25
β_{IV}

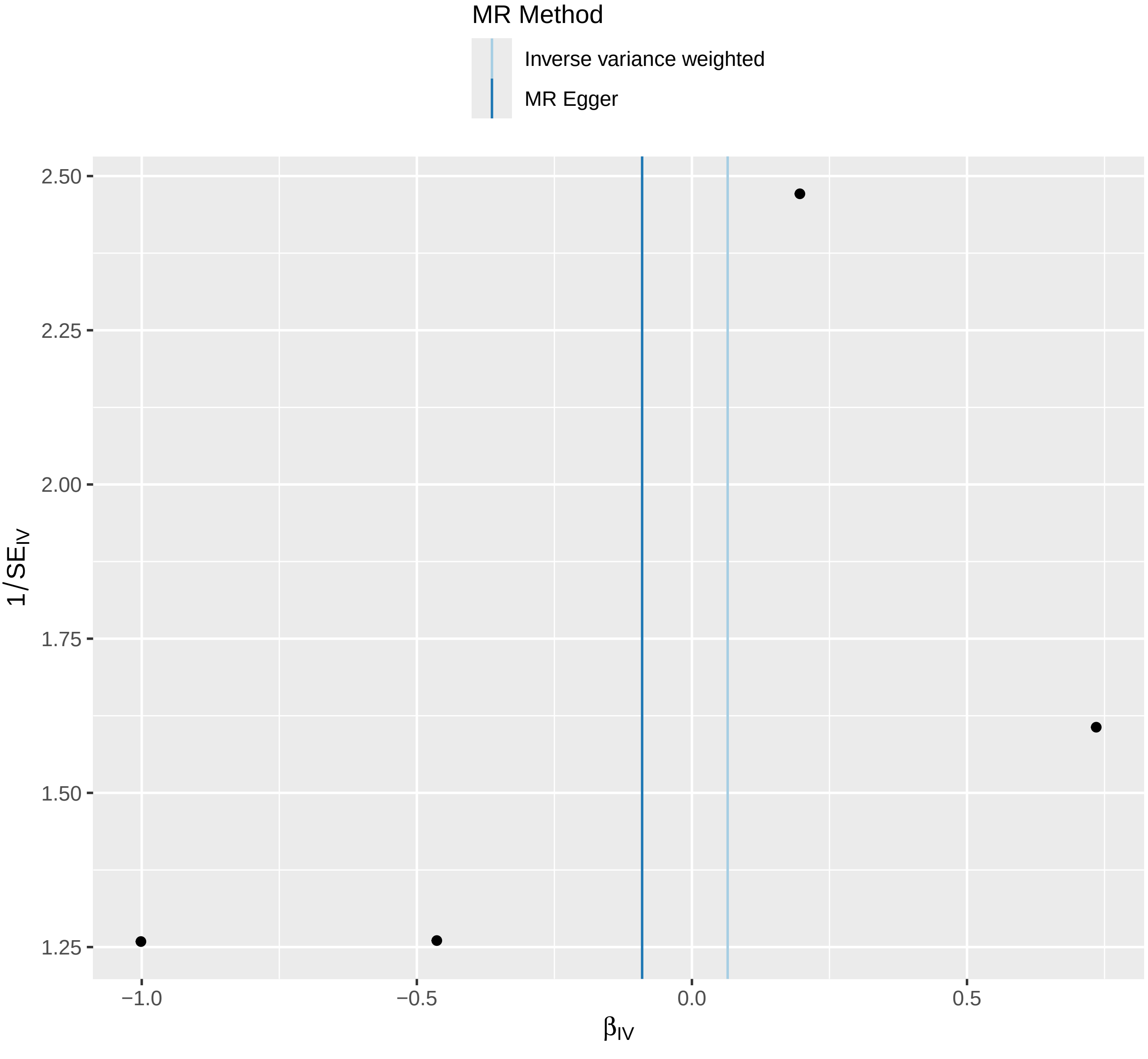
MR Method
Inverse variance weighted
MR Egger
2.50
2.25
2.00
1.75
1.50
1.25
1/SE$_{IV}$
−1.0
−0.5
0.0
0.5
β_{IV}

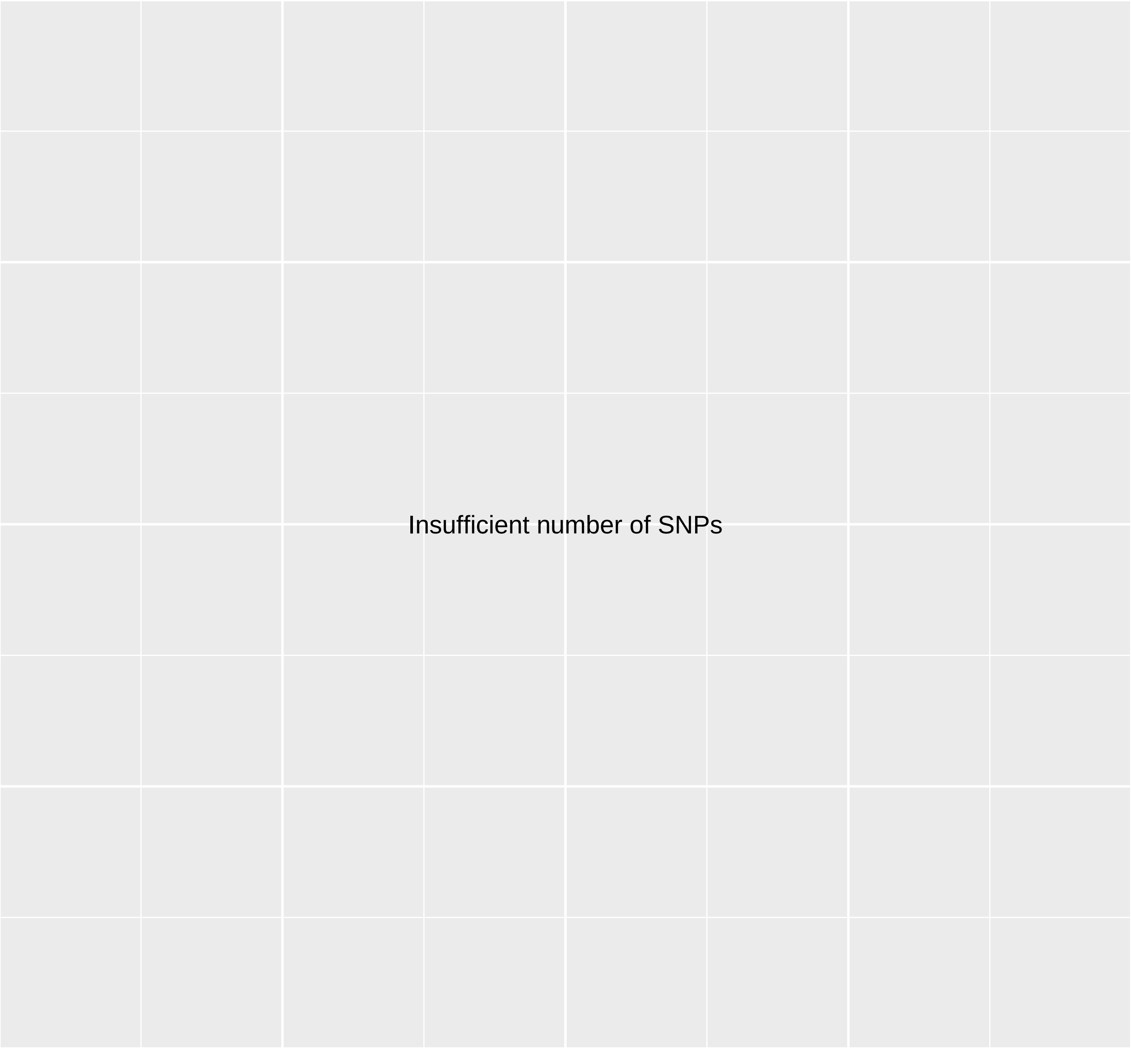
Insufficient number of SNPs

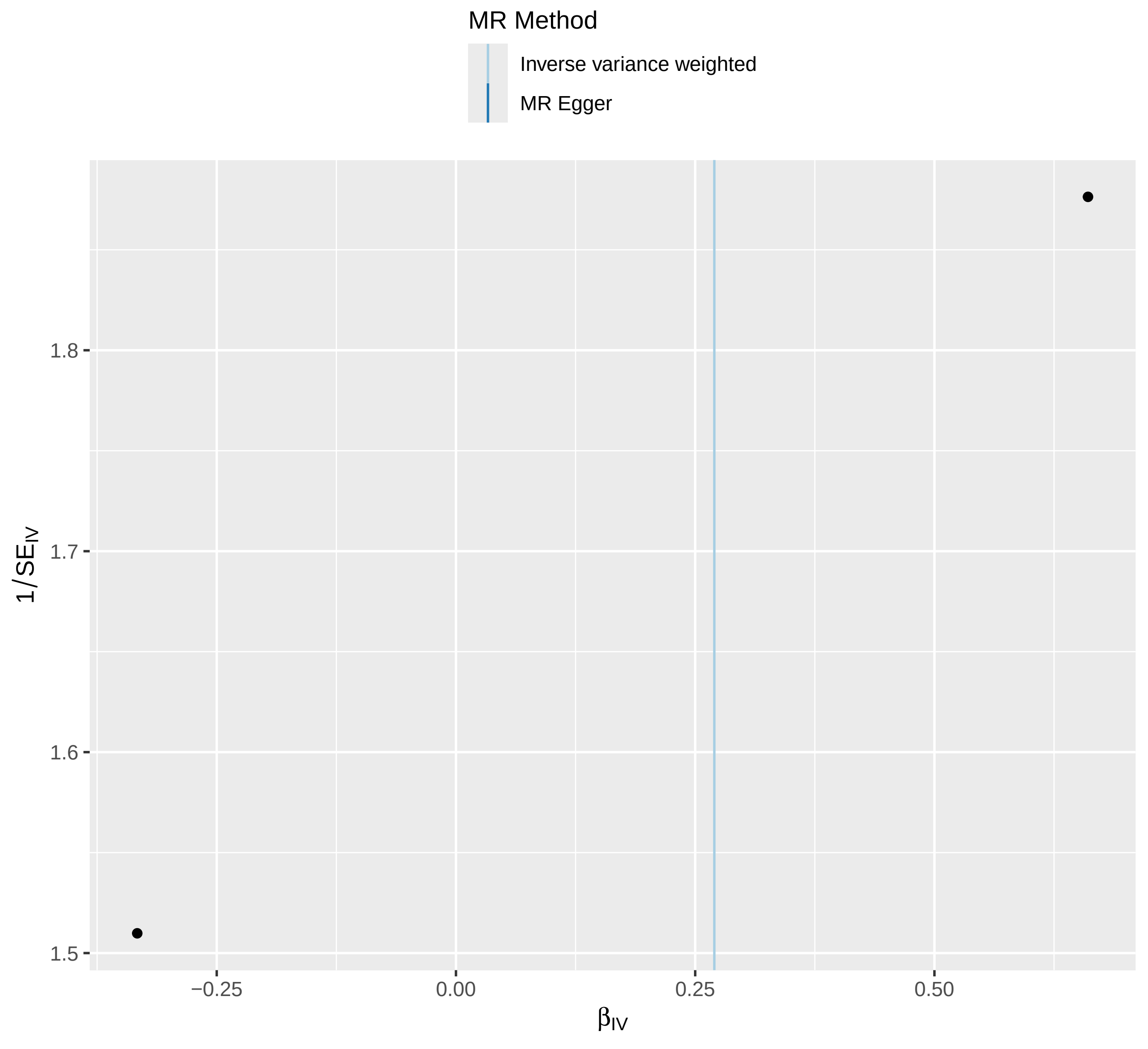
MR Method
Inverse variance weighted
MR Egger
1.8
1.7
1.6
1.5
1/SE_IV
−0.25
0.00
0.25
0.50
β_IV

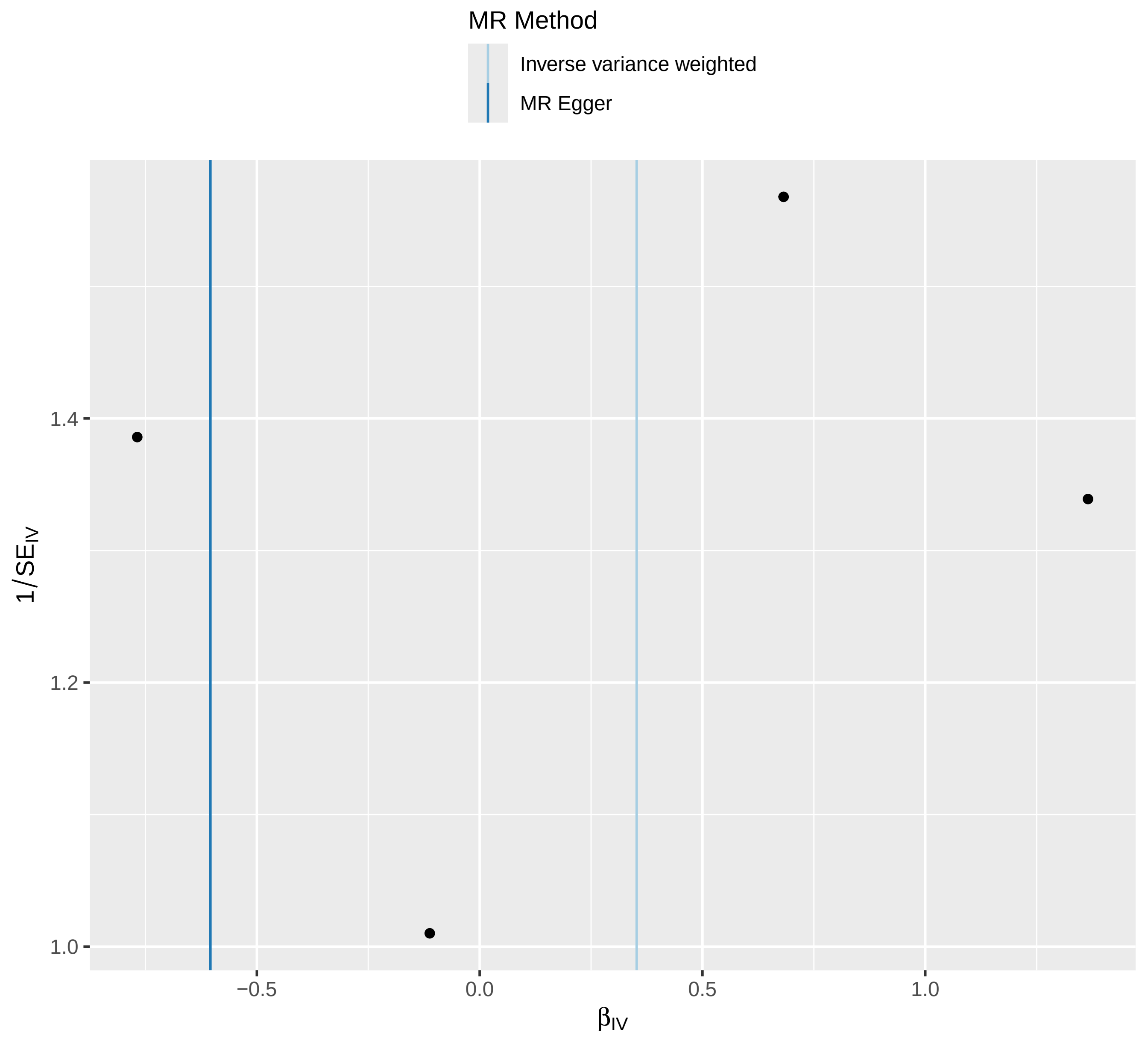
MR Method
Inverse variance weighted
MR Egger
1.4
1.2
1.0
1/SE$_{IV}$
−0.5
0.0
0.5
1.0
β$_{IV}$

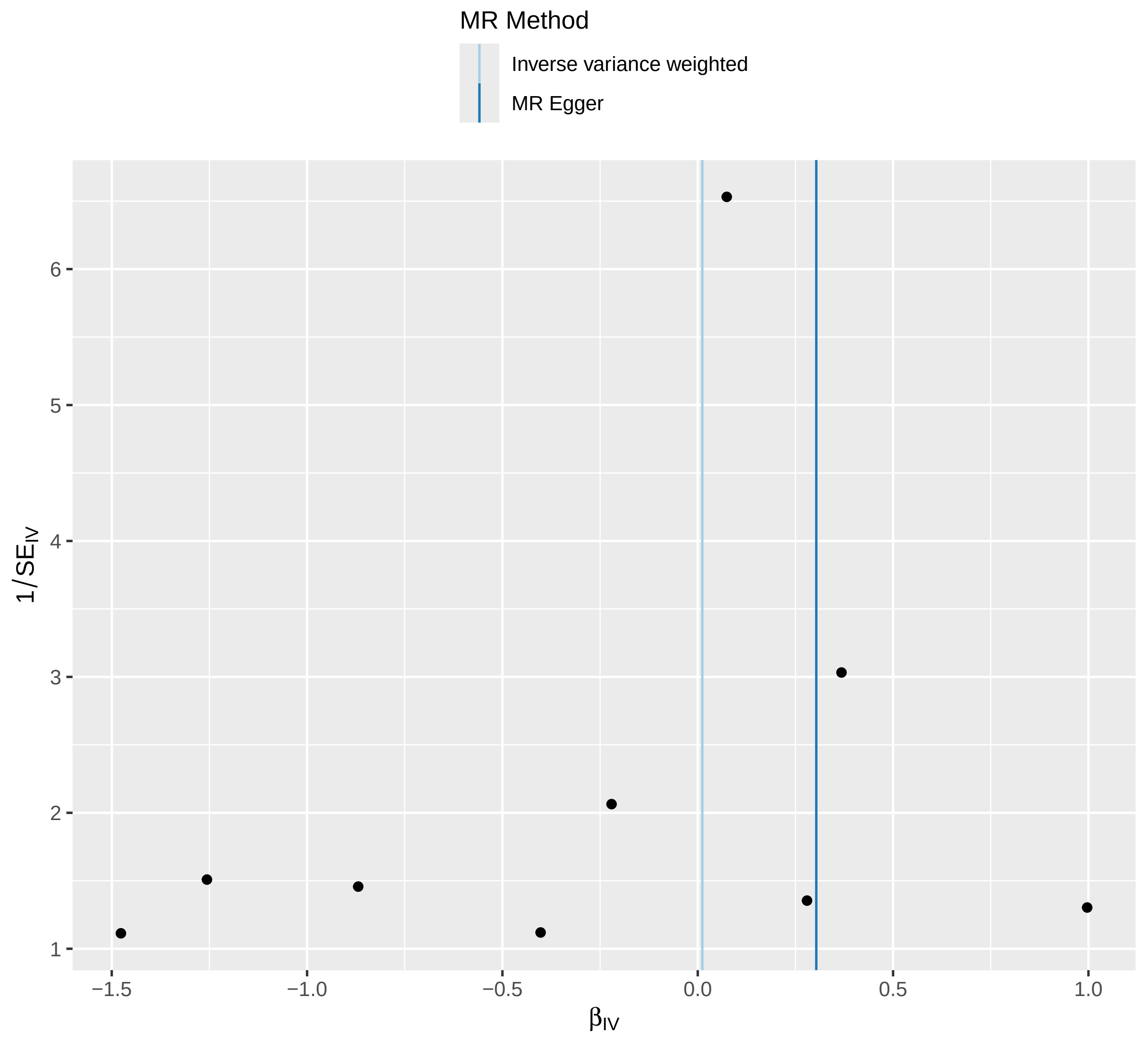

MR Method
Inverse variance weighted
MR Egger
1/SE_IV
6
5
4
3
2
1
−1.5
−1.0
−0.5
0.0
0.5
1.0
β_IV

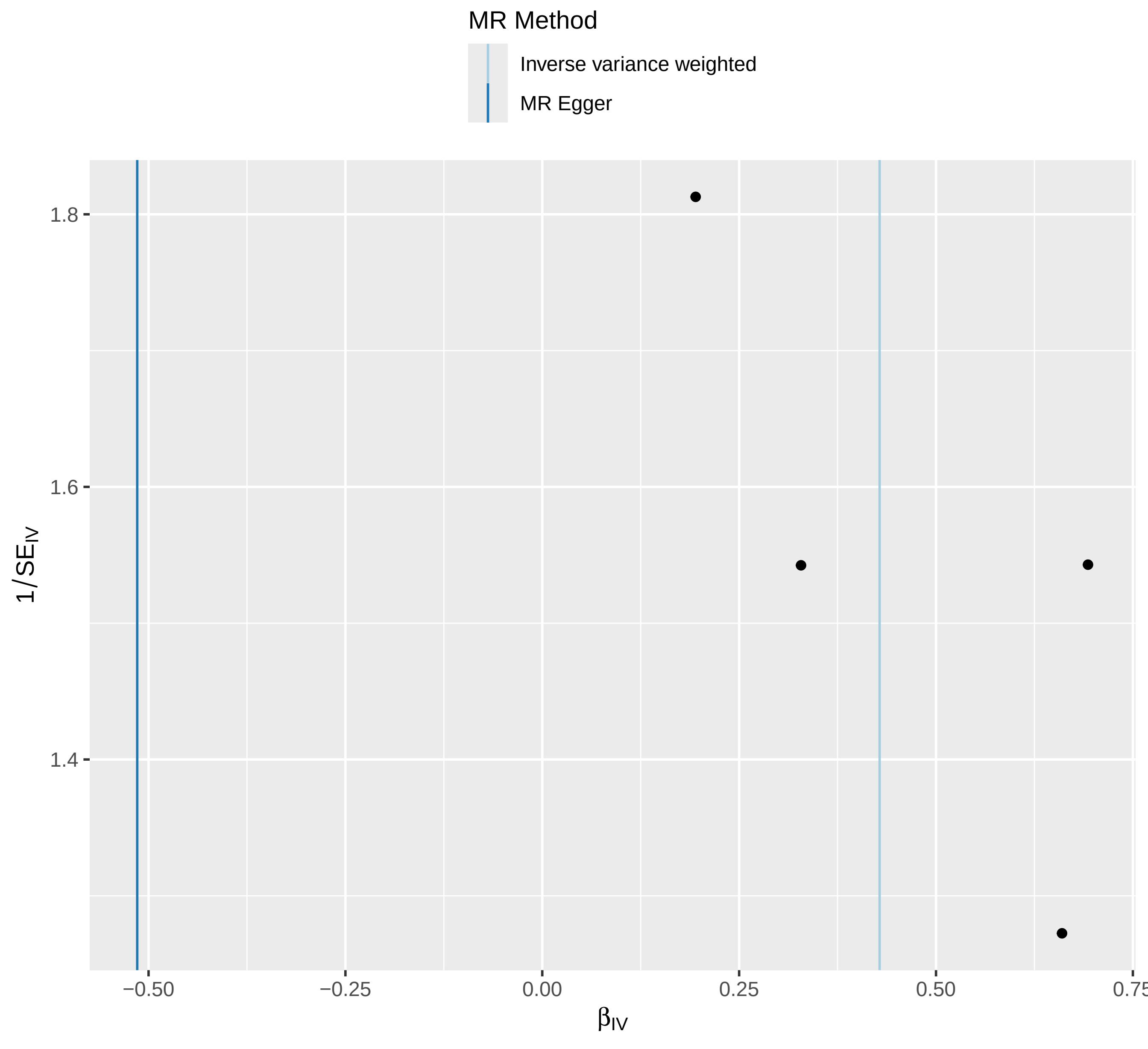

MR Method
Inverse variance weighted
MR Egger
1.8
1.6
1.4
1/SE_IV
−0.50
−0.25
0.00
0.25
0.50
0.75
β_IV

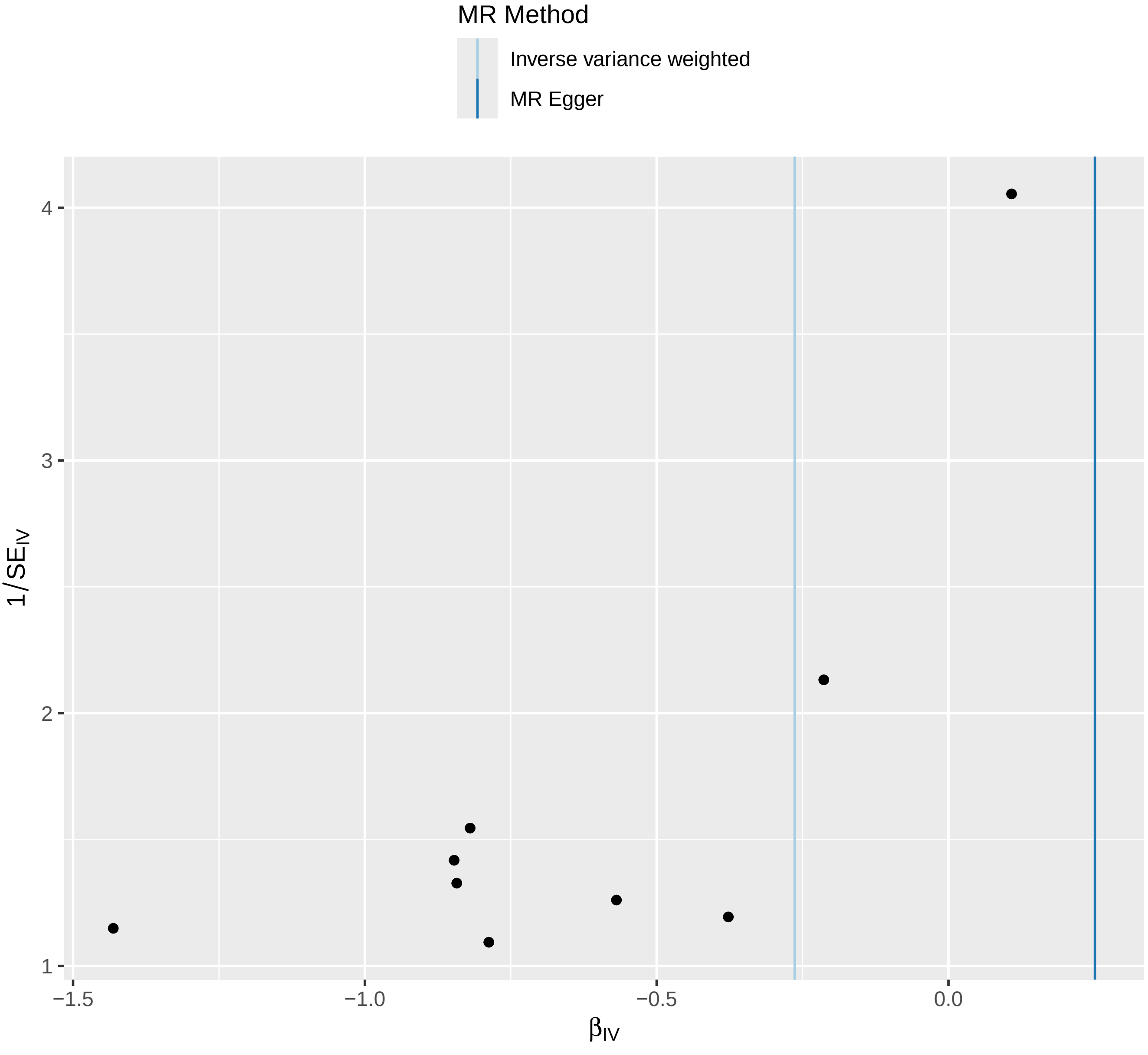

MR Method
Inverse variance weighted
MR Egger
4
3
2
1
1/SE$_{IV}$
−1.5
−1.0
−0.5
0.0
β$_{IV}$

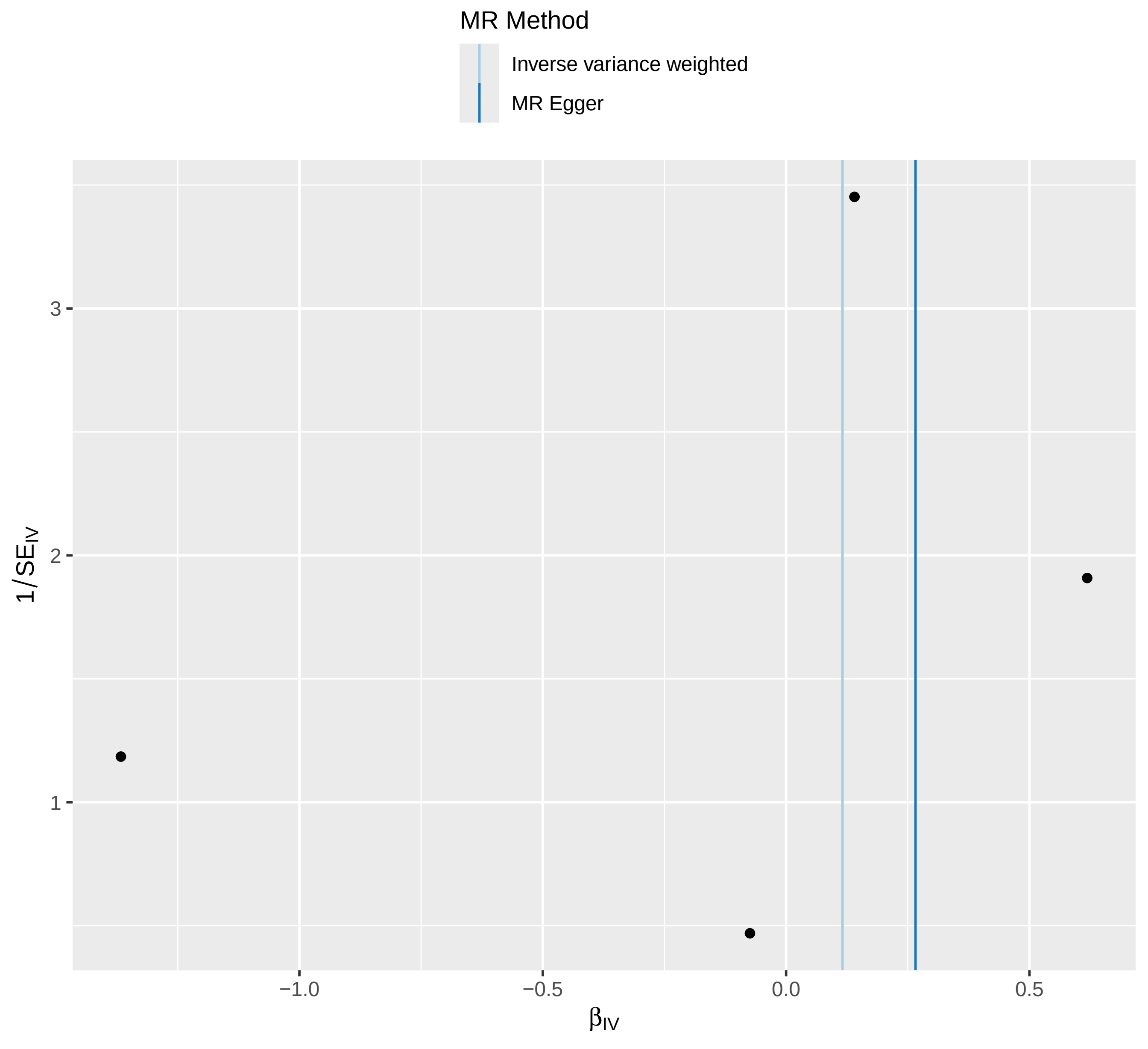
MR Method
Inverse variance weighted
MR Egger
3
2
1
1/SE_IV
−1.0
−0.5
0.0
0.5
β_IV

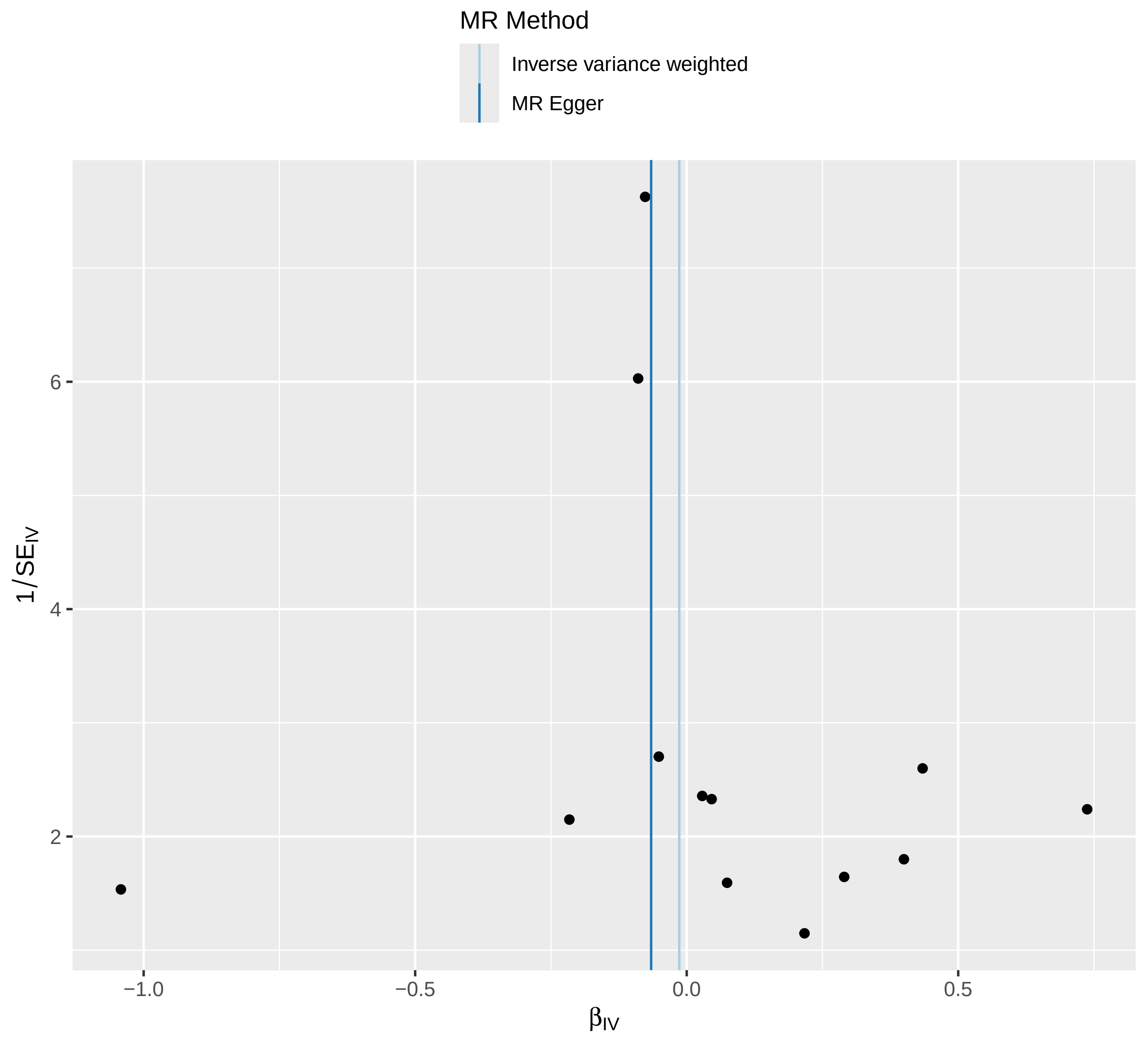

MR Method
Inverse variance weighted
MR Egger
1/SE_{IV}
6
4
2
−1.0
−0.5
0.0
0.5
β_{IV}

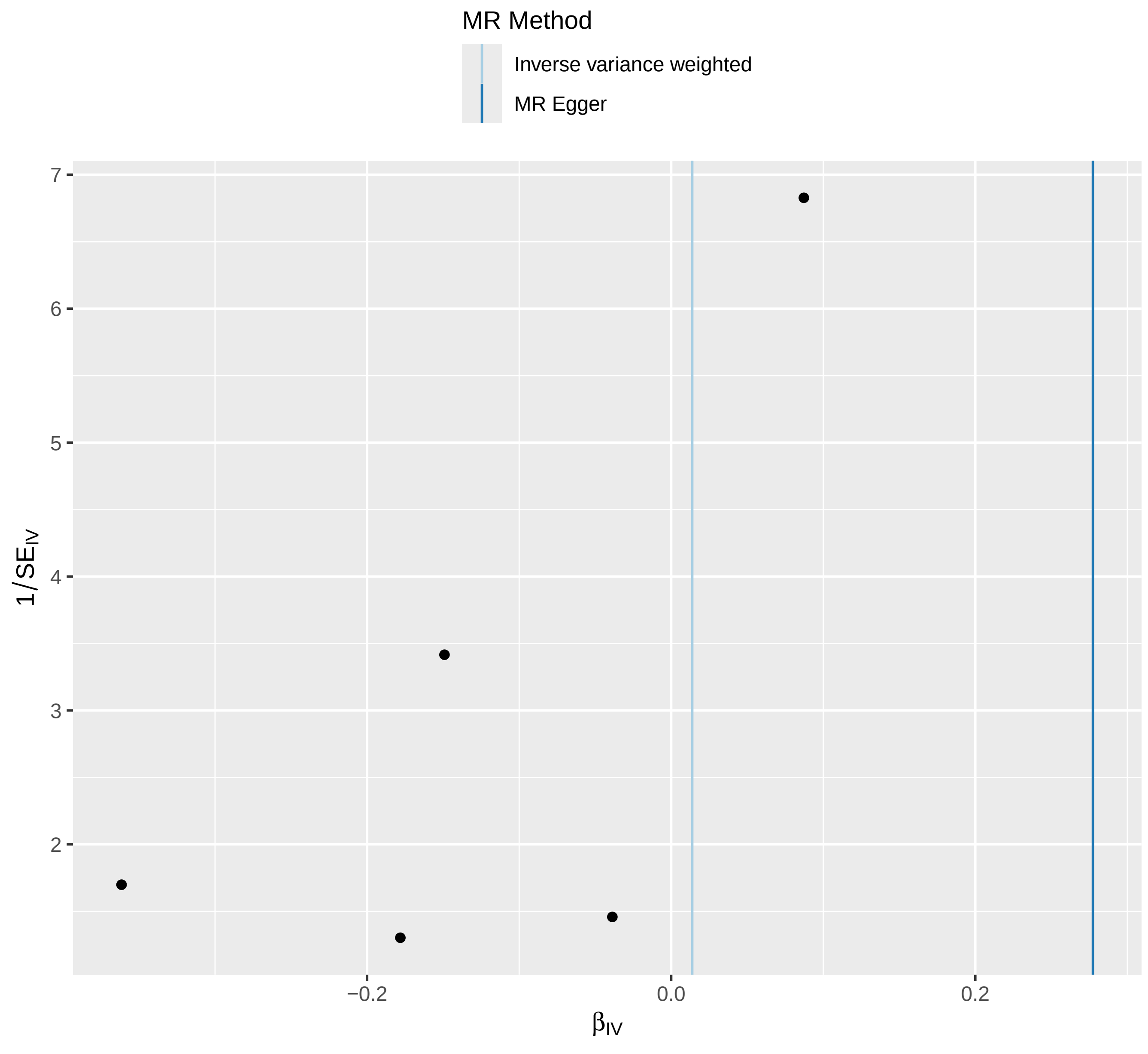
MR Method
Inverse variance weighted
MR Egger
1/SE_IV
β_IV
7
6
5
4
3
2
−0.2
0.0
0.2

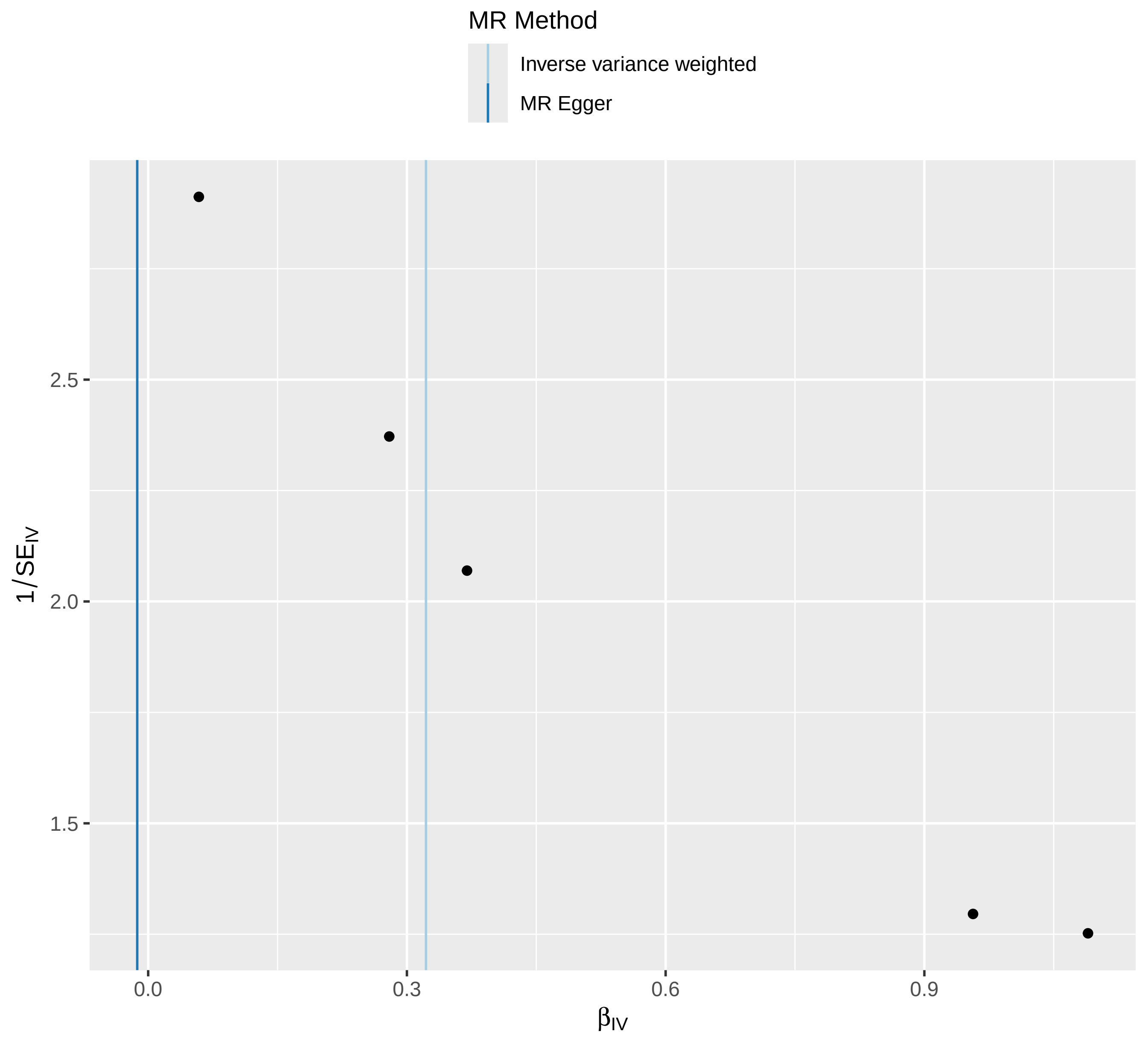
MR Method
Inverse variance weighted
MR Egger
2.5
2.0
1.5
1/SE_IV
0.0
0.3
0.6
0.9
β_IV

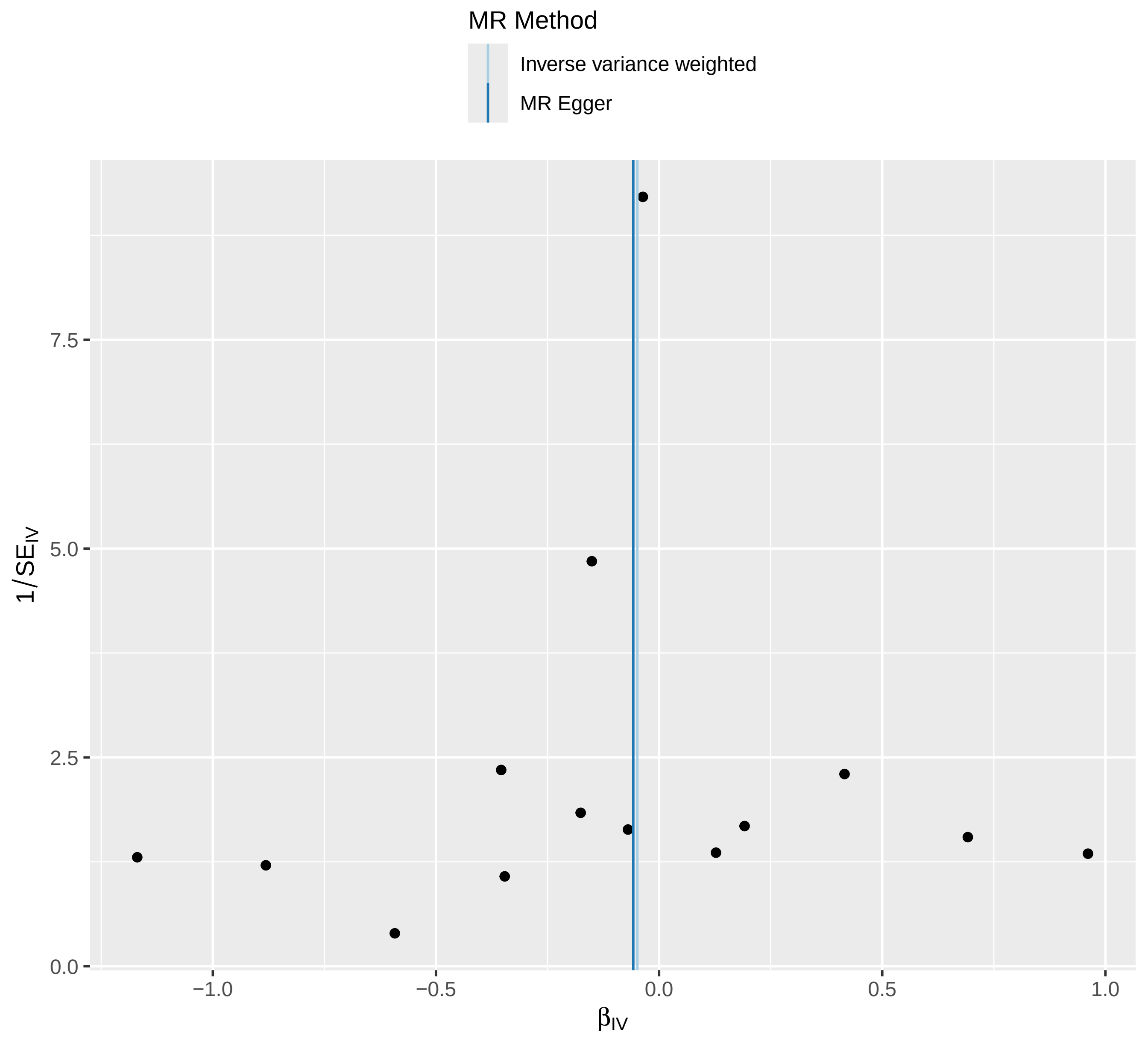
MR Method
Inverse variance weighted
MR Egger
1/SE_IV
7.5
5.0
2.5
0.0
−1.0
−0.5
0.0
0.5
1.0
β_IV

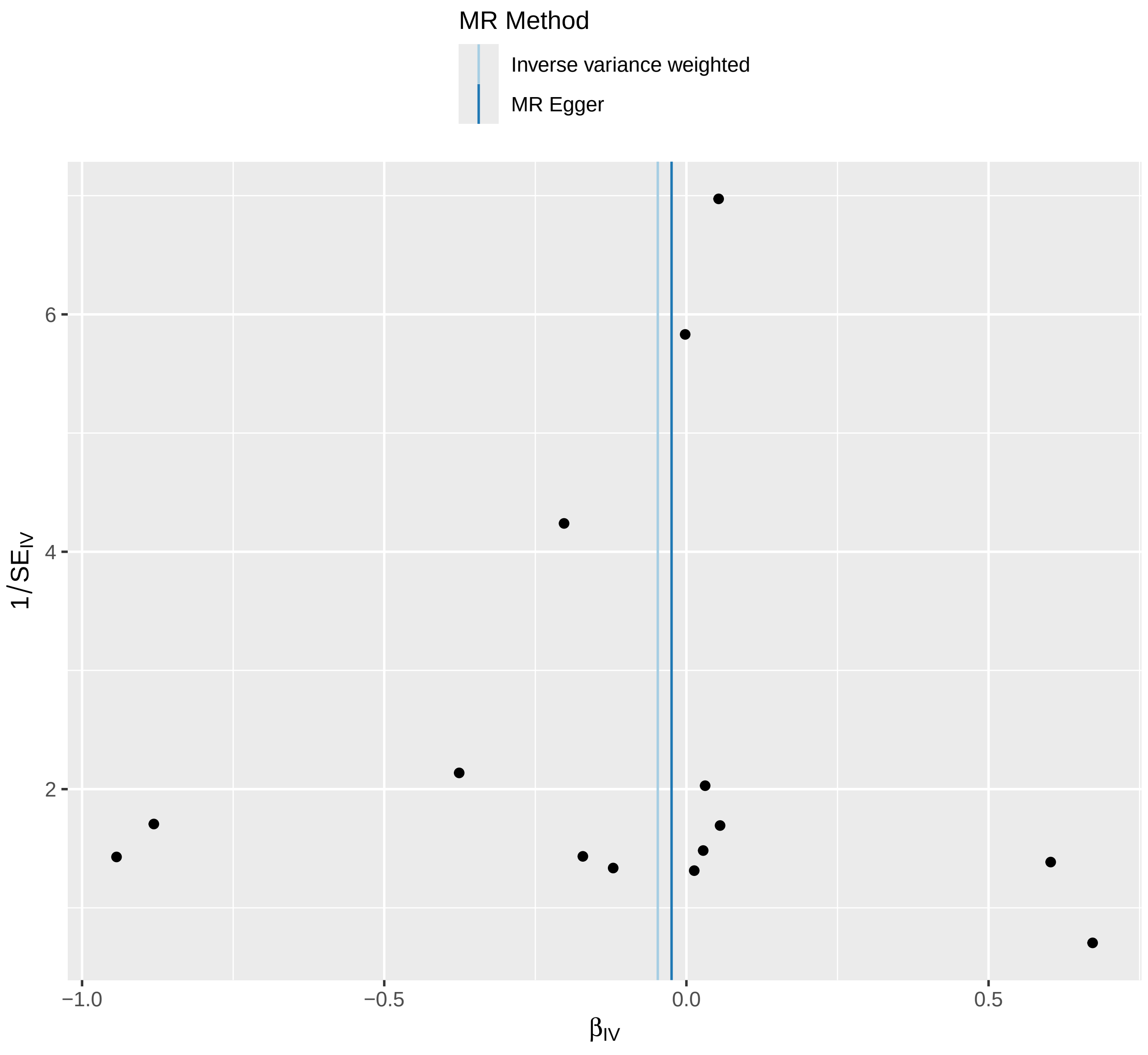
MR Method
Inverse variance weighted
MR Egger
1/SE$_{IV}$
6
4
2
−1.0
−0.5
0.0
0.5
β$_{IV}$

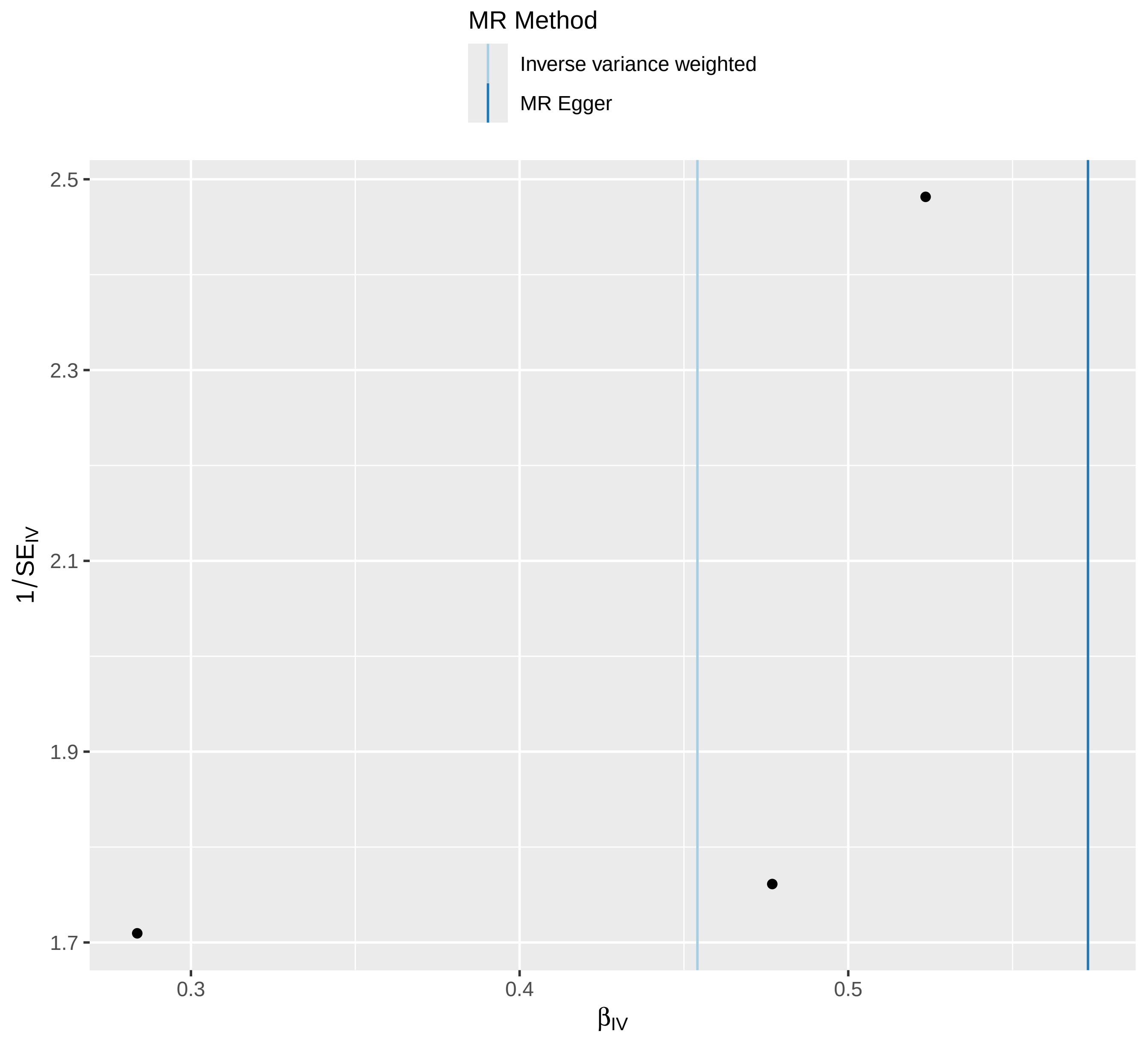
MR Method
Inverse variance weighted
MR Egger
2.5
2.3
2.1
1.9
1.7
1/SE_IV
0.3
0.4
0.5
β_IV

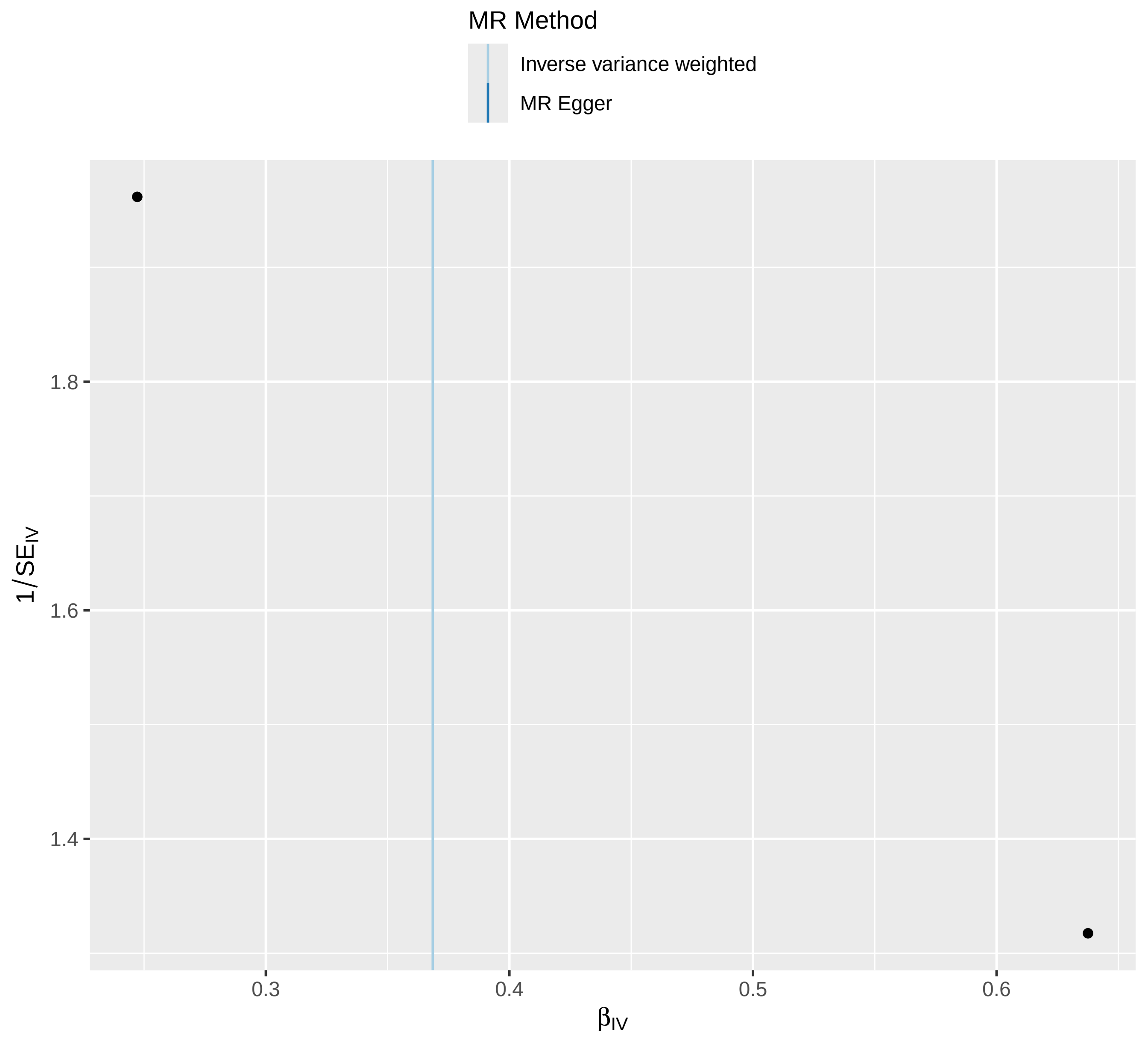

MR Method
Inverse variance weighted
MR Egger
1.8
1.6
1.4
1/SE$_{IV}$
0.3
0.4
0.5
0.6
β$_{IV}$

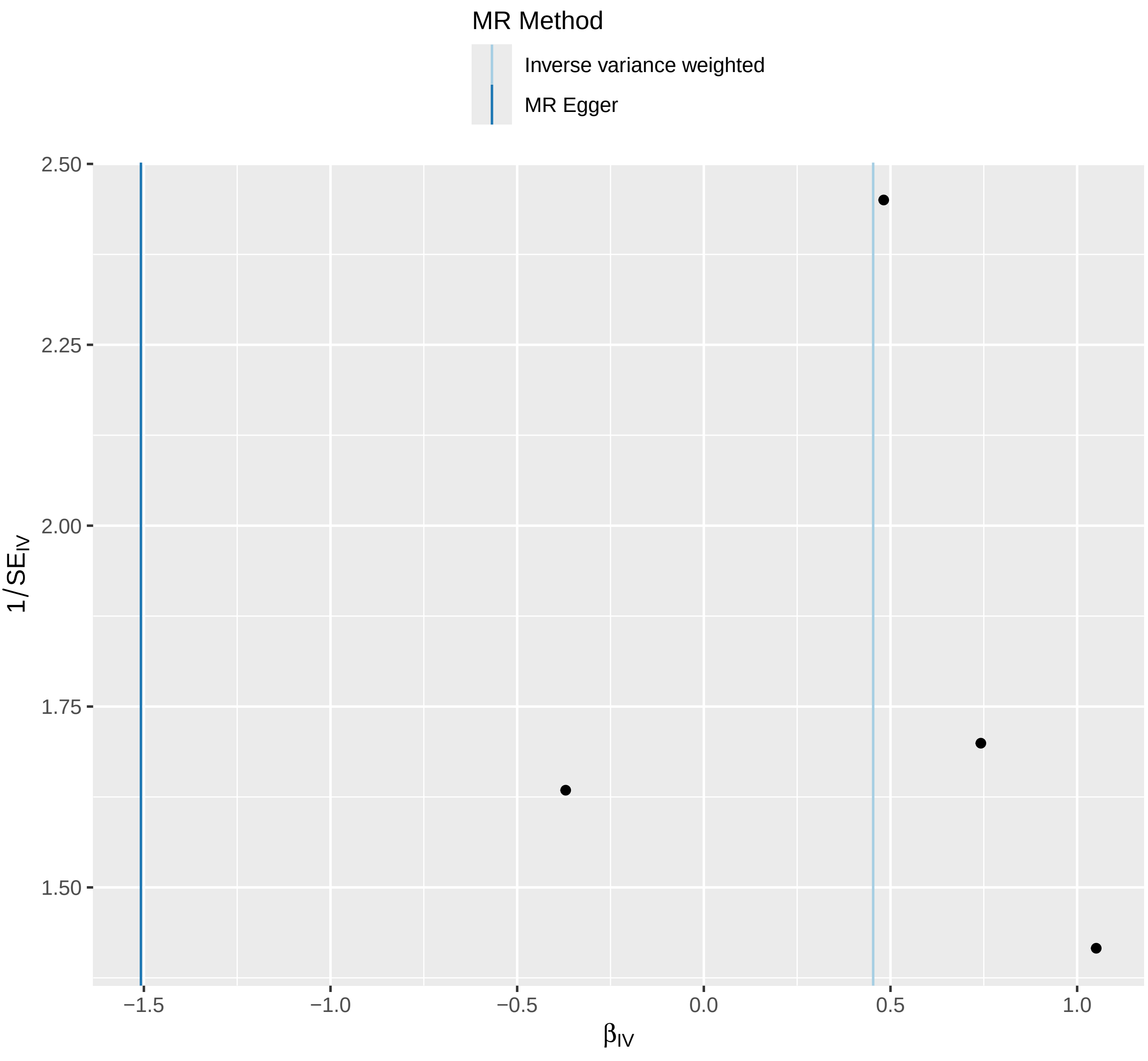
MR Method
Inverse variance weighted
MR Egger
2.50
2.25
2.00
1.75
1.50
1/SE$_{IV}$
−1.5
−1.0
−0.5
0.0
0.5
1.0
β$_{IV}$

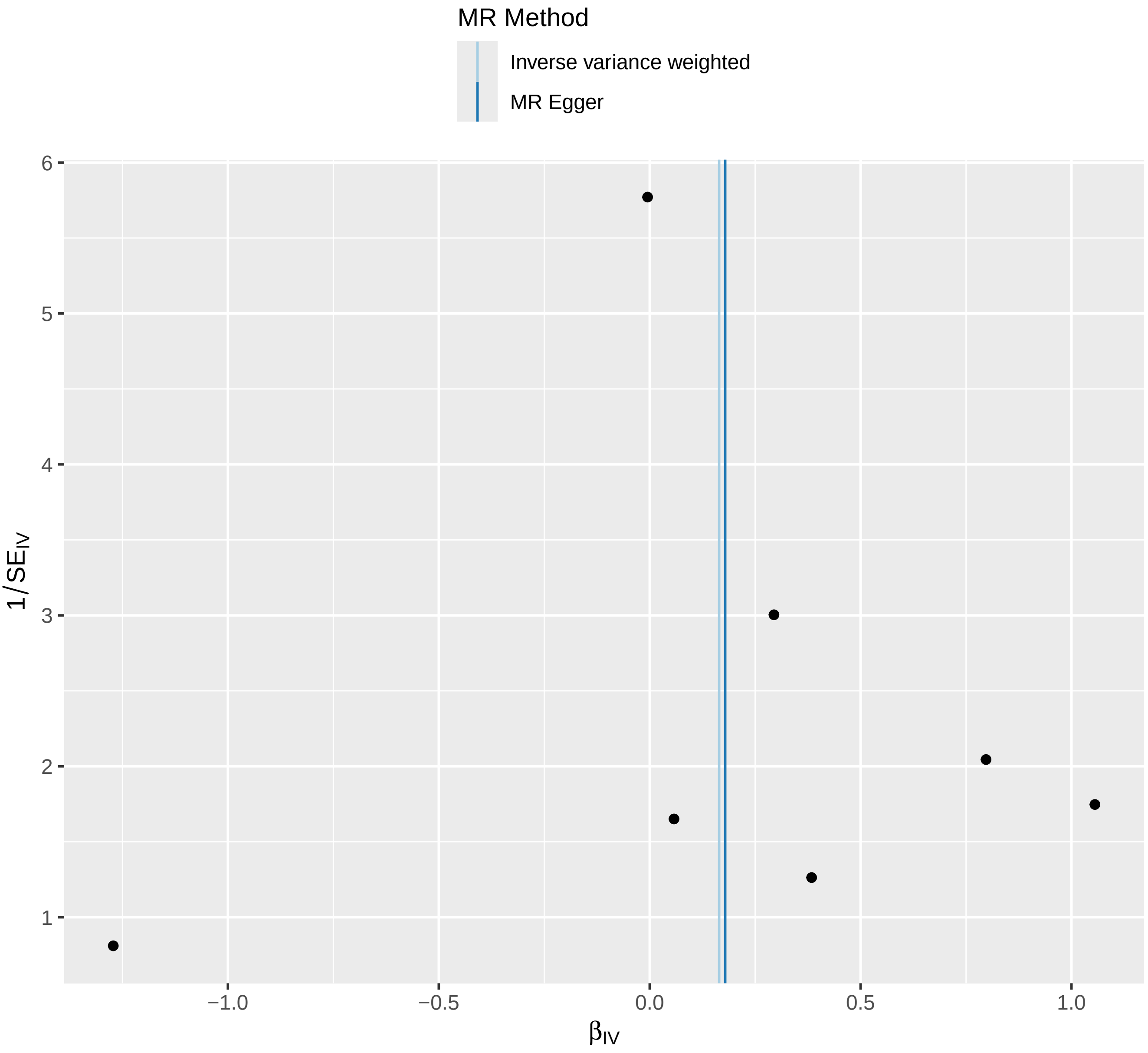

MR Method
Inverse variance weighted
MR Egger
6
5
4
3
2
1
1/SE_IV
−1.0
−0.5
0.0
0.5
1.0
β_IV

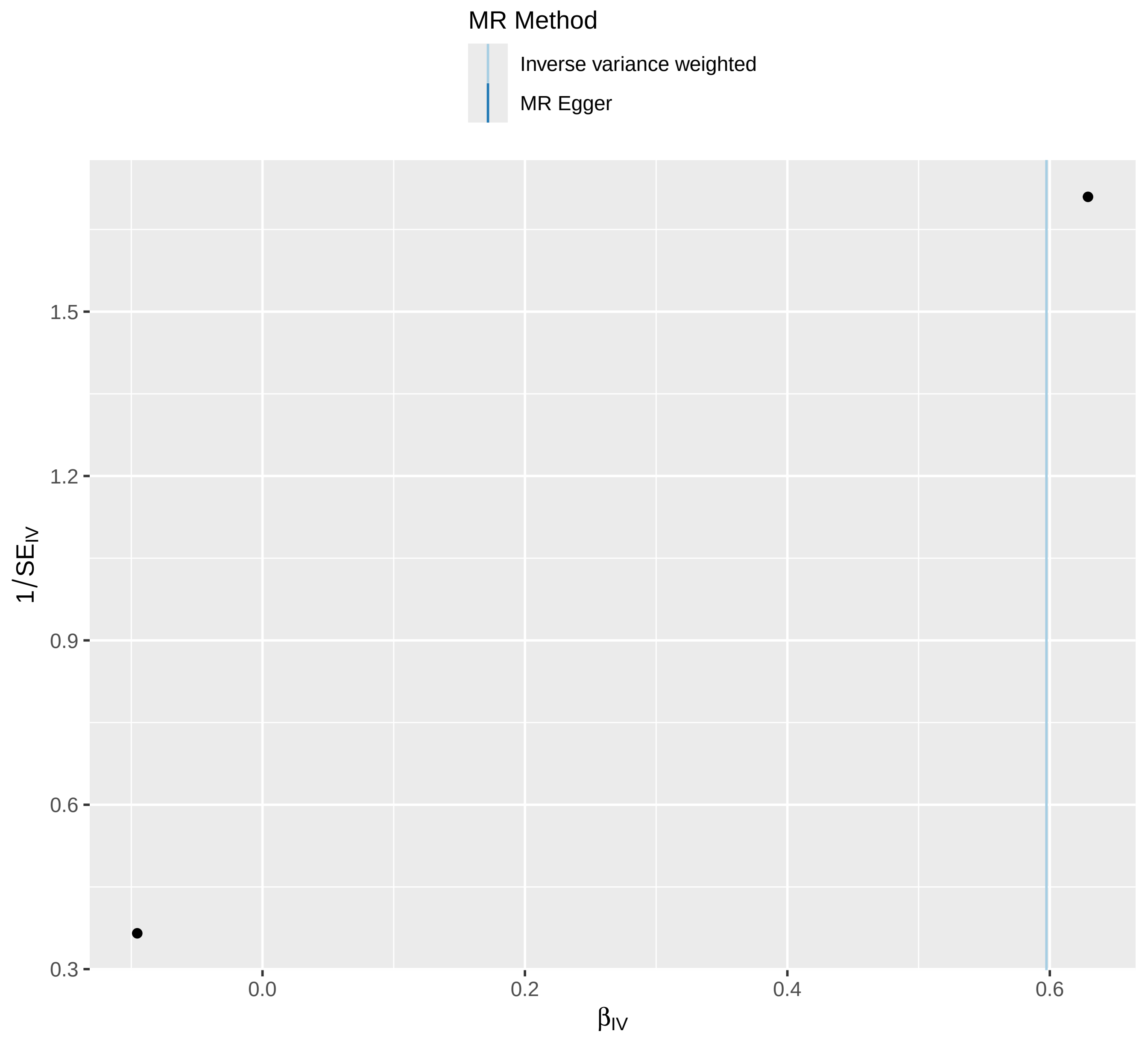
MR Method
Inverse variance weighted
MR Egger
1.5
1.2
0.9
0.6
0.3
1/SE_IV
0.0
0.2
0.4
0.6
β_IV

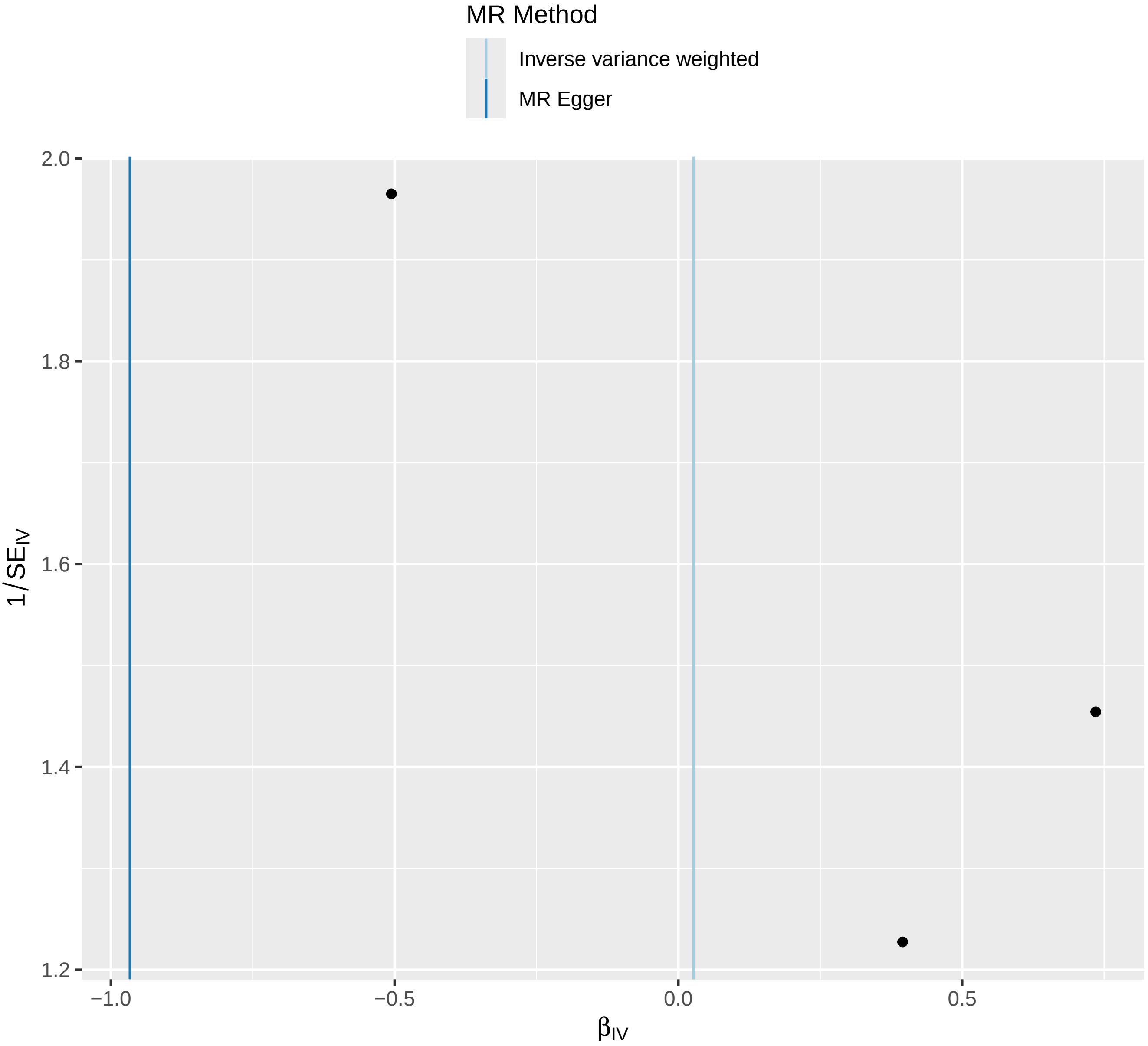
MR Method
Inverse variance weighted
MR Egger
2.0
1.8
1.6
1.4
1.2
$1/SE_{IV}$
−1.0
−0.5
0.0
0.5
β_{IV}

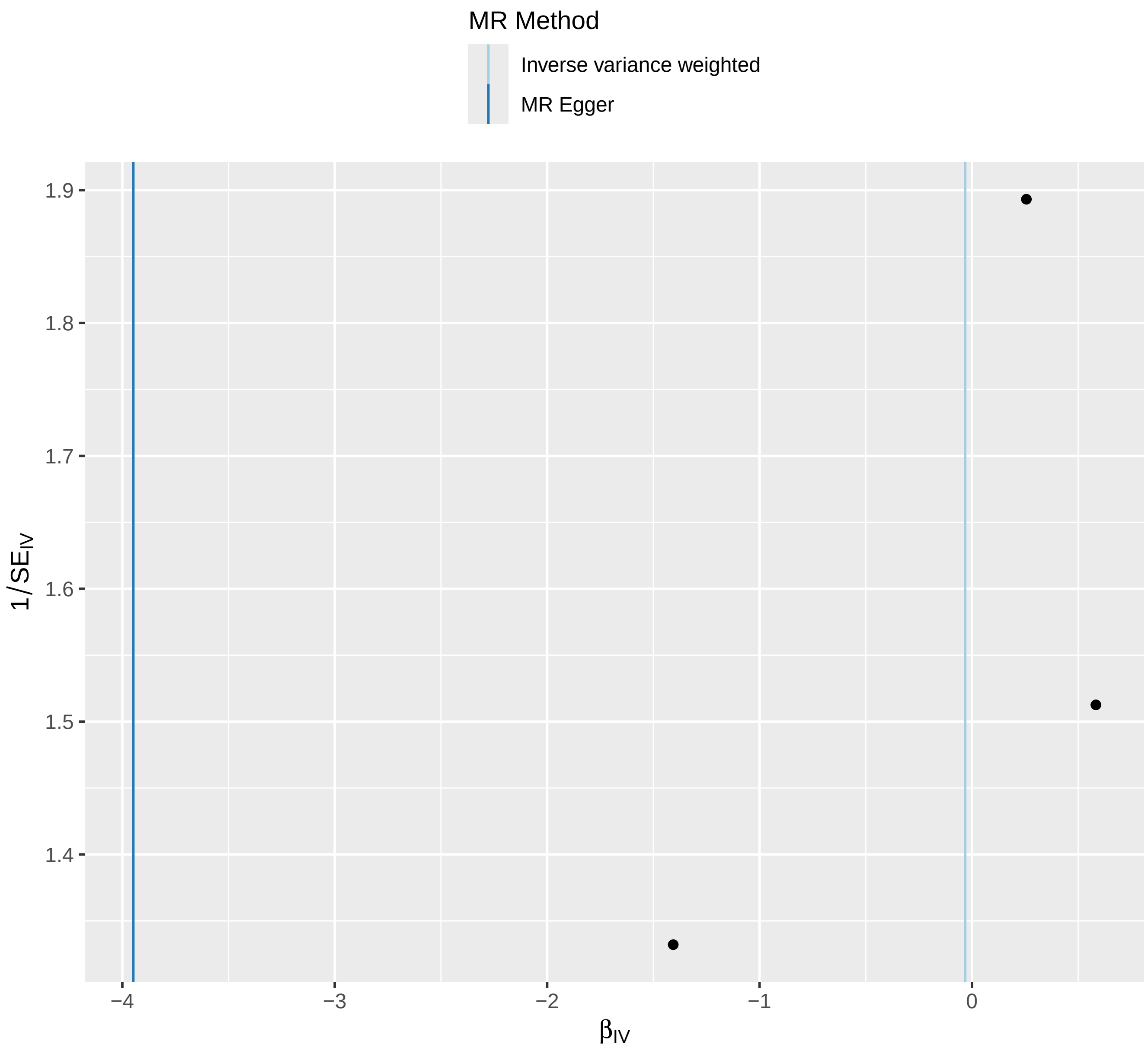

MR Method
Inverse variance weighted
MR Egger
1.9
1.8
1.7
1.6
1.5
1.4
1/SE$_{IV}$
−4
−3
−2
−1
0
β$_{IV}$

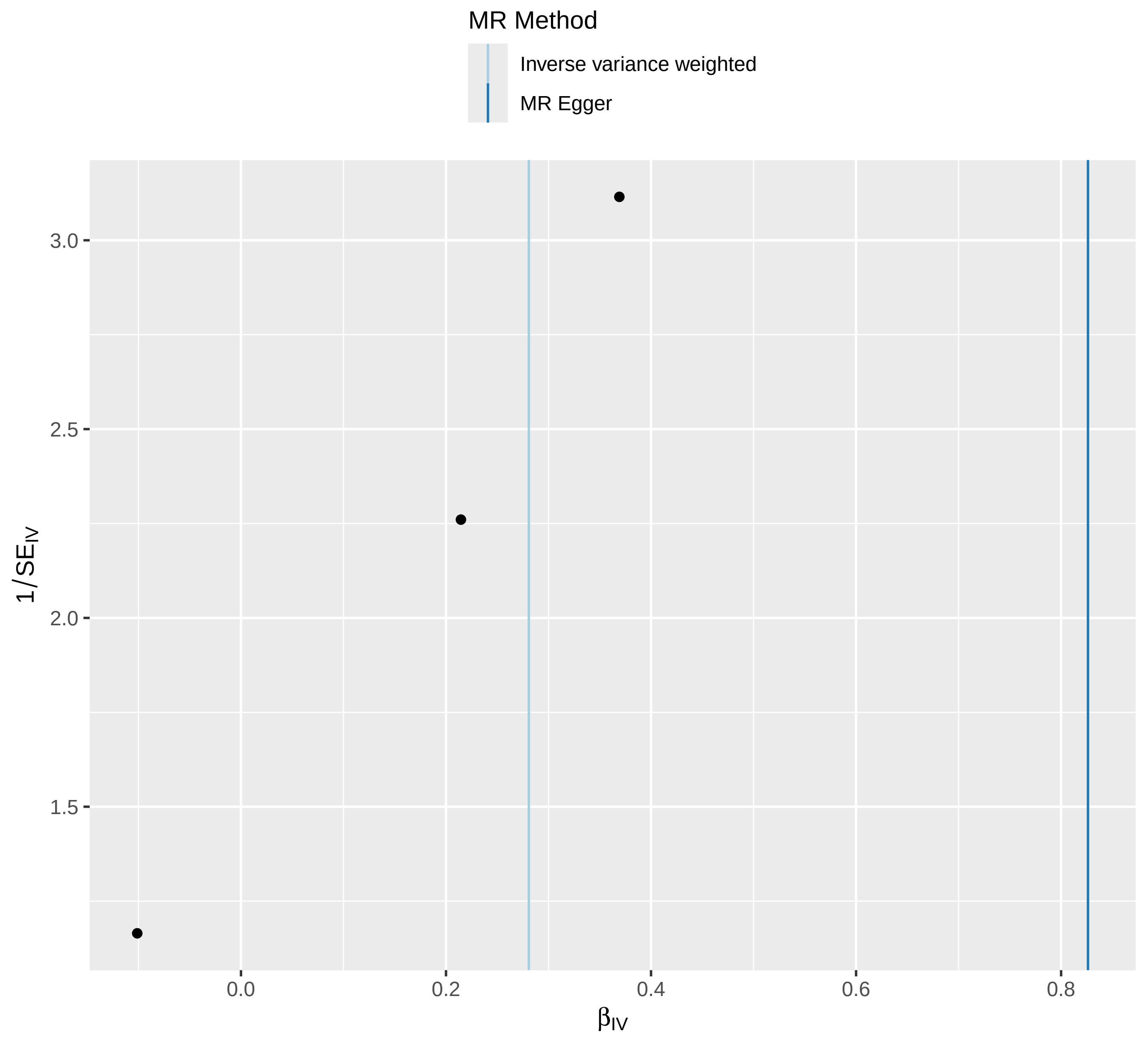
MR Method
Inverse variance weighted
MR Egger
1/SE$_{IV}$
3.0
2.5
2.0
1.5
0.0
0.2
0.4
0.6
0.8
β$_{IV}$

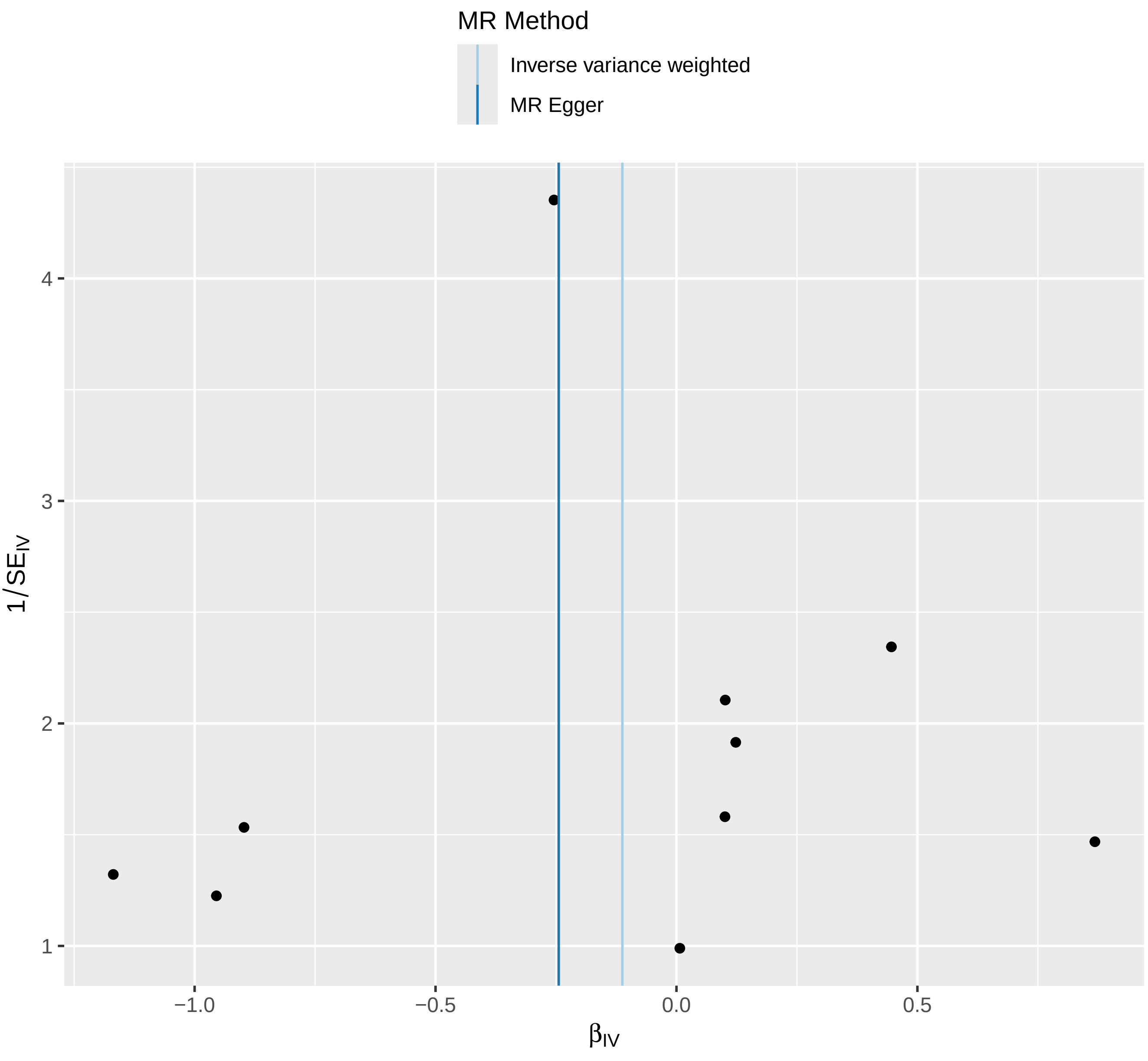
MR Method
Inverse variance weighted
MR Egger
1/SE_{IV}
4
3
2
1
−1.0
−0.5
0.0
0.5
β_{IV}

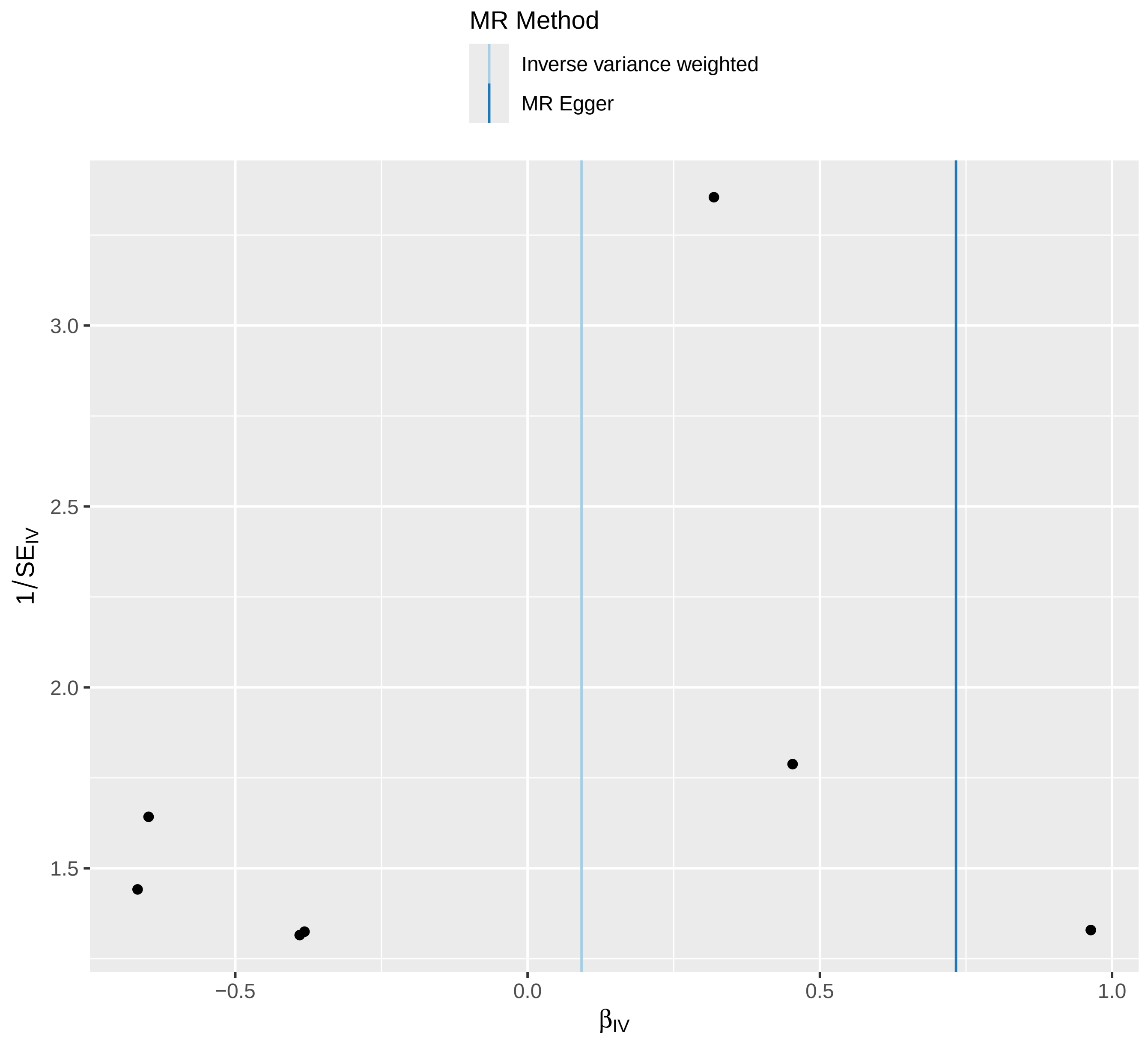

MR Method
Inverse variance weighted
MR Egger
3.0
2.5
2.0
1.5
$1/SE_{IV}$
−0.5
0.0
0.5
1.0
β_{IV}

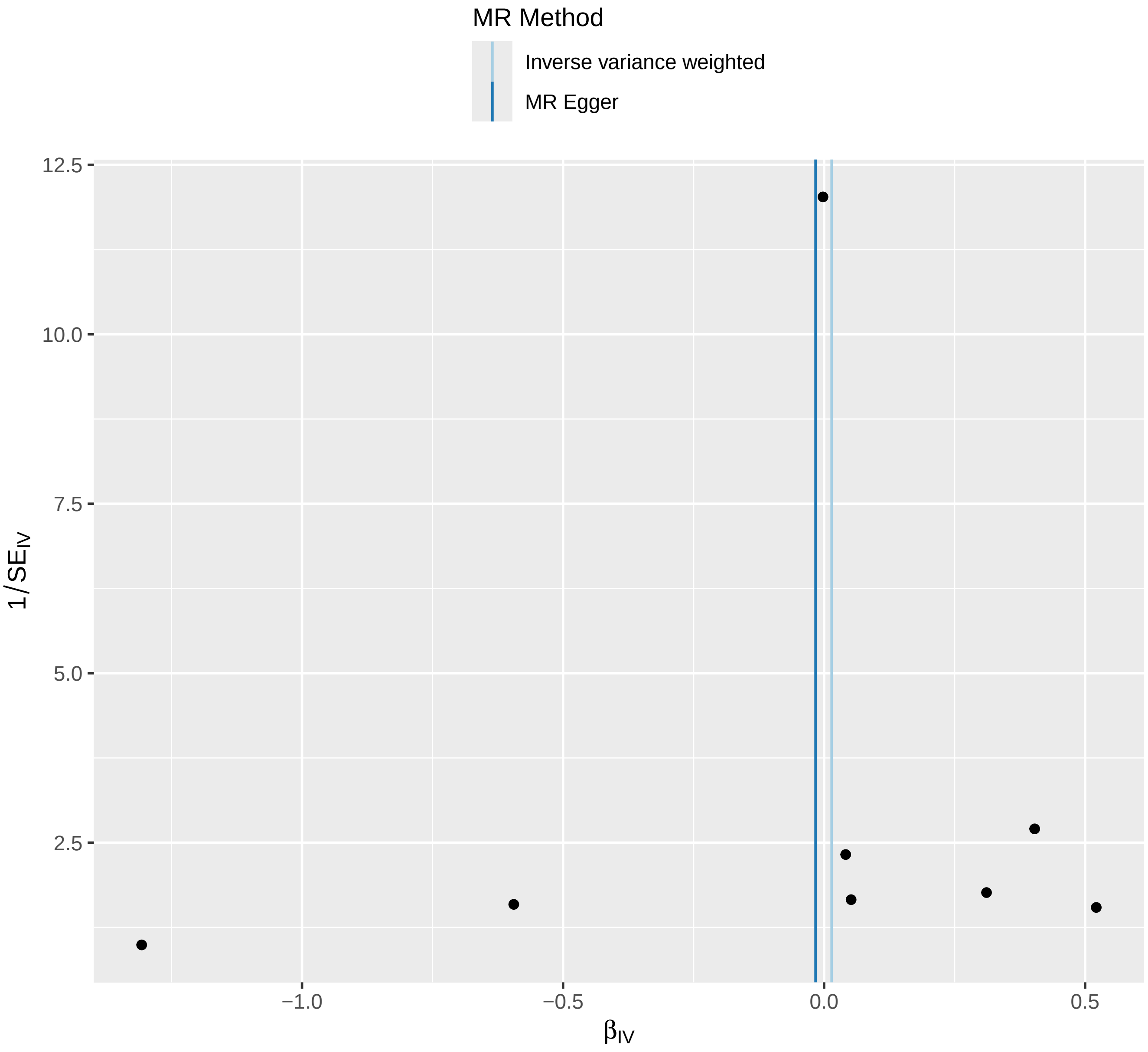

MR Method
Inverse variance weighted
MR Egger
12.5
10.0
7.5
5.0
2.5
1/SE$_{IV}$
−1.0
−0.5
0.0
0.5
β$_{IV}$

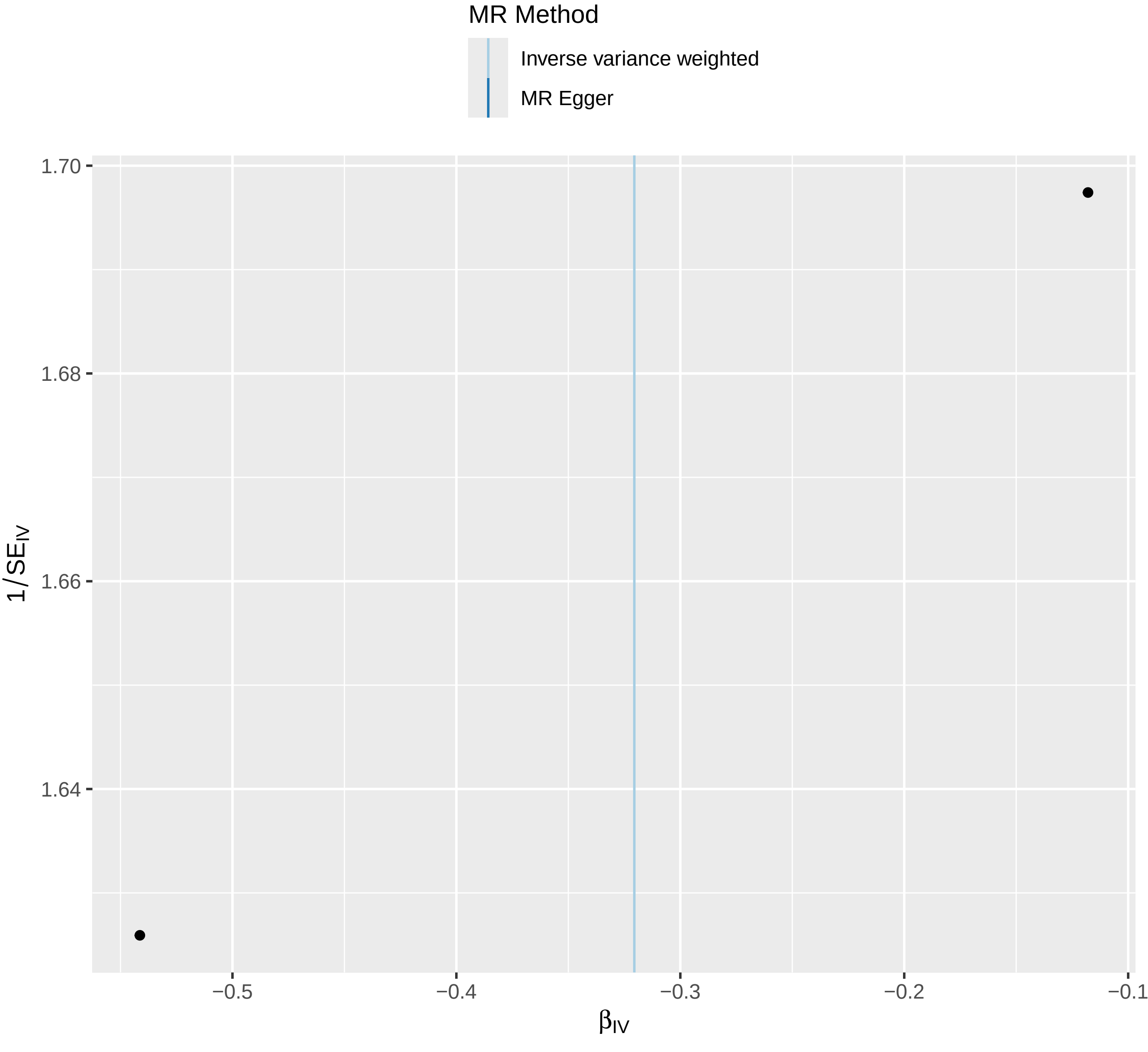

MR Method
Inverse variance weighted
MR Egger
1.70
1.68
1.66
1.64
1/SE$_{IV}$
−0.5
−0.4
−0.3
−0.2
−0.1
β_{IV}

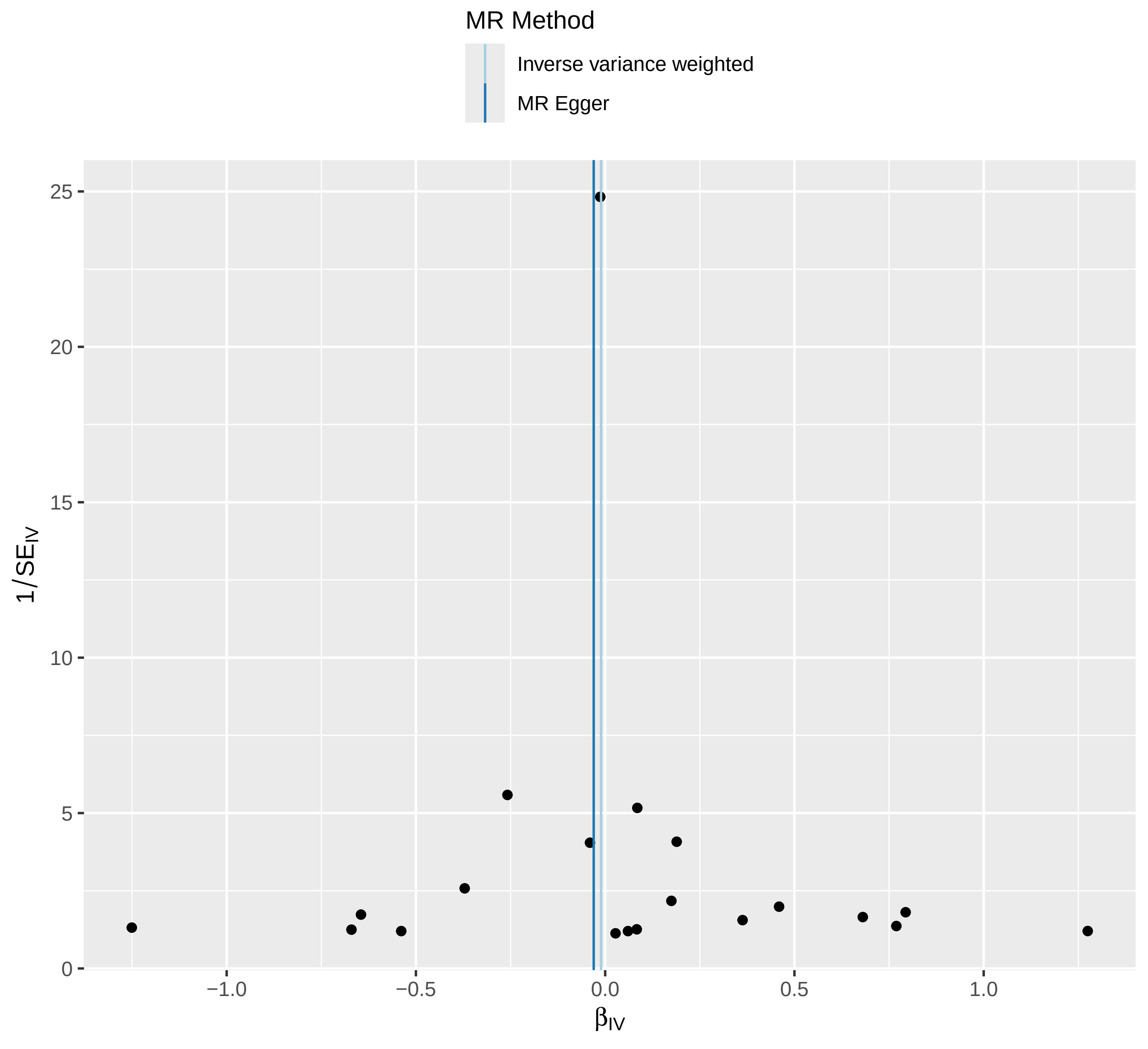
MR Method
Inverse variance weighted
MR Egger
25
20
15
10
5
0
1/SE_{IV}
−1.0
−0.5
0.0
0.5
1.0
β_{IV}

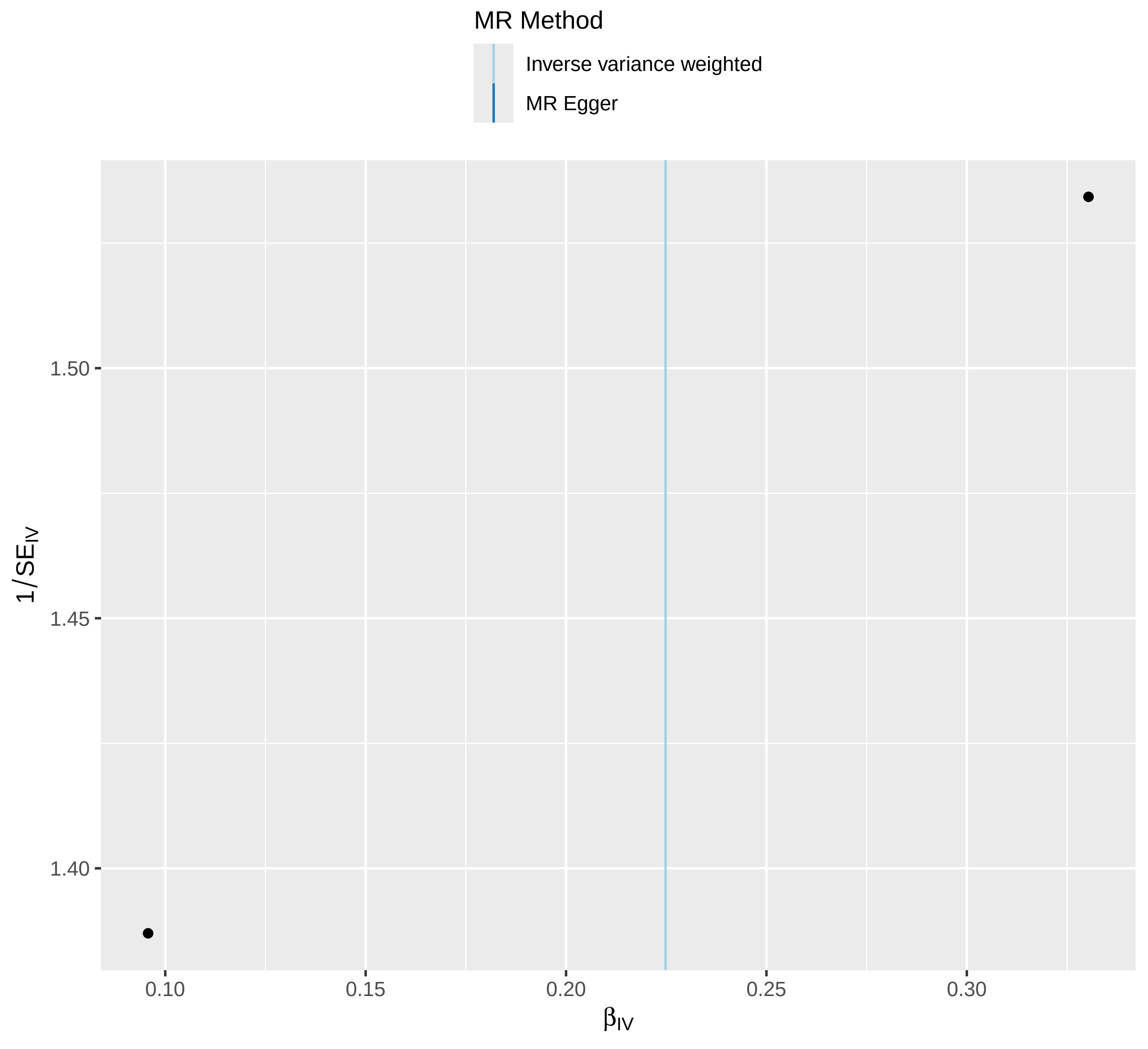
MR Method
Inverse variance weighted
MR Egger
1.50
1.45
1.40
1/SE$_{IV}$
0.10
0.15
0.20
0.25
0.30
β_{IV}

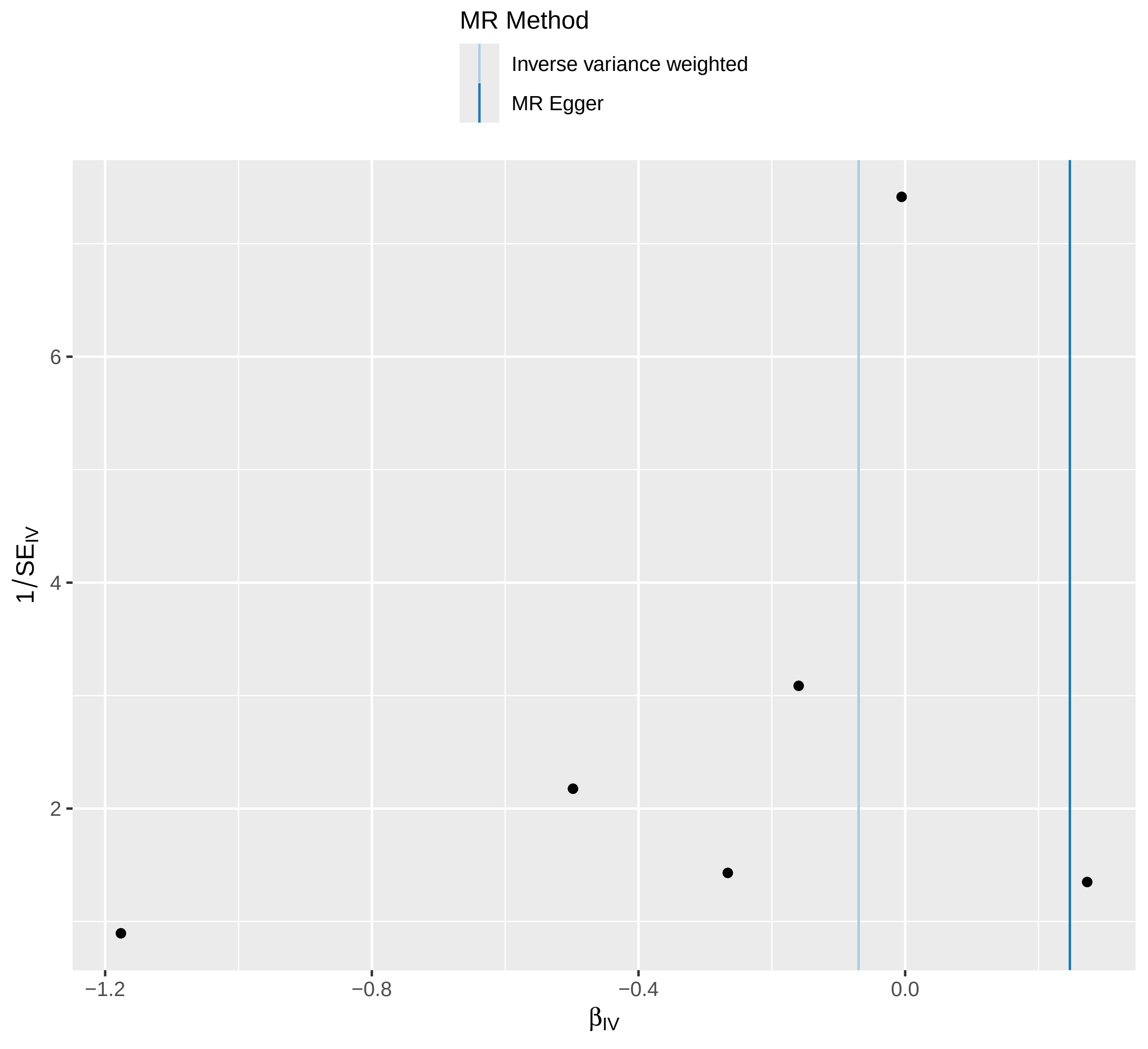

MR Method
Inverse variance weighted
MR Egger
1/SE$_{IV}$
6
4
2
−1.2
−0.8
−0.4
0.0
β$_{IV}$

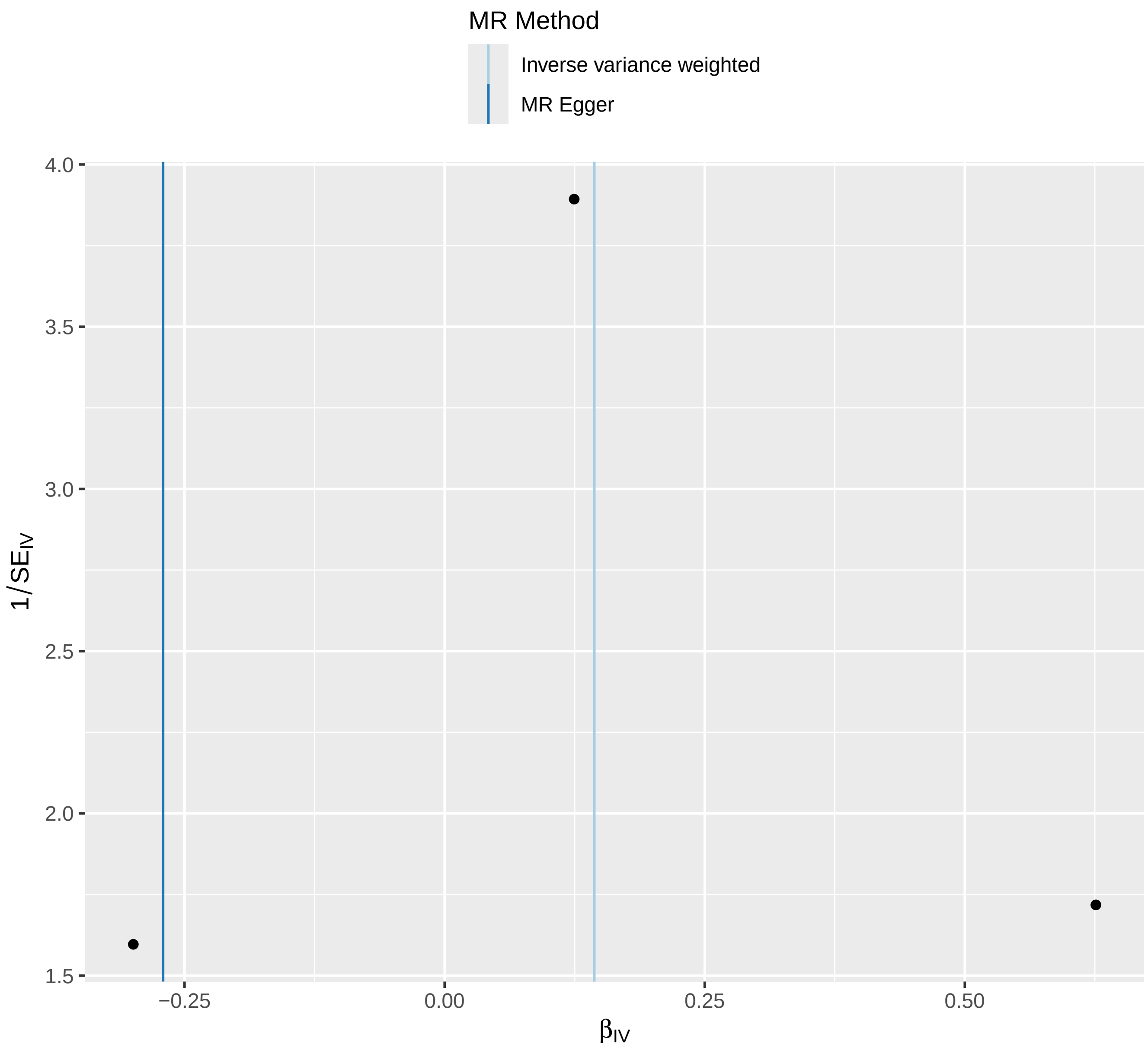

MR Method
Inverse variance weighted
MR Egger
4.0
3.5
3.0
2.5
2.0
1.5
1/SE$_{IV}$
−0.25
0.00
0.25
0.50
β$_{IV}$

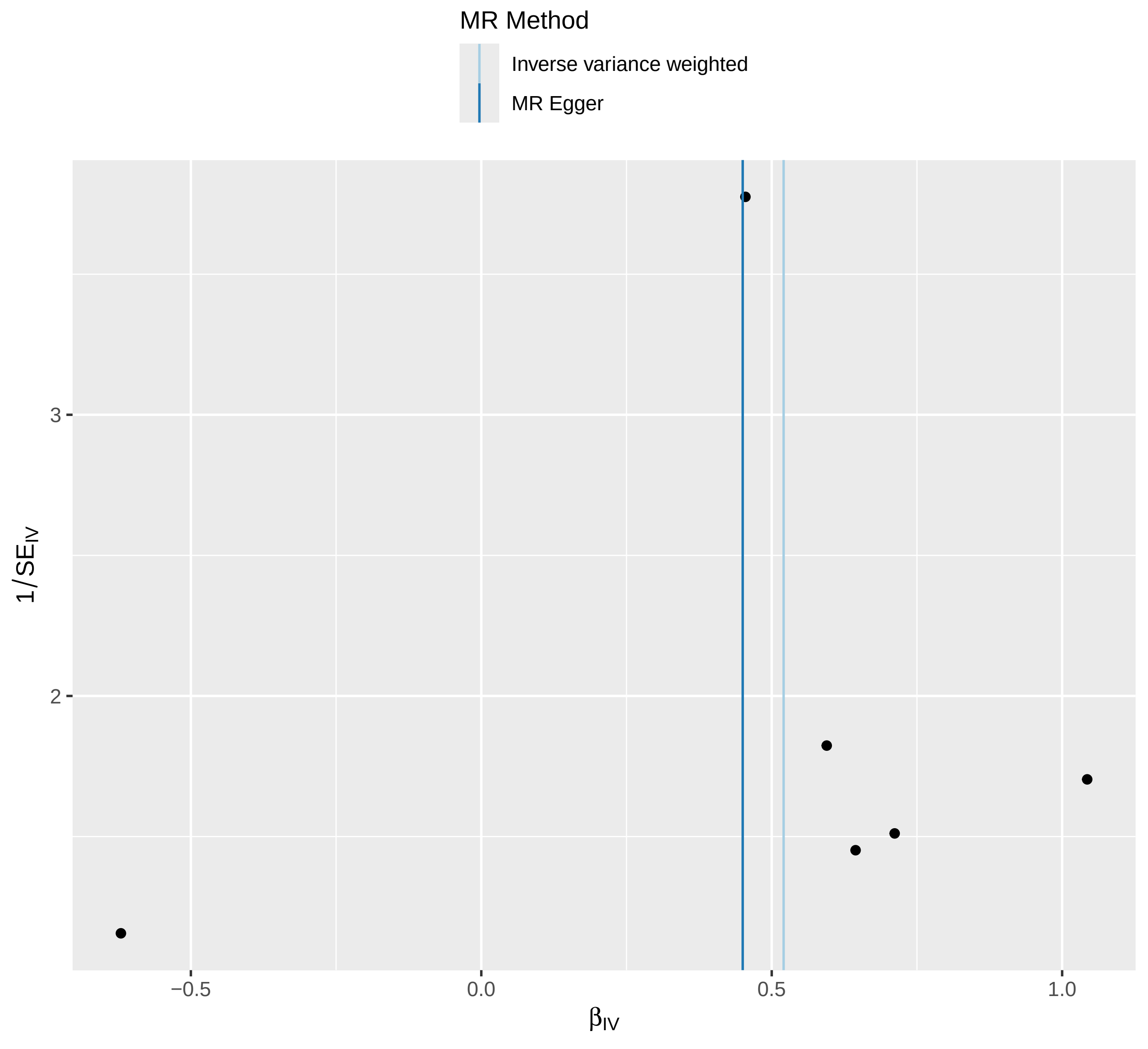

MR Method
Inverse variance weighted
MR Egger
1/SE_IV
3
2
−0.5
0.0
0.5
1.0
β_IV

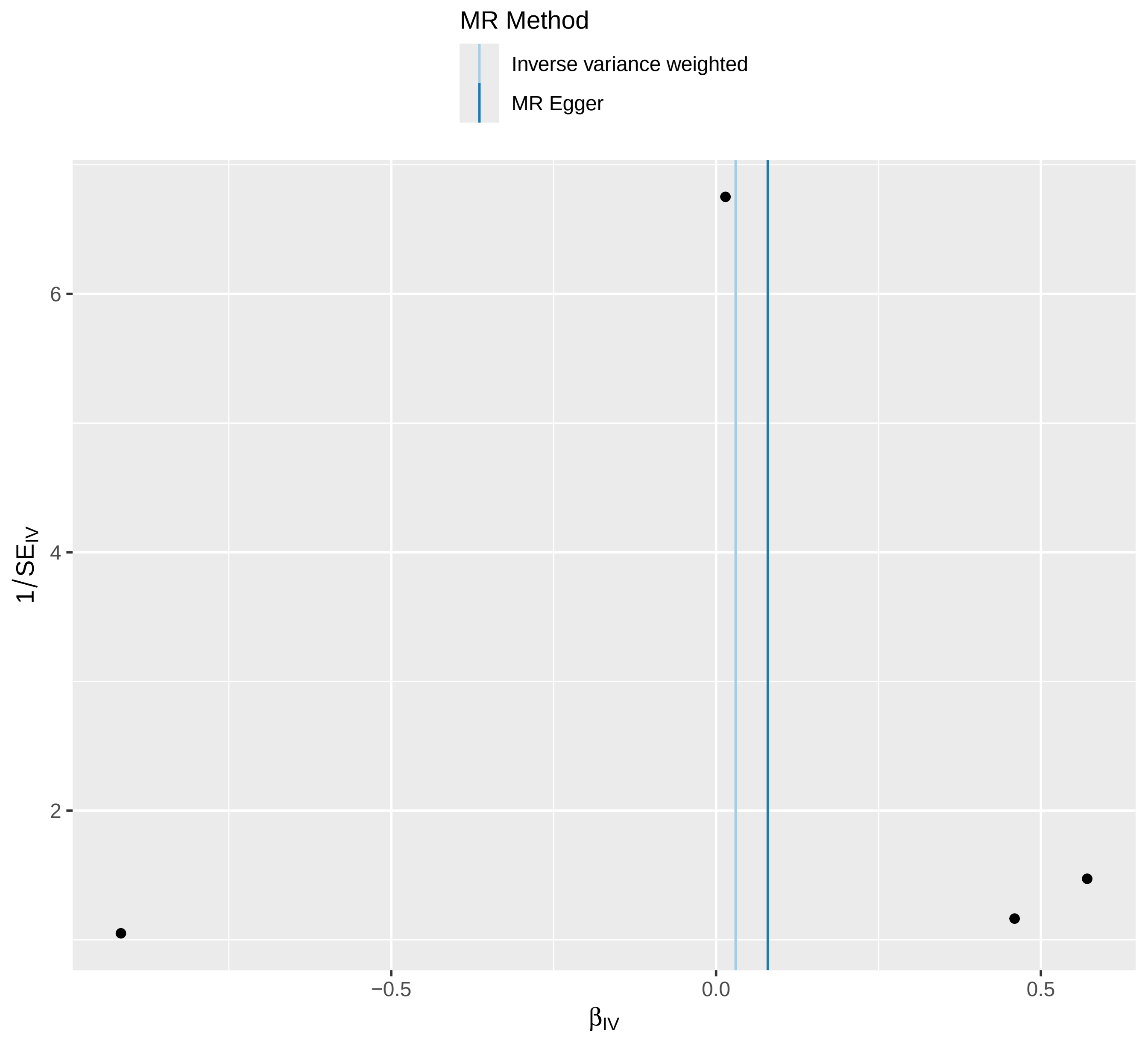

MR Method
Inverse variance weighted
MR Egger
1/SE_IV
6
4
2
−0.5
0.0
0.5
β_IV

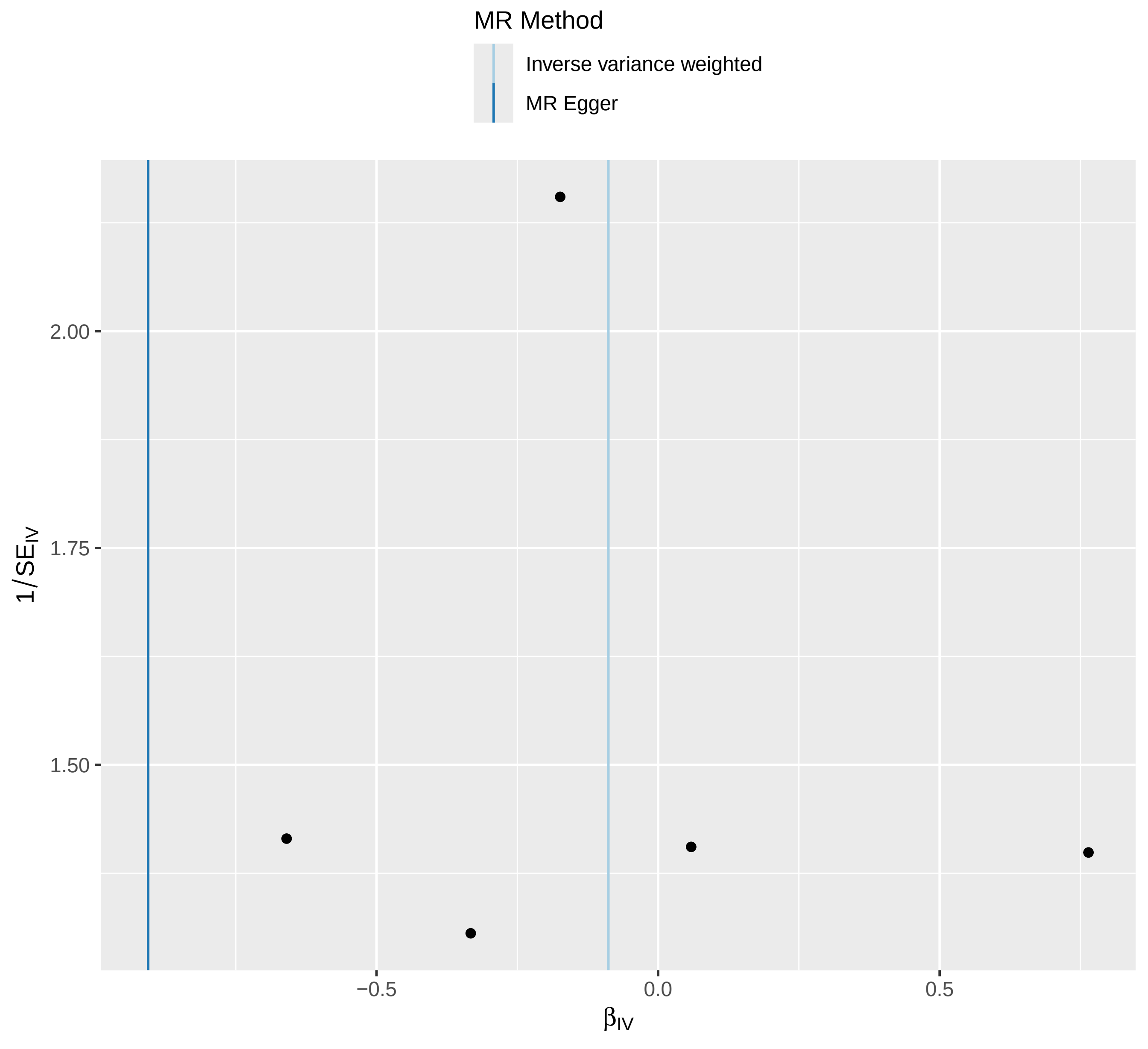

MR Method
Inverse variance weighted
MR Egger
2.00
1.75
1.50
1/SE$_{IV}$
−0.5
0.0
0.5
β_{IV}

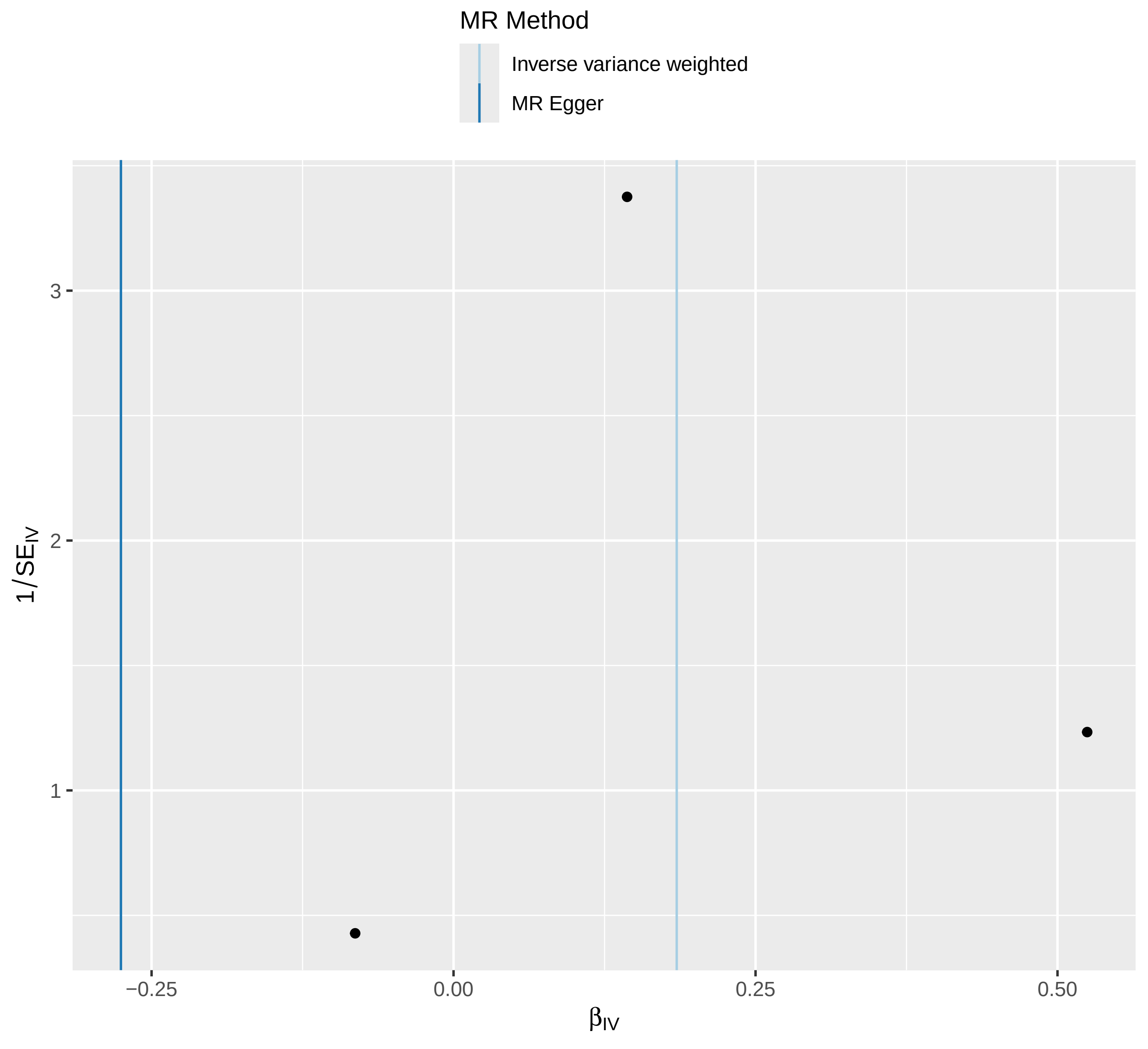

MR Method
Inverse variance weighted
MR Egger
3
2
1
1/SE_IV
−0.25
0.00
0.25
0.50
β_IV

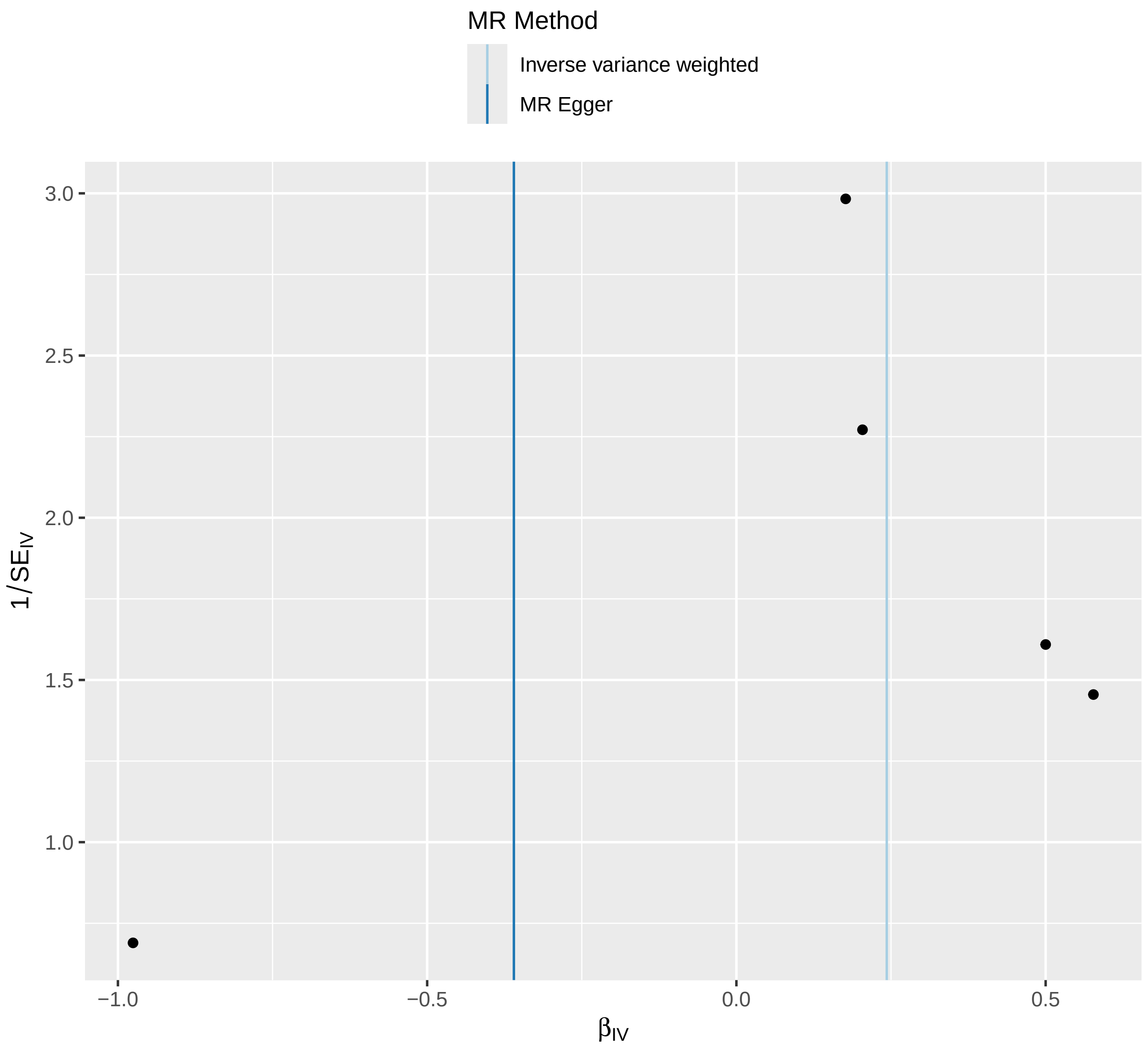

MR Method
Inverse variance weighted
MR Egger
3.0
2.5
2.0
1.5
1.0
1/SE$_{IV}$
−1.0
−0.5
0.0
0.5
β$_{IV}$

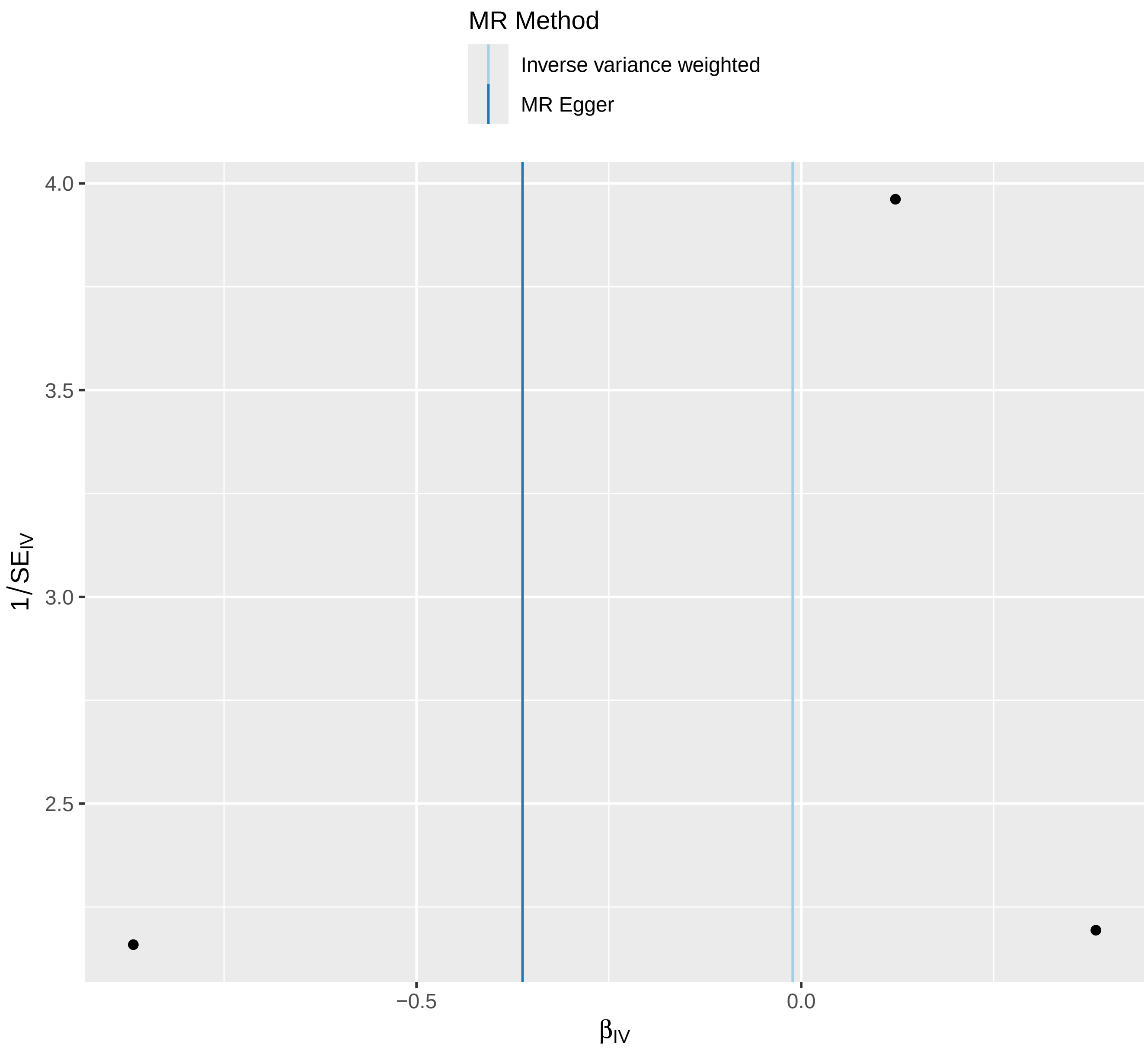

MR Method
Inverse variance weighted
MR Egger
4.0
3.5
3.0
2.5
$1/SE_{IV}$
−0.5
0.0
β_{IV}

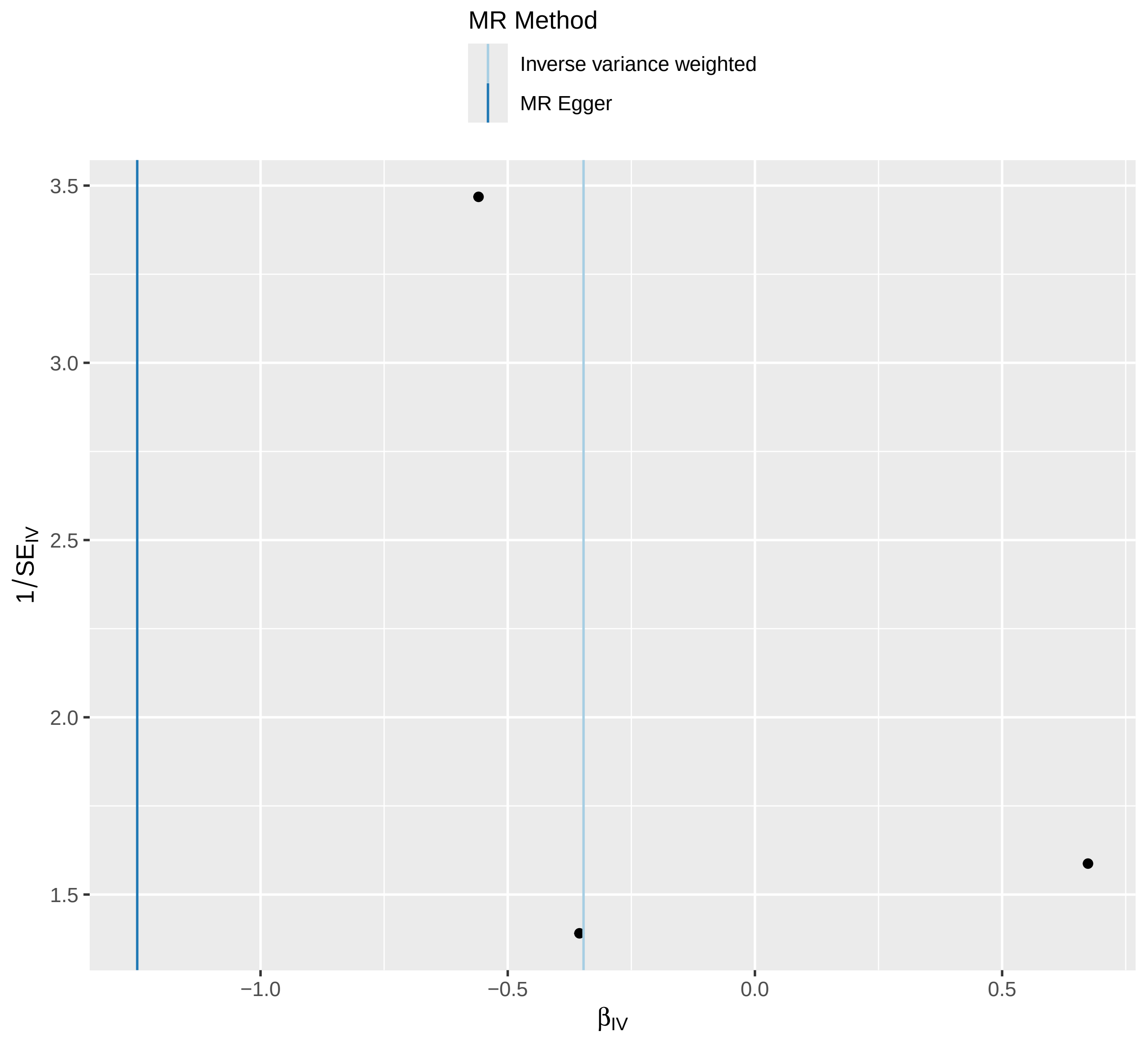
MR Method
Inverse variance weighted
MR Egger
3.5
3.0
2.5
2.0
1.5
1/SE$_{IV}$
−1.0
−0.5
0.0
0.5
β$_{IV}$

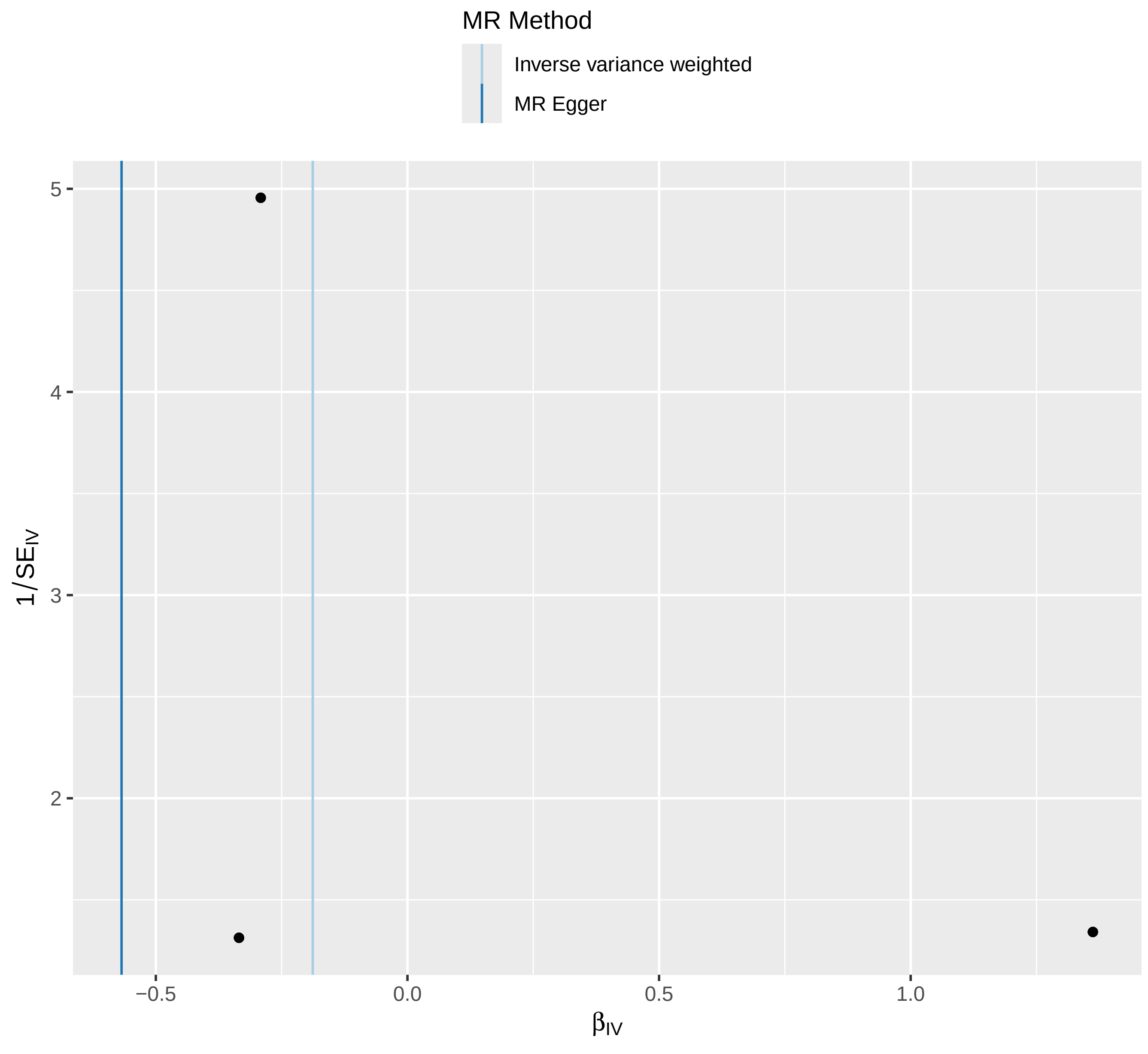

MR Method
Inverse variance weighted
MR Egger
5
4
3
2
1/SE_IV
−0.5
0.0
0.5
1.0
β_IV

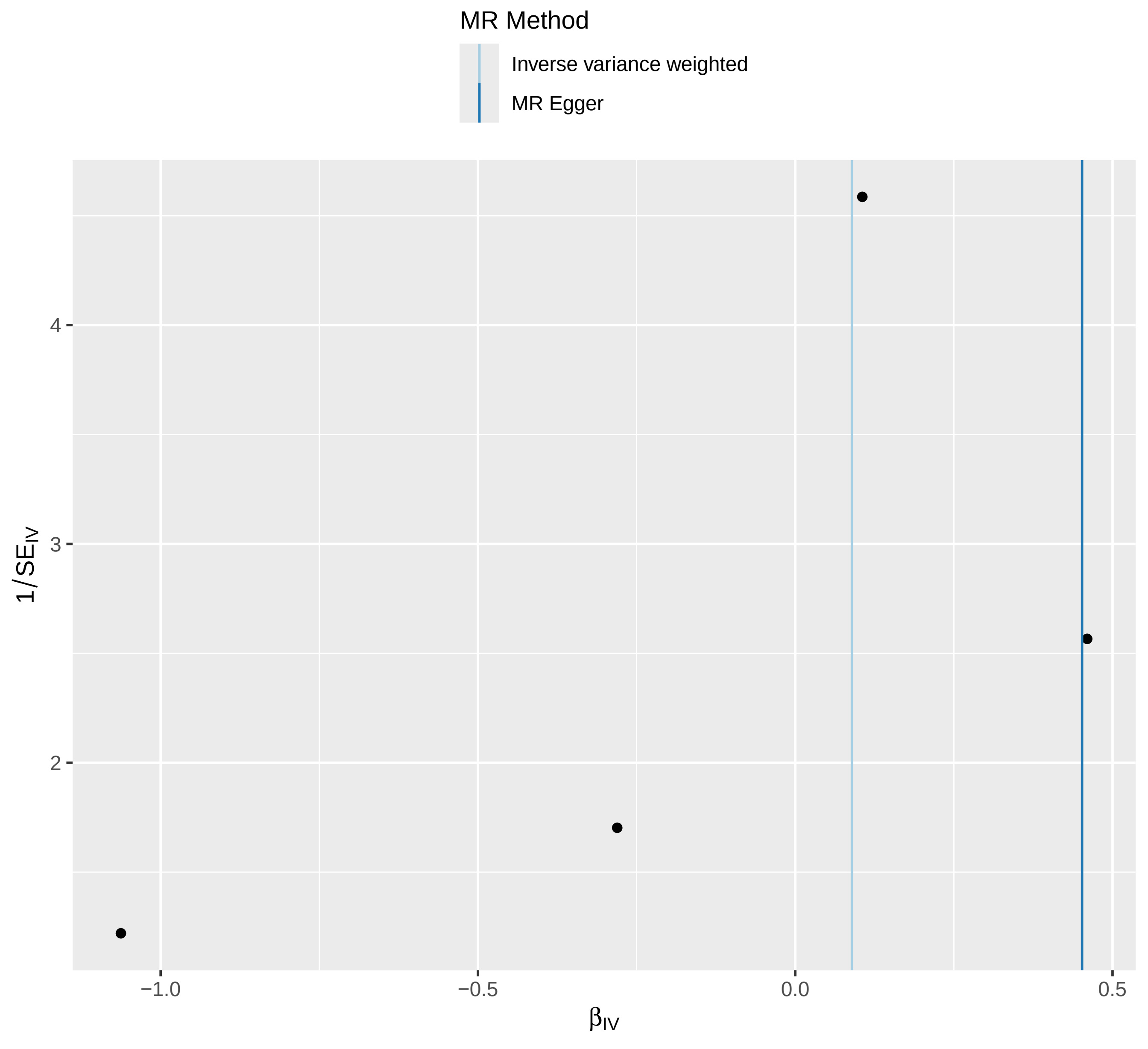
MR Method
Inverse variance weighted
MR Egger
4
3
2
1/SE$_{IV}$
−1.0
−0.5
0.0
0.5
β$_{IV}$

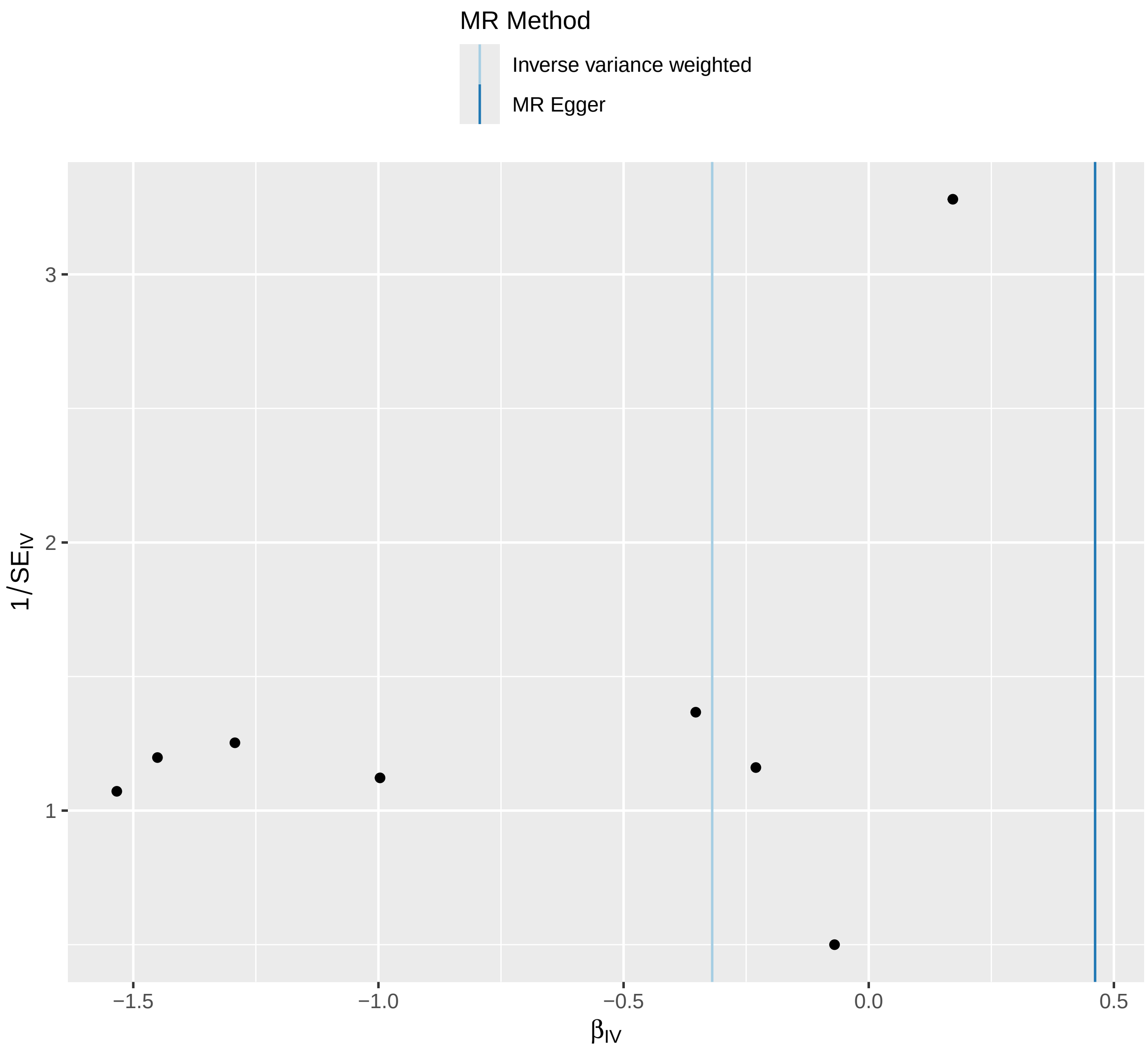
MR Method
Inverse variance weighted
MR Egger
1/SE_IV
3
2
1
−1.5
−1.0
−0.5
0.0
0.5
β_IV

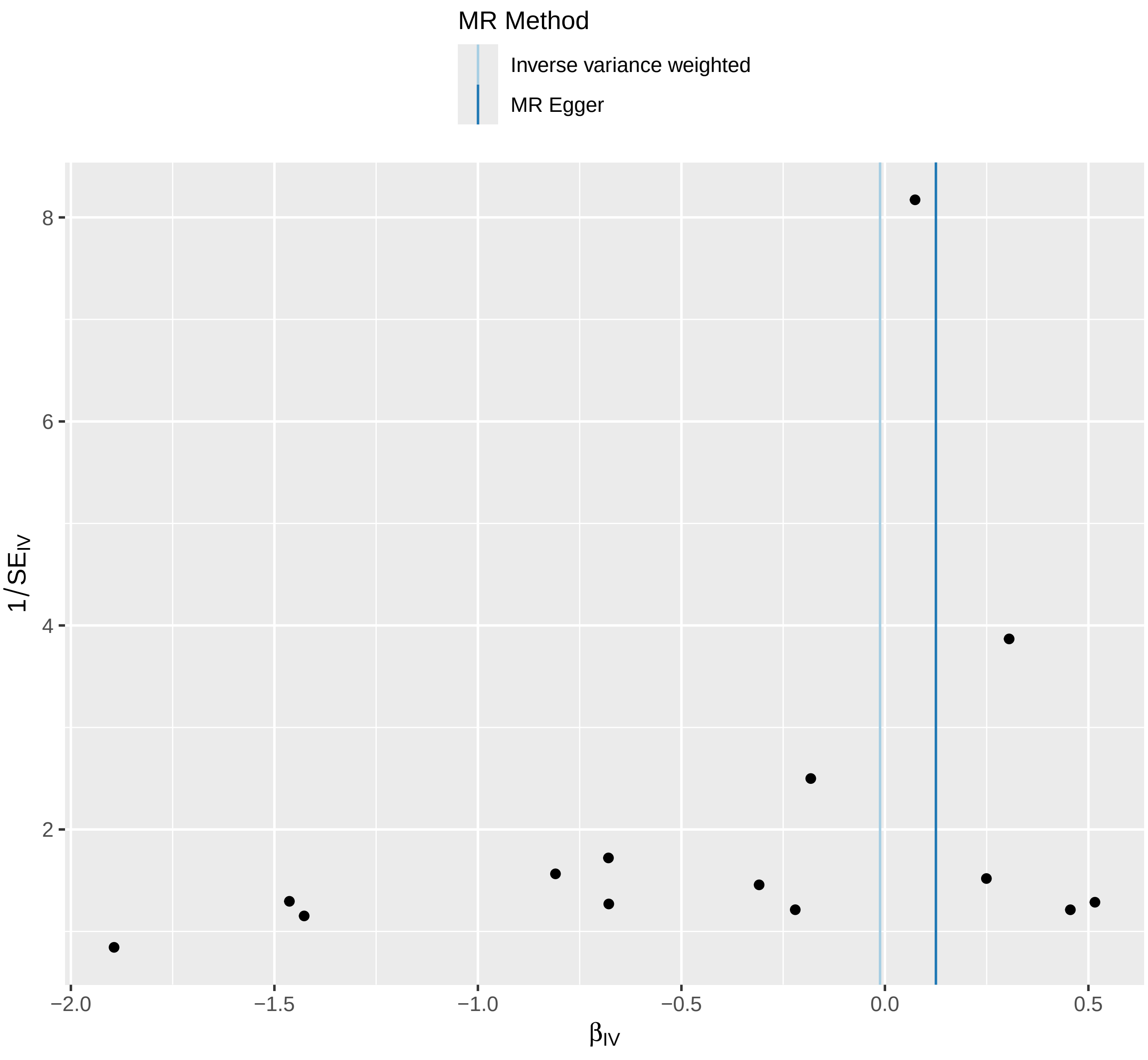
MR Method
Inverse variance weighted
MR Egger
8
6
4
2
1/SE$_{IV}$
−2.0
−1.5
−1.0
−0.5
0.0
0.5
β$_{IV}$

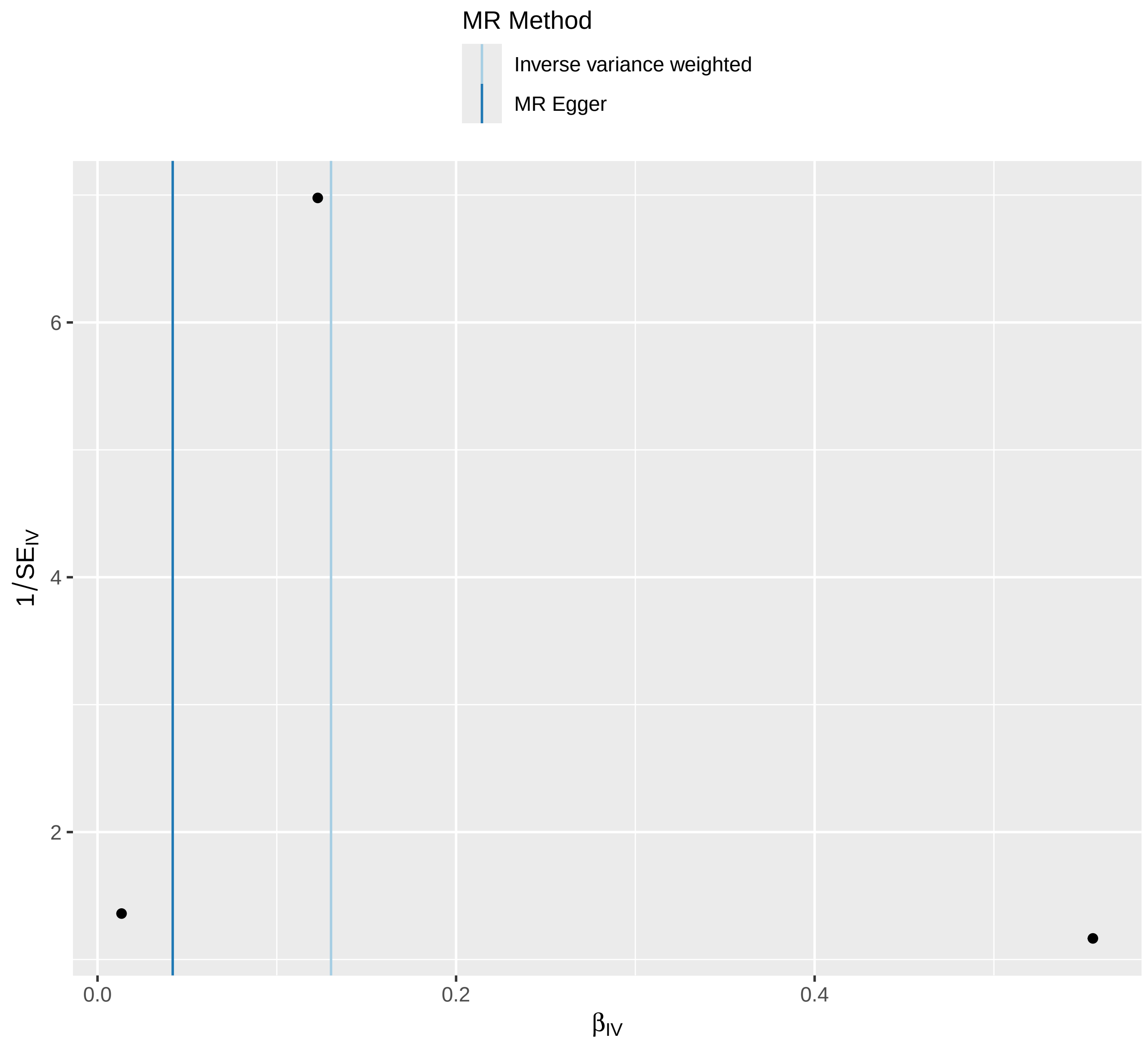

MR Method
Inverse variance weighted
MR Egger
6
4
2
1/SE$_{IV}$
0.0
0.2
0.4
β$_{IV}$

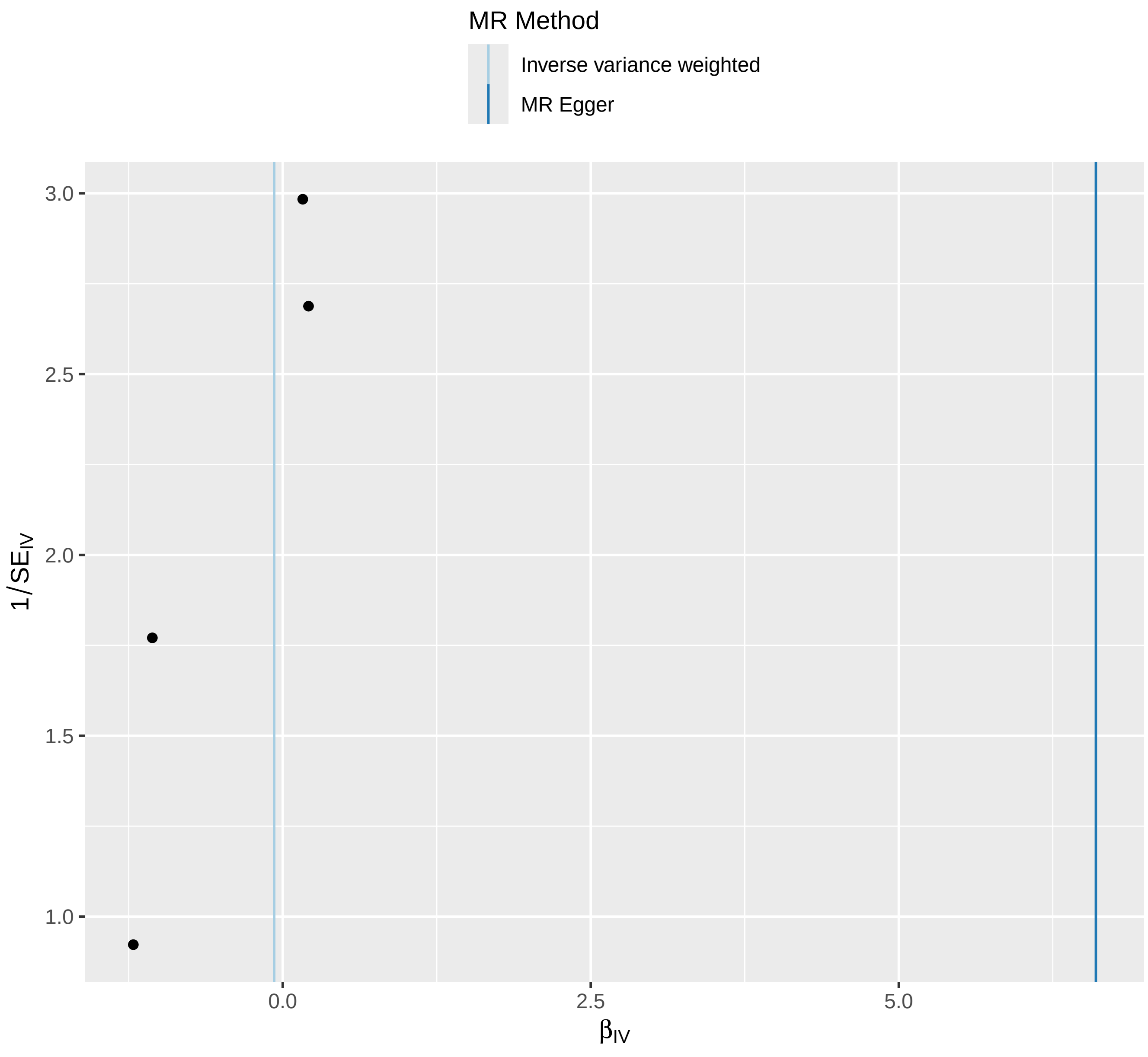

MR Method
Inverse variance weighted
MR Egger
3.0
2.5
2.0
1.5
1.0
$1/SE_{IV}$
0.0
2.5
5.0
β_{IV}

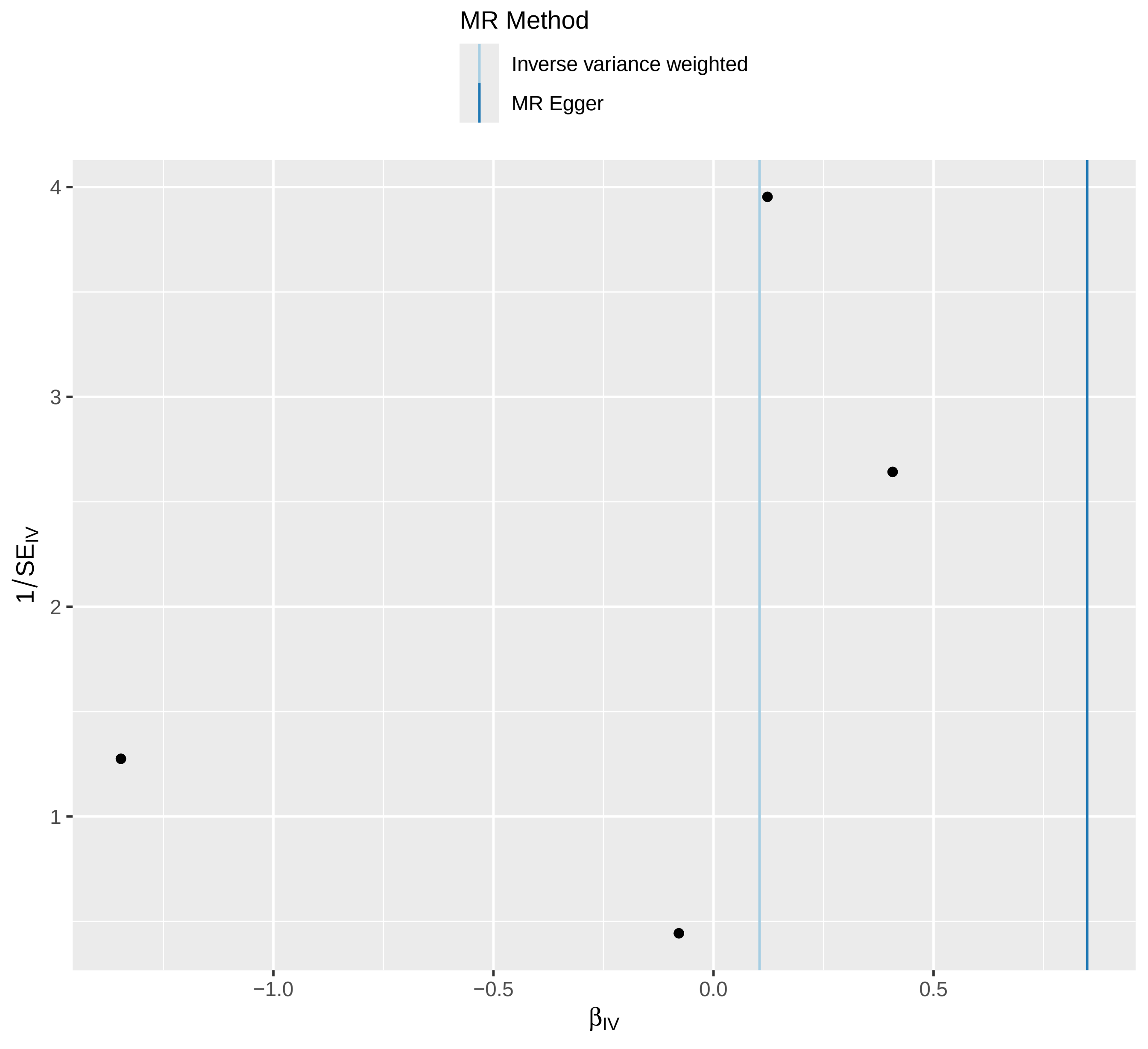
MR Method
Inverse variance weighted
MR Egger
4
3
2
1
1/SE_IV
−1.0
−0.5
0.0
0.5
β_IV

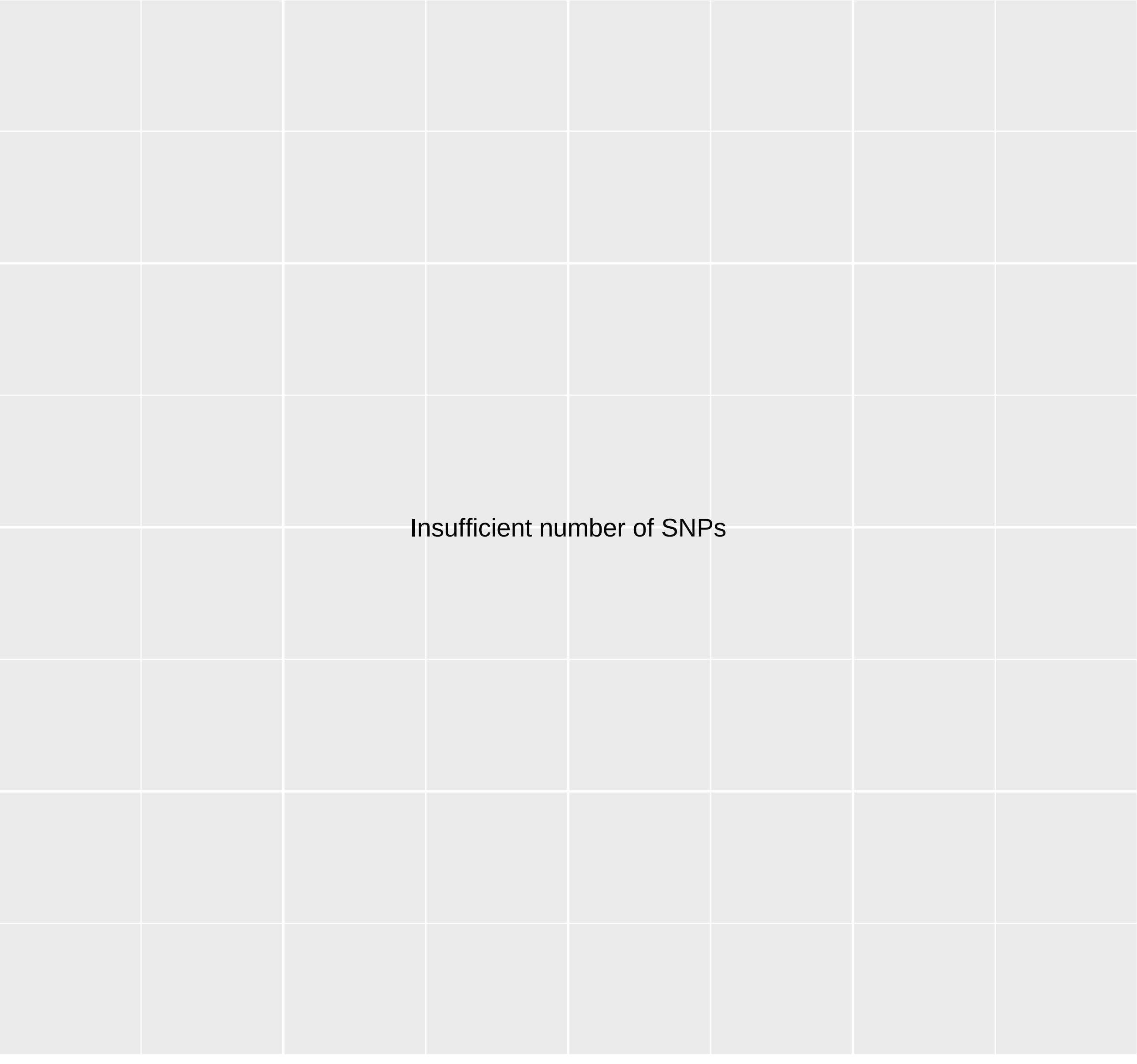
Insufficient number of SNPs

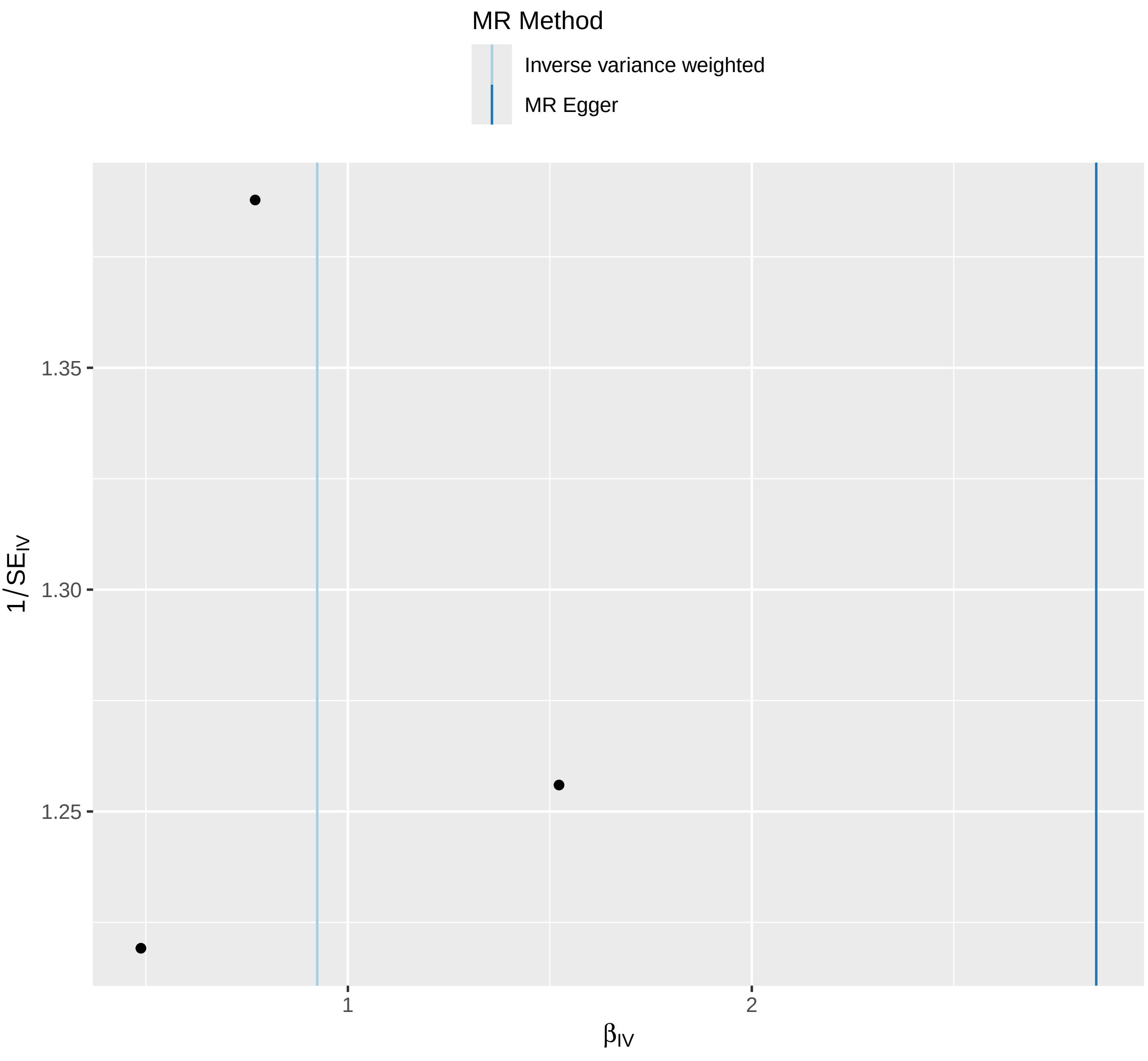

MR Method
Inverse variance weighted
MR Egger
1.35
1.30
1.25
1/SE_IV
1
2
β_IV

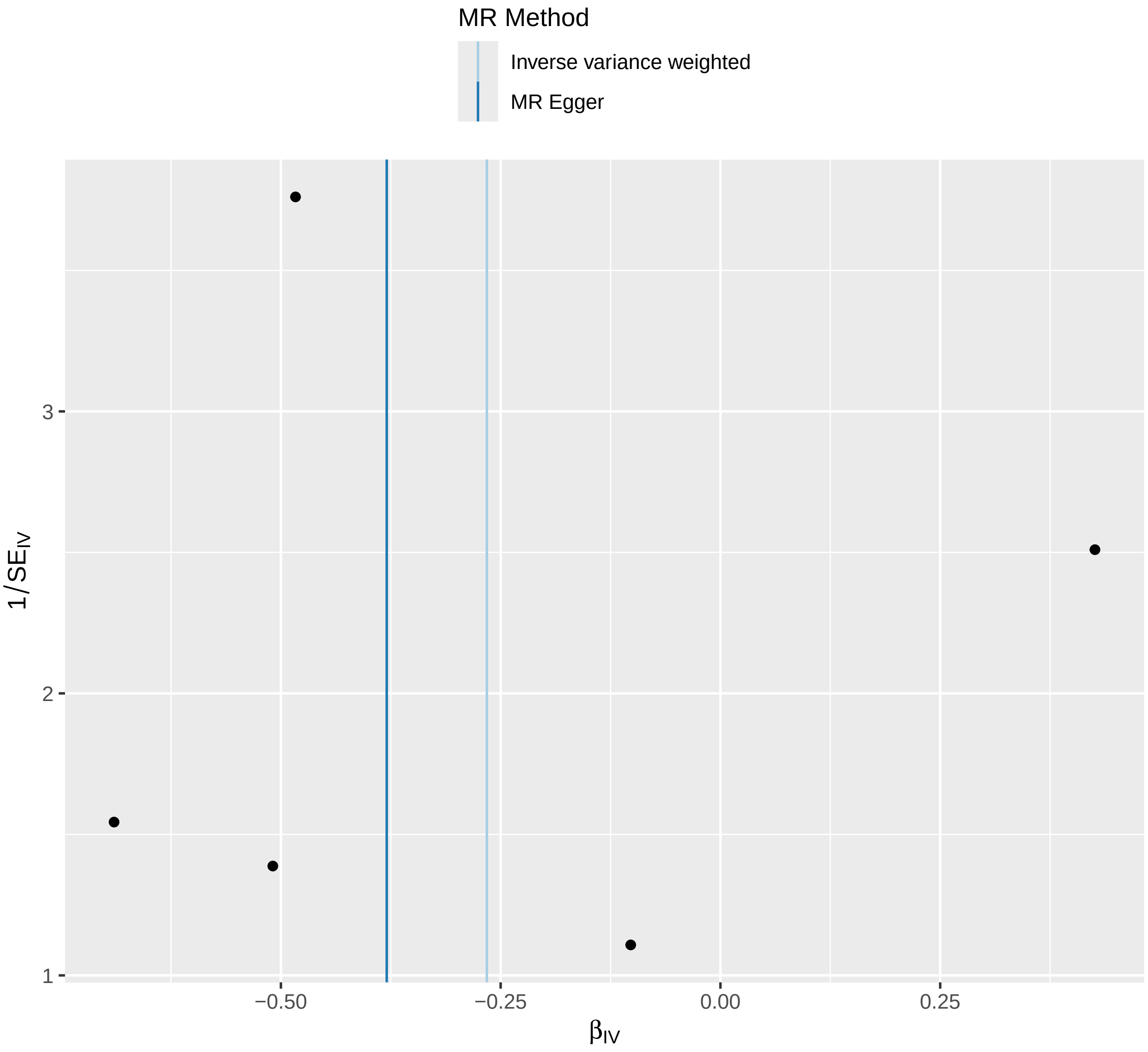
MR Method
Inverse variance weighted
MR Egger
1/SE_{IV}
3
2
1
−0.50
−0.25
0.00
0.25
β_{IV}

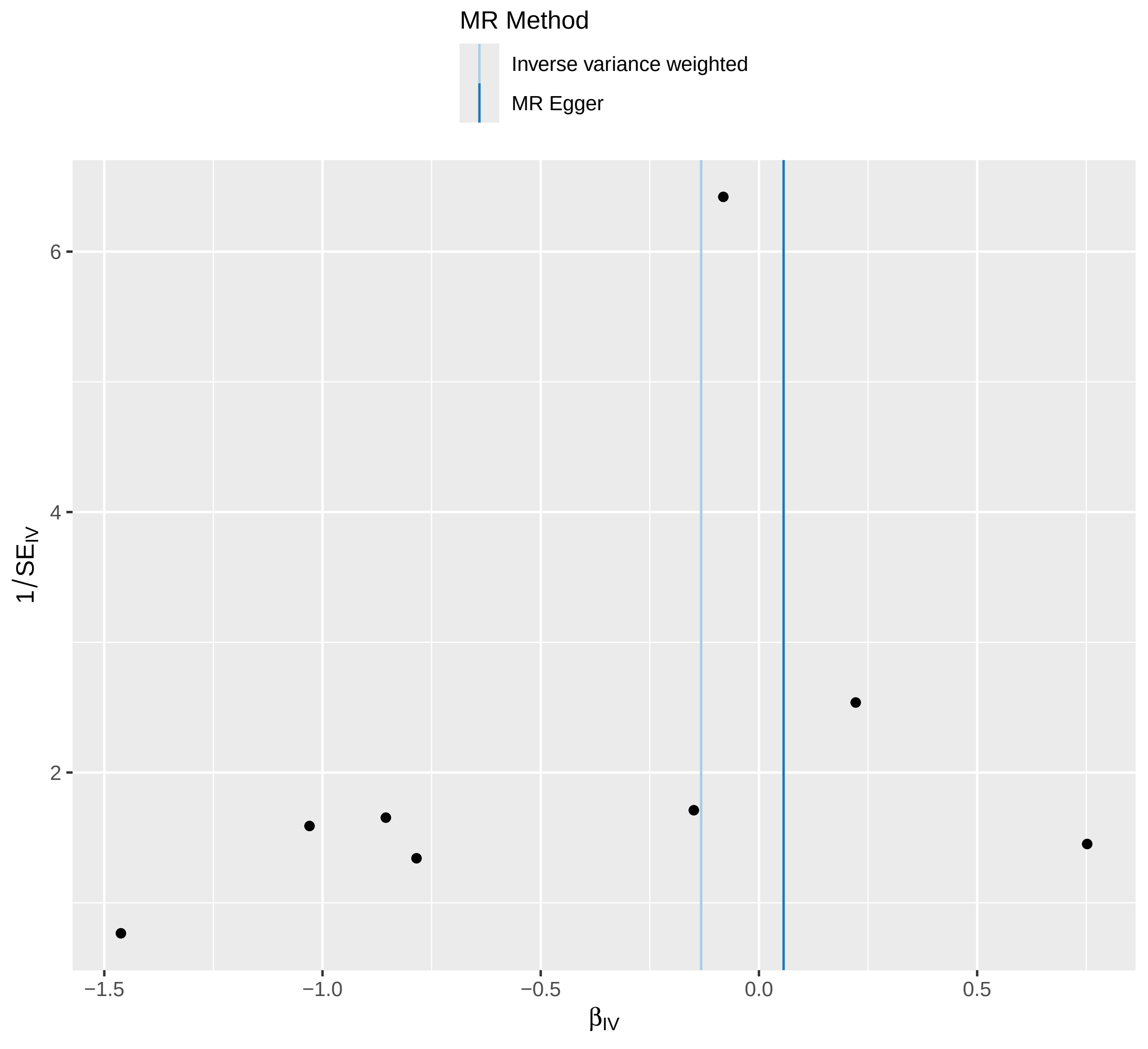

MR Method
Inverse variance weighted
MR Egger
6
4
2
1/SE_IV
−1.5
−1.0
−0.5
0.0
0.5
β_IV

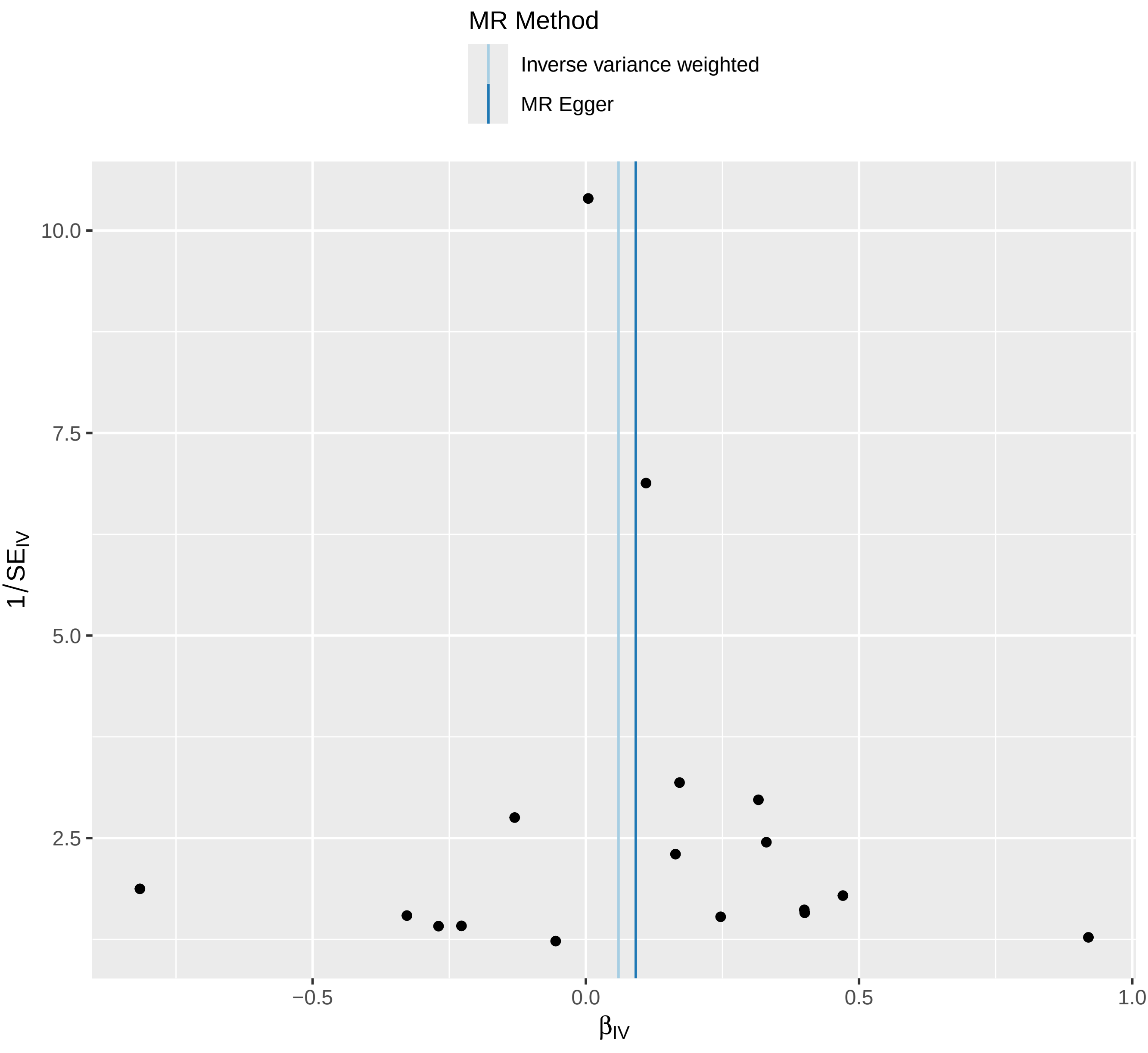
MR Method
Inverse variance weighted
MR Egger
1/SE_{IV}
10.0
7.5
5.0
2.5
−0.5
0.0
0.5
1.0
β_{IV}

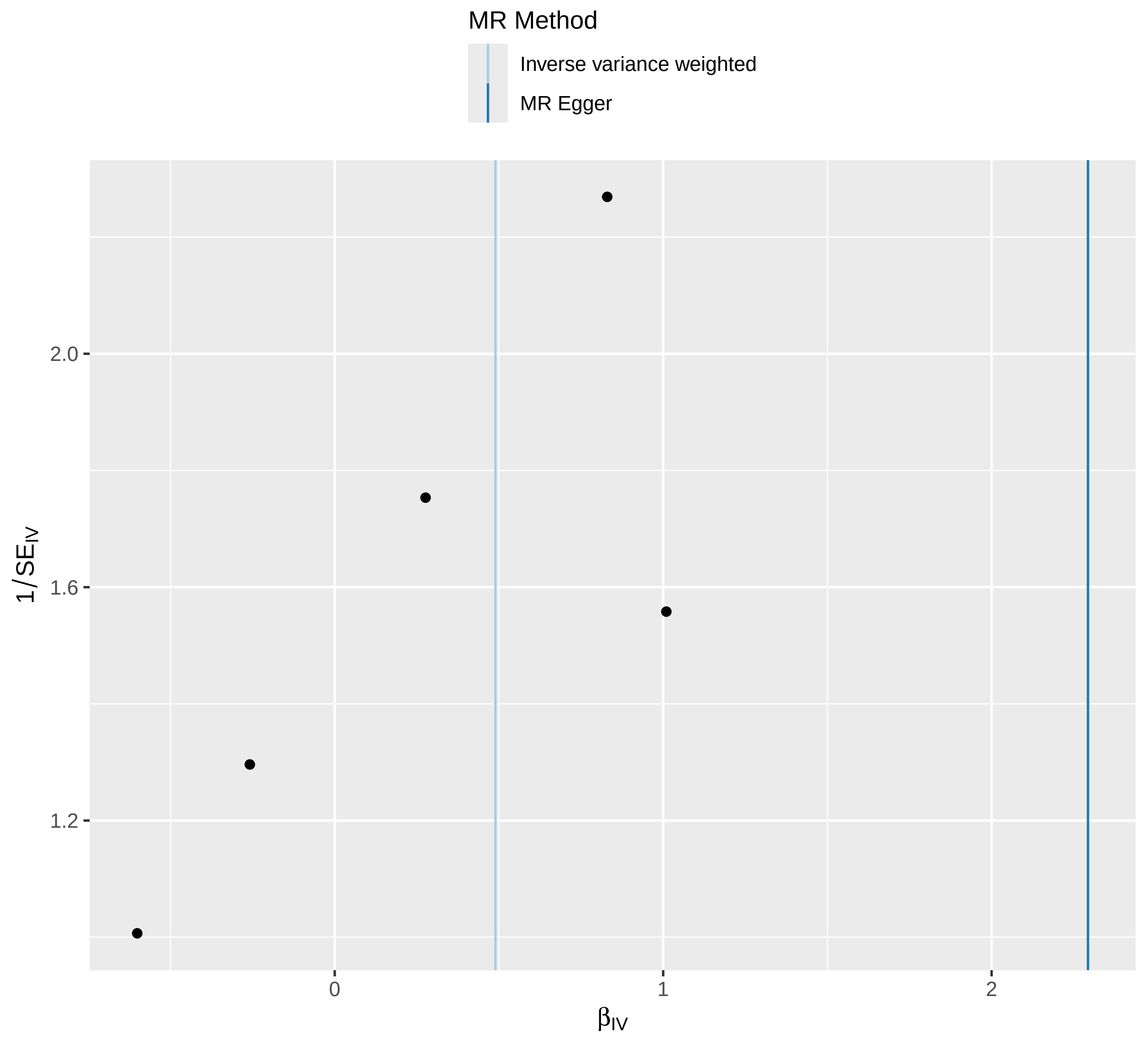

MR Method
Inverse variance weighted
MR Egger
2.0
1.6
1.2
0
1
2
1/SE_IV
β_IV

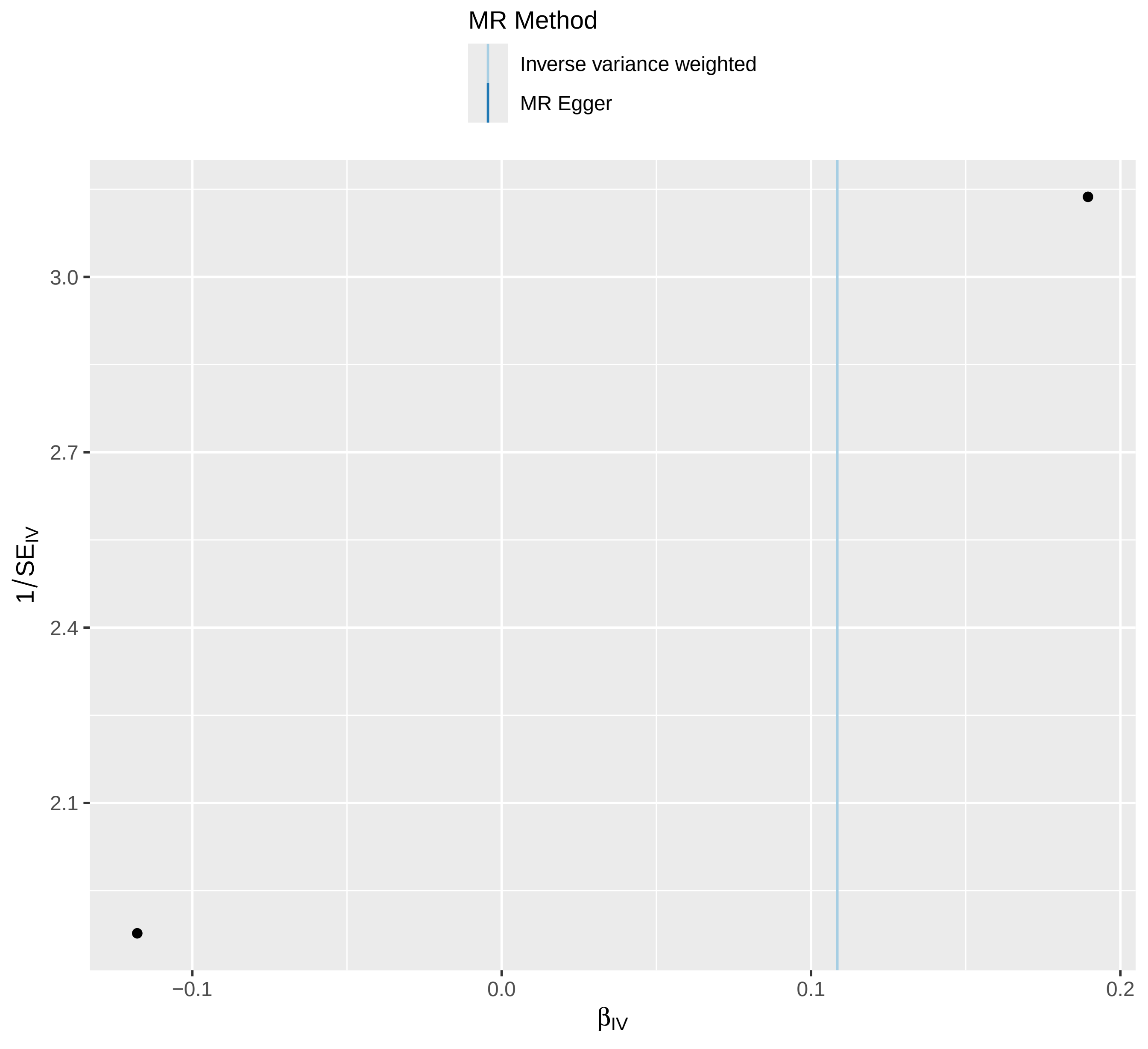
MR Method
Inverse variance weighted
MR Egger
3.0
2.7
2.4
2.1
1/SE_IV
−0.1
0.0
0.1
0.2
β_IV

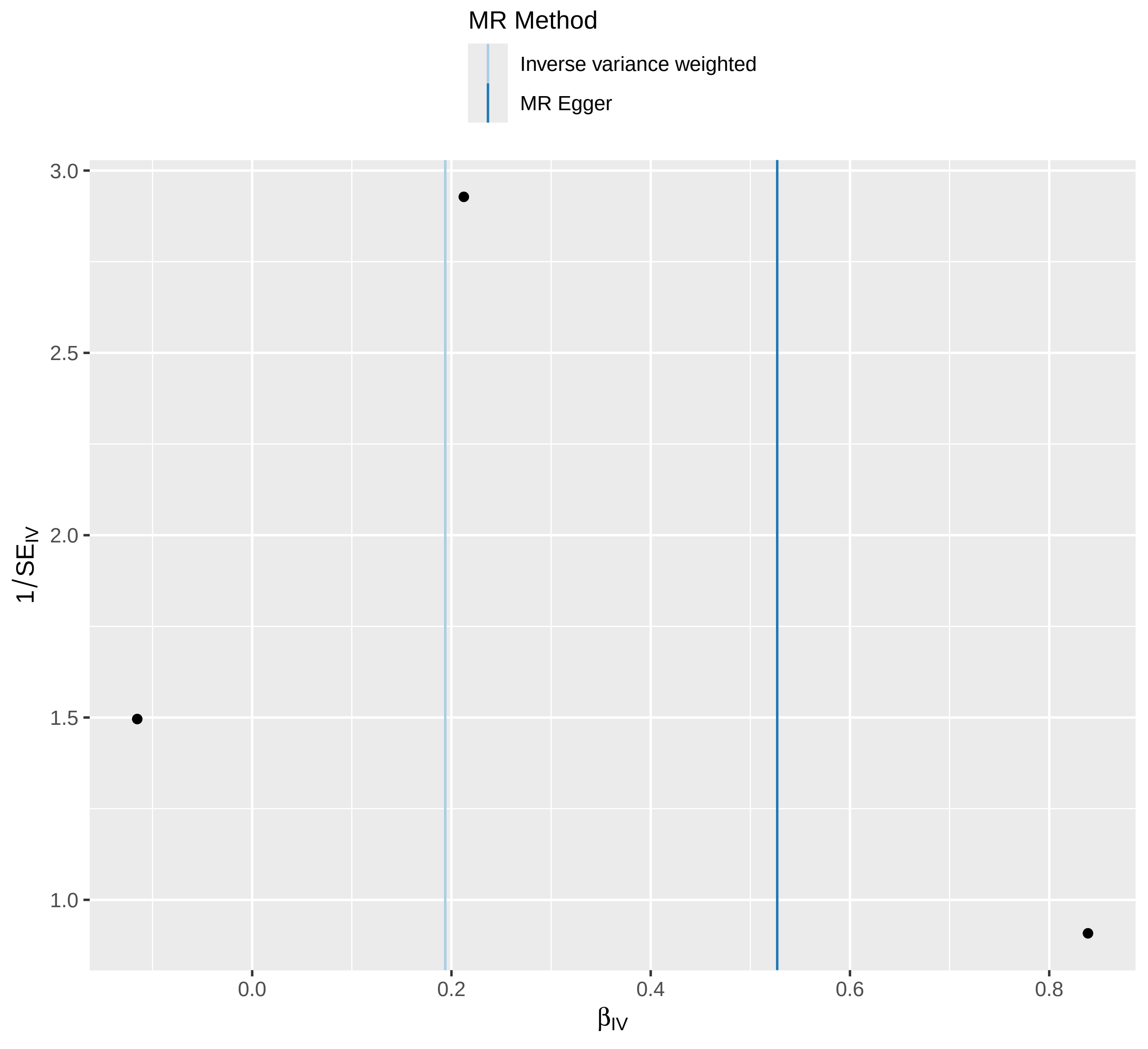

MR Method
Inverse variance weighted
MR Egger
3.0
2.5
2.0
1.5
1.0
1/SE_IV
0.0
0.2
0.4
0.6
0.8
β_IV

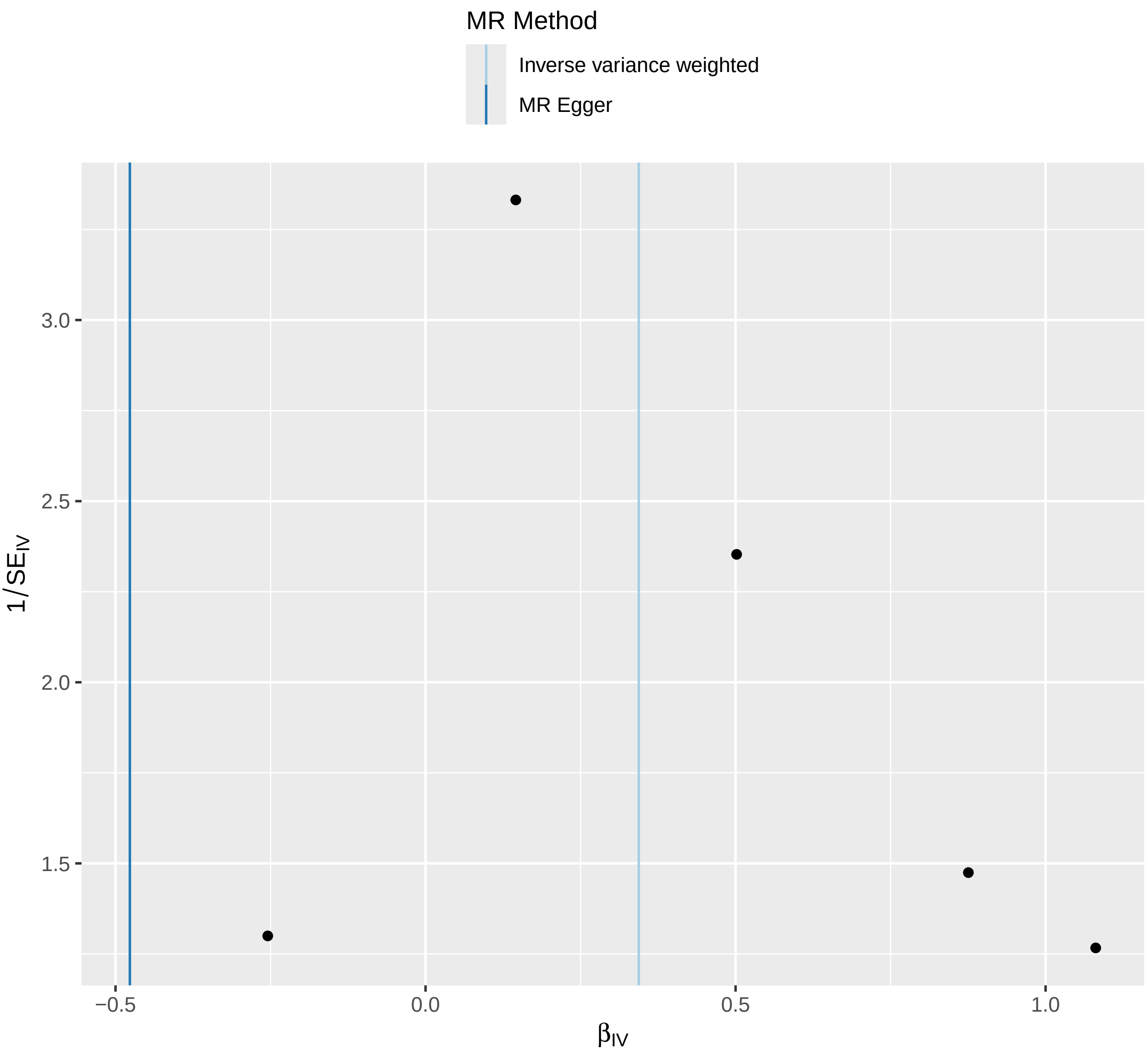

MR Method
Inverse variance weighted
MR Egger
3.0
2.5
2.0
1.5
1/SE$_{IV}$
−0.5
0.0
0.5
1.0
β$_{IV}$

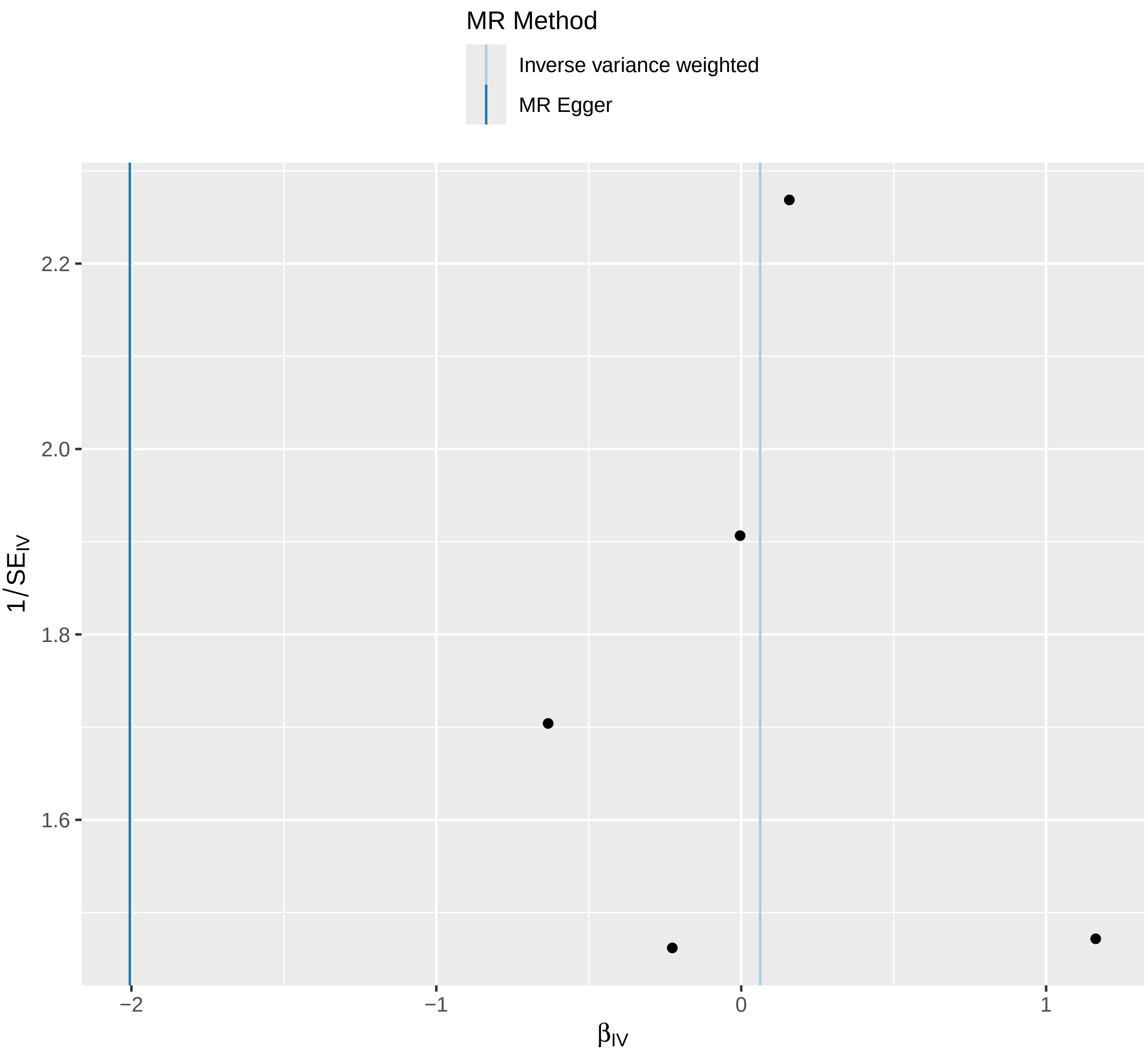
MR Method
Inverse variance weighted
MR Egger
2.2
2.0
1.8
1.6
1/SE_IV
−2
−1
0
1
β_IV

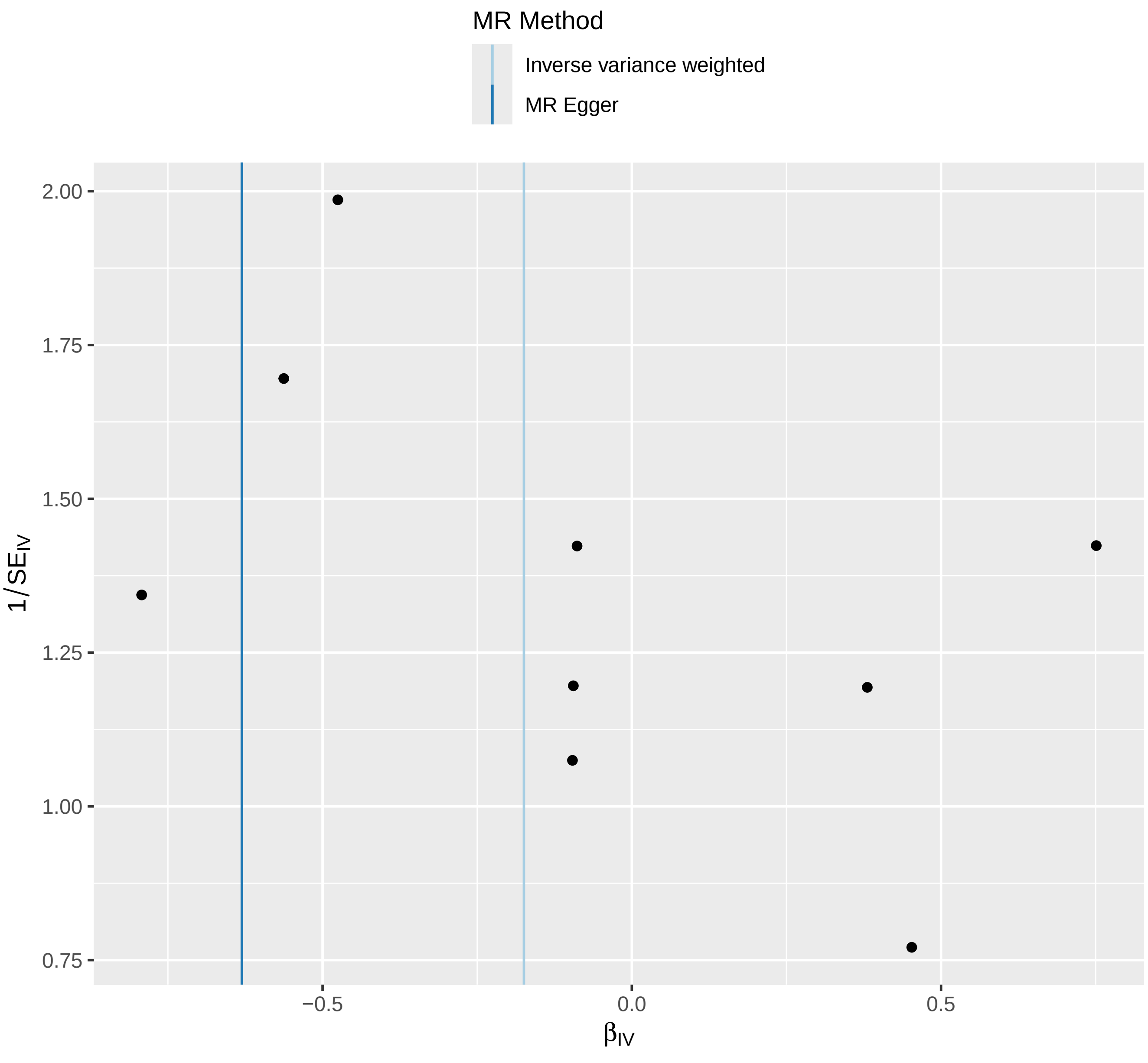
MR Method
Inverse variance weighted
MR Egger
2.00
1.75
1.50
1.25
1.00
0.75
1/SE_IV
−0.5
0.0
0.5
β_IV

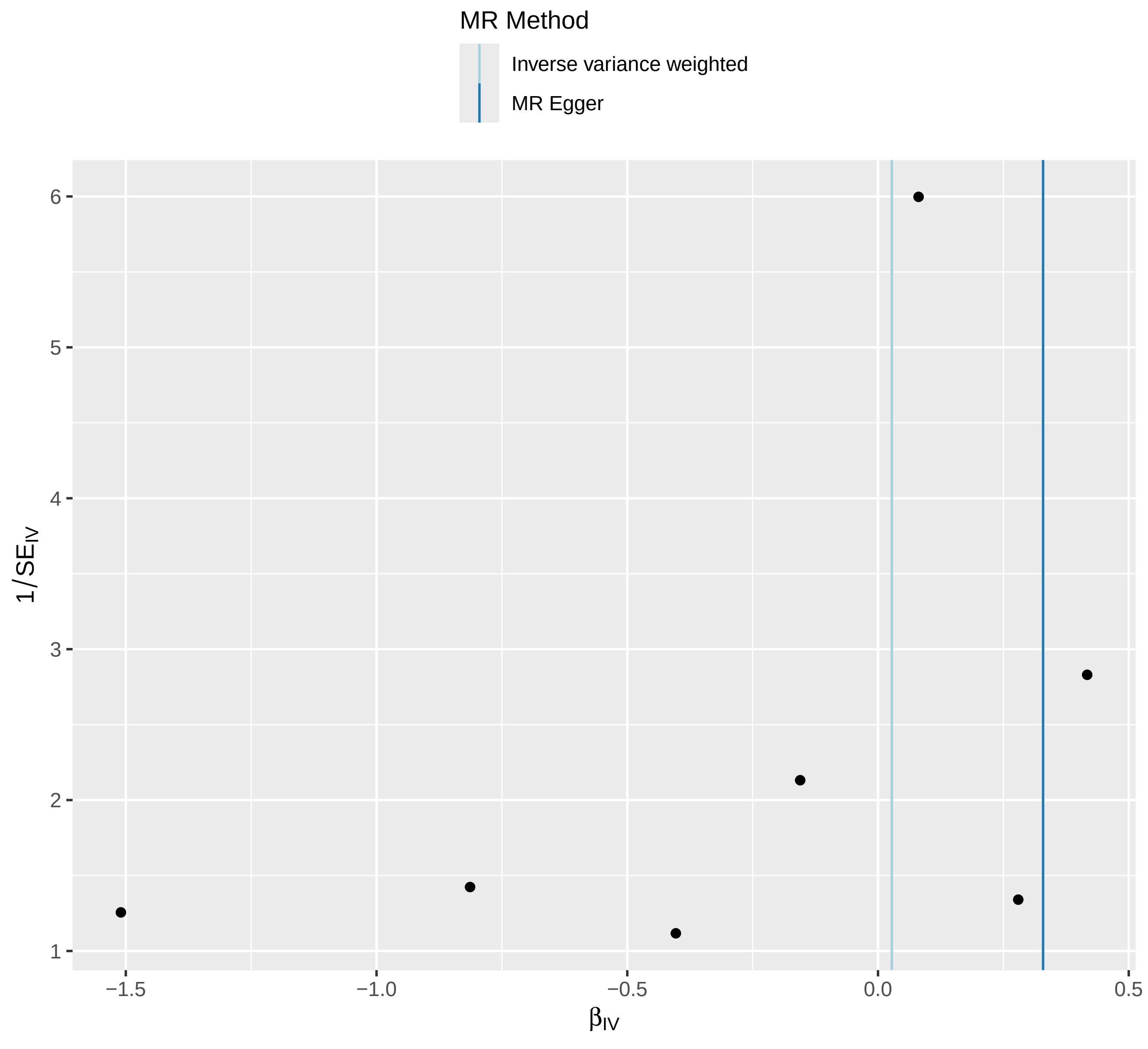
MR Method
Inverse variance weighted
MR Egger
1/SE_IV
β_IV
6
5
4
3
2
1
−1.5
−1.0
−0.5
0.0
0.5

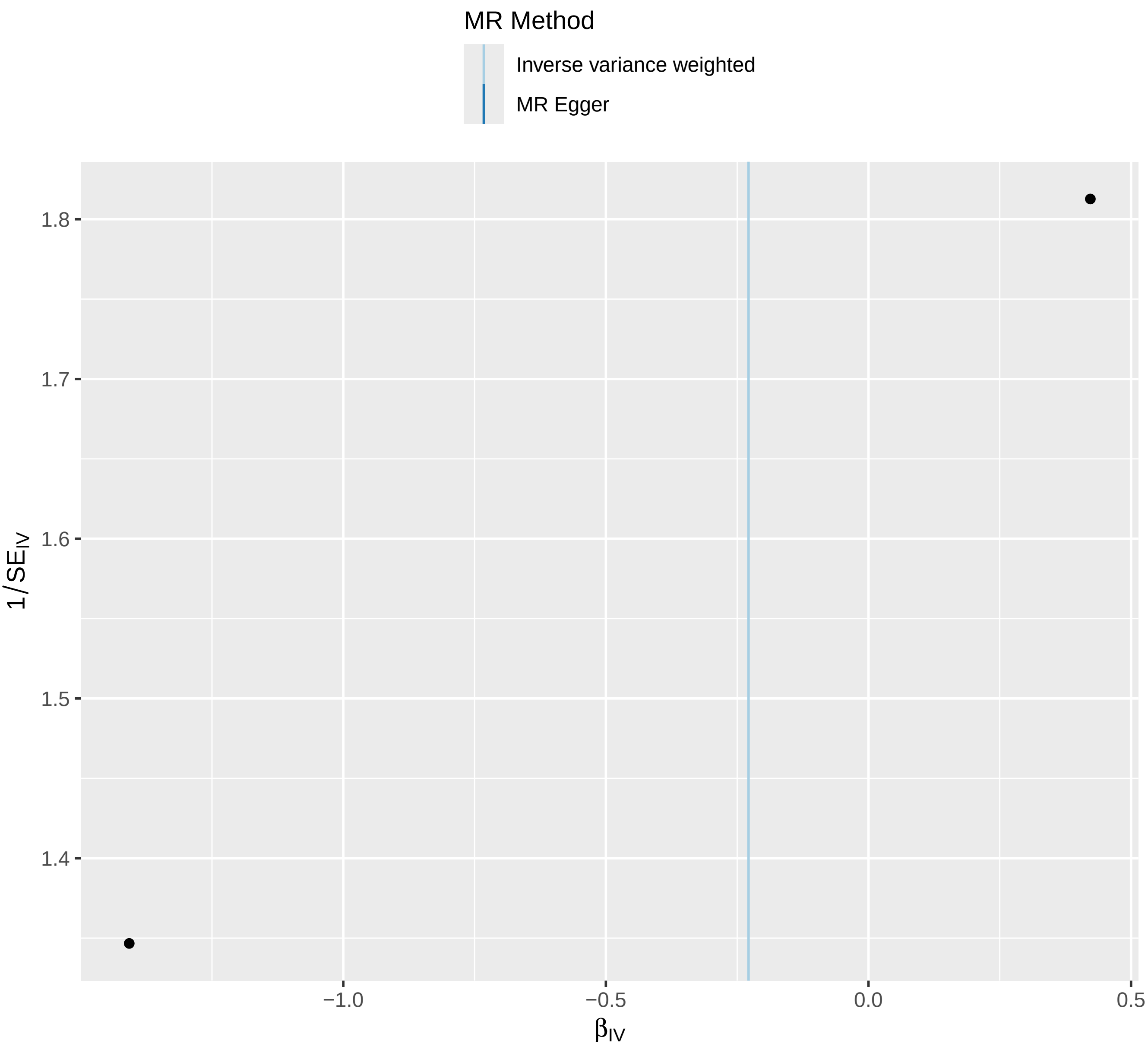
MR Method
Inverse variance weighted
MR Egger
1.8
1.7
1.6
1.5
1.4
1/SE_IV
−1.0
−0.5
0.0
0.5
β_IV

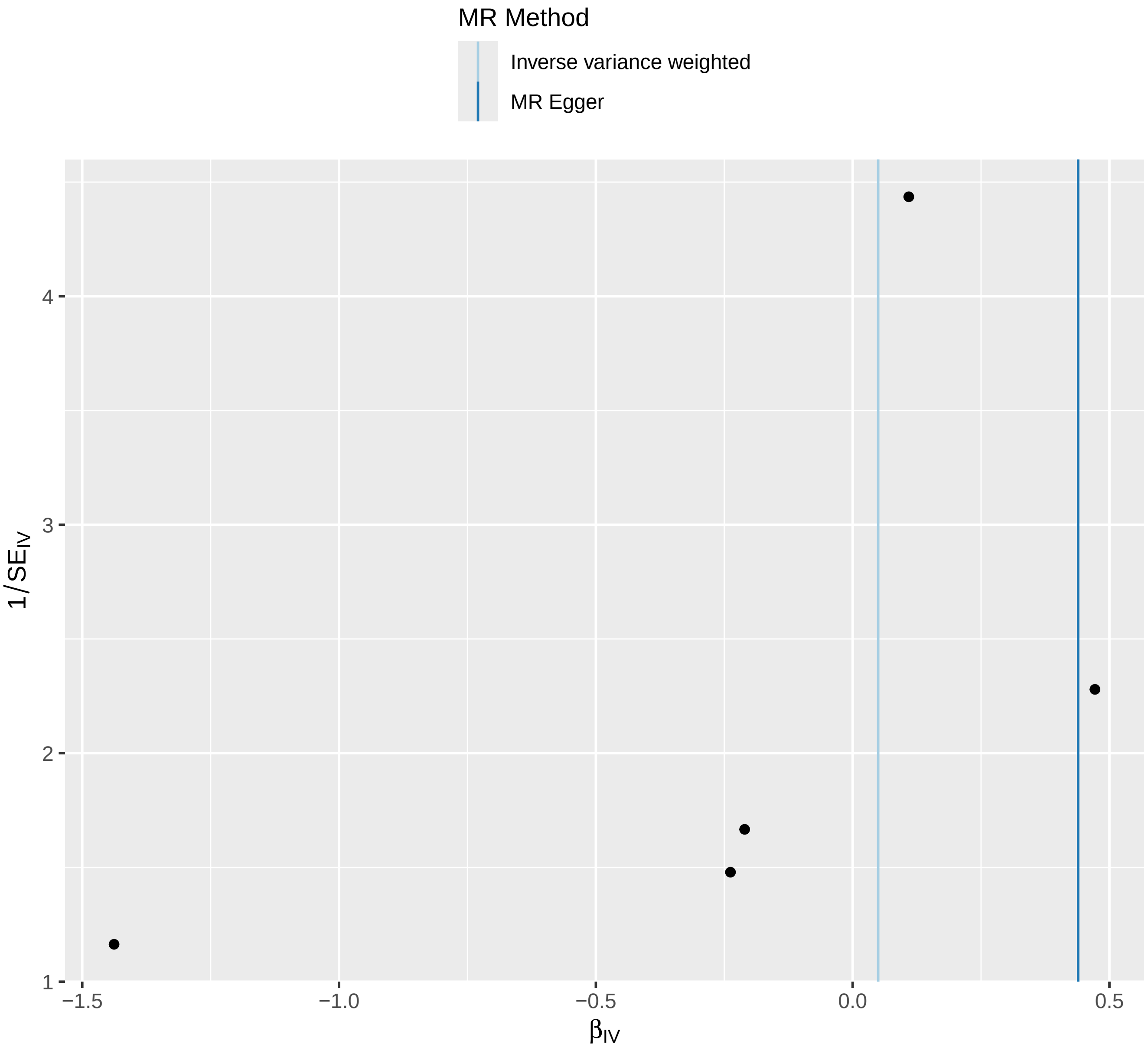
MR Method
Inverse variance weighted
MR Egger
1/SE_IV
4
3
2
1
−1.5
−1.0
−0.5
0.0
0.5
β_IV

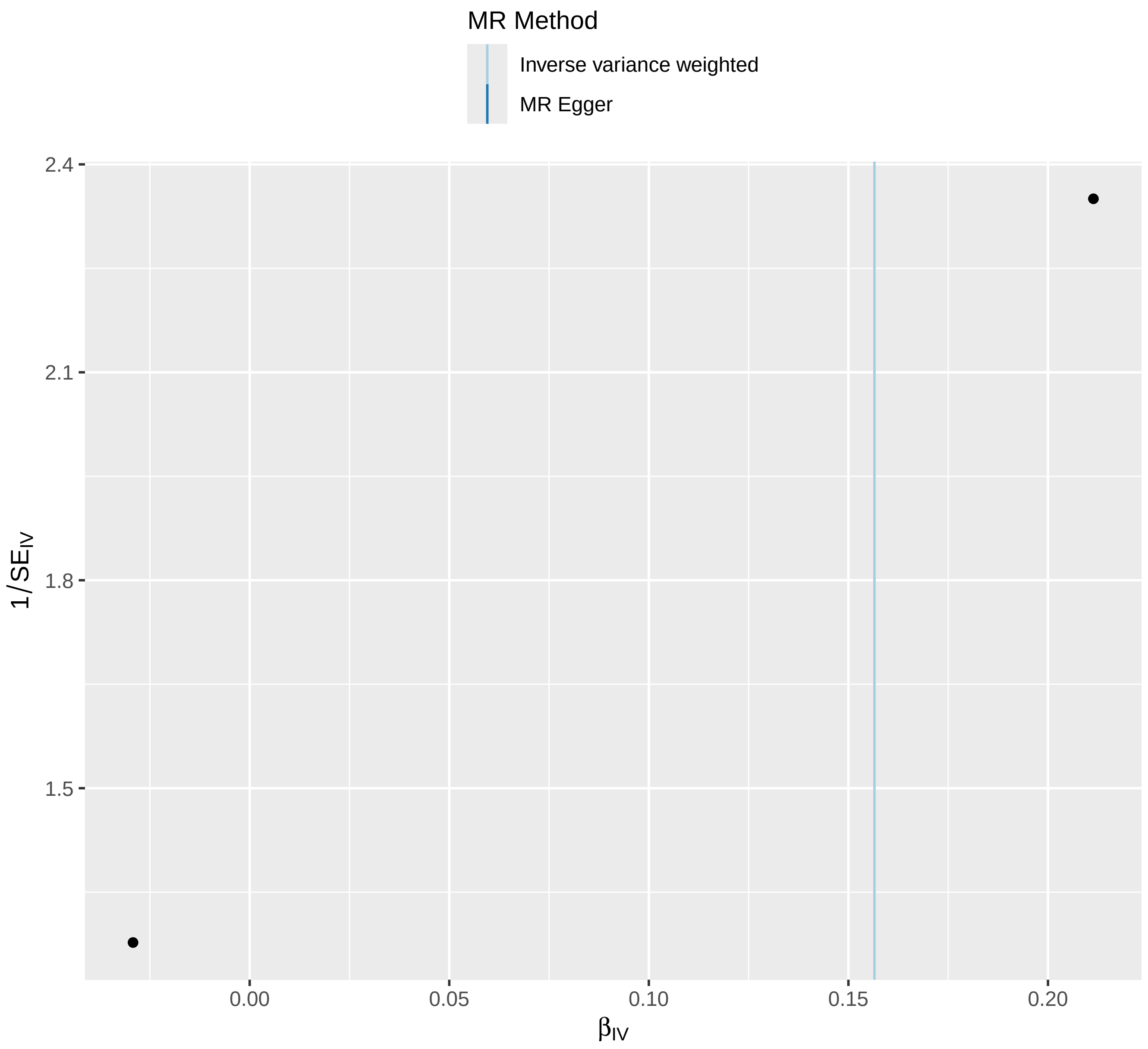

MR Method
Inverse variance weighted
MR Egger
2.4
2.1
1.8
1.5
$1/SE_{IV}$
0.00
0.05
0.10
0.15
0.20
β_{IV}

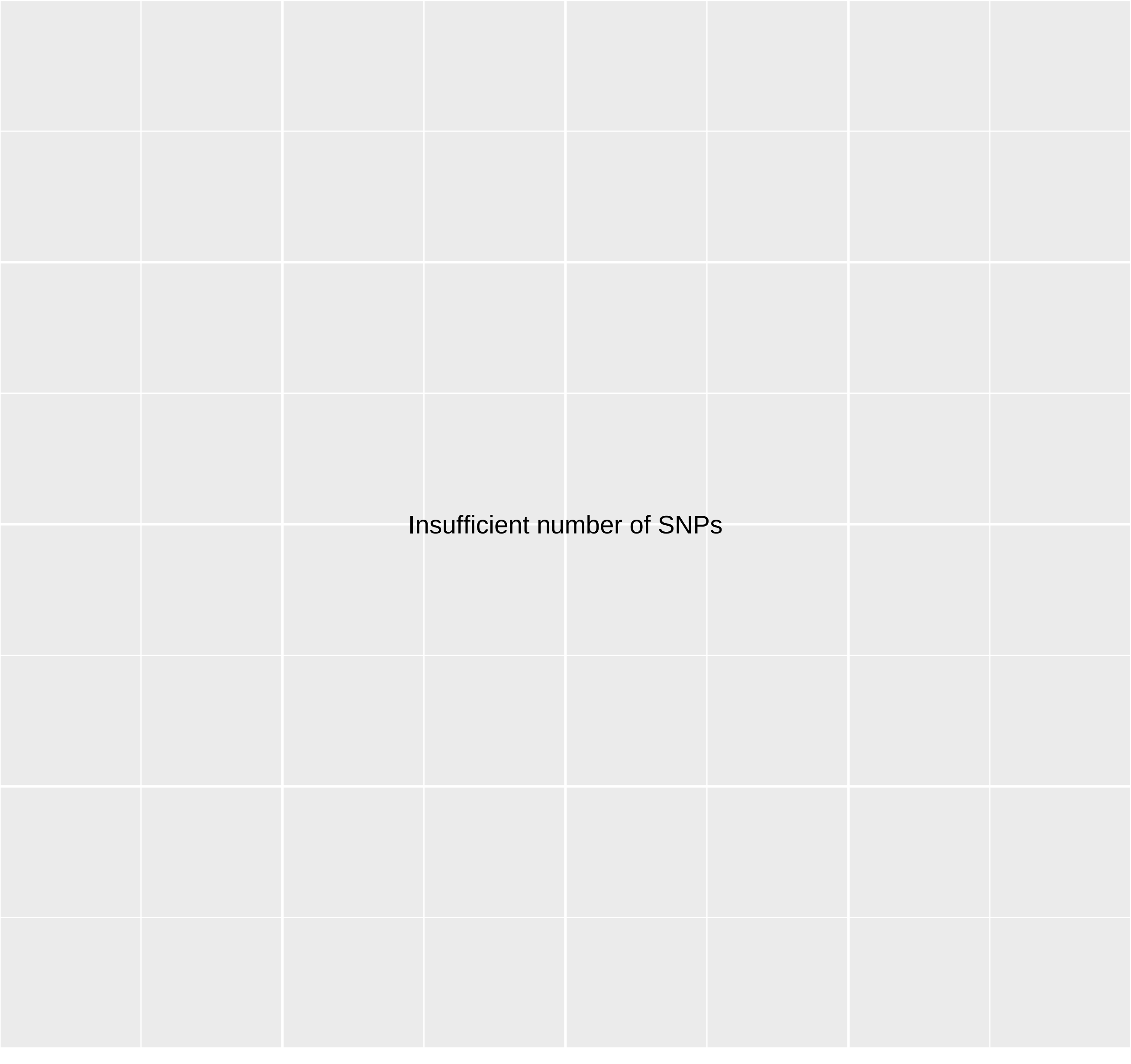
Insufficient number of SNPs

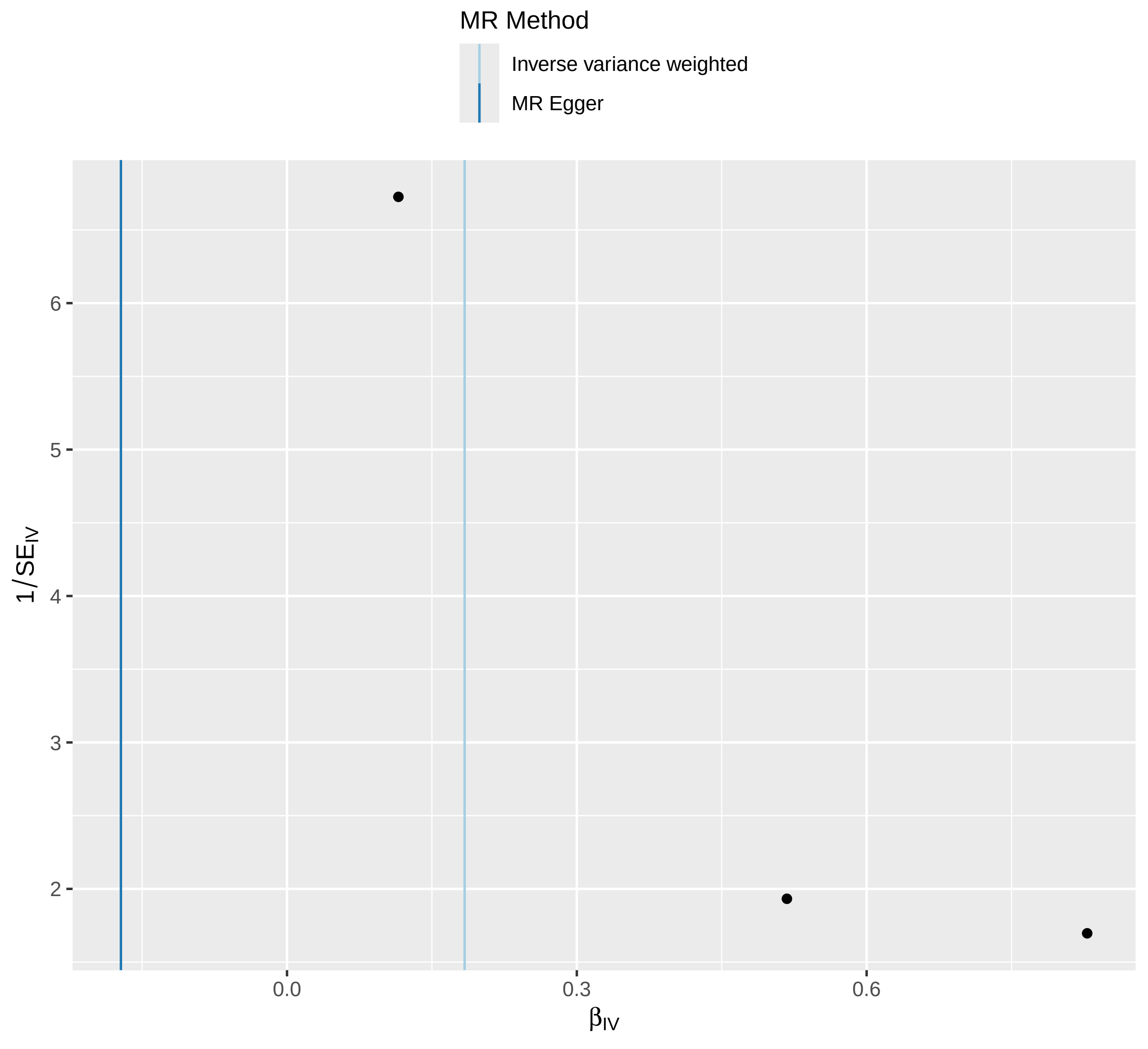
MR Method
Inverse variance weighted
MR Egger
6
5
4
3
2
$1/SE_{IV}$
0.0
0.3
0.6
β_{IV}

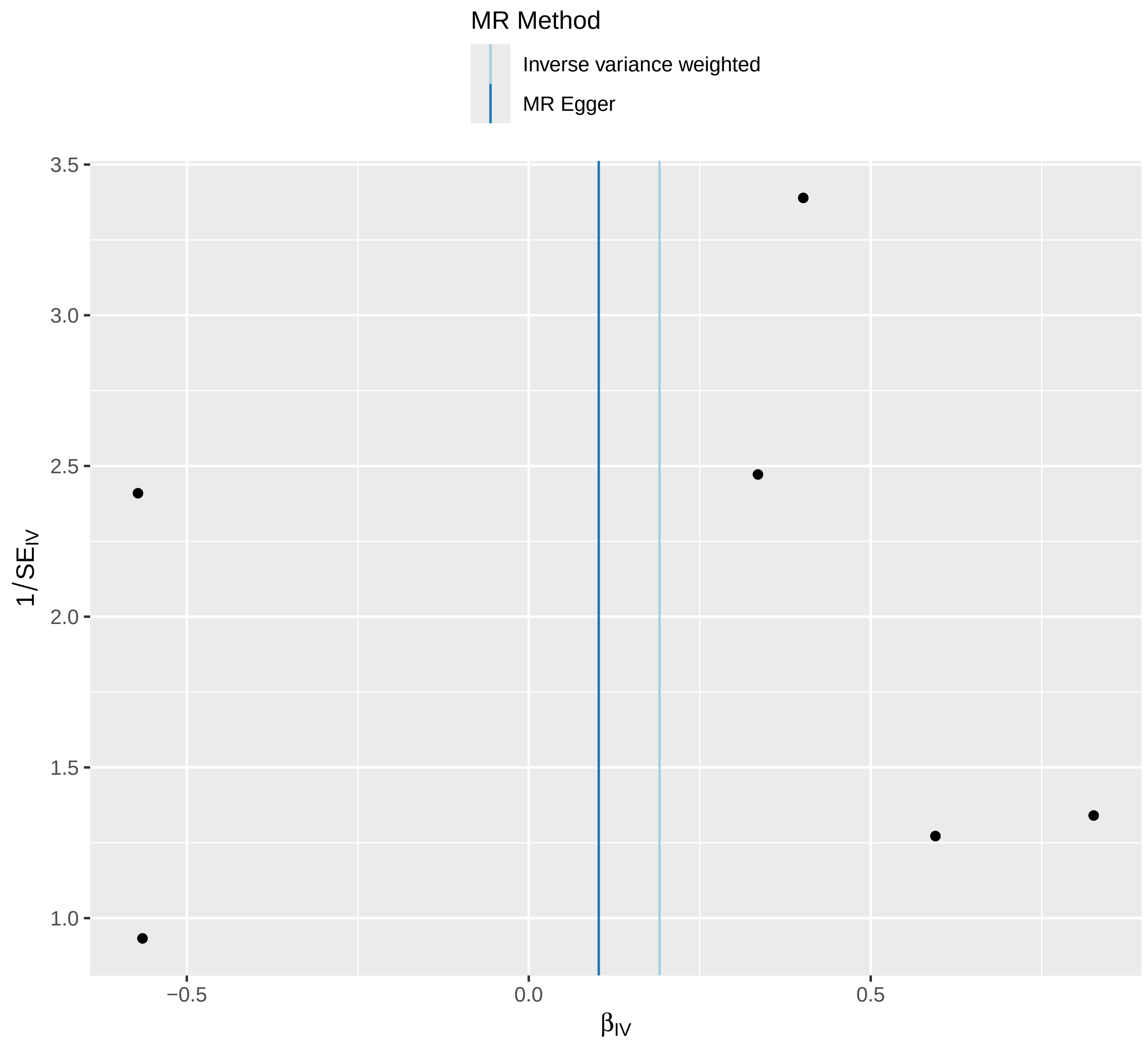
MR Method
Inverse variance weighted
MR Egger
3.5
3.0
2.5
2.0
1.5
1.0
1/SE$_{IV}$
−0.5
0.0
0.5
β$_{IV}$

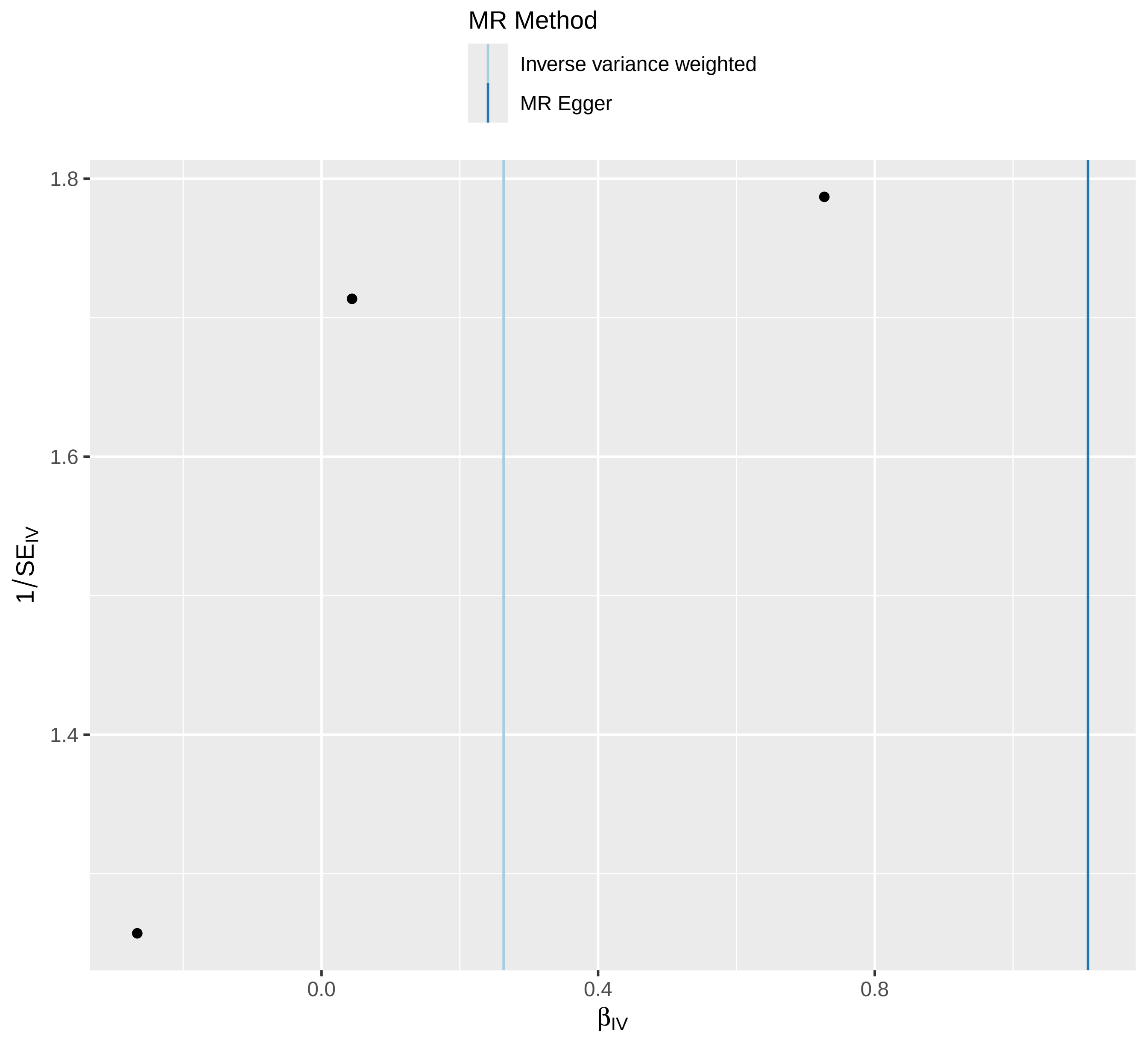

MR Method
Inverse variance weighted
MR Egger
1.8
1.6
1.4
$1/SE_{IV}$
0.0
0.4
0.8
β_{IV}

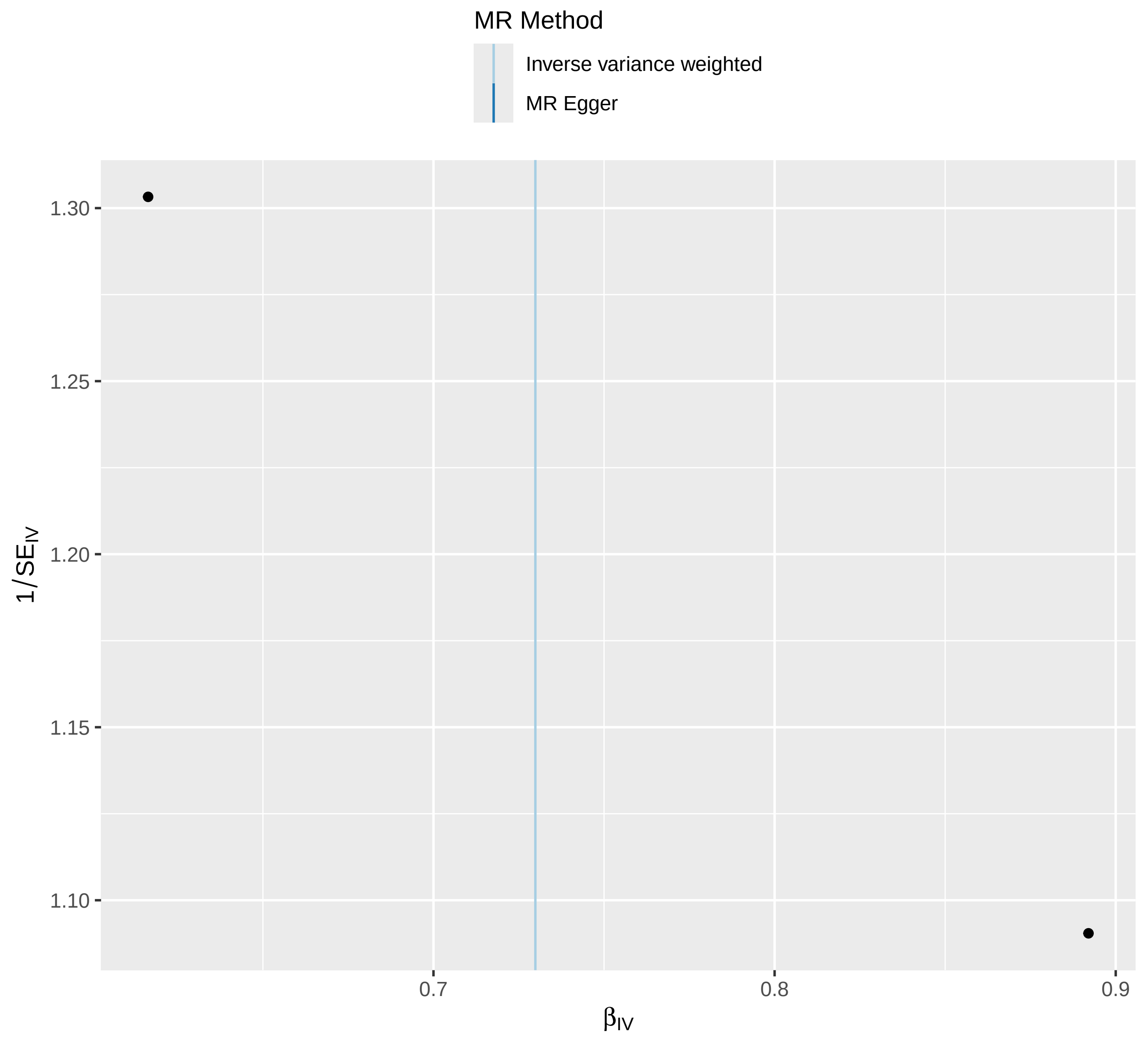
MR Method
Inverse variance weighted
MR Egger
1.30
1.25
1.20
1.15
1.10
1/SE_IV
0.7
0.8
0.9
β_IV

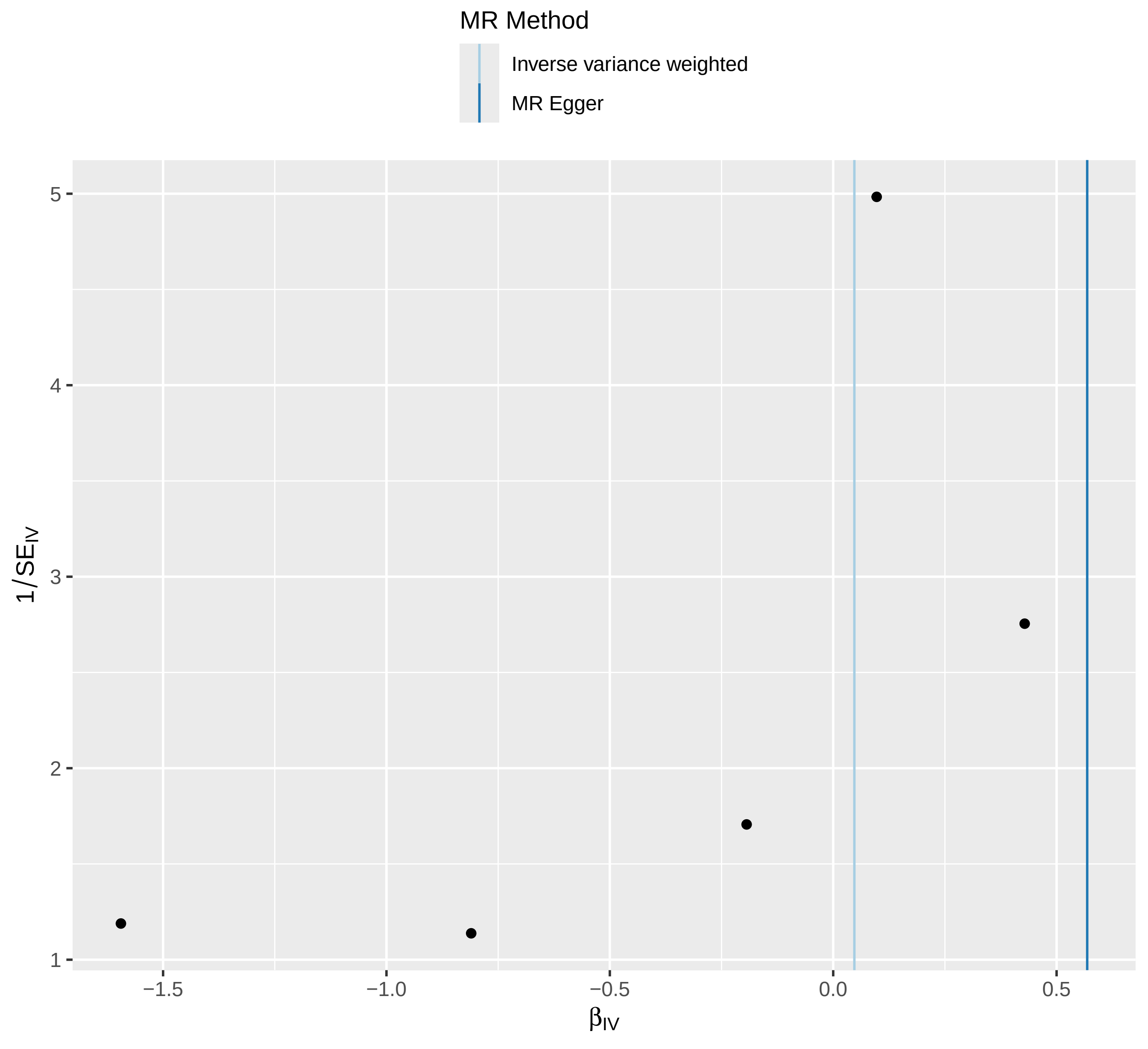

MR Method
Inverse variance weighted
MR Egger
5
4
3
2
1
$1/SE_{IV}$
−1.5
−1.0
−0.5
0.0
0.5
β_{IV}

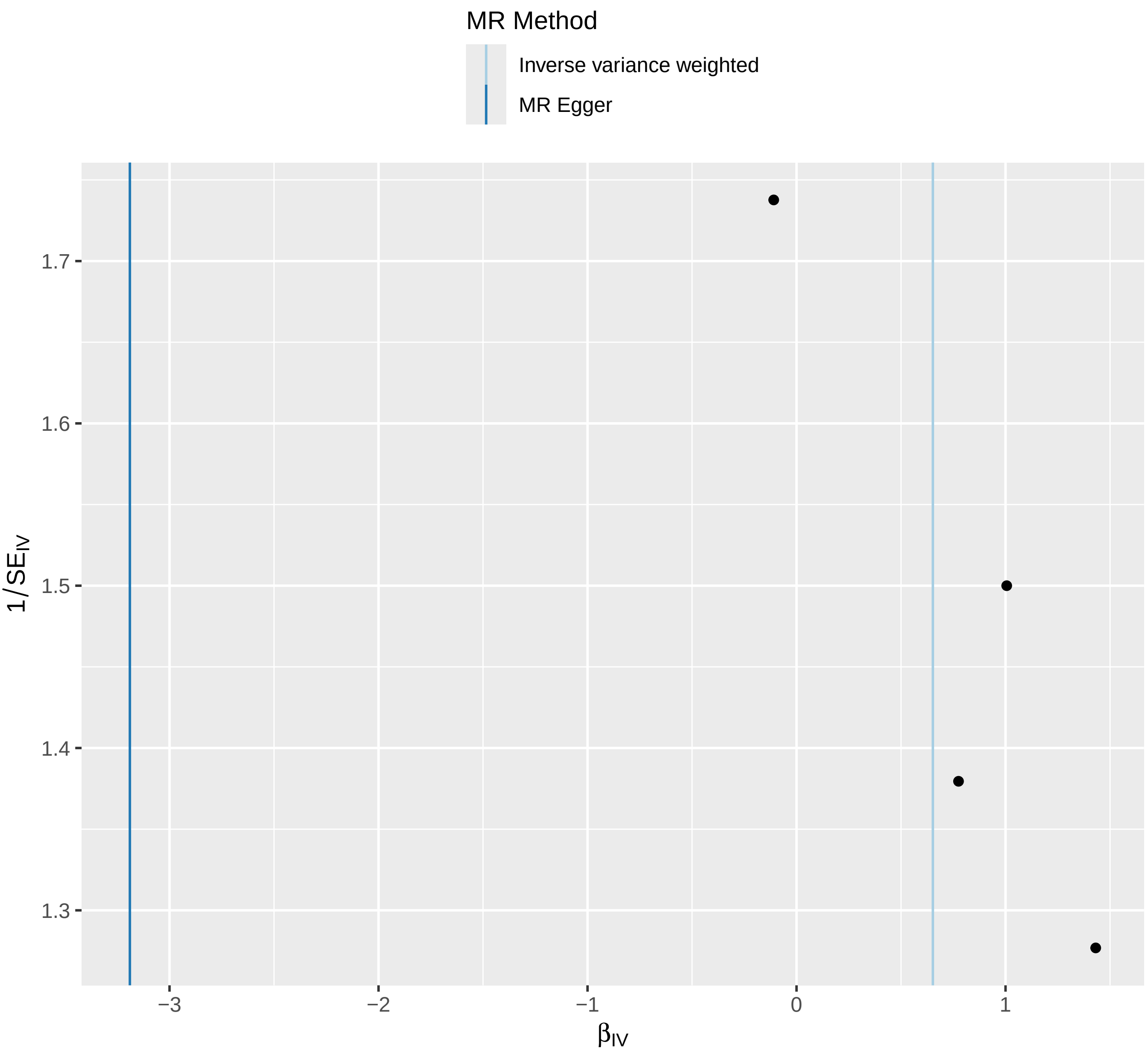

MR Method
Inverse variance weighted
MR Egger
1.7
1.6
1.5
1.4
1.3
1/SE_IV
−3
−2
−1
0
1
β_IV

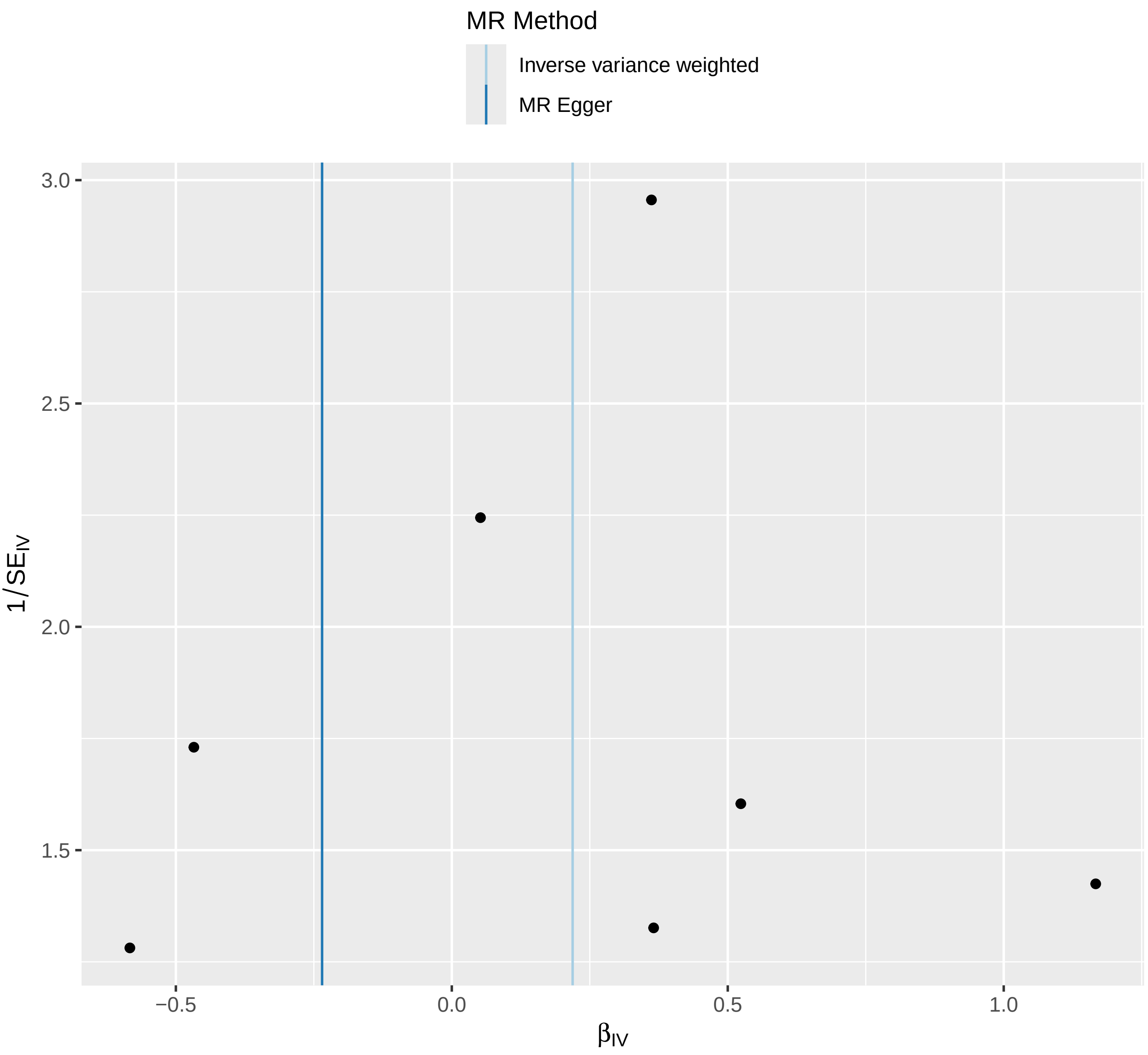
MR Method
Inverse variance weighted
MR Egger
3.0
2.5
2.0
1.5
$1/SE_{IV}$
−0.5
0.0
0.5
1.0
β_{IV}

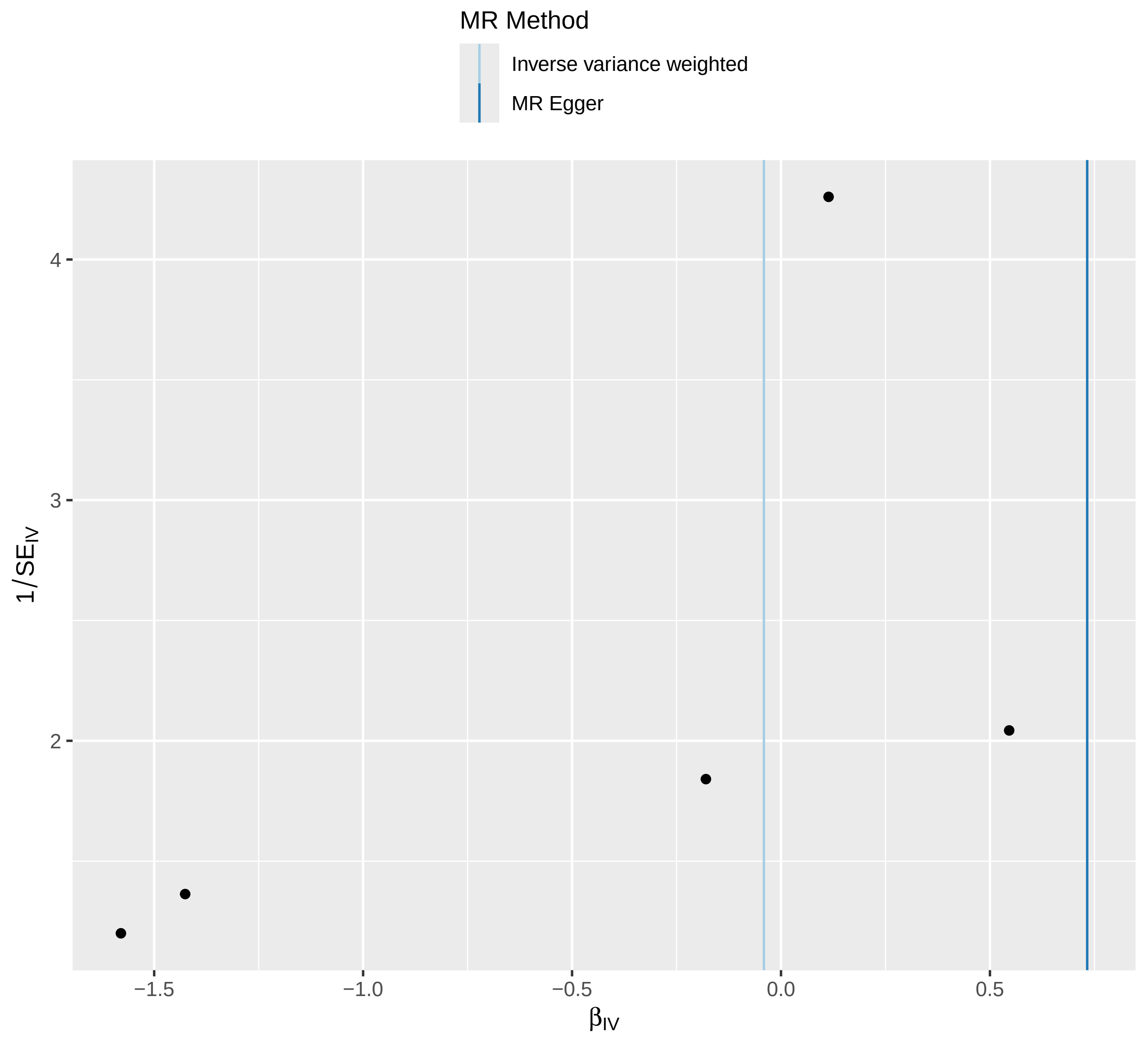
MR Method
Inverse variance weighted
MR Egger
4
3
2
1/SE_{IV}
−1.5
−1.0
−0.5
0.0
0.5
β_{IV}

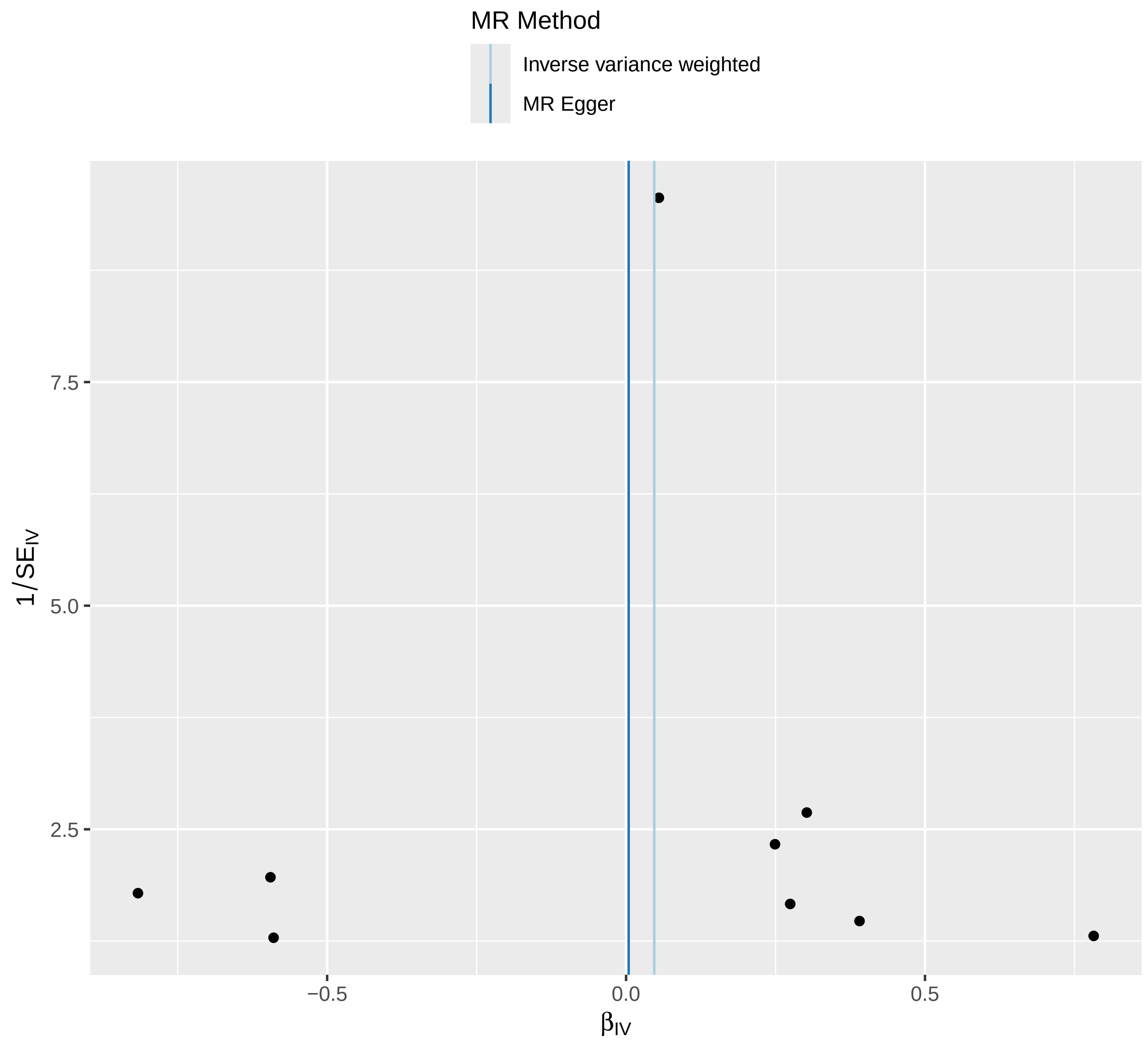

MR Method
Inverse variance weighted
MR Egger
7.5
5.0
2.5
1/SE$_{IV}$
−0.5
0.0
0.5
β_{IV}

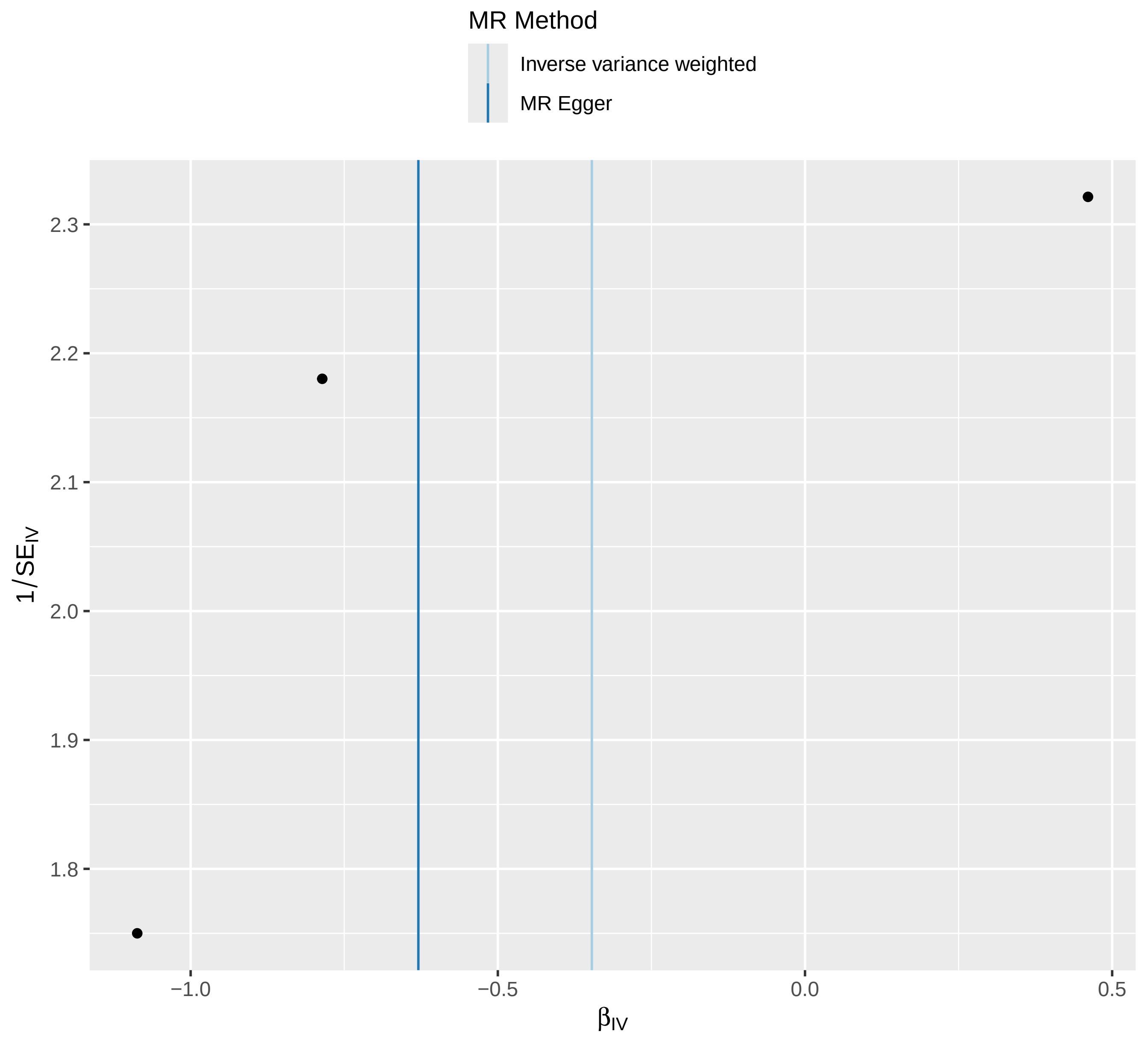

MR Method
Inverse variance weighted
MR Egger
2.3
2.2
2.1
2.0
1.9
1.8
$1/SE_{IV}$
−1.0
−0.5
0.0
0.5
β_{IV}

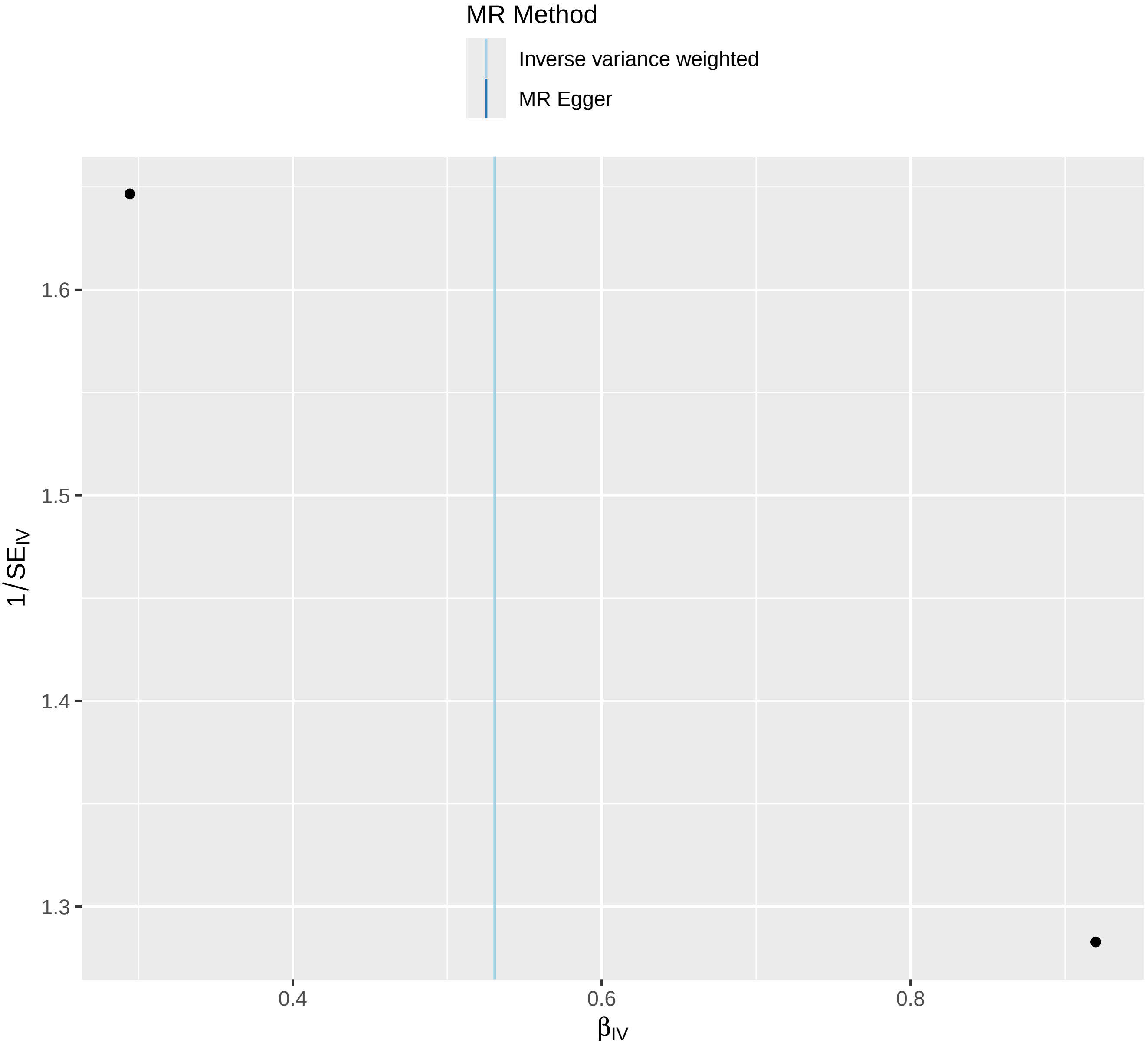

MR Method
Inverse variance weighted
MR Egger
1.6
1.5
1.4
1.3
$1/SE_{IV}$
0.4
0.6
0.8
β_{IV}

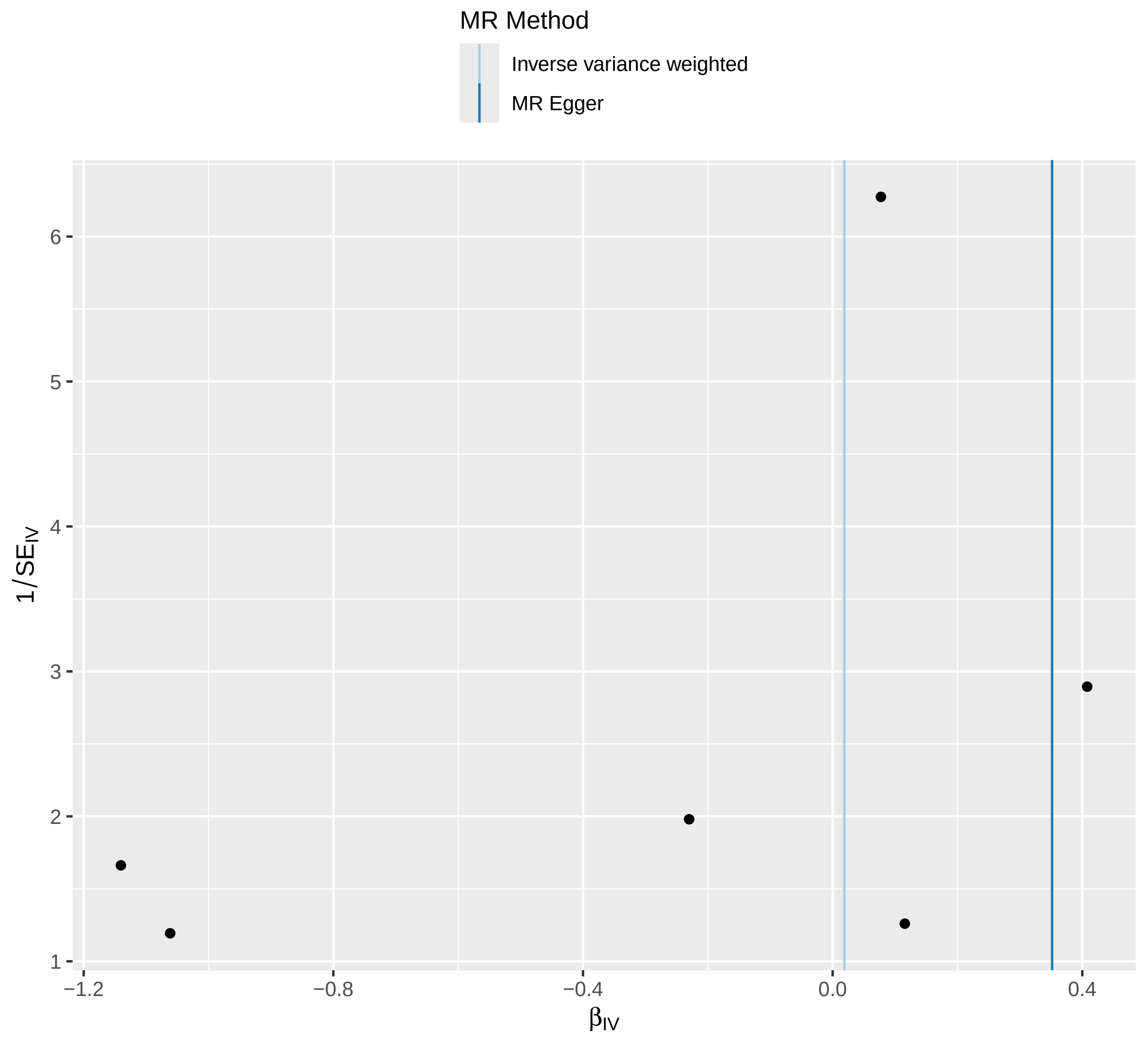
MR Method
Inverse variance weighted
MR Egger
$1/SE_{IV}$
β_{IV}
1
2
3
4
5
6
−1.2
−0.8
−0.4
0.0
0.4

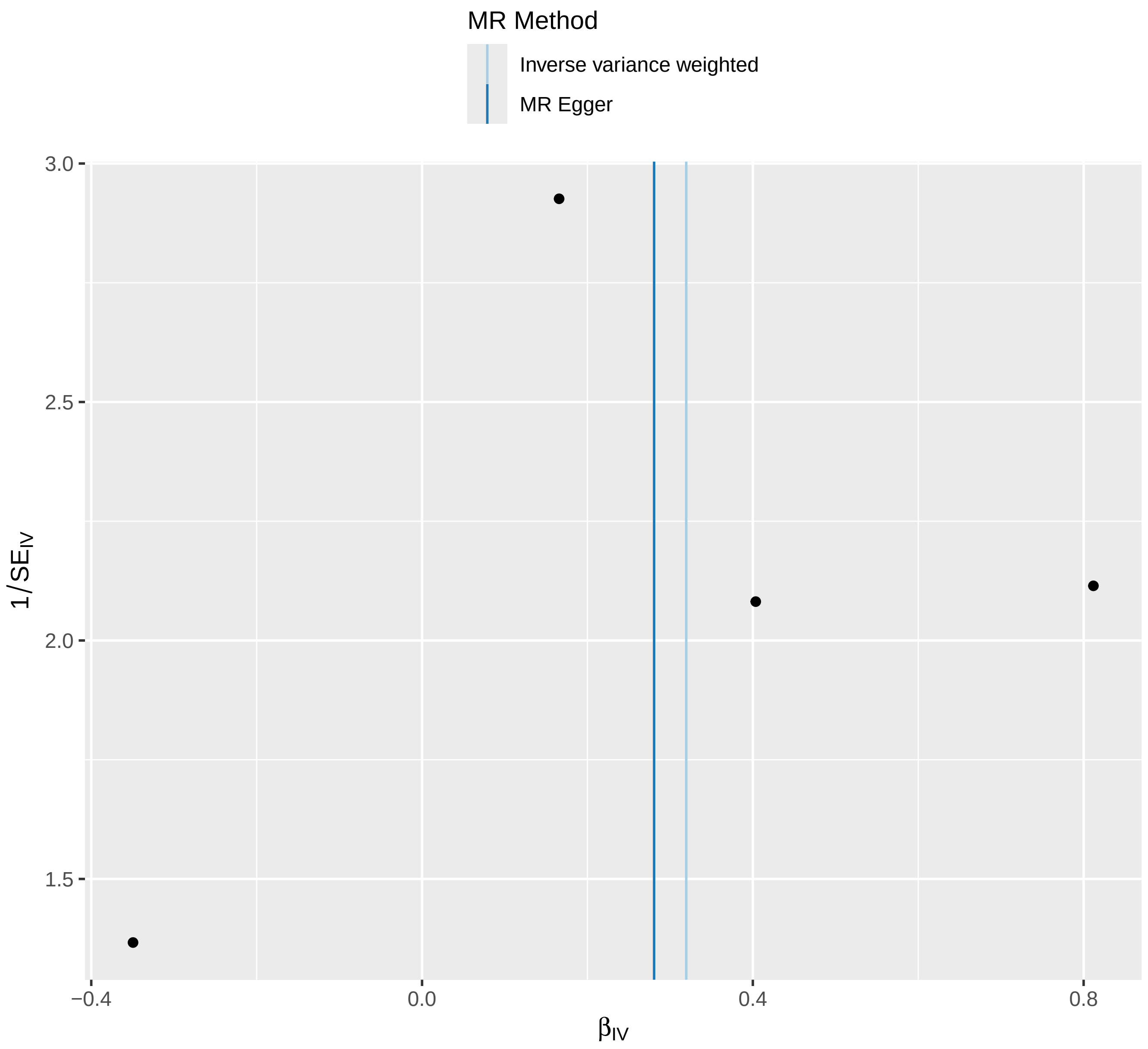

MR Method
Inverse variance weighted
MR Egger
3.0
2.5
2.0
1.5
$1/SE_{IV}$
−0.4
0.0
0.4
0.8
β_{IV}

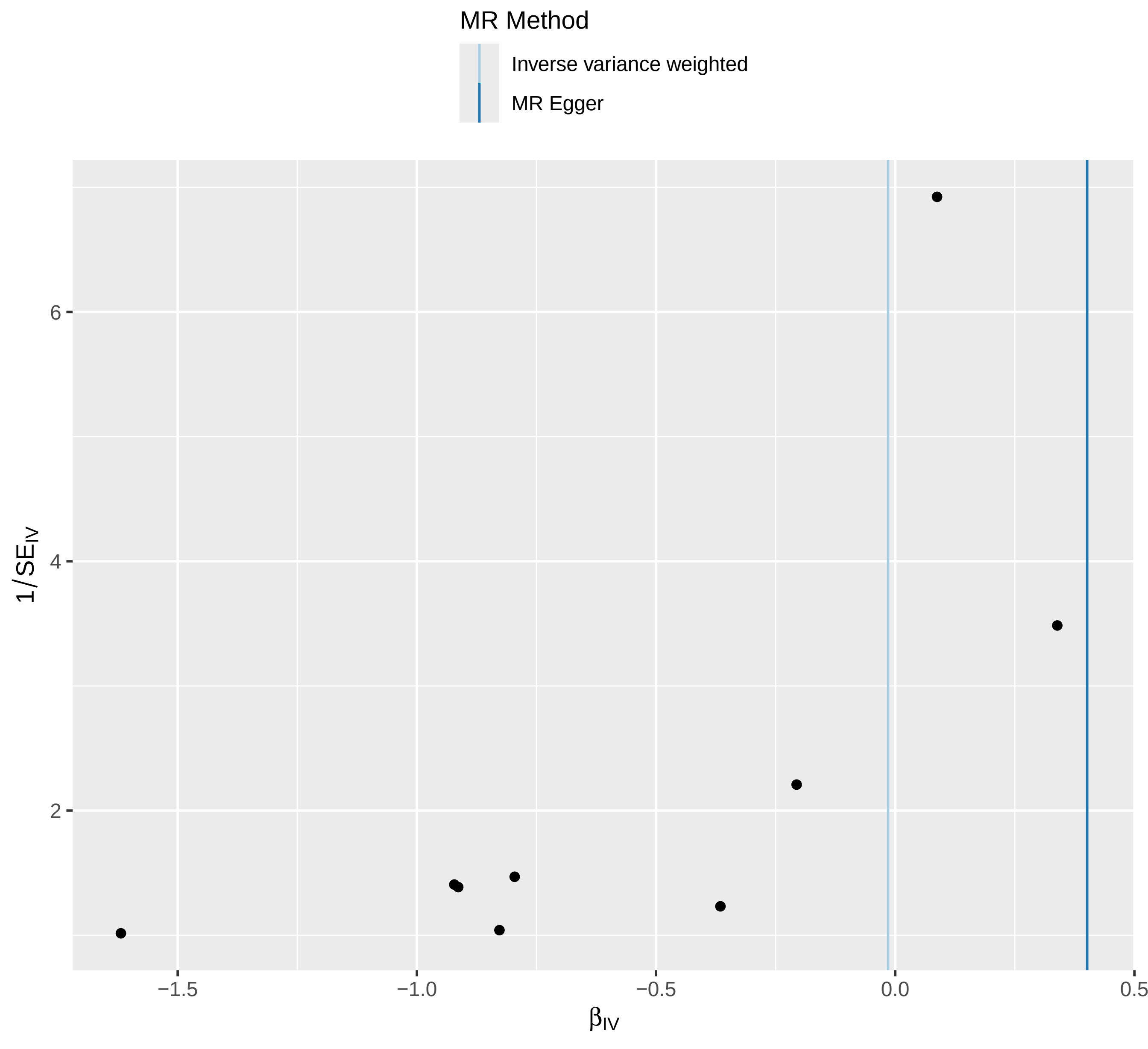
MR Method
Inverse variance weighted
MR Egger
1/SE$_{IV}$
6
4
2
−1.5
−1.0
−0.5
0.0
0.5
β$_{IV}$

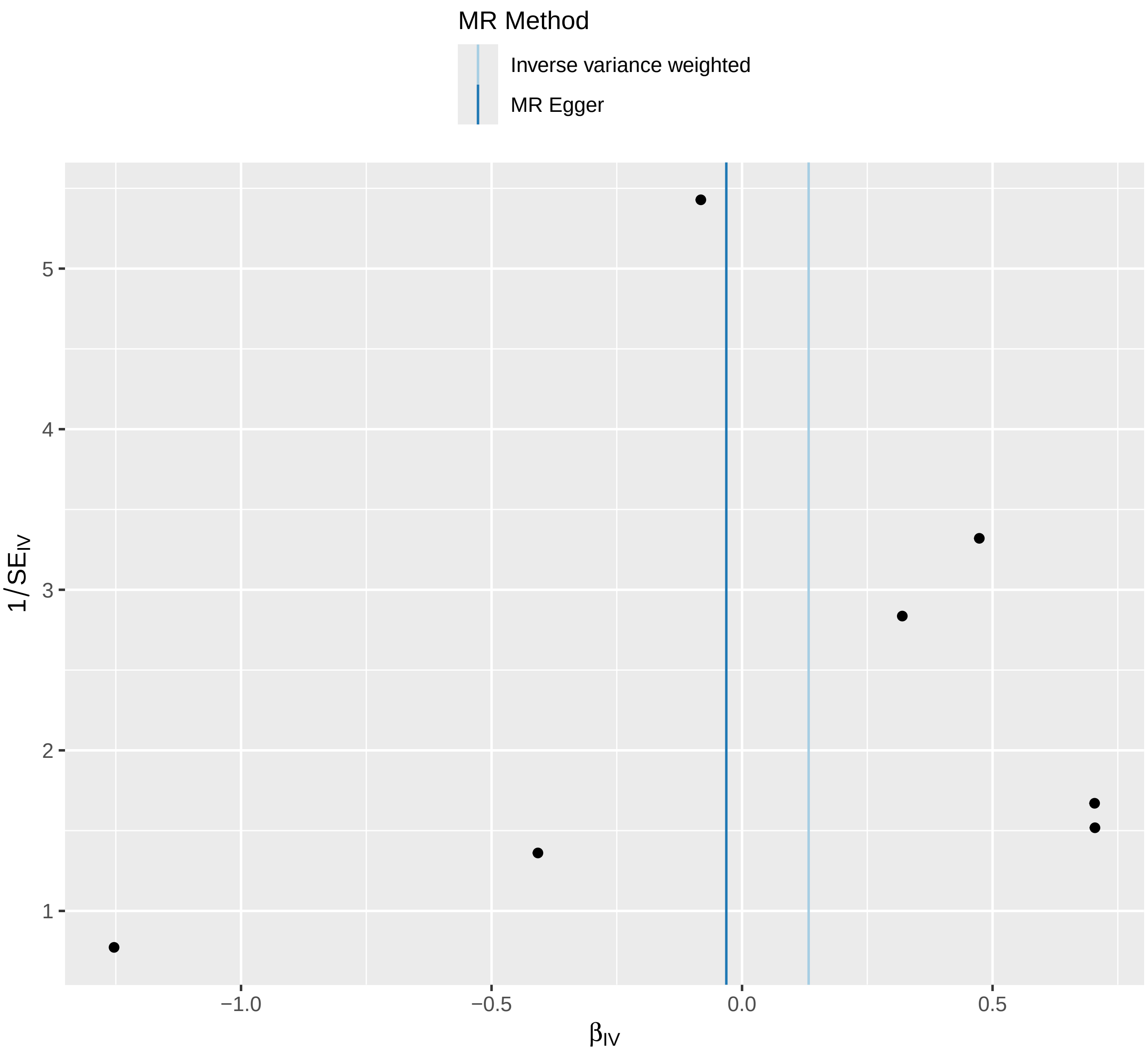

MR Method
Inverse variance weighted
MR Egger
1/SE$_{IV}$
5
4
3
2
1
−1.0
−0.5
0.0
0.5
β$_{IV}$

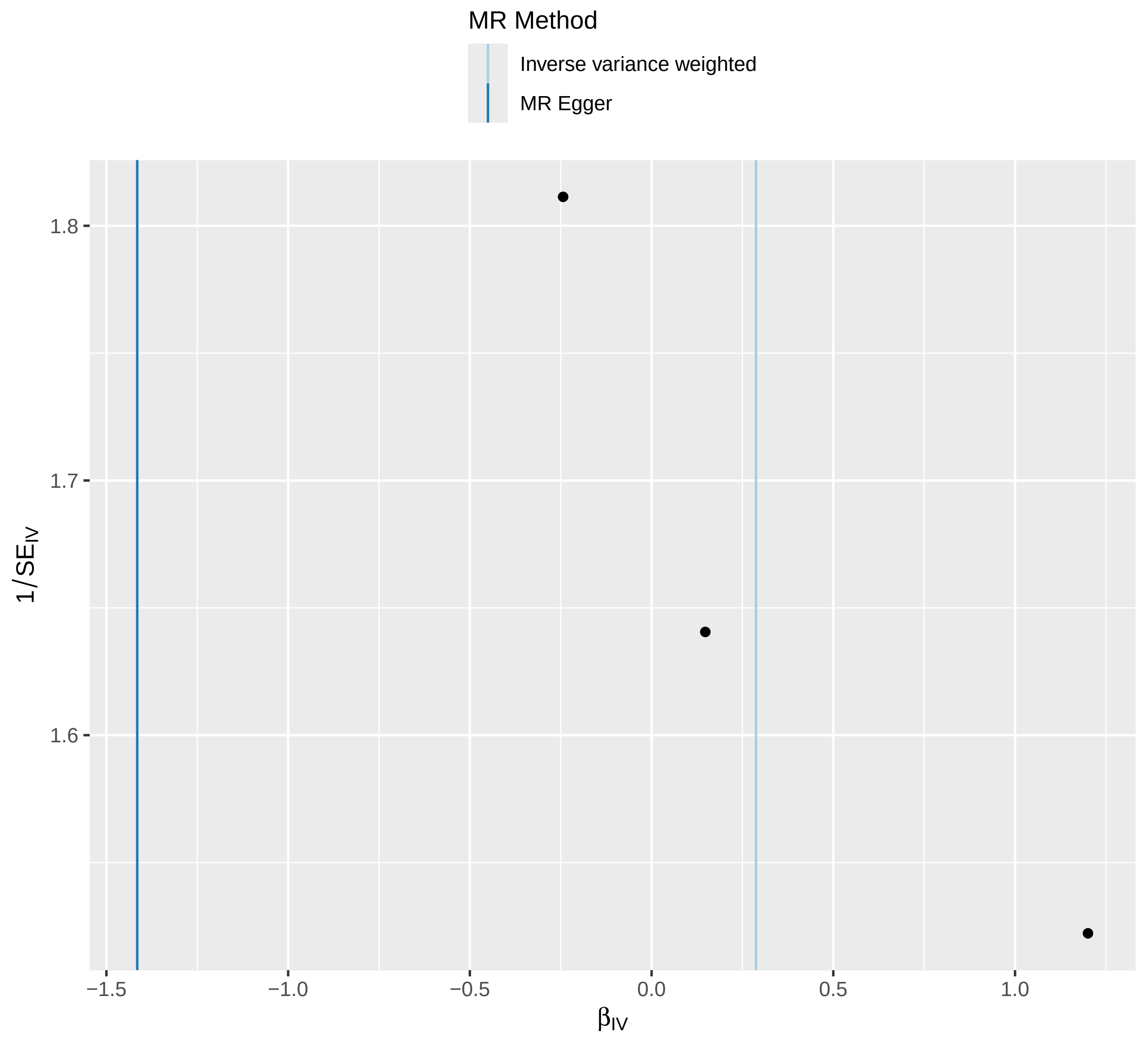

MR Method
Inverse variance weighted
MR Egger
1.8
1.7
1.6
1/SE_IV
−1.5
−1.0
−0.5
0.0
0.5
1.0
β_IV

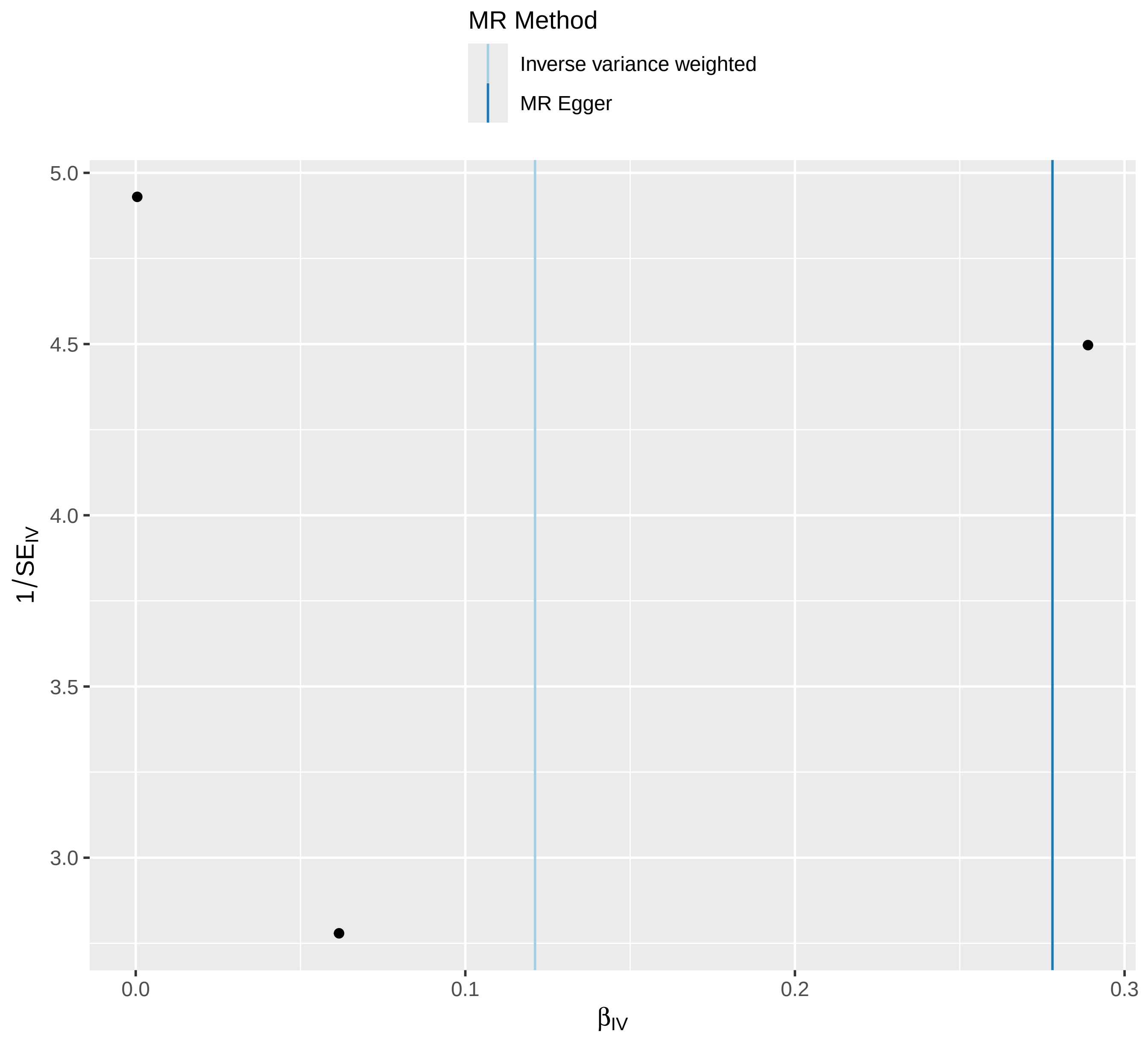

MR Method
Inverse variance weighted
MR Egger
5.0
4.5
4.0
3.5
3.0
1/SE_IV
0.0
0.1
0.2
0.3
β_IV

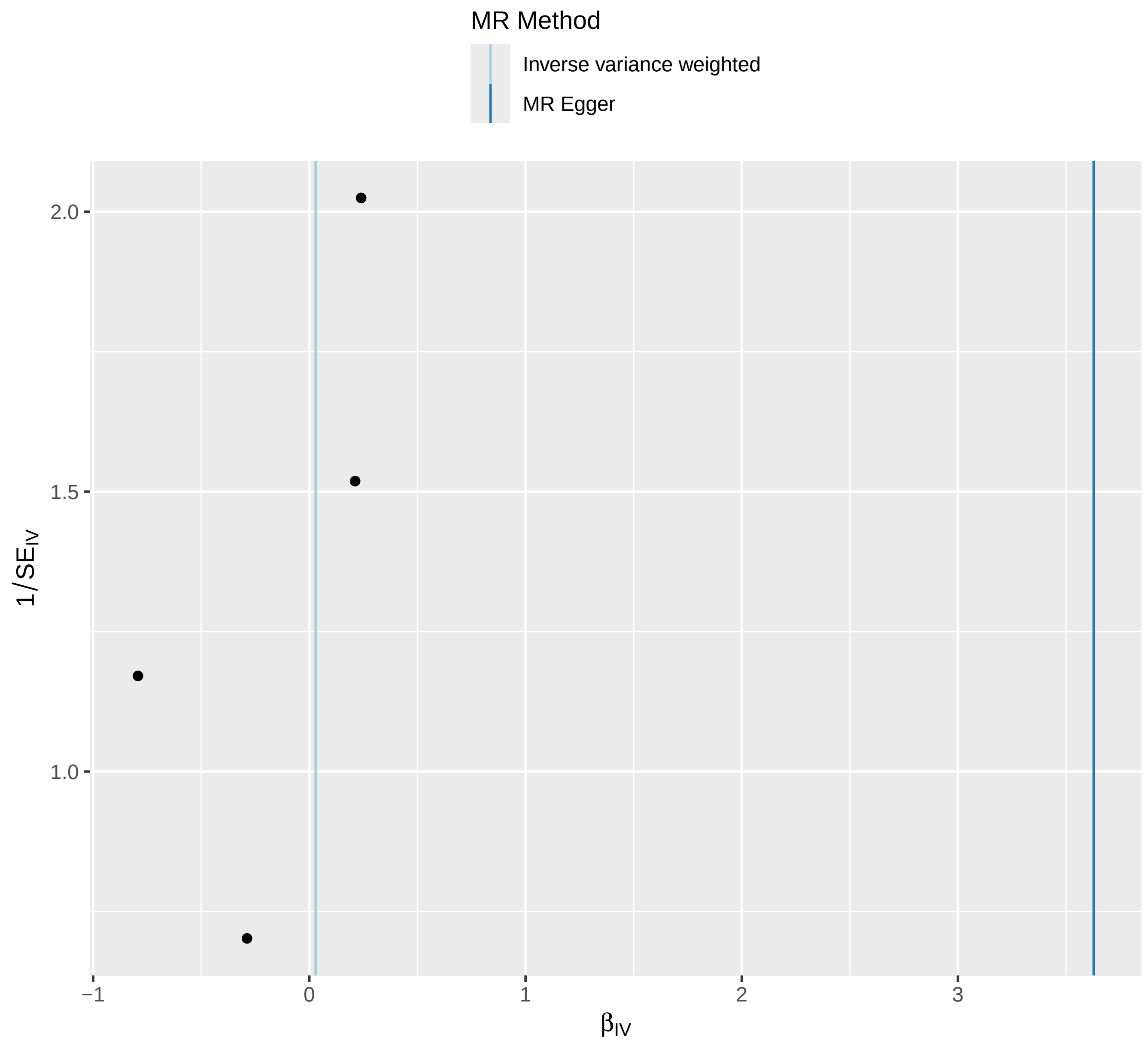

MR Method
Inverse variance weighted
MR Egger
2.0
1.5
1.0
1/SE$_{IV}$
−1
0
1
2
3
β$_{IV}$

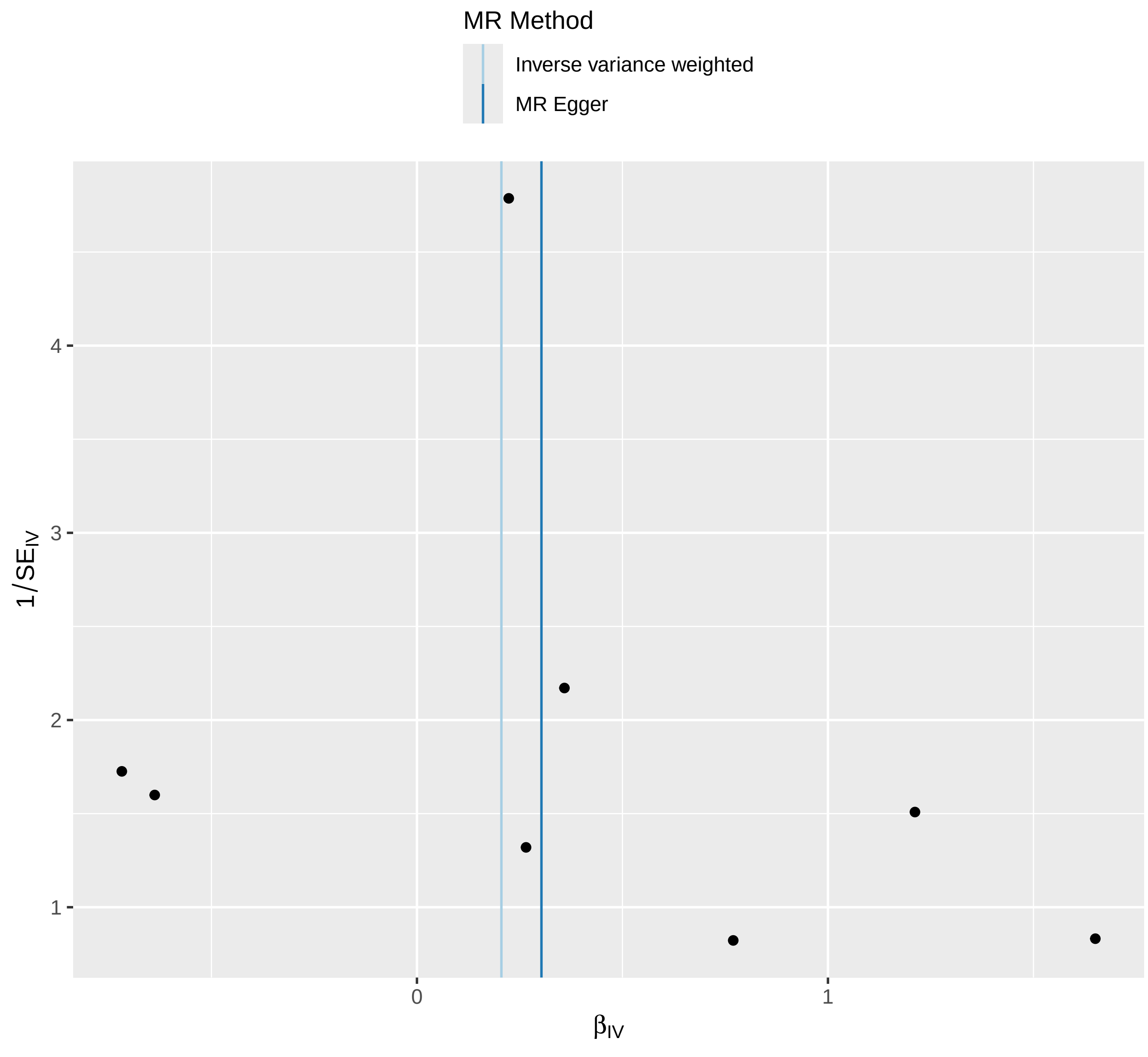
MR Method
Inverse variance weighted
MR Egger
1/SE$_{IV}$
4
3
2
1
0
1
β$_{IV}$

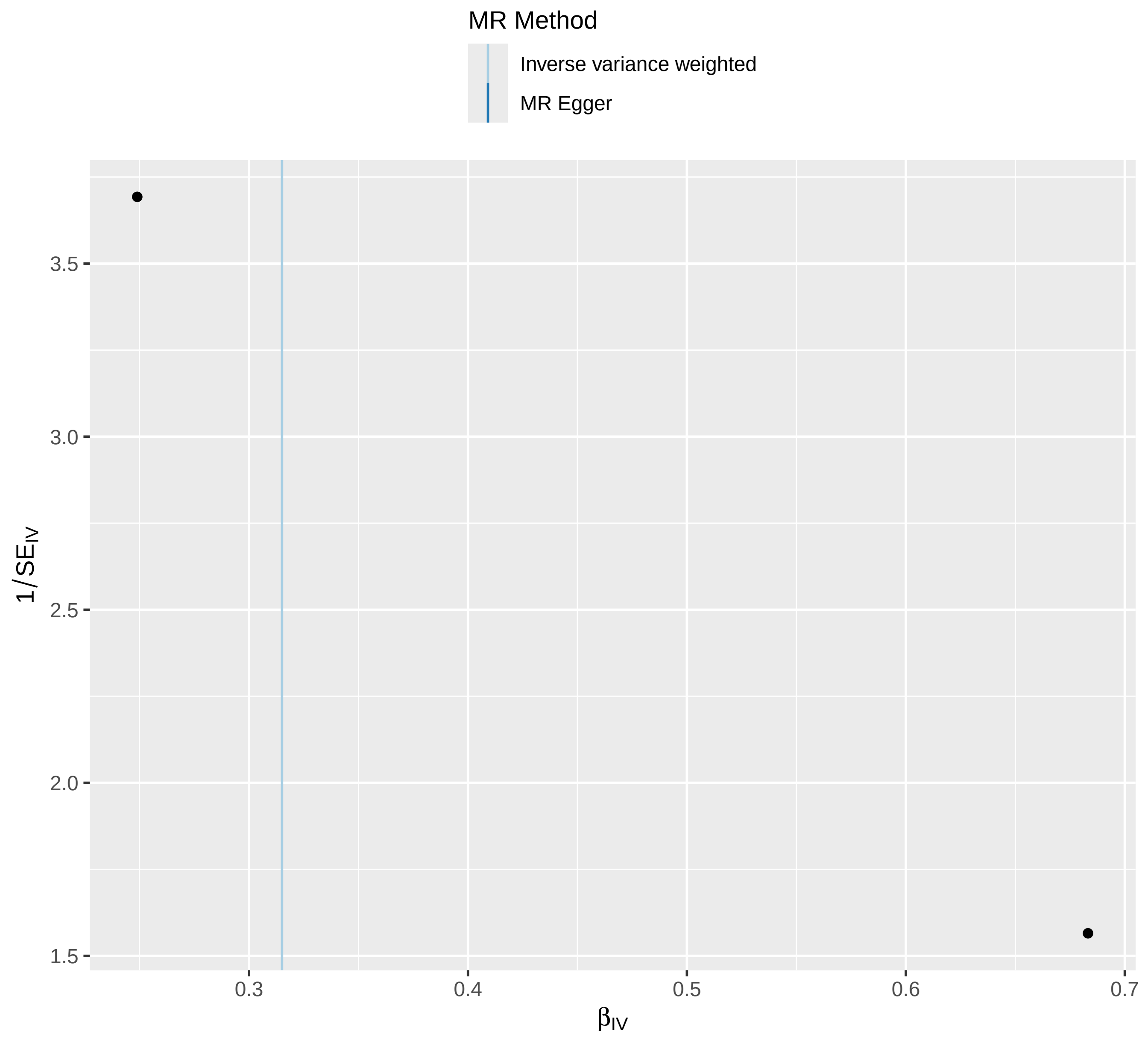

MR Method
Inverse variance weighted
MR Egger
3.5
3.0
2.5
2.0
1.5
1/SE$_{IV}$
0.3
0.4
0.5
0.6
0.7
β_{IV}

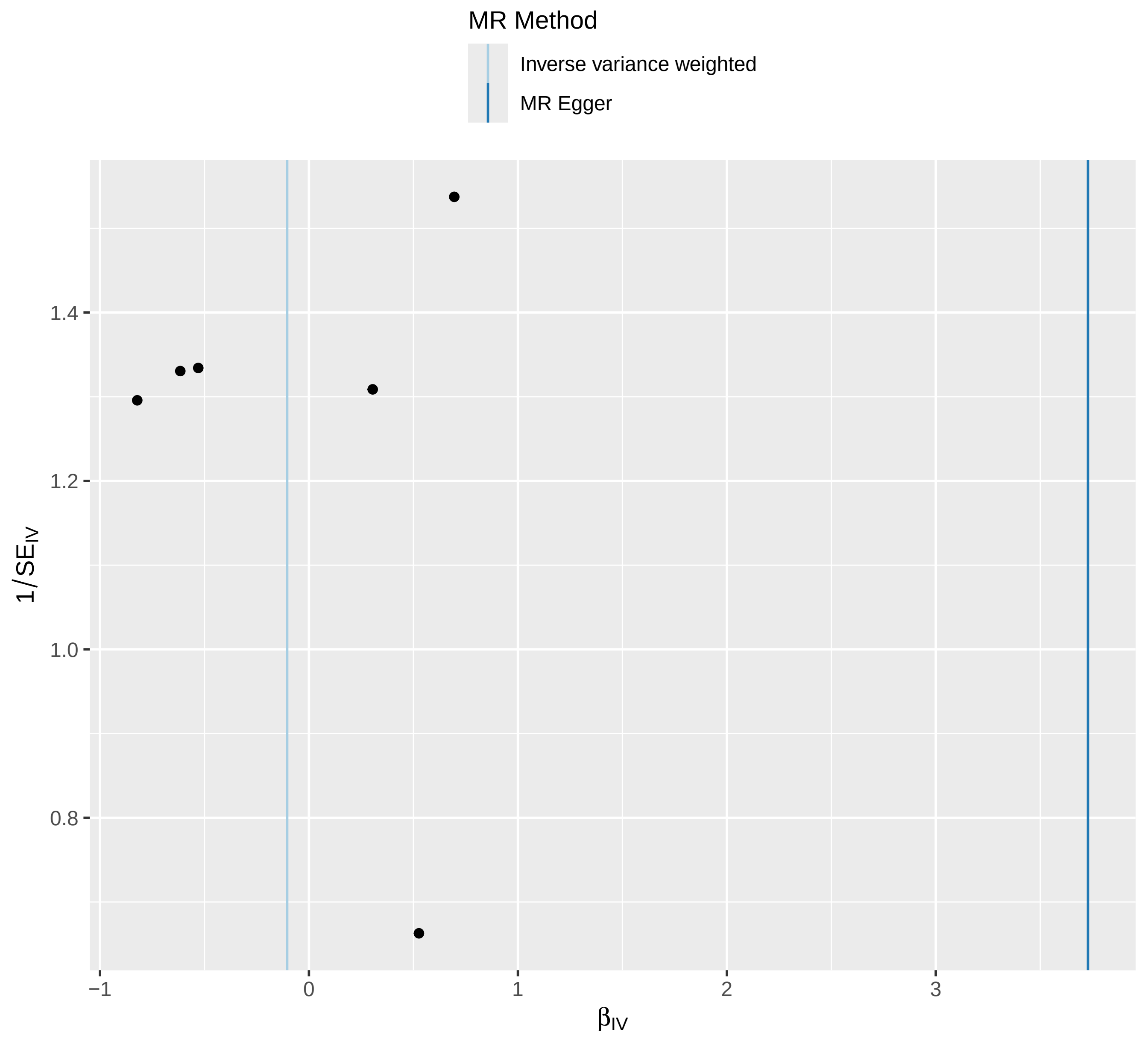

MR Method
Inverse variance weighted
MR Egger
1.4
1.2
1.0
0.8
1/SE_IV
−1
0
1
2
3
β_IV

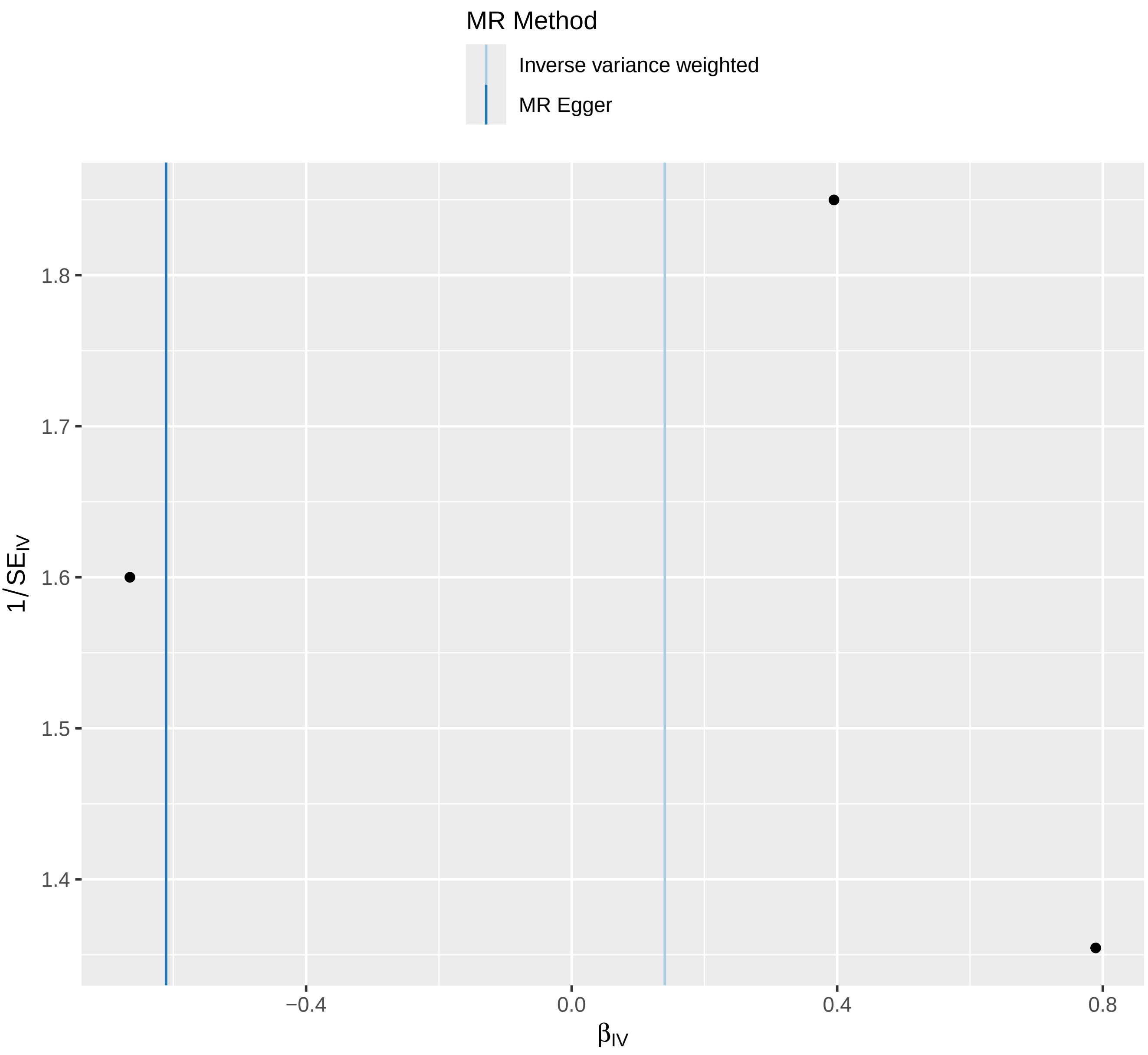
MR Method
Inverse variance weighted
MR Egger
1.8
1.7
1.6
1.5
1.4
−0.4
0.0
0.4
0.8
$1/SE_{IV}$
β_{IV}

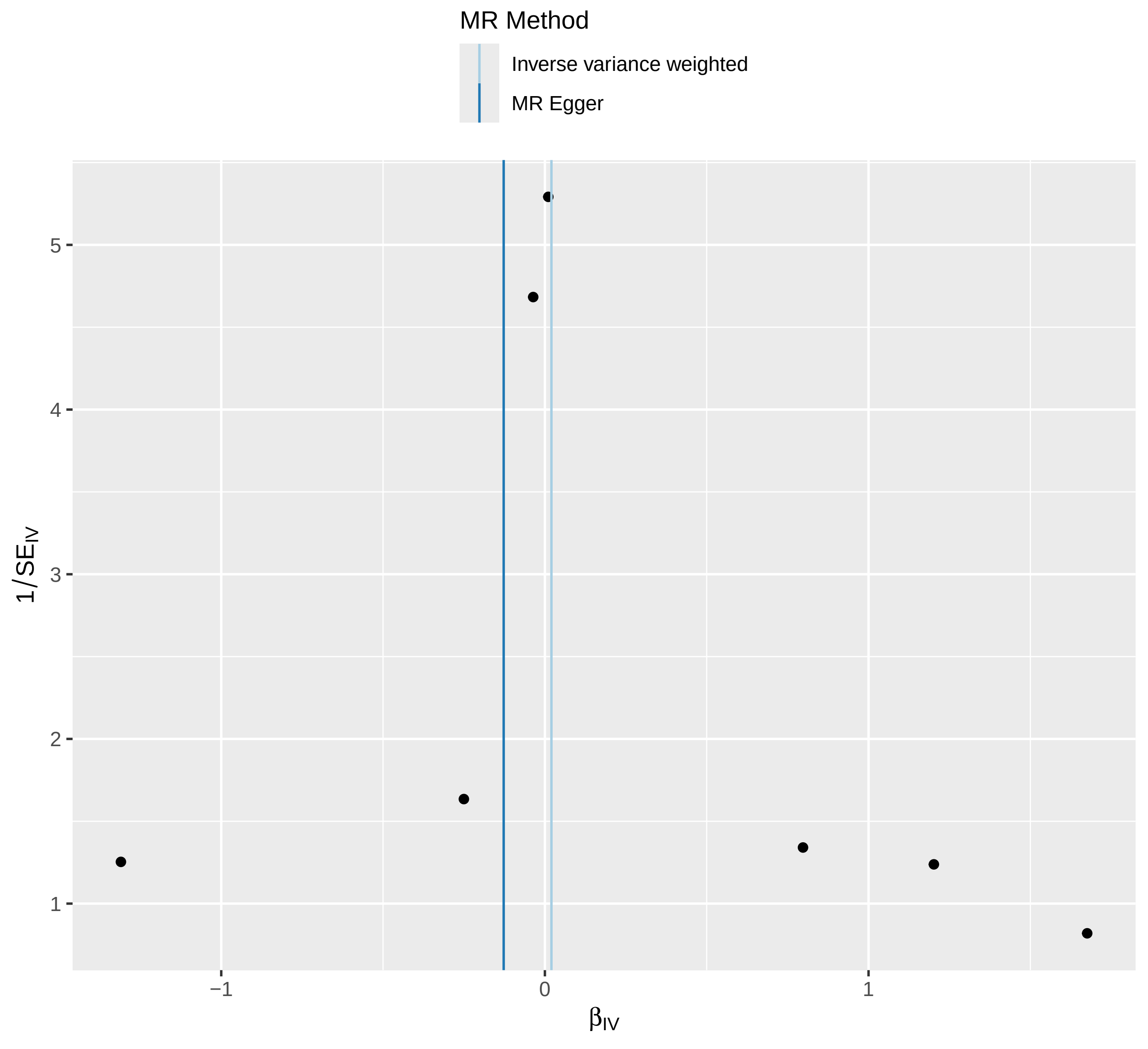
MR Method
Inverse variance weighted
MR Egger
1/SE_IV
5
4
3
2
1
−1
0
1
β_IV

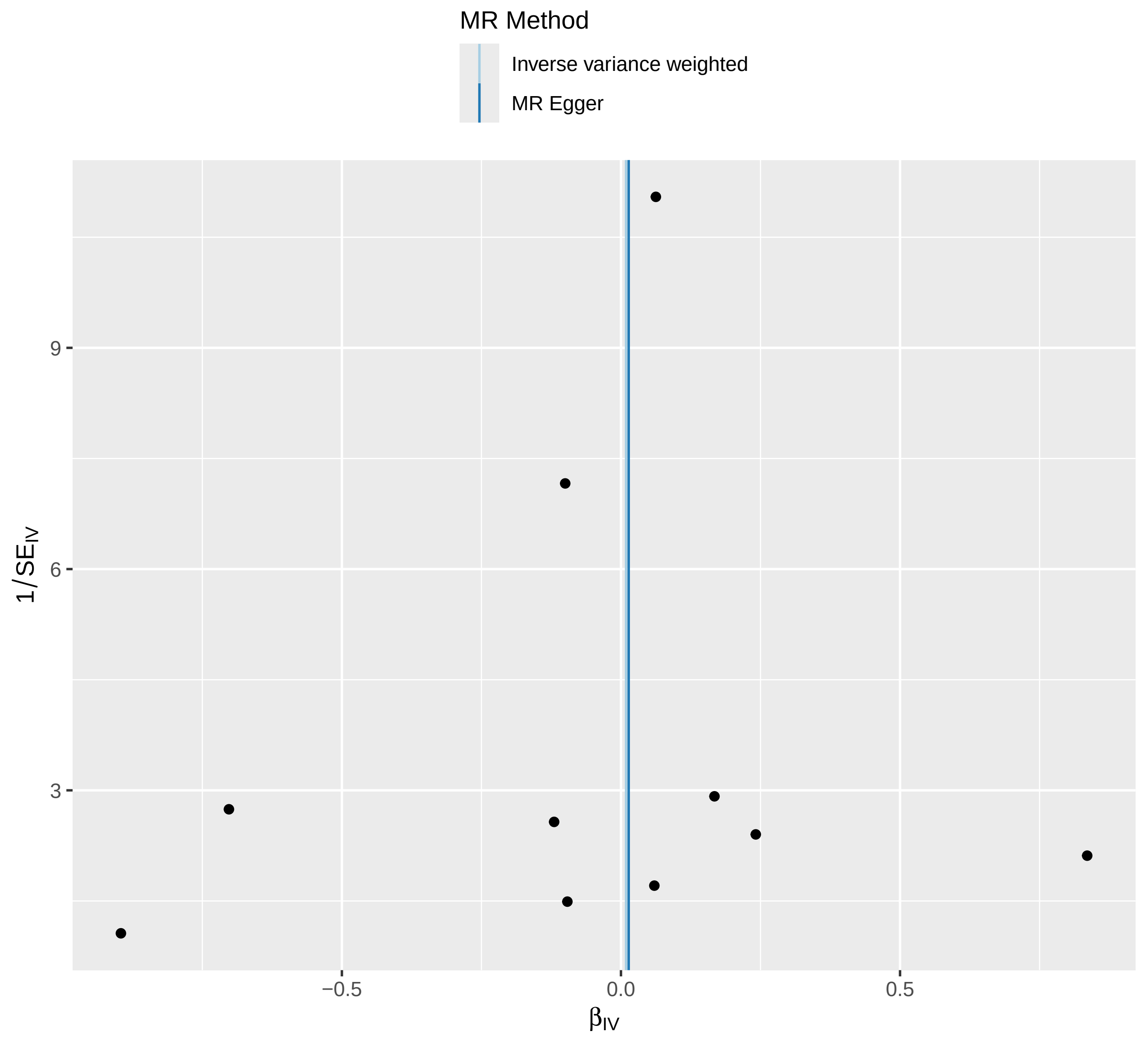
MR Method
Inverse variance weighted
MR Egger
1/SE$_{IV}$
9
6
3
−0.5
0.0
0.5
β_{IV}

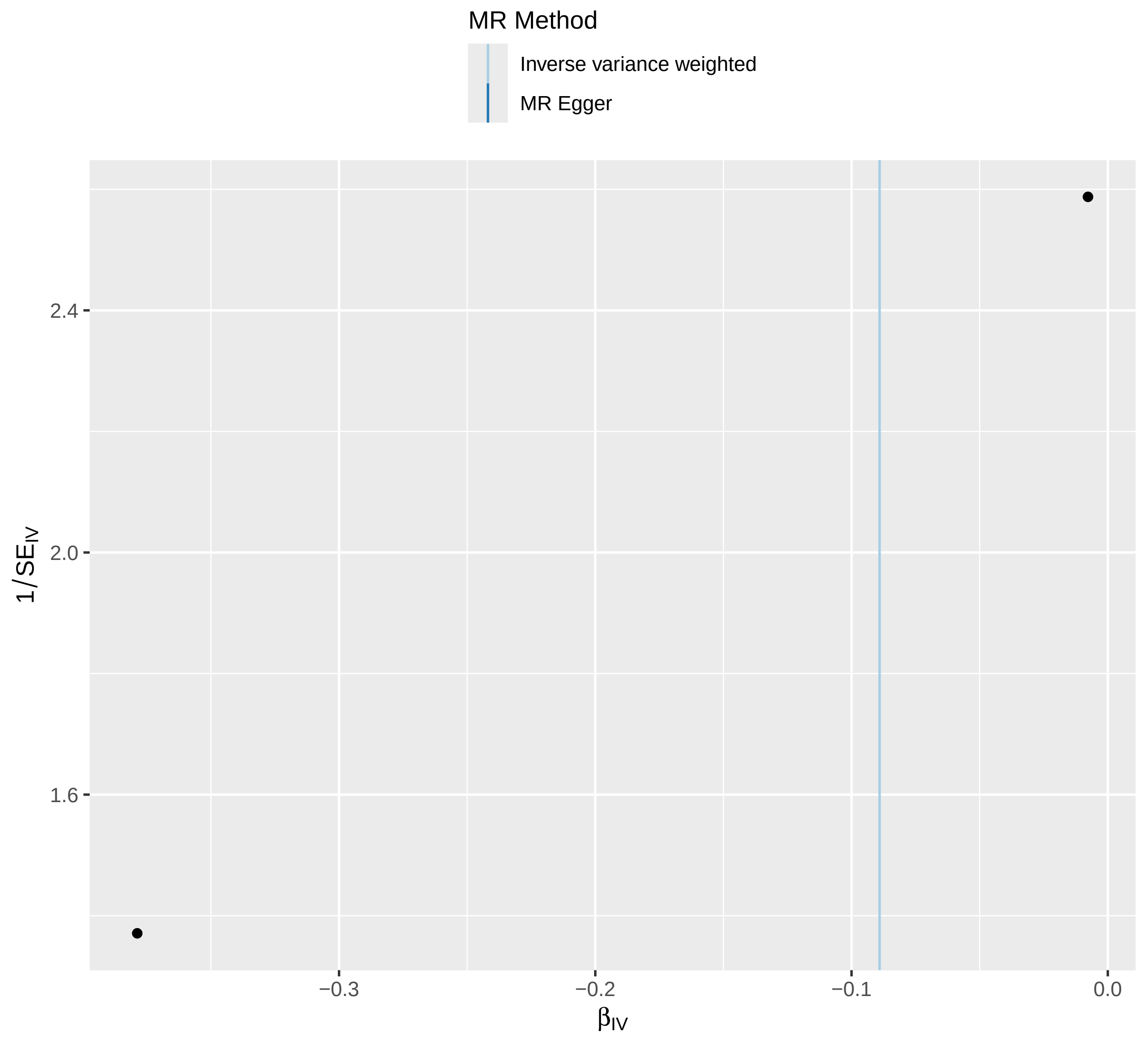
MR Method
Inverse variance weighted
MR Egger
2.4
2.0
1.6
1/SE_{IV}
−0.3
−0.2
−0.1
0.0
β_{IV}

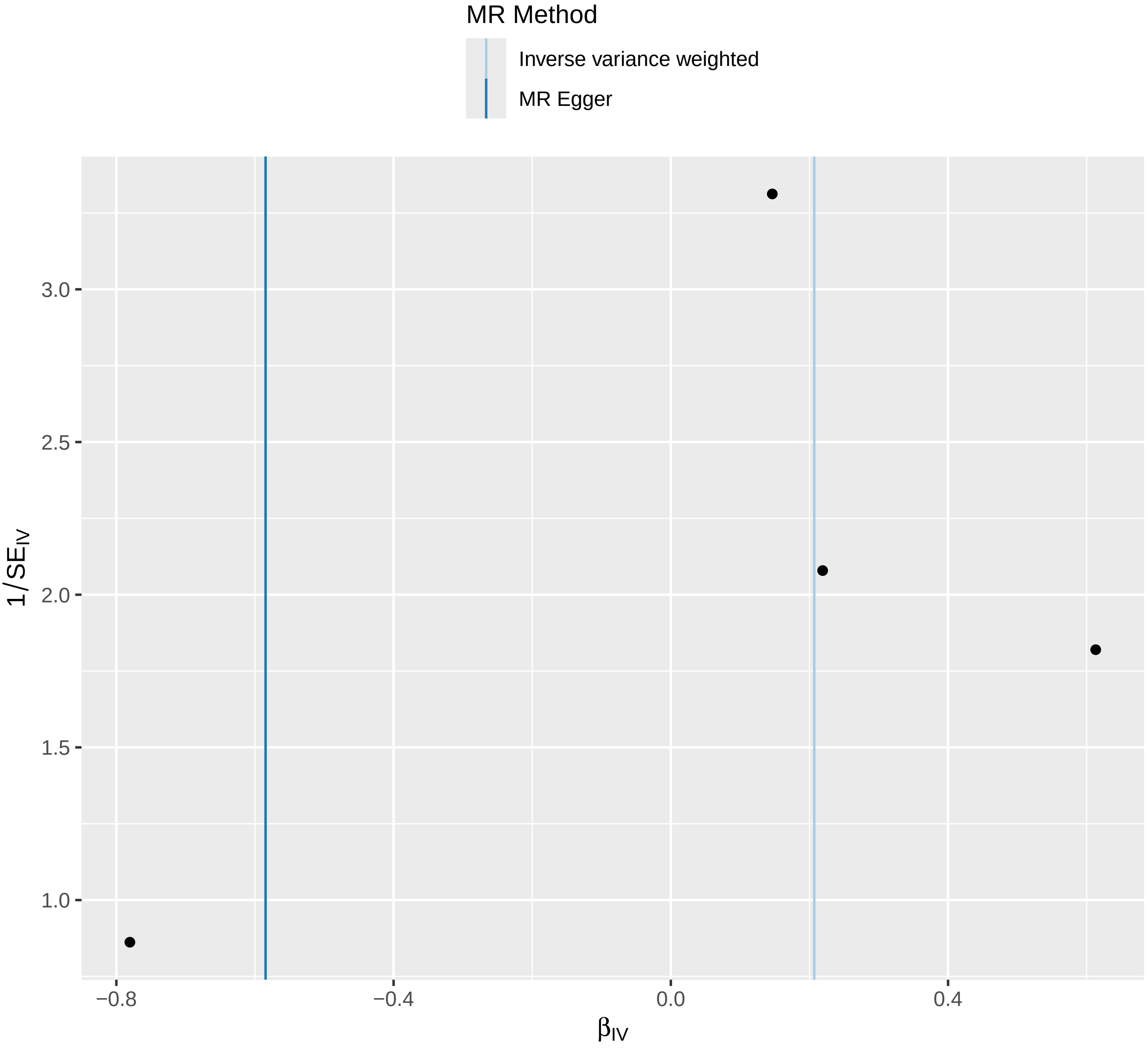
MR Method
Inverse variance weighted
MR Egger
3.0
2.5
2.0
1.5
1.0
$1/SE_{IV}$
−0.8
−0.4
0.0
0.4
β_{IV}

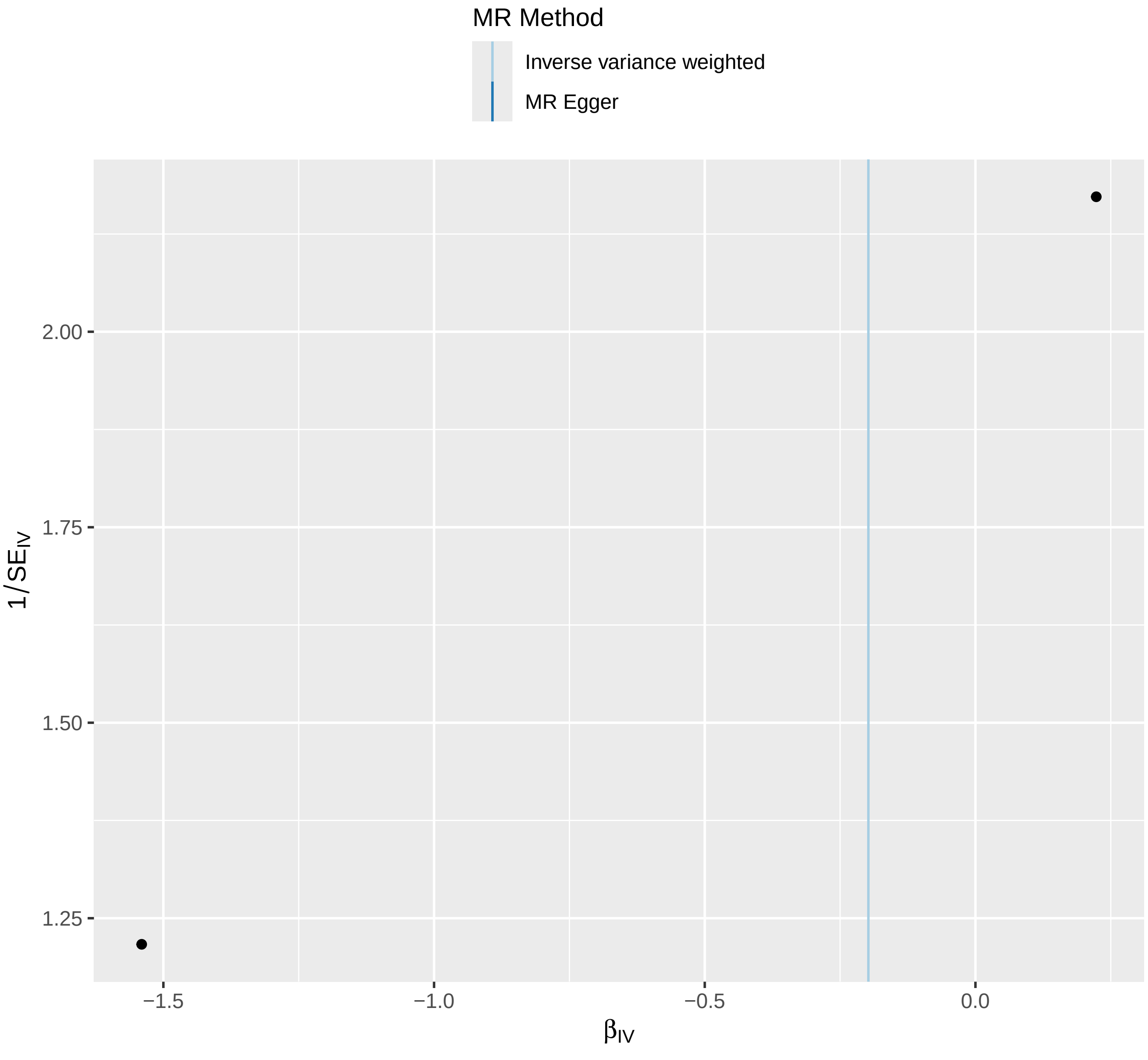

MR Method
Inverse variance weighted
MR Egger
2.00
1.75
1.50
1.25
1/SE_IV
−1.5
−1.0
−0.5
0.0
β_IV

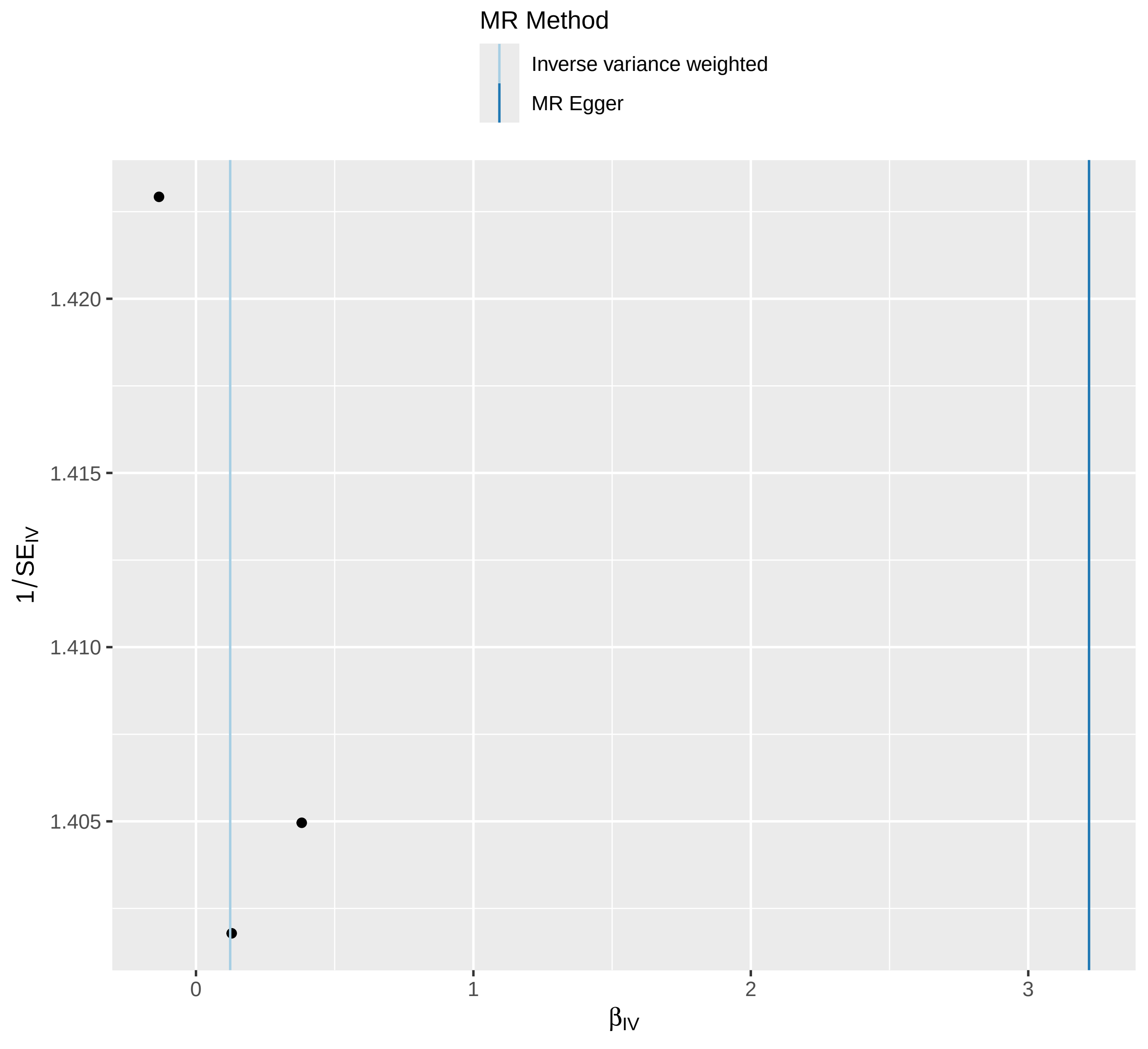
MR Method
Inverse variance weighted
MR Egger
1.420
1.415
1.410
1.405
1/SE_IV
0
1
2
3
β_IV

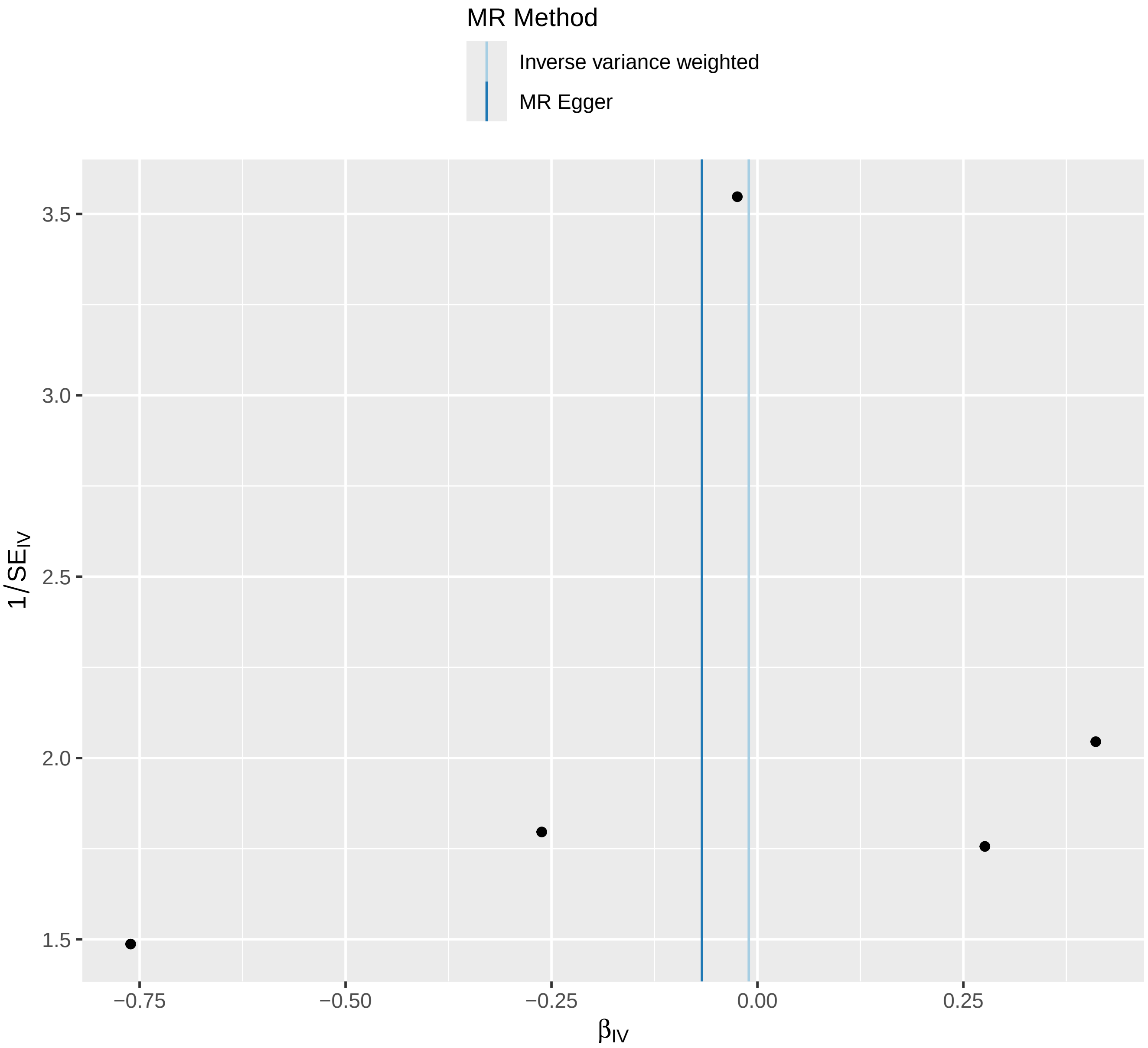
MR Method
Inverse variance weighted
MR Egger
3.5
3.0
2.5
2.0
1.5
1/SE_IV
−0.75
−0.50
−0.25
0.00
0.25
β_IV

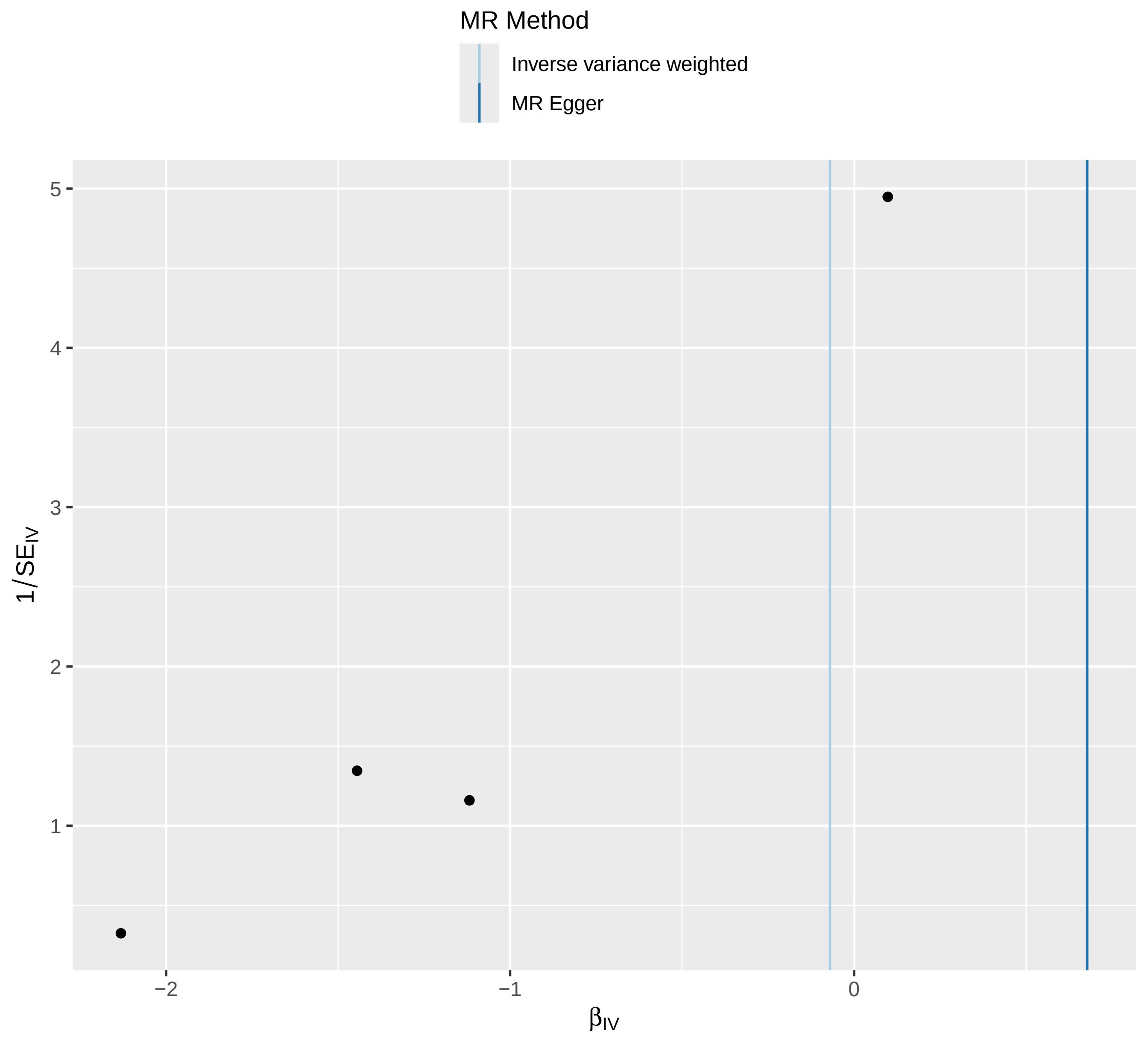

MR Method
Inverse variance weighted
MR Egger
5
4
3
2
1
1/SE_IV
−2
−1
0
β_IV

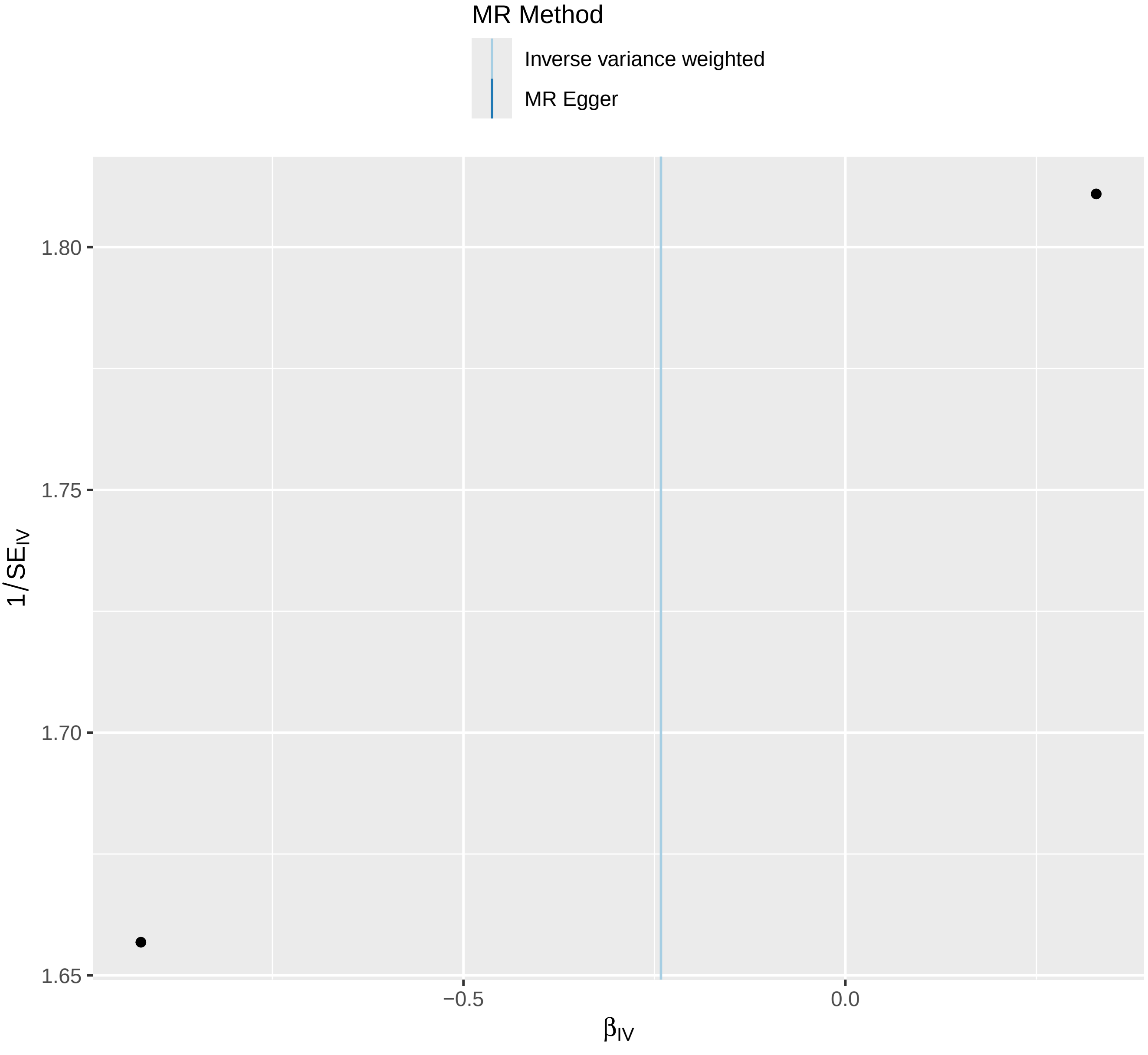
MR Method
Inverse variance weighted
MR Egger
1.80
1.75
1.70
1.65
1/SE_IV
−0.5
0.0
β_IV

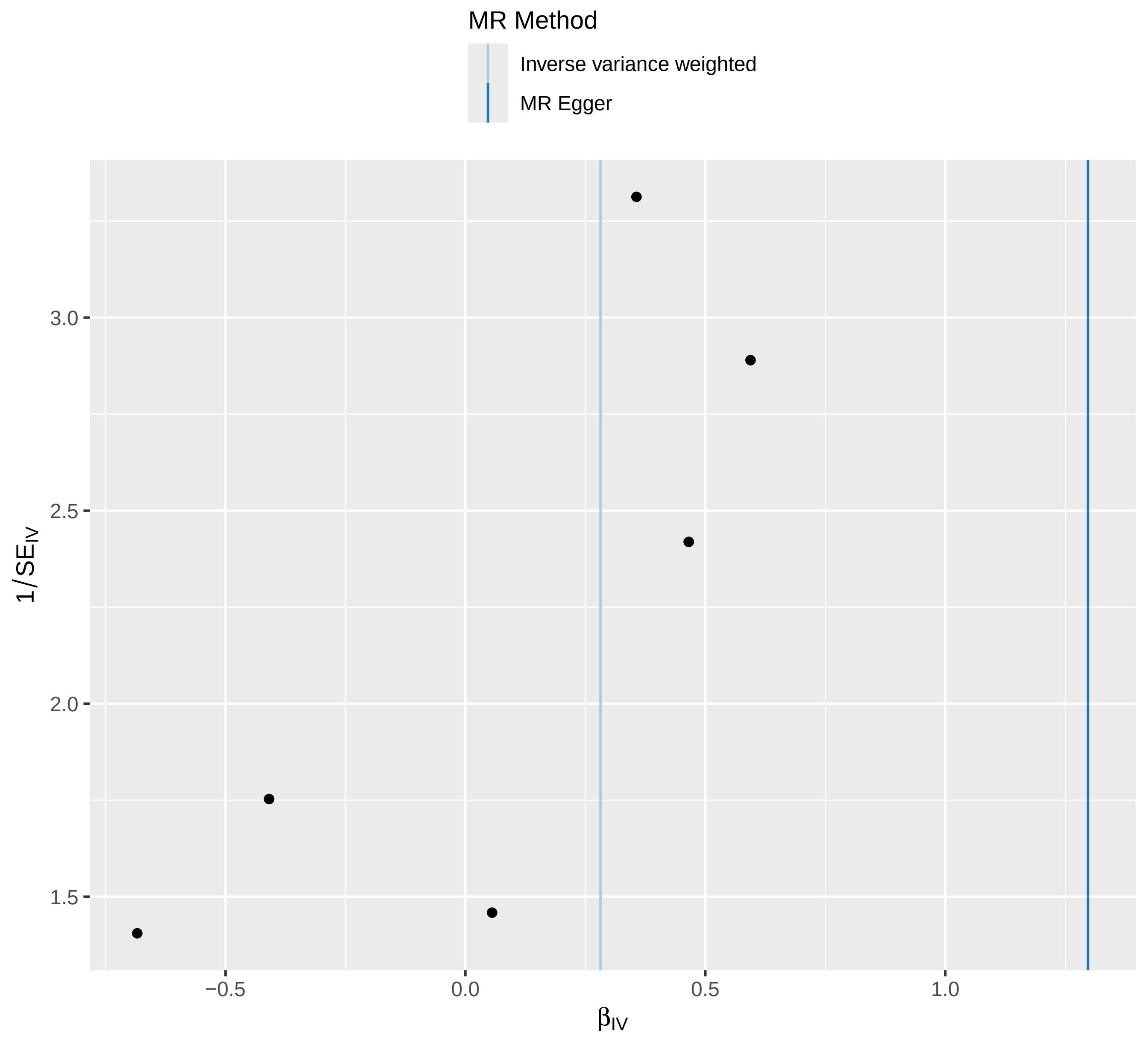

MR Method
Inverse variance weighted
MR Egger
1/SE_IV
3.0
2.5
2.0
1.5
−0.5
0.0
0.5
1.0
β_IV

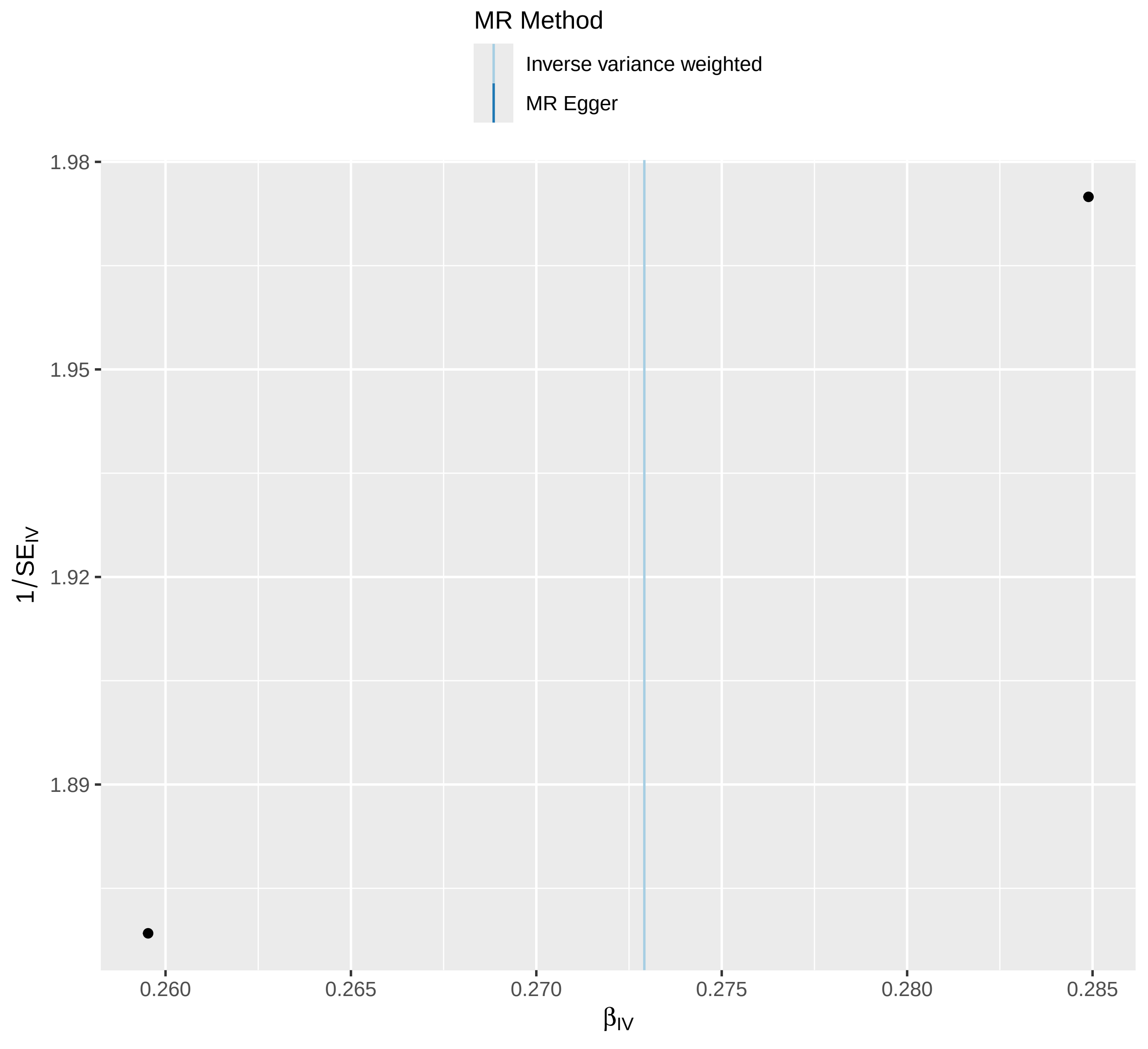
MR Method
Inverse variance weighted
MR Egger
1.98
1.95
1.92
1.89
1/SE_IV
0.260
0.265
0.270
0.275
0.280
0.285
β_IV

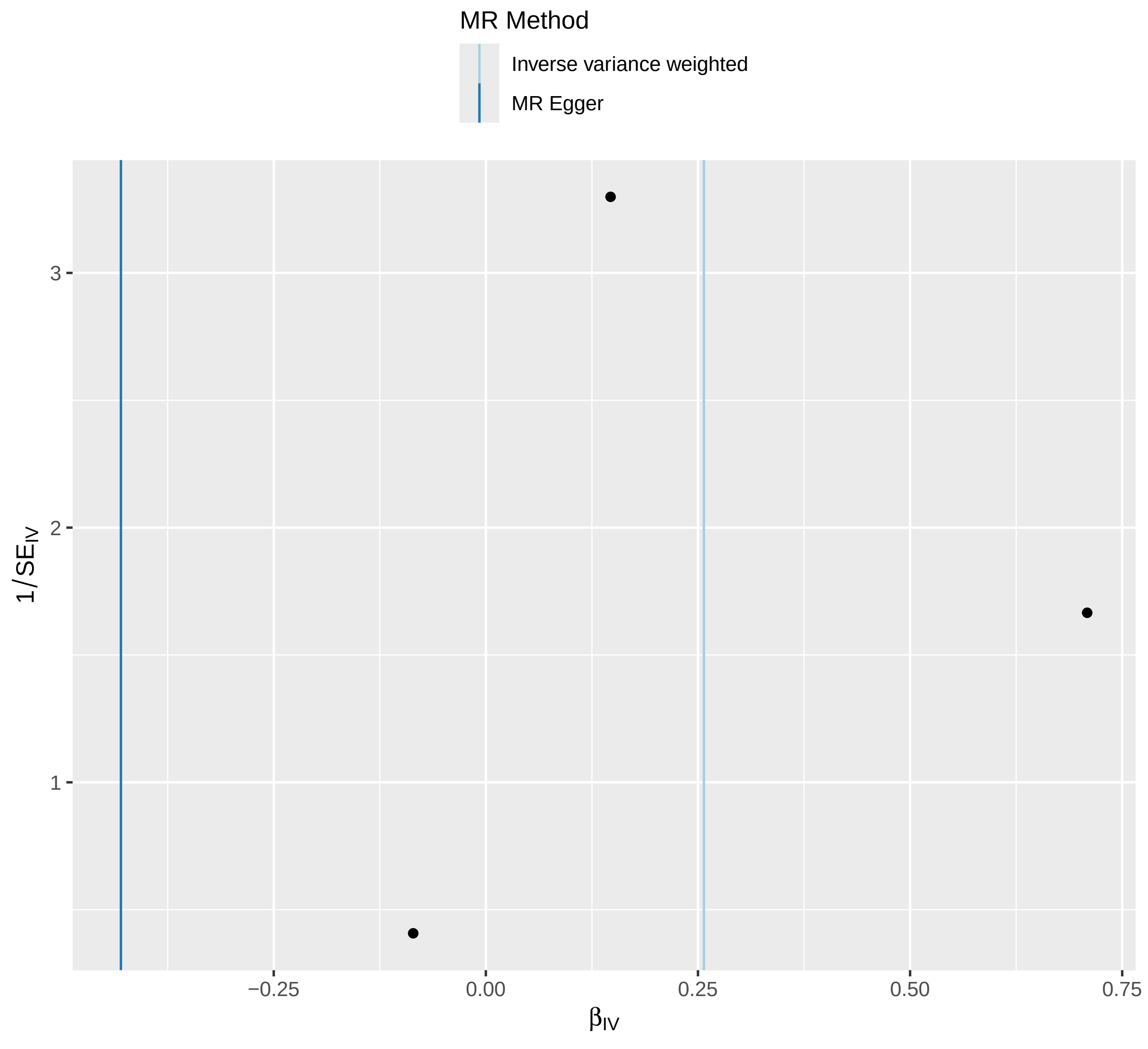

MR Method
Inverse variance weighted
MR Egger
3
2
1
1/SE_IV
−0.25
0.00
0.25
0.50
0.75
β_IV

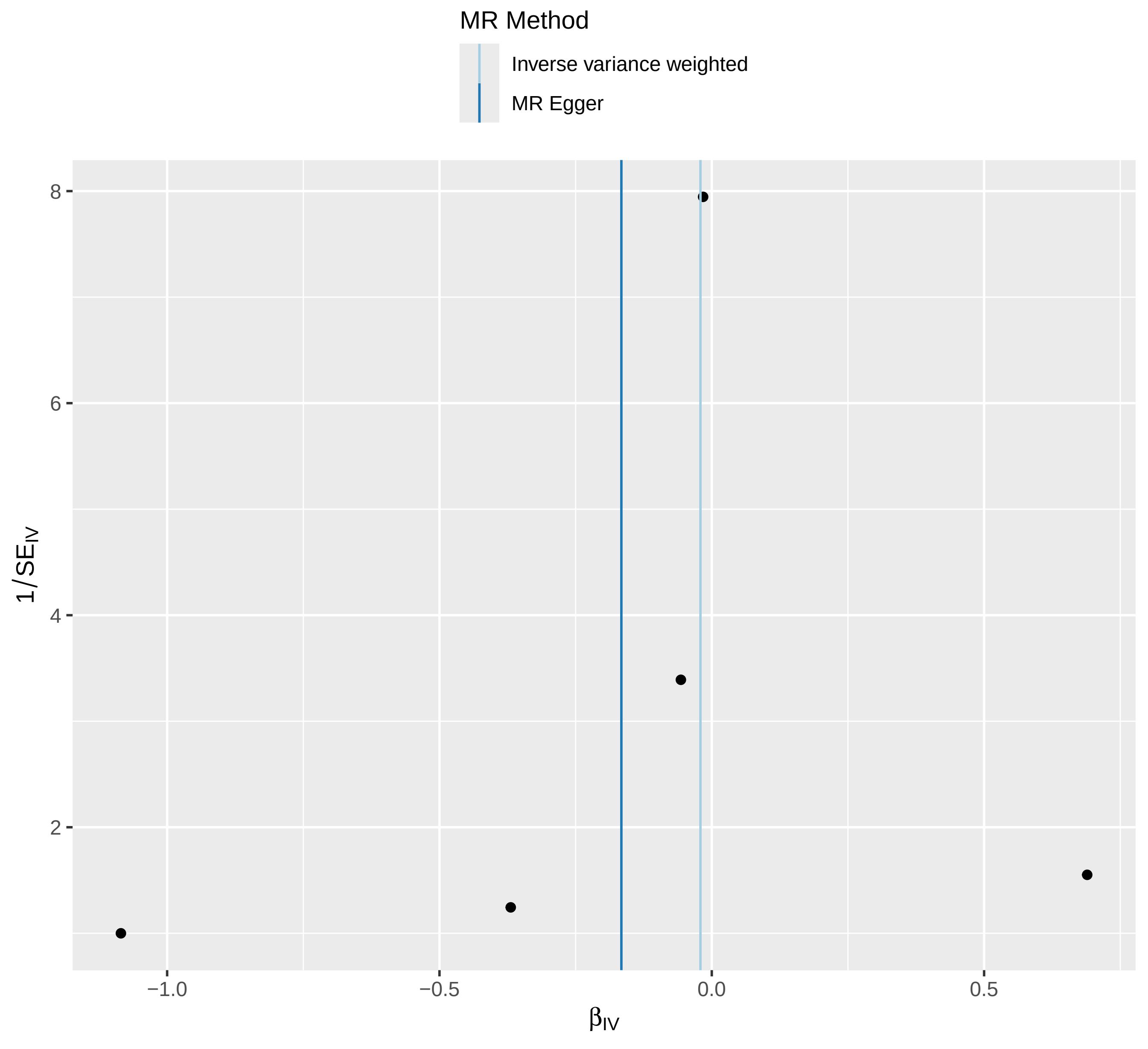

MR Method
Inverse variance weighted
MR Egger
8
6
4
2
1/SE_IV
−1.0
−0.5
0.0
0.5
β_IV

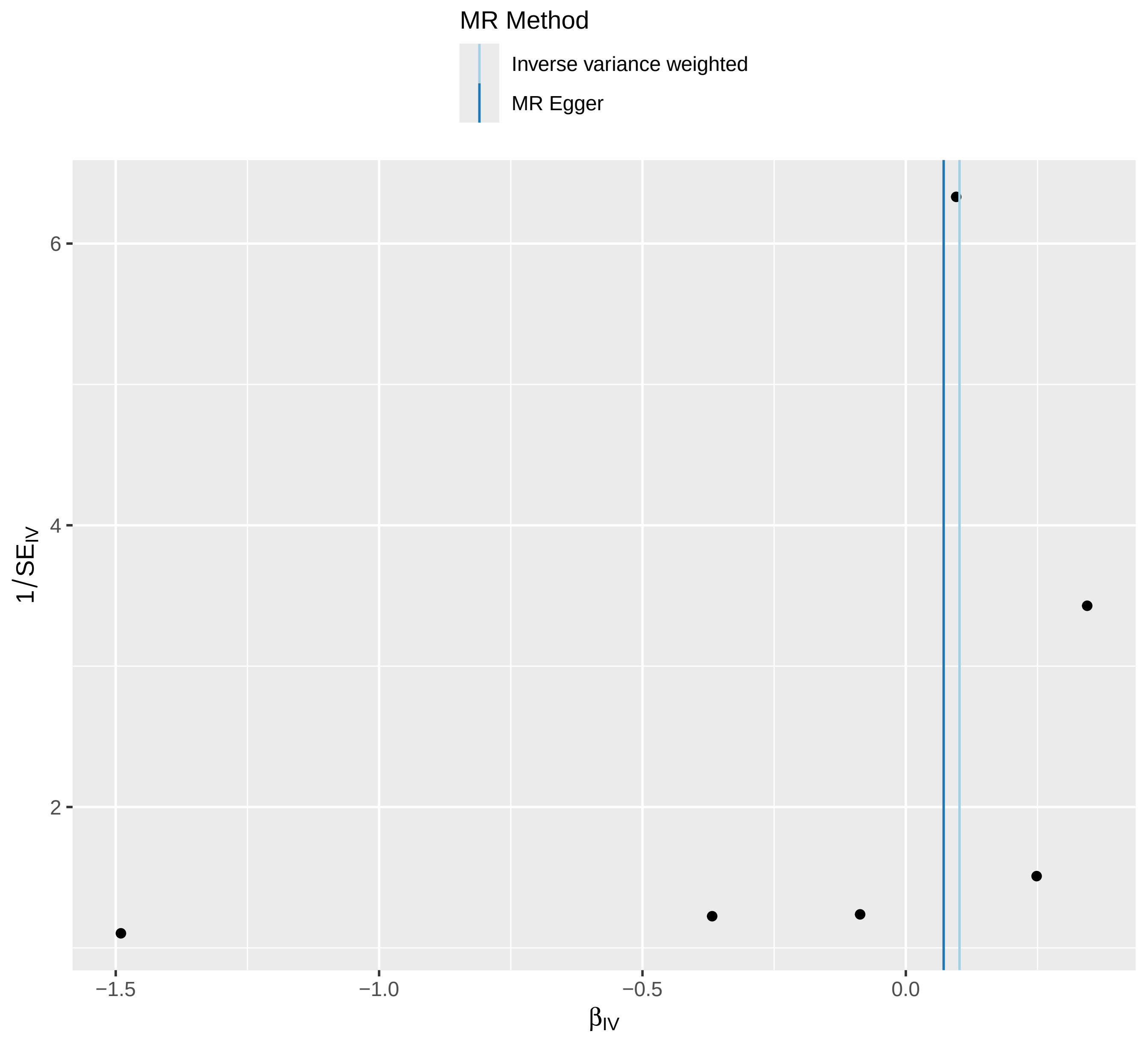

MR Method
Inverse variance weighted
MR Egger
6
4
2
1/SE_IV
−1.5
−1.0
−0.5
0.0
β_IV

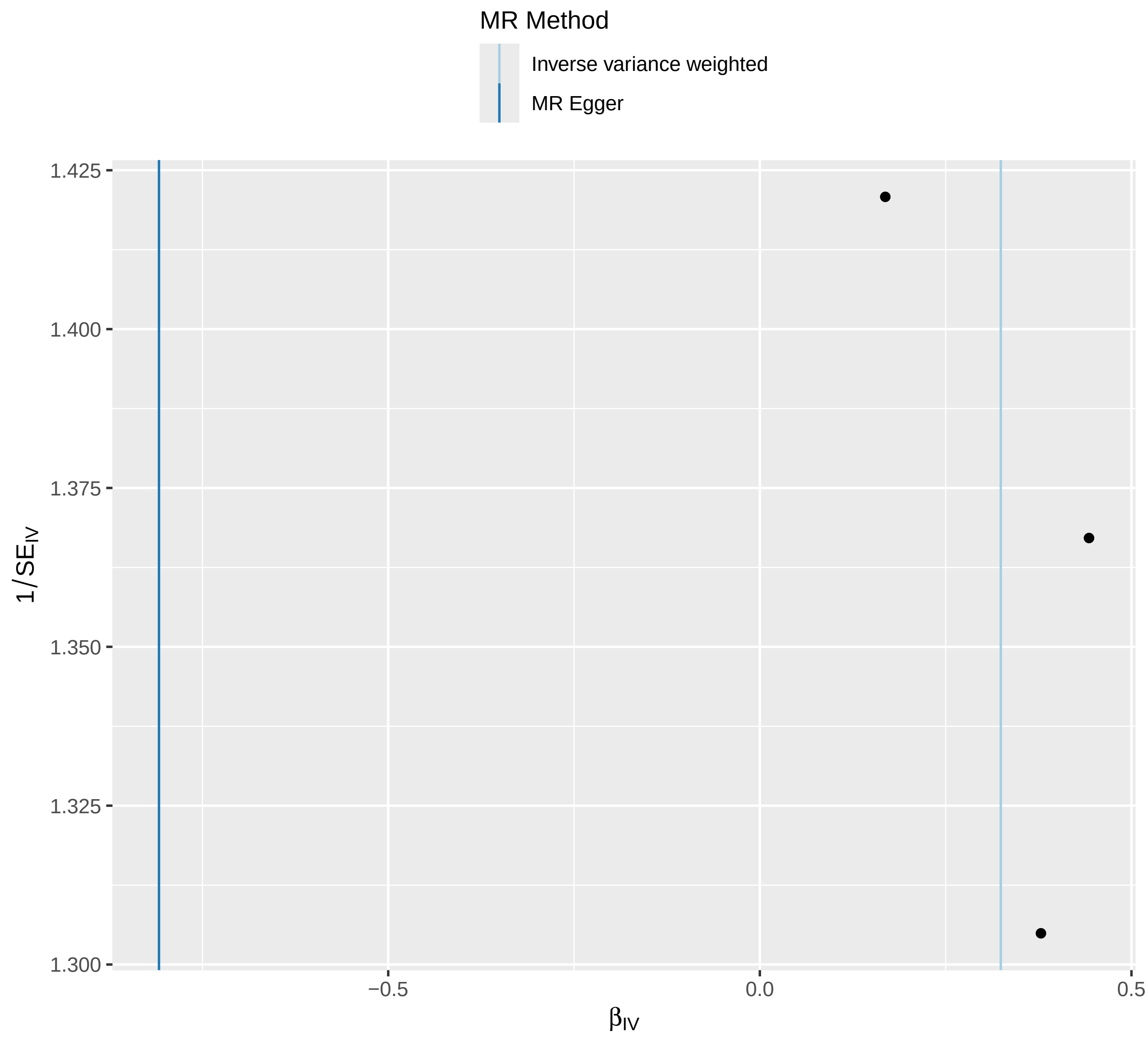
MR Method
Inverse variance weighted
MR Egger
1.425
1.400
1.375
1.350
1.325
1.300
1/SE_IV
−0.5
0.0
0.5
β_IV

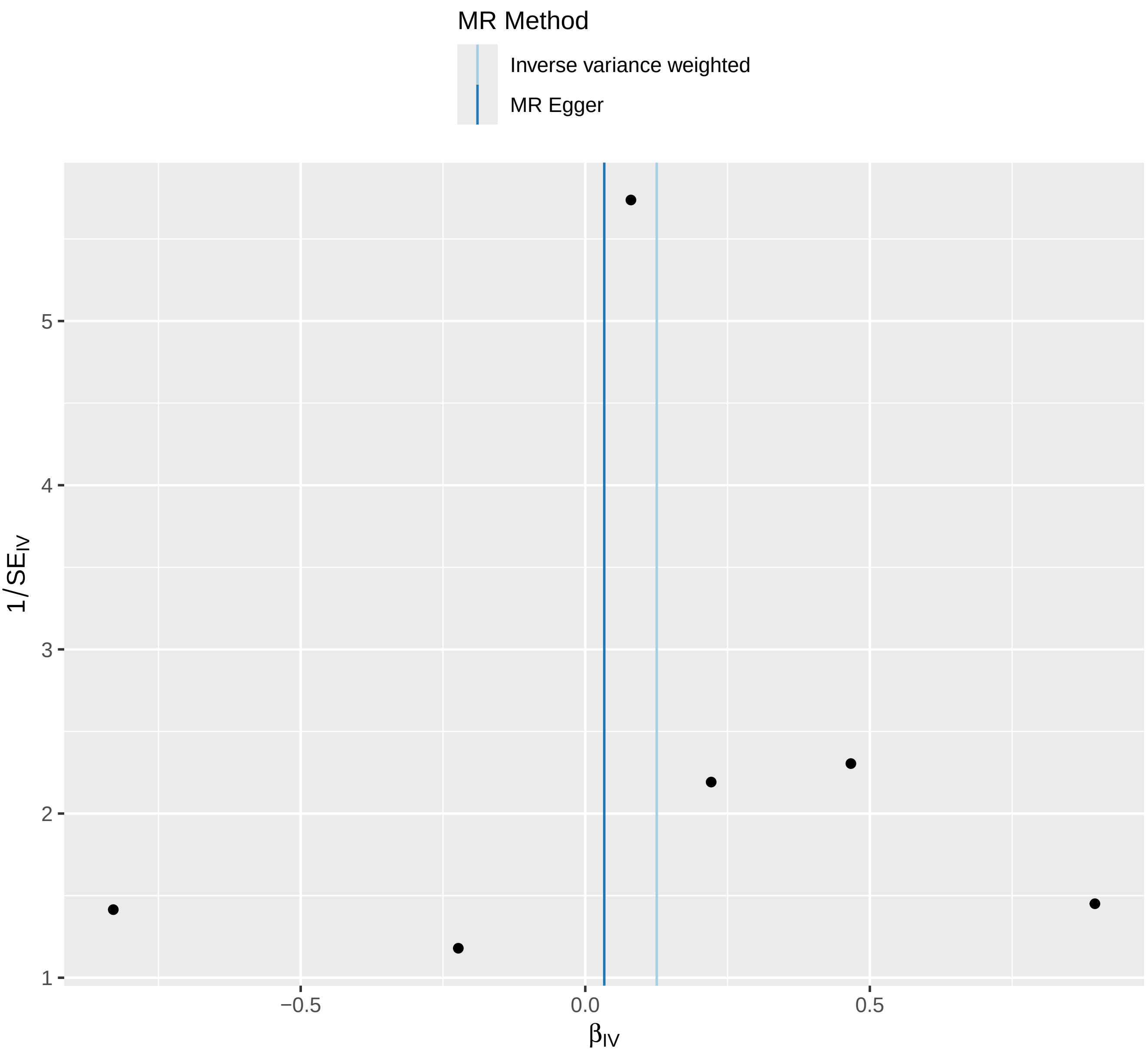

MR Method
Inverse variance weighted
MR Egger
5
4
3
2
1
−0.5
0.0
0.5
$1/SE_{IV}$
β_{IV}

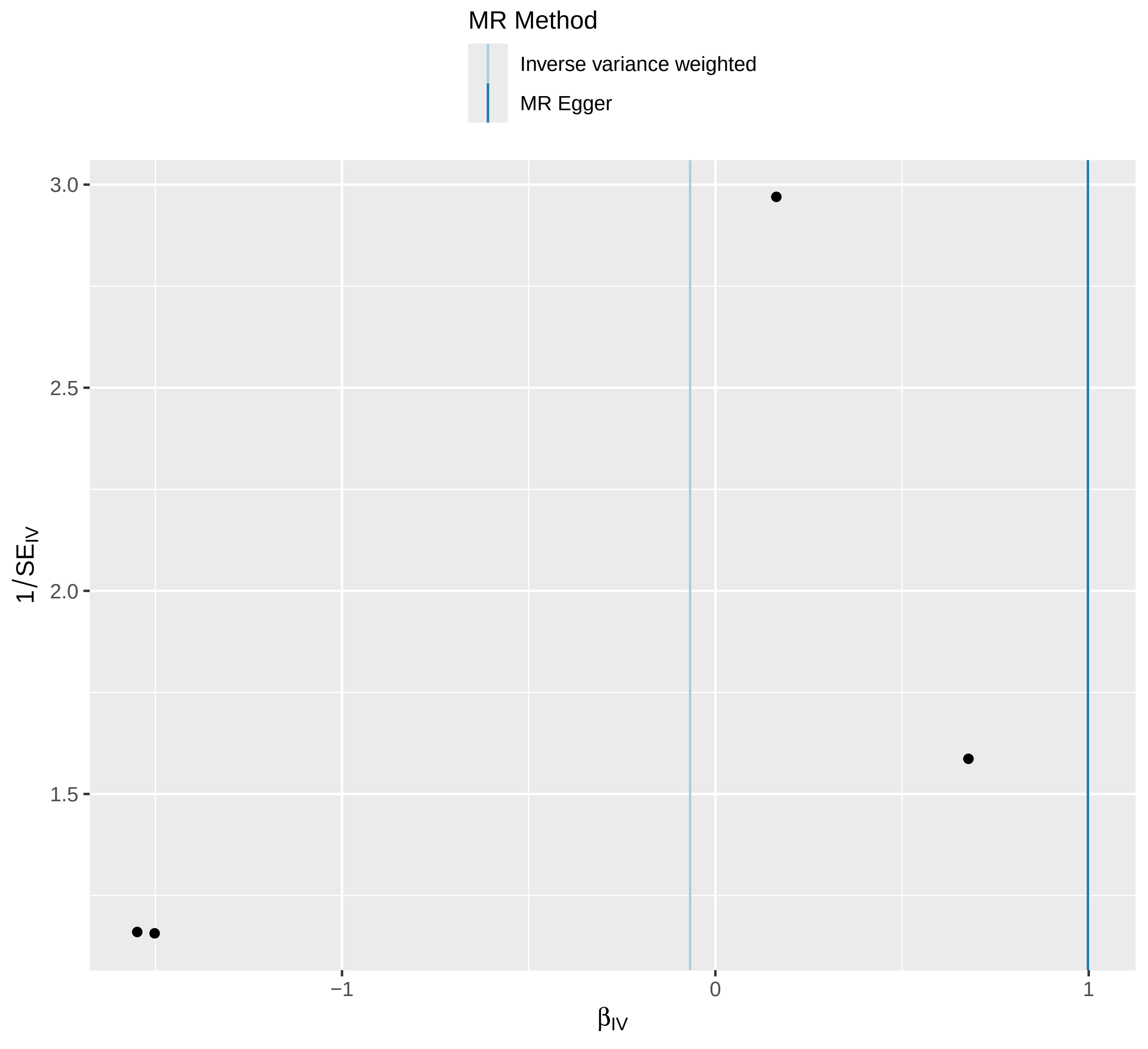

MR Method
Inverse variance weighted
MR Egger
3.0
2.5
2.0
1.5
1/SE$_{IV}$
−1
0
1
β$_{IV}$

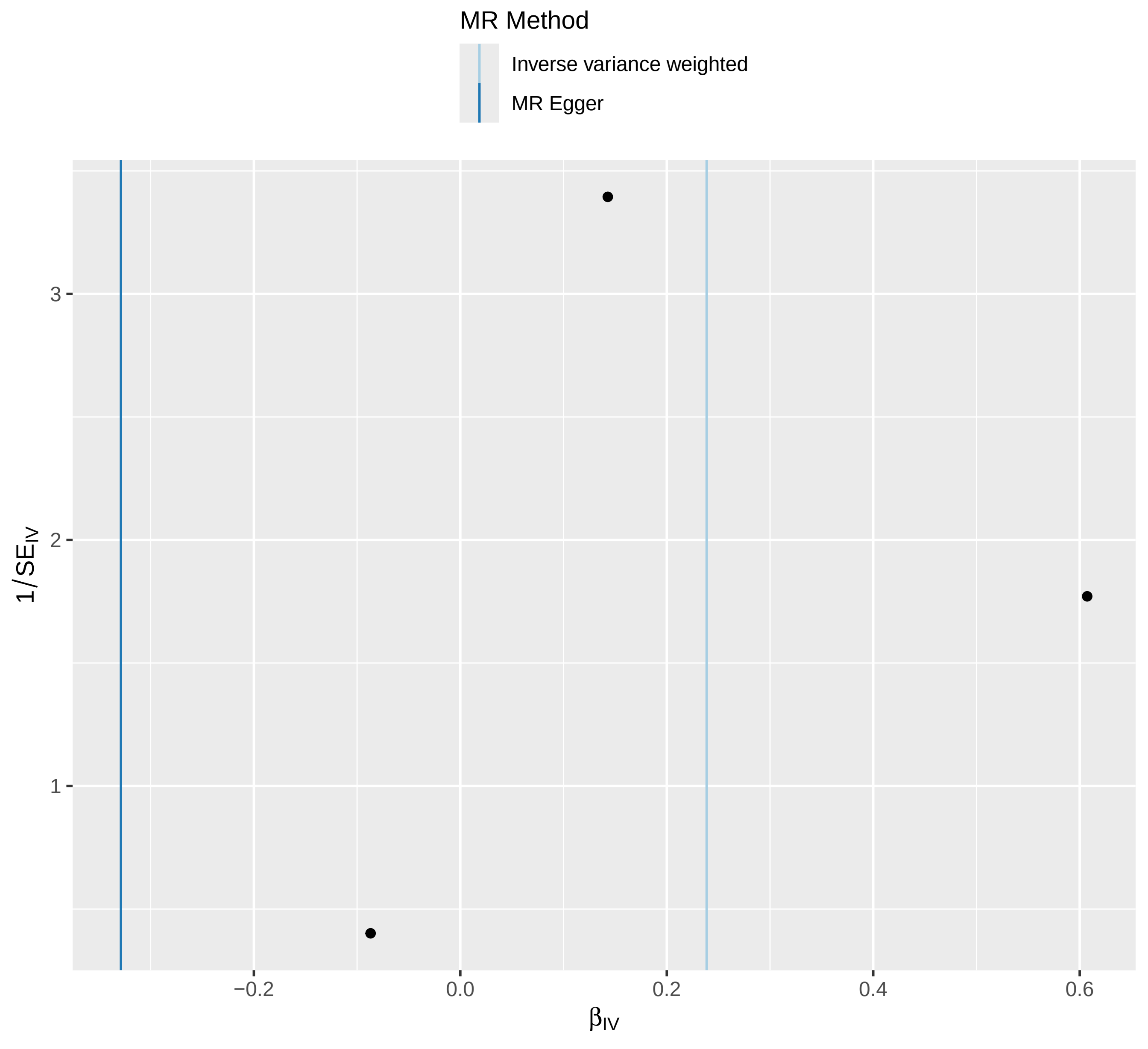
MR Method
Inverse variance weighted
MR Egger
3
2
1
1/SE_IV
−0.2
0.0
0.2
0.4
0.6
β_IV

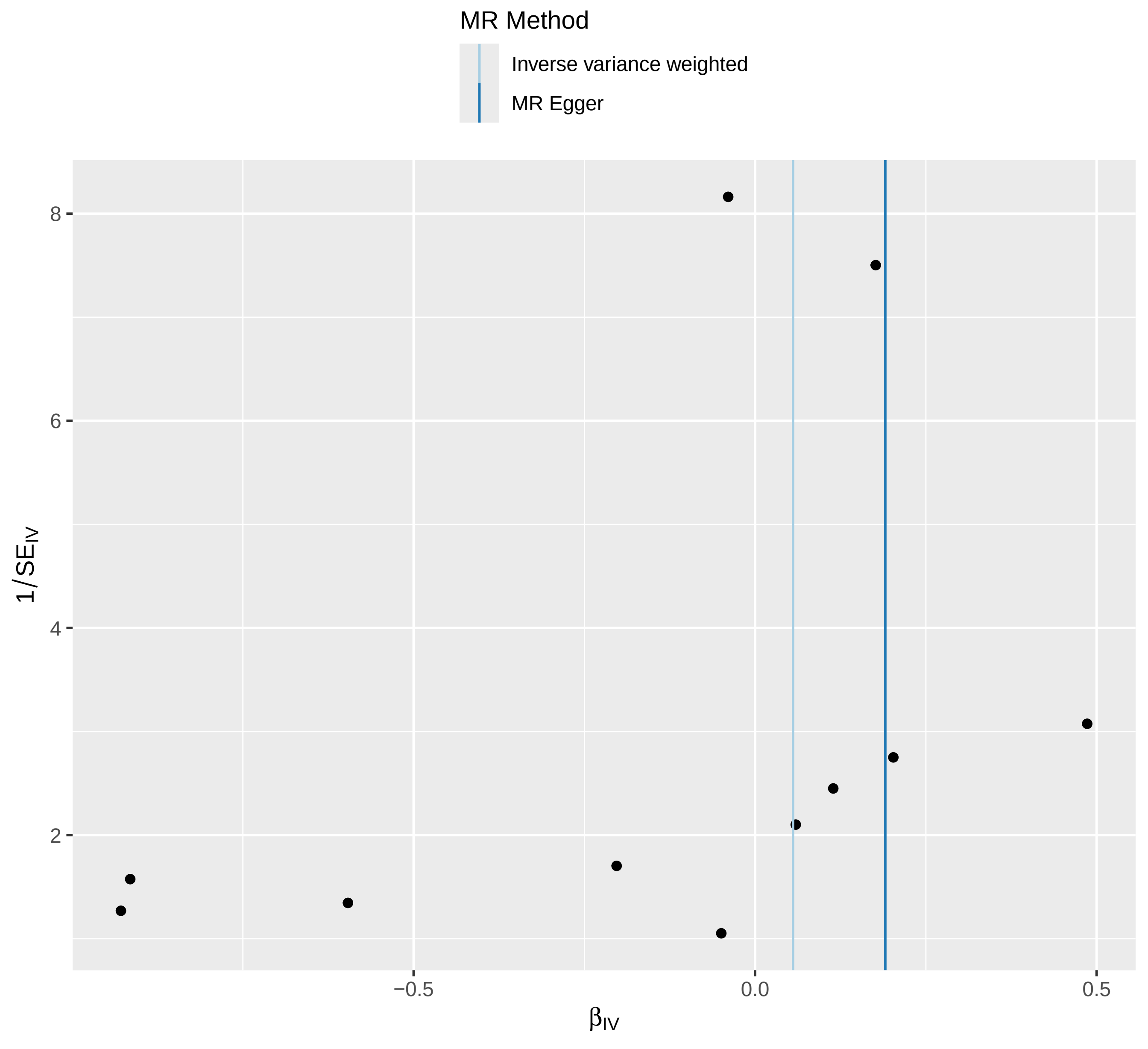
MR Method
Inverse variance weighted
MR Egger
8
6
4
2
−0.5
0.0
0.5
$1/SE_{IV}$
β_{IV}

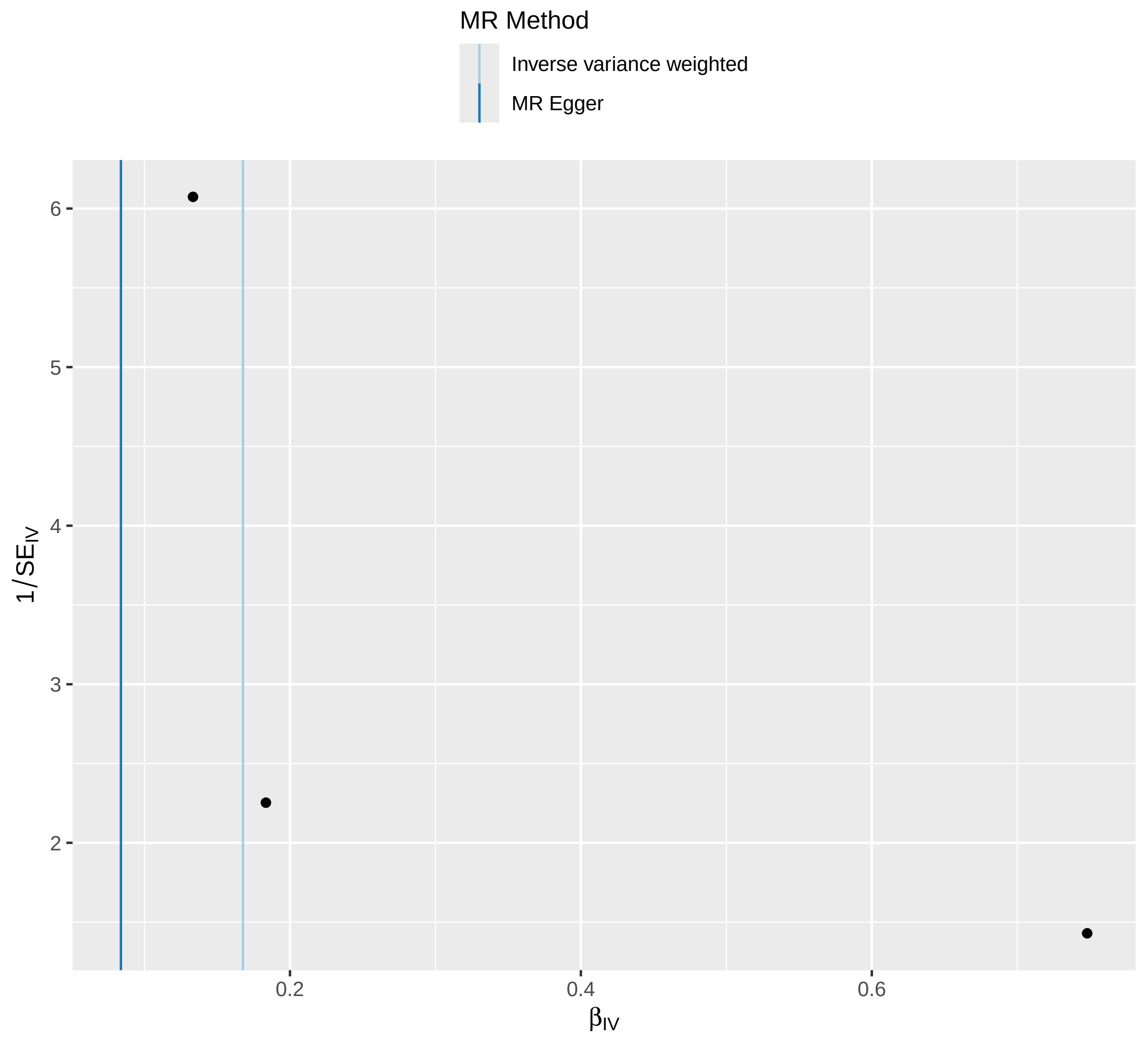

MR Method
Inverse variance weighted
MR Egger
1/SE$_{IV}$
β_{IV}
6
5
4
3
2
0.2
0.4
0.6

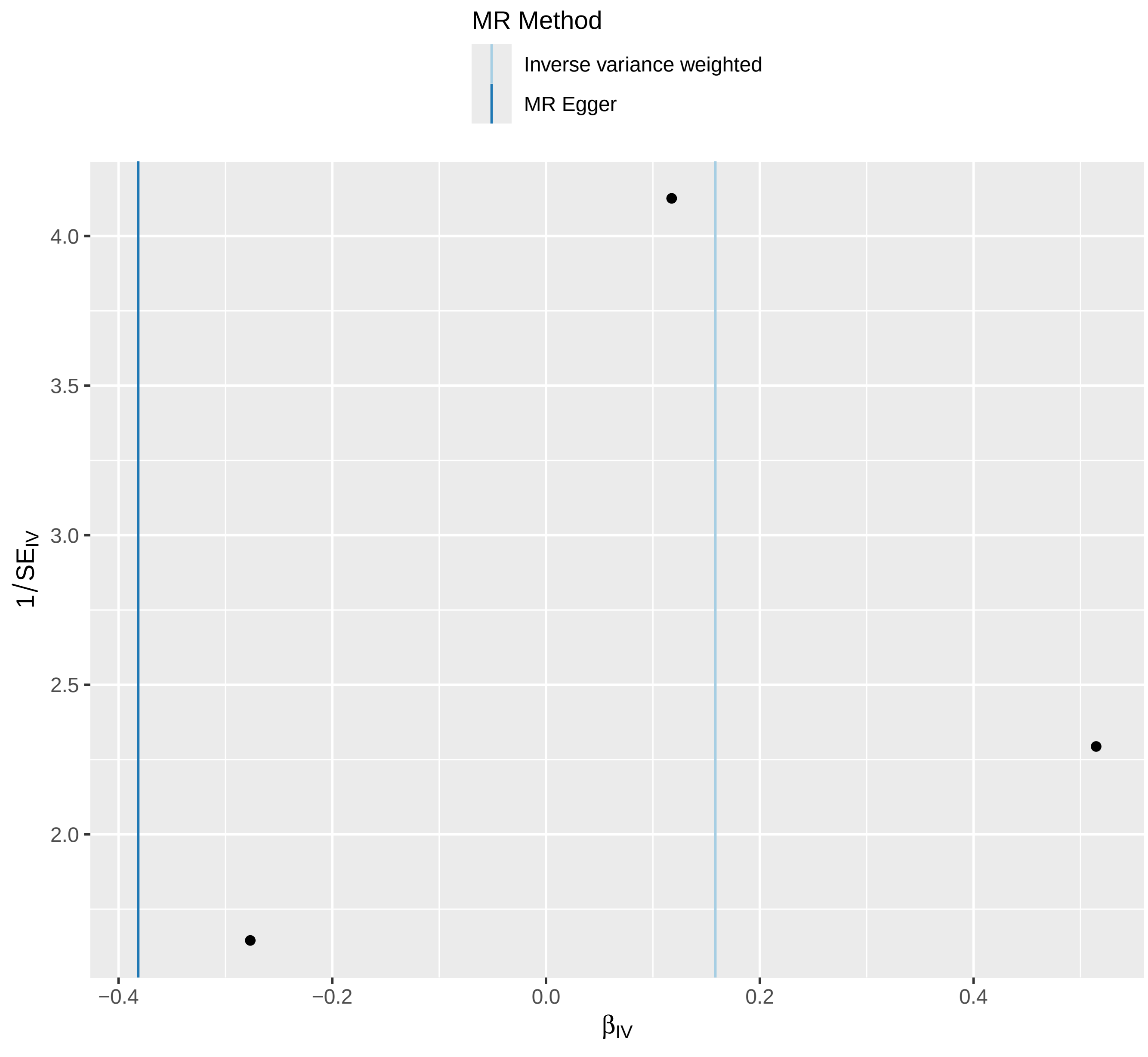
MR Method
Inverse variance weighted
MR Egger
4.0
3.5
3.0
2.5
2.0
1/SE_IV
−0.4
−0.2
0.0
0.2
0.4
β_IV

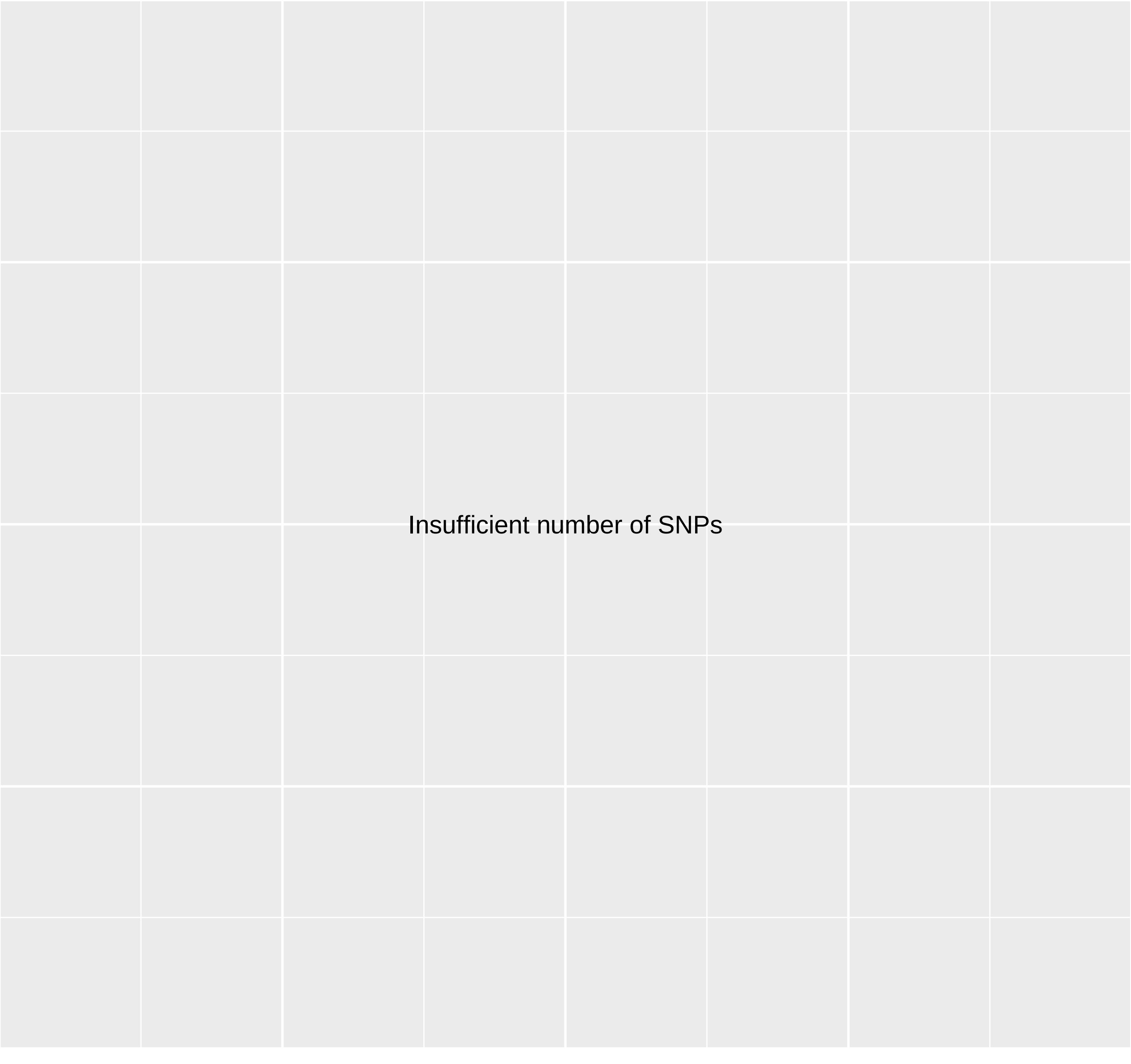
Insufficient number of SNPs

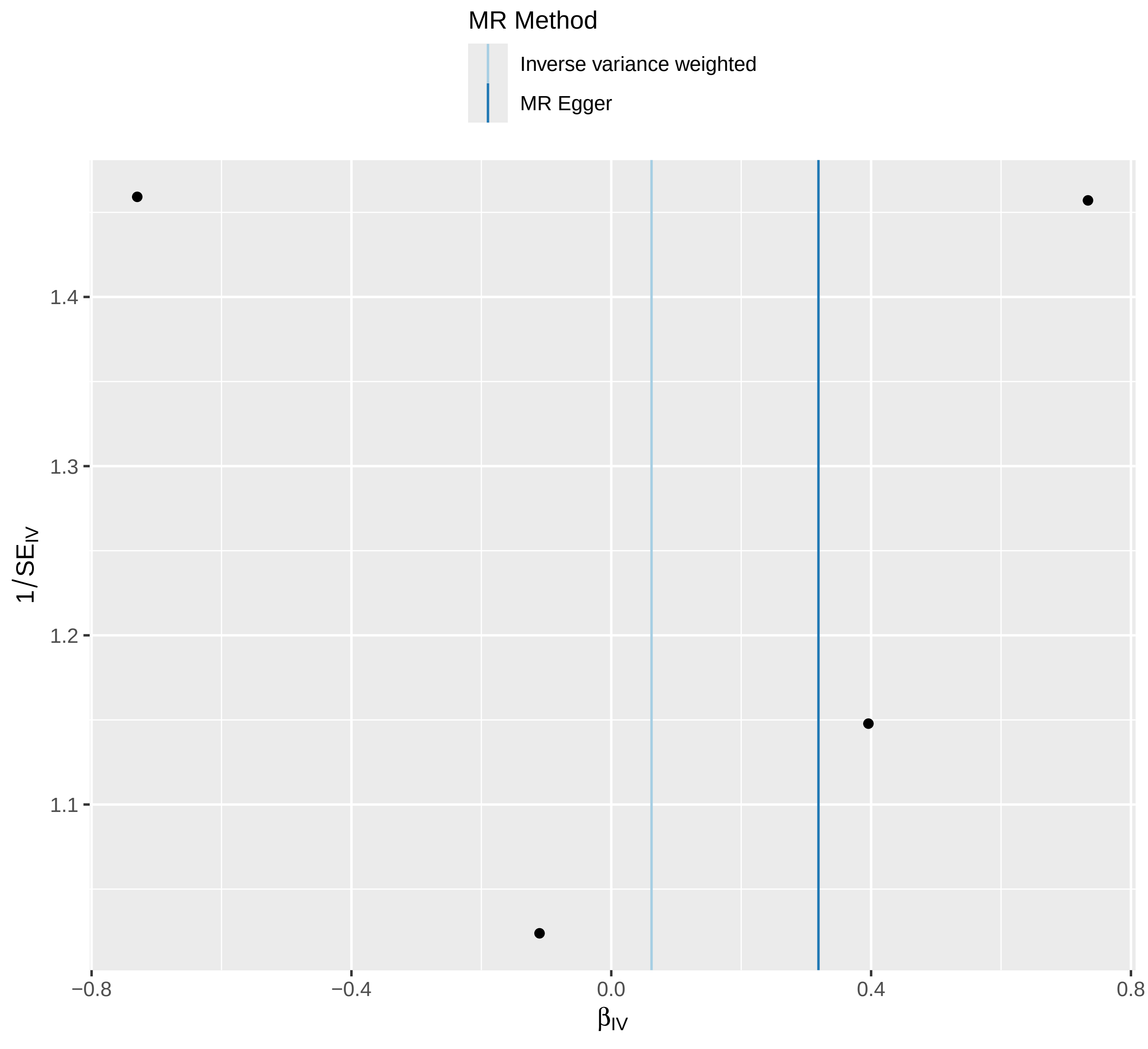
MR Method
Inverse variance weighted
MR Egger
1.4
1.3
1.2
1.1
1/SE_IV
−0.8
−0.4
0.0
0.4
0.8
β_IV

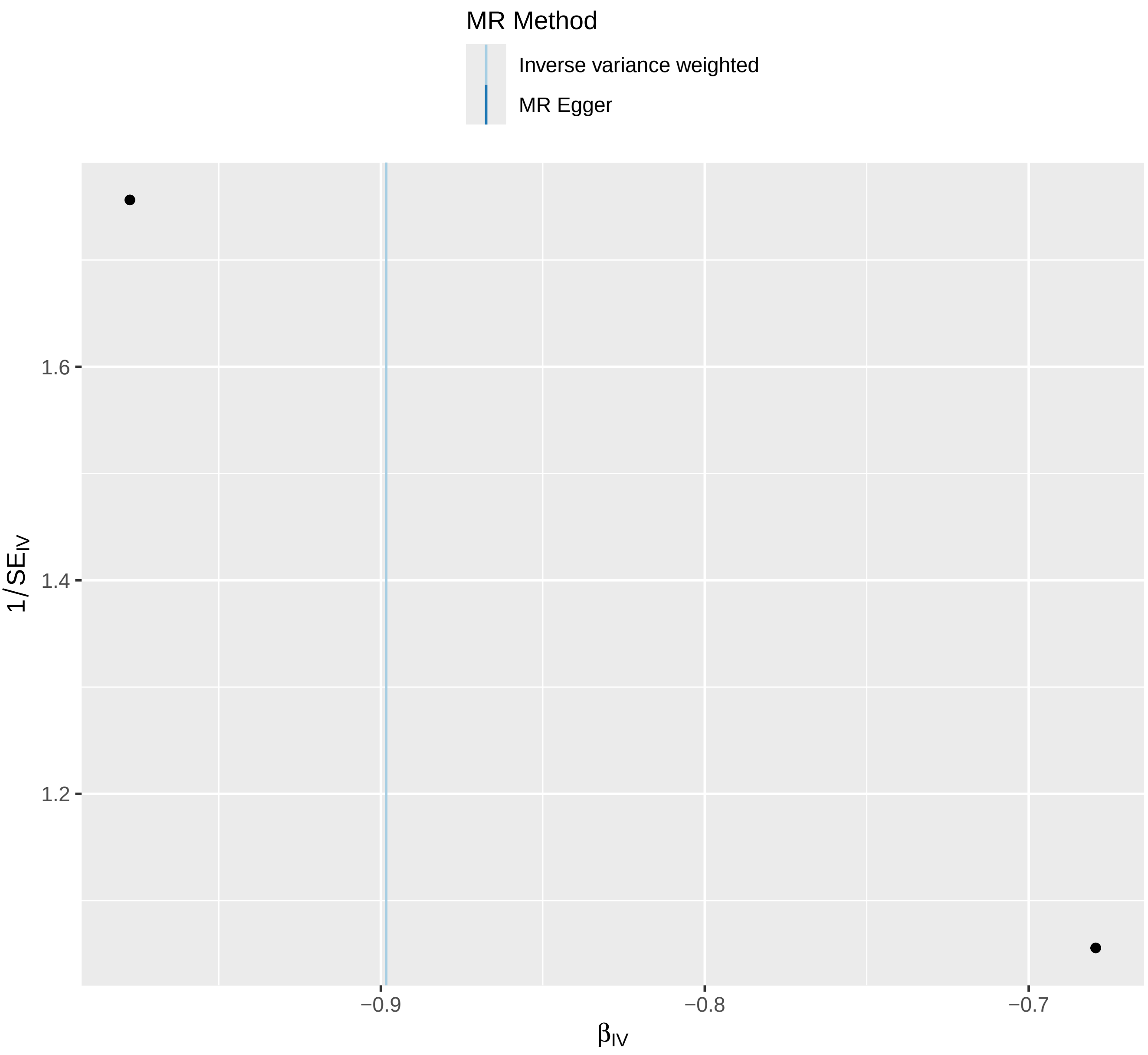
MR Method
Inverse variance weighted
MR Egger
1.6
1.4
1.2
1/SE_IV
−0.9
−0.8
−0.7
β_IV

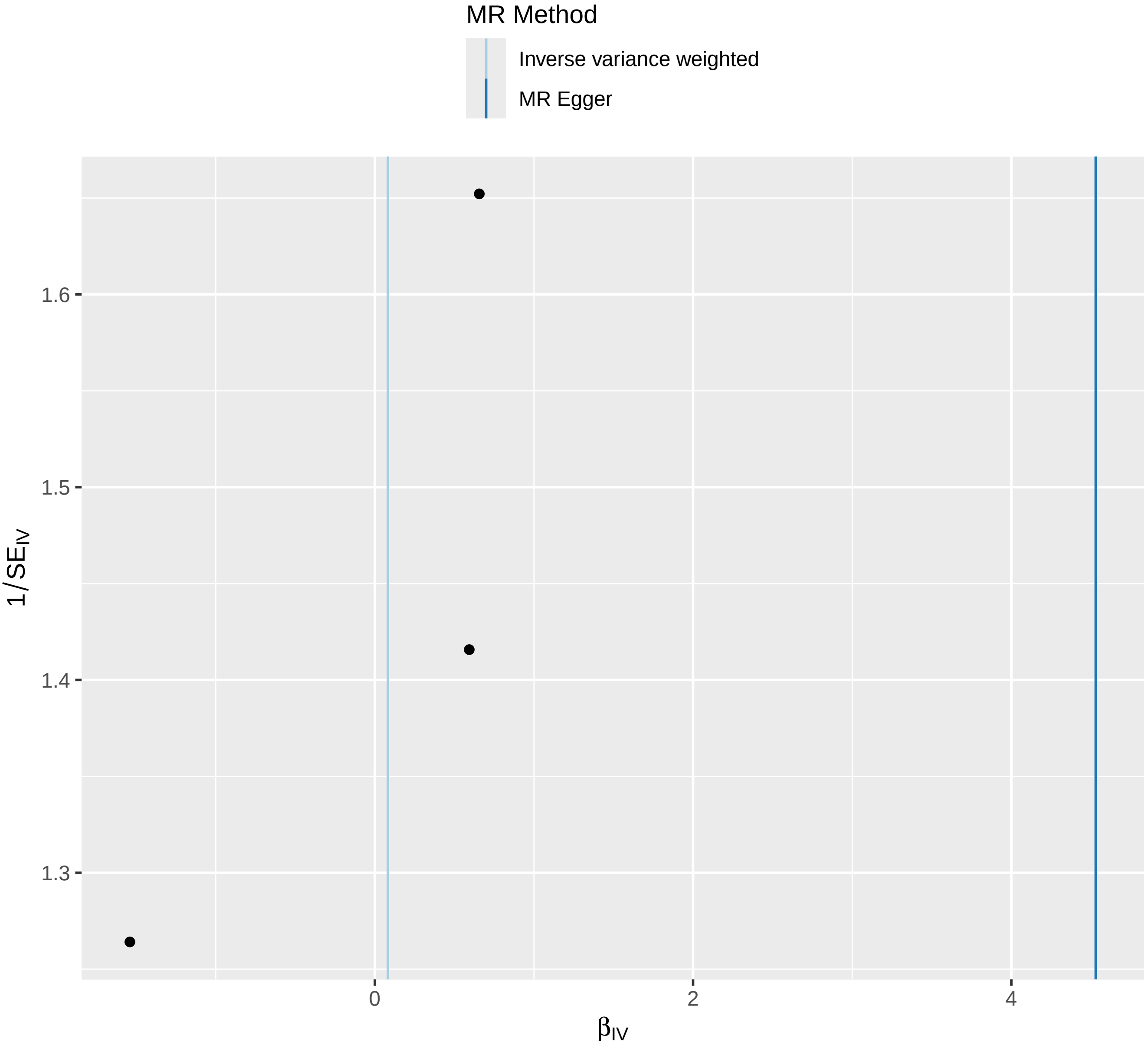
MR Method
Inverse variance weighted
MR Egger
1.6
1.5
1.4
1.3
$1/SE_{IV}$
0
2
4
β_{IV}

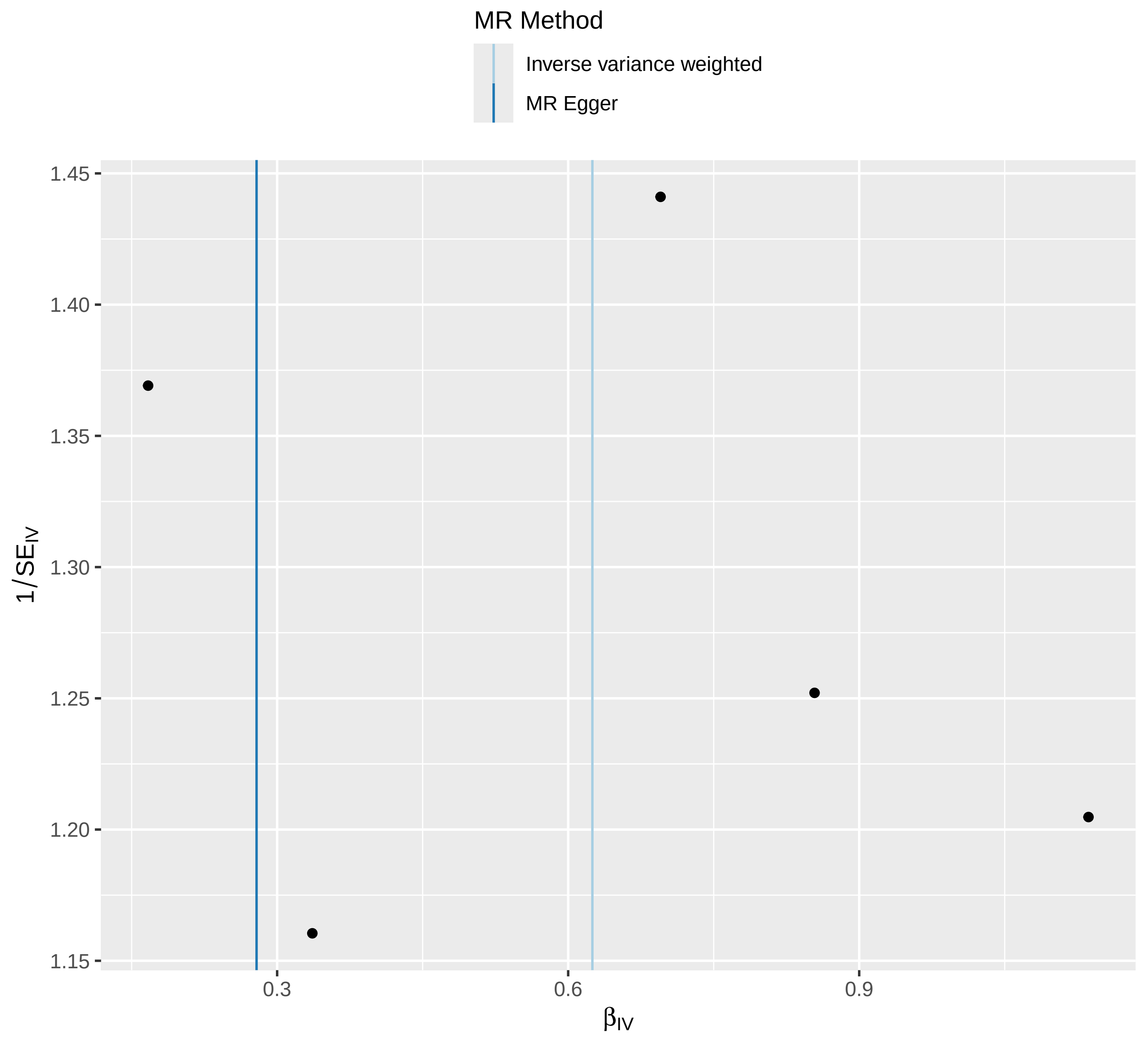

MR Method
Inverse variance weighted
MR Egger
1.45
1.40
1.35
1.30
1.25
1.20
1.15
0.3
0.6
0.9
$1/SE_{IV}$
β_{IV}

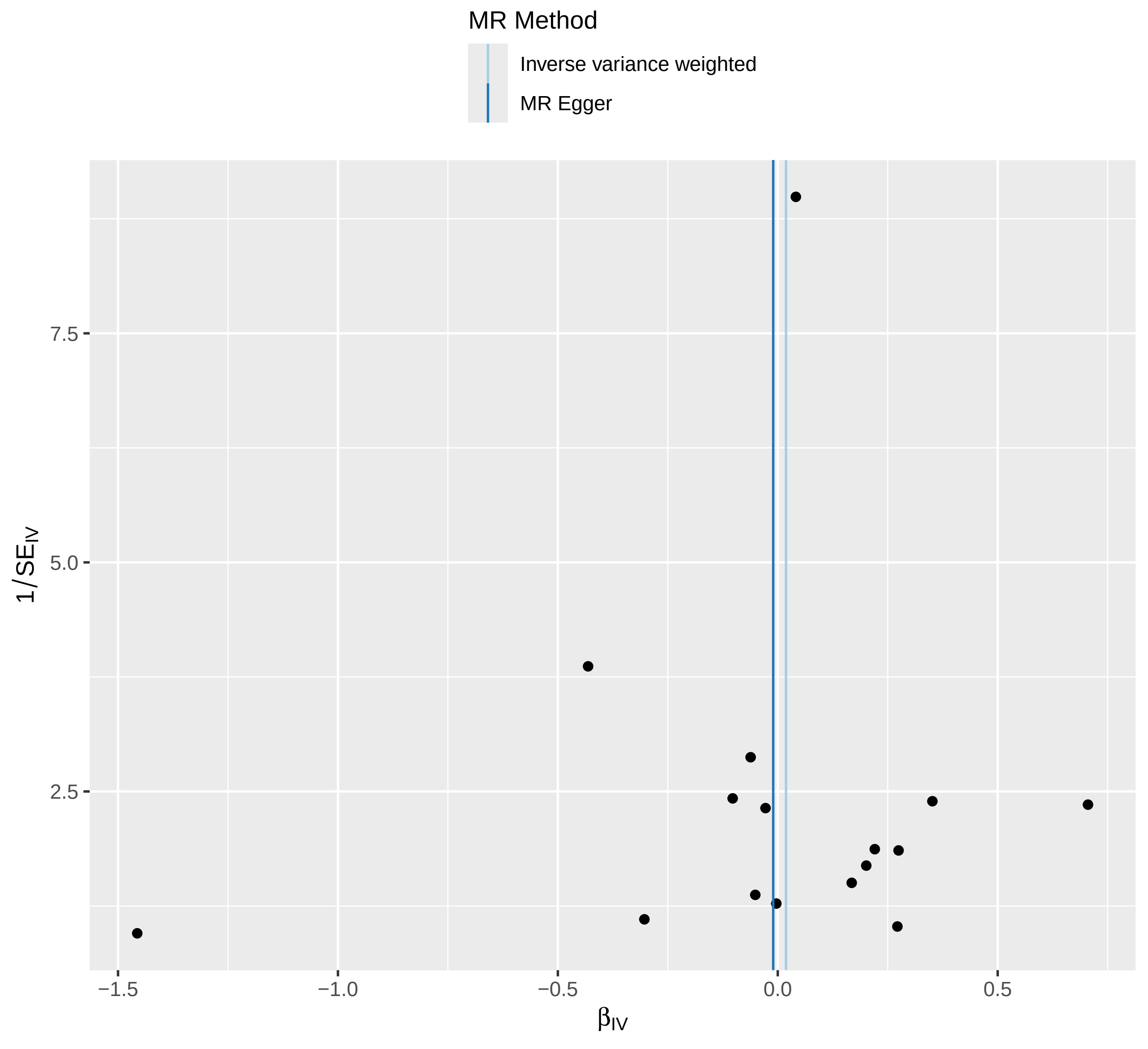

MR Method
Inverse variance weighted
MR Egger
1/SE$_{IV}$
7.5
5.0
2.5
−1.5
−1.0
−0.5
0.0
0.5
β$_{IV}$

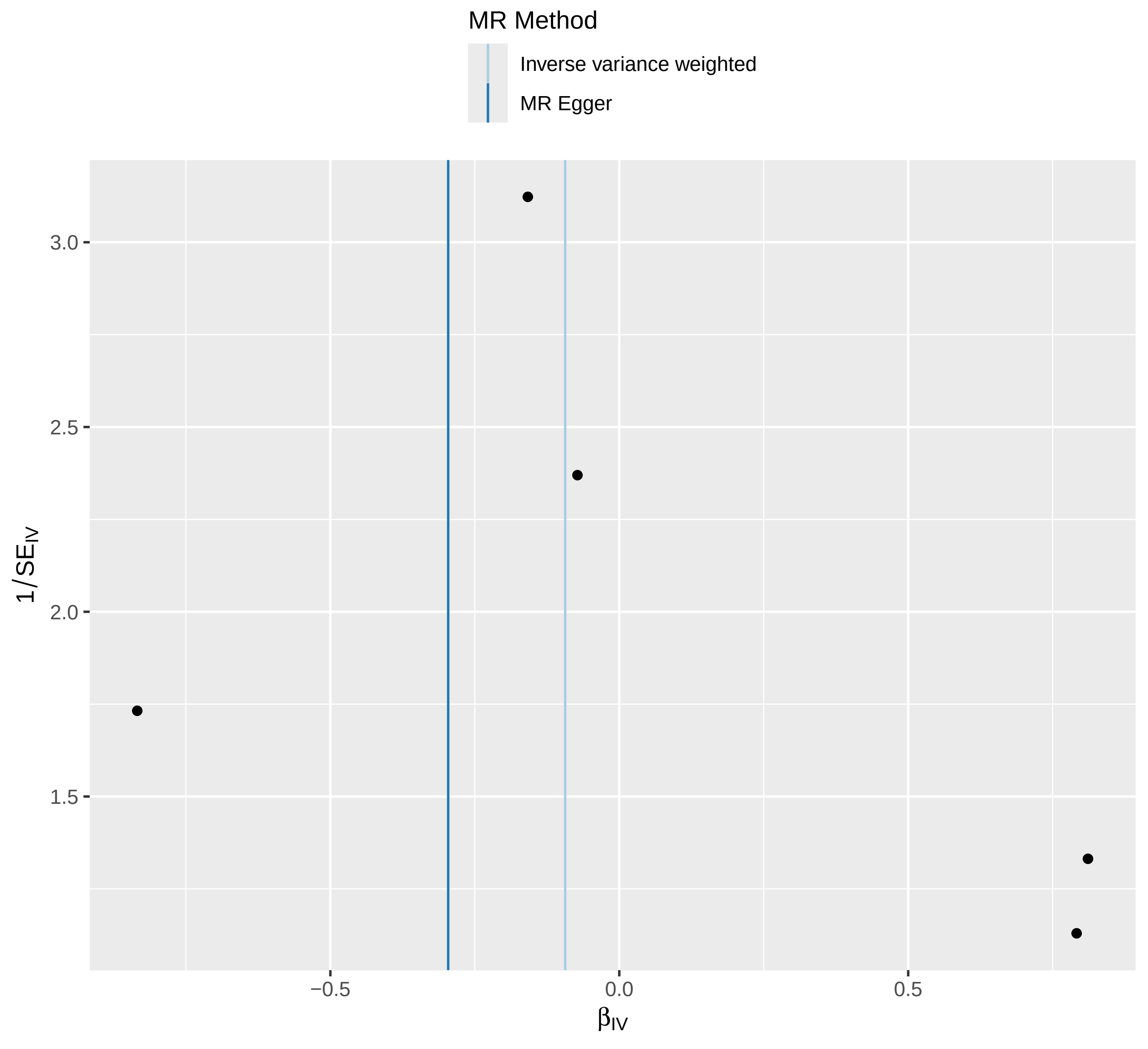

MR Method
Inverse variance weighted
MR Egger
3.0
2.5
2.0
1.5
1/SE$_{IV}$
−0.5
0.0
0.5
β$_{IV}$

Insufficient number of SNPs

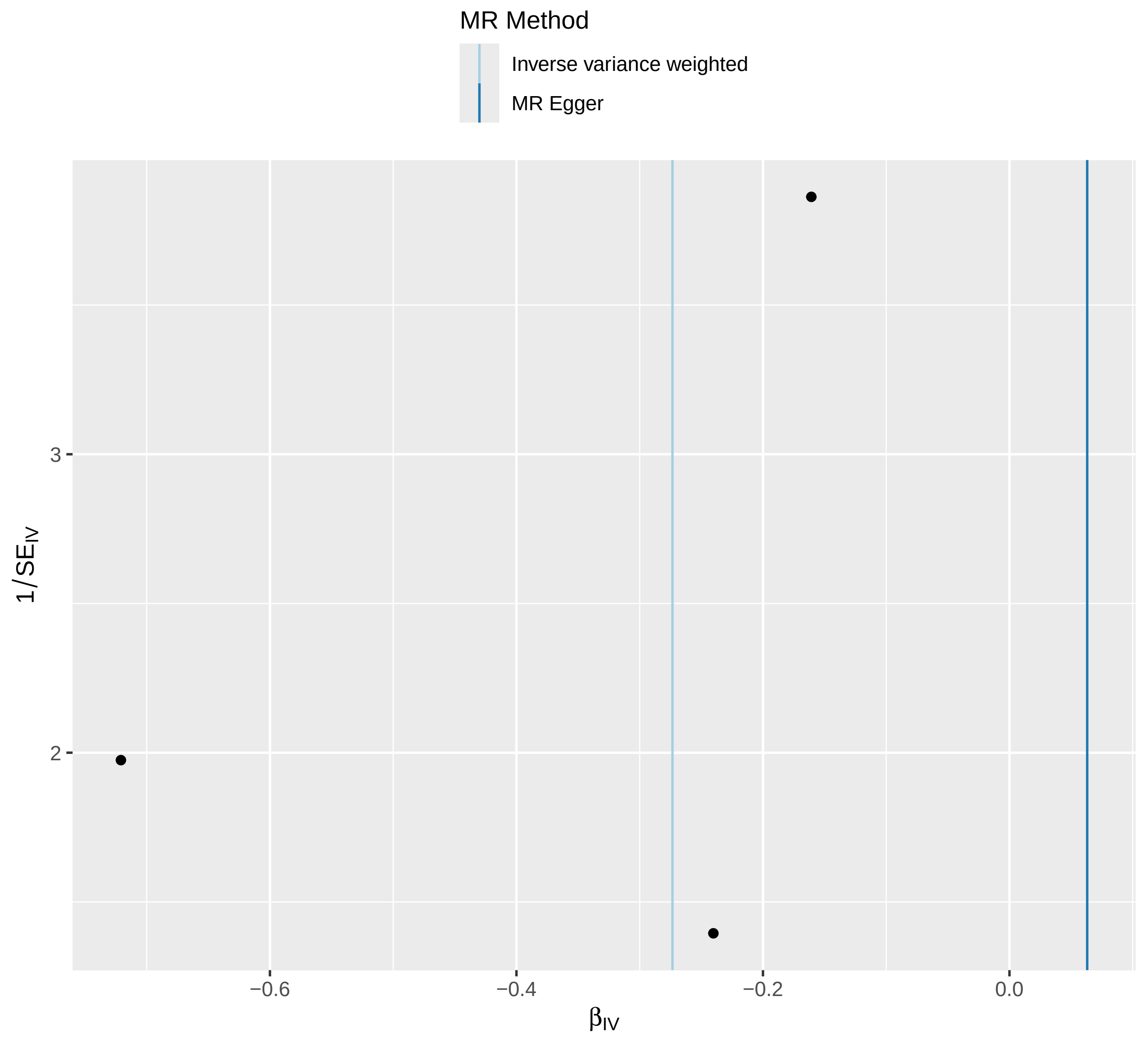

MR Method
Inverse variance weighted
MR Egger
3
2
$1/SE_{IV}$
−0.6
−0.4
−0.2
0.0
β_{IV}

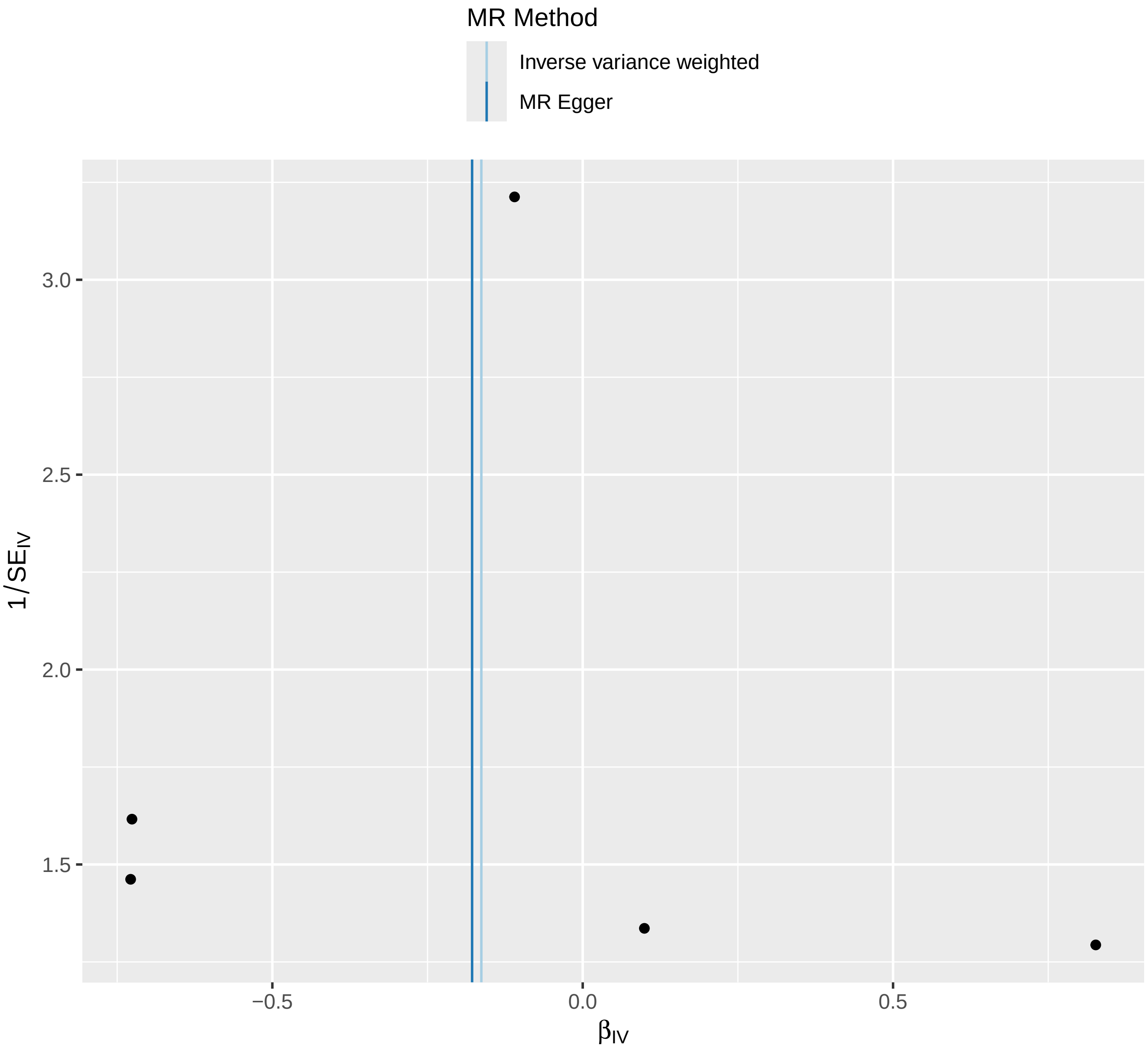
MR Method
Inverse variance weighted
MR Egger
3.0
2.5
2.0
1.5
$1/SE_{IV}$
−0.5
0.0
0.5
β_{IV}

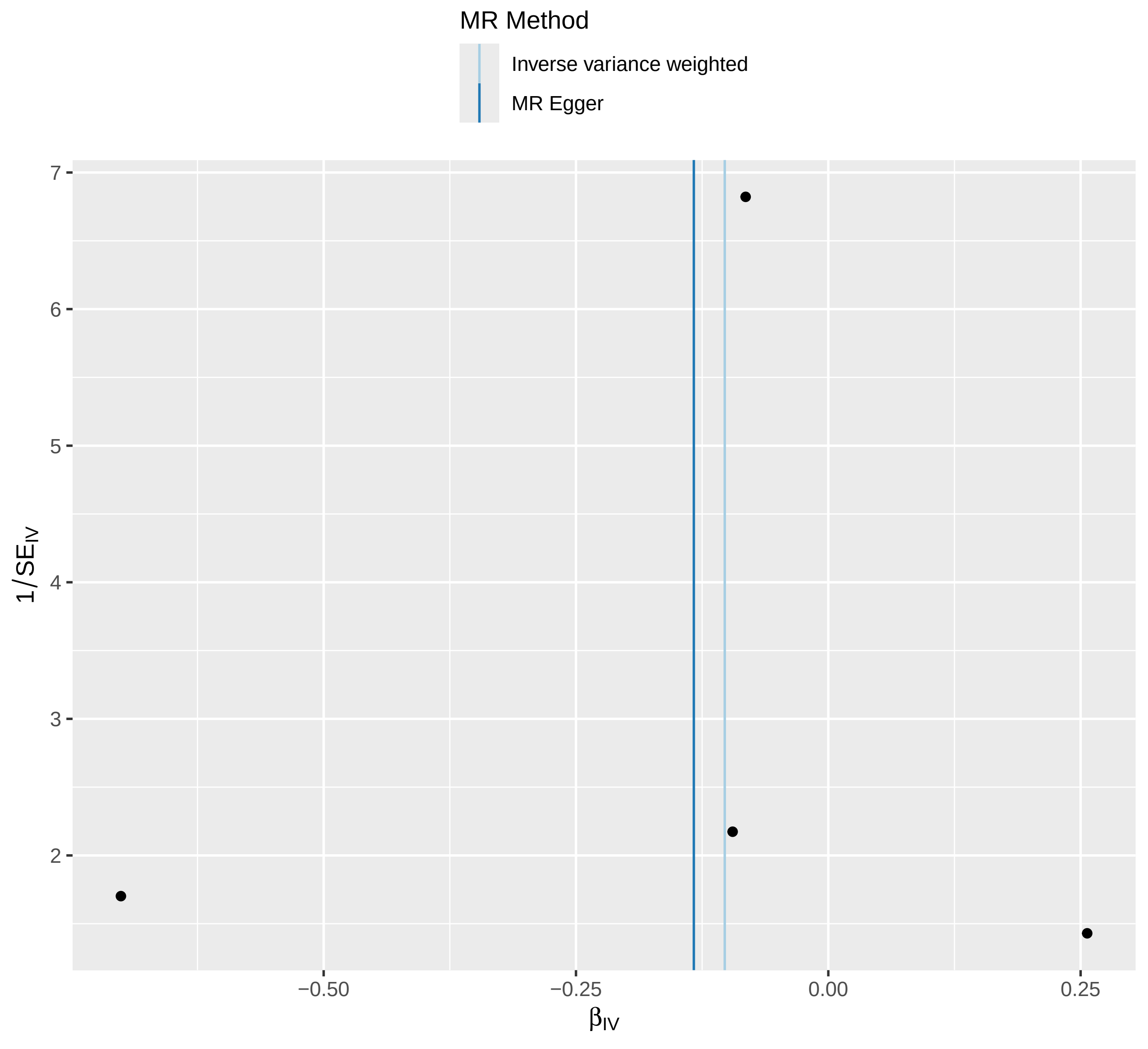
MR Method
Inverse variance weighted
MR Egger
1/SE_IV
7
6
5
4
3
2
−0.50
−0.25
0.00
0.25
β_IV

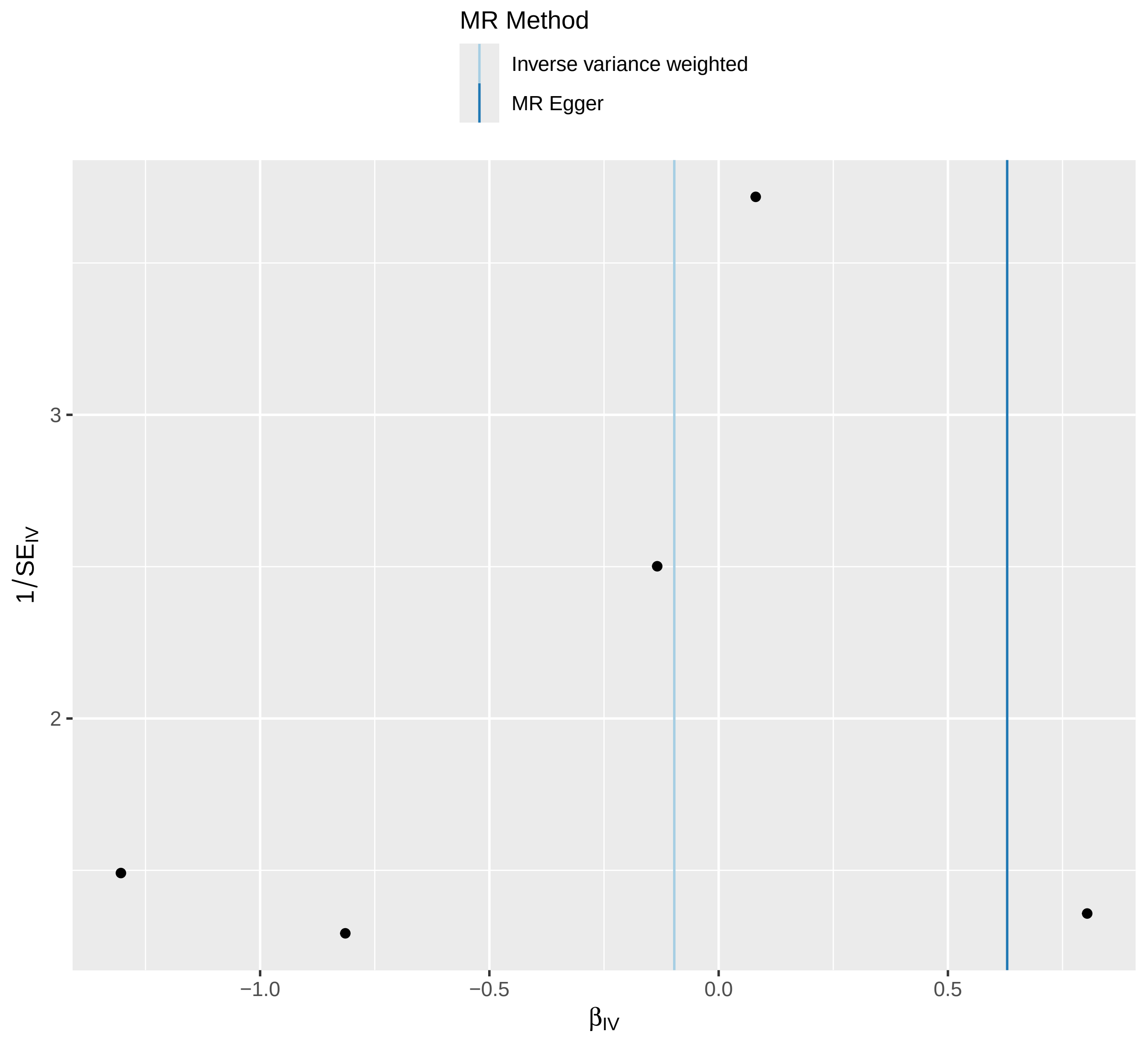
MR Method
Inverse variance weighted
MR Egger
1/SE_IV
3
2
−1.0
−0.5
0.0
0.5
β_IV

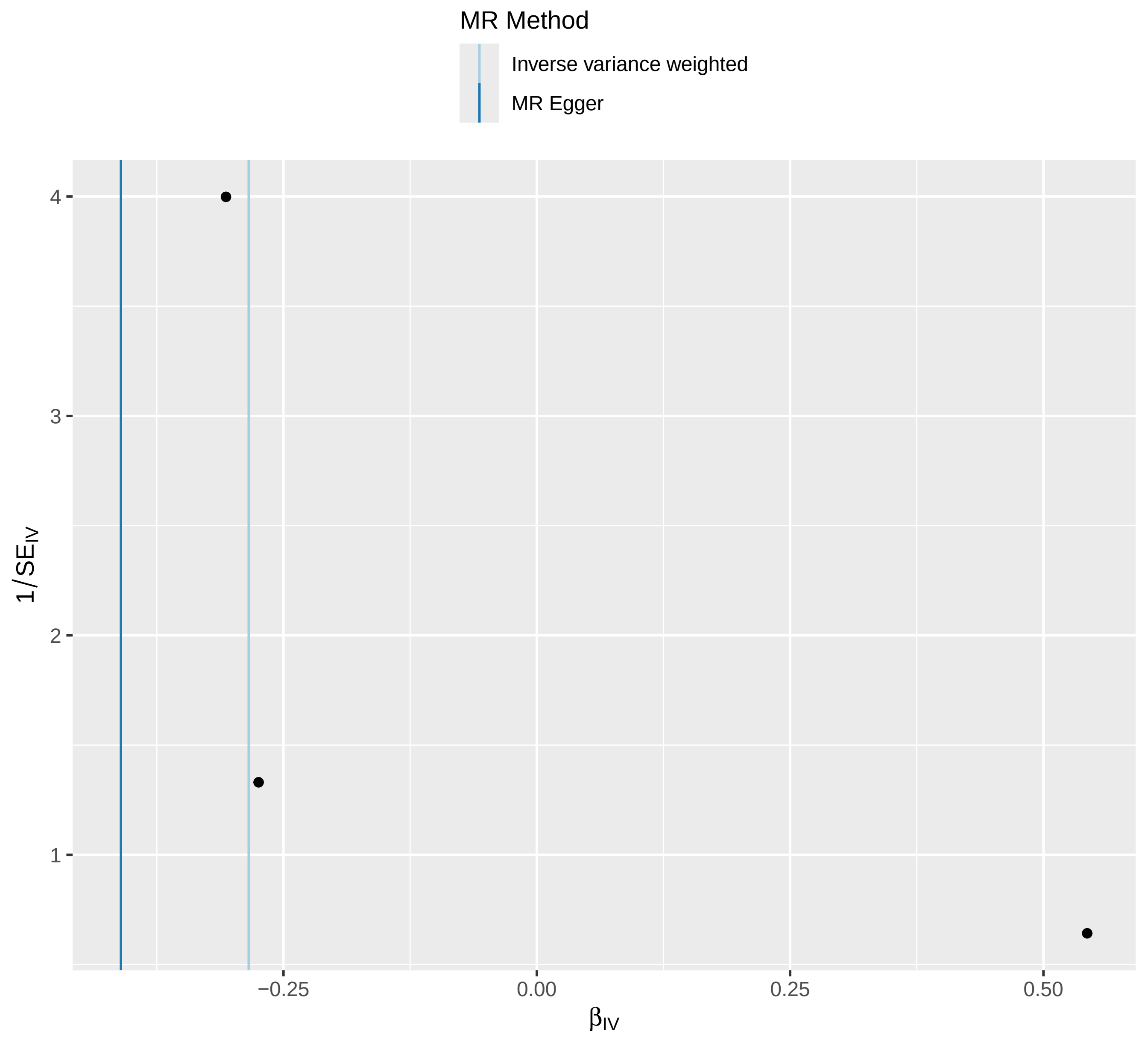
MR Method
Inverse variance weighted
MR Egger
4
3
2
1
1/SE_IV
−0.25
0.00
0.25
0.50
β_IV

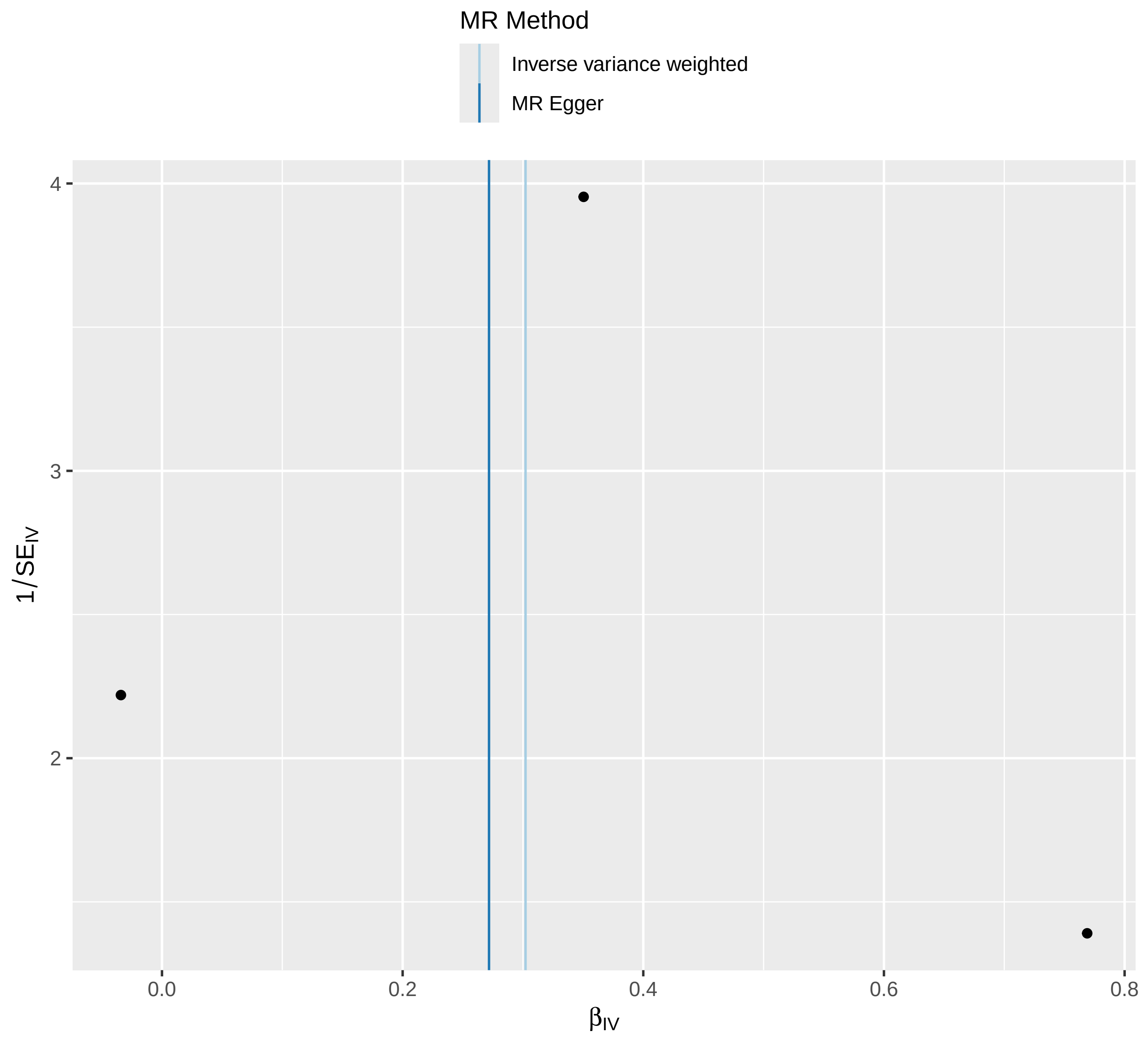
MR Method
Inverse variance weighted
MR Egger
4
3
2
1/SE_IV
0.0
0.2
0.4
0.6
0.8
β_IV

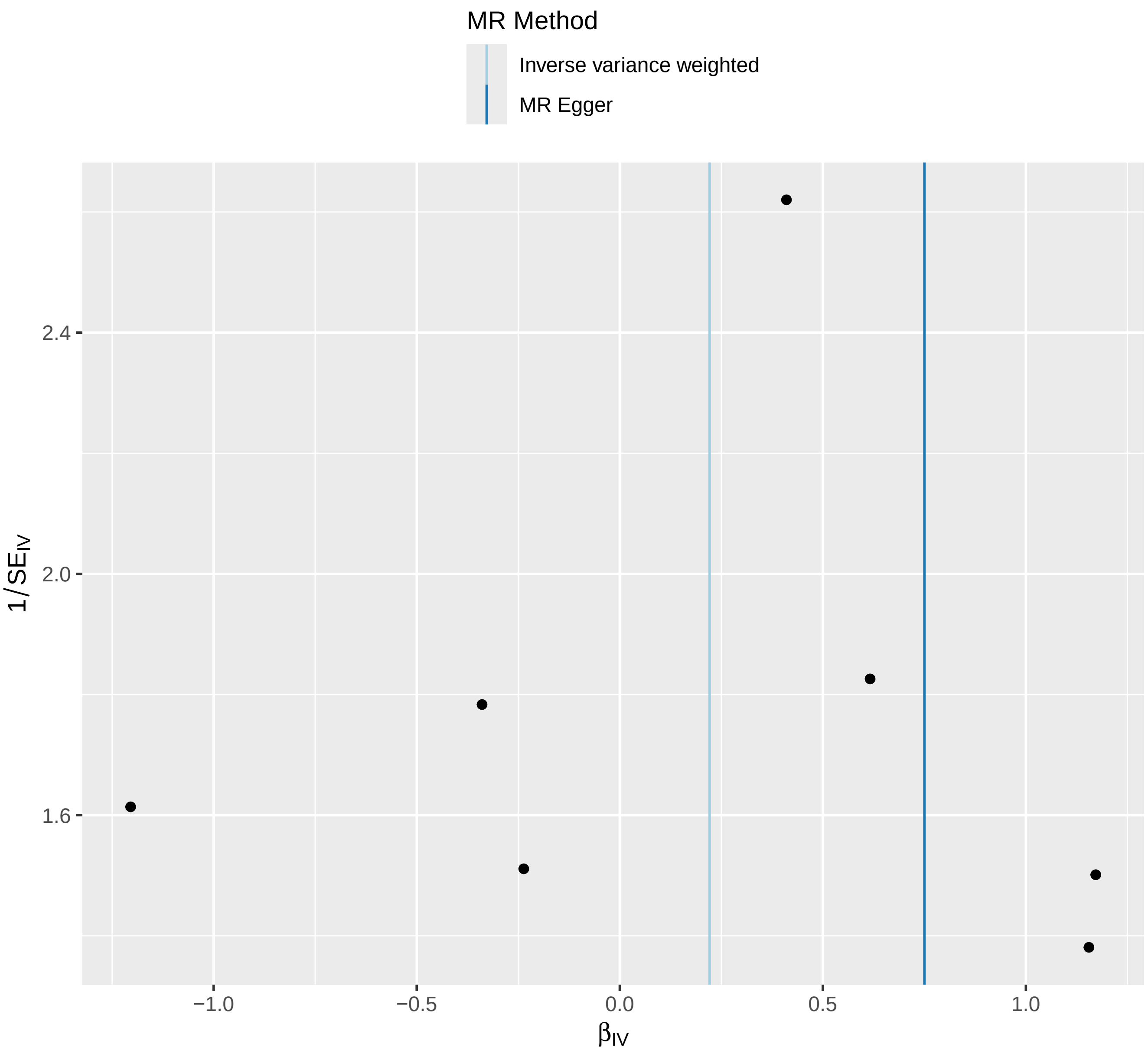

MR Method
Inverse variance weighted
MR Egger
2.4
2.0
1.6
1/SE$_{IV}$
−1.0
−0.5
0.0
0.5
1.0
β$_{IV}$

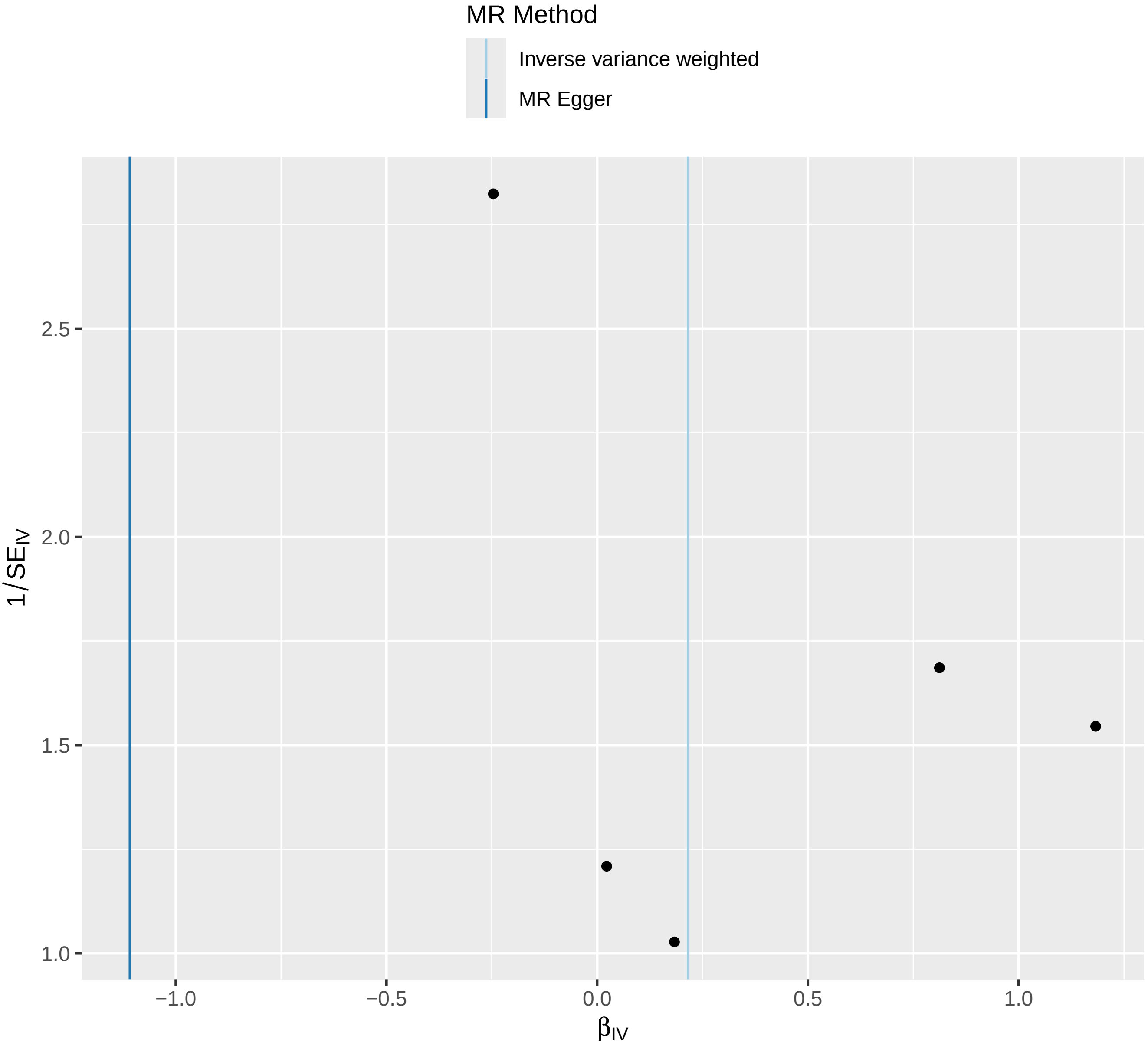
MR Method
Inverse variance weighted
MR Egger
2.5
2.0
1.5
1.0
1/SE_IV
−1.0
−0.5
0.0
0.5
1.0
β_IV

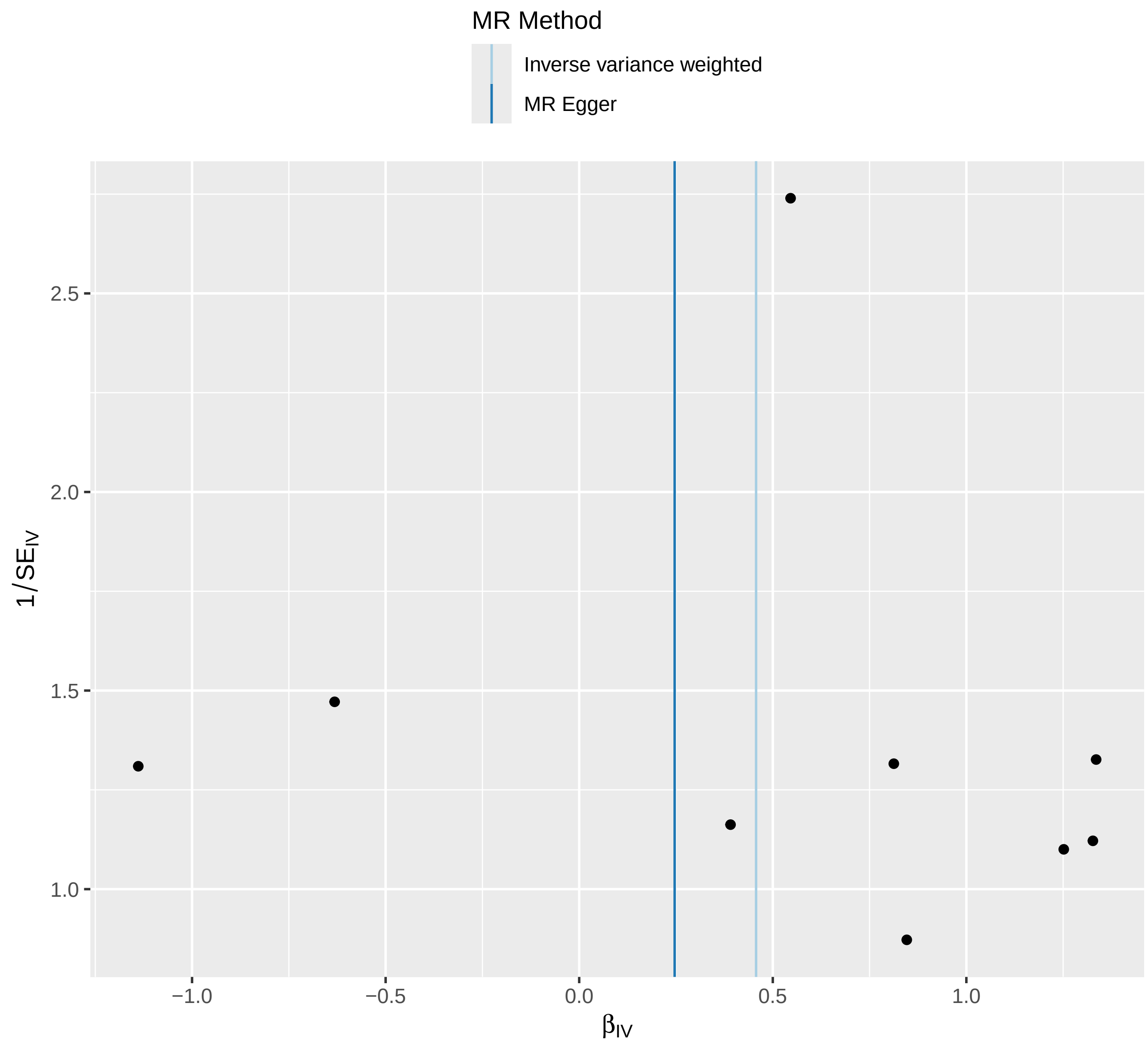
MR Method
Inverse variance weighted
MR Egger
2.5
2.0
1.5
1.0
$1/SE_{IV}$
−1.0
−0.5
0.0
0.5
1.0
β_{IV}

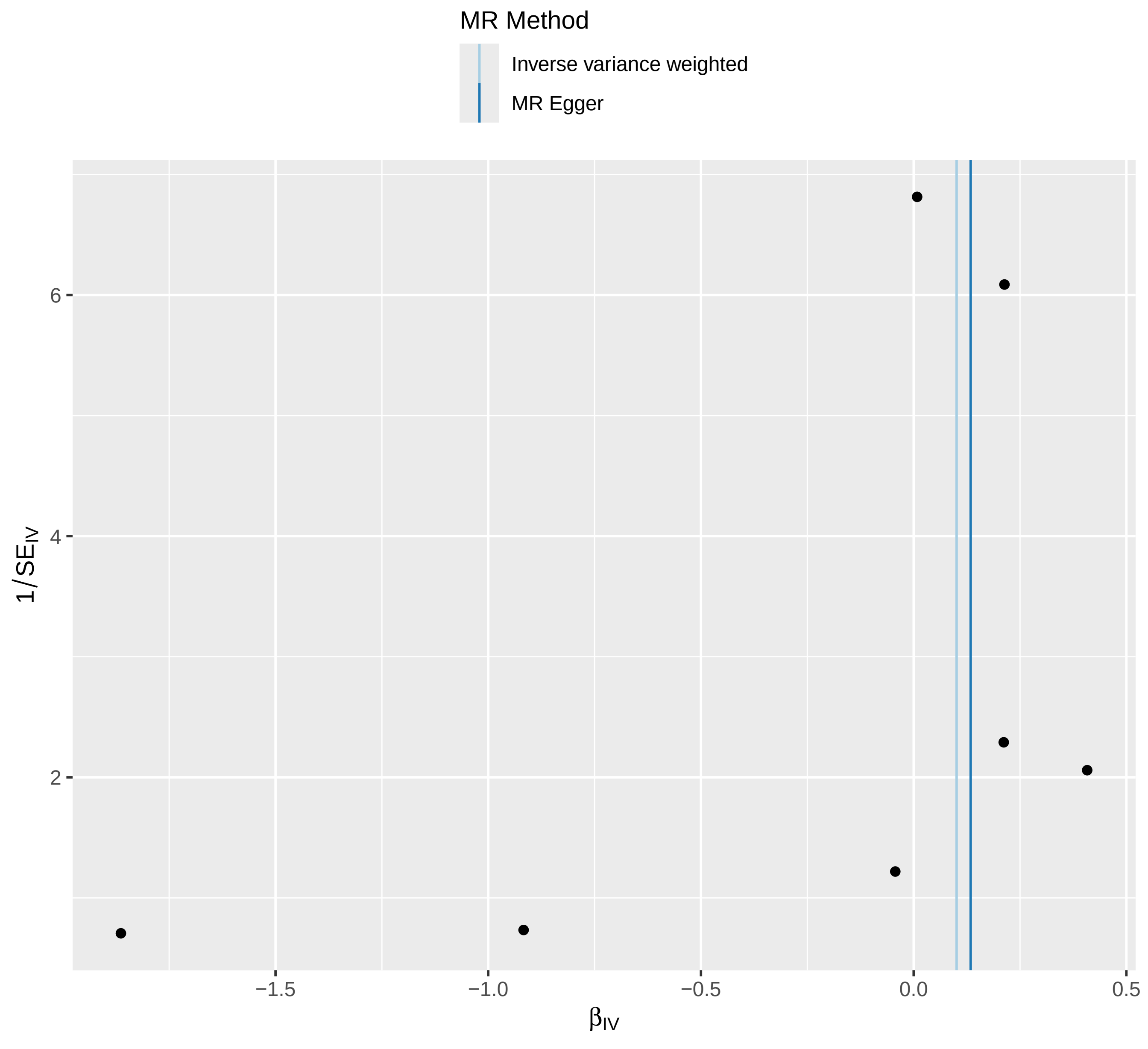

MR Method
Inverse variance weighted
MR Egger
6
4
2
1/SE_IV
−1.5
−1.0
−0.5
0.0
0.5
β_IV

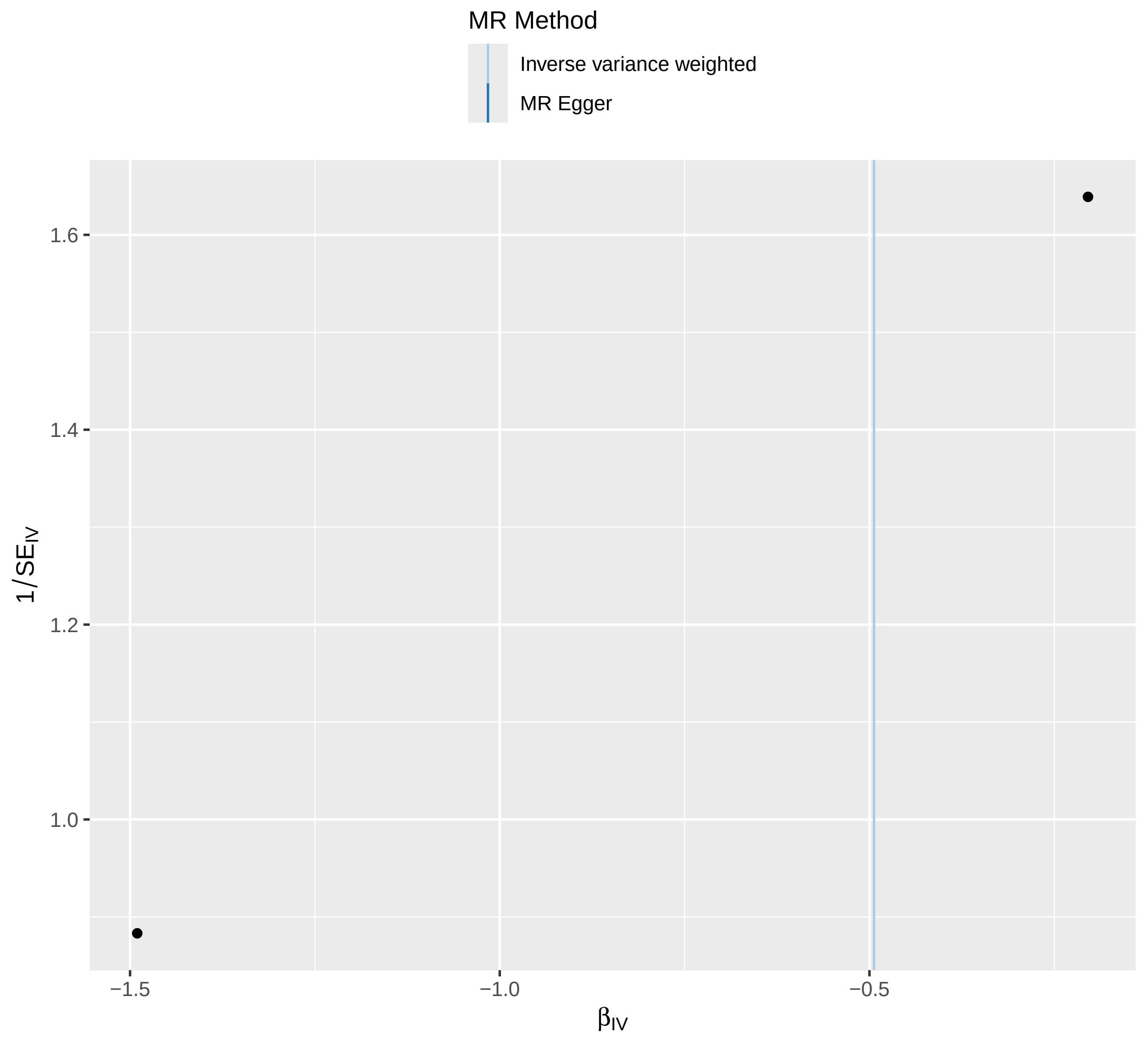
MR Method
Inverse variance weighted
MR Egger
1/SE_IV
1.6
1.4
1.2
1.0
−1.5
−1.0
−0.5
β_IV

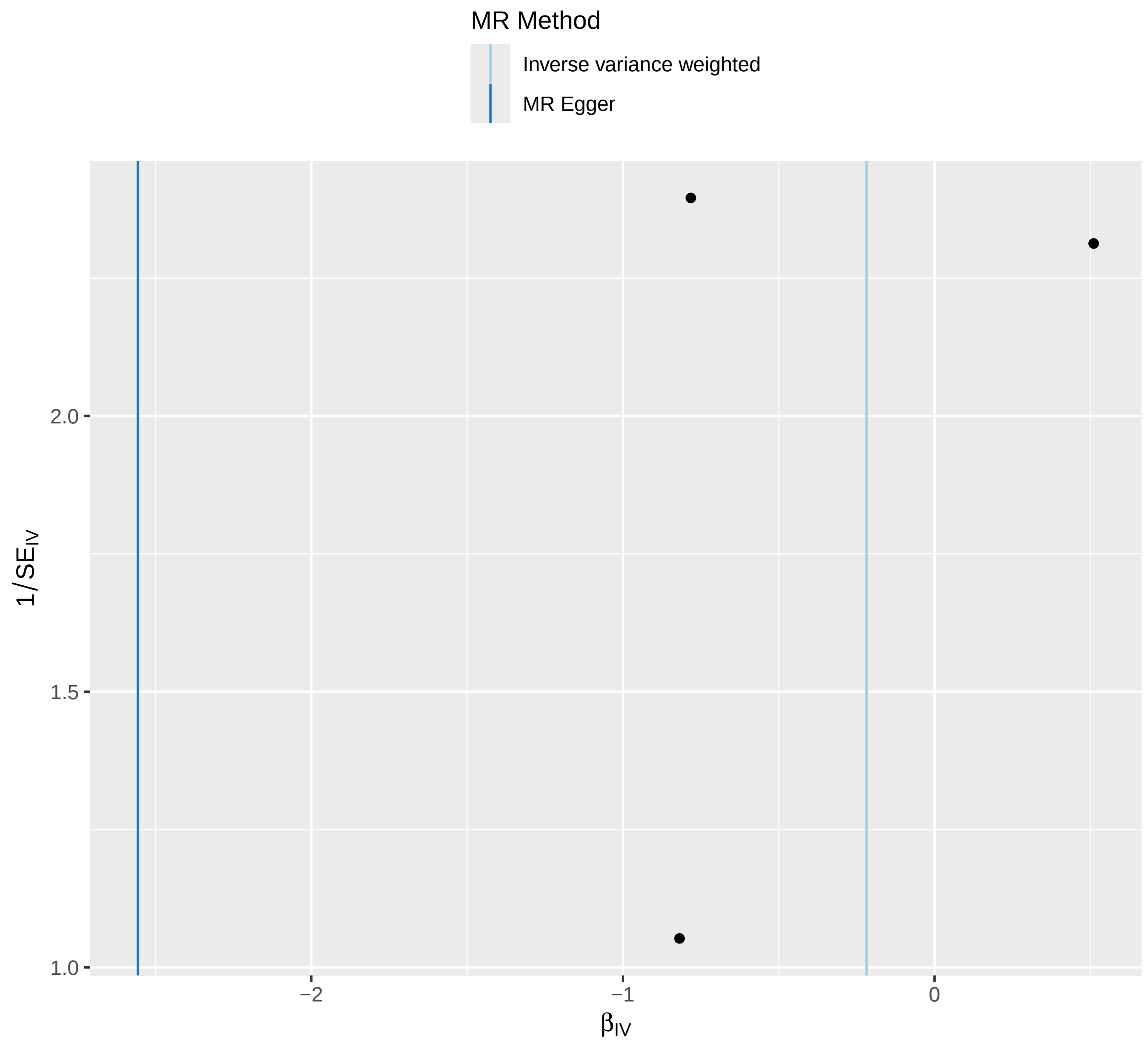
MR Method
Inverse variance weighted
MR Egger
2.0
1.5
1.0
−2
−1
0
$1/SE_{IV}$
β_{IV}

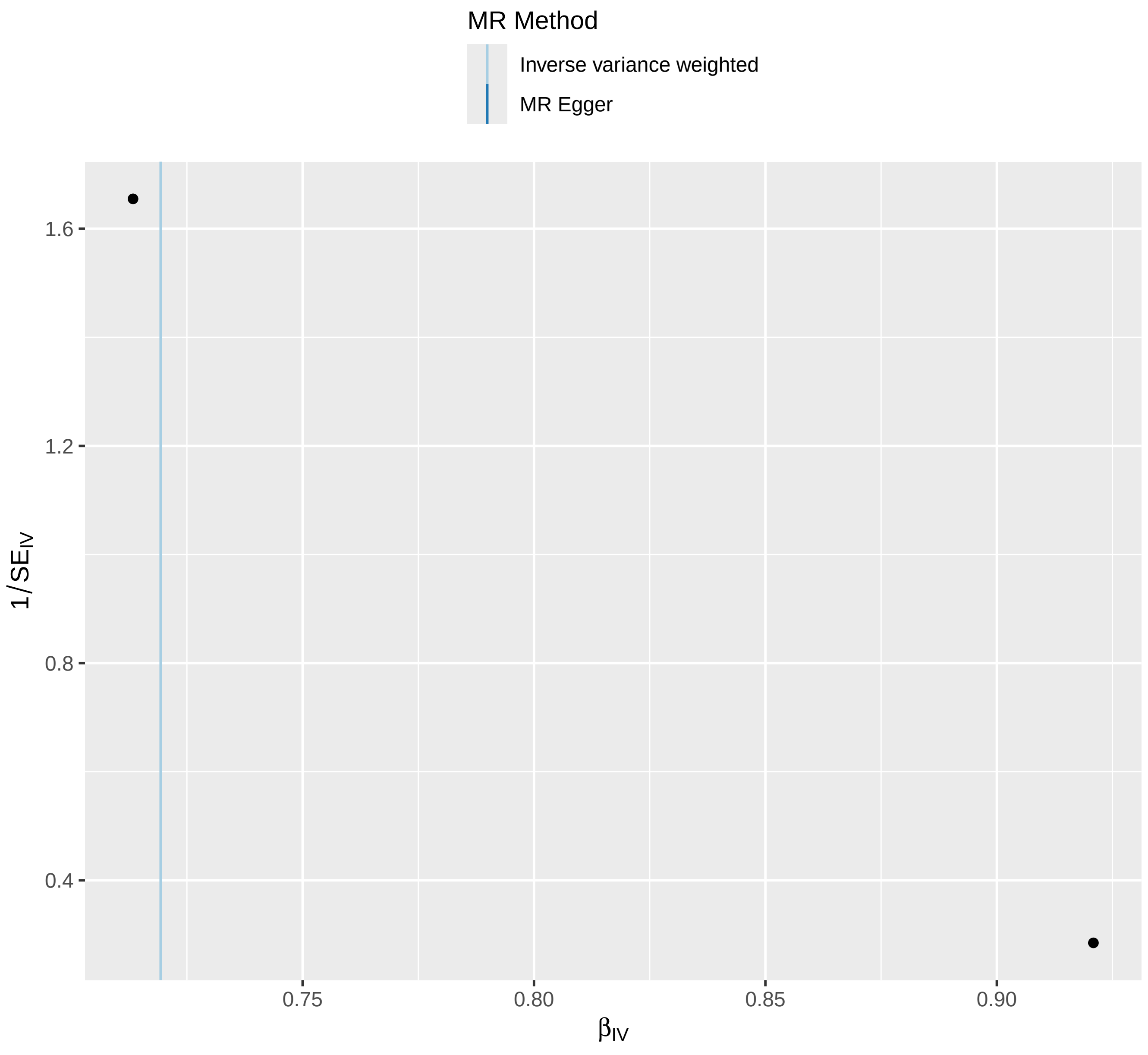

MR Method
Inverse variance weighted
MR Egger
1.6
1.2
0.8
0.4
0.75
0.80
0.85
0.90
β_{IV}
$1/SE_{IV}$

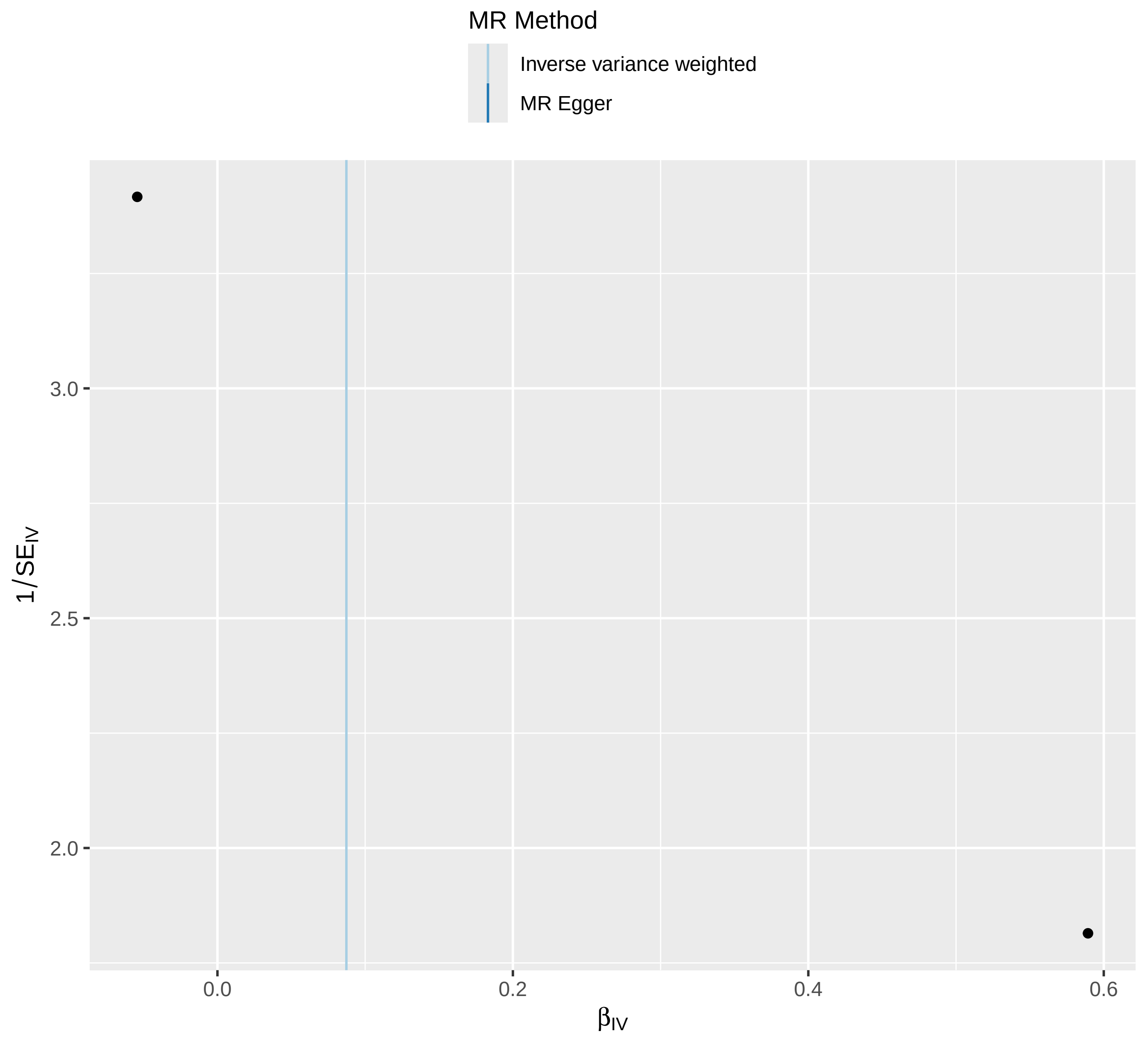
MR Method
Inverse variance weighted
MR Egger
3.0
2.5
2.0
1/SE_IV
0.0
0.2
0.4
0.6
β_IV

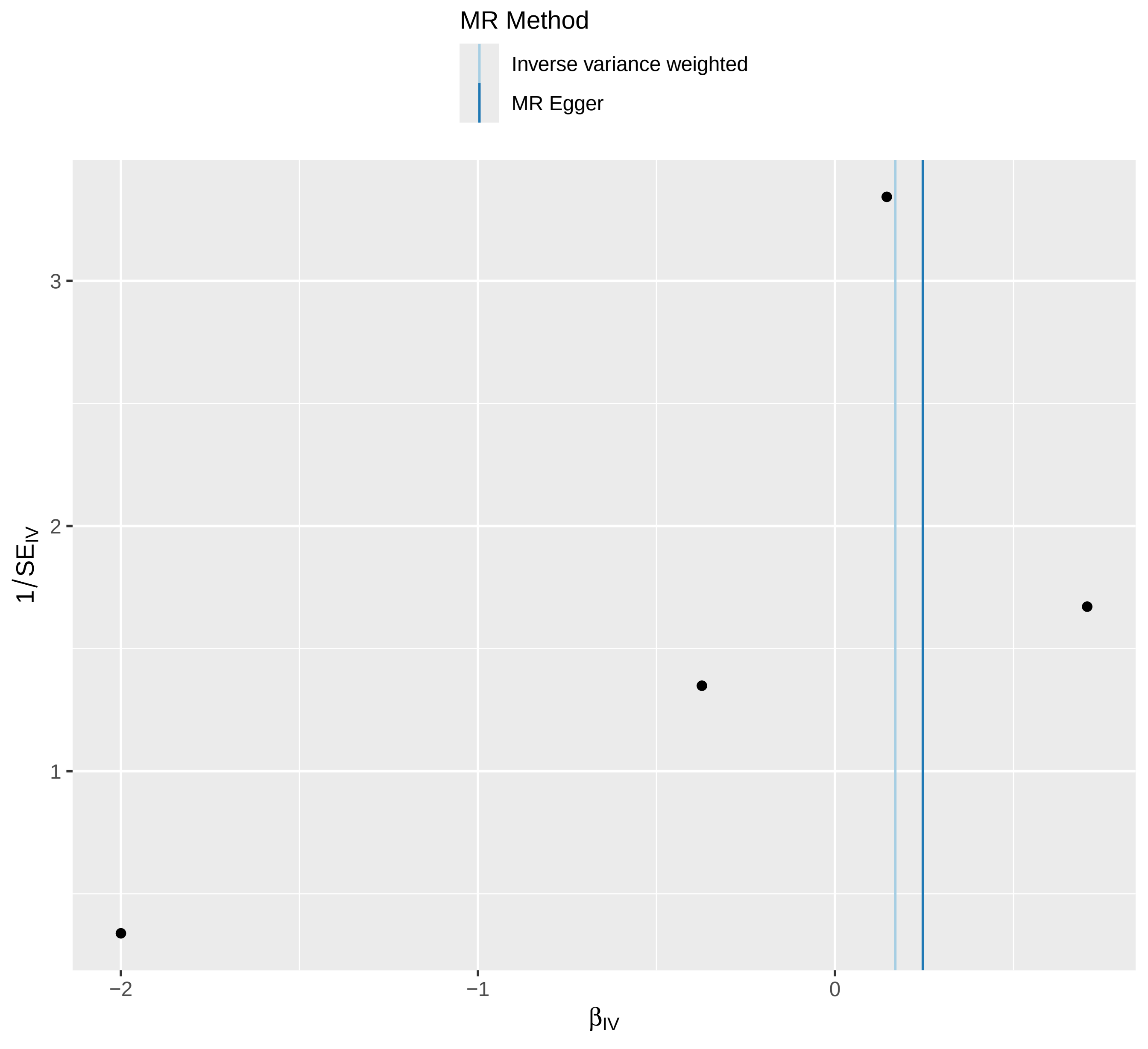
MR Method
Inverse variance weighted
MR Egger
3
2
1
$1/SE_{IV}$
−2
−1
0
β_{IV}

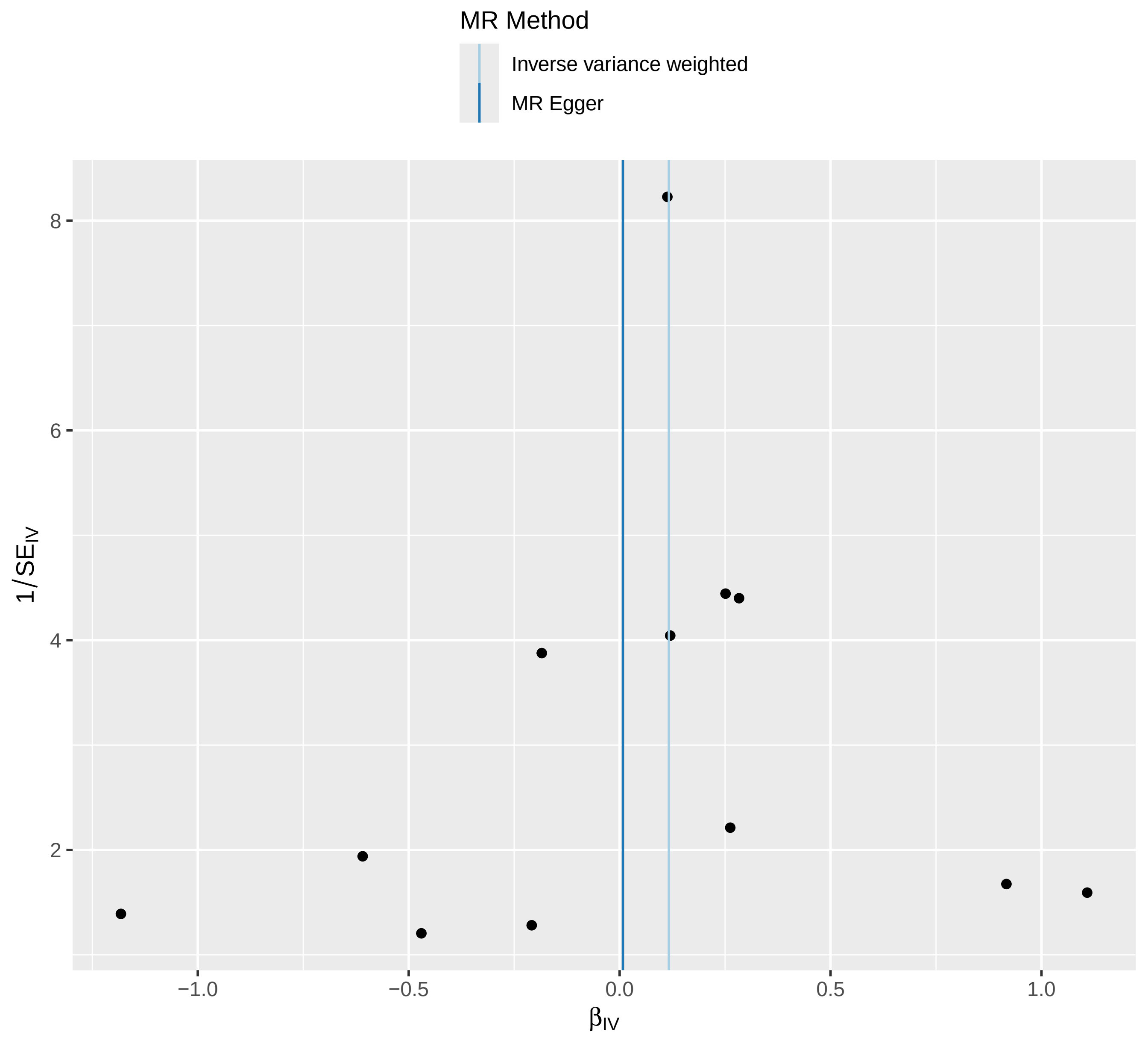

MR Method
Inverse variance weighted
MR Egger
8
6
4
2
1/SE$_{IV}$
−1.0
−0.5
0.0
0.5
1.0
β_{IV}

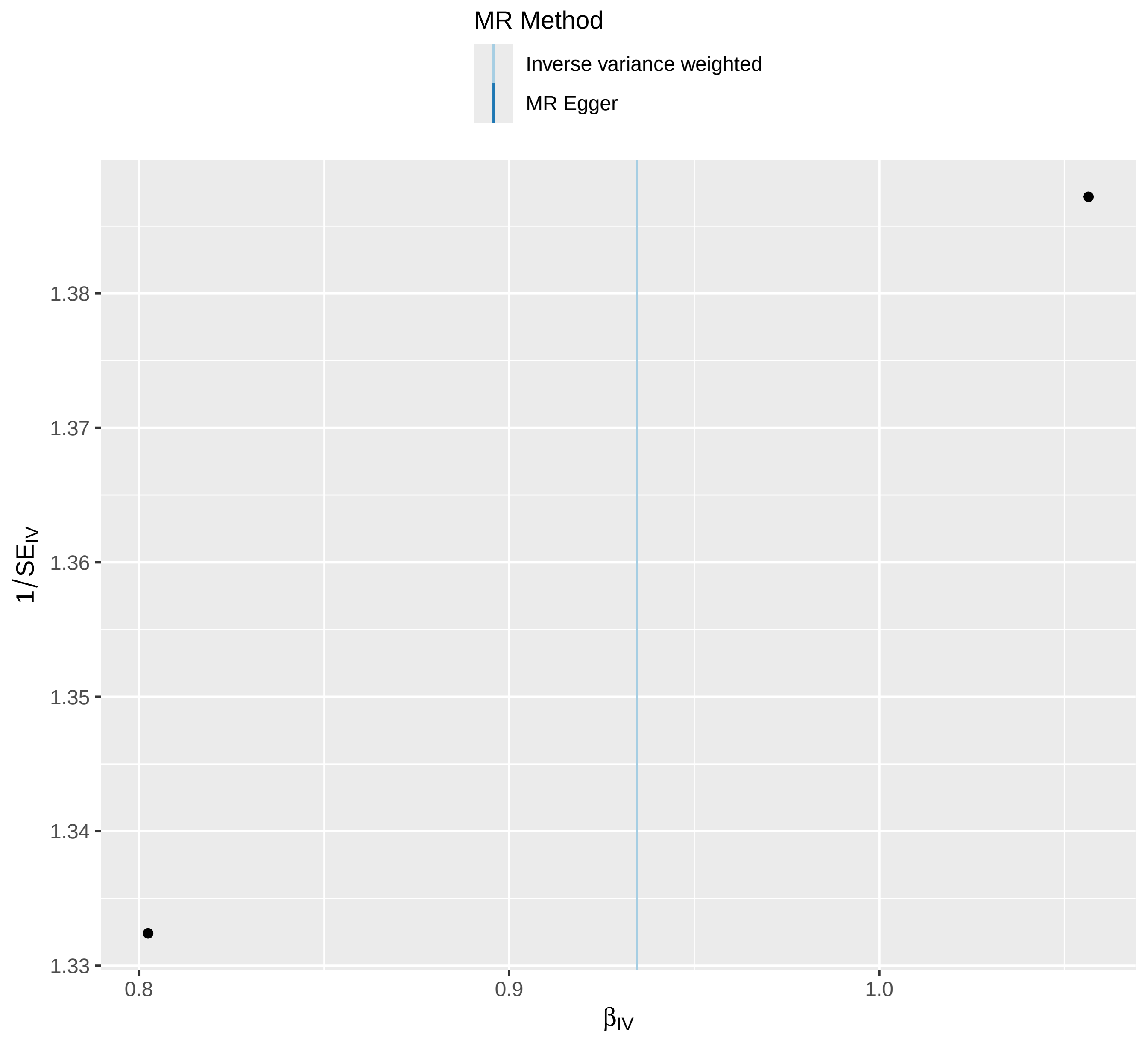

MR Method
Inverse variance weighted
MR Egger
1.38
1.37
1.36
1.35
1.34
1.33
1/SE_IV
0.8
0.9
1.0
β_IV

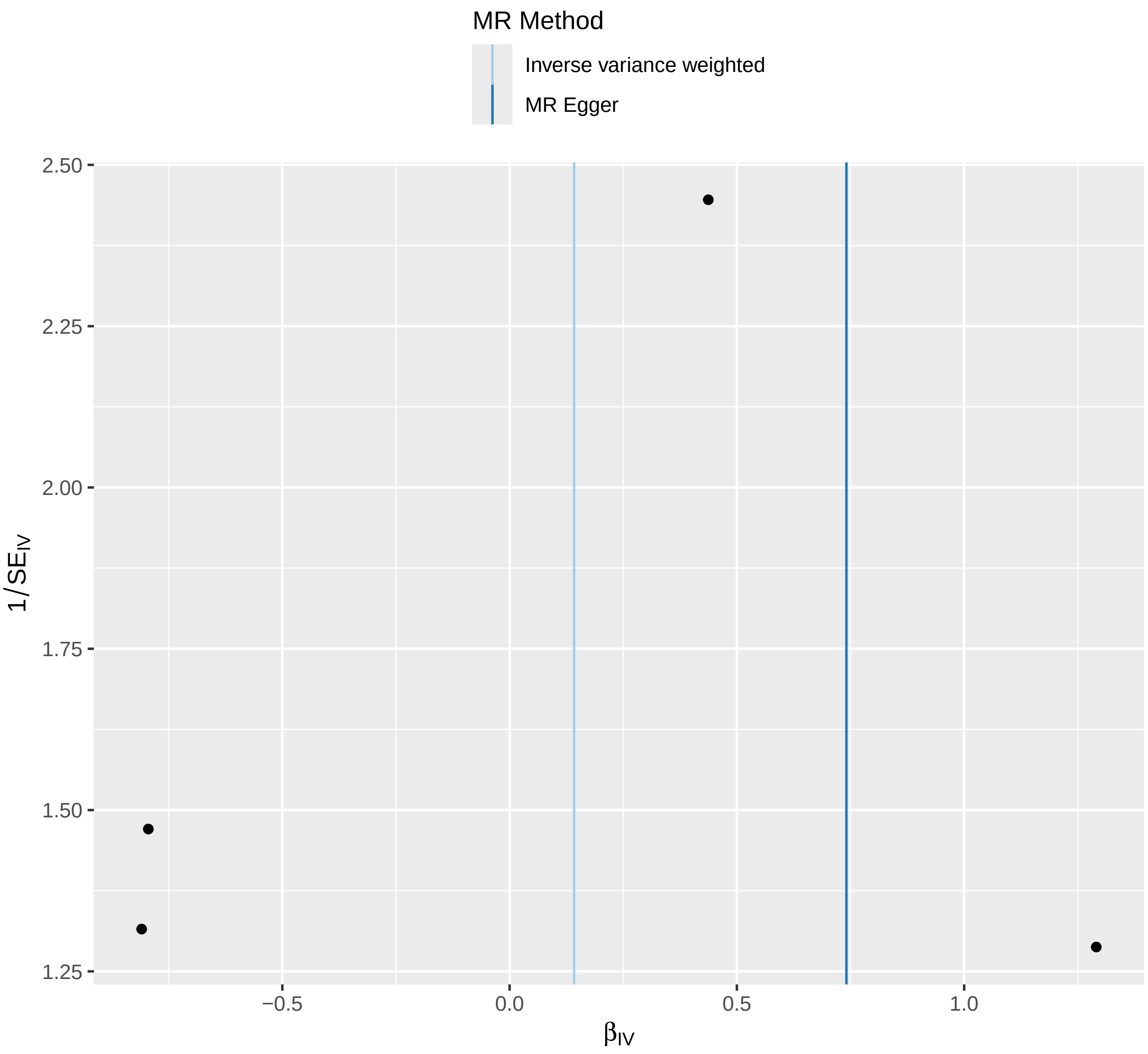
MR Method
Inverse variance weighted
MR Egger
2.50
2.25
2.00
1.75
1.50
1.25
$1/SE_{IV}$
−0.5
0.0
0.5
1.0
β_{IV}

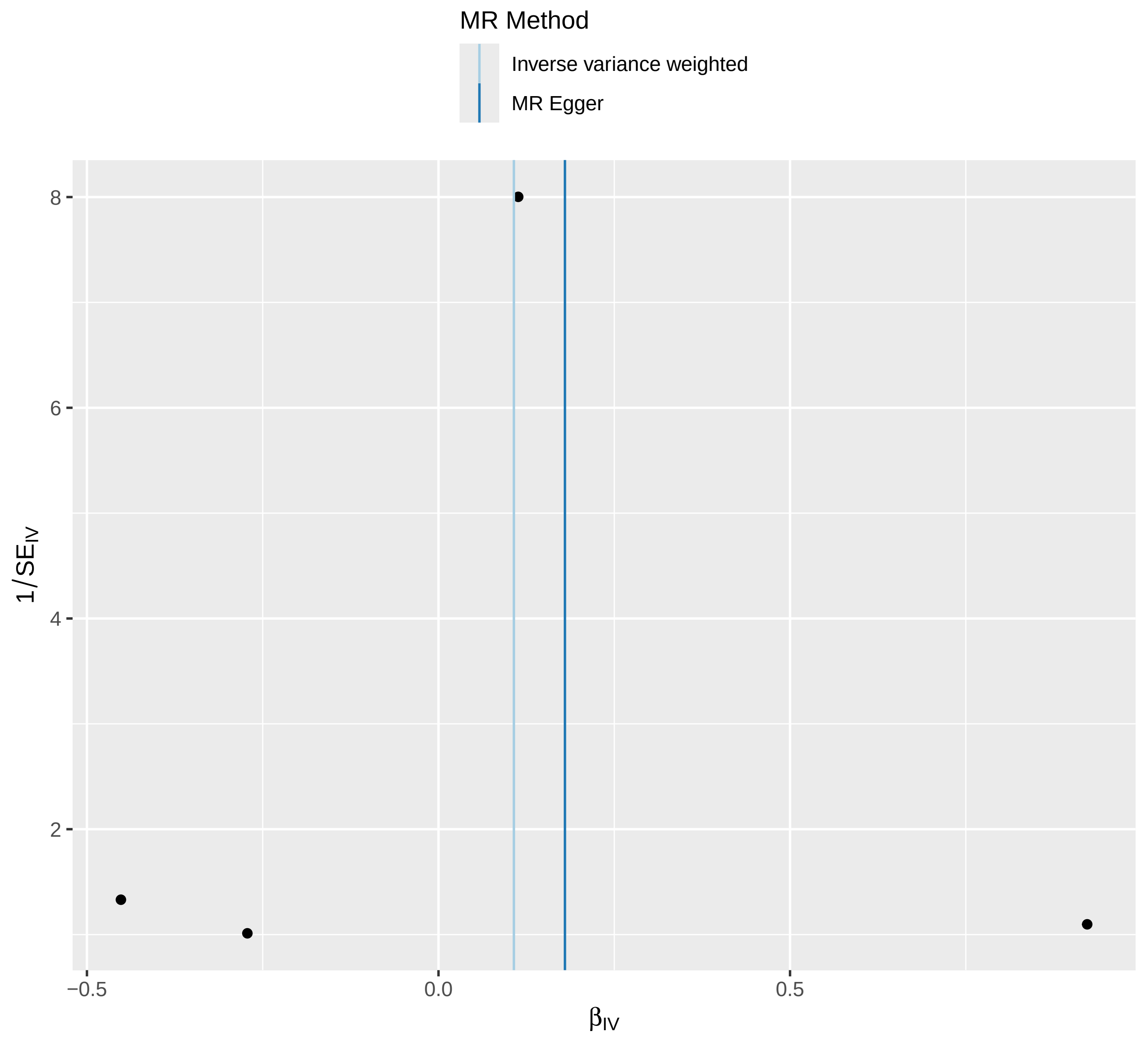
MR Method
Inverse variance weighted
MR Egger
8
6
4
2
1/SE_IV
−0.5
0.0
0.5
β_IV

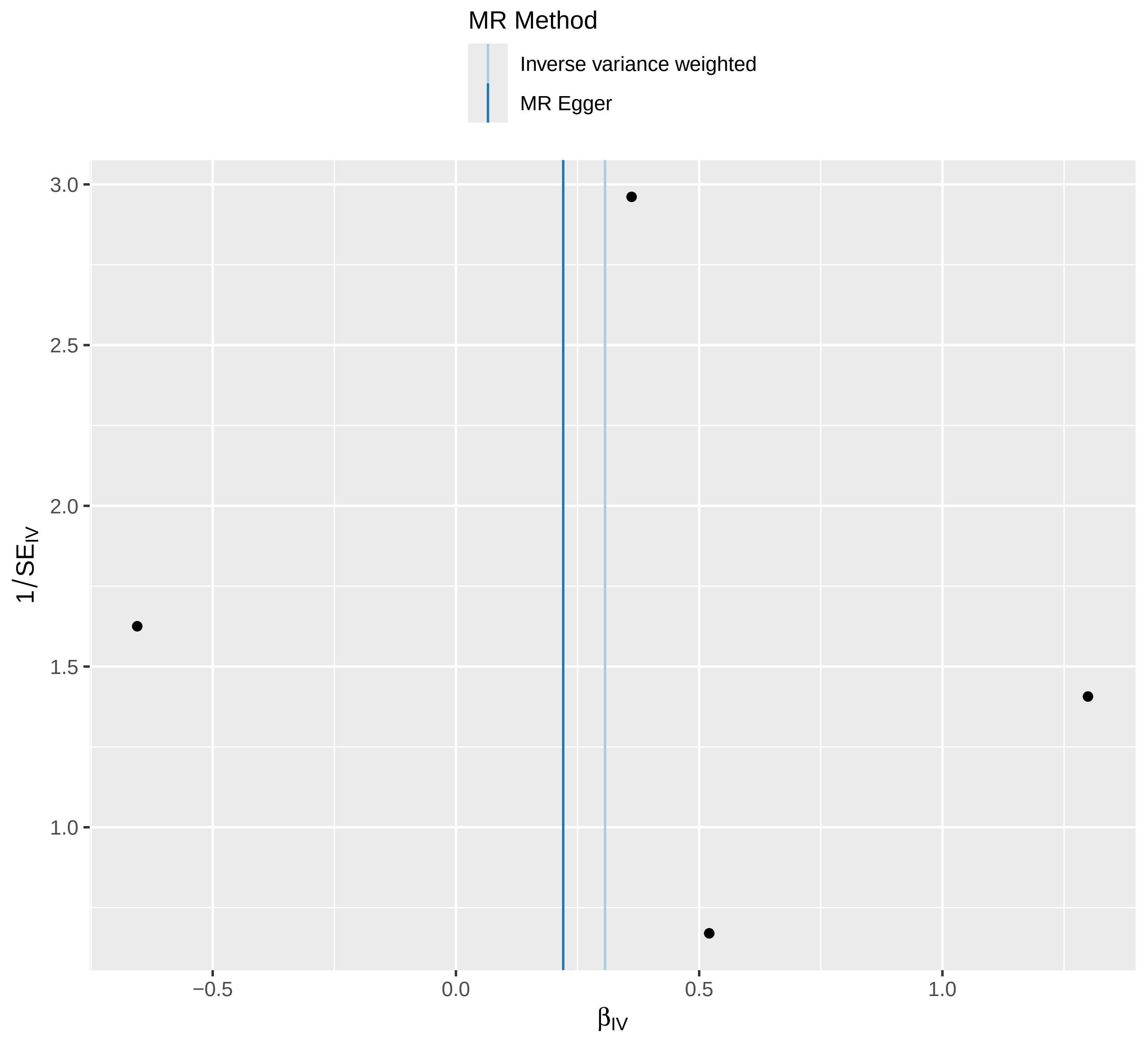
MR Method
Inverse variance weighted
MR Egger
3.0
2.5
2.0
1.5
1.0
1/SE_IV
−0.5
0.0
0.5
1.0
β_IV

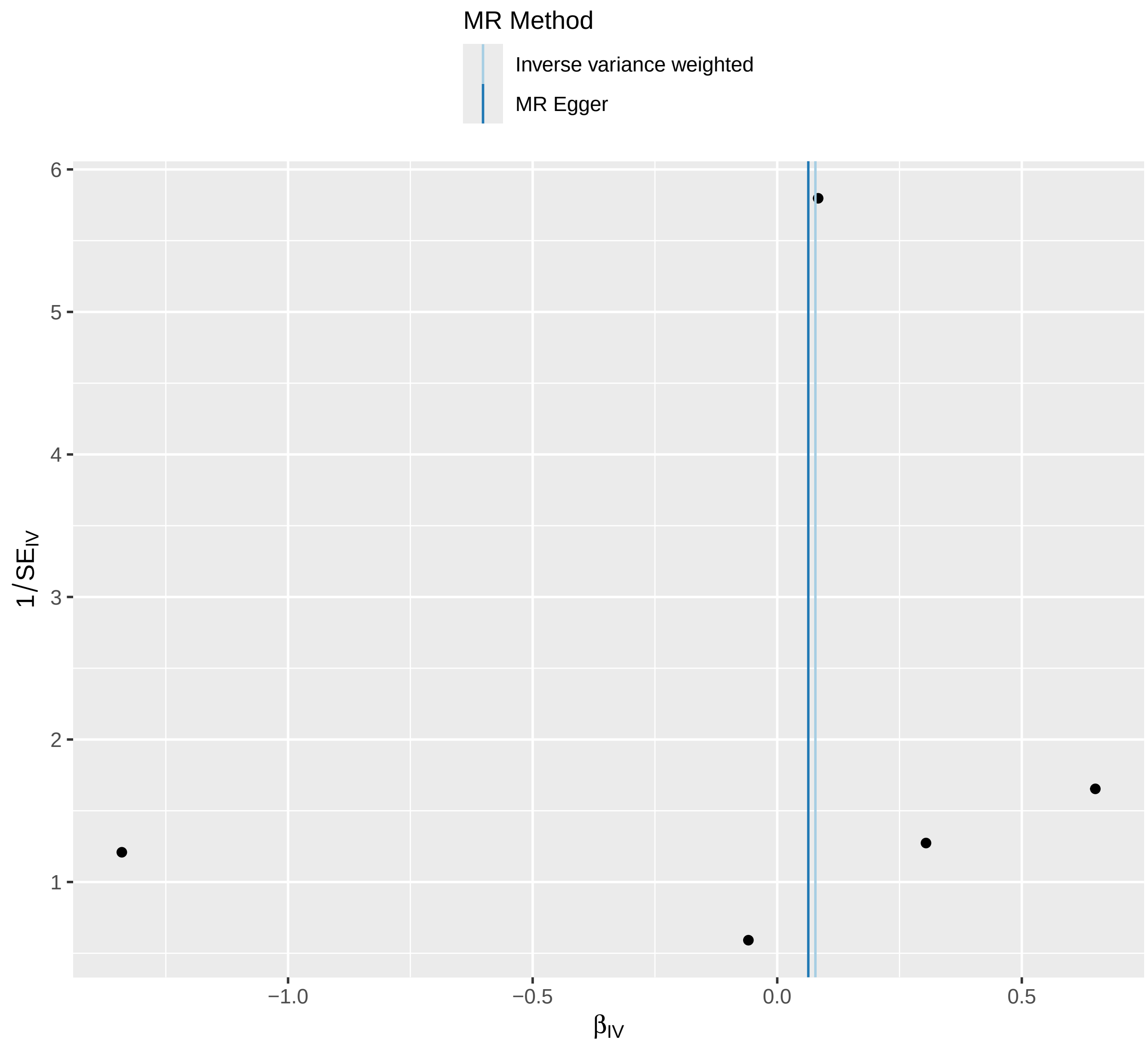

MR Method
Inverse variance weighted
MR Egger
1/SE$_{IV}$
1
2
3
4
5
6
−1.0
−0.5
0.0
0.5
β$_{IV}$

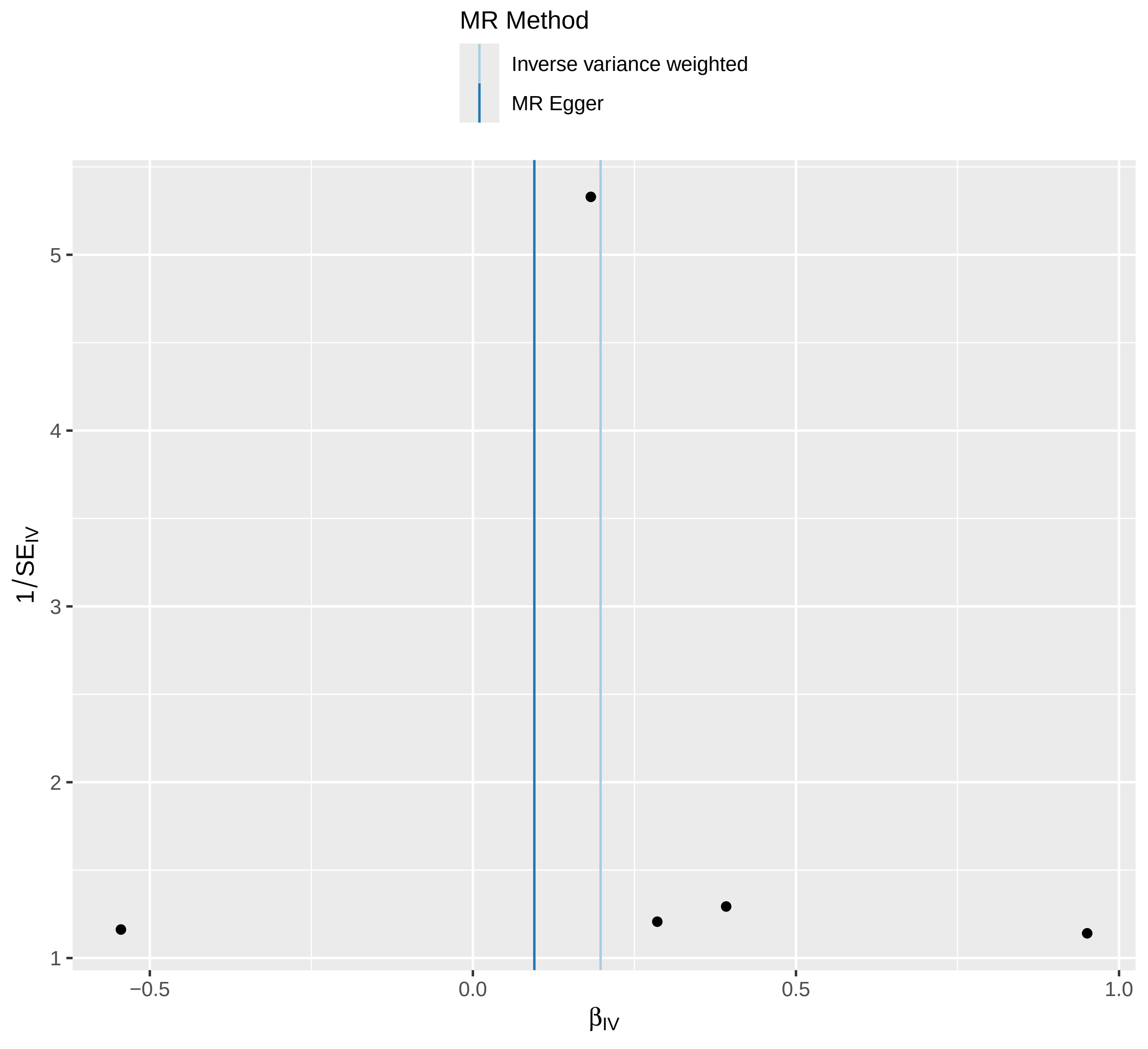
MR Method
Inverse variance weighted
MR Egger
5
4
3
2
1
1/SE_IV
−0.5
0.0
0.5
1.0
β_IV

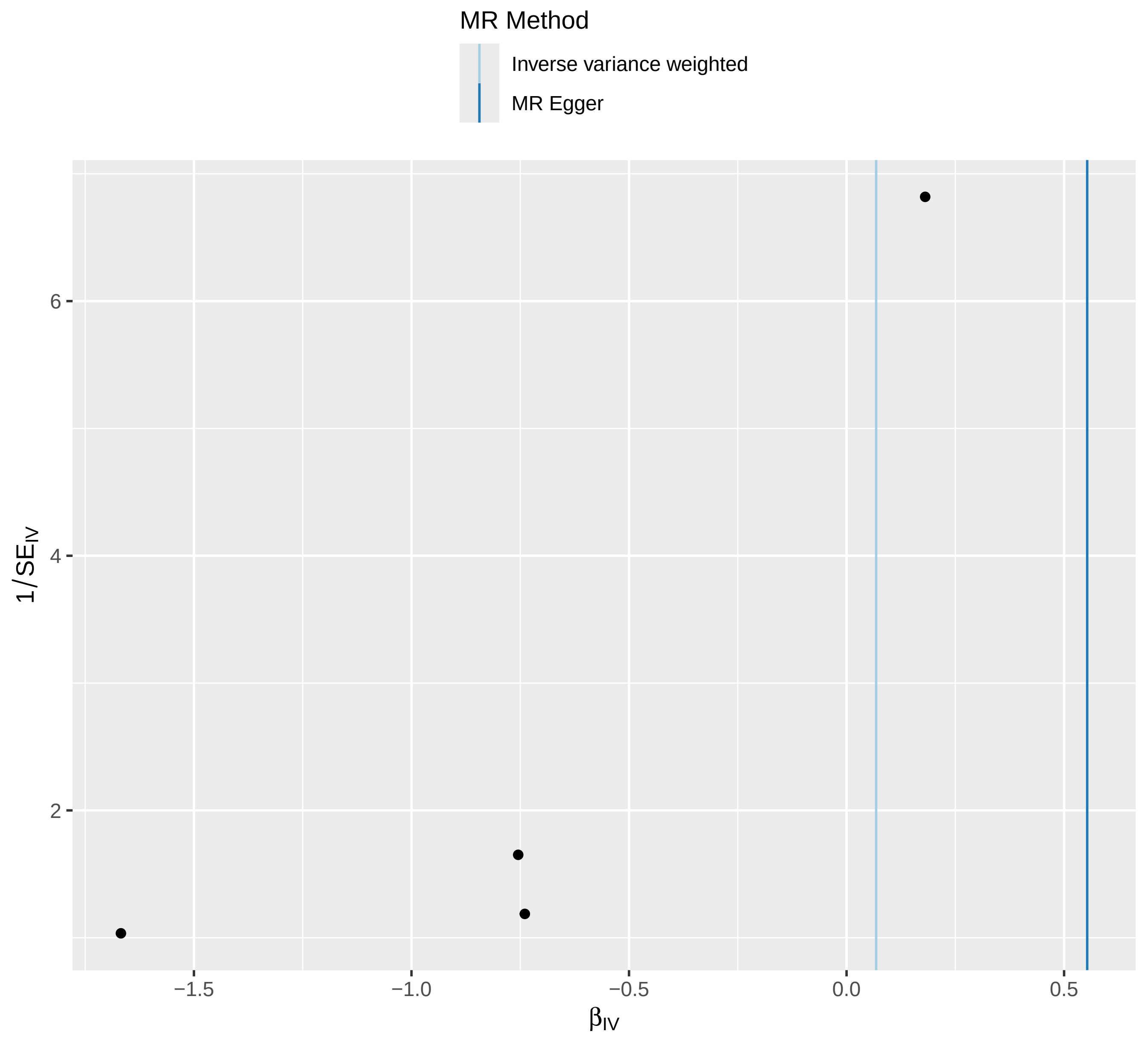
MR Method
Inverse variance weighted
MR Egger
6
4
2
$1/SE_{IV}$
−1.5
−1.0
−0.5
0.0
0.5
β_{IV}

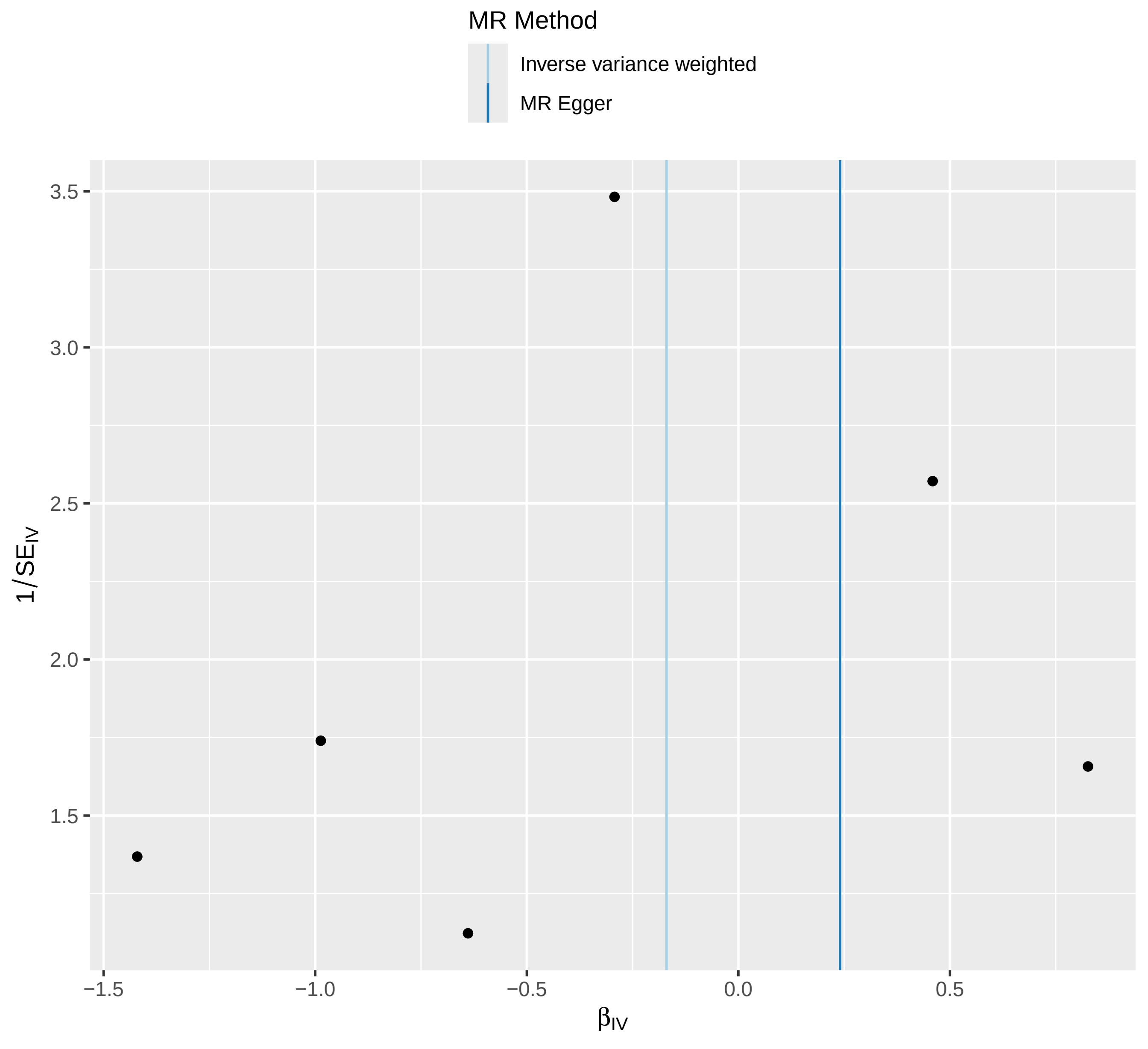

MR Method
Inverse variance weighted
MR Egger
3.5
3.0
2.5
2.0
1.5
$1/SE_{IV}$
−1.5
−1.0
−0.5
0.0
0.5
β_{IV}

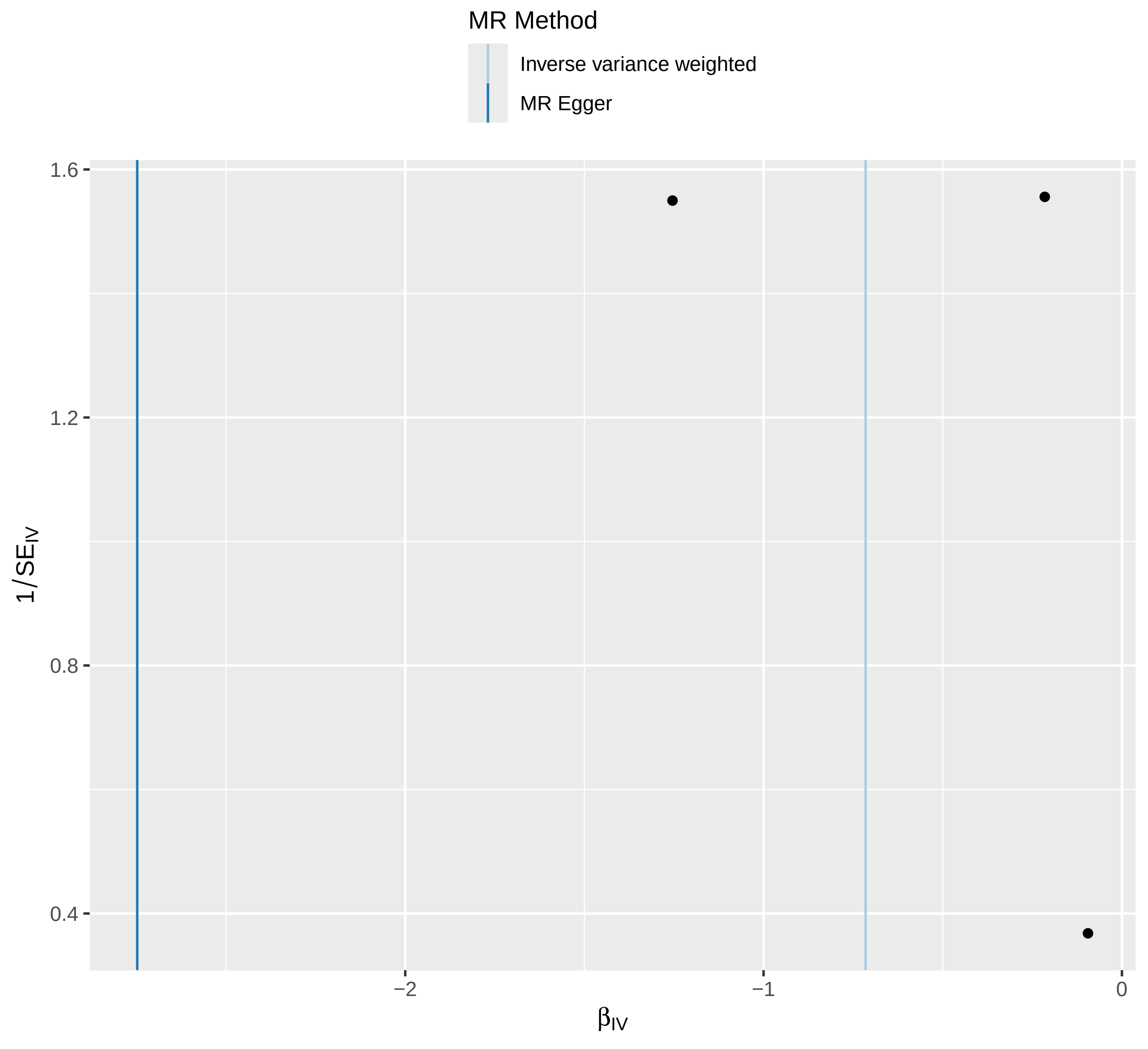
MR Method
Inverse variance weighted
MR Egger
1.6
1.2
0.8
0.4
1/SE_IV
−2
−1
0
β_IV

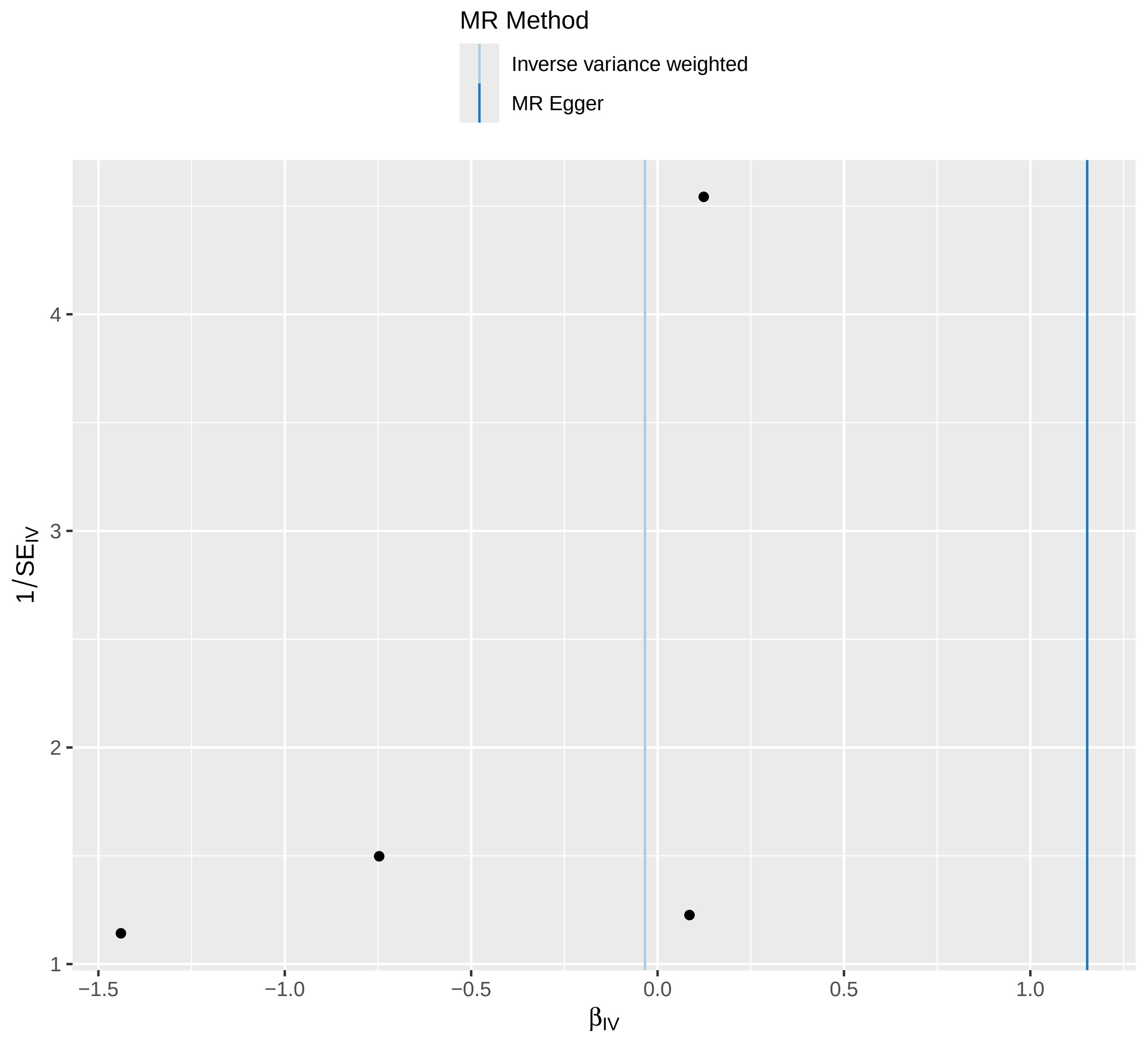
MR Method
Inverse variance weighted
MR Egger
1/SE_IV
β_IV
4
3
2
1
−1.5
−1.0
−0.5
0.0
0.5
1.0

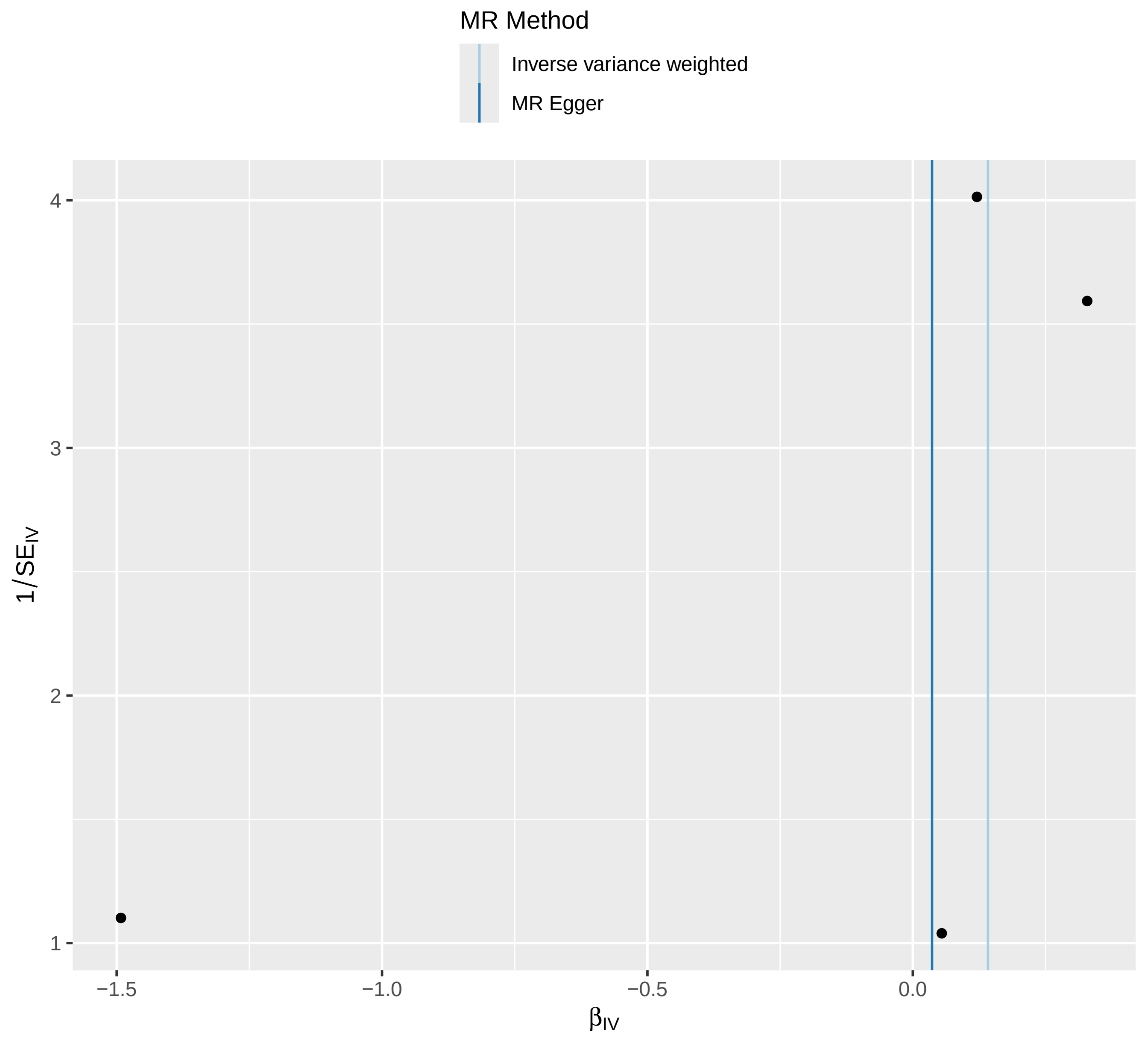
MR Method
Inverse variance weighted
MR Egger
1/SE_IV
β_IV
4
3
2
1
−1.5
−1.0
−0.5
0.0

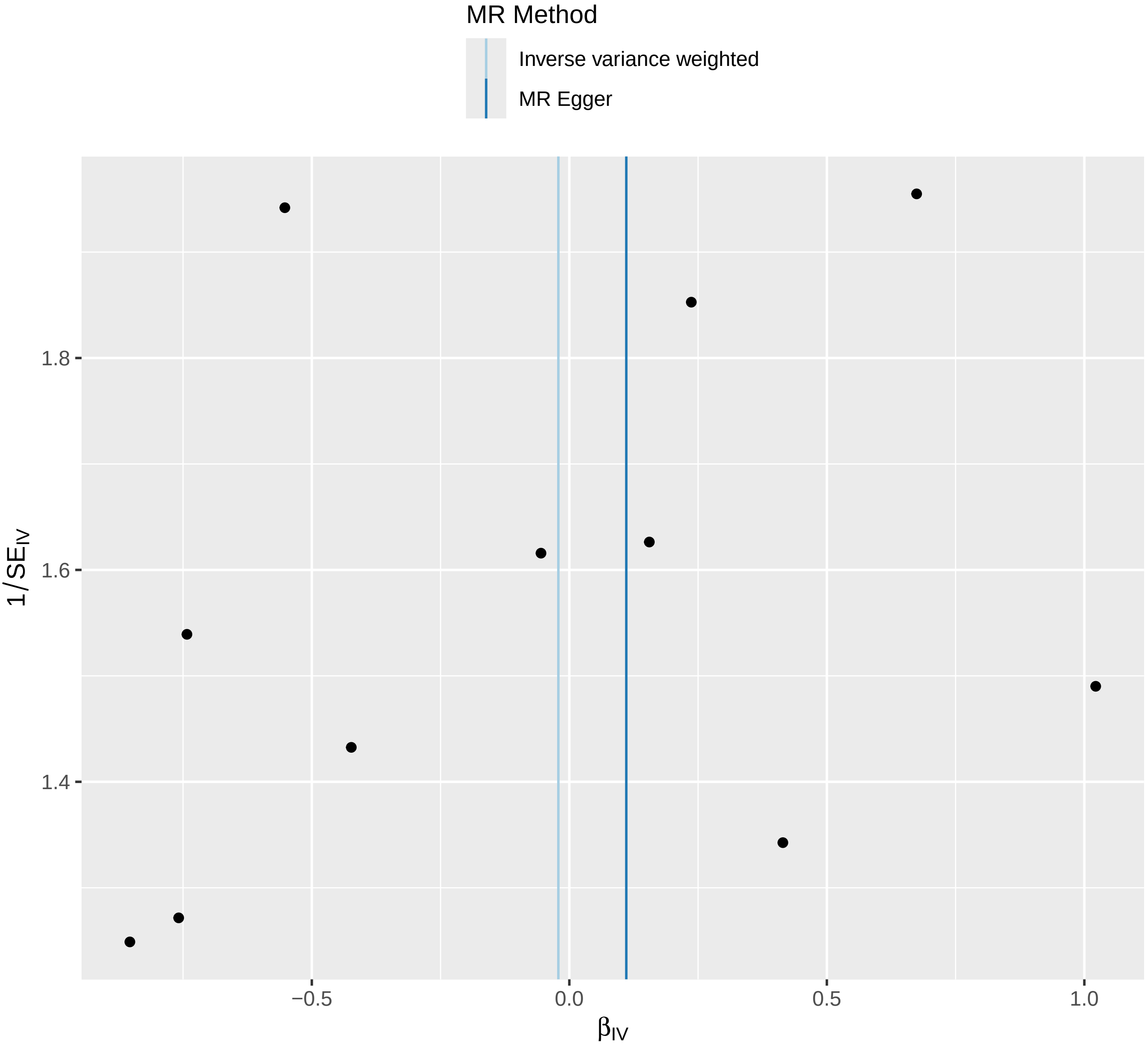

MR Method
Inverse variance weighted
MR Egger
1.8
1.6
1.4
$1/SE_{IV}$
−0.5
0.0
0.5
1.0
β_{IV}

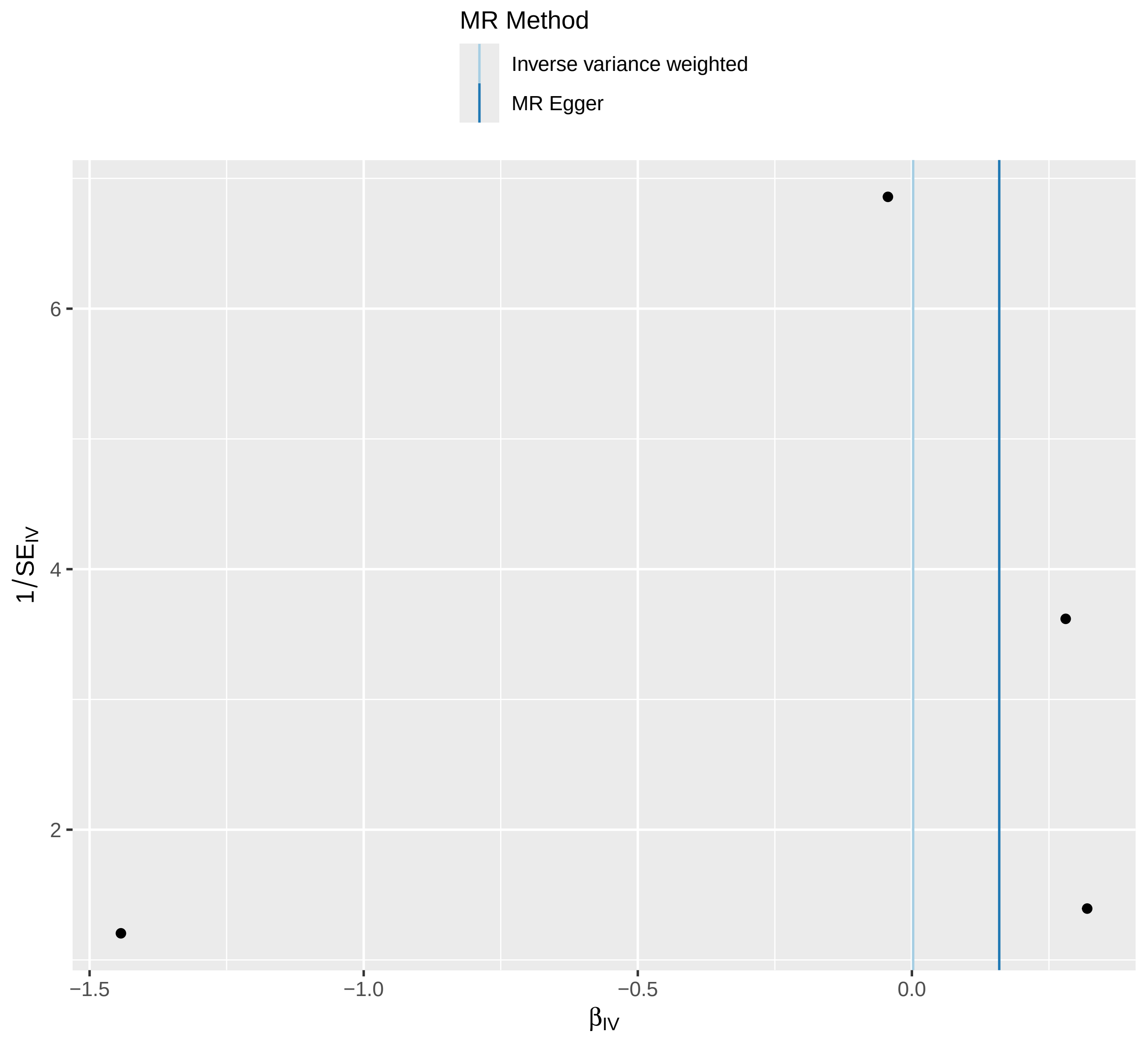

MR Method
Inverse variance weighted
MR Egger
6
4
2
1/SE_IV
−1.5
−1.0
−0.5
0.0
β_IV

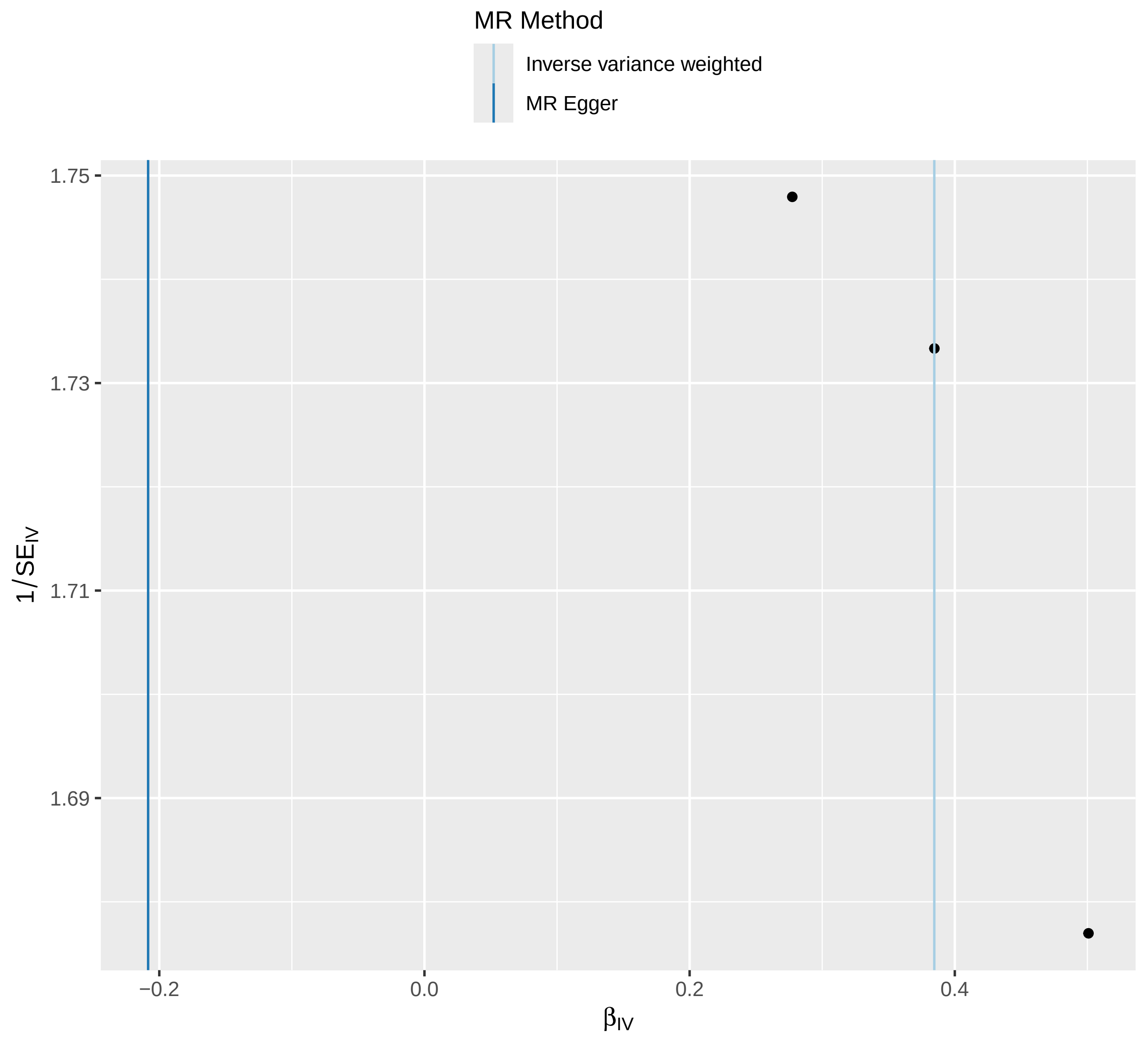

MR Method
Inverse variance weighted
MR Egger
1.75
1.73
1.71
1.69
$1/SE_{IV}$
−0.2
0.0
0.2
0.4
β_{IV}

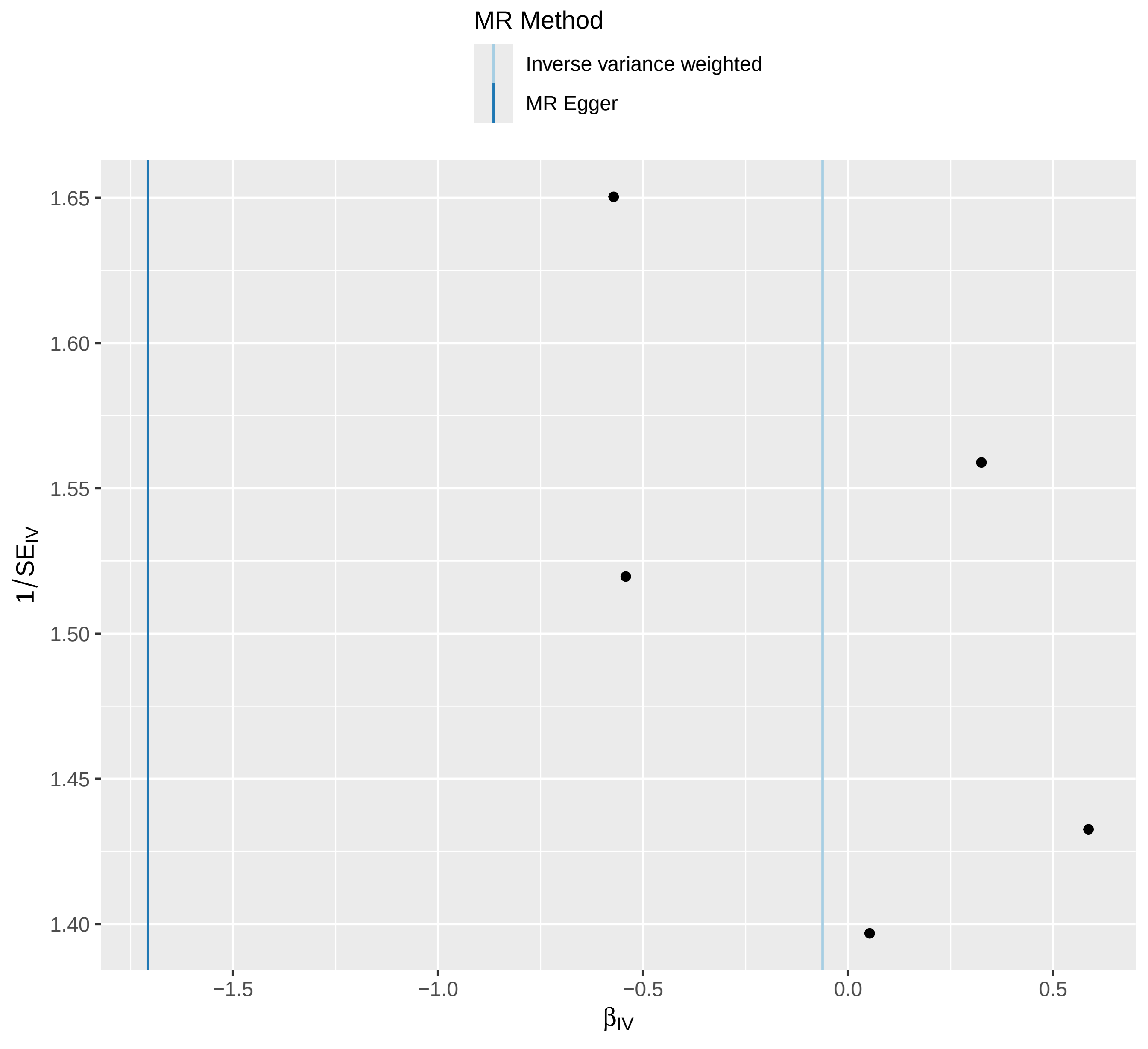
MR Method
Inverse variance weighted
MR Egger
1.65
1.60
1.55
1.50
1.45
1.40
1/SE$_{IV}$
−1.5
−1.0
−0.5
0.0
0.5
β$_{IV}$

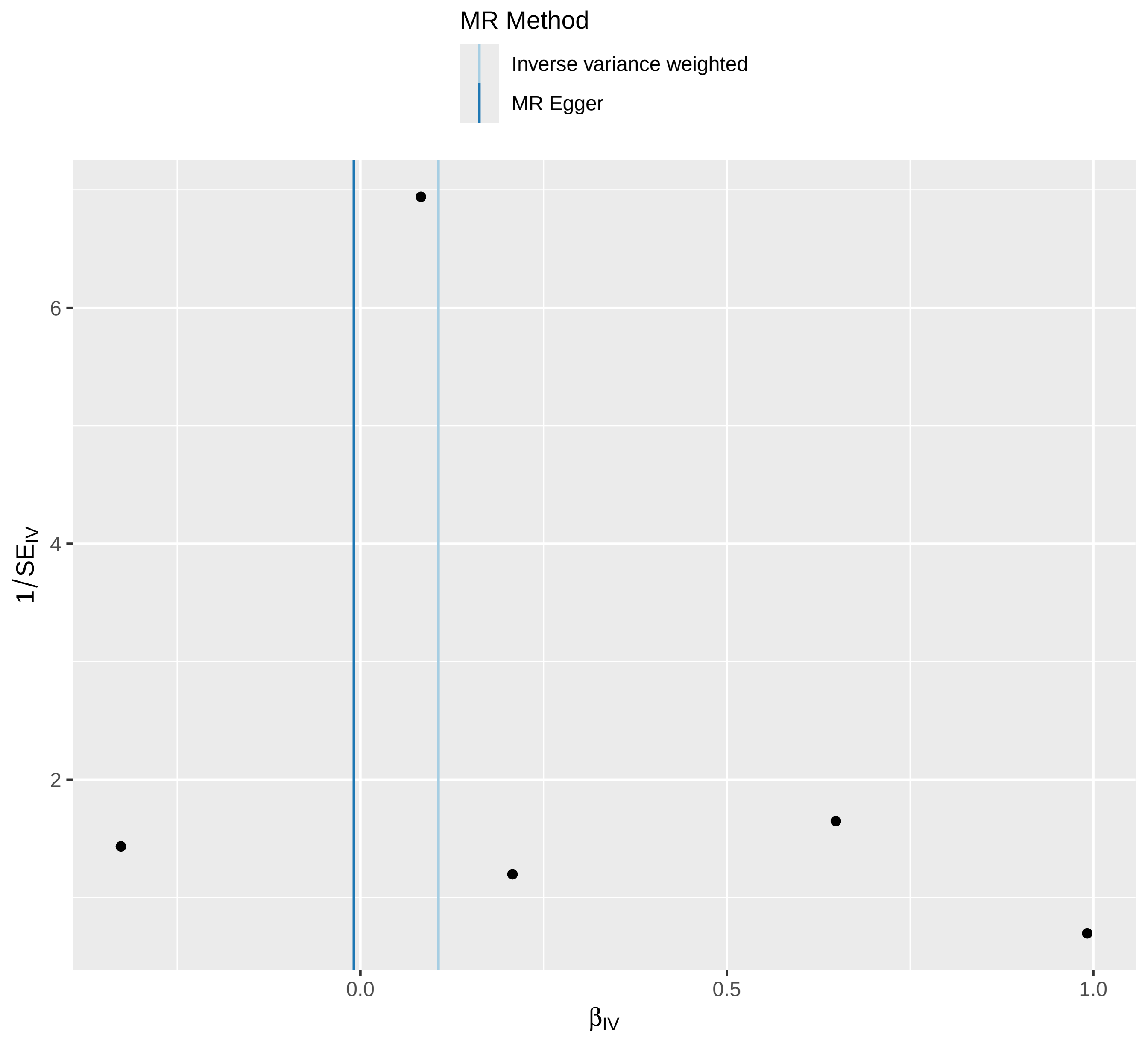
MR Method
Inverse variance weighted
MR Egger
6
4
2
1/SE$_{IV}$
0.0
0.5
1.0
β$_{IV}$

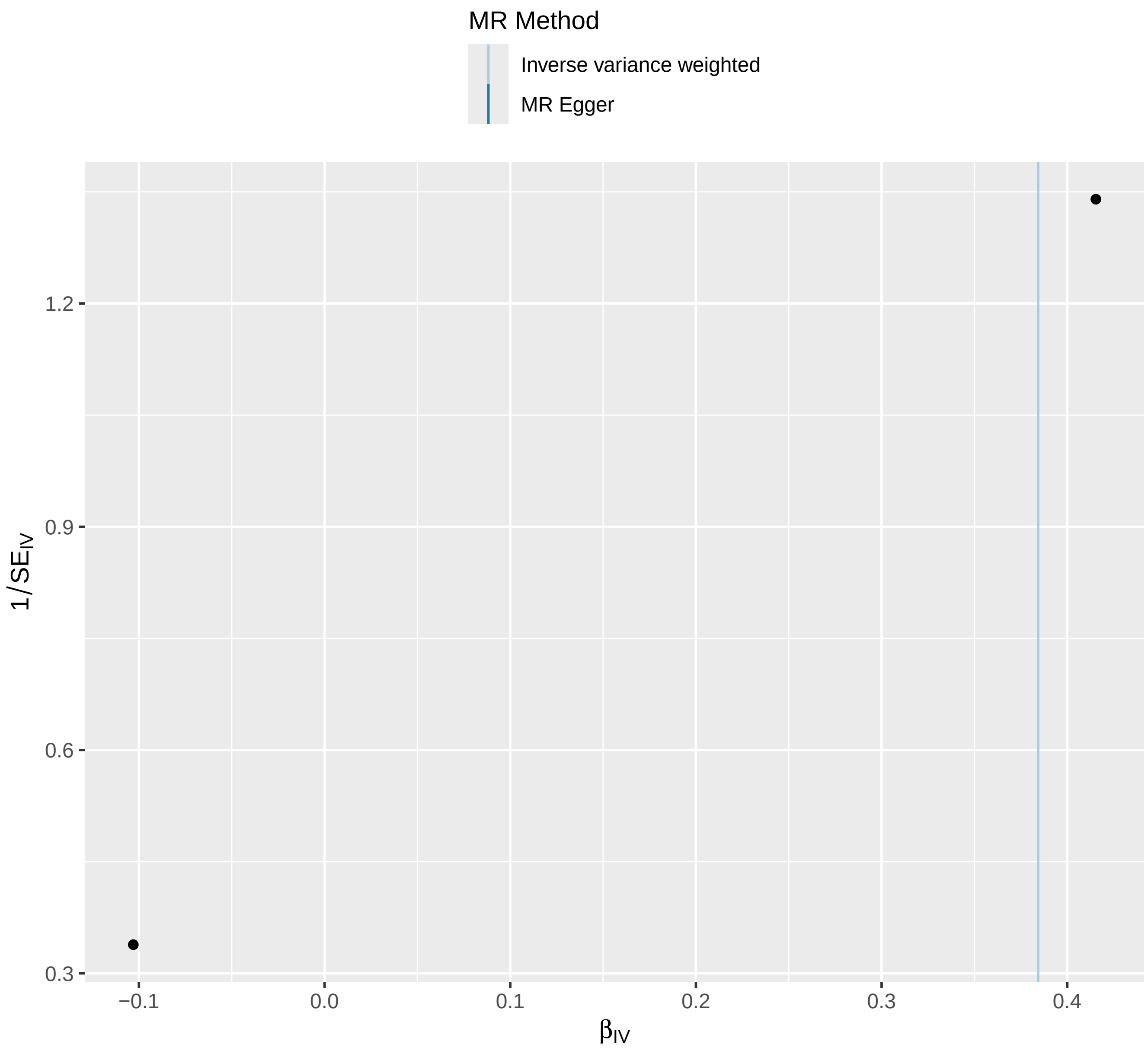
MR Method
Inverse variance weighted
MR Egger
1.2
0.9
0.6
0.3
1/SE_IV
−0.1
0.0
0.1
0.2
0.3
0.4
β_IV

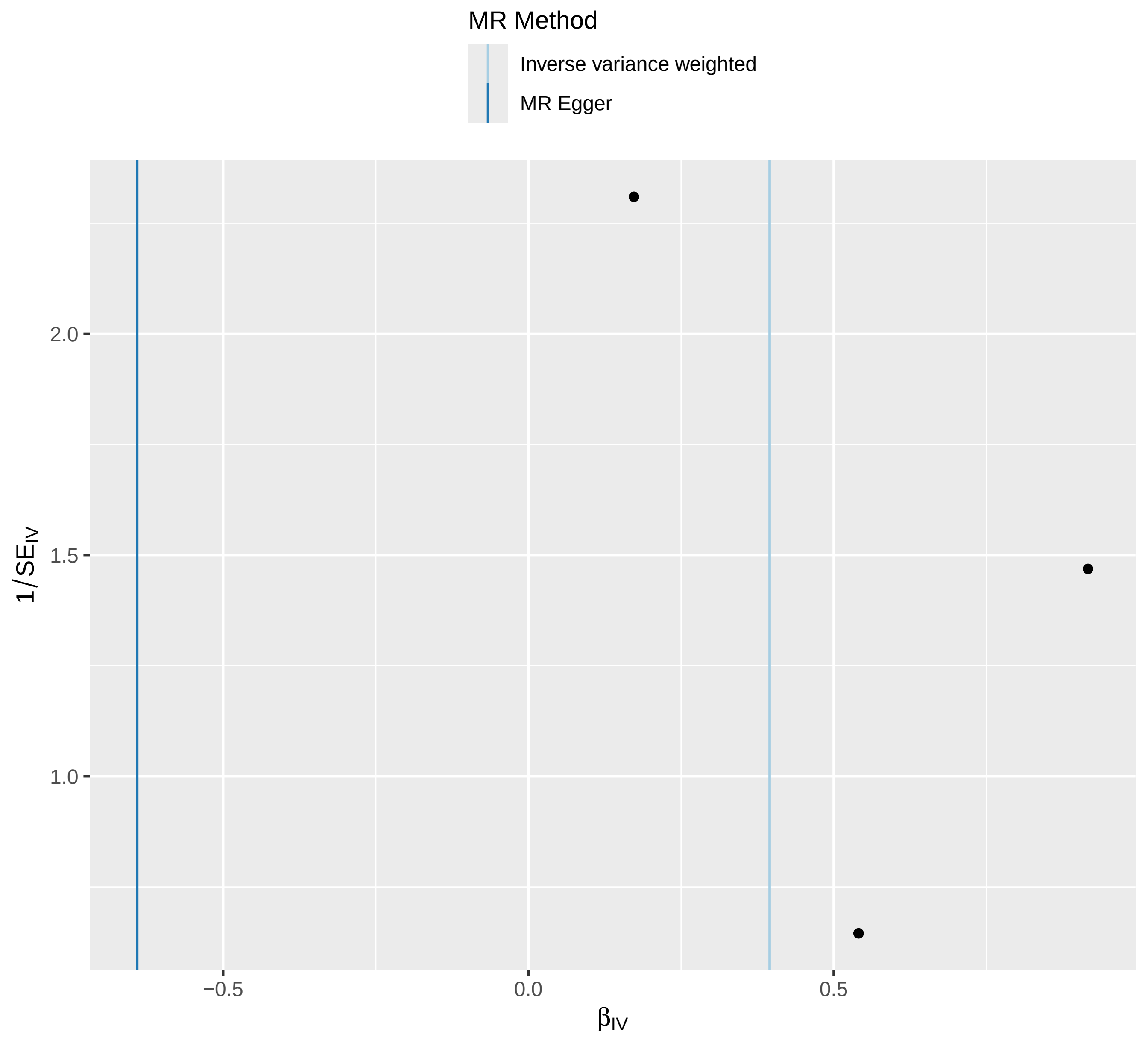
MR Method
Inverse variance weighted
MR Egger
2.0
1.5
1.0
1/SE$_{IV}$
−0.5
0.0
0.5
β$_{IV}$

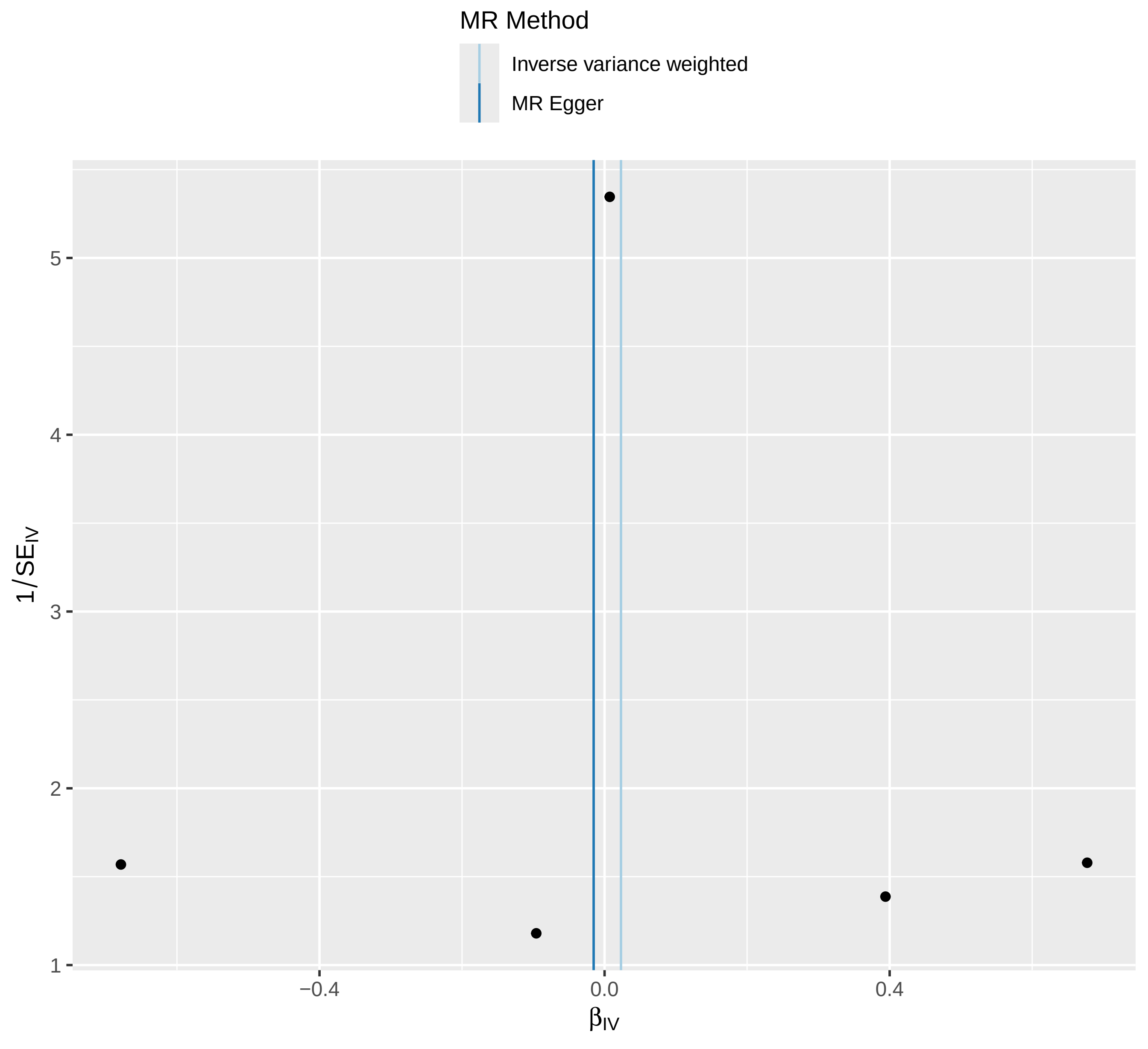
MR Method
Inverse variance weighted
MR Egger
1/SE_IV
5
4
3
2
1
−0.4
0.0
0.4
β_IV

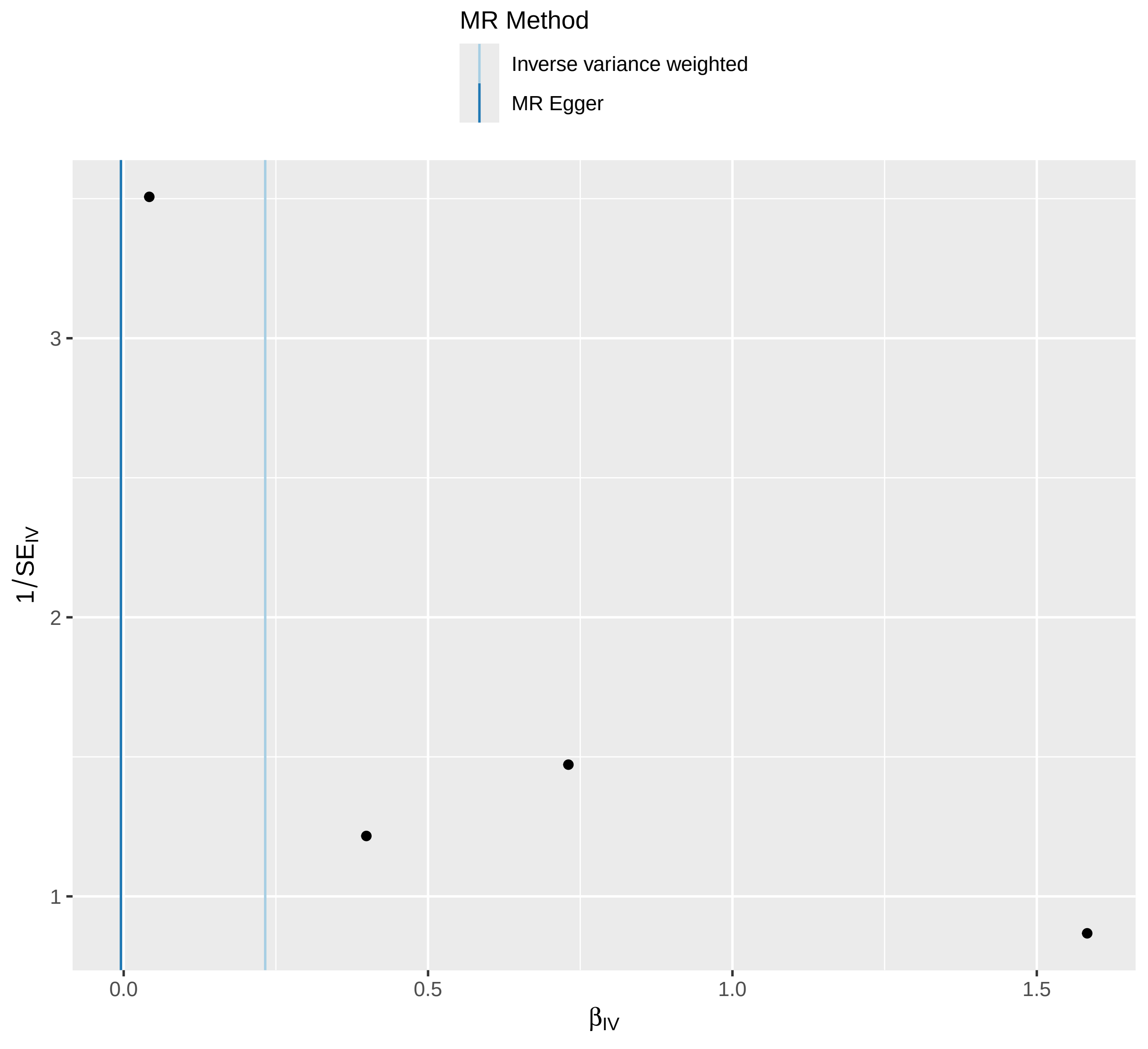
MR Method
Inverse variance weighted
MR Egger
1/SE_{IV}
3
2
1
0.0
0.5
1.0
1.5
β_{IV}

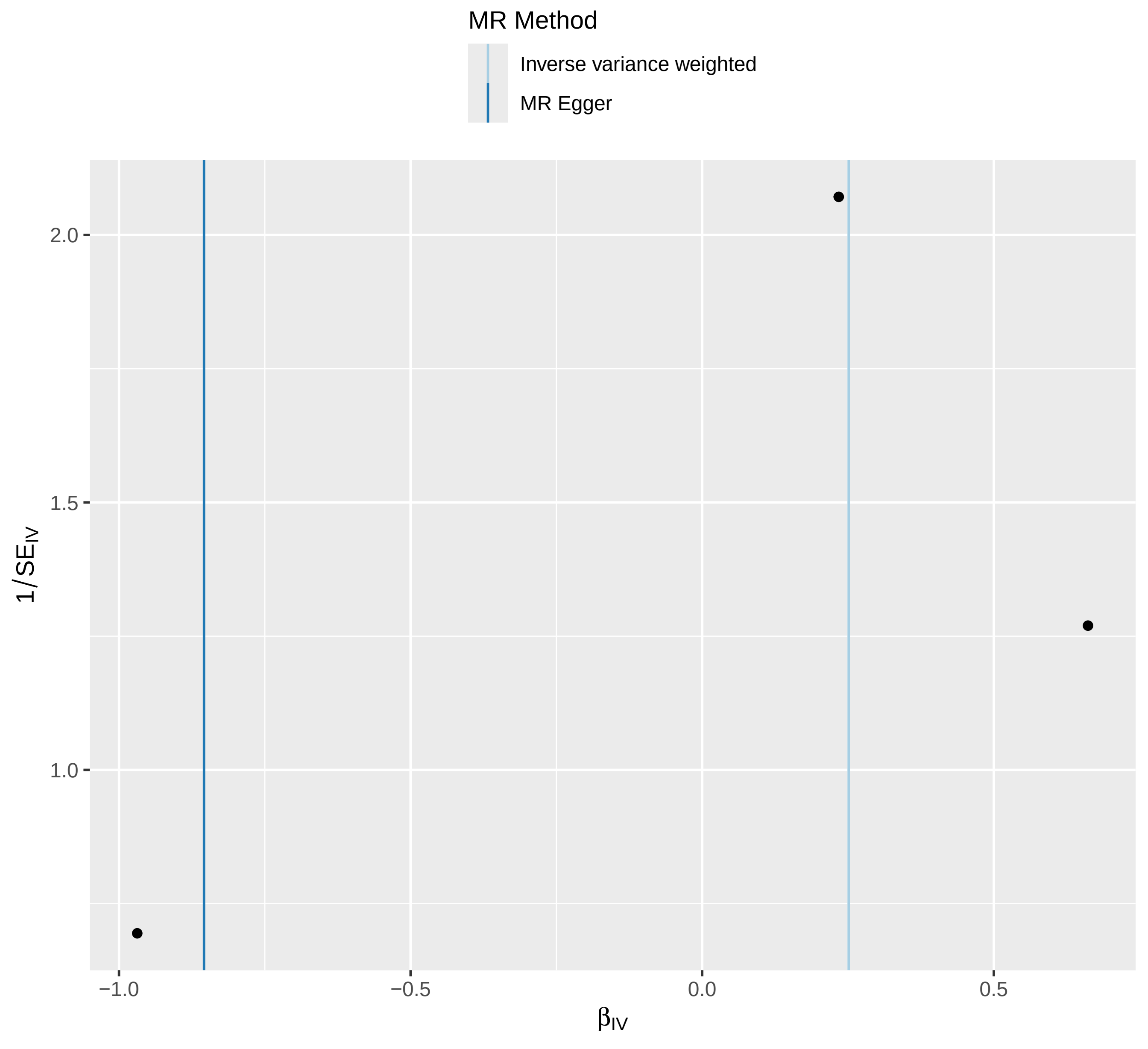

MR Method
Inverse variance weighted
MR Egger
2.0
1.5
1.0
1/SE$_{IV}$
−1.0
−0.5
0.0
0.5
β_{IV}

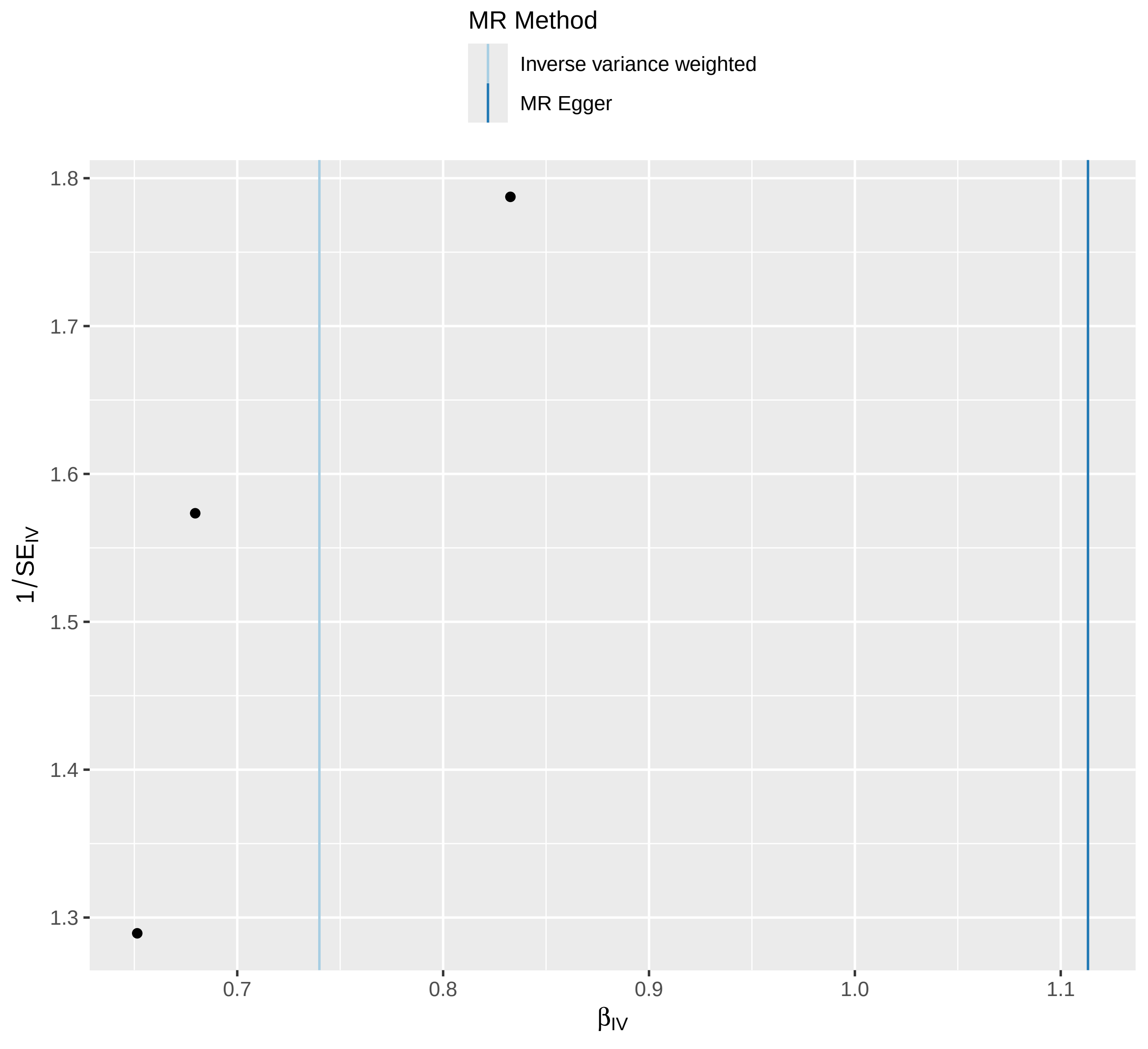

MR Method
Inverse variance weighted
MR Egger
1.8
1.7
1.6
1.5
1.4
1.3
1/SE_IV
0.7
0.8
0.9
1.0
1.1
β_IV

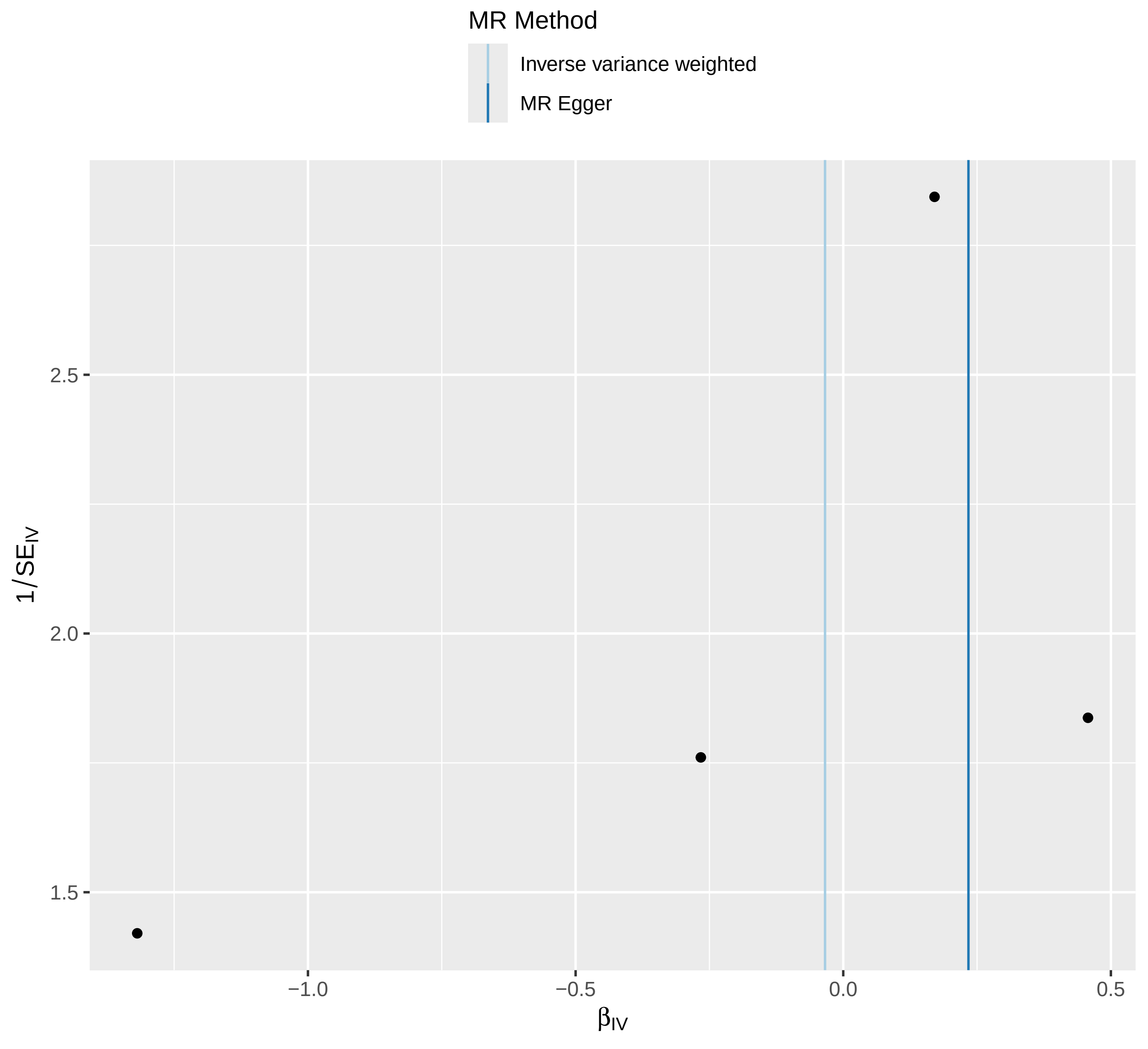
MR Method
Inverse variance weighted
MR Egger
2.5
2.0
1.5
1/SE$_{IV}$
−1.0
−0.5
0.0
0.5
β$_{IV}$

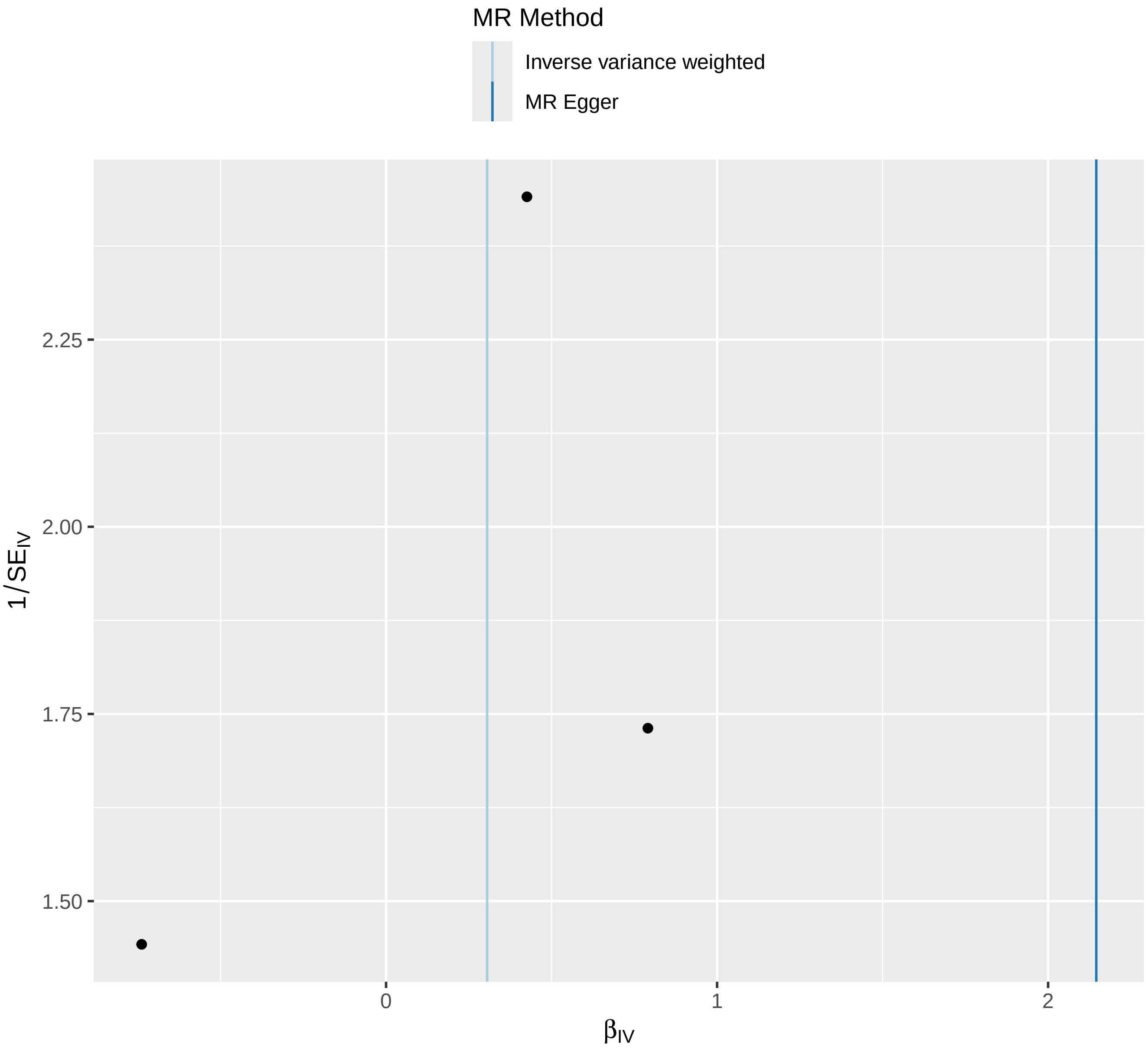
MR Method
Inverse variance weighted
MR Egger
2.25
2.00
1.75
1.50
$1/SE_{IV}$
0
1
2
β_{IV}

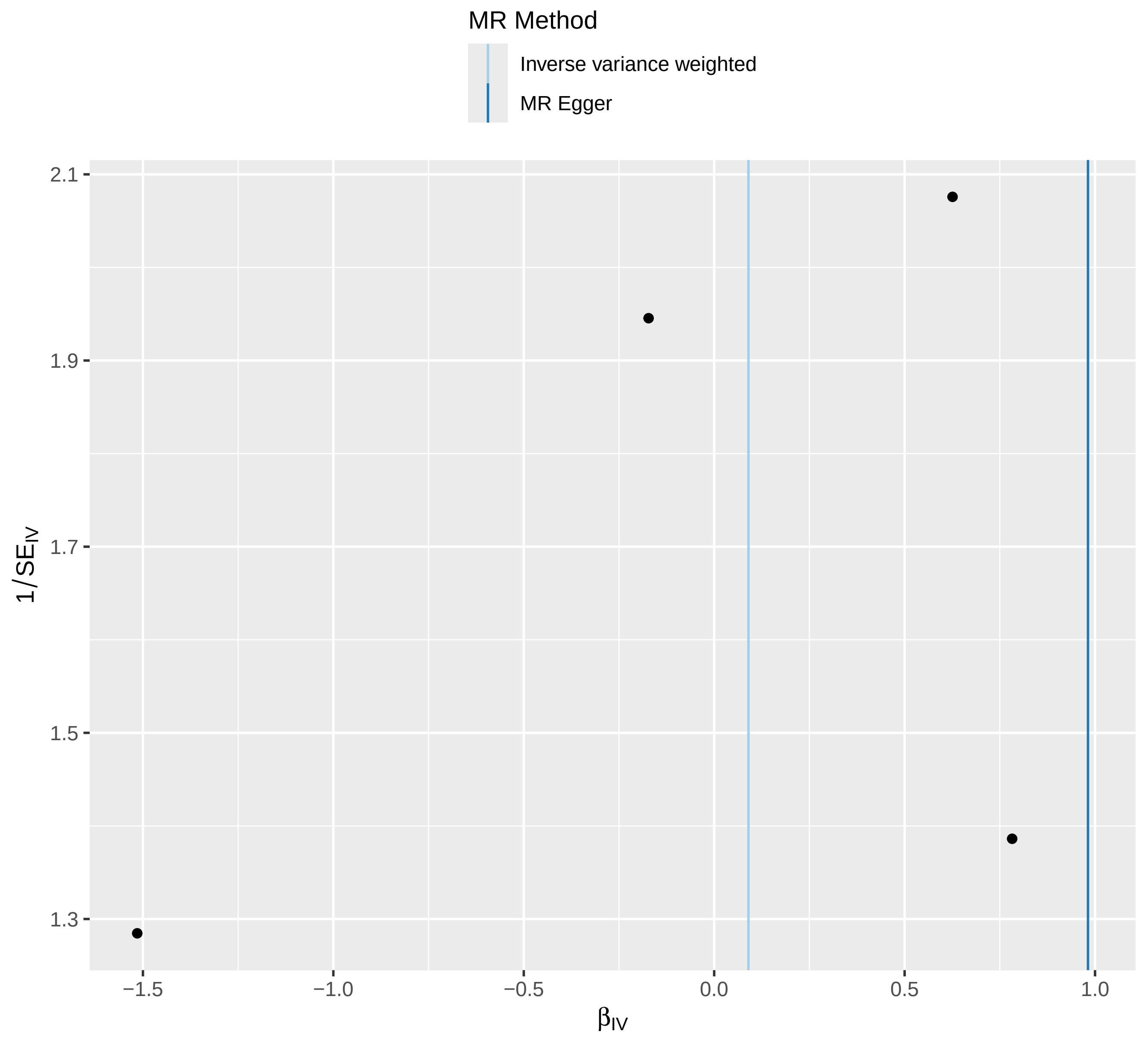
MR Method
Inverse variance weighted
MR Egger
2.1
1.9
1.7
1.5
1.3
1/SE_IV
−1.5
−1.0
−0.5
0.0
0.5
1.0
β_IV

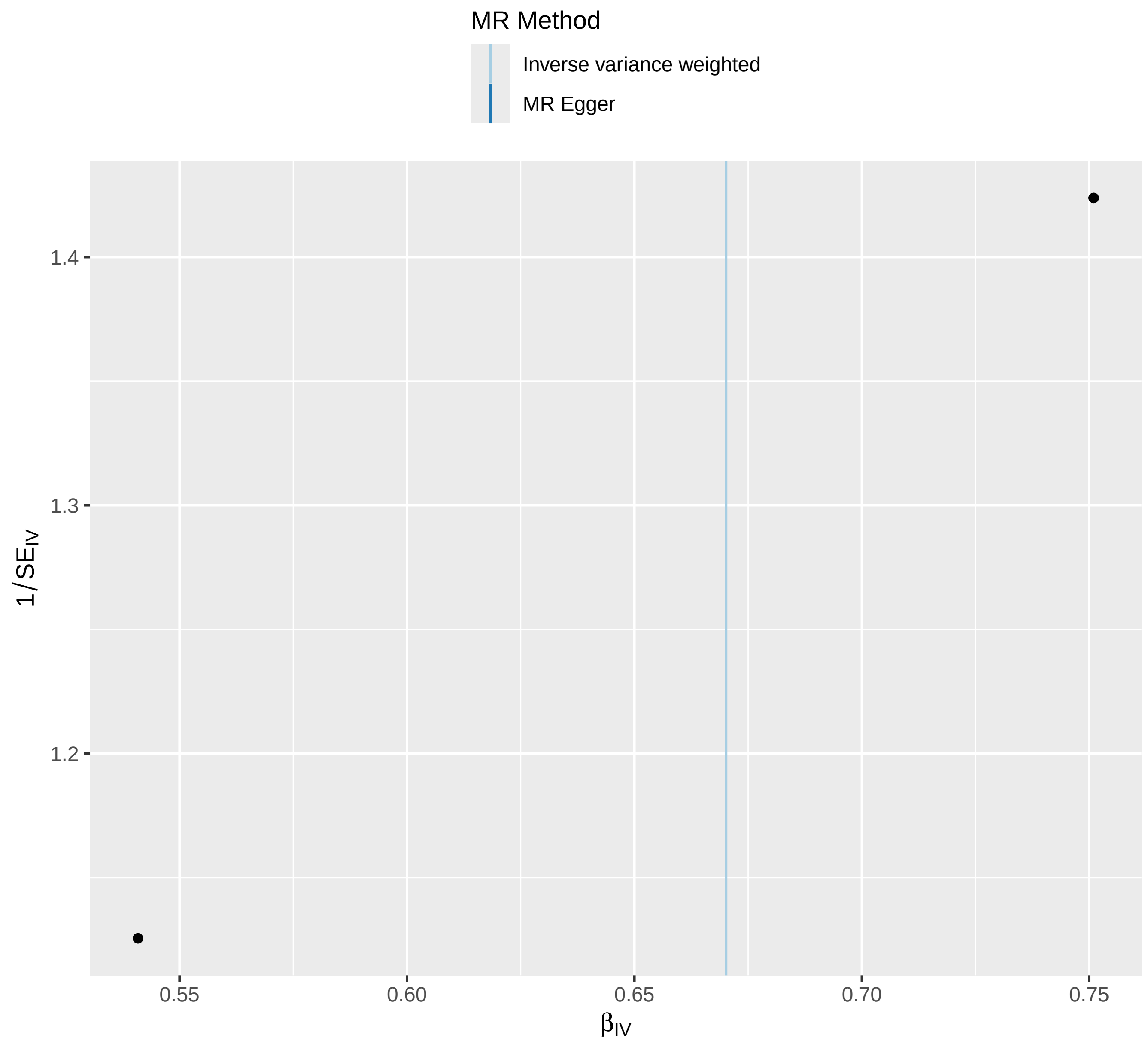
MR Method
Inverse variance weighted
MR Egger
1.4
1.3
1.2
0.55
0.60
0.65
0.70
0.75
$1/SE_{IV}$
β_{IV}

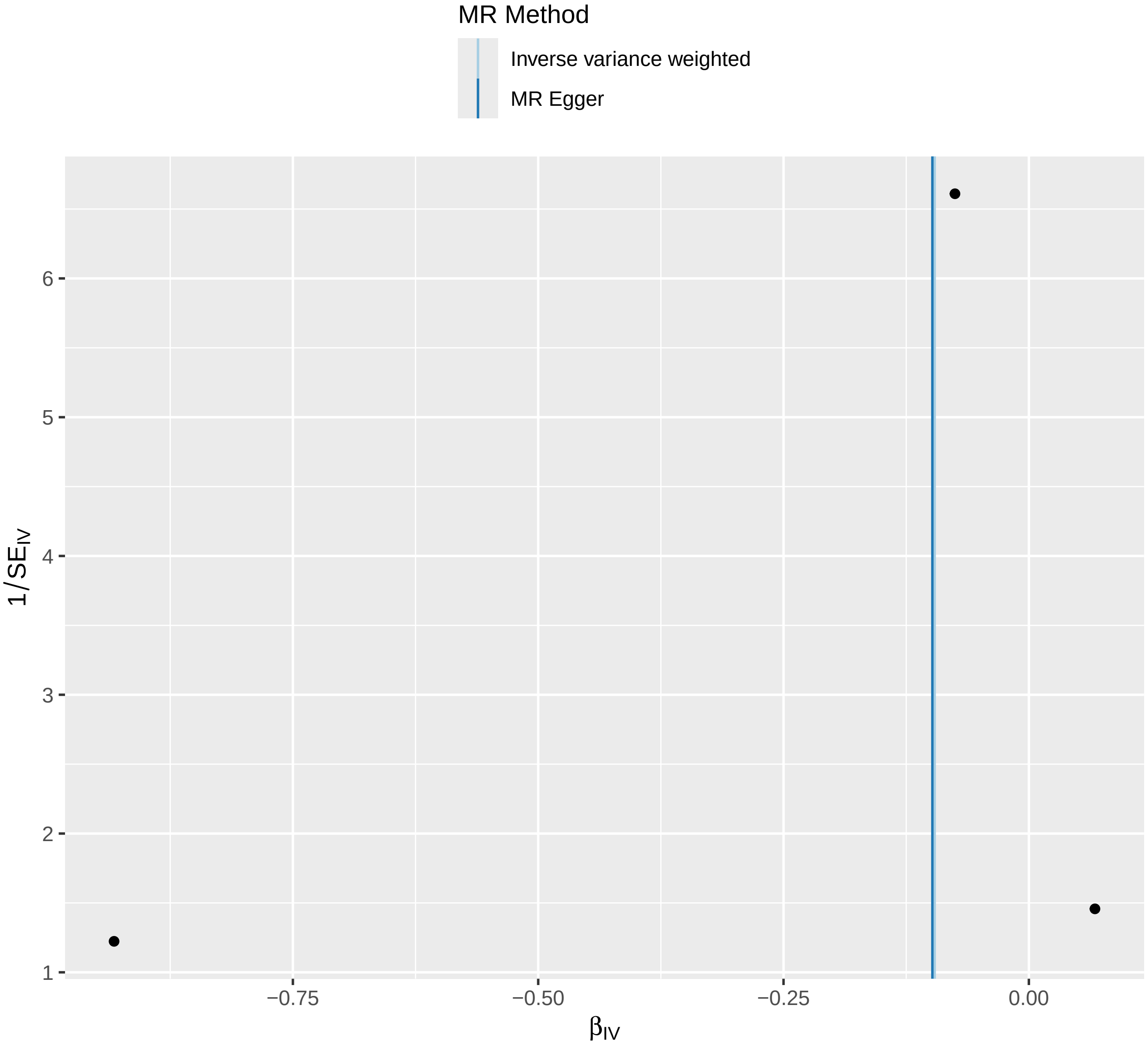
MR Method
Inverse variance weighted
MR Egger
1/SE_{IV}
6
5
4
3
2
1
−0.75
−0.50
−0.25
0.00
β_{IV}

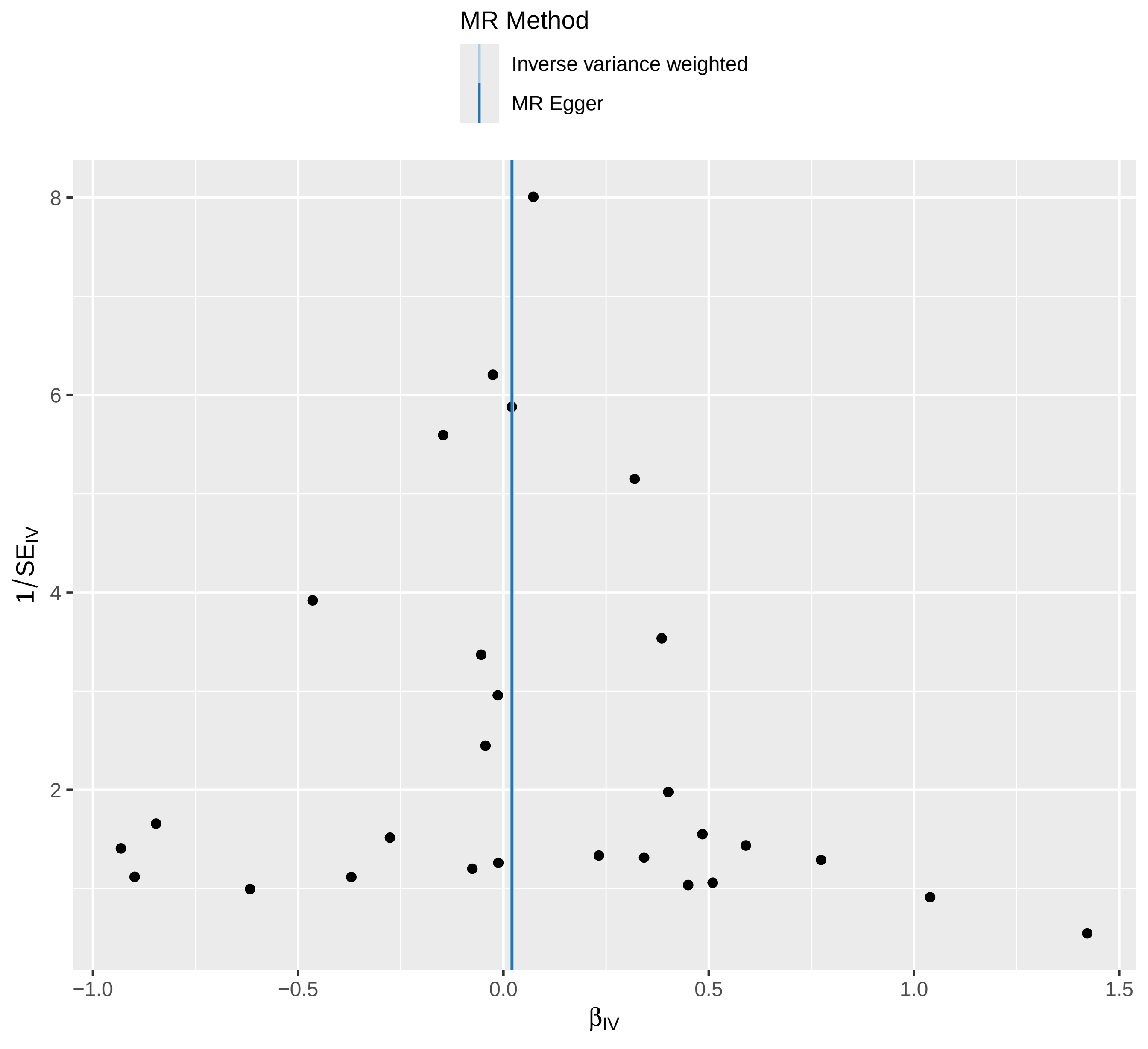
MR Method
Inverse variance weighted
MR Egger
8
6
4
2
1/SE_IV
−1.0
−0.5
0.0
0.5
1.0
1.5
β_IV

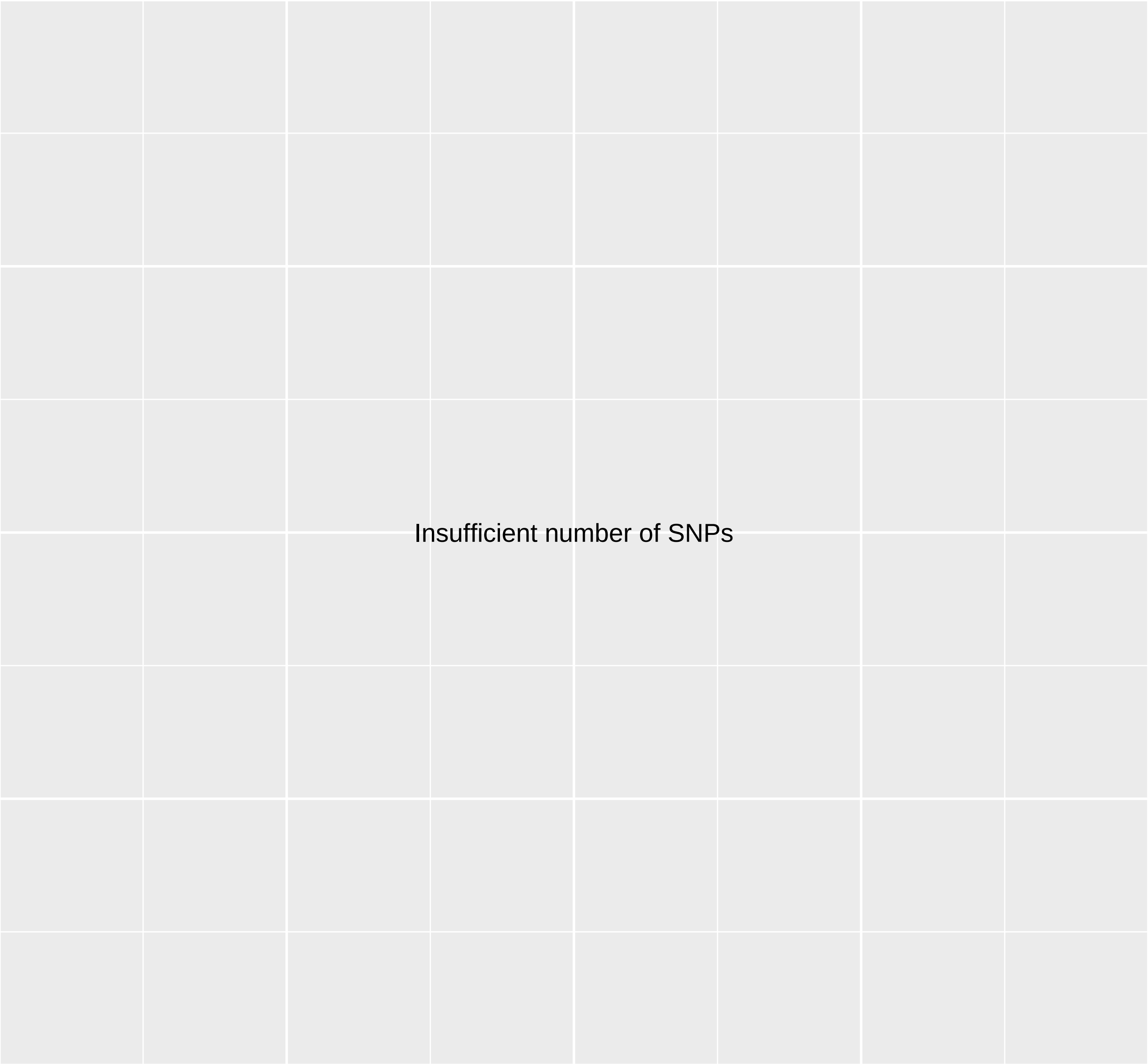
Insufficient number of SNPs

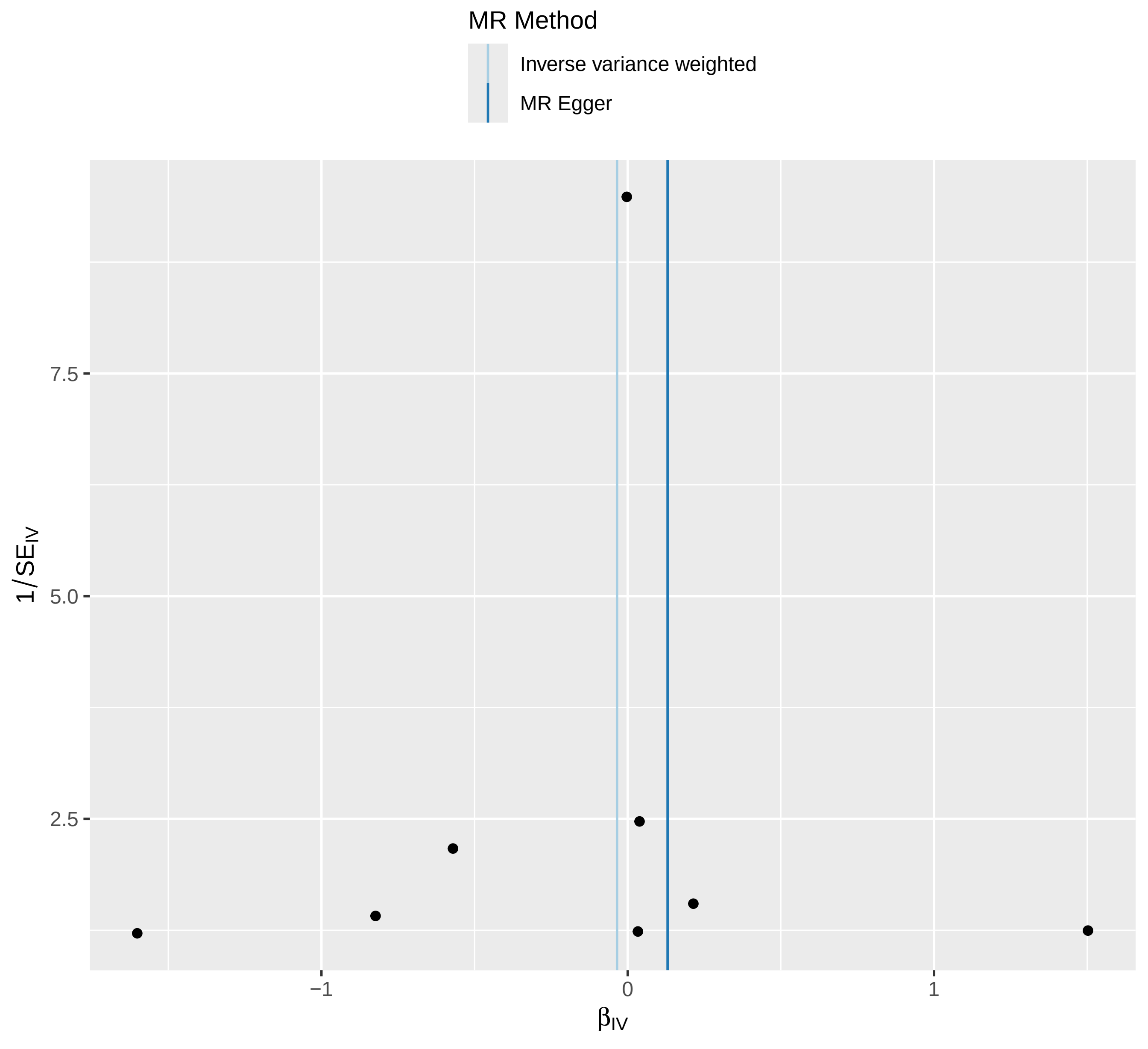
MR Method
Inverse variance weighted
MR Egger
7.5
5.0
2.5
−1
0
1
$1/SE_{IV}$
β_{IV}

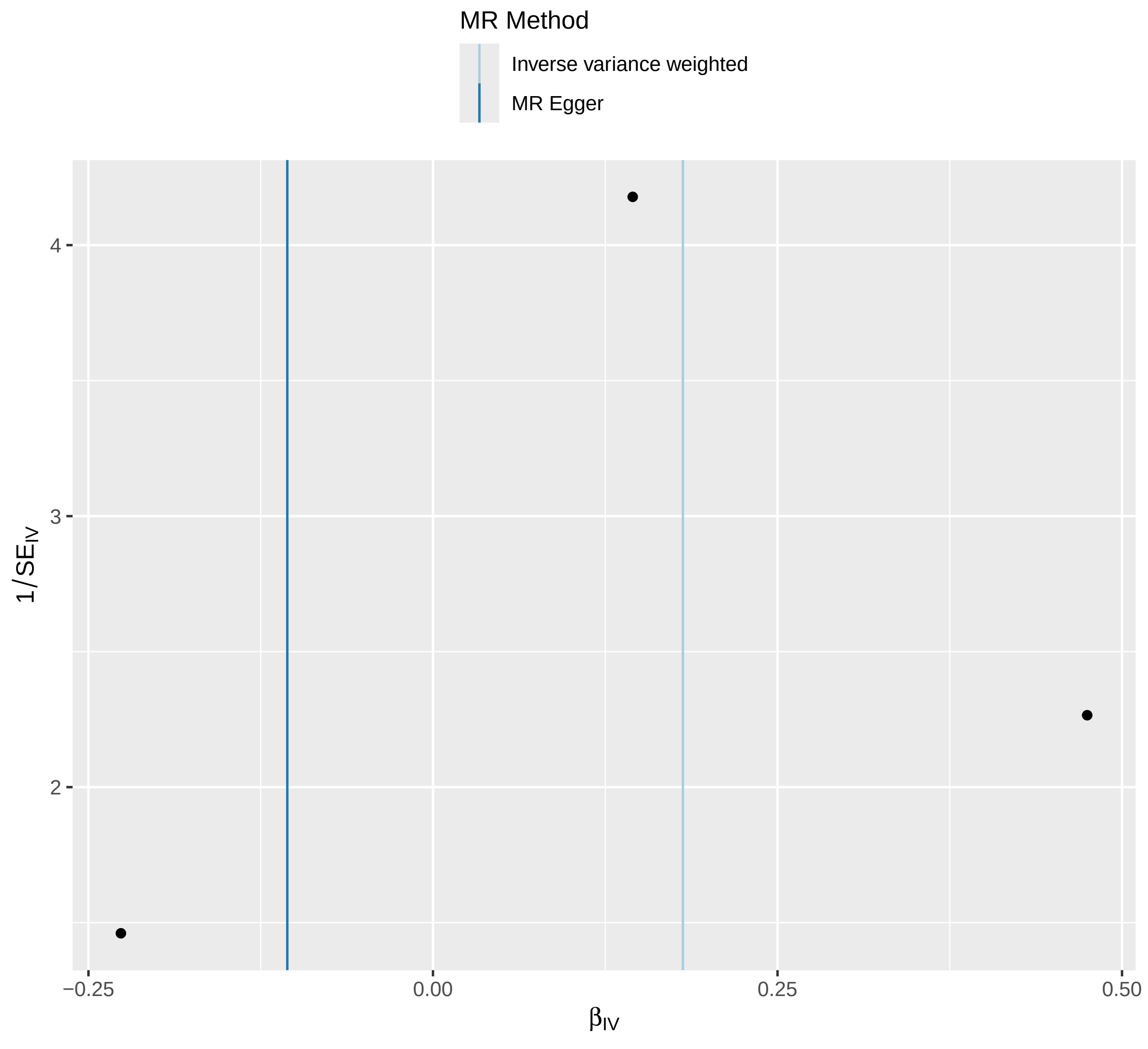

MR Method
Inverse variance weighted
MR Egger
4
3
2
1/SE_IV
−0.25
0.00
0.25
0.50
β_IV

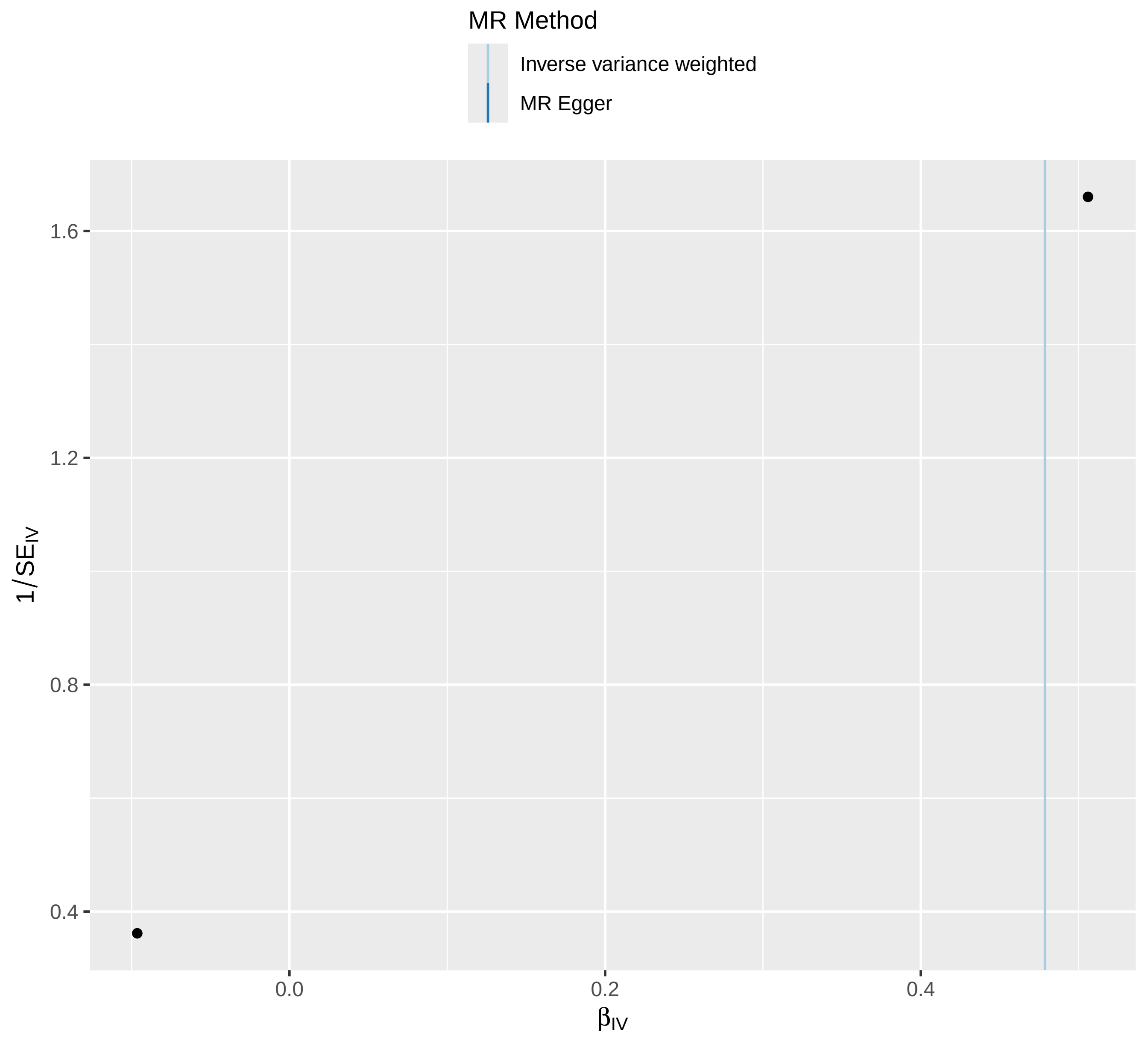

MR Method
Inverse variance weighted
MR Egger
1.6
1.2
0.8
0.4
1/SE_IV
0.0
0.2
0.4
β_IV

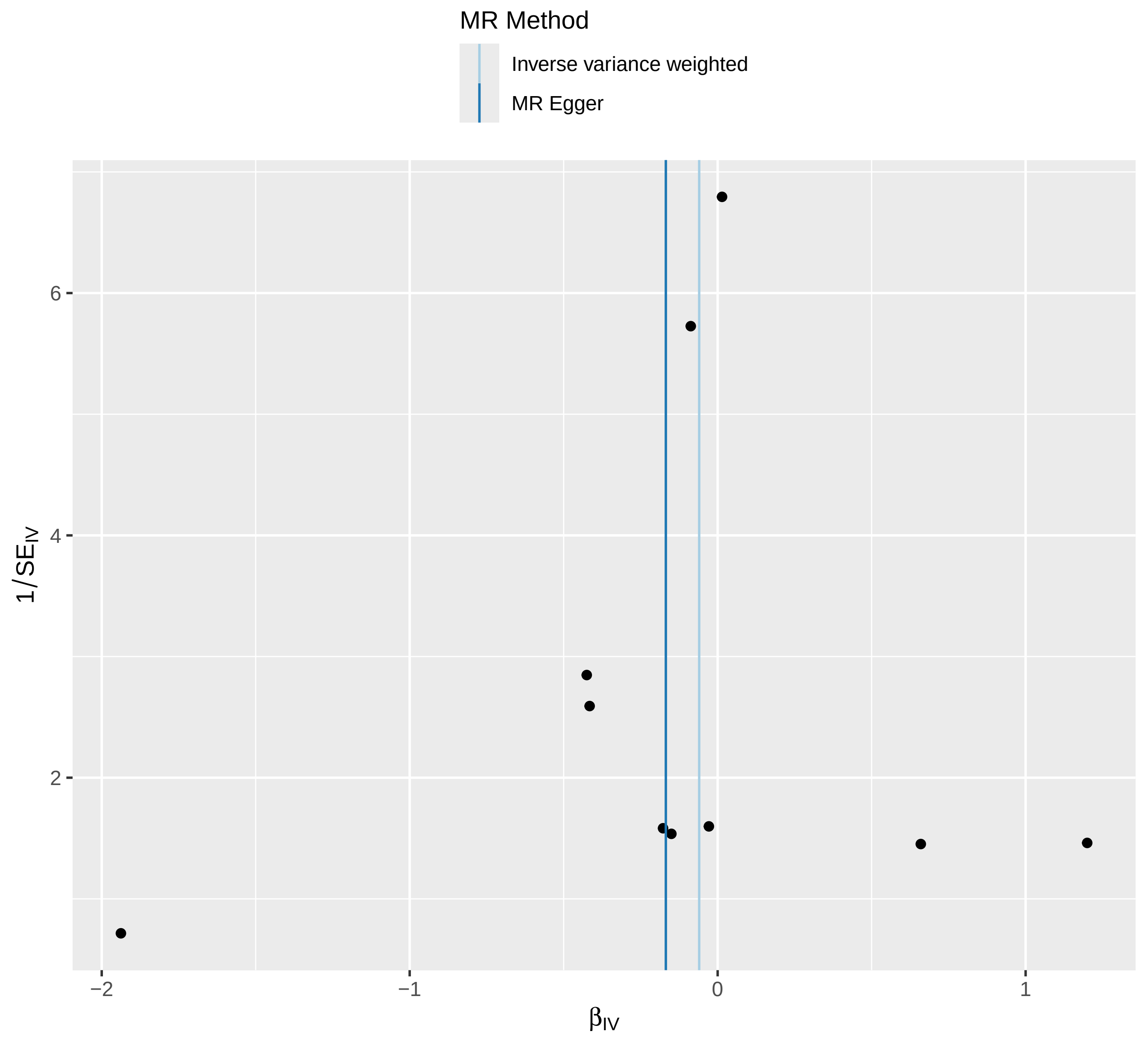
MR Method
Inverse variance weighted
MR Egger
6
4
2
1/SE_IV
−2
−1
0
1
β_IV

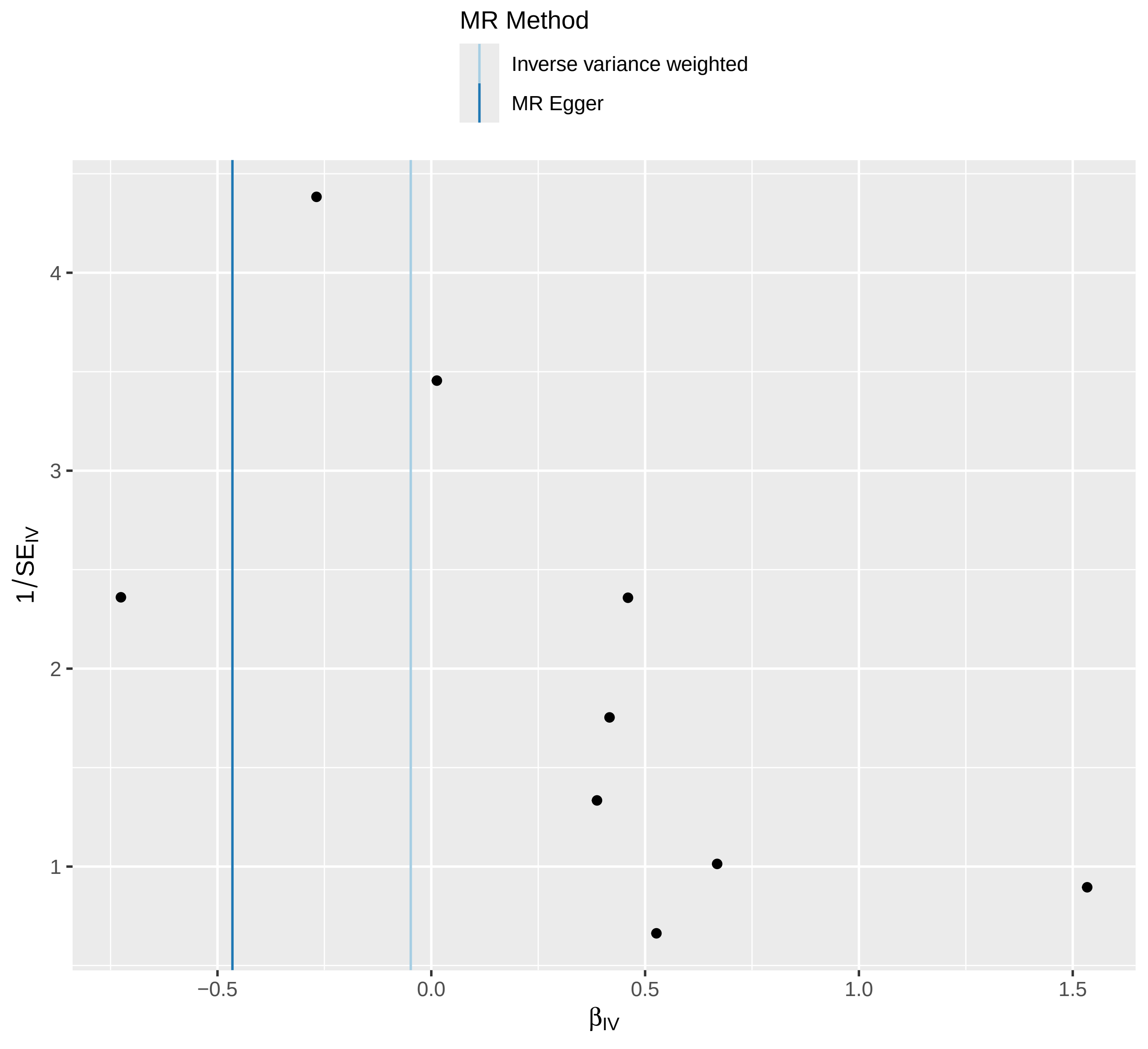

MR Method
Inverse variance weighted
MR Egger
1/SE_IV
β_IV
4
3
2
1
−0.5
0.0
0.5
1.0
1.5

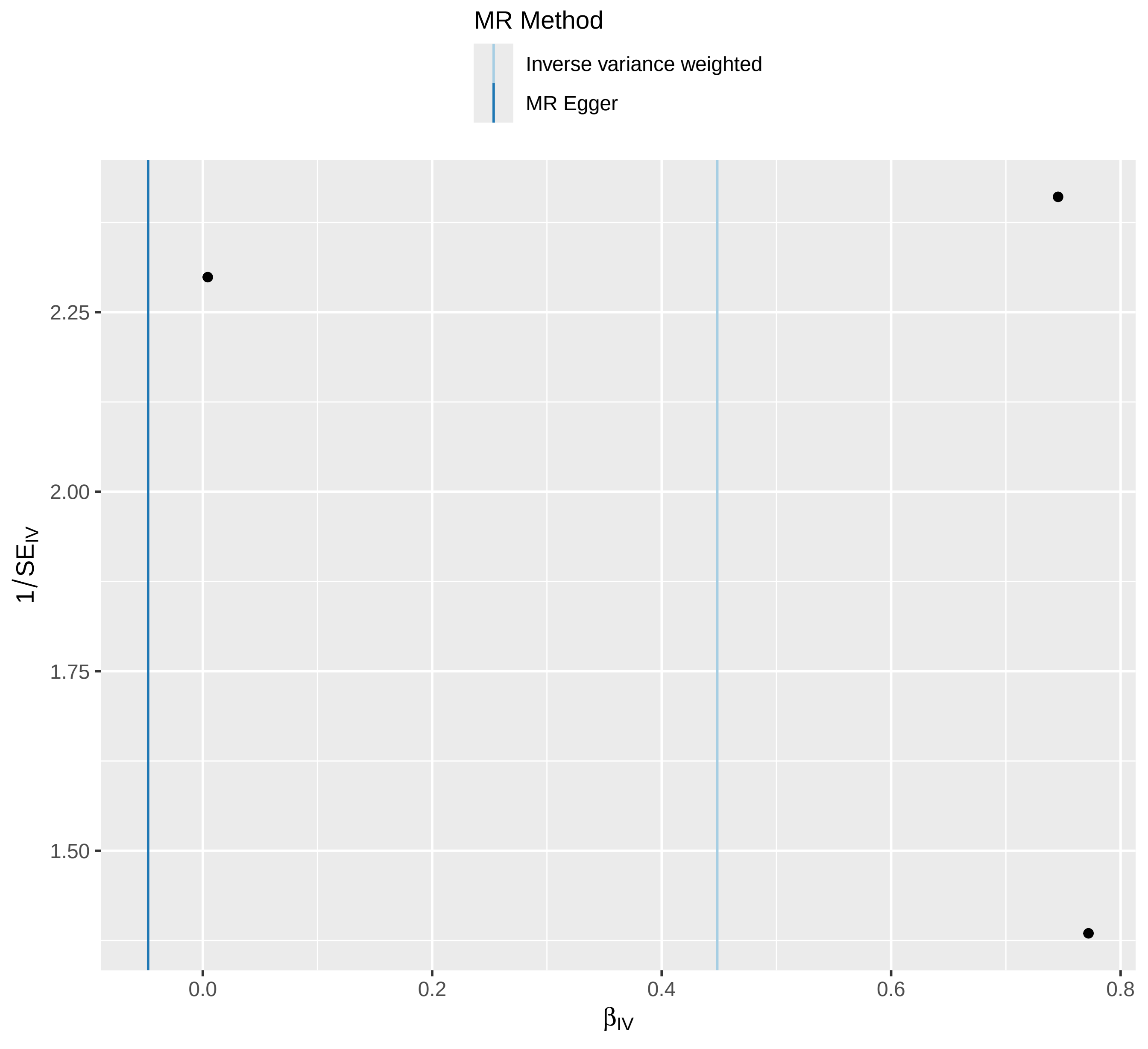

MR Method
Inverse variance weighted
MR Egger
2.25
2.00
1.75
1.50
1/SE_IV
0.0
0.2
0.4
0.6
0.8
β_IV

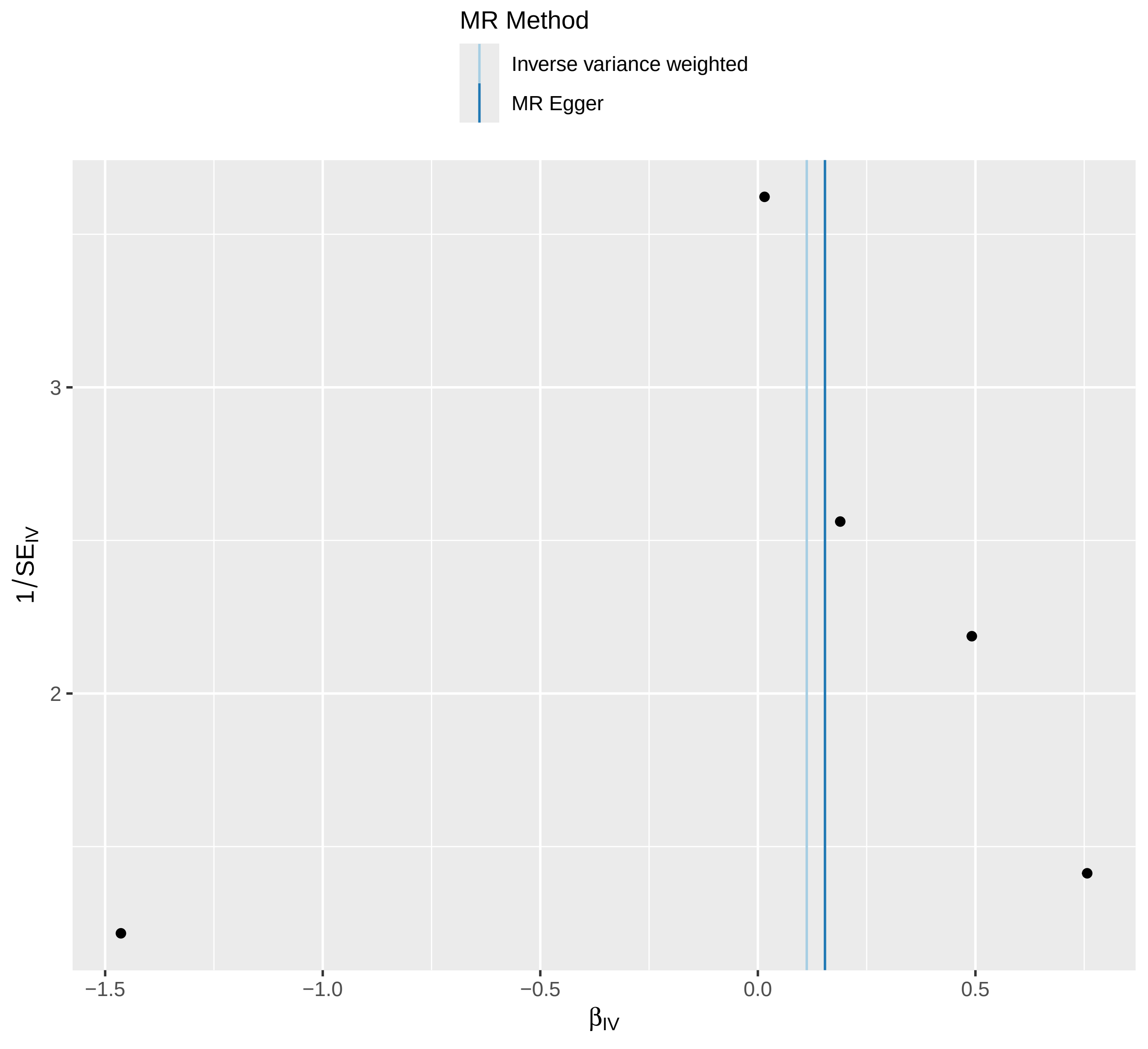
MR Method
Inverse variance weighted
MR Egger
3
2
1/SE_IV
−1.5
−1.0
−0.5
0.0
0.5
β_IV

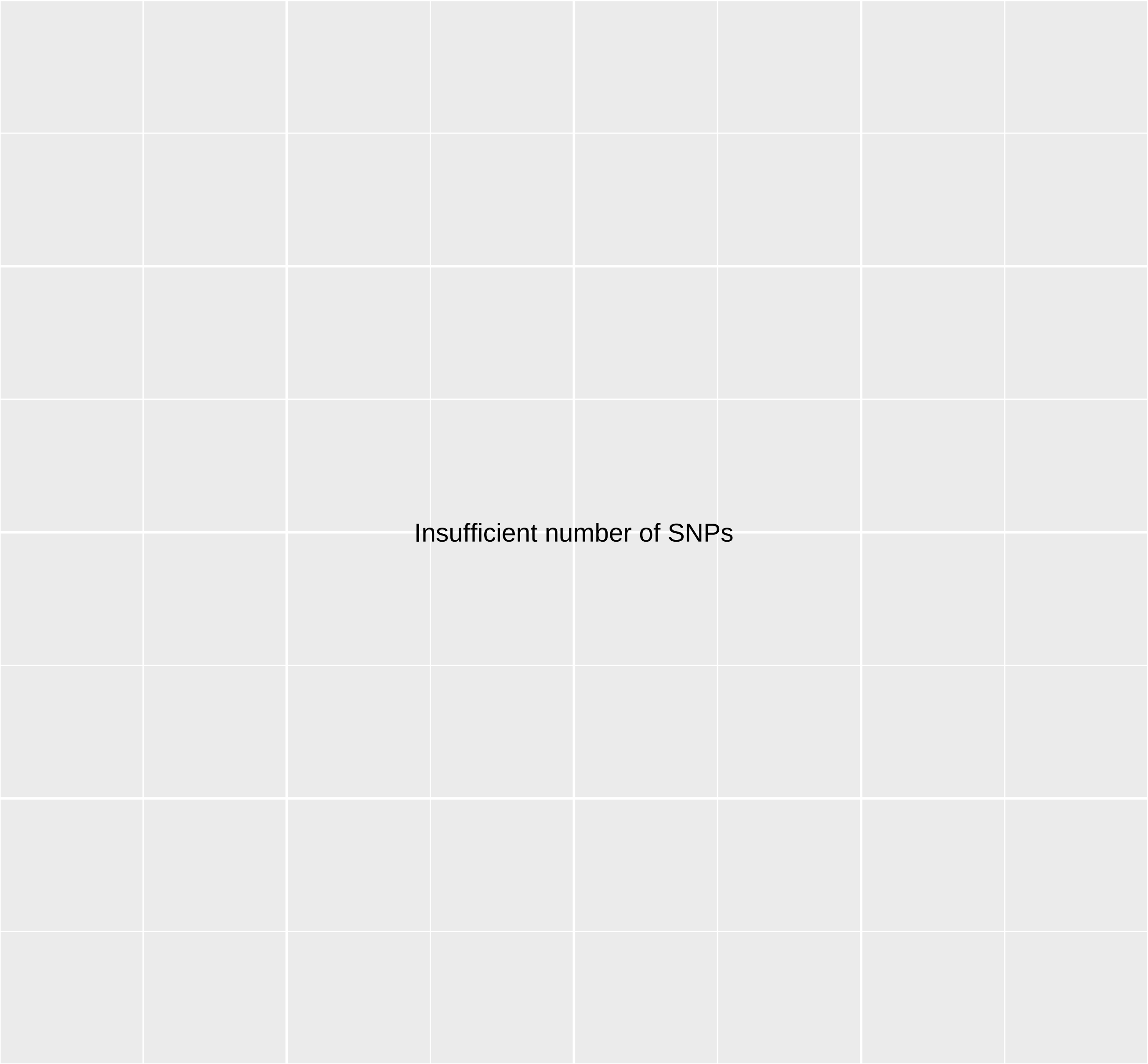
Insufficient number of SNPs

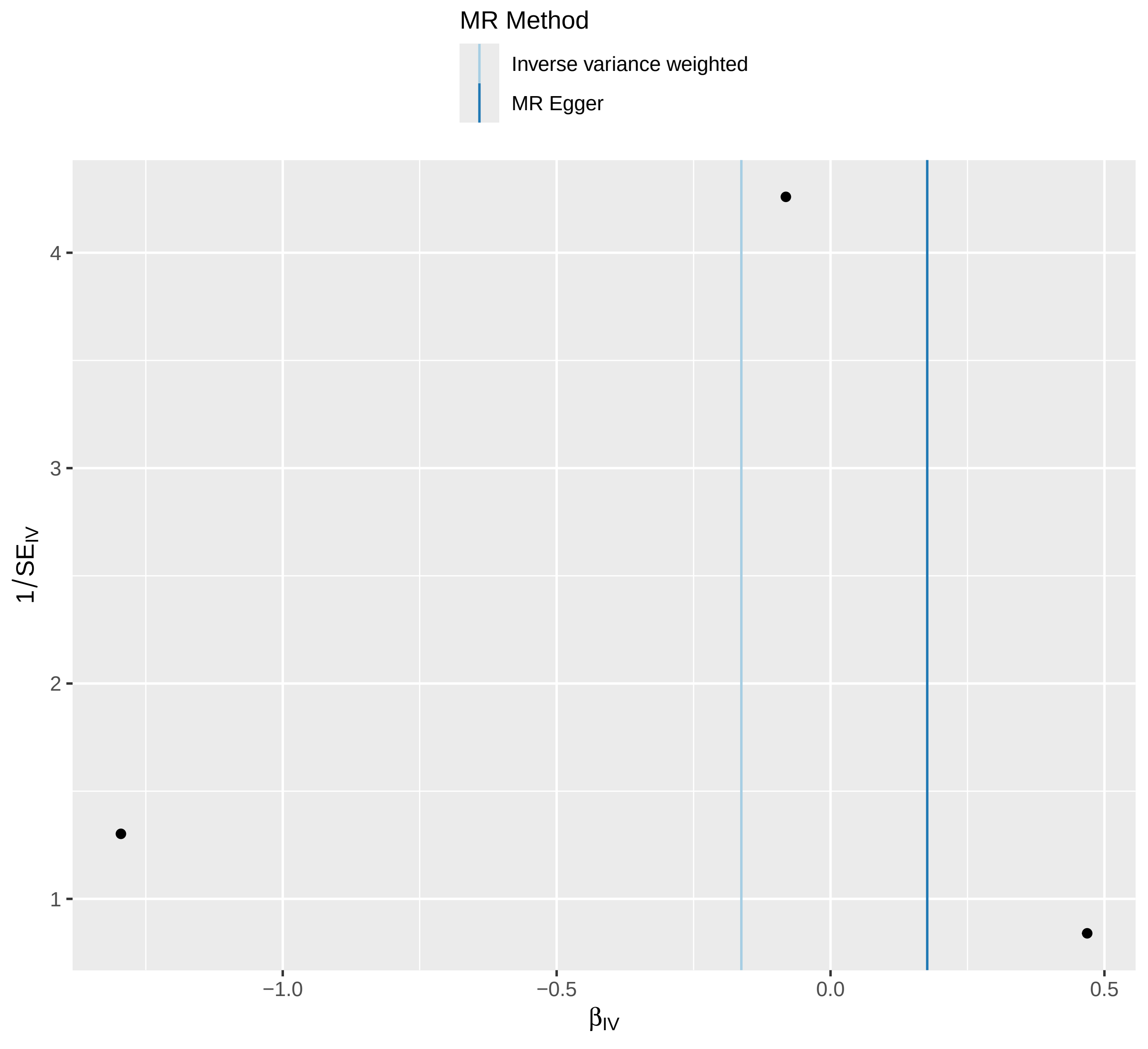

MR Method
Inverse variance weighted
MR Egger
1/SE$_{IV}$
4
3
2
1
−1.0
−0.5
0.0
0.5
β$_{IV}$

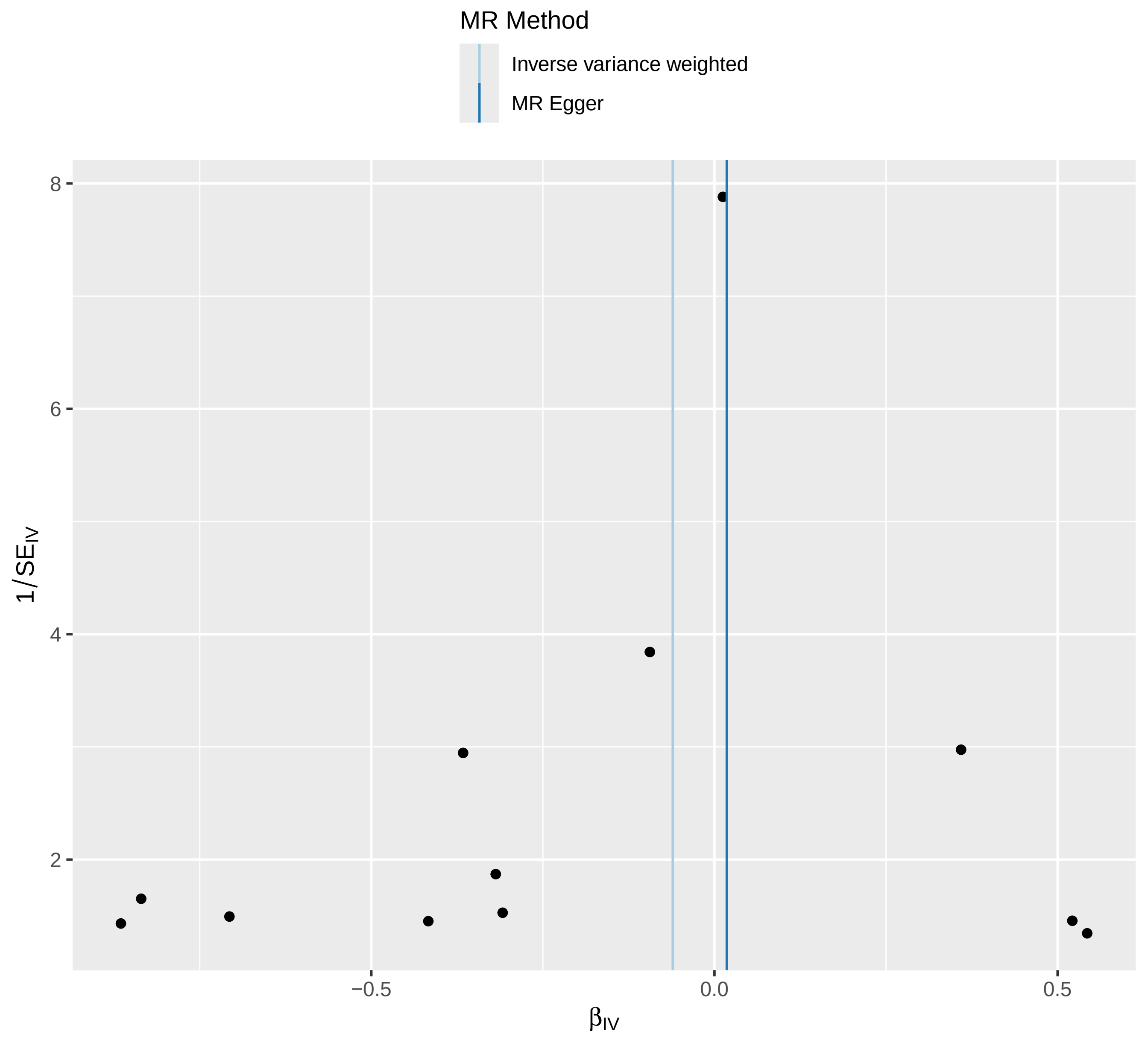

MR Method
Inverse variance weighted
MR Egger
8
6
4
2
1/SE$_{IV}$
−0.5
0.0
0.5
β$_{IV}$

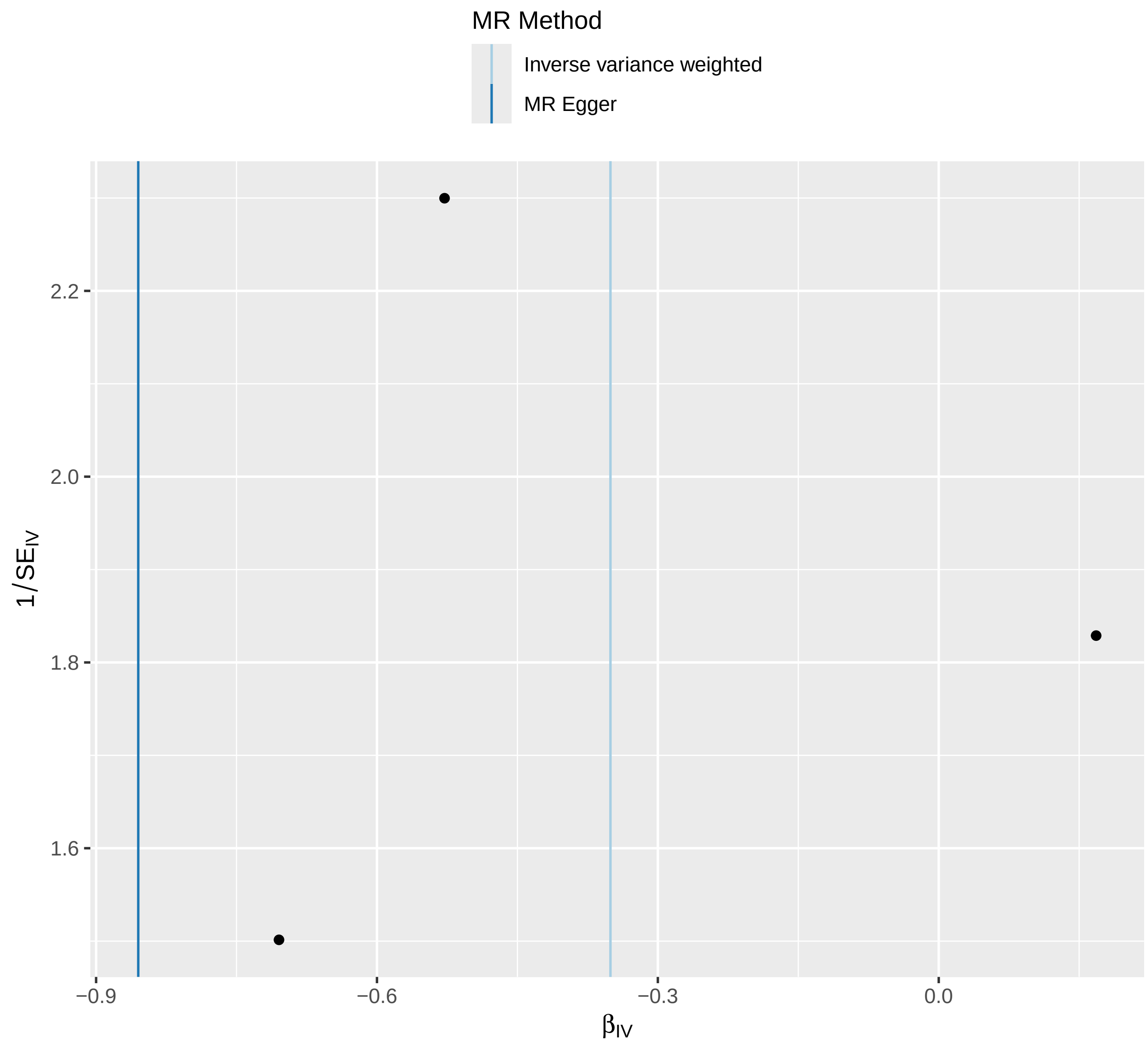
MR Method
Inverse variance weighted
MR Egger
2.2
2.0
1.8
1.6
1/SE_IV
−0.9
−0.6
−0.3
0.0
β_IV

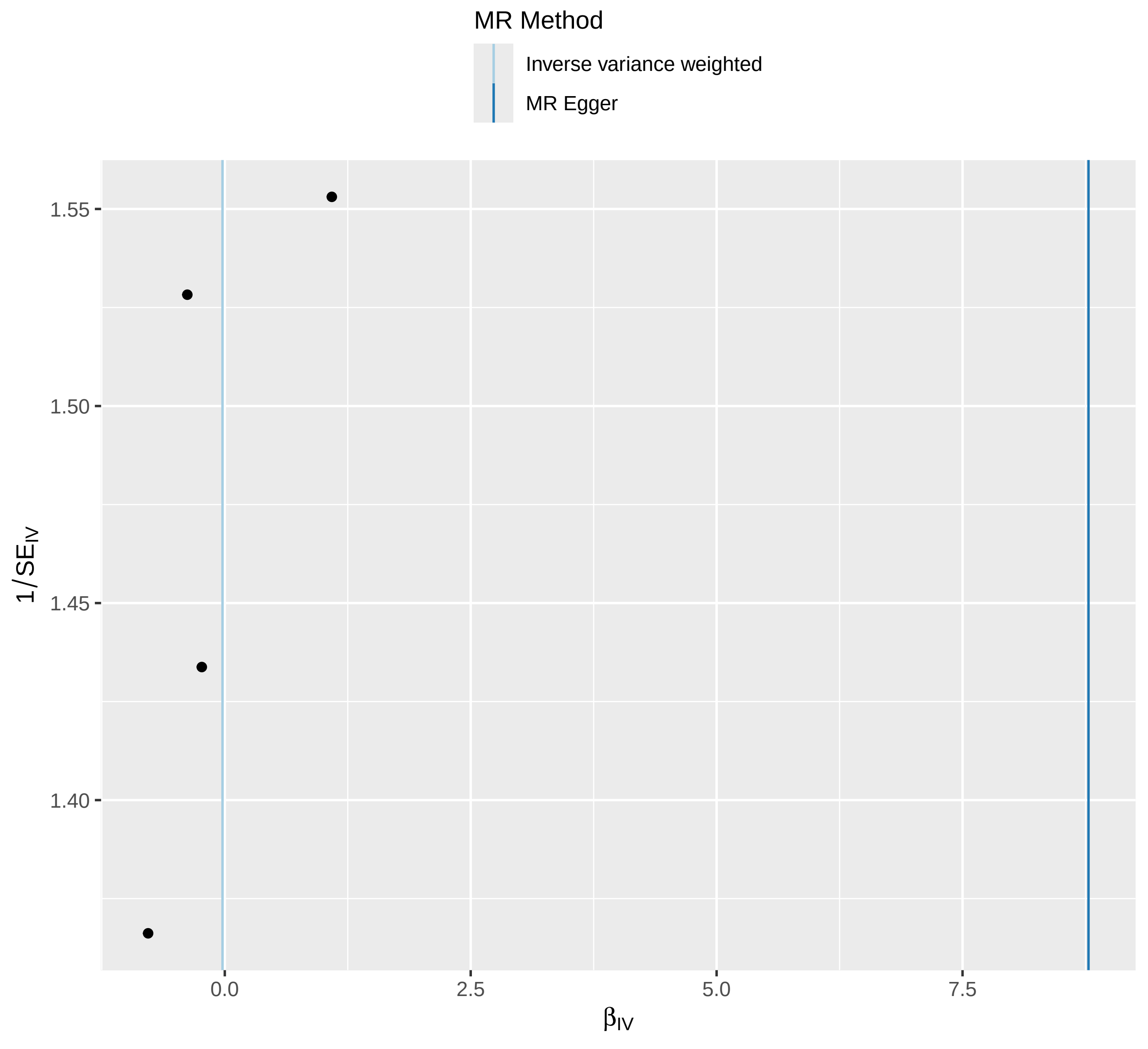
MR Method
Inverse variance weighted
MR Egger
1.55
1.50
1.45
1.40
1/SE_IV
0.0
2.5
5.0
7.5
β_IV

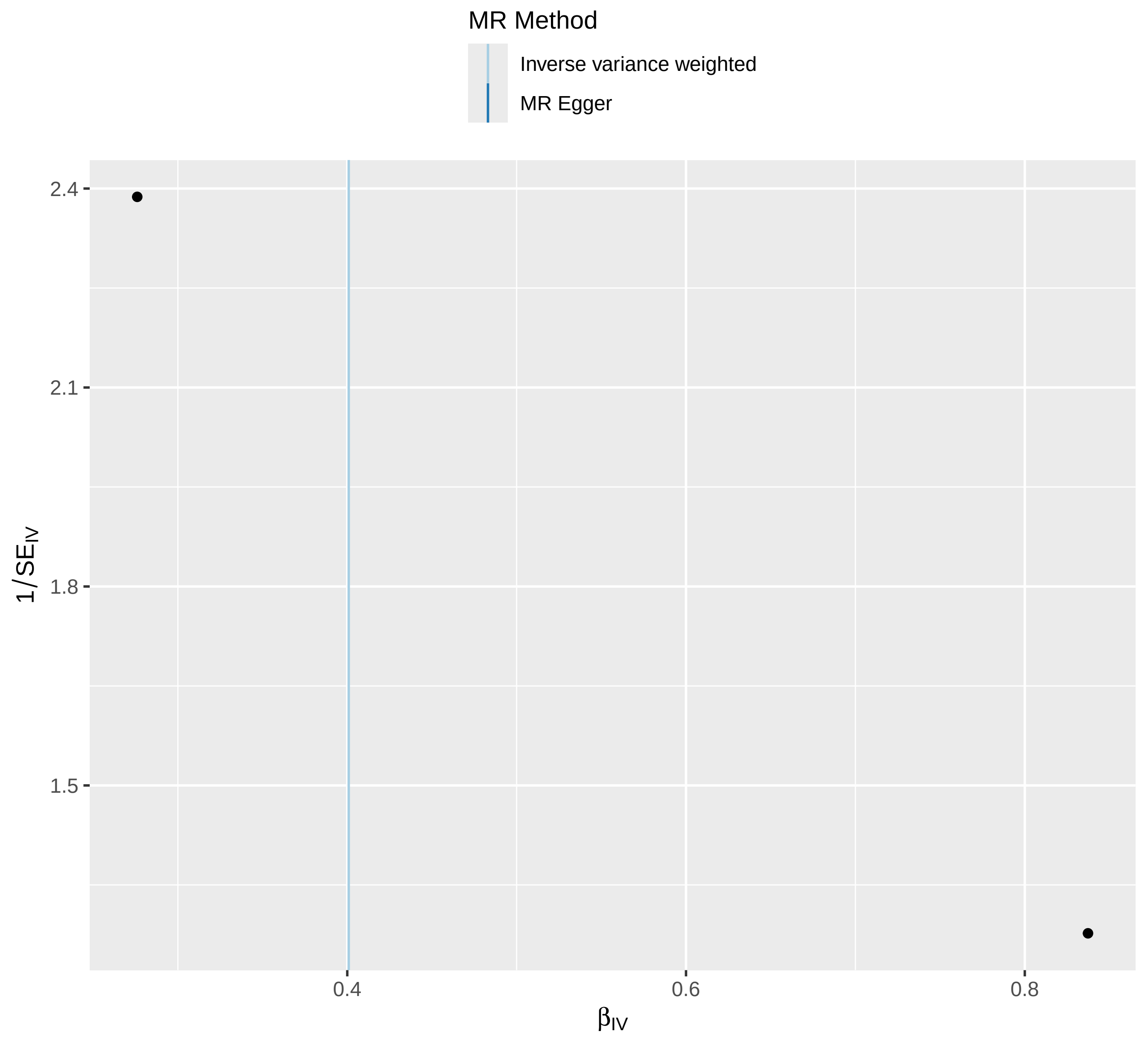

MR Method
Inverse variance weighted
MR Egger
2.4
2.1
1.8
1.5
$1/SE_{IV}$
0.4
0.6
0.8
β_{IV}

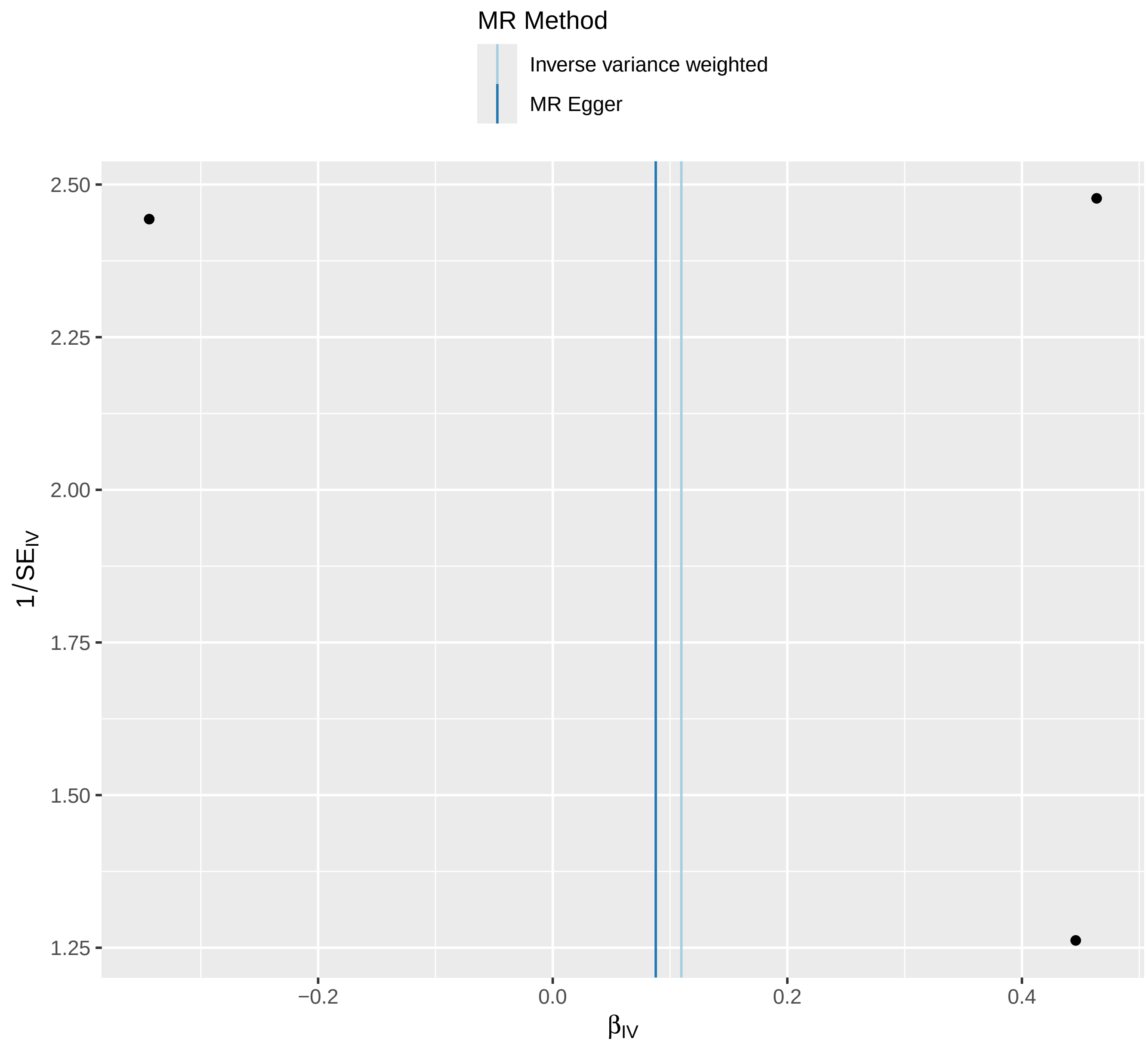
MR Method
Inverse variance weighted
MR Egger
2.50
2.25
2.00
1.75
1.50
1.25
1/SE_IV
−0.2
0.0
0.2
0.4
β_IV

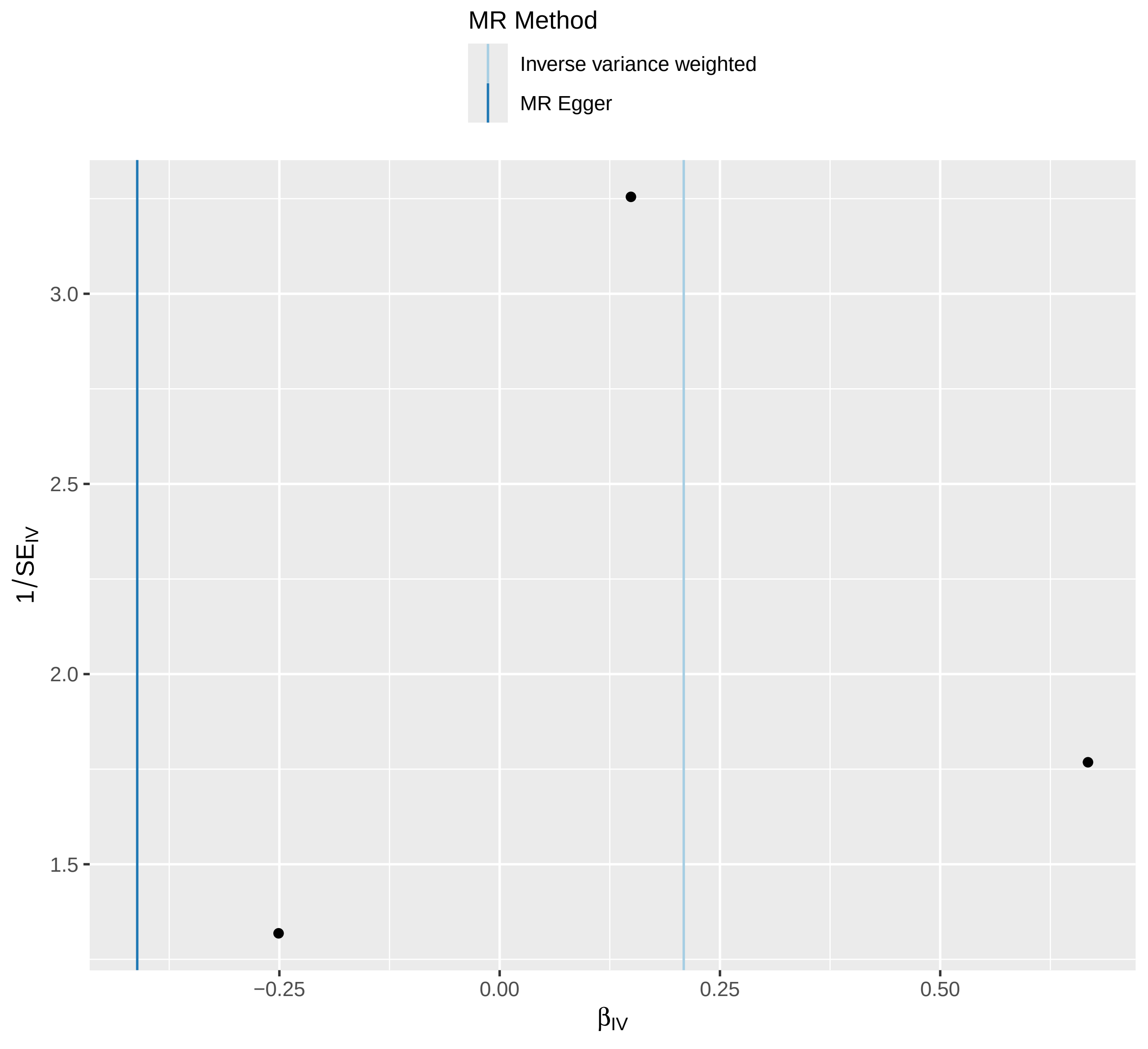

MR Method
Inverse variance weighted
MR Egger
3.0
2.5
2.0
1.5
1/SE$_{IV}$
−0.25
0.00
0.25
0.50
β$_{IV}$

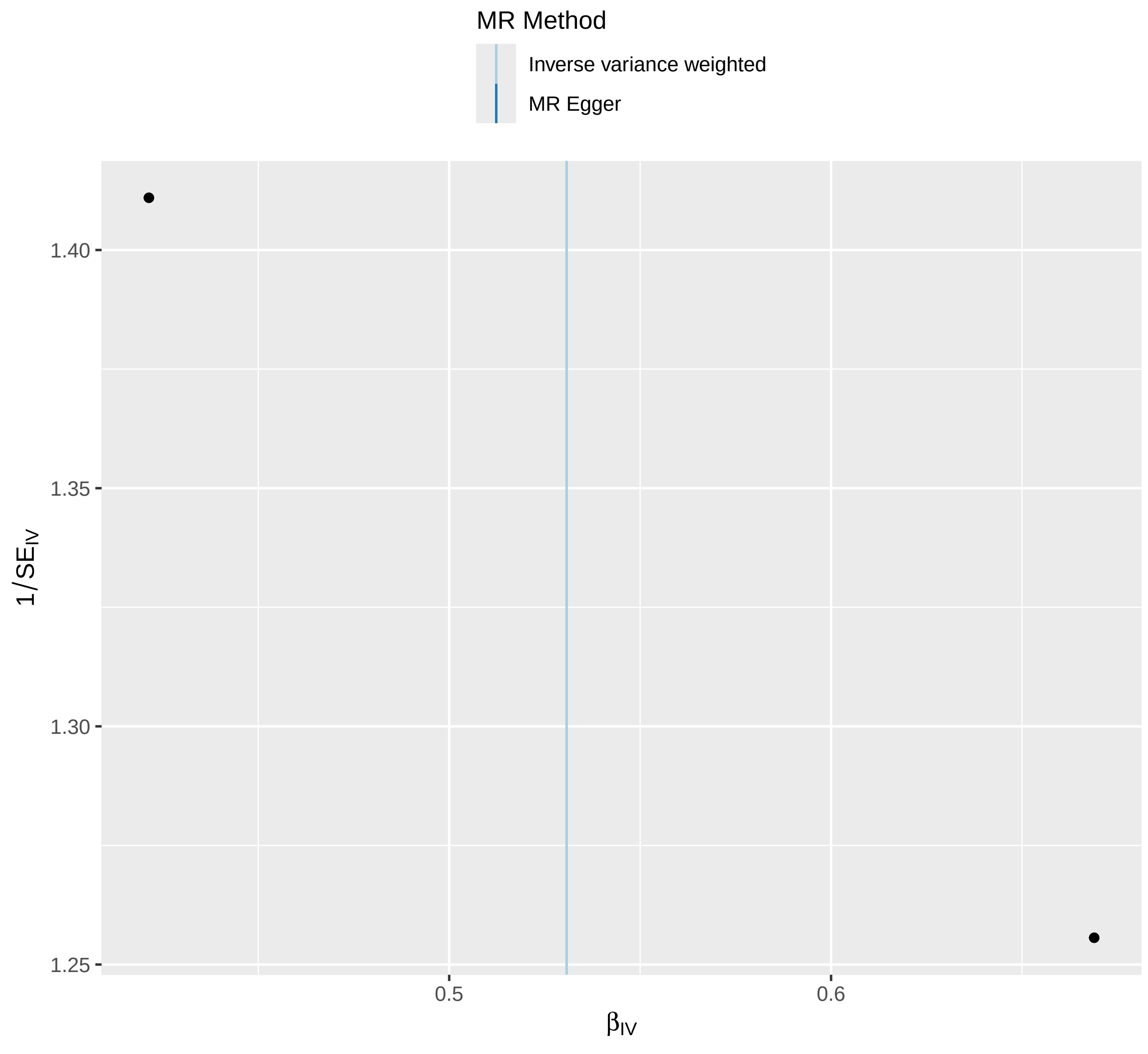
MR Method
Inverse variance weighted
MR Egger
1.40
1.35
1.30
1.25
$1/SE_{IV}$
0.5
0.6
β_{IV}

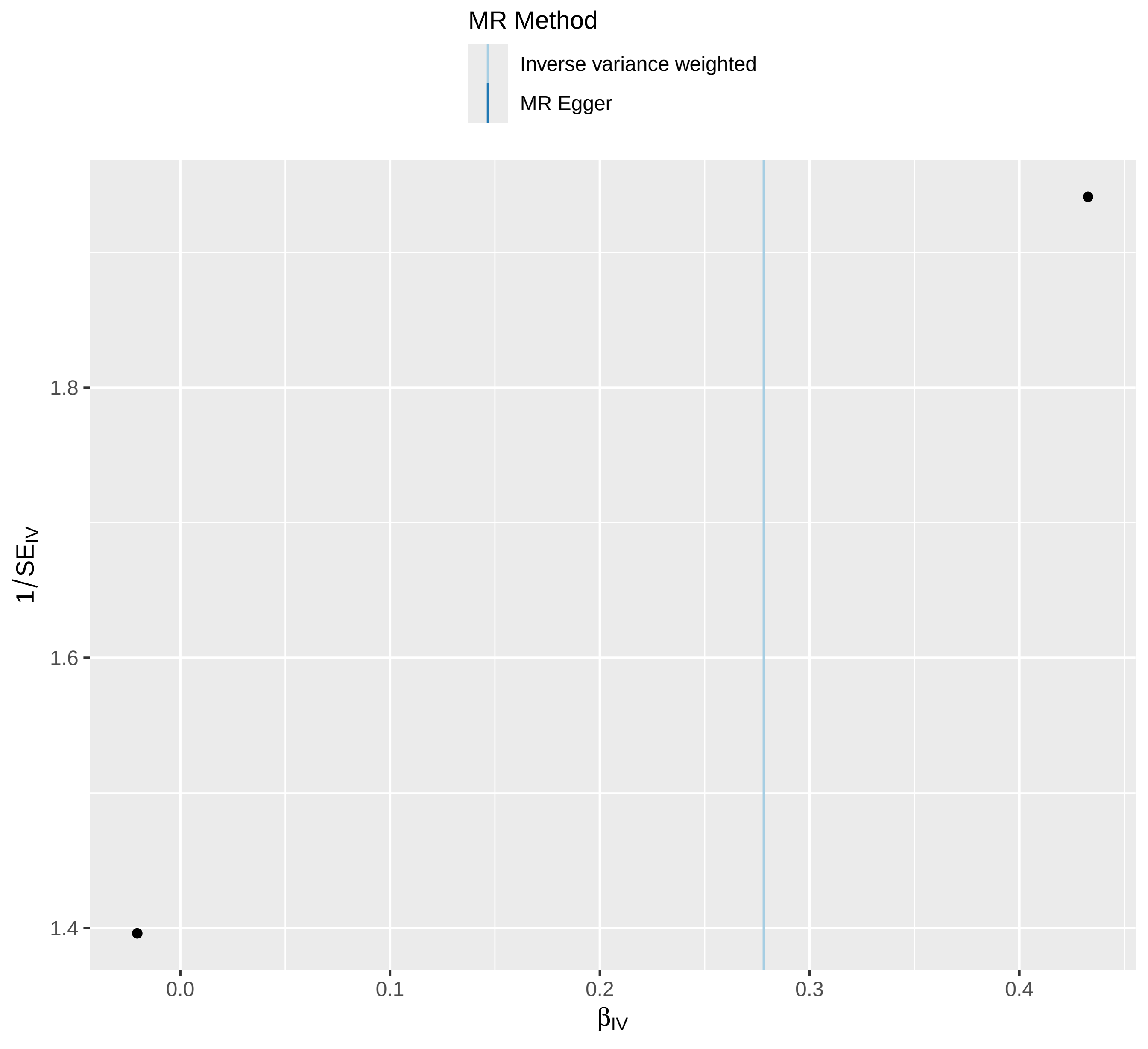

MR Method
Inverse variance weighted
MR Egger
1.8
1.6
1.4
1/SE_IV
0.0
0.1
0.2
0.3
0.4
β_IV

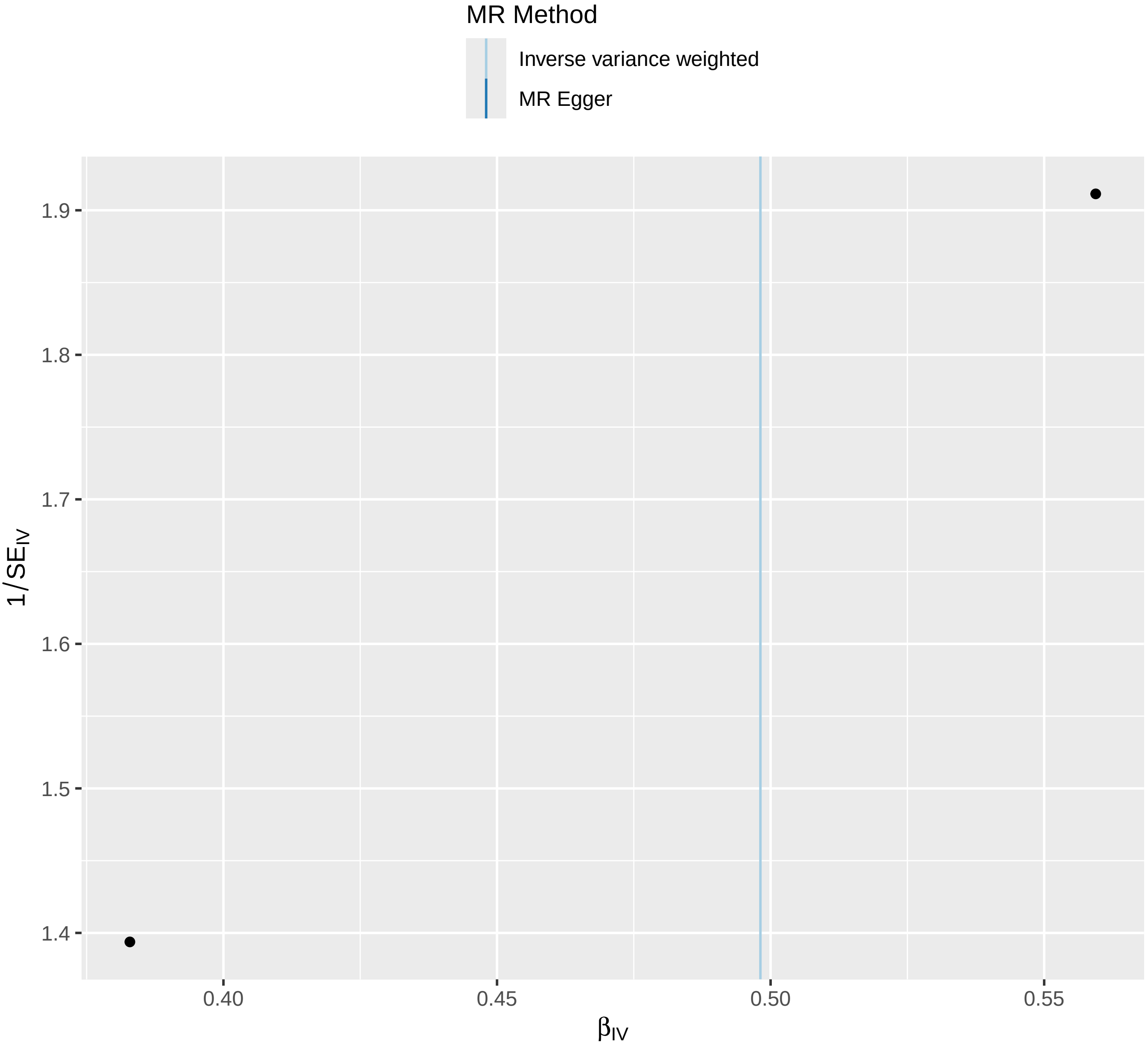
MR Method
Inverse variance weighted
MR Egger
1.9
1.8
1.7
1.6
1.5
1.4
1/SE_IV
0.40
0.45
0.50
0.55
β_IV

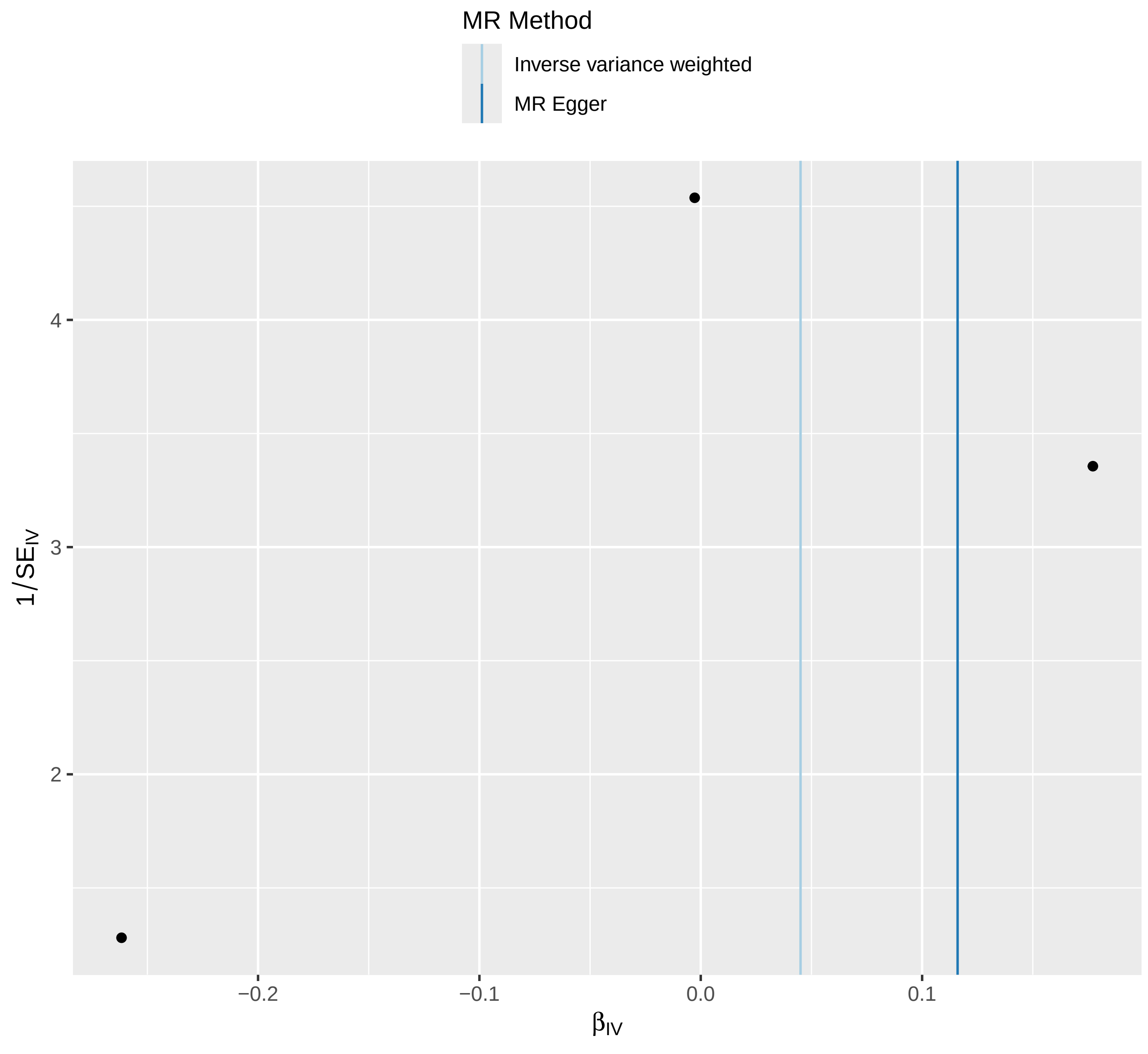

MR Method
Inverse variance weighted
MR Egger
4
3
2
$1/SE_{IV}$
−0.2
−0.1
0.0
0.1
β_{IV}

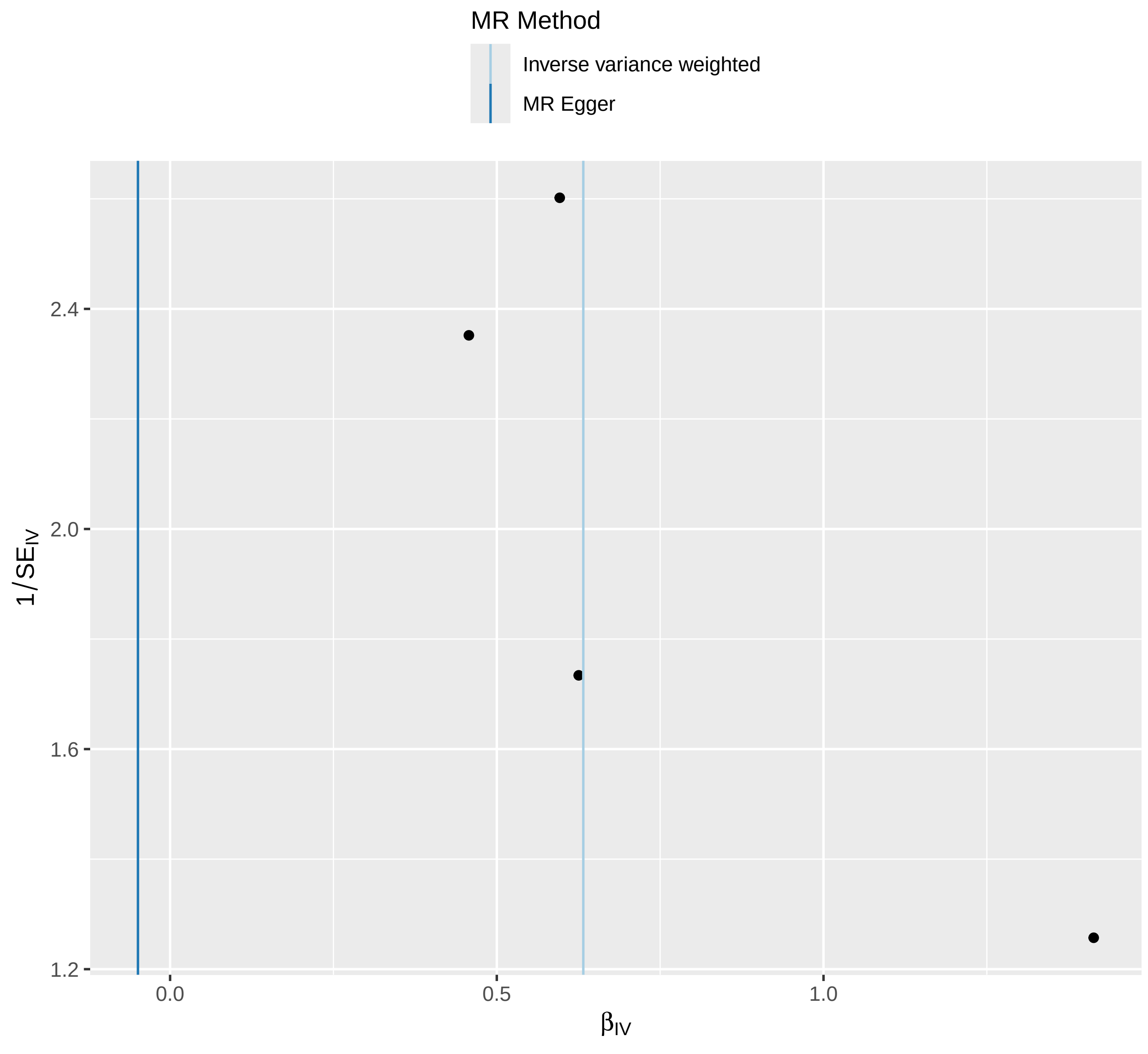
MR Method
Inverse variance weighted
MR Egger
2.4
2.0
1.6
1.2
1/SE_IV
0.0
0.5
1.0
β_IV

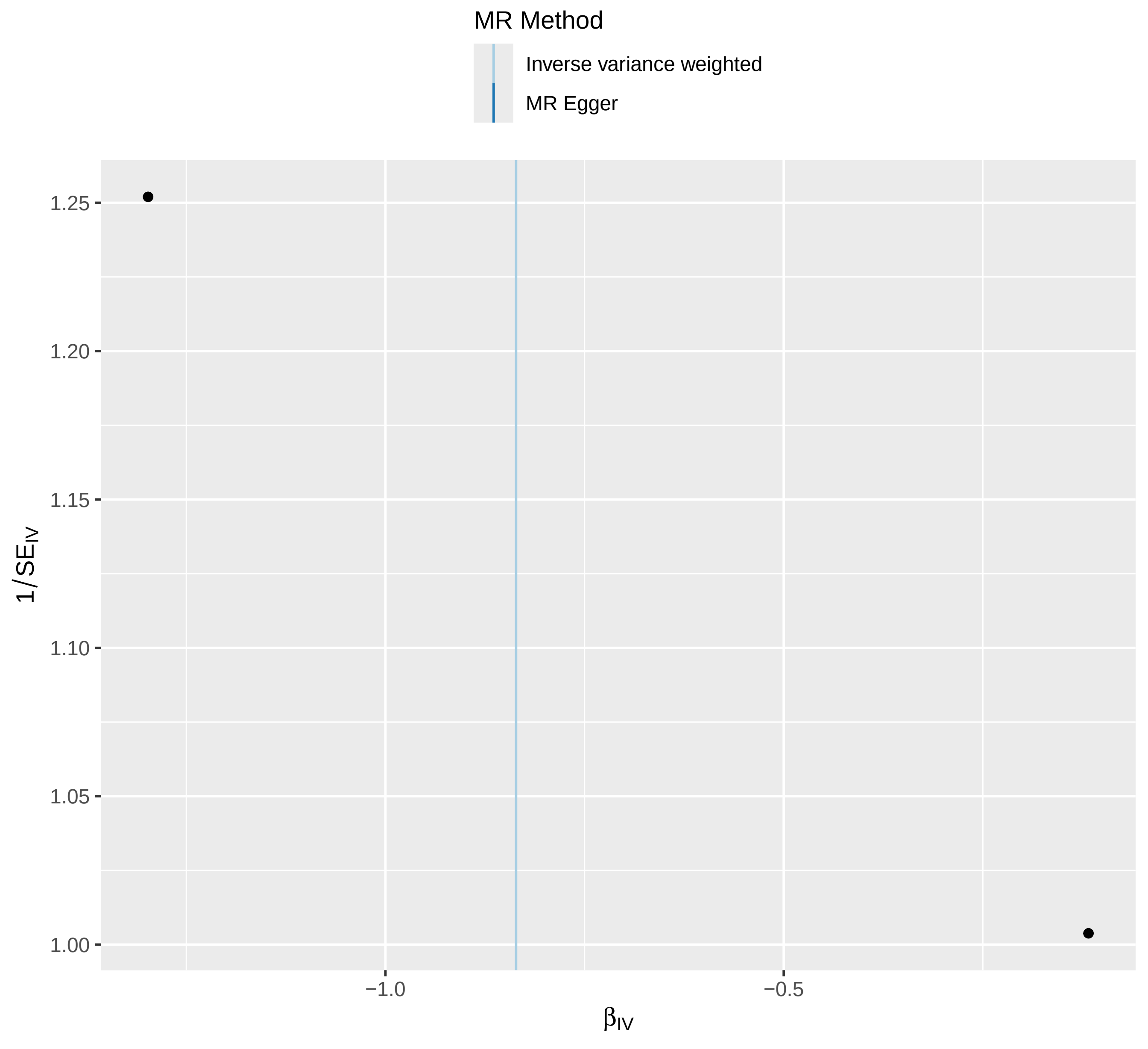

MR Method
Inverse variance weighted
MR Egger
1.25
1.20
1.15
1.10
1.05
1.00
1/SE_IV
−1.0
−0.5
β_IV

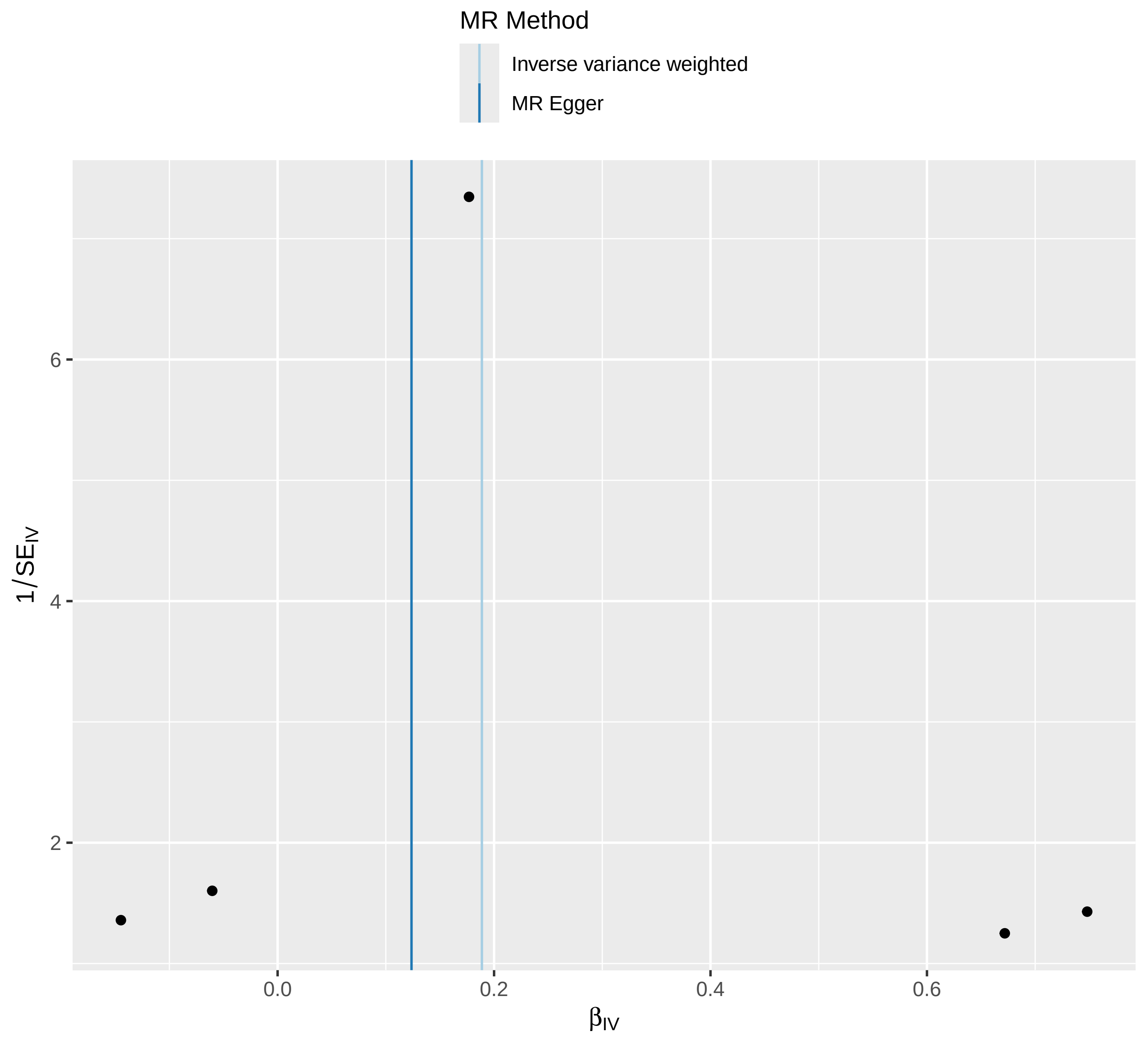

MR Method
Inverse variance weighted
MR Egger
6
4
2
$1/SE_{IV}$
0.0
0.2
0.4
0.6
β_{IV}

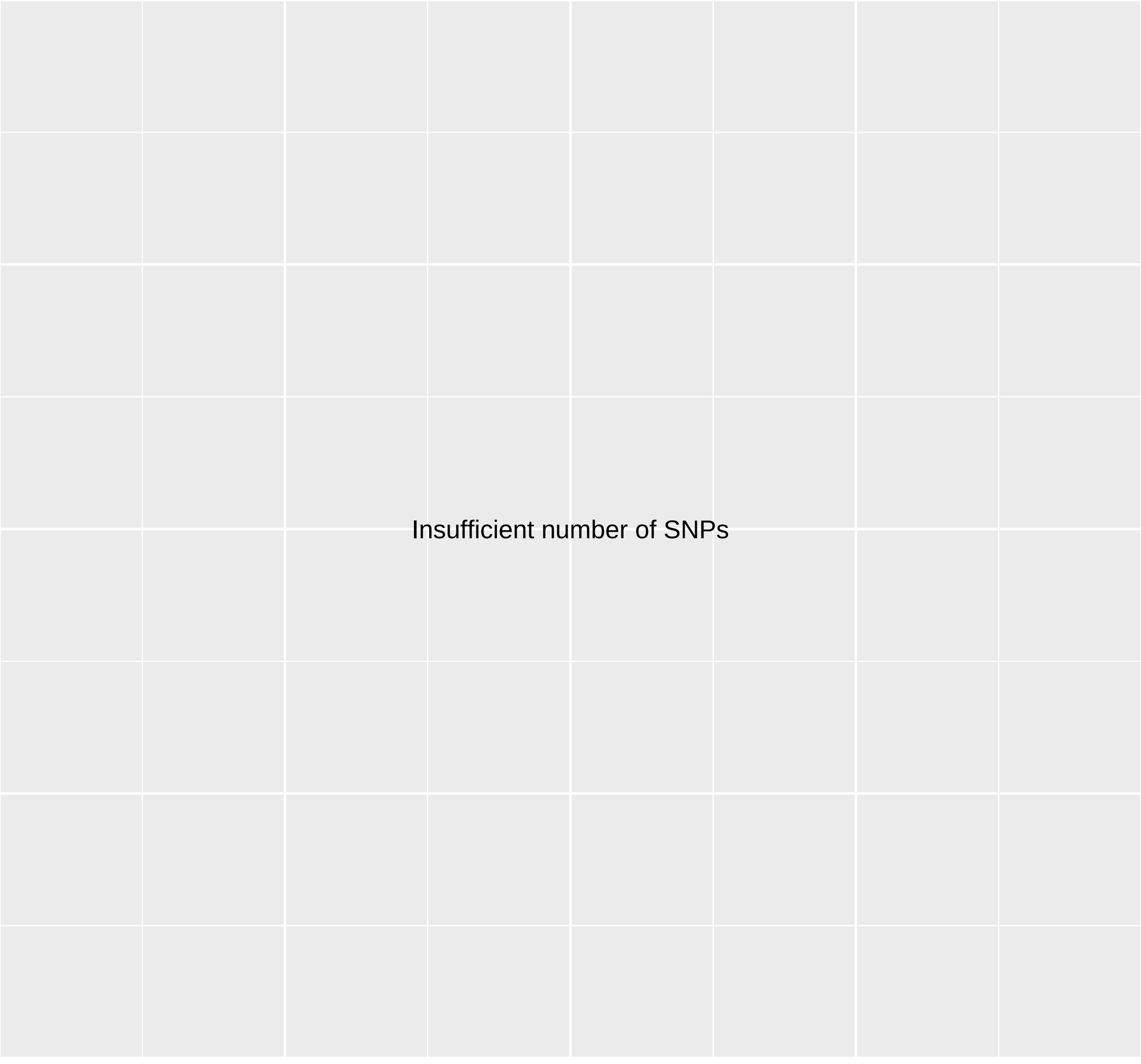
Insufficient number of SNPs

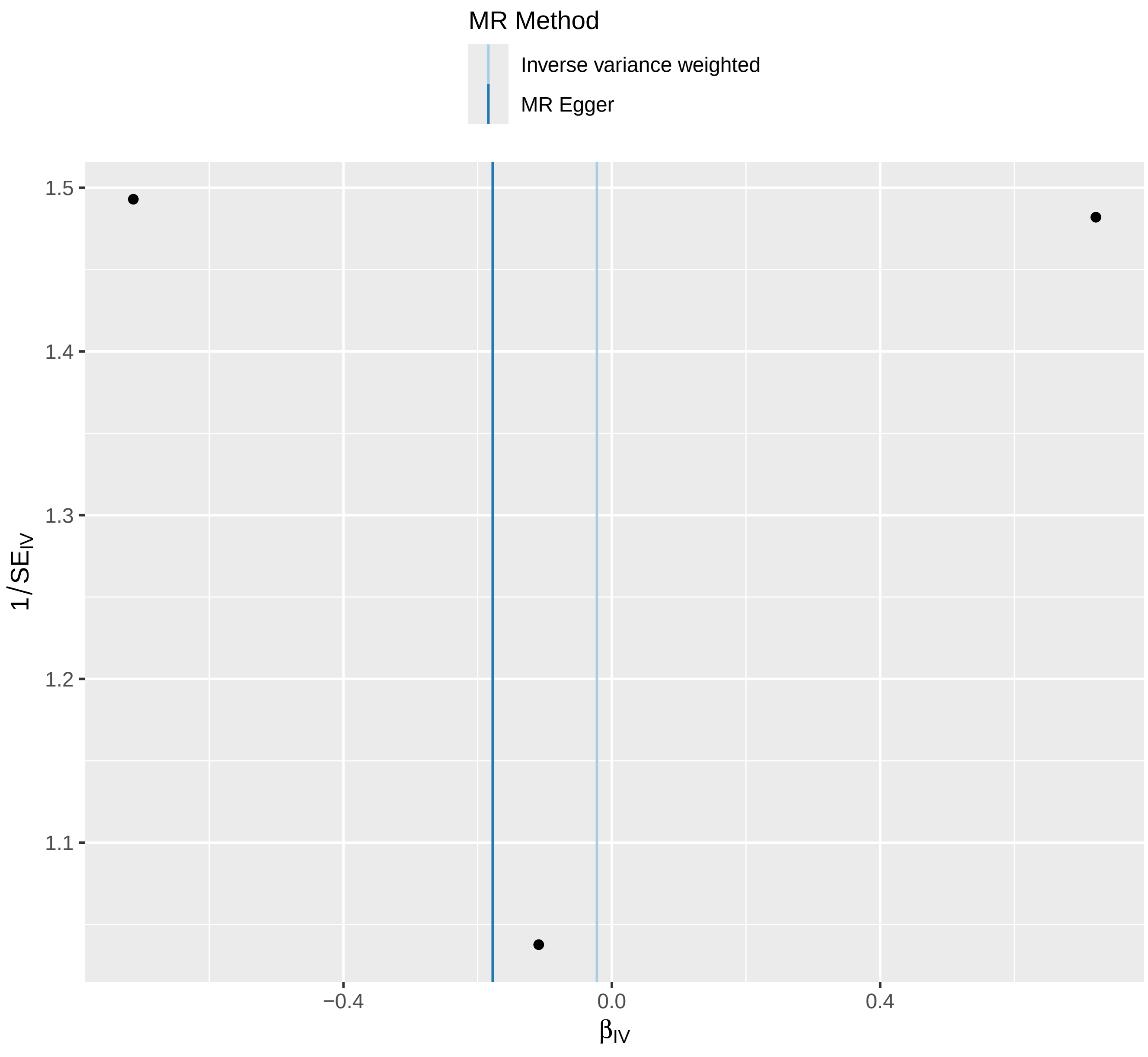
MR Method
Inverse variance weighted
MR Egger
1.5
1.4
1.3
1.2
1.1
1/SE_IV
−0.4
0.0
0.4
β_IV

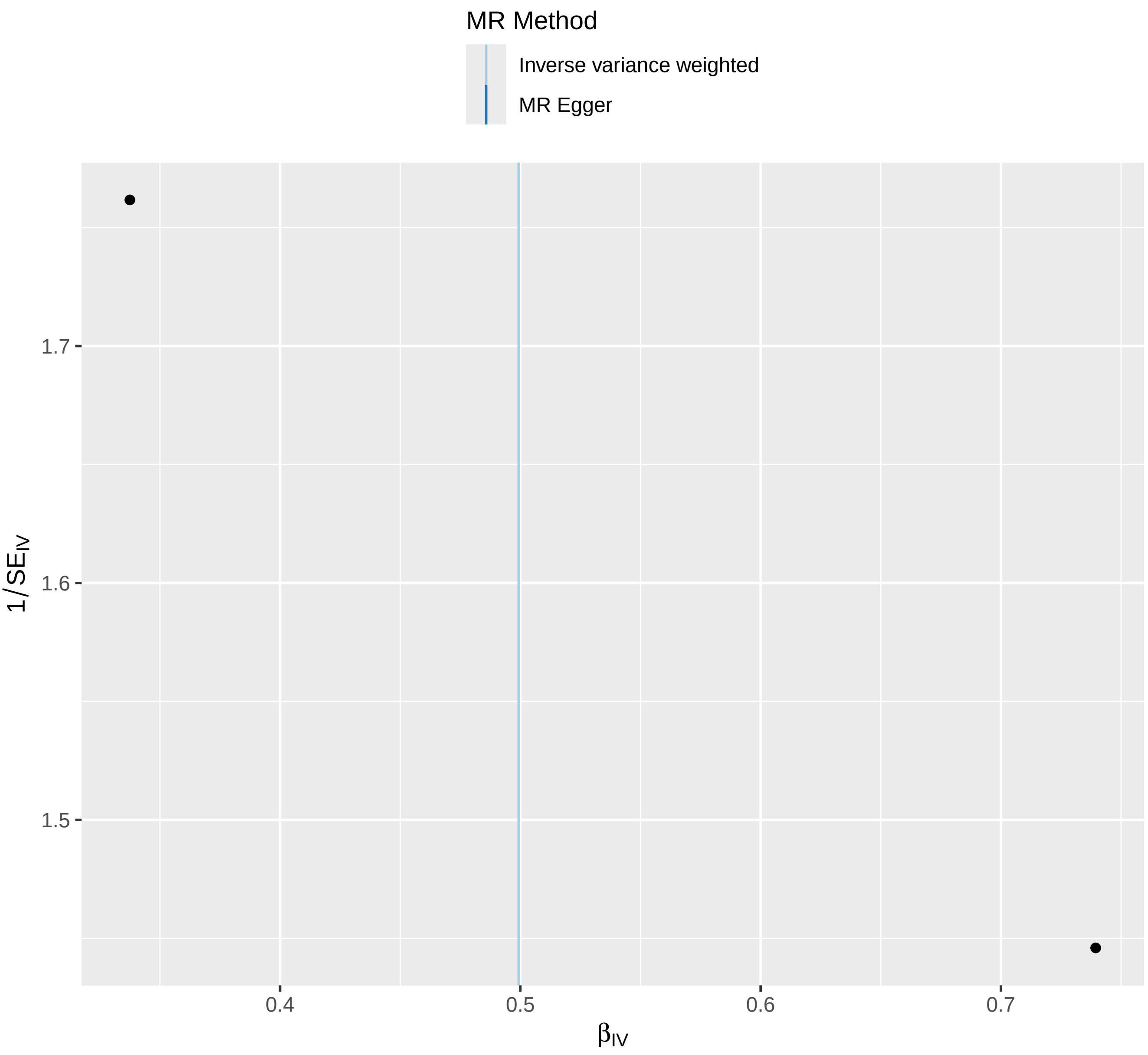
MR Method
Inverse variance weighted
MR Egger
1.7
1.6
1.5
0.4
0.5
0.6
0.7
β_{IV}
$1/SE_{IV}$

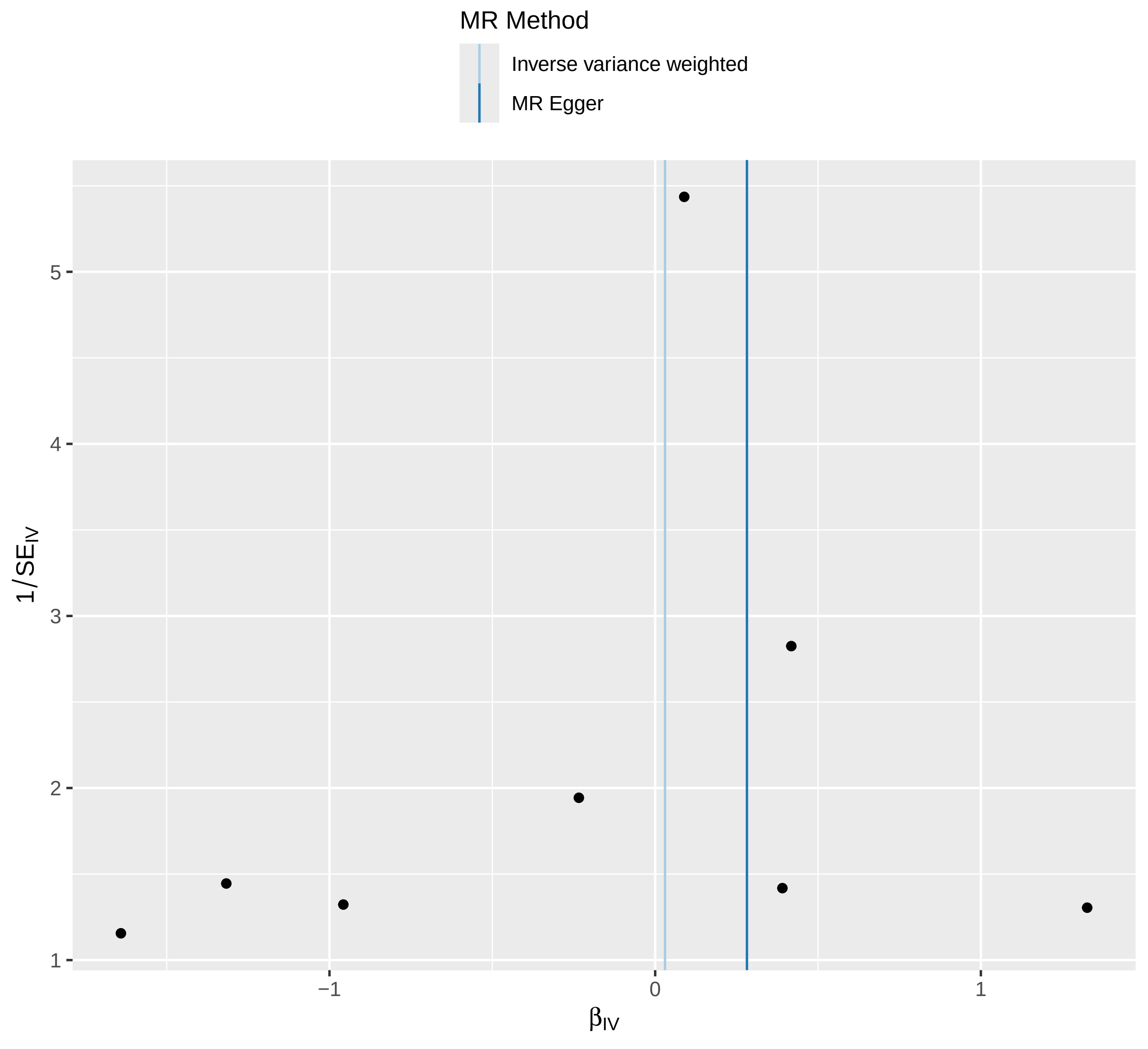
MR Method
Inverse variance weighted
MR Egger
1/SE_{IV}
5
4
3
2
1
−1
0
1
β_{IV}

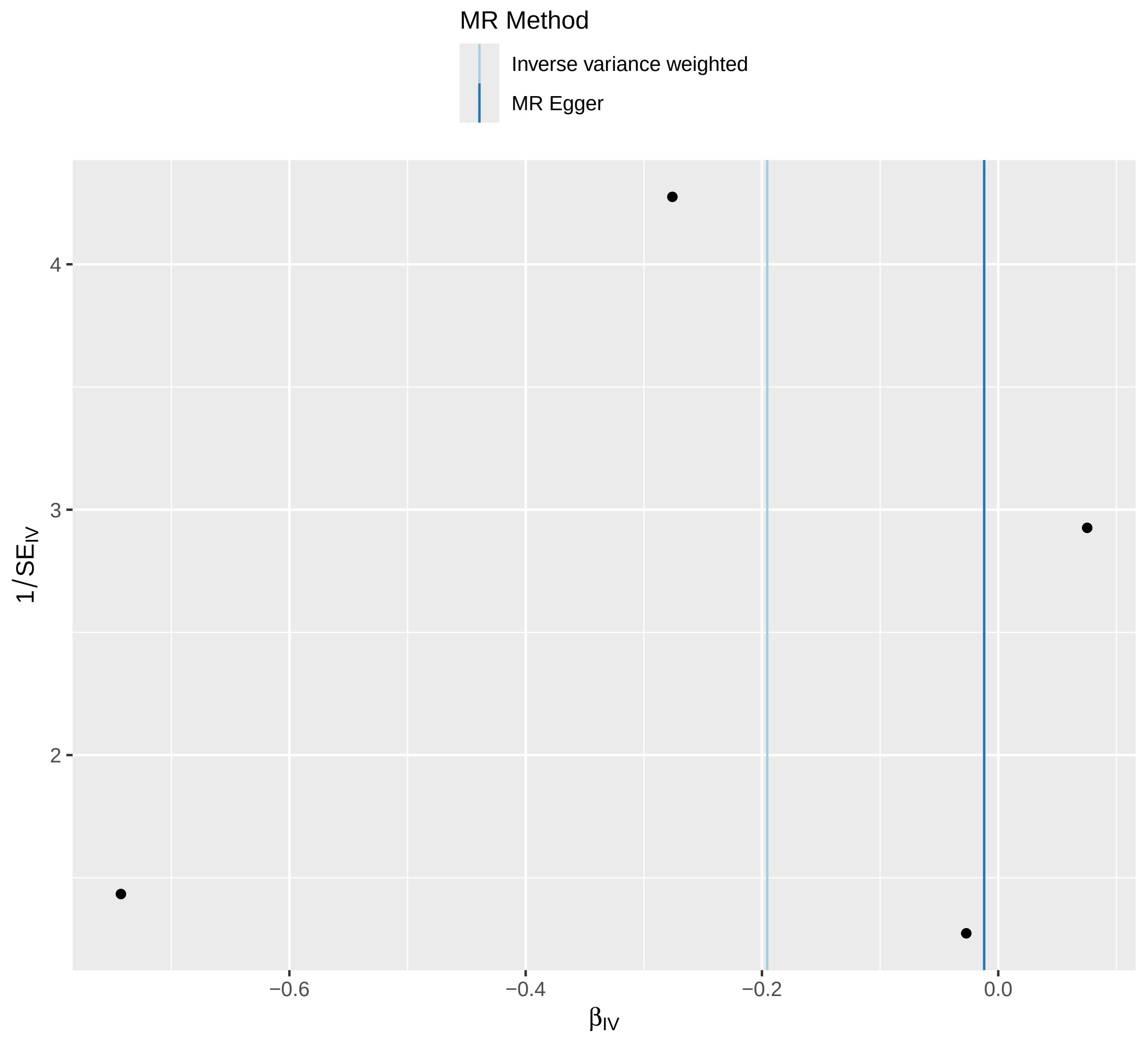

MR Method
Inverse variance weighted
MR Egger
4
3
2
1/SE_IV
−0.6
−0.4
−0.2
0.0
β_IV

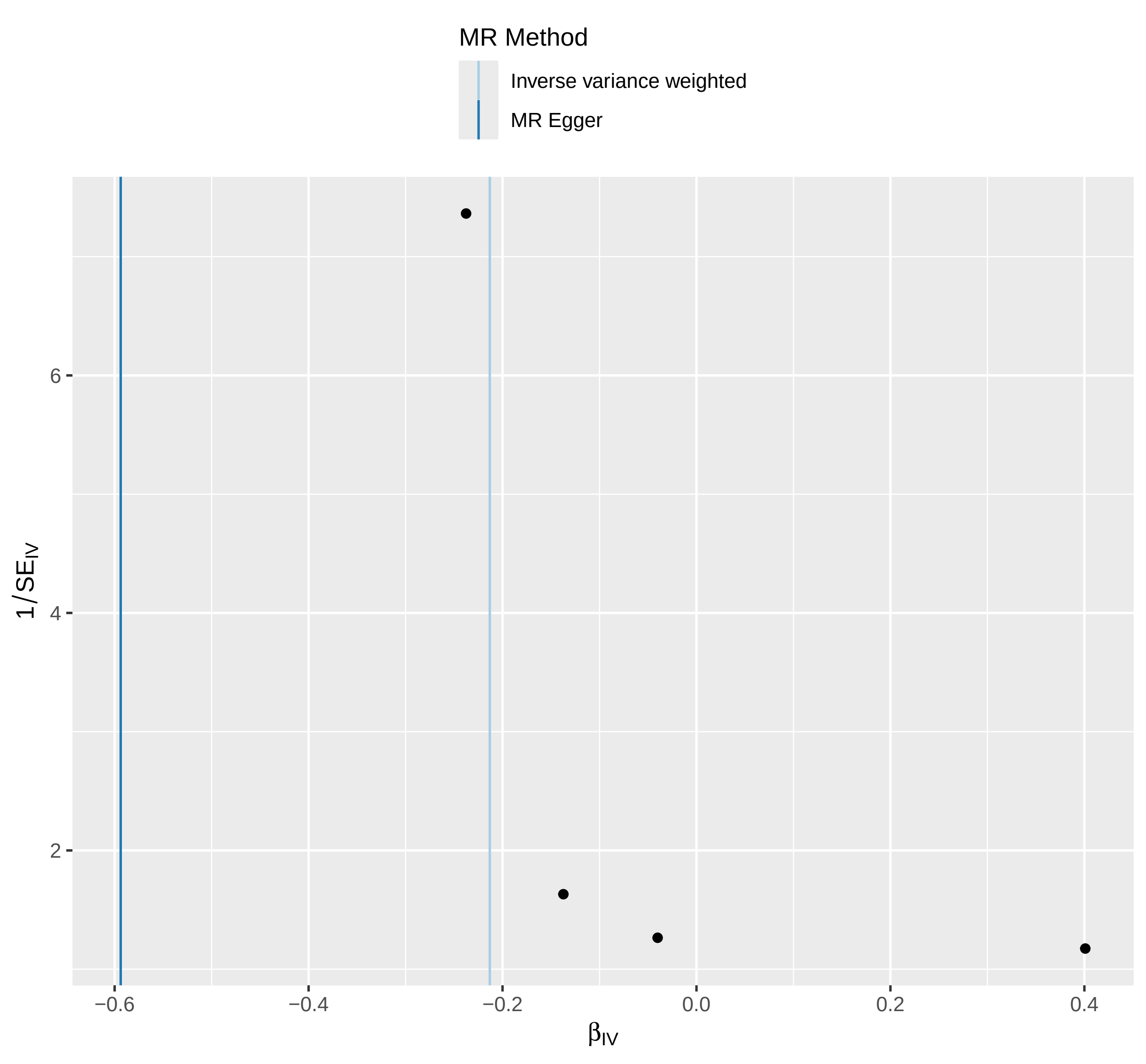

MR Method
Inverse variance weighted
MR Egger
6
4
2
1/SE_IV
−0.6
−0.4
−0.2
0.0
0.2
0.4
β_IV

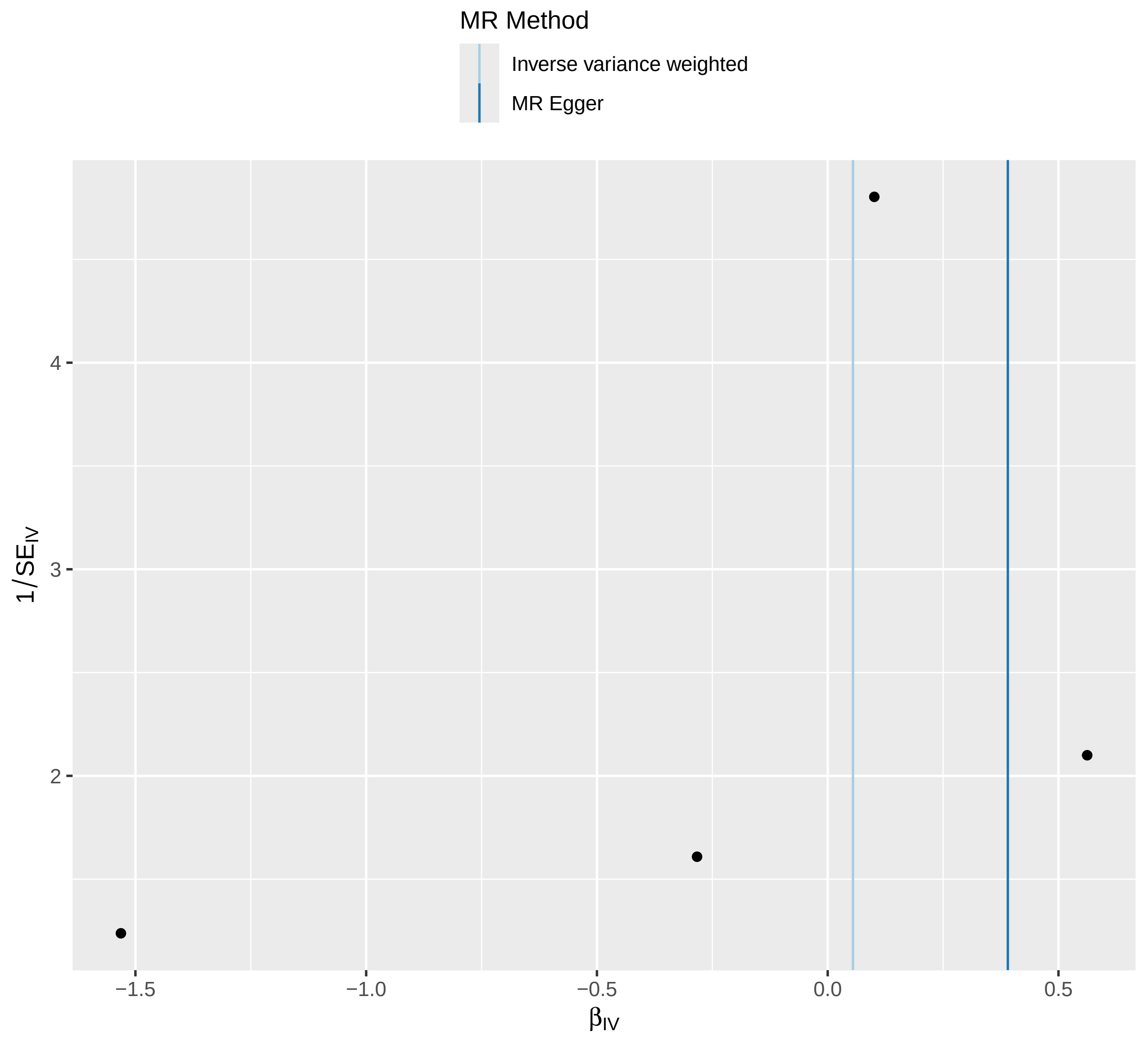

MR Method
Inverse variance weighted
MR Egger
4
3
2
1/SE_IV
−1.5
−1.0
−0.5
0.0
0.5
β_IV

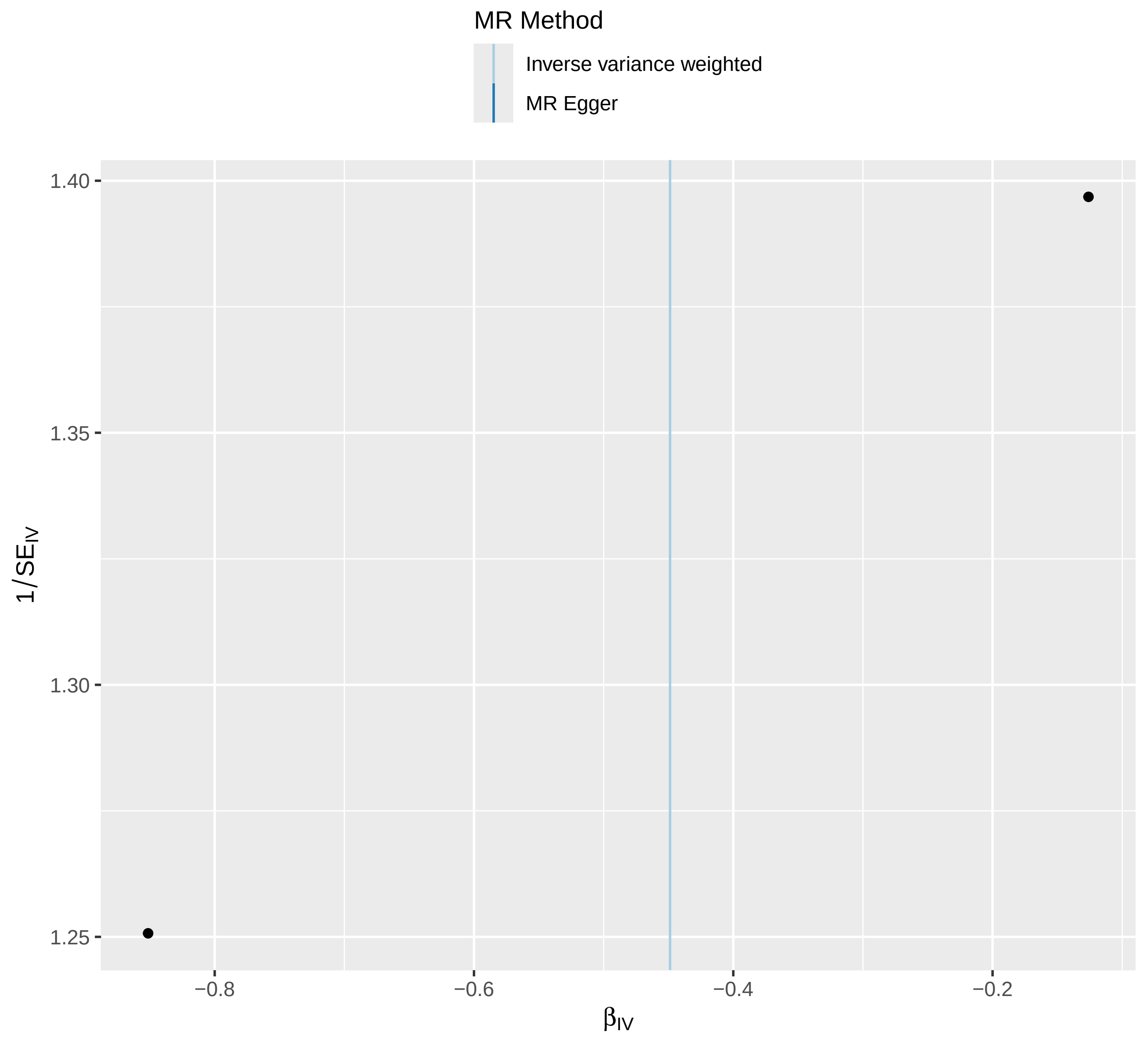

MR Method
Inverse variance weighted
MR Egger
1.40
1.35
1.30
1.25
1/SE_IV
−0.8
−0.6
−0.4
−0.2
β_IV

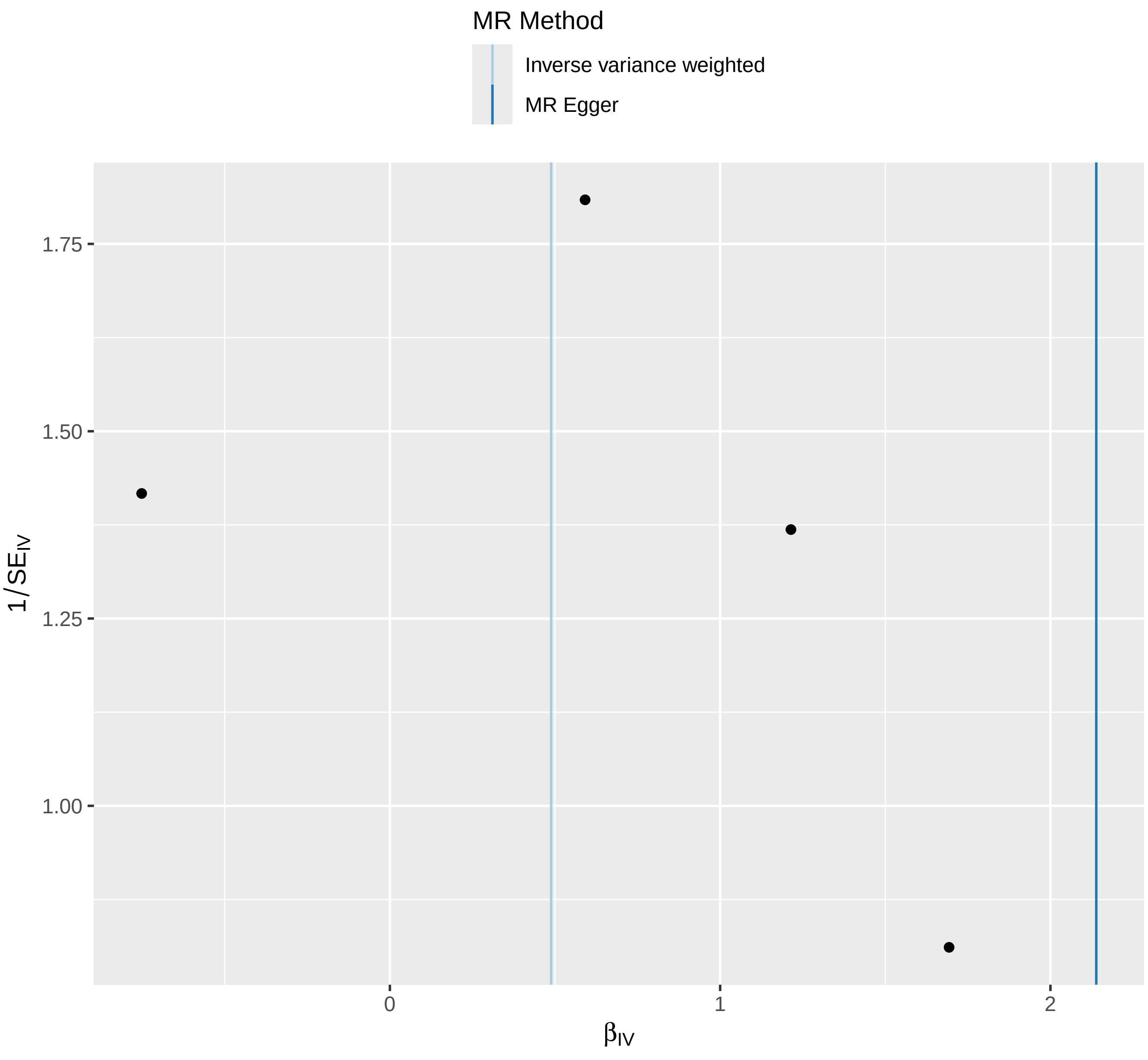
MR Method
Inverse variance weighted
MR Egger
1.75
1.50
1.25
1.00
1/SE_IV
0
1
2
β_IV

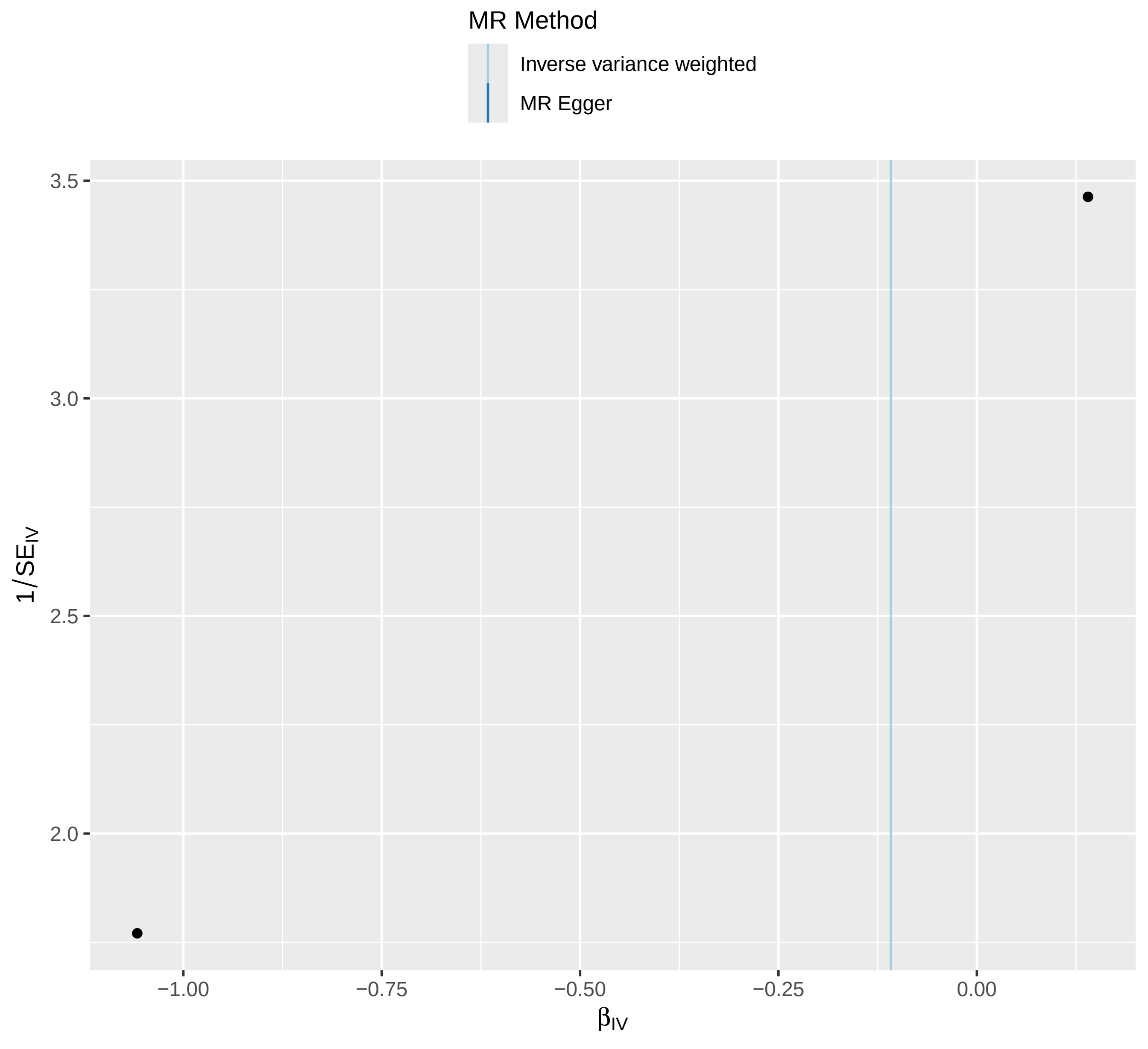
MR Method
Inverse variance weighted
MR Egger
3.5
3.0
2.5
2.0
1/SE_IV
−1.00
−0.75
−0.50
−0.25
0.00
β_IV

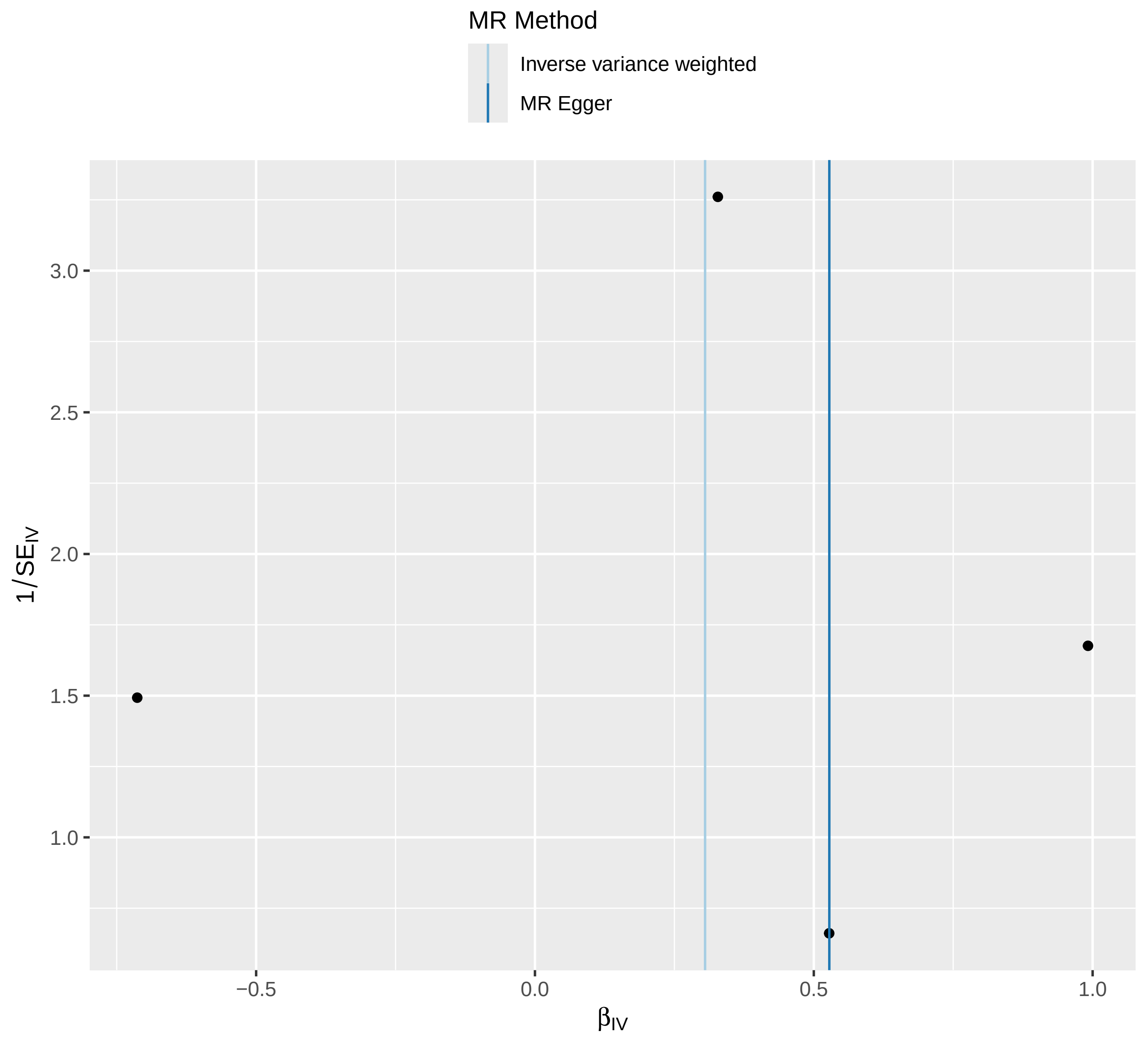
MR Method
Inverse variance weighted
MR Egger
3.0
2.5
2.0
1.5
1.0
$1/SE_{IV}$
−0.5
0.0
0.5
1.0
β_{IV}

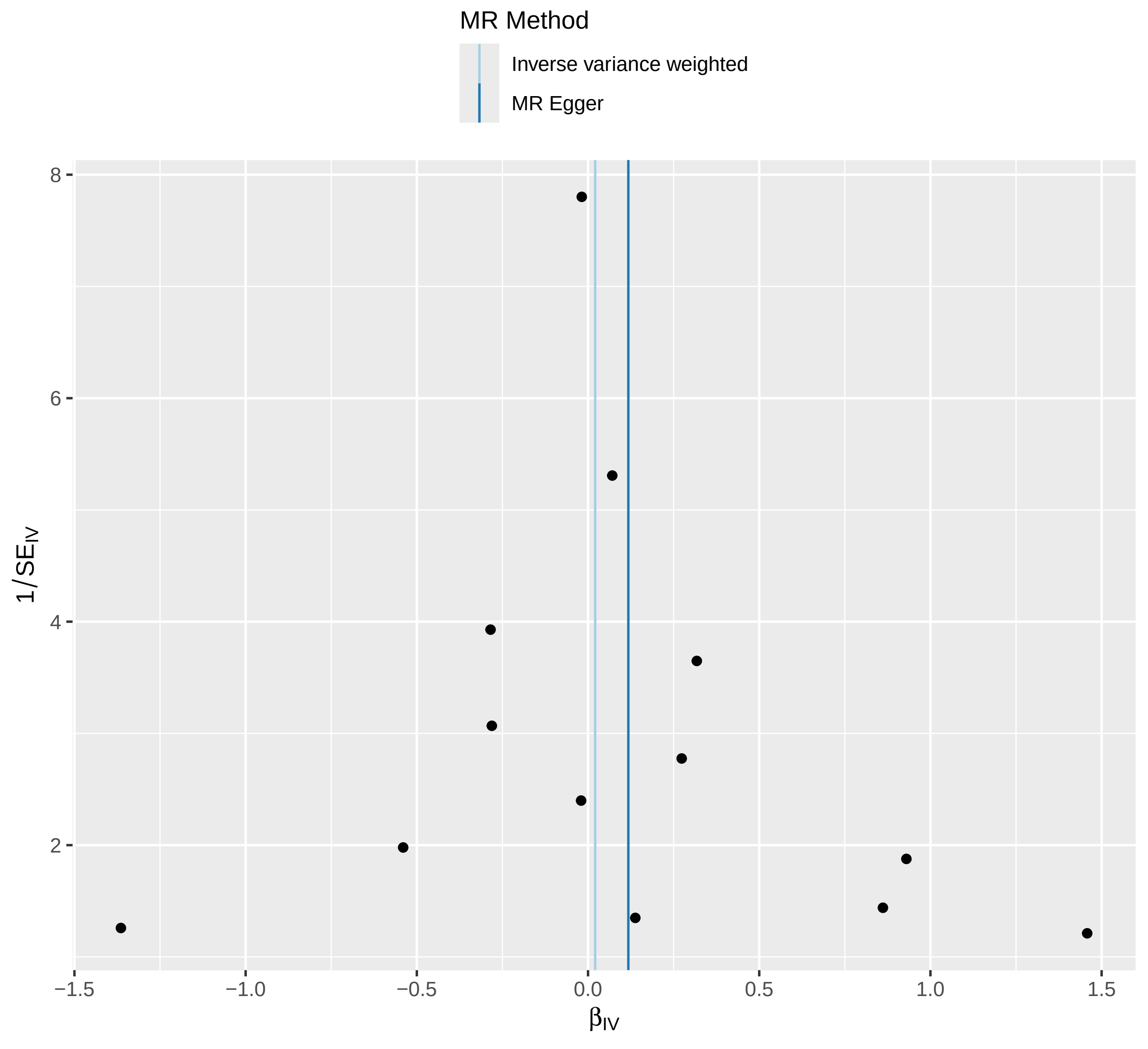

MR Method
Inverse variance weighted
MR Egger
8
6
4
2
1/SE_IV
−1.5
−1.0
−0.5
0.0
0.5
1.0
1.5
β_IV

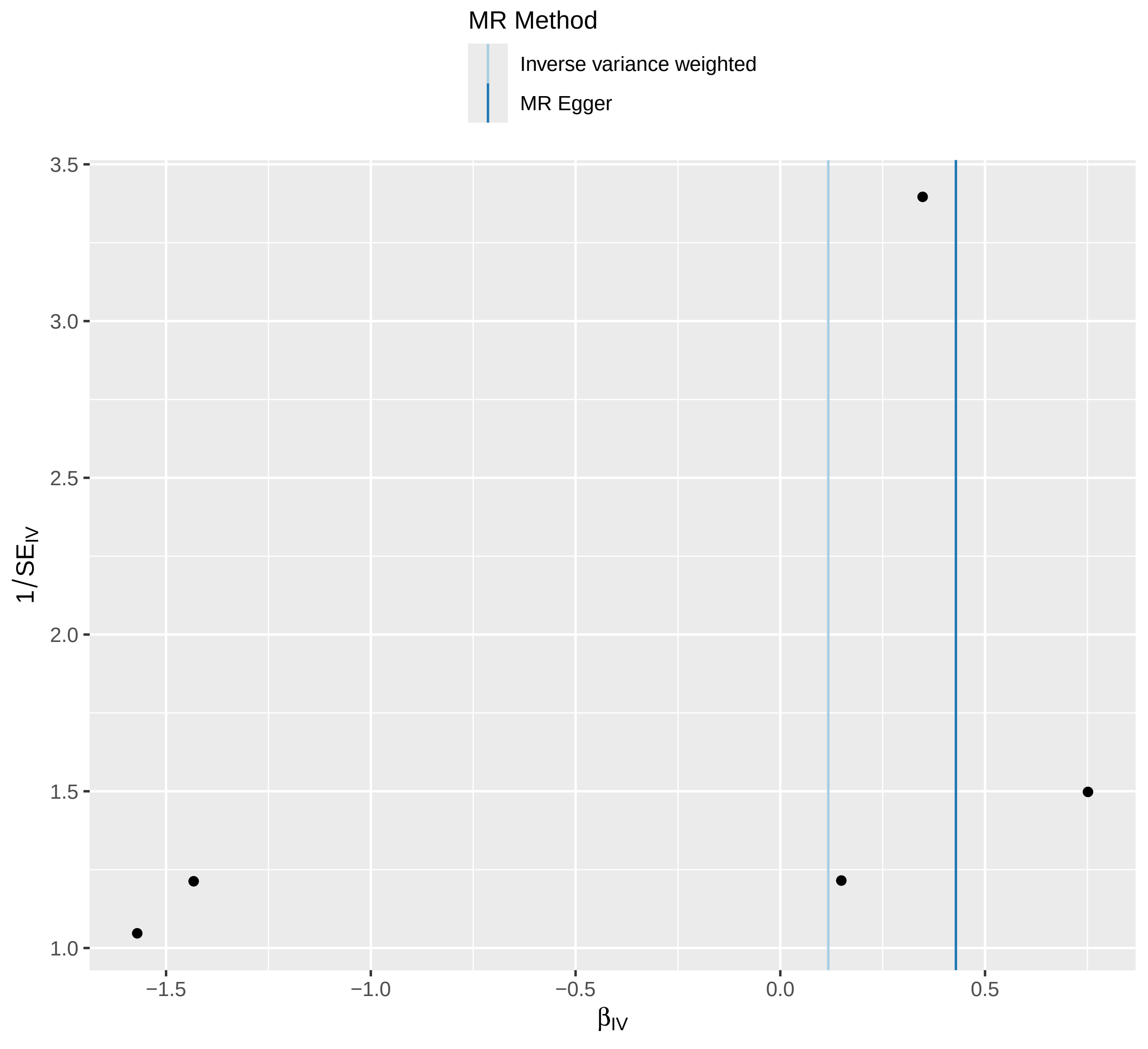

MR Method
Inverse variance weighted
MR Egger
3.5
3.0
2.5
2.0
1.5
1.0
1/SE_IV
−1.5
−1.0
−0.5
0.0
0.5
β_IV

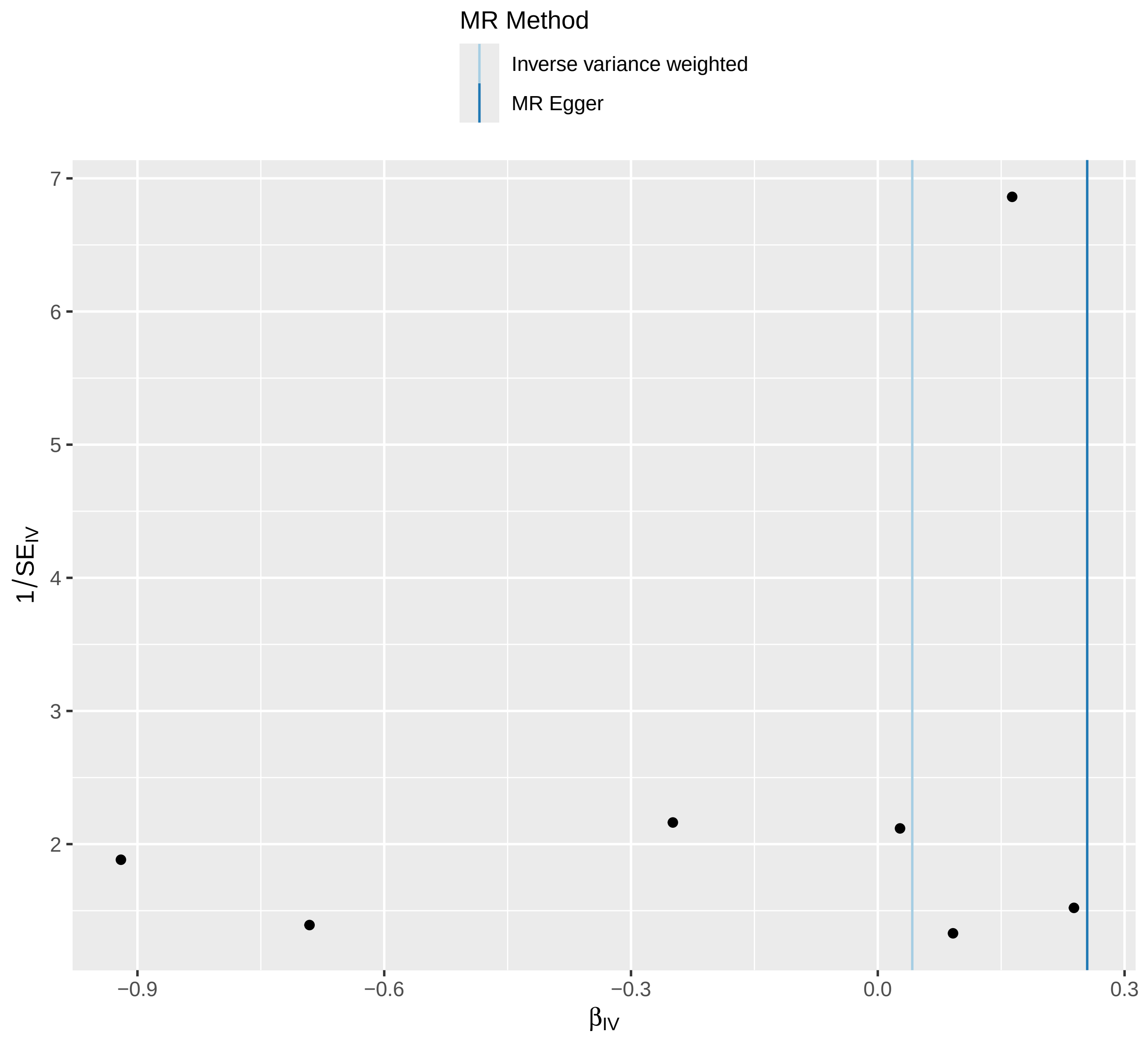
MR Method
Inverse variance weighted
MR Egger
1/SE_{IV}
7
6
5
4
3
2
−0.9
−0.6
−0.3
0.0
0.3
β_{IV}

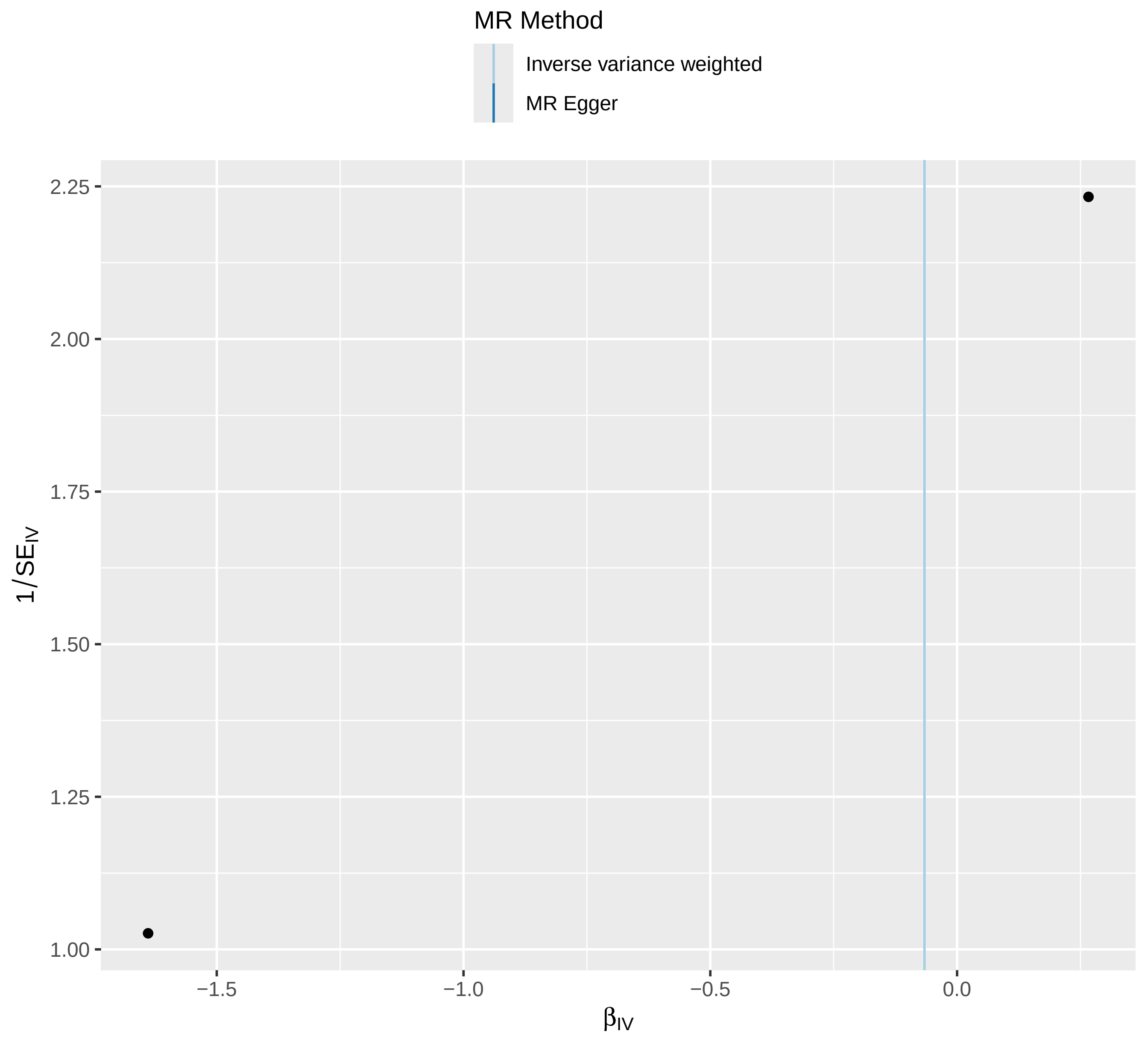

MR Method
Inverse variance weighted
MR Egger
2.25
2.00
1.75
1.50
1.25
1.00
1/SE_IV
−1.5
−1.0
−0.5
0.0
β_IV

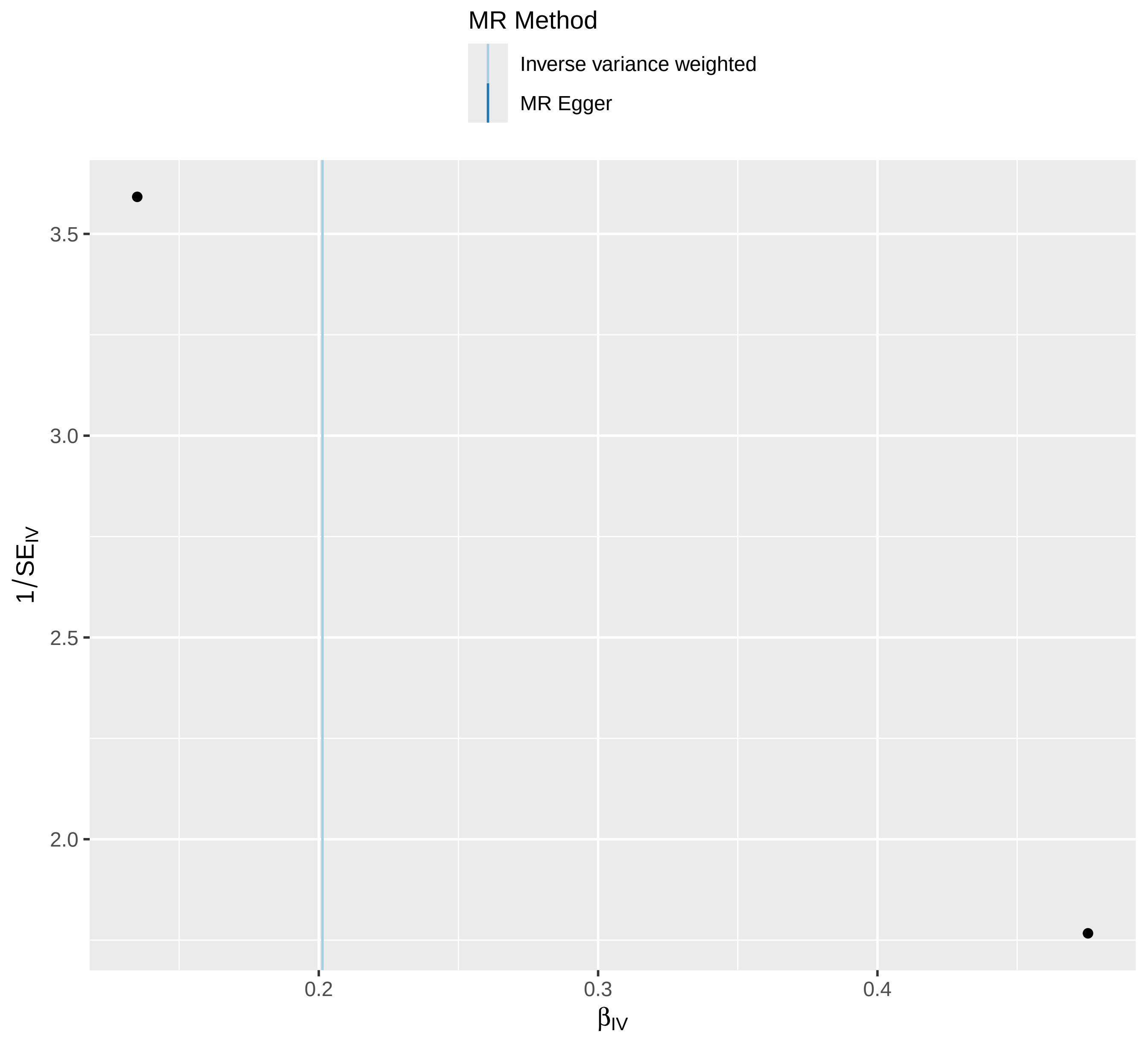

MR Method
Inverse variance weighted
MR Egger
3.5
3.0
2.5
2.0
1/SE_IV
0.2
0.3
0.4
β_IV

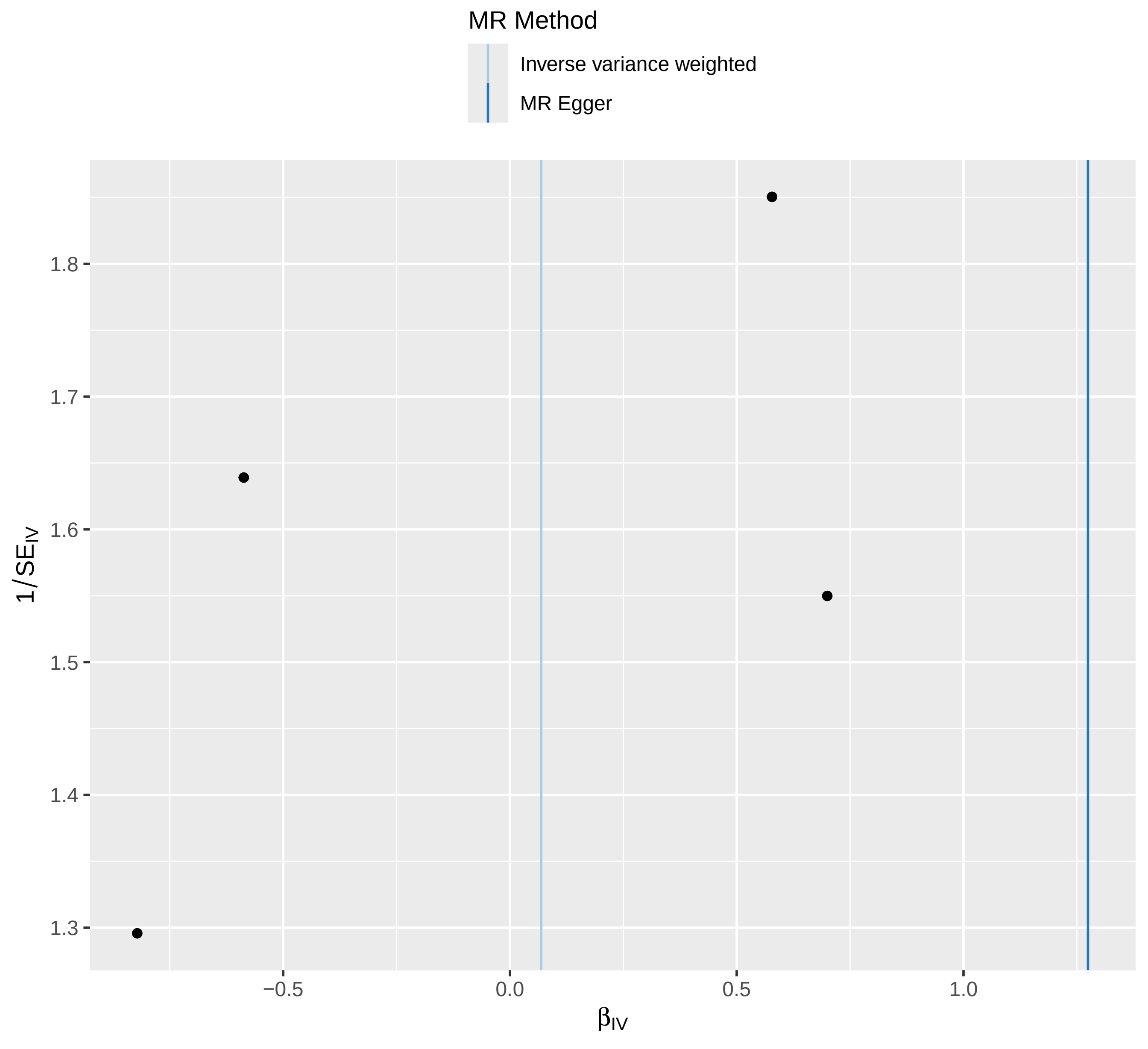
MR Method
Inverse variance weighted
MR Egger
1.8
1.7
1.6
1.5
1.4
1.3
1/SE_IV
−0.5
0.0
0.5
1.0
β_IV

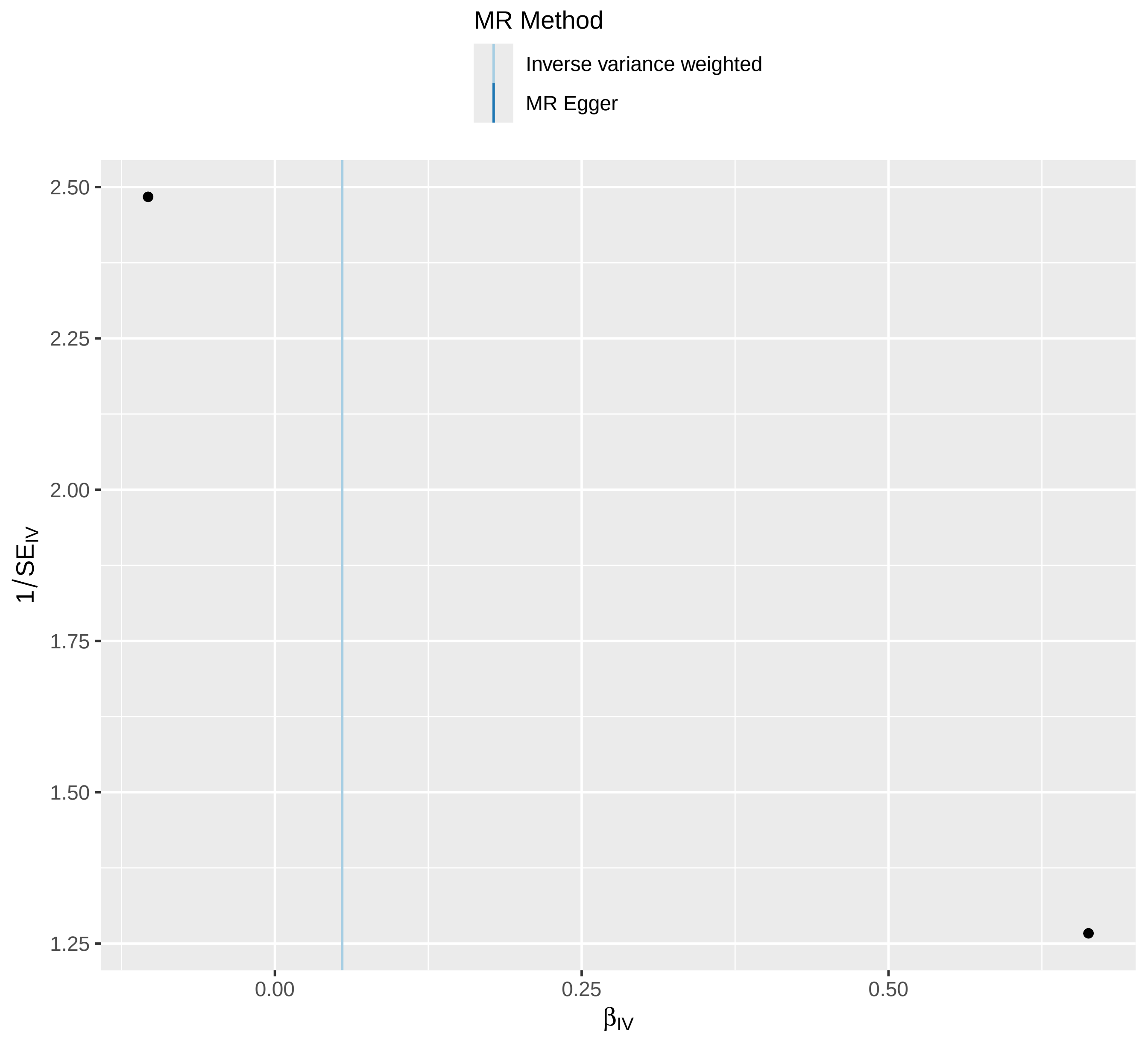
MR Method
Inverse variance weighted
MR Egger
2.50
2.25
2.00
1.75
1.50
1.25
$1/SE_{IV}$
0.00
0.25
0.50
β_{IV}

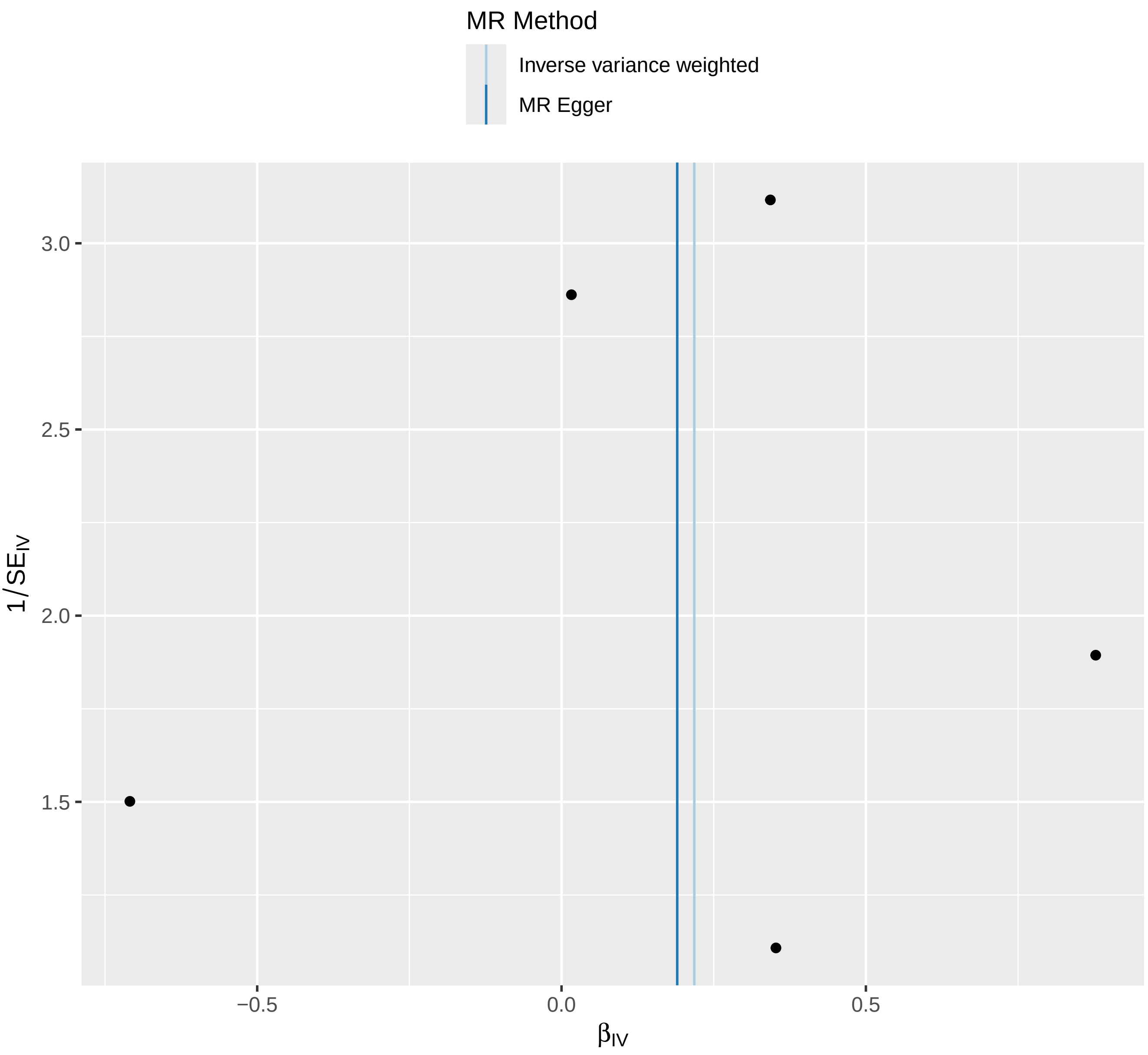

MR Method
Inverse variance weighted
MR Egger
3.0
2.5
2.0
1.5
1/SE$_{IV}$
−0.5
0.0
0.5
β$_{IV}$

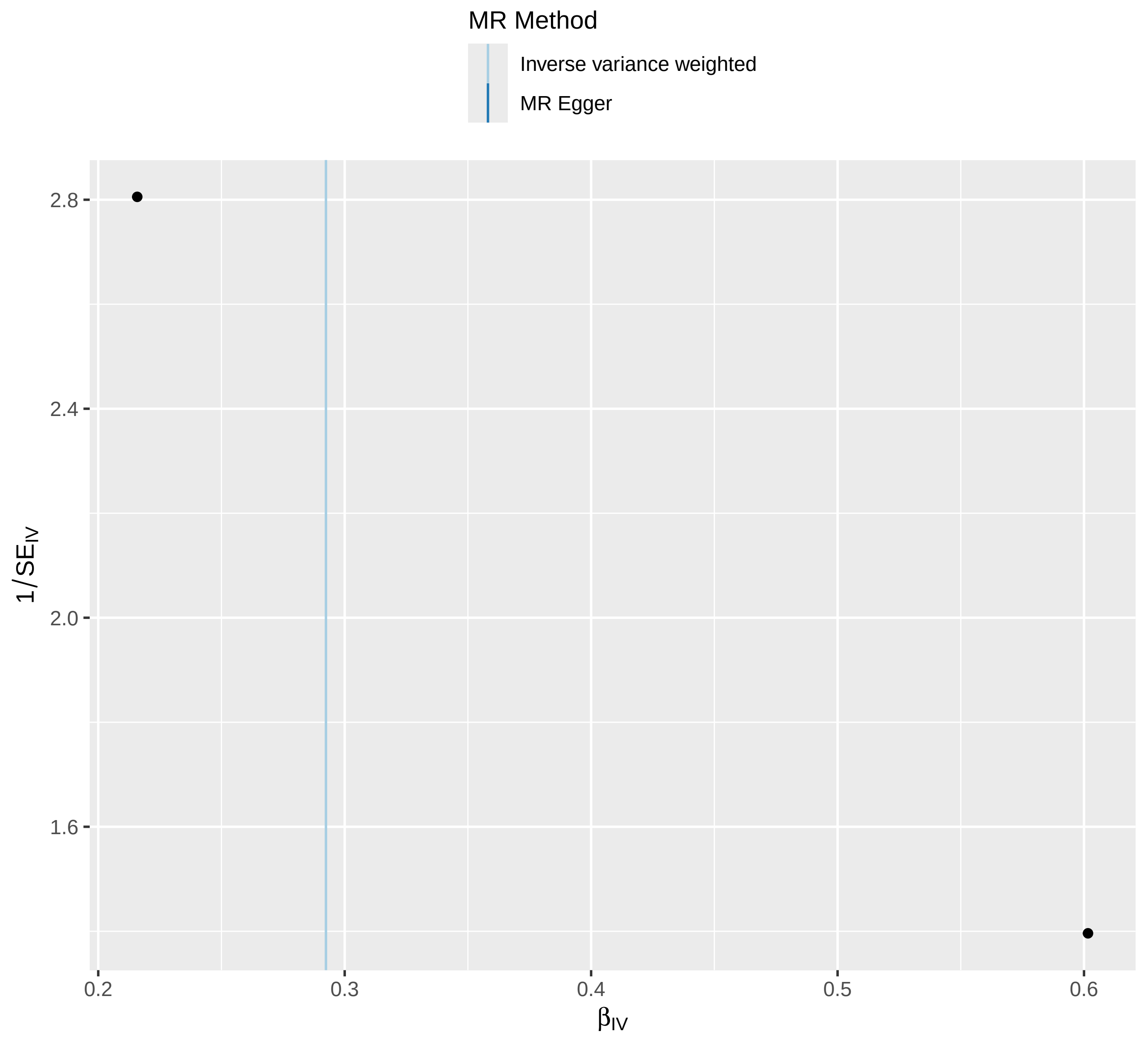

MR Method
Inverse variance weighted
MR Egger
2.8
2.4
2.0
1.6
1/SE_IV
0.2
0.3
0.4
0.5
0.6
β_IV

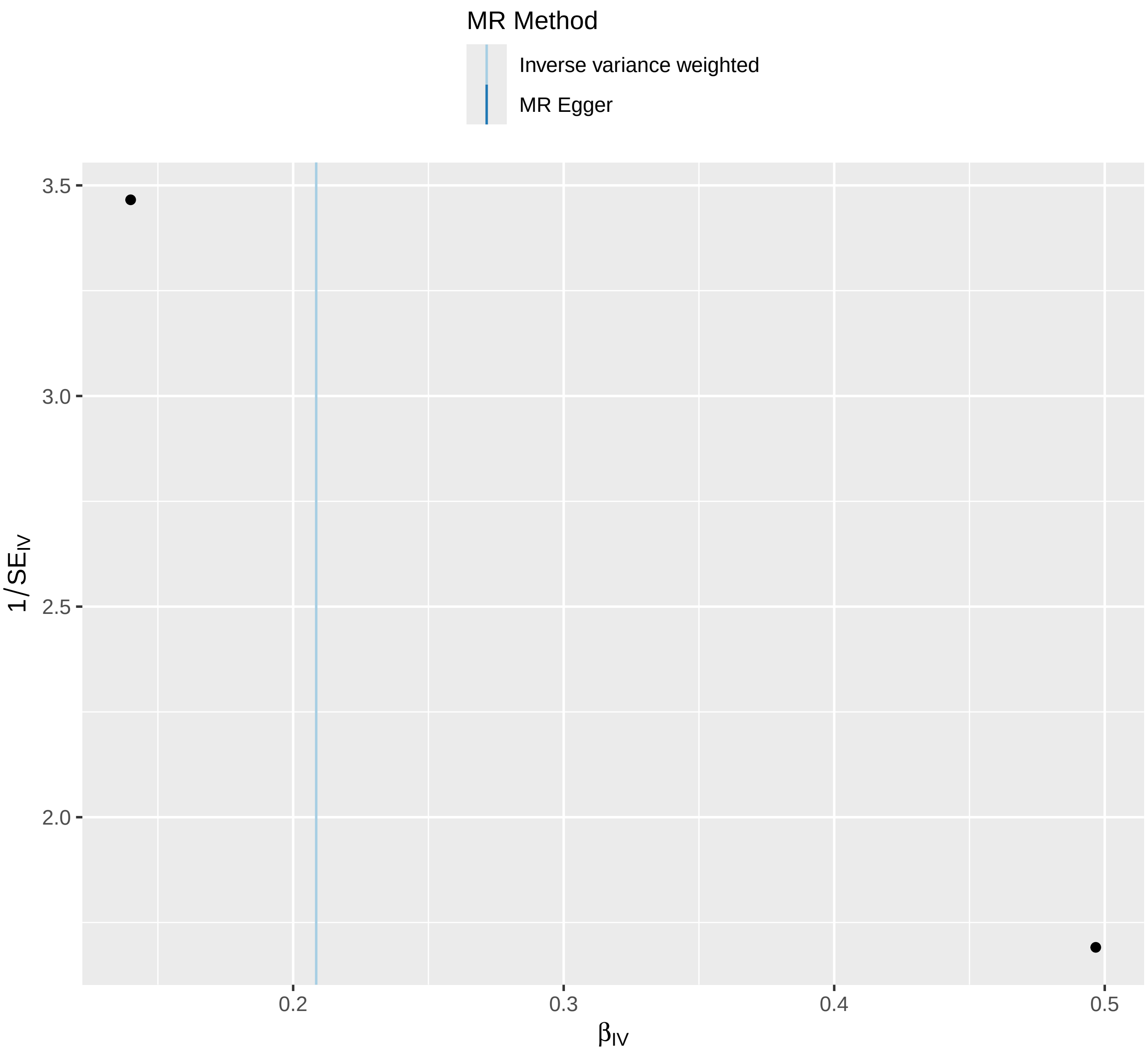
MR Method
Inverse variance weighted
MR Egger
3.5
3.0
2.5
2.0
$1/SE_{IV}$
0.2
0.3
0.4
0.5
β_{IV}

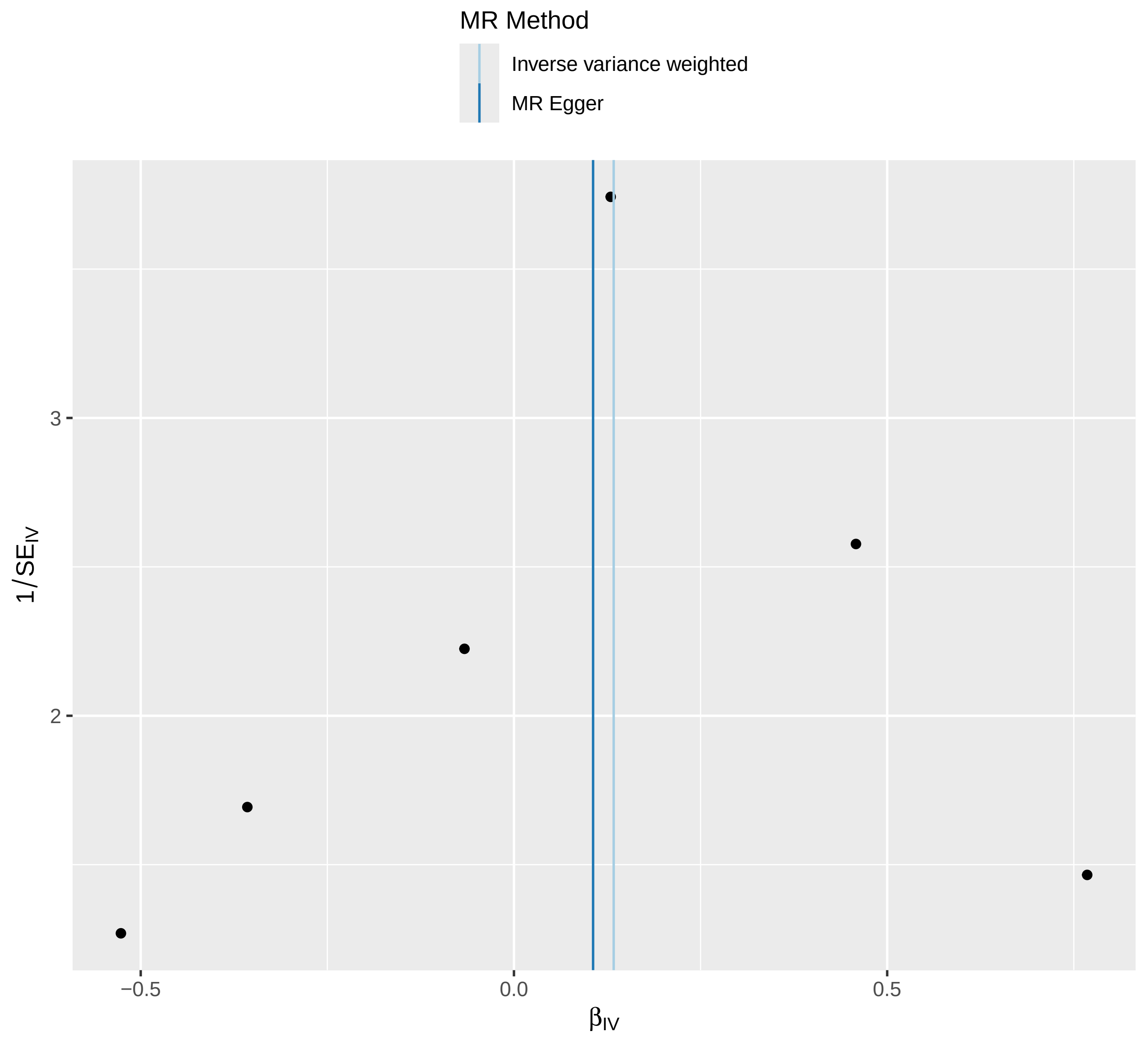

MR Method
Inverse variance weighted
MR Egger
3
2
1/SE_IV
−0.5
0.0
0.5
β_IV

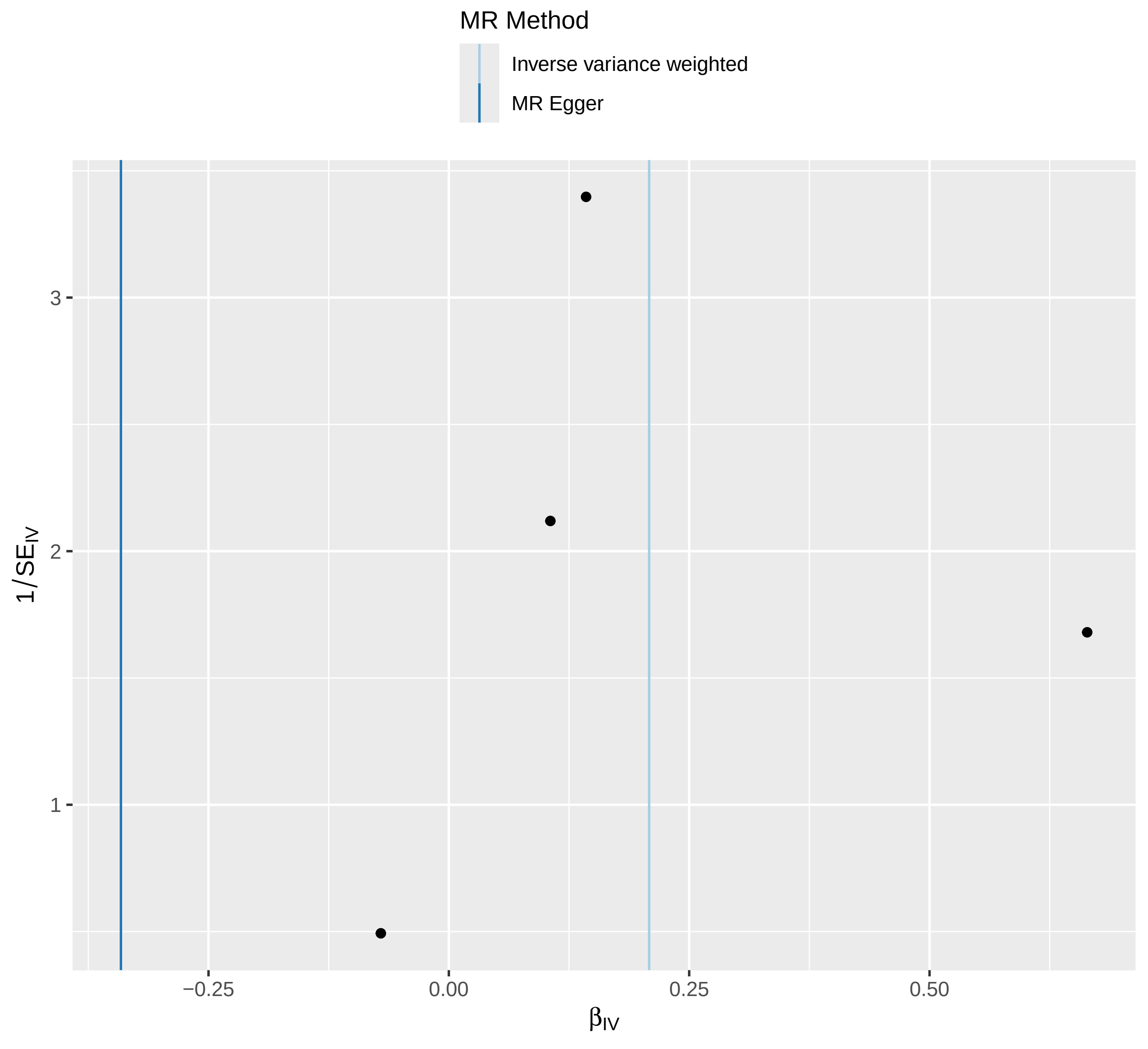

MR Method
Inverse variance weighted
MR Egger
3
2
1
$1/SE_{IV}$
−0.25
0.00
0.25
0.50
β_{IV}

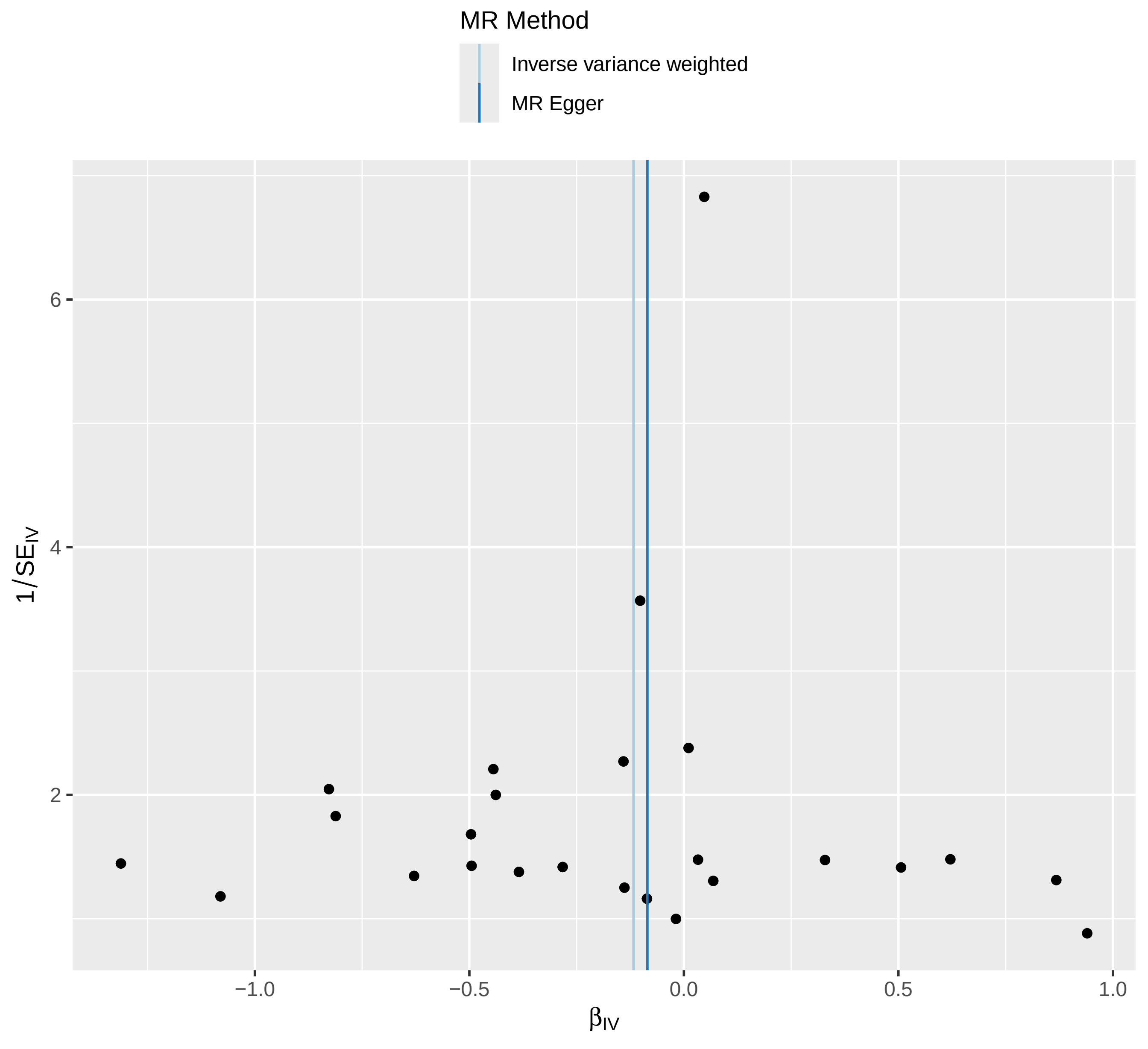
MR Method
Inverse variance weighted
MR Egger
6
4
2
−1.0
−0.5
0.0
0.5
1.0
$1/SE_{IV}$
β_{IV}

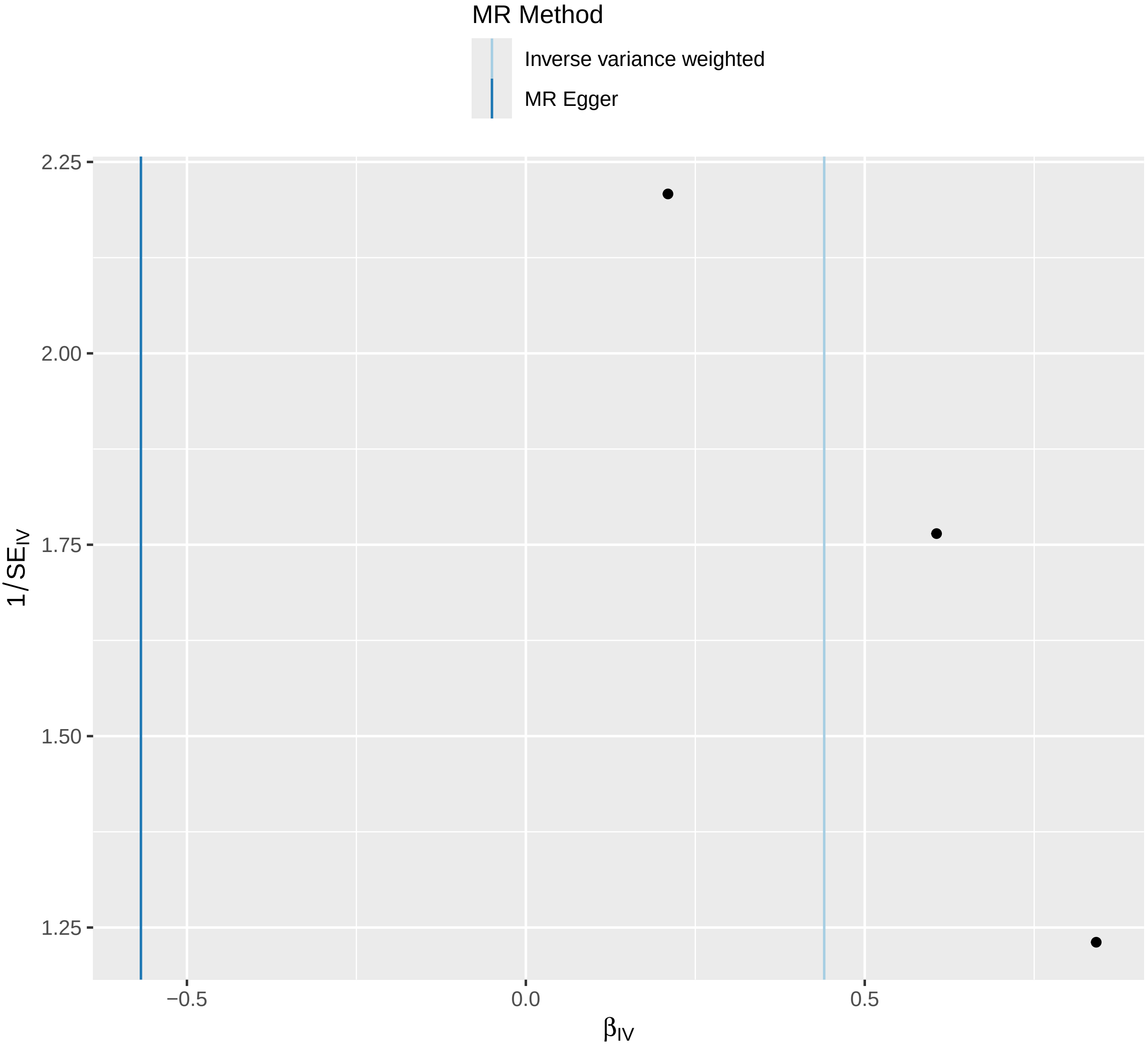
MR Method
Inverse variance weighted
MR Egger
2.25
2.00
1.75
1.50
1.25
$1/SE_{IV}$
−0.5
0.0
0.5
β_{IV}

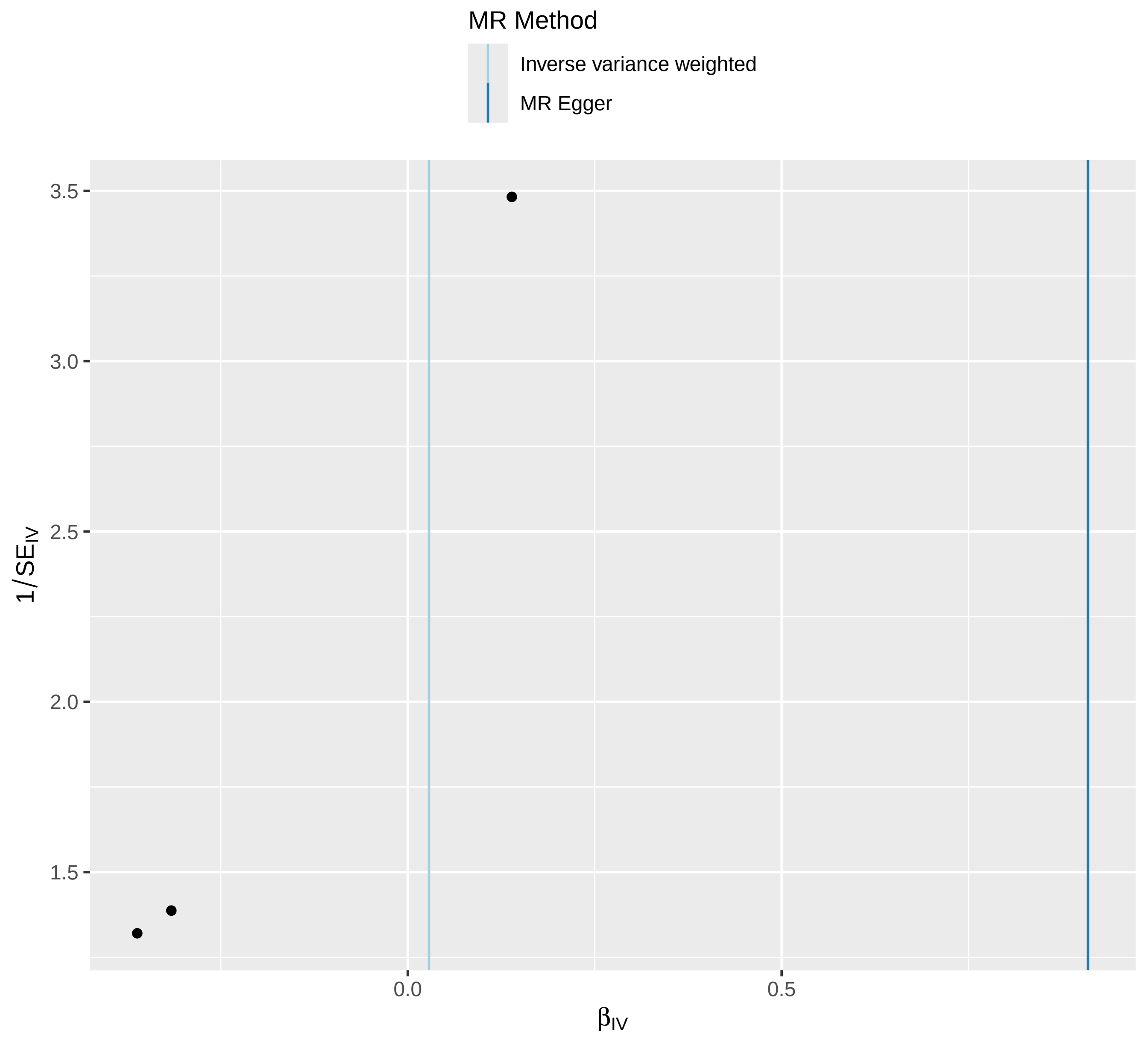

MR Method
Inverse variance weighted
MR Egger
3.5
3.0
2.5
2.0
1.5
1/SE$_{IV}$
0.0
0.5
β$_{IV}$

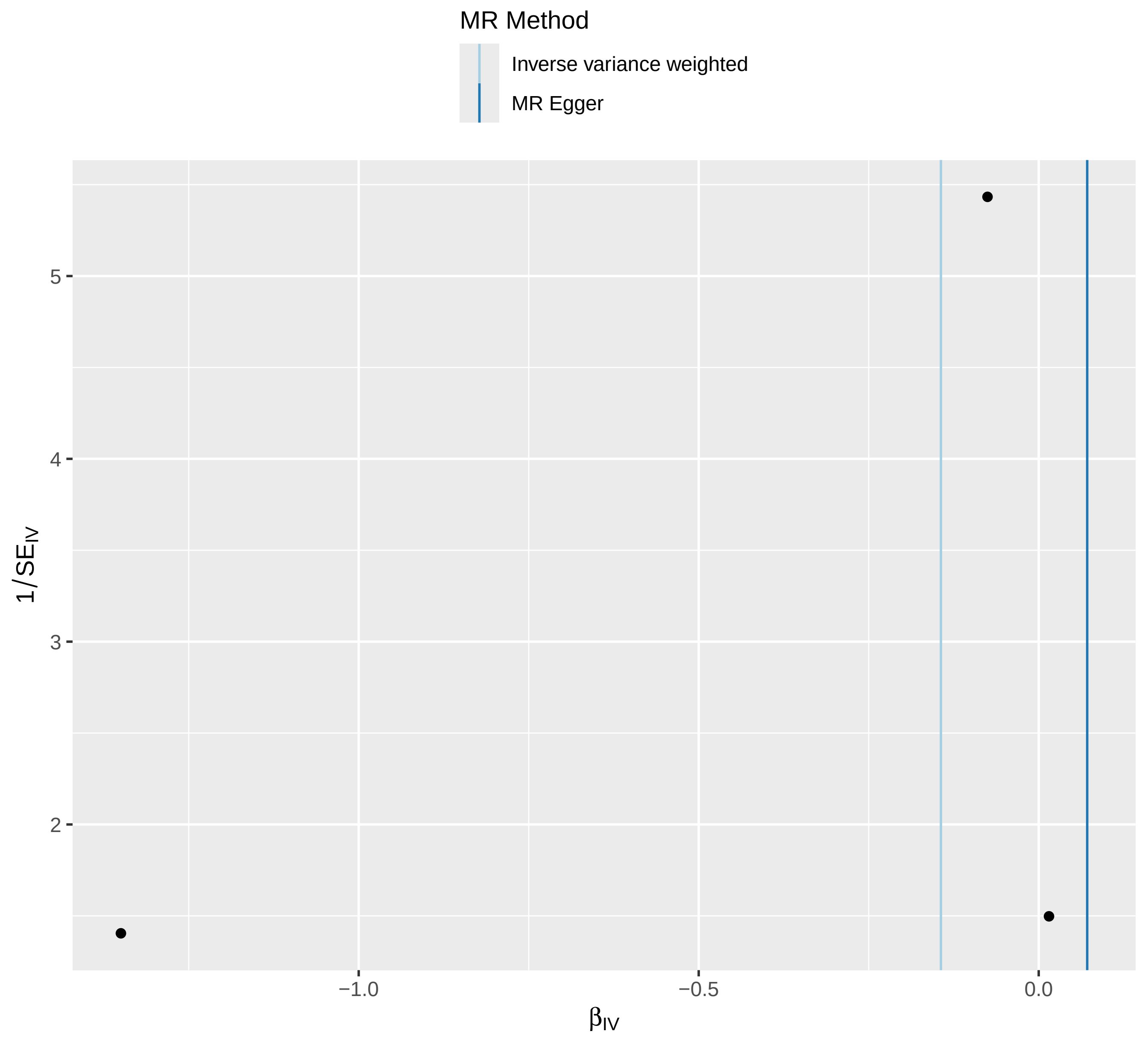
MR Method
Inverse variance weighted
MR Egger
1/SE_IV
5
4
3
2
−1.0
−0.5
0.0
β_IV

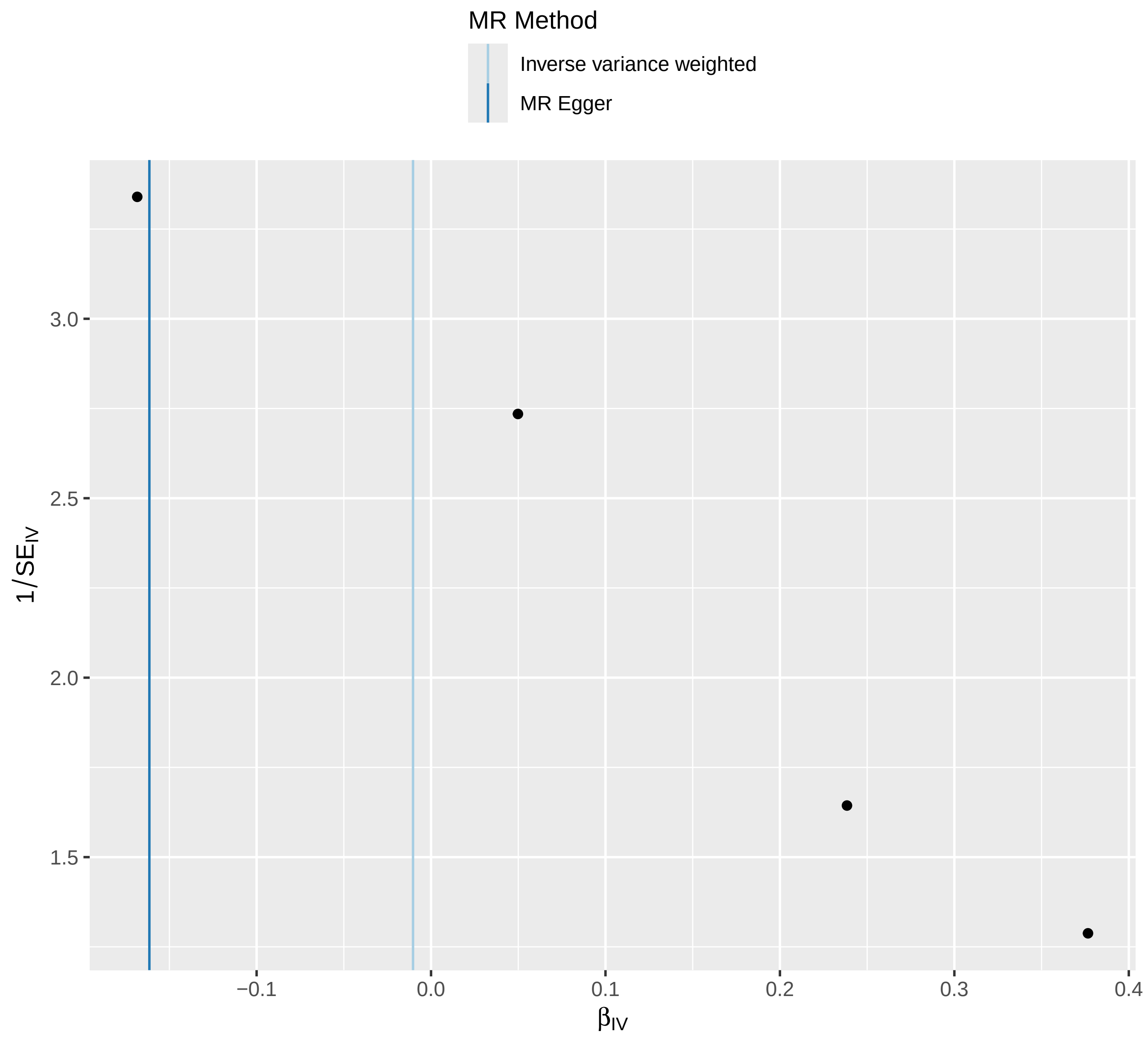
MR Method
Inverse variance weighted
MR Egger
3.0
2.5
2.0
1.5
1/SE_IV
−0.1
0.0
0.1
0.2
0.3
0.4
β_IV

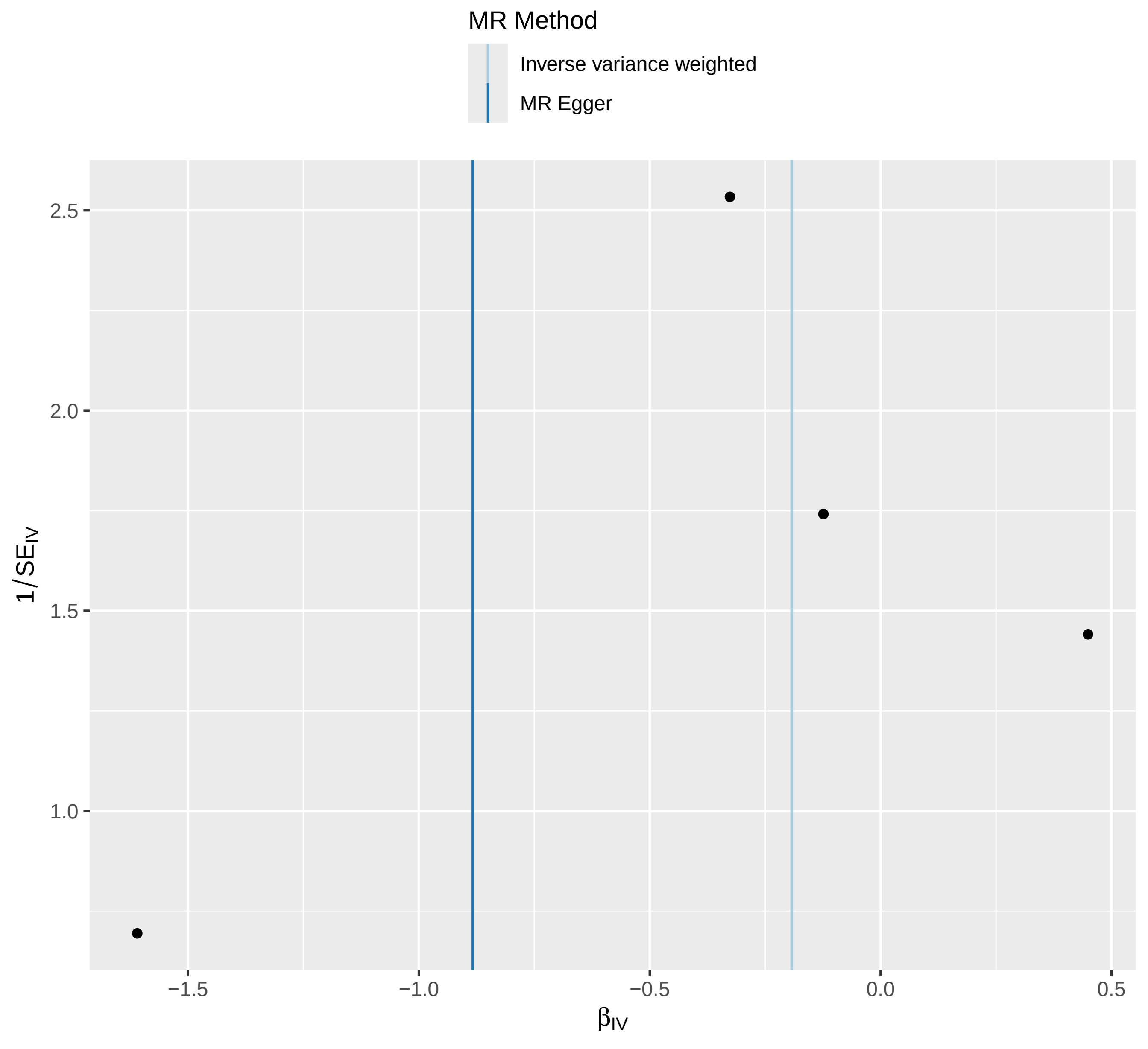

MR Method
Inverse variance weighted
MR Egger
2.5
2.0
1.5
1.0
1/SE_IV
−1.5
−1.0
−0.5
0.0
0.5
β_IV

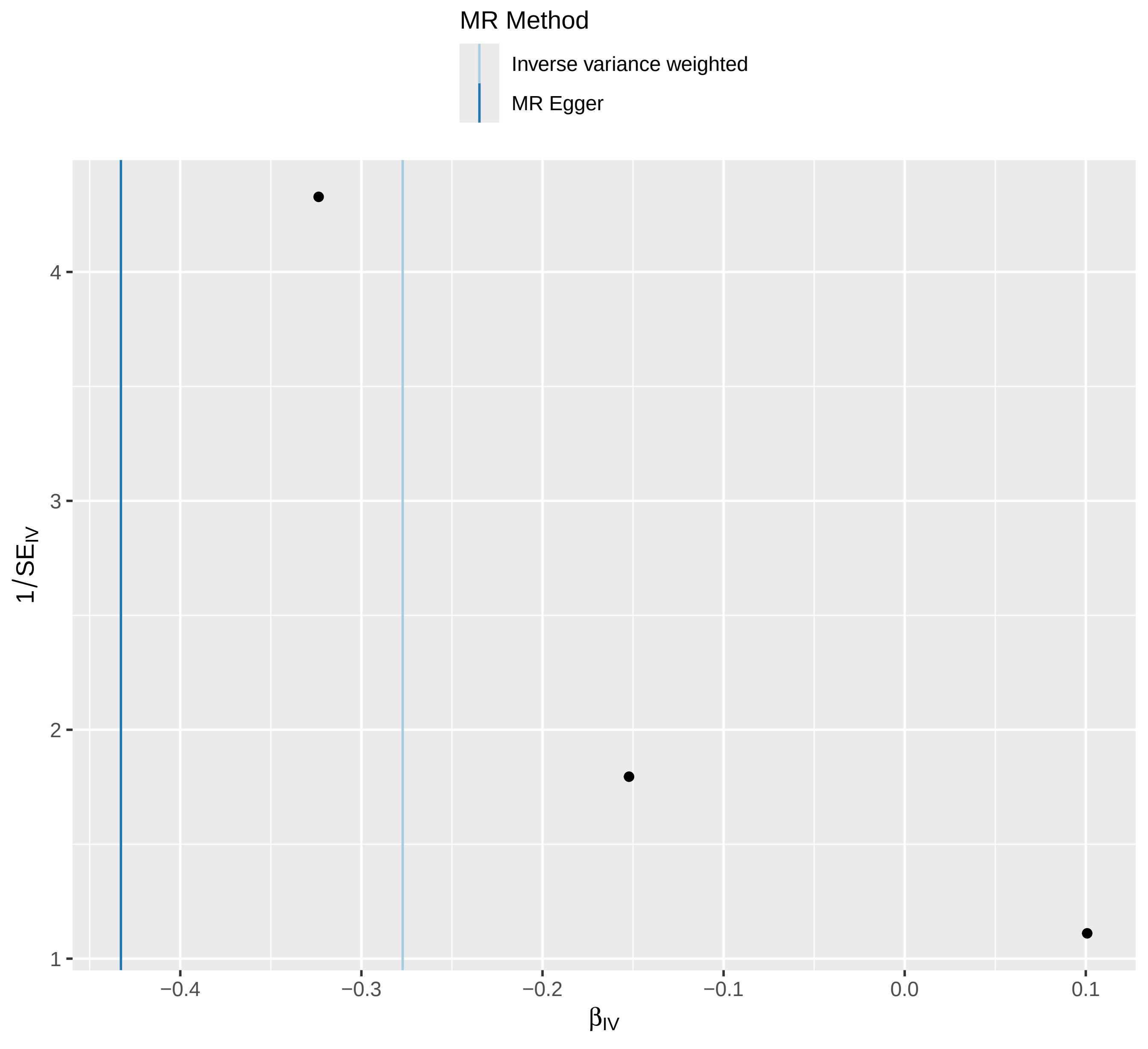

MR Method
Inverse variance weighted
MR Egger
4
3
2
1
1/SE$_{IV}$
−0.4
−0.3
−0.2
−0.1
0.0
0.1
β_{IV}

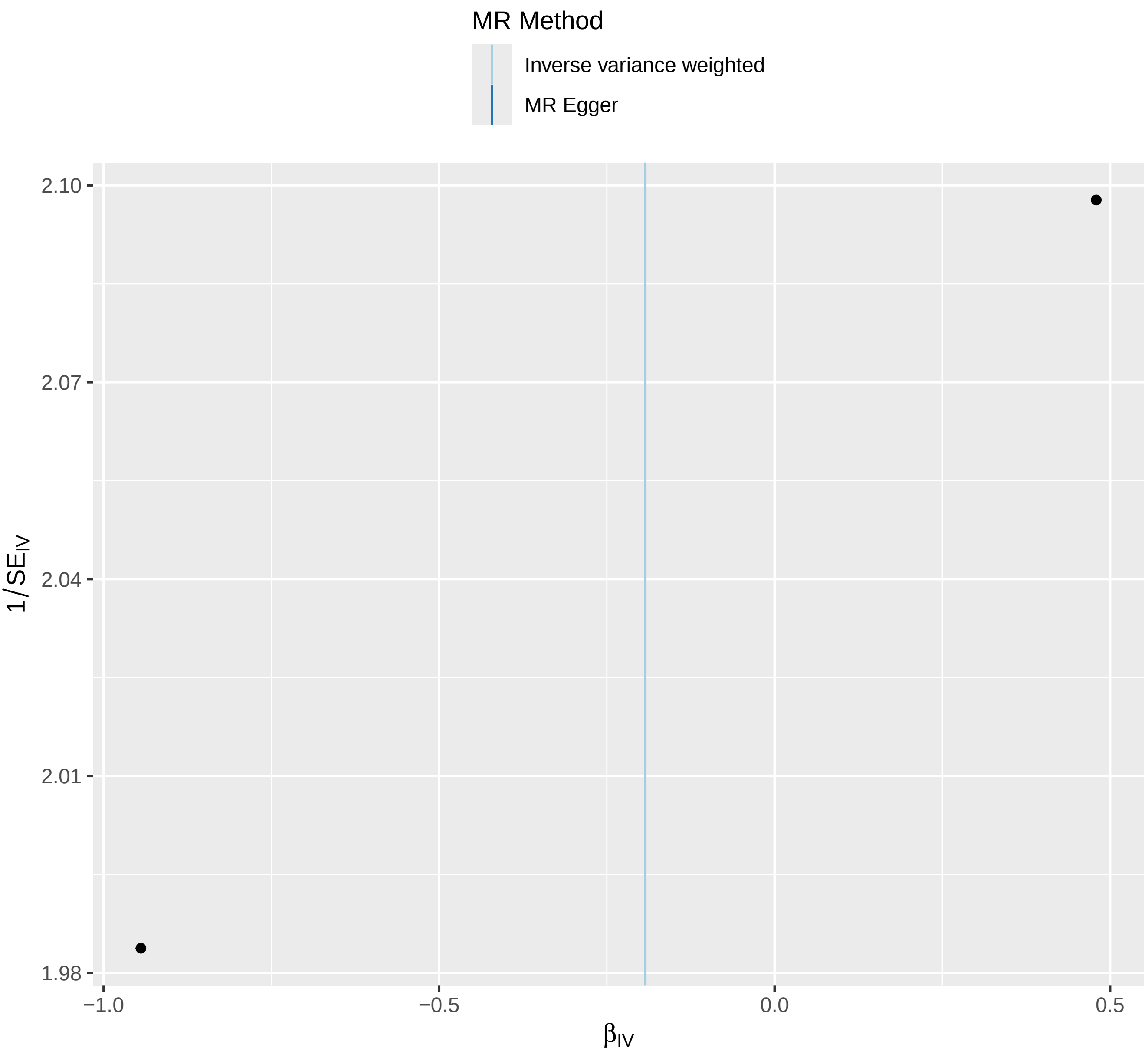

MR Method
Inverse variance weighted
MR Egger
2.10
2.07
2.04
2.01
1.98
1/SE_IV
−1.0
−0.5
0.0
0.5
β_IV

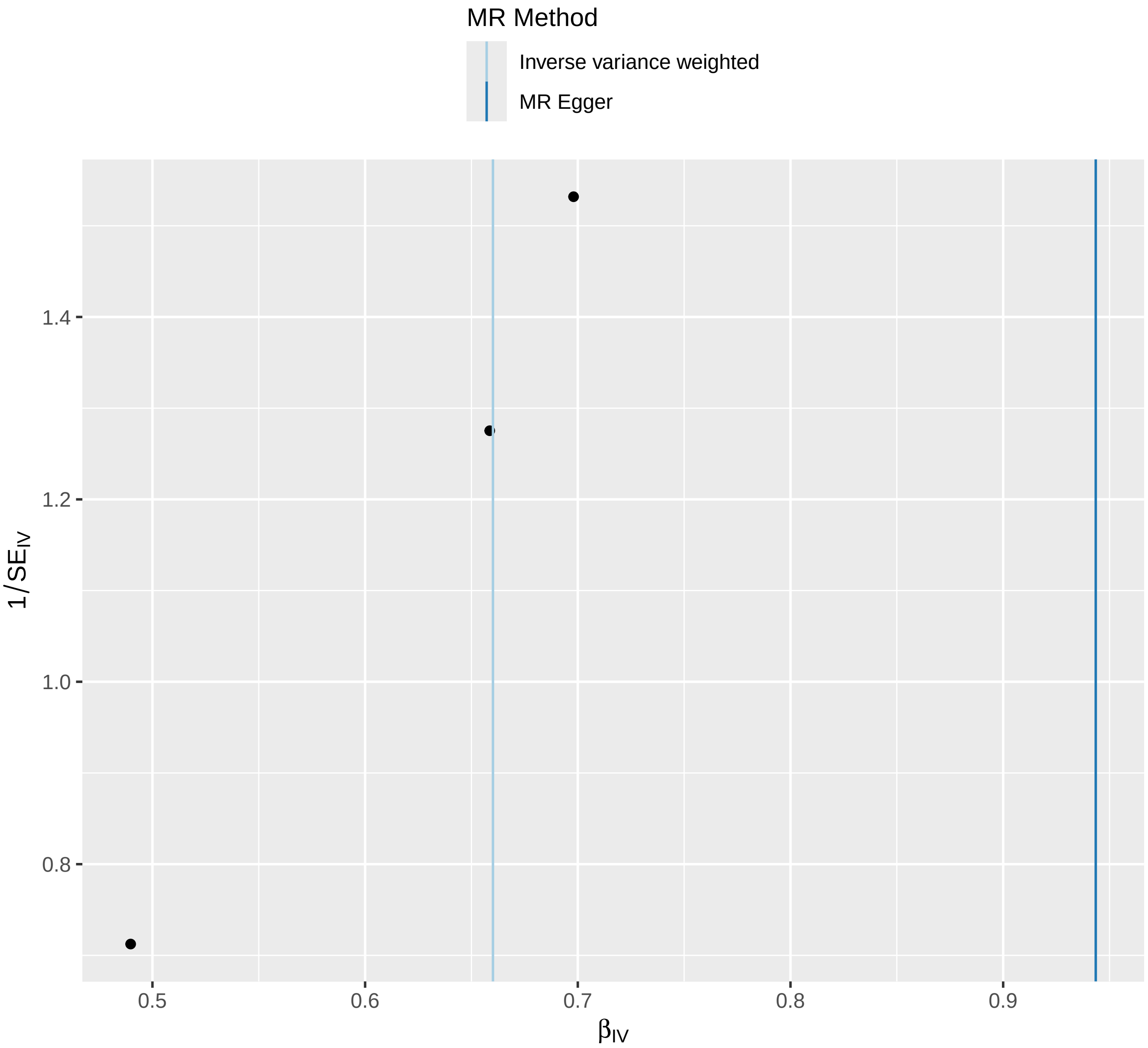

MR Method
Inverse variance weighted
MR Egger
1.4
1.2
1.0
0.8
0.5
0.6
0.7
0.8
0.9
$1/SE_{IV}$
β_{IV}

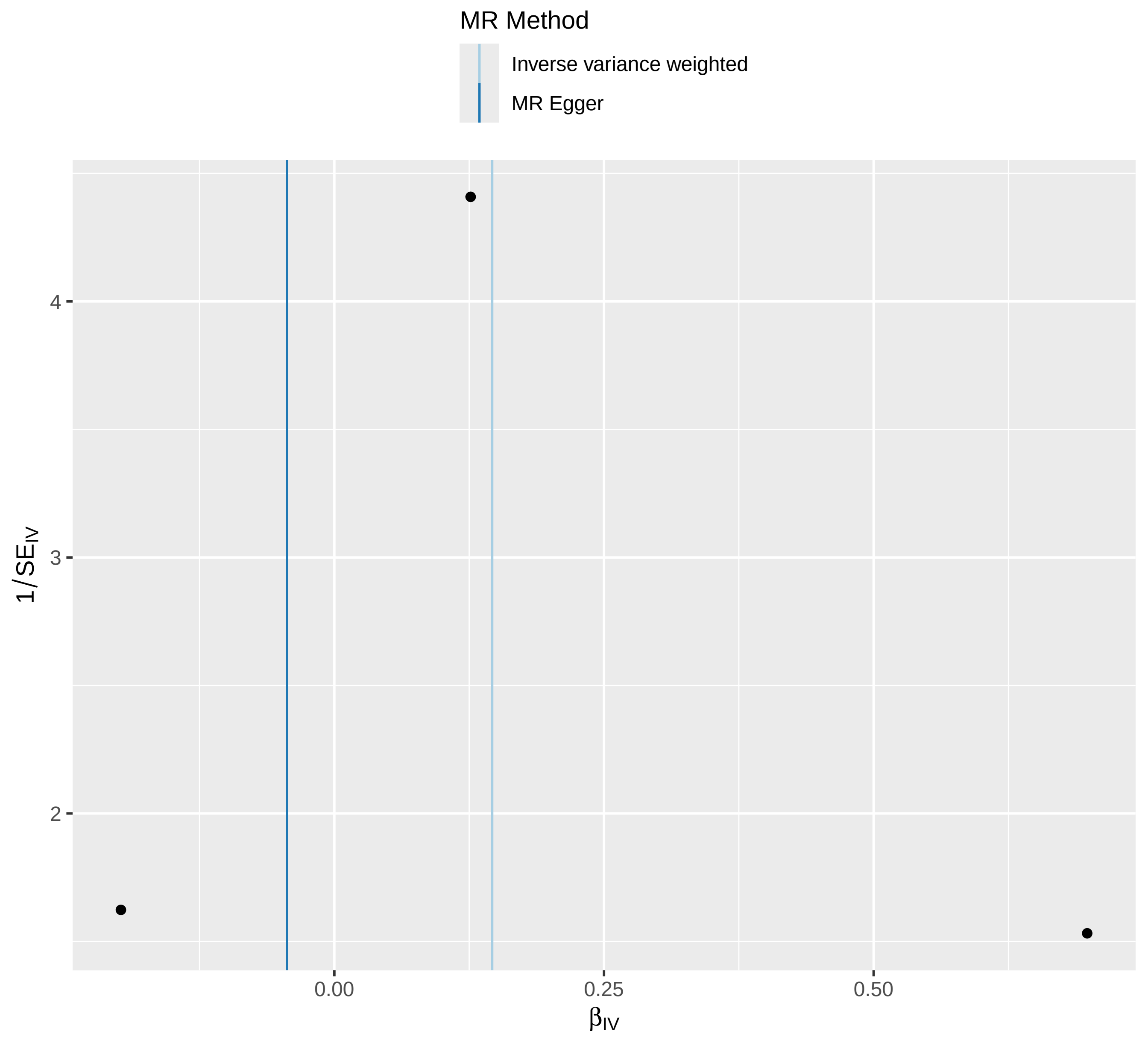
MR Method
Inverse variance weighted
MR Egger
4
3
2
1/SE_IV
0.00
0.25
0.50
β_IV

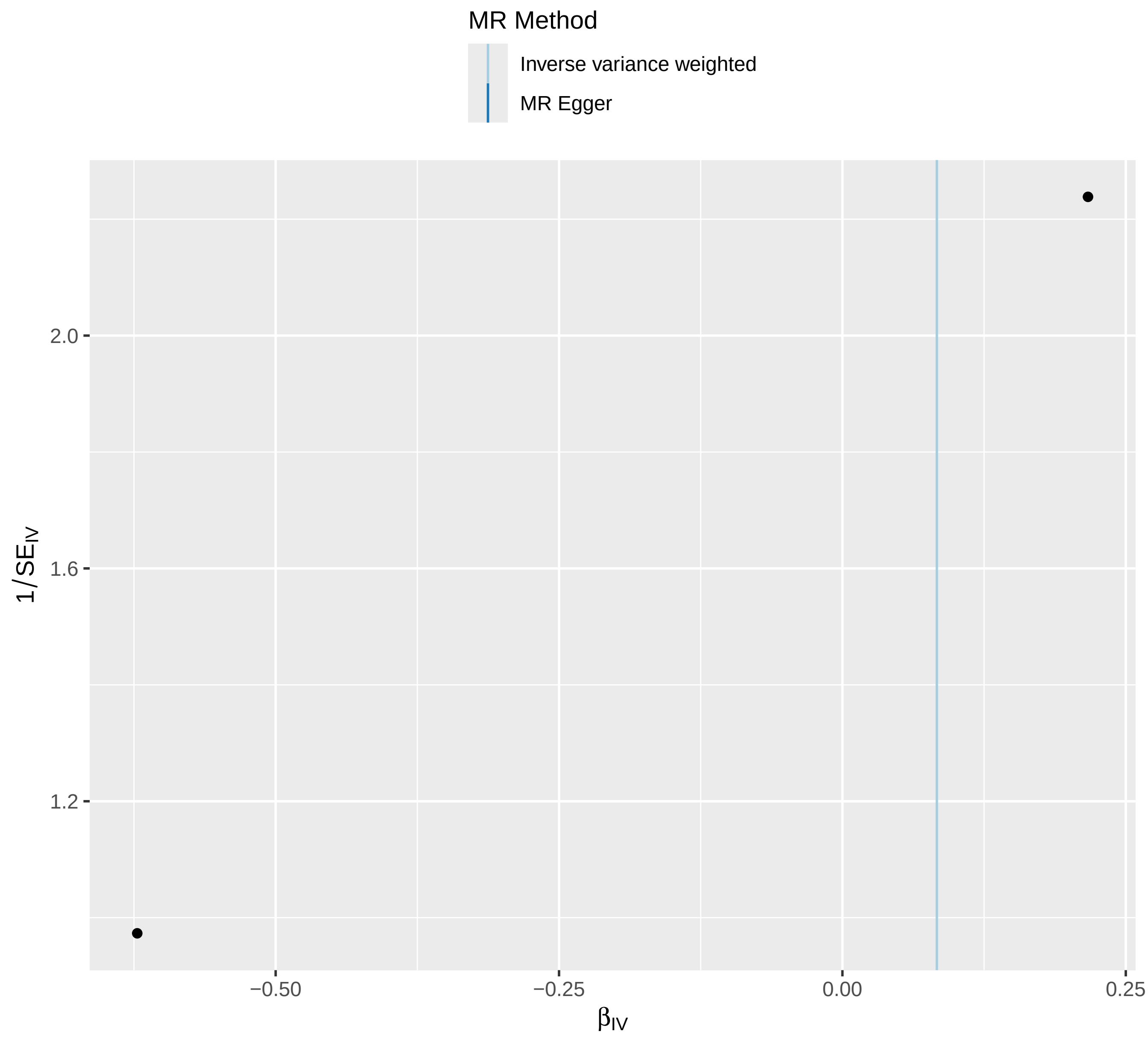
MR Method
Inverse variance weighted
MR Egger
2.0
1.6
1.2
1/SE$_{IV}$
−0.50
−0.25
0.00
0.25
β$_{IV}$

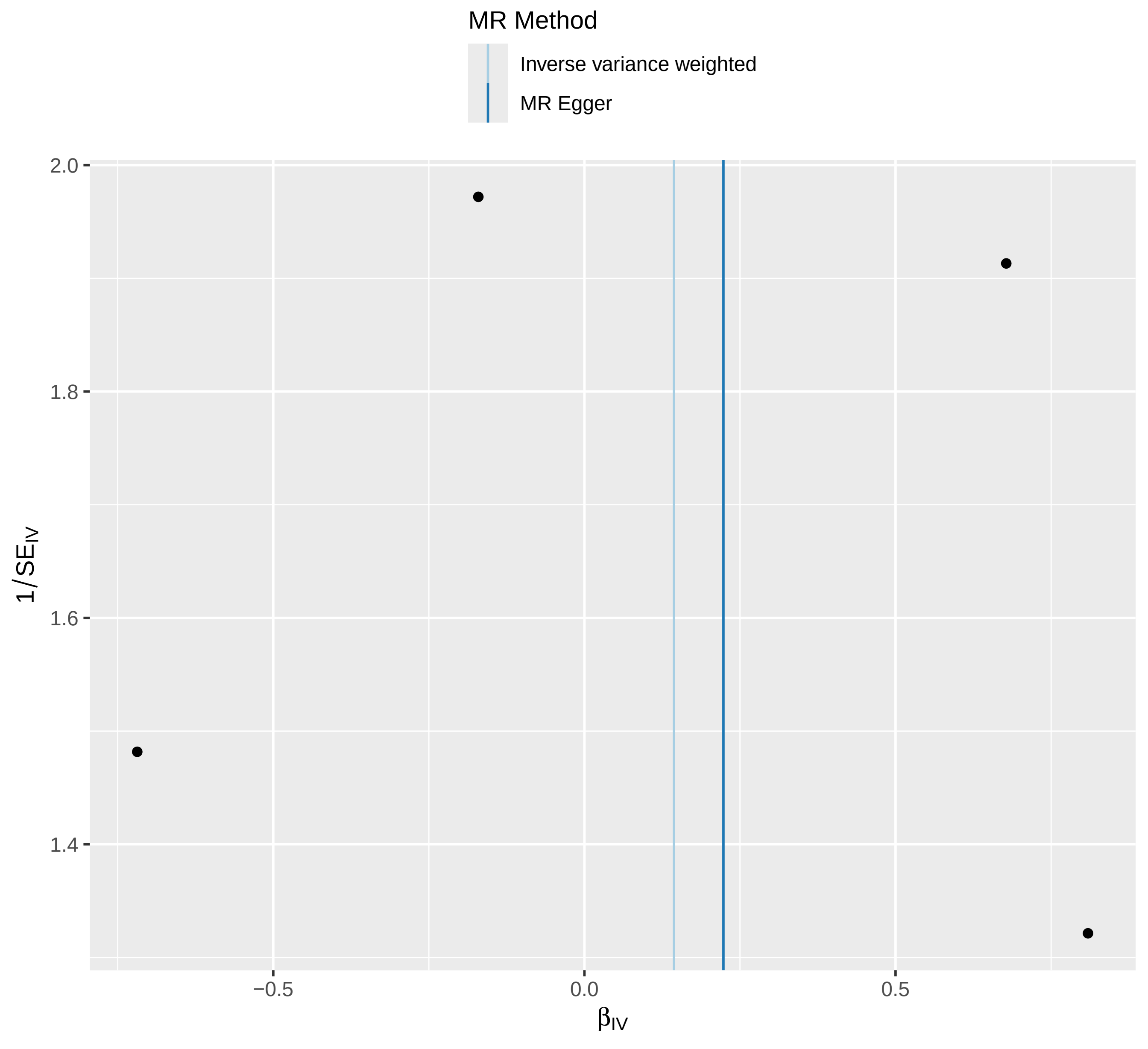
MR Method
Inverse variance weighted
MR Egger
2.0
1.8
1.6
1.4
1/SE$_{IV}$
−0.5
0.0
0.5
β$_{IV}$

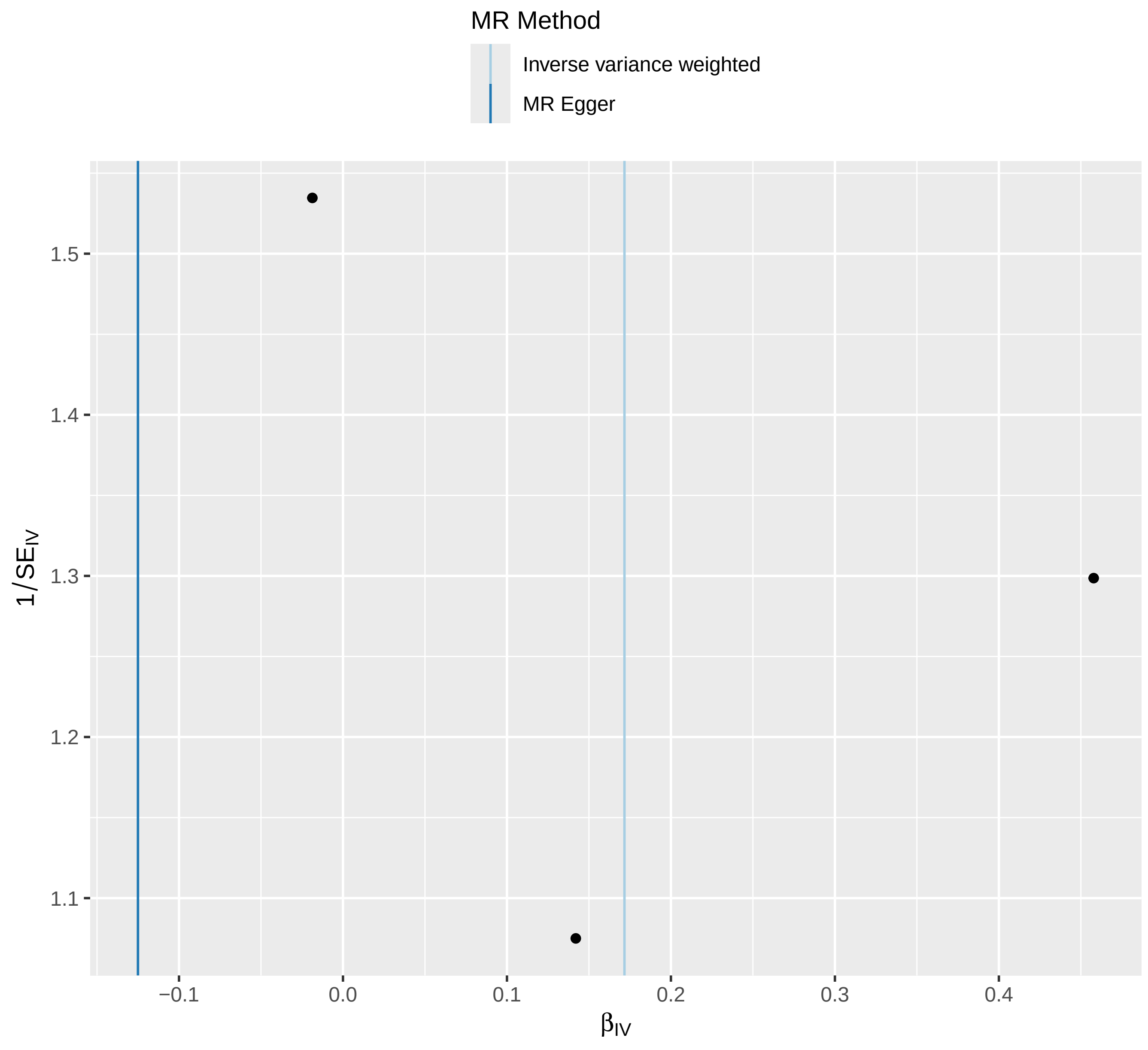
MR Method
Inverse variance weighted
MR Egger
1.5
1.4
1.3
1.2
1.1
1/SE_IV
−0.1
0.0
0.1
0.2
0.3
0.4
β_IV

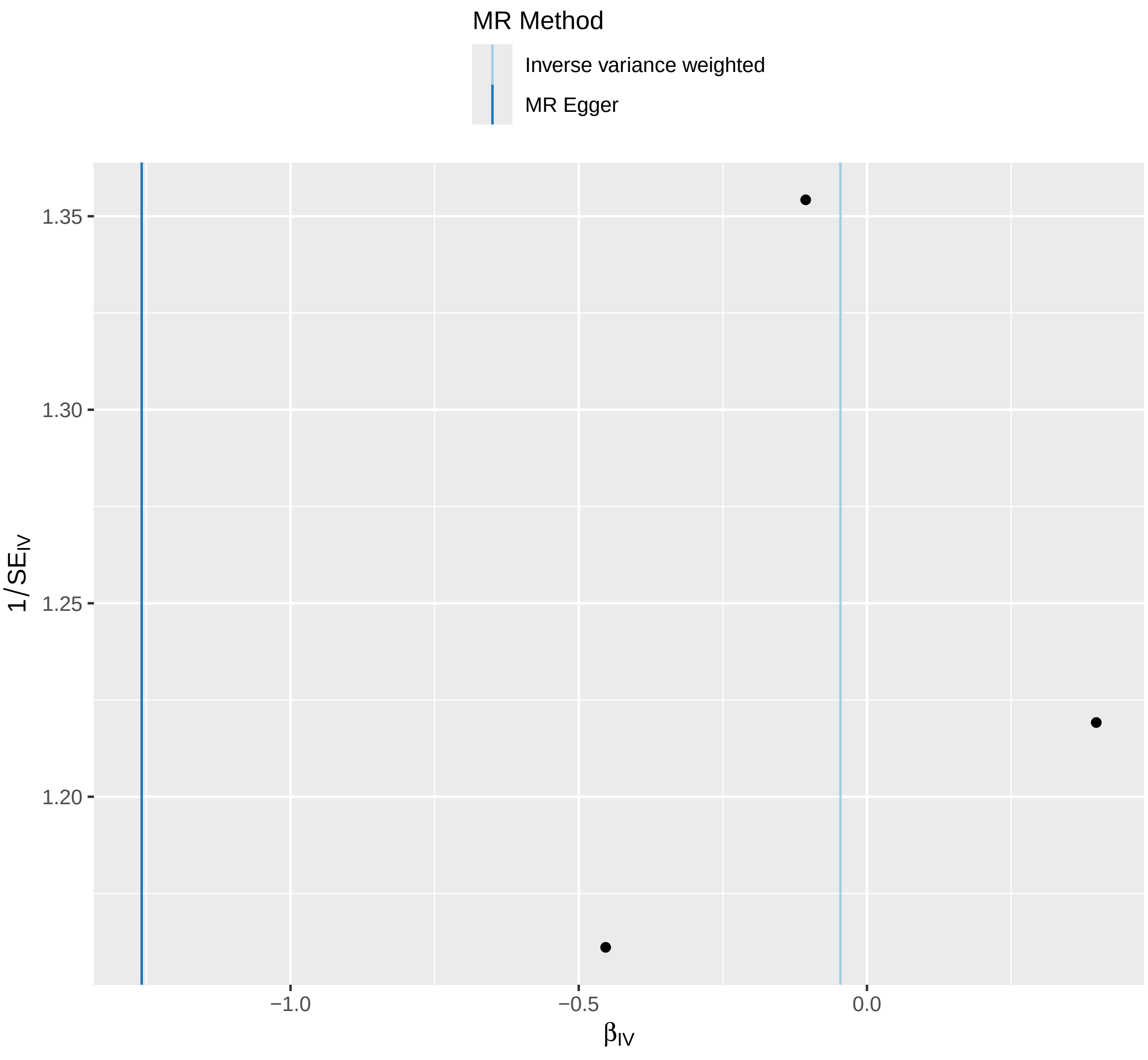

MR Method
Inverse variance weighted
MR Egger
1.35
1.30
1.25
1.20
−1.0
−0.5
0.0
1/SE_IV
β_IV

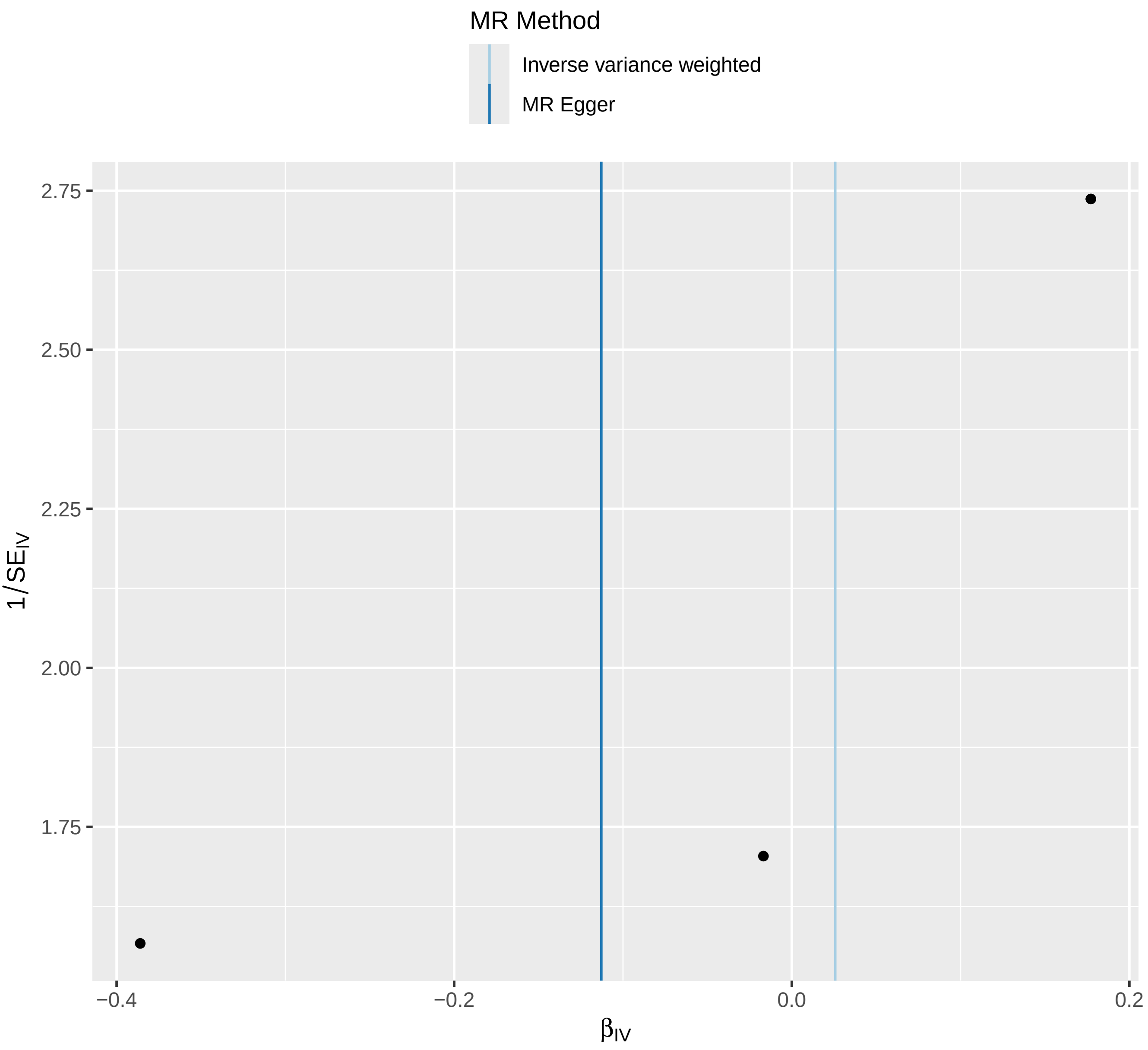

MR Method
Inverse variance weighted
MR Egger
2.75
2.50
2.25
2.00
1.75
$1/SE_{IV}$
−0.4
−0.2
0.0
0.2
β_{IV}

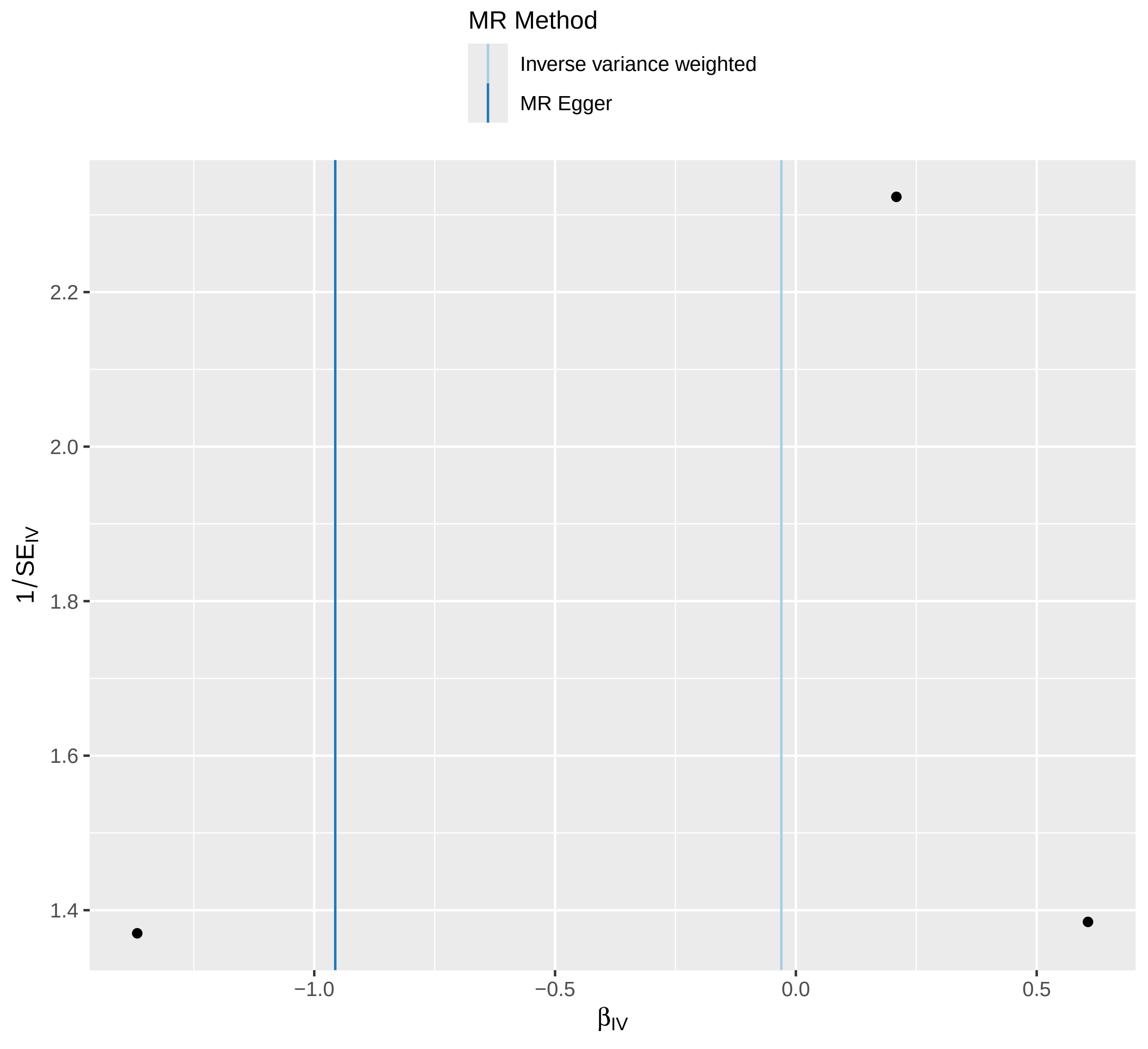

MR Method
Inverse variance weighted
MR Egger
1/SE_IV
2.2
2.0
1.8
1.6
1.4
−1.0
−0.5
0.0
0.5
β_IV

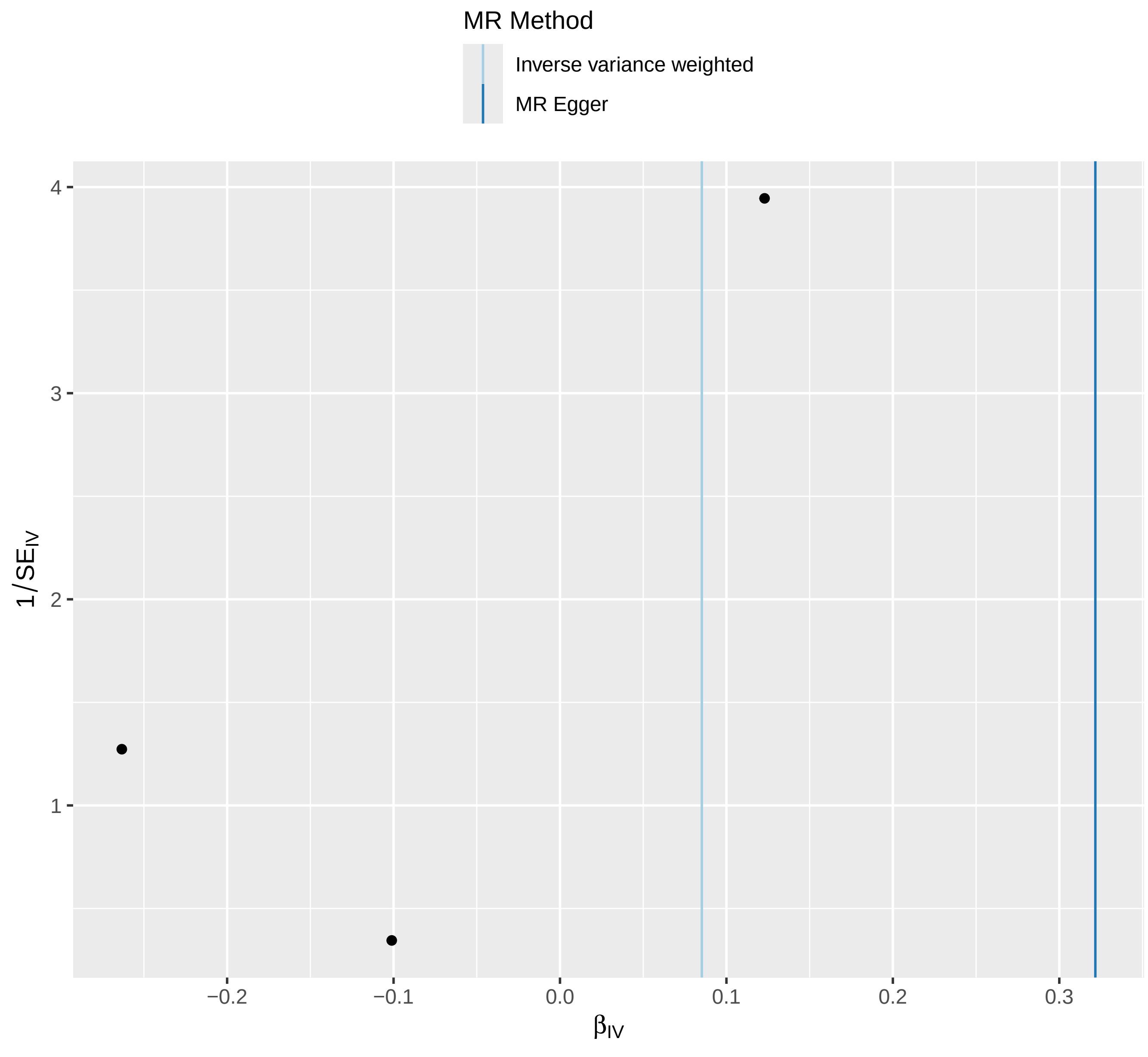

MR Method
Inverse variance weighted
MR Egger
4
3
2
1
−0.2
−0.1
0.0
0.1
0.2
0.3
$1/SE_{IV}$
β_{IV}

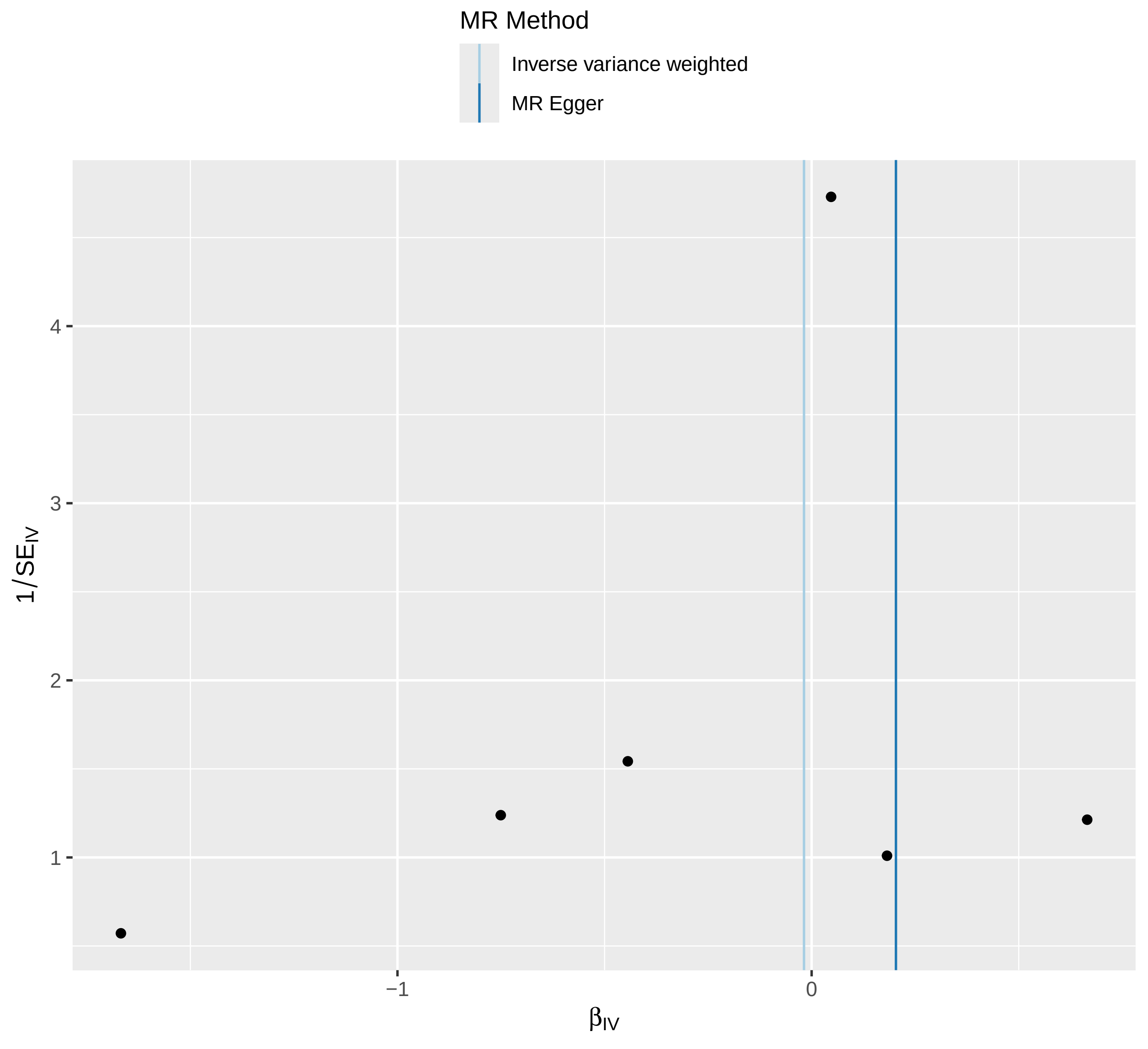
MR Method
Inverse variance weighted
MR Egger
4
3
2
1
1/SE_IV
−1
0
β_IV

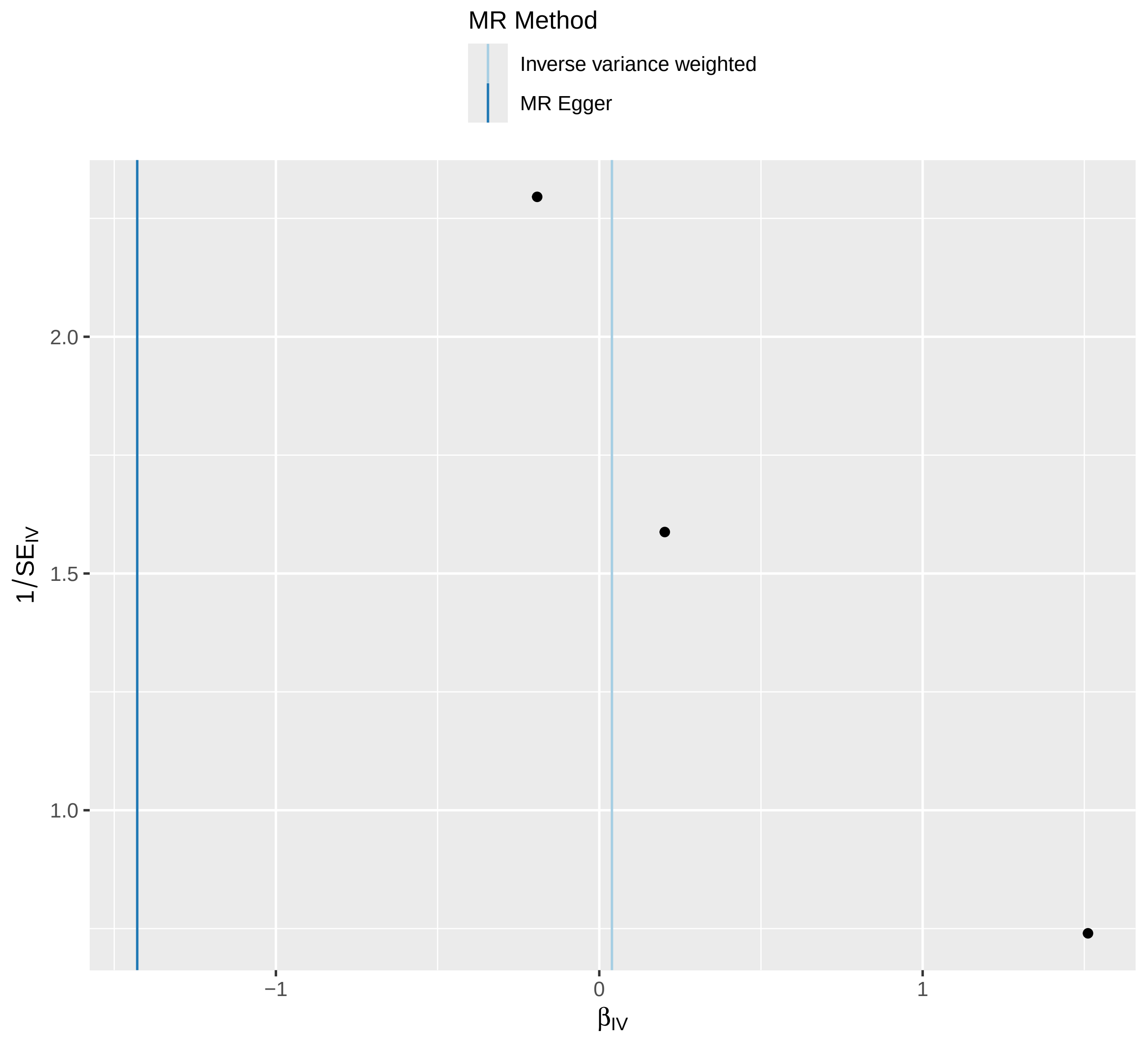
MR Method
Inverse variance weighted
MR Egger
2.0
1.5
1.0
1/SE_IV
−1
0
1
β_IV

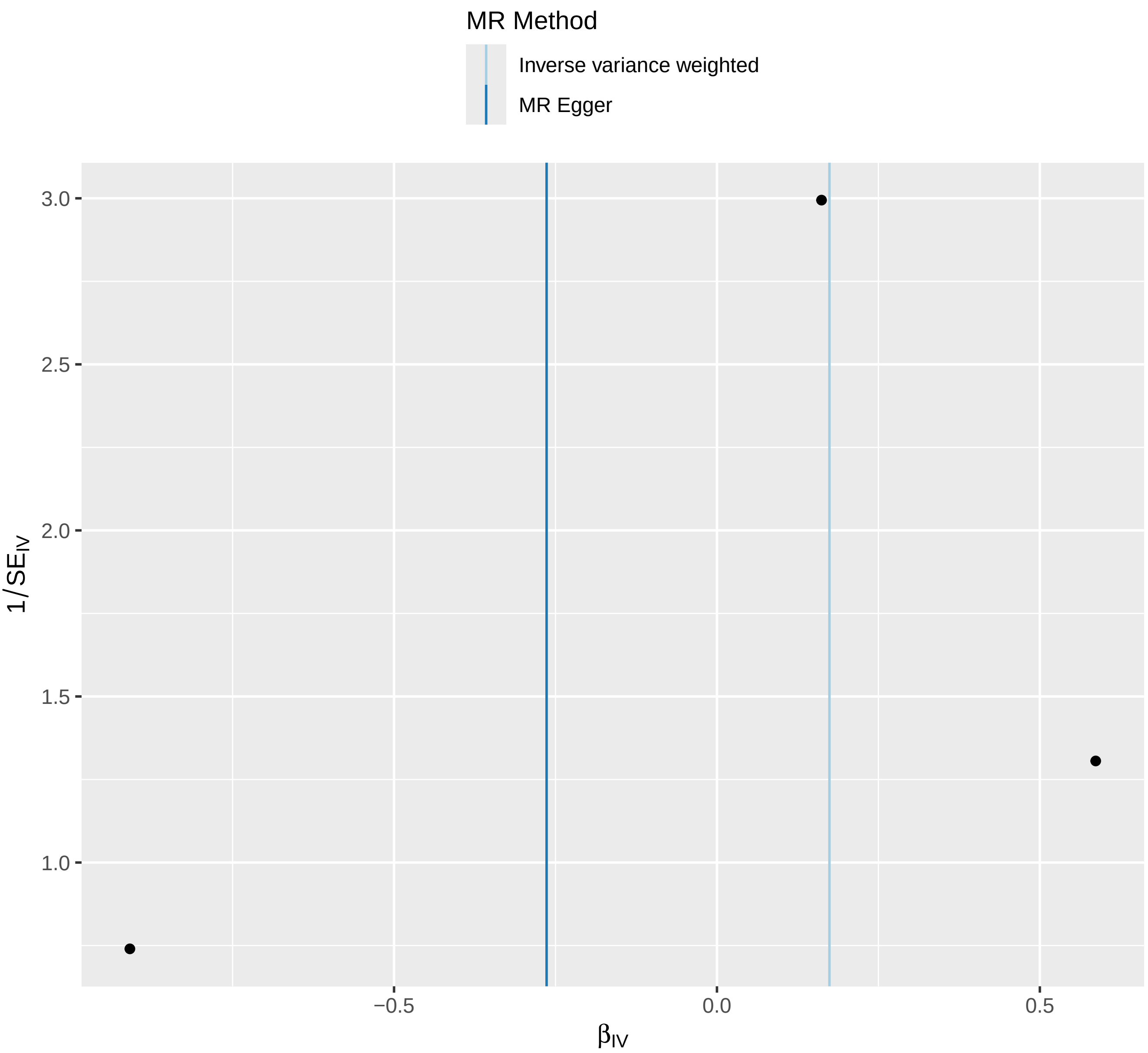

MR Method
Inverse variance weighted
MR Egger
3.0
2.5
2.0
1.5
1.0
1/SE$_{IV}$
−0.5
0.0
0.5
β$_{IV}$

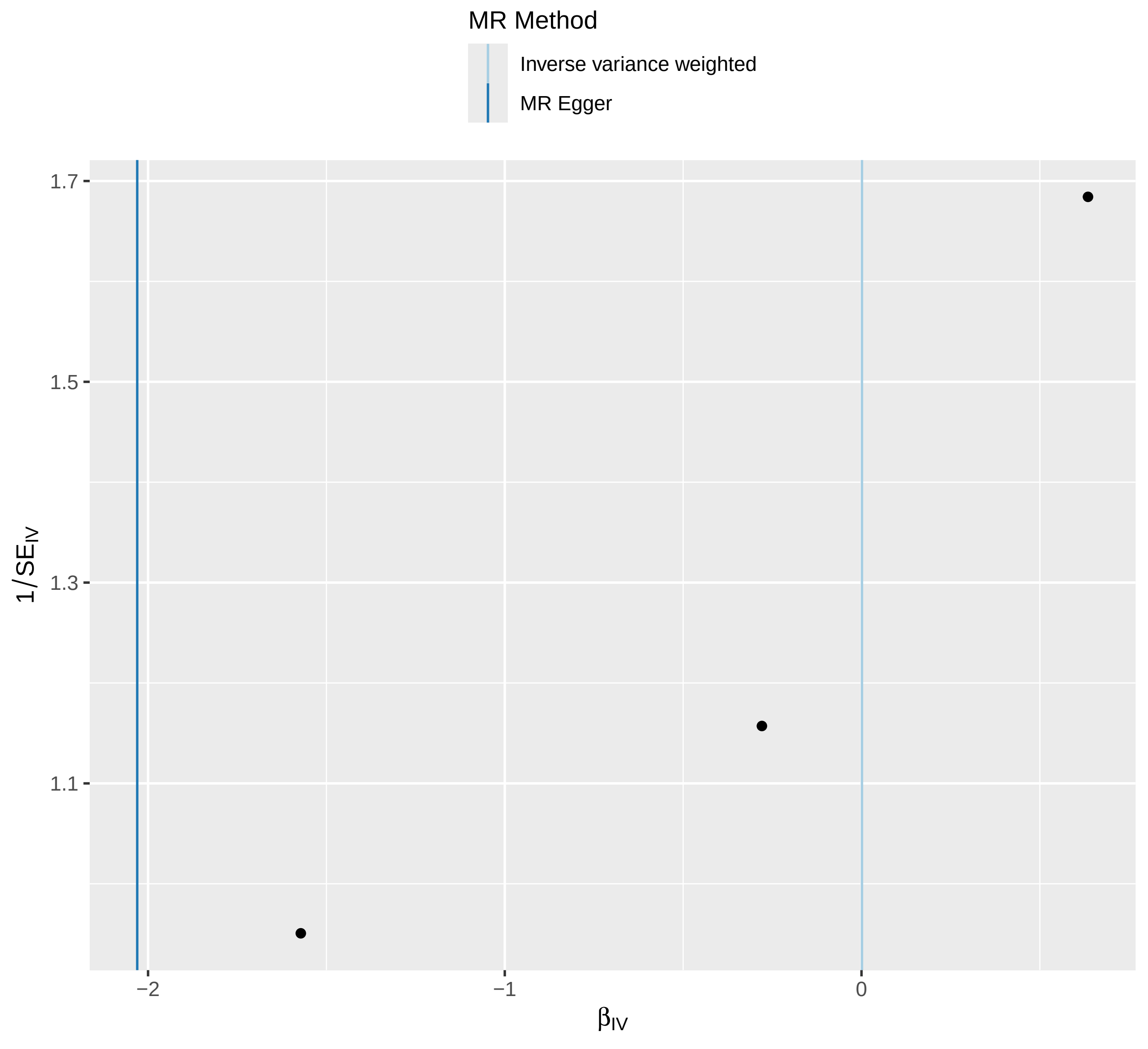
MR Method
Inverse variance weighted
MR Egger
1.7
1.5
1.3
1.1
1/SE_IV
−2
−1
0
β_IV

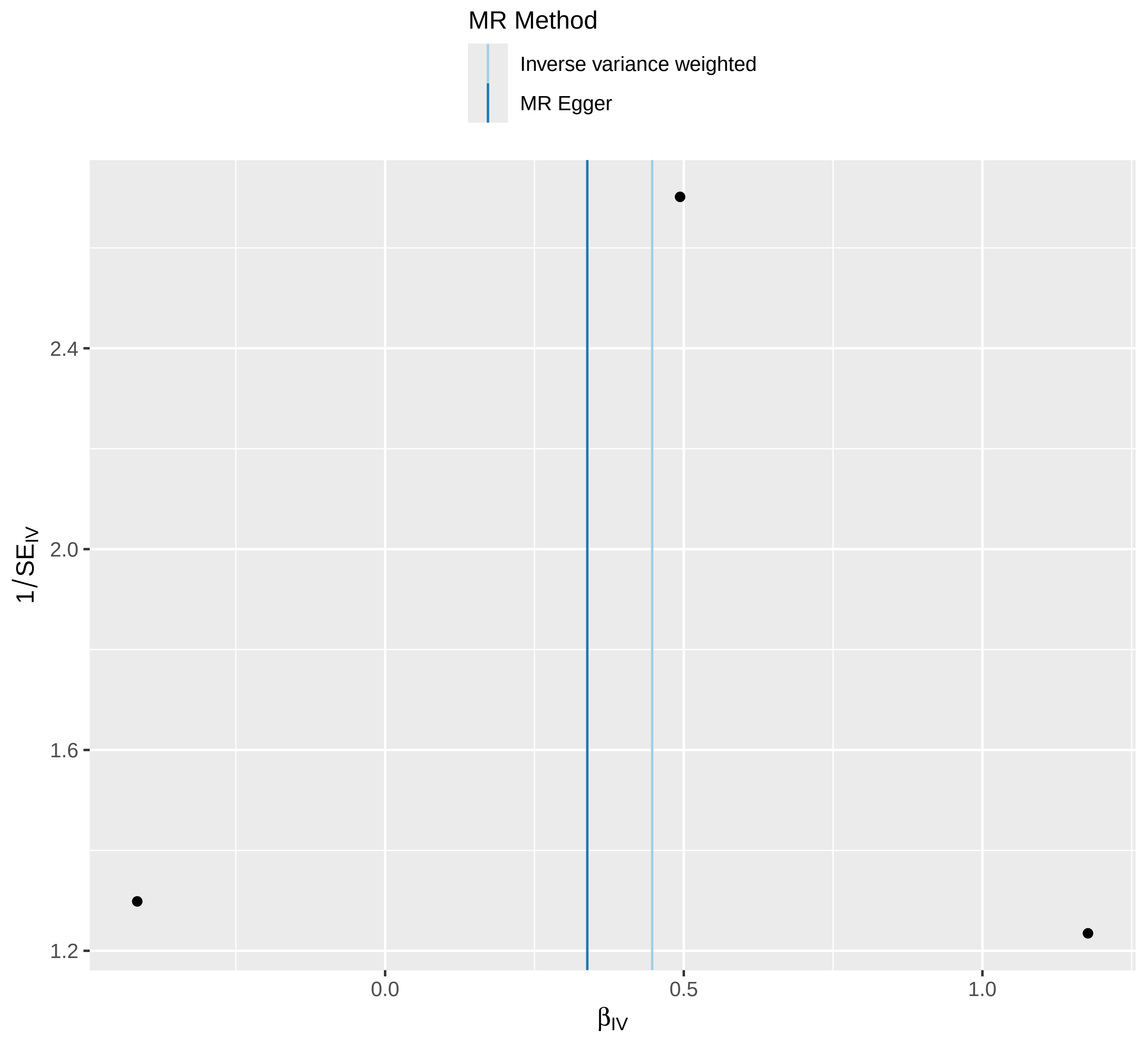

MR Method
Inverse variance weighted
MR Egger
2.4
2.0
1.6
1.2
1/SE_IV
0.0
0.5
1.0
β_IV

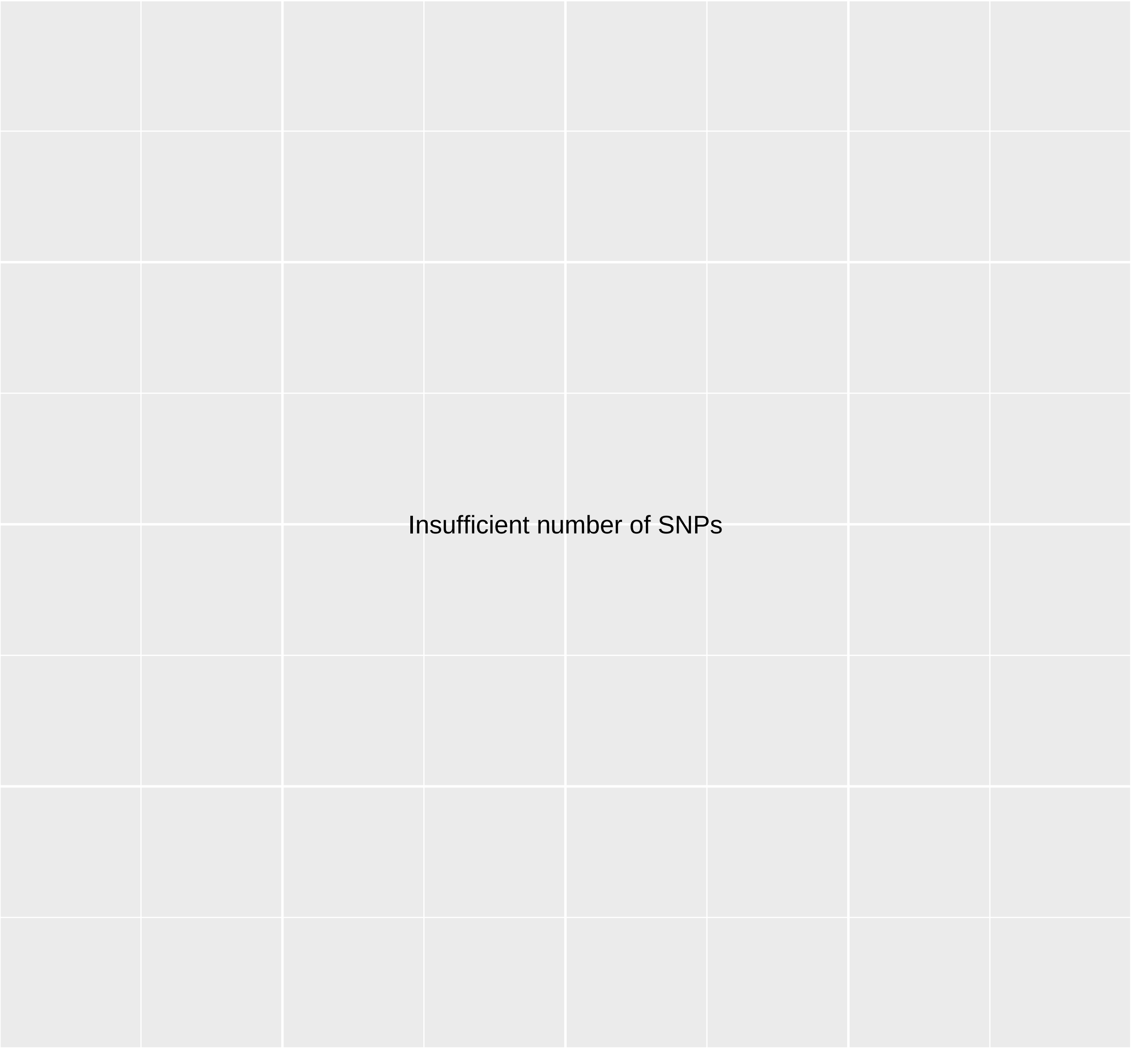
Insufficient number of SNPs

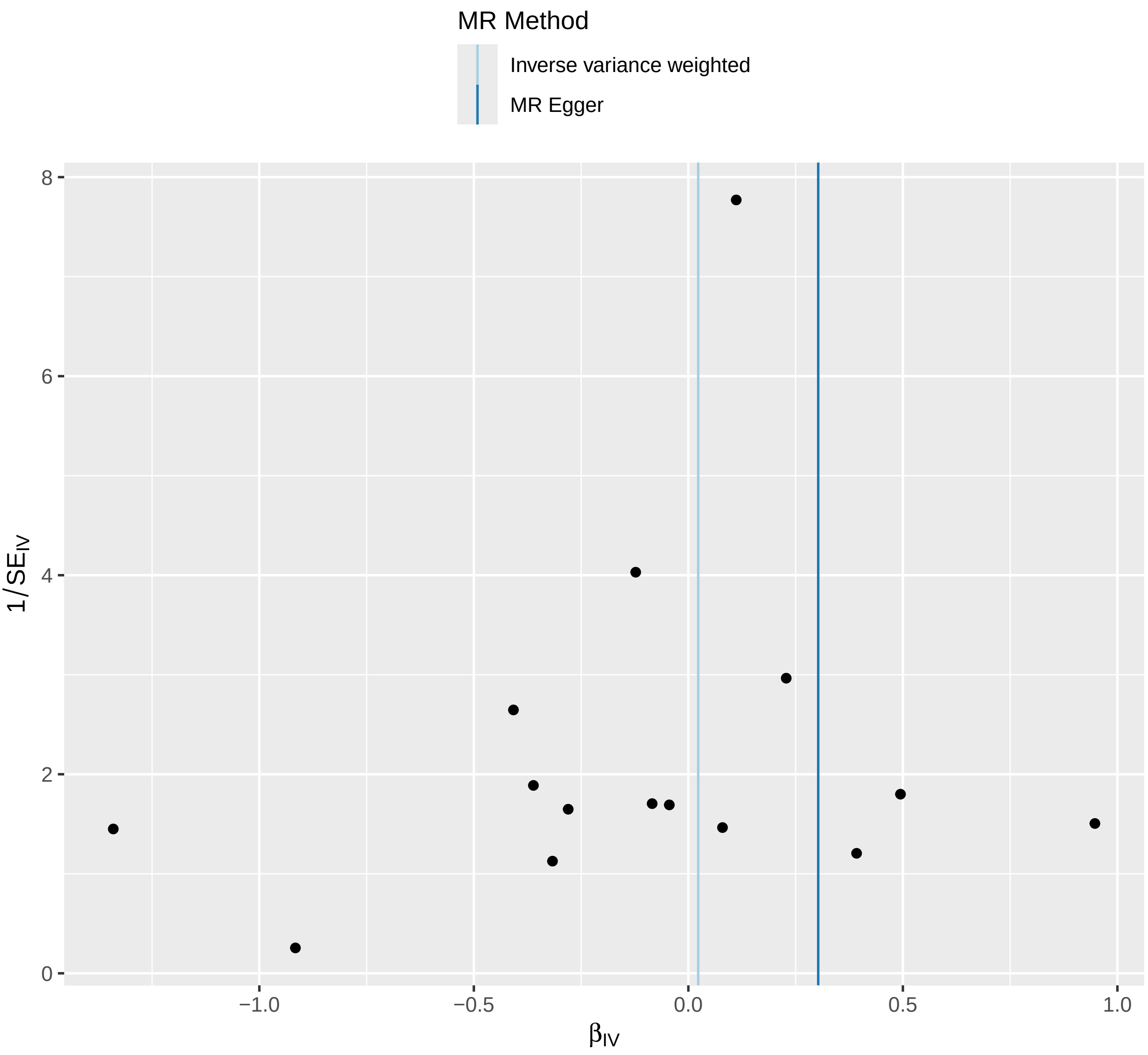
MR Method
Inverse variance weighted
MR Egger
8
6
4
2
0
1/SE$_{IV}$
−1.0
−0.5
0.0
0.5
1.0
β$_{IV}$

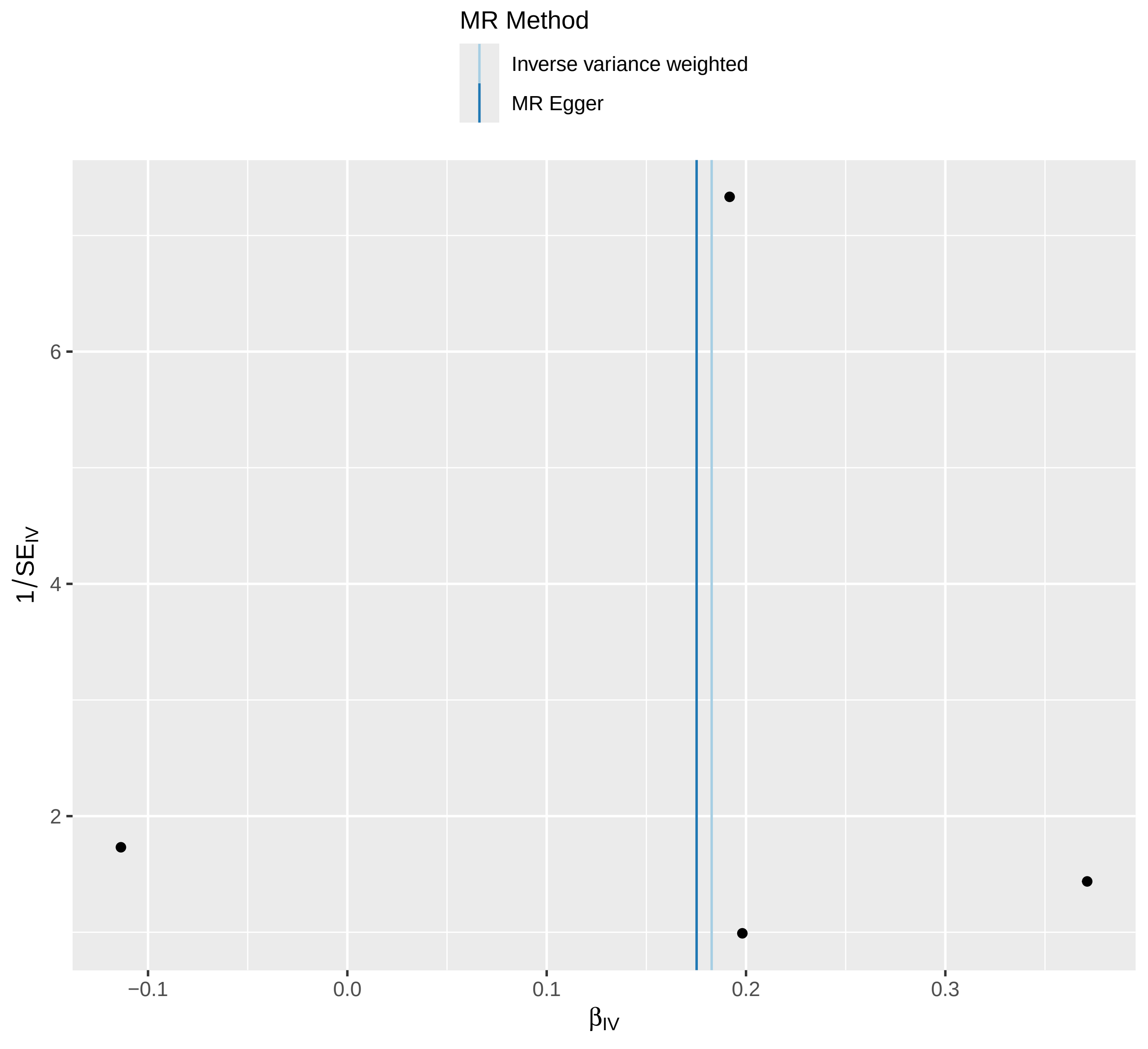

MR Method
Inverse variance weighted
MR Egger
6
4
2
1/SE$_{IV}$
−0.1
0.0
0.1
0.2
0.3
β$_{IV}$

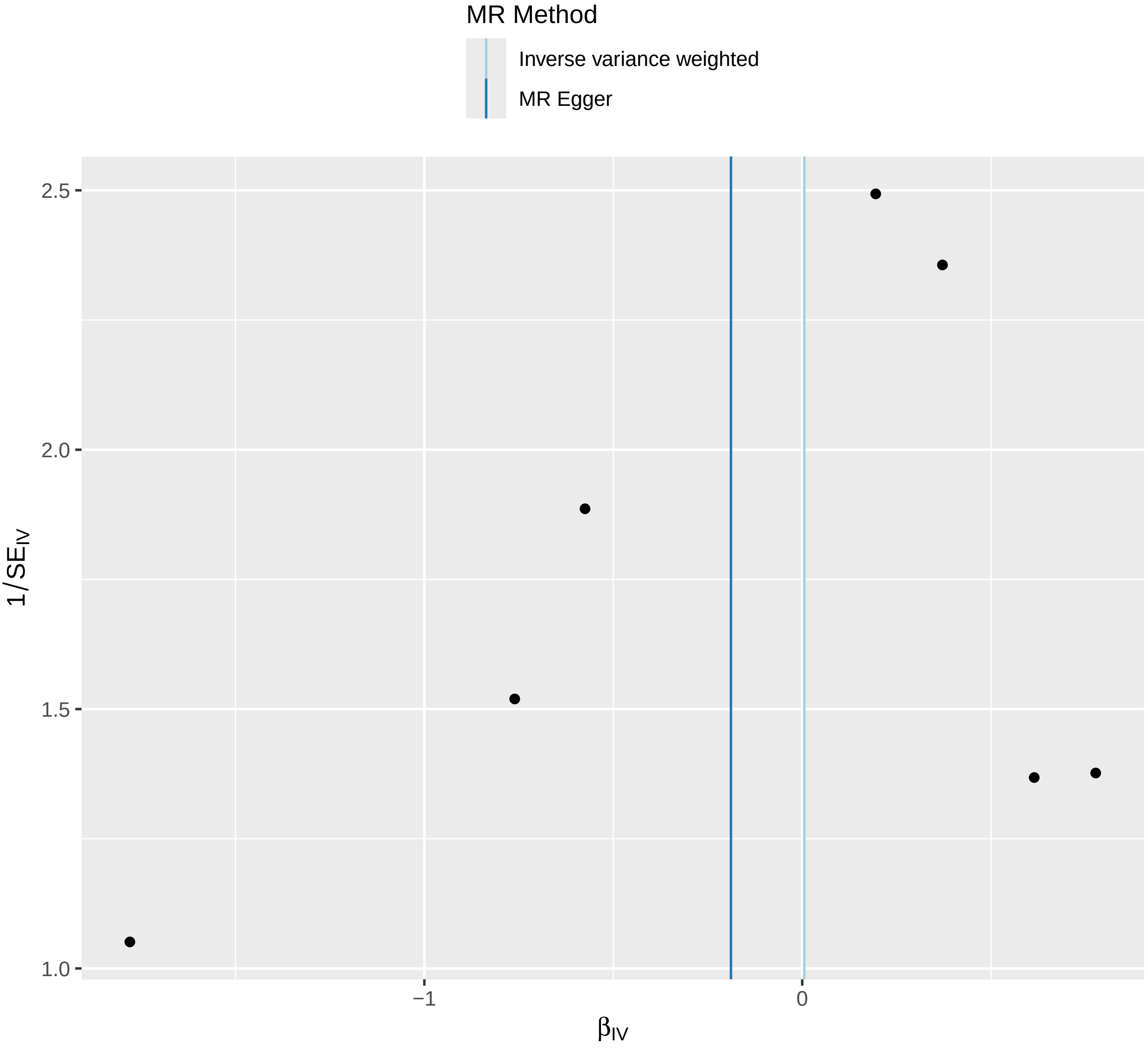
MR Method
Inverse variance weighted
MR Egger
2.5
2.0
1.5
1.0
1/SE_IV
−1
0
β_IV

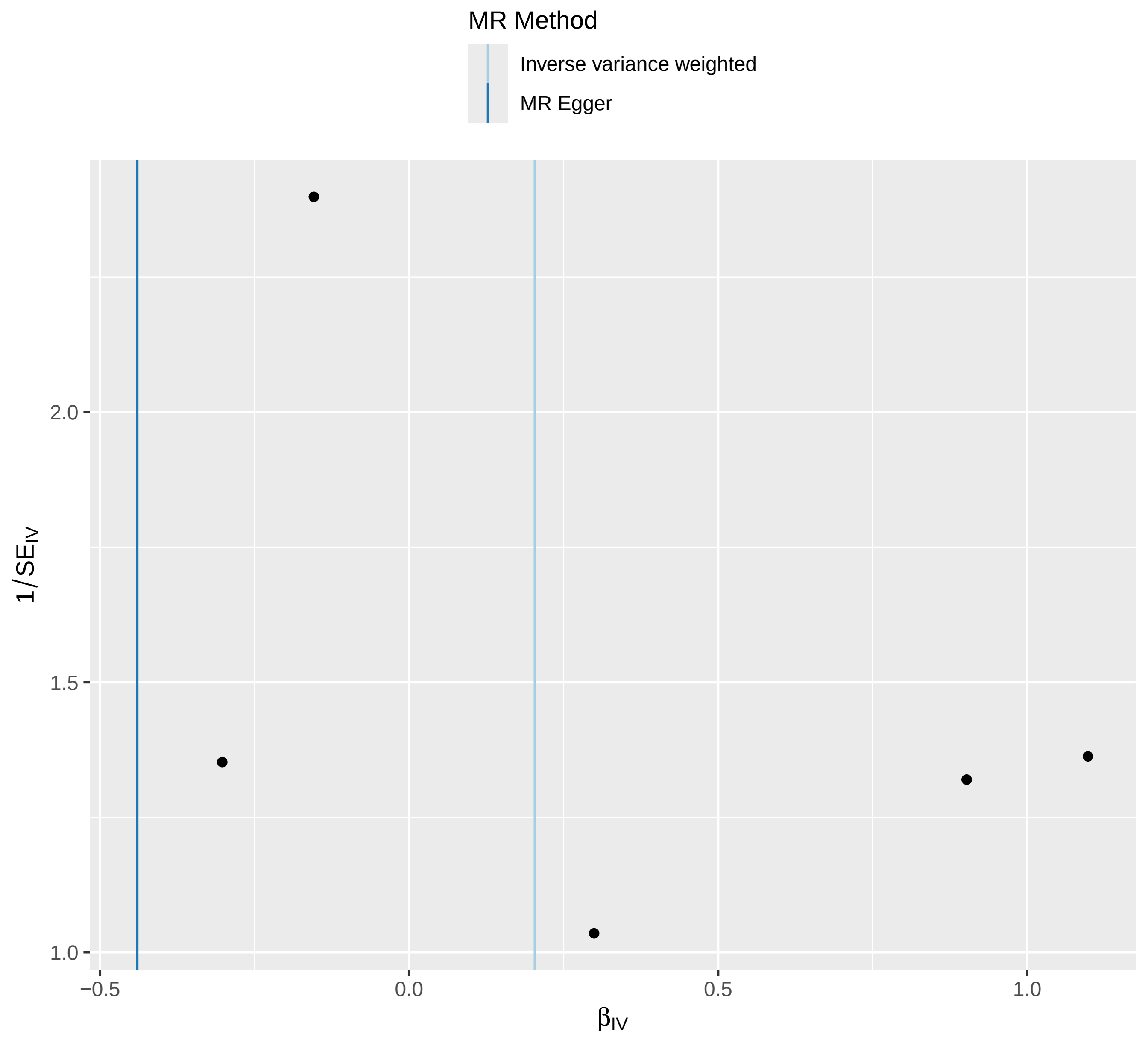
MR Method
Inverse variance weighted
MR Egger
2.0
1.5
1.0
1/SE_IV
−0.5
0.0
0.5
1.0
β_IV

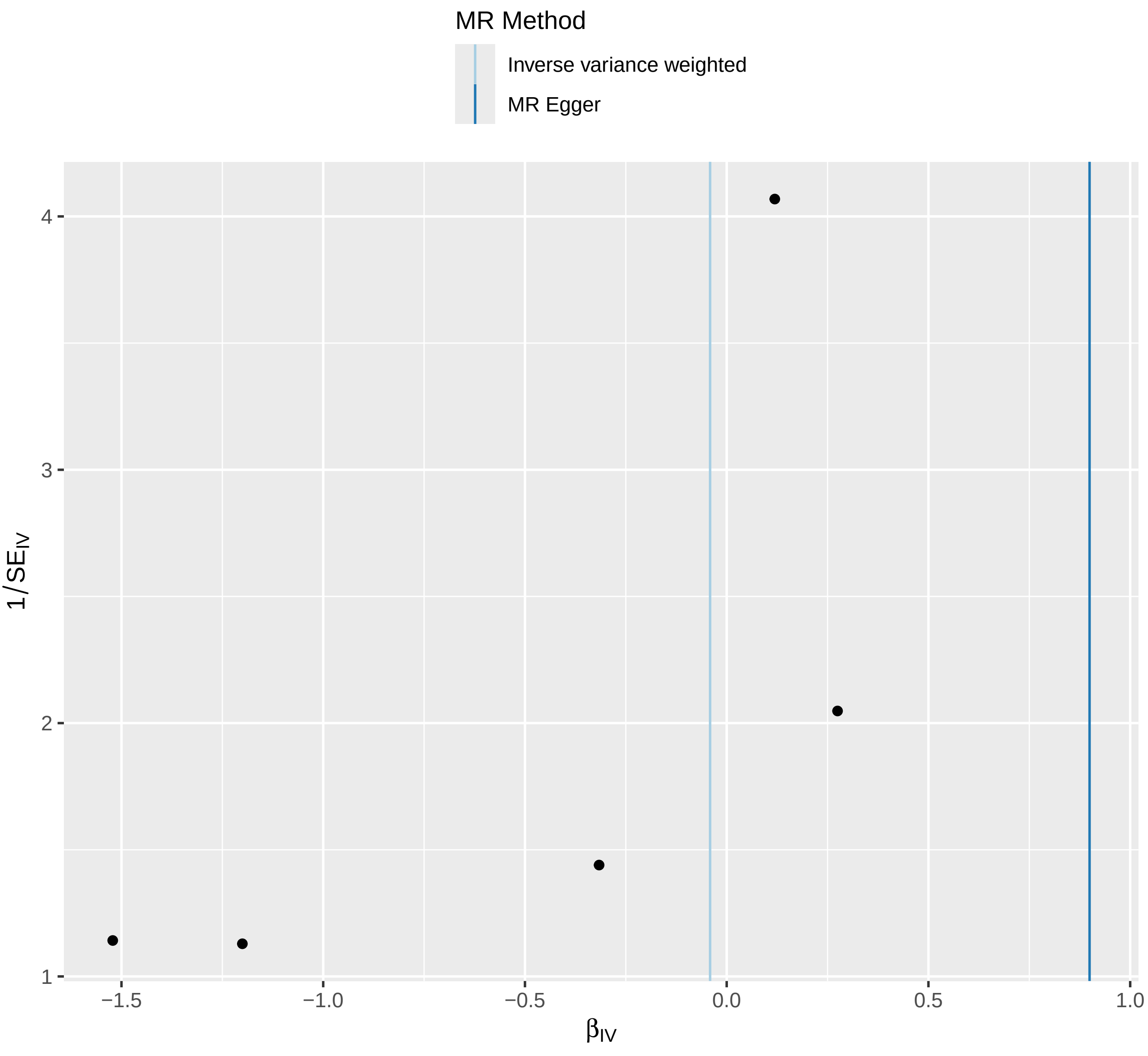

MR Method
Inverse variance weighted
MR Egger
4
3
2
1
1/SE$_{IV}$
−1.5
−1.0
−0.5
0.0
0.5
1.0
β$_{IV}$

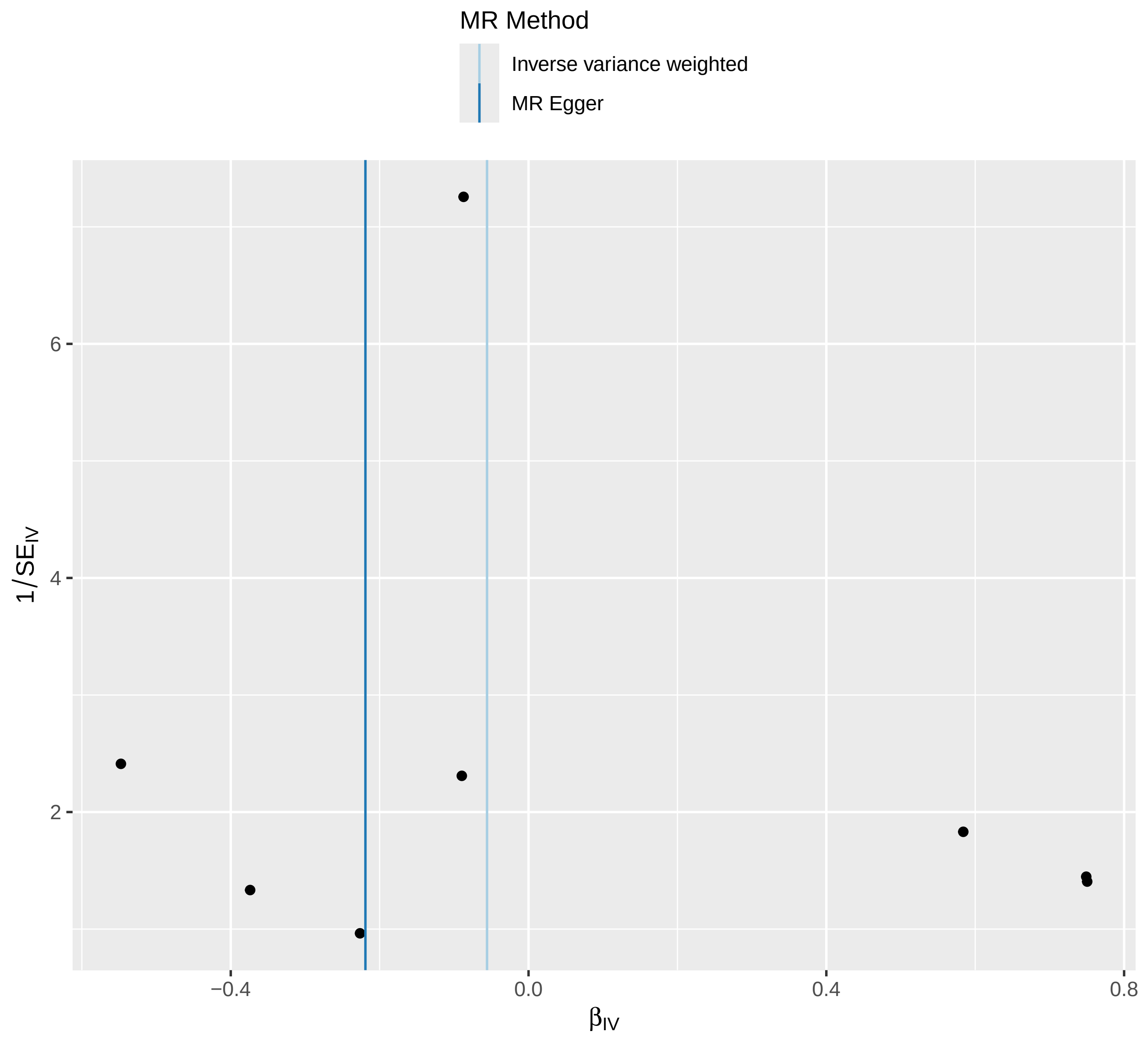
MR Method
Inverse variance weighted
MR Egger
1/SE_IV
6
4
2
−0.4
0.0
0.4
0.8
β_IV

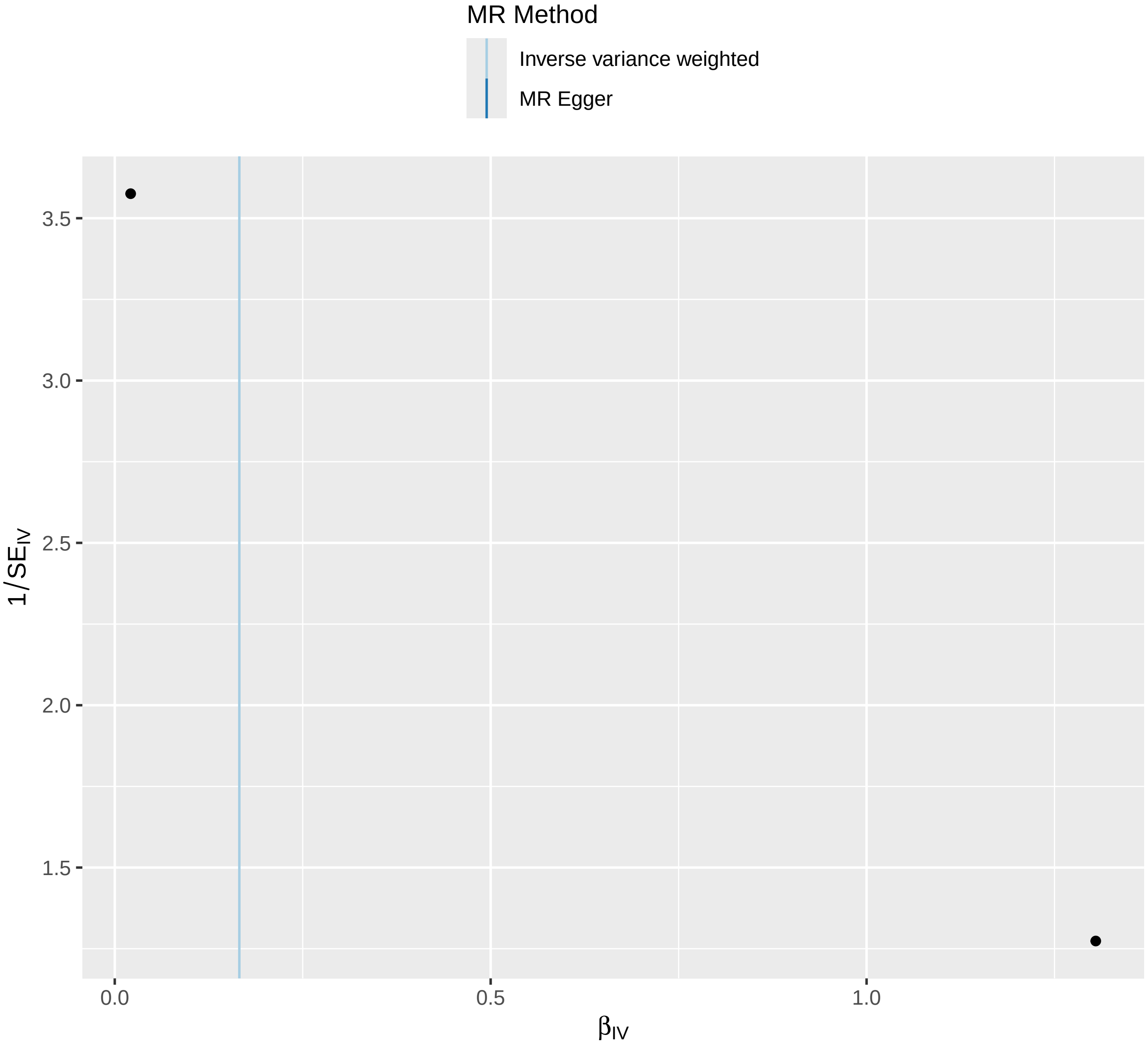

MR Method
Inverse variance weighted
MR Egger
3.5
3.0
2.5
2.0
1.5
$1/SE_{IV}$
0.0
0.5
1.0
β_{IV}

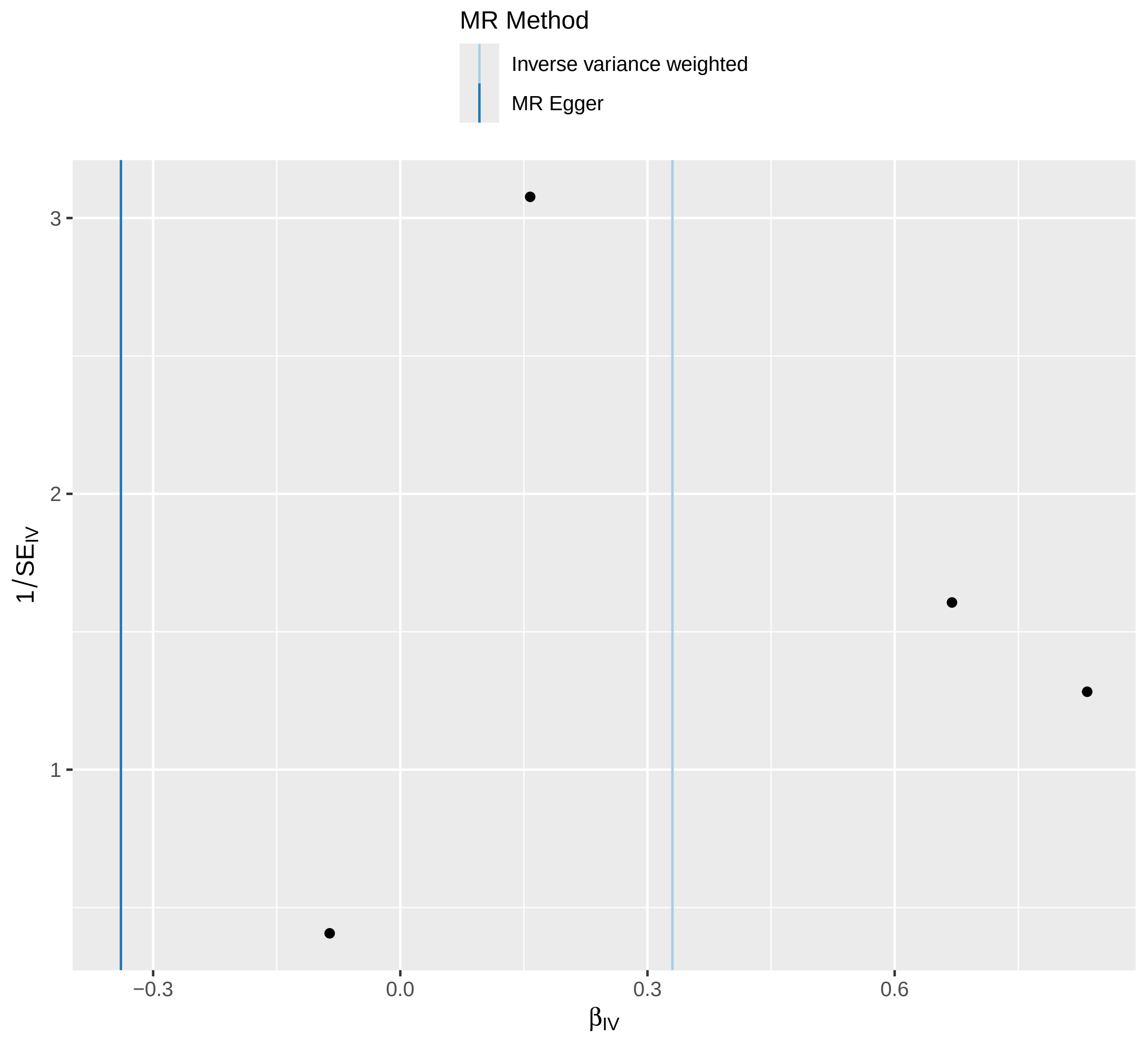

MR Method
Inverse variance weighted
MR Egger
3
2
1
1/SE_IV
−0.3
0.0
0.3
0.6
β_IV

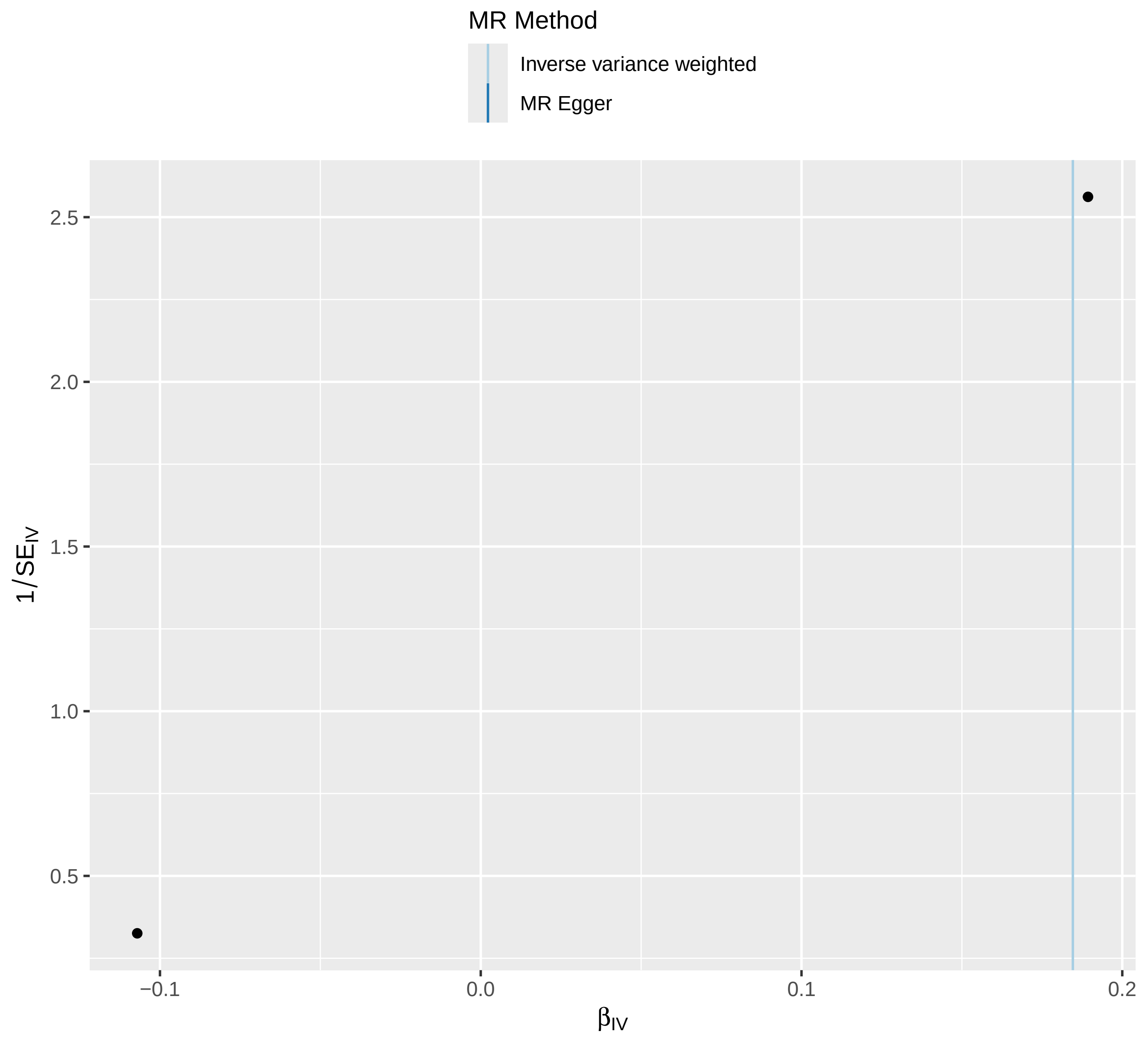

MR Method
Inverse variance weighted
MR Egger
2.5
2.0
1.5
1.0
0.5
$1/SE_{IV}$
−0.1
0.0
0.1
0.2
β_{IV}

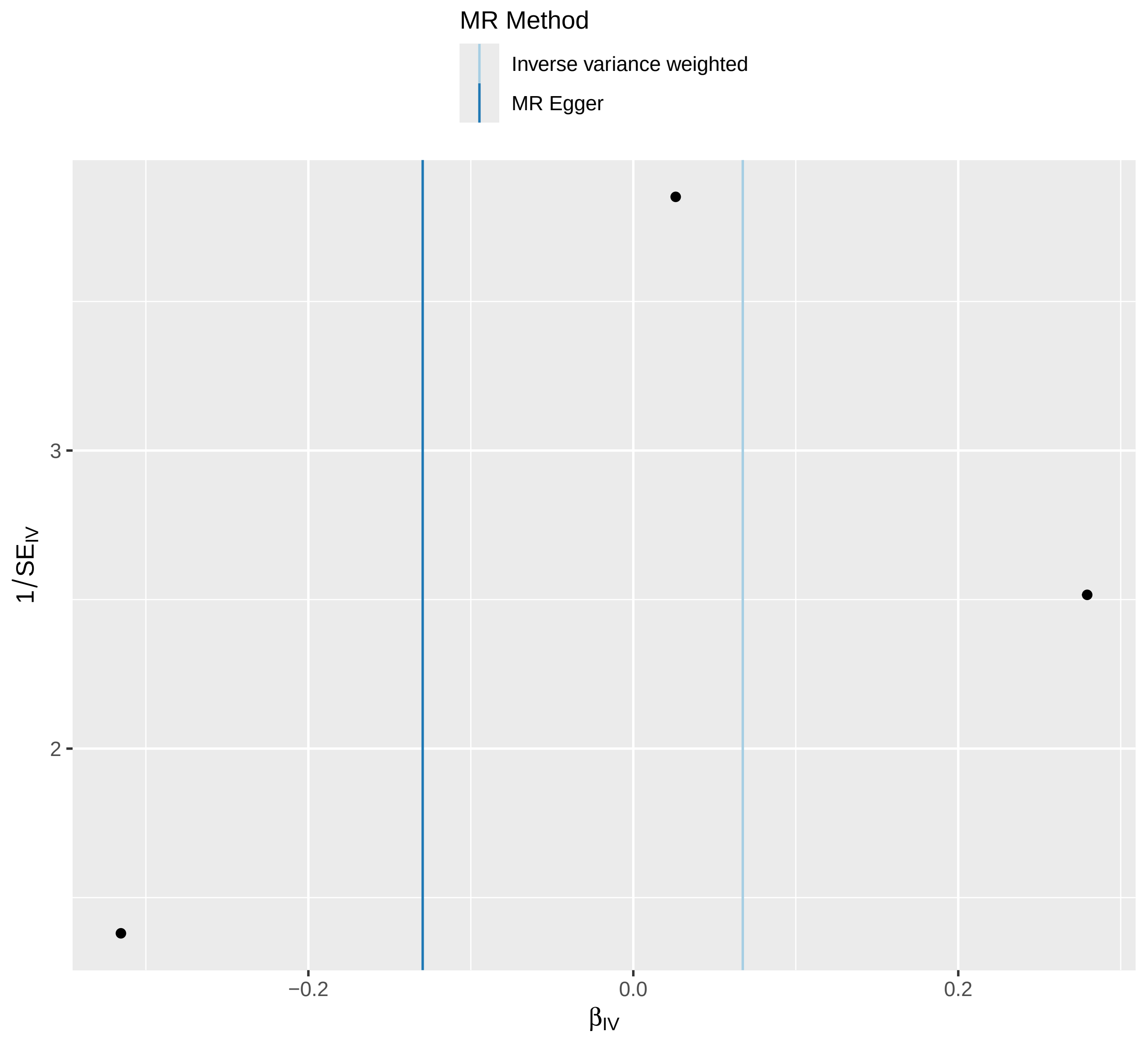
MR Method
Inverse variance weighted
MR Egger
1/SE$_{IV}$
3
2
−0.2
0.0
0.2
β$_{IV}$

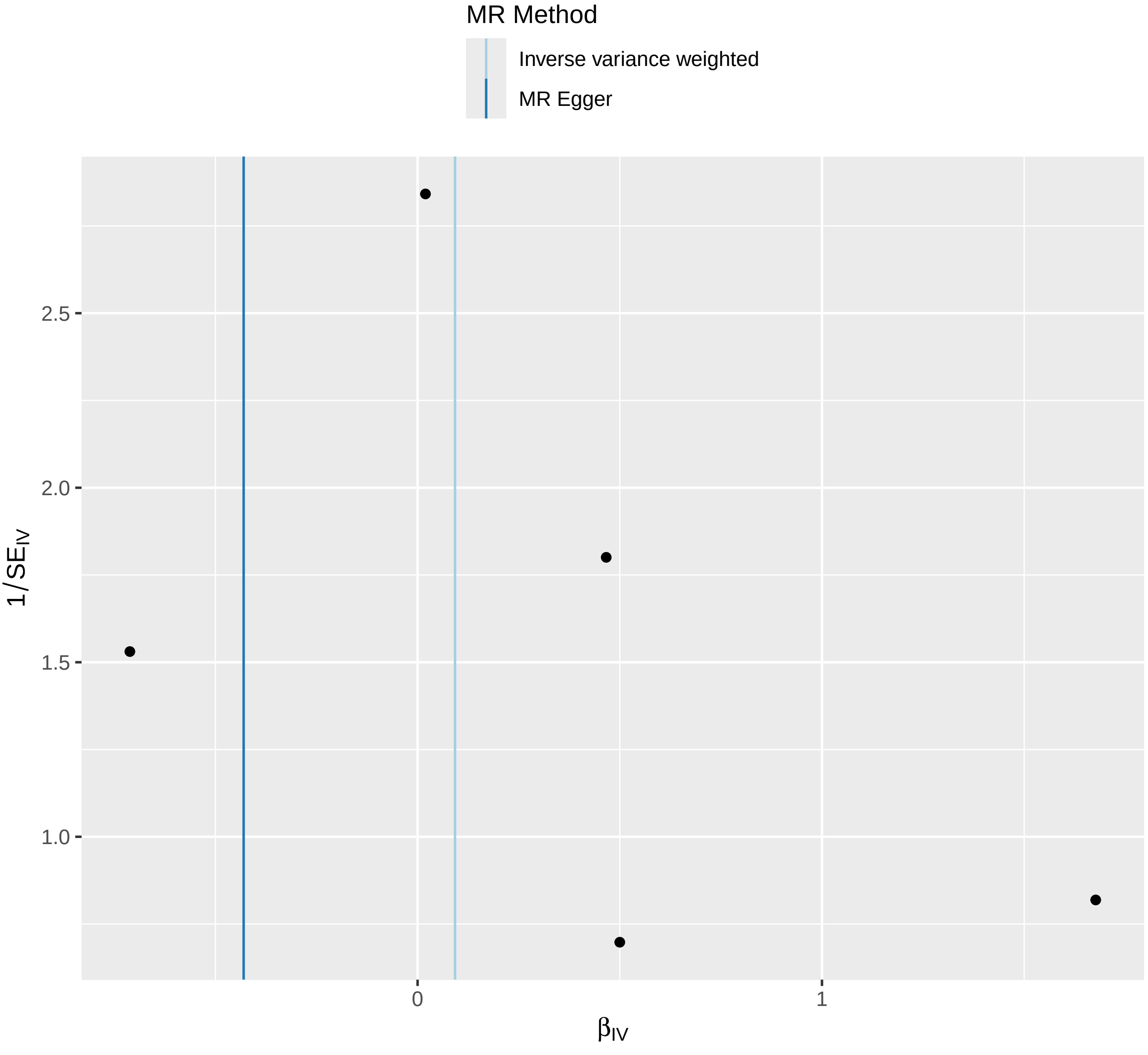

MR Method
Inverse variance weighted
MR Egger
2.5
2.0
1.5
1.0
$1/SE_{IV}$
0
1
β_{IV}

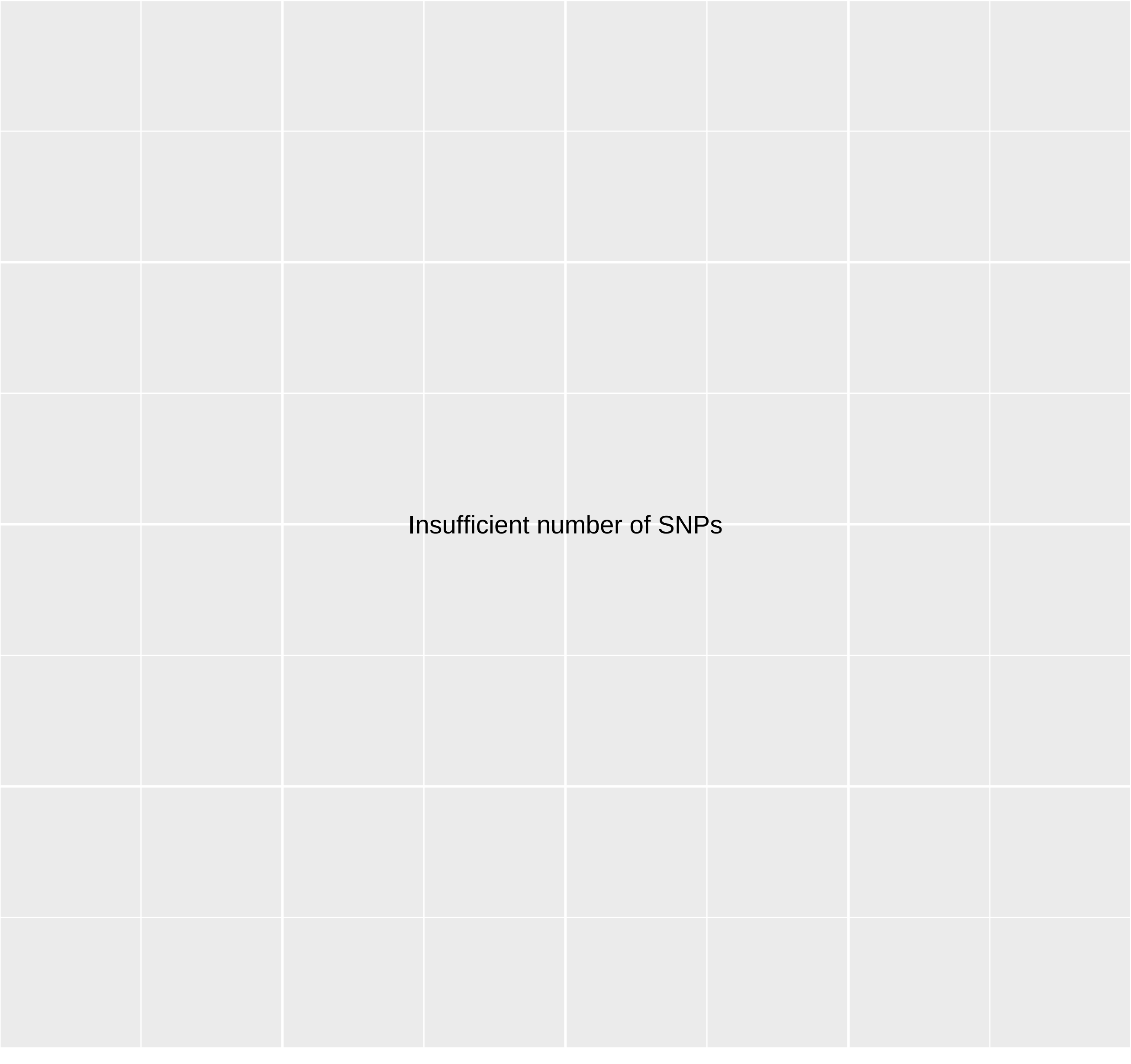
Insufficient number of SNPs

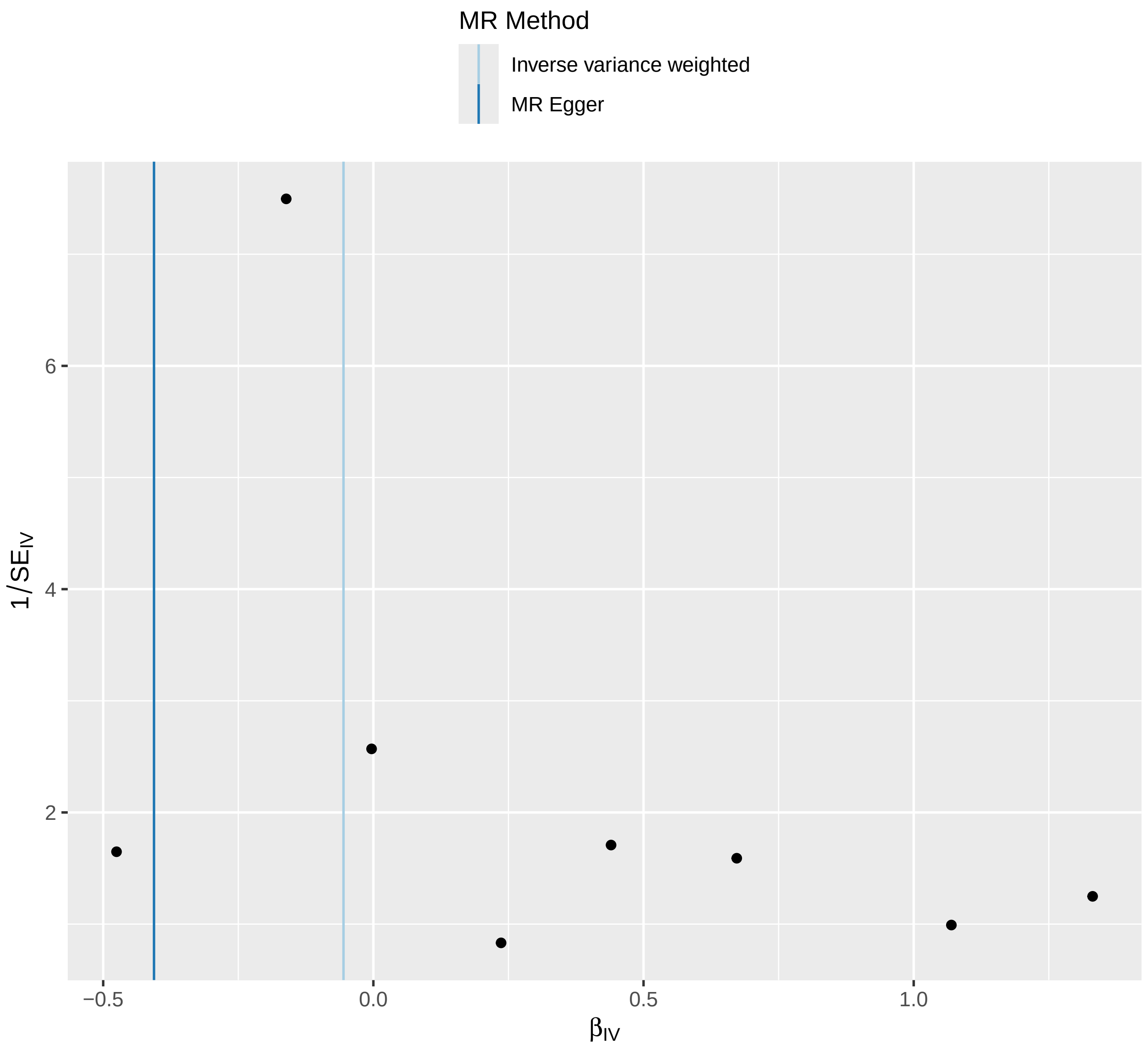
MR Method
Inverse variance weighted
MR Egger
1/SE_IV
6
4
2
−0.5
0.0
0.5
1.0
β_IV

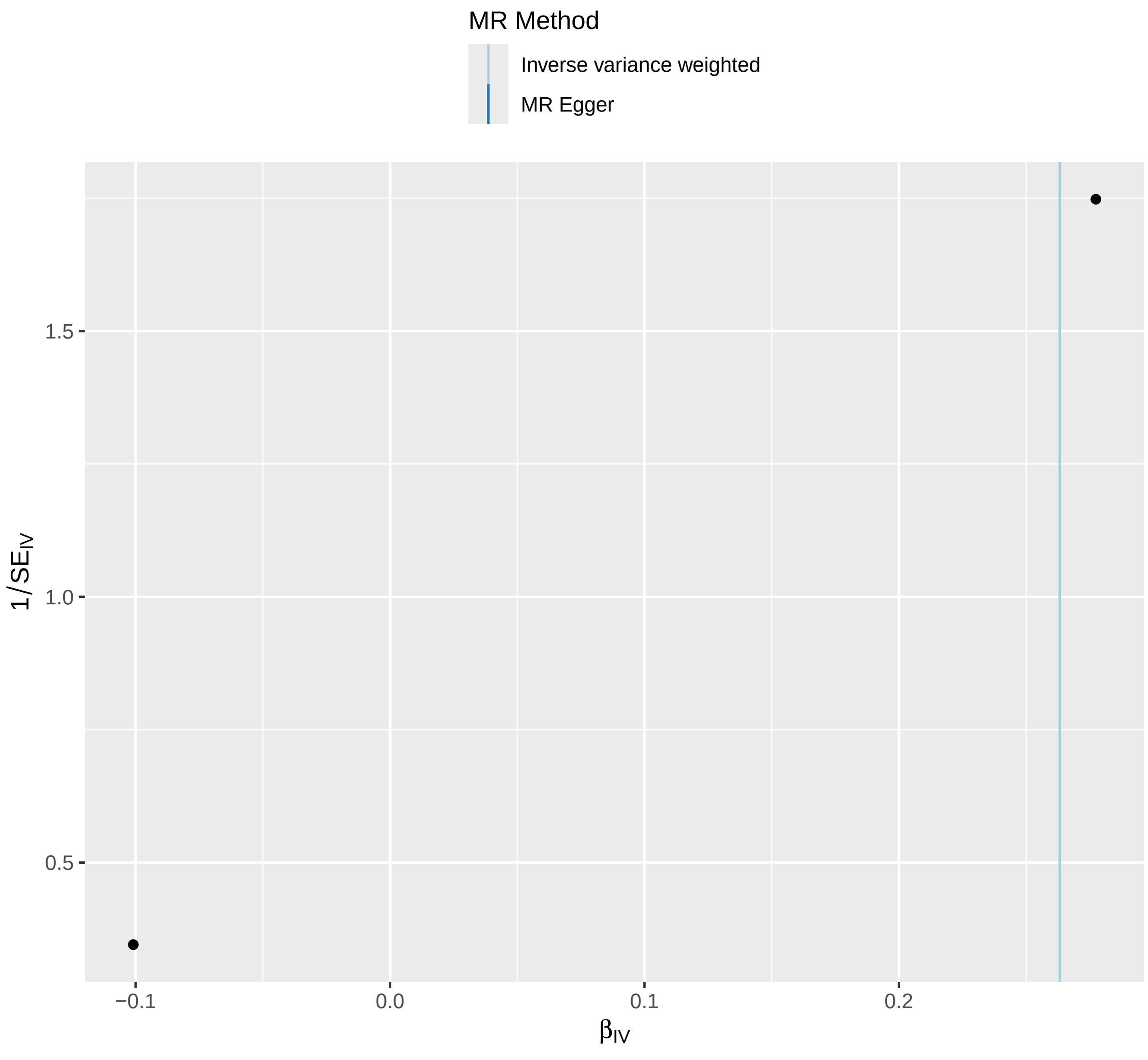

MR Method
Inverse variance weighted
MR Egger
1.5
1.0
0.5
1/SE_IV
−0.1
0.0
0.1
0.2
β_IV

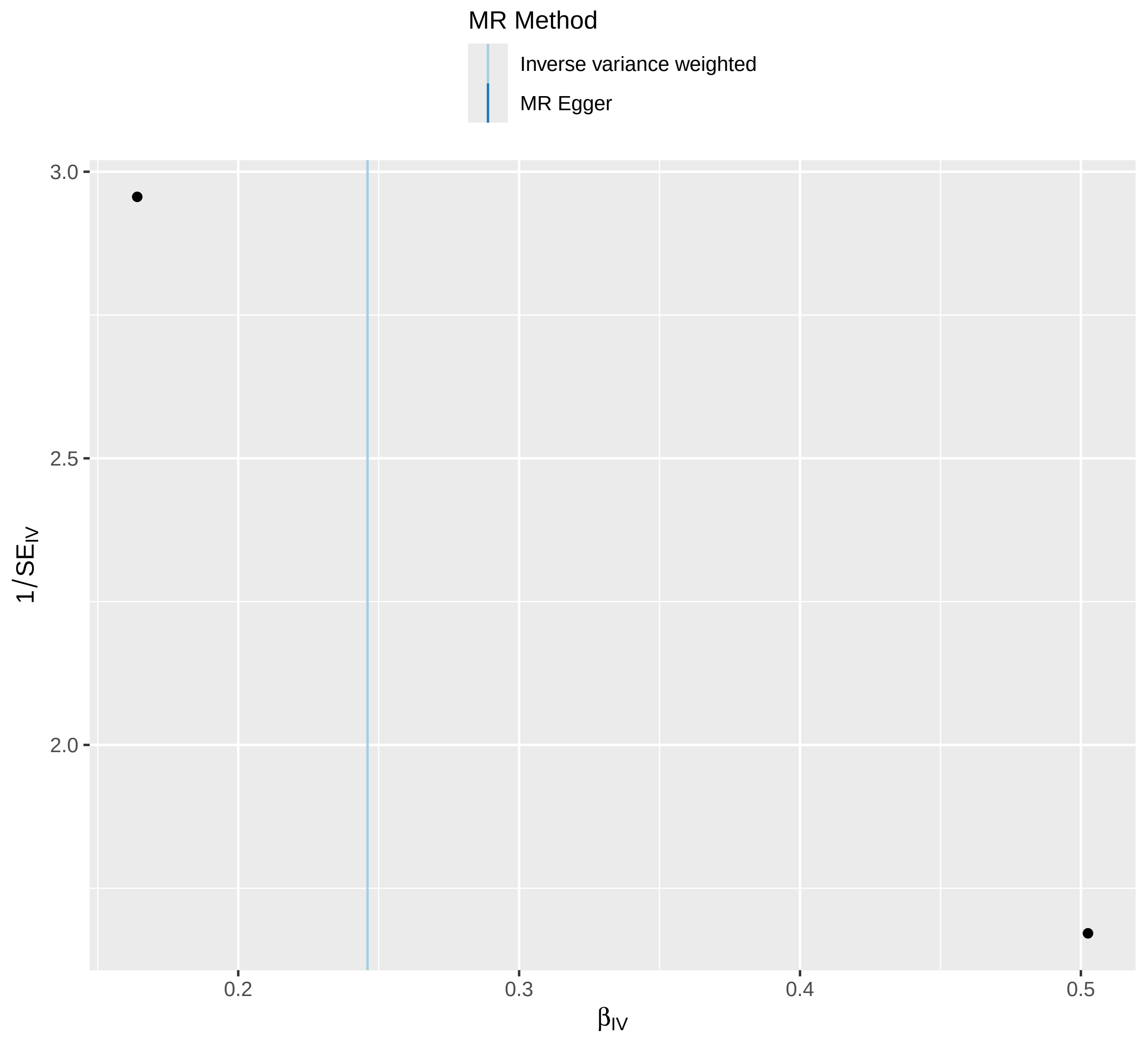

MR Method
Inverse variance weighted
MR Egger
3.0
2.5
2.0
1/SE_IV
0.2
0.3
0.4
0.5
β_IV

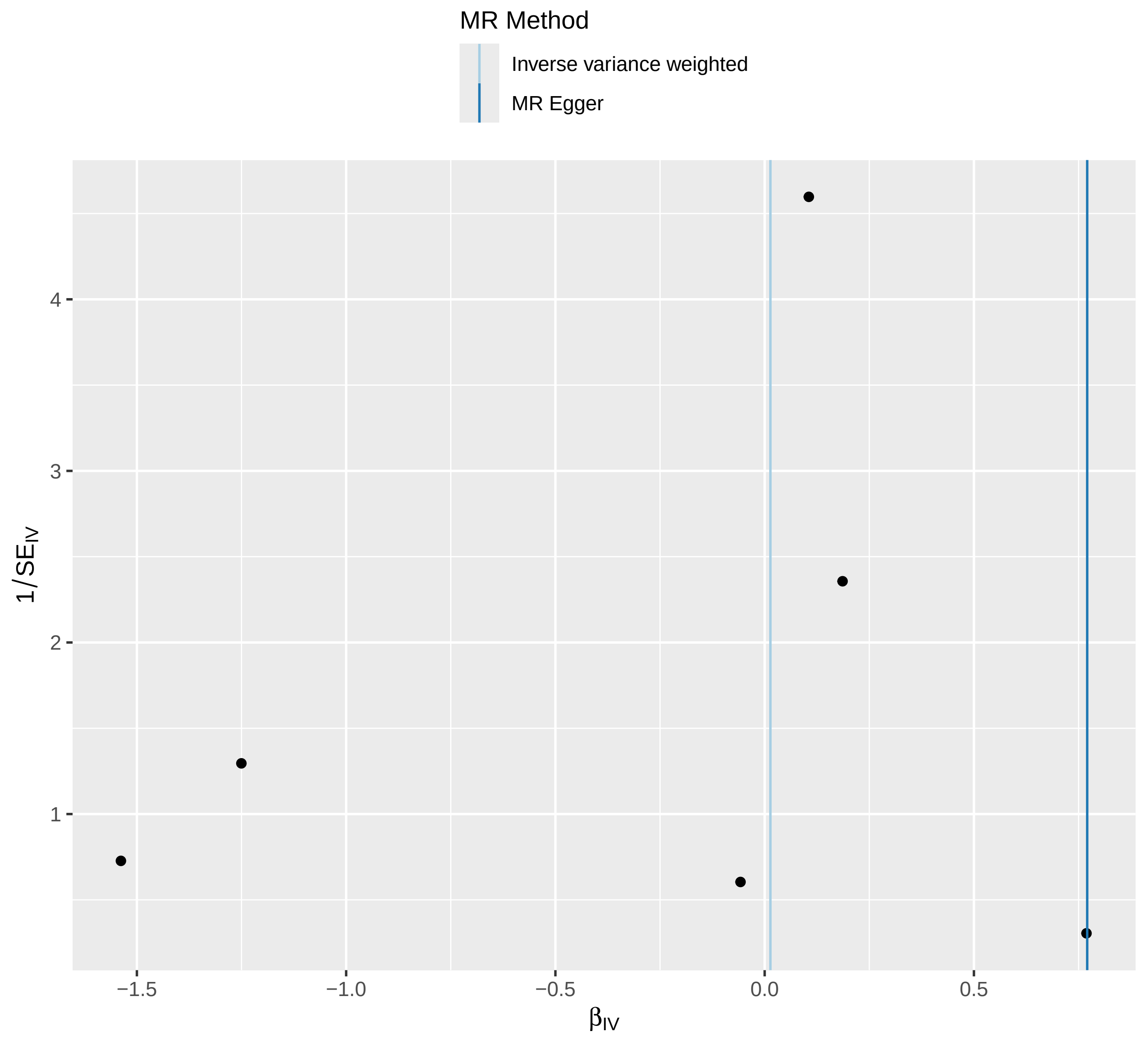

MR Method
Inverse variance weighted
MR Egger
4
3
2
1
−1.5
−1.0
−0.5
0.0
0.5
1/SE_IV
β_IV

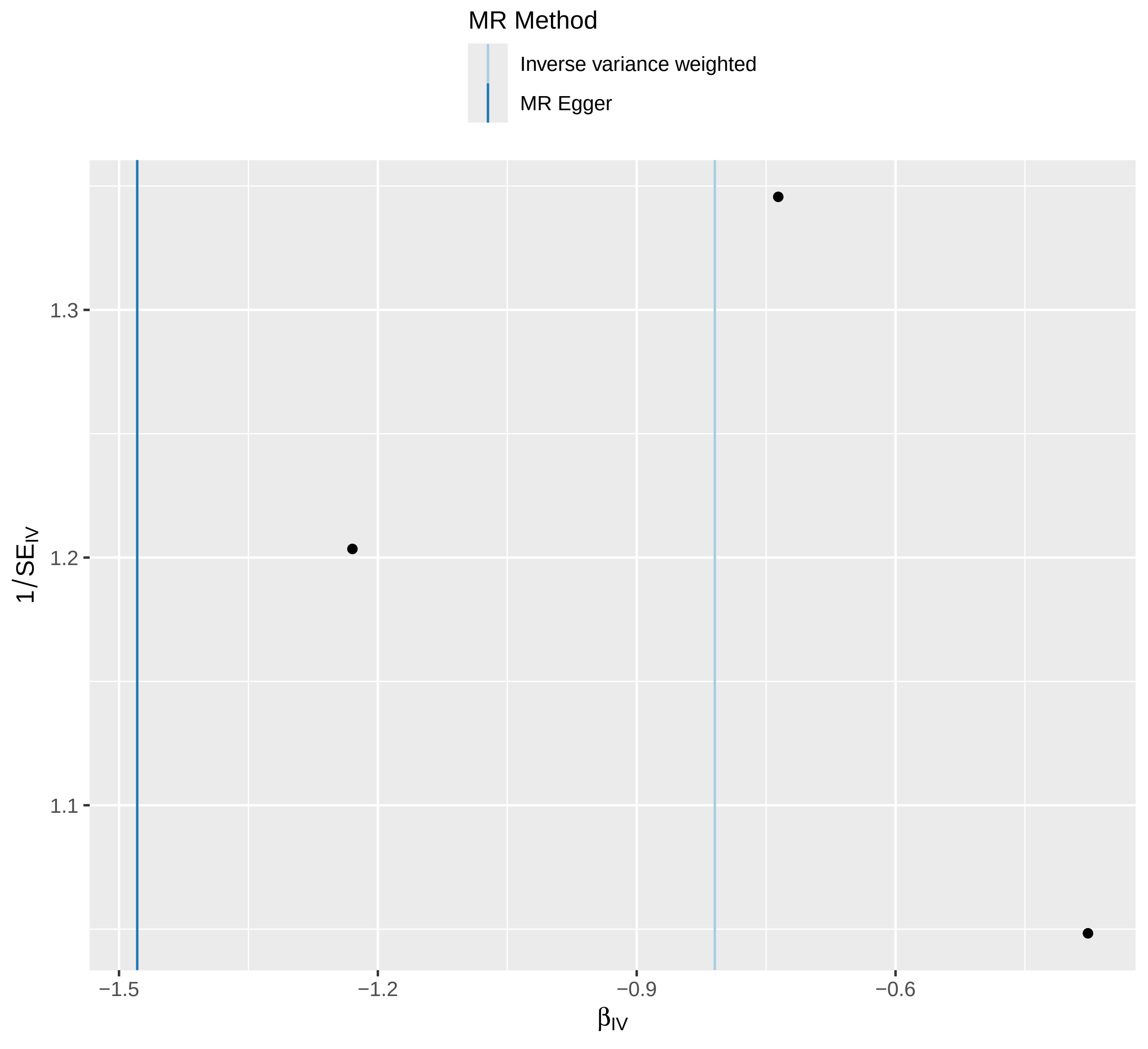

MR Method
Inverse variance weighted
MR Egger
1.3
1.2
1.1
−1.5
−1.2
−0.9
−0.6
1/SE_IV
β_IV

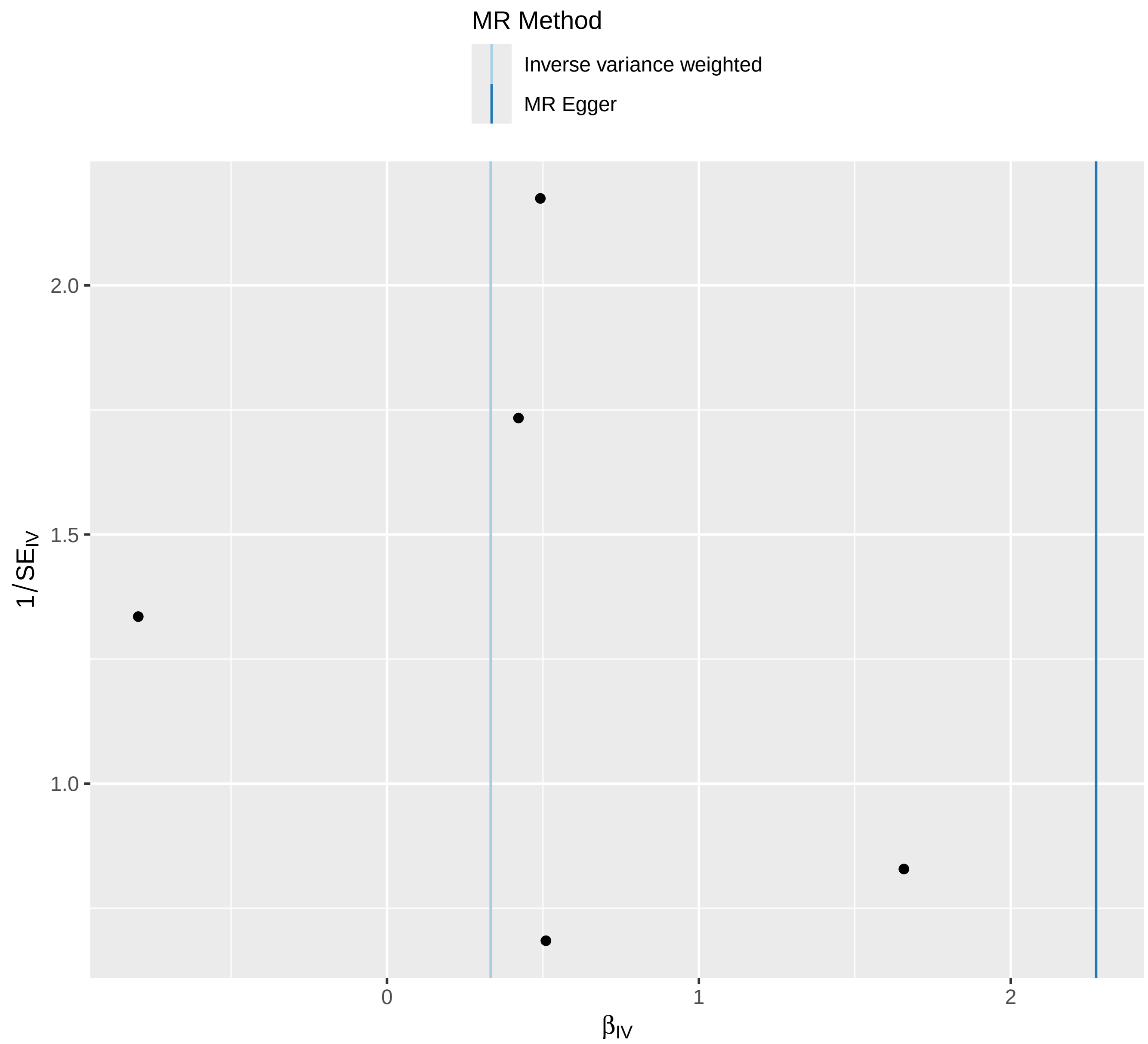
MR Method
Inverse variance weighted
MR Egger
2.0
1.5
1.0
1/SE_IV
0
1
2
β_IV

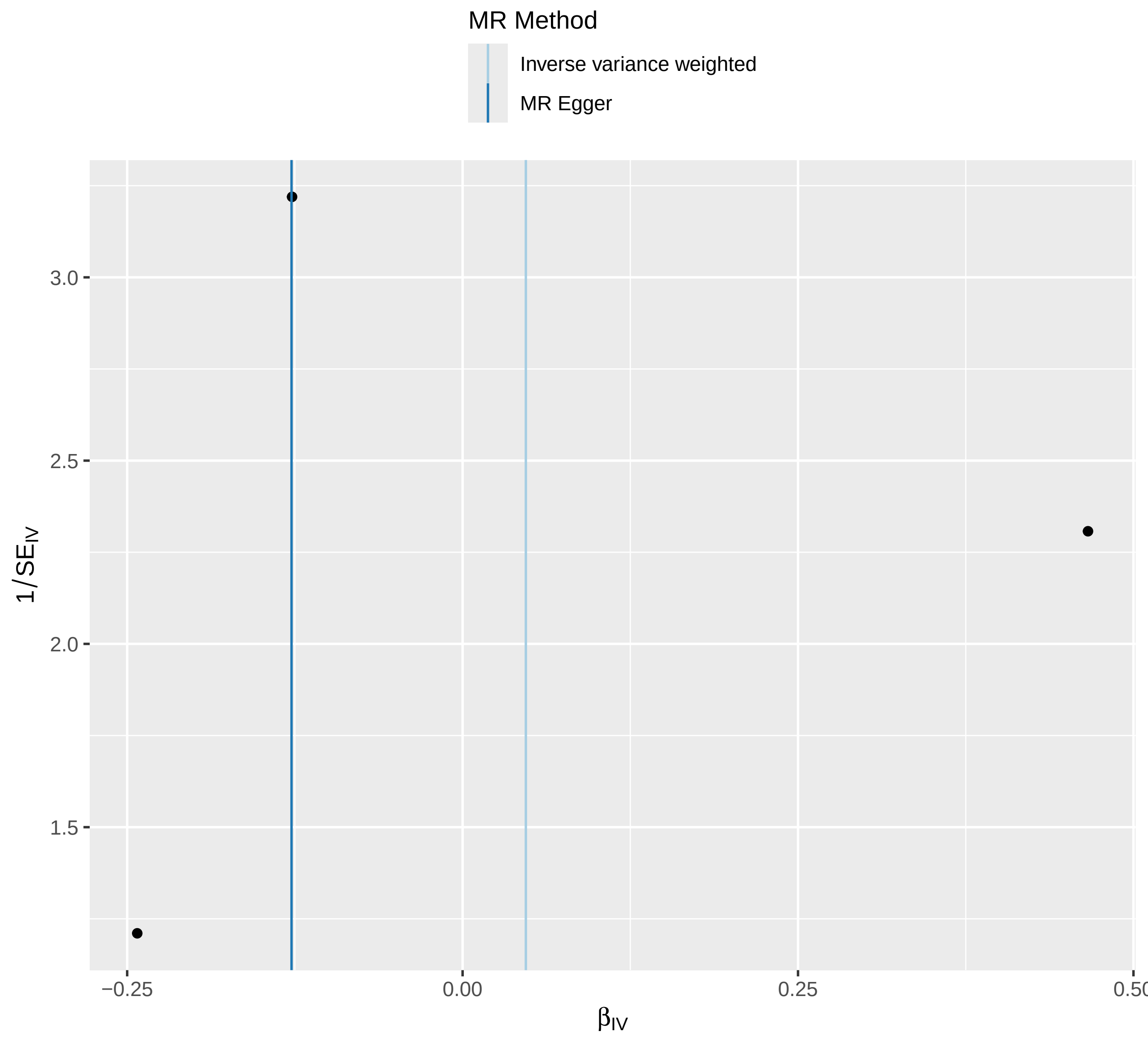

MR Method
Inverse variance weighted
MR Egger
3.0
2.5
2.0
1.5
1/SE_IV
−0.25
0.00
0.25
0.50
β_IV

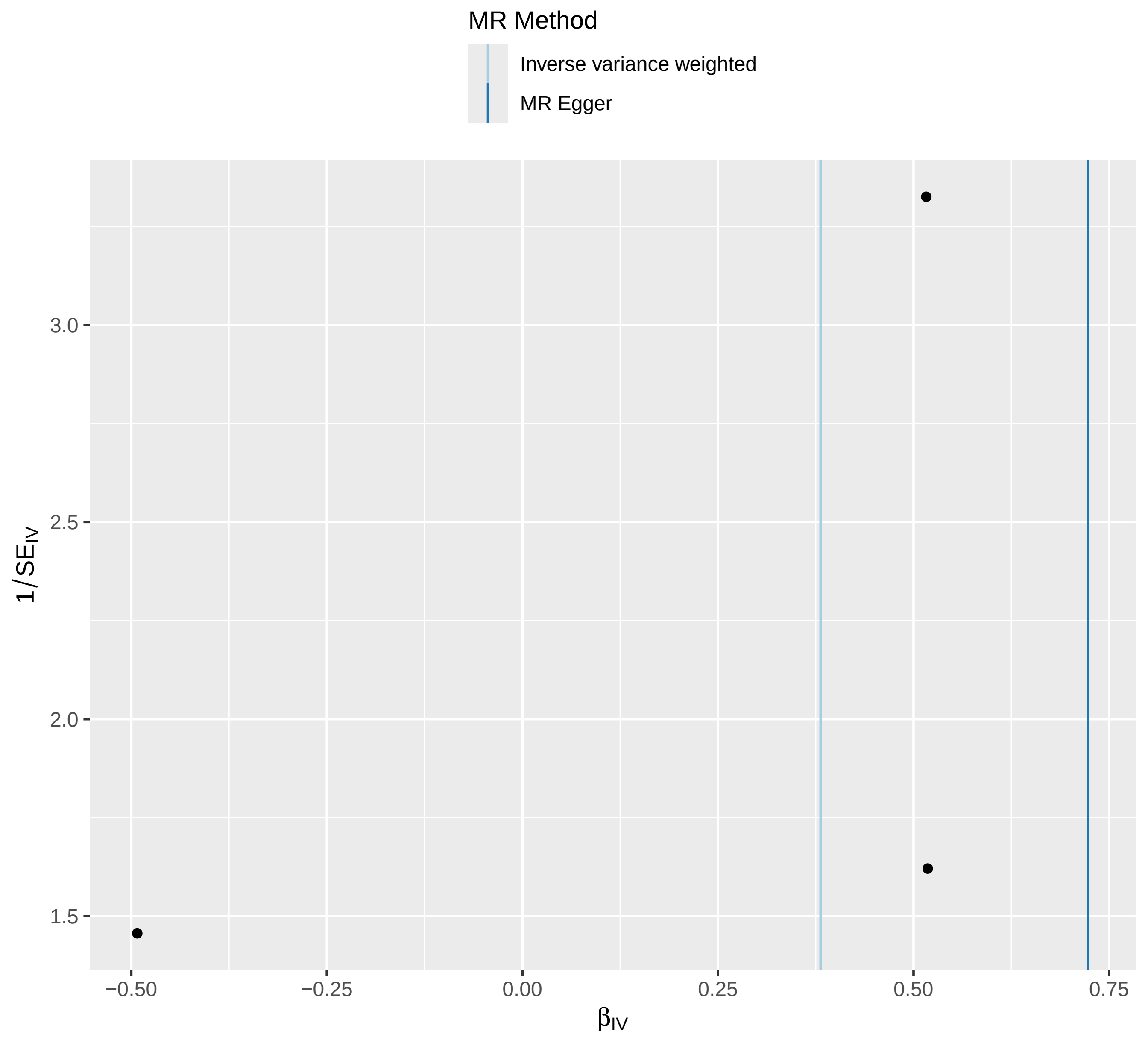
MR Method
Inverse variance weighted
MR Egger
3.0
2.5
2.0
1.5
1/SE_IV
−0.50
−0.25
0.00
0.25
0.50
0.75
β_IV

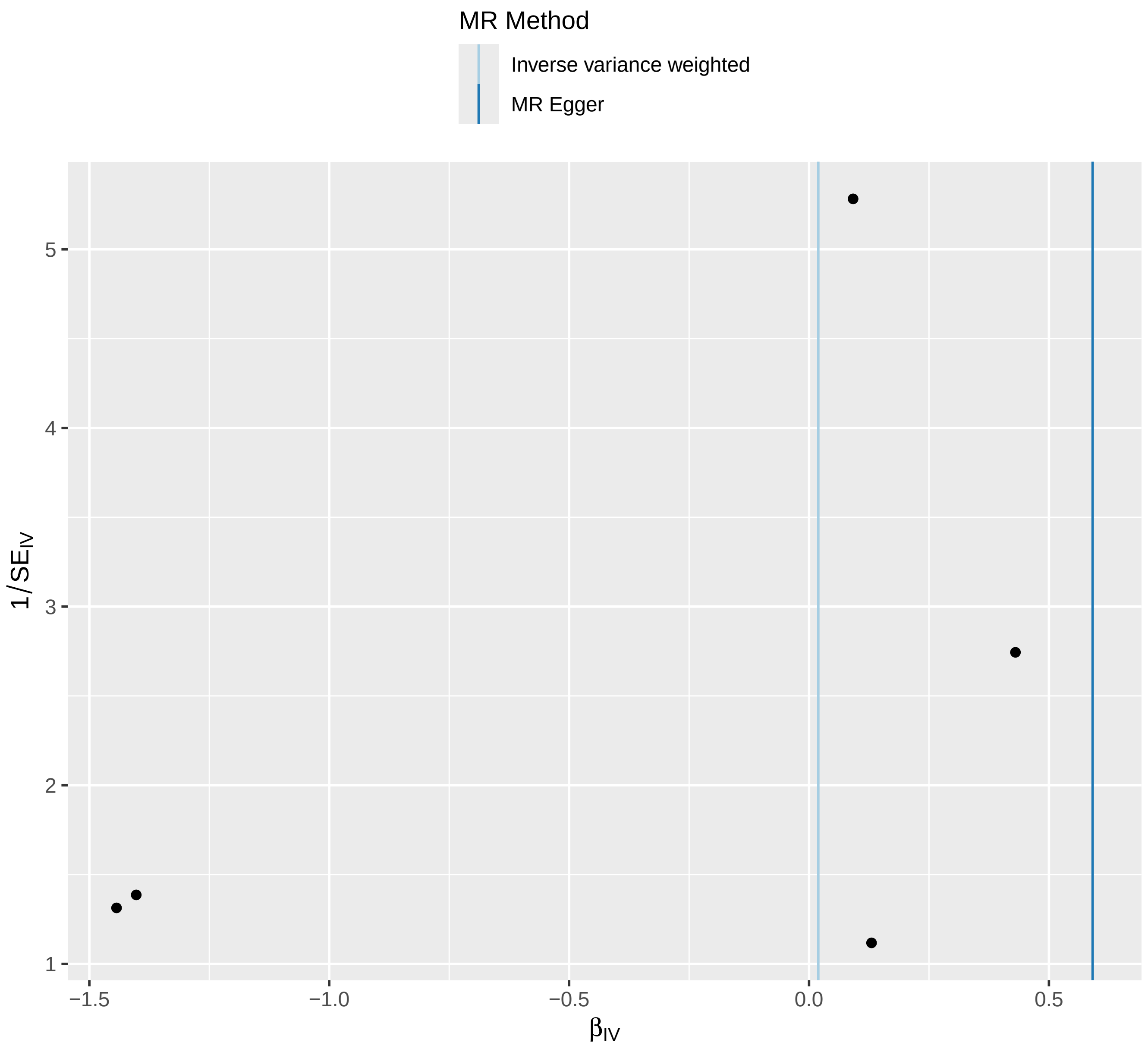

MR Method
Inverse variance weighted
MR Egger
1/SE$_{IV}$
5
4
3
2
1
−1.5
−1.0
−0.5
0.0
0.5
β$_{IV}$

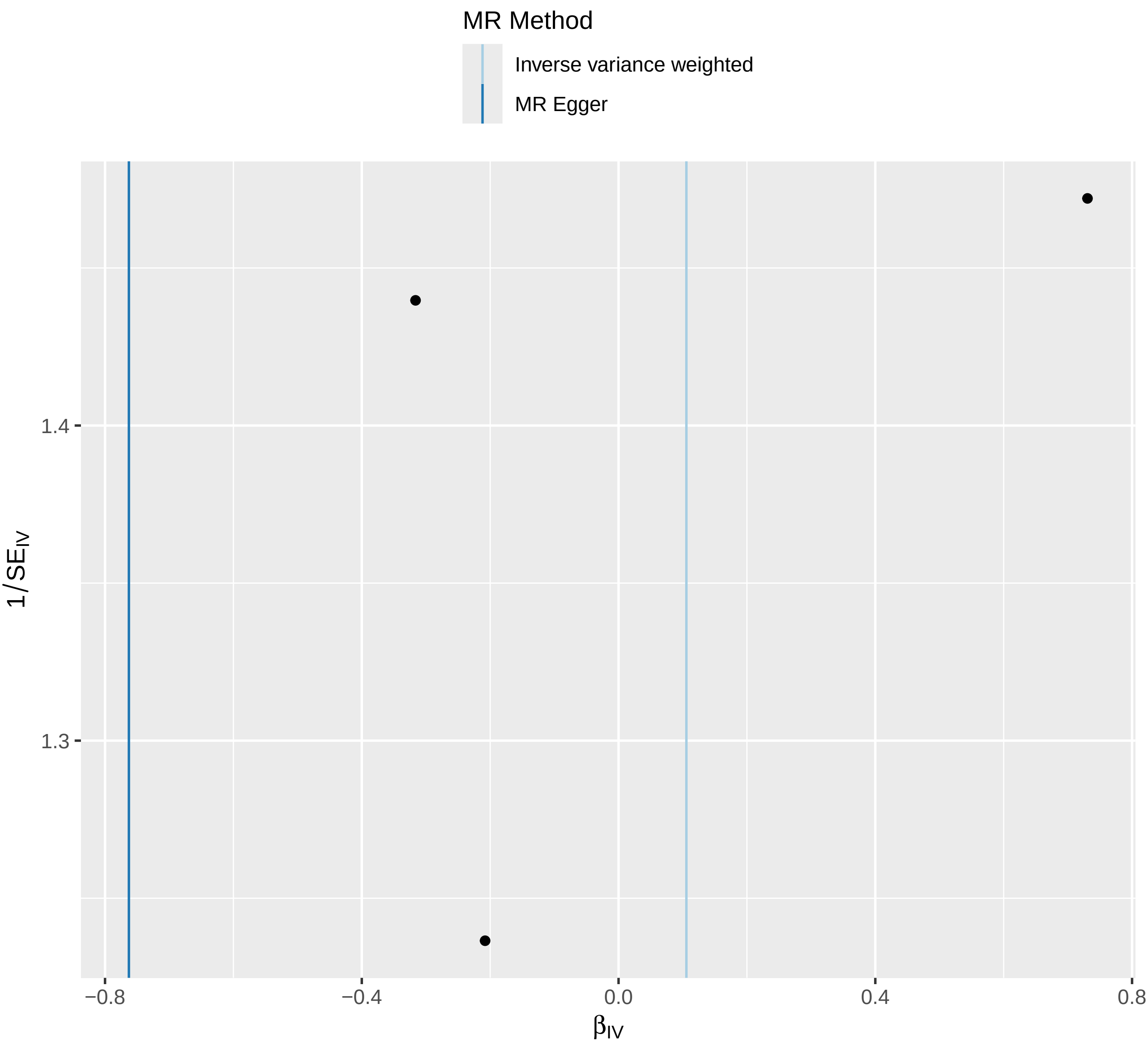
MR Method
Inverse variance weighted
MR Egger
1.4
1.3
1/SE_IV
−0.8
−0.4
0.0
0.4
0.8
β_IV

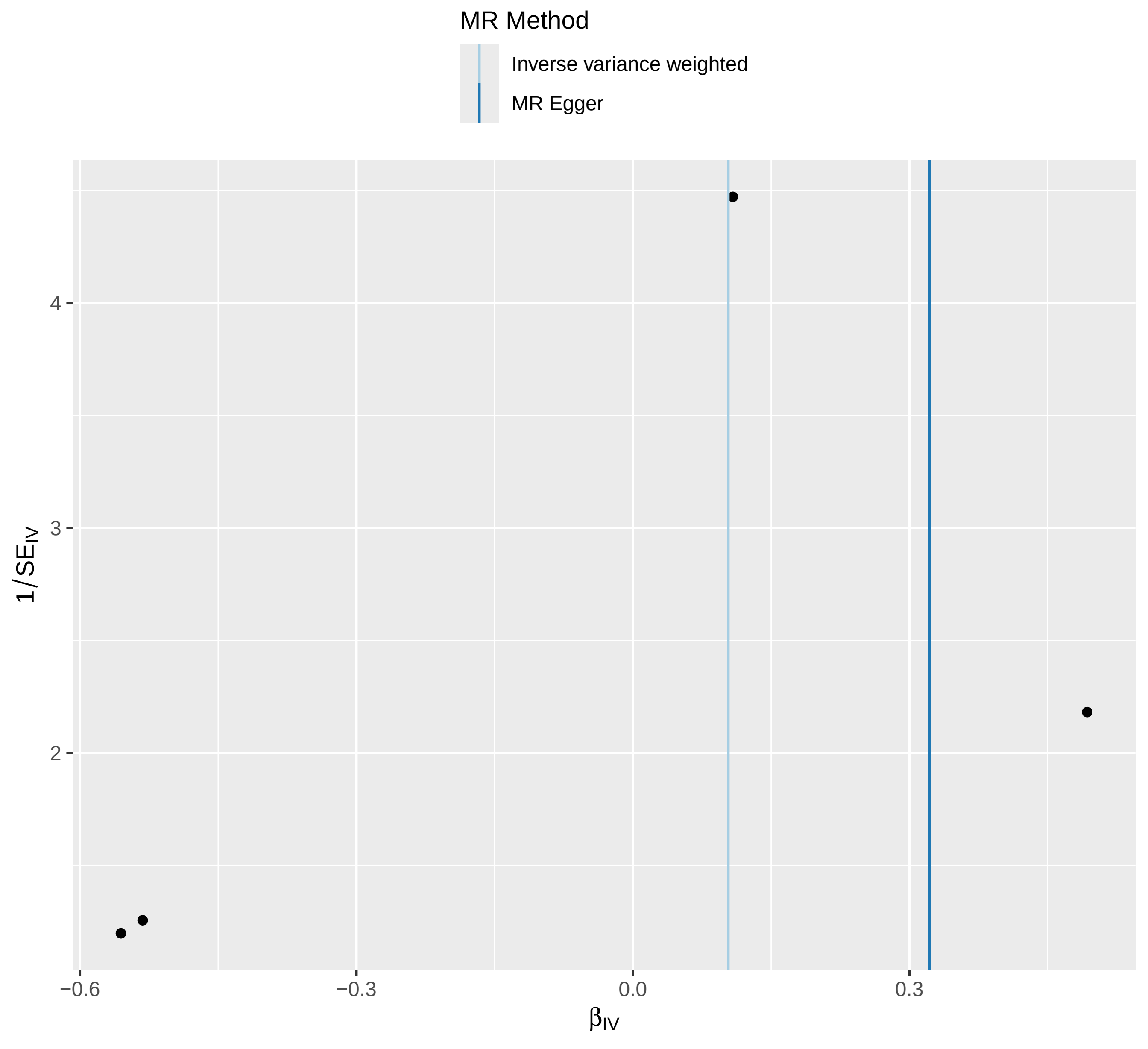
MR Method
Inverse variance weighted
MR Egger
4
3
2
1/SE_IV
−0.6
−0.3
0.0
0.3
β_IV

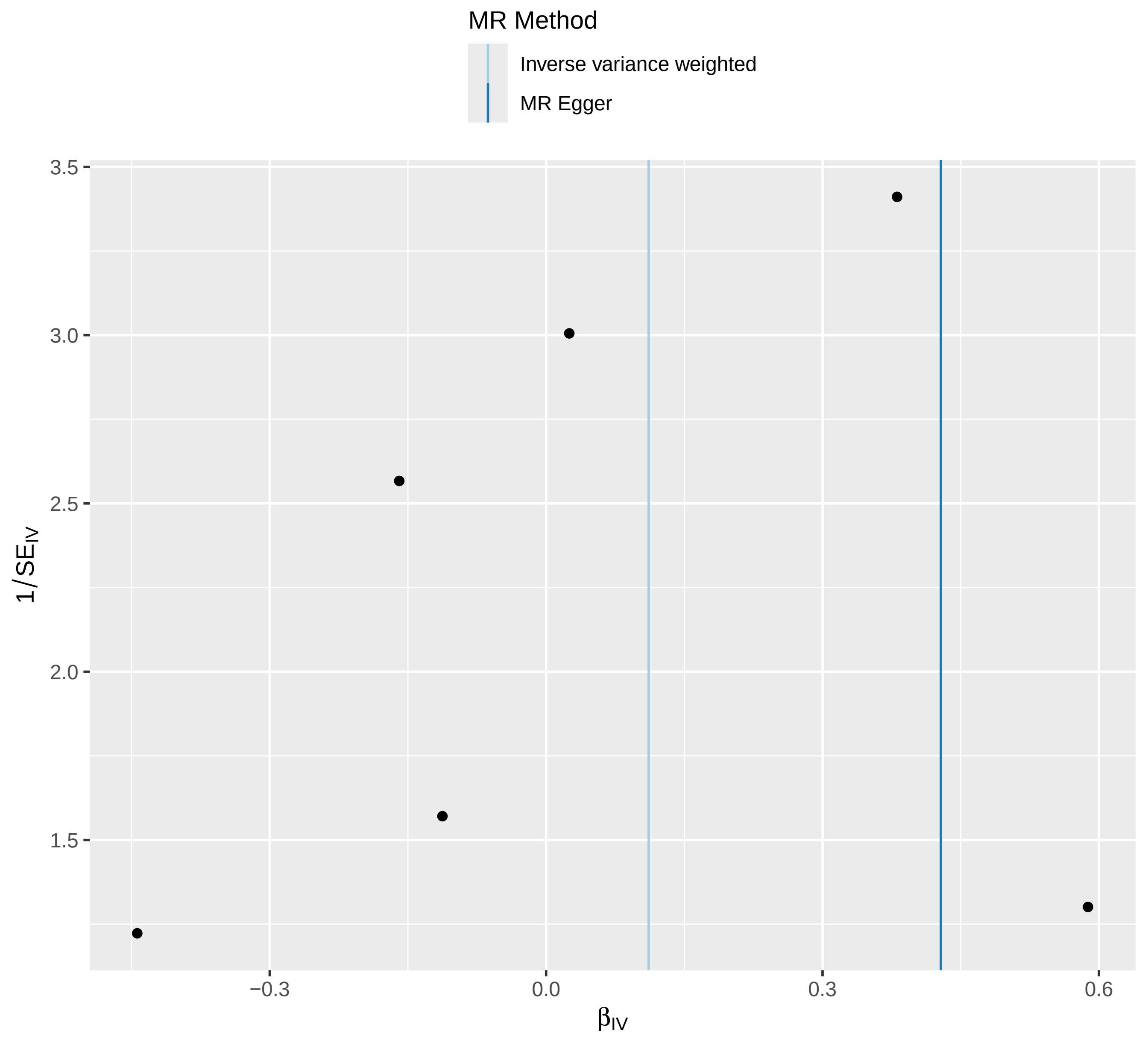

MR Method
Inverse variance weighted
MR Egger
3.5
3.0
2.5
2.0
1.5
1/SE$_{IV}$
−0.3
0.0
0.3
0.6
β$_{IV}$

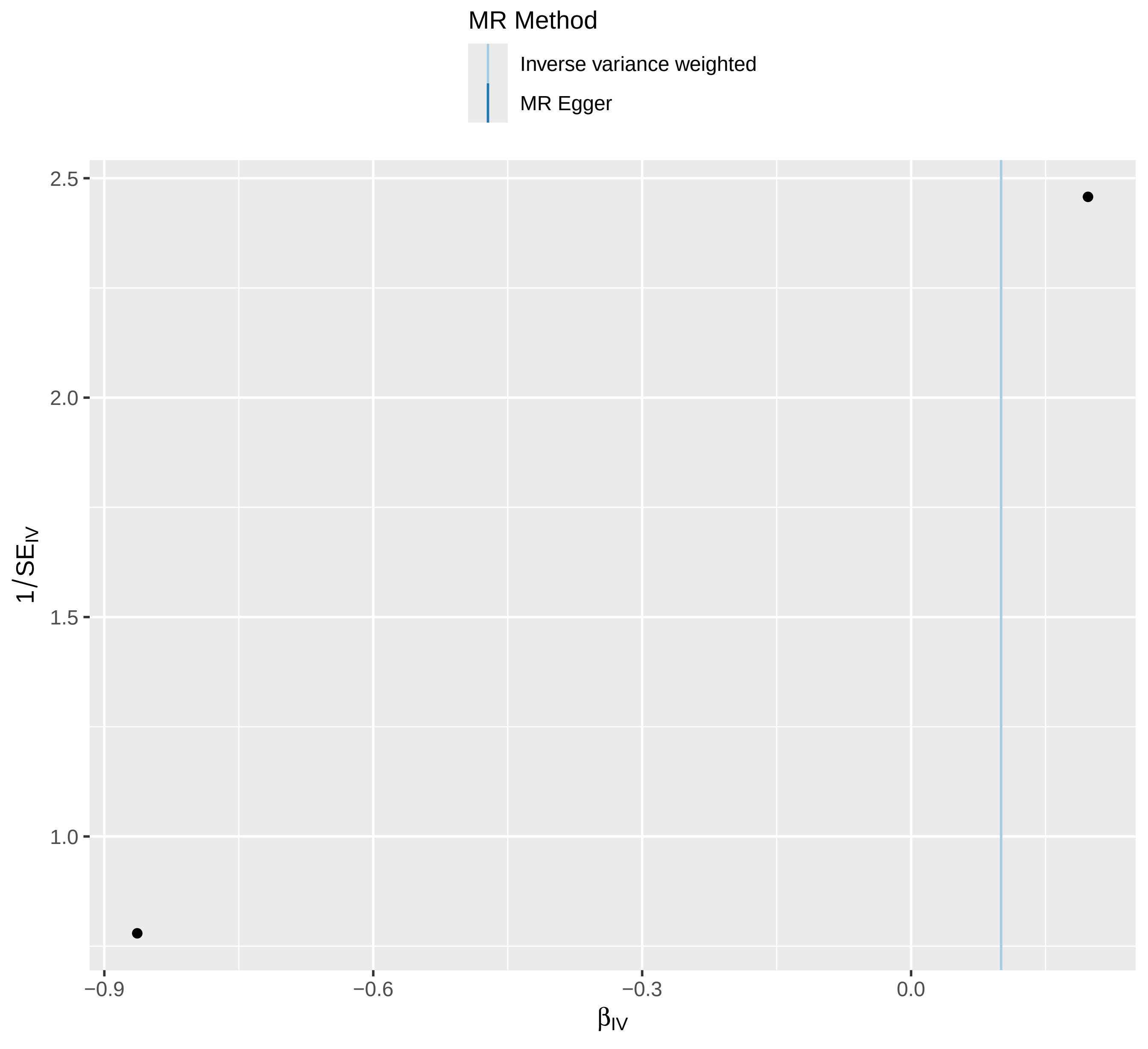
MR Method
Inverse variance weighted
MR Egger
2.5
2.0
1.5
1.0
1/SE_IV
−0.9
−0.6
−0.3
0.0
β_IV

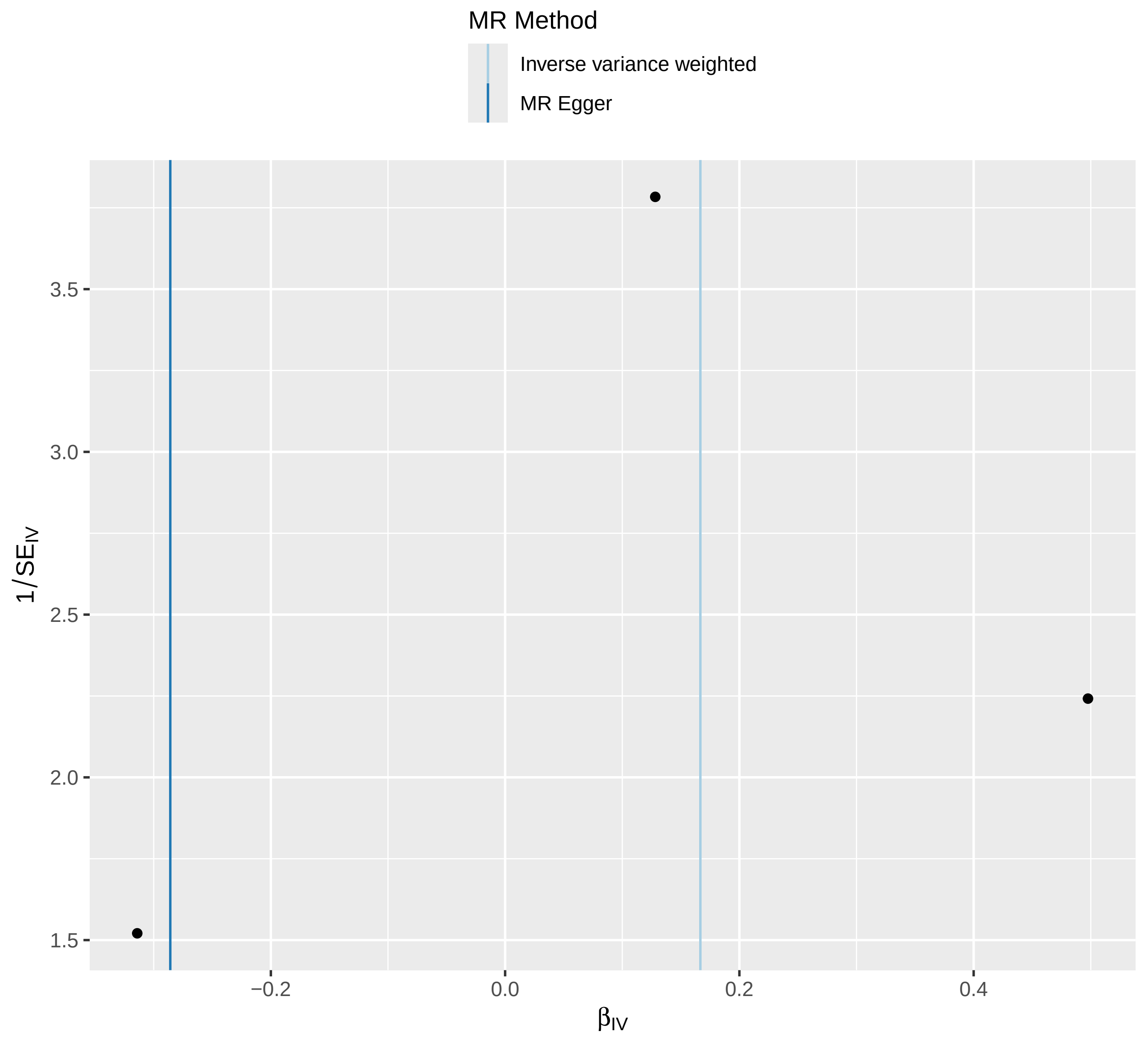
MR Method
Inverse variance weighted
MR Egger
3.5
3.0
2.5
2.0
1.5
1/SE_IV
−0.2
0.0
0.2
0.4
β_IV

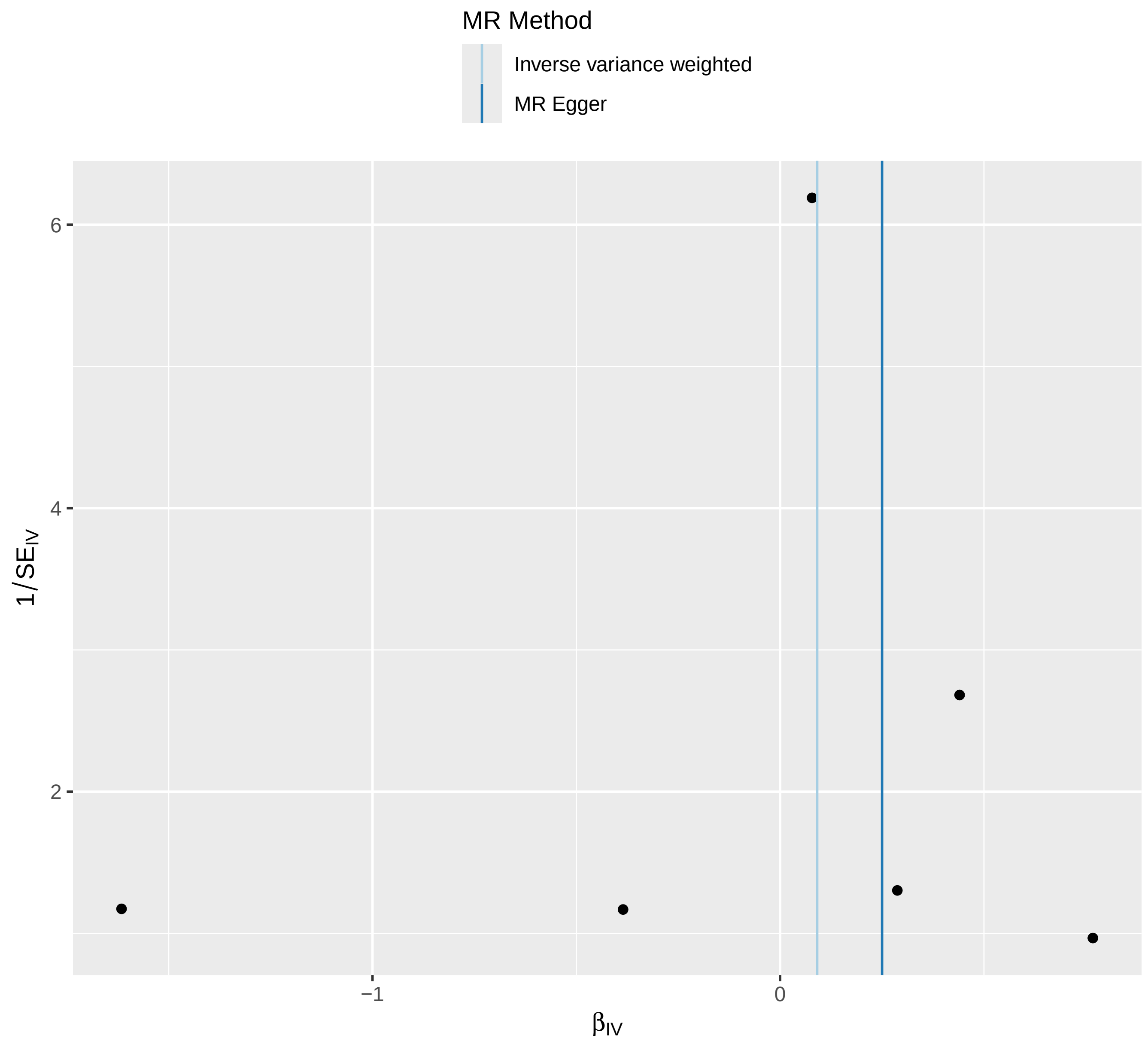

MR Method
Inverse variance weighted
MR Egger
6
4
2
1/SE_IV
−1
0
β_IV

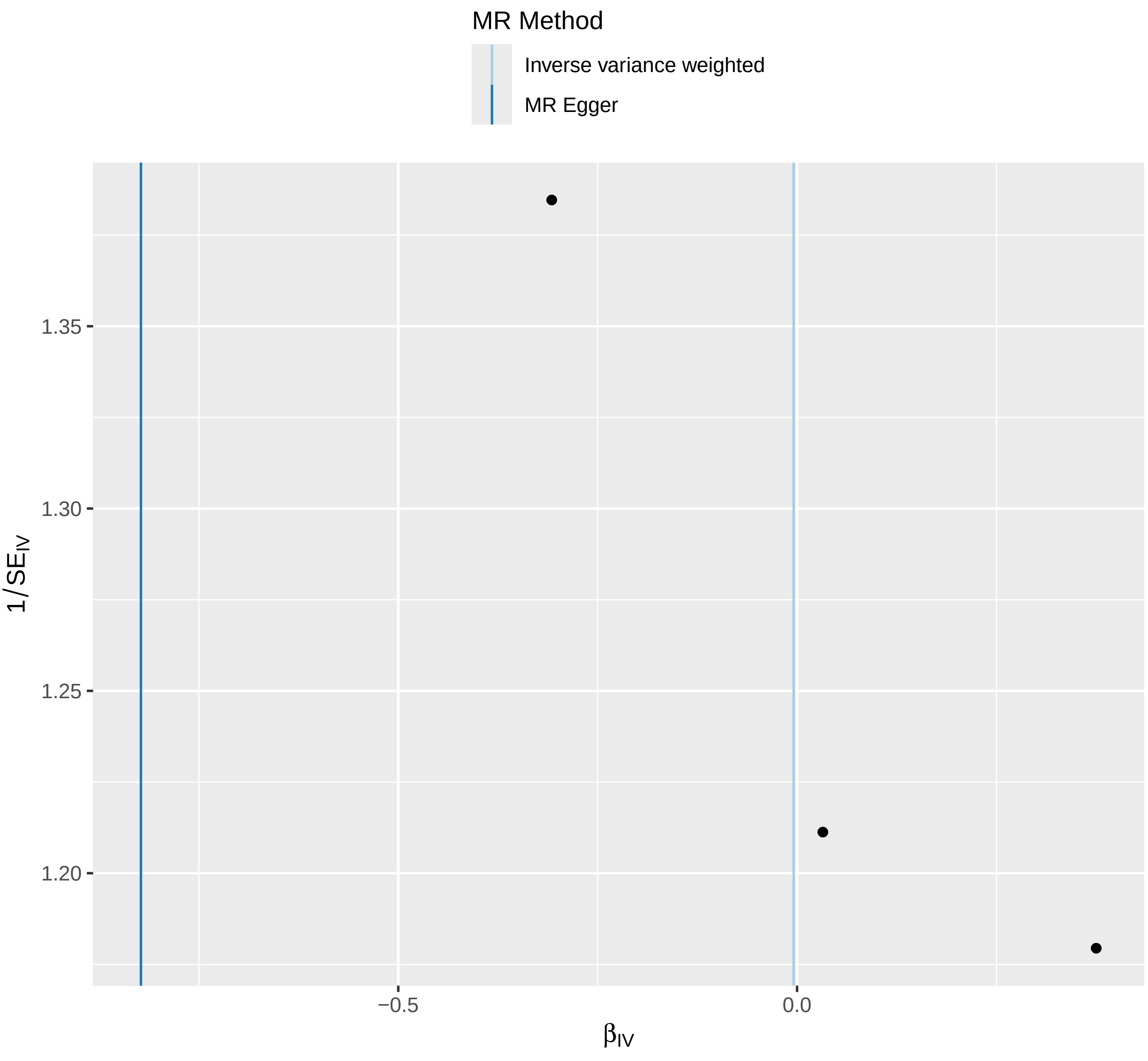
MR Method
Inverse variance weighted
MR Egger
1.35
1.30
1.25
1.20
1/SE_IV
−0.5
0.0
β_IV

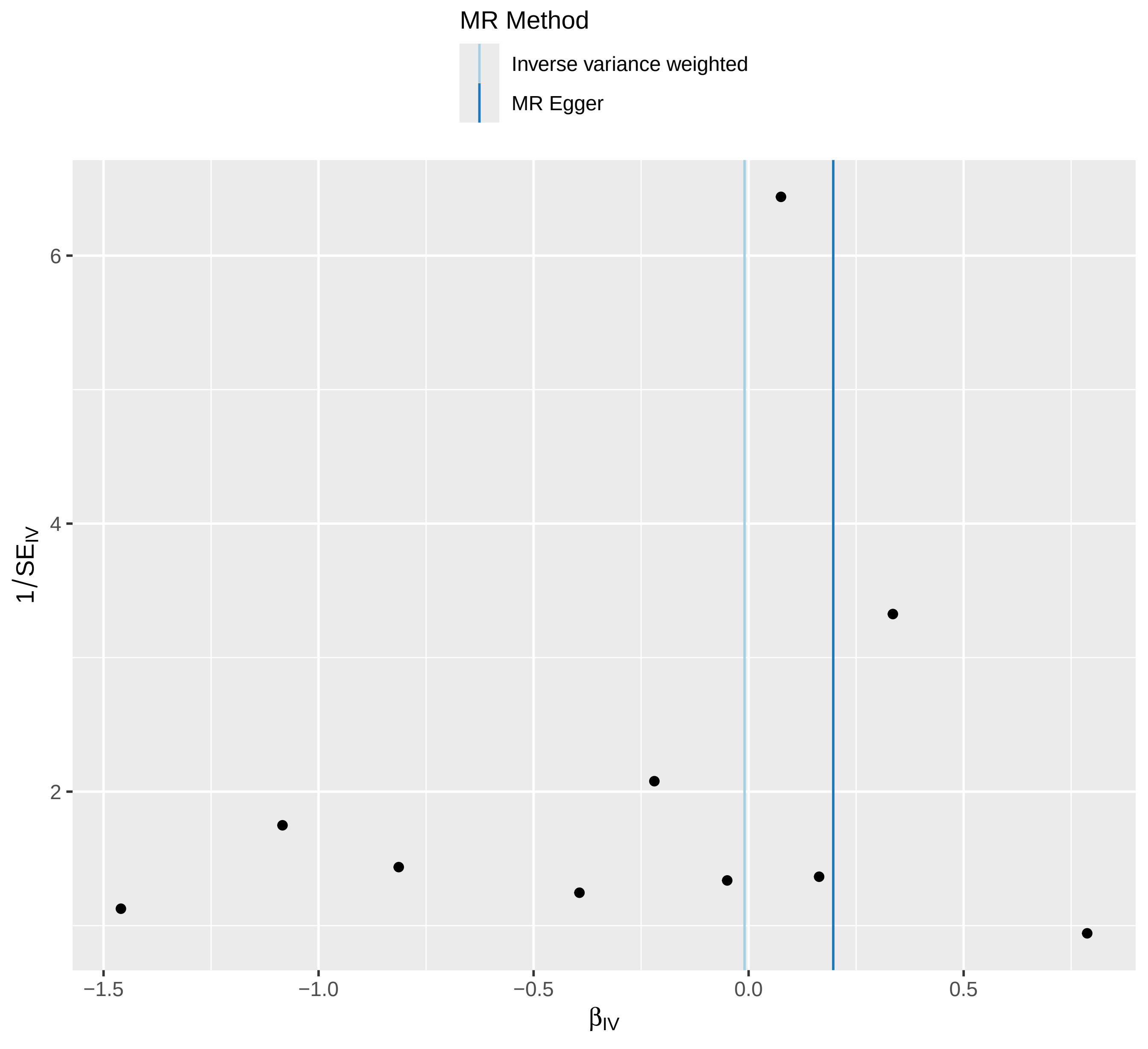

MR Method
Inverse variance weighted
MR Egger
6
4
2
1/SE$_{IV}$
−1.5
−1.0
−0.5
0.0
0.5
β$_{IV}$

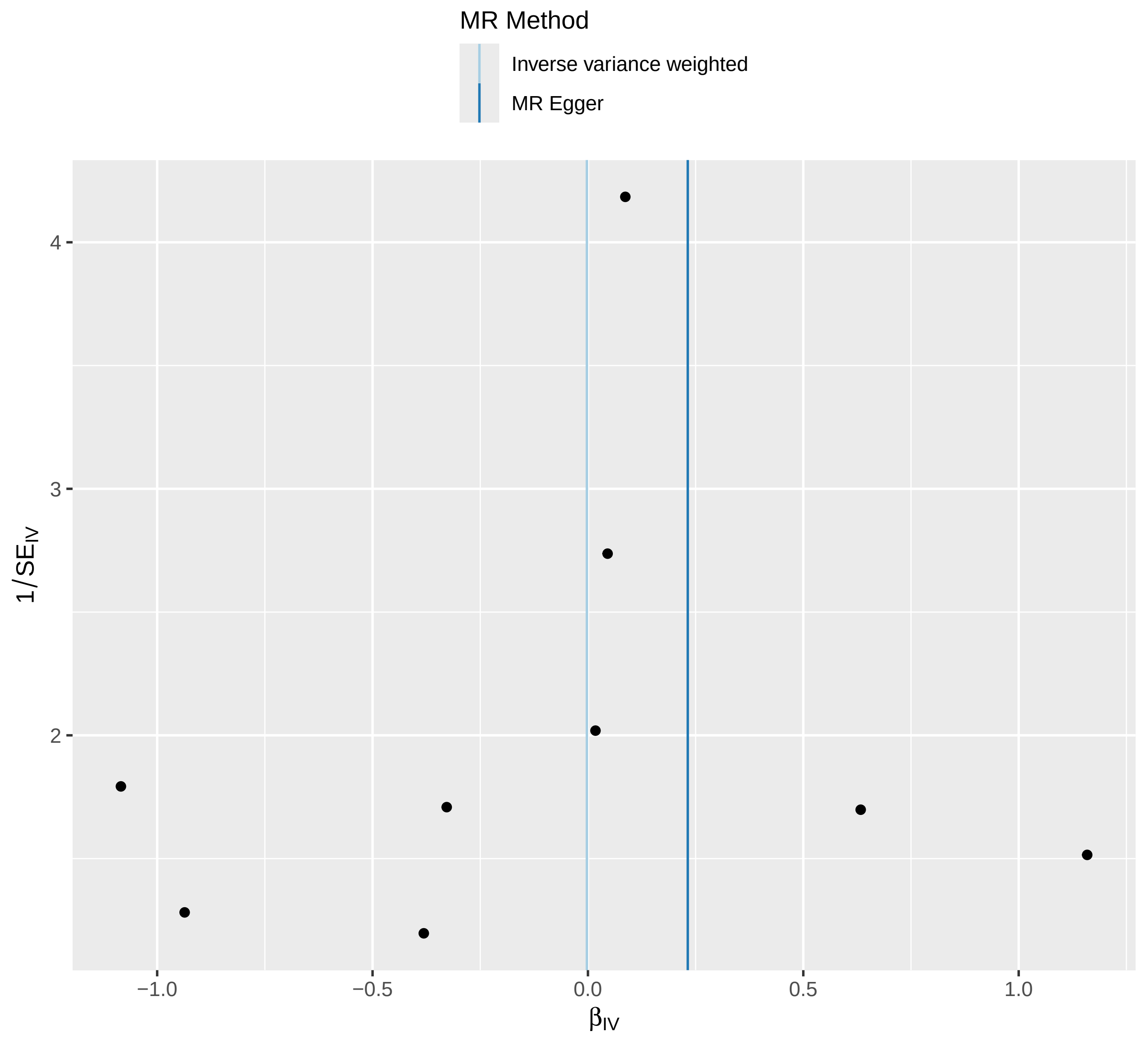

MR Method
Inverse variance weighted
MR Egger
4
3
2
1/SE_IV
−1.0
−0.5
0.0
0.5
1.0
β_IV

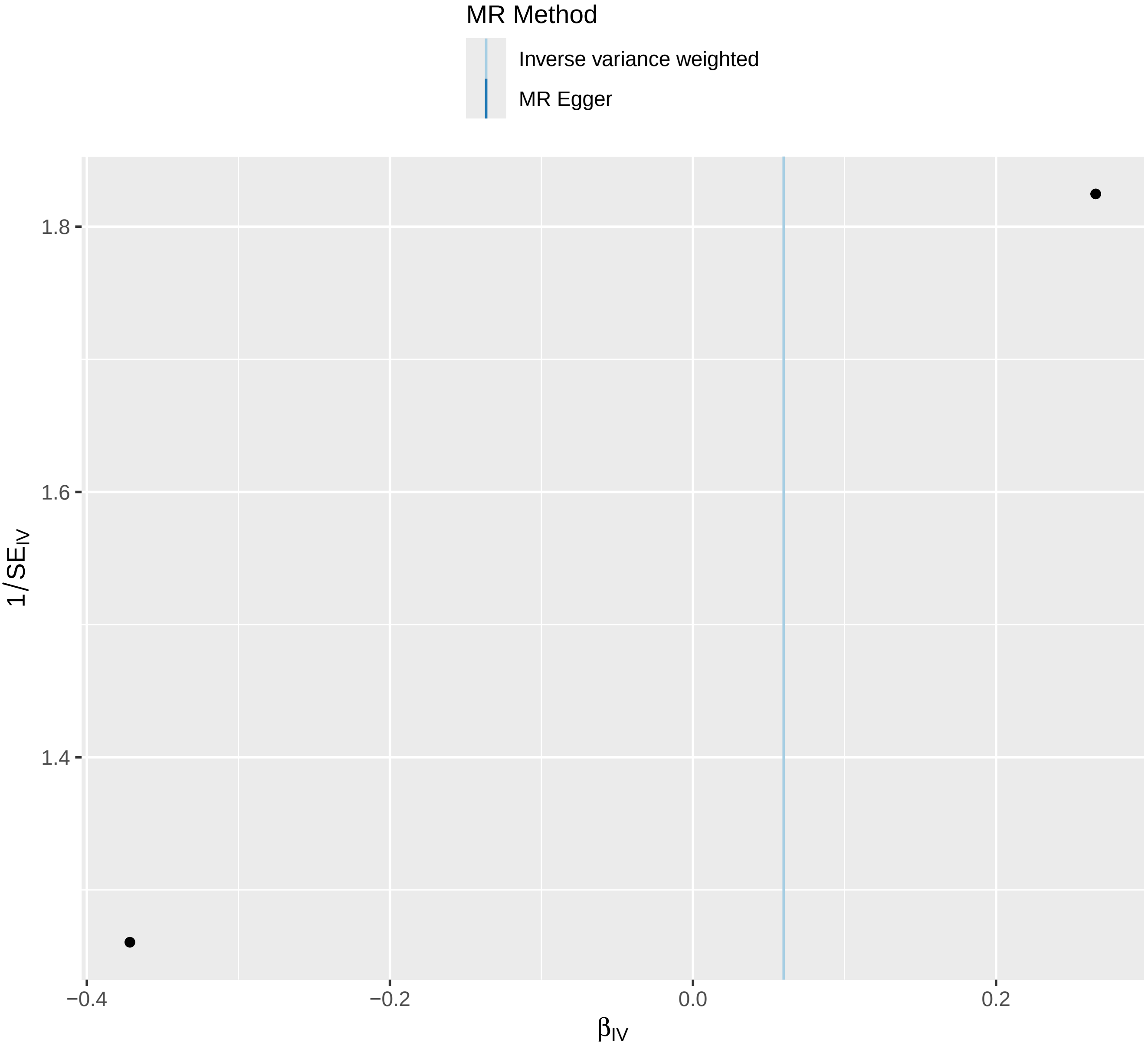

MR Method
Inverse variance weighted
MR Egger
1.8
1.6
1.4
1/SE_IV
−0.4
−0.2
0.0
0.2
β_IV

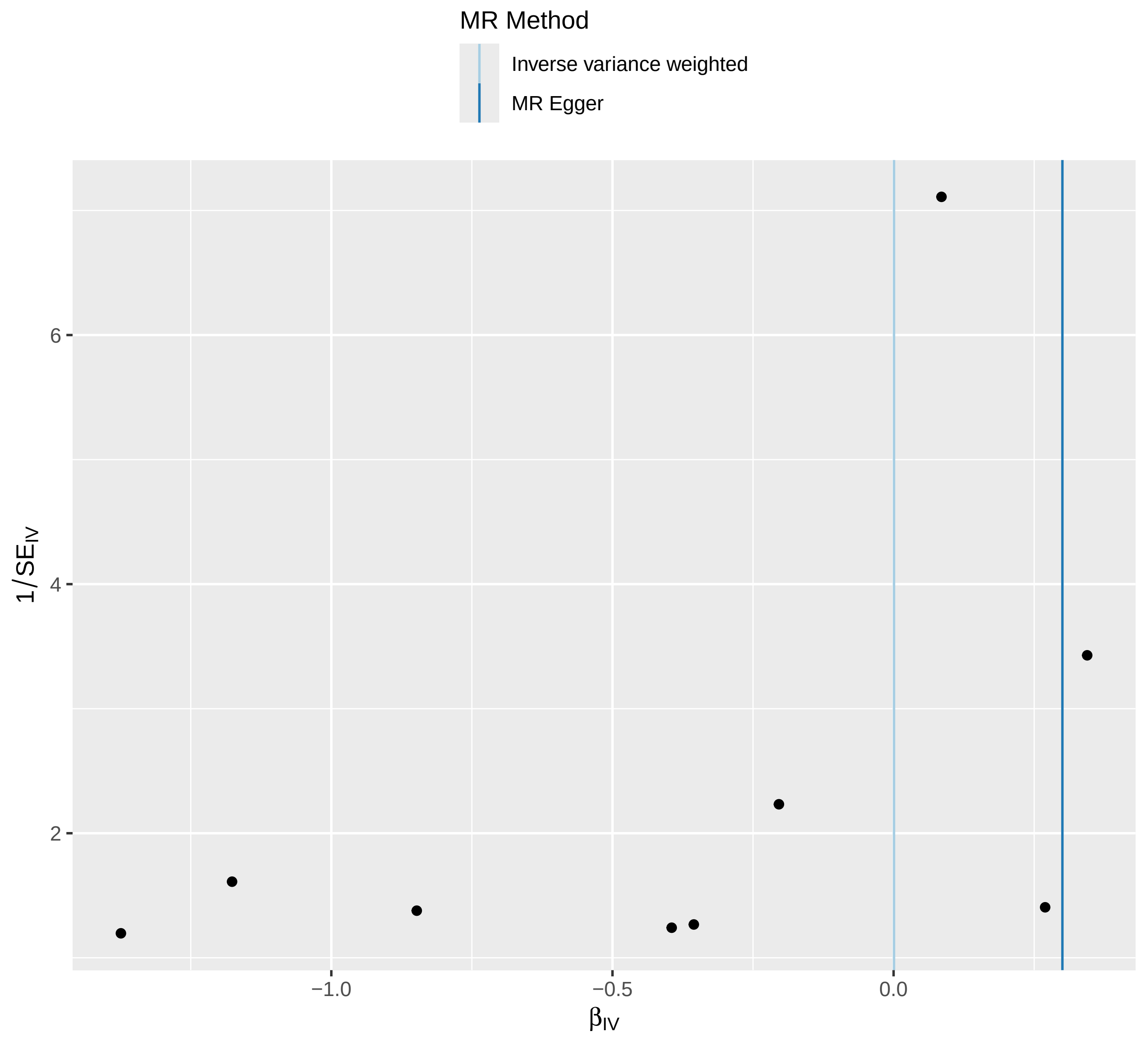
MR Method
Inverse variance weighted
MR Egger
6
4
2
1/SE_IV
−1.0
−0.5
0.0
β_IV

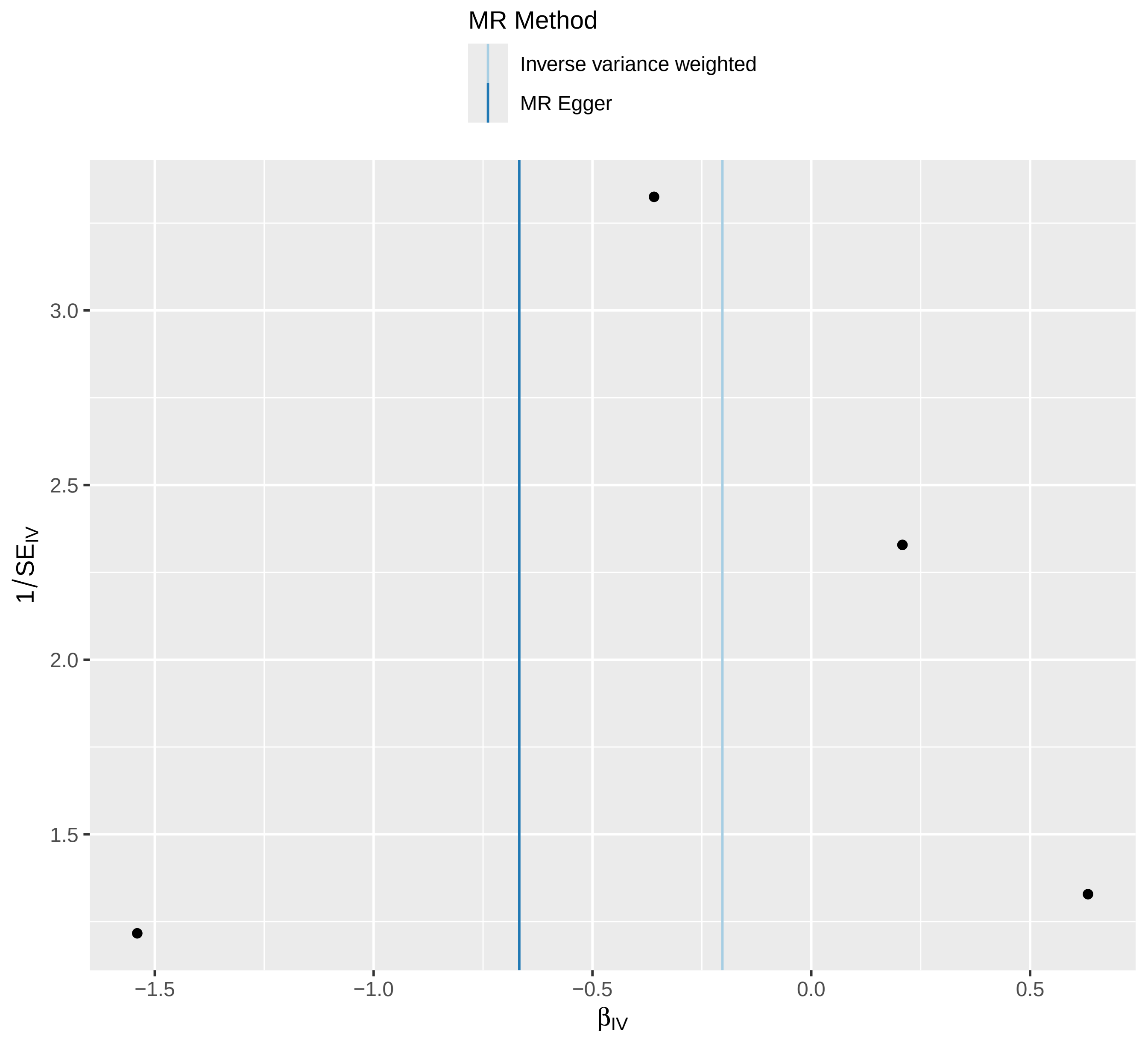

MR Method
Inverse variance weighted
MR Egger
3.0
2.5
2.0
1.5
1/SE_IV
−1.5
−1.0
−0.5
0.0
0.5
β_IV

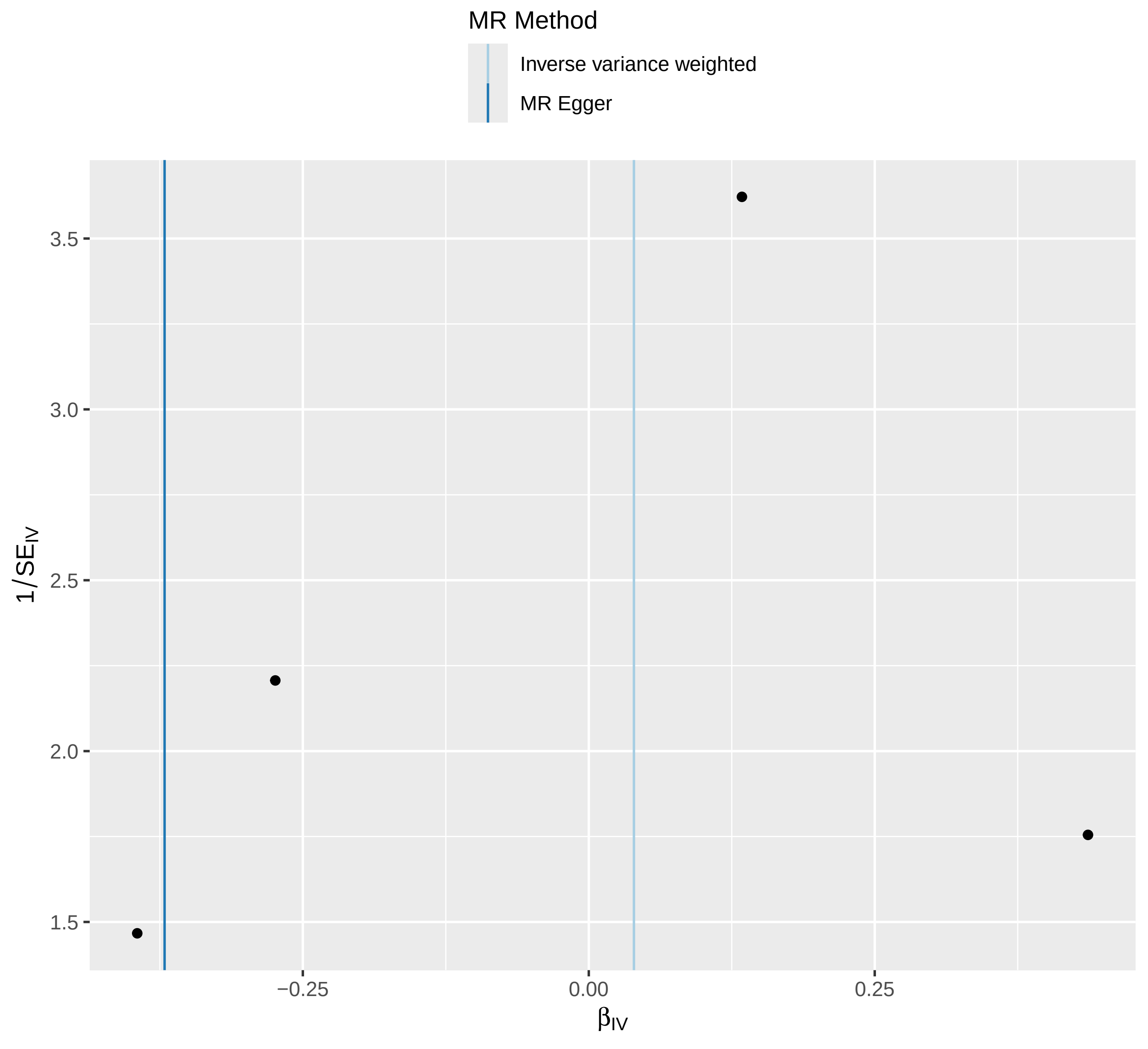
MR Method
Inverse variance weighted
MR Egger
3.5
3.0
2.5
2.0
1.5
1/SE_IV
−0.25
0.00
0.25
β_IV

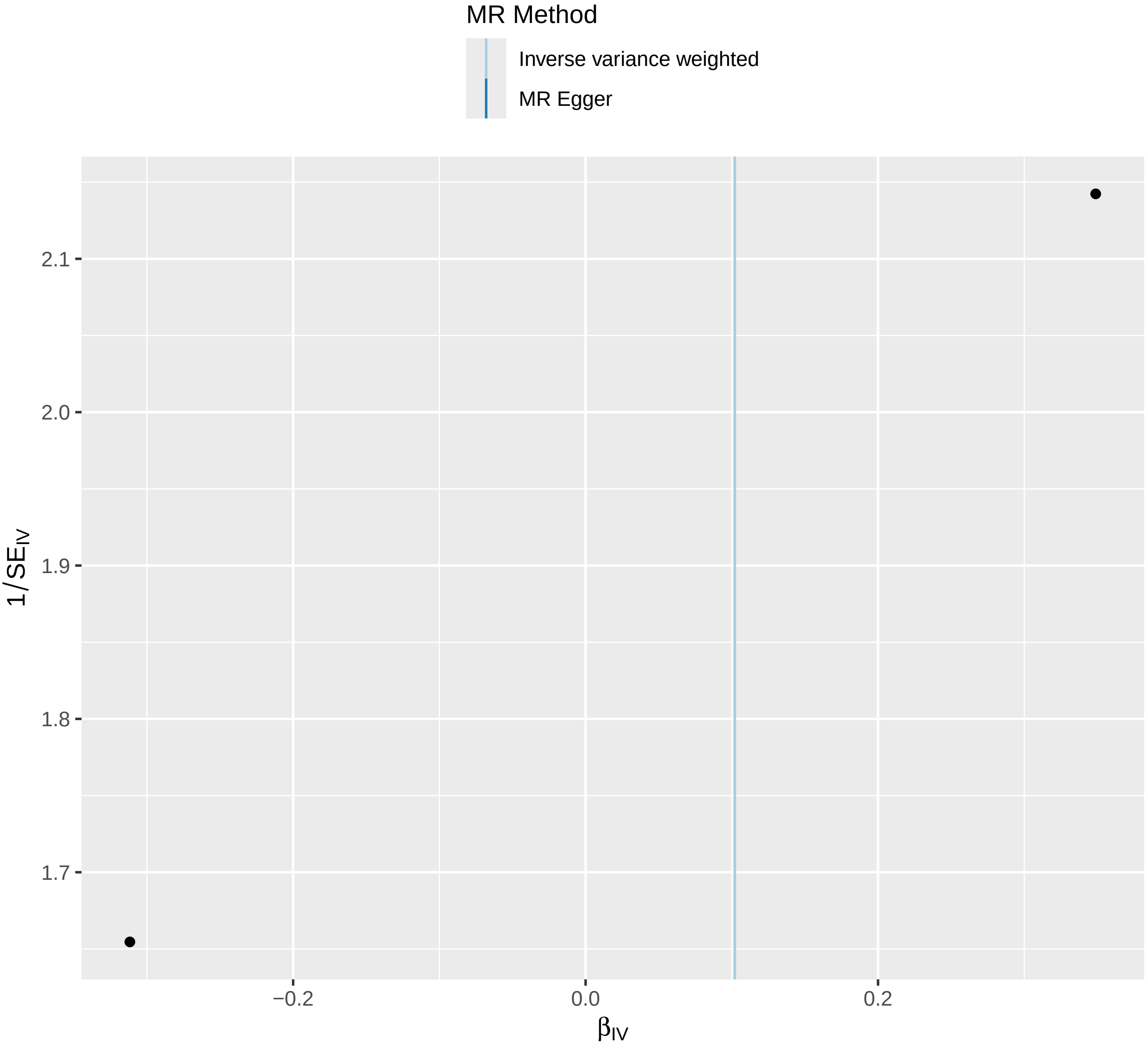
MR Method
Inverse variance weighted
MR Egger
2.1
2.0
1.9
1.8
1.7
1/SE$_{IV}$
−0.2
0.0
0.2
β$_{IV}$

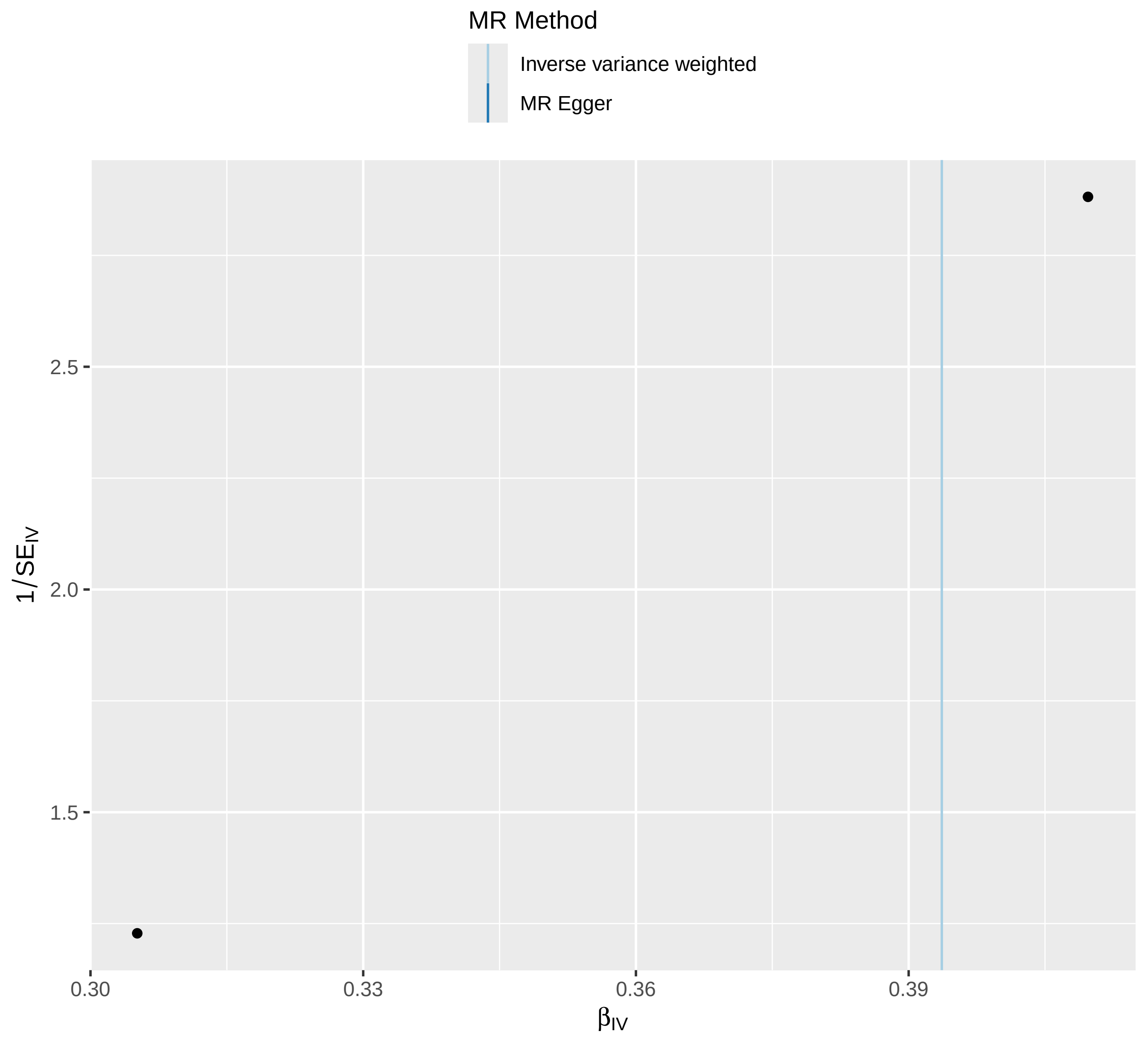
MR Method
Inverse variance weighted
MR Egger
2.5
2.0
1.5
1/SE$_{IV}$
0.30
0.33
0.36
0.39
β$_{IV}$

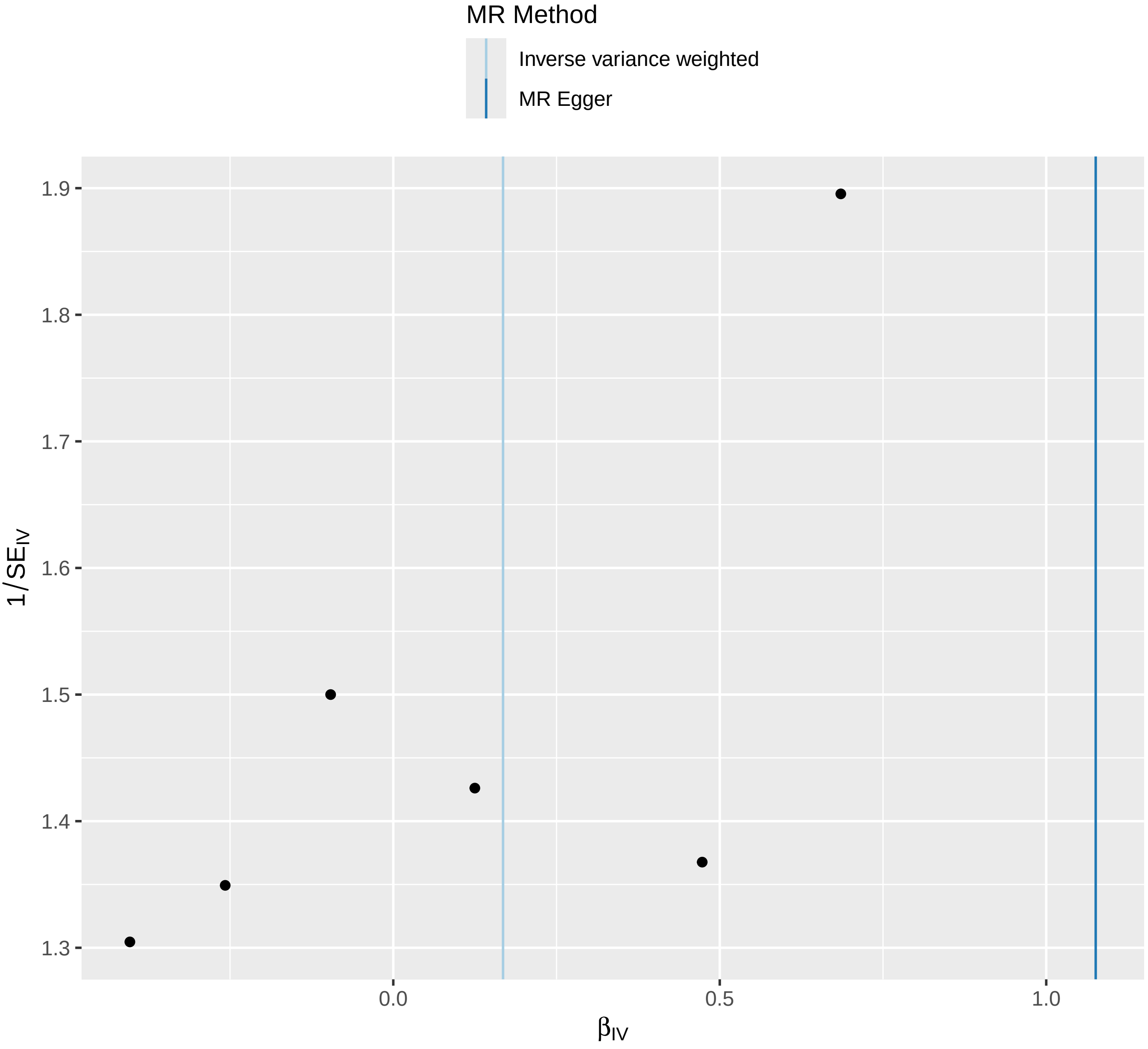
MR Method
Inverse variance weighted
MR Egger
1.9
1.8
1.7
1.6
1.5
1.4
1.3
$1/SE_{IV}$
0.0
0.5
1.0
β_{IV}

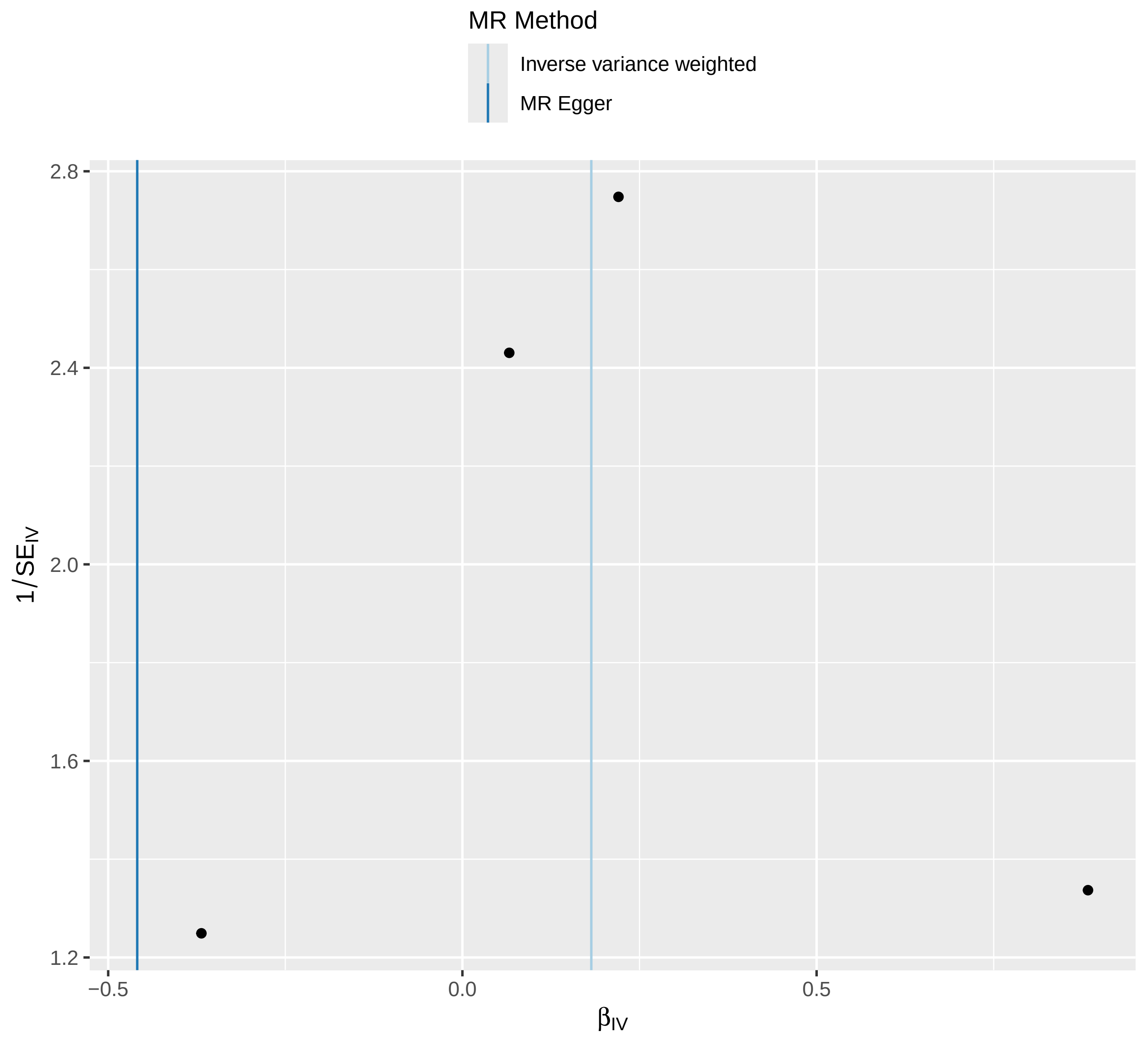
MR Method
Inverse variance weighted
MR Egger
2.8
2.4
2.0
1.6
1.2
$1/SE_{IV}$
−0.5
0.0
0.5
β_{IV}

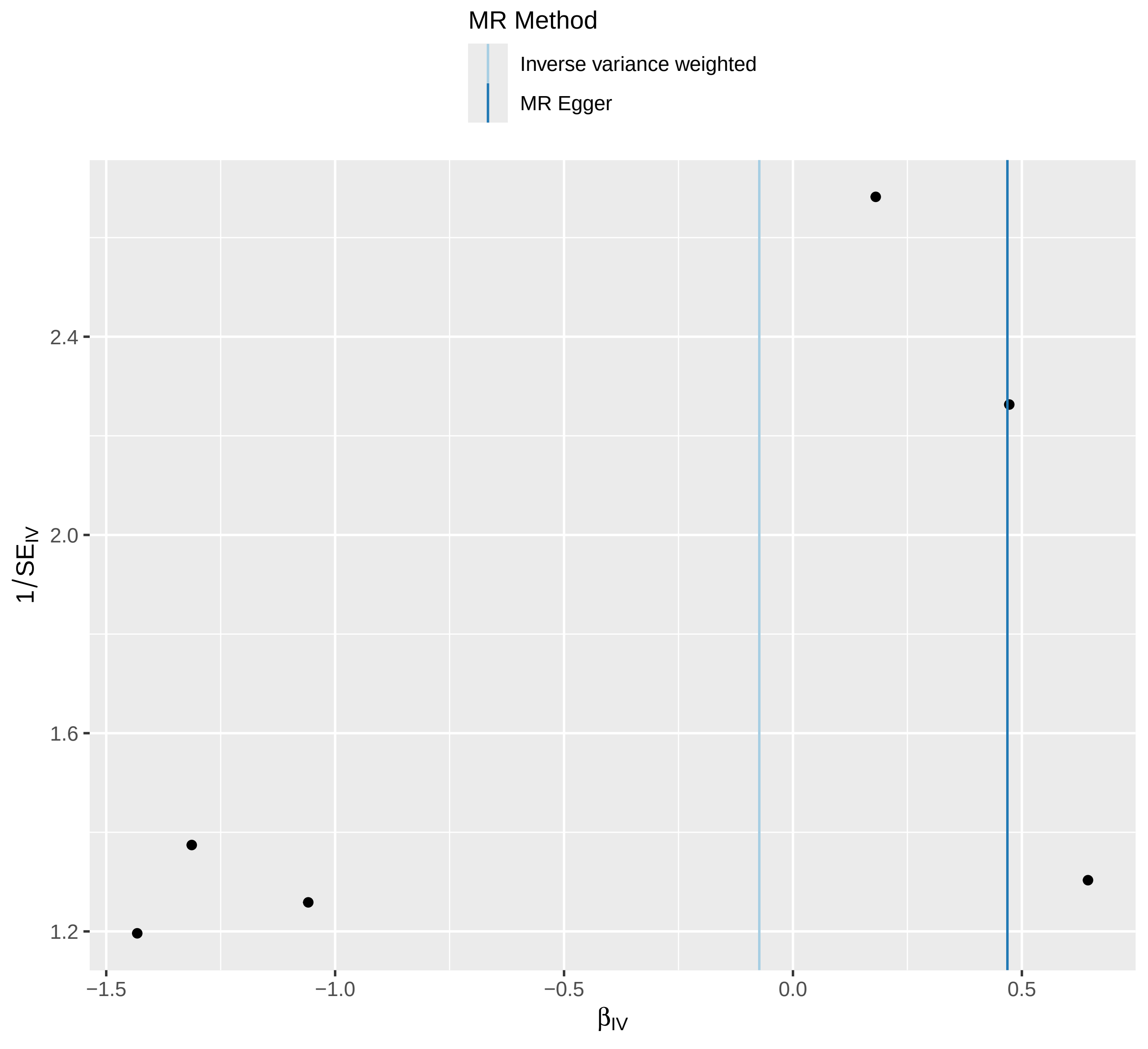

MR Method
Inverse variance weighted
MR Egger
2.4
2.0
1.6
1.2
1/SE$_{IV}$
−1.5
−1.0
−0.5
0.0
0.5
β$_{IV}$

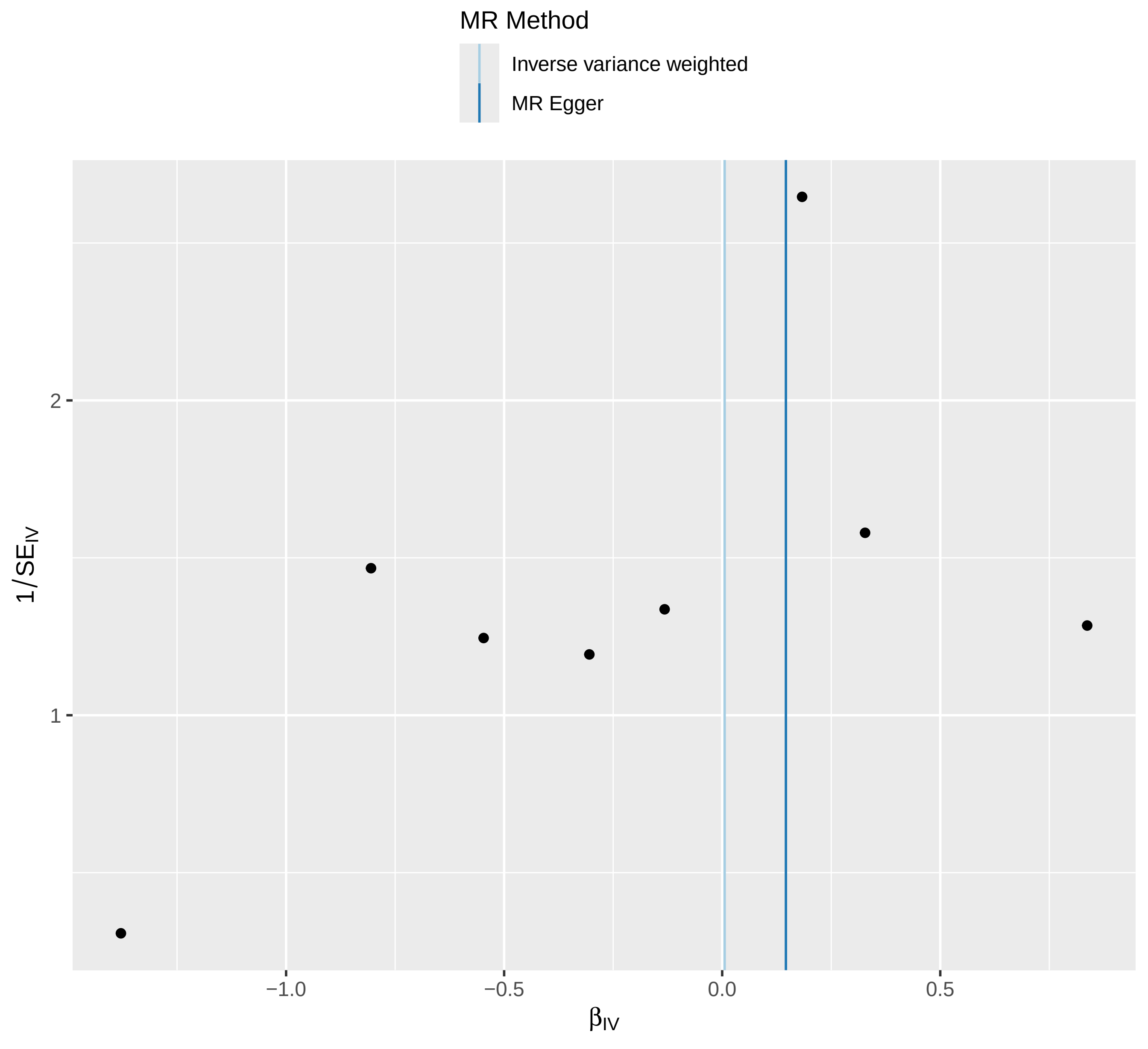
MR Method
Inverse variance weighted
MR Egger
2
1
−1.0
−0.5
0.0
0.5
βIV
1/SEIV

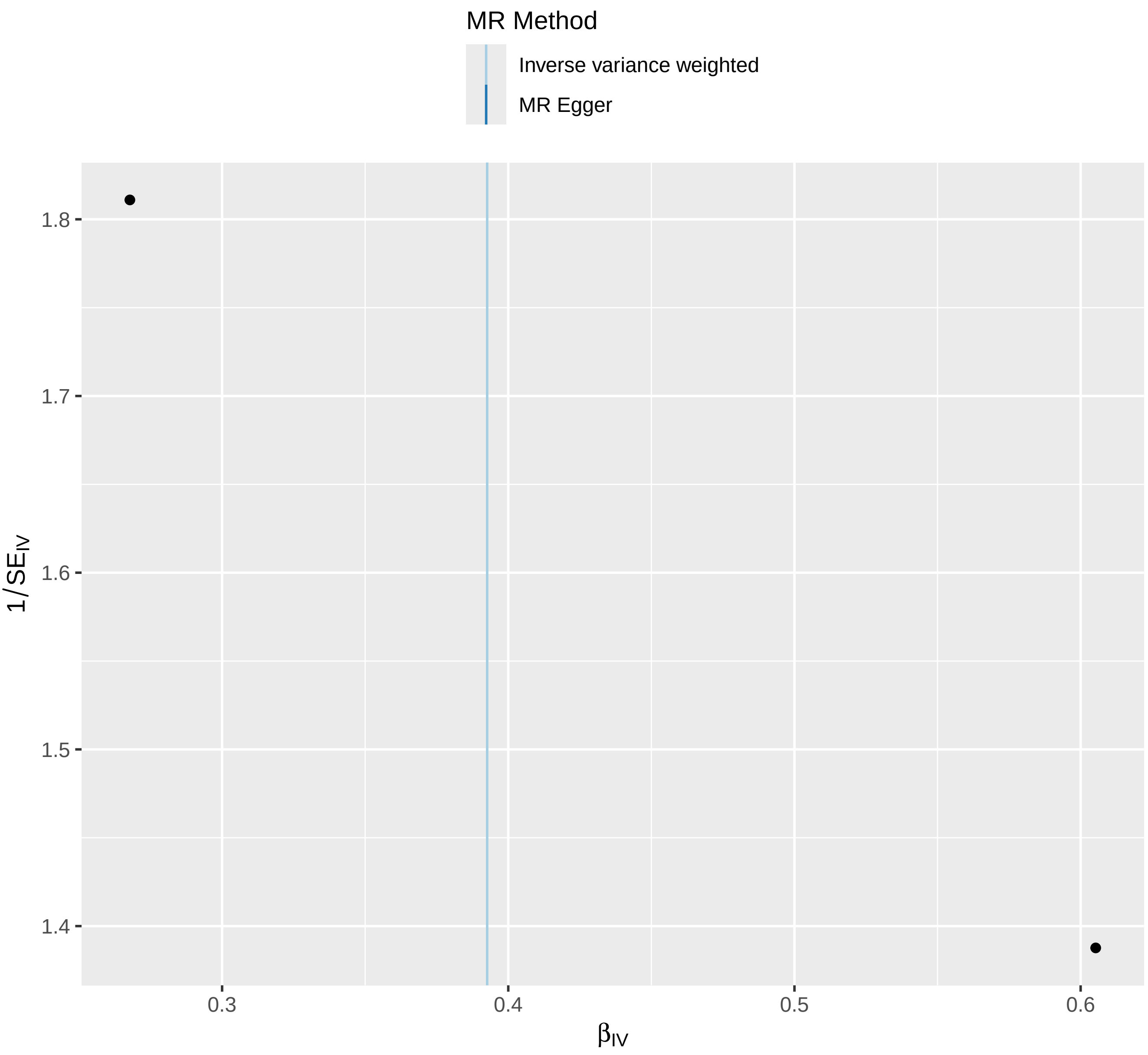

MR Method
Inverse variance weighted
MR Egger
1.8
1.7
1.6
1.5
1.4
$1/SE_{IV}$
0.3
0.4
0.5
0.6
β_{IV}

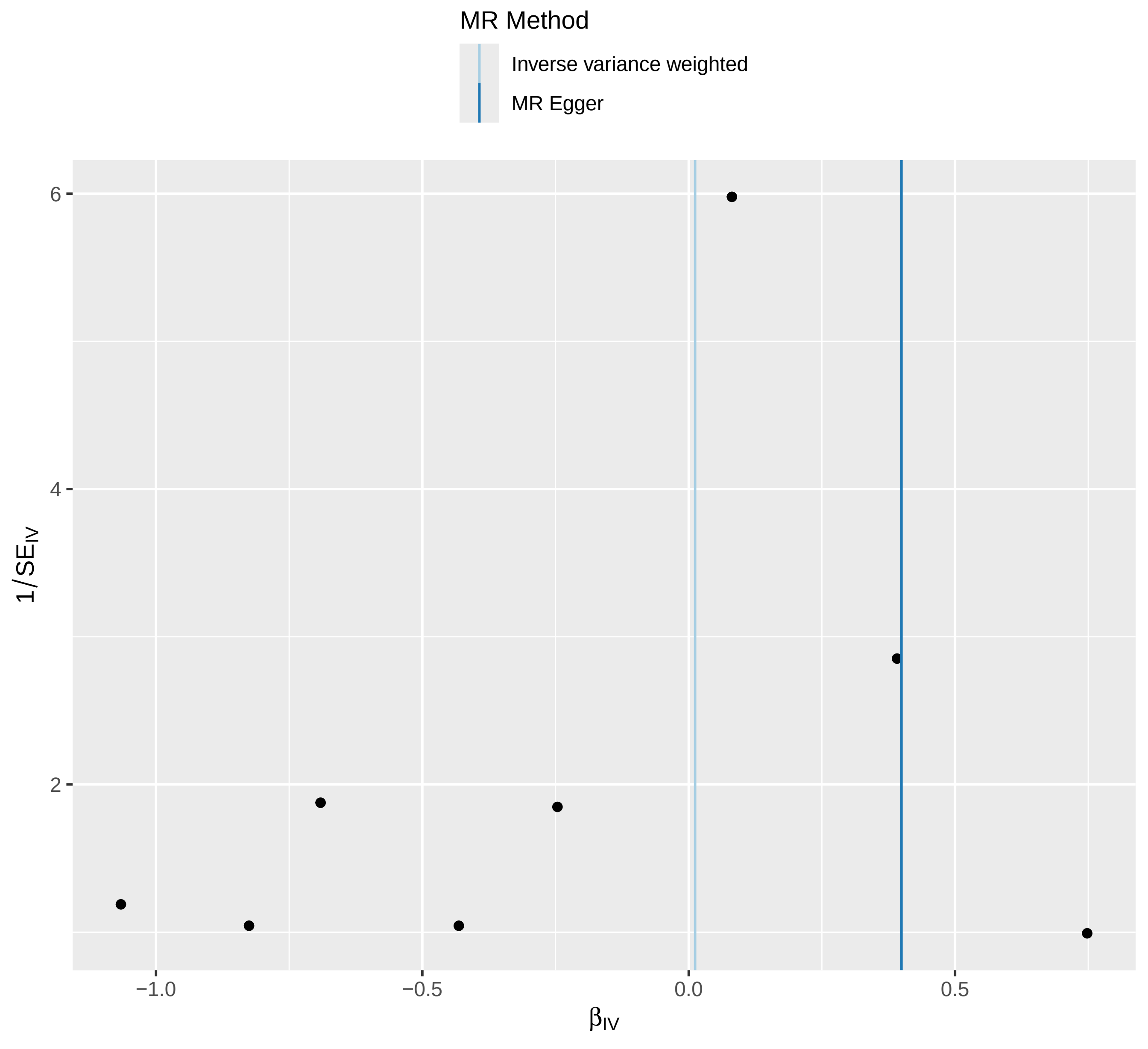

MR Method
Inverse variance weighted
MR Egger
6
4
2
1/SE_IV
−1.0
−0.5
0.0
0.5
β_IV

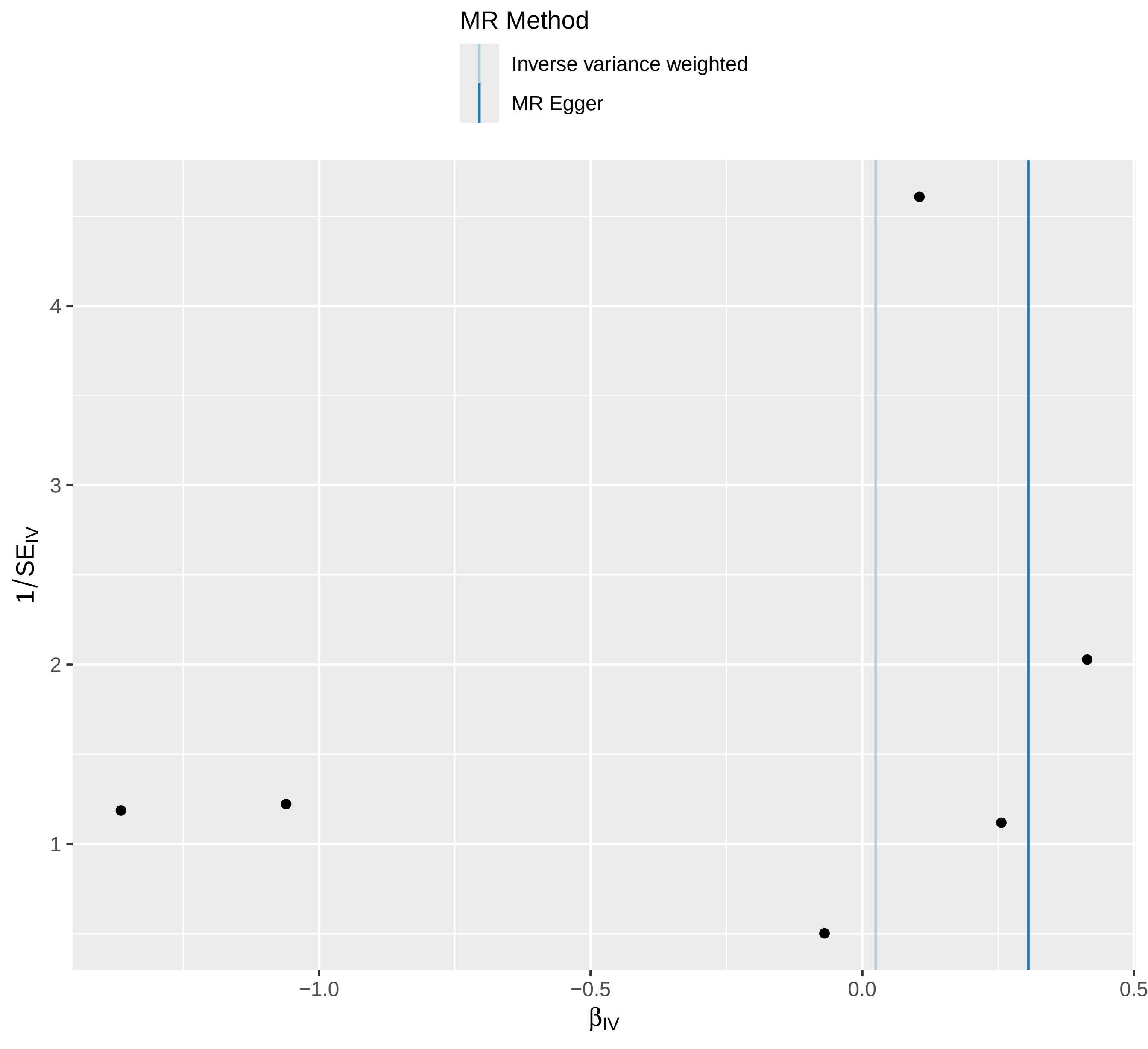

MR Method
Inverse variance weighted
MR Egger
1/SE$_{IV}$
4
3
2
1
−1.0
−0.5
0.0
0.5
β$_{IV}$

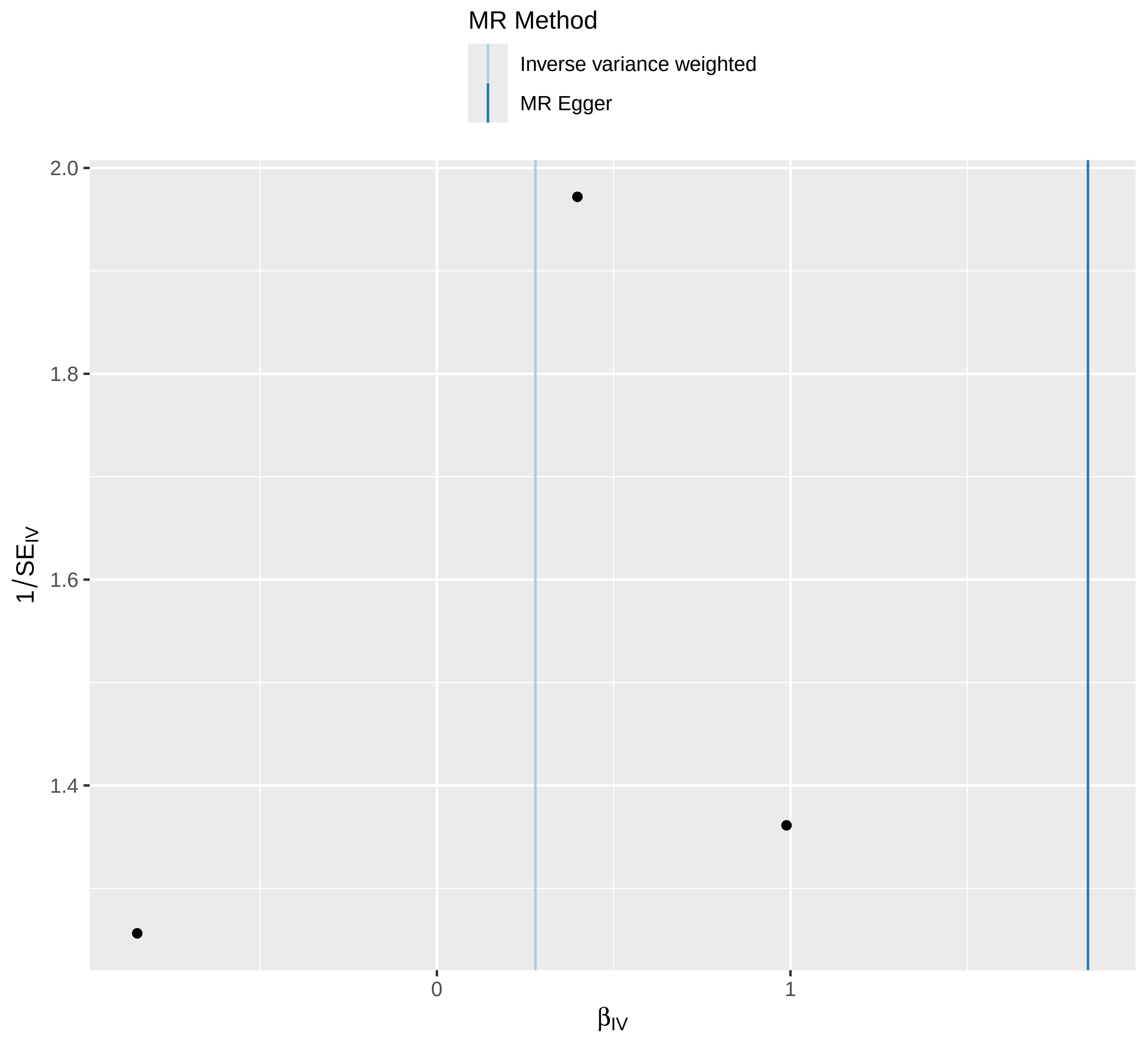

MR Method
Inverse variance weighted
MR Egger
2.0
1.8
1.6
1.4
$1/SE_{IV}$
0
1
β_{IV}

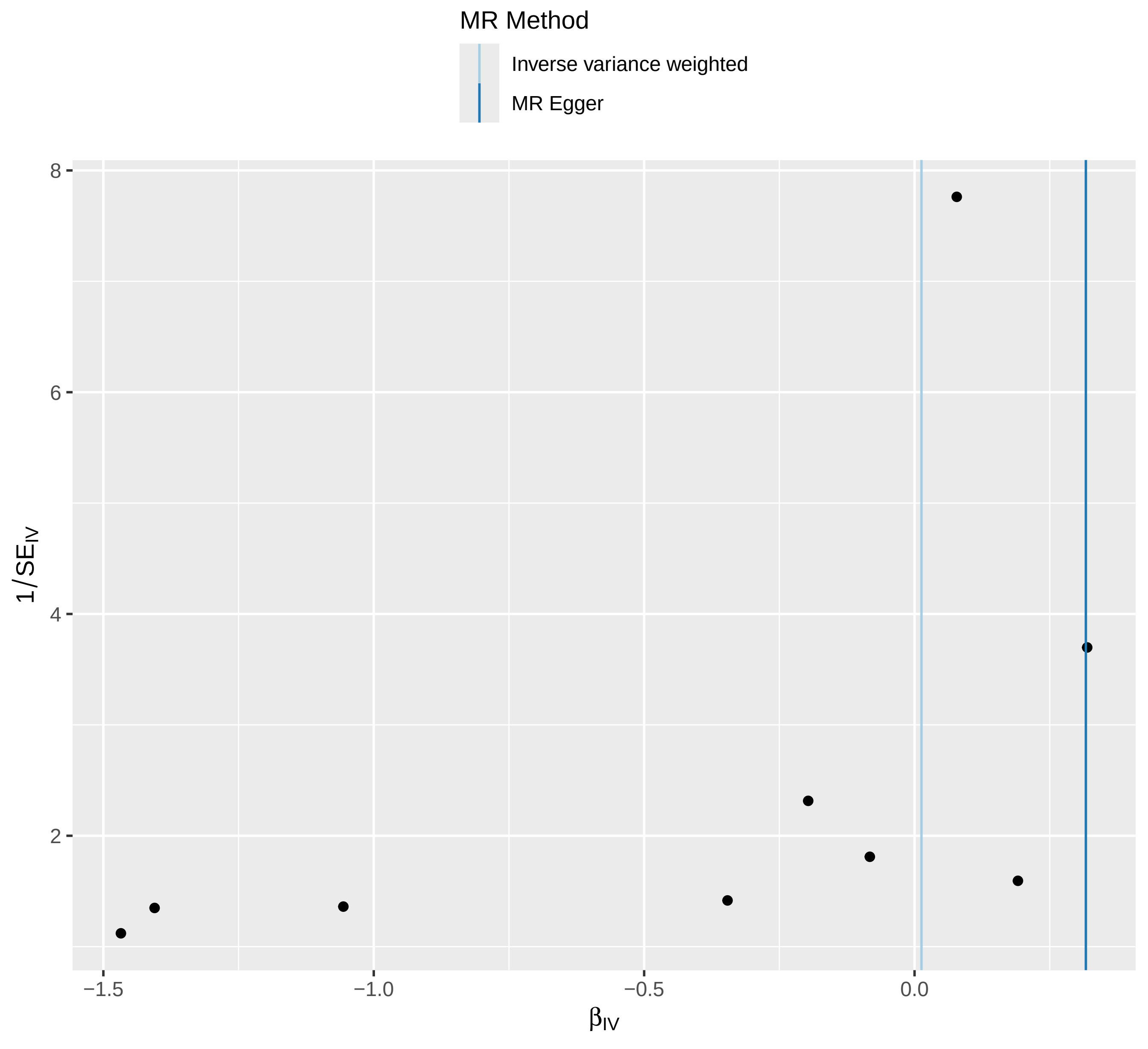

MR Method
Inverse variance weighted
MR Egger
8
6
4
2
1/SE$_{IV}$
−1.5
−1.0
−0.5
0.0
β$_{IV}$

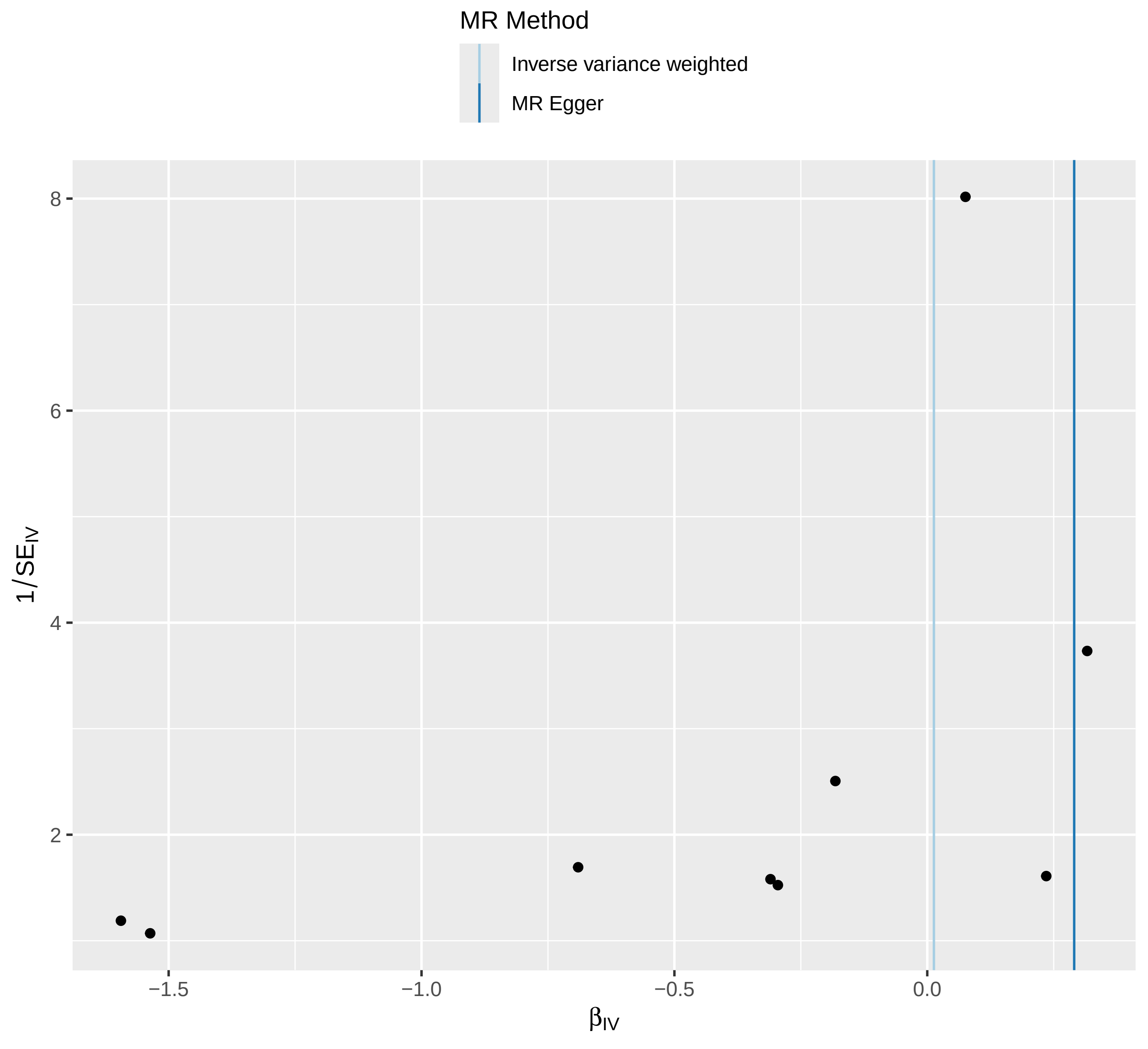
MR Method
Inverse variance weighted
MR Egger
8
6
4
2
1/SE$_{IV}$
−1.5
−1.0
−0.5
0.0
β$_{IV}$

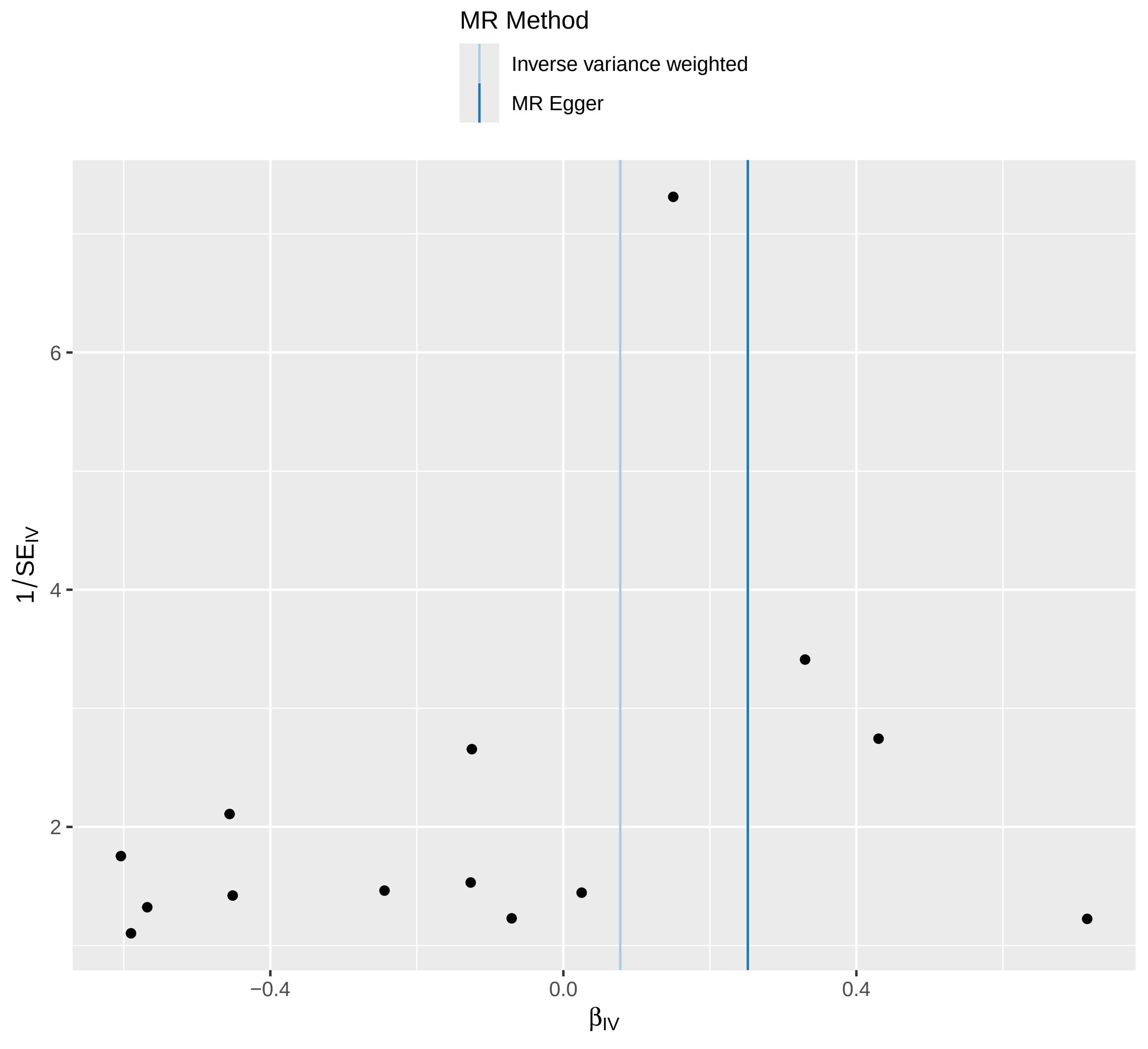

MR Method
Inverse variance weighted
MR Egger
1/SE$_{IV}$
6
4
2
−0.4
0.0
0.4
β$_{IV}$

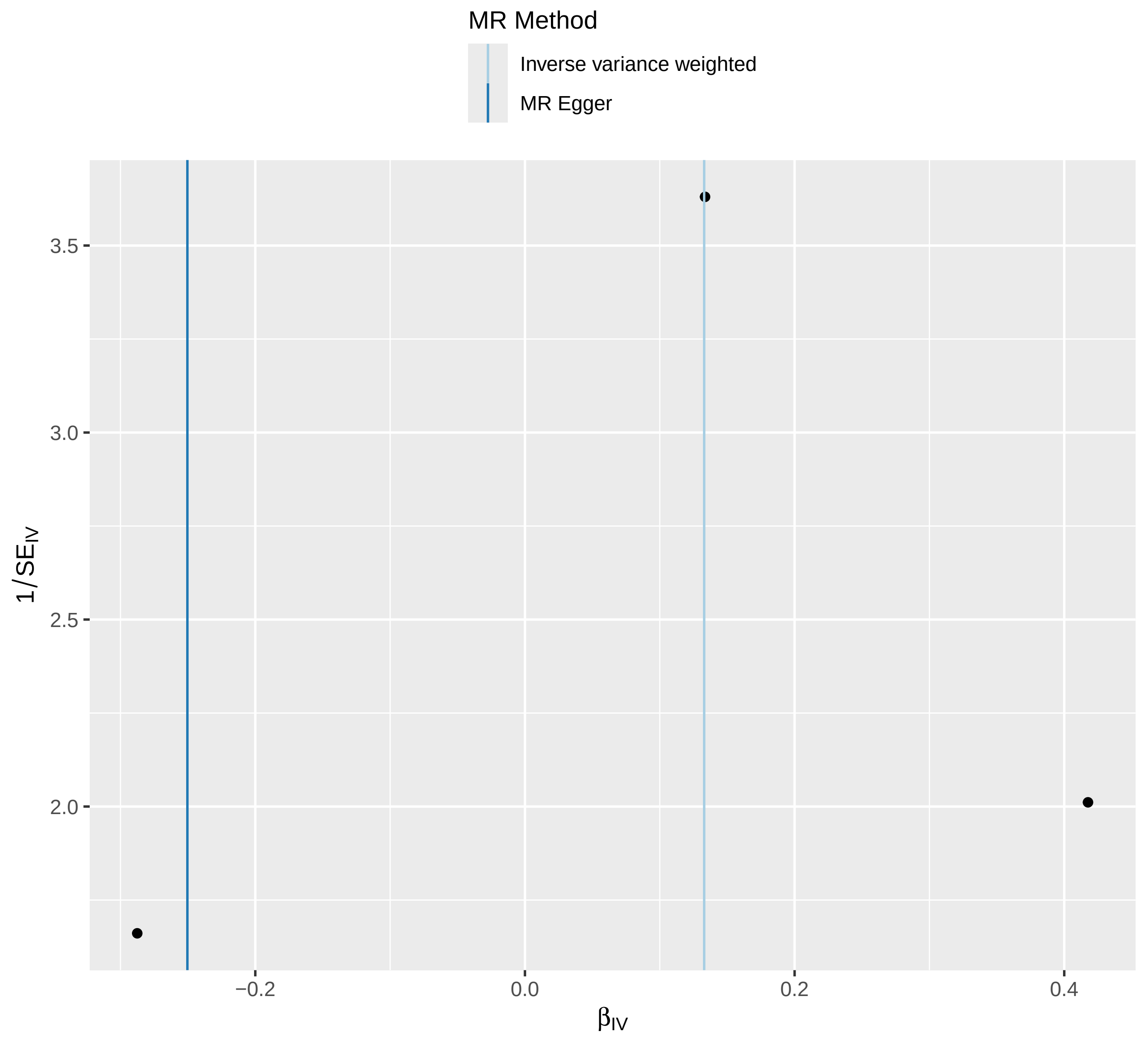
MR Method
Inverse variance weighted
MR Egger
3.5
3.0
2.5
2.0
1/SE_IV
−0.2
0.0
0.2
0.4
β_IV

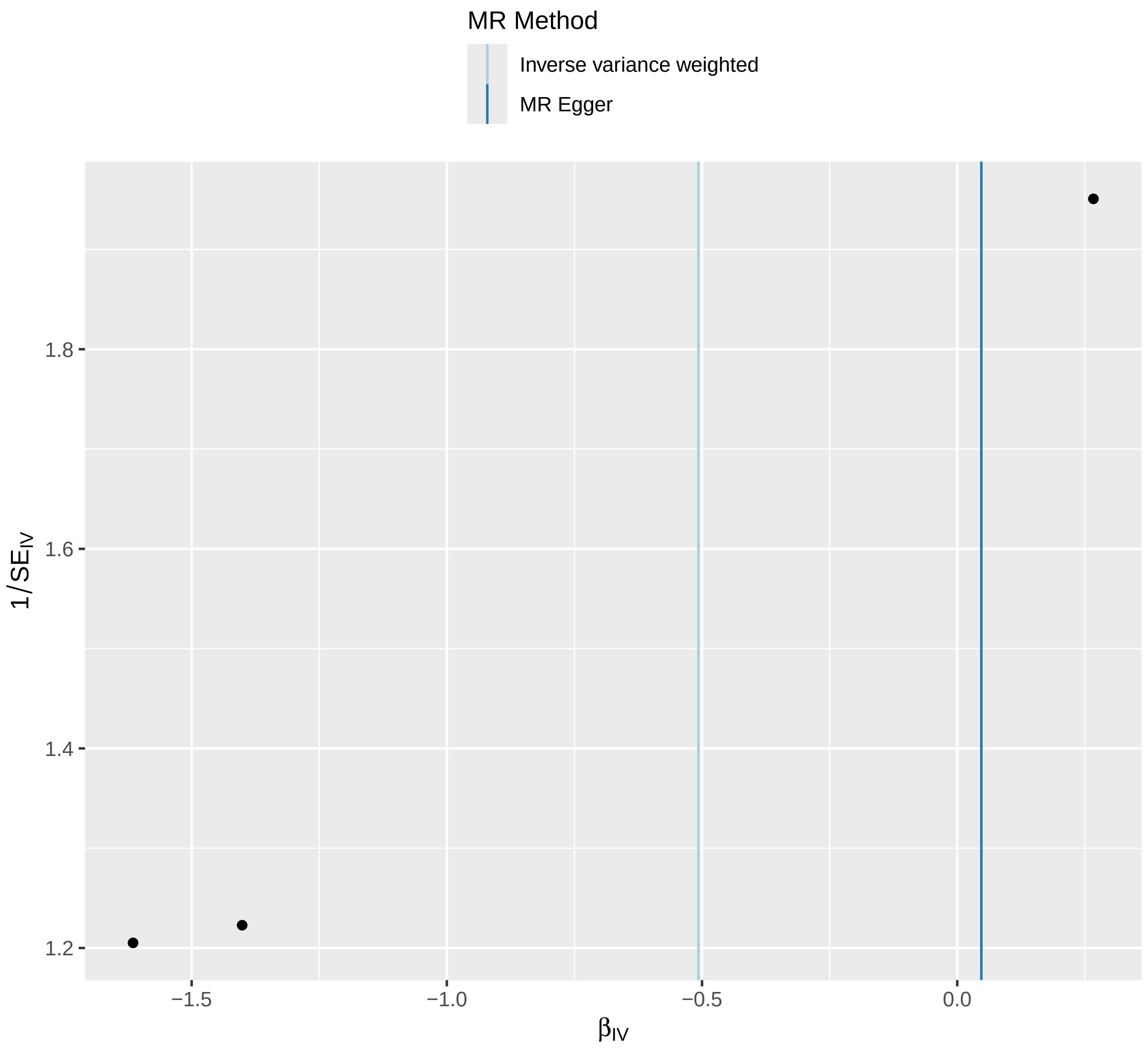
MR Method
Inverse variance weighted
MR Egger
1.8
1.6
1.4
1.2
1/SE_IV
−1.5
−1.0
−0.5
0.0
β_IV

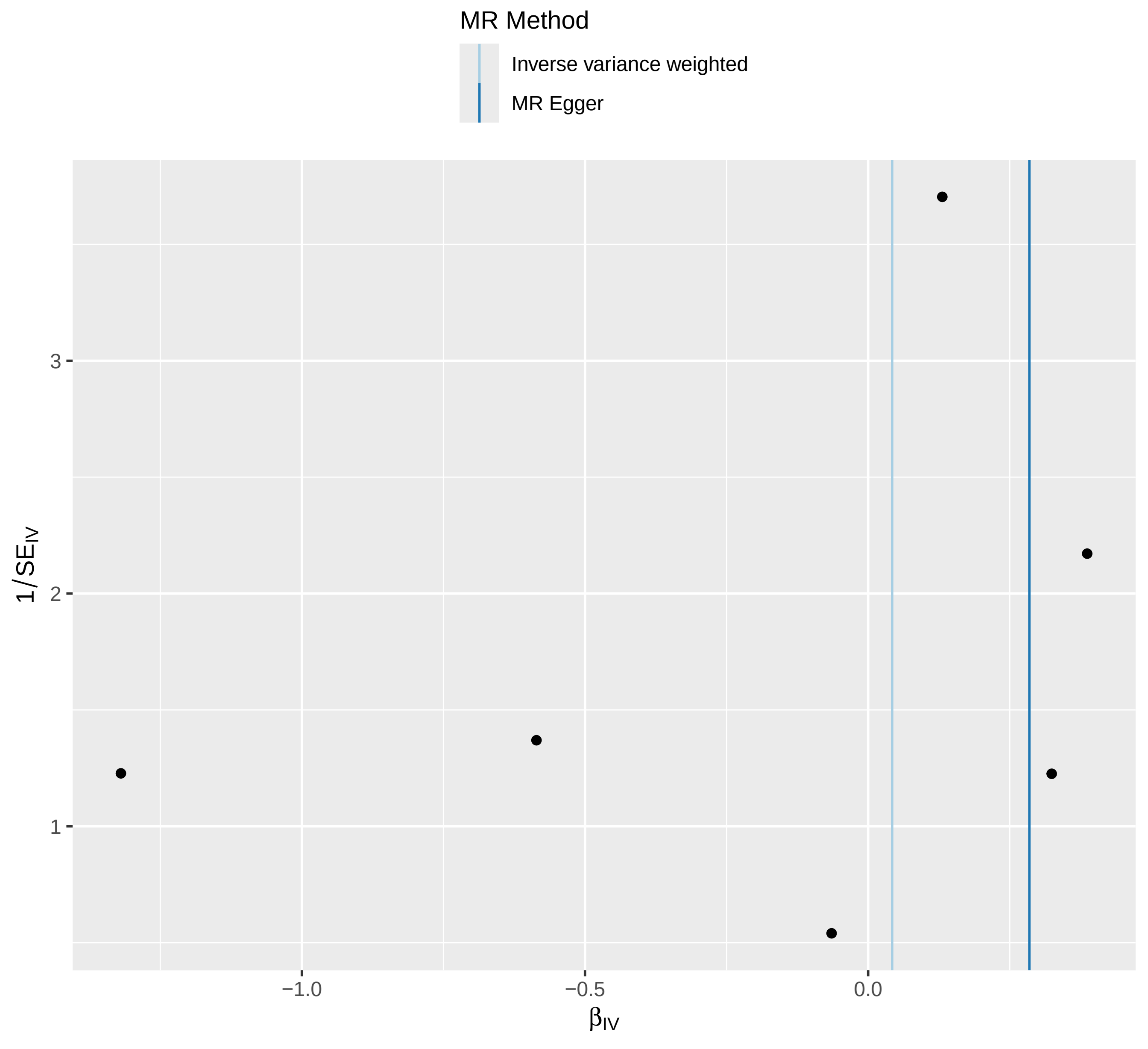
MR Method
Inverse variance weighted
MR Egger
1/SE_IV
3
2
1
−1.0
−0.5
0.0
β_IV

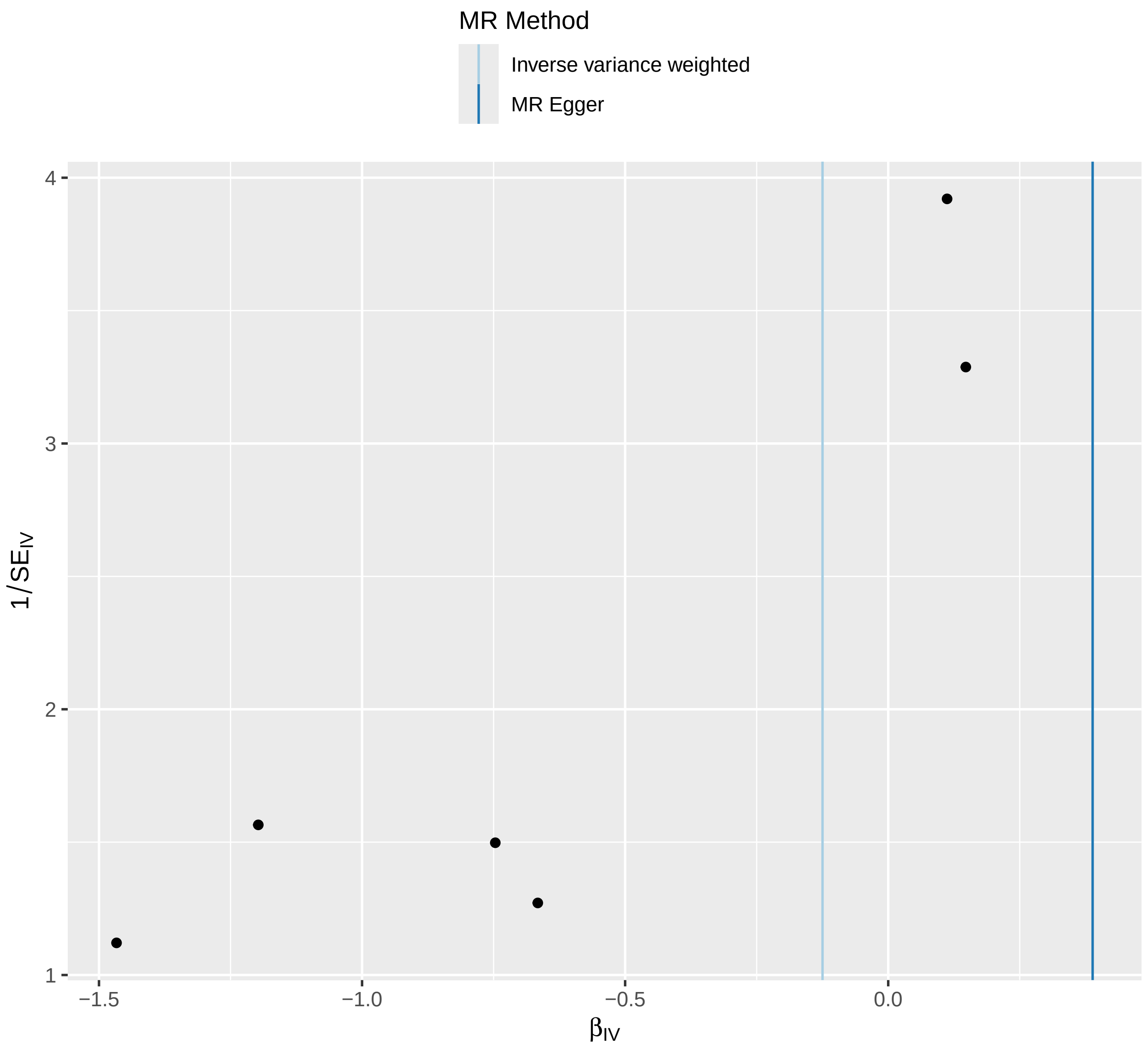

MR Method
Inverse variance weighted
MR Egger
4
3
2
1
1/SE$_{IV}$
−1.5
−1.0
−0.5
0.0
β$_{IV}$

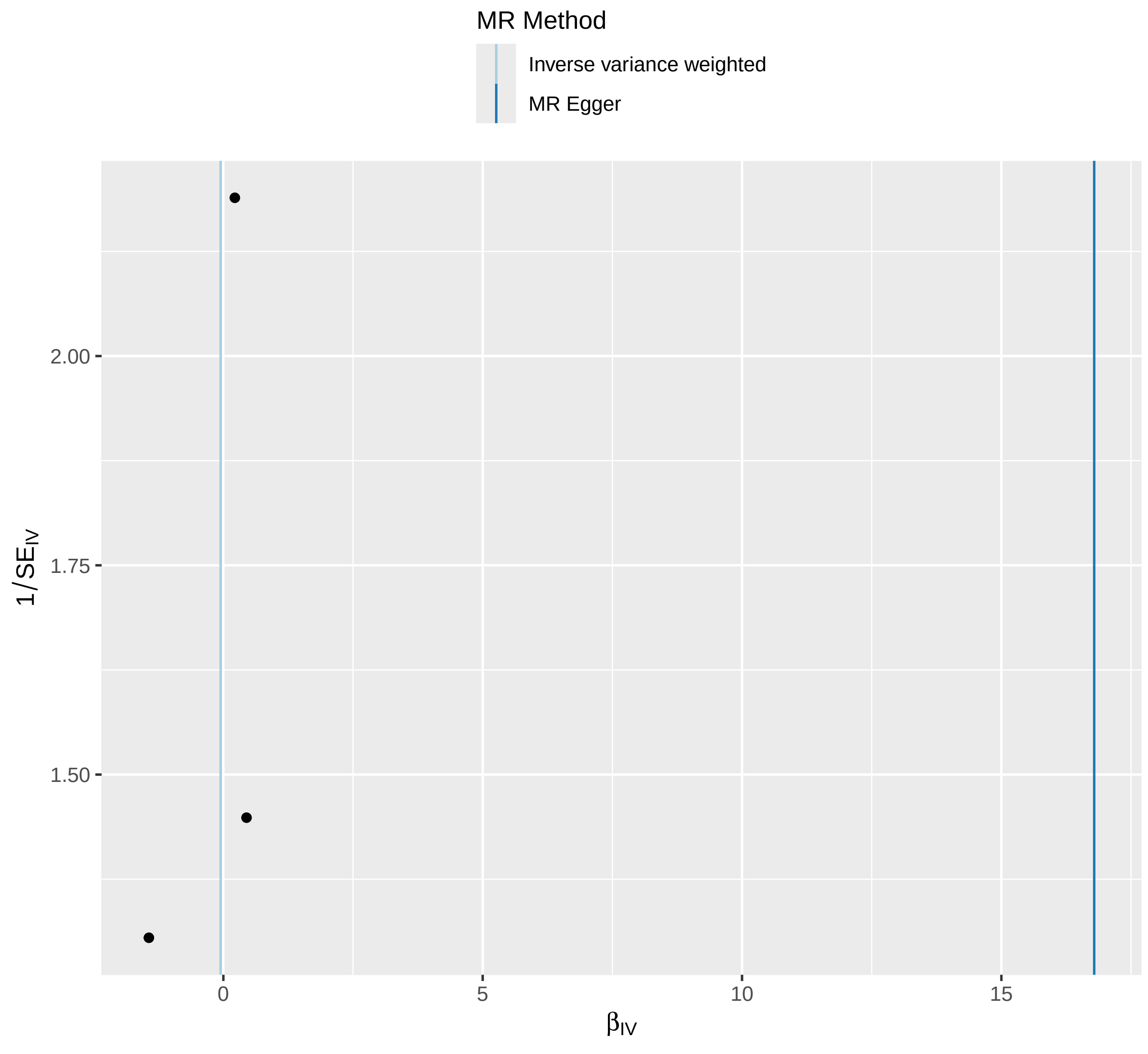
MR Method
Inverse variance weighted
MR Egger
2.00
1.75
1.50
1/SE_IV
0
5
10
15
β_IV

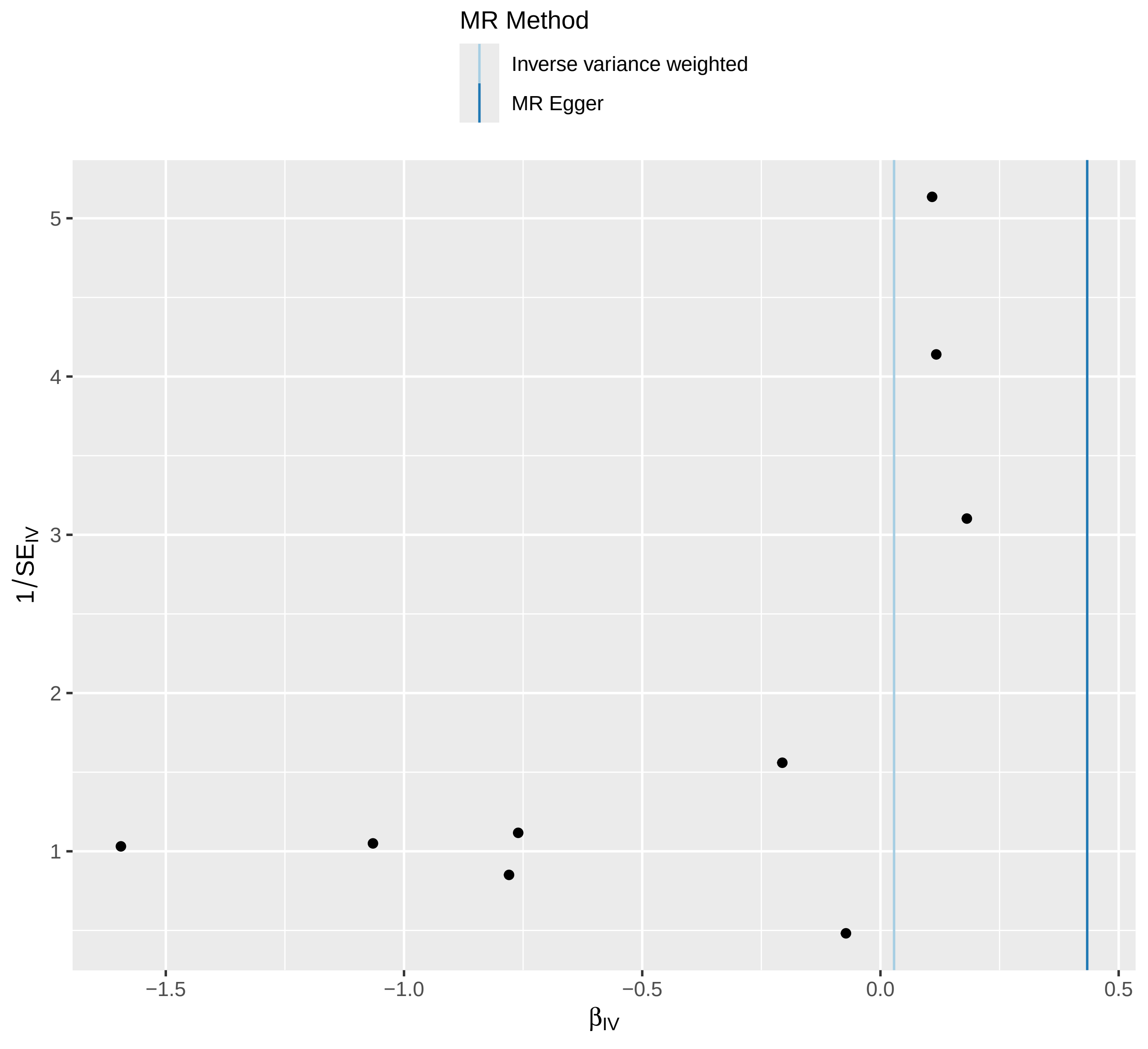
MR Method
Inverse variance weighted
MR Egger
1/SE_IV
β_IV
5
4
3
2
1
−1.5
−1.0
−0.5
0.0
0.5

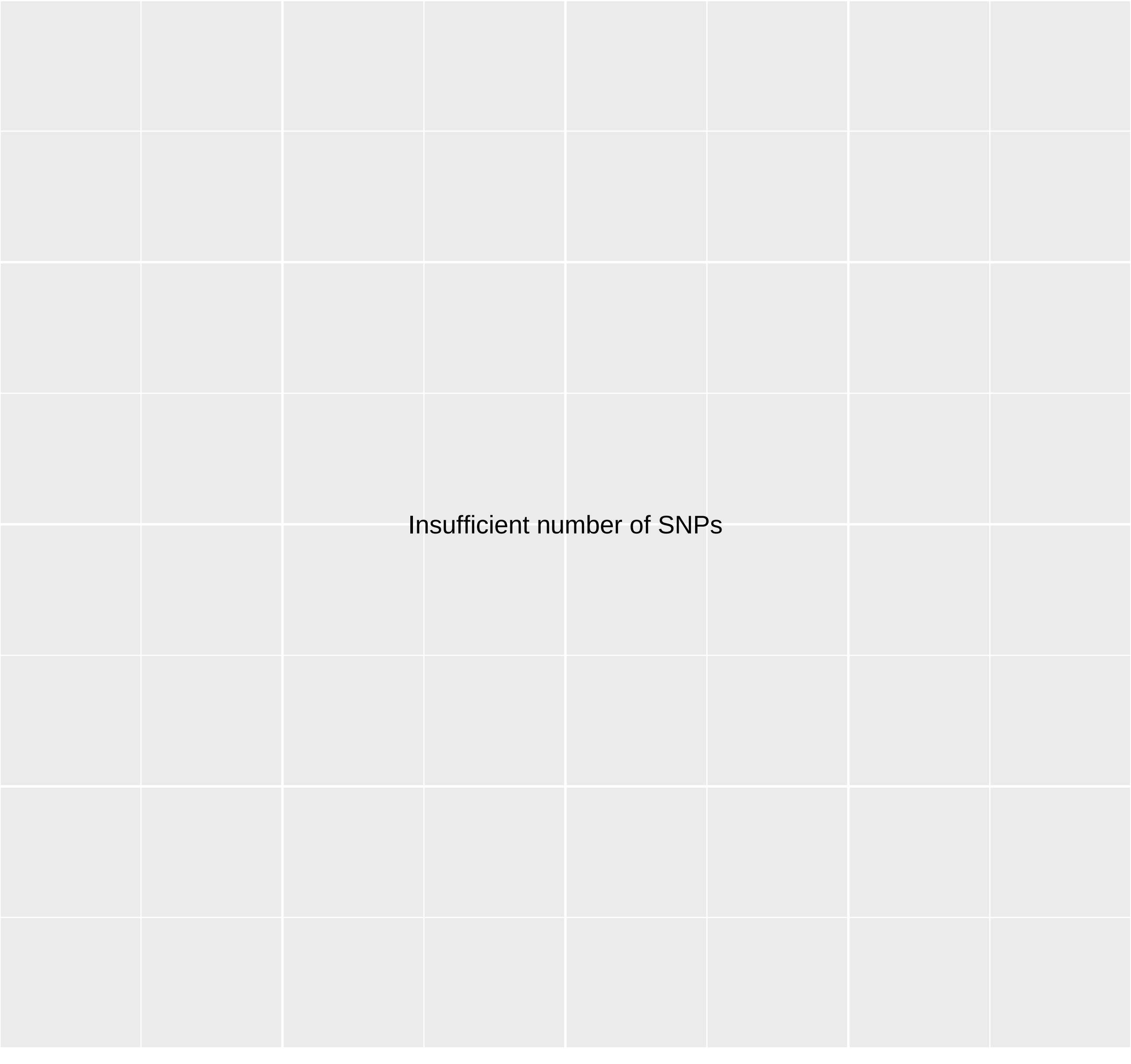
Insufficient number of SNPs

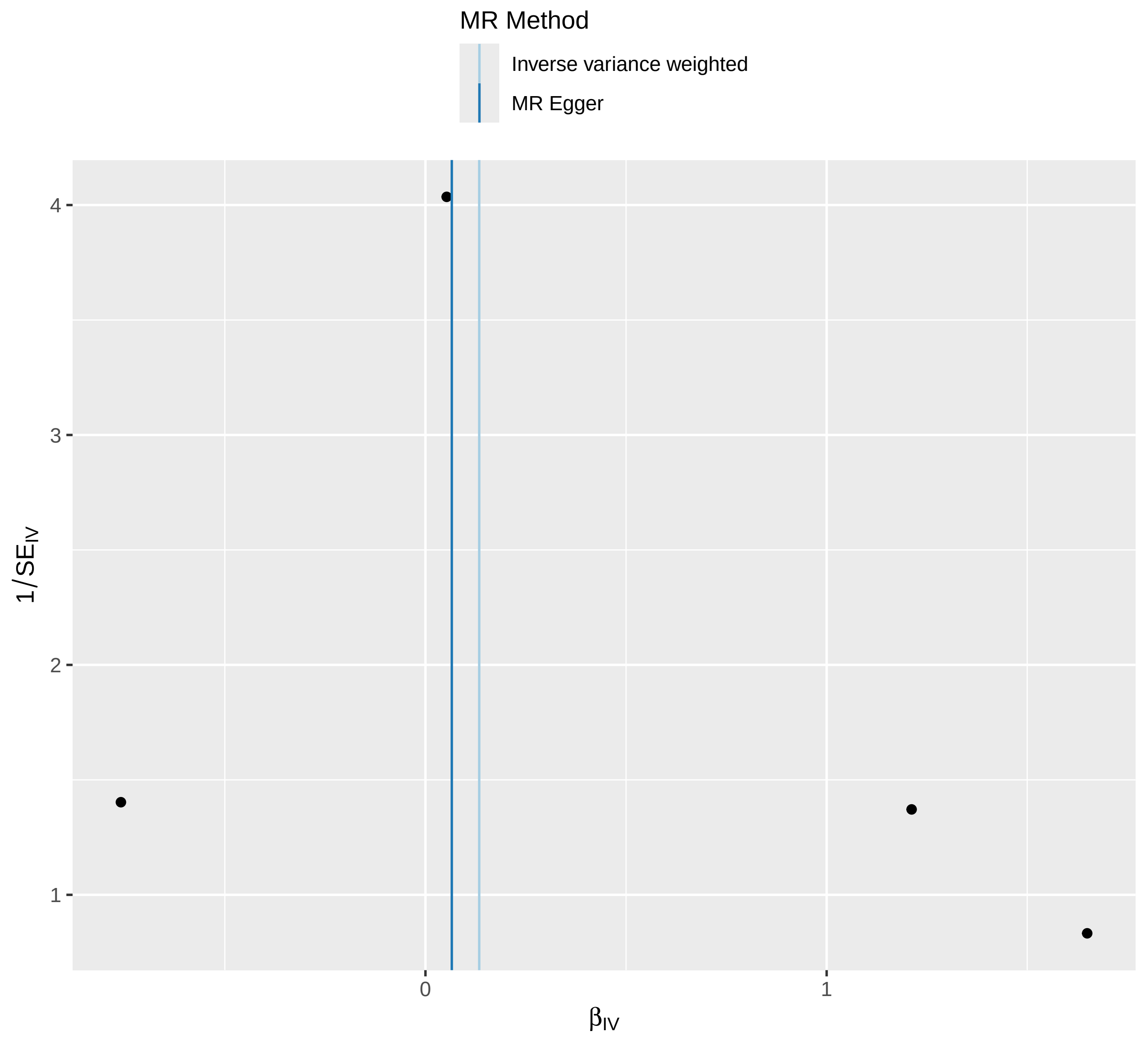

MR Method
Inverse variance weighted
MR Egger
4
3
2
1
0
1
1/SE_IV
β_IV

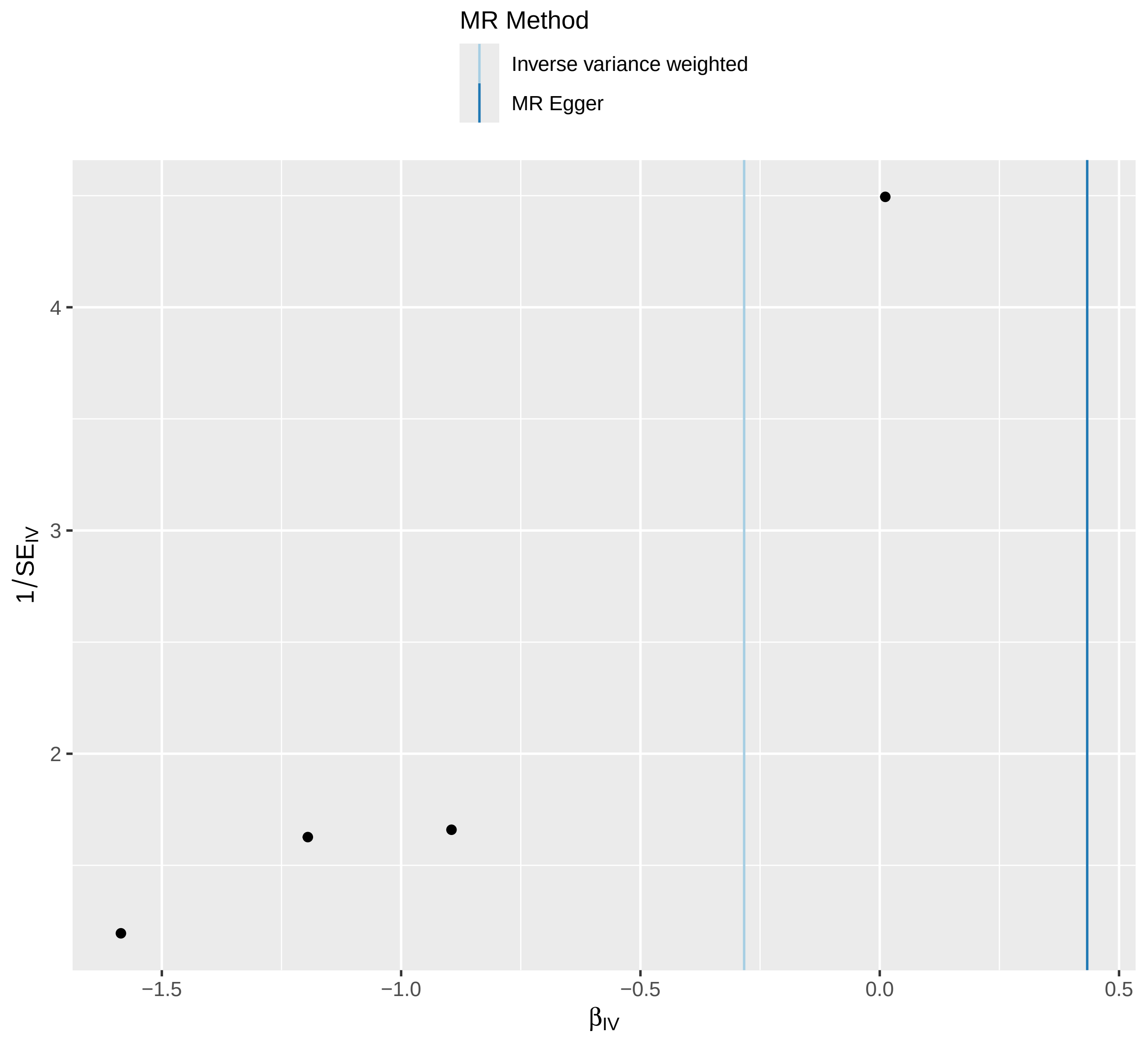

MR Method
Inverse variance weighted
MR Egger
1/SE$_{IV}$
4
3
2
−1.5
−1.0
−0.5
0.0
0.5
β$_{IV}$

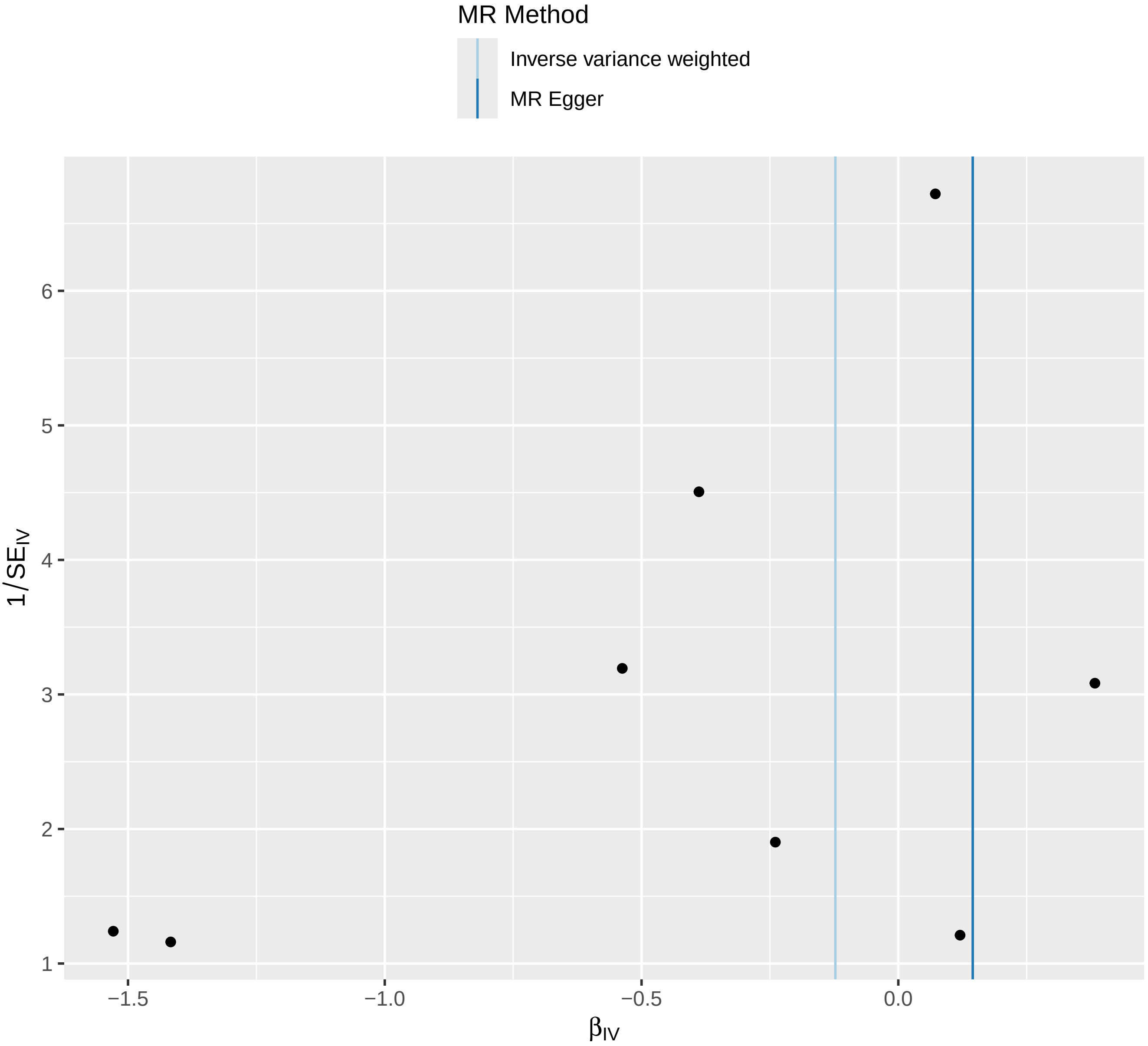
MR Method
Inverse variance weighted
MR Egger
1/SE$_{IV}$
6
5
4
3
2
1
−1.5
−1.0
−0.5
0.0
β$_{IV}$

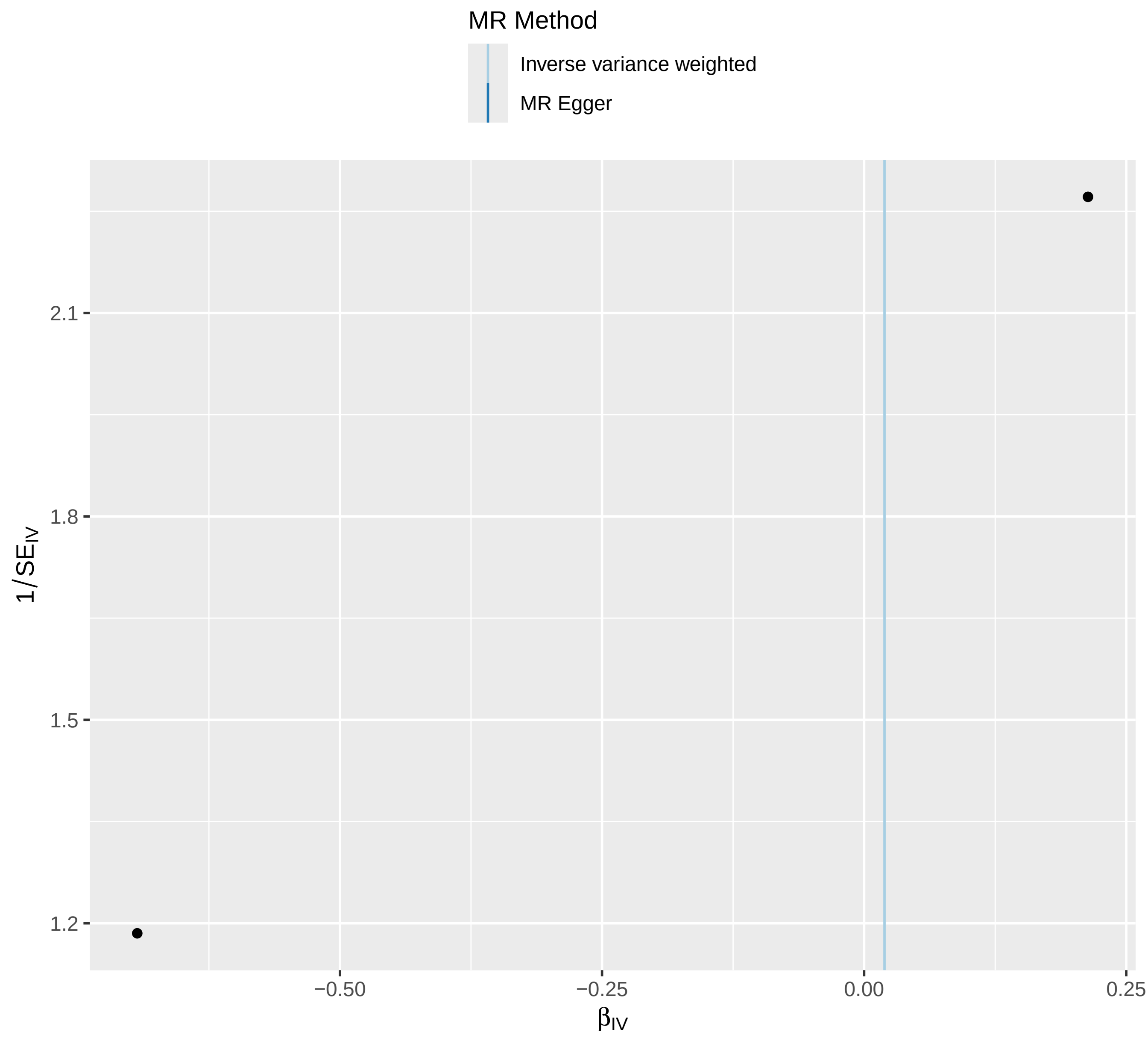
MR Method
Inverse variance weighted
MR Egger
2.1
1.8
1.5
1.2
1/SE_IV
−0.50
−0.25
0.00
0.25
β_IV

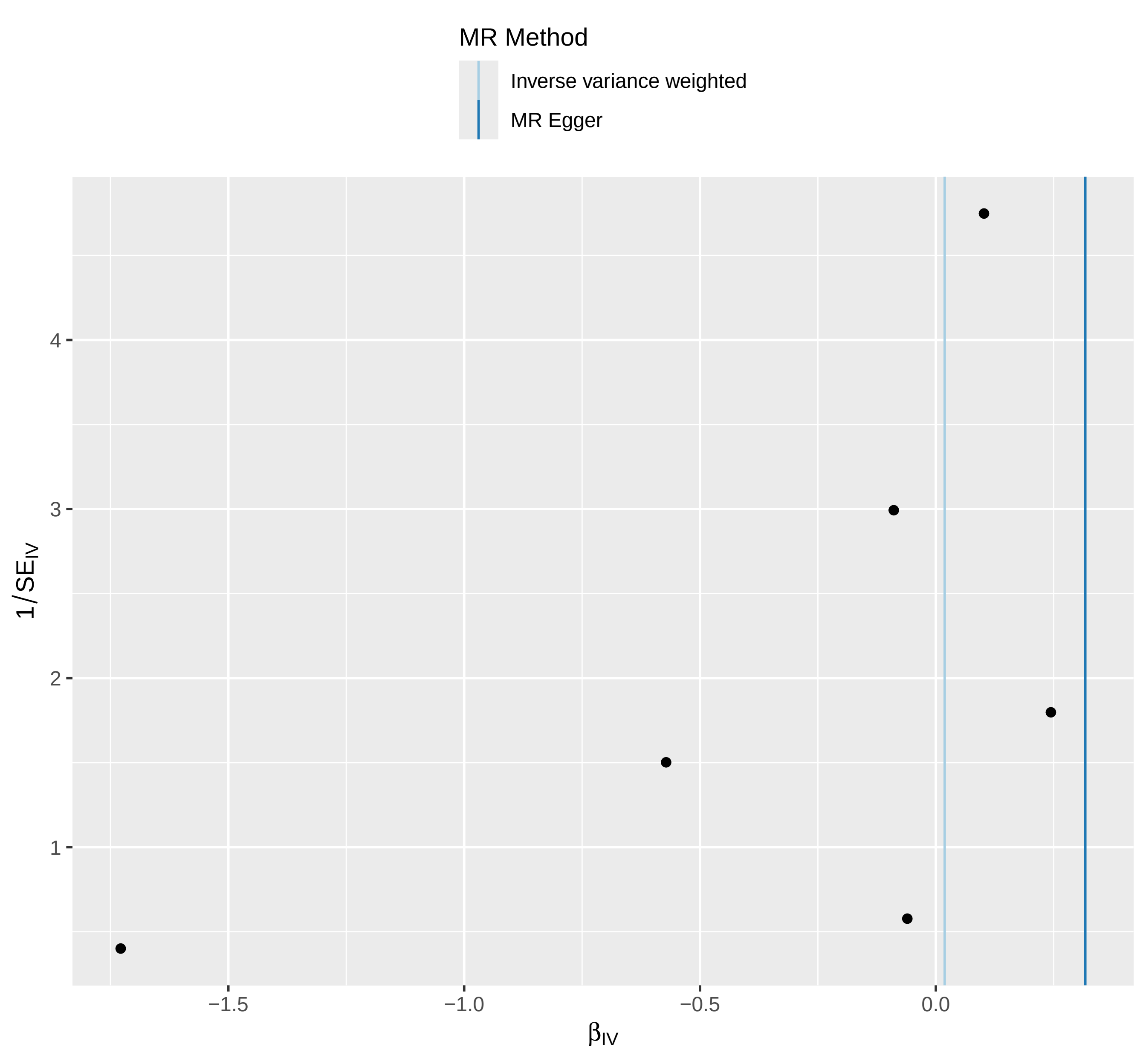
MR Method
Inverse variance weighted
MR Egger
1/SE$_{IV}$
4
3
2
1
−1.5
−1.0
−0.5
0.0
β$_{IV}$

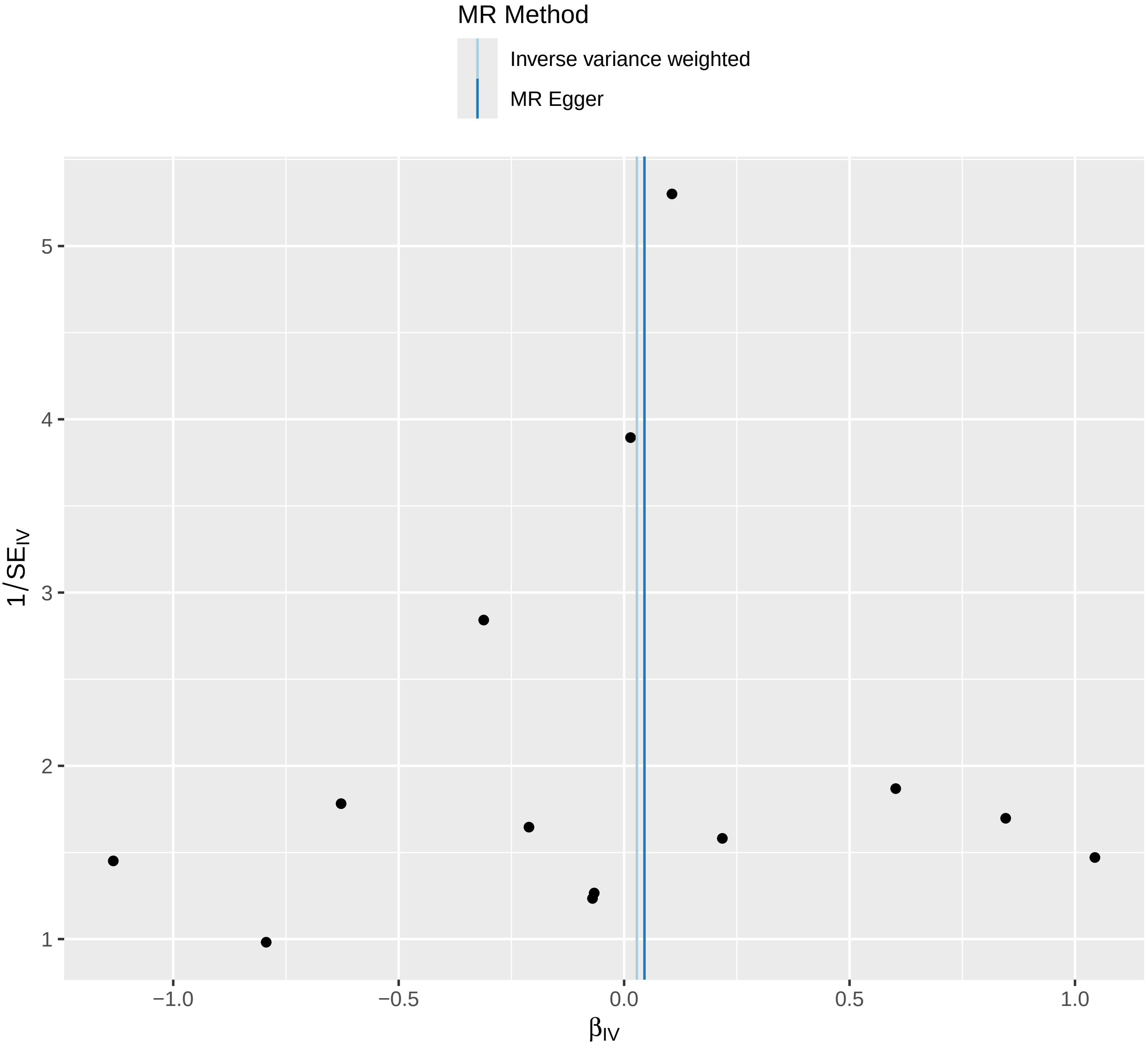

MR Method
Inverse variance weighted
MR Egger
5
4
3
2
1
$1/SE_{IV}$
−1.0
−0.5
0.0
0.5
1.0
β_{IV}

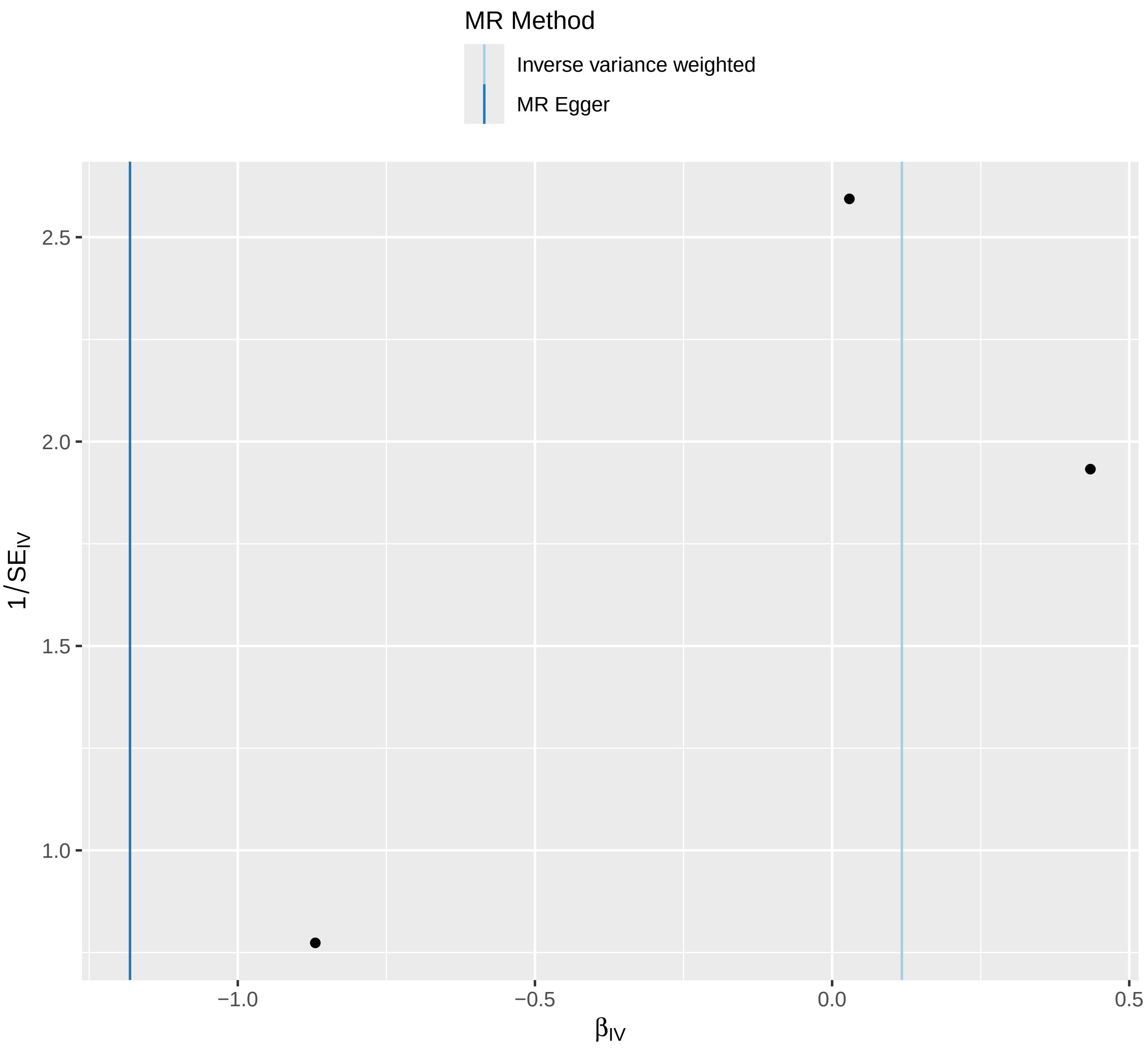
MR Method
Inverse variance weighted
MR Egger
2.5
2.0
1.5
1.0
1/SE$_{IV}$
−1.0
−0.5
0.0
0.5
β$_{IV}$

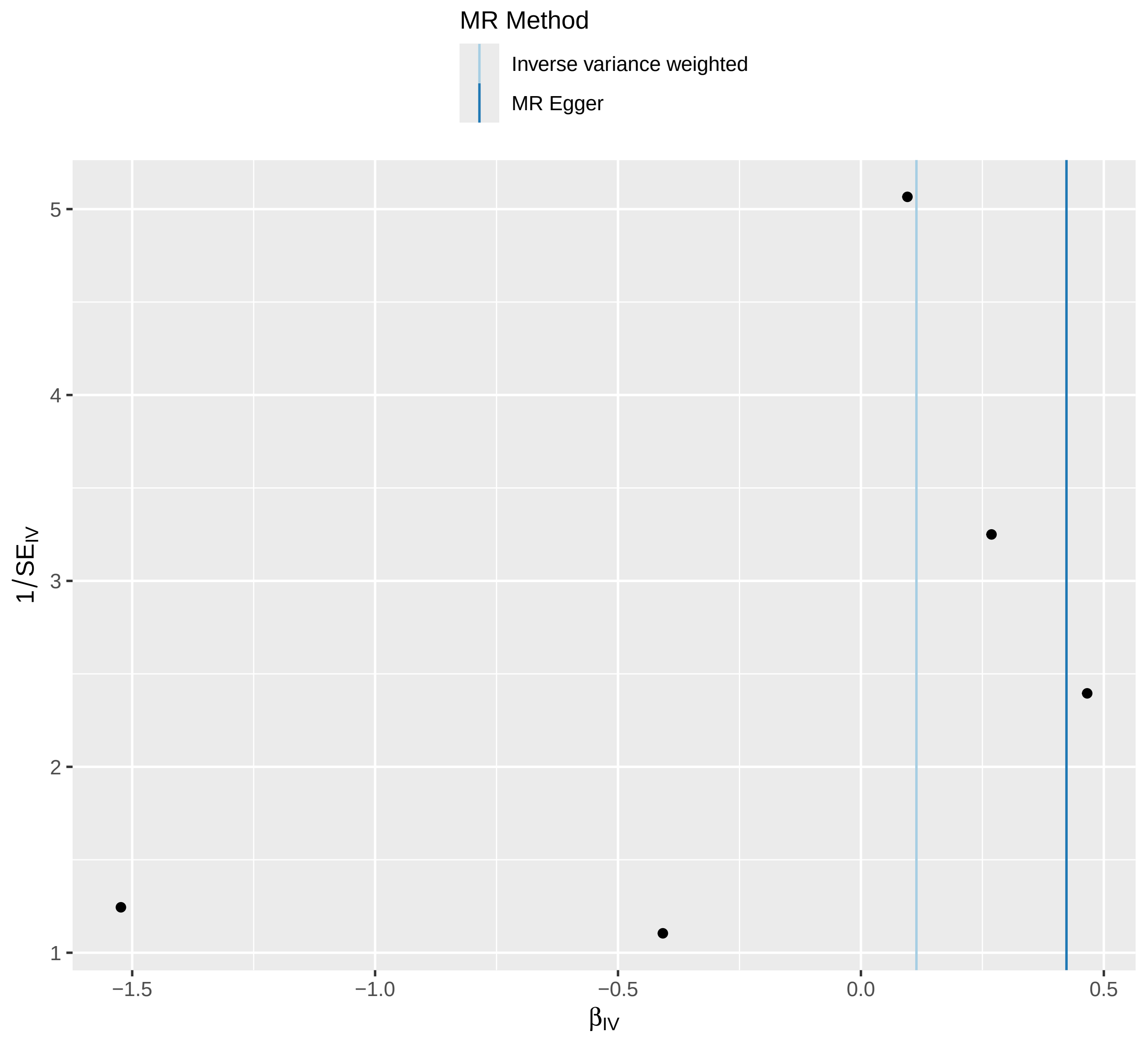

MR Method
Inverse variance weighted
MR Egger
5
4
3
2
1
1/SE_IV
−1.5
−1.0
−0.5
0.0
0.5
β_IV

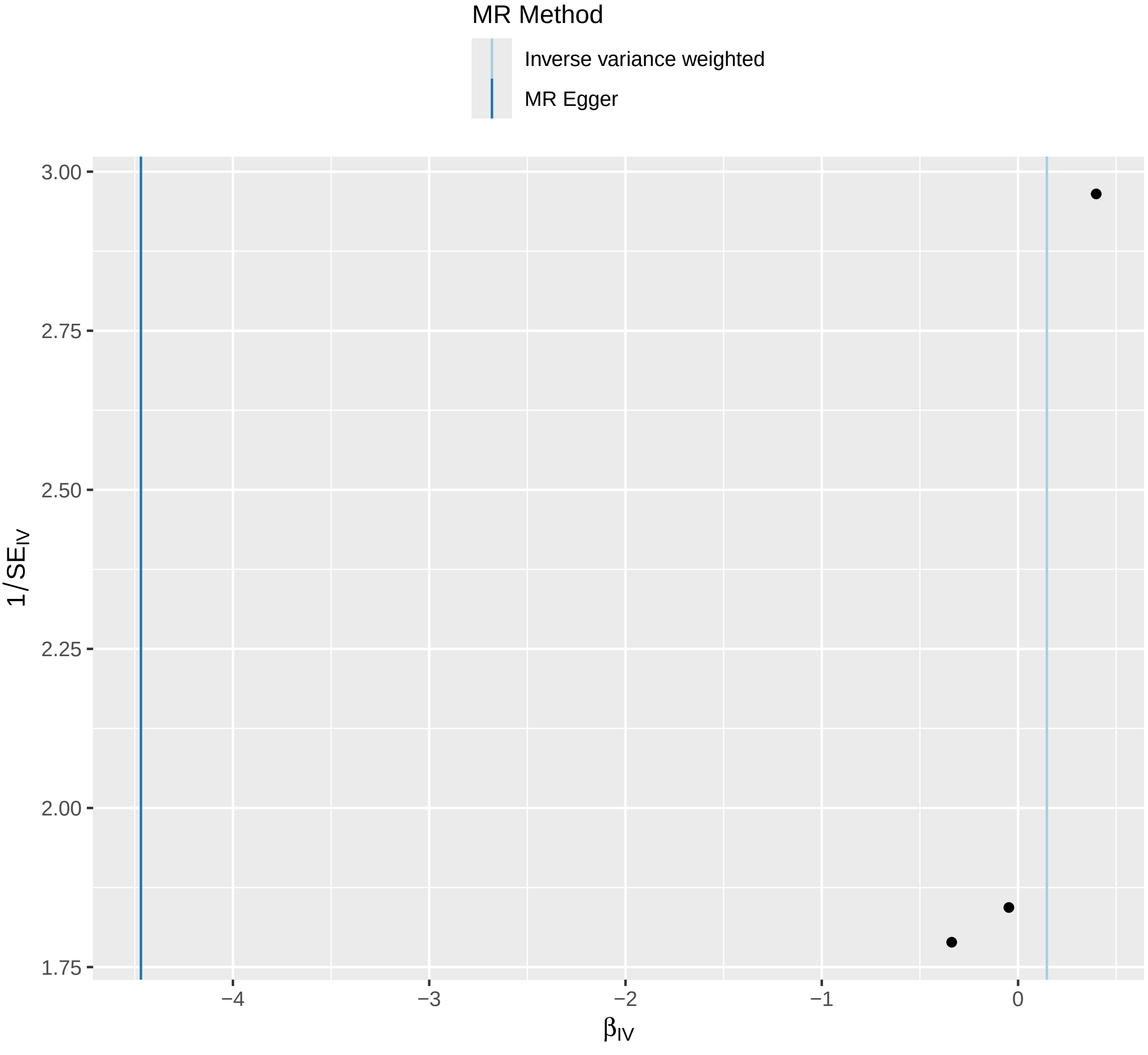
MR Method
Inverse variance weighted
MR Egger
3.00
2.75
2.50
2.25
2.00
1.75
1/SE$_{IV}$
−4
−3
−2
−1
0
β$_{IV}$

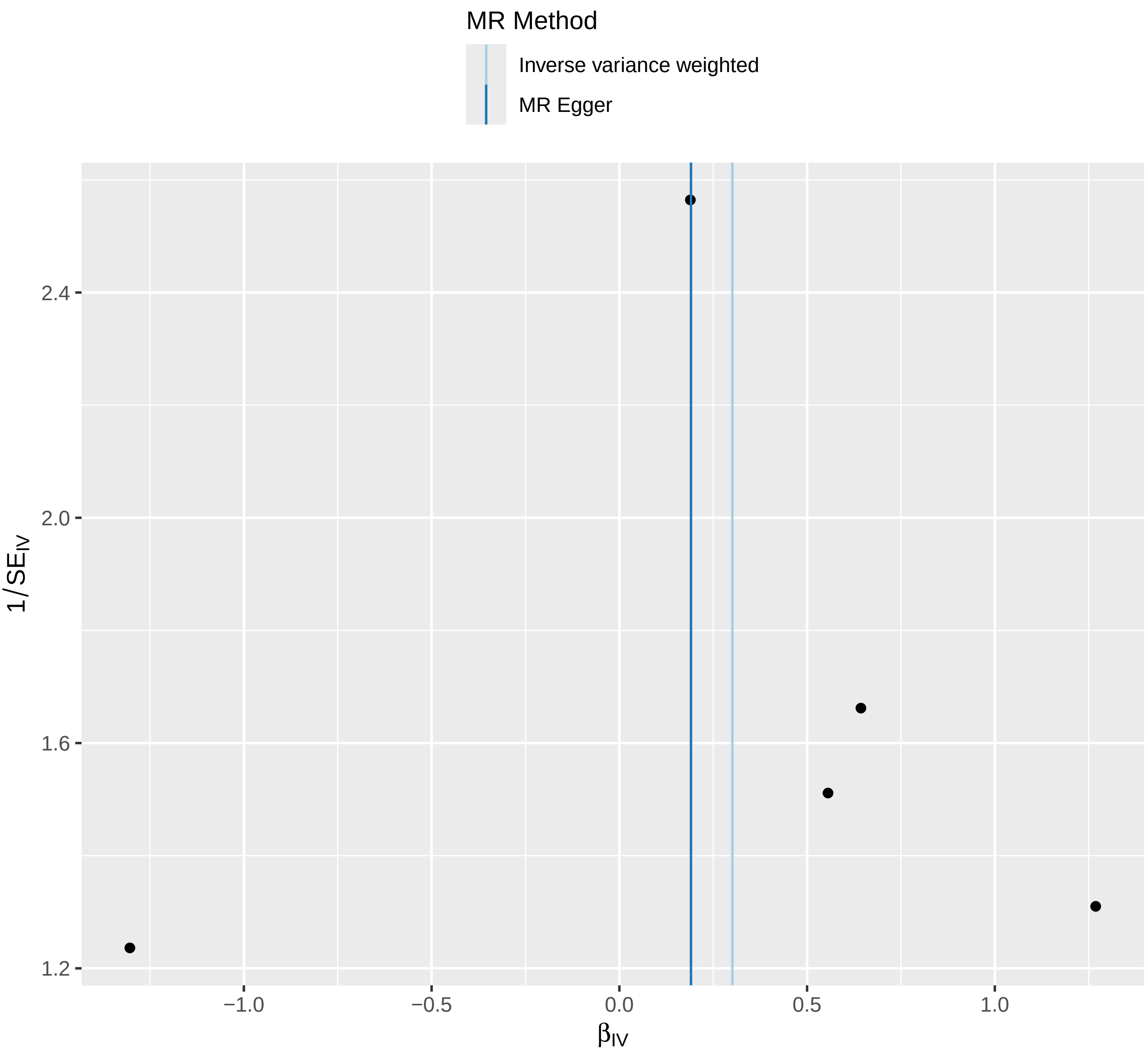
MR Method
Inverse variance weighted
MR Egger
2.4
2.0
1.6
1.2
1/SE$_{IV}$
−1.0
−0.5
0.0
0.5
1.0
β$_{IV}$

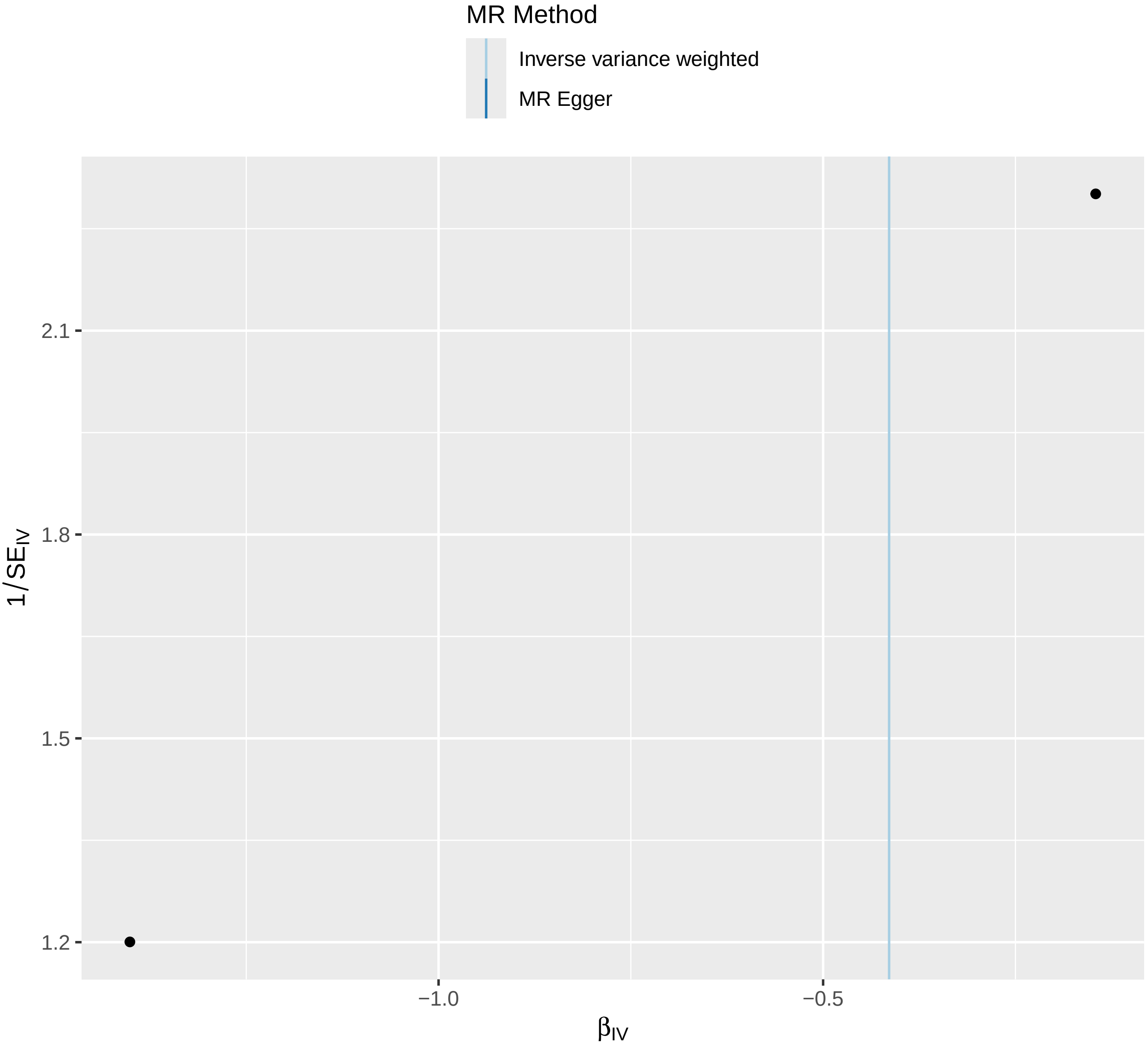
MR Method
Inverse variance weighted
MR Egger
2.1
1.8
1.5
1.2
$1/SE_{IV}$
−1.0
−0.5
β_{IV}

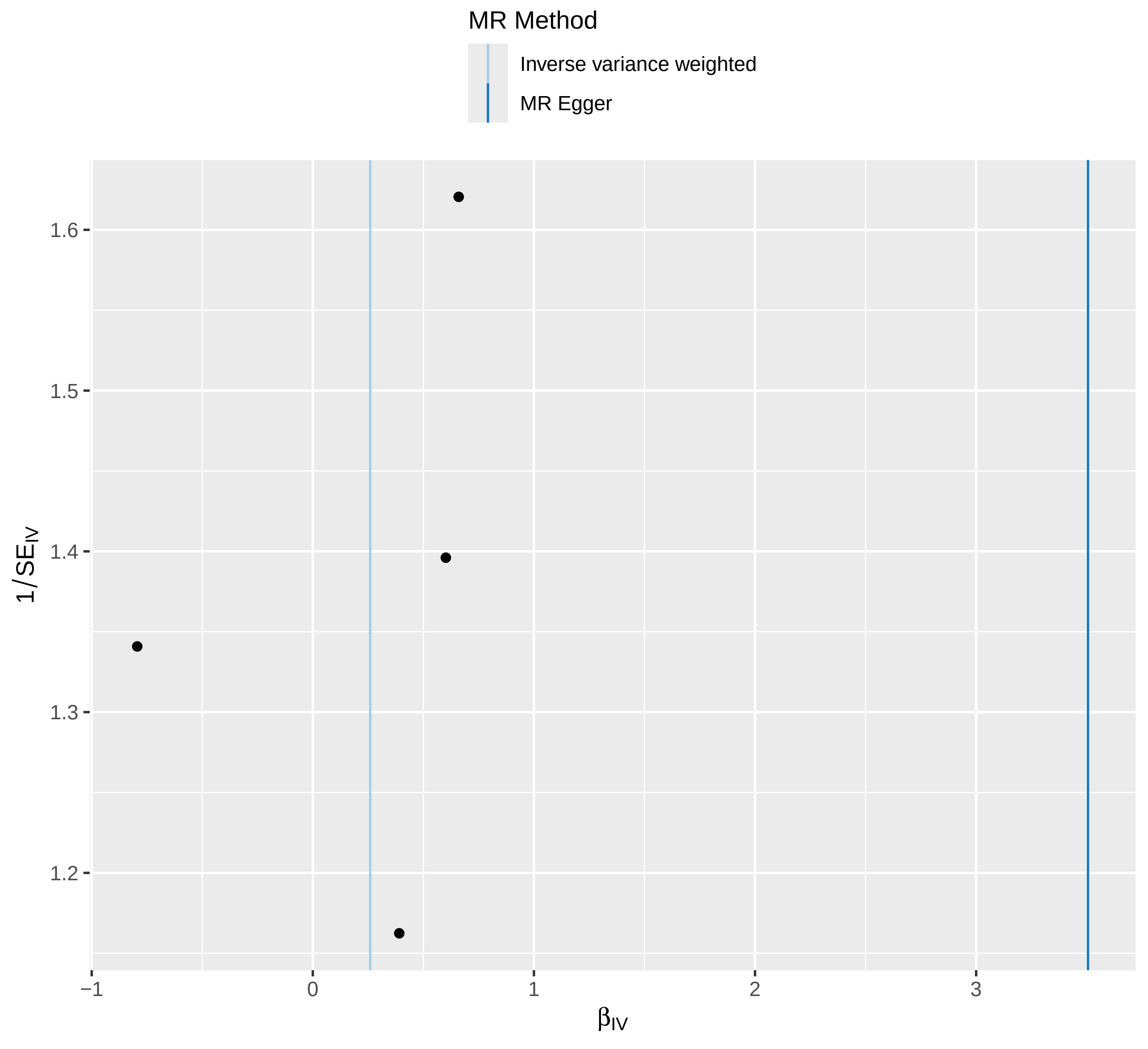

MR Method
Inverse variance weighted
MR Egger
1.6
1.5
1.4
1.3
1.2
1/SE_IV
−1
0
1
2
3
β_IV

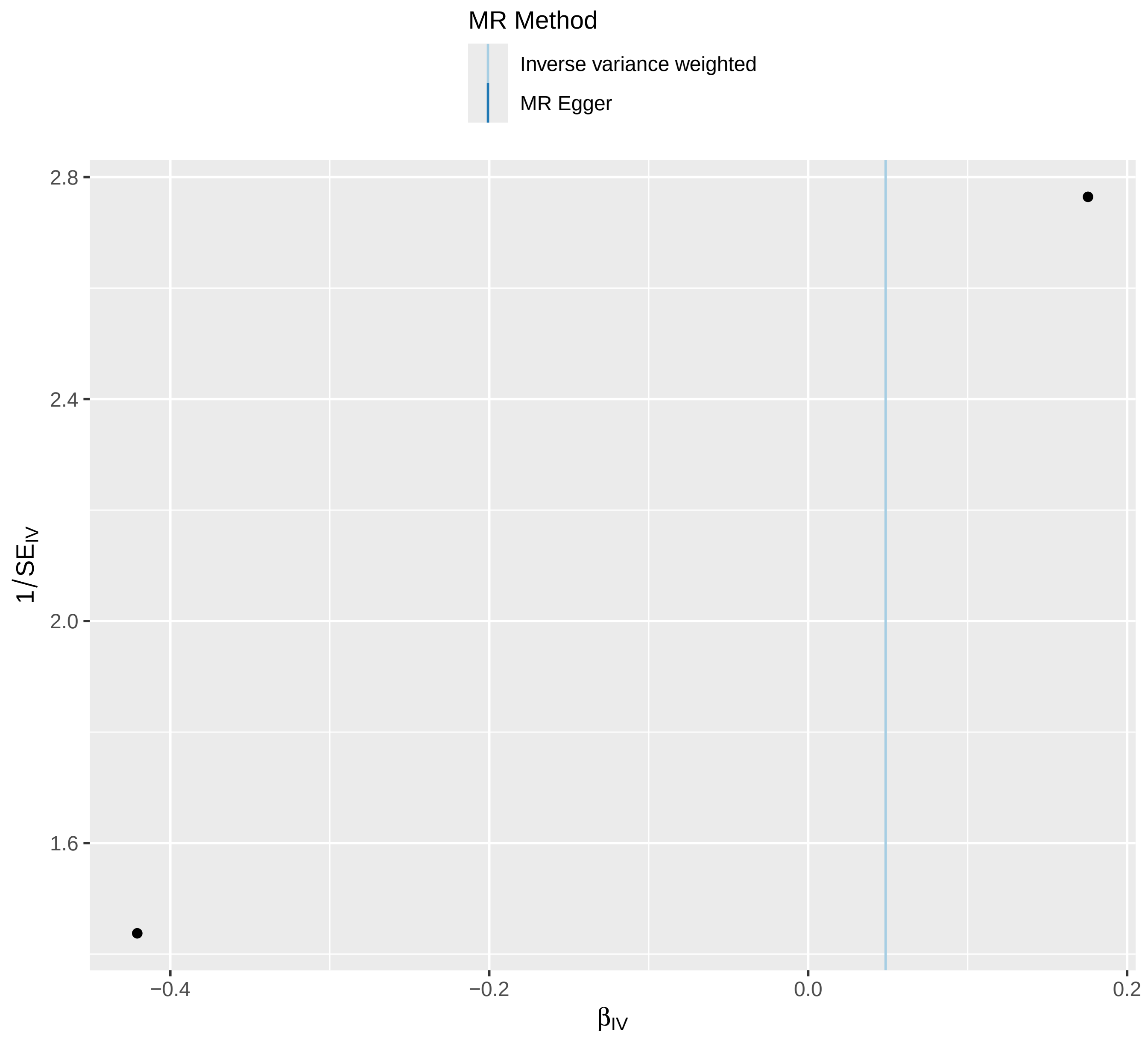
MR Method
Inverse variance weighted
MR Egger
2.8
2.4
2.0
1.6
1/SE_IV
−0.4
−0.2
0.0
0.2
β_IV

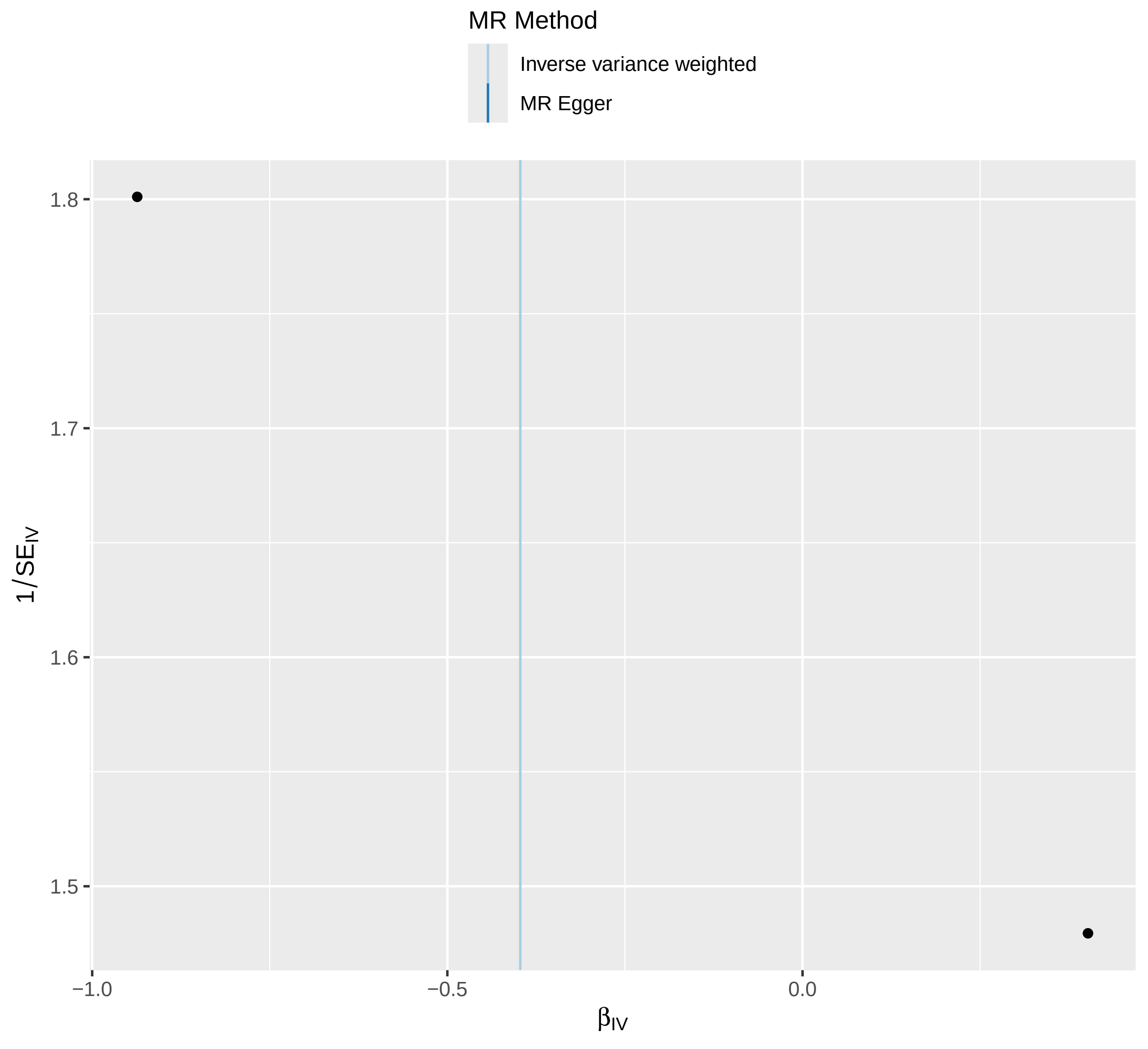
MR Method
Inverse variance weighted
MR Egger
1.8
1.7
1.6
1.5
$1/SE_{IV}$
−1.0
−0.5
0.0
β_{IV}

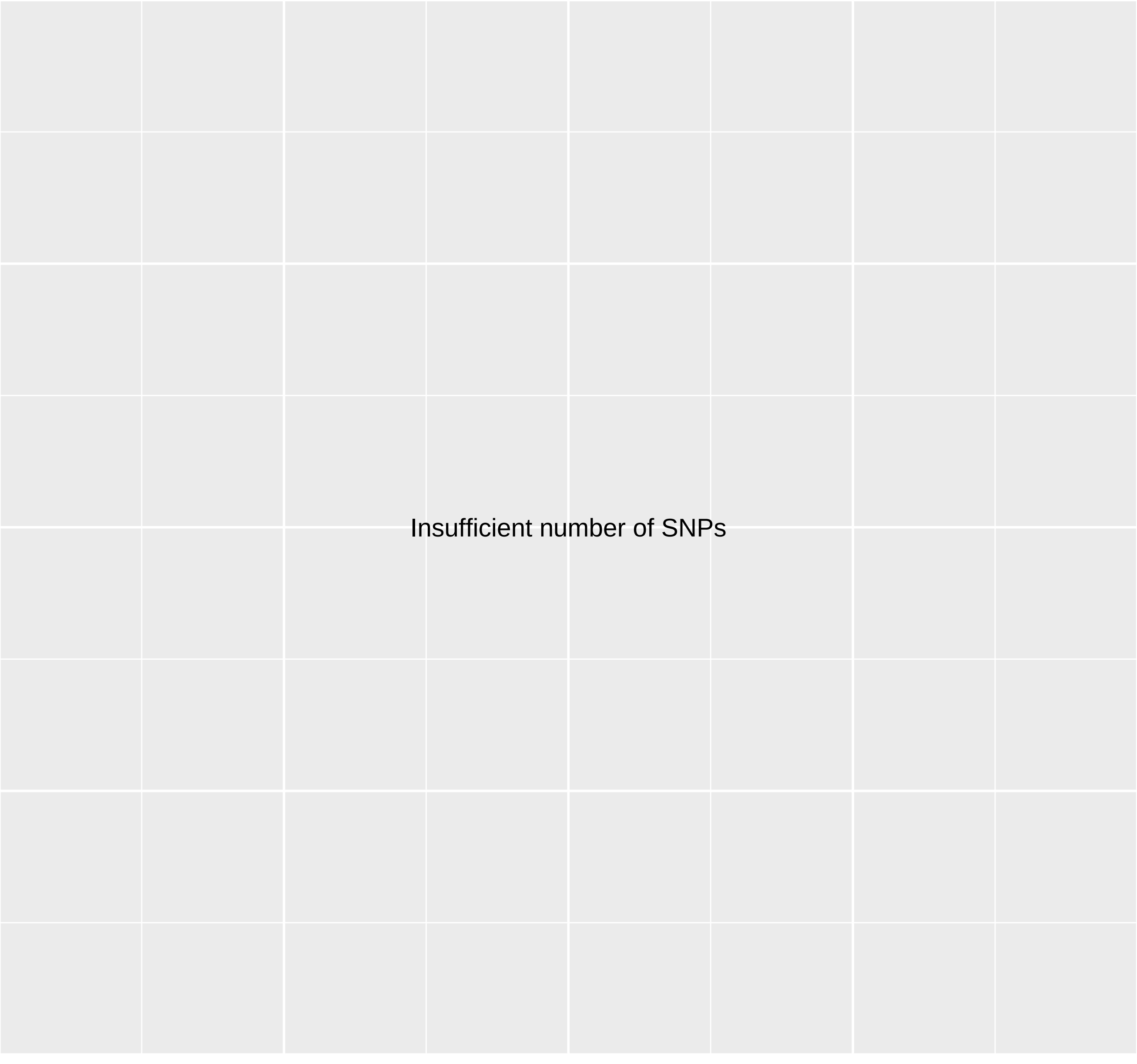
Insufficient number of SNPs

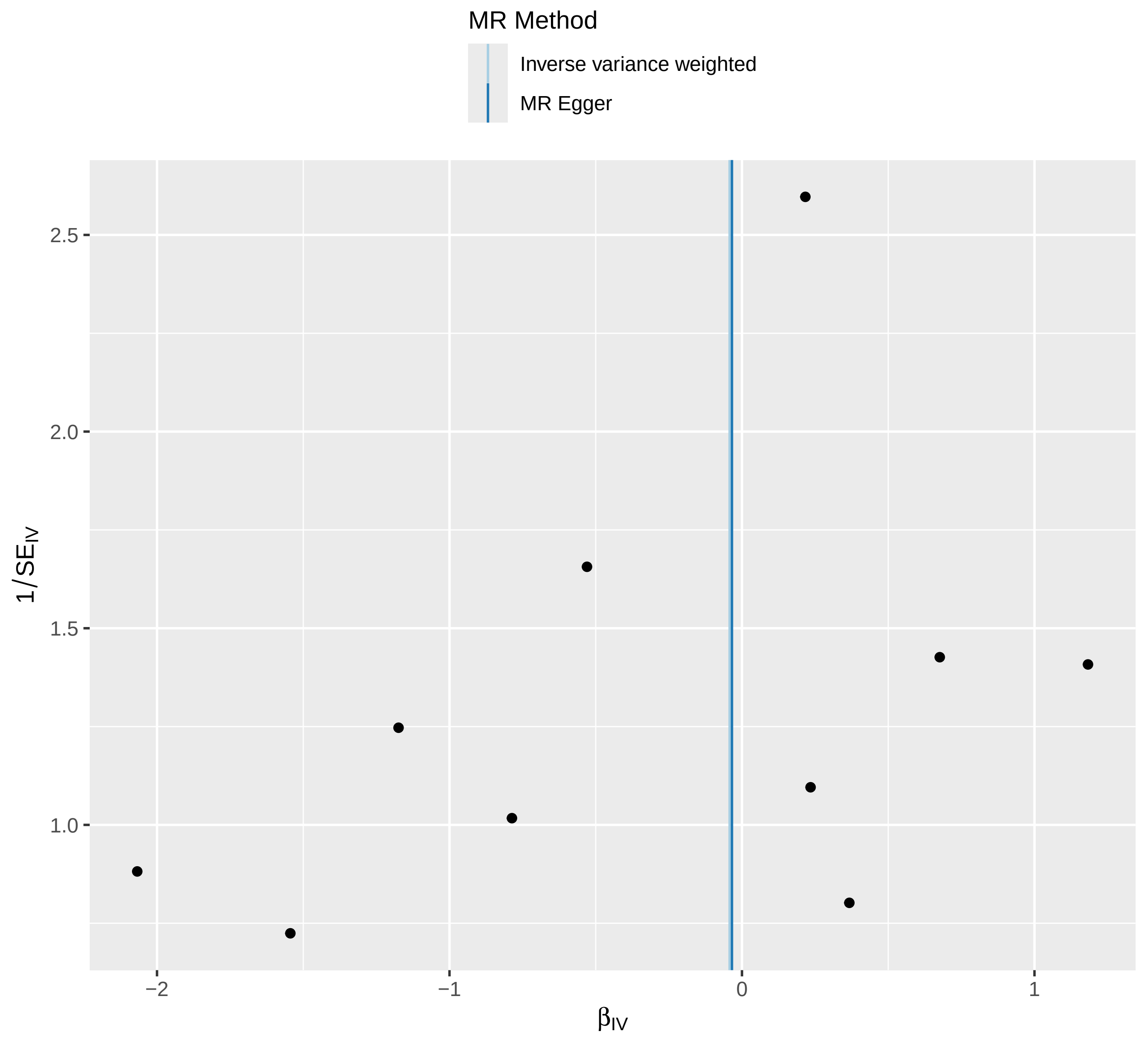
MR Method
Inverse variance weighted
MR Egger
2.5
2.0
1.5
1.0
1/SE_{IV}
−2
−1
0
1
β_{IV}

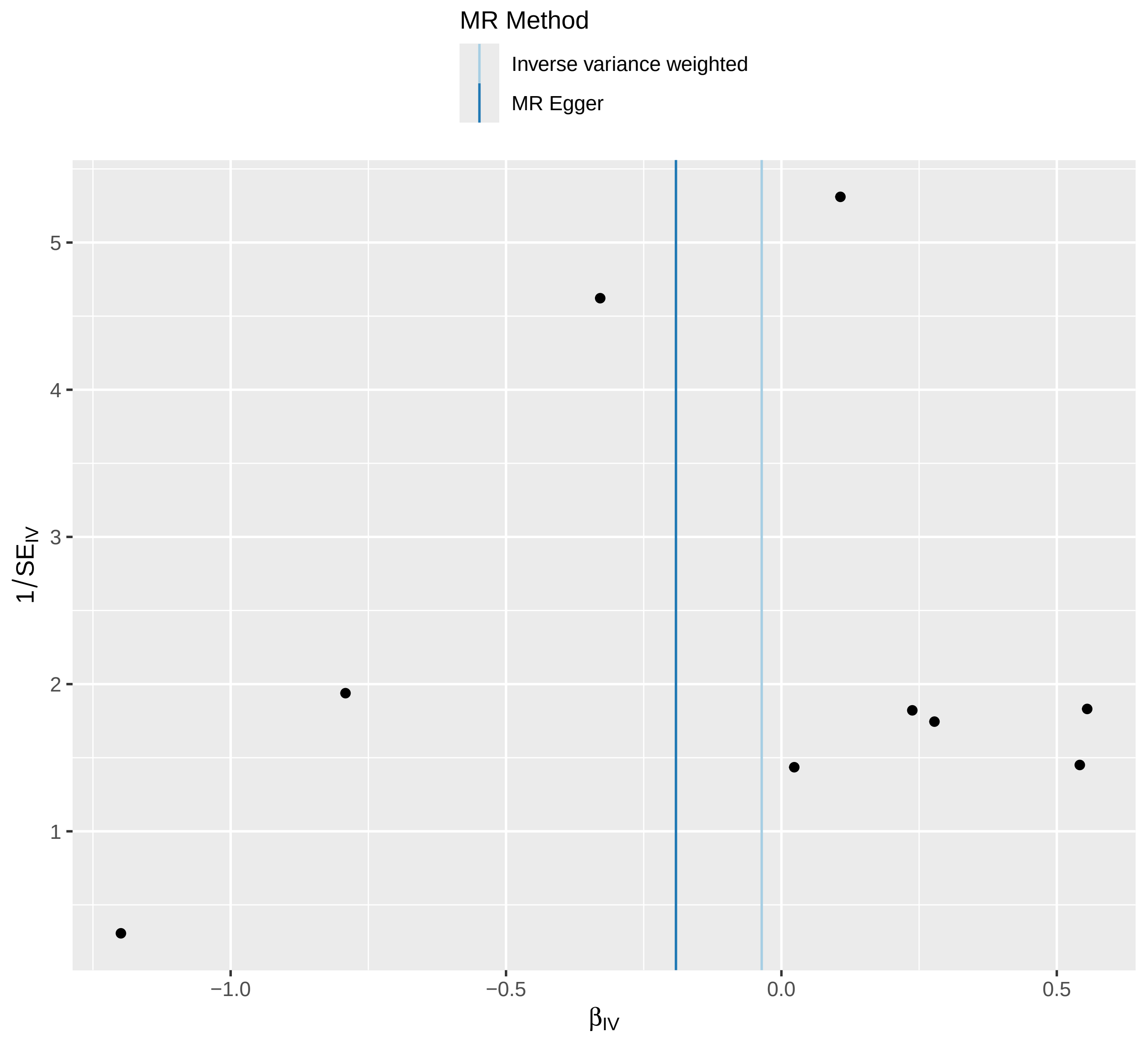
MR Method
Inverse variance weighted
MR Egger
1/SE_IV
β_IV
−1.0
−0.5
0.0
0.5
1
2
3
4
5

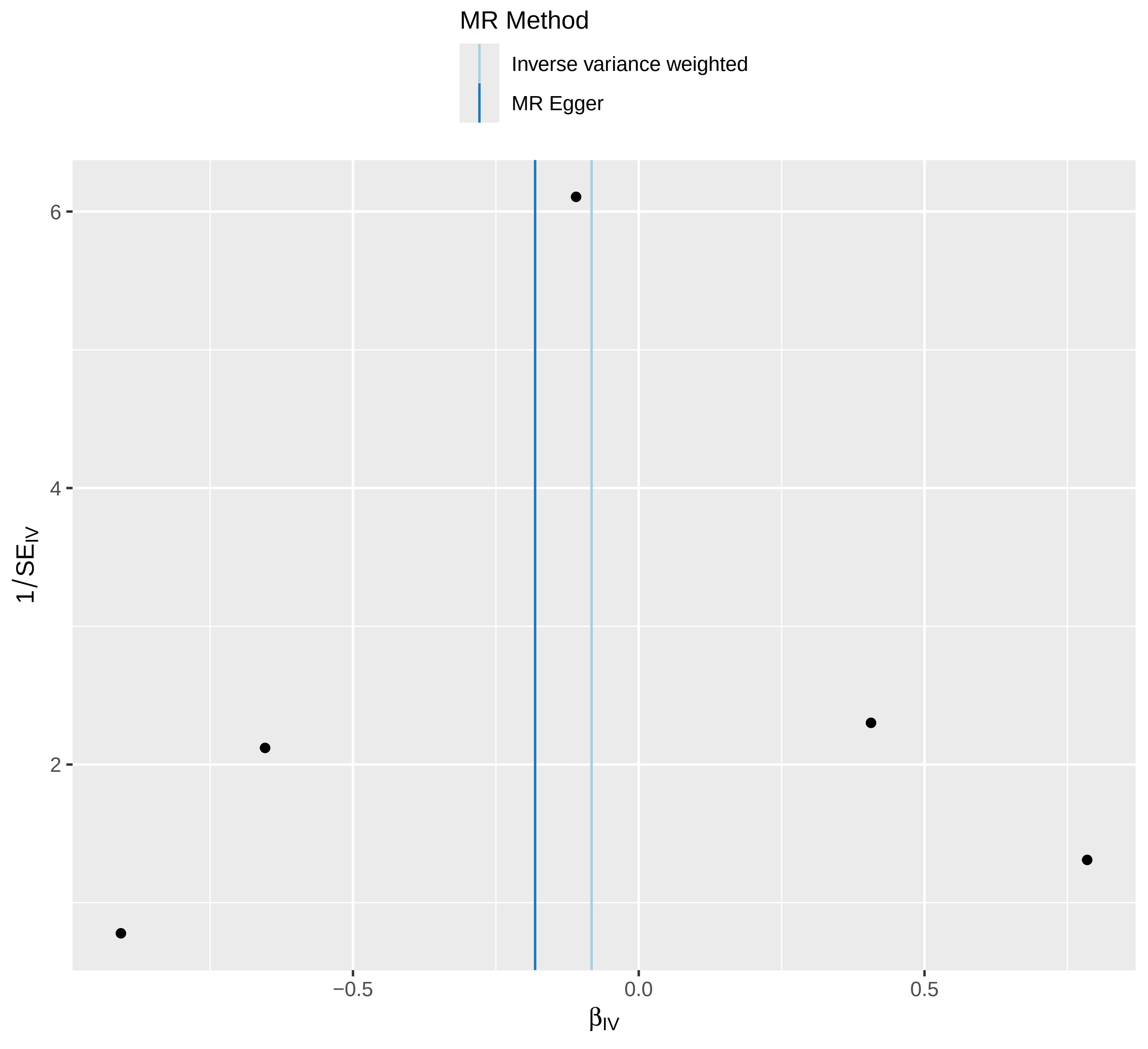
MR Method
Inverse variance weighted
MR Egger
6
4
2
1/SE_IV
−0.5
0.0
0.5
β_IV

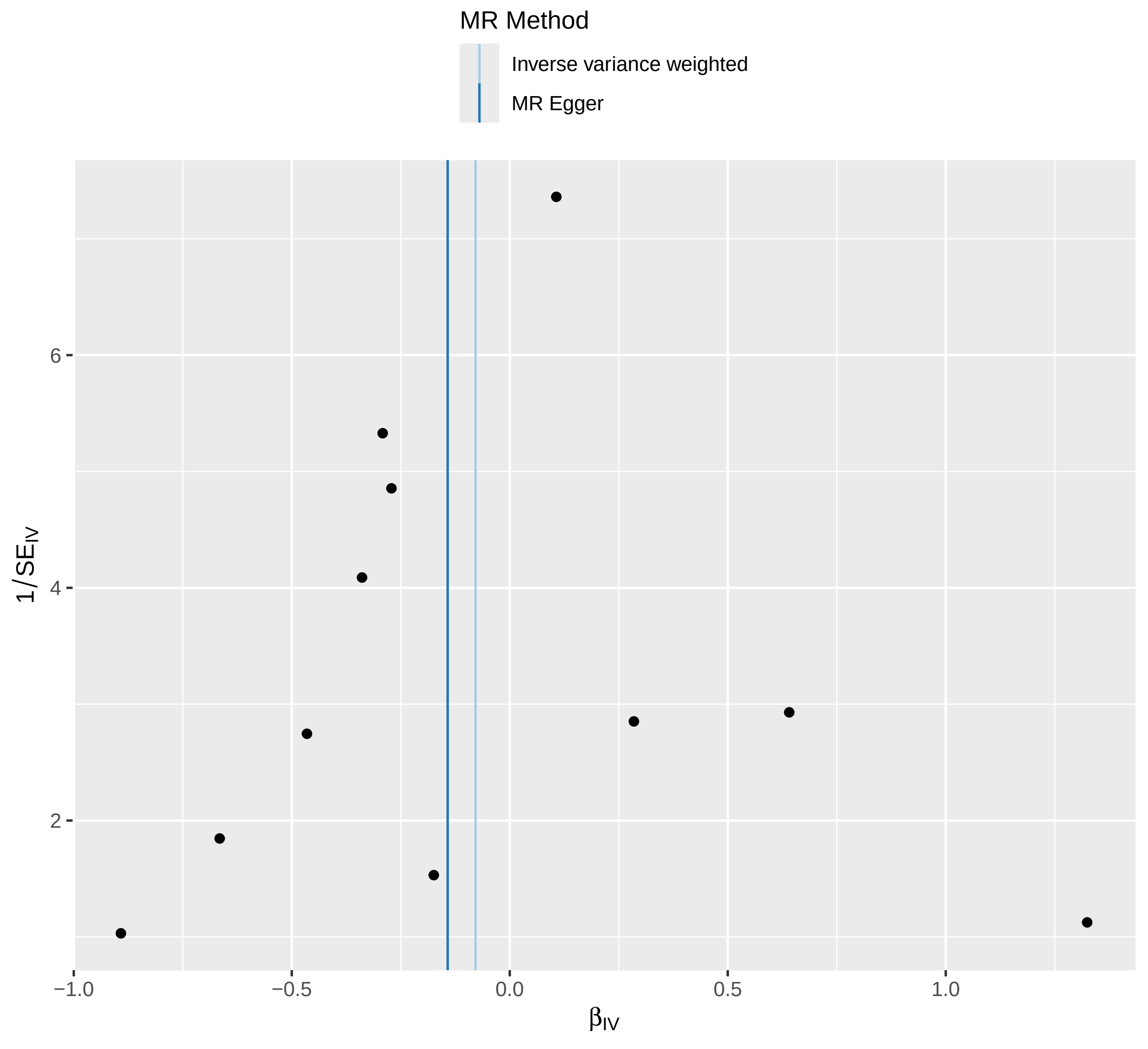

MR Method
Inverse variance weighted
MR Egger
1/SE_IV
6
4
2
−1.0
−0.5
0.0
0.5
1.0
β_IV

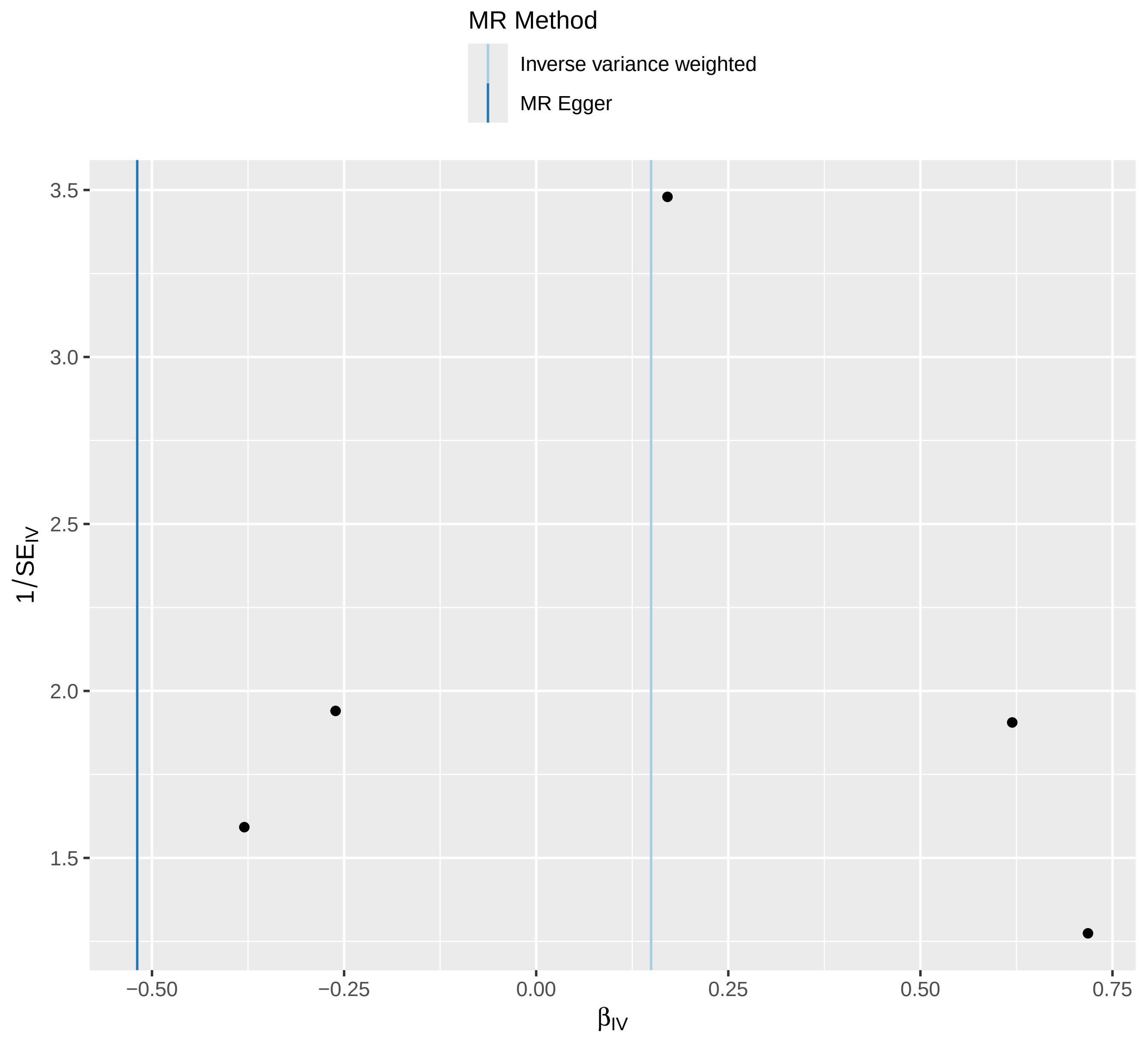
MR Method
Inverse variance weighted
MR Egger
3.5
3.0
2.5
2.0
1.5
1/SE_IV
−0.50
−0.25
0.00
0.25
0.50
0.75
β_IV

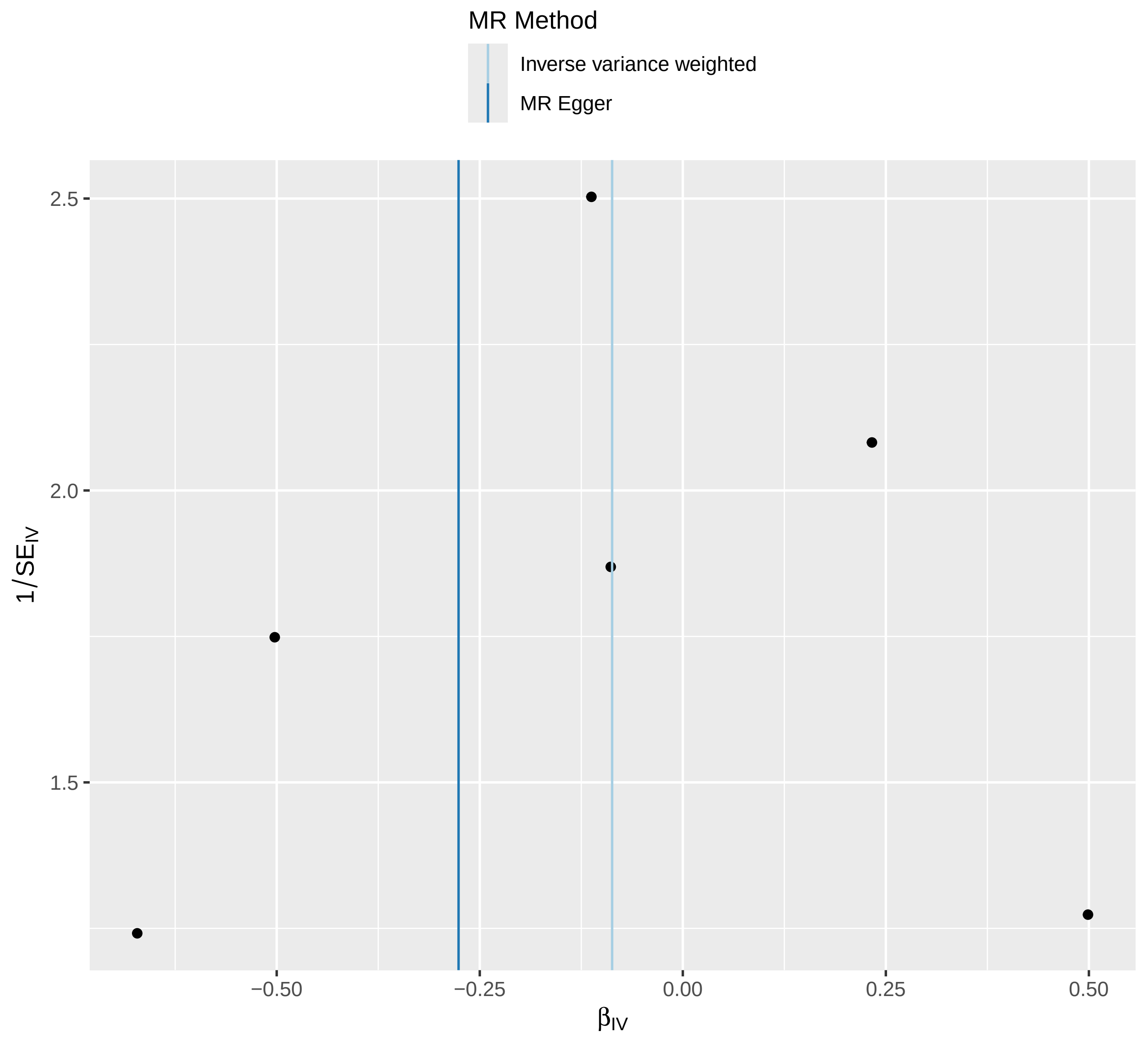

MR Method
Inverse variance weighted
MR Egger
2.5
2.0
1.5
$1/SE_{IV}$
−0.50
−0.25
0.00
0.25
0.50
β_{IV}

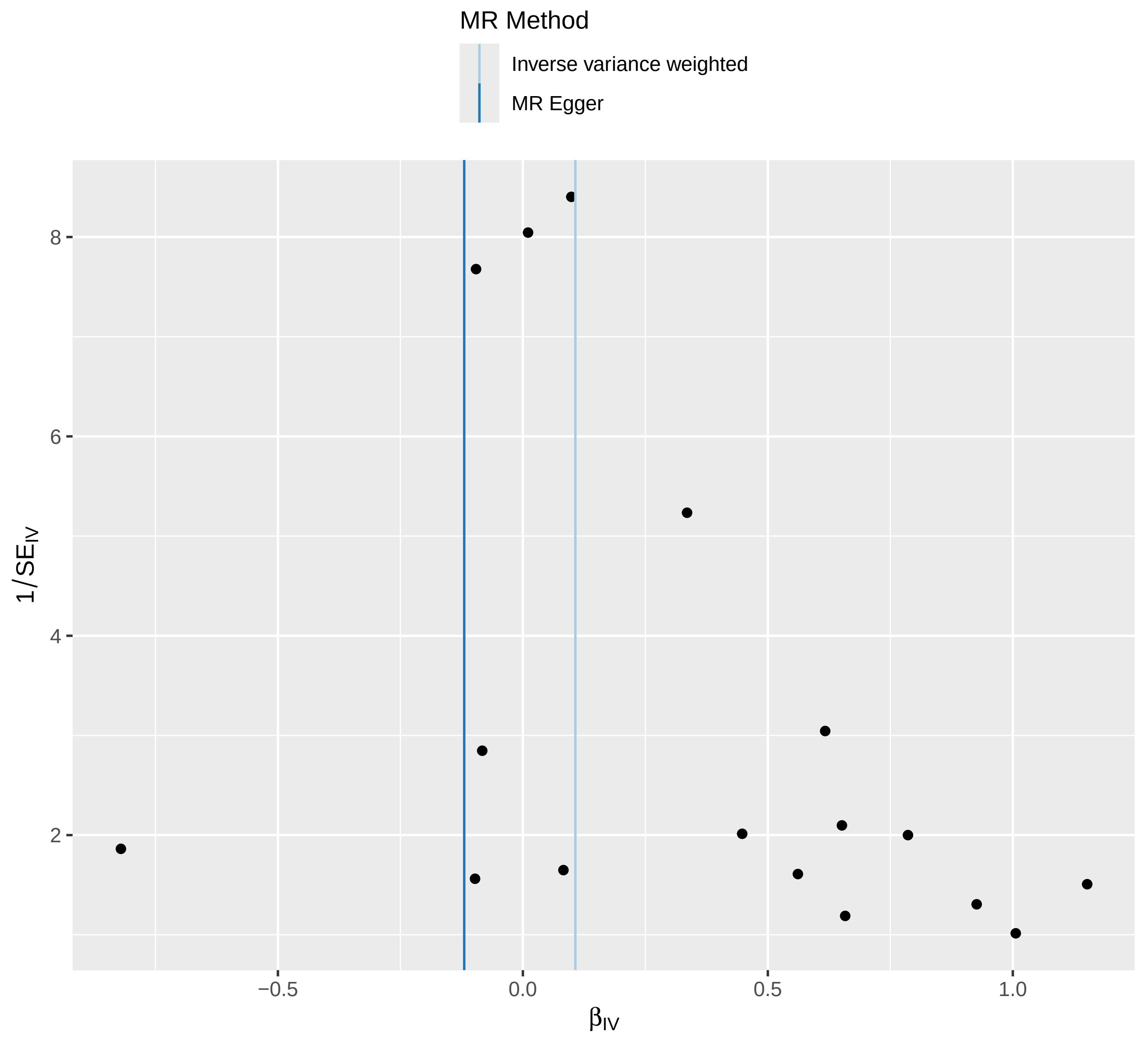
MR Method
Inverse variance weighted
MR Egger
8
6
4
2
1/SE_IV
−0.5
0.0
0.5
1.0
β_IV

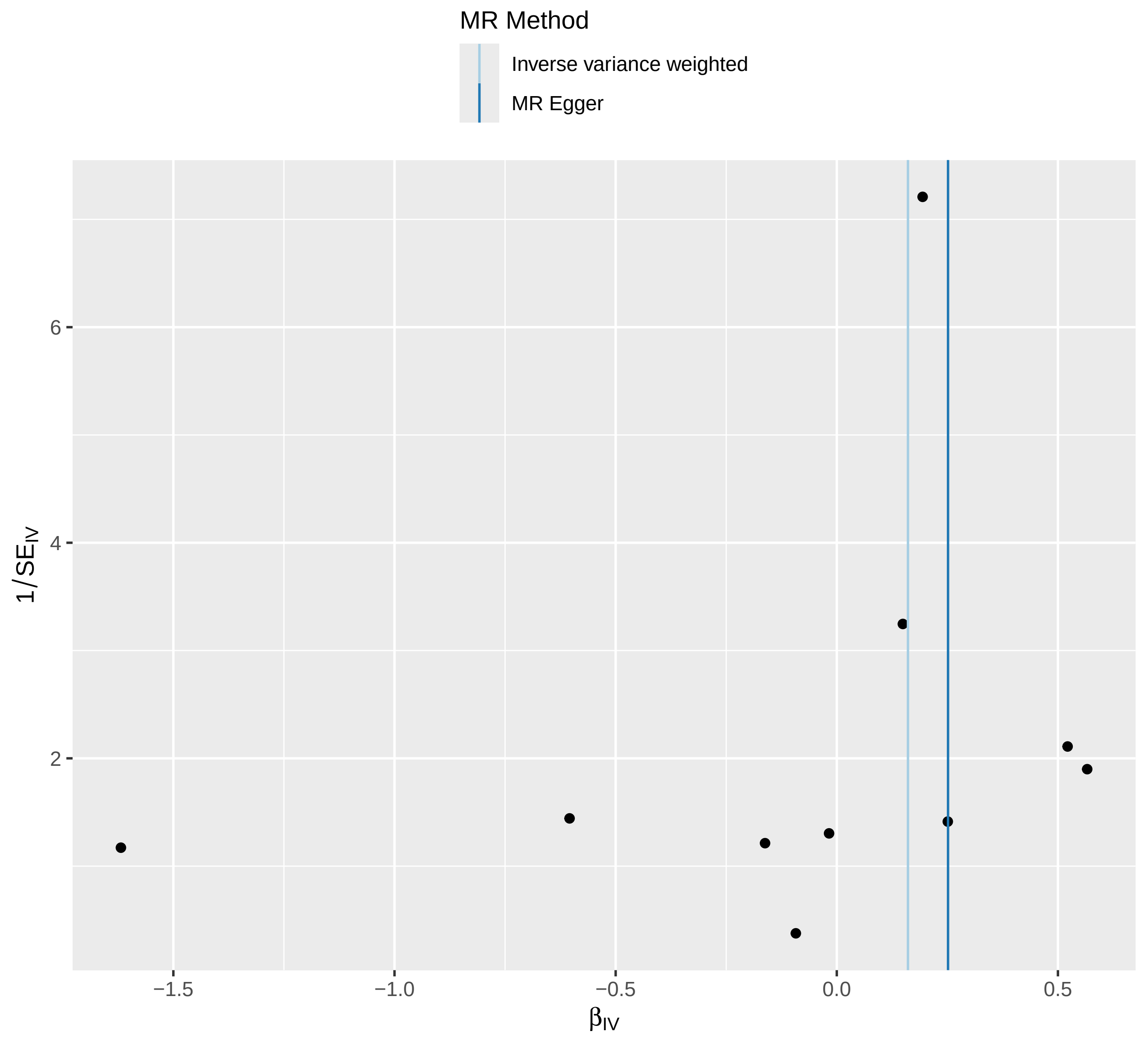

MR Method
Inverse variance weighted
MR Egger
6
4
2
1/SE_IV
−1.5
−1.0
−0.5
0.0
0.5
β_IV

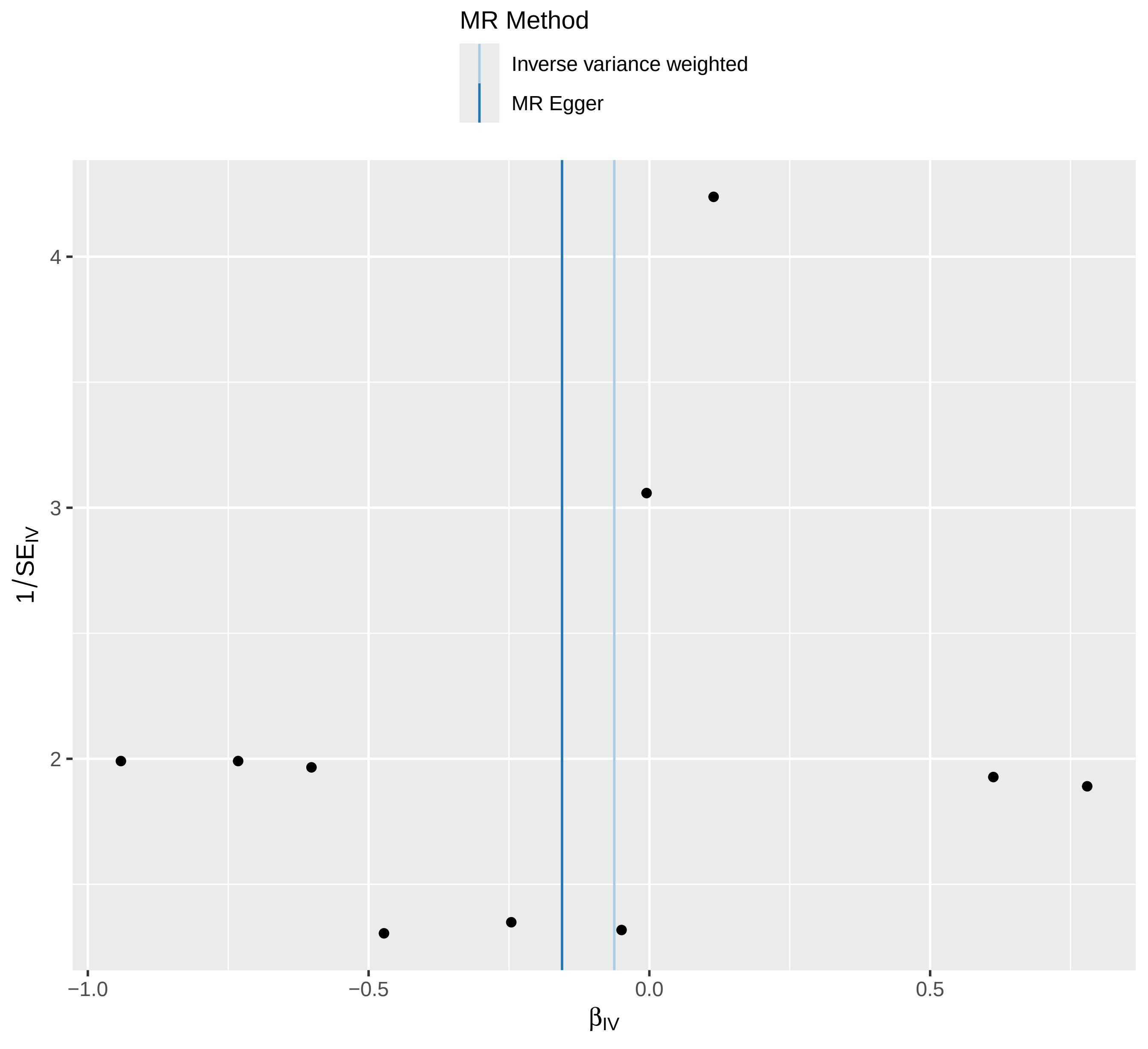
MR Method
Inverse variance weighted
MR Egger
4
3
2
1/SE_IV
−1.0
−0.5
0.0
0.5
β_IV

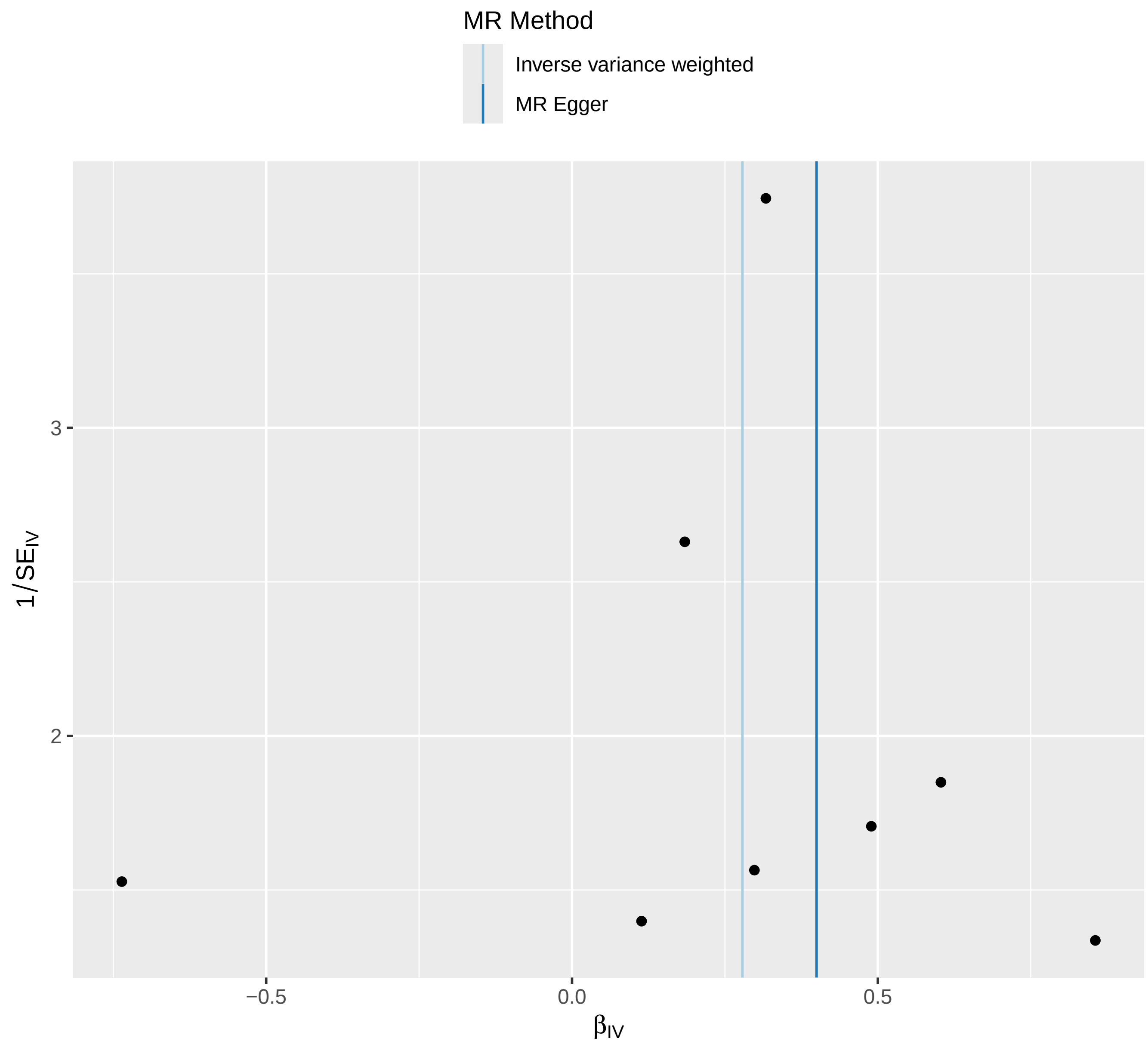
MR Method
Inverse variance weighted
MR Egger
3
2
1/SE_IV
−0.5
0.0
0.5
β_IV

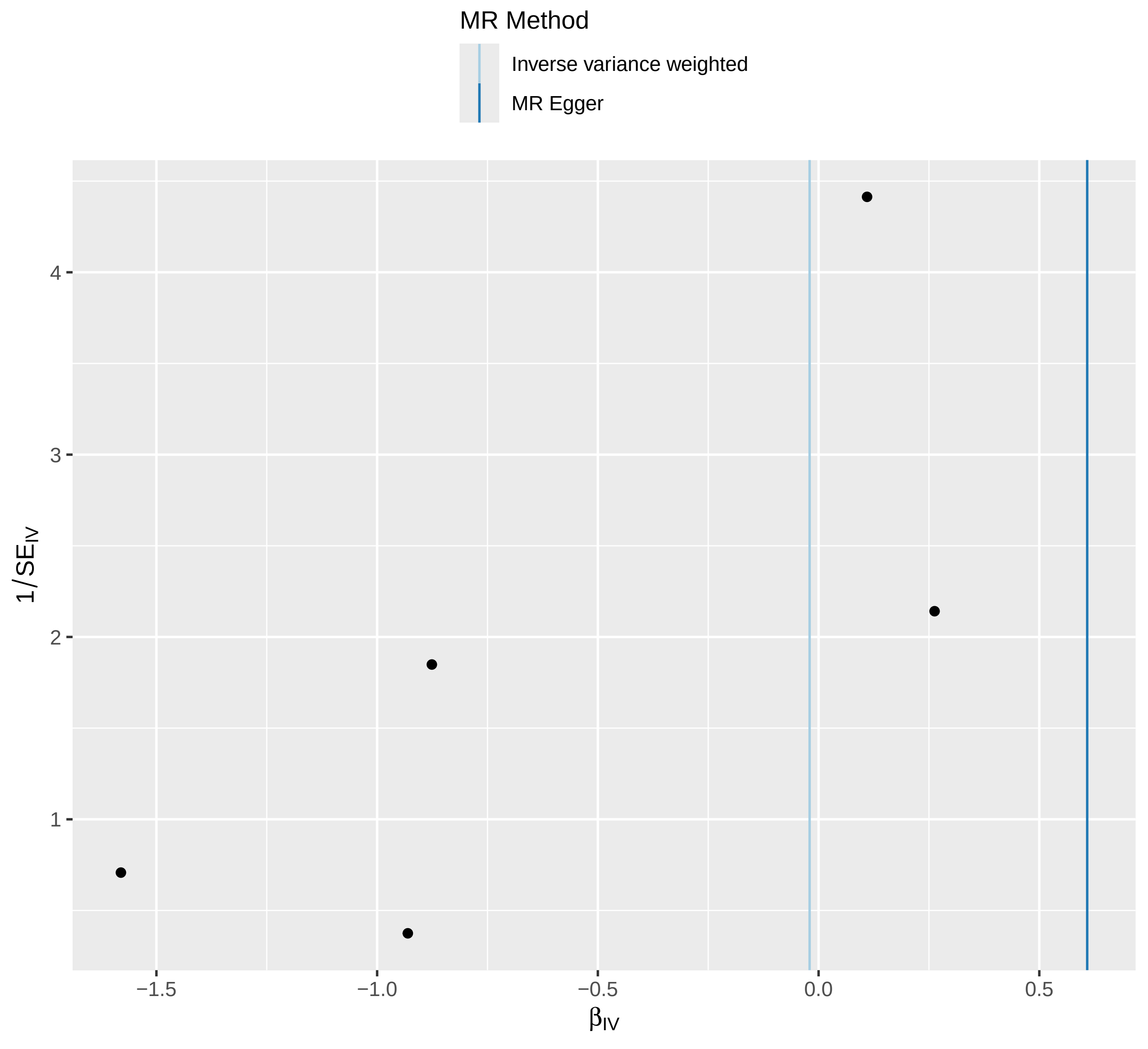

MR Method
Inverse variance weighted
MR Egger
1/SE_IV
4
3
2
1
−1.5
−1.0
−0.5
0.0
0.5
β_IV

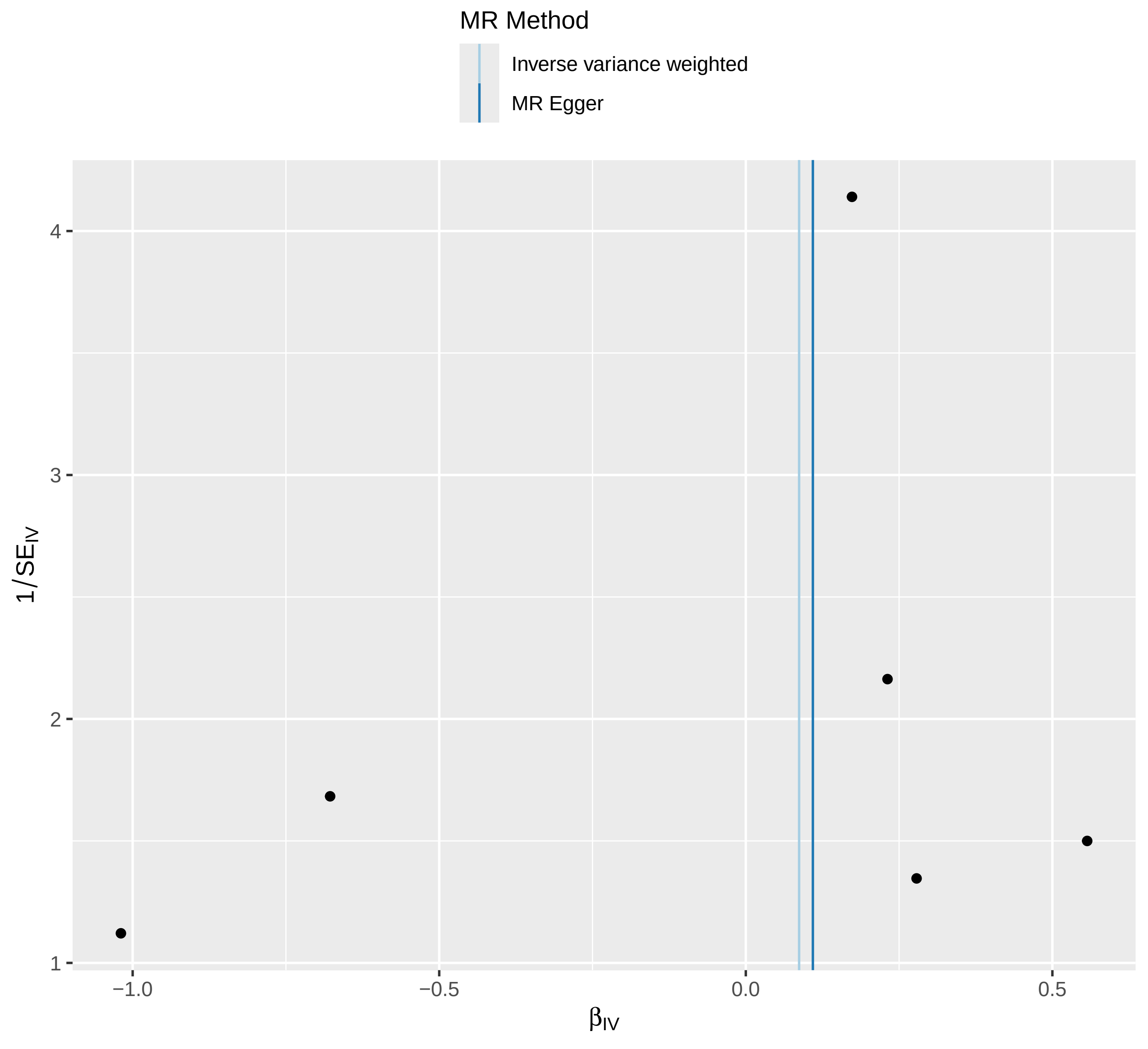
MR Method
Inverse variance weighted
MR Egger
4
3
2
1
1/SE_IV
−1.0
−0.5
0.0
0.5
β_IV

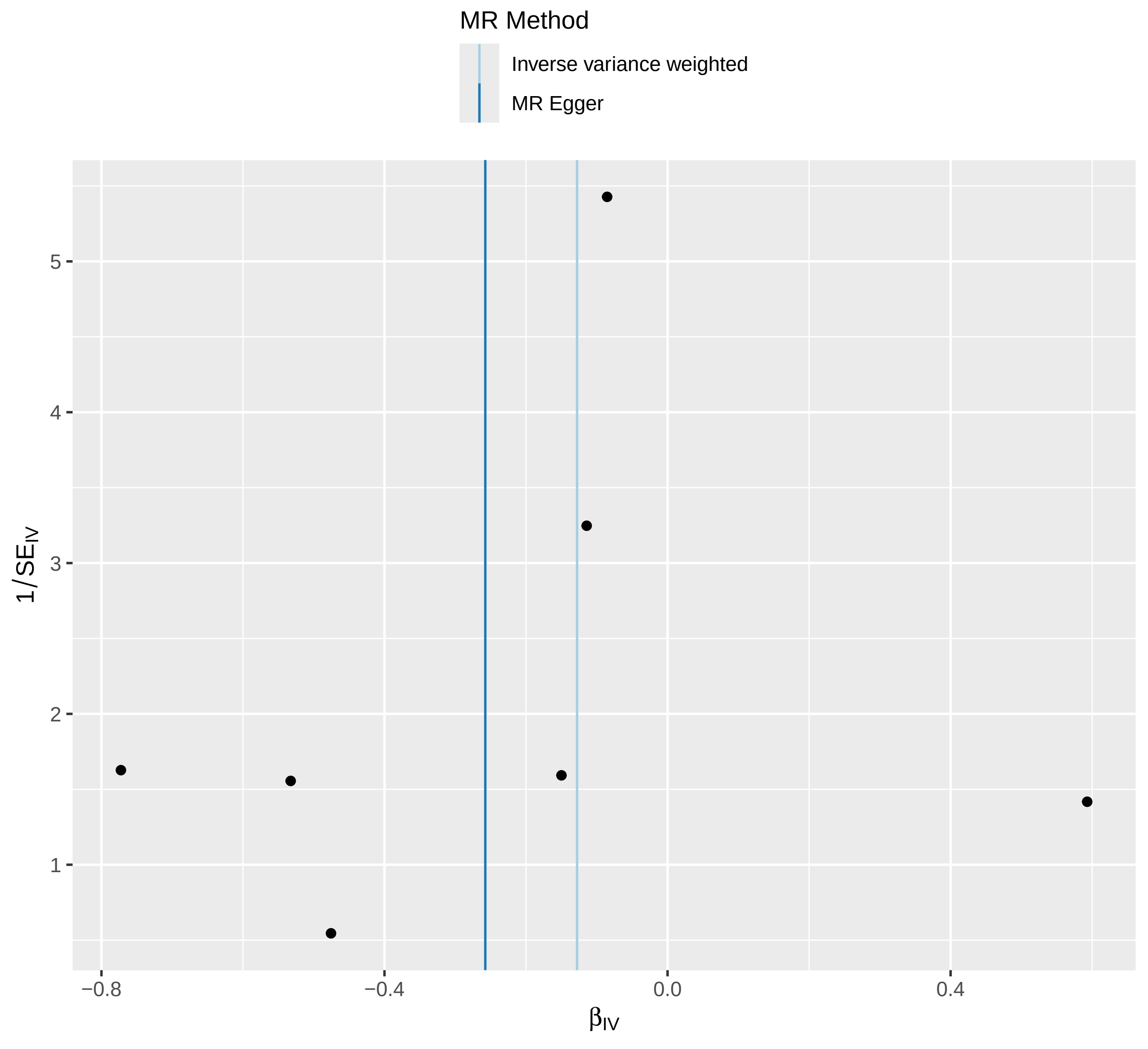

MR Method
Inverse variance weighted
MR Egger
5
4
3
2
1
1/SE_IV
−0.8
−0.4
0.0
0.4
β_IV

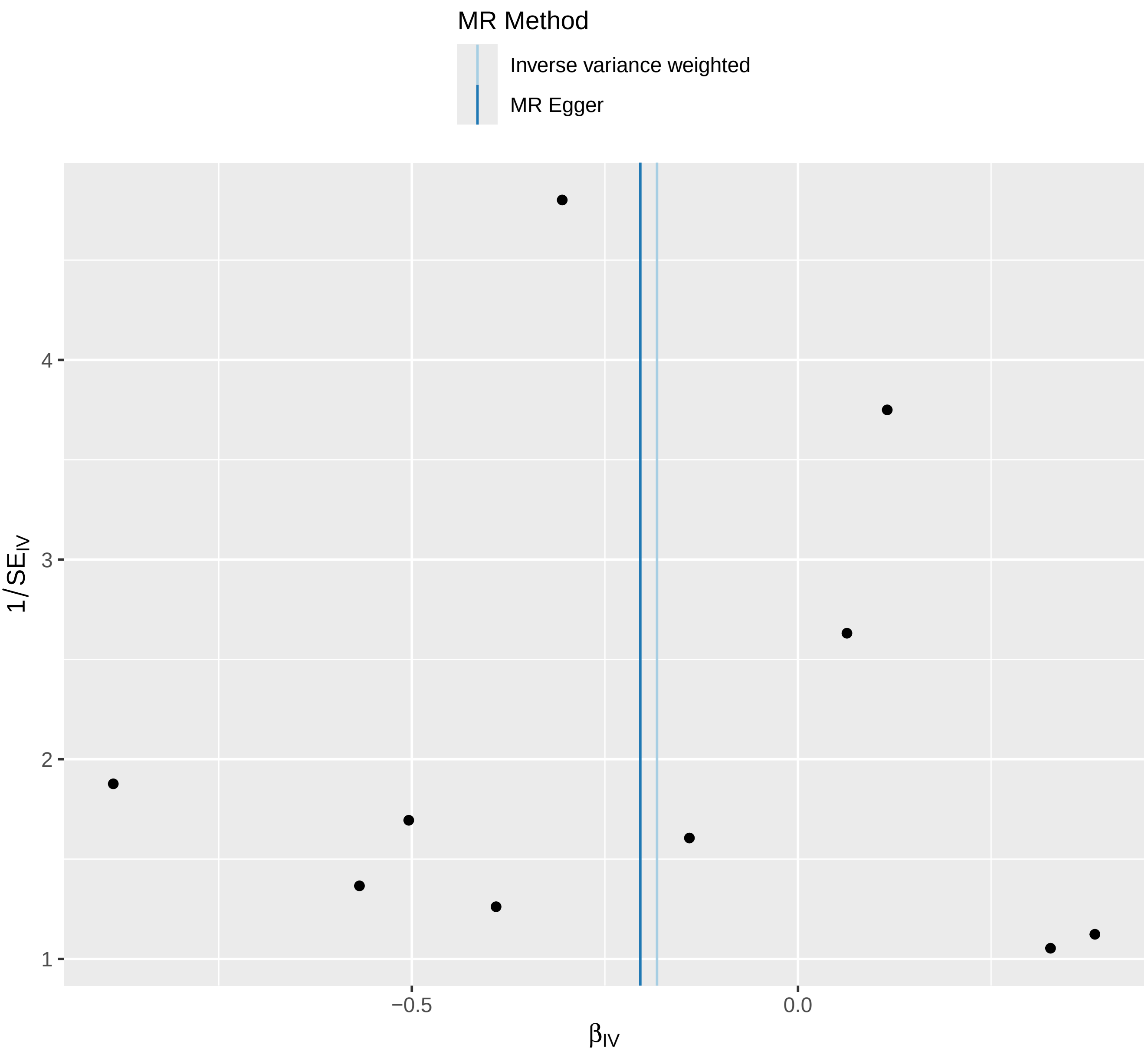

MR Method
Inverse variance weighted
MR Egger
4
3
2
1
1/SE_{IV}
−0.5
0.0
β_{IV}

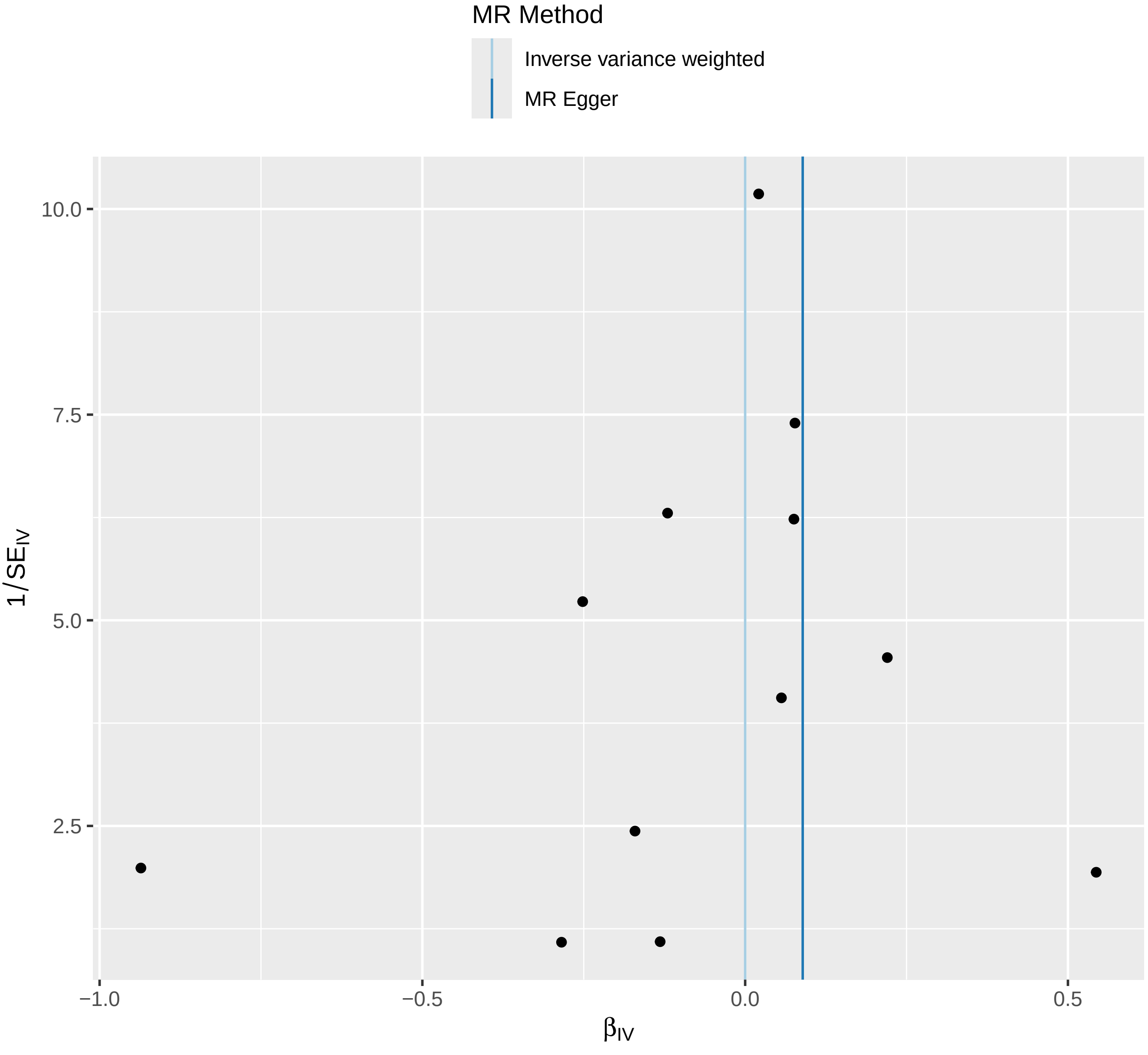
MR Method
Inverse variance weighted
MR Egger
10.0
7.5
5.0
2.5
1/SE$_{IV}$
−1.0
−0.5
0.0
0.5
β$_{IV}$

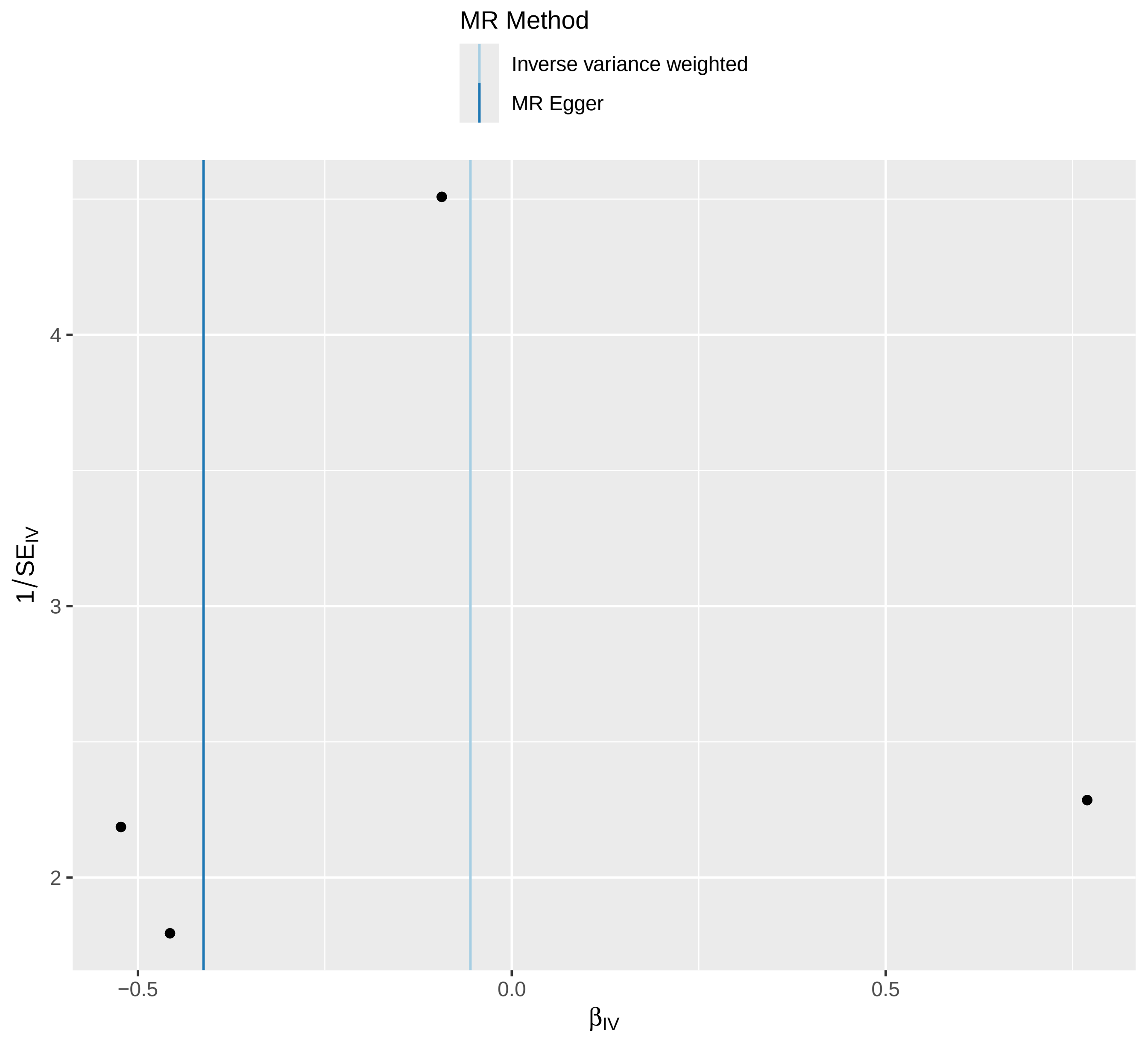

MR Method
Inverse variance weighted
MR Egger
4
3
2
1/SE_IV
−0.5
0.0
0.5
β_IV

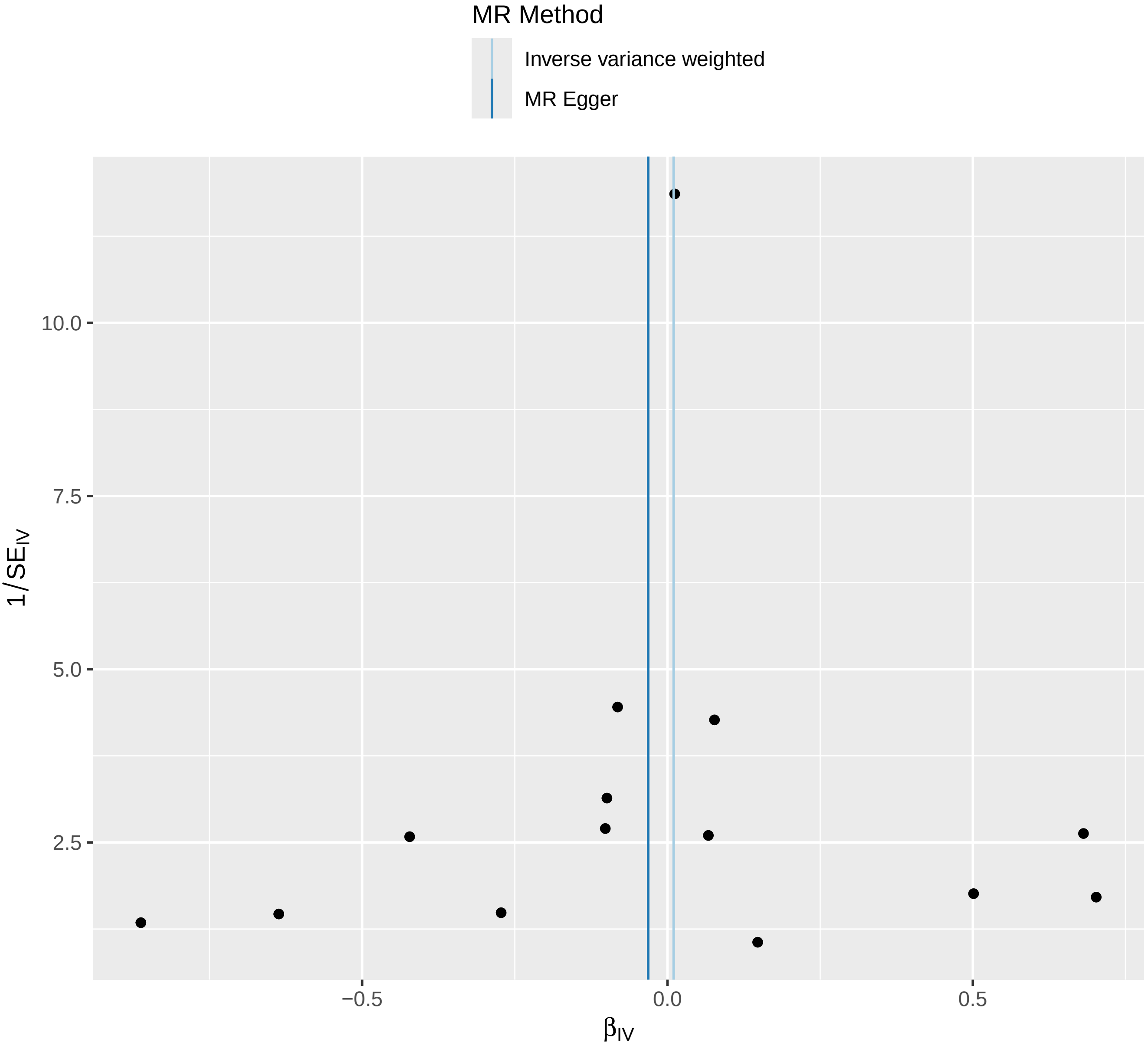

MR Method
Inverse variance weighted
MR Egger
1/SE_IV
10.0
7.5
5.0
2.5
−0.5
0.0
0.5
β_IV

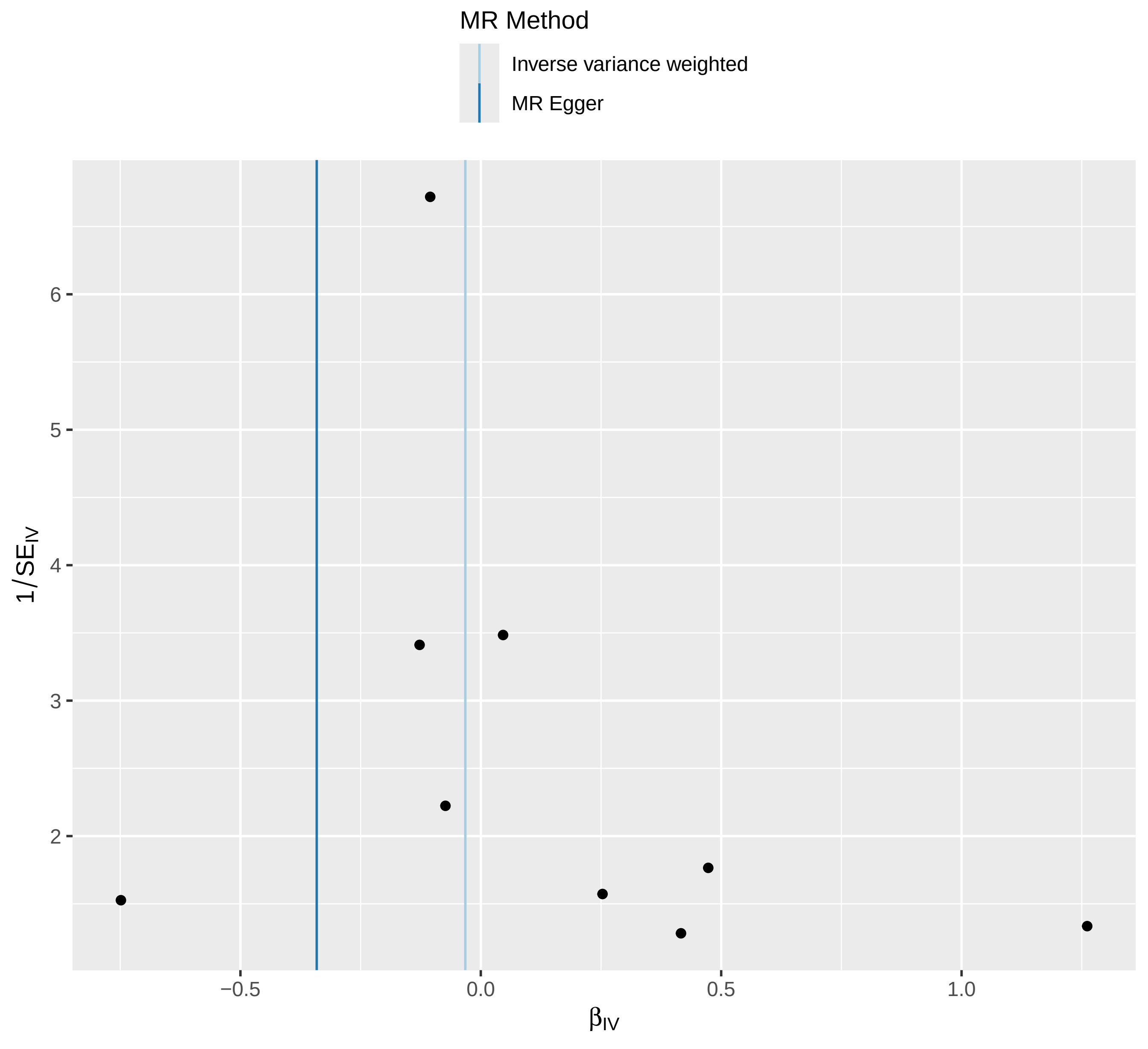

MR Method
Inverse variance weighted
MR Egger
1/SE_IV
6
5
4
3
2
−0.5
0.0
0.5
1.0
β_IV

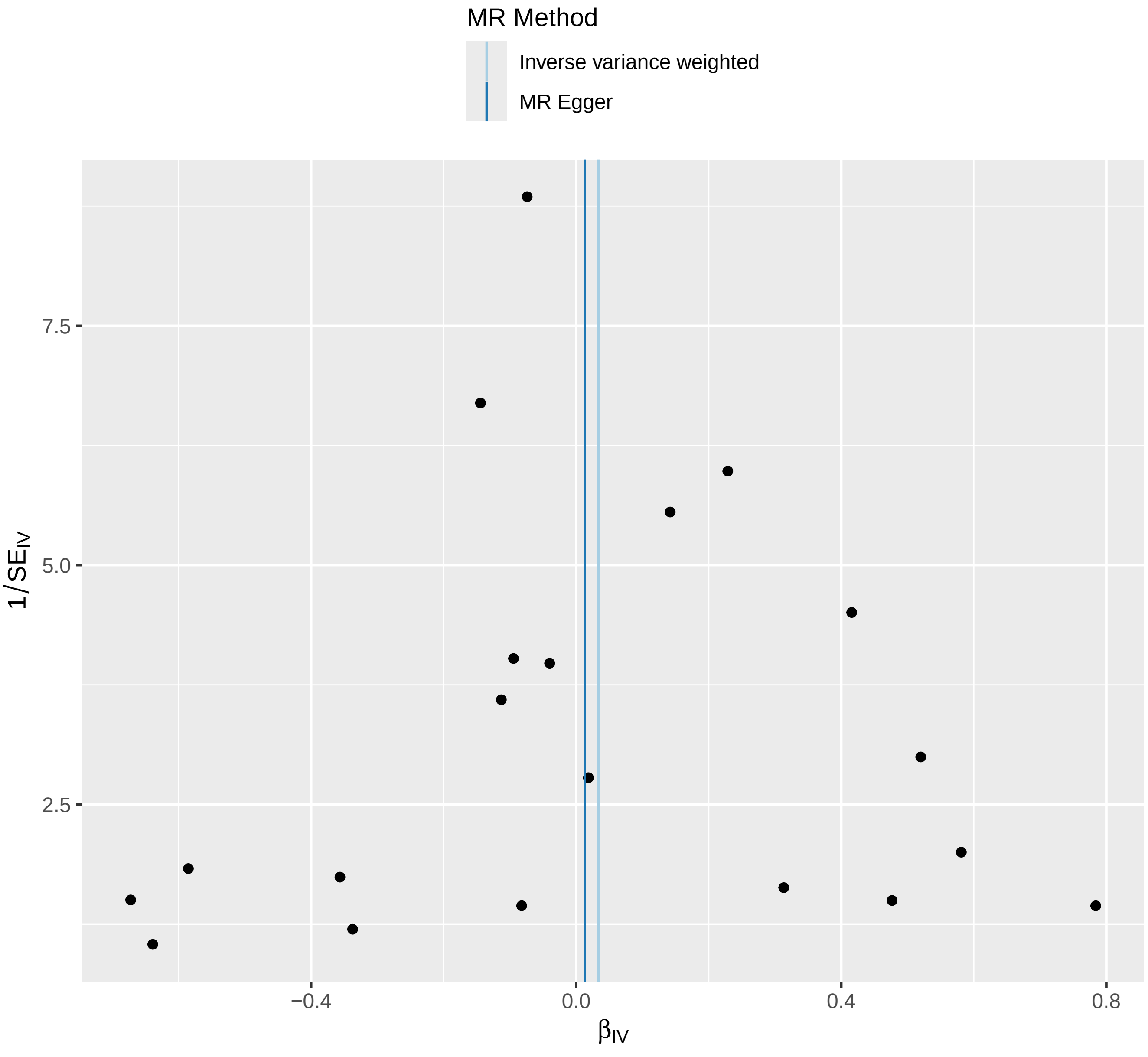

MR Method
Inverse variance weighted
MR Egger
7.5
5.0
2.5
−0.4
0.0
0.4
0.8
1/SE$_{IV}$
β$_{IV}$

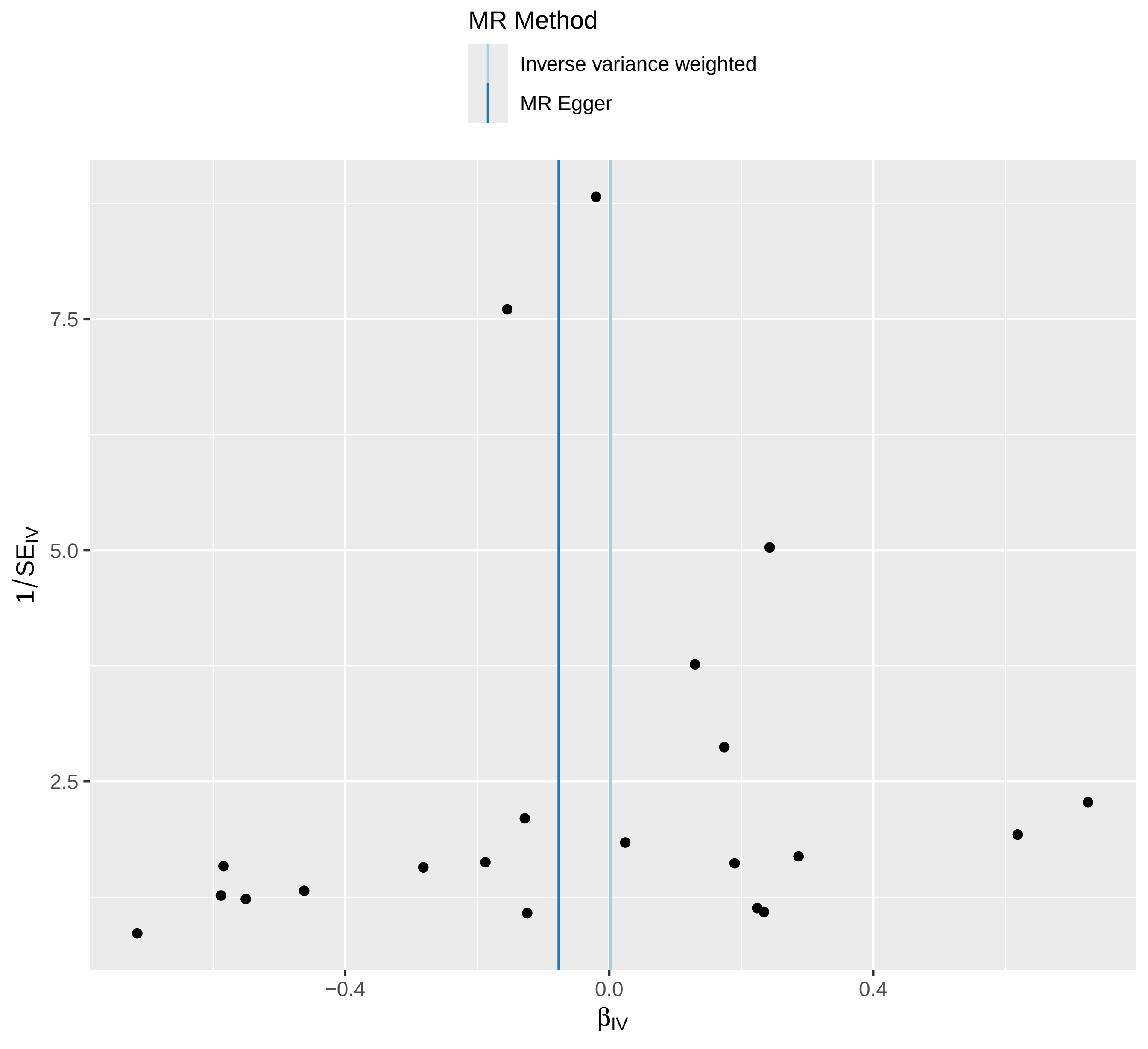
MR Method
Inverse variance weighted
MR Egger
7.5
5.0
2.5
1/SE$_{IV}$
−0.4
0.0
0.4
β$_{IV}$

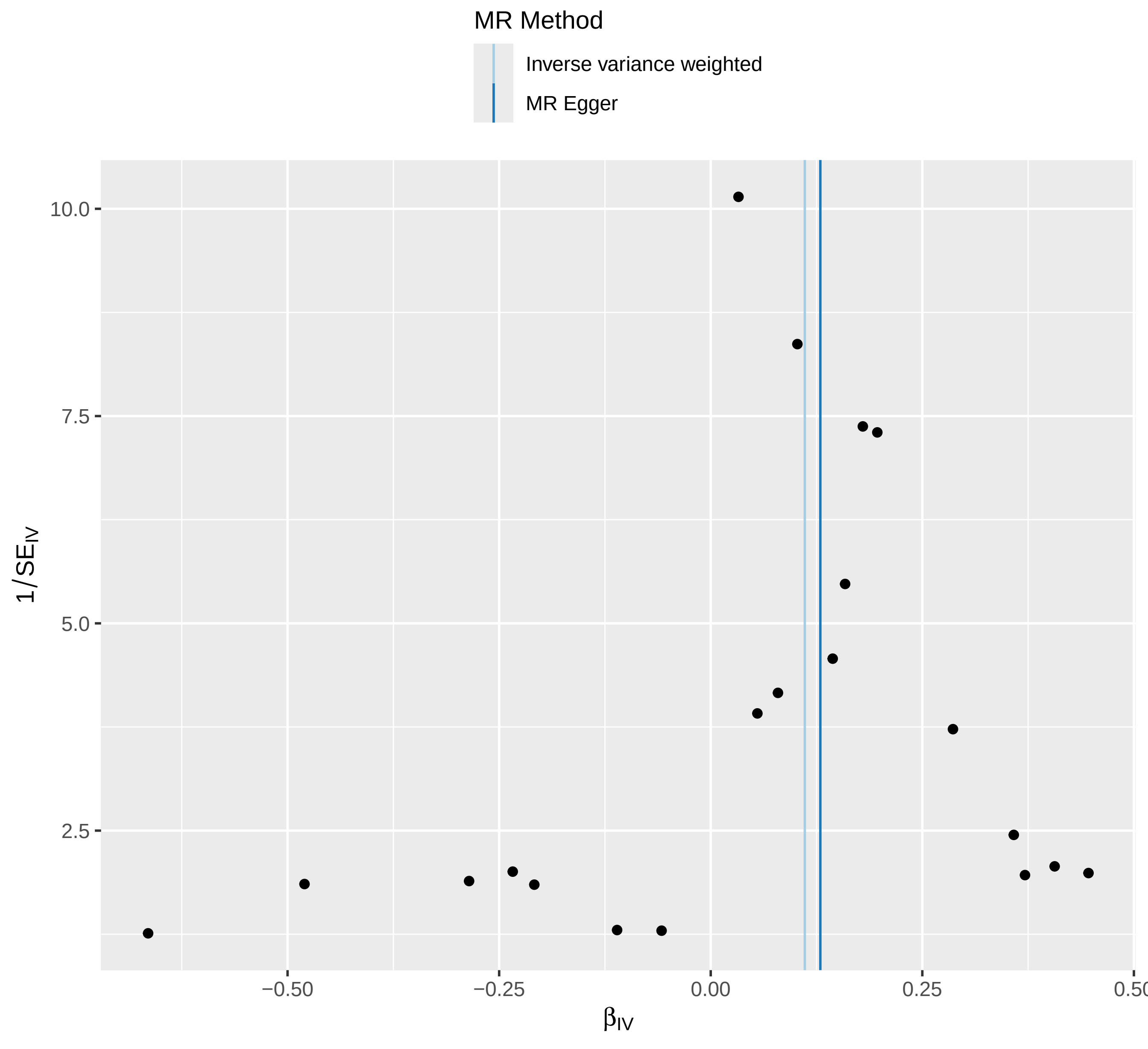

MR Method
Inverse variance weighted
MR Egger
10.0
7.5
5.0
2.5
1/SE_IV
−0.50
−0.25
0.00
0.25
0.50
β_IV

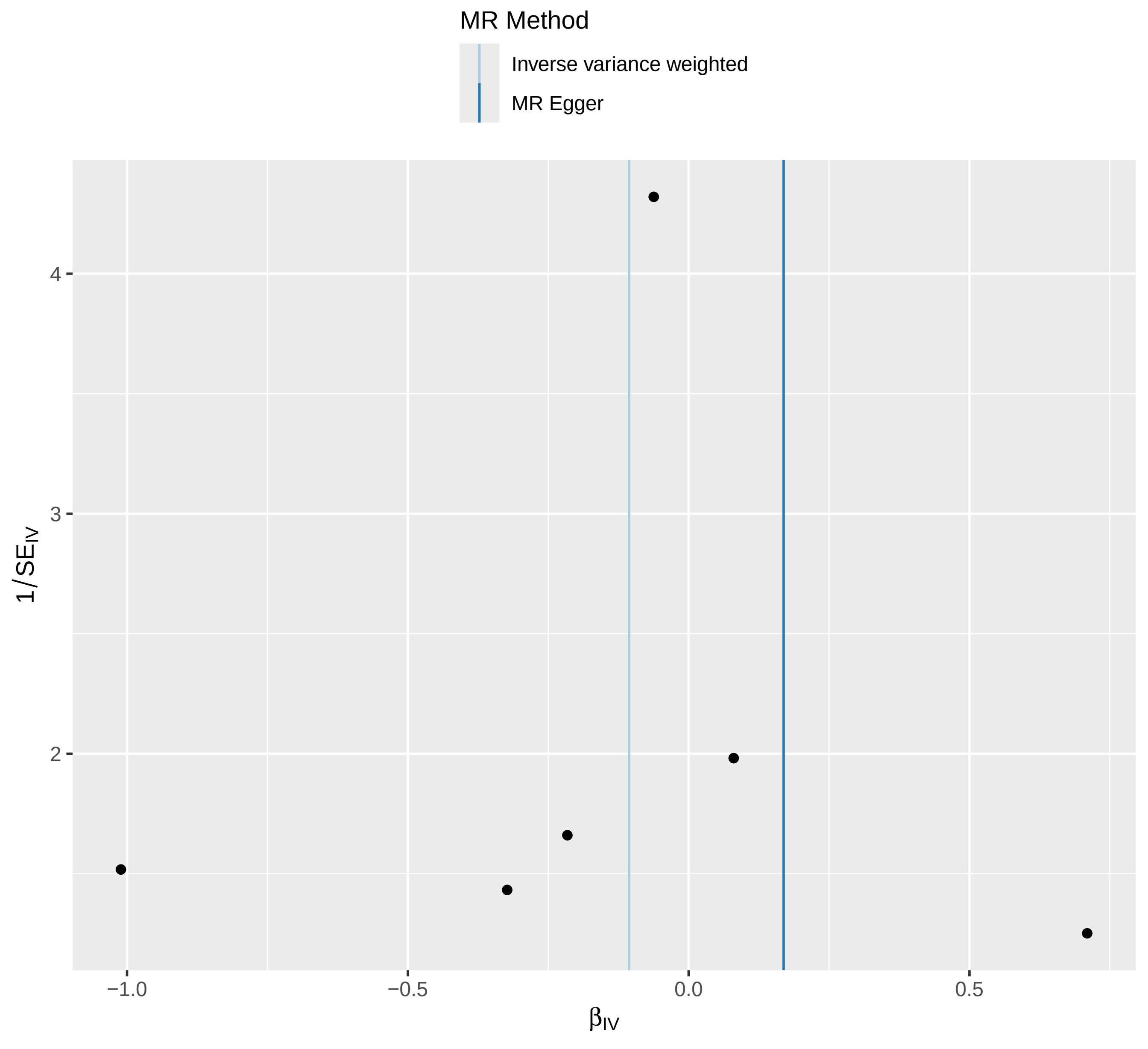
MR Method
Inverse variance weighted
MR Egger
4
3
2
1/SE_IV
−1.0
−0.5
0.0
0.5
β_IV

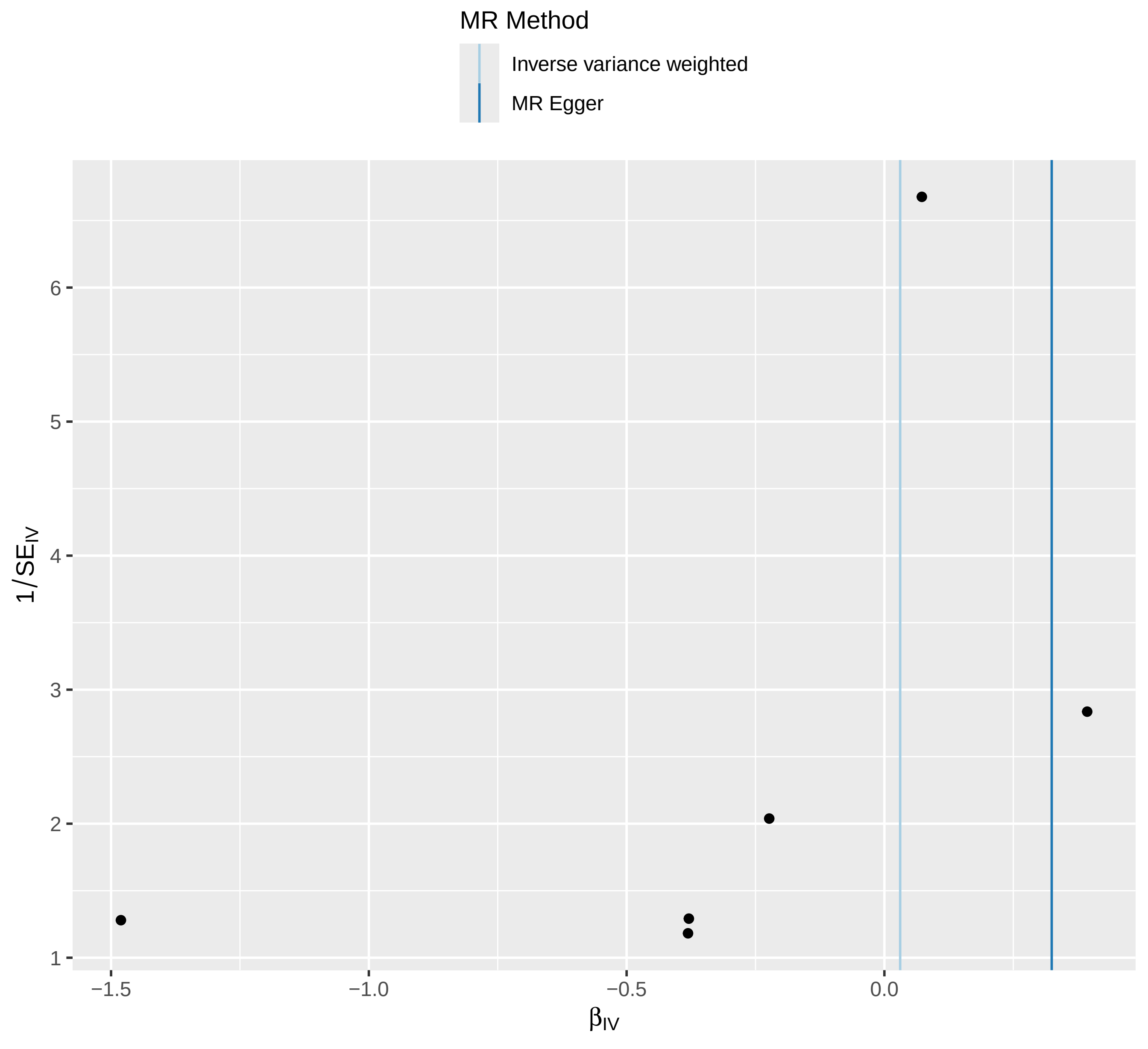

MR Method
Inverse variance weighted
MR Egger
1/SE_IV
β_IV
6
5
4
3
2
1
−1.5
−1.0
−0.5
0.0

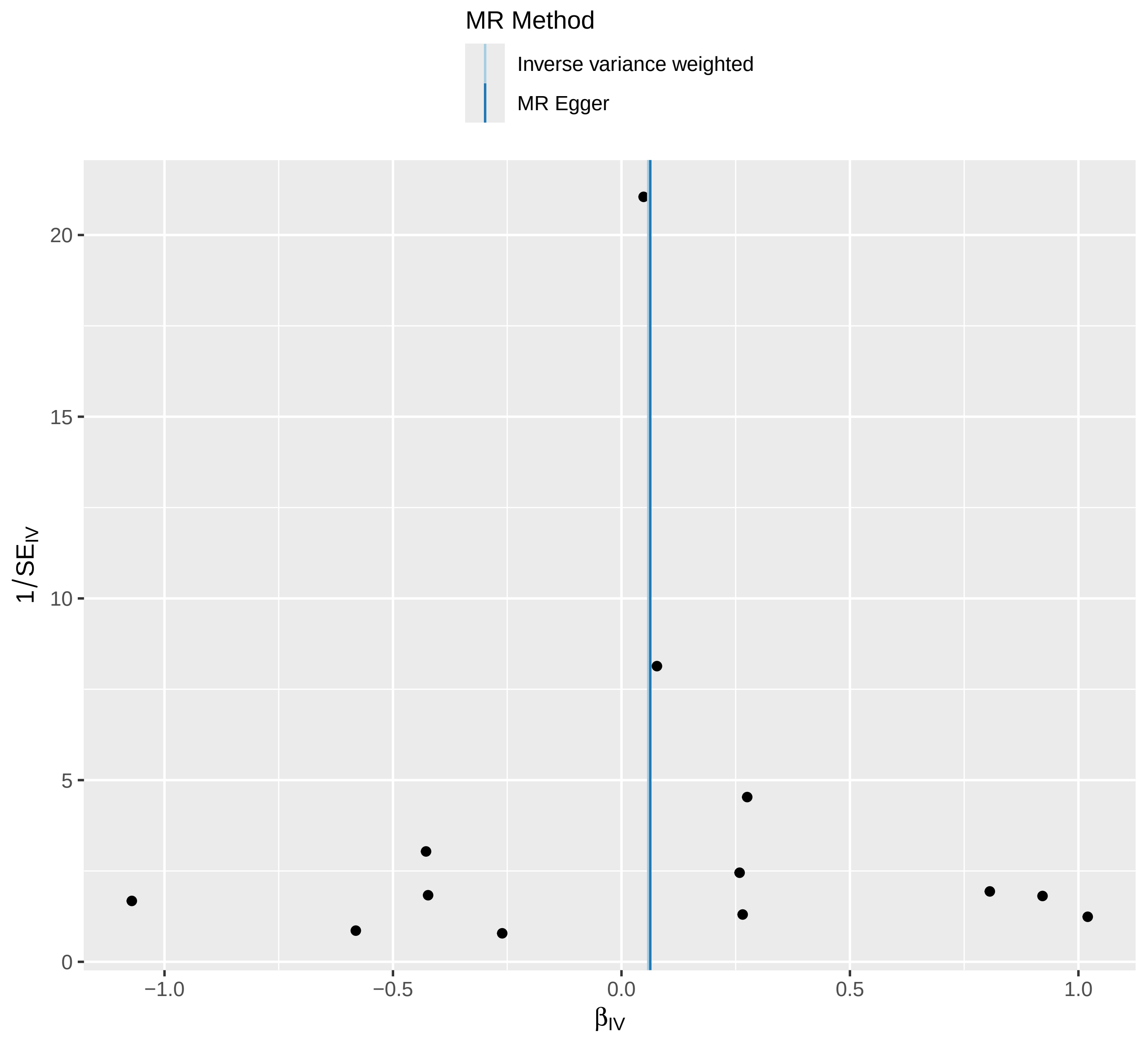

MR Method
Inverse variance weighted
MR Egger
20
15
10
5
0
1/SE_IV
−1.0
−0.5
0.0
0.5
1.0
β_IV

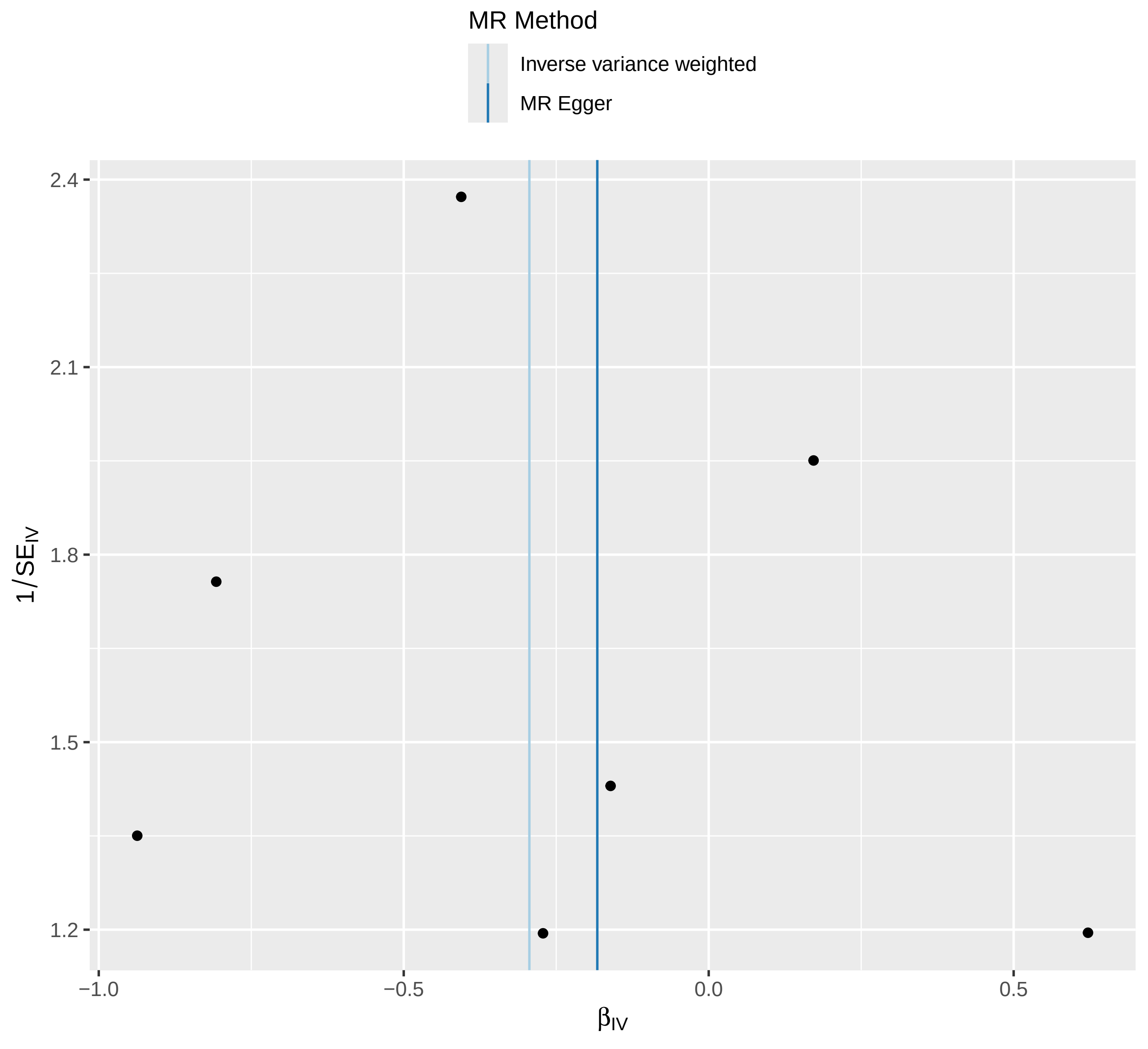

MR Method
Inverse variance weighted
MR Egger
2.4
2.1
1.8
1.5
1.2
1/SE_IV
−1.0
−0.5
0.0
0.5
β_IV

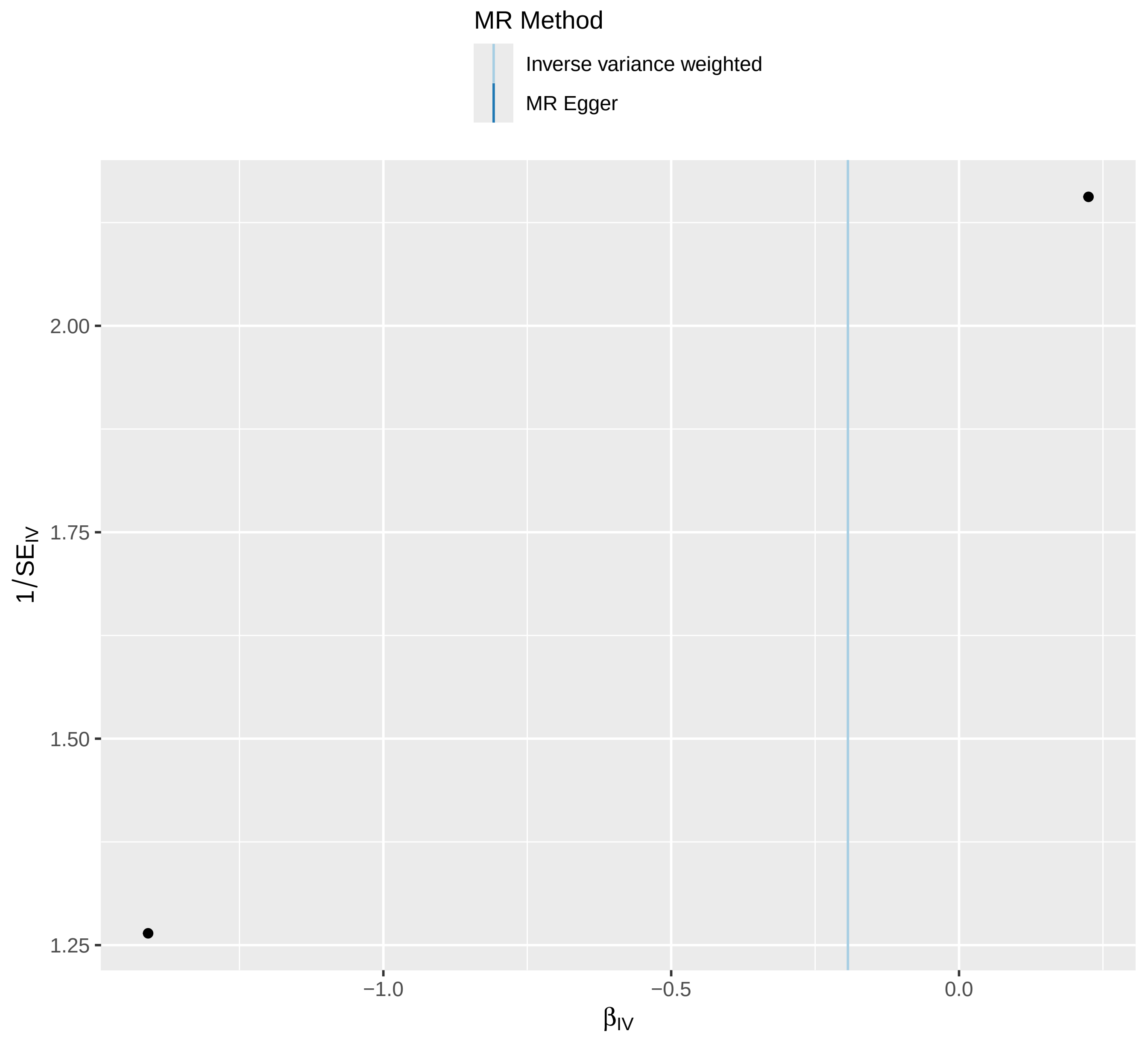
MR Method
Inverse variance weighted
MR Egger
2.00
1.75
1.50
1.25
1/SE_IV
−1.0
−0.5
0.0
β_IV

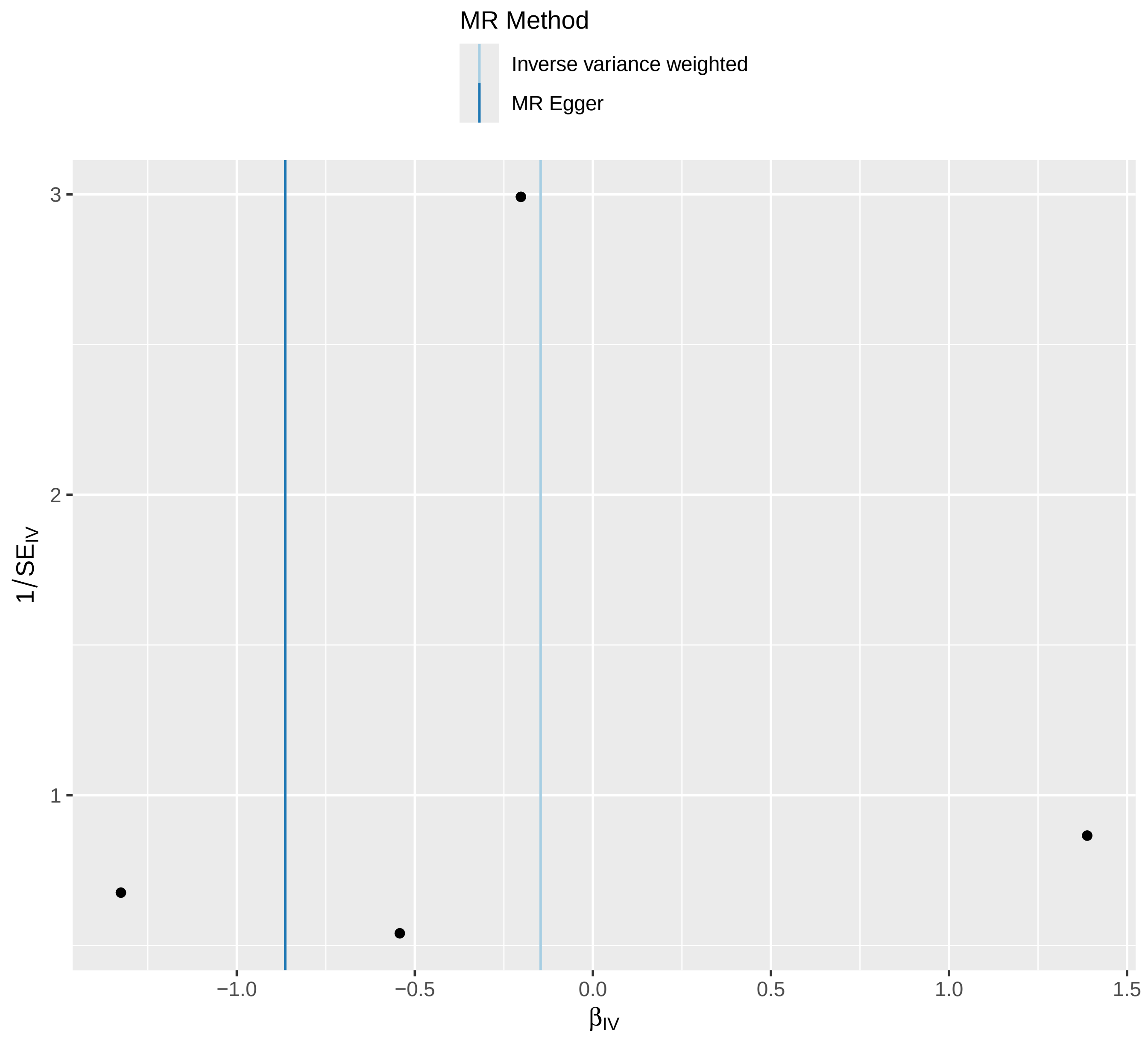

MR Method
Inverse variance weighted
MR Egger
3
2
1
1/SE_IV
−1.0
−0.5
0.0
0.5
1.0
1.5
β_IV

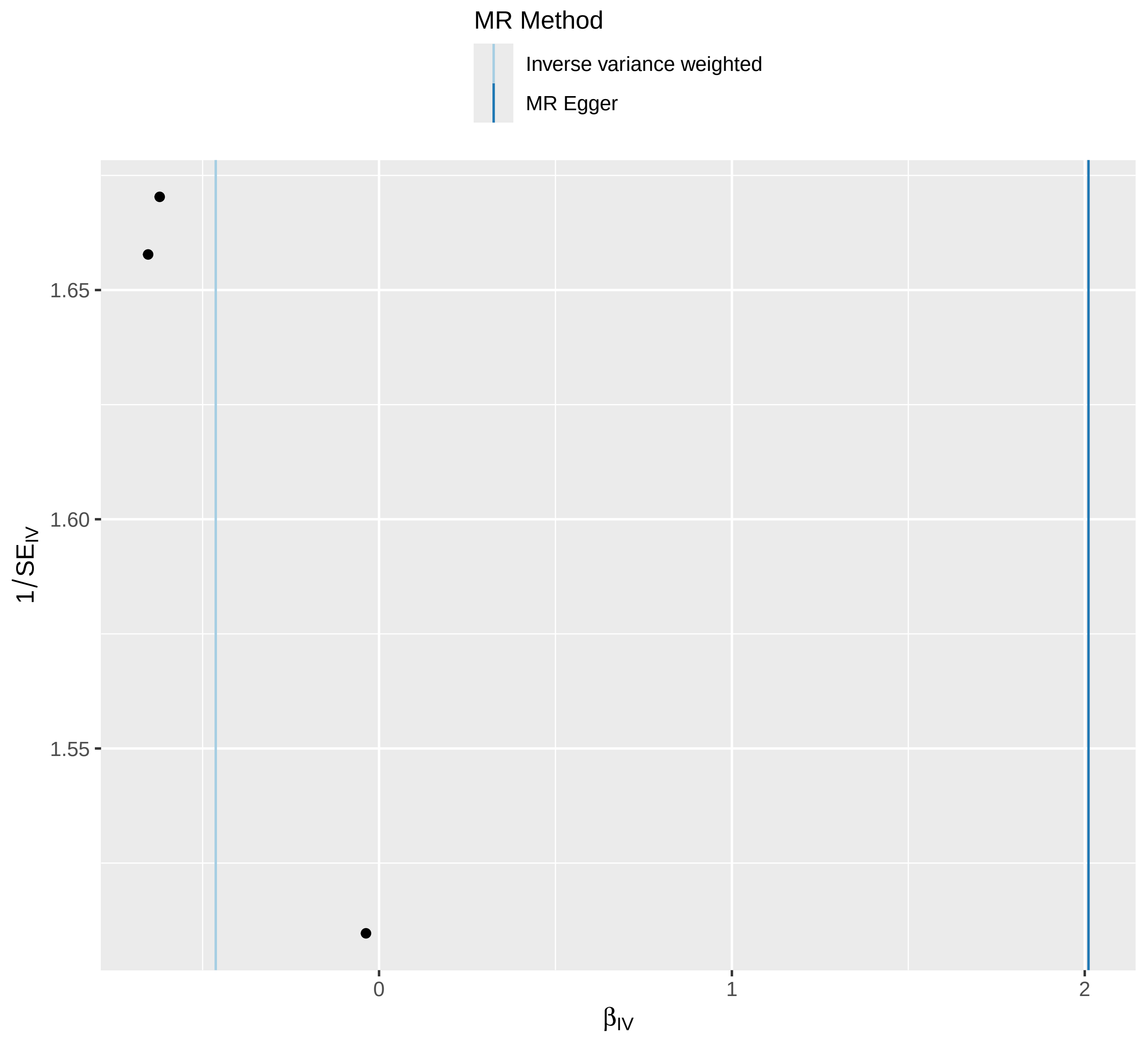

MR Method
Inverse variance weighted
MR Egger
1.65
1.60
1.55
$1/SE_{IV}$
0
1
2
β_{IV}

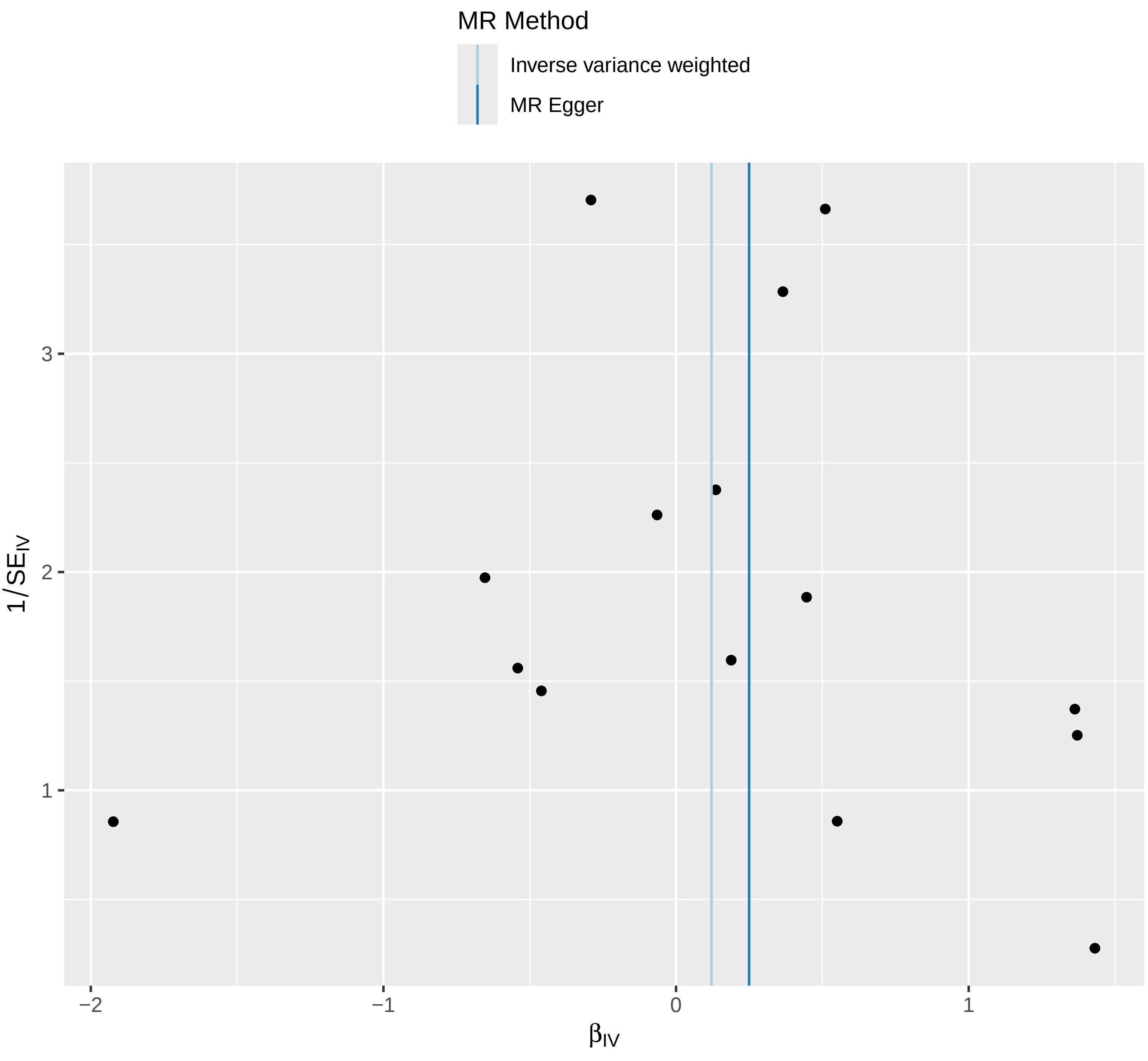
MR Method
Inverse variance weighted
MR Egger
1/SE_IV
3
2
1
−2
−1
0
1
β_IV

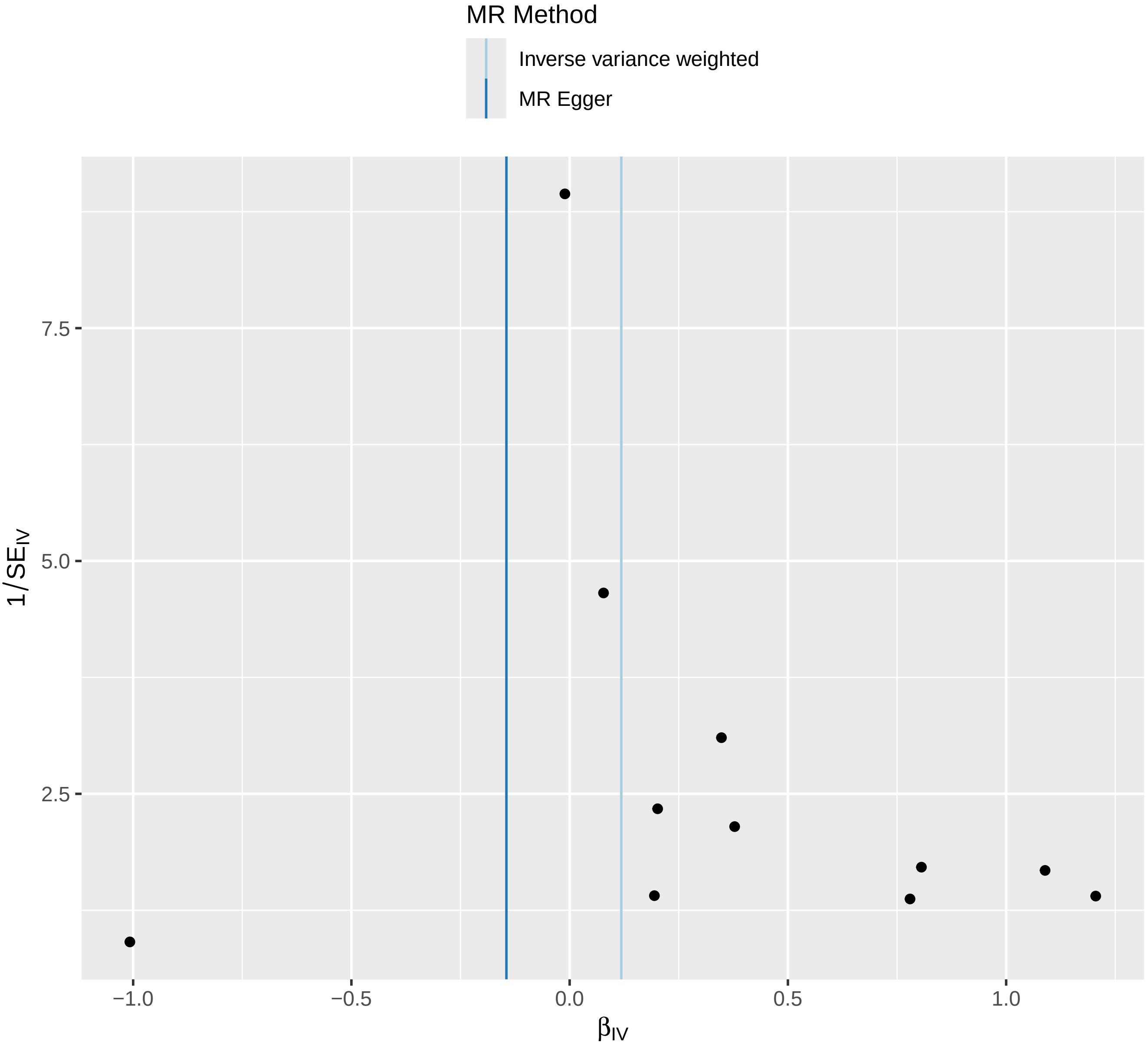
MR Method
Inverse variance weighted
MR Egger
7.5
5.0
2.5
1/SE_IV
−1.0
−0.5
0.0
0.5
1.0
β_IV

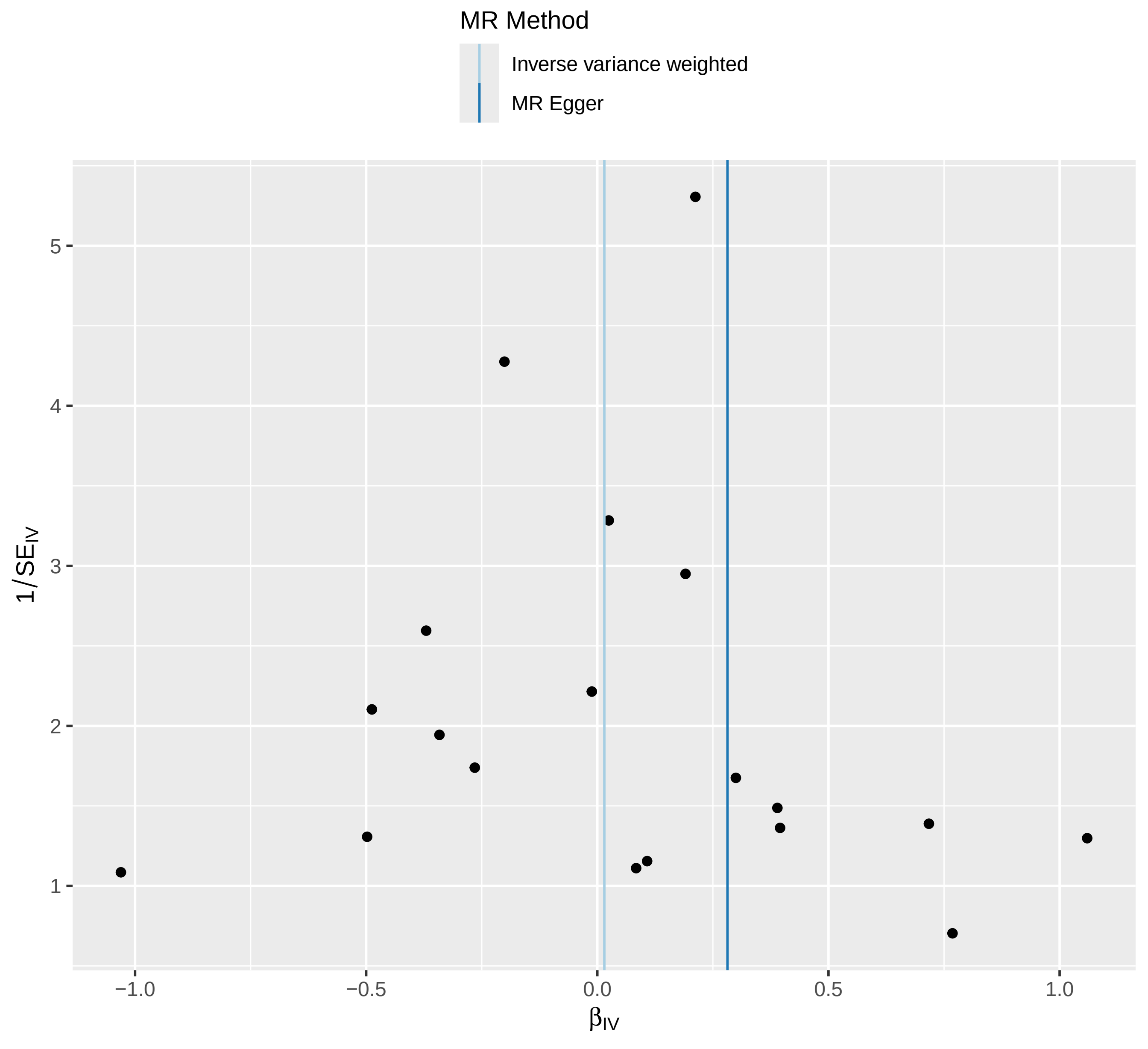
MR Method
Inverse variance weighted
MR Egger
1/SE_IV
5
4
3
2
1
−1.0
−0.5
0.0
0.5
1.0
β_IV

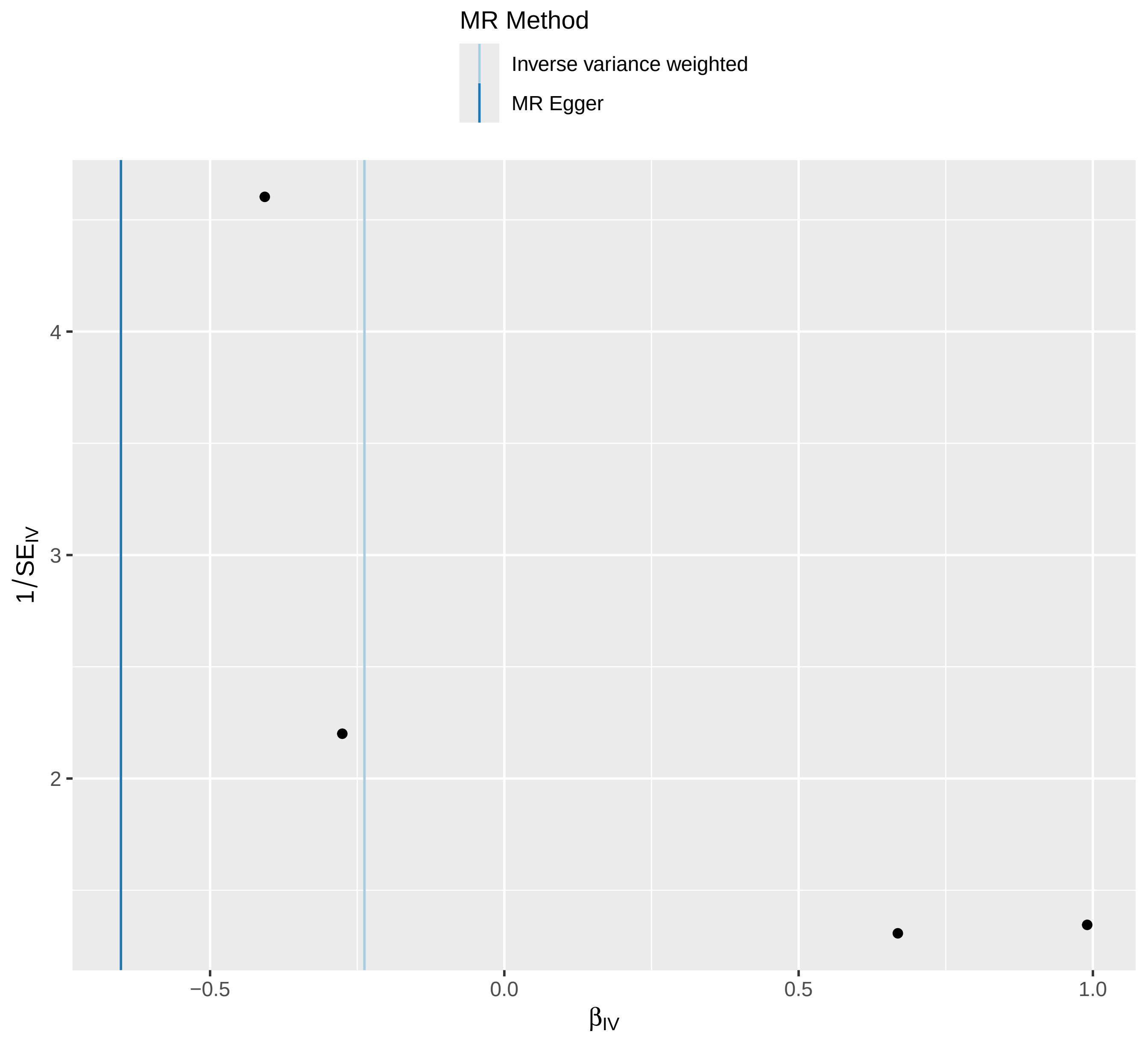
MR Method
Inverse variance weighted
MR Egger
1/SE_{IV}
β_{IV}
4
3
2
−0.5
0.0
0.5
1.0

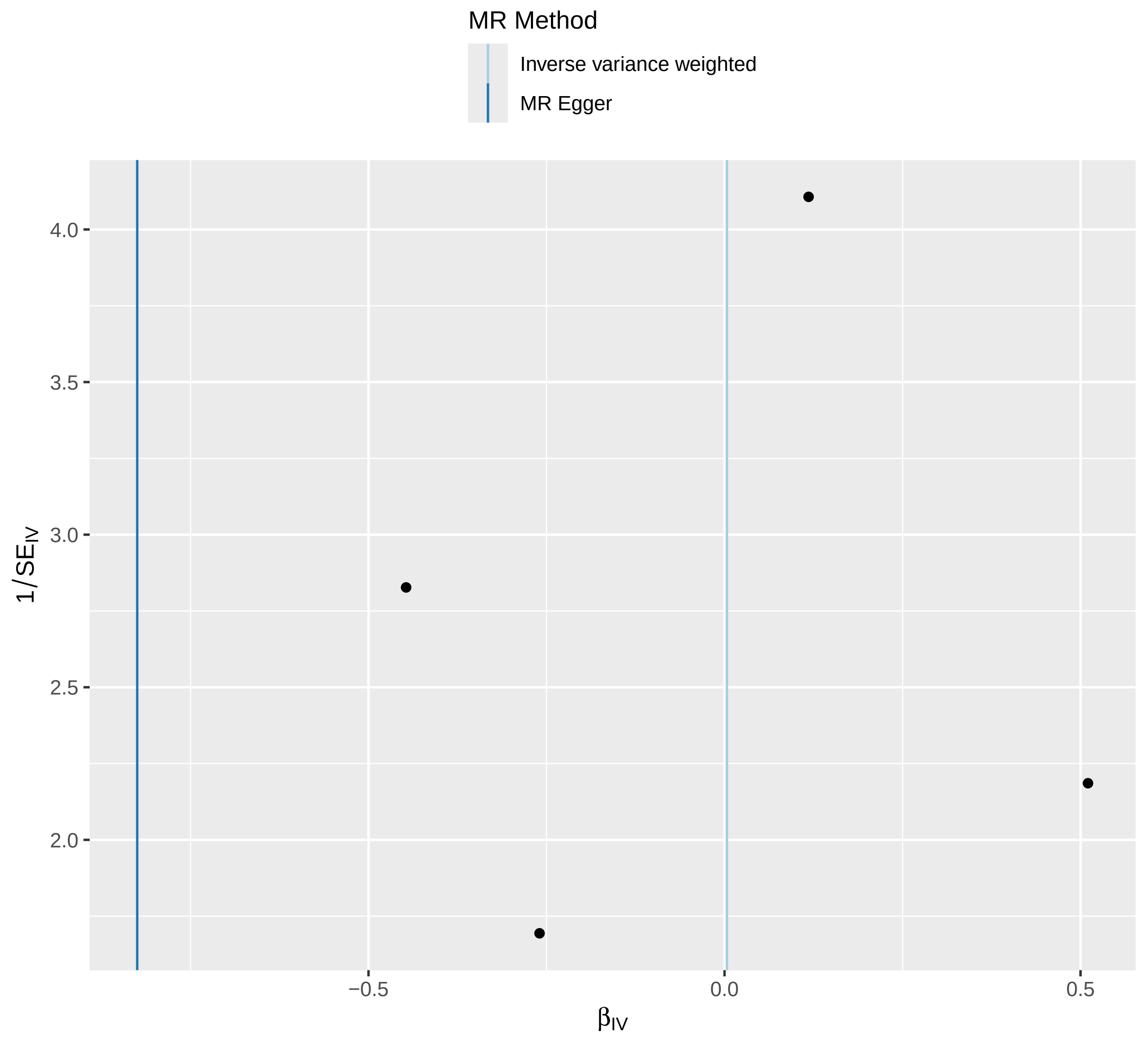
MR Method
Inverse variance weighted
MR Egger
4.0
3.5
3.0
2.5
2.0
$1/SE_{IV}$
−0.5
0.0
0.5
β_{IV}

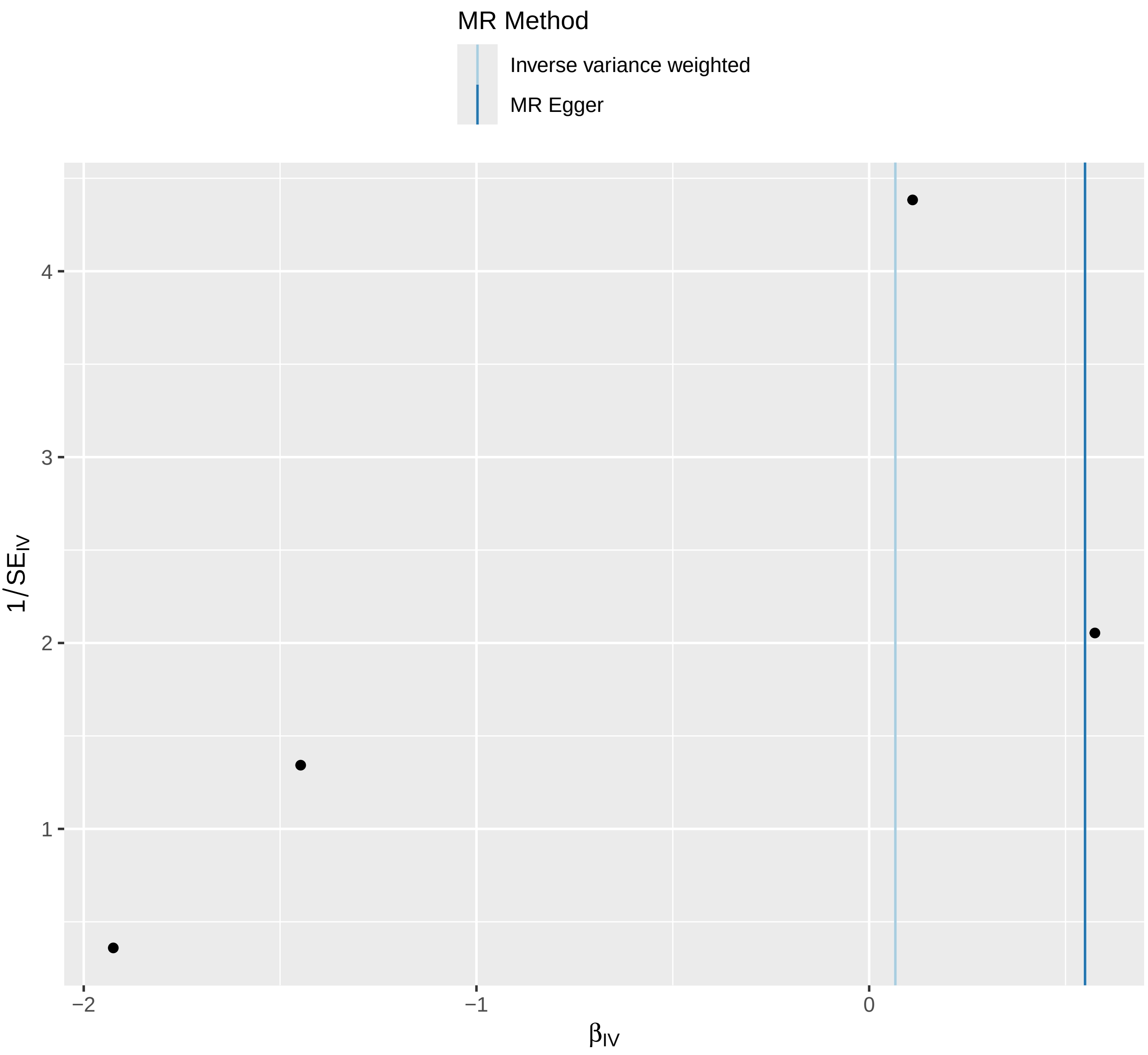
MR Method
Inverse variance weighted
MR Egger
1/SE_IV
4
3
2
1
−2
−1
0
β_IV

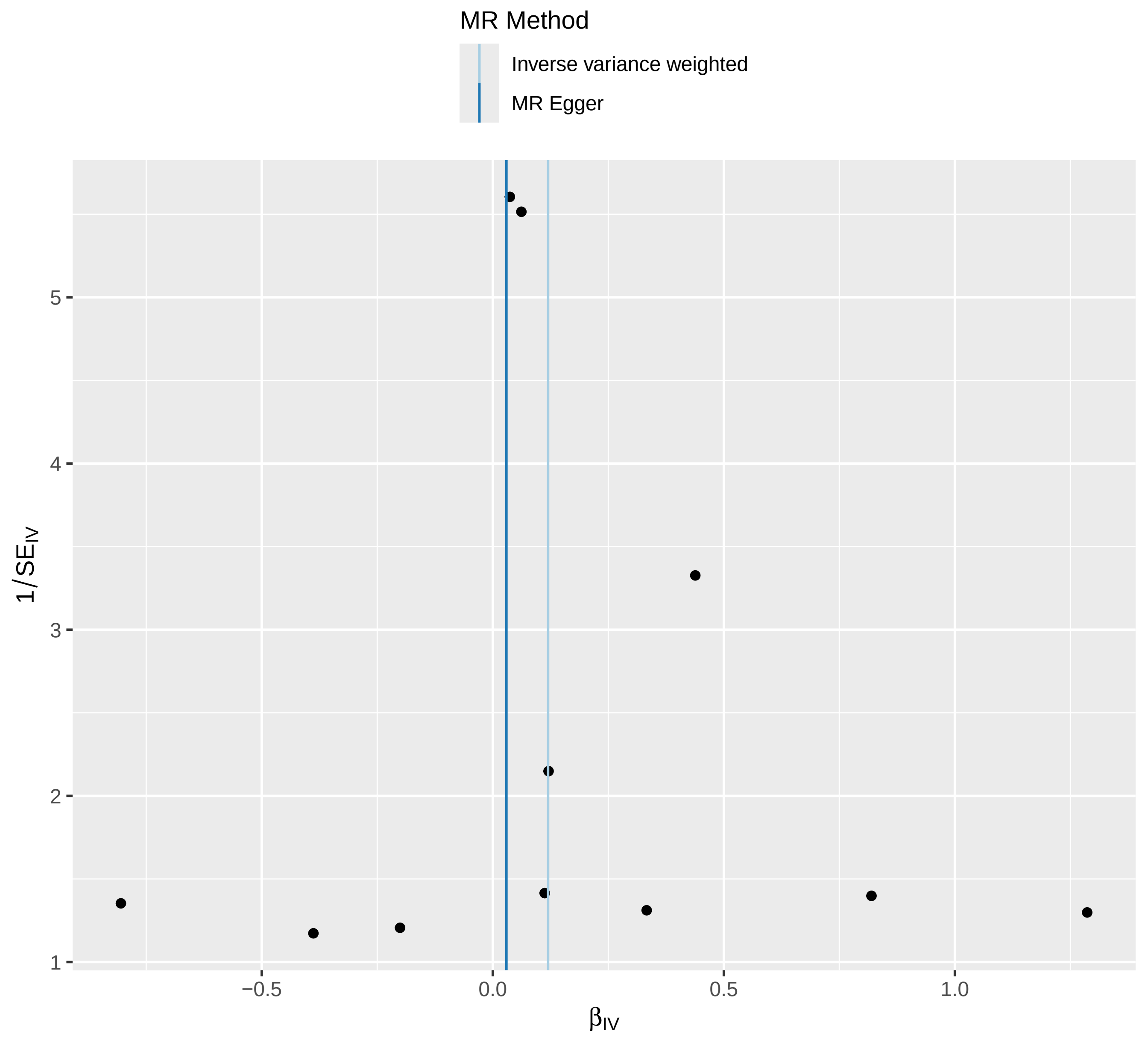
MR Method
Inverse variance weighted
MR Egger
1/SE_IV
β_IV
−0.5
0.0
0.5
1.0
1
2
3
4
5

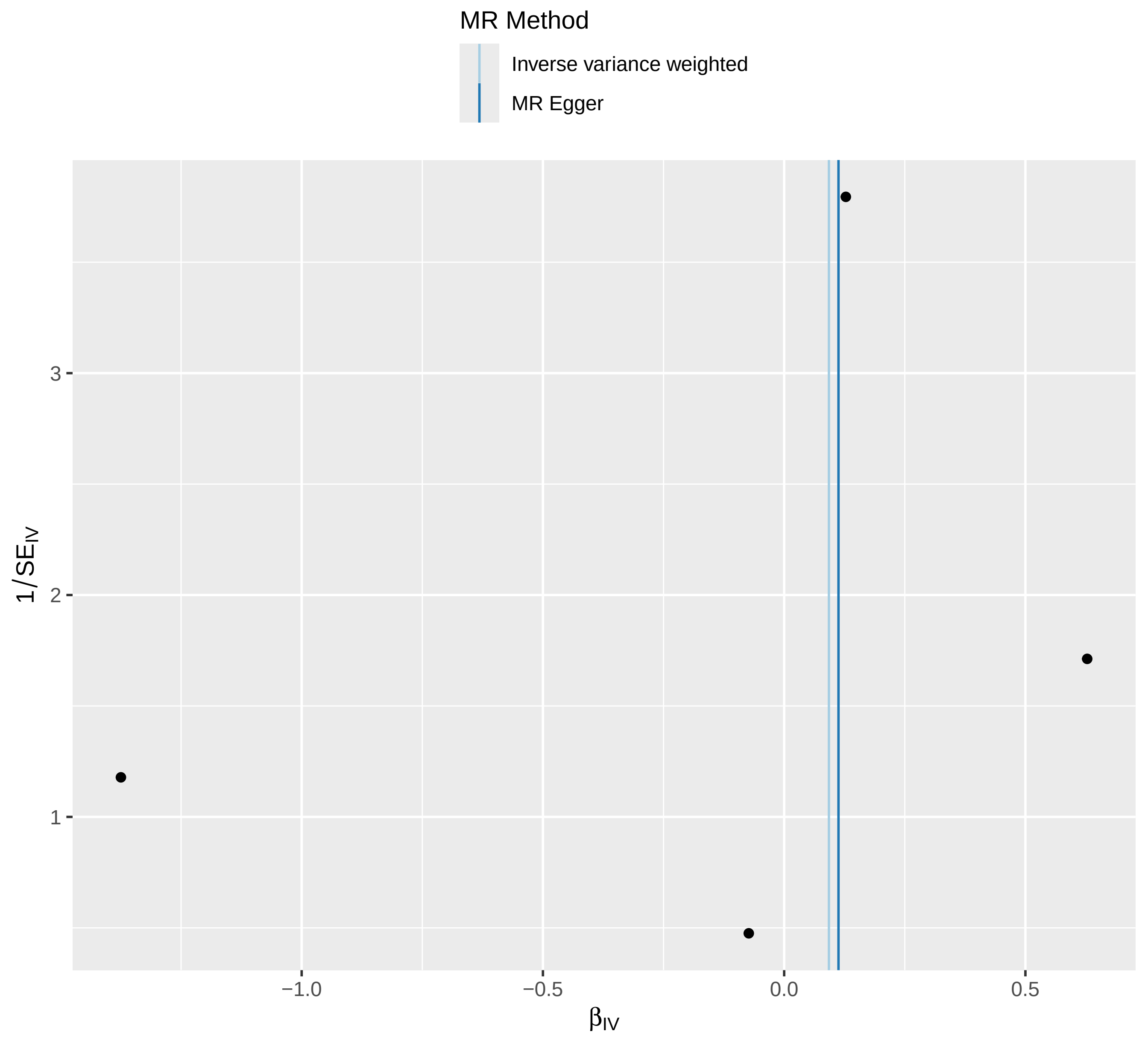
MR Method
Inverse variance weighted
MR Egger
1/SE_IV
3
2
1
−1.0
−0.5
0.0
0.5
β_IV

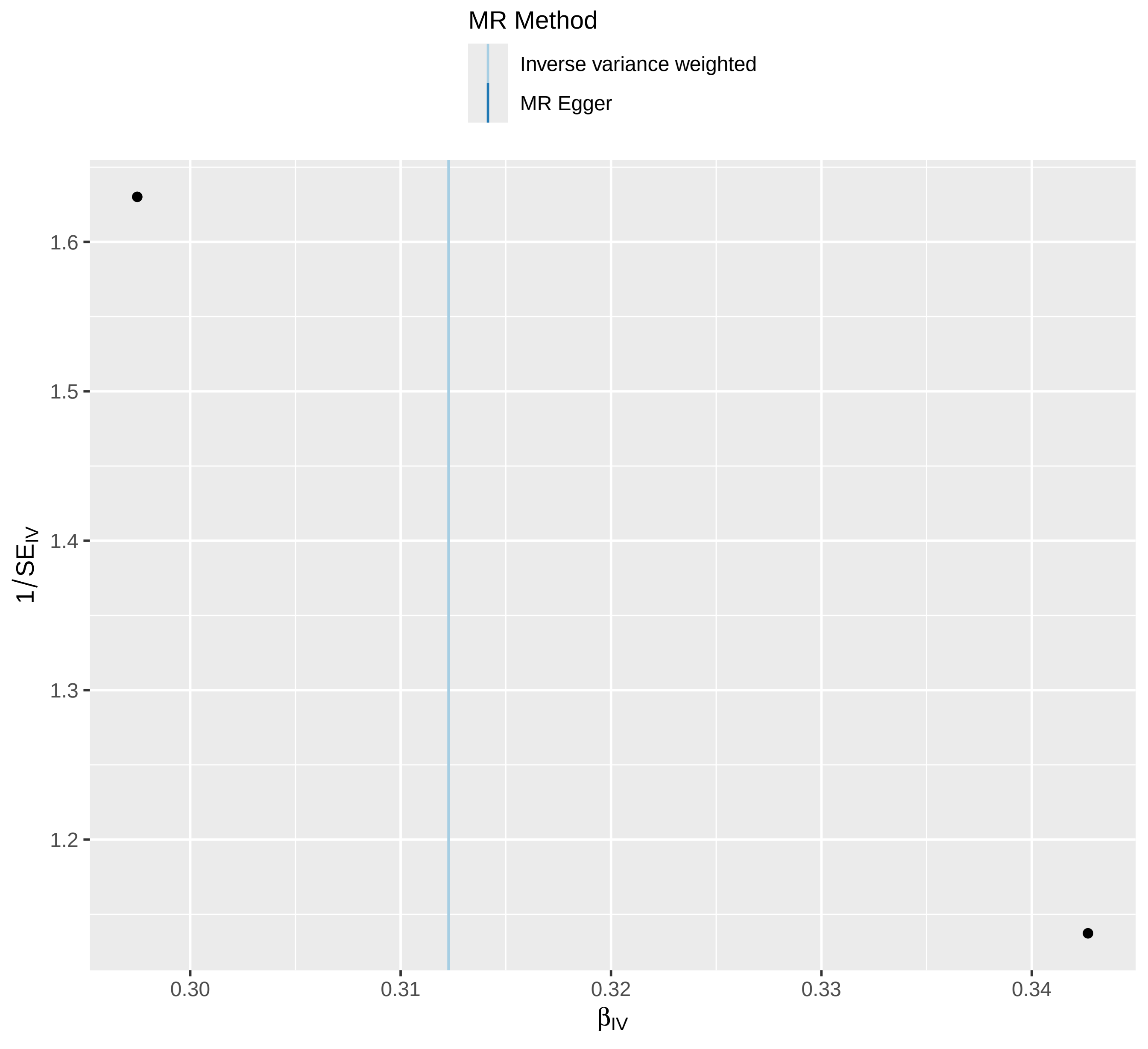

MR Method
Inverse variance weighted
MR Egger
1.6
1.5
1.4
1.3
1.2
1/SE_IV
0.30
0.31
0.32
0.33
0.34
β_IV

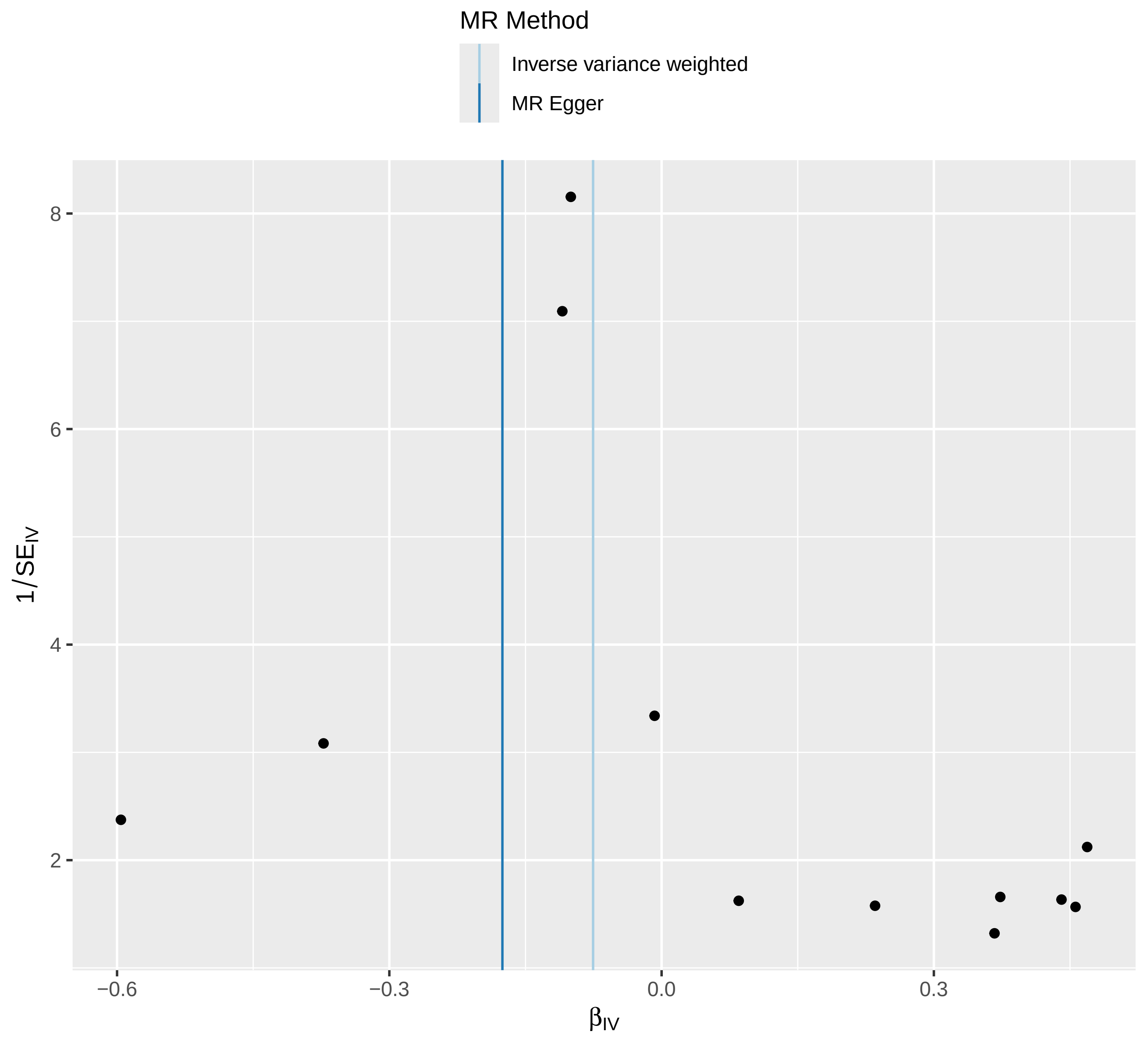
MR Method
Inverse variance weighted
MR Egger
1/SE_IV
8
6
4
2
−0.6
−0.3
0.0
0.3
β_IV

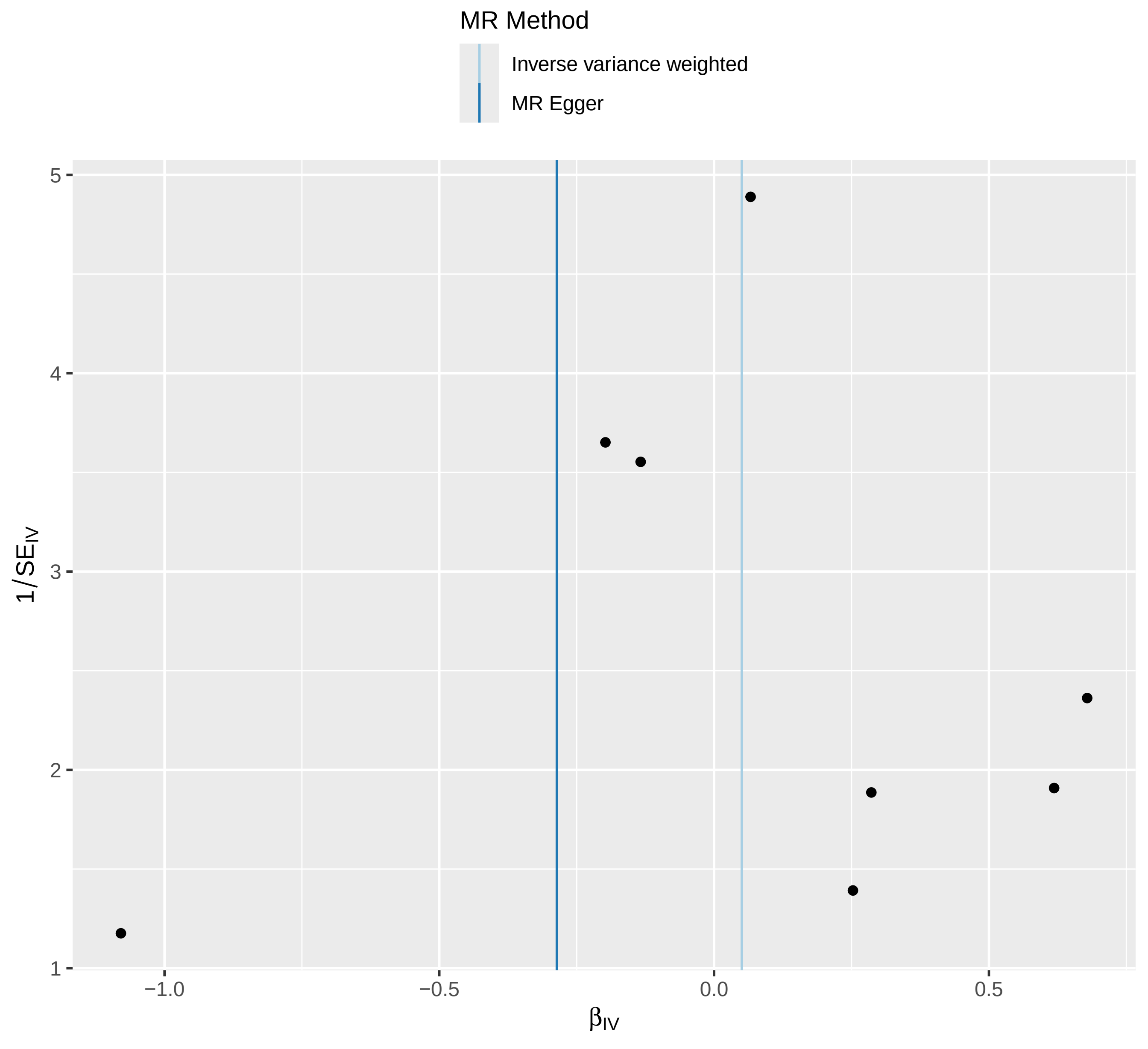

MR Method
Inverse variance weighted
MR Egger
5
4
3
2
1
$1/SE_{IV}$
−1.0
−0.5
0.0
0.5
β_{IV}

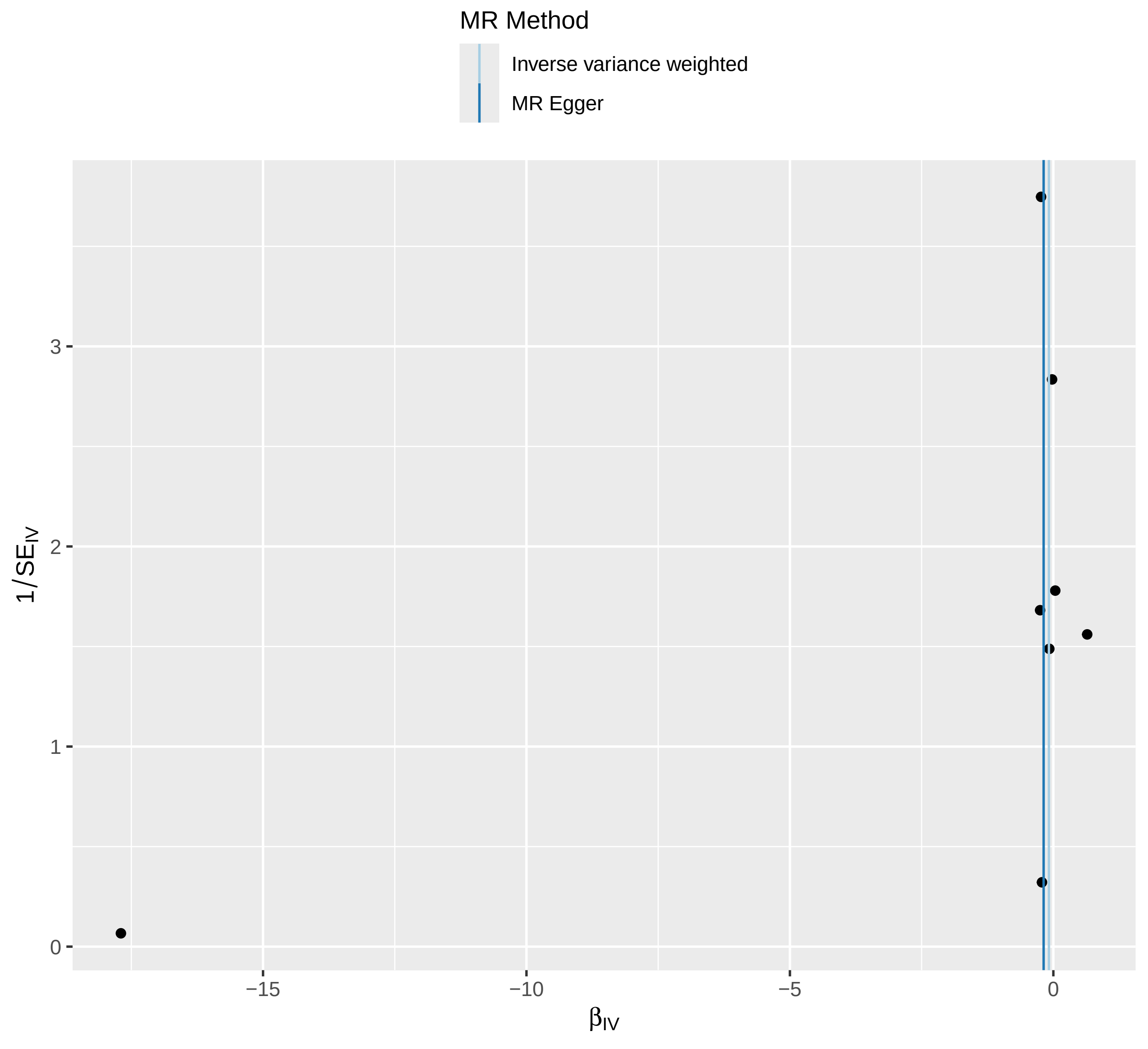
MR Method
Inverse variance weighted
MR Egger
1/SE_IV
3
2
1
0
−15
−10
−5
0
β_IV

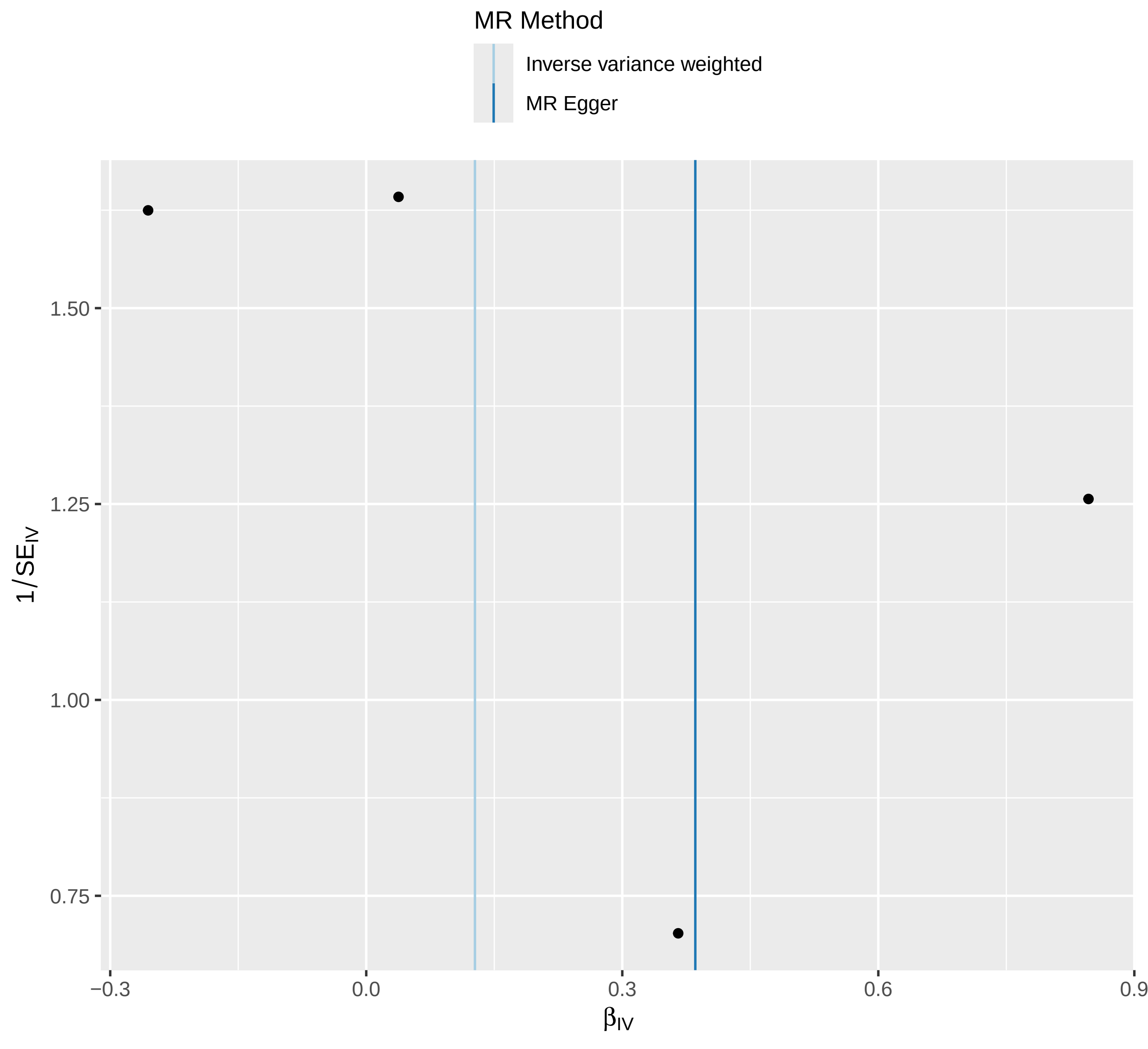
MR Method
Inverse variance weighted
MR Egger
1.50
1.25
1.00
0.75
1/SE_IV
−0.3
0.0
0.3
0.6
0.9
β_IV

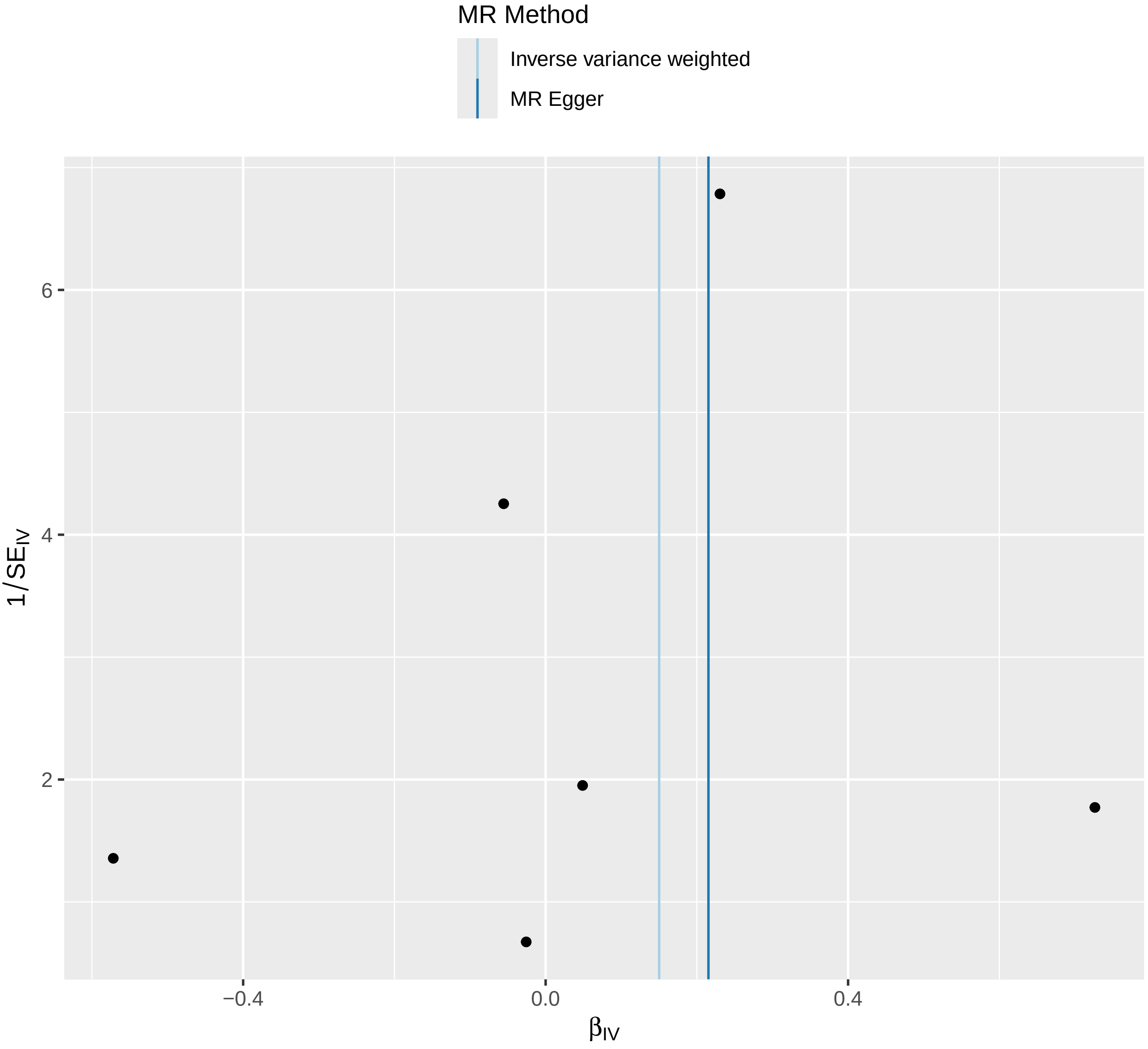
MR Method
Inverse variance weighted
MR Egger
6
4
2
1/SE_IV
−0.4
0.0
0.4
β_IV

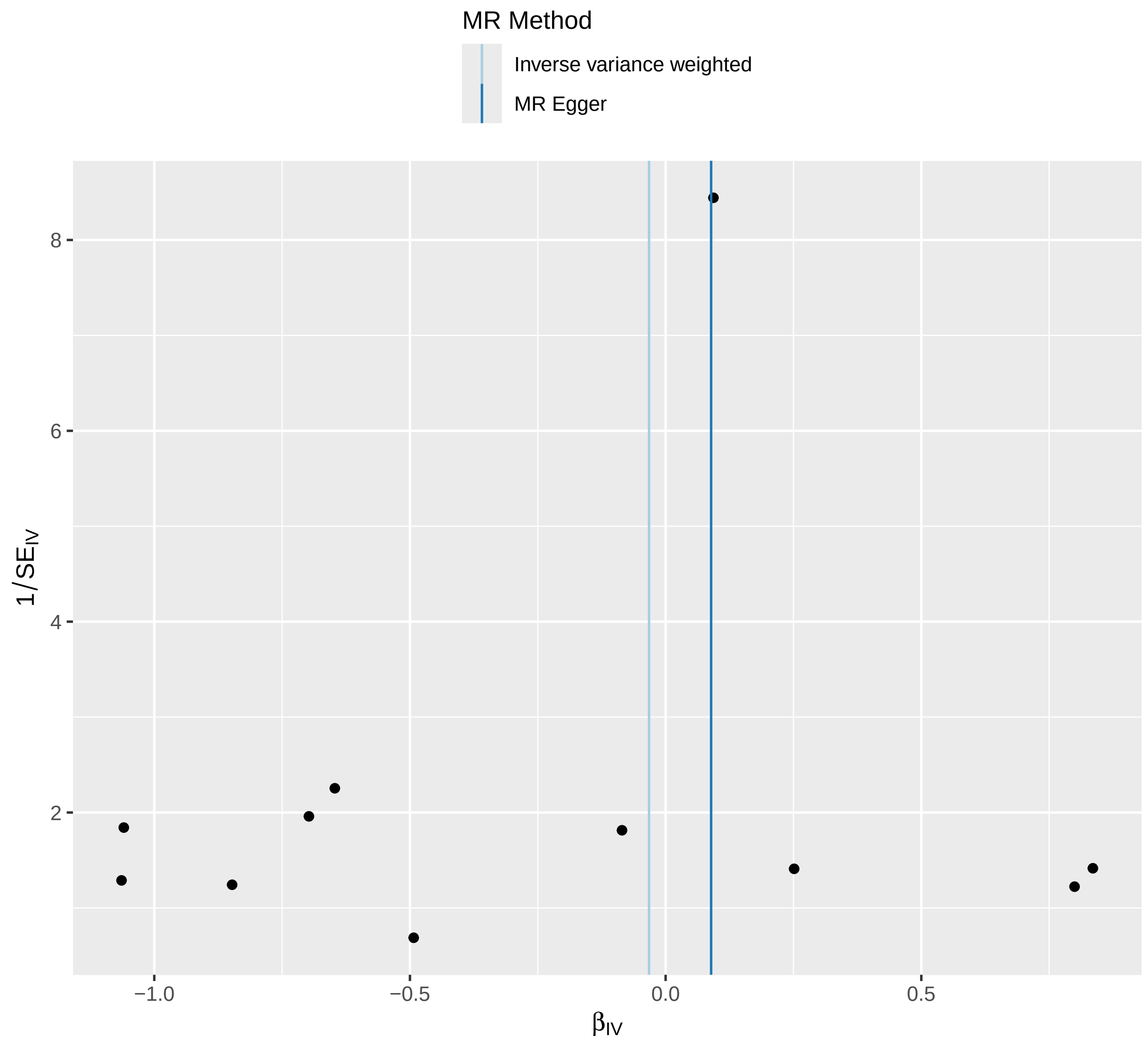

MR Method
Inverse variance weighted
MR Egger
1/SE_IV
β_IV
8
6
4
2
−1.0
−0.5
0.0
0.5

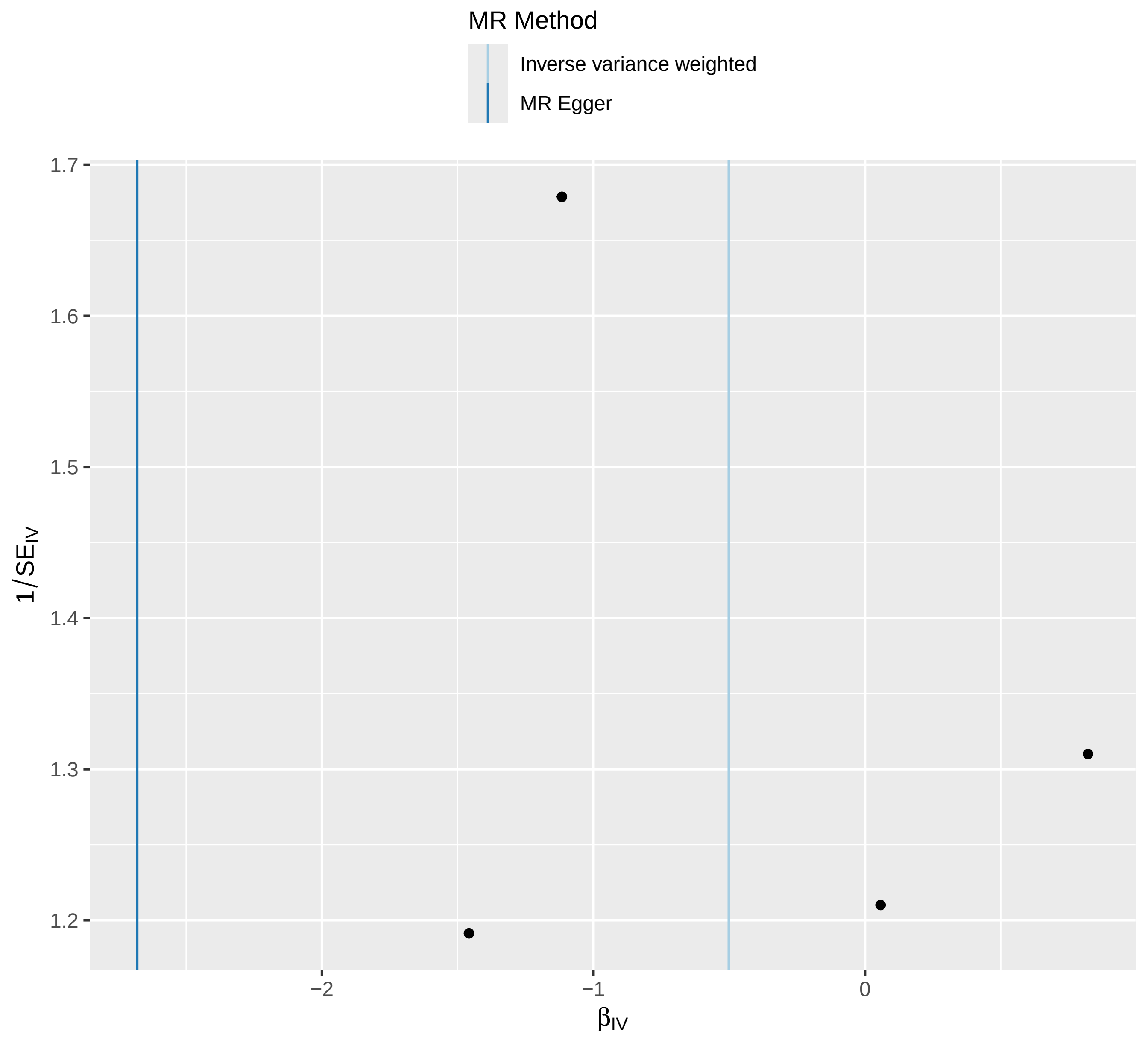

MR Method
Inverse variance weighted
MR Egger
1.7
1.6
1.5
1.4
1.3
1.2
1/SE$_{IV}$
−2
−1
0
β$_{IV}$

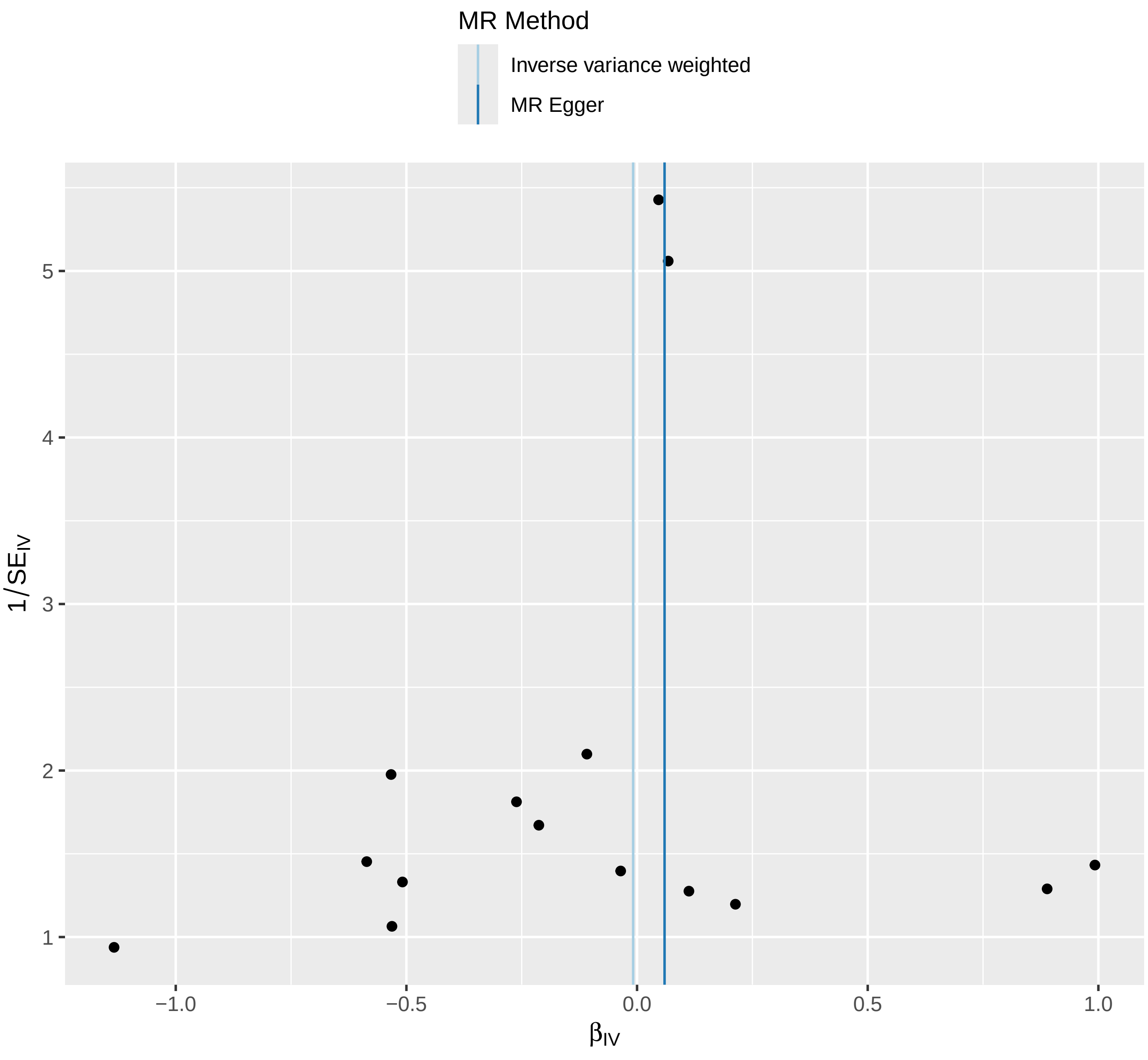

MR Method
Inverse variance weighted
MR Egger
1/SE_IV
5
4
3
2
1
−1.0
−0.5
0.0
0.5
1.0
β_IV

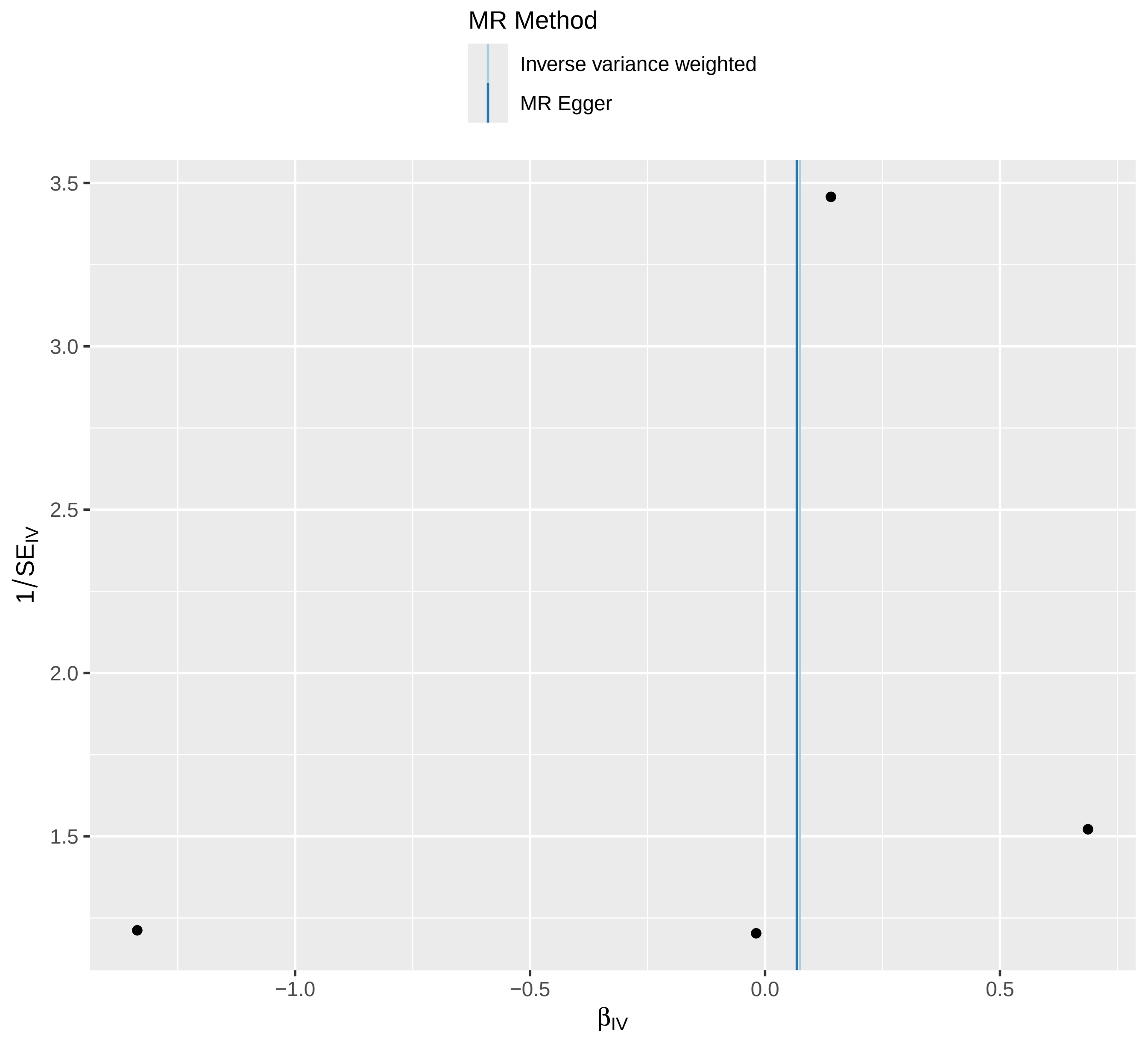
MR Method
Inverse variance weighted
MR Egger
3.5
3.0
2.5
2.0
1.5
1/SE_IV
−1.0
−0.5
0.0
0.5
β_IV

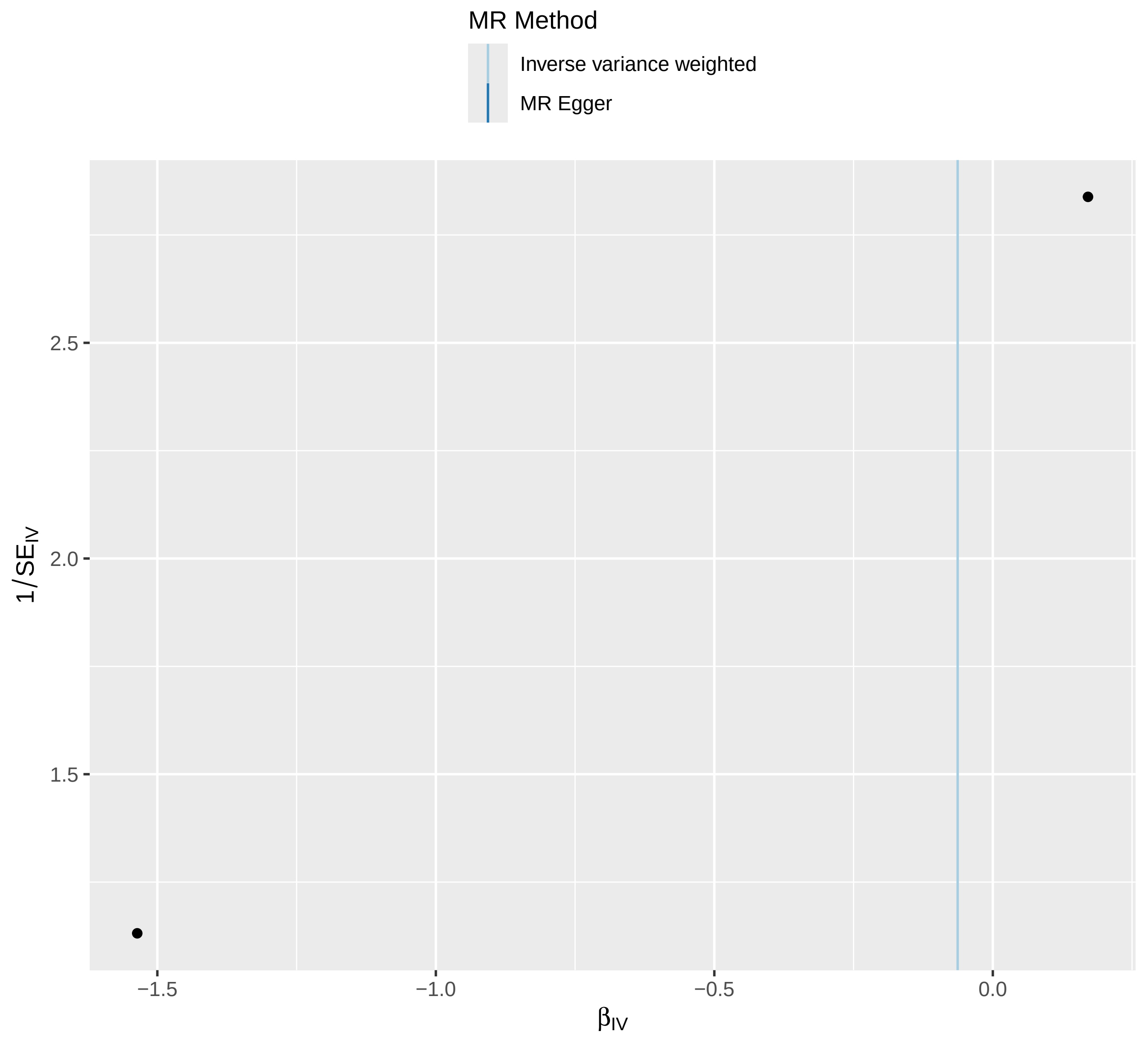
MR Method
Inverse variance weighted
MR Egger
2.5
2.0
1.5
1/SE$_{IV}$
−1.5
−1.0
−0.5
0.0
β_{IV}

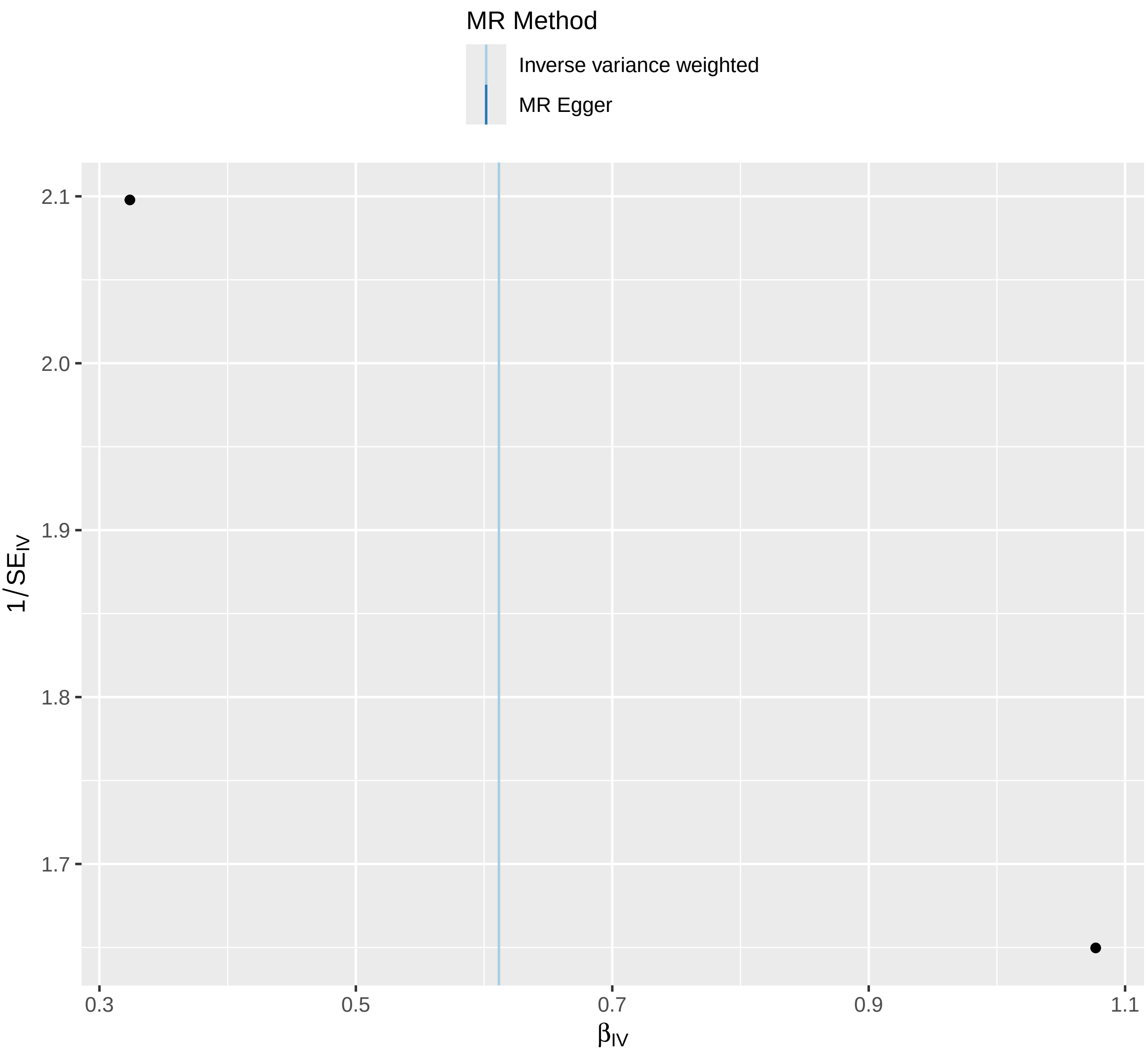
MR Method
Inverse variance weighted
MR Egger
2.1
2.0
1.9
1.8
1.7
0.3
0.5
0.7
0.9
1.1
1/SE_IV
β_IV

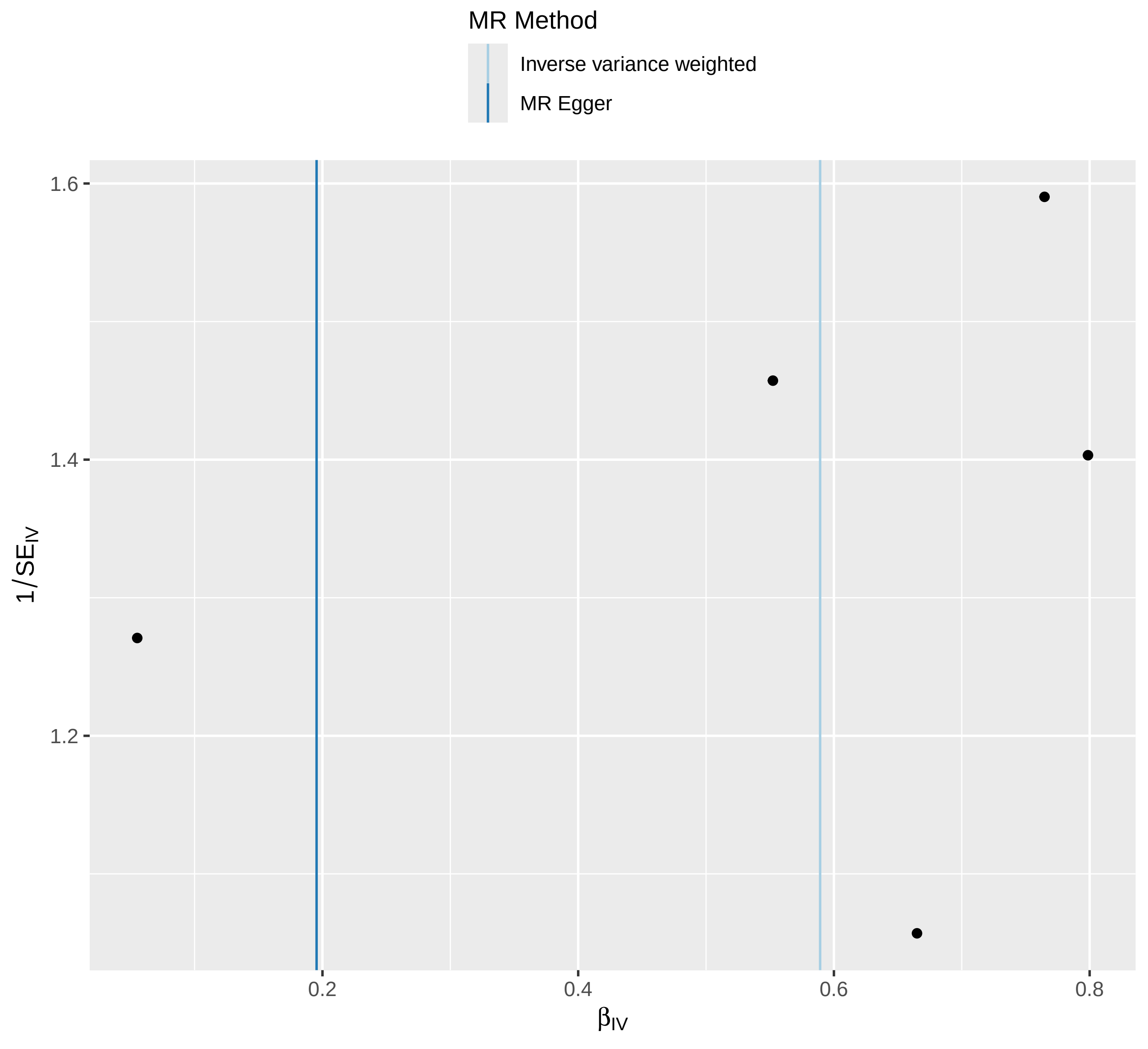
MR Method
Inverse variance weighted
MR Egger
1.6
1.4
1.2
$1/SE_{IV}$
0.2
0.4
0.6
0.8
β_{IV}

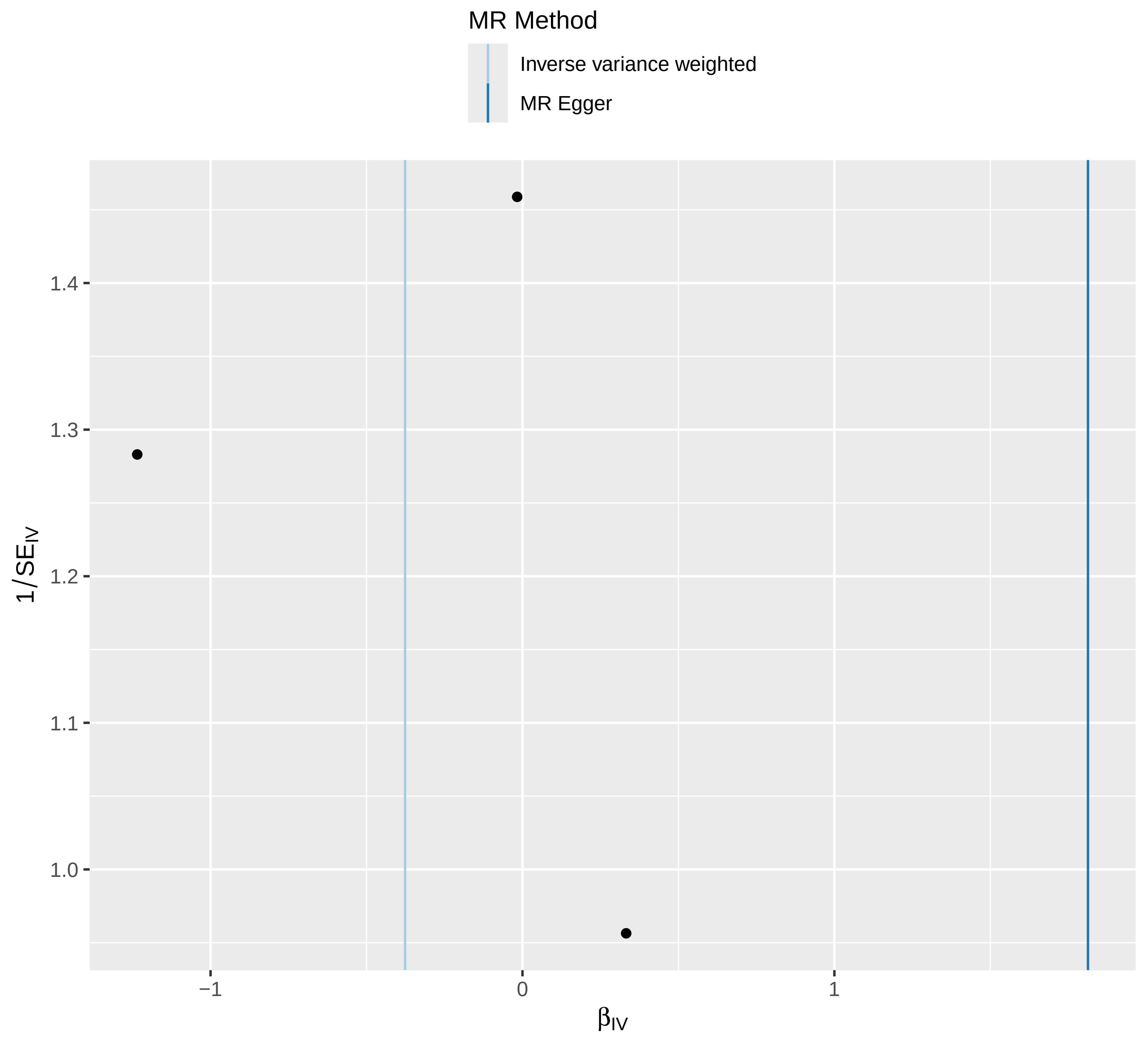
MR Method
Inverse variance weighted
MR Egger
1.4
1.3
1.2
1.1
1.0
$1/SE_{IV}$
−1
0
1
β_{IV}

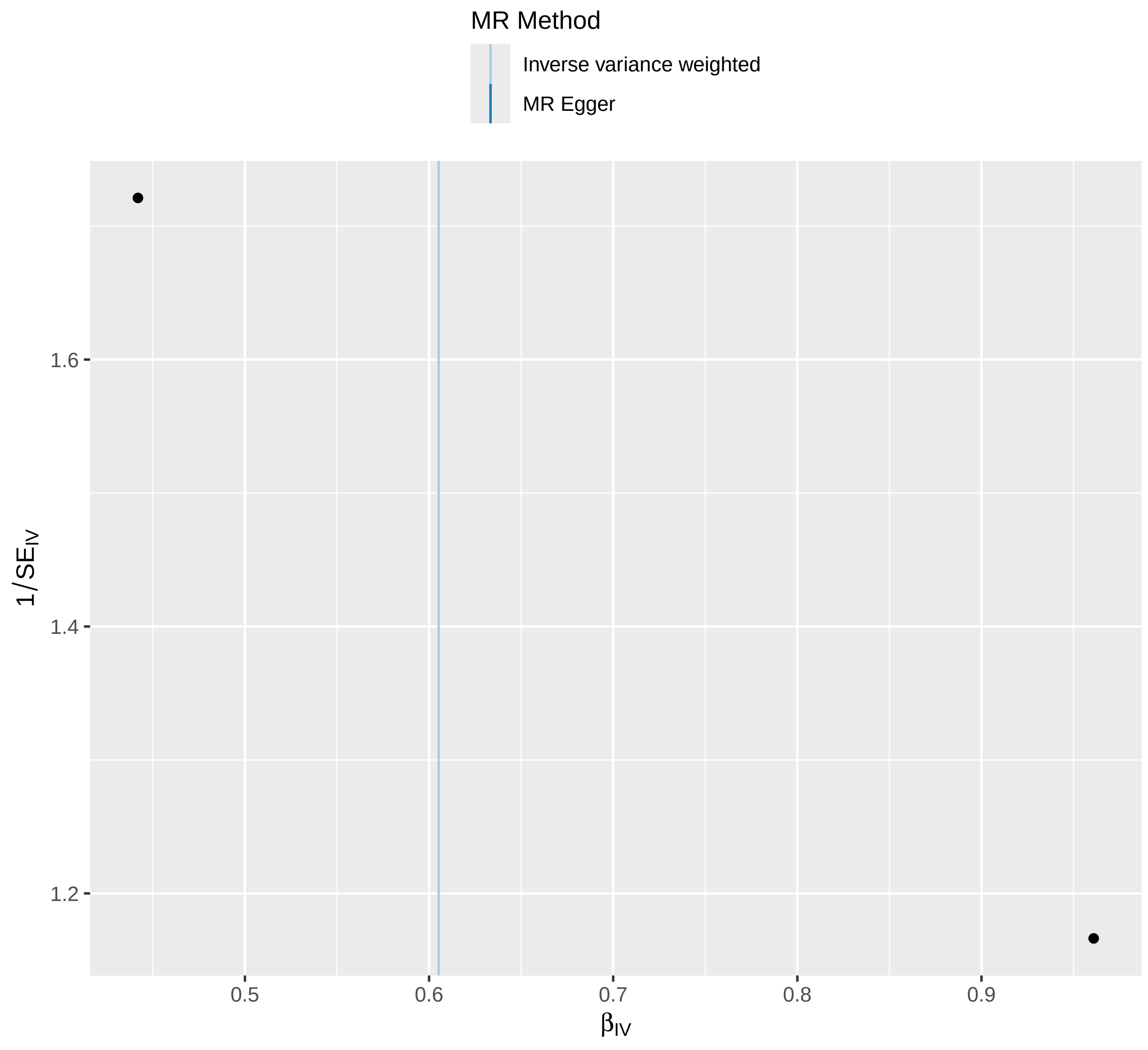
MR Method
Inverse variance weighted
MR Egger
1/SE_{IV}
1.6
1.4
1.2
0.5
0.6
0.7
0.8
0.9
β_{IV}

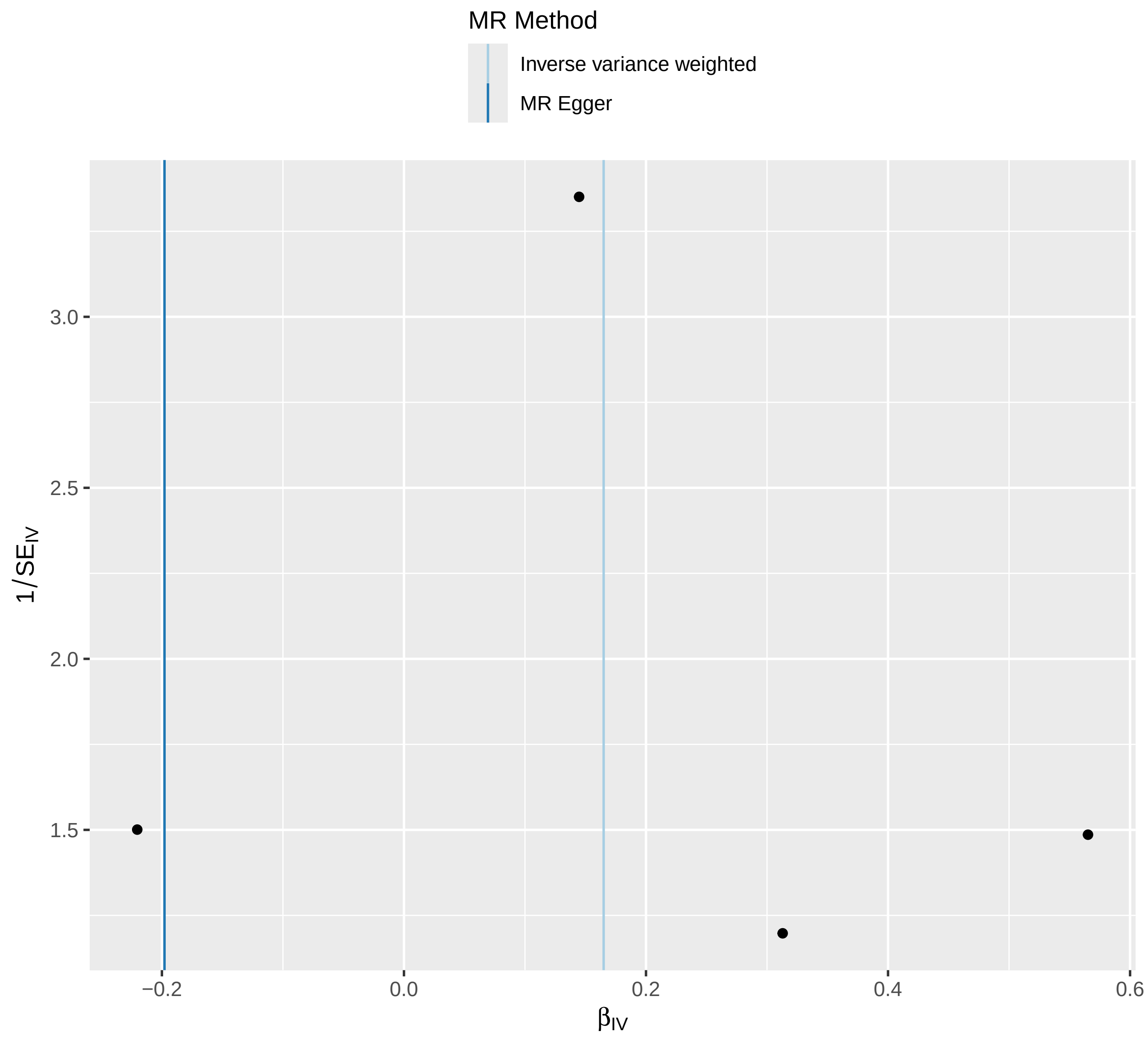

MR Method
Inverse variance weighted
MR Egger
1/SE_IV
3.0
2.5
2.0
1.5
−0.2
0.0
0.2
0.4
0.6
β_IV

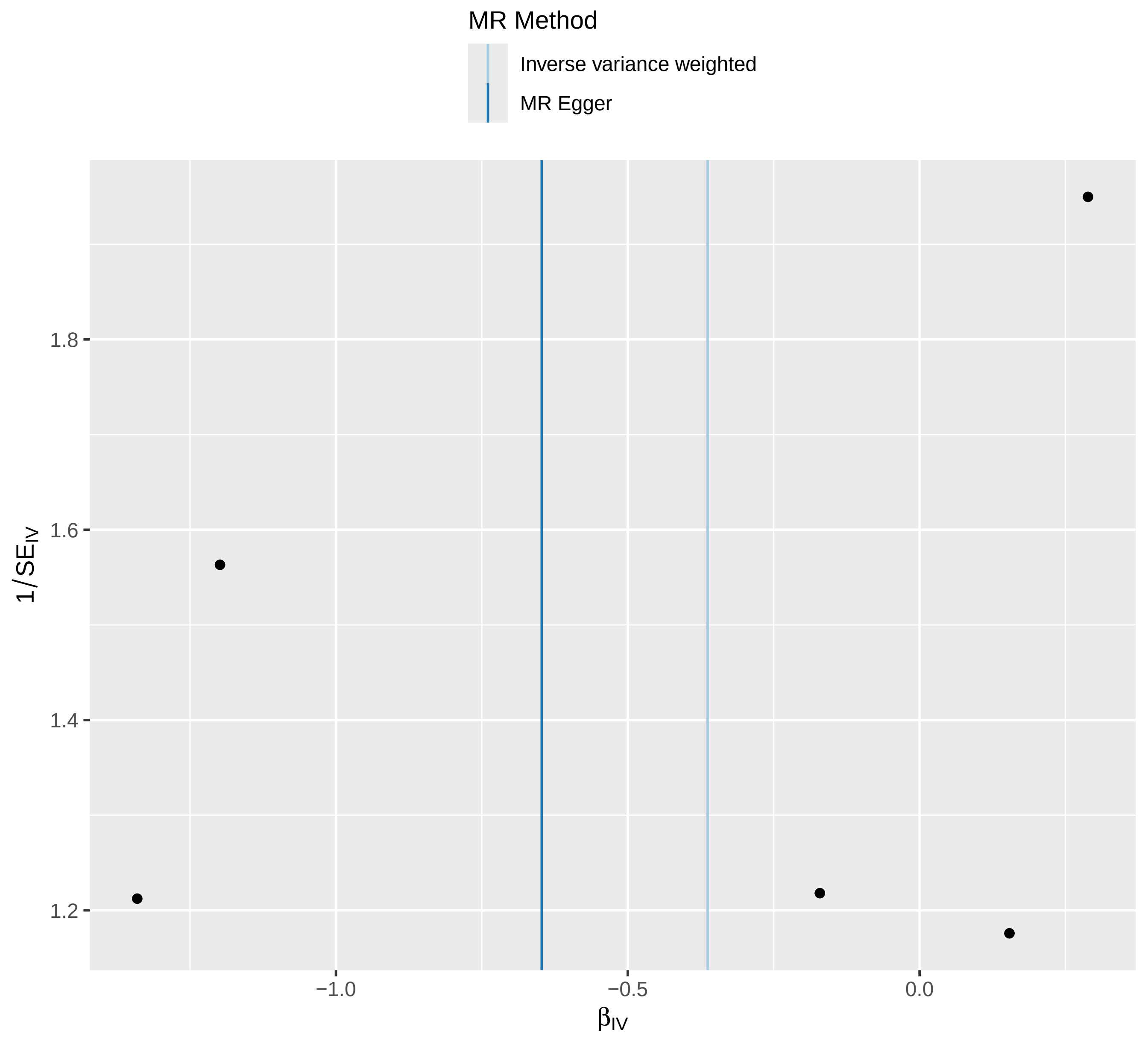
MR Method
Inverse variance weighted
MR Egger
1.8
1.6
1.4
1.2
−1.0
−0.5
0.0
$1/SE_{IV}$
β_{IV}

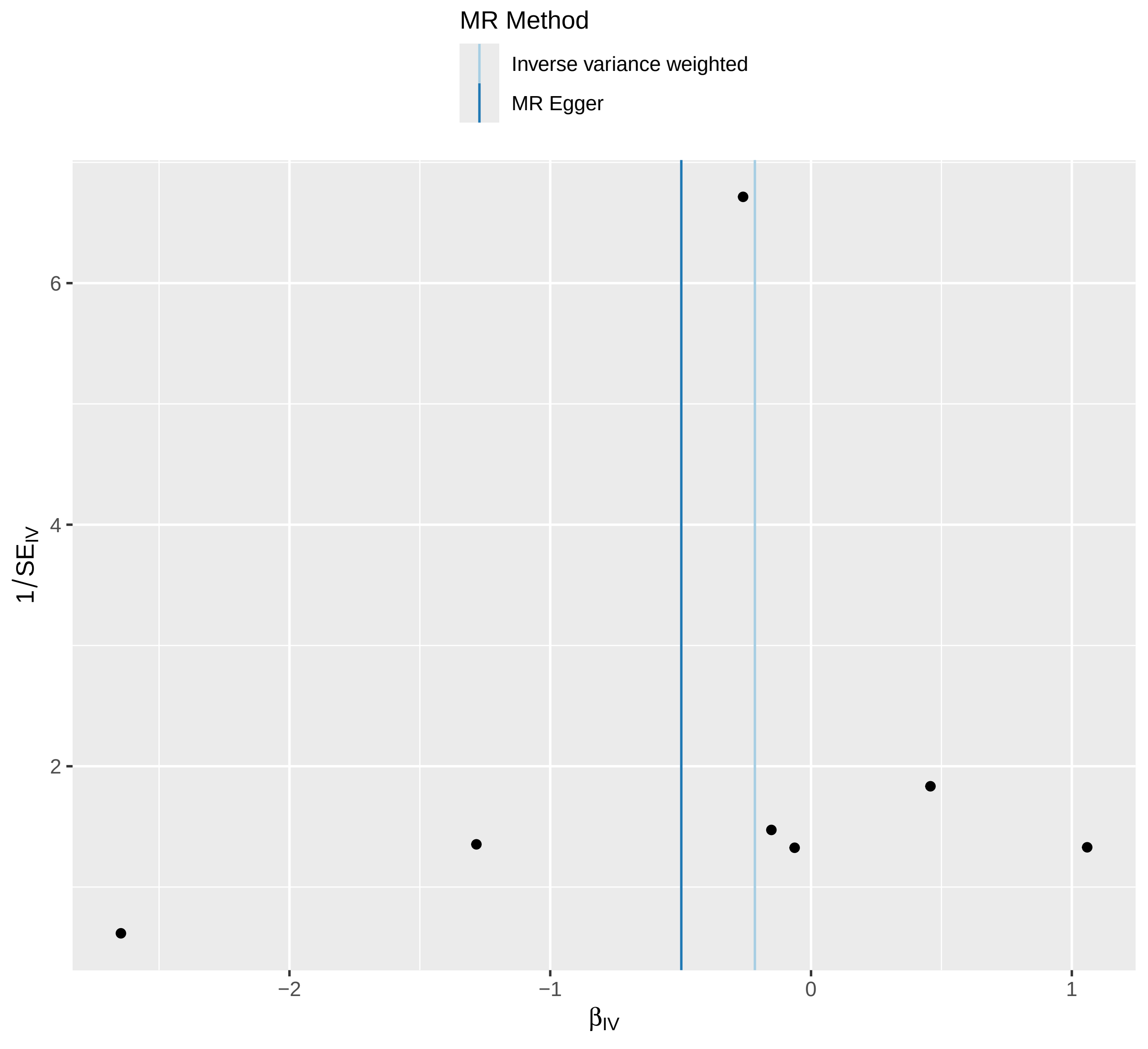
MR Method
Inverse variance weighted
MR Egger
6
4
2
1/SE_IV
−2
−1
0
1
β_IV

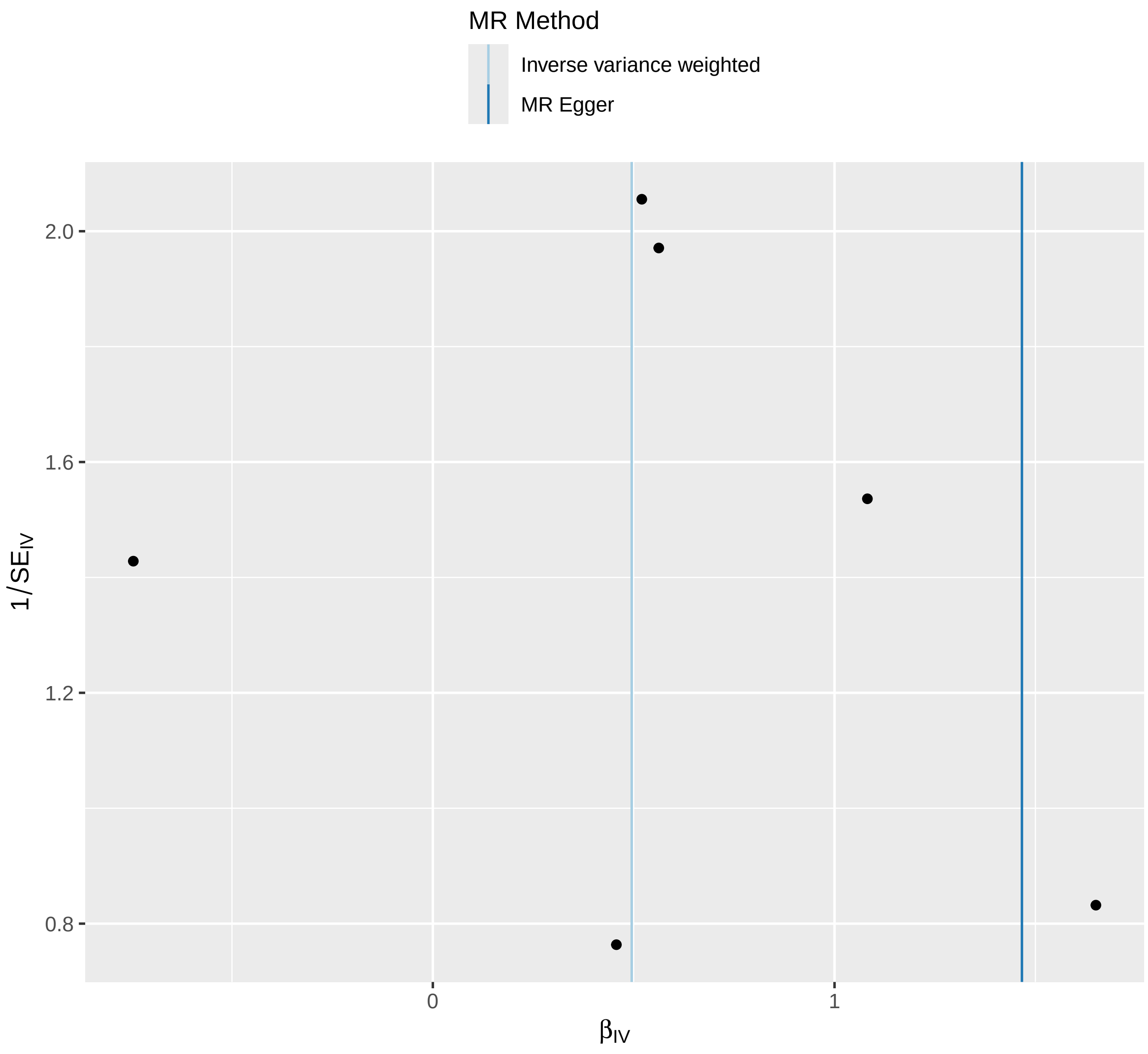

MR Method
Inverse variance weighted
MR Egger
2.0
1.6
1.2
0.8
$1/SE_{IV}$
0
1
β_{IV}

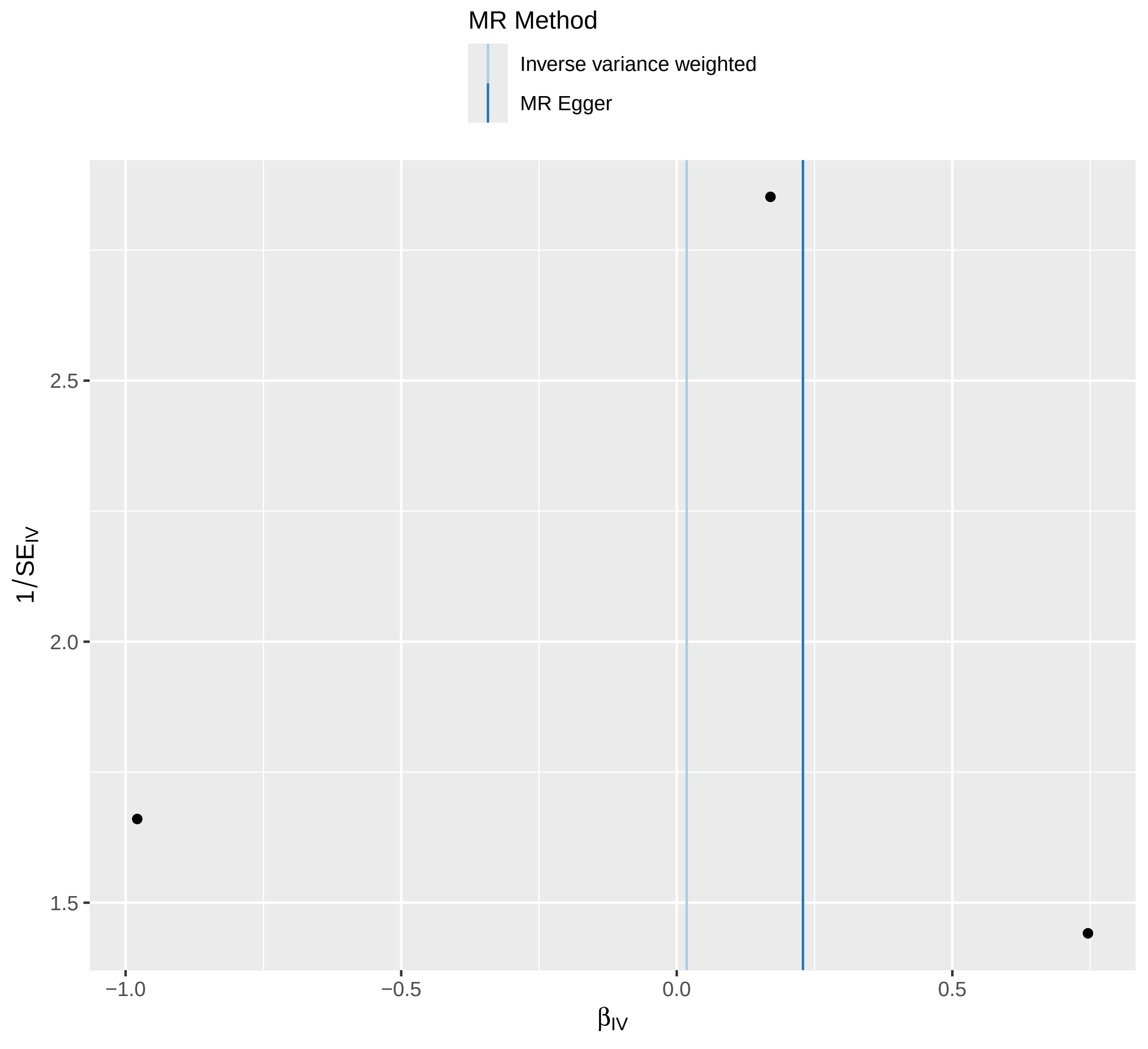

MR Method
Inverse variance weighted
MR Egger
1/SE_IV
2.5
2.0
1.5
−1.0
−0.5
0.0
0.5
β_IV

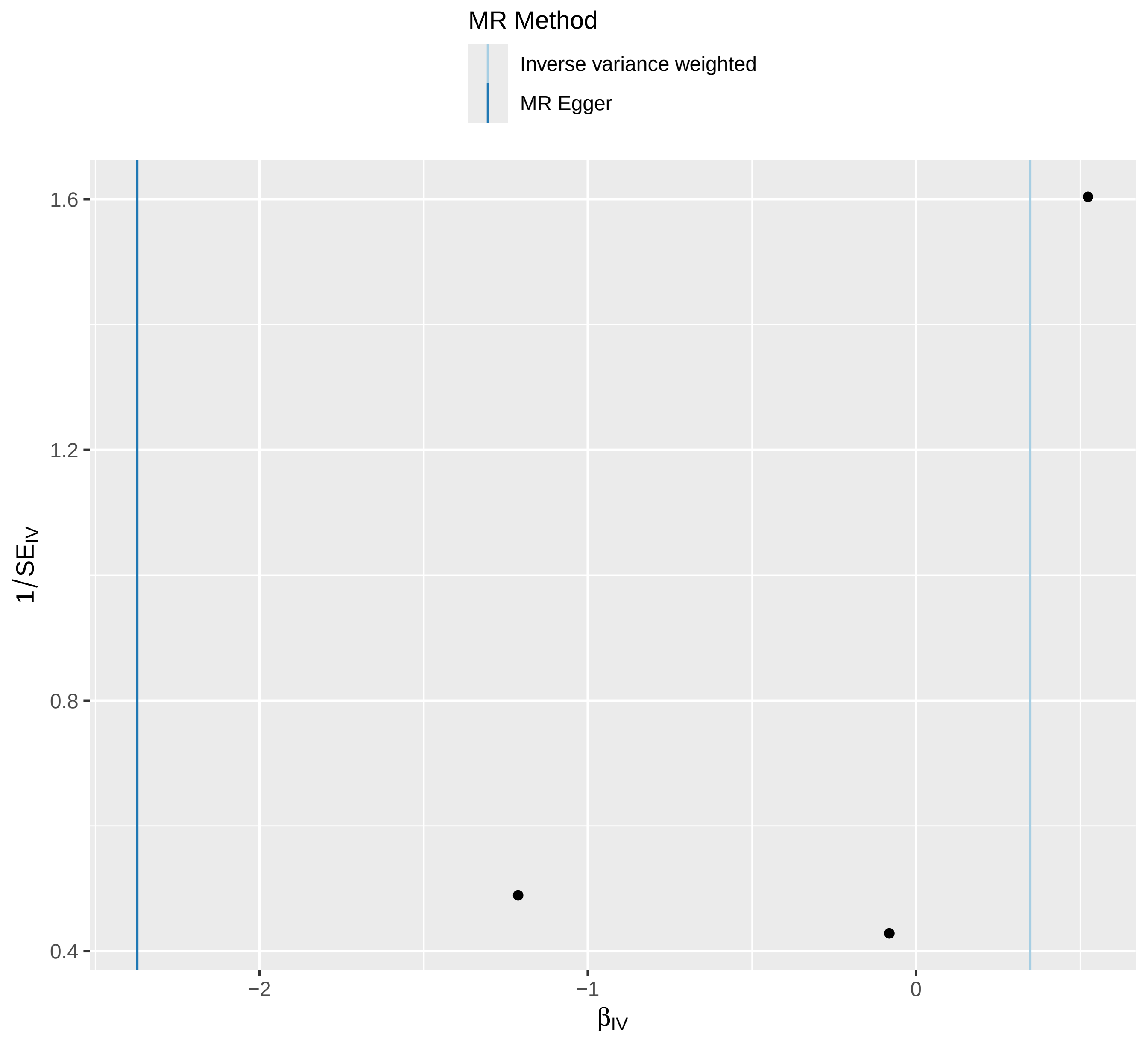
MR Method
Inverse variance weighted
MR Egger
1.6
1.2
0.8
0.4
−2
−1
0
$1/SE_{IV}$
β_{IV}

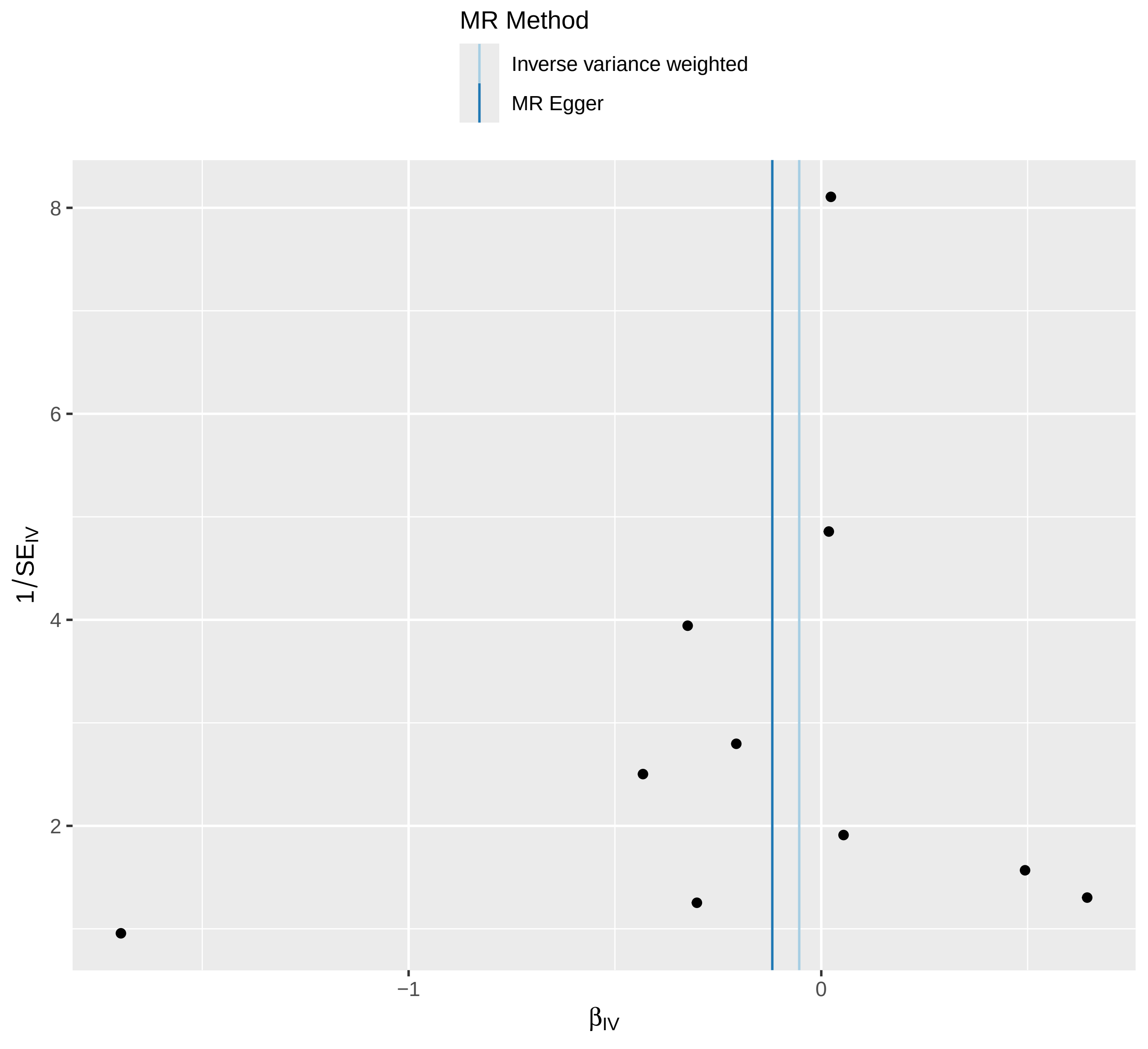

MR Method
Inverse variance weighted
MR Egger
8
6
4
2
1/SE_IV
−1
0
β_IV

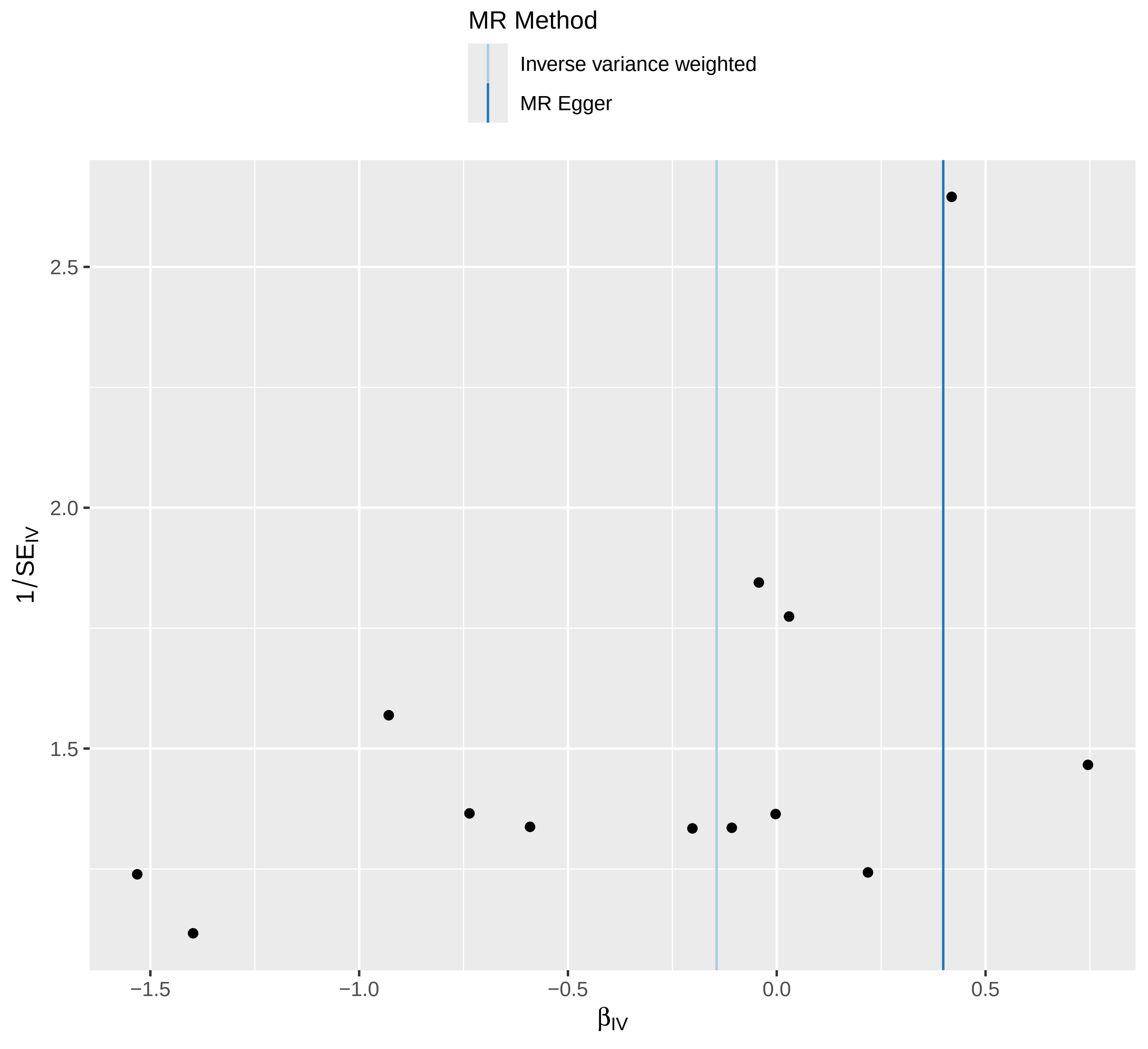

MR Method
Inverse variance weighted
MR Egger
2.5
2.0
1.5
1/SE_IV
−1.5
−1.0
−0.5
0.0
0.5
β_IV

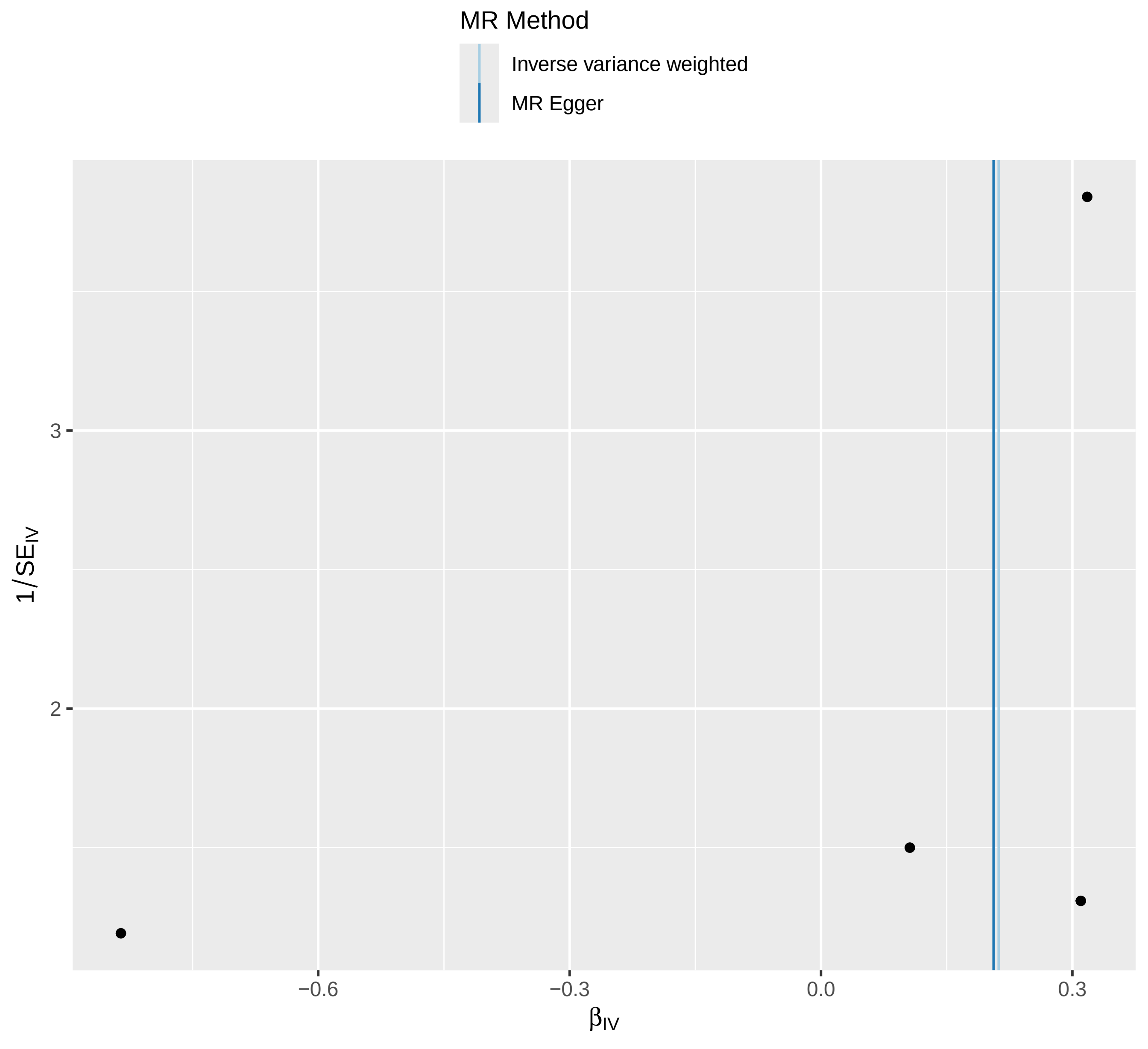

MR Method
Inverse variance weighted
MR Egger
3
2
1/SE_IV
−0.6
−0.3
0.0
0.3
β_IV

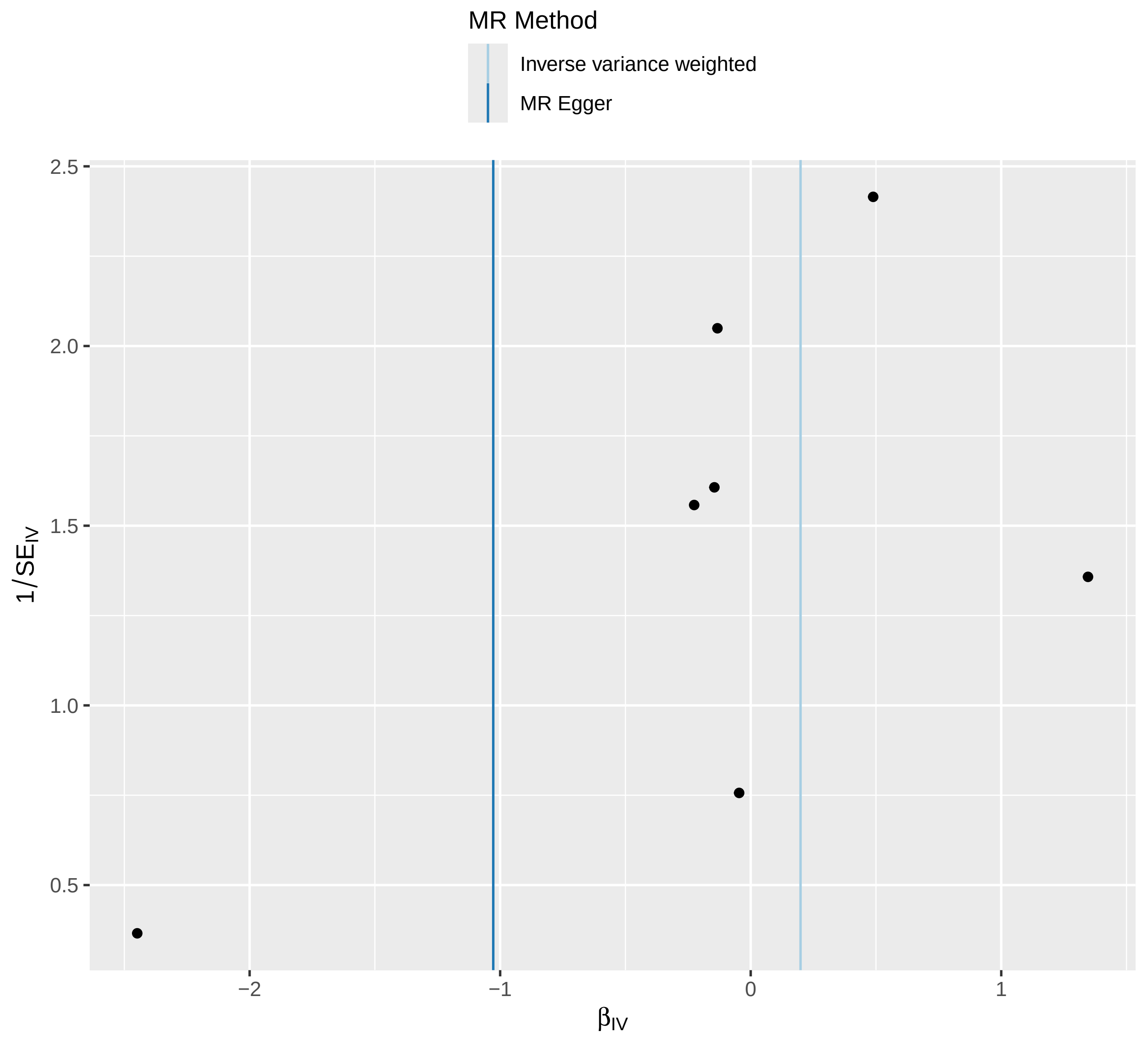
MR Method
Inverse variance weighted
MR Egger
2.5
2.0
1.5
1.0
0.5
$1/SE_{IV}$
−2
−1
0
1
β_{IV}

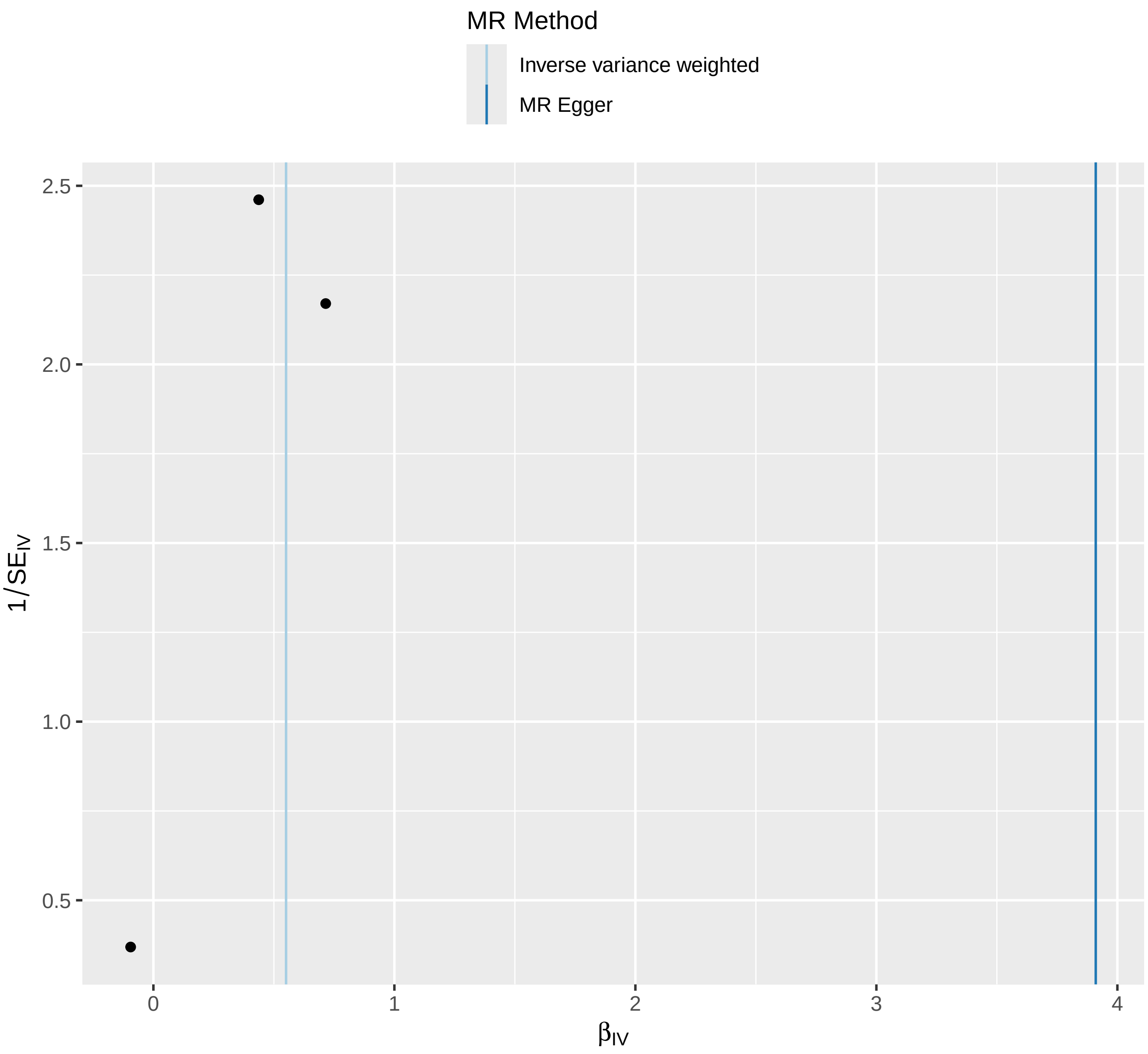

MR Method
Inverse variance weighted
MR Egger
2.5
2.0
1.5
1.0
0.5
1/SE_IV
0
1
2
3
4
β_IV

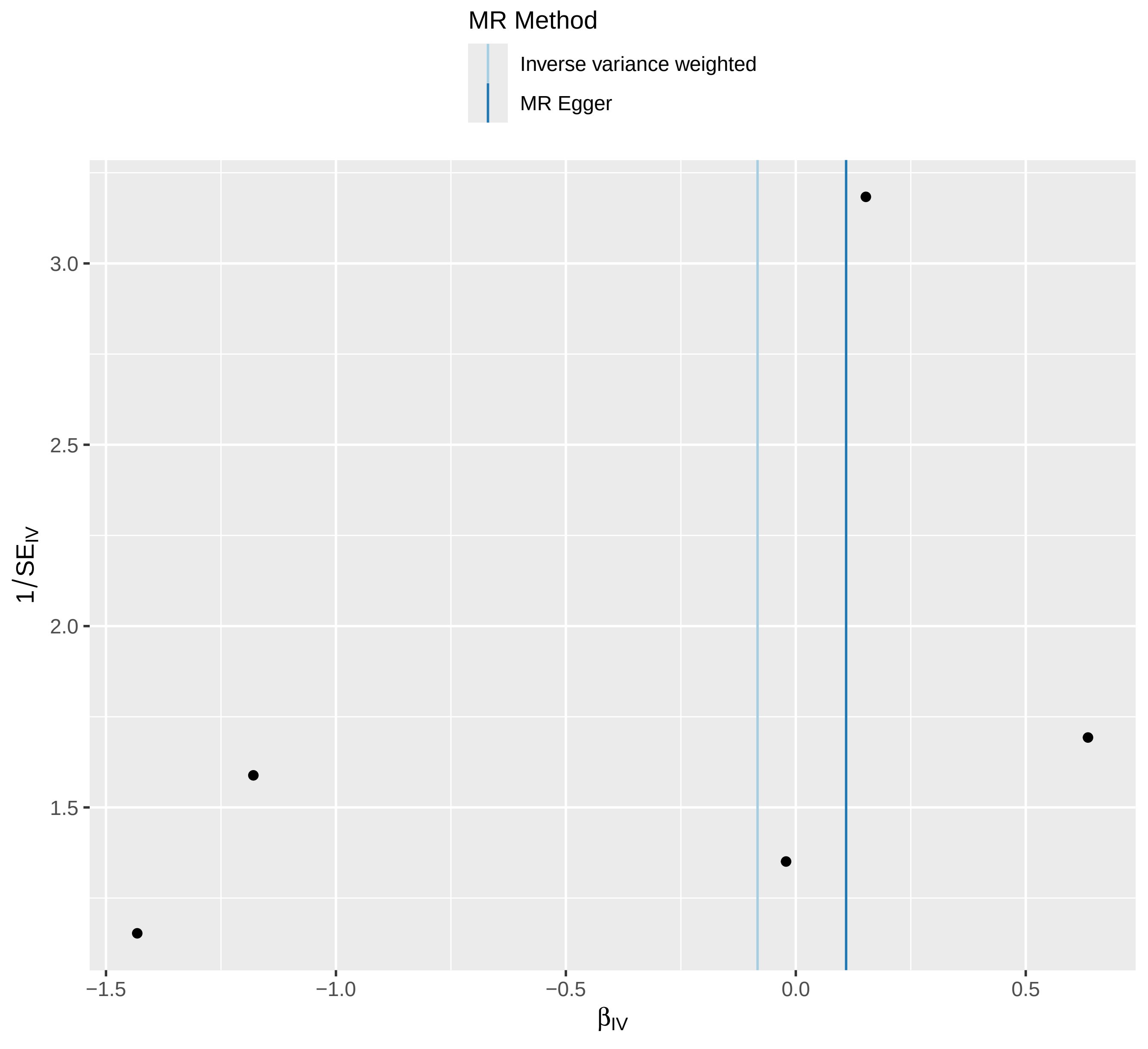
MR Method
Inverse variance weighted
MR Egger
3.0
2.5
2.0
1.5
1/SE_IV
−1.5
−1.0
−0.5
0.0
0.5
β_IV

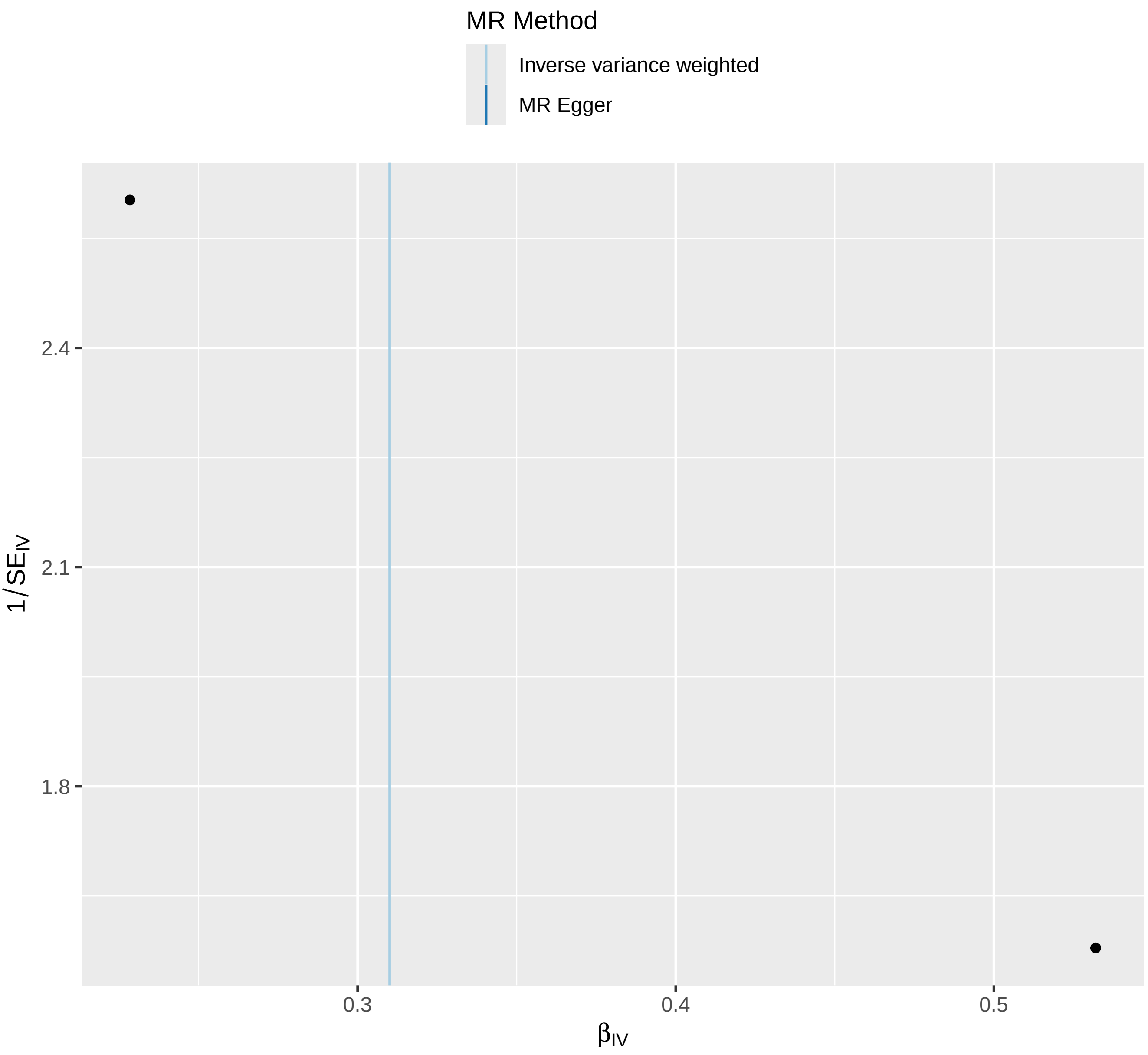
MR Method
Inverse variance weighted
MR Egger
2.4
2.1
1.8
1/SE$_{IV}$
0.3
0.4
0.5
β$_{IV}$

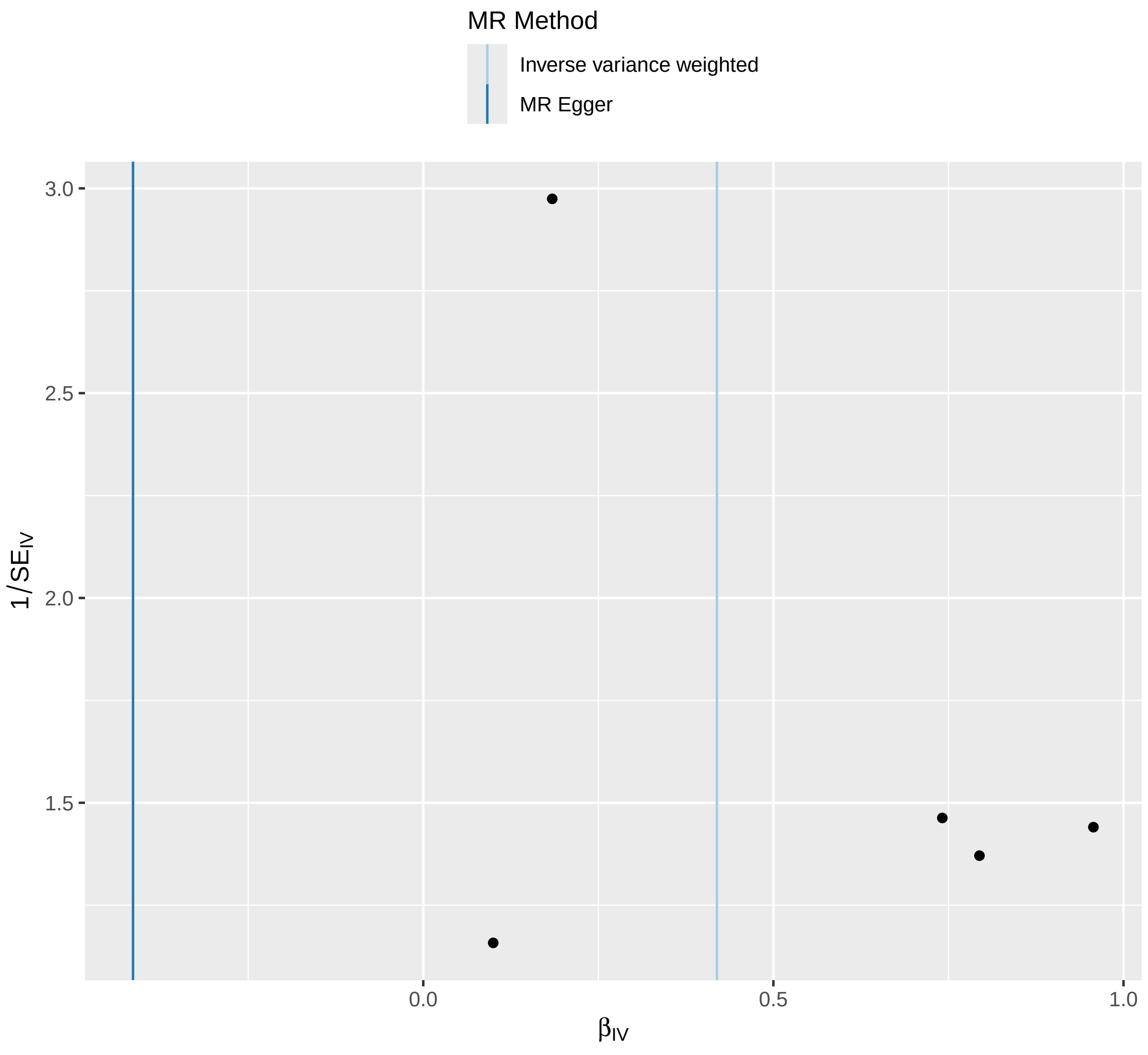
MR Method
Inverse variance weighted
MR Egger
3.0
2.5
2.0
1.5
1/SE$_{IV}$
0.0
0.5
1.0
β$_{IV}$

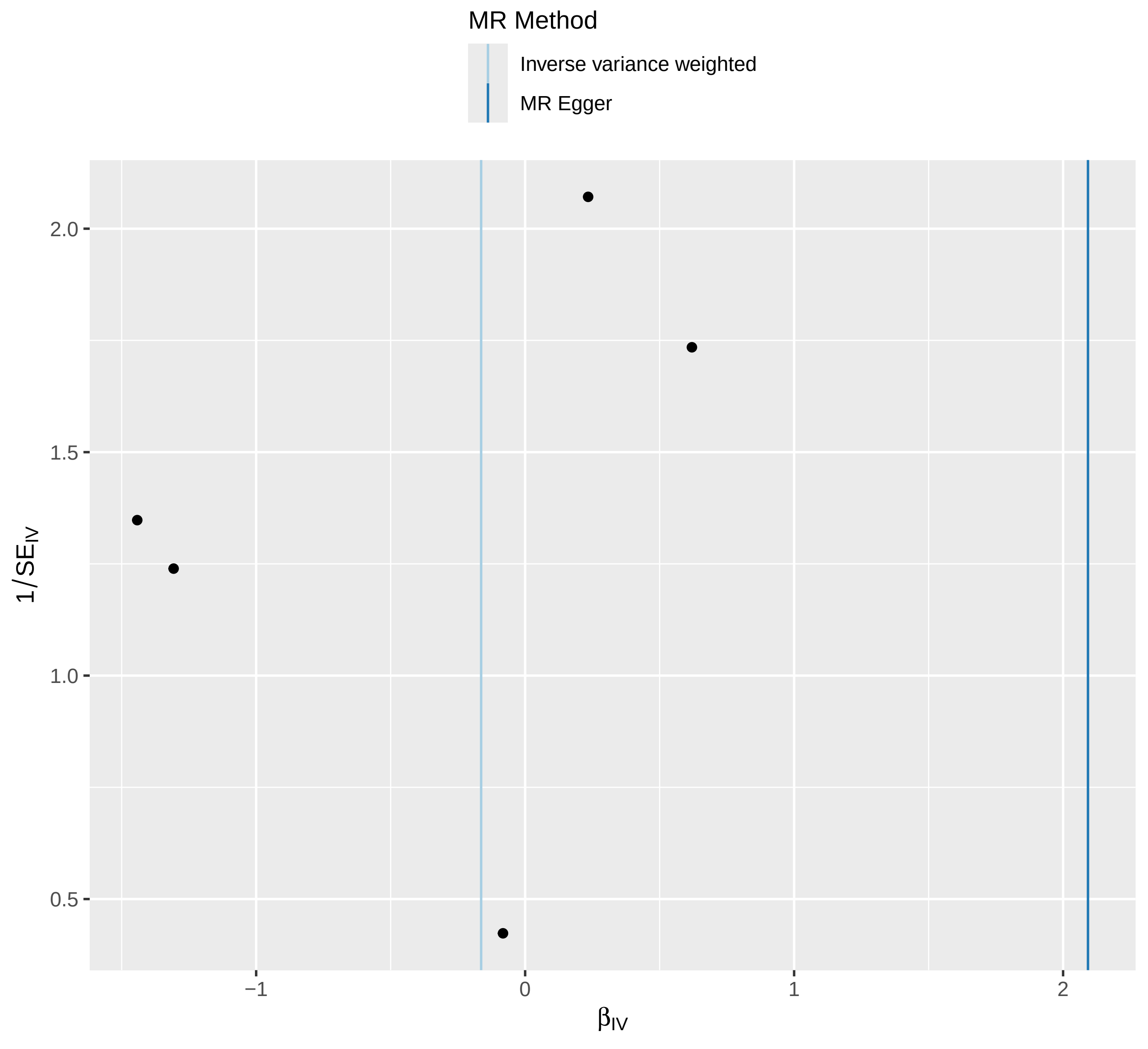

MR Method
Inverse variance weighted
MR Egger
2.0
1.5
1.0
0.5
1/SE_IV
−1
0
1
2
β_IV

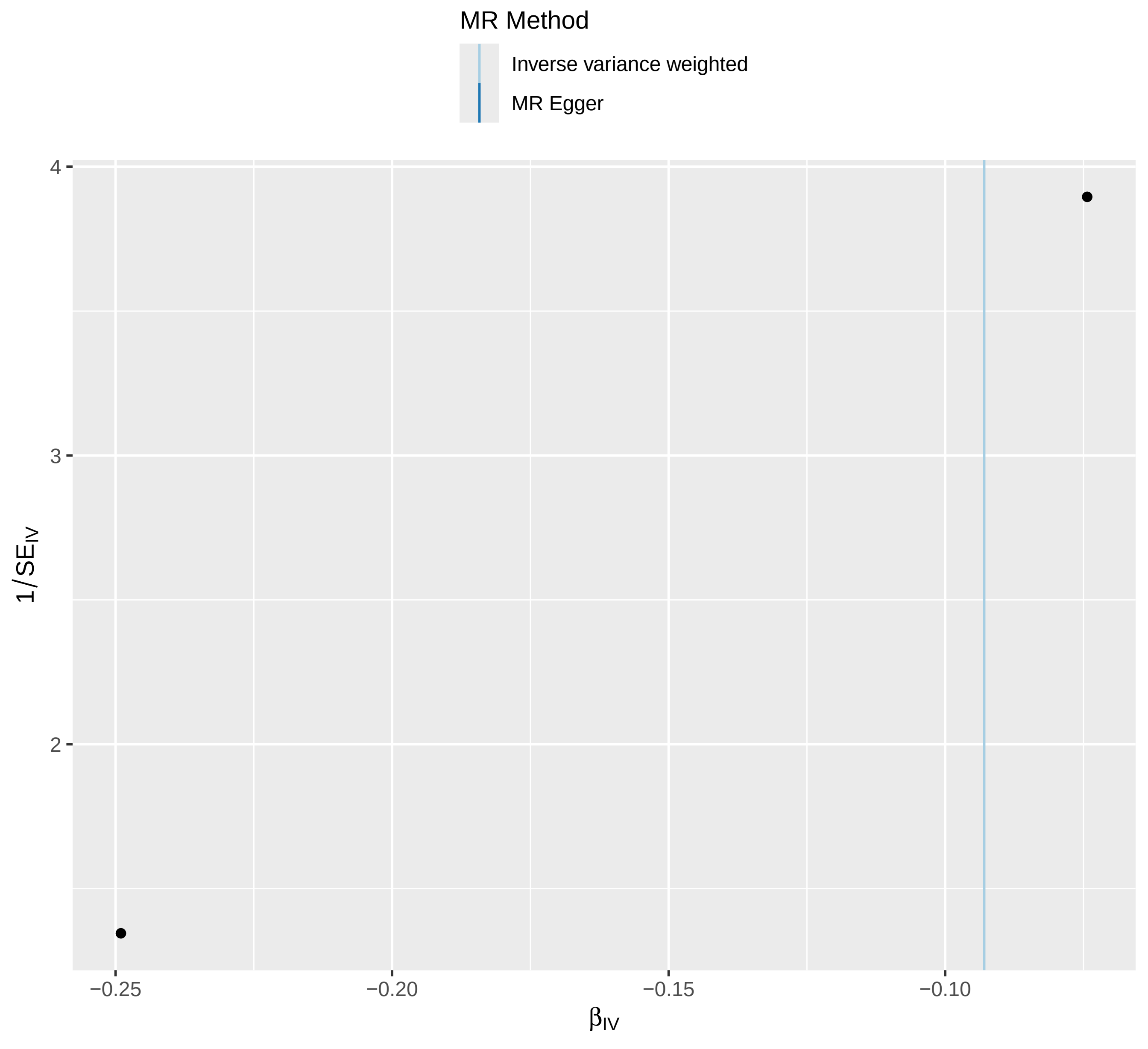
MR Method
Inverse variance weighted
MR Egger
4
3
2
1/SE_IV
−0.25
−0.20
−0.15
−0.10
β_IV

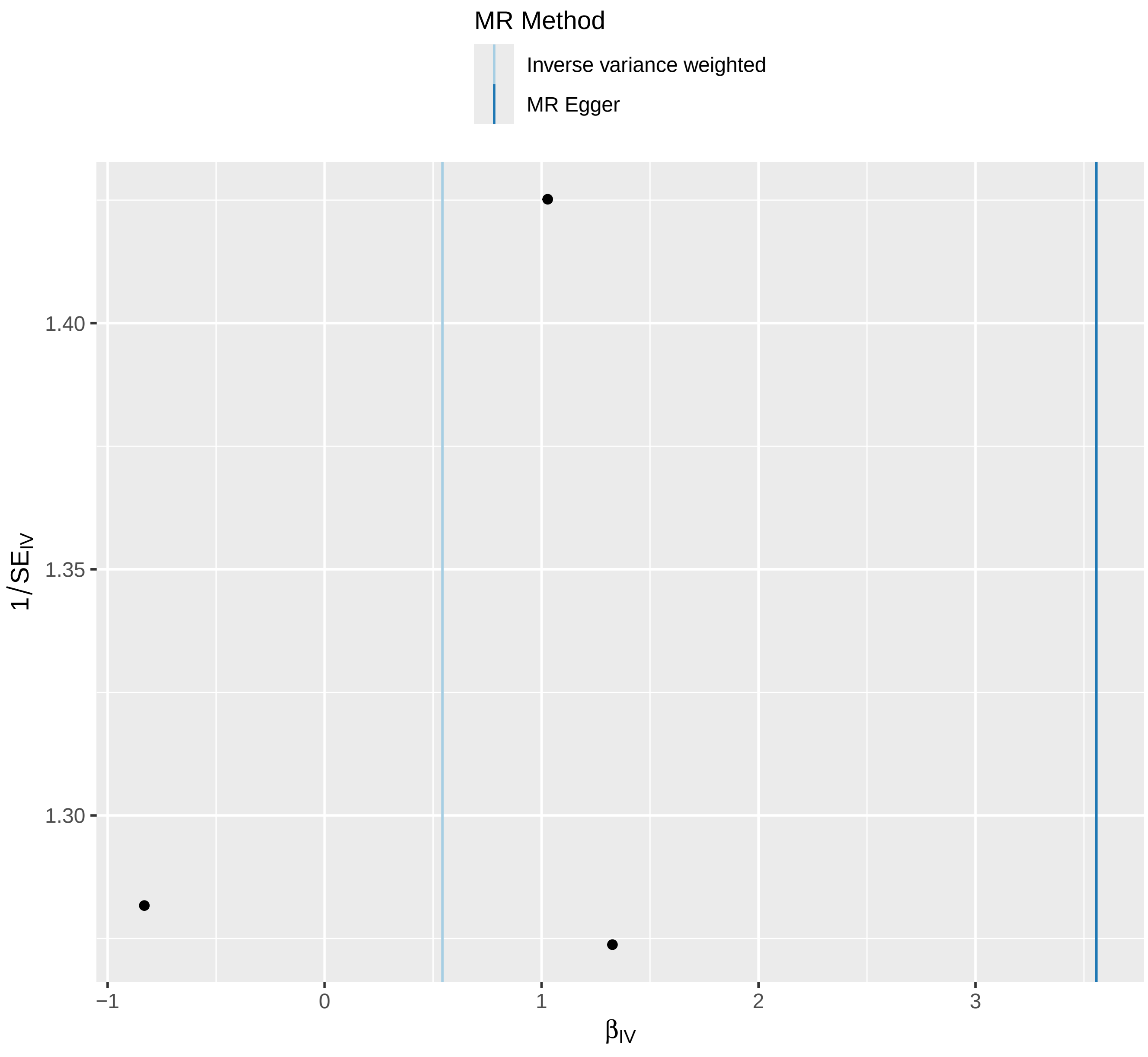
MR Method
Inverse variance weighted
MR Egger
1/SE_IV
1.40
1.35
1.30
−1
0
1
2
3
β_IV

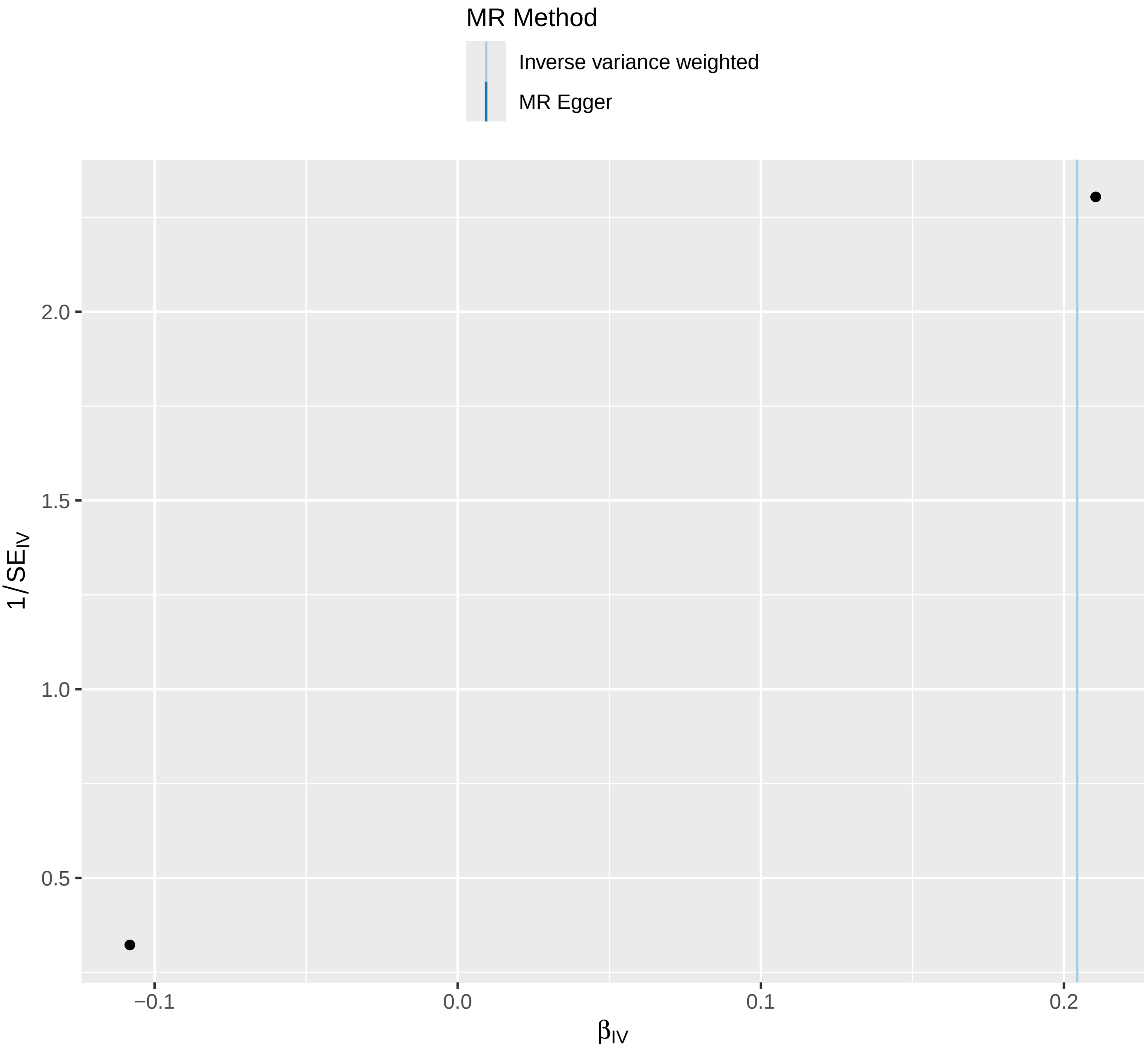

MR Method
Inverse variance weighted
MR Egger
2.0
1.5
1.0
0.5
$1/SE_{IV}$
−0.1
0.0
0.1
0.2
β_{IV}

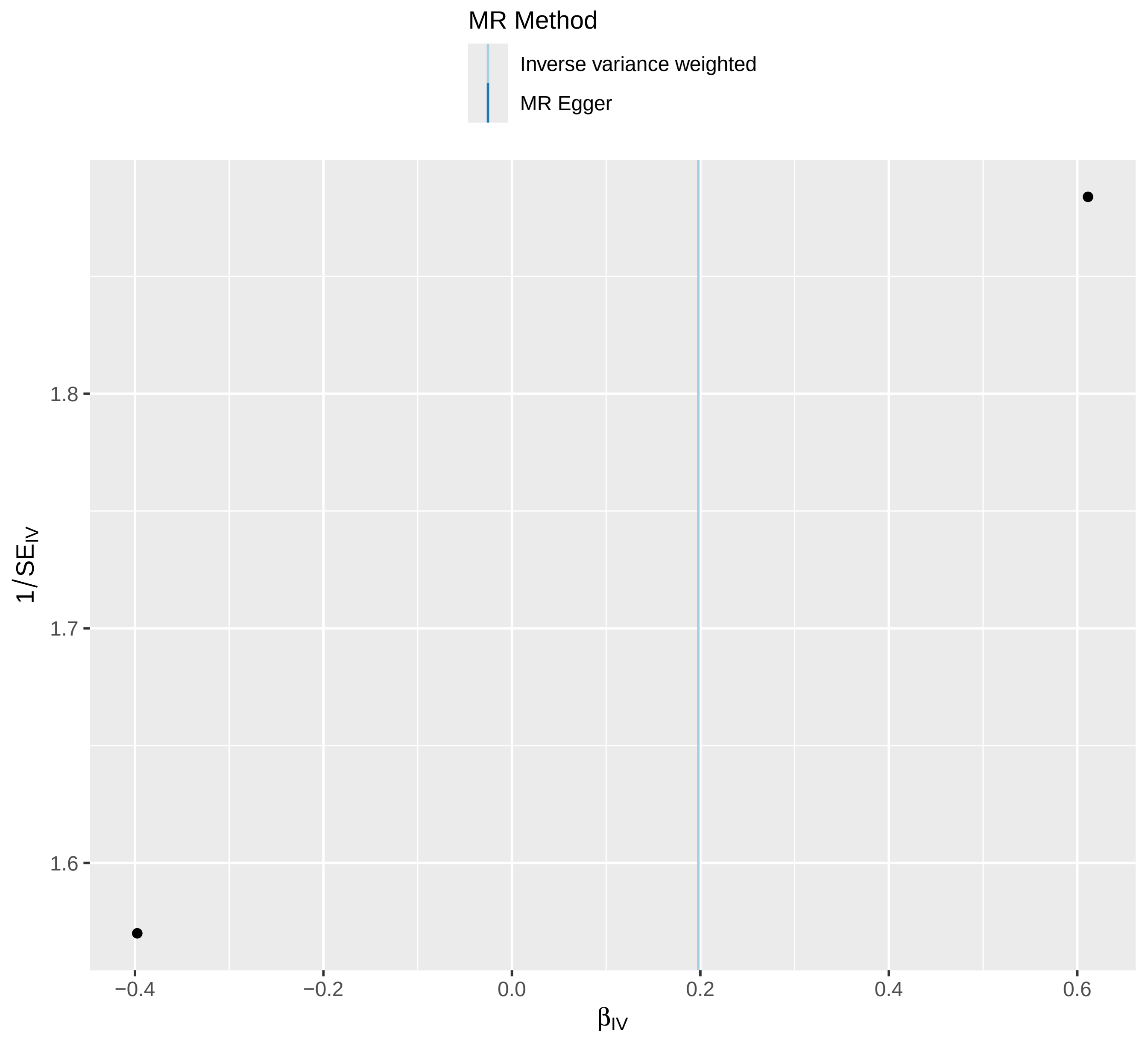

MR Method
Inverse variance weighted
MR Egger
1.8
1.7
1.6
1/SE_IV
−0.4
−0.2
0.0
0.2
0.4
0.6
β_IV

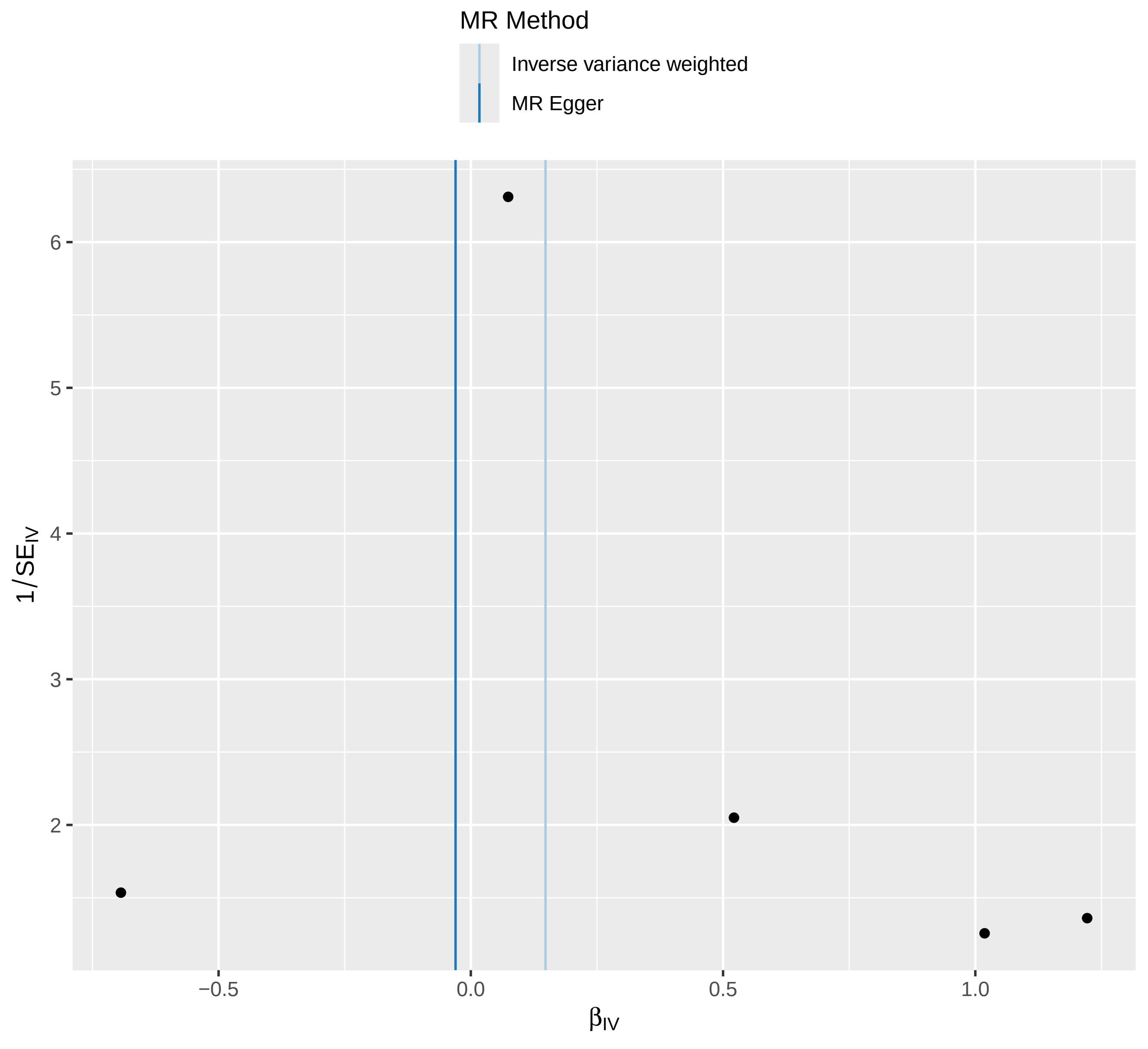

MR Method
Inverse variance weighted
MR Egger
6
5
4
3
2
1/SE$_{IV}$
−0.5
0.0
0.5
1.0
β$_{IV}$

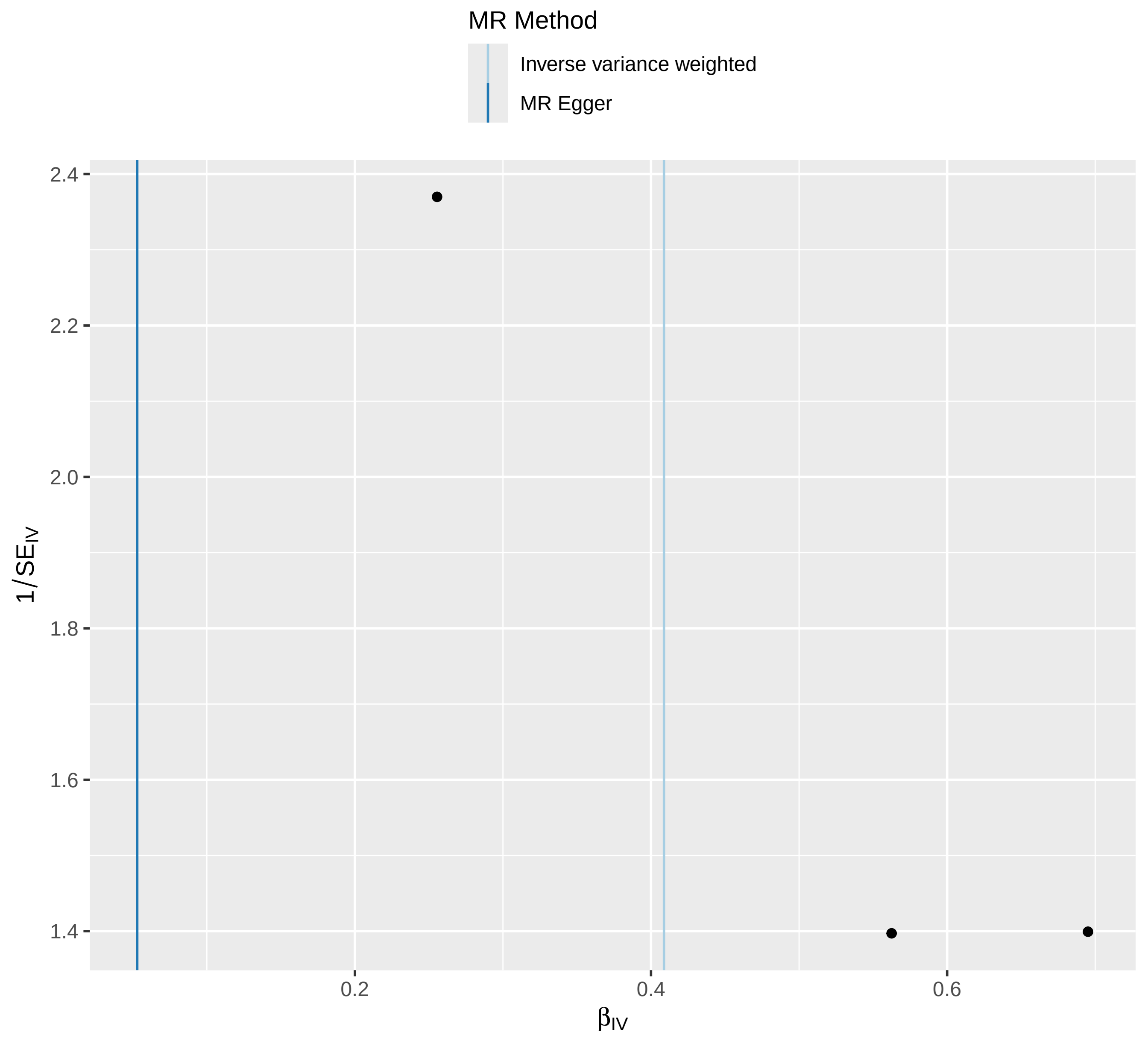

MR Method
Inverse variance weighted
MR Egger
2.4
2.2
2.0
1.8
1.6
1.4
1/SE$_{IV}$
0.2
0.4
0.6
β$_{IV}$

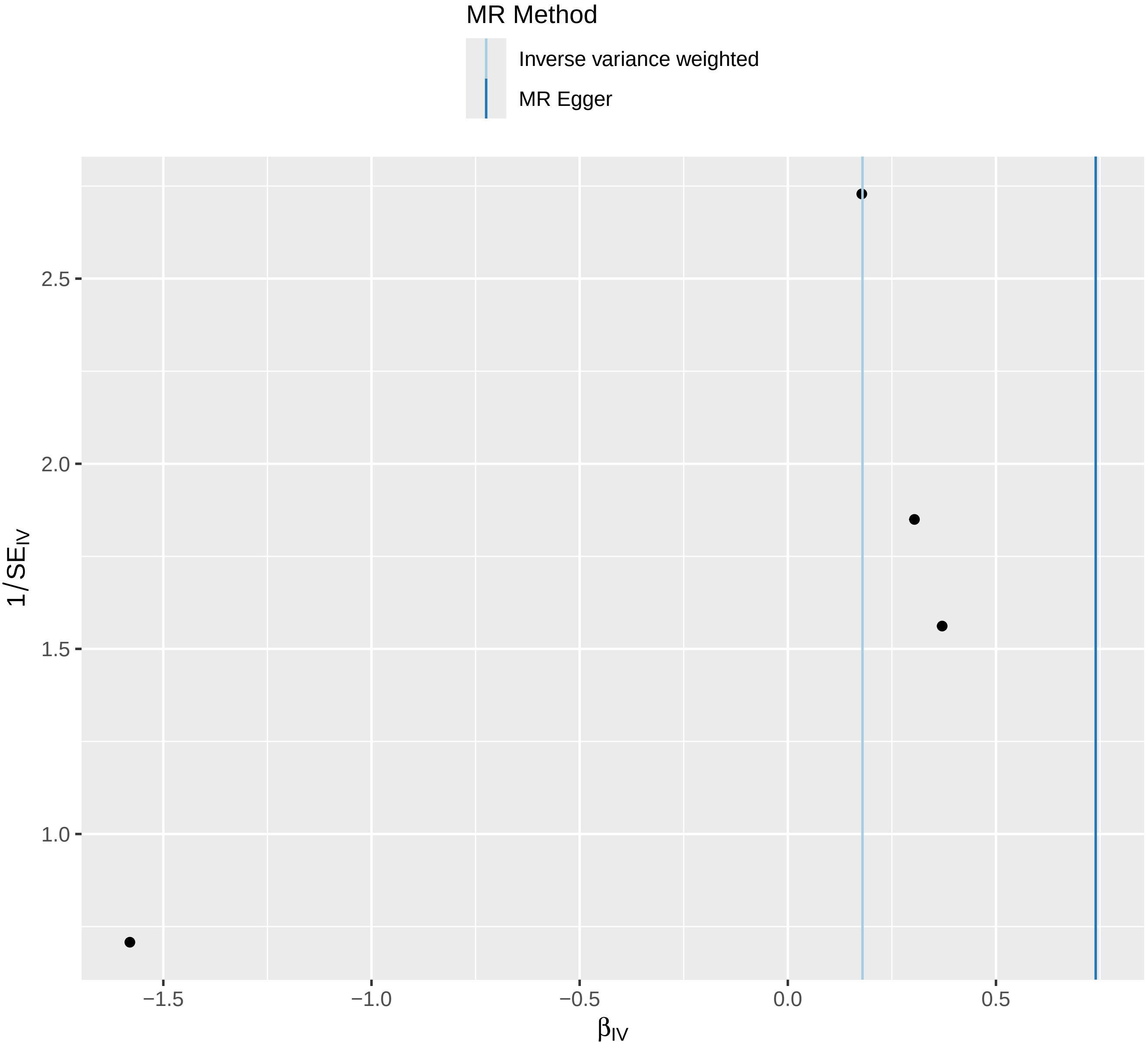
MR Method
Inverse variance weighted
MR Egger
2.5
2.0
1.5
1.0
1/SE_IV
−1.5
−1.0
−0.5
0.0
0.5
β_IV

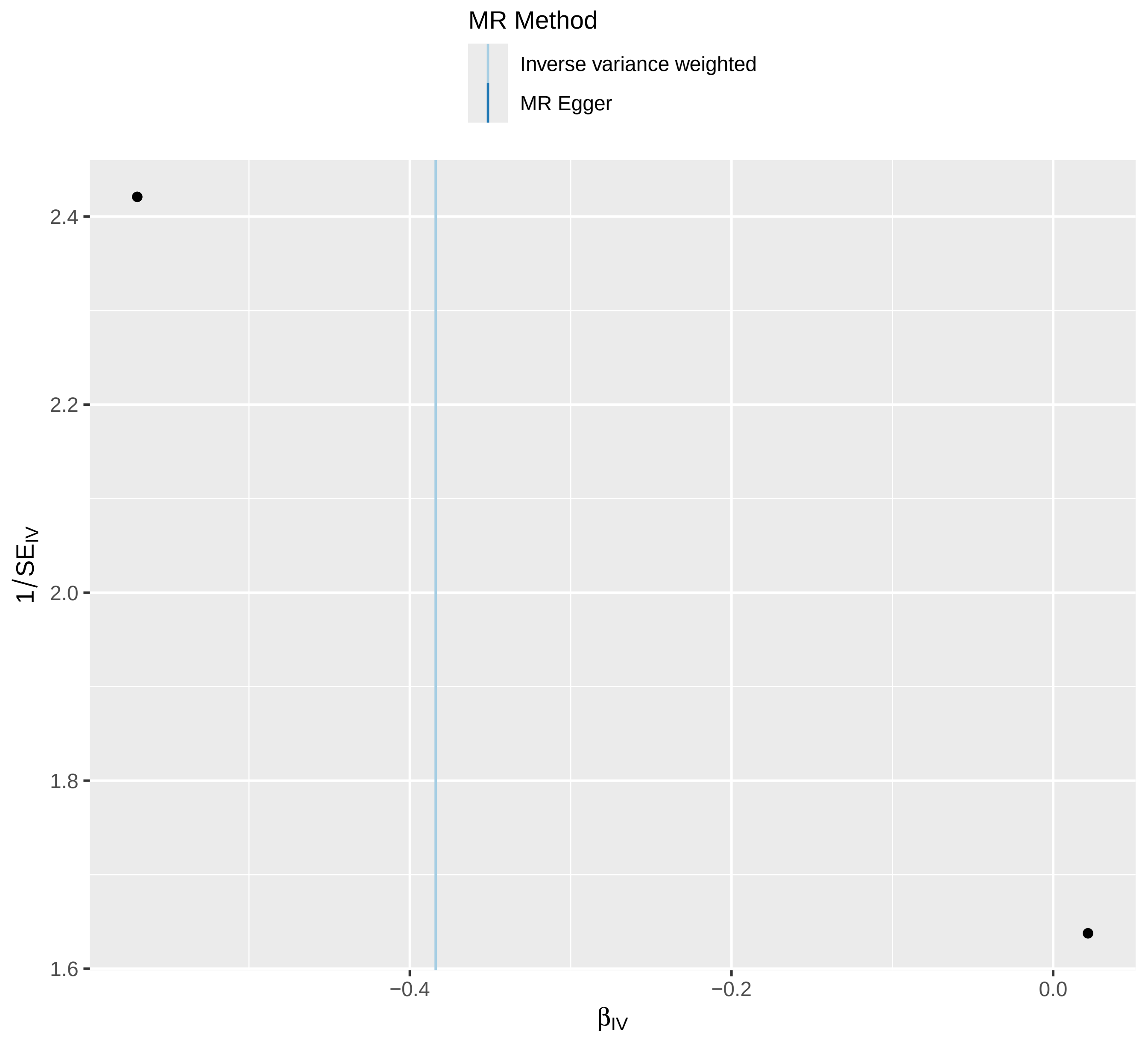
MR Method
Inverse variance weighted
MR Egger
2.4
2.2
2.0
1.8
1.6
1/SE_IV
−0.4
−0.2
0.0
β_IV

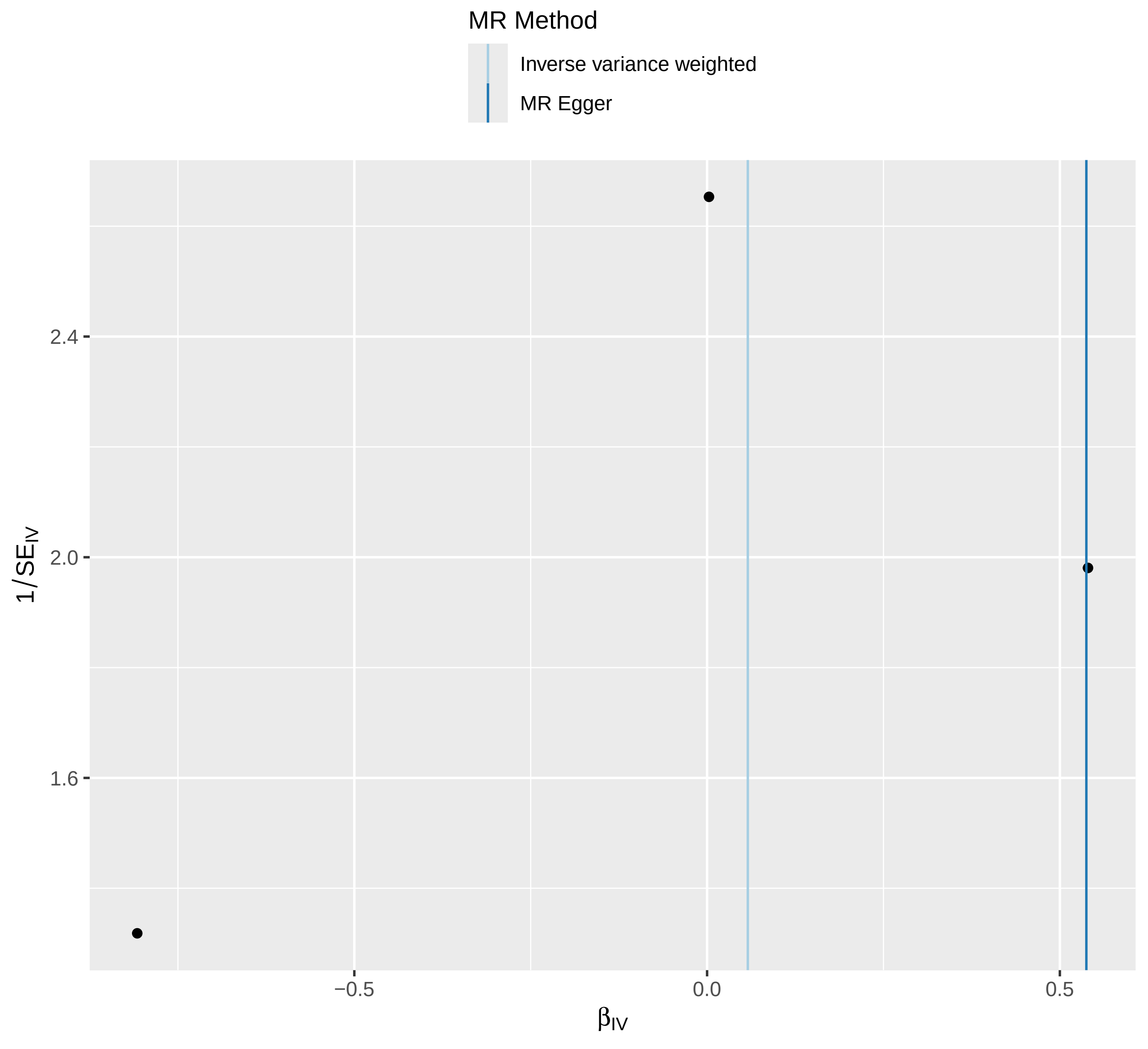

MR Method
Inverse variance weighted
MR Egger
2.4
2.0
1.6
1/SE_IV
−0.5
0.0
0.5
β_IV

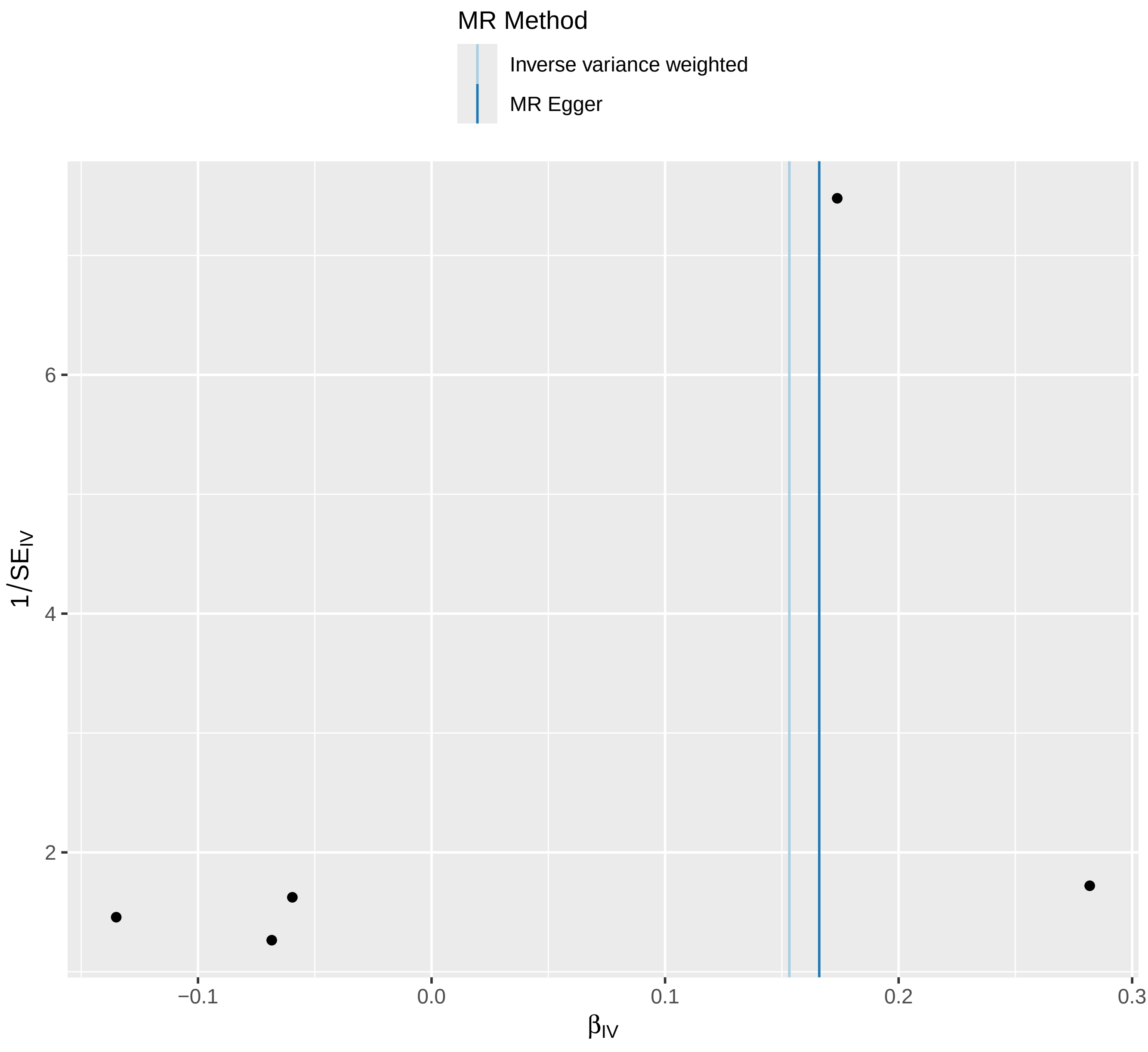
MR Method
Inverse variance weighted
MR Egger
6
4
2
1/SE_IV
−0.1
0.0
0.1
0.2
0.3
β_IV

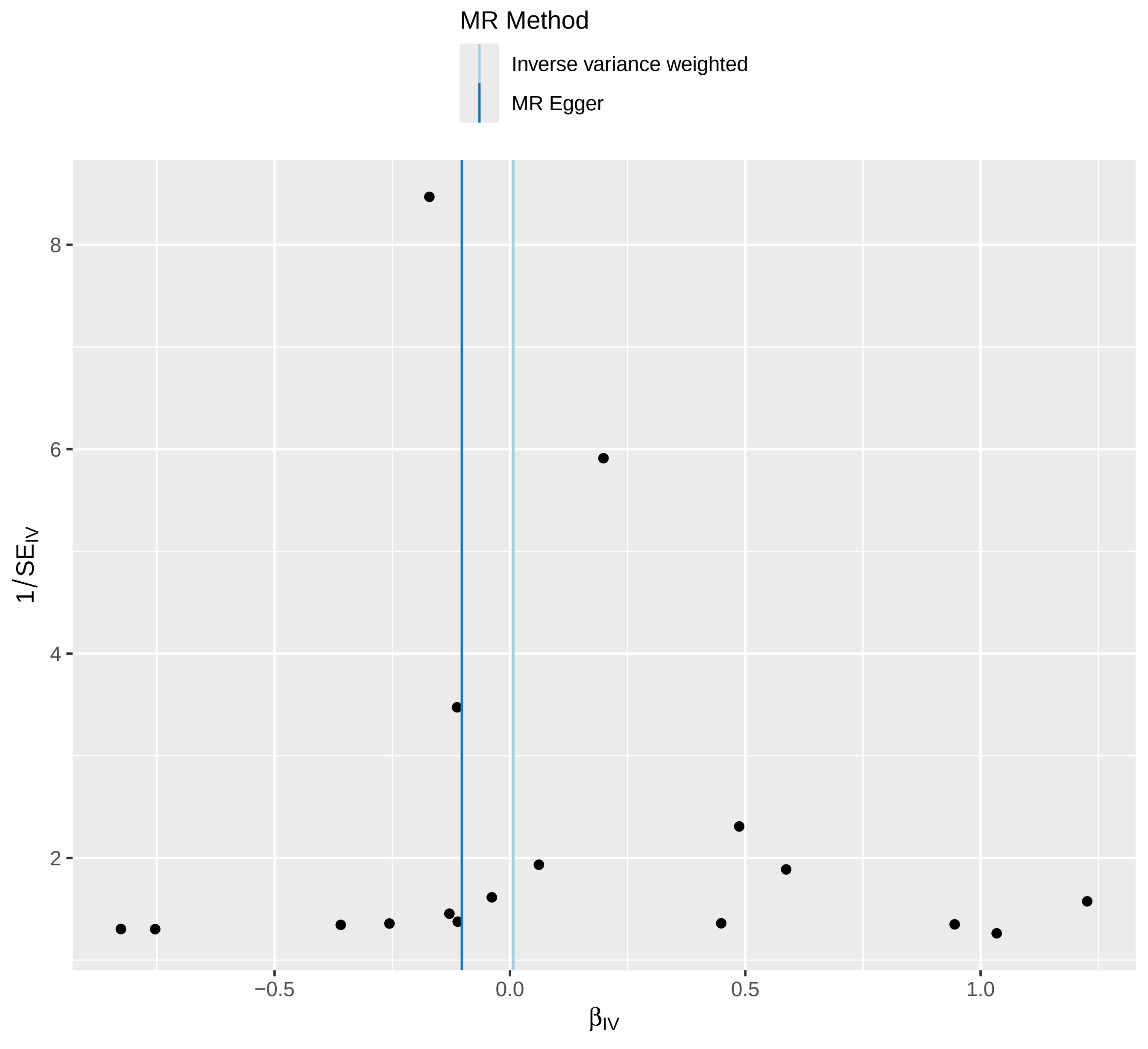

MR Method
Inverse variance weighted
MR Egger
8
6
4
2
1/SE$_{IV}$
−0.5
0.0
0.5
1.0
β$_{IV}$

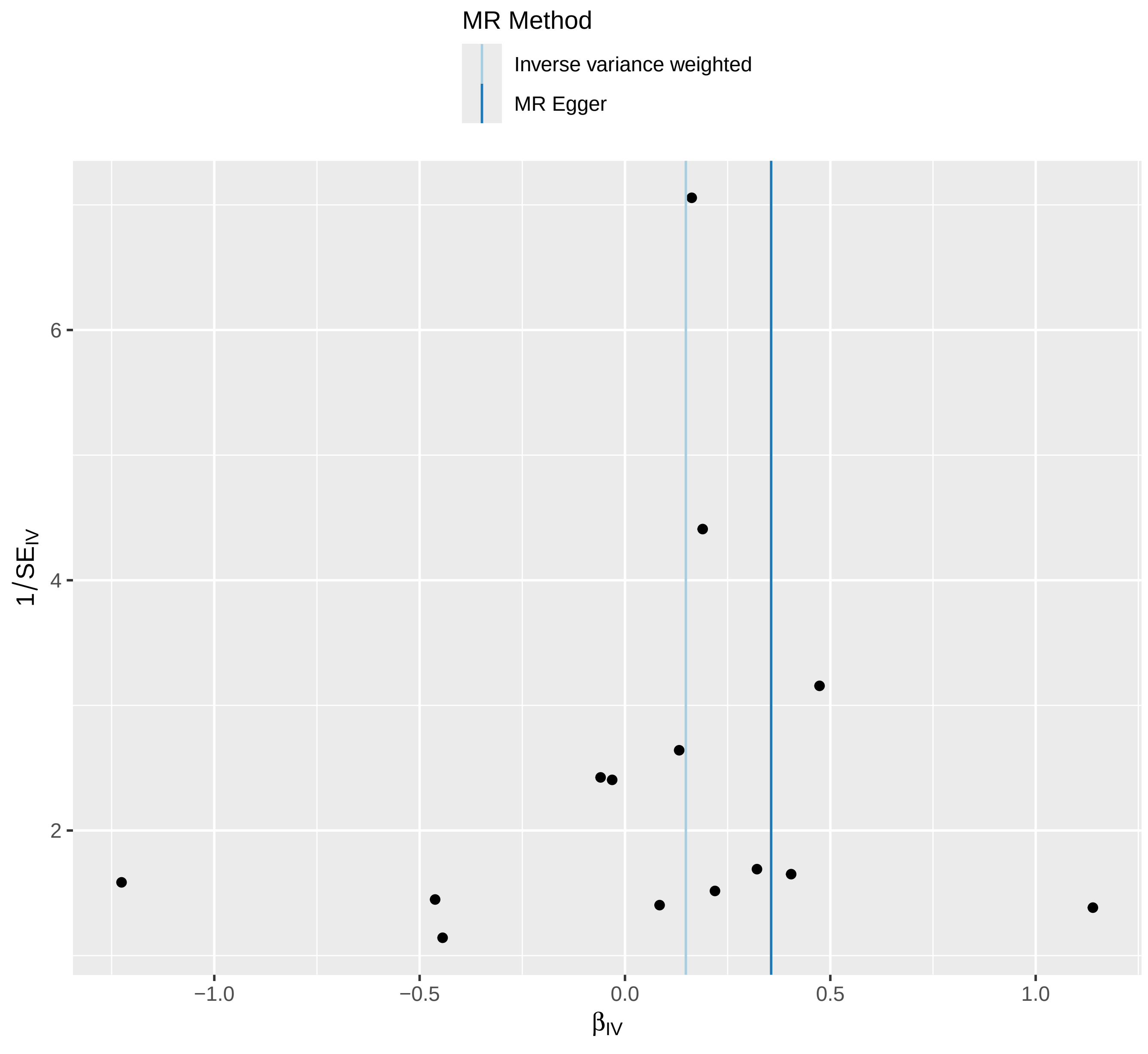
MR Method
Inverse variance weighted
MR Egger
6
4
2
1/SE$_{IV}$
−1.0
−0.5
0.0
0.5
1.0
β_{IV}

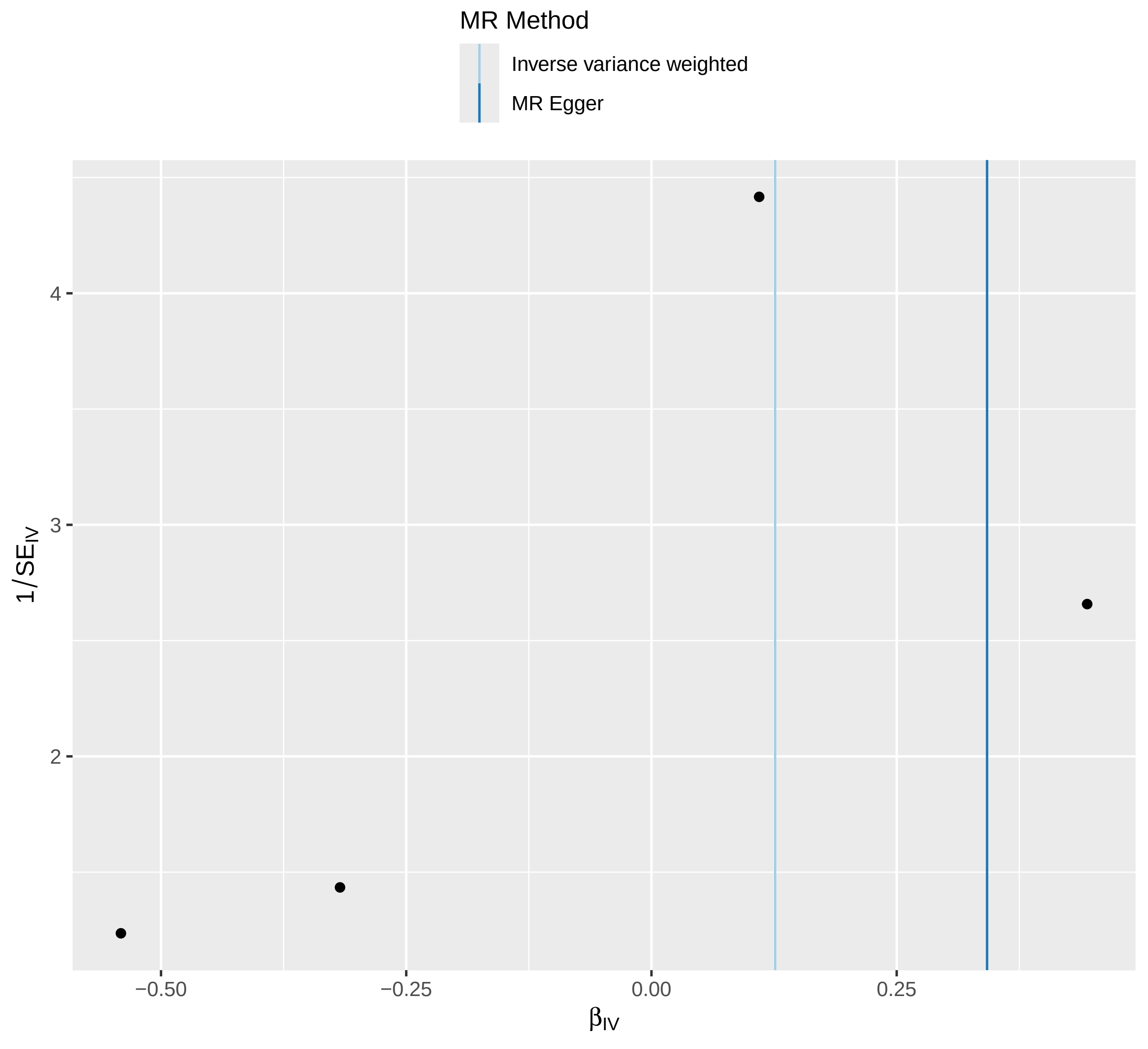

MR Method
Inverse variance weighted
MR Egger
4
3
2
1/SE$_{IV}$
−0.50
−0.25
0.00
0.25
β$_{IV}$

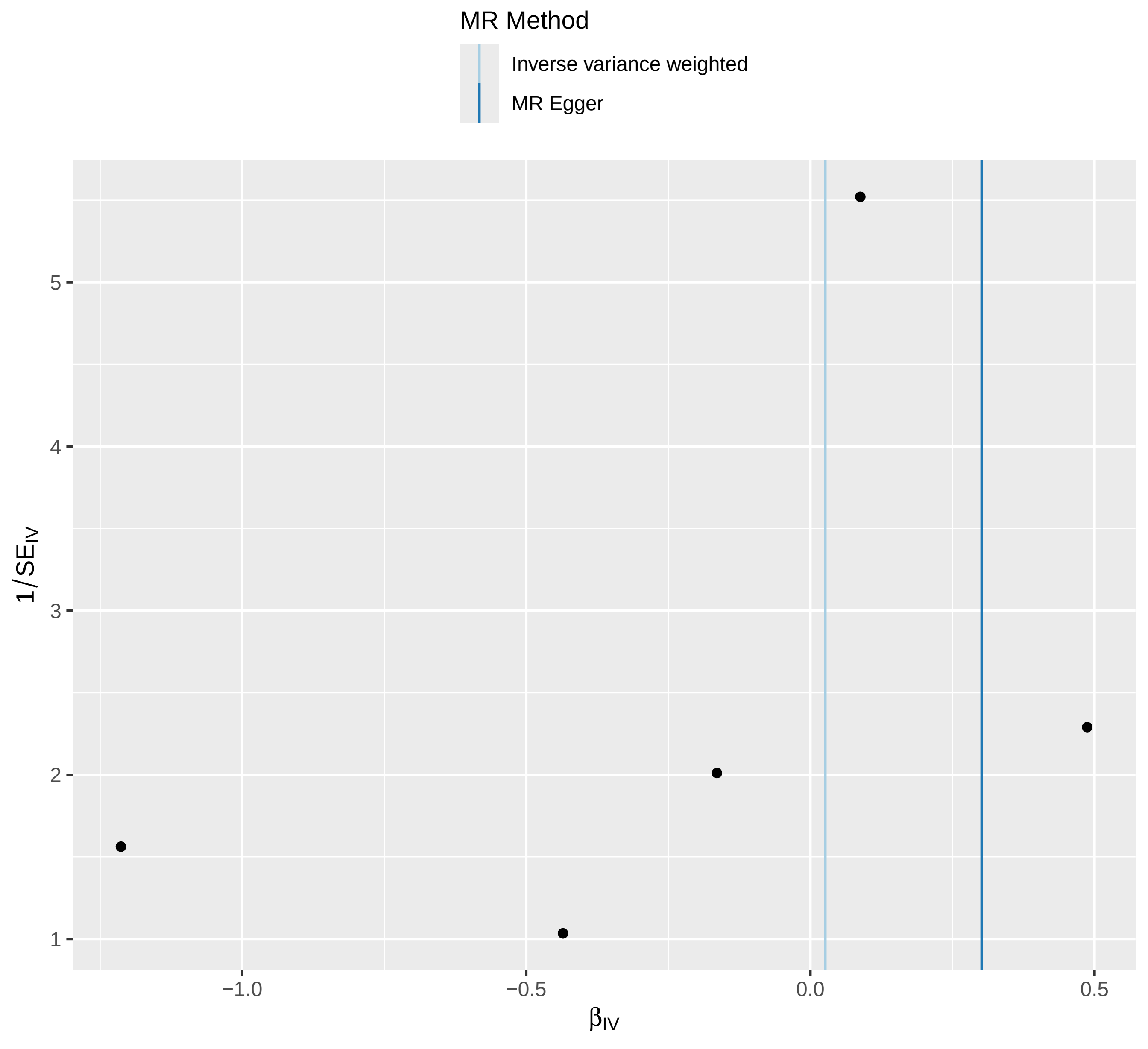
MR Method
Inverse variance weighted
MR Egger
5
4
3
2
1
1/SE_IV
−1.0
−0.5
0.0
0.5
β_IV

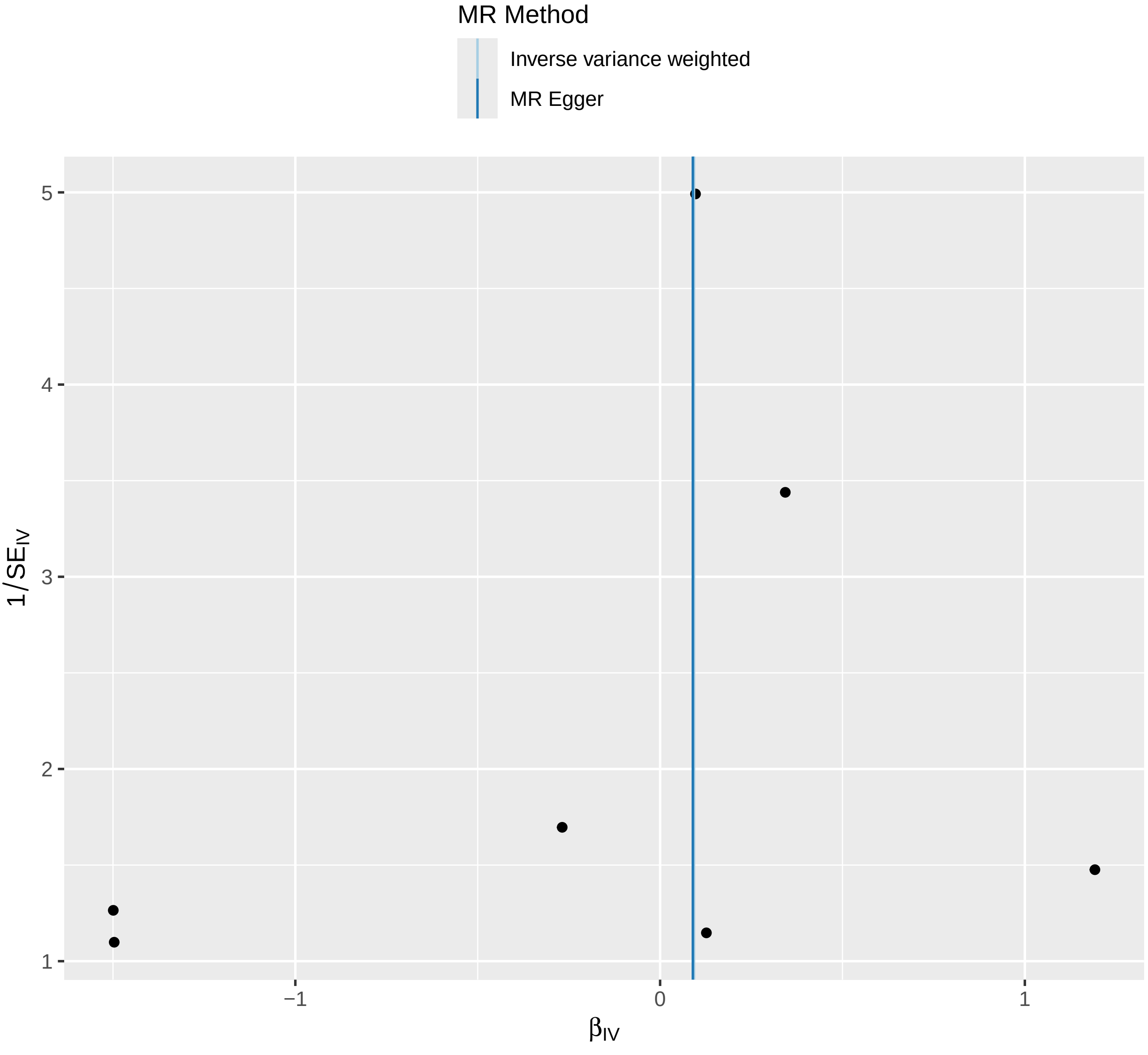

MR Method
Inverse variance weighted
MR Egger
5
4
3
2
1
−1
0
1
$1/SE_{IV}$
β_{IV}

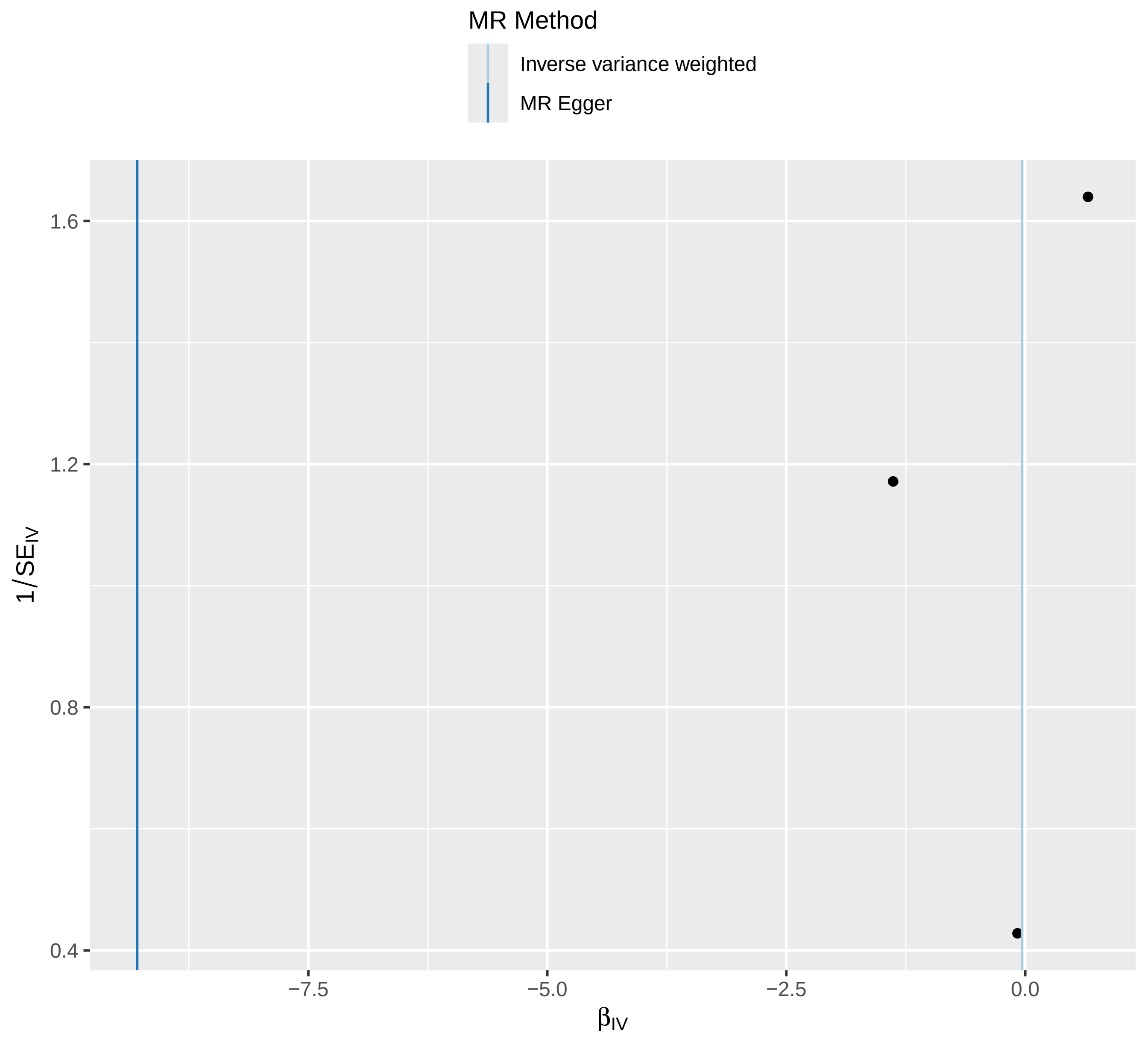

MR Method
Inverse variance weighted
MR Egger
1.6
1.2
0.8
0.4
$1/SE_{IV}$
−7.5
−5.0
−2.5
0.0
β_{IV}

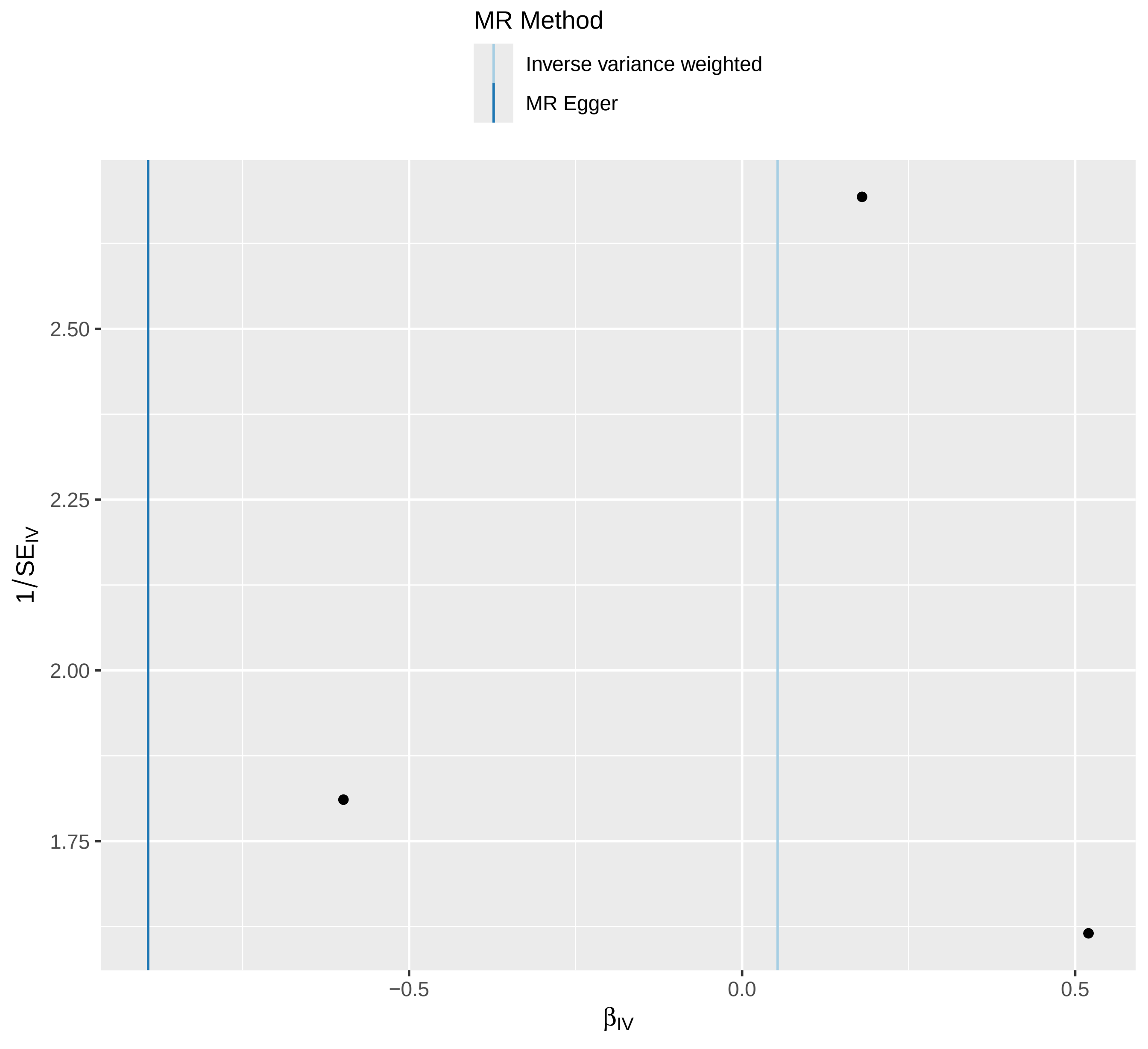

MR Method
Inverse variance weighted
MR Egger
2.50
2.25
2.00
1.75
1/SE$_{IV}$
−0.5
0.0
0.5
β$_{IV}$

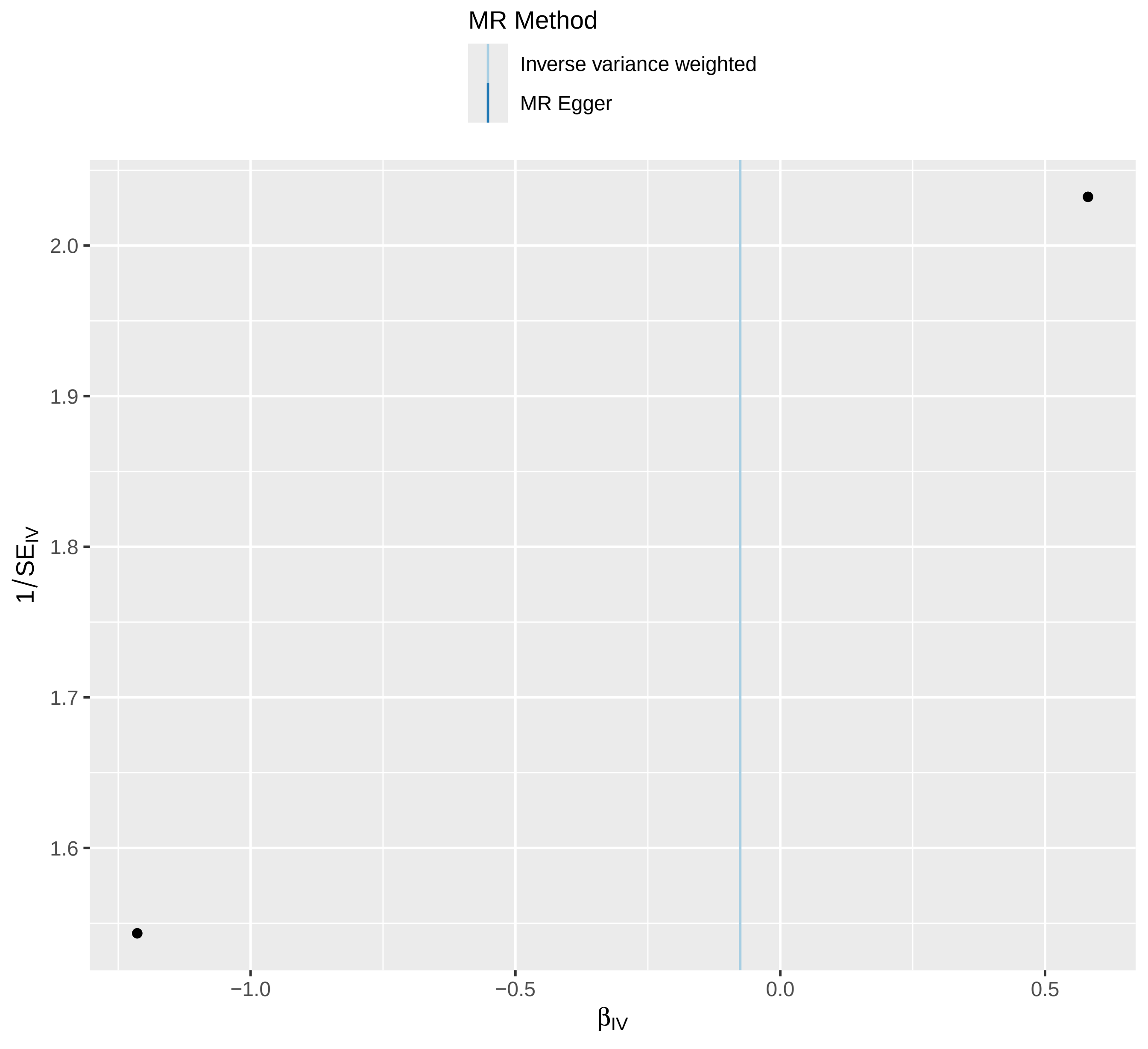
MR Method
Inverse variance weighted
MR Egger
2.0
1.9
1.8
1.7
1.6
$1/SE_{IV}$
−1.0
−0.5
0.0
0.5
β_{IV}

Insufficient number of SNPs

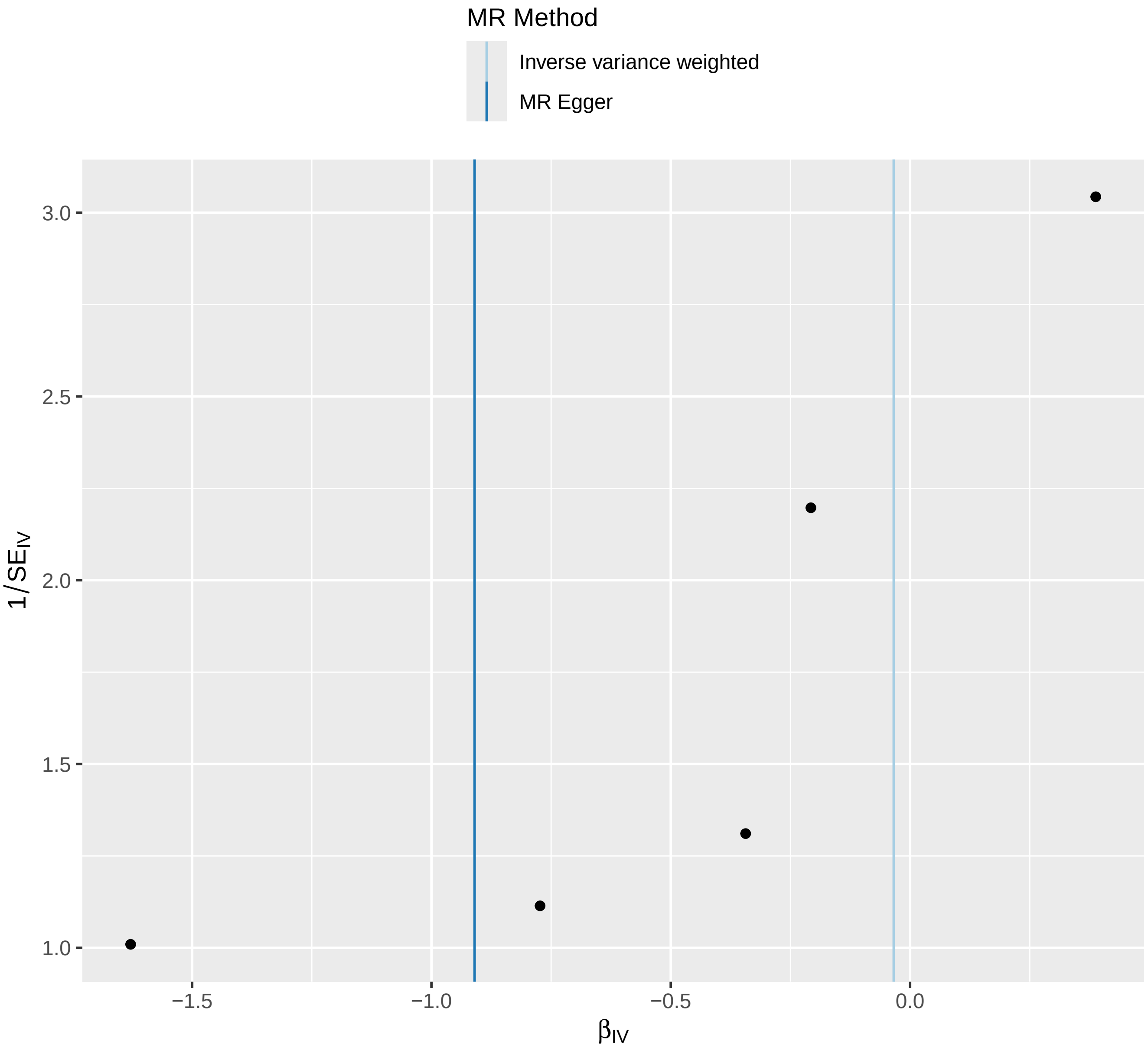

MR Method
Inverse variance weighted
MR Egger
3.0
2.5
2.0
1.5
1.0
1/SE_IV
−1.5
−1.0
−0.5
0.0
β_IV

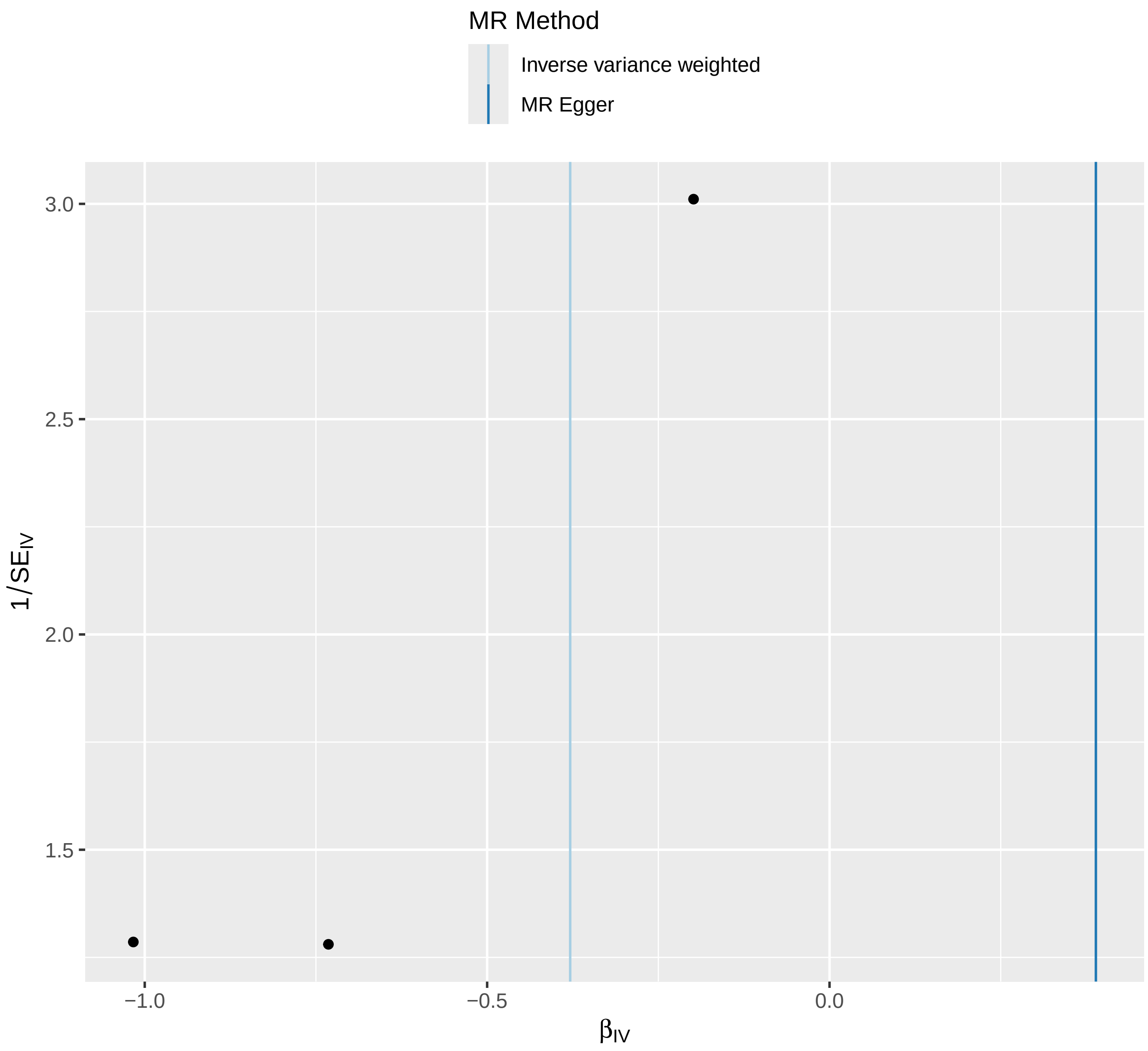
MR Method
Inverse variance weighted
MR Egger
3.0
2.5
2.0
1.5
1/SE_IV
−1.0
−0.5
0.0
β_IV

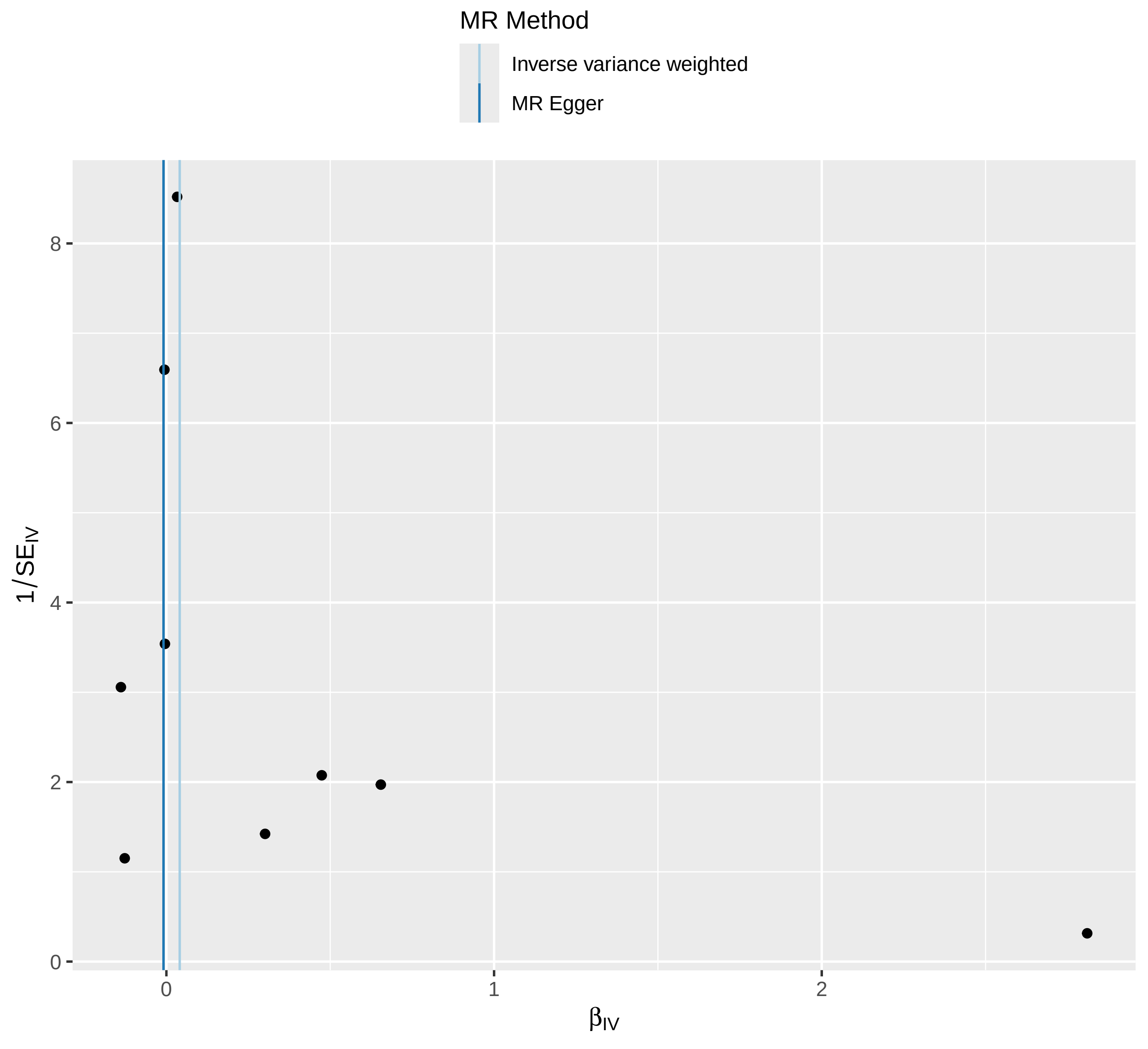

MR Method
Inverse variance weighted
MR Egger
1/SE_IV
β_IV
0
2
4
6
8
0
1
2

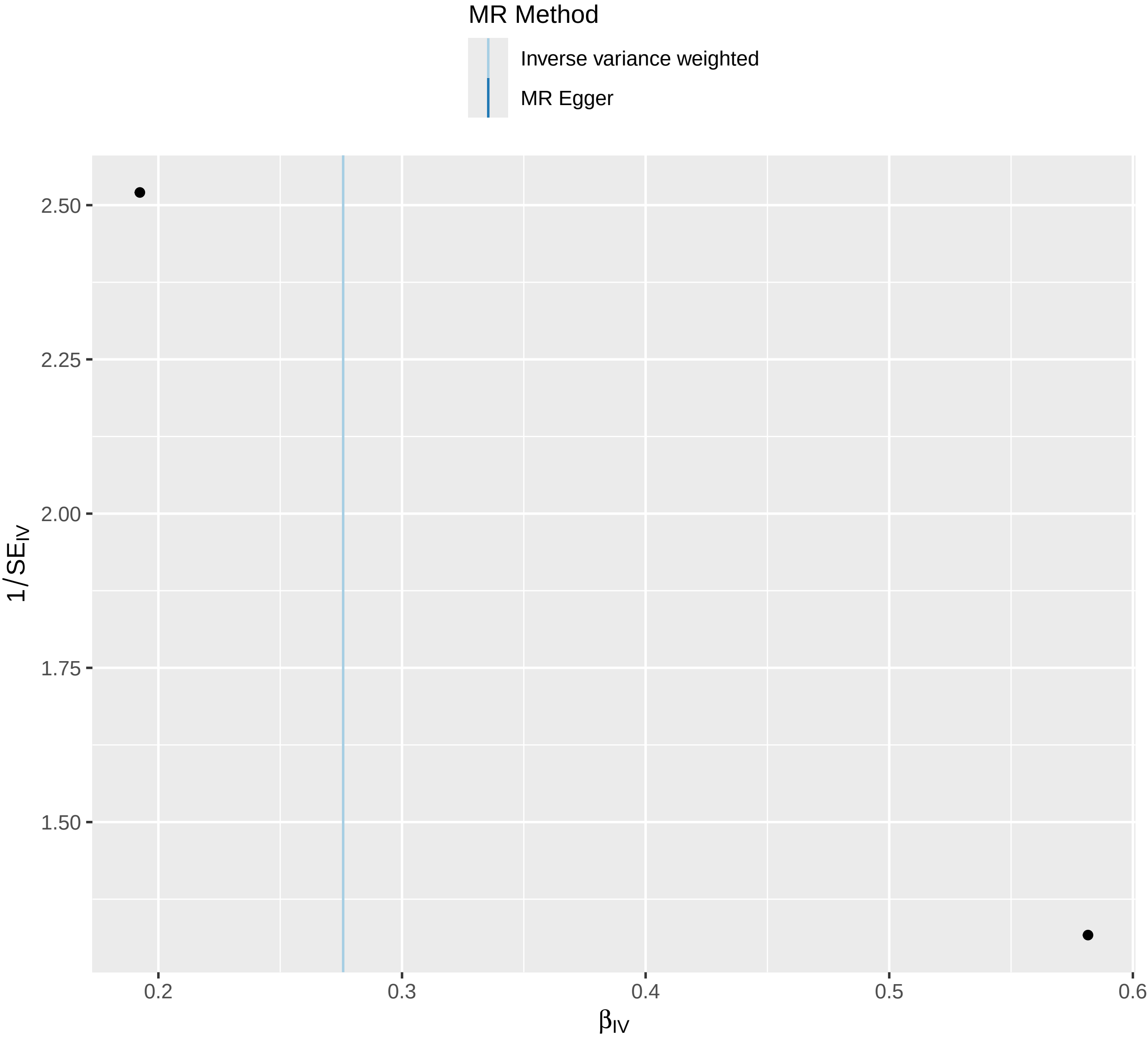
MR Method
Inverse variance weighted
MR Egger
2.50
2.25
2.00
1.75
1.50
1/SE_IV
0.2
0.3
0.4
0.5
0.6
β_IV

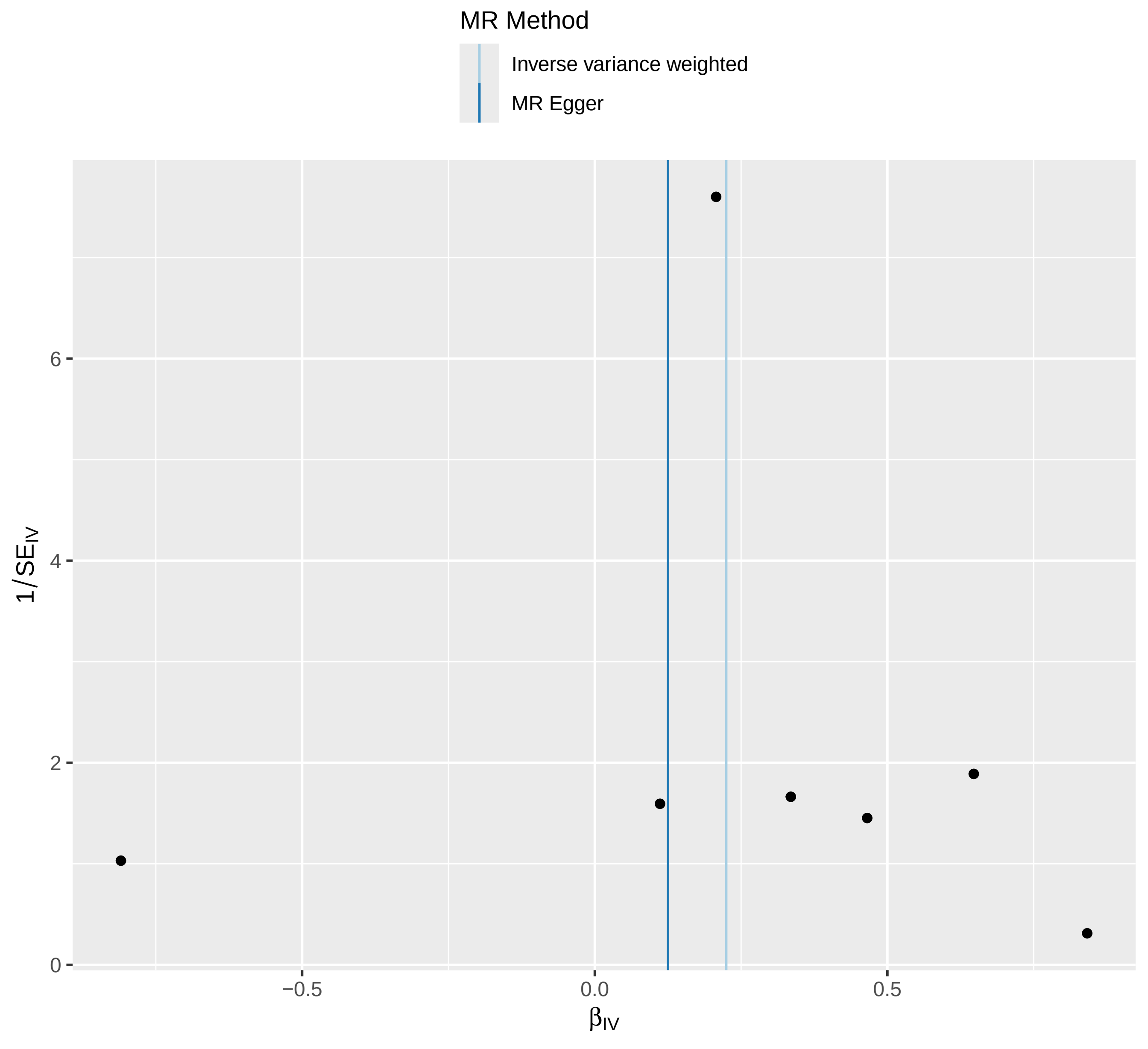
MR Method
Inverse variance weighted
MR Egger
6
4
2
0
1/SE_IV
−0.5
0.0
0.5
β_IV

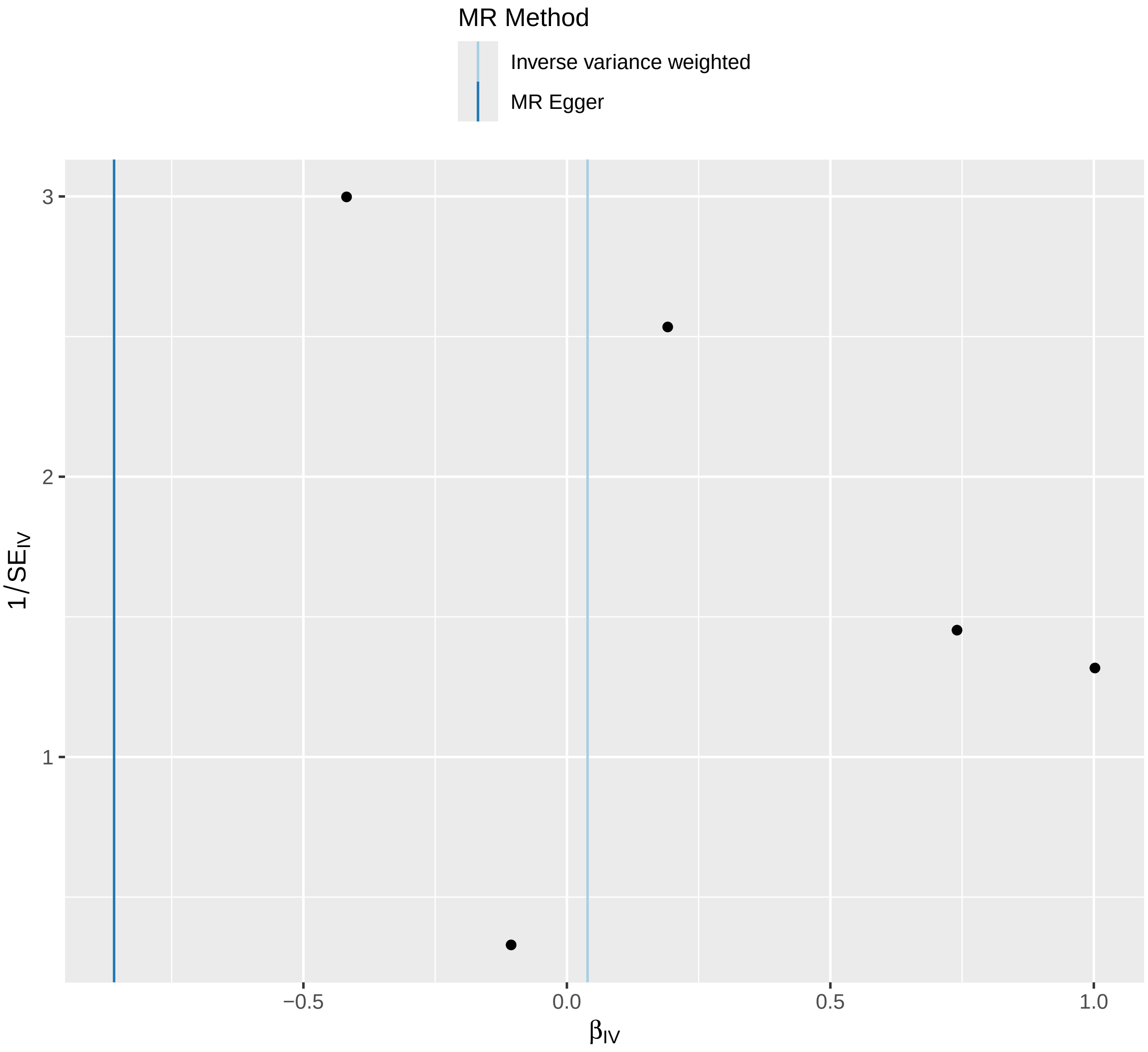
MR Method
Inverse variance weighted
MR Egger
1/SE$_{IV}$
3
2
1
−0.5
0.0
0.5
1.0
β$_{IV}$

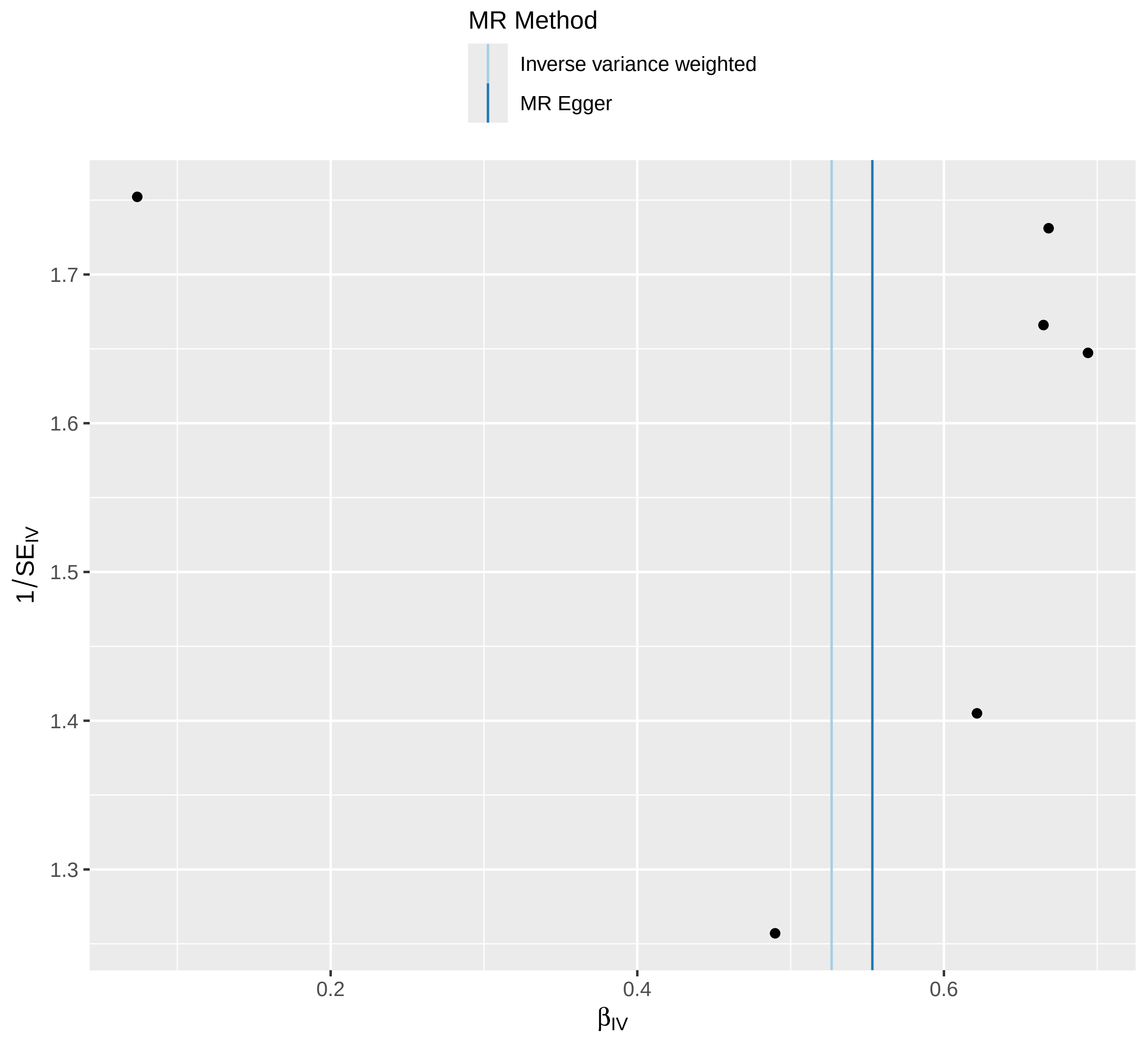
MR Method
Inverse variance weighted
MR Egger
1.7
1.6
1.5
1.4
1.3
1/SE_{IV}
0.2
0.4
0.6
β_{IV}

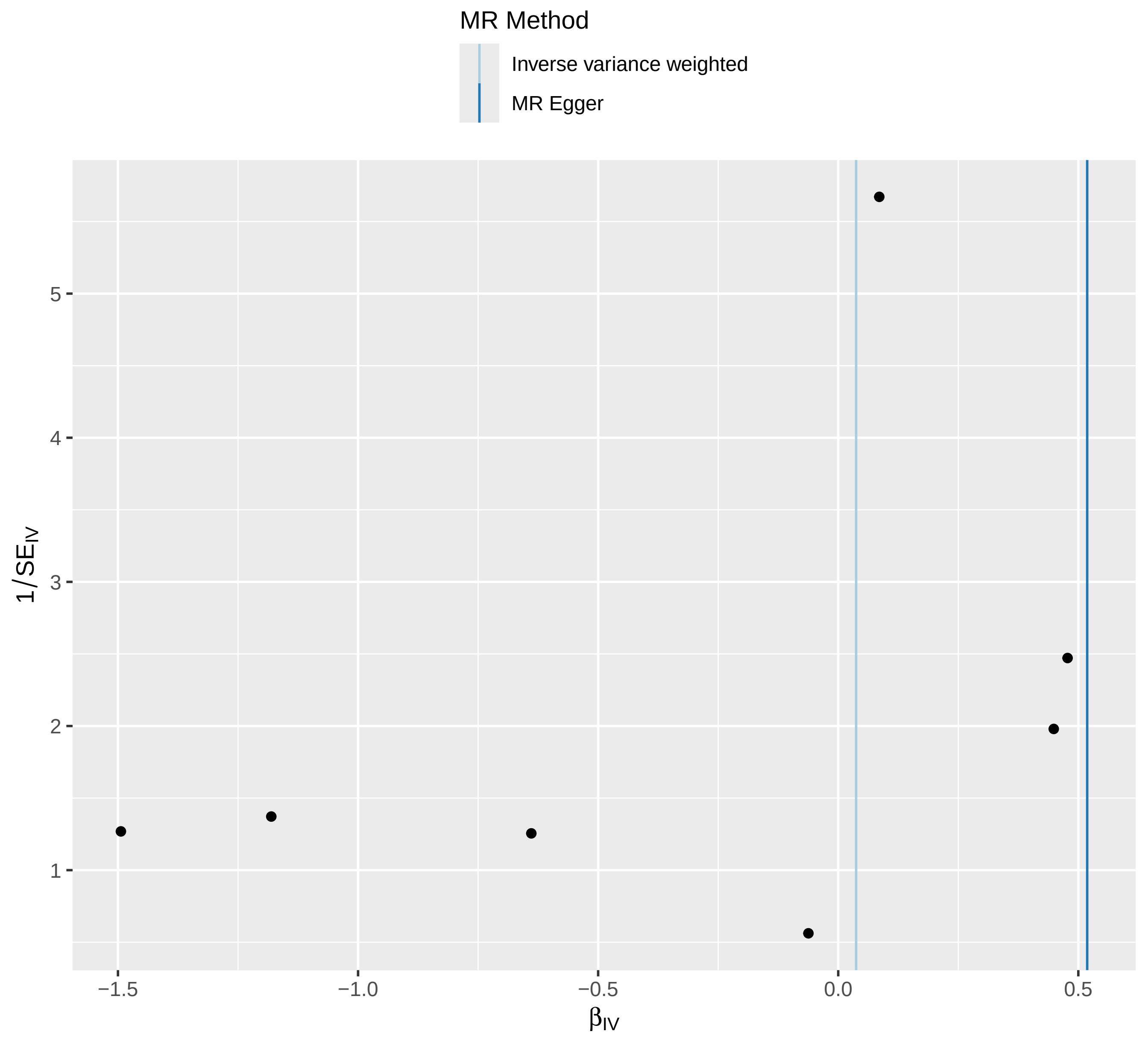

MR Method
Inverse variance weighted
MR Egger
1/SE_IV
5
4
3
2
1
−1.5
−1.0
−0.5
0.0
0.5
β_IV

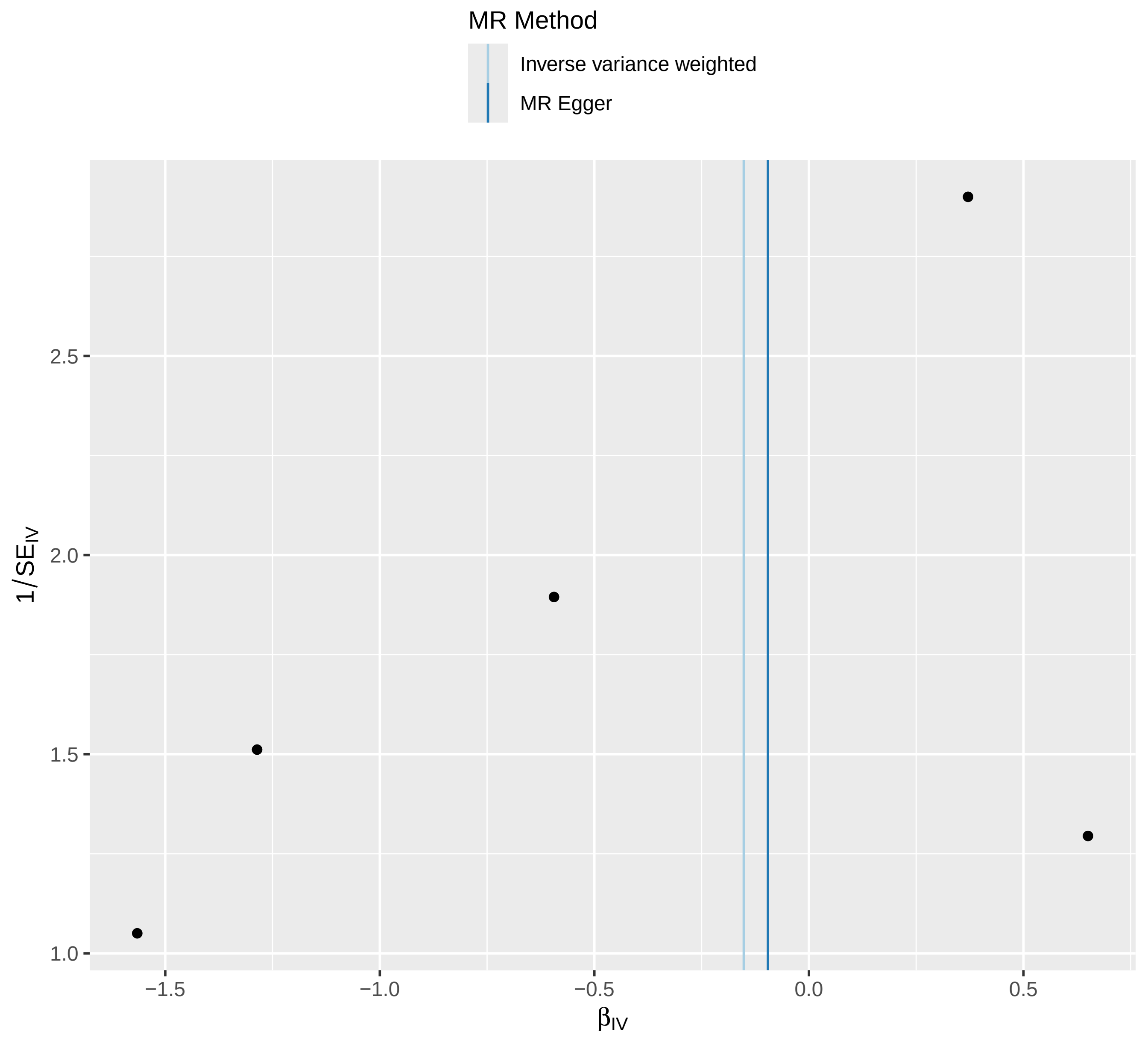
MR Method
Inverse variance weighted
MR Egger
1/SE_IV
2.5
2.0
1.5
1.0
−1.5
−1.0
−0.5
0.0
0.5
β_IV

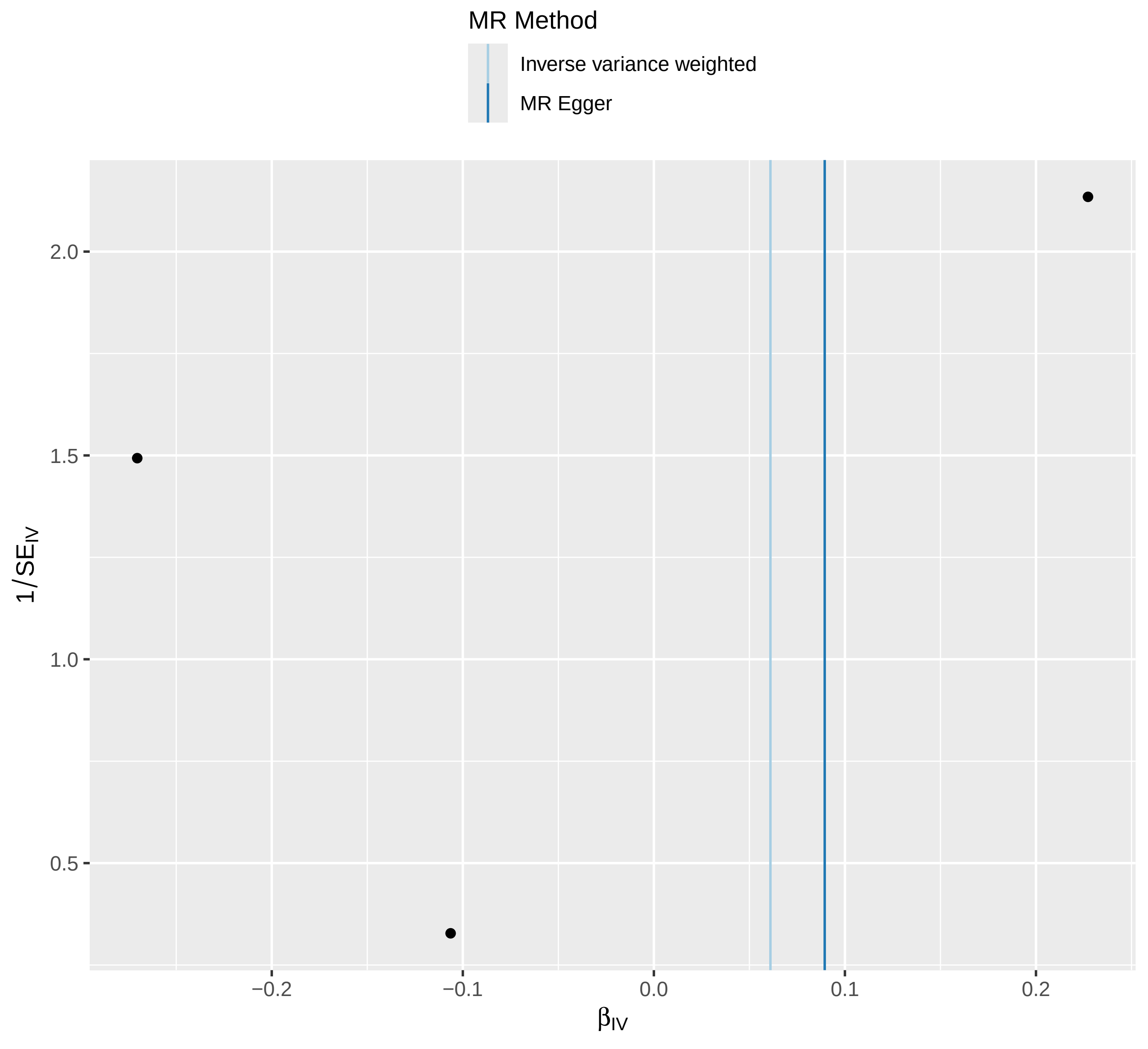
MR Method
Inverse variance weighted
MR Egger
2.0
1.5
1.0
0.5
−0.2
−0.1
0.0
0.1
0.2
$1/SE_{IV}$
β_{IV}

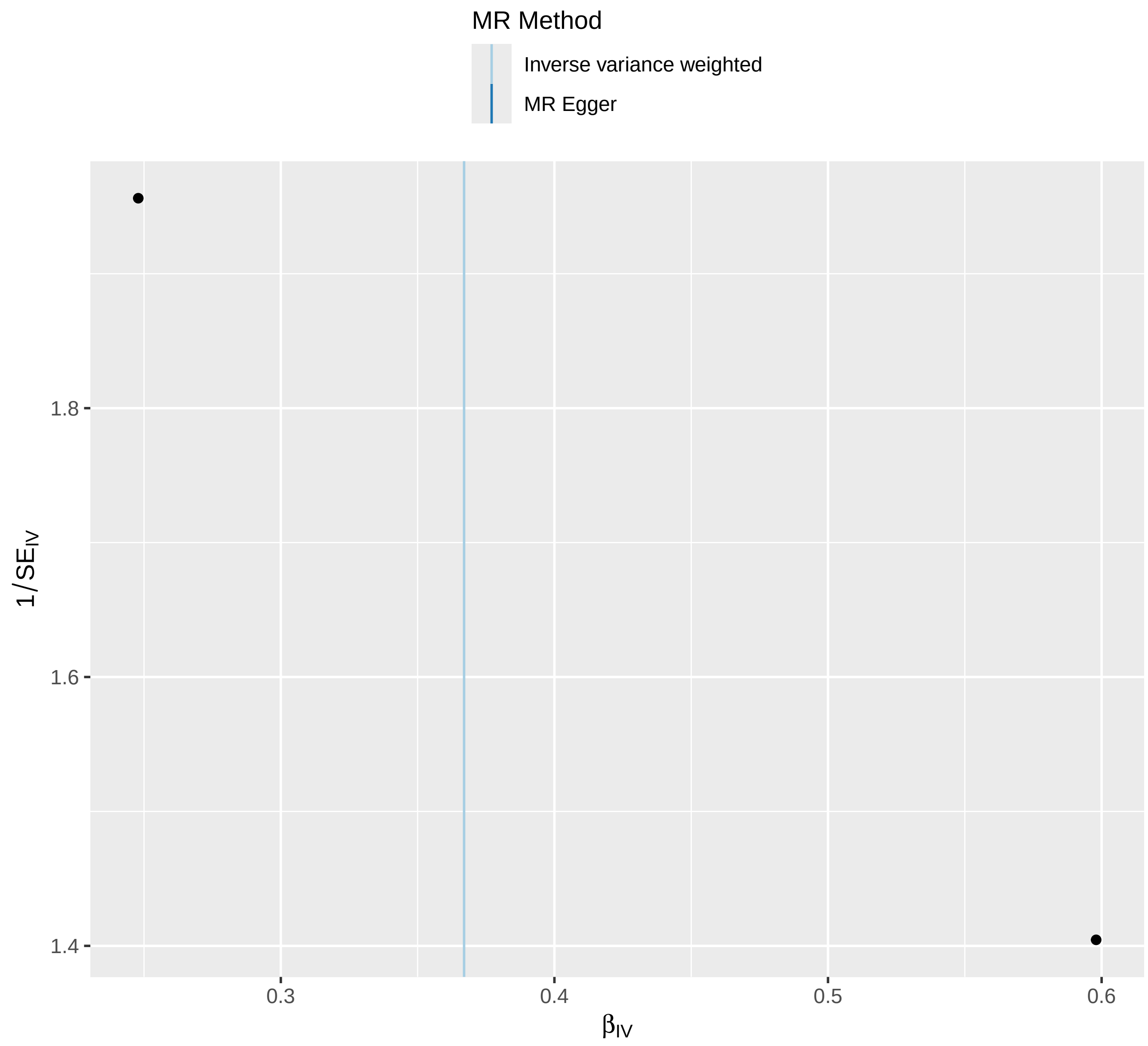
MR Method
Inverse variance weighted
MR Egger
1.8
1.6
1.4
$1/SE_{IV}$
0.3
0.4
0.5
0.6
β_{IV}

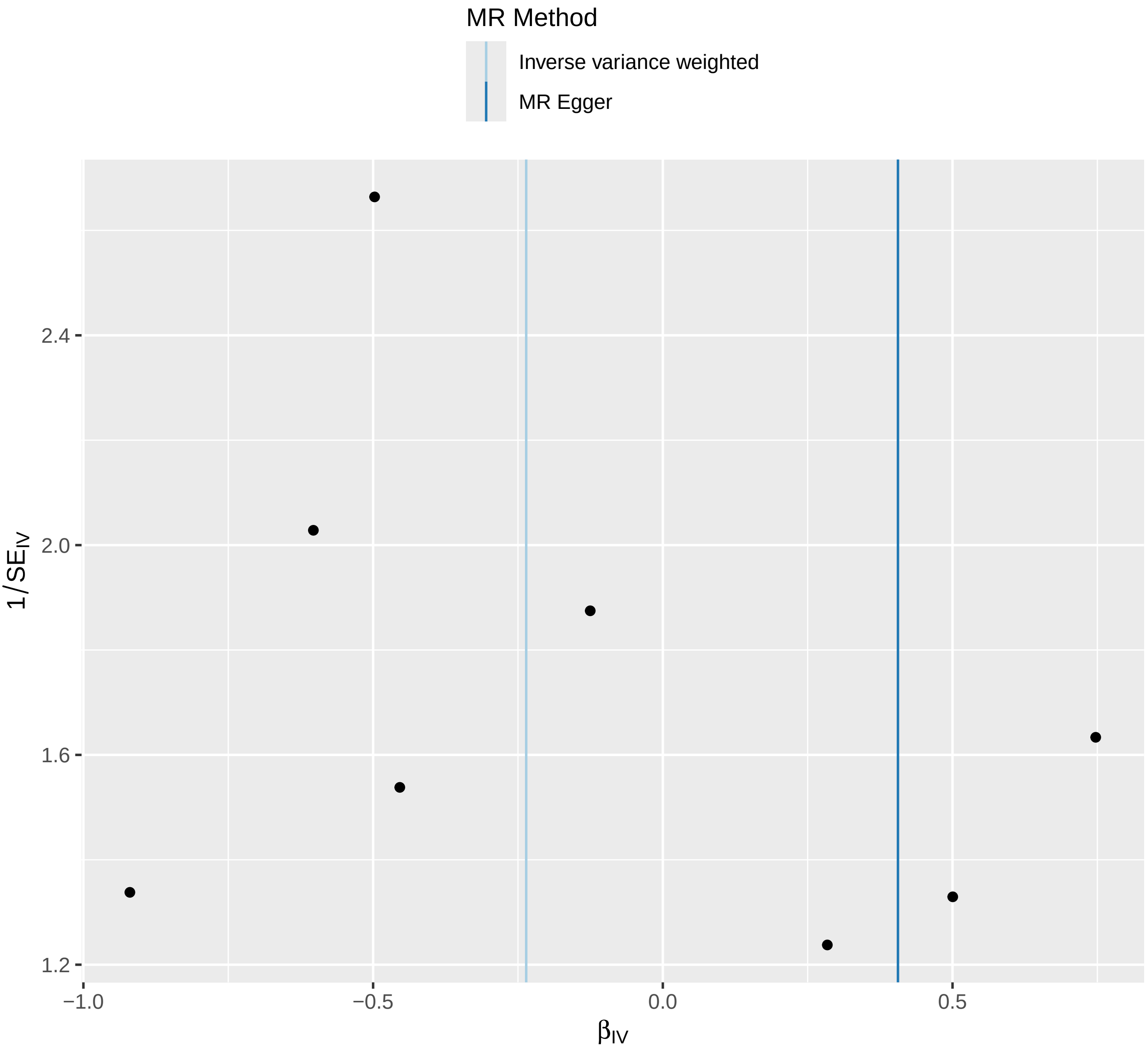
MR Method
Inverse variance weighted
MR Egger
2.4
2.0
1.6
1.2
$1/SE_{IV}$
−1.0
−0.5
0.0
0.5
β_{IV}

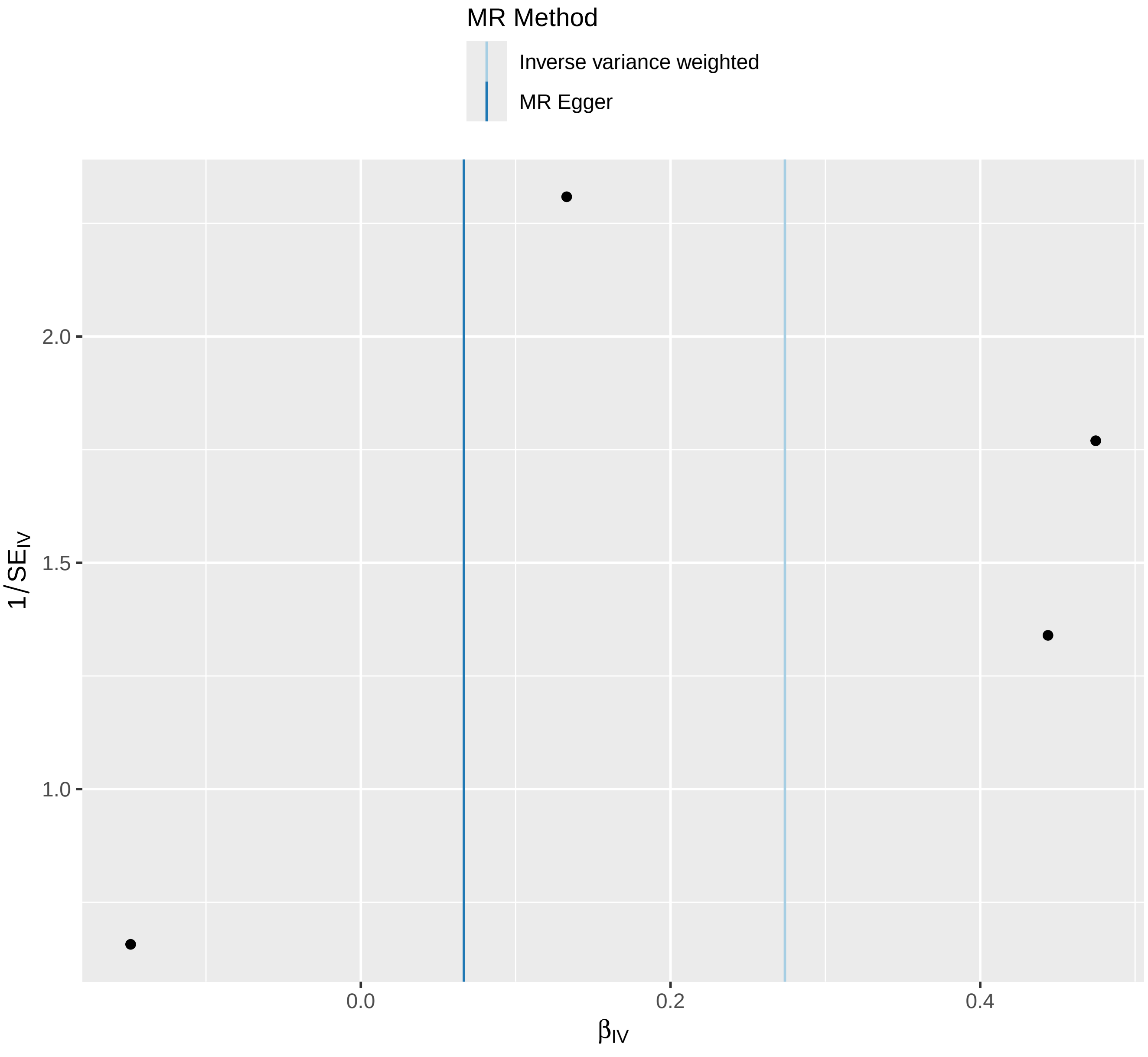

MR Method
Inverse variance weighted
MR Egger
2.0
1.5
1.0
1/SE$_{IV}$
0.0
0.2
0.4
β_{IV}

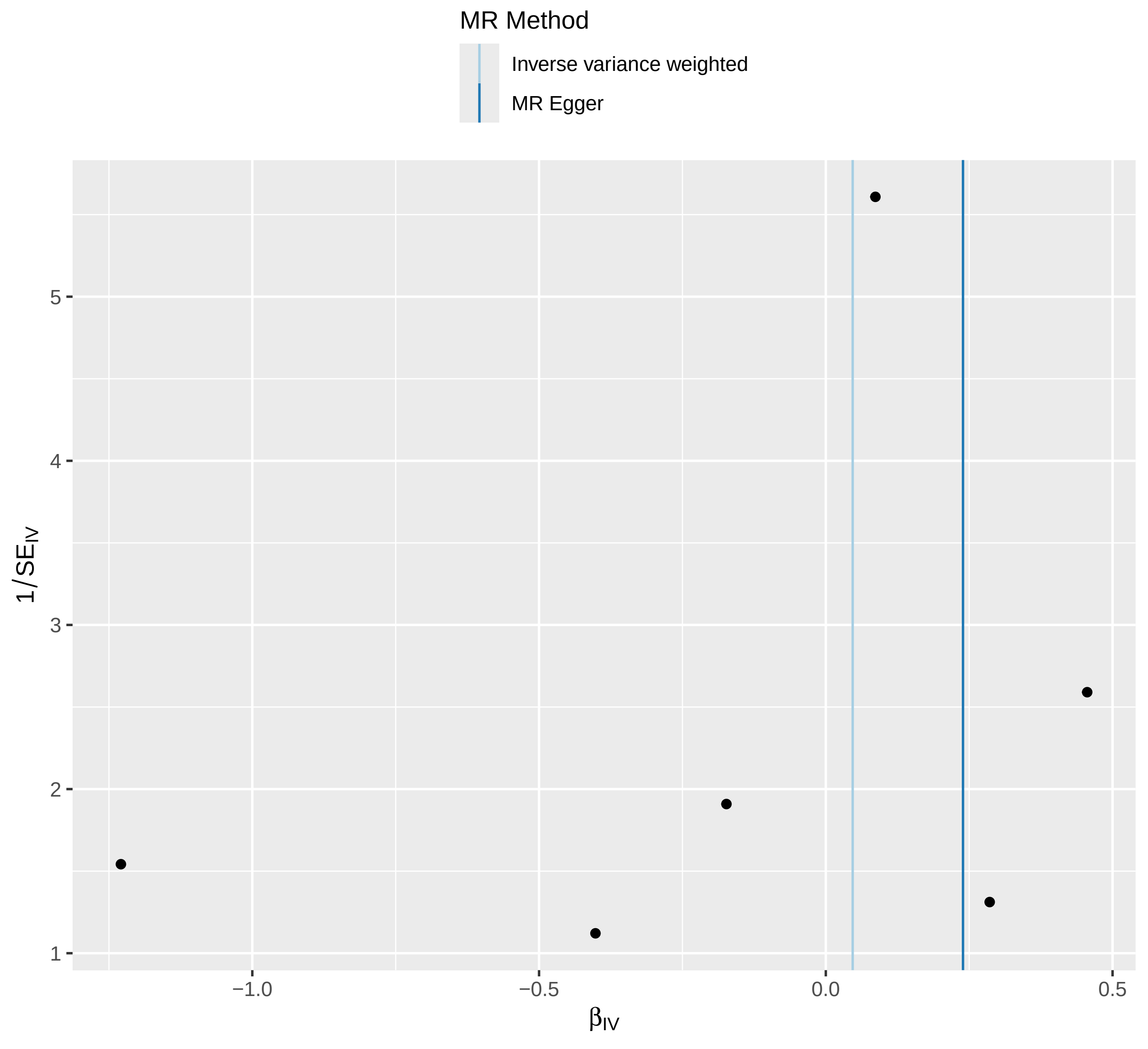
MR Method
Inverse variance weighted
MR Egger
1/SE$_{IV}$
1
2
3
4
5
−1.0
−0.5
0.0
0.5
β$_{IV}$

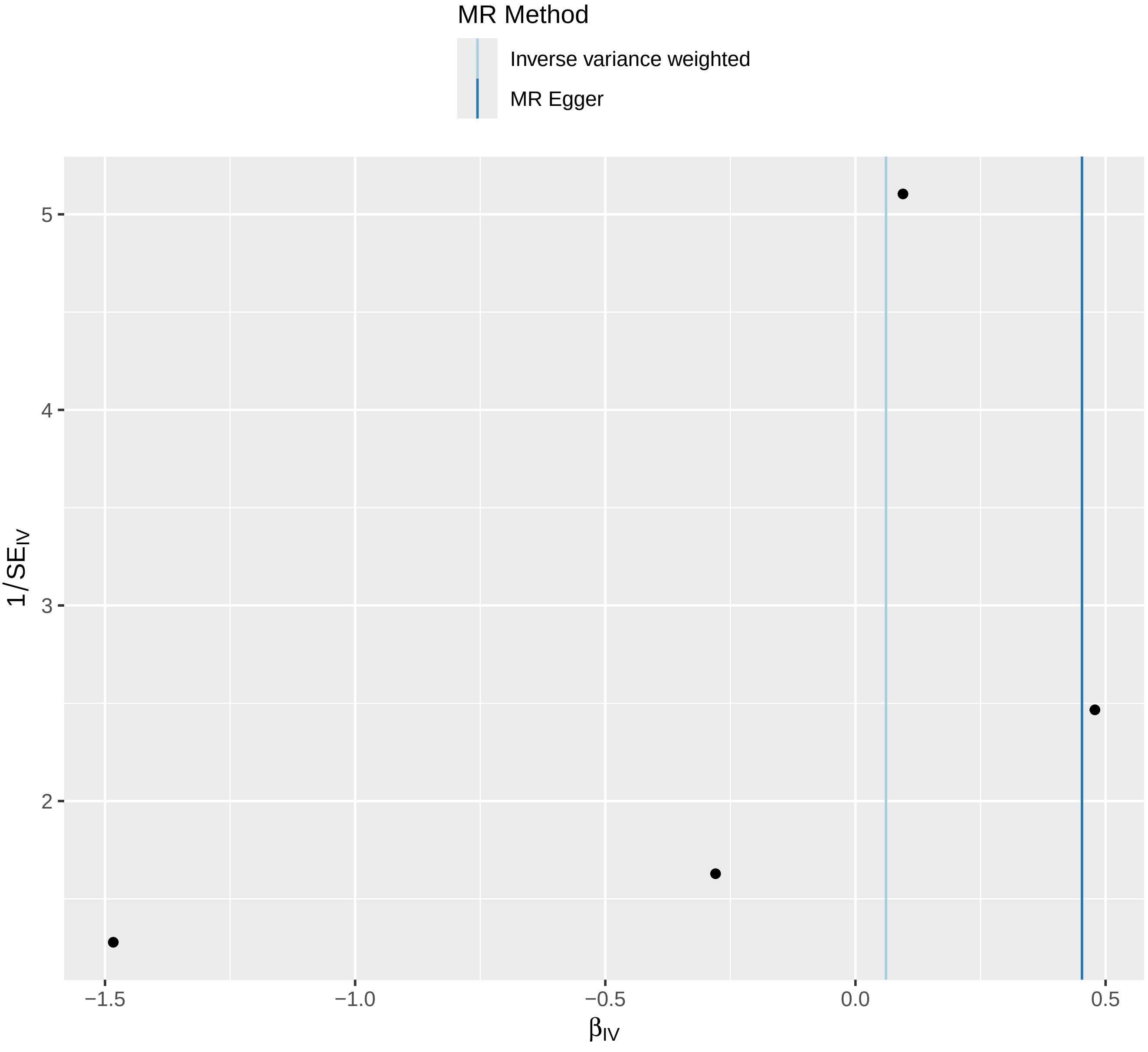
MR Method
Inverse variance weighted
MR Egger
5
4
3
2
1/SE_IV
−1.5
−1.0
−0.5
0.0
0.5
β_IV

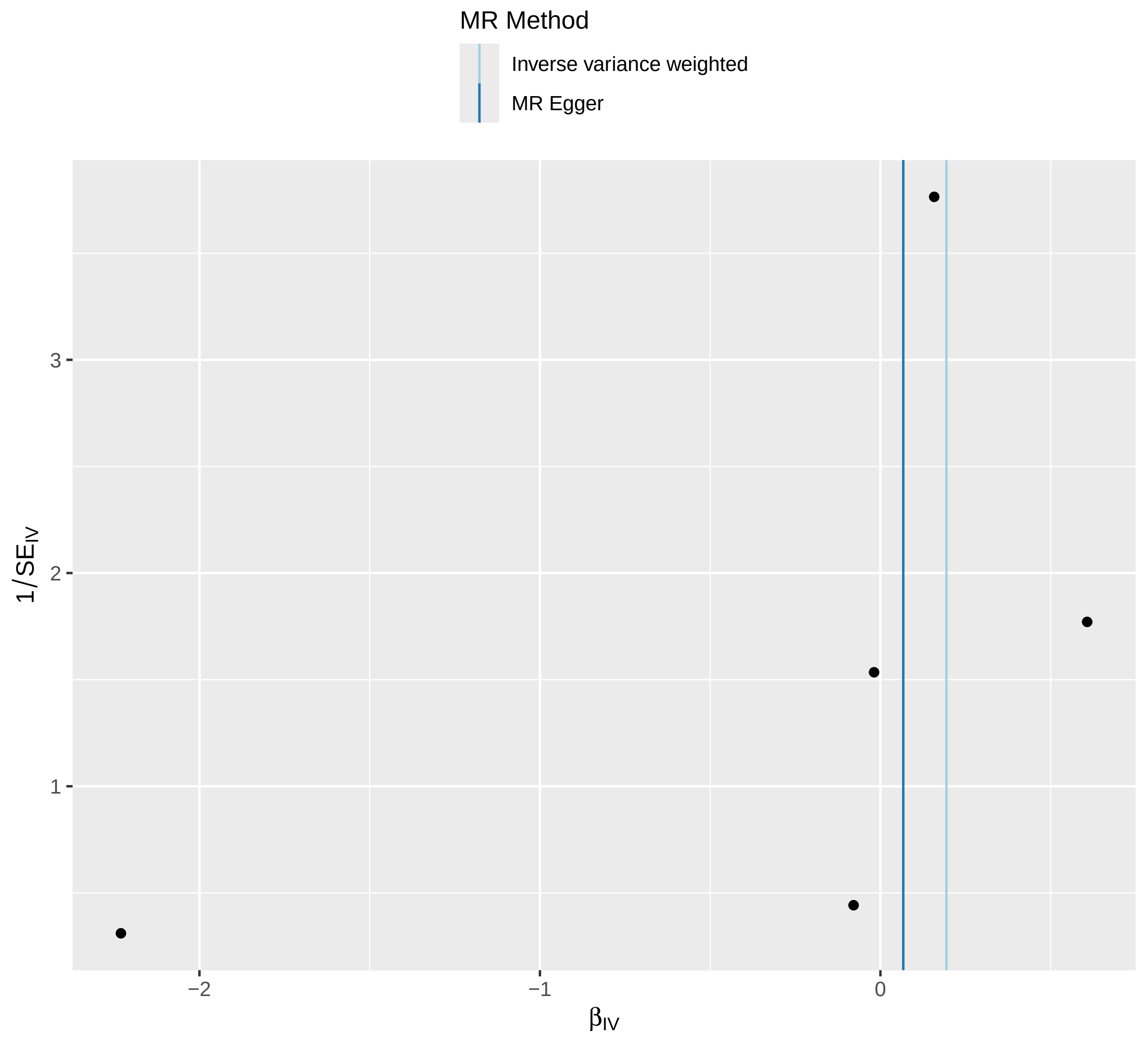

MR Method
Inverse variance weighted
MR Egger
3
2
1
1/SE_IV
−2
−1
0
β_IV

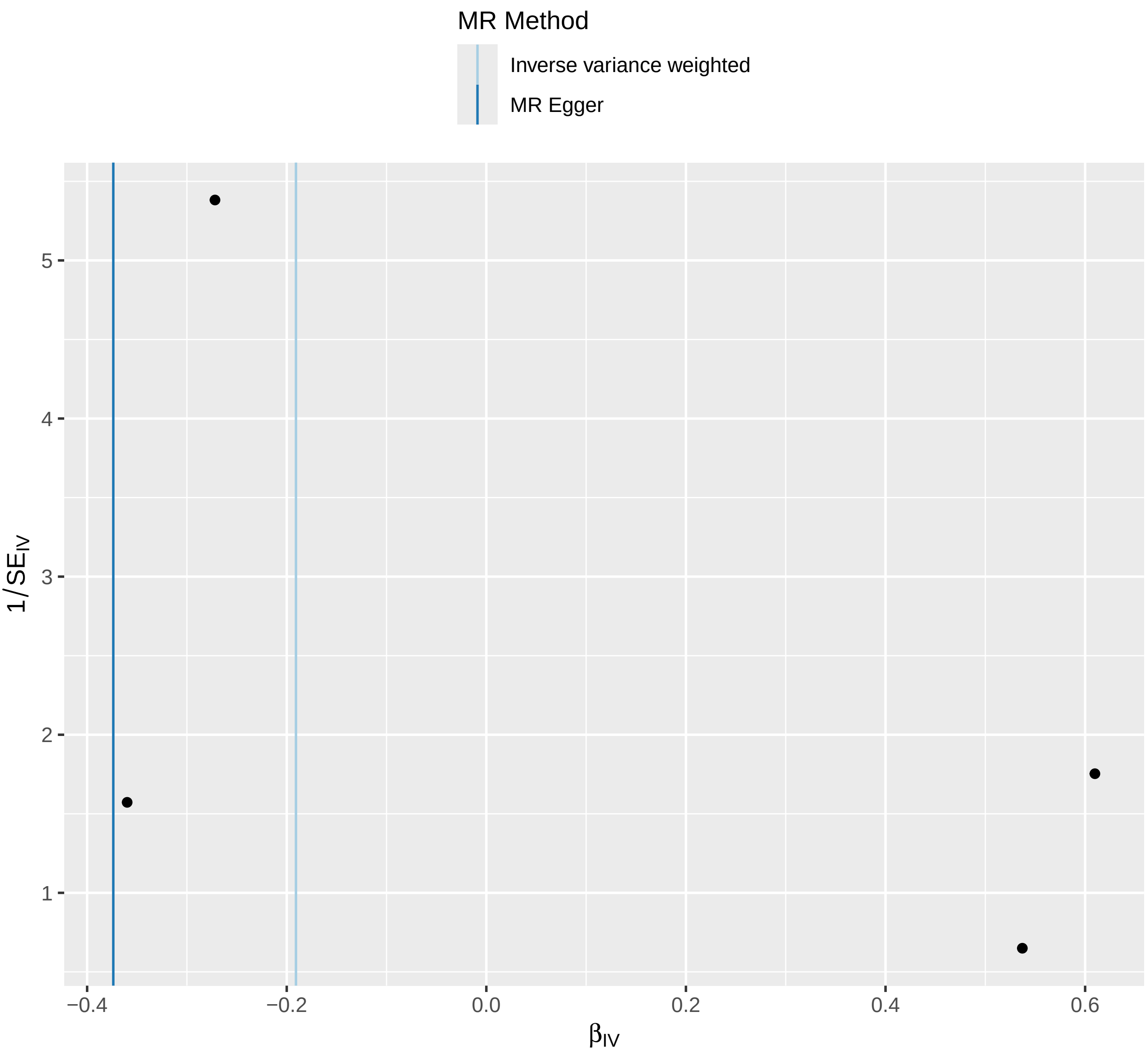
MR Method
Inverse variance weighted
MR Egger
5
4
3
2
1
1/SE$_{IV}$
−0.4
−0.2
0.0
0.2
0.4
0.6
β$_{IV}$

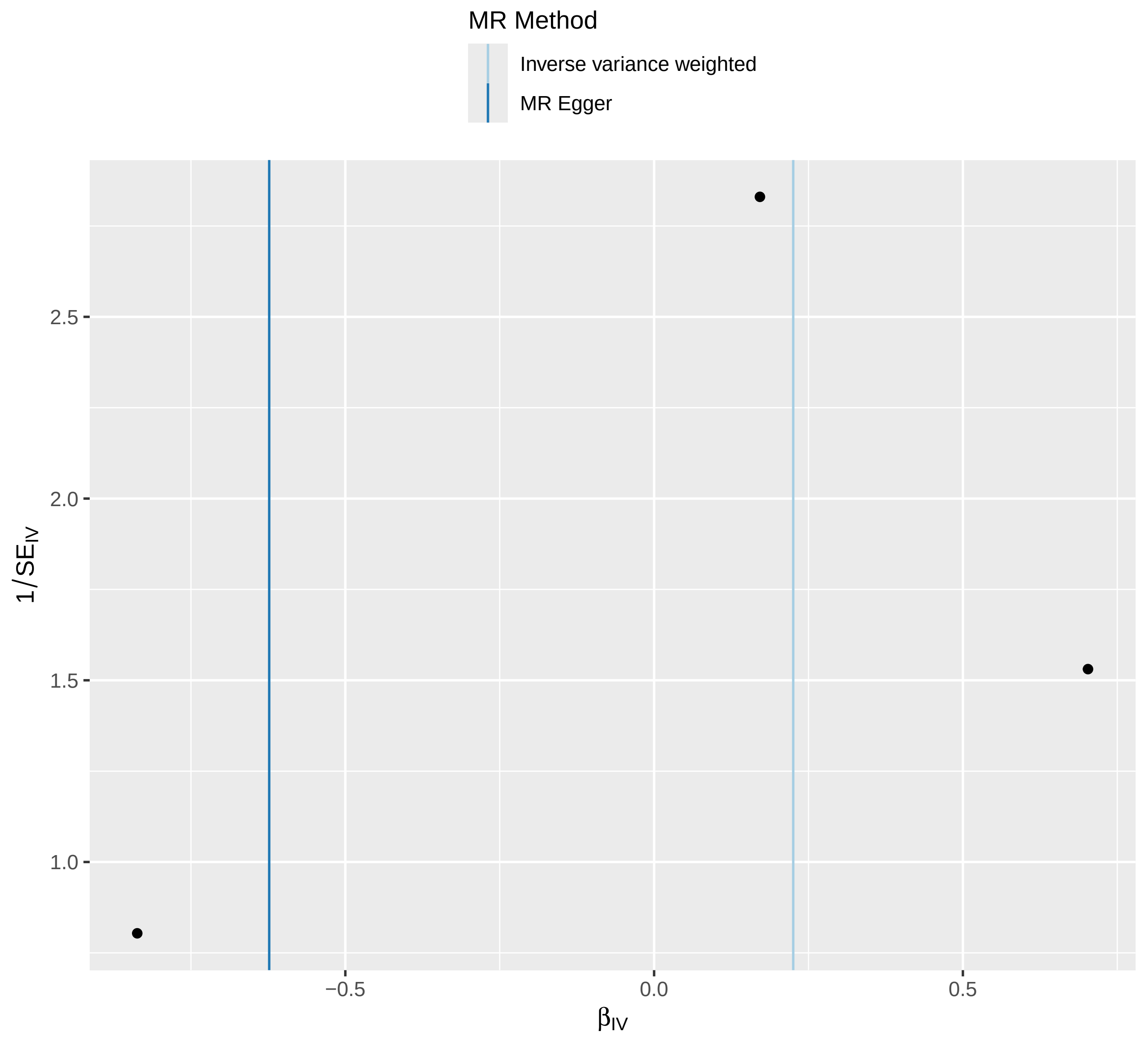
MR Method
Inverse variance weighted
MR Egger
2.5
2.0
1.5
1.0
1/SE_IV
−0.5
0.0
0.5
β_IV

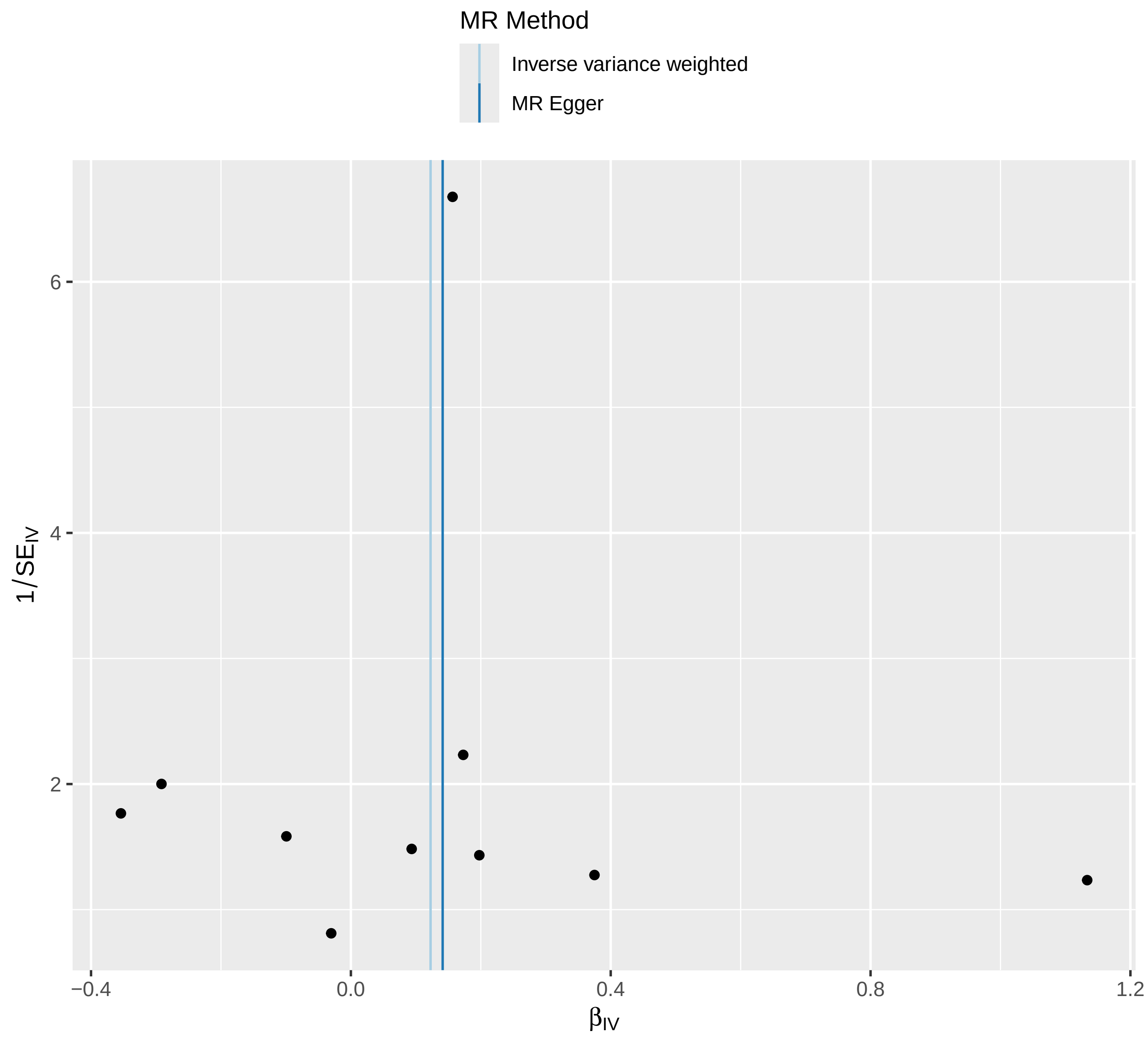

MR Method
Inverse variance weighted
MR Egger
6
4
2
$1/SE_{IV}$
−0.4
0.0
0.4
0.8
1.2
β_{IV}

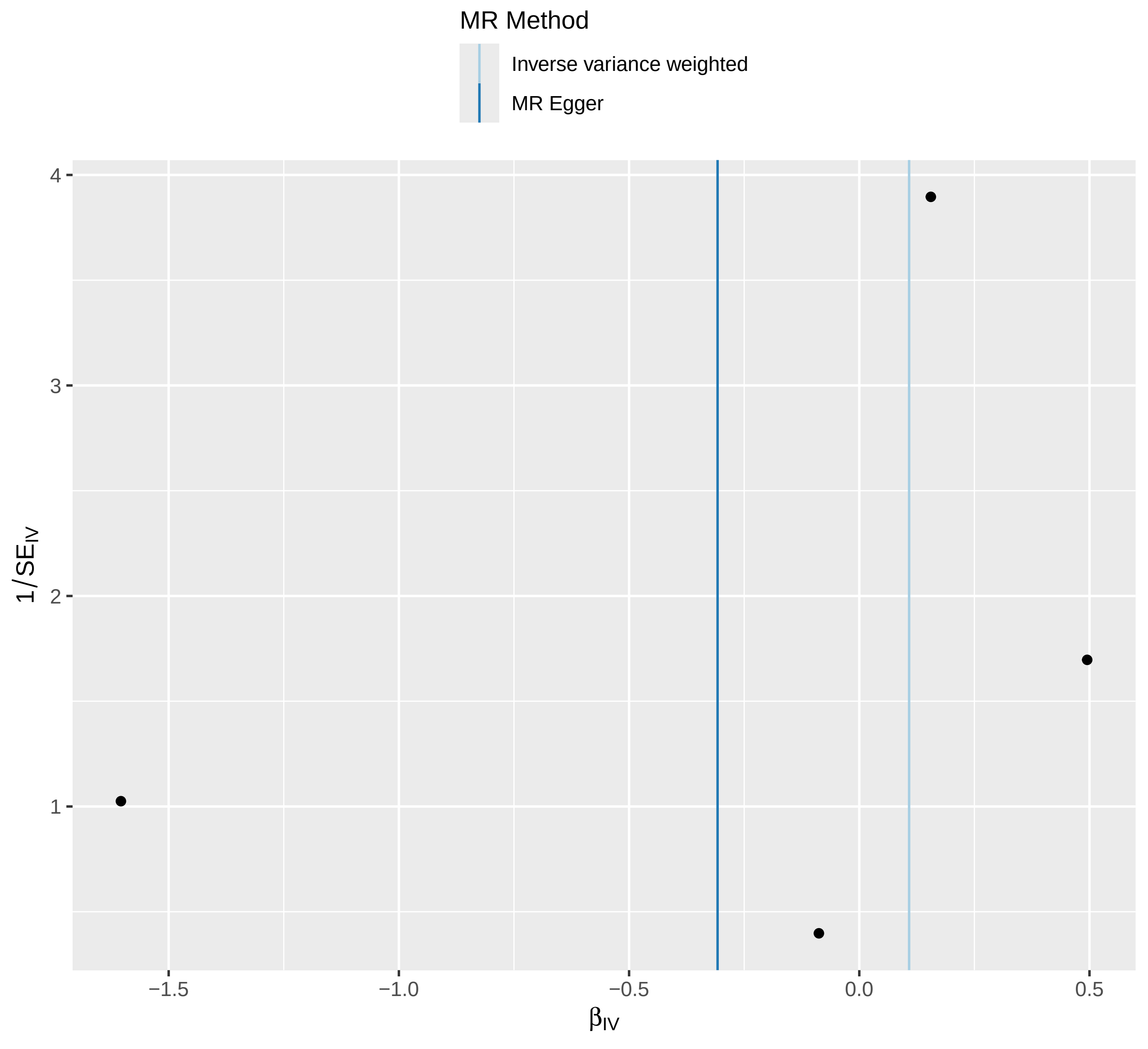
MR Method
Inverse variance weighted
MR Egger
4
3
2
1
$1/SE_{IV}$
−1.5
−1.0
−0.5
0.0
0.5
β_{IV}

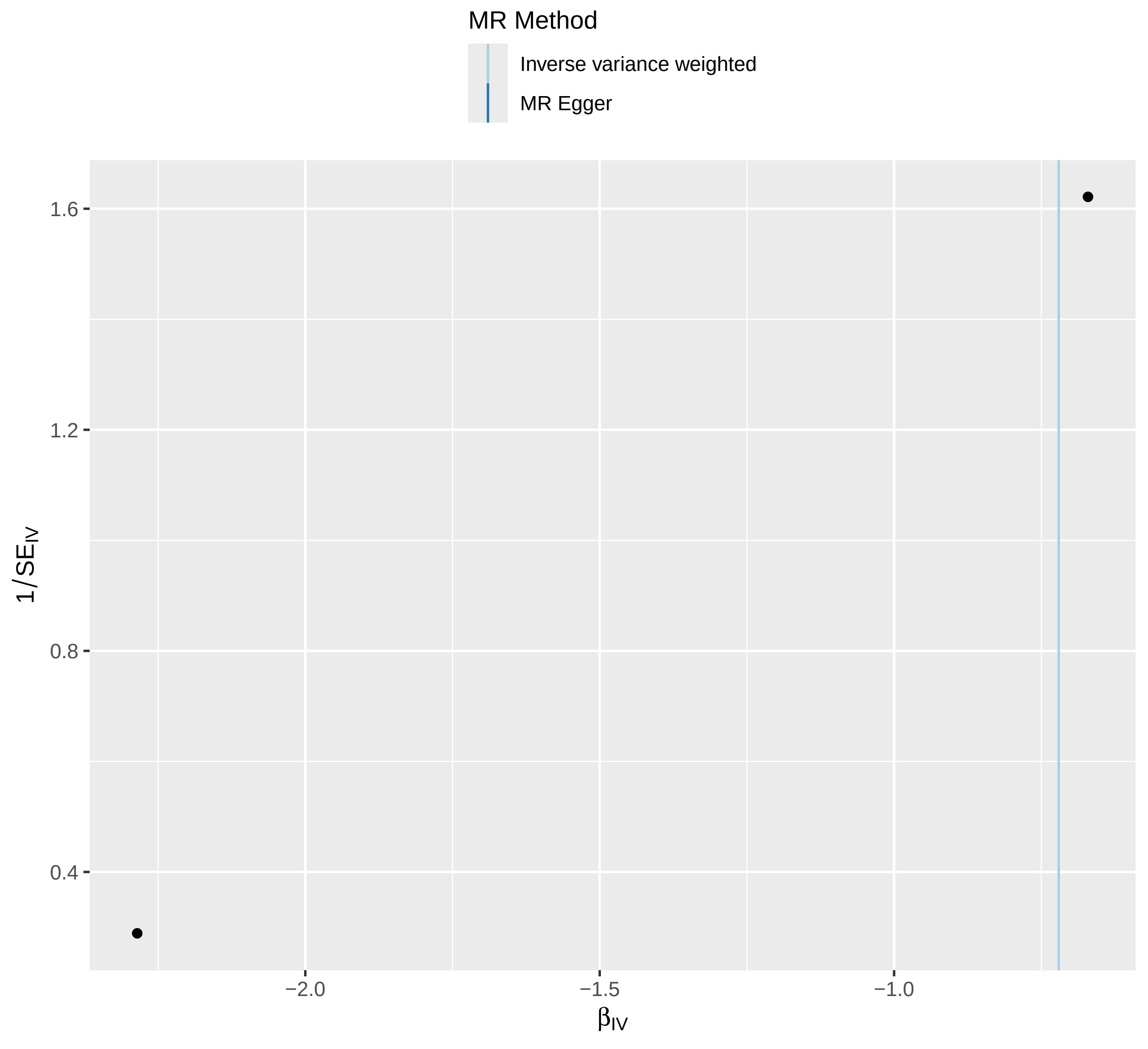
MR Method
Inverse variance weighted
MR Egger
1.6
1.2
0.8
0.4
1/SE_IV
−2.0
−1.5
−1.0
β_IV

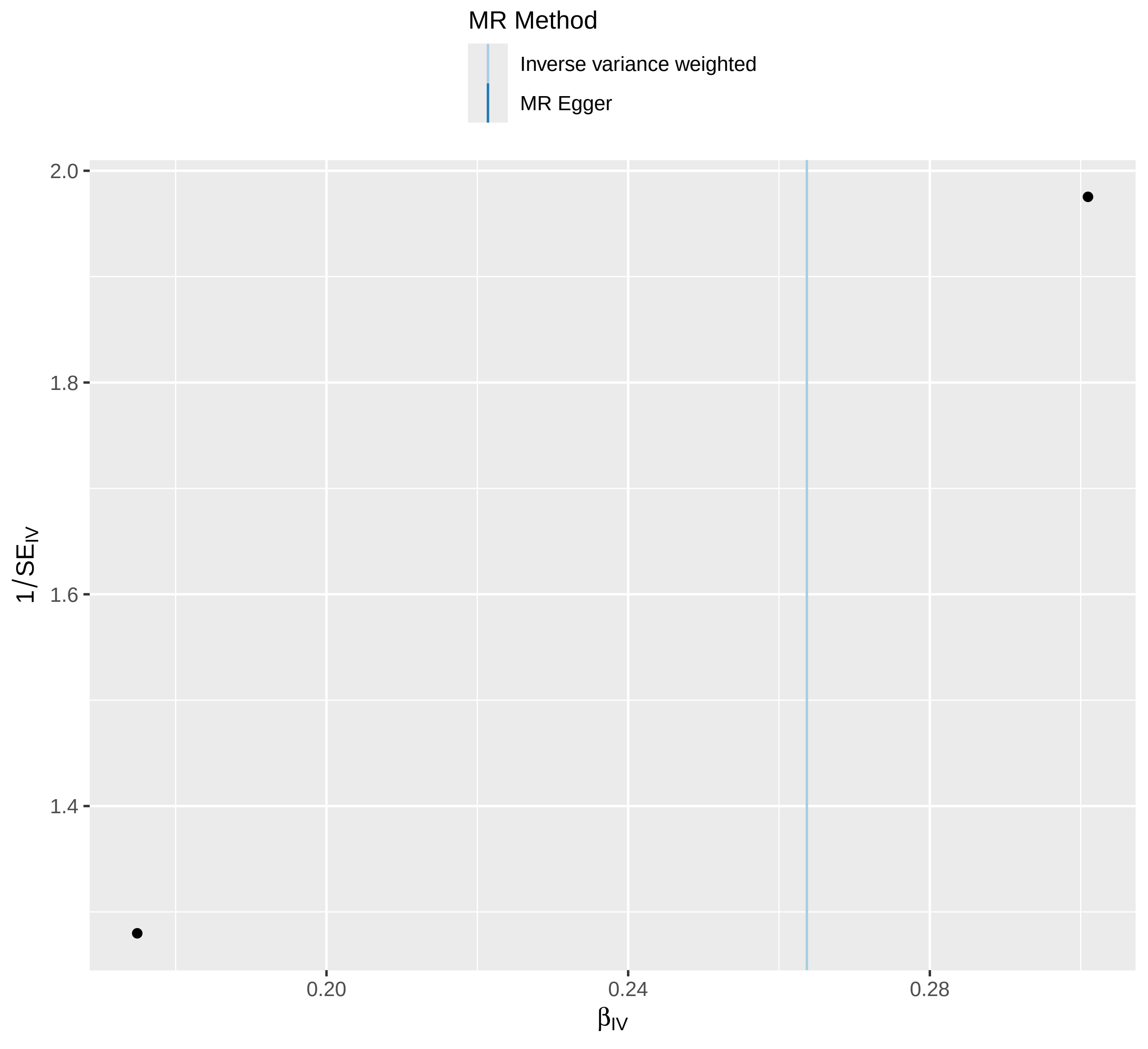
MR Method
Inverse variance weighted
MR Egger
2.0
1.8
1.6
1.4
1/SE_IV
0.20
0.24
0.28
β_IV

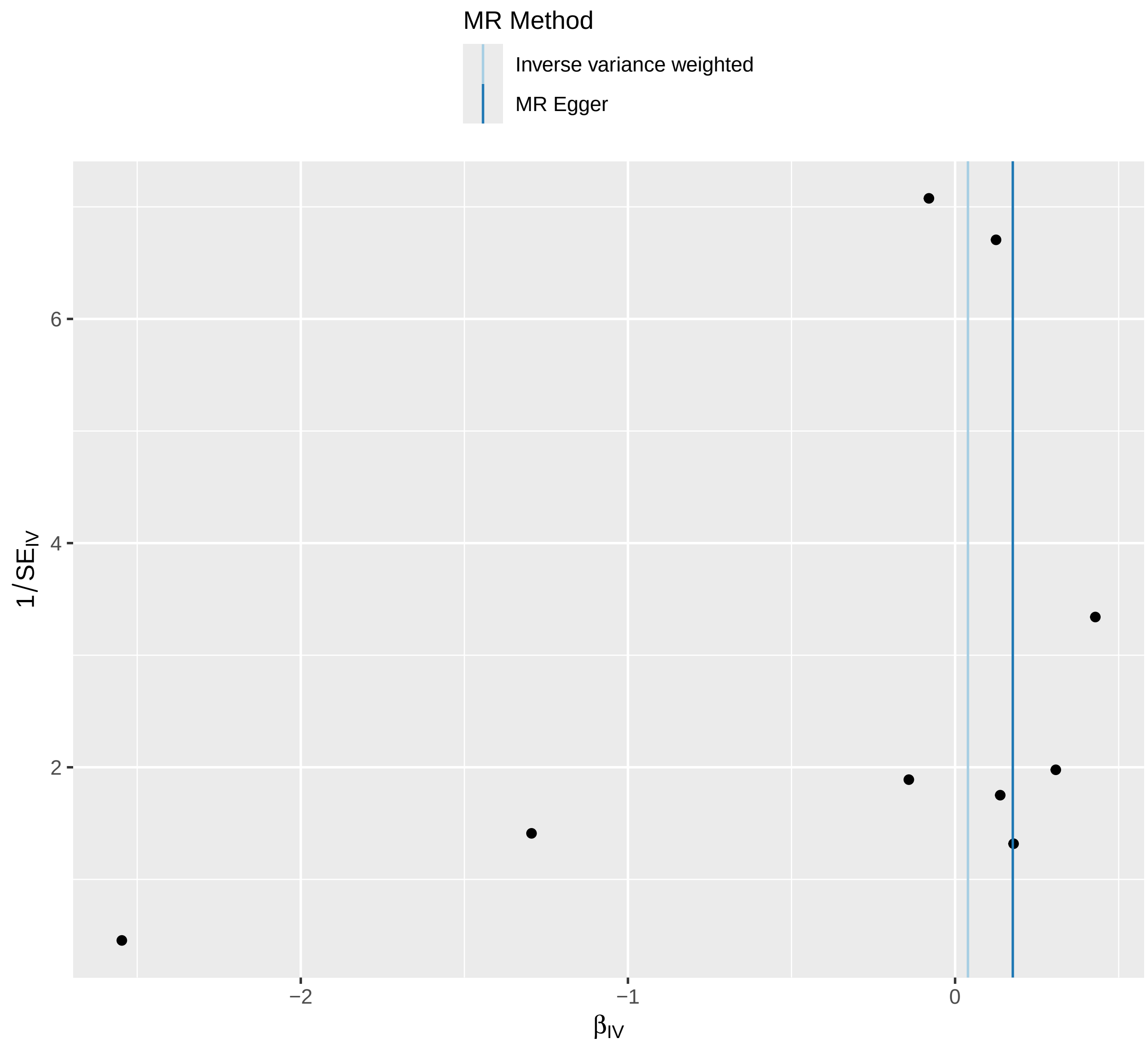
MR Method
Inverse variance weighted
MR Egger
6
4
2
−2
−1
0
$1/SE_{IV}$
β_{IV}

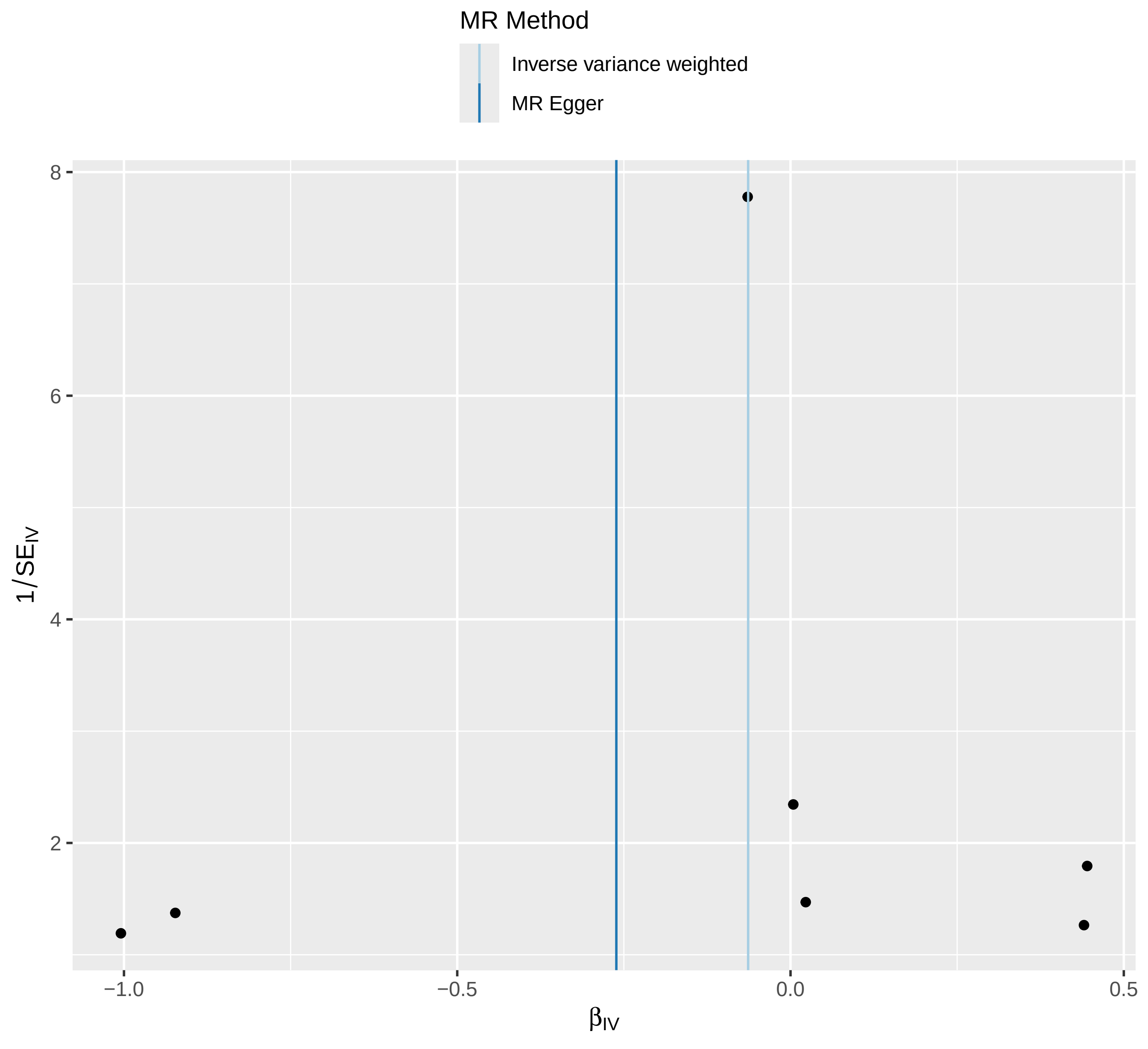
MR Method
Inverse variance weighted
MR Egger
8
6
4
2
1/SE$_{IV}$
−1.0
−0.5
0.0
0.5
β$_{IV}$

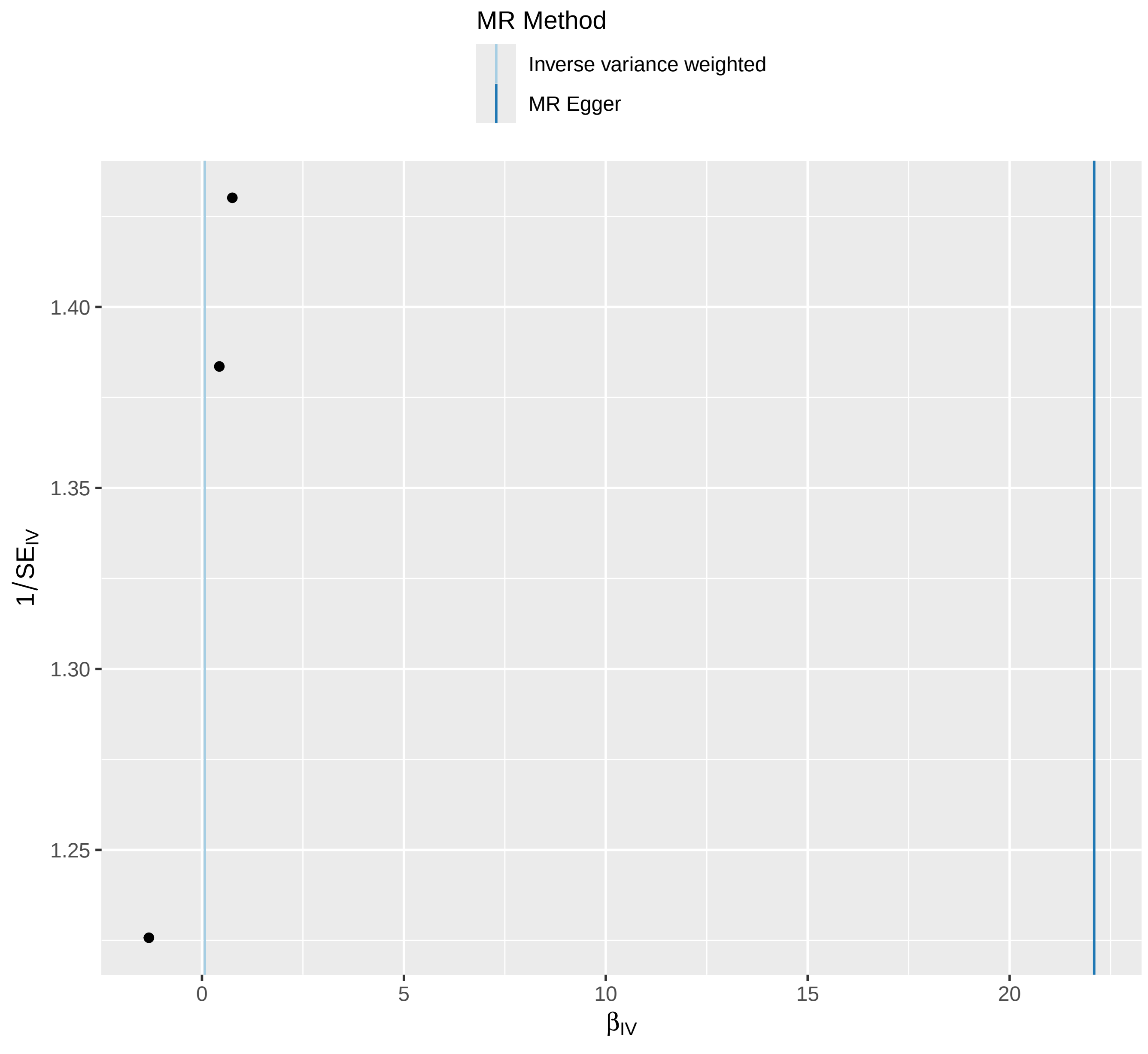
MR Method
Inverse variance weighted
MR Egger
1.40
1.35
1.30
1.25
$1/SE_{IV}$
0
5
10
15
20
β_{IV}

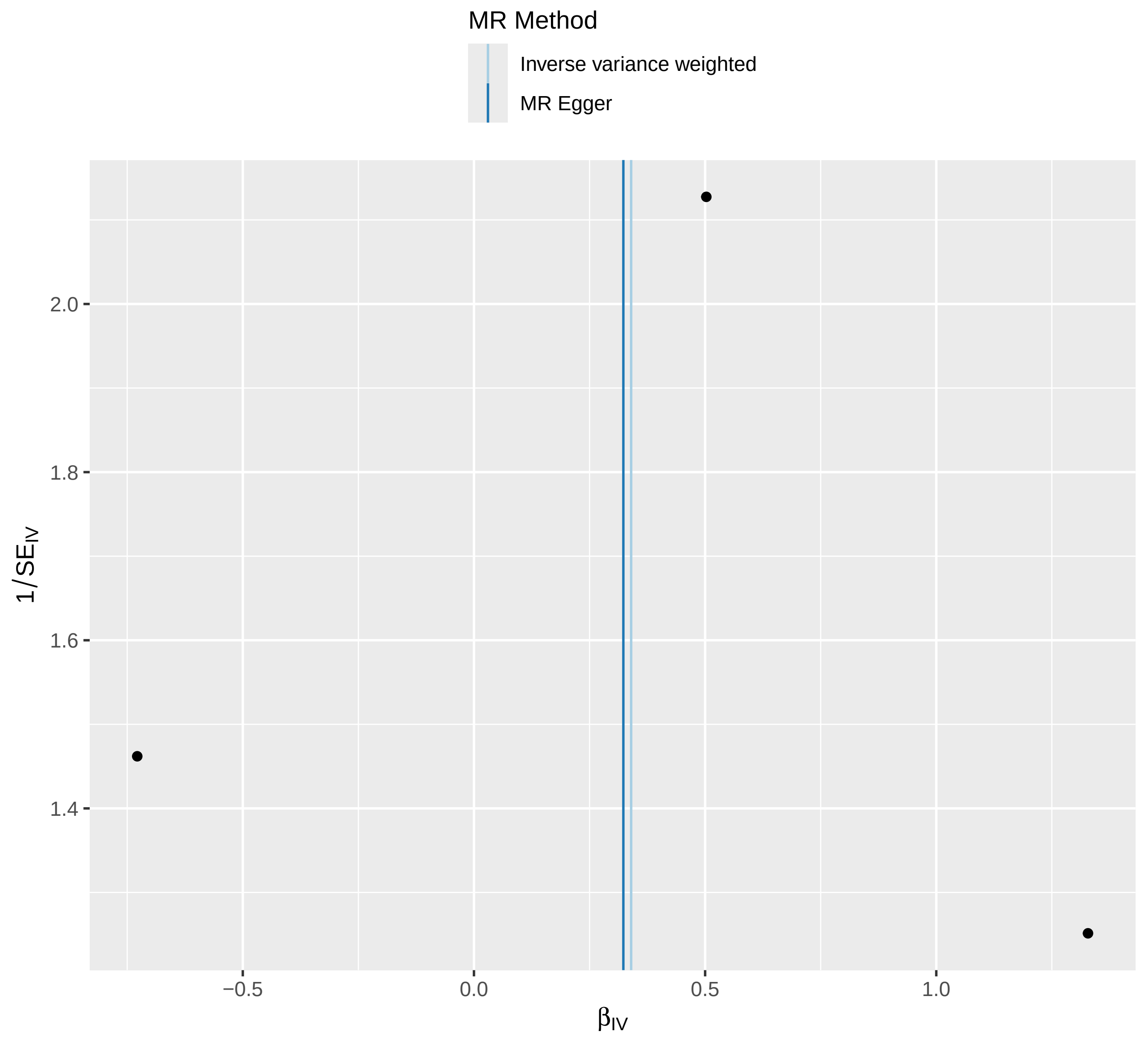
MR Method
Inverse variance weighted
MR Egger
2.0
1.8
1.6
1.4
1/SE_IV
−0.5
0.0
0.5
1.0
β_IV

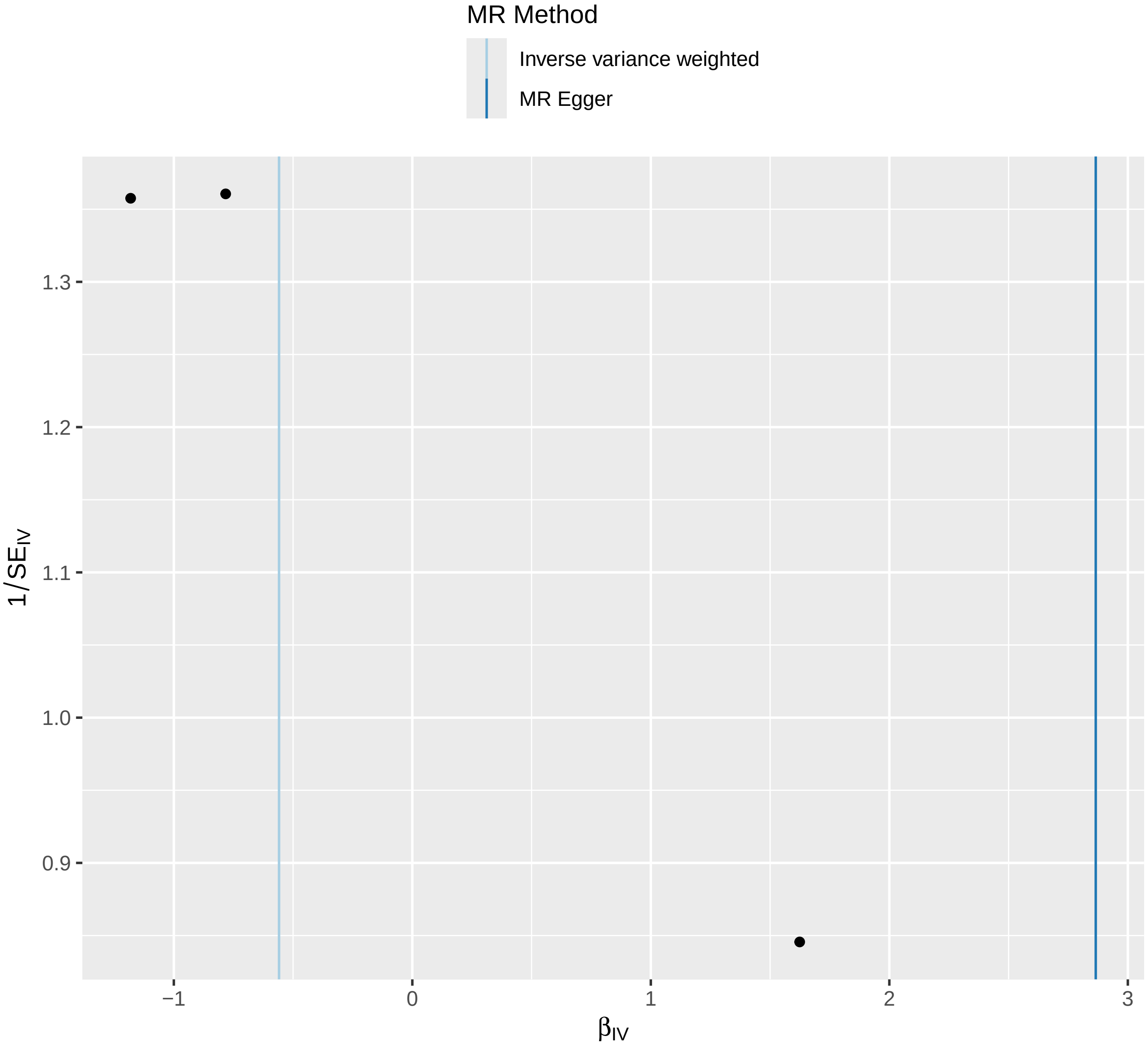

MR Method
Inverse variance weighted
MR Egger
1.3
1.2
1.1
1.0
0.9
$1/SE_{IV}$
−1
0
1
2
3
β_{IV}

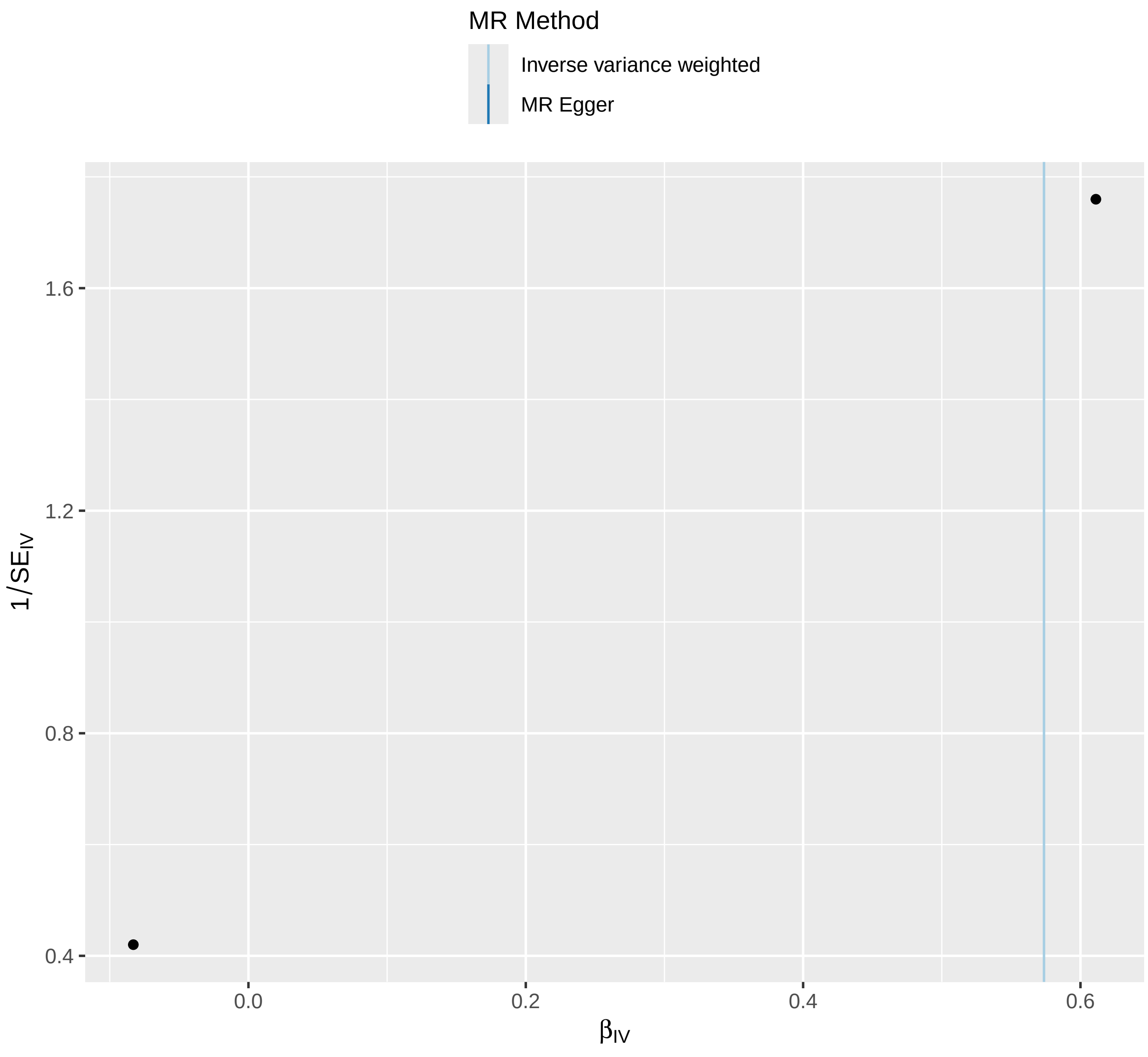

MR Method
Inverse variance weighted
MR Egger
1.6
1.2
0.8
0.4
0.0
0.2
0.4
0.6
$1/SE_{IV}$
β_{IV}

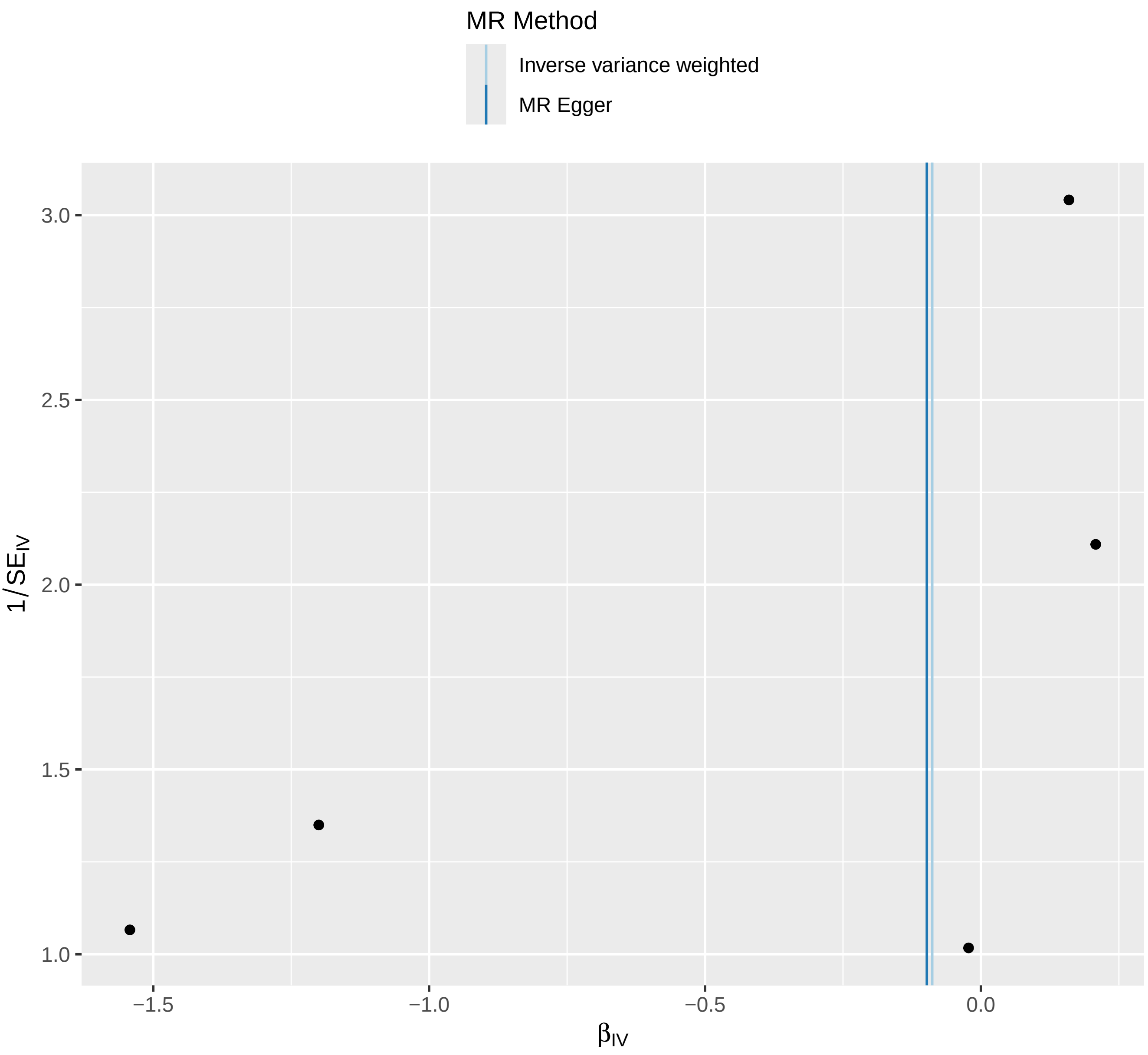
MR Method
Inverse variance weighted
MR Egger
1/SE_IV
3.0
2.5
2.0
1.5
1.0
−1.5
−1.0
−0.5
0.0
β_IV

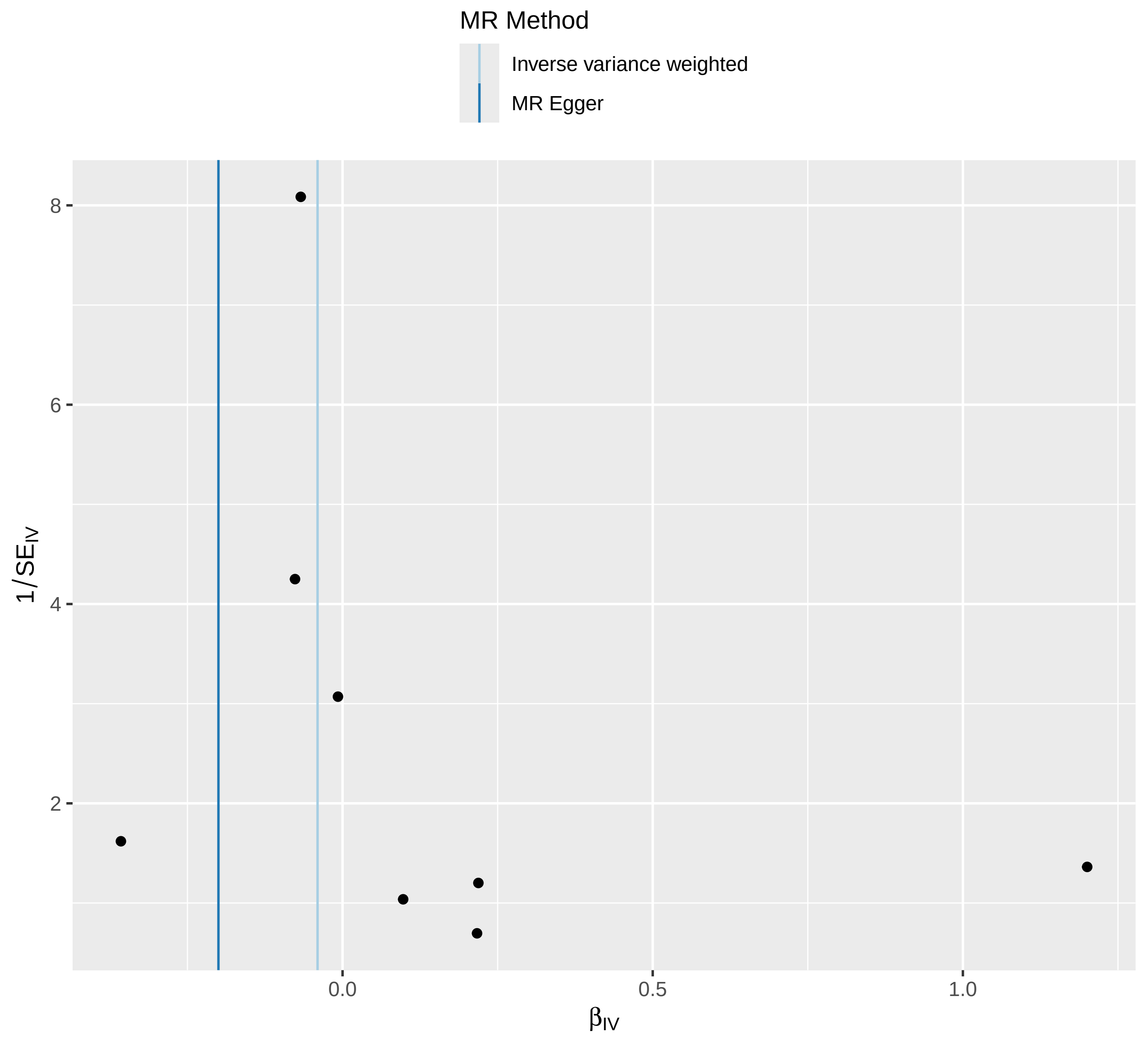
MR Method
Inverse variance weighted
MR Egger
8
6
4
2
1/SE$_{IV}$
0.0
0.5
1.0
β$_{IV}$

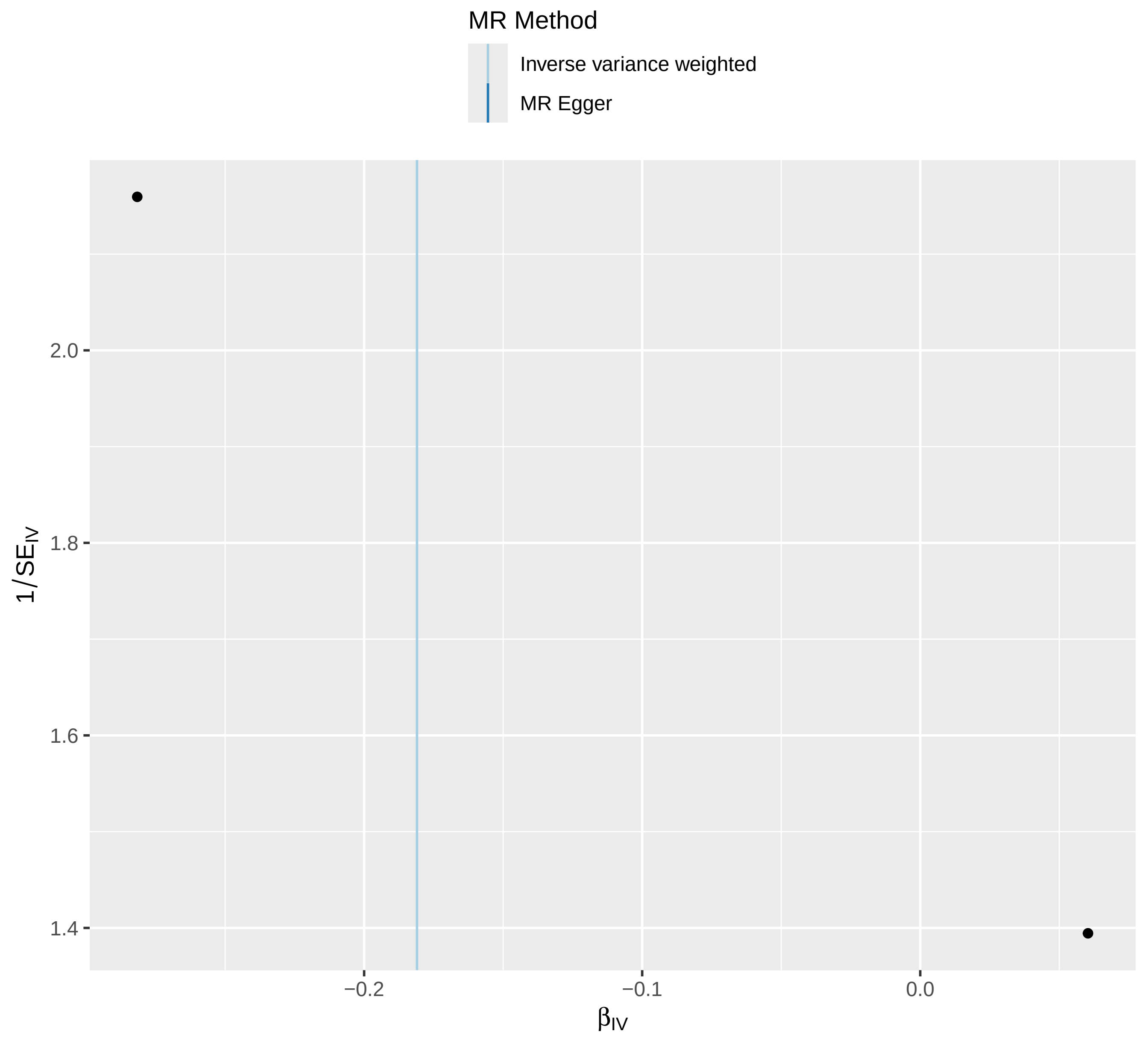

MR Method
Inverse variance weighted
MR Egger
2.0
1.8
1.6
1.4
1/SE_IV
−0.2
−0.1
0.0
β_IV

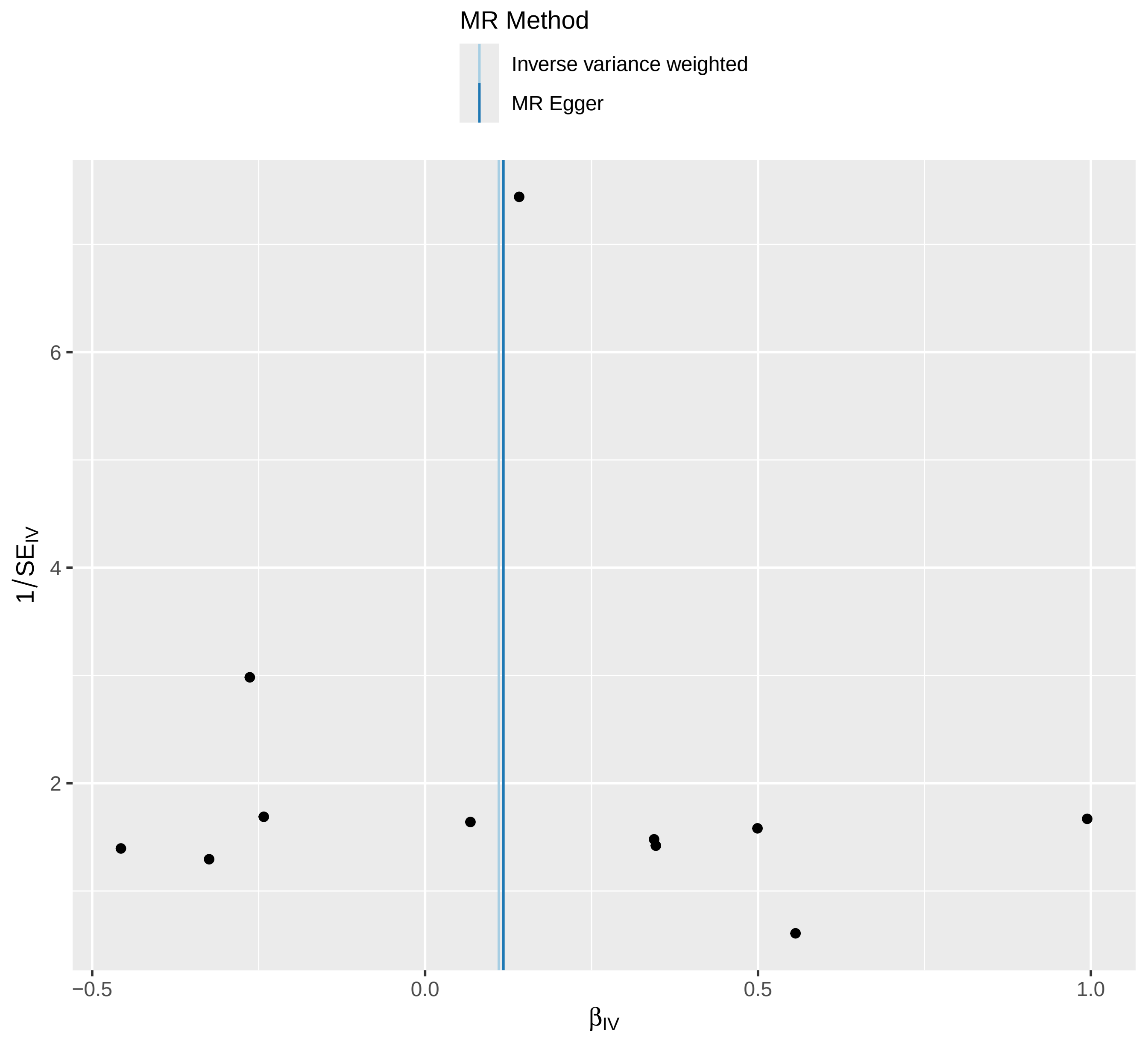
MR Method
Inverse variance weighted
MR Egger
6
4
2
1/SE_IV
−0.5
0.0
0.5
1.0
β_IV

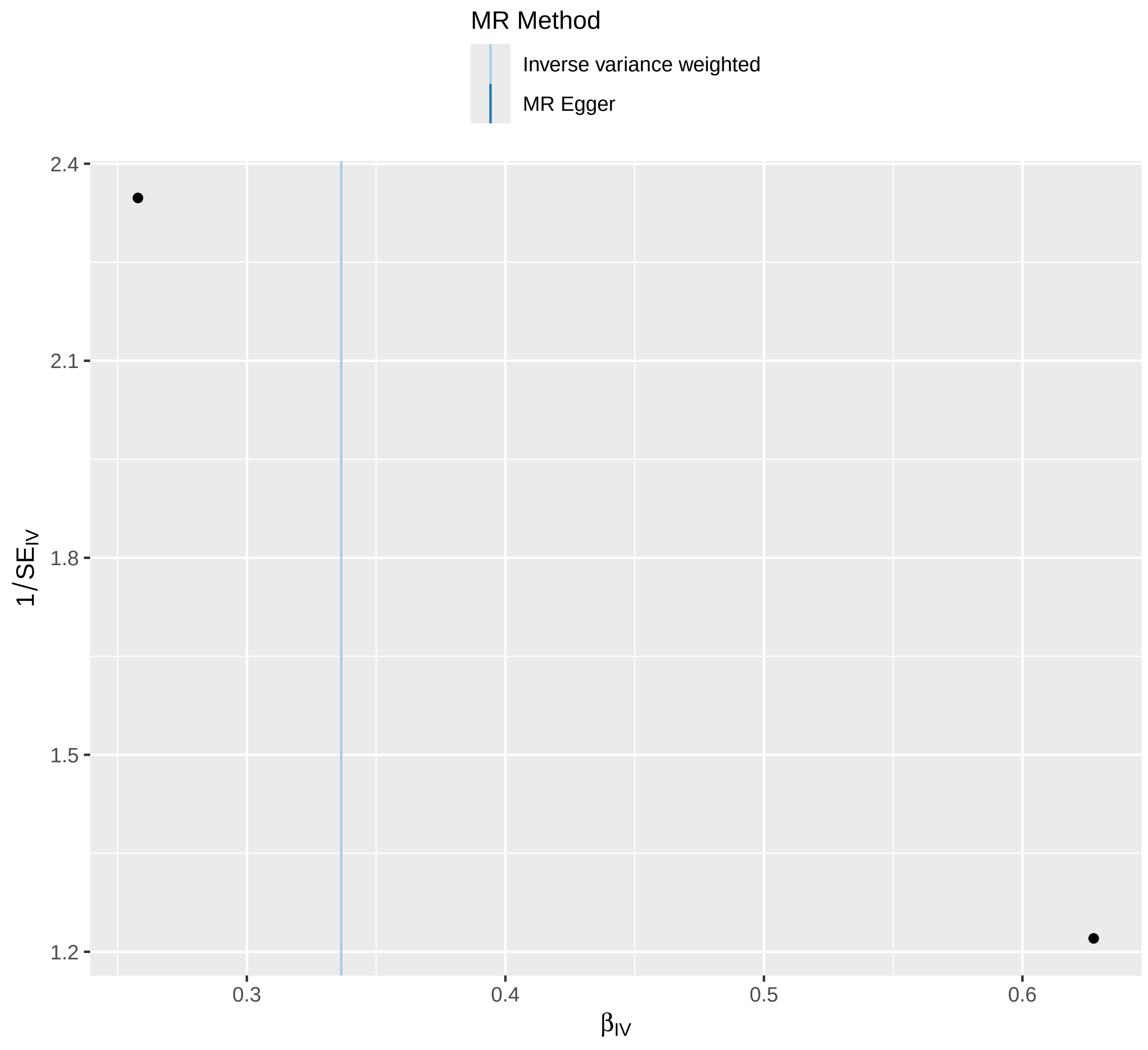
MR Method
Inverse variance weighted
MR Egger
2.4
2.1
1.8
1.5
1.2
$1/SE_{IV}$
0.3
0.4
0.5
0.6
β_{IV}

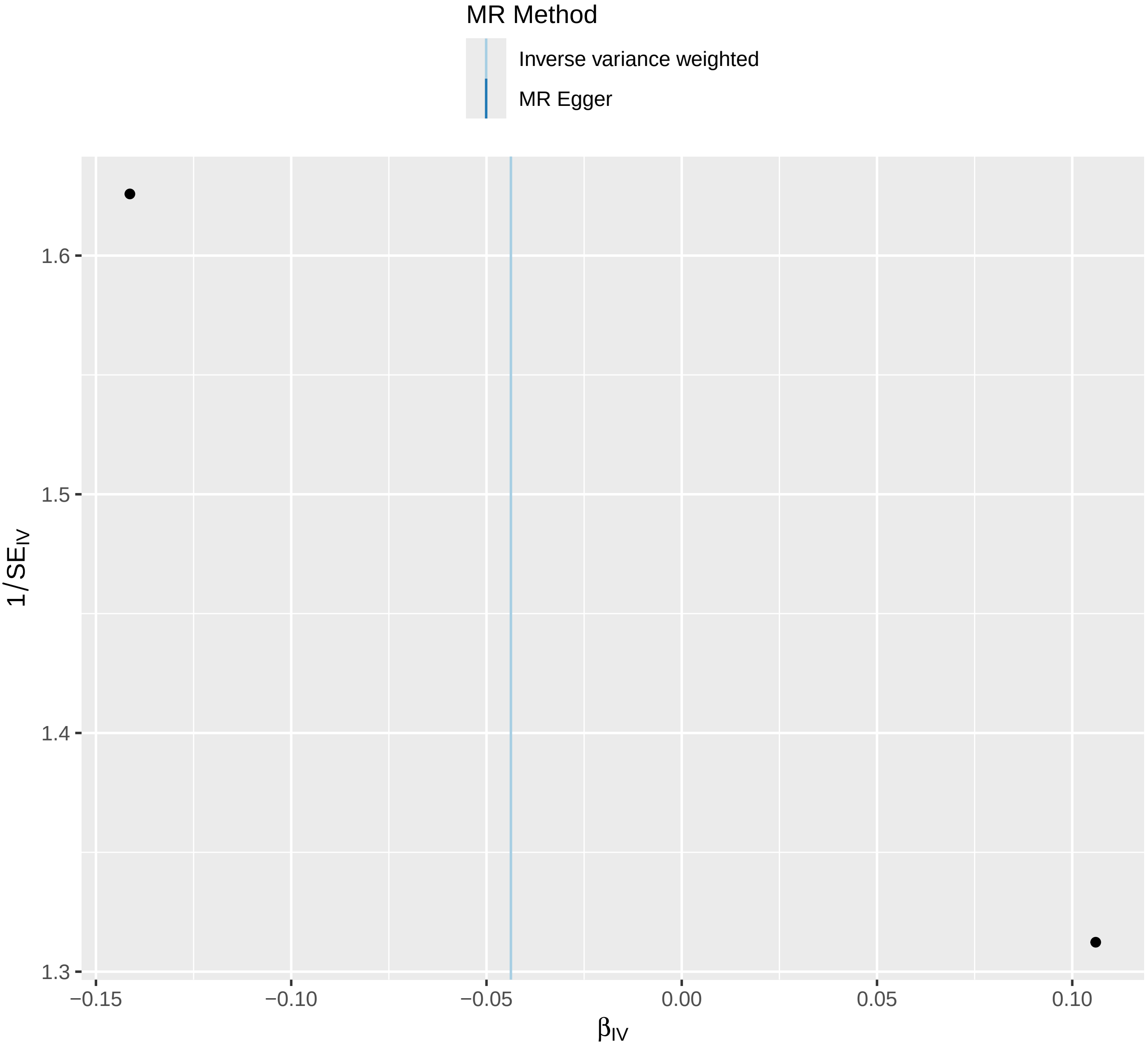
MR Method
Inverse variance weighted
MR Egger
1.6
1.5
1.4
1.3
1/SE$_{IV}$
−0.15
−0.10
−0.05
0.00
0.05
0.10
β$_{IV}$

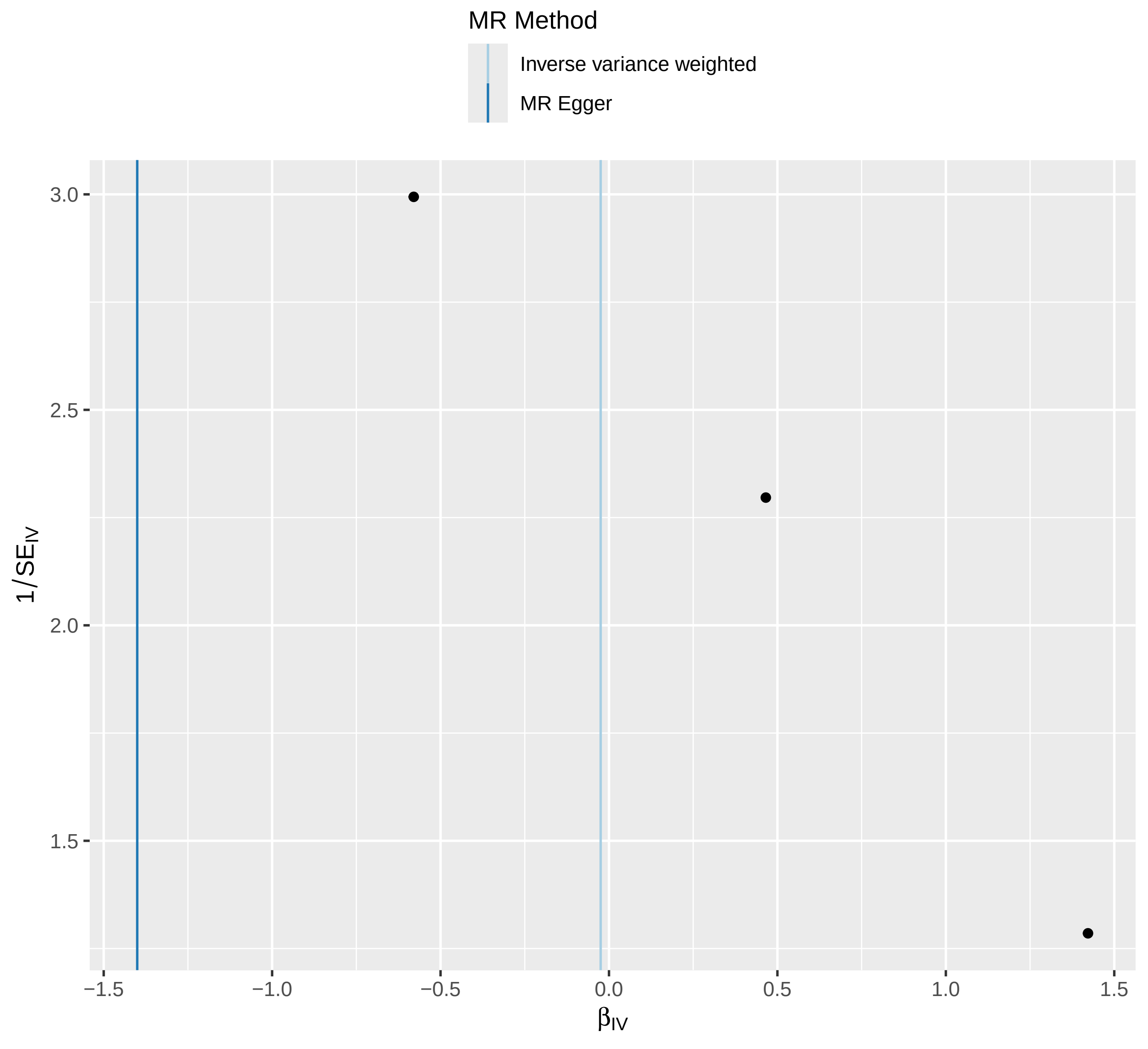
MR Method
Inverse variance weighted
MR Egger
3.0
2.5
2.0
1.5
1/SE_IV
−1.5
−1.0
−0.5
0.0
0.5
1.0
1.5
β_IV

Insufficient number of SNPs

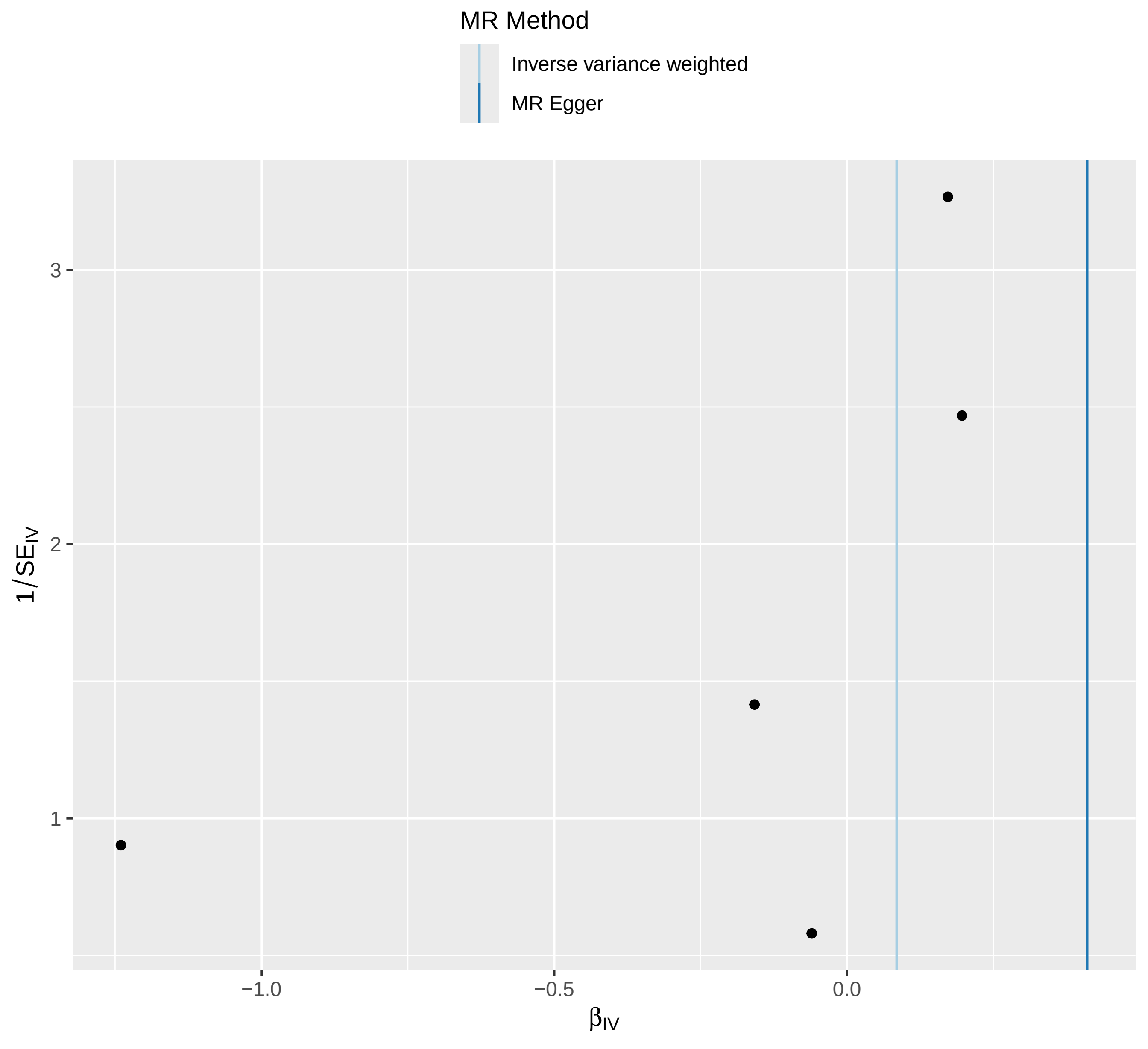

MR Method
Inverse variance weighted
MR Egger
3
2
1
1/SE$_{IV}$
−1.0
−0.5
0.0
β$_{IV}$

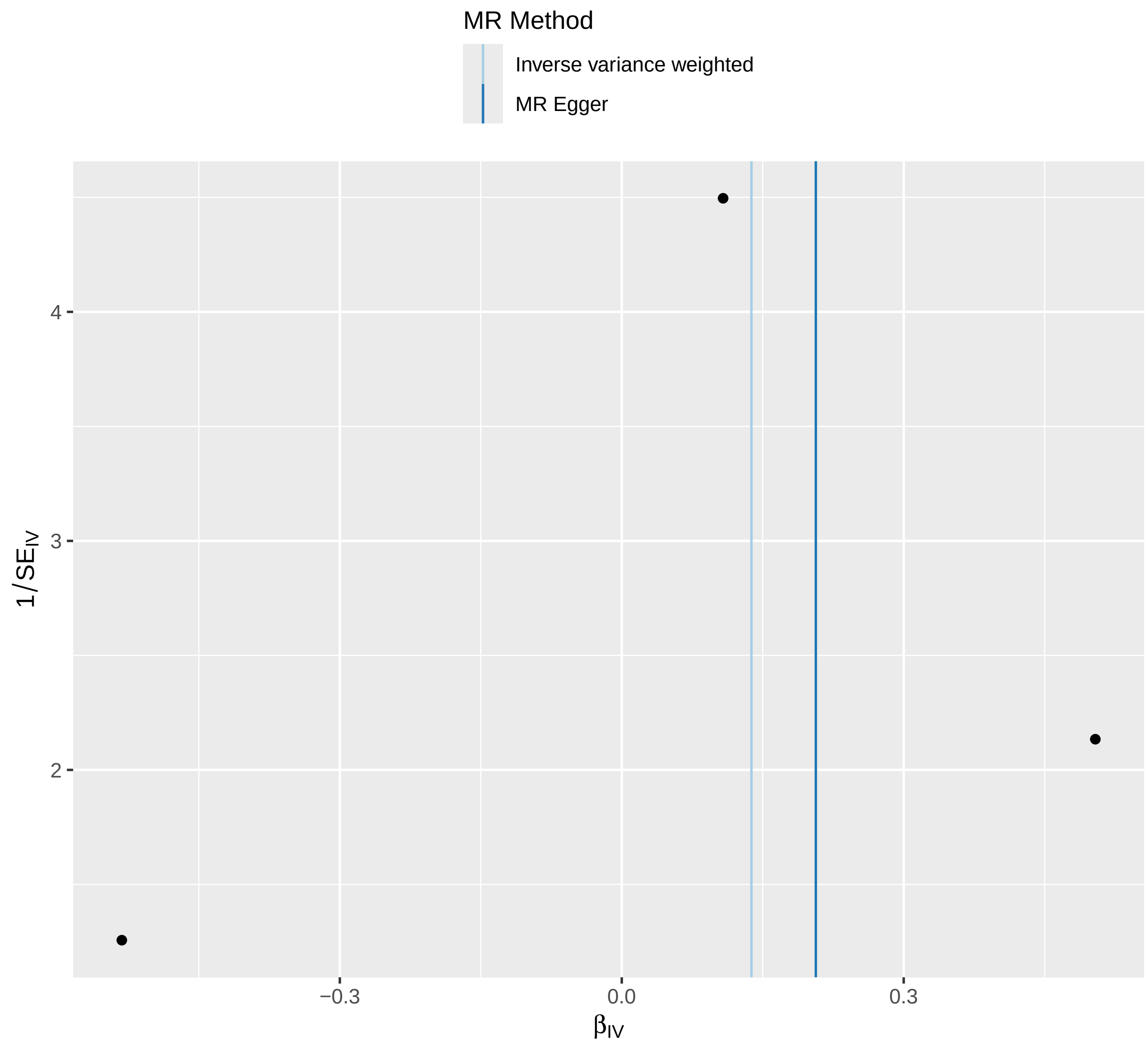
MR Method
Inverse variance weighted
MR Egger
4
3
2
1/SE_IV
−0.3
0.0
0.3
β_IV

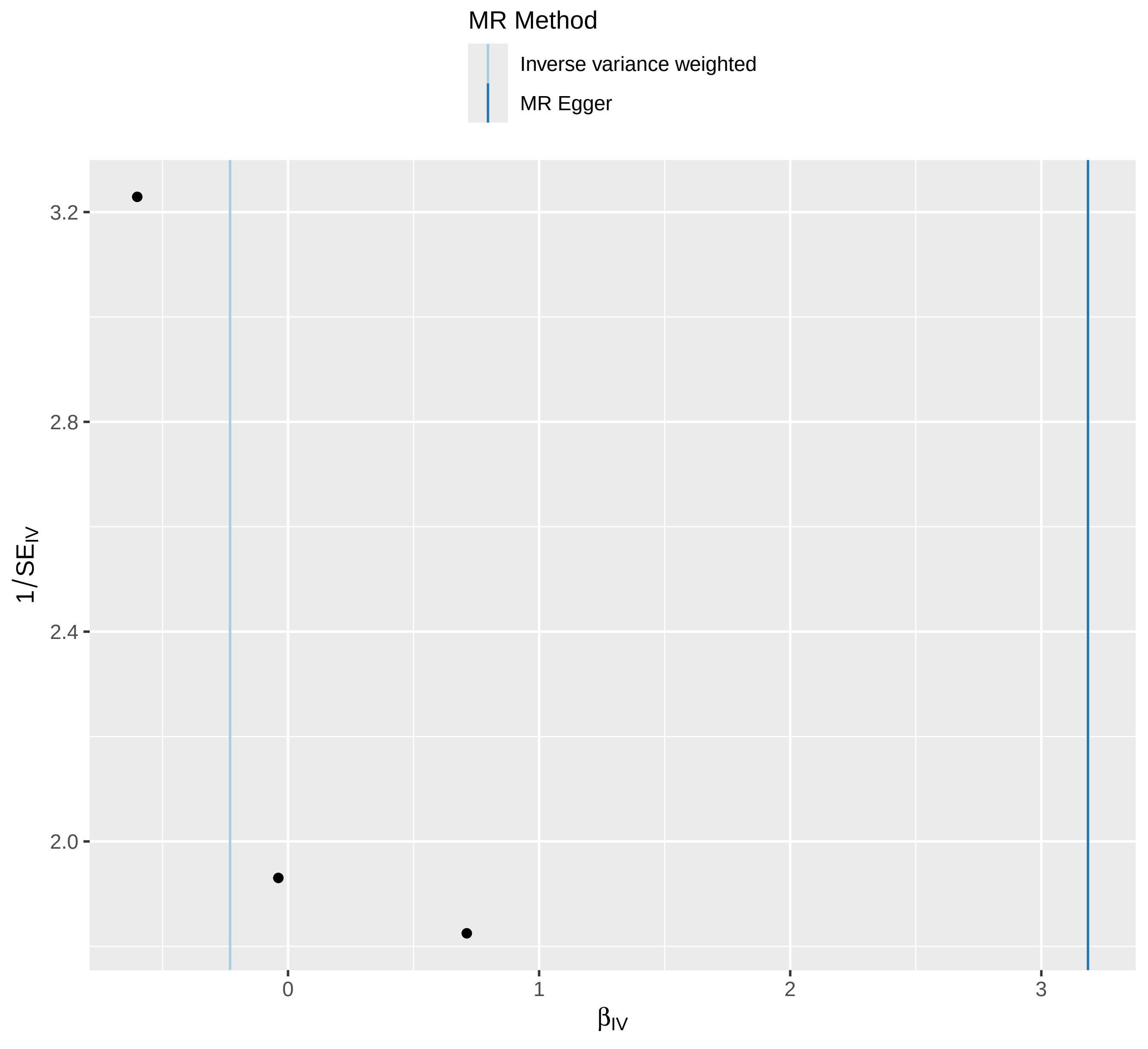

MR Method
Inverse variance weighted
MR Egger
3.2
2.8
2.4
2.0
1/SE$_{IV}$
0
1
2
3
β$_{IV}$

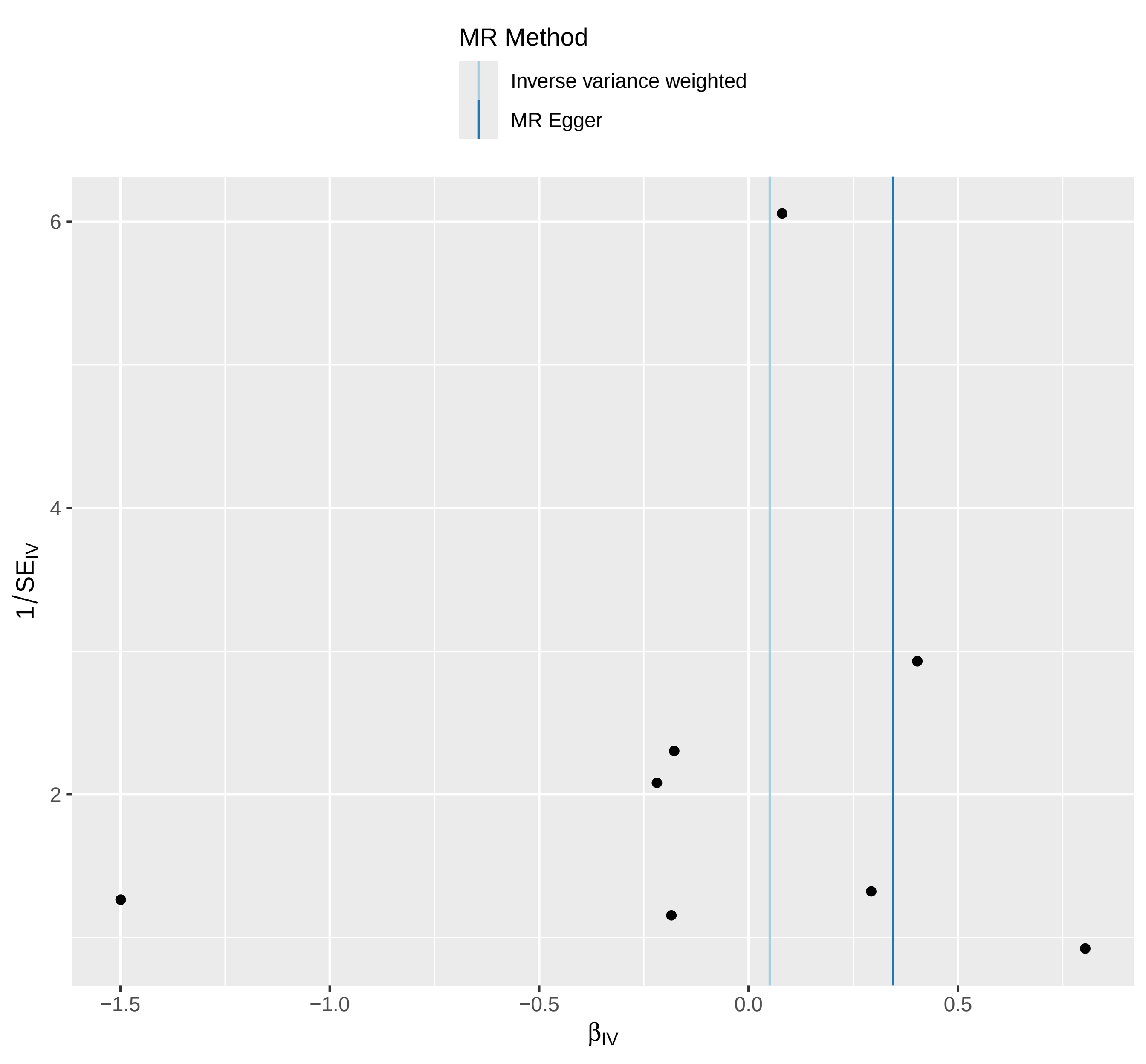

MR Method
Inverse variance weighted
MR Egger
6
4
2
1/SE_IV
−1.5
−1.0
−0.5
0.0
0.5
β_IV

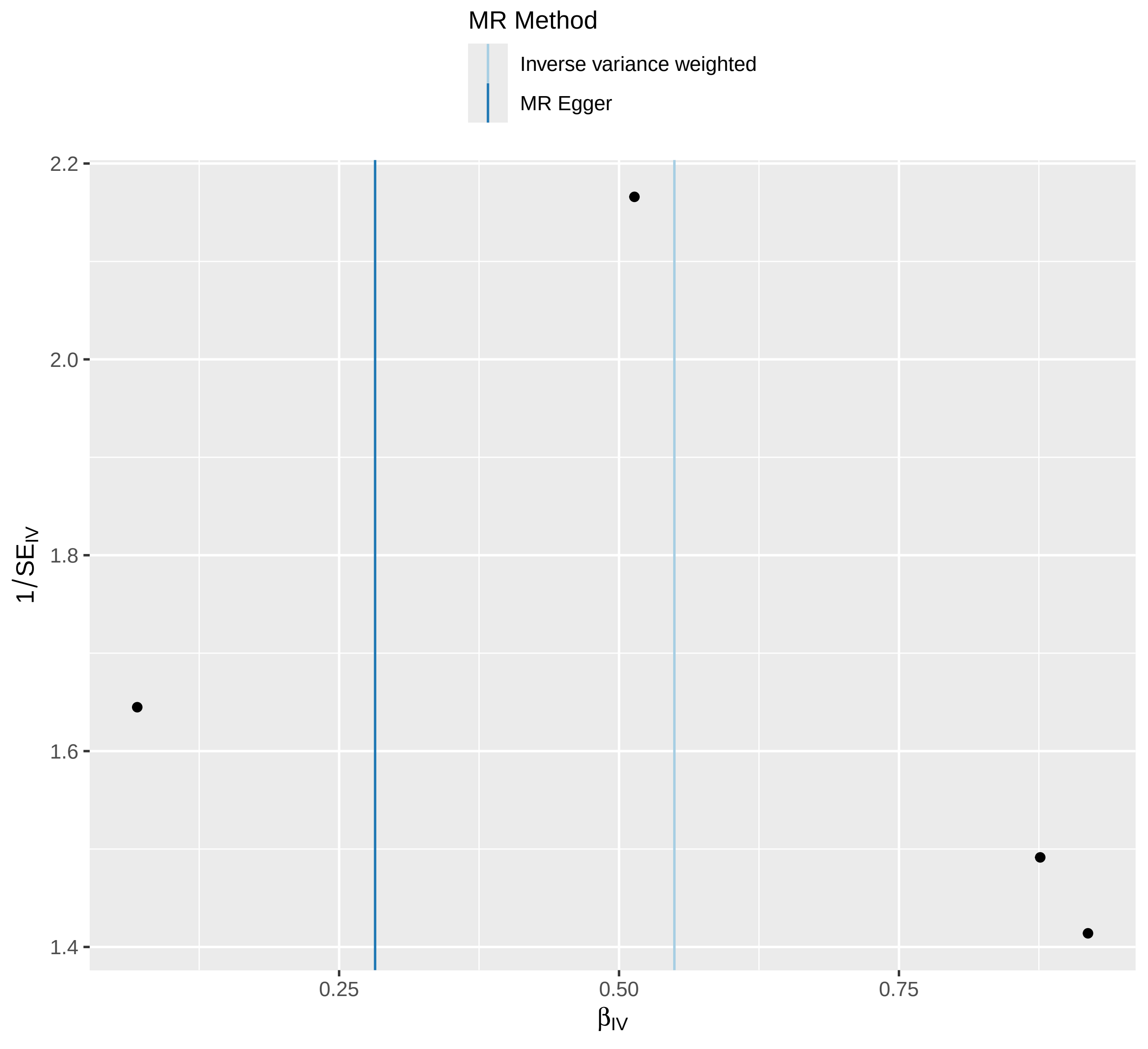

MR Method
Inverse variance weighted
MR Egger
2.2
2.0
1.8
1.6
1.4
1/SE_IV
0.25
0.50
0.75
β_IV

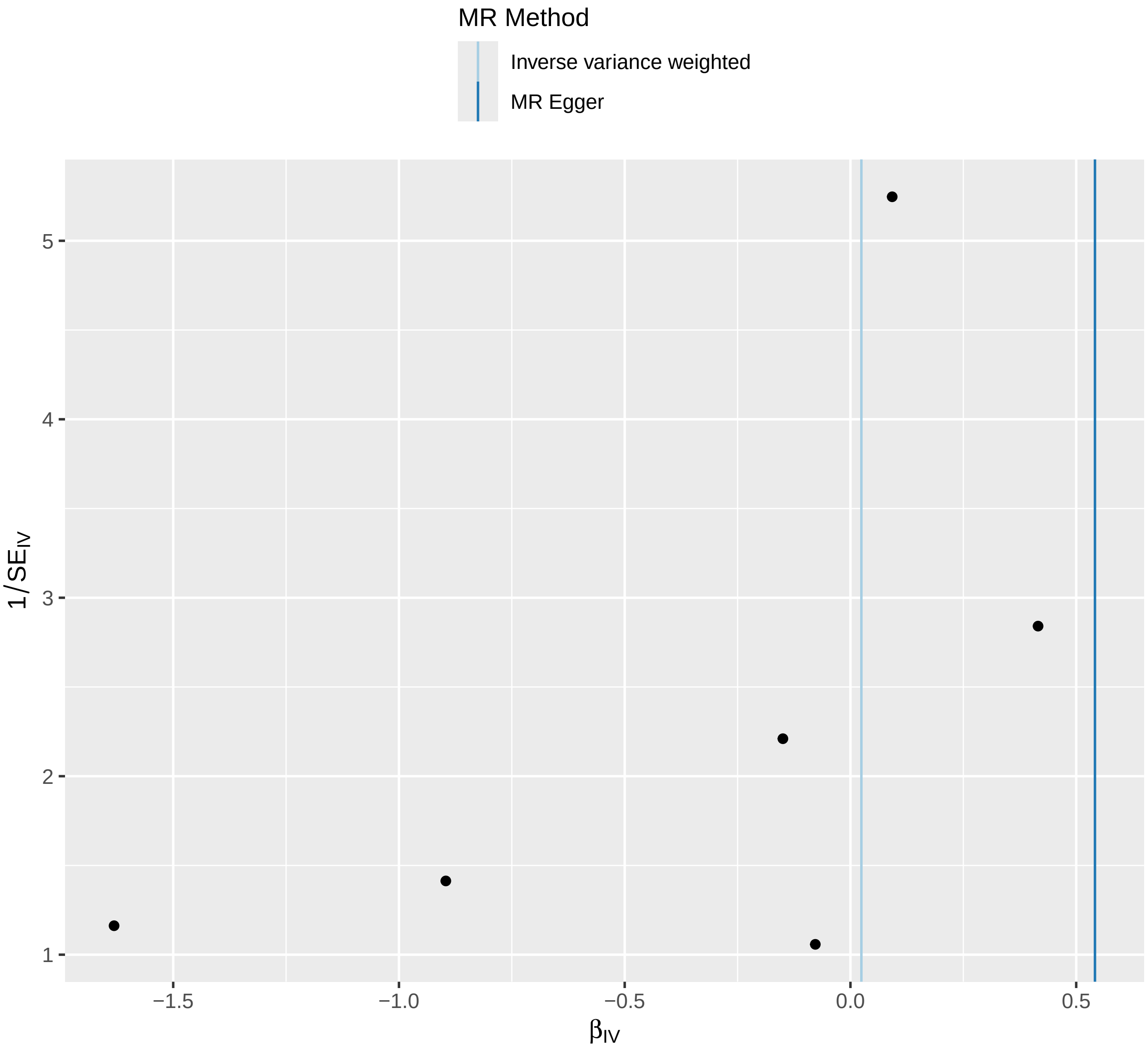

MR Method
Inverse variance weighted
MR Egger
1/SE_IV
5
4
3
2
1
−1.5
−1.0
−0.5
0.0
0.5
β_IV

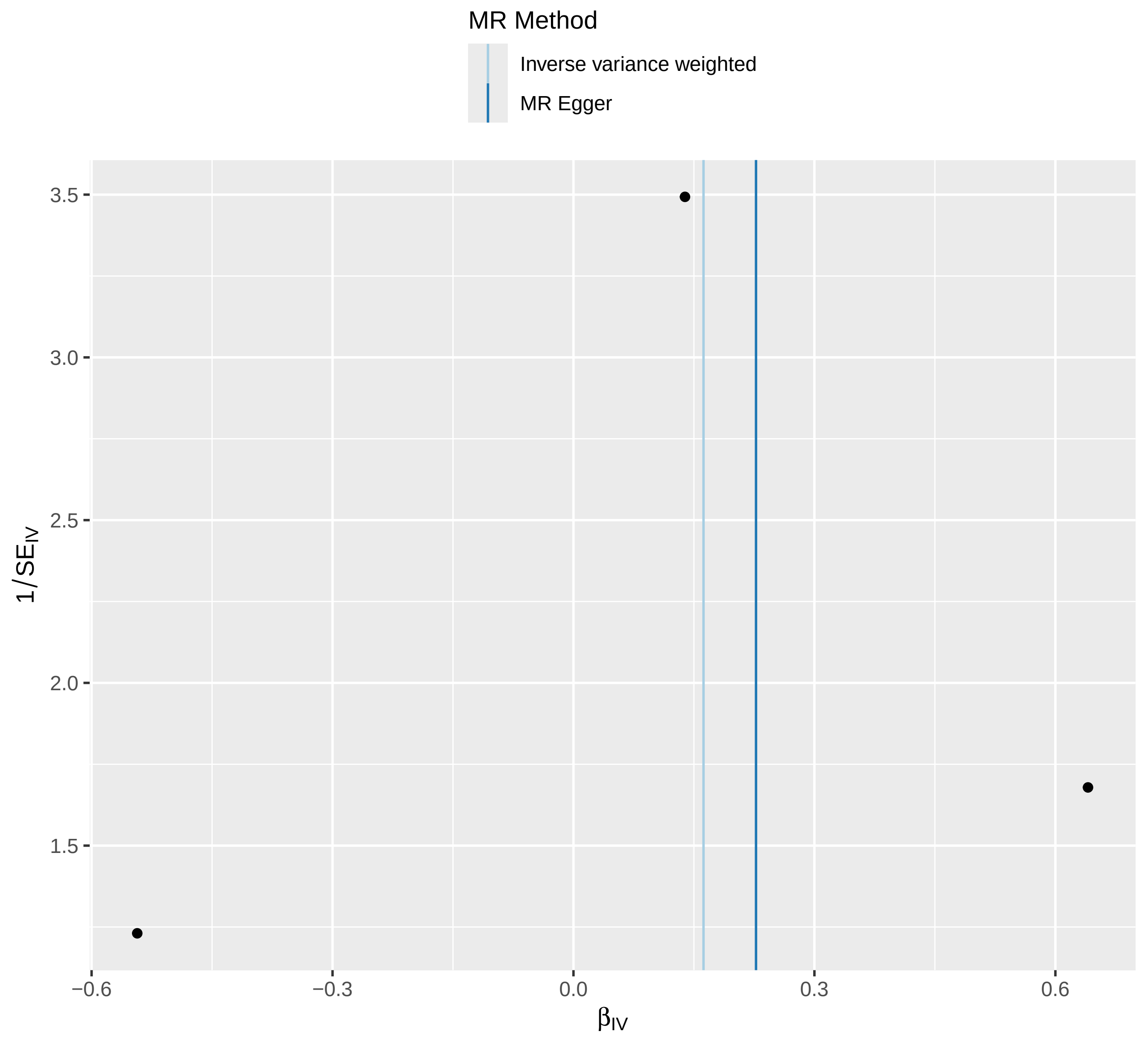

MR Method
Inverse variance weighted
MR Egger
3.5
3.0
2.5
2.0
1.5
1/SE$_{IV}$
−0.6
−0.3
0.0
0.3
0.6
β$_{IV}$

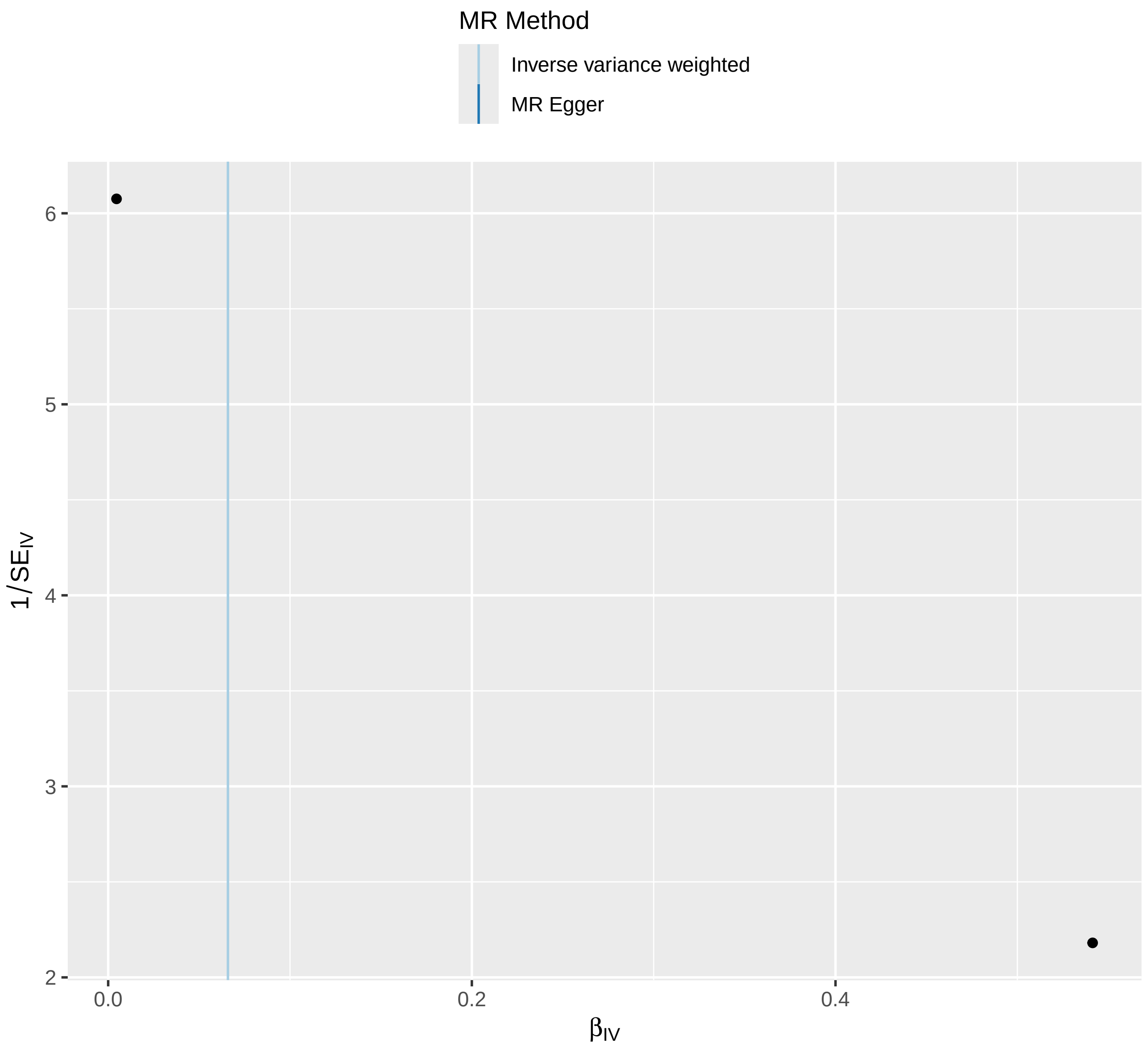
MR Method
Inverse variance weighted
MR Egger
6
5
4
3
2
1/SE_IV
0.0
0.2
0.4
β_IV

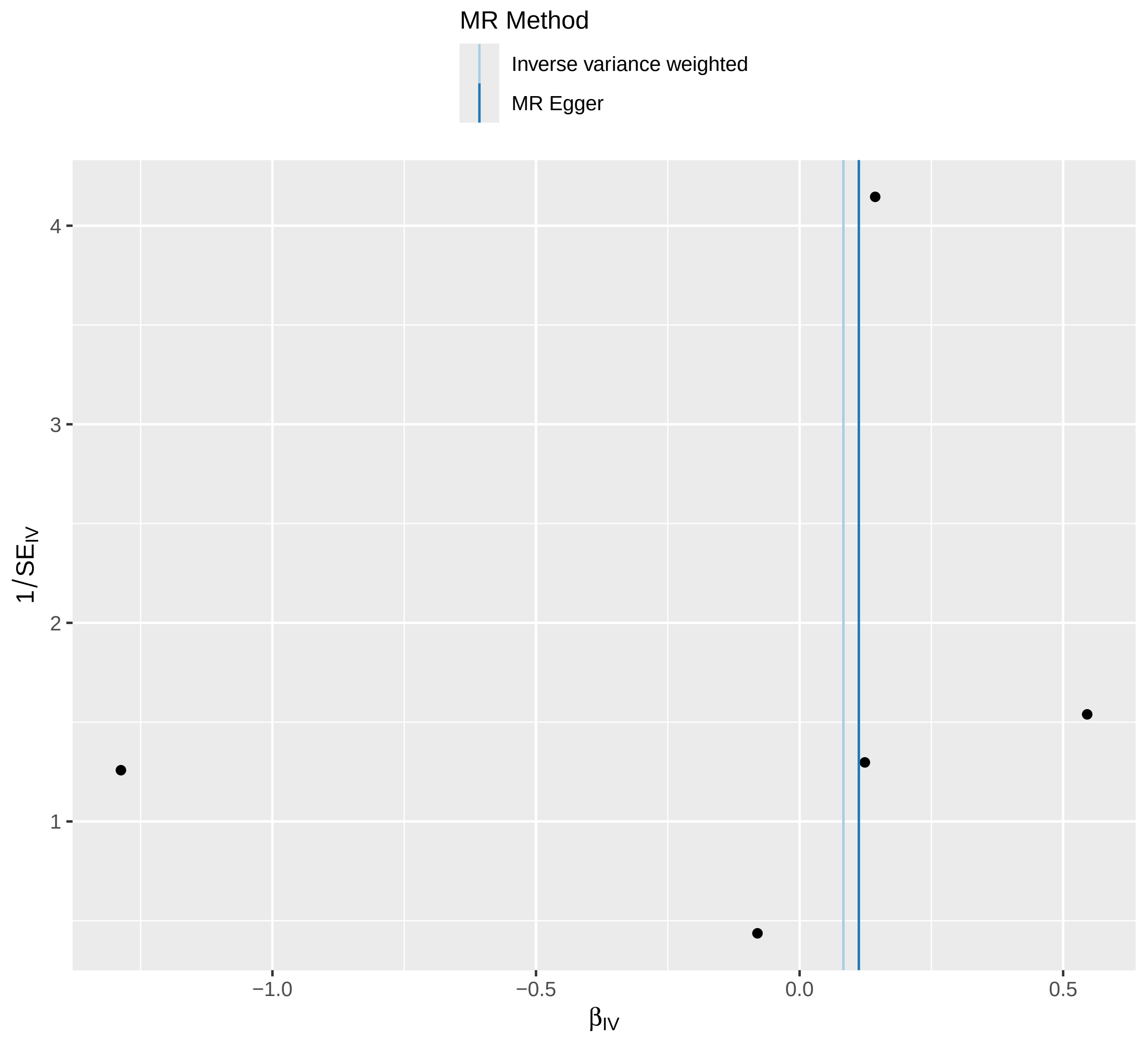

MR Method
Inverse variance weighted
MR Egger
4
3
2
1
1/SE_IV
−1.0
−0.5
0.0
0.5
β_IV

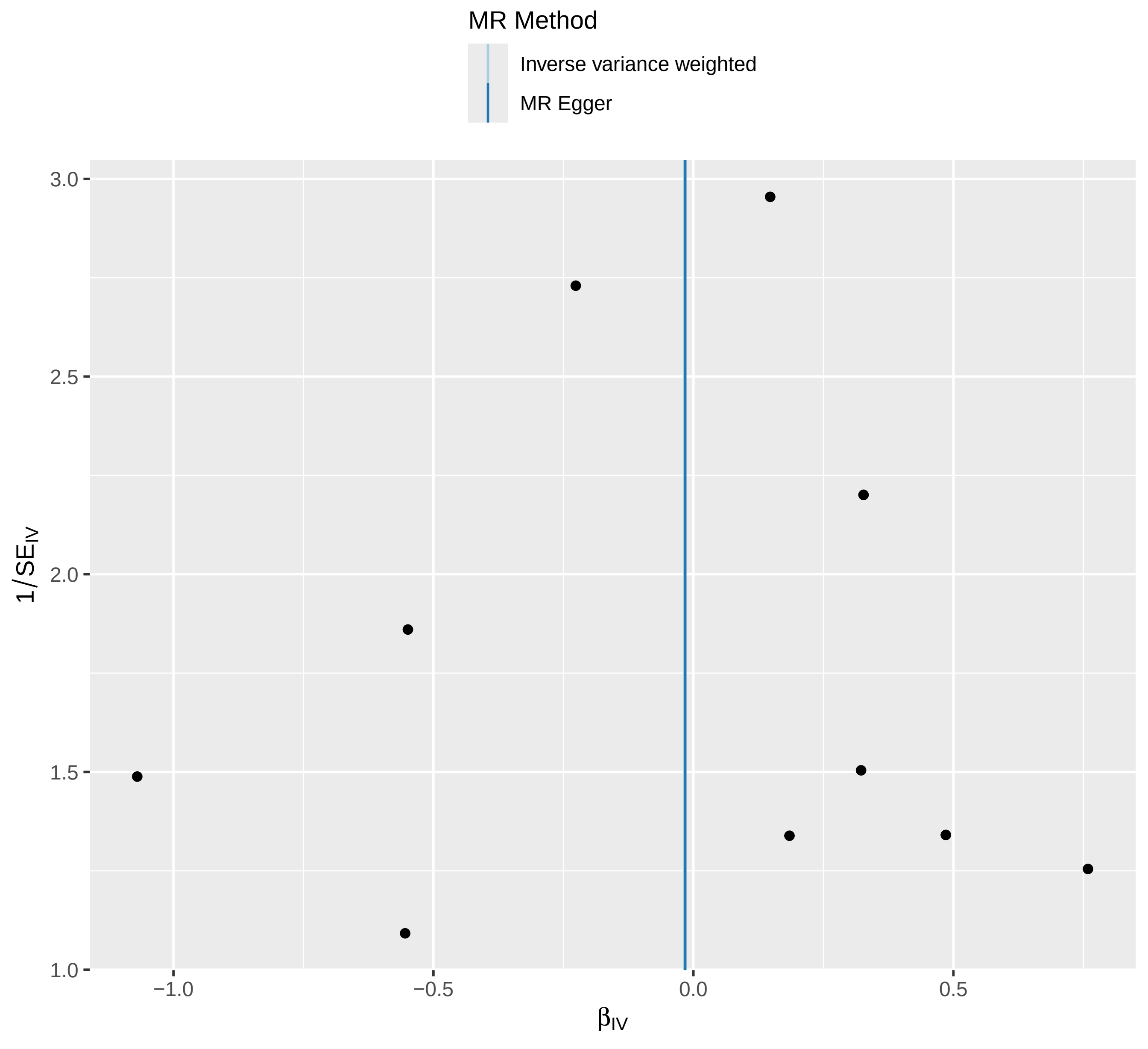

MR Method
Inverse variance weighted
MR Egger
3.0
2.5
2.0
1.5
1.0
−1.0
−0.5
0.0
0.5
$1/SE_{IV}$
β_{IV}

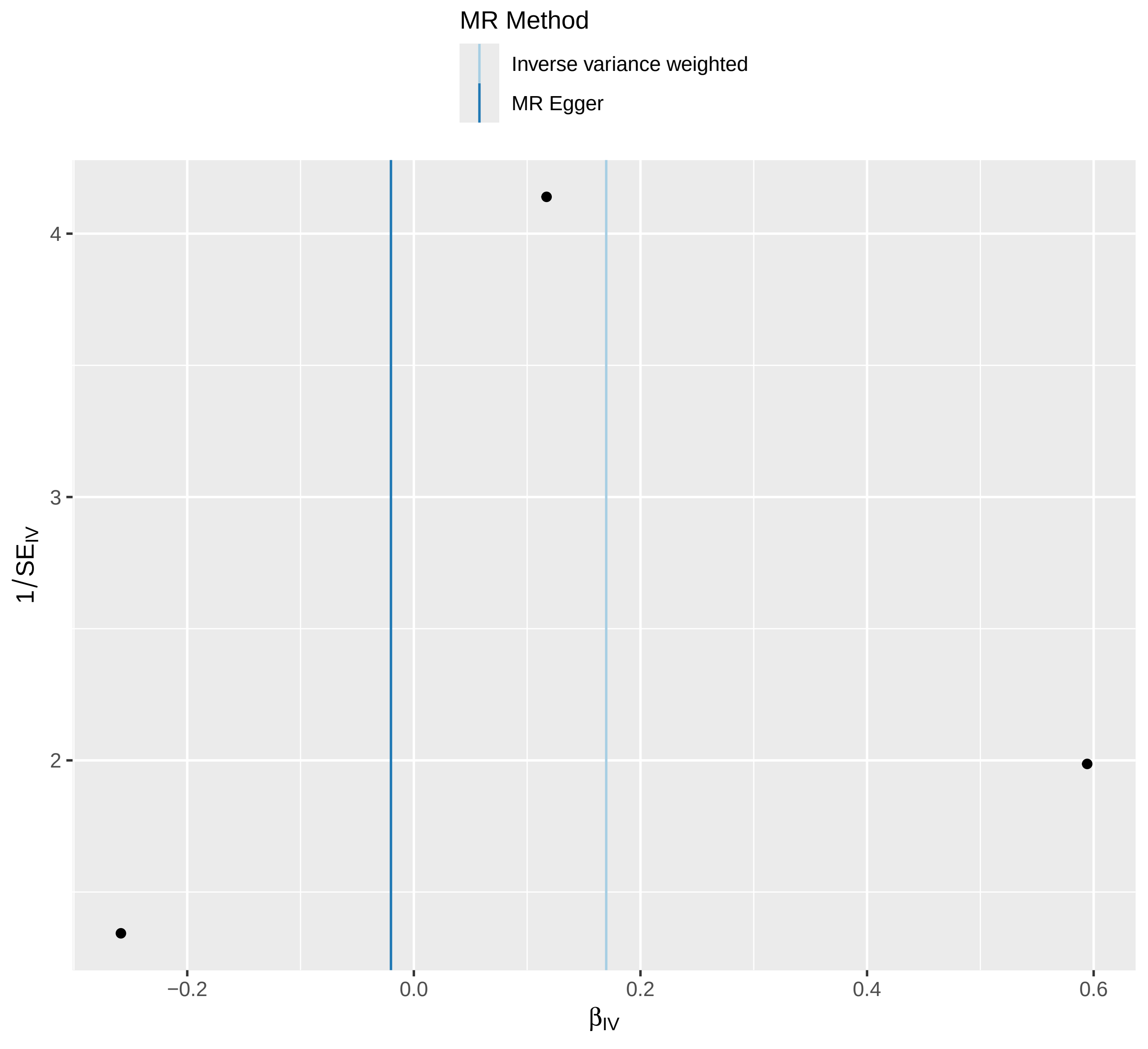

MR Method
Inverse variance weighted
MR Egger
4
3
2
1/SE_IV
−0.2
0.0
0.2
0.4
0.6
β_IV

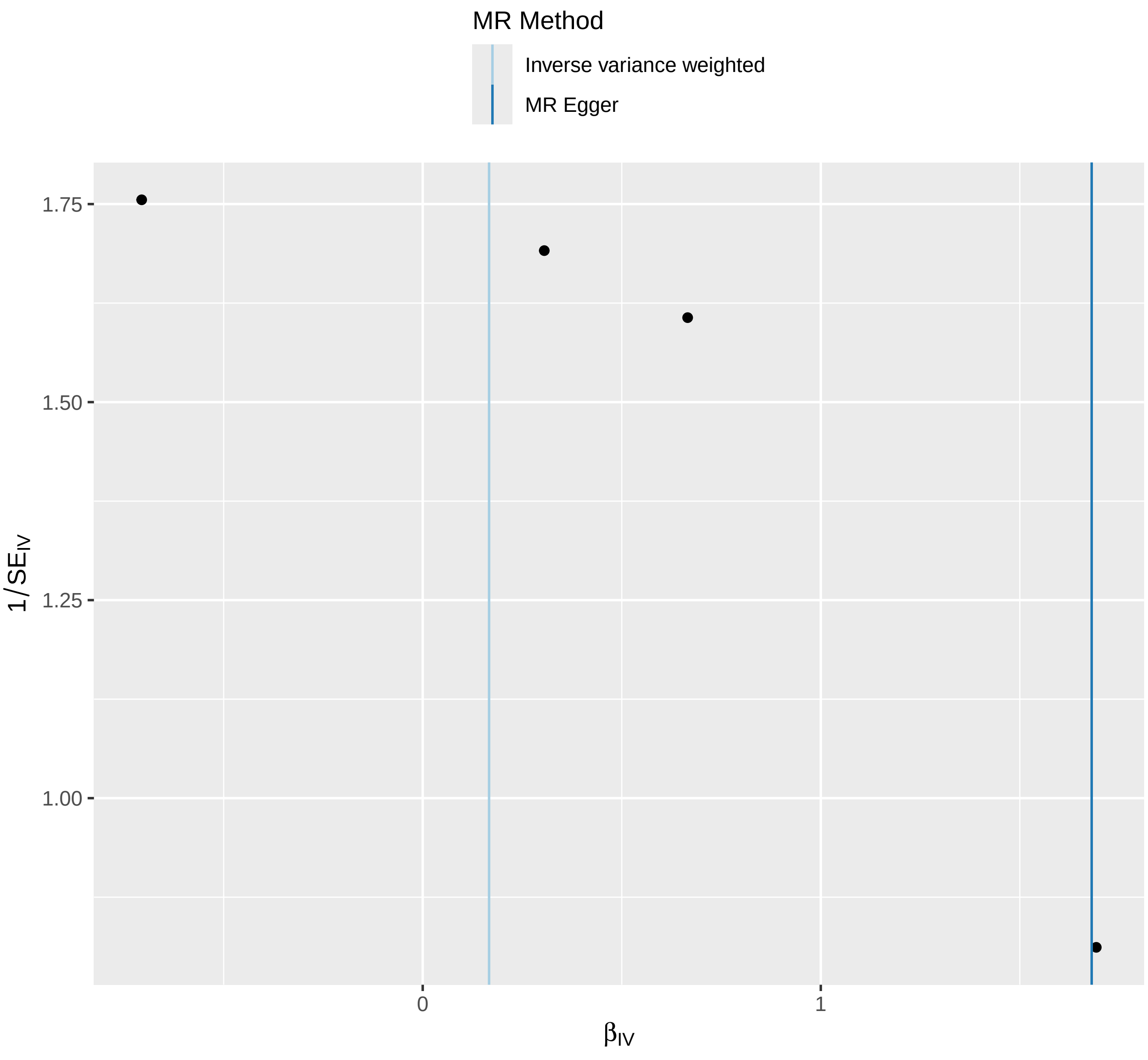

MR Method
Inverse variance weighted
MR Egger
1.75
1.50
1.25
1.00
1/SE$_{IV}$
0
1
β$_{IV}$

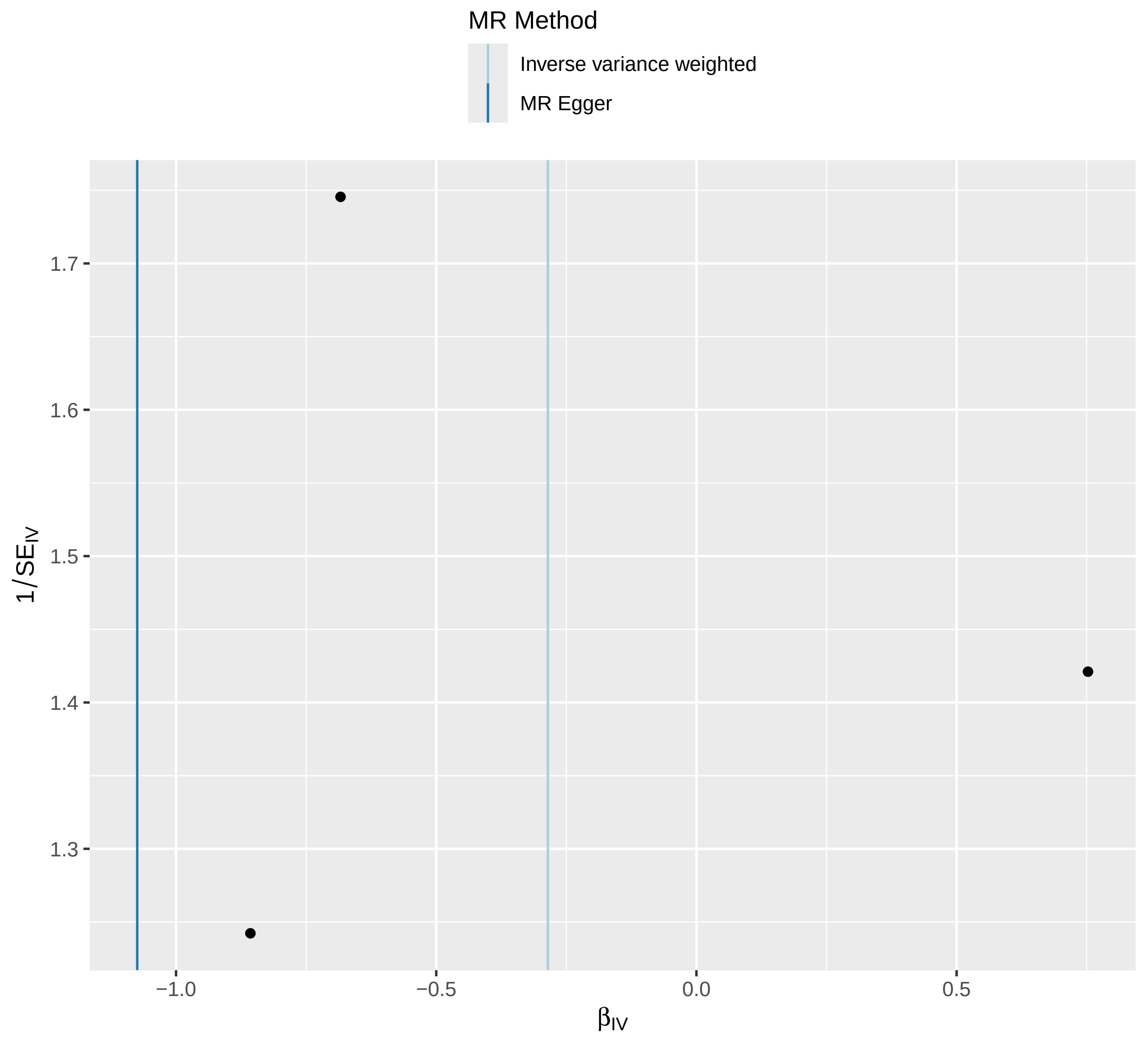
MR Method
Inverse variance weighted
MR Egger
1.7
1.6
1.5
1.4
1.3
$1/SE_{IV}$
−1.0
−0.5
0.0
0.5
β_{IV}

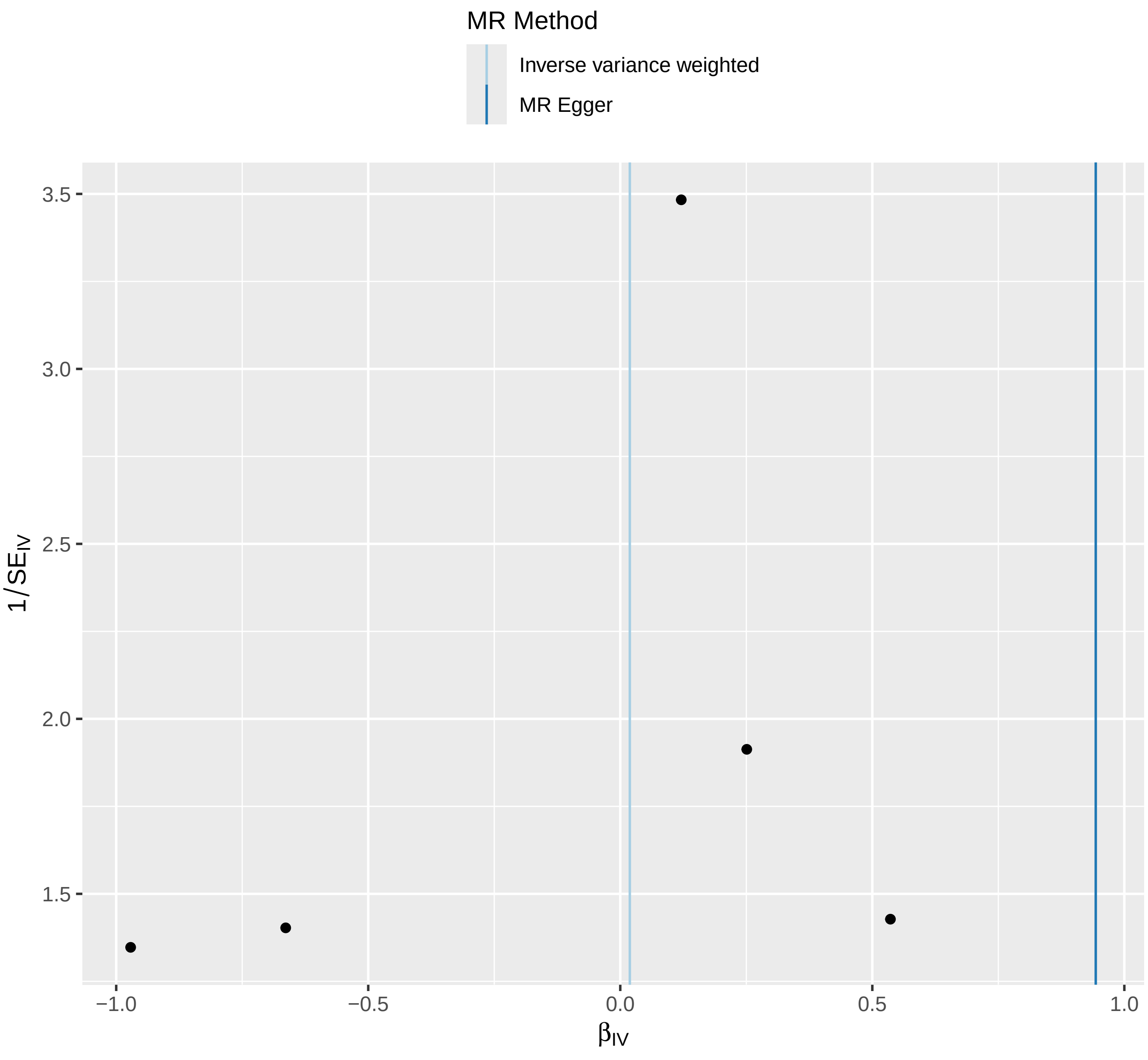

MR Method
Inverse variance weighted
MR Egger
3.5
3.0
2.5
2.0
1.5
1/SE_IV
−1.0
−0.5
0.0
0.5
1.0
β_IV

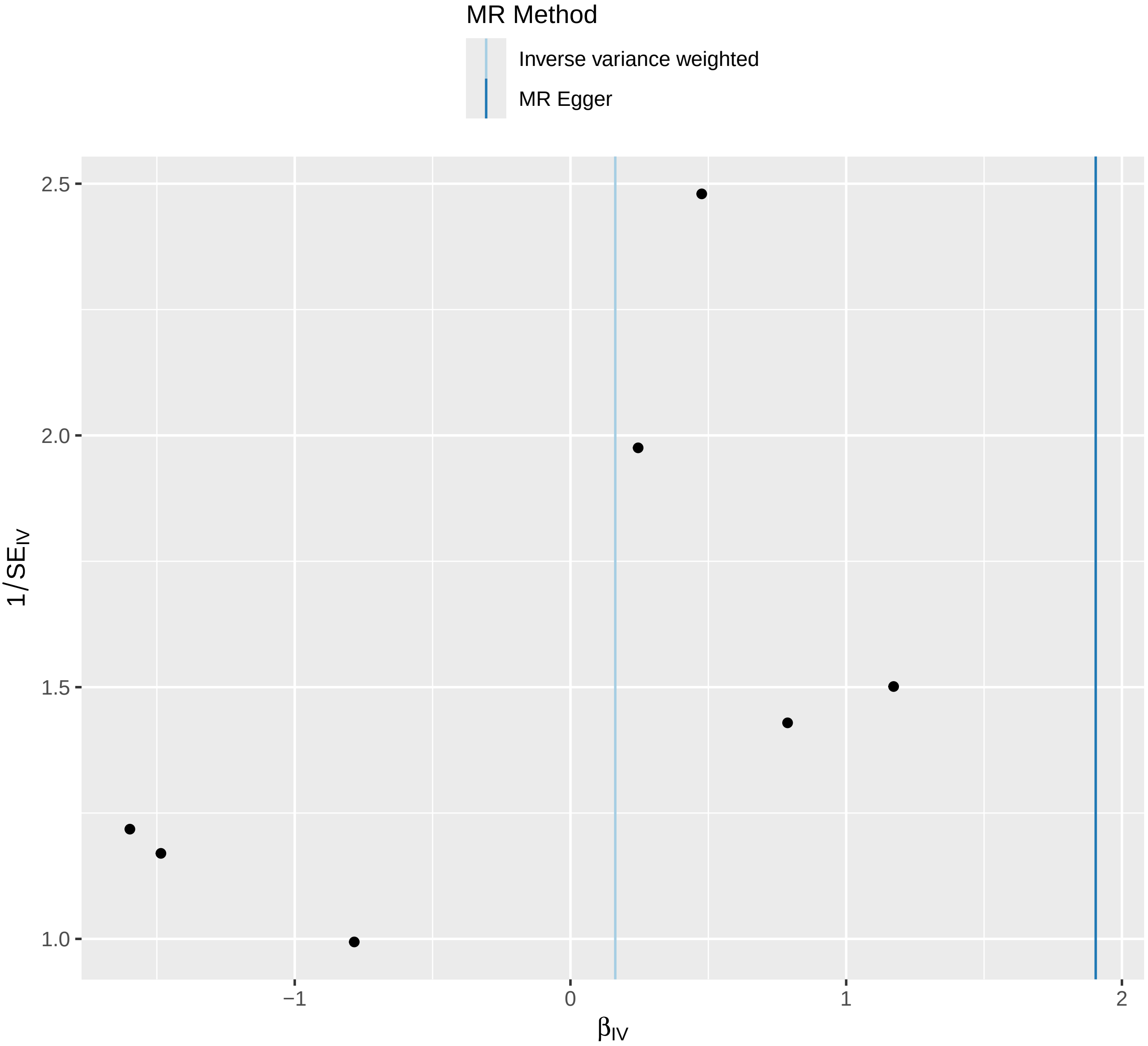

MR Method
Inverse variance weighted
MR Egger
2.5
2.0
1.5
1.0
−1
0
1
2
1/SE_IV
β_IV

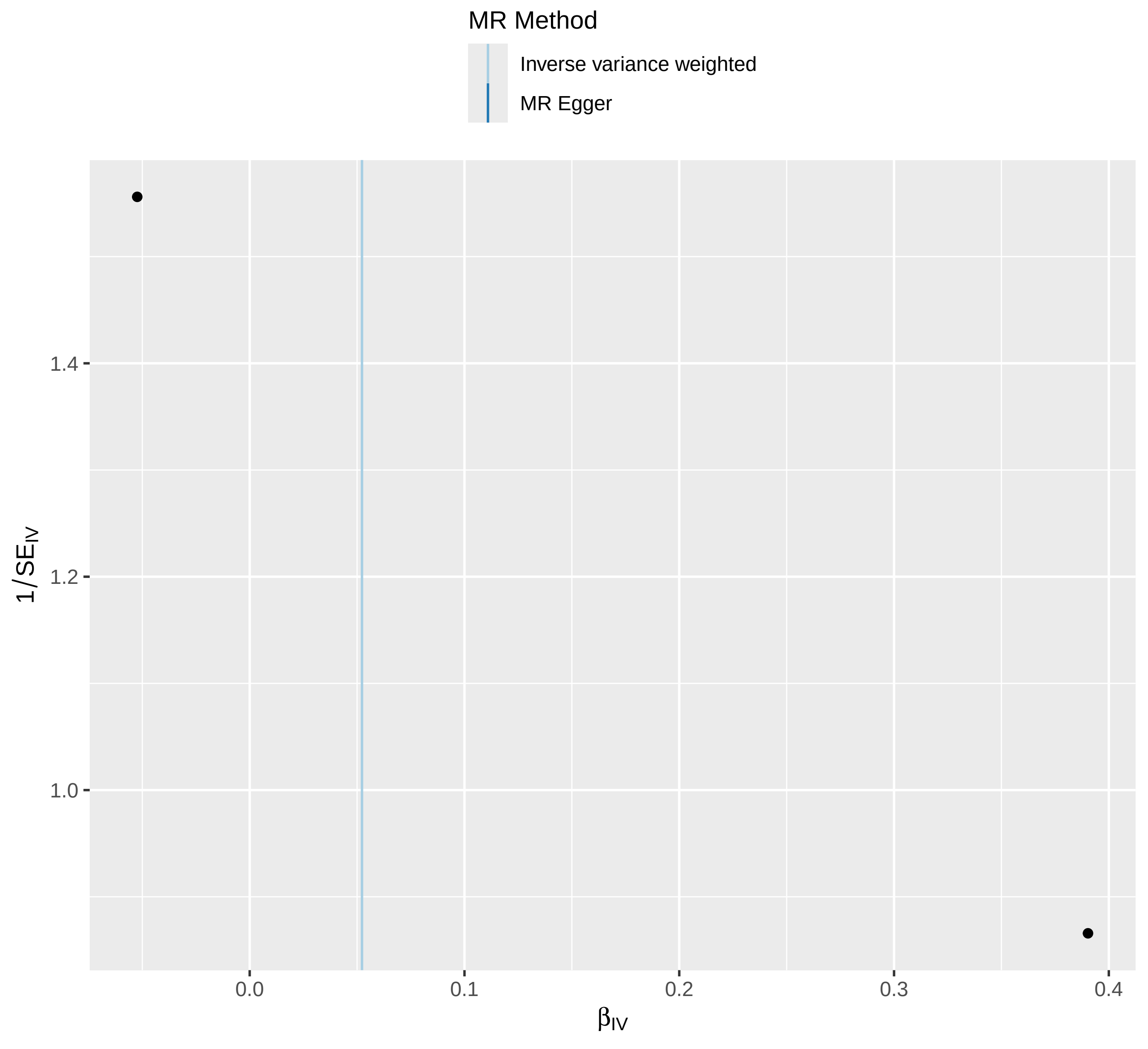

MR Method
Inverse variance weighted
MR Egger
1.4
1.2
1.0
1/SE_IV
0.0
0.1
0.2
0.3
0.4
β_IV

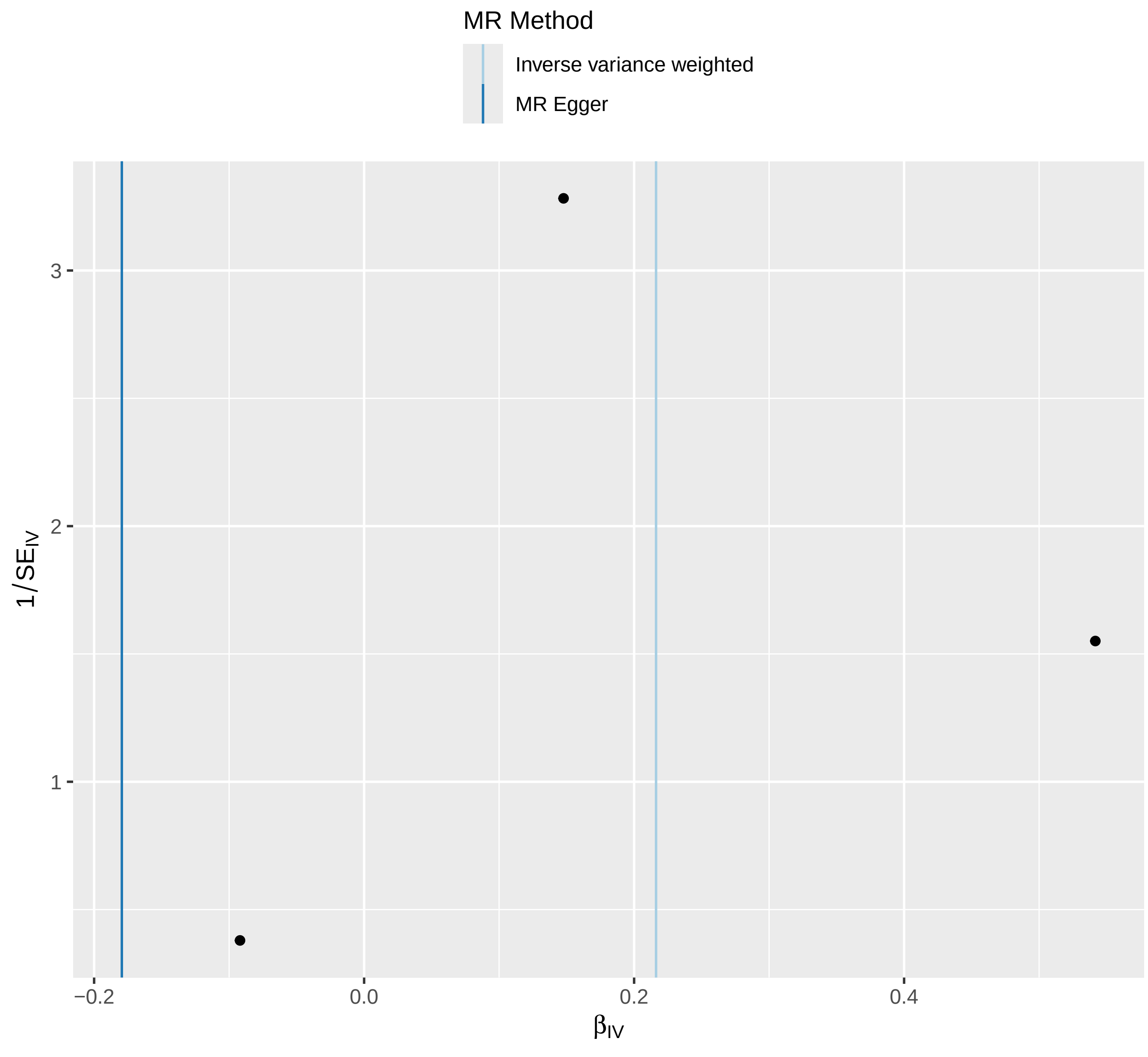
MR Method
Inverse variance weighted
MR Egger
3
2
1
1/SE_IV
−0.2
0.0
0.2
0.4
β_IV

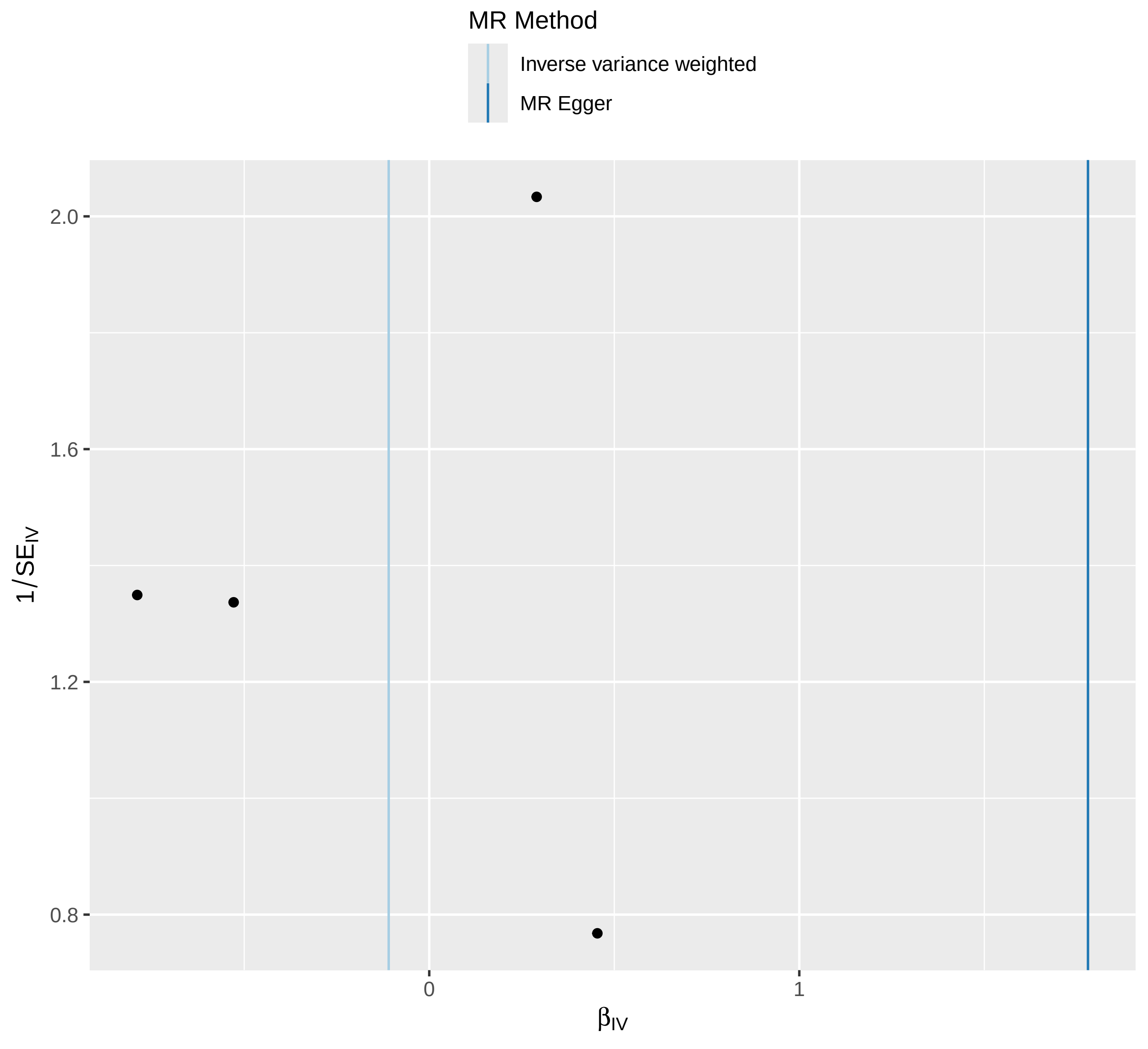
MR Method
Inverse variance weighted
MR Egger
2.0
1.6
1.2
0.8
0
1
1/SE_IV
β_IV

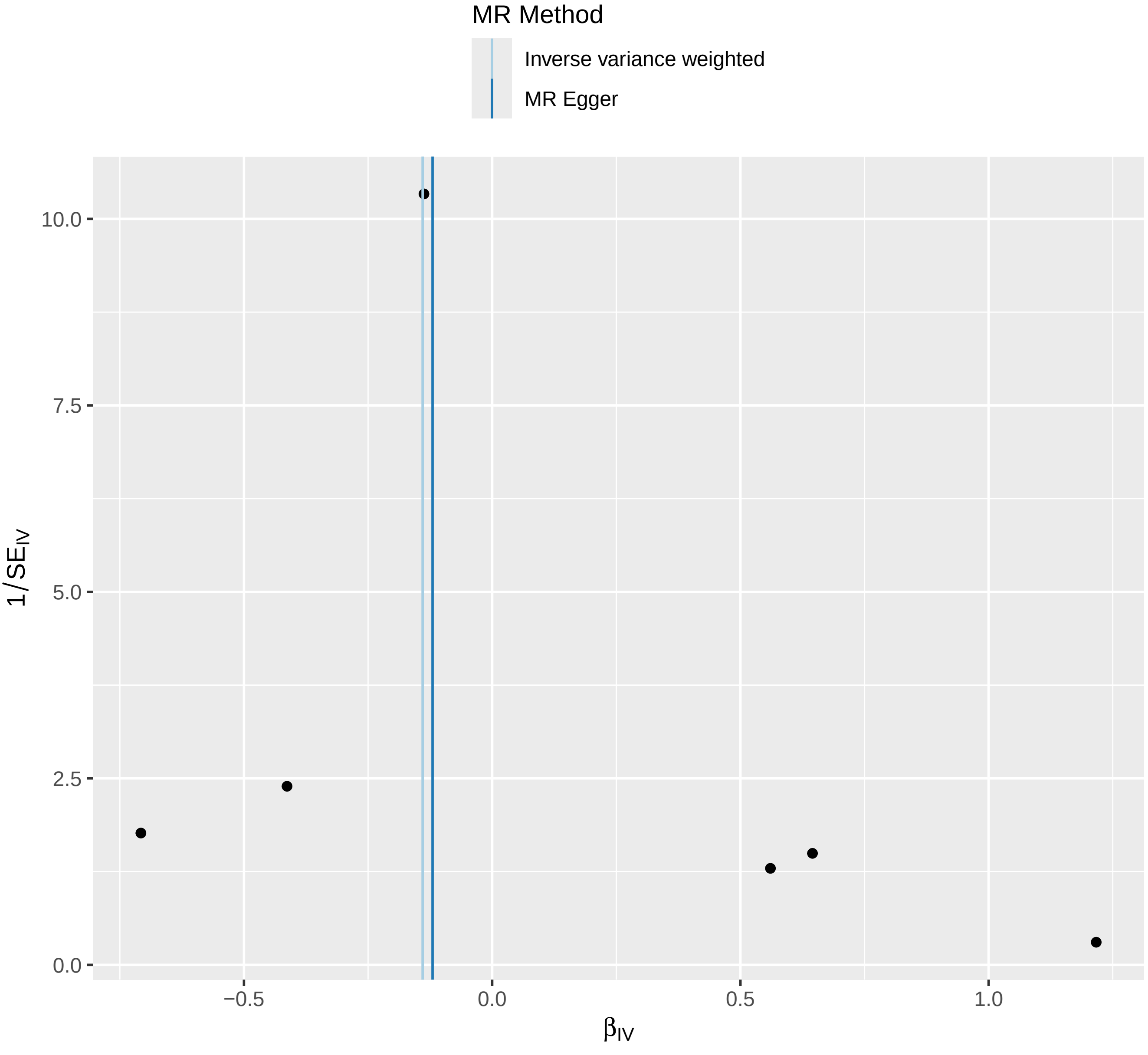
MR Method
Inverse variance weighted
MR Egger
1/SE_IV
β_IV
10.0
7.5
5.0
2.5
0.0
−0.5
0.0
0.5
1.0

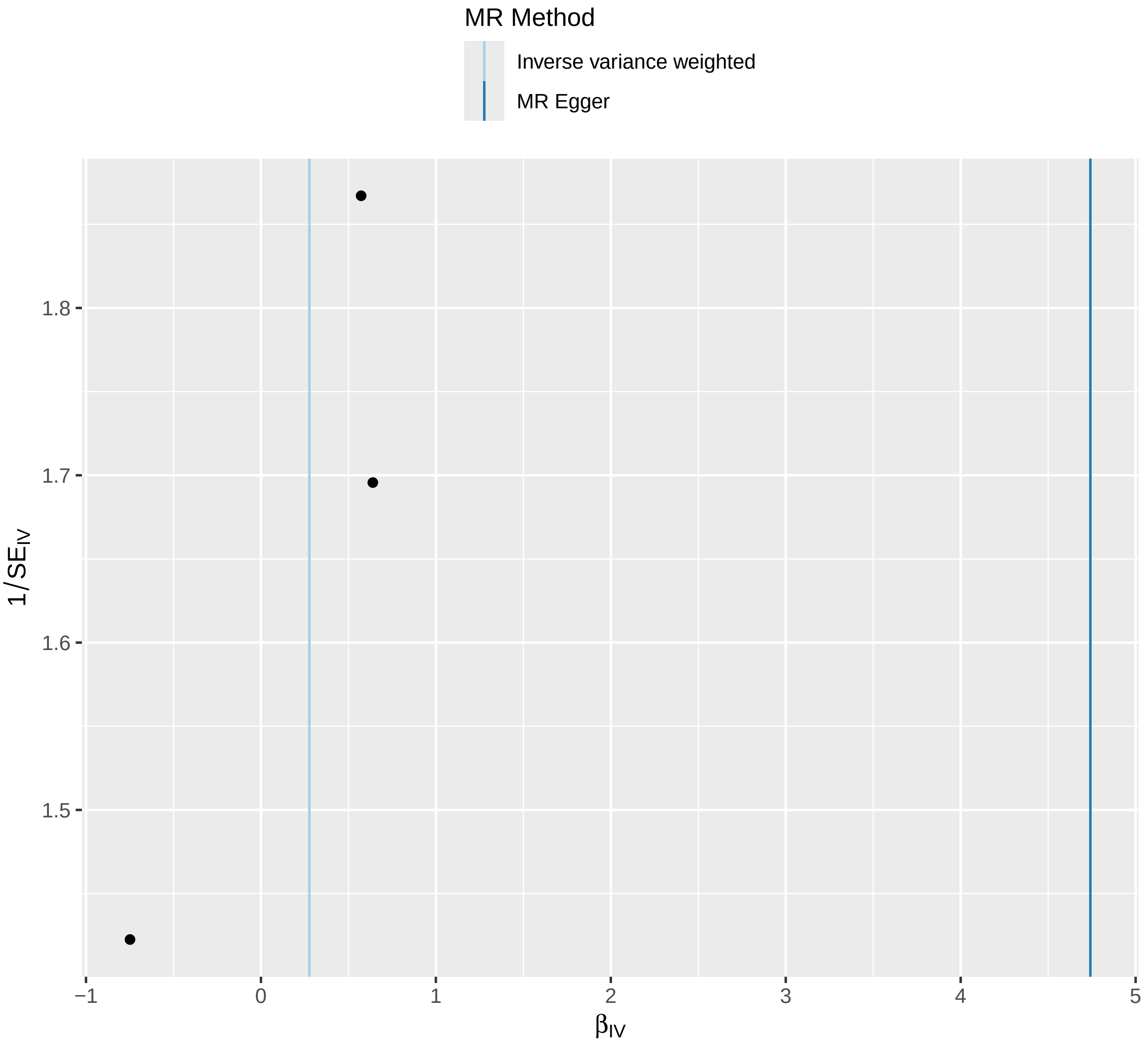

MR Method
Inverse variance weighted
MR Egger
1.8
1.7
1.6
1.5
1/SE_IV
−1
0
1
2
3
4
5
β_IV

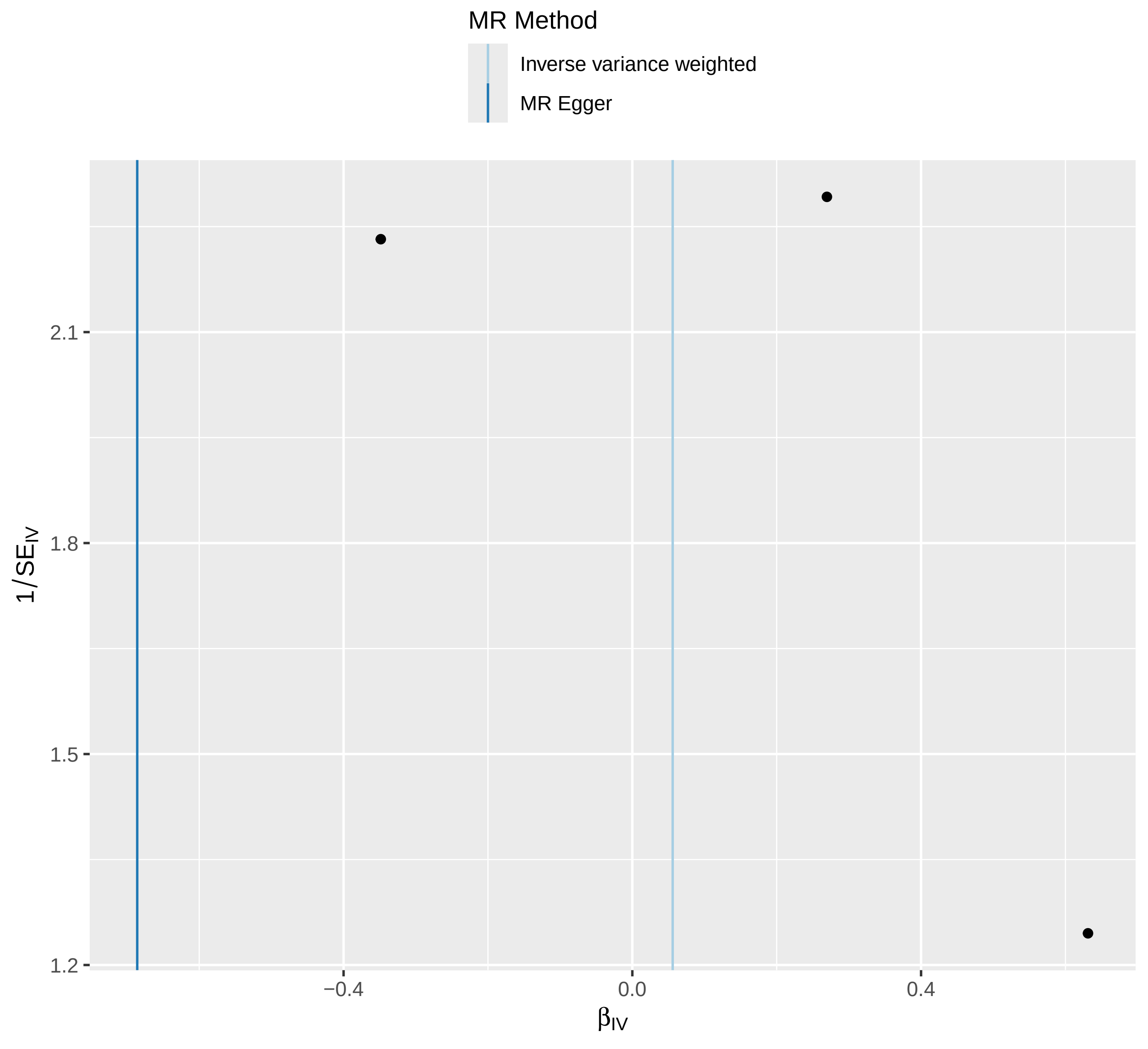

MR Method
Inverse variance weighted
MR Egger
2.1
1.8
1.5
1.2
1/SE_IV
−0.4
0.0
0.4
β_IV

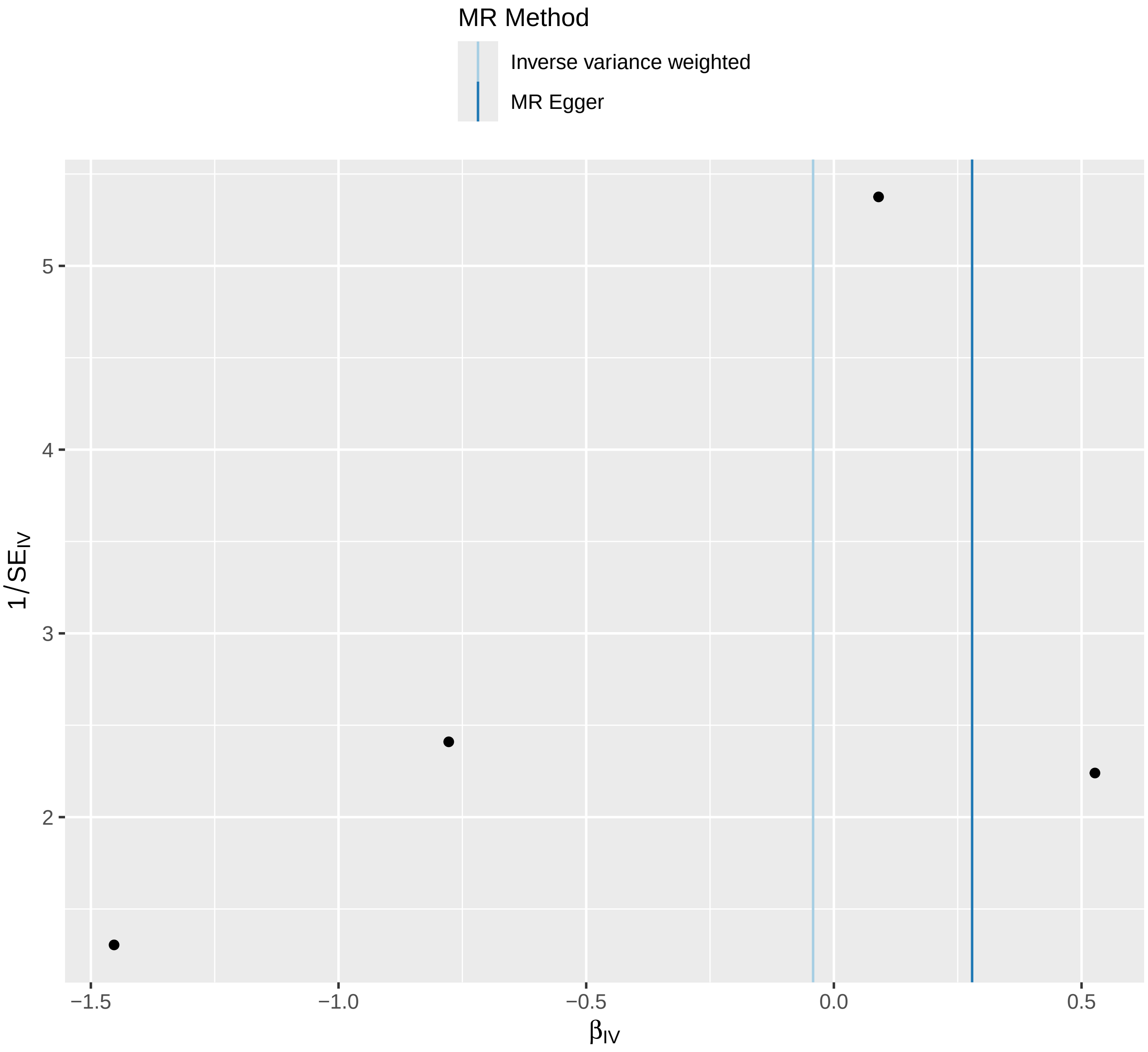

MR Method
Inverse variance weighted
MR Egger
1/SE_{IV}
β_{IV}
5
4
3
2
−1.5
−1.0
−0.5
0.0
0.5

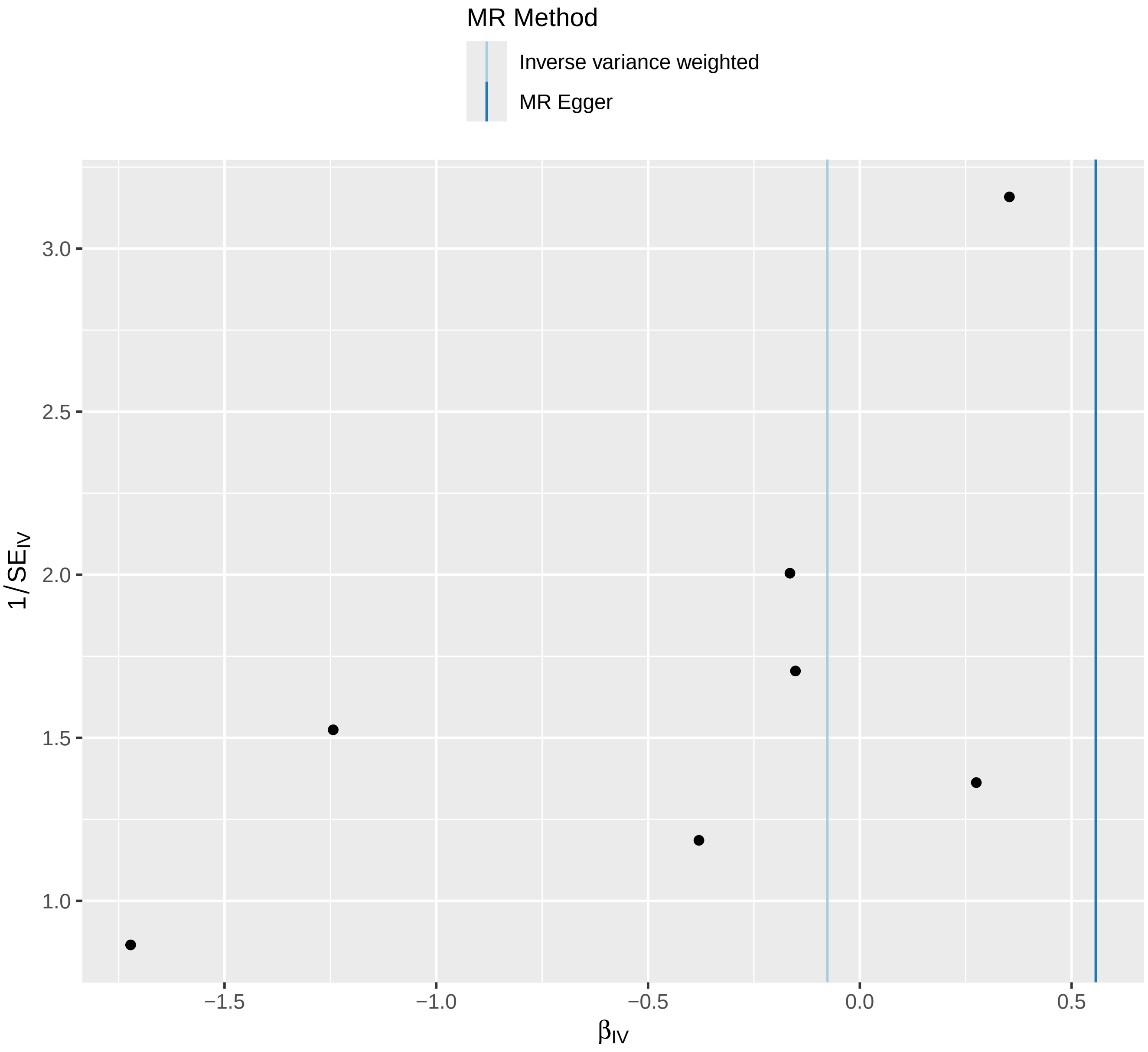

MR Method
Inverse variance weighted
MR Egger
$1/SE_{IV}$
3.0
2.5
2.0
1.5
1.0
−1.5
−1.0
−0.5
0.0
0.5
β_{IV}

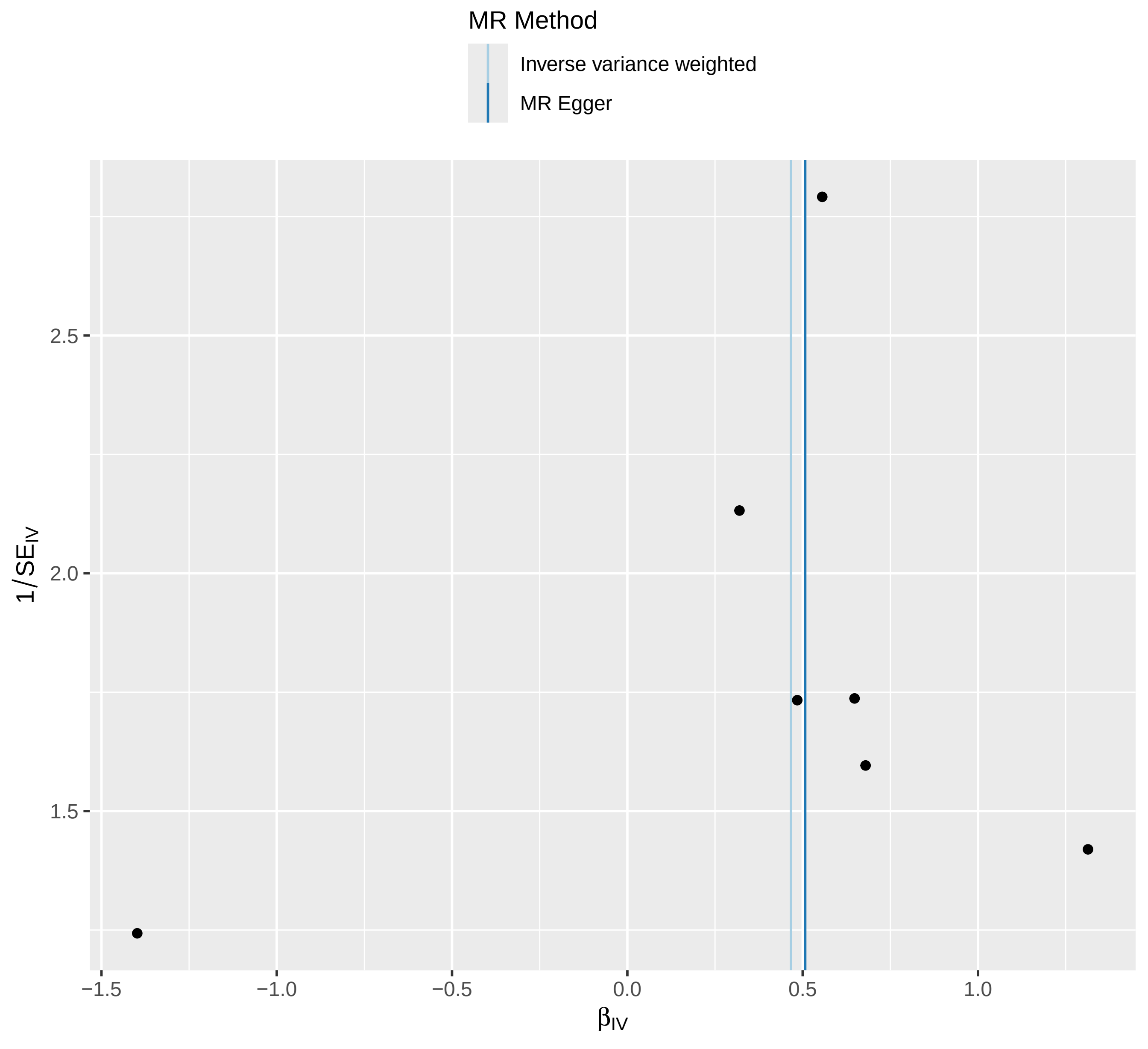
MR Method
Inverse variance weighted
MR Egger
1/SE$_{IV}$
2.5
2.0
1.5
−1.5
−1.0
−0.5
0.0
0.5
1.0
β$_{IV}$

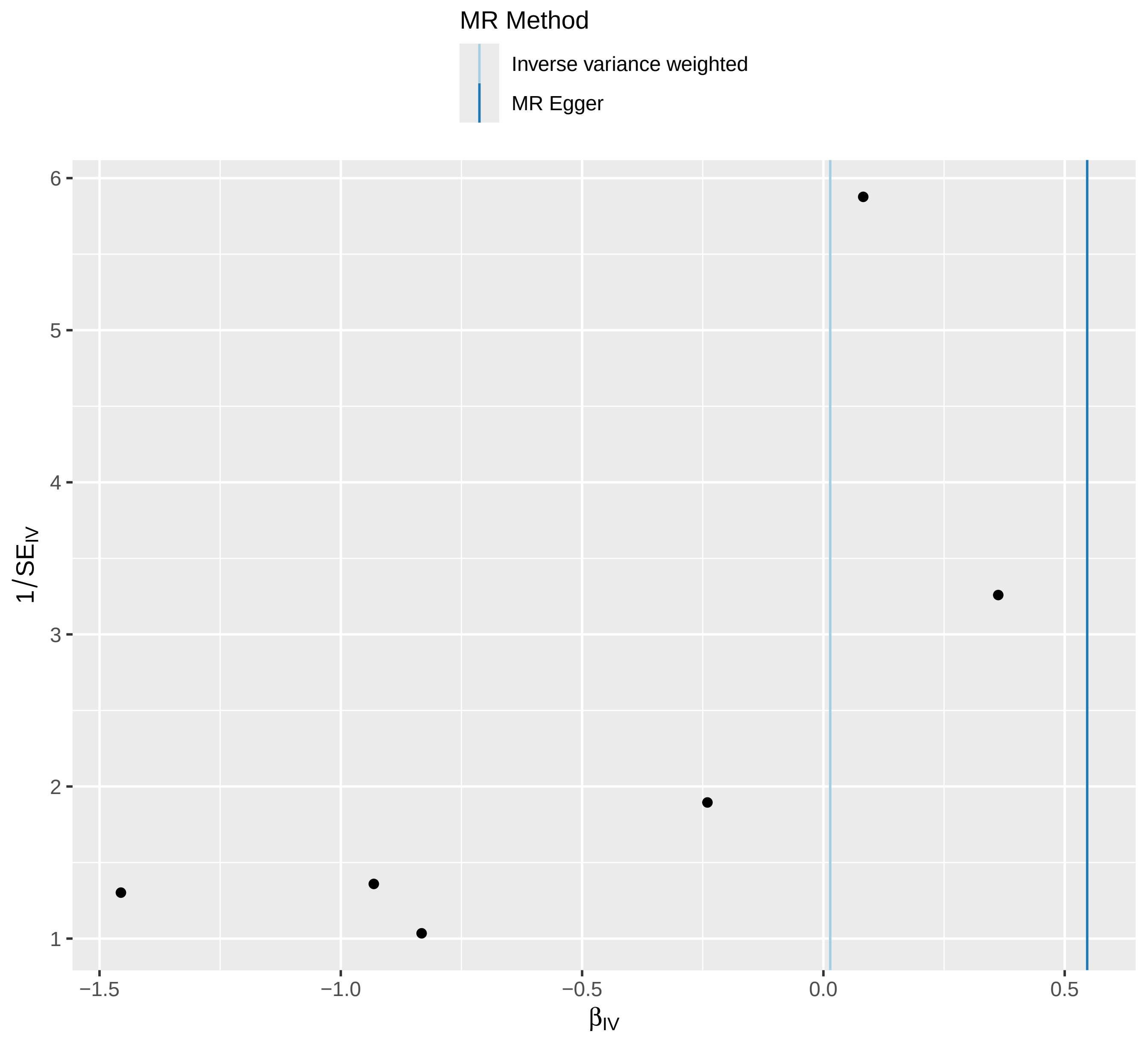
MR Method
Inverse variance weighted
MR Egger
1/SE_IV
β_IV
6
5
4
3
2
1
−1.5
−1.0
−0.5
0.0
0.5

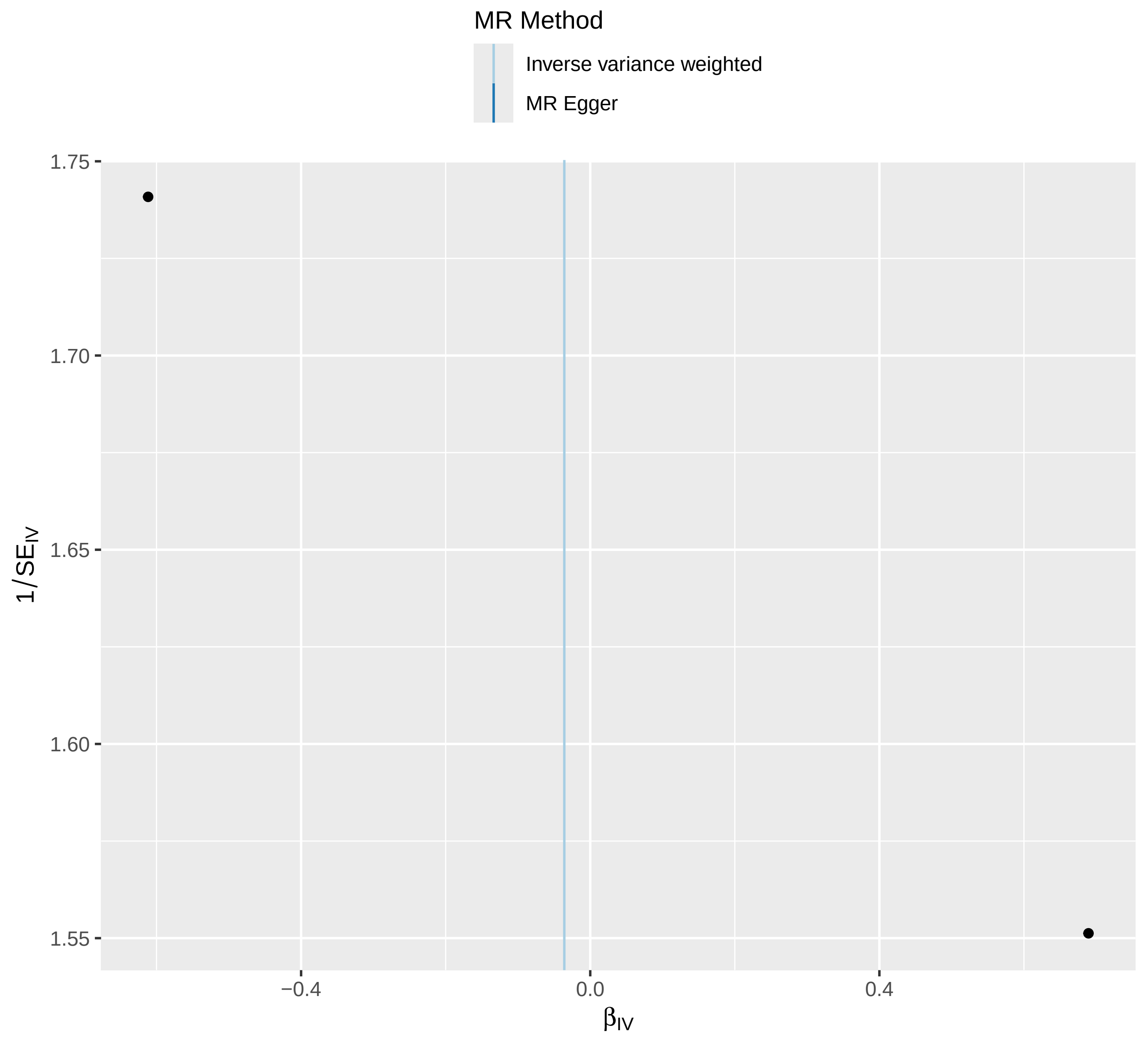

MR Method
Inverse variance weighted
MR Egger
1.75
1.70
1.65
1.60
1.55
1/SE_IV
−0.4
0.0
0.4
β_IV

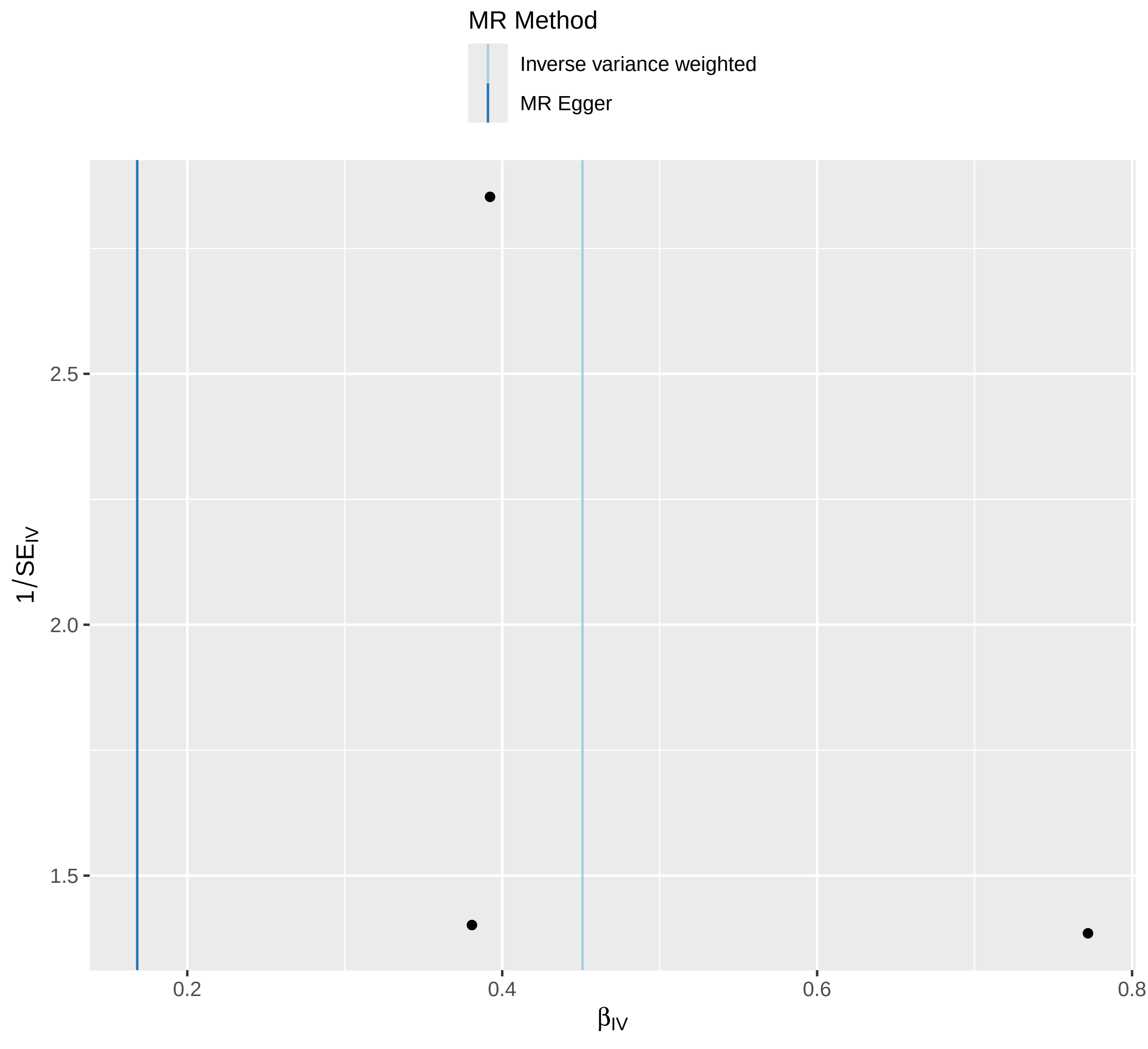
MR Method
Inverse variance weighted
MR Egger
2.5
2.0
1.5
1/SE_IV
0.2
0.4
0.6
0.8
β_IV

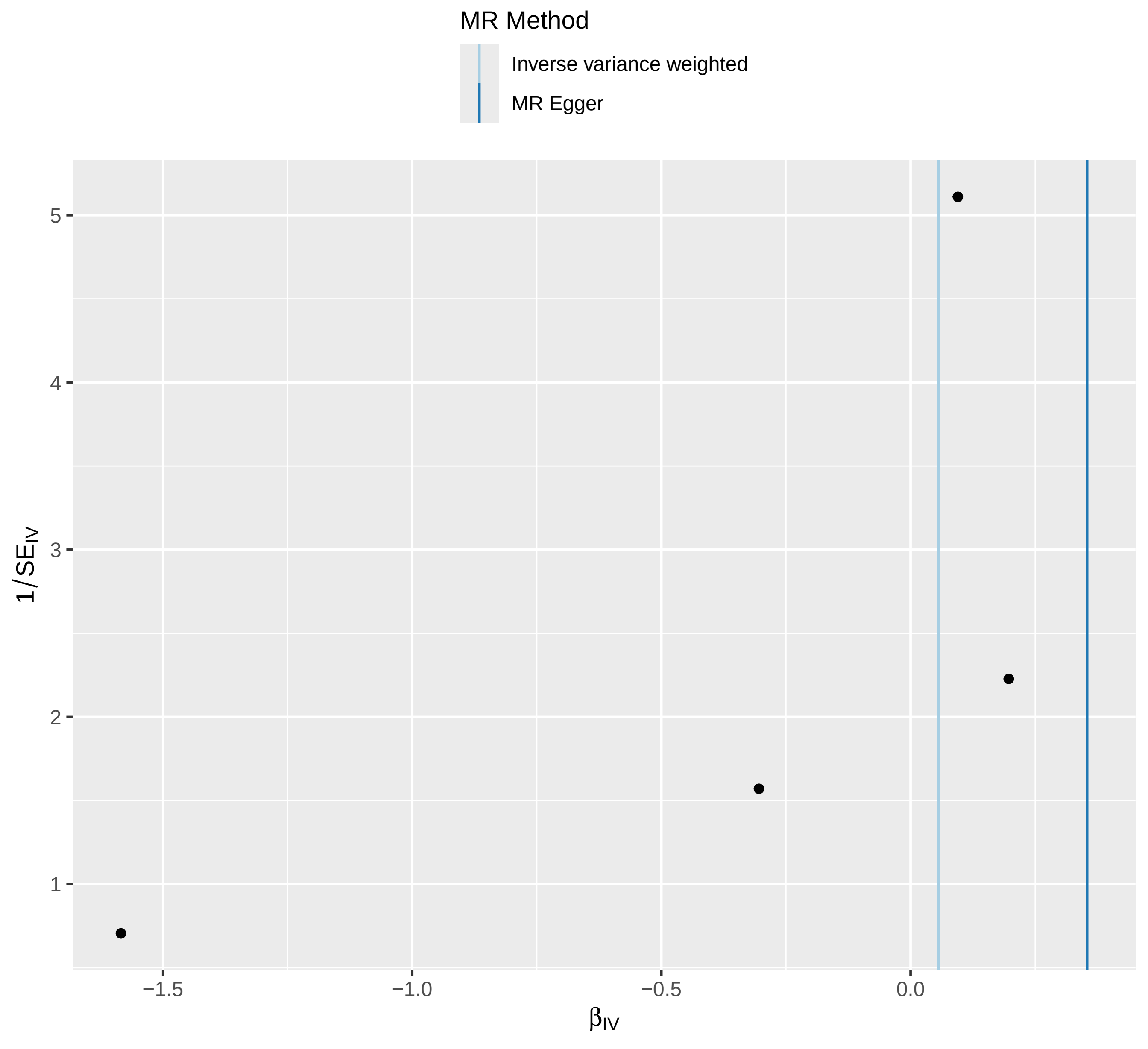

MR Method
Inverse variance weighted
MR Egger
5
4
3
2
1
$1/SE_{IV}$
−1.5
−1.0
−0.5
0.0
β_{IV}

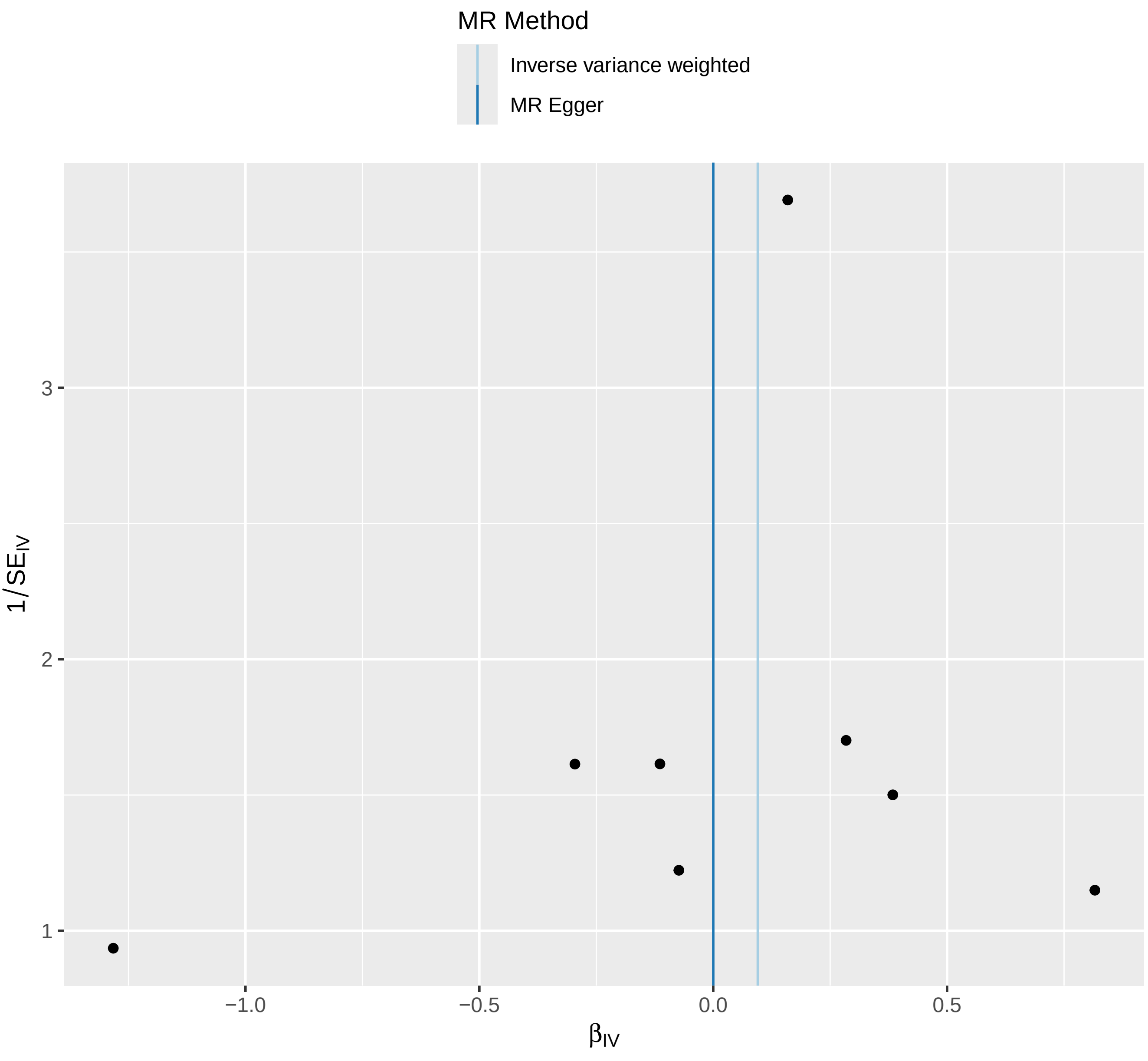
MR Method
Inverse variance weighted
MR Egger
1/SE$_{IV}$
3
2
1
−1.0
−0.5
0.0
0.5
β$_{IV}$

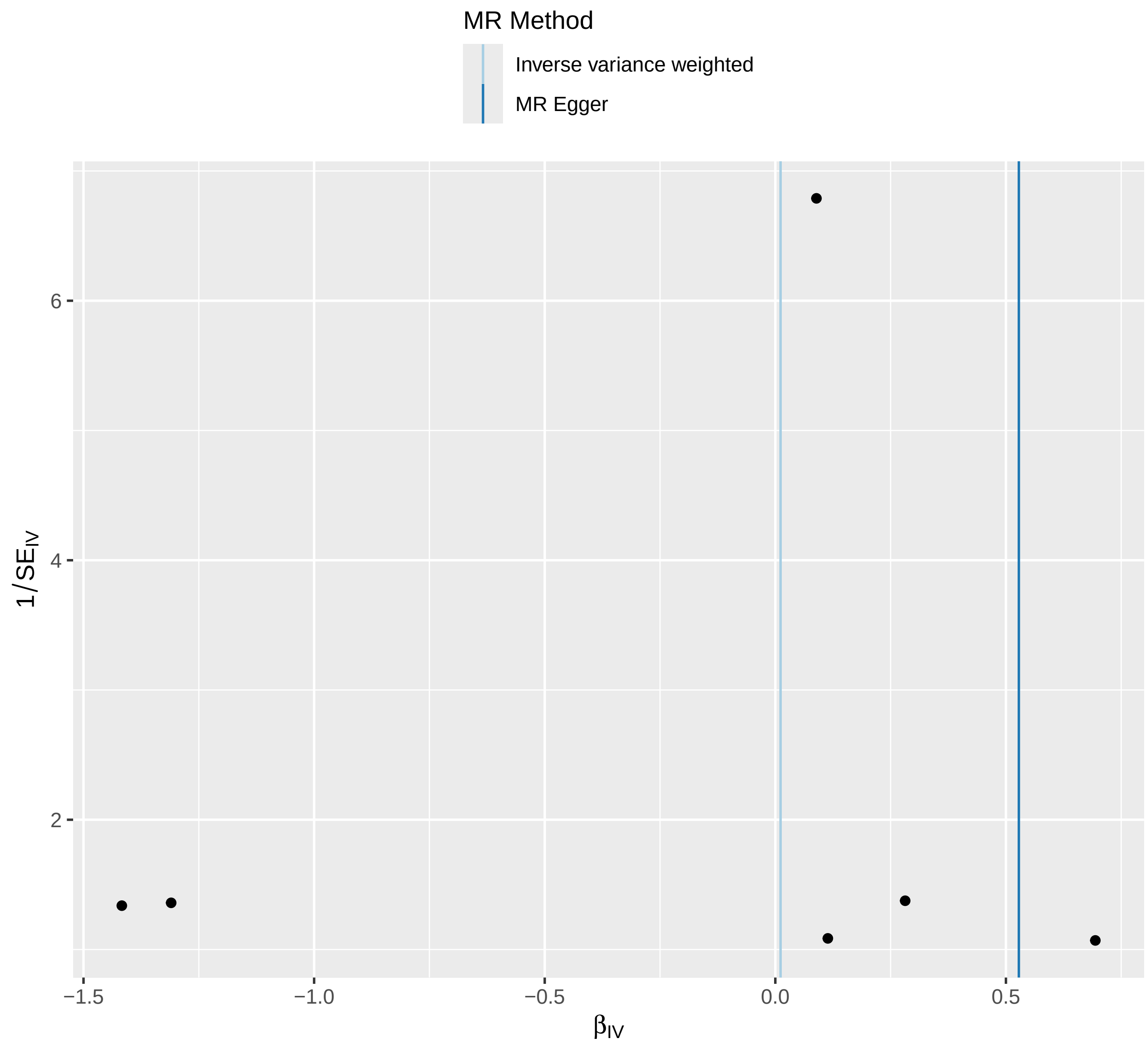
MR Method
Inverse variance weighted
MR Egger
6
4
2
1/SE_IV
−1.5
−1.0
−0.5
0.0
0.5
β_IV

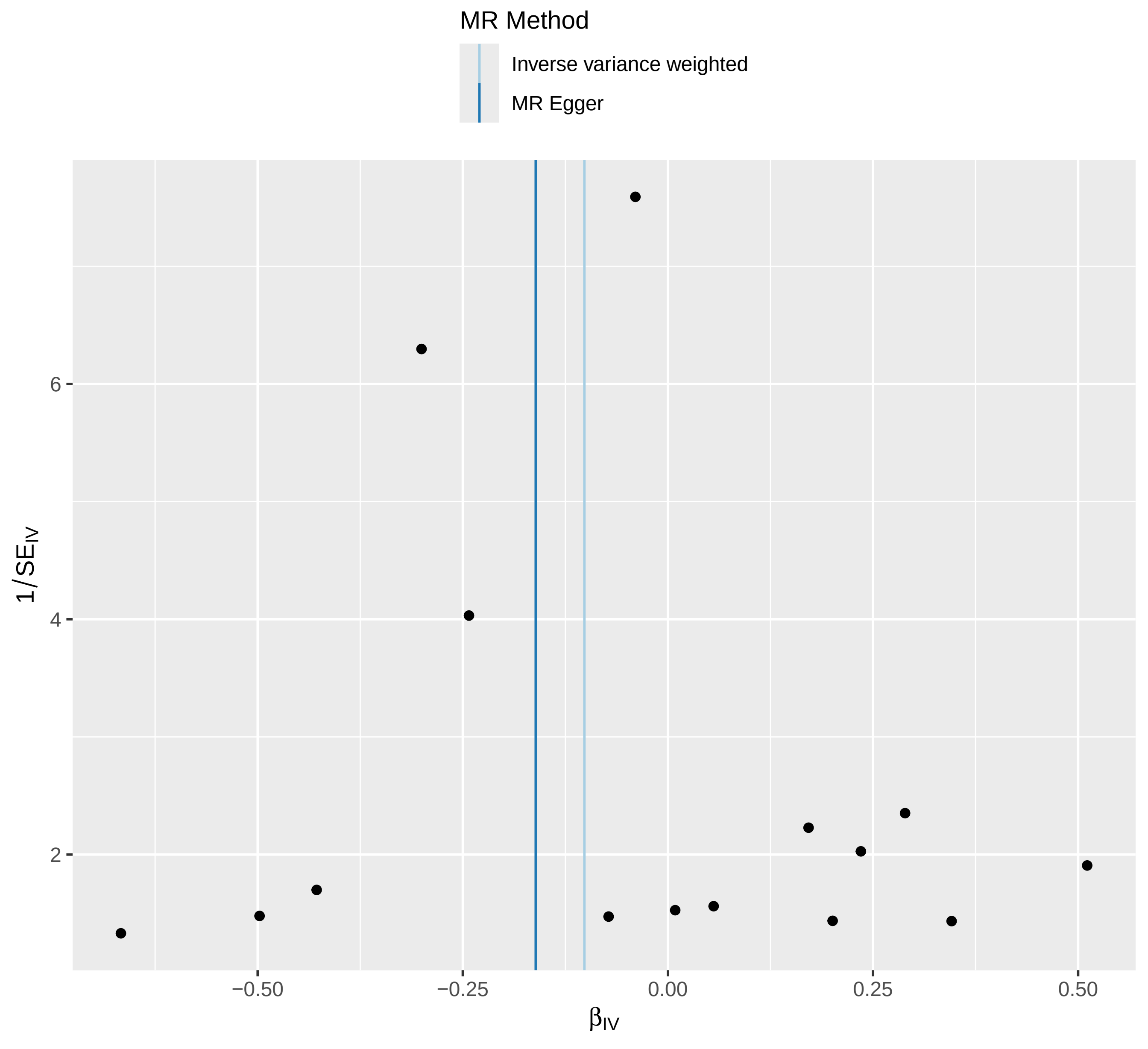

MR Method
Inverse variance weighted
MR Egger
1/SE_IV
6
4
2
−0.50
−0.25
0.00
0.25
0.50
β_IV

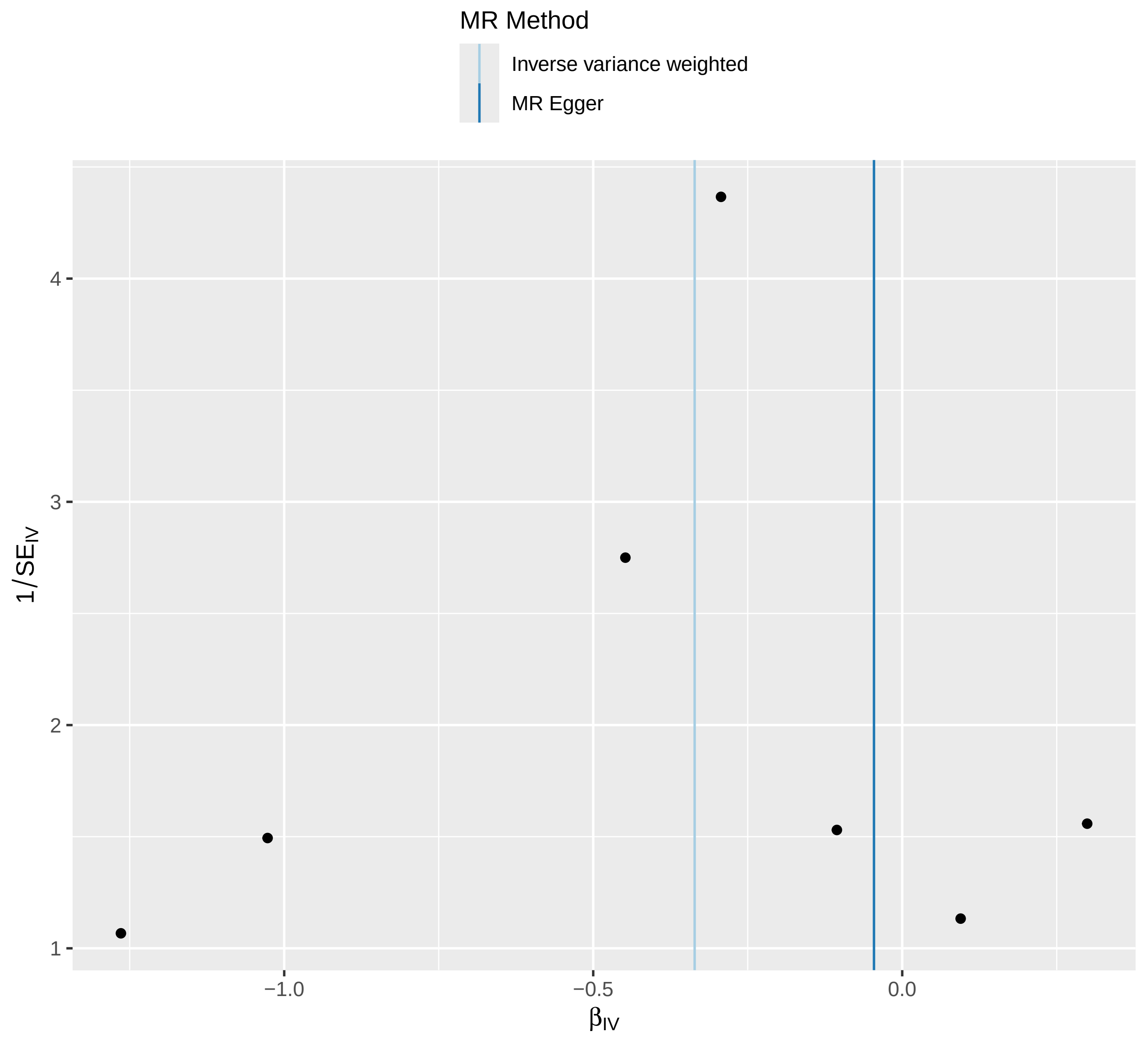
MR Method
Inverse variance weighted
MR Egger
4
3
2
1
1/SE$_{IV}$
−1.0
−0.5
0.0
β$_{IV}$

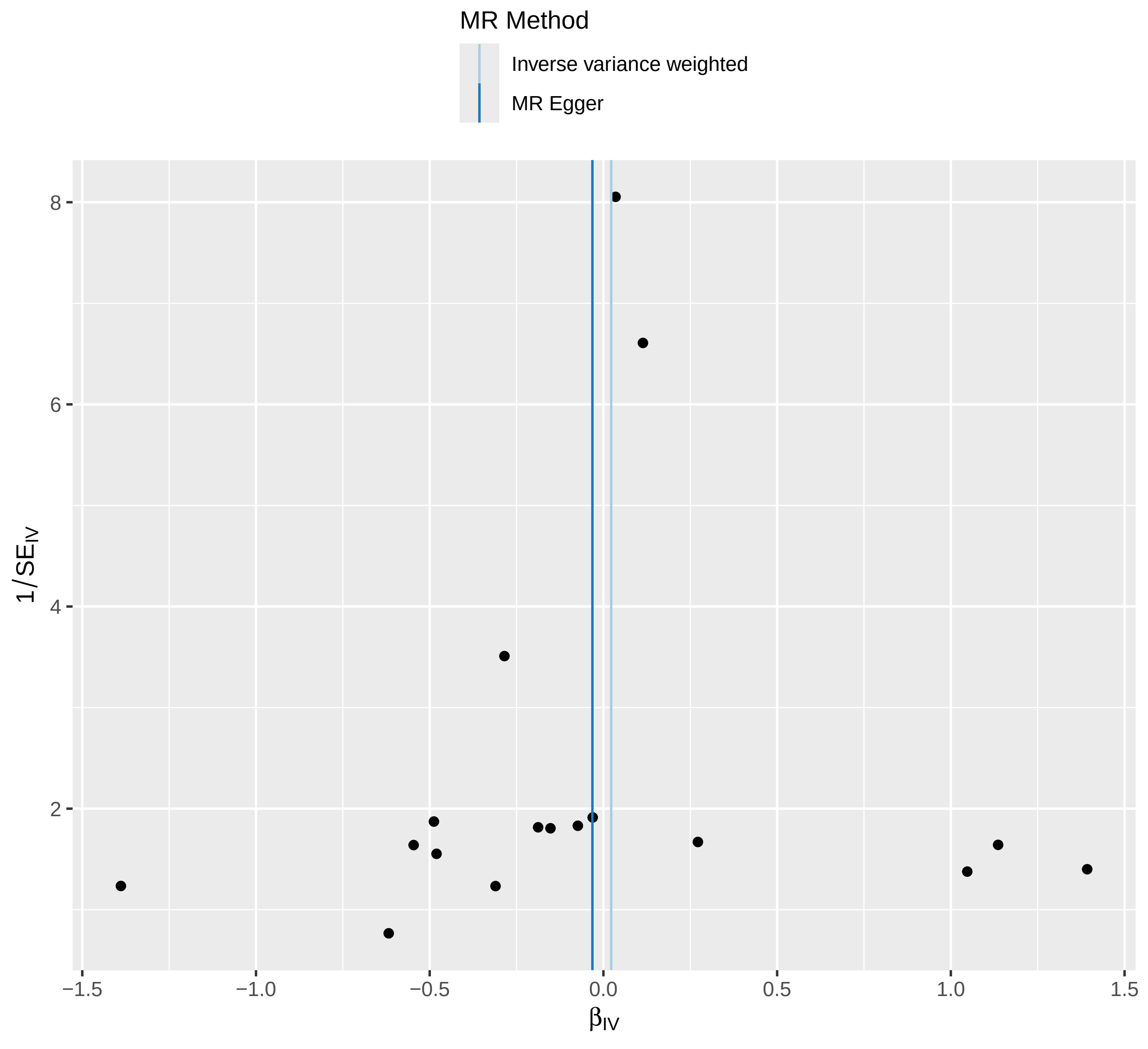

MR Method
Inverse variance weighted
MR Egger
8
6
4
2
1/SE$_{IV}$
−1.5
−1.0
−0.5
0.0
0.5
1.0
1.5
β$_{IV}$

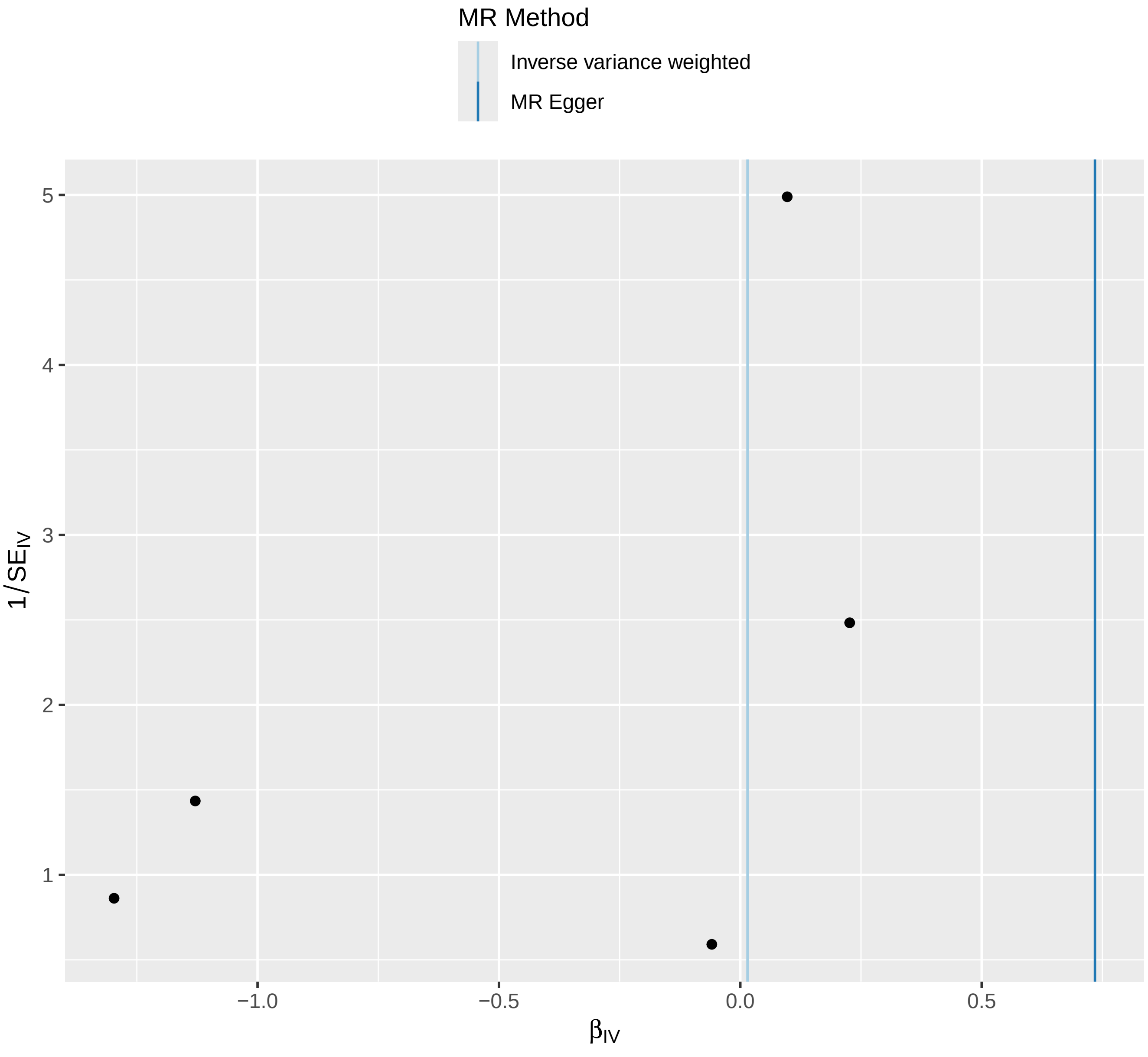

MR Method
Inverse variance weighted
MR Egger
1/SE_IV
β_IV
5
4
3
2
1
−1.0
−0.5
0.0
0.5

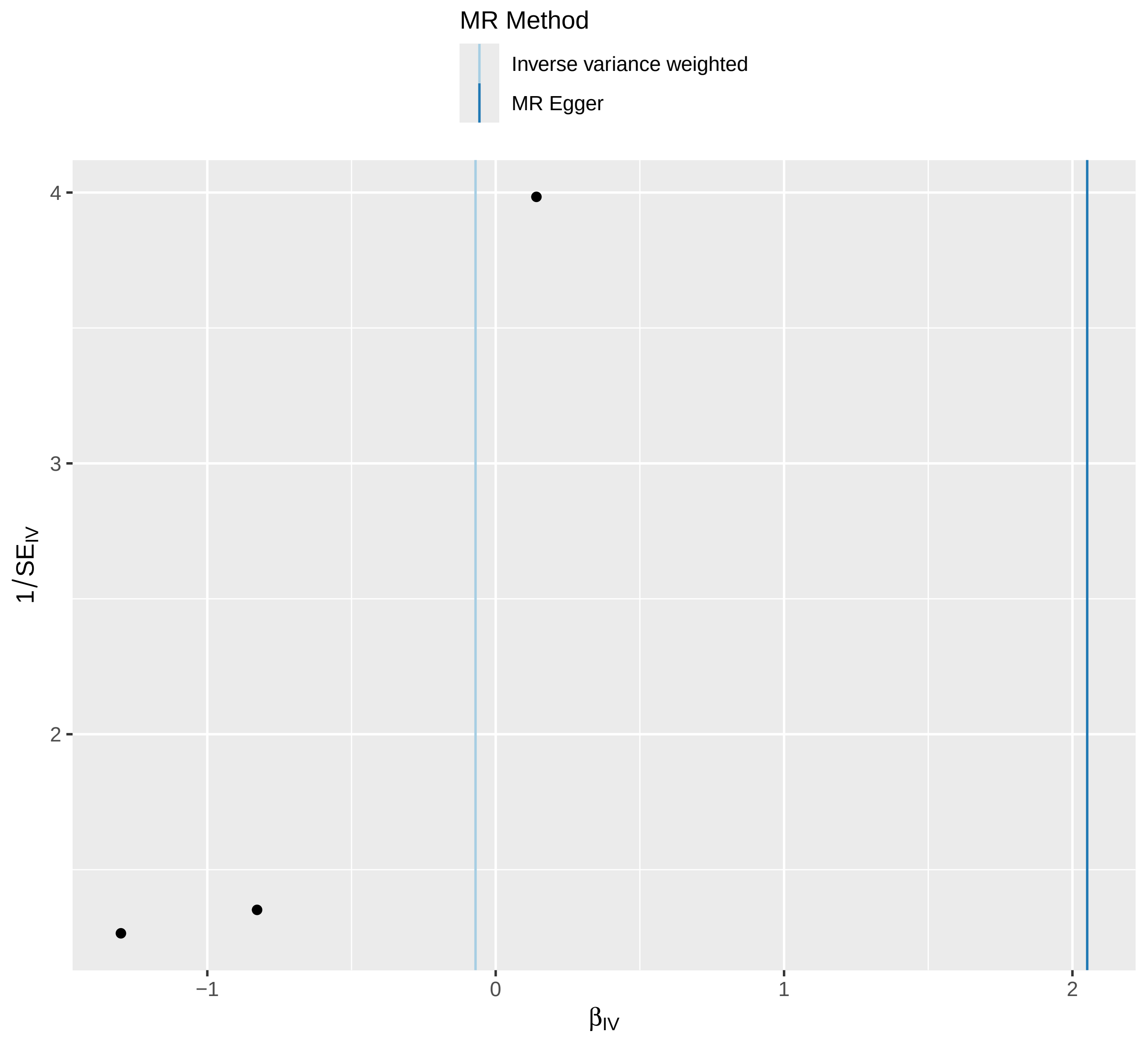

MR Method
Inverse variance weighted
MR Egger
4
3
2
1/SE_IV
−1
0
1
2
β_IV

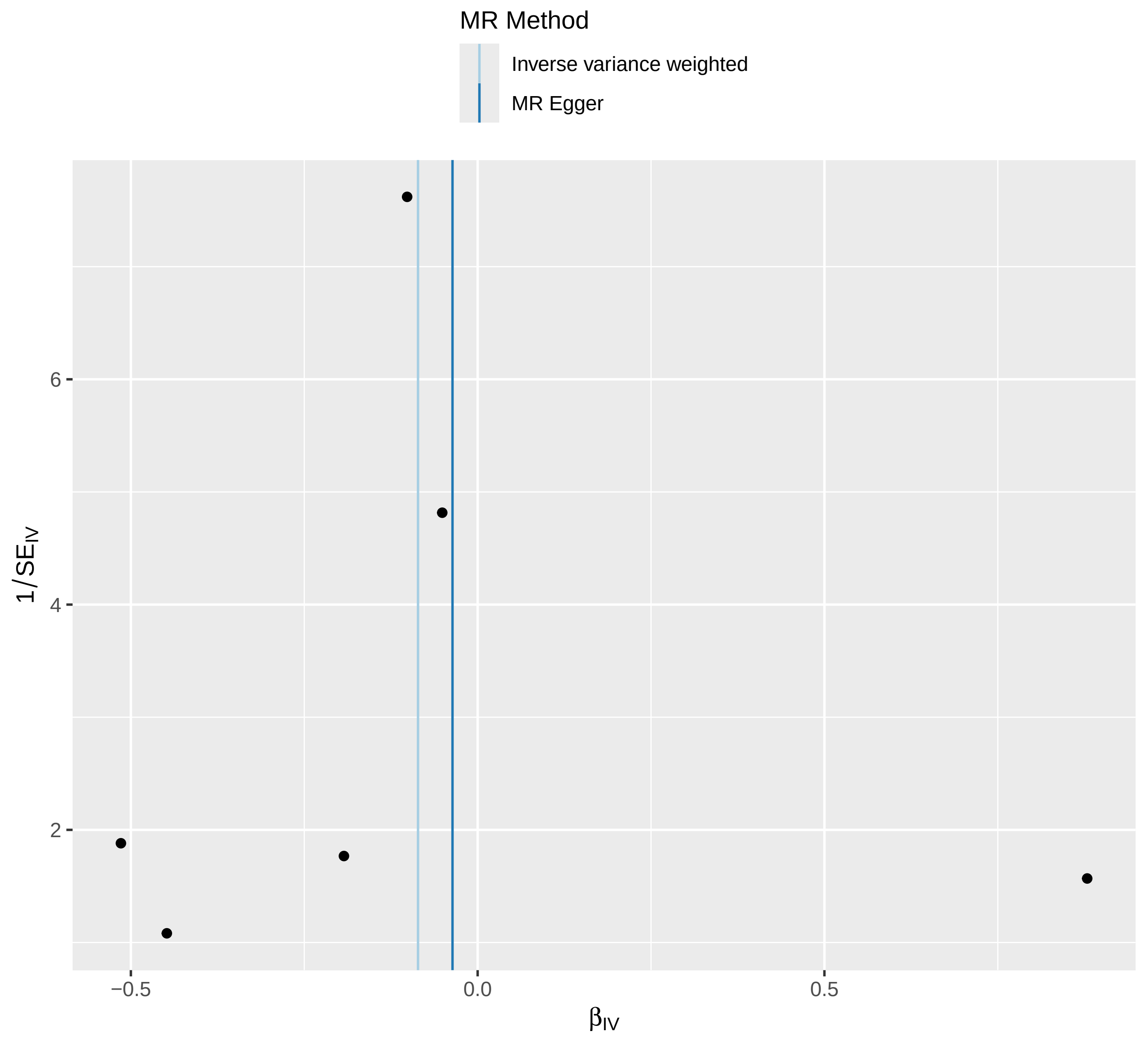
MR Method
Inverse variance weighted
MR Egger
6
4
2
1/SE$_{IV}$
−0.5
0.0
0.5
β$_{IV}$

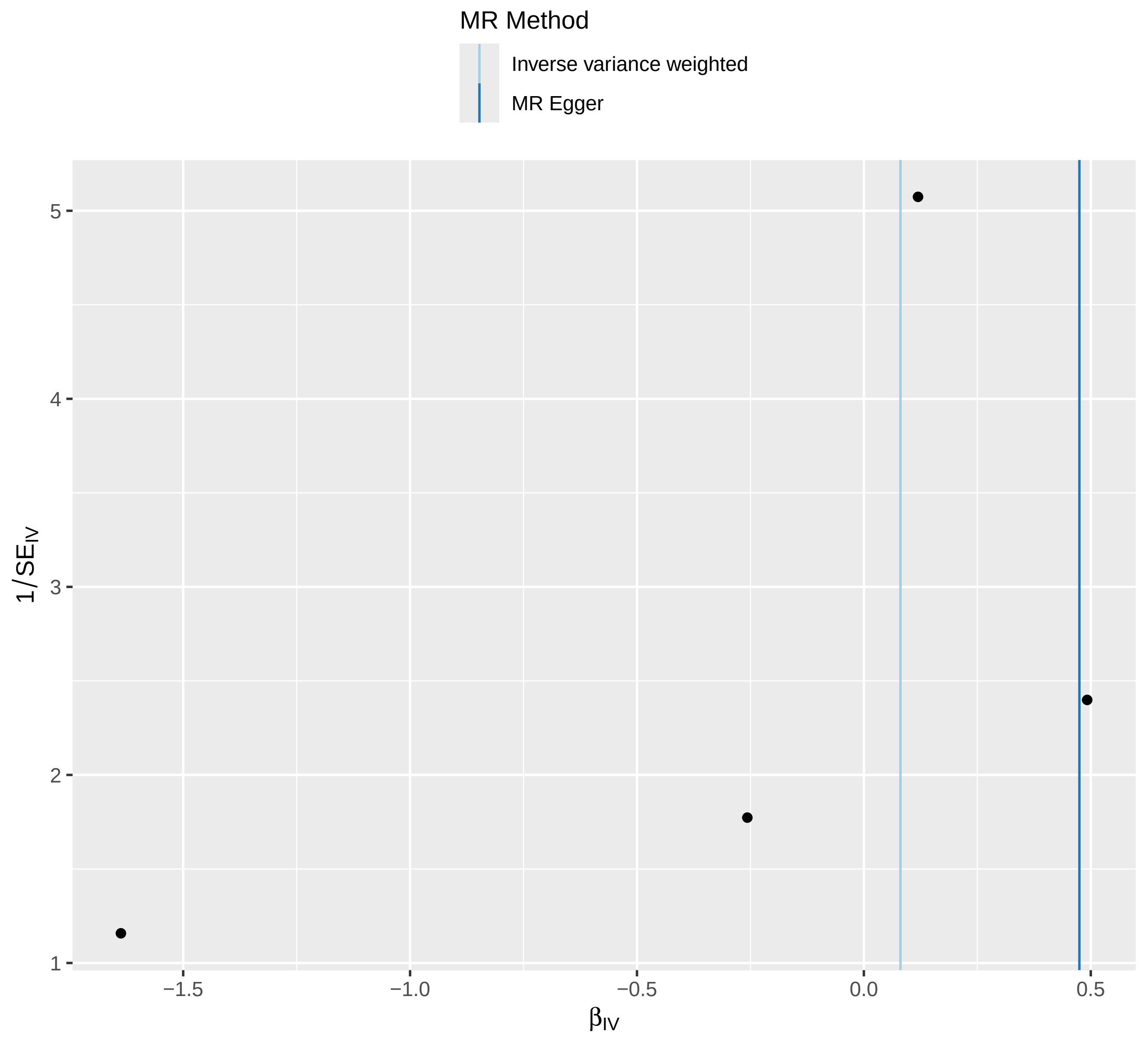

MR Method
Inverse variance weighted
MR Egger
5
4
3
2
1
1/SE_IV
−1.5
−1.0
−0.5
0.0
0.5
β_IV

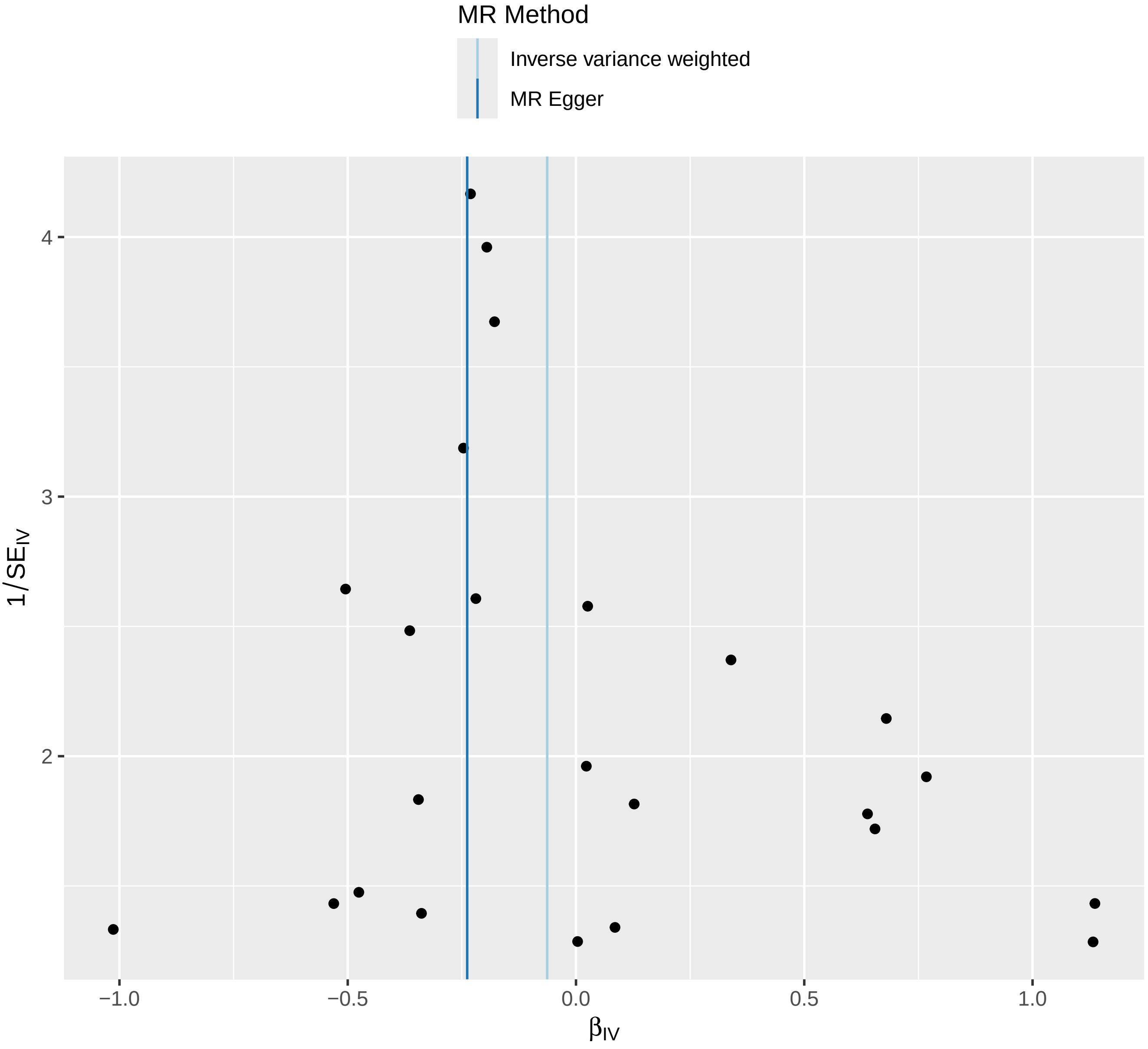
MR Method
Inverse variance weighted
MR Egger
4
3
2
1/SE_IV
−1.0
−0.5
0.0
0.5
1.0
β_IV

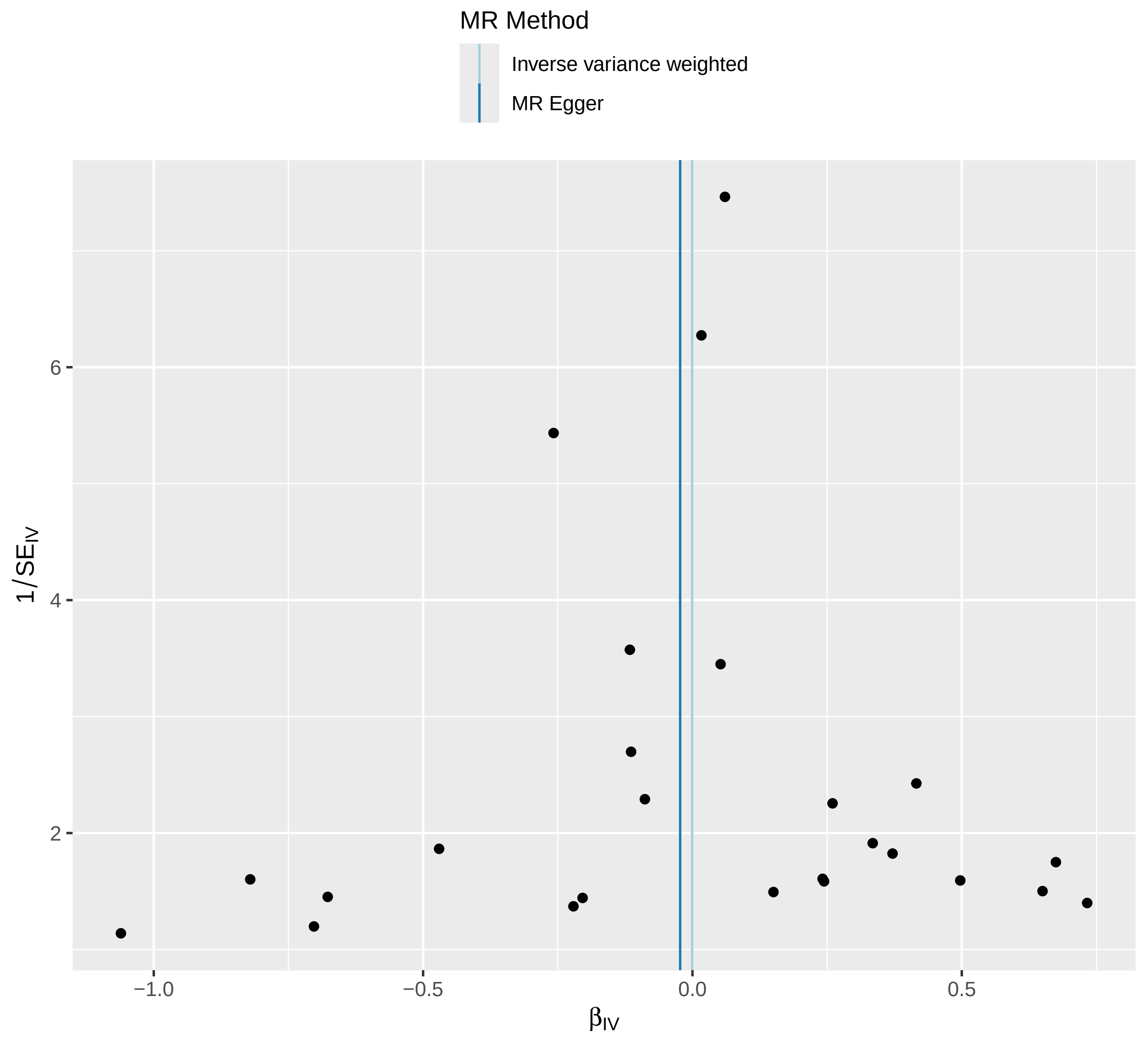
MR Method
Inverse variance weighted
MR Egger
1/SE_IV
6
4
2
−1.0
−0.5
0.0
0.5
β_IV

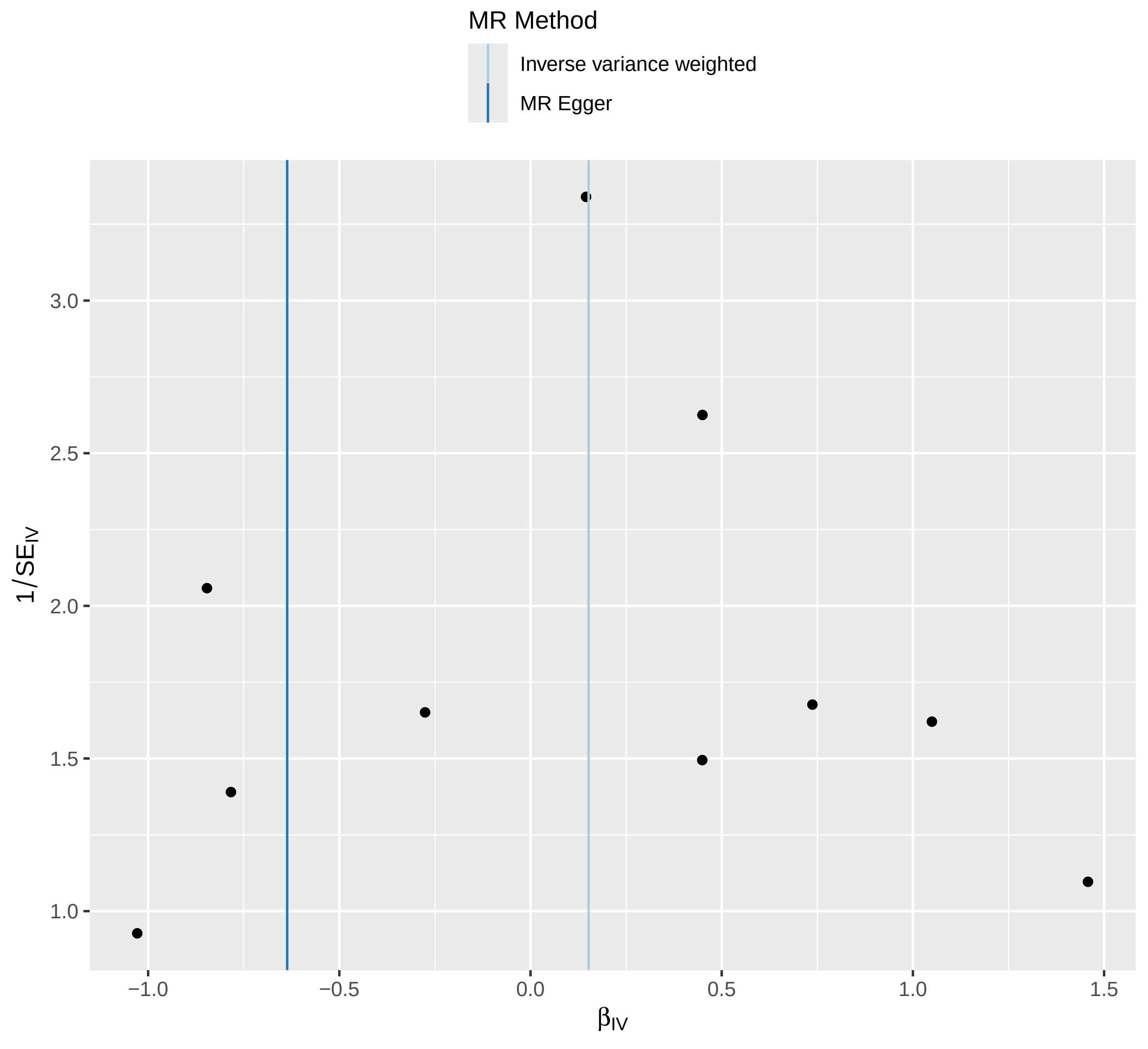

MR Method
Inverse variance weighted
MR Egger
3.0
2.5
2.0
1.5
1.0
1/SE$_{IV}$
−1.0
−0.5
0.0
0.5
1.0
1.5
β$_{IV}$

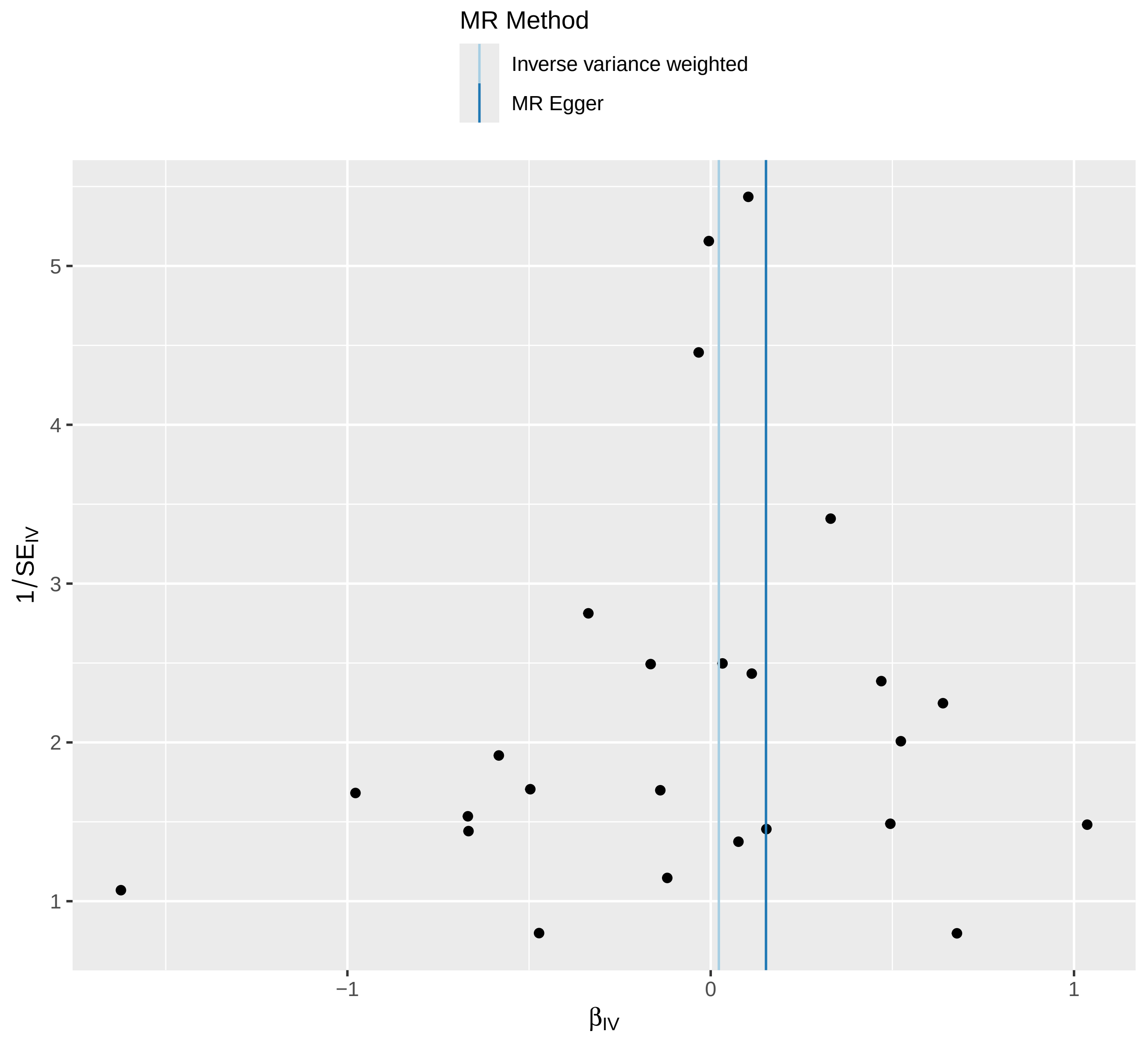
MR Method
Inverse variance weighted
MR Egger
1/SE_IV
5
4
3
2
1
−1
0
1
β_IV

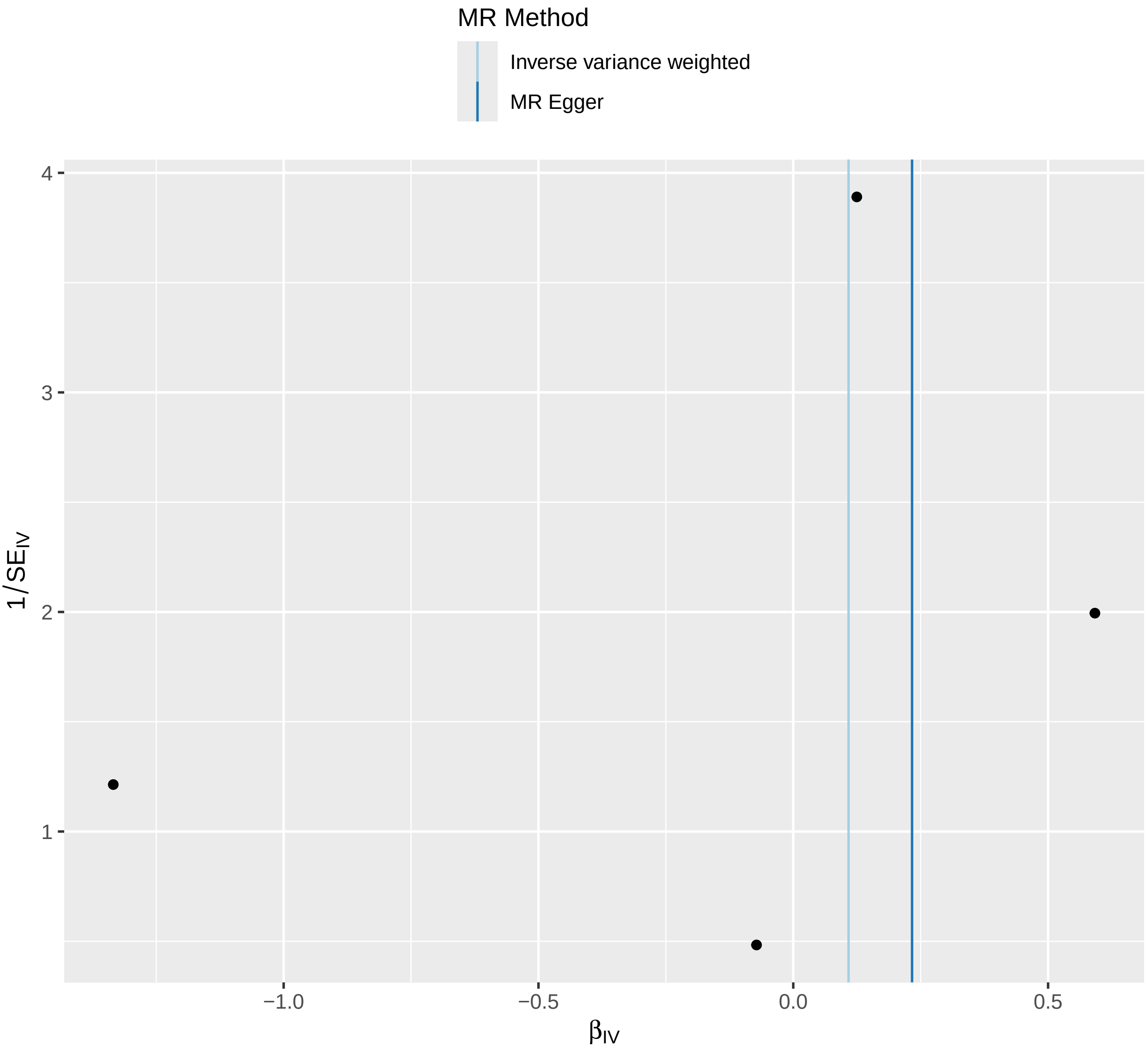

MR Method
Inverse variance weighted
MR Egger
4
3
2
1
1/SE_IV
−1.0
−0.5
0.0
0.5
β_IV

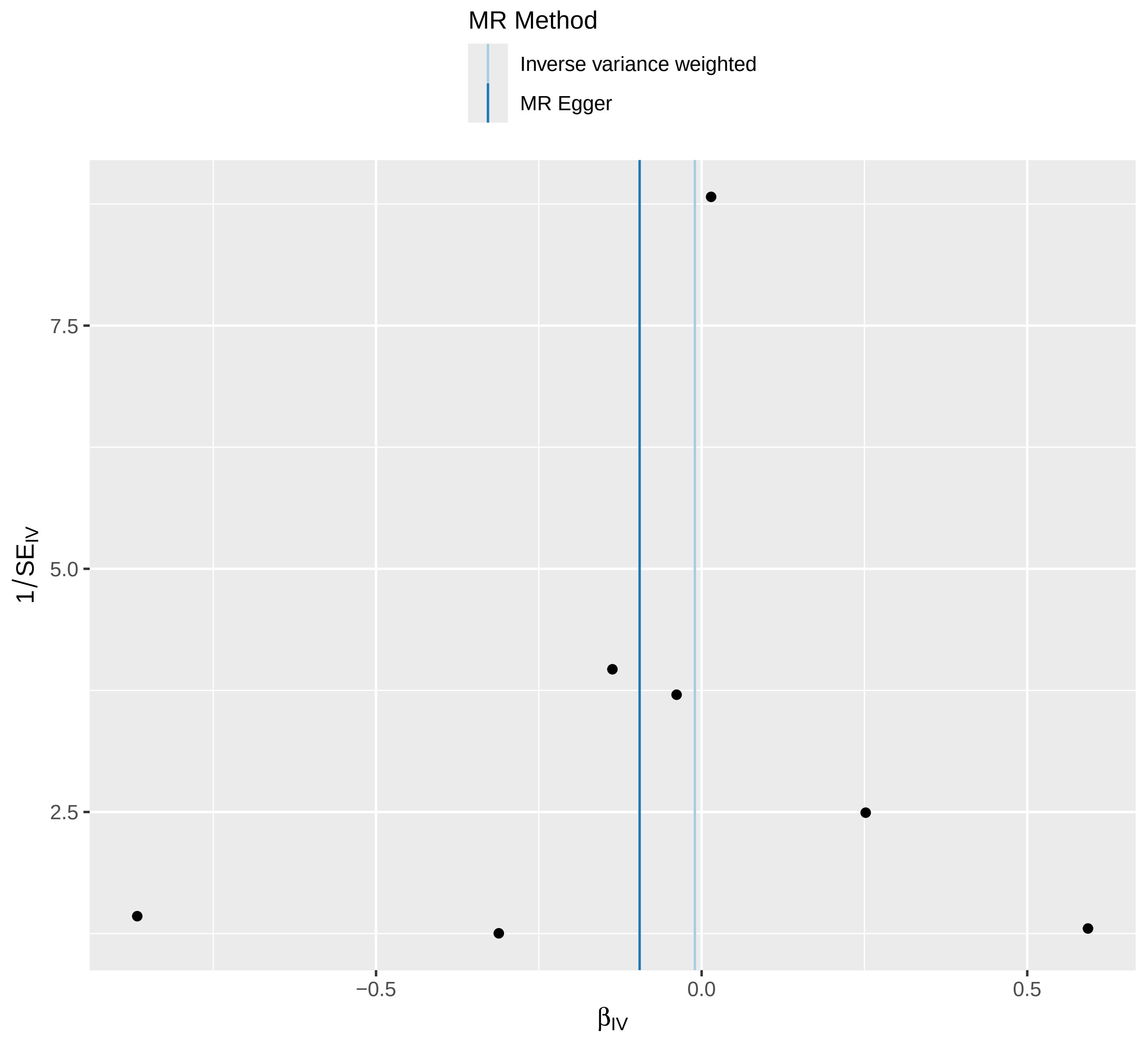

MR Method
Inverse variance weighted
MR Egger
7.5
5.0
2.5
$1/SE_{IV}$
−0.5
0.0
0.5
β_{IV}

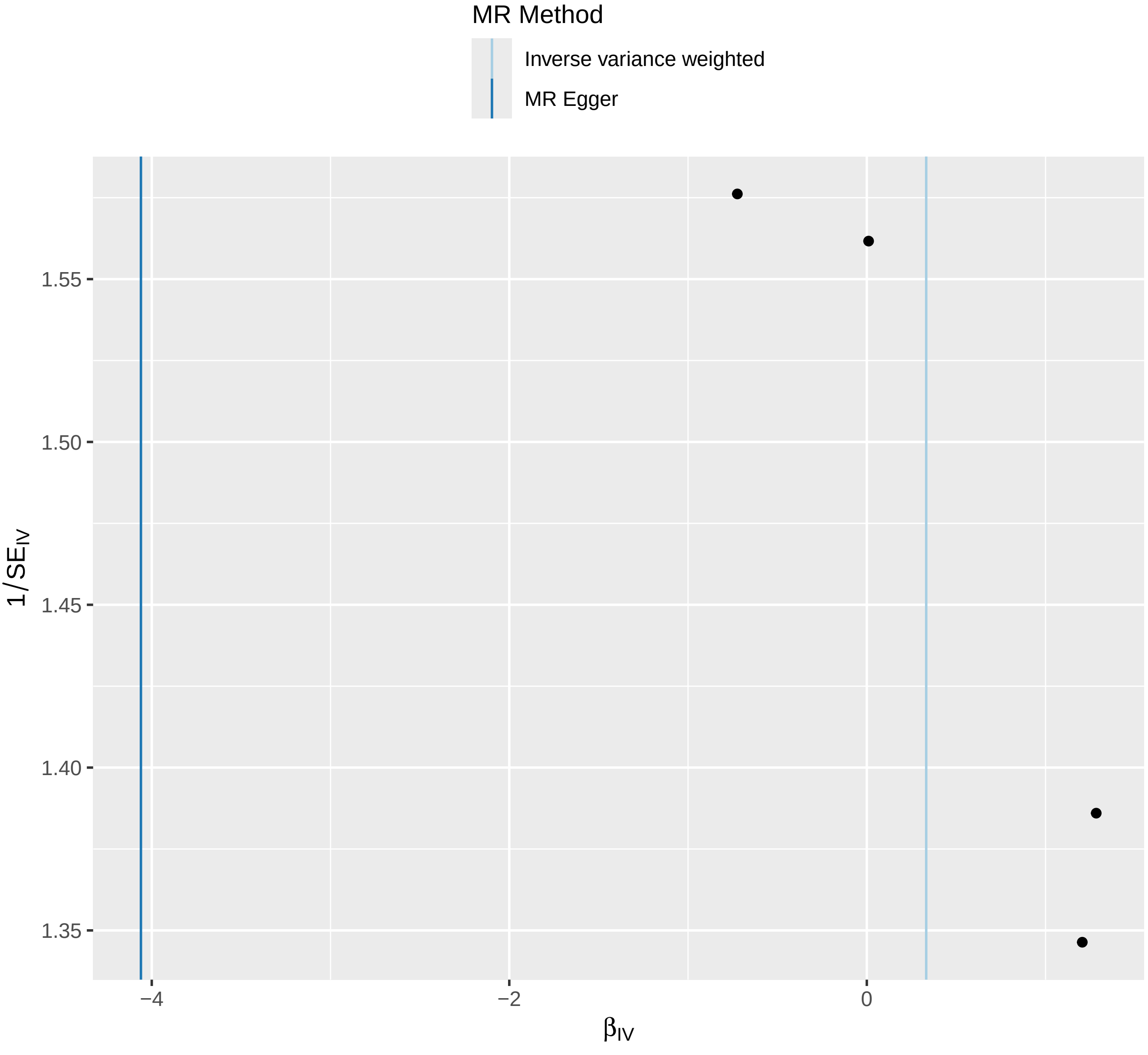
MR Method
Inverse variance weighted
MR Egger
1.55
1.50
1.45
1.40
1.35
$1/SE_{IV}$
−4
−2
0
β_{IV}

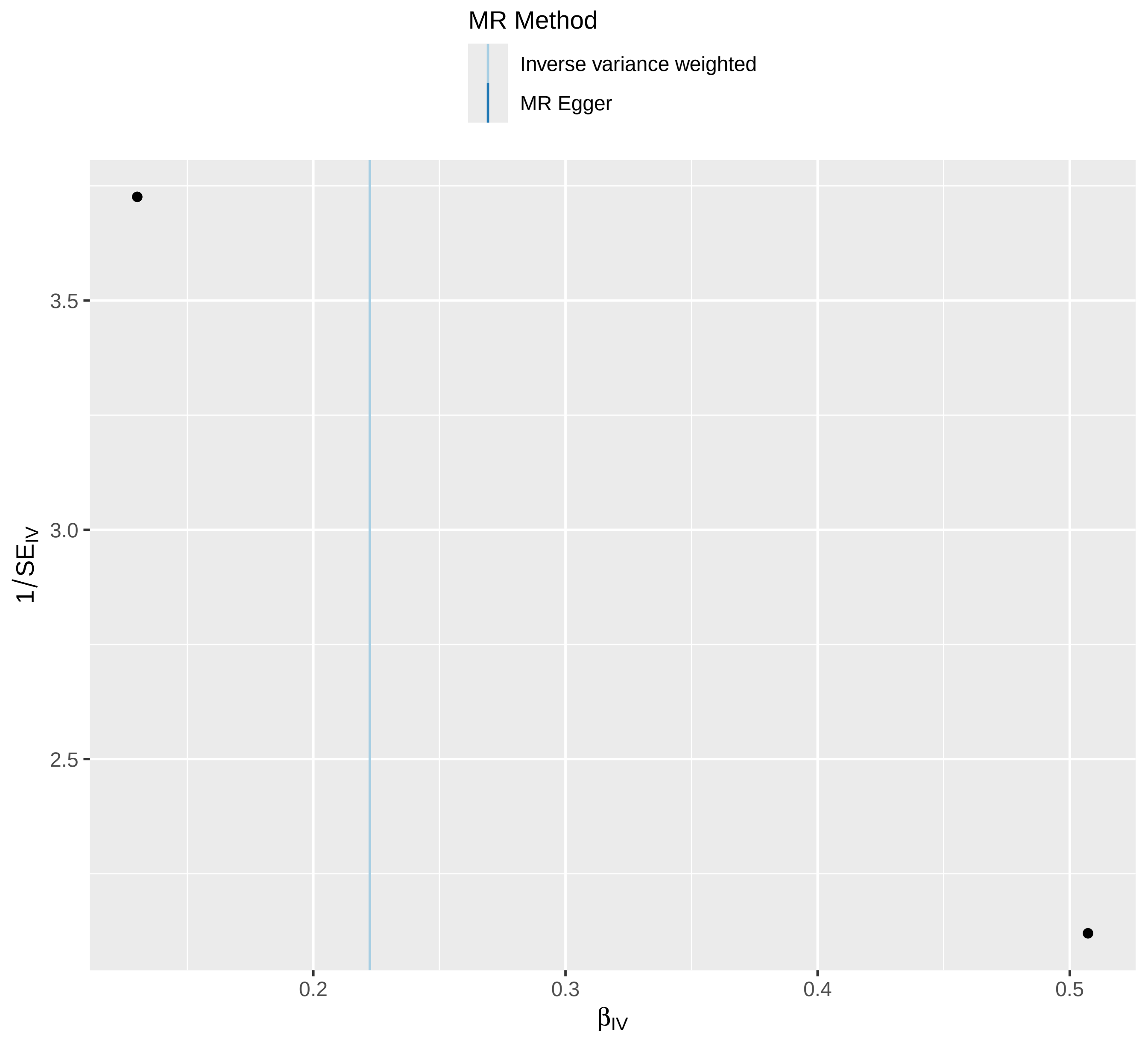
MR Method
Inverse variance weighted
MR Egger
3.5
3.0
2.5
1/SE_IV
0.2
0.3
0.4
0.5
β_IV

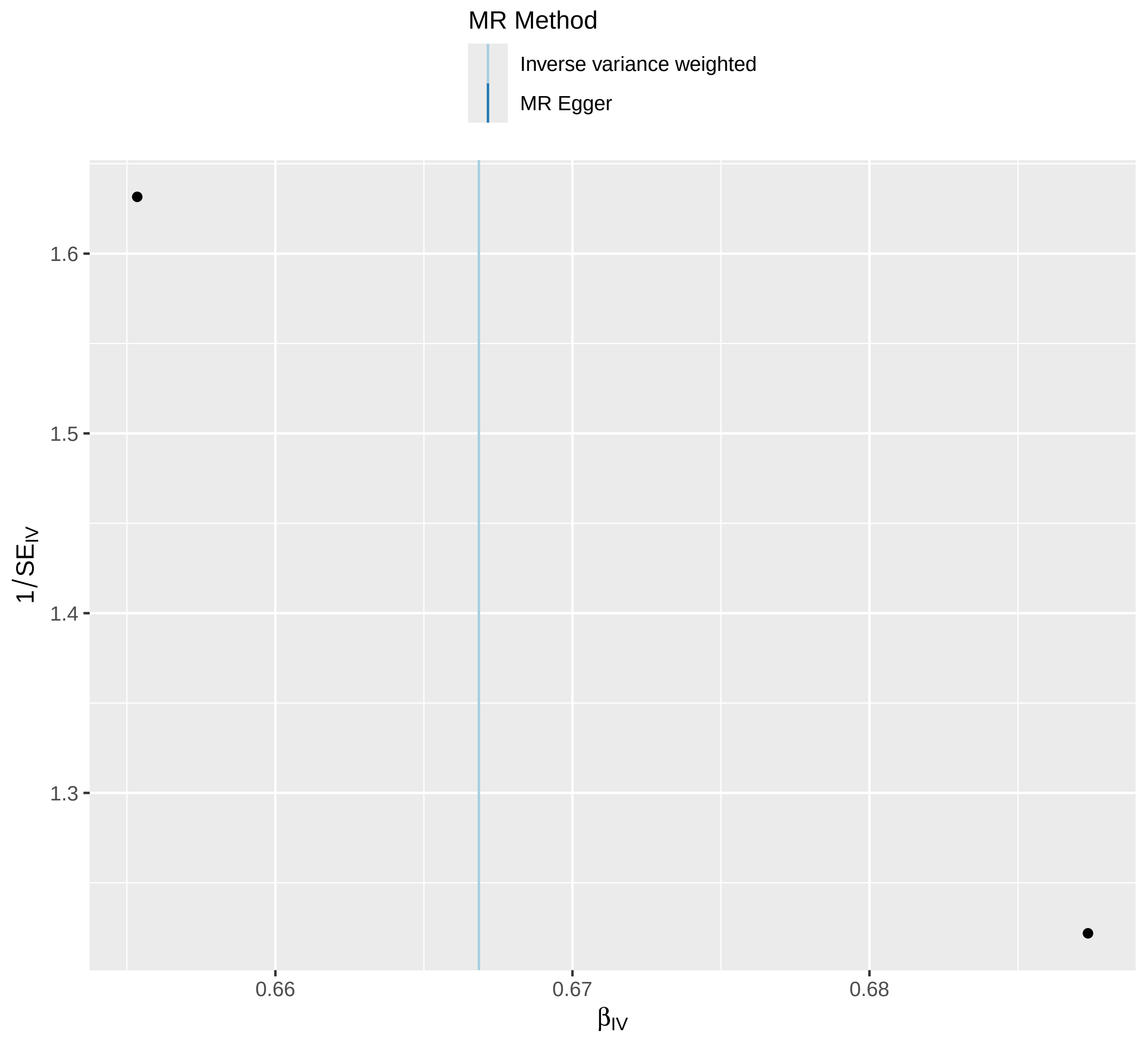

MR Method
Inverse variance weighted
MR Egger
1.6
1.5
1.4
1.3
1/SE_IV
0.66
0.67
0.68
β_IV

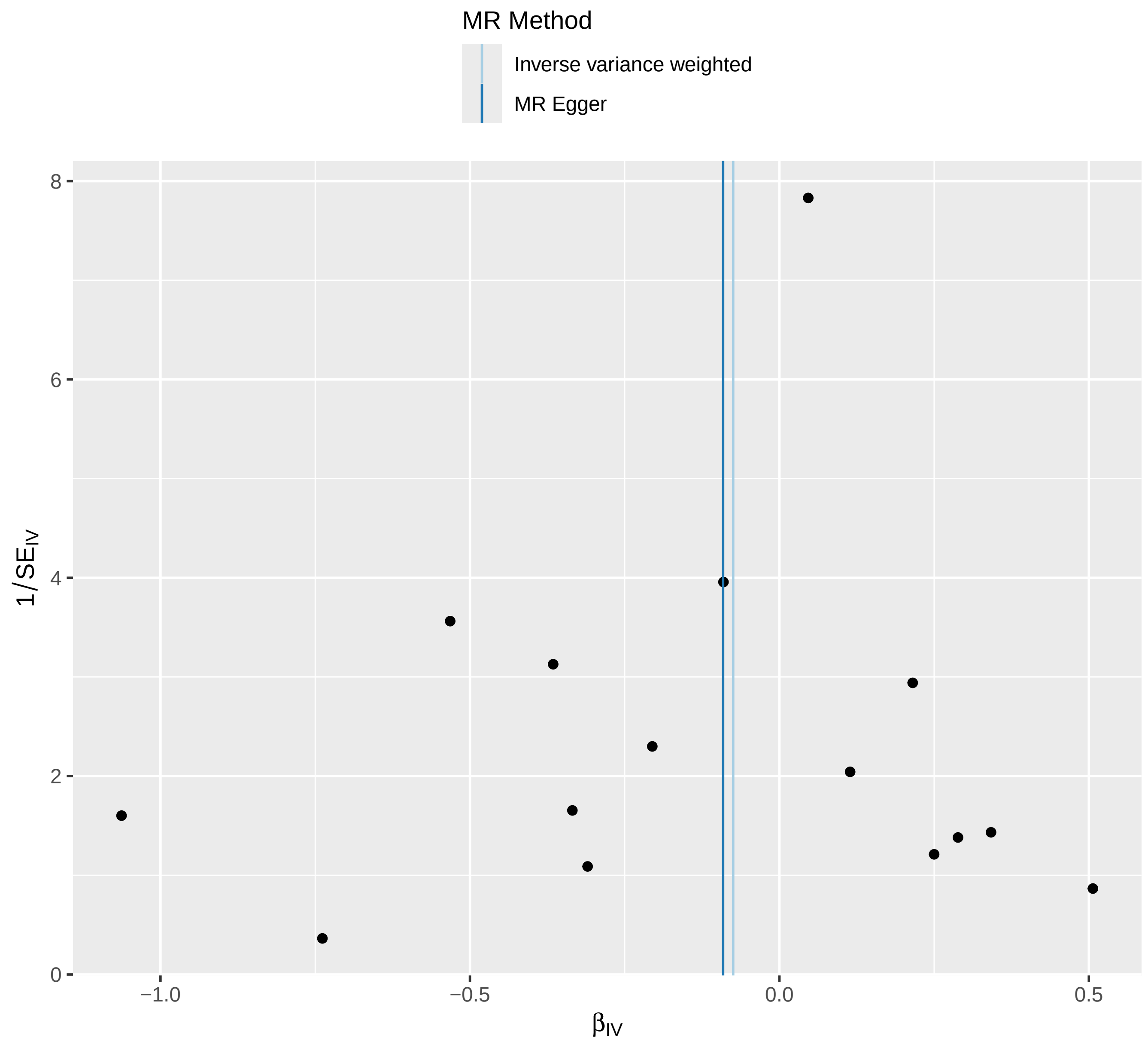
MR Method
Inverse variance weighted
MR Egger
8
6
4
2
0
1/SE$_{IV}$
−1.0
−0.5
0.0
0.5
β$_{IV}$

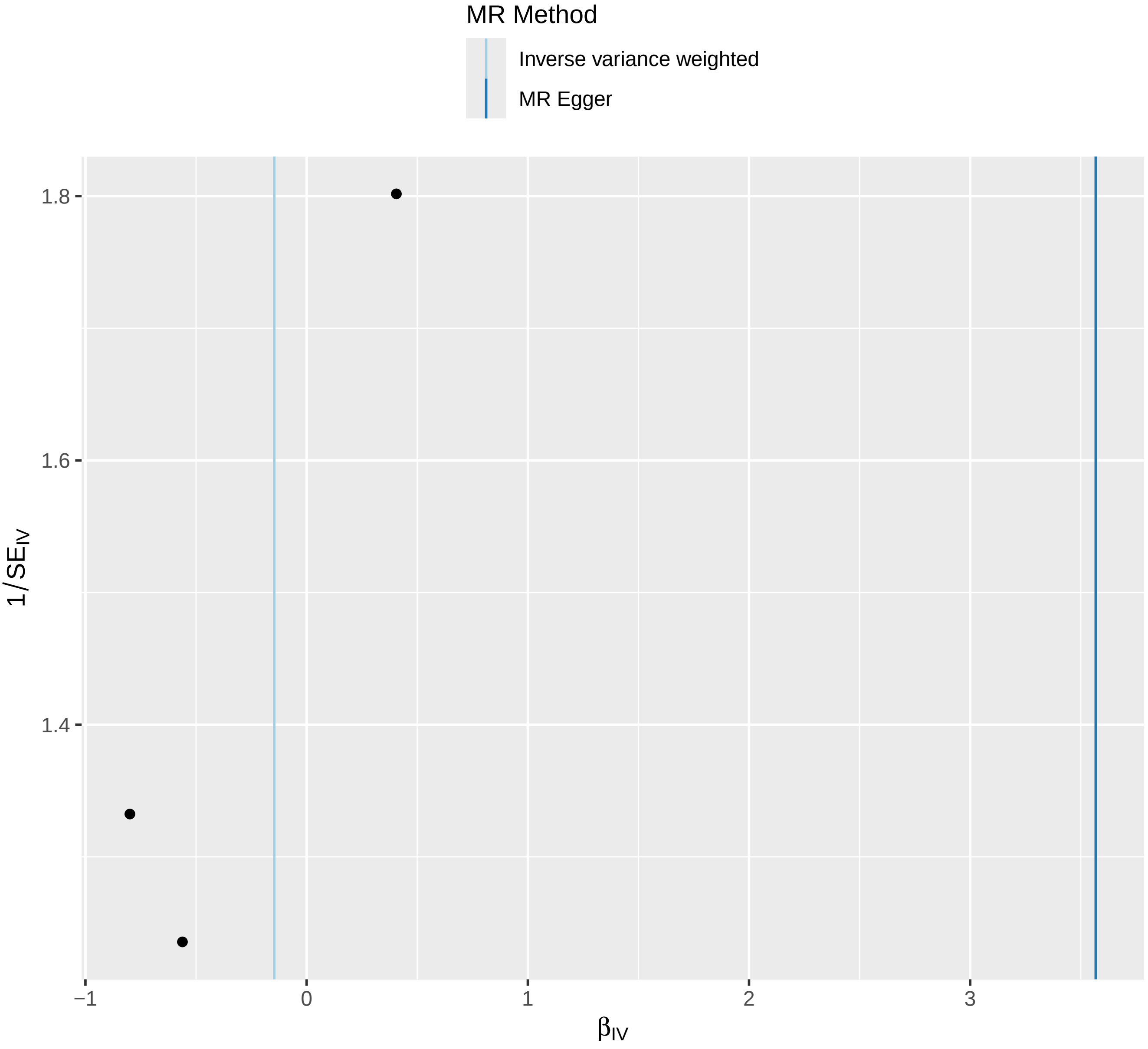
MR Method
Inverse variance weighted
MR Egger
1.8
1.6
1.4
1/SE_IV
−1
0
1
2
3
β_IV

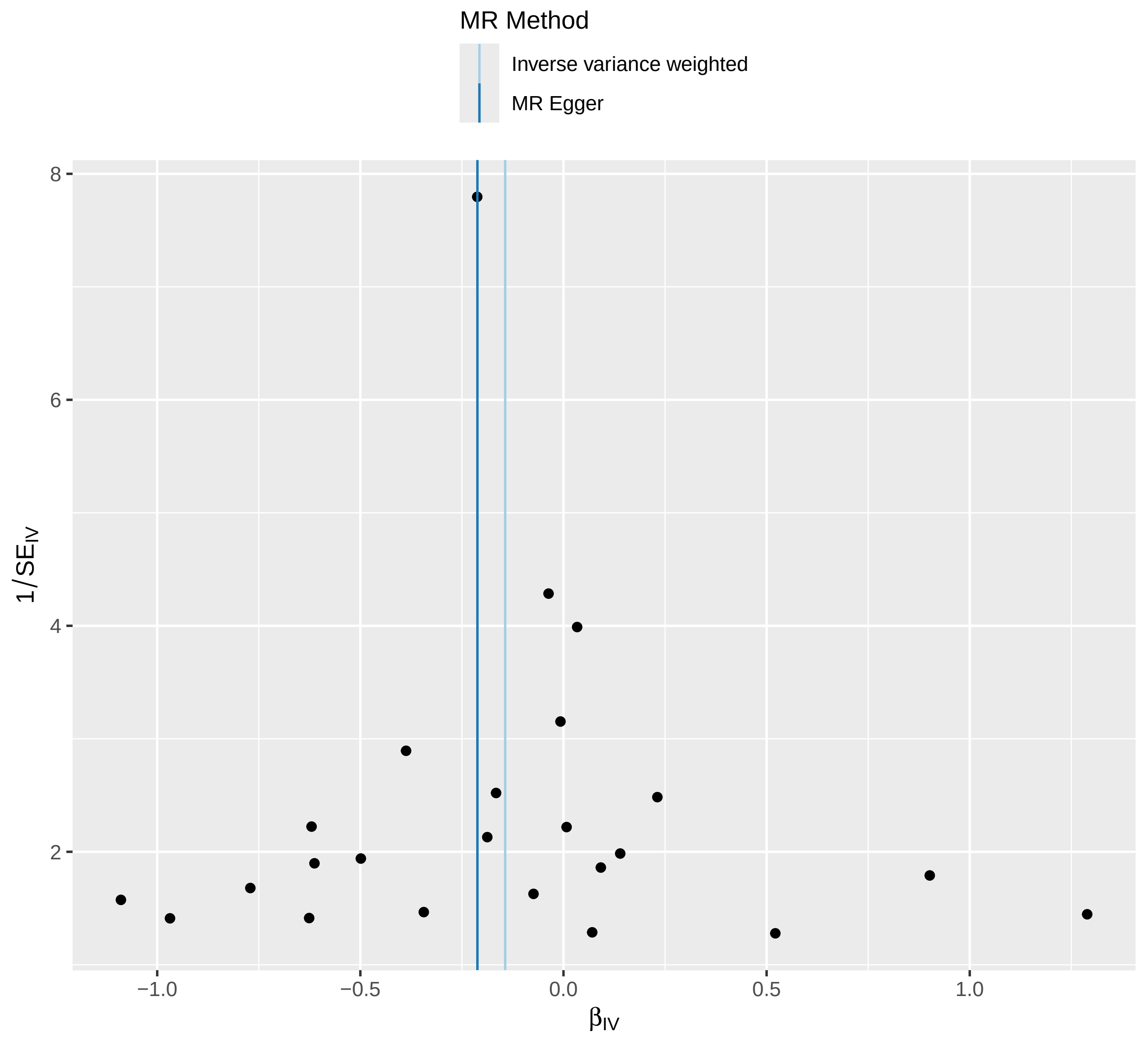

MR Method
Inverse variance weighted
MR Egger
8
6
4
2
1/SE_IV
−1.0
−0.5
0.0
0.5
1.0
β_IV

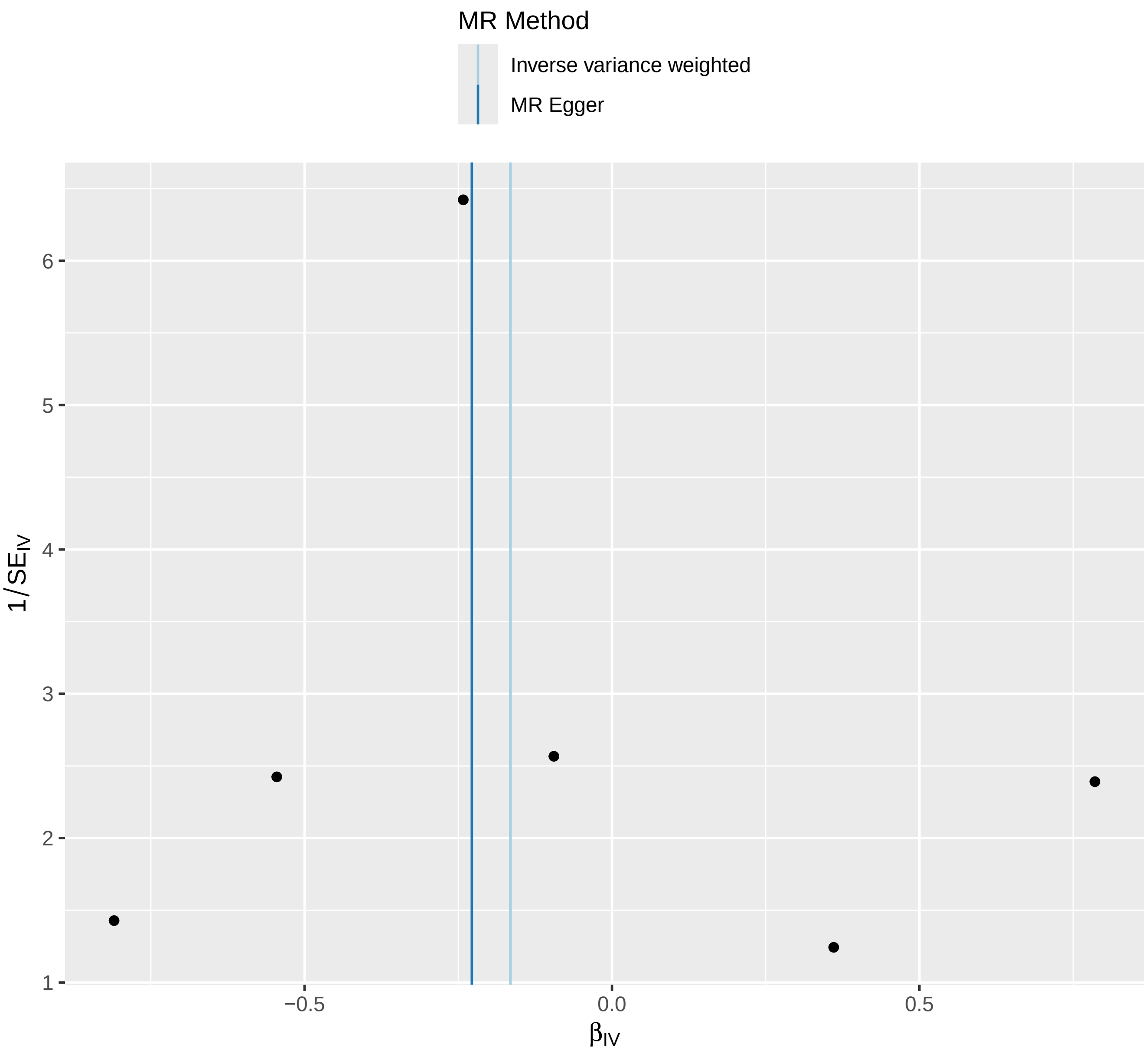
MR Method
Inverse variance weighted
MR Egger
6
5
4
3
2
1
$1/SE_{IV}$
−0.5
0.0
0.5
β_{IV}

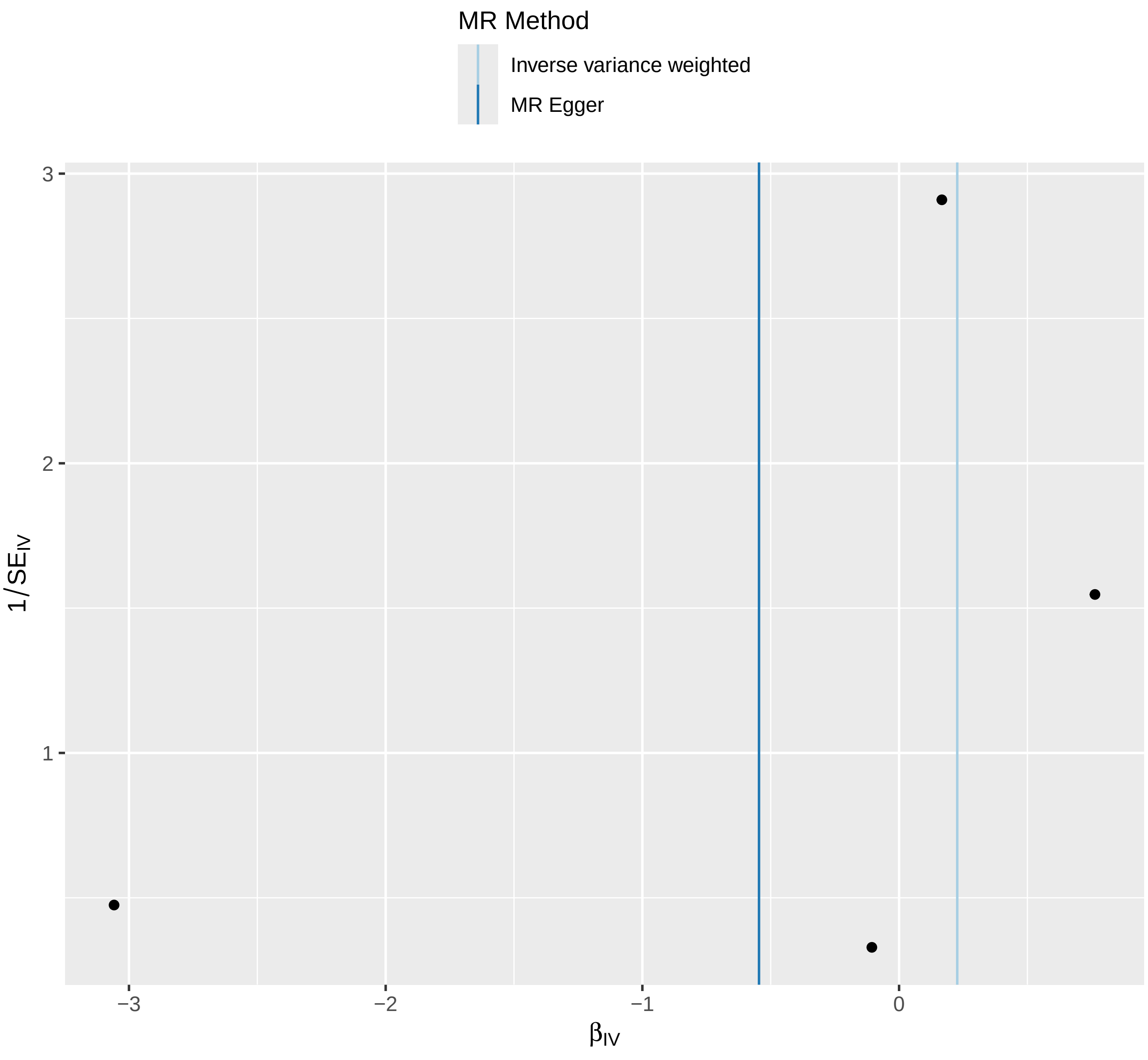

MR Method
Inverse variance weighted
MR Egger
3
2
1
1/SE$_{IV}$
−3
−2
−1
0
β$_{IV}$

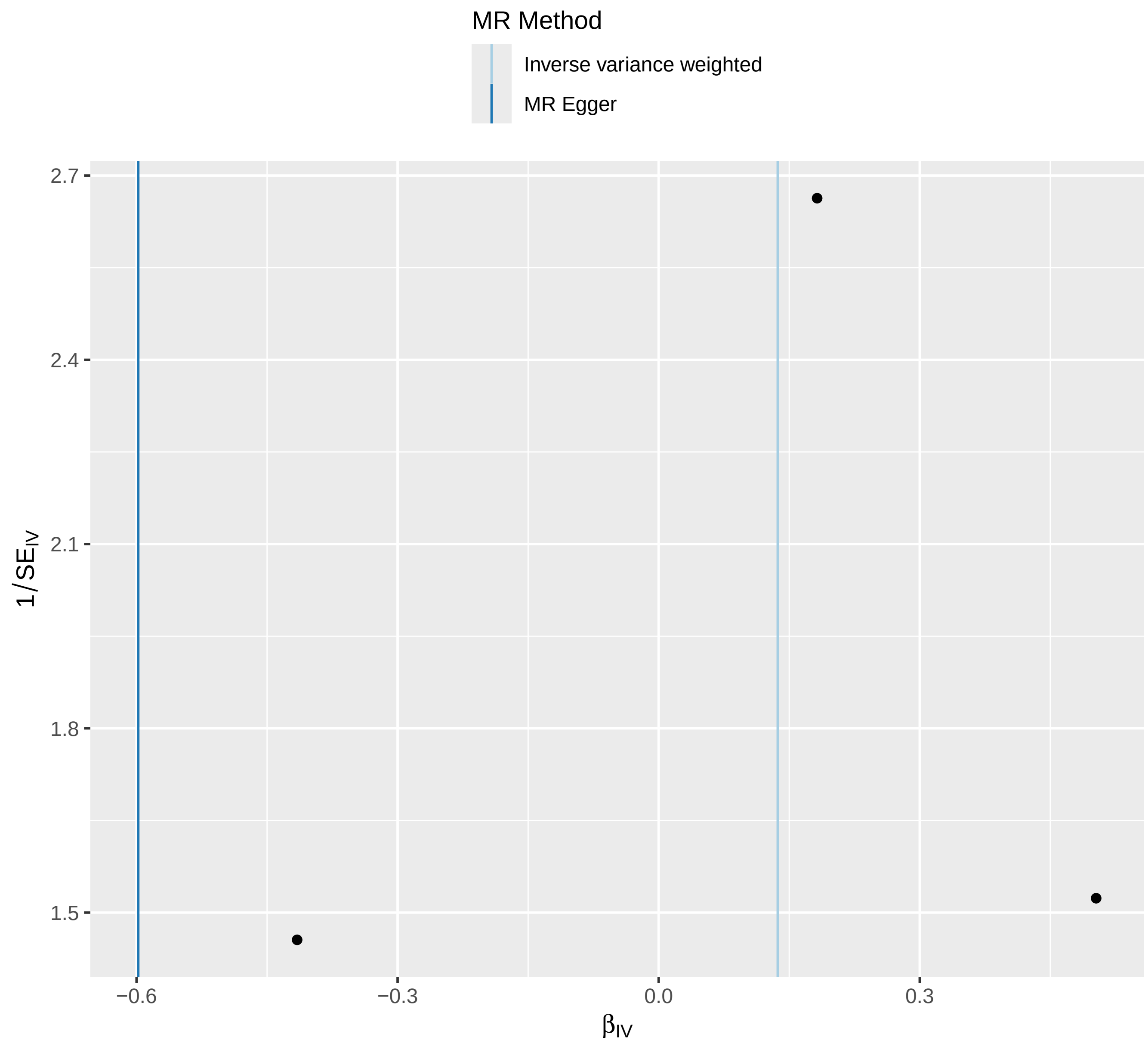

MR Method
Inverse variance weighted
MR Egger
2.7
2.4
2.1
1.8
1.5
1/SE_IV
−0.6
−0.3
0.0
0.3
β_IV

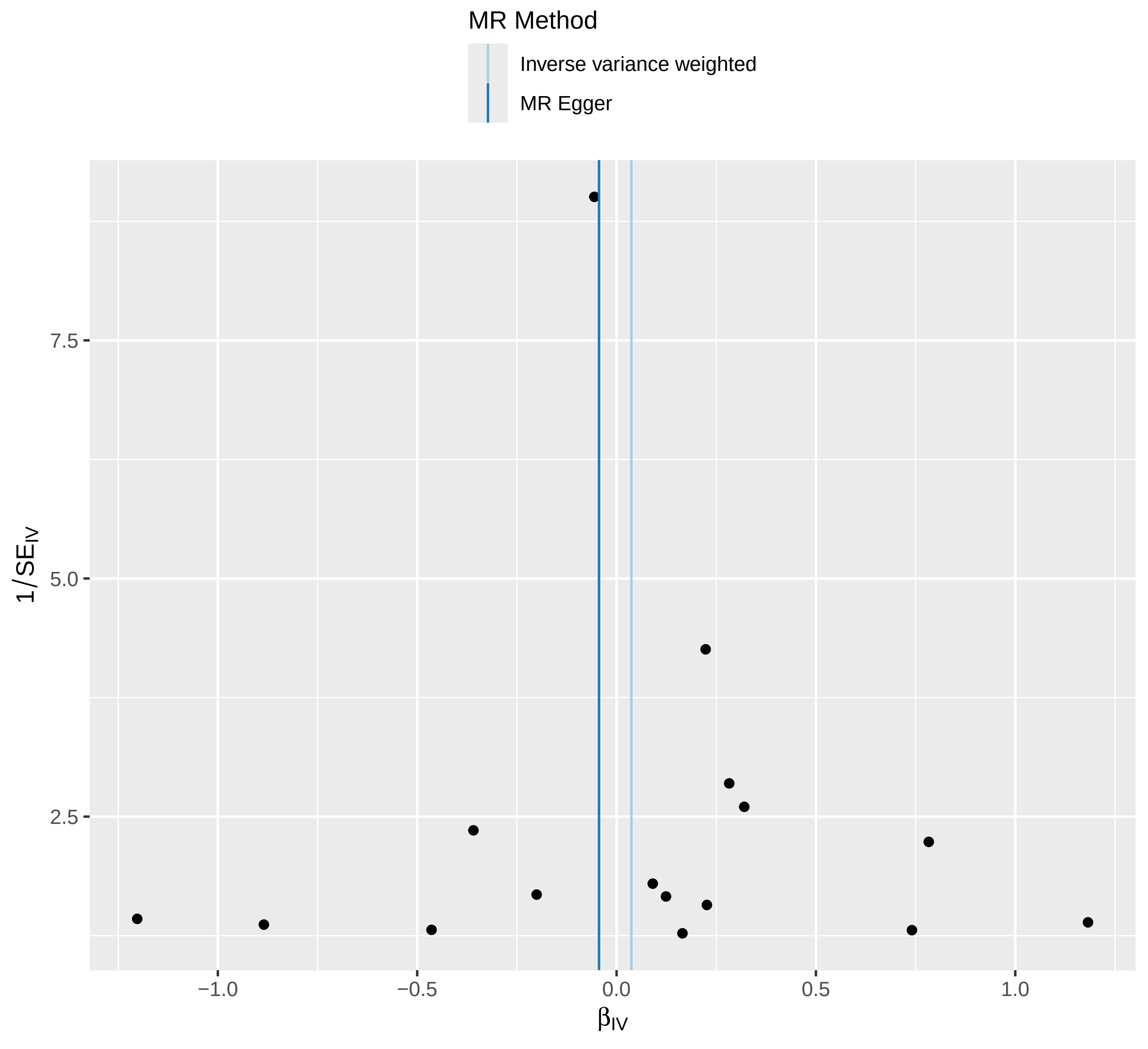

MR Method
Inverse variance weighted
MR Egger
7.5
5.0
2.5
1/SE$_{IV}$
−1.0
−0.5
0.0
0.5
1.0
β$_{IV}$

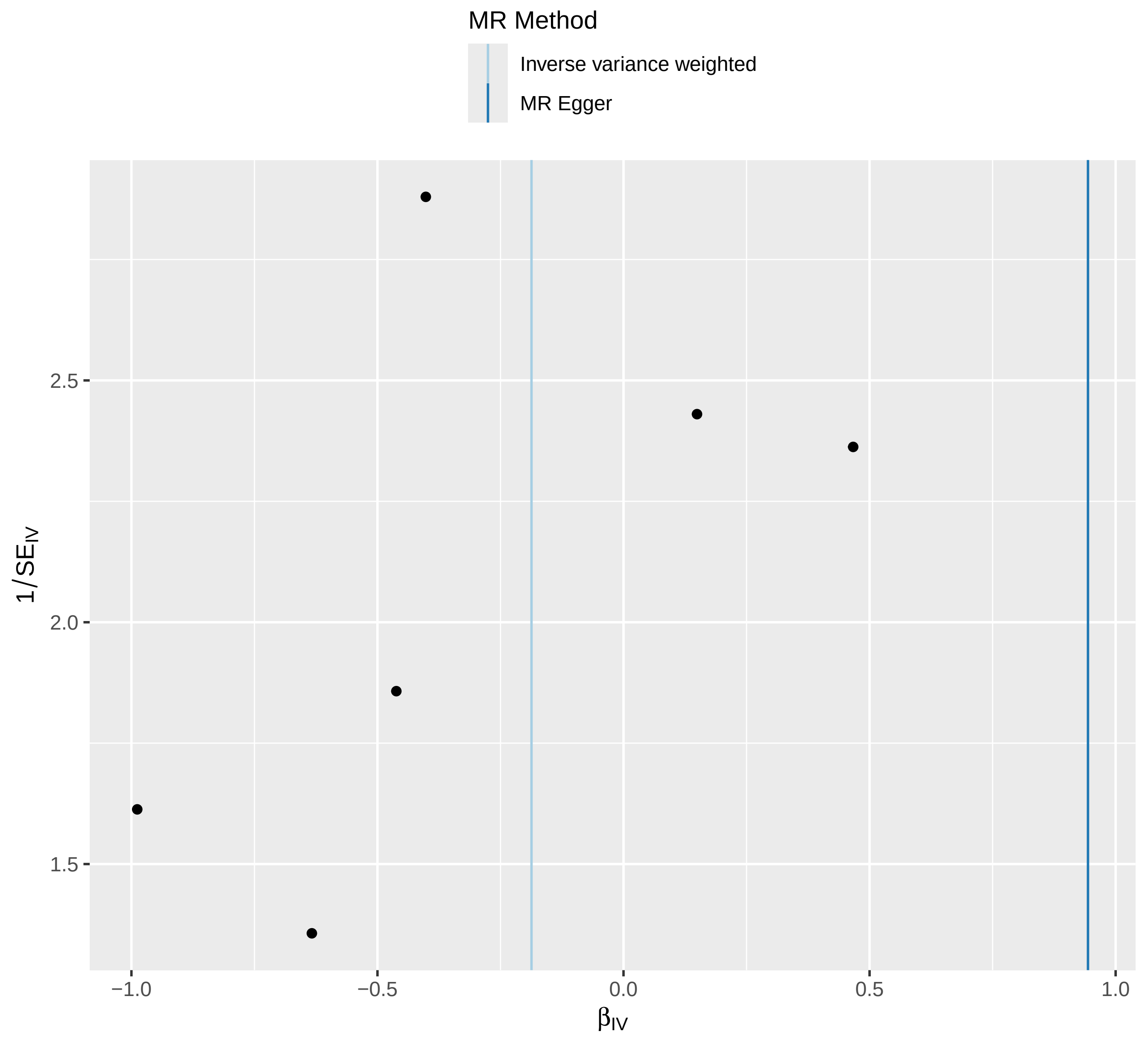

MR Method
Inverse variance weighted
MR Egger
2.5
2.0
1.5
1/SE_IV
−1.0
−0.5
0.0
0.5
1.0
β_IV

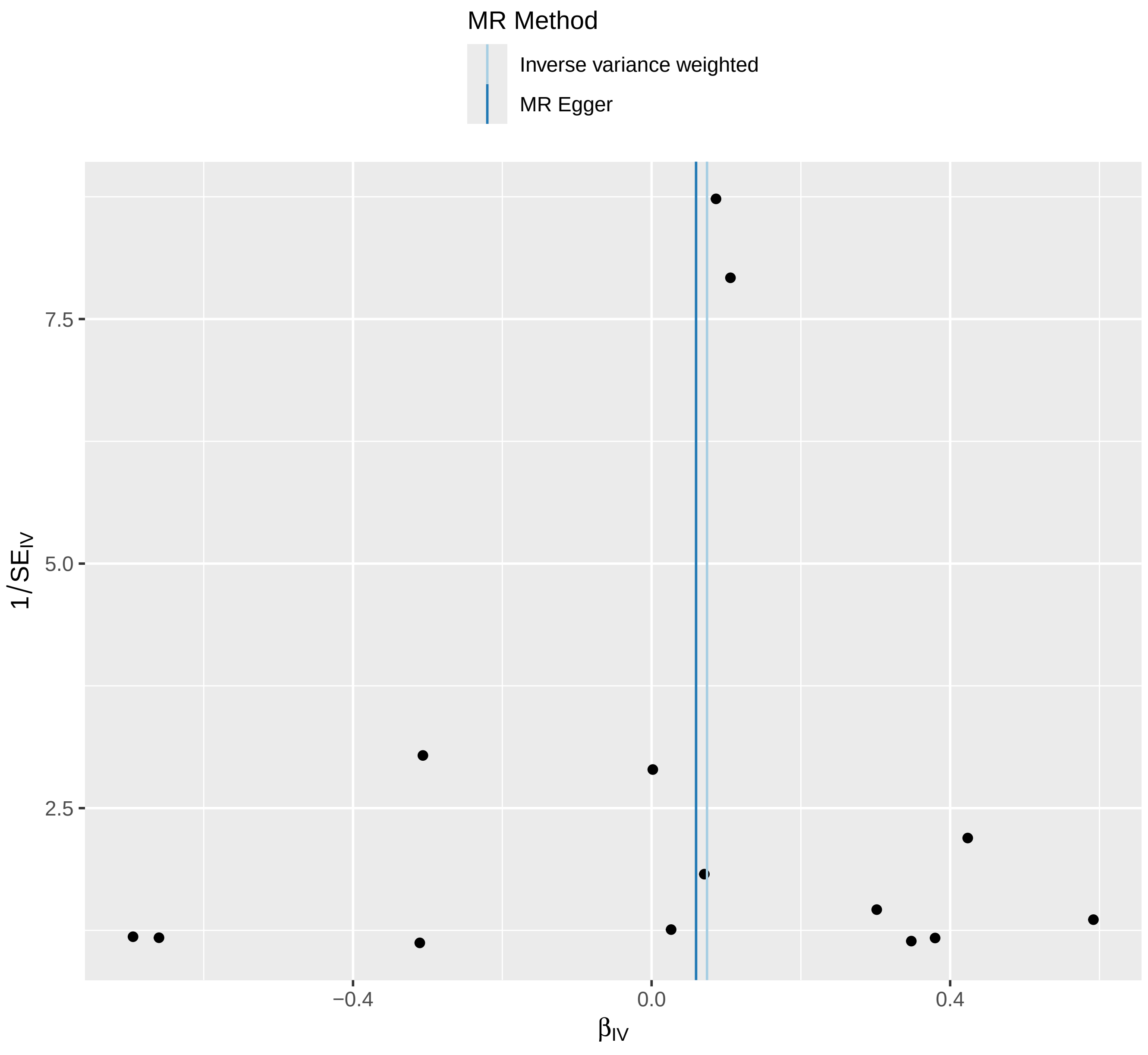

MR Method
Inverse variance weighted
MR Egger
7.5
5.0
2.5
1/SE$_{IV}$
−0.4
0.0
0.4
β$_{IV}$

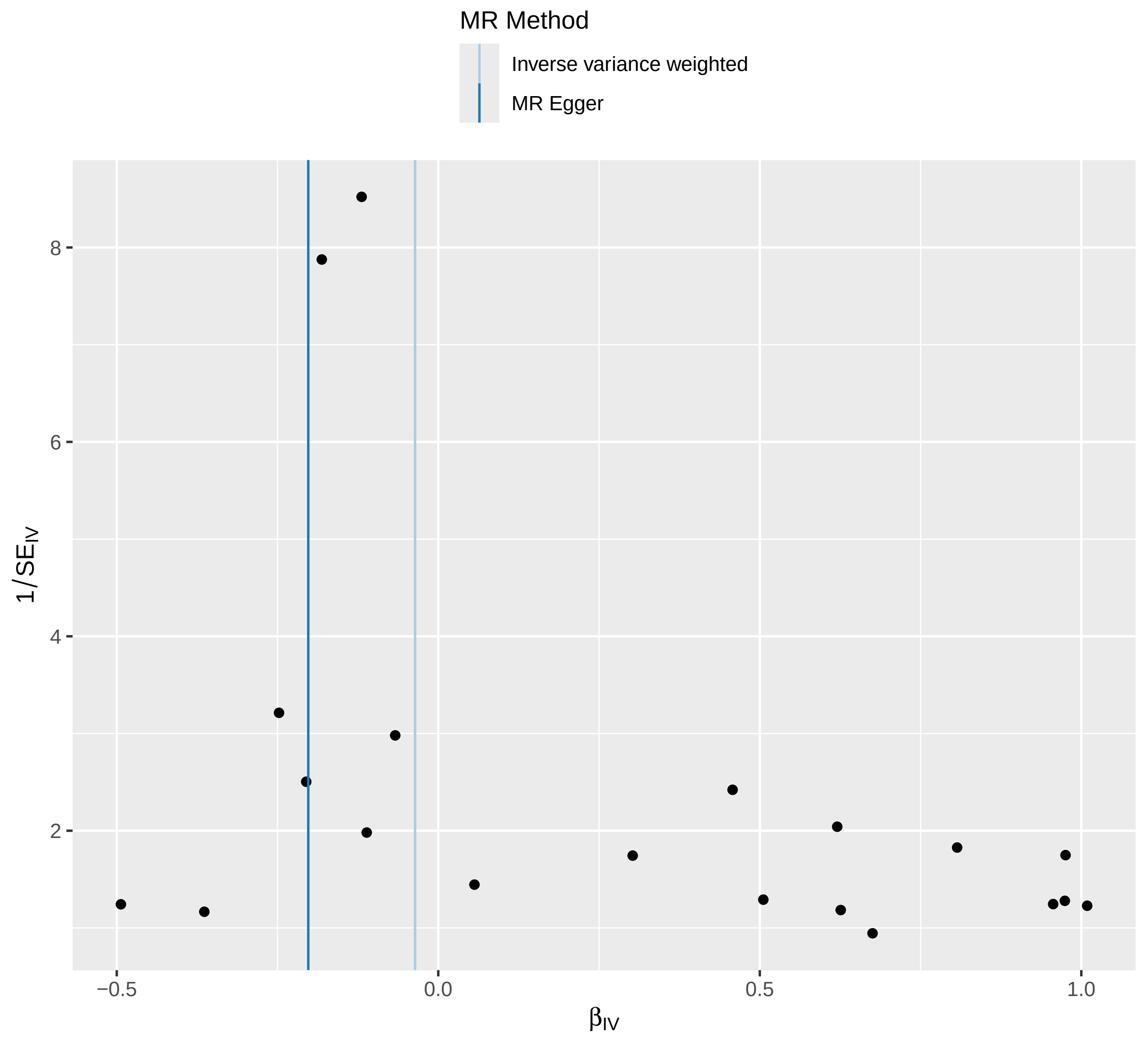
MR Method
Inverse variance weighted
MR Egger
1/SE_IV
β_IV
8
6
4
2
−0.5
0.0
0.5
1.0

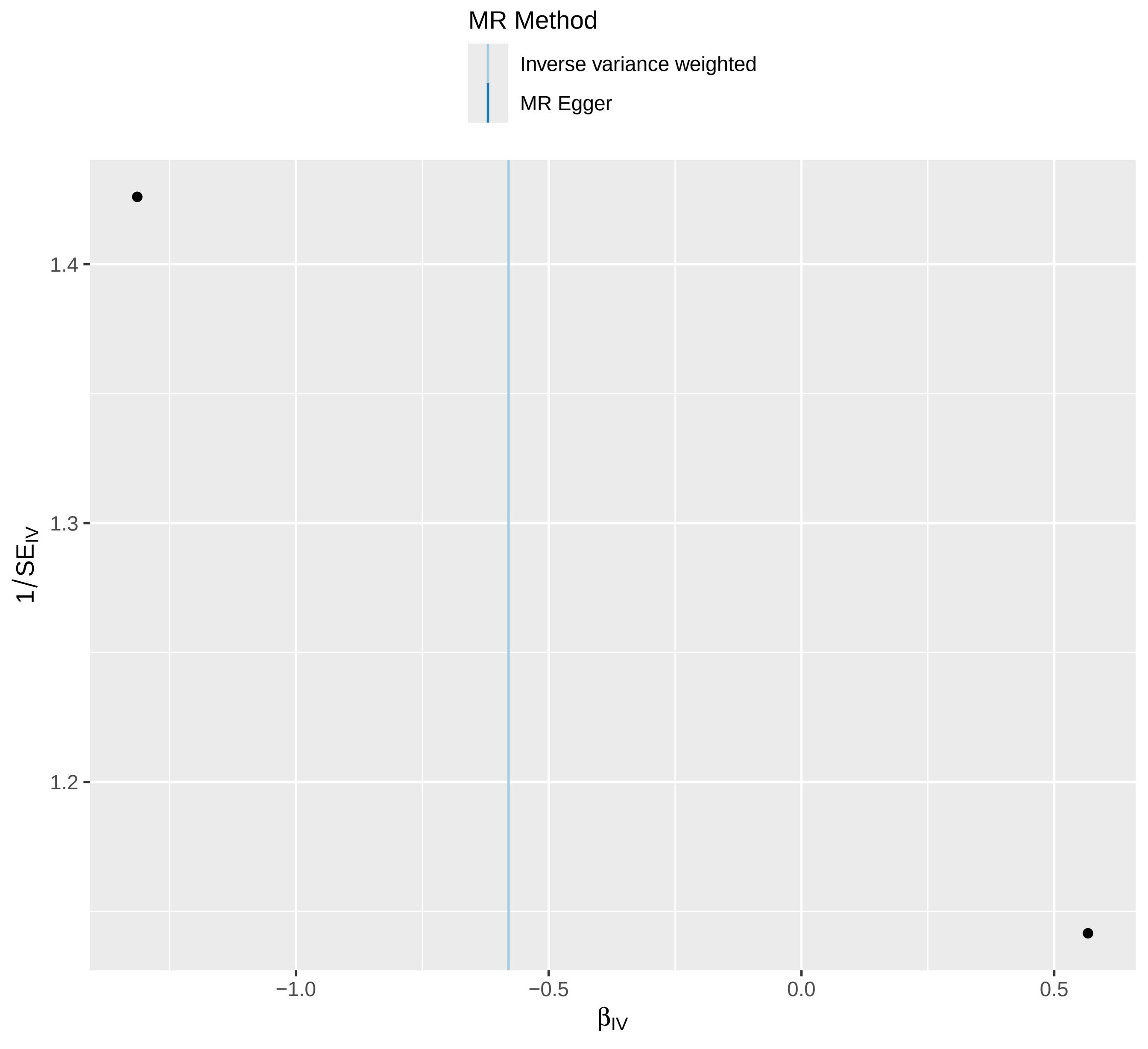
MR Method
Inverse variance weighted
MR Egger
1.4
1.3
1.2
1/SE$_{IV}$
−1.0
−0.5
0.0
0.5
β$_{IV}$

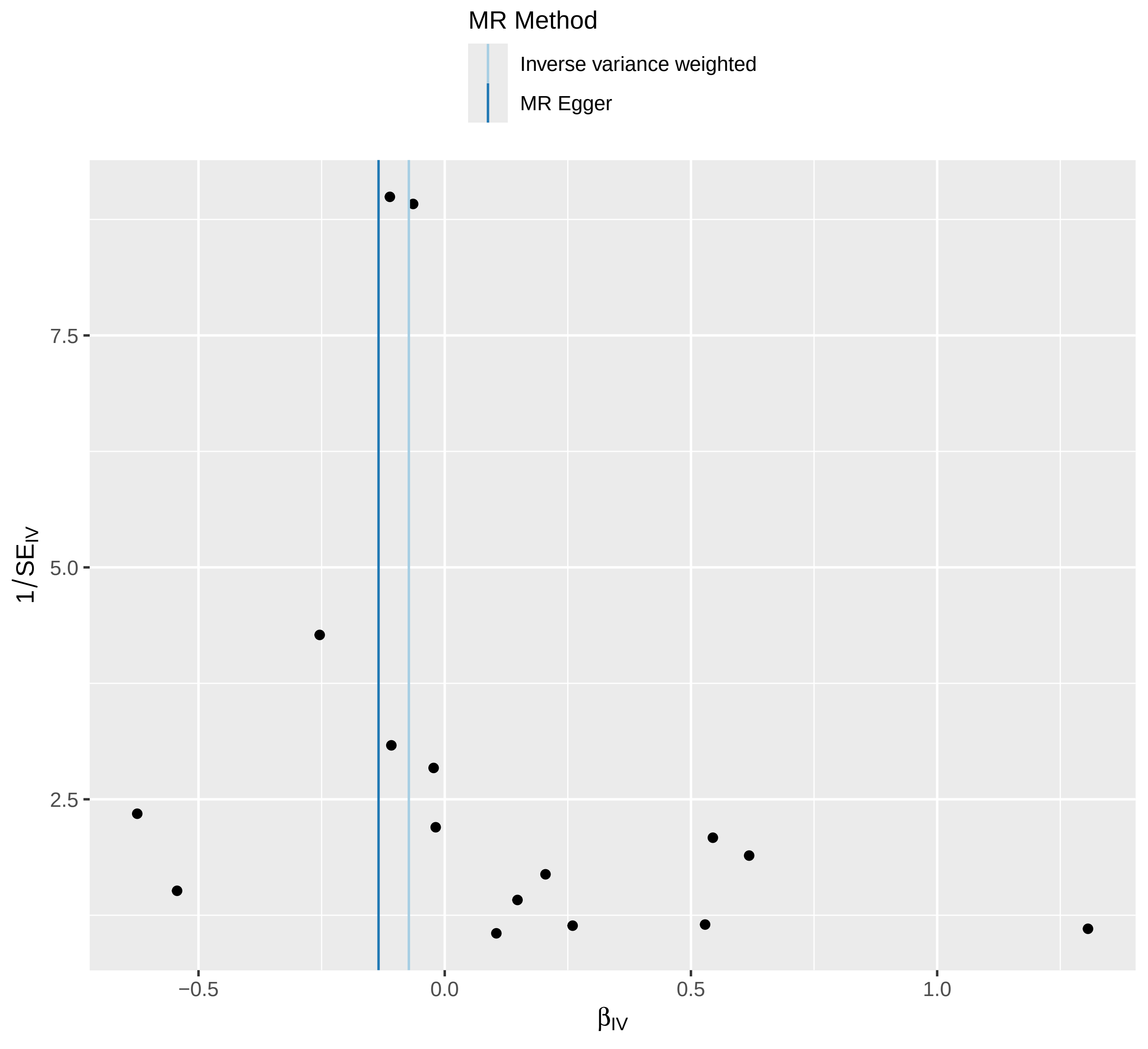
MR Method
Inverse variance weighted
MR Egger
7.5
5.0
2.5
1/SE_IV
−0.5
0.0
0.5
1.0
β_IV

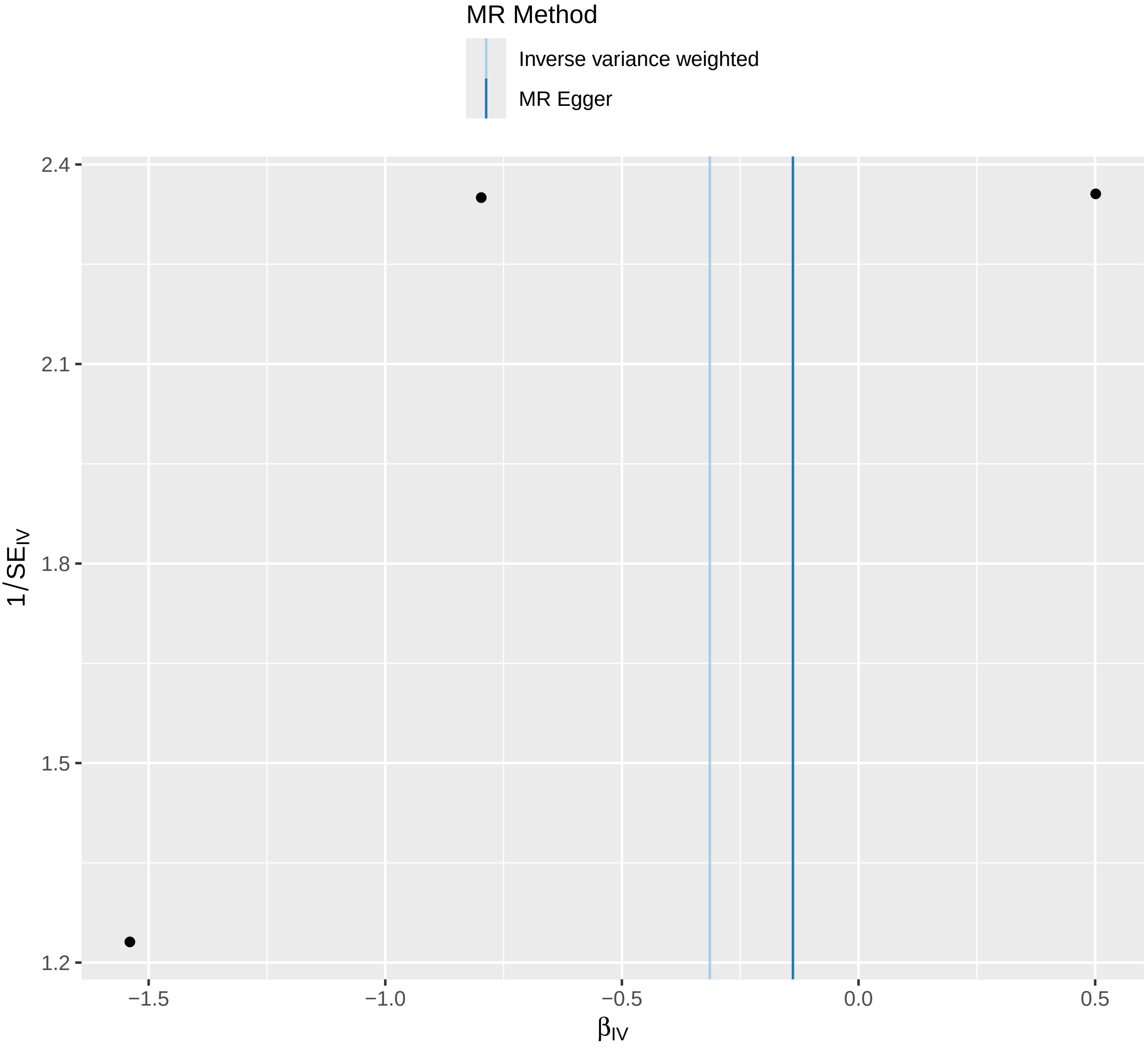

MR Method
Inverse variance weighted
MR Egger
2.4
2.1
1.8
1.5
1.2
1/SE_IV
−1.5
−1.0
−0.5
0.0
0.5
β_IV

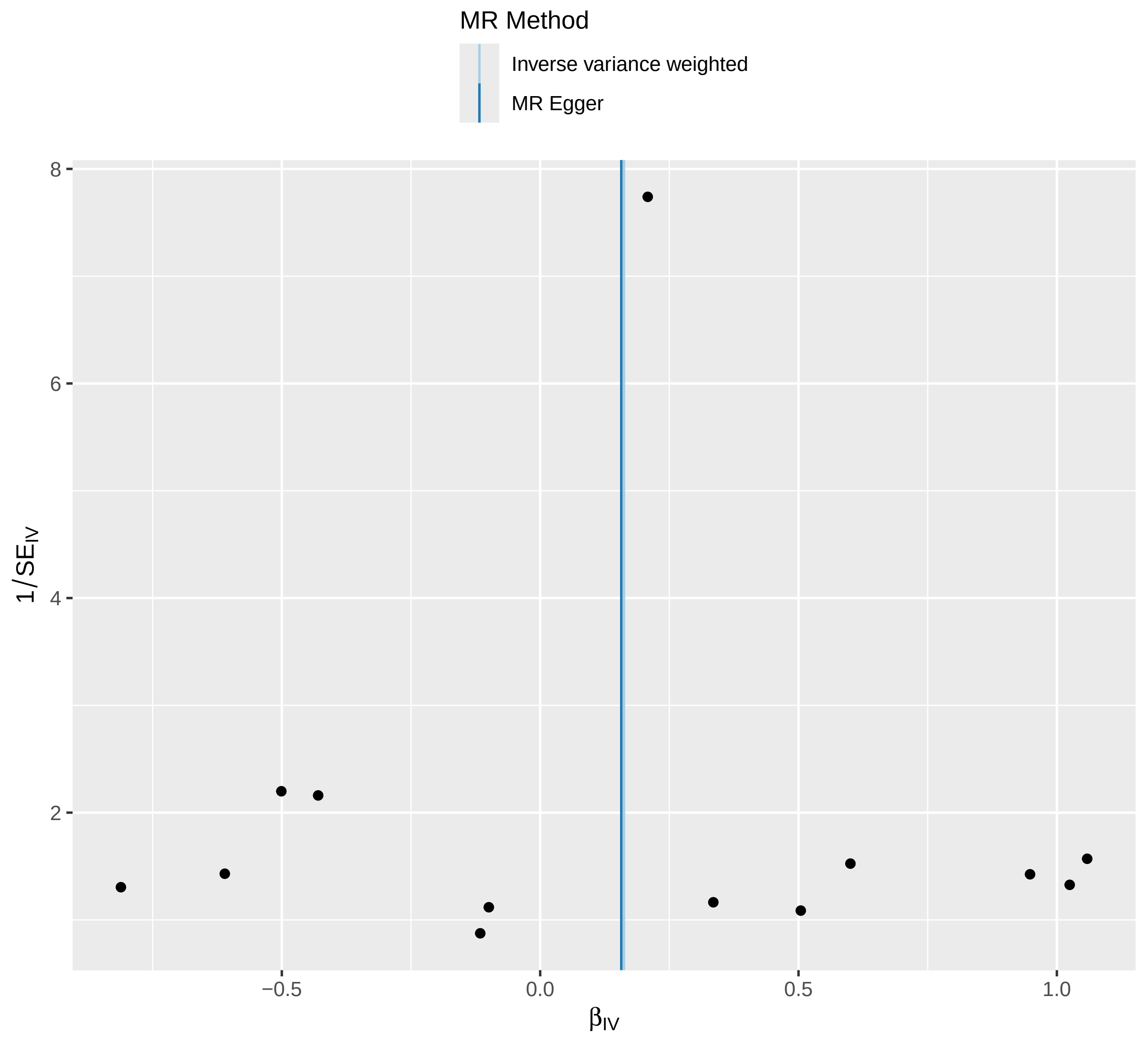

MR Method
Inverse variance weighted
MR Egger
8
6
4
2
1/SE_IV
−0.5
0.0
0.5
1.0
β_IV

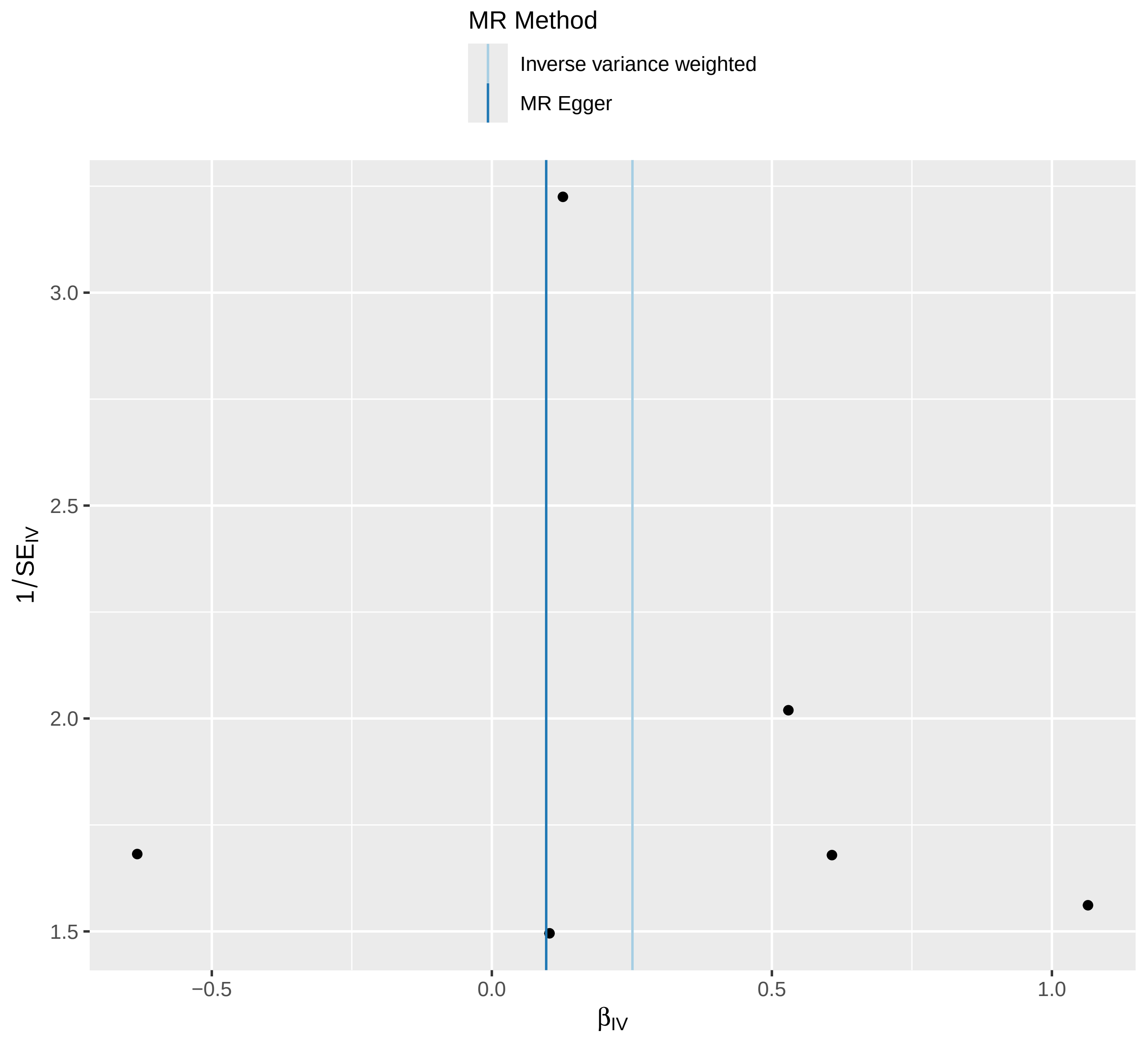
MR Method
Inverse variance weighted
MR Egger
3.0
2.5
2.0
1.5
1/SE$_{IV}$
−0.5
0.0
0.5
1.0
β$_{IV}$

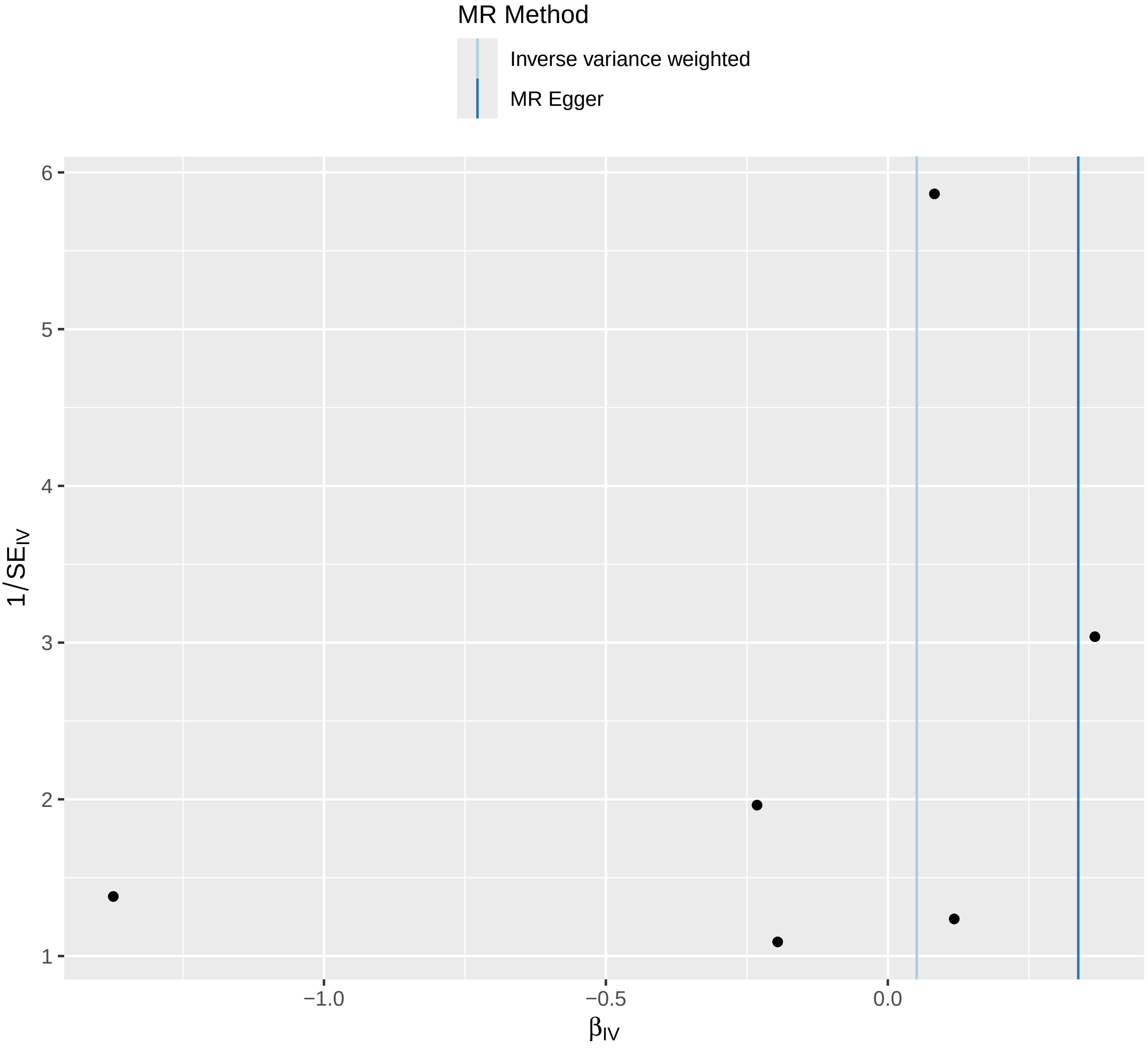
MR Method
Inverse variance weighted
MR Egger
1/SE$_{IV}$
6
5
4
3
2
1
−1.0
−0.5
0.0
β$_{IV}$

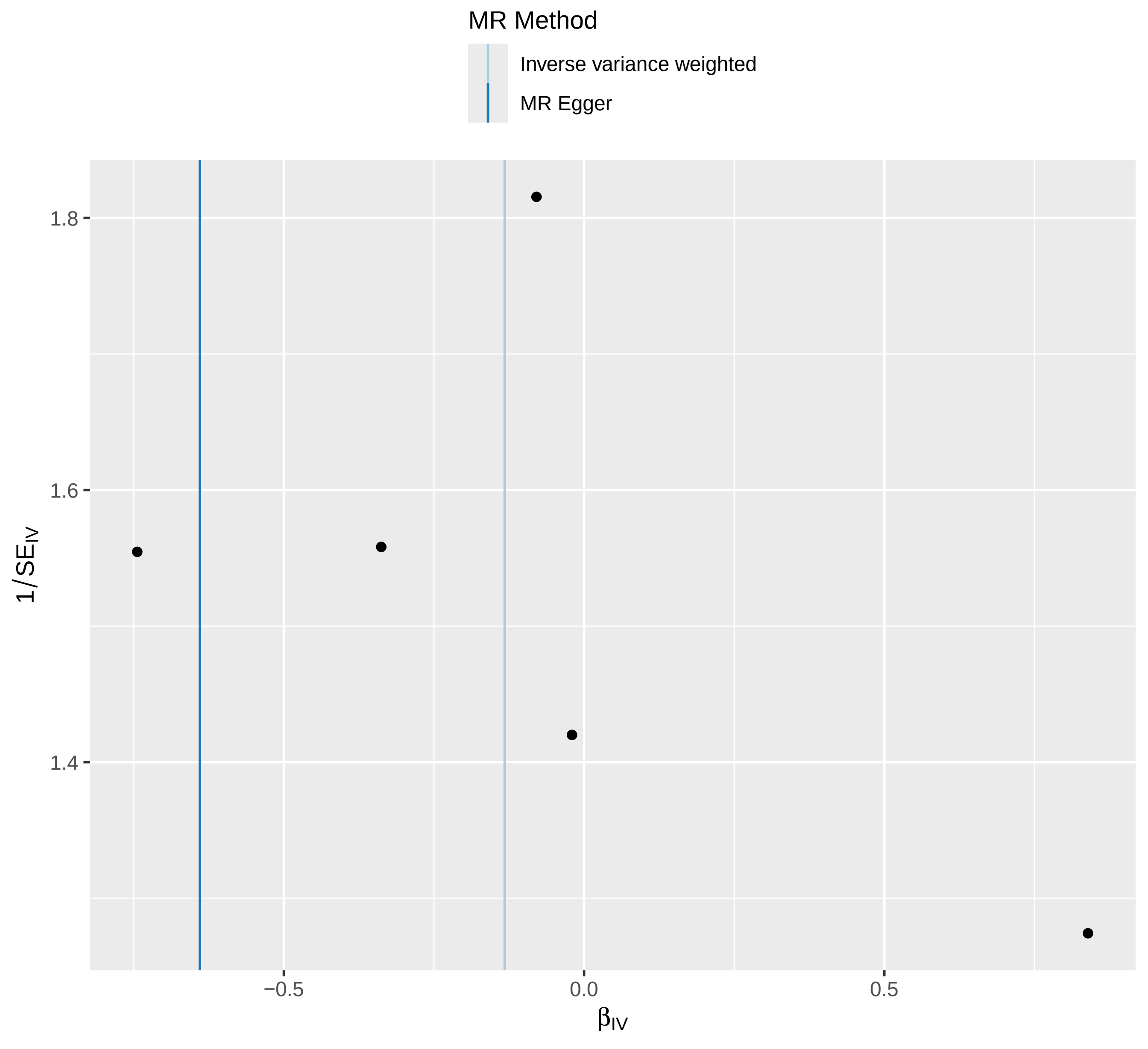

MR Method
Inverse variance weighted
MR Egger
1.8
1.6
1.4
1/SE_IV
−0.5
0.0
0.5
β_IV

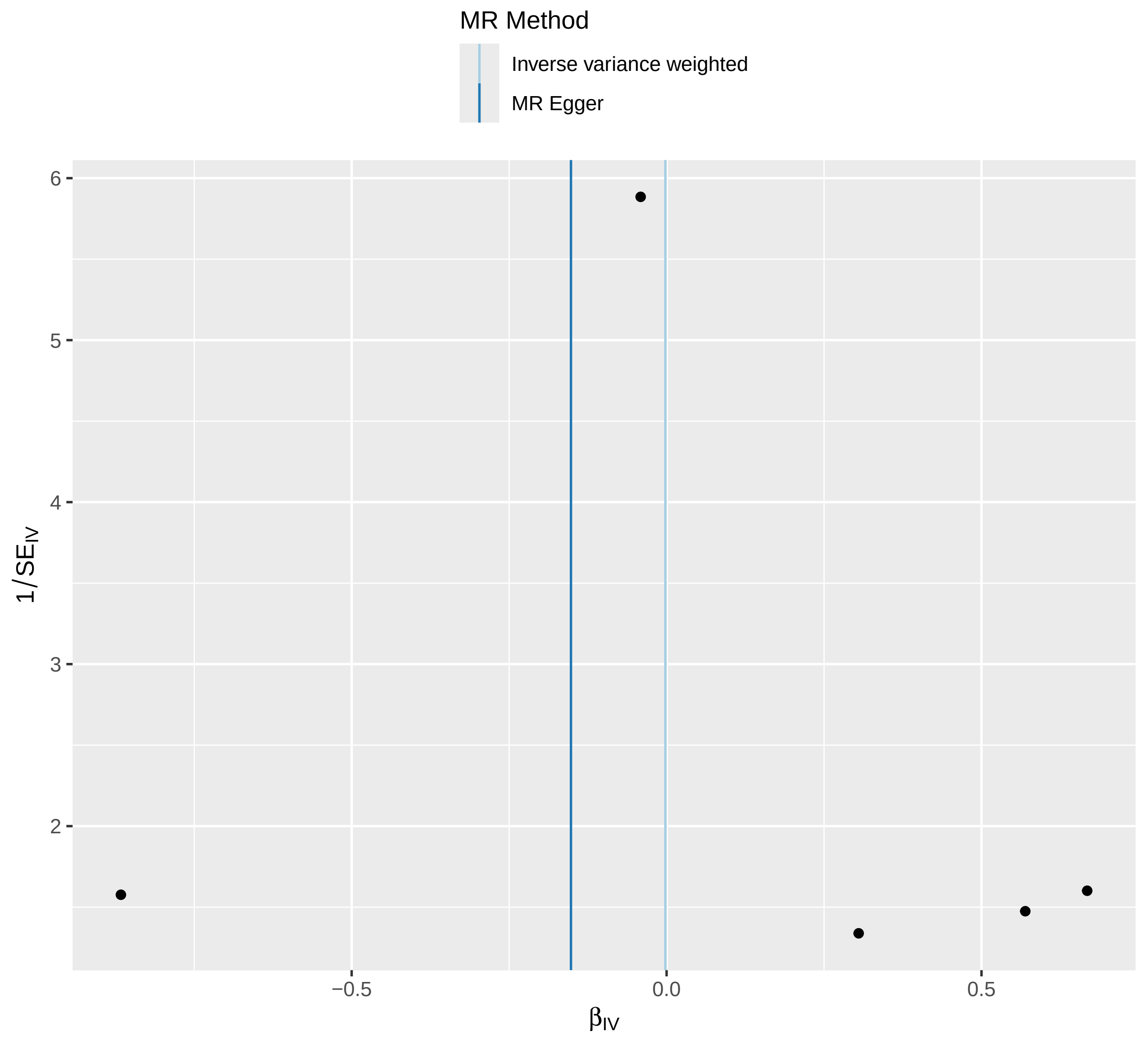
MR Method
Inverse variance weighted
MR Egger
6
5
4
3
2
$1/SE_{IV}$
−0.5
0.0
0.5
β_{IV}

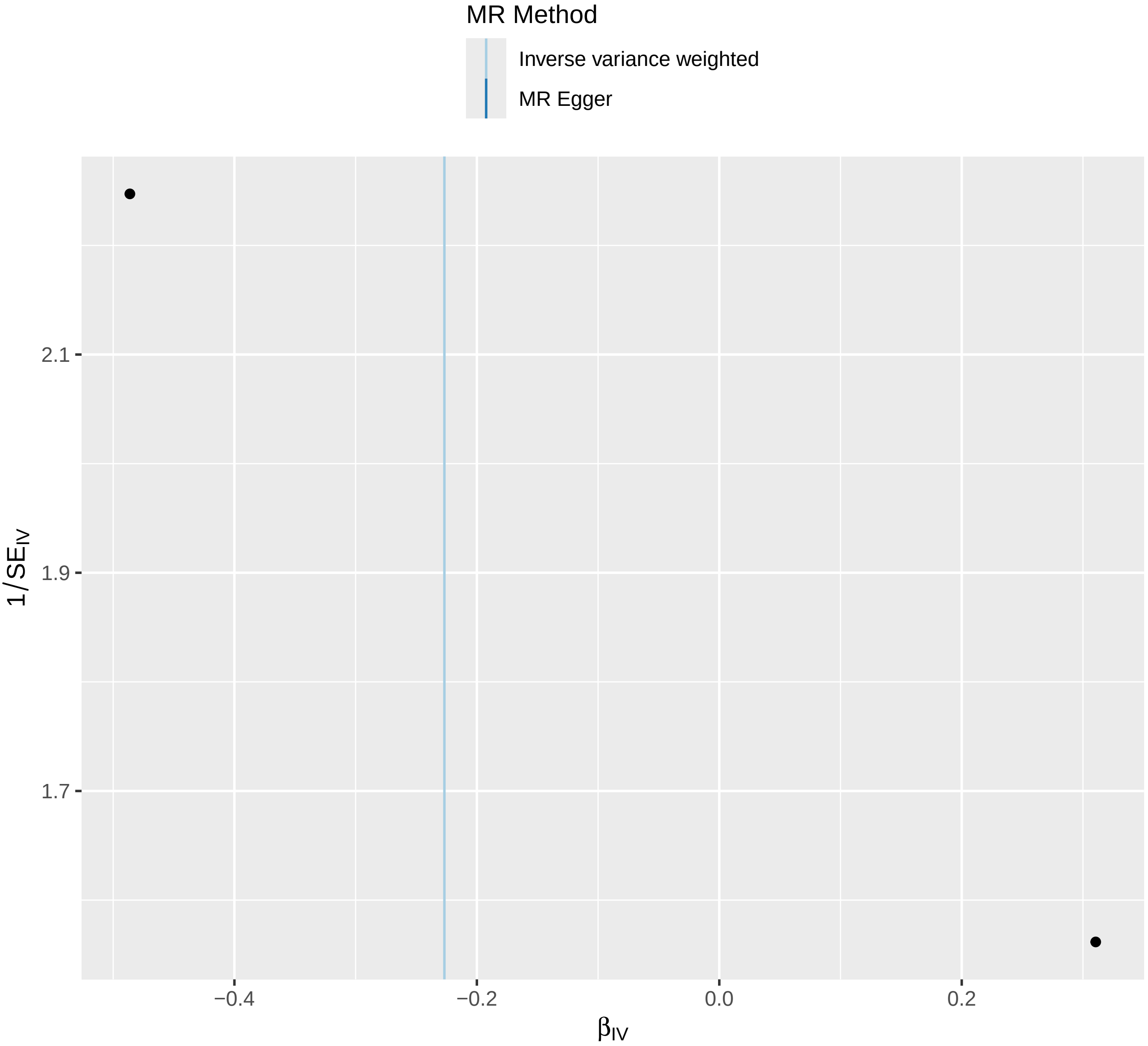
MR Method
Inverse variance weighted
MR Egger
2.1
1.9
1.7
−0.4
−0.2
0.0
0.2
$1/SE_{IV}$
β_{IV}

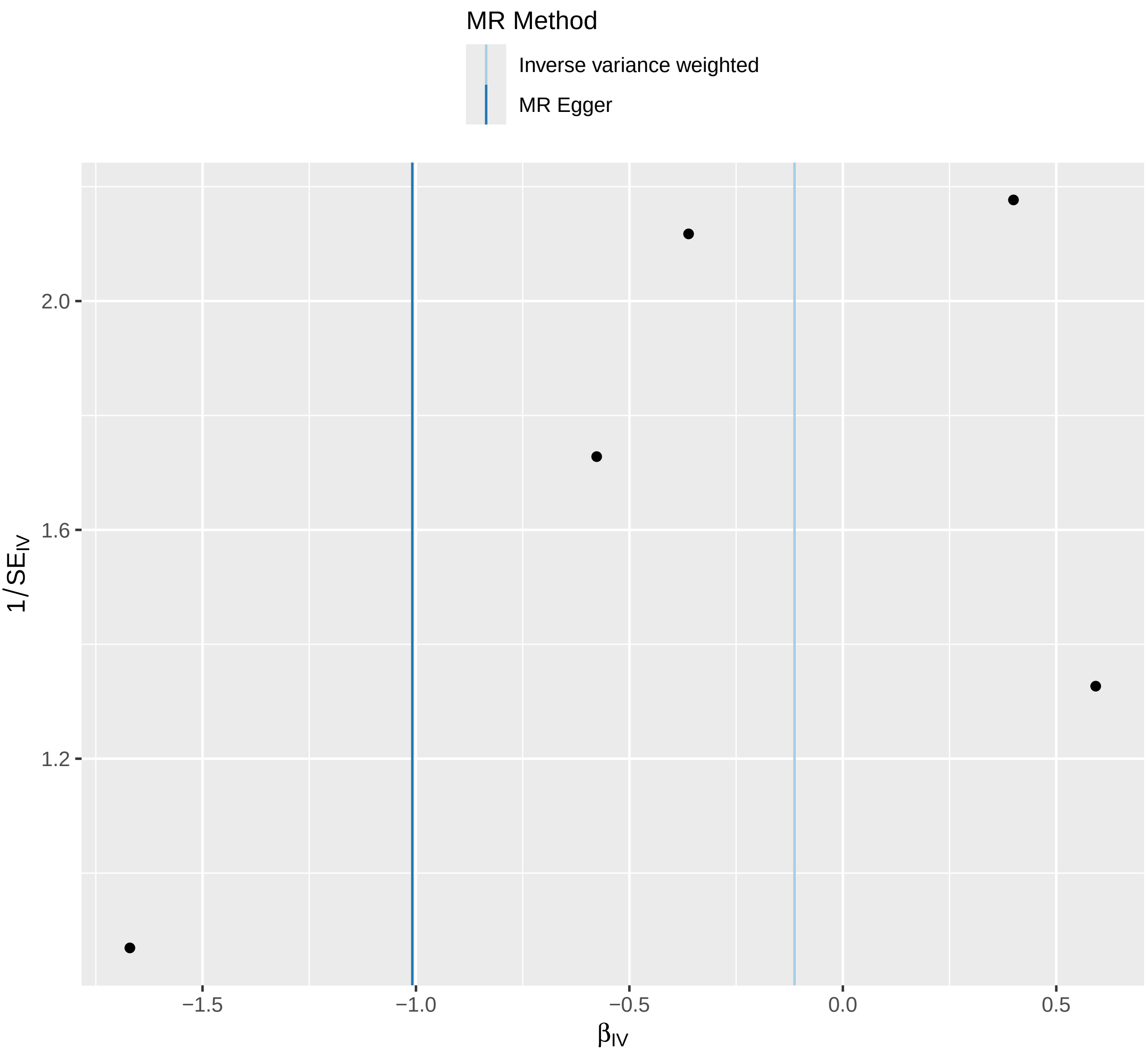

MR Method
Inverse variance weighted
MR Egger
1/SE_IV
2.0
1.6
1.2
−1.5
−1.0
−0.5
0.0
0.5
β_IV

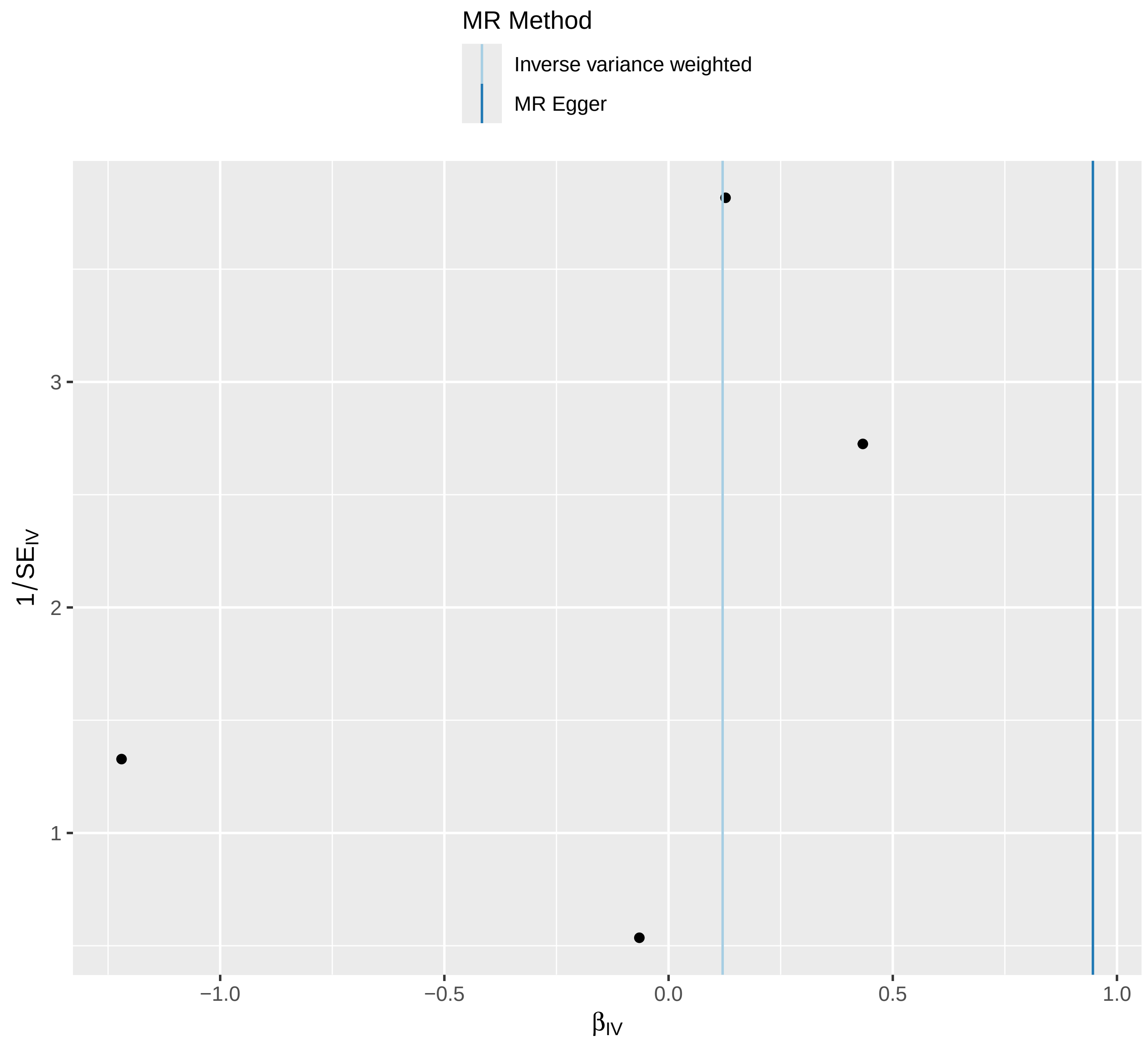

MR Method
Inverse variance weighted
MR Egger
1/SE$_{IV}$
1
2
3
−1.0
−0.5
0.0
0.5
1.0
β_{IV}

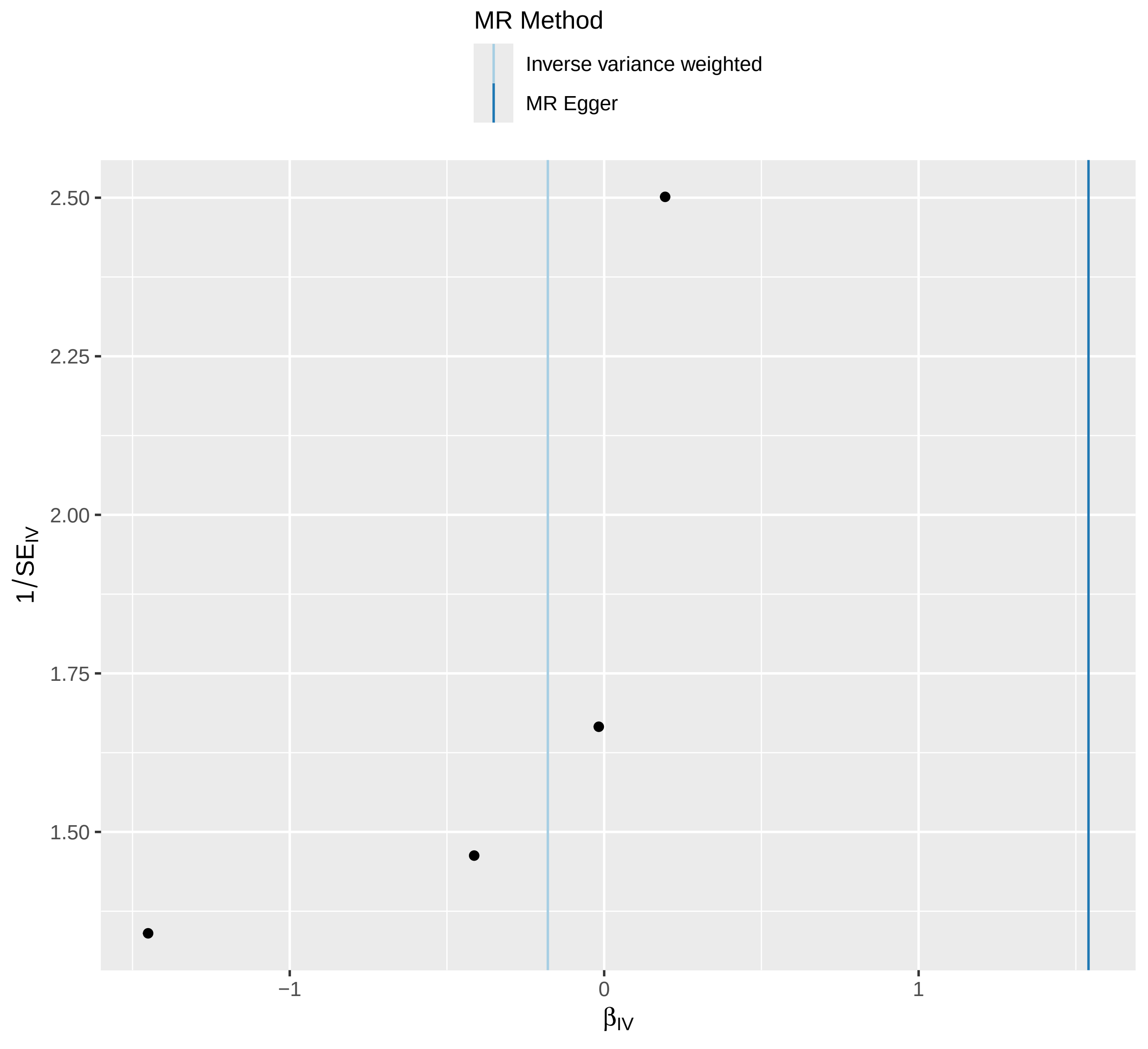
MR Method
Inverse variance weighted
MR Egger
2.50
2.25
2.00
1.75
1.50
1/SE_IV
−1
0
1
β_IV

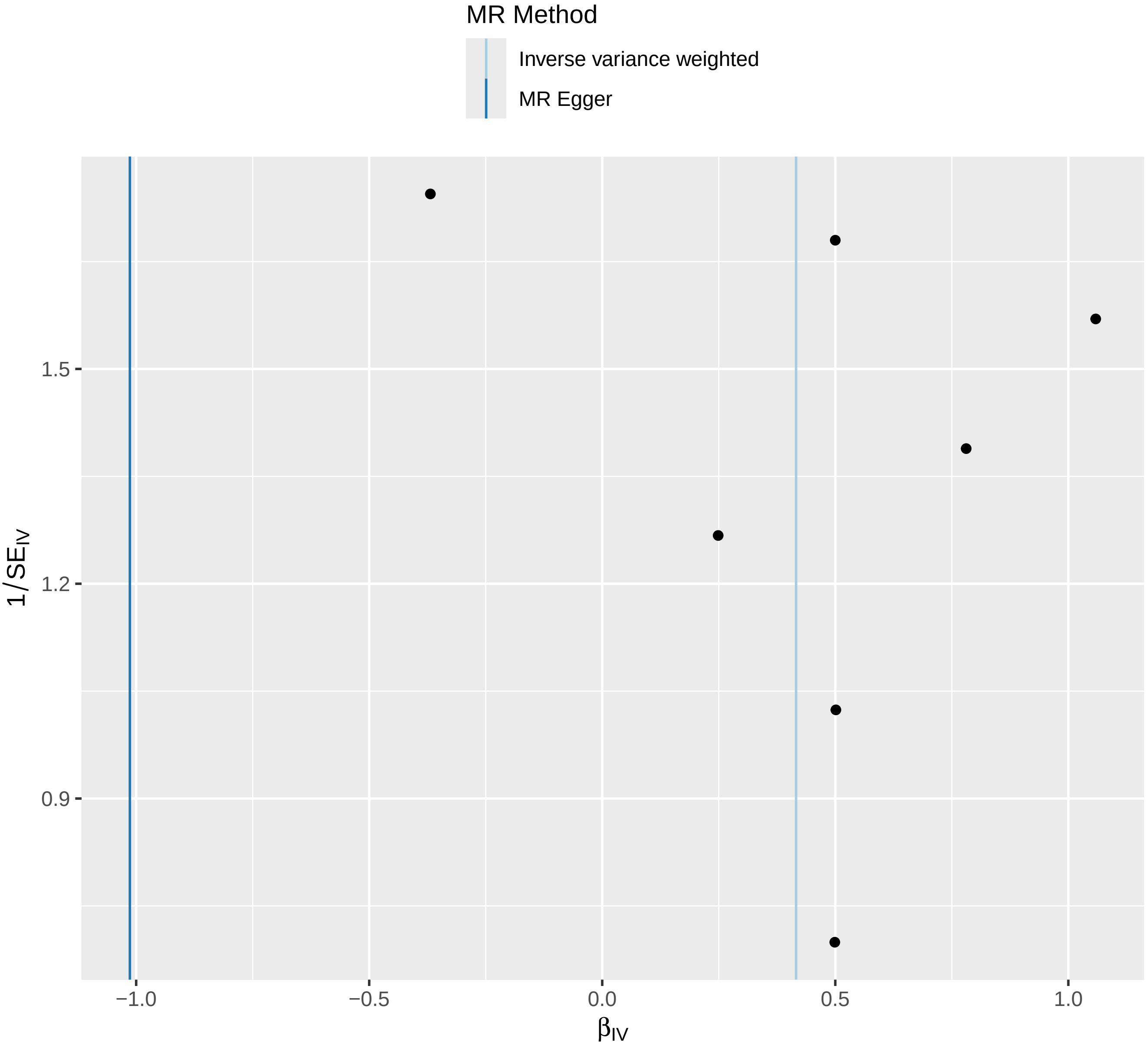

MR Method
Inverse variance weighted
MR Egger
1/SE_{IV}
1.5
1.2
0.9
−1.0
−0.5
0.0
0.5
1.0
β_{IV}

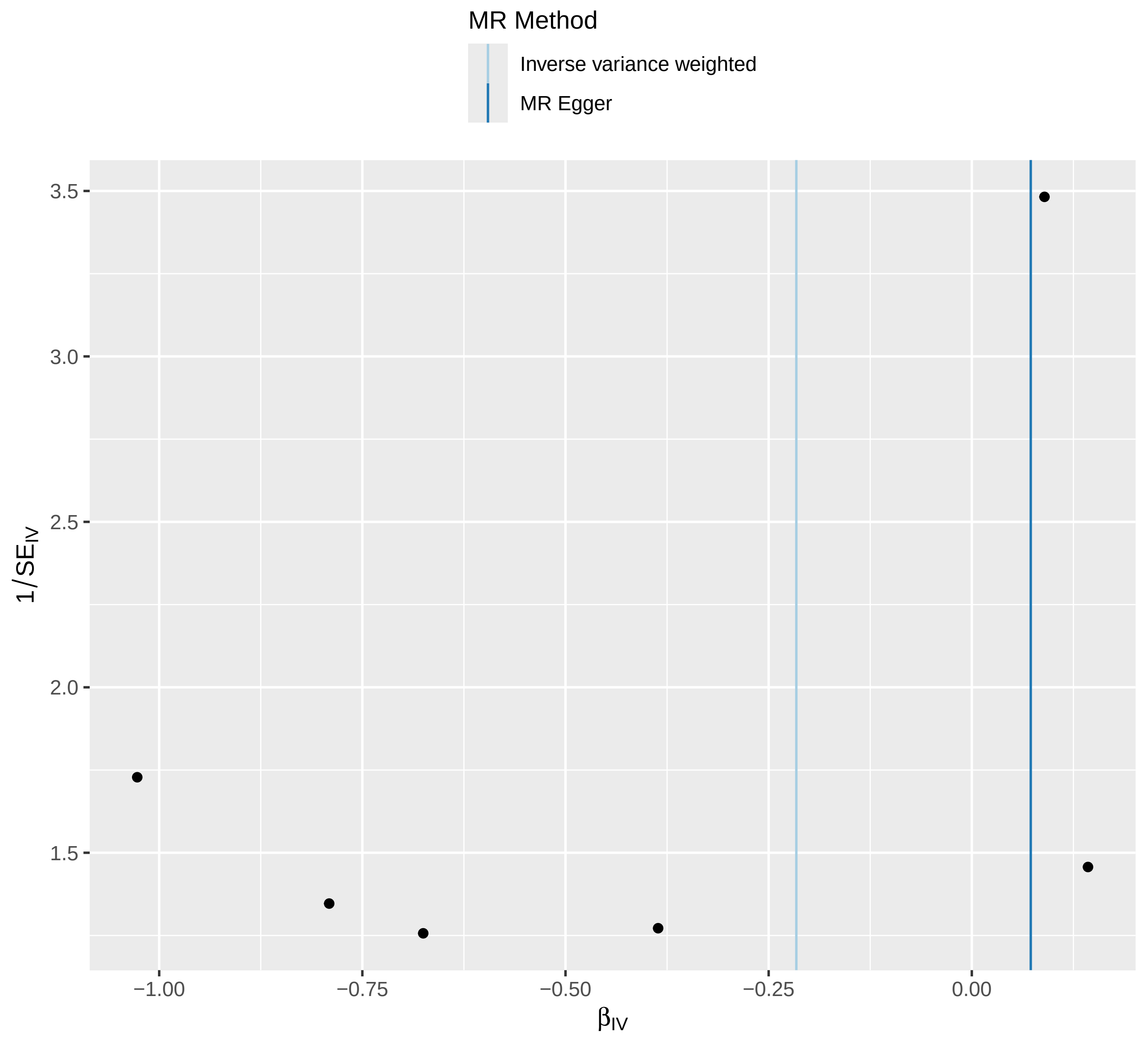

MR Method
Inverse variance weighted
MR Egger
3.5
3.0
2.5
2.0
1.5
$1/SE_{IV}$
−1.00
−0.75
−0.50
−0.25
0.00
β_{IV}

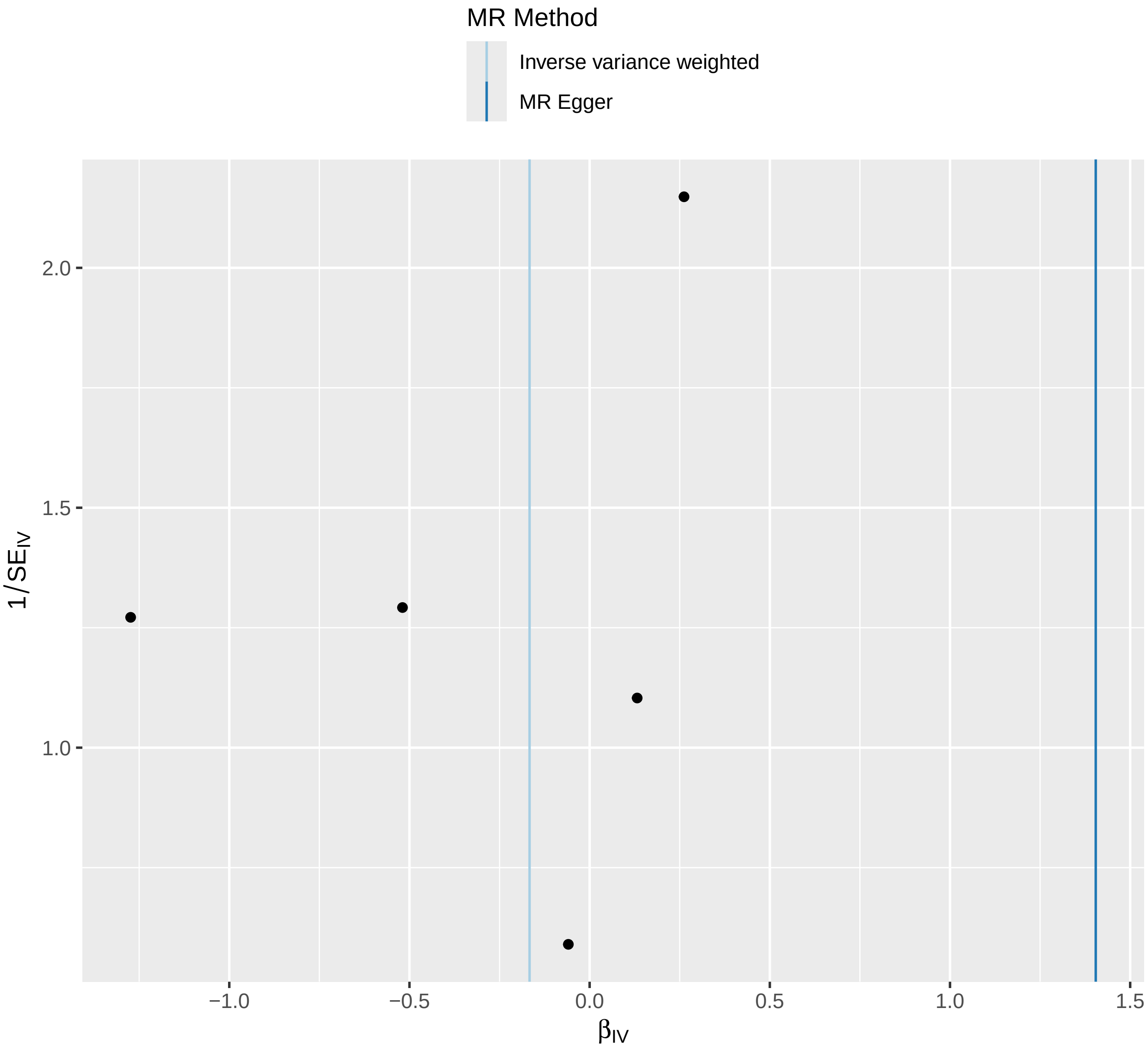

MR Method
Inverse variance weighted
MR Egger
2.0
1.5
1.0
1/SE$_{IV}$
−1.0
−0.5
0.0
0.5
1.0
1.5
β$_{IV}$

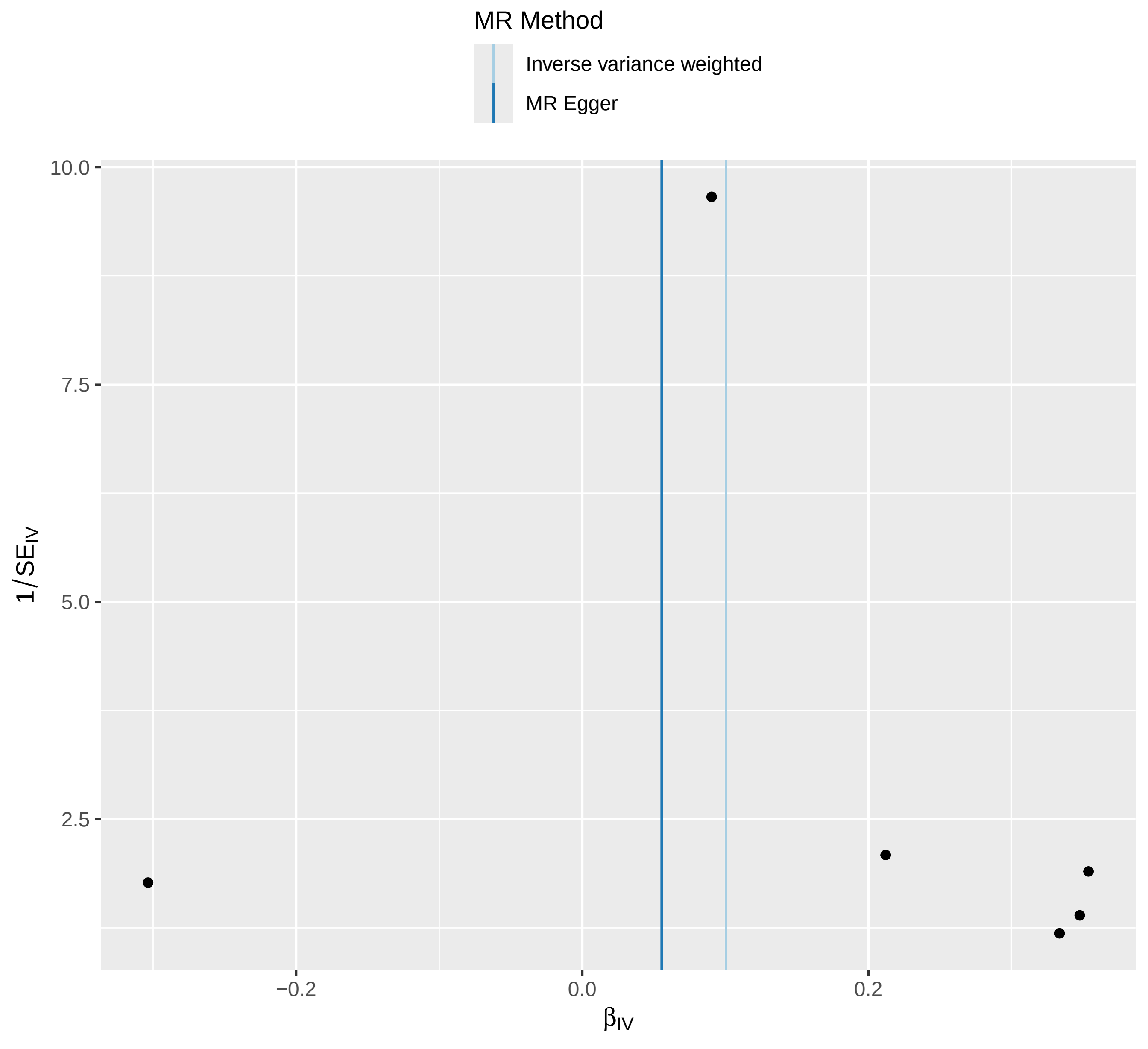
MR Method
Inverse variance weighted
MR Egger
10.0
7.5
5.0
2.5
1/SE_IV
−0.2
0.0
0.2
β_IV

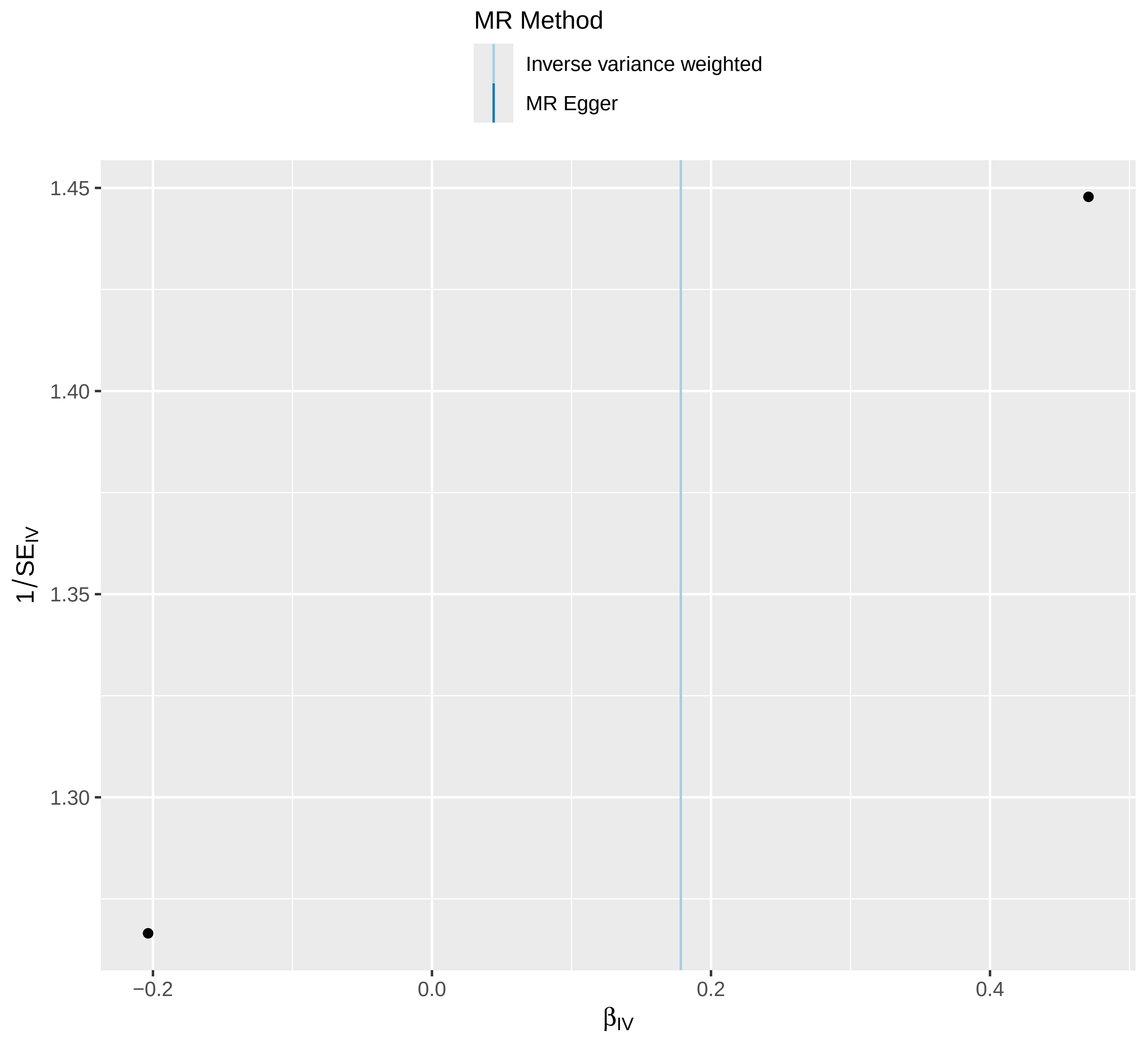

MR Method
Inverse variance weighted
MR Egger
1.45
1.40
1.35
1.30
1/SE_IV
−0.2
0.0
0.2
0.4
β_IV

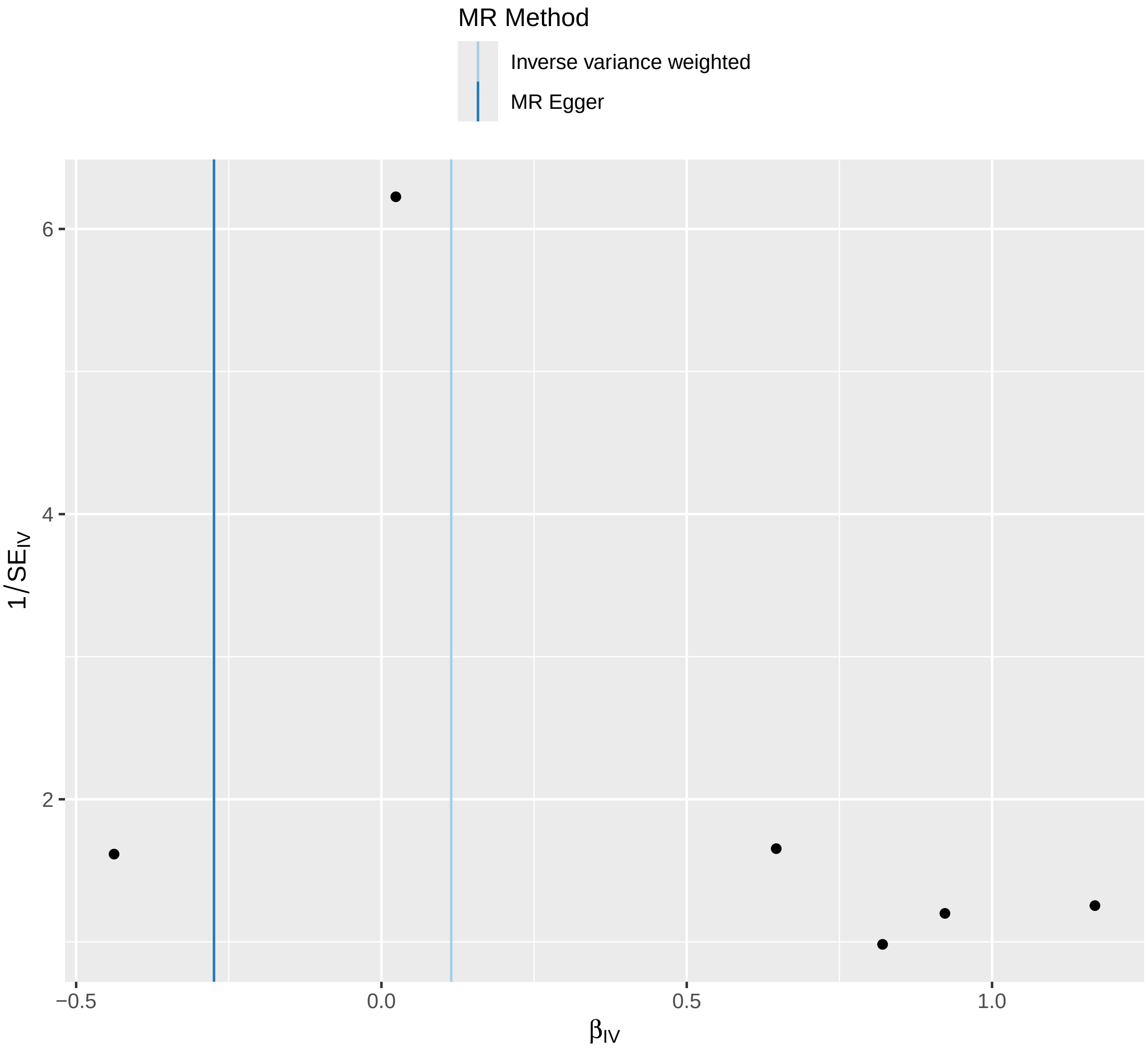

MR Method
Inverse variance weighted
MR Egger
6
4
2
1/SE$_{IV}$
−0.5
0.0
0.5
1.0
β$_{IV}$

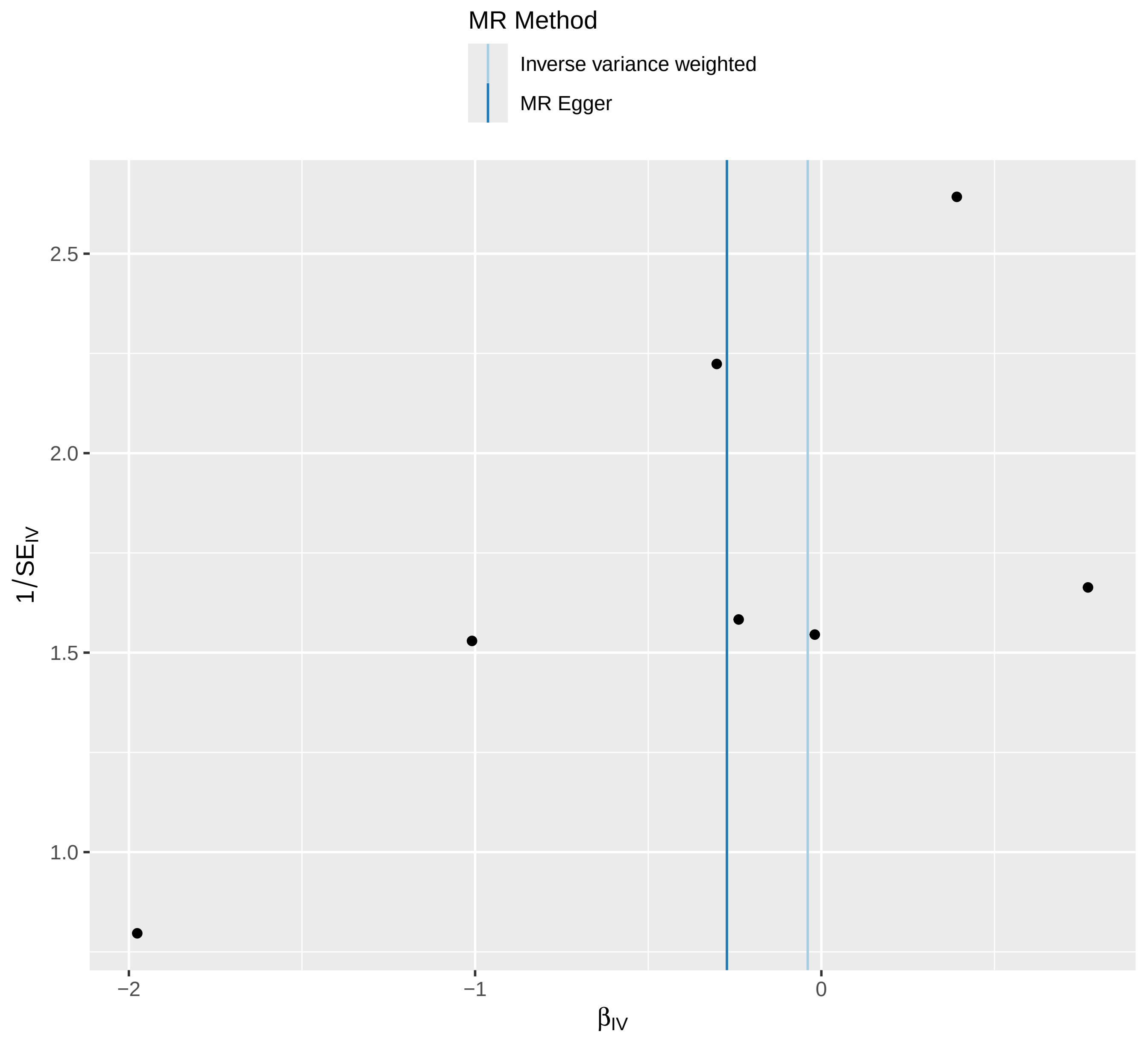
MR Method
Inverse variance weighted
MR Egger
2.5
2.0
1.5
1.0
−2
−1
0
1/SE_IV
β_IV

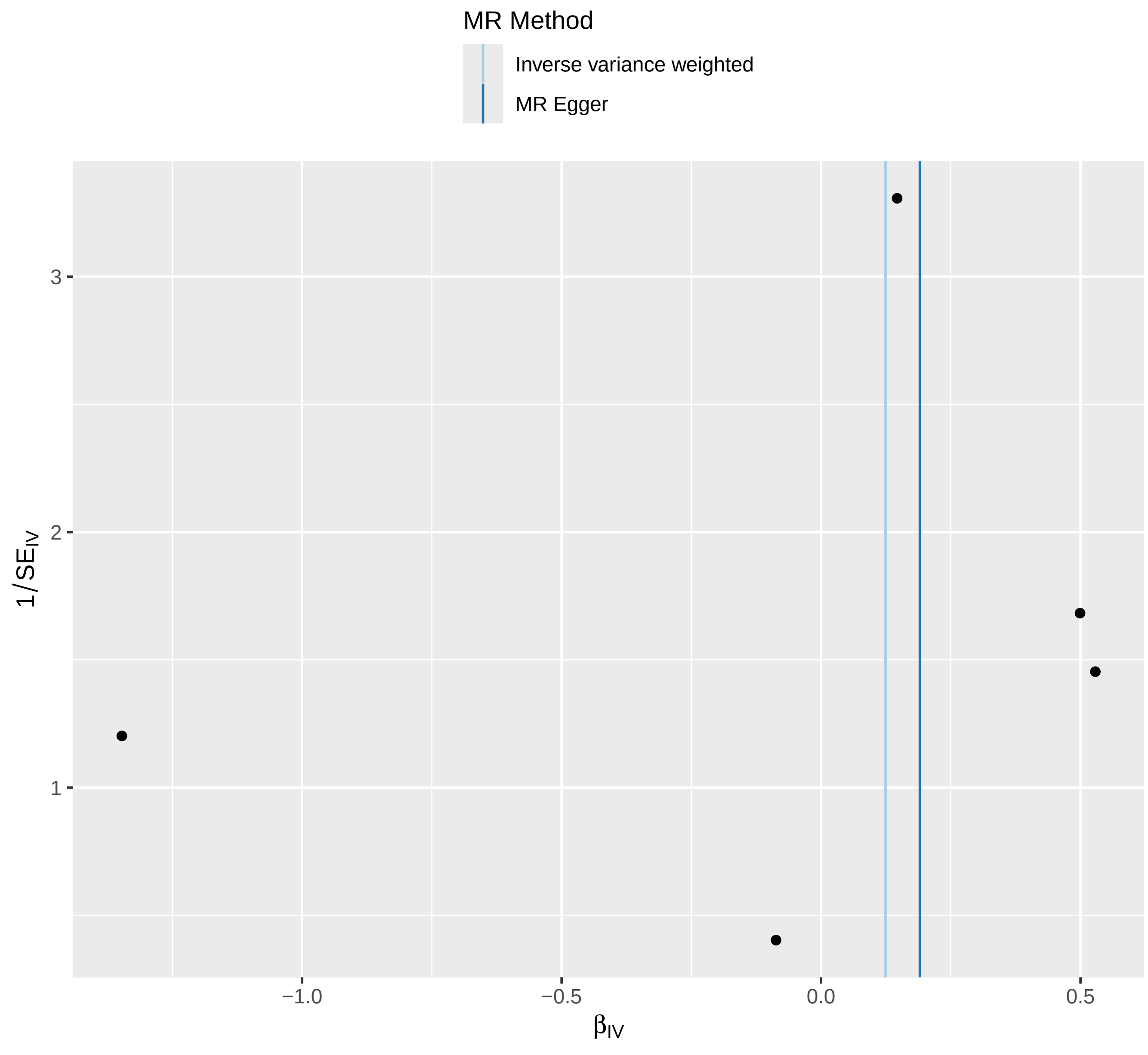

MR Method
Inverse variance weighted
MR Egger
3
2
1
1/SE$_{IV}$
−1.0
−0.5
0.0
0.5
β$_{IV}$

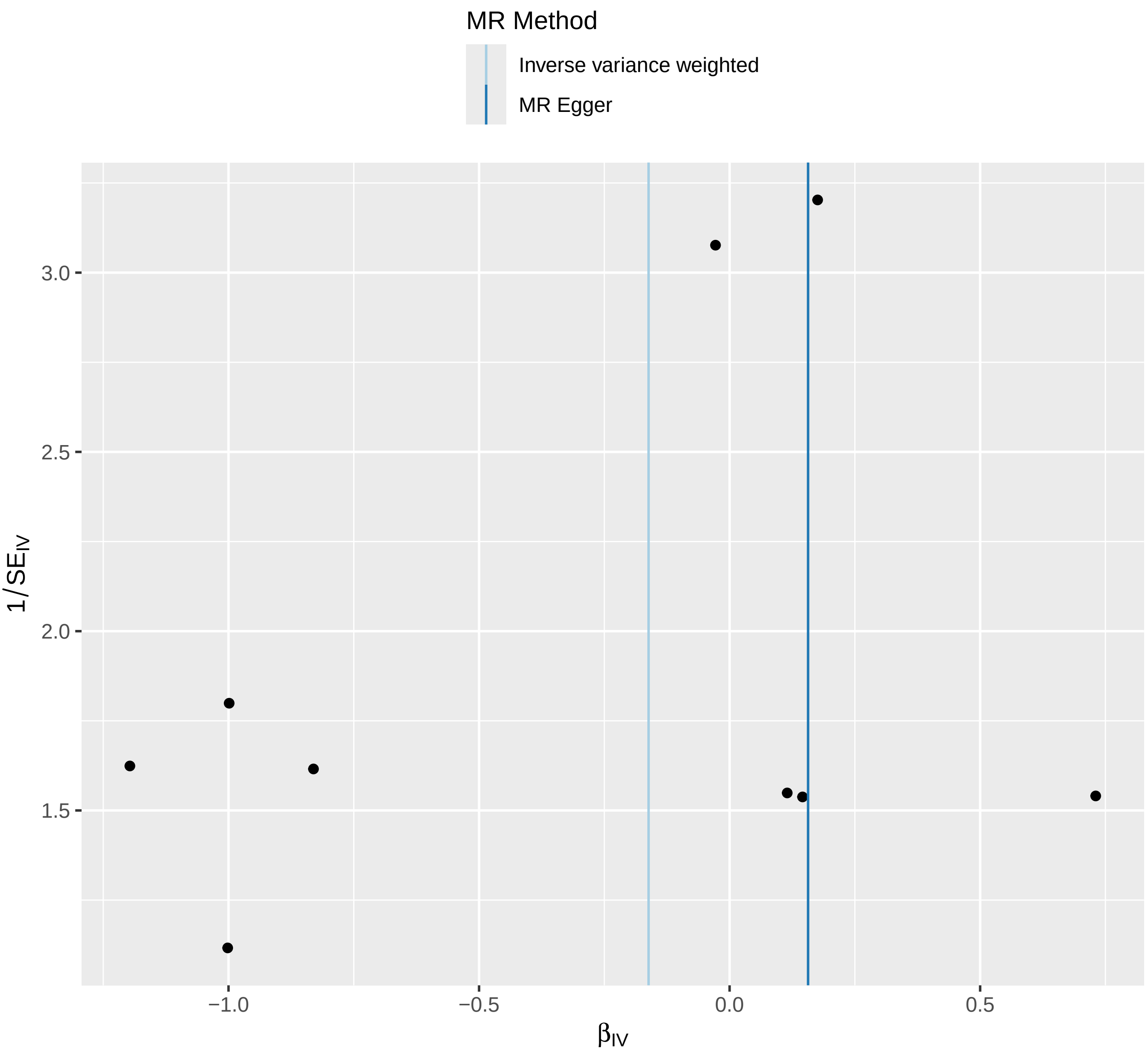
MR Method
Inverse variance weighted
MR Egger
3.0
2.5
2.0
1.5
1/SE_{IV}
−1.0
−0.5
0.0
0.5
$β_{IV}$

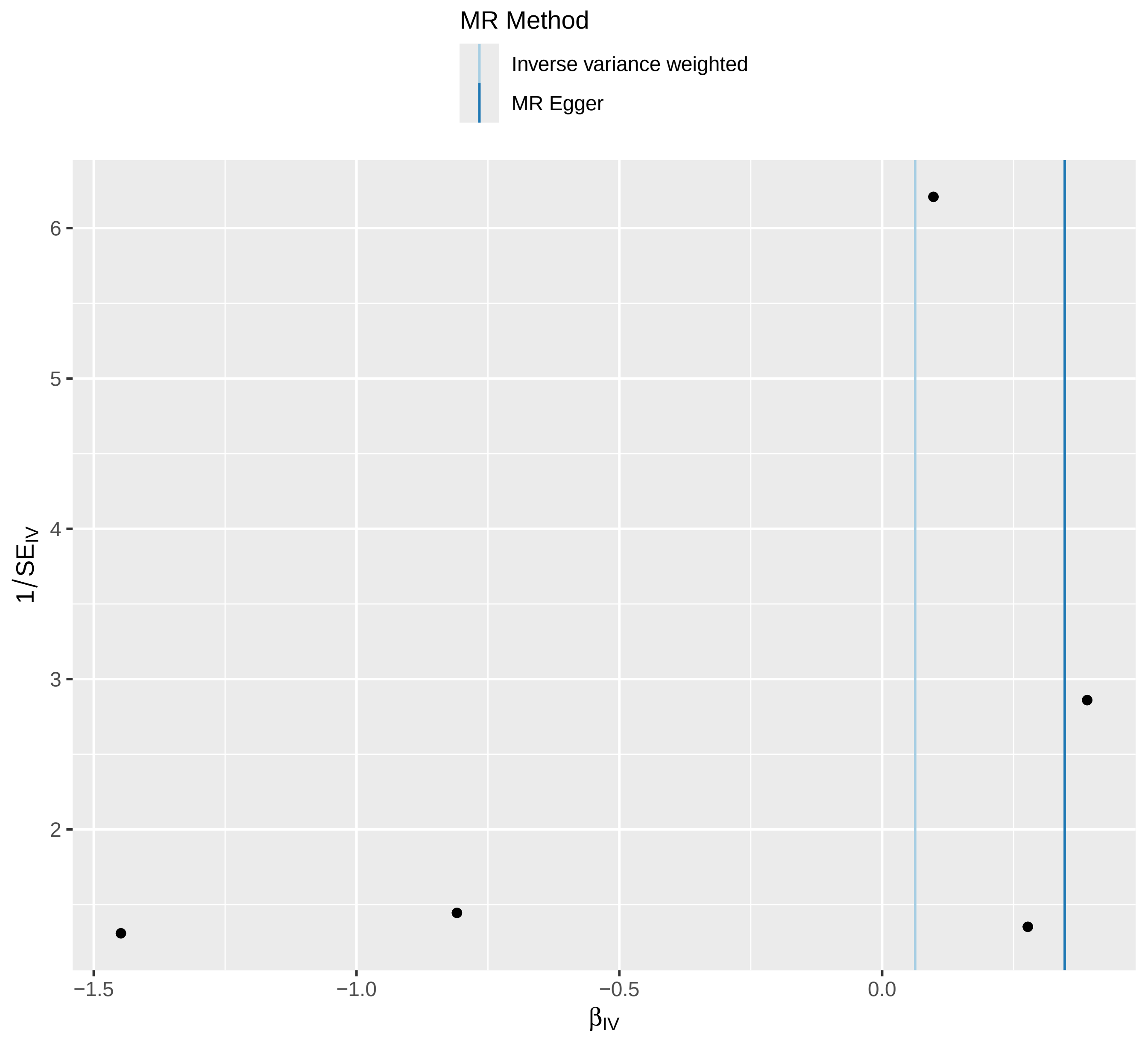
MR Method
Inverse variance weighted
MR Egger
6
5
4
3
2
1/SE_IV
−1.5
−1.0
−0.5
0.0
β_IV

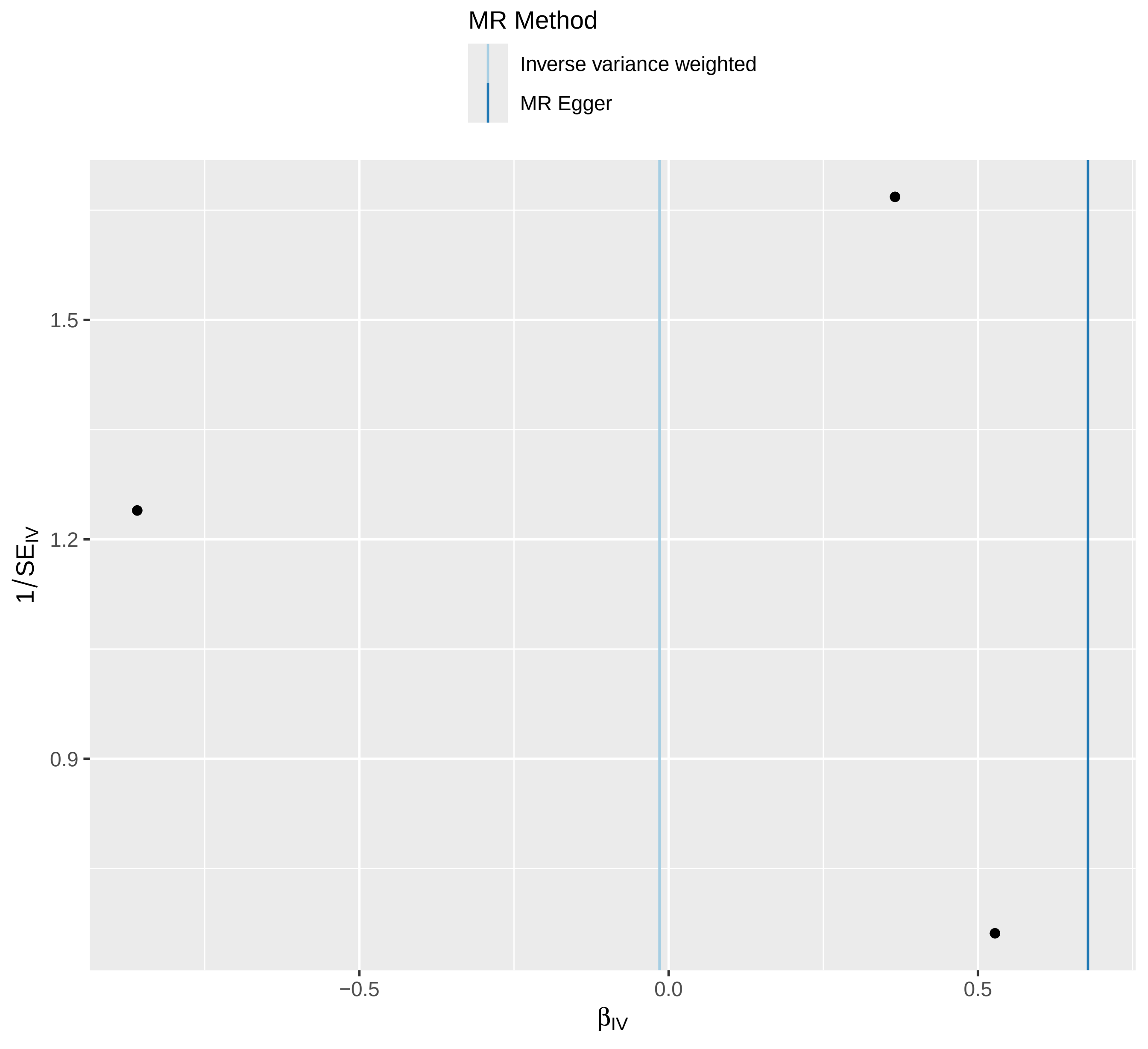

MR Method
Inverse variance weighted
MR Egger
1.5
1.2
0.9
1/SE_IV
−0.5
0.0
0.5
β_IV

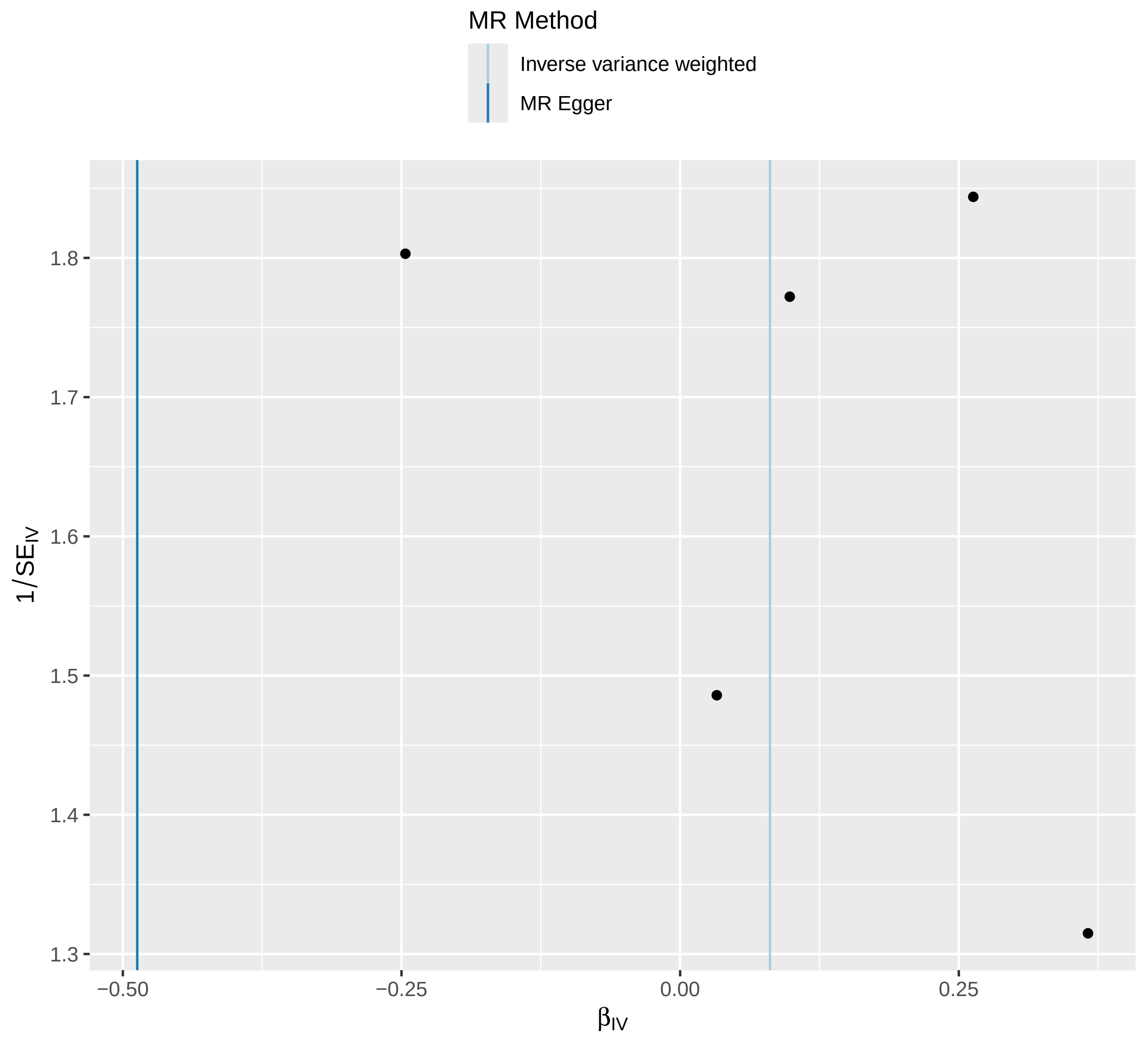
MR Method
Inverse variance weighted
MR Egger
1.8
1.7
1.6
1.5
1.4
1.3
1/SE$_{IV}$
−0.50
−0.25
0.00
0.25
β$_{IV}$

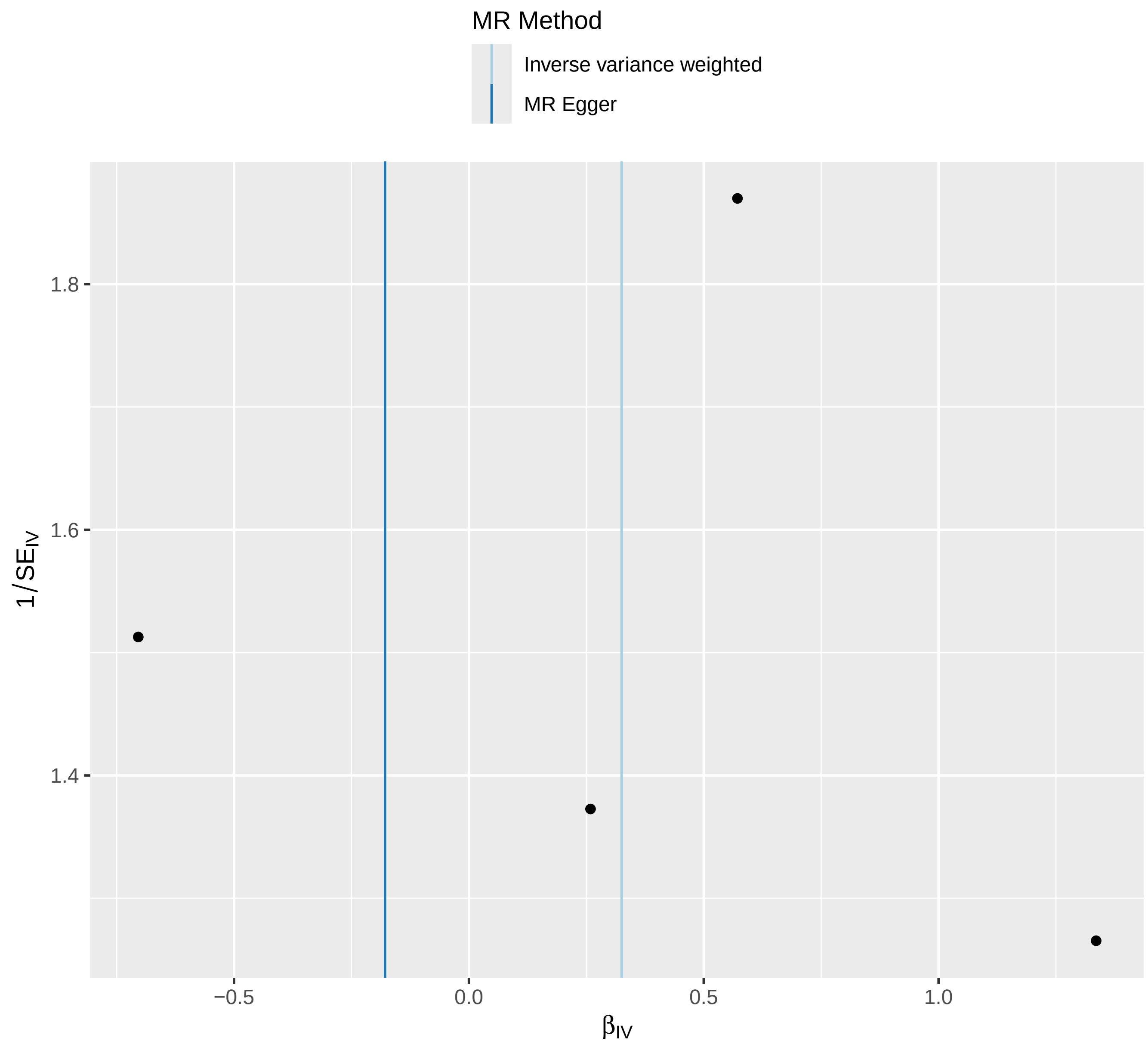

MR Method
Inverse variance weighted
MR Egger
1.8
1.6
1.4
$1/SE_{IV}$
−0.5
0.0
0.5
1.0
β_{IV}

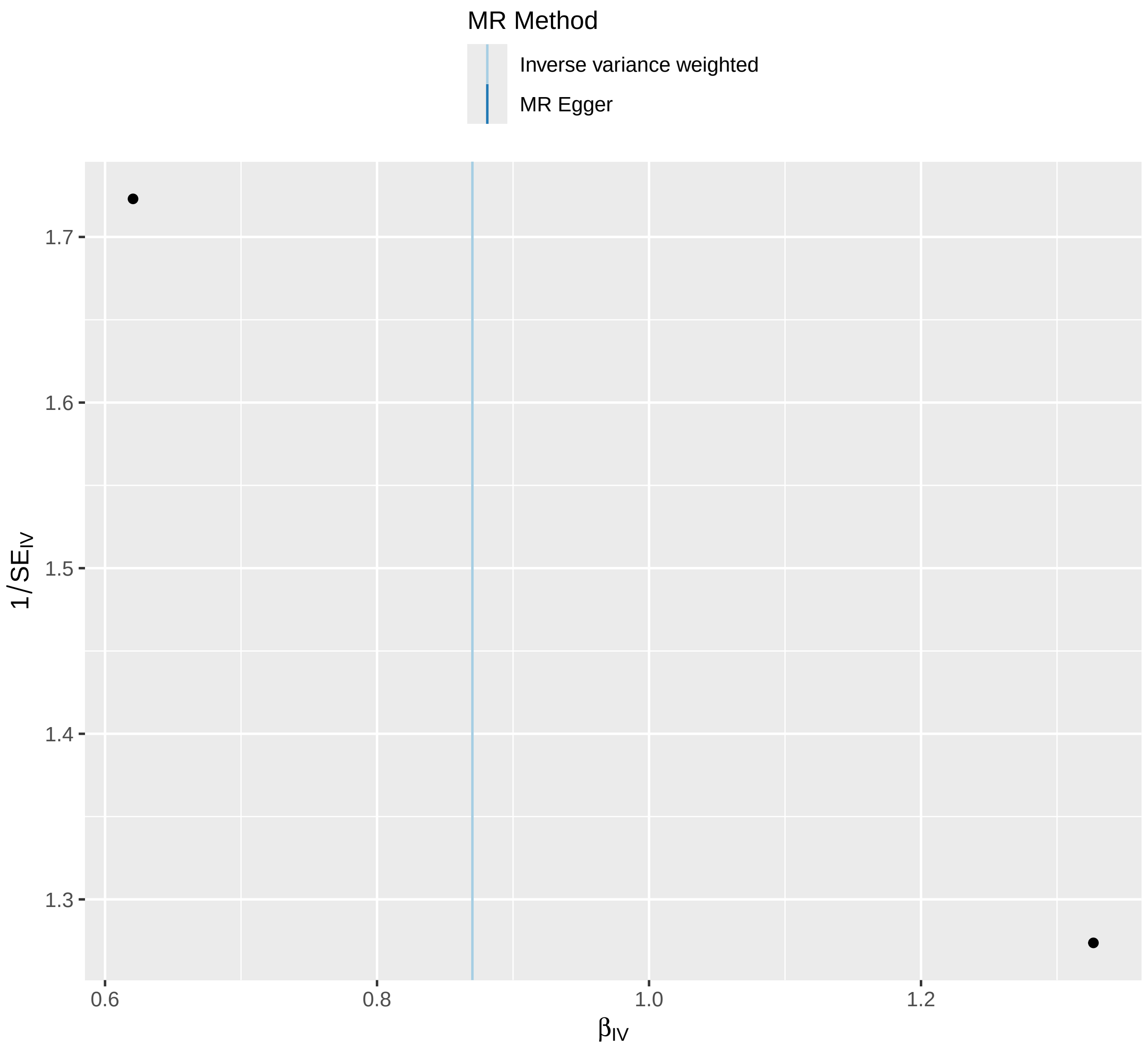
MR Method
Inverse variance weighted
MR Egger
1.7
1.6
1.5
1.4
1.3
$1/SE_{IV}$
0.6
0.8
1.0
1.2
β_{IV}

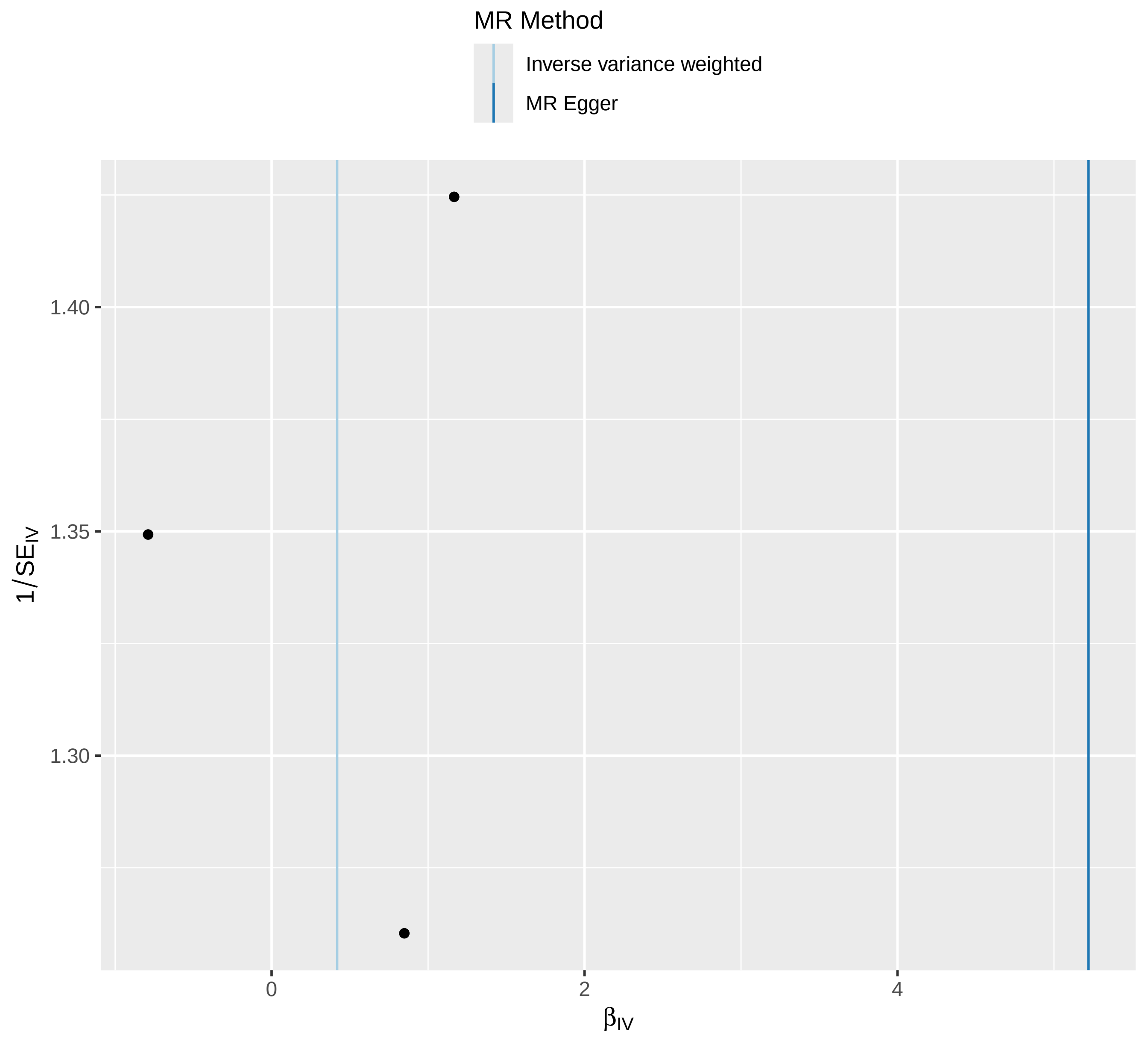
MR Method
Inverse variance weighted
MR Egger
1.40
1.35
1.30
1/SE_IV
0
2
4
β_IV

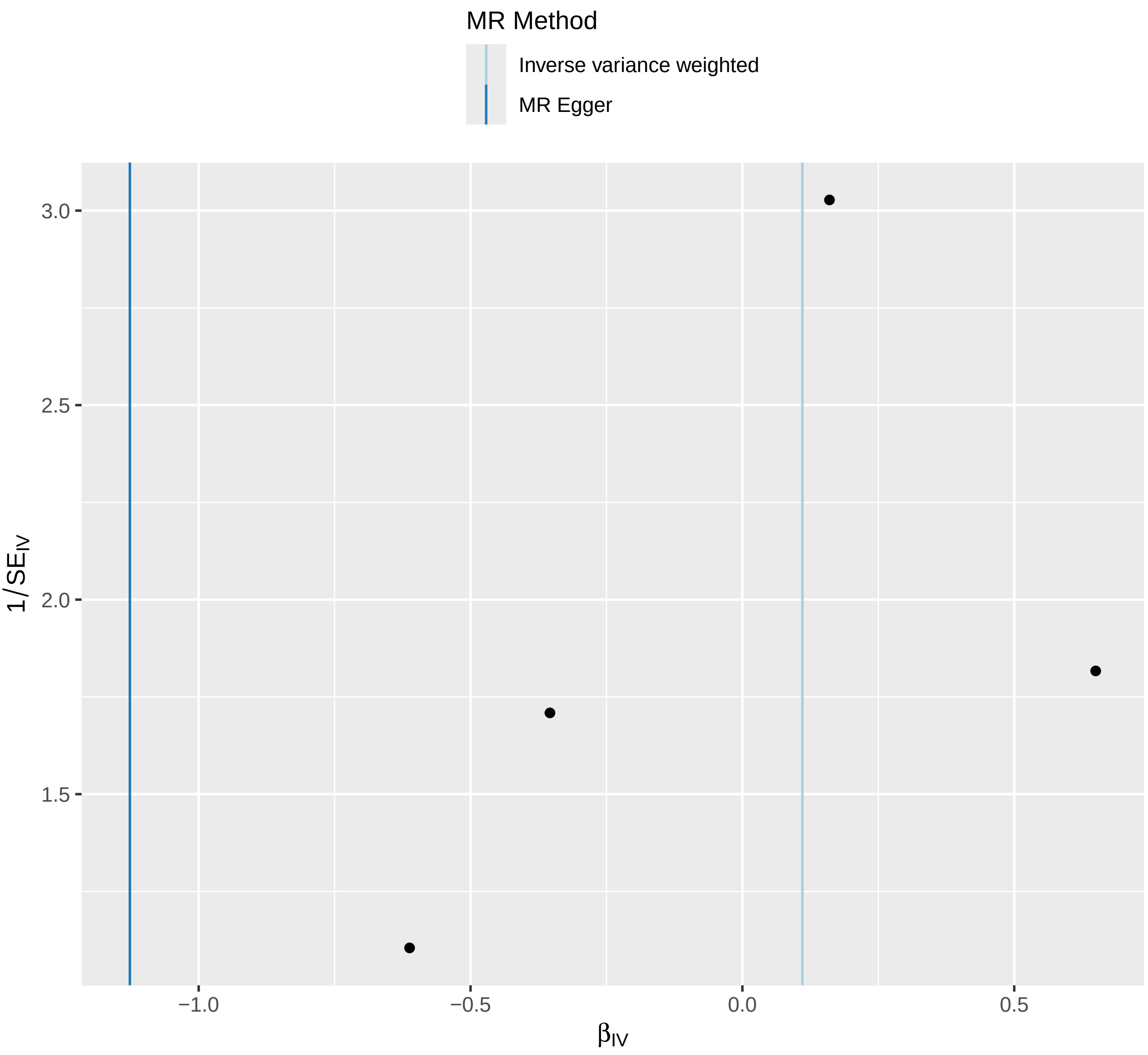

MR Method
Inverse variance weighted
MR Egger
3.0
2.5
2.0
1.5
1/SE_IV
−1.0
−0.5
0.0
0.5
β_IV

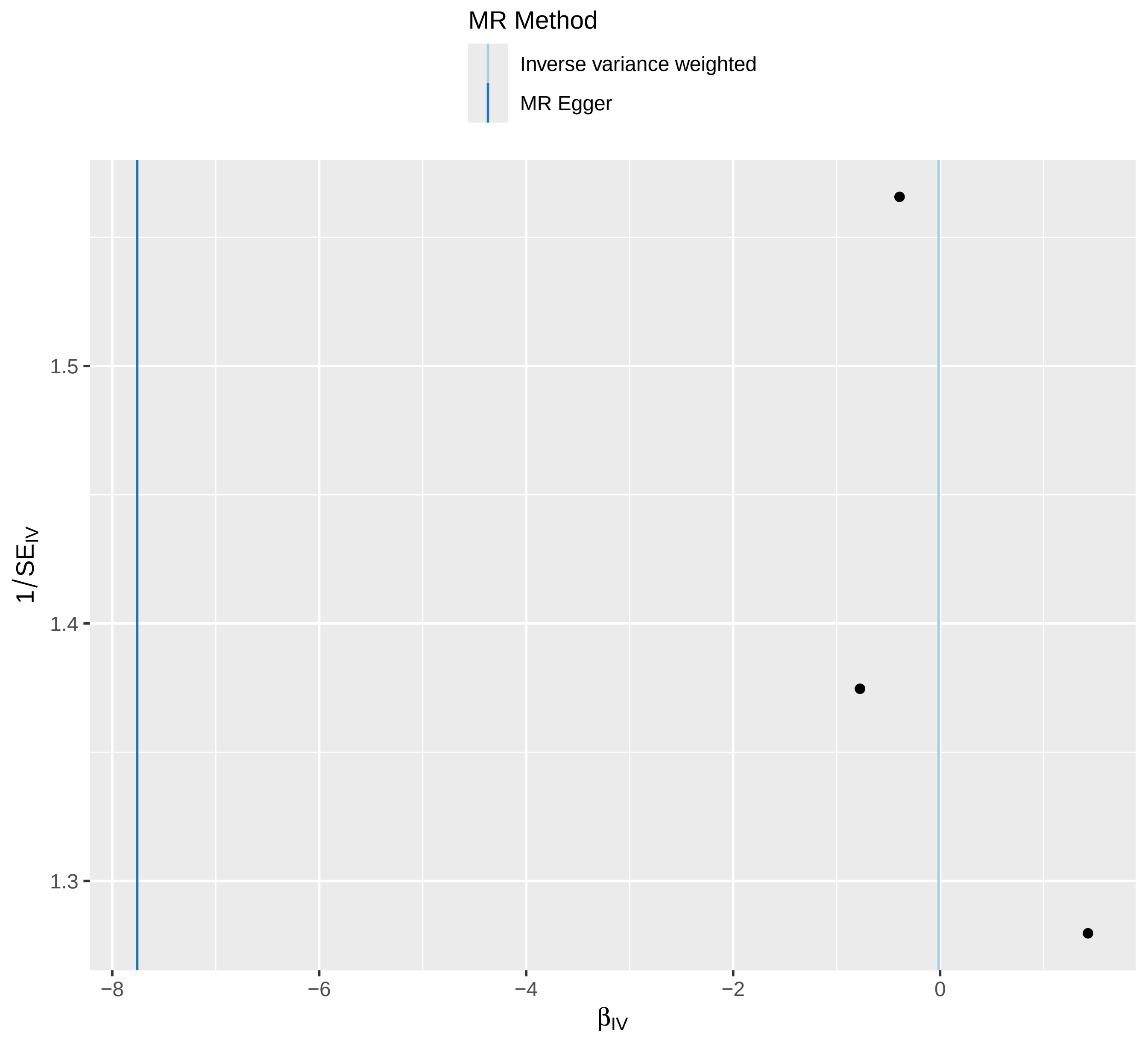
MR Method
Inverse variance weighted
MR Egger
1/SE$_{IV}$
1.5
1.4
1.3
−8
−6
−4
−2
0
β$_{IV}$

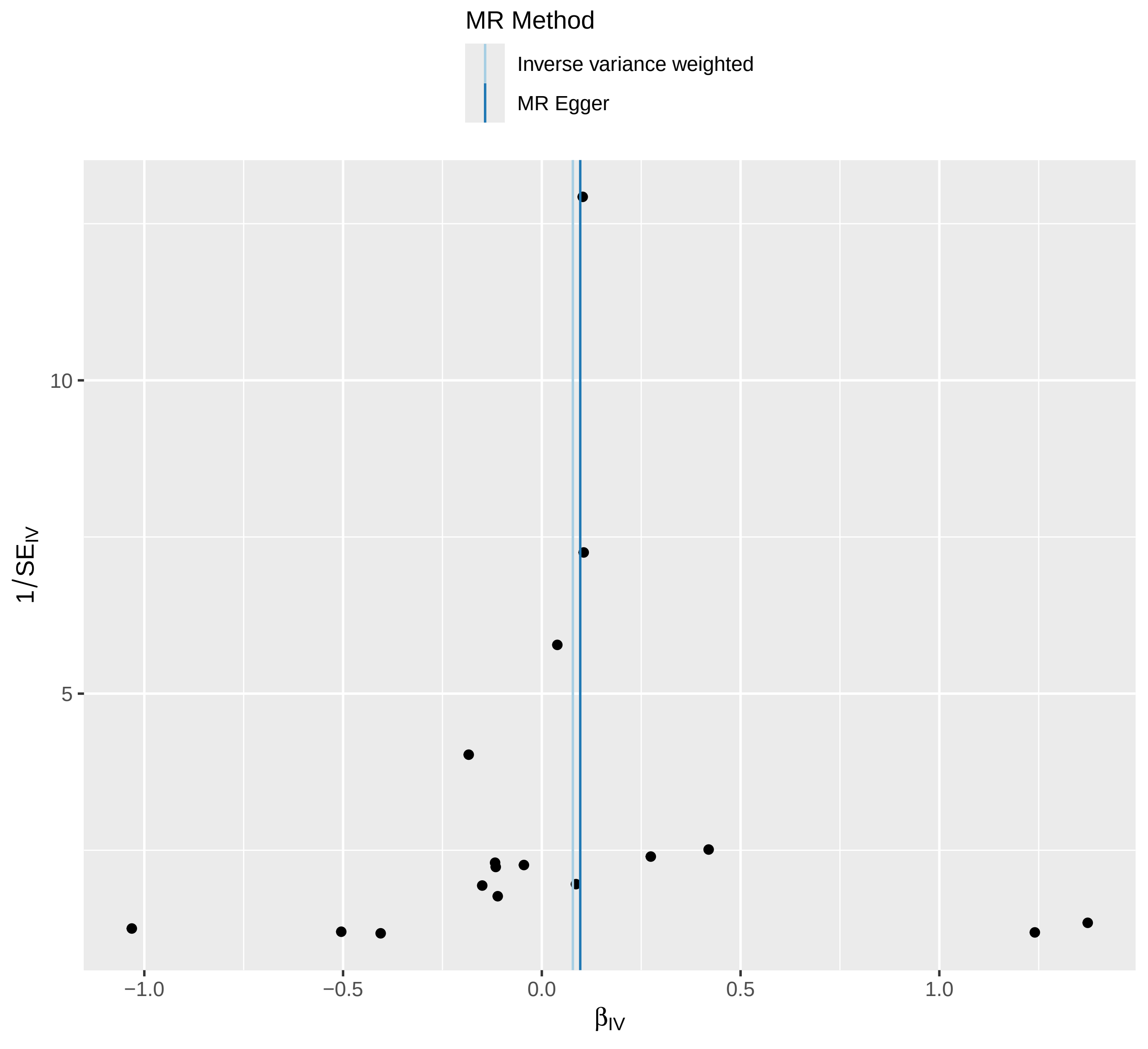

MR Method
Inverse variance weighted
MR Egger
1/SE_IV
10
5
−1.0
−0.5
0.0
0.5
1.0
β_IV

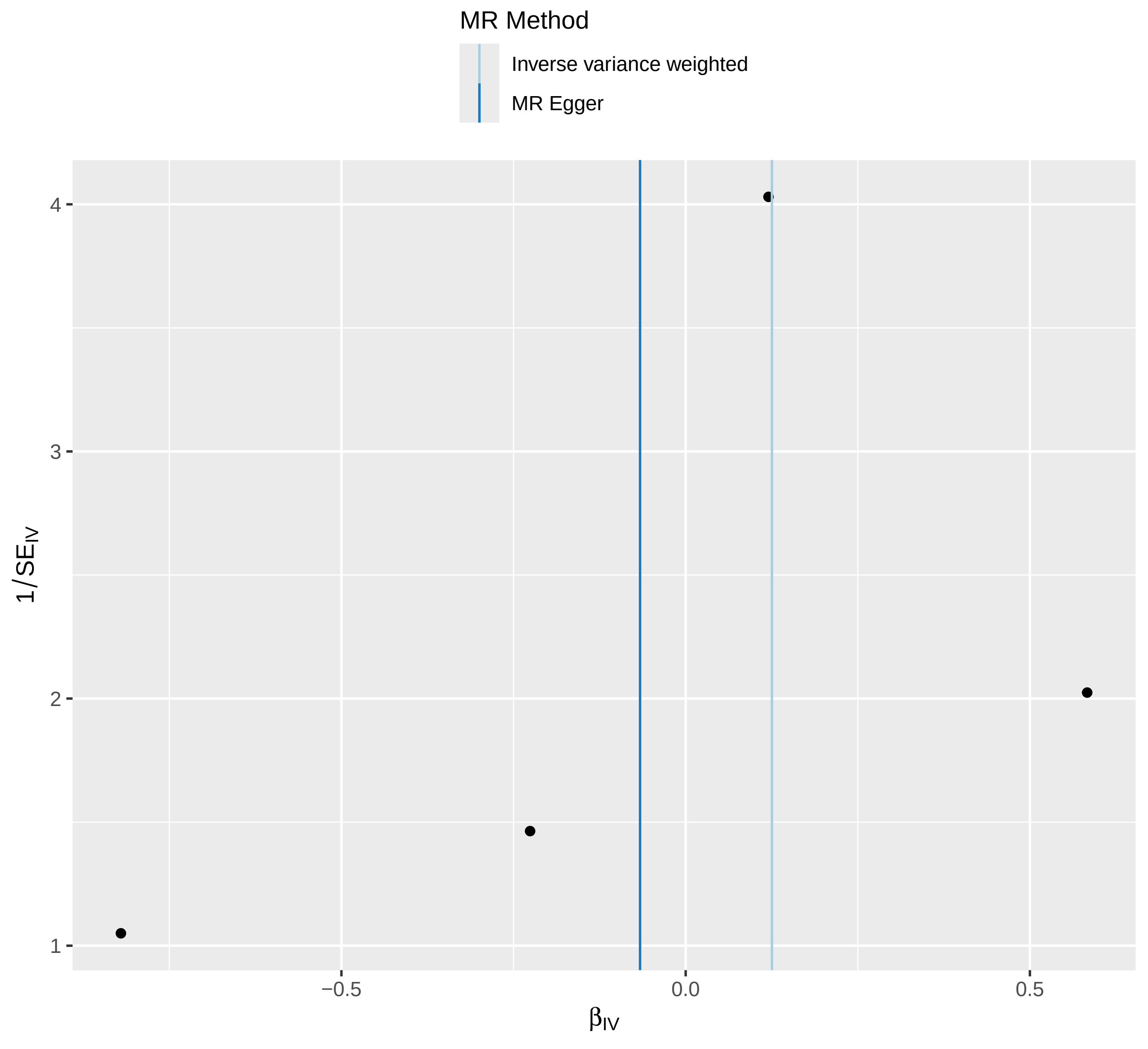
MR Method
Inverse variance weighted
MR Egger
1/SE_{IV}
4
3
2
1
−0.5
0.0
0.5
β_{IV}

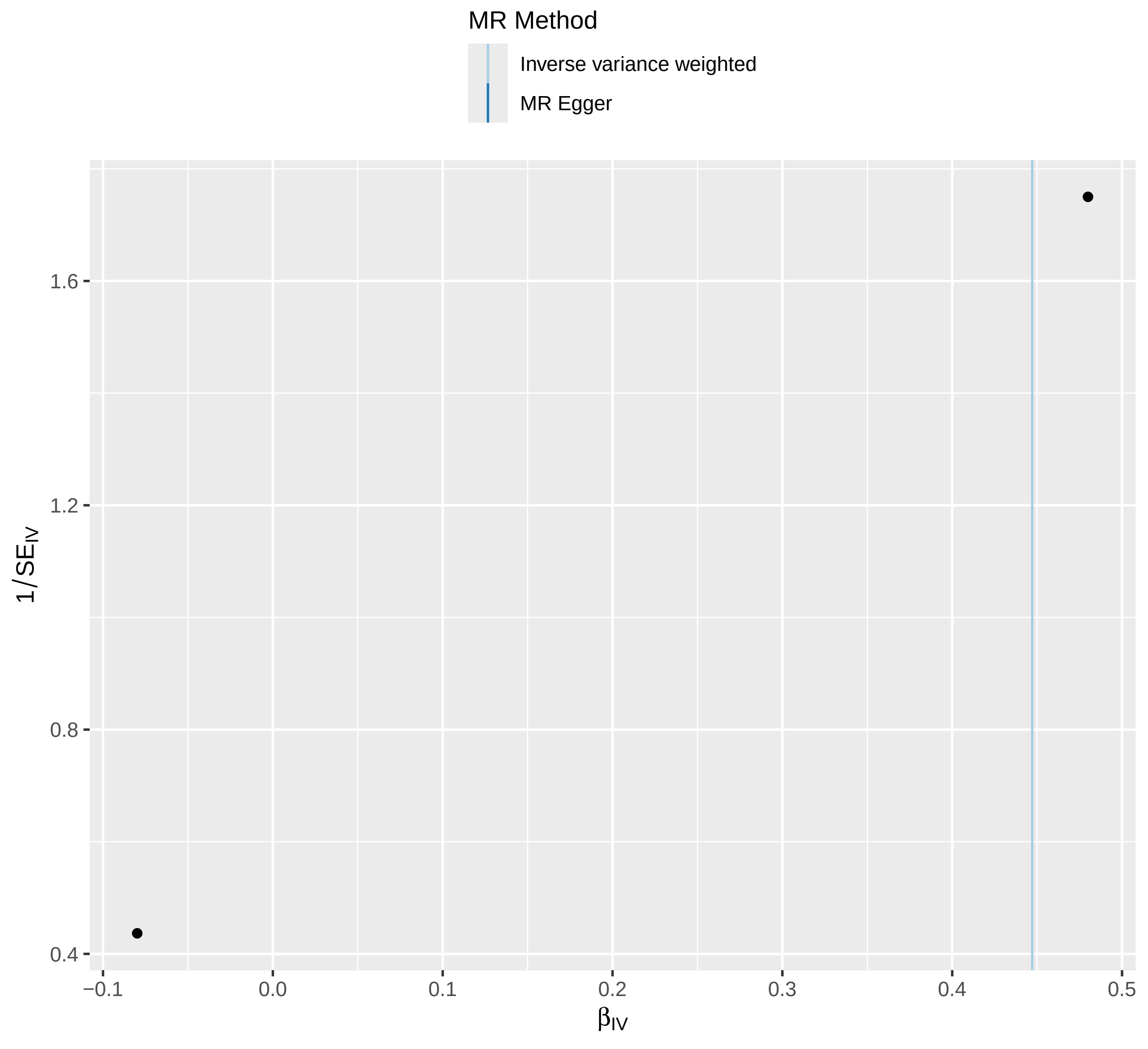

MR Method
Inverse variance weighted
MR Egger
1.6
1.2
0.8
0.4
1/SE_IV
−0.1
0.0
0.1
0.2
0.3
0.4
0.5
β_IV

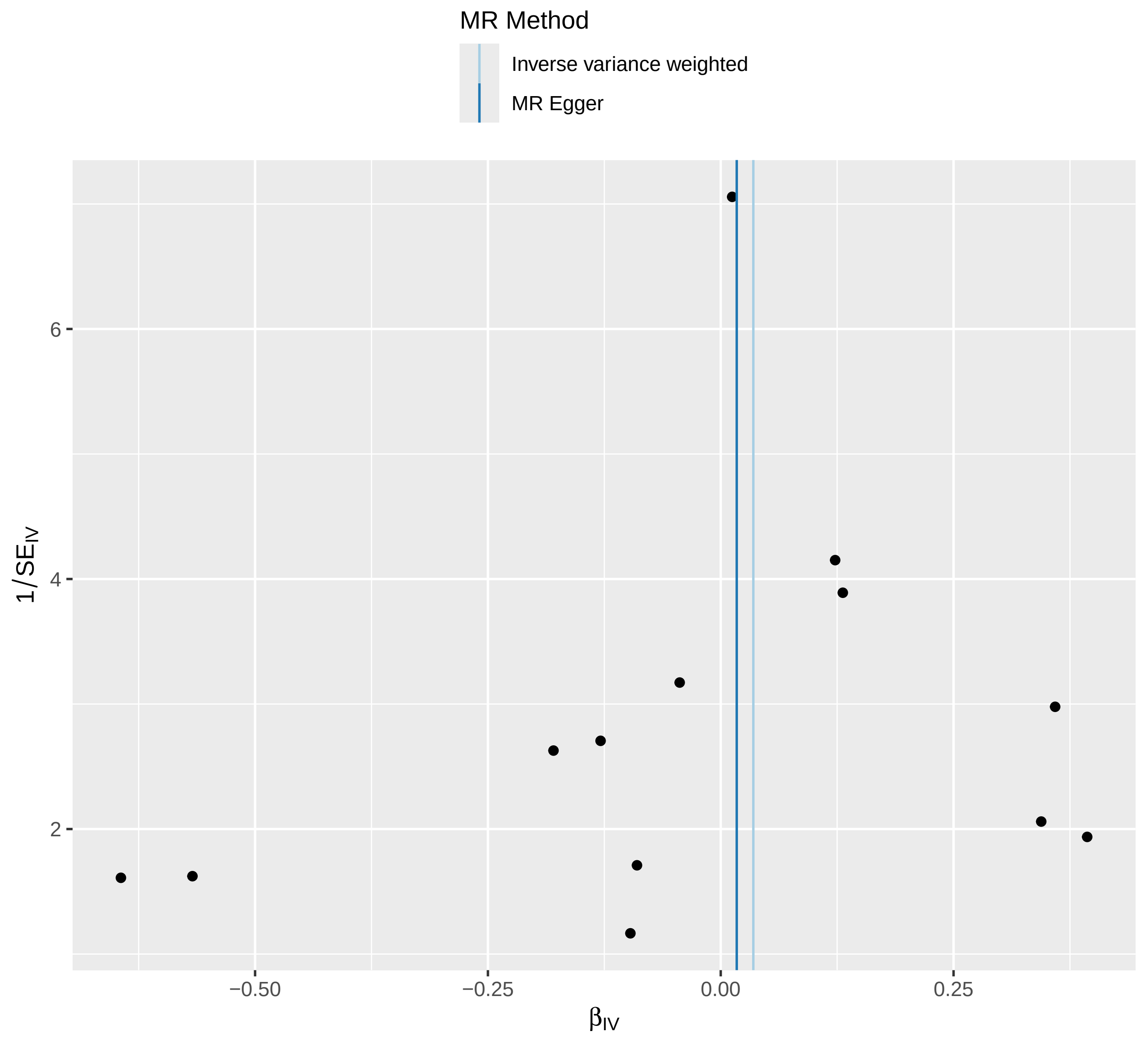
MR Method
Inverse variance weighted
MR Egger
1/SE_IV
6
4
2
−0.50
−0.25
0.00
0.25
β_IV

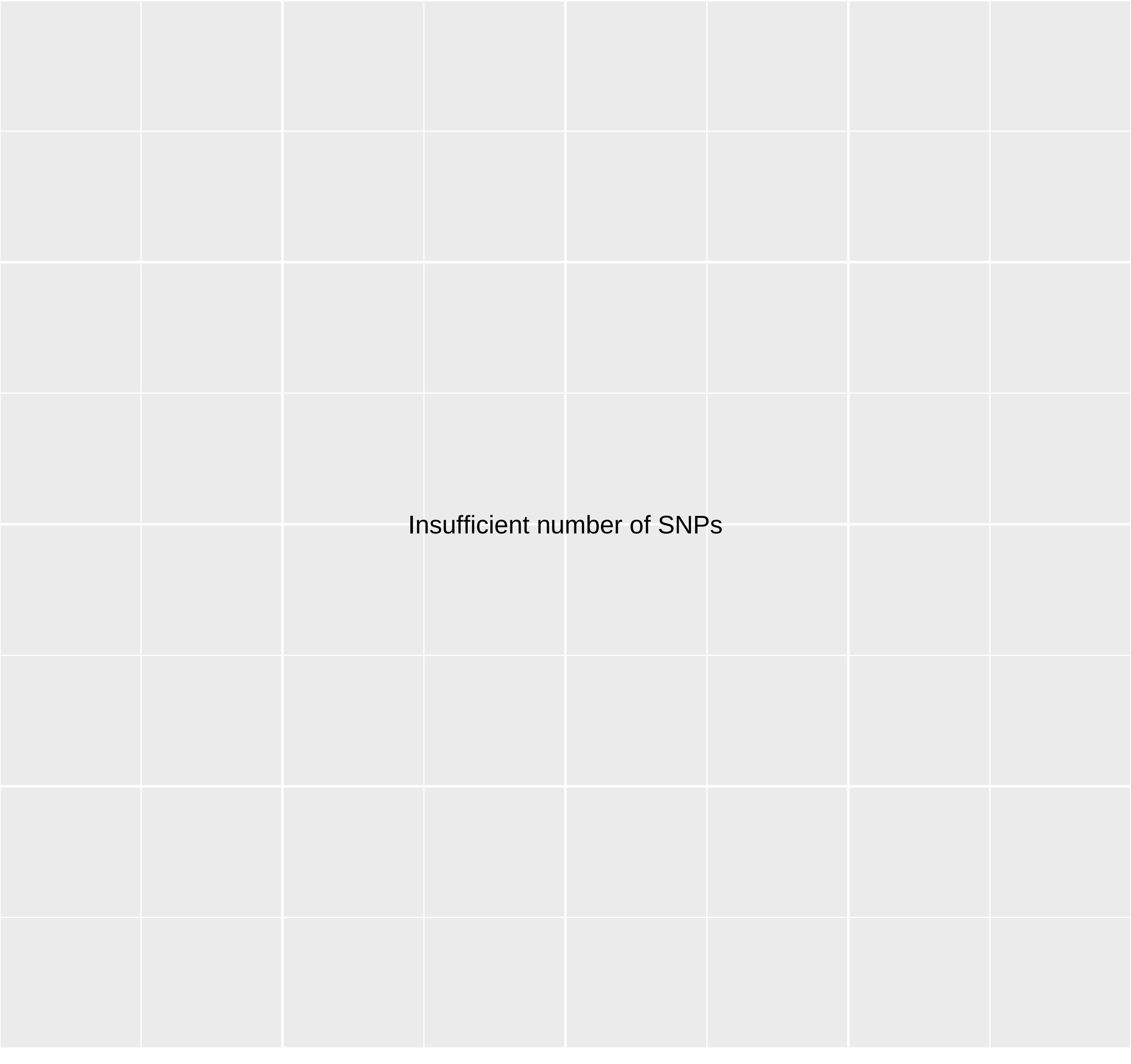
Insufficient number of SNPs

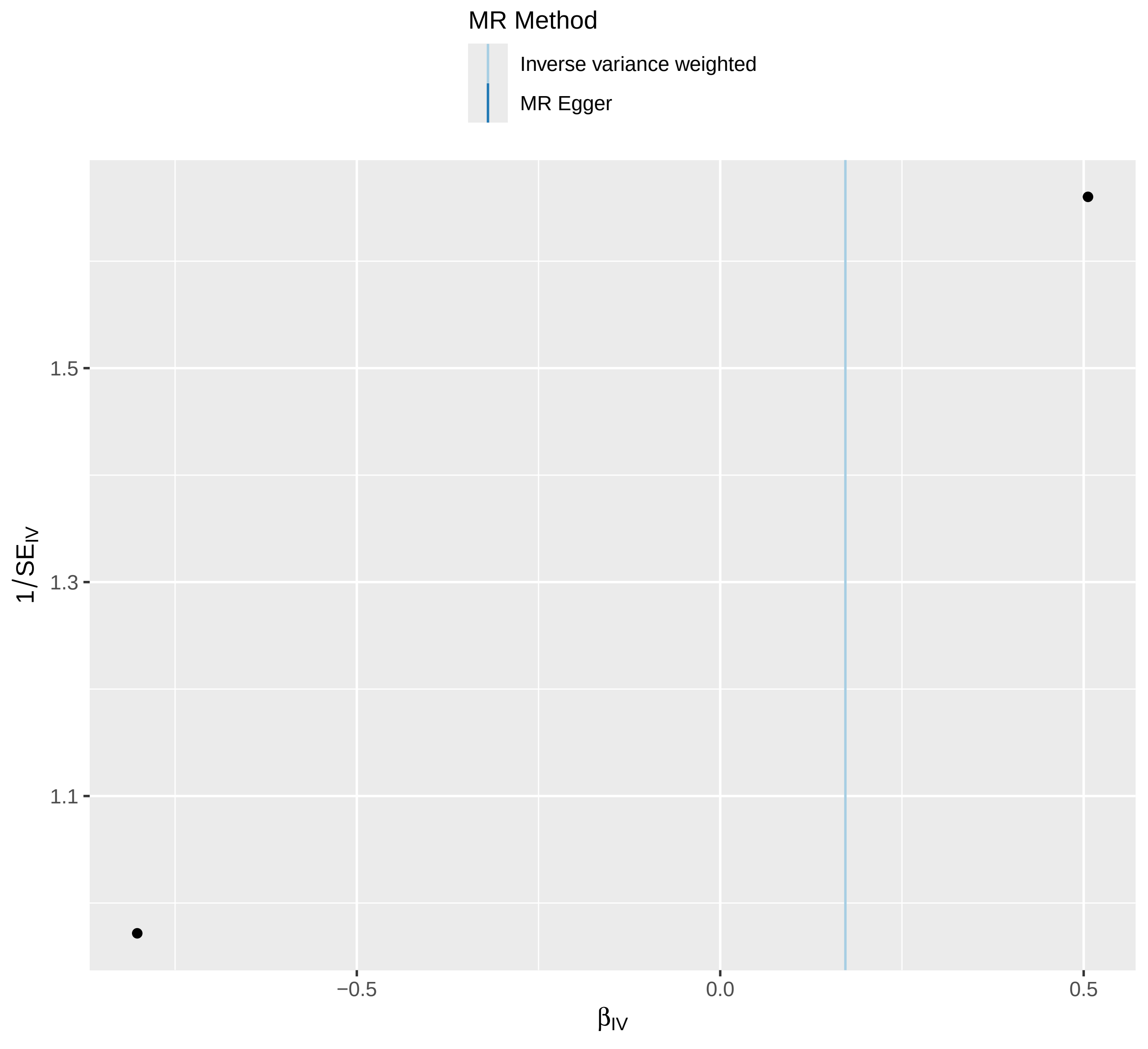
MR Method
Inverse variance weighted
MR Egger
1.5
1.3
1.1
1/SE_IV
−0.5
0.0
0.5
β_IV

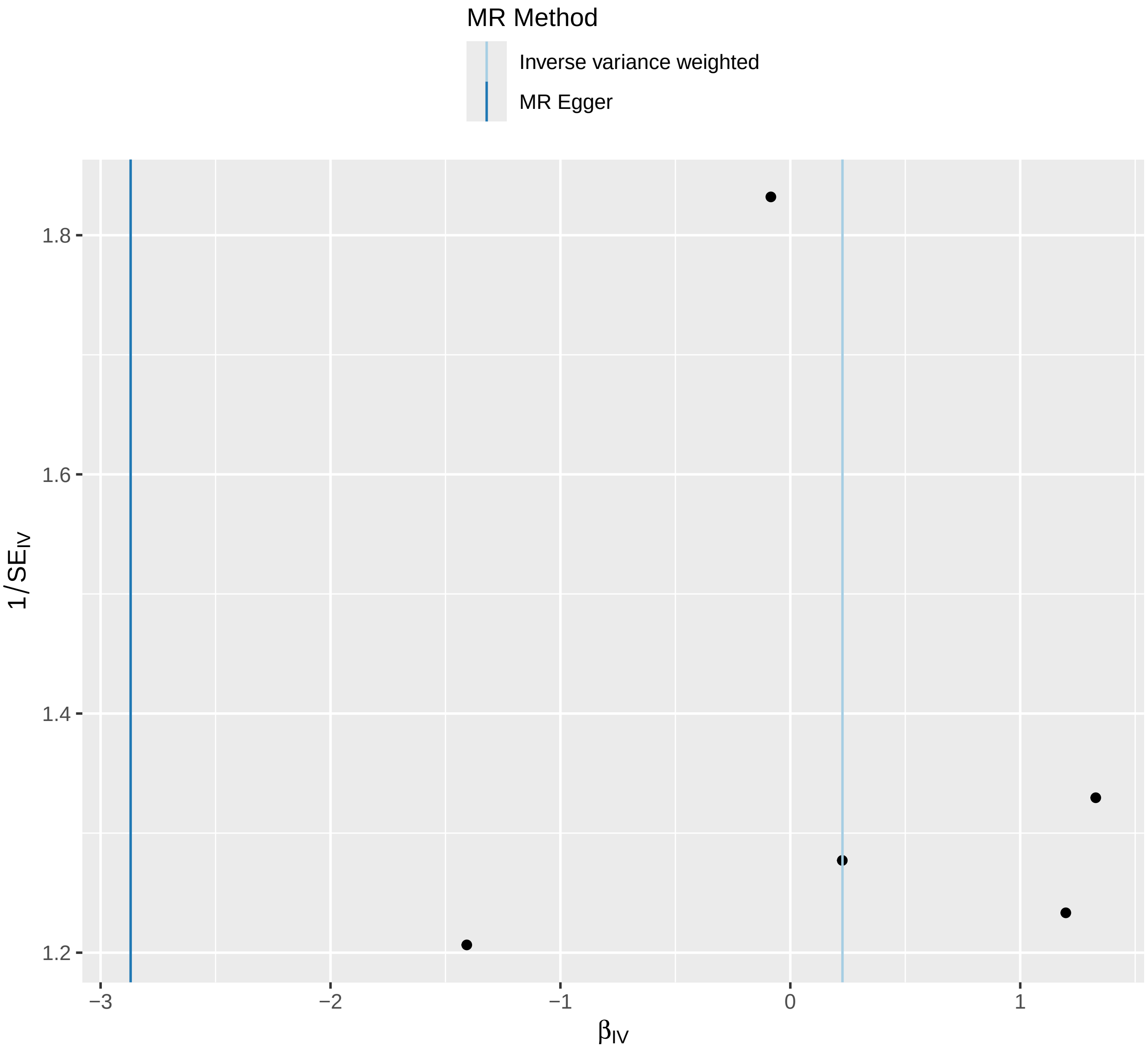
MR Method
Inverse variance weighted
MR Egger
1.8
1.6
1.4
1.2
1/SE_IV
−3
−2
−1
0
1
β_IV

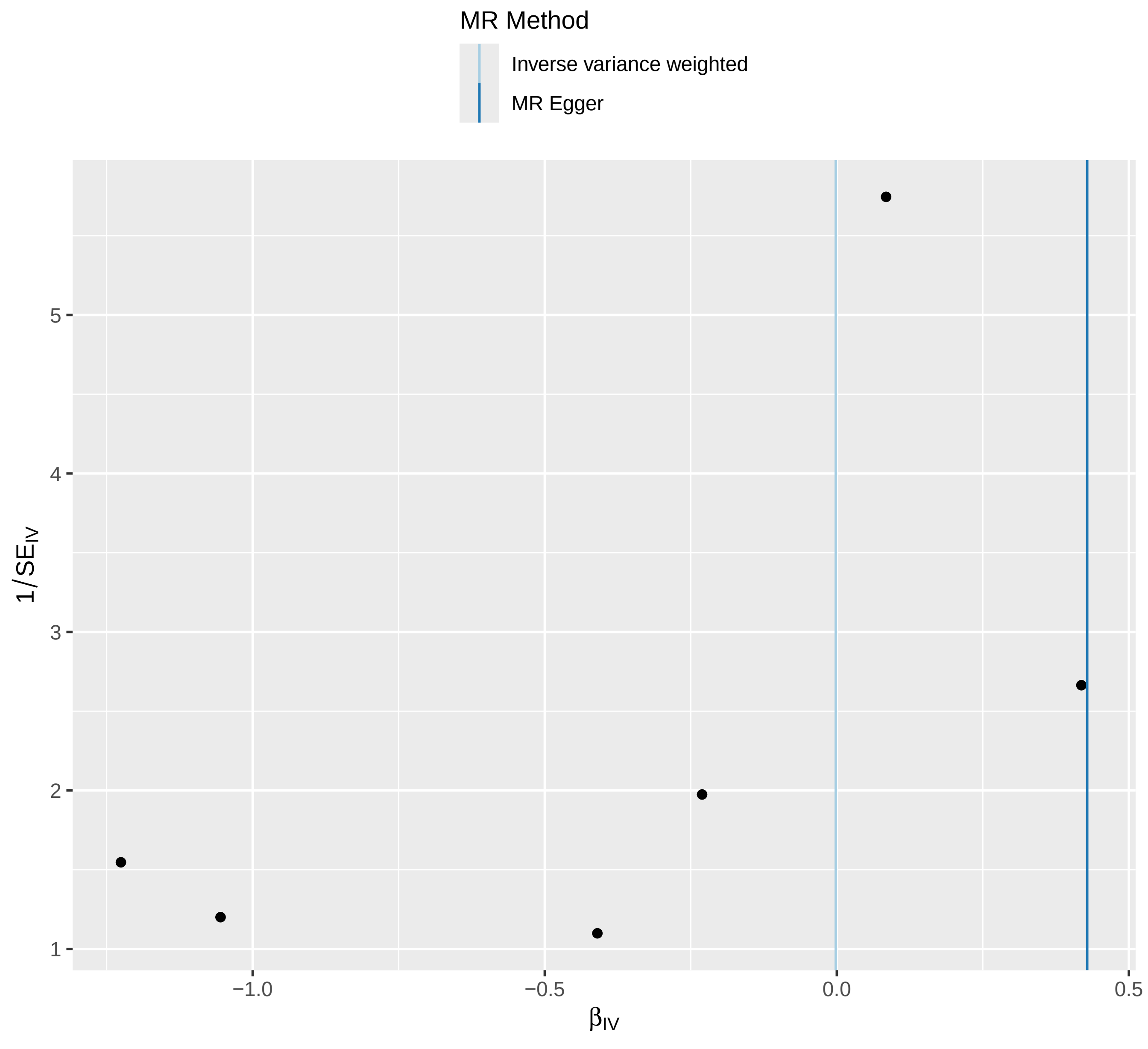
MR Method
Inverse variance weighted
MR Egger
1/SE_IV
5
4
3
2
1
−1.0
−0.5
0.0
0.5
β_IV

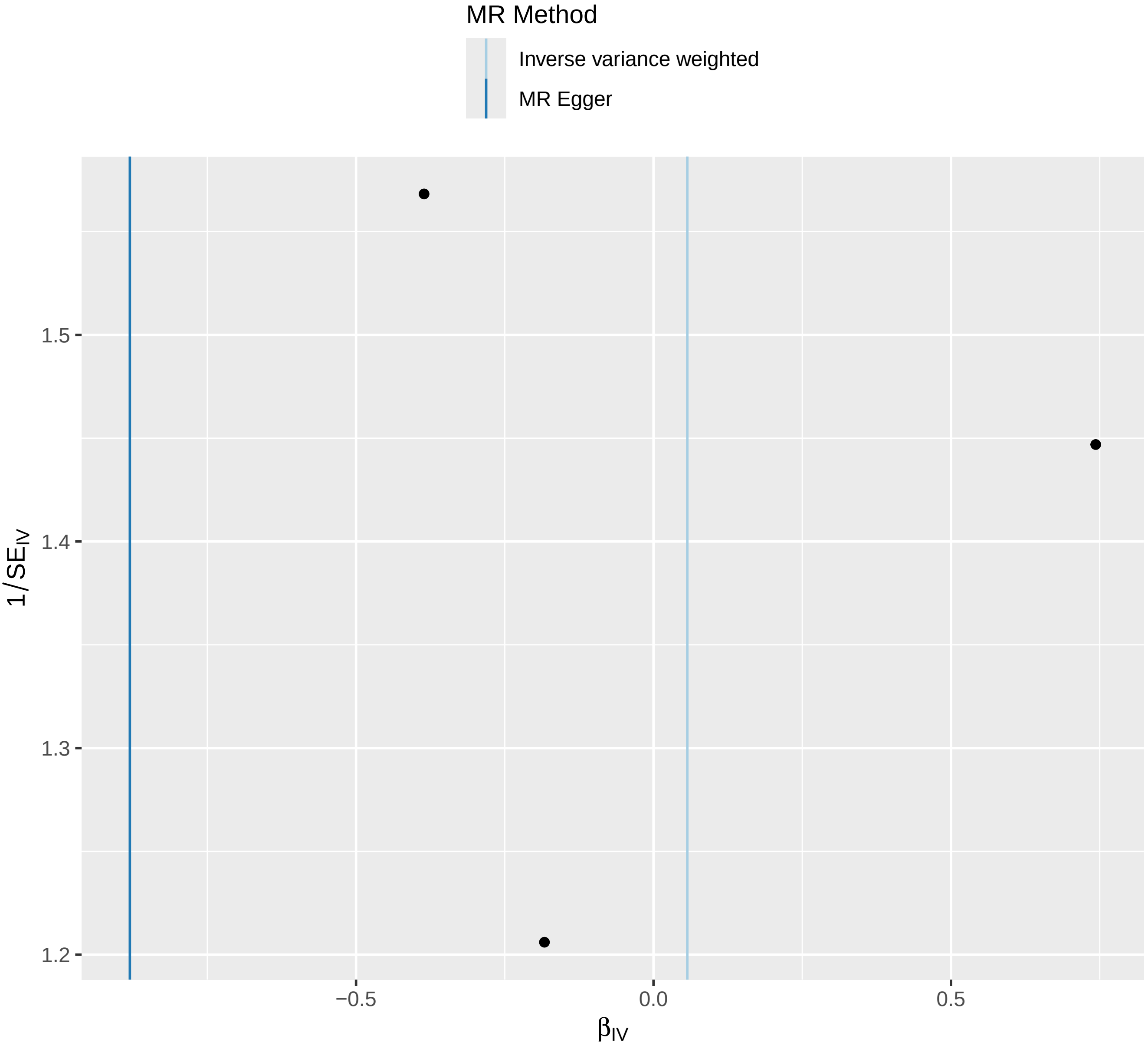

MR Method
Inverse variance weighted
MR Egger
1.5
1.4
1.3
1.2
$1/SE_{IV}$
−0.5
0.0
0.5
β_{IV}

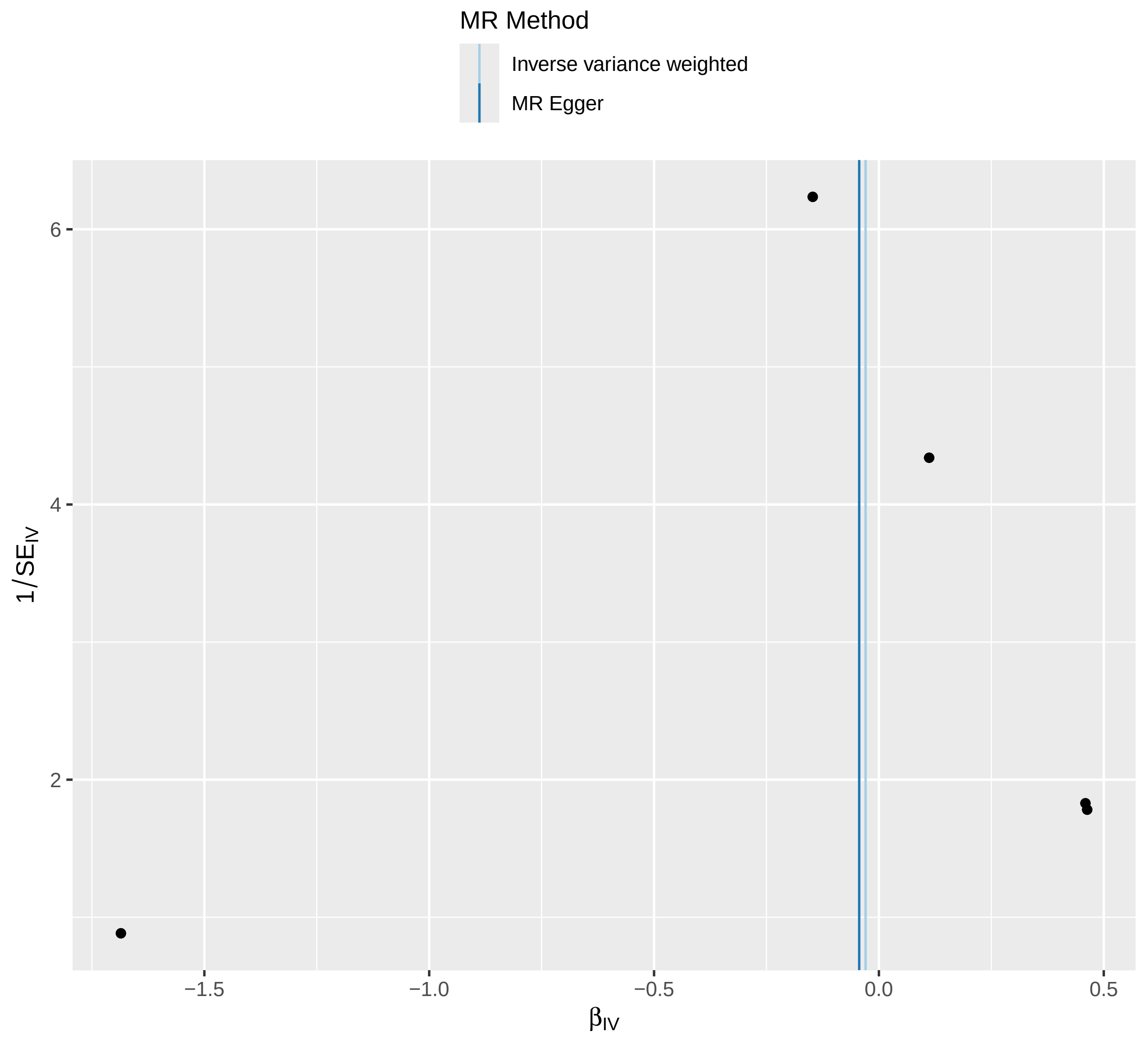
MR Method
Inverse variance weighted
MR Egger
6
4
2
$1/SE_{IV}$
−1.5
−1.0
−0.5
0.0
0.5
β_{IV}

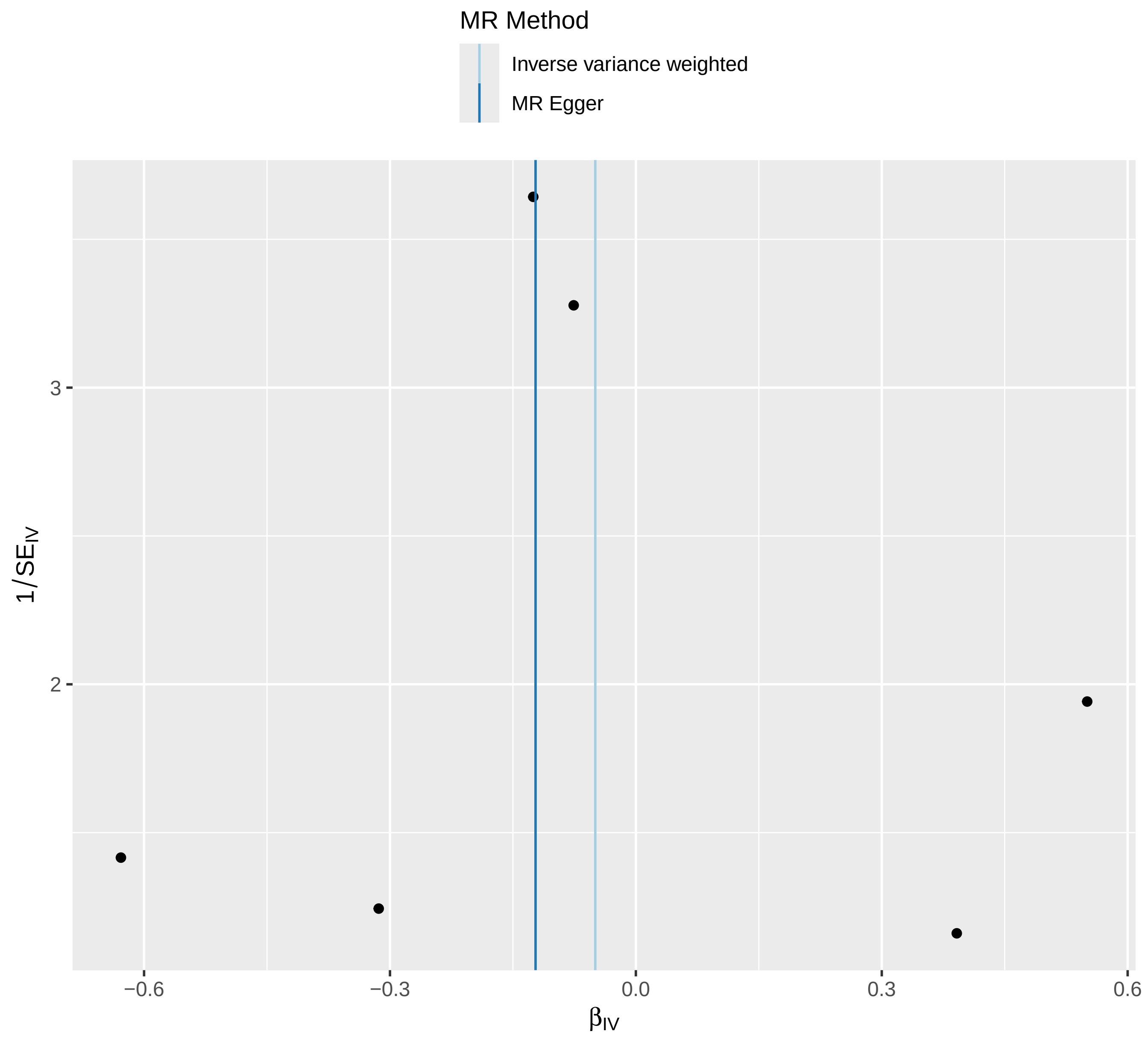
MR Method
Inverse variance weighted
MR Egger
1/SE$_{IV}$
3
2
−0.6
−0.3
0.0
0.3
0.6
β$_{IV}$

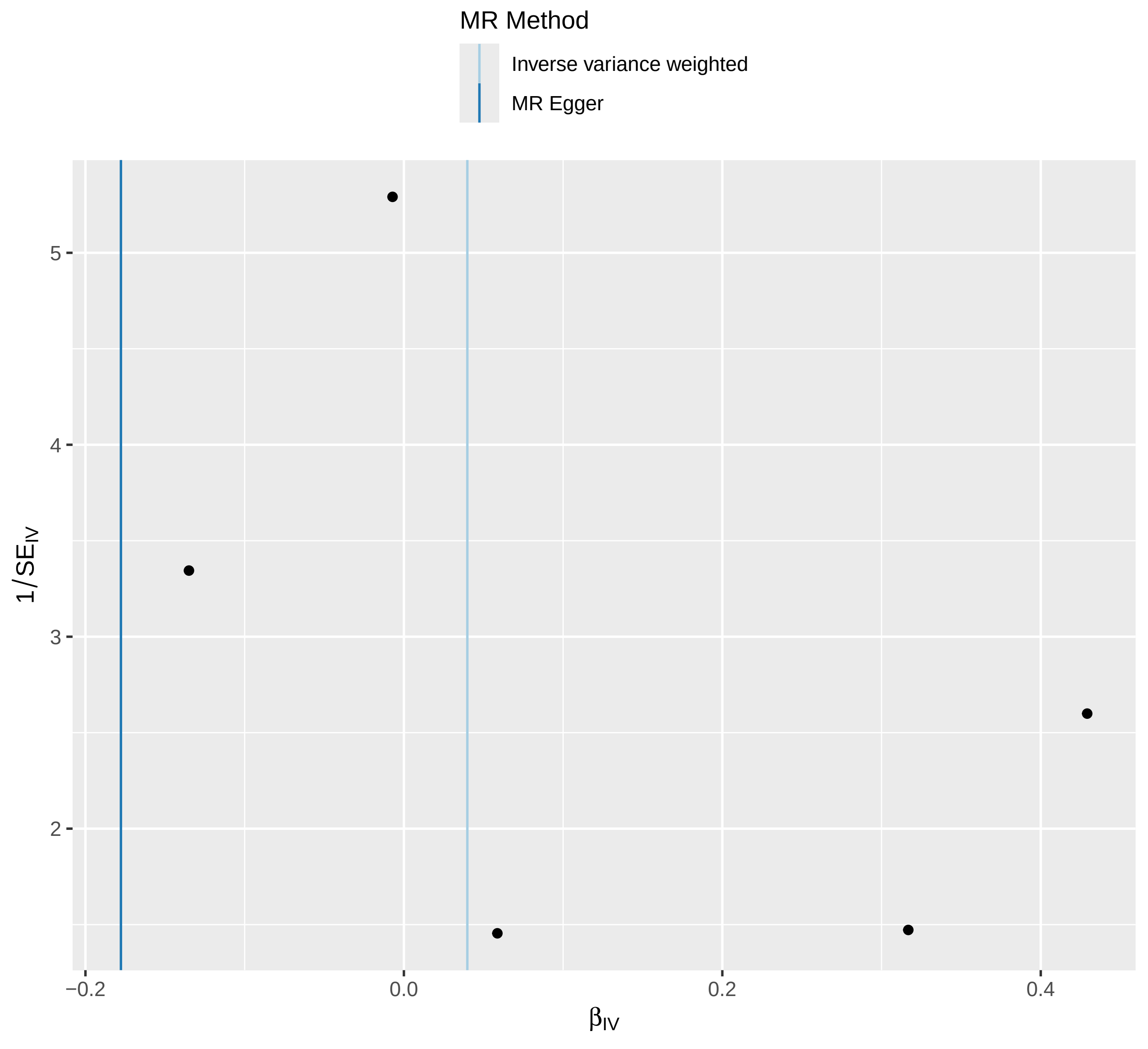
MR Method
Inverse variance weighted
MR Egger
5
4
3
2
1/SE_IV
−0.2
0.0
0.2
0.4
β_IV

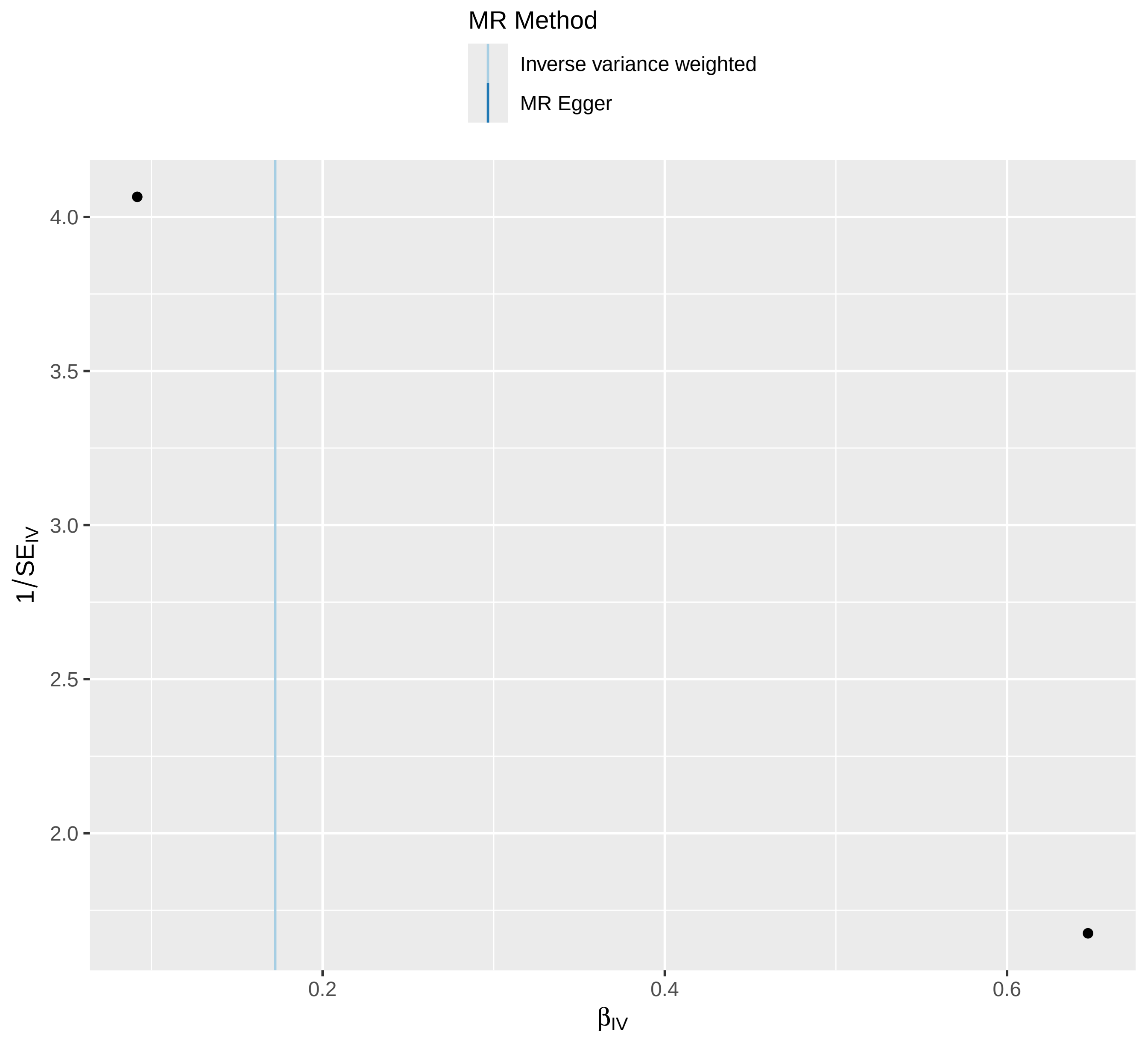

MR Method
Inverse variance weighted
MR Egger
4.0
3.5
3.0
2.5
2.0
1/SE_IV
0.2
0.4
0.6
β_IV

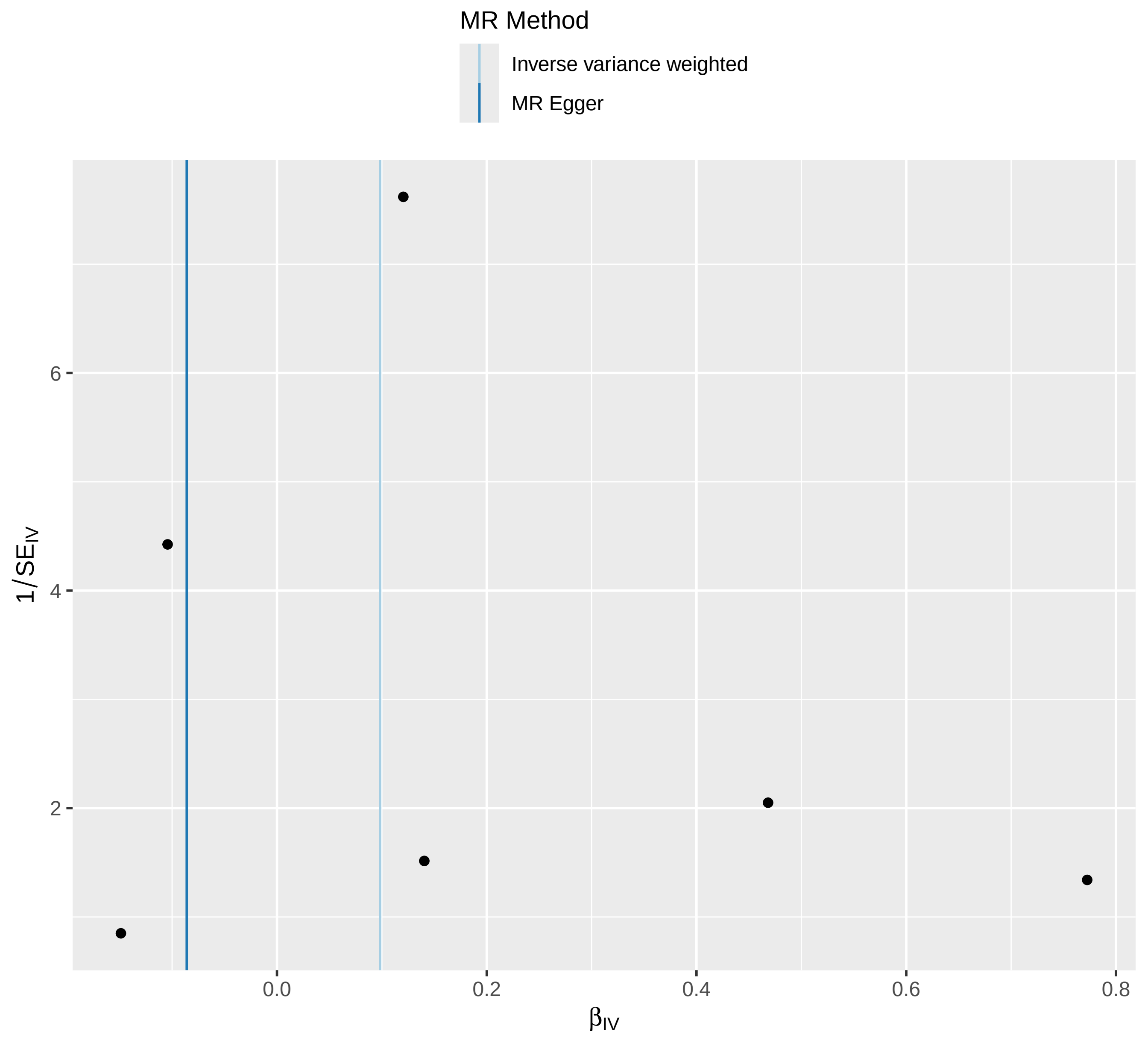

MR Method
Inverse variance weighted
MR Egger
6
4
2
1/SE_IV
0.0
0.2
0.4
0.6
0.8
β_IV

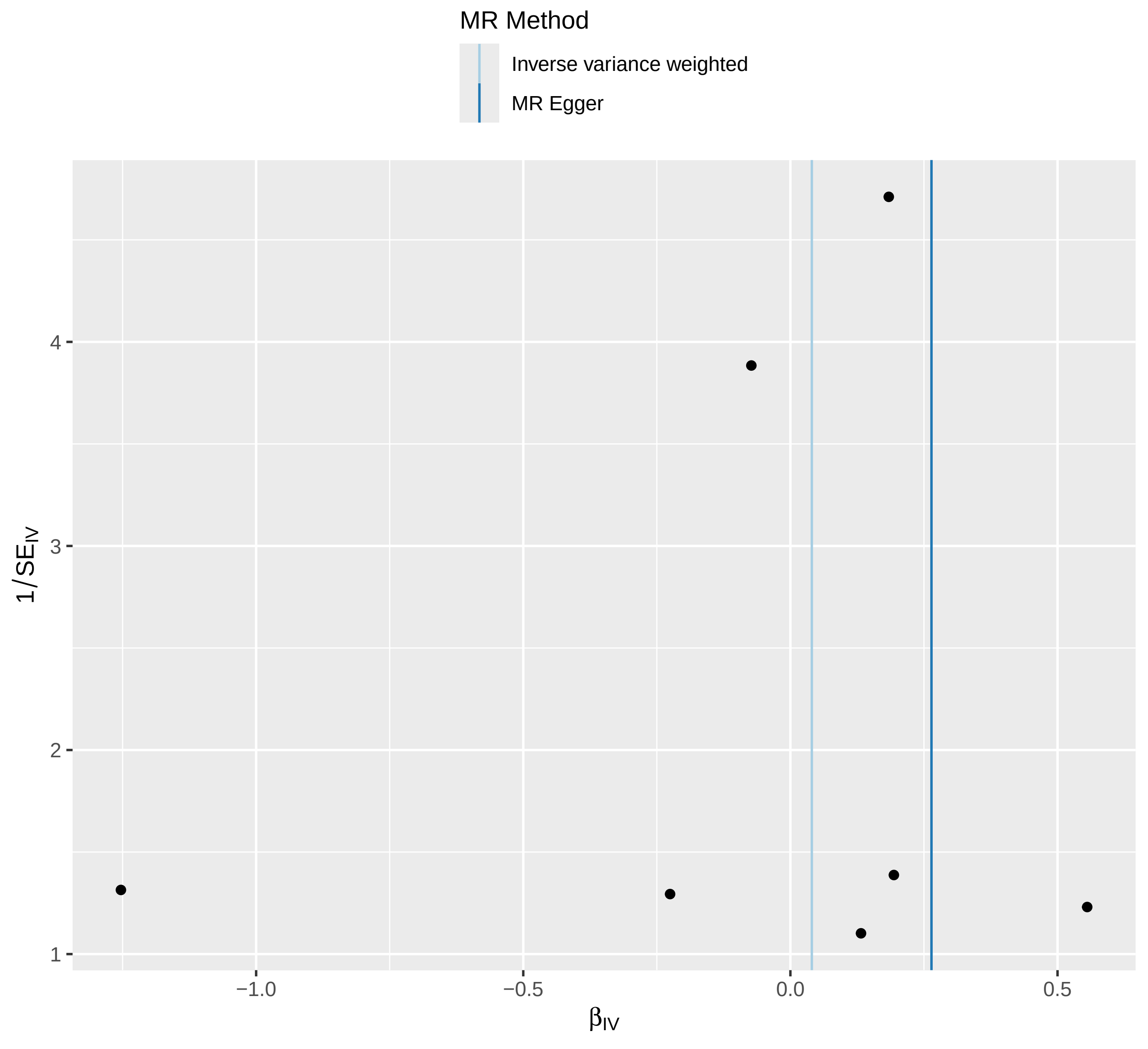
MR Method
Inverse variance weighted
MR Egger
1/SE_IV
β_IV
4
3
2
1
−1.0
−0.5
0.0
0.5

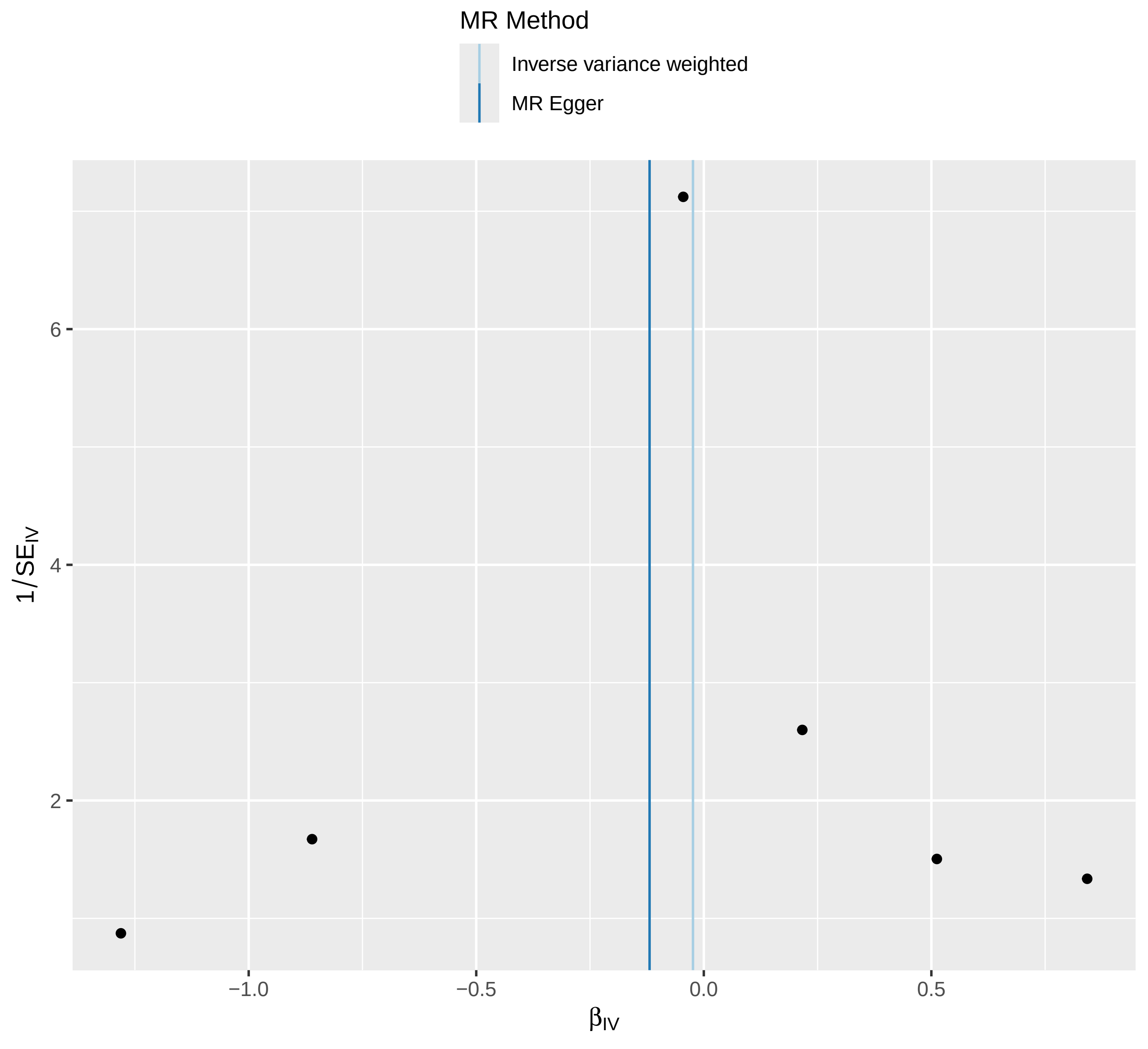
MR Method
Inverse variance weighted
MR Egger
6
4
2
−1.0
−0.5
0.0
0.5
1/SE_IV
β_IV

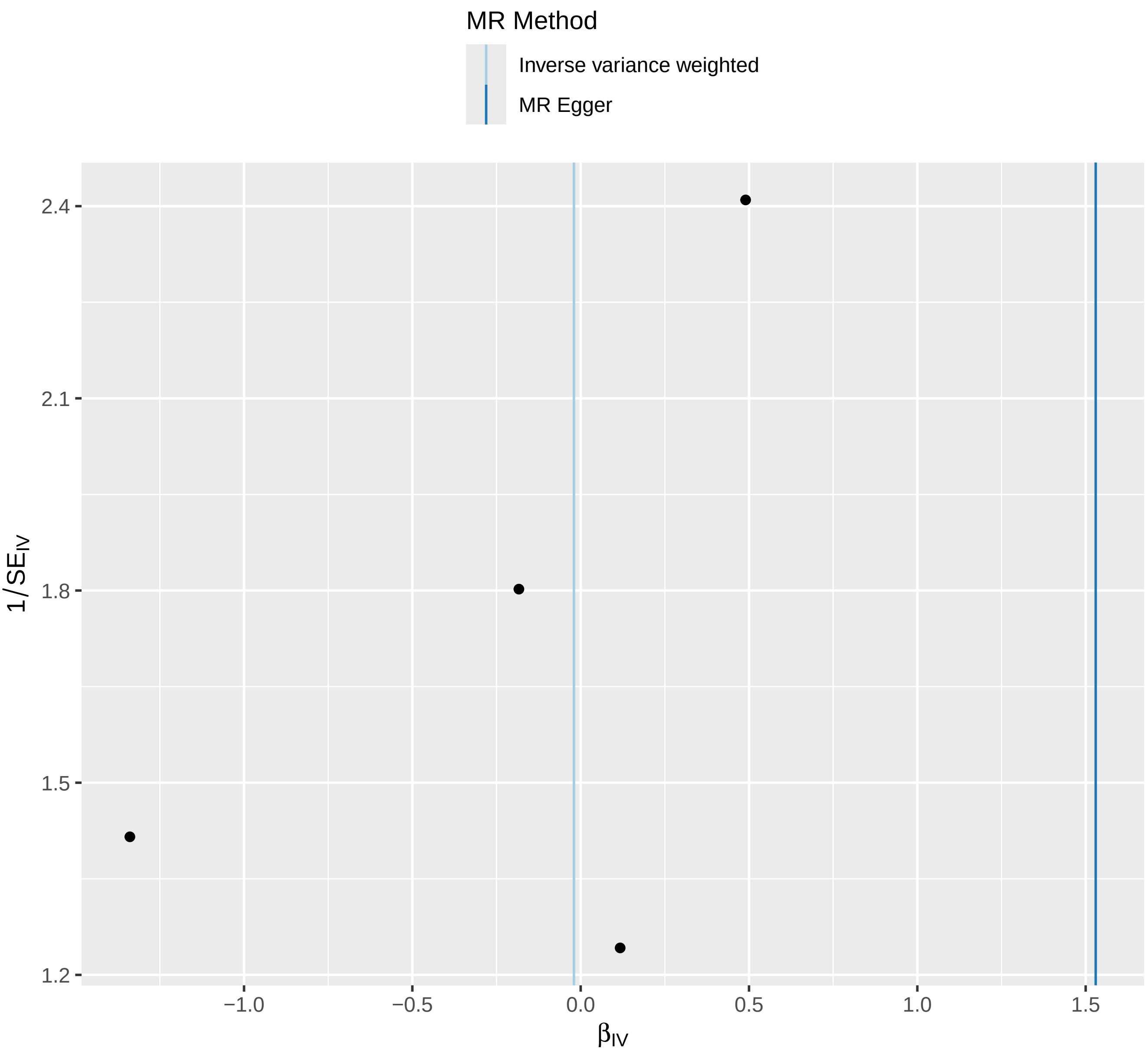

MR Method
Inverse variance weighted
MR Egger
2.4
2.1
1.8
1.5
1.2
1/SE_IV
−1.0
−0.5
0.0
0.5
1.0
1.5
β_IV

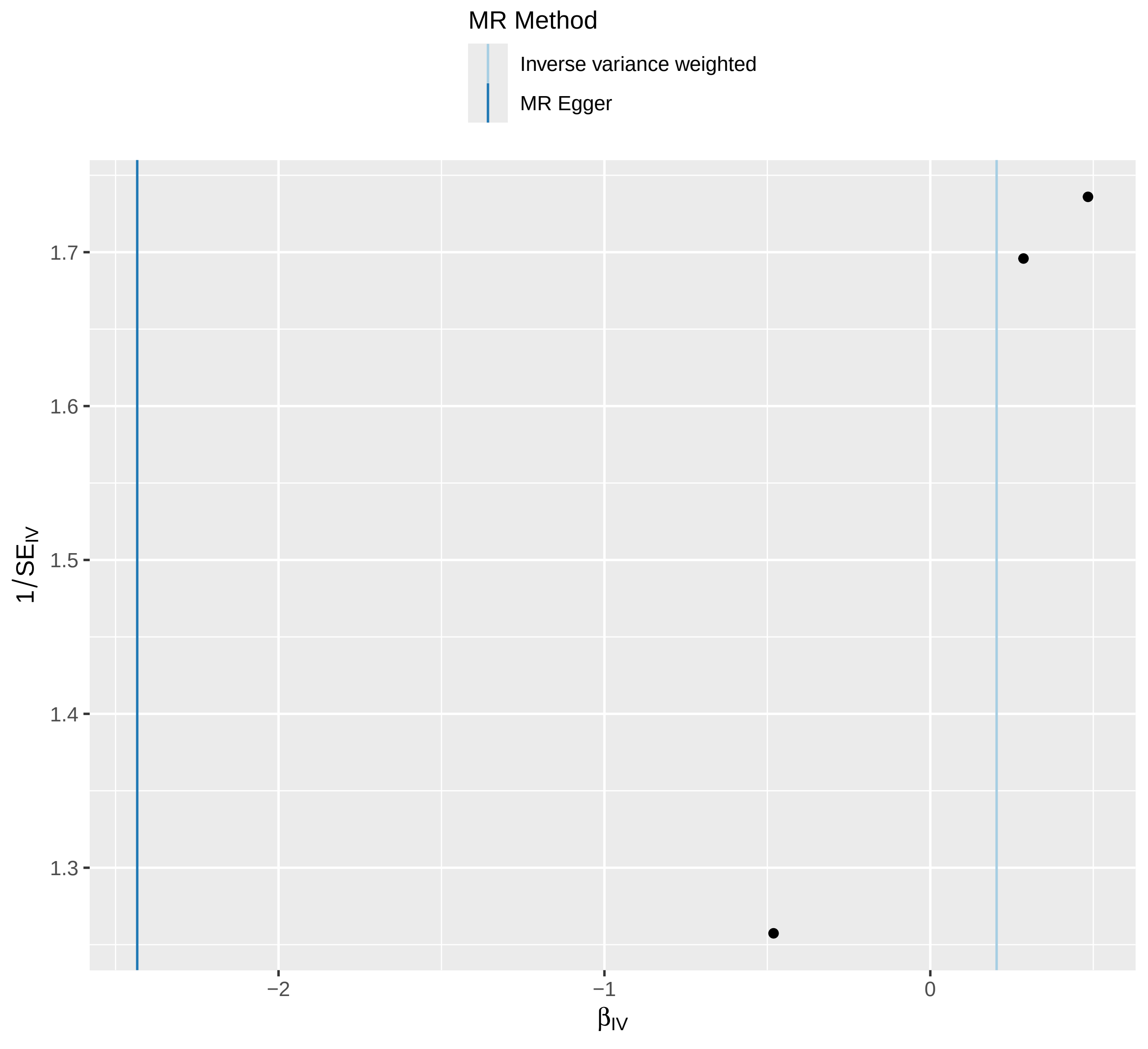
MR Method
Inverse variance weighted
MR Egger
1.7
1.6
1.5
1.4
1.3
1/SE_IV
−2
−1
0
β_IV

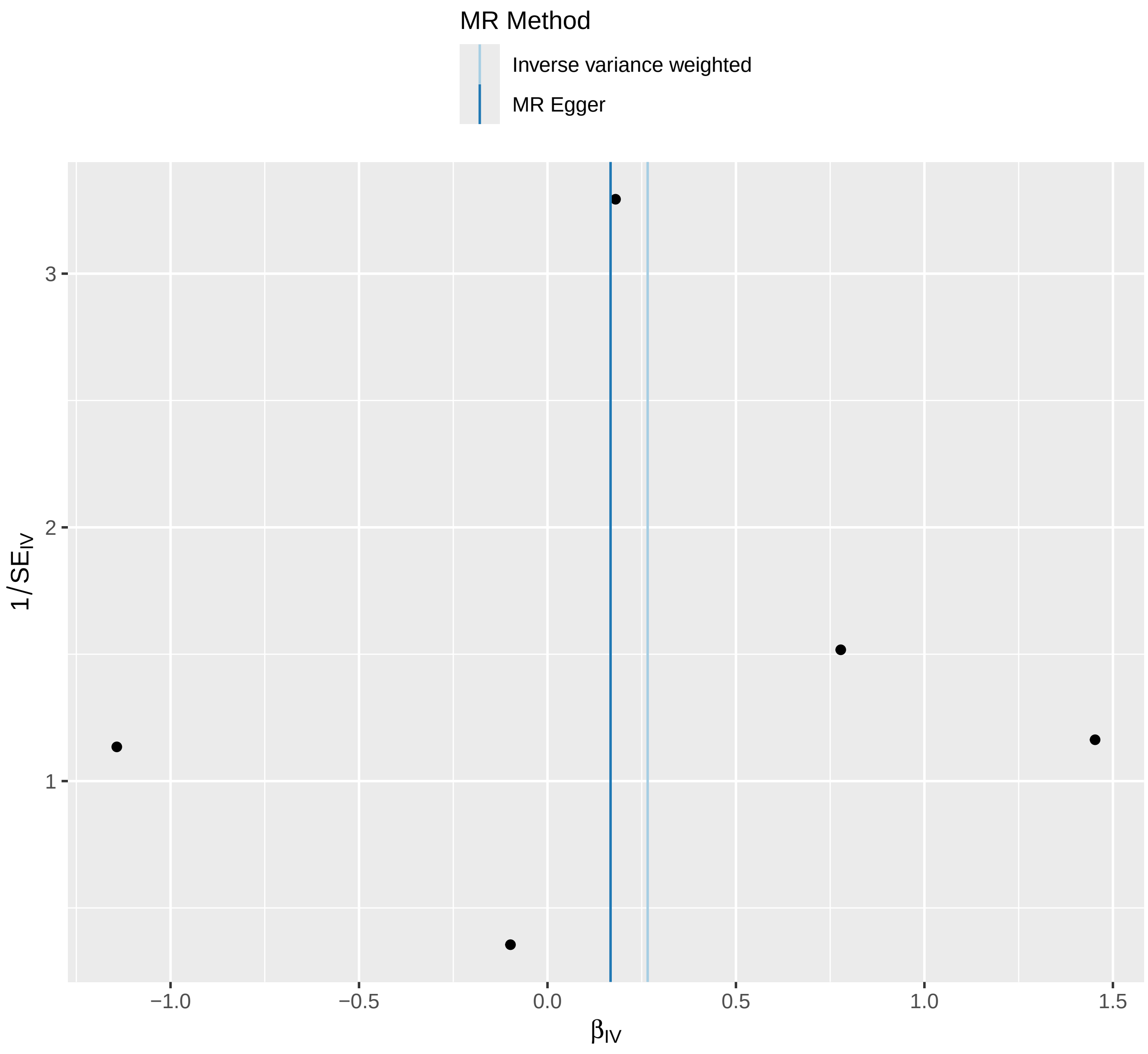

MR Method
Inverse variance weighted
MR Egger
1/SE$_{IV}$
3
2
1
−1.0
−0.5
0.0
0.5
1.0
1.5
β$_{IV}$

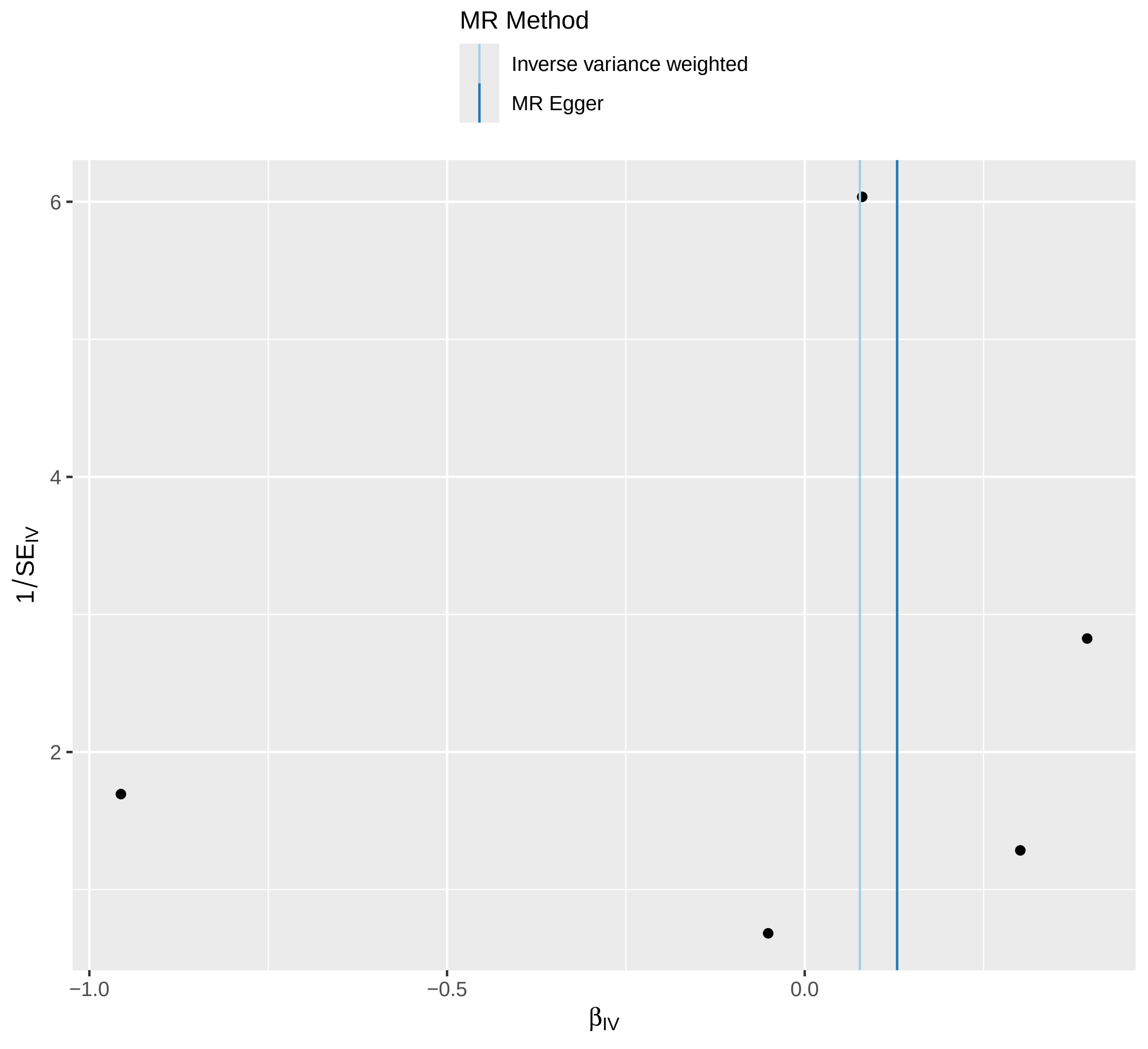

MR Method
Inverse variance weighted
MR Egger
6
4
2
1/SE$_{IV}$
−1.0
−0.5
0.0
β$_{IV}$

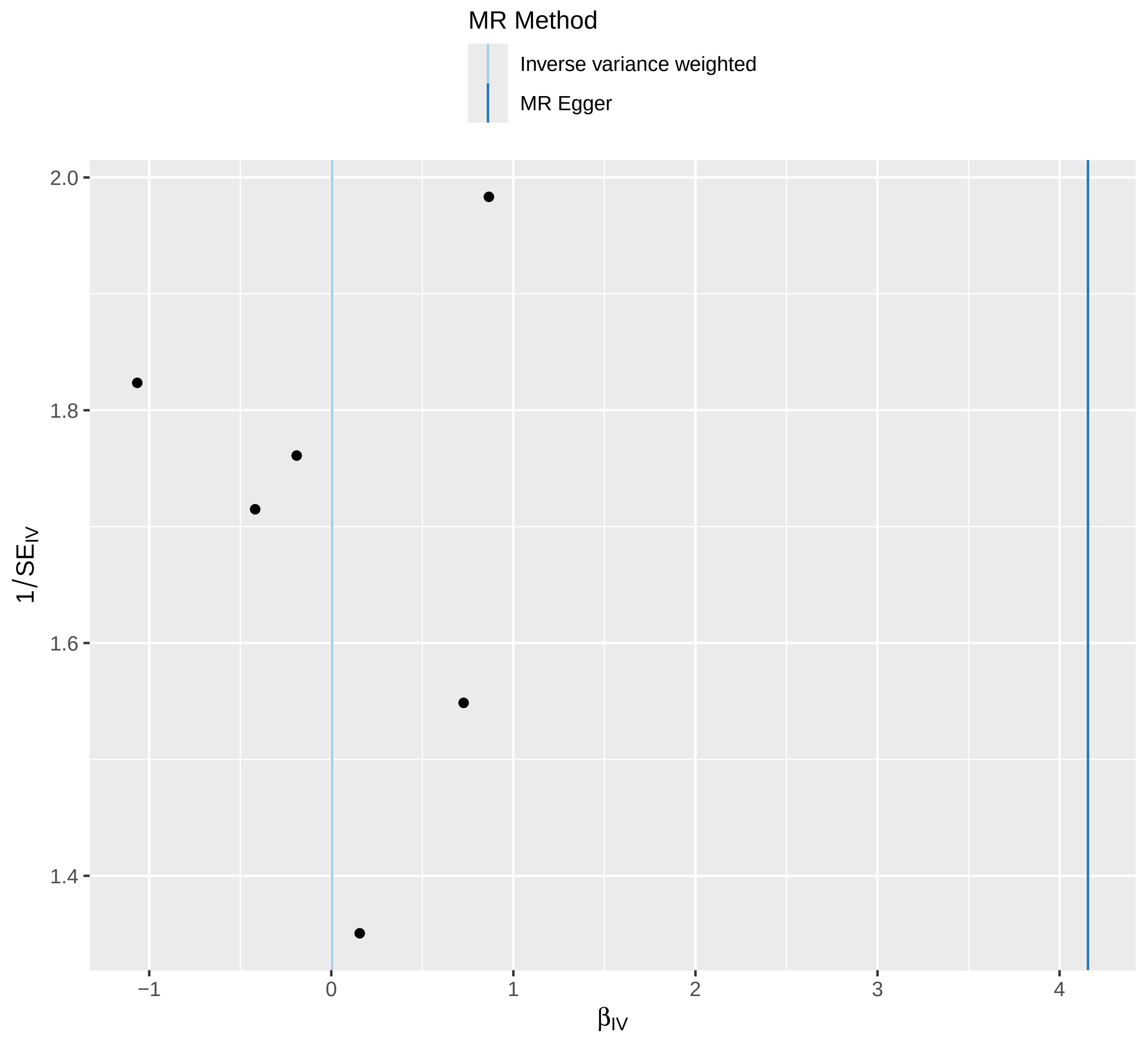
MR Method
Inverse variance weighted
MR Egger
2.0
1.8
1.6
1.4
$1/SE_{IV}$
−1
0
1
2
3
4
β_{IV}

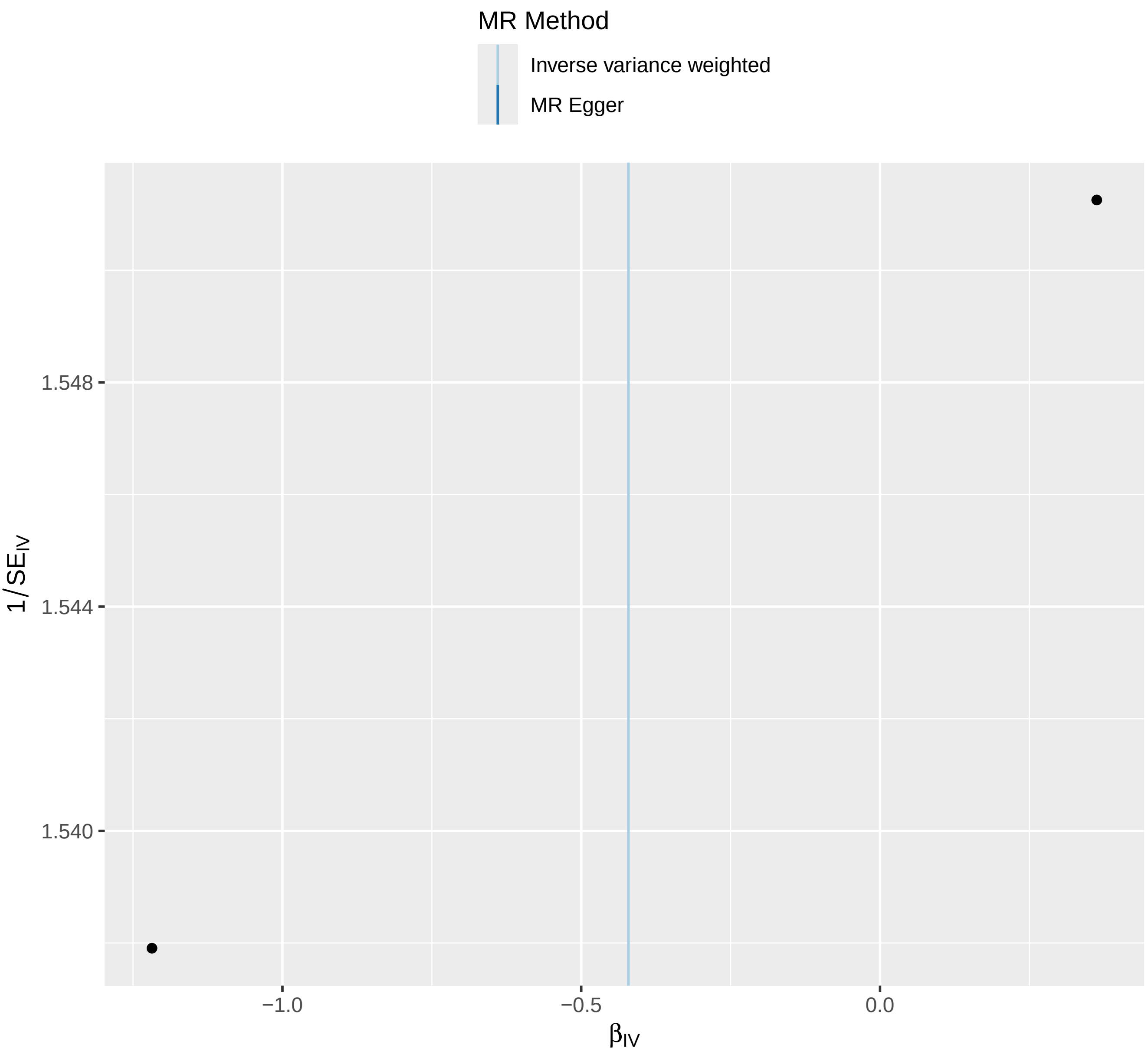

MR Method
Inverse variance weighted
MR Egger
1.548
1.544
1.540
1/SE_IV
−1.0
−0.5
0.0
β_IV

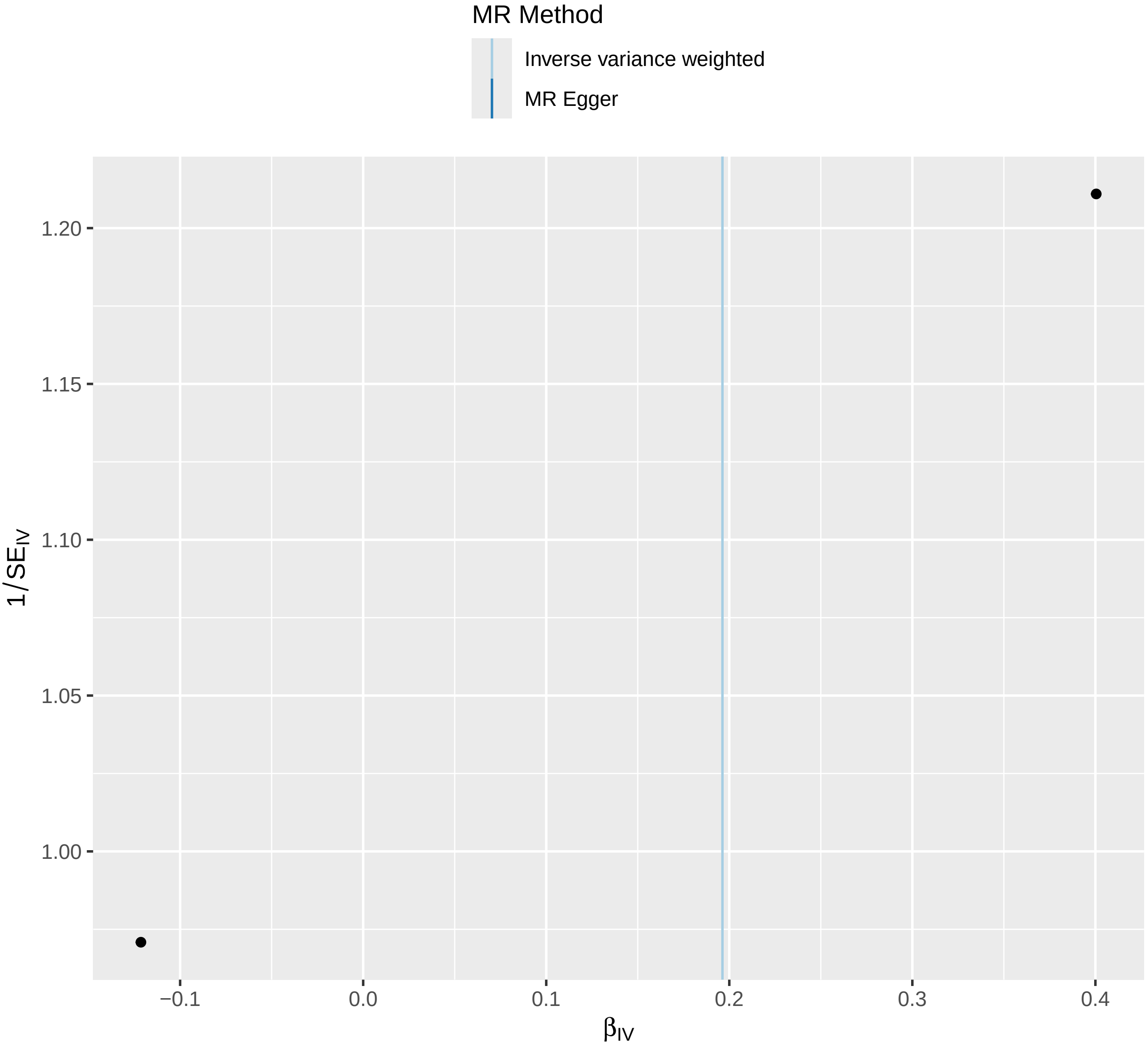

MR Method
Inverse variance weighted
MR Egger
1.20
1.15
1.10
1.05
1.00
1/SE_IV
−0.1
0.0
0.1
0.2
0.3
0.4
β_IV

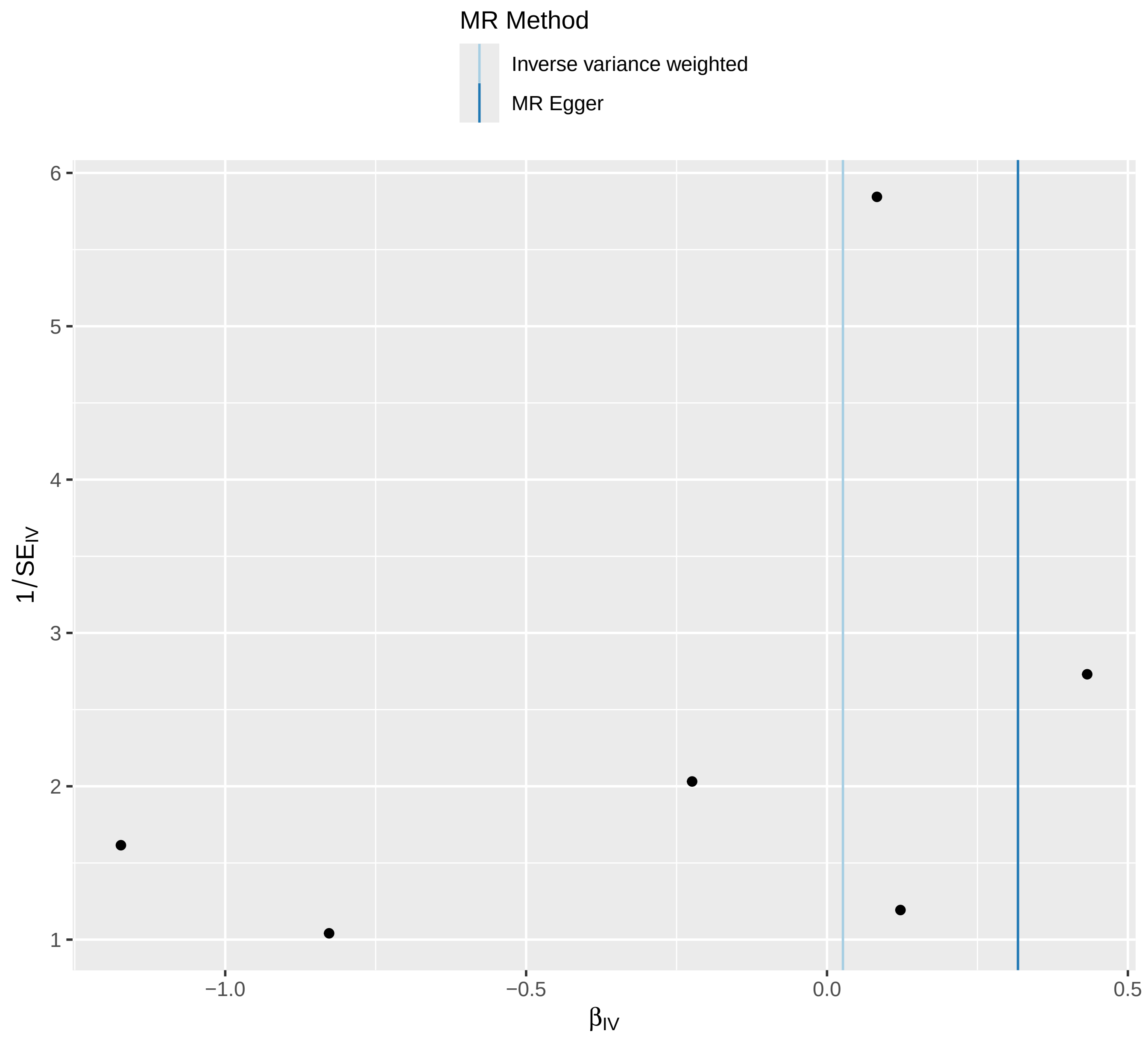
MR Method
Inverse variance weighted
MR Egger
6
5
4
3
2
1
1/SE_IV
−1.0
−0.5
0.0
0.5
β_IV

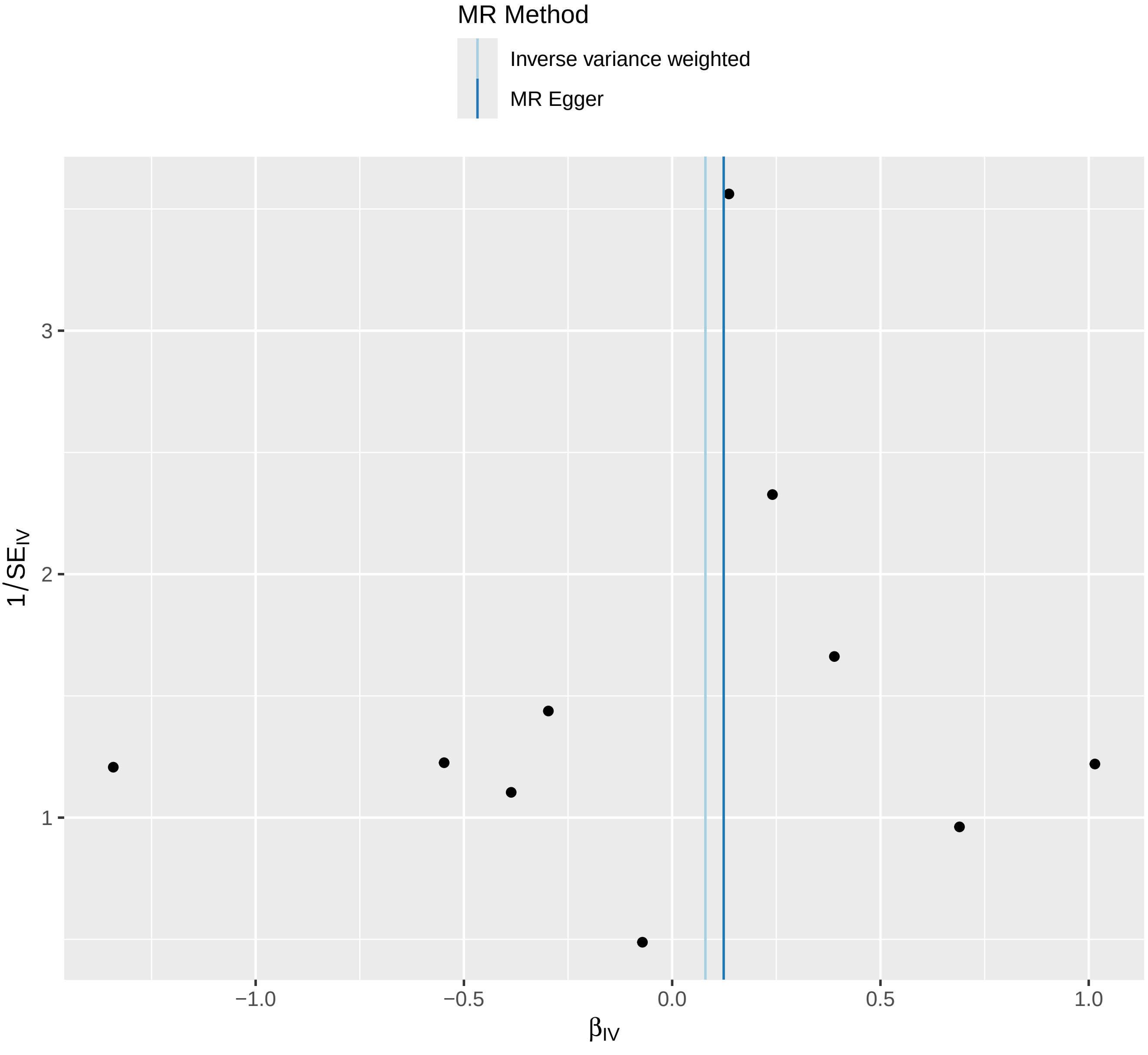

MR Method
Inverse variance weighted
MR Egger
1/SE_IV
3
2
1
−1.0
−0.5
0.0
0.5
1.0
β_IV

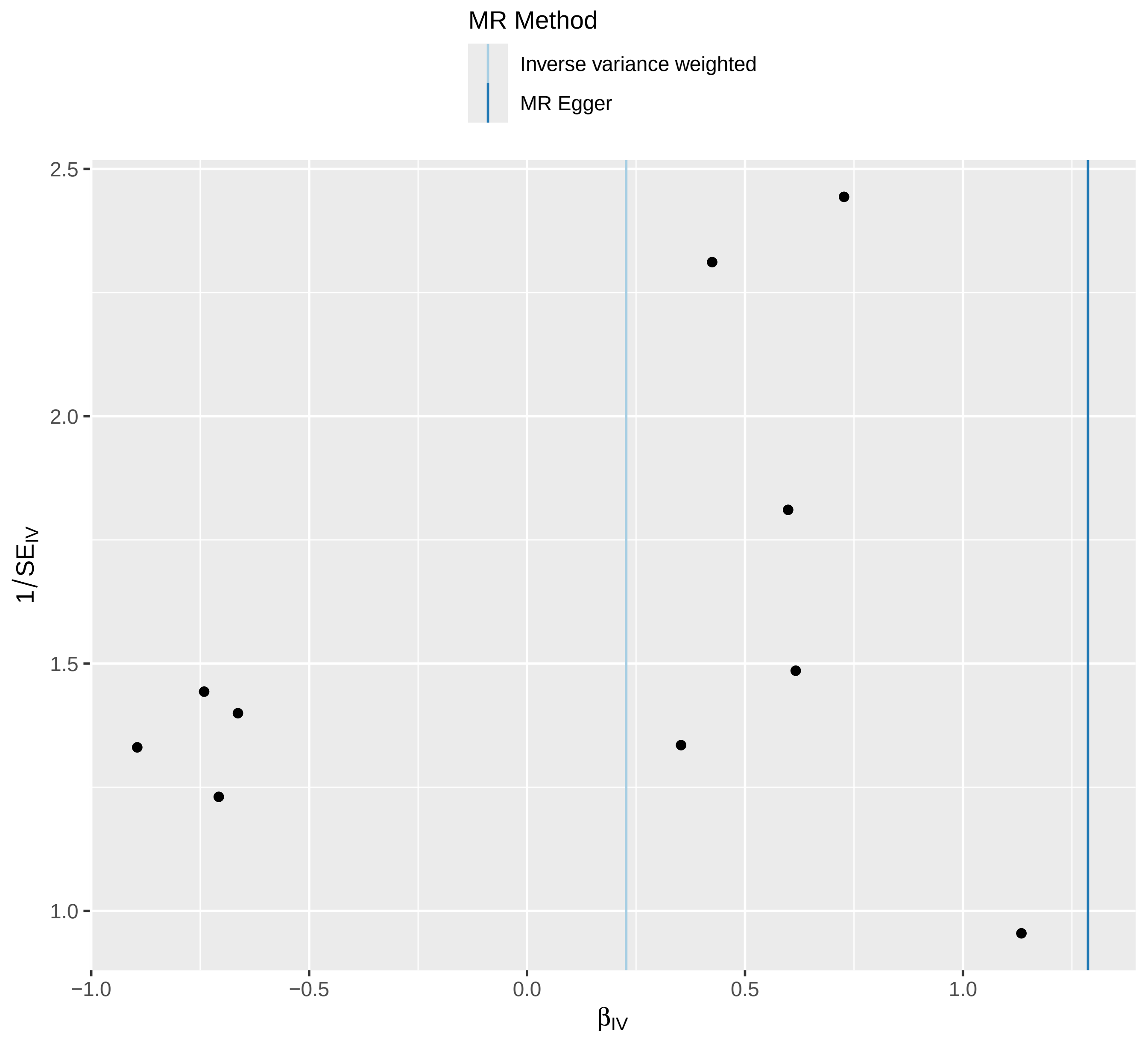

MR Method
Inverse variance weighted
MR Egger
2.5
2.0
1.5
1.0
1/SE_IV
−1.0
−0.5
0.0
0.5
1.0
β_IV

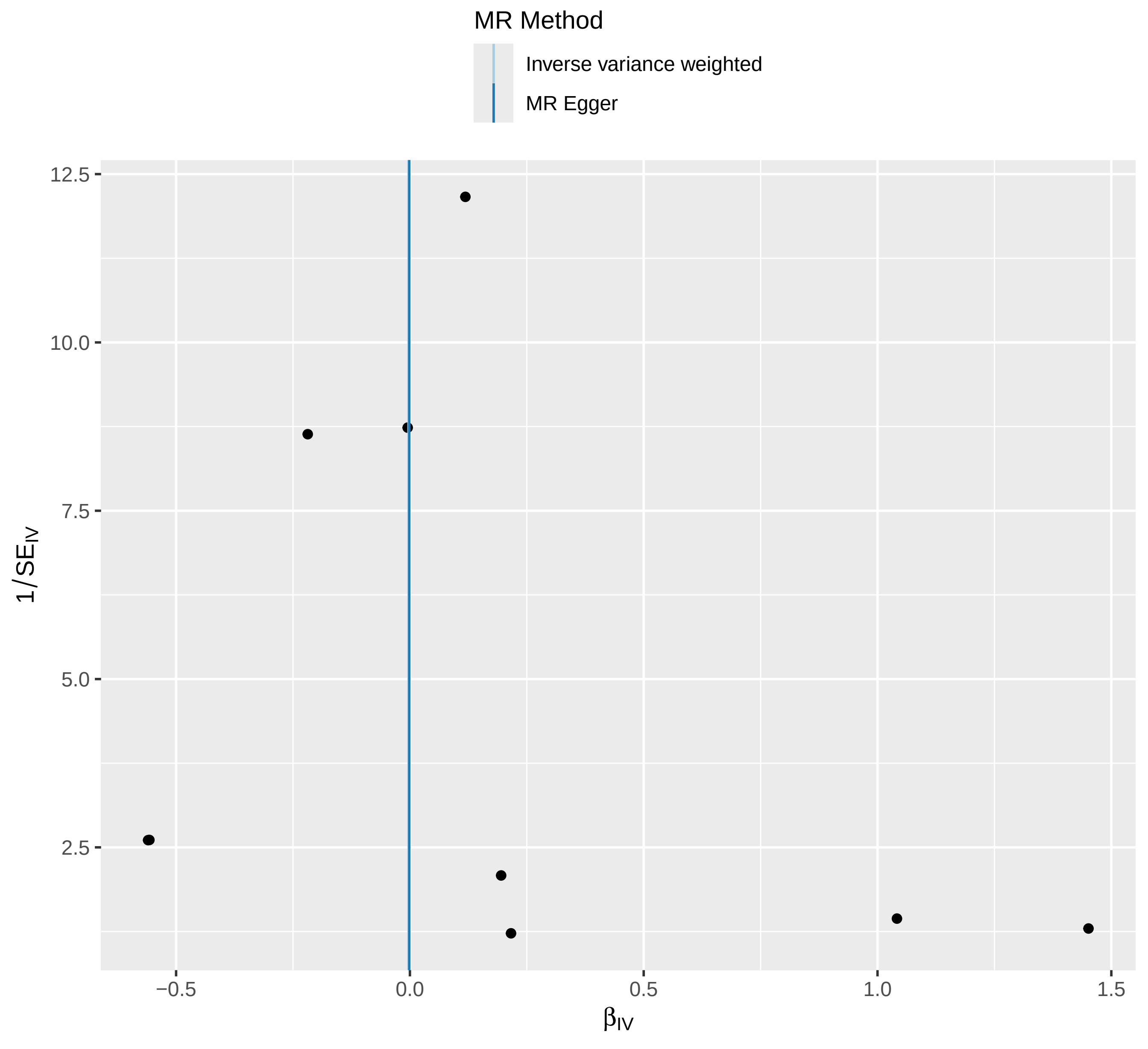
MR Method
Inverse variance weighted
MR Egger
12.5
10.0
7.5
5.0
2.5
−0.5
0.0
0.5
1.0
1.5
$1/SE_{IV}$
β_{IV}

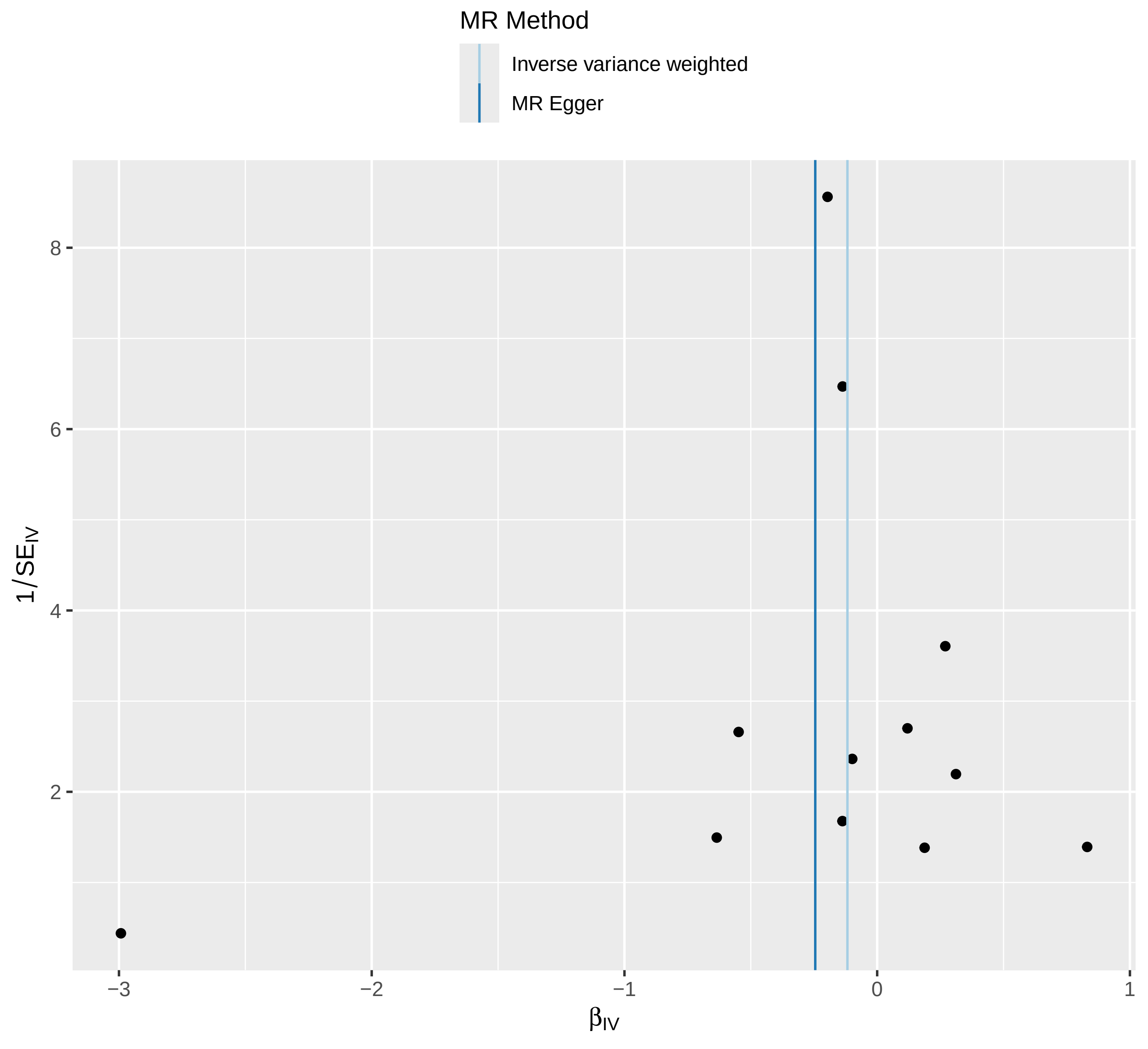
MR Method
Inverse variance weighted
MR Egger
1/SE_IV
β_IV
8
6
4
2
−3
−2
−1
0
1

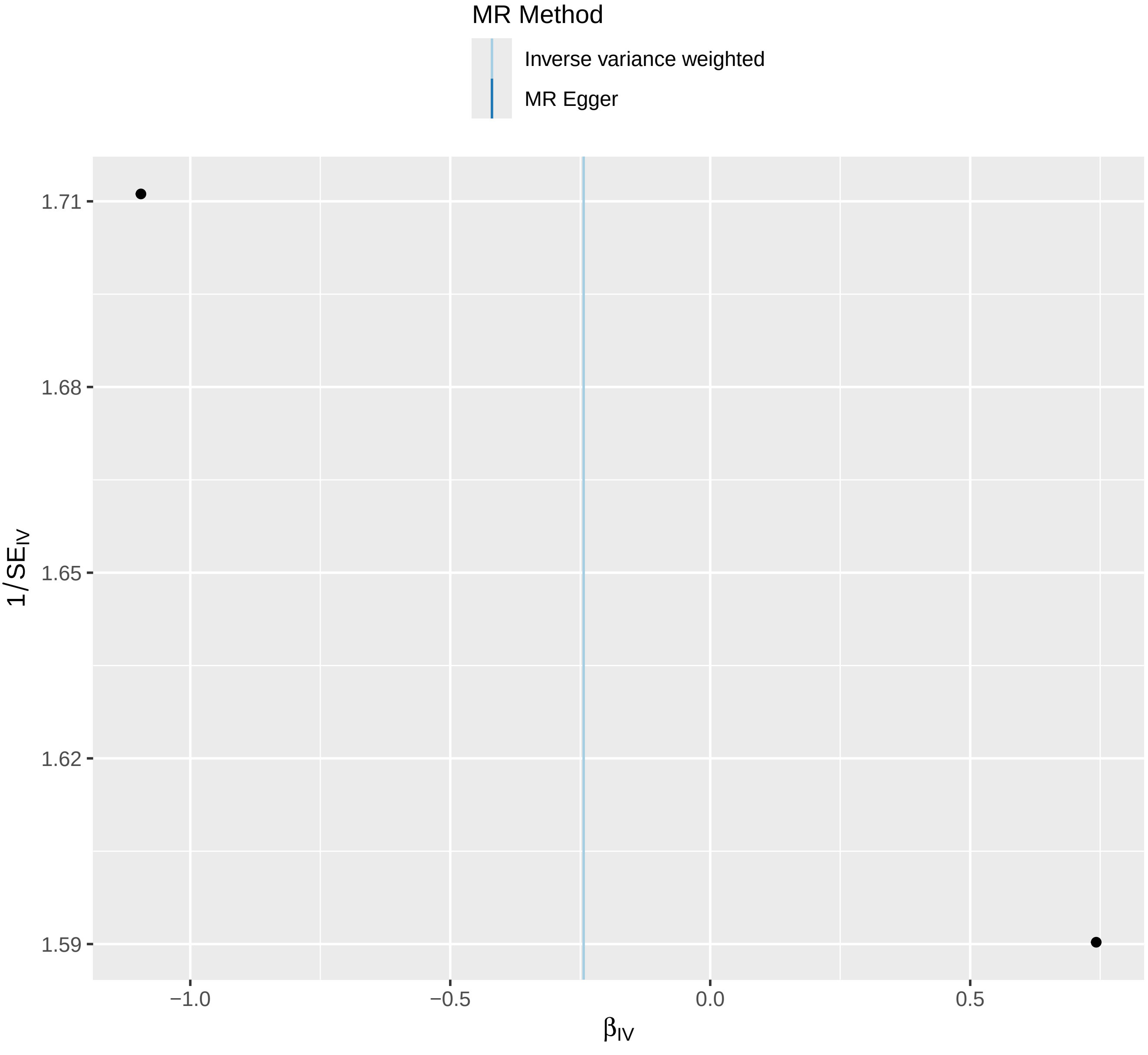
MR Method
Inverse variance weighted
MR Egger
1.71
1.68
1.65
1.62
1.59
$1/SE_{IV}$
−1.0
−0.5
0.0
0.5
β_{IV}

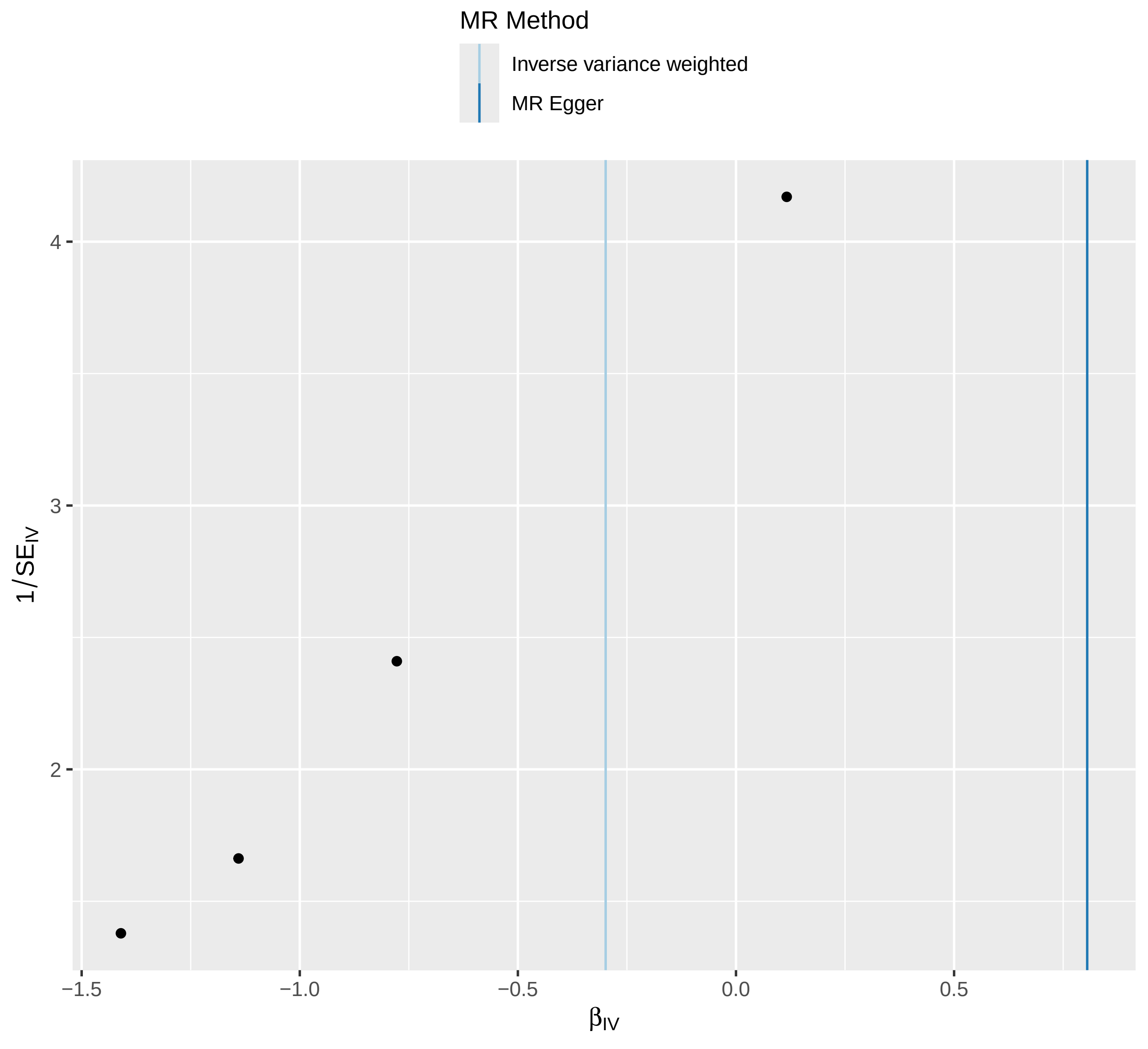
MR Method
Inverse variance weighted
MR Egger
4
3
2
1/SE_IV
−1.5
−1.0
−0.5
0.0
0.5
β_IV

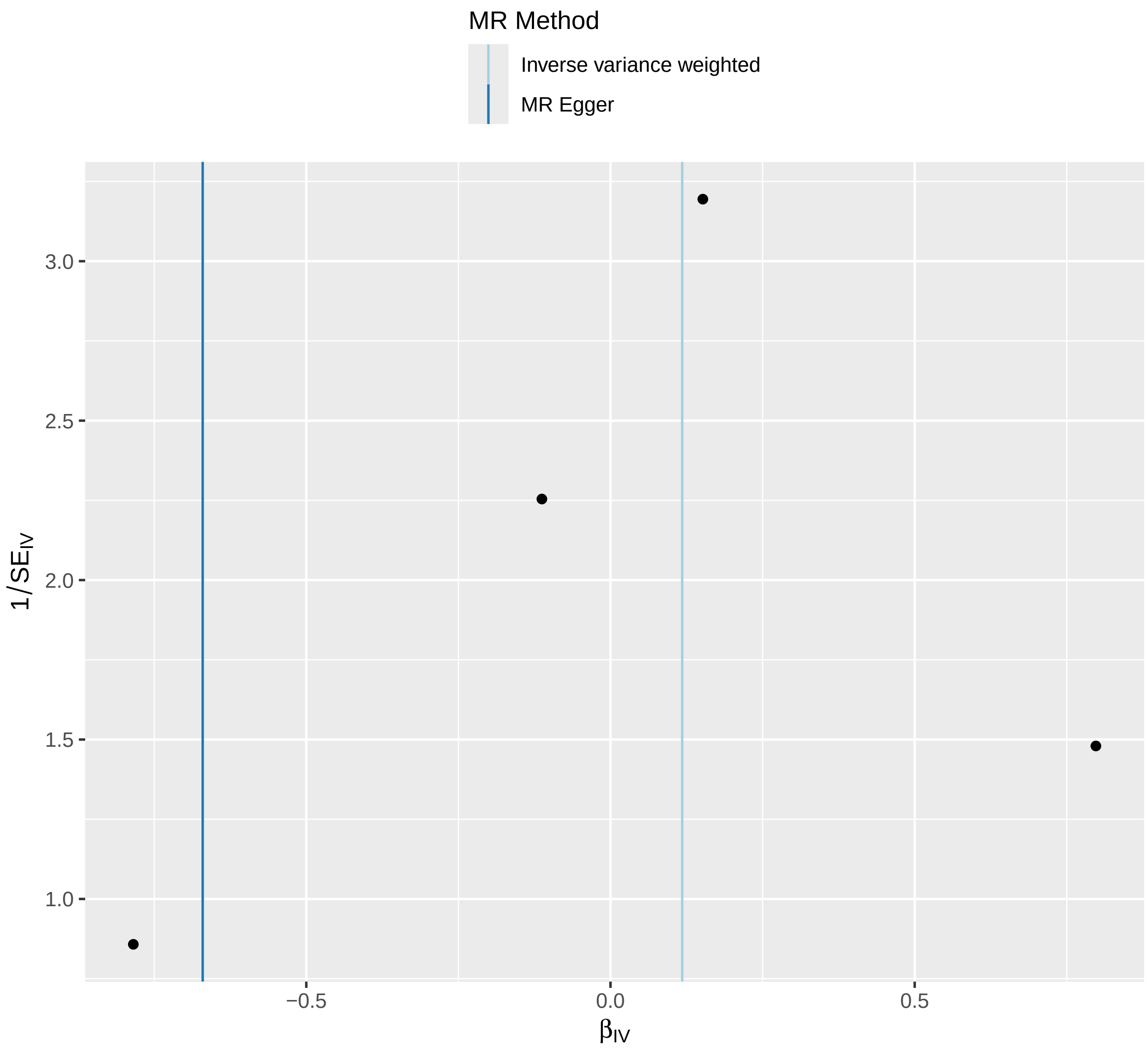
MR Method
Inverse variance weighted
MR Egger
3.0
2.5
2.0
1.5
1.0
1/SE_IV
−0.5
0.0
0.5
β_IV

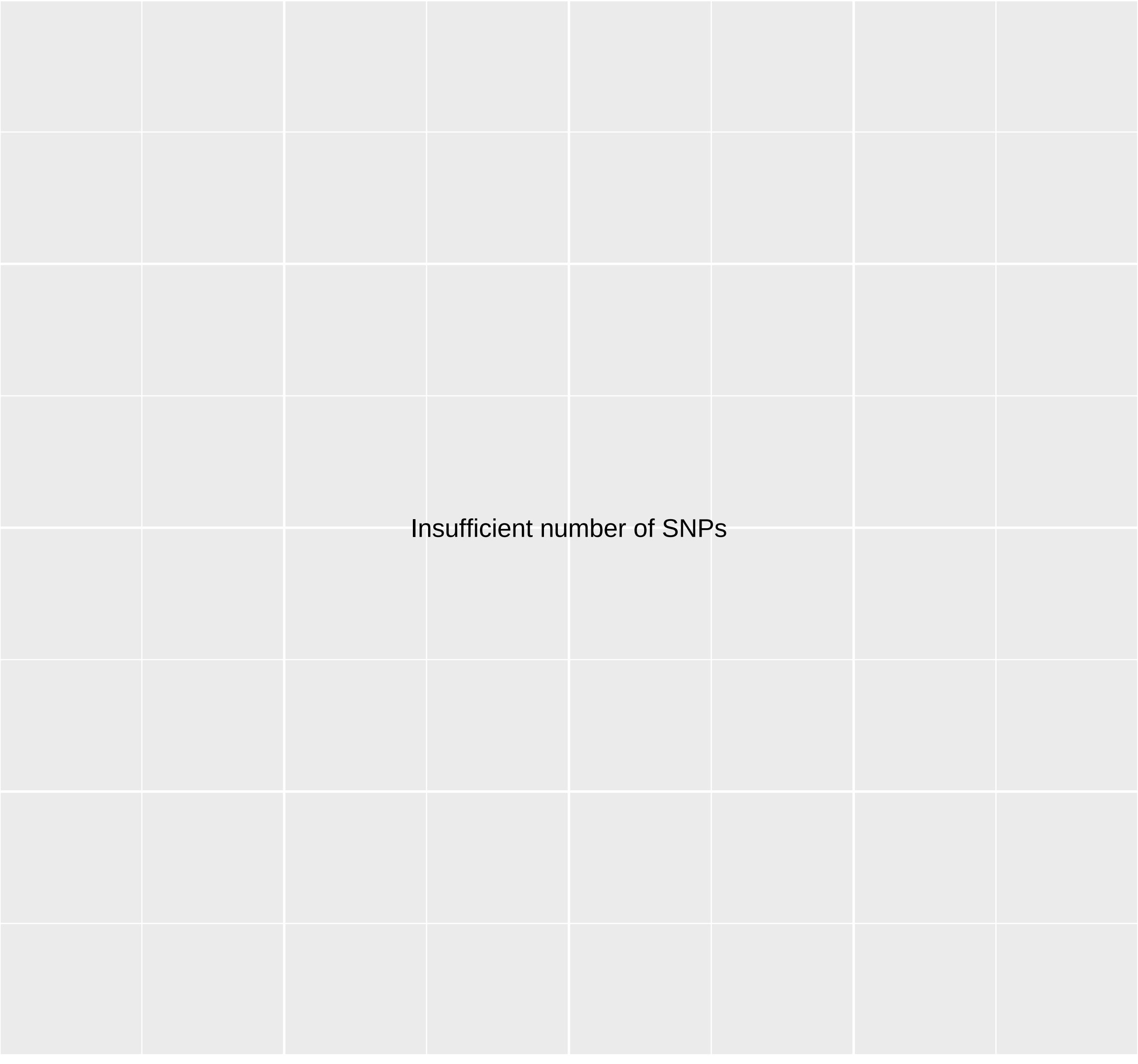
Insufficient number of SNPs

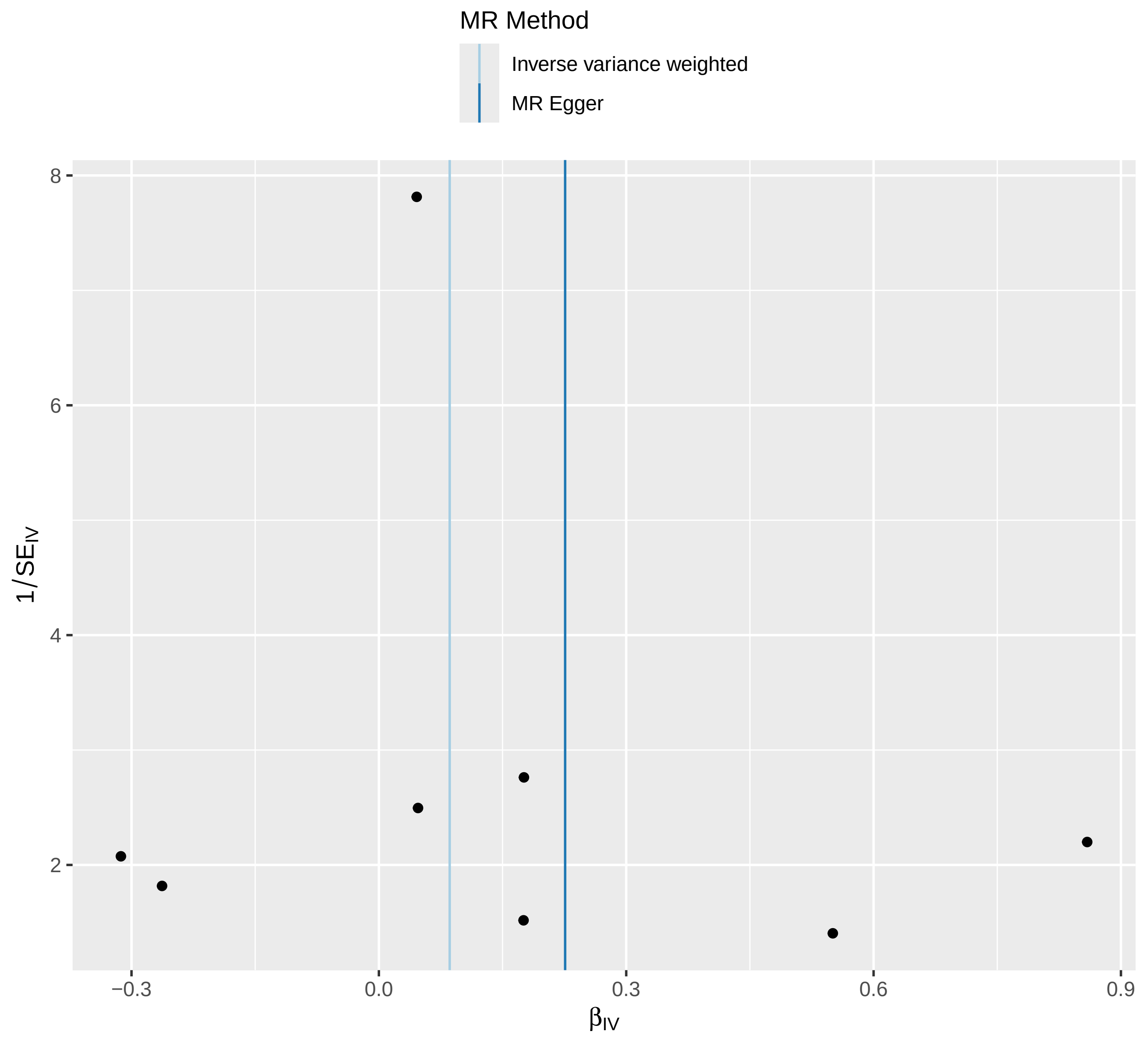
MR Method
Inverse variance weighted
MR Egger
1/SE$_{IV}$
8
6
4
2
−0.3
0.0
0.3
0.6
0.9
β$_{IV}$

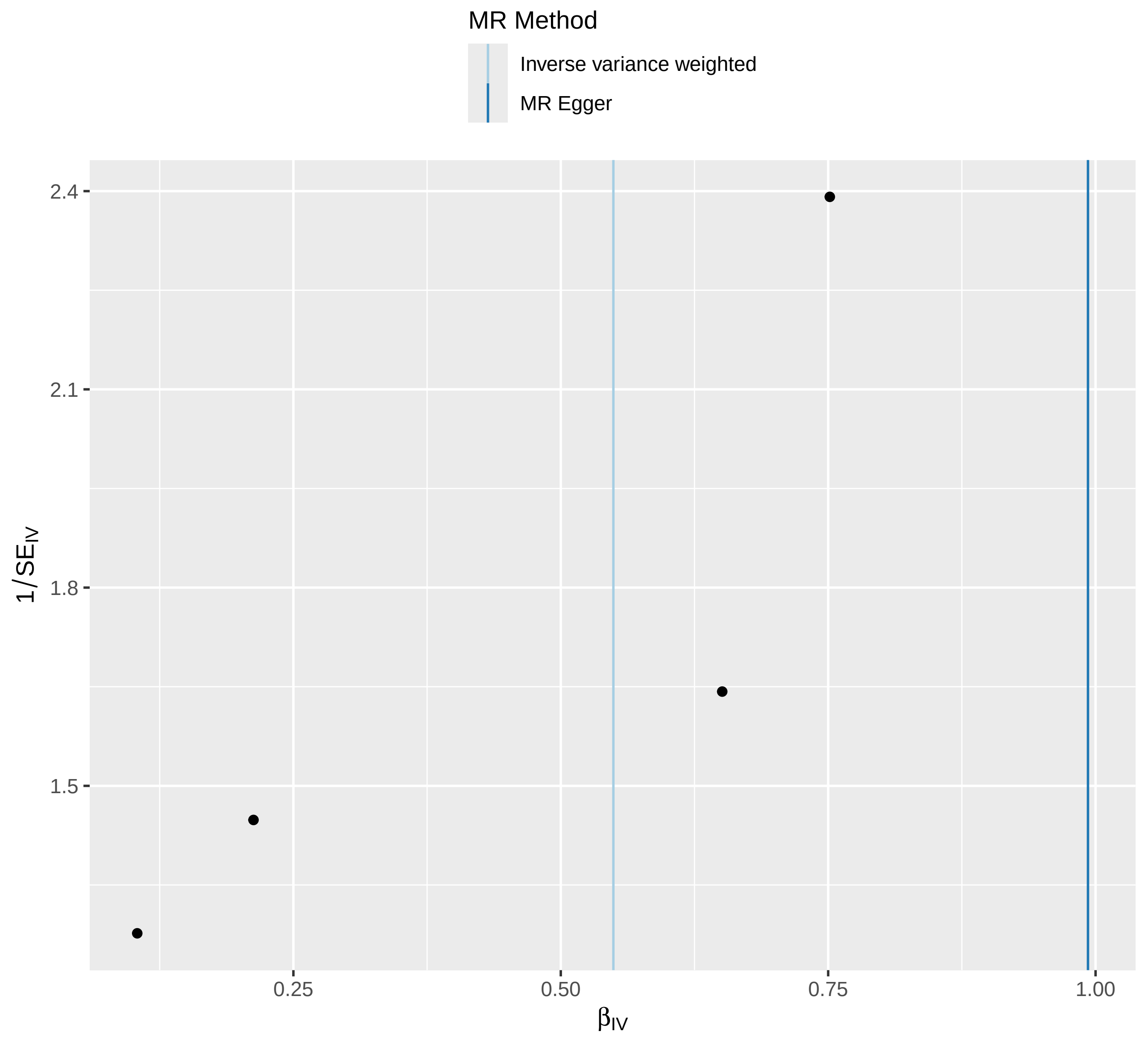

MR Method
Inverse variance weighted
MR Egger
2.4
2.1
1.8
1.5
1/SE_IV
0.25
0.50
0.75
1.00
β_IV

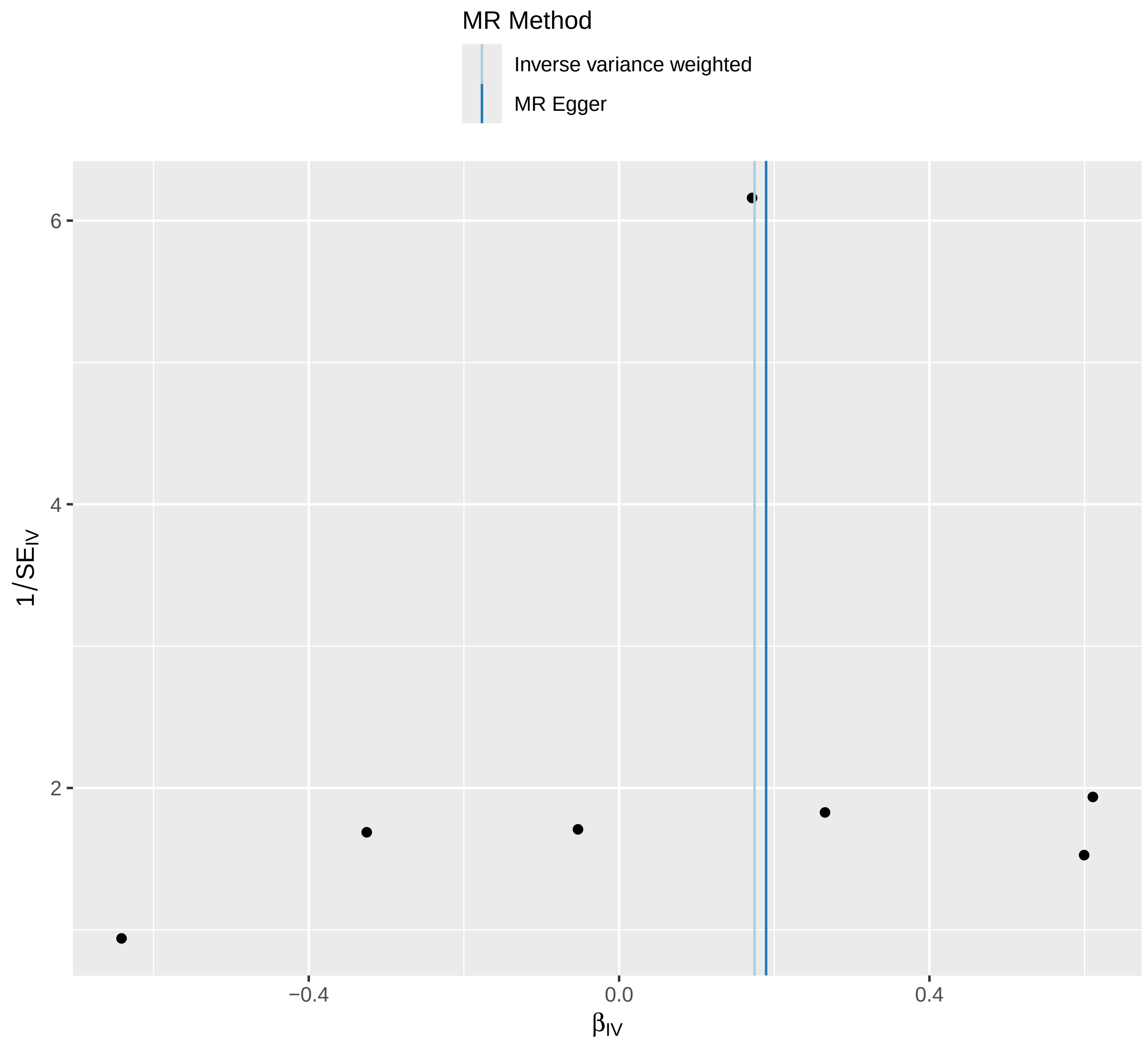
MR Method
Inverse variance weighted
MR Egger
6
4
2
1/SE$_{IV}$
−0.4
0.0
0.4
β$_{IV}$

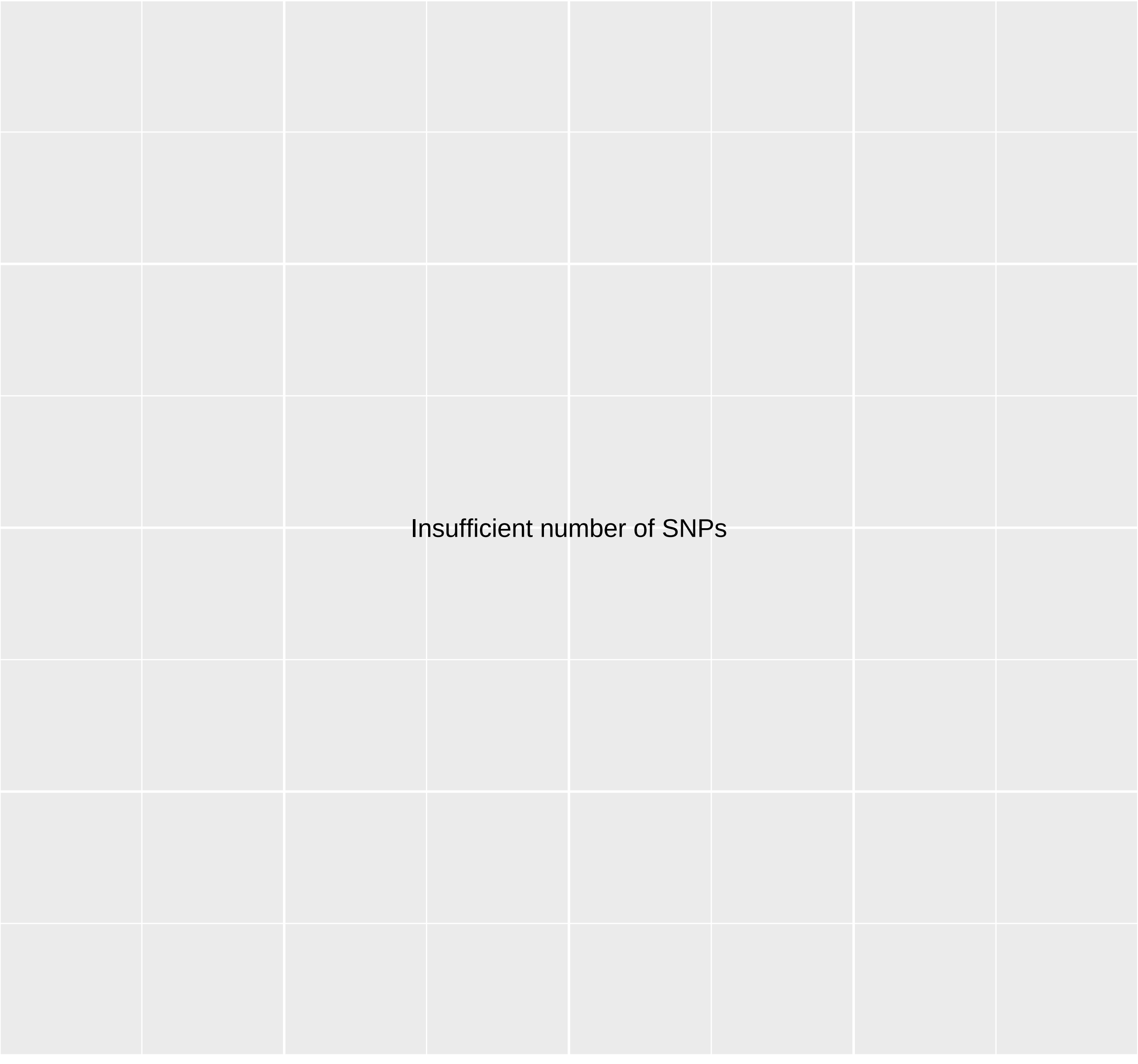
Insufficient number of SNPs

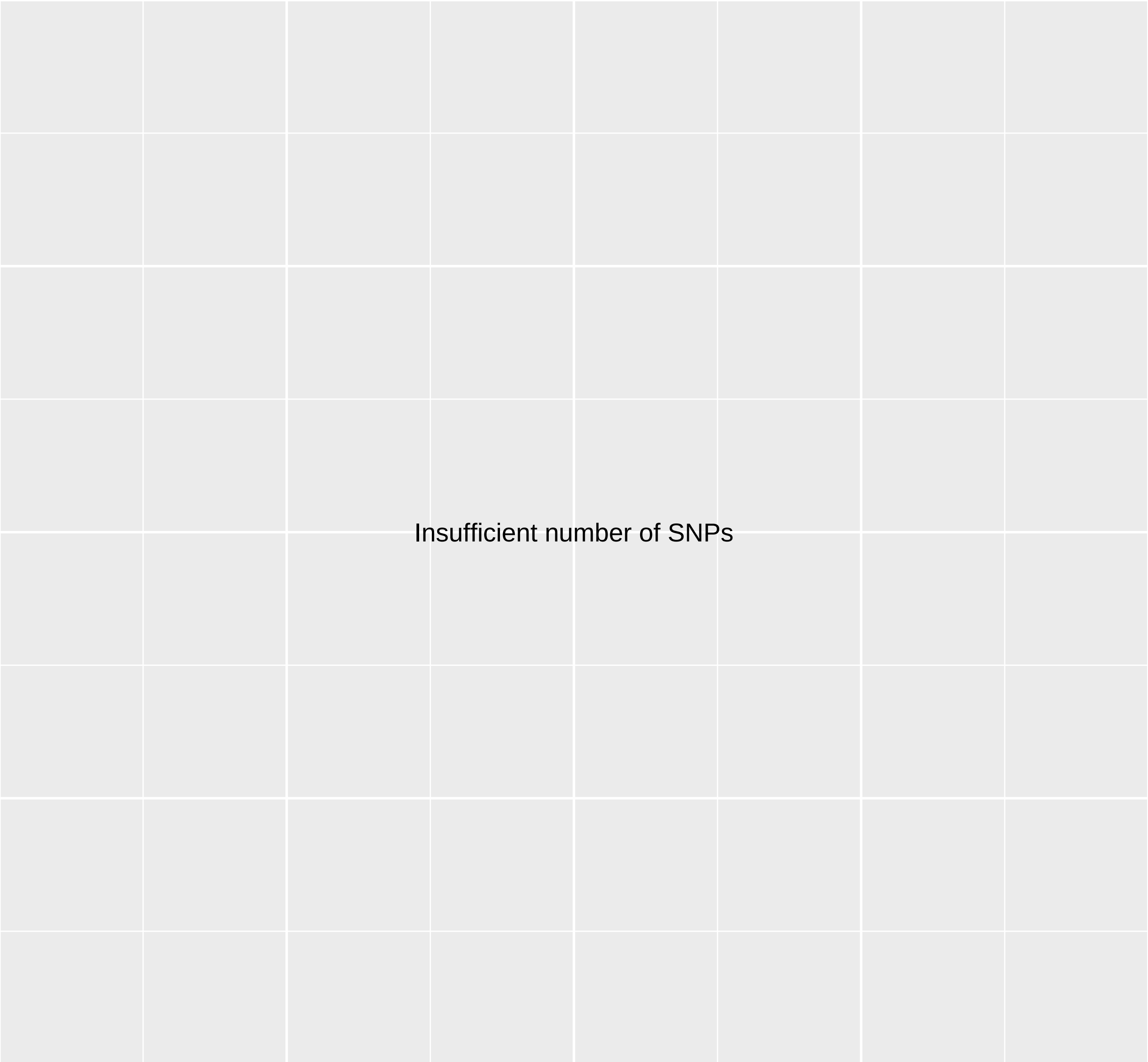
Insufficient number of SNPs

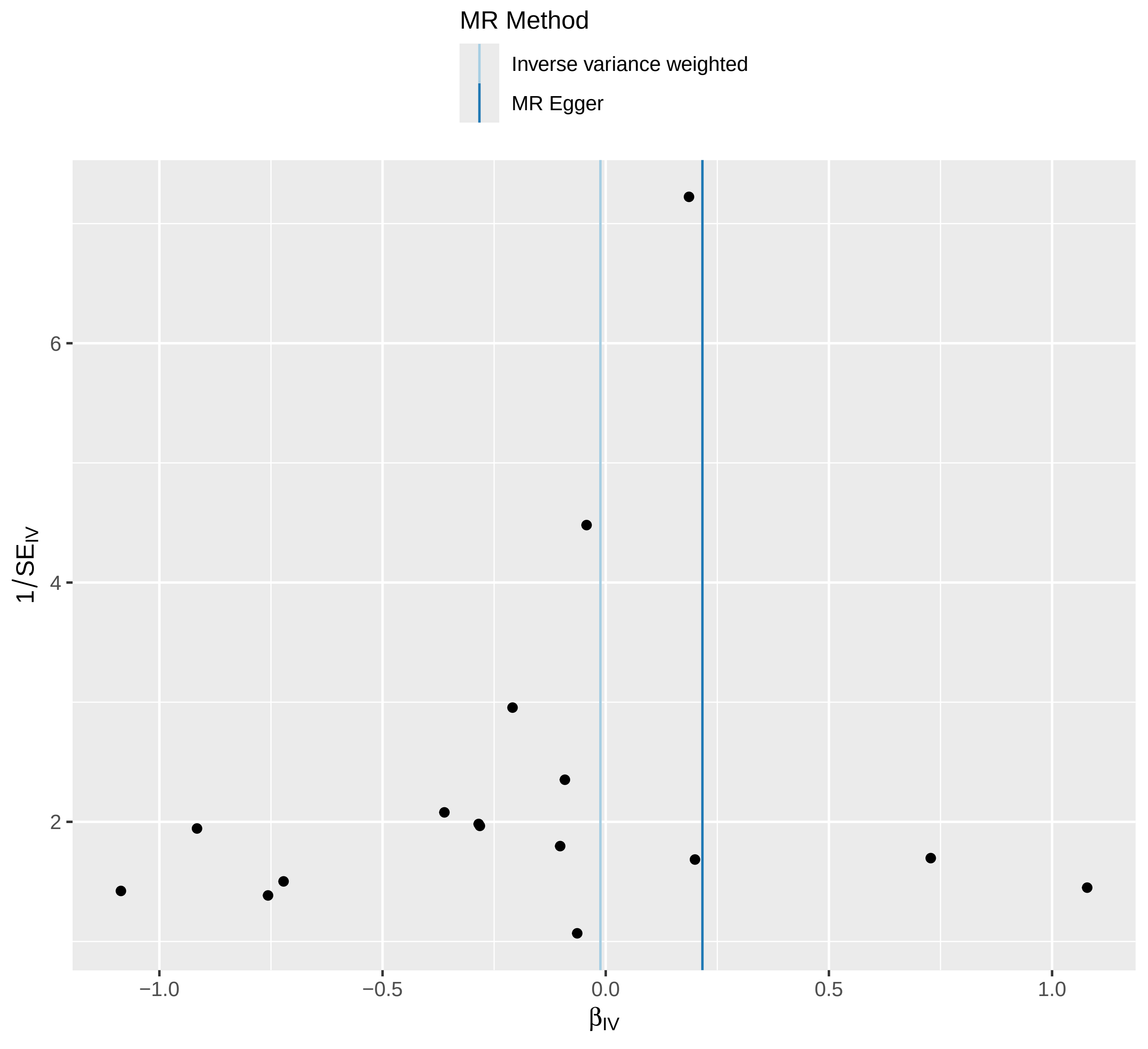

MR Method
Inverse variance weighted
MR Egger
6
4
2
1/SE_IV
−1.0
−0.5
0.0
0.5
1.0
β_IV

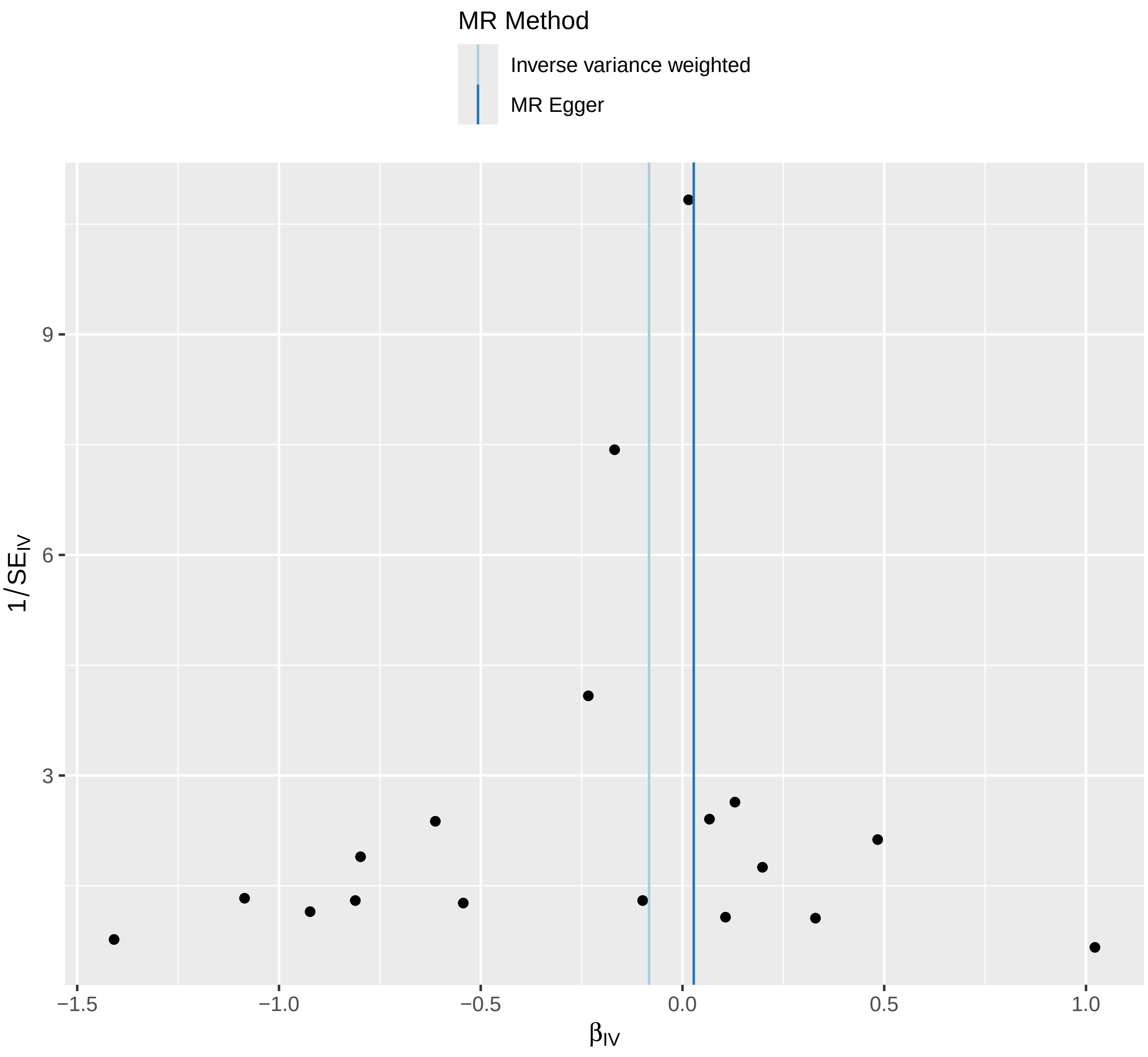

MR Method
Inverse variance weighted
MR Egger
9
6
3
1/SE_IV
−1.5
−1.0
−0.5
0.0
0.5
1.0
β_IV

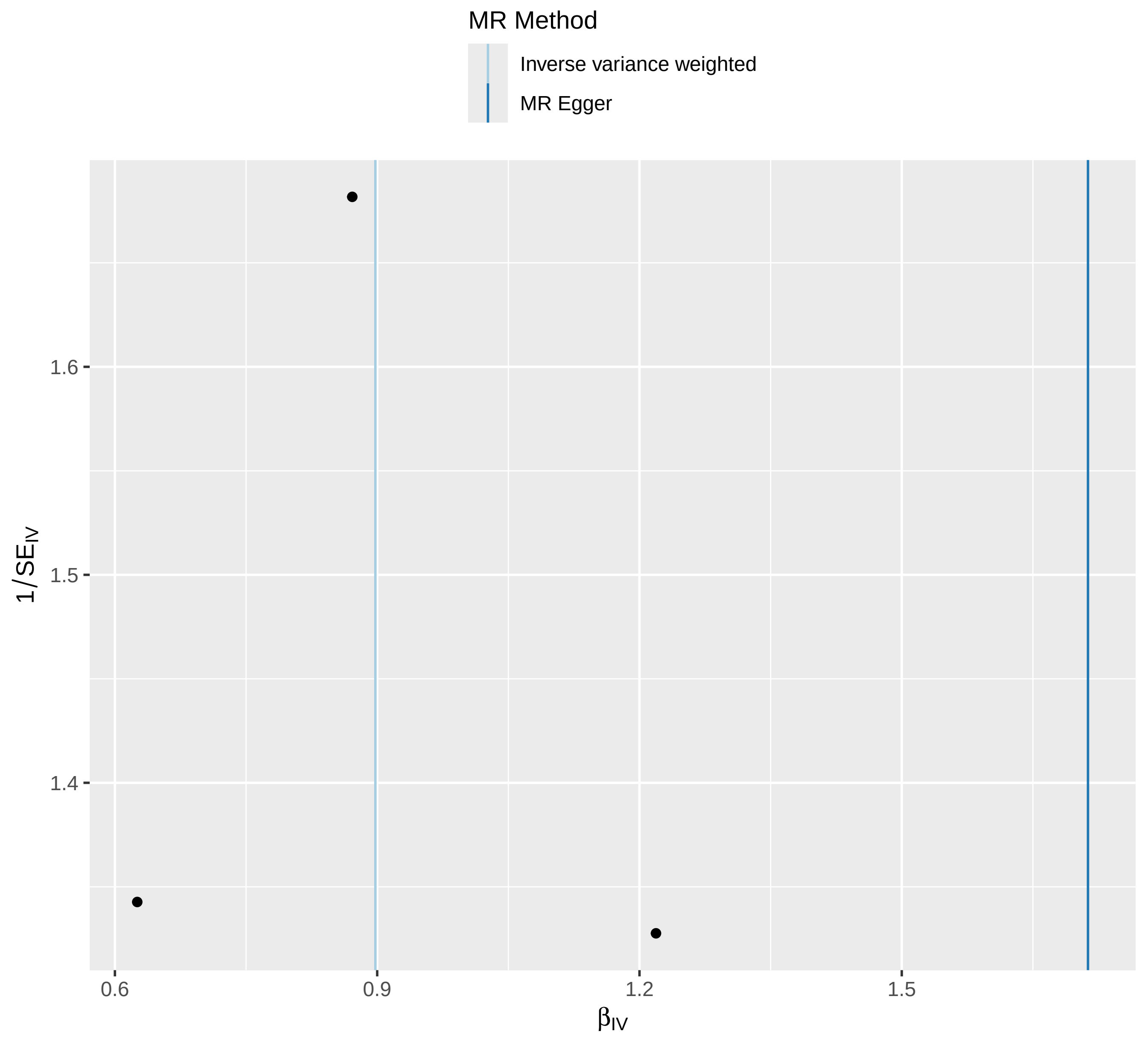
MR Method
Inverse variance weighted
MR Egger
1/SE_IV
1.4
1.5
1.6
0.6
0.9
1.2
1.5
β_IV

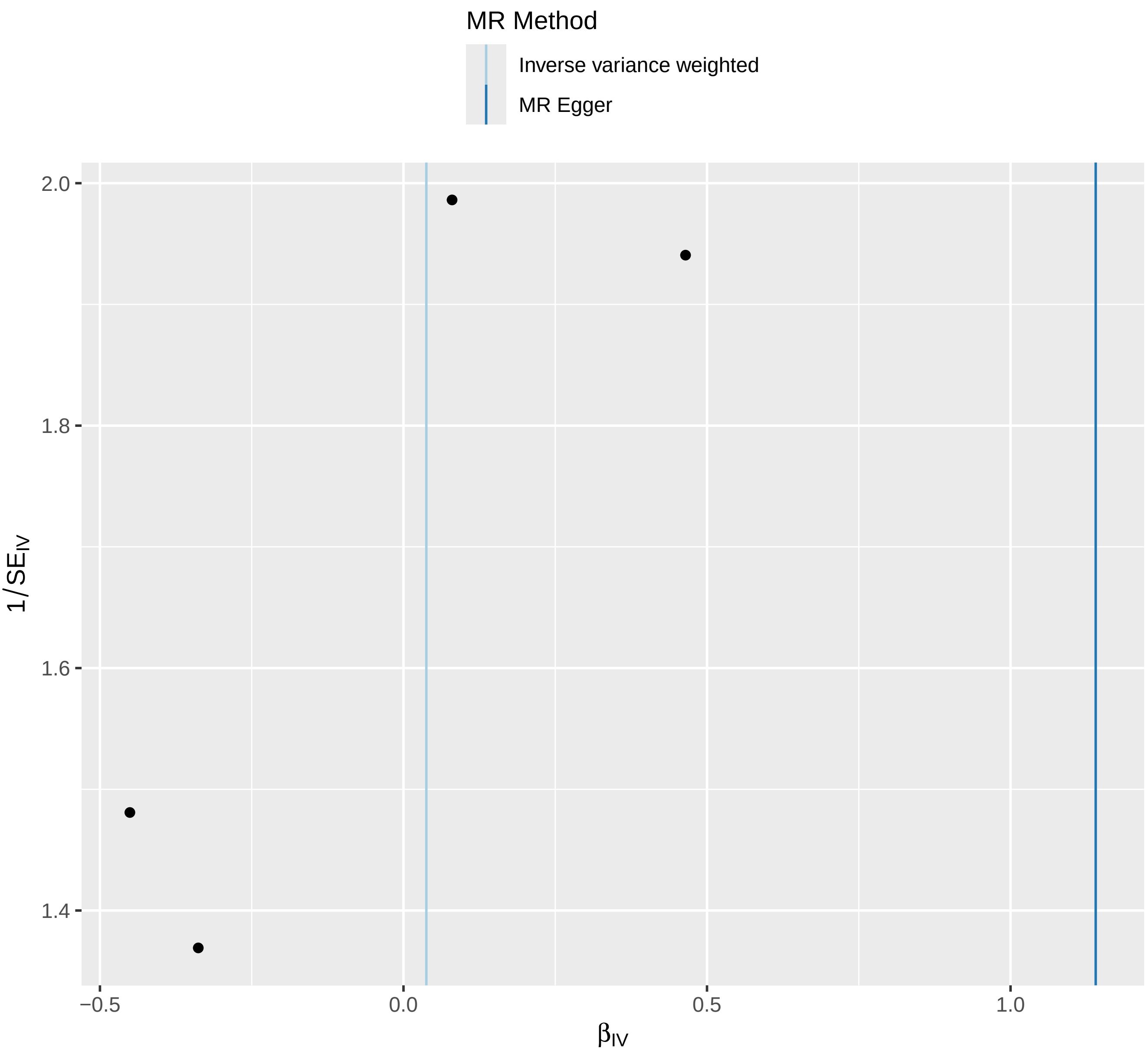
MR Method
Inverse variance weighted
MR Egger
2.0
1.8
1.6
1.4
1/SE_IV
−0.5
0.0
0.5
1.0
β_IV

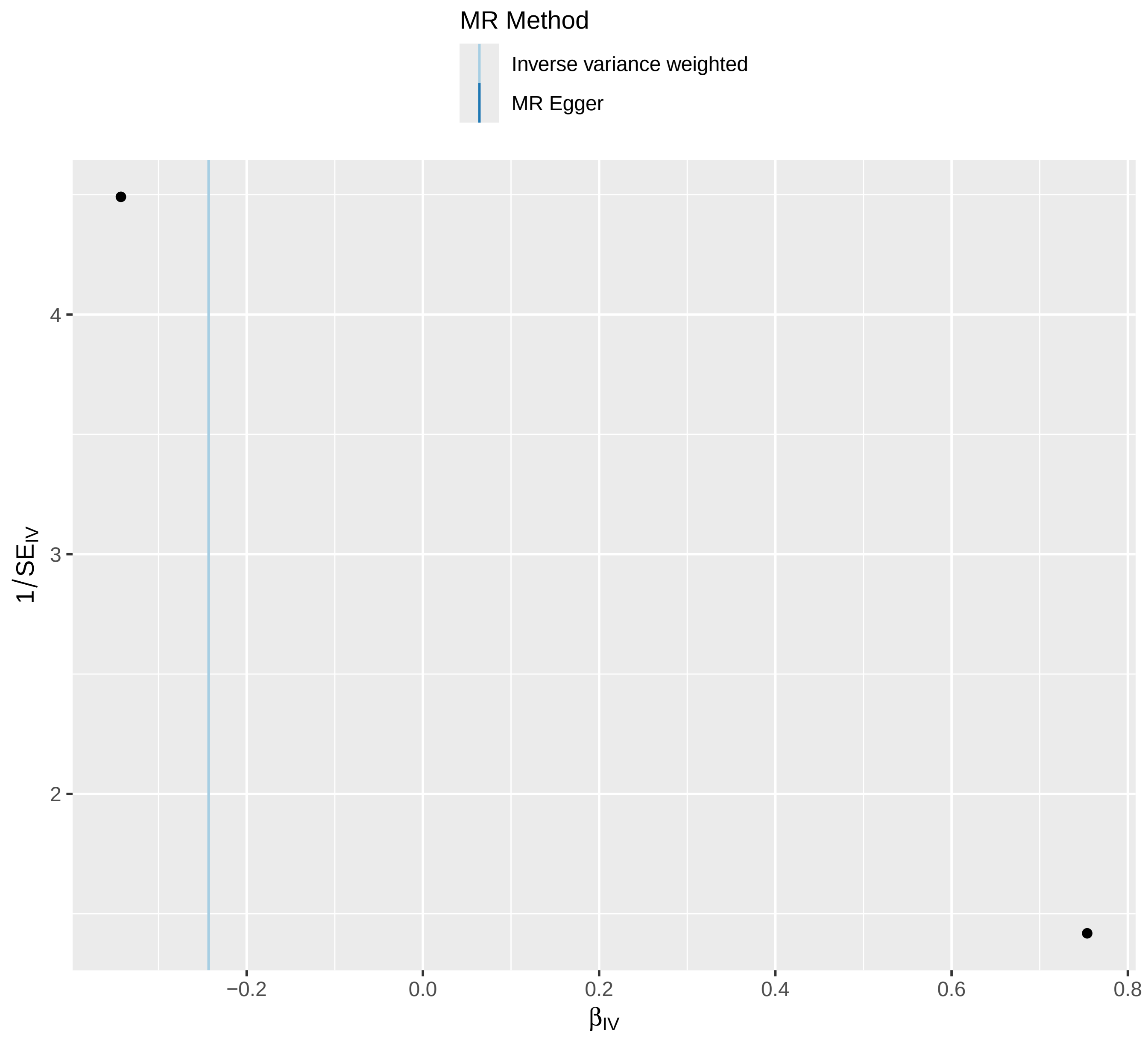
MR Method
Inverse variance weighted
MR Egger
4
3
2
1/SE_IV
−0.2
0.0
0.2
0.4
0.6
0.8
β_IV

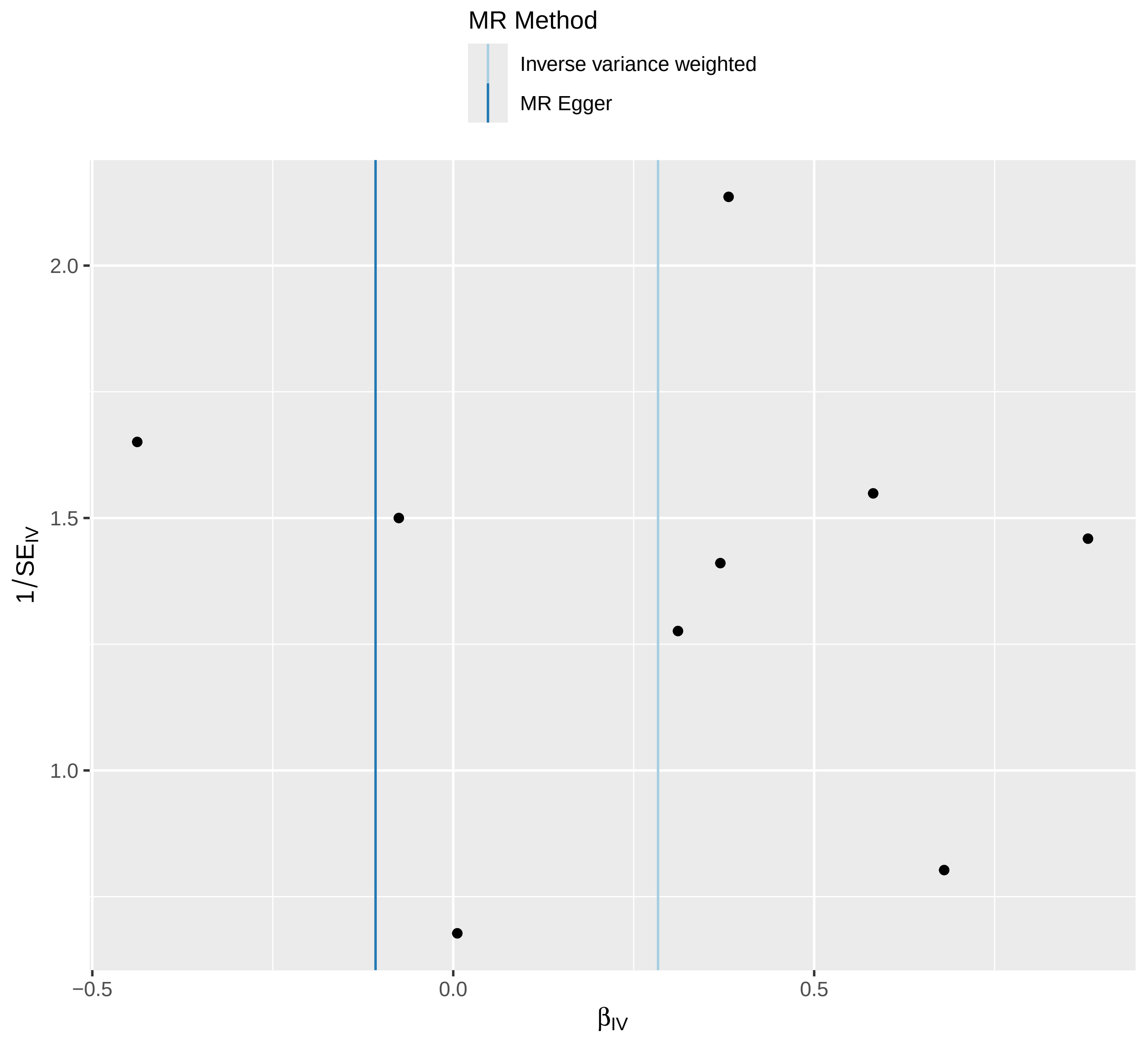
MR Method
Inverse variance weighted
MR Egger
2.0
1.5
1.0
1/SE_IV
−0.5
0.0
0.5
β_IV

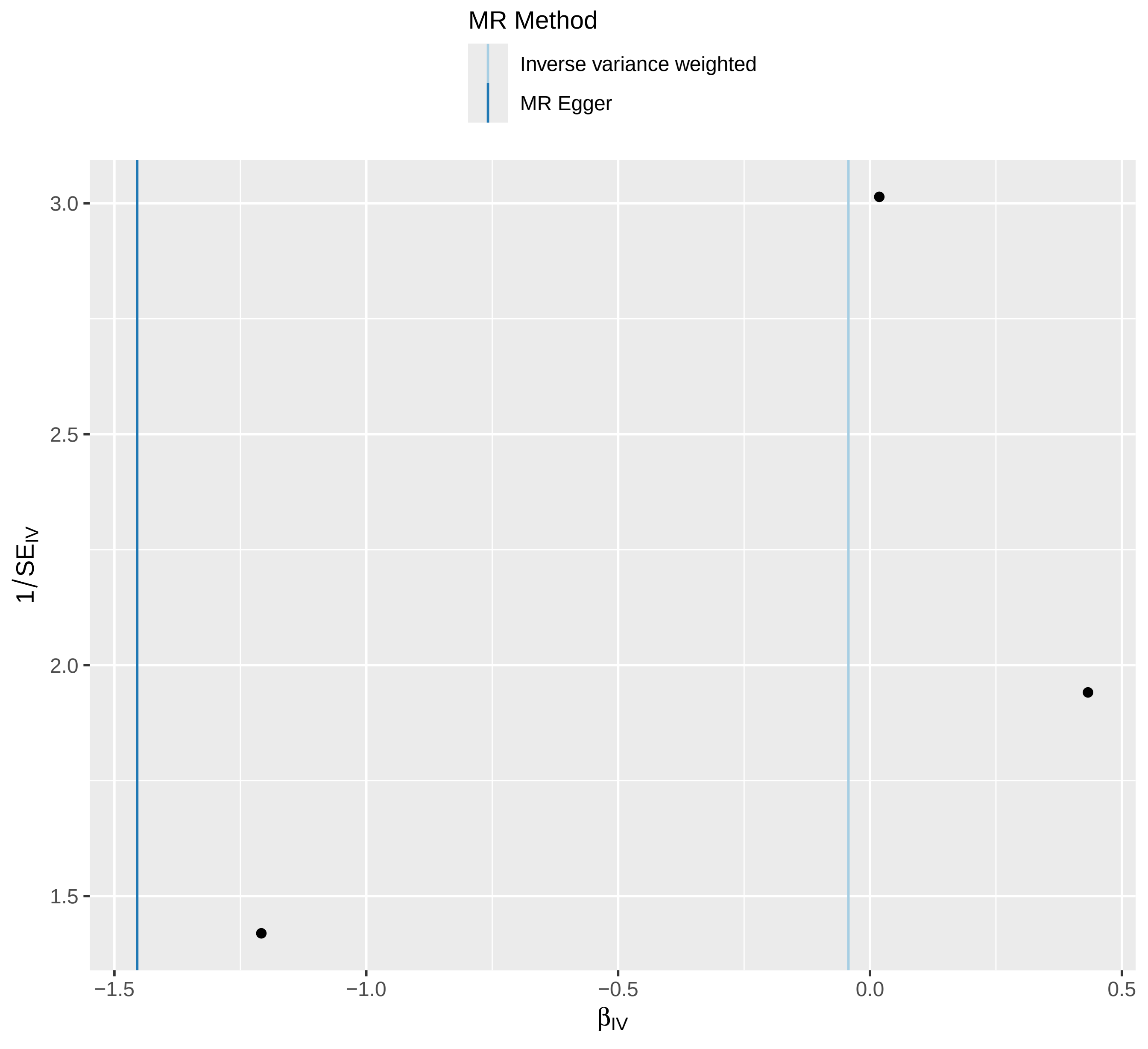

MR Method
Inverse variance weighted
MR Egger
1/SE$_{IV}$
3.0
2.5
2.0
1.5
−1.5
−1.0
−0.5
0.0
0.5
β$_{IV}$

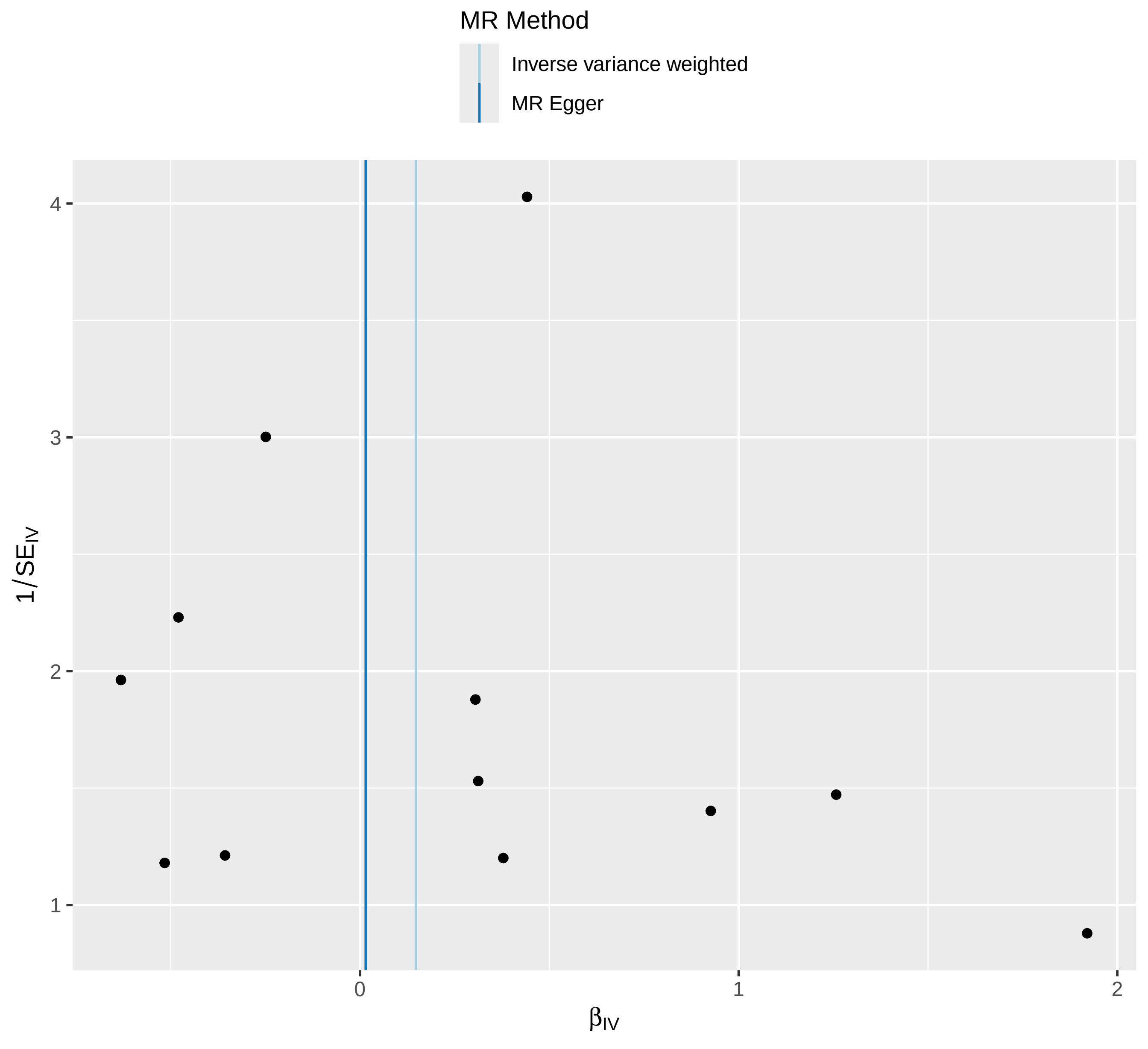

MR Method
Inverse variance weighted
MR Egger
4
3
2
1
1/SE_IV
0
1
2
β_IV

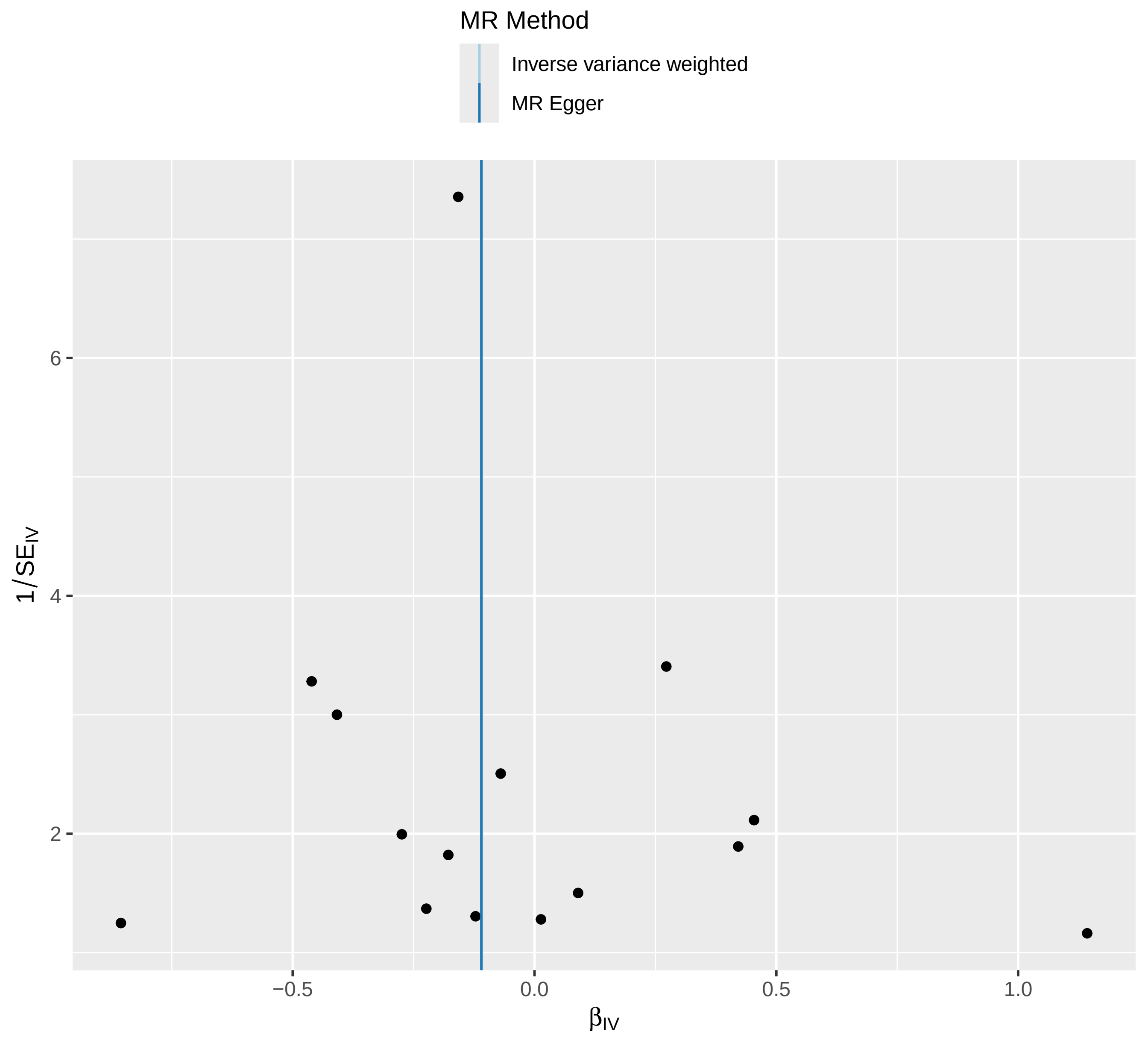
MR Method
Inverse variance weighted
MR Egger
6
4
2
1/SE$_{IV}$
−0.5
0.0
0.5
1.0
β$_{IV}$

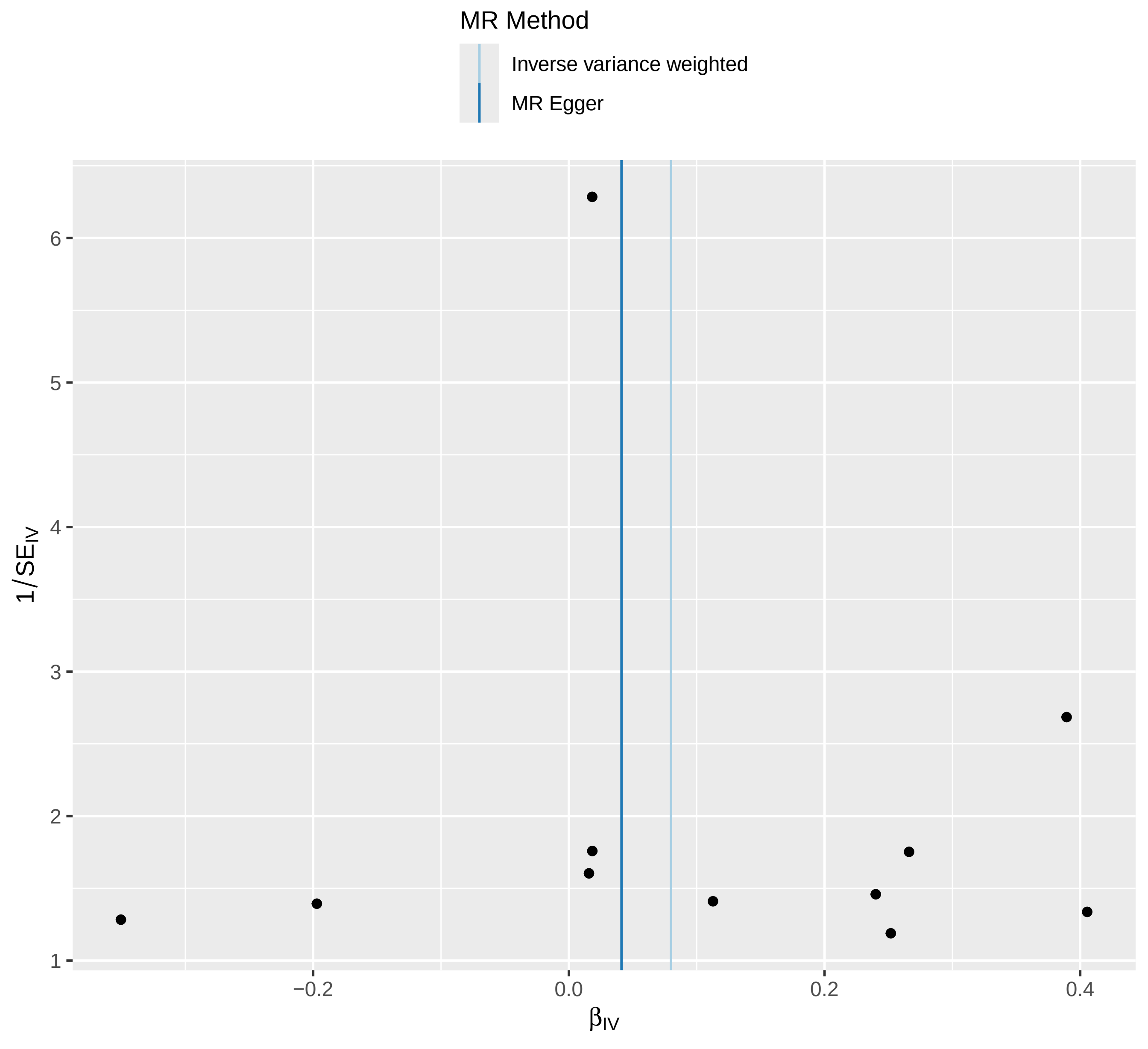

MR Method
Inverse variance weighted
MR Egger
6
5
4
3
2
1
1/SE_IV
−0.2
0.0
0.2
0.4
β_IV

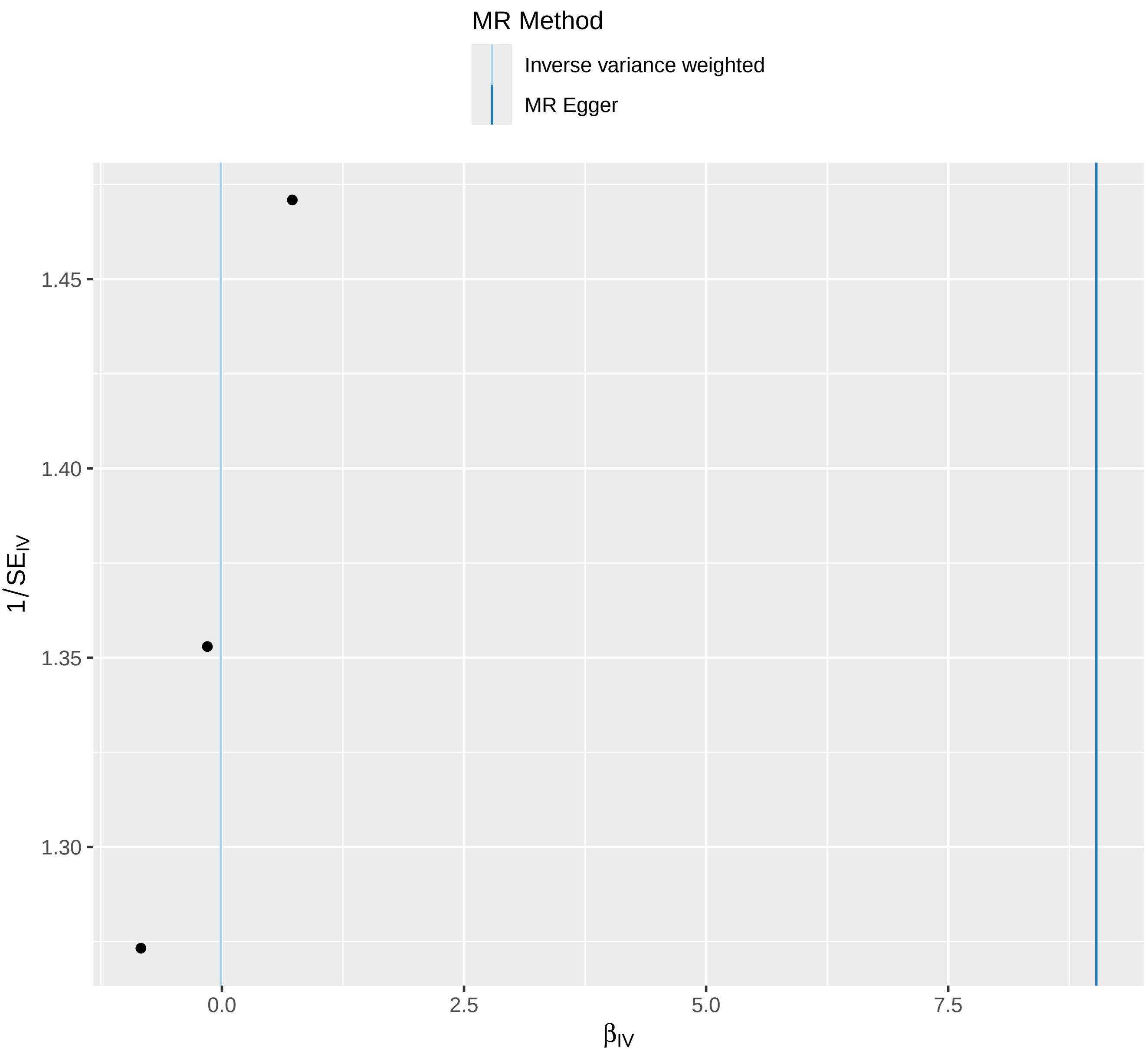
MR Method
Inverse variance weighted
MR Egger
1.45
1.40
1.35
1.30
1/SE_IV
0.0
2.5
5.0
7.5
β_IV

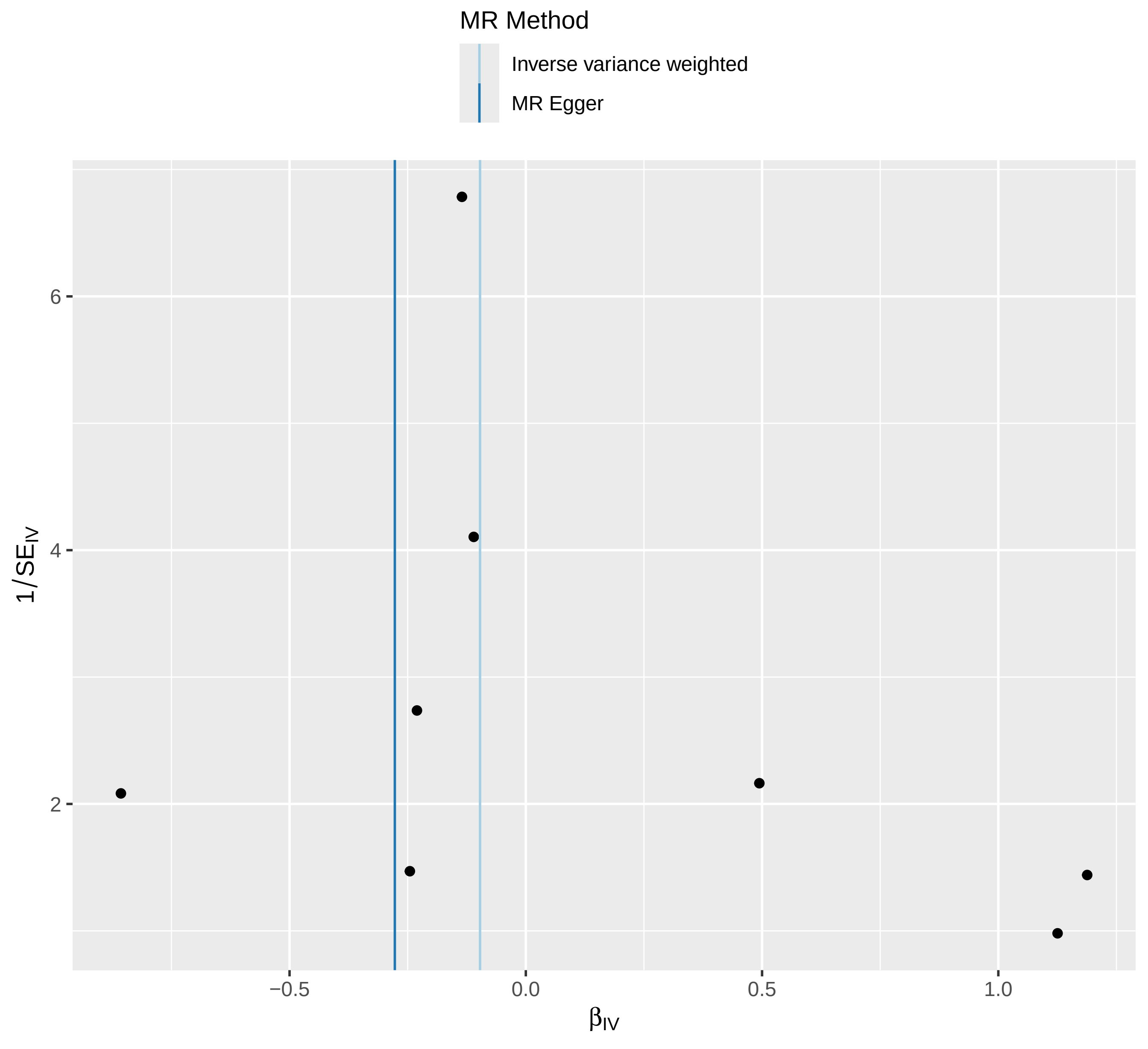

MR Method
Inverse variance weighted
MR Egger
6
4
2
$1/SE_{IV}$
−0.5
0.0
0.5
1.0
β_{IV}

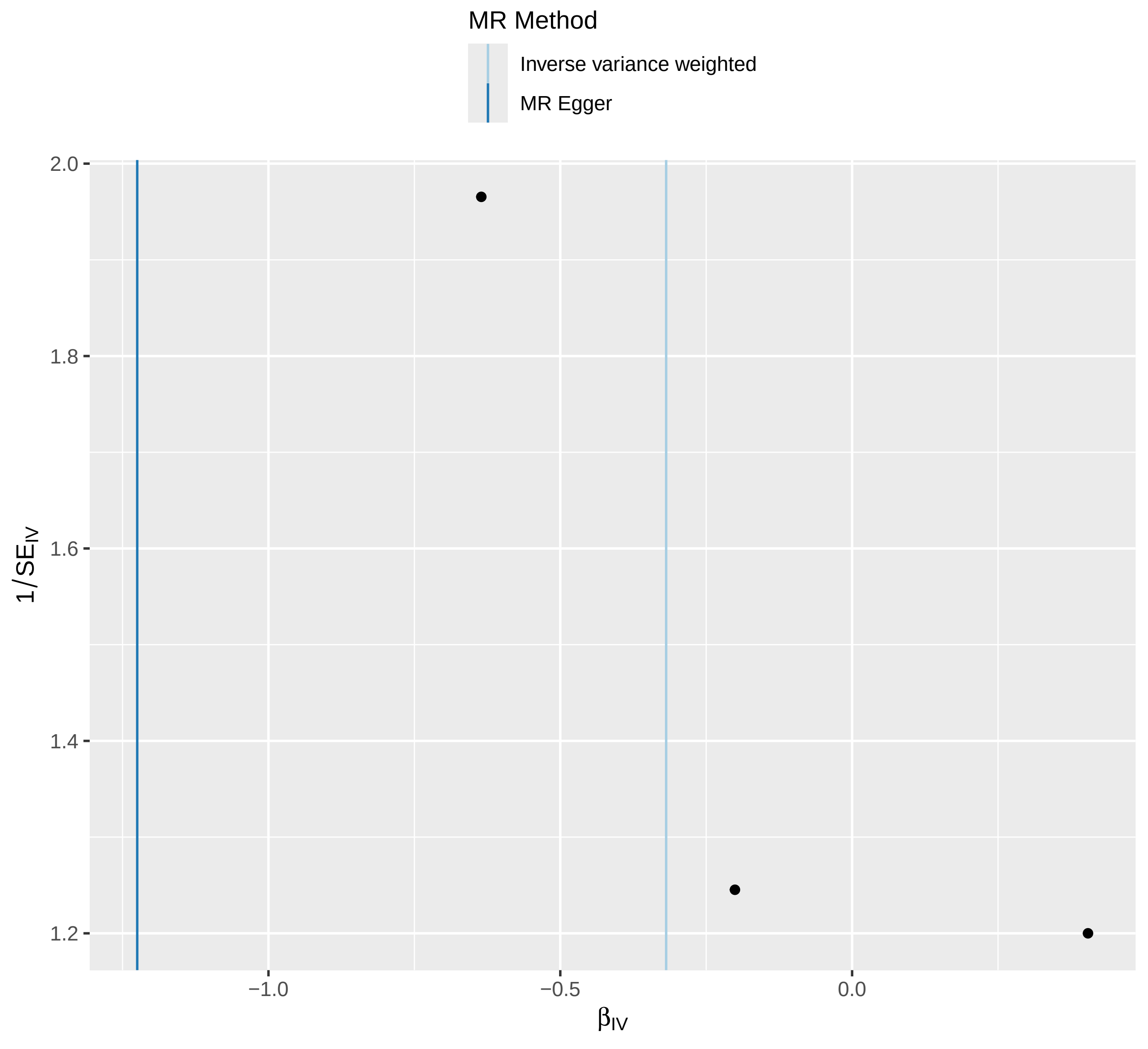

MR Method
Inverse variance weighted
MR Egger
2.0
1.8
1.6
1.4
1.2
1/SE_IV
−1.0
−0.5
0.0
β_IV

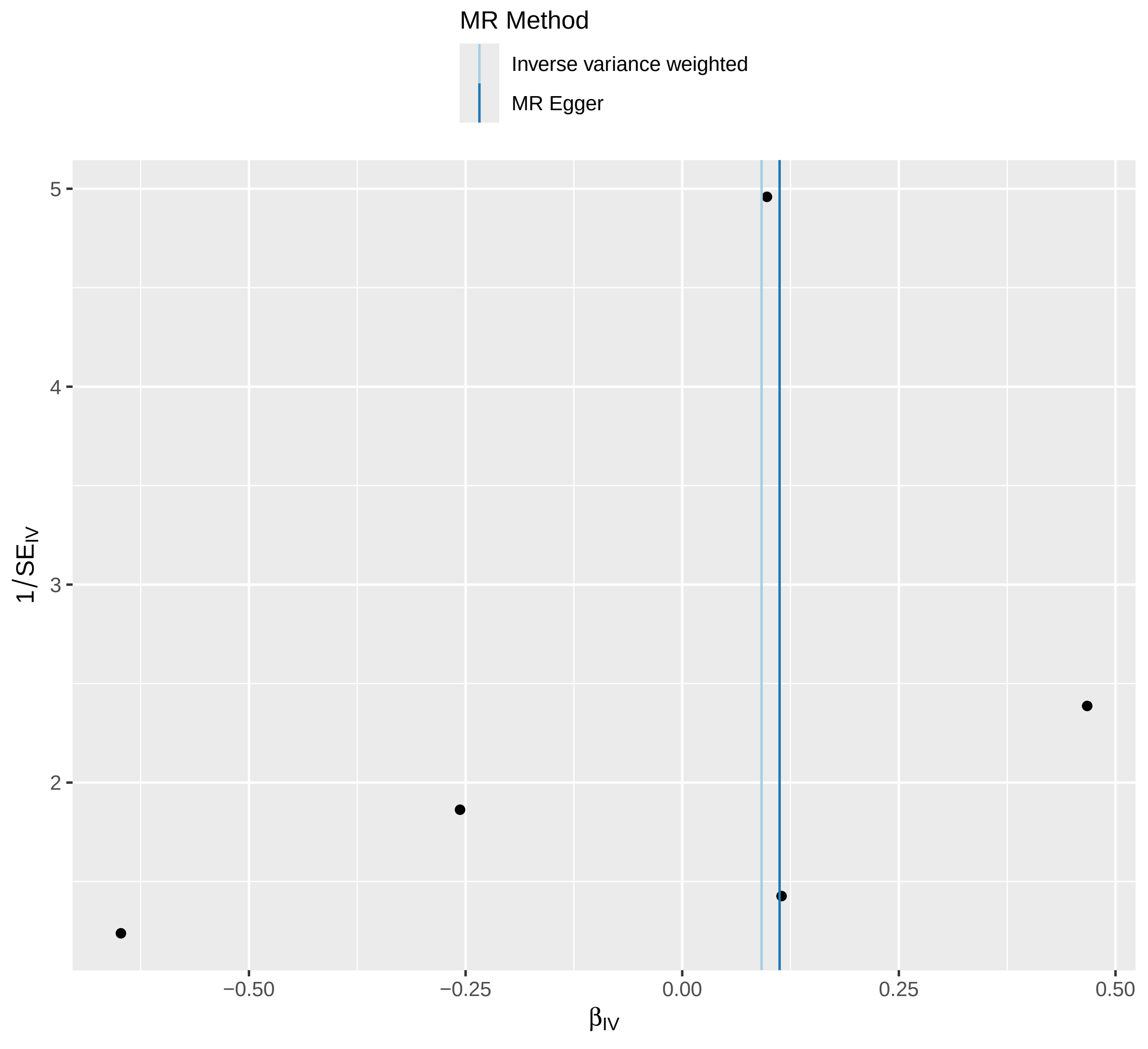

MR Method
Inverse variance weighted
MR Egger
5
4
3
2
1/SE_IV
−0.50
−0.25
0.00
0.25
0.50
β_IV

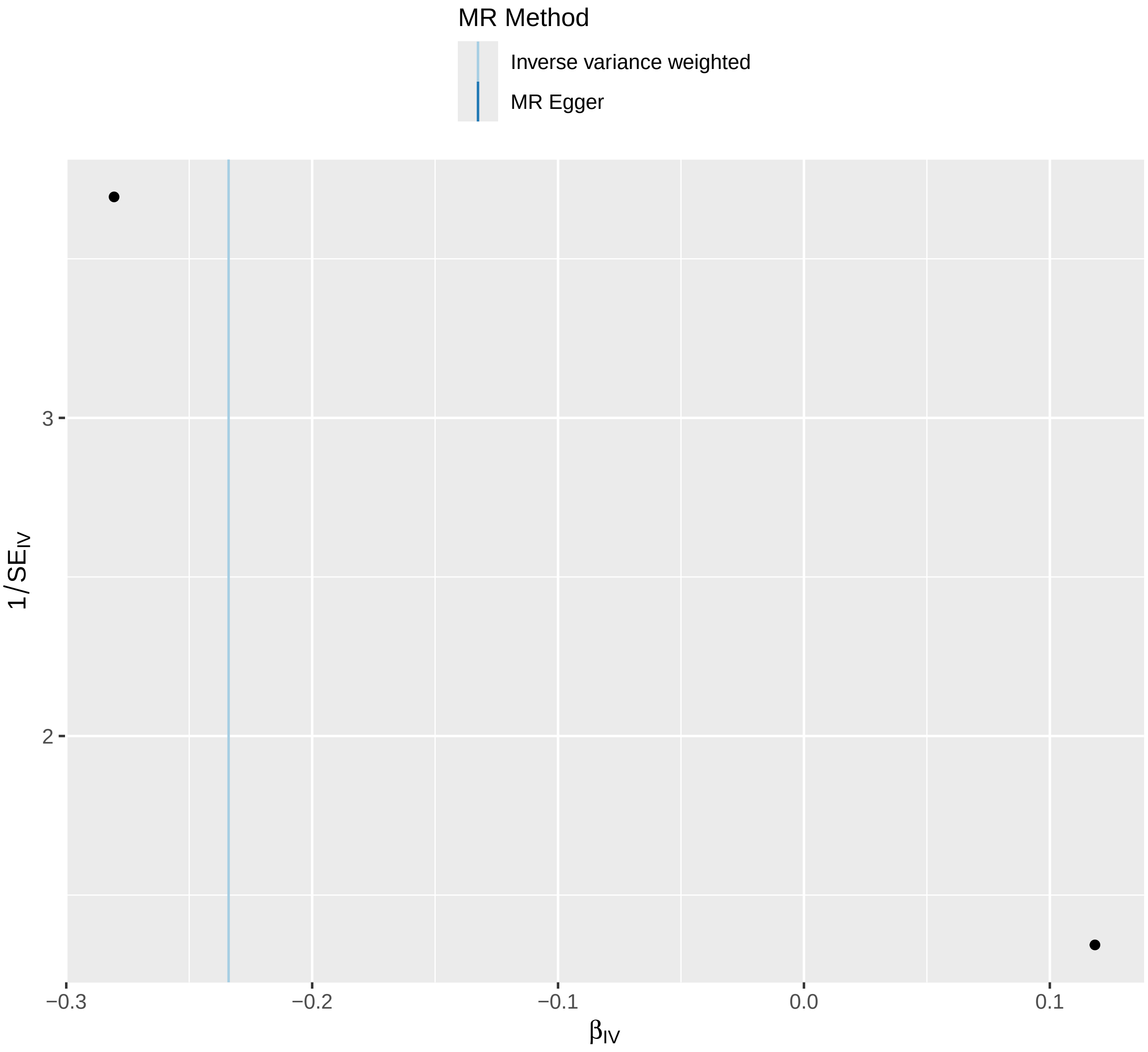
MR Method
Inverse variance weighted
MR Egger
1/SE$_{IV}$
3
2
−0.3
−0.2
−0.1
0.0
0.1
β$_{IV}$

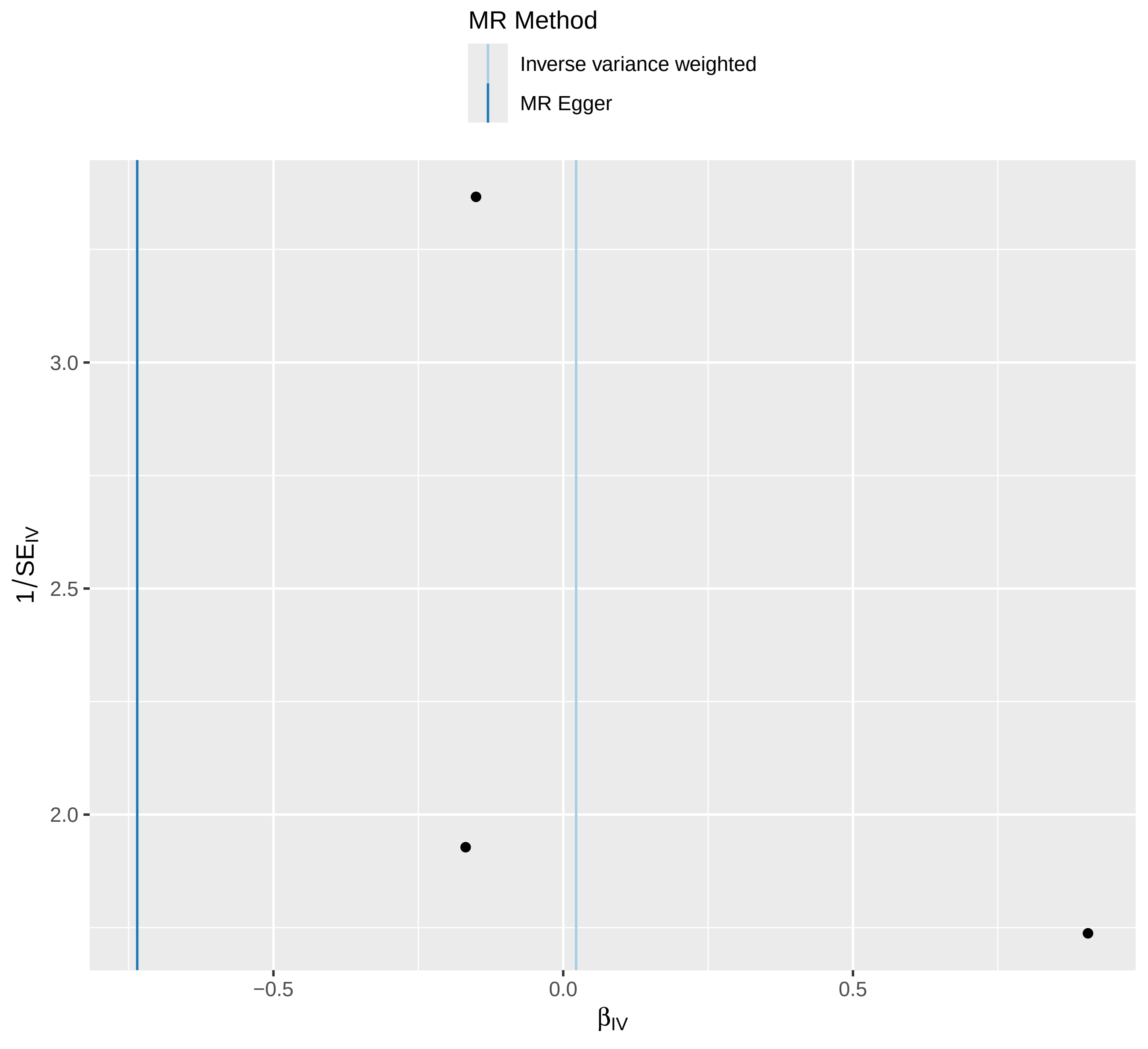
MR Method
Inverse variance weighted
MR Egger
3.0
2.5
2.0
1/SE_IV
−0.5
0.0
0.5
β_IV

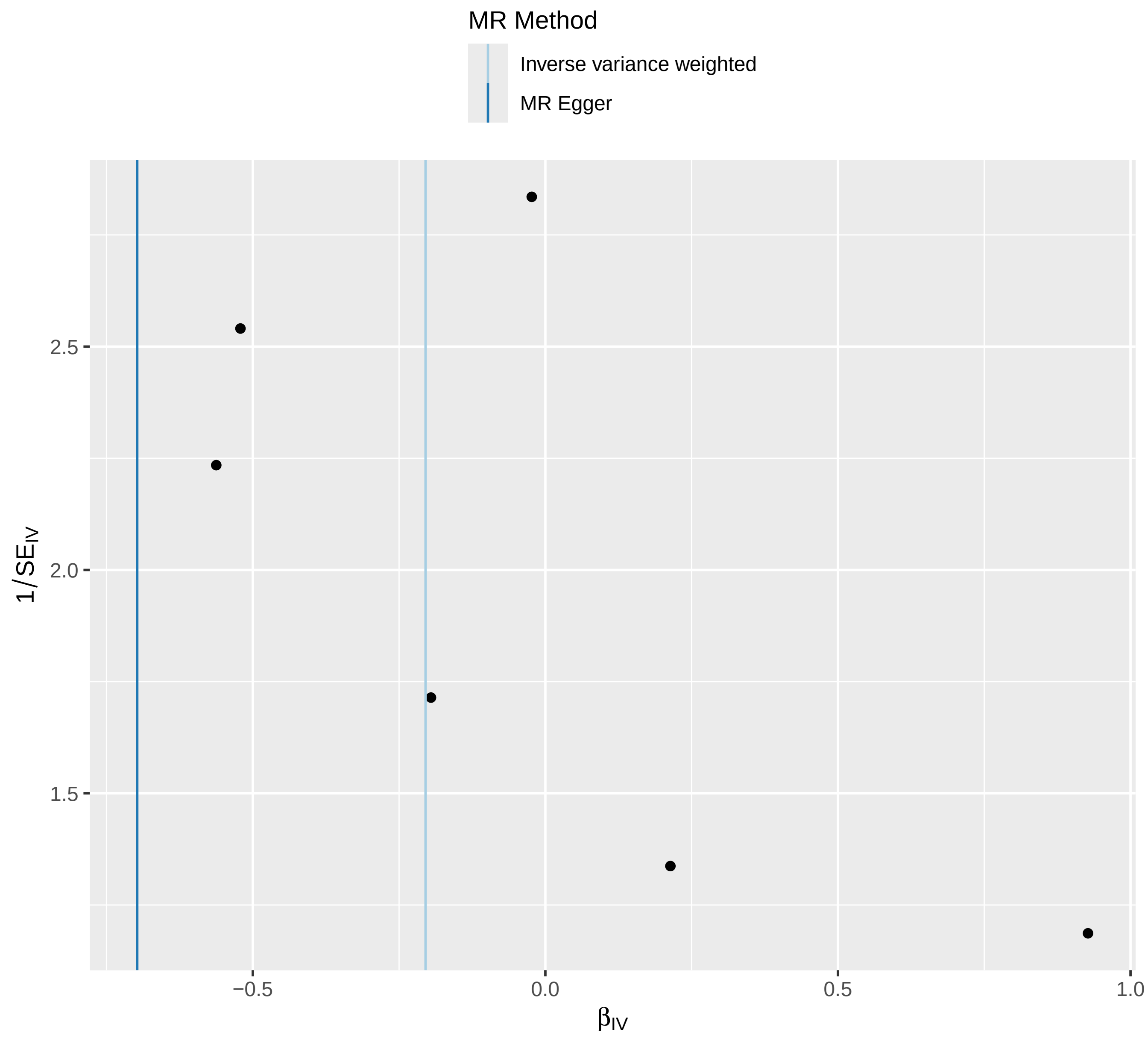

MR Method
Inverse variance weighted
MR Egger
2.5
2.0
1.5
1/SE_IV
−0.5
0.0
0.5
1.0
β_IV

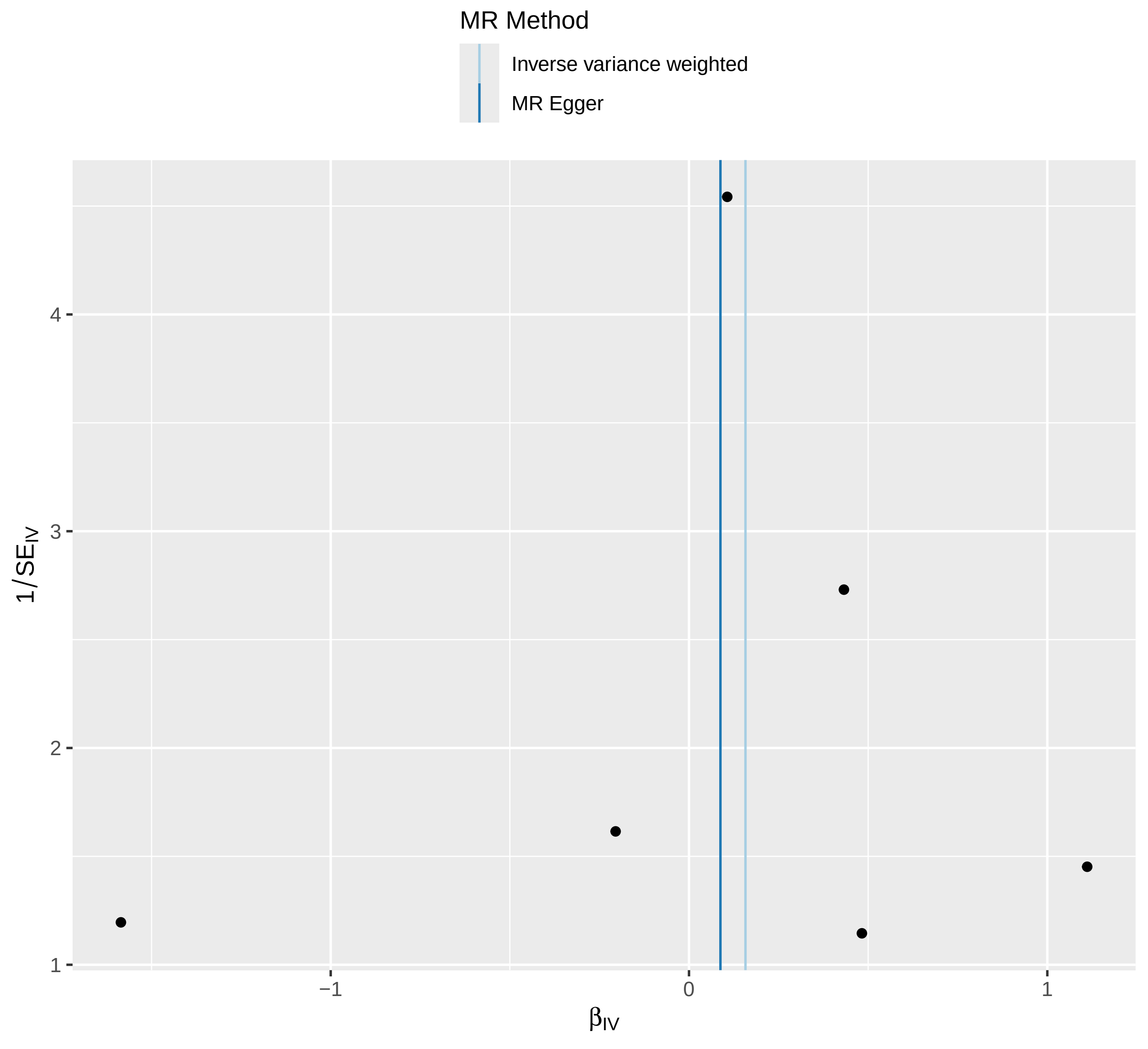

MR Method
Inverse variance weighted
MR Egger
1/SE_IV
4
3
2
1
−1
0
1
β_IV

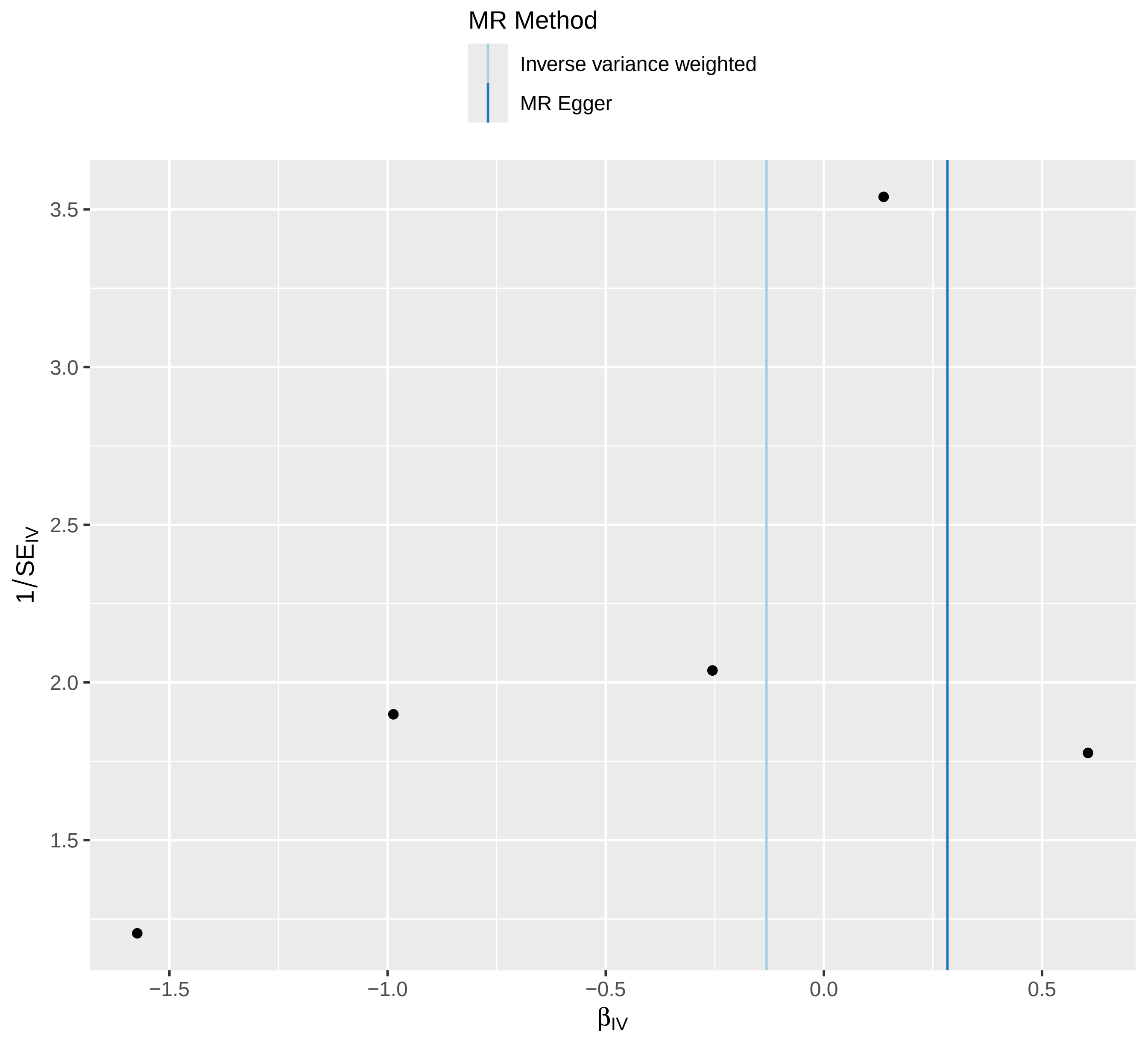

MR Method
Inverse variance weighted
MR Egger
1/SE_IV
3.5
3.0
2.5
2.0
1.5
−1.5
−1.0
−0.5
0.0
0.5
β_IV

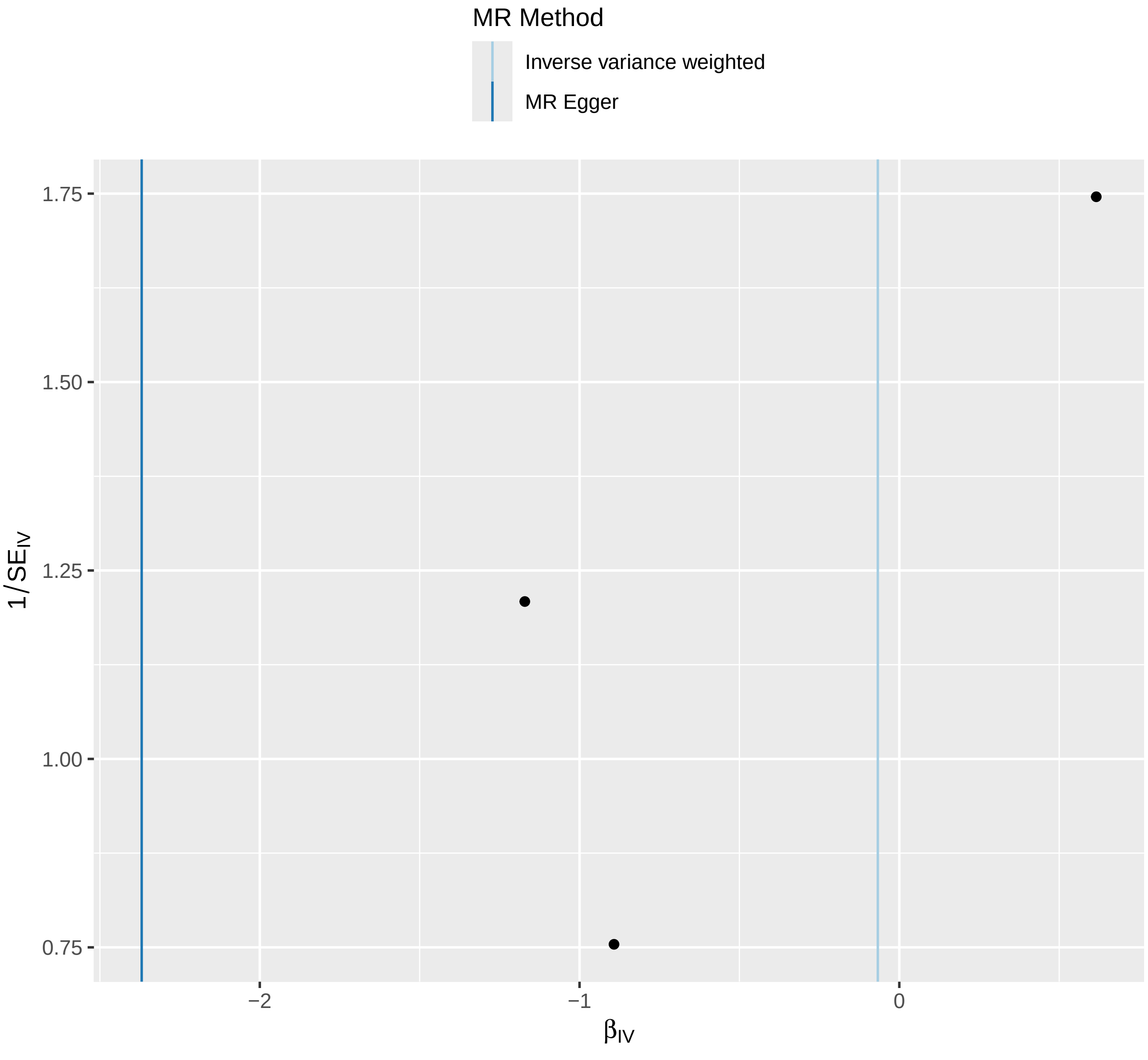

MR Method
Inverse variance weighted
MR Egger
1.75
1.50
1.25
1.00
0.75
$1/SE_{IV}$
−2
−1
0
β_{IV}

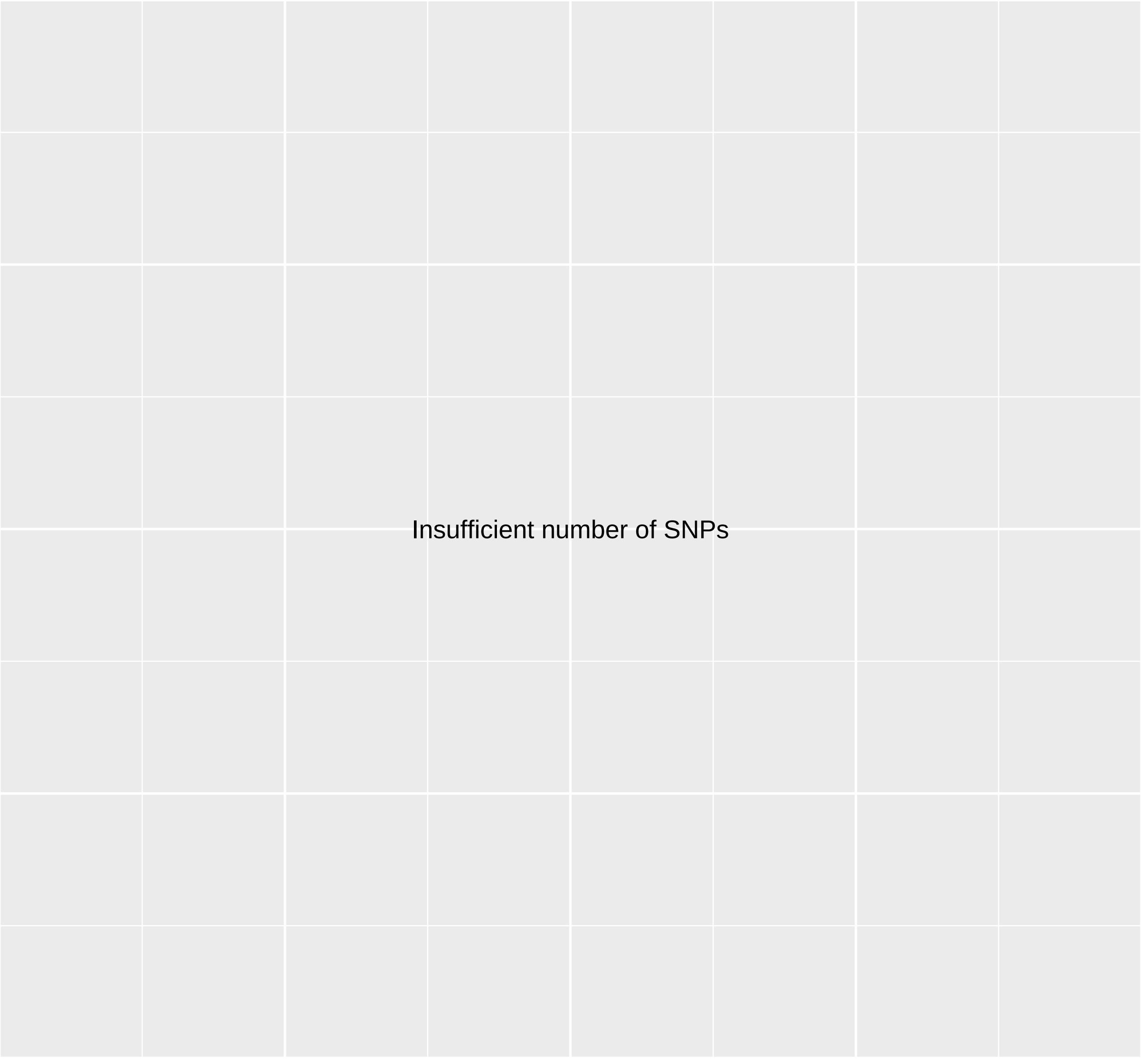
Insufficient number of SNPs

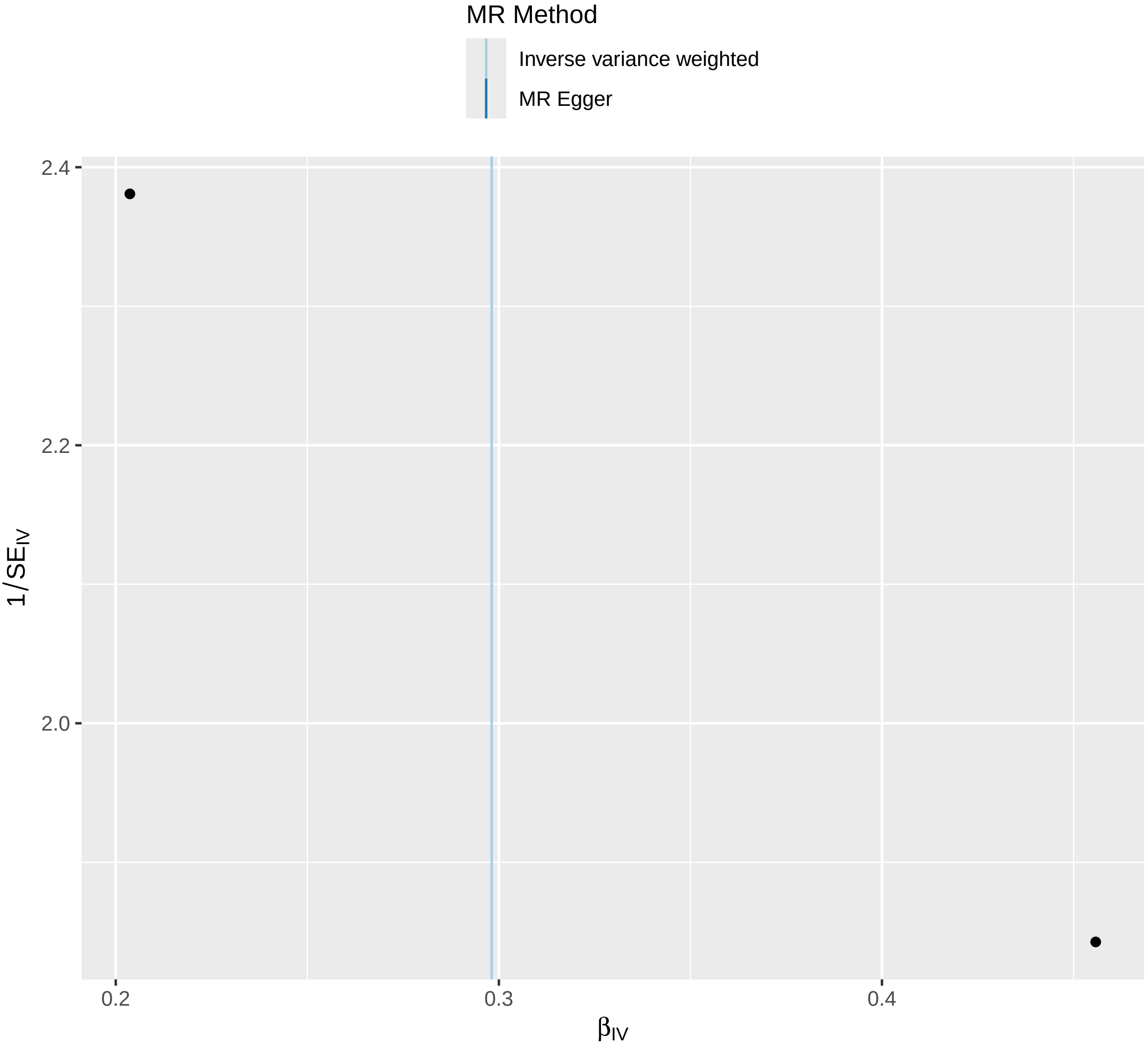
MR Method
Inverse variance weighted
MR Egger
2.4
2.2
2.0
1/SE$_{IV}$
0.2
0.3
0.4
β$_{IV}$

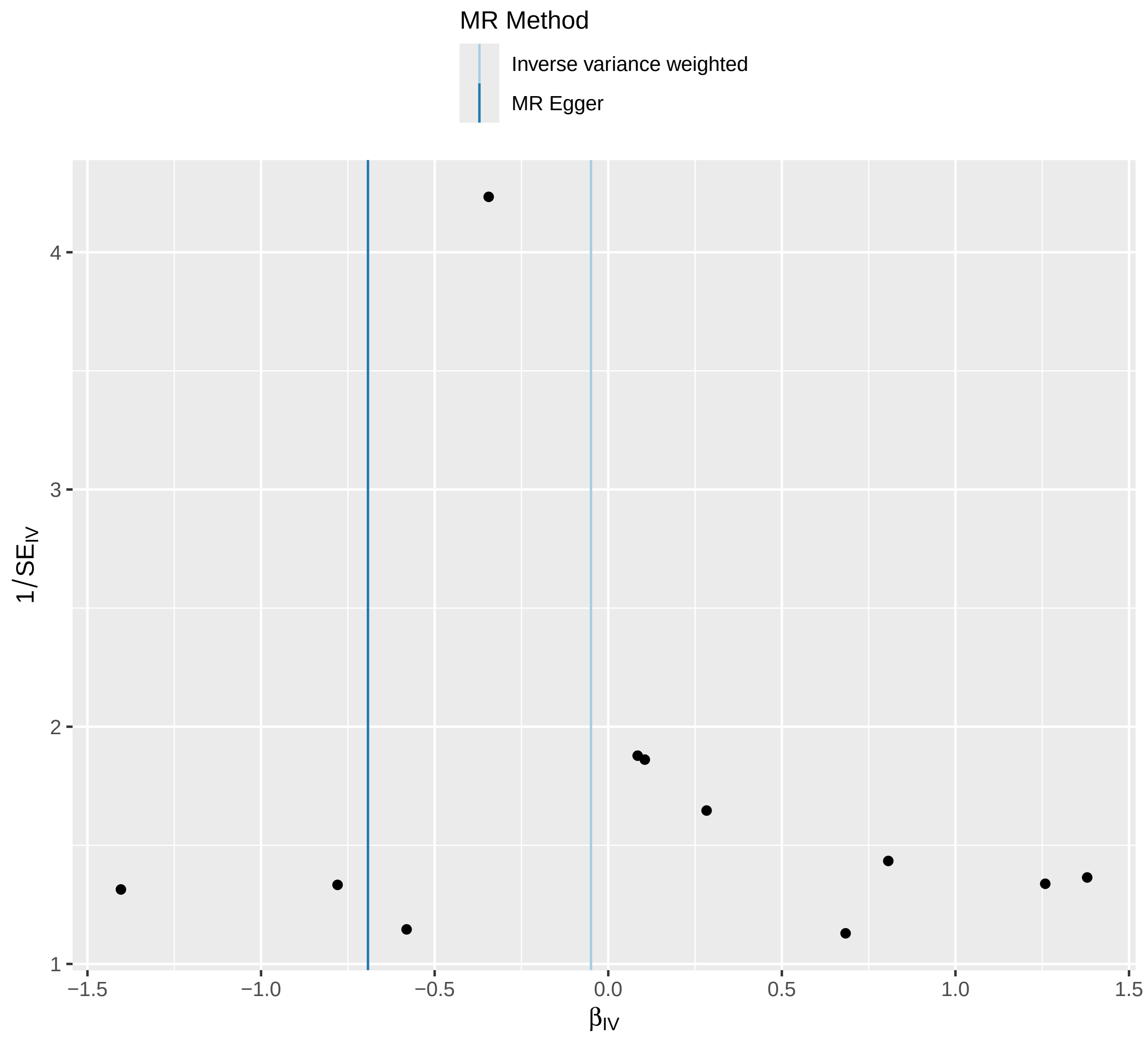
MR Method
Inverse variance weighted
MR Egger
1/SE_IV
β_IV
4
3
2
1
−1.5
−1.0
−0.5
0.0
0.5
1.0
1.5

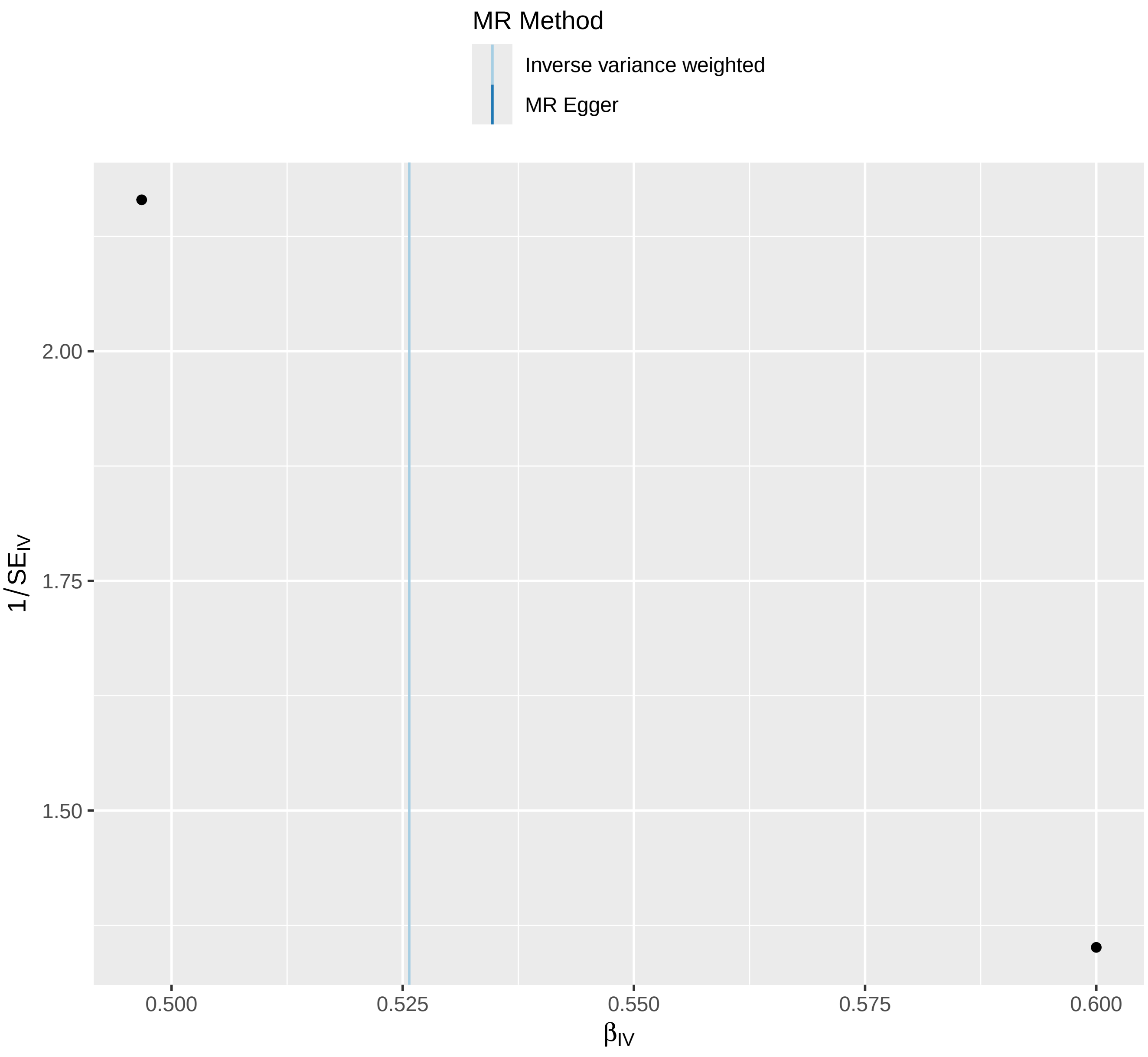

MR Method
Inverse variance weighted
MR Egger
2.00
1.75
1.50
1/SE_IV
0.500
0.525
0.550
0.575
0.600
β_IV

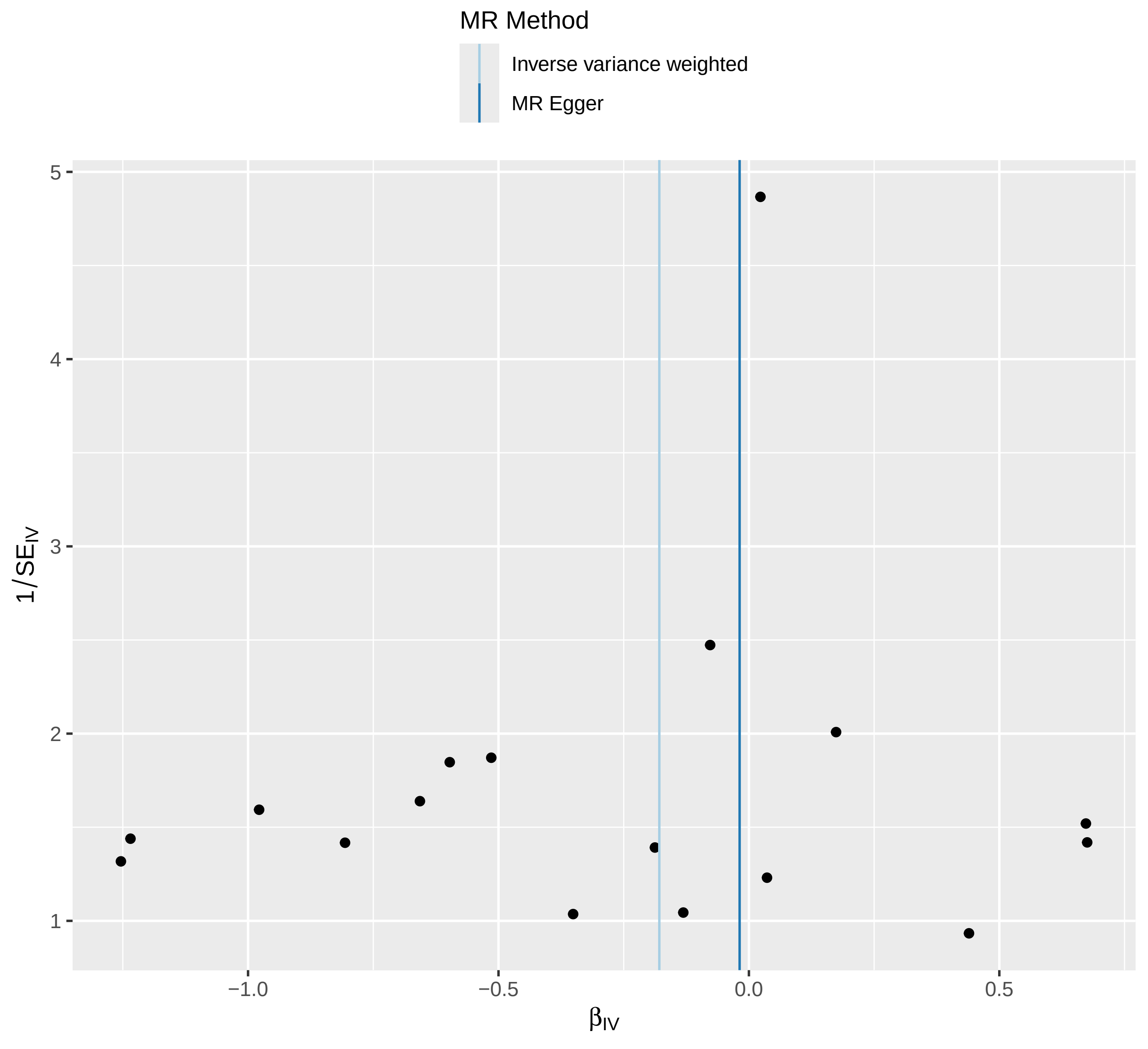

MR Method
Inverse variance weighted
MR Egger
5
4
3
2
1
$1/SE_{IV}$
−1.0
−0.5
0.0
0.5
β_{IV}

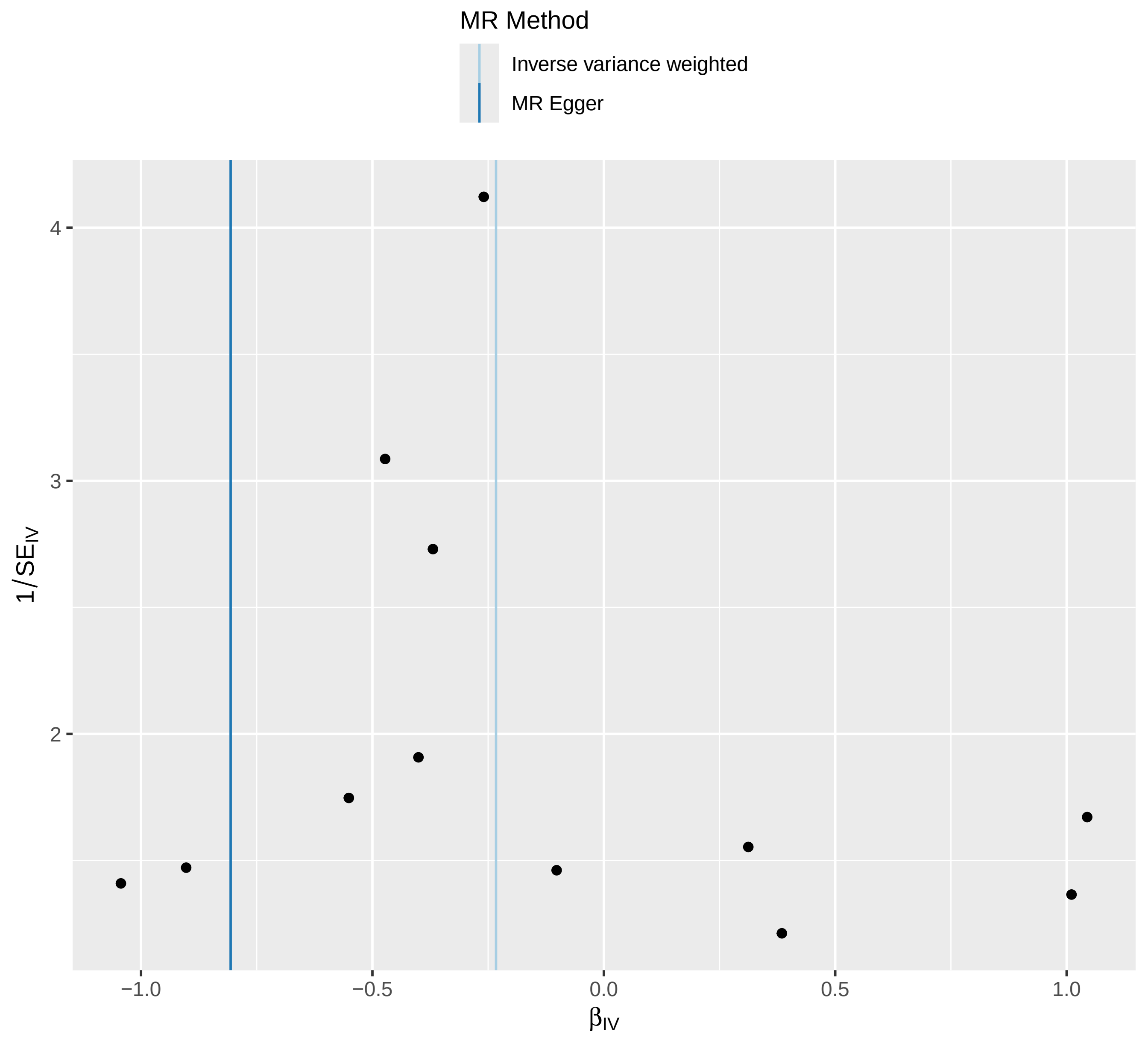

MR Method
Inverse variance weighted
MR Egger
4
3
2
1/SE_IV
−1.0
−0.5
0.0
0.5
1.0
β_IV

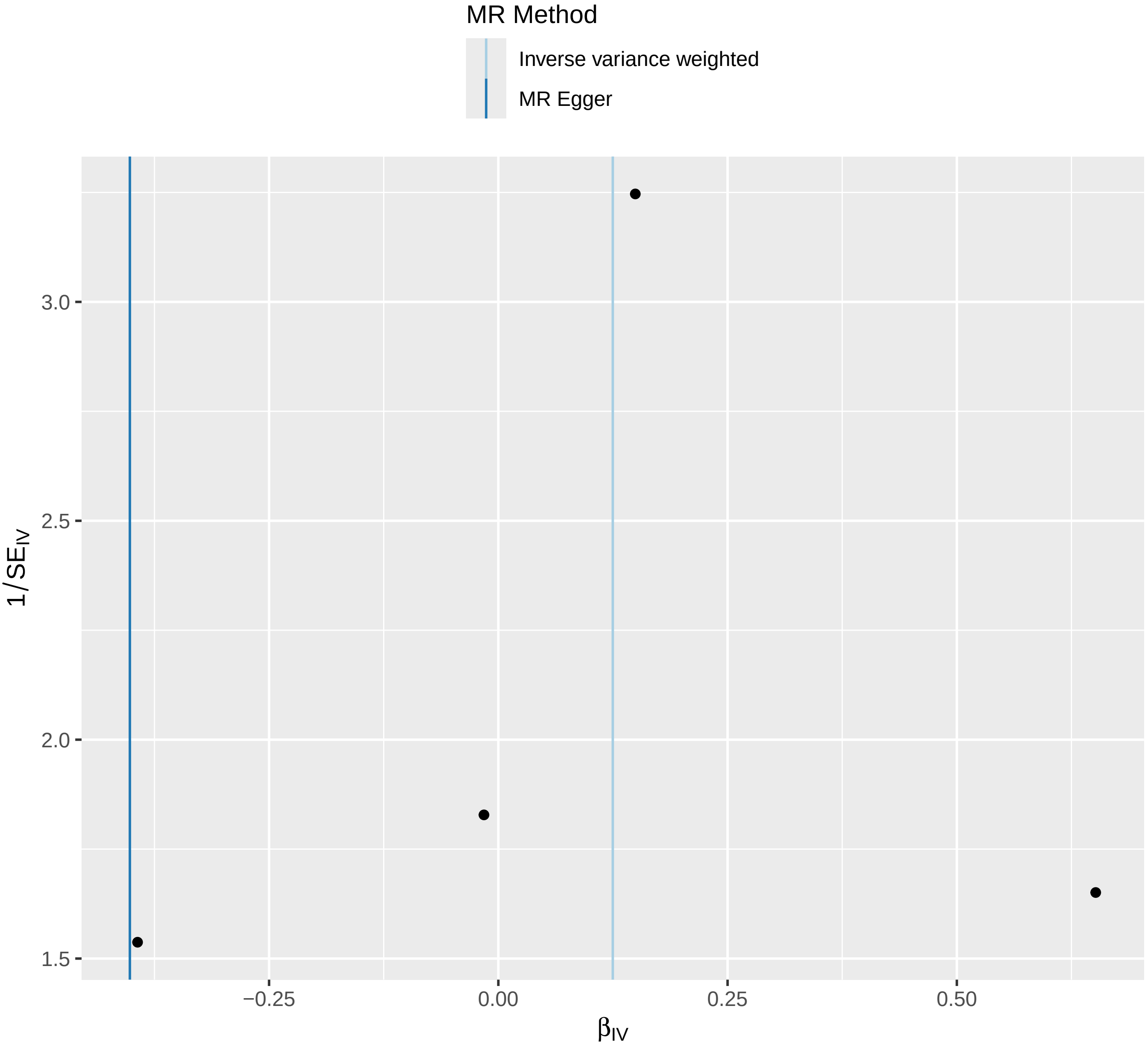
MR Method
Inverse variance weighted
MR Egger
3.0
2.5
2.0
1.5
1/SE$_{IV}$
−0.25
0.00
0.25
0.50
β$_{IV}$

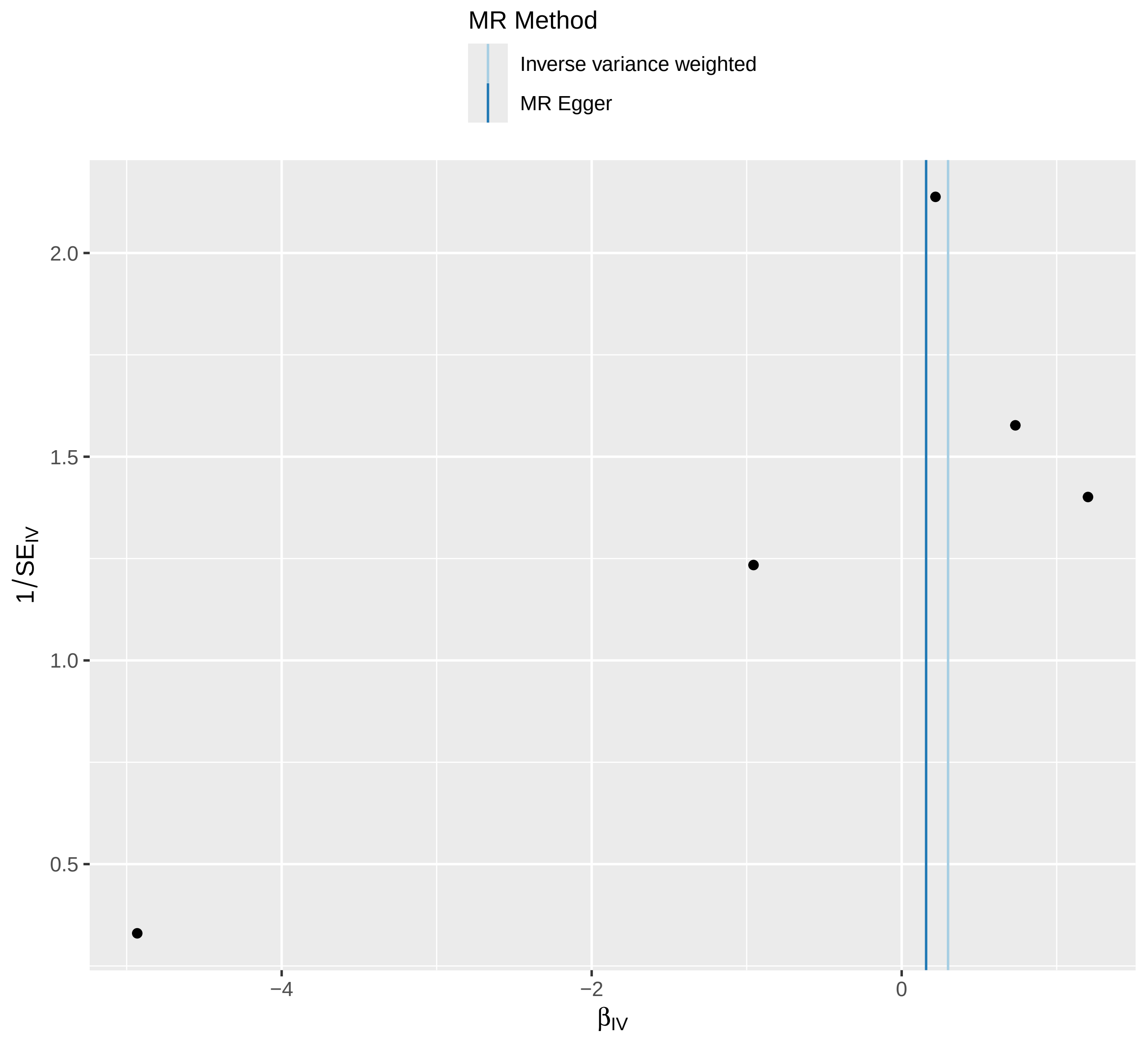
MR Method
Inverse variance weighted
MR Egger
2.0
1.5
1.0
0.5
1/SE$_{IV}$
−4
−2
0
β$_{IV}$

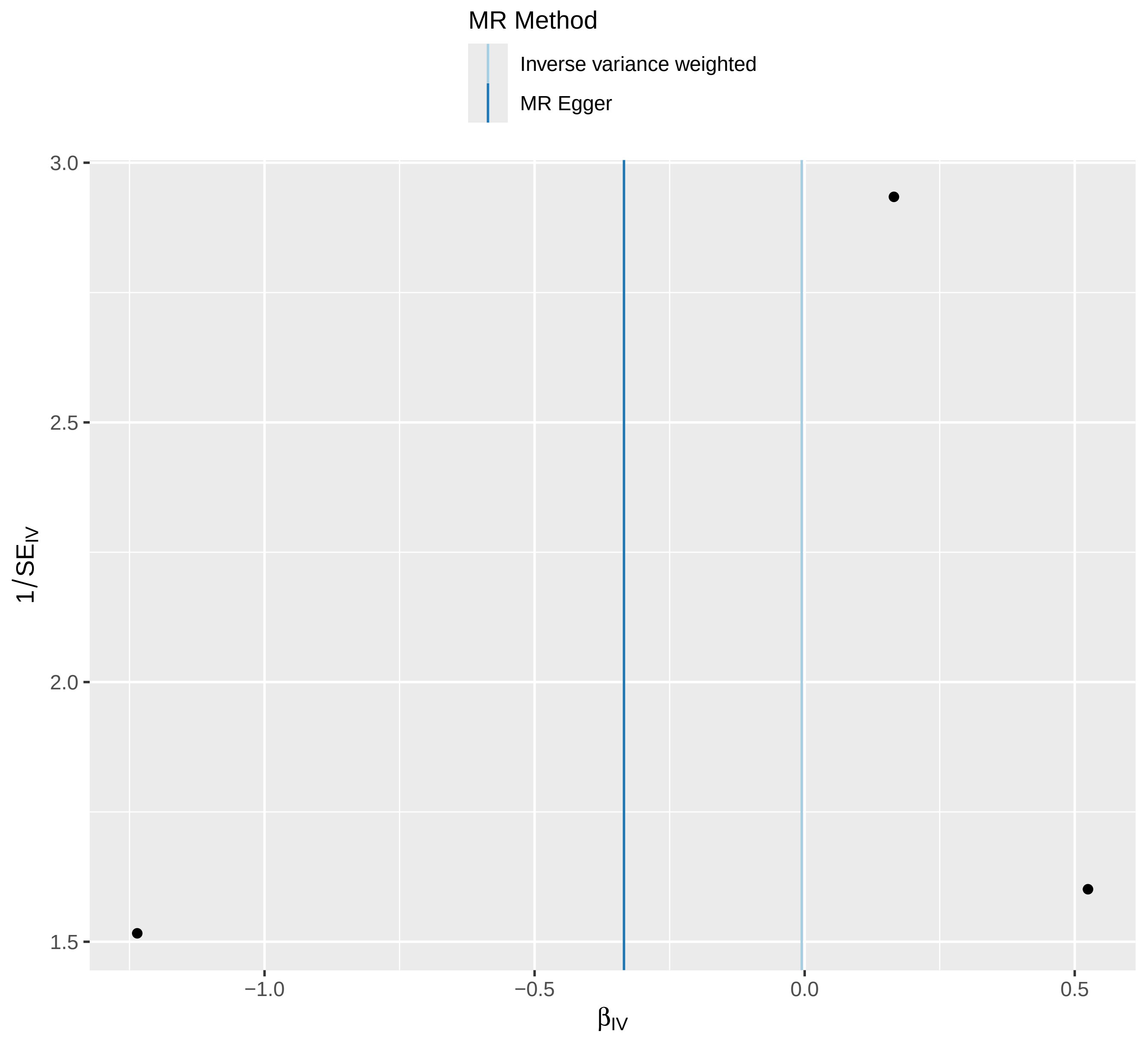
MR Method
Inverse variance weighted
MR Egger
3.0
2.5
2.0
1.5
1/SE_IV
−1.0
−0.5
0.0
0.5
β_IV

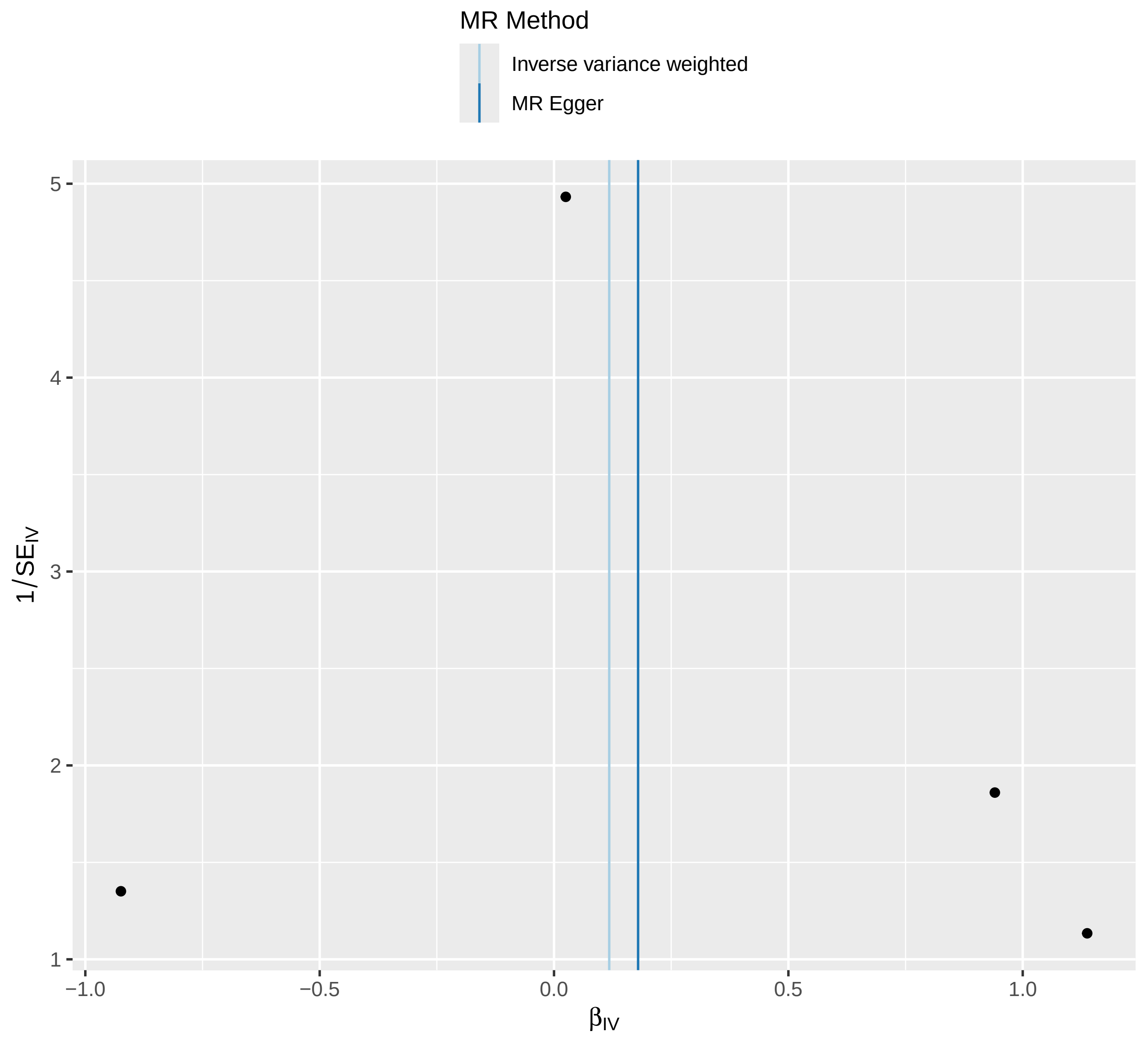
MR Method
Inverse variance weighted
MR Egger
5
4
3
2
1
1/SE$_{IV}$
−1.0
−0.5
0.0
0.5
1.0
β$_{IV}$

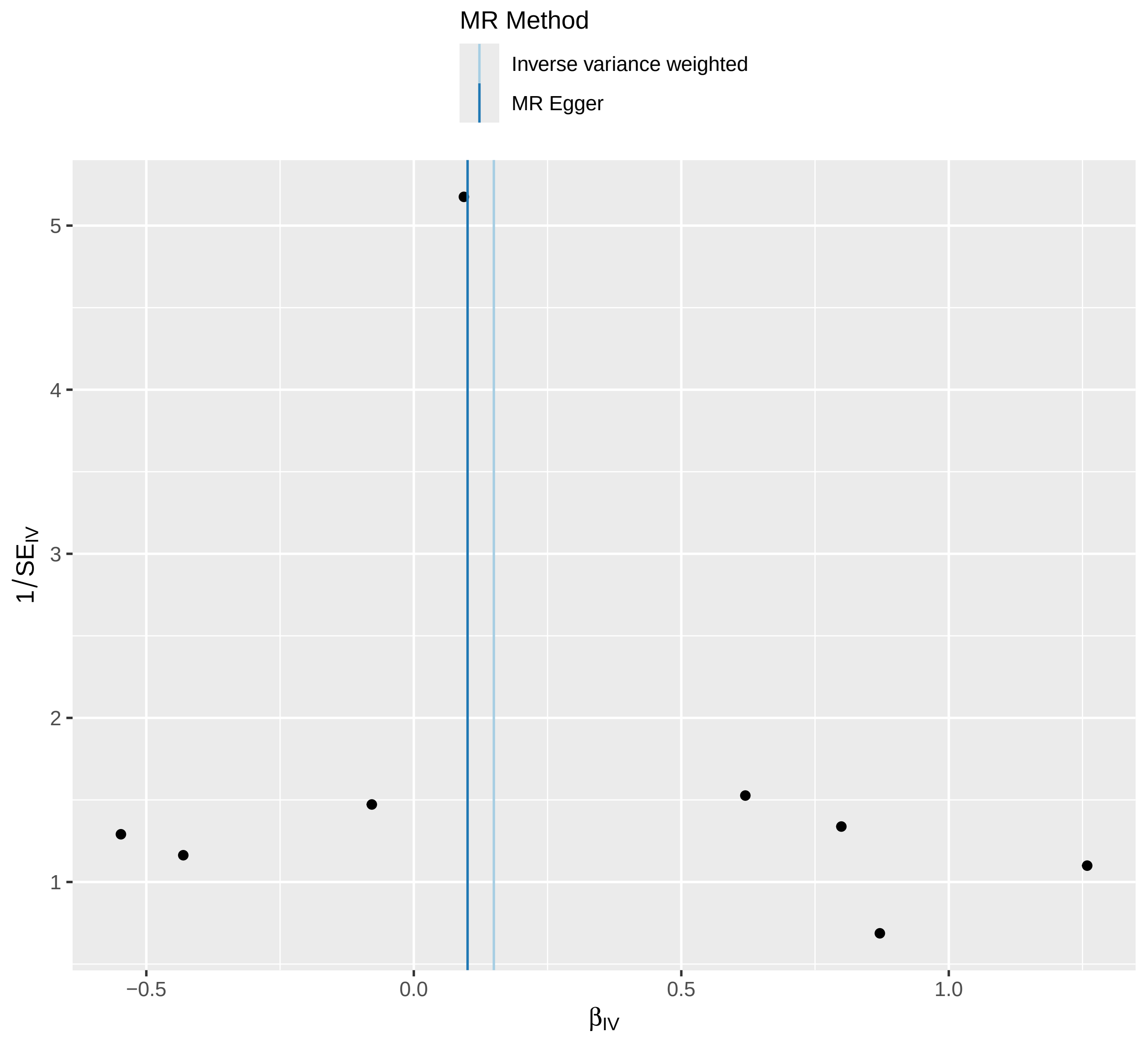

MR Method
Inverse variance weighted
MR Egger
1/SE_IV
5
4
3
2
1
−0.5
0.0
0.5
1.0
β_IV

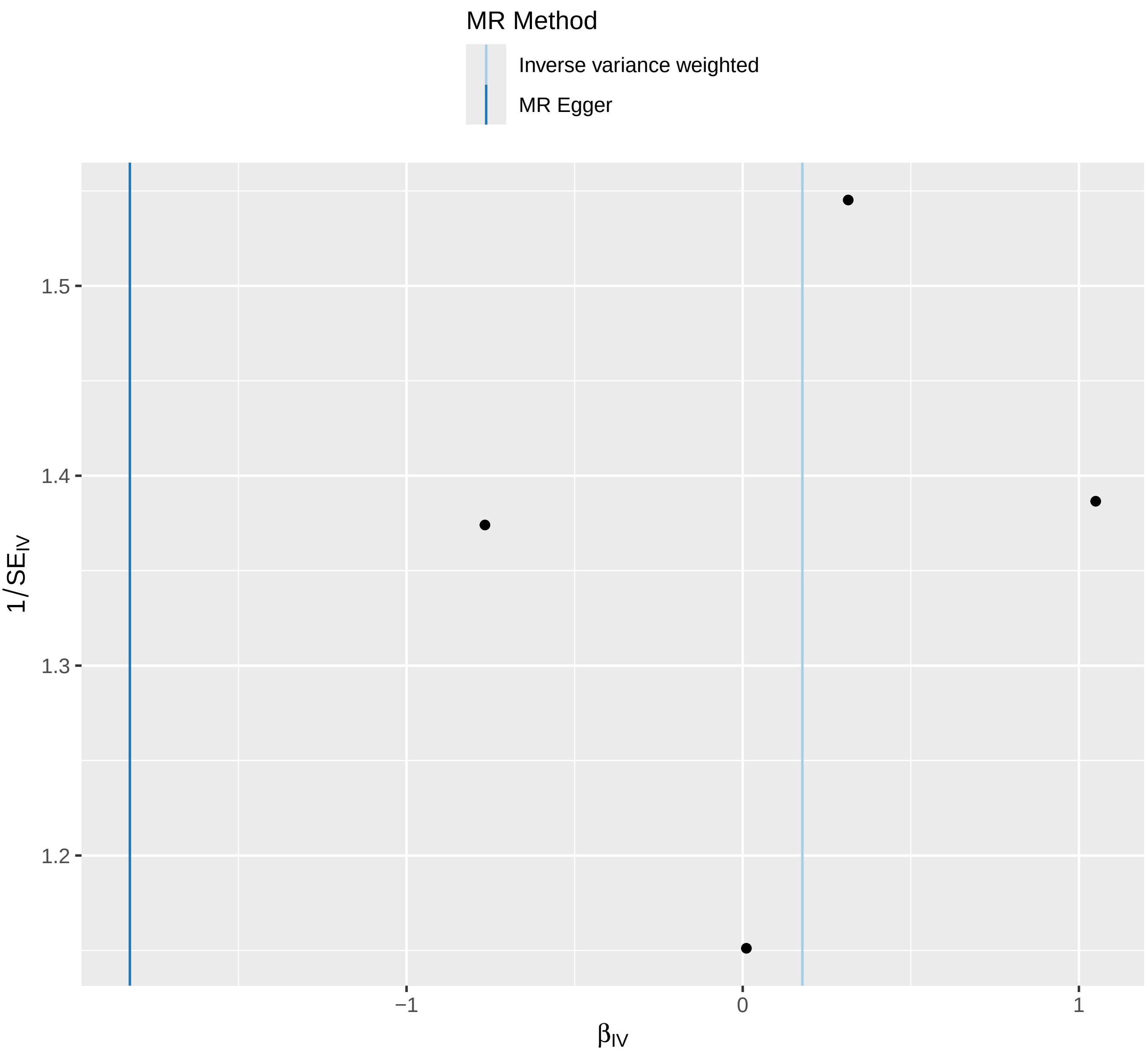

MR Method
Inverse variance weighted
MR Egger
1.5
1.4
1.3
1.2
$1/SE_{IV}$
−1
0
1
β_{IV}

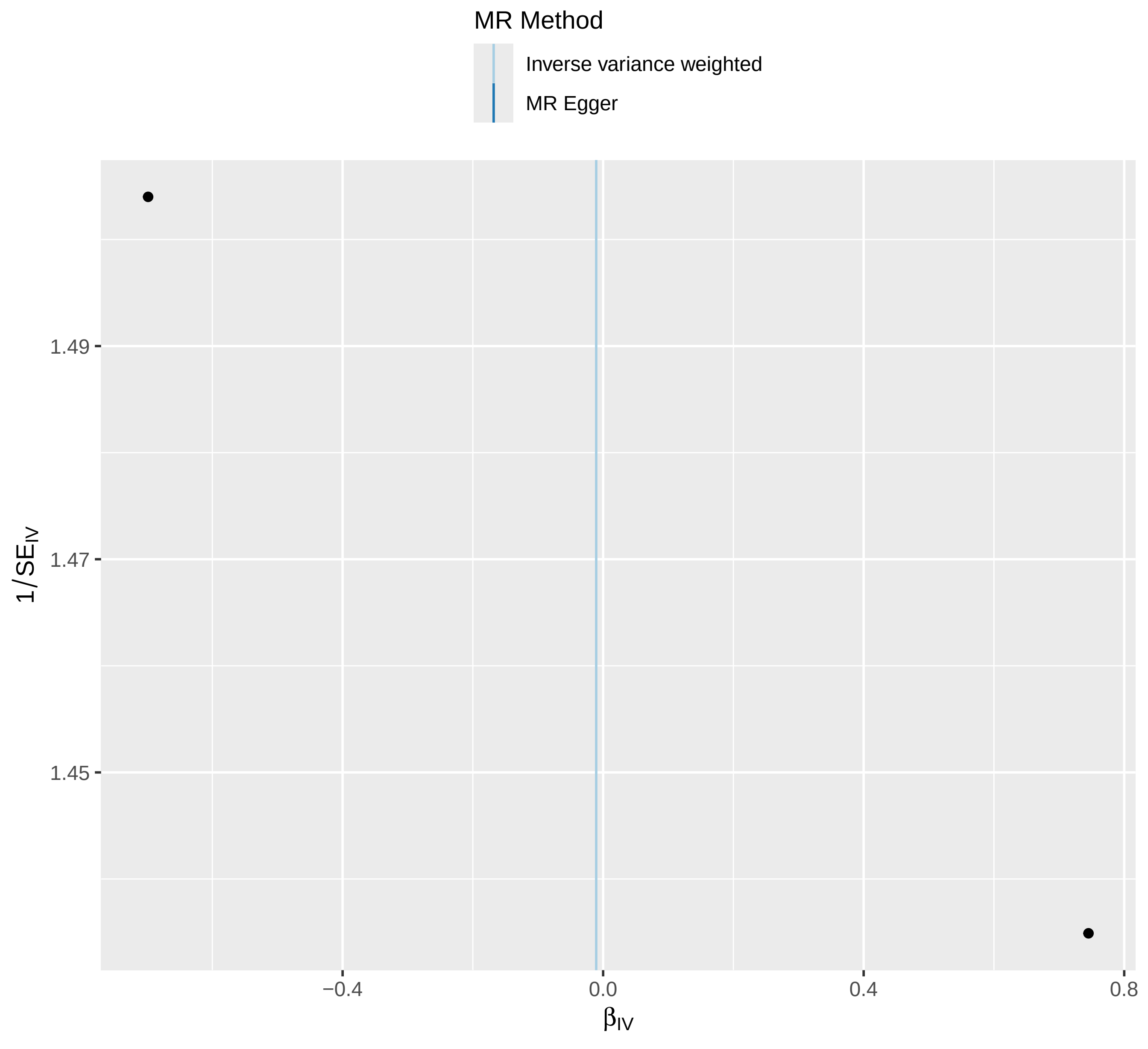
MR Method
Inverse variance weighted
MR Egger
1.49
1.47
1.45
1/SE_IV
−0.4
0.0
0.4
0.8
β_IV

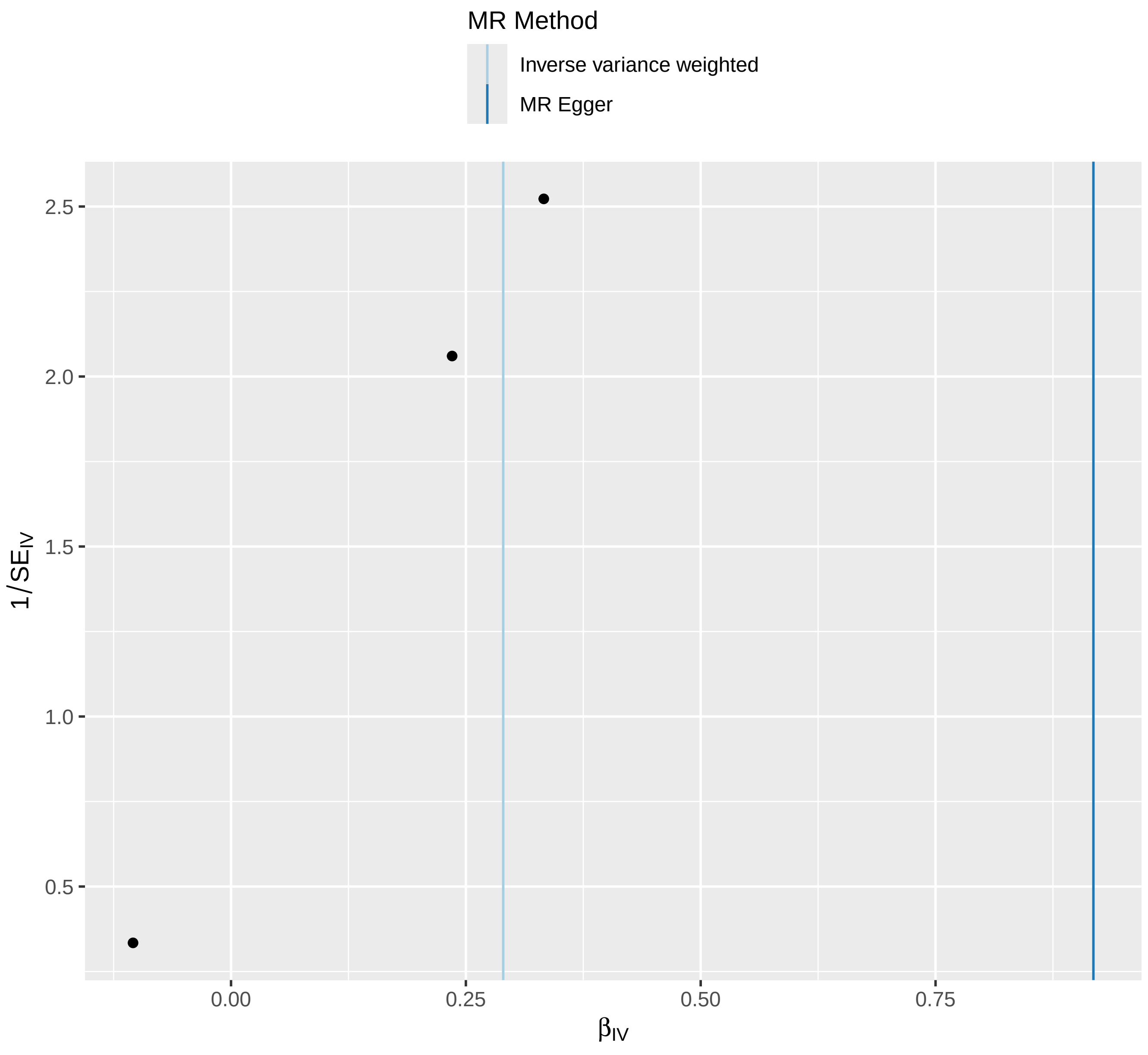

MR Method
Inverse variance weighted
MR Egger
2.5
2.0
1.5
1.0
0.5
$1/SE_{IV}$
0.00
0.25
0.50
0.75
β_{IV}

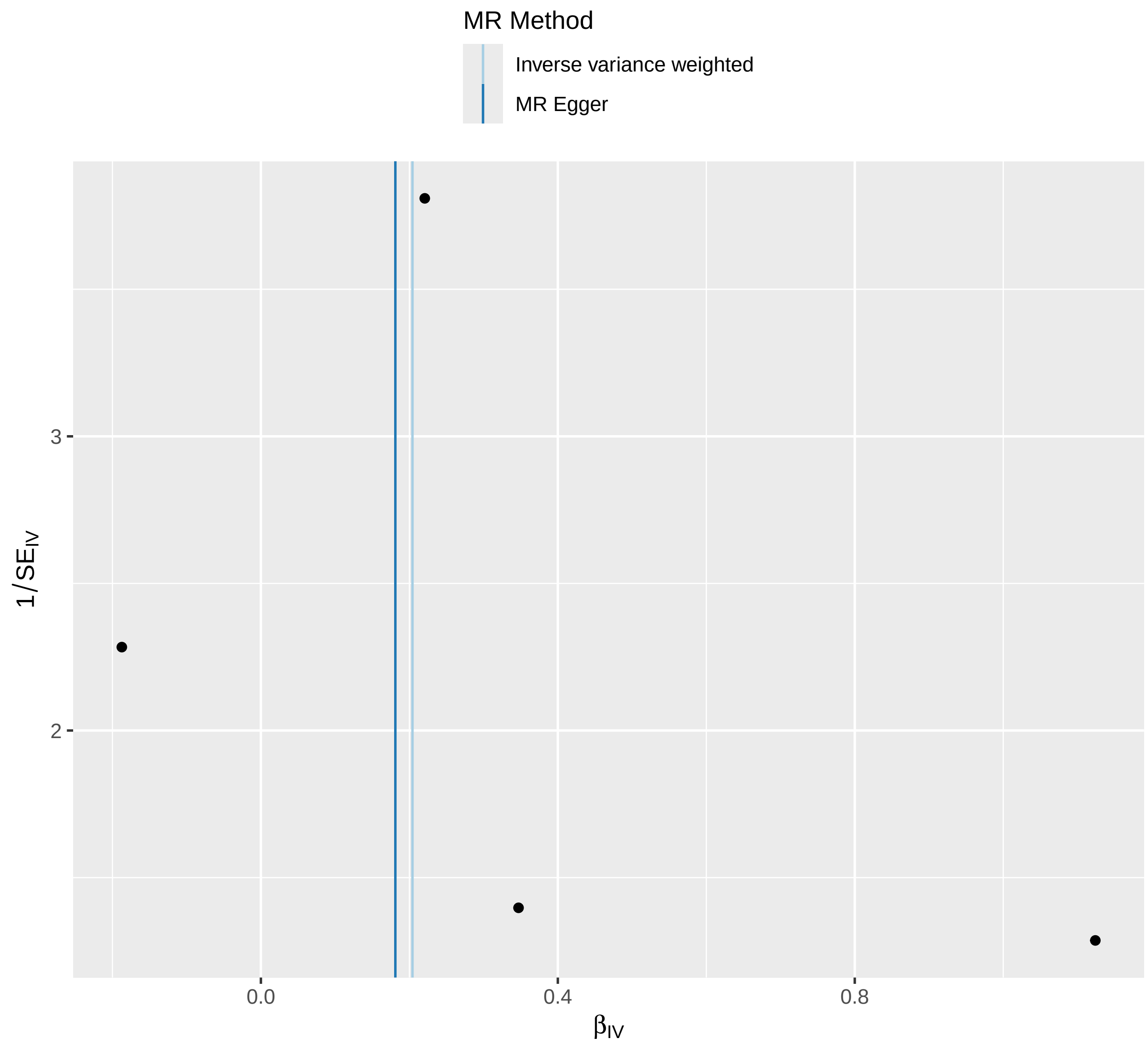

MR Method
Inverse variance weighted
MR Egger
1/SE_IV
3
2
0.0
0.4
0.8
β_IV

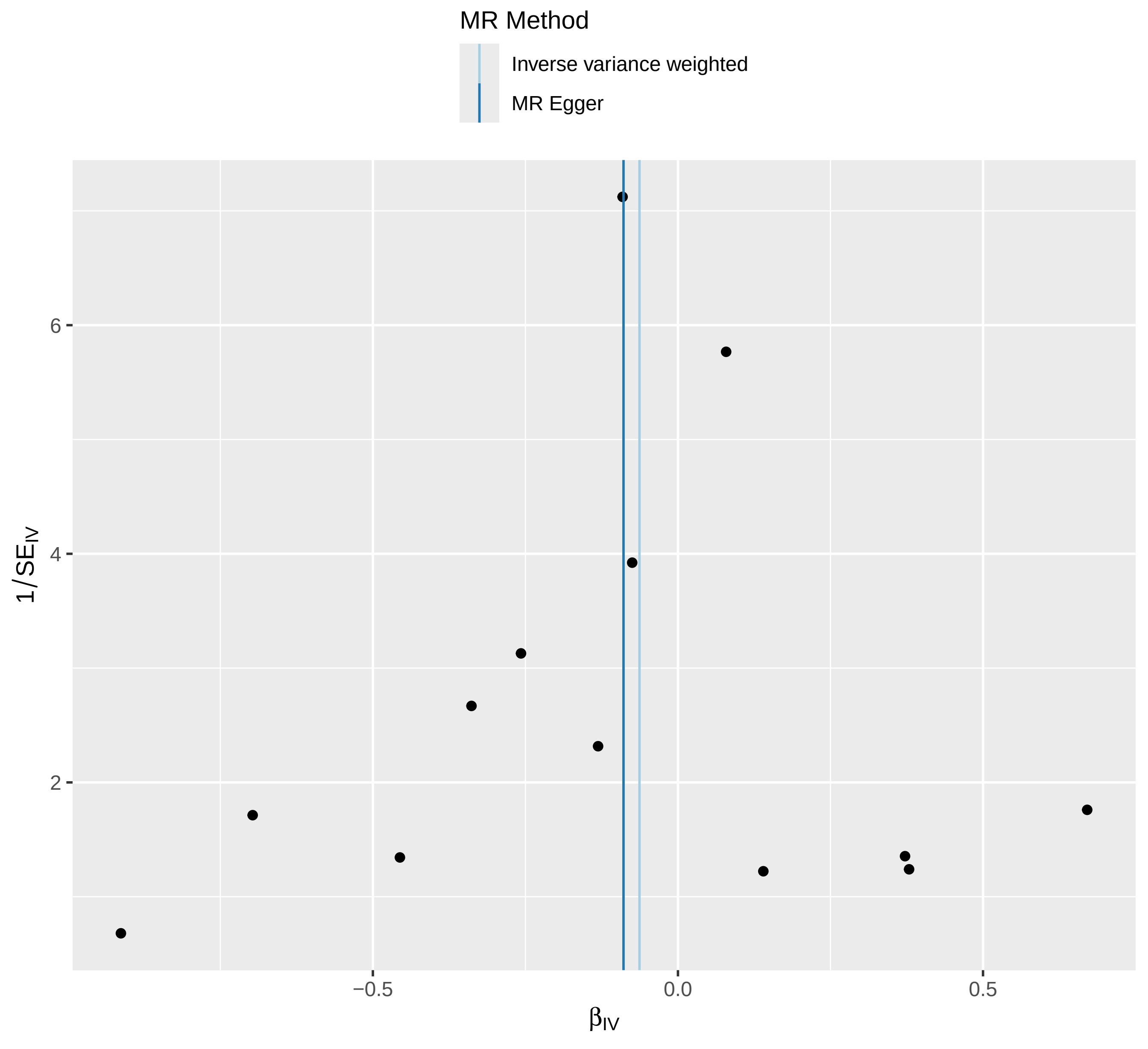
MR Method
Inverse variance weighted
MR Egger
6
4
2
1/SE$_{IV}$
−0.5
0.0
0.5
β$_{IV}$

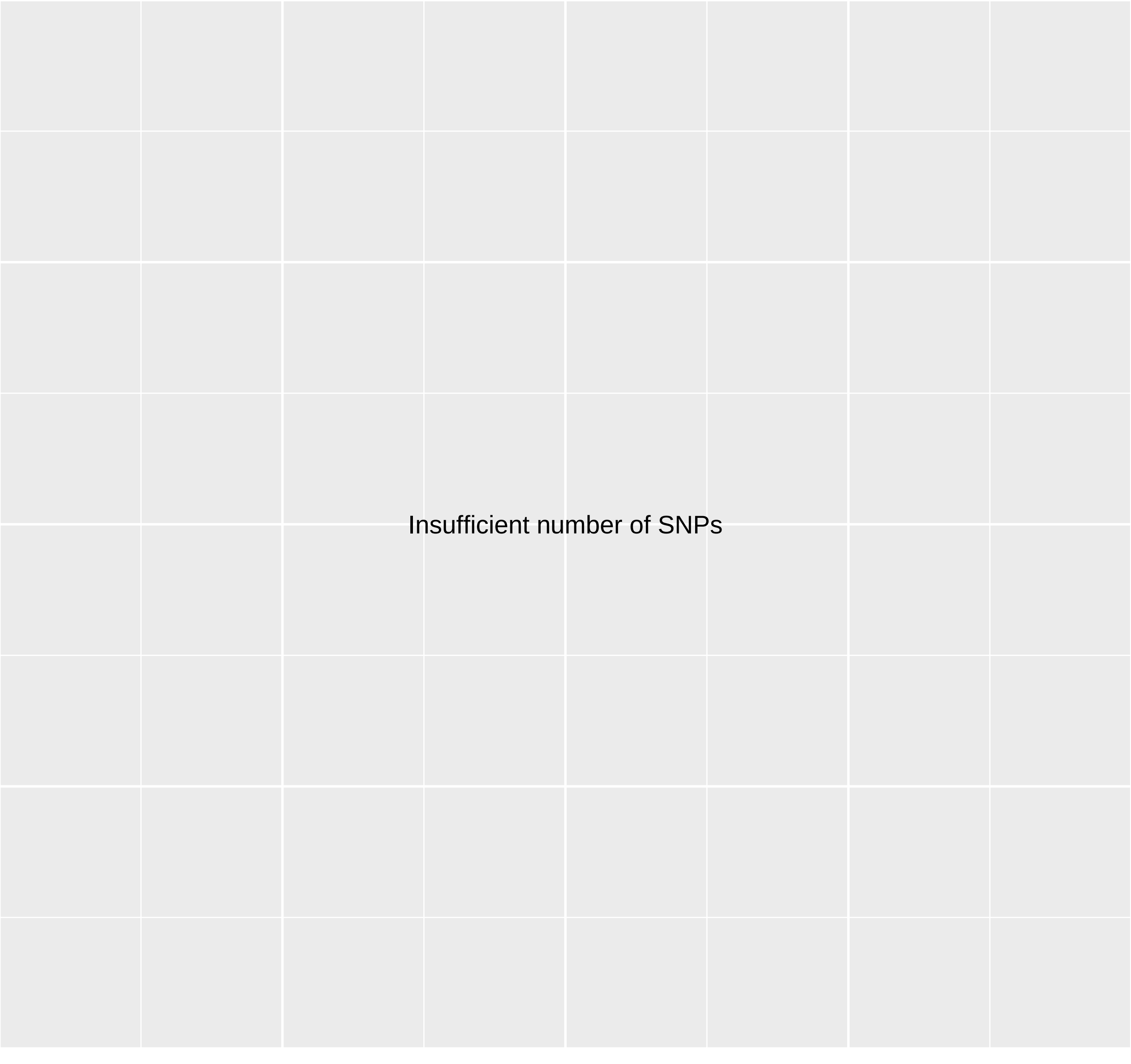
Insufficient number of SNPs

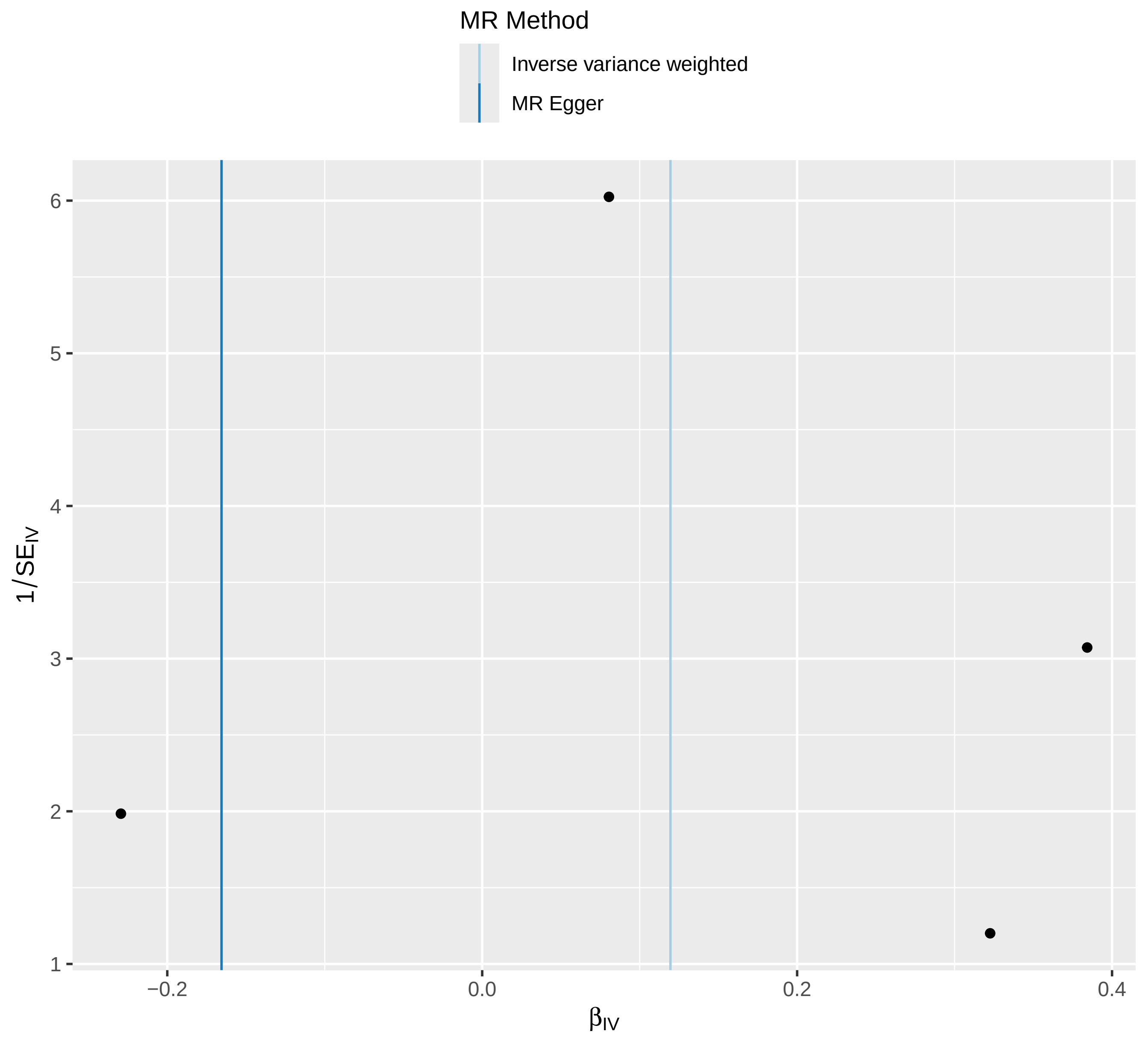
MR Method
Inverse variance weighted
MR Egger
$1/SE_{IV}$
β_{IV}
6
5
4
3
2
1
−0.2
0.0
0.2
0.4

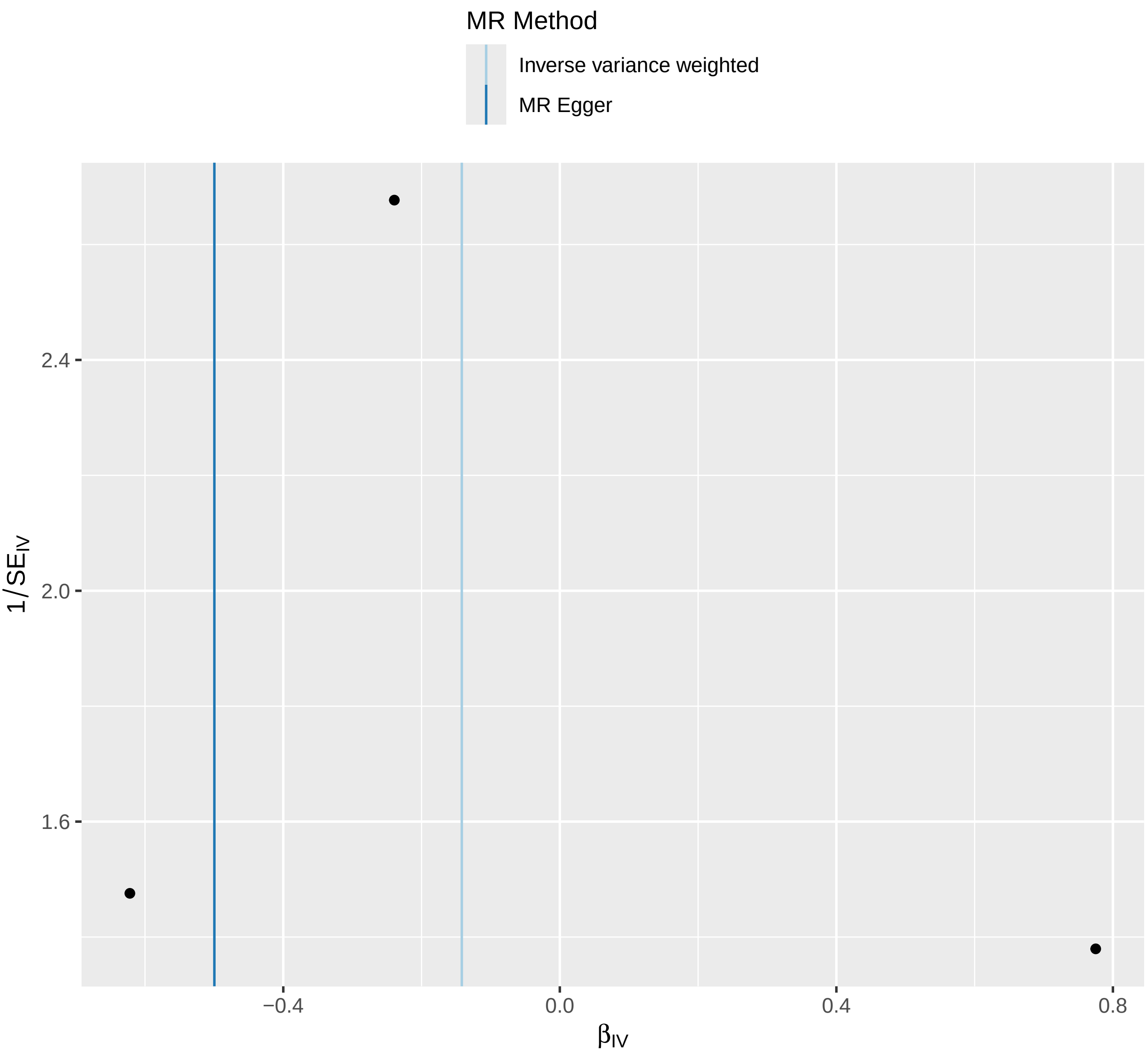
MR Method
Inverse variance weighted
MR Egger
2.4
2.0
1.6
1/SE_IV
−0.4
0.0
0.4
0.8
β_IV

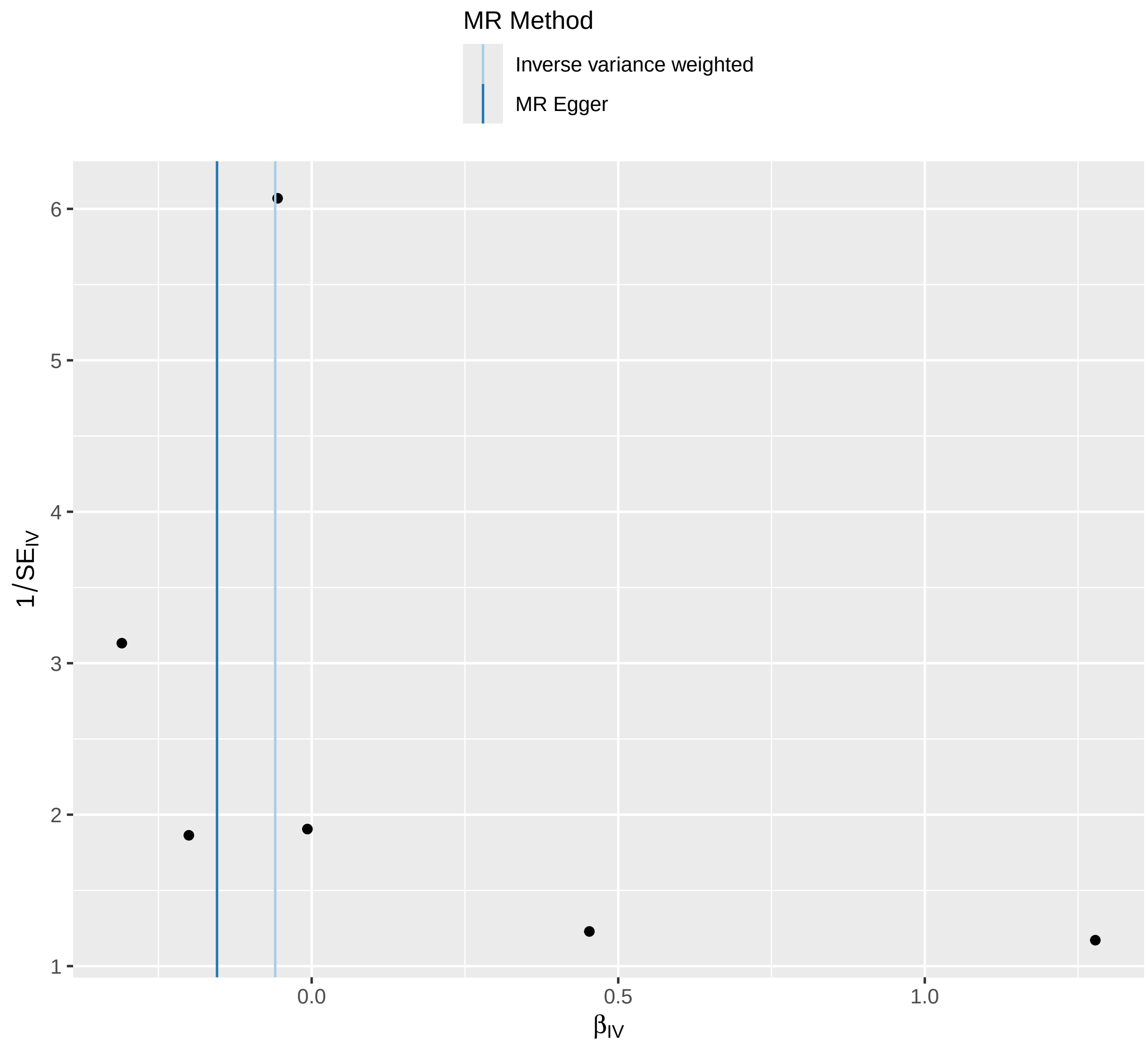
MR Method
Inverse variance weighted
MR Egger
1/SE_IV
β_IV
6
5
4
3
2
1
0.0
0.5
1.0

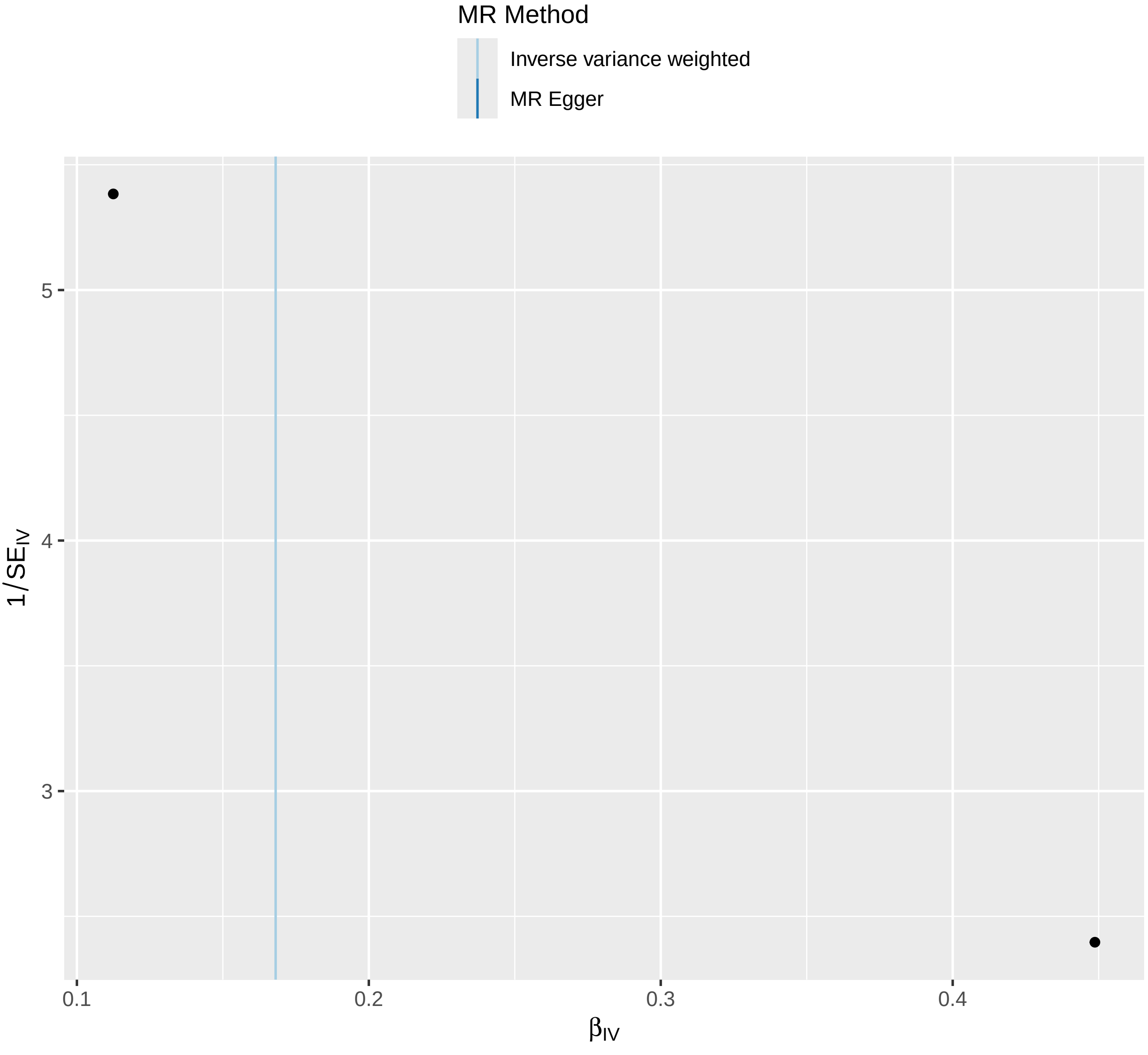

MR Method
Inverse variance weighted
MR Egger
5
4
3
$1/SE_{IV}$
0.1
0.2
0.3
0.4
β_{IV}

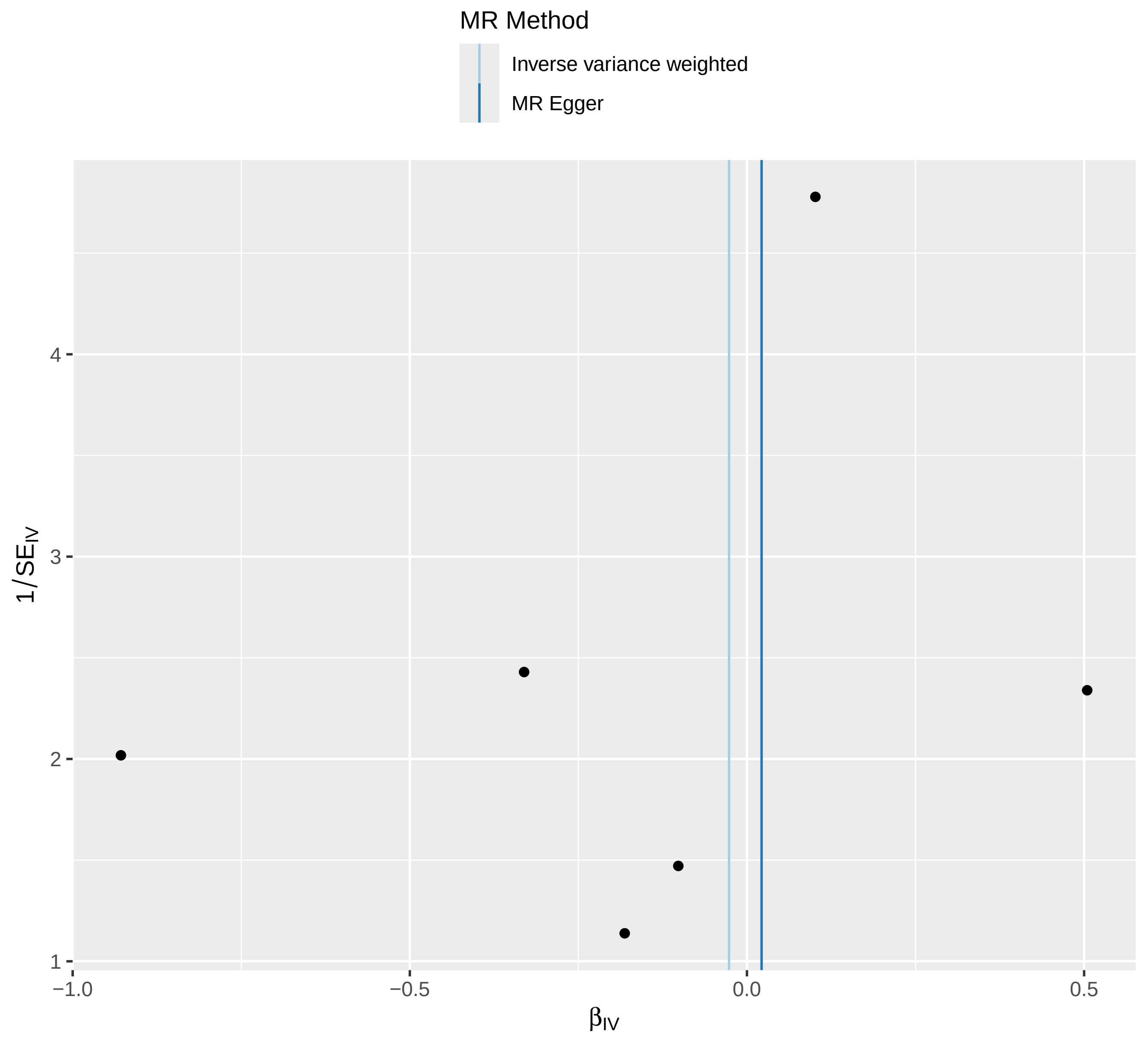

MR Method
Inverse variance weighted
MR Egger
1/SE$_{IV}$
β$_{IV}$
4
3
2
1
−1.0
−0.5
0.0
0.5

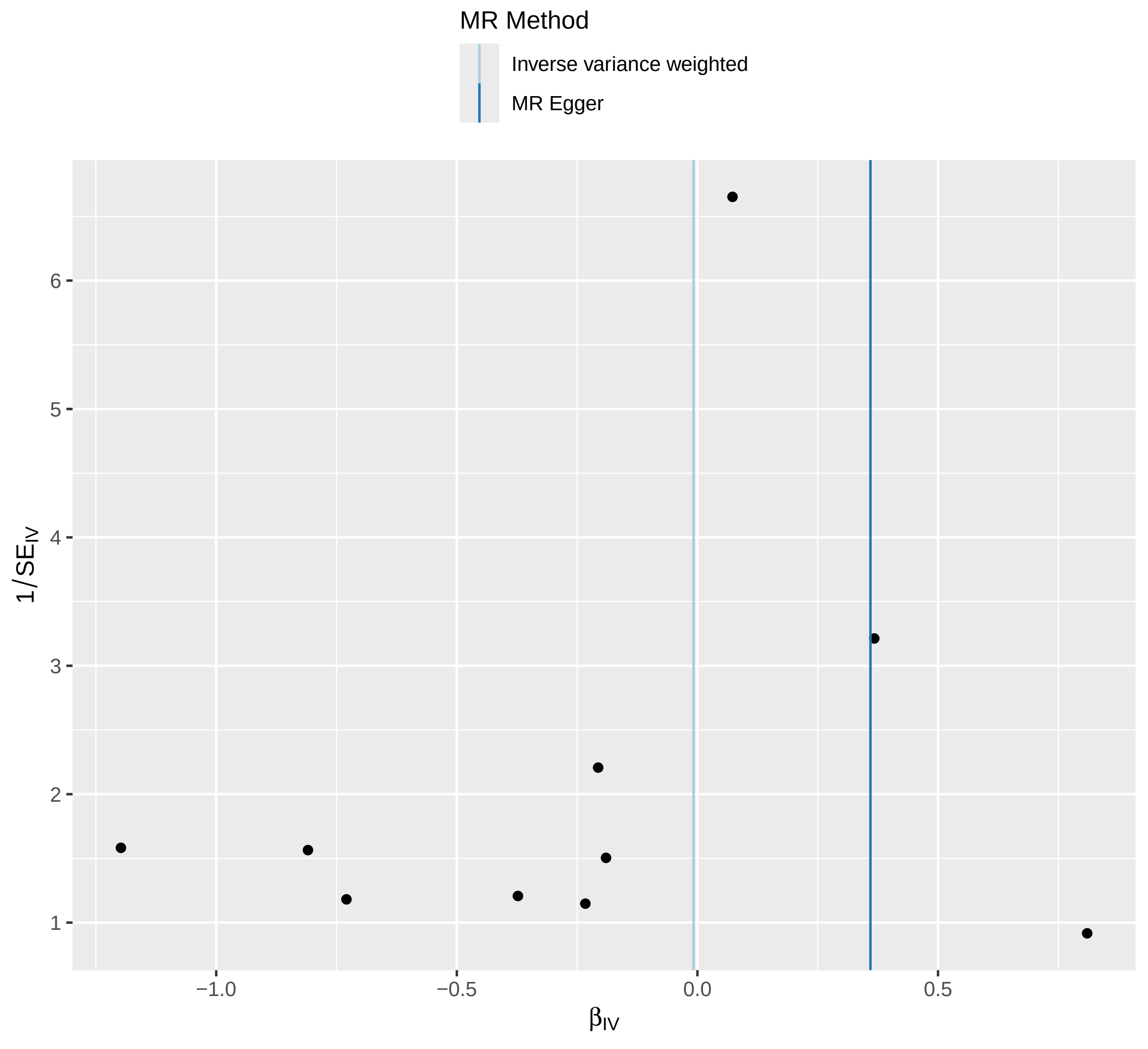

MR Method
Inverse variance weighted
MR Egger
1/SE_IV
6
5
4
3
2
1
−1.0
−0.5
0.0
0.5
β_IV

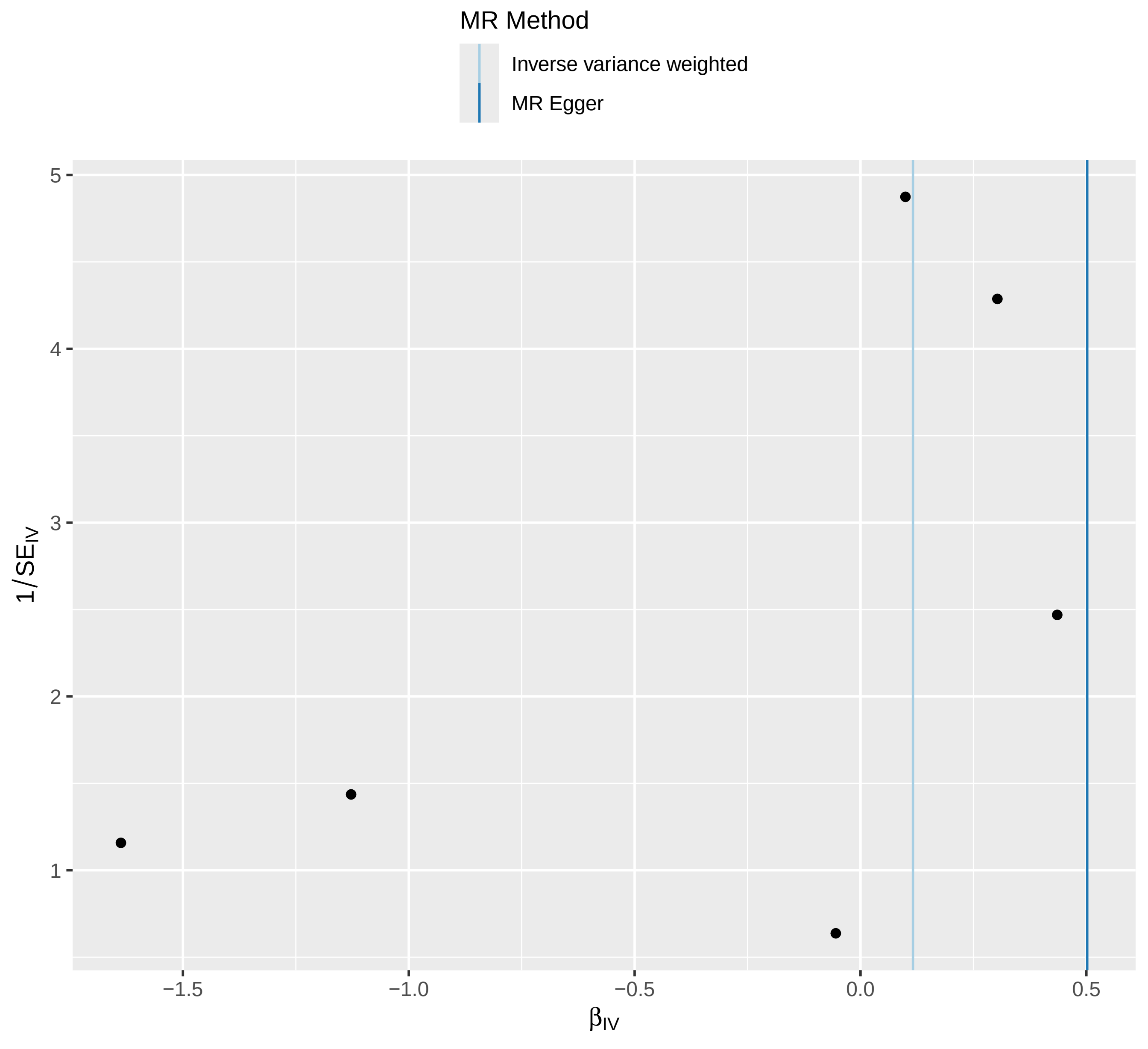
MR Method
Inverse variance weighted
MR Egger
5
4
3
2
1
$1/SE_{IV}$
−1.5
−1.0
−0.5
0.0
0.5
β_{IV}

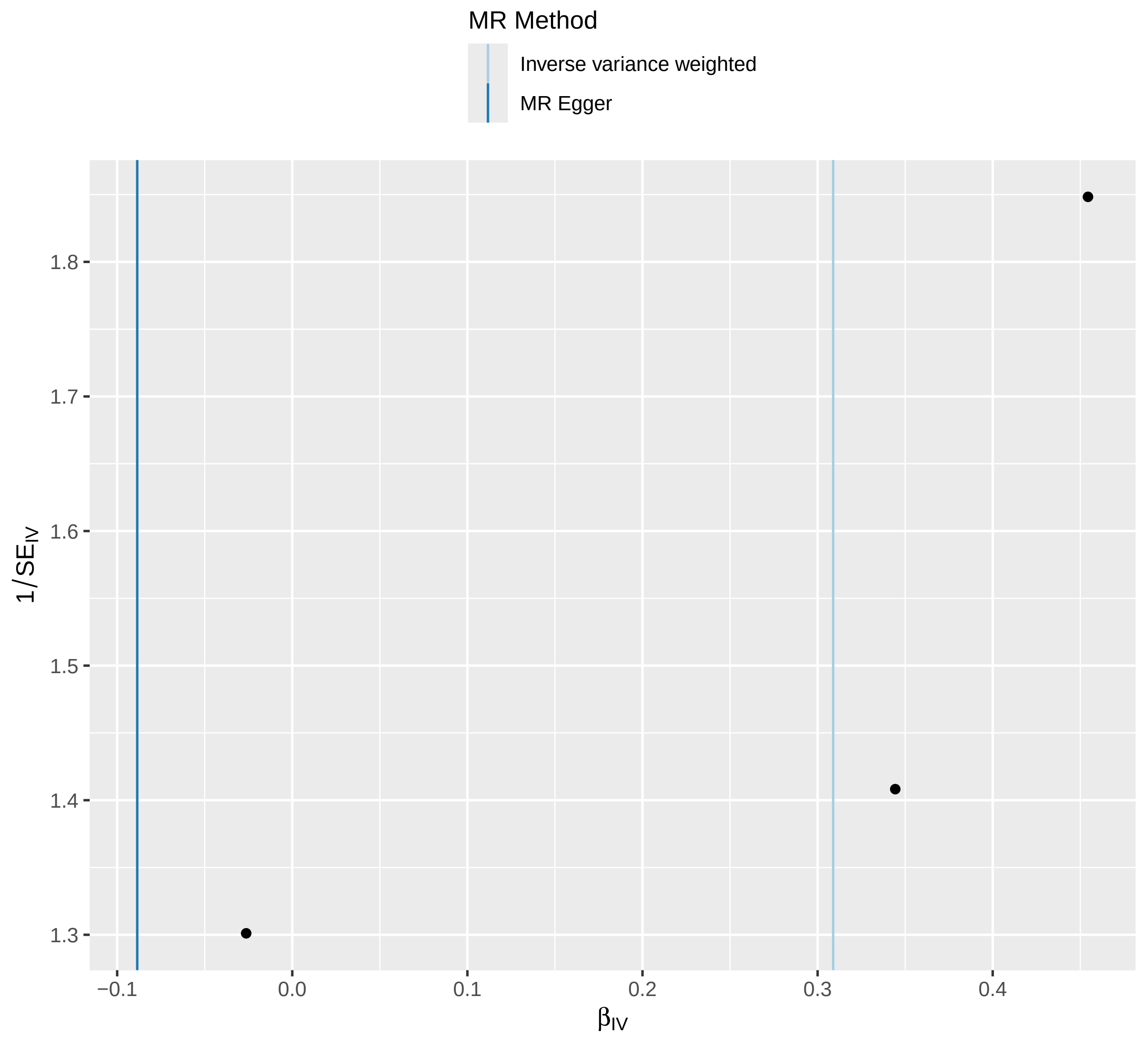
MR Method
Inverse variance weighted
MR Egger
1.8
1.7
1.6
1.5
1.4
1.3
1/SE_IV
−0.1
0.0
0.1
0.2
0.3
0.4
β_IV

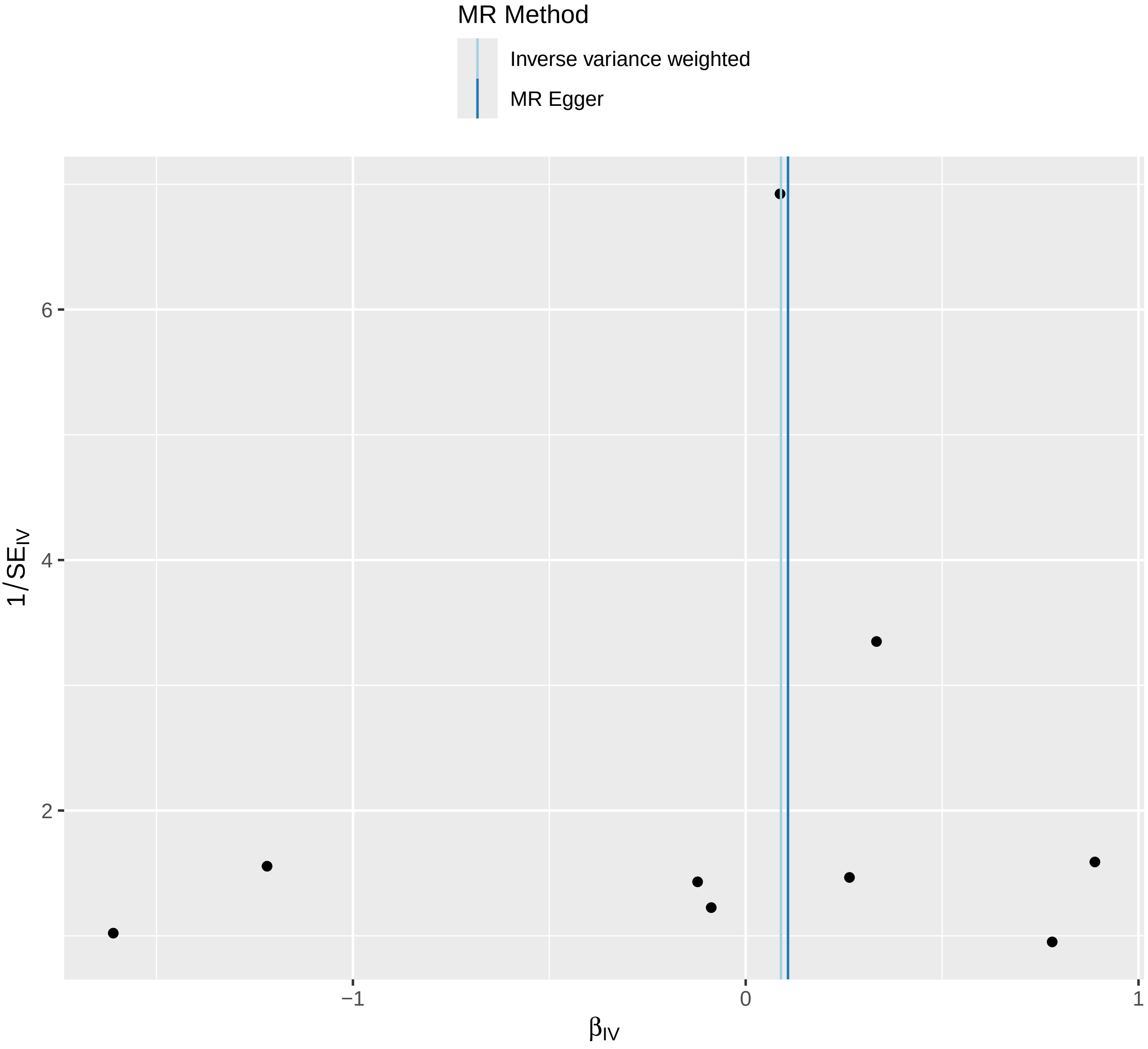

MR Method
Inverse variance weighted
MR Egger
6
4
2
1/SE_IV
−1
0
1
β_IV

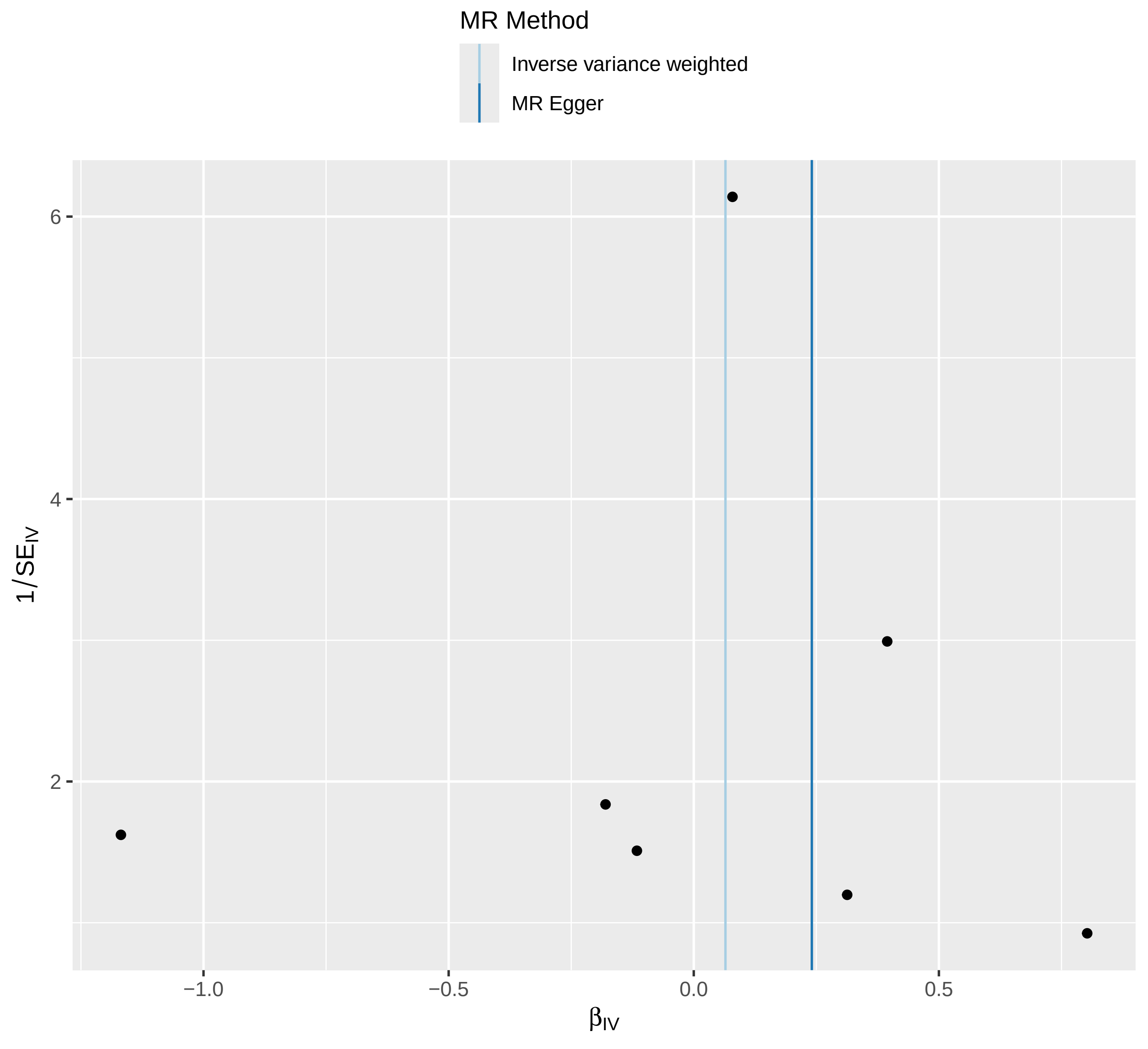

MR Method
Inverse variance weighted
MR Egger
6
4
2
1/SE$_{IV}$
−1.0
−0.5
0.0
0.5
β$_{IV}$

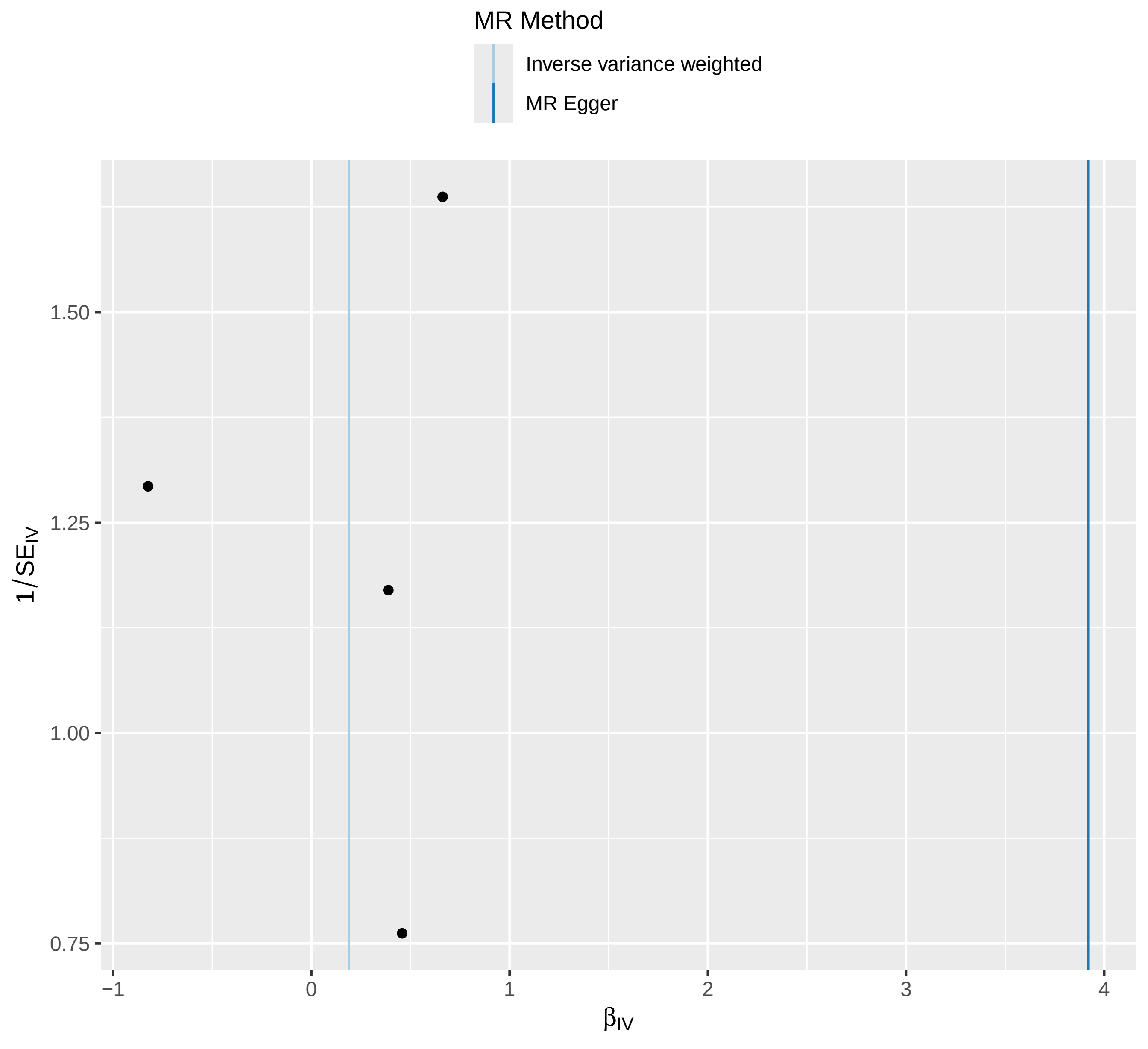
MR Method
Inverse variance weighted
MR Egger
1.50
1.25
1.00
0.75
$1/SE_{IV}$
−1
0
1
2
3
4
β_{IV}

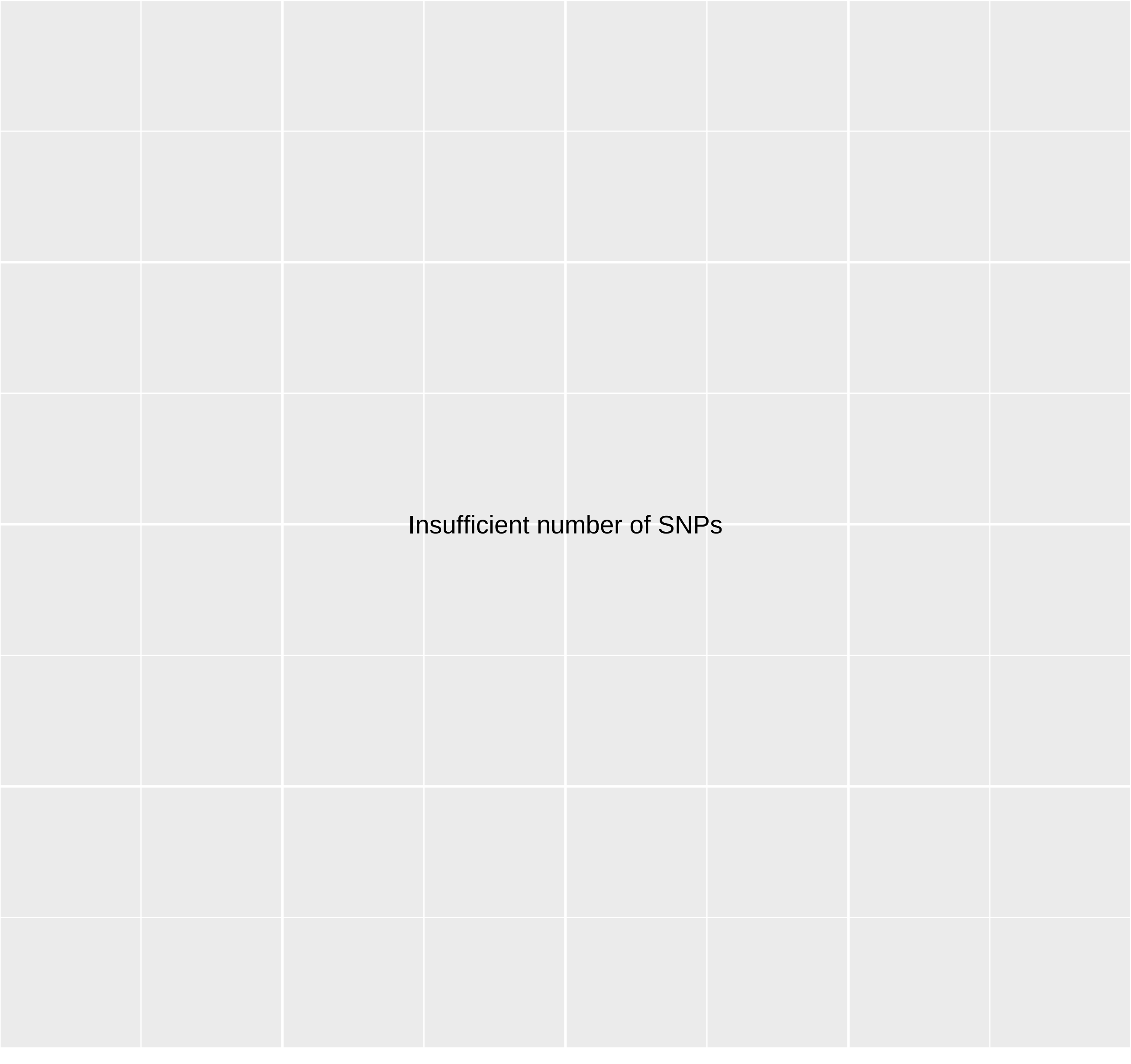
Insufficient number of SNPs

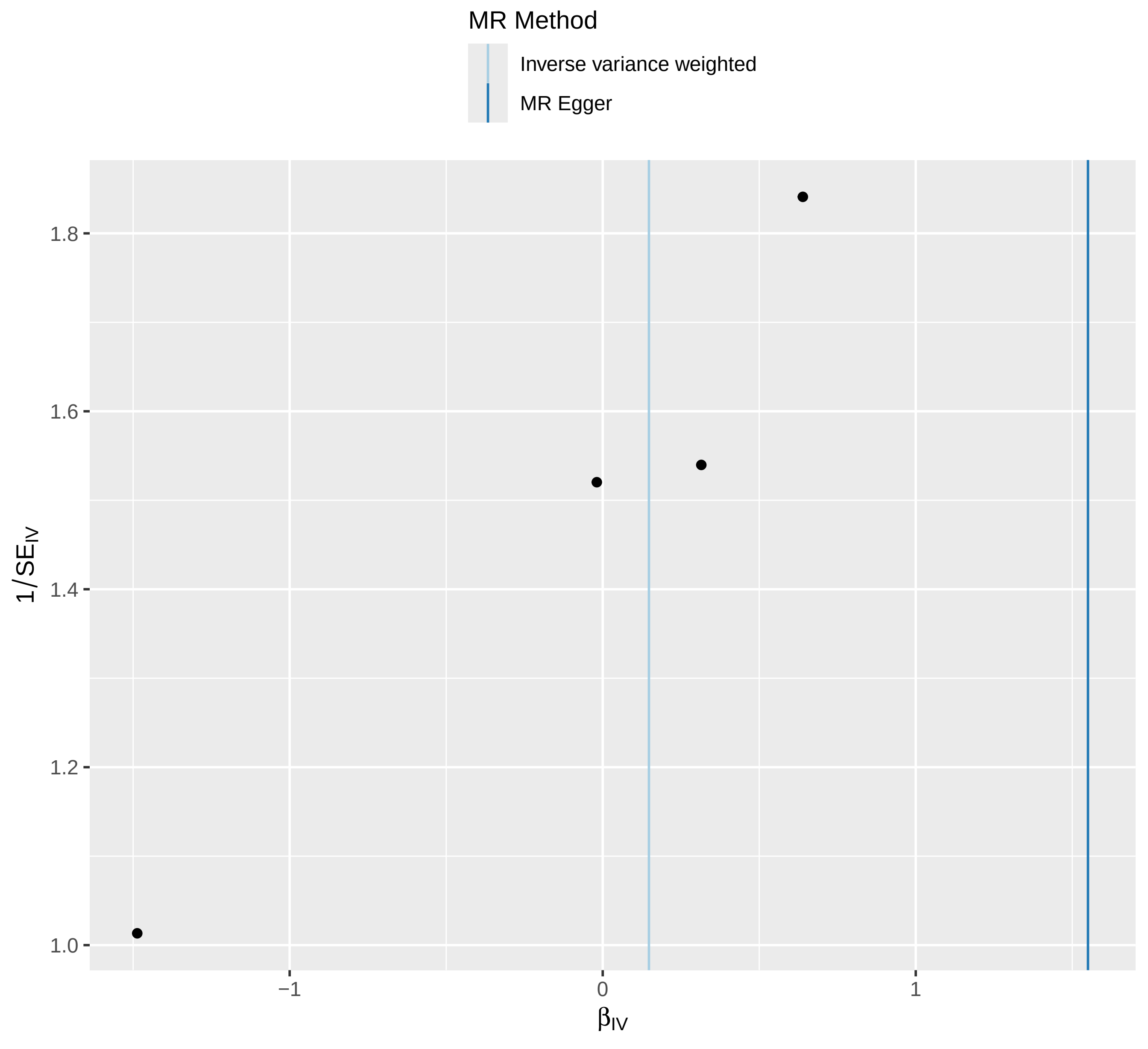

MR Method
Inverse variance weighted
MR Egger
1.8
1.6
1.4
1.2
1.0
1/SE_IV
−1
0
1
β_IV

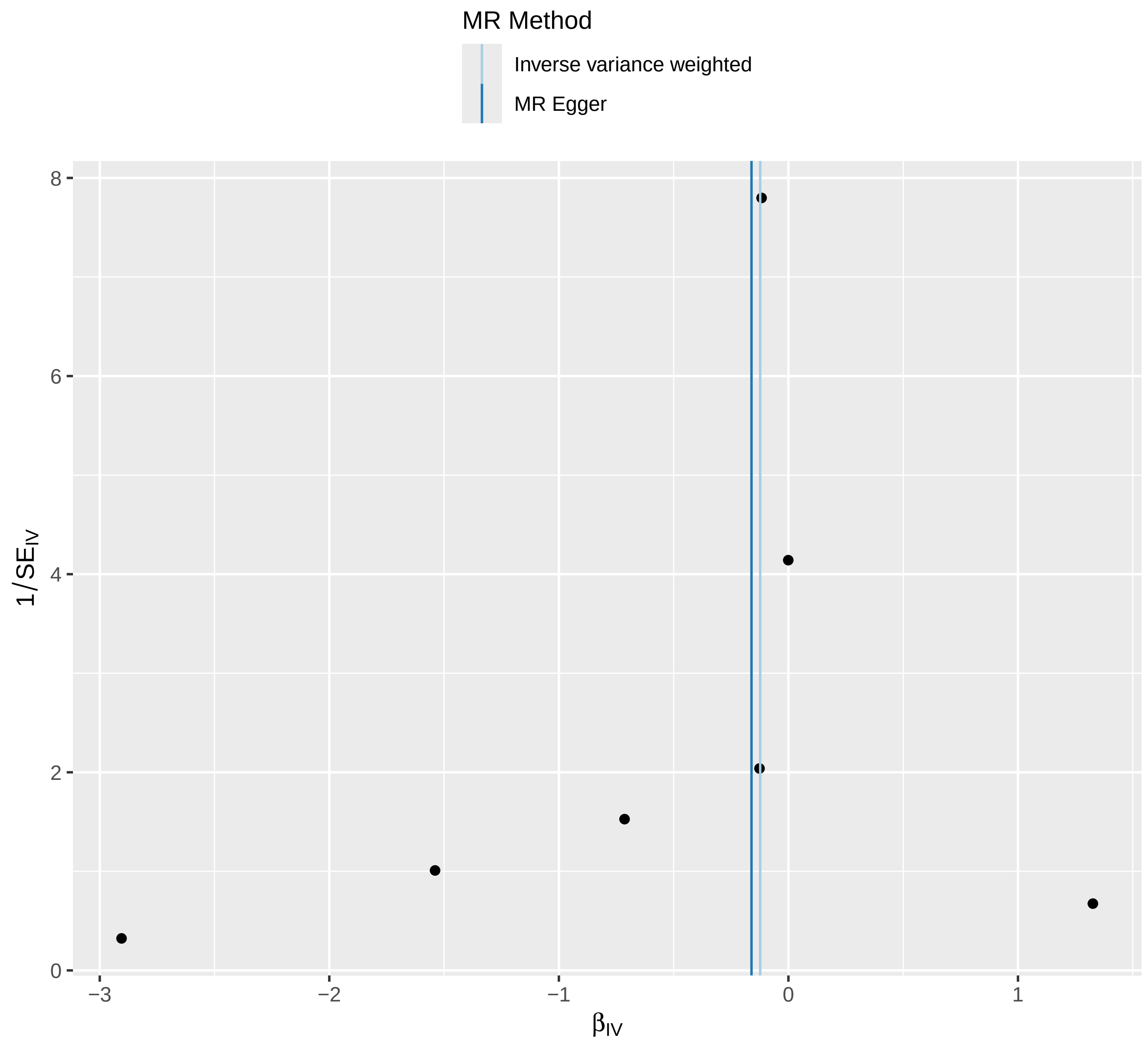

MR Method
Inverse variance weighted
MR Egger
1/SE_IV
β_IV
8
6
4
2
0
−3
−2
−1
0
1

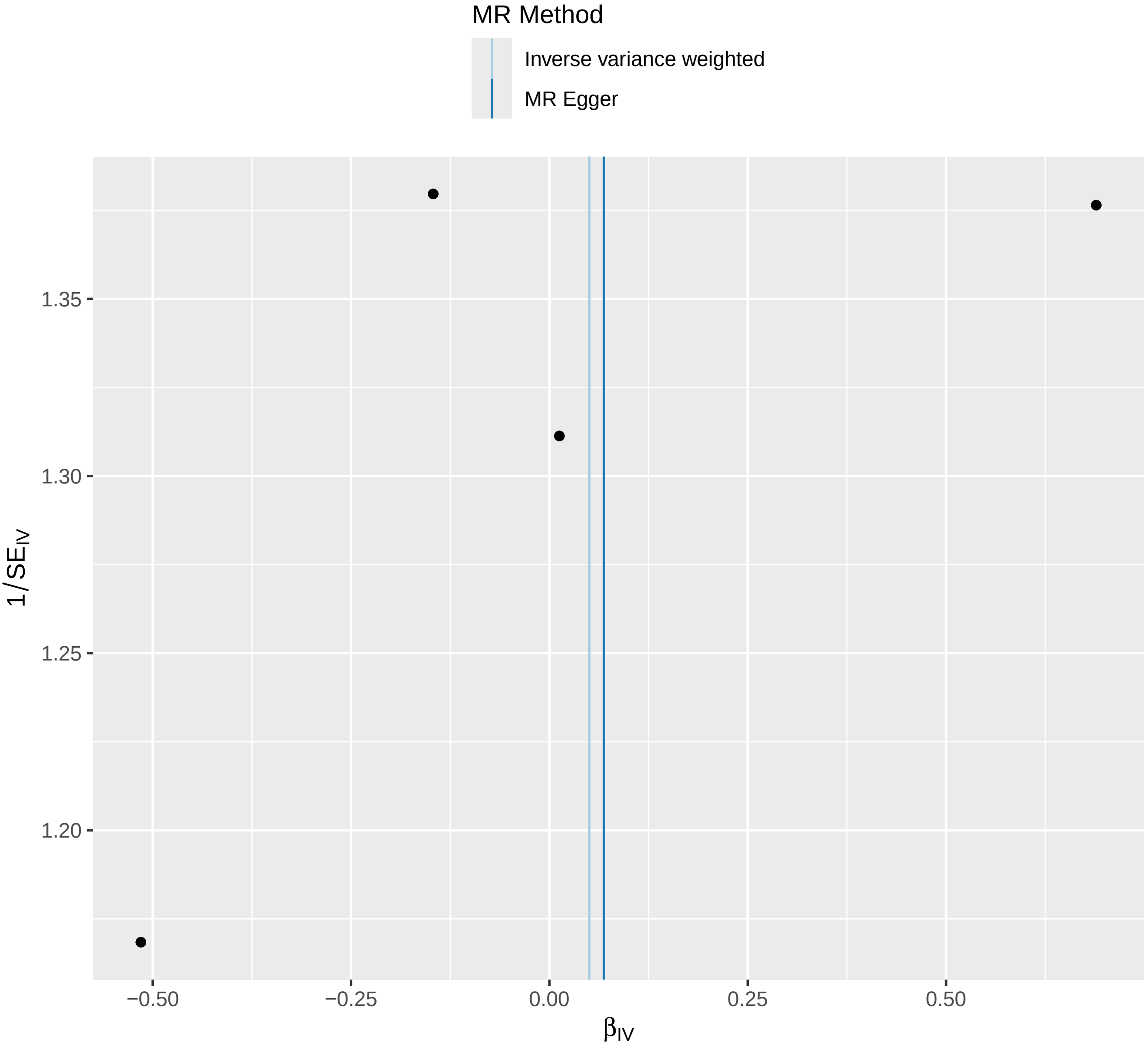

MR Method
Inverse variance weighted
MR Egger
1.35
1.30
1.25
1.20
1/SE_IV
−0.50
−0.25
0.00
0.25
0.50
β_IV

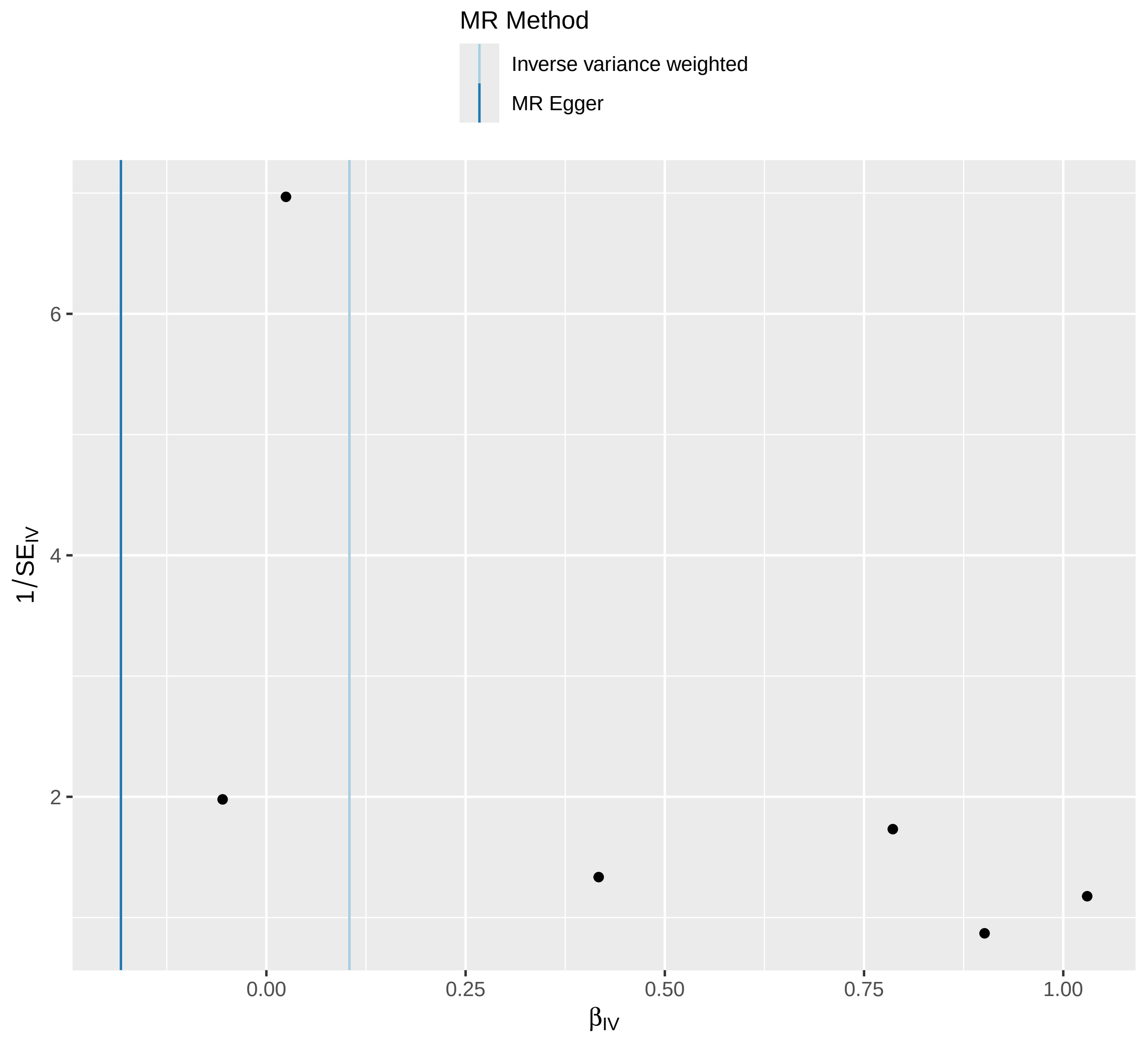
MR Method
Inverse variance weighted
MR Egger
6
4
2
1/SE_IV
0.00
0.25
0.50
0.75
1.00
β_IV

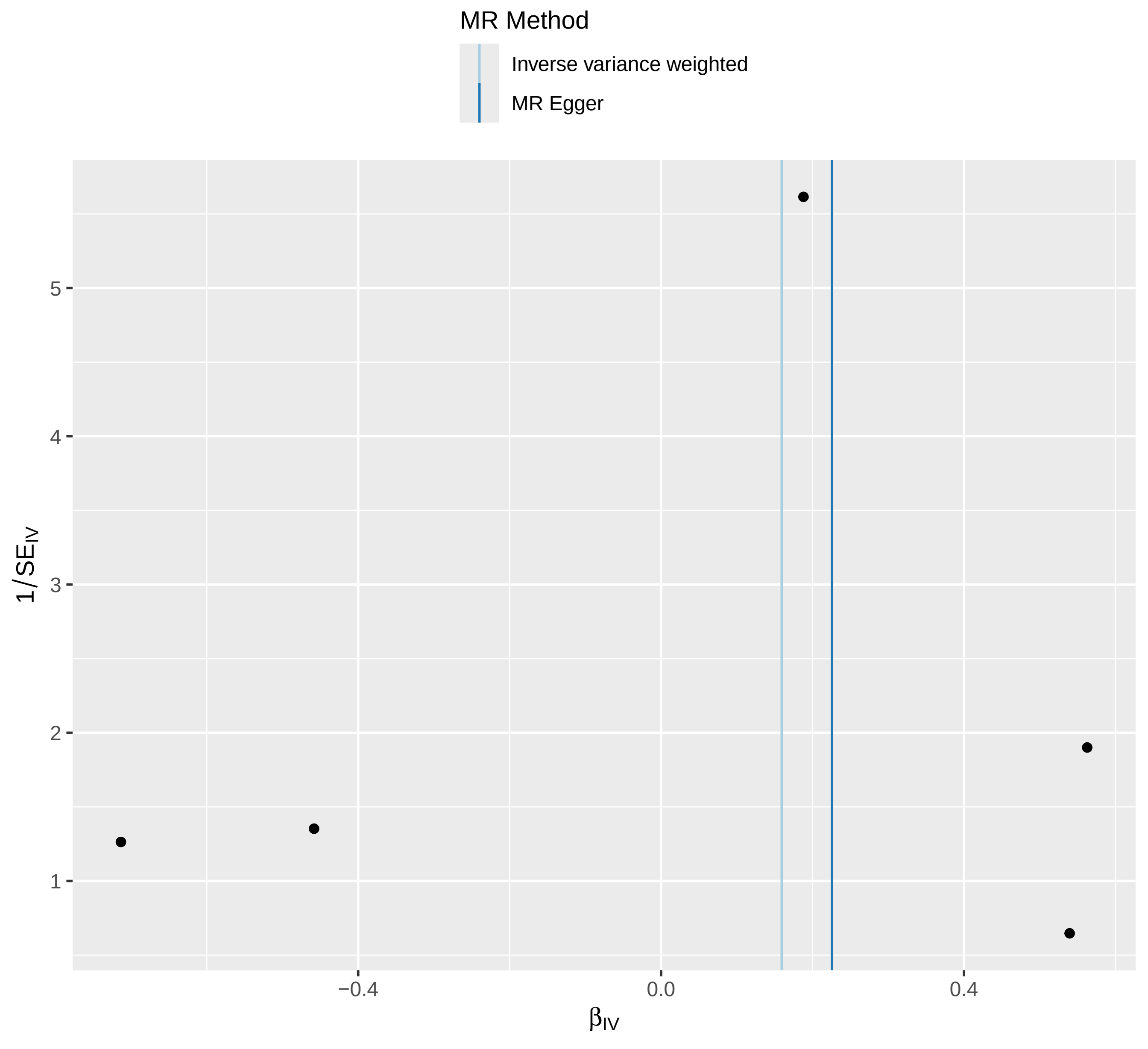

MR Method
Inverse variance weighted
MR Egger
5
4
3
2
1
1/SE_IV
−0.4
0.0
0.4
β_IV

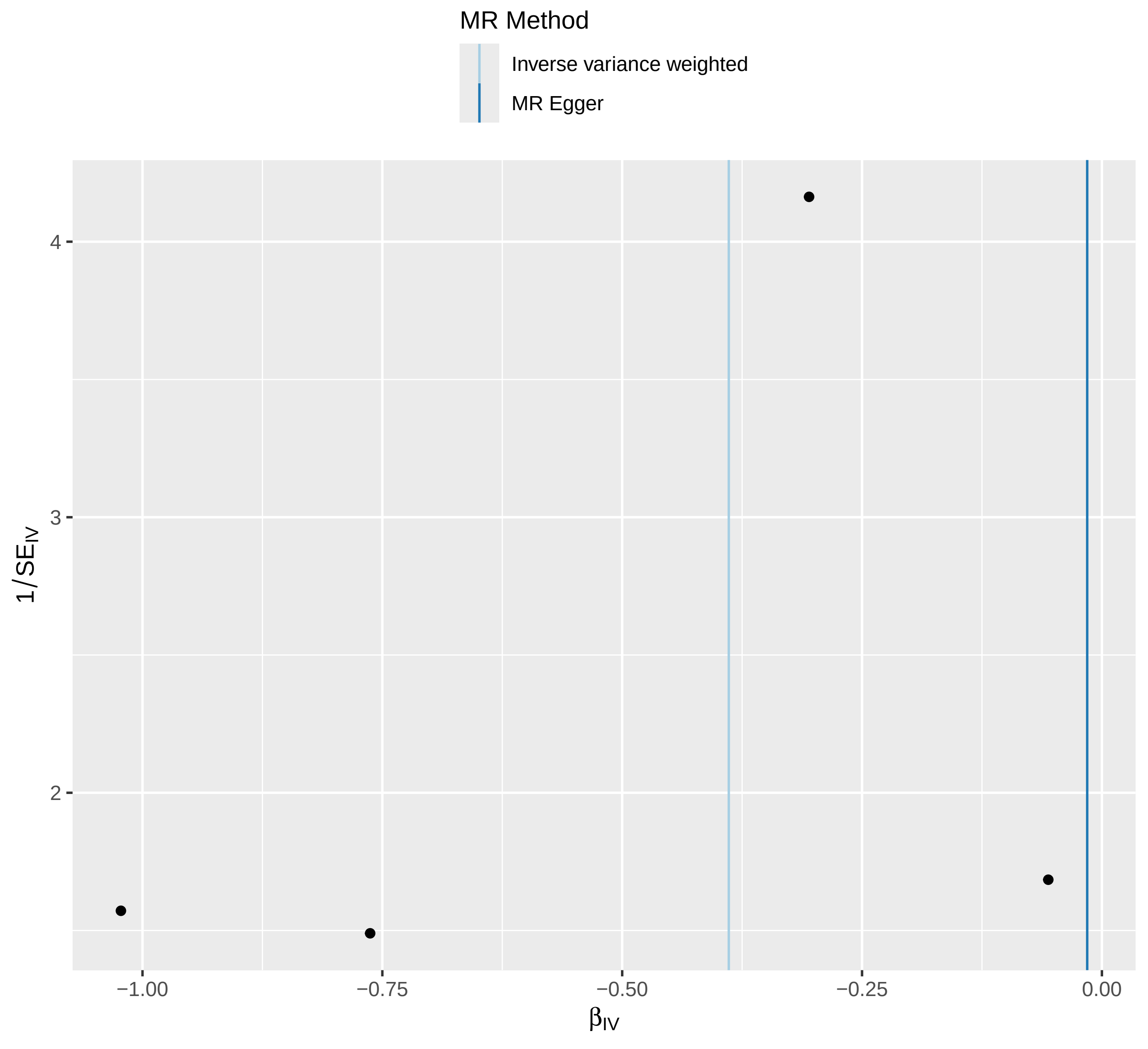
MR Method
Inverse variance weighted
MR Egger
4
3
2
1/SE_IV
−1.00
−0.75
−0.50
−0.25
0.00
β_IV

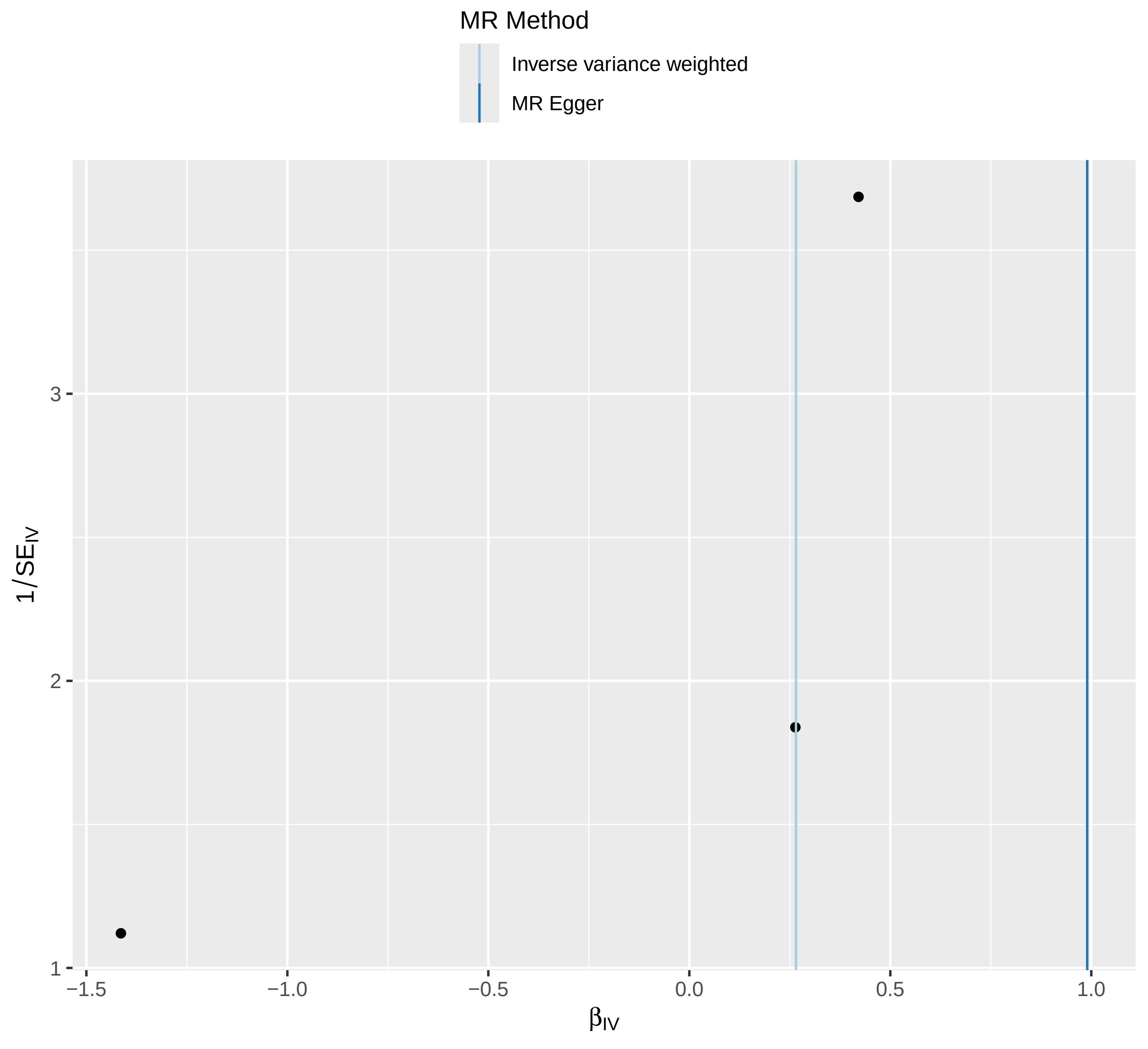
MR Method
Inverse variance weighted
MR Egger
1/SE_IV
3
2
1
−1.5
−1.0
−0.5
0.0
0.5
1.0
β_IV

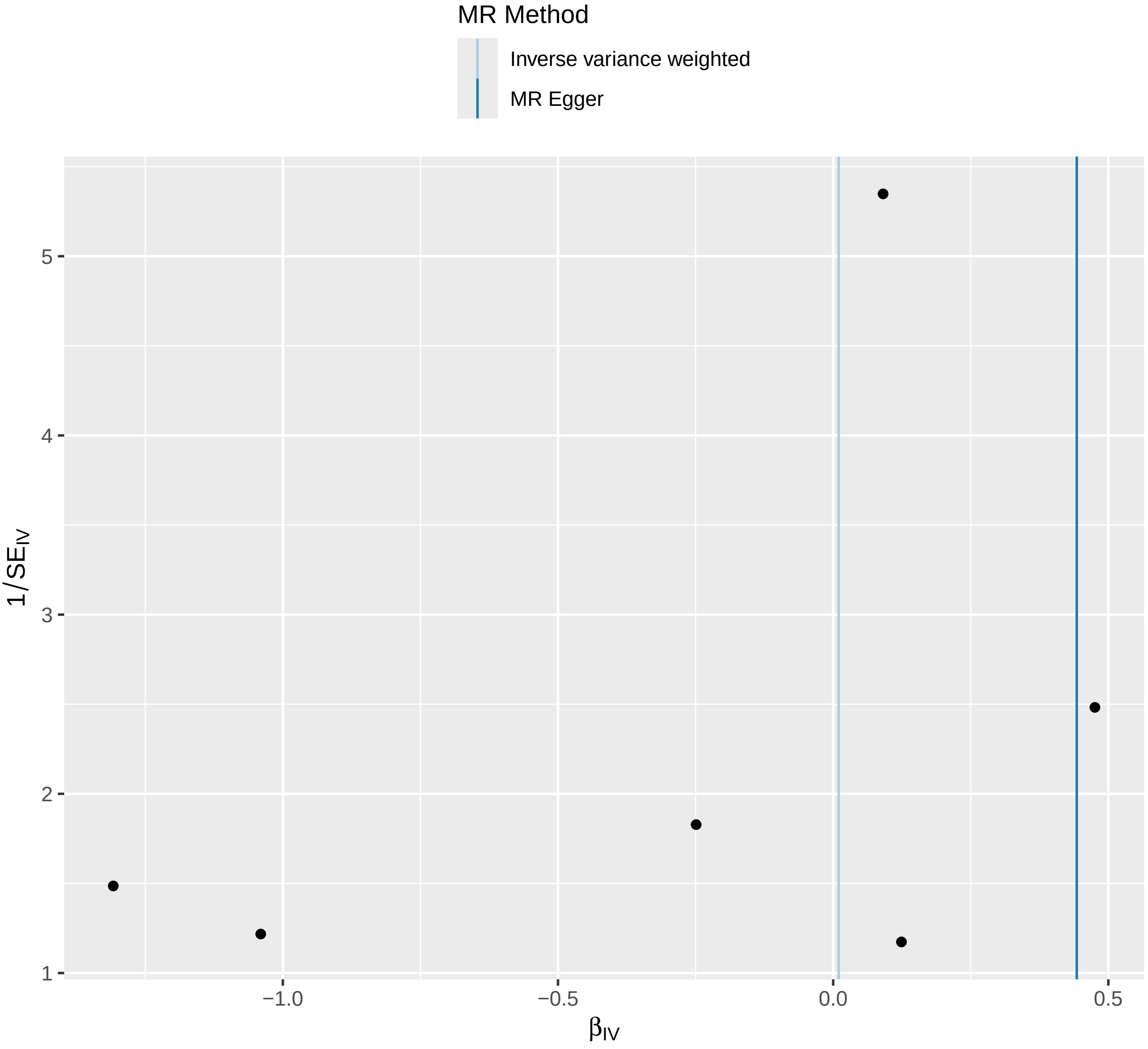
MR Method
Inverse variance weighted
MR Egger
1/SE_IV
5
4
3
2
1
−1.0
−0.5
0.0
0.5
β_IV

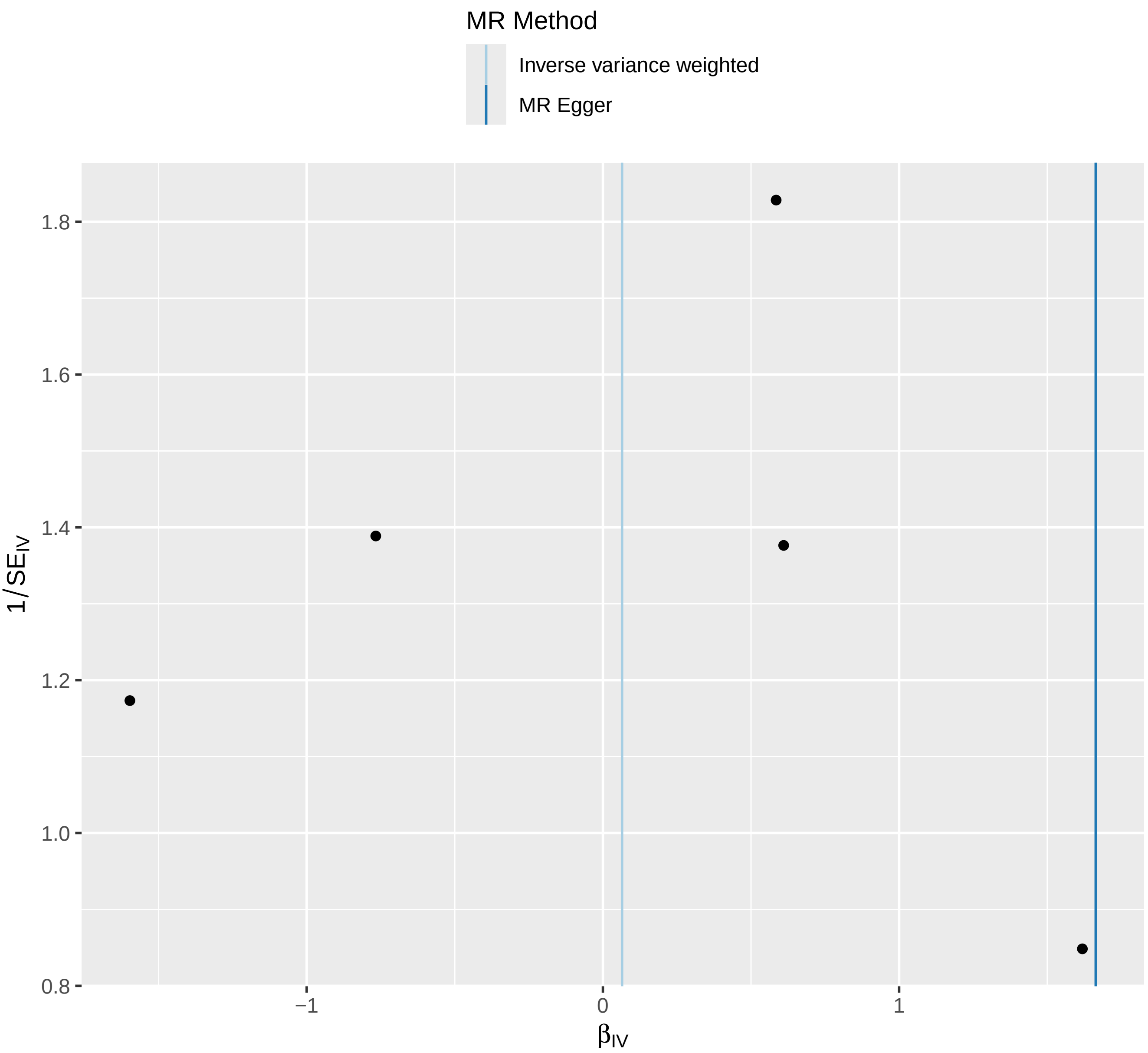

MR Method
Inverse variance weighted
MR Egger
1.8
1.6
1.4
1.2
1.0
0.8
1/SE_IV
−1
0
1
β_IV

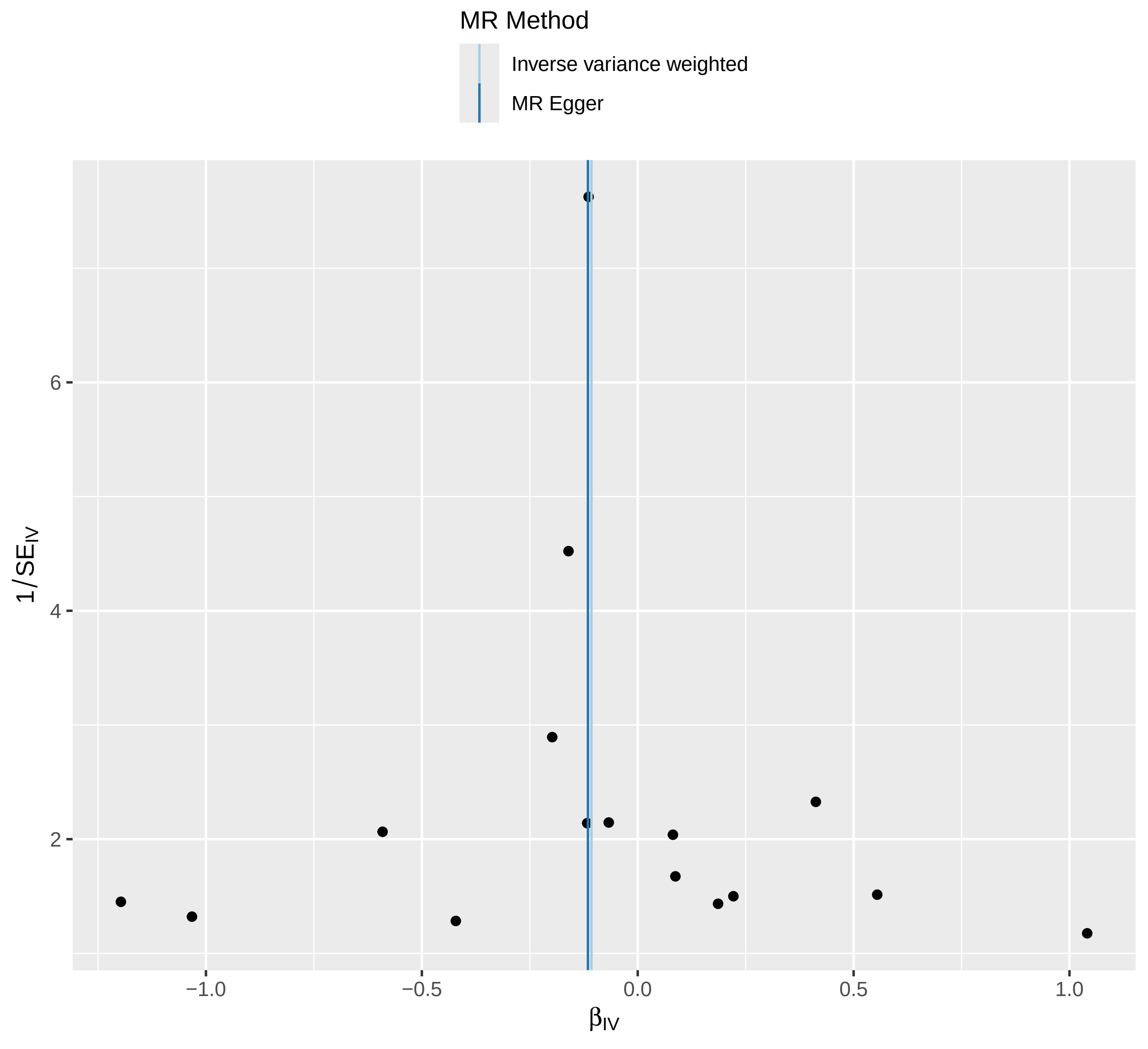

MR Method
Inverse variance weighted
MR Egger
6
4
2
1/SE_IV
−1.0
−0.5
0.0
0.5
1.0
β_IV

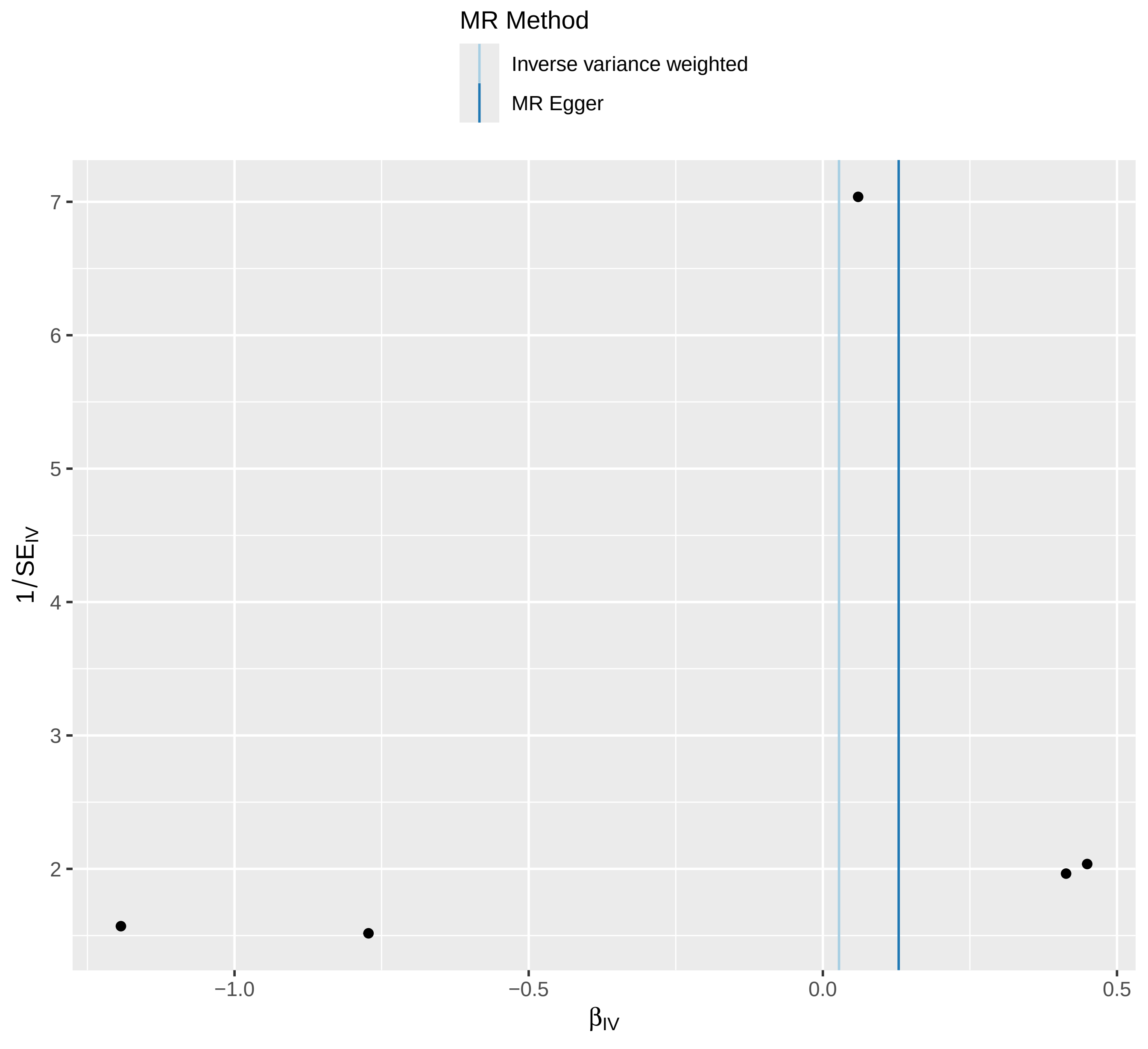

MR Method
Inverse variance weighted
MR Egger
7
6
5
4
3
2
1/SE_IV
−1.0
−0.5
0.0
0.5
β_IV

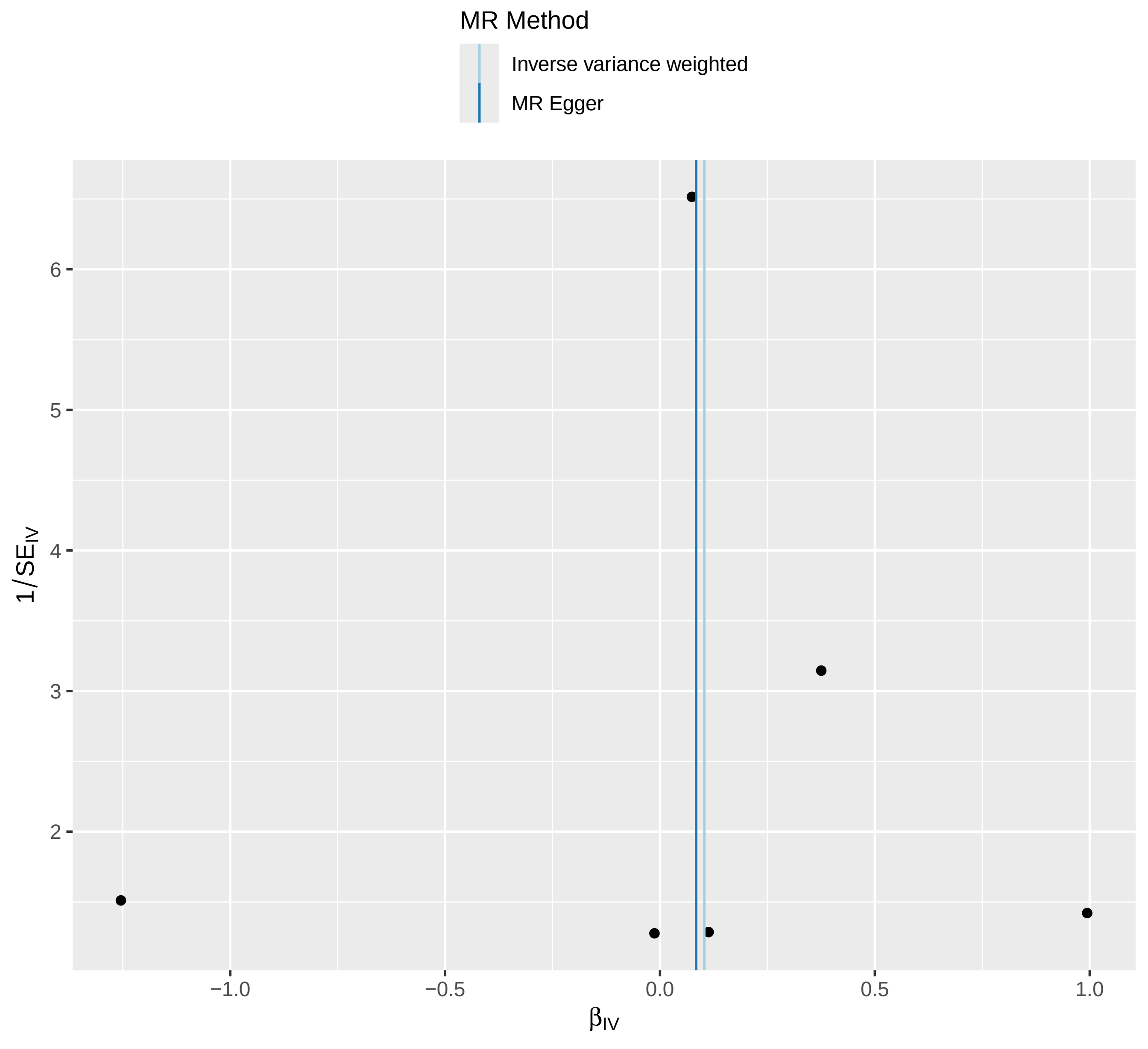
MR Method
Inverse variance weighted
MR Egger
1/SE_{IV}
6
5
4
3
2
−1.0
−0.5
0.0
0.5
1.0
β_{IV}

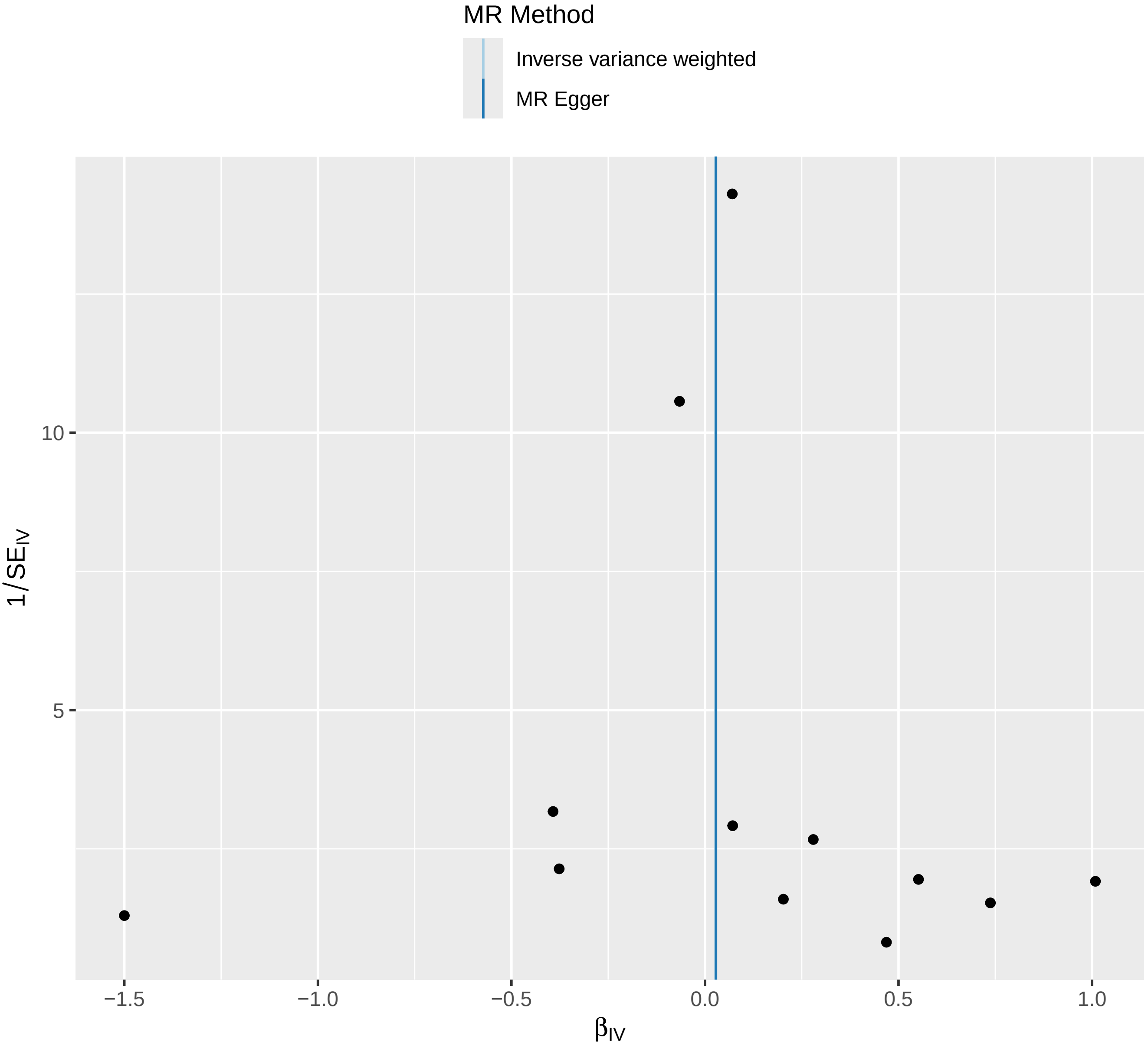
MR Method
Inverse variance weighted
MR Egger
10
5
1/SE_IV
−1.5
−1.0
−0.5
0.0
0.5
1.0
β_IV

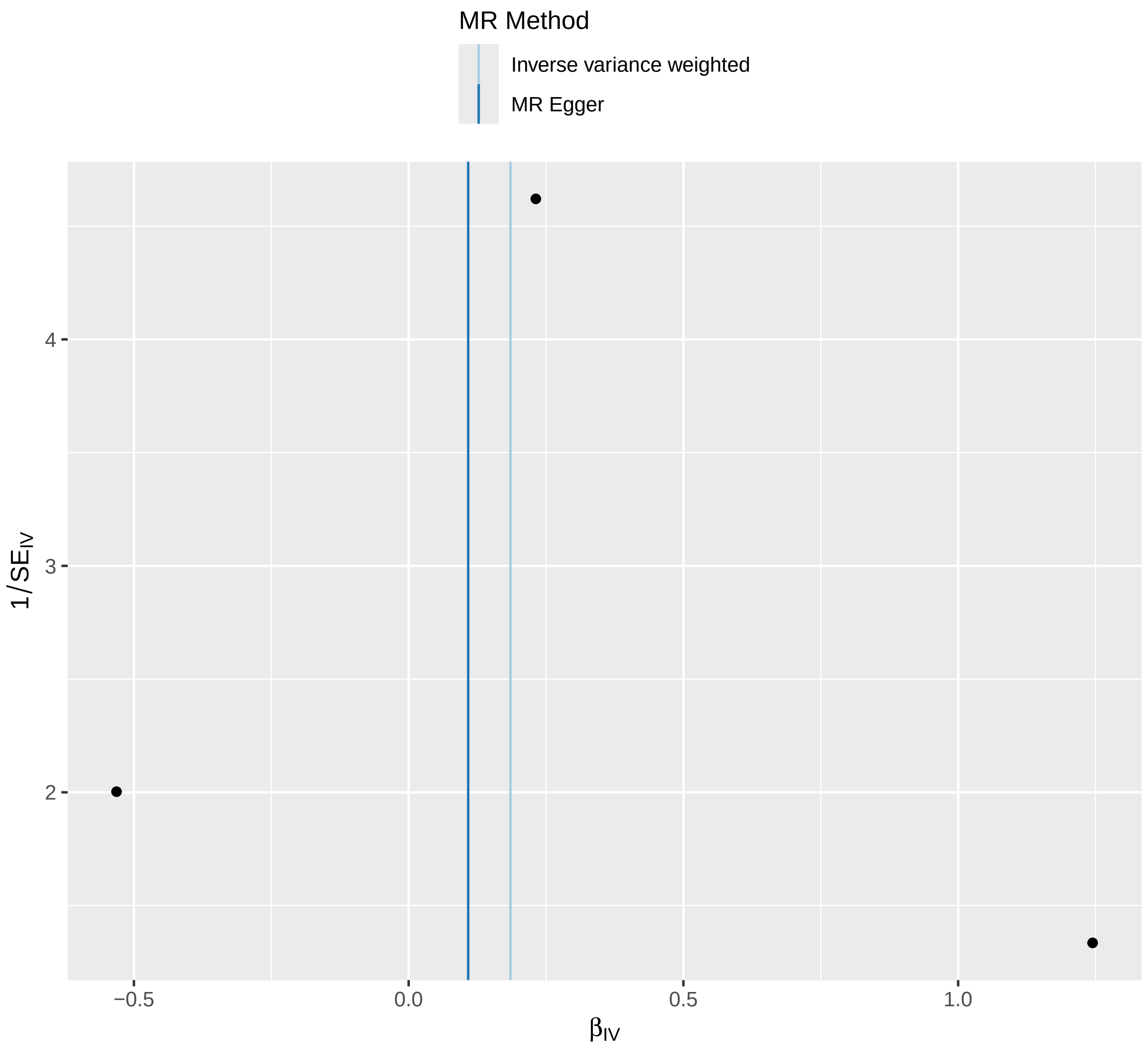
MR Method
Inverse variance weighted
MR Egger
1/SE$_{IV}$
4
3
2
−0.5
0.0
0.5
1.0
β_{IV}

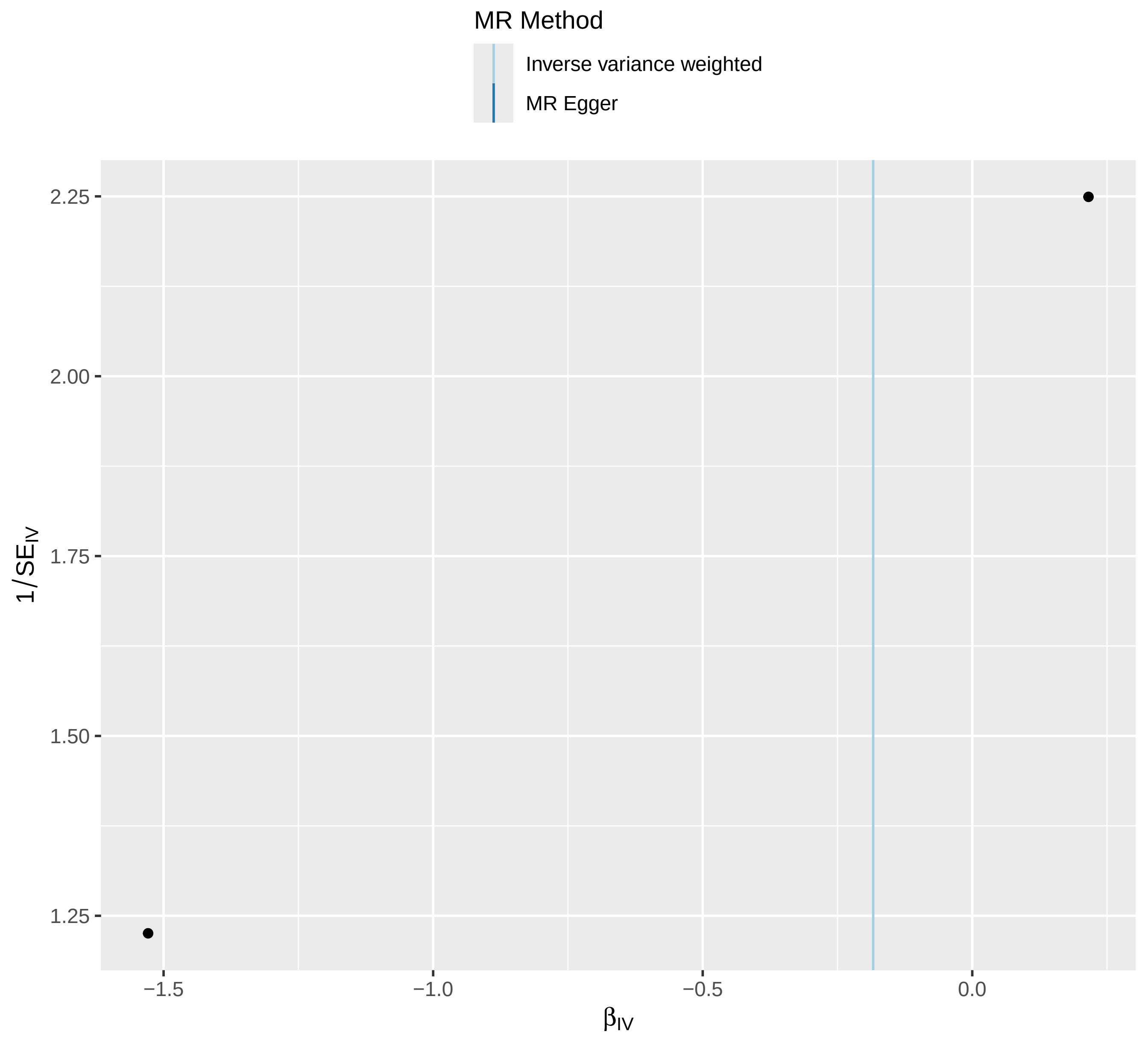

MR Method
Inverse variance weighted
MR Egger
2.25
2.00
1.75
1.50
1.25
1/SE$_{IV}$
−1.5
−1.0
−0.5
0.0
β$_{IV}$

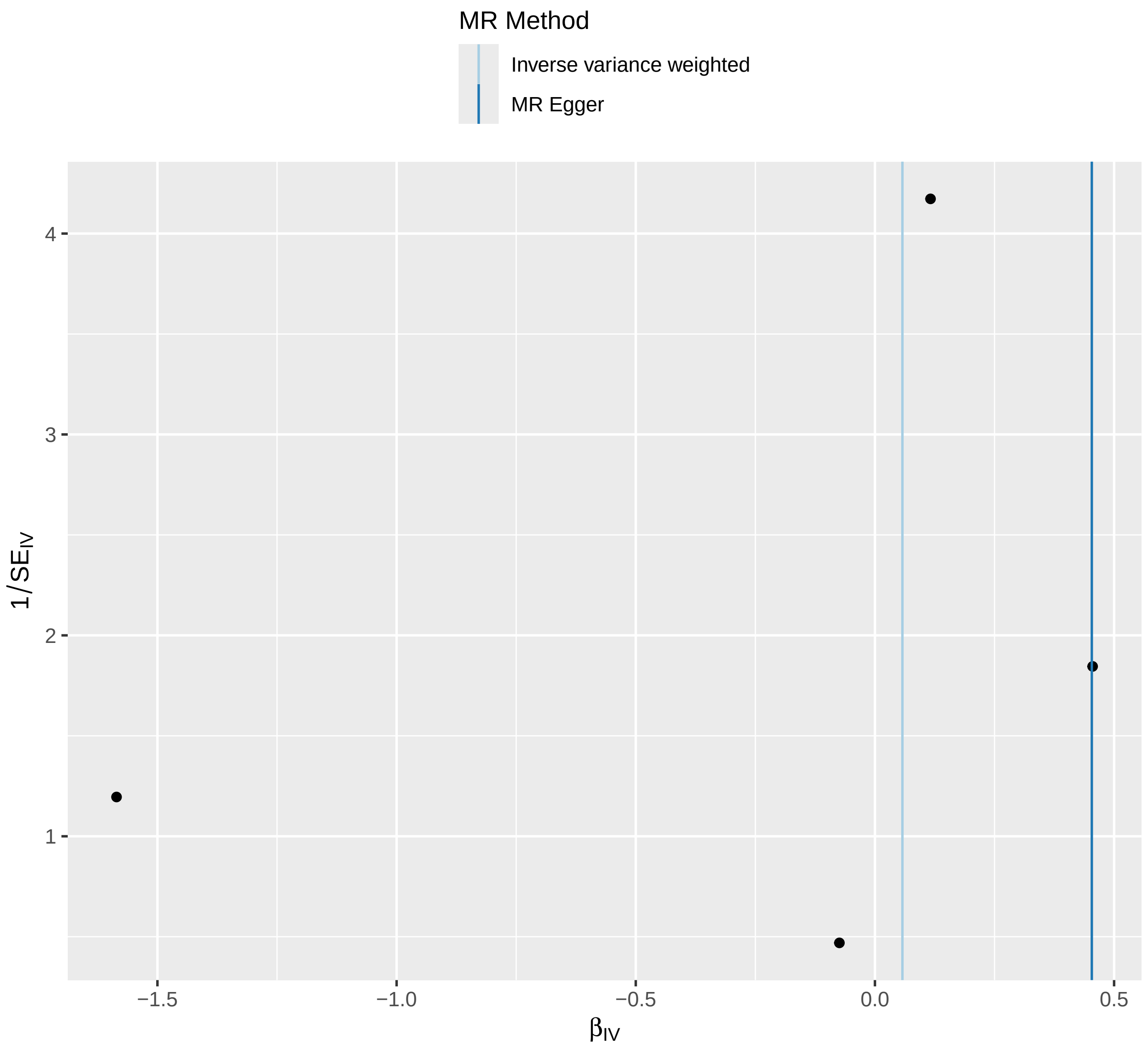
MR Method
Inverse variance weighted
MR Egger
4
3
2
1
1/SE_IV
−1.5
−1.0
−0.5
0.0
0.5
β_IV

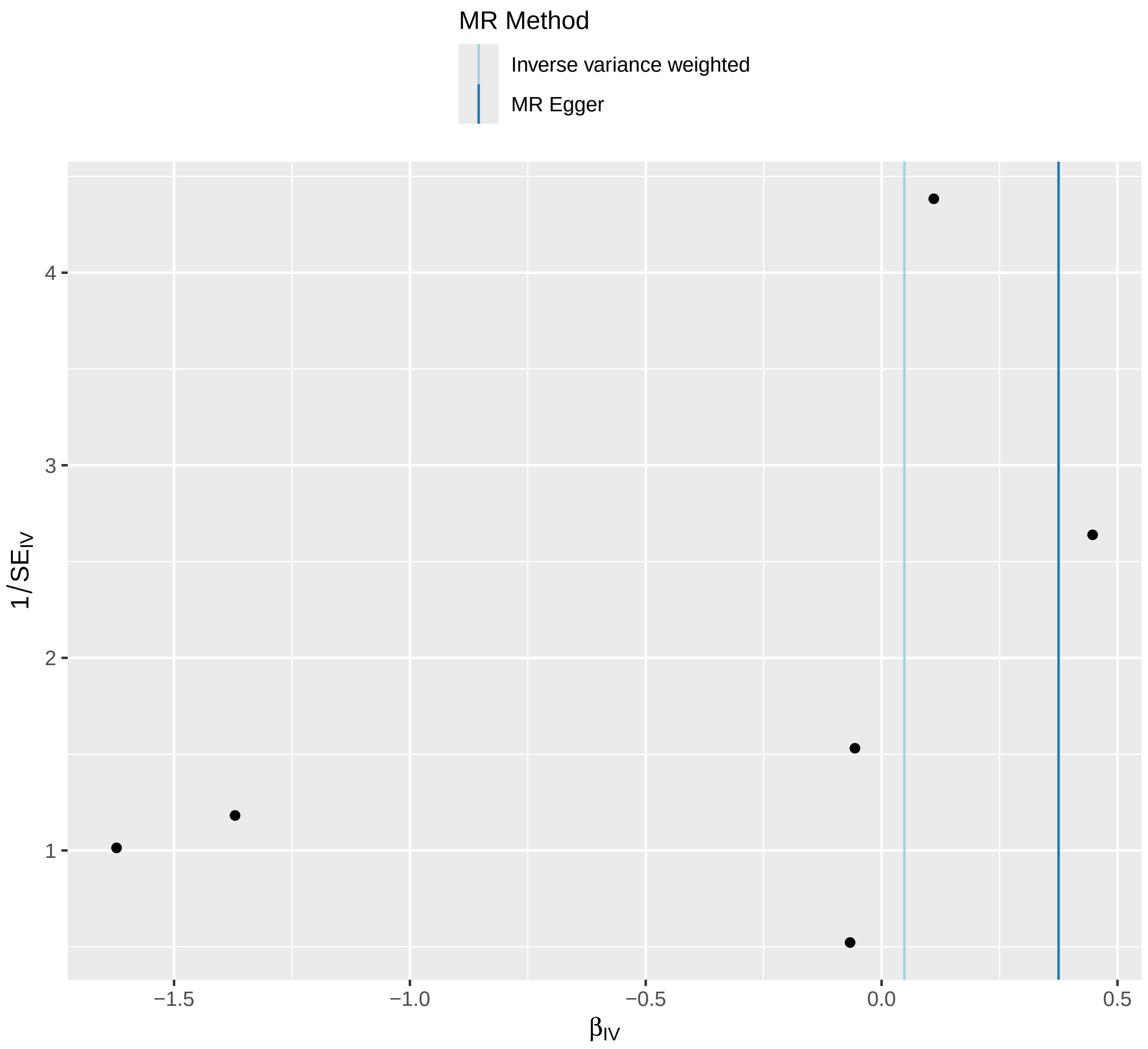
MR Method
Inverse variance weighted
MR Egger
1/SE$_{IV}$
4
3
2
1
−1.5
−1.0
−0.5
0.0
0.5
β$_{IV}$

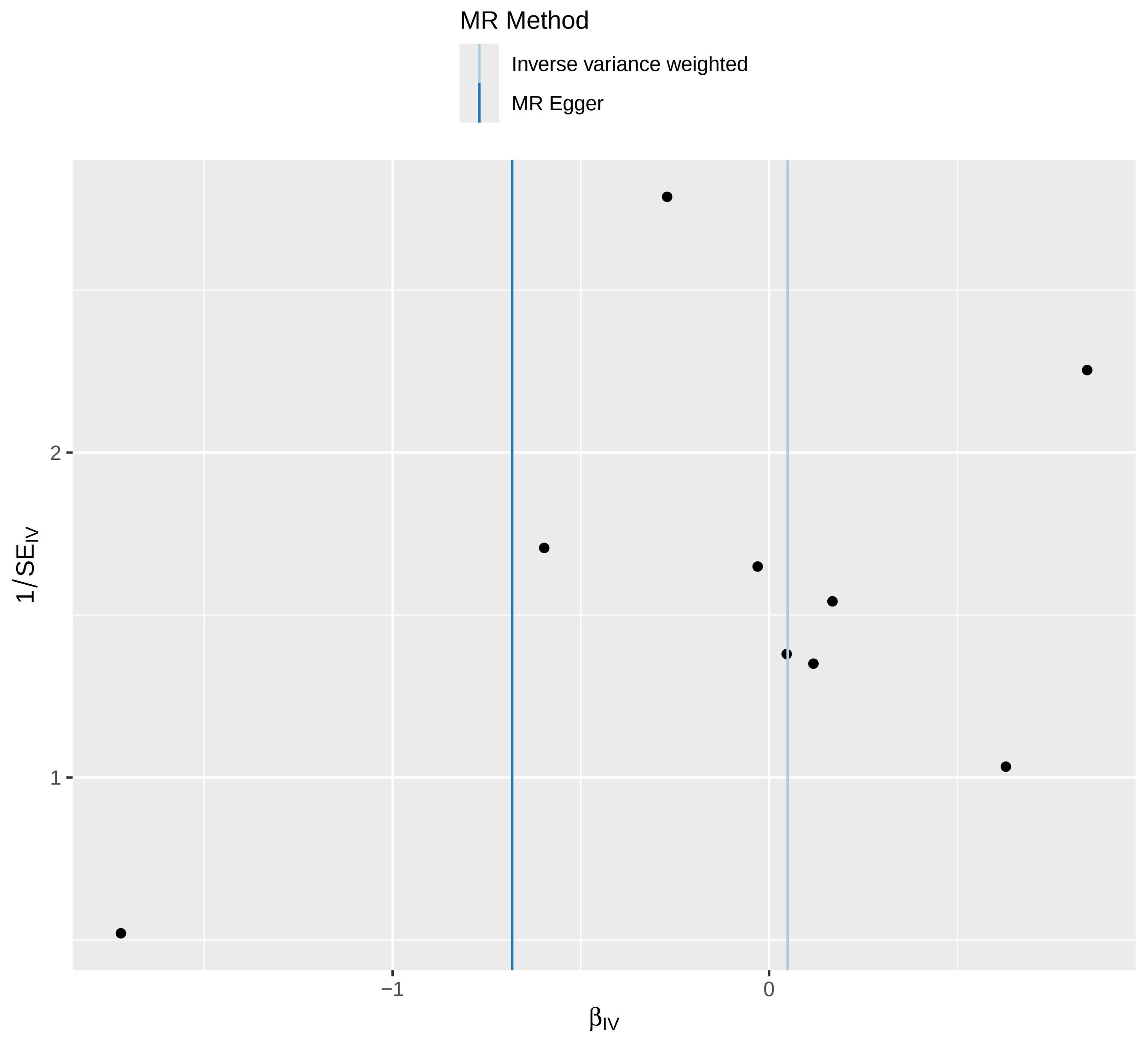
MR Method
Inverse variance weighted
MR Egger
2
1
1/SE_IV
−1
0
β_IV

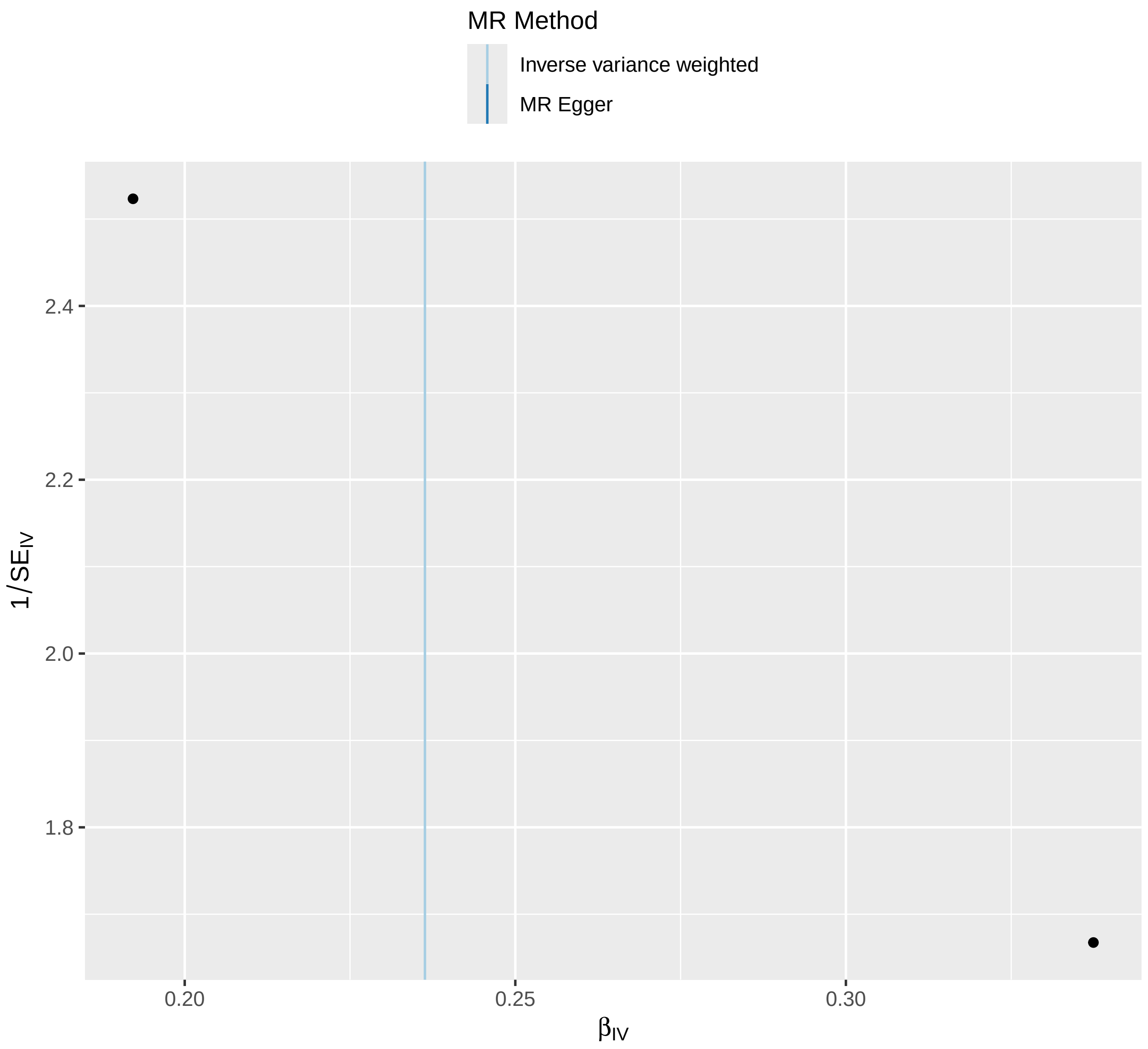

MR Method
Inverse variance weighted
MR Egger
2.4
2.2
2.0
1.8
1/SE_IV
0.20
0.25
0.30
β_IV

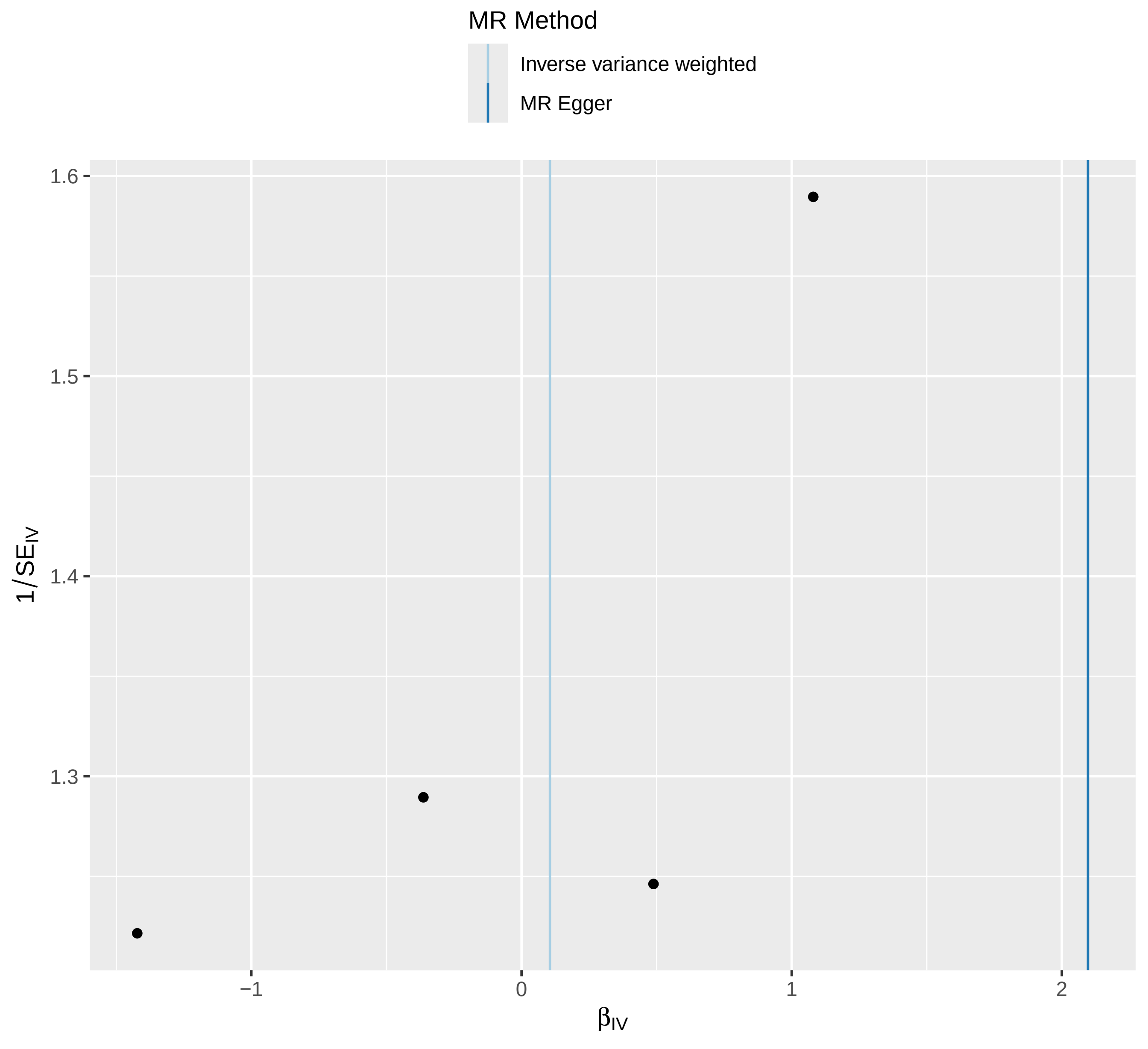
MR Method
Inverse variance weighted
MR Egger
1.6
1.5
1.4
1.3
1/SE$_{IV}$
−1
0
1
2
β$_{IV}$

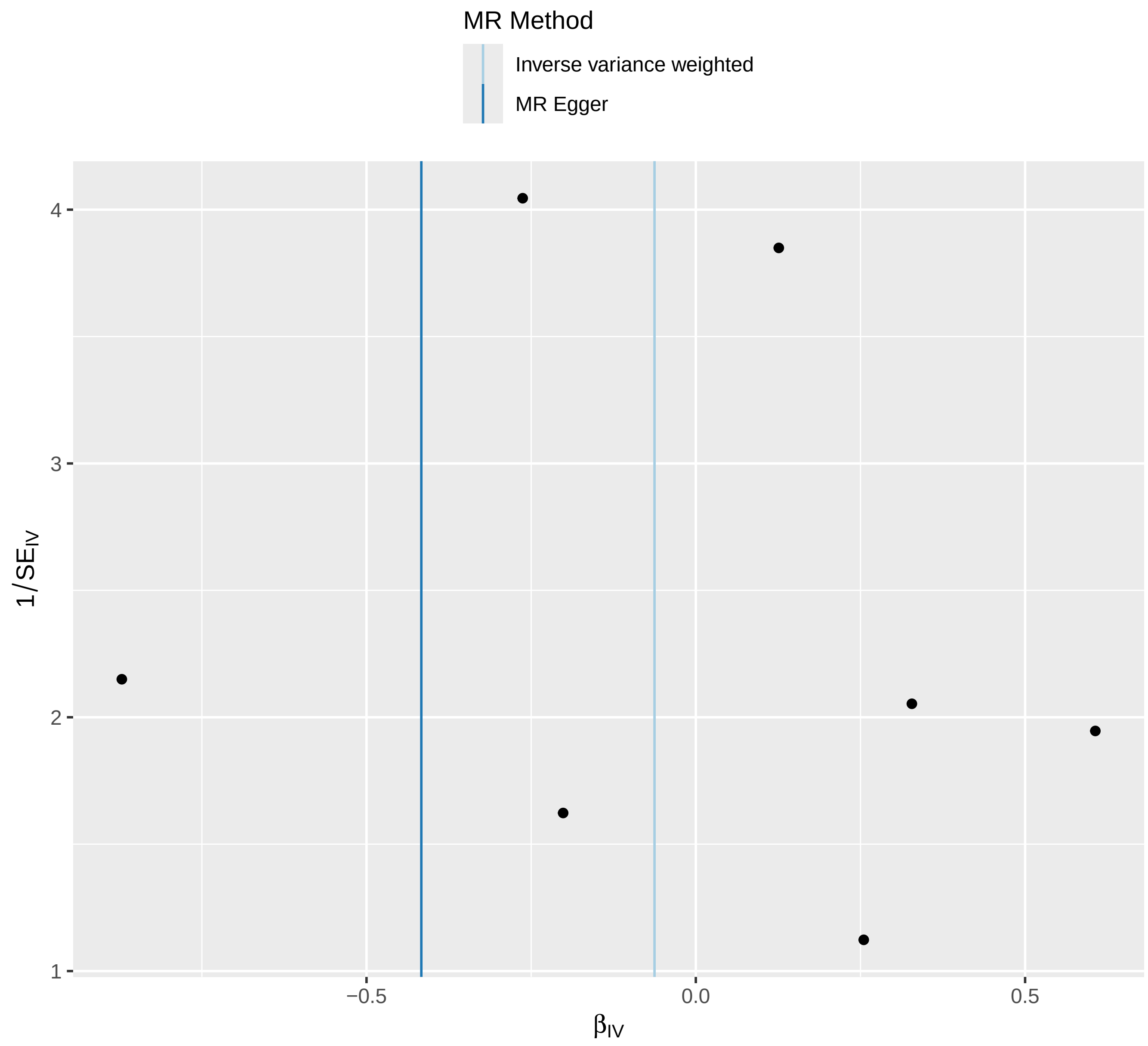

MR Method
Inverse variance weighted
MR Egger
4
3
2
1
1/SE$_{IV}$
−0.5
0.0
0.5
β$_{IV}$

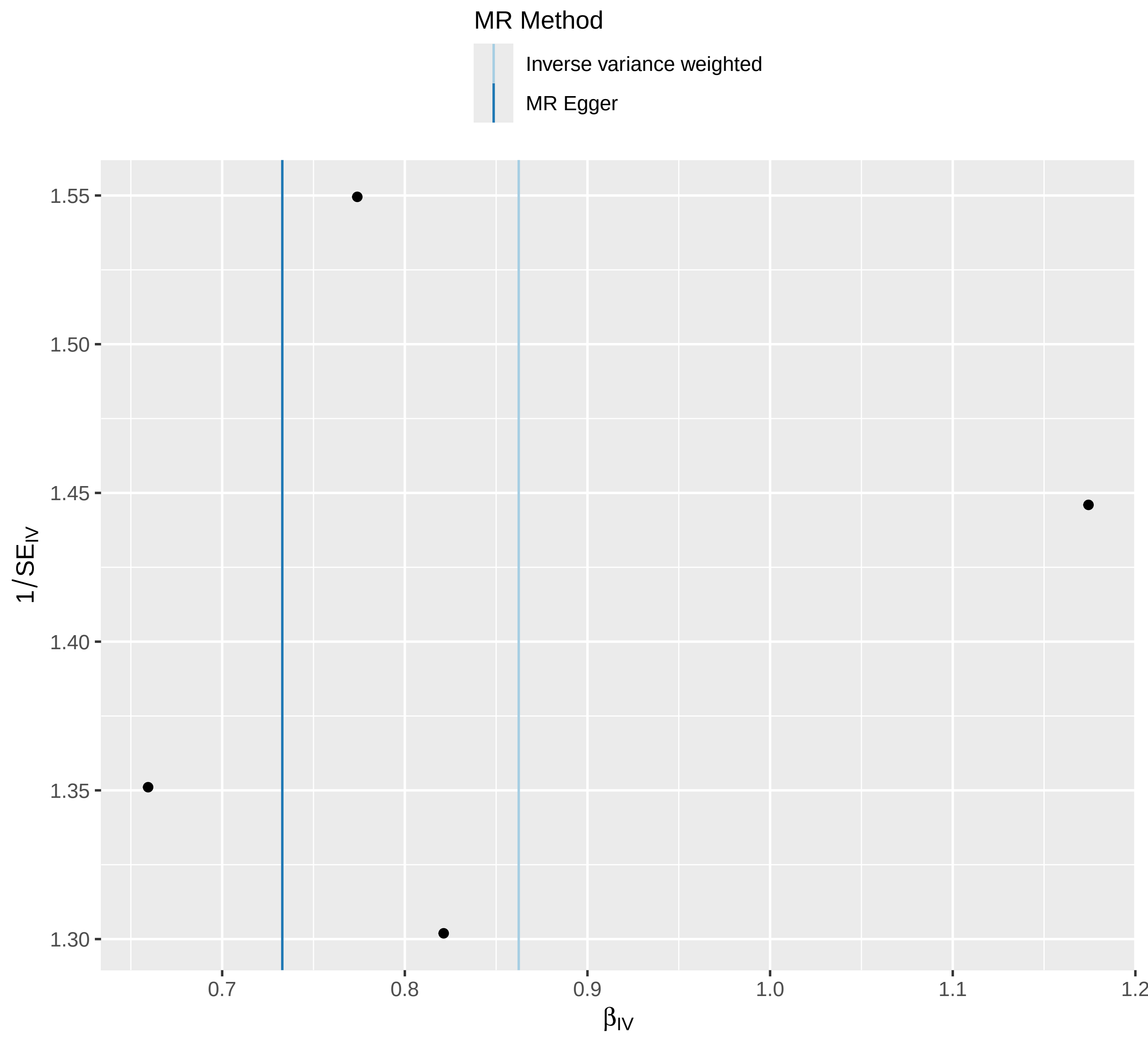

MR Method
Inverse variance weighted
MR Egger
1.55
1.50
1.45
1.40
1.35
1.30
$1/SE_{IV}$
0.7
0.8
0.9
1.0
1.1
1.2
β_{IV}

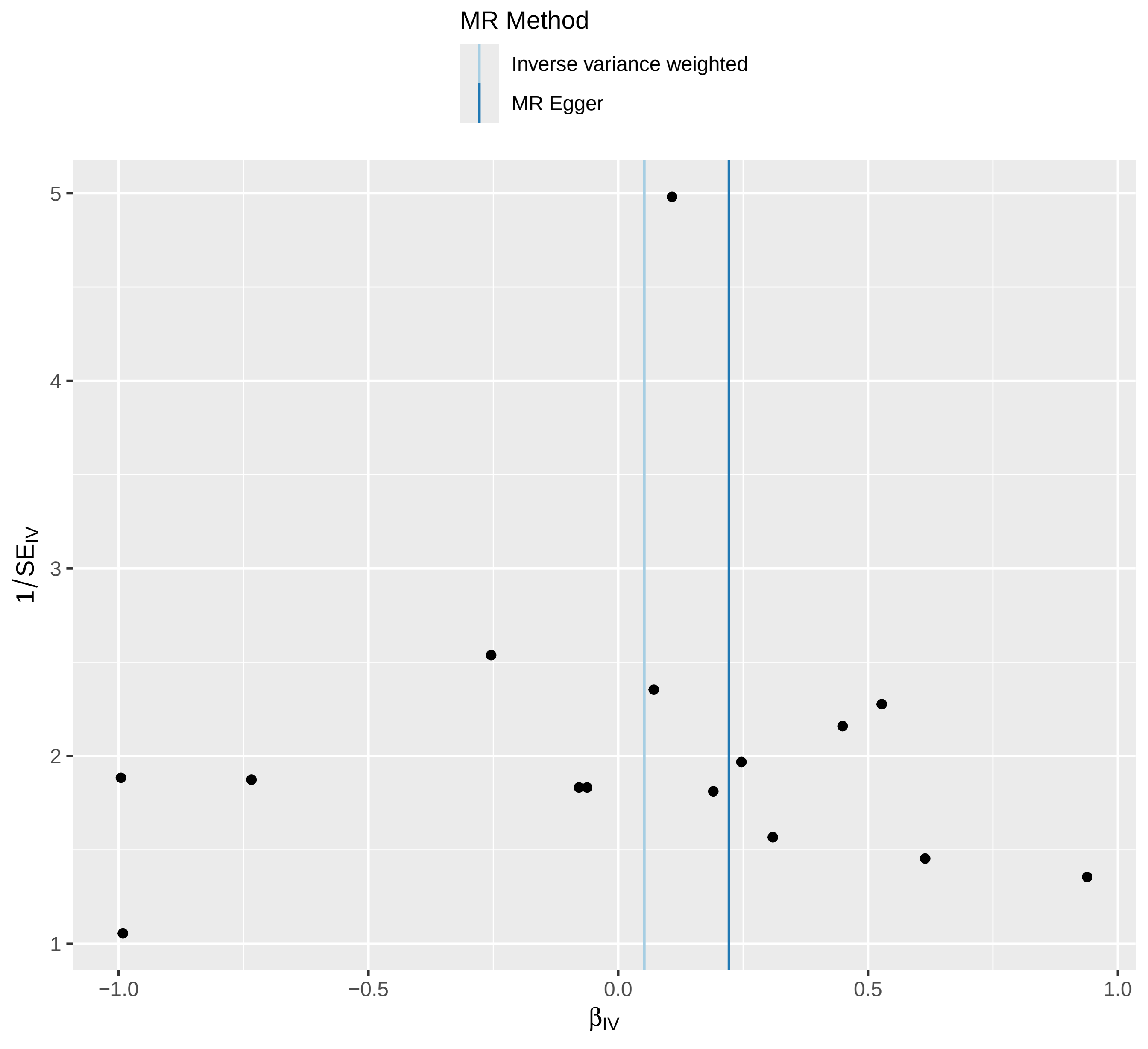
MR Method
Inverse variance weighted
MR Egger
5
4
3
2
1
1/SE$_{IV}$
−1.0
−0.5
0.0
0.5
1.0
β$_{IV}$

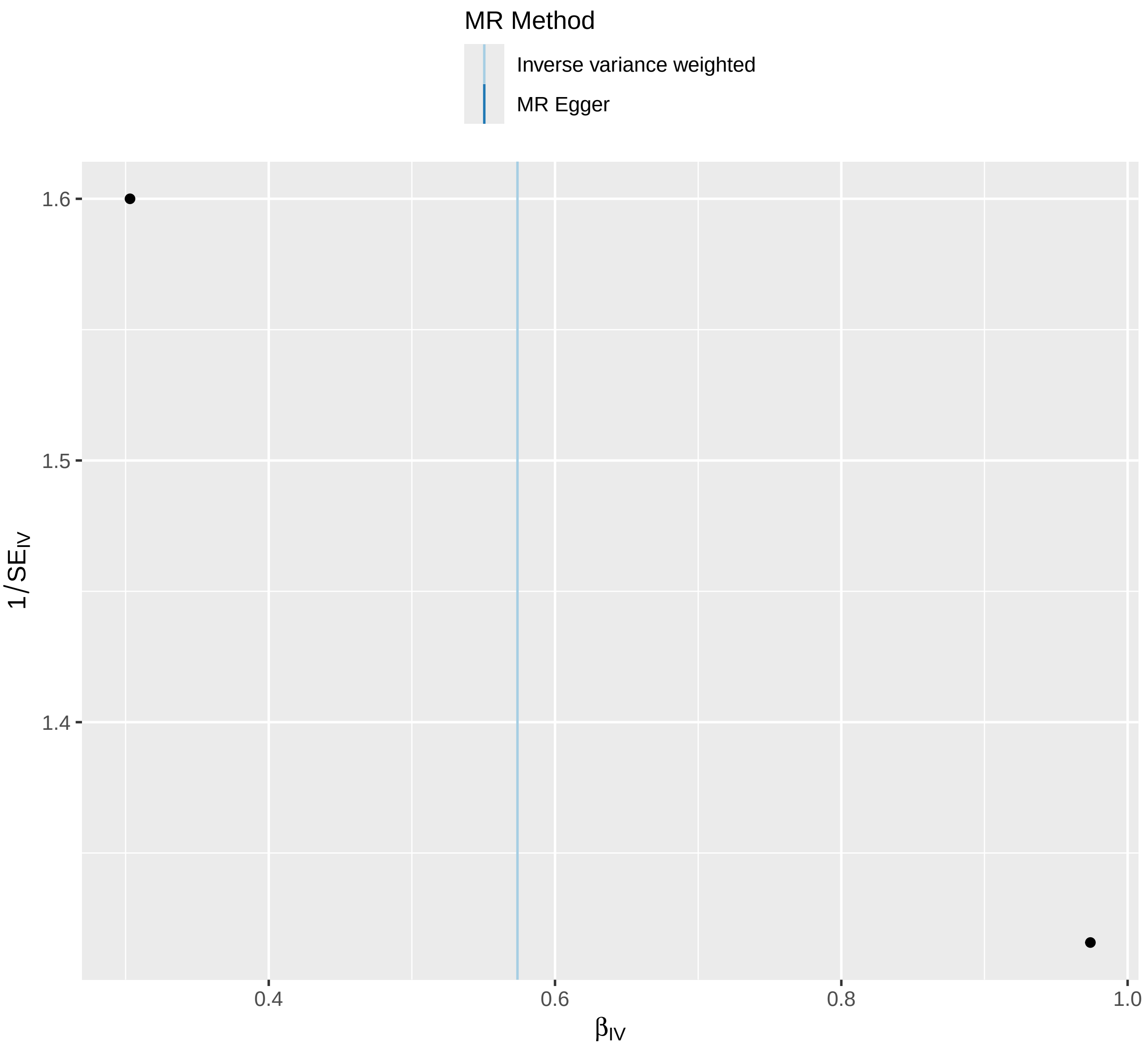
MR Method
Inverse variance weighted
MR Egger
1.6
1.5
1.4
1/SE_IV
0.4
0.6
0.8
1.0
β_IV

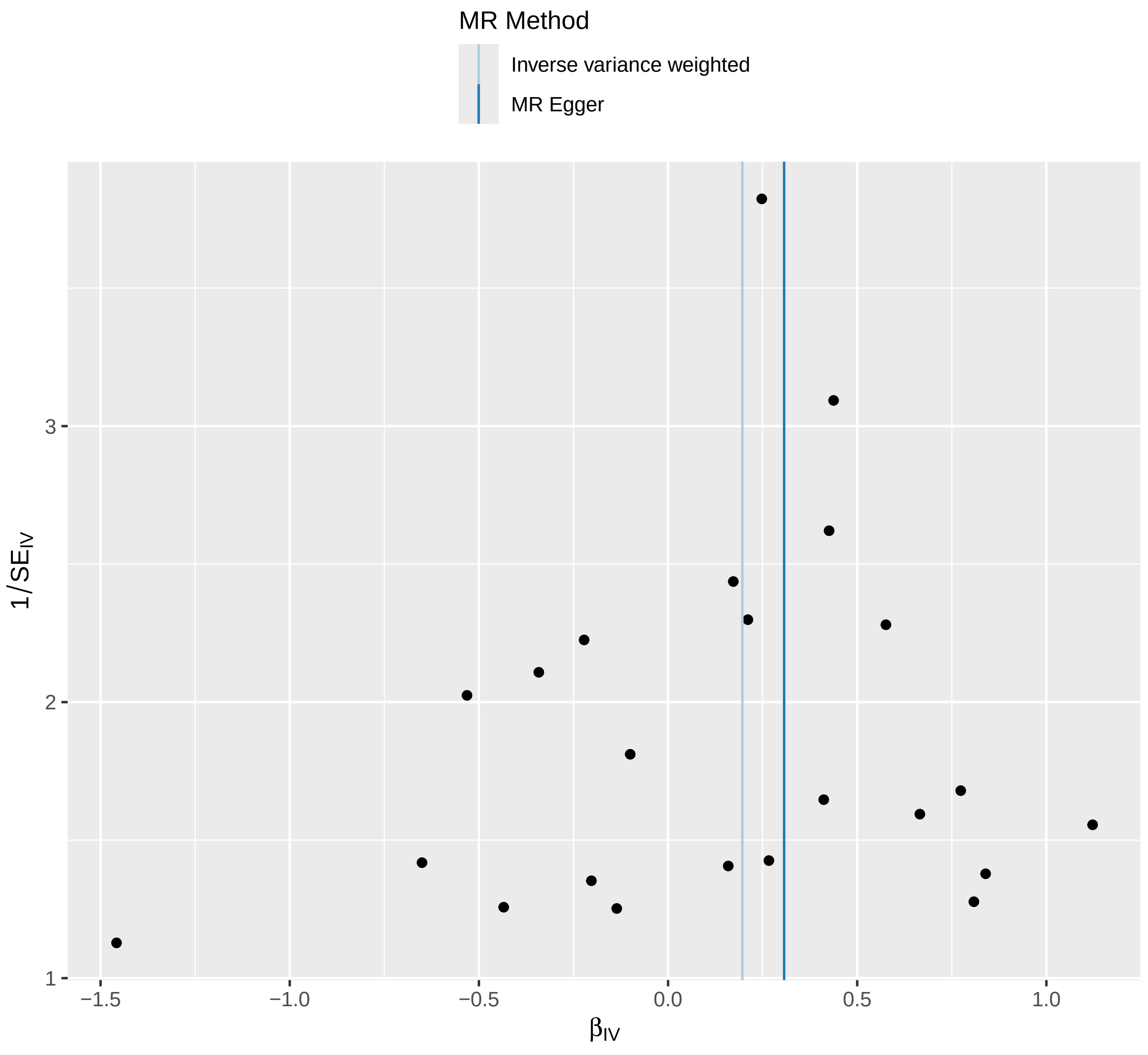

MR Method
Inverse variance weighted
MR Egger
1/SE$_{IV}$
3
2
1
−1.5
−1.0
−0.5
0.0
0.5
1.0
β$_{IV}$

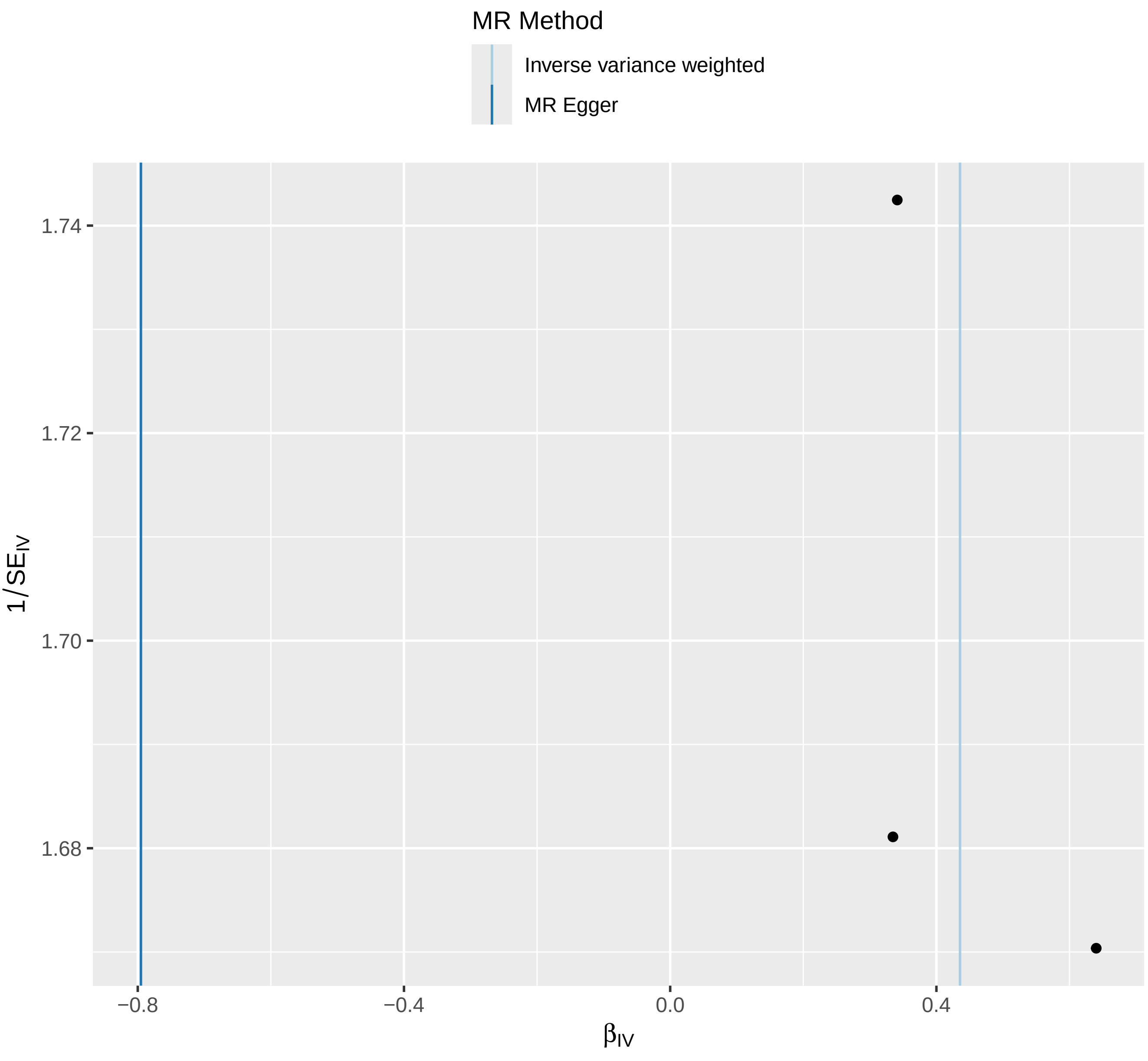
MR Method
Inverse variance weighted
MR Egger
1.74
1.72
1.70
1.68
1/SE_IV
−0.8
−0.4
0.0
0.4
β_IV

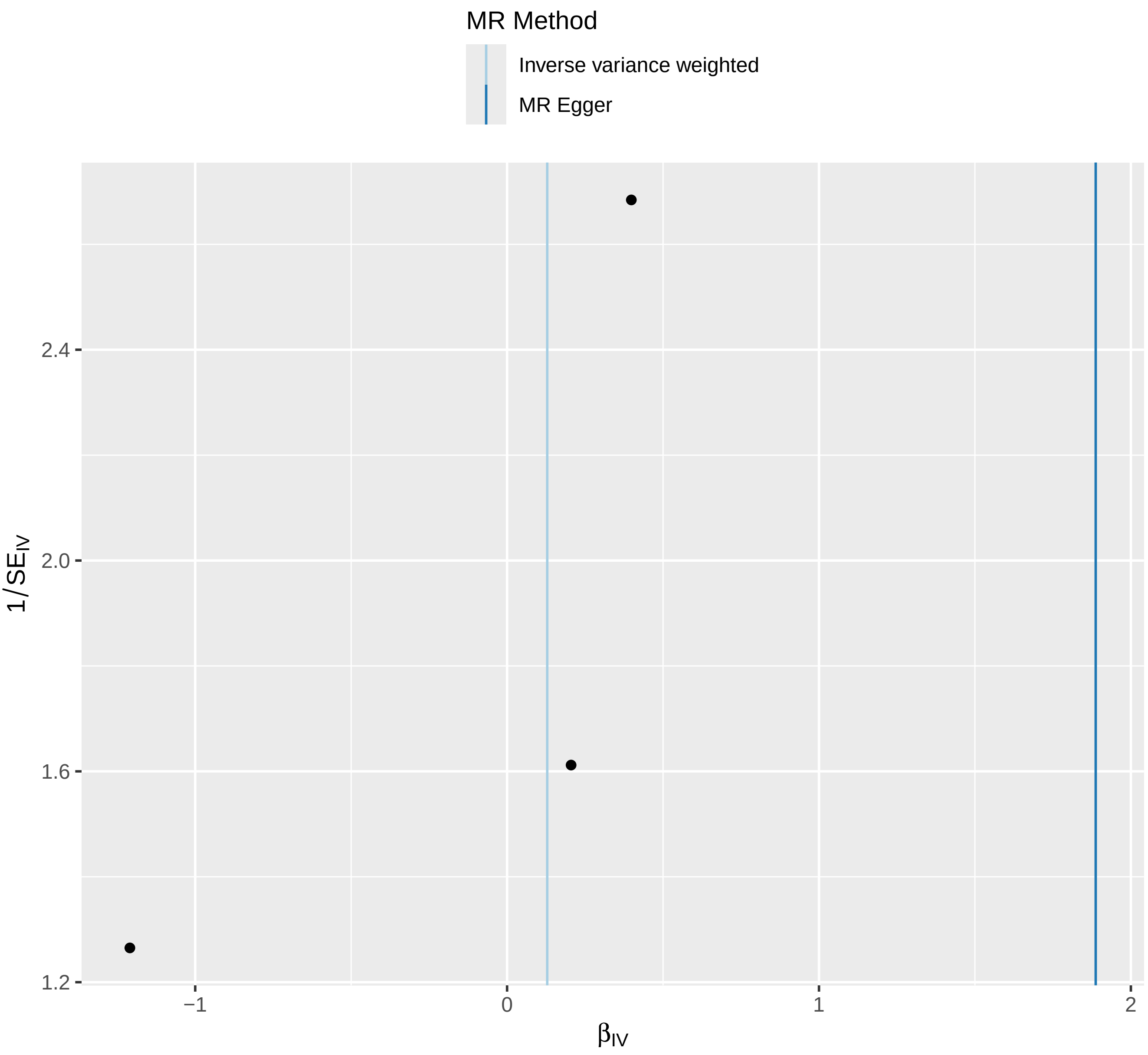
MR Method
Inverse variance weighted
MR Egger
2.4
2.0
1.6
1.2
−1
0
1
2
1/SE_IV
β_IV

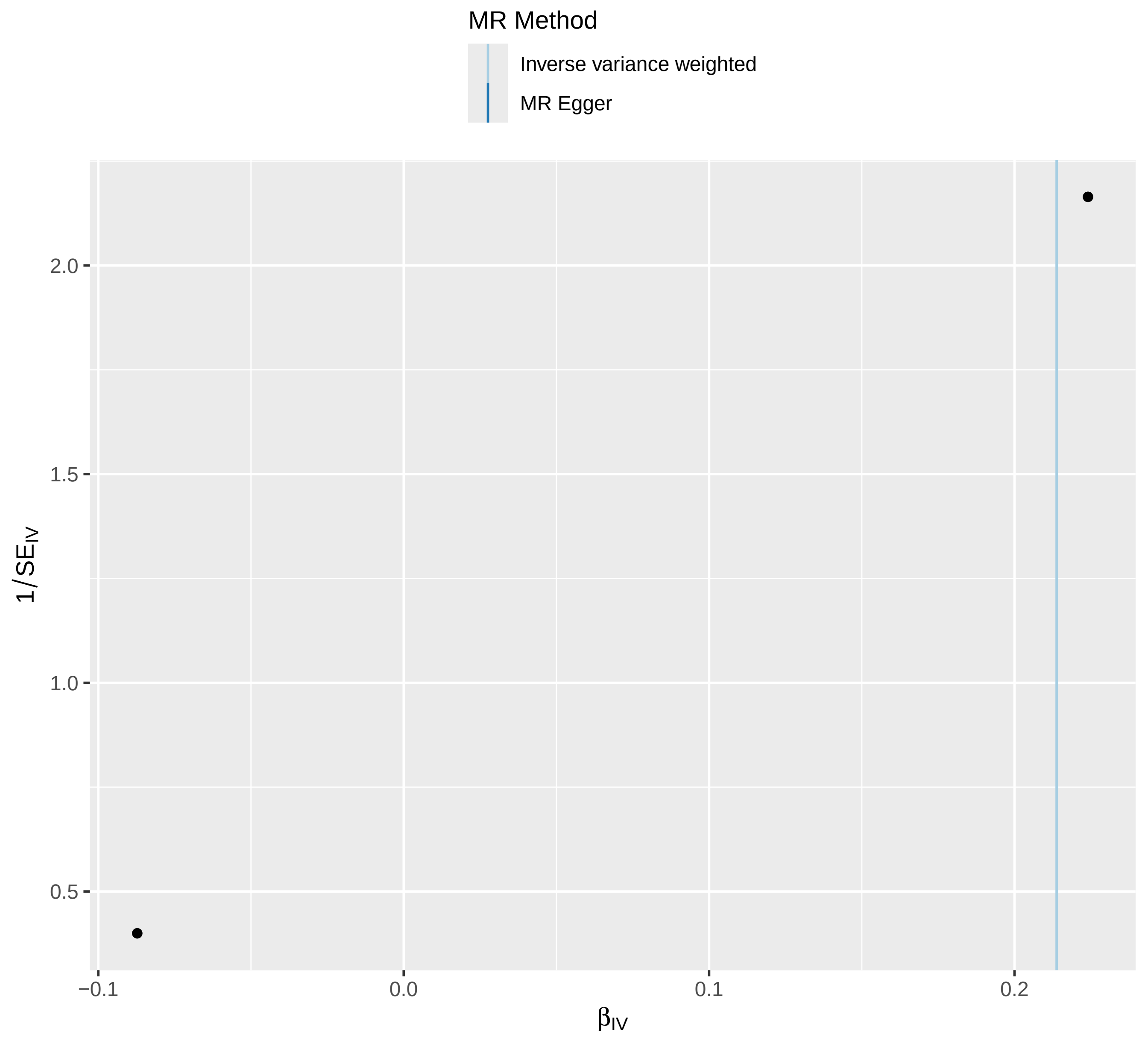
MR Method
Inverse variance weighted
MR Egger
2.0
1.5
1.0
0.5
1/SE_IV
−0.1
0.0
0.1
0.2
β_IV

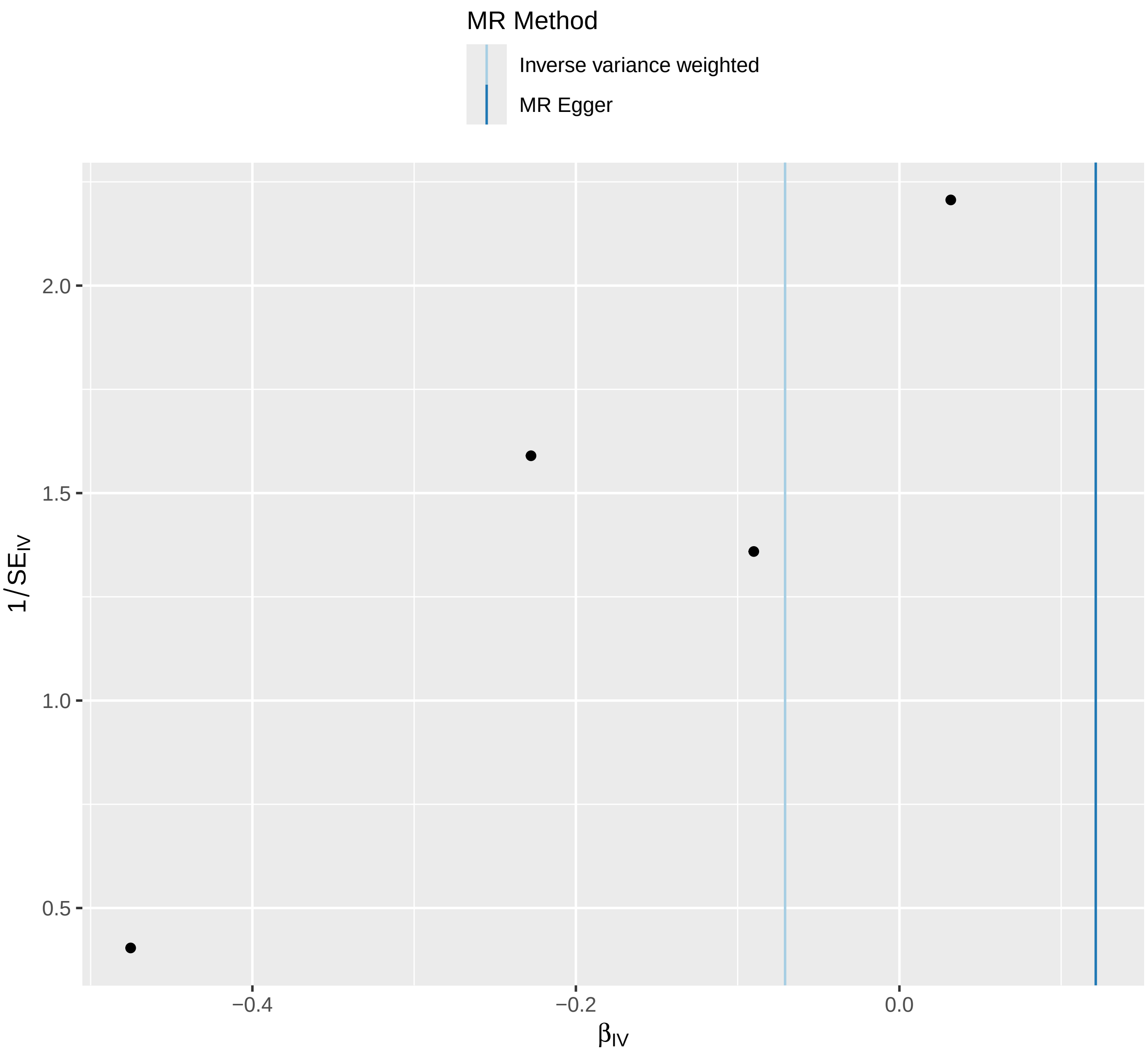

MR Method
Inverse variance weighted
MR Egger
2.0
1.5
1.0
0.5
$1/SE_{IV}$
−0.4
−0.2
0.0
β_{IV}

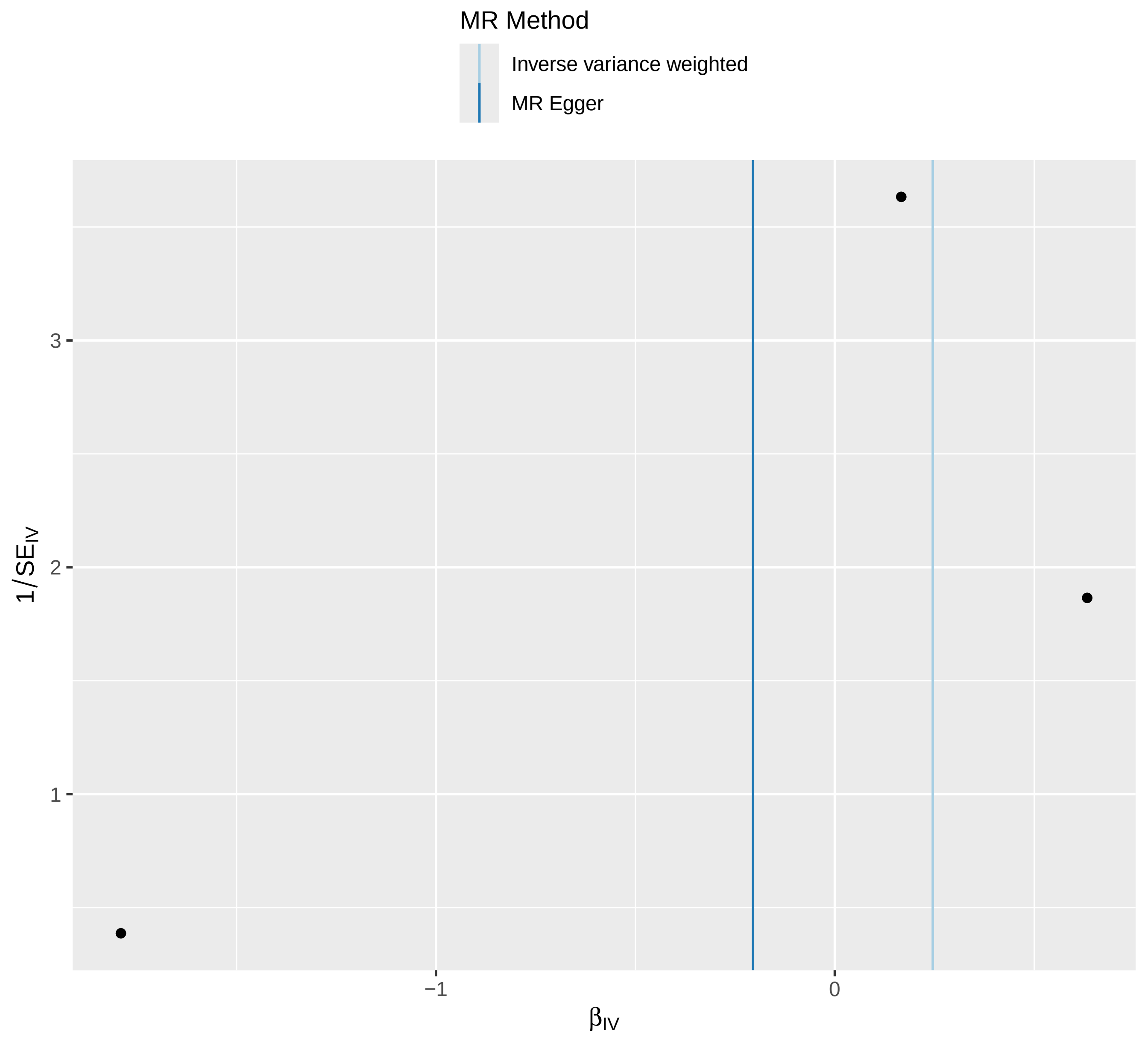

MR Method
Inverse variance weighted
MR Egger
3
2
1
1/SE$_{IV}$
−1
0
β$_{IV}$

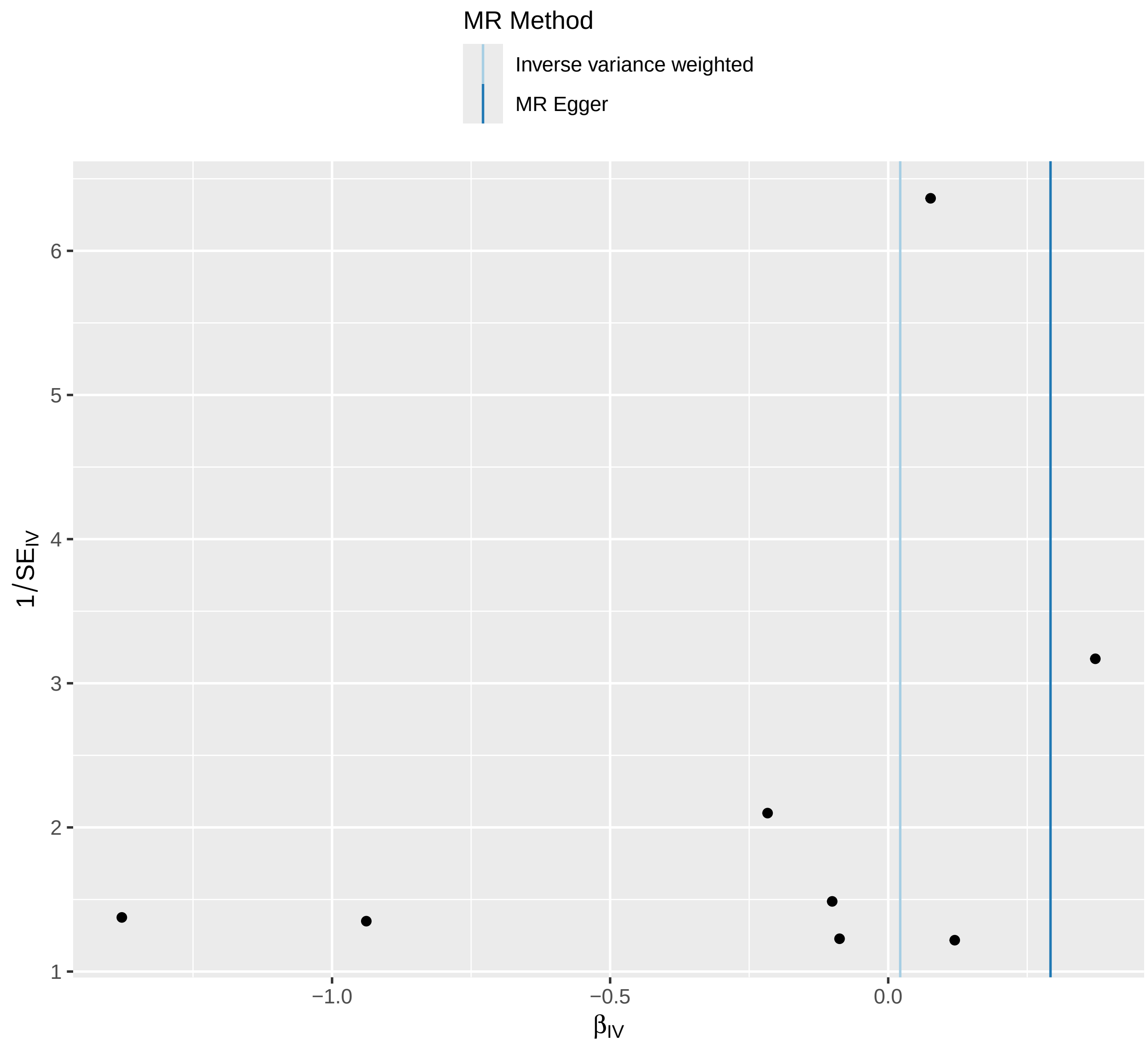
MR Method
Inverse variance weighted
MR Egger
6
5
4
3
2
1
$1/SE_{IV}$
−1.0
−0.5
0.0
β_{IV}

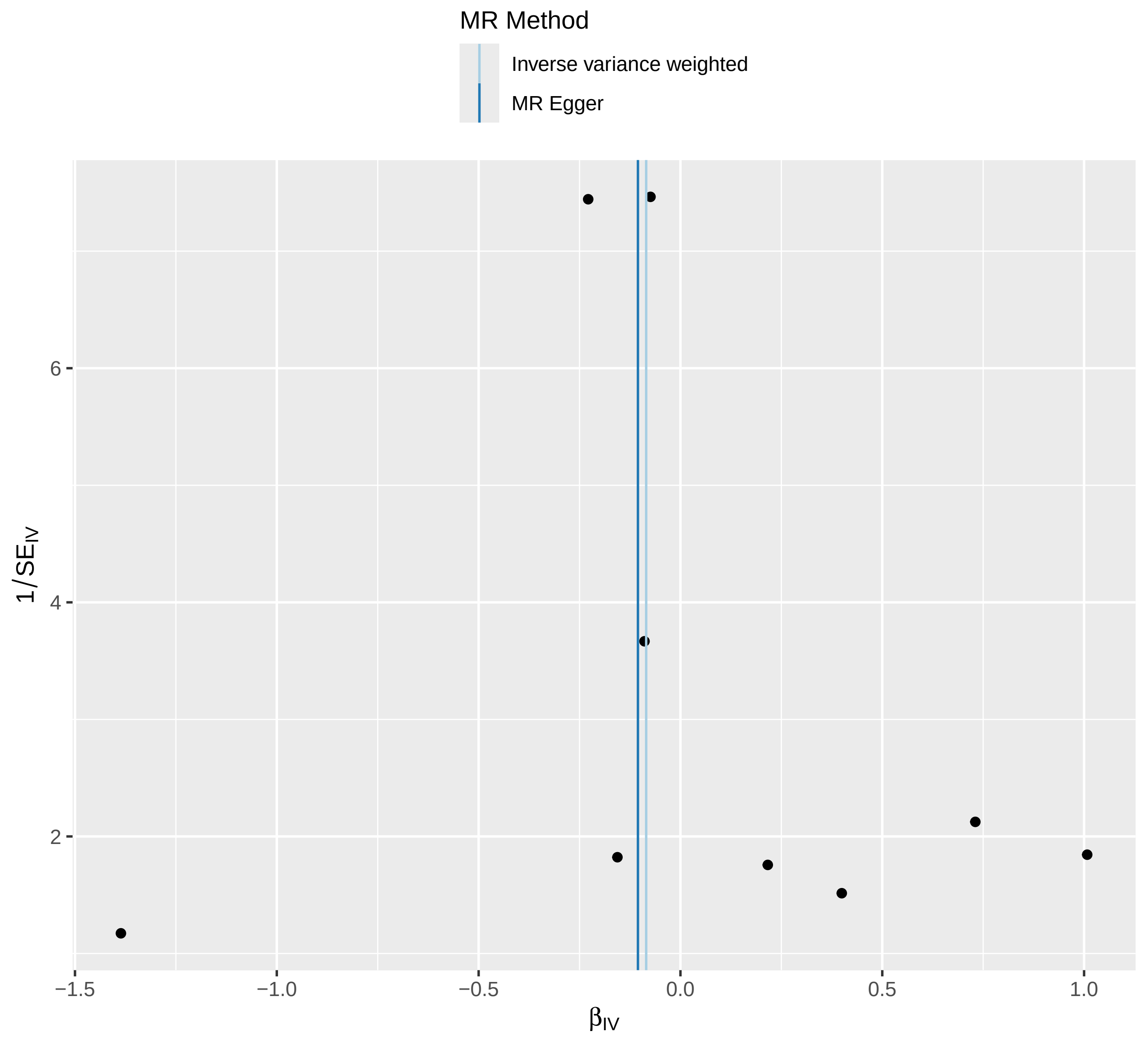
MR Method
Inverse variance weighted
MR Egger
1/SE$_{IV}$
6
4
2
−1.5
−1.0
−0.5
0.0
0.5
1.0
β$_{IV}$

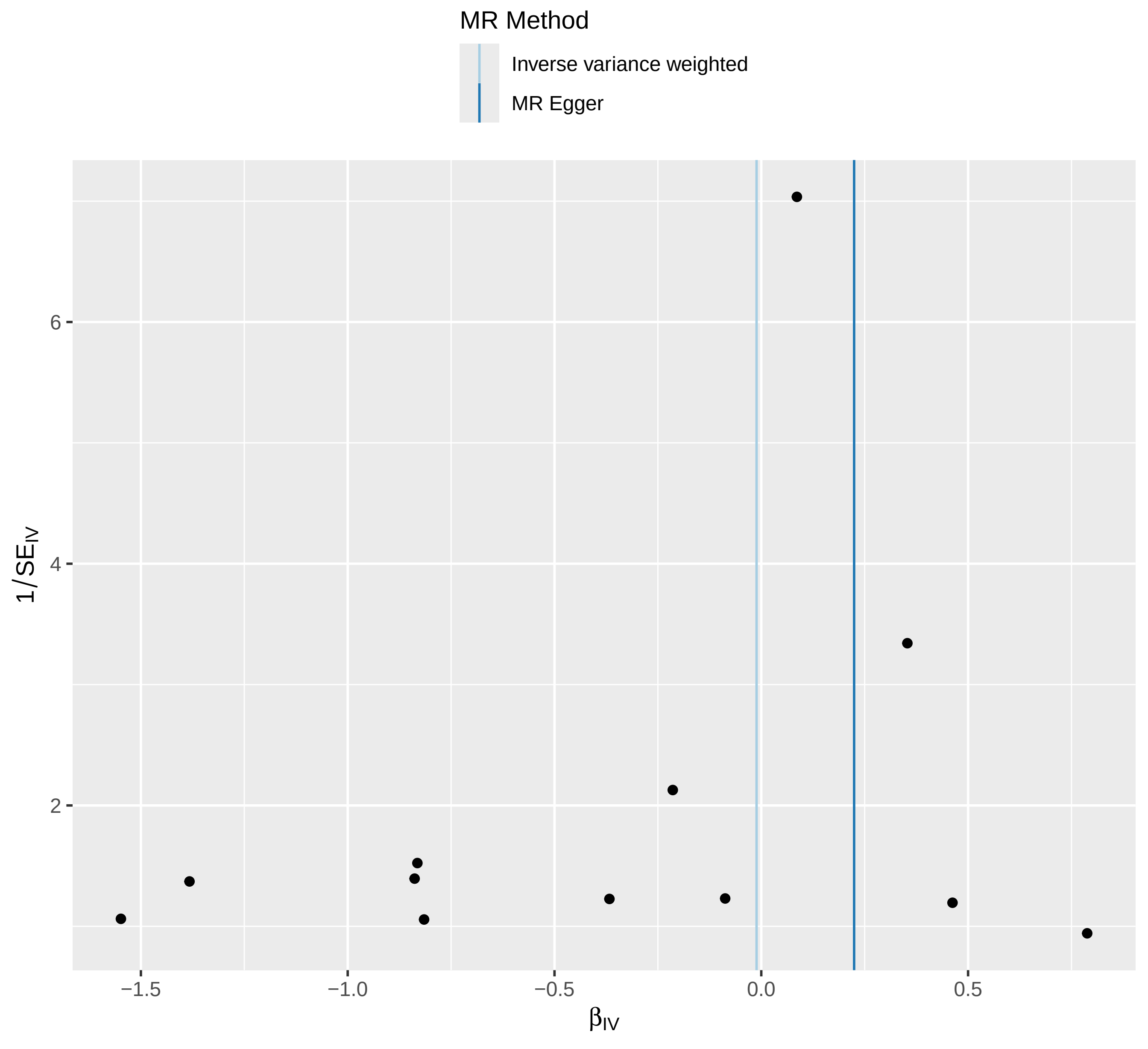
MR Method
Inverse variance weighted
MR Egger
1/SE_IV
6
4
2
−1.5
−1.0
−0.5
0.0
0.5
β_IV

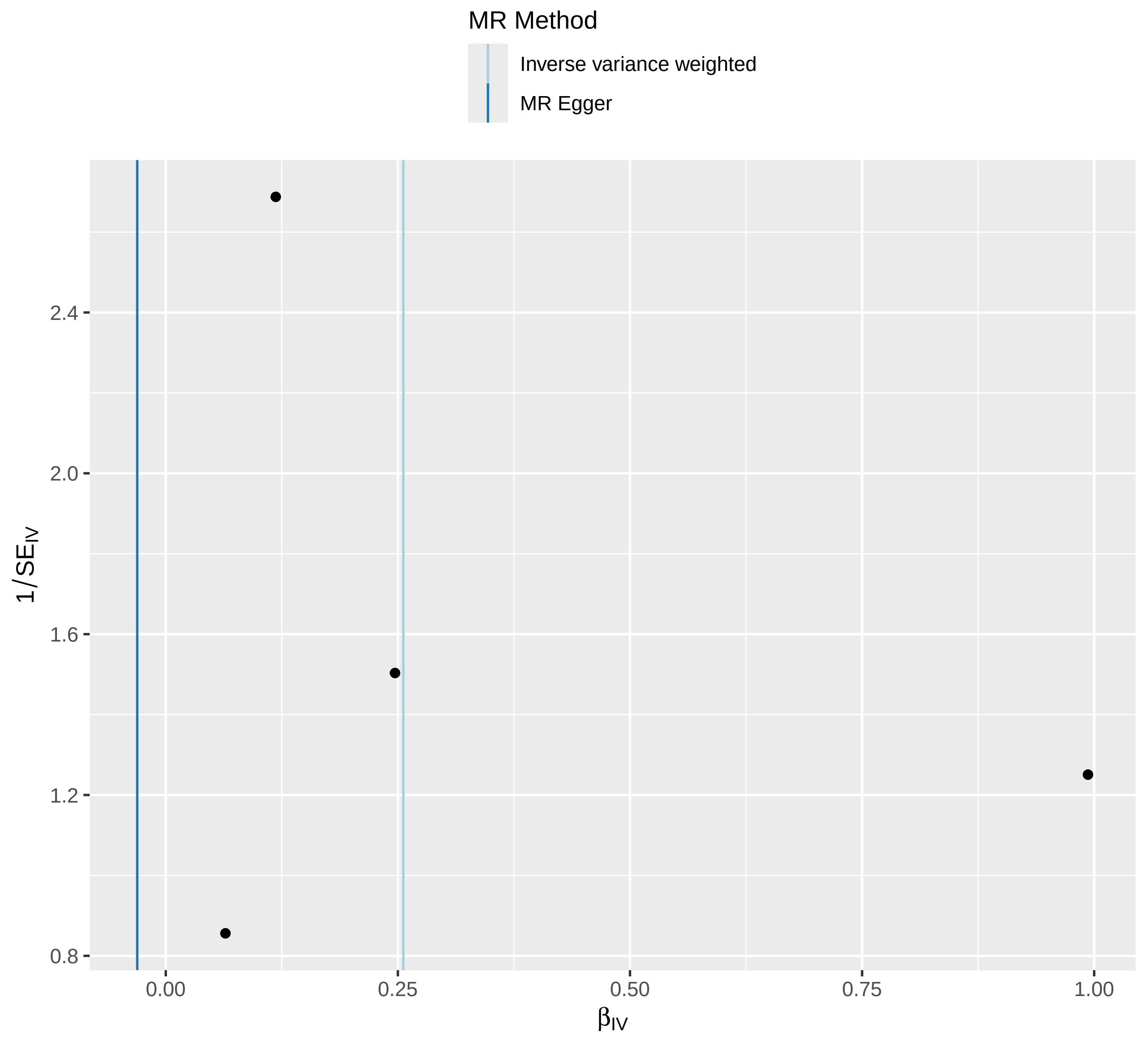
MR Method
Inverse variance weighted
MR Egger
2.4
2.0
1.6
1.2
0.8
$1/SE_{IV}$
0.00
0.25
0.50
0.75
1.00
β_{IV}

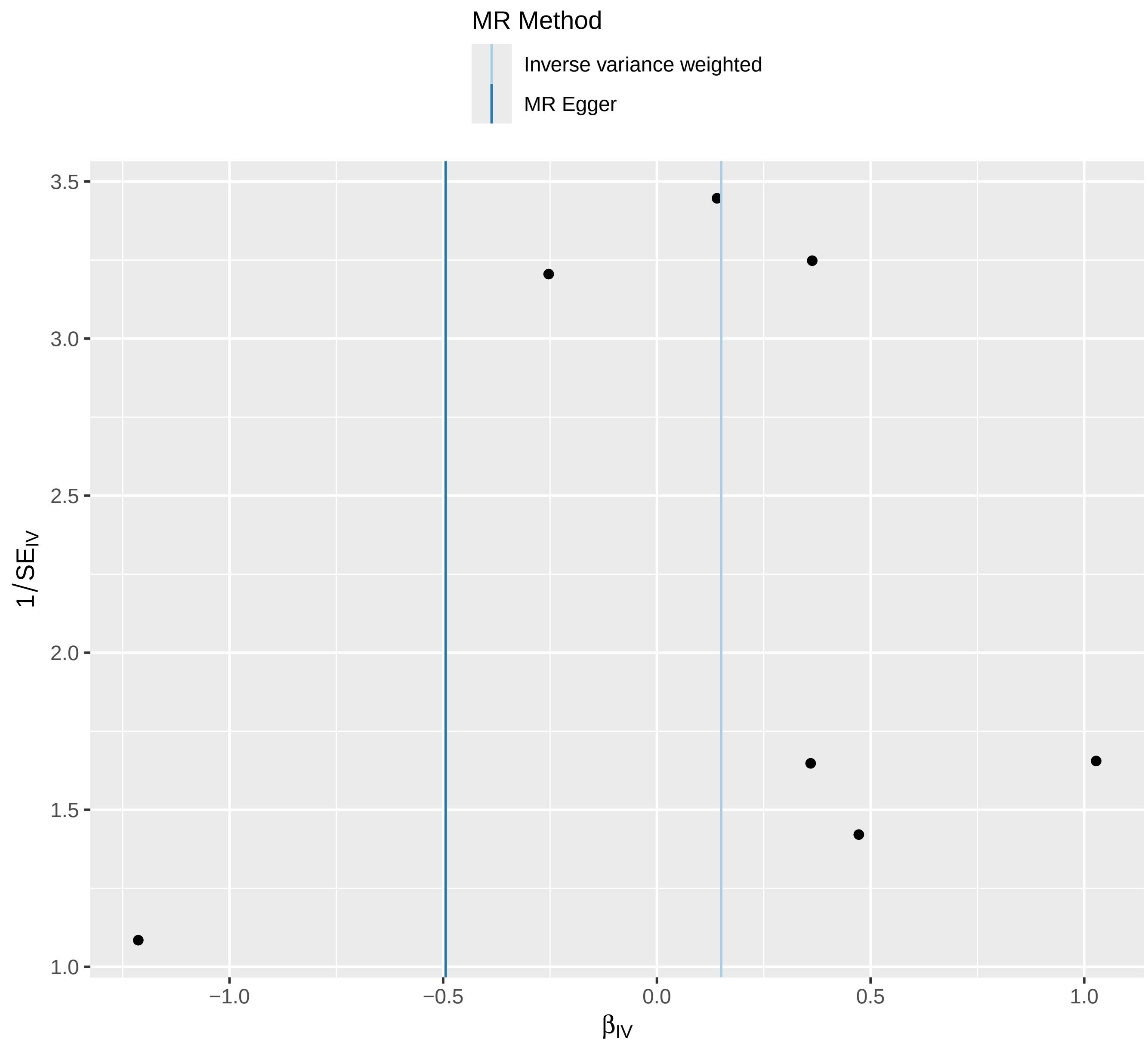
MR Method
Inverse variance weighted
MR Egger
3.5
3.0
2.5
2.0
1.5
1.0
1/SE$_{IV}$
−1.0
−0.5
0.0
0.5
1.0
β$_{IV}$

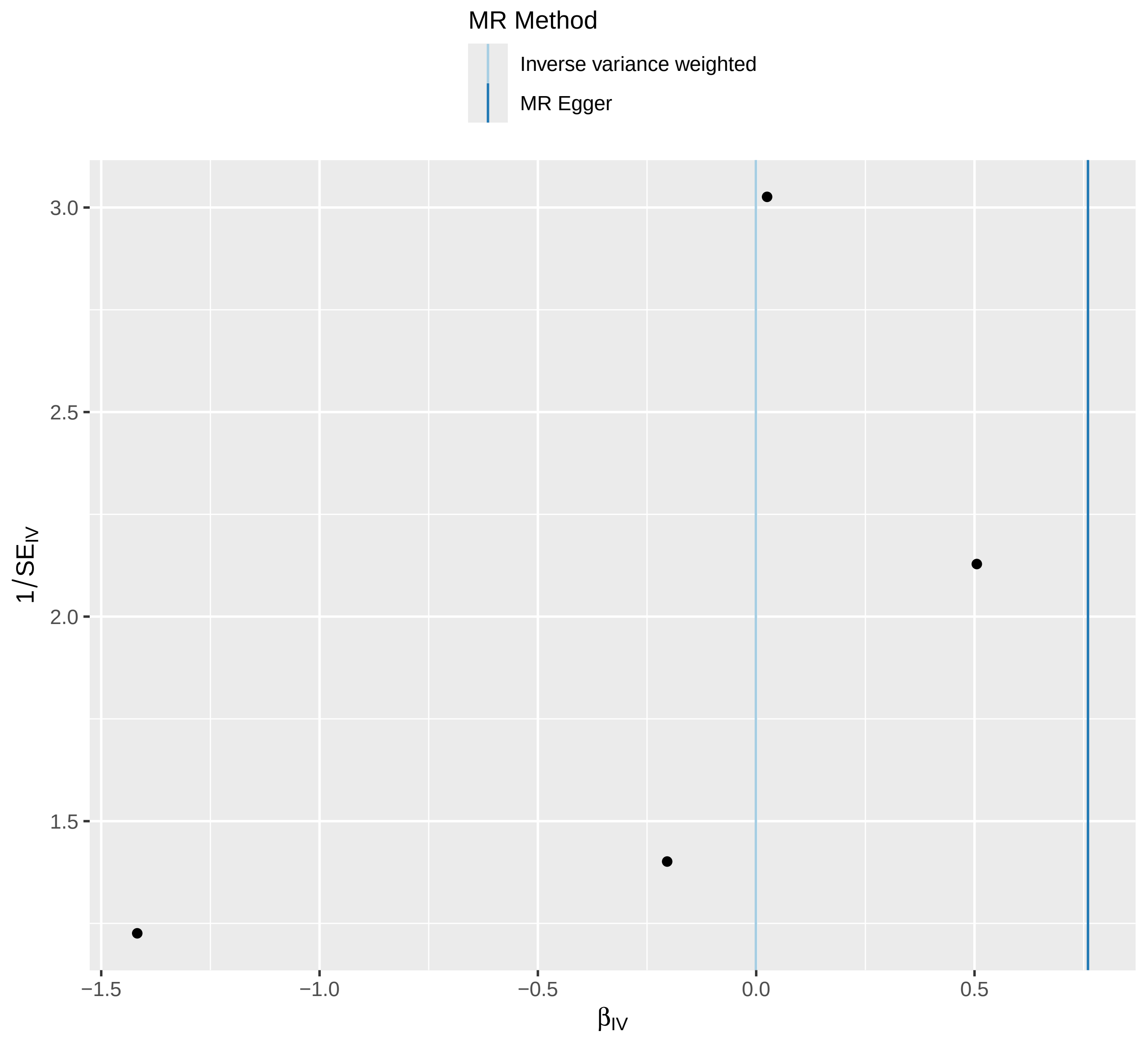
MR Method
Inverse variance weighted
MR Egger
3.0
2.5
2.0
1.5
1/SE_IV
−1.5
−1.0
−0.5
0.0
0.5
β_IV

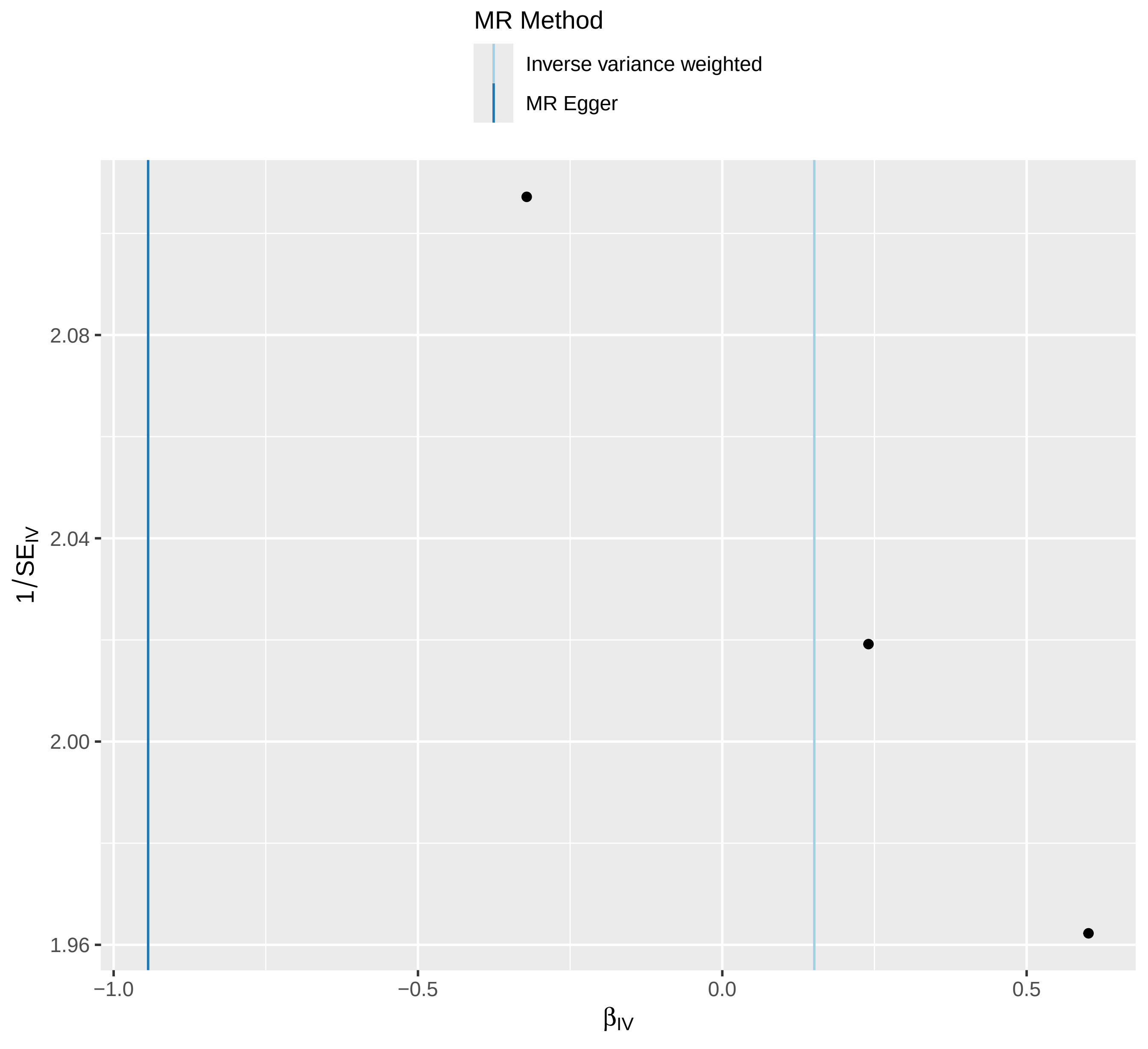
MR Method
Inverse variance weighted
MR Egger
1/SE_IV
2.08
2.04
2.00
1.96
−1.0
−0.5
0.0
0.5
β_IV

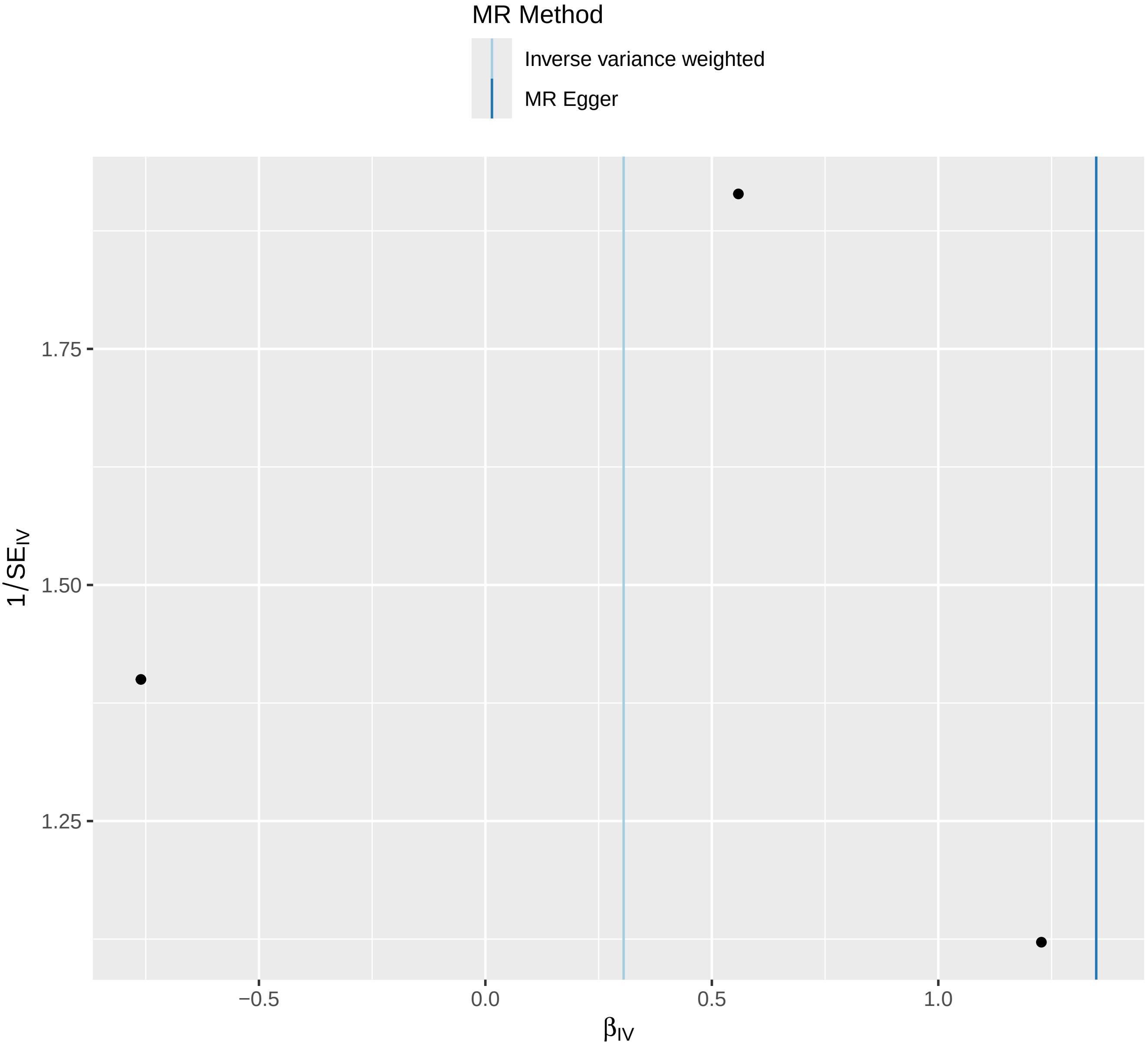
MR Method
Inverse variance weighted
MR Egger
1.75
1.50
1.25
$1/SE_{IV}$
−0.5
0.0
0.5
1.0
β_{IV}

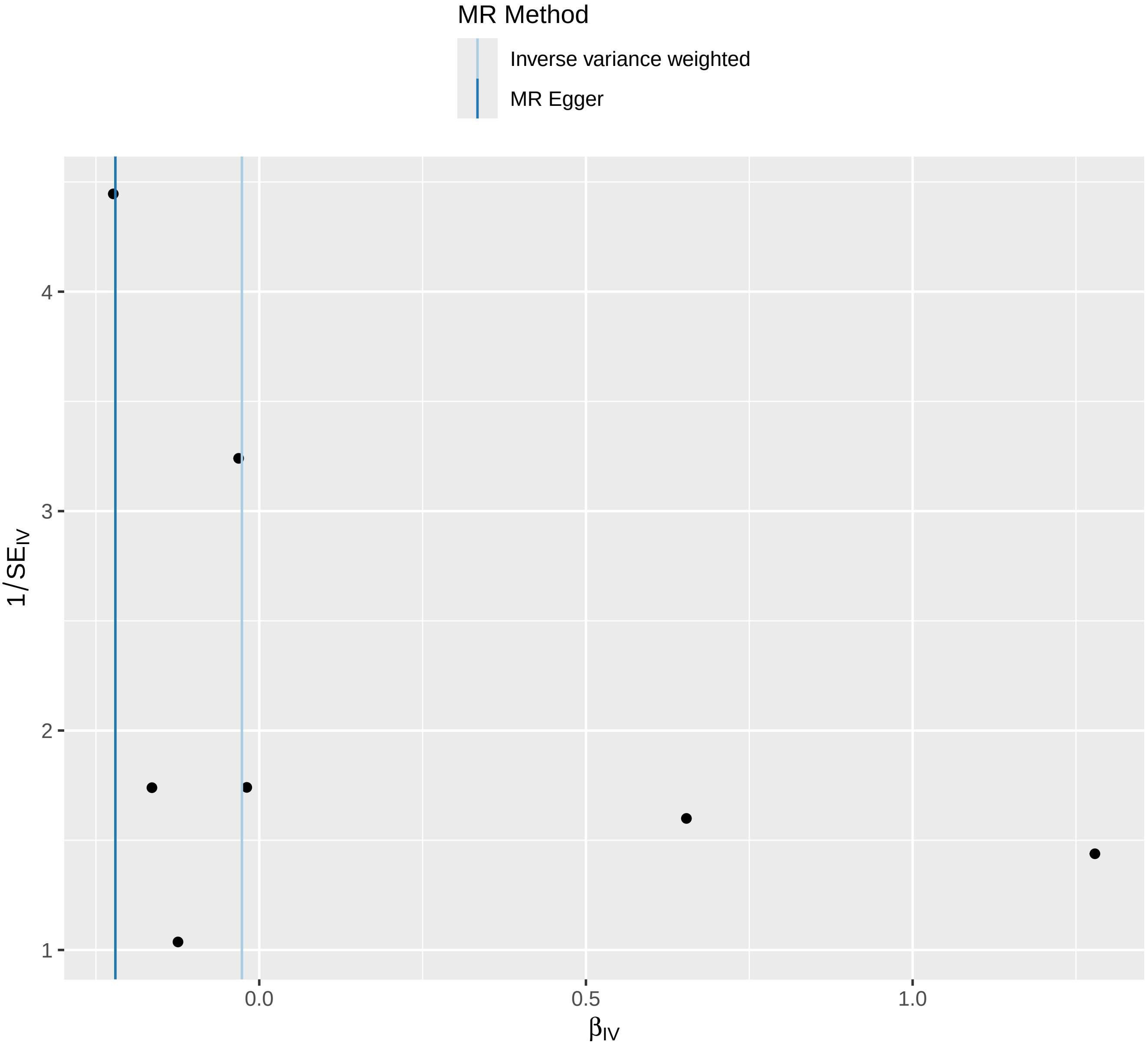
MR Method
Inverse variance weighted
MR Egger
1
2
3
4
0.0
0.5
1.0
β_IV
1/SE_IV

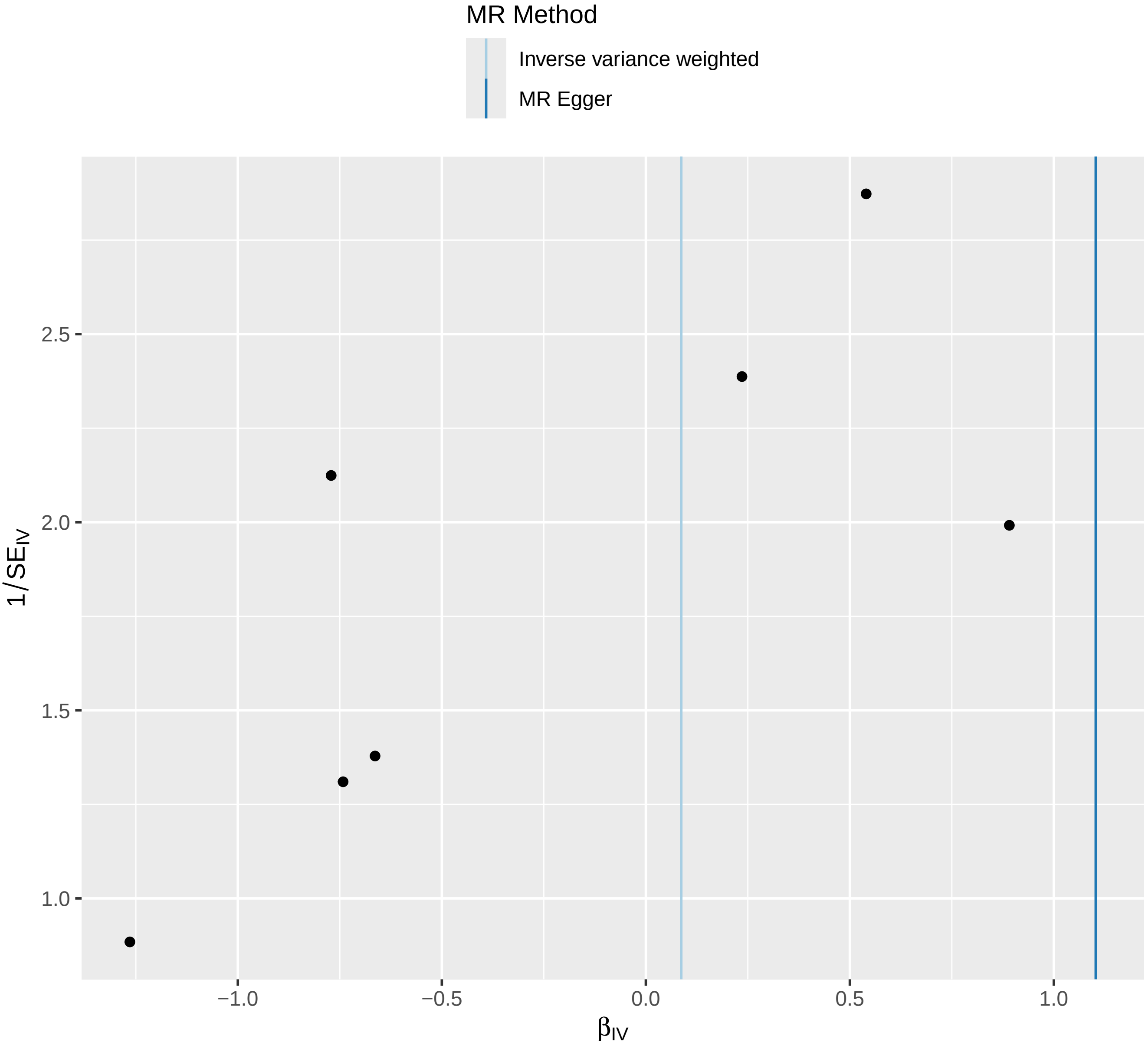

MR Method
Inverse variance weighted
MR Egger
2.5
2.0
1.5
1.0
1/SE_IV
−1.0
−0.5
0.0
0.5
1.0
β_IV

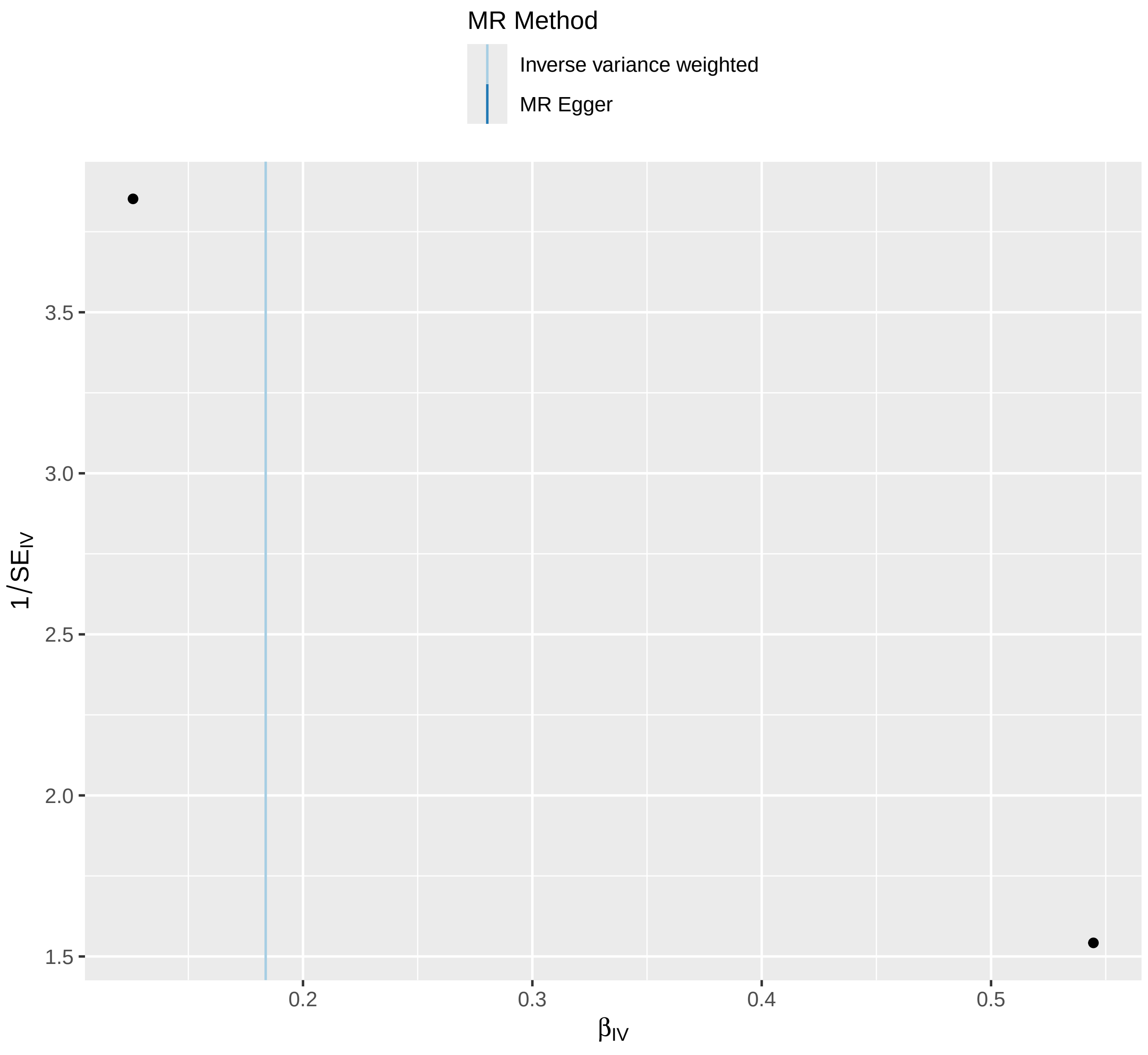

MR Method
Inverse variance weighted
MR Egger
3.5
3.0
2.5
2.0
1.5
1/SE_IV
0.2
0.3
0.4
0.5
β_IV

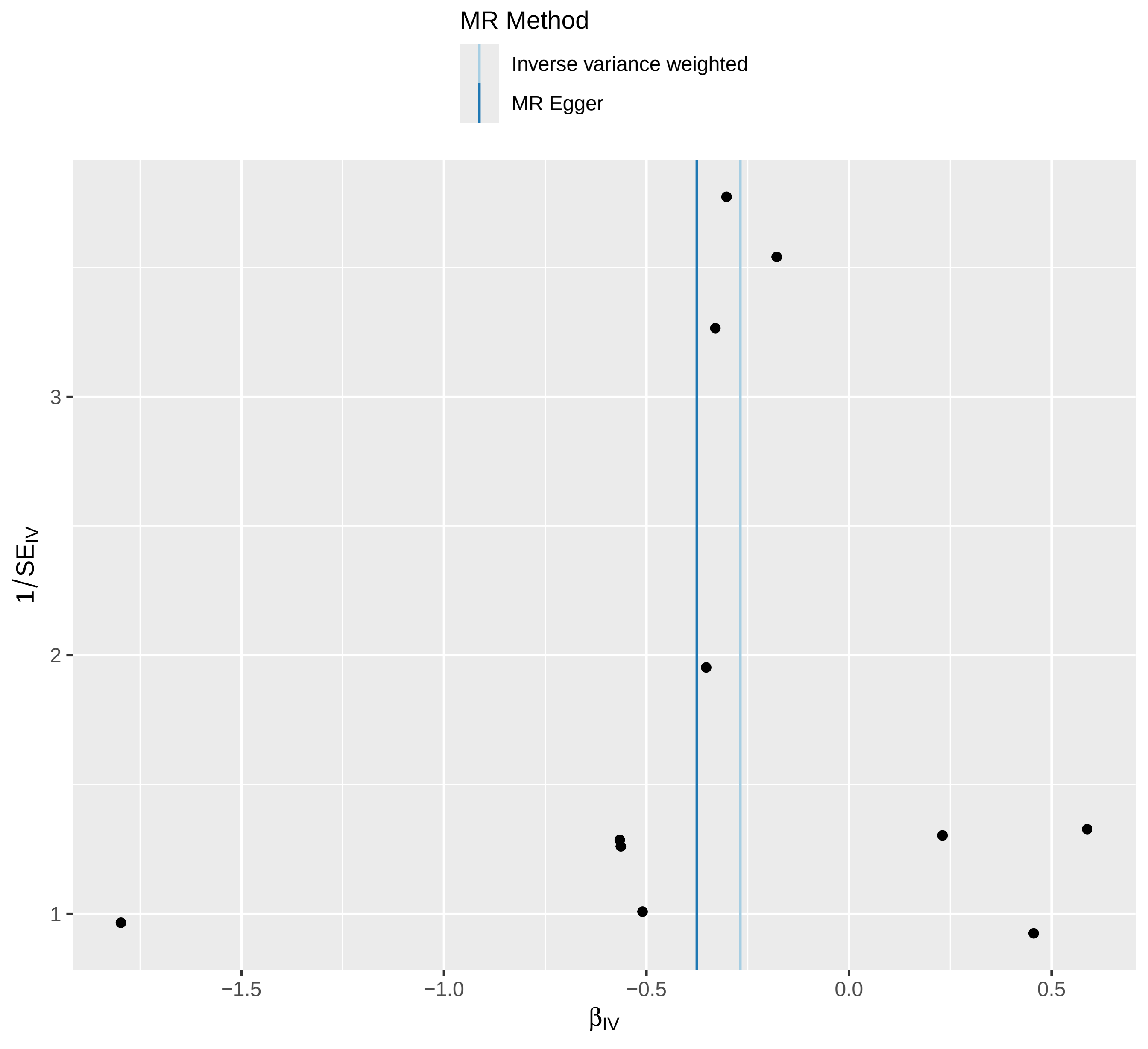

MR Method
Inverse variance weighted
MR Egger
$1/SE_{IV}$
3
2
1
−1.5
−1.0
−0.5
0.0
0.5
β_{IV}

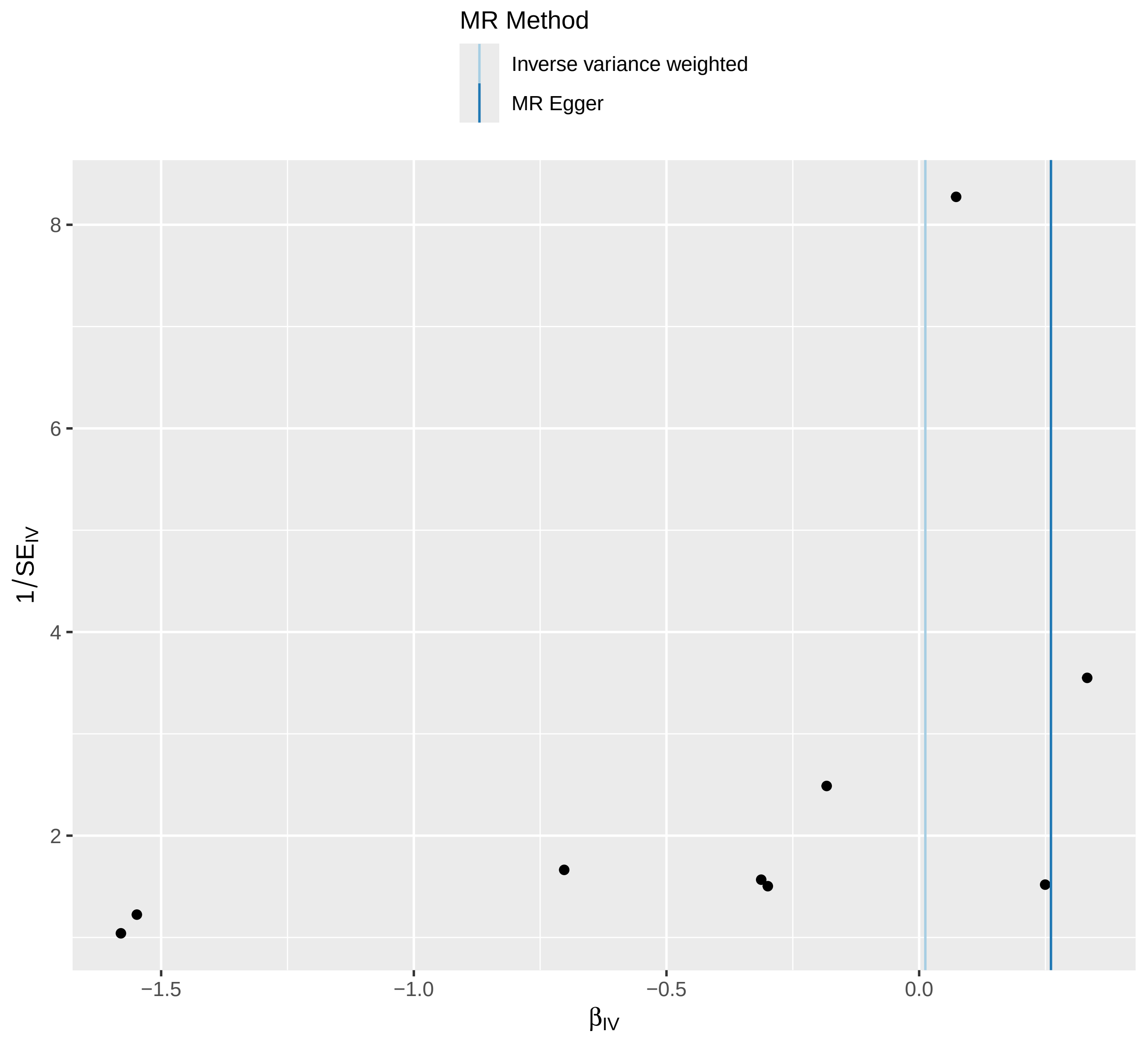

MR Method
Inverse variance weighted
MR Egger
8
6
4
2
1/SE_IV
−1.5
−1.0
−0.5
0.0
β_IV

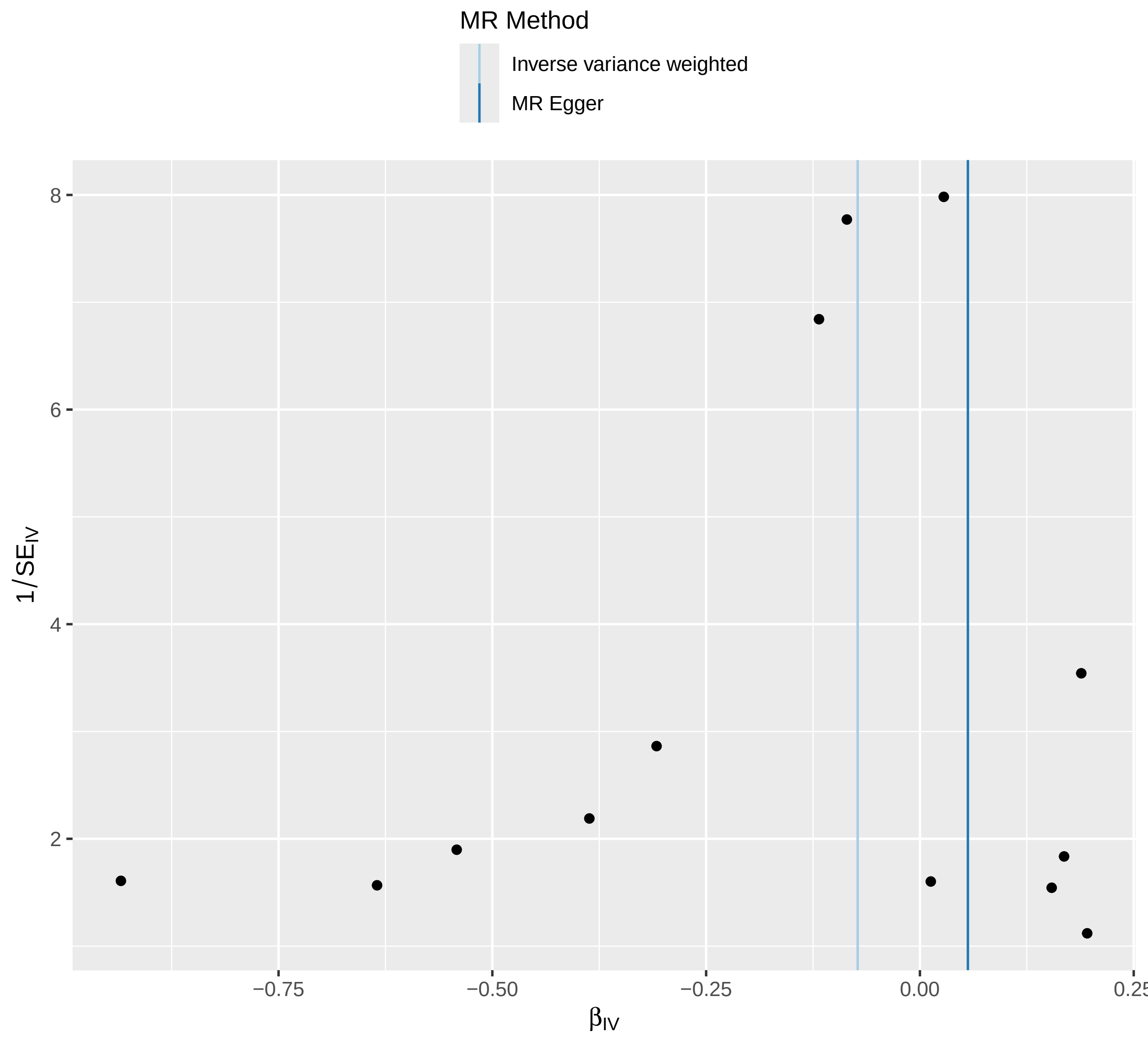
MR Method
Inverse variance weighted
MR Egger
8
6
4
2
1/SE_IV
−0.75
−0.50
−0.25
0.00
0.25
β_IV

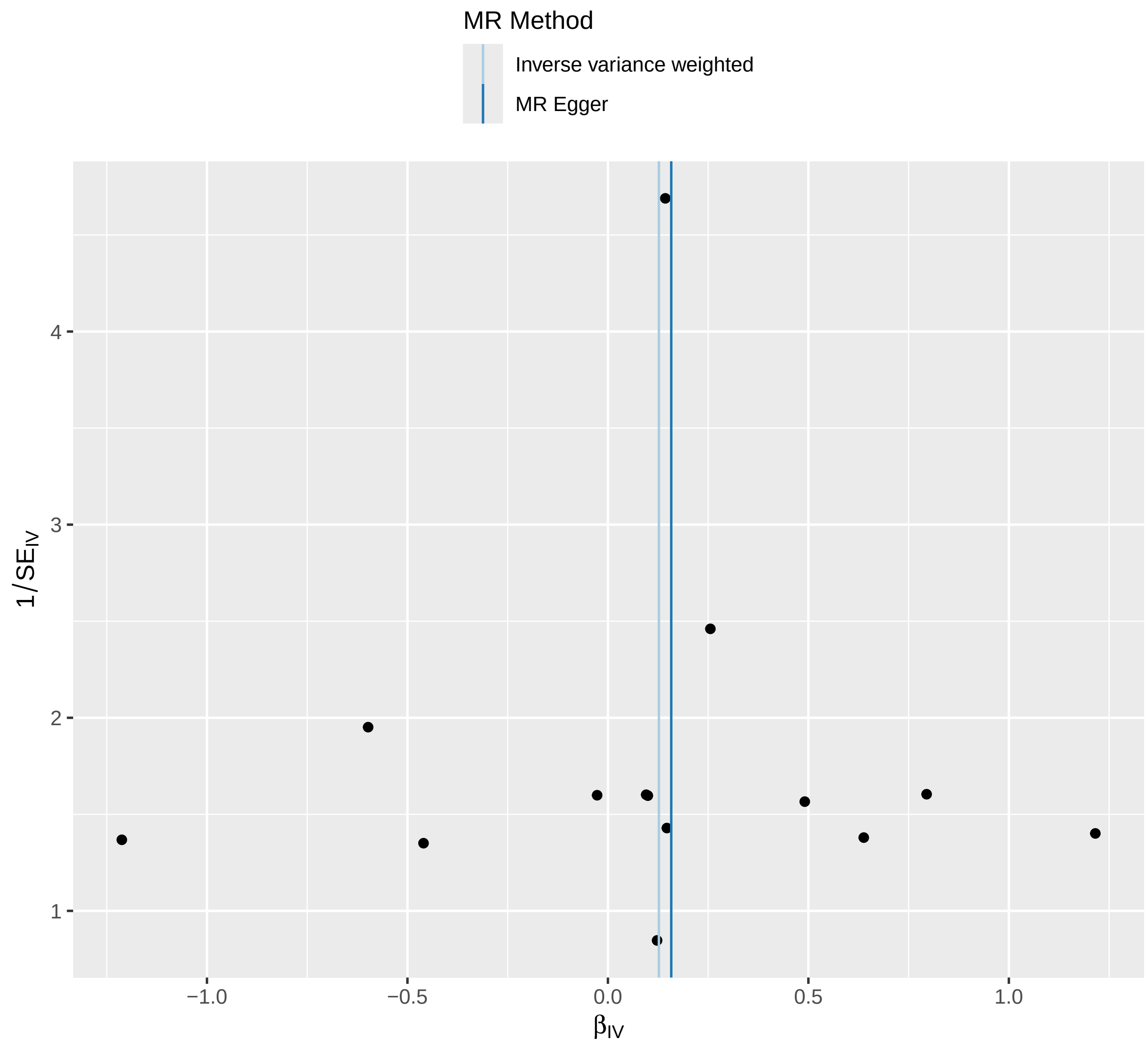
MR Method
Inverse variance weighted
MR Egger
4
3
2
1
1/SE$_{IV}$
−1.0
−0.5
0.0
0.5
1.0
β$_{IV}$

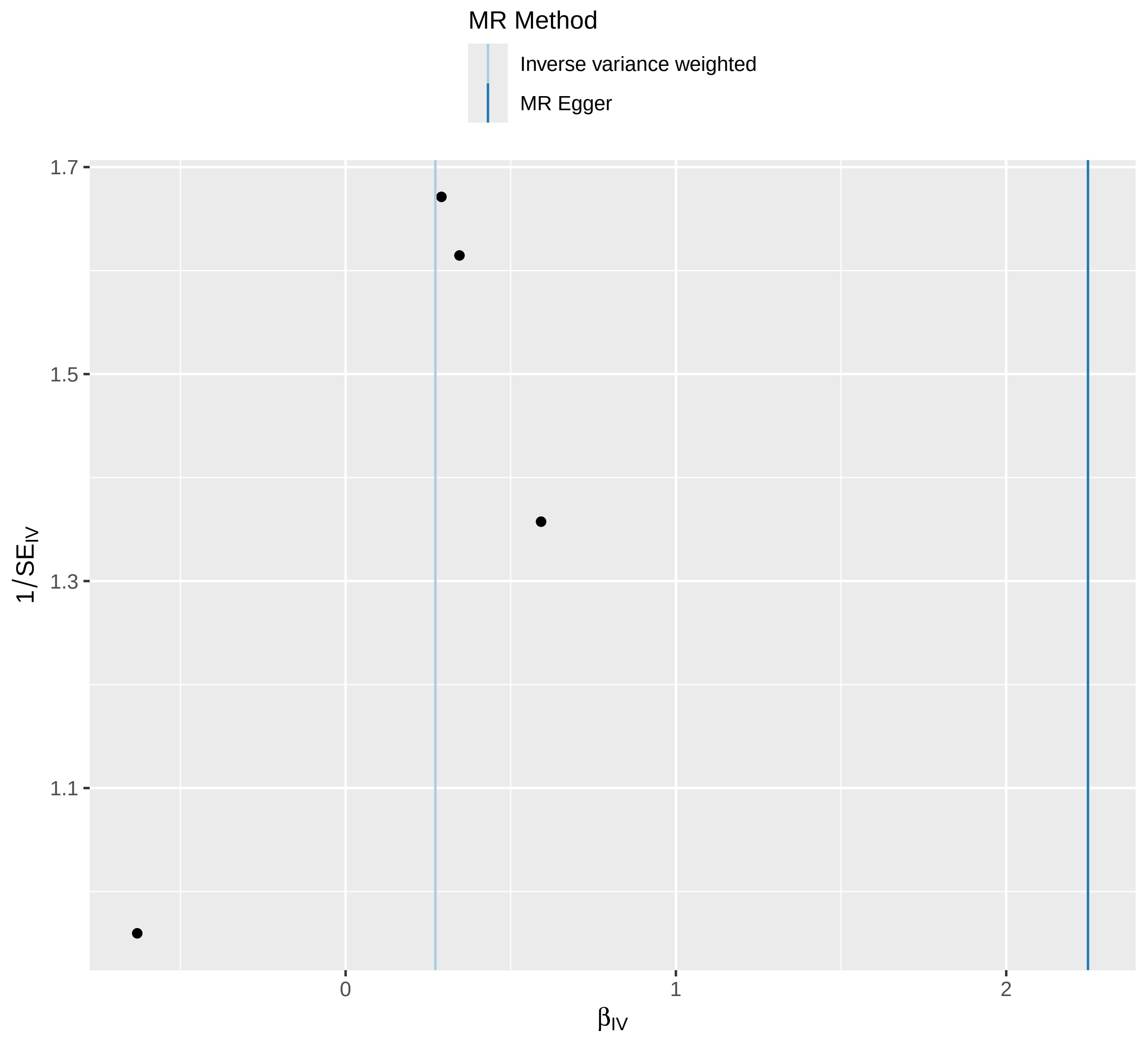
MR Method
Inverse variance weighted
MR Egger
1.7
1.5
1.3
1.1
0
1
2
1/SE$_{IV}$
β$_{IV}$

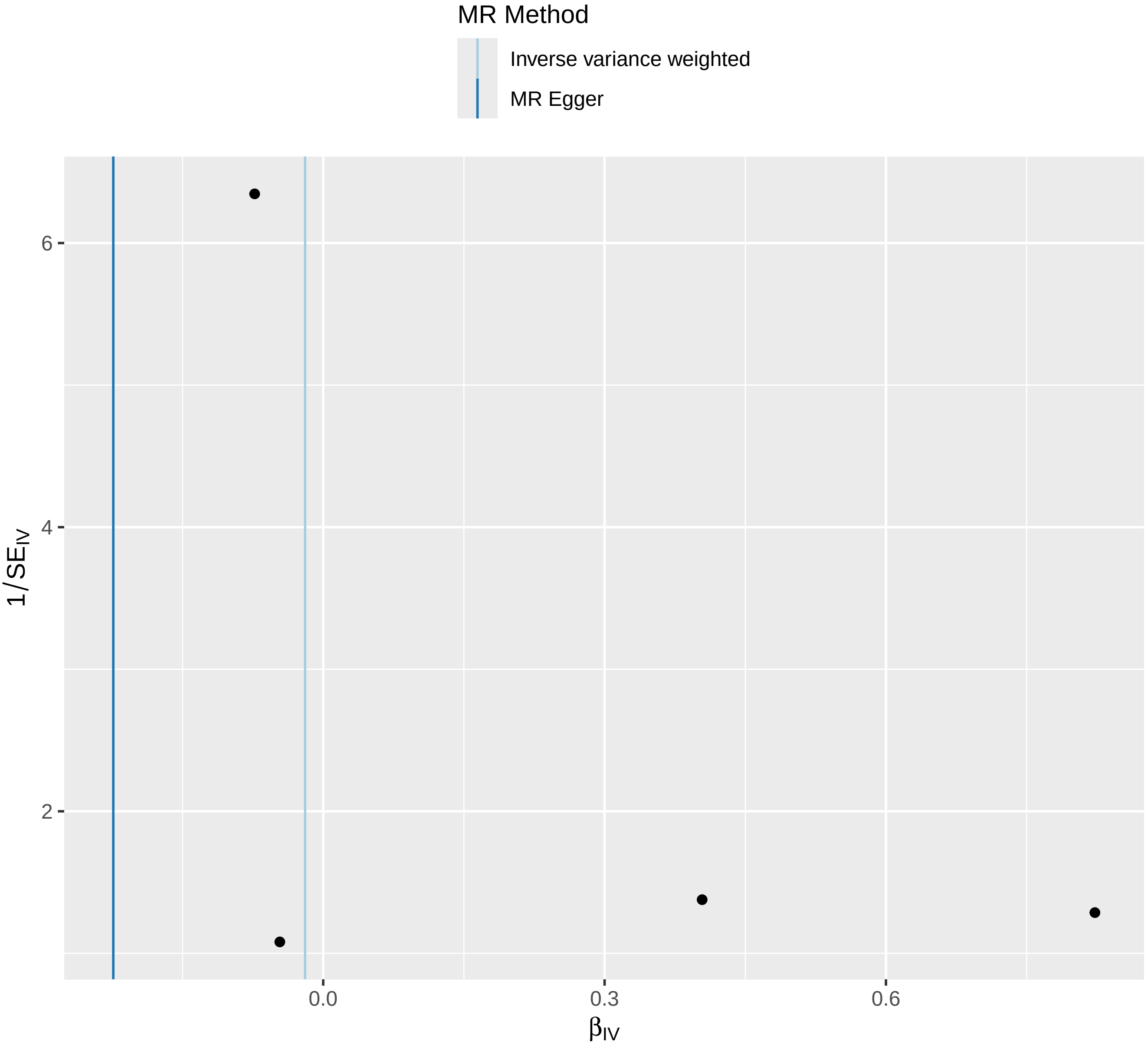
MR Method
Inverse variance weighted
MR Egger
6
4
2
1/SE_IV
0.0
0.3
0.6
β_IV

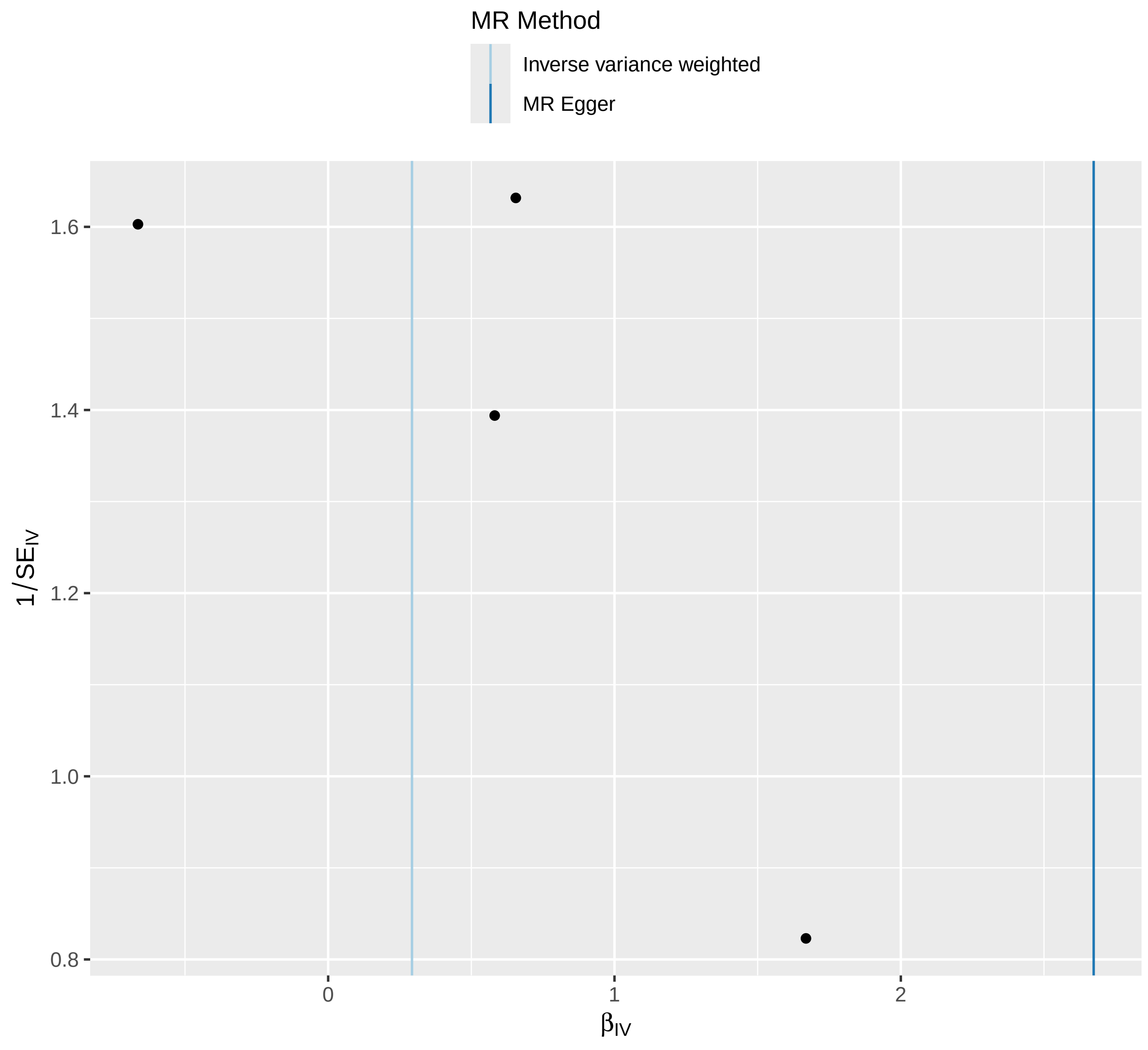

MR Method
Inverse variance weighted
MR Egger
1.6
1.4
1.2
1.0
0.8
$1/SE_{IV}$
0
1
2
β_{IV}

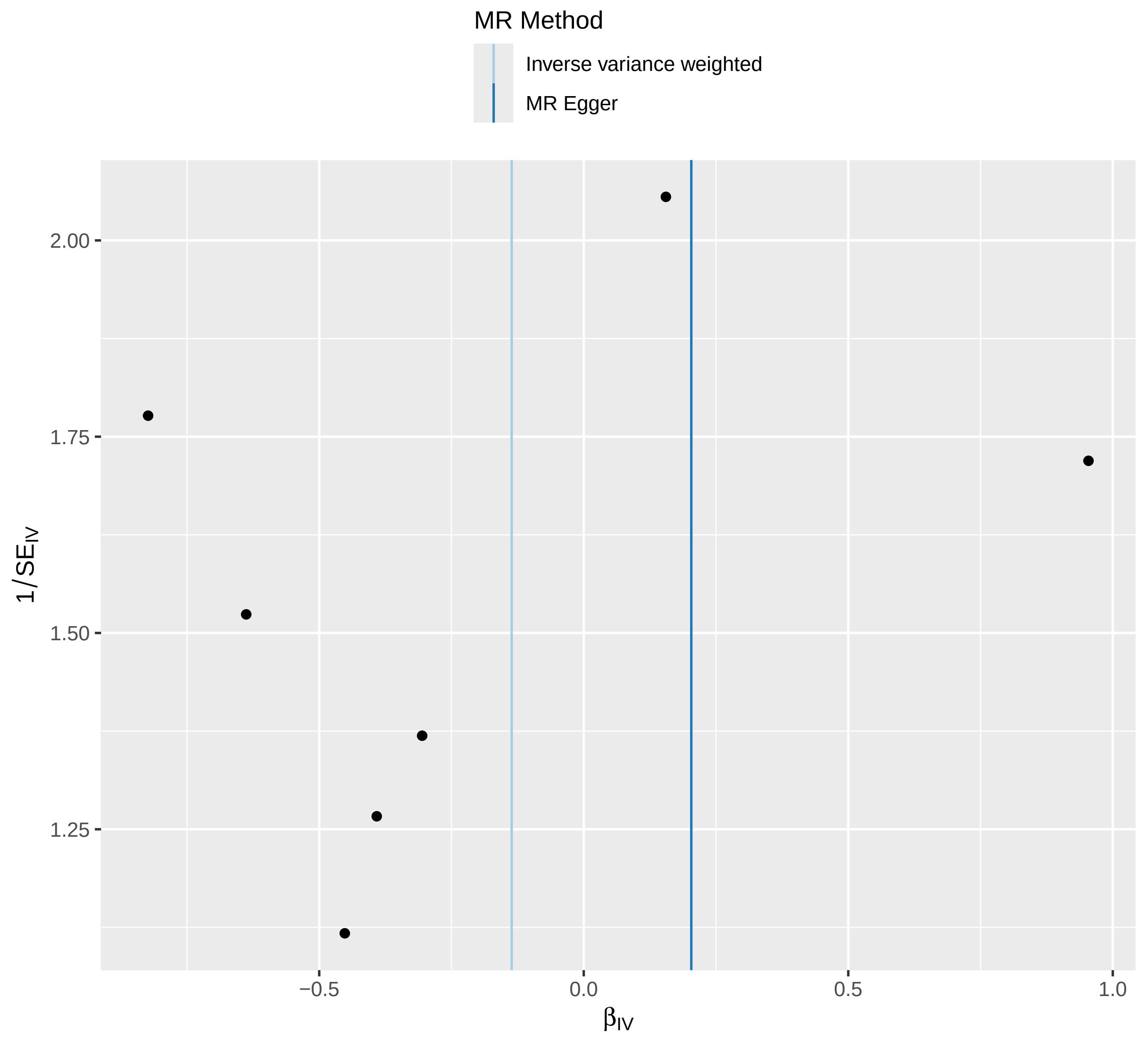

MR Method
Inverse variance weighted
MR Egger
2.00
1.75
1.50
1.25
1/SE_IV
−0.5
0.0
0.5
1.0
β_IV

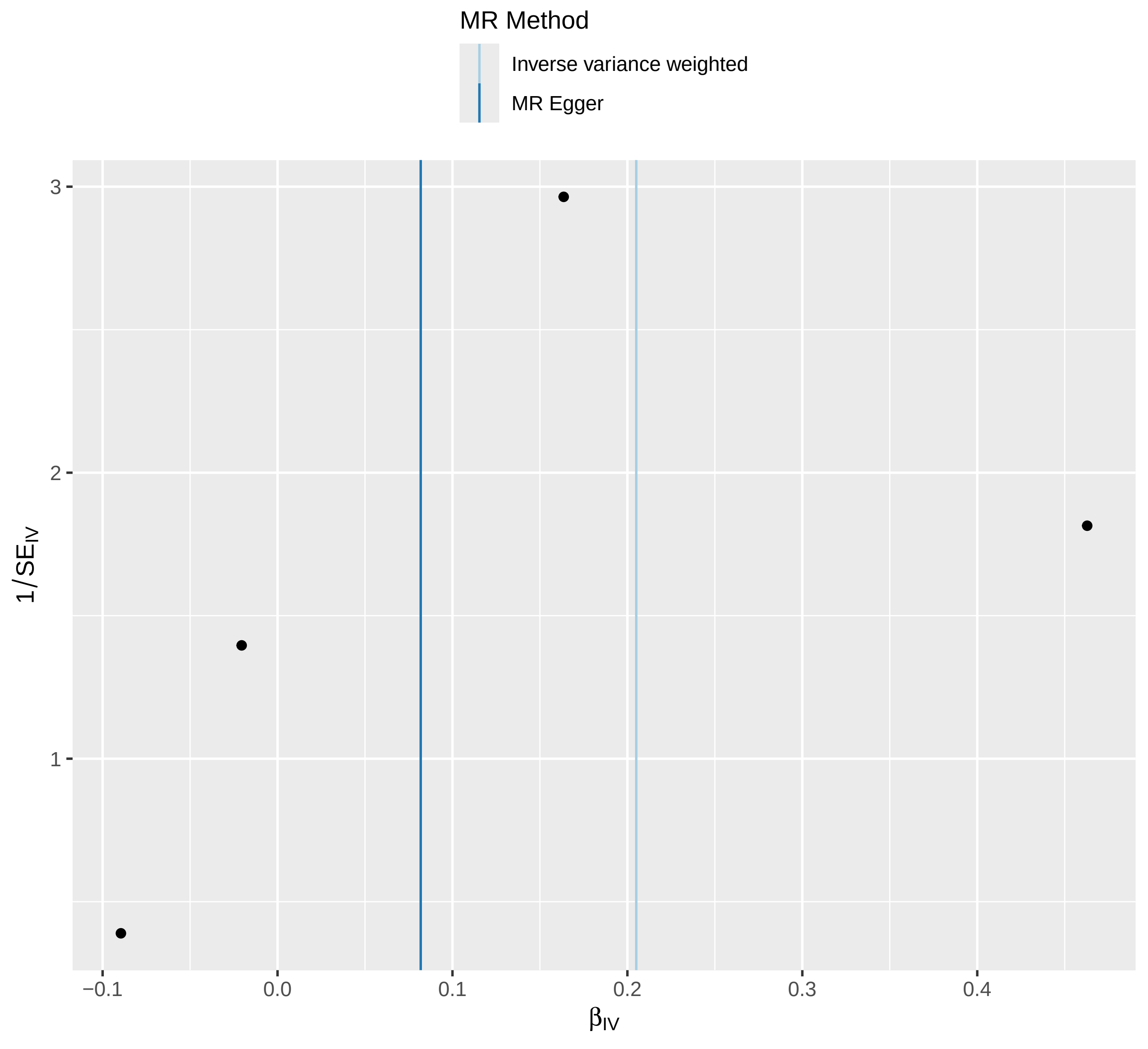

MR Method
Inverse variance weighted
MR Egger
3
2
1
1/SE_IV
−0.1
0.0
0.1
0.2
0.3
0.4
β_IV

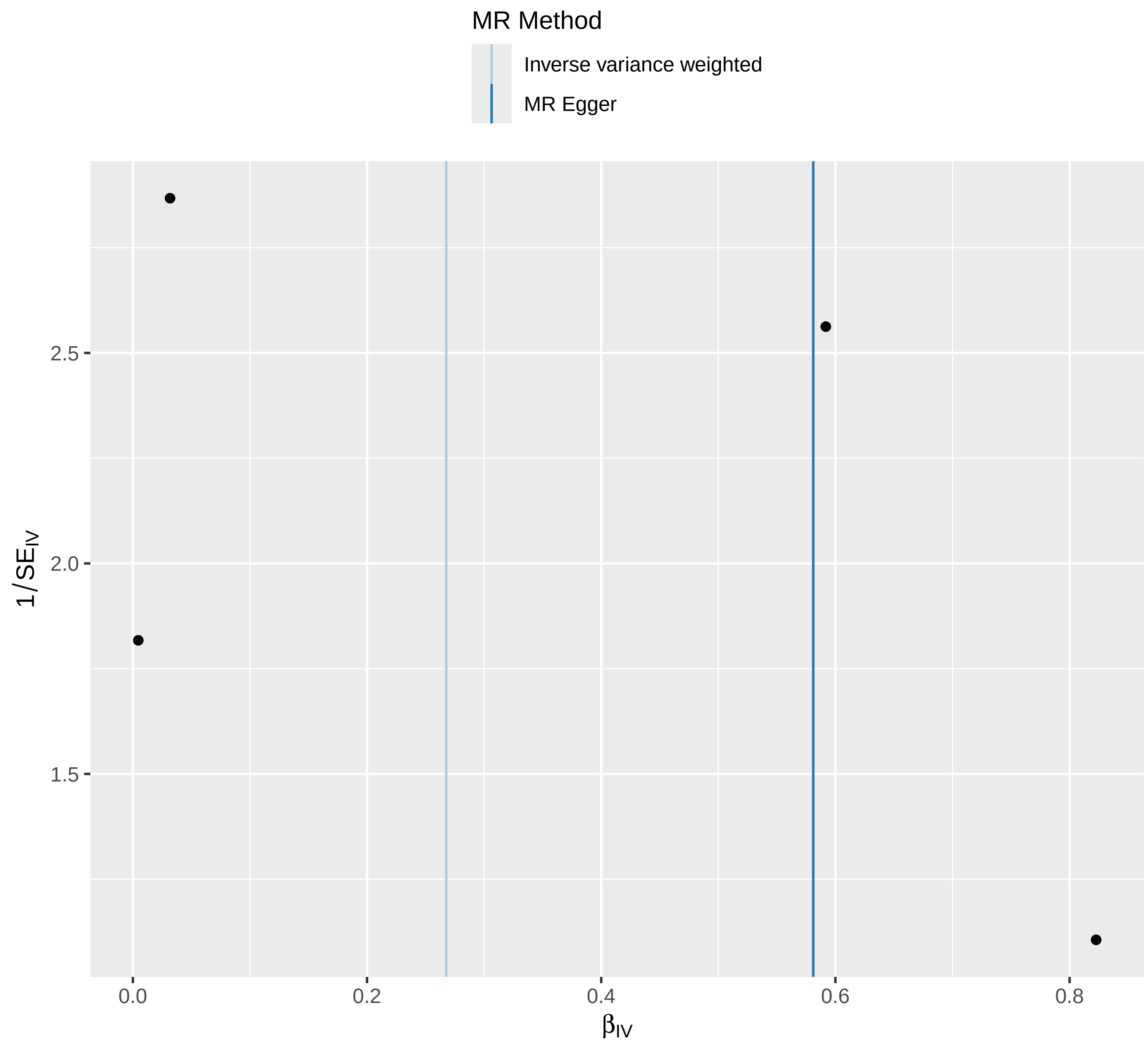
MR Method
Inverse variance weighted
MR Egger
1/SE$_{IV}$
2.5
2.0
1.5
0.0
0.2
0.4
0.6
0.8
β_{IV}

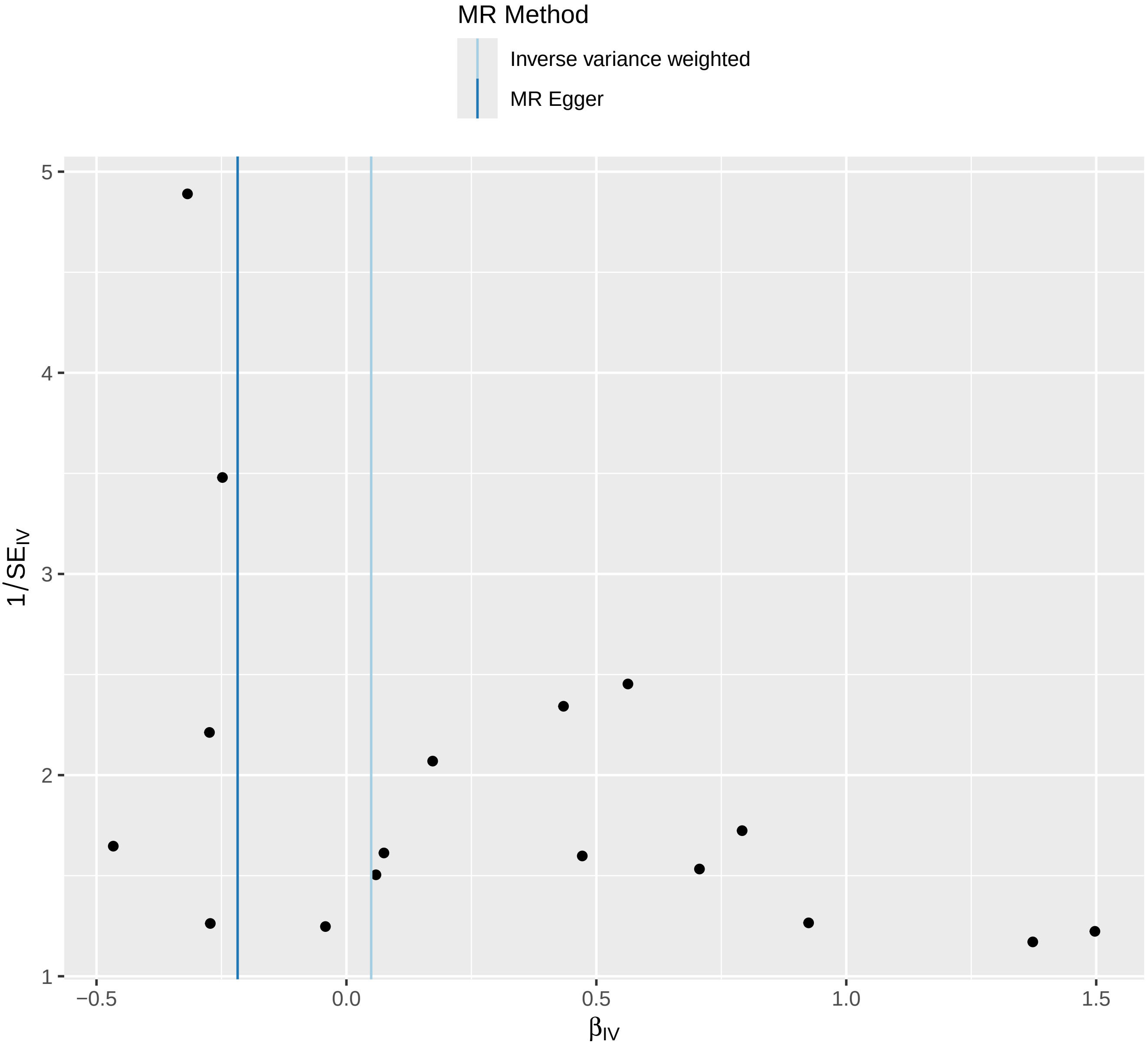

MR Method
Inverse variance weighted
MR Egger
5
4
3
2
1
$1/SE_{IV}$
−0.5
0.0
0.5
1.0
1.5
β_{IV}

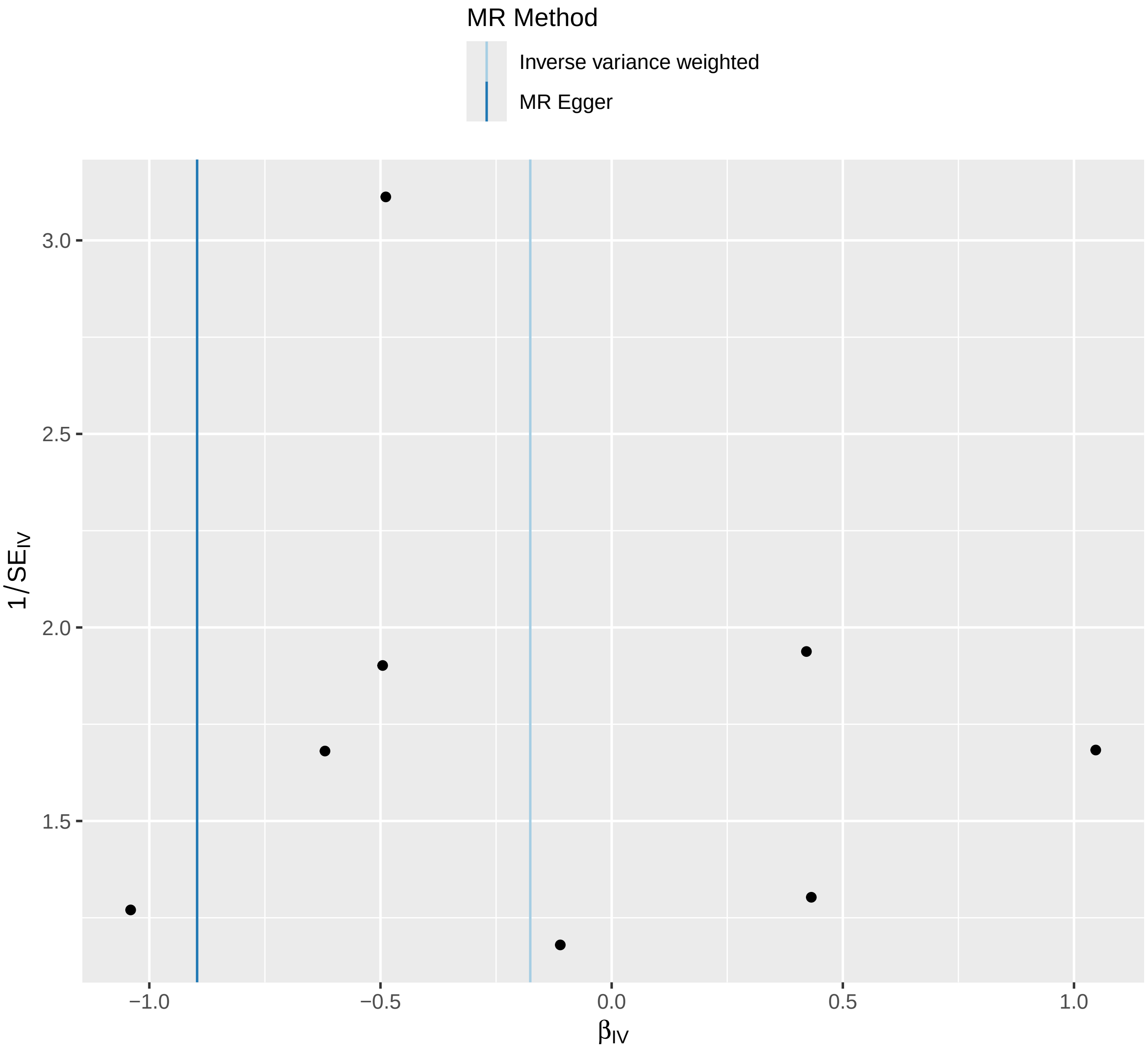
MR Method
Inverse variance weighted
MR Egger
3.0
2.5
2.0
1.5
1/SE_IV
−1.0
−0.5
0.0
0.5
1.0
β_IV

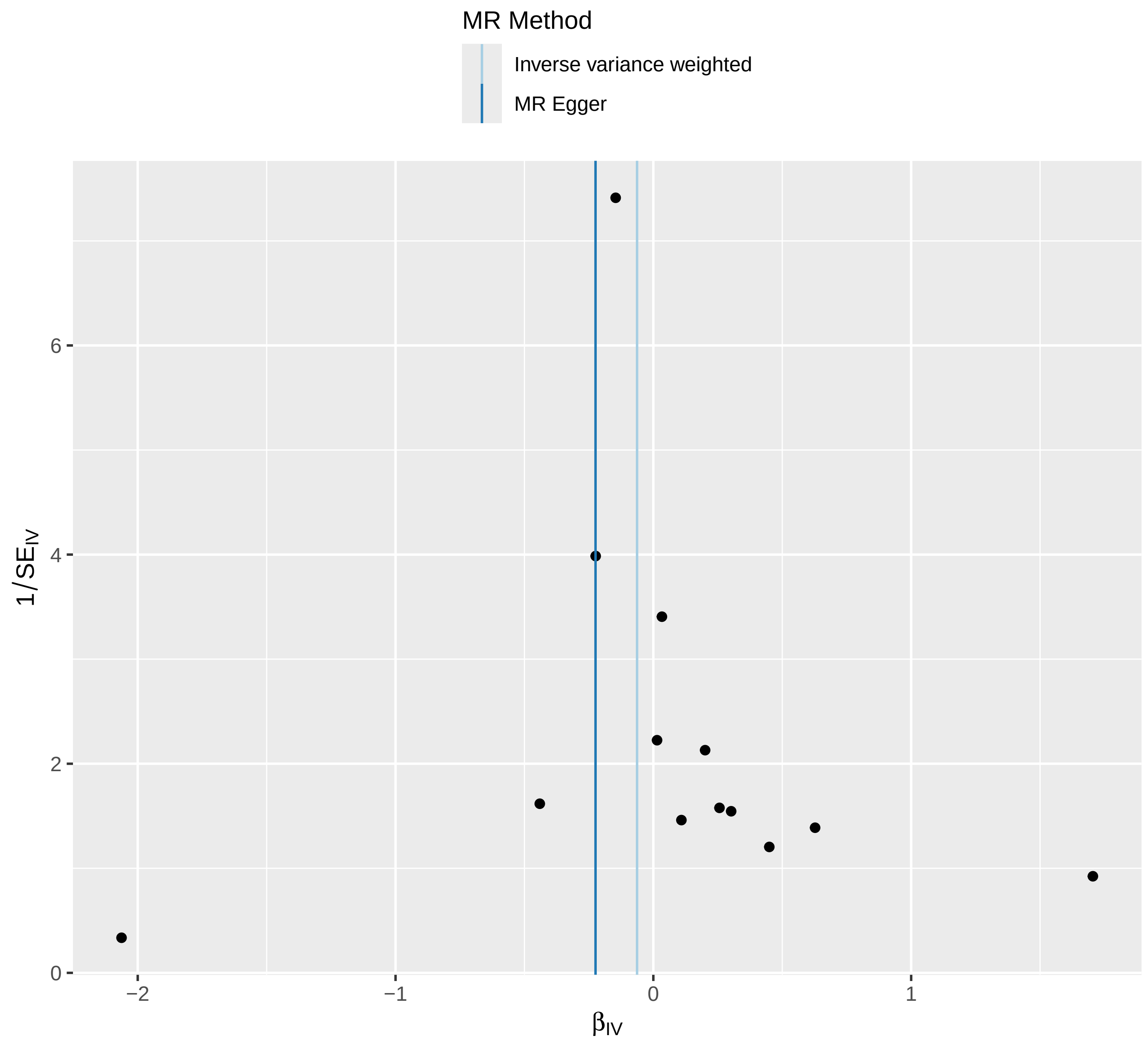
MR Method
Inverse variance weighted
MR Egger
1/SE$_{IV}$
6
4
2
0
−2
−1
0
1
β$_{IV}$

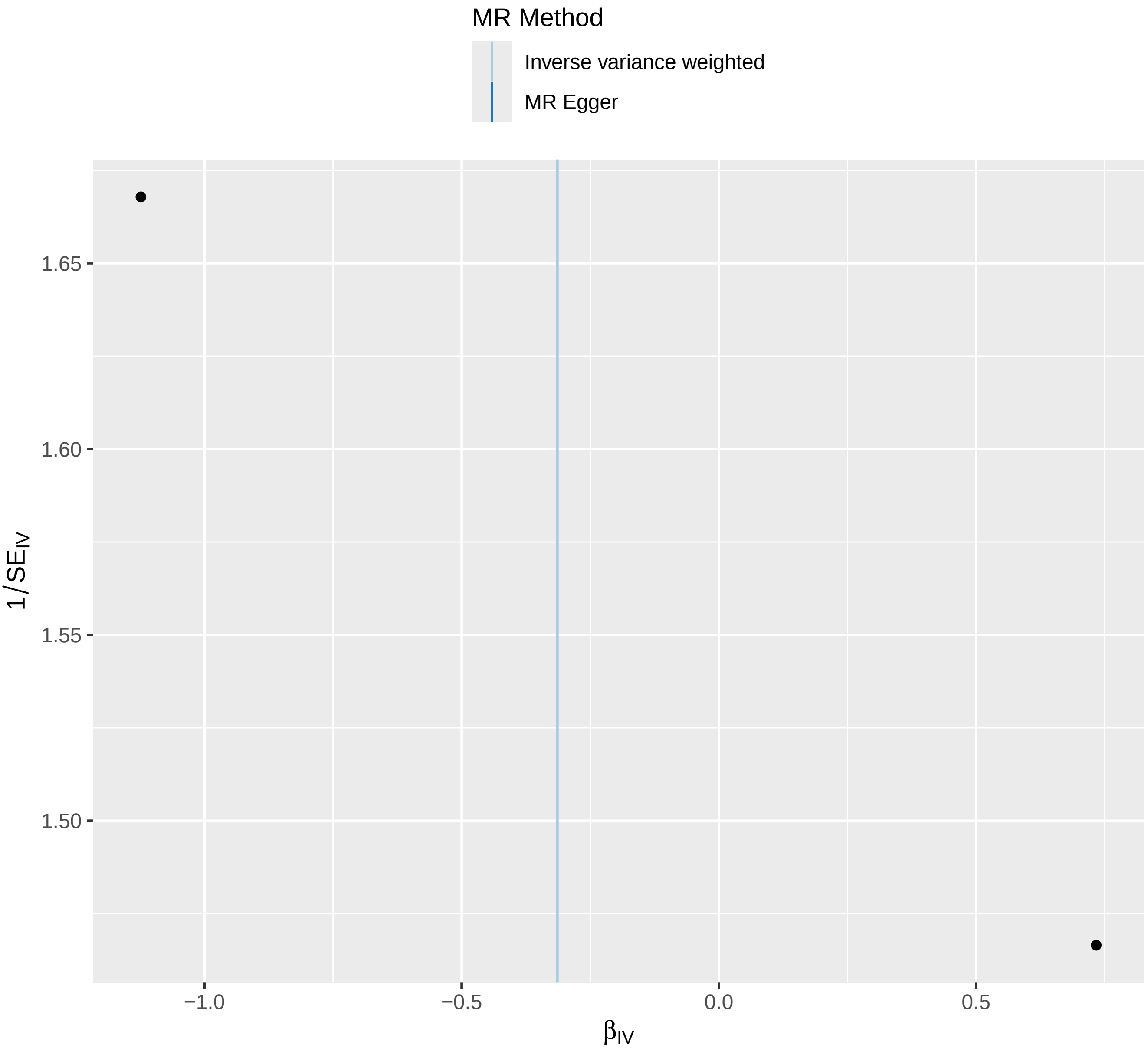
MR Method
Inverse variance weighted
MR Egger
1.65
1.60
1.55
1.50
1/SE_IV
−1.0
−0.5
0.0
0.5
β_IV

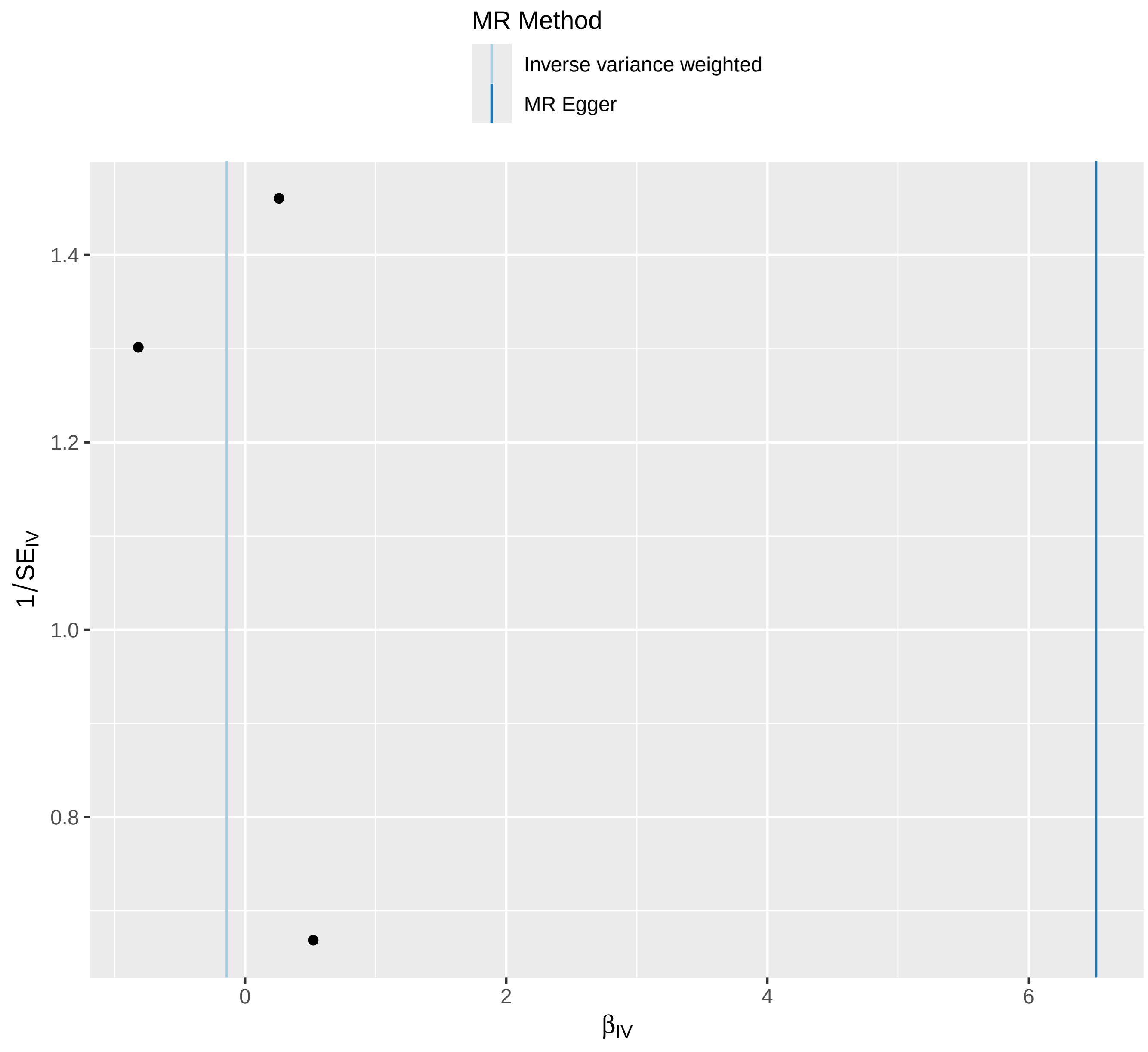
MR Method
Inverse variance weighted
MR Egger
1.4
1.2
1.0
0.8
$1/SE_{IV}$
0
2
4
6
β_{IV}

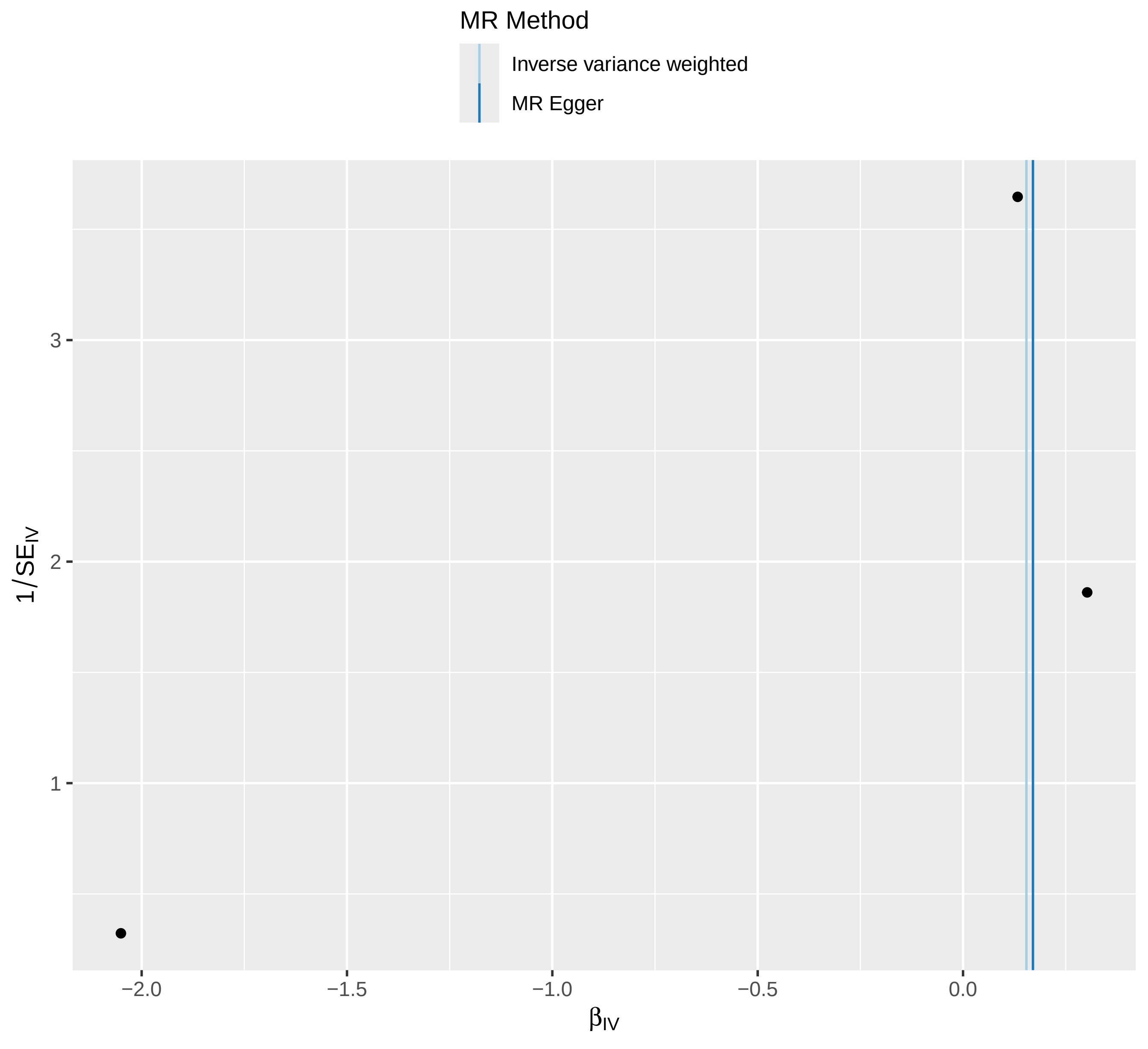

MR Method
Inverse variance weighted
MR Egger
3
2
1
1/SE$_{IV}$
−2.0
−1.5
−1.0
−0.5
0.0
β$_{IV}$

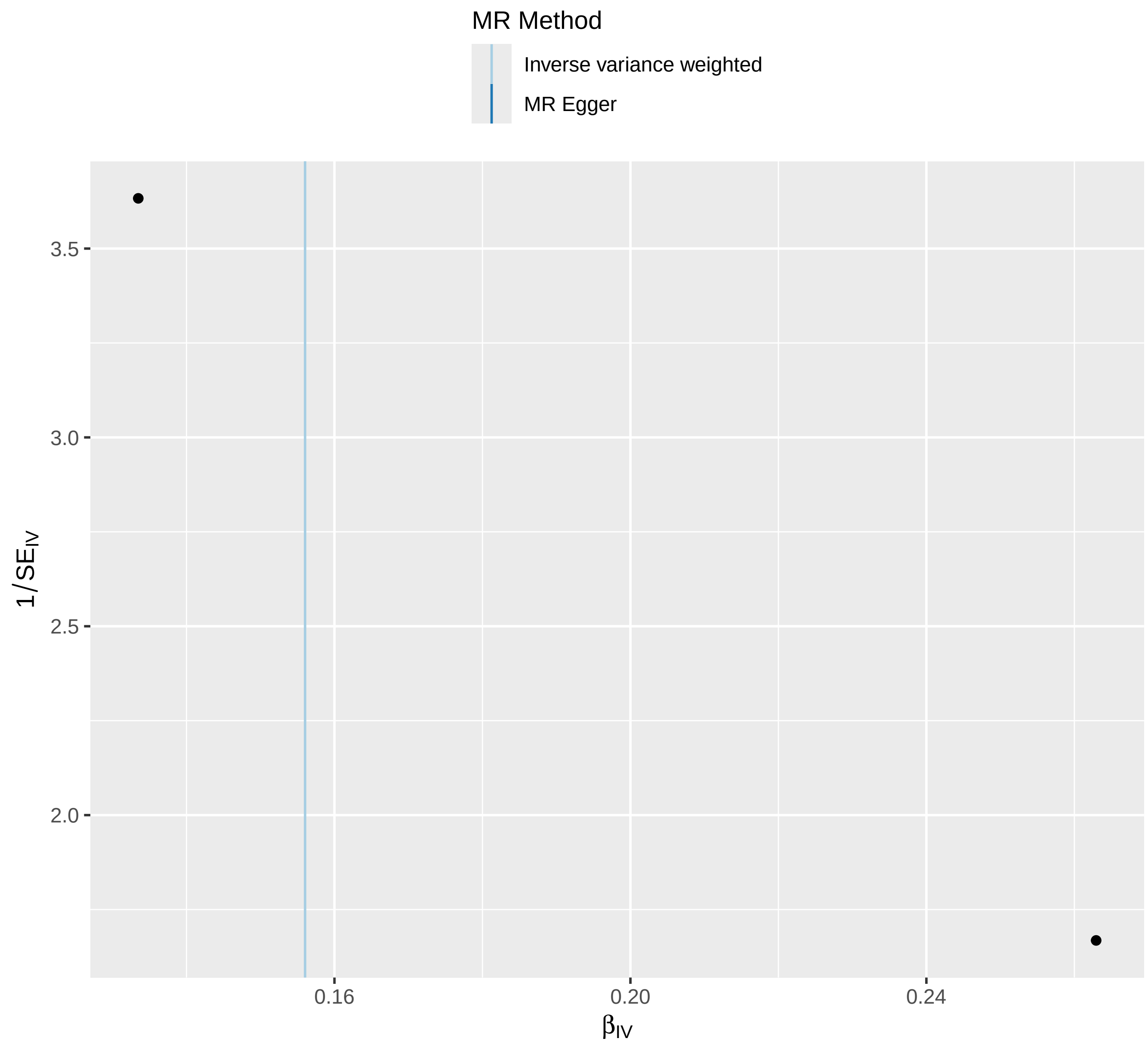

MR Method
Inverse variance weighted
MR Egger
3.5
3.0
2.5
2.0
1/SE_IV
0.16
0.20
0.24
β_IV

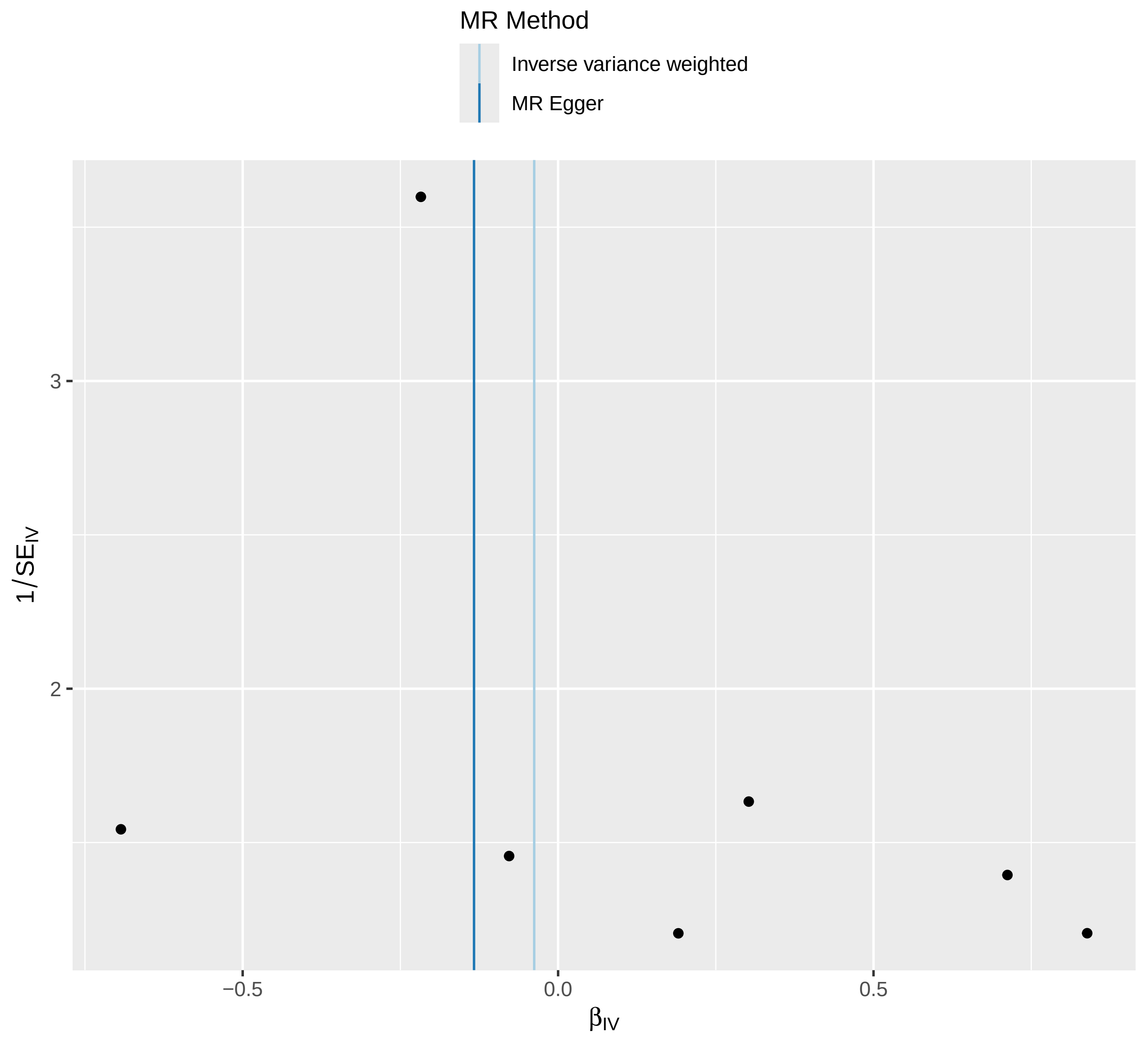
MR Method
Inverse variance weighted
MR Egger
3
2
1/SE_IV
−0.5
0.0
0.5
β_IV

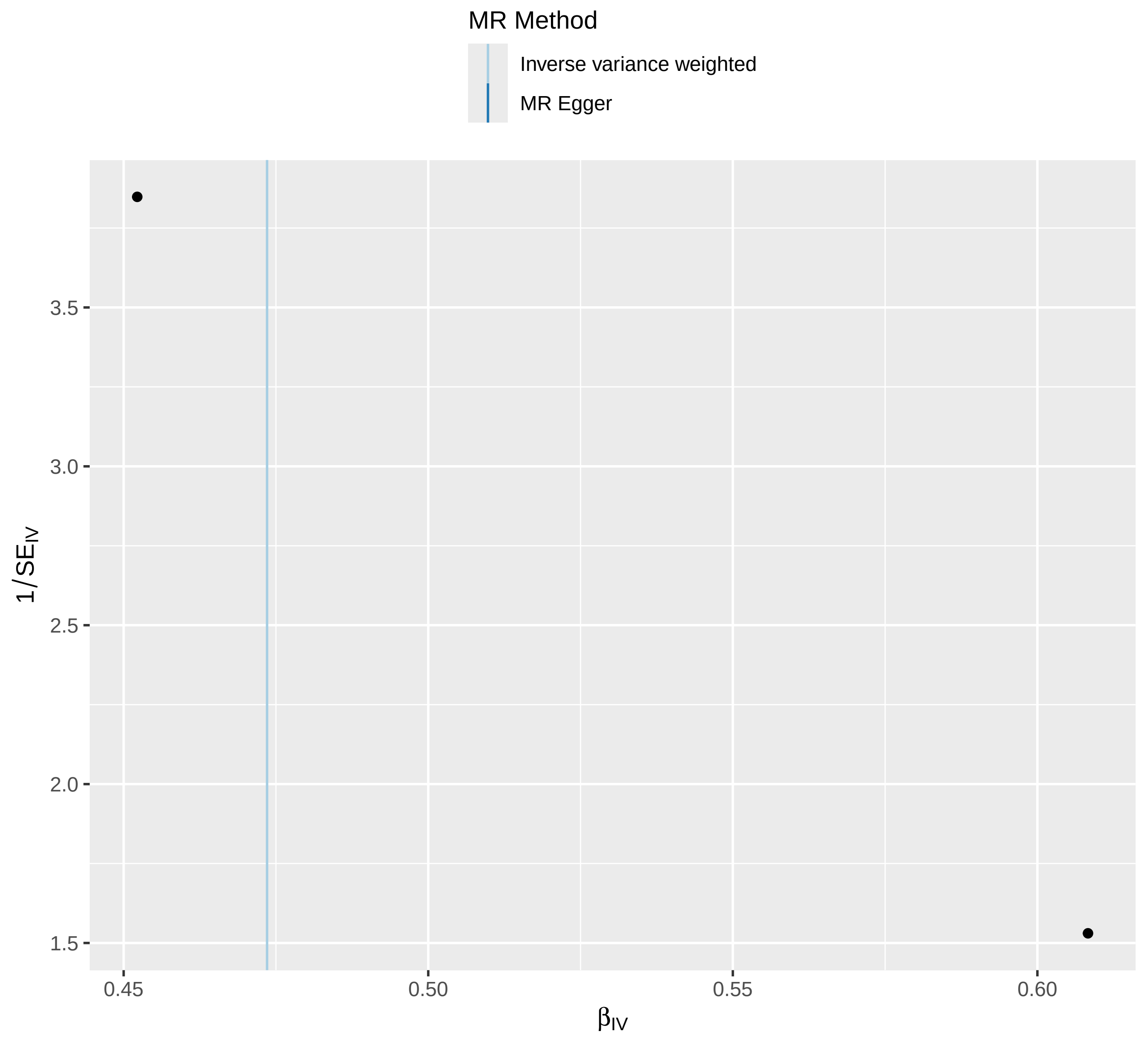
MR Method
Inverse variance weighted
MR Egger
1/SE_IV
3.5
3.0
2.5
2.0
1.5
0.45
0.50
0.55
0.60
β_IV

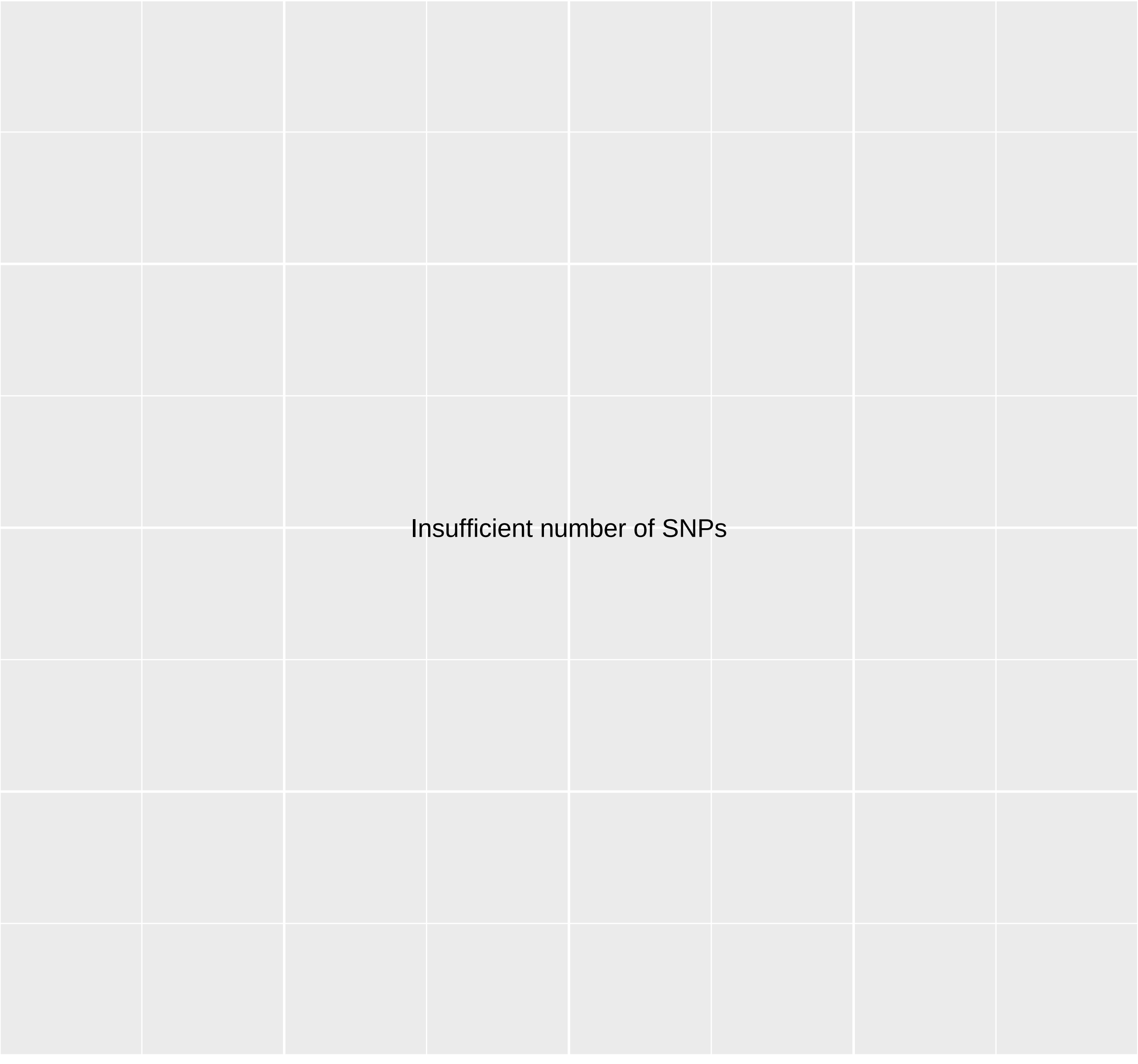
Insufficient number of SNPs

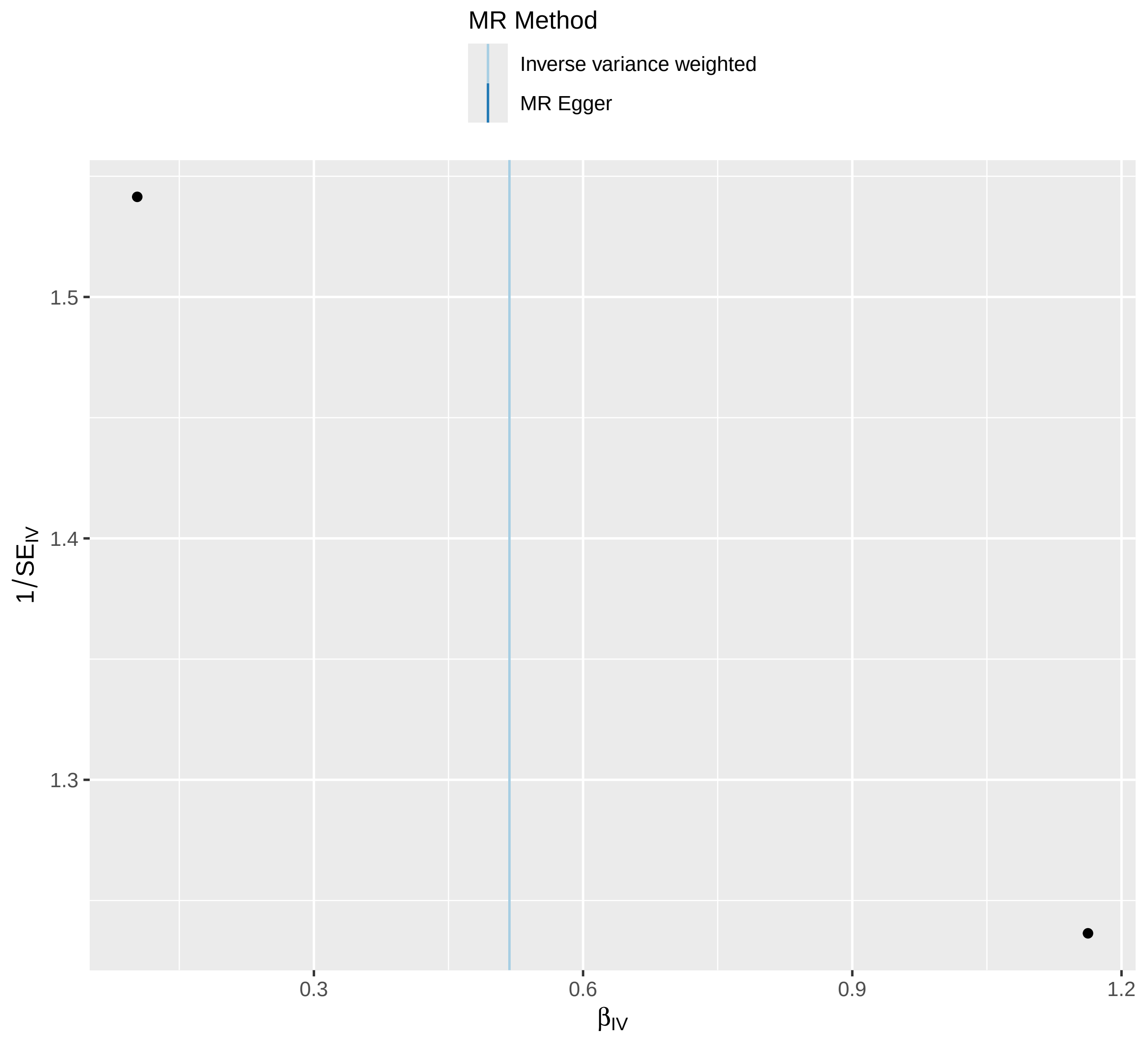
MR Method
Inverse variance weighted
MR Egger
1.5
1.4
1.3
$1/SE_{IV}$
0.3
0.6
0.9
1.2
β_{IV}

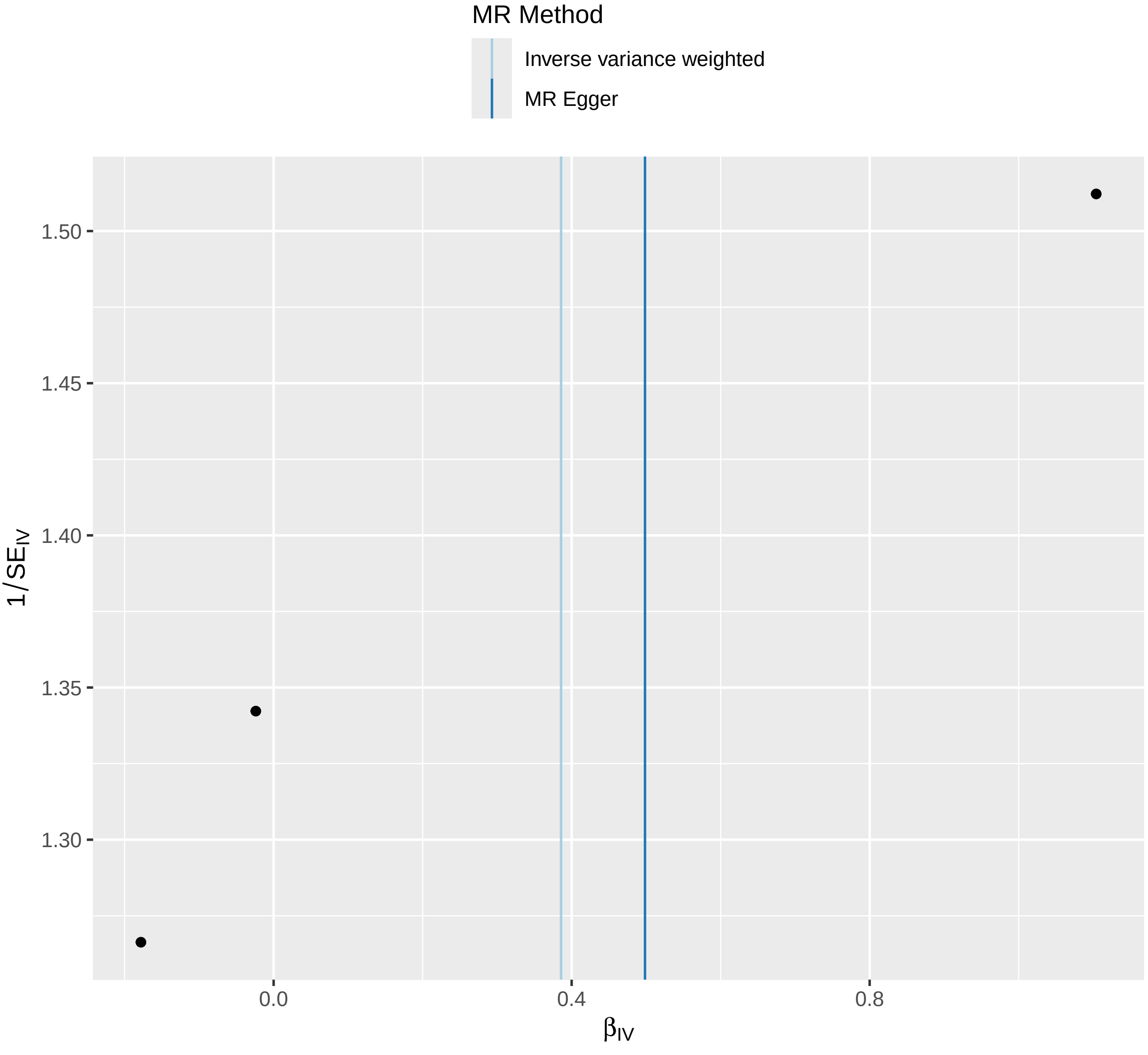

MR Method
Inverse variance weighted
MR Egger
1.50
1.45
1.40
1.35
1.30
$1/SE_{IV}$
0.0
0.4
0.8
β_{IV}

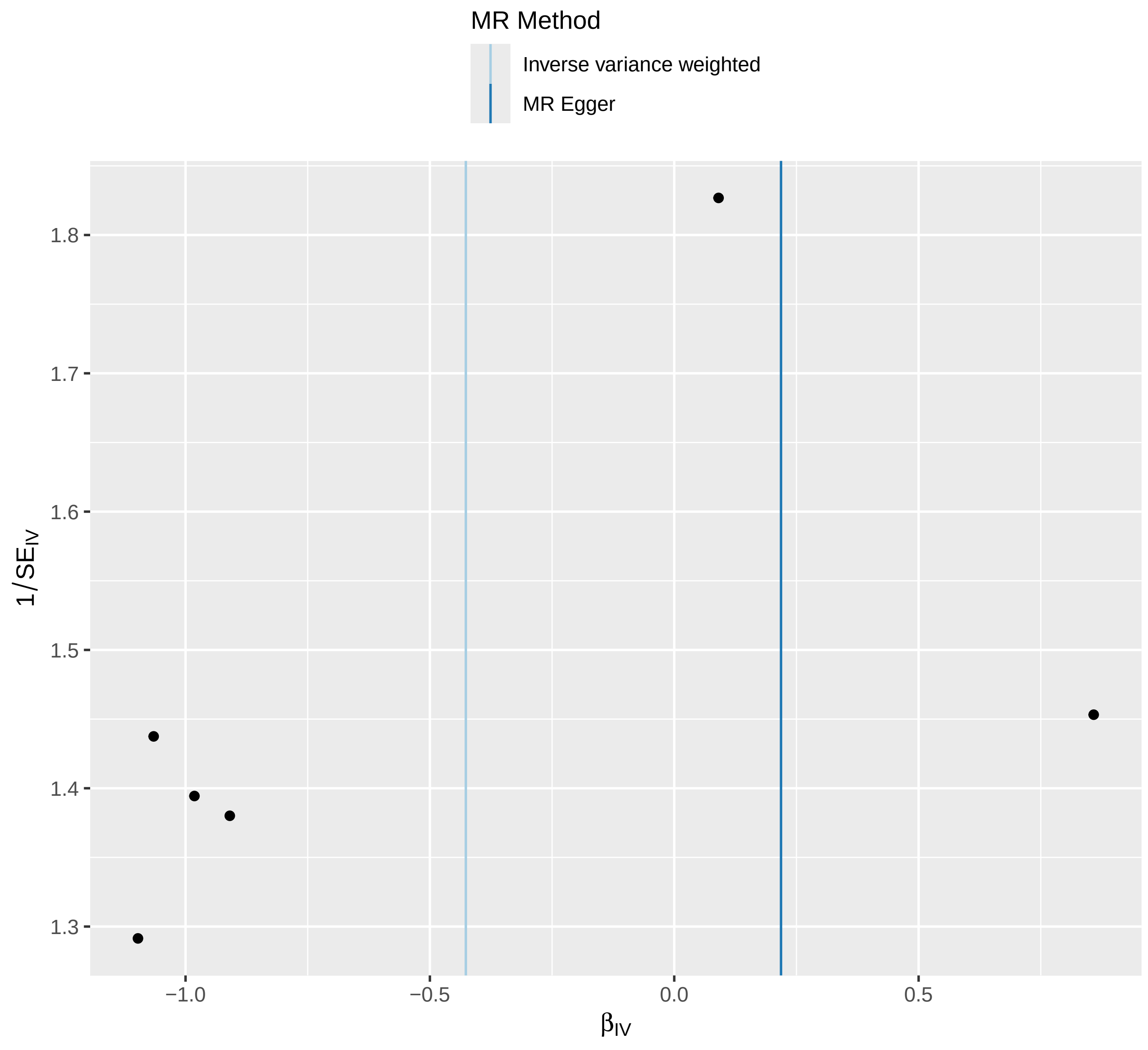
MR Method
Inverse variance weighted
MR Egger
1.8
1.7
1.6
1.5
1.4
1.3
1/SE$_{IV}$
−1.0
−0.5
0.0
0.5
β$_{IV}$

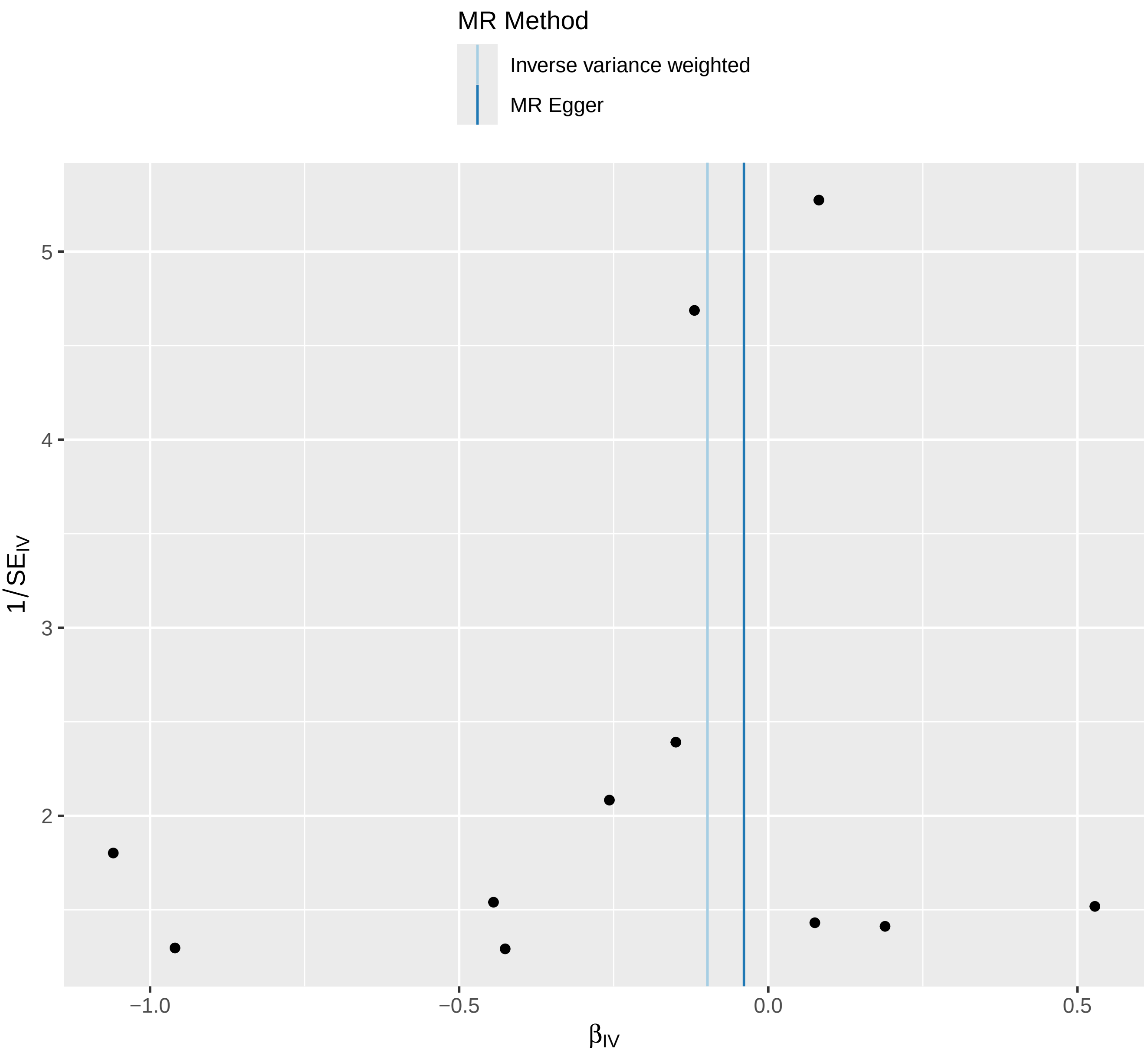

MR Method
Inverse variance weighted
MR Egger
5
4
3
2
1/SE$_{IV}$
−1.0
−0.5
0.0
0.5
β$_{IV}$

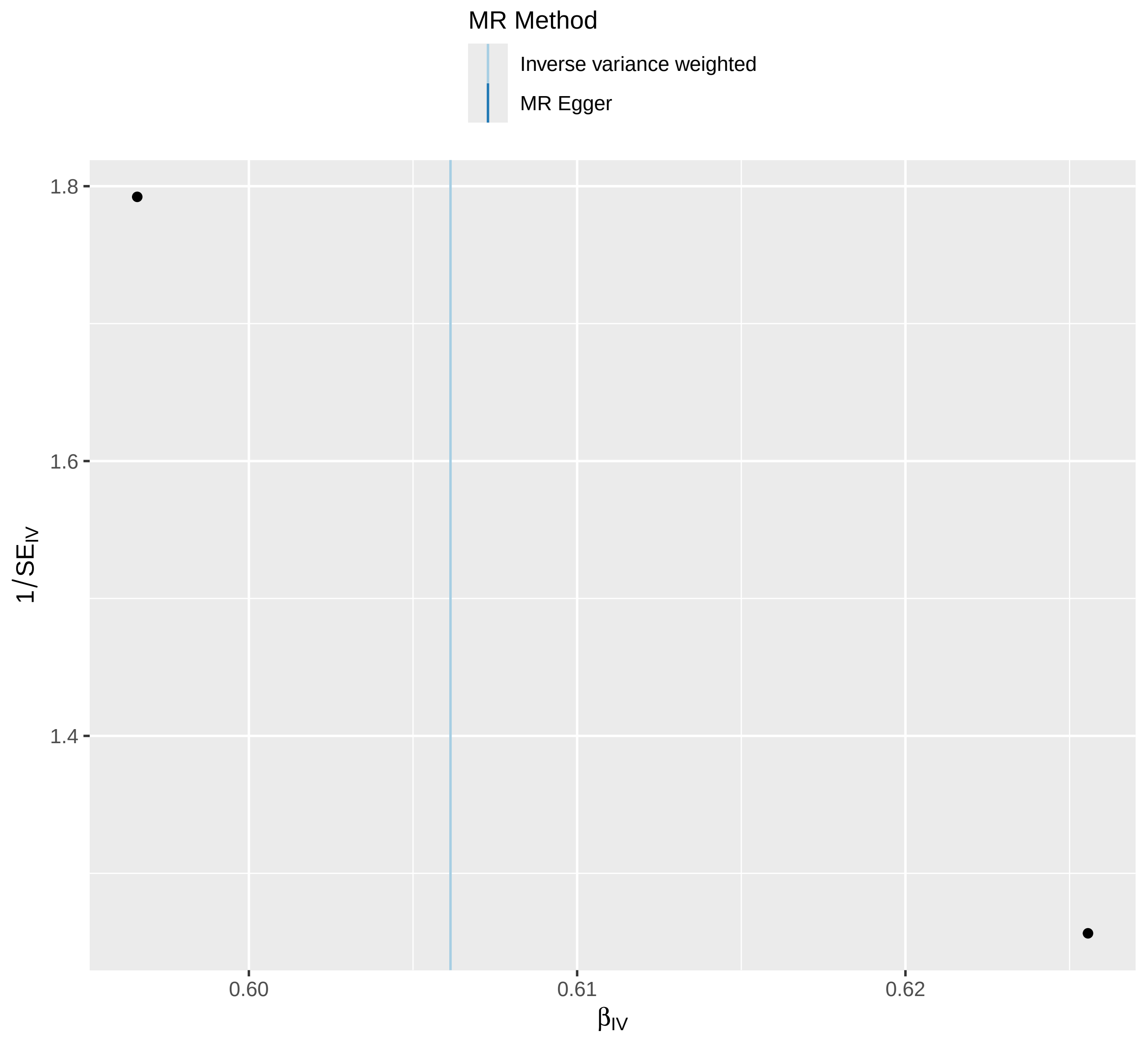
MR Method
Inverse variance weighted
MR Egger
1.8
1.6
1.4
1/SE$_{IV}$
0.60
0.61
0.62
β$_{IV}$

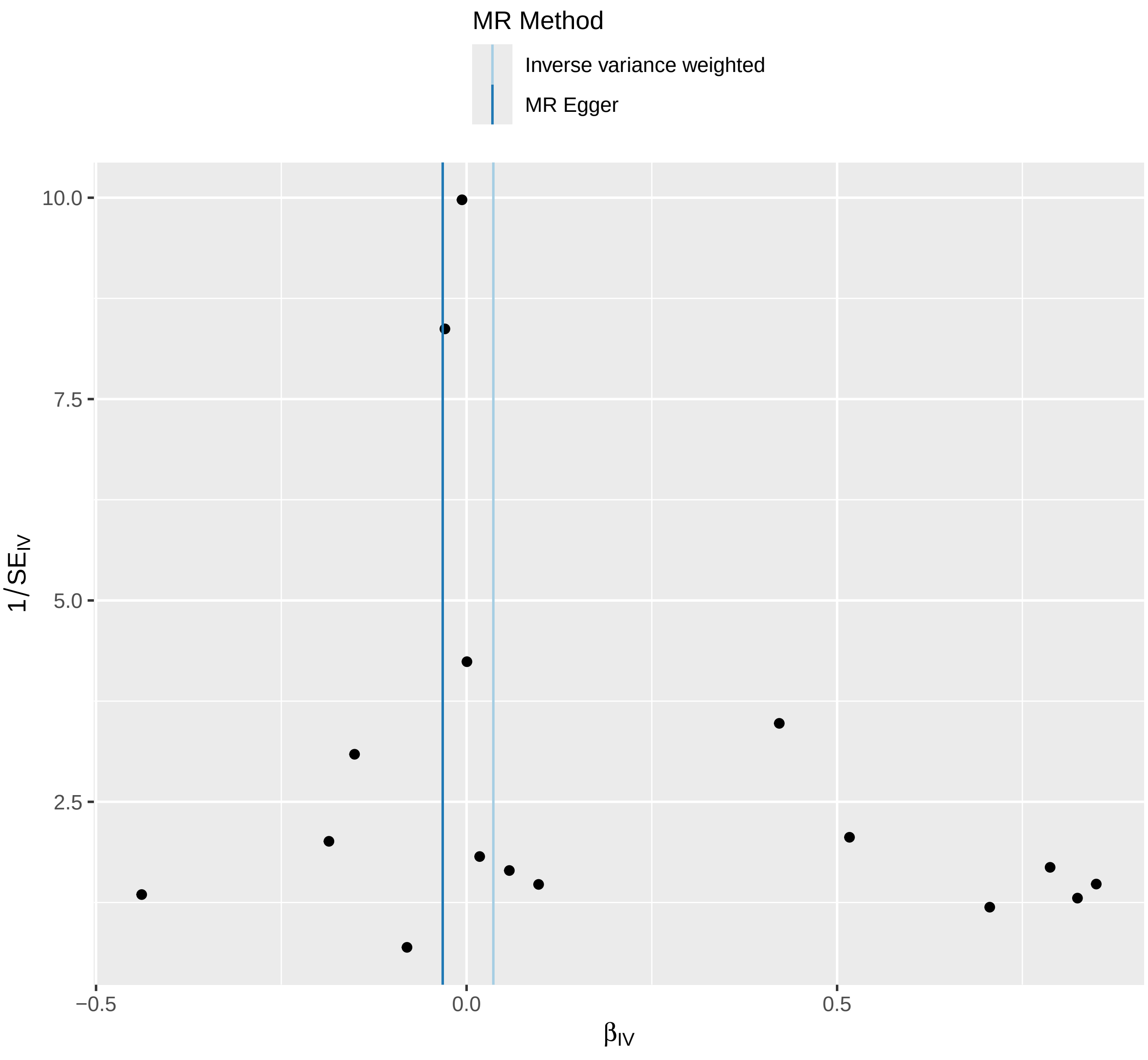
MR Method
Inverse variance weighted
MR Egger
10.0
7.5
5.0
2.5
$1/SE_{IV}$
−0.5
0.0
0.5
β_{IV}

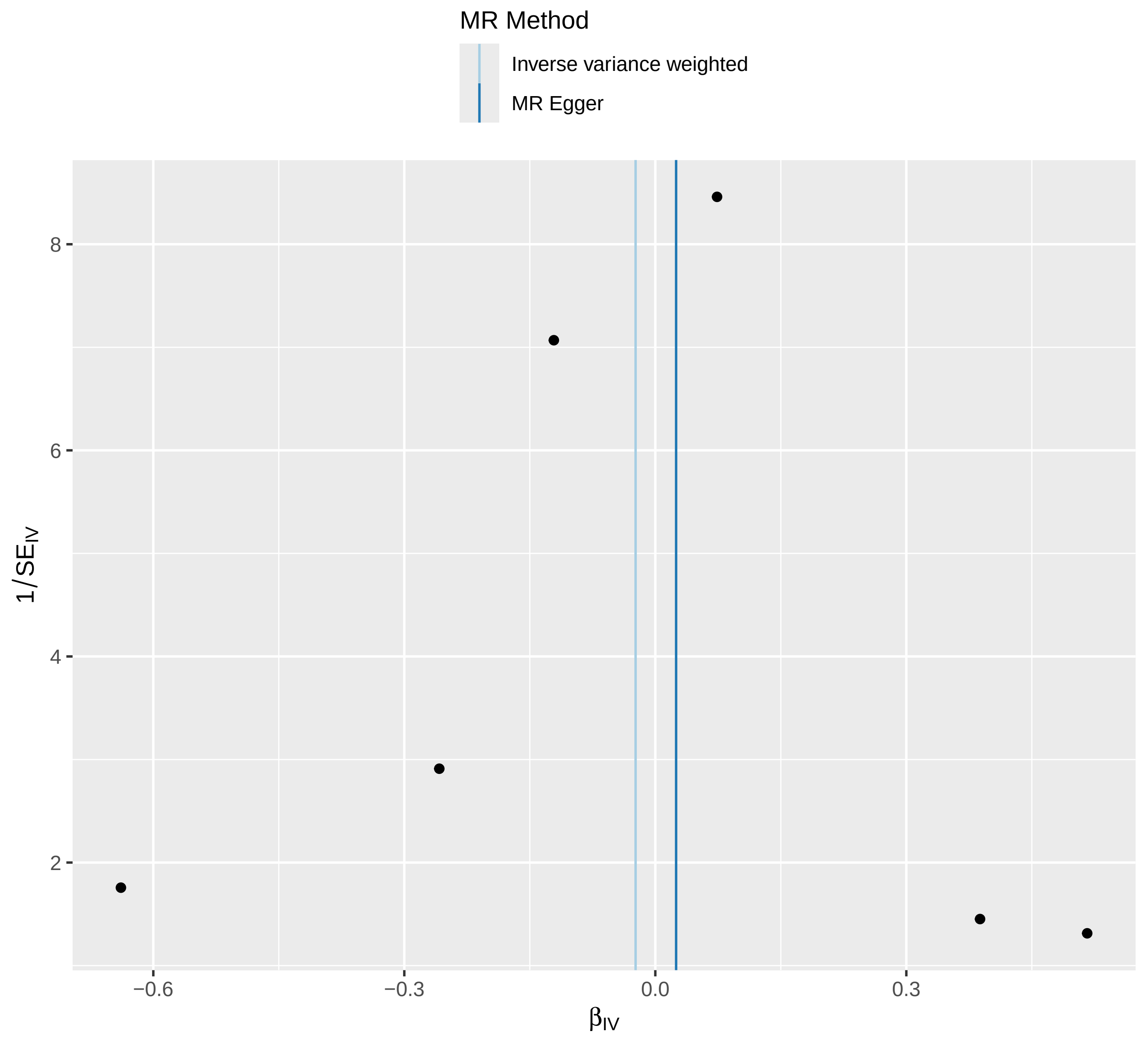

MR Method
Inverse variance weighted
MR Egger
8
6
4
2
1/SE$_{IV}$
−0.6
−0.3
0.0
0.3
β$_{IV}$

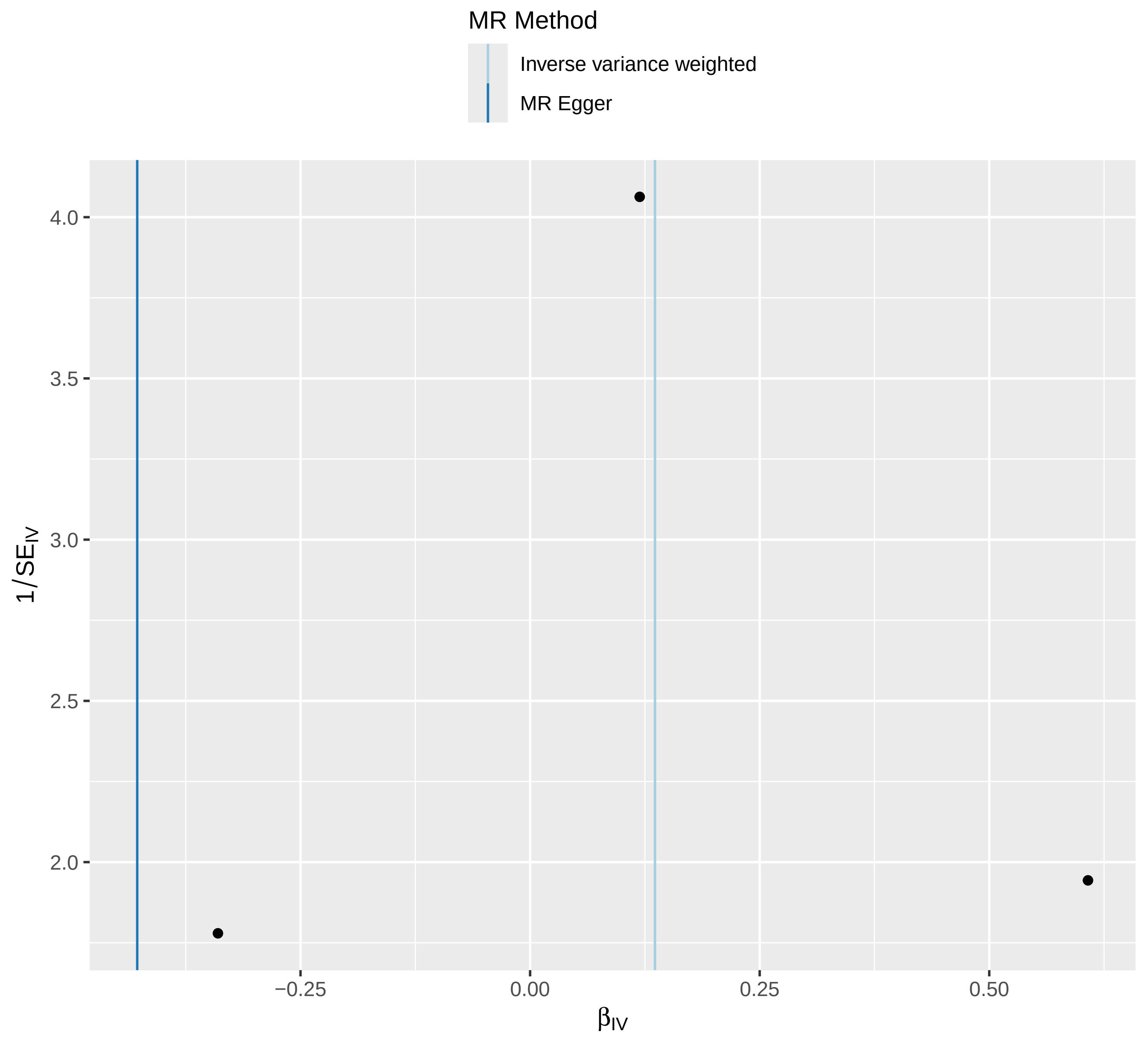
MR Method
Inverse variance weighted
MR Egger
4.0
3.5
3.0
2.5
2.0
$1/SE_{IV}$
−0.25
0.00
0.25
0.50
β_{IV}

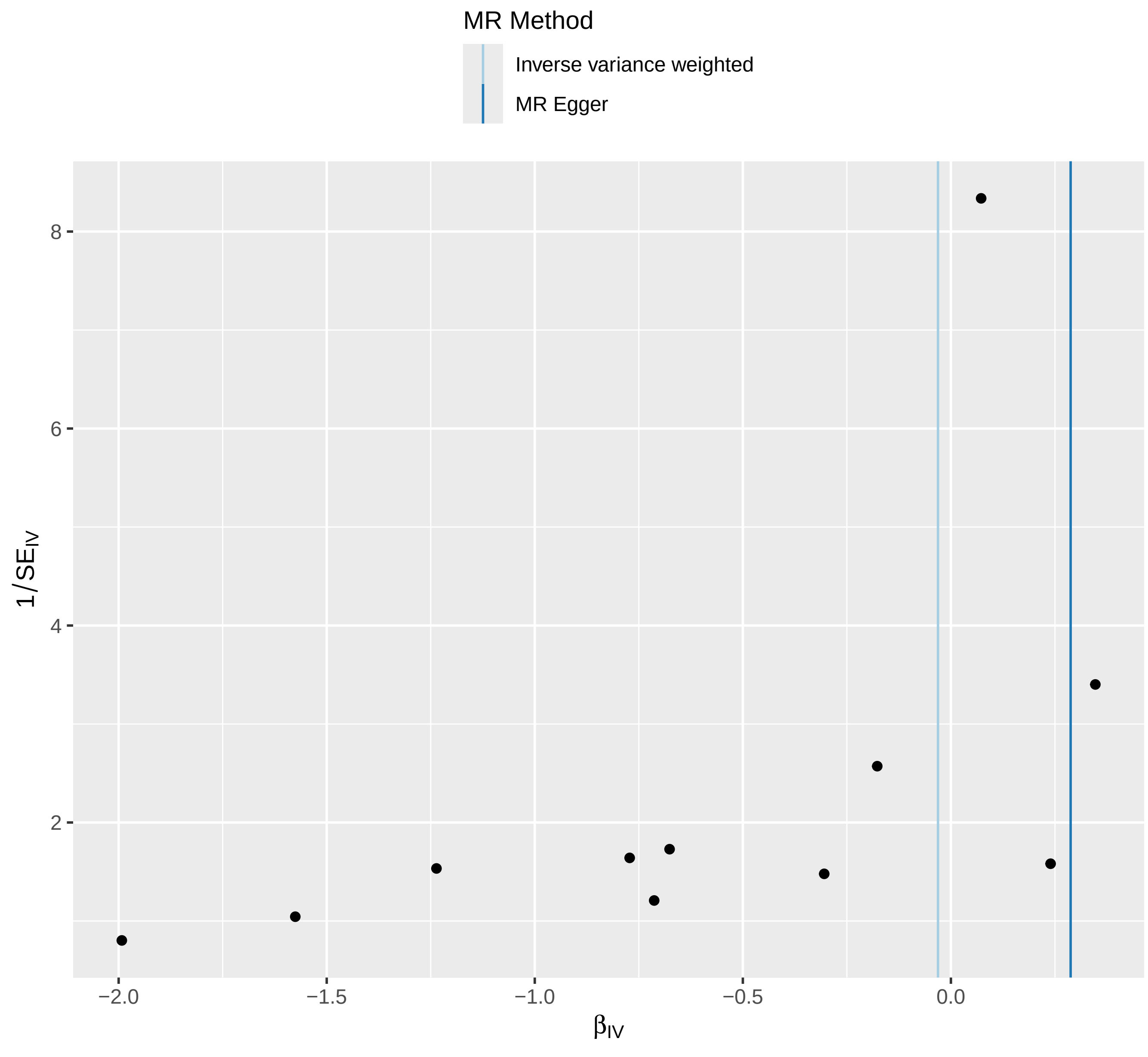
MR Method
Inverse variance weighted
MR Egger
$1/SE_{IV}$
8
6
4
2
−2.0
−1.5
−1.0
−0.5
0.0
β_{IV}

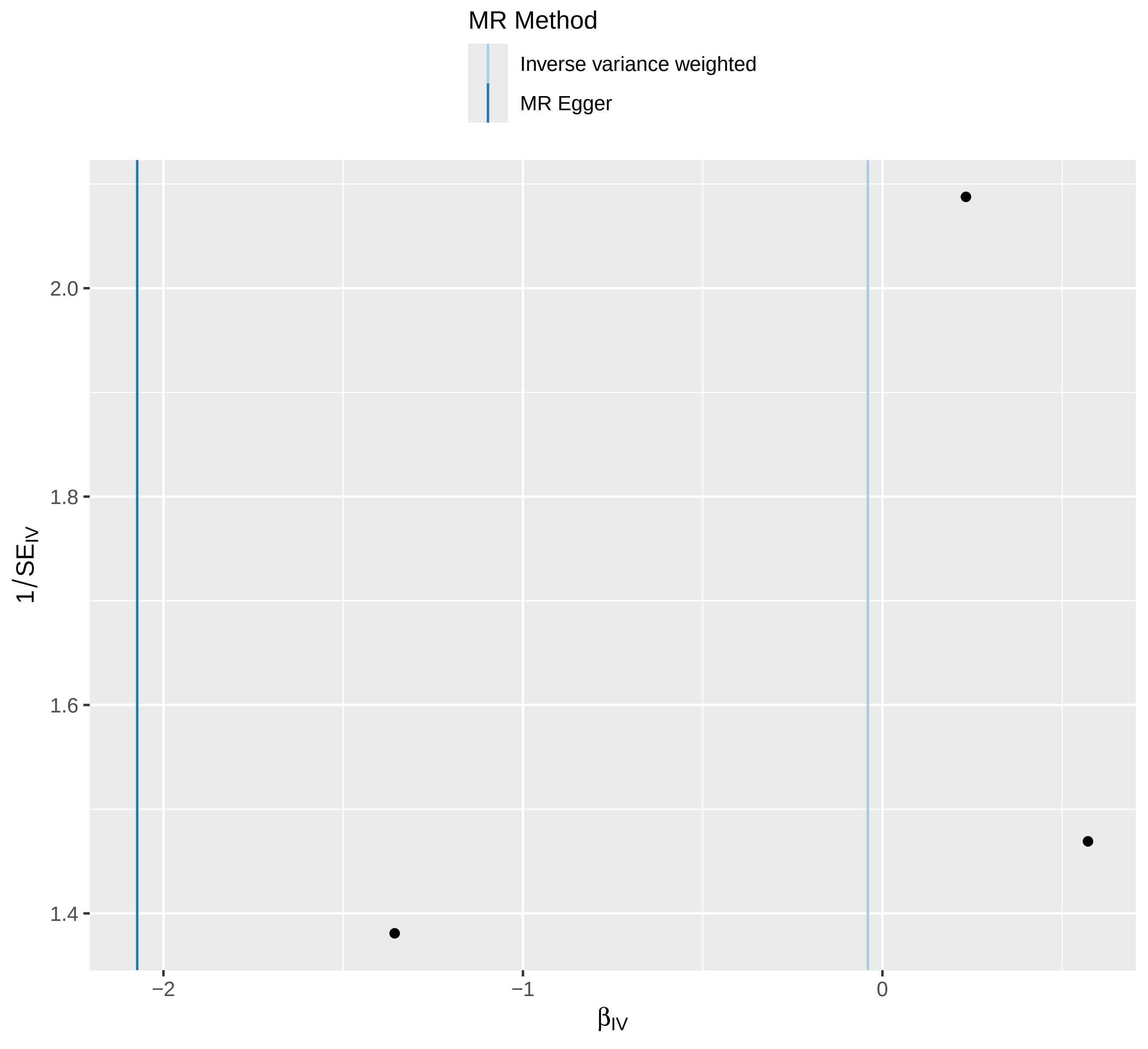
MR Method
Inverse variance weighted
MR Egger
2.0
1.8
1.6
1.4
1/SE_IV
−2
−1
0
β_IV

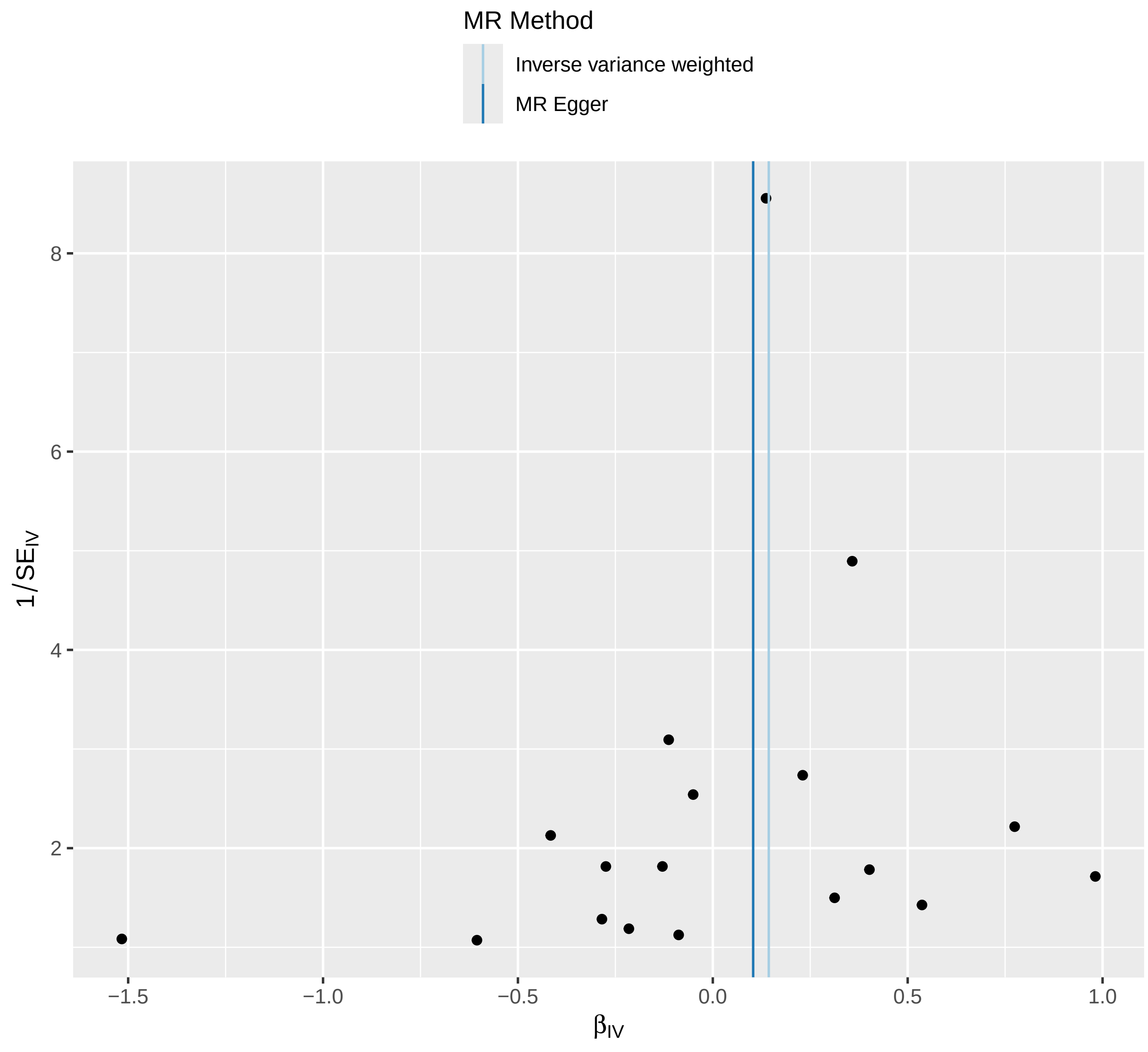
MR Method
Inverse variance weighted
MR Egger
1/SE_IV
8
6
4
2
β_IV
−1.5
−1.0
−0.5
0.0
0.5
1.0

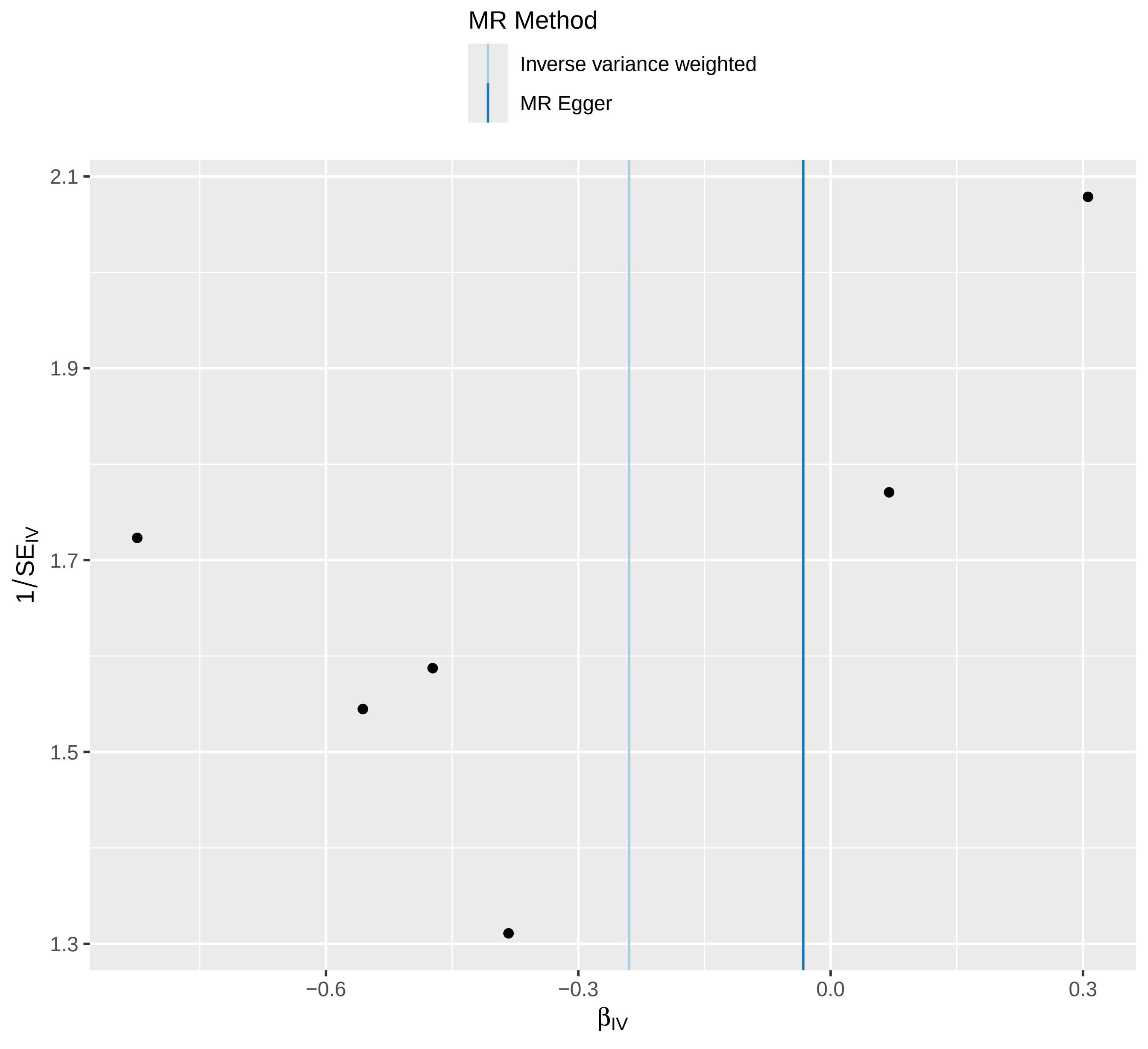
MR Method
Inverse variance weighted
MR Egger
2.1
1.9
1.7
1.5
1.3
1/SE$_{IV}$
−0.6
−0.3
0.0
0.3
β$_{IV}$

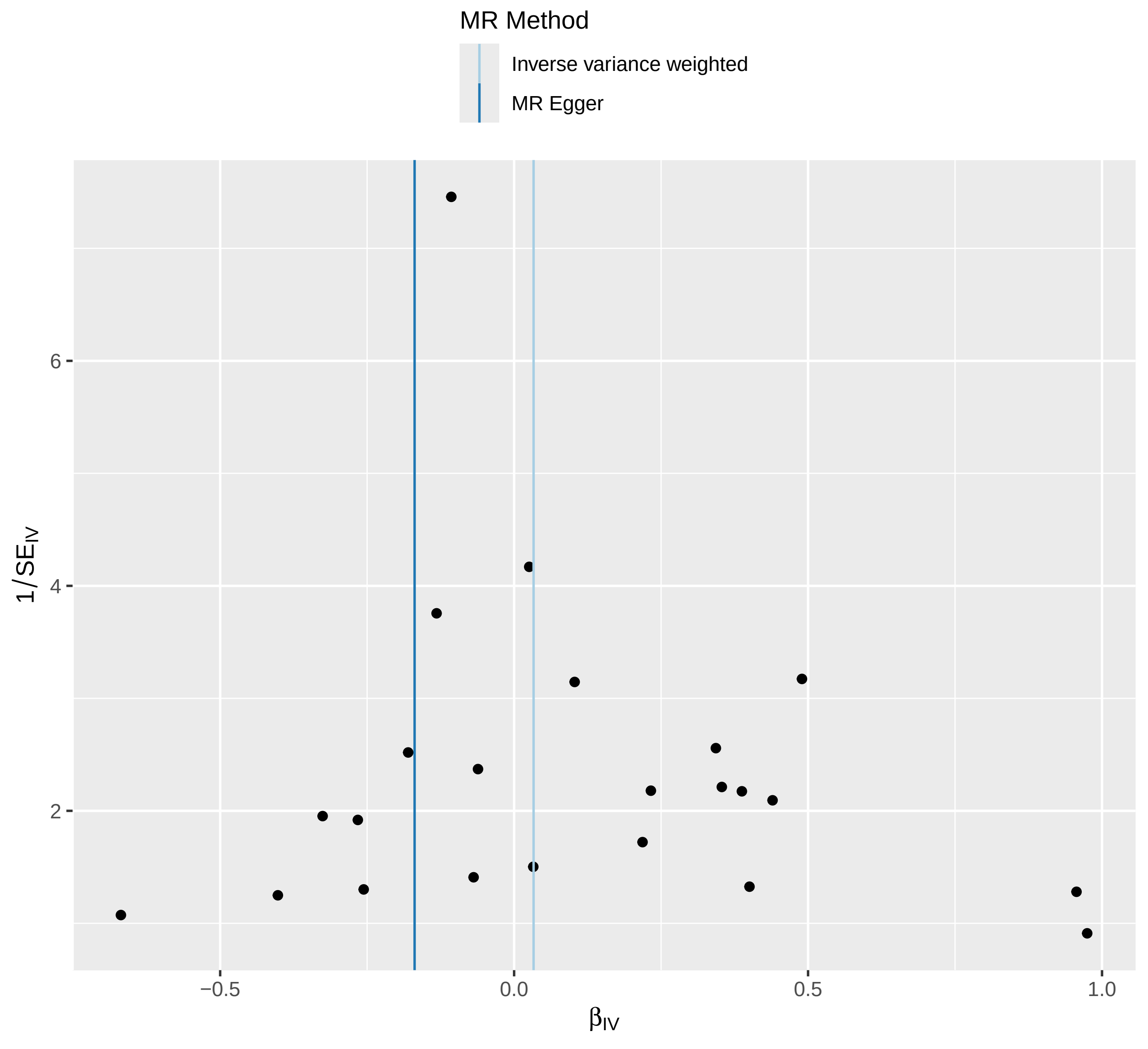
MR Method
Inverse variance weighted
MR Egger
6
4
2
1/SE_IV
−0.5
0.0
0.5
1.0
β_IV

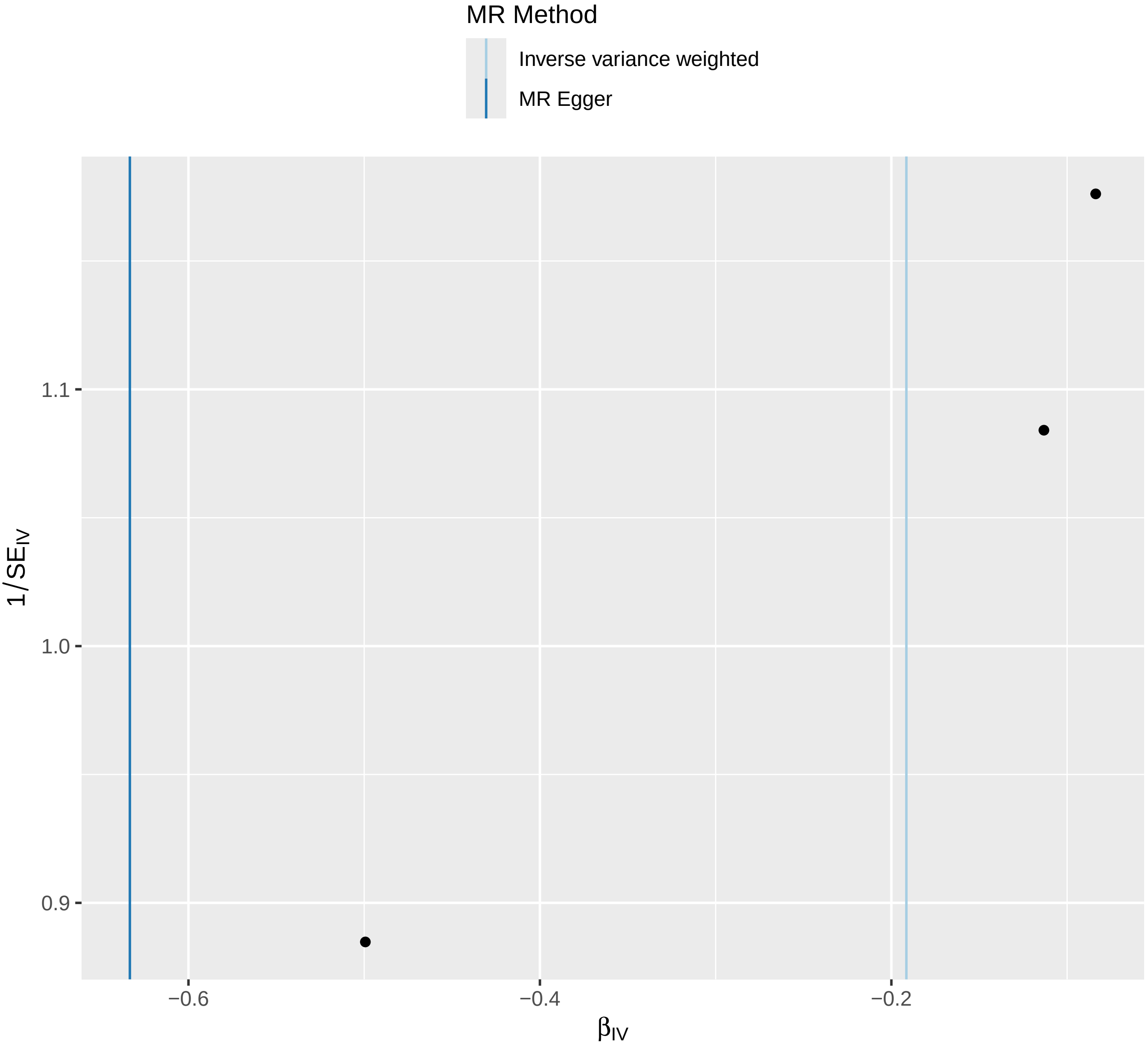
MR Method
Inverse variance weighted
MR Egger
1.1
1.0
0.9
1/SE$_{IV}$
−0.6
−0.4
−0.2
β$_{IV}$

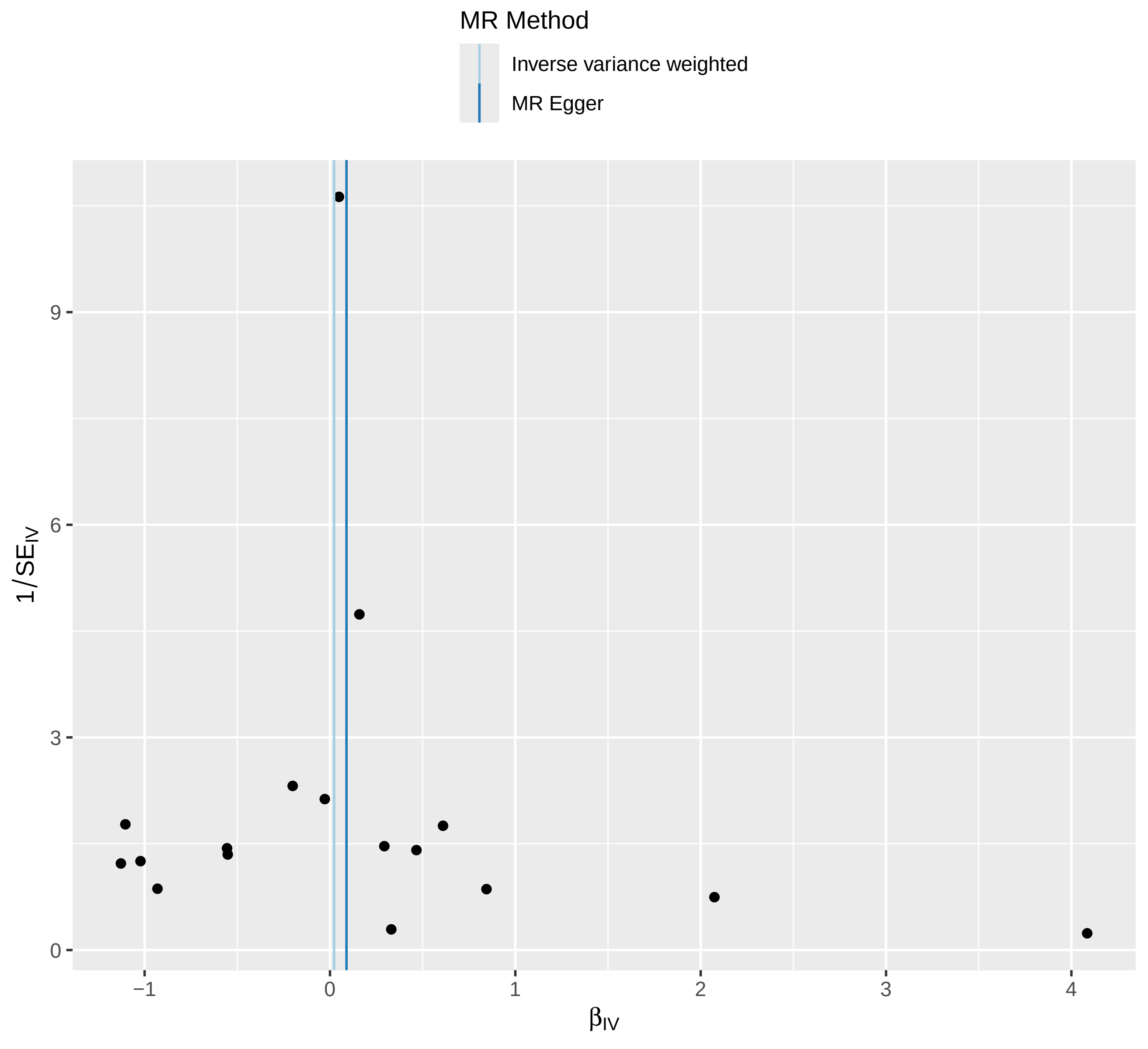
MR Method
Inverse variance weighted
MR Egger
9
6
3
0
1/SE$_{IV}$
−1
0
1
2
3
4
β$_{IV}$

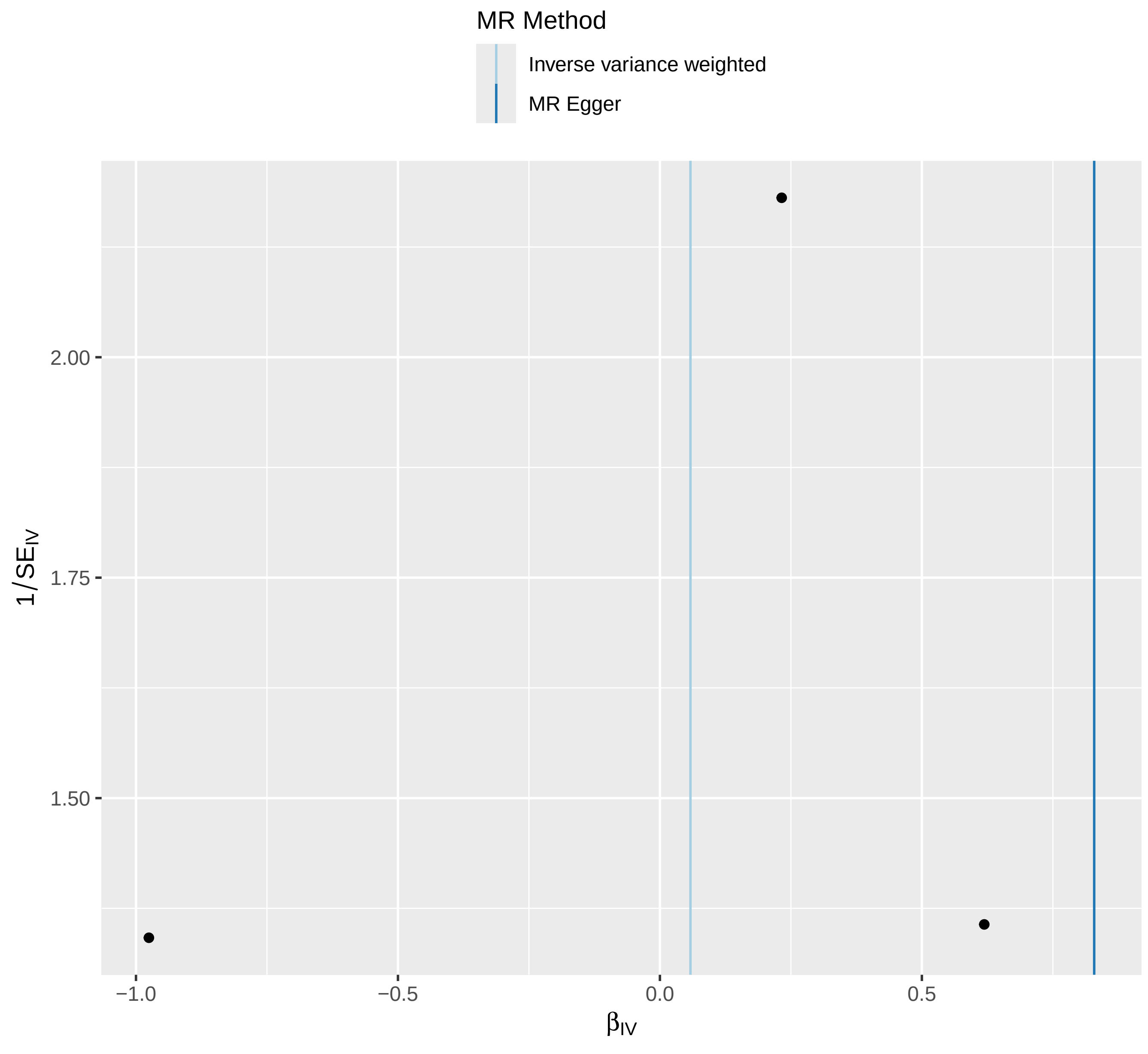
MR Method
Inverse variance weighted
MR Egger
2.00
1.75
1.50
1/SE_IV
−1.0
−0.5
0.0
0.5
β_IV

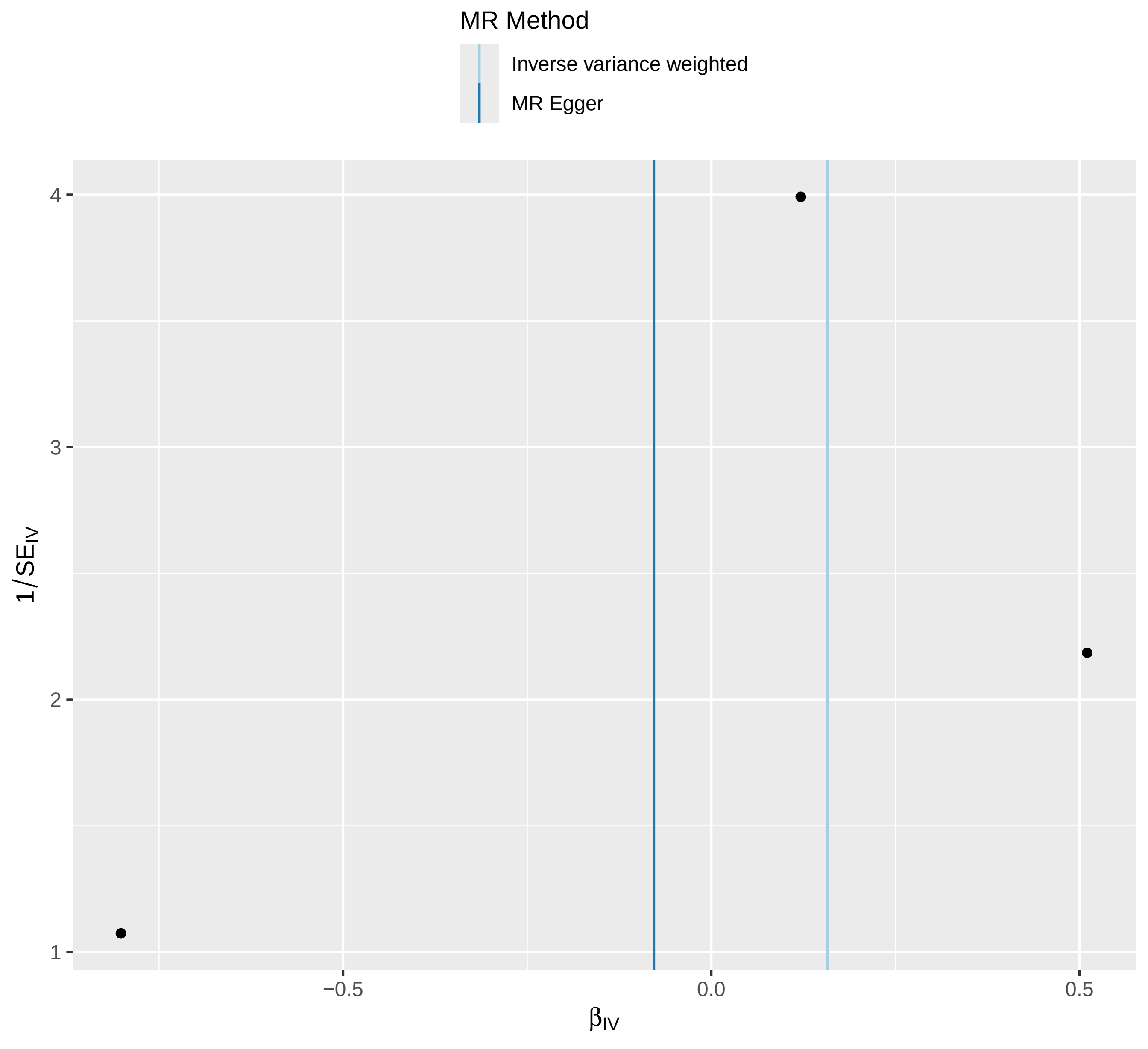

MR Method
Inverse variance weighted
MR Egger
4
3
2
1
1/SE_IV
−0.5
0.0
0.5
β_IV

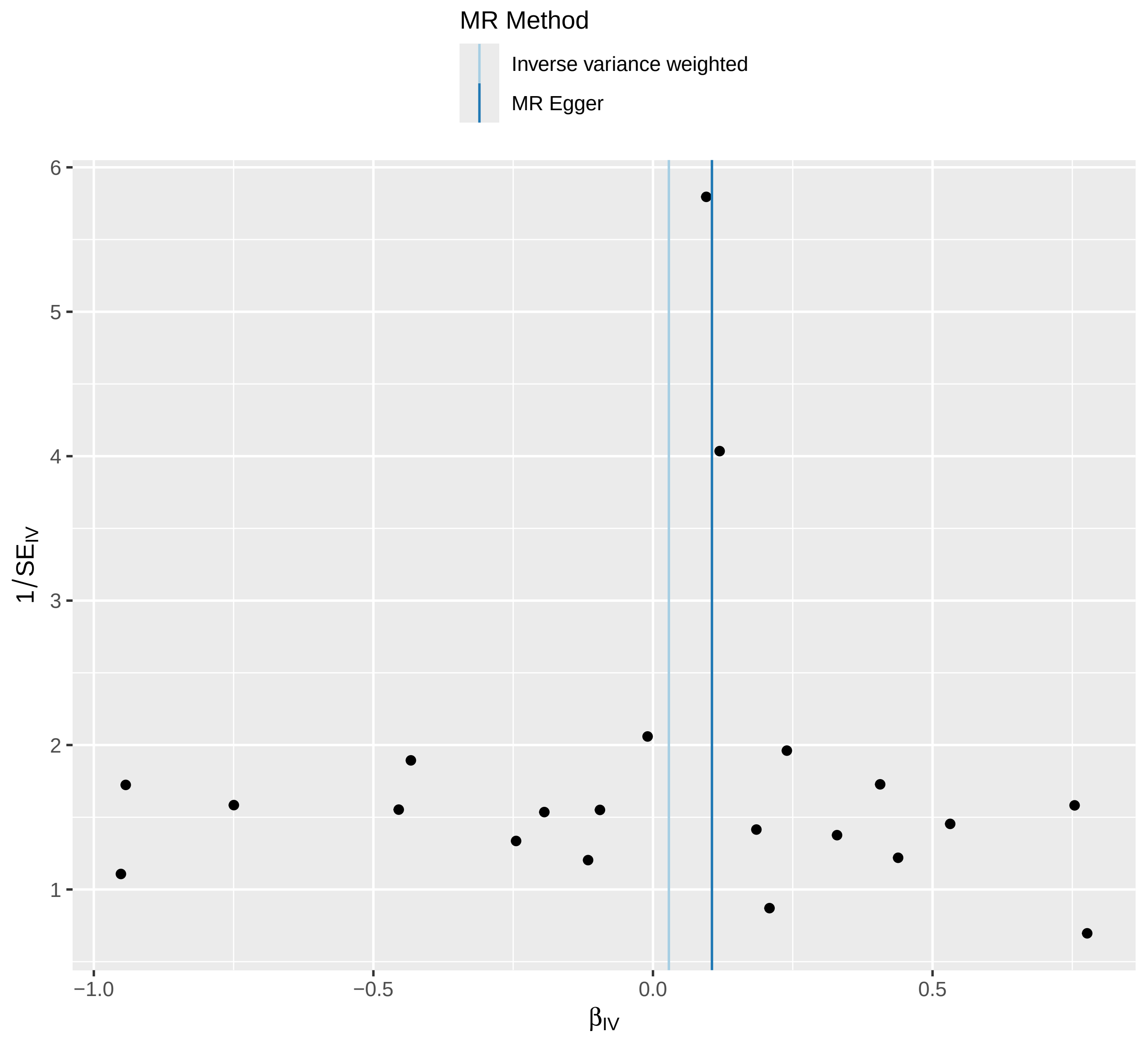
MR Method
Inverse variance weighted
MR Egger
1/SE_IV
β_IV
6
5
4
3
2
1
−1.0
−0.5
0.0
0.5

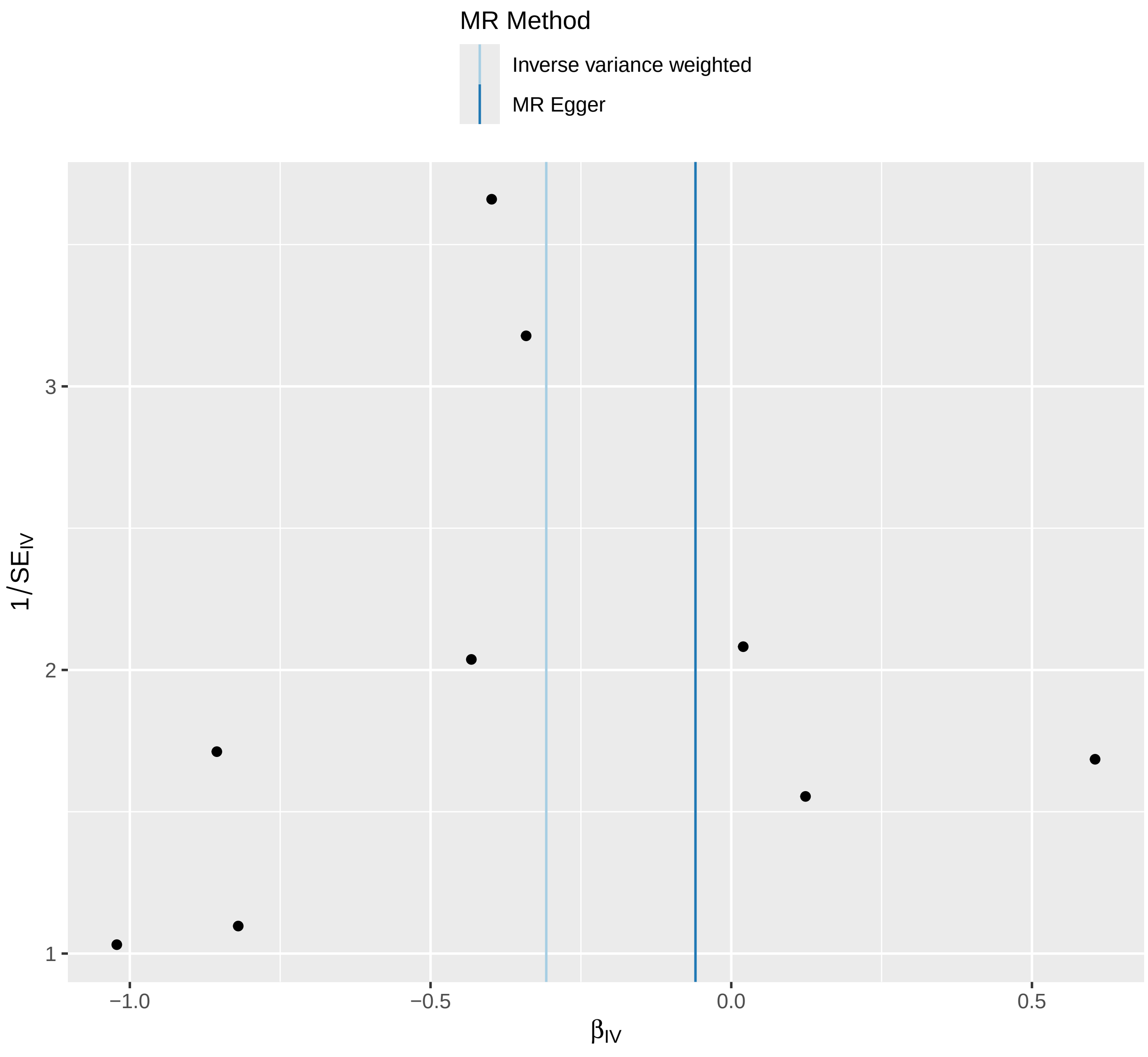
MR Method
Inverse variance weighted
MR Egger
1/SE_IV
3
2
1
−1.0
−0.5
0.0
0.5
β_IV

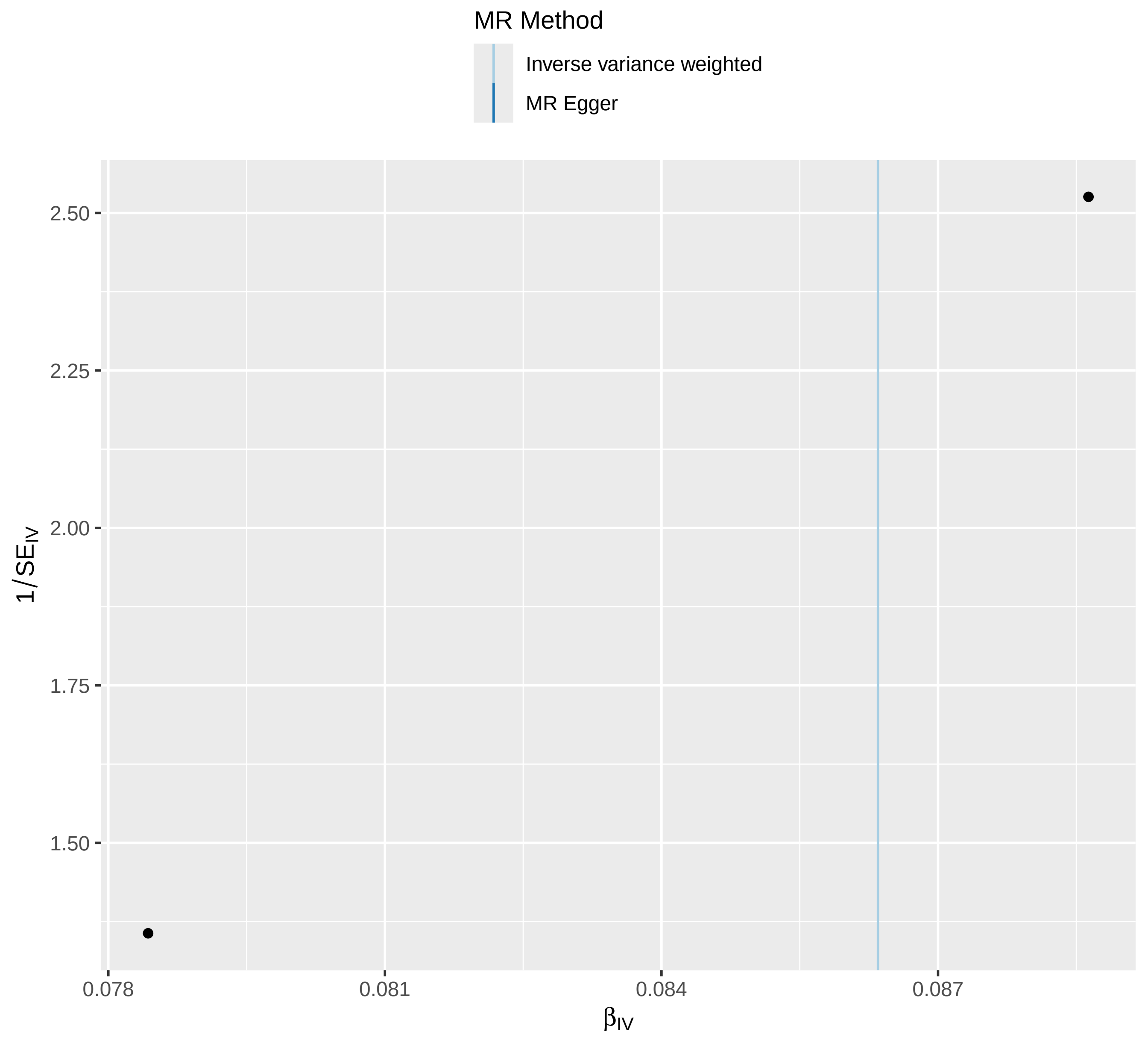

MR Method
Inverse variance weighted
MR Egger
2.50
2.25
2.00
1.75
1.50
$1/SE_{IV}$
0.078
0.081
0.084
0.087
β_{IV}

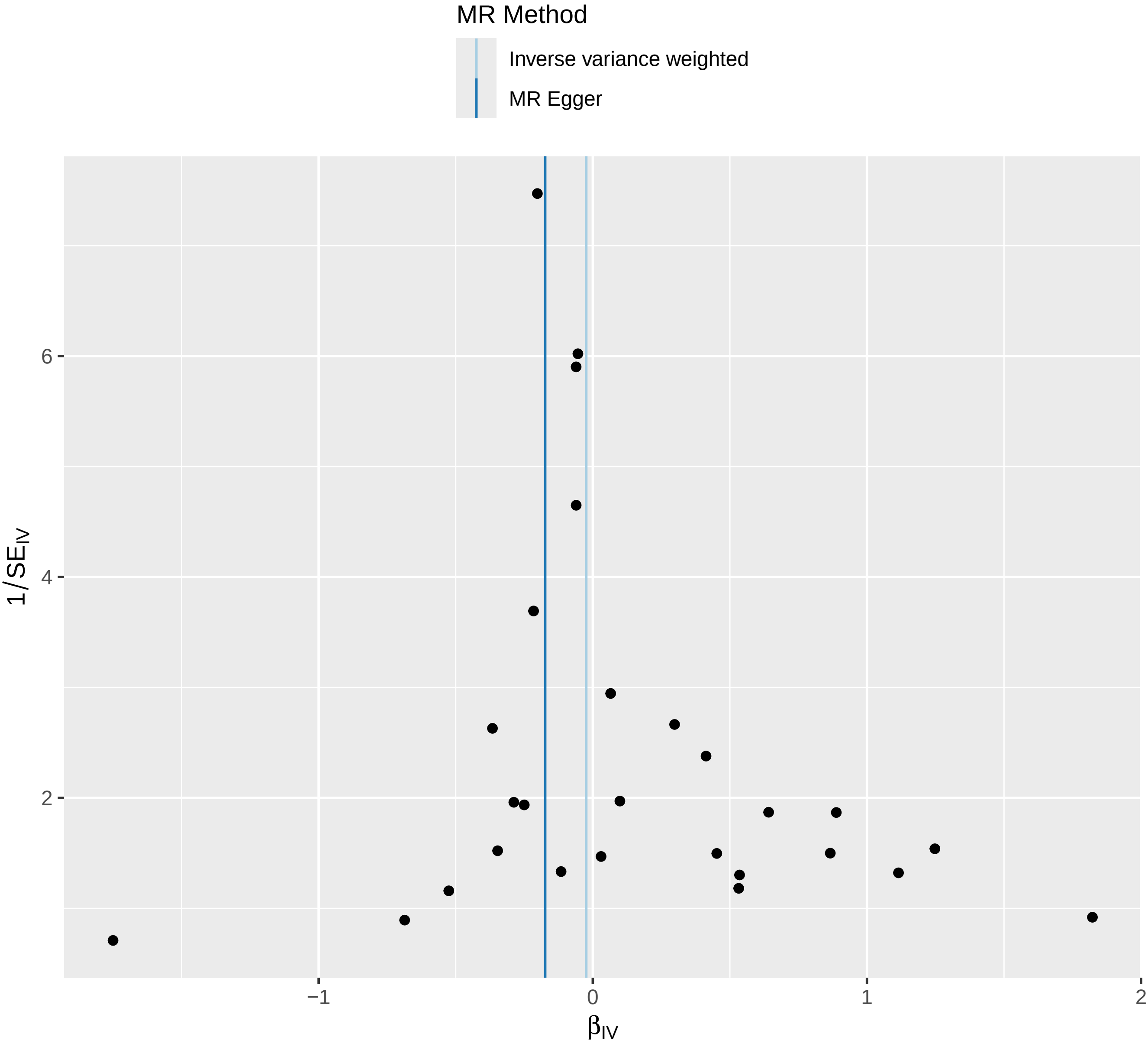
MR Method
Inverse variance weighted
MR Egger
1/SE$_{IV}$
6
4
2
−1
0
1
2
β$_{IV}$

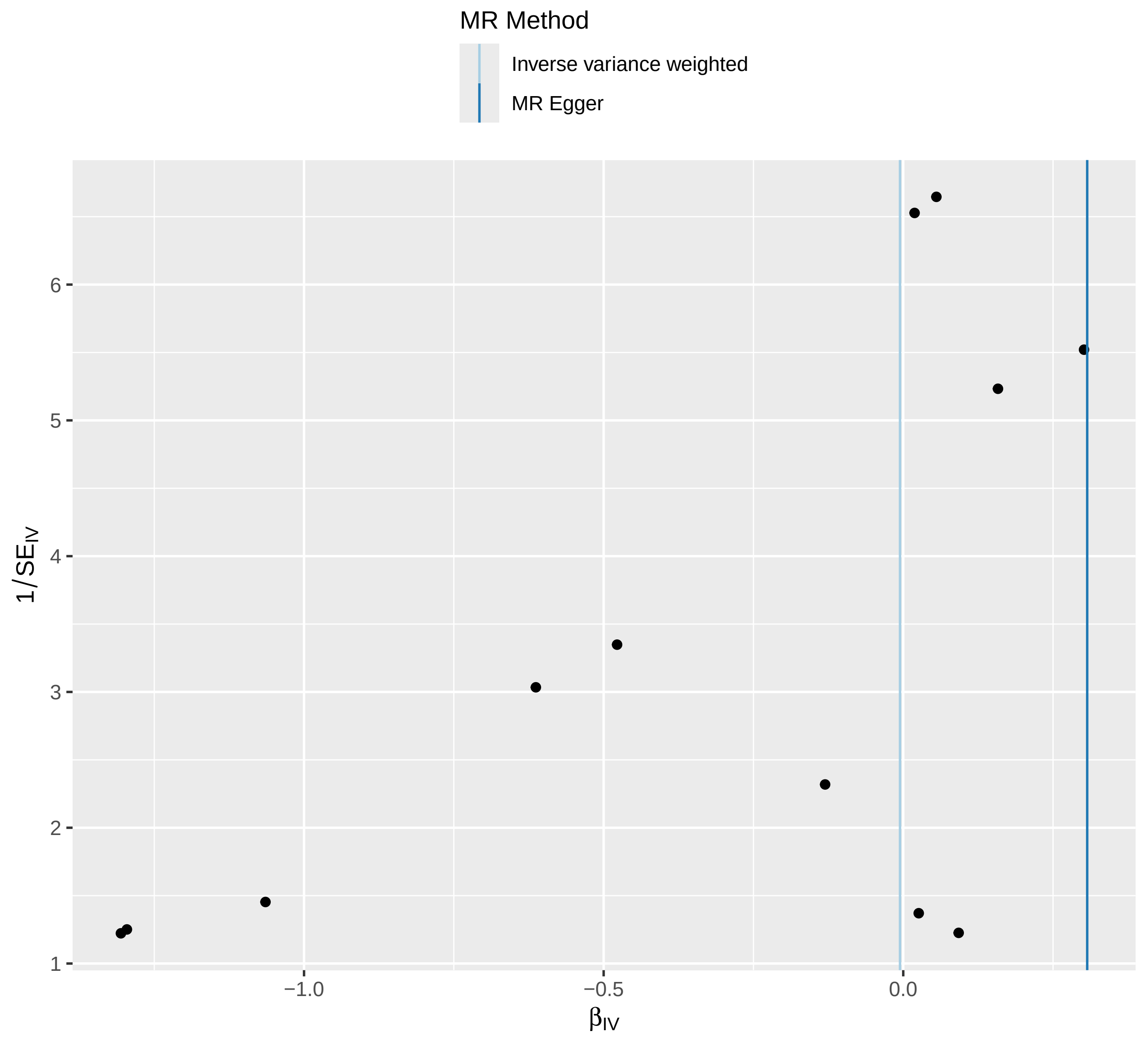
MR Method
Inverse variance weighted
MR Egger
6
5
4
3
2
1
1/SE_IV
−1.0
−0.5
0.0
β_IV

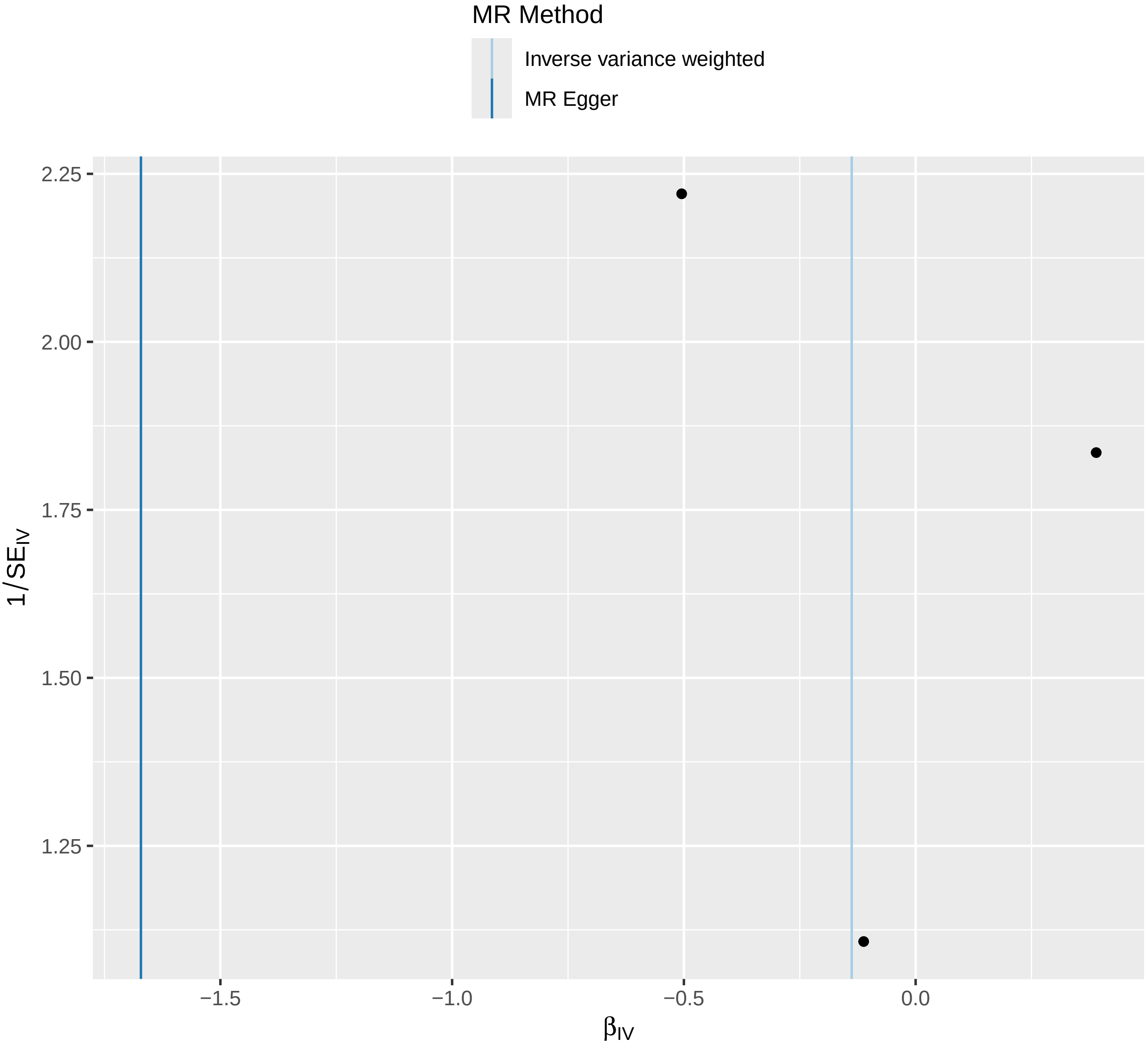

MR Method
Inverse variance weighted
MR Egger
2.25
2.00
1.75
1.50
1.25
1/SE_IV
−1.5
−1.0
−0.5
0.0
β_IV

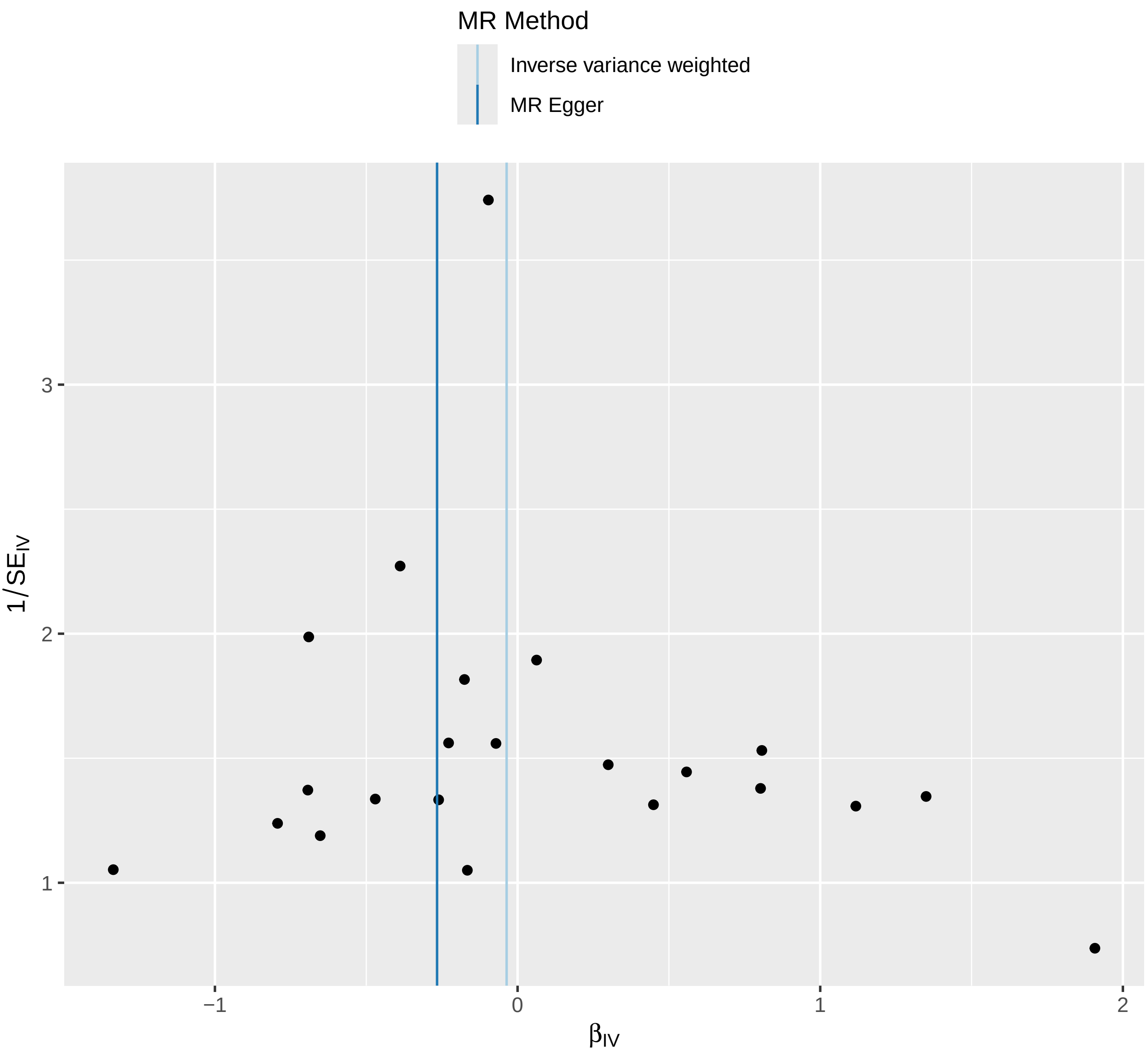
MR Method
Inverse variance weighted
MR Egger
1/SE_{IV}
3
2
1
−1
0
1
2
β_{IV}

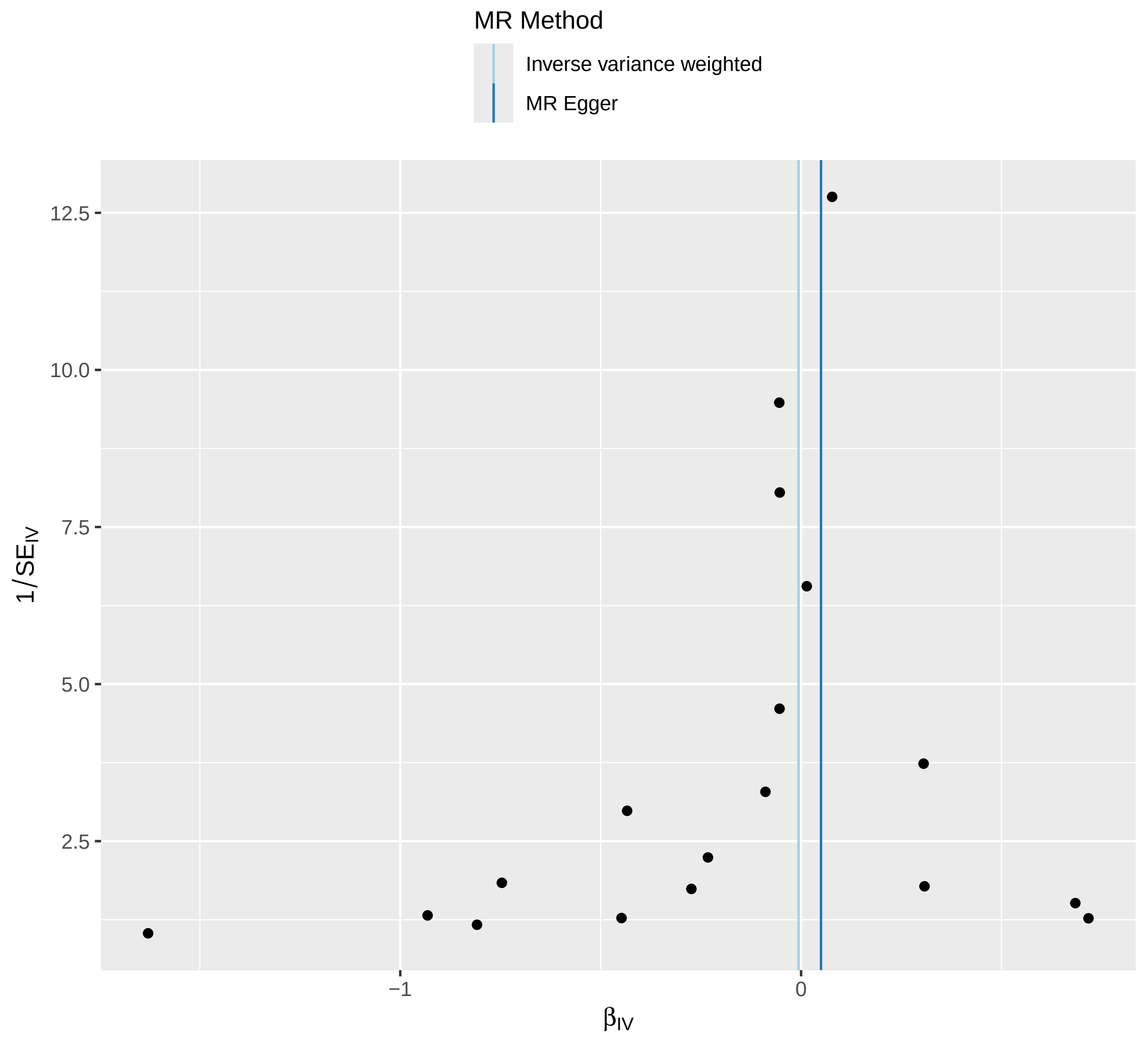

MR Method
Inverse variance weighted
MR Egger
12.5
10.0
7.5
5.0
2.5
$1/SE_{IV}$
−1
0
β_{IV}

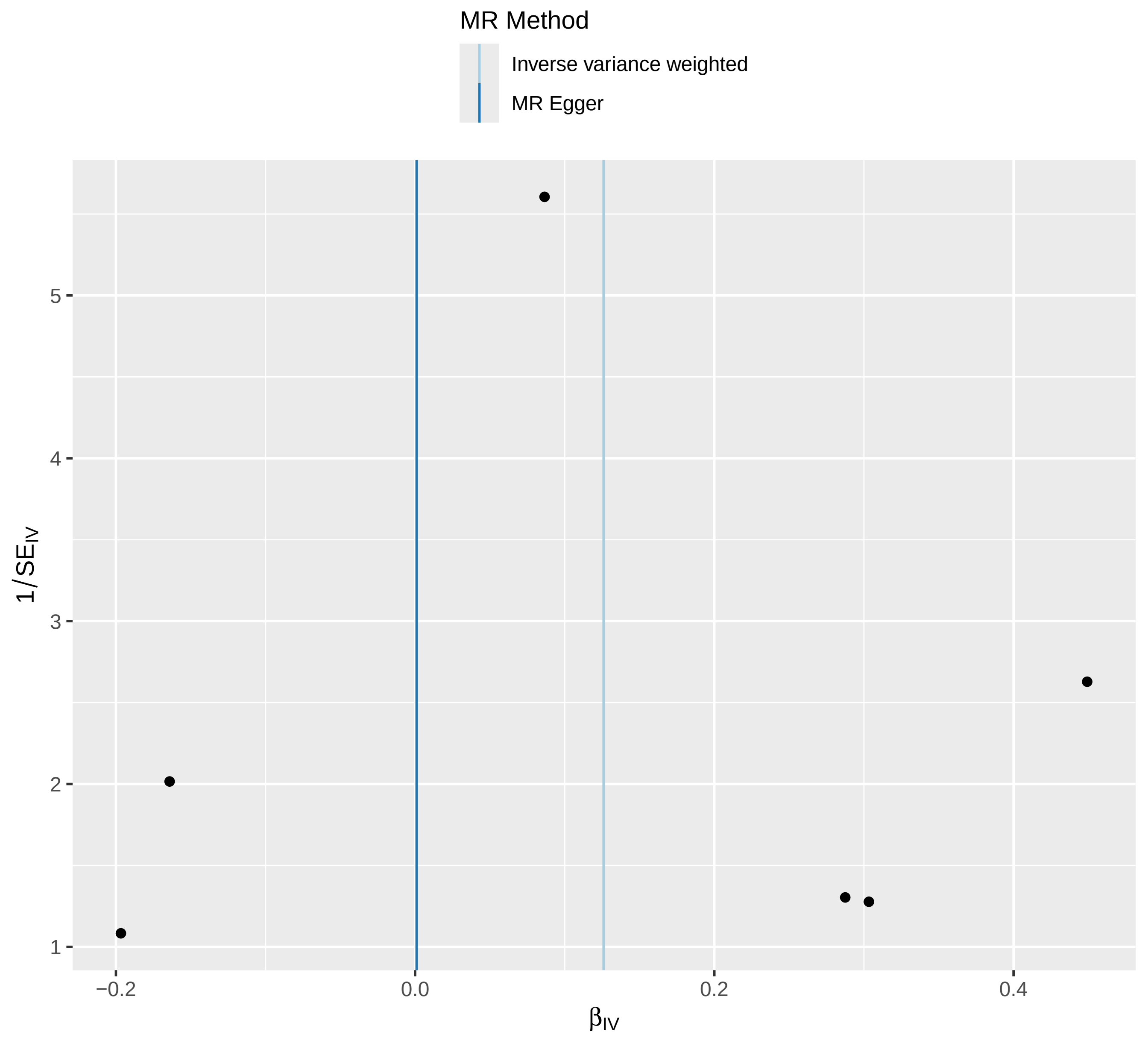

MR Method
Inverse variance weighted
MR Egger
5
4
3
2
1
1/SE_IV
−0.2
0.0
0.2
0.4
β_IV

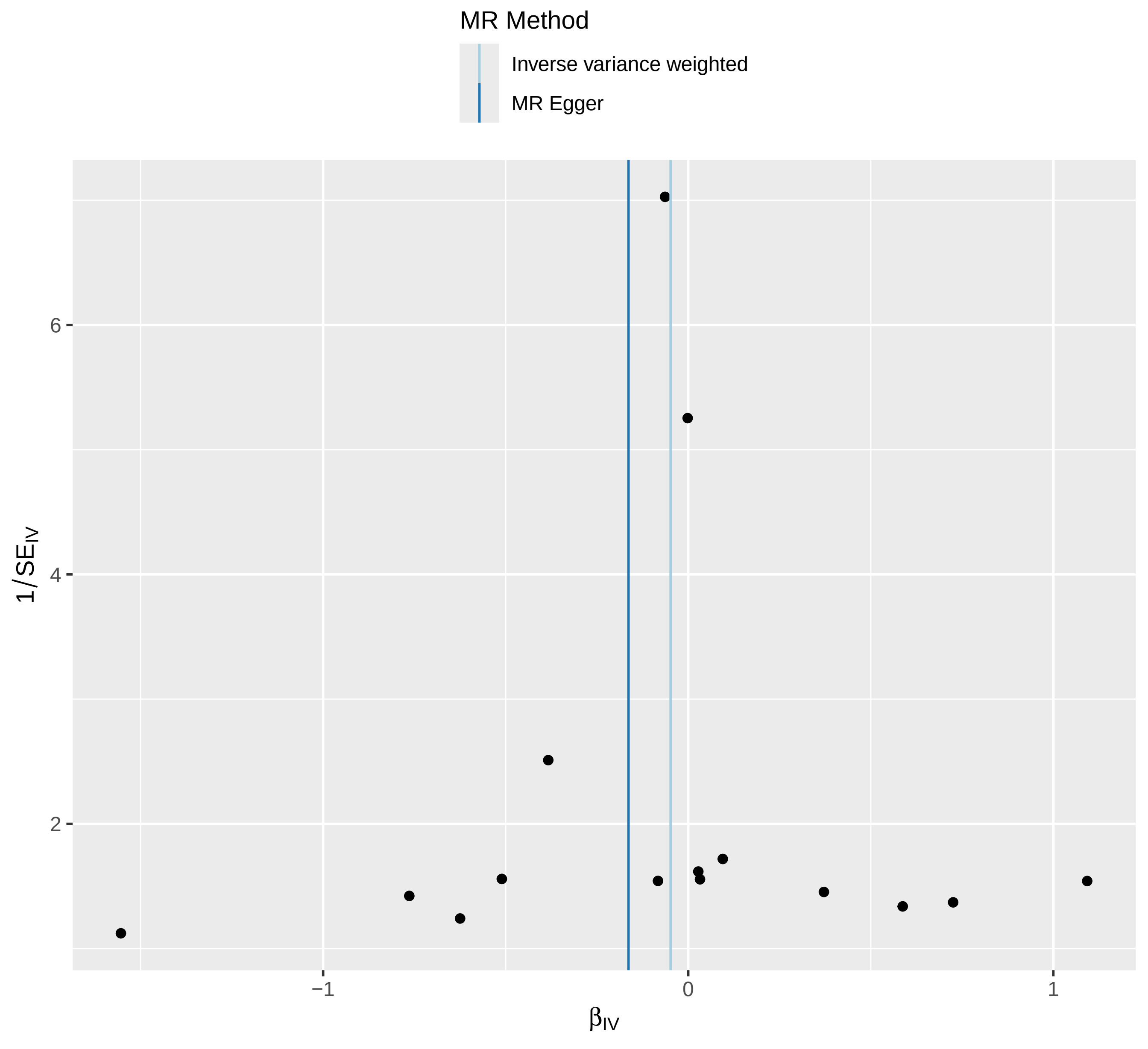
MR Method
Inverse variance weighted
MR Egger
6
4
2
1/SE$_{IV}$
−1
0
1
β$_{IV}$

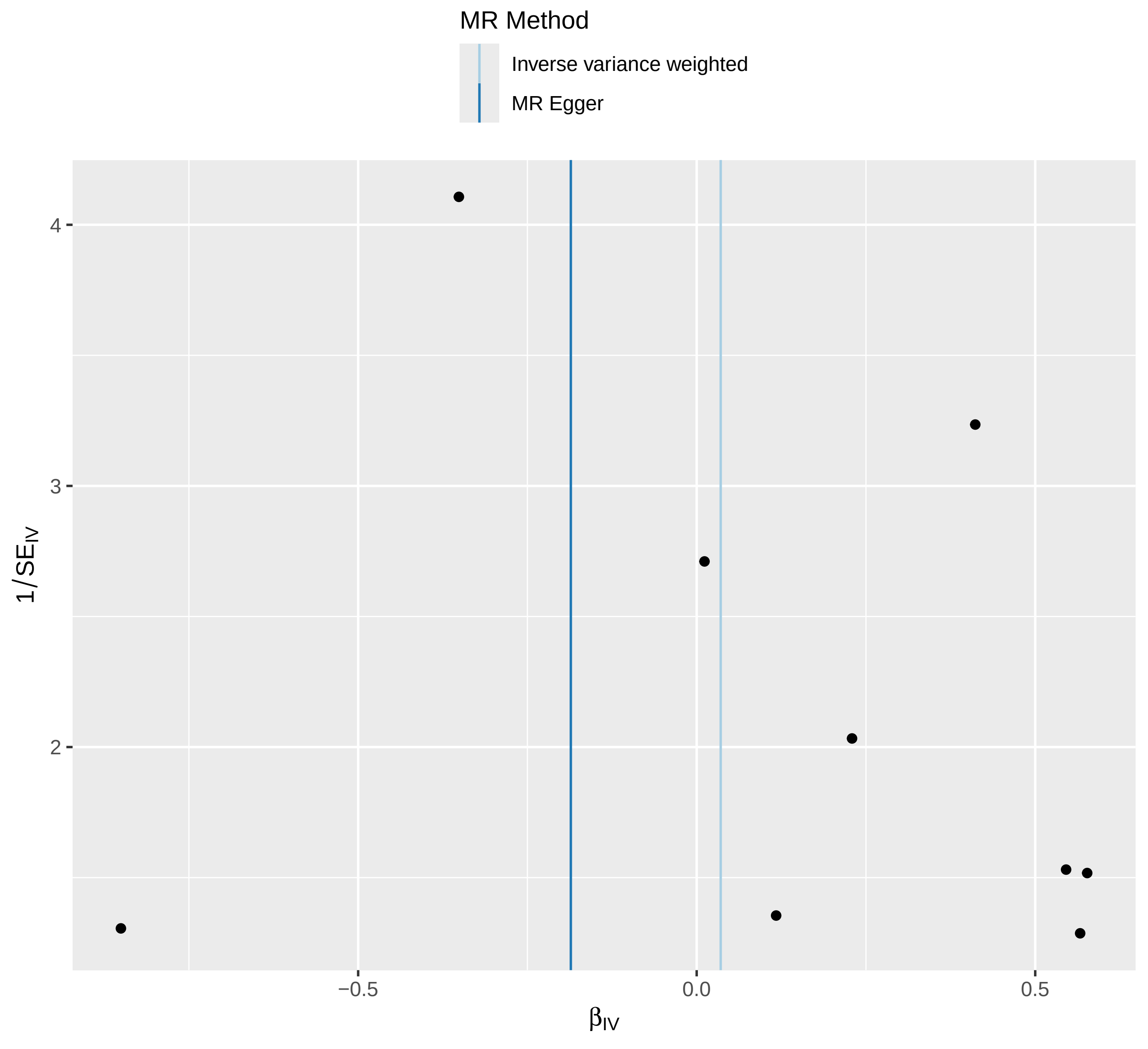
MR Method
Inverse variance weighted
MR Egger
4
3
2
1/SE_IV
−0.5
0.0
0.5
β_IV

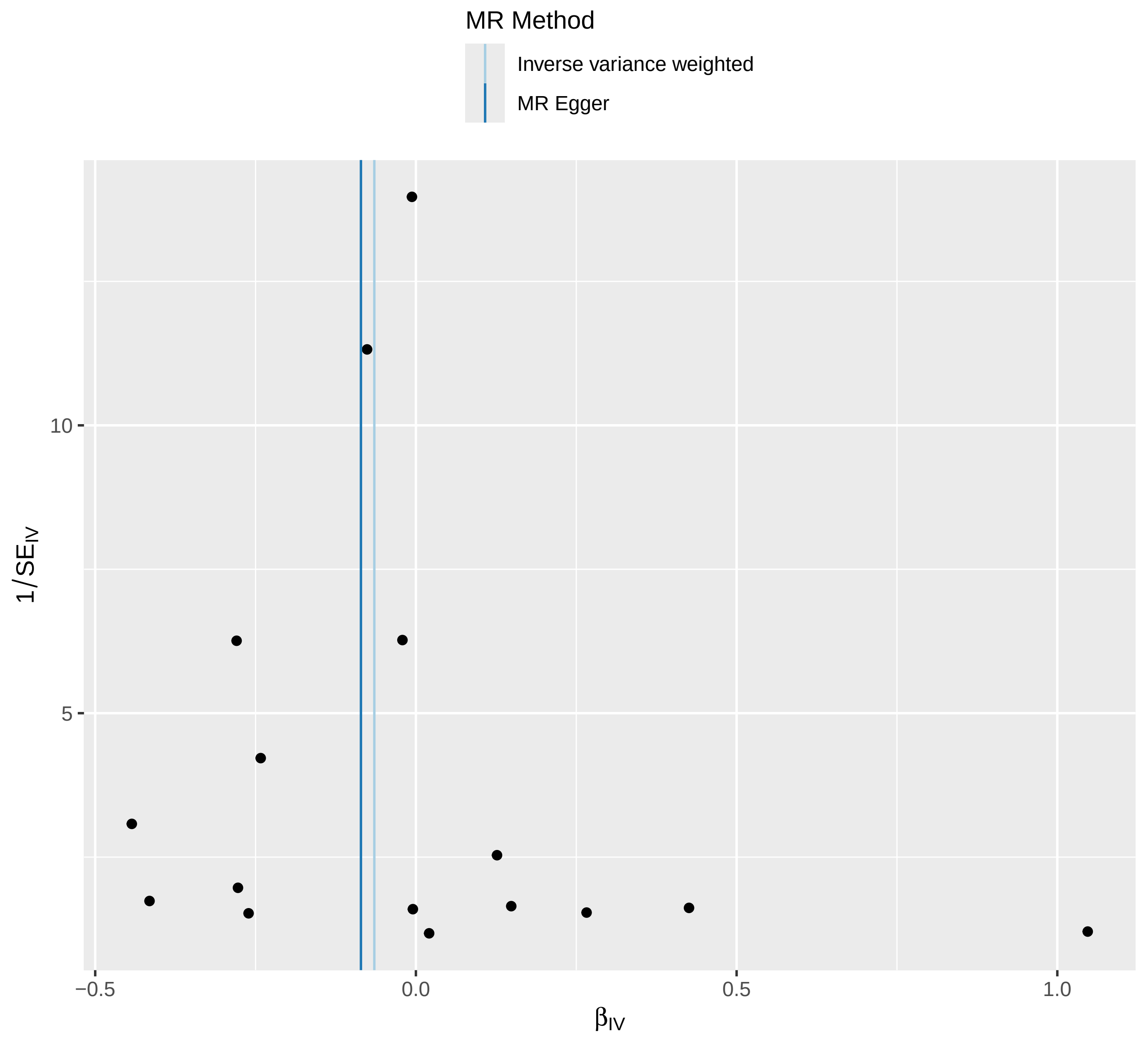
MR Method
Inverse variance weighted
MR Egger
10
5
1/SE$_{IV}$
−0.5
0.0
0.5
1.0
β$_{IV}$

Insufficient number of SNPs

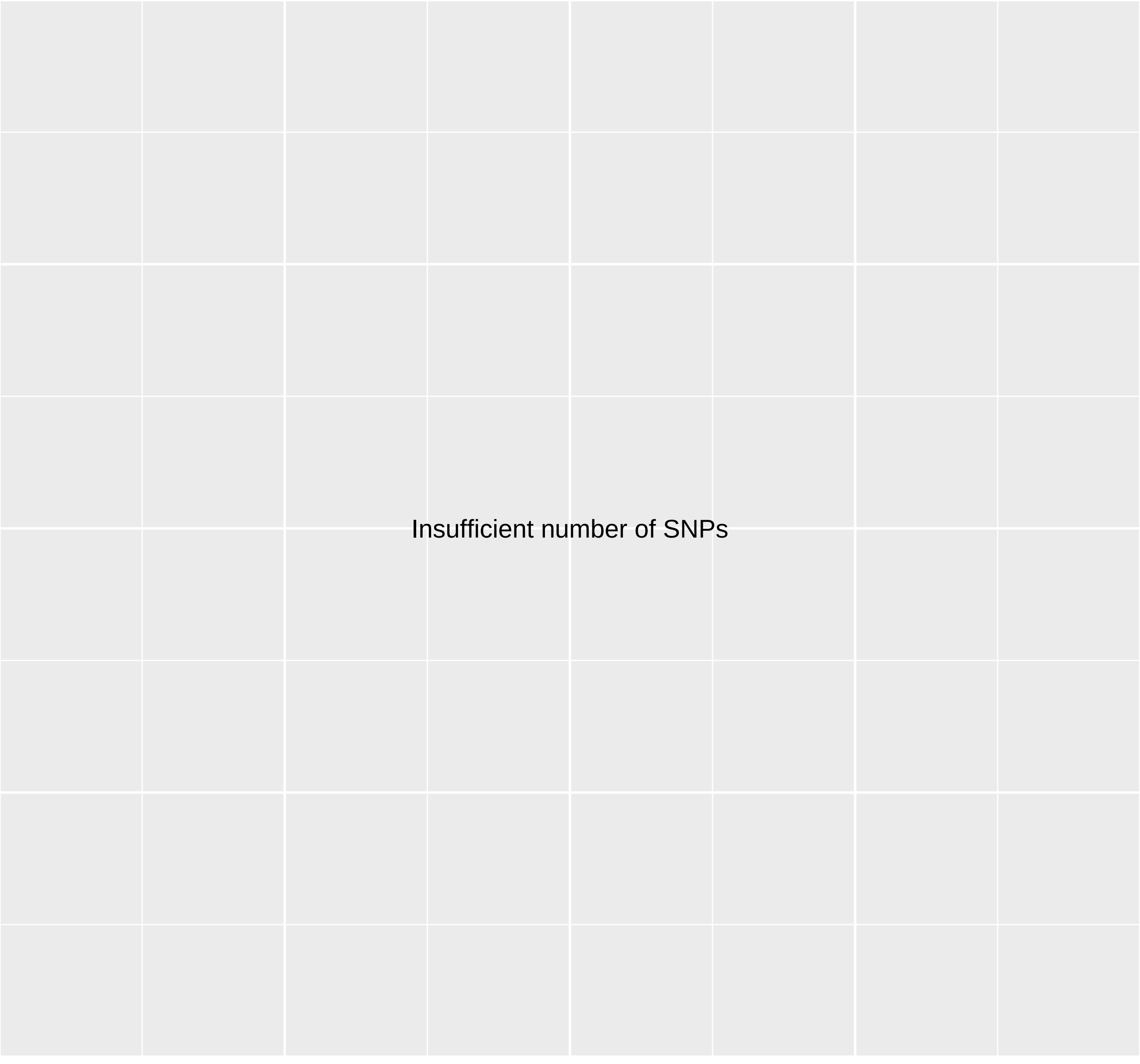
Insufficient number of SNPs

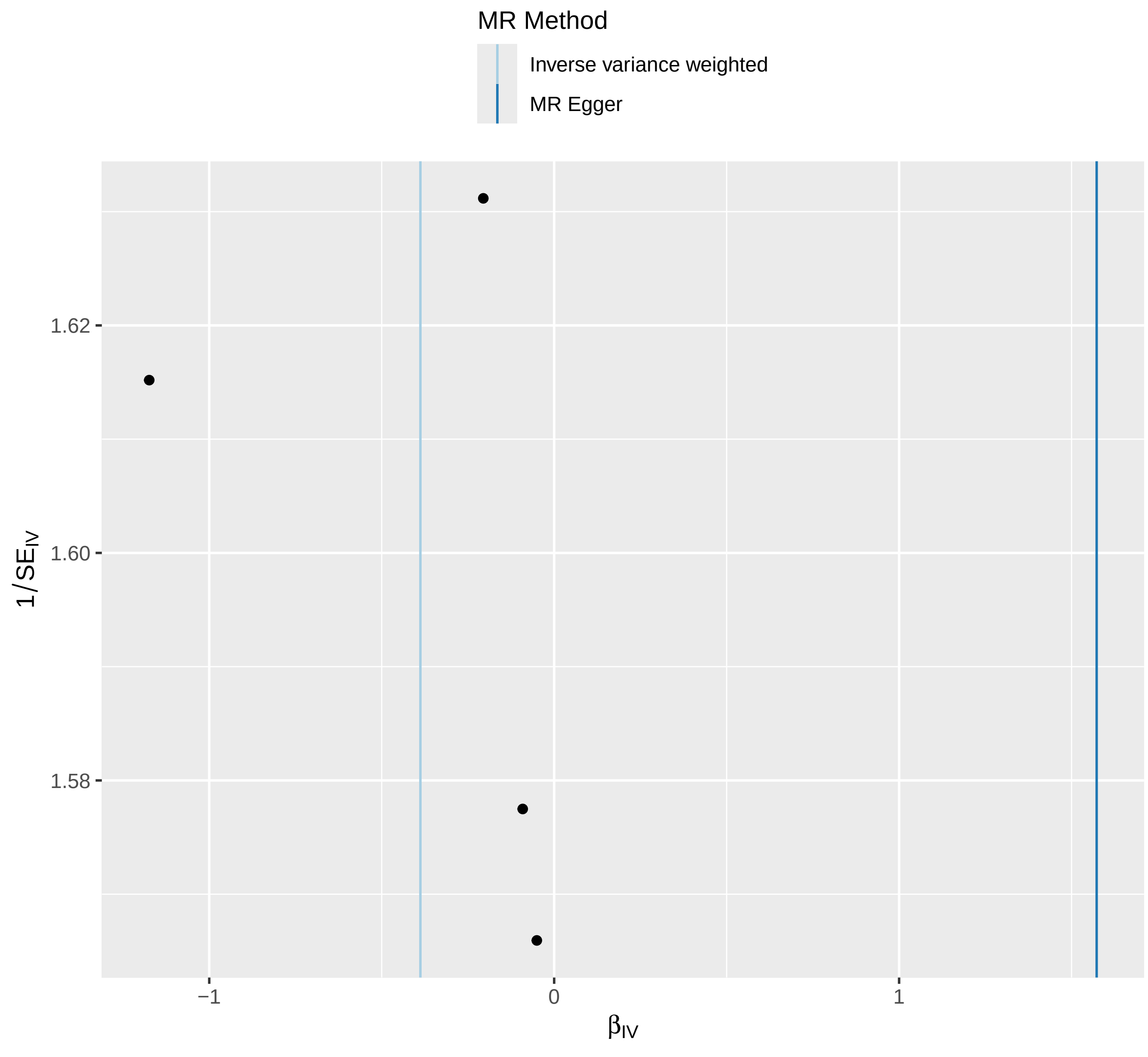
MR Method
Inverse variance weighted
MR Egger
1.62
1.60
1.58
$1/SE_{IV}$
−1
0
1
β_{IV}

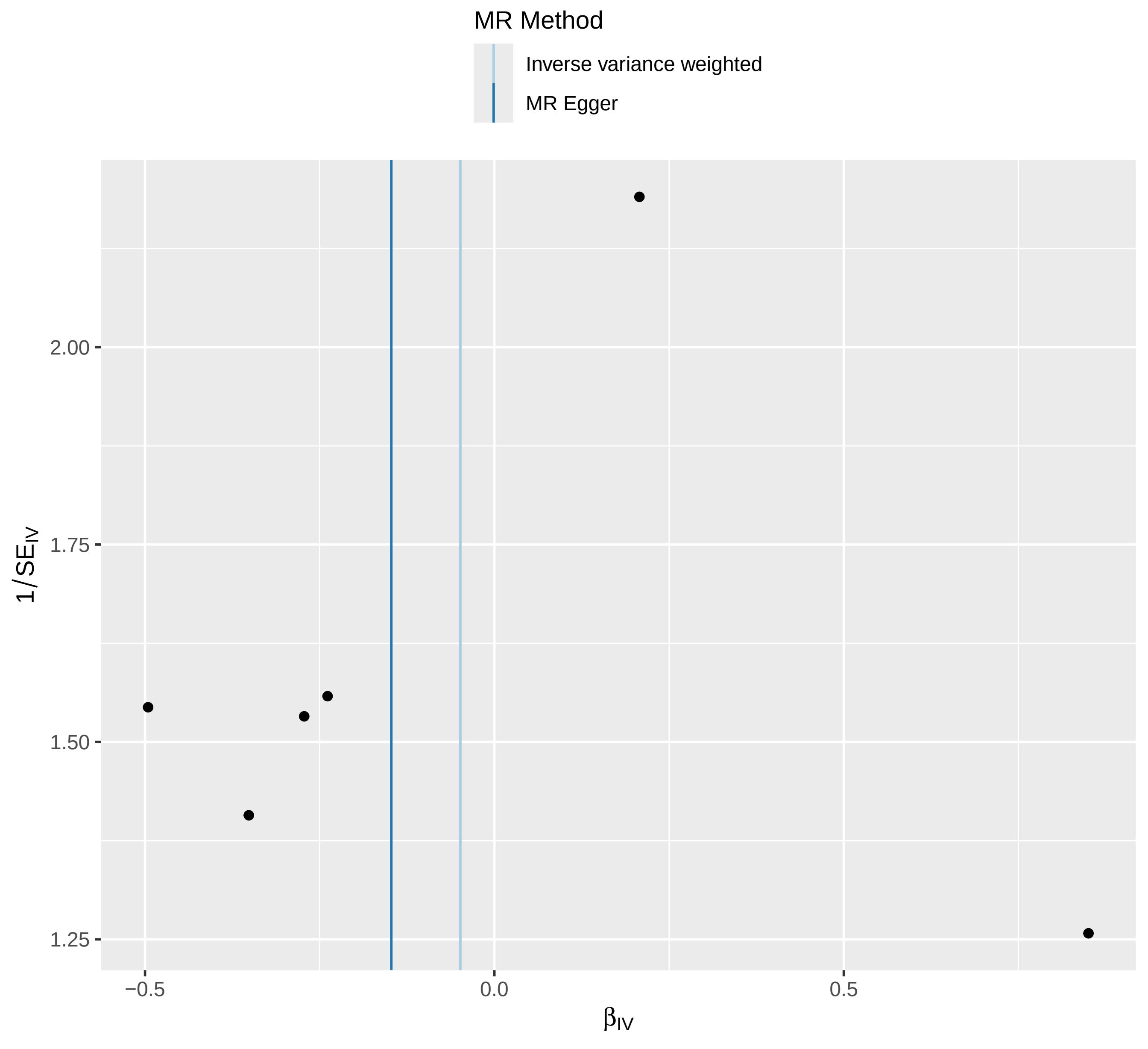

MR Method
Inverse variance weighted
MR Egger
2.00
1.75
1.50
1.25
1/SE$_{IV}$
−0.5
0.0
0.5
β$_{IV}$

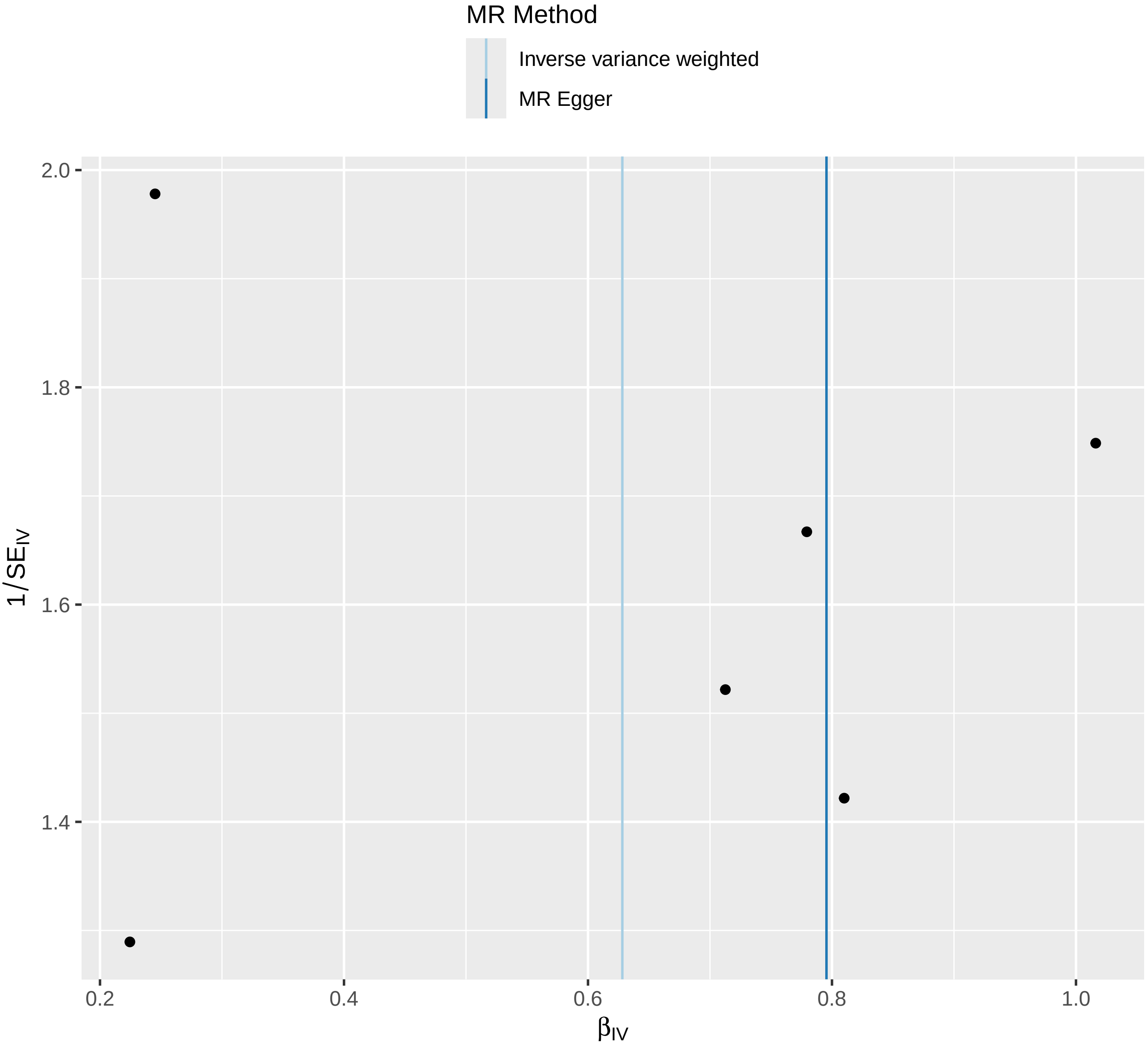

MR Method
Inverse variance weighted
MR Egger
2.0
1.8
1.6
1.4
1/SE_IV
0.2
0.4
0.6
0.8
1.0
β_IV

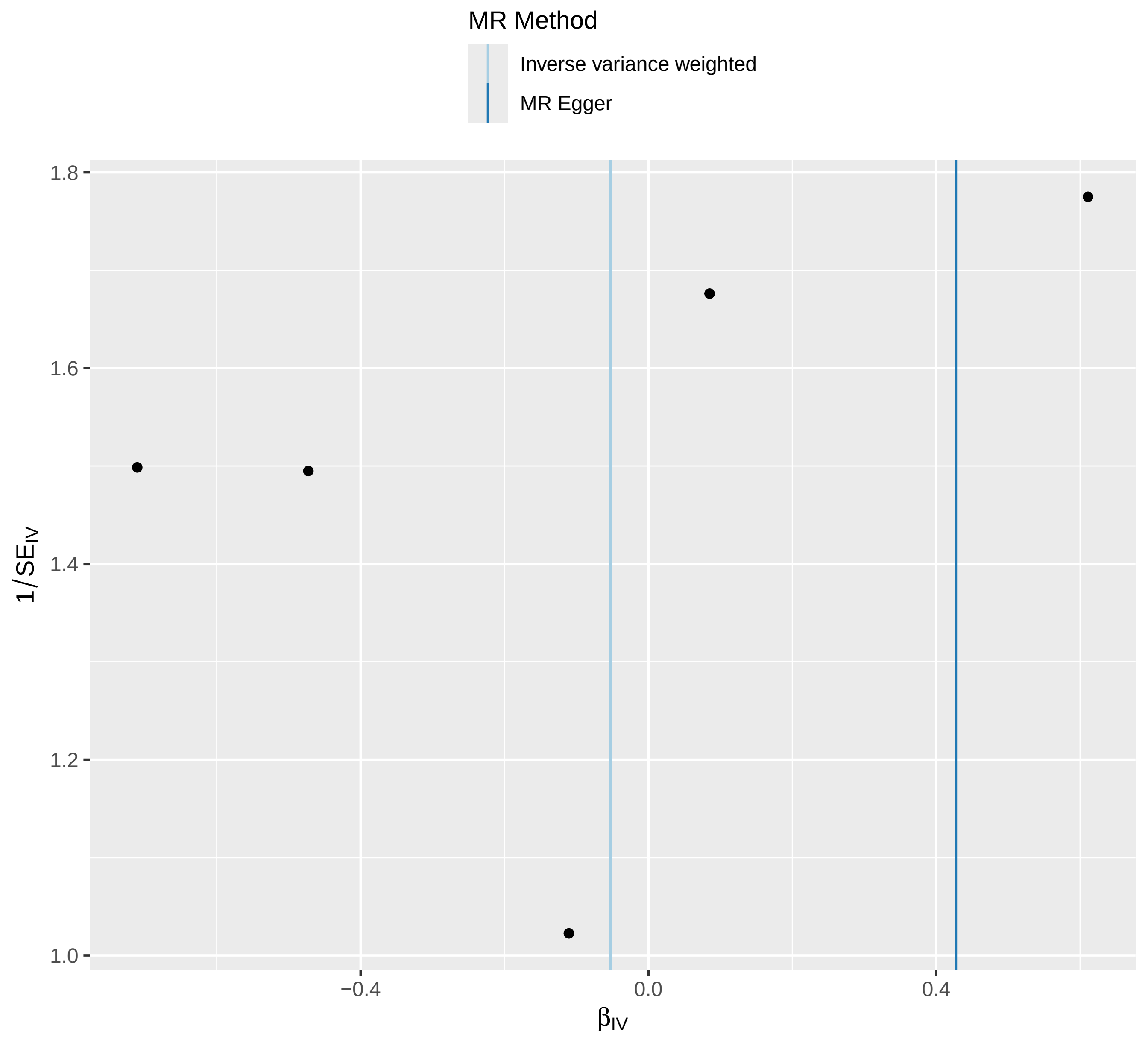

MR Method
Inverse variance weighted
MR Egger
1.8
1.6
1.4
1.2
1.0
1/SE_IV
−0.4
0.0
0.4
β_IV

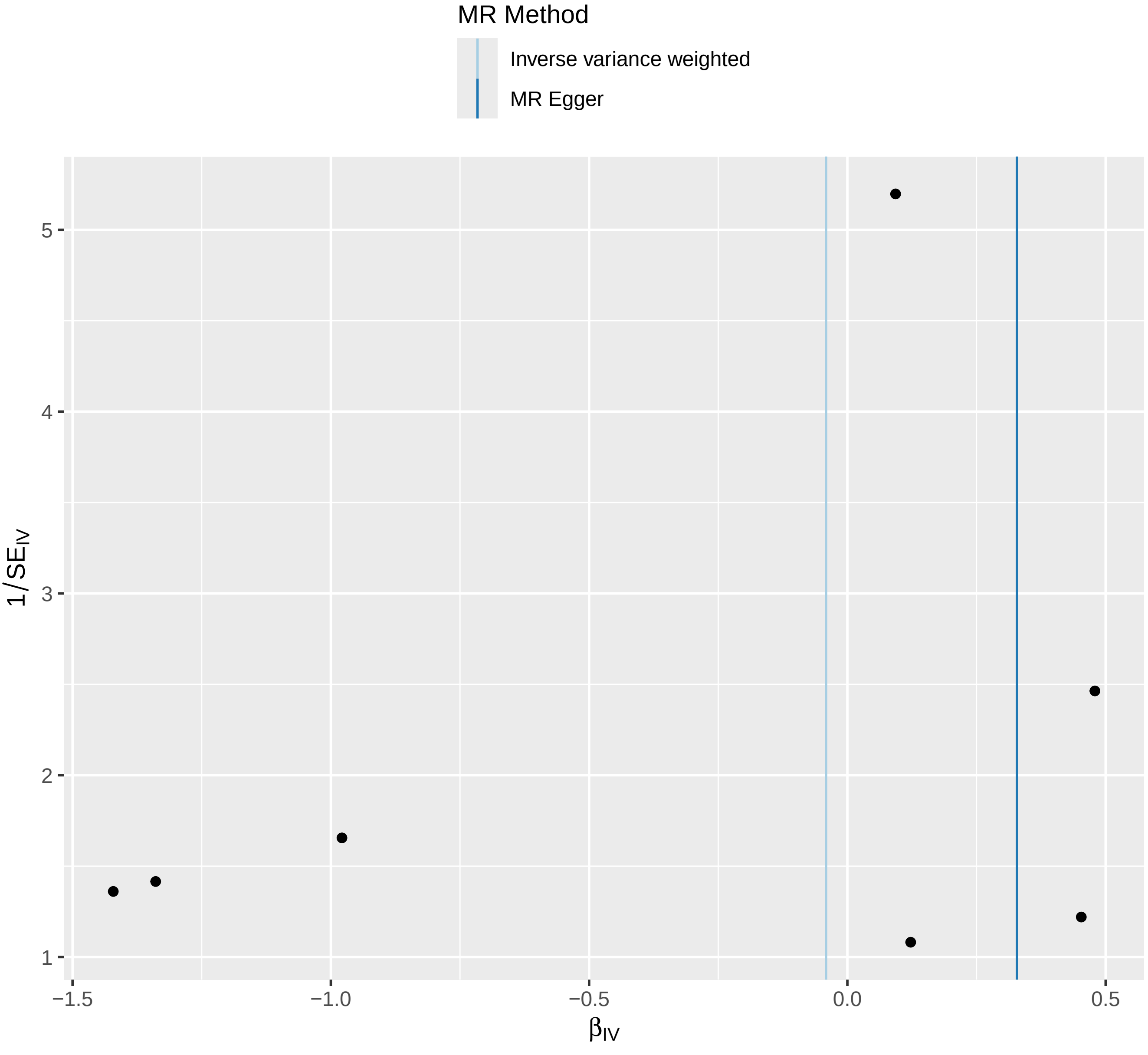

MR Method
Inverse variance weighted
MR Egger
5
4
3
2
1
1/SE_IV
−1.5
−1.0
−0.5
0.0
0.5
β_IV

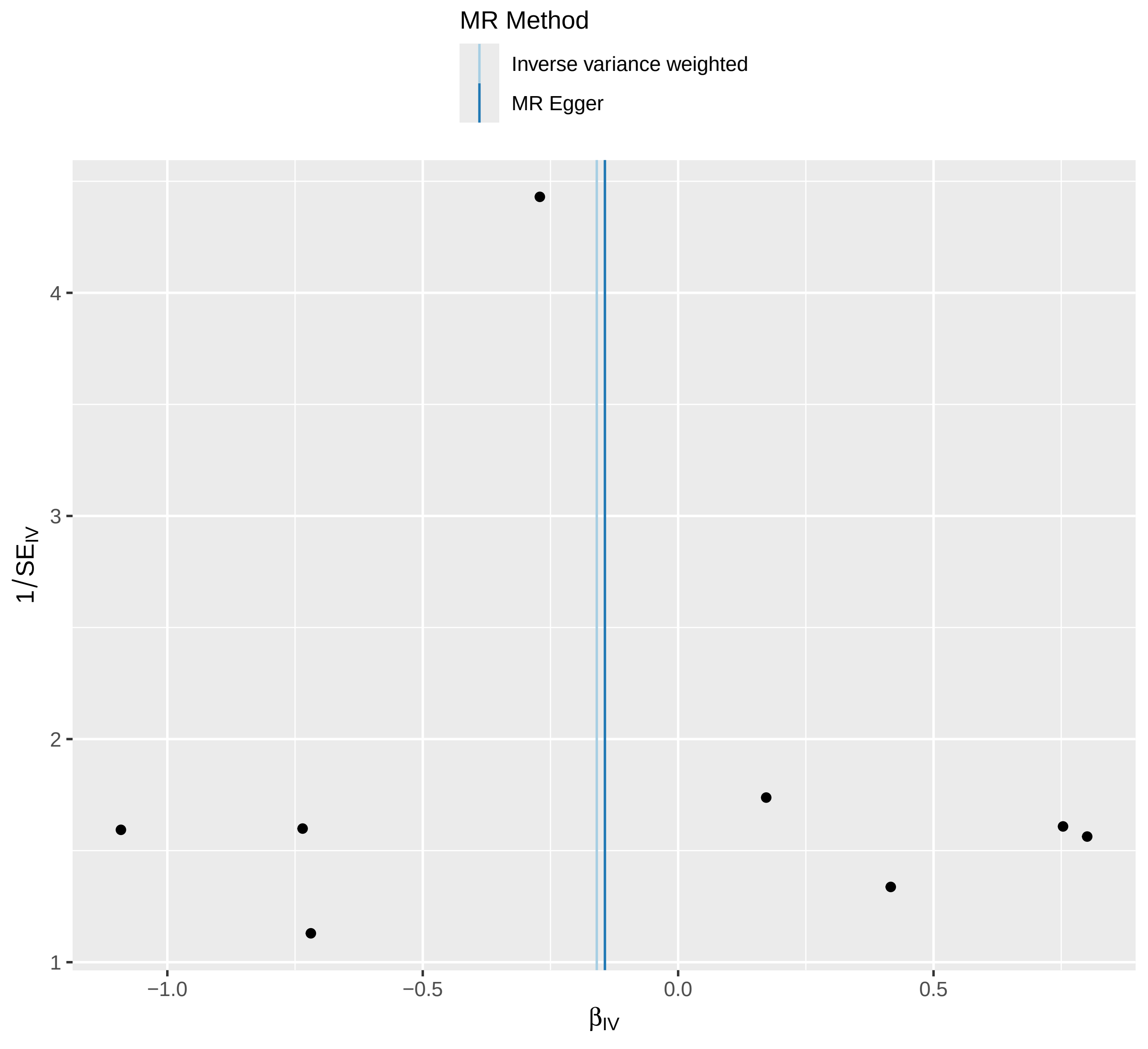

MR Method
Inverse variance weighted
MR Egger
4
3
2
1
$1/SE_{IV}$
−1.0
−0.5
0.0
0.5
β_{IV}

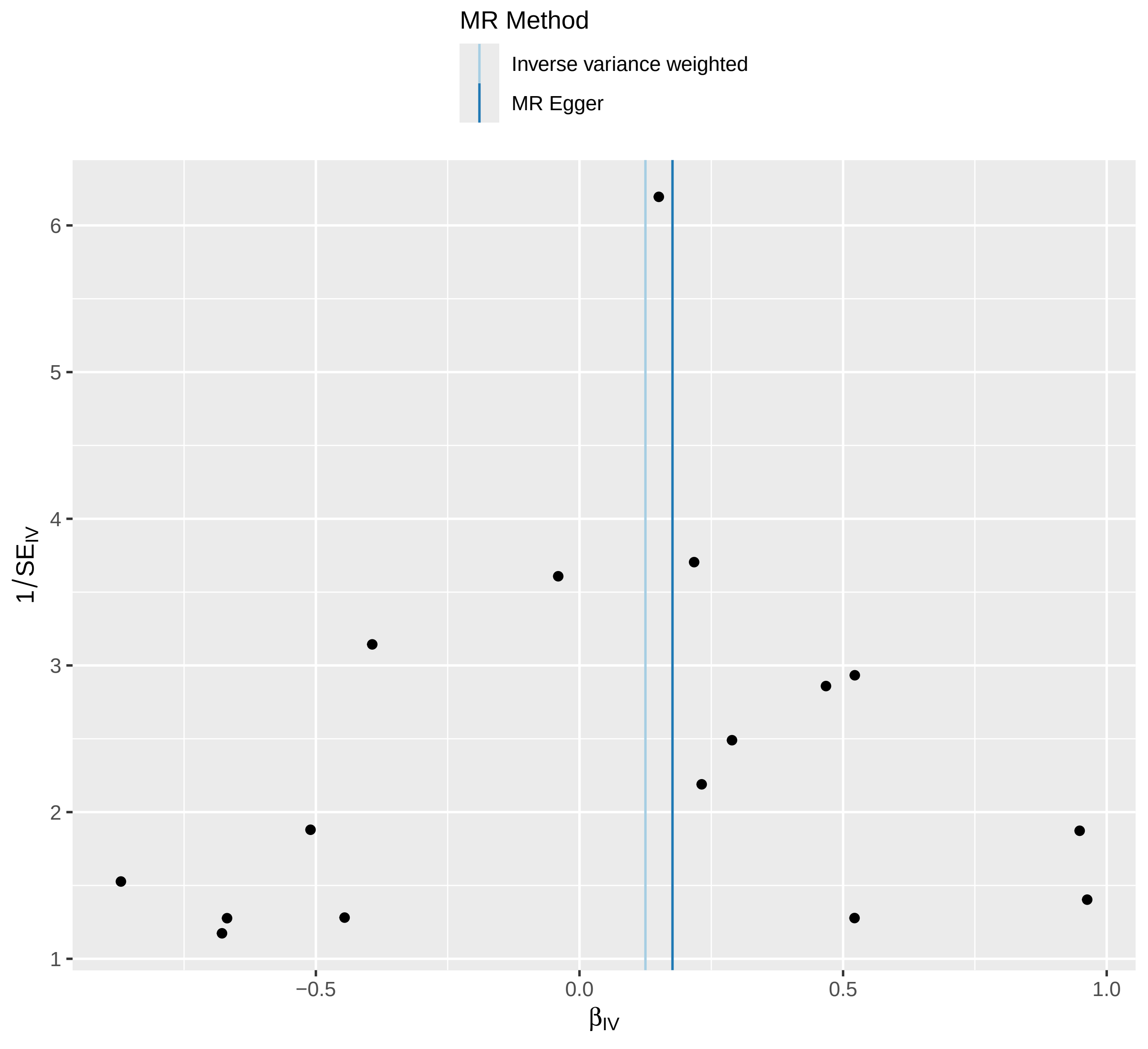

MR Method
Inverse variance weighted
MR Egger
6
5
4
3
2
1
1/SE_IV
−0.5
0.0
0.5
1.0
β_IV

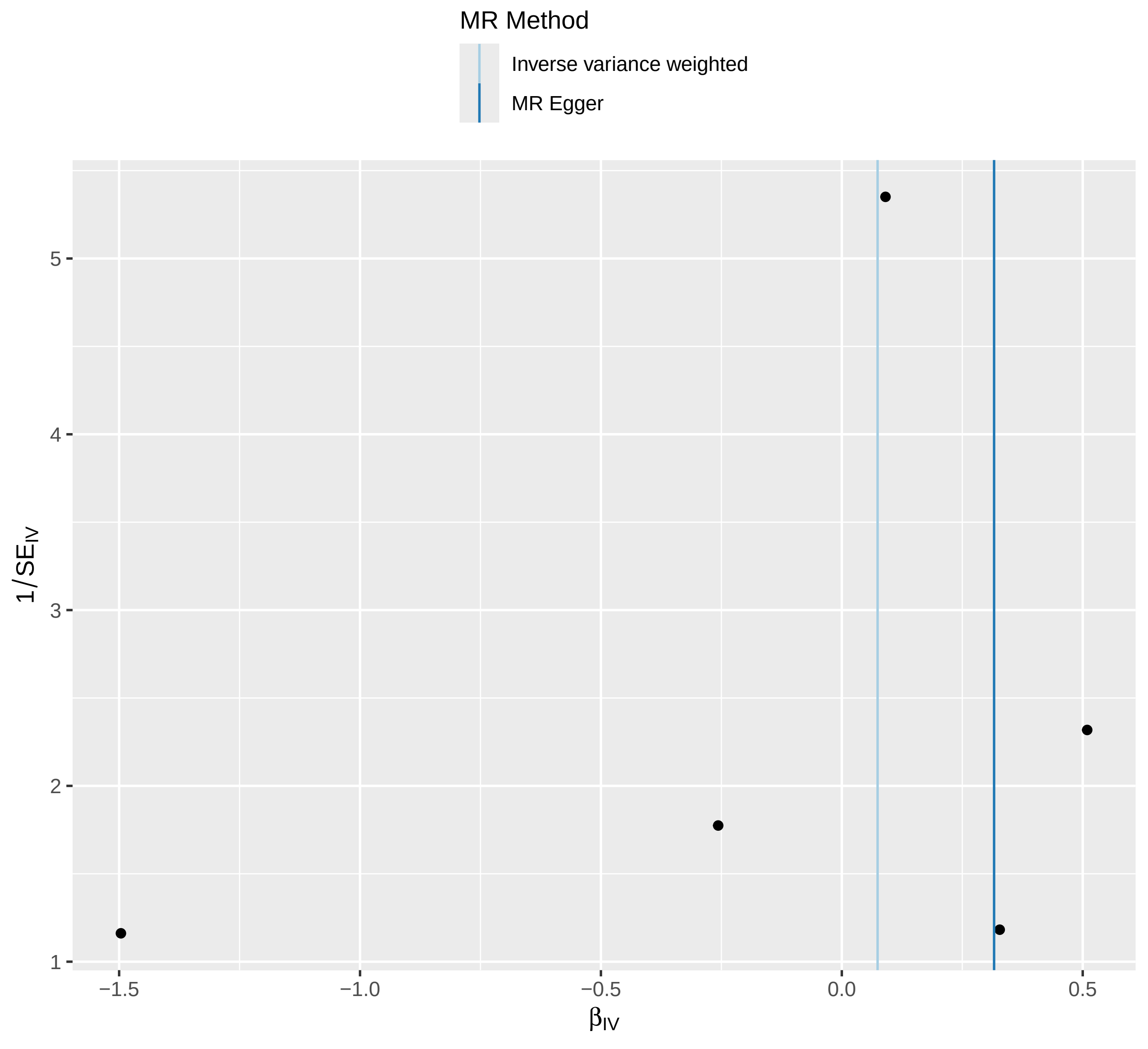
MR Method
Inverse variance weighted
MR Egger
1/SE$_{IV}$
5
4
3
2
1
−1.5
−1.0
−0.5
0.0
0.5
β$_{IV}$

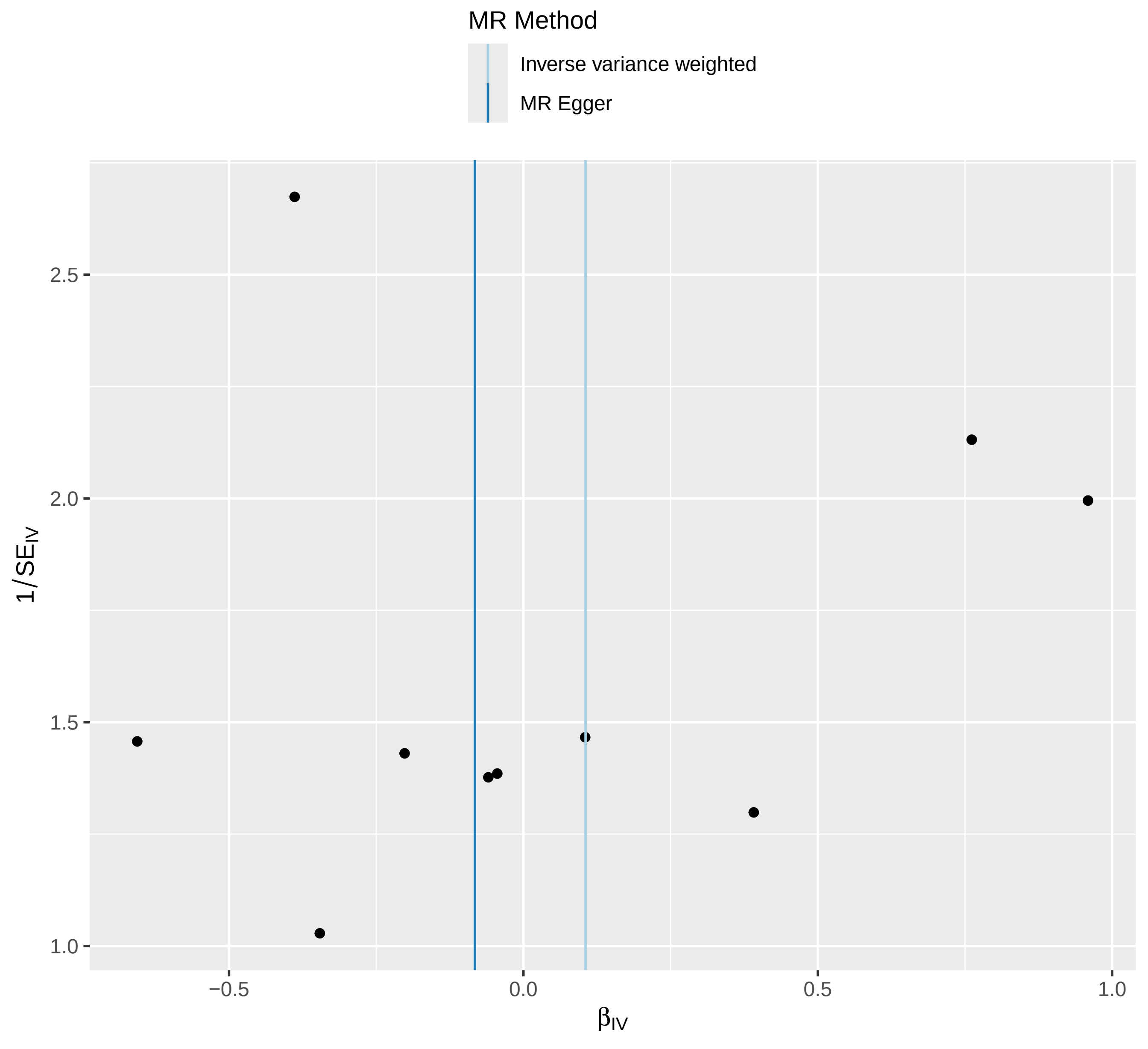
MR Method
Inverse variance weighted
MR Egger
1/SE_IV
2.5
2.0
1.5
1.0
−0.5
0.0
0.5
1.0
β_IV

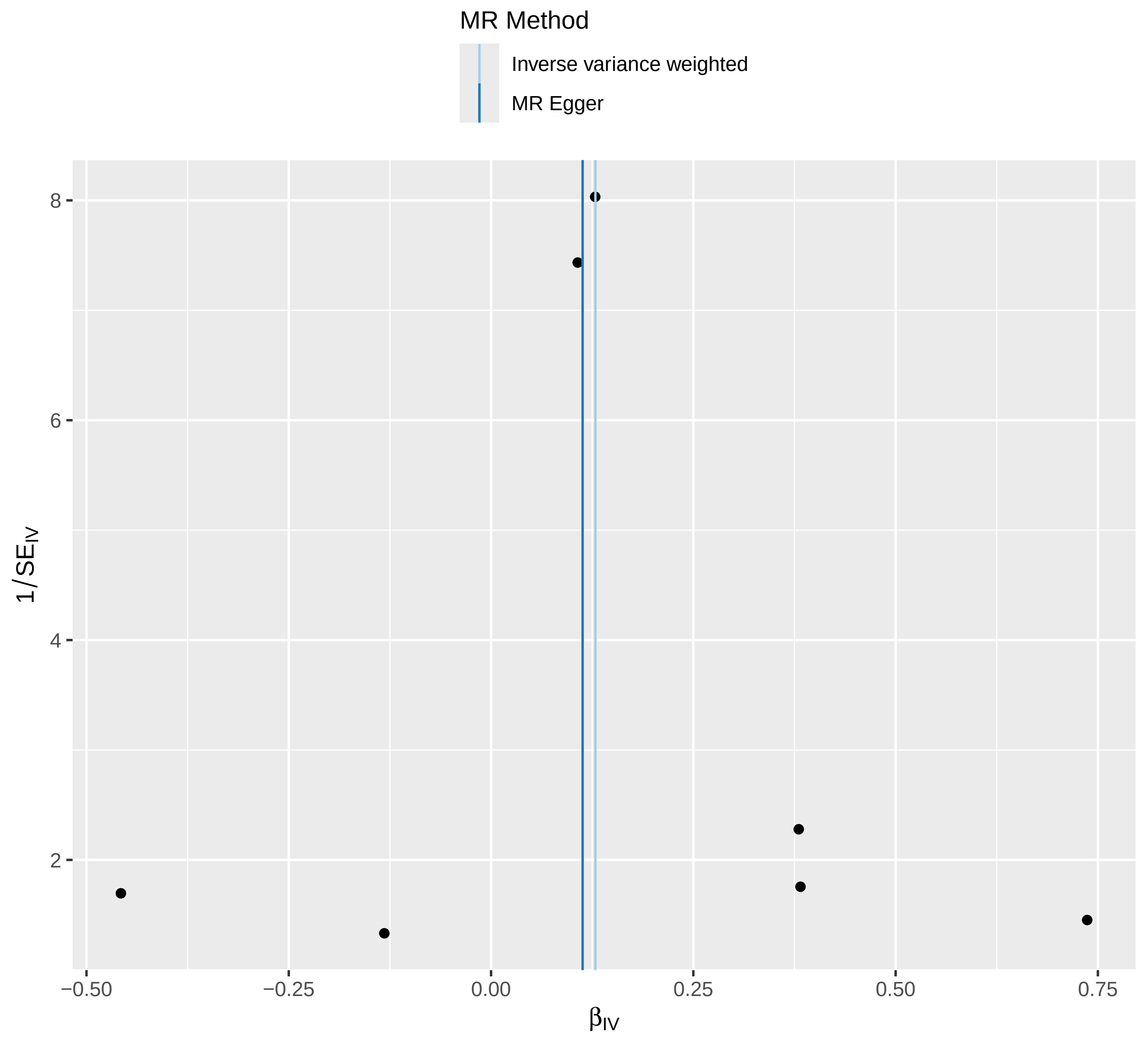

MR Method
Inverse variance weighted
MR Egger
8
6
4
2
1/SE$_{IV}$
−0.50
−0.25
0.00
0.25
0.50
0.75
β$_{IV}$

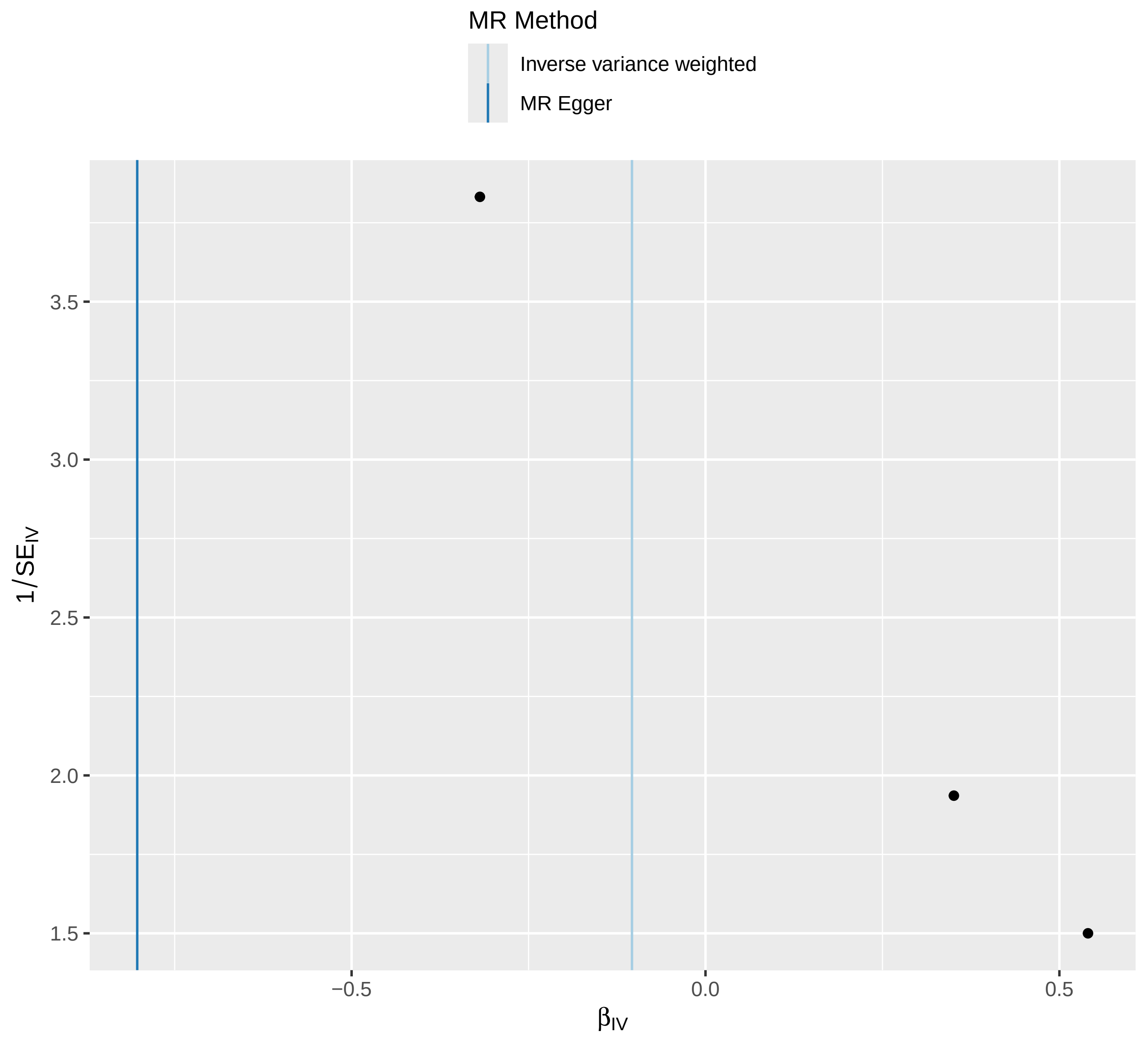
MR Method
Inverse variance weighted
MR Egger
3.5
3.0
2.5
2.0
1.5
$1/SE_{IV}$
−0.5
0.0
0.5
β_{IV}

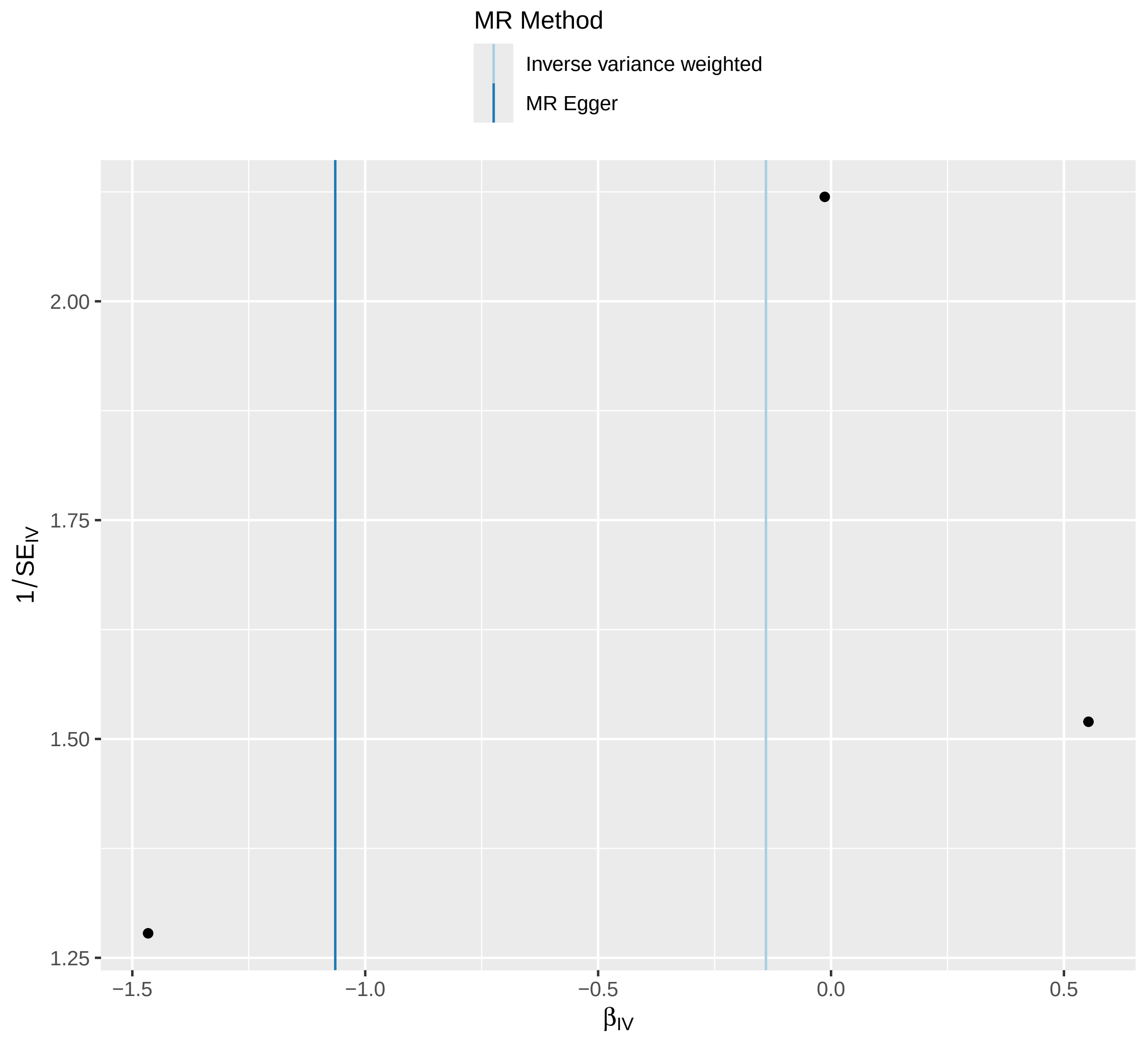
MR Method
Inverse variance weighted
MR Egger
2.00
1.75
1.50
1.25
1/SE_IV
−1.5
−1.0
−0.5
0.0
0.5
β_IV

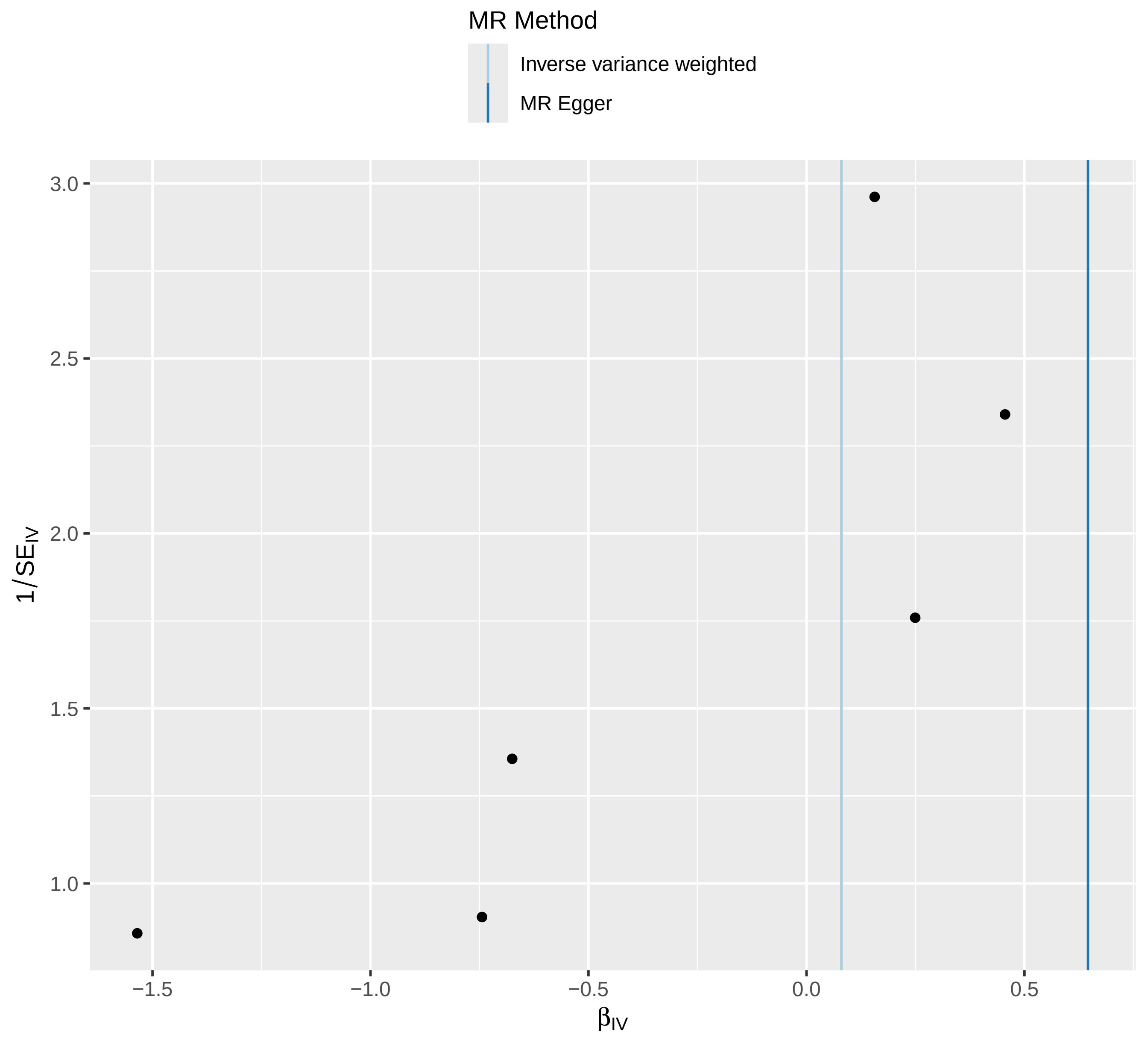
MR Method
Inverse variance weighted
MR Egger
3.0
2.5
2.0
1.5
1.0
1/SE_IV
−1.5
−1.0
−0.5
0.0
0.5
β_IV

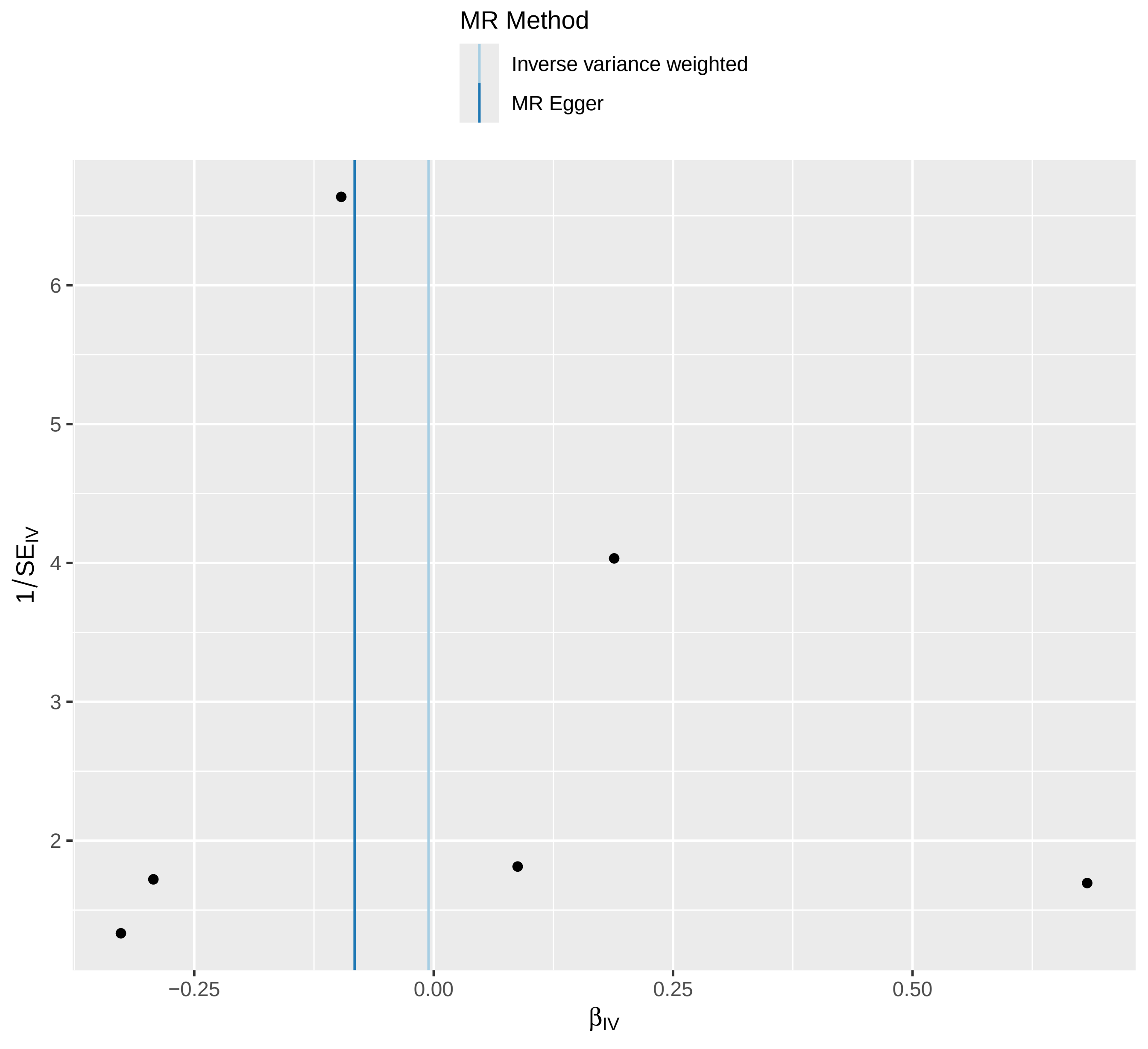

MR Method
Inverse variance weighted
MR Egger
6
5
4
3
2
1/SE$_{IV}$
−0.25
0.00
0.25
0.50
β$_{IV}$

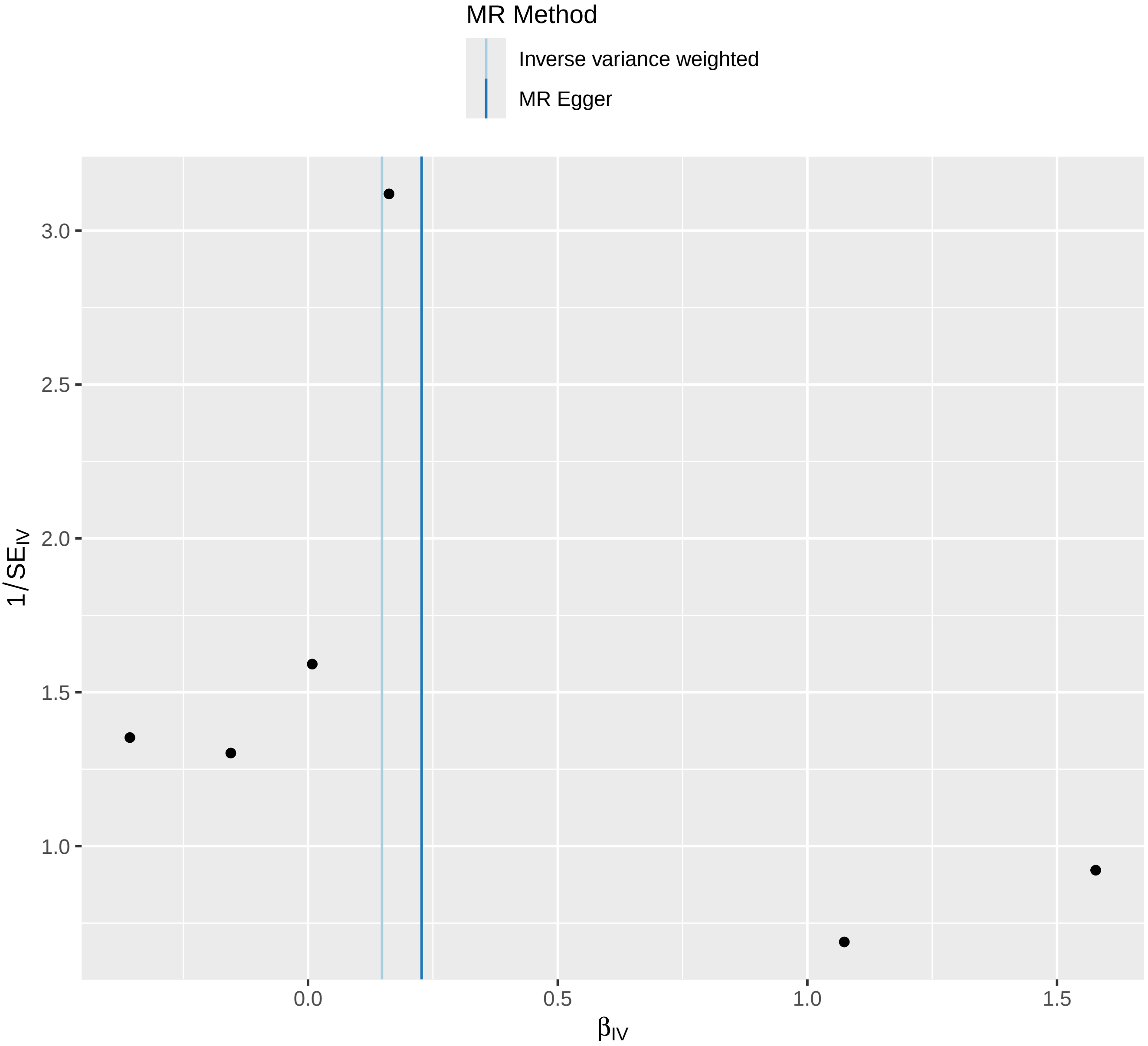

MR Method
Inverse variance weighted
MR Egger
3.0
2.5
2.0
1.5
1.0
$1/SE_{IV}$
0.0
0.5
1.0
1.5
β_{IV}

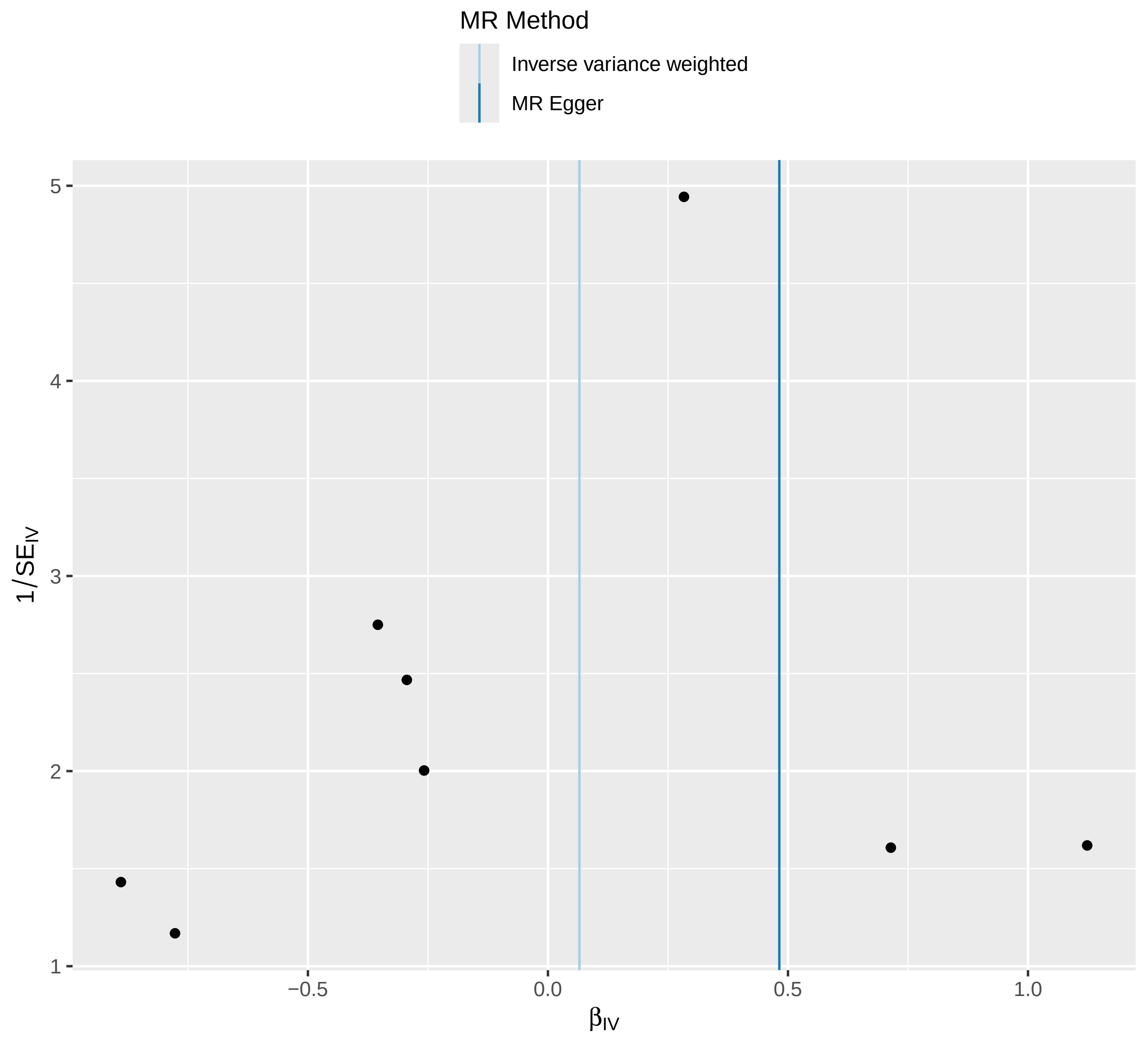

MR Method
Inverse variance weighted
MR Egger
5
4
3
2
1
1/SE_IV
−0.5
0.0
0.5
1.0
β_IV

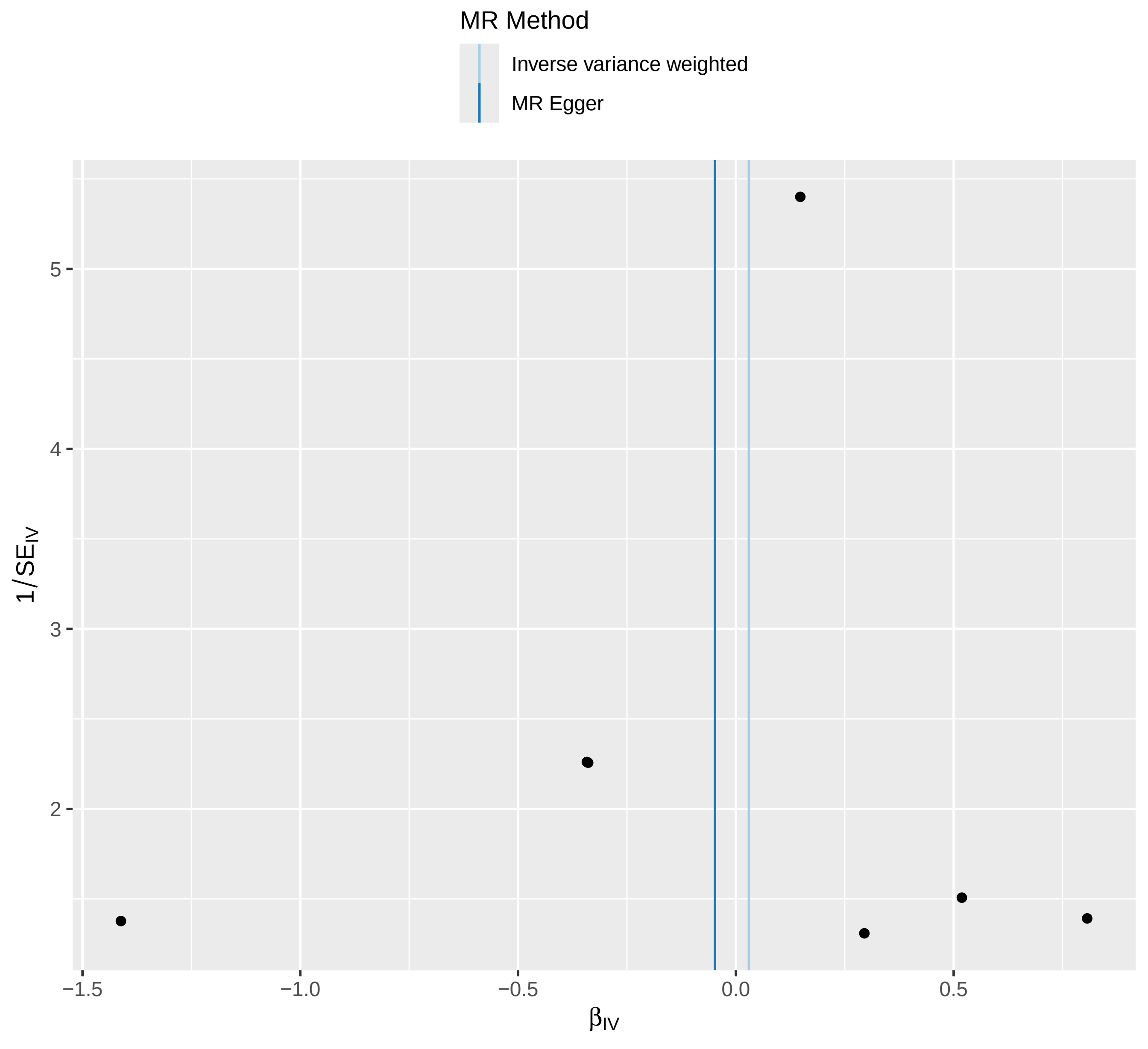
MR Method
Inverse variance weighted
MR Egger
5
4
3
2
1/SE_IV
−1.5
−1.0
−0.5
0.0
0.5
β_IV

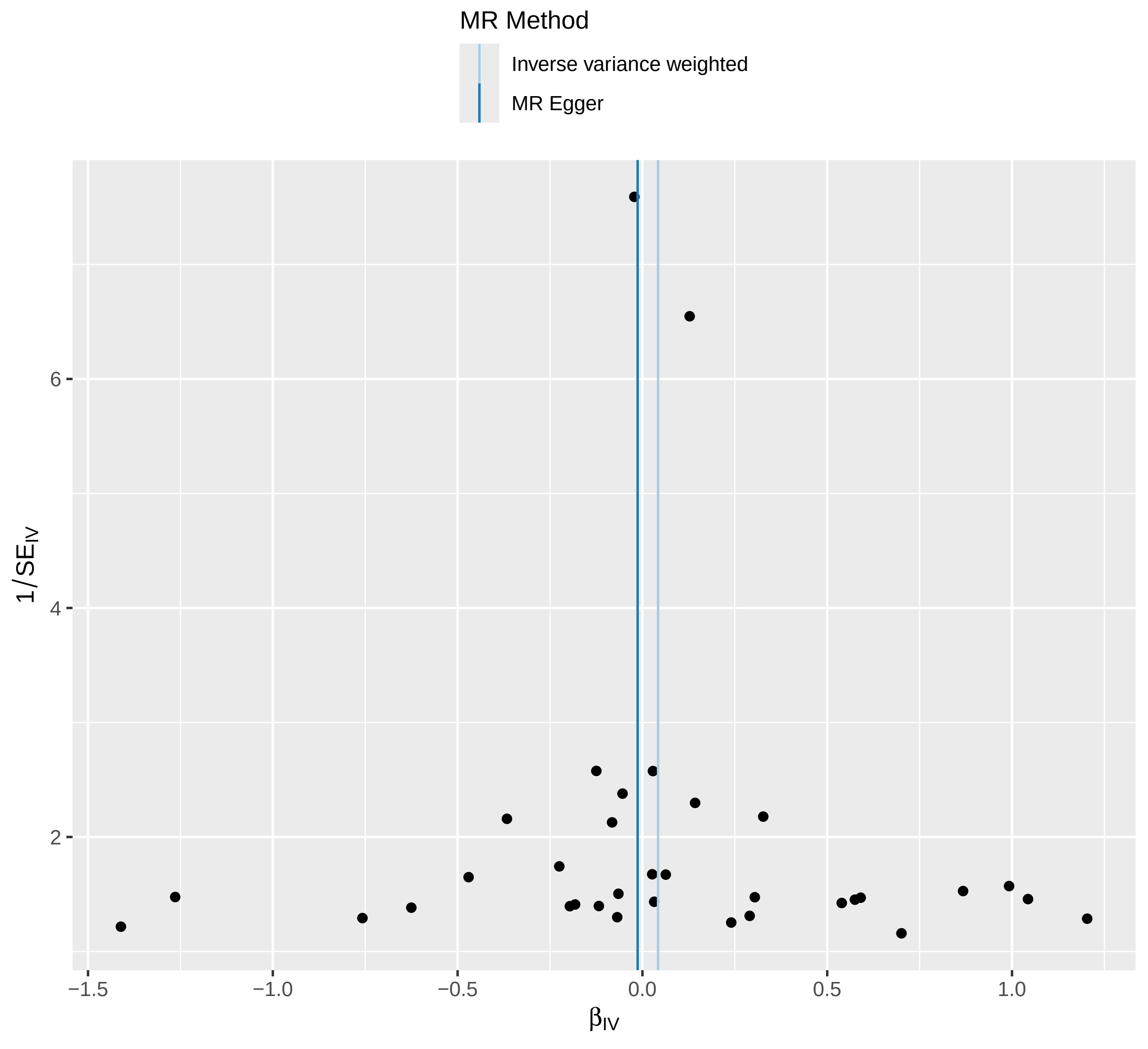
MR Method
Inverse variance weighted
MR Egger
6
4
2
1/SE_IV
−1.5
−1.0
−0.5
0.0
0.5
1.0
β_IV

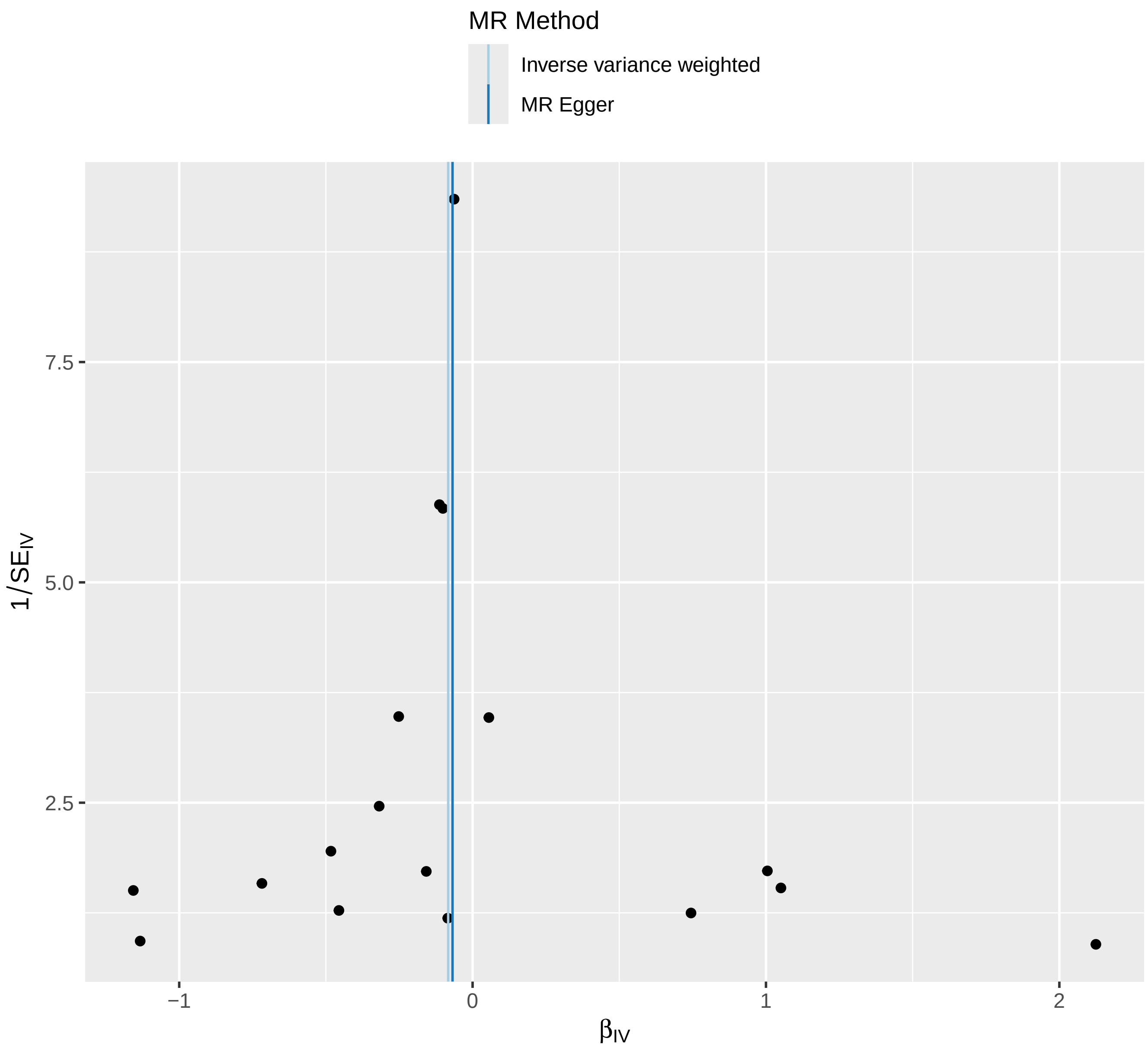

MR Method
Inverse variance weighted
MR Egger
7.5
5.0
2.5
1/SE_IV
−1
0
1
2
β_IV

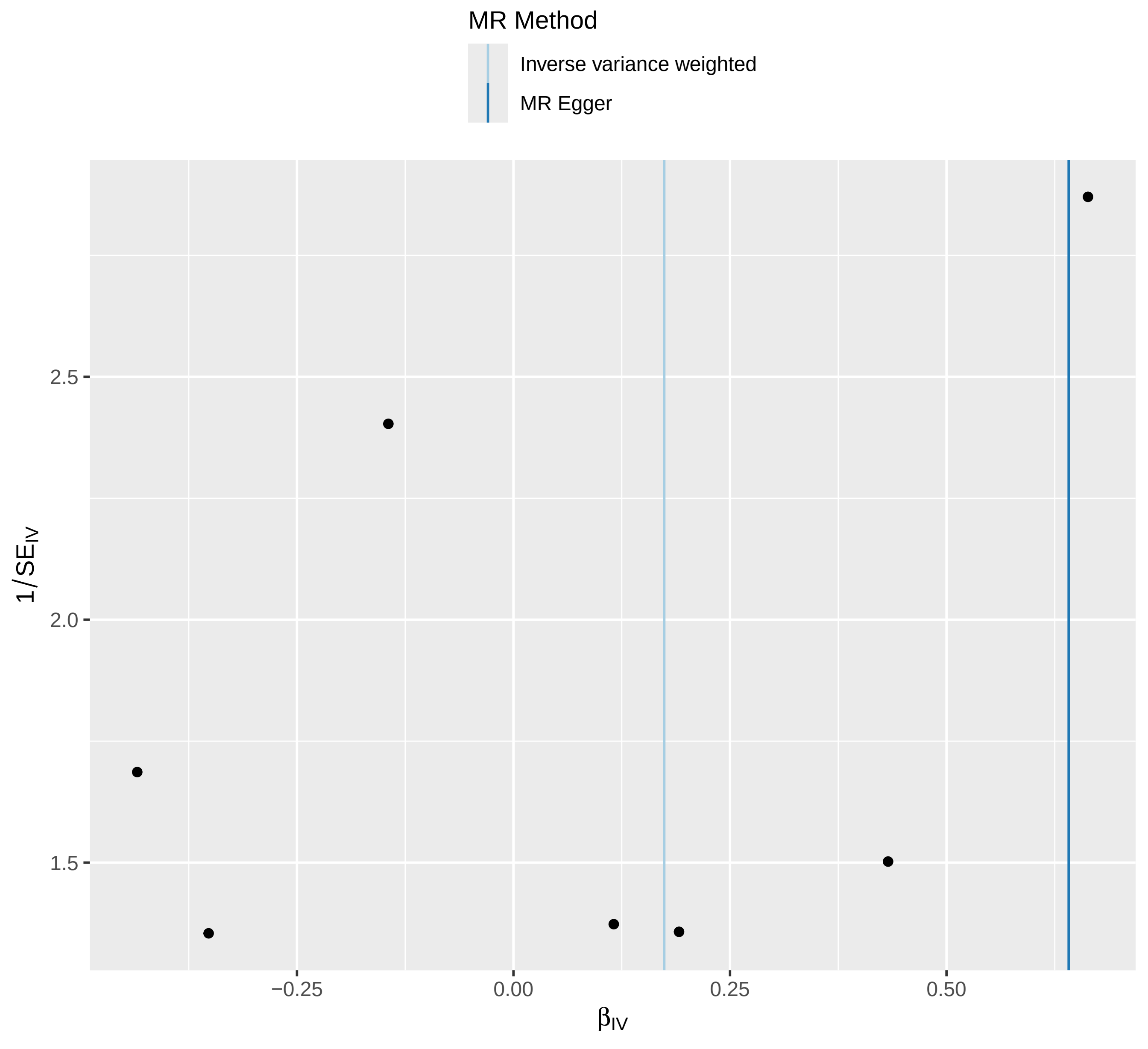
MR Method
Inverse variance weighted
MR Egger
2.5
2.0
1.5
1/SE_IV
−0.25
0.00
0.25
0.50
β_IV

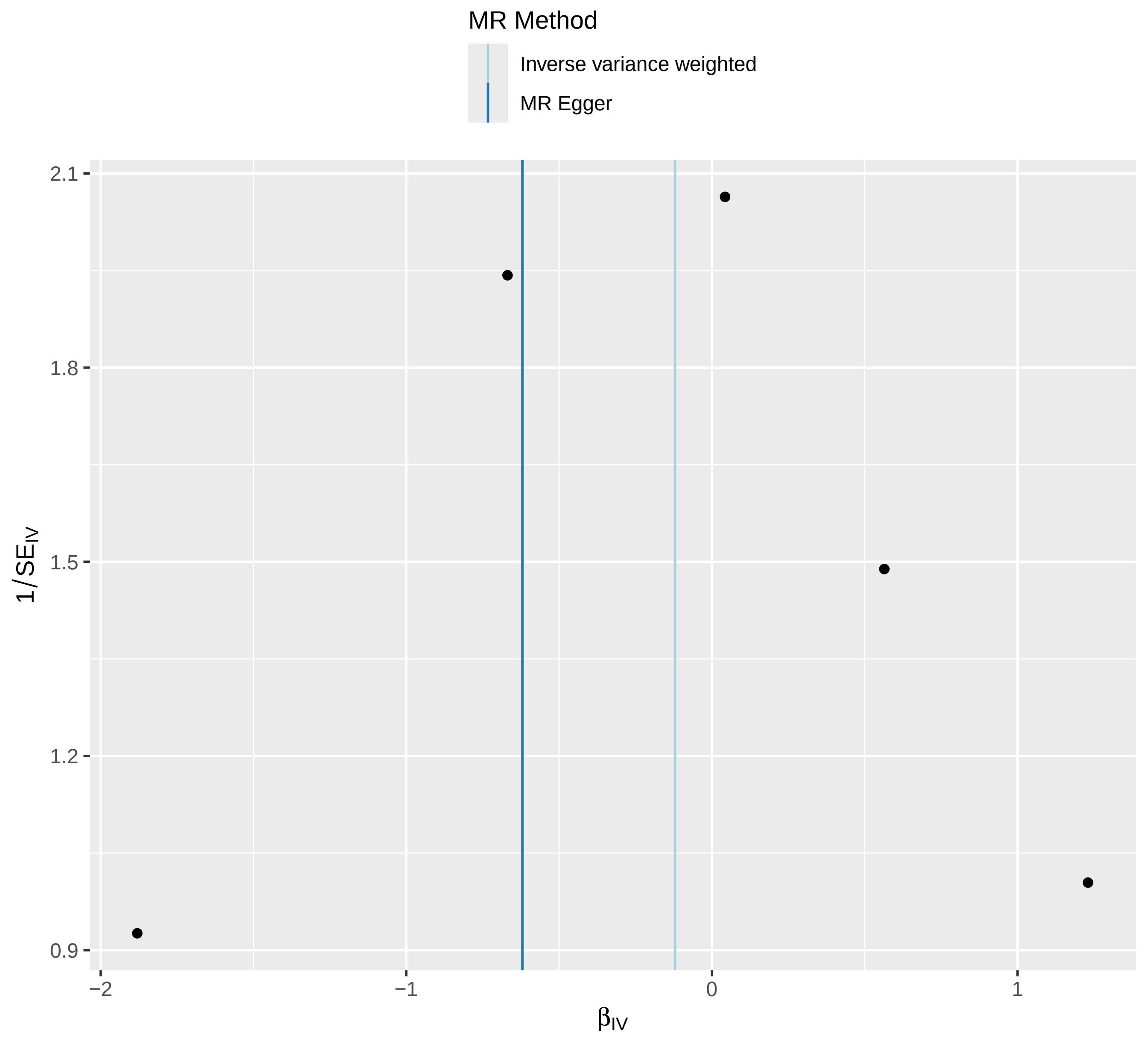

MR Method
Inverse variance weighted
MR Egger
2.1
1.8
1.5
1.2
0.9
1/SE_IV
−2
−1
0
1
β_IV

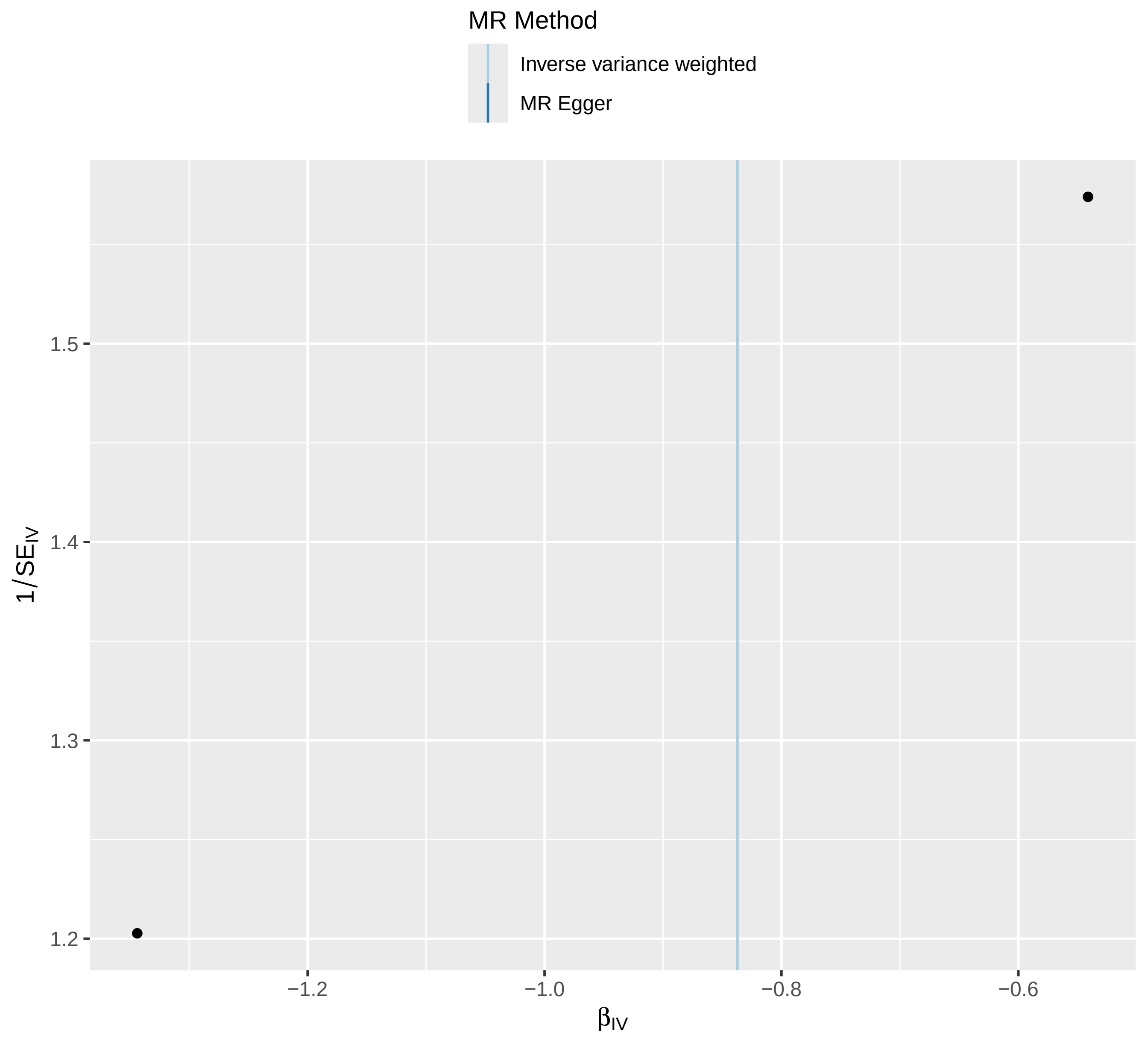

MR Method
Inverse variance weighted
MR Egger
1.5
1.4
1.3
1.2
−1.2
−1.0
−0.8
−0.6
$1/SE_{IV}$
β_{IV}

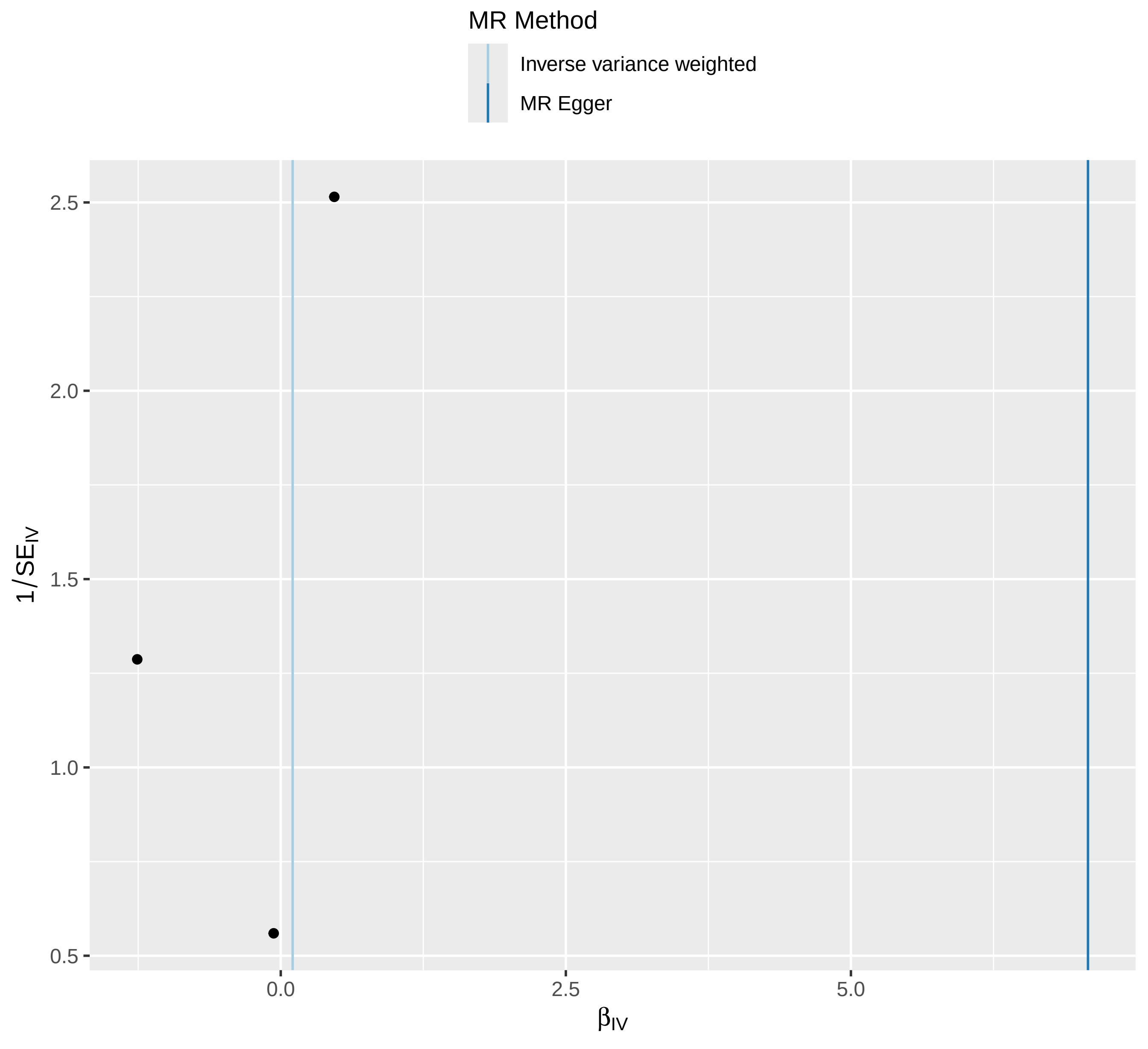

MR Method
Inverse variance weighted
MR Egger
1/SE_{IV}
2.5
2.0
1.5
1.0
0.5
0.0
2.5
5.0
β_{IV}

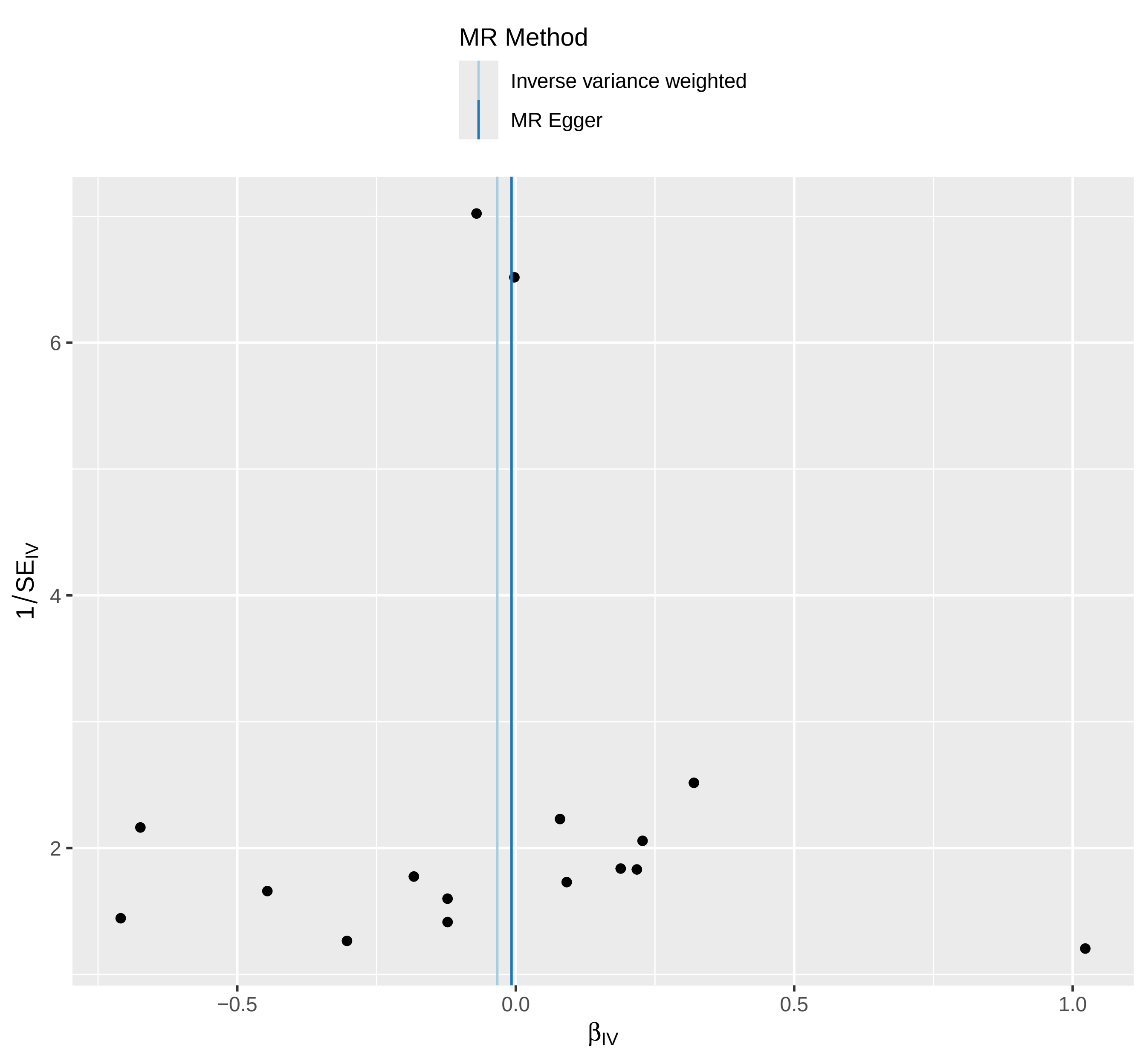
MR Method
Inverse variance weighted
MR Egger
6
4
2
−0.5
0.0
0.5
1.0
$1/SE_{IV}$
β_{IV}

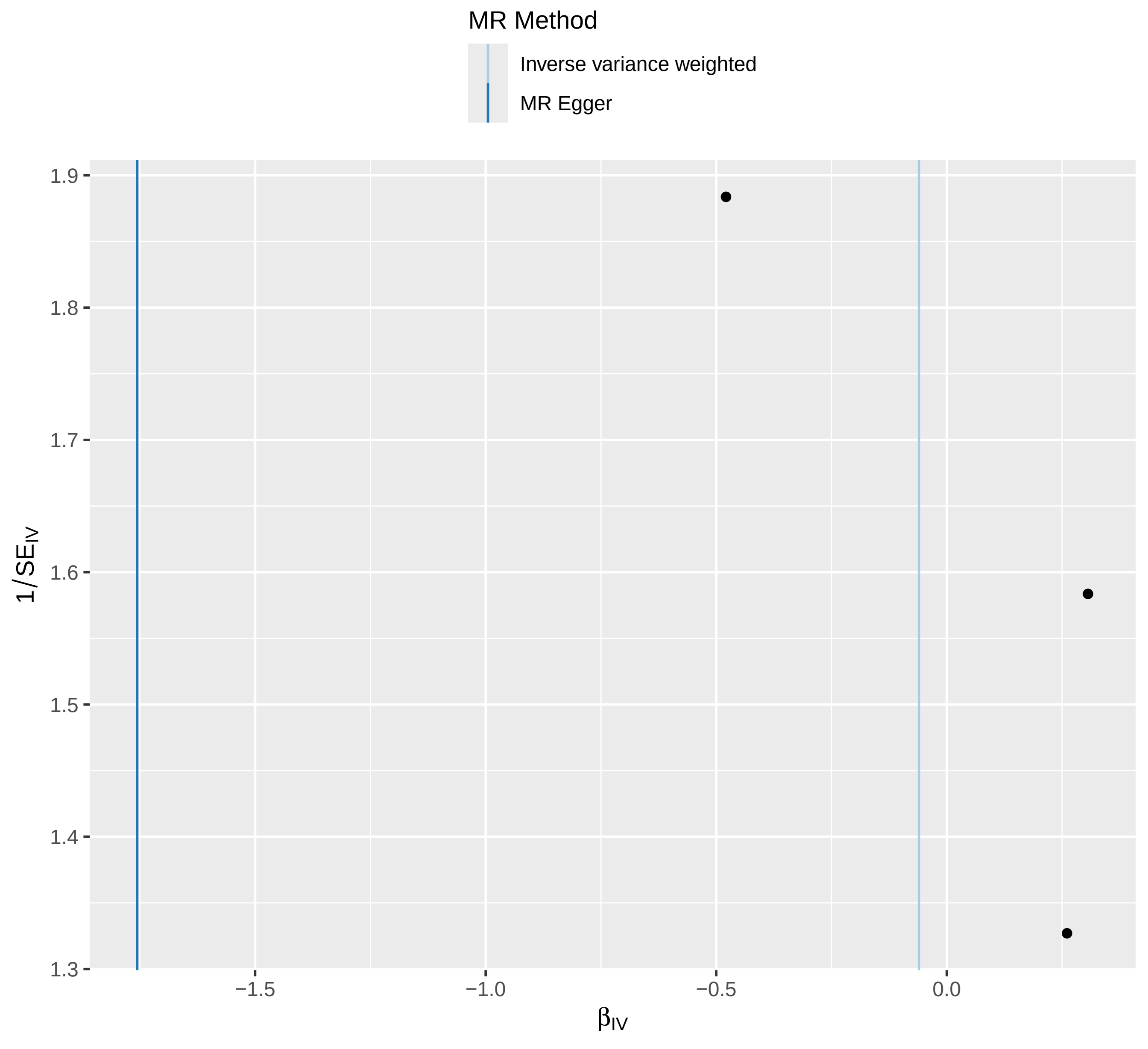
MR Method
Inverse variance weighted
MR Egger
1.9
1.8
1.7
1.6
1.5
1.4
1.3
1/SE_IV
−1.5
−1.0
−0.5
0.0
β_IV

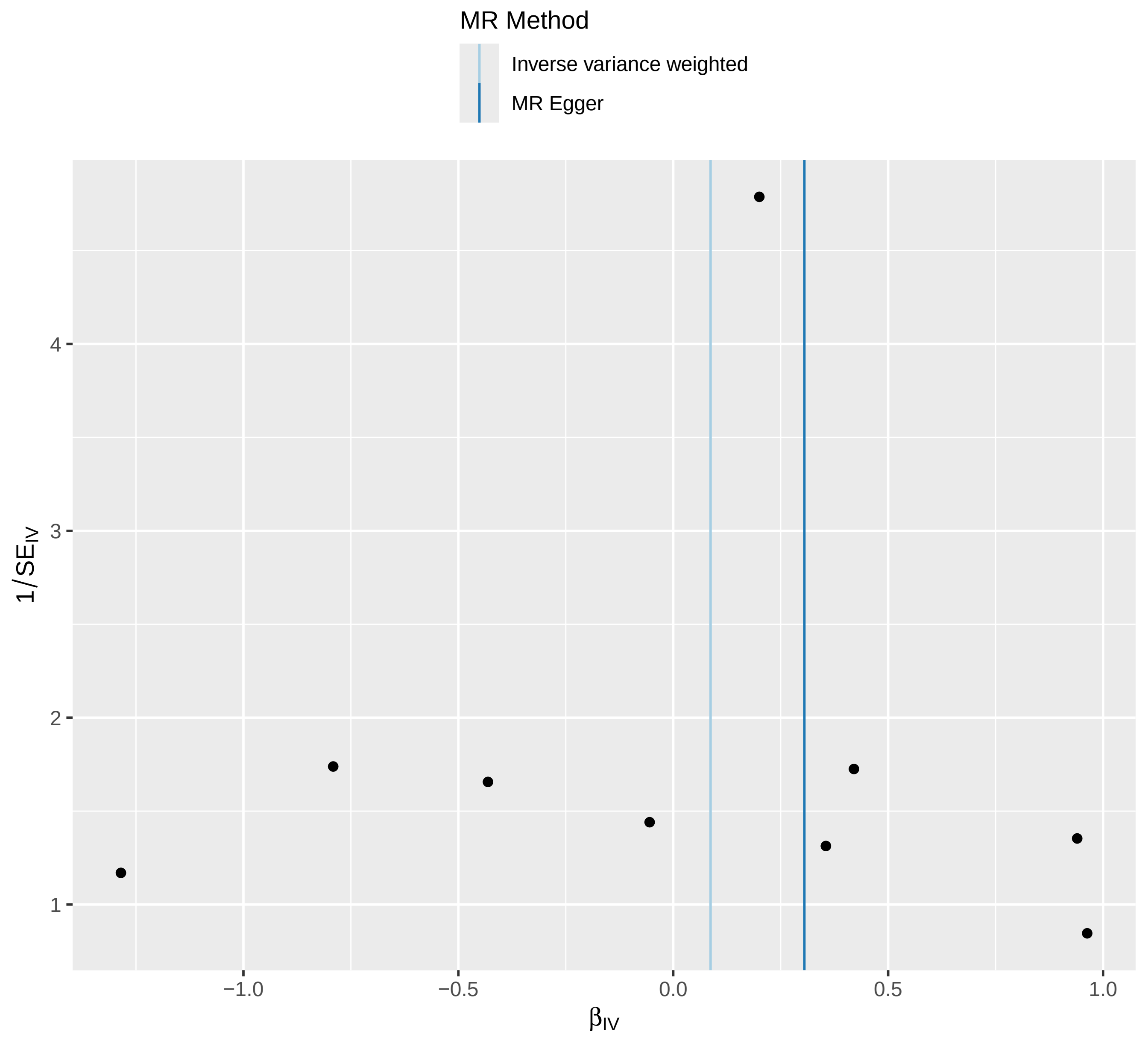

MR Method
Inverse variance weighted
MR Egger
1/SE_IV
4
3
2
1
−1.0
−0.5
0.0
0.5
1.0
β_IV

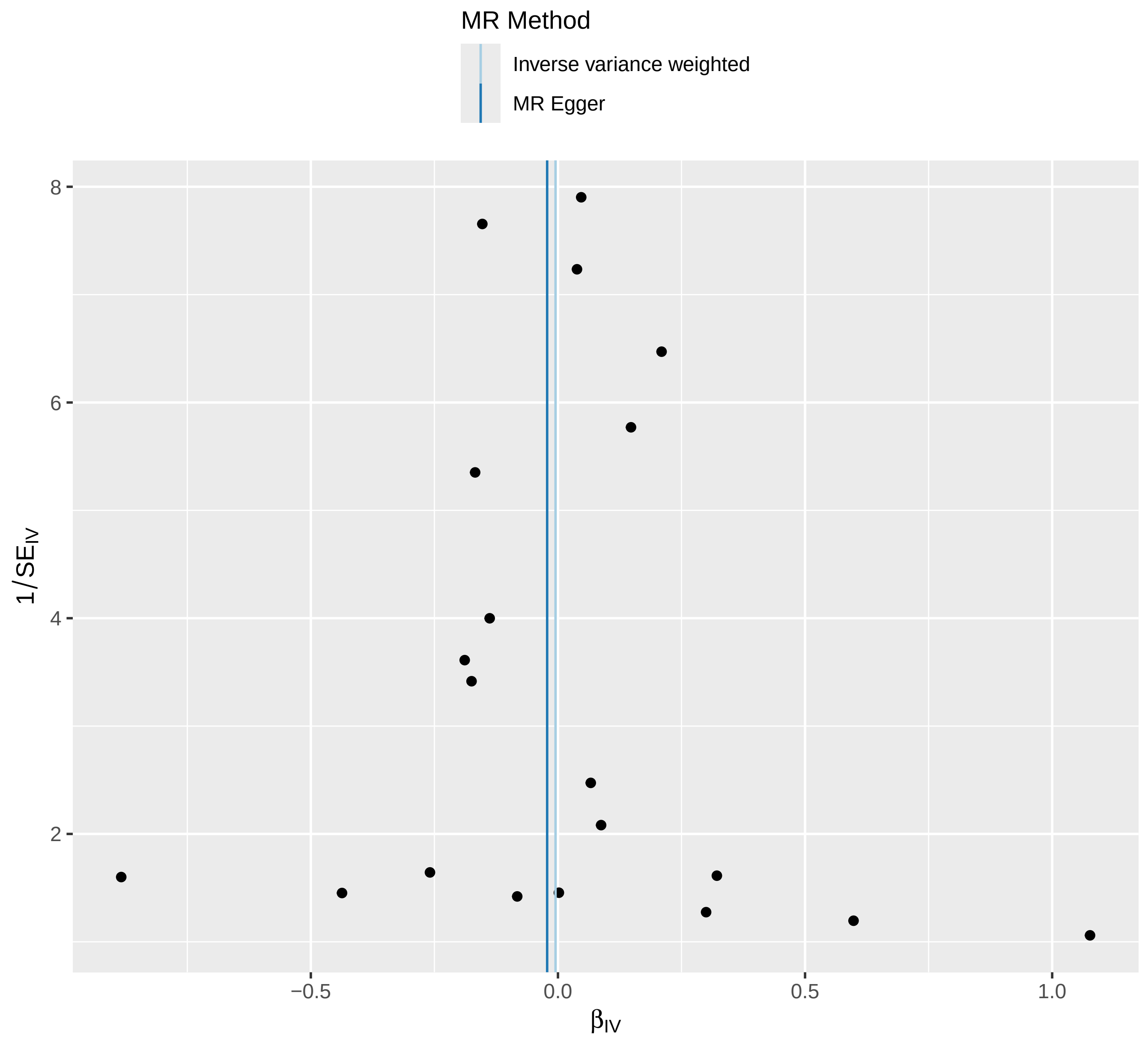
MR Method
Inverse variance weighted
MR Egger
8
6
4
2
1/SE_IV
−0.5
0.0
0.5
1.0
β_IV

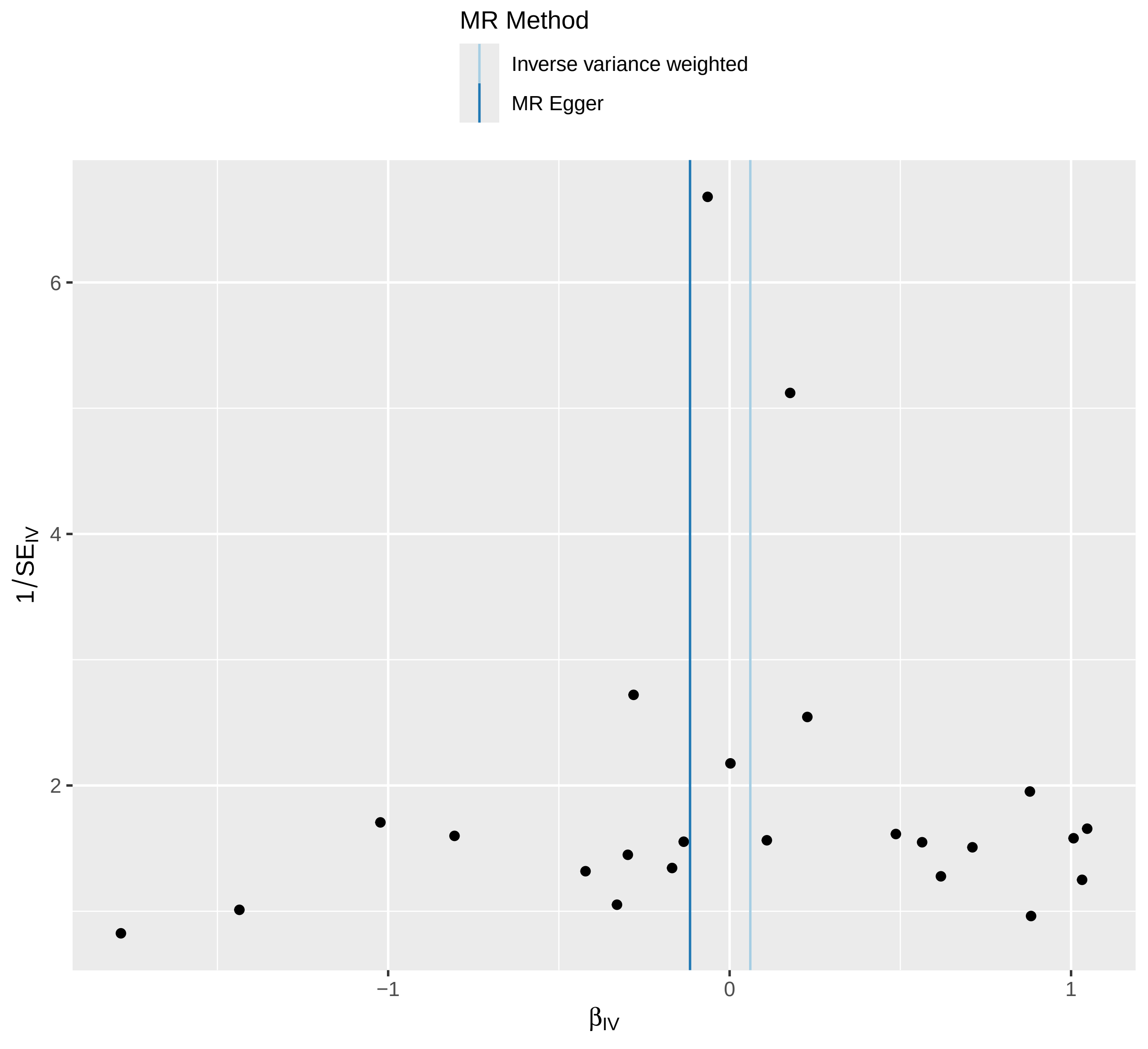
MR Method
Inverse variance weighted
MR Egger
6
4
2
1/SE_IV
−1
0
1
β_IV

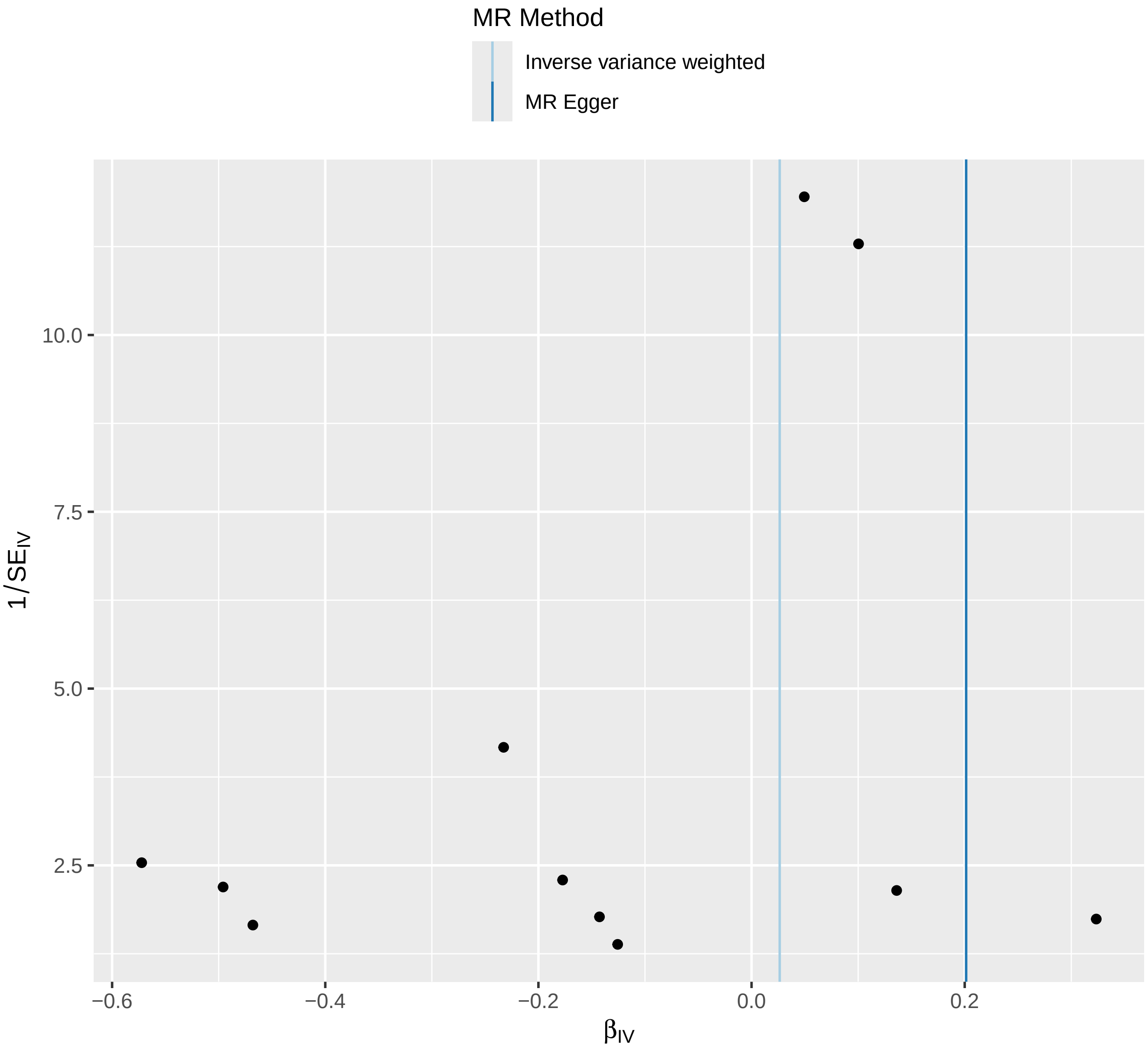
MR Method
Inverse variance weighted
MR Egger
1/SE_IV
β_IV
10.0
7.5
5.0
2.5
−0.6
−0.4
−0.2
0.0
0.2

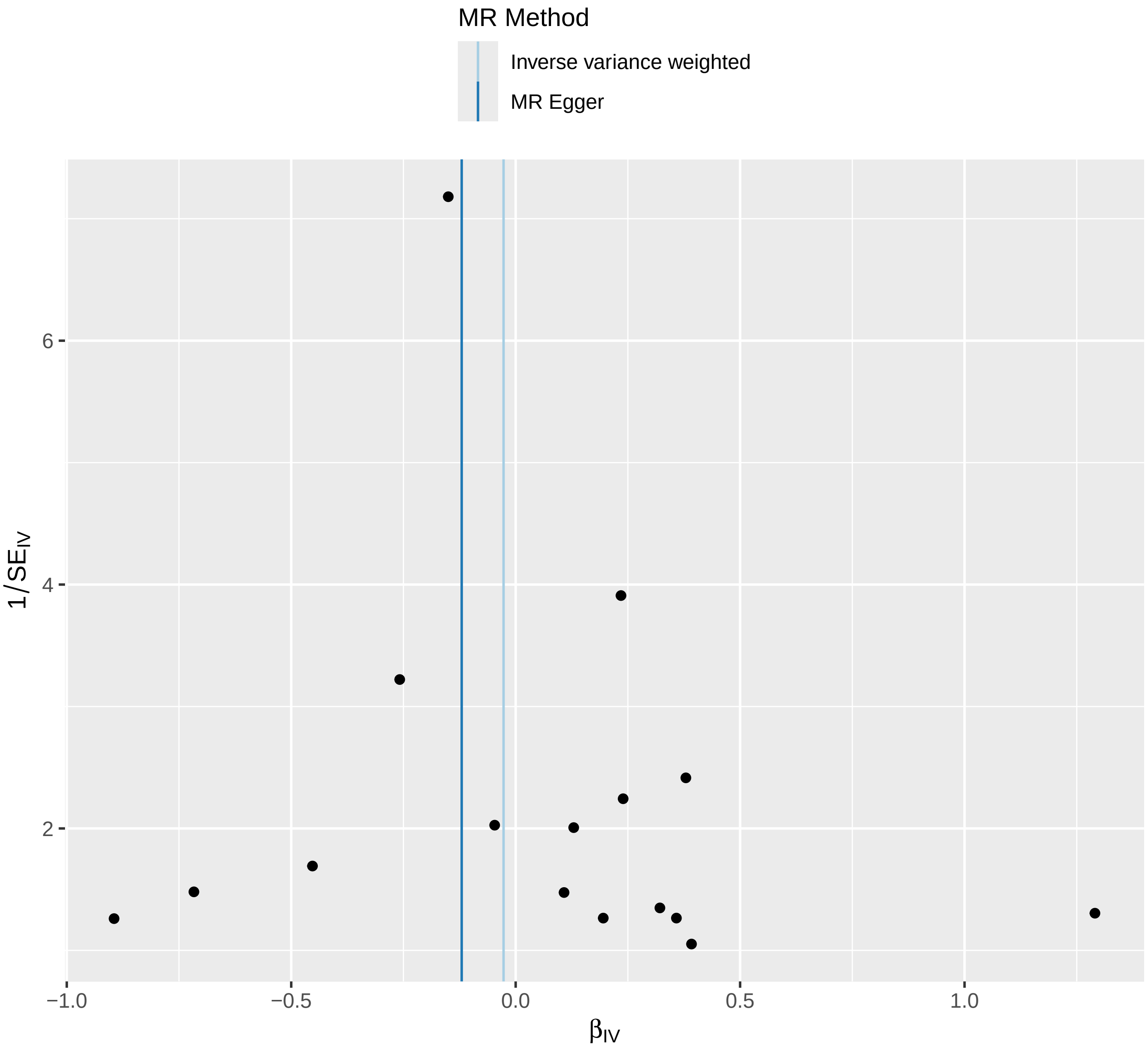

MR Method
Inverse variance weighted
MR Egger
6
4
2
1/SE_IV
−1.0
−0.5
0.0
0.5
1.0
β_IV

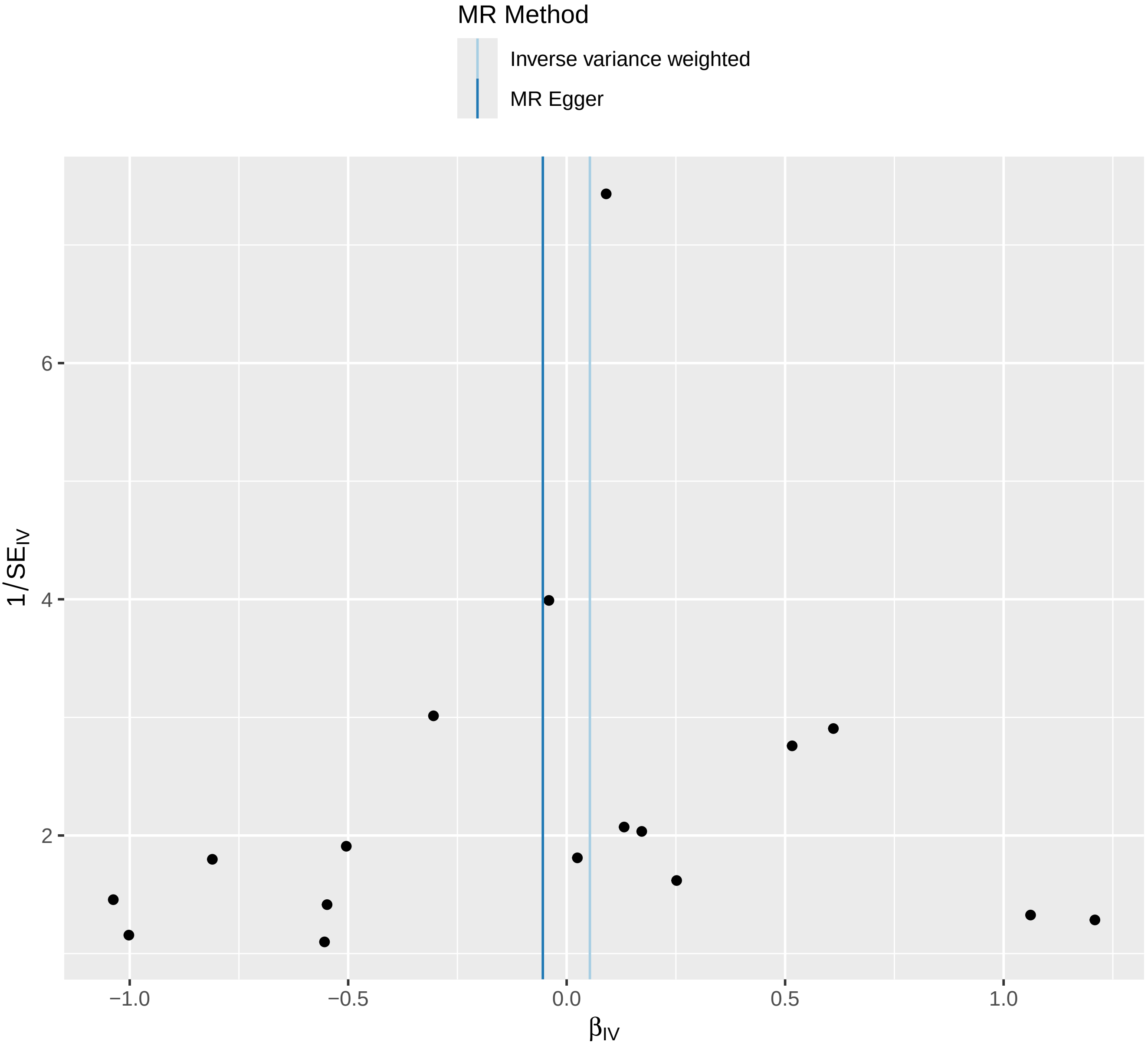
MR Method
Inverse variance weighted
MR Egger
1/SE$_{IV}$
6
4
2
−1.0
−0.5
0.0
0.5
1.0
β$_{IV}$

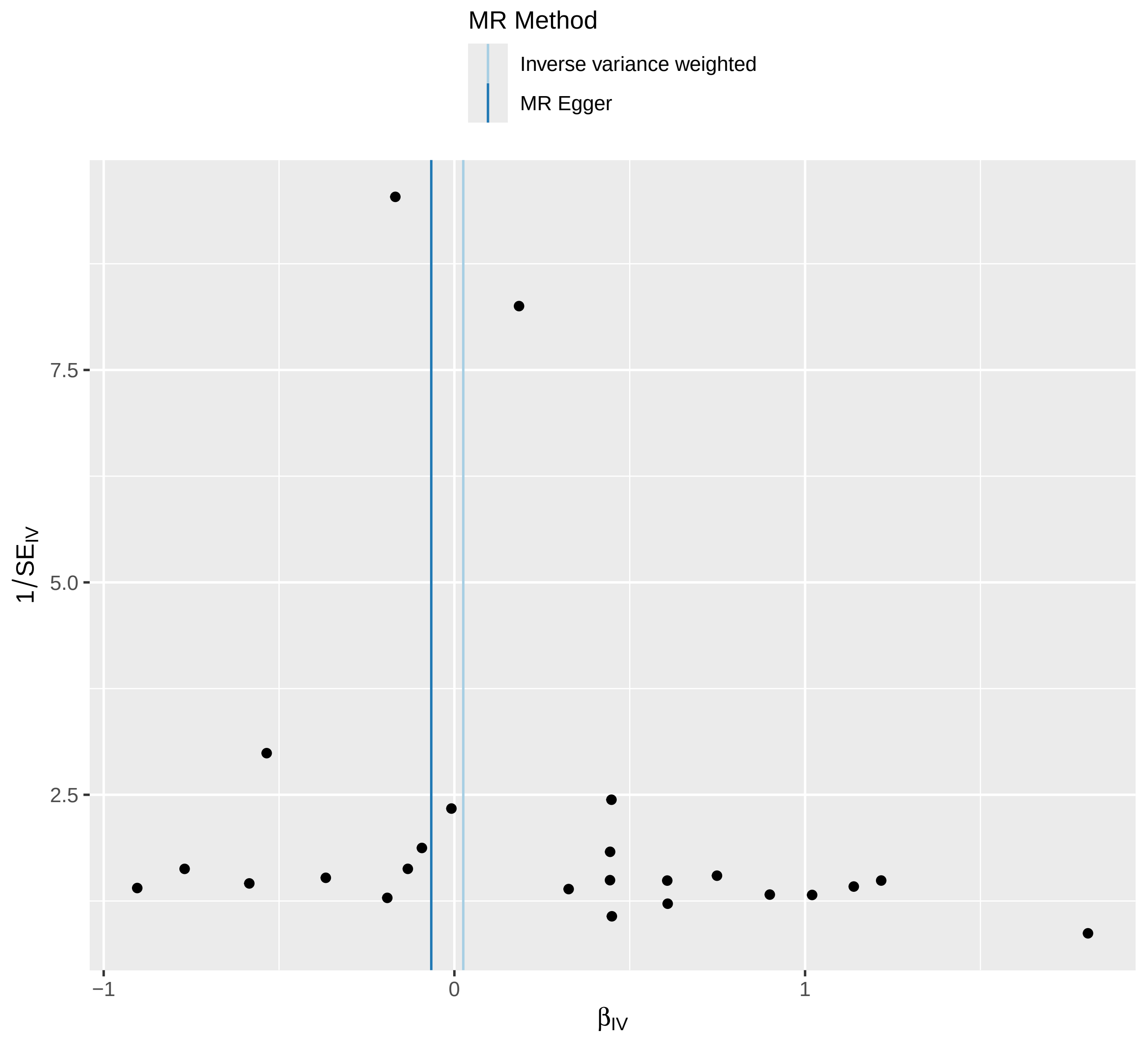
MR Method
Inverse variance weighted
MR Egger
7.5
5.0
2.5
1/SE_IV
−1
0
1
β_IV

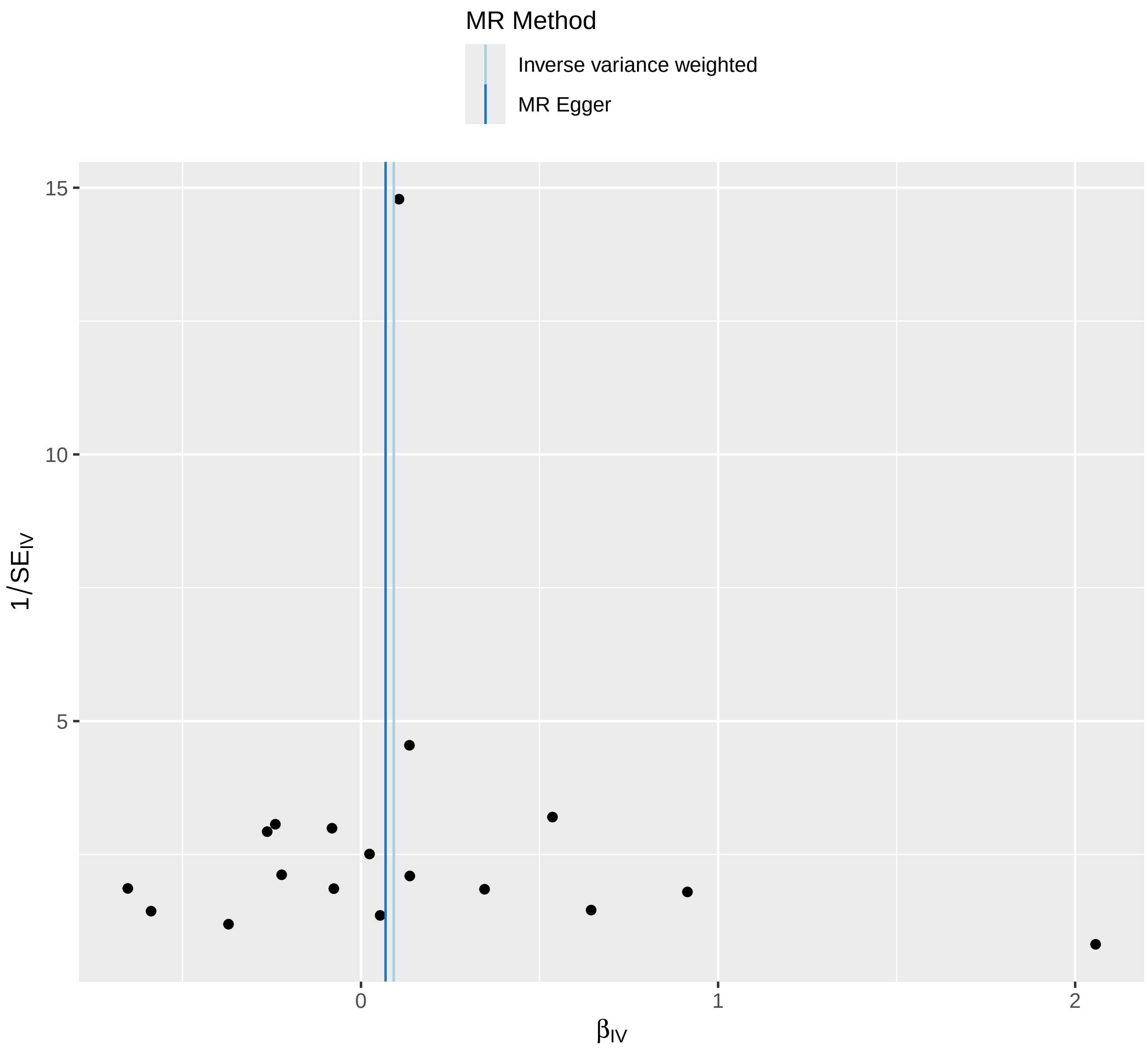

MR Method
Inverse variance weighted
MR Egger
15
10
5
1/SE_IV
0
1
2
β_IV

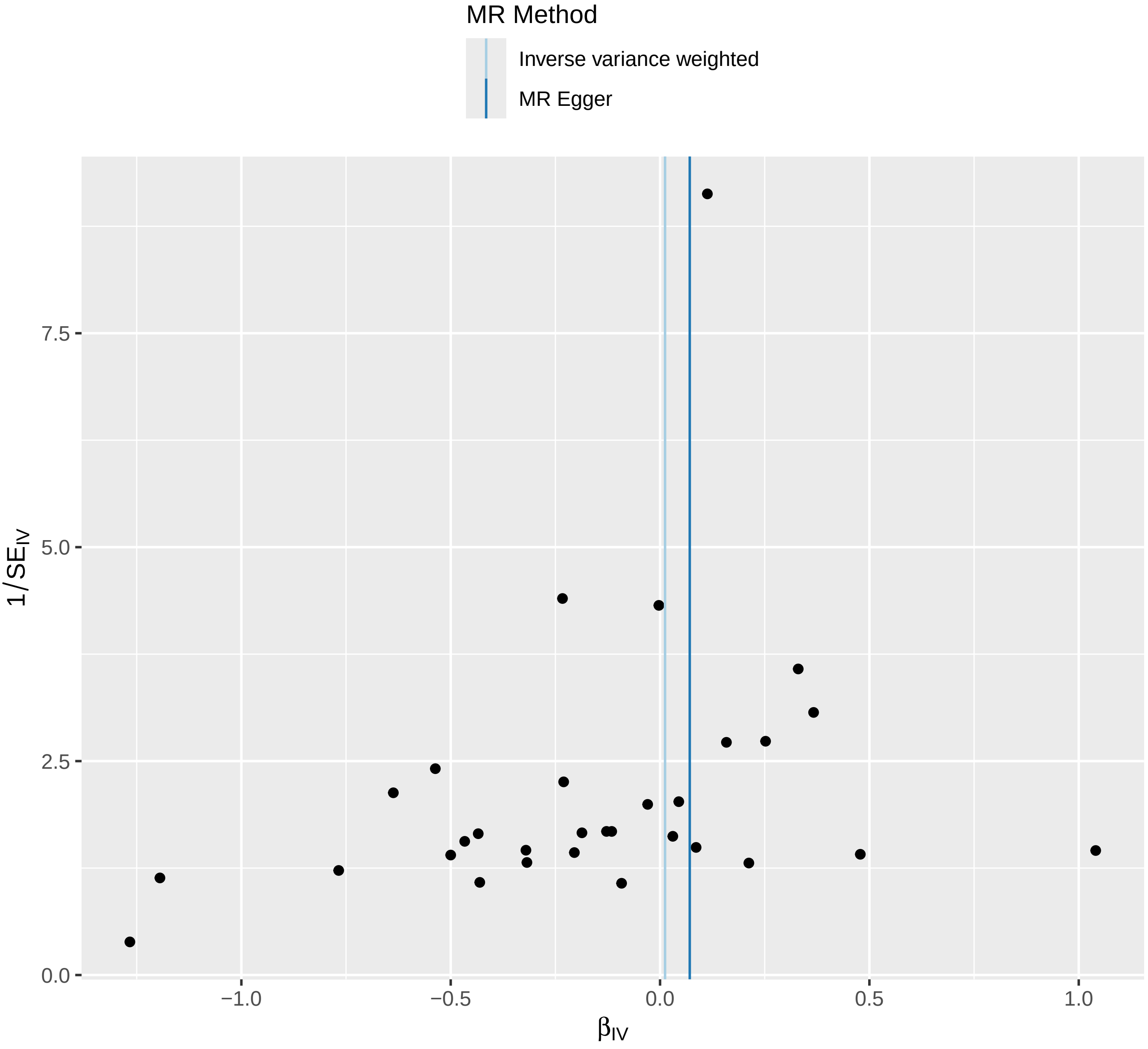
MR Method
Inverse variance weighted
MR Egger
7.5
5.0
2.5
0.0
1/SE$_{IV}$
−1.0
−0.5
0.0
0.5
1.0
β$_{IV}$

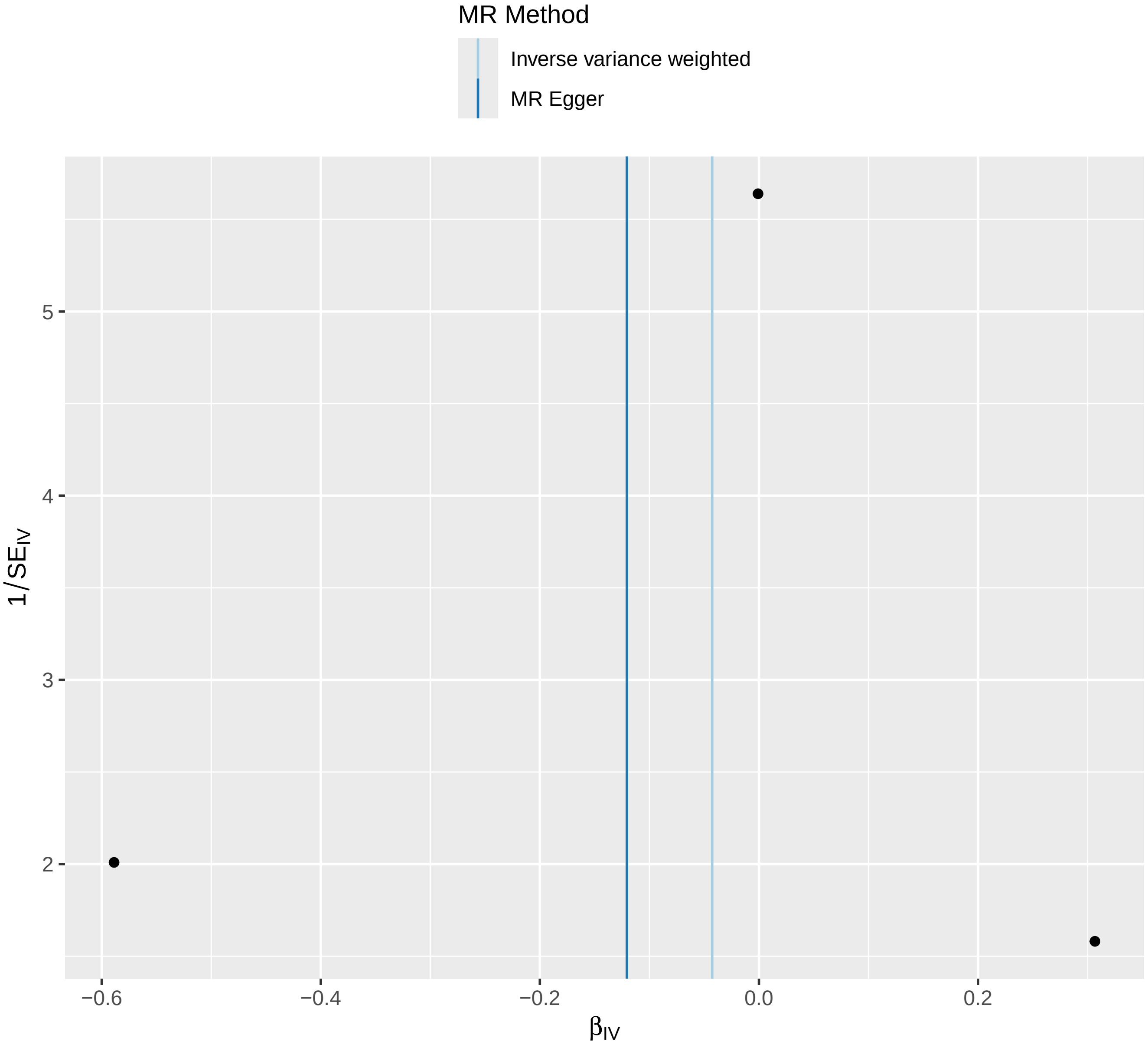
MR Method
Inverse variance weighted
MR Egger
5
4
3
2
1/SE_IV
−0.6
−0.4
−0.2
0.0
0.2
β_IV

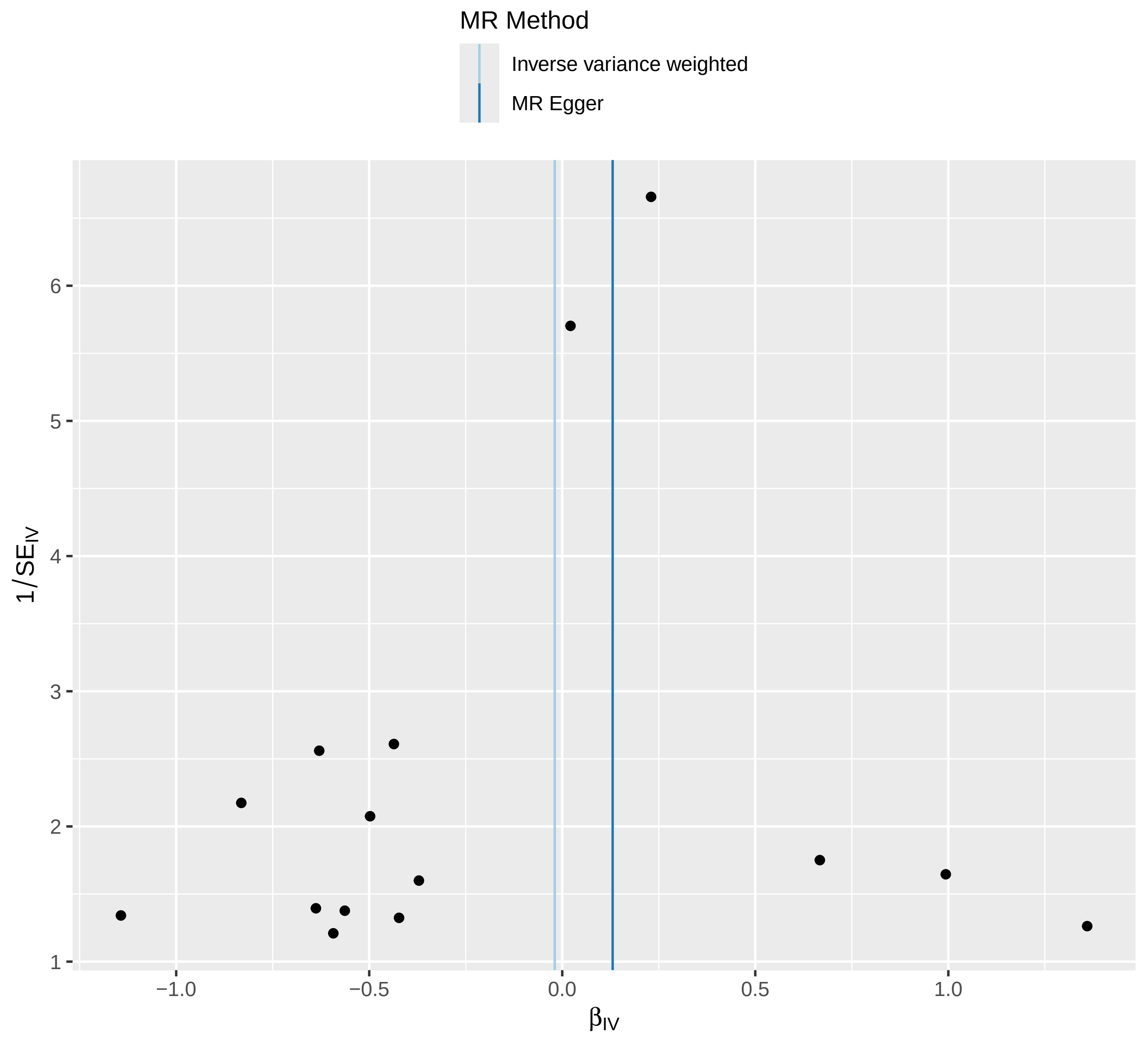

MR Method
Inverse variance weighted
MR Egger
1/SE_IV
6
5
4
3
2
1
−1.0
−0.5
0.0
0.5
1.0
β_IV

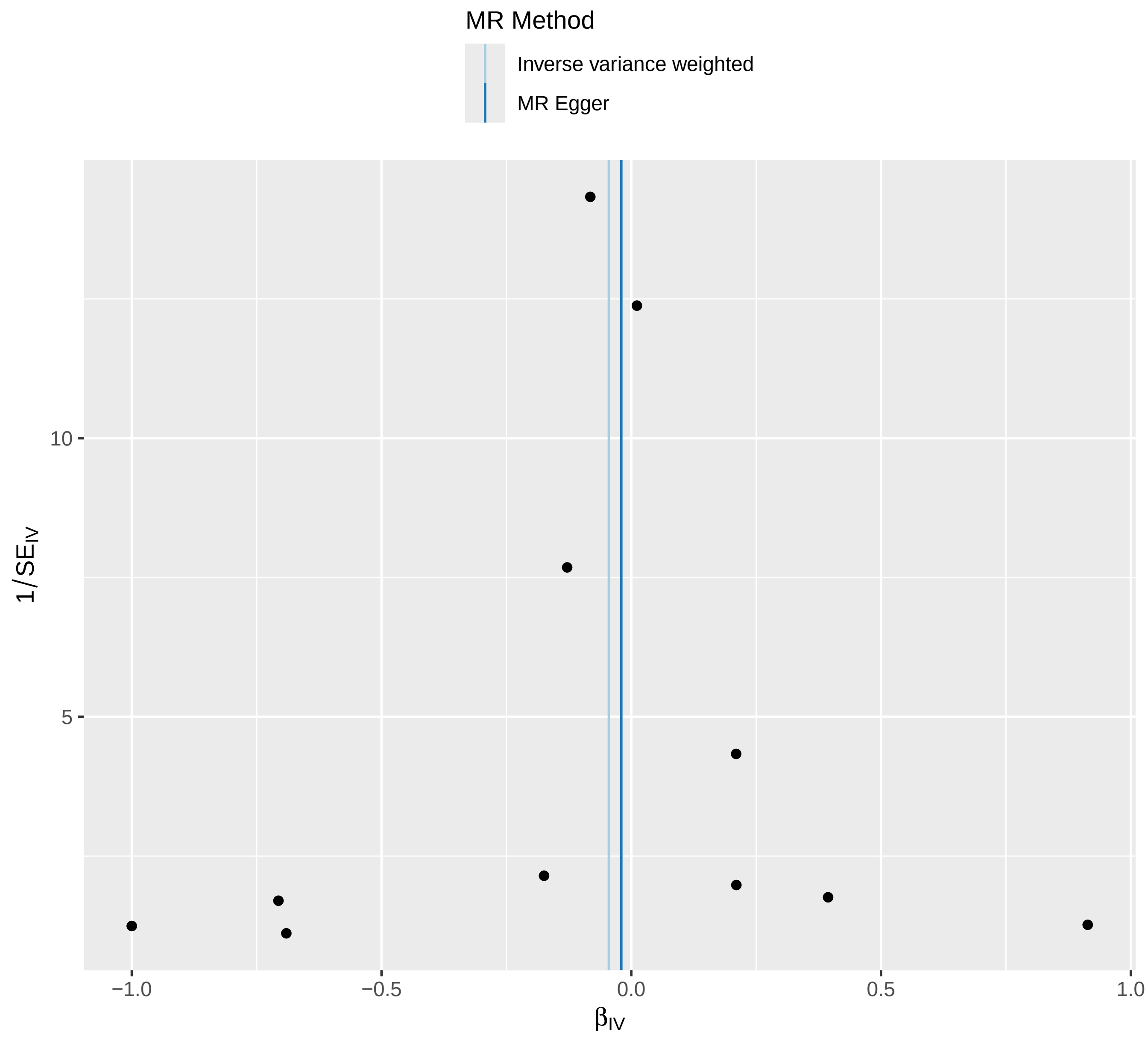

MR Method
Inverse variance weighted
MR Egger
10
5
1/SE$_{IV}$
−1.0
−0.5
0.0
0.5
1.0
β$_{IV}$

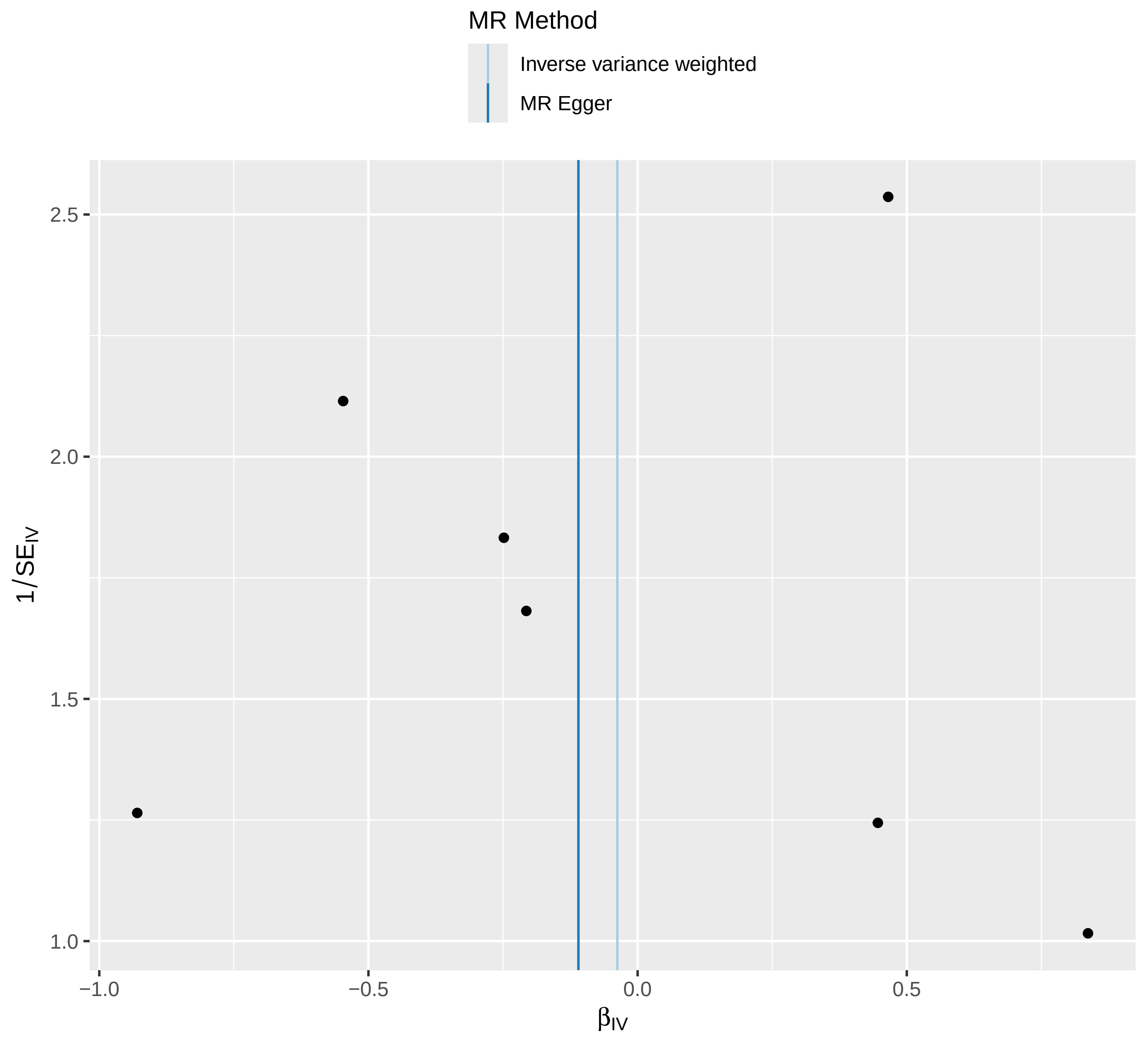

MR Method
Inverse variance weighted
MR Egger
2.5
2.0
1.5
1.0
1/SE$_{IV}$
−1.0
−0.5
0.0
0.5
β$_{IV}$

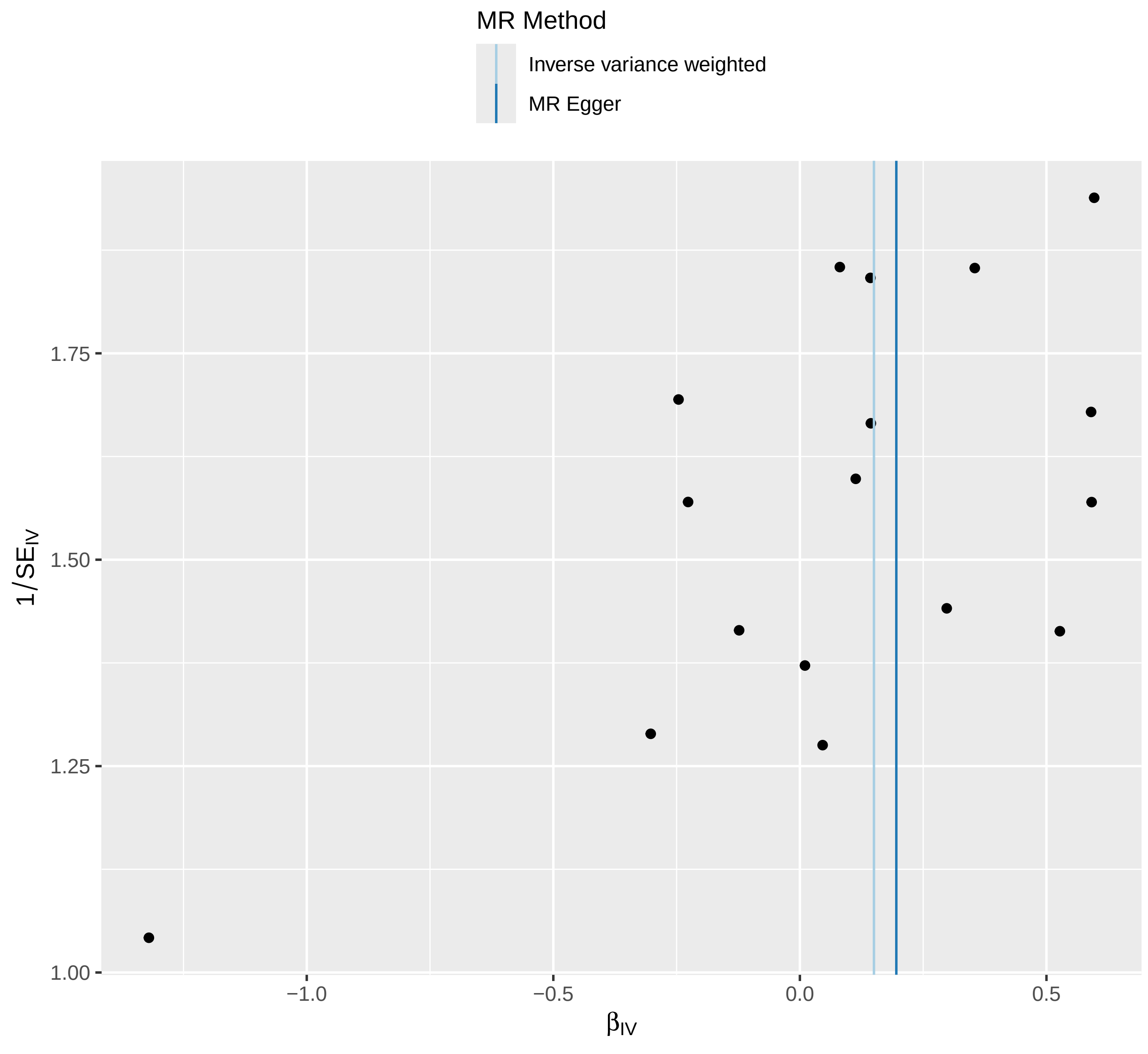

MR Method
Inverse variance weighted
MR Egger
1.75
1.50
1.25
1.00
1/SE_IV
−1.0
−0.5
0.0
0.5
β_IV

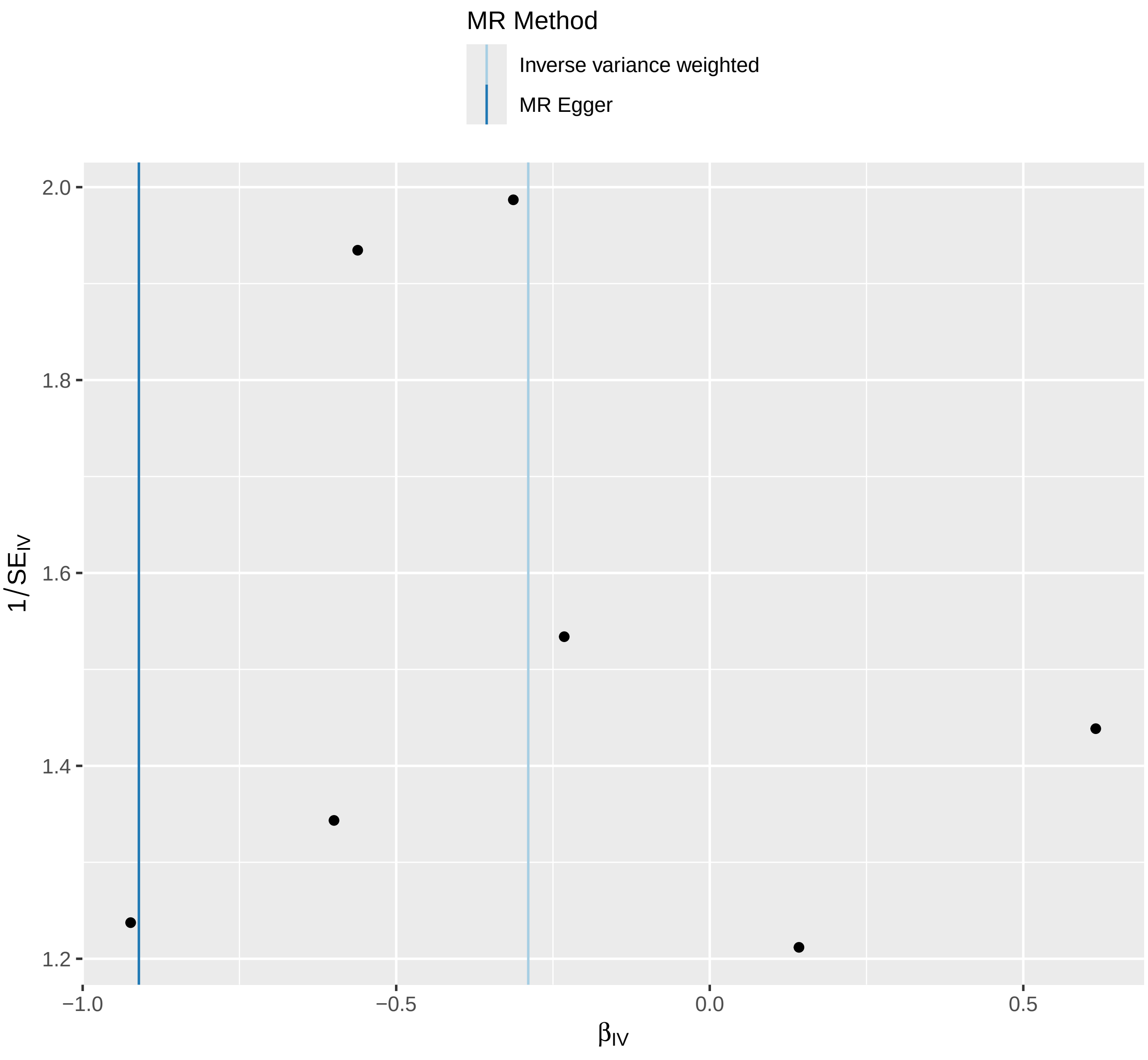

MR Method
Inverse variance weighted
MR Egger
2.0
1.8
1.6
1.4
1.2
1/SE_IV
−1.0
−0.5
0.0
0.5
β_IV

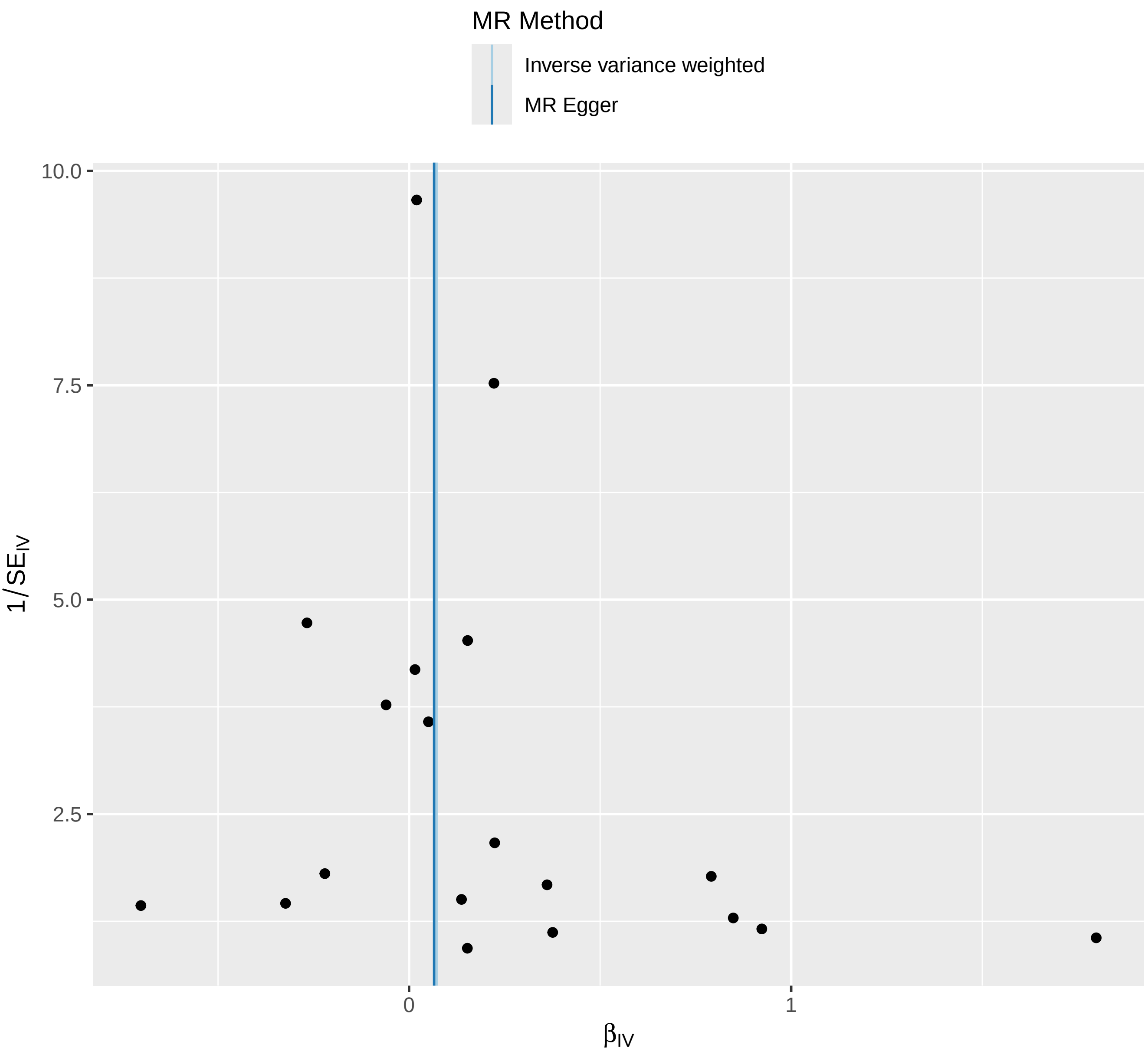

MR Method
Inverse variance weighted
MR Egger
10.0
7.5
5.0
2.5
1/SE_IV
0
1
β_IV

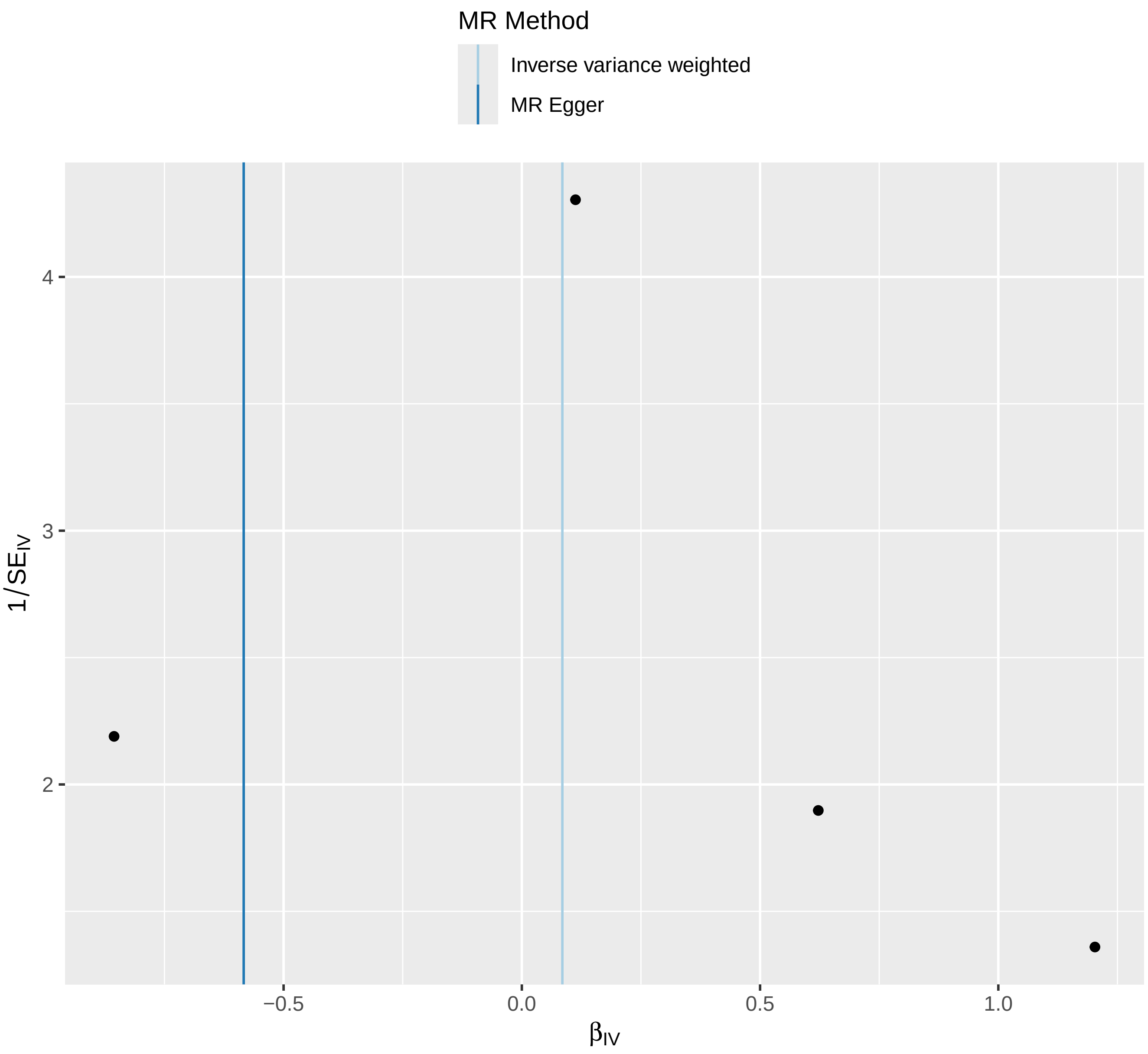

MR Method
Inverse variance weighted
MR Egger
4
3
2
1/SE$_{IV}$
−0.5
0.0
0.5
1.0
β$_{IV}$

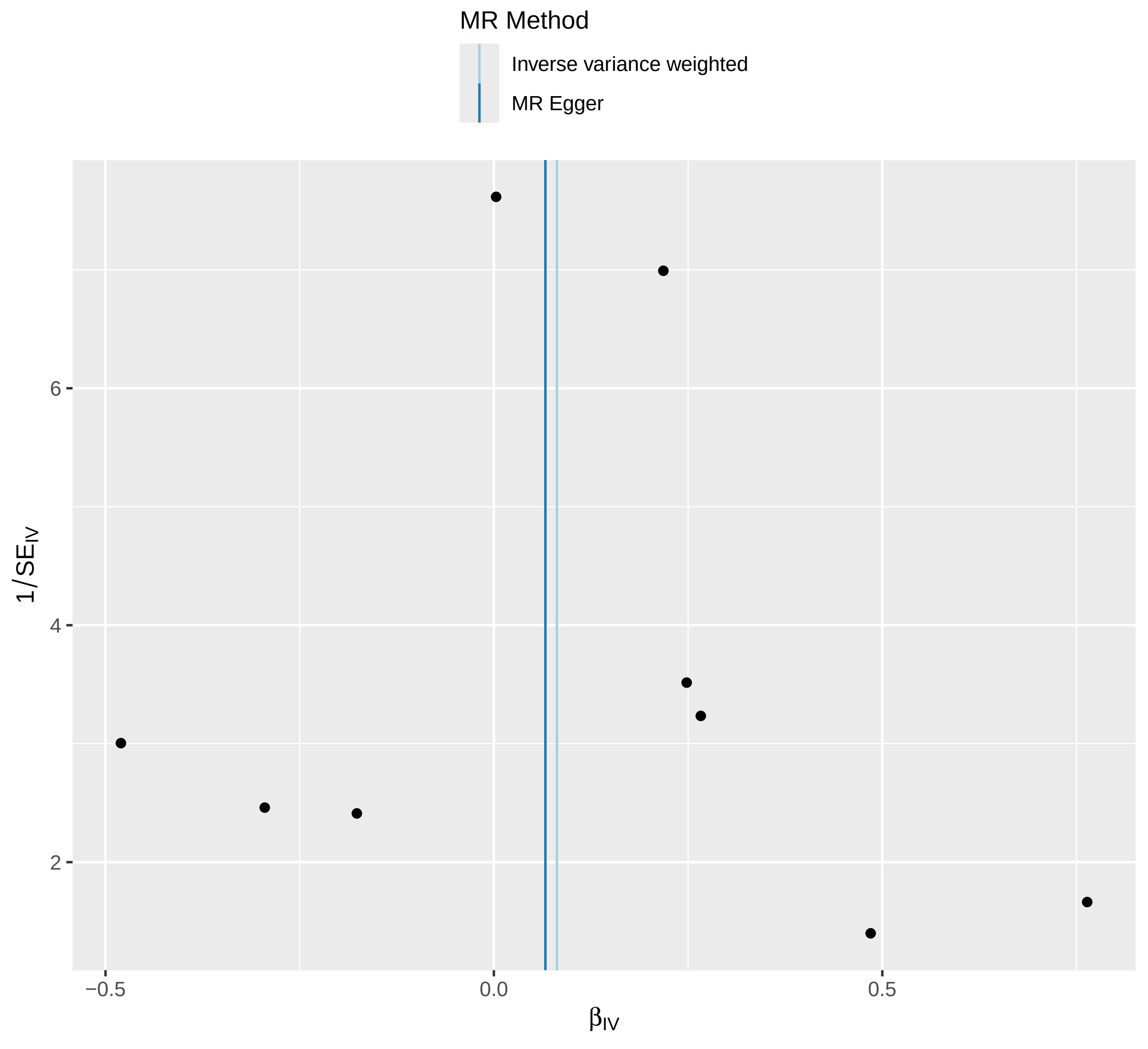

MR Method
Inverse variance weighted
MR Egger
6
4
2
1/SE$_{IV}$
−0.5
0.0
0.5
β$_{IV}$

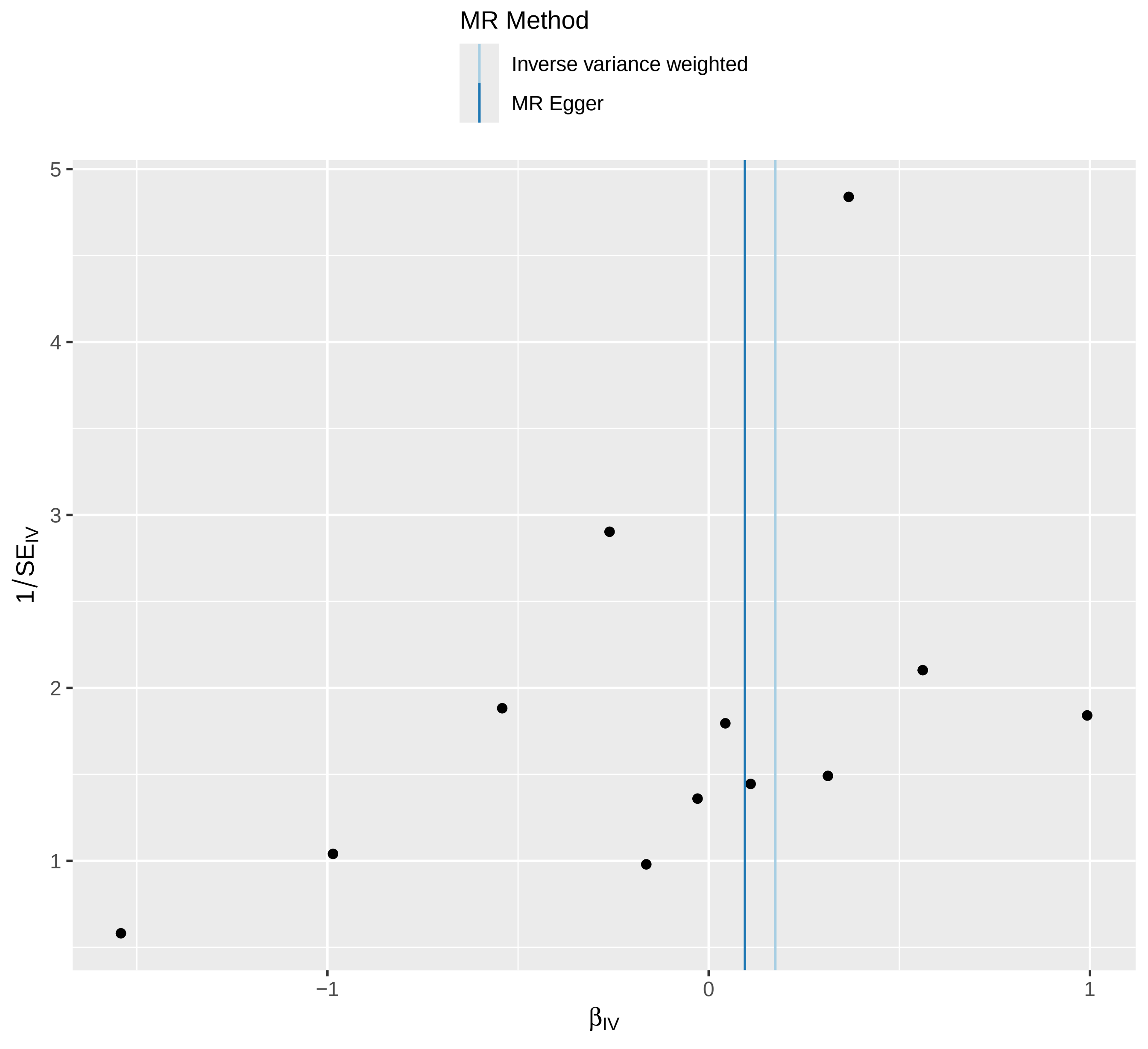
MR Method
Inverse variance weighted
MR Egger
5
4
3
2
1
1/SE_IV
−1
0
1
β_IV

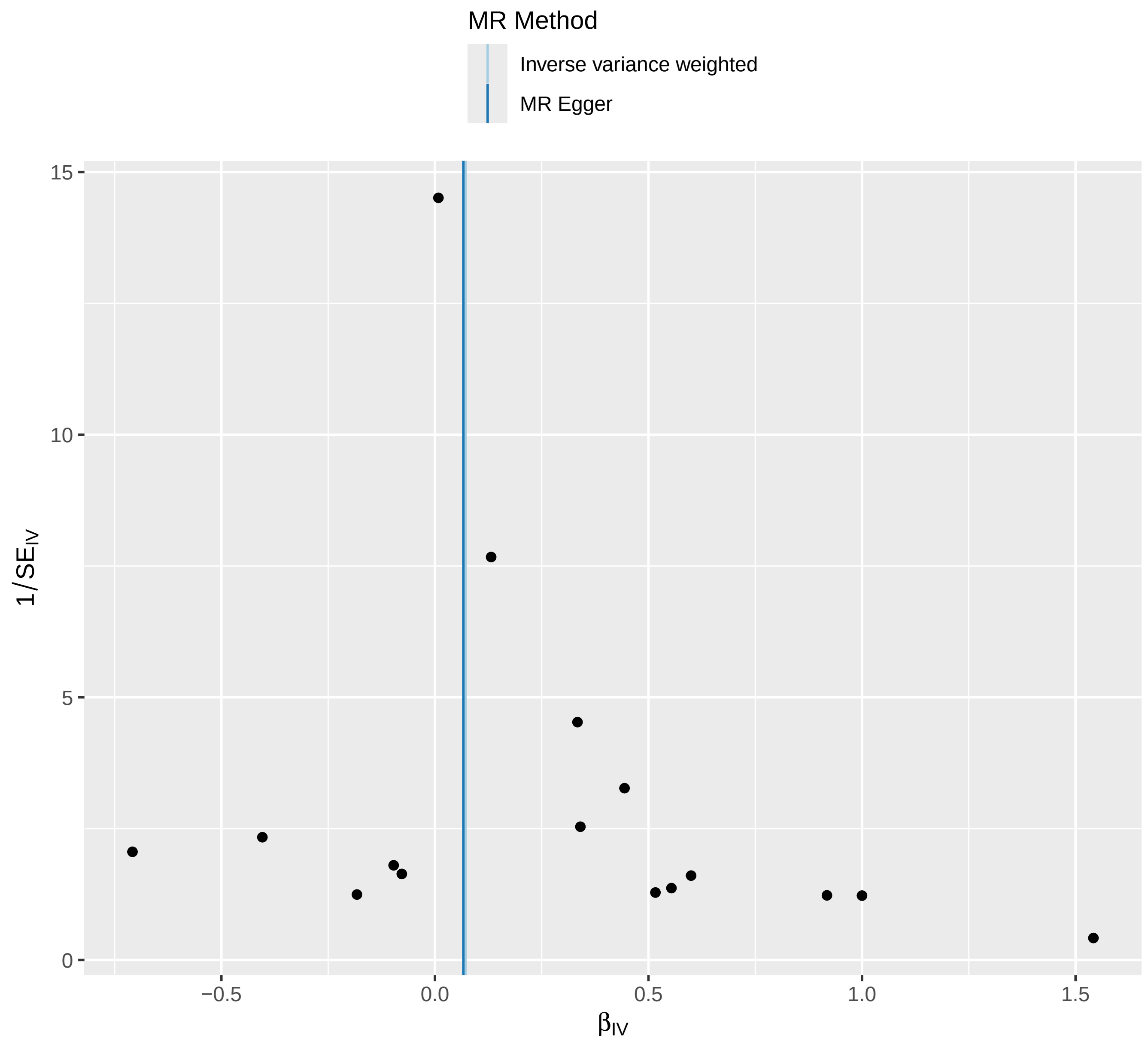

MR Method
Inverse variance weighted
MR Egger
15
10
5
0
−0.5
0.0
0.5
1.0
1.5
$1/SE_{IV}$
β_{IV}

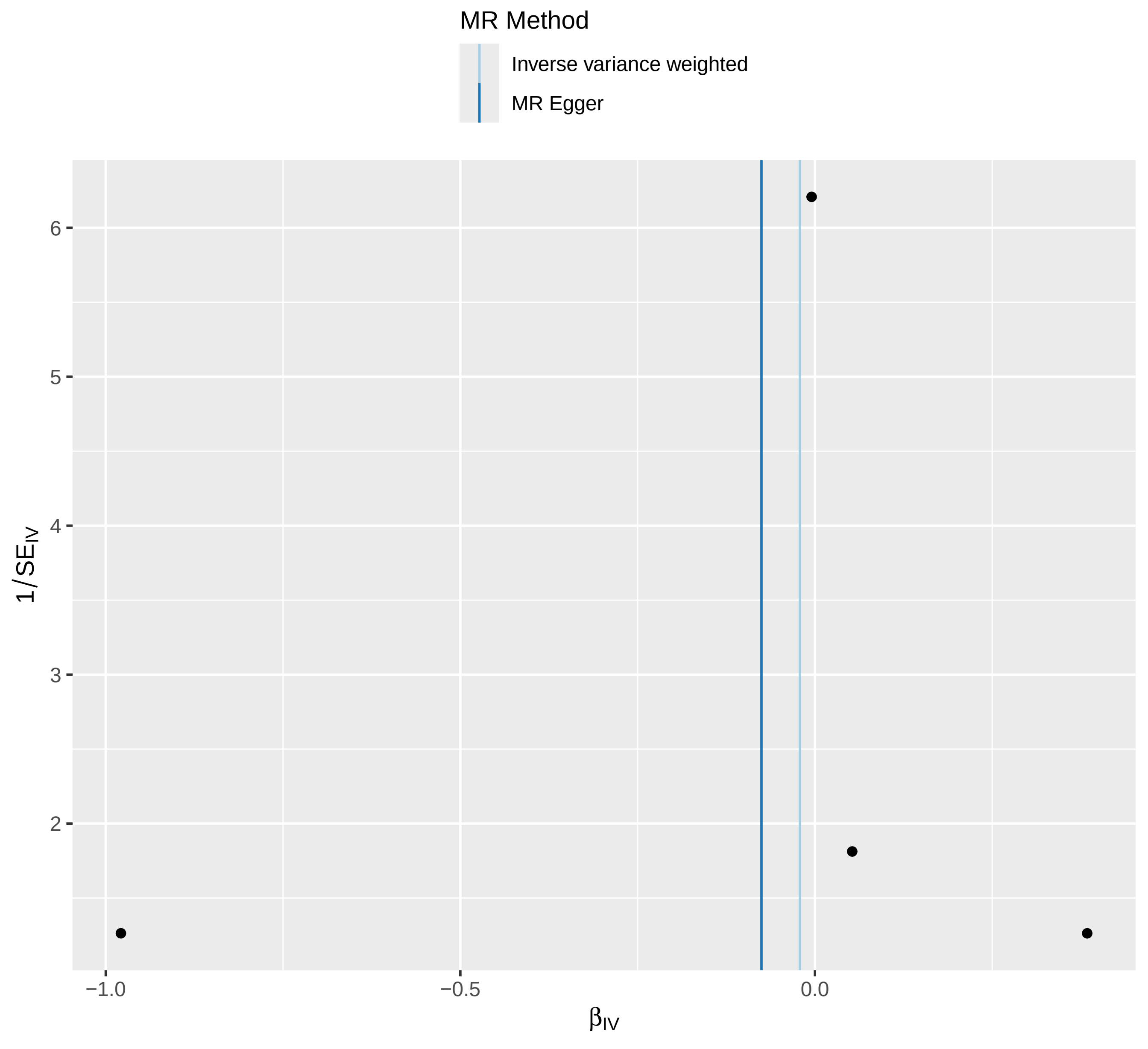

MR Method
Inverse variance weighted
MR Egger
6
5
4
3
2
1/SE_IV
−1.0
−0.5
0.0
β_IV

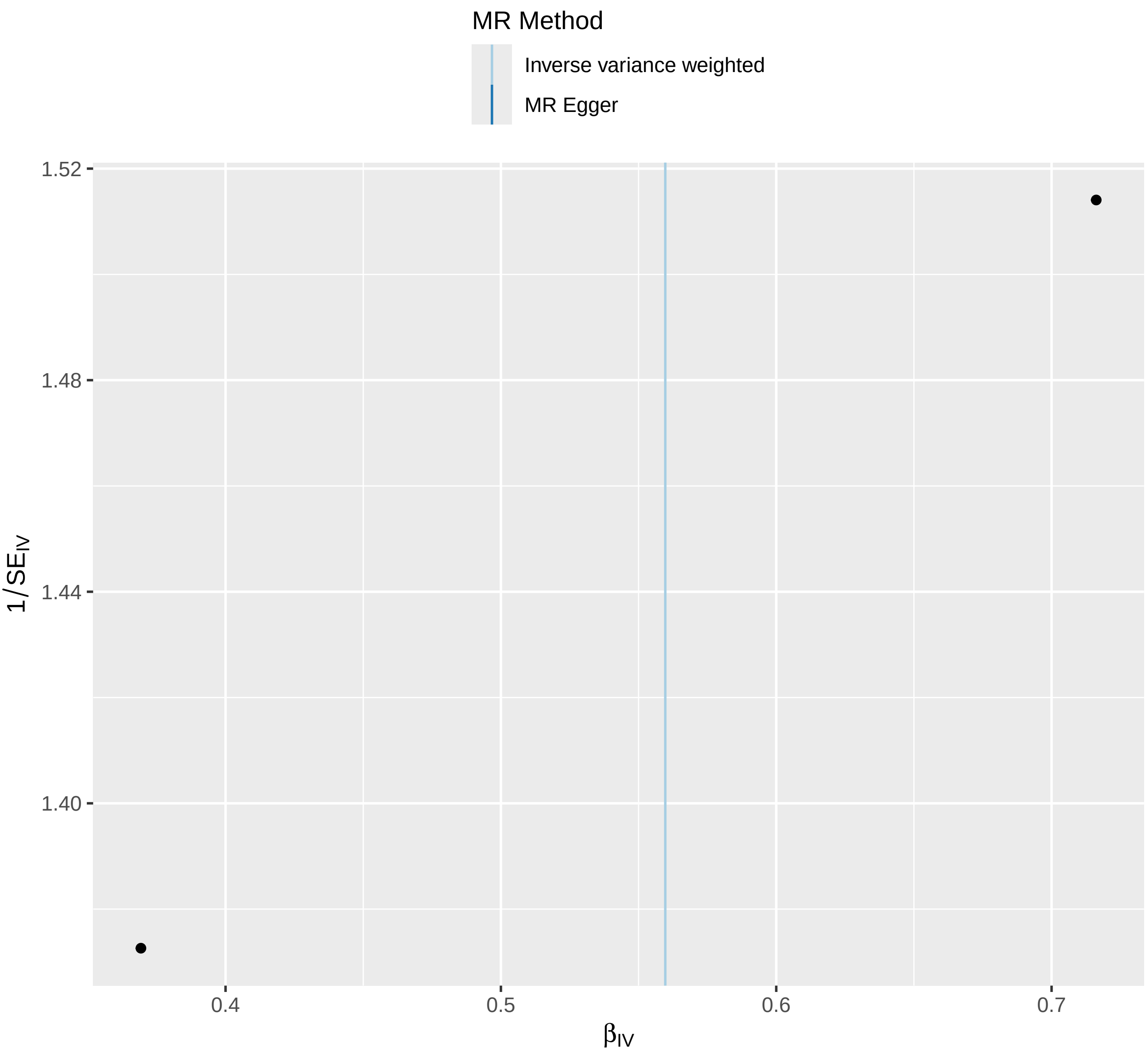
MR Method
Inverse variance weighted
MR Egger
1.52
1.48
1.44
1.40
0.4
0.5
0.6
0.7
$1/SE_{IV}$
β_{IV}

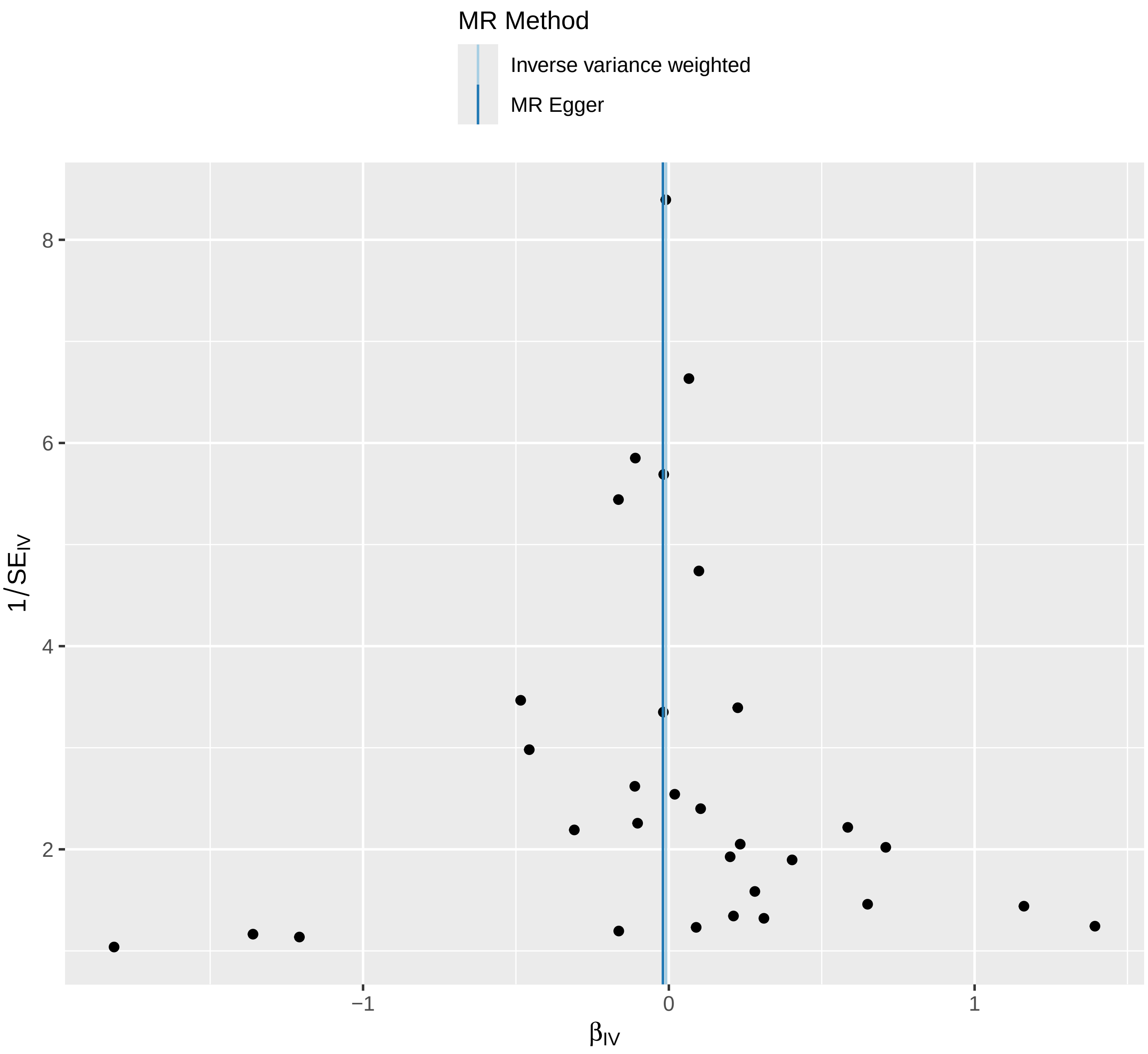
MR Method
Inverse variance weighted
MR Egger
8
6
4
2
1/SE_IV
−1
0
1
β_IV

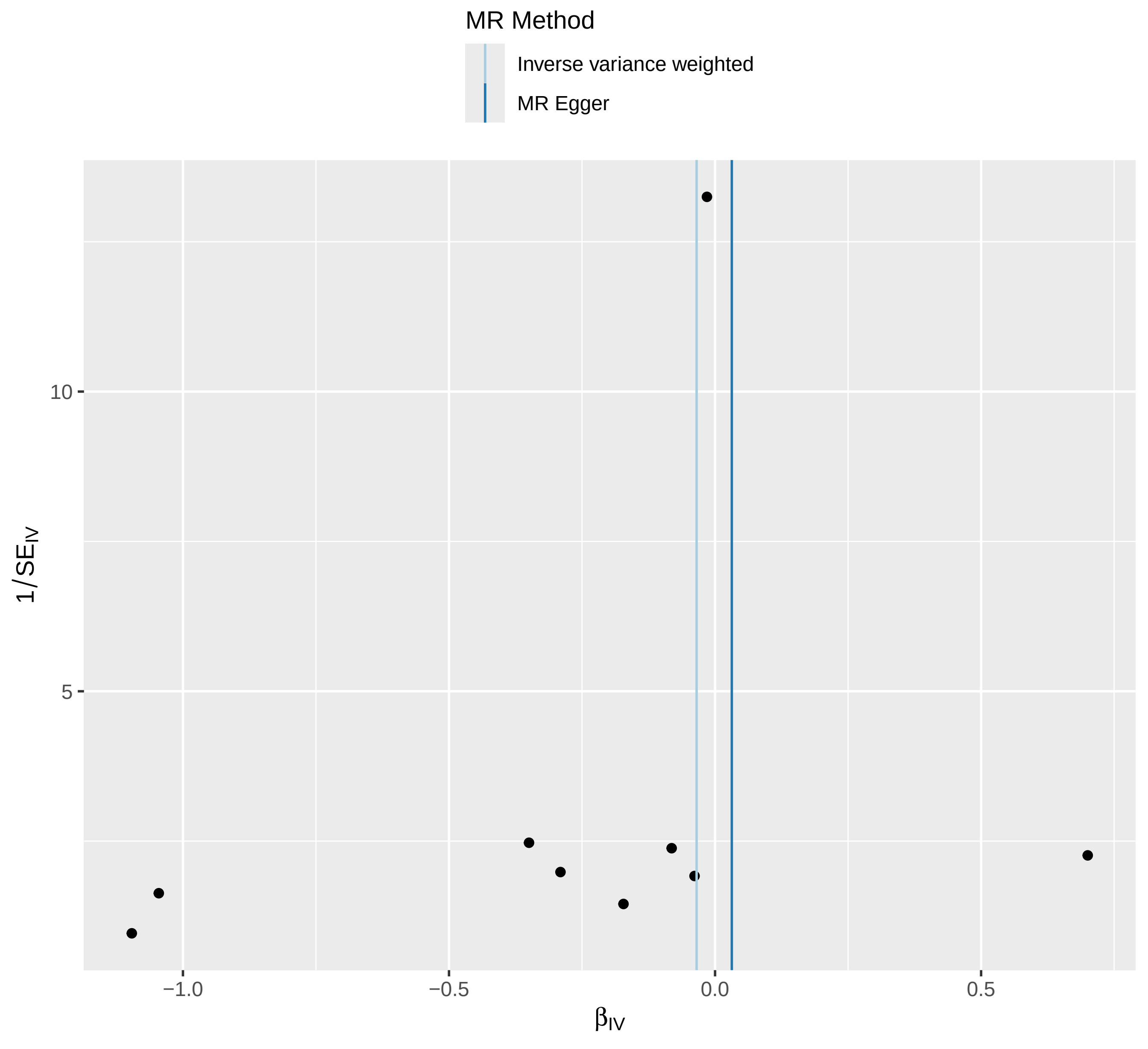
MR Method
Inverse variance weighted
MR Egger
10
5
1/SE_IV
−1.0
−0.5
0.0
0.5
β_IV

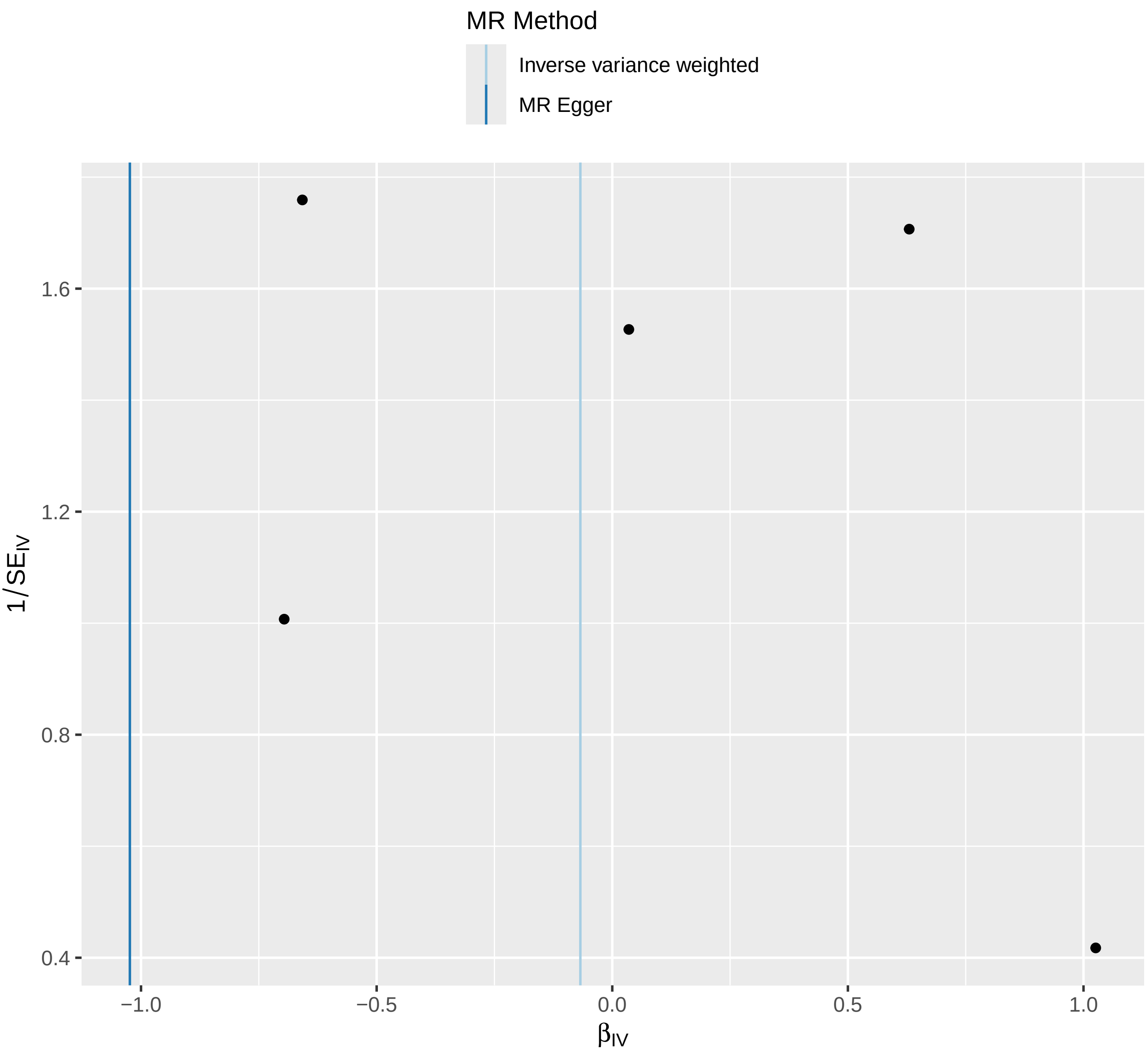
MR Method
Inverse variance weighted
MR Egger
1.6
1.2
0.8
0.4
1/SE$_{IV}$
−1.0
−0.5
0.0
0.5
1.0
β$_{IV}$

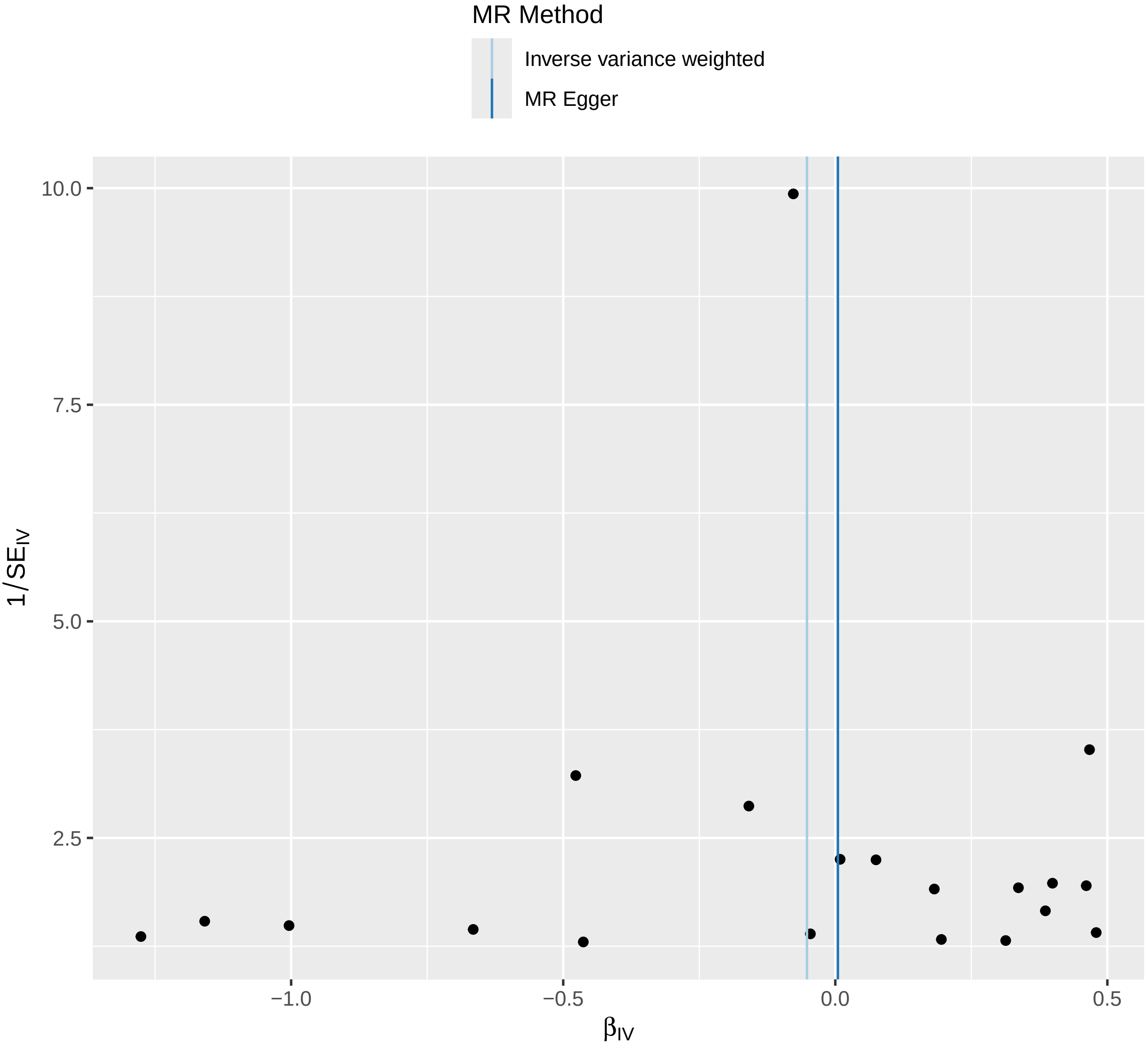

MR Method
Inverse variance weighted
MR Egger
10.0
7.5
5.0
2.5
1/SE$_{IV}$
−1.0
−0.5
0.0
0.5
β$_{IV}$

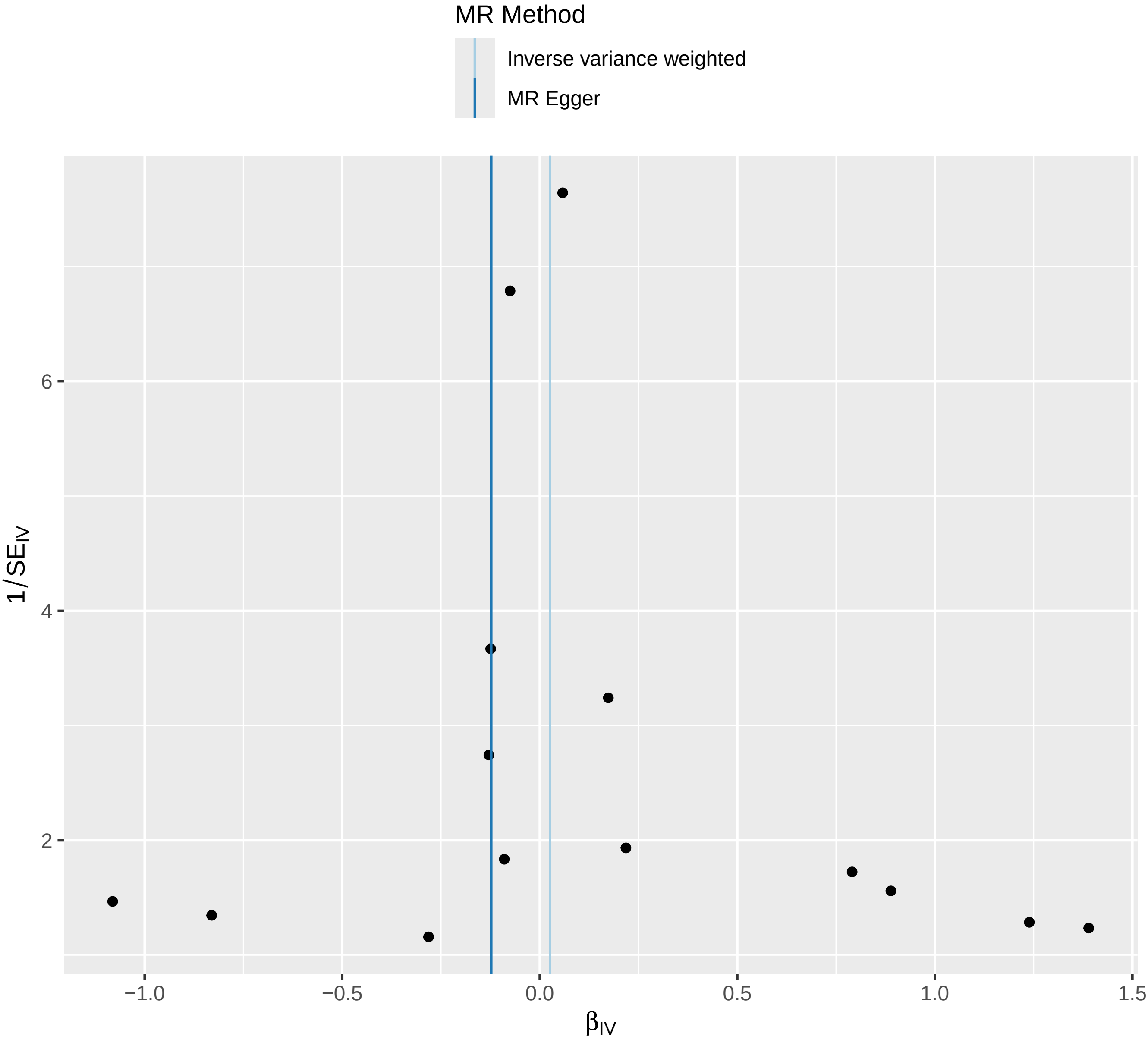

MR Method
Inverse variance weighted
MR Egger
6
4
2
$1/SE_{IV}$
−1.0
−0.5
0.0
0.5
1.0
1.5
β_{IV}

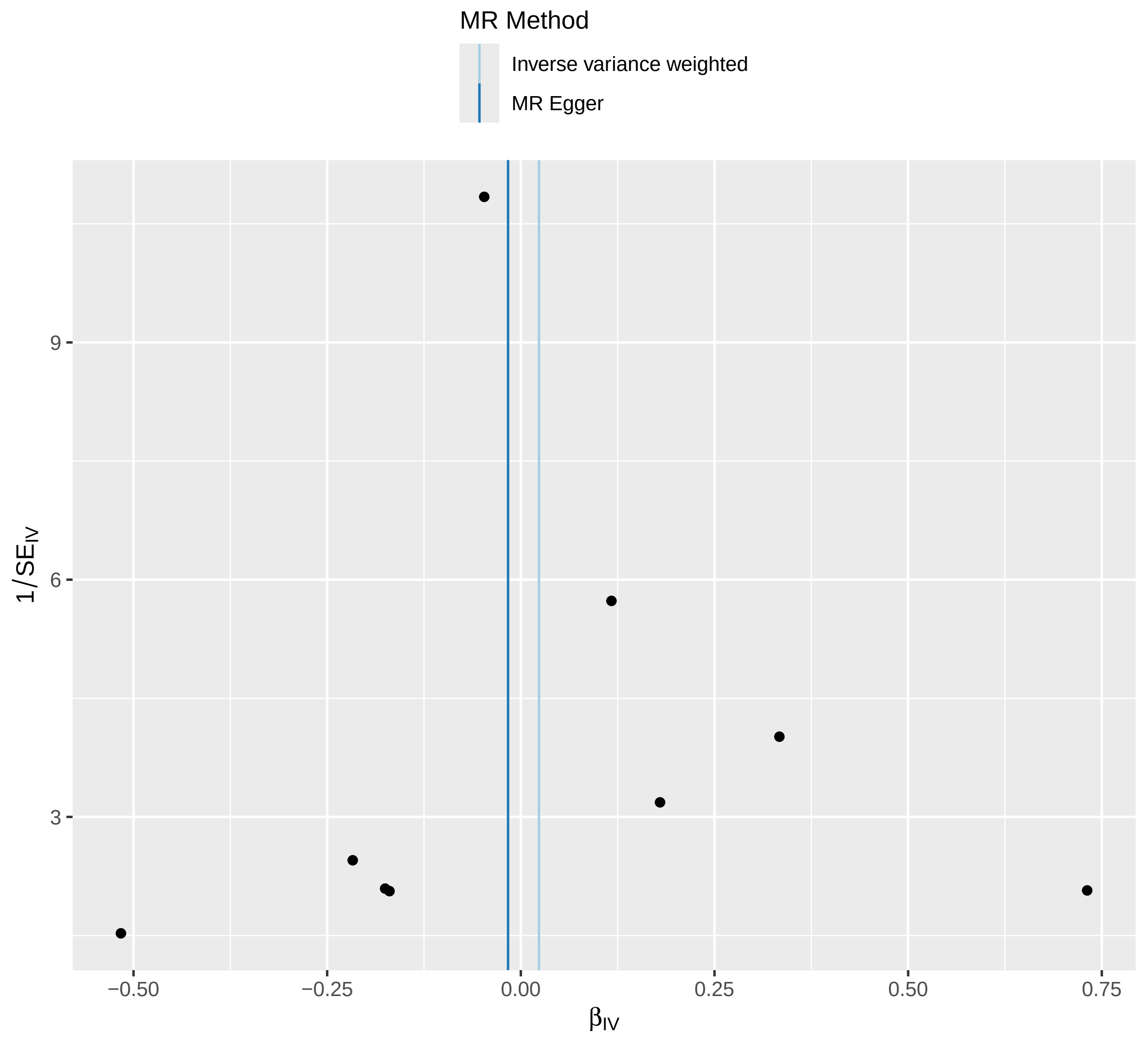
MR Method
Inverse variance weighted
MR Egger
1/SE_IV
9
6
3
−0.50
−0.25
0.00
0.25
0.50
0.75
β_IV

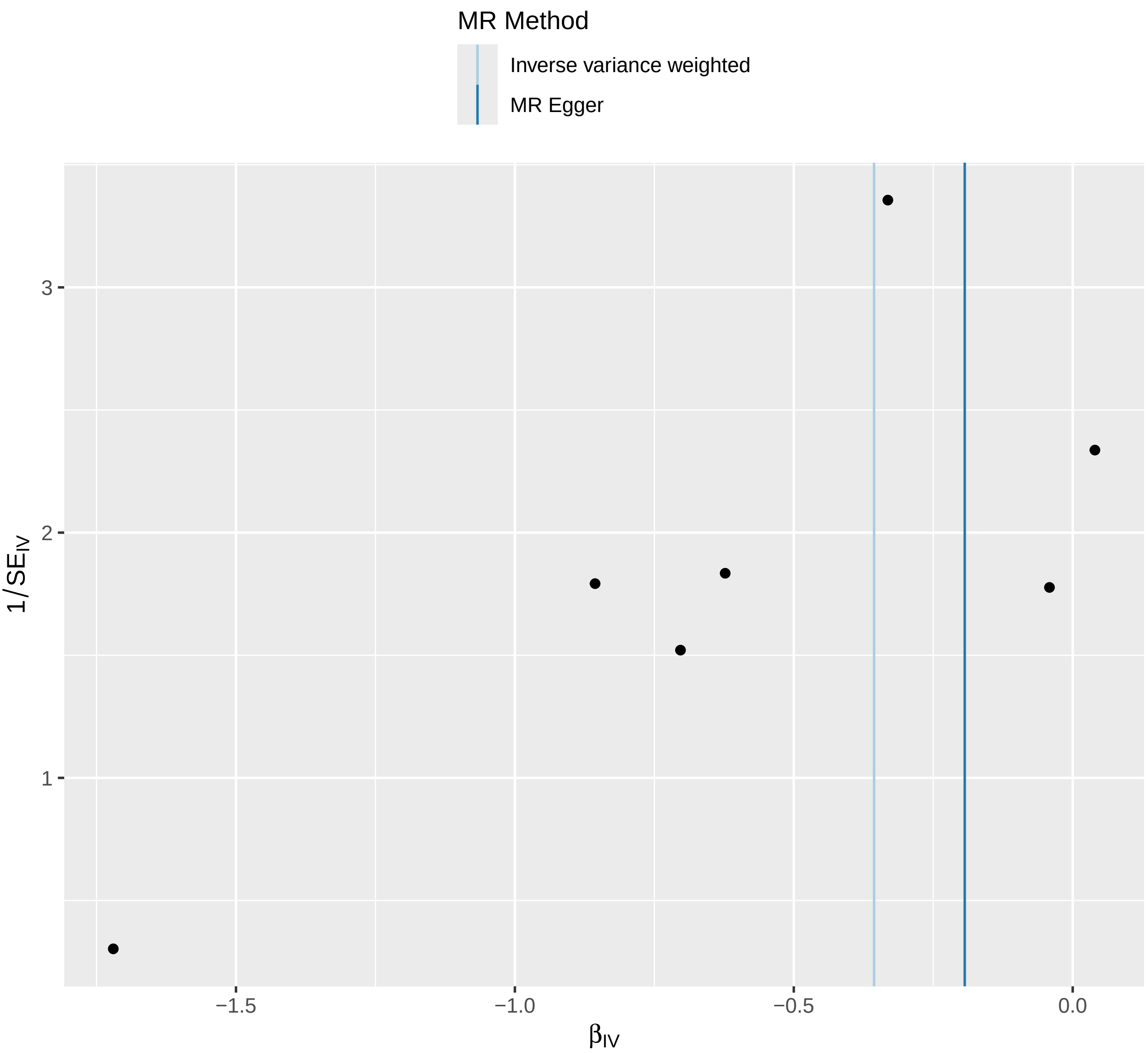
MR Method
Inverse variance weighted
MR Egger
3
2
1
1/SE_IV
−1.5
−1.0
−0.5
0.0
β_IV

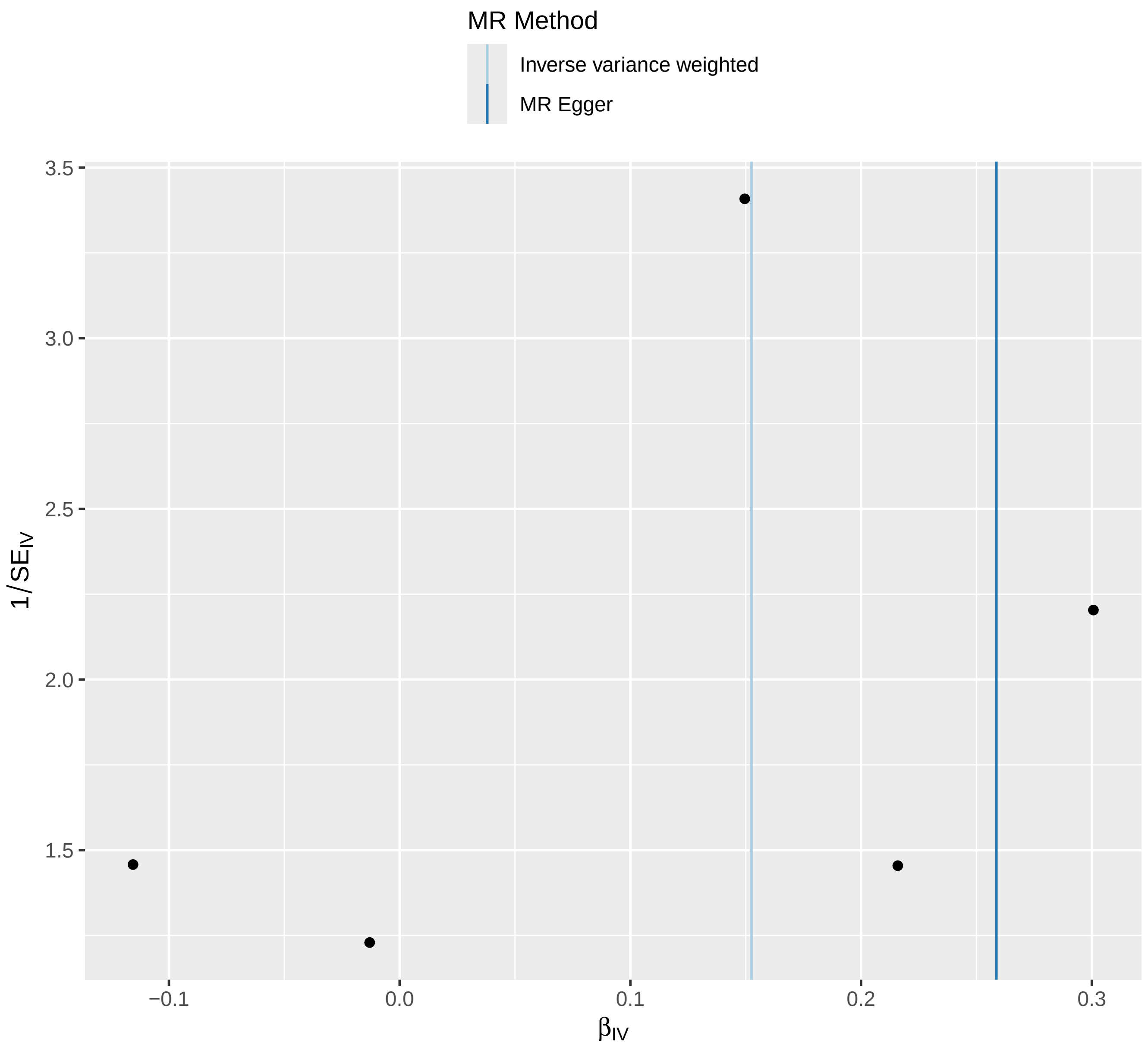

MR Method
Inverse variance weighted
MR Egger
3.5
3.0
2.5
2.0
1.5
1/SE_IV
−0.1
0.0
0.1
0.2
0.3
β_IV

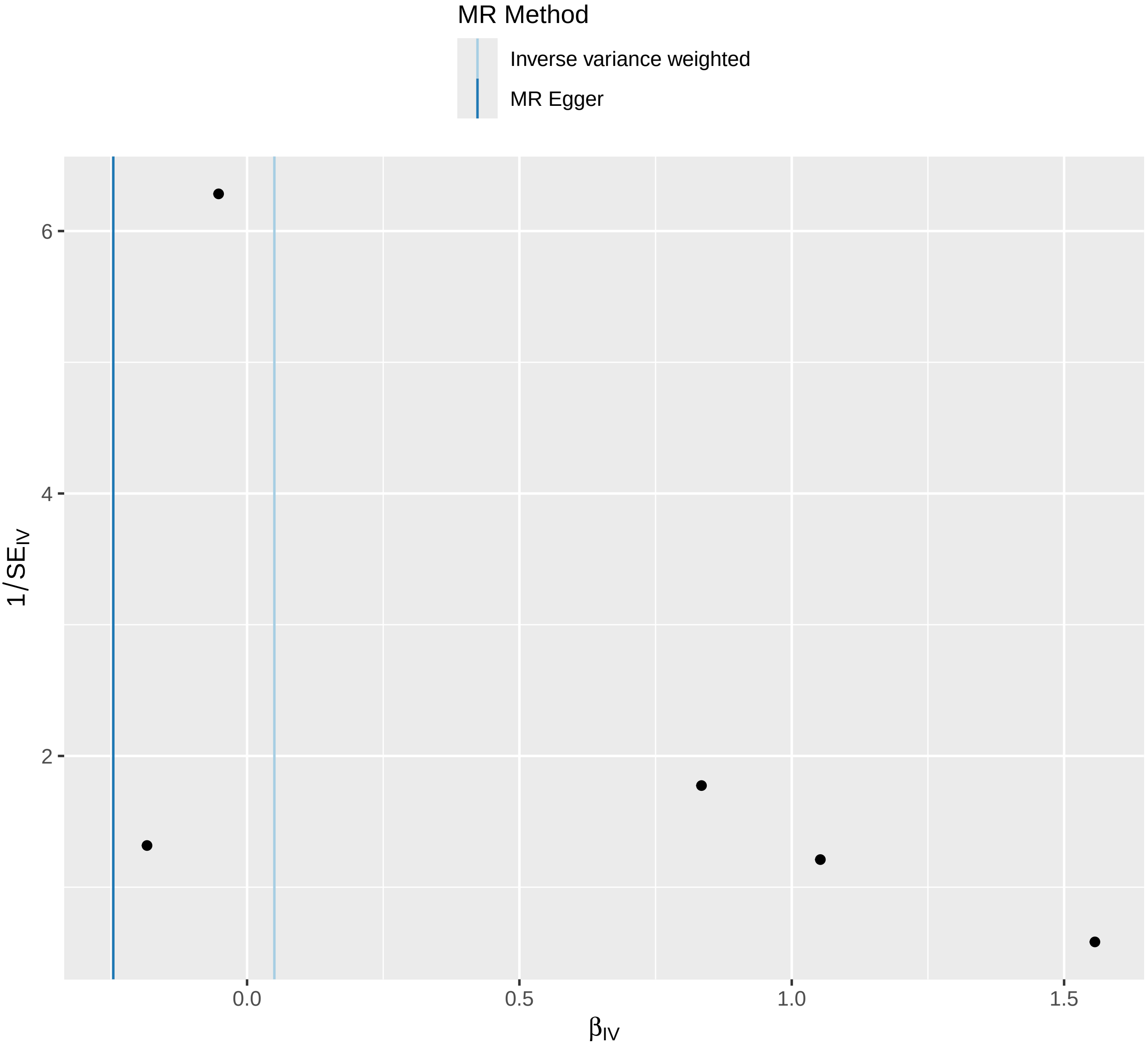

MR Method
Inverse variance weighted
MR Egger
6
4
2
$1/SE_{IV}$
0.0
0.5
1.0
1.5
β_{IV}

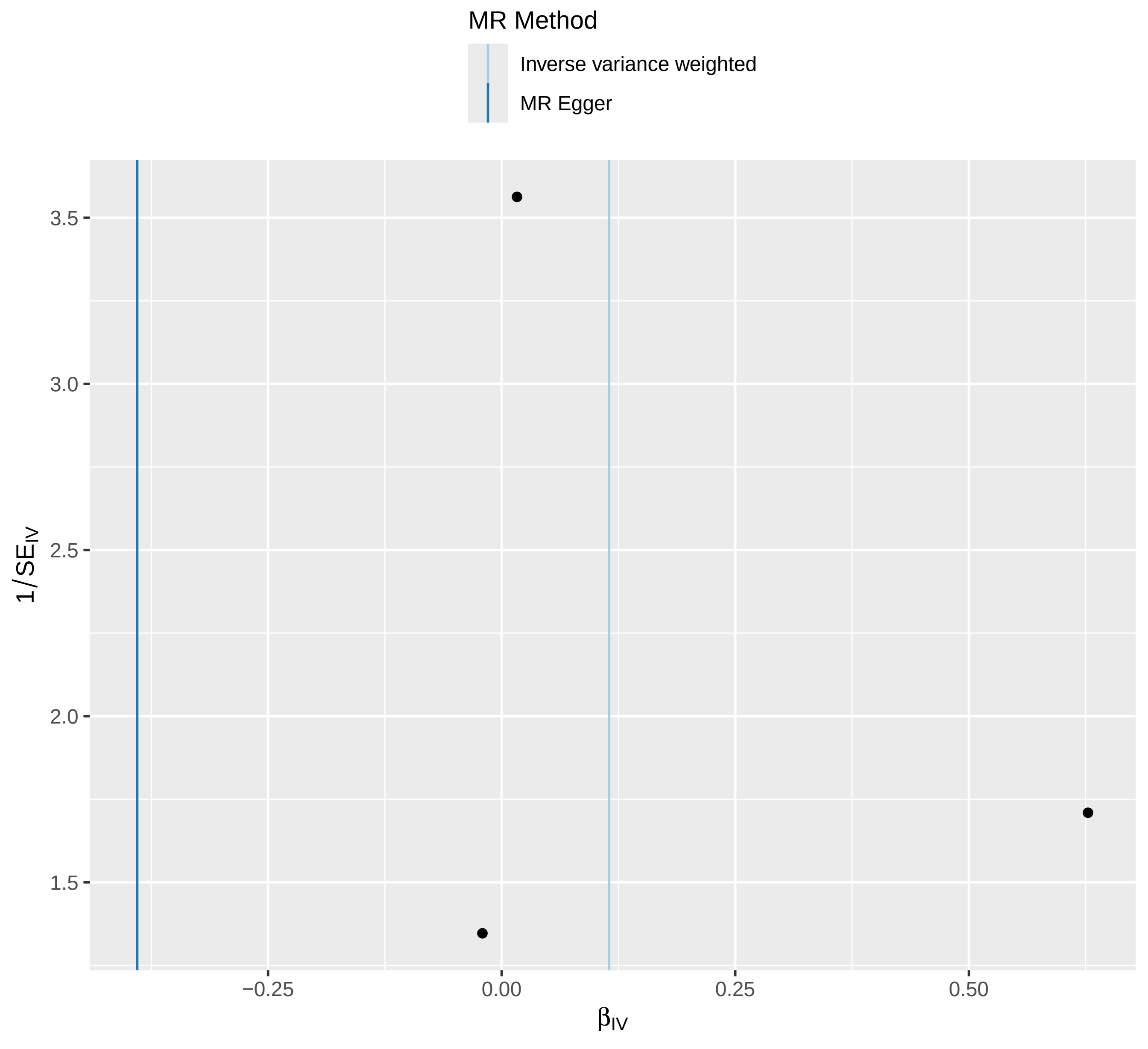
MR Method
Inverse variance weighted
MR Egger
3.5
3.0
2.5
2.0
1.5
1/SE$_{IV}$
−0.25
0.00
0.25
0.50
β$_{IV}$

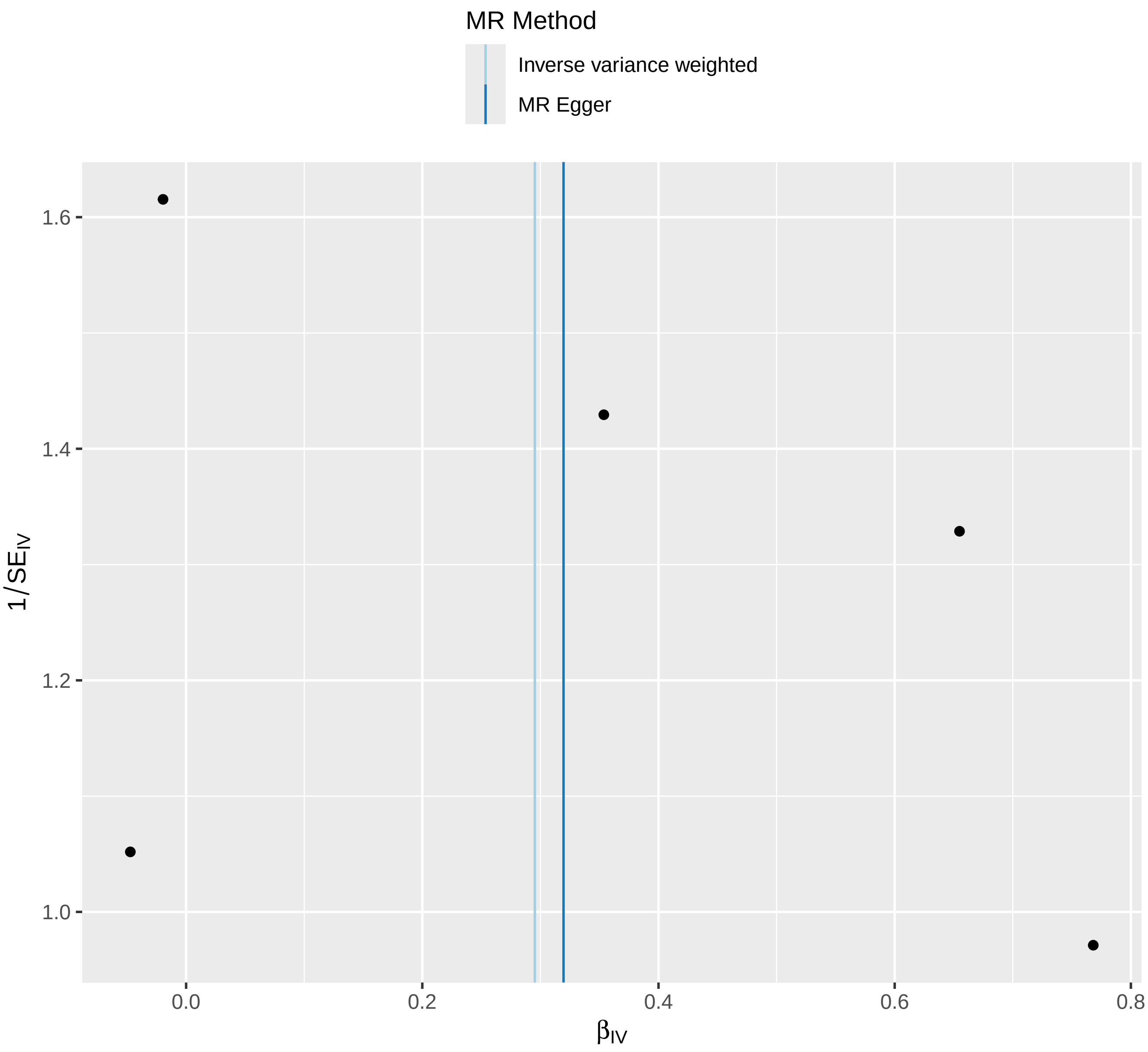

MR Method
Inverse variance weighted
MR Egger
1.6
1.4
1.2
1.0
0.0
0.2
0.4
0.6
0.8
$1/SE_{IV}$
β_{IV}

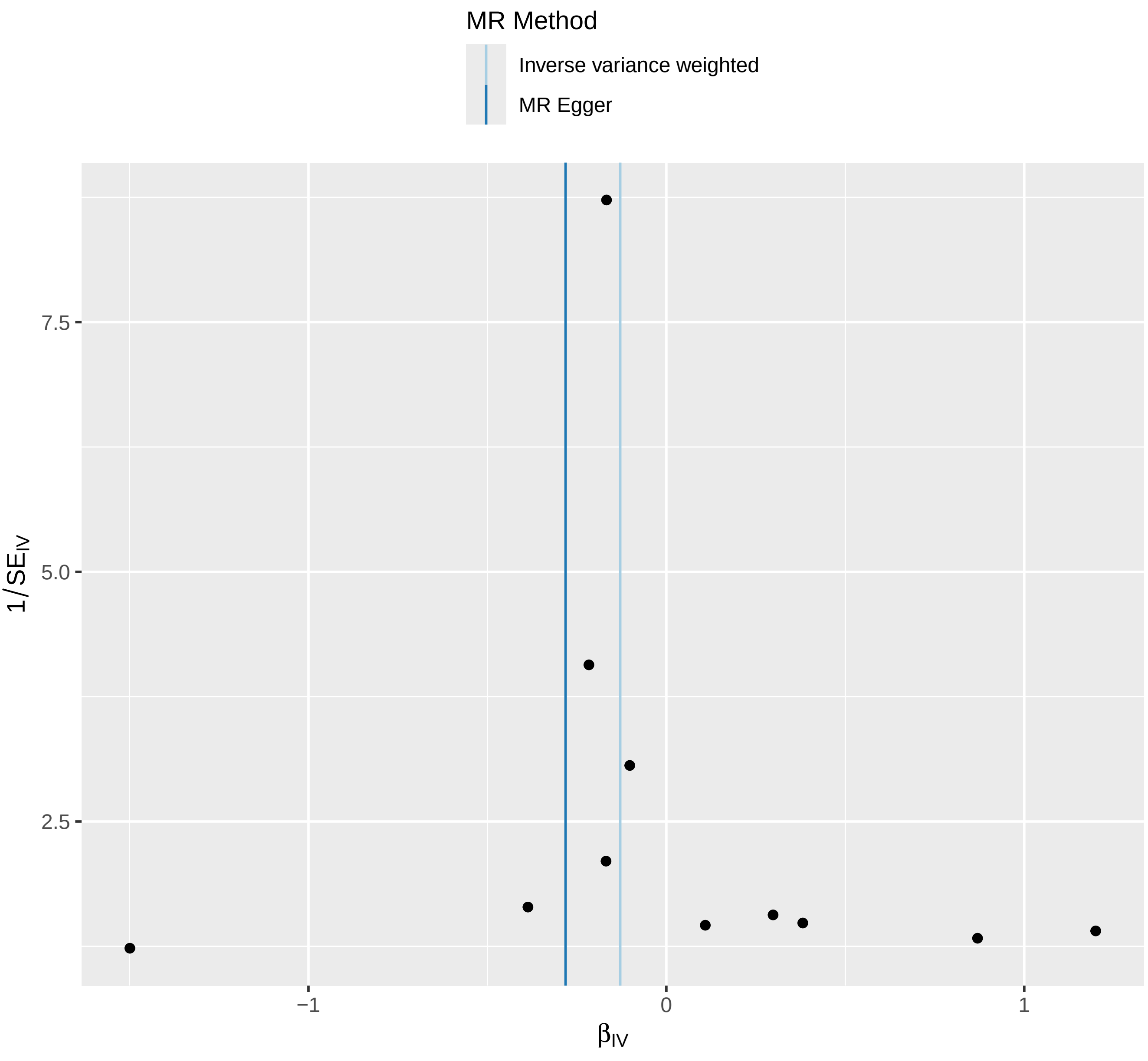

MR Method
Inverse variance weighted
MR Egger
7.5
5.0
2.5
1/SE$_{IV}$
−1
0
1
β$_{IV}$

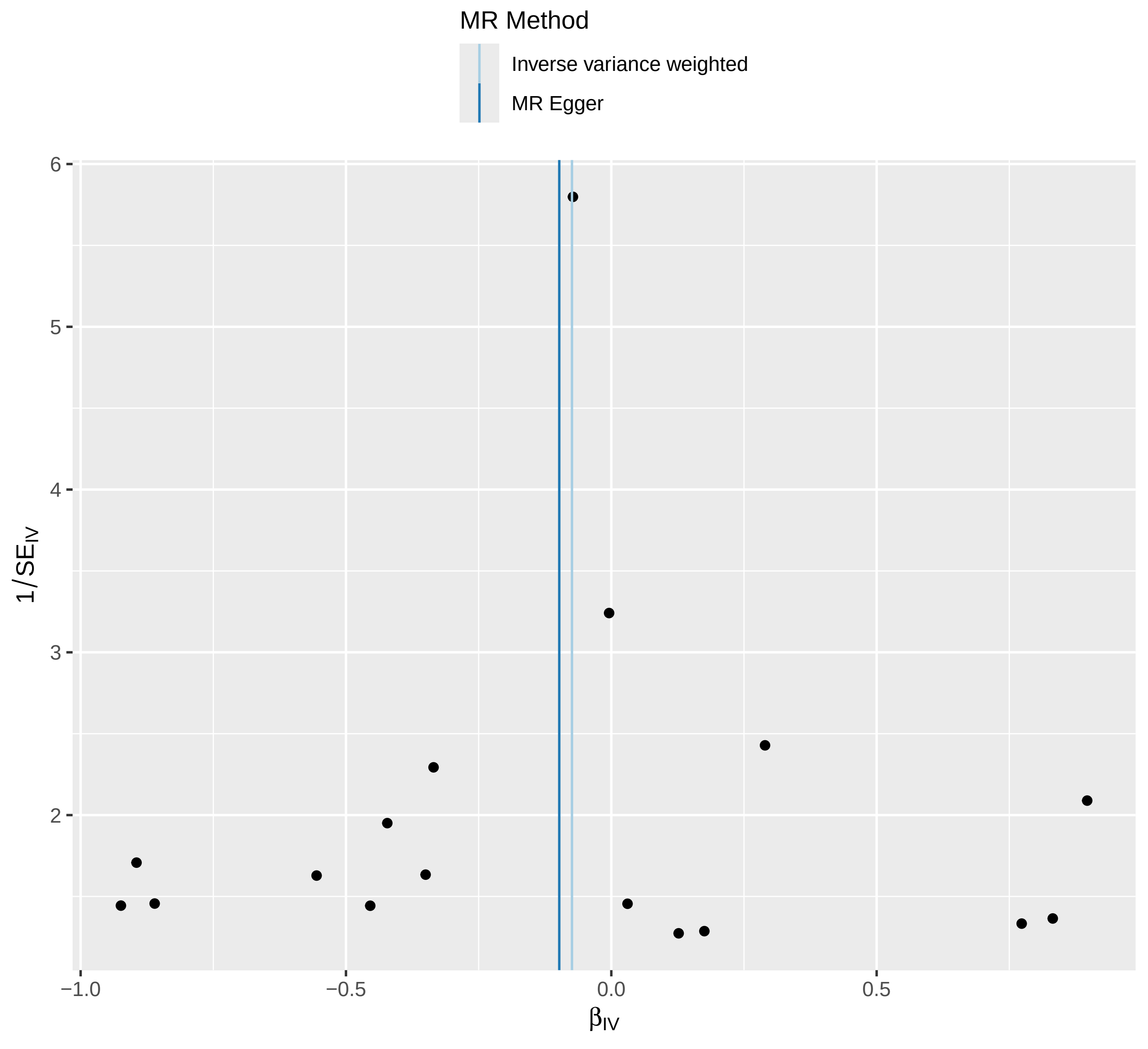
MR Method
Inverse variance weighted
MR Egger
6
5
4
3
2
1/SE_IV
−1.0
−0.5
0.0
0.5
β_IV

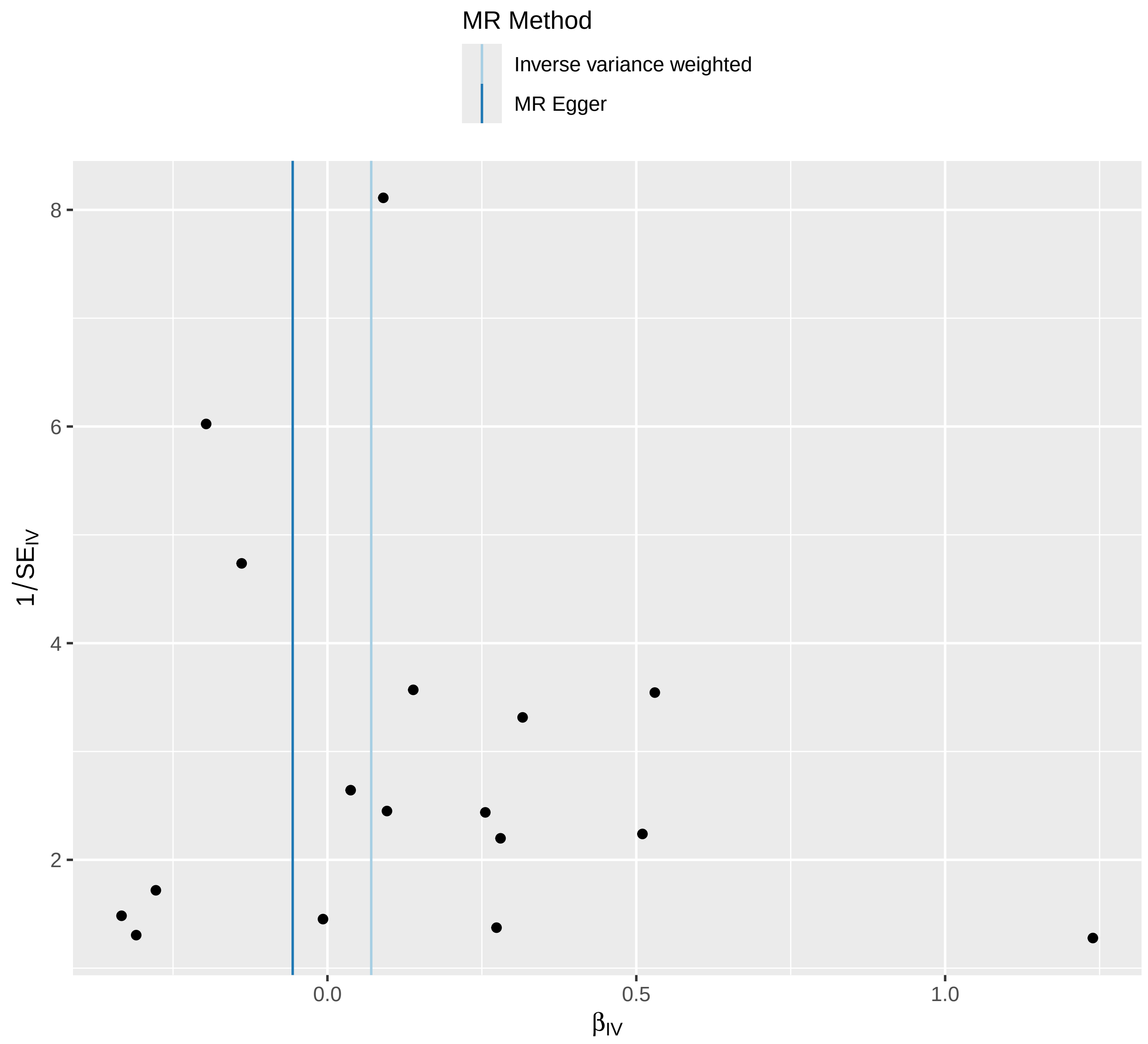

MR Method
Inverse variance weighted
MR Egger
8
6
4
2
1/SE$_{IV}$
0.0
0.5
1.0
β_{IV}

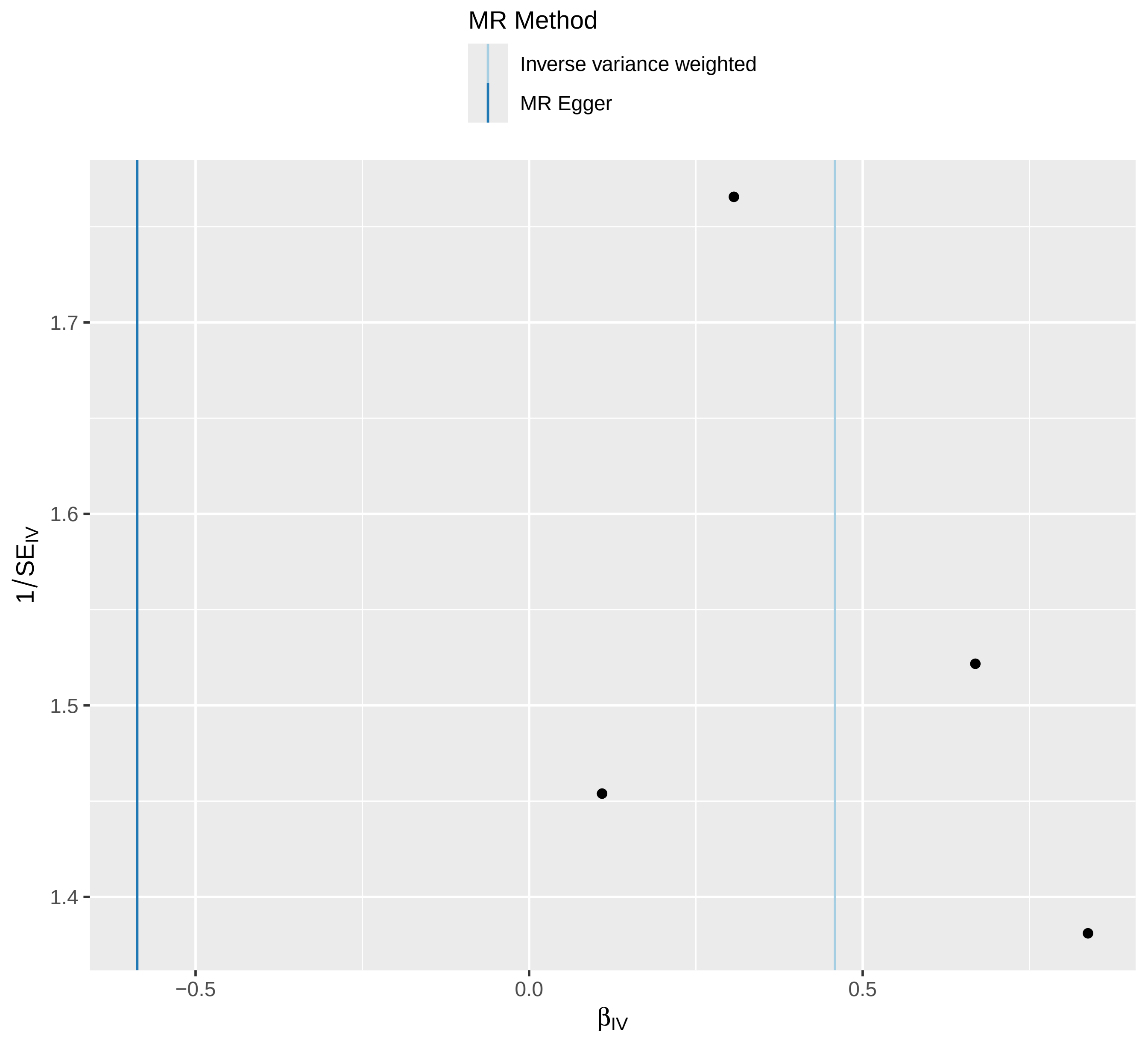

MR Method
Inverse variance weighted
MR Egger
1.7
1.6
1.5
1.4
1/SE_IV
−0.5
0.0
0.5
β_IV

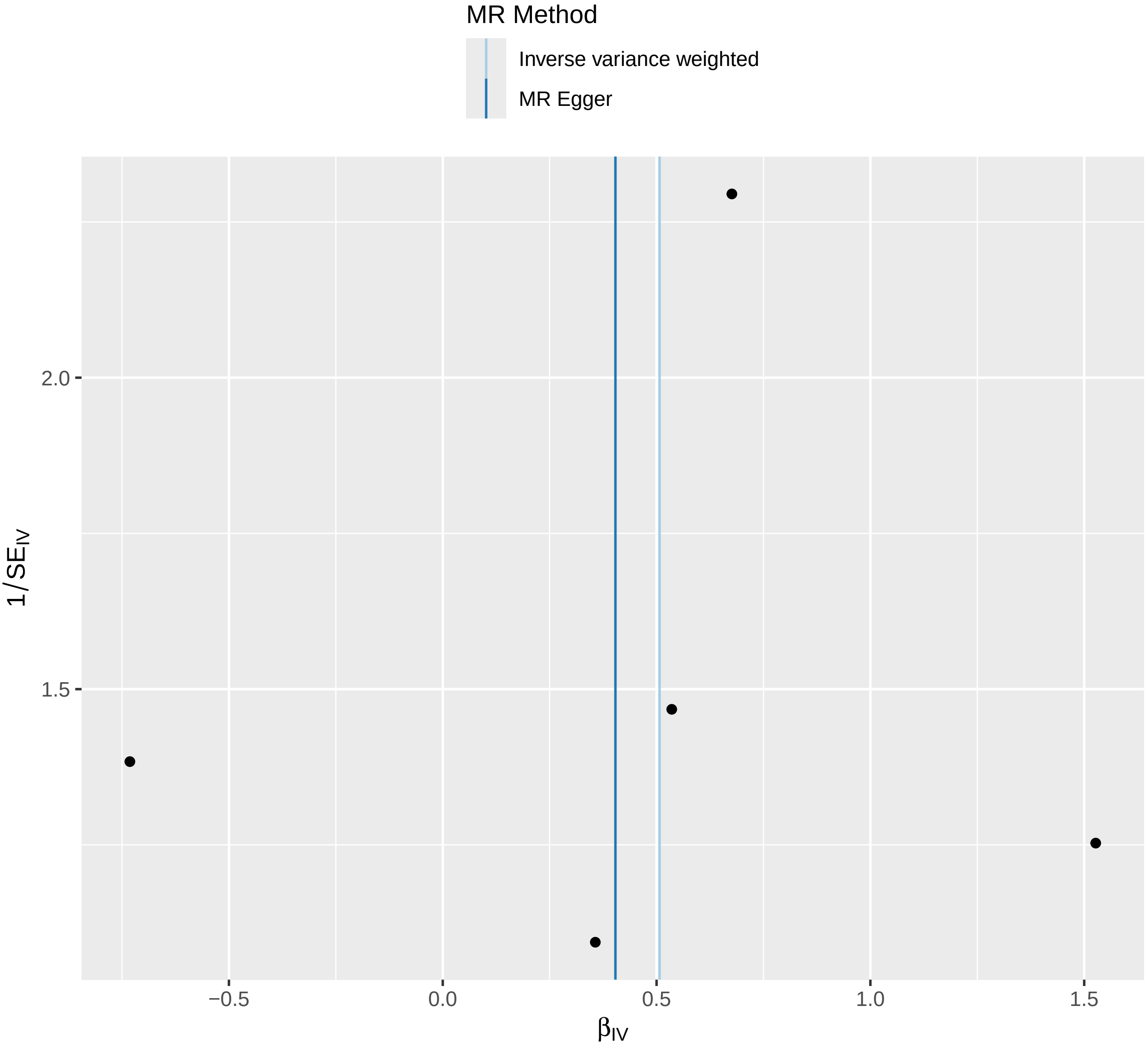
MR Method
Inverse variance weighted
MR Egger
2.0
1.5
1/SE$_{IV}$
−0.5
0.0
0.5
1.0
1.5
β$_{IV}$

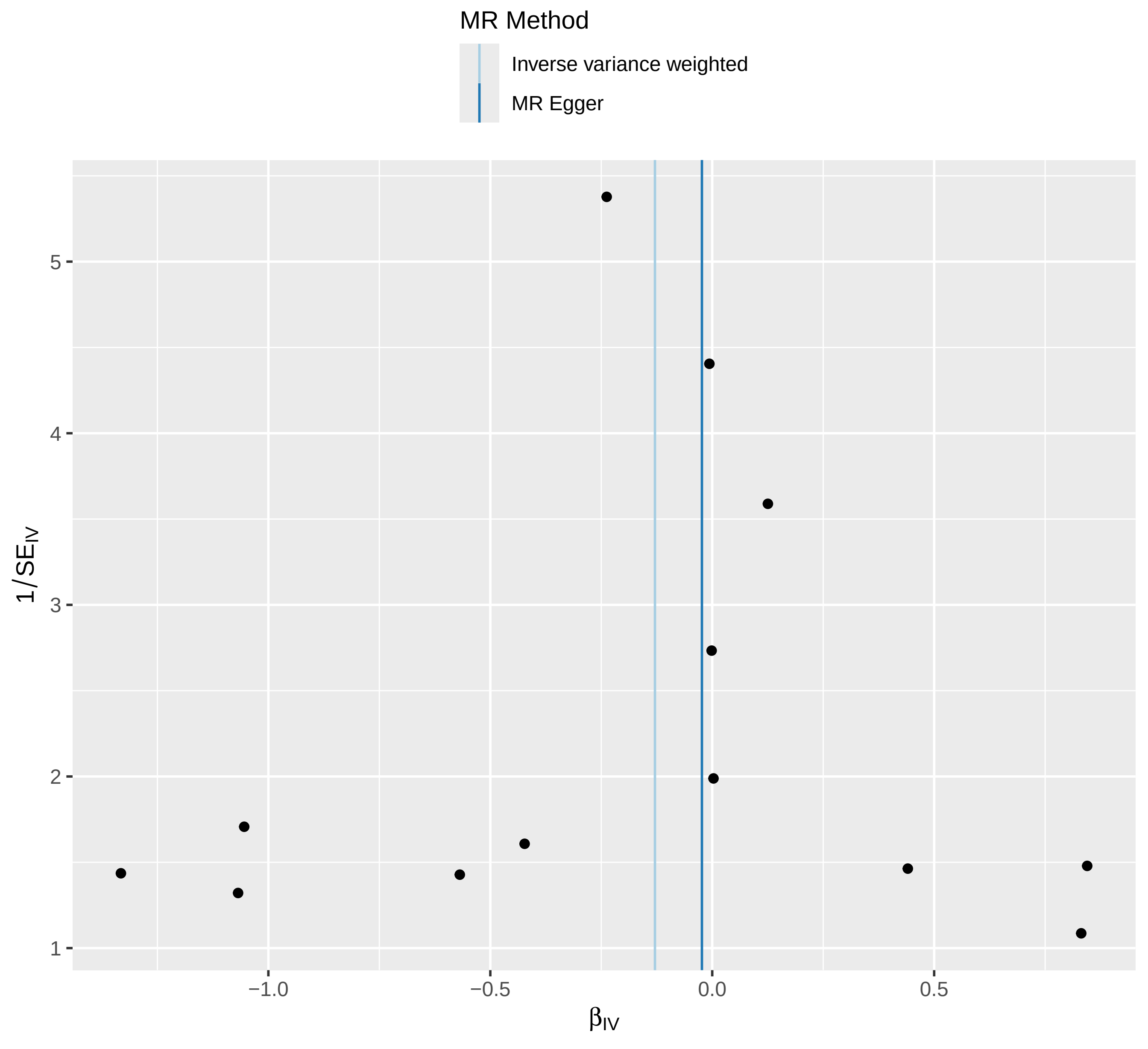

MR Method
Inverse variance weighted
MR Egger
5
4
3
2
1
1/SE$_{IV}$
−1.0
−0.5
0.0
0.5
β$_{IV}$

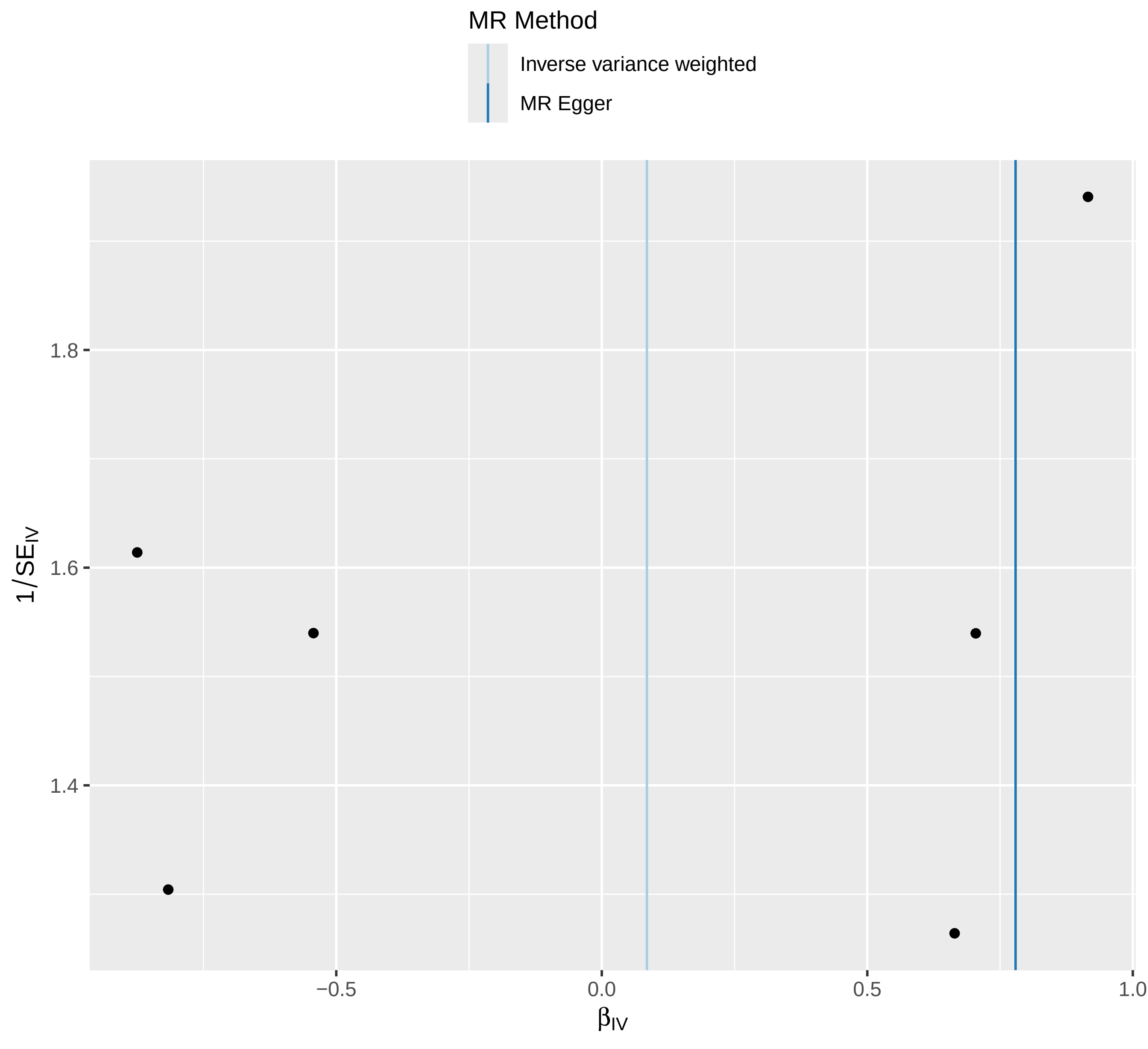
MR Method
Inverse variance weighted
MR Egger
1.8
1.6
1.4
1/SE_IV
−0.5
0.0
0.5
1.0
β_IV

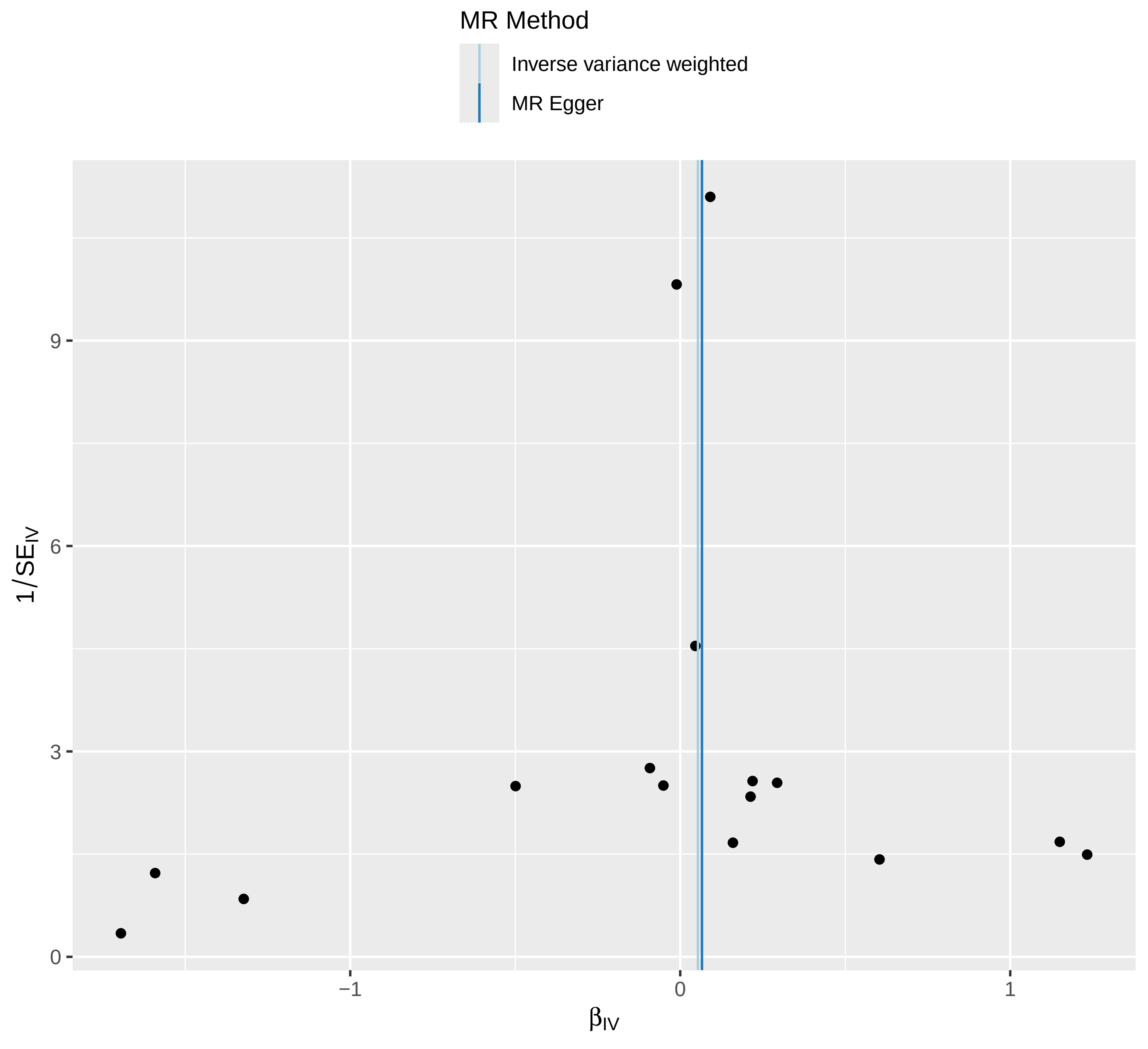
MR Method
Inverse variance weighted
MR Egger
1/SE_IV
β_IV
9
6
3
0
−1
0
1

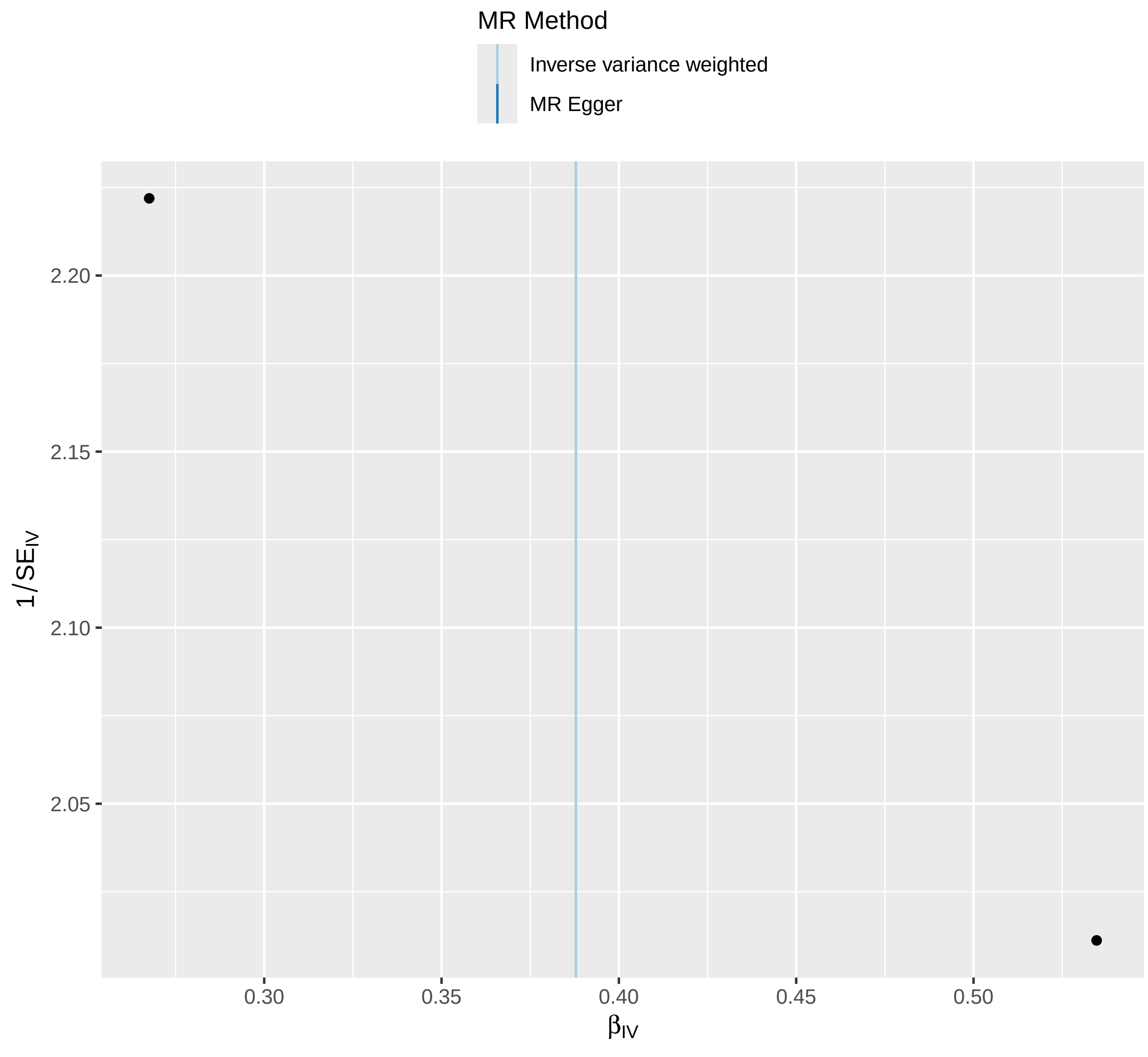
MR Method
Inverse variance weighted
MR Egger
2.20
2.15
2.10
2.05
$1/SE_{IV}$
0.30
0.35
0.40
0.45
0.50
β_{IV}

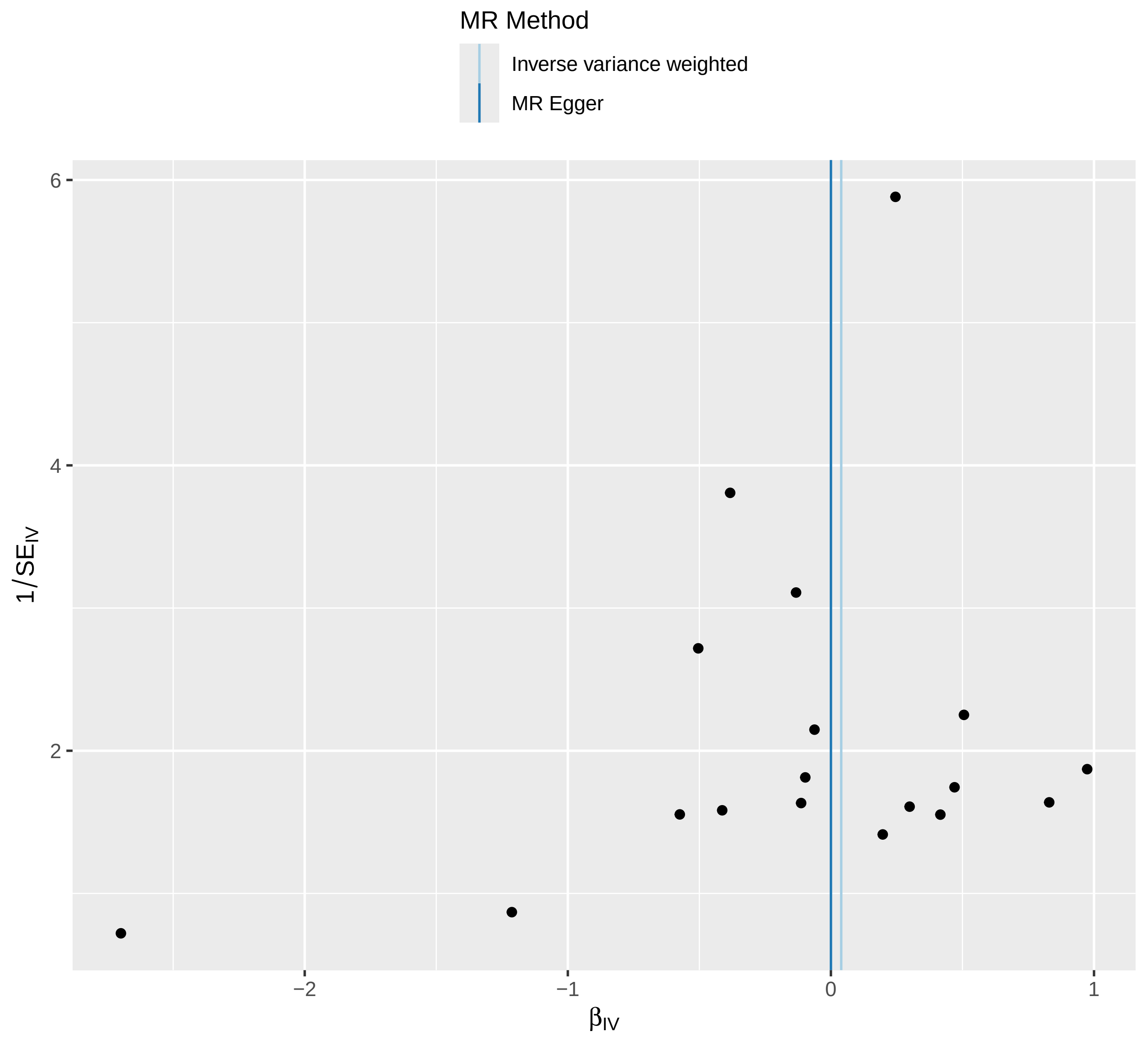

MR Method
Inverse variance weighted
MR Egger
6
4
2
1/SE_IV
−2
−1
0
1
β_IV

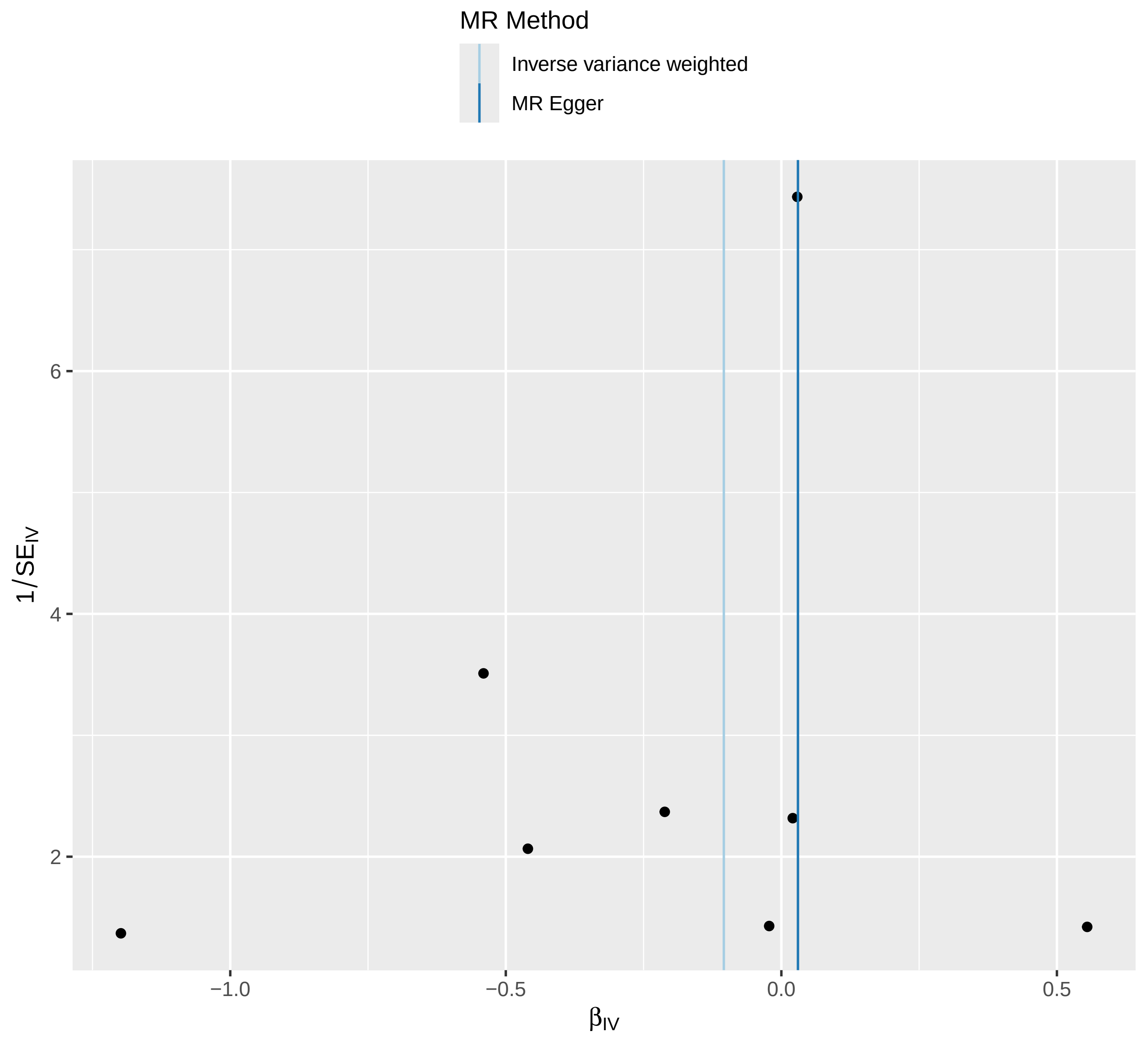

MR Method
Inverse variance weighted
MR Egger
6
4
2
1/SE$_{IV}$
−1.0
−0.5
0.0
0.5
β$_{IV}$

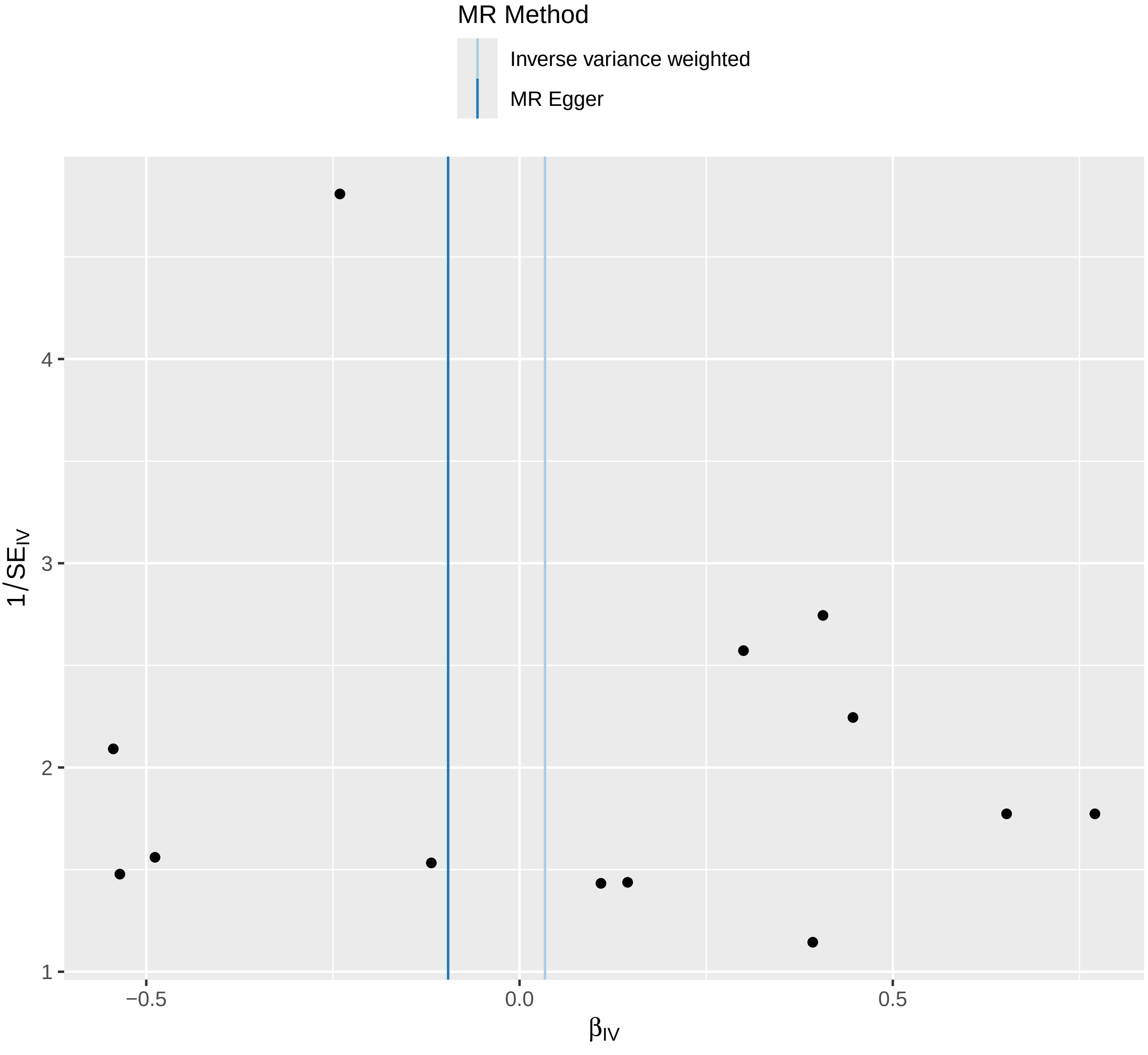

MR Method
Inverse variance weighted
MR Egger
1/SE_IV
4
3
2
1
−0.5
0.0
0.5
β_IV

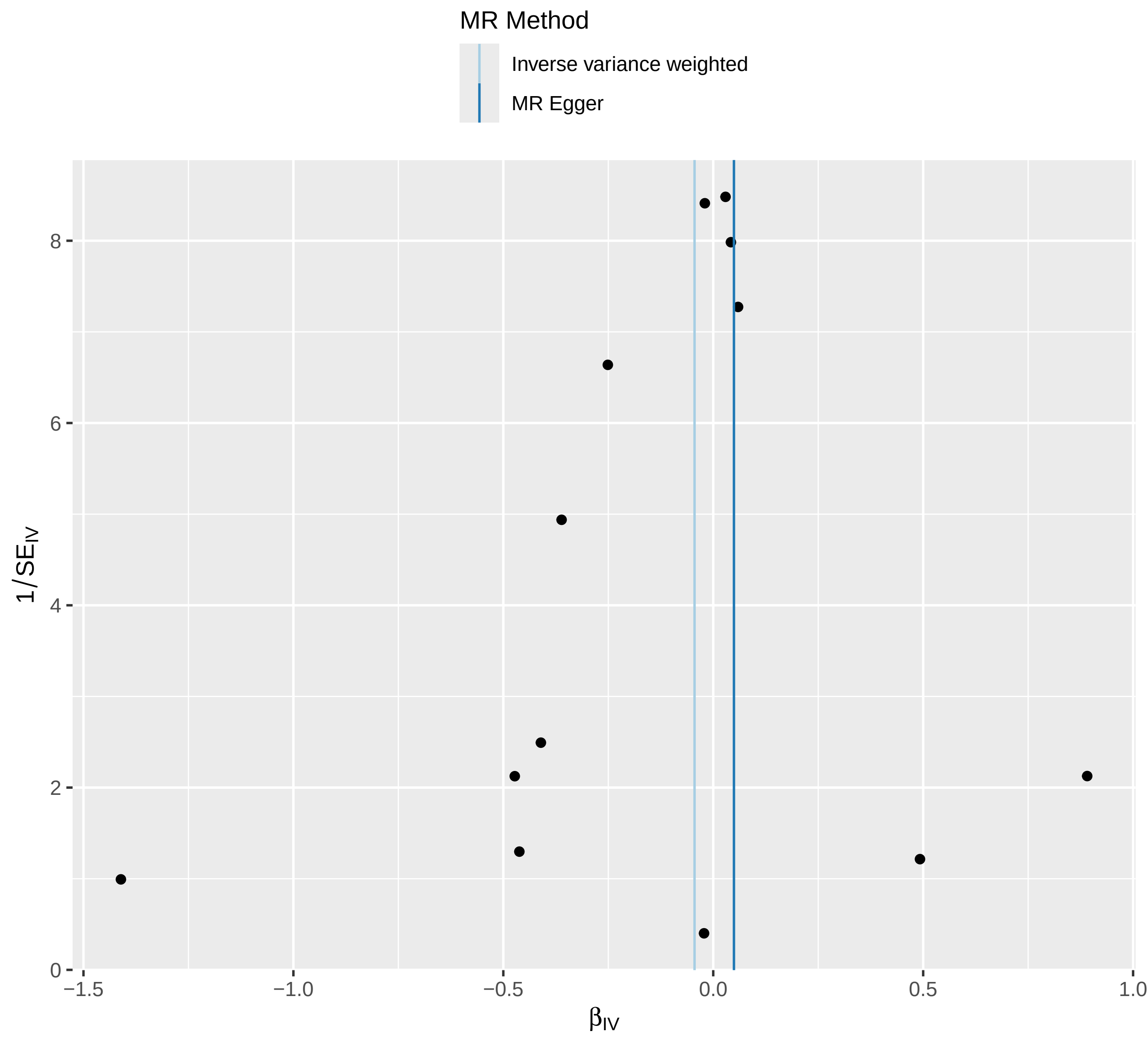
MR Method
Inverse variance weighted
MR Egger
1/SE_IV
8
6
4
2
0
−1.5
−1.0
−0.5
0.0
0.5
1.0
β_IV

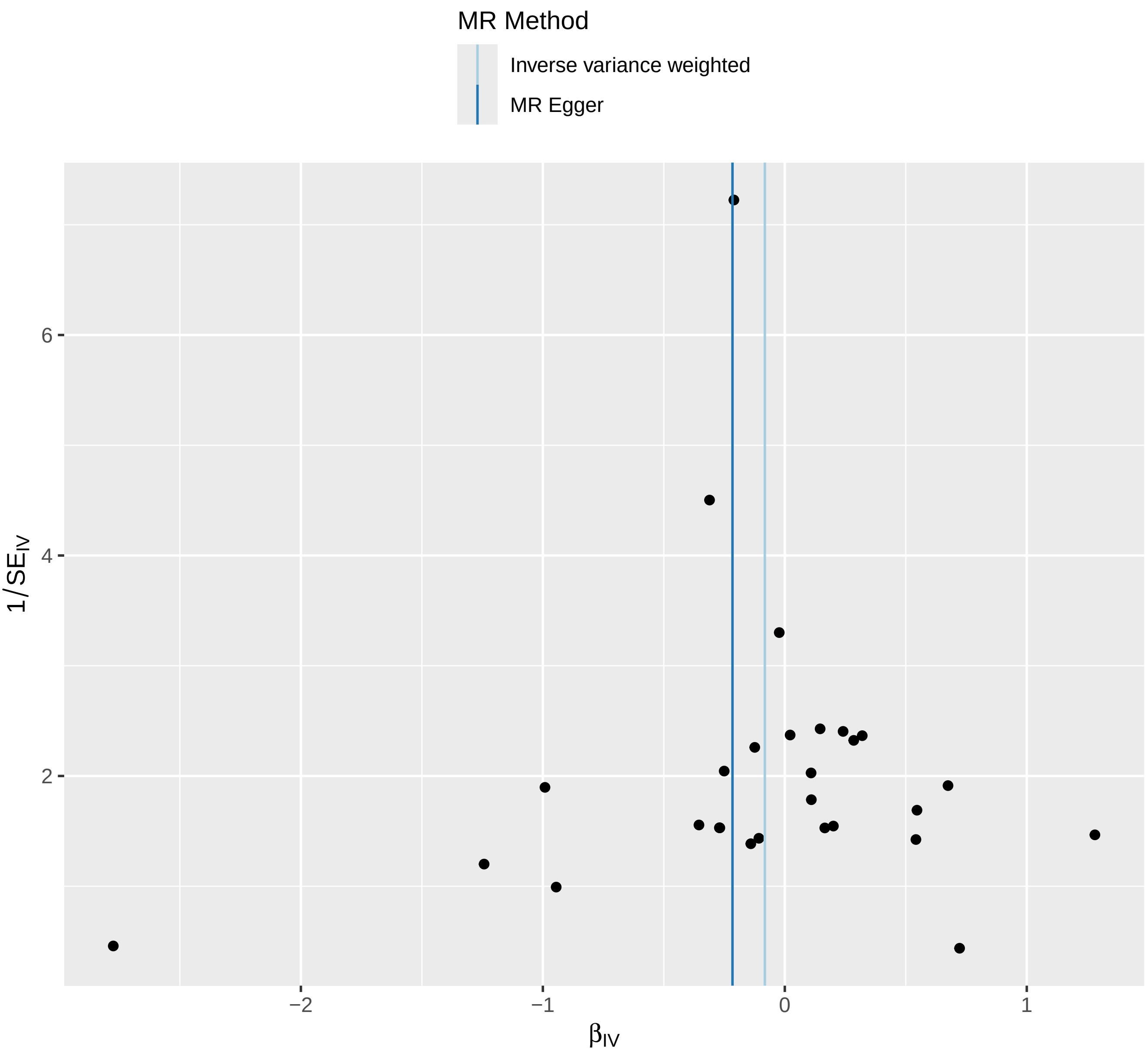

MR Method
Inverse variance weighted
MR Egger
6
4
2
1/SE$_{IV}$
−2
−1
0
1
β$_{IV}$

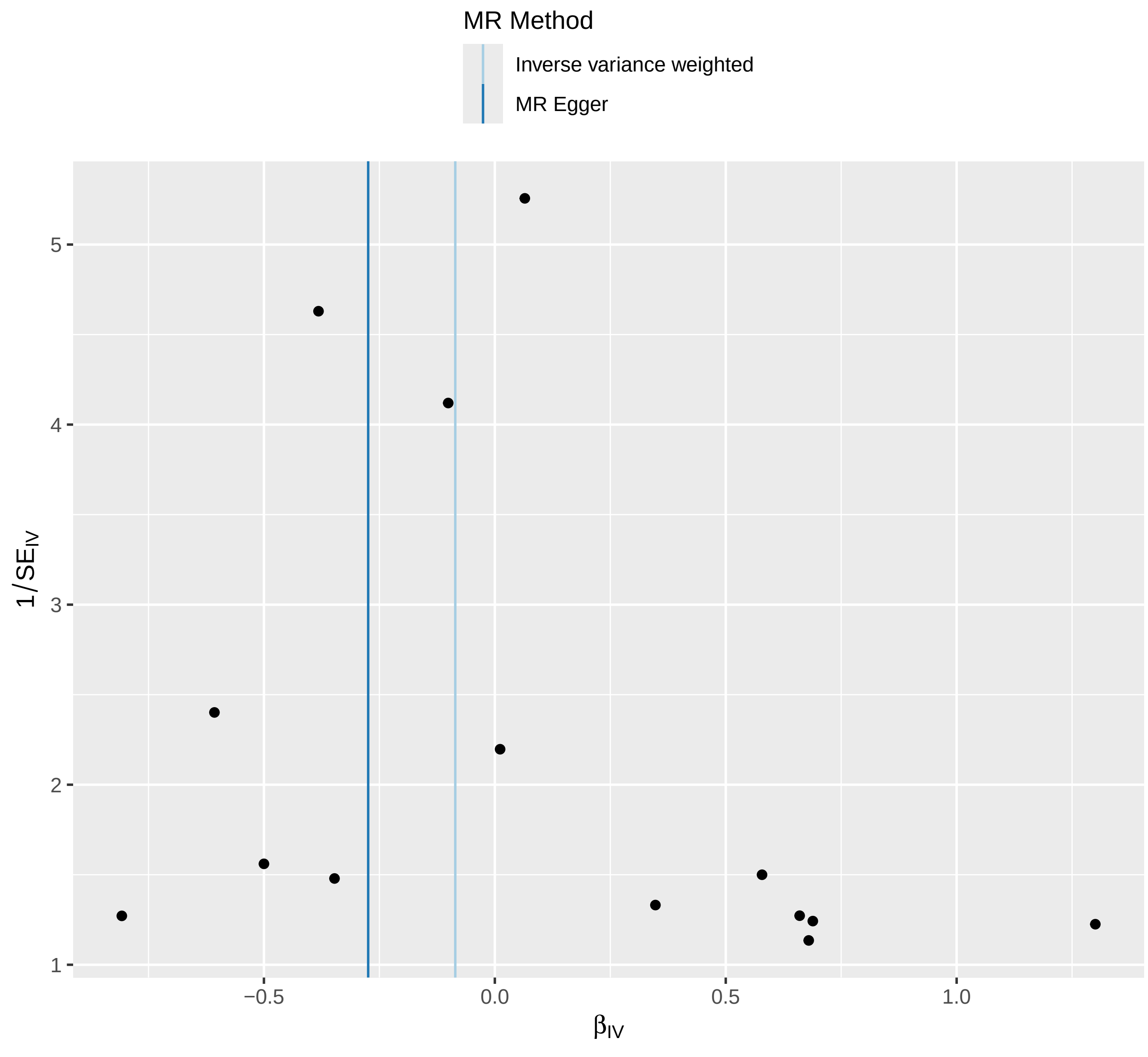
MR Method
Inverse variance weighted
MR Egger
5
4
3
2
1
1/SE_IV
−0.5
0.0
0.5
1.0
β_IV

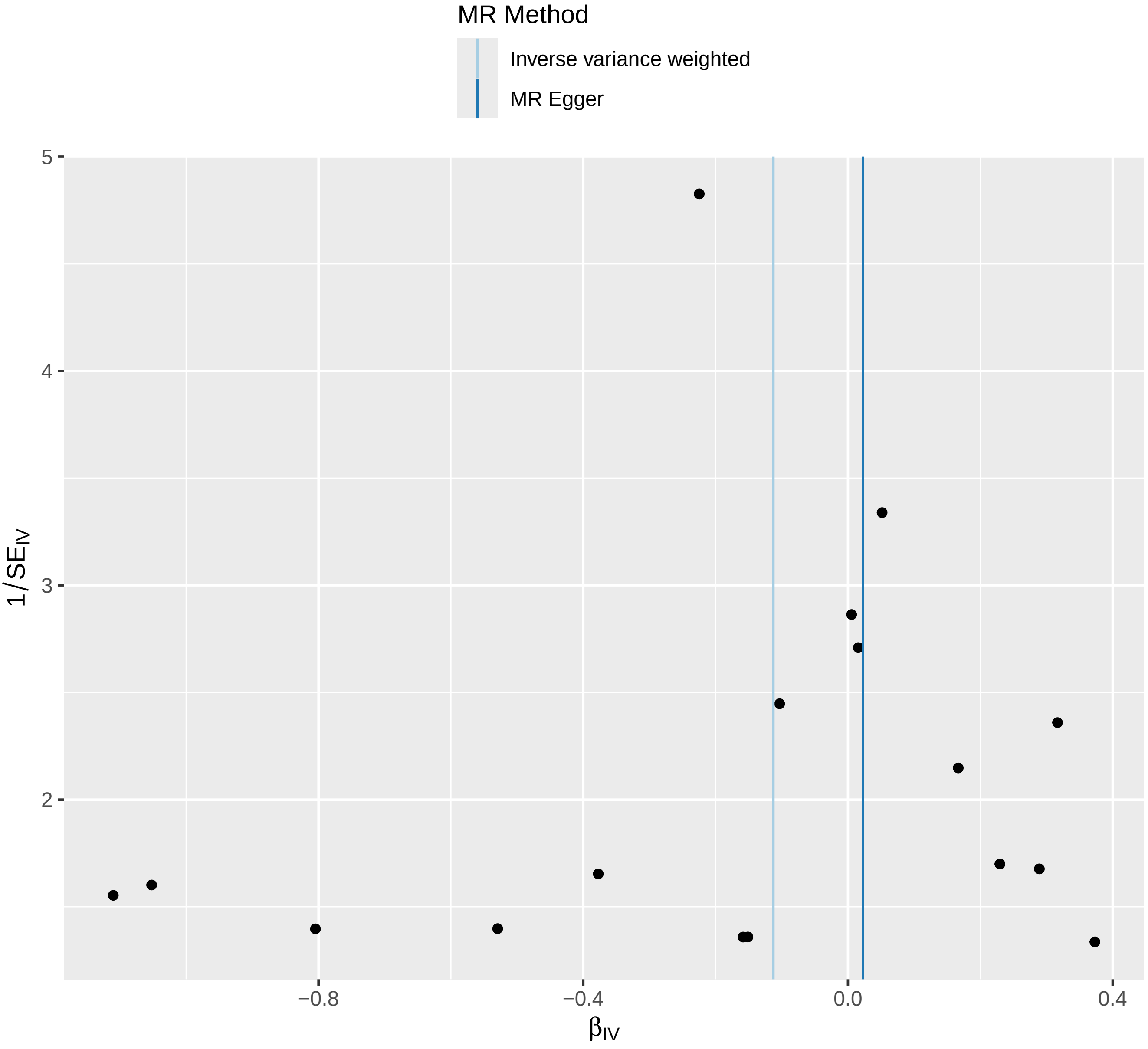

MR Method
Inverse variance weighted
MR Egger
5
4
3
2
1/SE$_{IV}$
−0.8
−0.4
0.0
0.4
β$_{IV}$

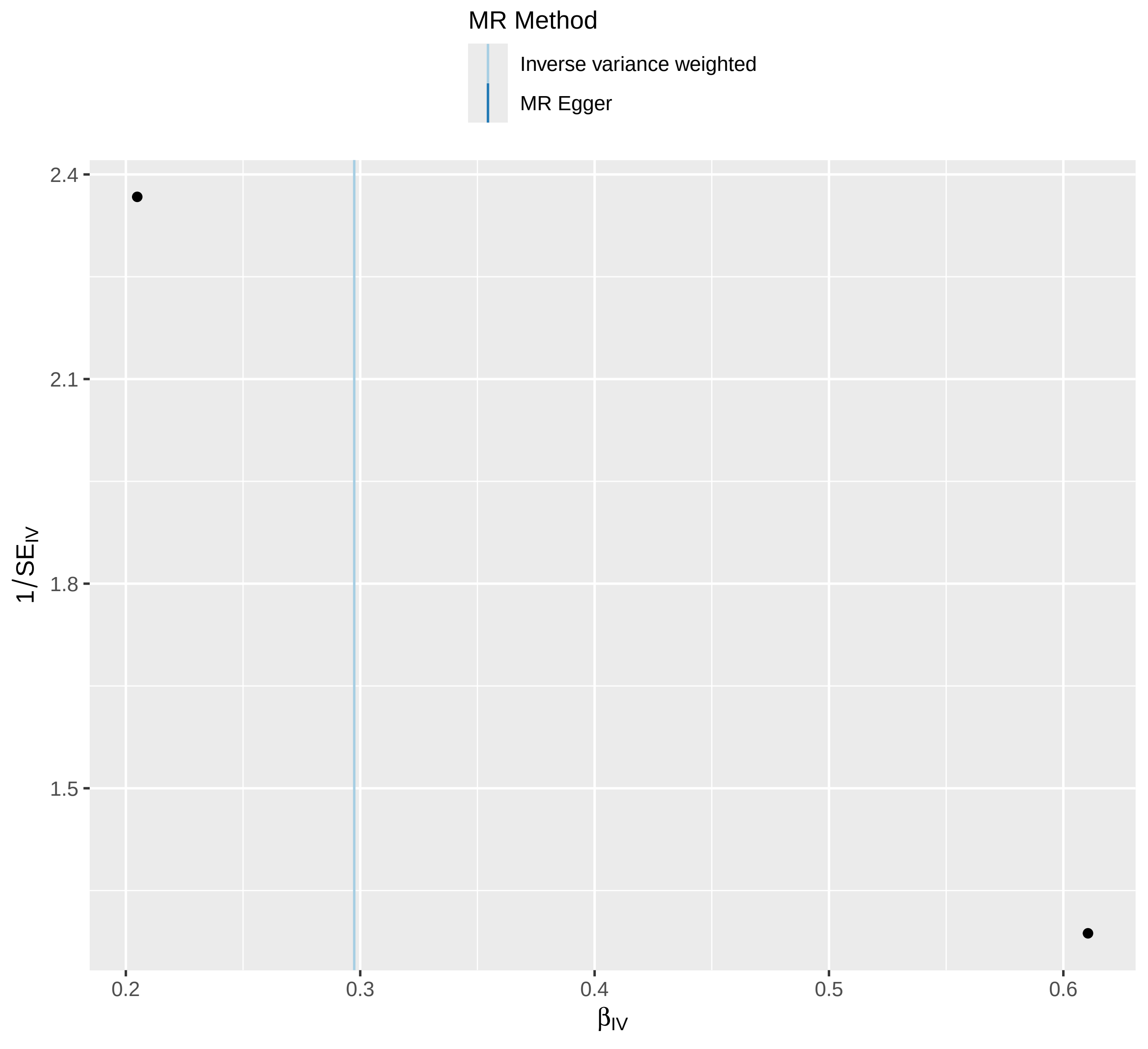
MR Method
Inverse variance weighted
MR Egger
2.4
2.1
1.8
1.5
1/SE_IV
0.2
0.3
0.4
0.5
0.6
β_IV

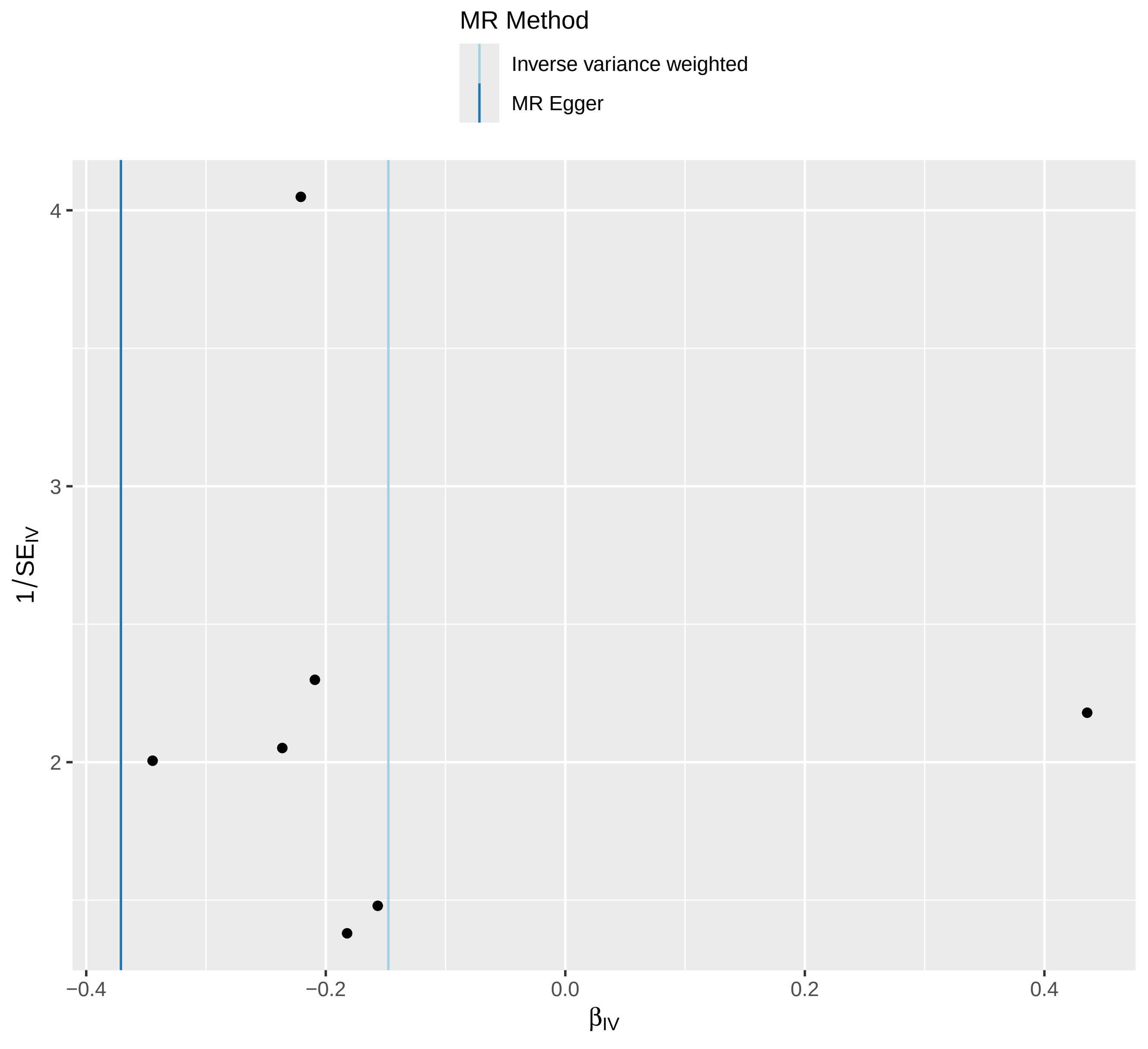

MR Method
Inverse variance weighted
MR Egger
4
3
2
1/SE_IV
−0.4
−0.2
0.0
0.2
0.4
β_IV

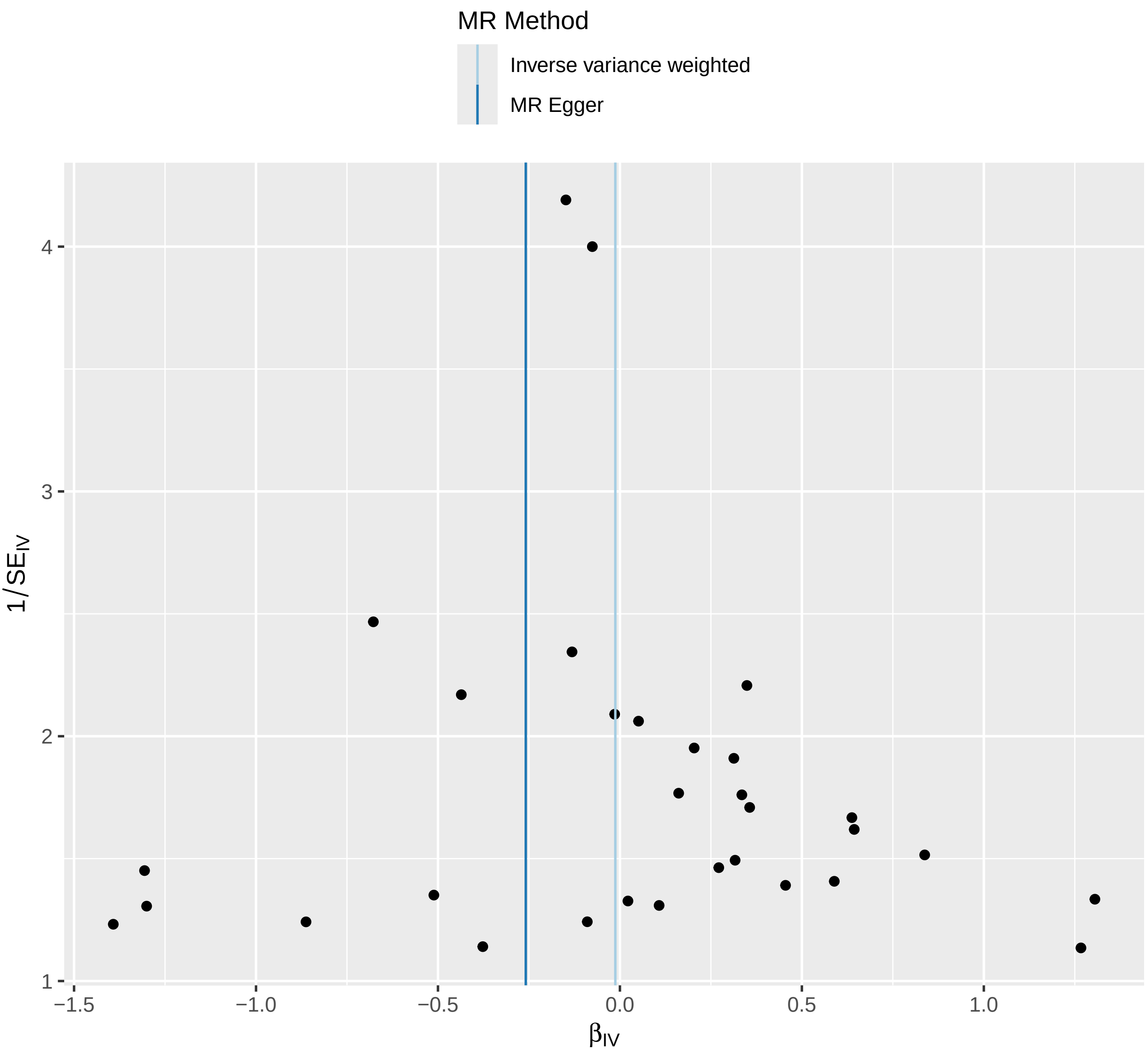
MR Method
Inverse variance weighted
MR Egger
1/SE$_{IV}$
β$_{IV}$
1
2
3
4
−1.5
−1.0
−0.5
0.0
0.5
1.0

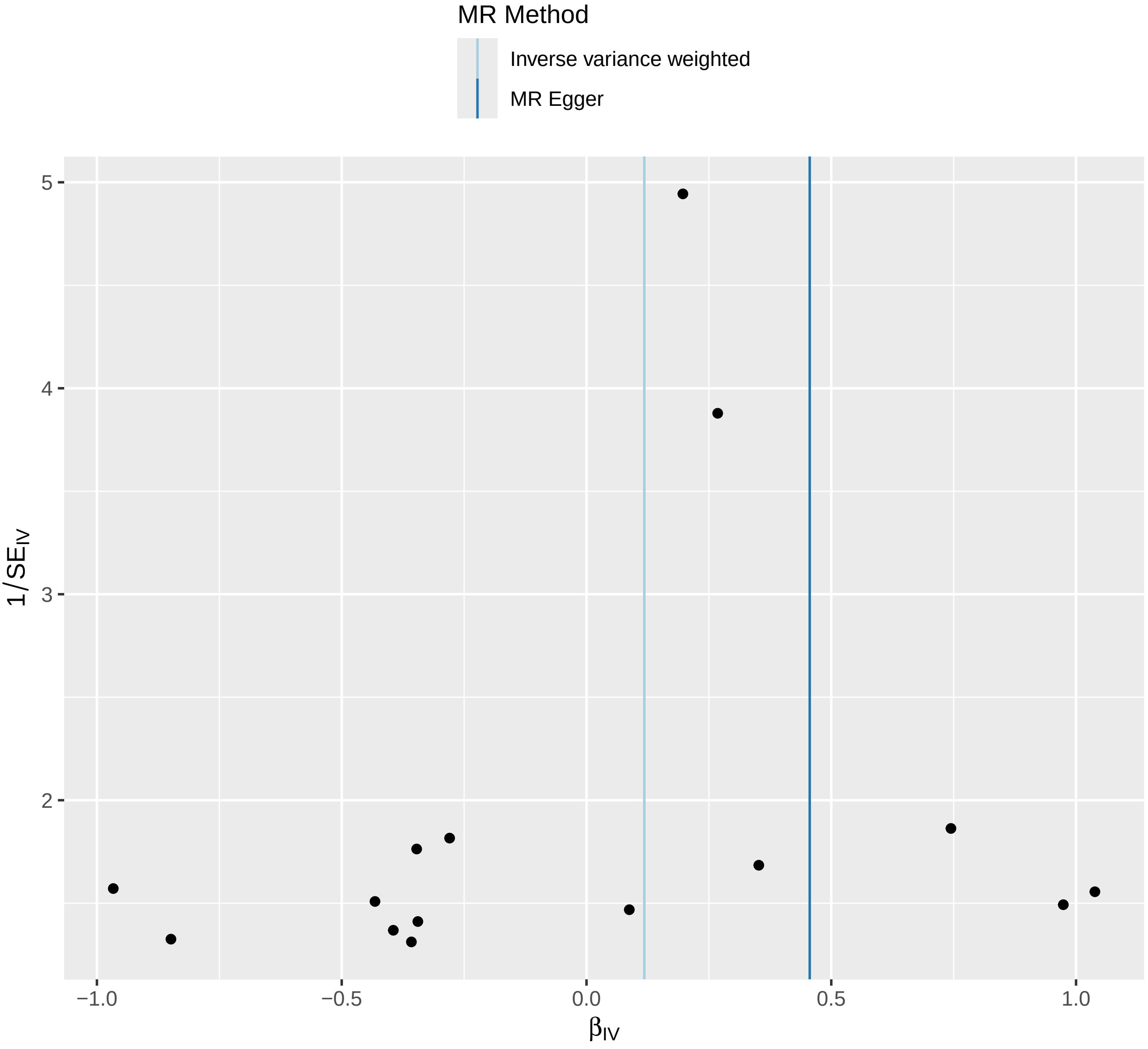

MR Method
Inverse variance weighted
MR Egger
5
4
3
2
$1/SE_{IV}$
−1.0
−0.5
0.0
0.5
1.0
β_{IV}

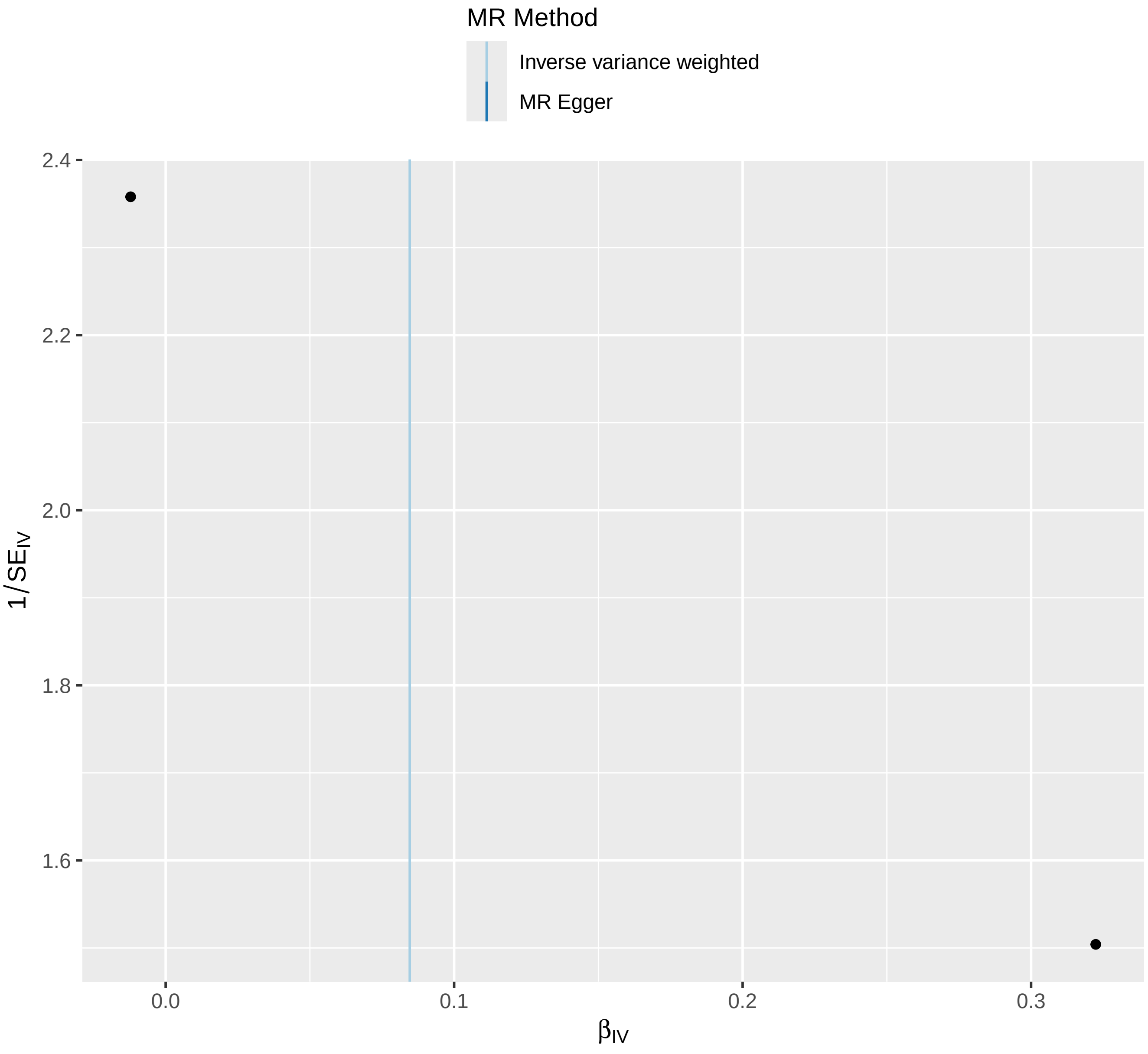
MR Method
Inverse variance weighted
MR Egger
2.4
2.2
2.0
1.8
1.6
1/SE_IV
0.0
0.1
0.2
0.3
β_IV

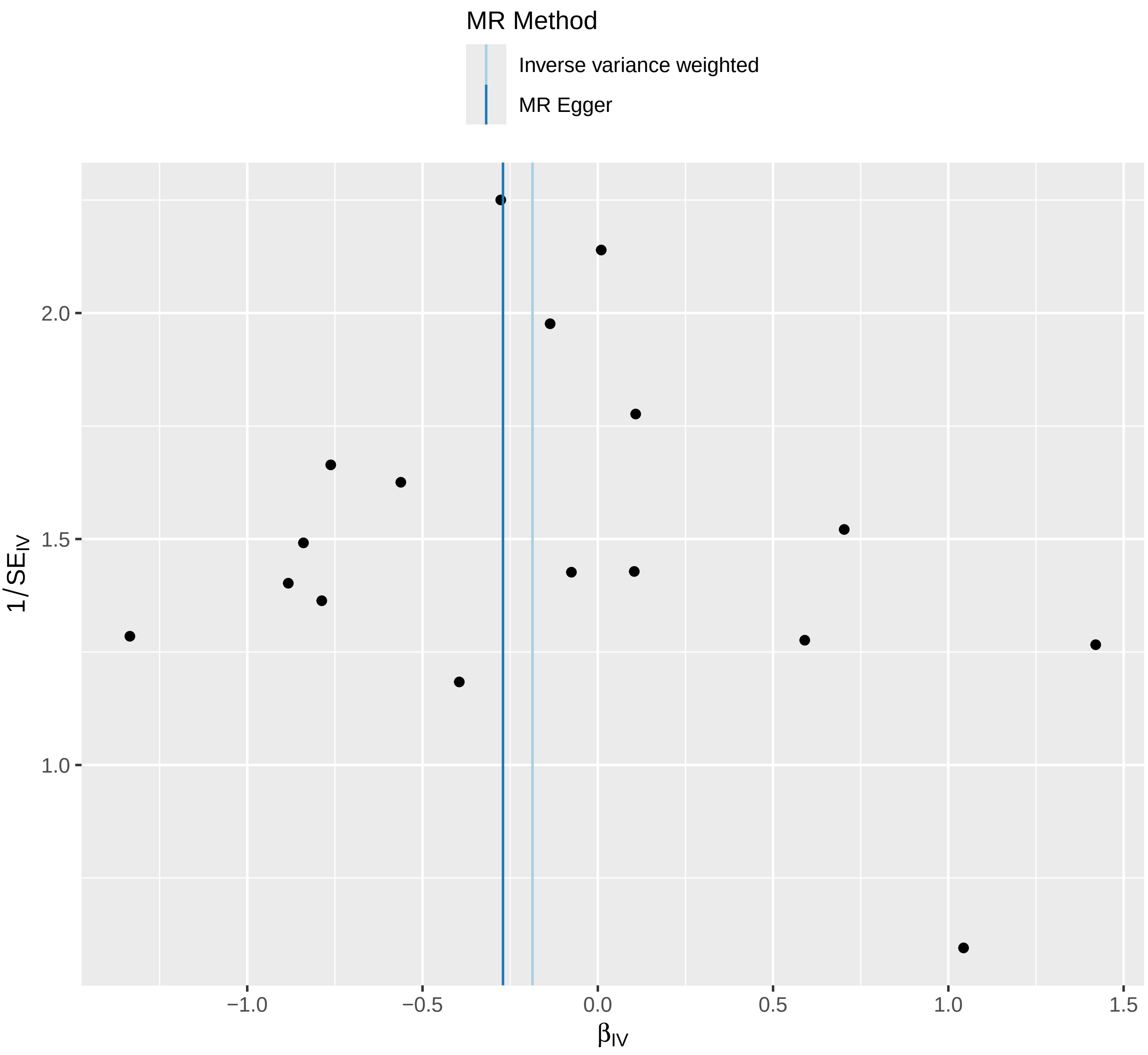
MR Method
Inverse variance weighted
MR Egger
2.0
1.5
1.0
1/SE$_{IV}$
−1.0
−0.5
0.0
0.5
1.0
1.5
β$_{IV}$

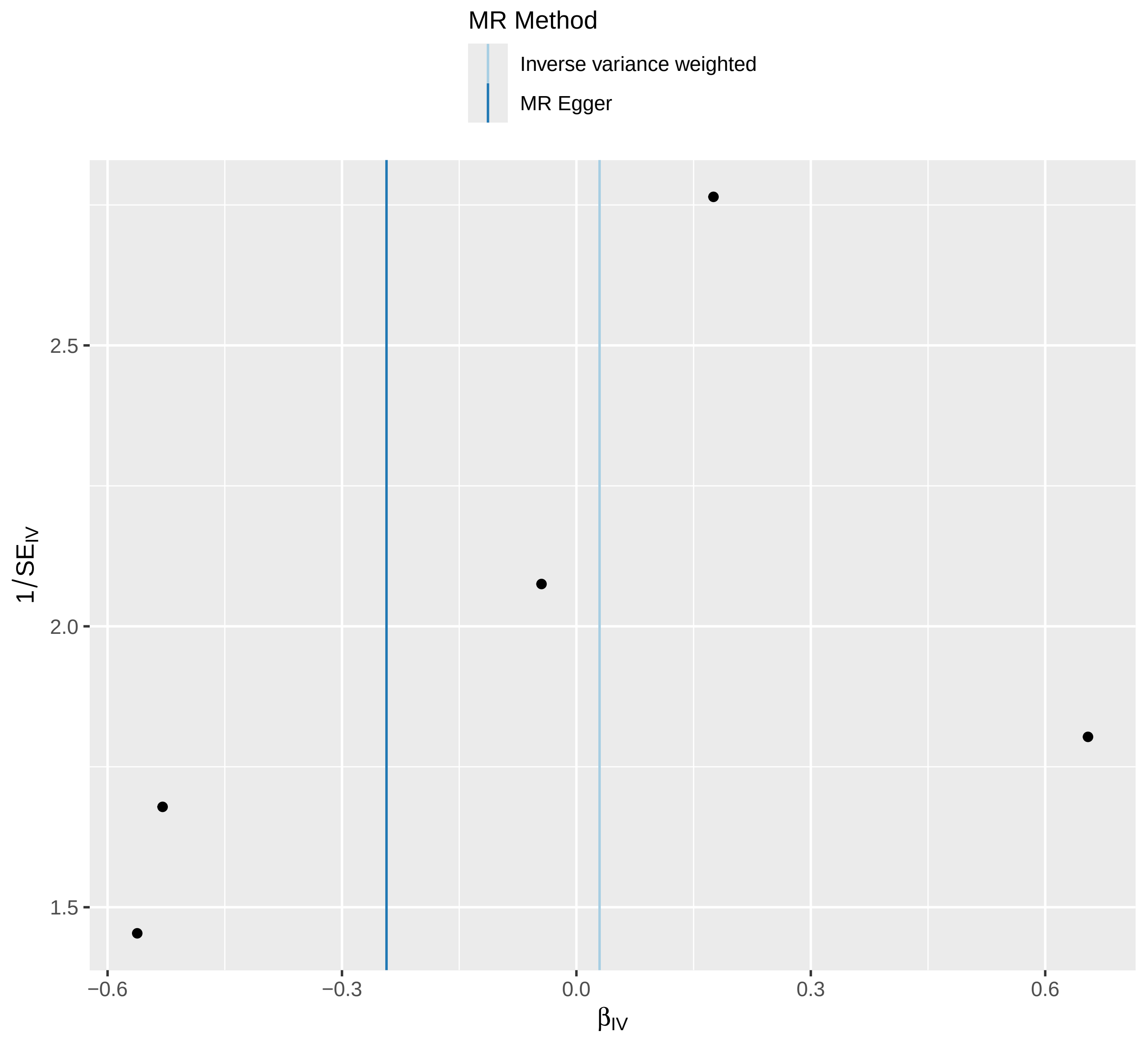
MR Method
Inverse variance weighted
MR Egger
1/SE_IV
2.5
2.0
1.5
−0.6
−0.3
0.0
0.3
0.6
β_IV

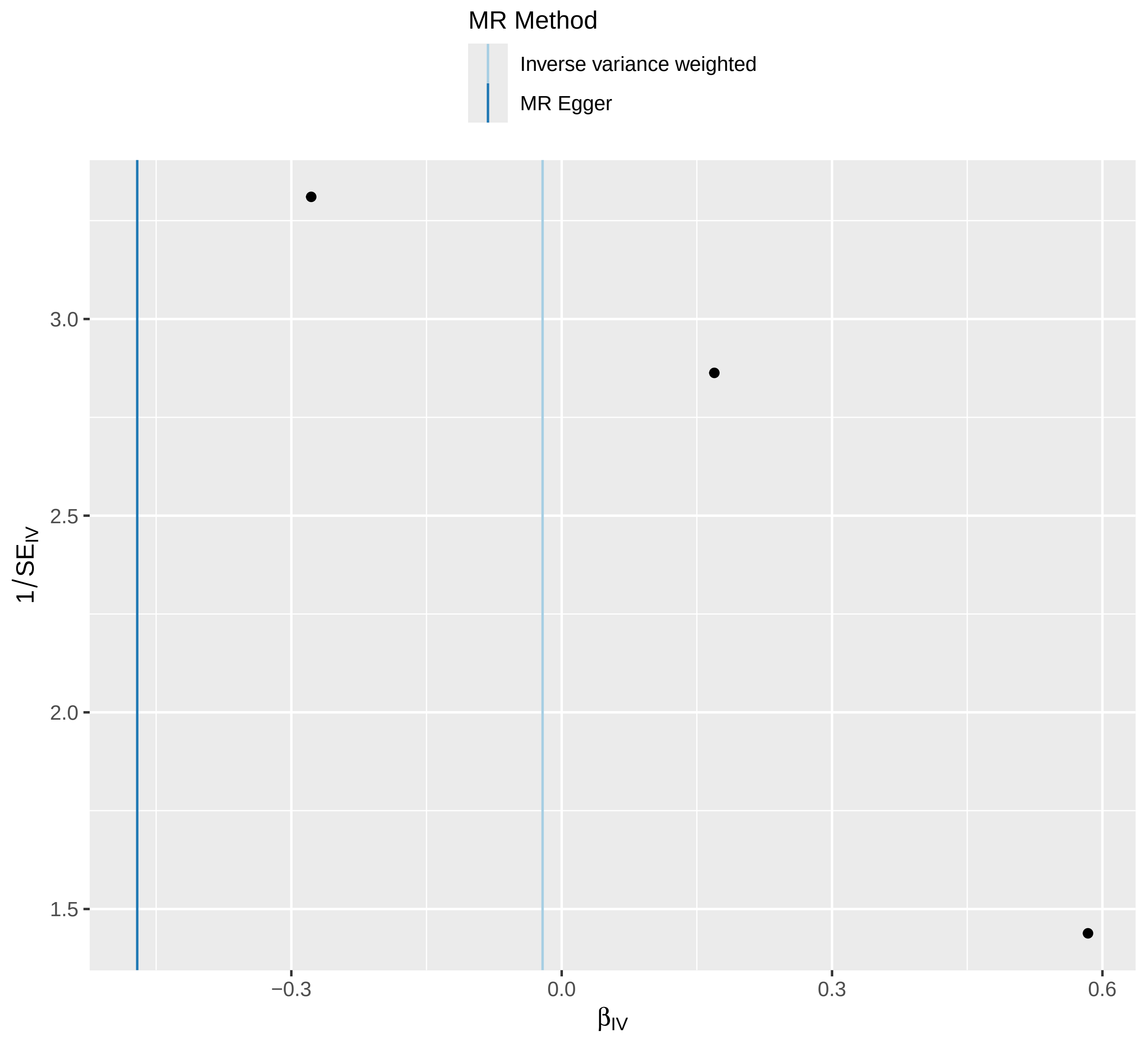

MR Method
Inverse variance weighted
MR Egger
1/SE_IV
3.0
2.5
2.0
1.5
−0.3
0.0
0.3
0.6
β_IV

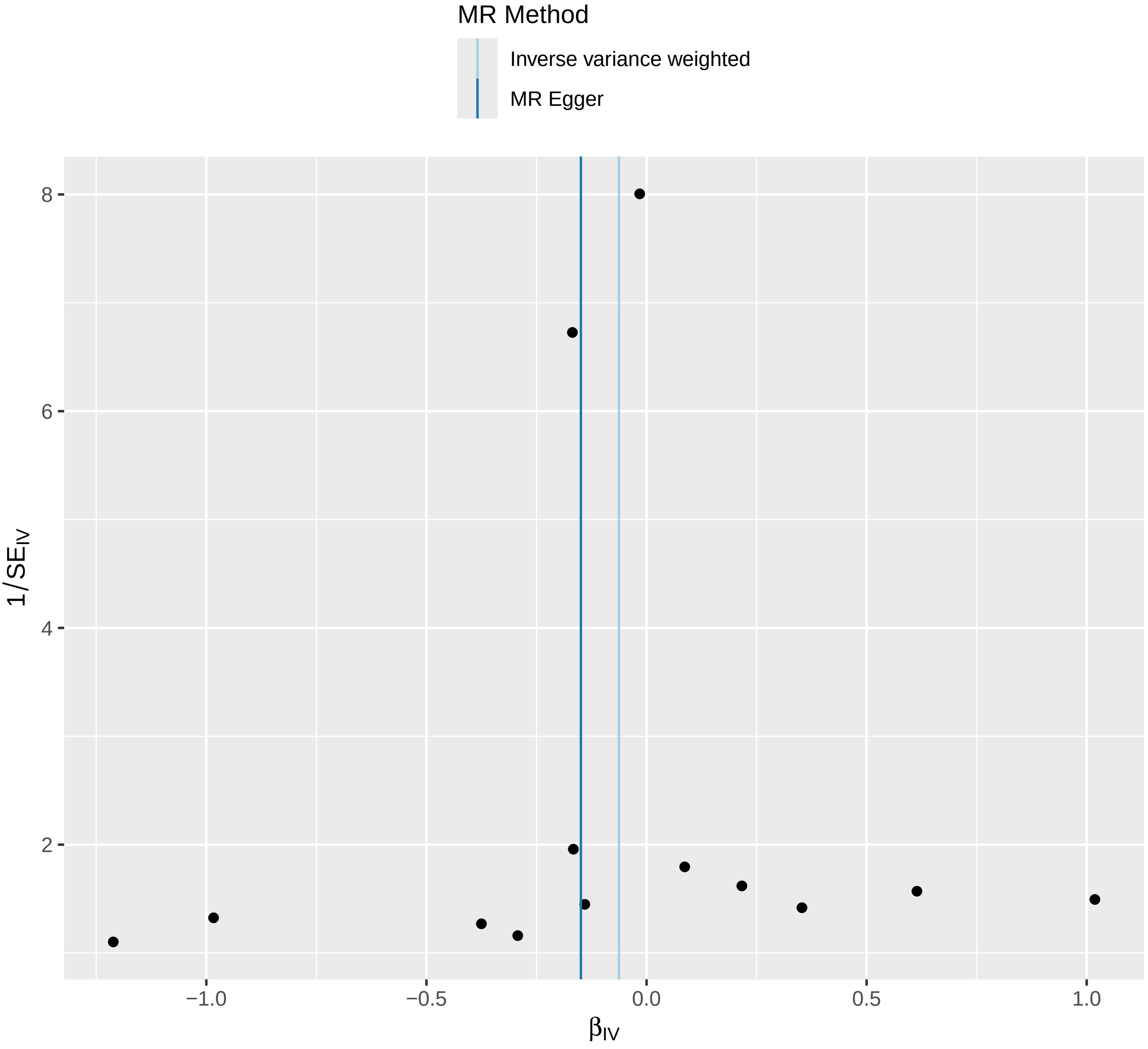
MR Method
Inverse variance weighted
MR Egger
8
6
4
2
$1/SE_{IV}$
−1.0
−0.5
0.0
0.5
1.0
β_{IV}

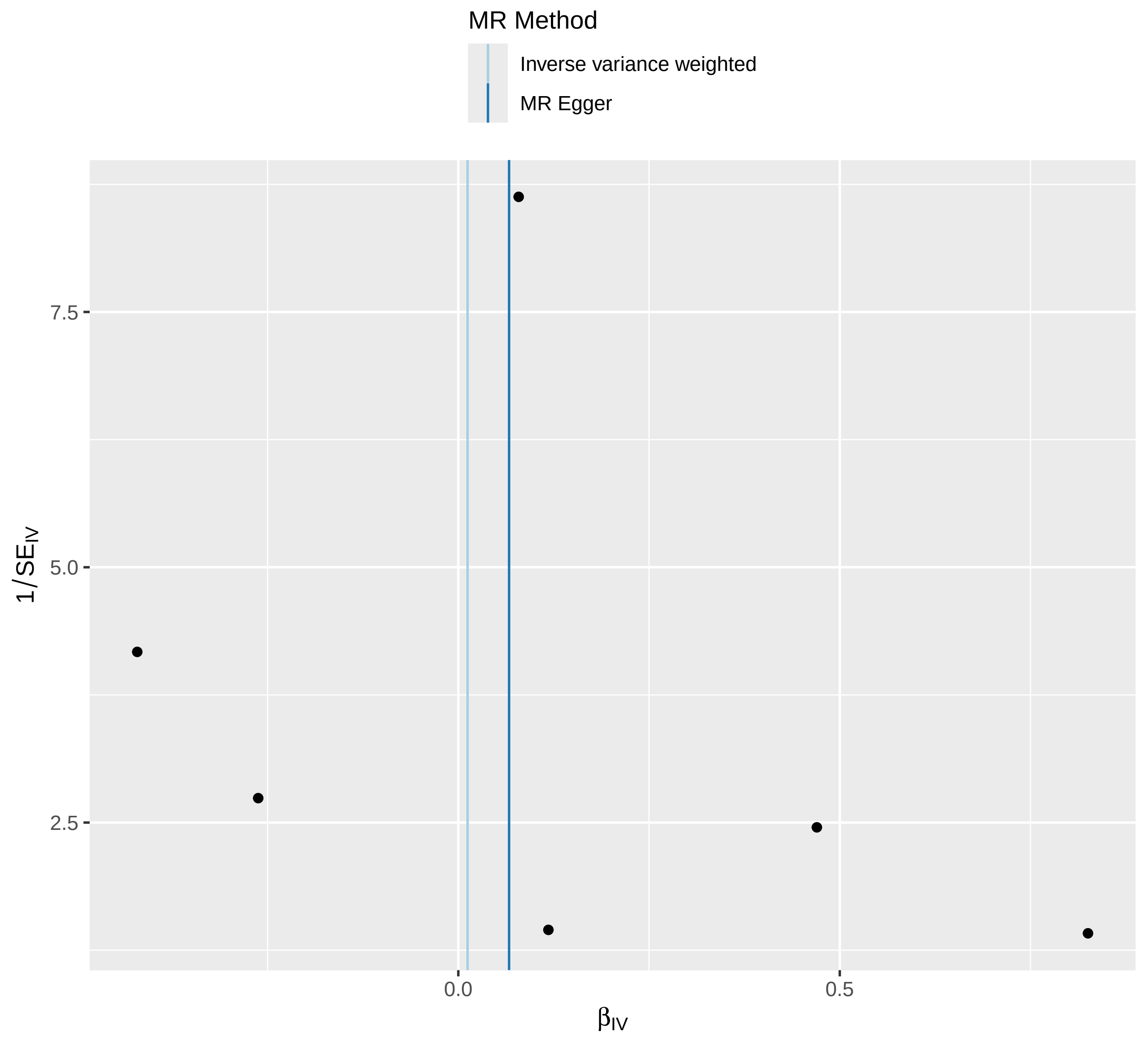

MR Method
Inverse variance weighted
MR Egger
7.5
5.0
2.5
1/SE_IV
0.0
0.5
β_IV

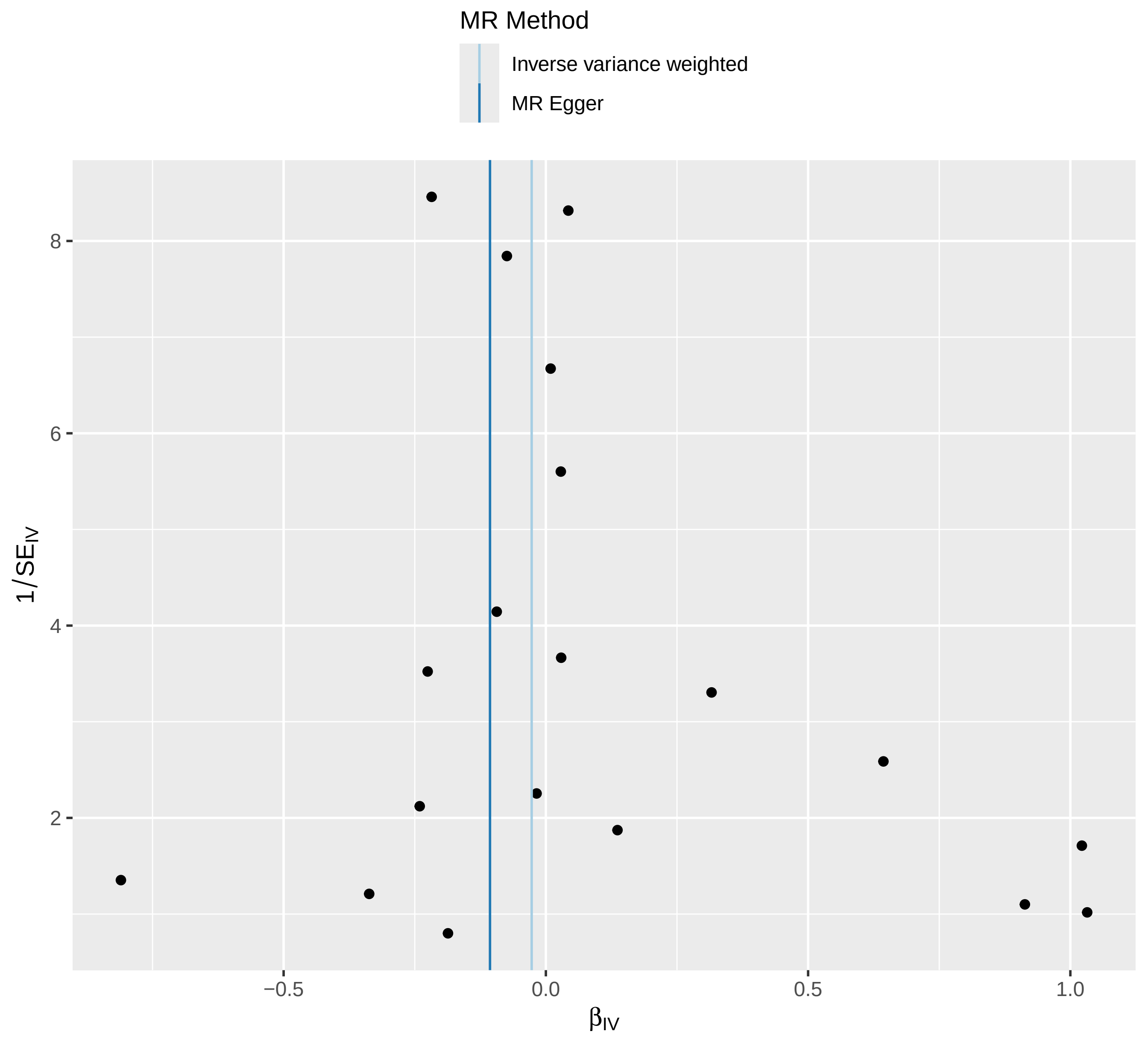

MR Method
Inverse variance weighted
MR Egger
8
6
4
2
1/SE$_{IV}$
−0.5
0.0
0.5
1.0
β$_{IV}$

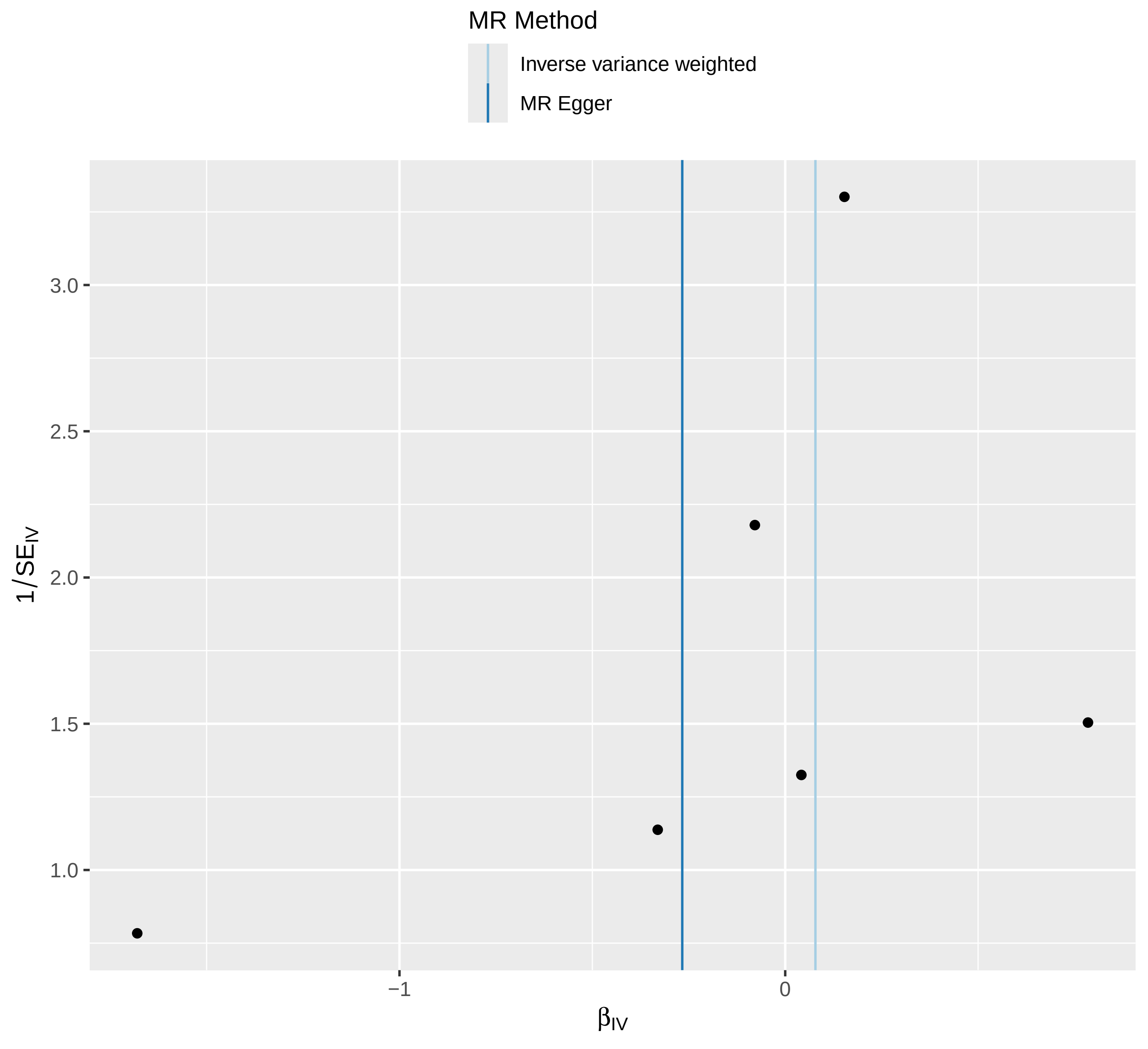
MR Method
Inverse variance weighted
MR Egger
3.0
2.5
2.0
1.5
1.0
1/SE$_{IV}$
−1
0
β$_{IV}$

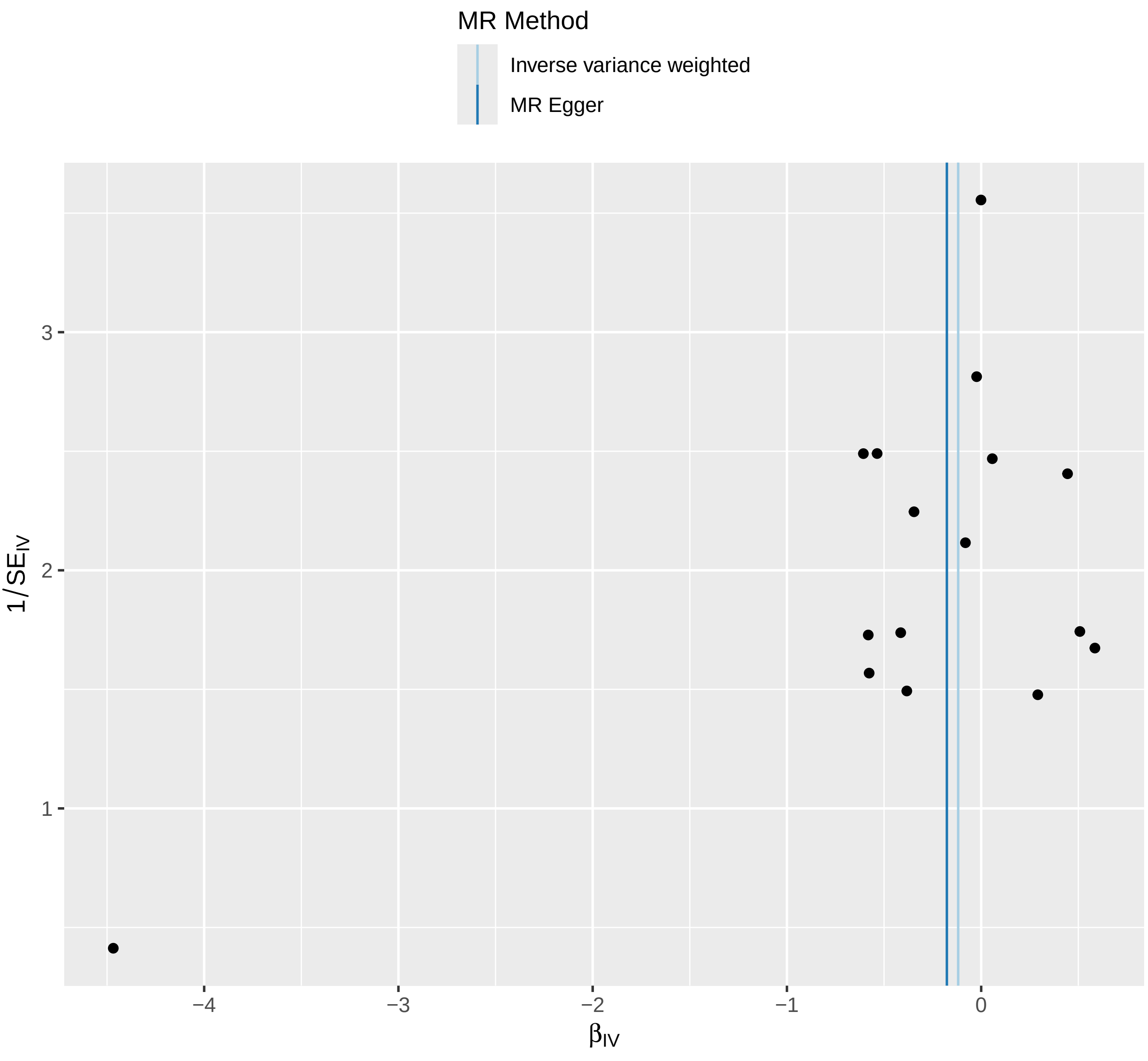
MR Method
Inverse variance weighted
MR Egger
3
2
1
1/SE_IV
−4
−3
−2
−1
0
β_IV

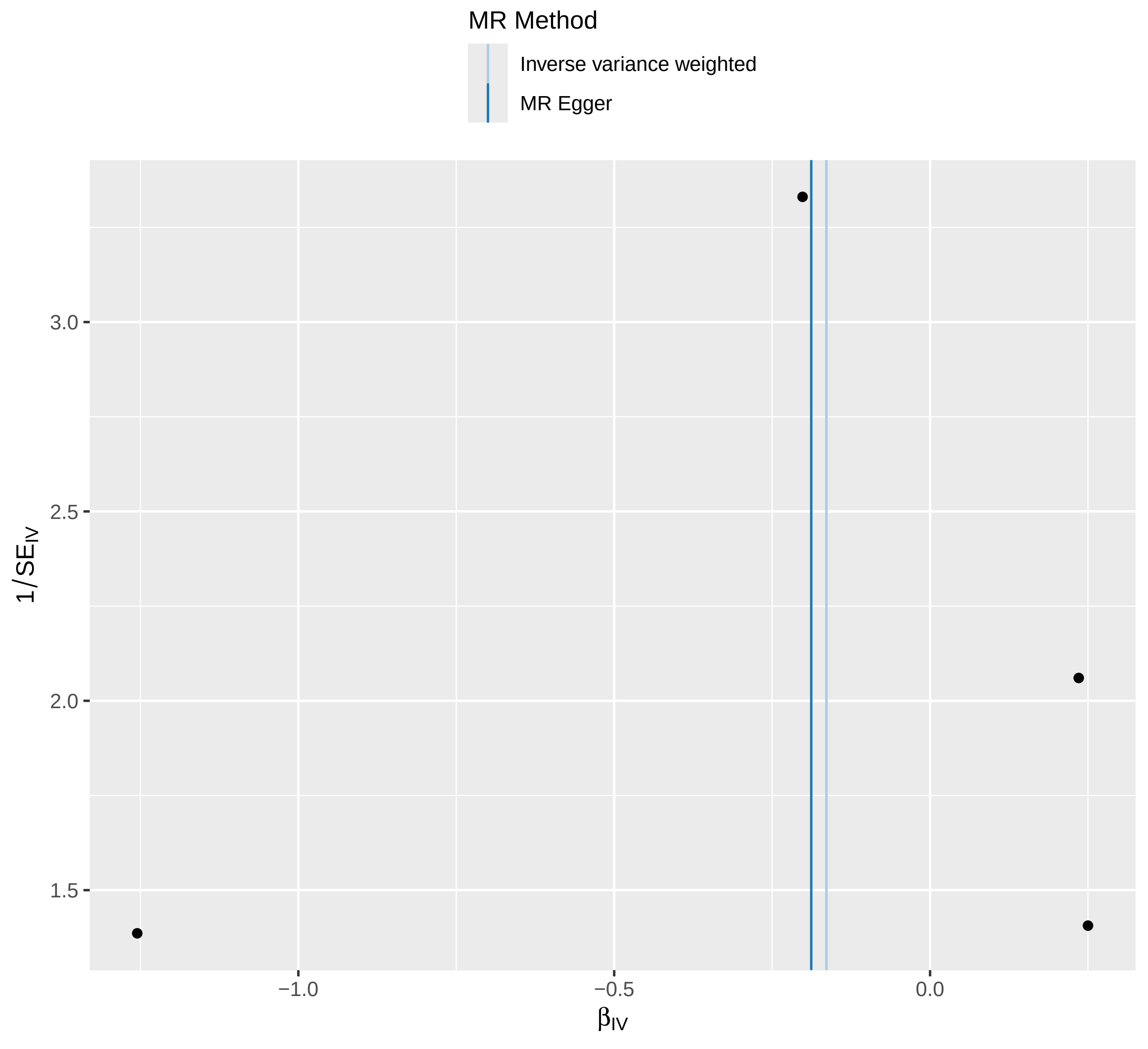
MR Method
Inverse variance weighted
MR Egger
1/SE$_{IV}$
3.0
2.5
2.0
1.5
−1.0
−0.5
0.0
β$_{IV}$

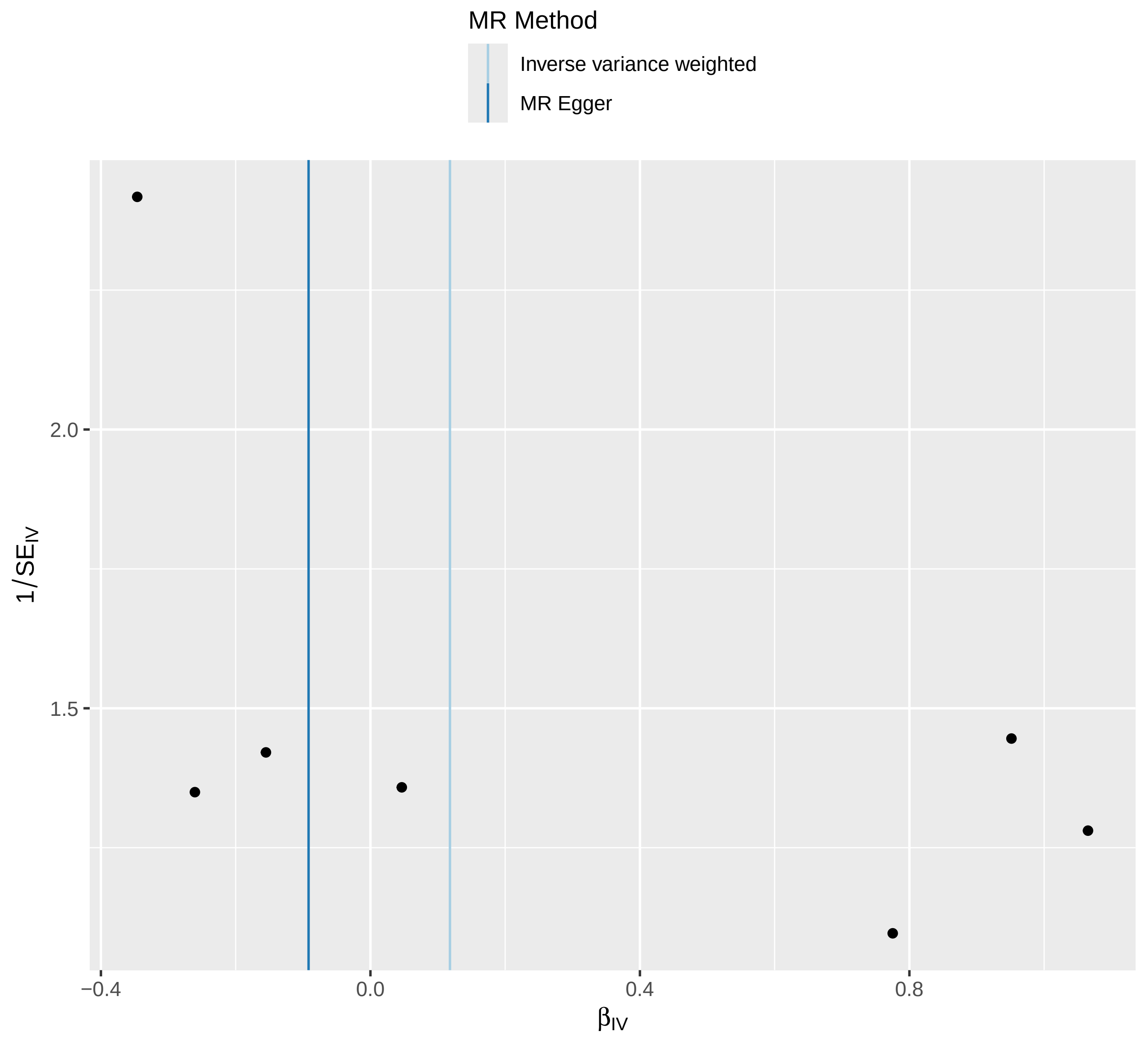

MR Method
Inverse variance weighted
MR Egger
2.0
1.5
$1/SE_{IV}$
−0.4
0.0
0.4
0.8
β_{IV}

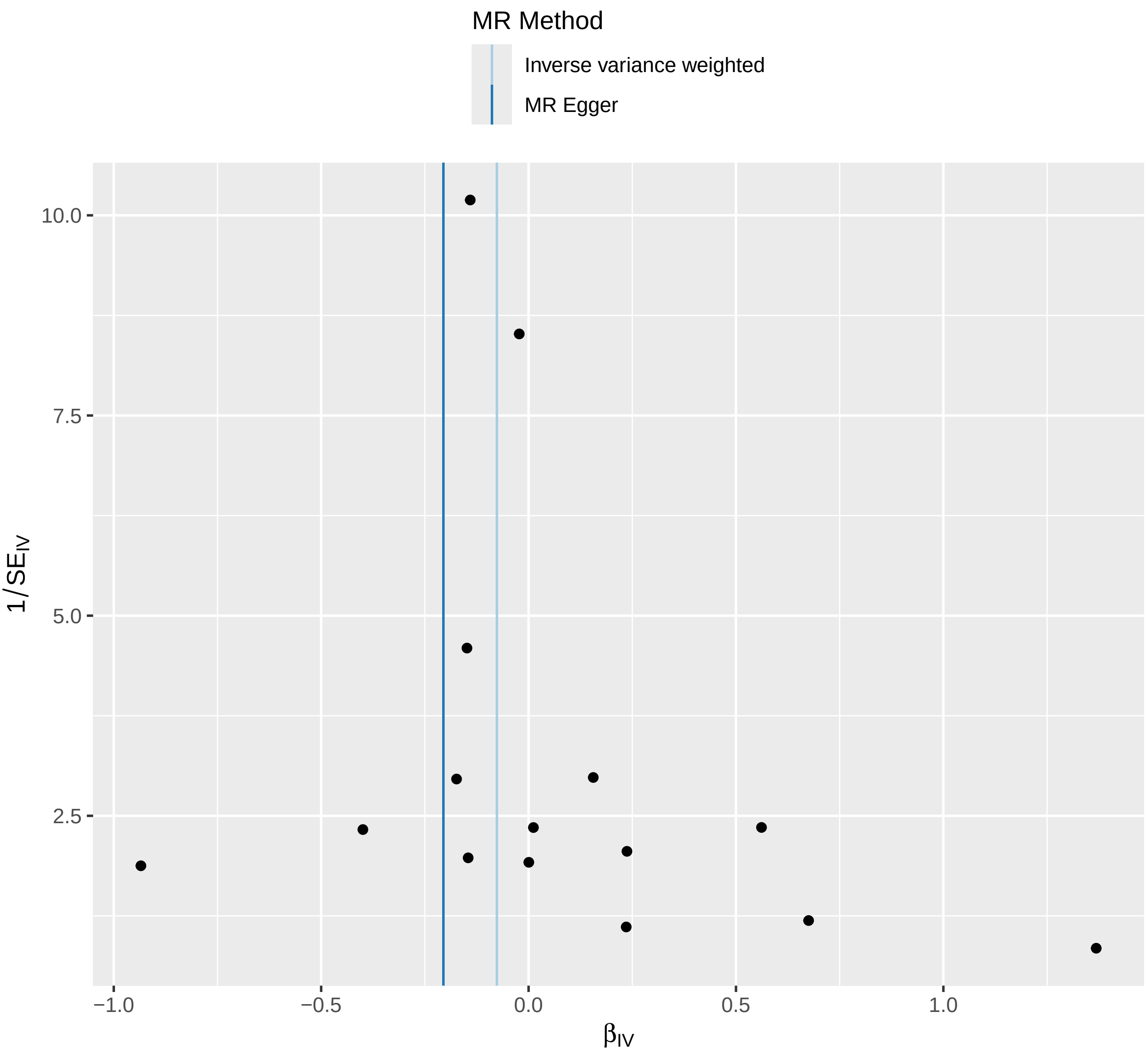

MR Method
Inverse variance weighted
MR Egger
10.0
7.5
5.0
2.5
1/SE_IV
−1.0
−0.5
0.0
0.5
1.0
β_IV

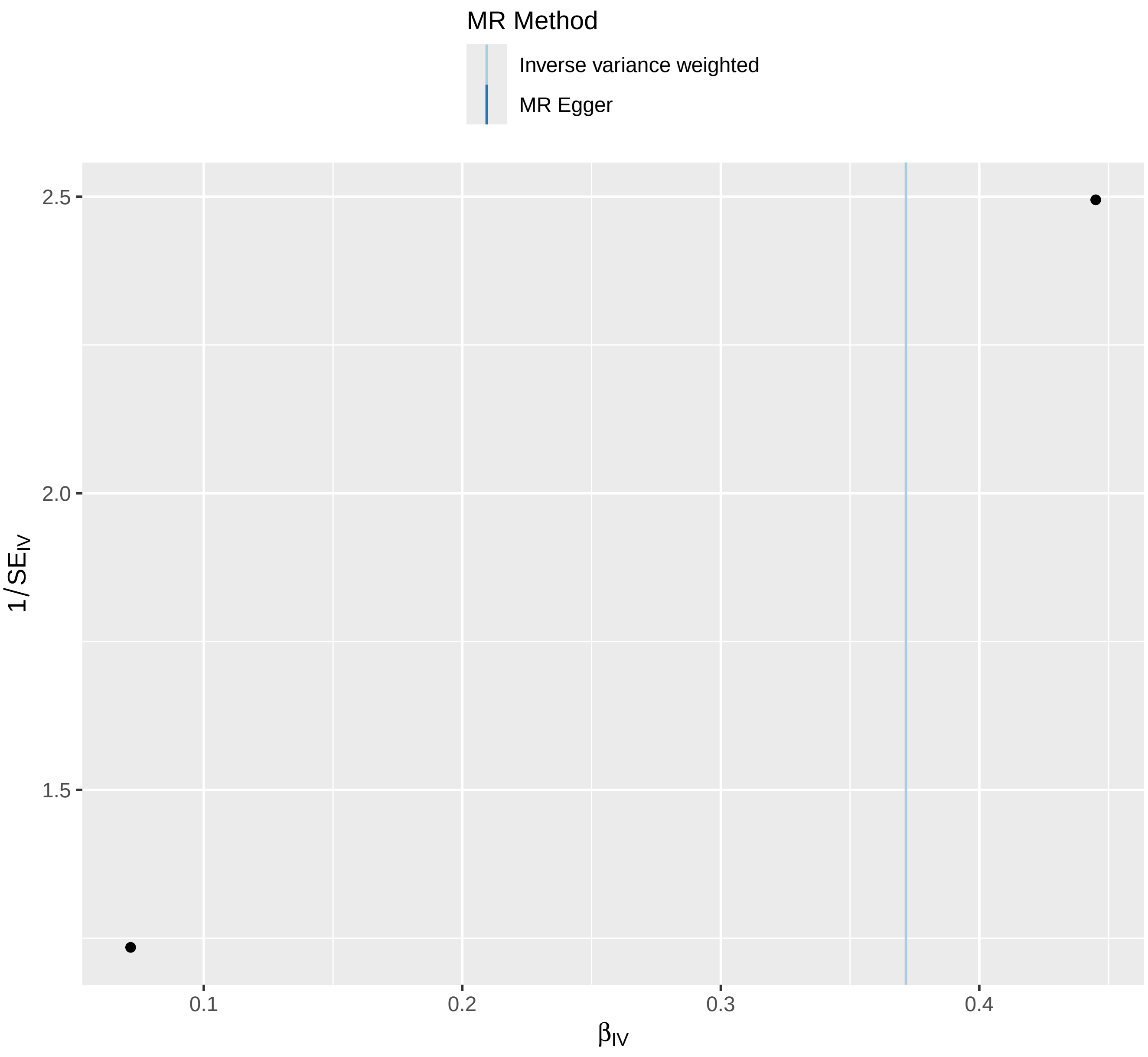

MR Method
Inverse variance weighted
MR Egger
2.5
2.0
1.5
1/SE_IV
0.1
0.2
0.3
0.4
β_IV

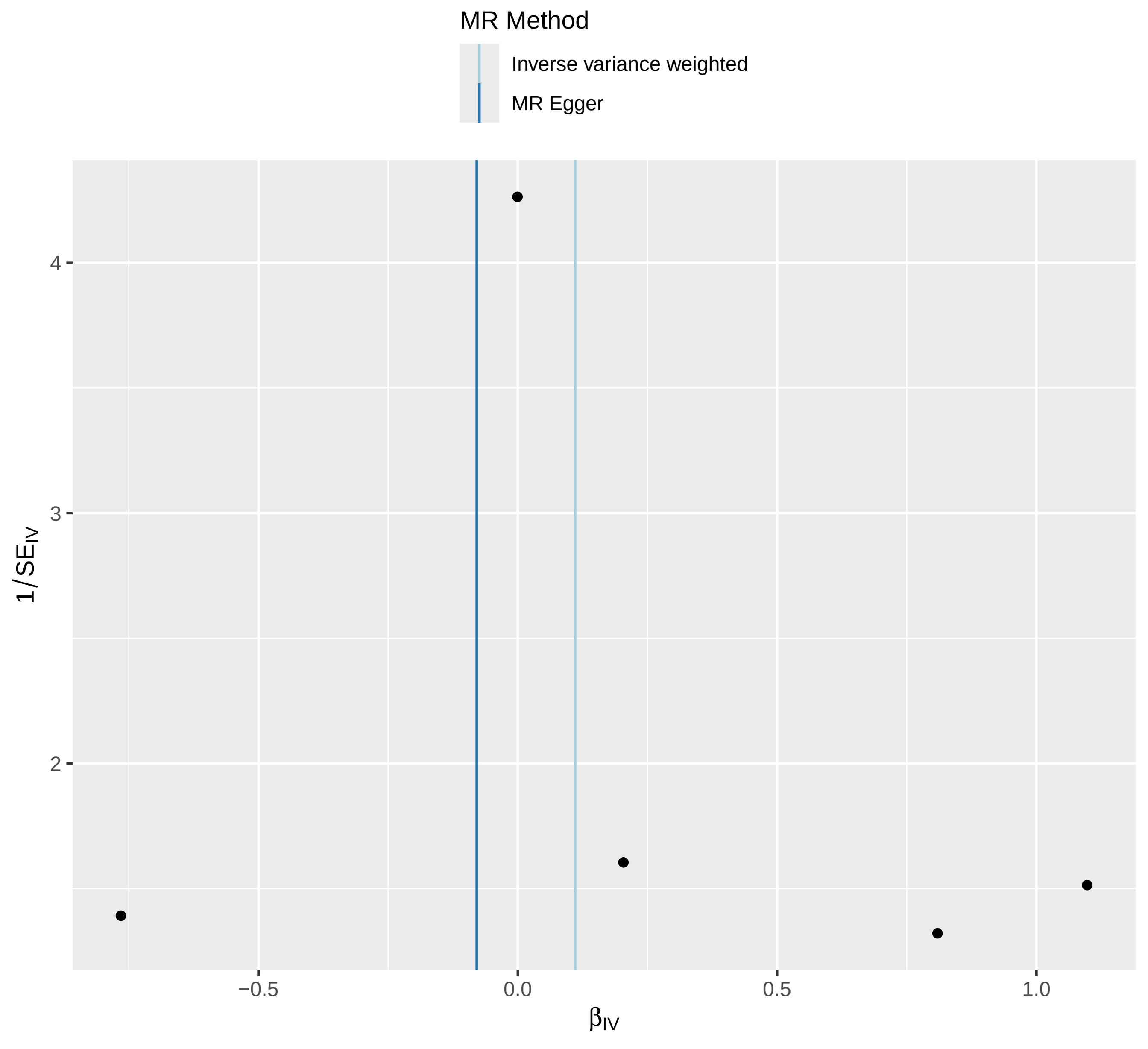
MR Method
Inverse variance weighted
MR Egger
4
3
2
1/SE_IV
−0.5
0.0
0.5
1.0
β_IV

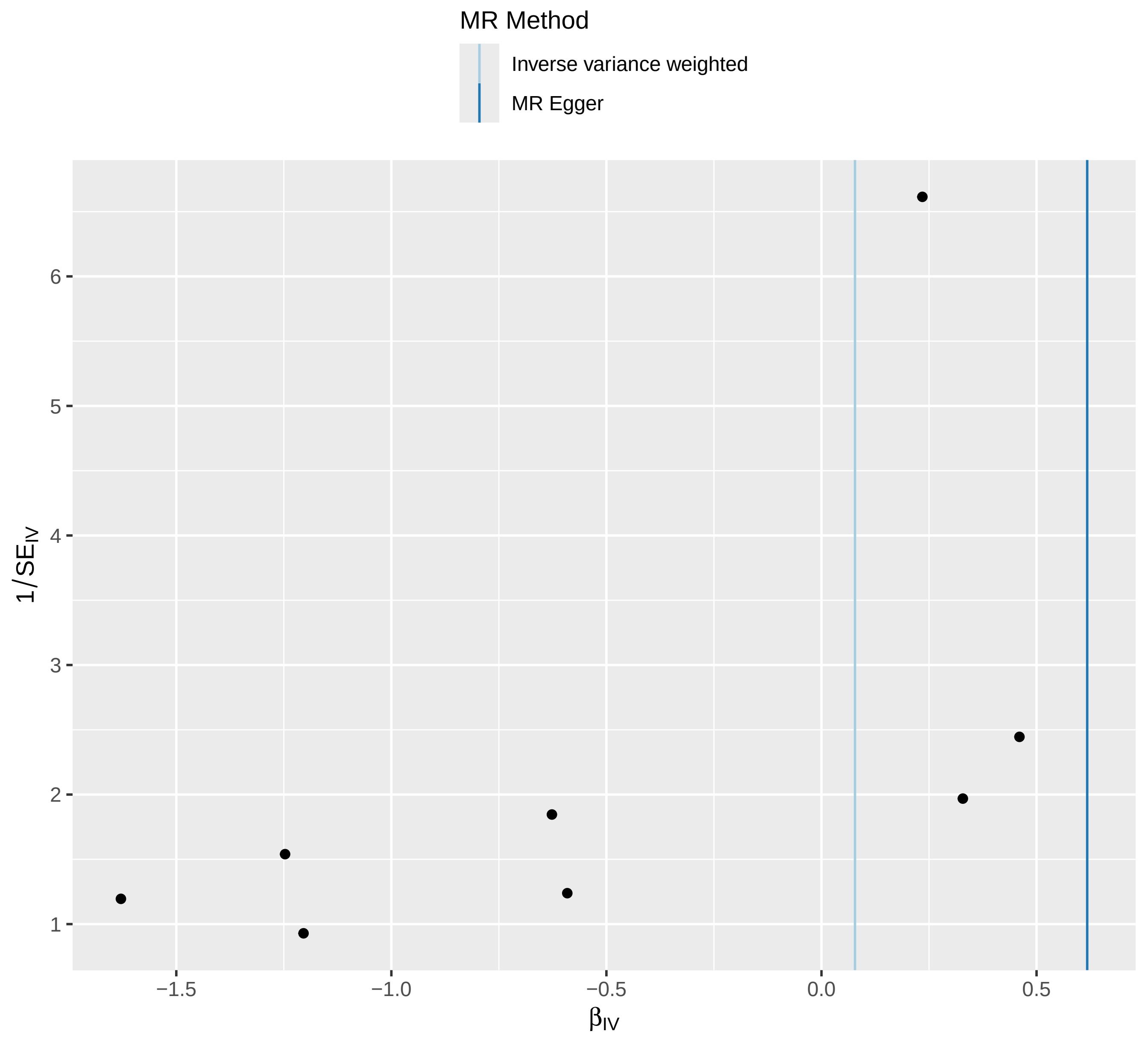

MR Method
Inverse variance weighted
MR Egger
1/SE$_{IV}$
β_{IV}
−1.5
−1.0
−0.5
0.0
0.5
1
2
3
4
5
6

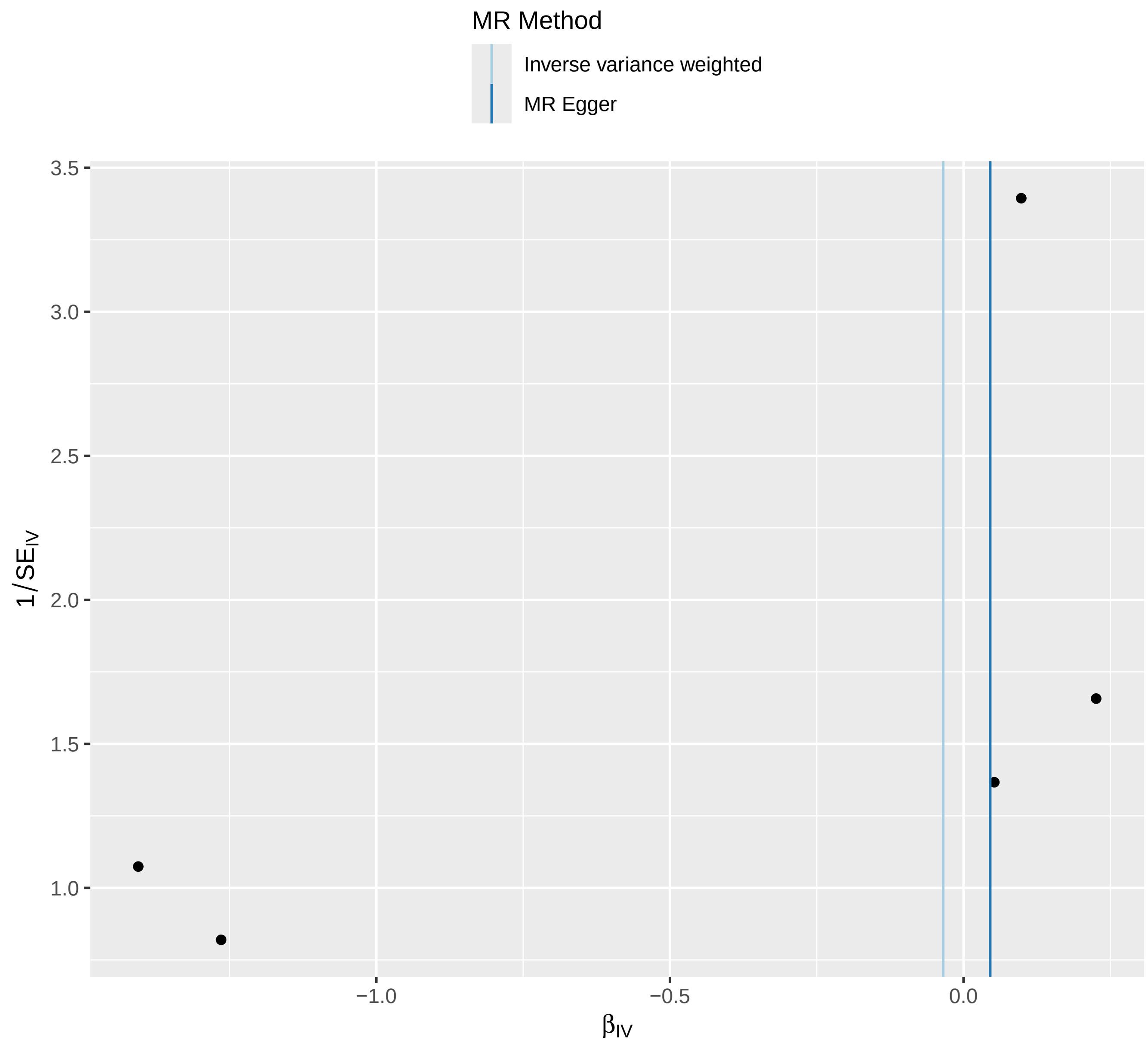

MR Method
Inverse variance weighted
MR Egger
3.5
3.0
2.5
2.0
1.5
1.0
1/SE$_{IV}$
−1.0
−0.5
0.0
β$_{IV}$

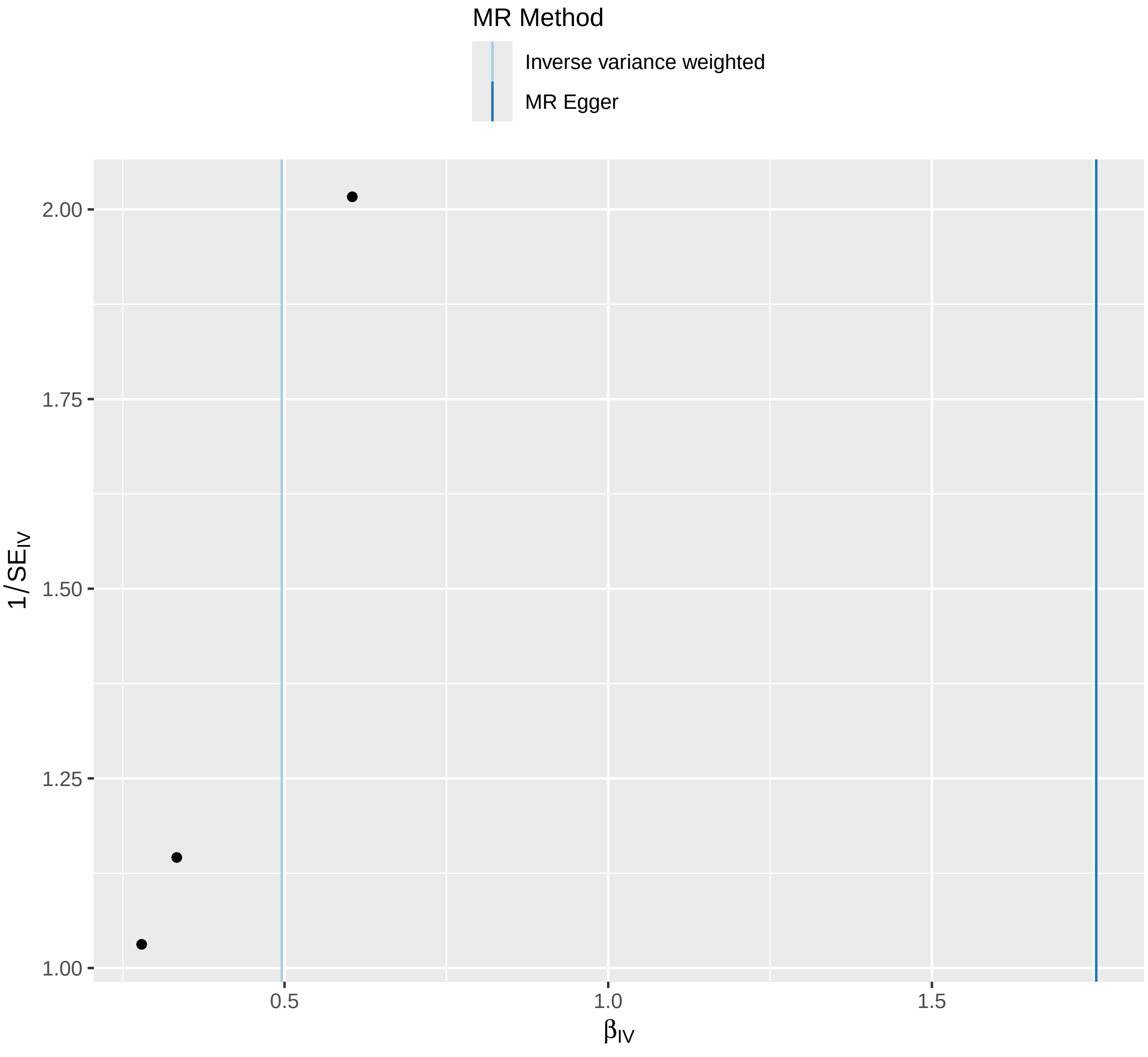
MR Method
Inverse variance weighted
MR Egger
2.00
1.75
1.50
1.25
1.00
1/SE_IV
0.5
1.0
1.5
β_IV

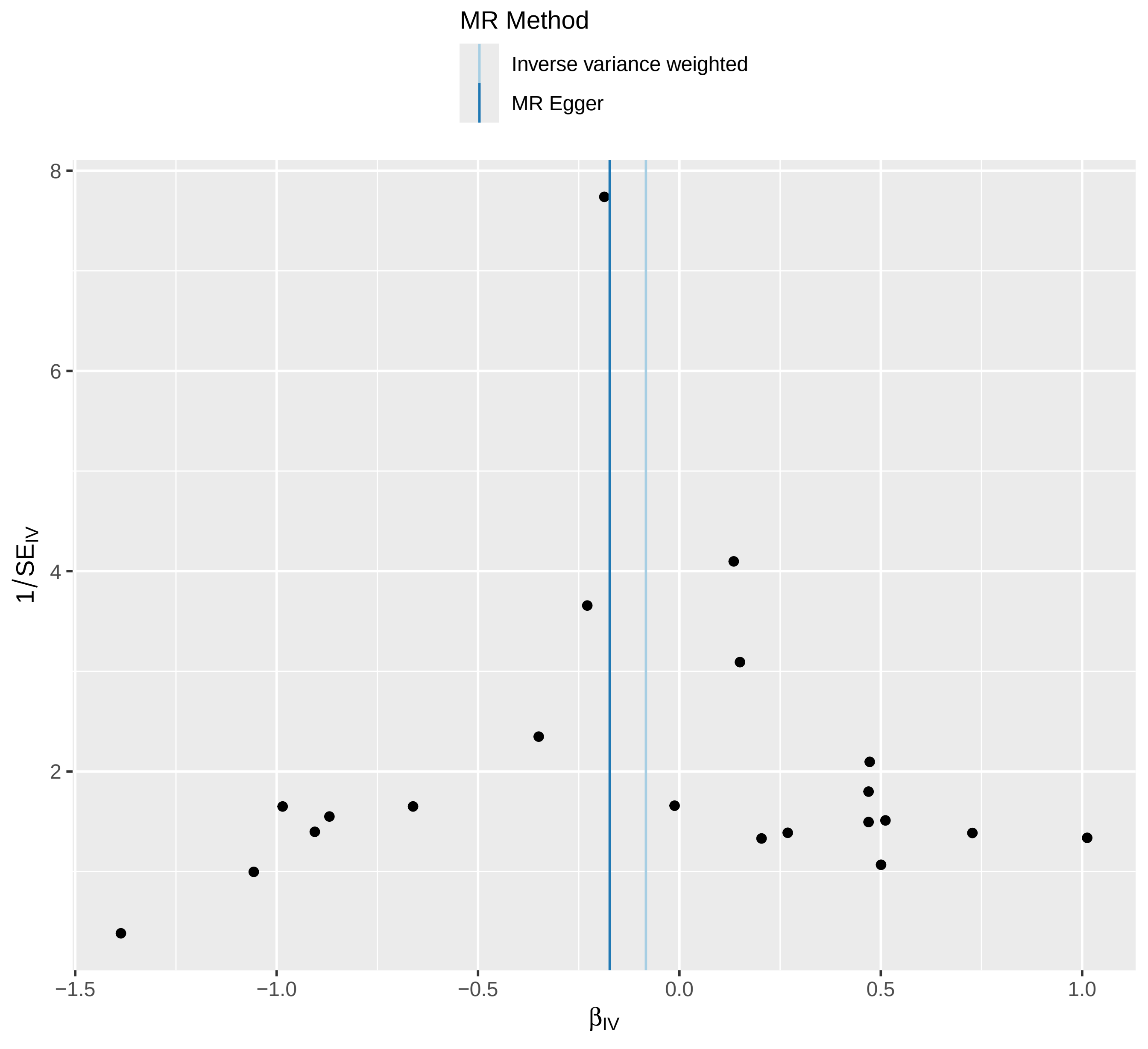

MR Method
Inverse variance weighted
MR Egger
8
6
4
2
1/SE_IV
−1.5
−1.0
−0.5
0.0
0.5
1.0
β_IV

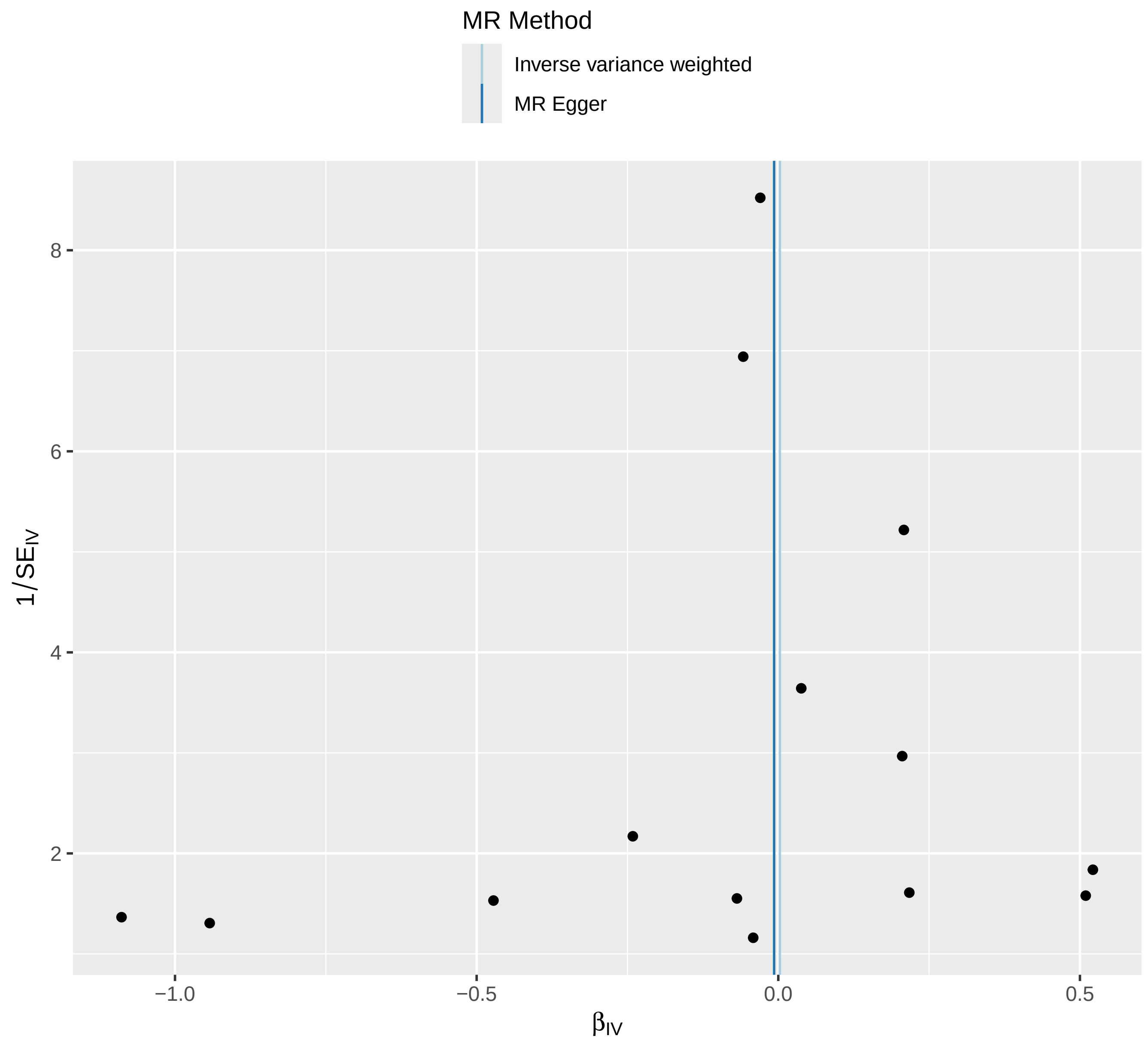

MR Method
Inverse variance weighted
MR Egger
8
6
4
2
1/SE_IV
−1.0
−0.5
0.0
0.5
β_IV

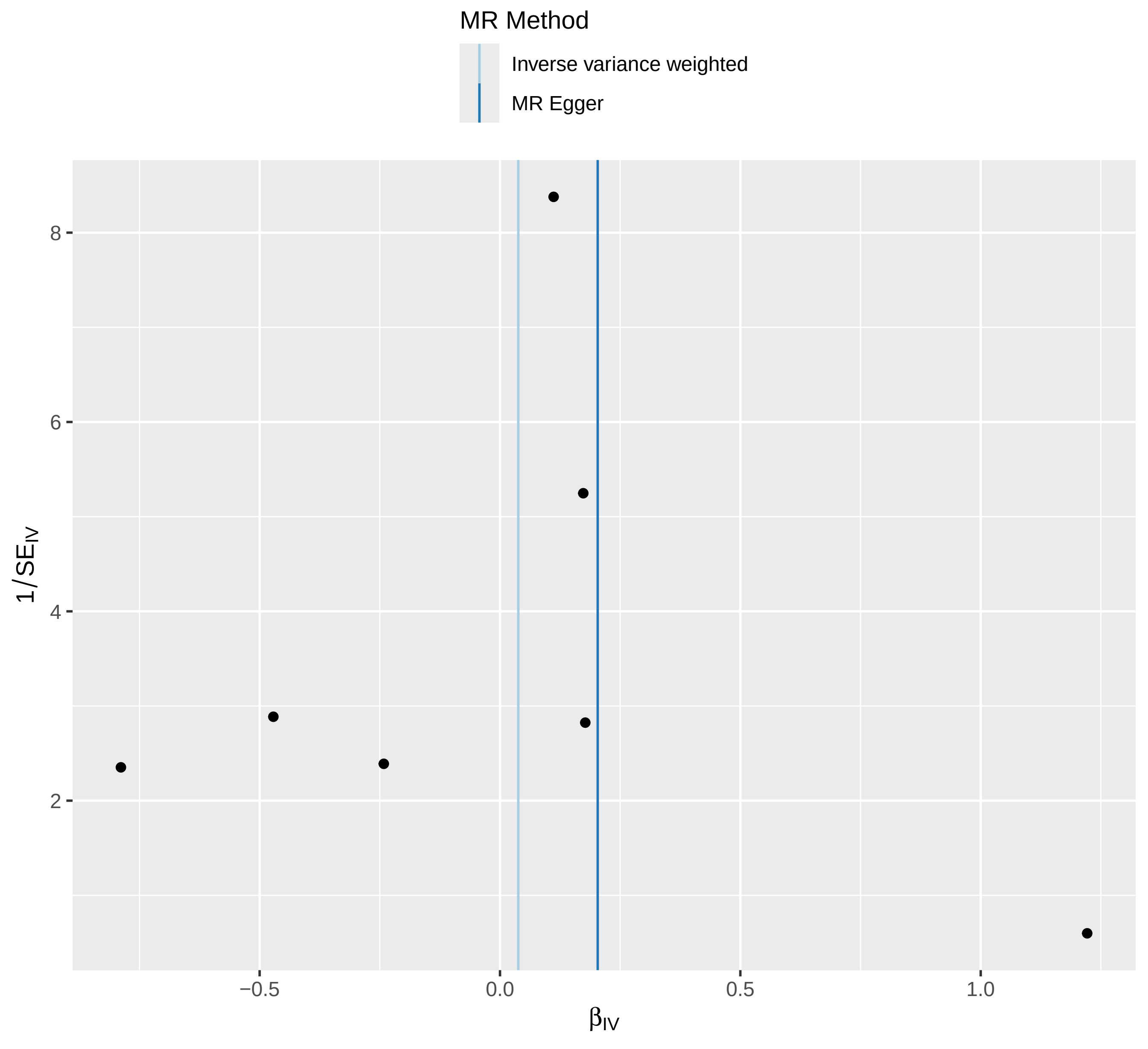
MR Method
Inverse variance weighted
MR Egger
8
6
4
2
$1/SE_{IV}$
−0.5
0.0
0.5
1.0
β_{IV}

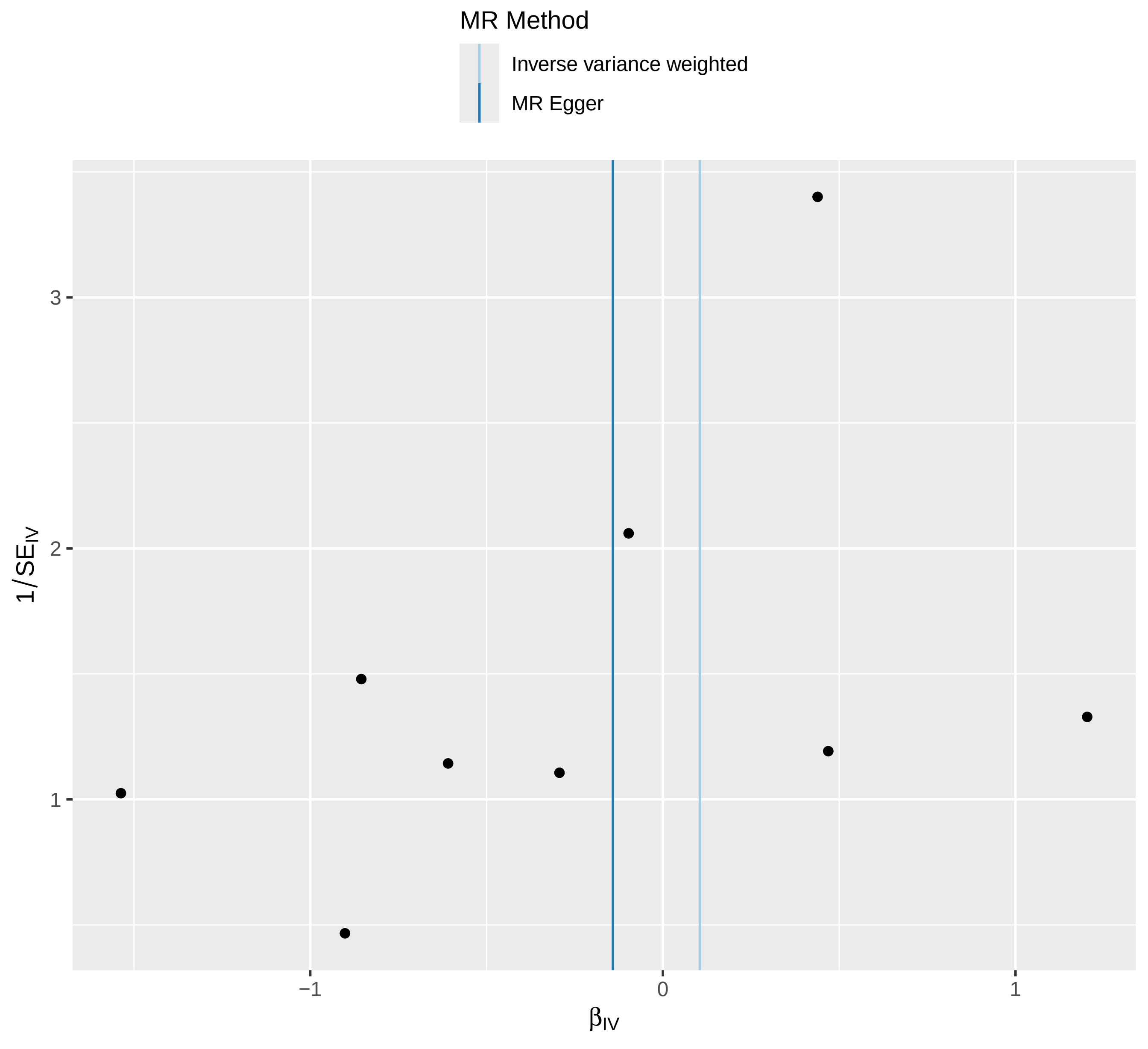

MR Method
Inverse variance weighted
MR Egger
3
2
1
−1
0
1
$1/SE_{IV}$
β_{IV}

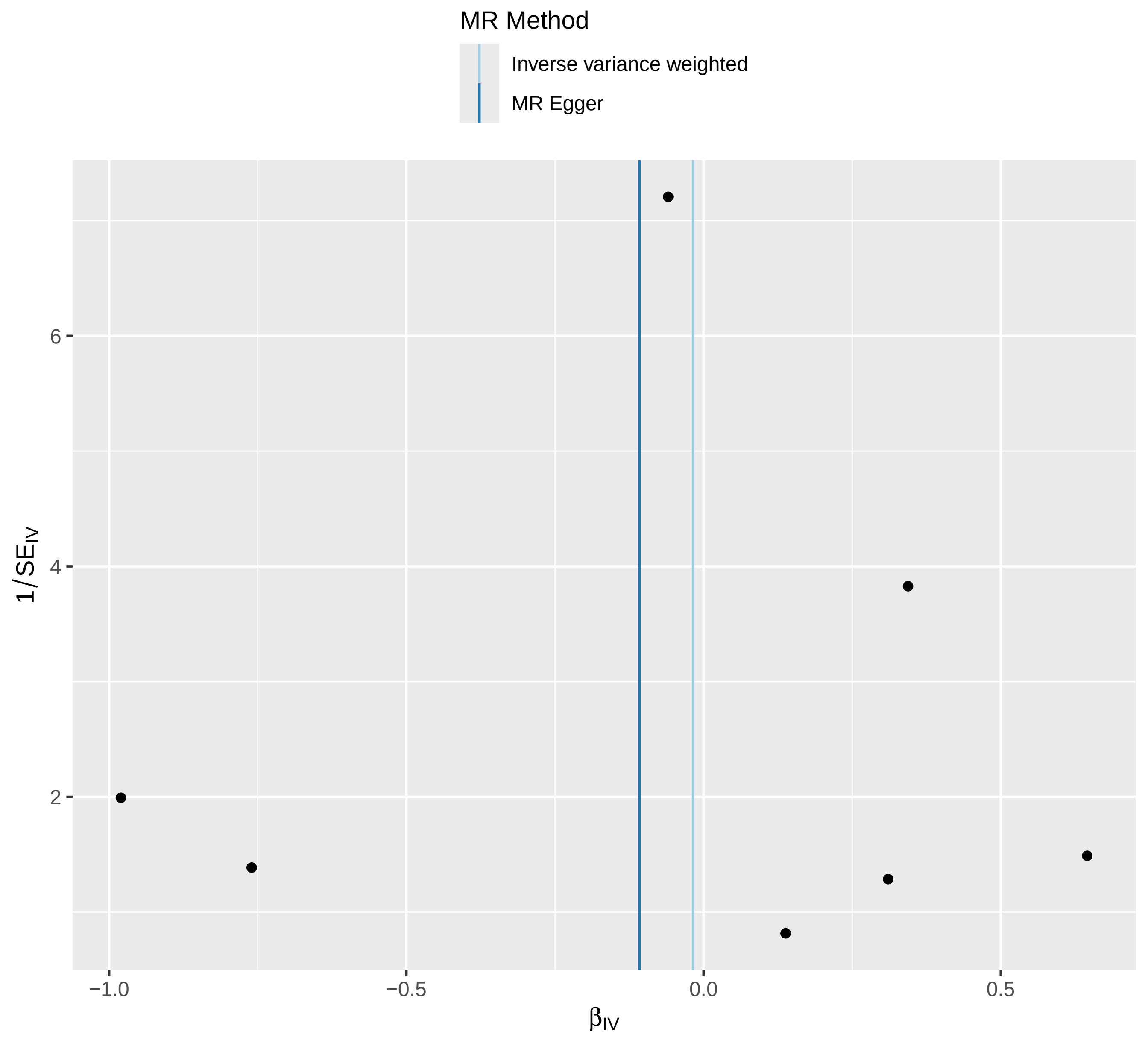

MR Method
Inverse variance weighted
MR Egger
6
4
2
1/SE_IV
−1.0
−0.5
0.0
0.5
β_IV

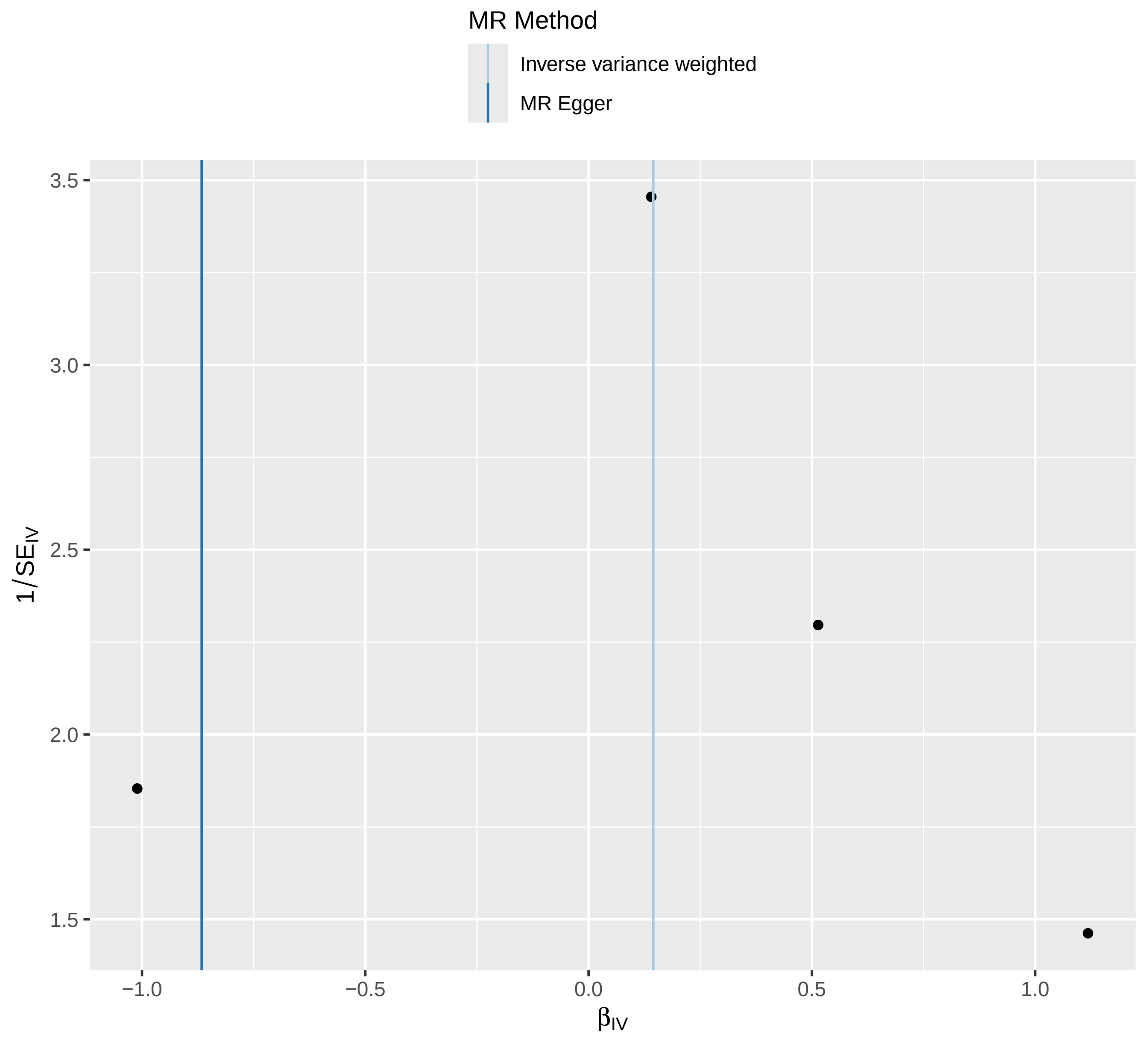

MR Method
Inverse variance weighted
MR Egger
3.5
3.0
2.5
2.0
1.5
$1/SE_{IV}$
−1.0
−0.5
0.0
0.5
1.0
β_{IV}

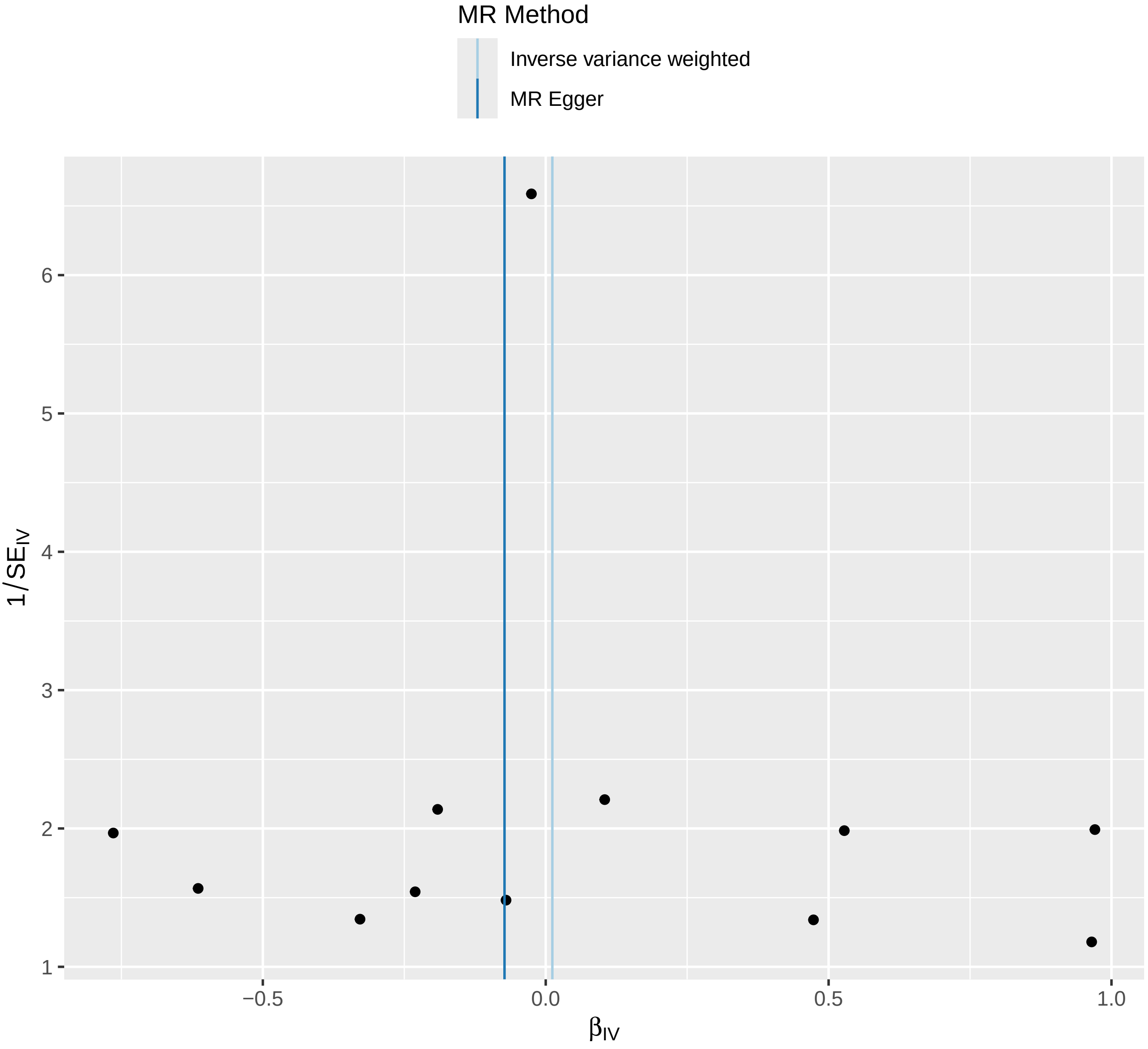
MR Method
Inverse variance weighted
MR Egger
6
5
4
3
2
1
1/SE_IV
−0.5
0.0
0.5
1.0
β_IV

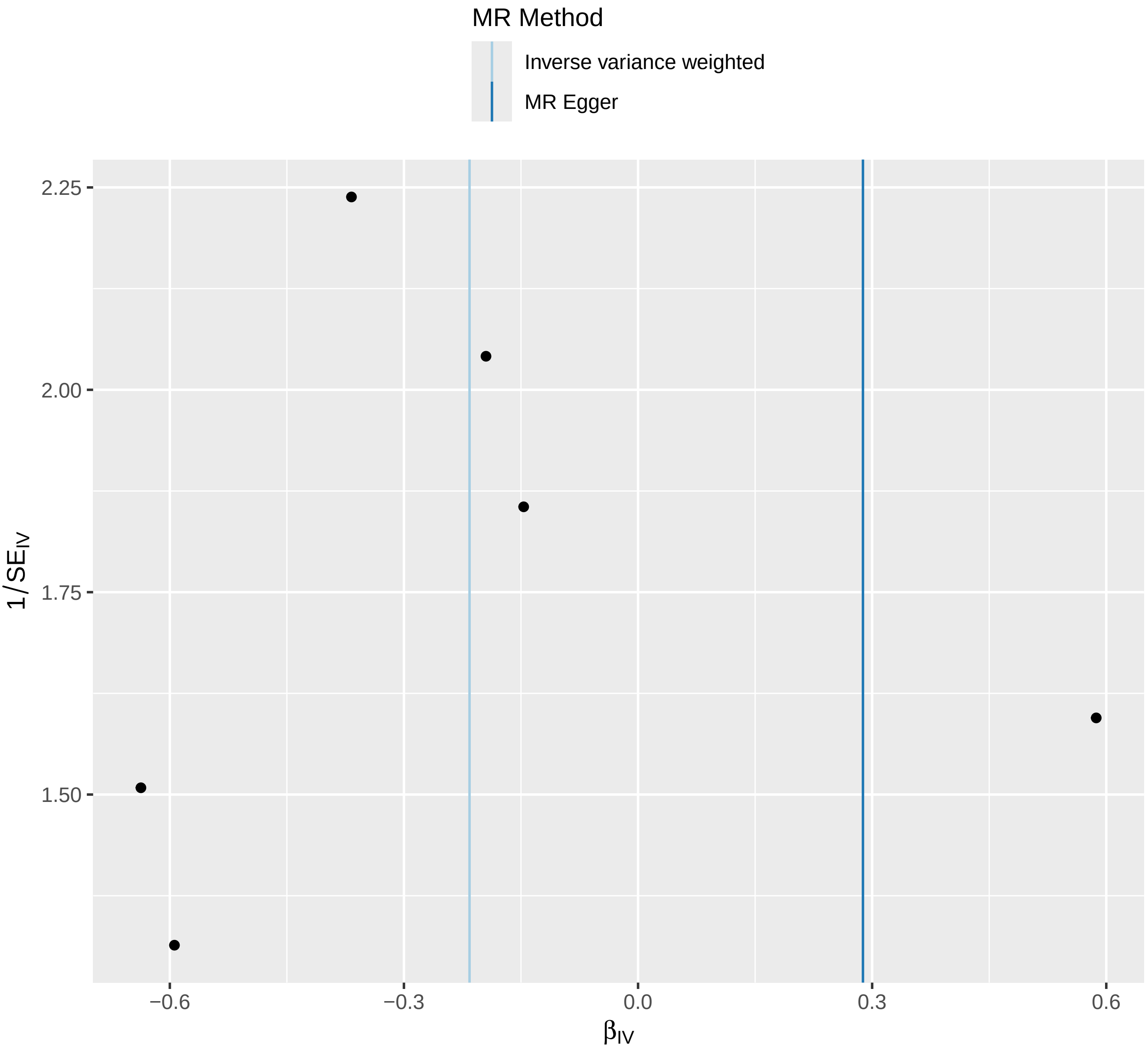

MR Method
Inverse variance weighted
MR Egger
2.25
2.00
1.75
1.50
1/SE_IV
−0.6
−0.3
0.0
0.3
0.6
β_IV

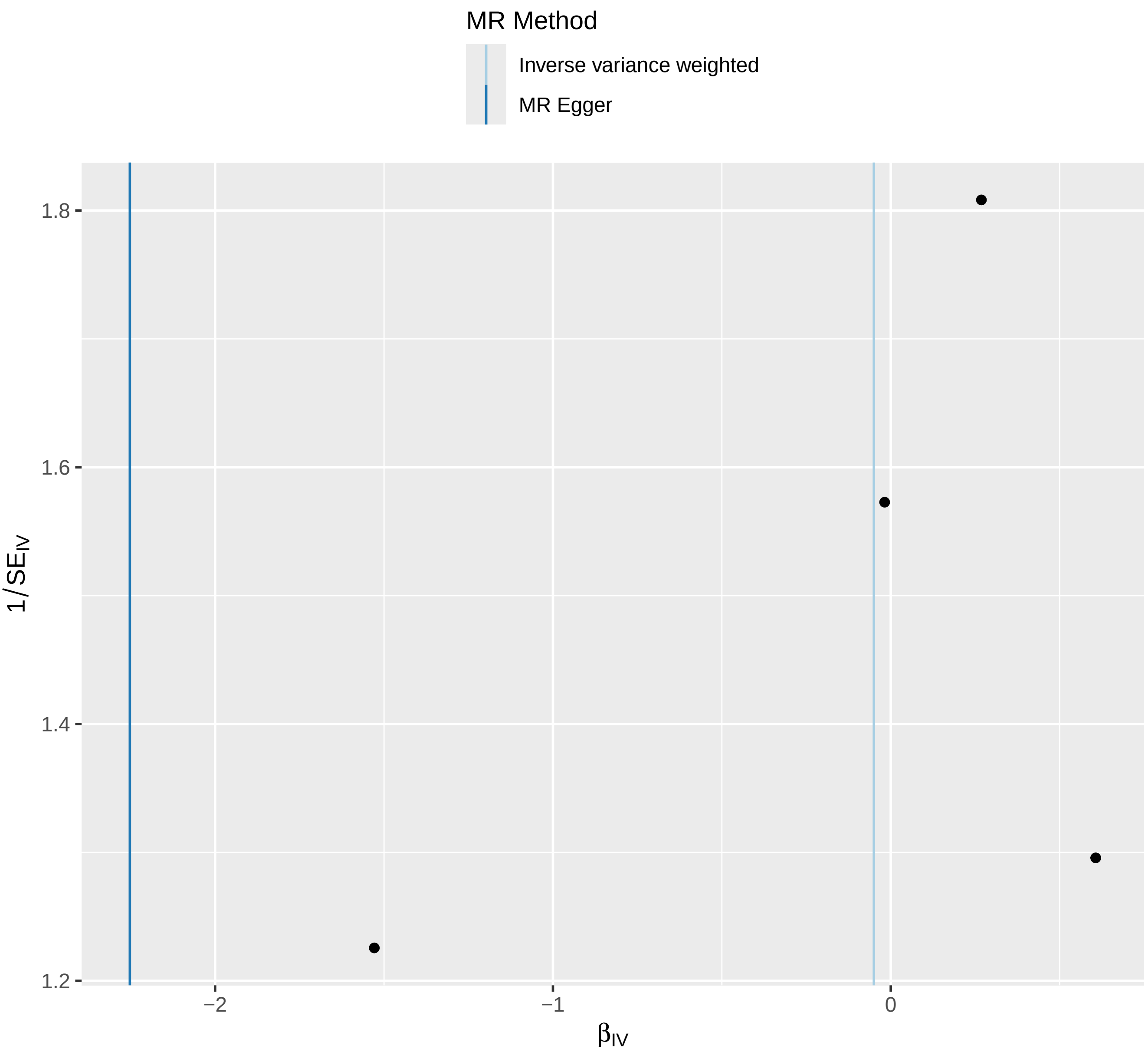

MR Method
Inverse variance weighted
MR Egger
1.8
1.6
1.4
1.2
1/SE$_{IV}$
−2
−1
0
β$_{IV}$

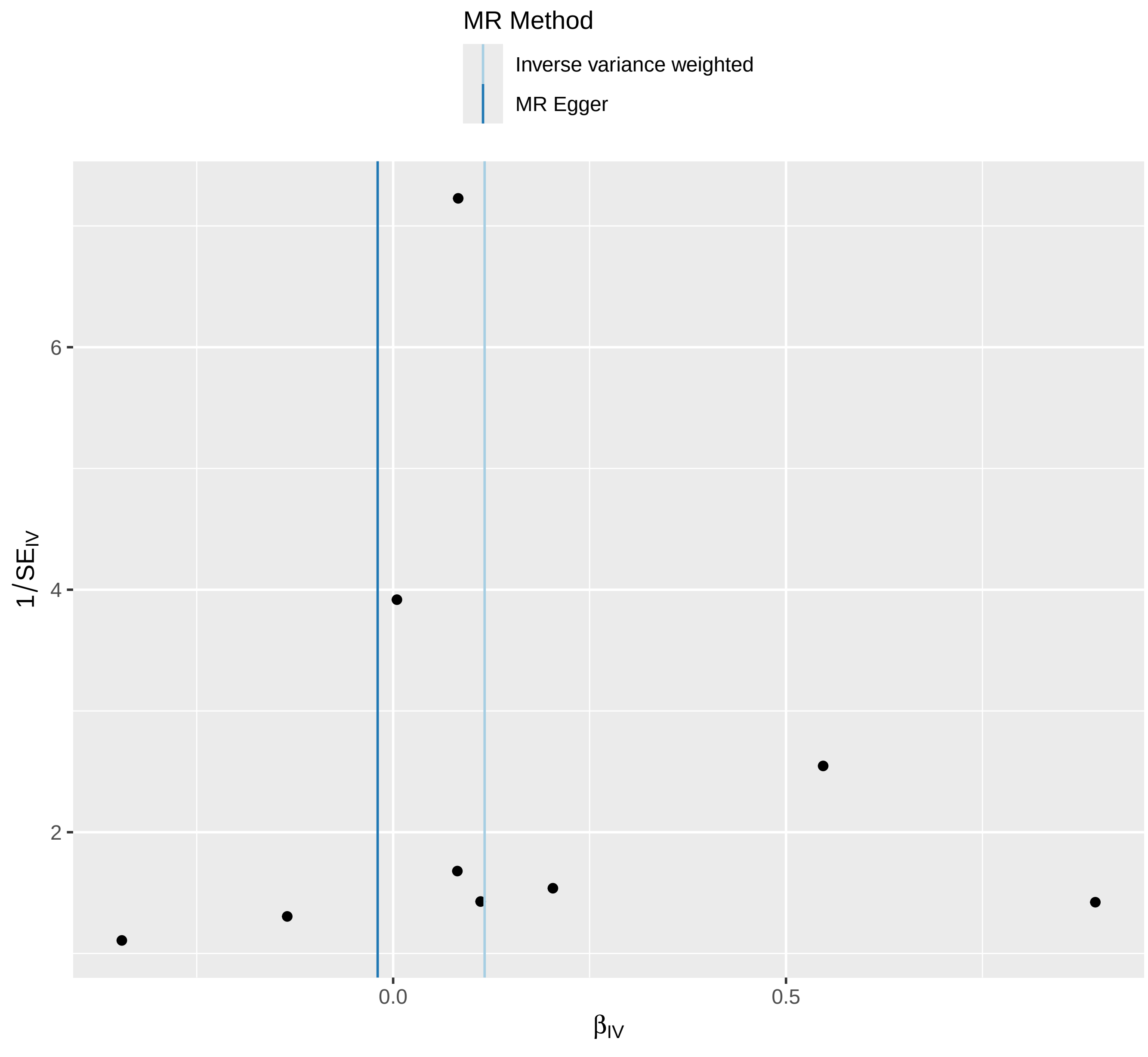

MR Method
Inverse variance weighted
MR Egger
6
4
2
1/SE$_{IV}$
0.0
0.5
β$_{IV}$

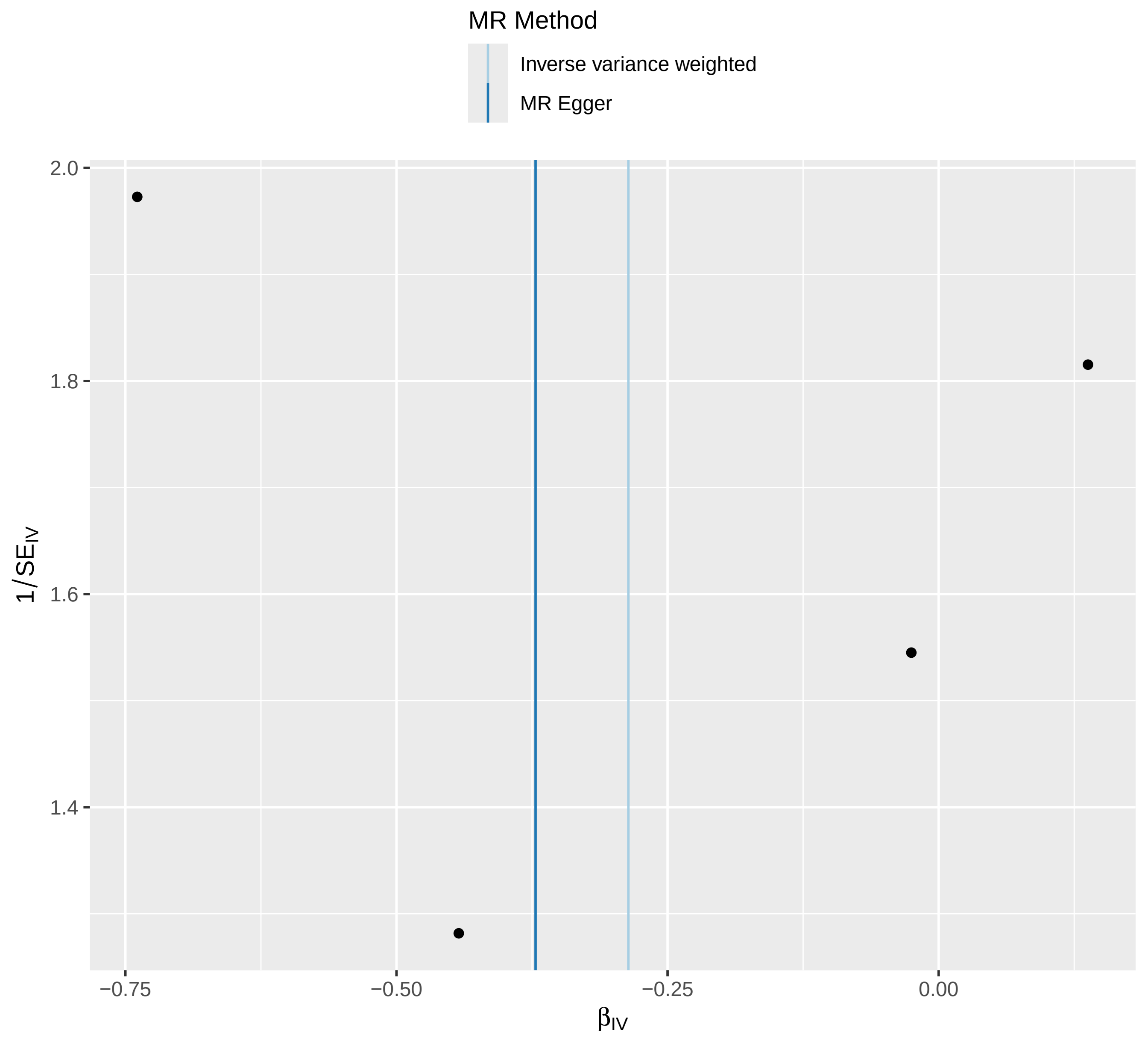
MR Method
Inverse variance weighted
MR Egger
2.0
1.8
1.6
1.4
$1/SE_{IV}$
−0.75
−0.50
−0.25
0.00
β_{IV}

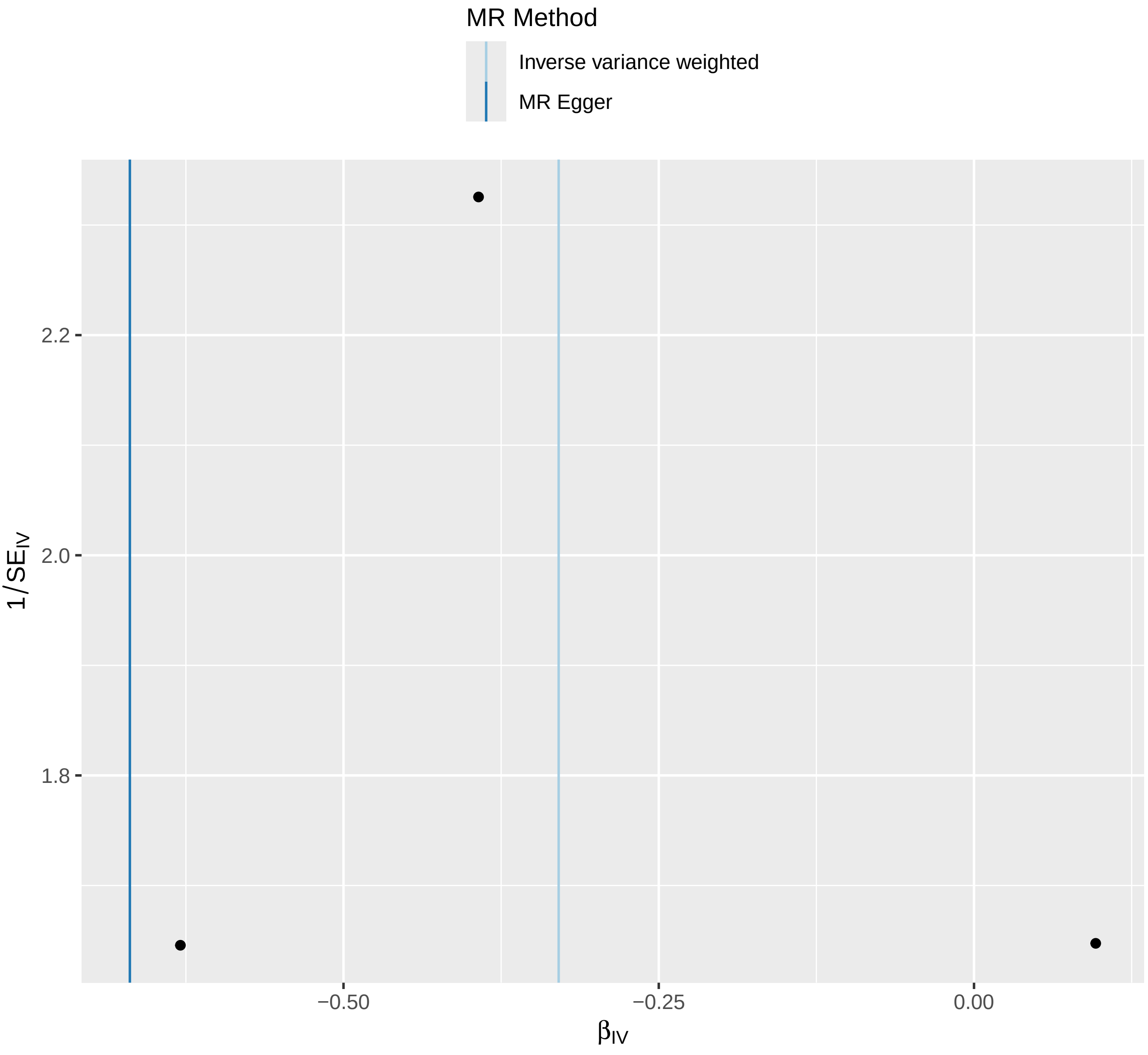
MR Method
Inverse variance weighted
MR Egger
2.2
2.0
1.8
1/SE_IV
−0.50
−0.25
0.00
β_IV

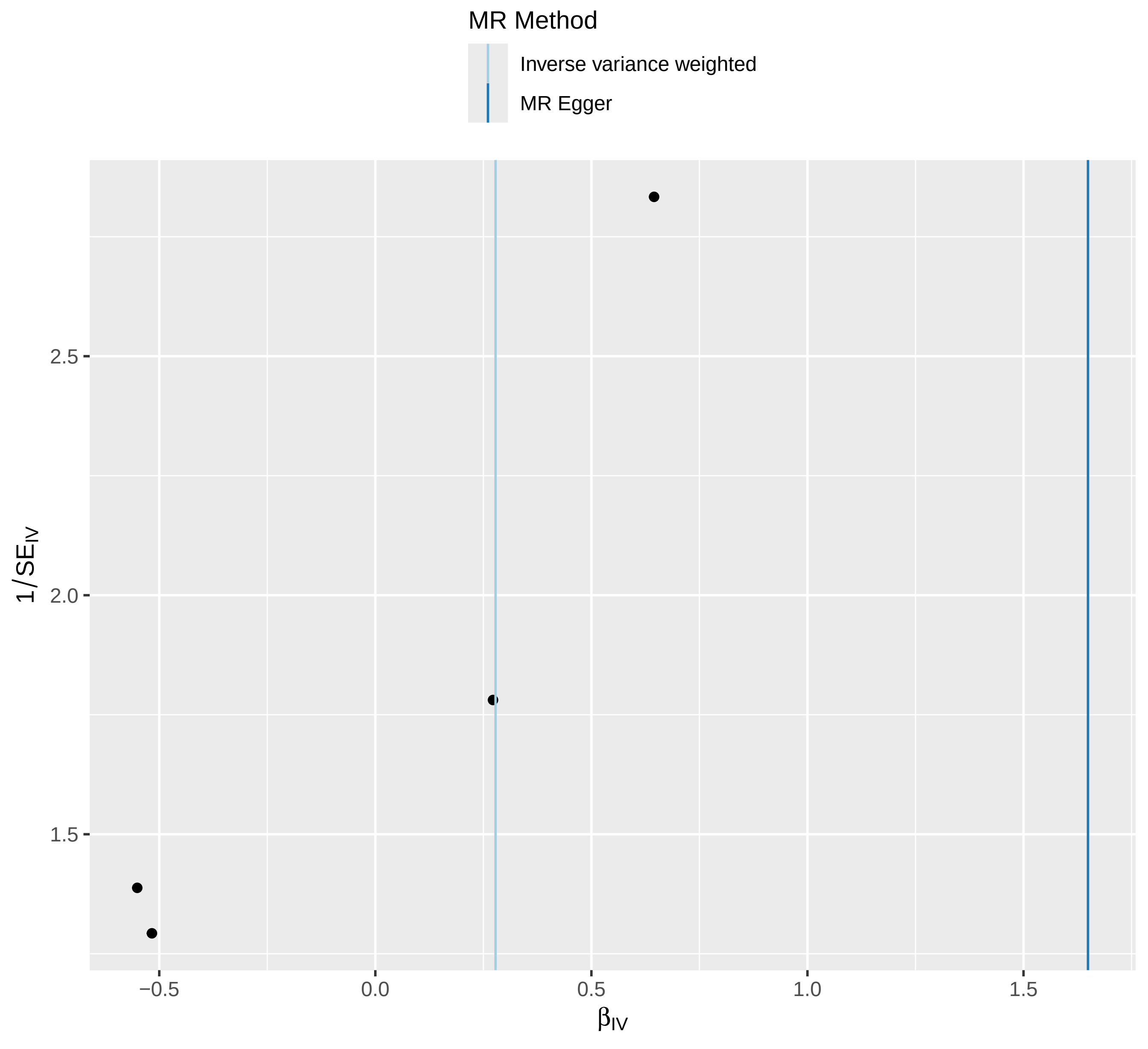
MR Method
Inverse variance weighted
MR Egger
1/SE_IV
2.5
2.0
1.5
−0.5
0.0
0.5
1.0
1.5
β_IV

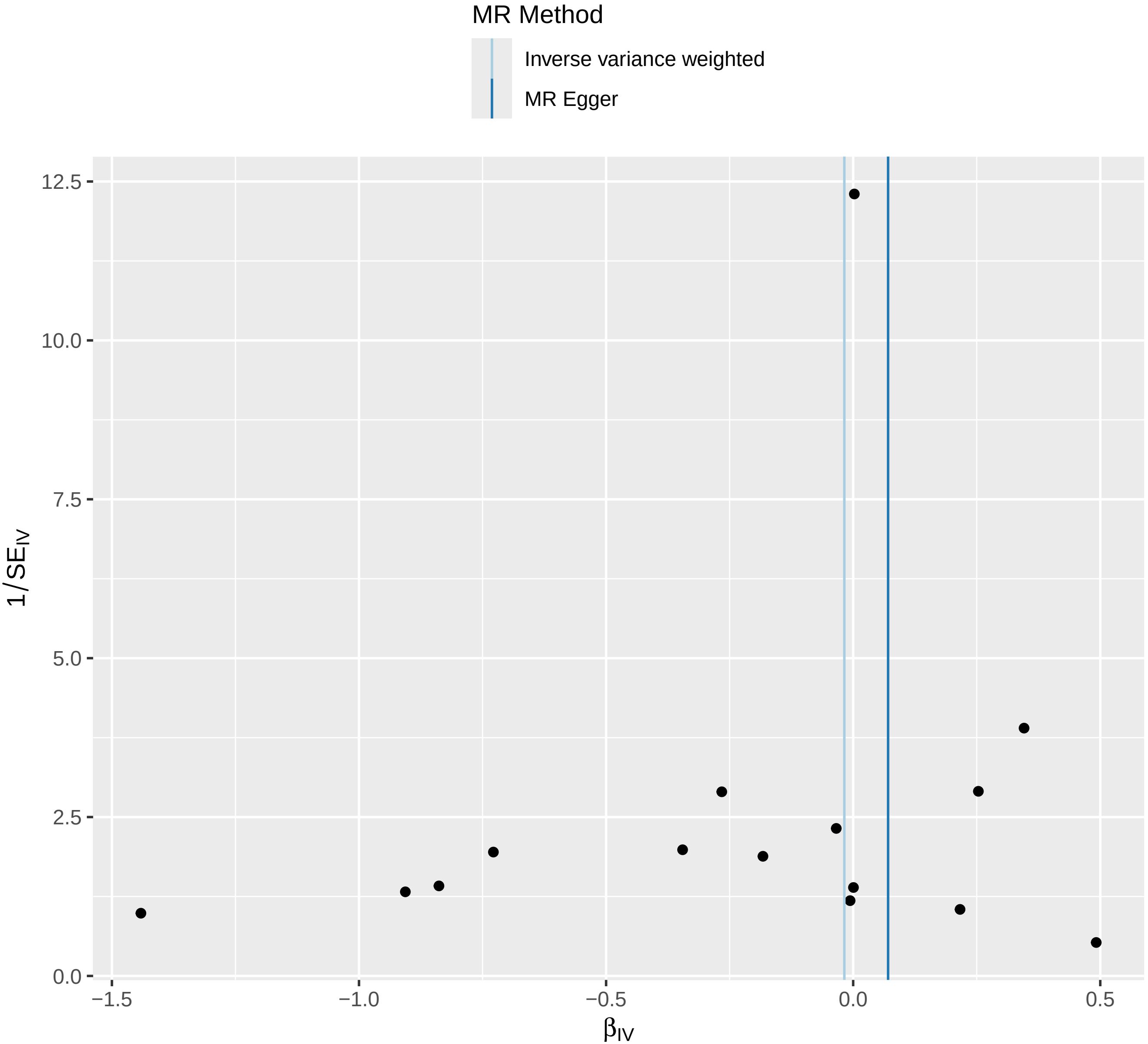

MR Method
Inverse variance weighted
MR Egger
12.5
10.0
7.5
5.0
2.5
0.0
1/SE_IV
−1.5
−1.0
−0.5
0.0
0.5
β_IV

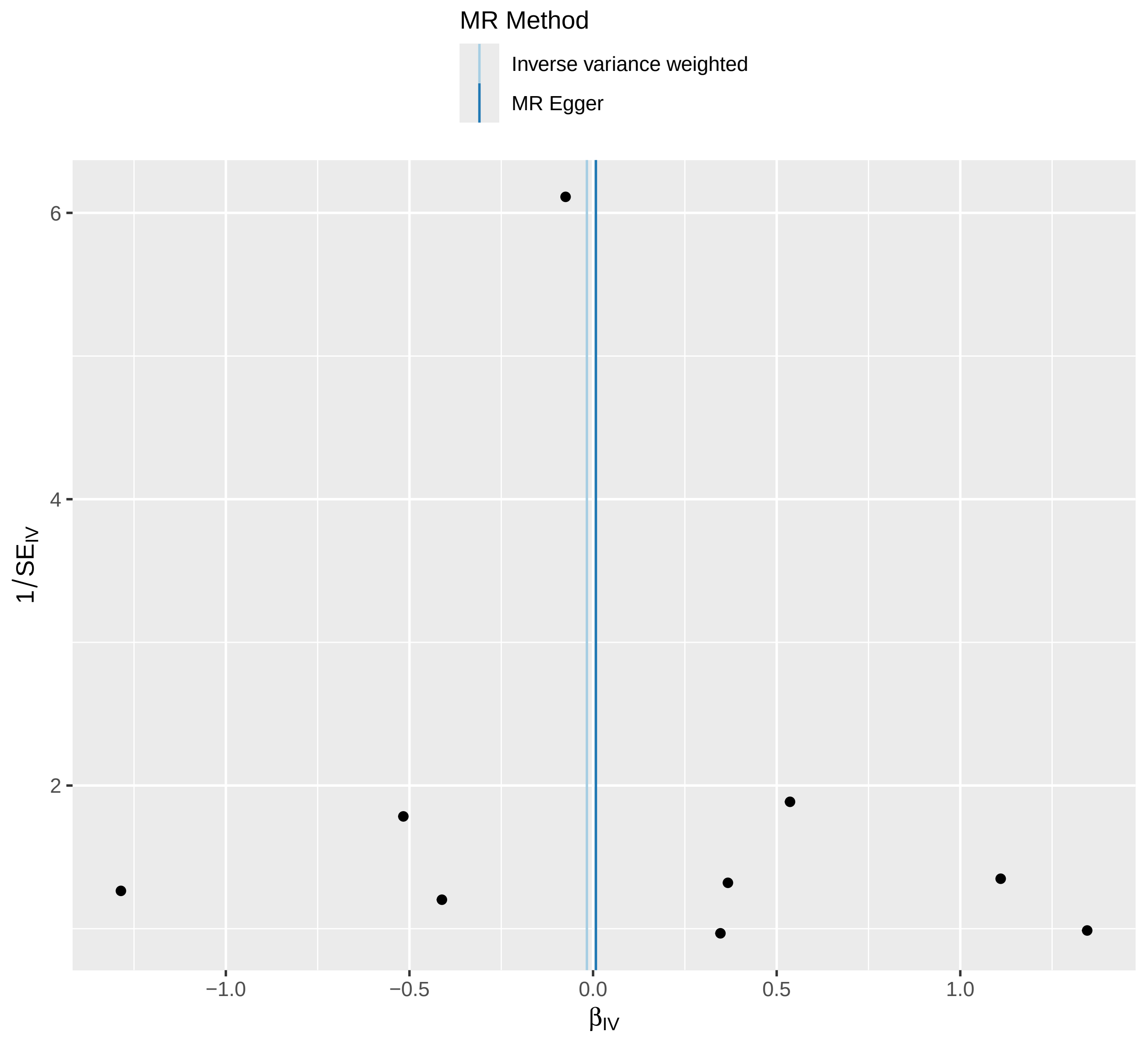

MR Method
Inverse variance weighted
MR Egger
6
4
2
$1/SE_{IV}$
−1.0
−0.5
0.0
0.5
1.0
β_{IV}

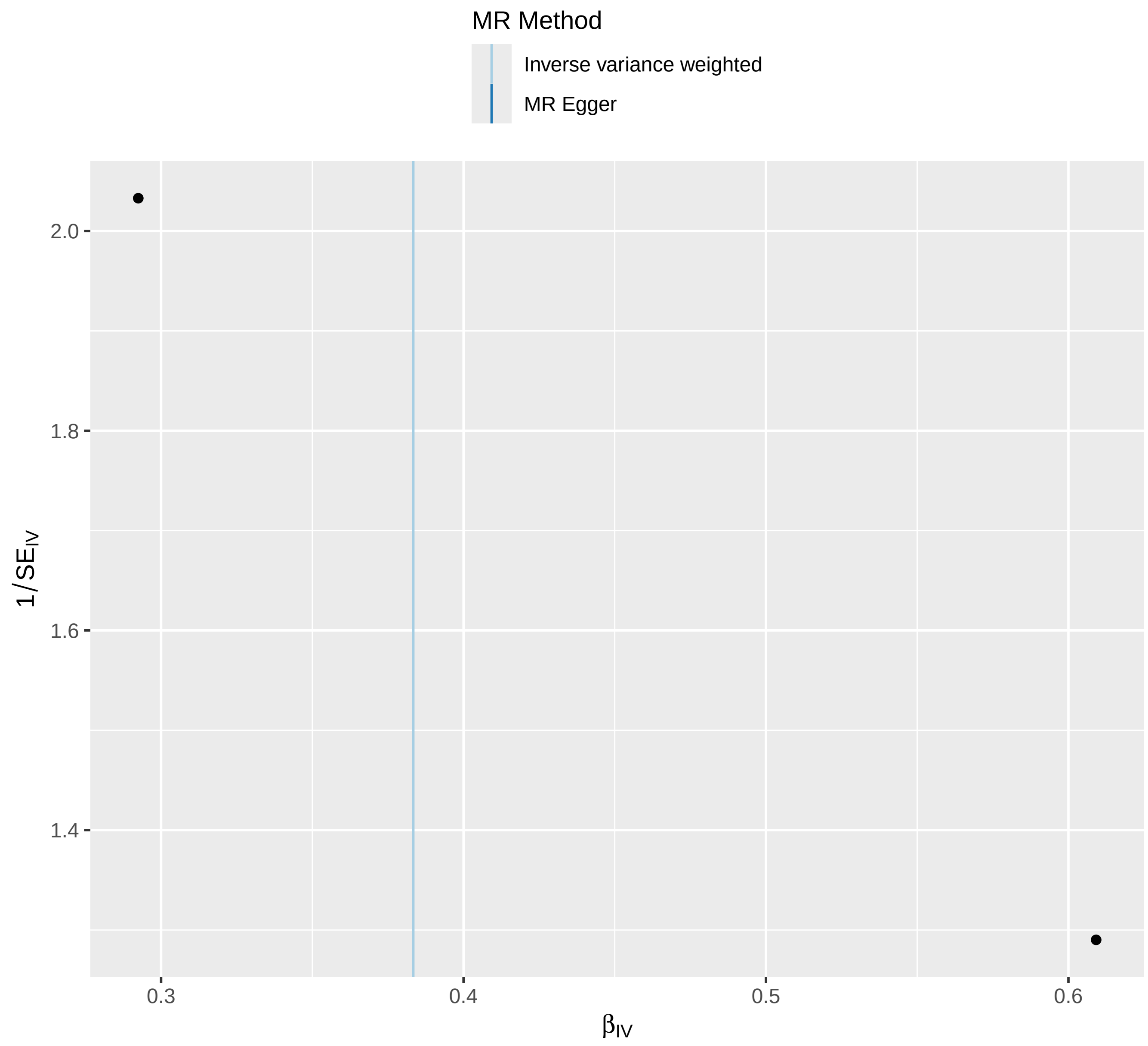
MR Method
Inverse variance weighted
MR Egger
2.0
1.8
1.6
1.4
0.3
0.4
0.5
0.6
$1/SE_{IV}$
β_{IV}

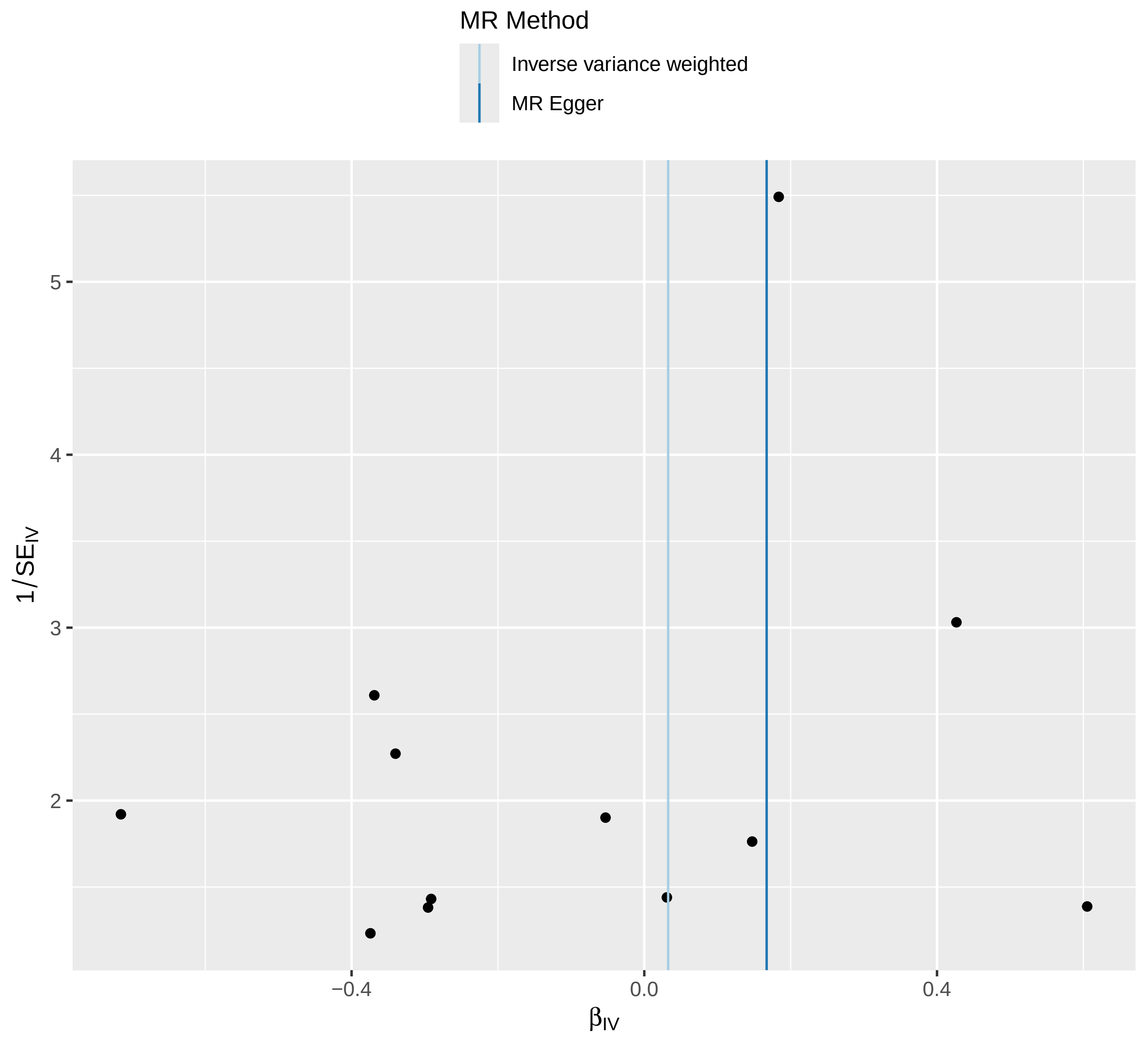
MR Method
Inverse variance weighted
MR Egger
5
4
3
2
1/SE$_{IV}$
−0.4
0.0
0.4
β$_{IV}$

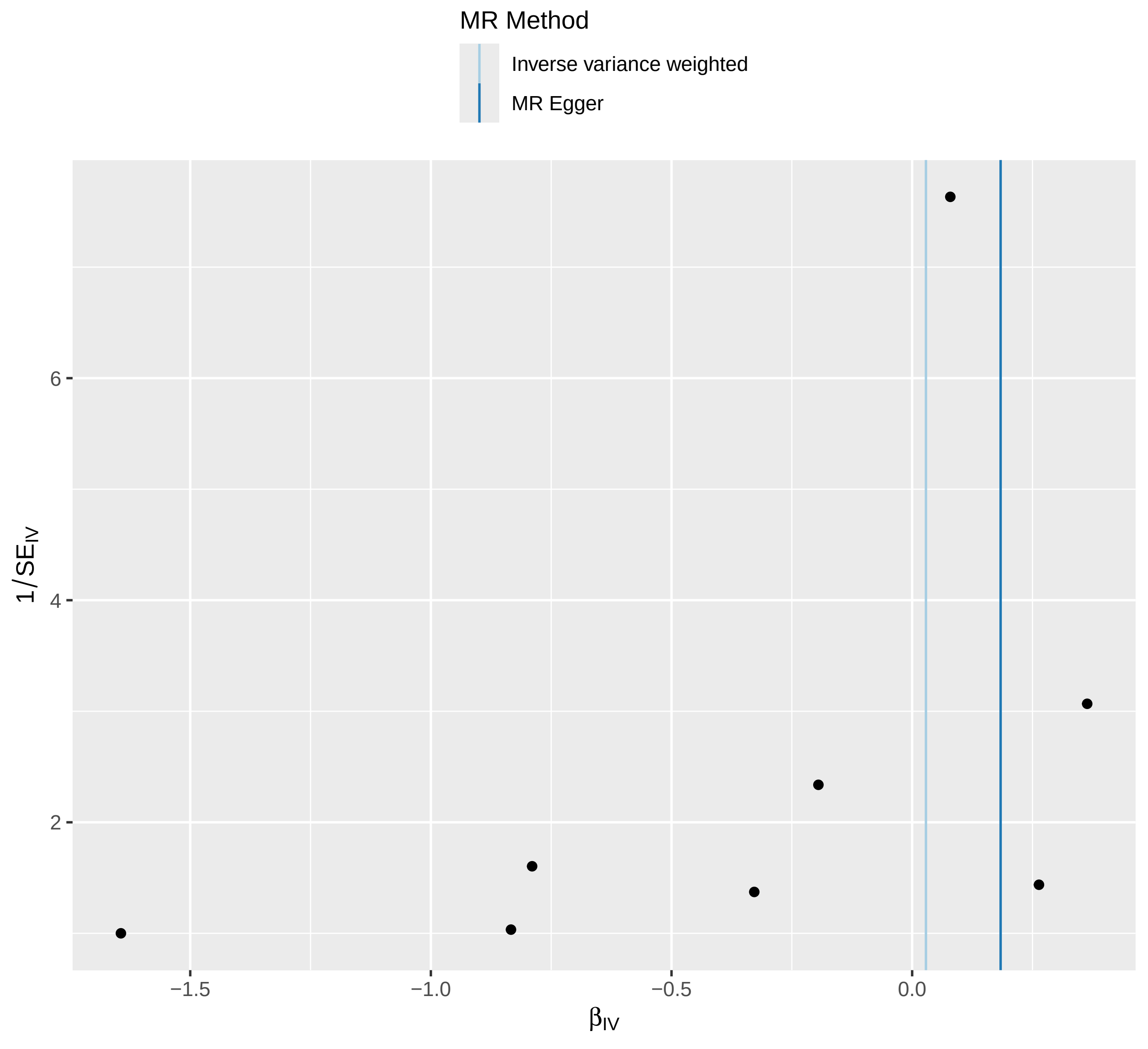
MR Method
Inverse variance weighted
MR Egger
1/SE_IV
6
4
2
−1.5
−1.0
−0.5
0.0
β_IV

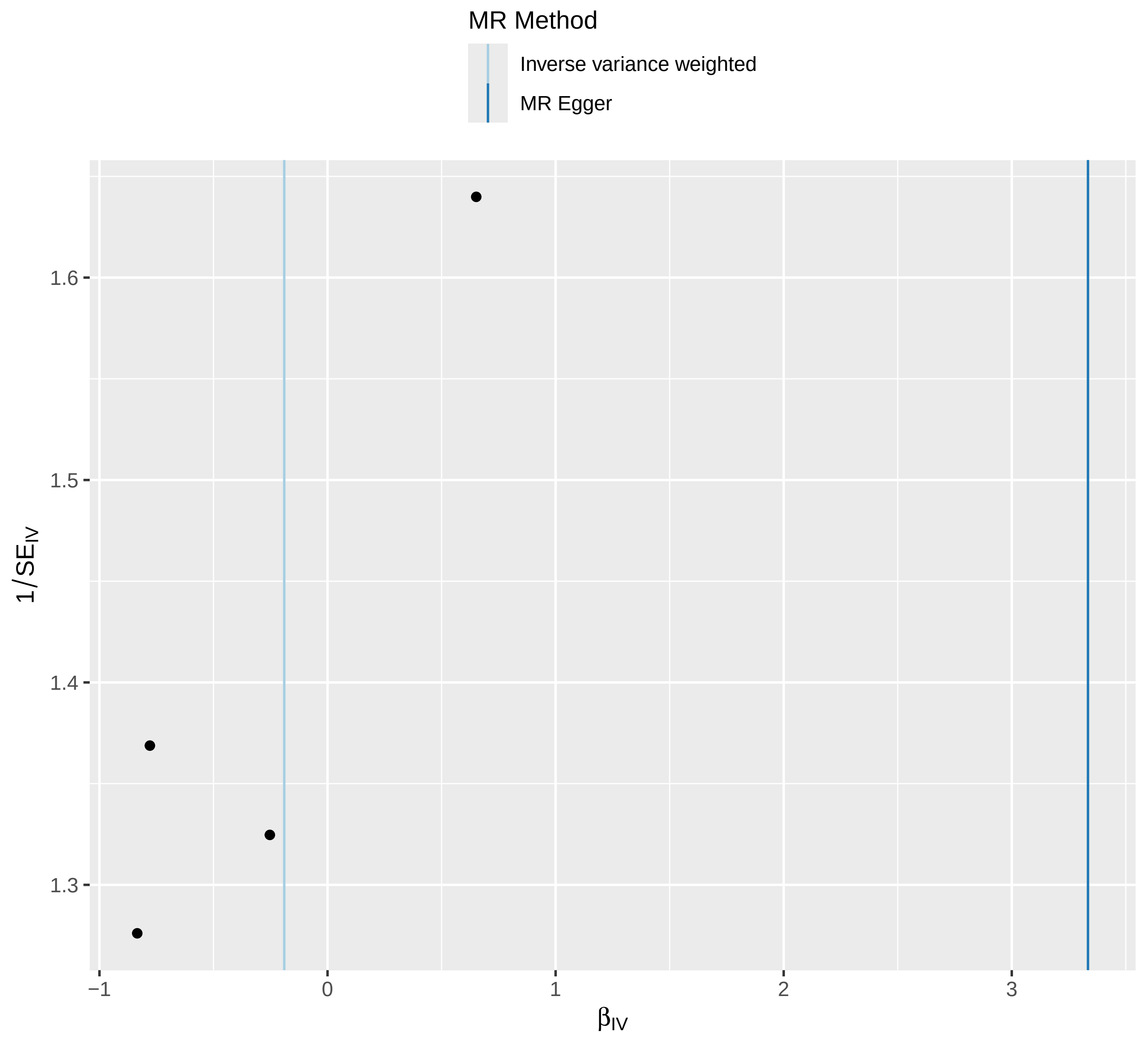

MR Method
Inverse variance weighted
MR Egger
1/SE_IV
1.6
1.5
1.4
1.3
−1
0
1
2
3
β_IV

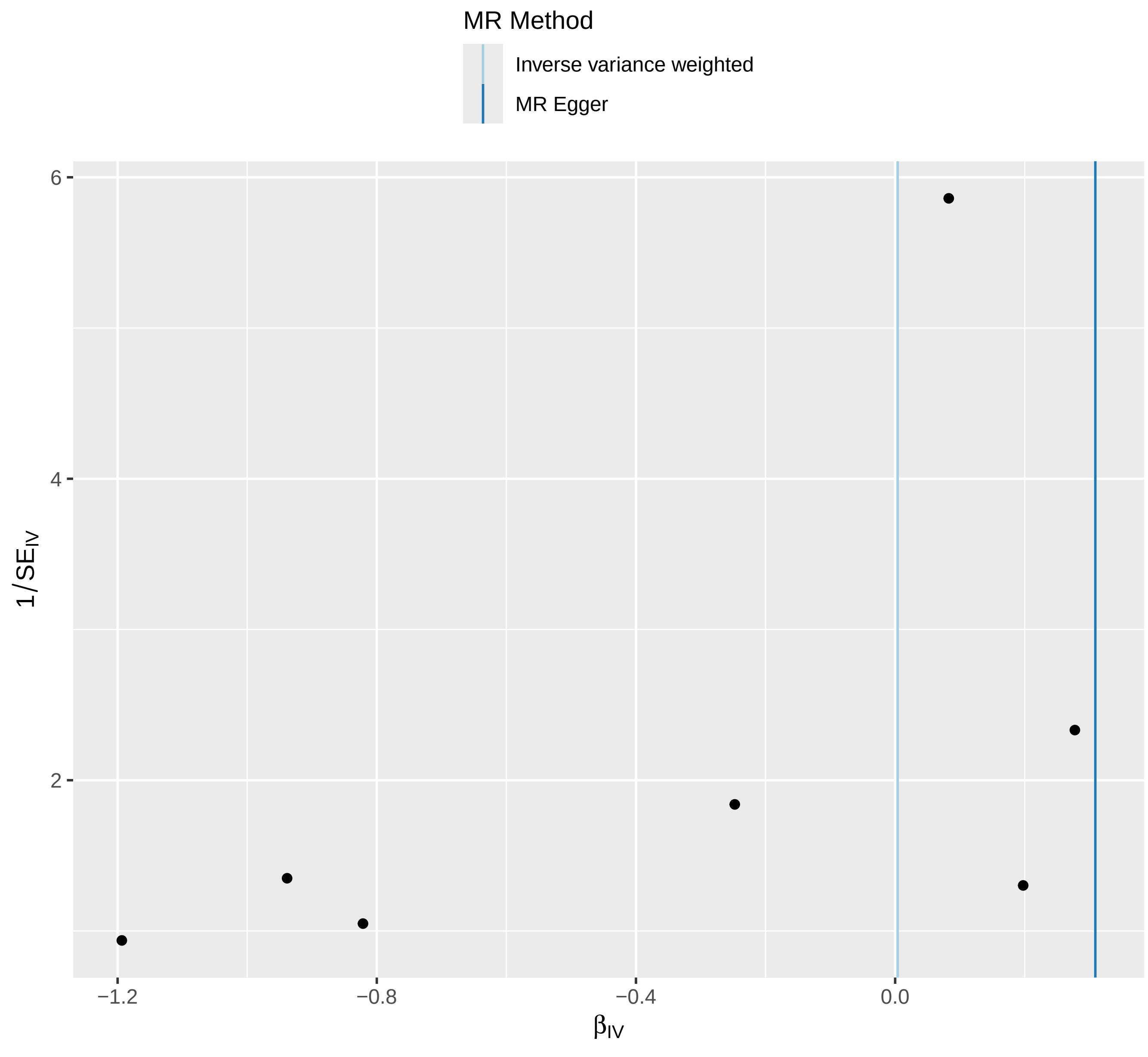
MR Method
Inverse variance weighted
MR Egger
6
4
2
1/SE$_{IV}$
−1.2
−0.8
−0.4
0.0
β$_{IV}$

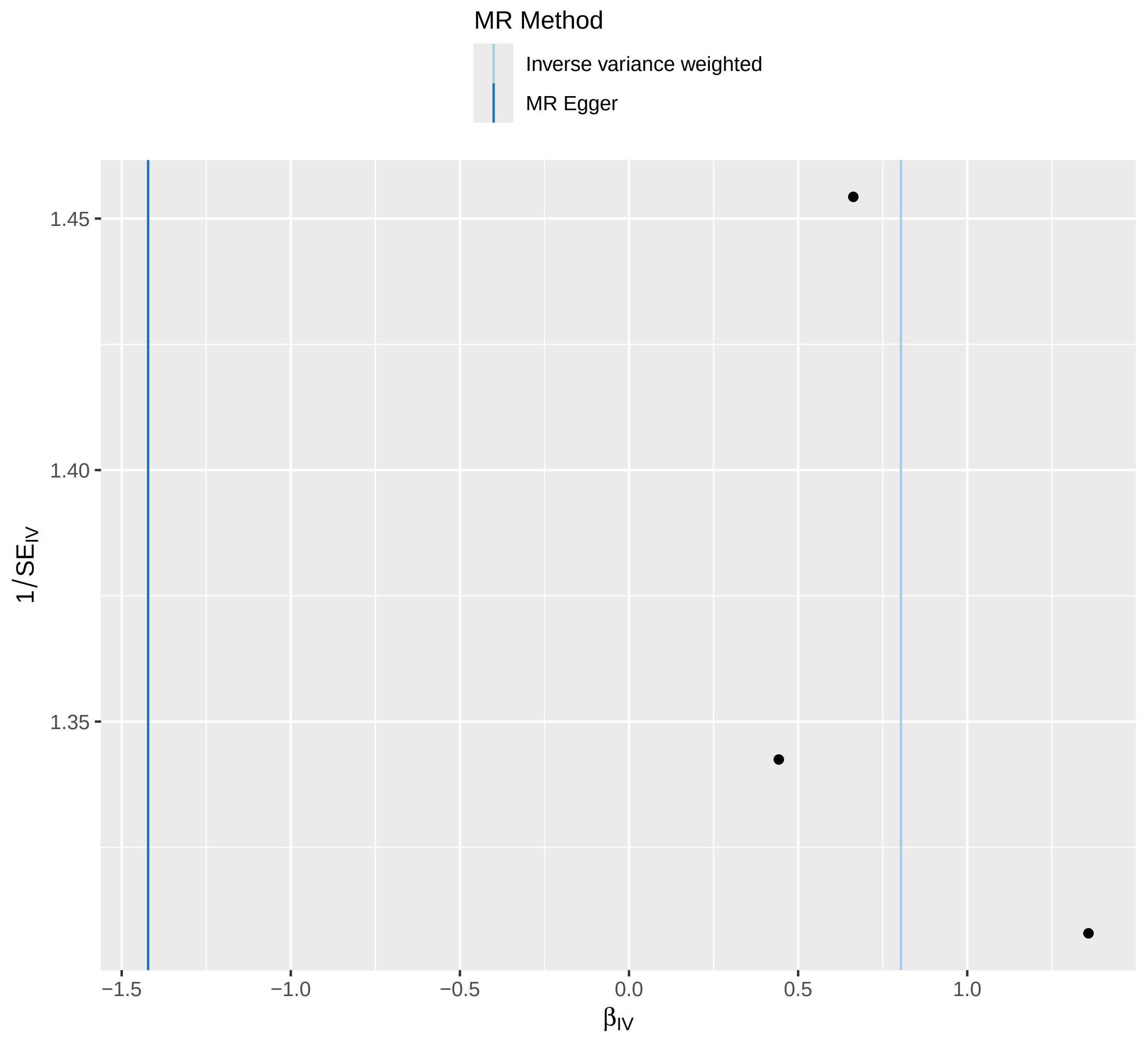

MR Method
Inverse variance weighted
MR Egger
1.45
1.40
1.35
$1/SE_{IV}$
−1.5
−1.0
−0.5
0.0
0.5
1.0
β_{IV}

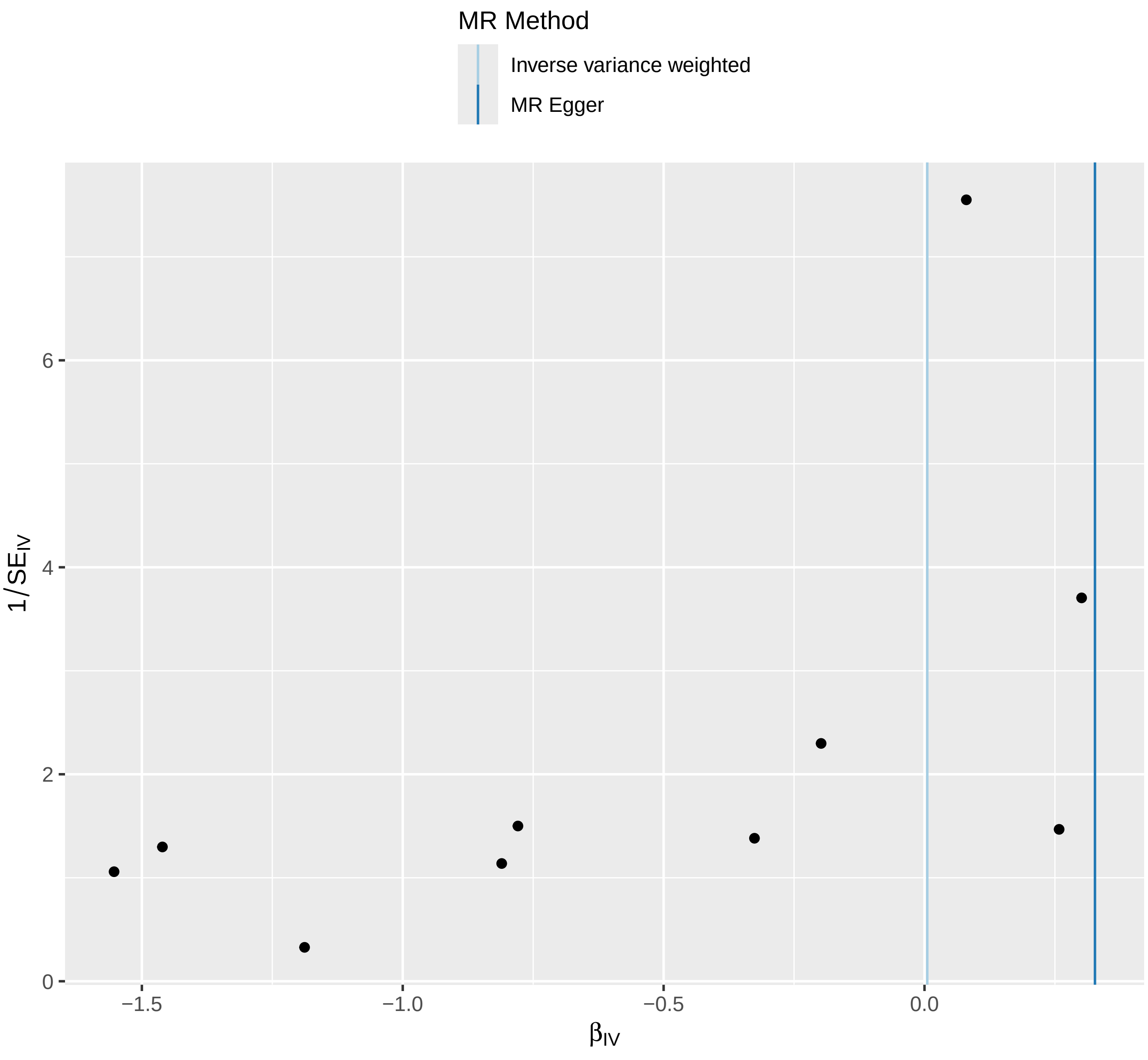
MR Method
Inverse variance weighted
MR Egger
0
2
4
6
1/SE_IV
−1.5
−1.0
−0.5
0.0
β_IV

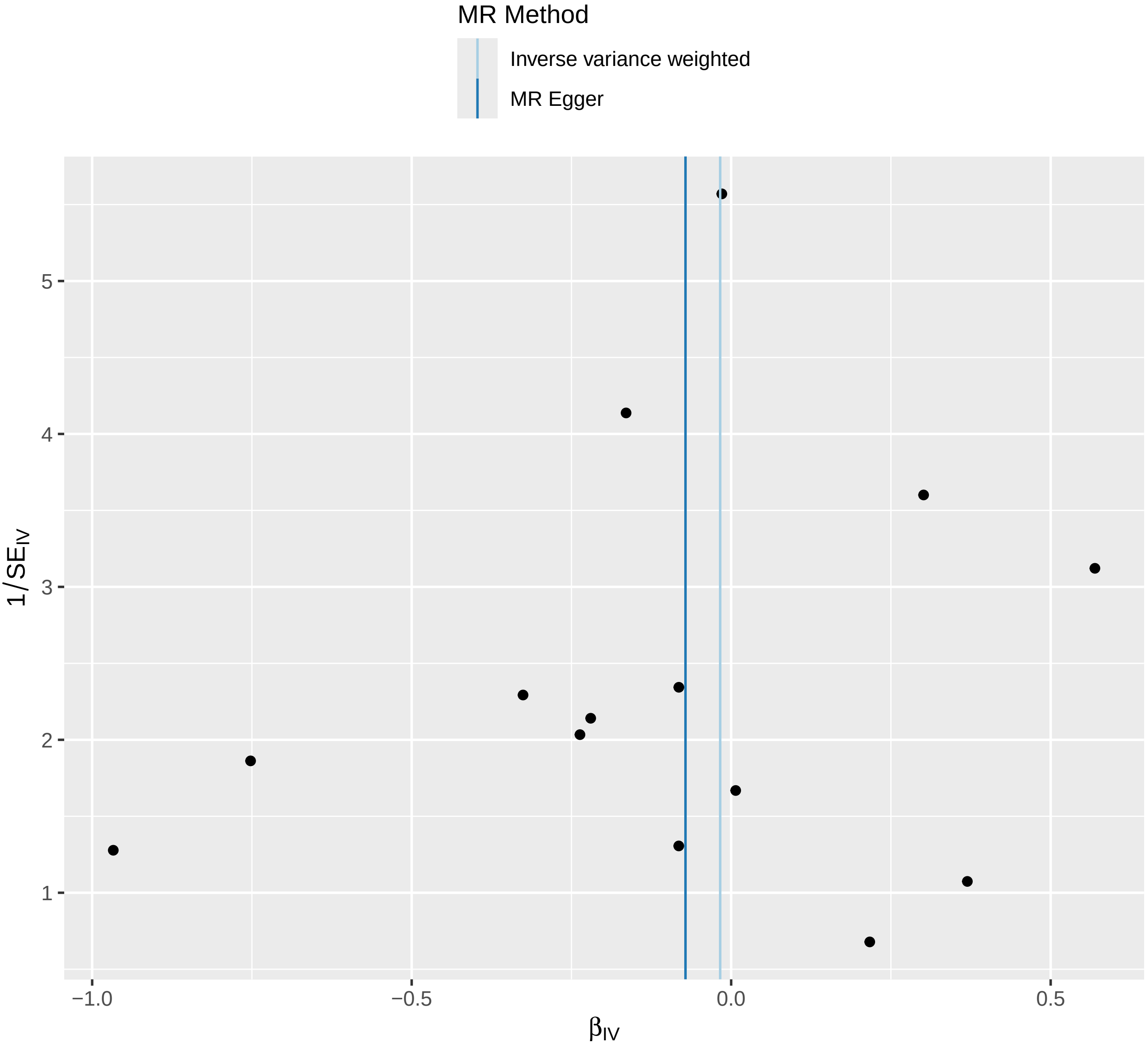
MR Method
Inverse variance weighted
MR Egger
1/SE_IV
β_IV
5
4
3
2
1
−1.0
−0.5
0.0
0.5

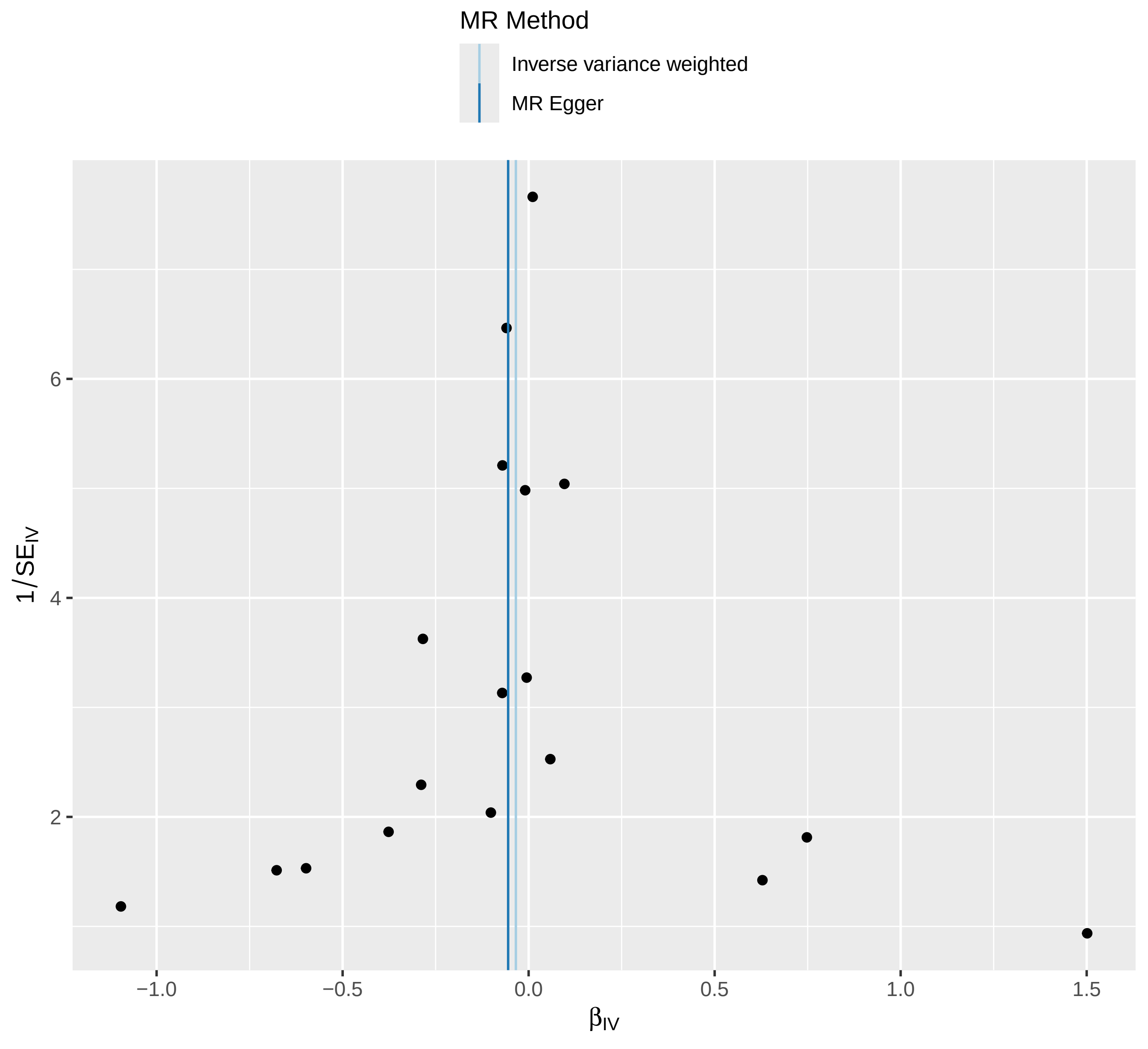
MR Method
Inverse variance weighted
MR Egger
1/SE_IV
6
4
2
−1.0
−0.5
0.0
0.5
1.0
1.5
β_IV

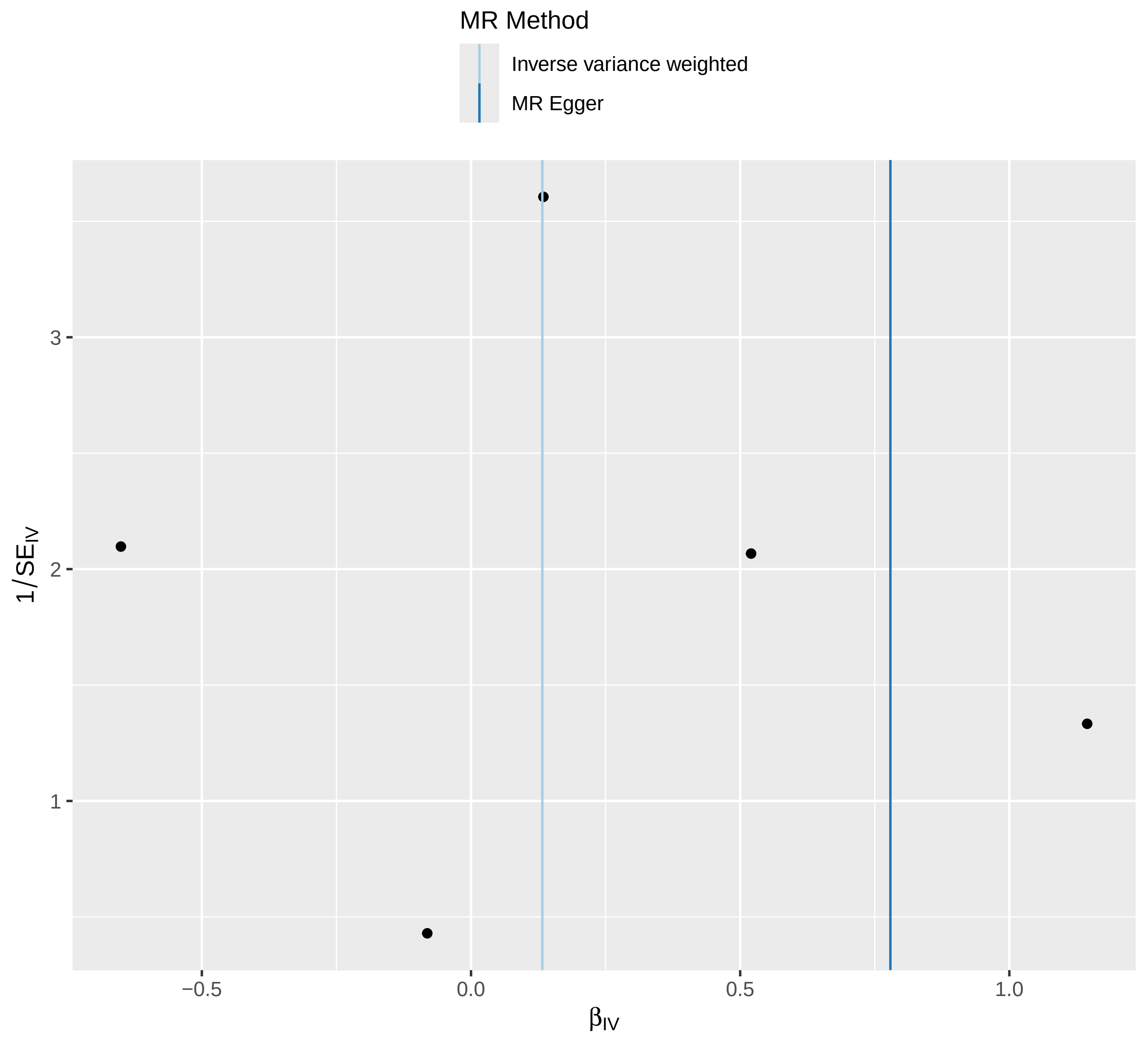
MR Method
Inverse variance weighted
MR Egger
1/SE$_{IV}$
3
2
1
−0.5
0.0
0.5
1.0
β$_{IV}$

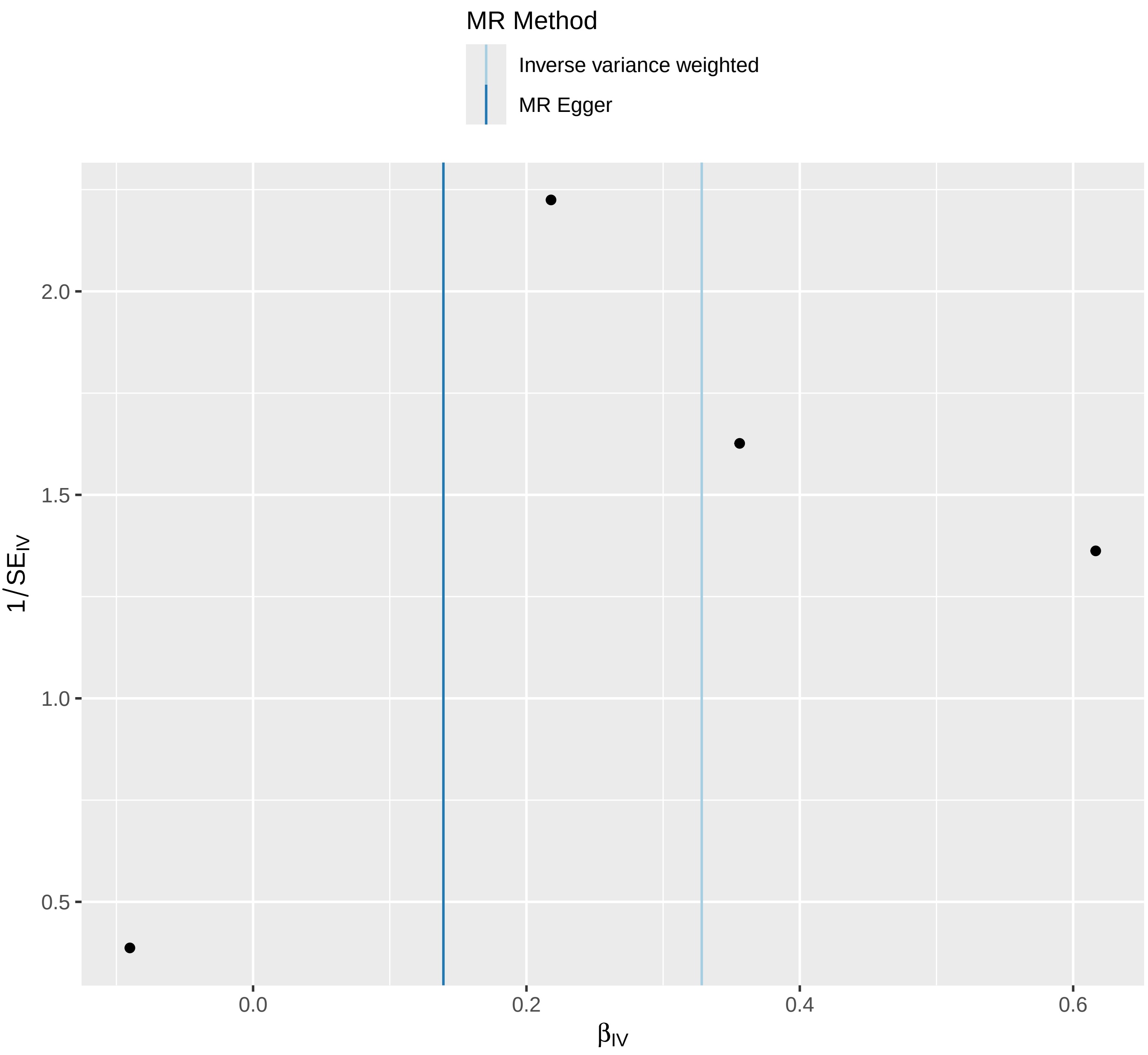
MR Method
Inverse variance weighted
MR Egger
2.0
1.5
1.0
0.5
1/SE_IV
0.0
0.2
0.4
0.6
β_IV

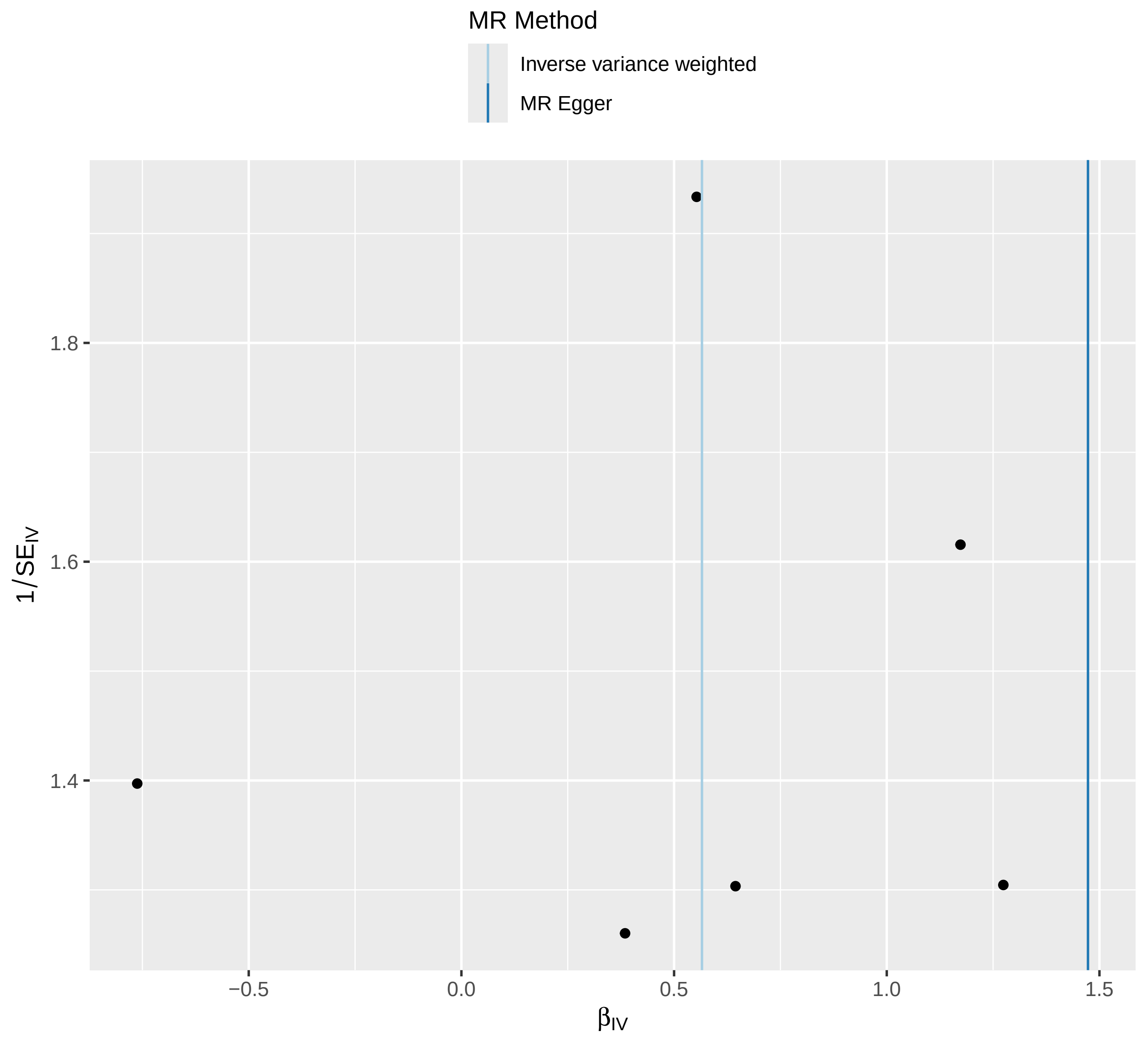

MR Method
Inverse variance weighted
MR Egger
1.8
1.6
1.4
1/SE_IV
−0.5
0.0
0.5
1.0
1.5
β_IV

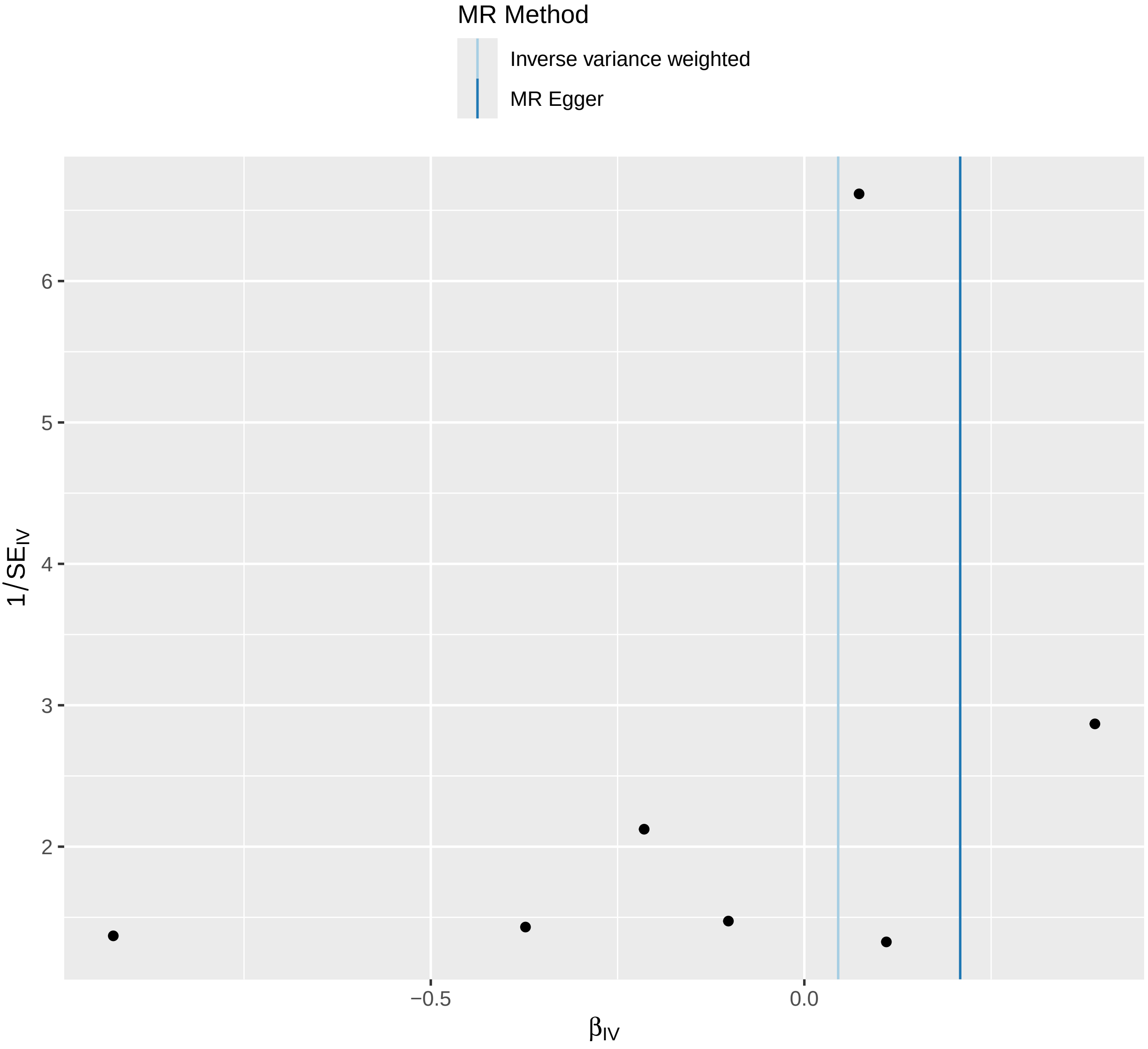
MR Method
Inverse variance weighted
MR Egger
1/SE_IV
6
5
4
3
2
−0.5
0.0
β_IV

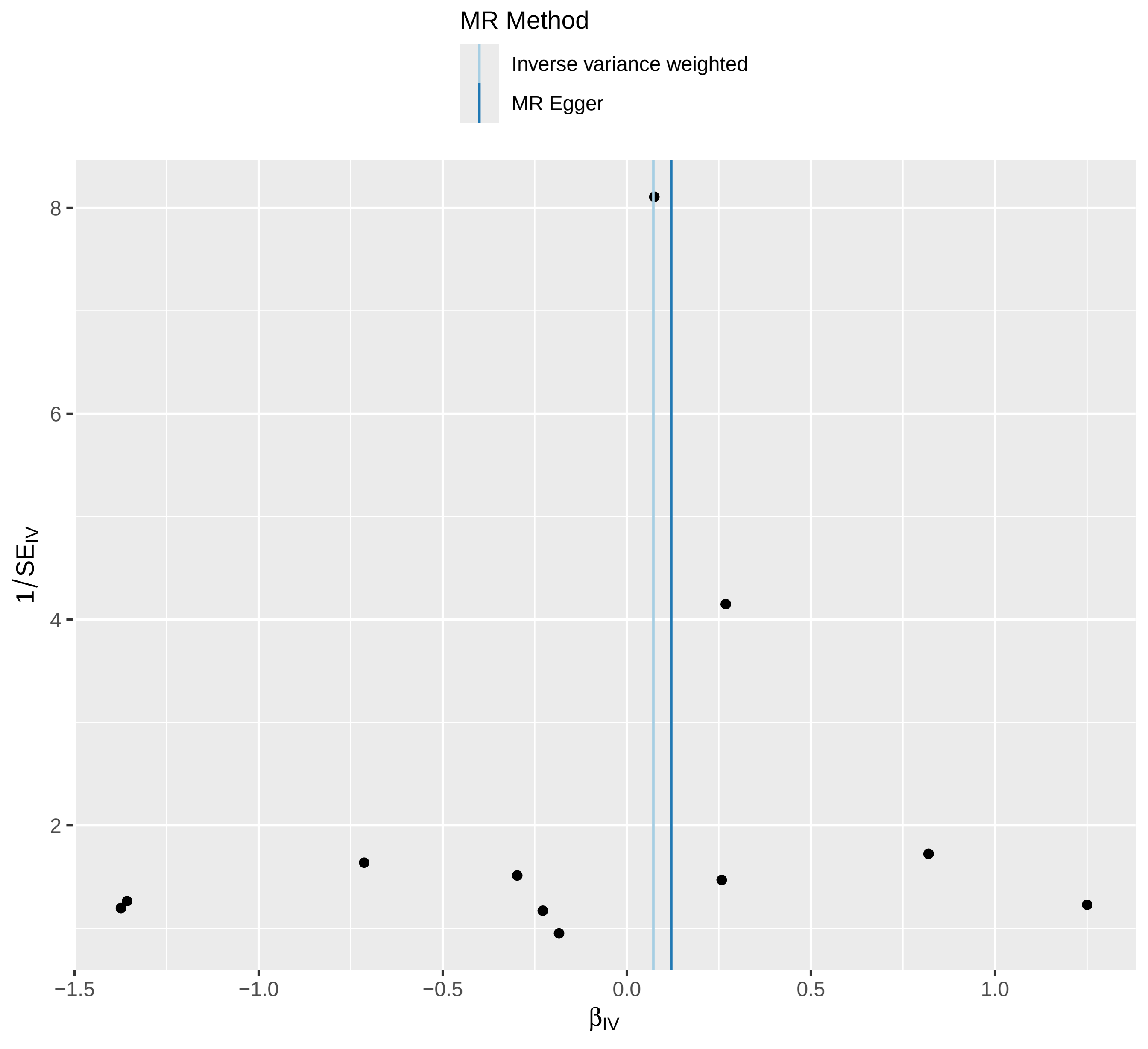

MR Method
Inverse variance weighted
MR Egger
8
6
4
2
1/SE_IV
−1.5
−1.0
−0.5
0.0
0.5
1.0
β_IV

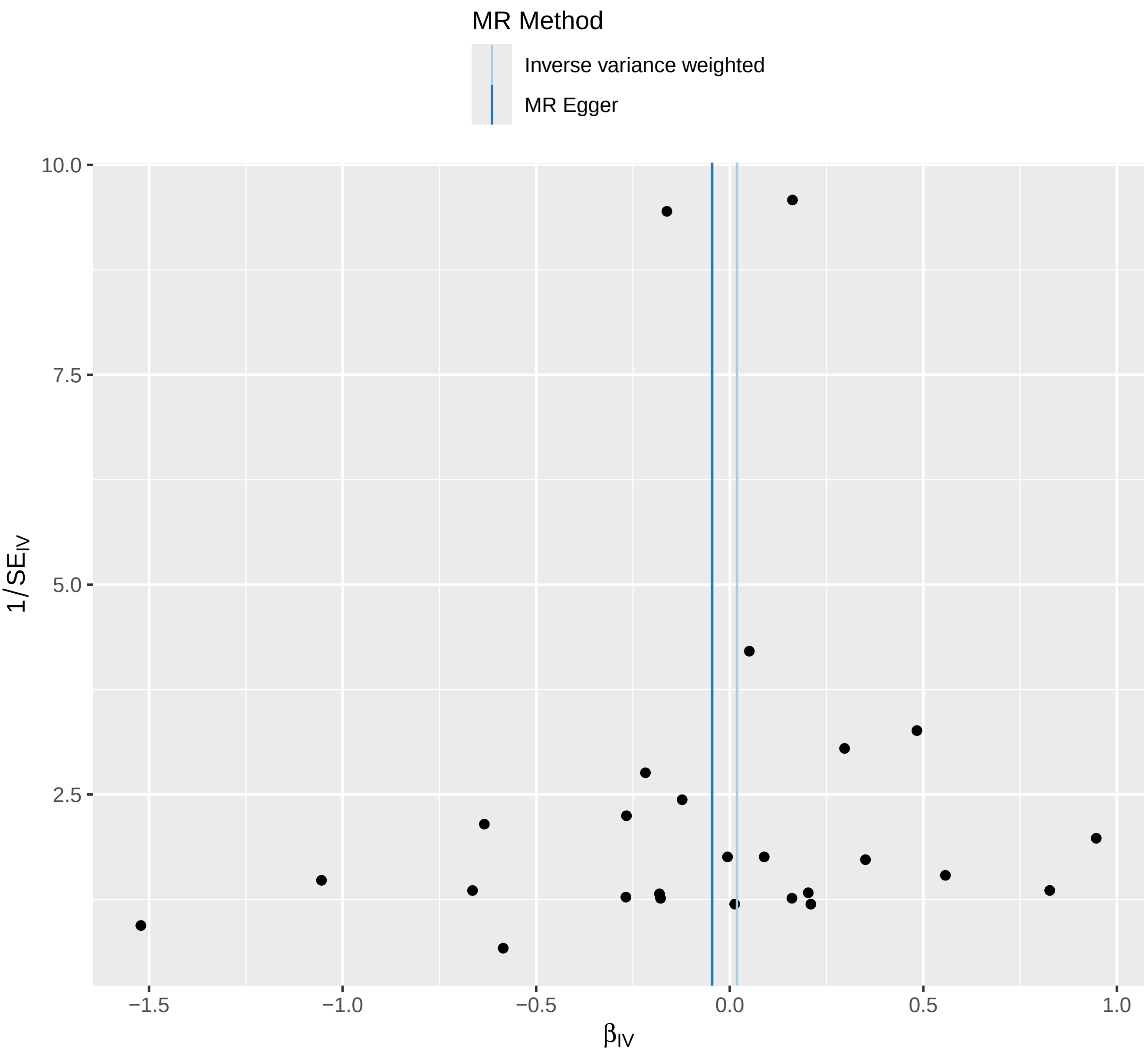

MR Method
Inverse variance weighted
MR Egger
10.0
7.5
5.0
2.5
1/SE_IV
−1.5
−1.0
−0.5
0.0
0.5
1.0
β_IV

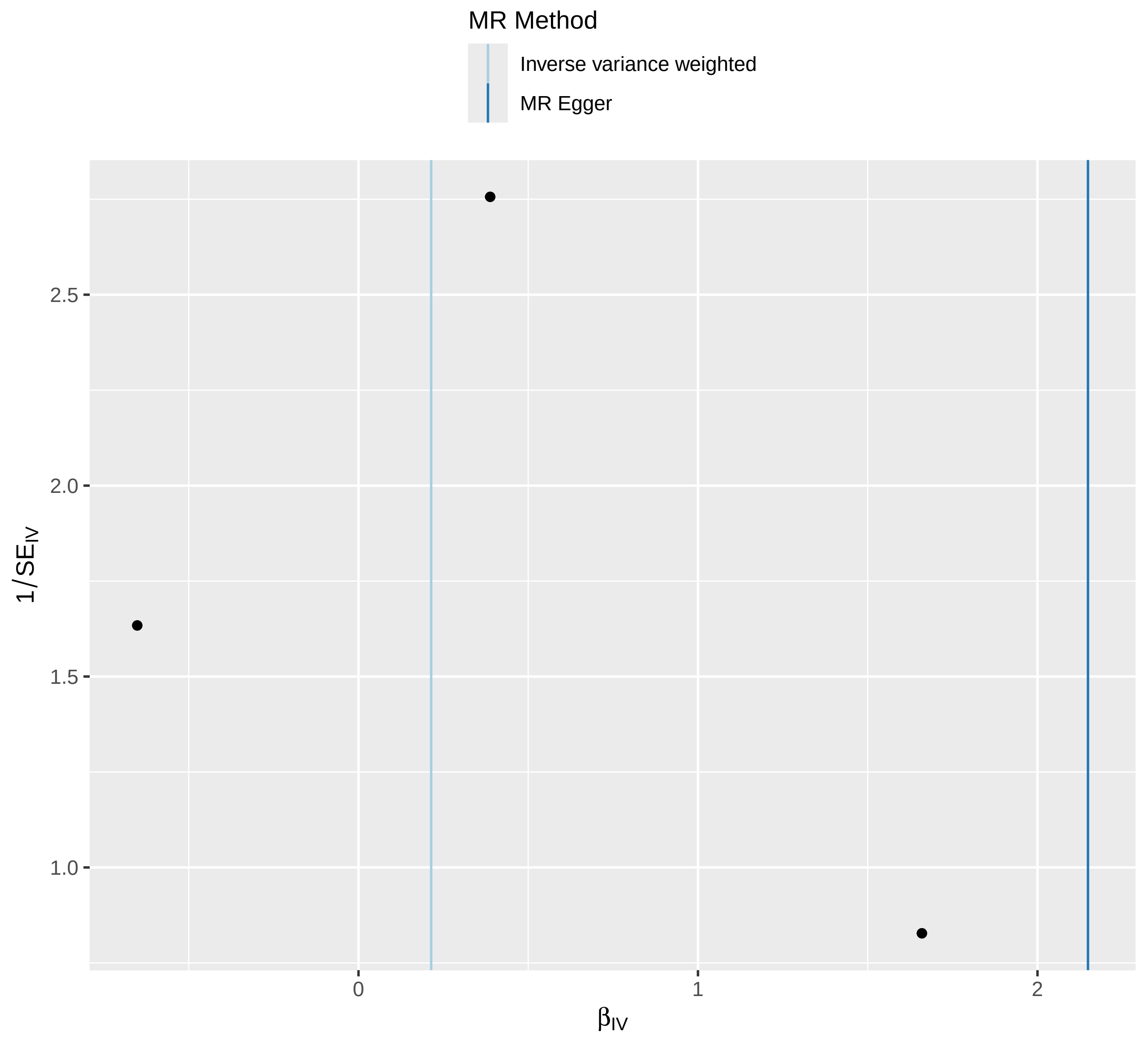

MR Method
Inverse variance weighted
MR Egger
2.5
2.0
1.5
1.0
$1/SE_{IV}$
0
1
2
β_{IV}

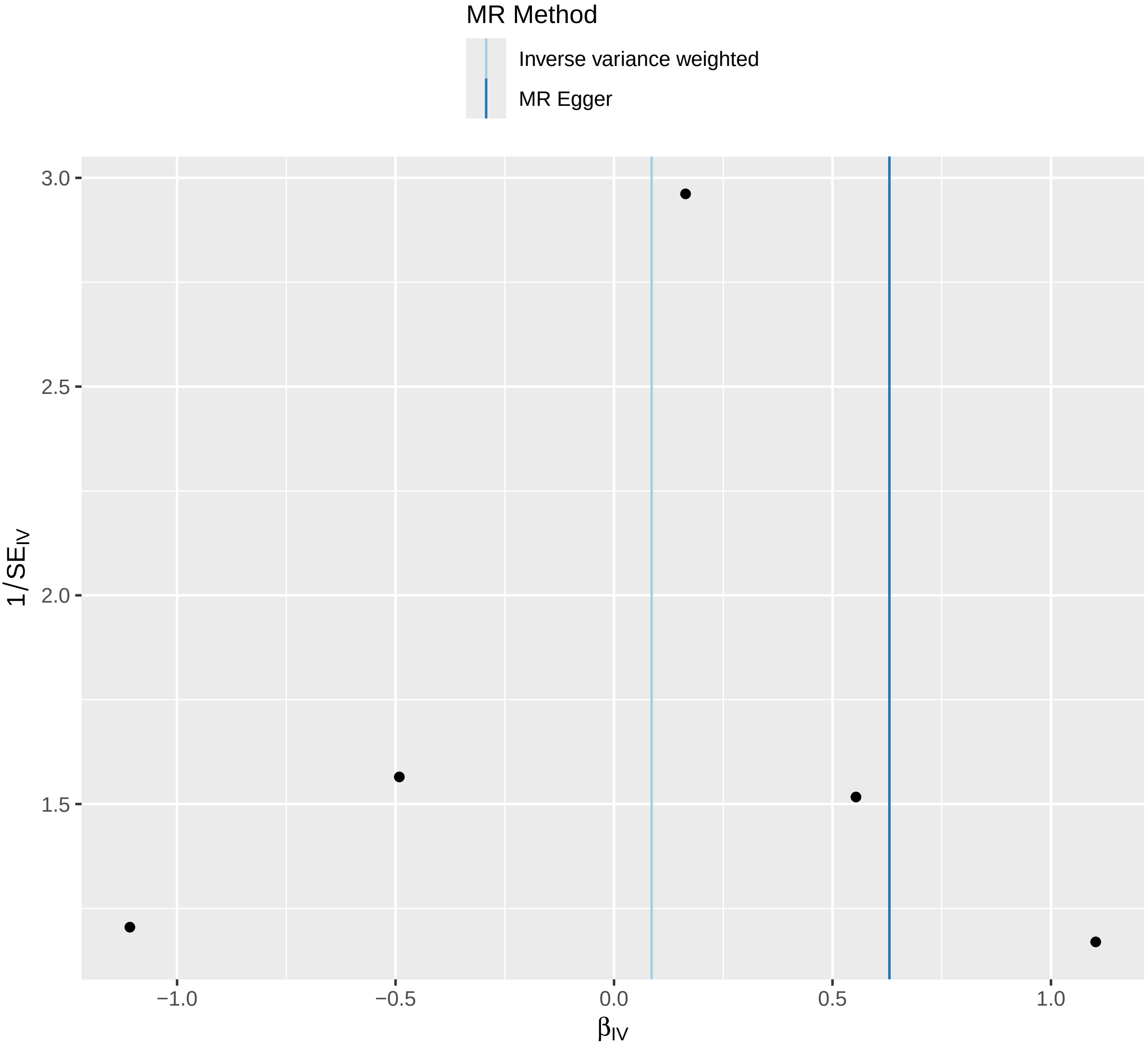

MR Method
Inverse variance weighted
MR Egger
3.0
2.5
2.0
1.5
1/SE_IV
−1.0
−0.5
0.0
0.5
1.0
β_IV

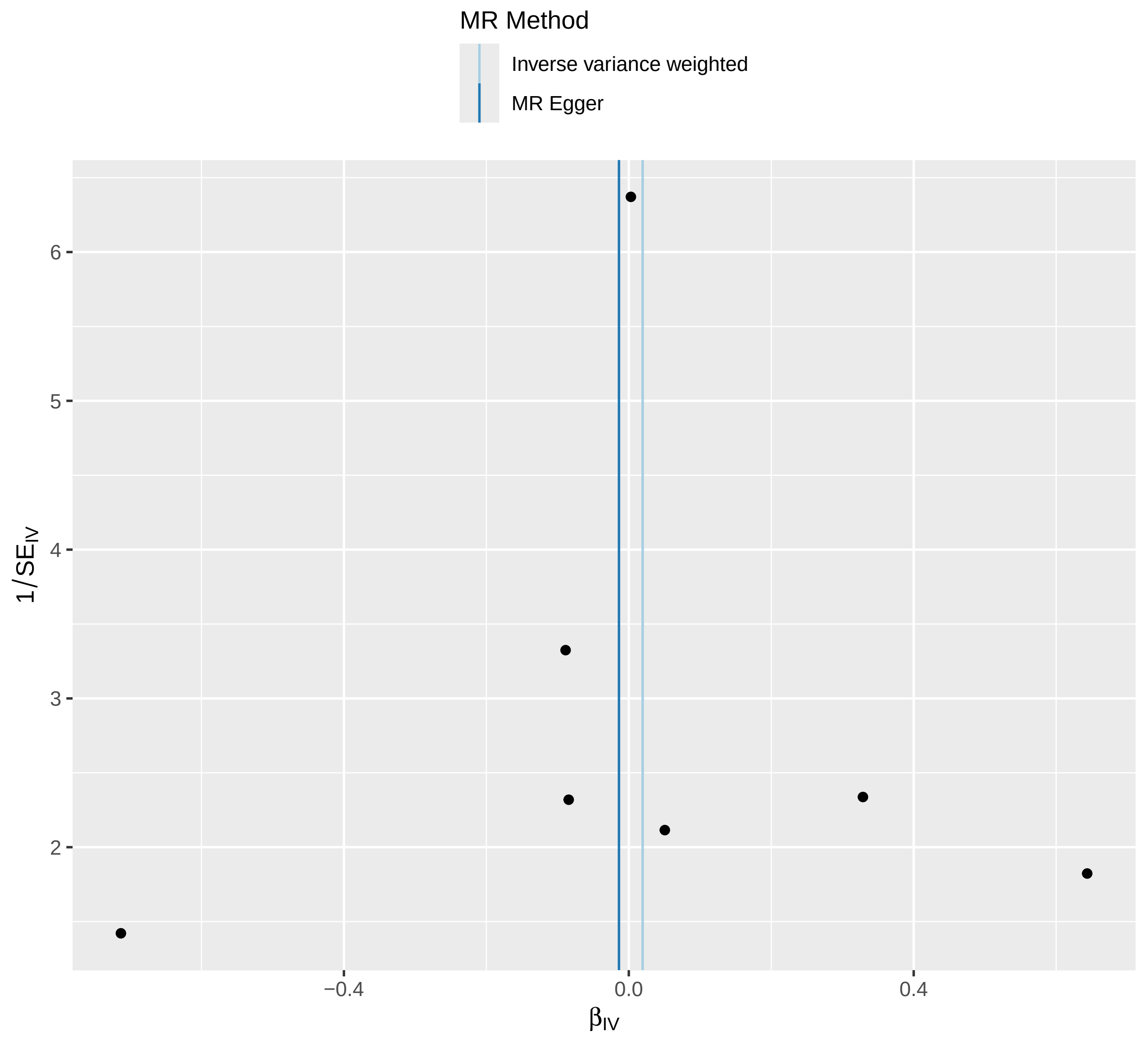
MR Method
Inverse variance weighted
MR Egger
6
5
4
3
2
$1/SE_{IV}$
−0.4
0.0
0.4
β_{IV}

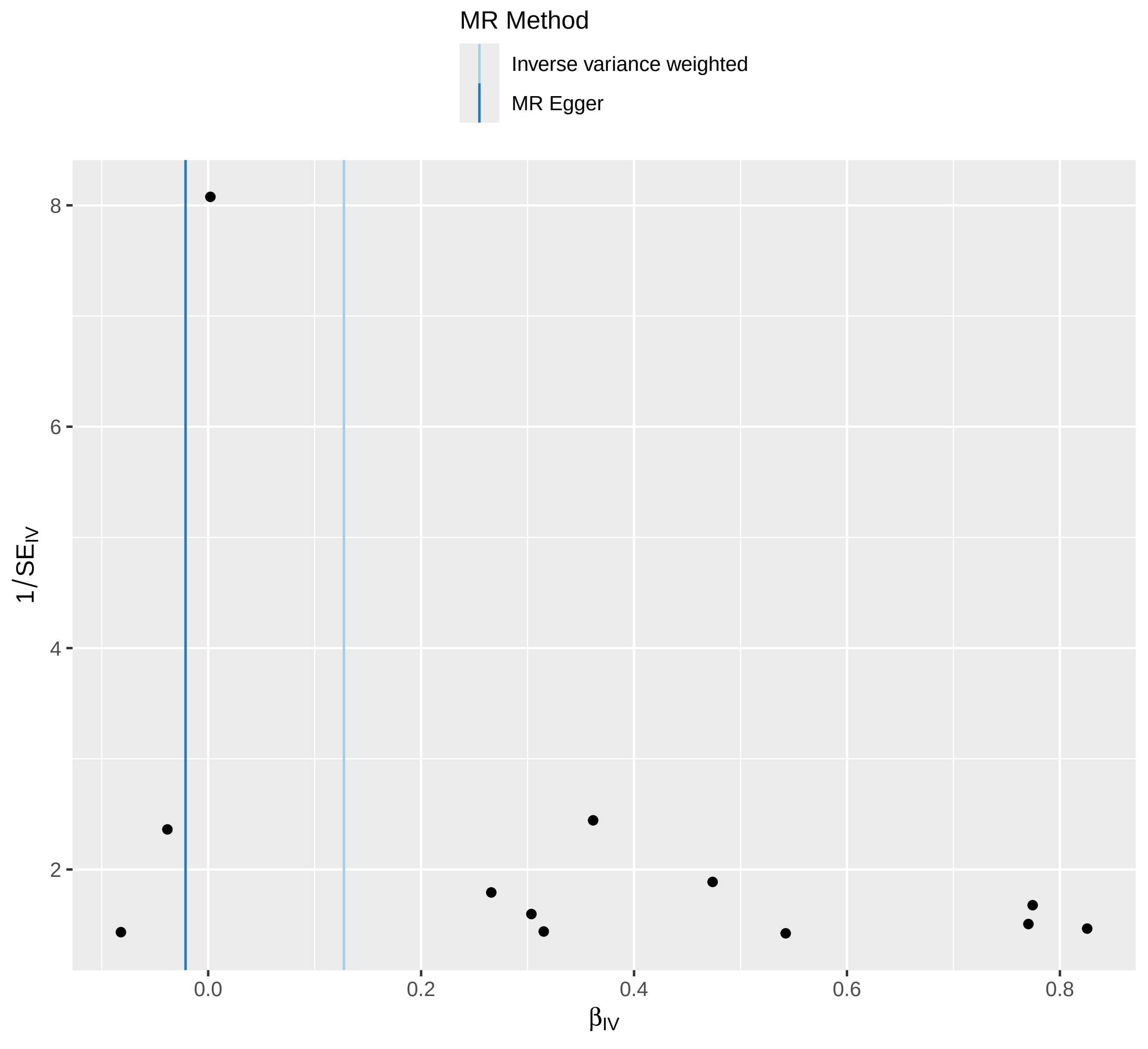

MR Method
Inverse variance weighted
MR Egger
8
6
4
2
1/SE$_{IV}$
0.0
0.2
0.4
0.6
0.8
β$_{IV}$

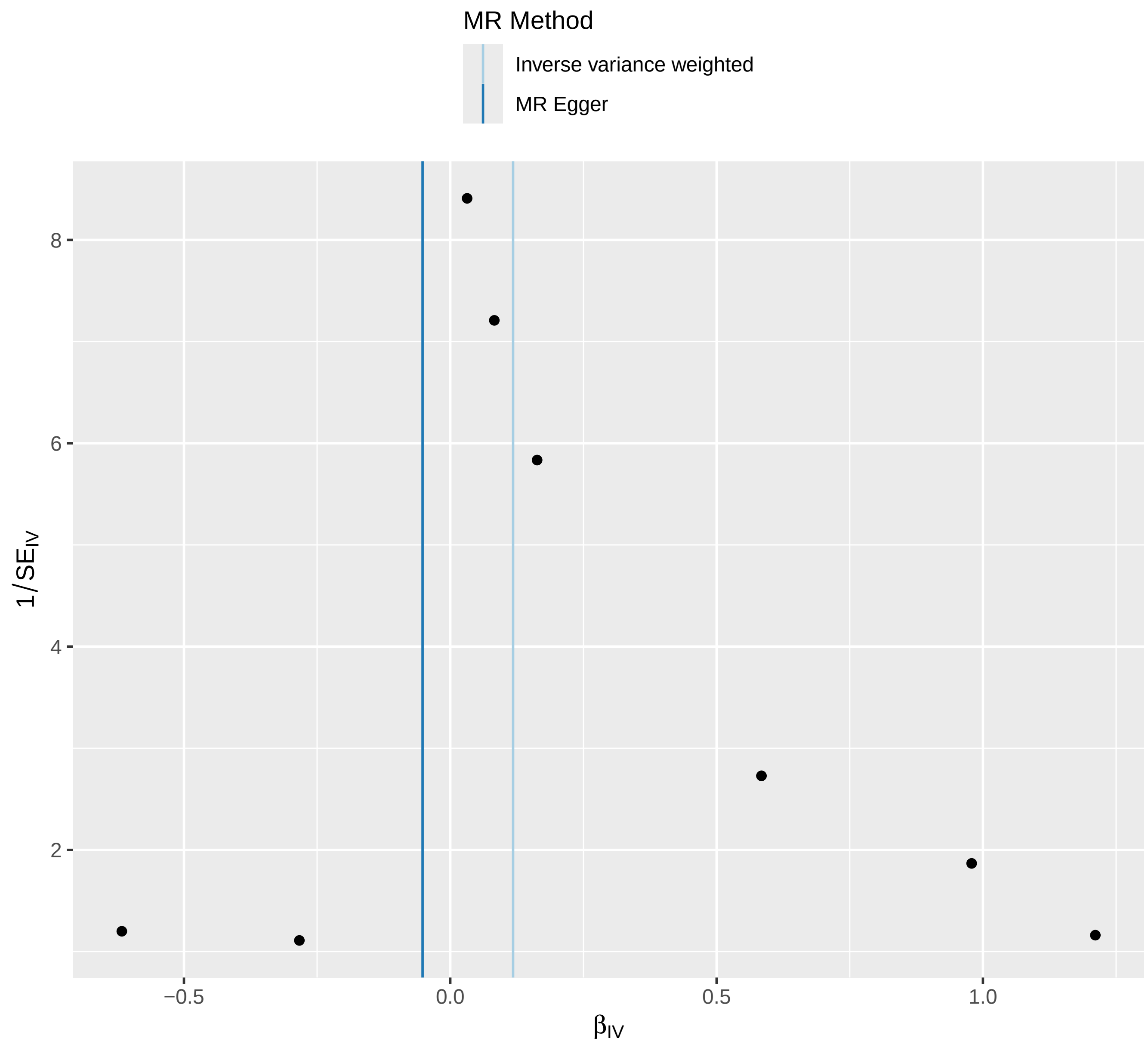

MR Method
Inverse variance weighted
MR Egger
8
6
4
2
1/SE_IV
−0.5
0.0
0.5
1.0
β_IV

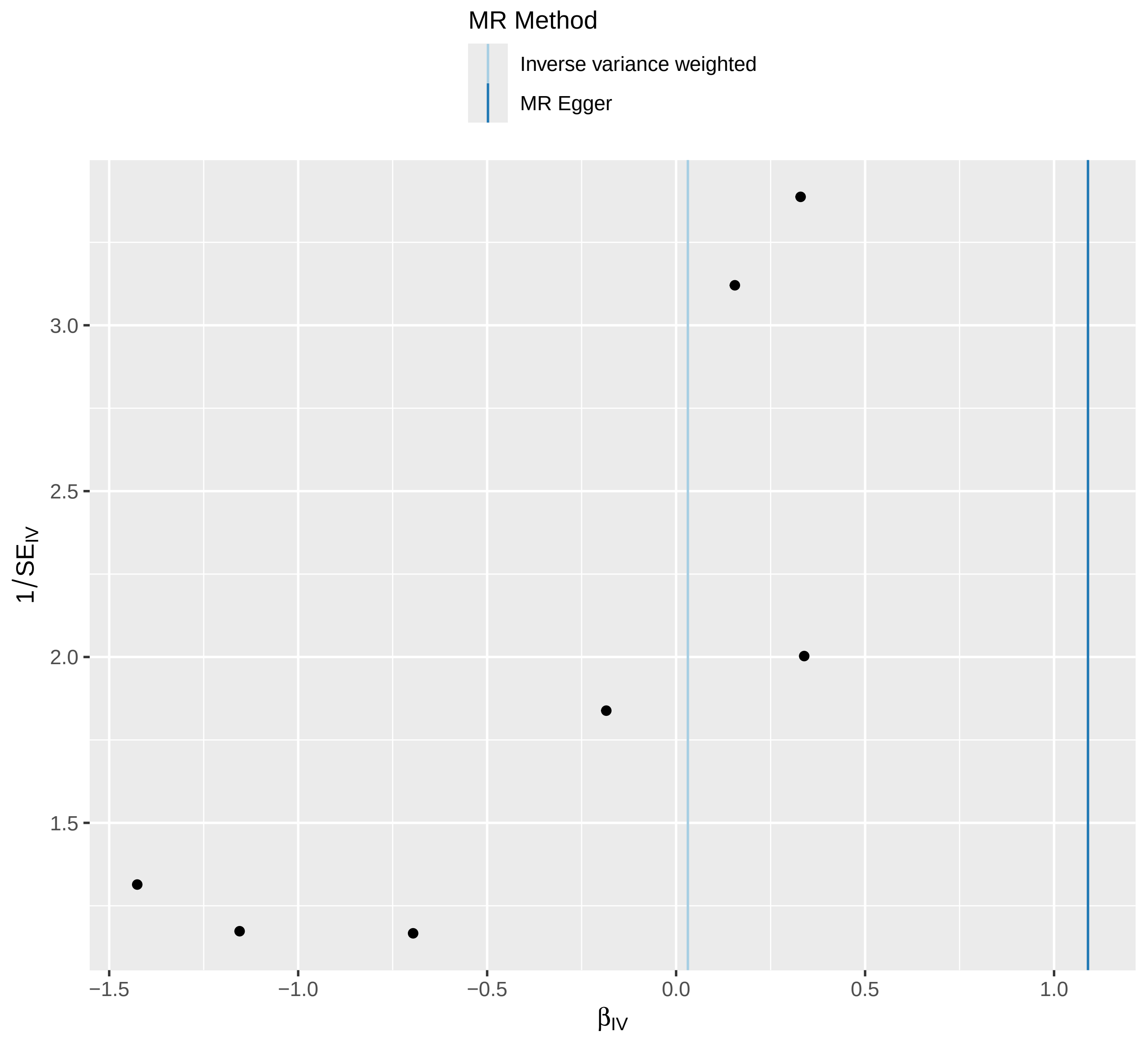
MR Method
Inverse variance weighted
MR Egger
3.0
2.5
2.0
1.5
$1/SE_{IV}$
−1.5
−1.0
−0.5
0.0
0.5
1.0
β_{IV}

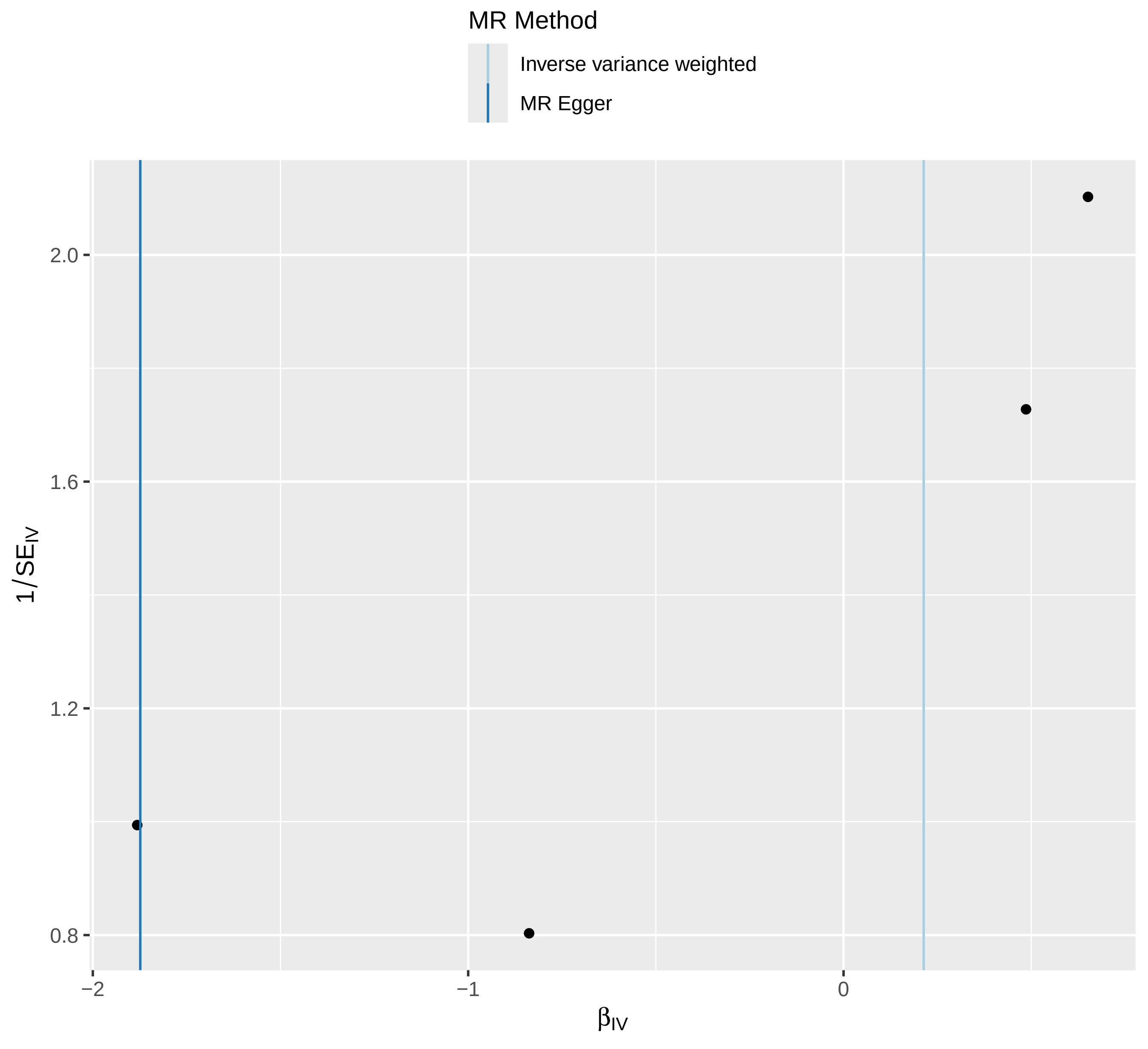
MR Method
Inverse variance weighted
MR Egger
2.0
1.6
1.2
0.8
$1/SE_{IV}$
−2
−1
0
β_{IV}

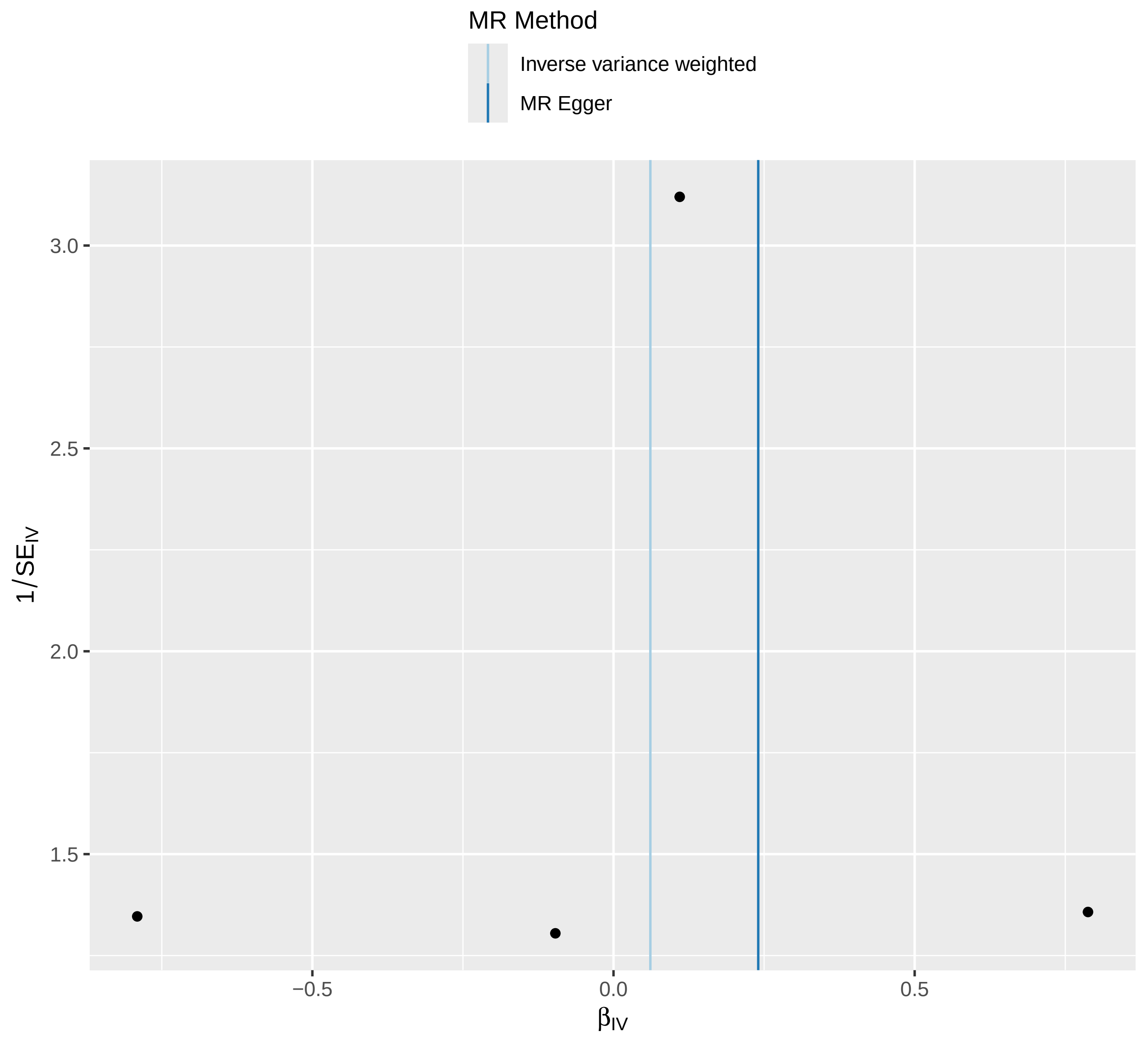

MR Method
Inverse variance weighted
MR Egger
3.0
2.5
2.0
1.5
1/SE_IV
−0.5
0.0
0.5
β_IV

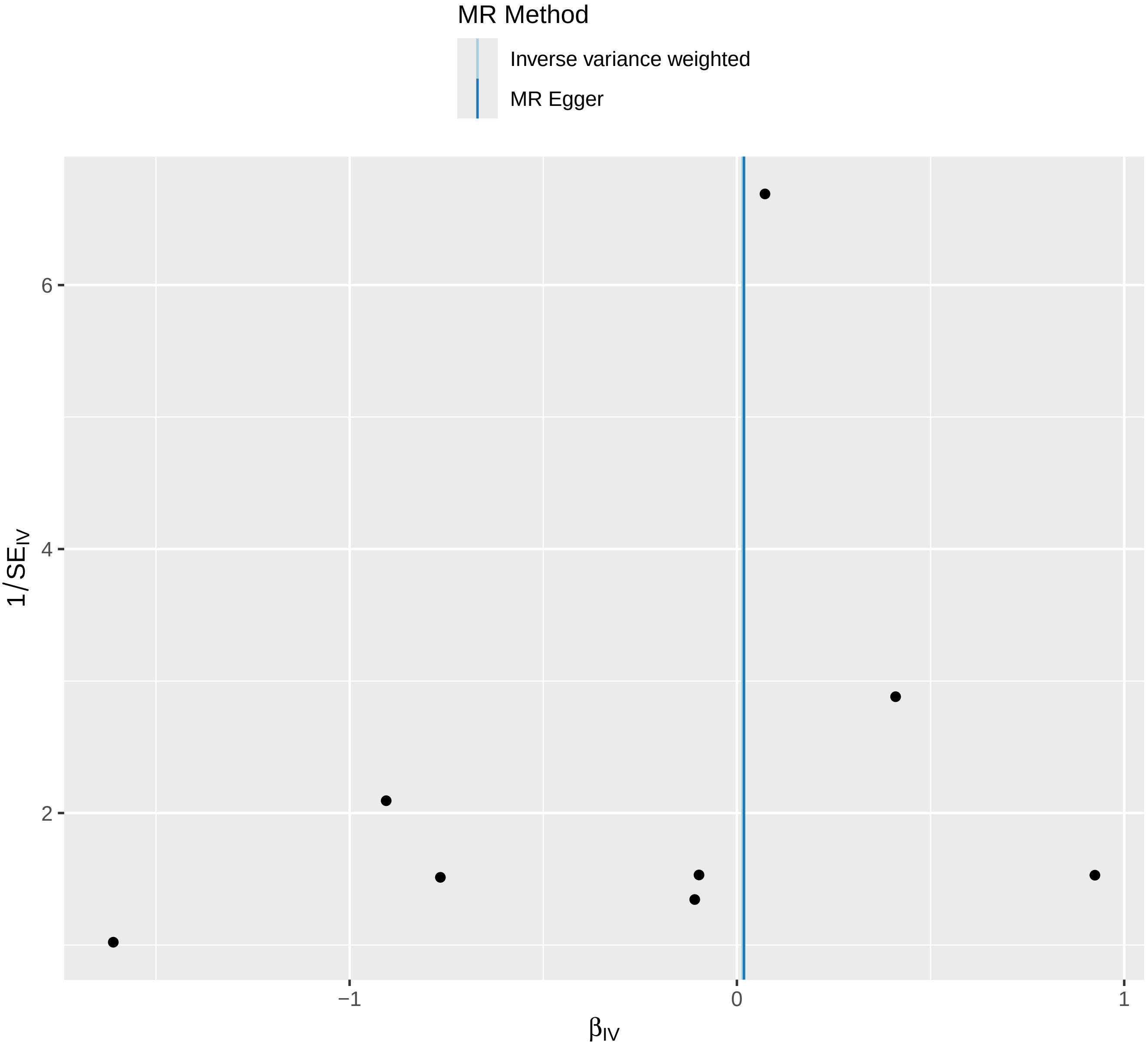
MR Method
Inverse variance weighted
MR Egger
1/SE$_{IV}$
6
4
2
−1
0
1
β$_{IV}$

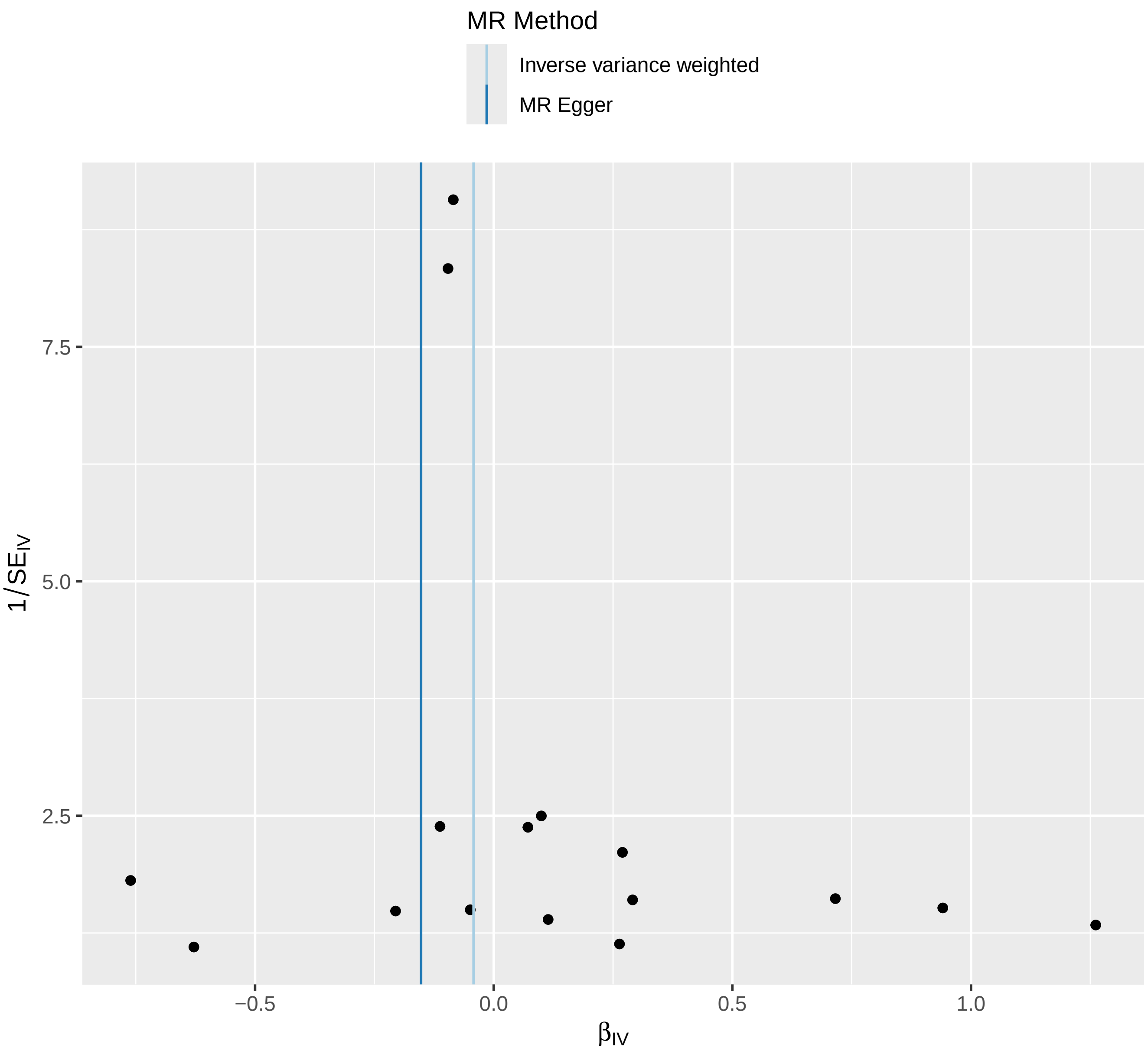
MR Method
Inverse variance weighted
MR Egger
7.5
5.0
2.5
1/SE_IV
−0.5
0.0
0.5
1.0
β_IV

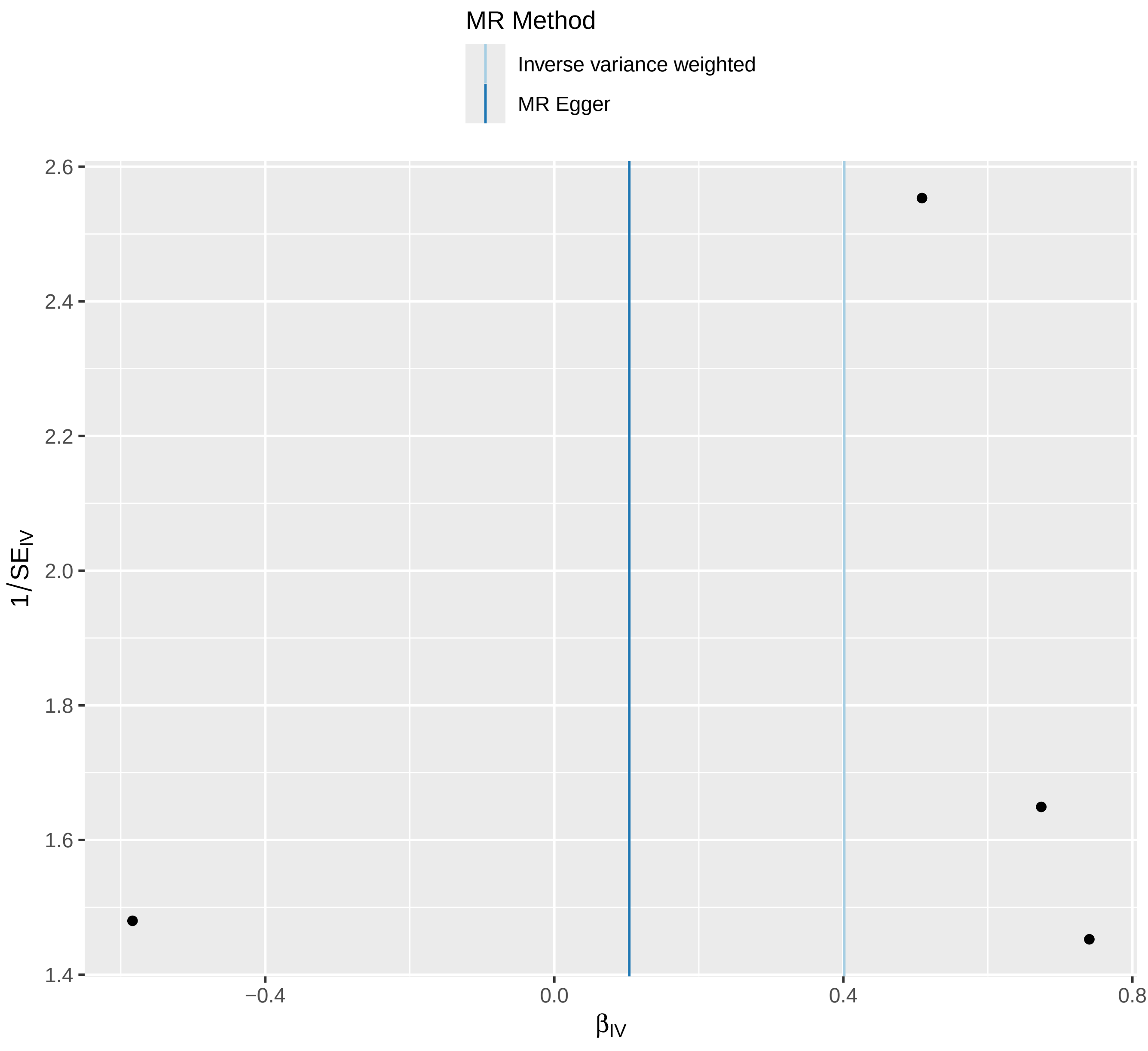
MR Method
Inverse variance weighted
MR Egger
2.6
2.4
2.2
2.0
1.8
1.6
1.4
1/SE_IV
−0.4
0.0
0.4
0.8
β_IV

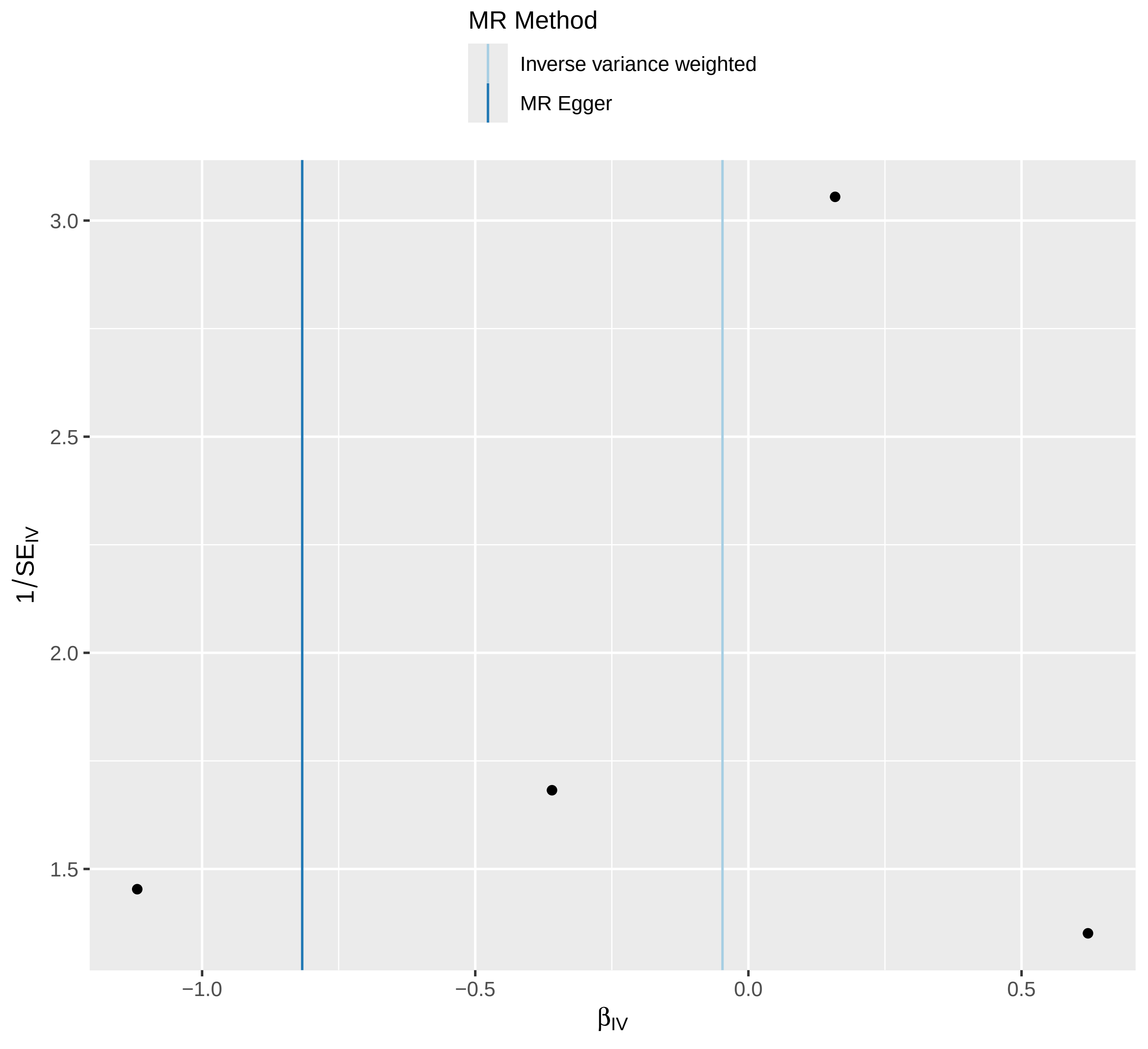
MR Method
Inverse variance weighted
MR Egger
1/SE$_{IV}$
3.0
2.5
2.0
1.5
−1.0
−0.5
0.0
0.5
β$_{IV}$

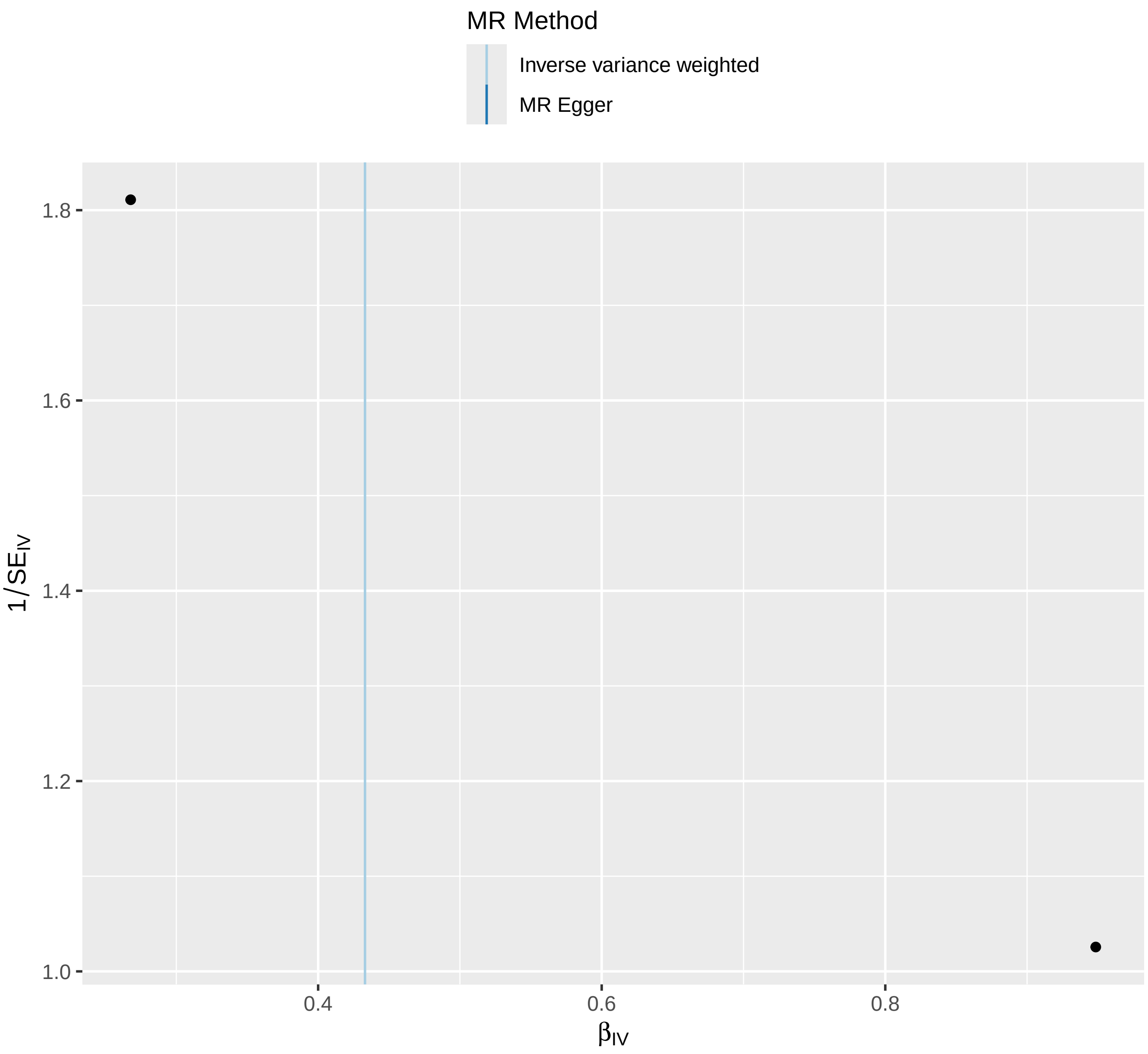

MR Method
Inverse variance weighted
MR Egger
1/SE_IV
β_IV
1.8
1.6
1.4
1.2
1.0
0.4
0.6
0.8

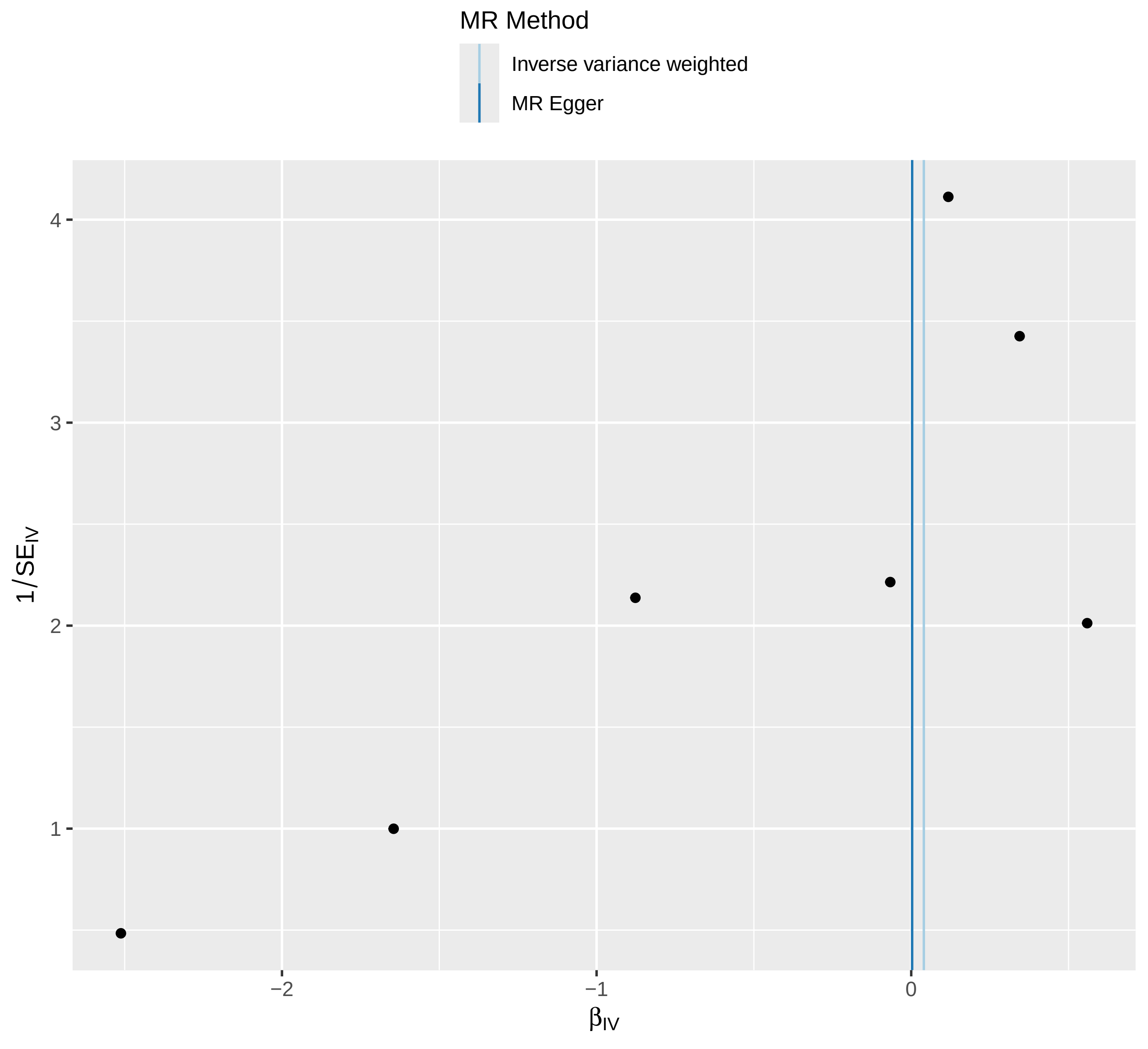

MR Method
Inverse variance weighted
MR Egger
1/SE_IV
4
3
2
1
−2
−1
0
β_IV

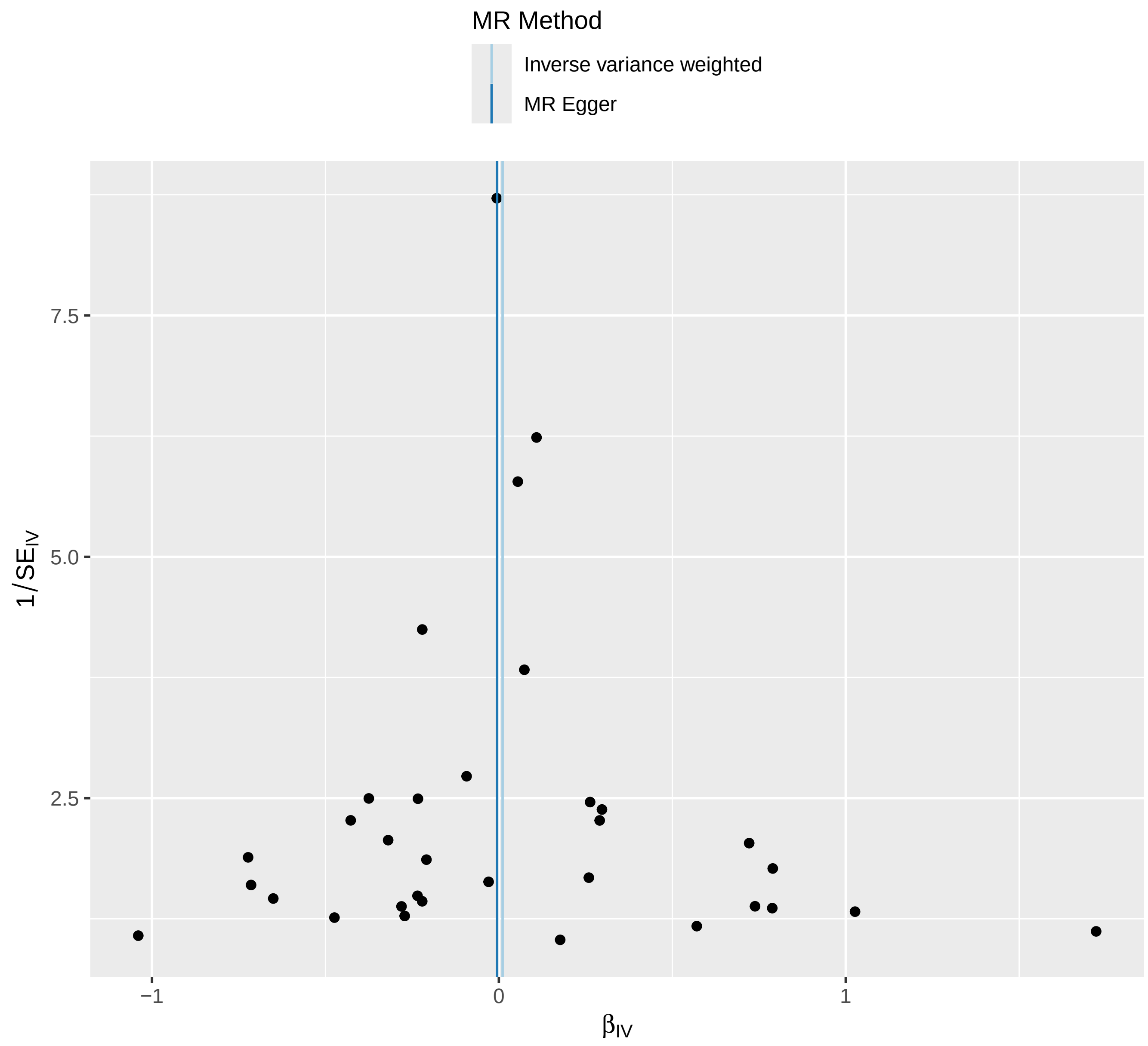

MR Method
Inverse variance weighted
MR Egger
7.5
5.0
2.5
$1/SE_{IV}$
−1
0
1
β_{IV}

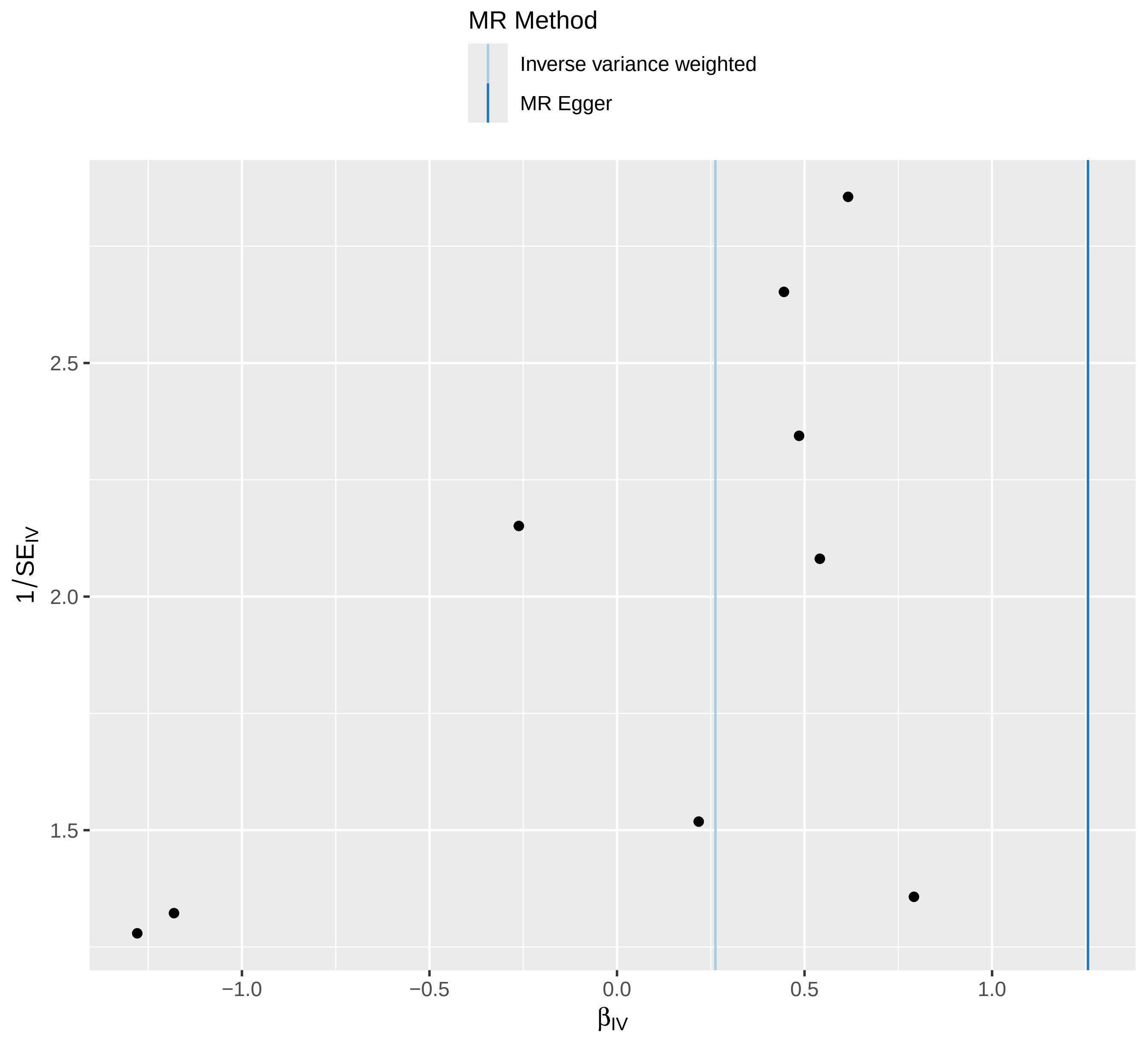

MR Method
Inverse variance weighted
MR Egger
2.5
2.0
1.5
1/SE_IV
−1.0
−0.5
0.0
0.5
1.0
β_IV

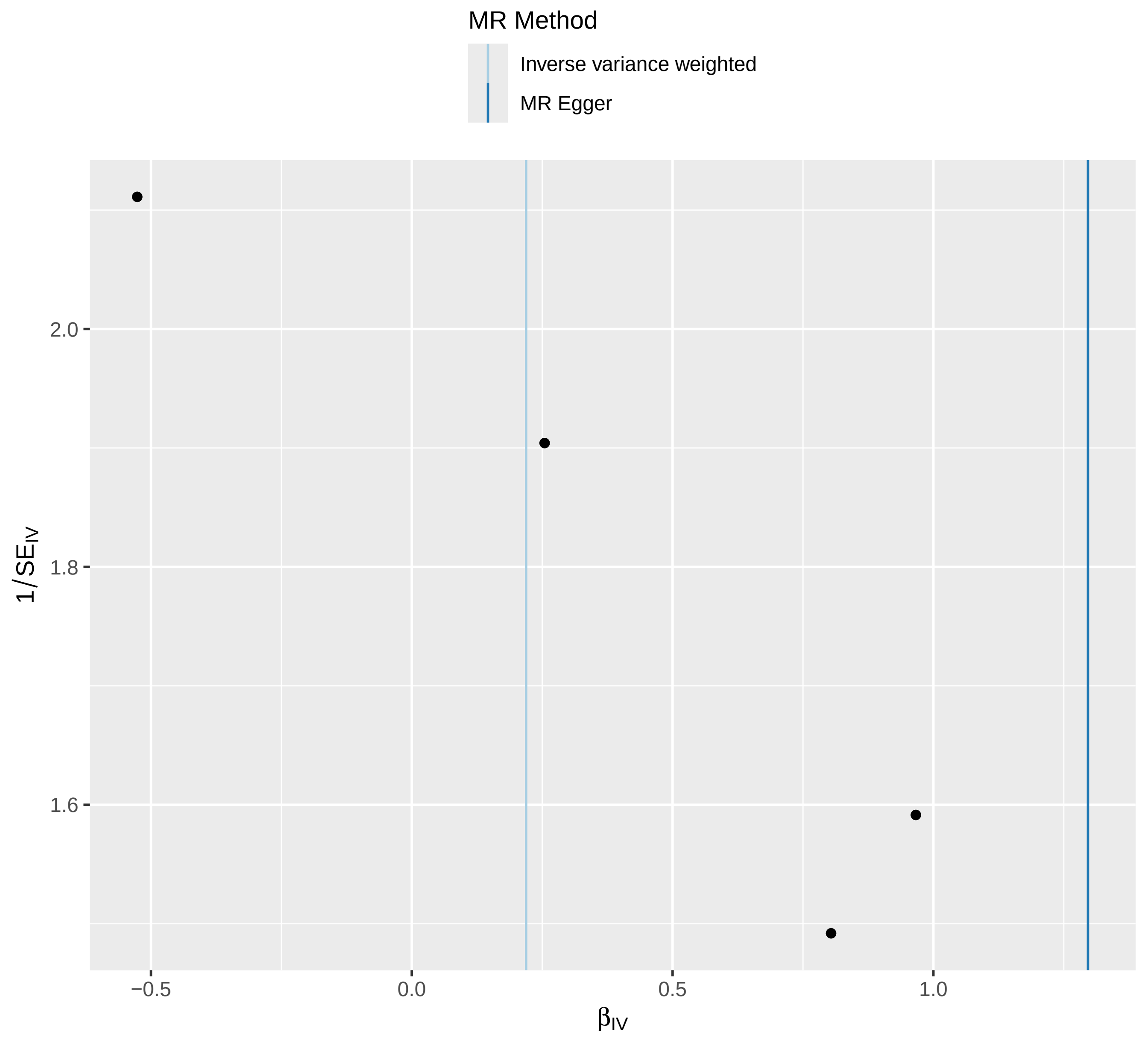
MR Method
Inverse variance weighted
MR Egger
2.0
1.8
1.6
−0.5
0.0
0.5
1.0
$1/SE_{IV}$
β_{IV}

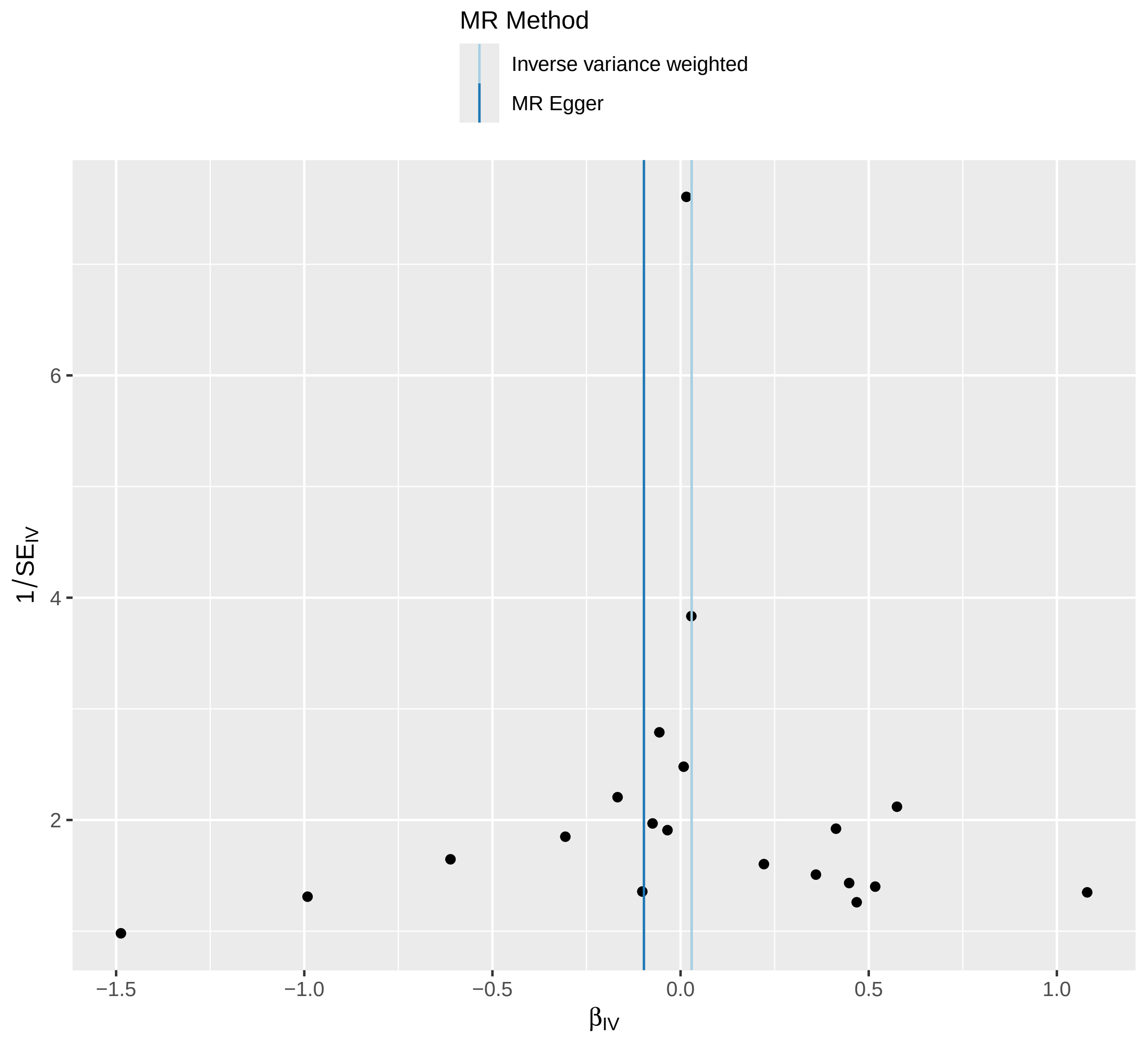
MR Method
Inverse variance weighted
MR Egger
1/SE_{IV}
6
4
2
−1.5
−1.0
−0.5
0.0
0.5
1.0
β_{IV}

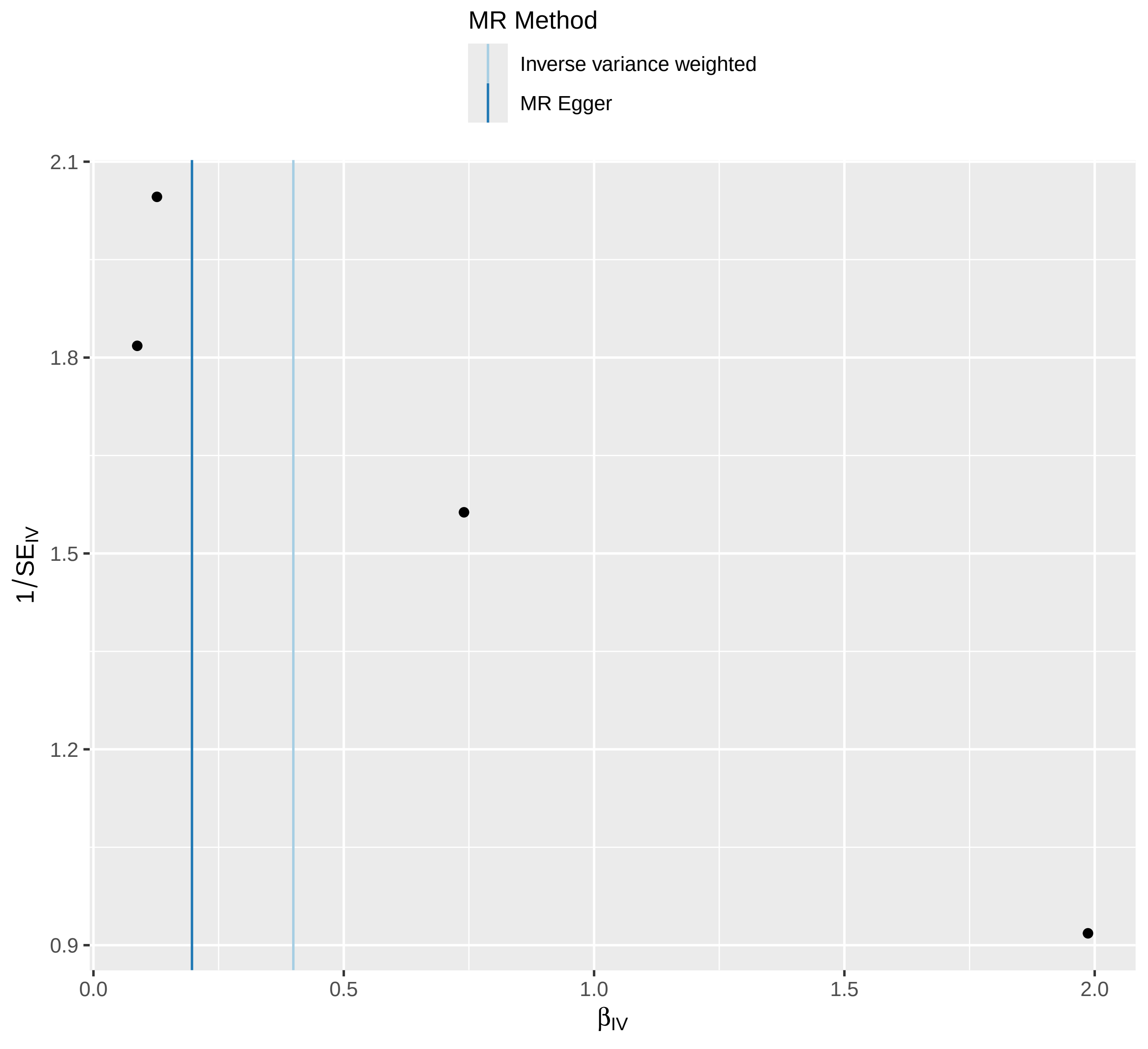

MR Method
Inverse variance weighted
MR Egger
2.1
1.8
1.5
1.2
0.9
1/SE_IV
0.0
0.5
1.0
1.5
2.0
β_IV

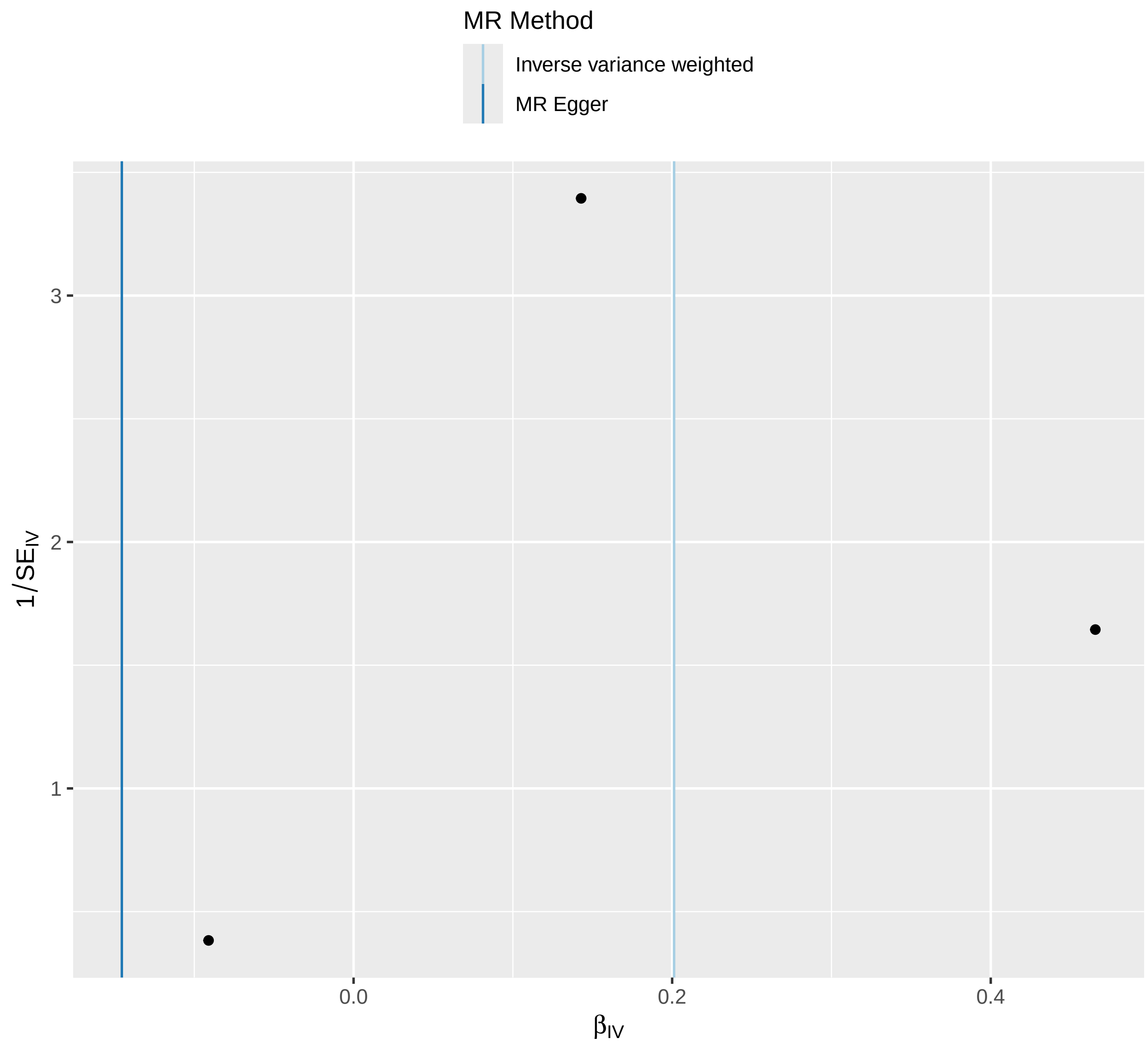

MR Method
Inverse variance weighted
MR Egger
3
2
1
1/SE_IV
0.0
0.2
0.4
β_IV

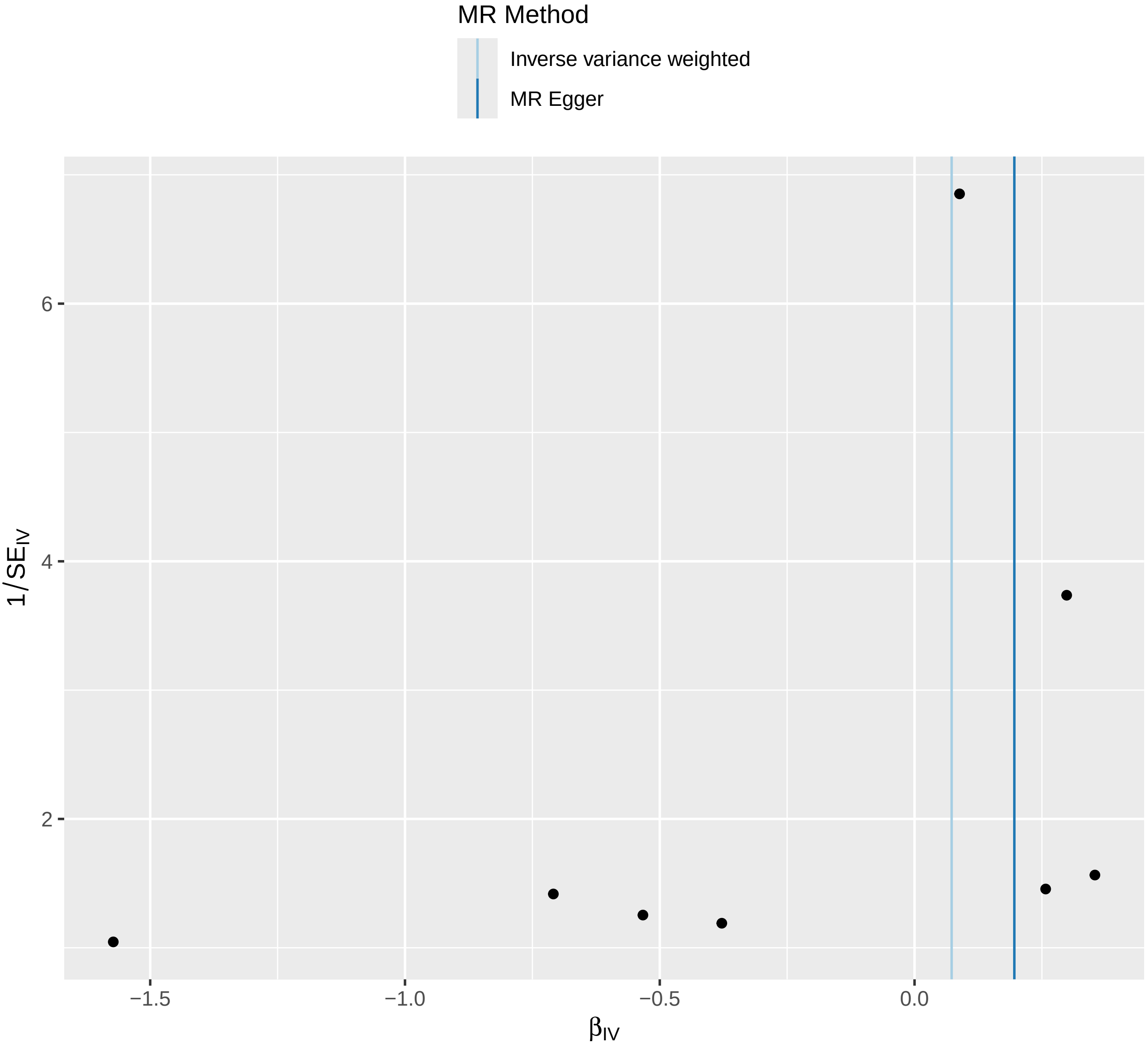
MR Method
Inverse variance weighted
MR Egger
6
4
2
$1/SE_{IV}$
−1.5
−1.0
−0.5
0.0
β_{IV}

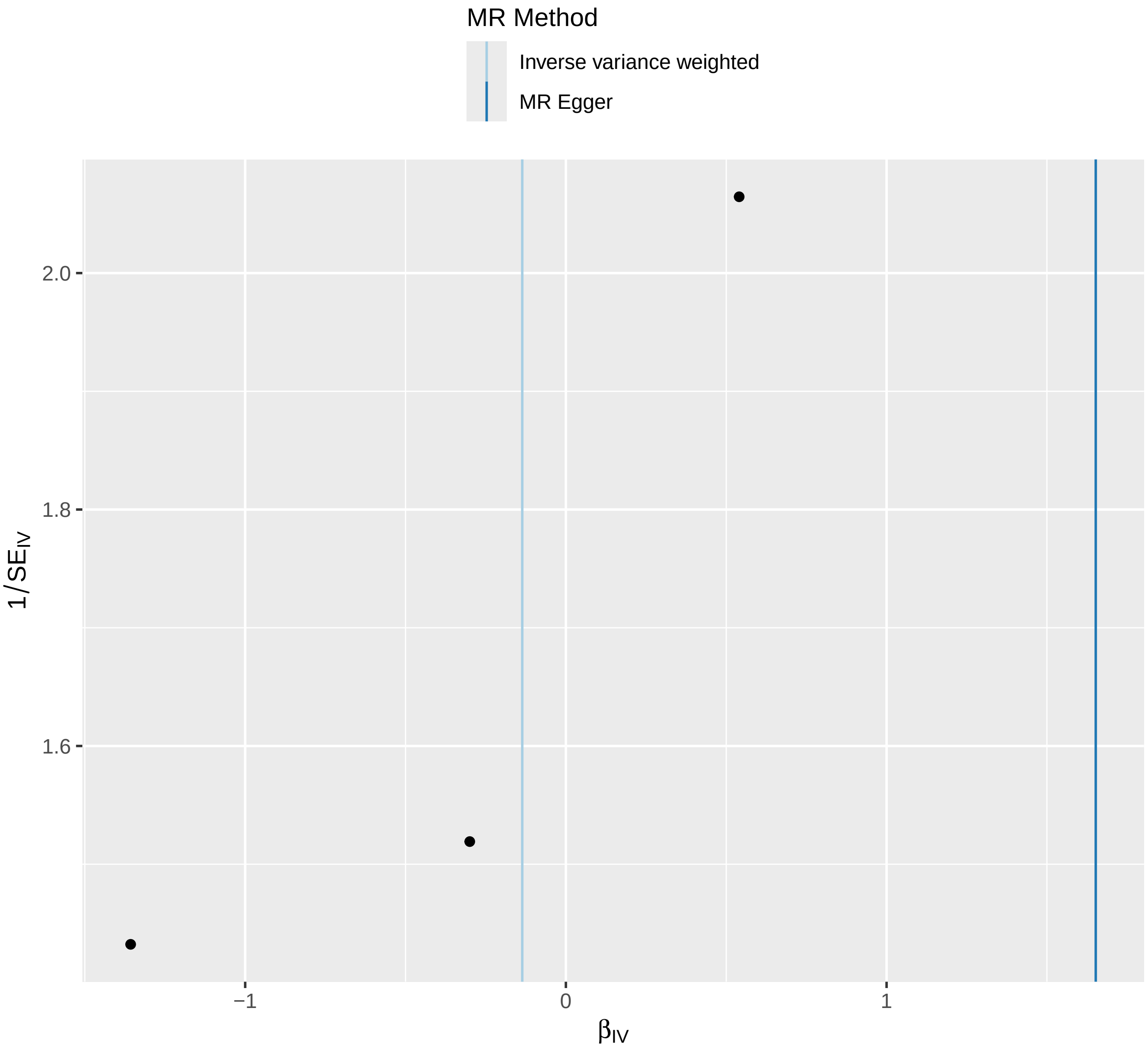

MR Method
Inverse variance weighted
MR Egger
2.0
1.8
1.6
1/SE_IV
−1
0
1
β_IV

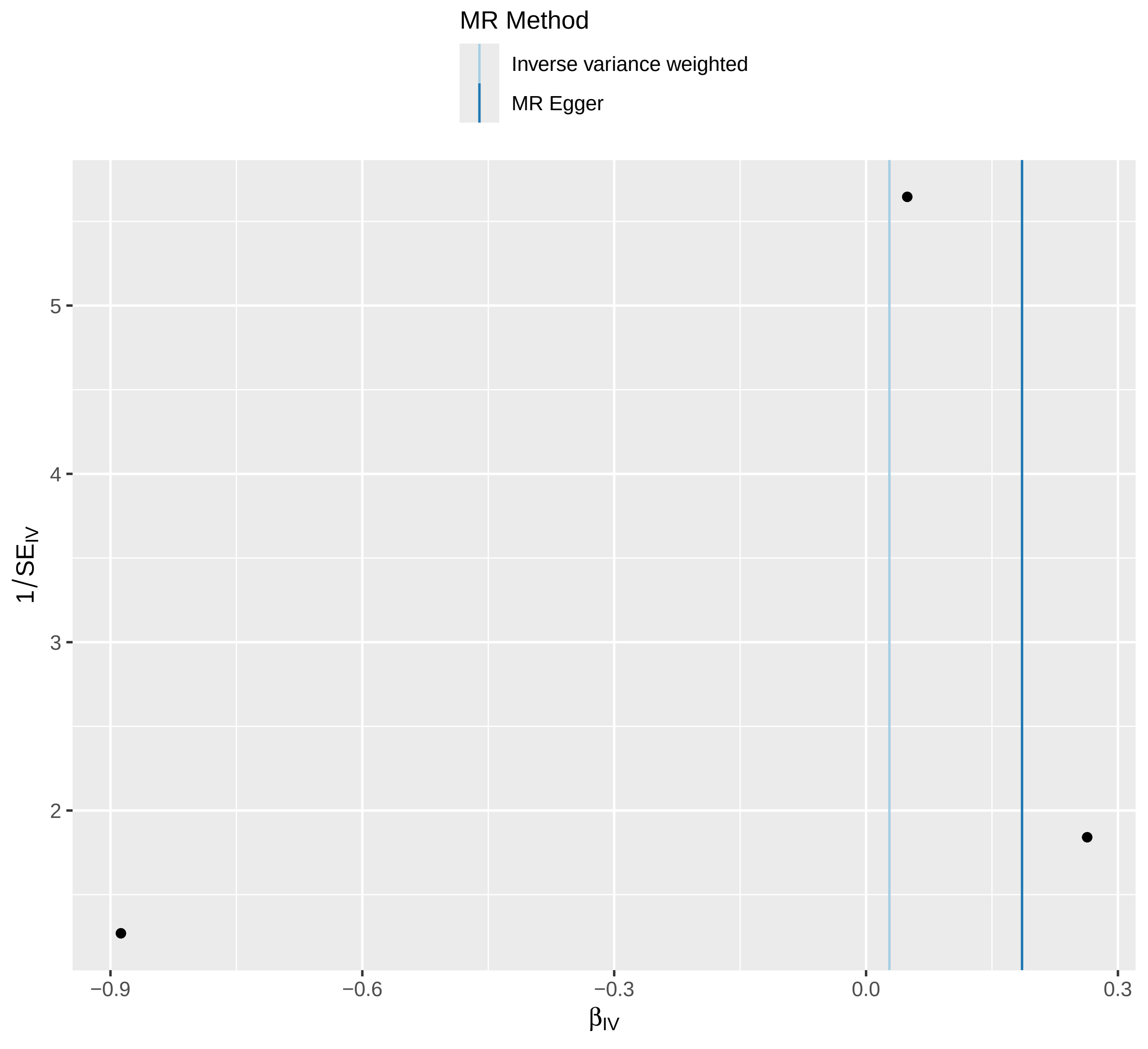
MR Method
Inverse variance weighted
MR Egger
1/SE$_{IV}$
5
4
3
2
−0.9
−0.6
−0.3
0.0
0.3
β$_{IV}$

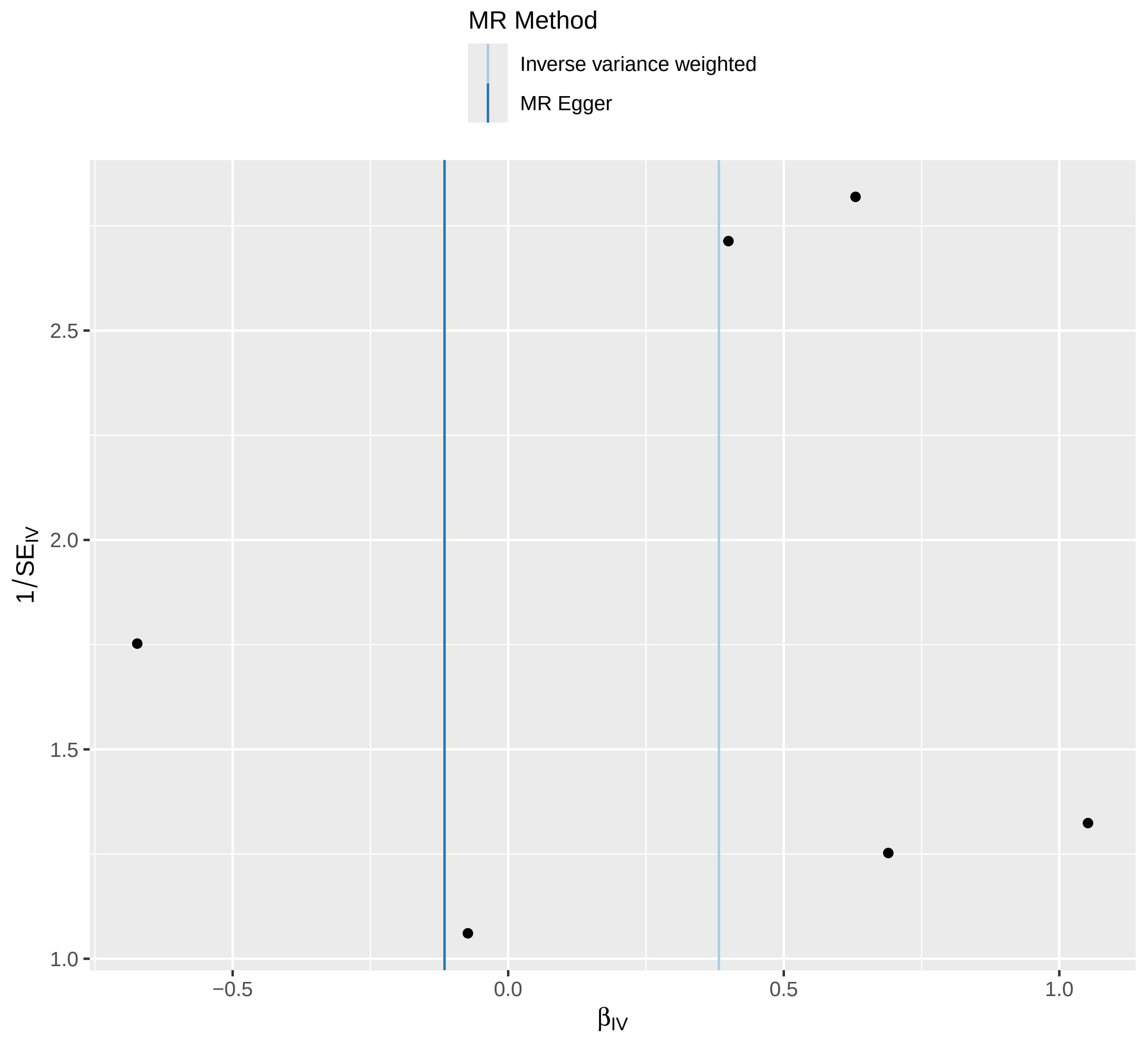
MR Method
Inverse variance weighted
MR Egger
2.5
2.0
1.5
1.0
1/SE$_{IV}$
−0.5
0.0
0.5
1.0
β$_{IV}$

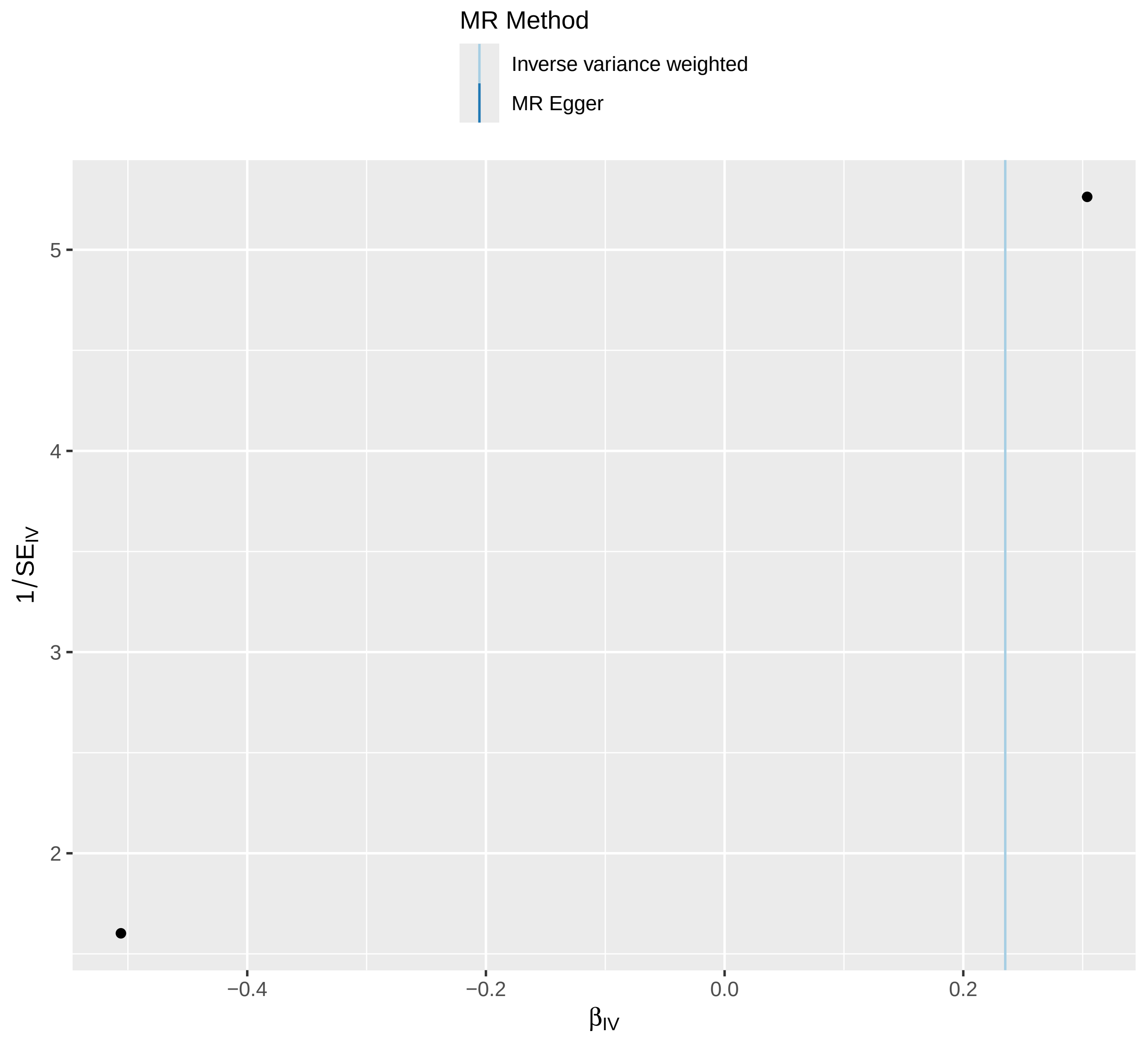
MR Method
Inverse variance weighted
MR Egger
5
4
3
2
1/SE_{IV}
−0.4
−0.2
0.0
0.2
β_{IV}

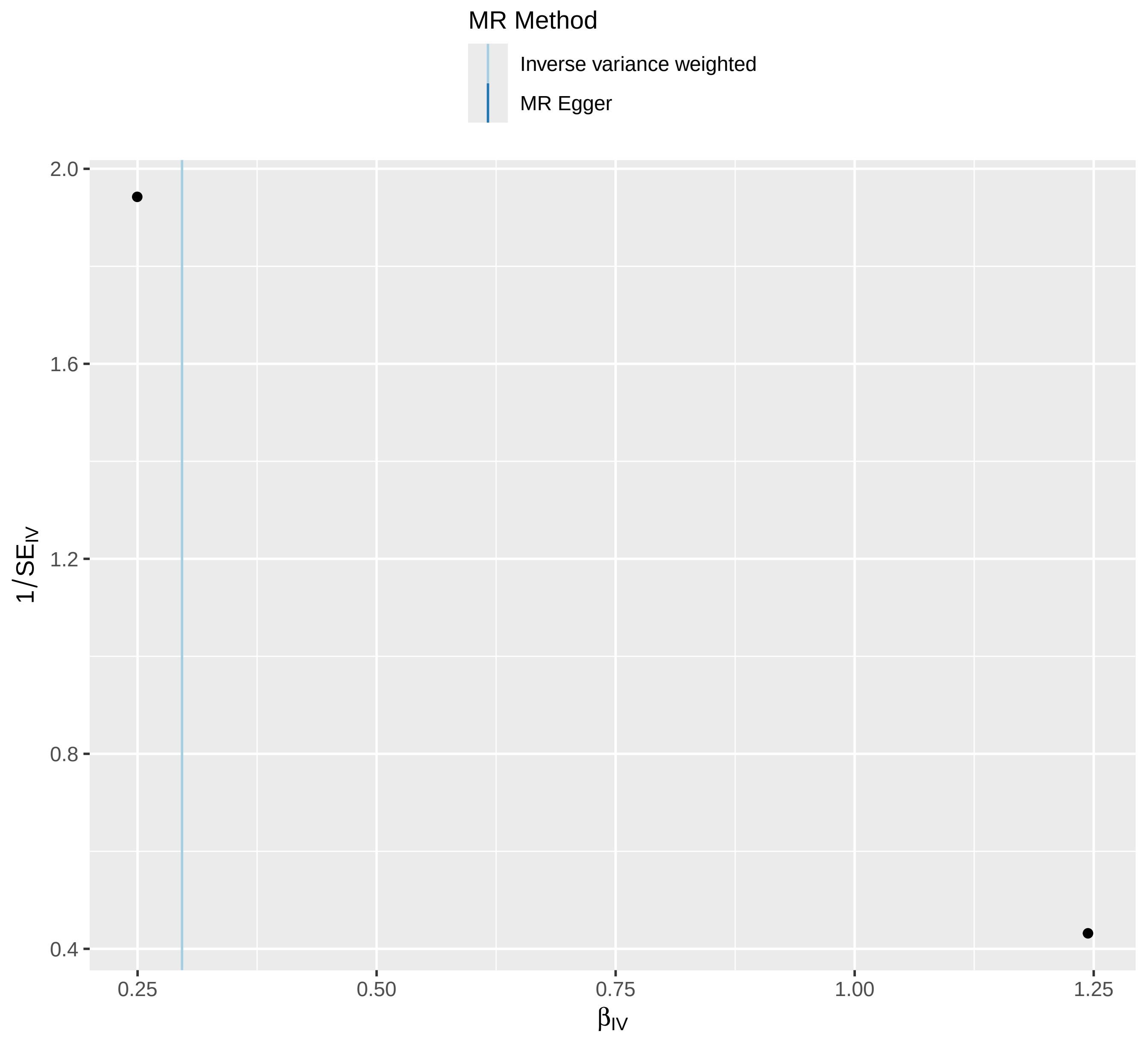

MR Method
Inverse variance weighted
MR Egger
2.0
1.6
1.2
0.8
0.4
$1/SE_{IV}$
0.25
0.50
0.75
1.00
1.25
β_{IV}

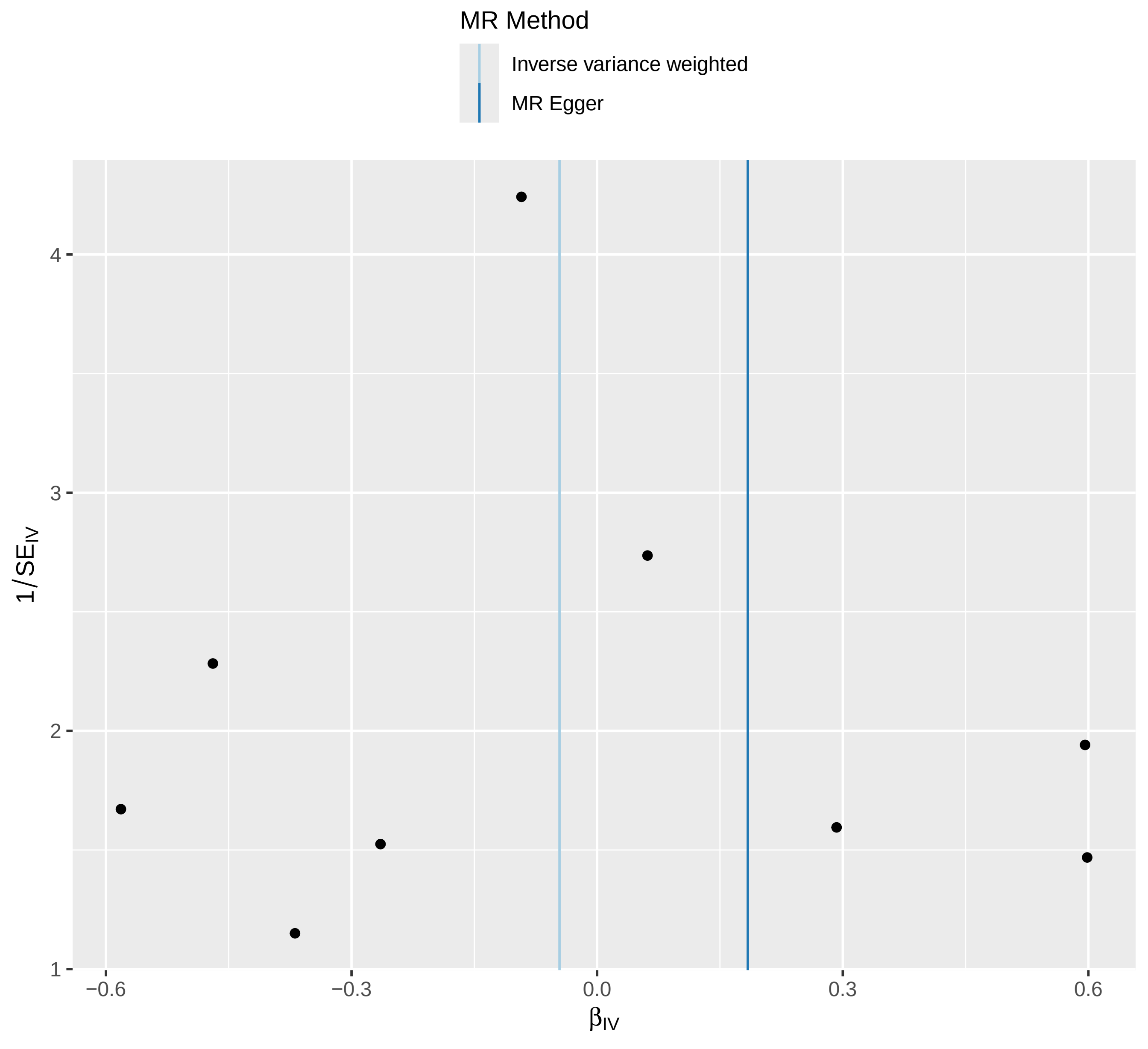

MR Method
Inverse variance weighted
MR Egger
4
3
2
1
1/SE_IV
−0.6
−0.3
0.0
0.3
0.6
β_IV

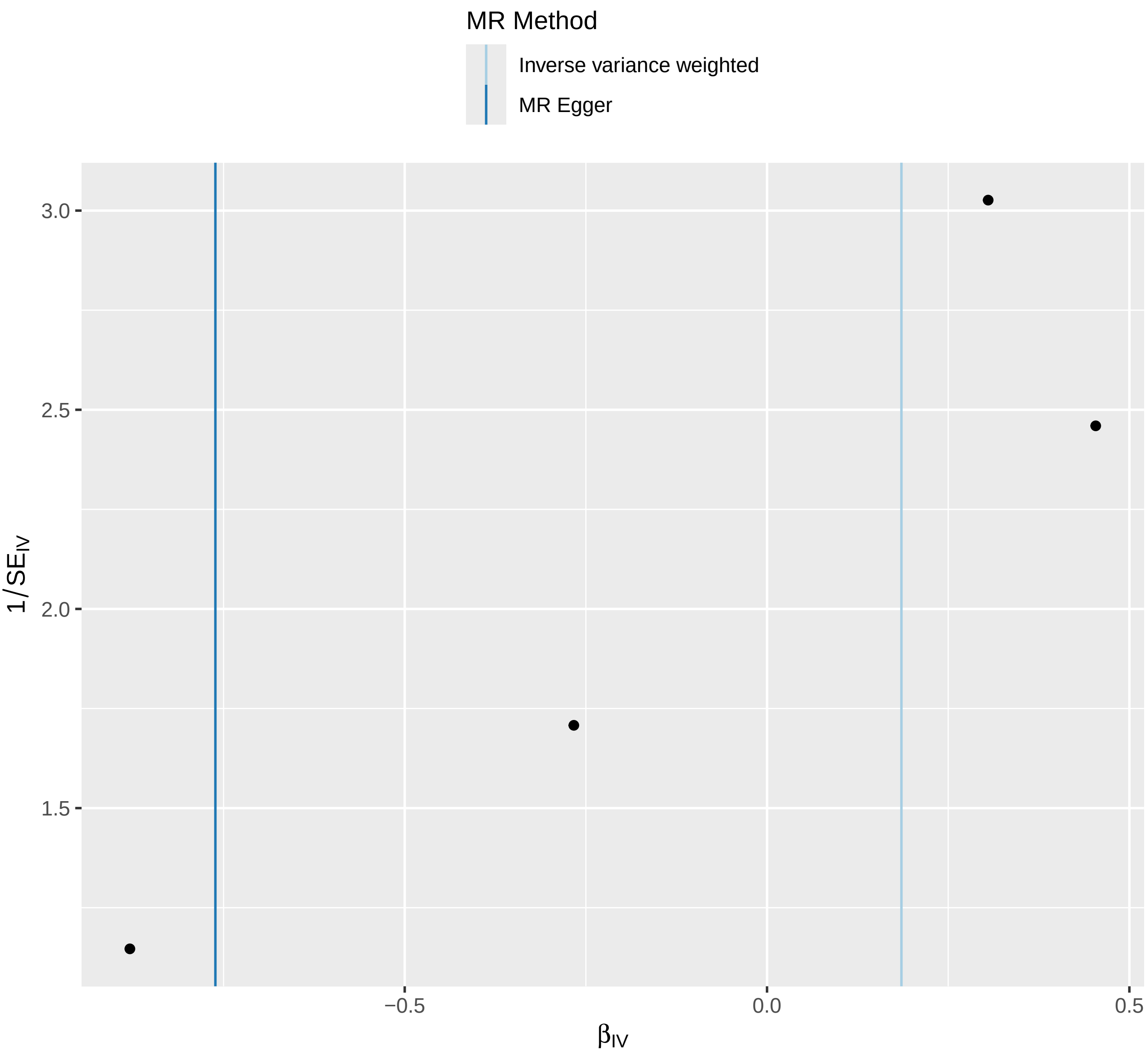
MR Method
Inverse variance weighted
MR Egger
3.0
2.5
2.0
1.5
1/SE_IV
−0.5
0.0
0.5
β_IV

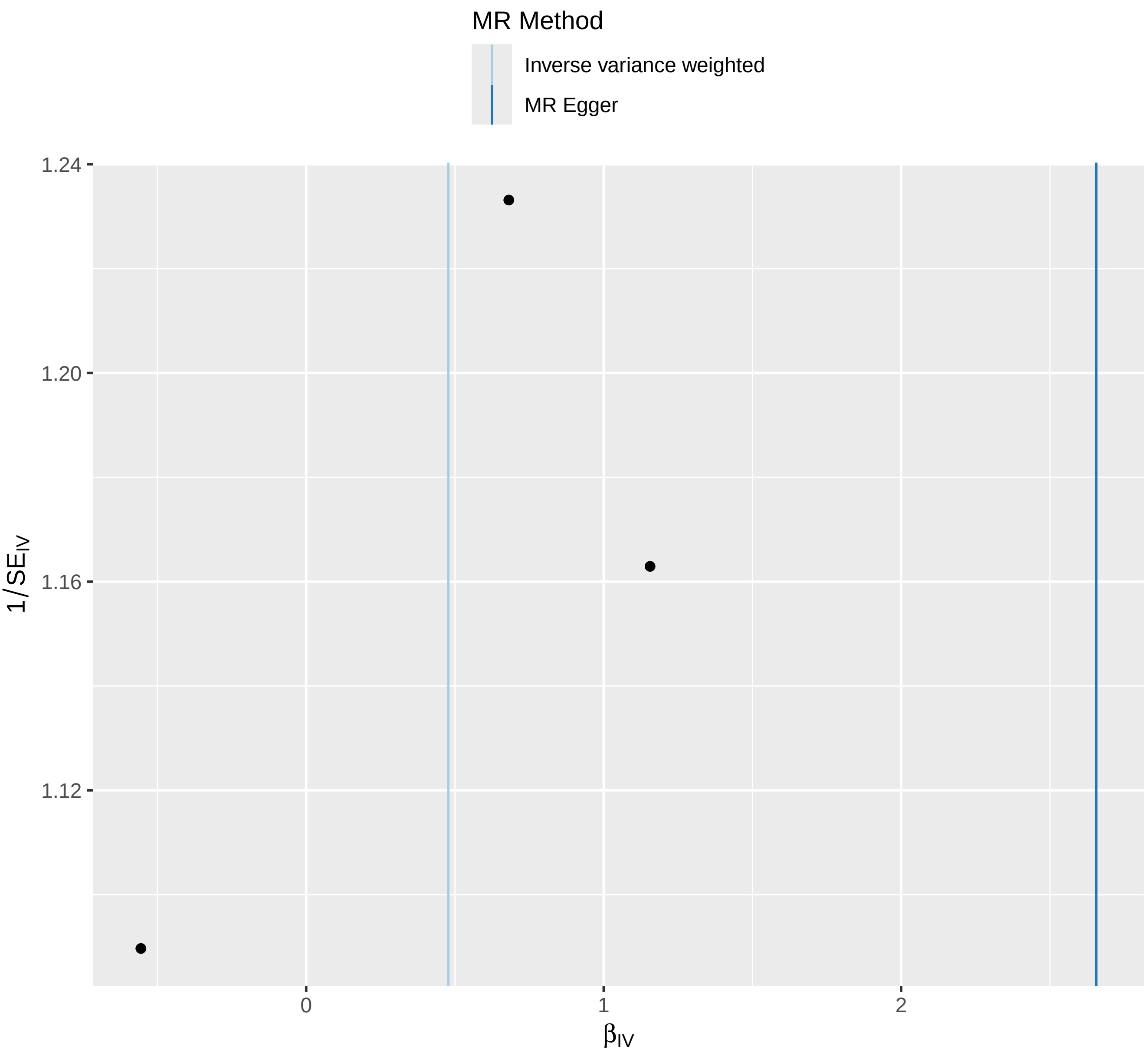
MR Method
Inverse variance weighted
MR Egger
1.24
1.20
1.16
1.12
1/SE_IV
0
1
2
β_IV

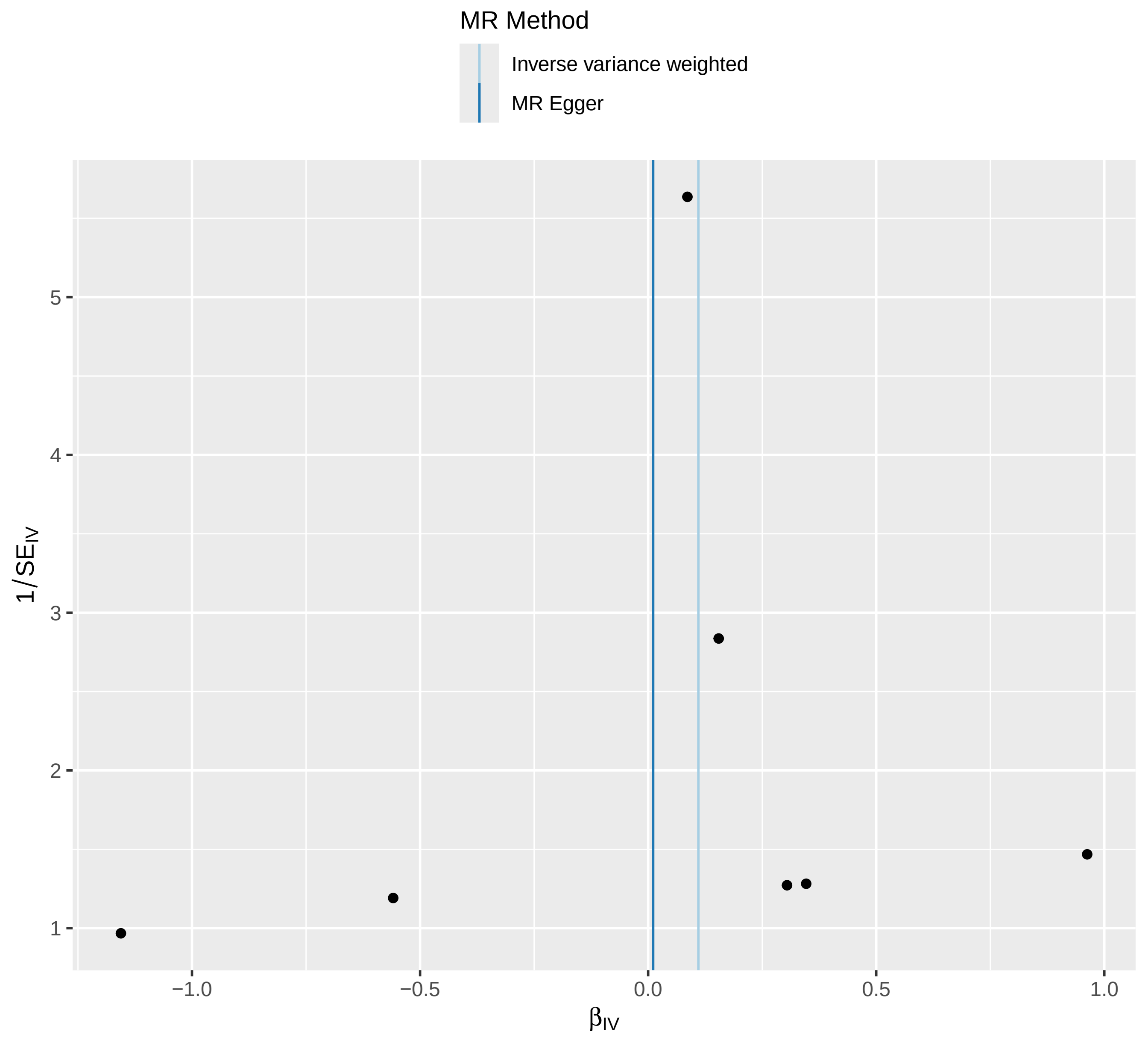
MR Method
Inverse variance weighted
MR Egger
1/SE_IV
5
4
3
2
1
−1.0
−0.5
0.0
0.5
1.0
β_IV

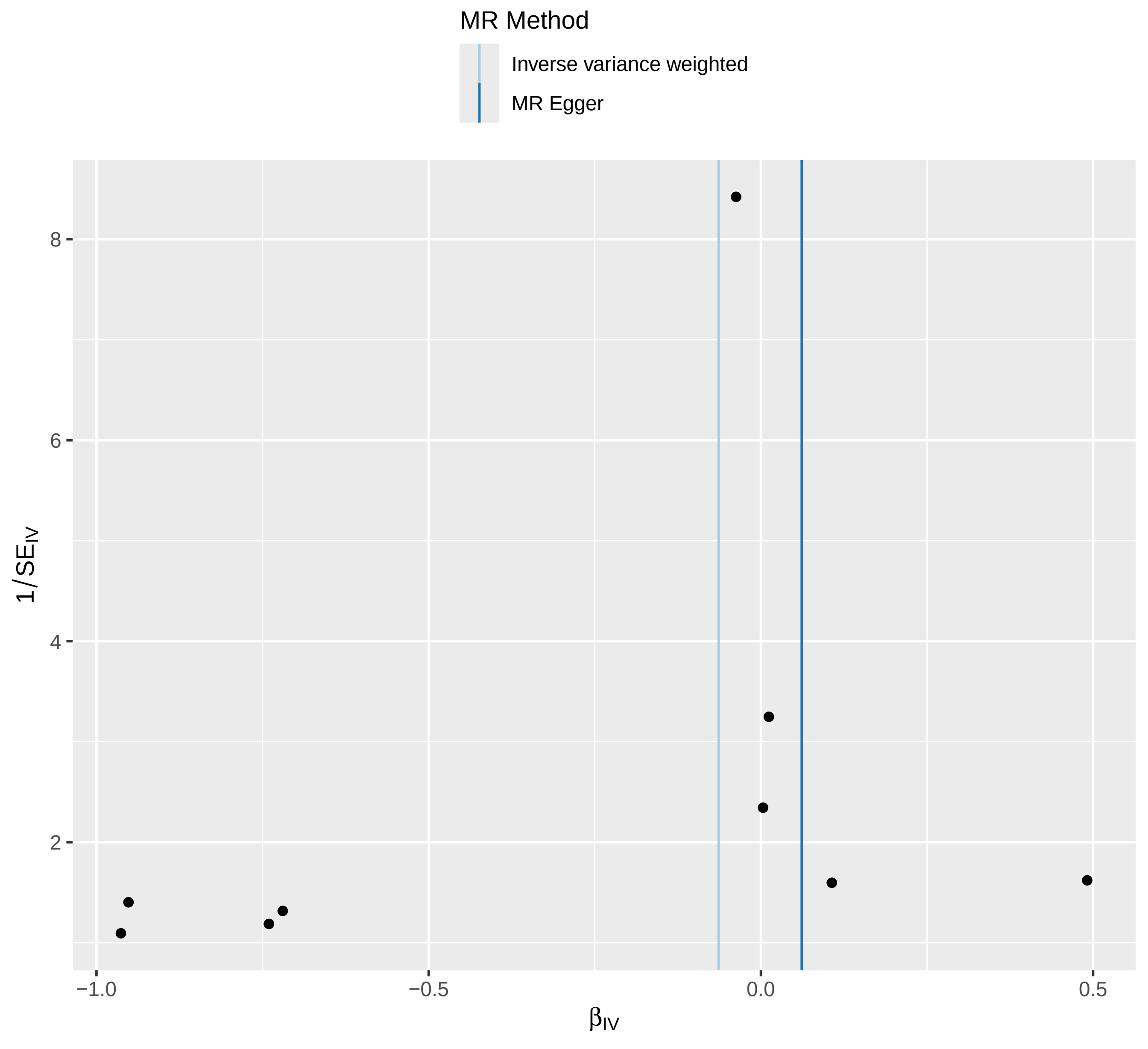
MR Method
Inverse variance weighted
MR Egger
8
6
4
2
1/SE_IV
−1.0
−0.5
0.0
0.5
β_IV

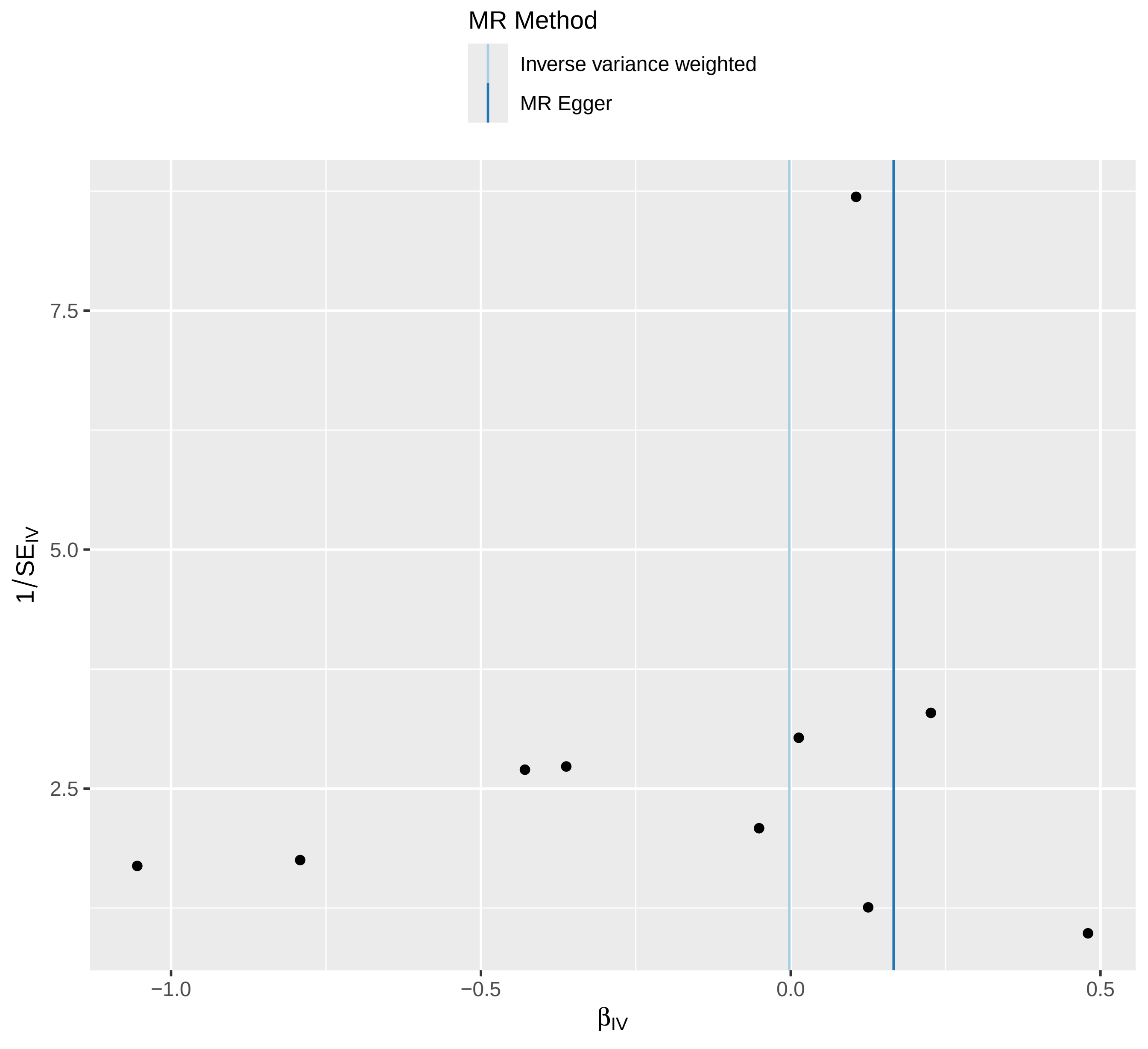

MR Method
Inverse variance weighted
MR Egger
7.5
5.0
2.5
1/SE_IV
−1.0
−0.5
0.0
0.5
β_IV

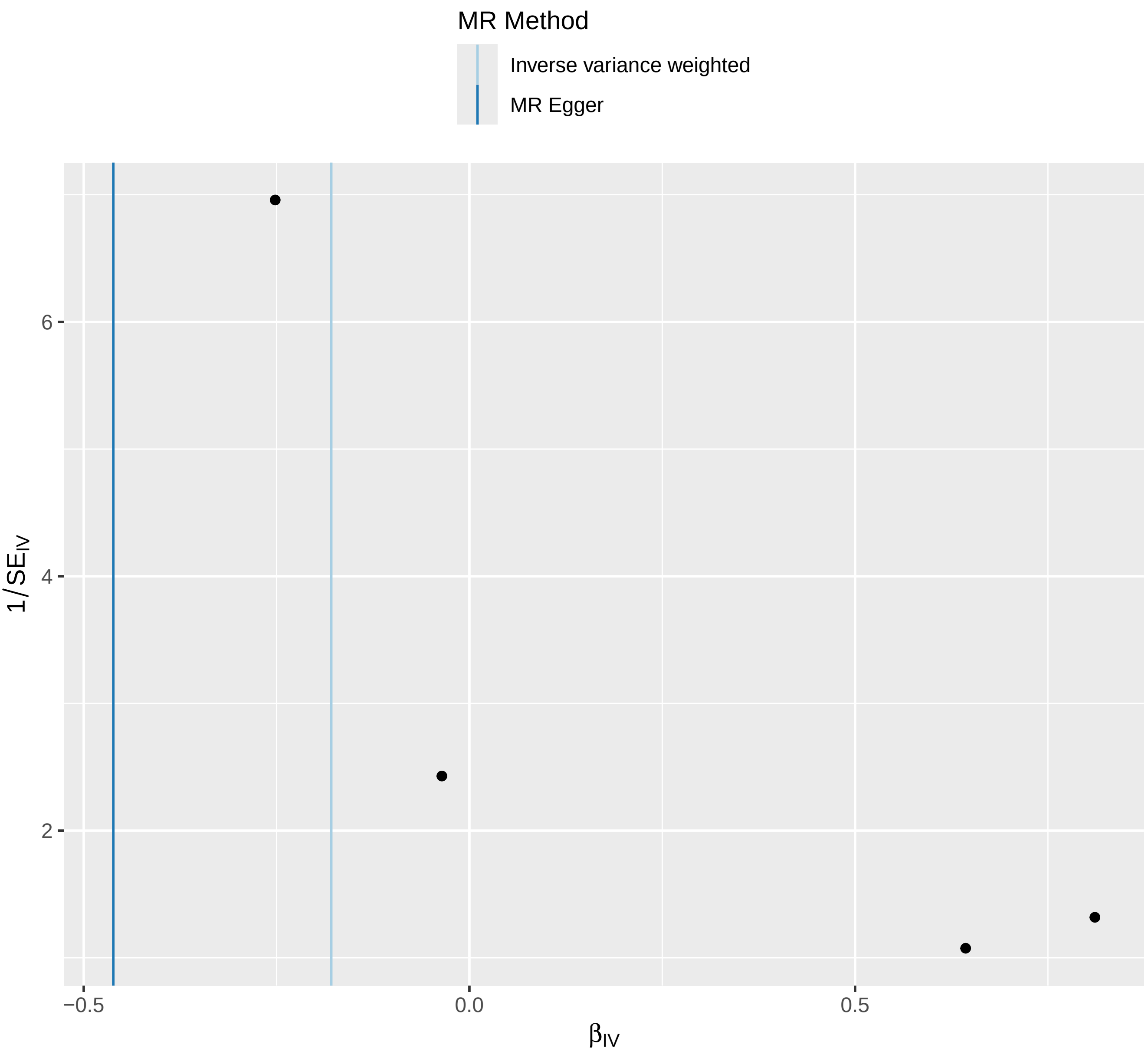
MR Method
Inverse variance weighted
MR Egger
6
4
2
1/SE$_{IV}$
−0.5
0.0
0.5
β$_{IV}$

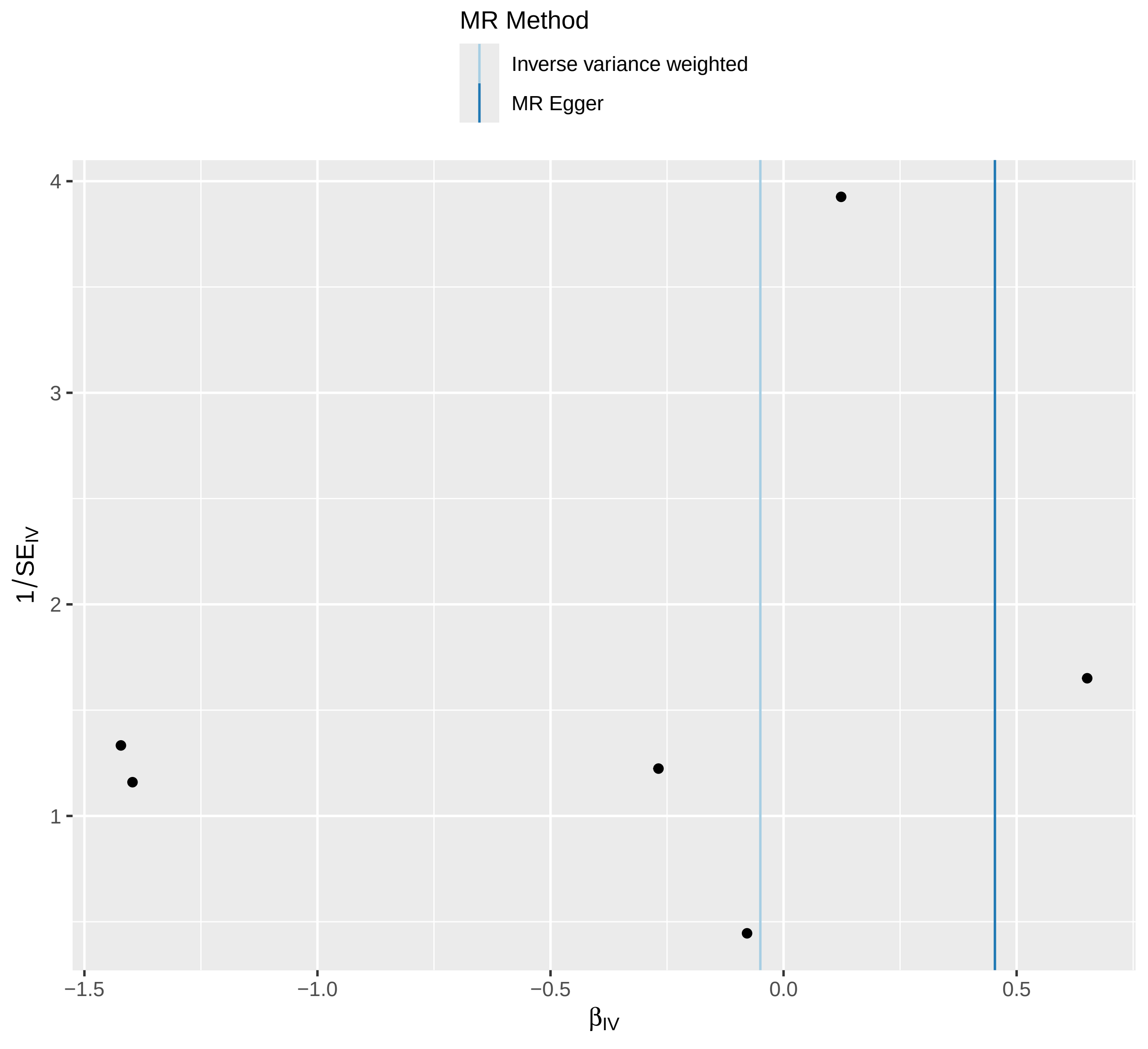
MR Method
Inverse variance weighted
MR Egger
4
3
2
1
1/SE_IV
−1.5
−1.0
−0.5
0.0
0.5
β_IV

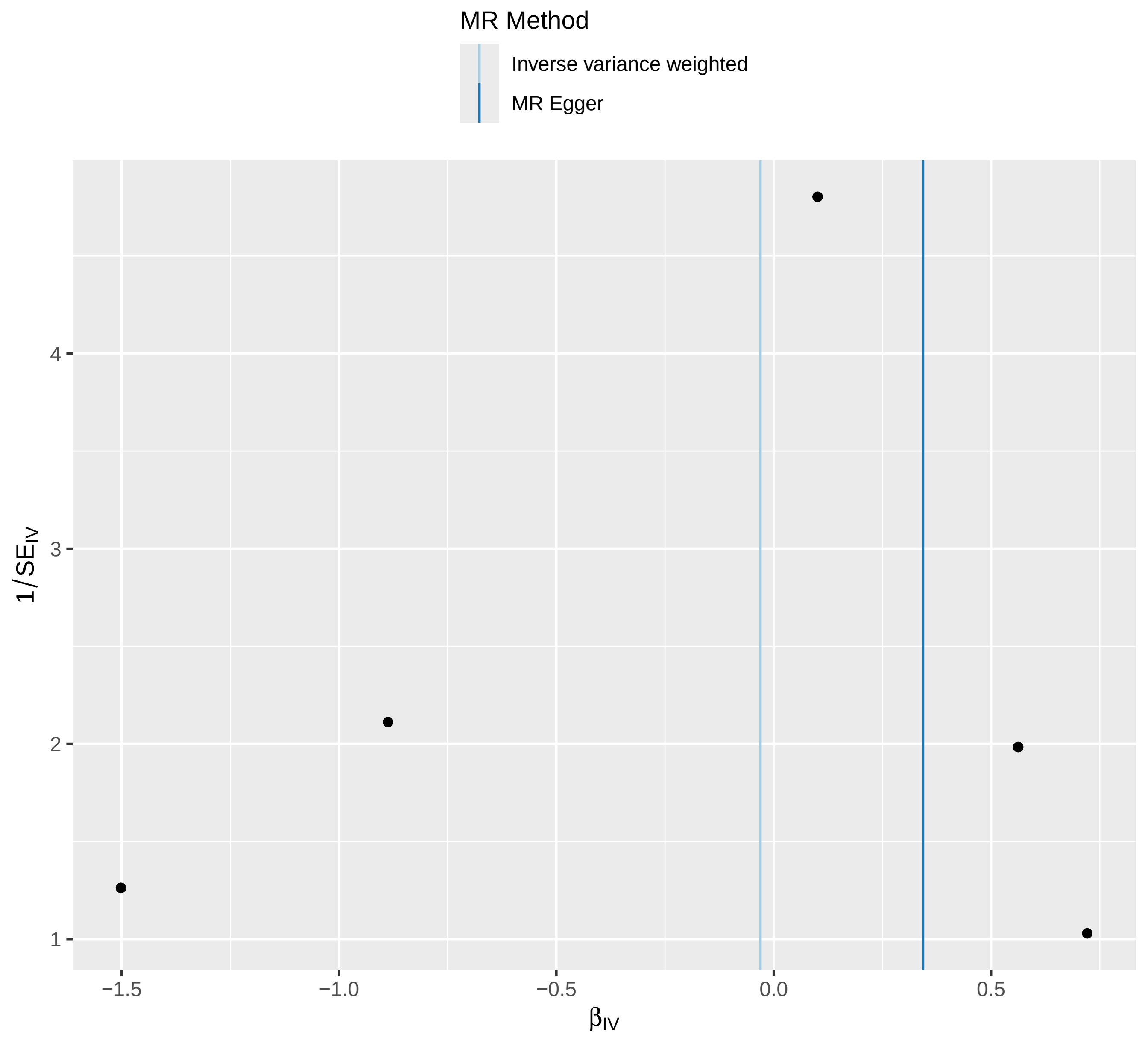

MR Method
Inverse variance weighted
MR Egger
1/SE_IV
4
3
2
1
−1.5
−1.0
−0.5
0.0
0.5
β_IV

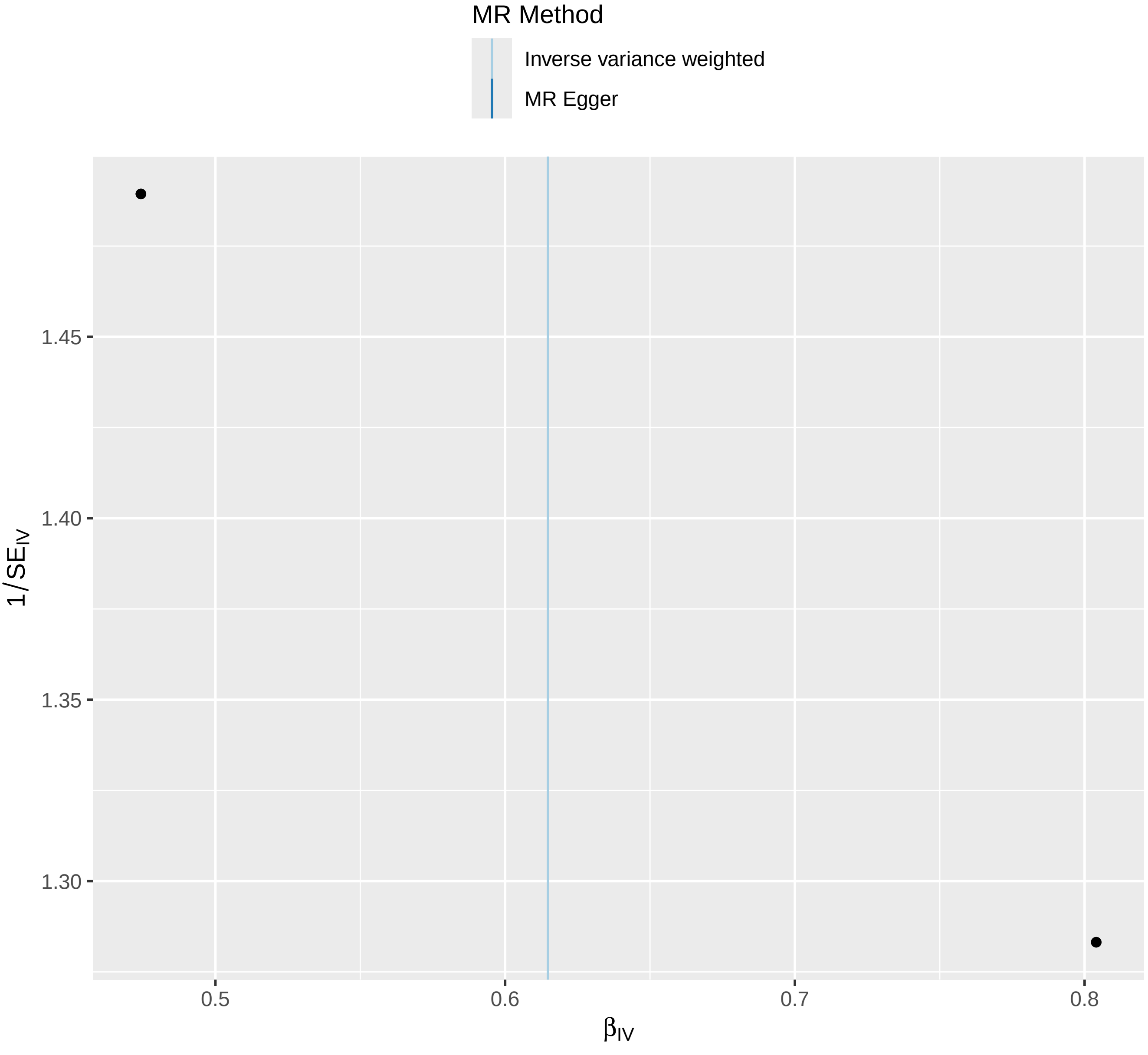

MR Method
Inverse variance weighted
MR Egger
1.45
1.40
1.35
1.30
$1/SE_{IV}$
0.5
0.6
0.7
0.8
β_{IV}

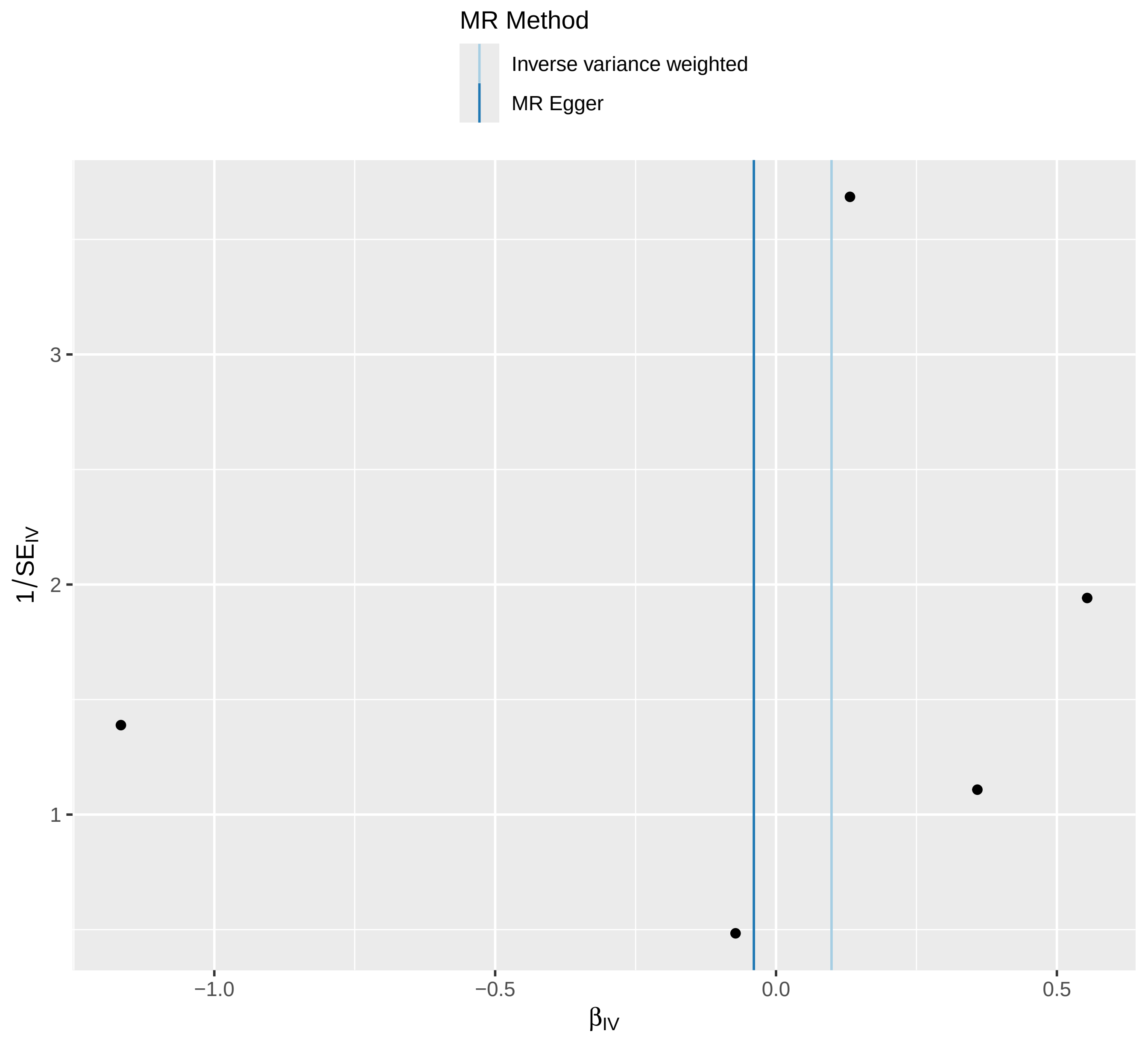
MR Method
Inverse variance weighted
MR Egger
1/SE_IV
3
2
1
−1.0
−0.5
0.0
0.5
β_IV

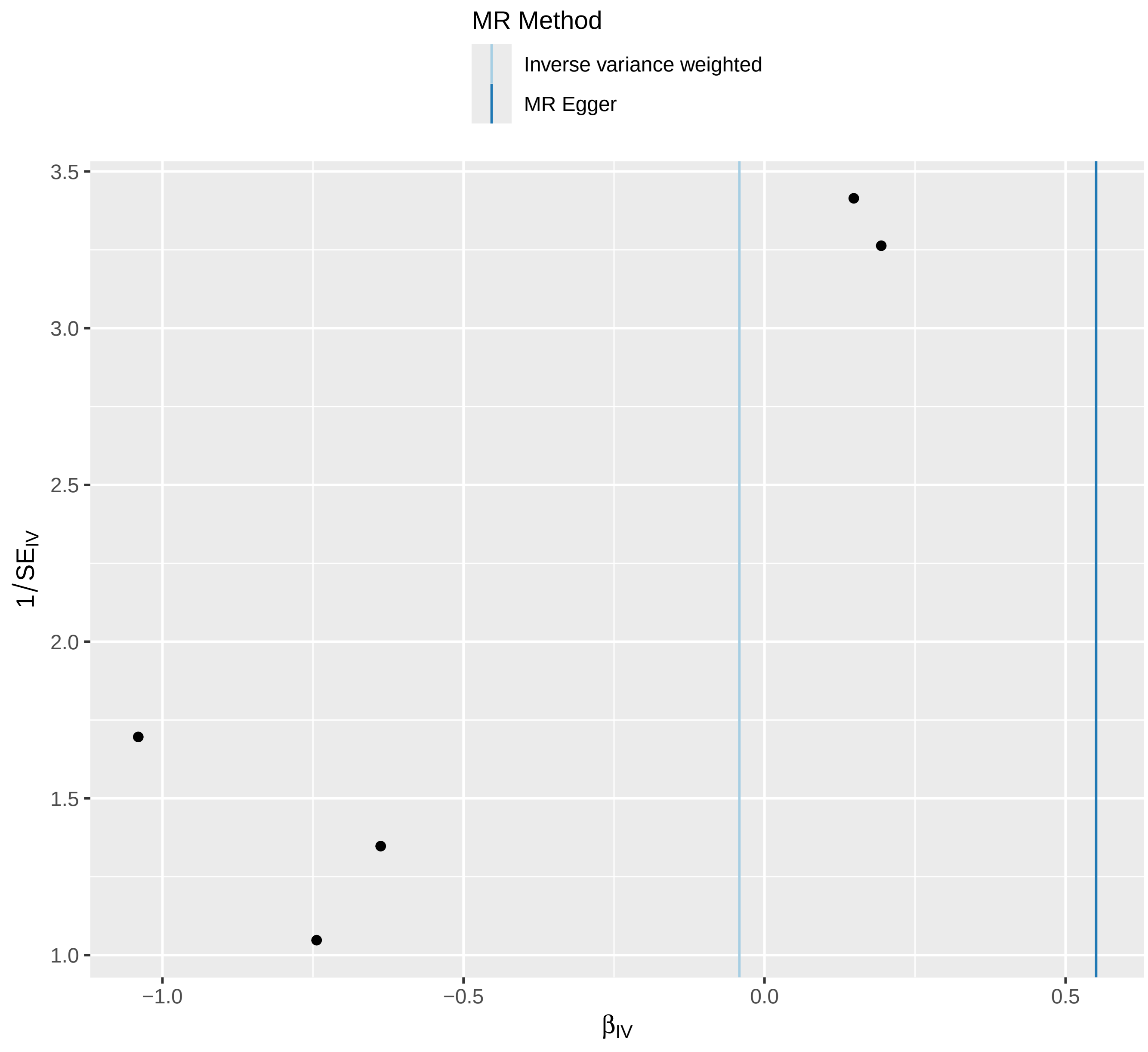

MR Method
Inverse variance weighted
MR Egger
3.5
3.0
2.5
2.0
1.5
1.0
1/SE$_{IV}$
−1.0
−0.5
0.0
0.5
β$_{IV}$

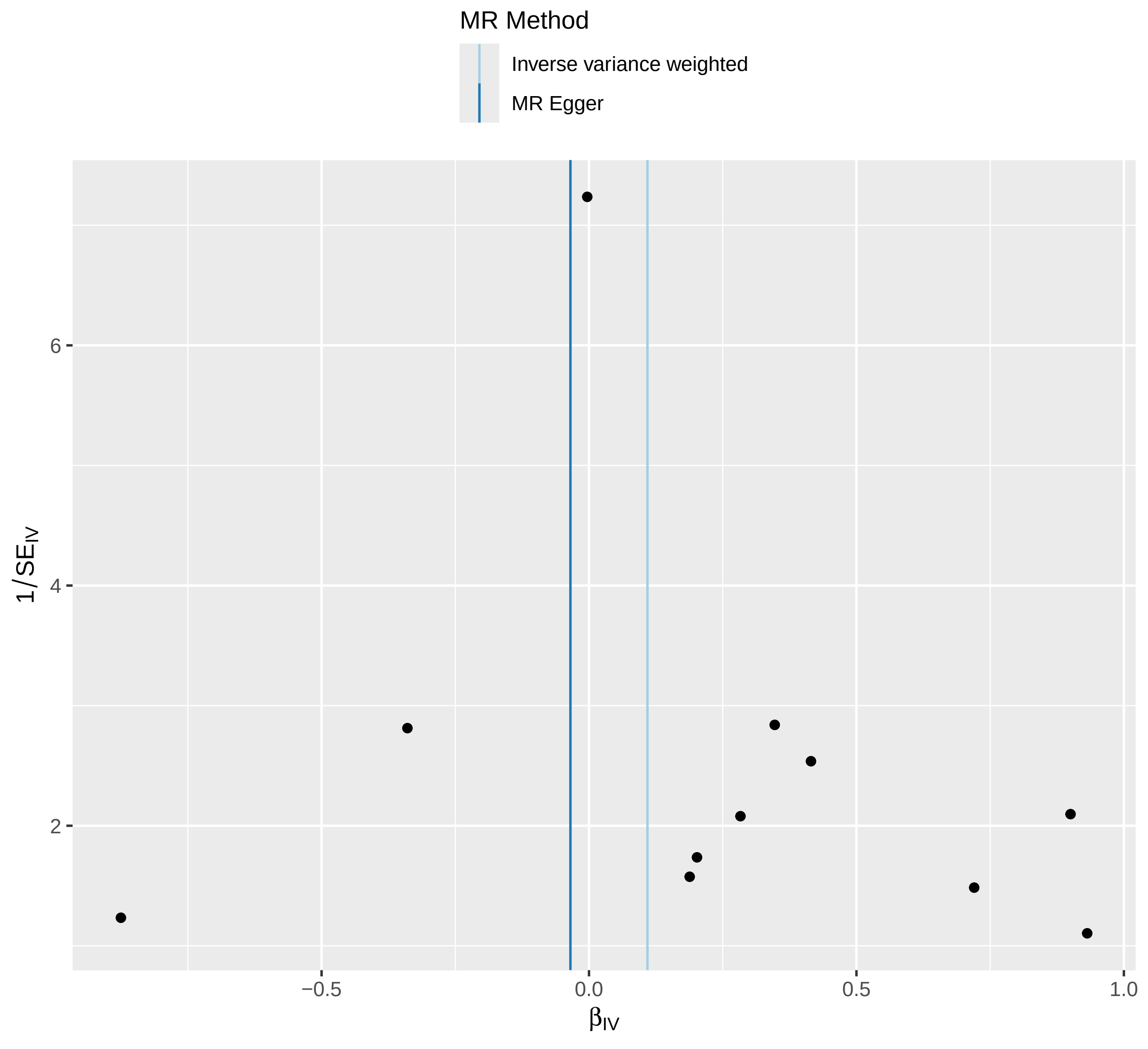
MR Method
Inverse variance weighted
MR Egger
6
4
2
1/SE_{IV}
−0.5
0.0
0.5
1.0
β_{IV}

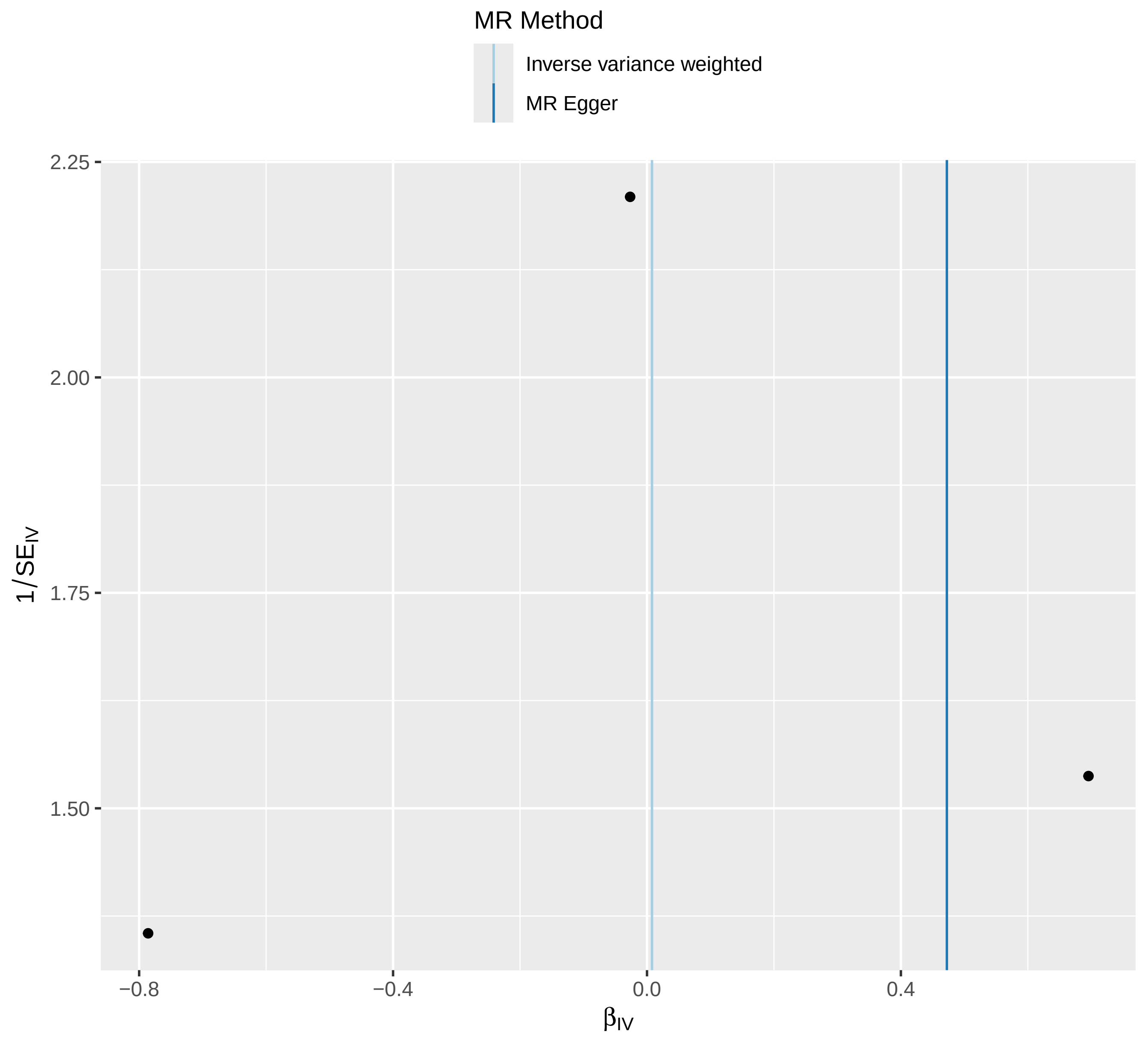

MR Method
Inverse variance weighted
MR Egger
2.25
2.00
1.75
1.50
1/SE$_{IV}$
−0.8
−0.4
0.0
0.4
β$_{IV}$

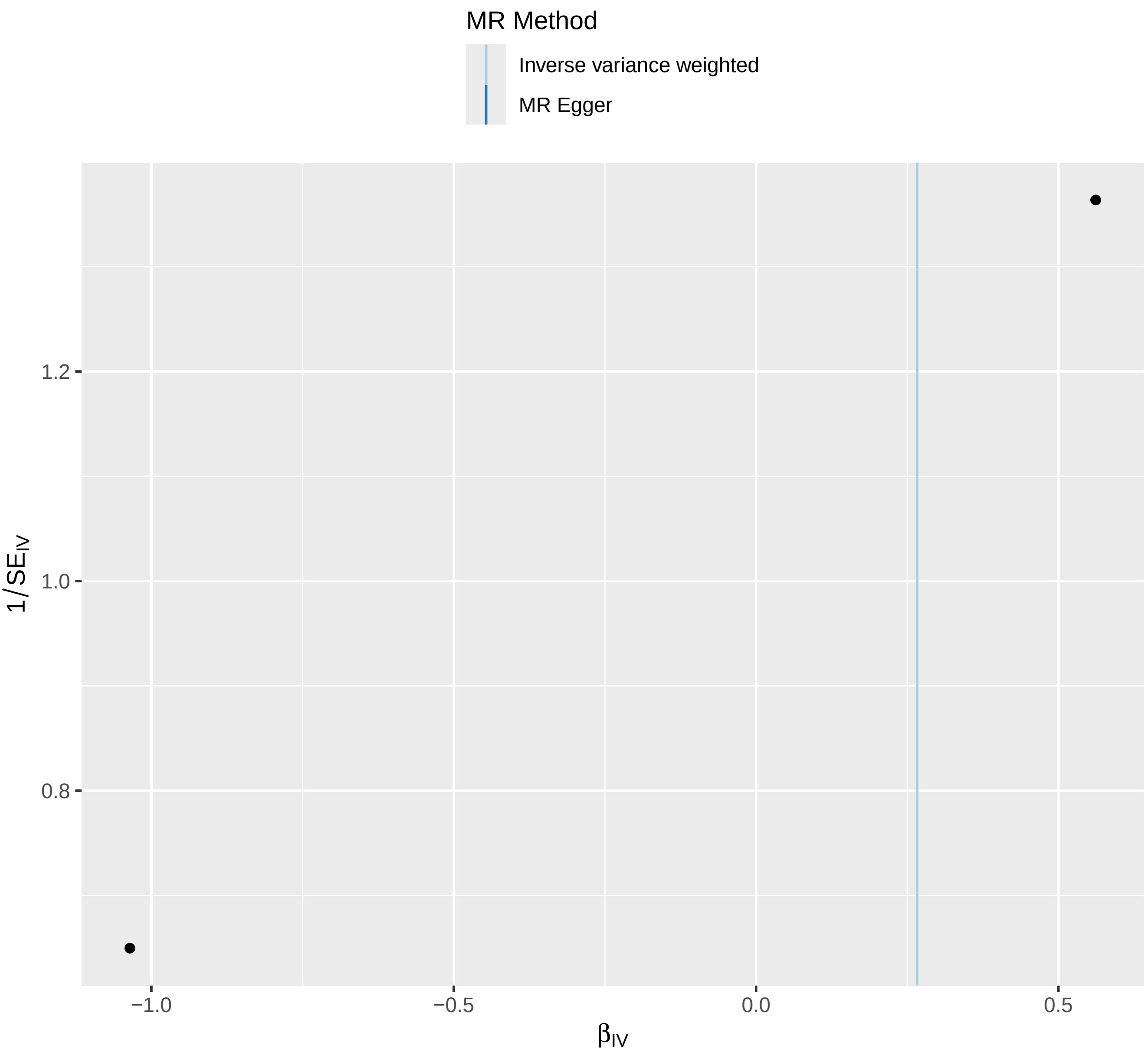

MR Method
Inverse variance weighted
MR Egger
1.2
1.0
0.8
1/SE_IV
−1.0
−0.5
0.0
0.5
β_IV

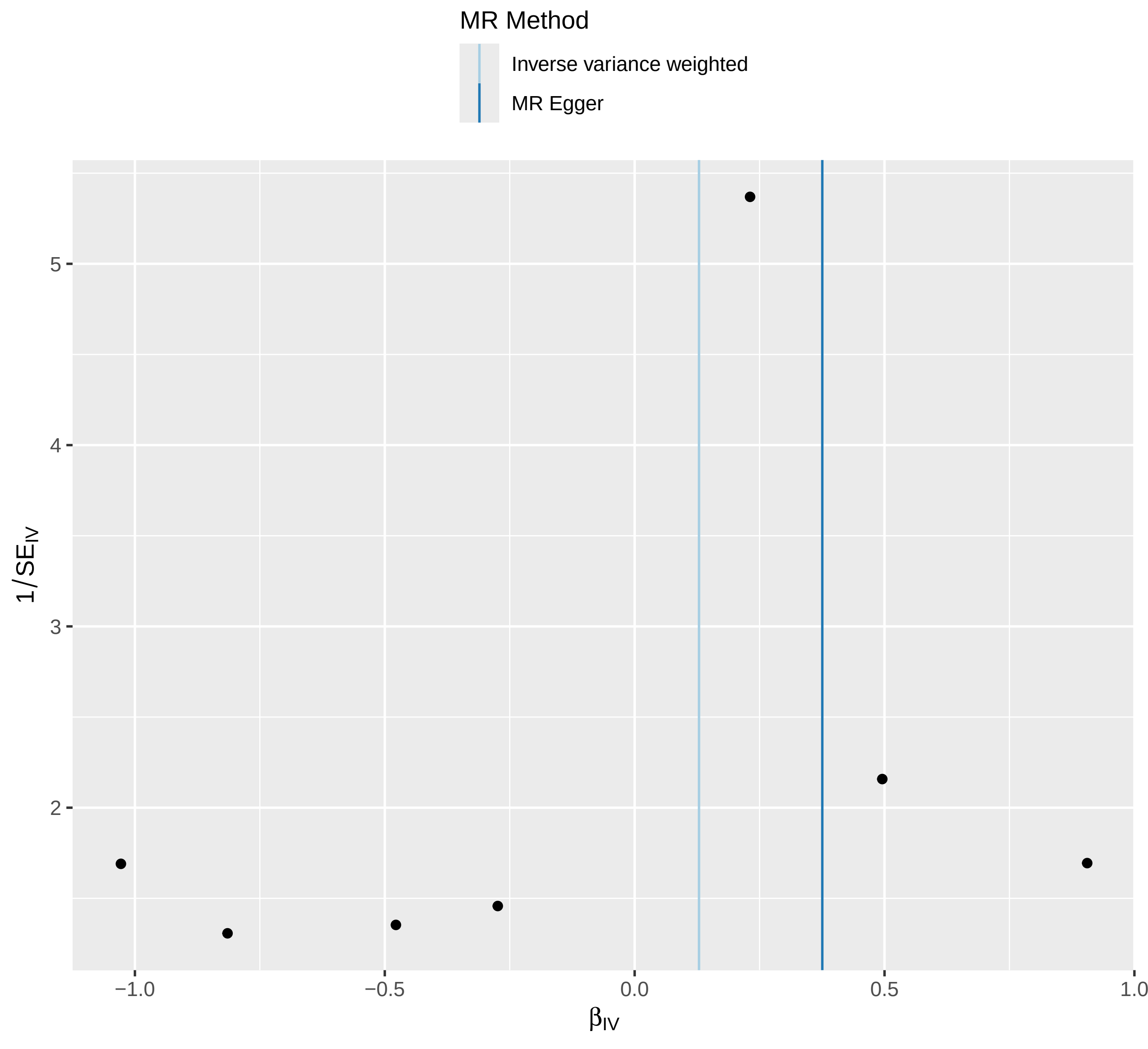
MR Method
Inverse variance weighted
MR Egger
1/SE_IV
5
4
3
2
−1.0
−0.5
0.0
0.5
1.0
β_IV

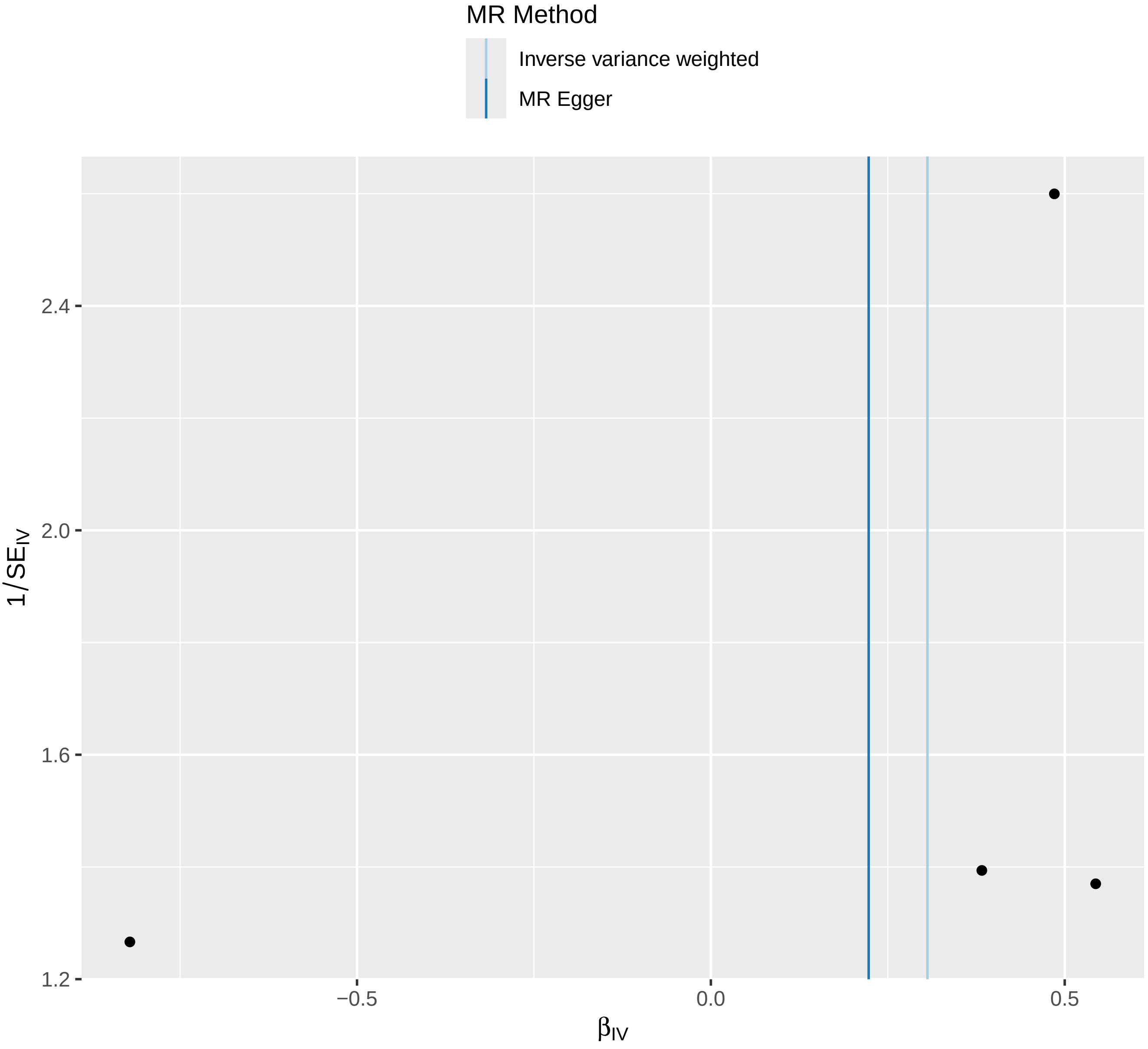
MR Method
Inverse variance weighted
MR Egger
2.4
2.0
1.6
1.2
1/SE$_{IV}$
−0.5
0.0
0.5
β$_{IV}$

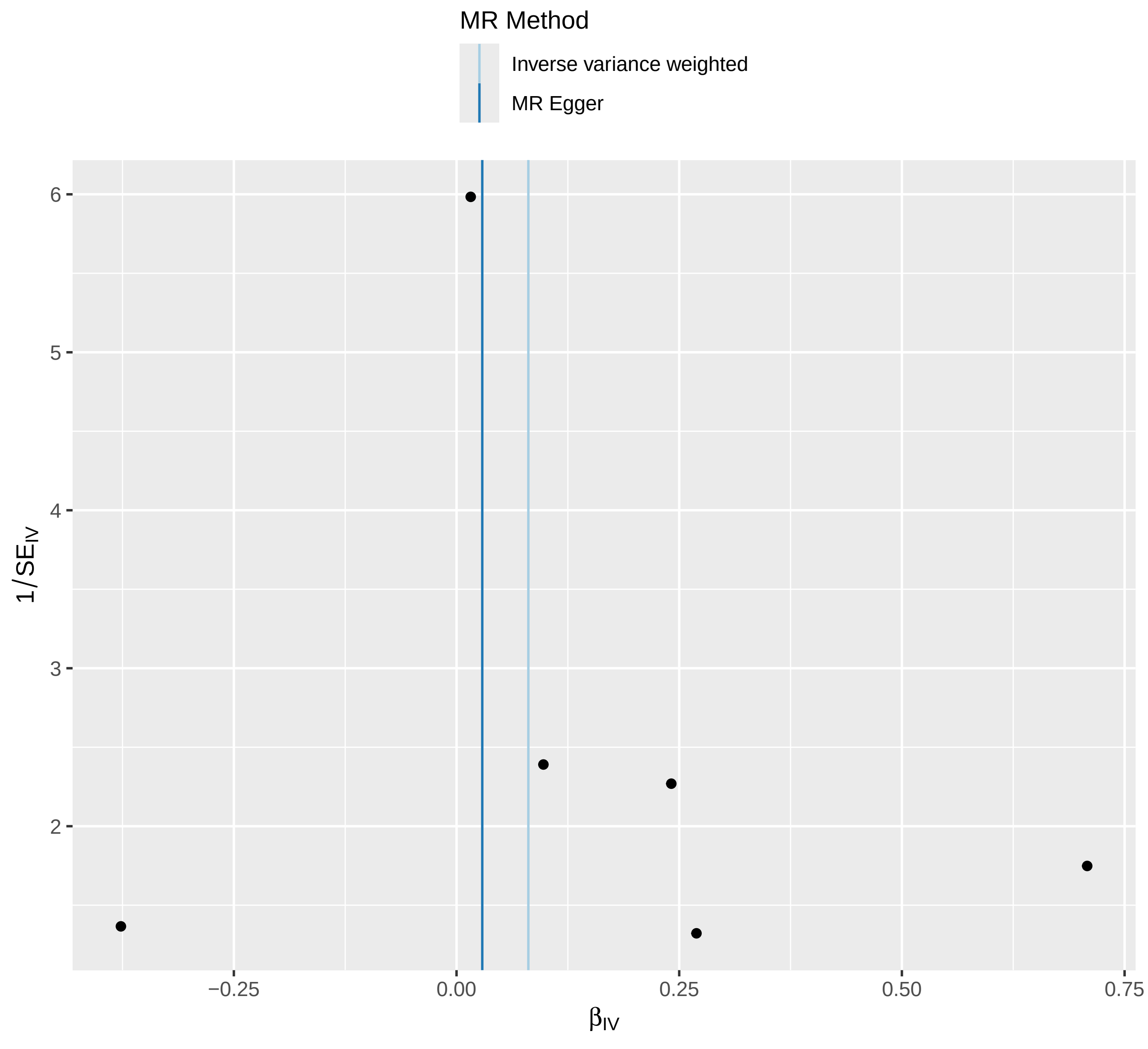
MR Method
Inverse variance weighted
MR Egger
6
5
4
3
2
1/SE_IV
−0.25
0.00
0.25
0.50
0.75
β_IV

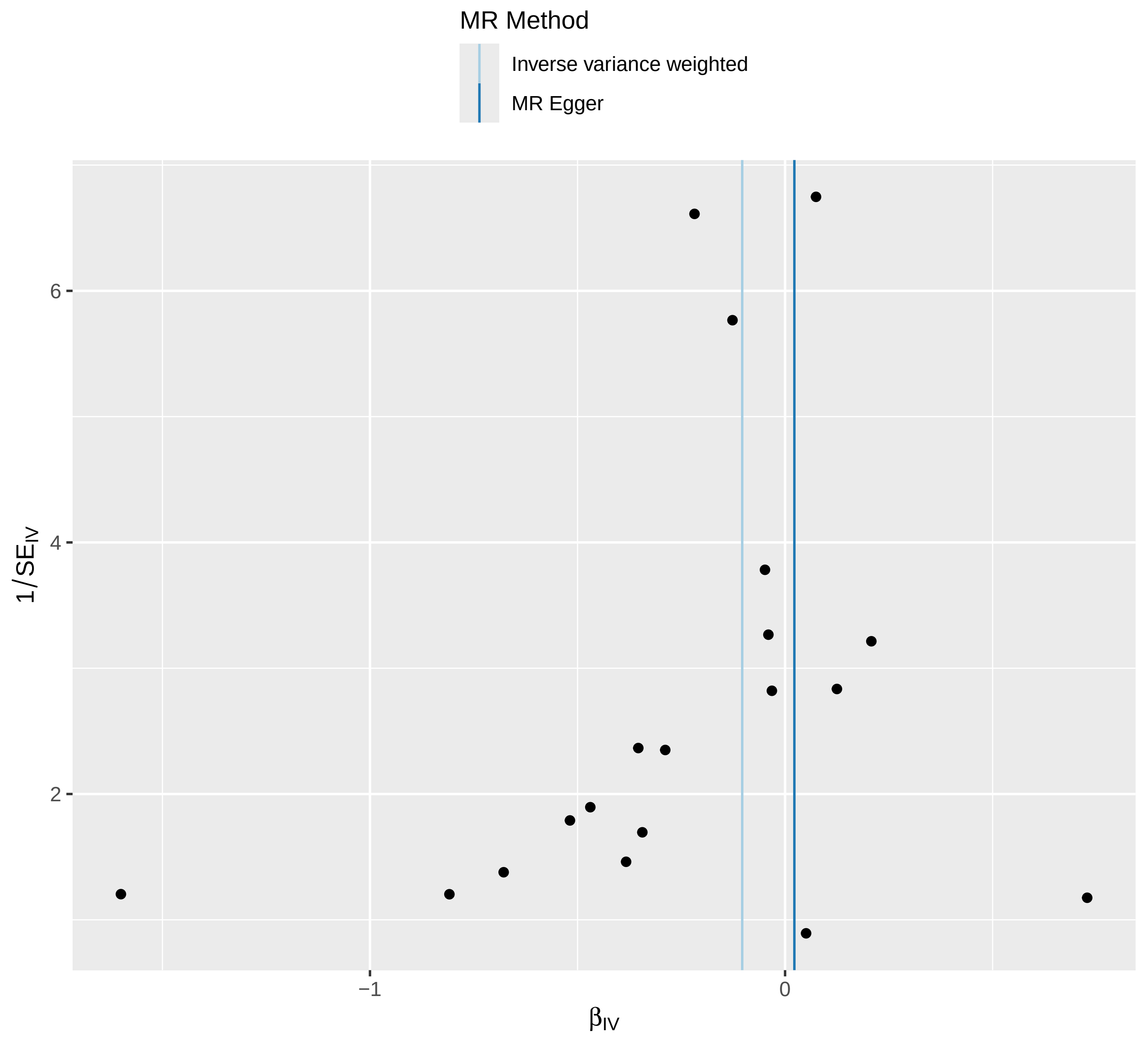

MR Method
Inverse variance weighted
MR Egger
6
4
2
1/SE_IV
−1
0
β_IV

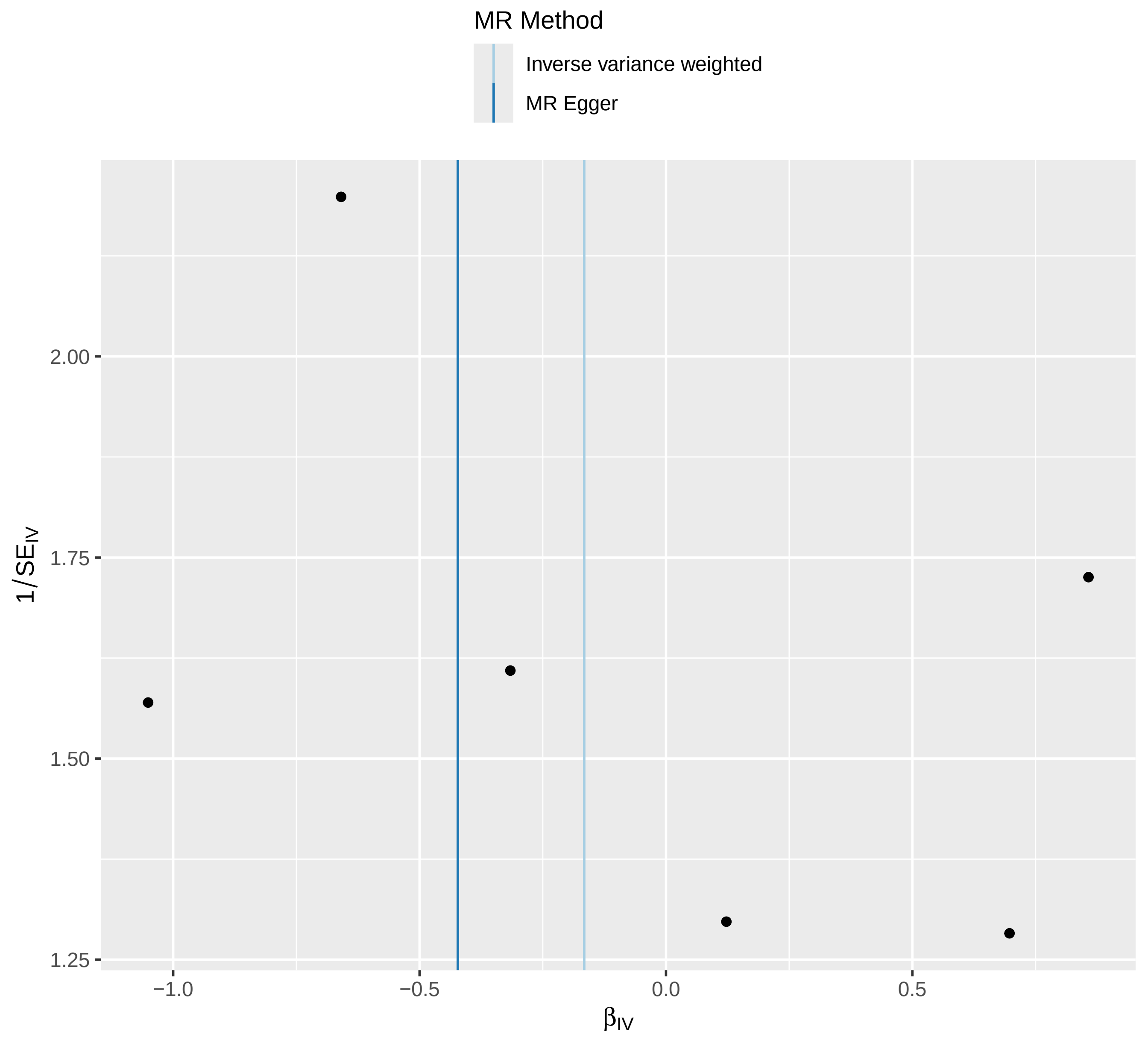
MR Method
Inverse variance weighted
MR Egger
2.00
1.75
1.50
1.25
−1.0
−0.5
0.0
0.5
$1/SE_{IV}$
β_{IV}

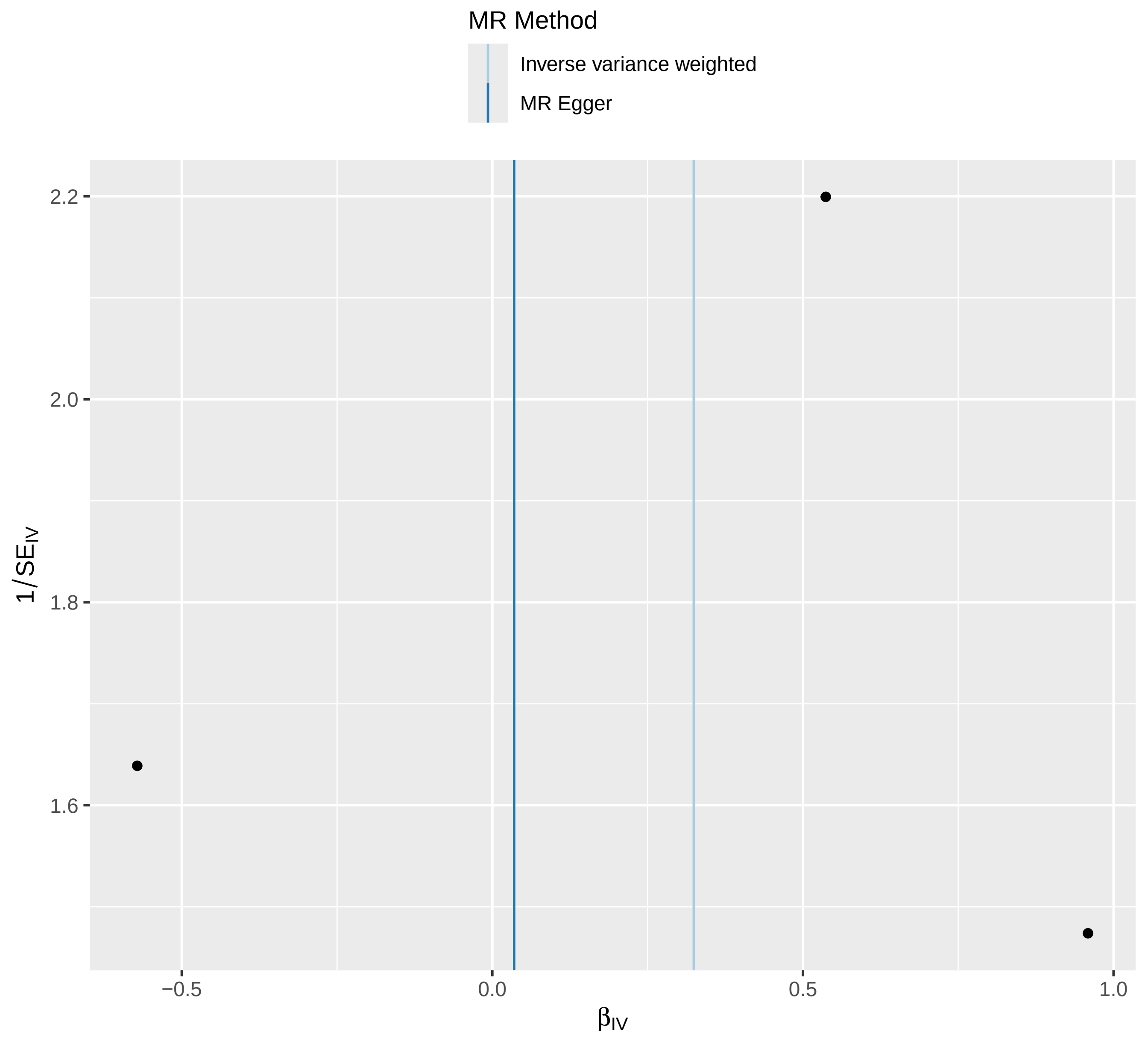

MR Method
Inverse variance weighted
MR Egger
2.2
2.0
1.8
1.6
1/SE_IV
−0.5
0.0
0.5
1.0
β_IV

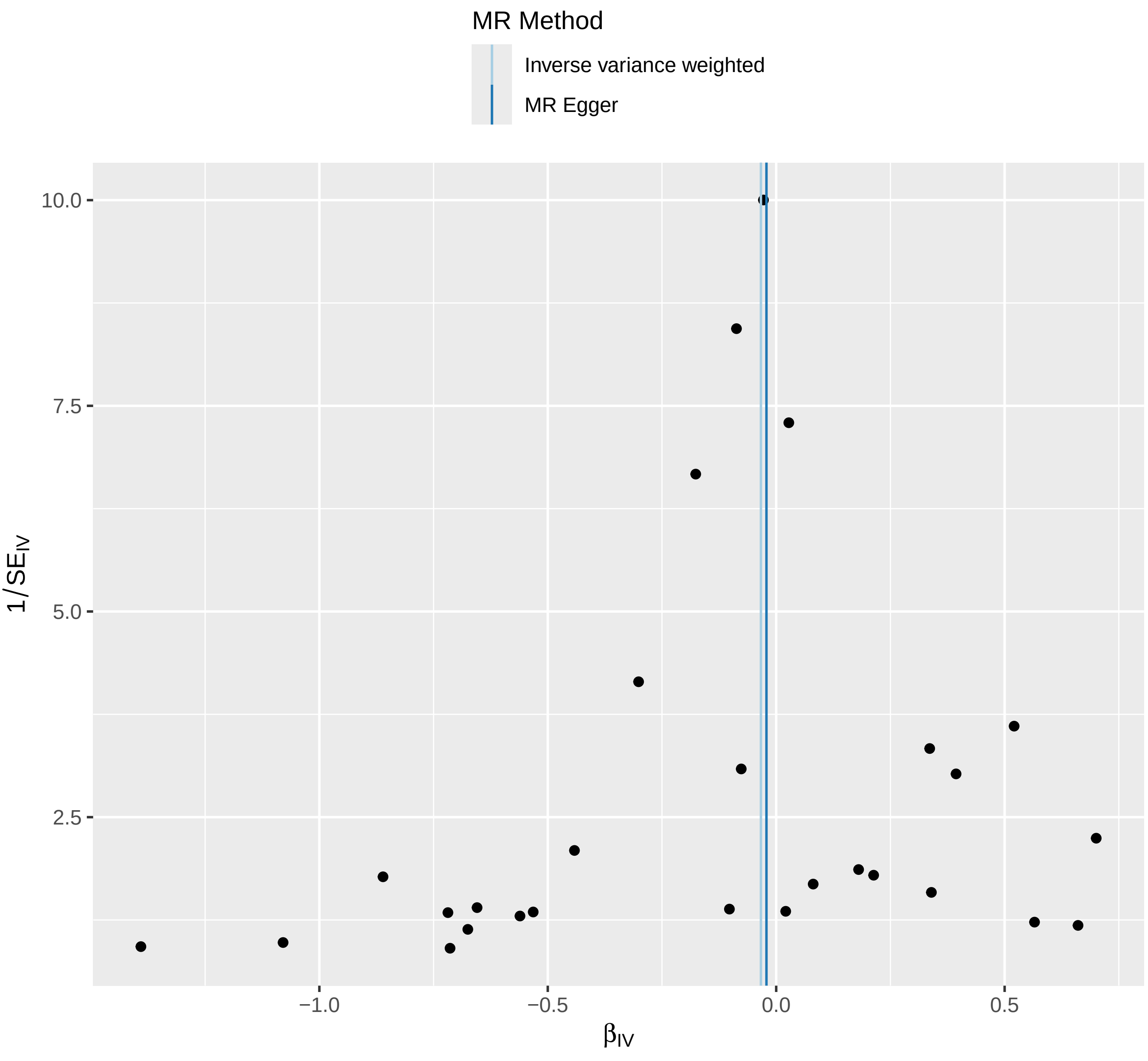

MR Method
Inverse variance weighted
MR Egger
10.0
7.5
5.0
2.5
$1/SE_{IV}$
−1.0
−0.5
0.0
0.5
β_{IV}

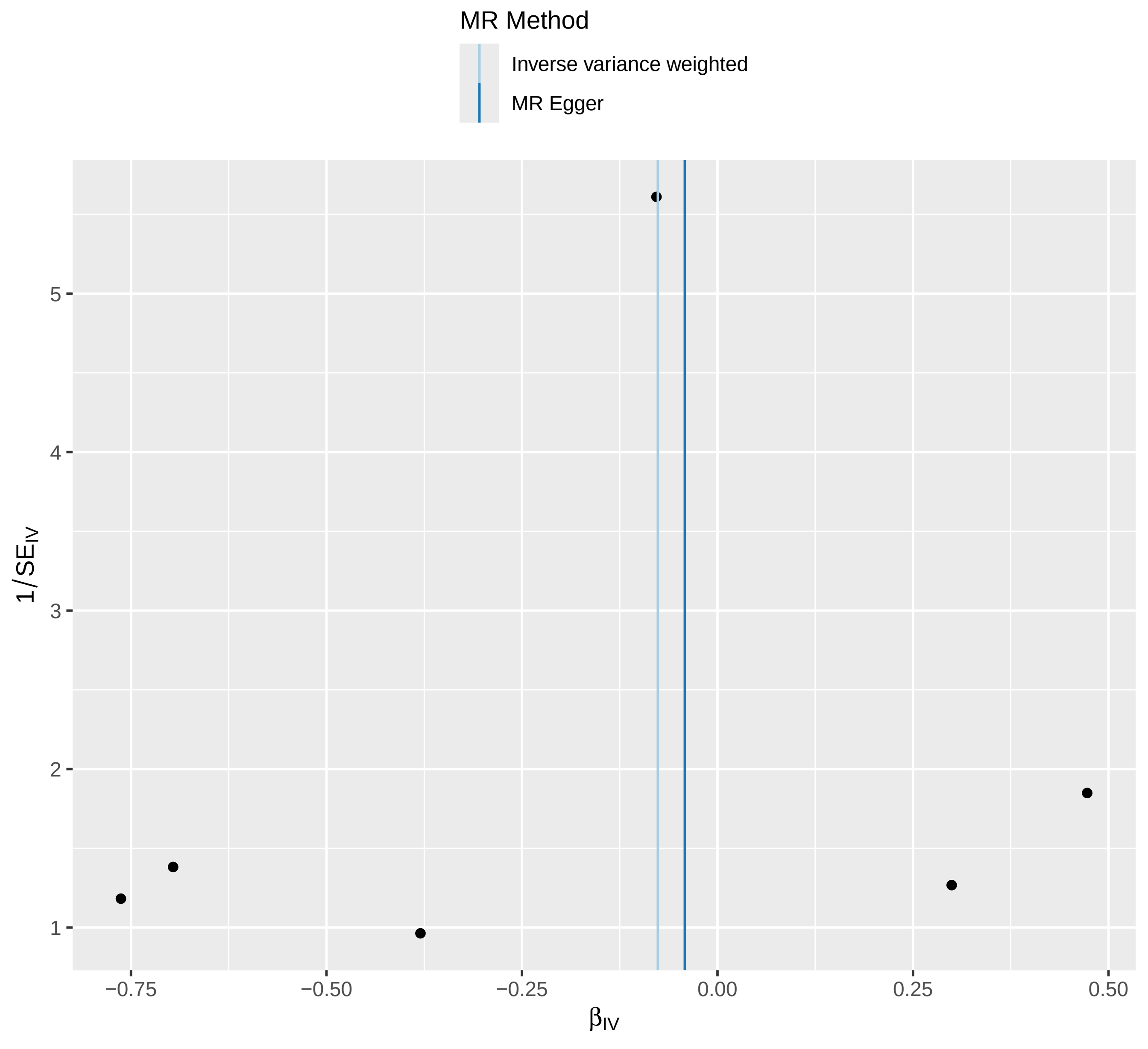
MR Method
Inverse variance weighted
MR Egger
$1/SE_{IV}$
5
4
3
2
1
−0.75
−0.50
−0.25
0.00
0.25
0.50
β_{IV}

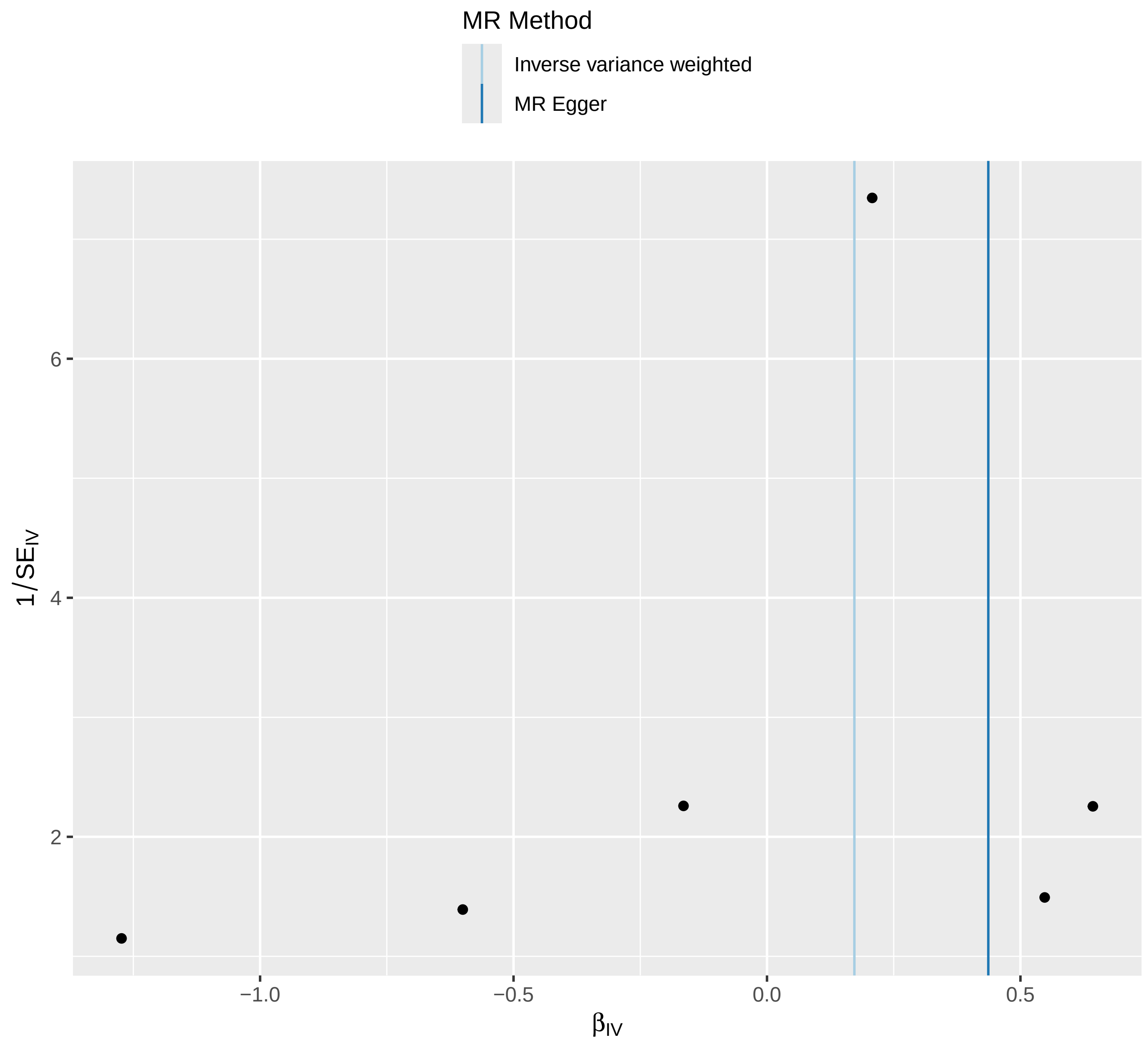

MR Method
Inverse variance weighted
MR Egger
1/SE_IV
6
4
2
−1.0
−0.5
0.0
0.5
β_IV

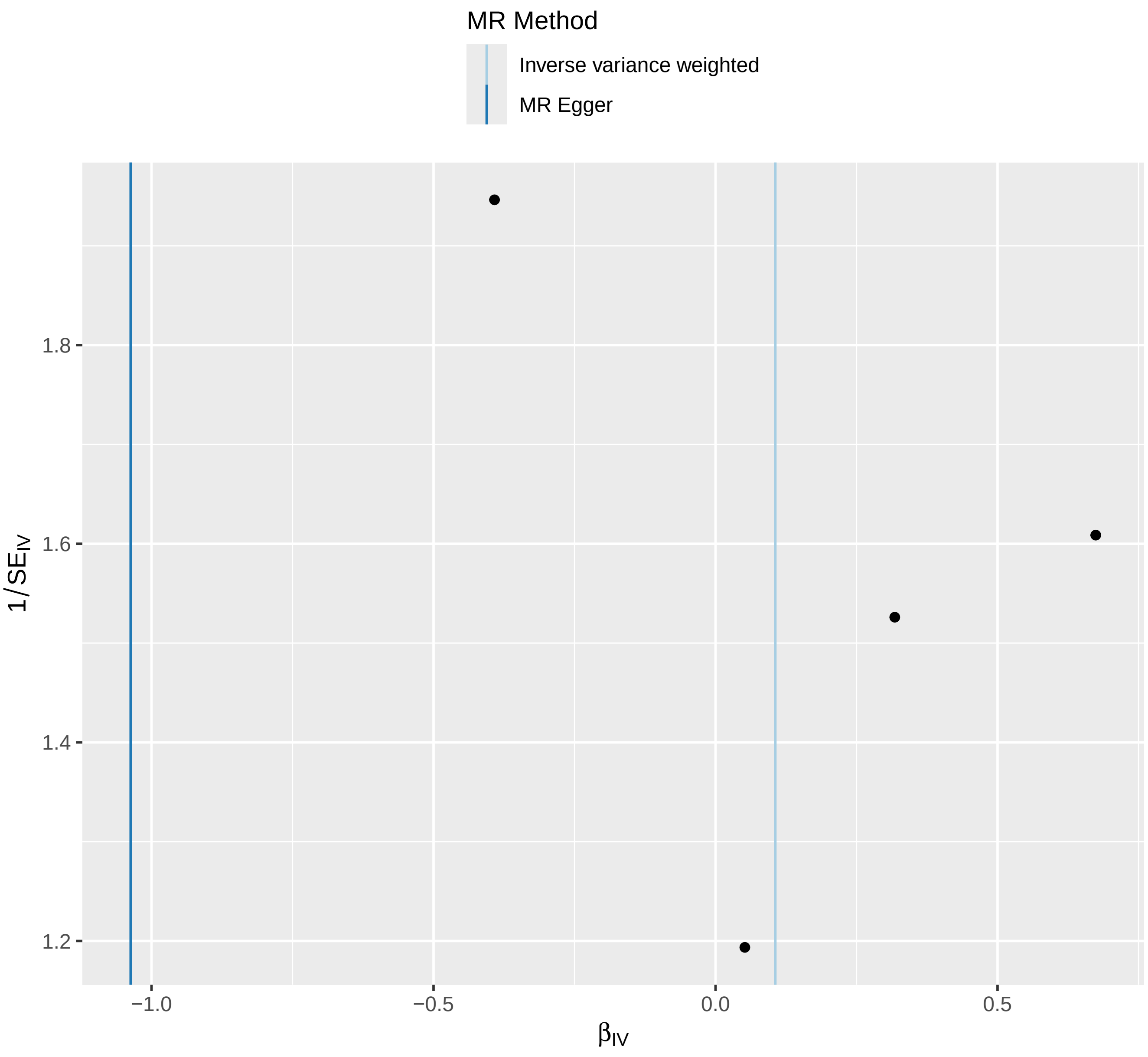

MR Method
Inverse variance weighted
MR Egger
1.8
1.6
1.4
1.2
1/SE_IV
−1.0
−0.5
0.0
0.5
β_IV

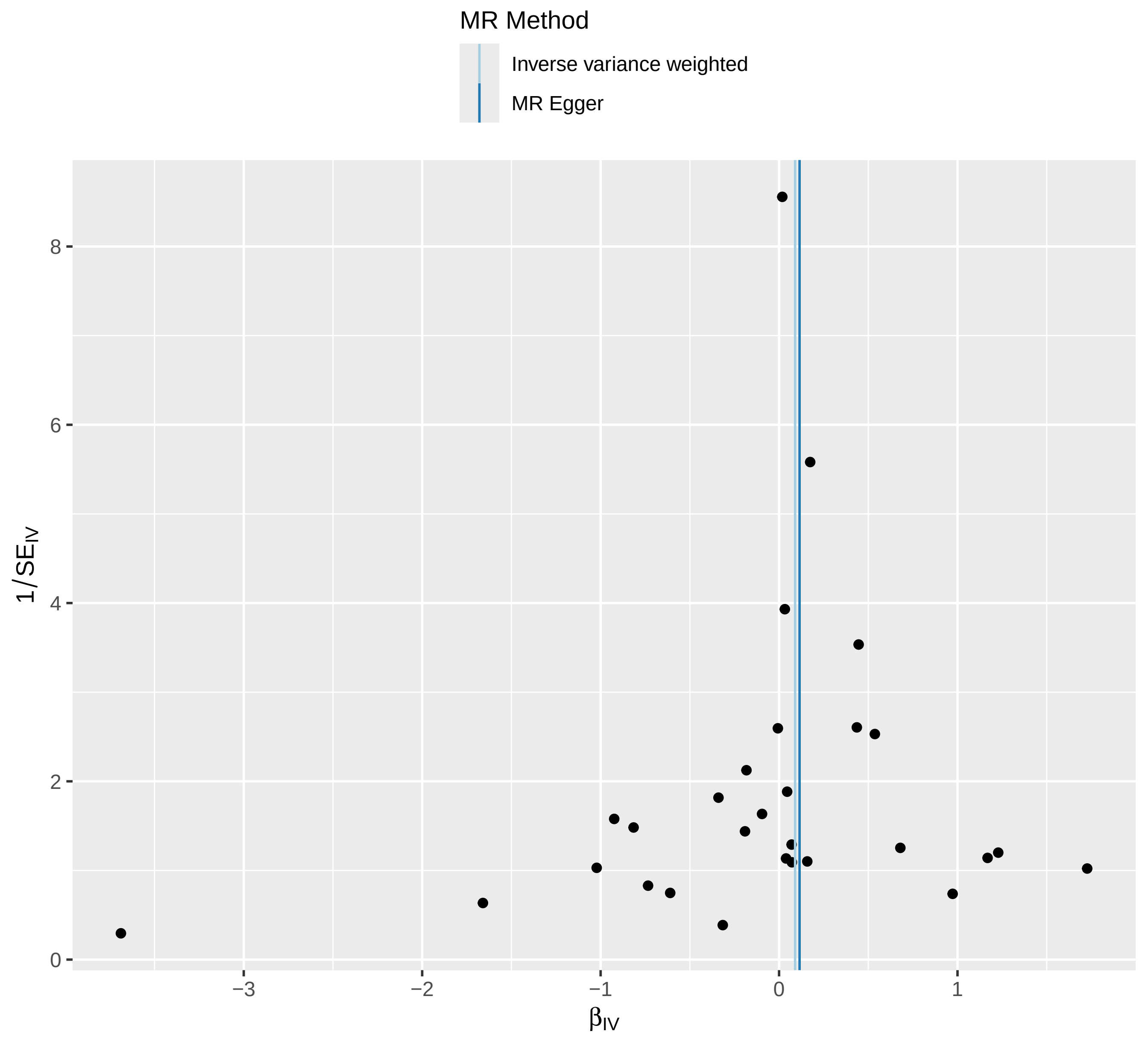

MR Method
Inverse variance weighted
MR Egger
8
6
4
2
0
1/SE$_{IV}$
−3
−2
−1
0
1
β_{IV}

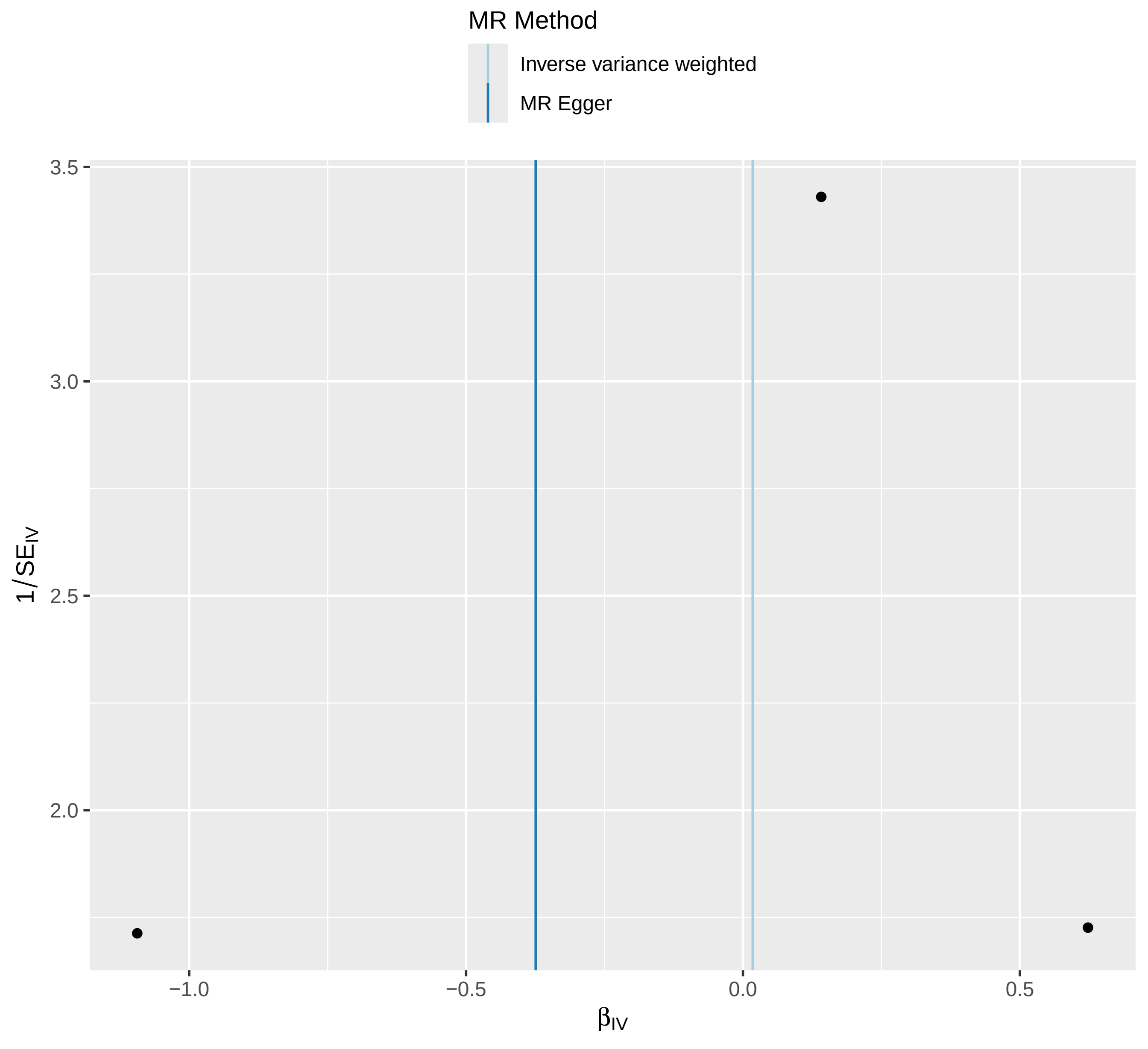
MR Method
Inverse variance weighted
MR Egger
3.5
3.0
2.5
2.0
1/SE_IV
−1.0
−0.5
0.0
0.5
β_IV

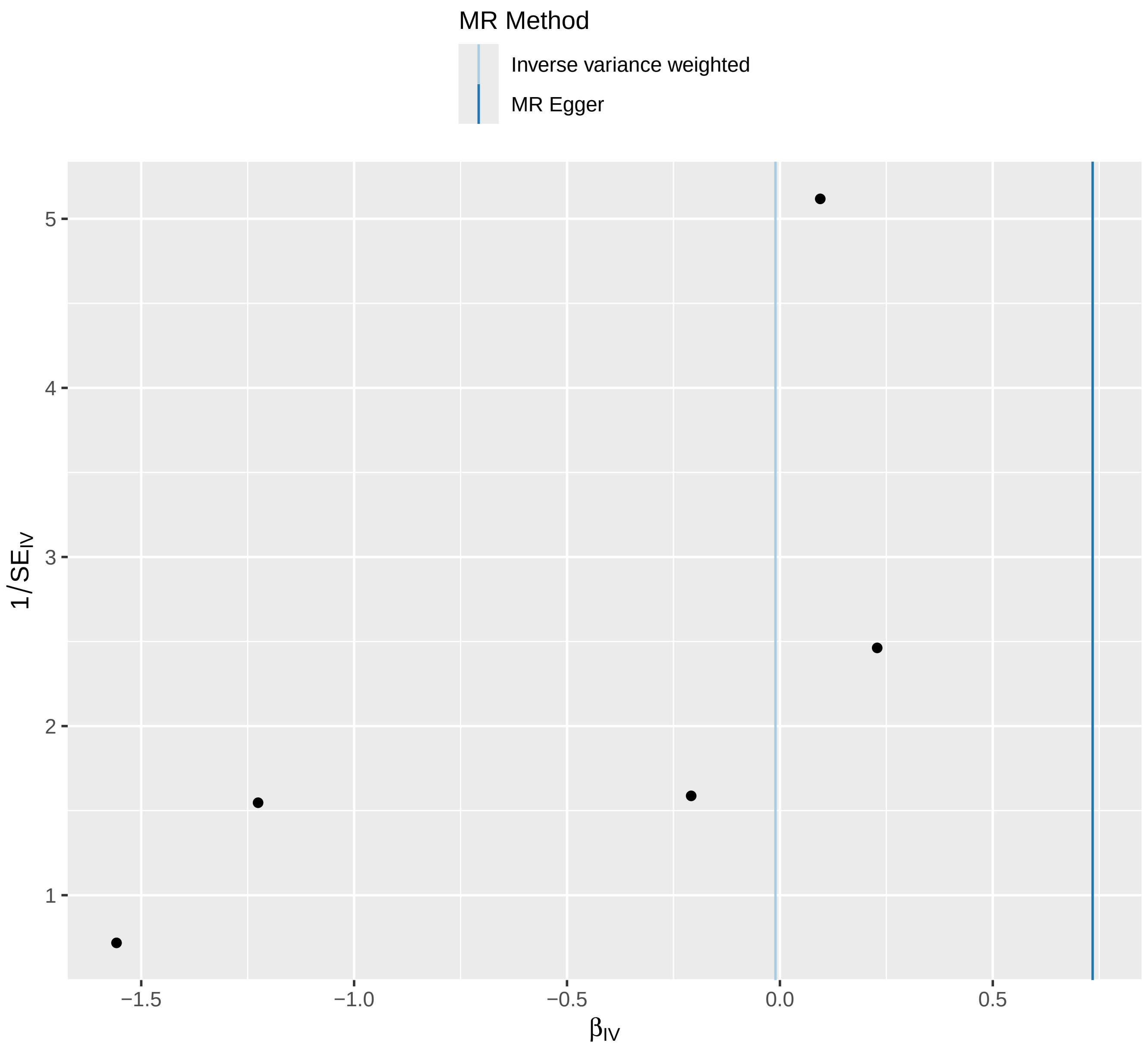

MR Method
Inverse variance weighted
MR Egger
5
4
3
2
1
1/SE$_{IV}$
−1.5
−1.0
−0.5
0.0
0.5
β$_{IV}$

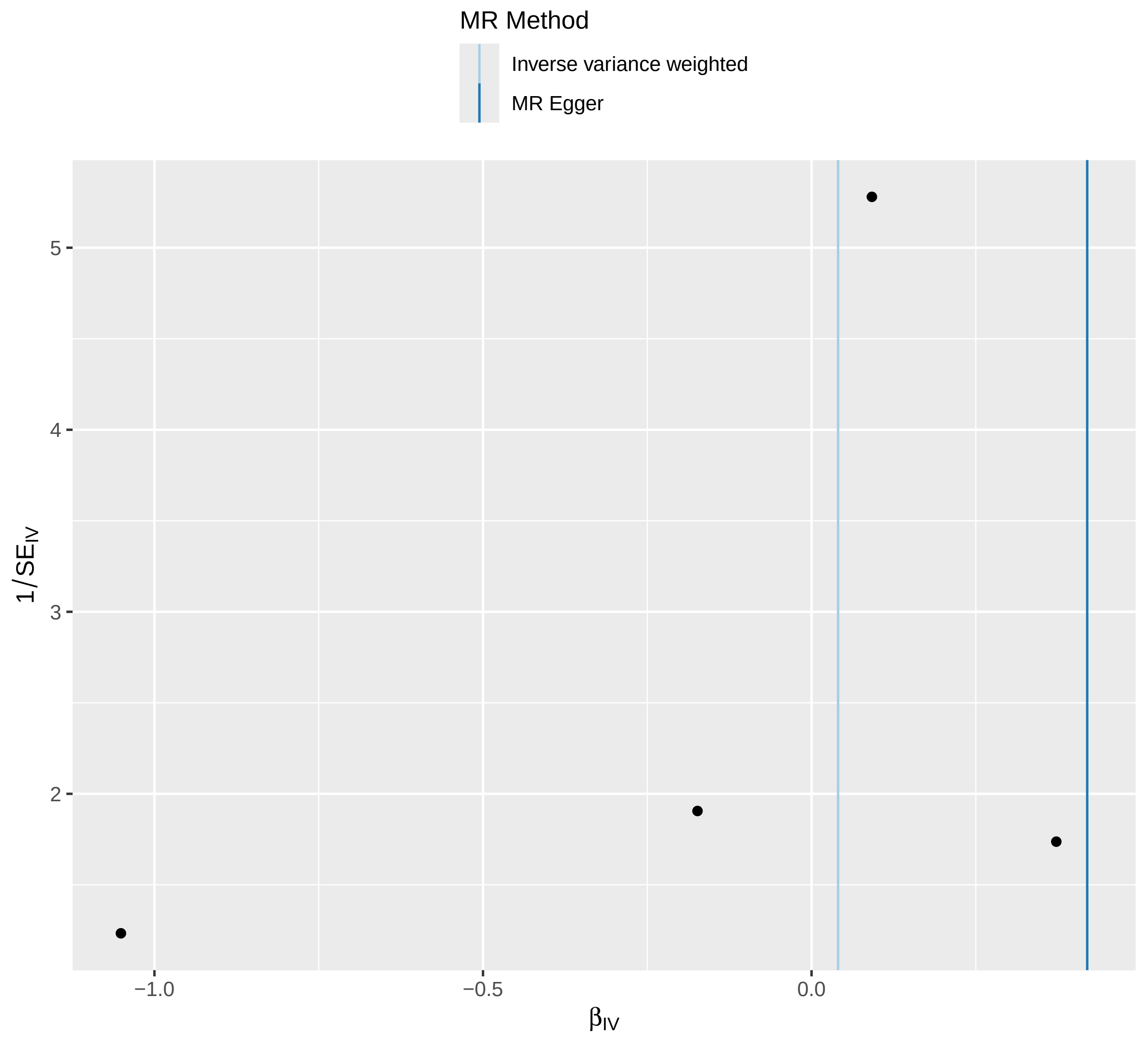

MR Method
Inverse variance weighted
MR Egger
1/SE_IV
5
4
3
2
−1.0
−0.5
0.0
β_IV

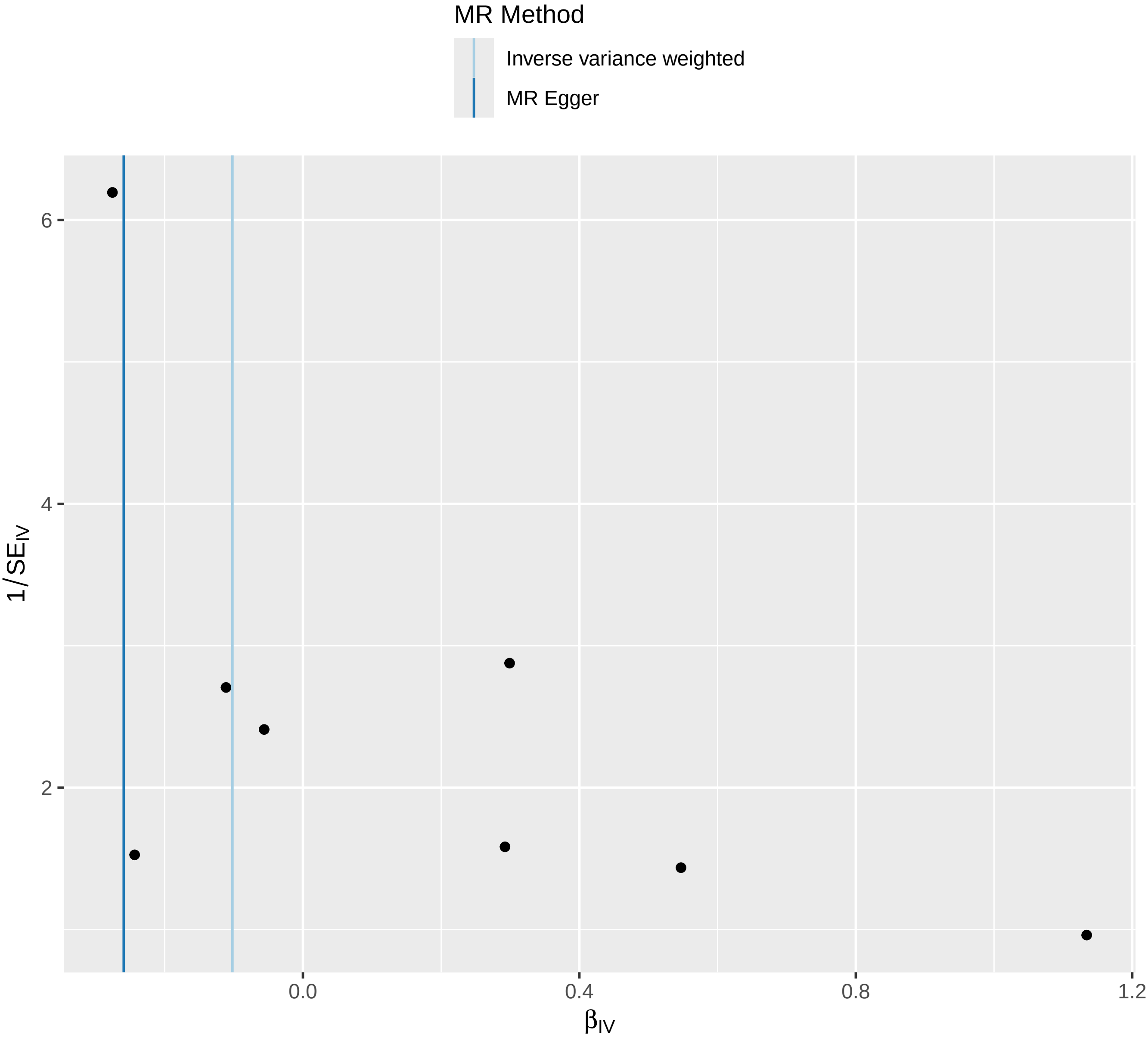

MR Method
Inverse variance weighted
MR Egger
6
4
2
1/SE_IV
0.0
0.4
0.8
1.2
β_IV

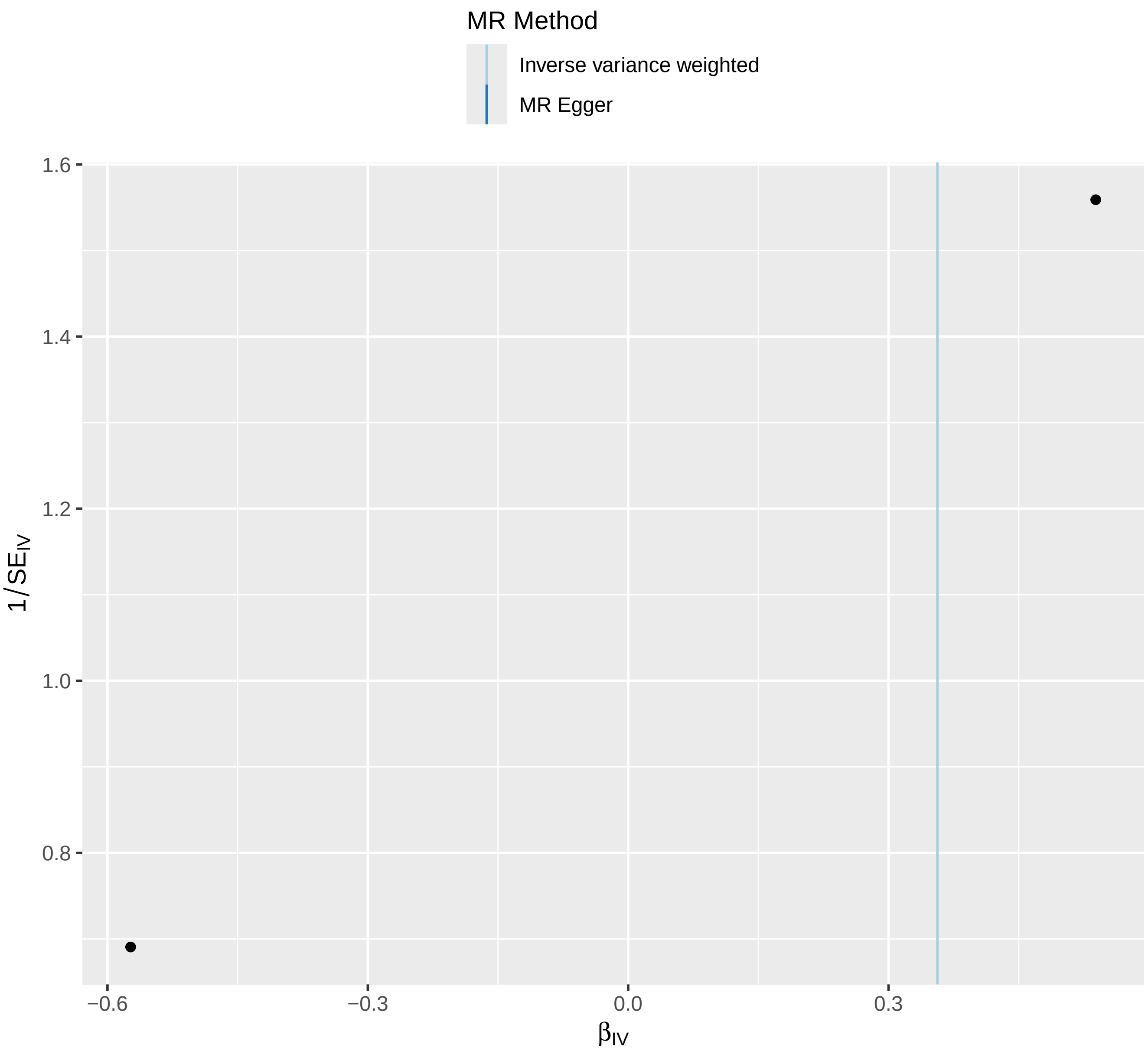
MR Method
Inverse variance weighted
MR Egger
1.6
1.4
1.2
1.0
0.8
1/SE_IV
−0.6
−0.3
0.0
0.3
β_IV

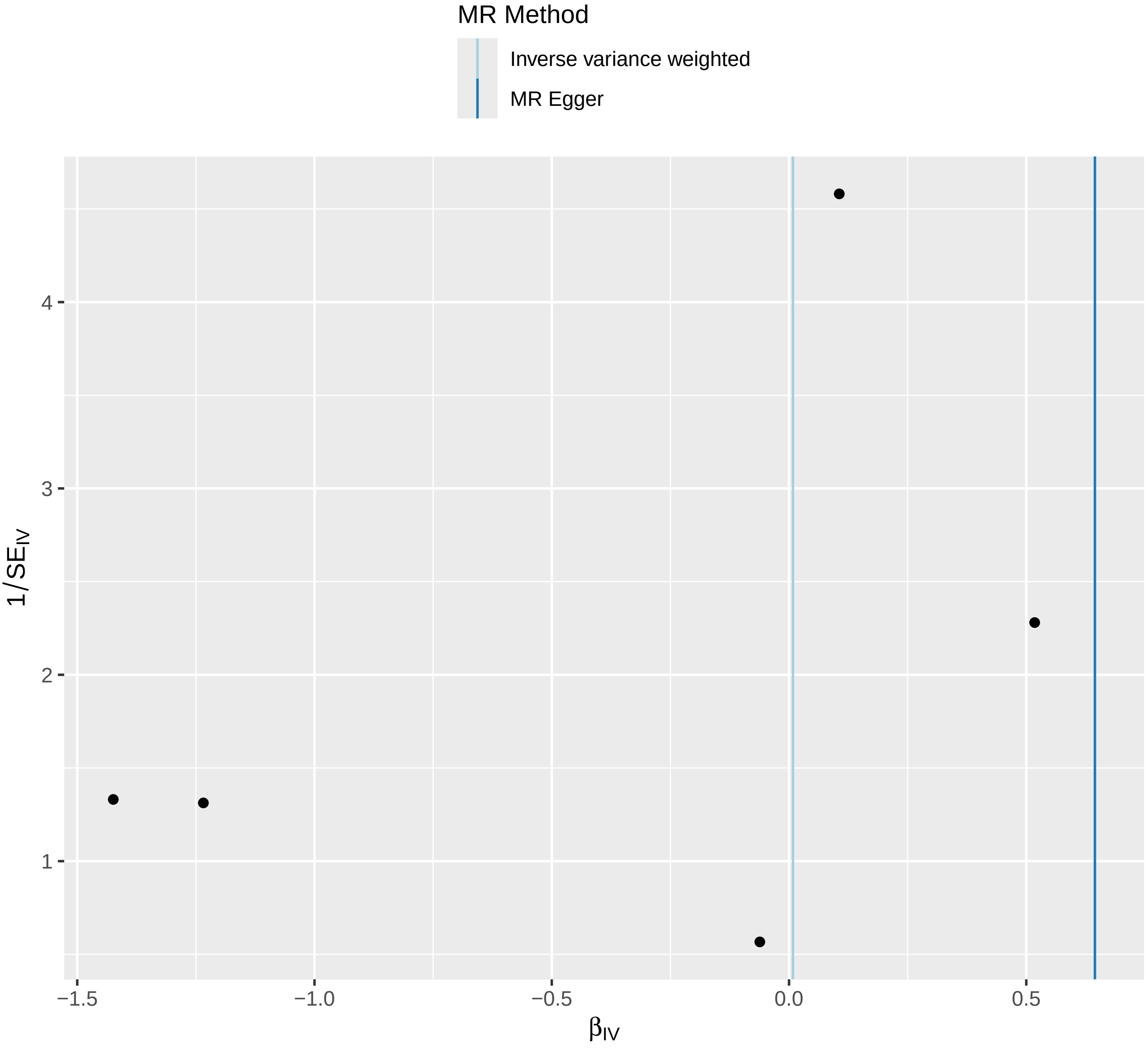

MR Method
Inverse variance weighted
MR Egger
4
3
2
1
$1/SE_{IV}$
−1.5
−1.0
−0.5
0.0
0.5
β_{IV}

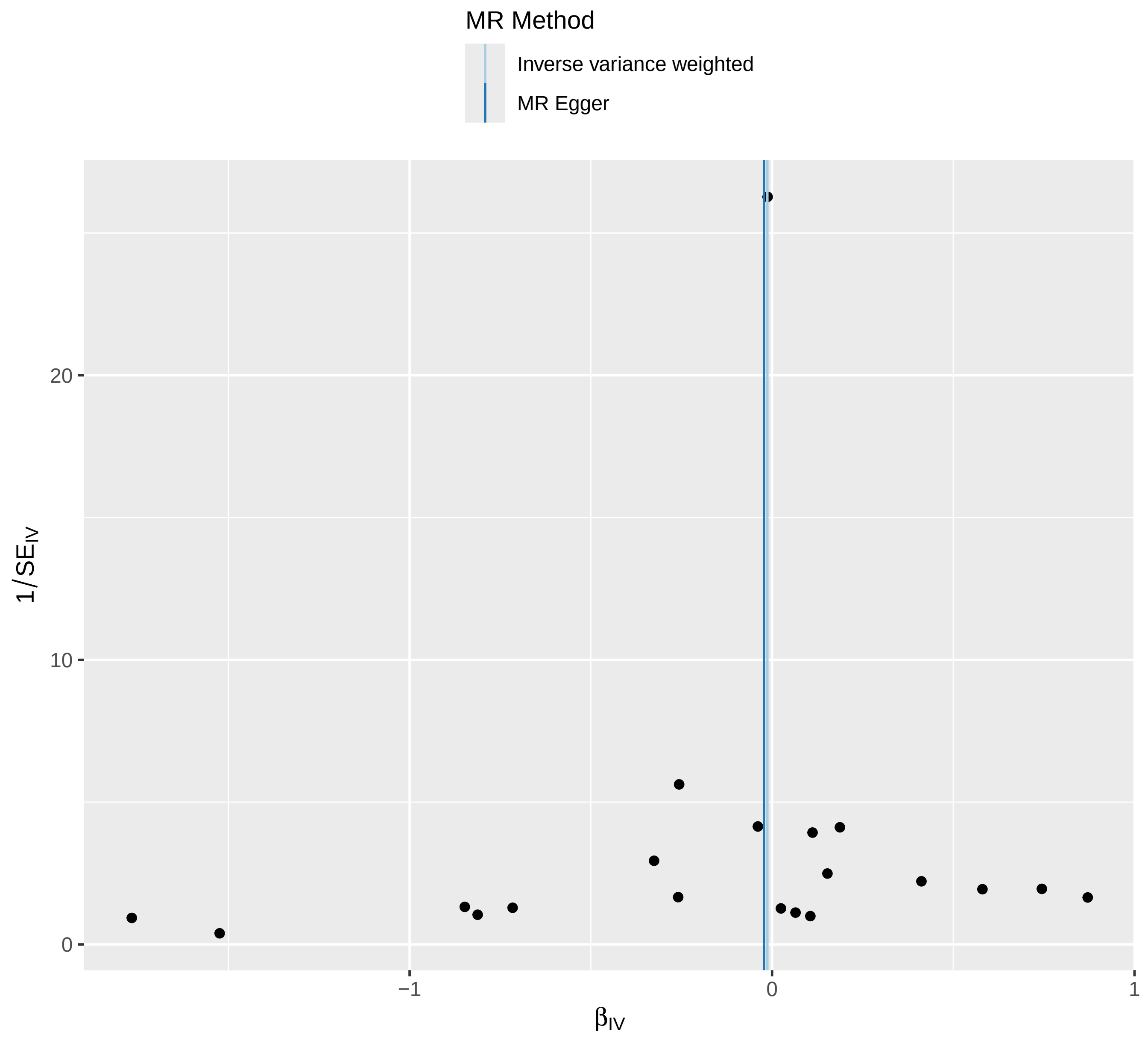

MR Method
Inverse variance weighted
MR Egger
20
10
0
$1/SE_{IV}$
−1
0
1
β_{IV}

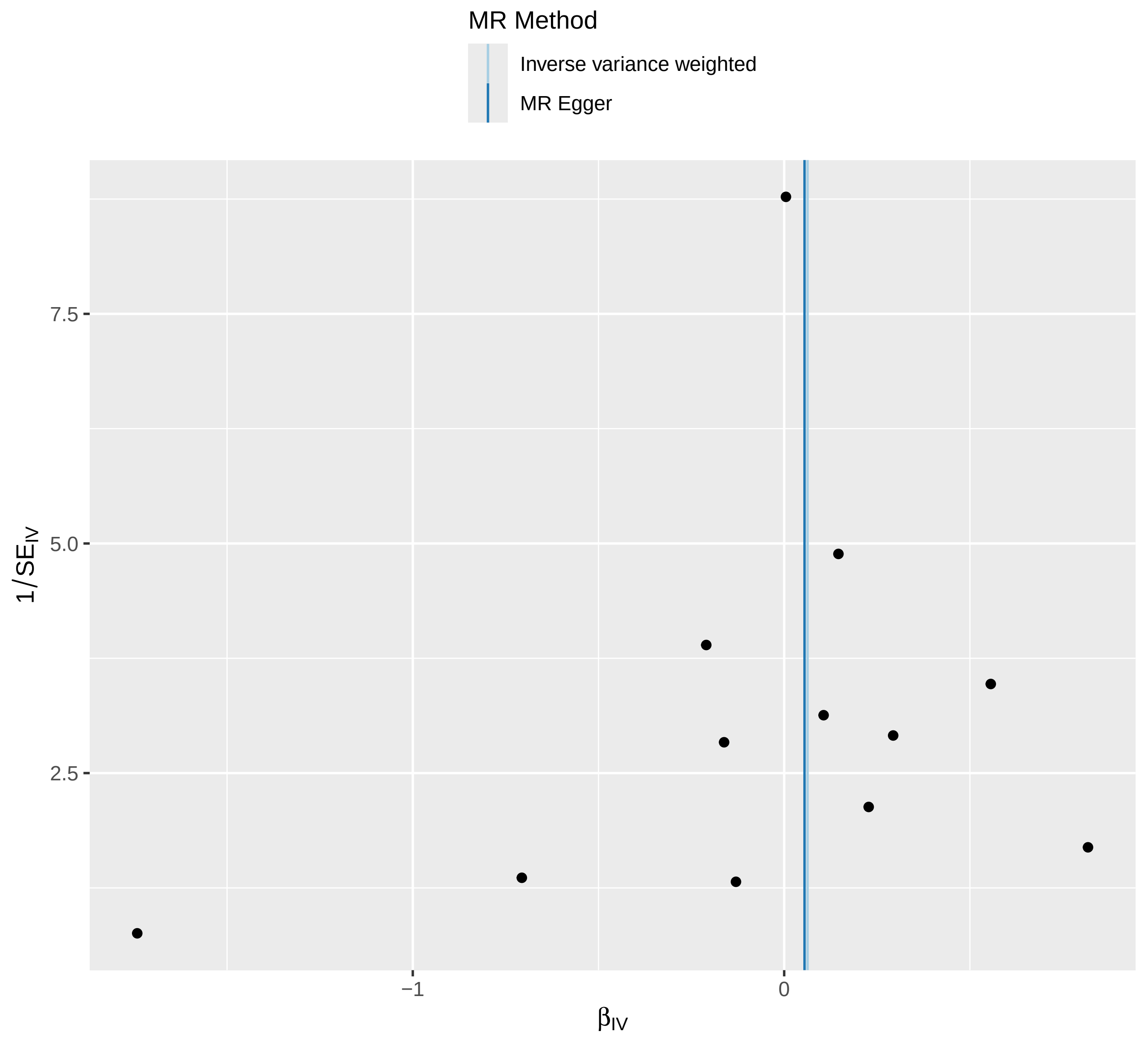
MR Method
Inverse variance weighted
MR Egger
7.5
5.0
2.5
1/SE_IV
−1
0
β_IV

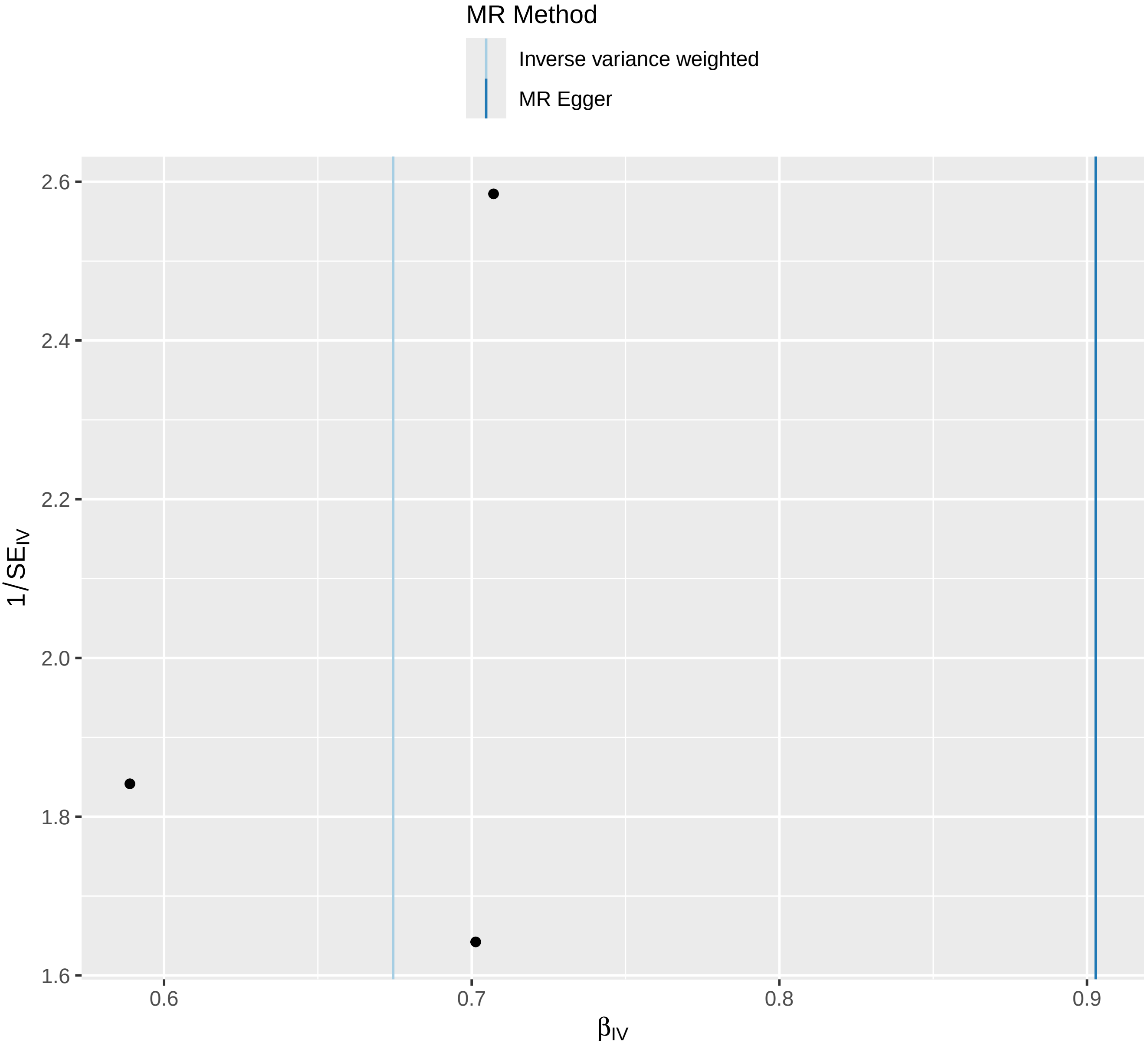

MR Method
Inverse variance weighted
MR Egger
2.6
2.4
2.2
2.0
1.8
1.6
$1/SE_{IV}$
0.6
0.7
0.8
0.9
β_{IV}

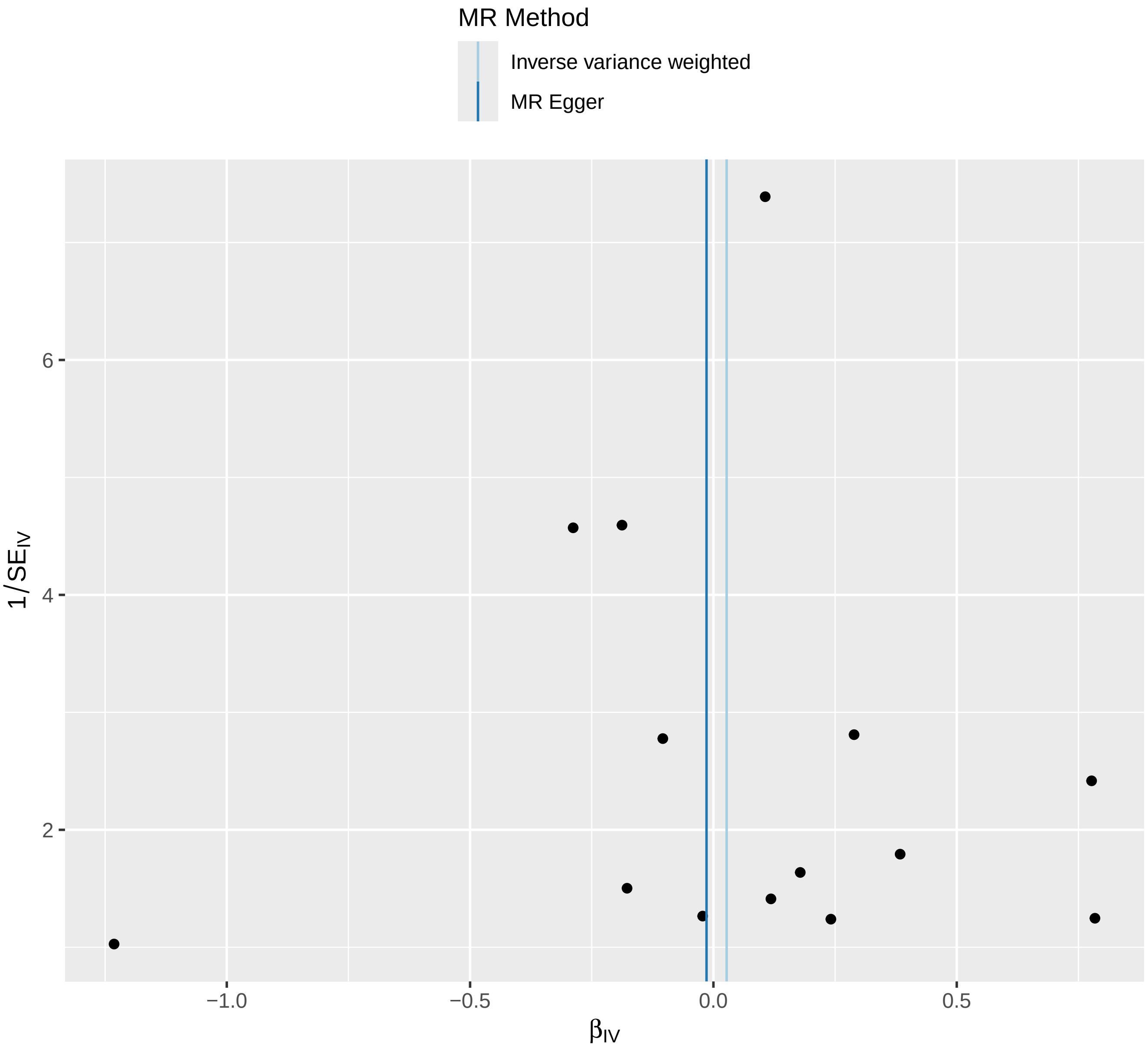

MR Method
Inverse variance weighted
MR Egger
6
4
2
1/SE_IV
−1.0
−0.5
0.0
0.5
β_IV

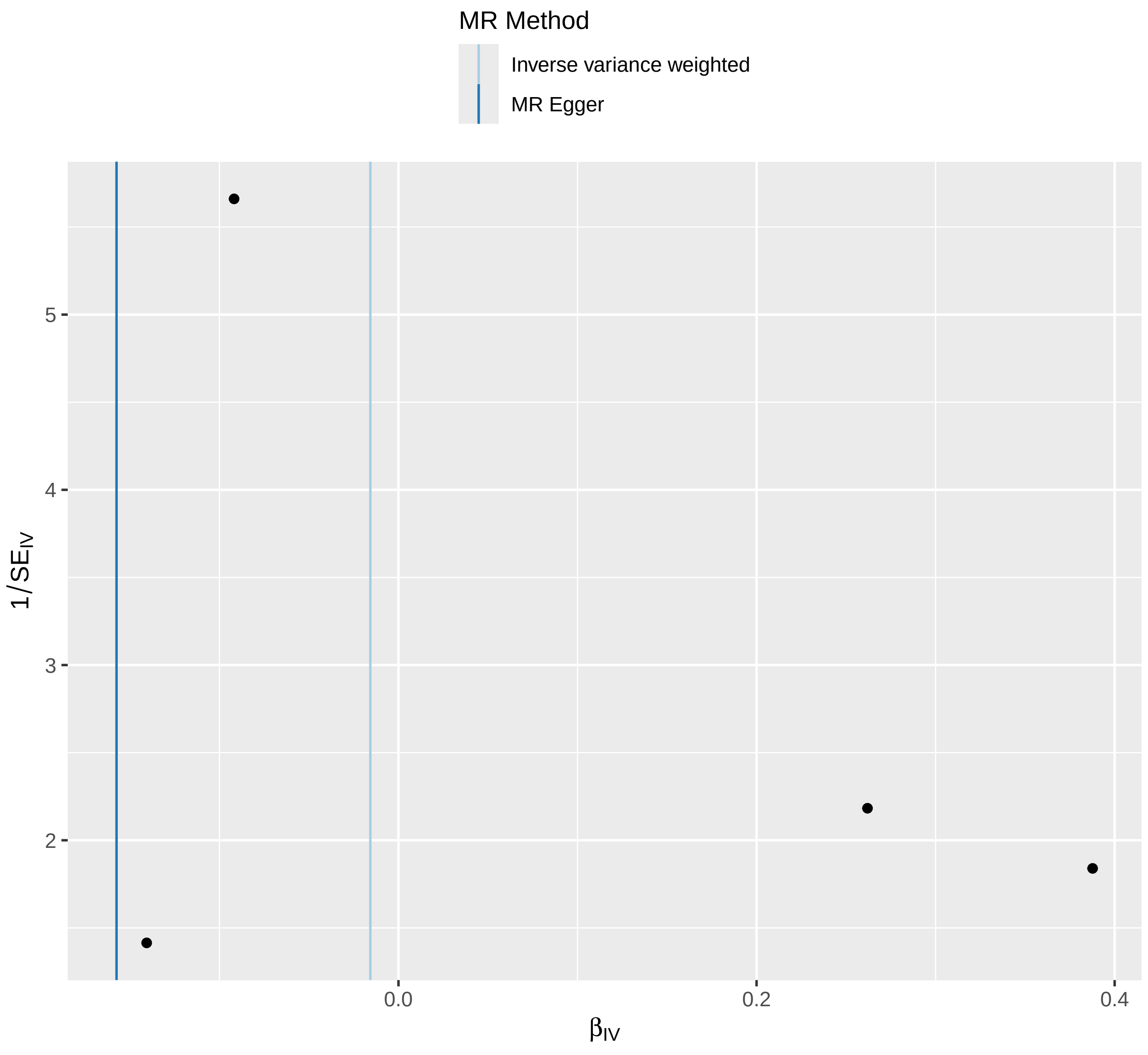

MR Method
Inverse variance weighted
MR Egger
5
4
3
2
1/SE_IV
0.0
0.2
0.4
β_IV

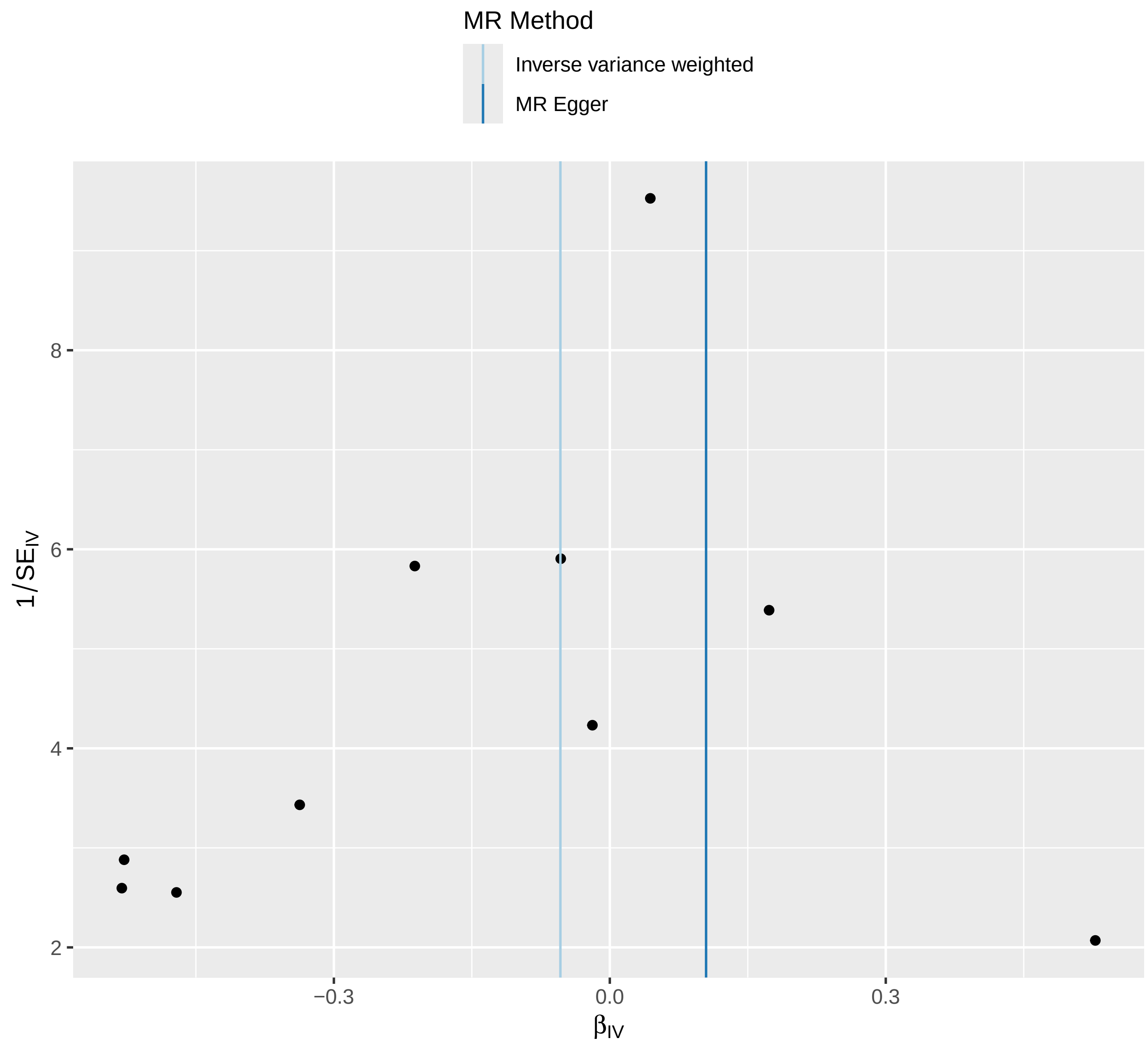
MR Method
Inverse variance weighted
MR Egger
8
6
4
2
1/SE$_{IV}$
−0.3
0.0
0.3
β$_{IV}$

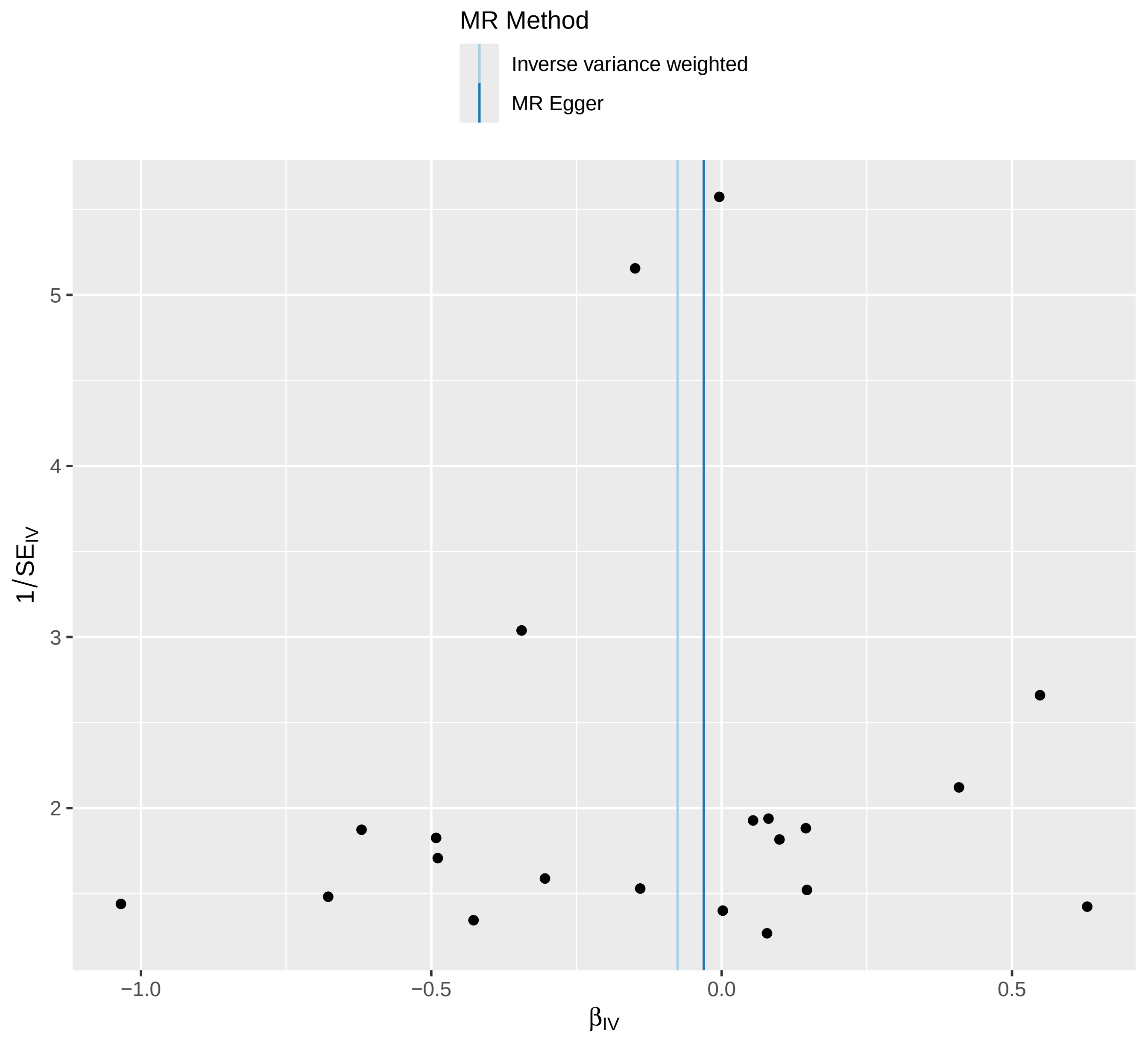

MR Method
Inverse variance weighted
MR Egger
1/SE$_{IV}$
5
4
3
2
−1.0
−0.5
0.0
0.5
β_{IV}

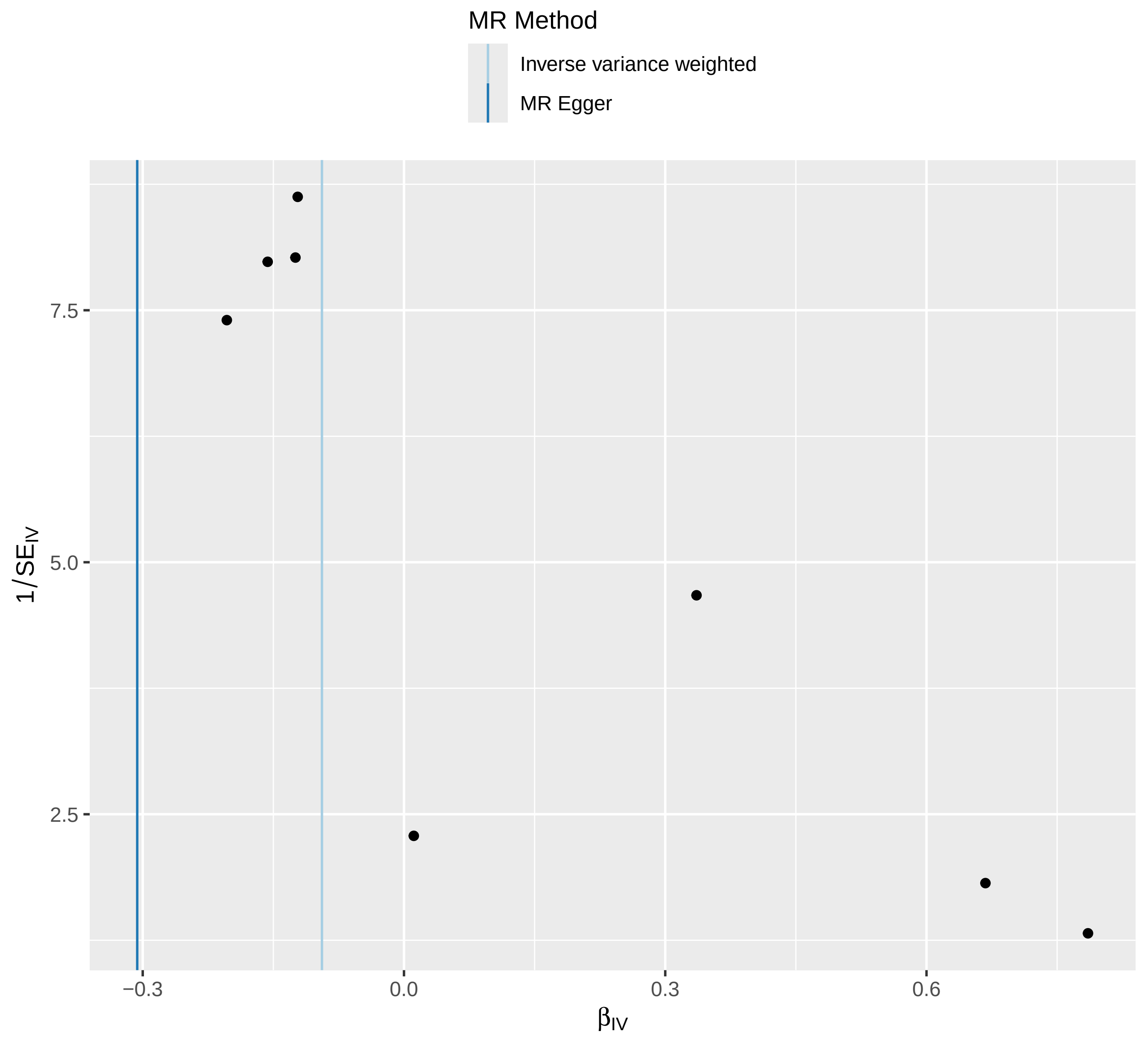
MR Method
Inverse variance weighted
MR Egger
7.5
5.0
2.5
1/SE_IV
−0.3
0.0
0.3
0.6
β_IV

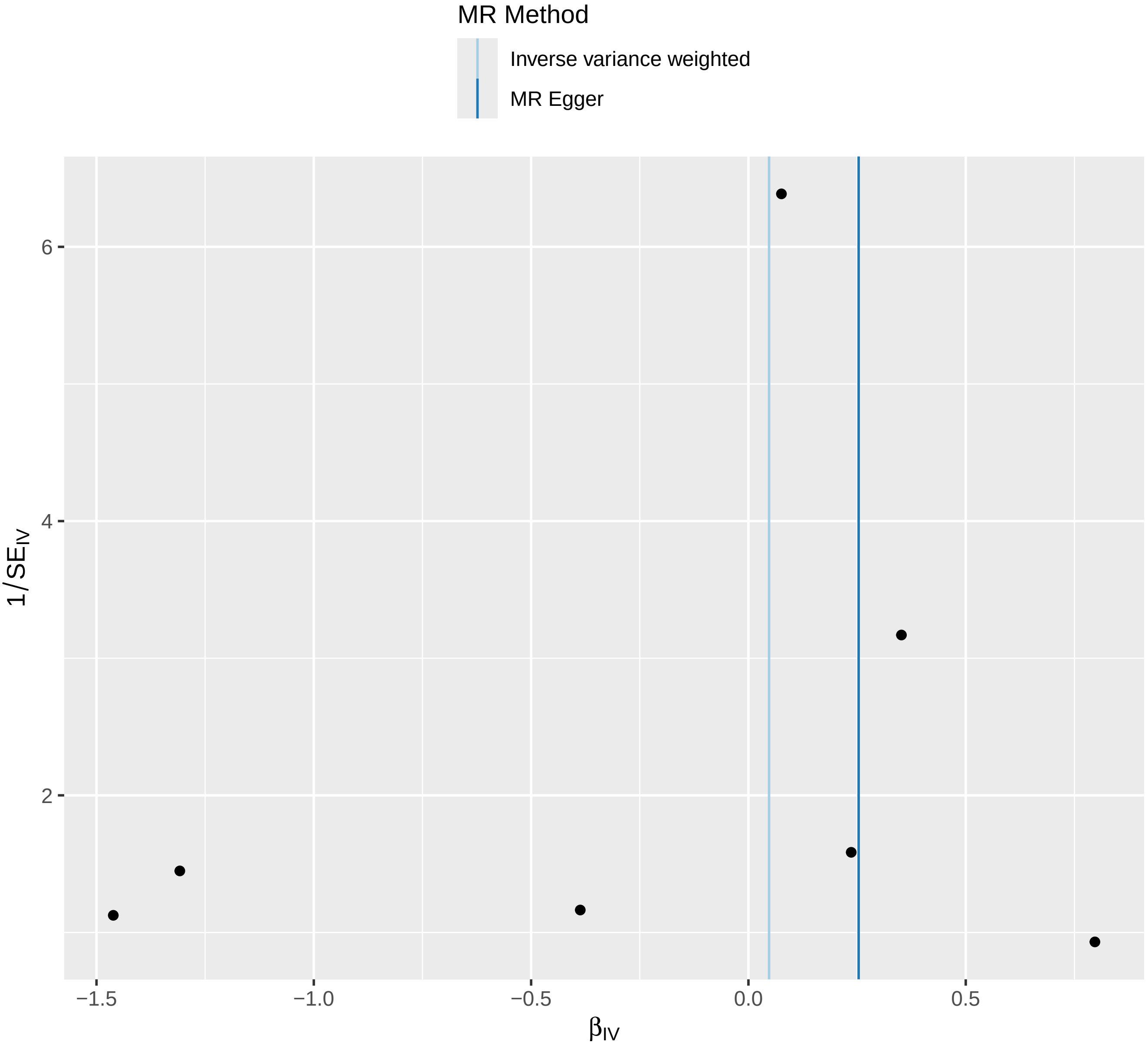

MR Method
Inverse variance weighted
MR Egger
6
4
2
1/SE_IV
−1.5
−1.0
−0.5
0.0
0.5
β_IV

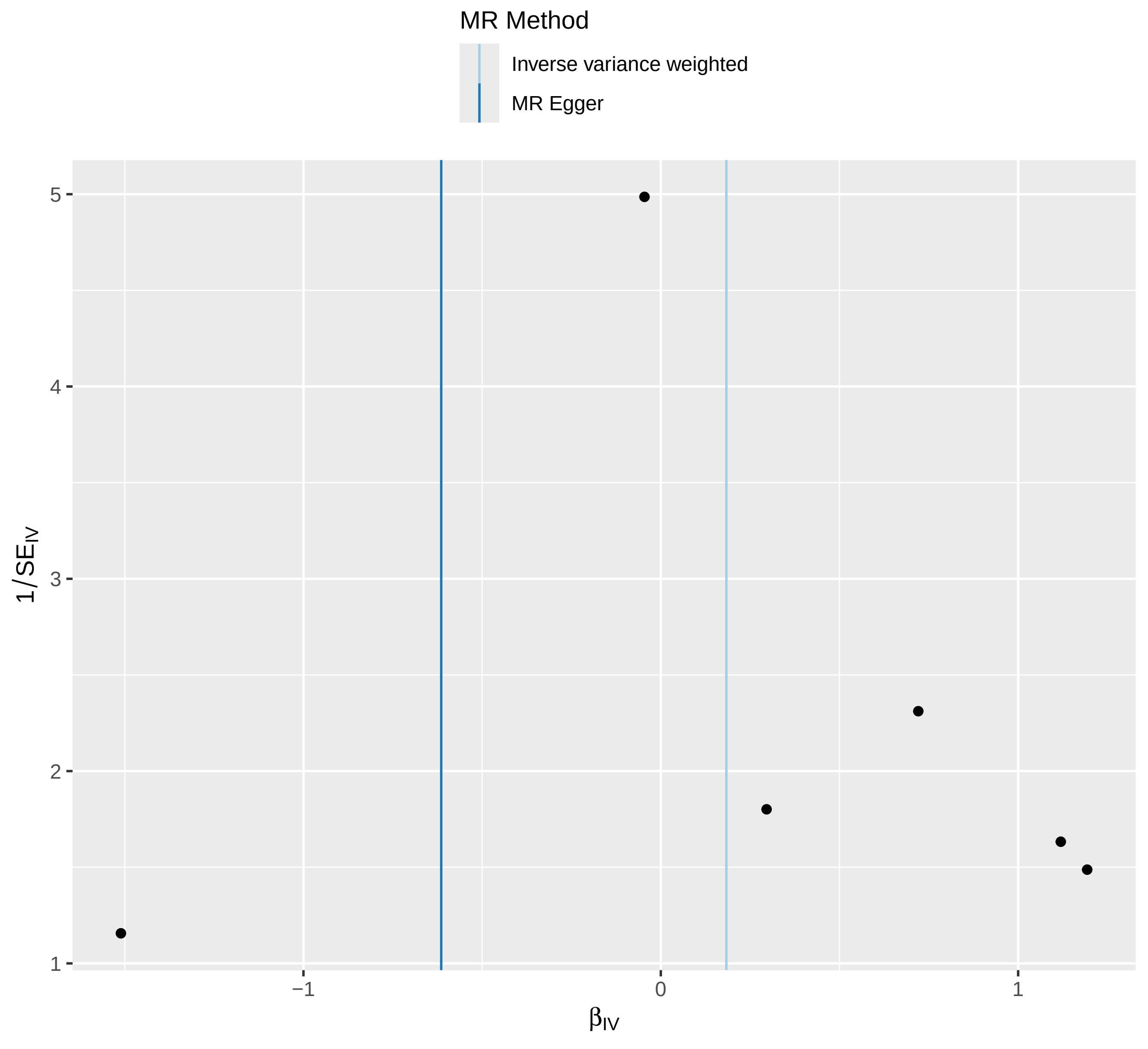

MR Method
Inverse variance weighted
MR Egger
5
4
3
2
1
−1
0
1
1/SE_IV
β_IV

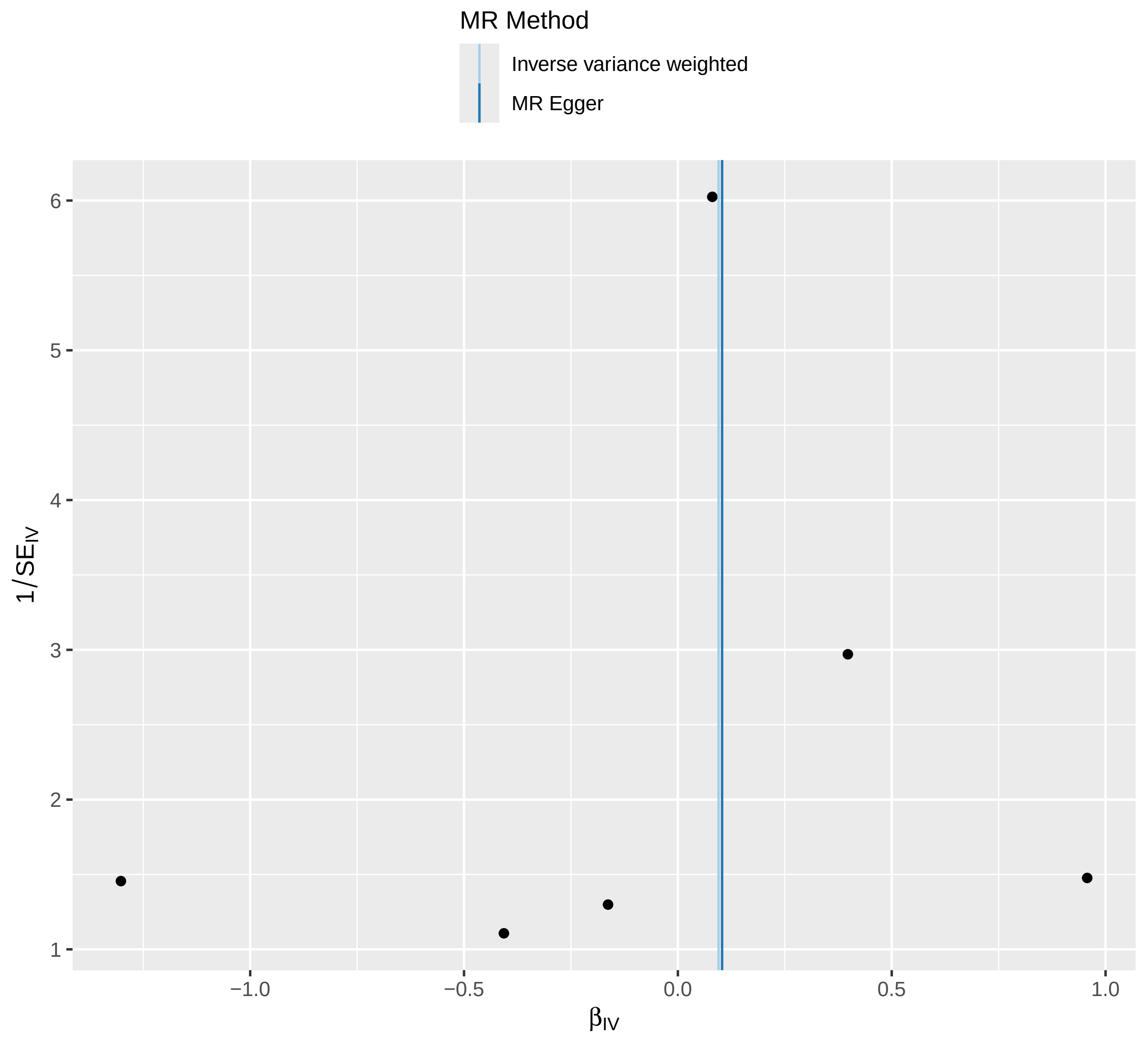
MR Method
Inverse variance weighted
MR Egger
1/SE_IV
6
5
4
3
2
1
−1.0
−0.5
0.0
0.5
1.0
β_IV

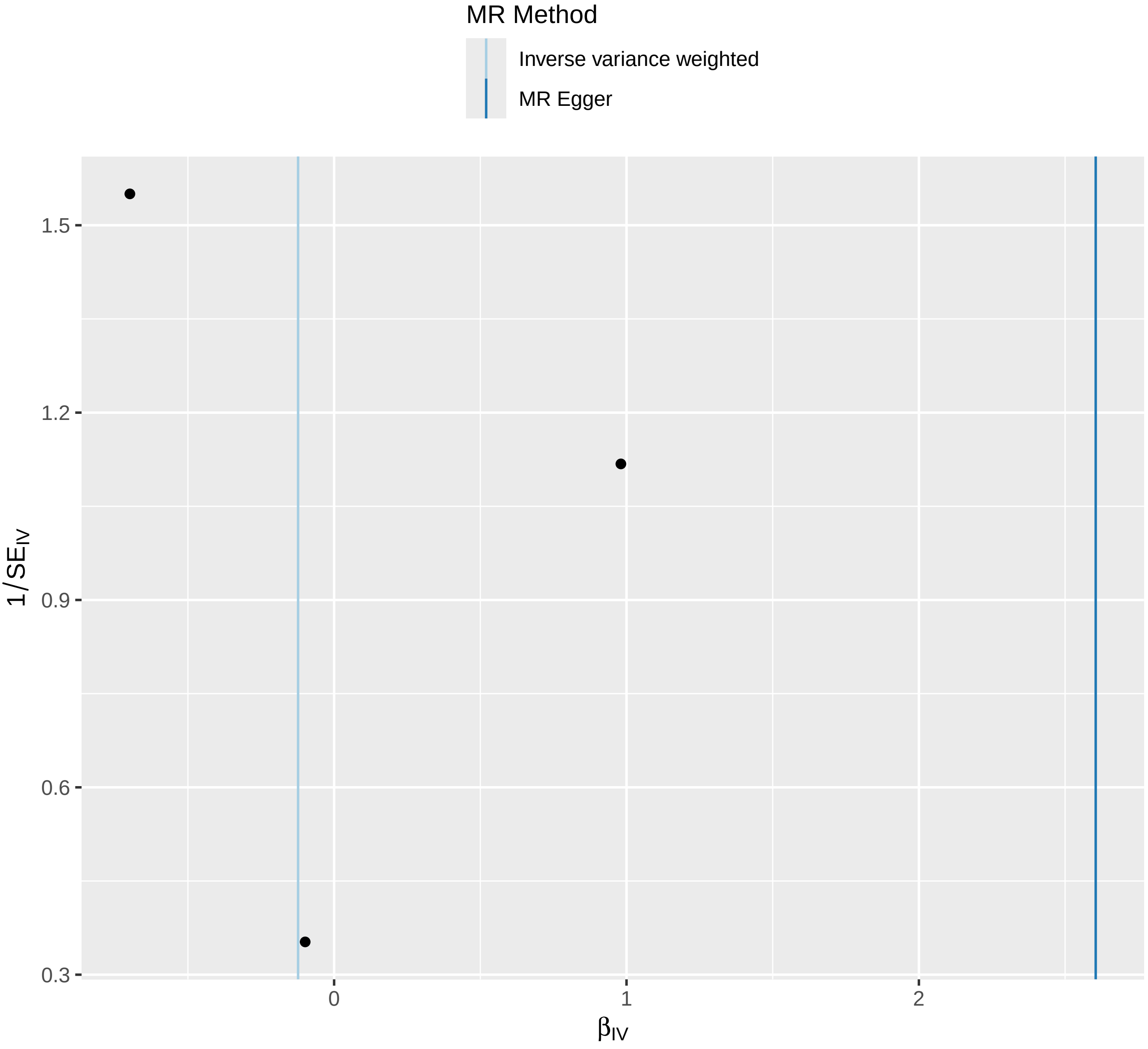

MR Method
Inverse variance weighted
MR Egger
1.5
1.2
0.9
0.6
0.3
0
1
2
$1/SE_{IV}$
β_{IV}

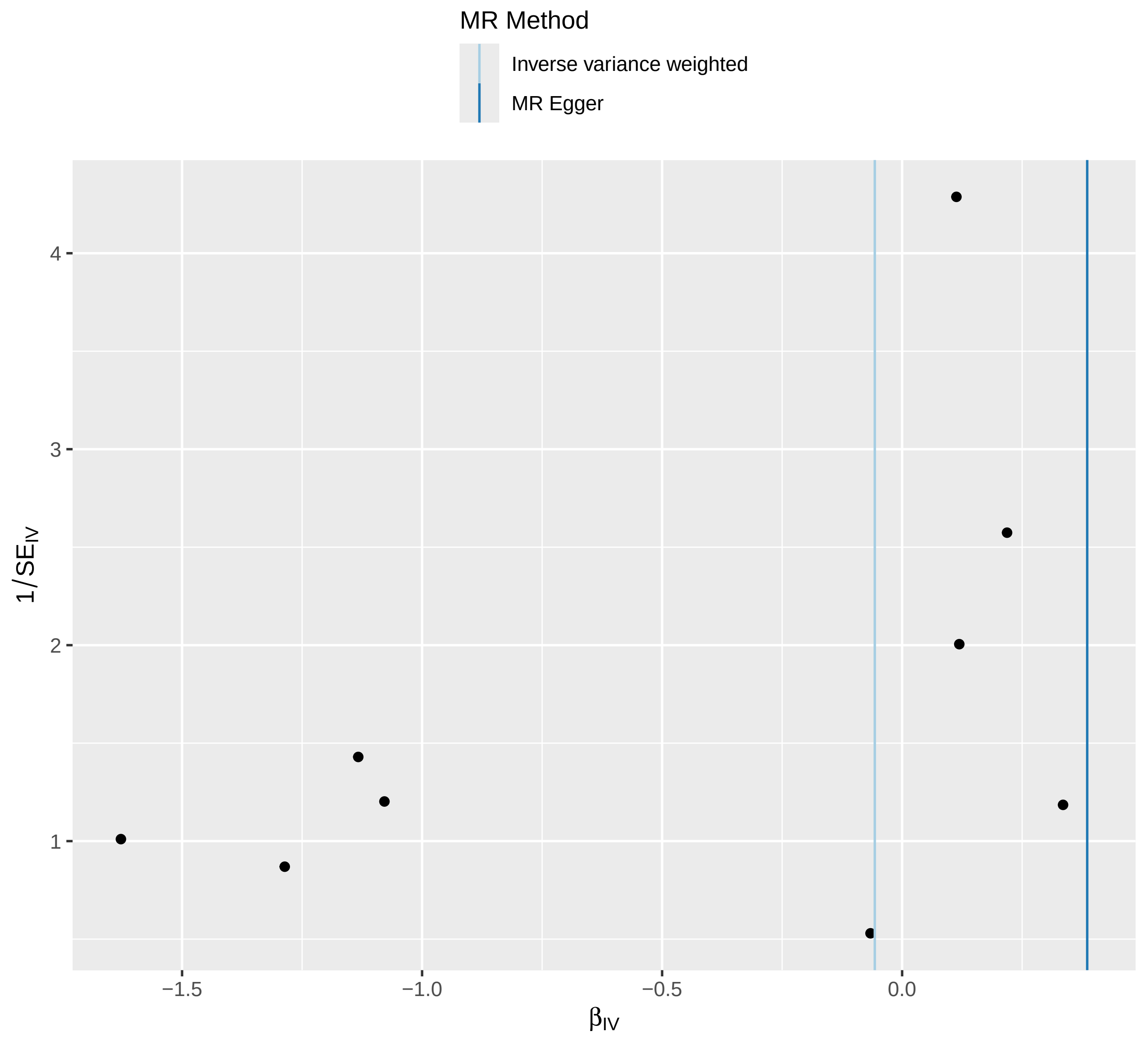
MR Method
Inverse variance weighted
MR Egger
4
3
2
1
1/SE$_{IV}$
−1.5
−1.0
−0.5
0.0
β$_{IV}$

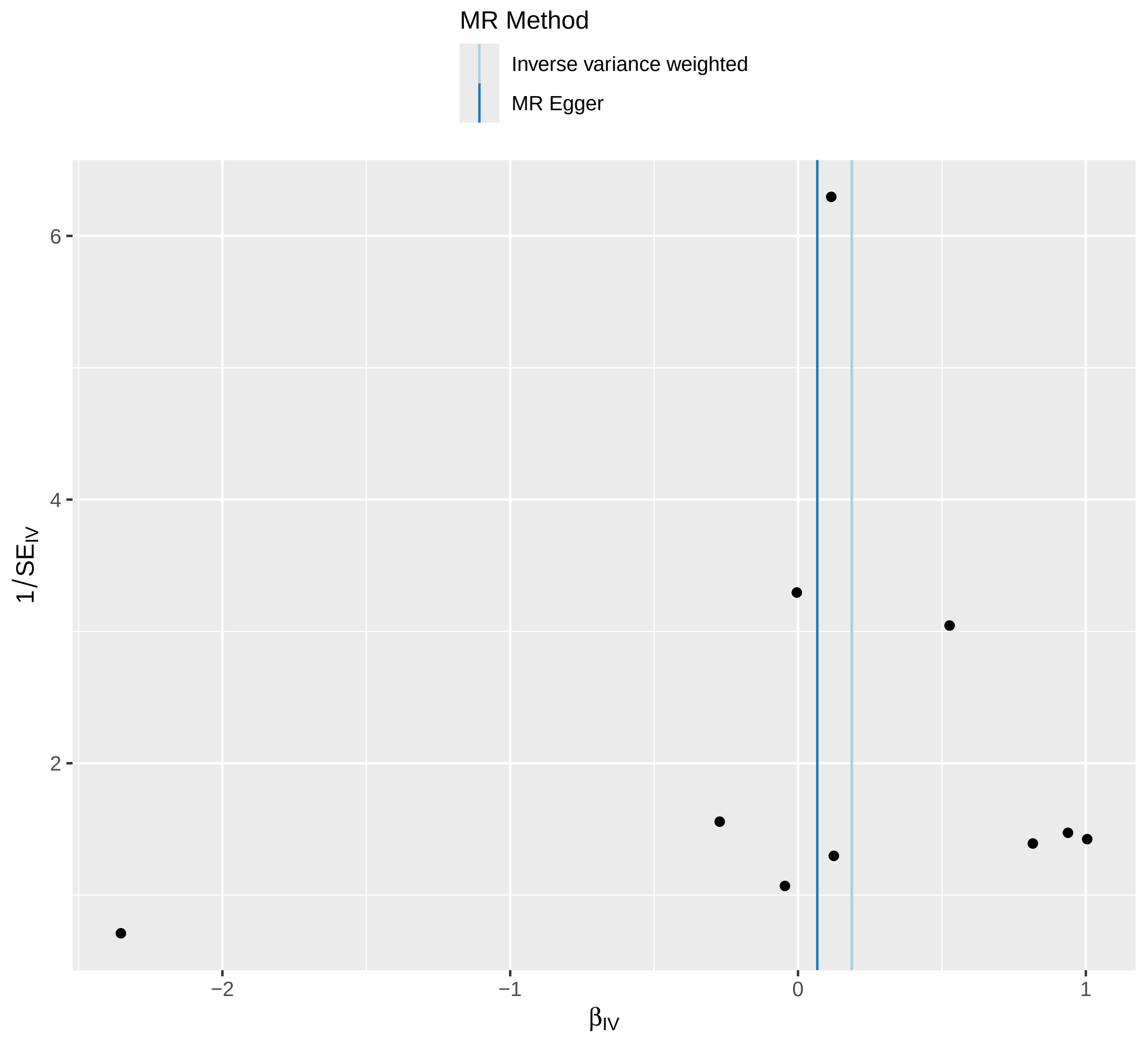
MR Method
Inverse variance weighted
MR Egger
6
4
2
1/SE_IV
−2
−1
0
1
β_IV

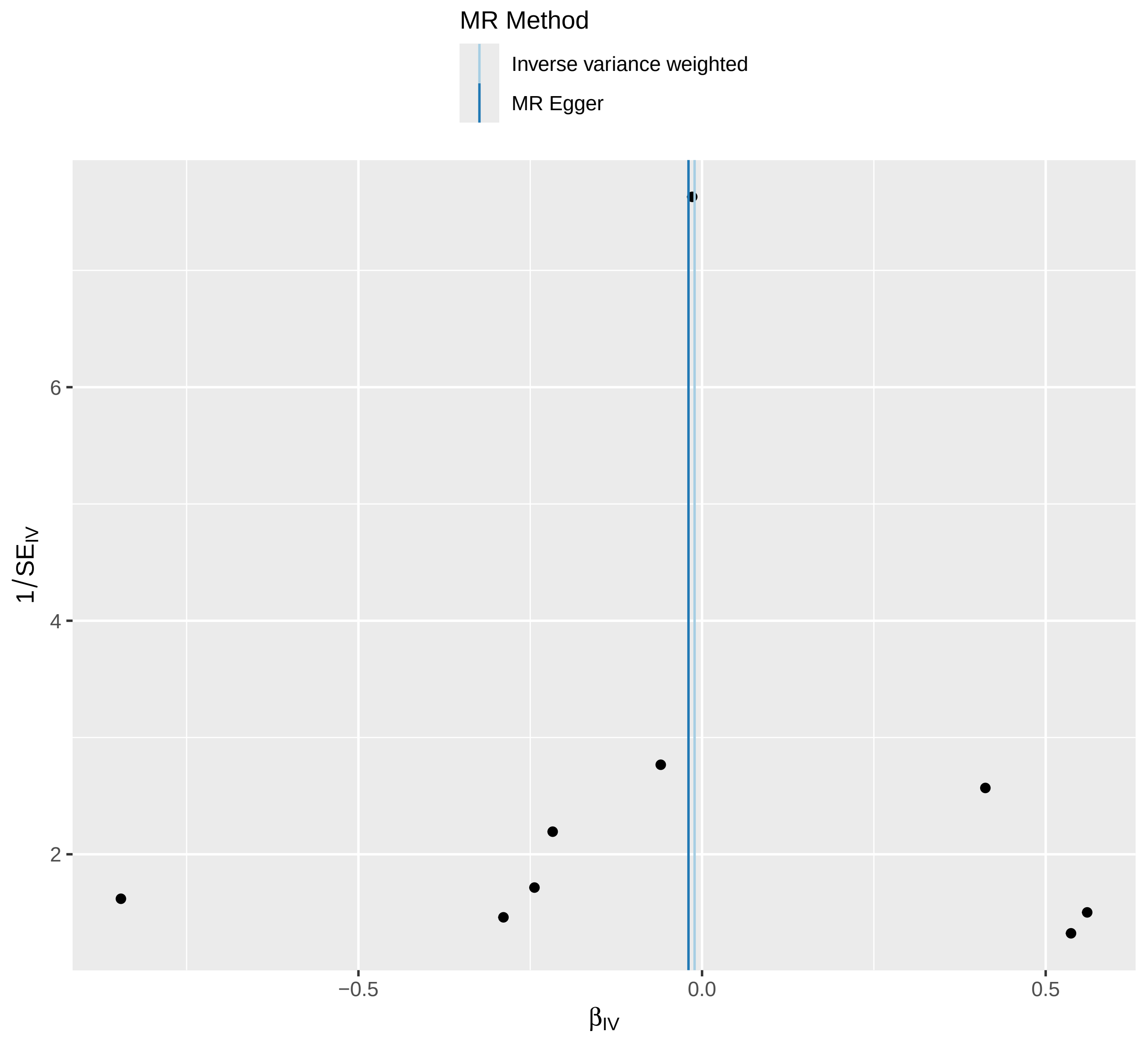

MR Method
Inverse variance weighted
MR Egger
6
4
2
1/SE_IV
−0.5
0.0
0.5
β_IV

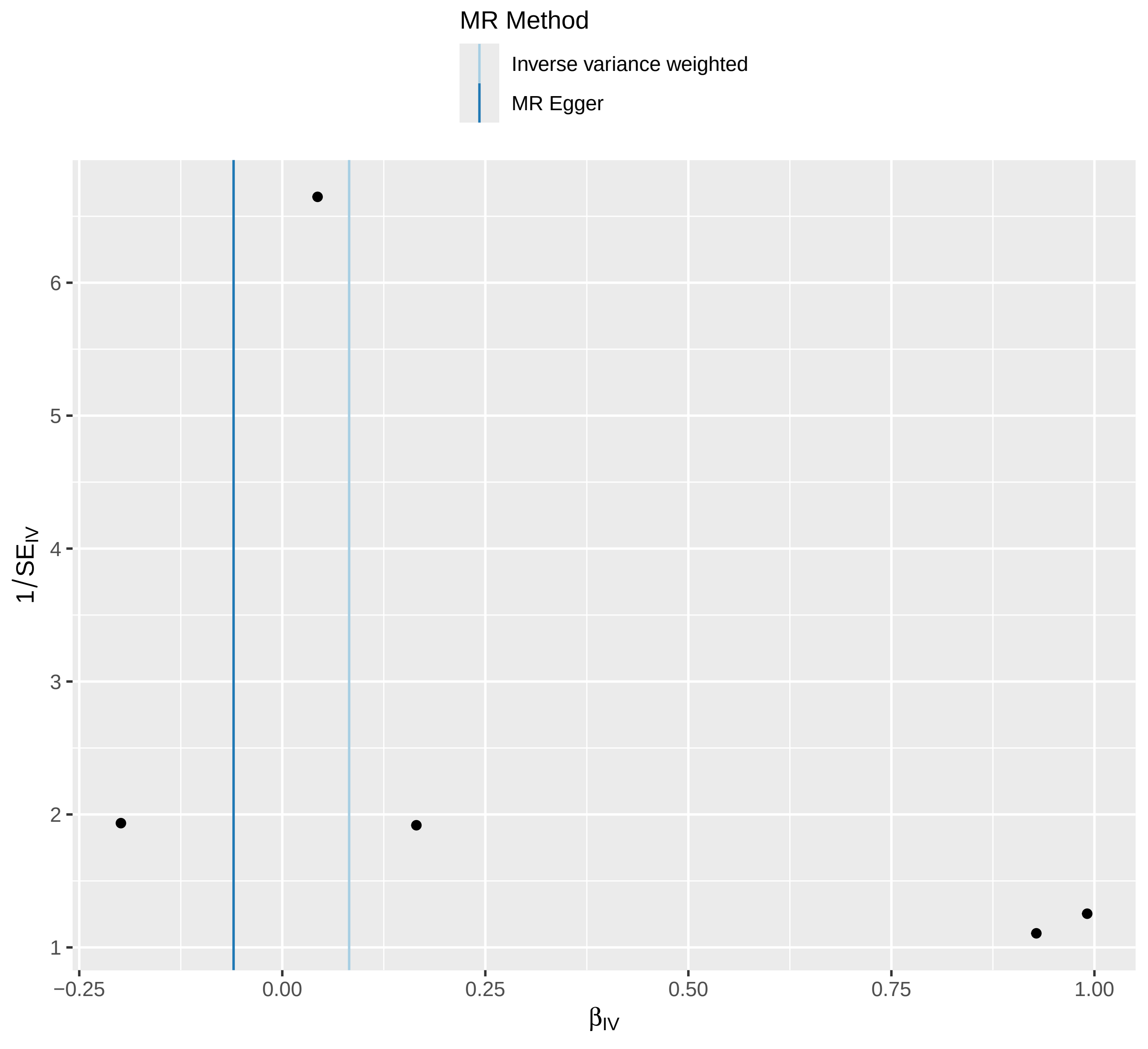
MR Method
Inverse variance weighted
MR Egger
1/SE$_{IV}$
β_{IV}
1
2
3
4
5
6
−0.25
0.00
0.25
0.50
0.75
1.00

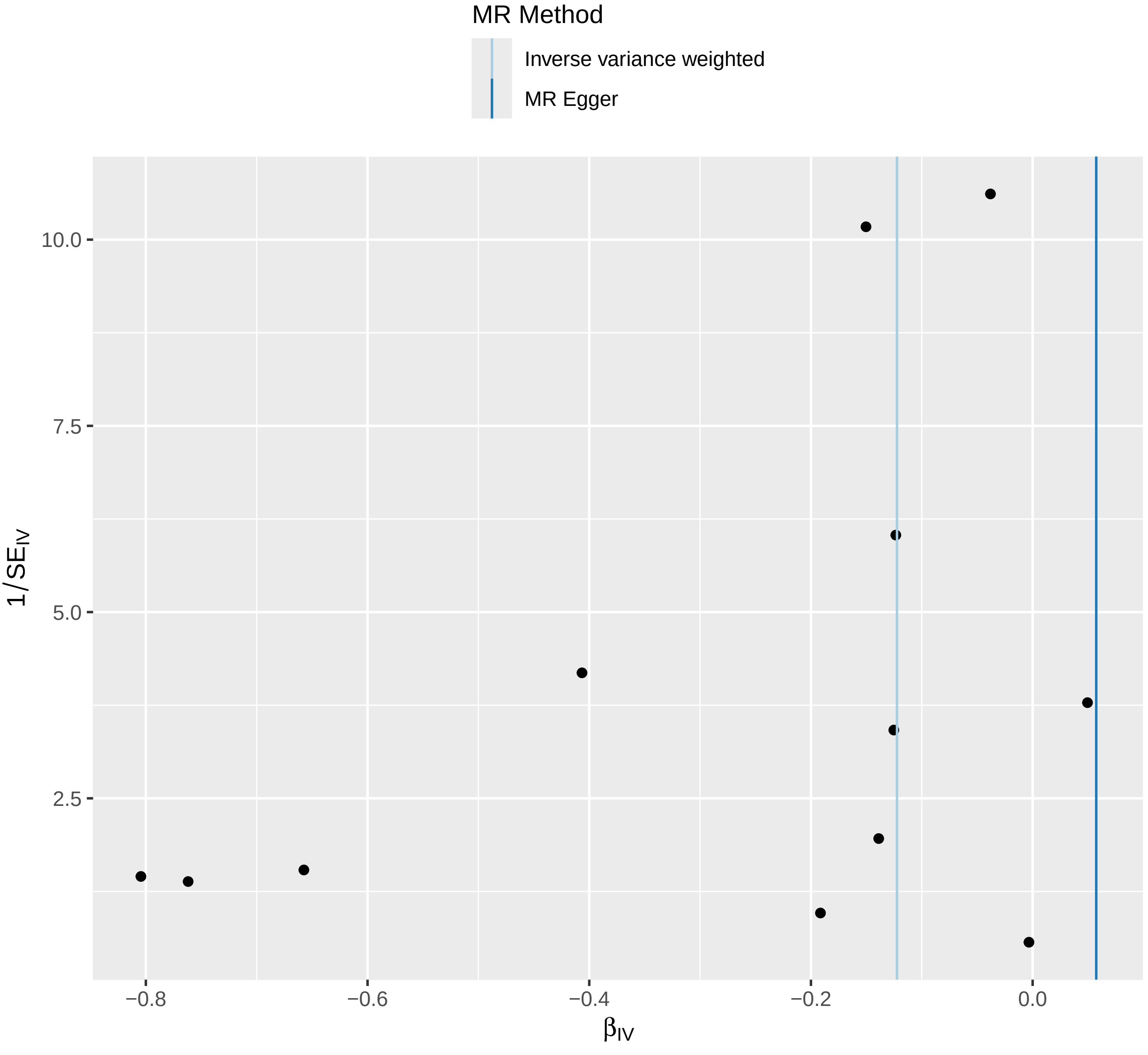
MR Method
Inverse variance weighted
MR Egger
10.0
7.5
5.0
2.5
1/SE_IV
−0.8
−0.6
−0.4
−0.2
0.0
β_IV

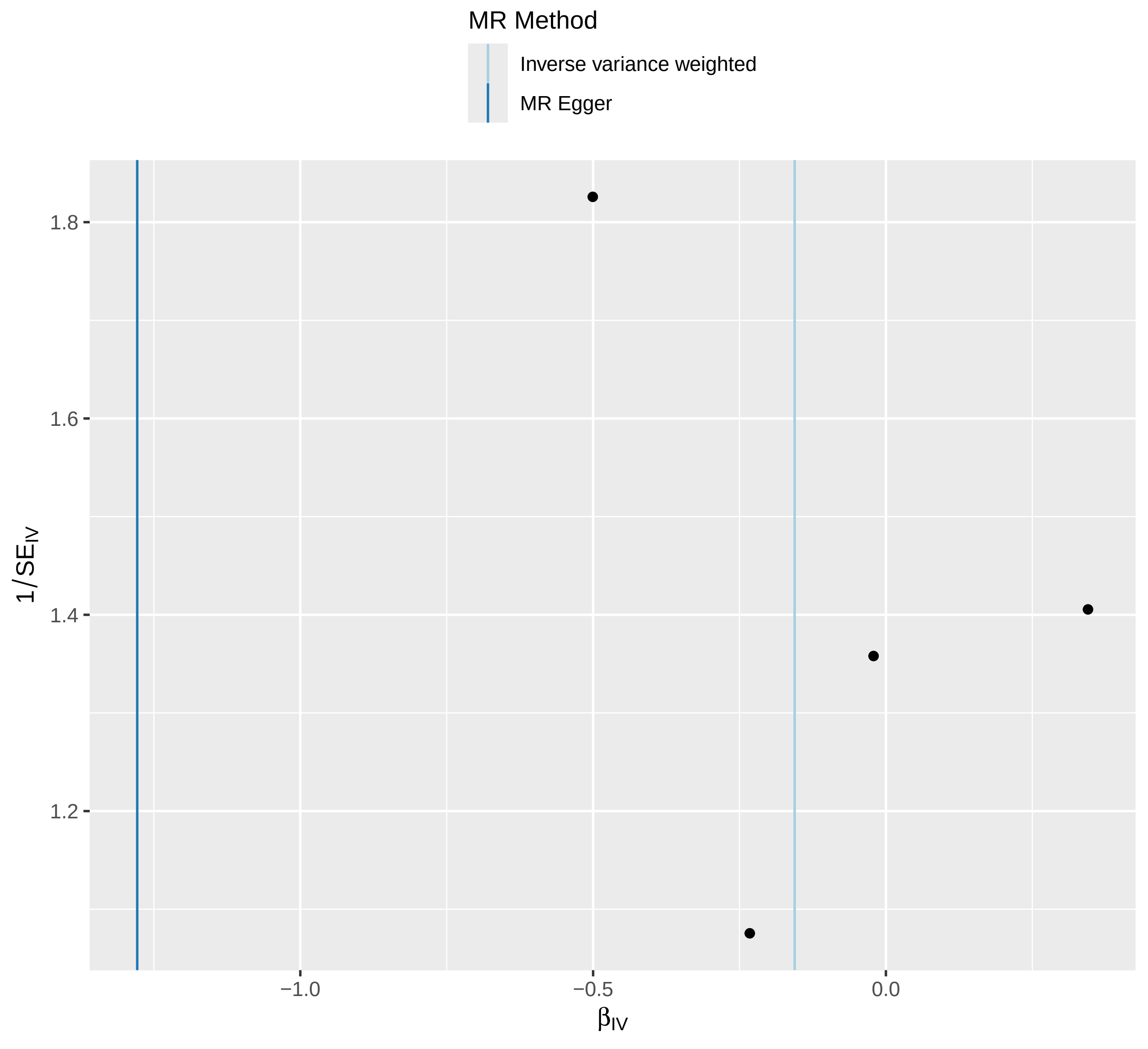

MR Method
Inverse variance weighted
MR Egger
1.8
1.6
1.4
1.2
1/SE$_{IV}$
−1.0
−0.5
0.0
β$_{IV}$

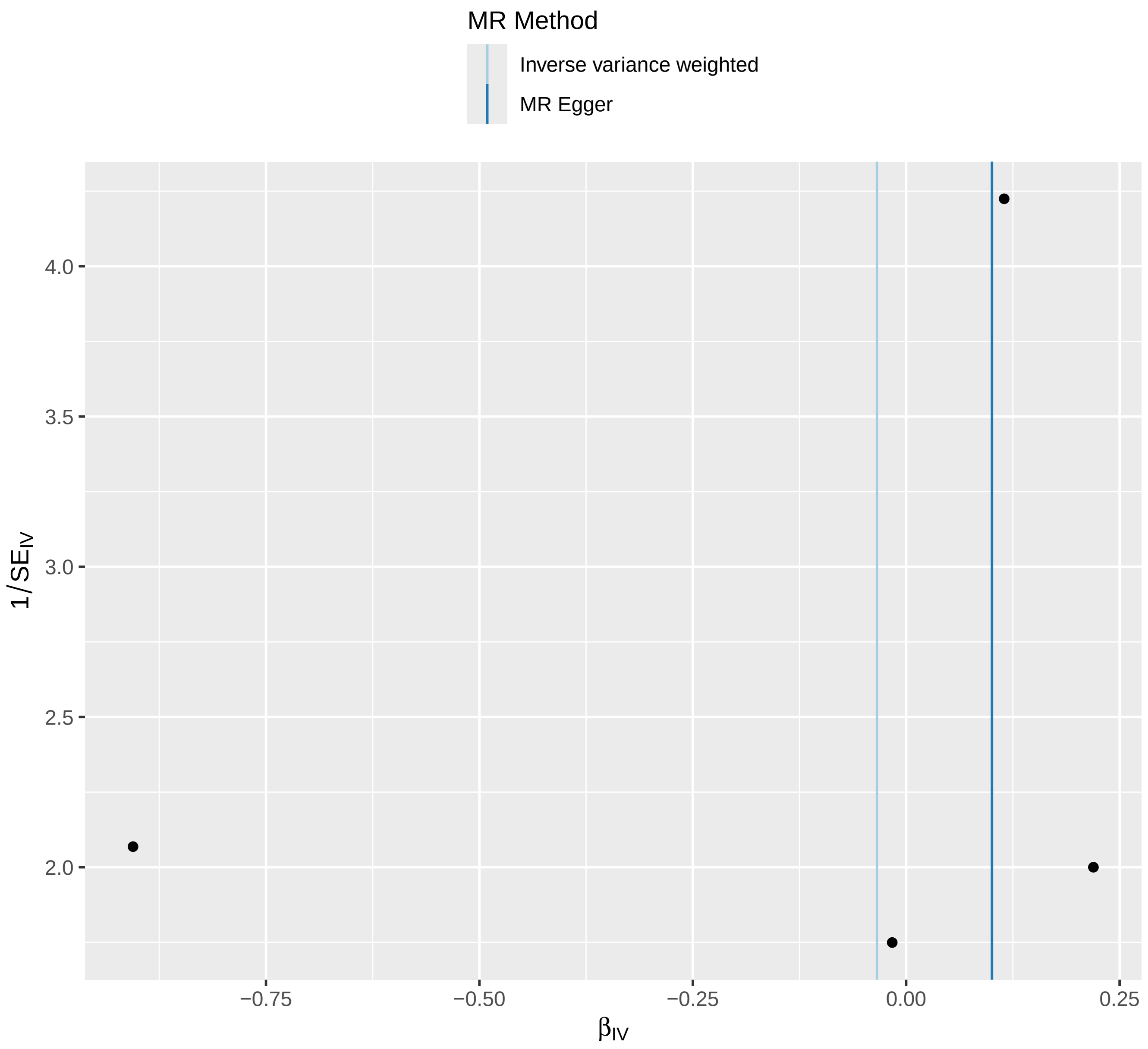

MR Method
Inverse variance weighted
MR Egger
1/SE_IV
4.0
3.5
3.0
2.5
2.0
−0.75
−0.50
−0.25
0.00
0.25
β_IV

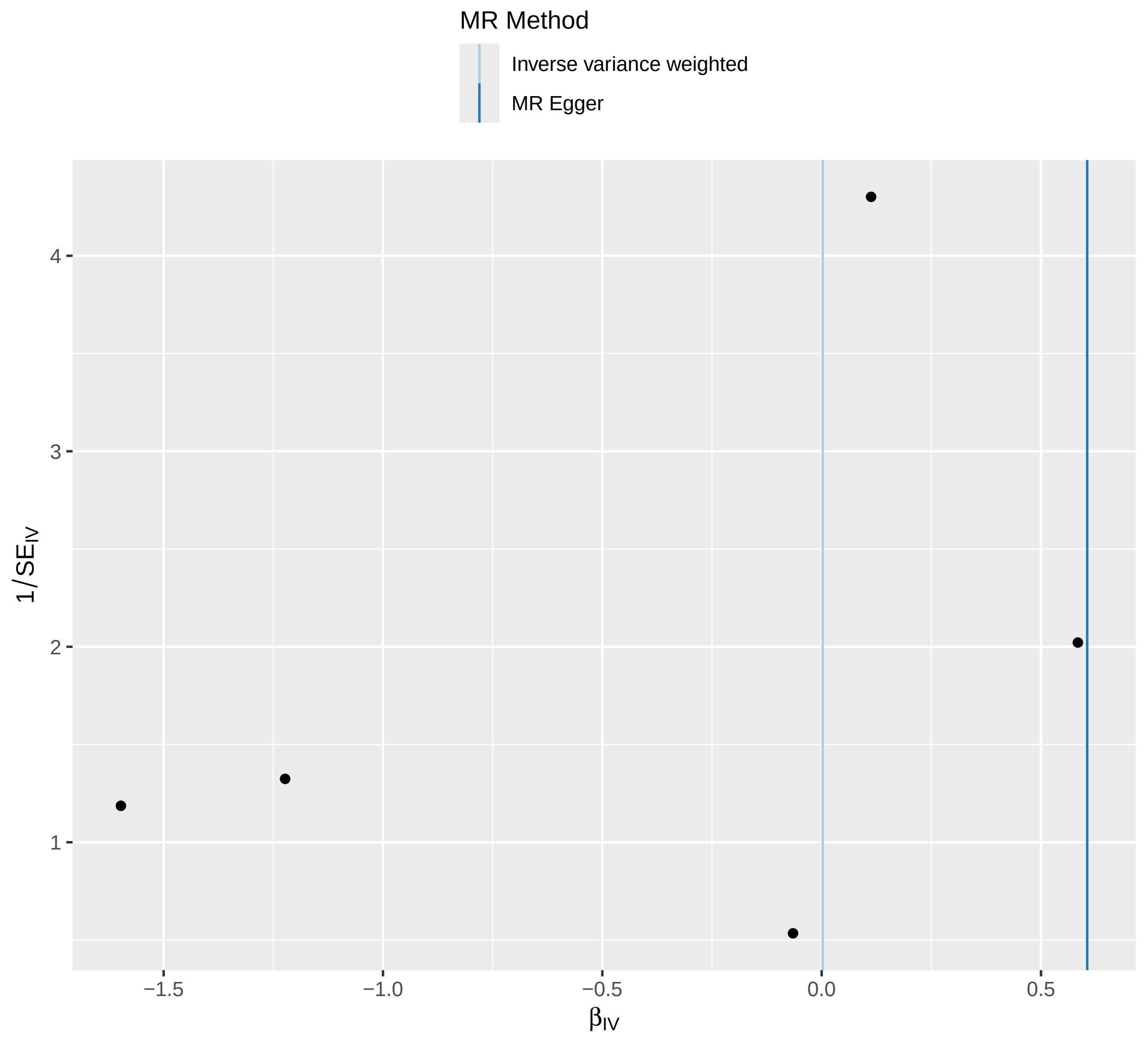
MR Method
Inverse variance weighted
MR Egger
1/SE$_{IV}$
4
3
2
1
−1.5
−1.0
−0.5
0.0
0.5
β$_{IV}$

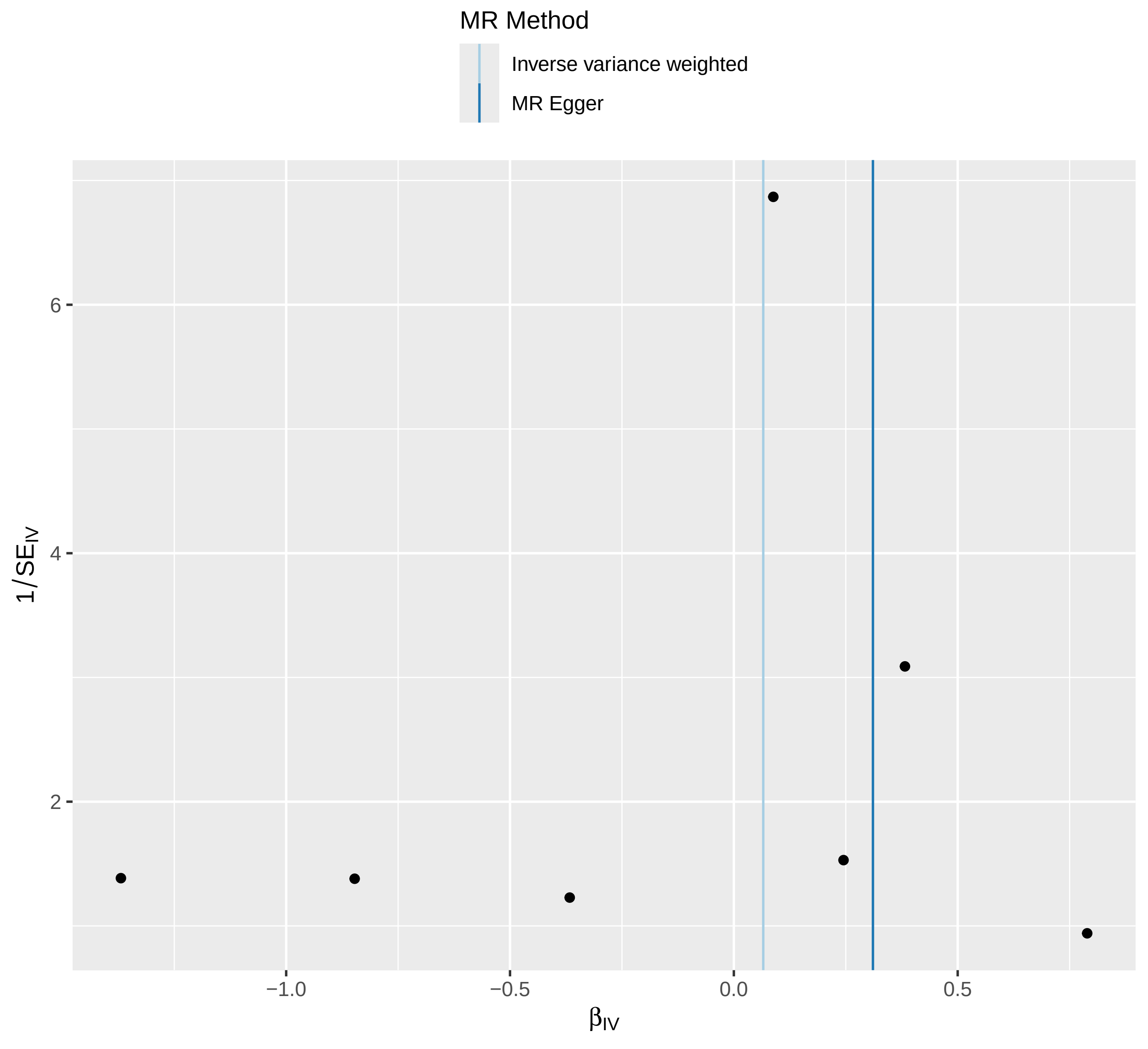
MR Method
Inverse variance weighted
MR Egger
1/SE_IV
6
4
2
−1.0
−0.5
0.0
0.5
β_IV

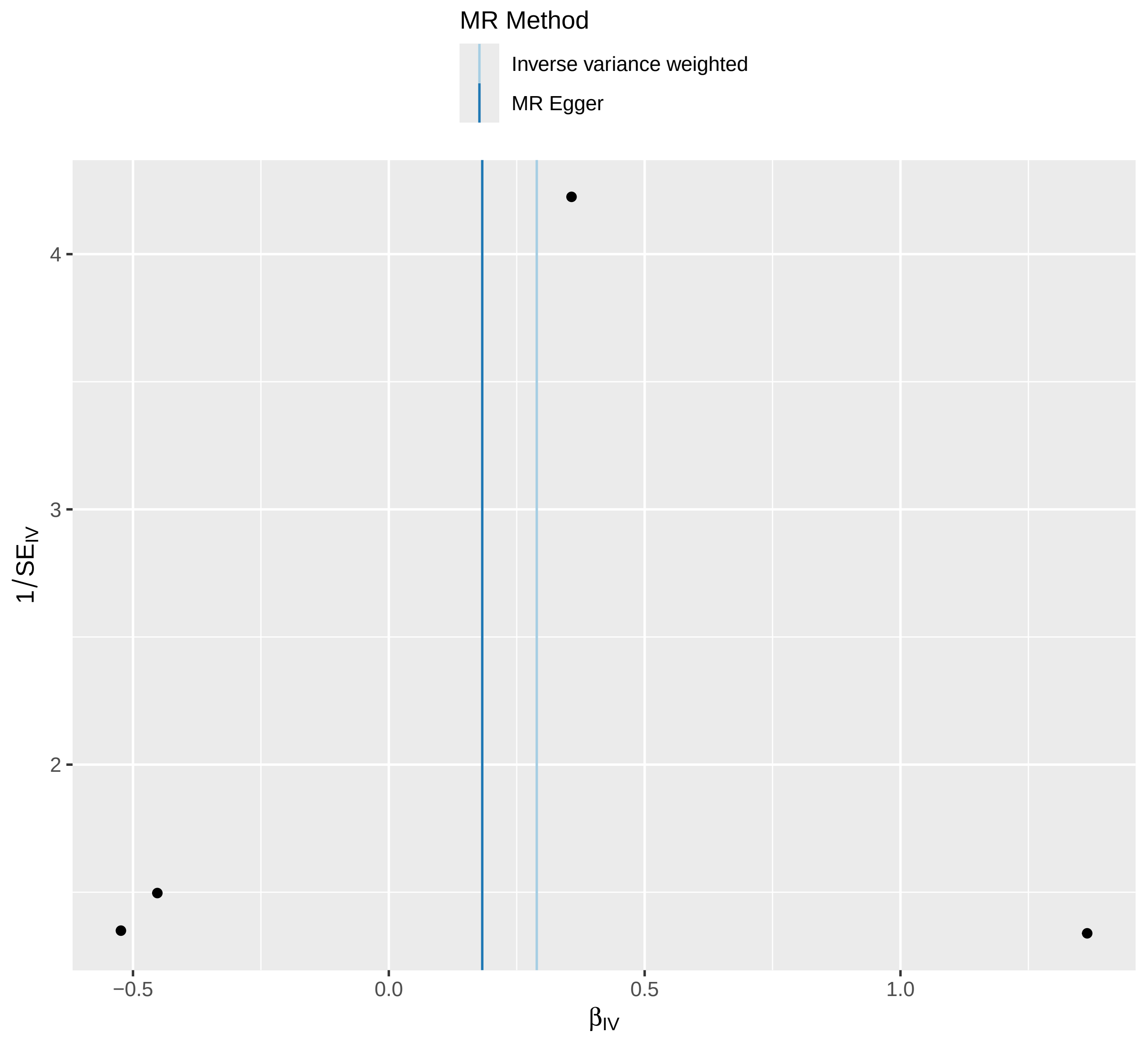
MR Method
Inverse variance weighted
MR Egger
4
3
2
1/SE_IV
−0.5
0.0
0.5
1.0
β_IV

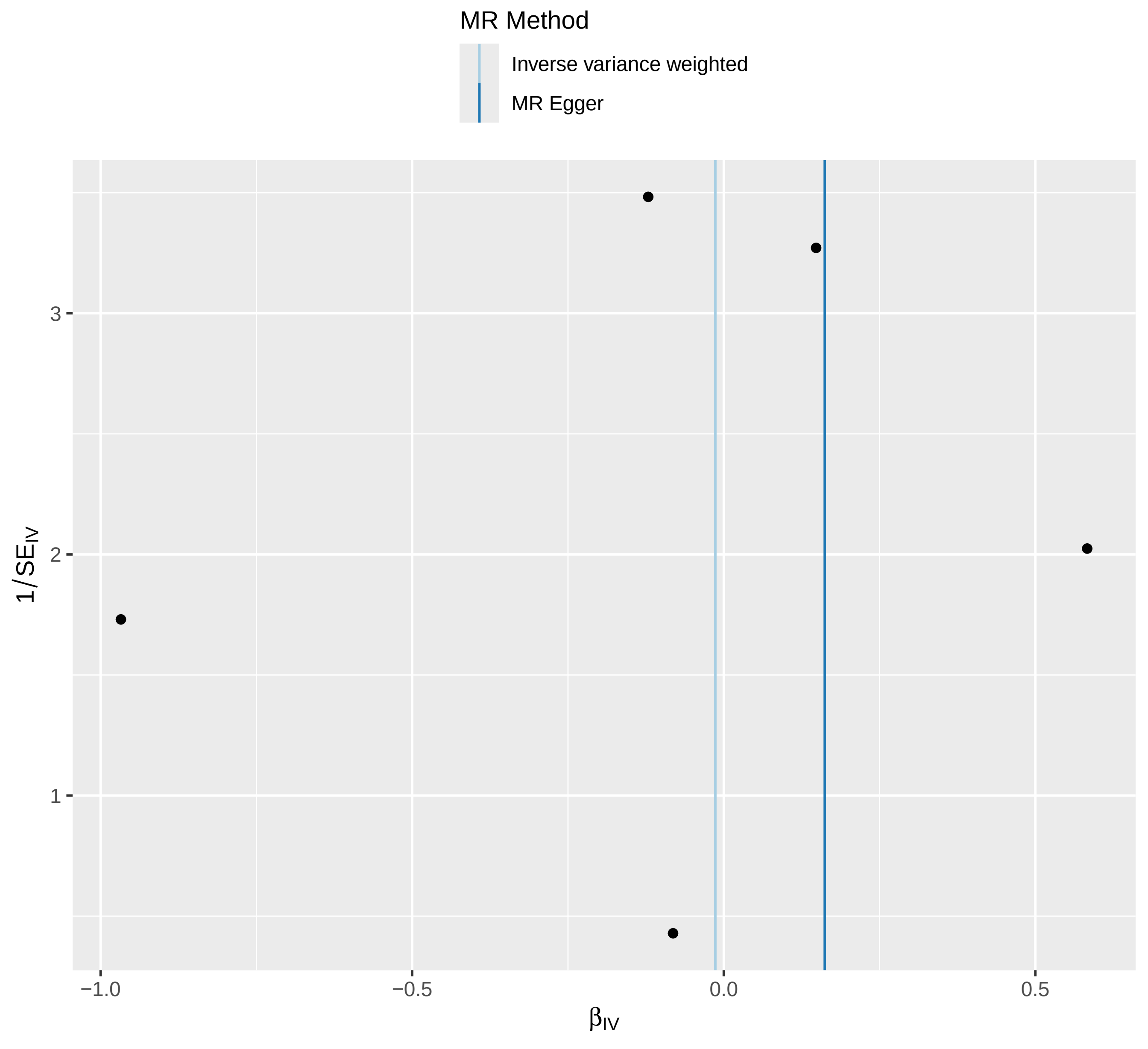

MR Method
Inverse variance weighted
MR Egger
3
2
1
1/SE_IV
−1.0
−0.5
0.0
0.5
β_IV

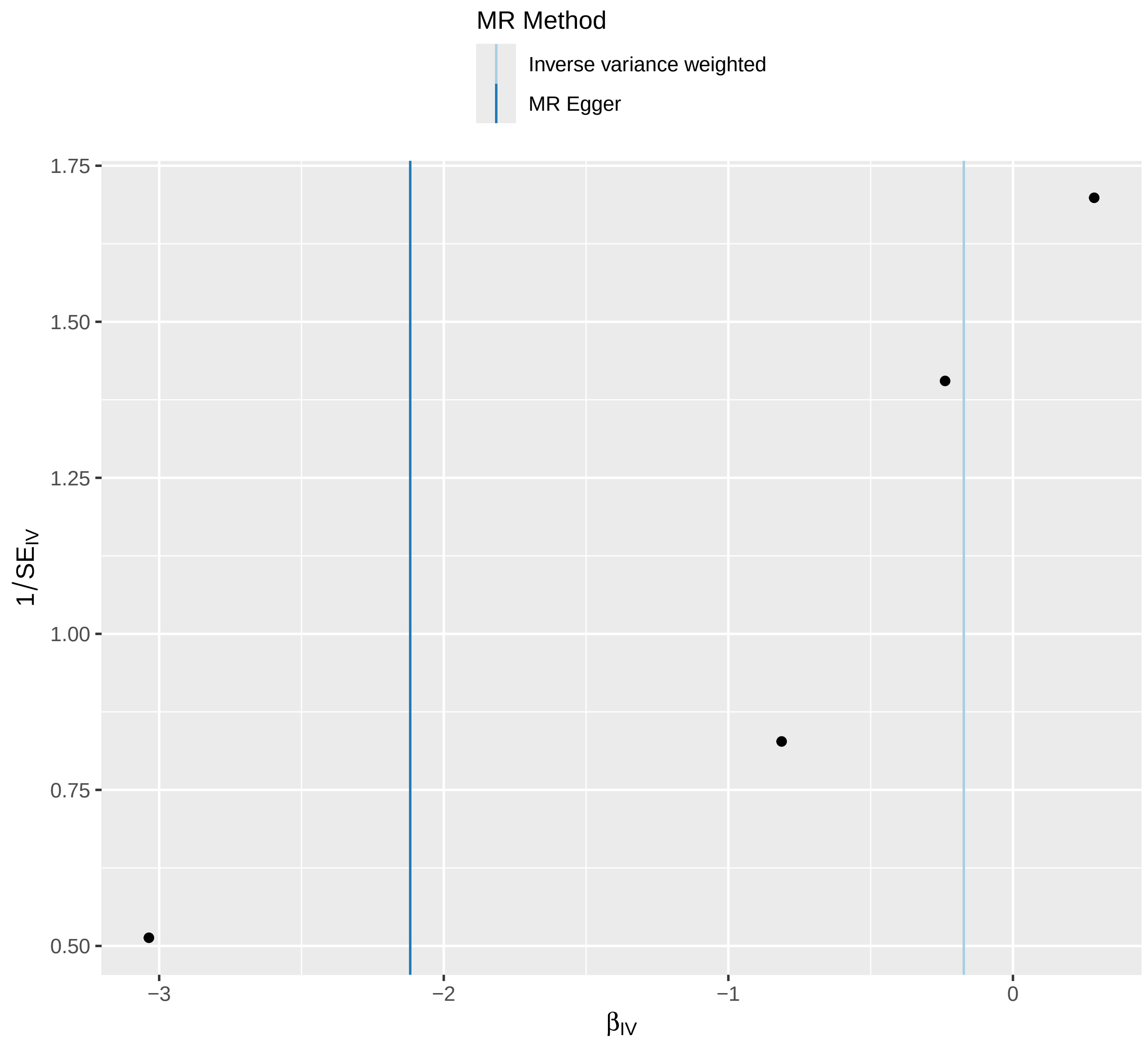
MR Method
Inverse variance weighted
MR Egger
1.75
1.50
1.25
1.00
0.75
0.50
−3
−2
−1
0
β_{IV}
$1/SE_{IV}$

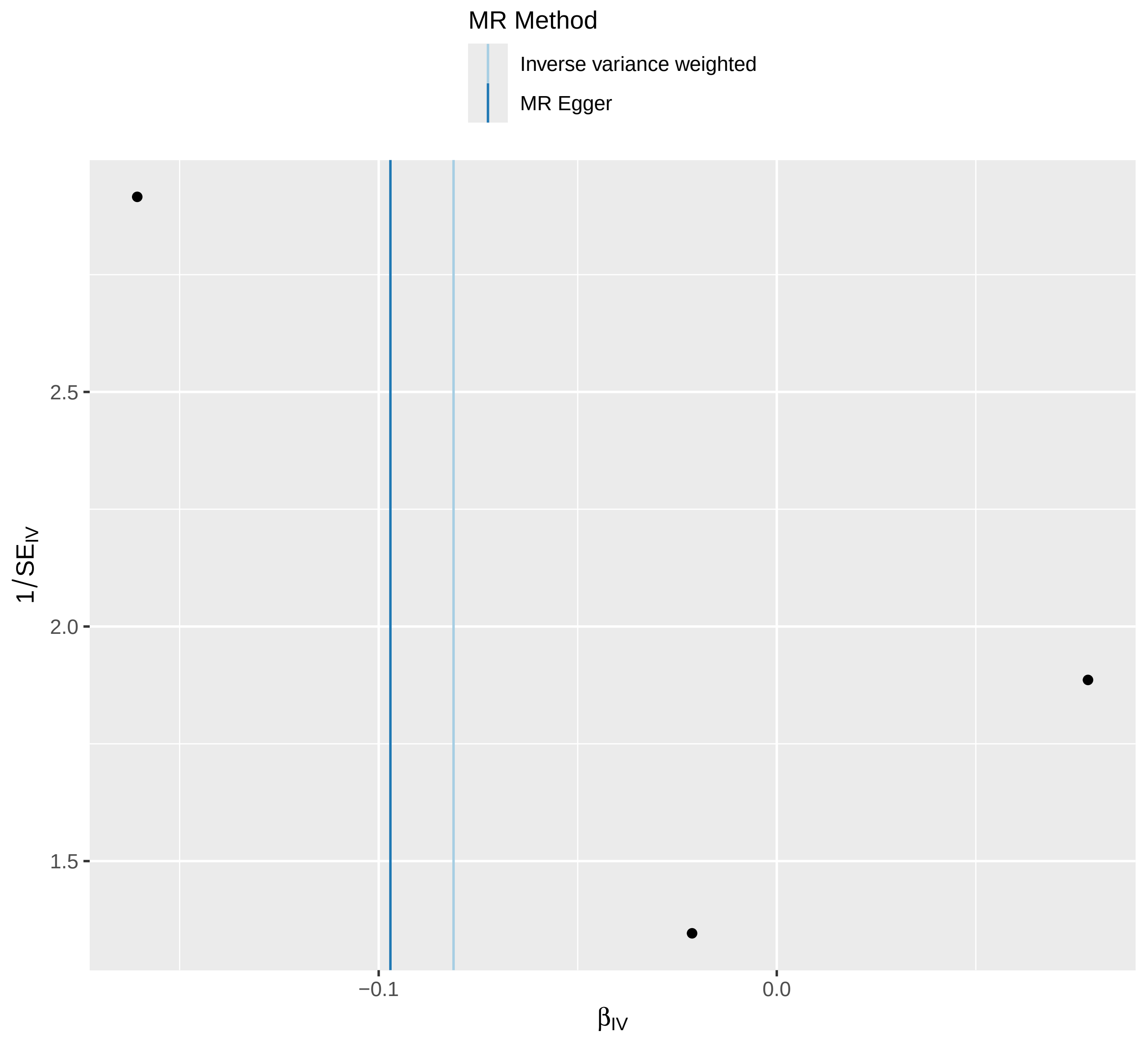
MR Method
Inverse variance weighted
MR Egger
2.5
2.0
1.5
1/SE_IV
−0.1
0.0
β_IV

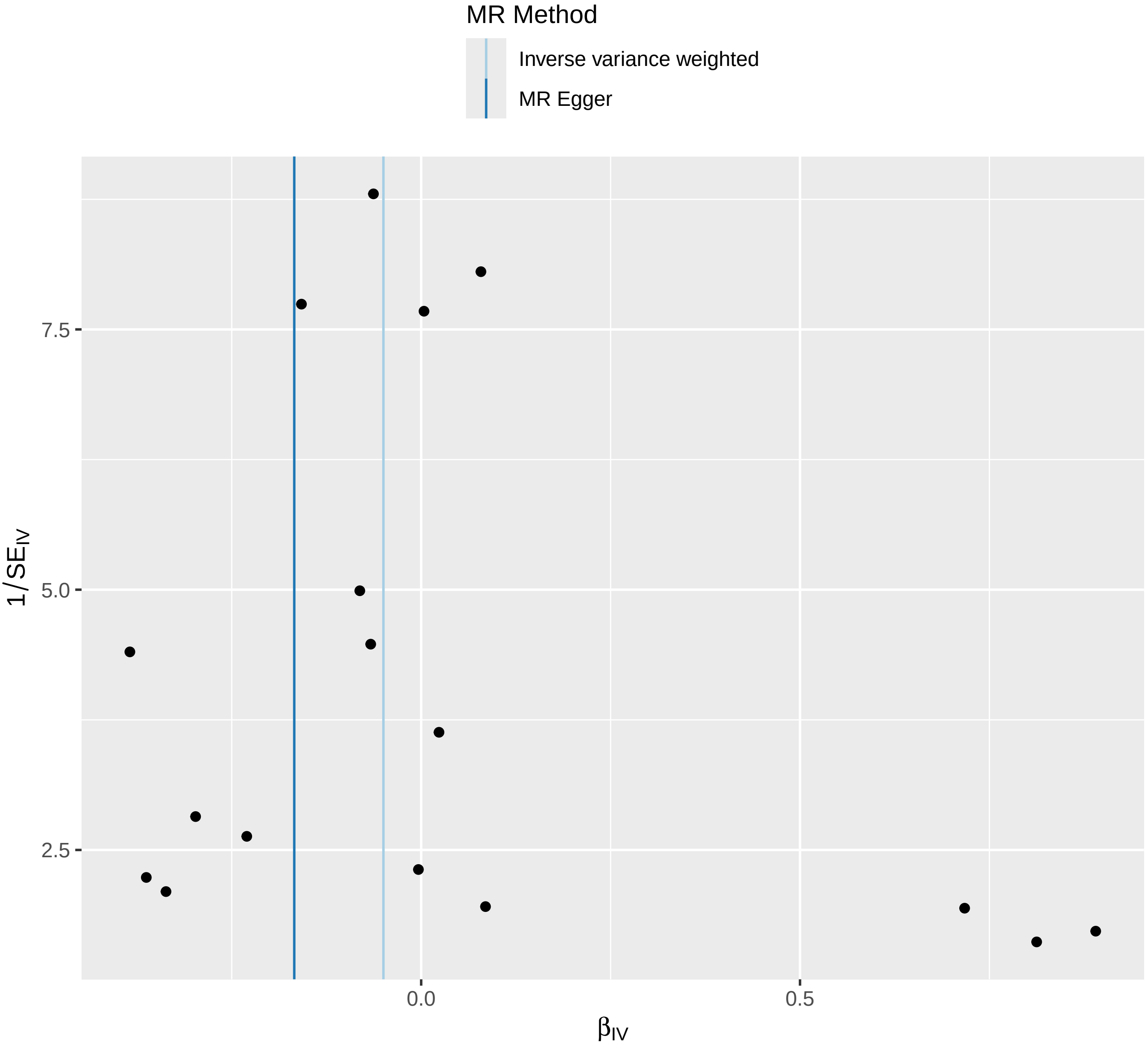
MR Method
Inverse variance weighted
MR Egger
7.5
5.0
2.5
$1/SE_{IV}$
0.0
0.5
β_{IV}

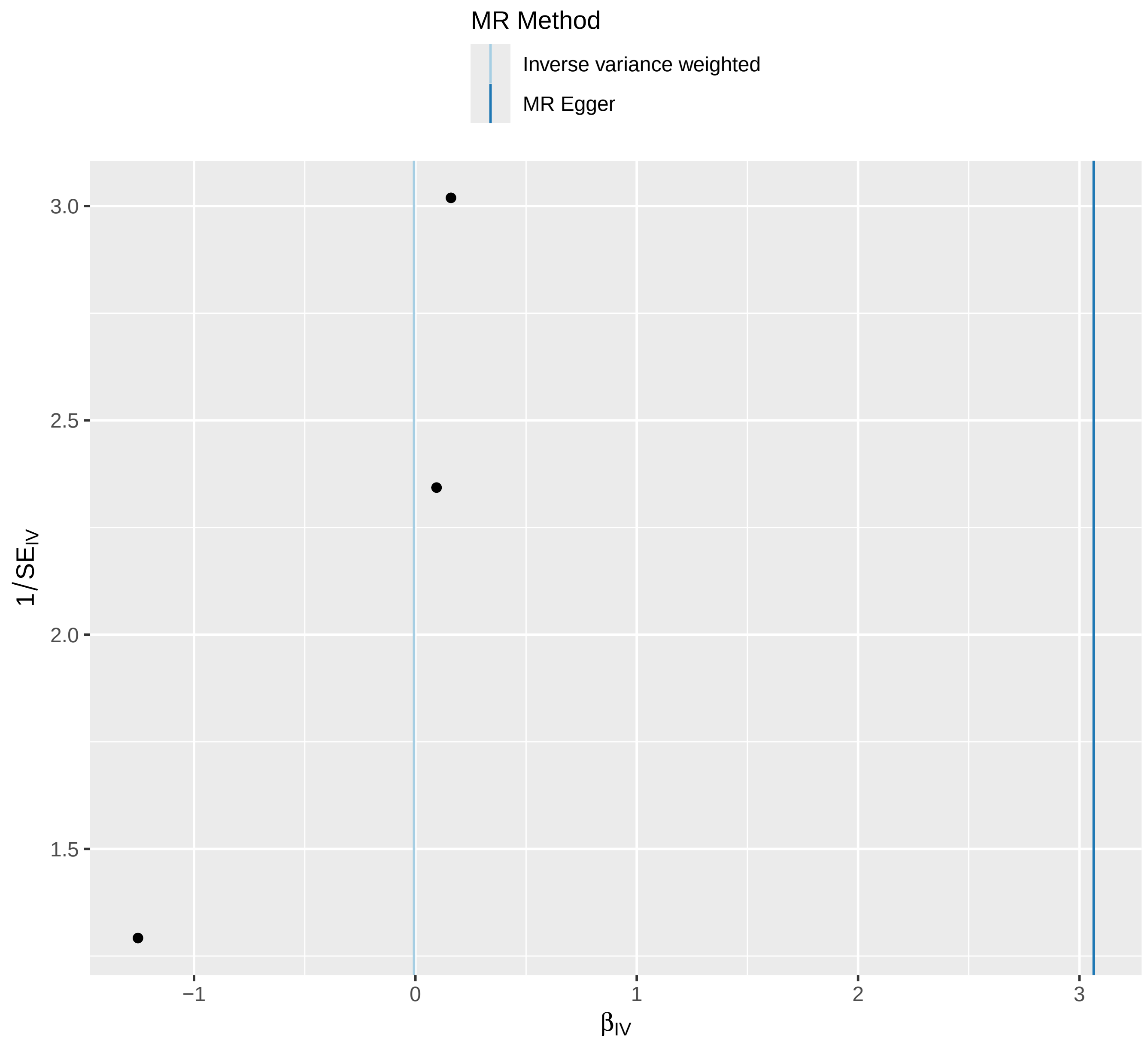

MR Method
Inverse variance weighted
MR Egger
3.0
2.5
2.0
1.5
1/SE_IV
−1
0
1
2
3
β_IV

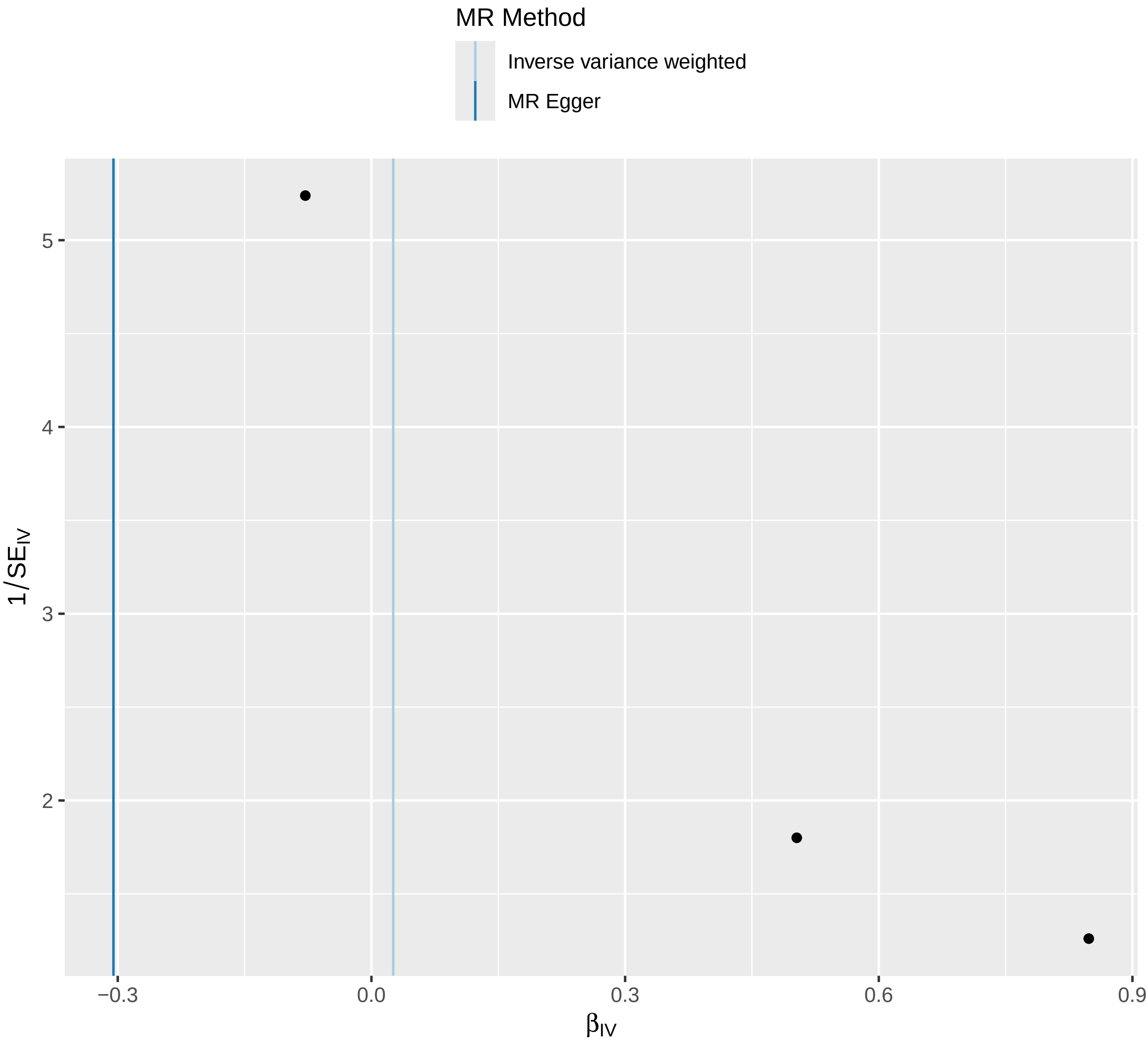
MR Method
Inverse variance weighted
MR Egger
5
4
3
2
1/SE_IV
−0.3
0.0
0.3
0.6
0.9
β_IV

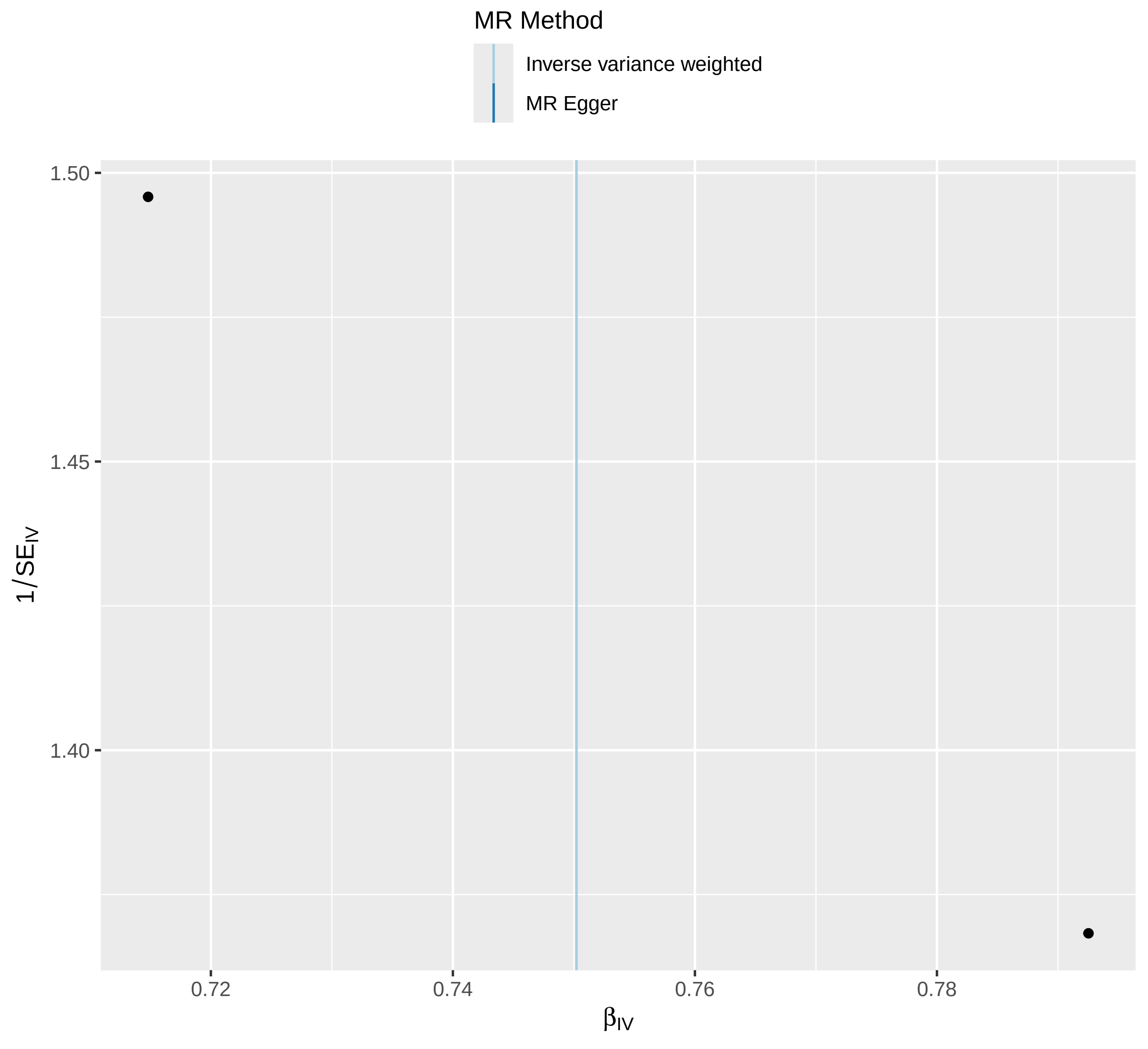

MR Method
Inverse variance weighted
MR Egger
1.50
1.45
1.40
1/SE$_{IV}$
0.72
0.74
0.76
0.78
β$_{IV}$

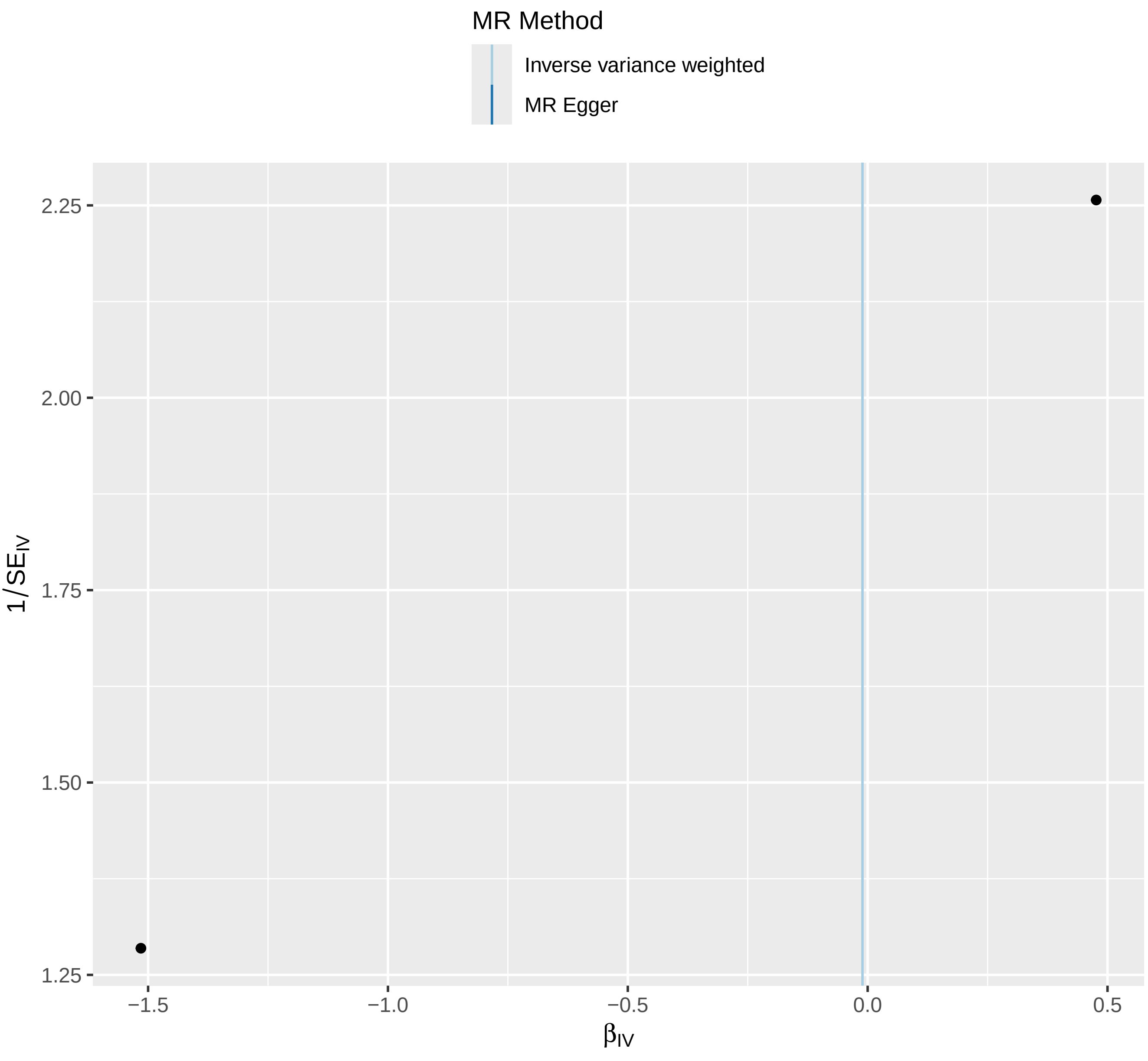
MR Method
Inverse variance weighted
MR Egger
2.25
2.00
1.75
1.50
1.25
1/SE_IV
−1.5
−1.0
−0.5
0.0
0.5
β_IV

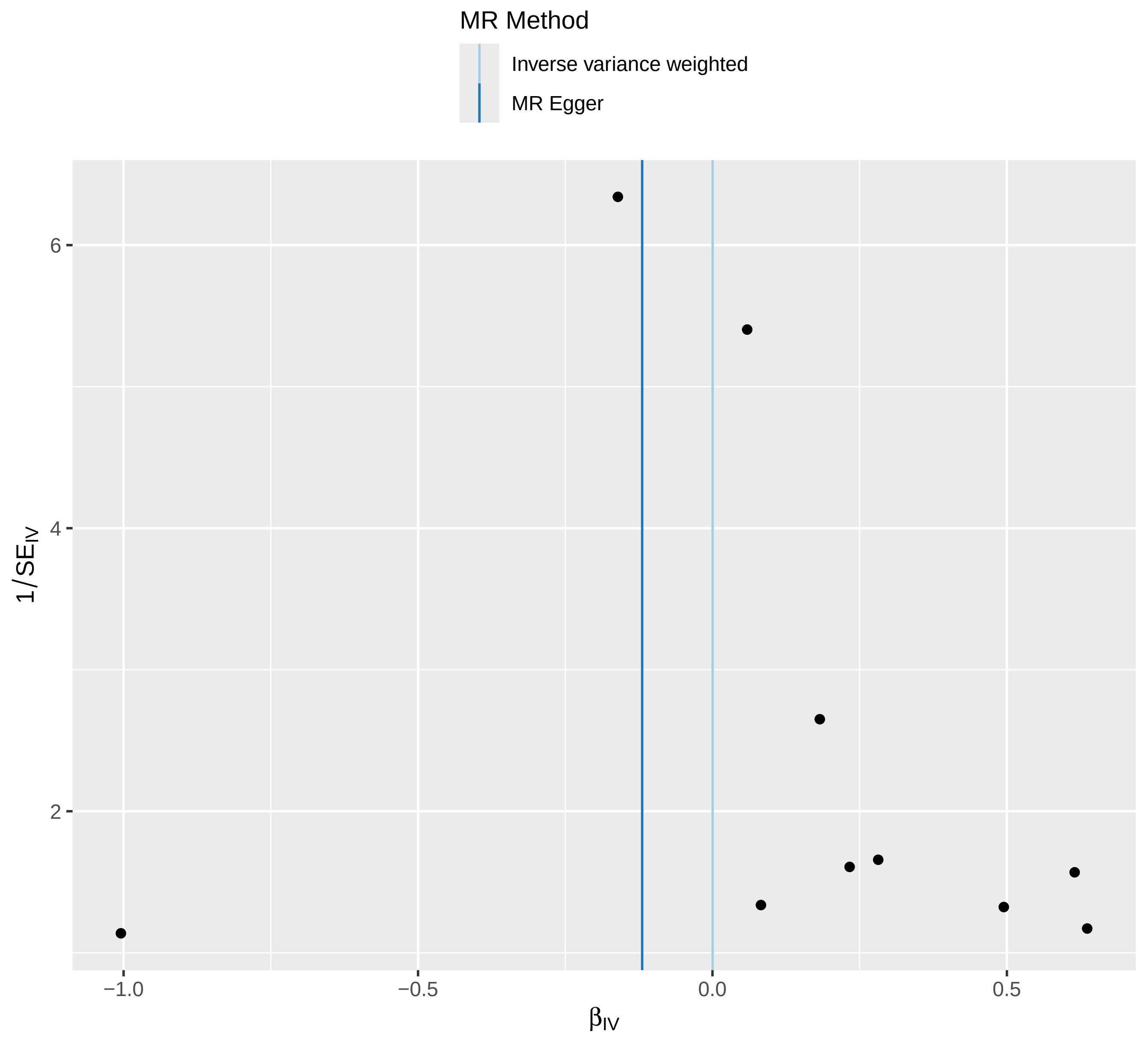
MR Method
Inverse variance weighted
MR Egger
6
4
2
$1/SE_{IV}$
−1.0
−0.5
0.0
0.5
β_{IV}

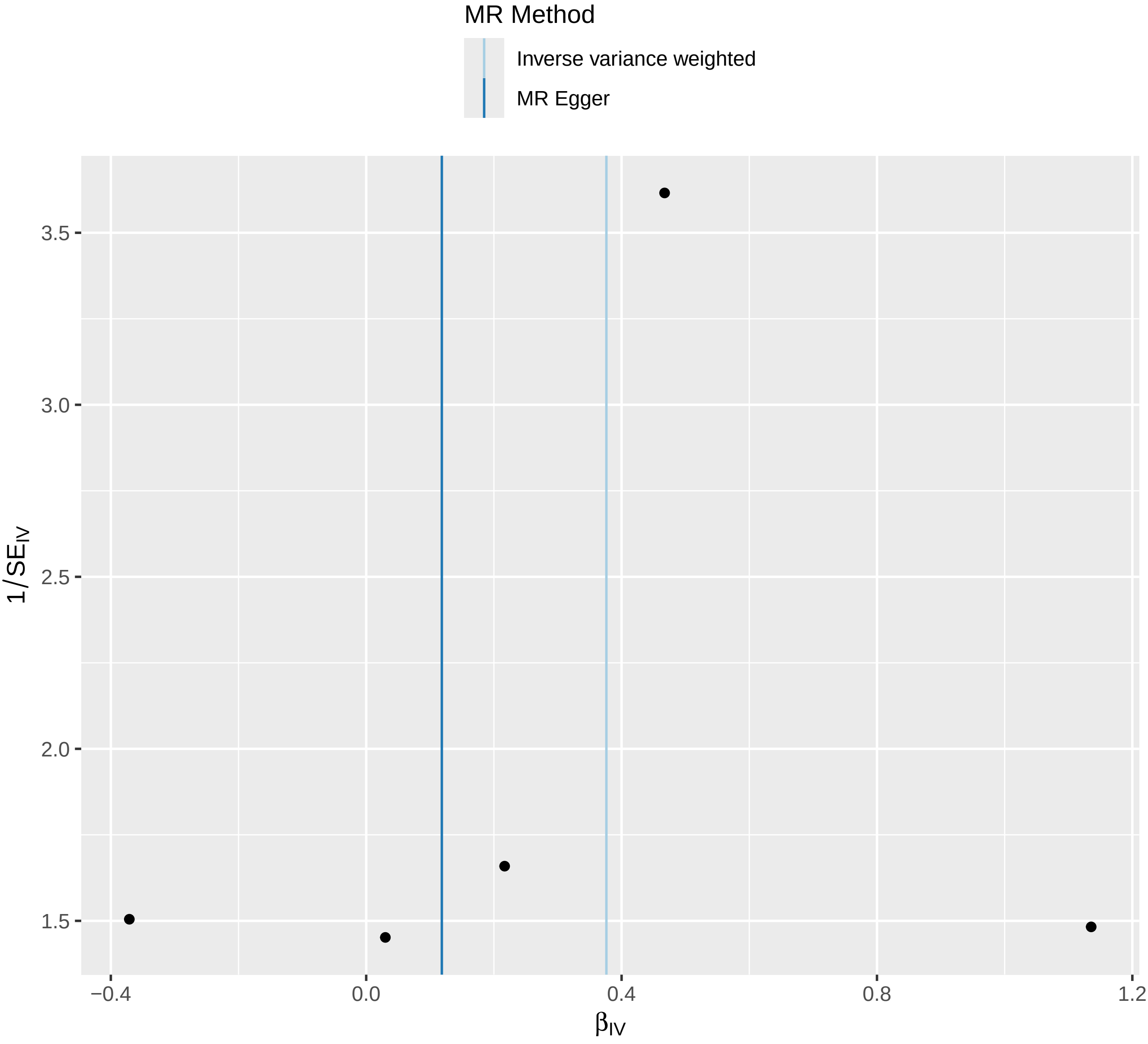
MR Method
Inverse variance weighted
MR Egger
3.5
3.0
2.5
2.0
1.5
1/SE_IV
−0.4
0.0
0.4
0.8
1.2
β_IV

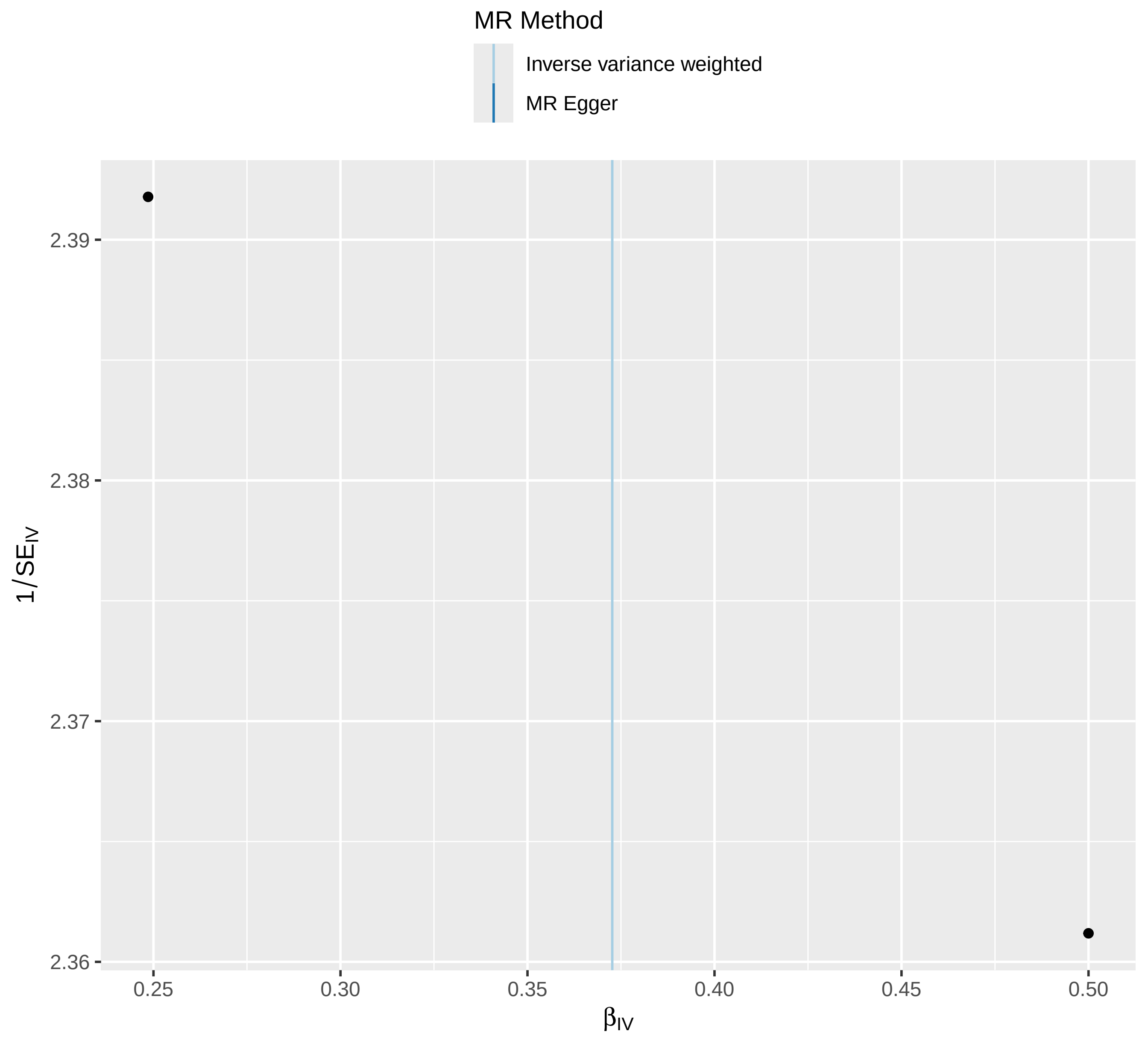
MR Method
Inverse variance weighted
MR Egger
2.39
2.38
2.37
2.36
1/SE_IV
0.25
0.30
0.35
0.40
0.45
0.50
β_IV

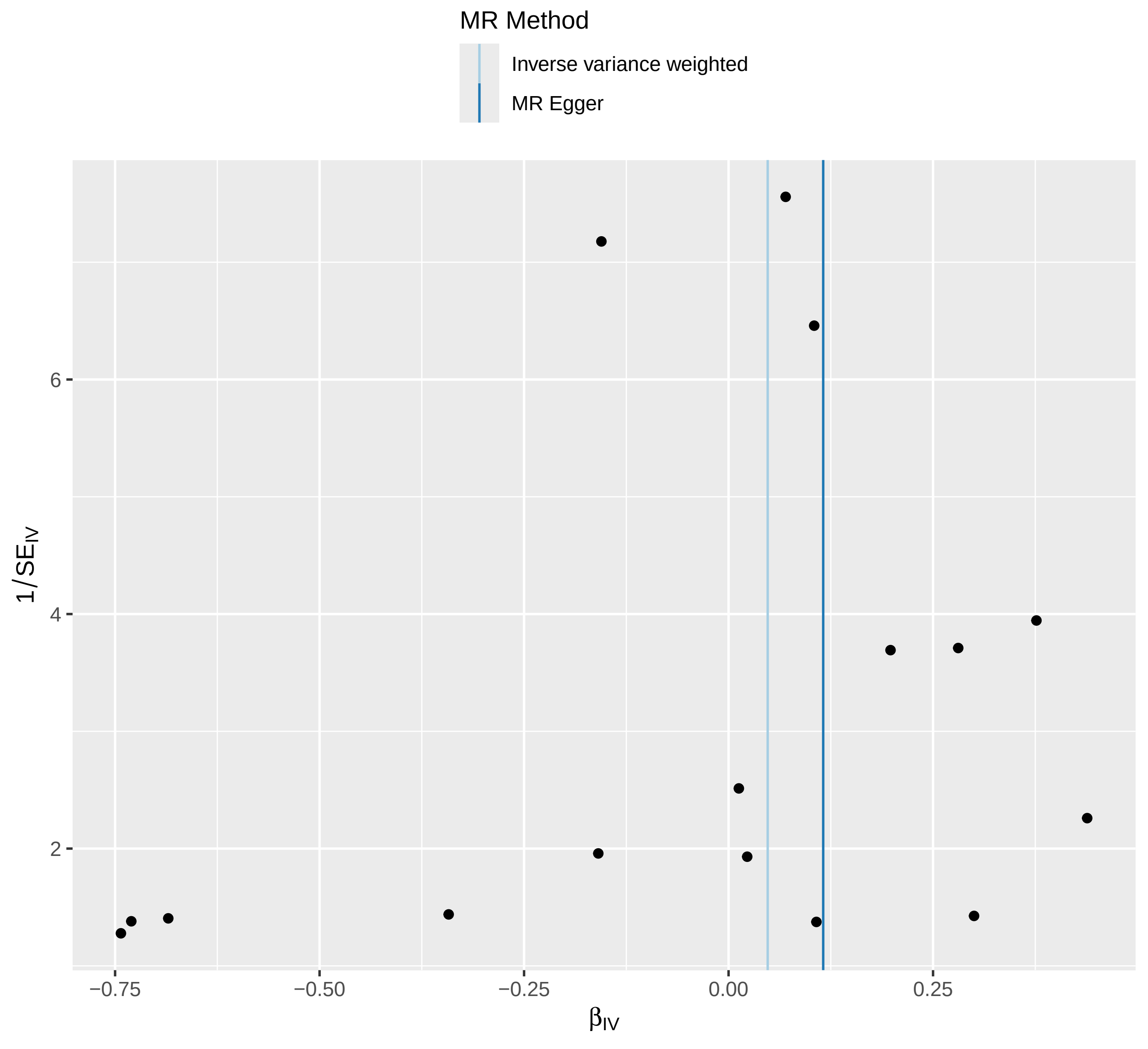

MR Method
Inverse variance weighted
MR Egger
6
4
2
1/SE$_{IV}$
−0.75
−0.50
−0.25
0.00
0.25
β$_{IV}$

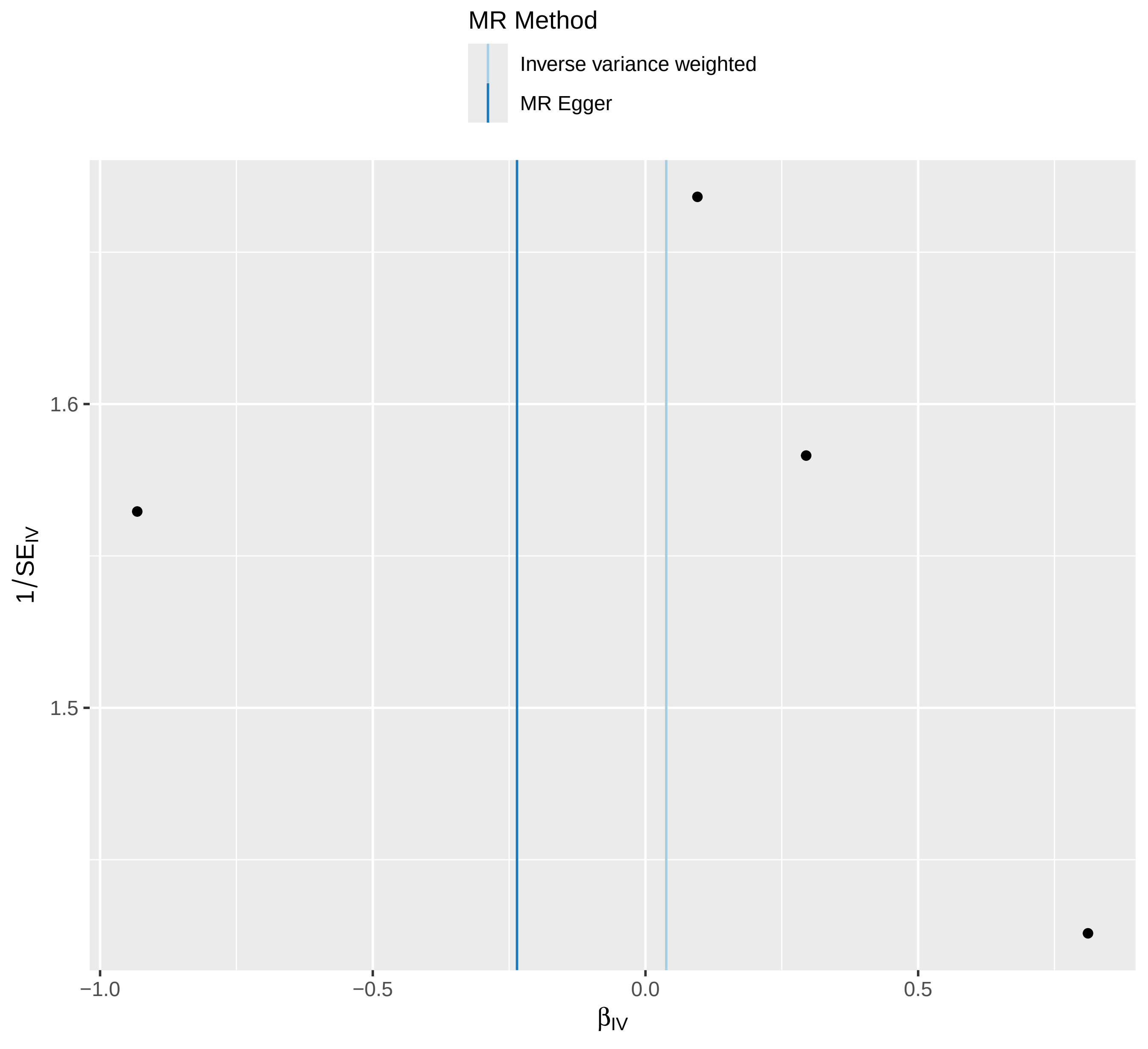

MR Method
Inverse variance weighted
MR Egger
1.6
1.5
$1/SE_{IV}$
−1.0
−0.5
0.0
0.5
β_{IV}

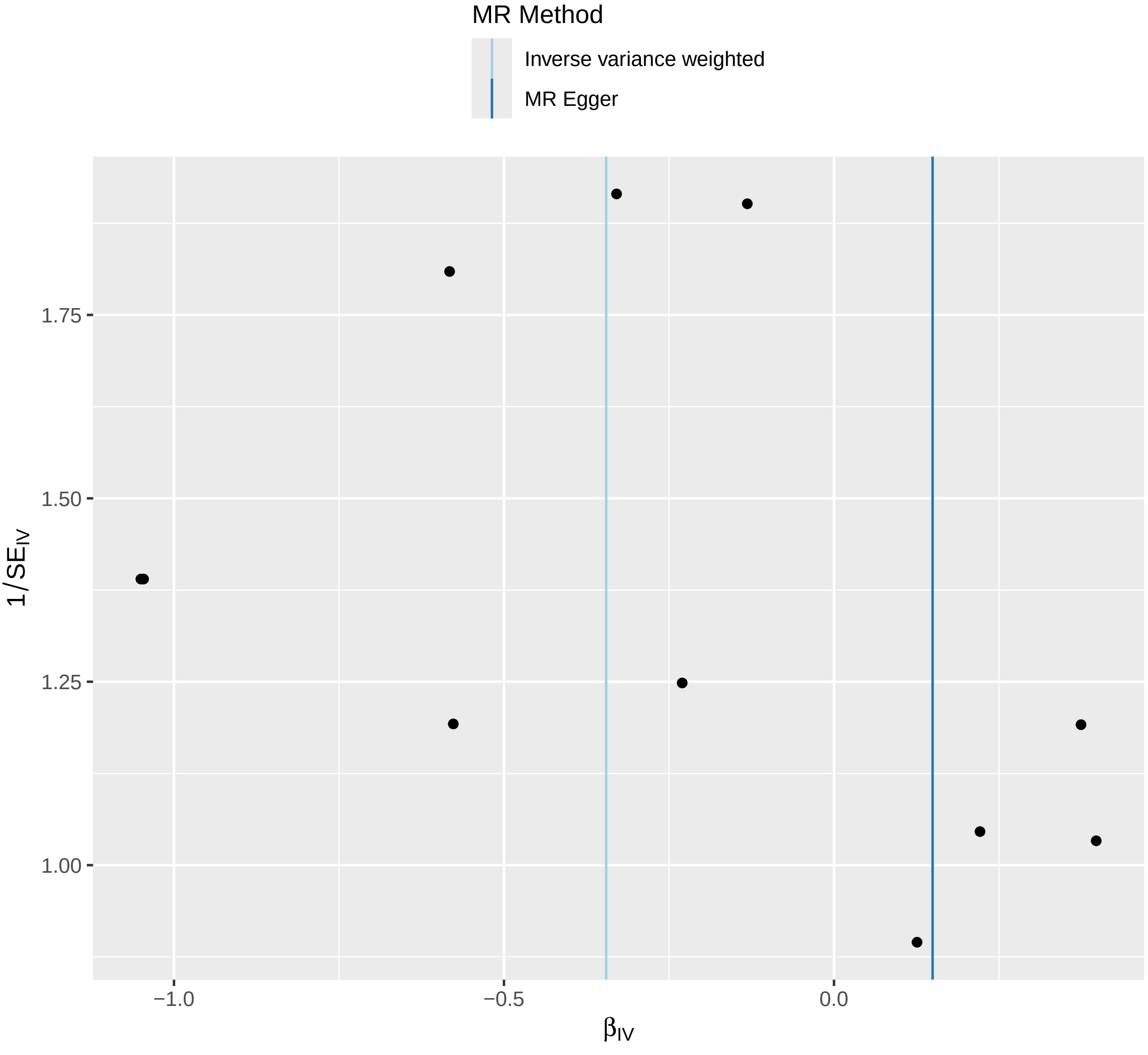

MR Method
Inverse variance weighted
MR Egger
1.75
1.50
1.25
1.00
1/SE$_{IV}$
−1.0
−0.5
0.0
β_{IV}

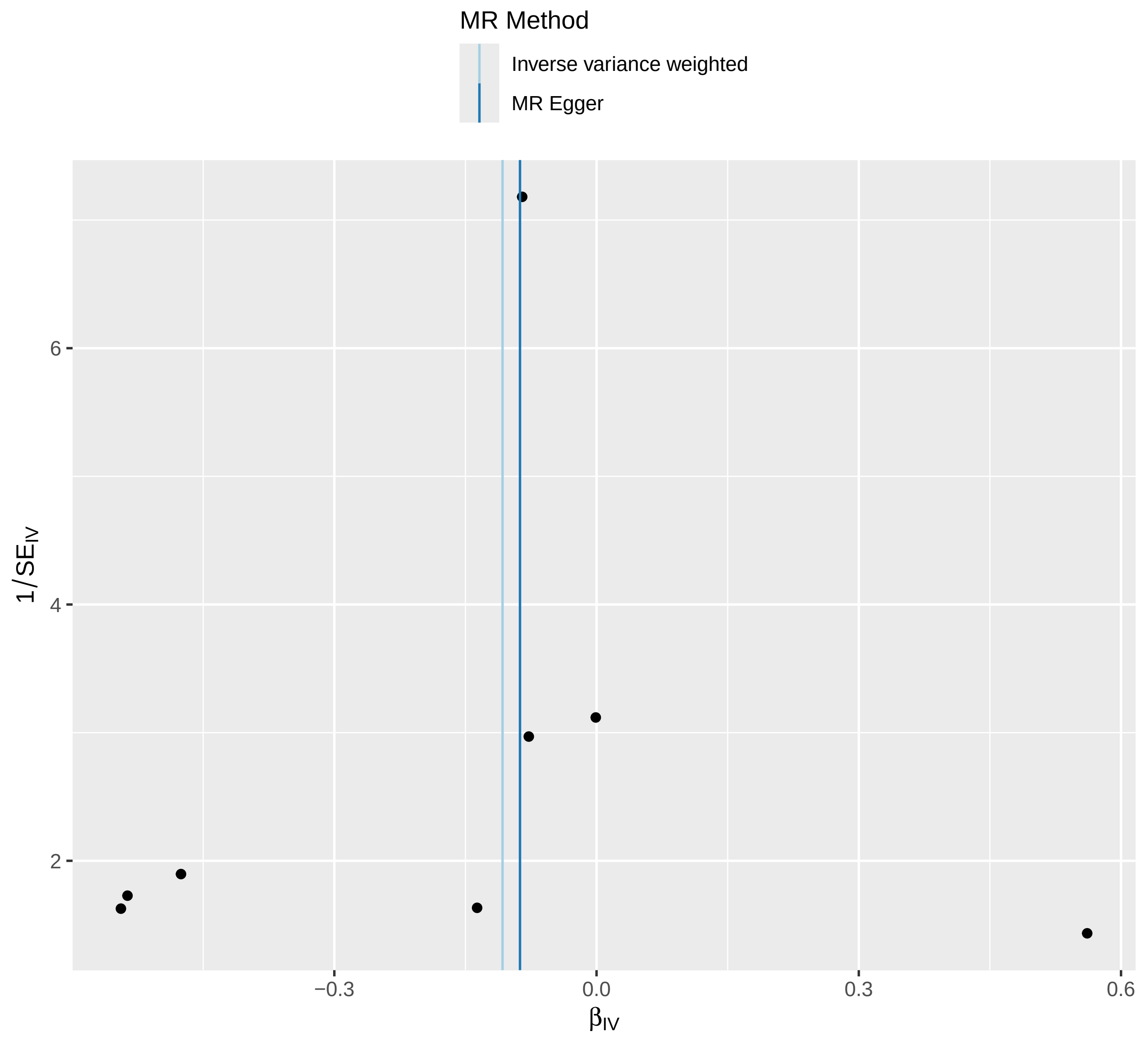

MR Method
Inverse variance weighted
MR Egger
1/SE_IV
6
4
2
−0.3
0.0
0.3
0.6
β_IV

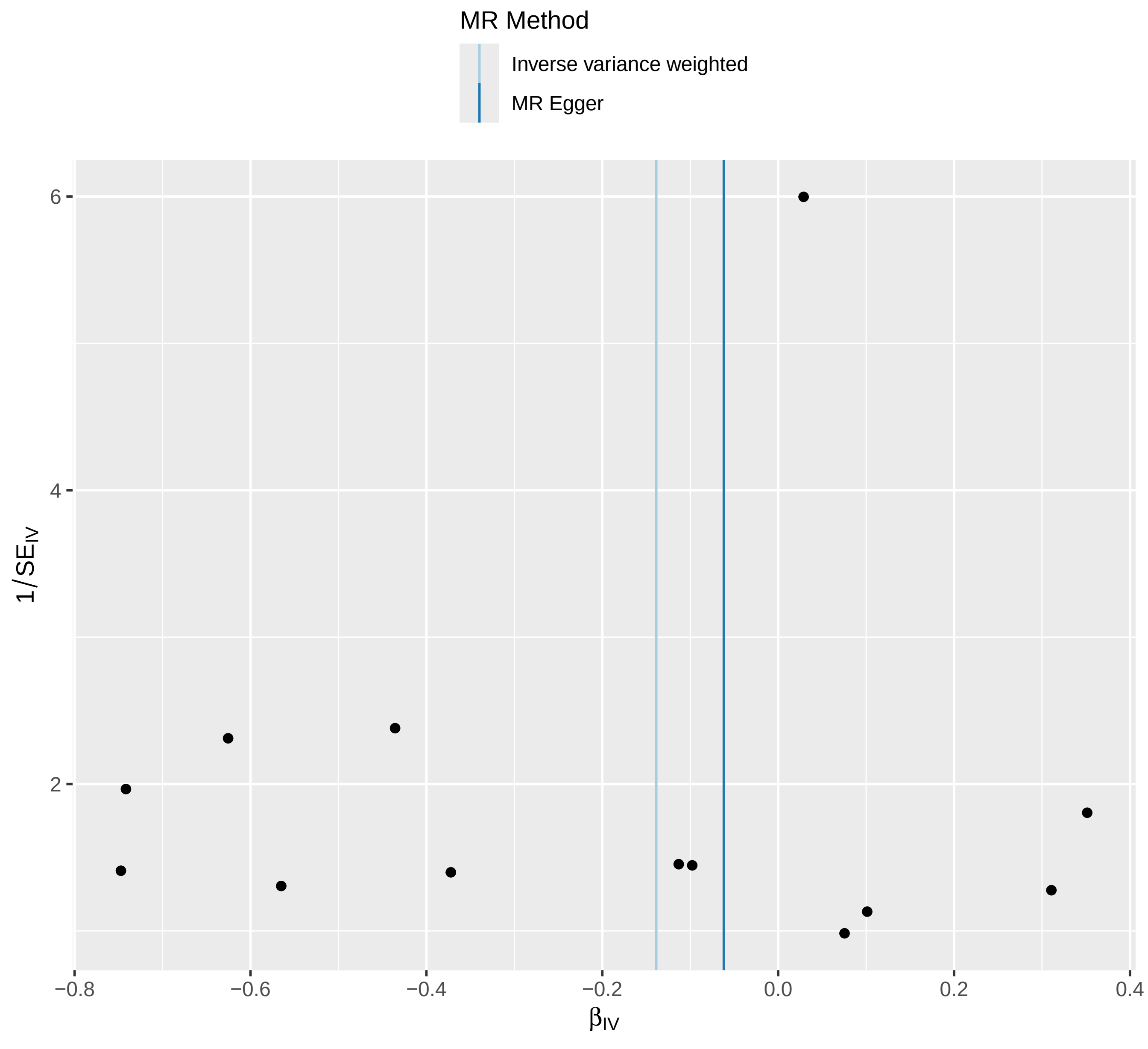
MR Method
Inverse variance weighted
MR Egger
6
4
2
1/SE_IV
−0.8
−0.6
−0.4
−0.2
0.0
0.2
0.4
β_IV

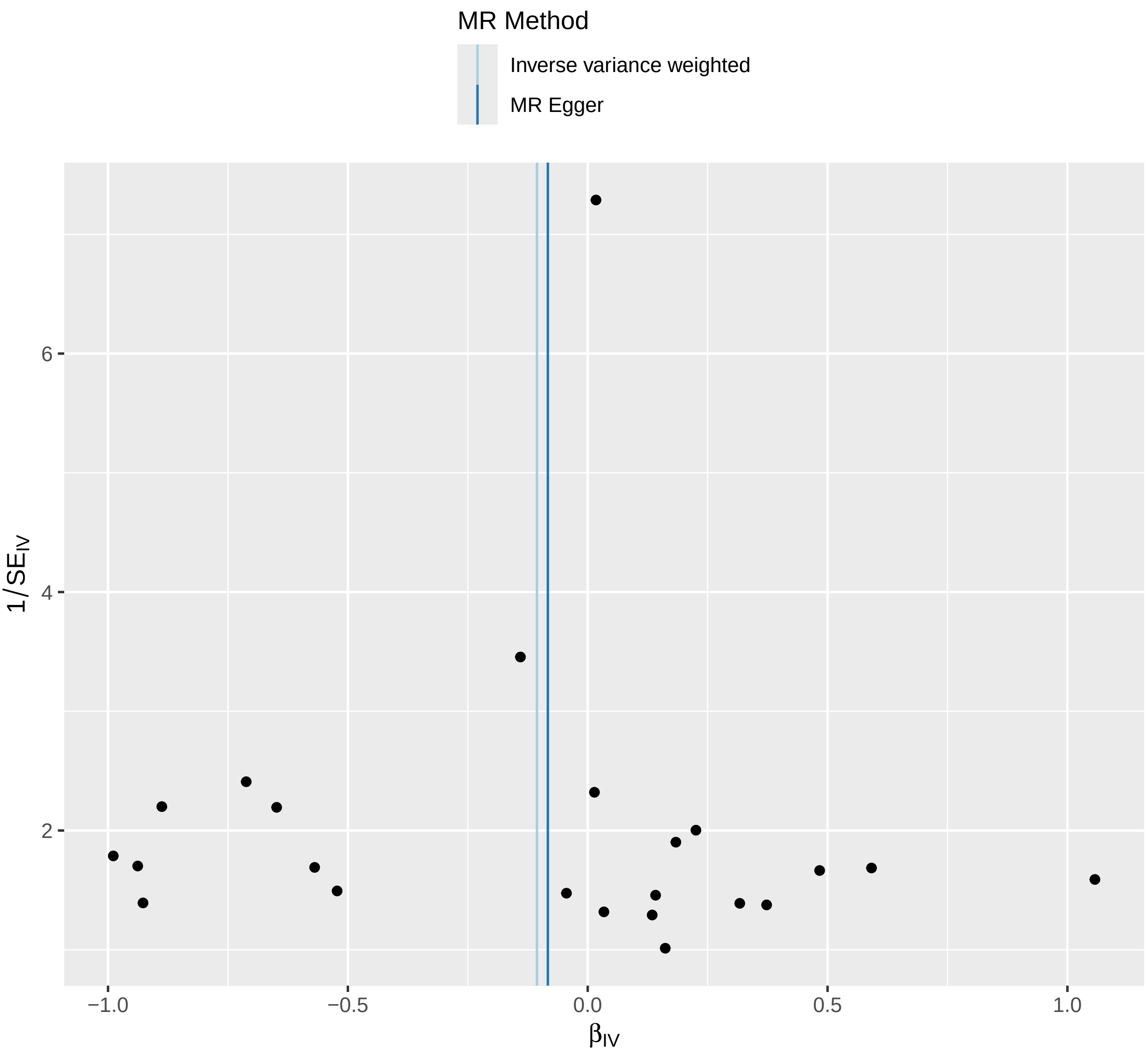
MR Method
Inverse variance weighted
MR Egger
$1/SE_{IV}$
6
4
2
−1.0
−0.5
0.0
0.5
1.0
β_{IV}

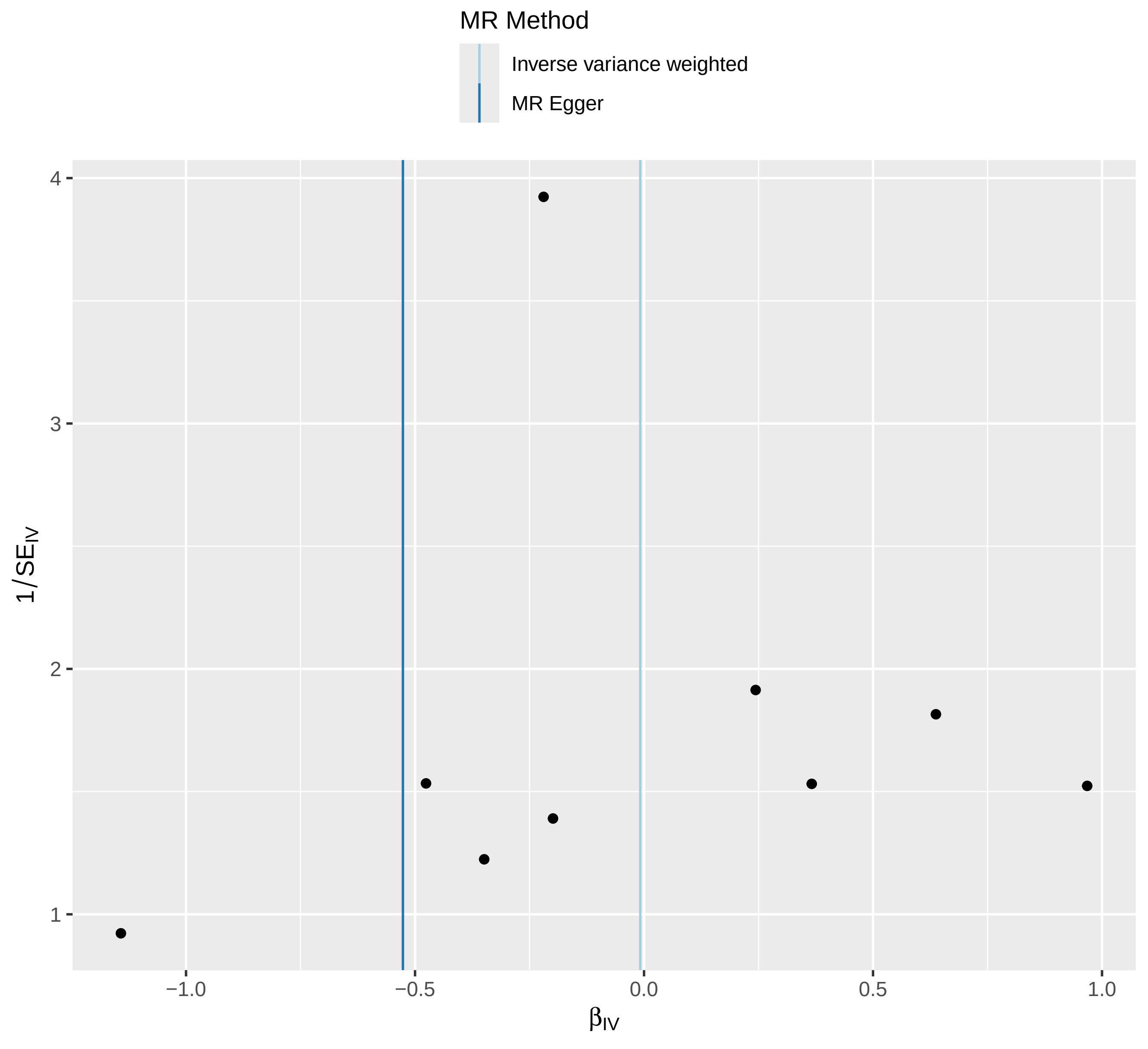
MR Method
Inverse variance weighted
MR Egger
4
3
2
1
$1/SE_{IV}$
−1.0
−0.5
0.0
0.5
1.0
β_{IV}

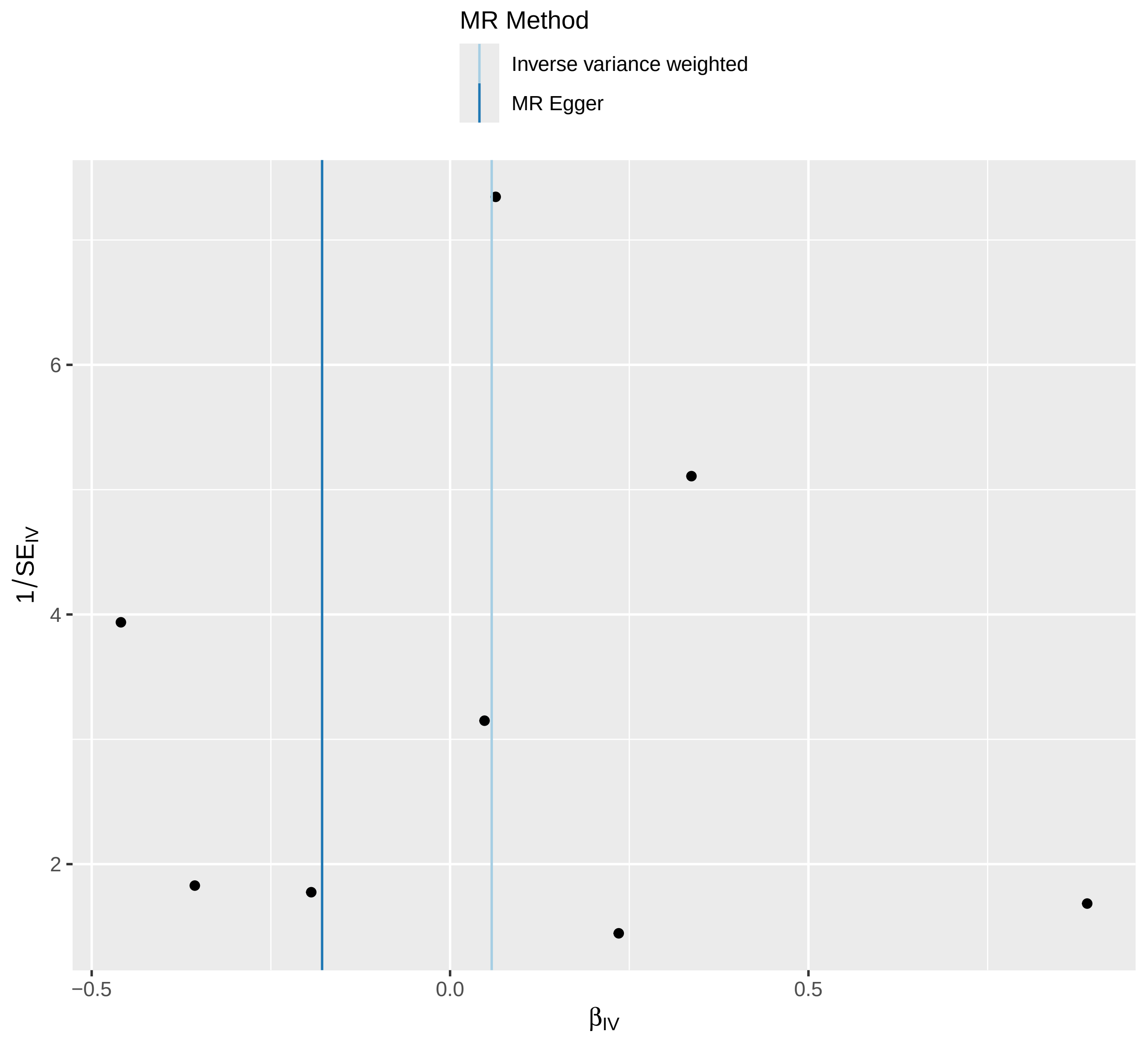
MR Method
Inverse variance weighted
MR Egger
1/SE$_{IV}$
6
4
2
−0.5
0.0
0.5
β$_{IV}$

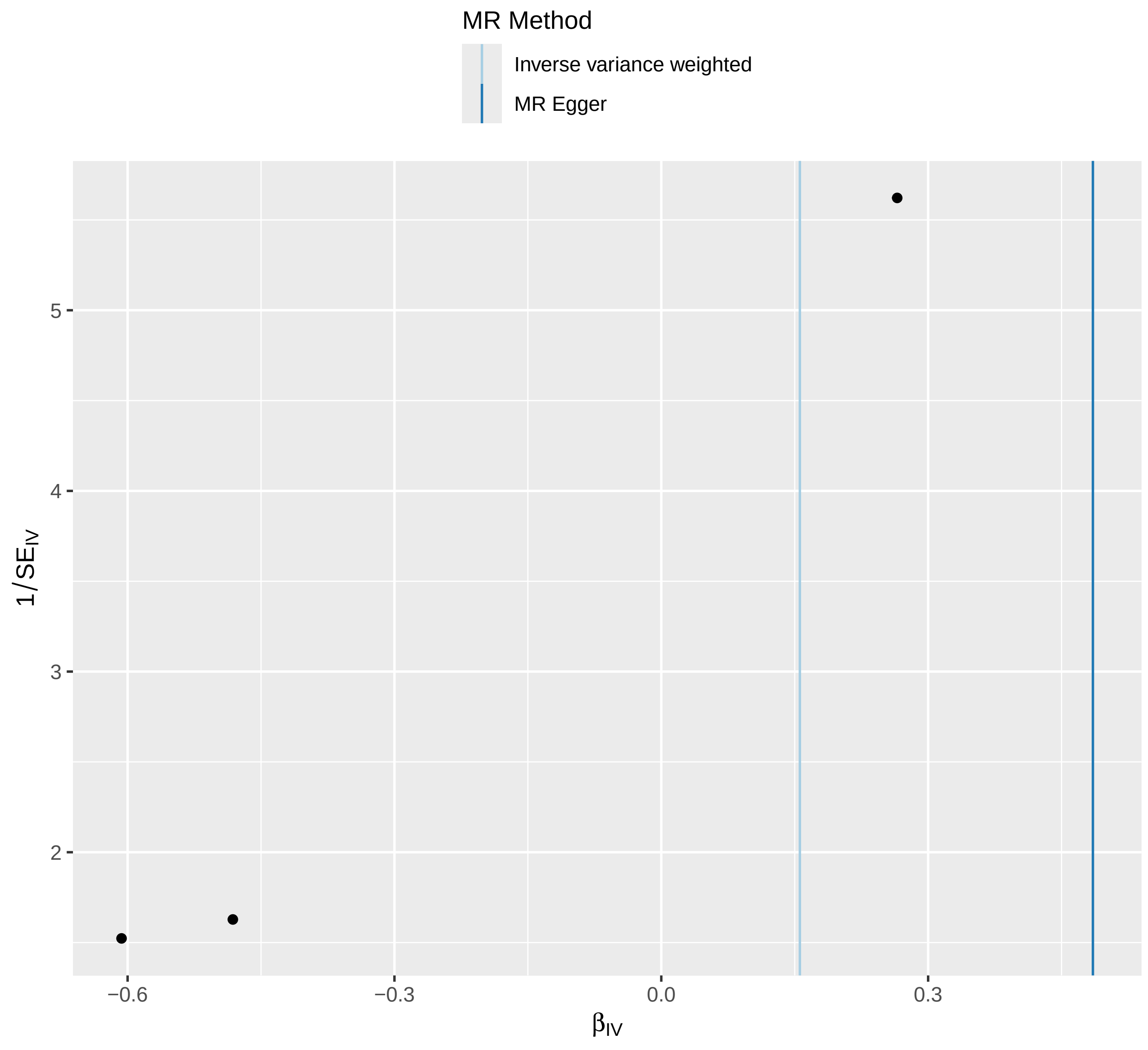
MR Method
Inverse variance weighted
MR Egger
5
4
3
2
1/SE_IV
−0.6
−0.3
0.0
0.3
β_IV

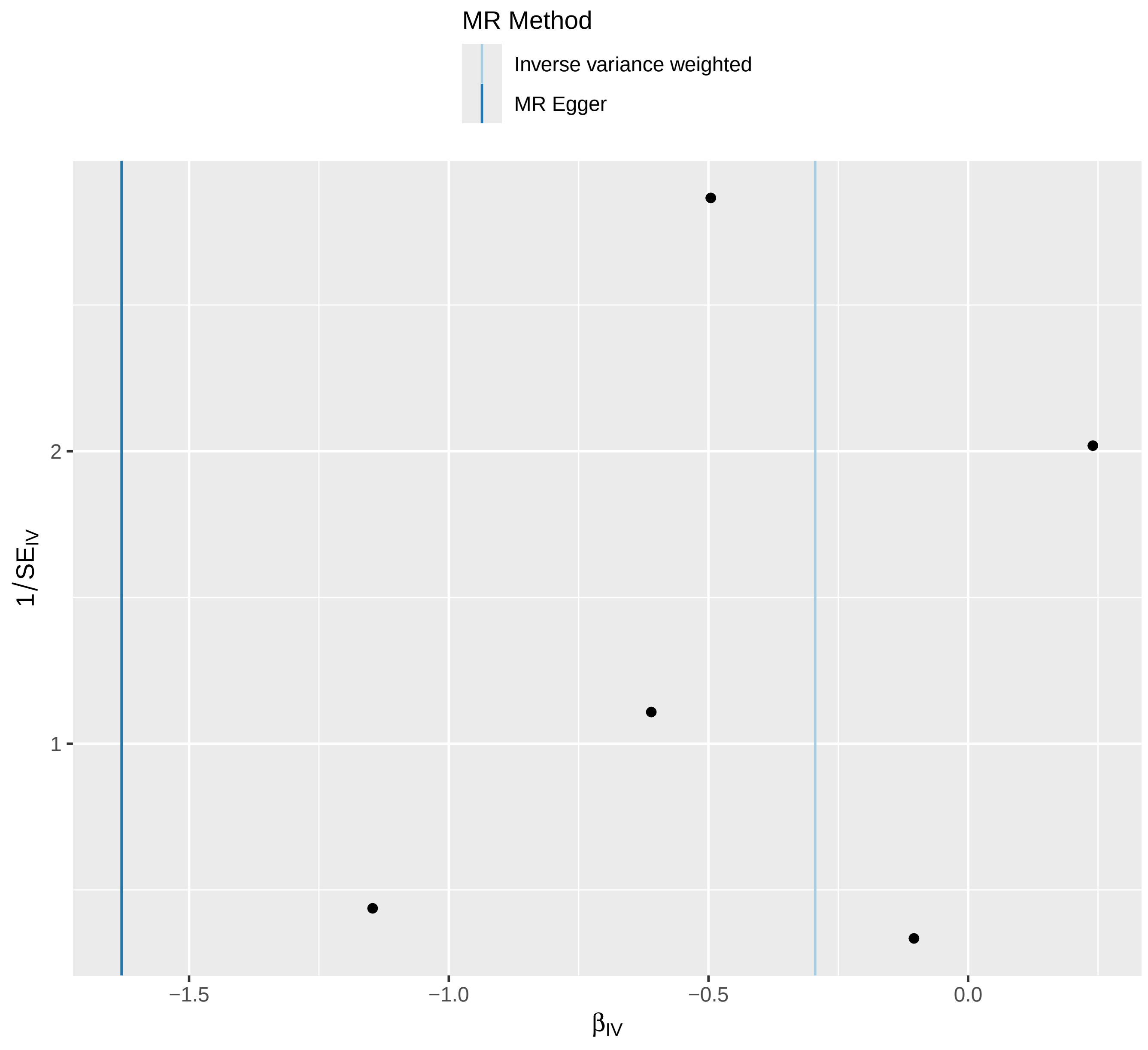
MR Method
Inverse variance weighted
MR Egger
2
1
1/SE$_{IV}$
−1.5
−1.0
−0.5
0.0
β$_{IV}$

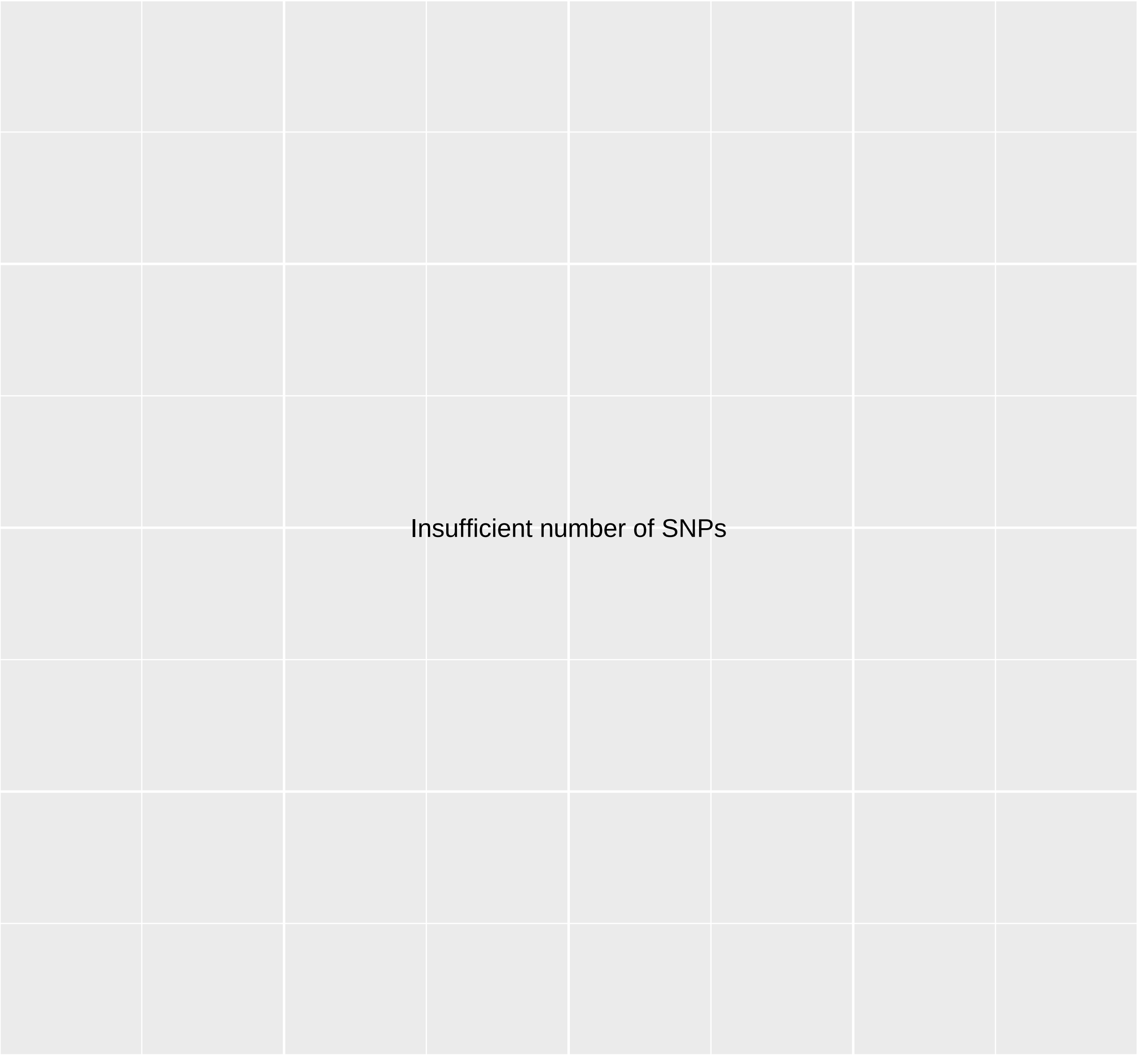
Insufficient number of SNPs

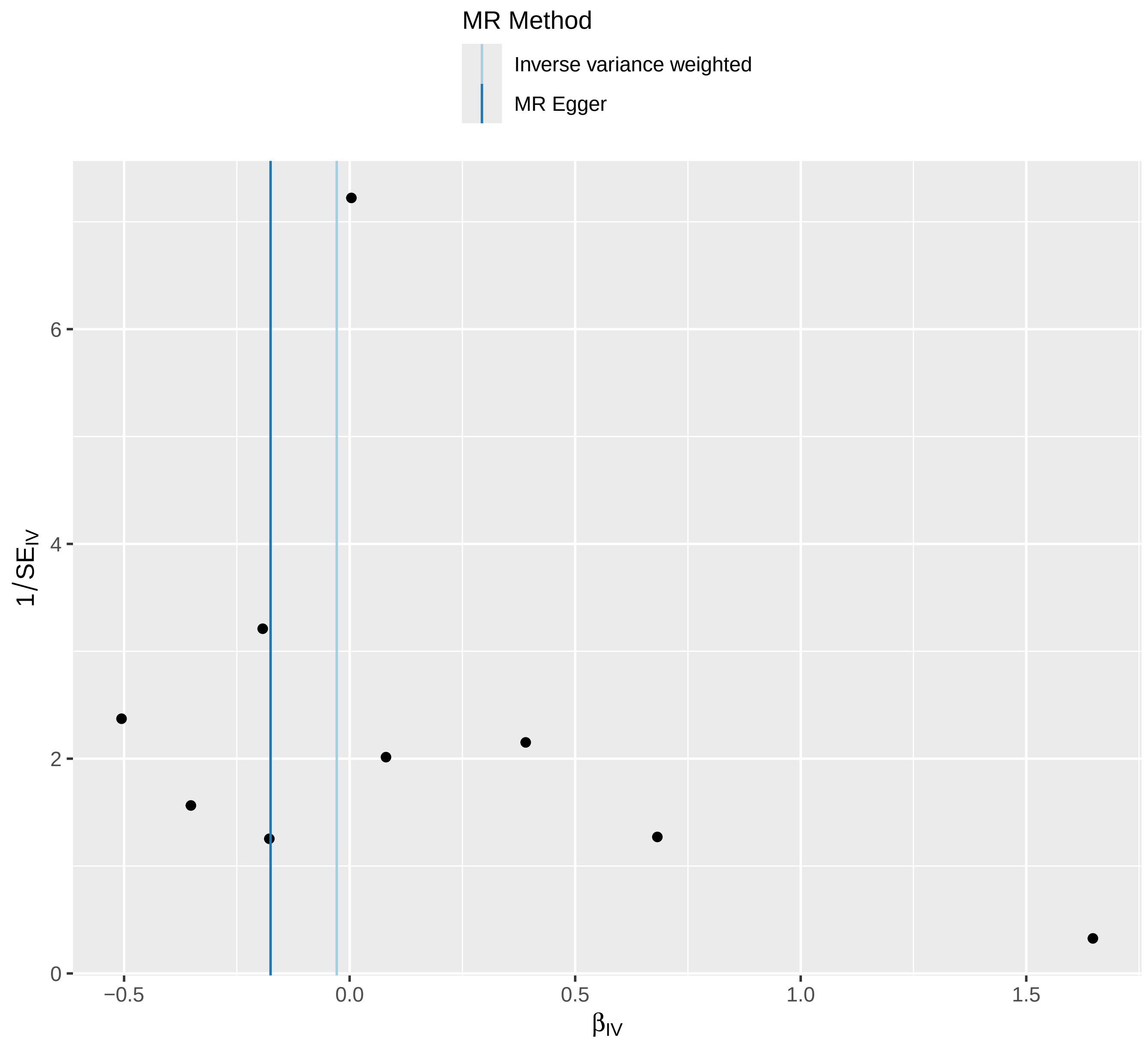
MR Method
Inverse variance weighted
MR Egger
1/SE_IV
6
4
2
0
−0.5
0.0
0.5
1.0
1.5
β_IV

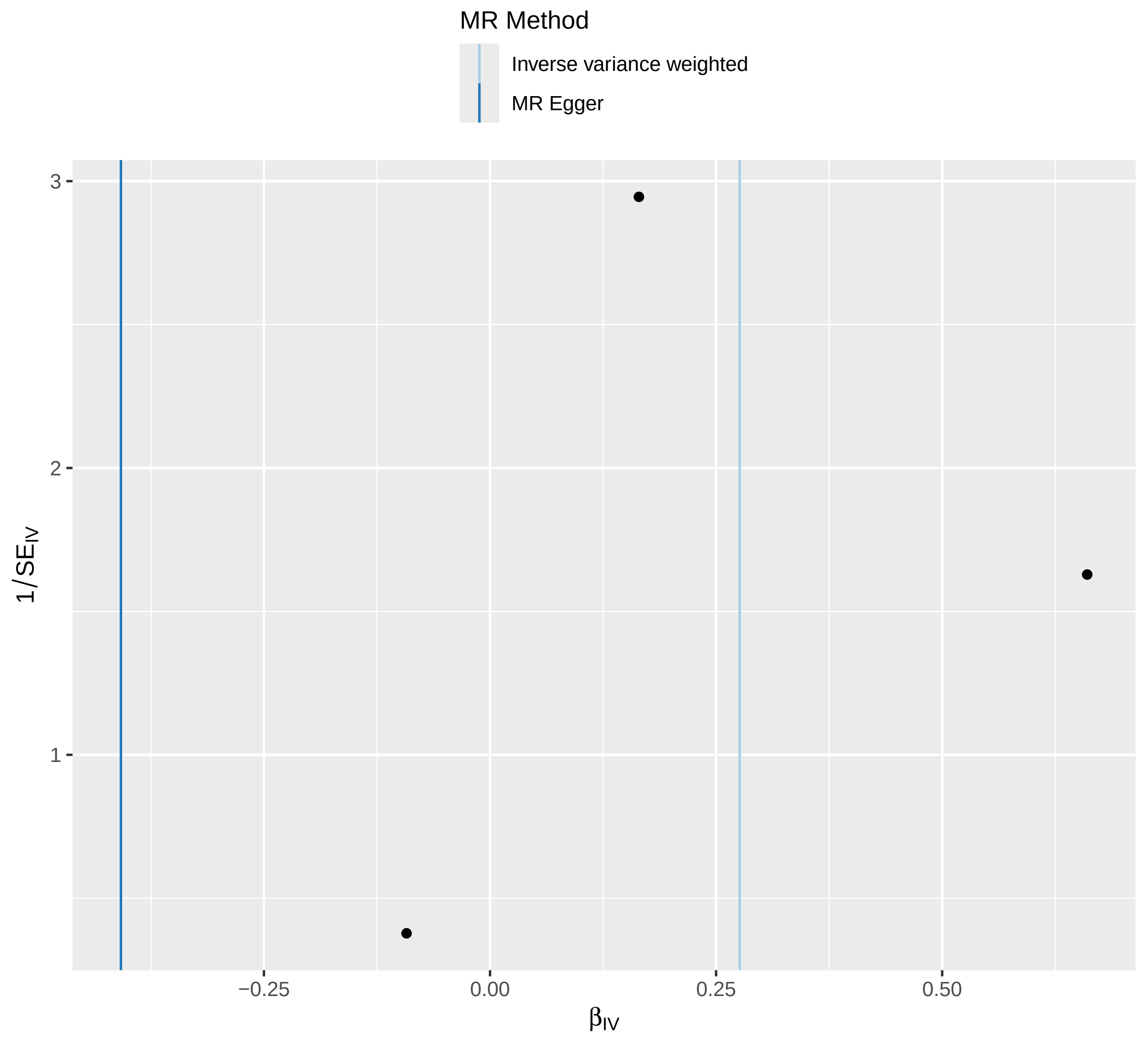
MR Method
Inverse variance weighted
MR Egger
3
2
1
1/SE_IV
−0.25
0.00
0.25
0.50
β_IV

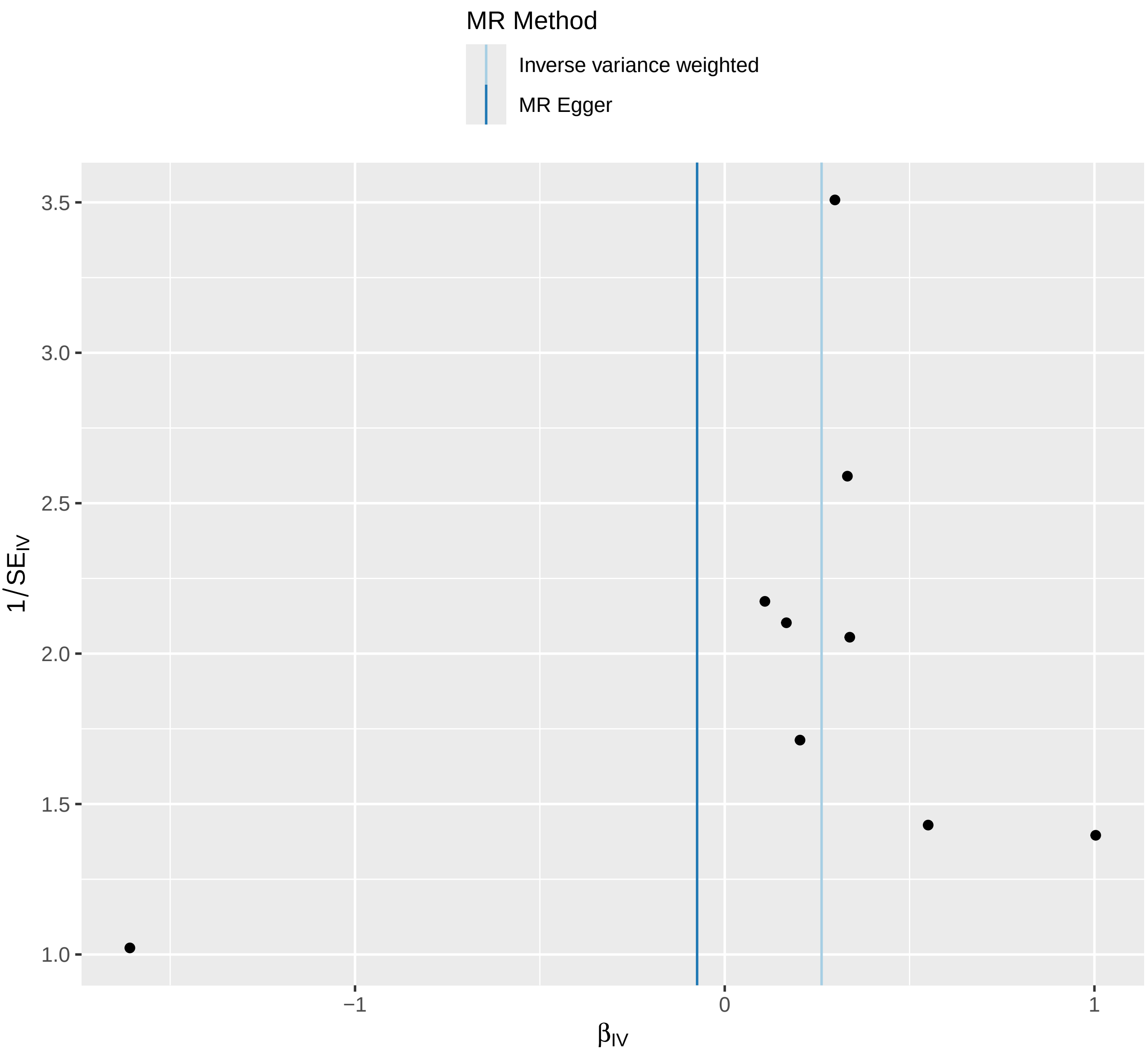
MR Method
Inverse variance weighted
MR Egger
3.5
3.0
2.5
2.0
1.5
1.0
1/SE_IV
−1
0
1
β_IV

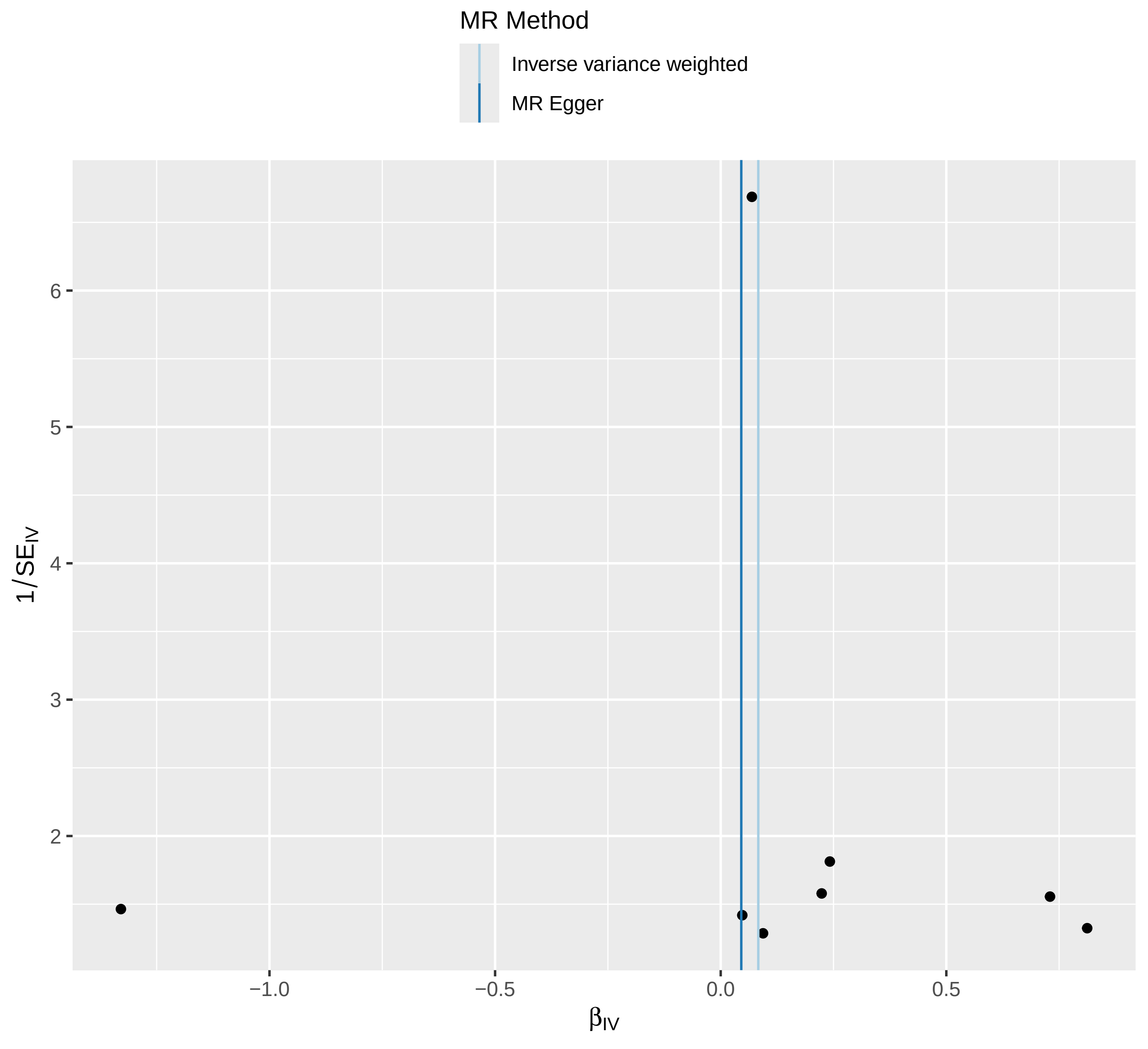
MR Method
Inverse variance weighted
MR Egger
6
5
4
3
2
1/SE$_{IV}$
−1.0
−0.5
0.0
0.5
β$_{IV}$

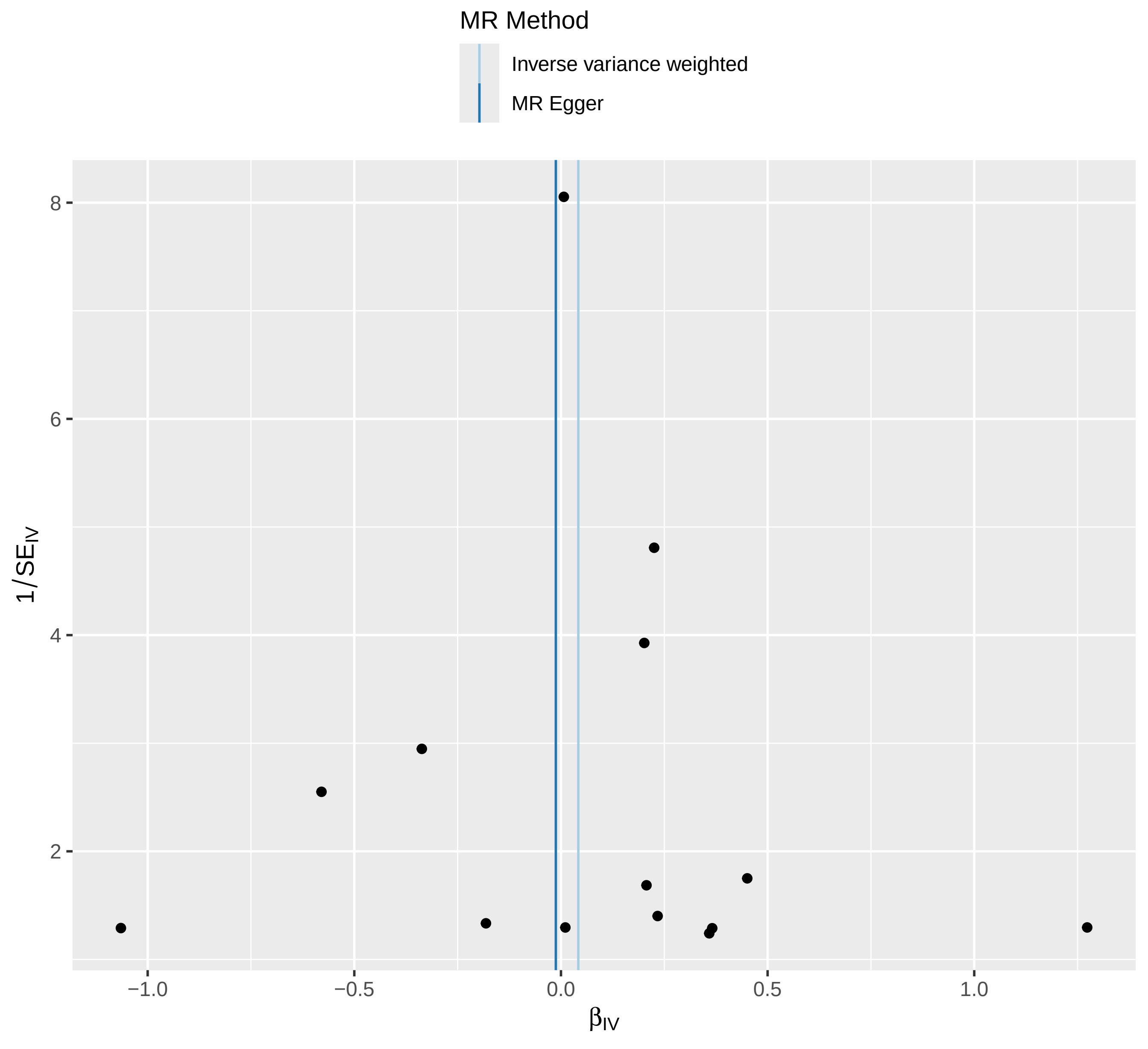
MR Method
Inverse variance weighted
MR Egger
8
6
4
2
1/SE_IV
−1.0
−0.5
0.0
0.5
1.0
β_IV

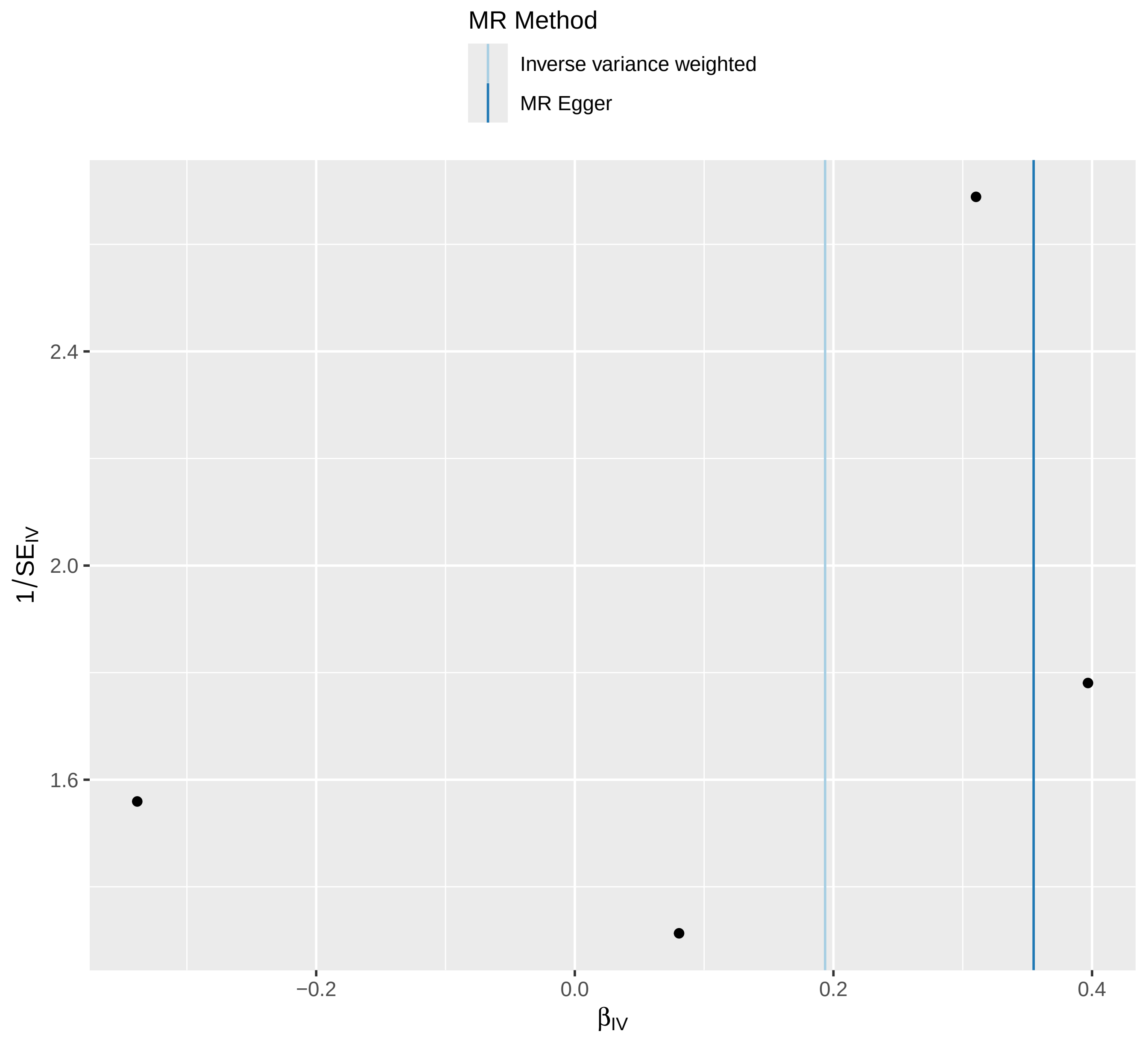
MR Method
Inverse variance weighted
MR Egger
1/SE_IV
2.4
2.0
1.6
−0.2
0.0
0.2
0.4
β_IV

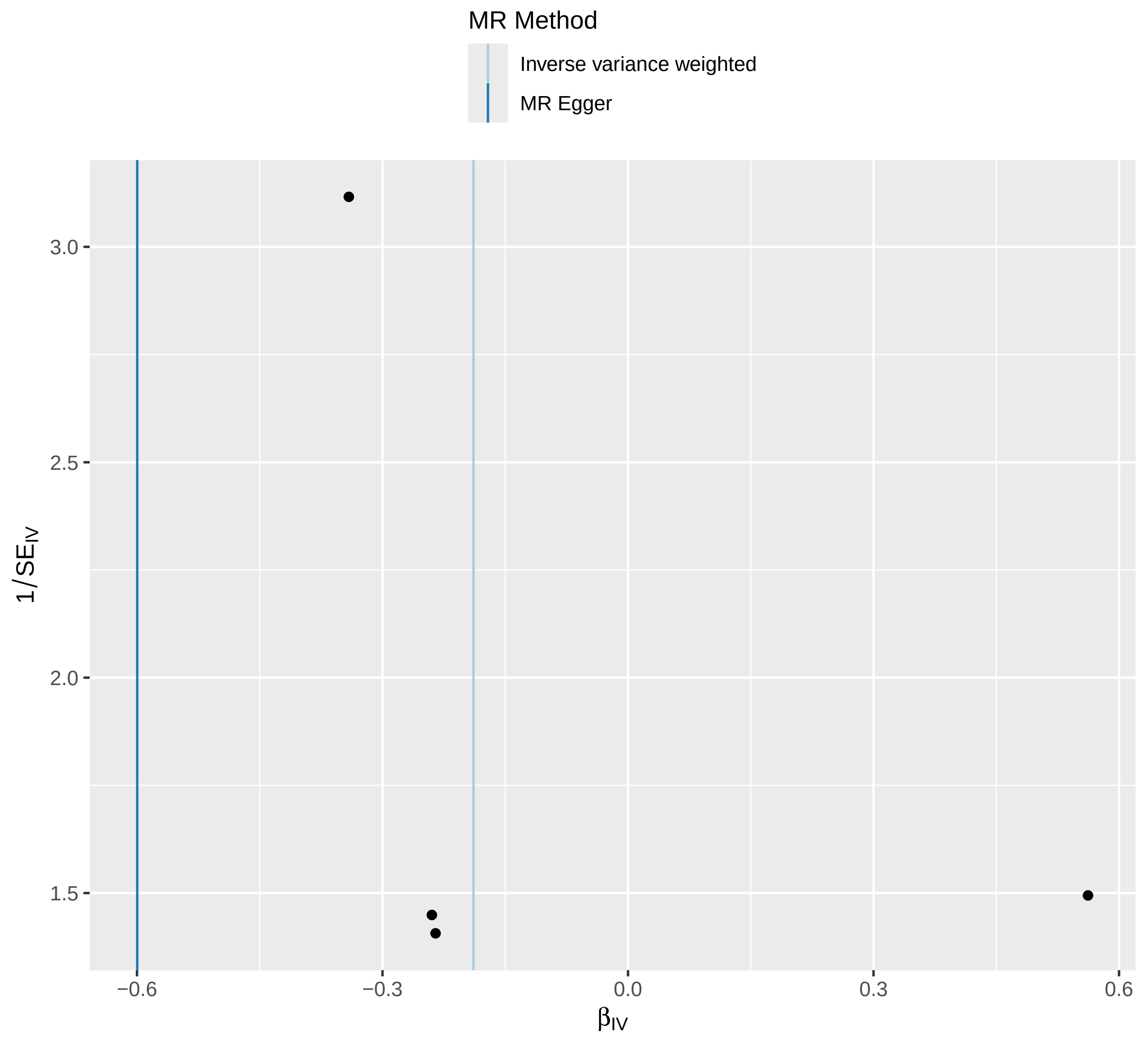

MR Method
Inverse variance weighted
MR Egger
3.0
2.5
2.0
1.5
1/SE_IV
−0.6
−0.3
0.0
0.3
0.6
β_IV

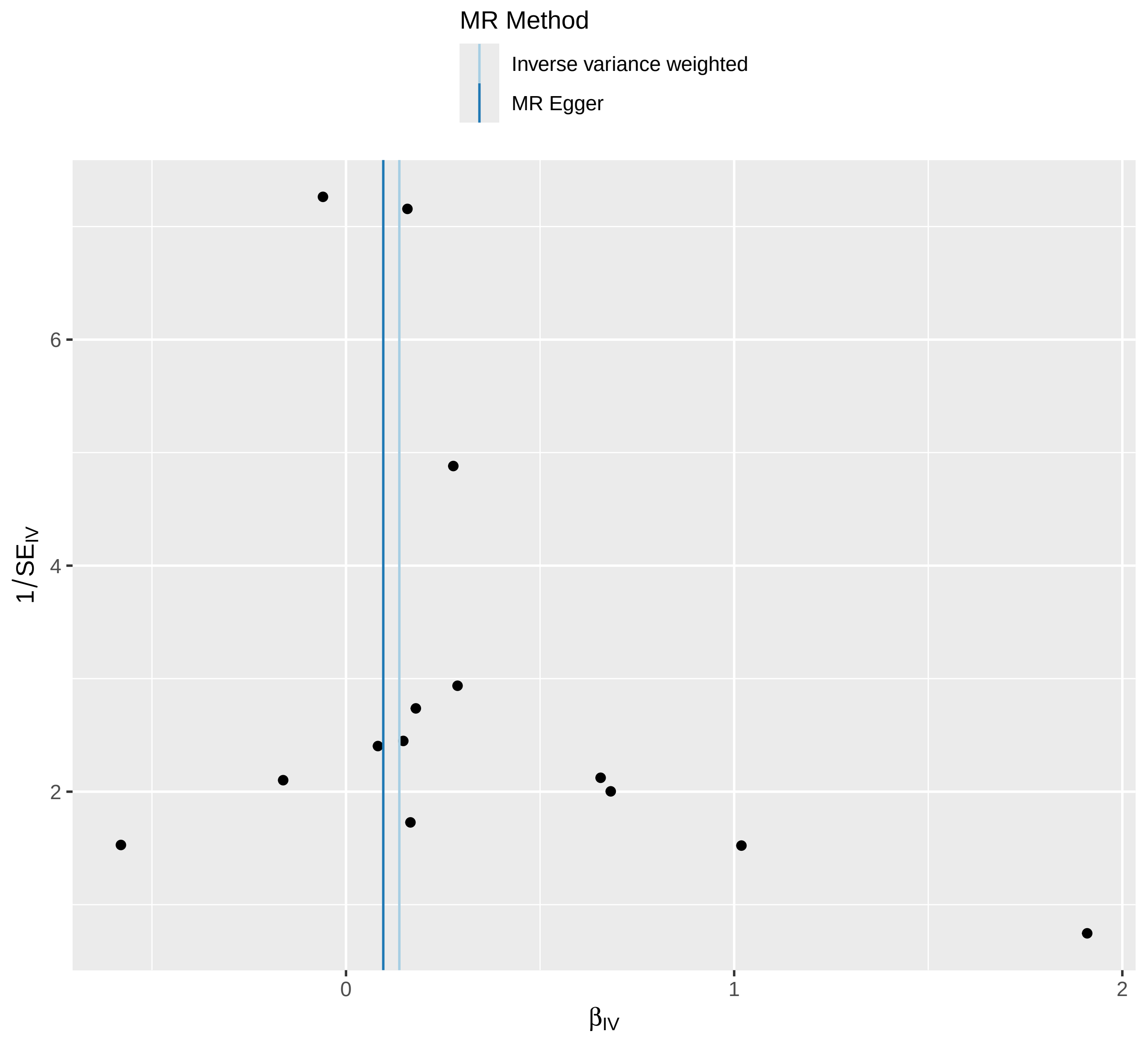
MR Method
Inverse variance weighted
MR Egger
6
4
2
1/SE_IV
0
1
2
β_IV

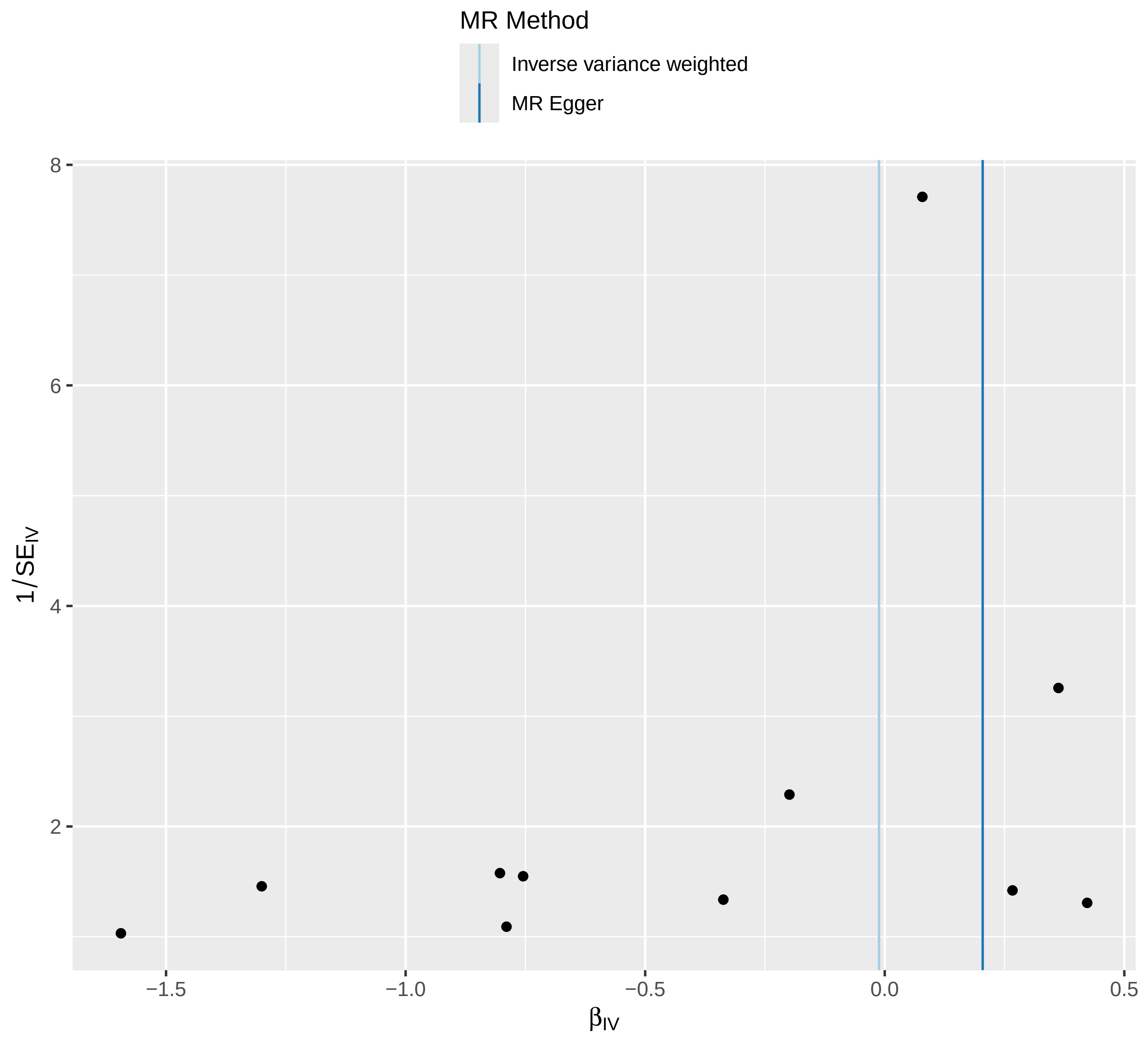
MR Method
Inverse variance weighted
MR Egger
8
6
4
2
1/SE$_{IV}$
−1.5
−1.0
−0.5
0.0
0.5
β$_{IV}$

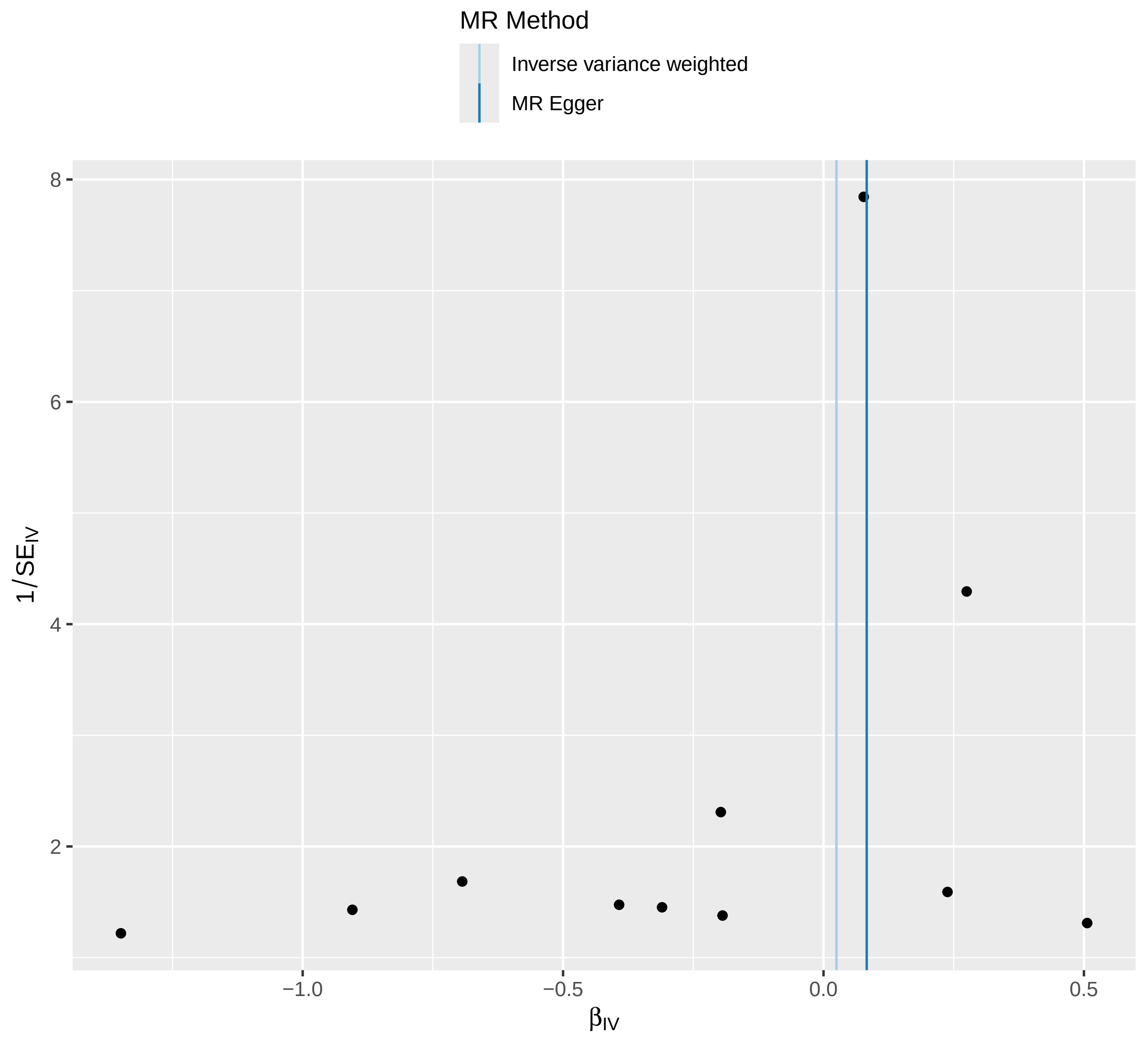
MR Method
Inverse variance weighted
MR Egger
8
6
4
2
1/SE_IV
−1.0
−0.5
0.0
0.5
β_IV

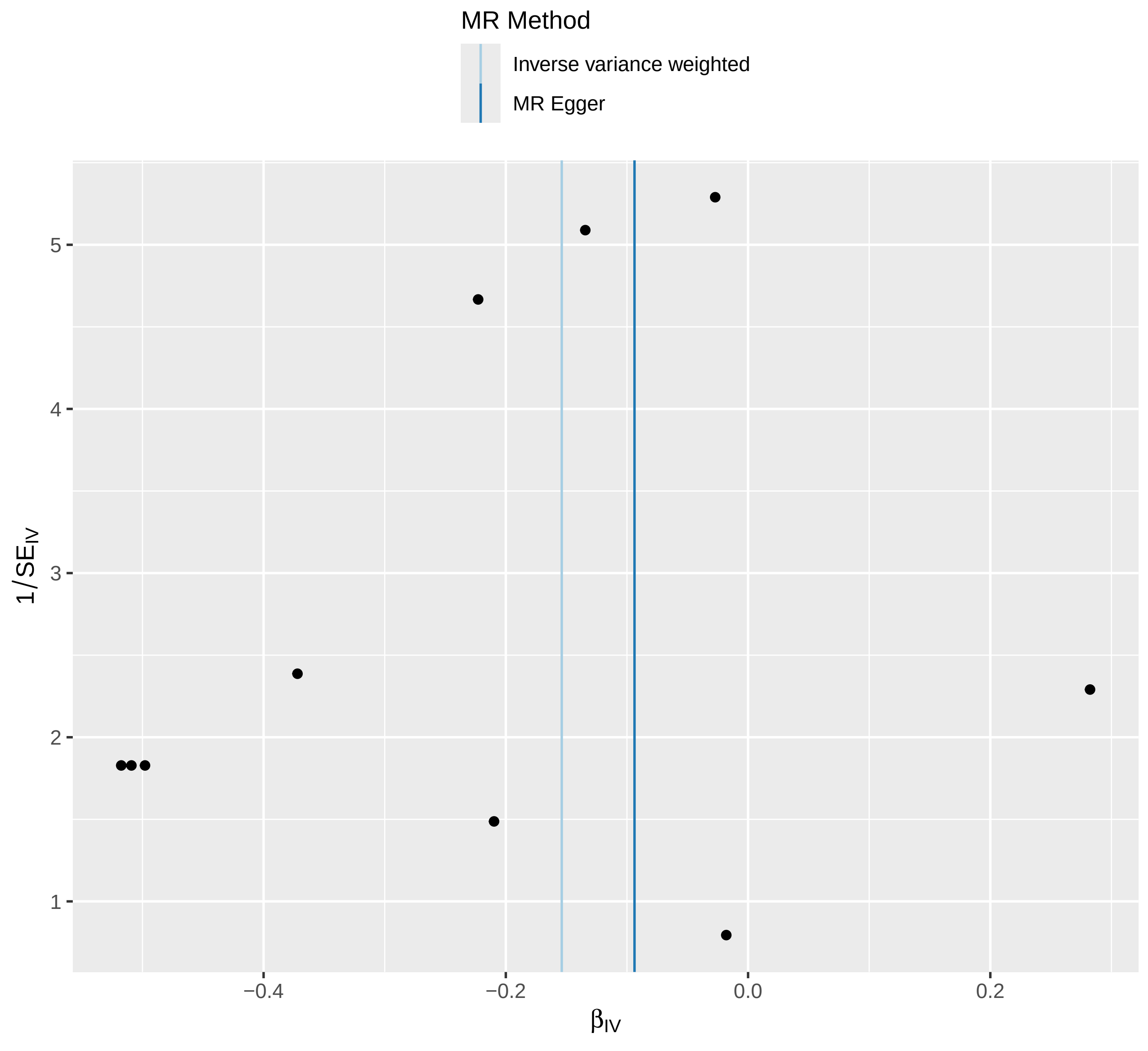
MR Method
Inverse variance weighted
MR Egger
1/SE_{IV}
5
4
3
2
1
−0.4
−0.2
0.0
0.2
β_{IV}

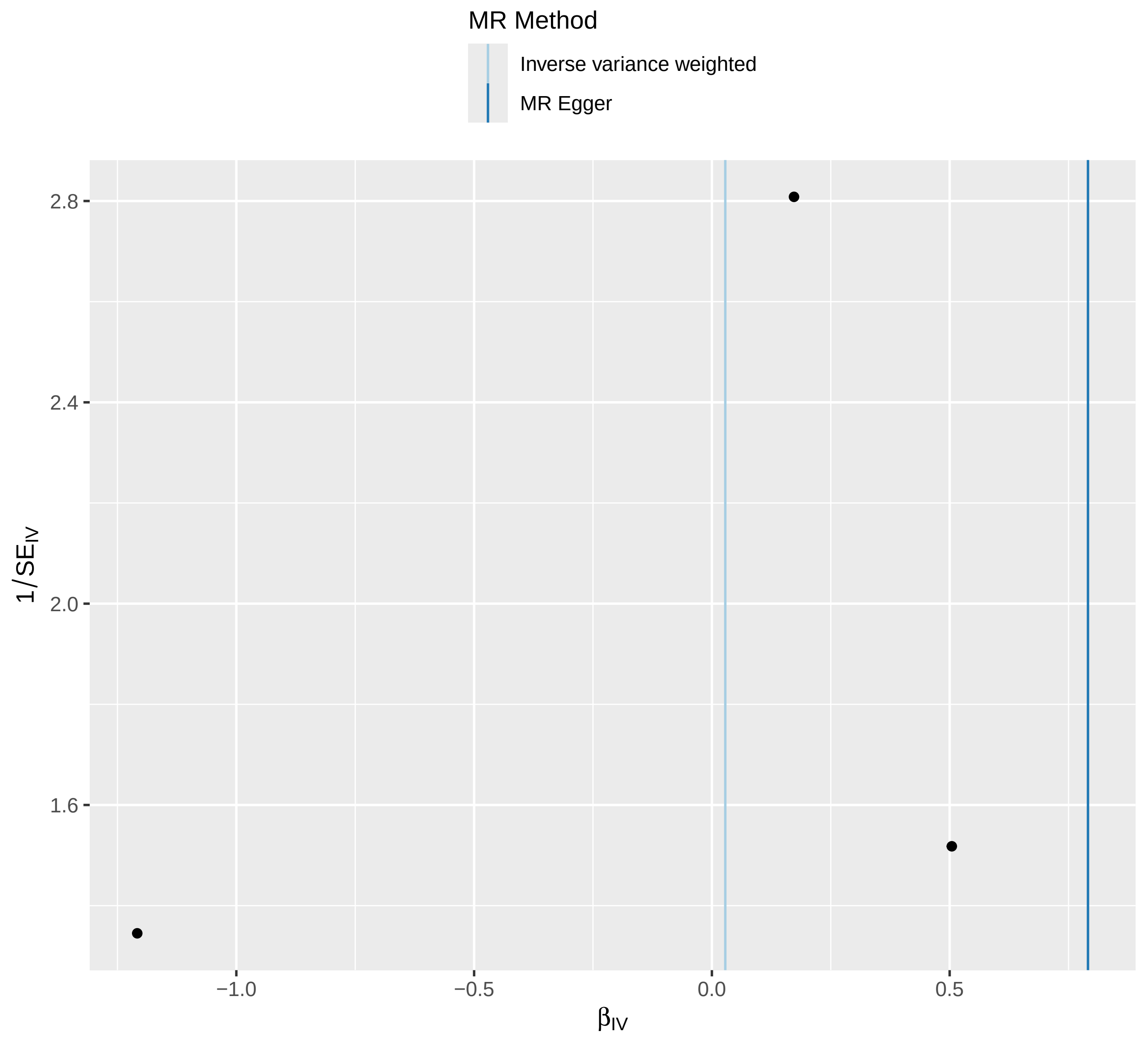

MR Method
Inverse variance weighted
MR Egger
2.8
2.4
2.0
1.6
$1/SE_{IV}$
−1.0
−0.5
0.0
0.5
β_{IV}

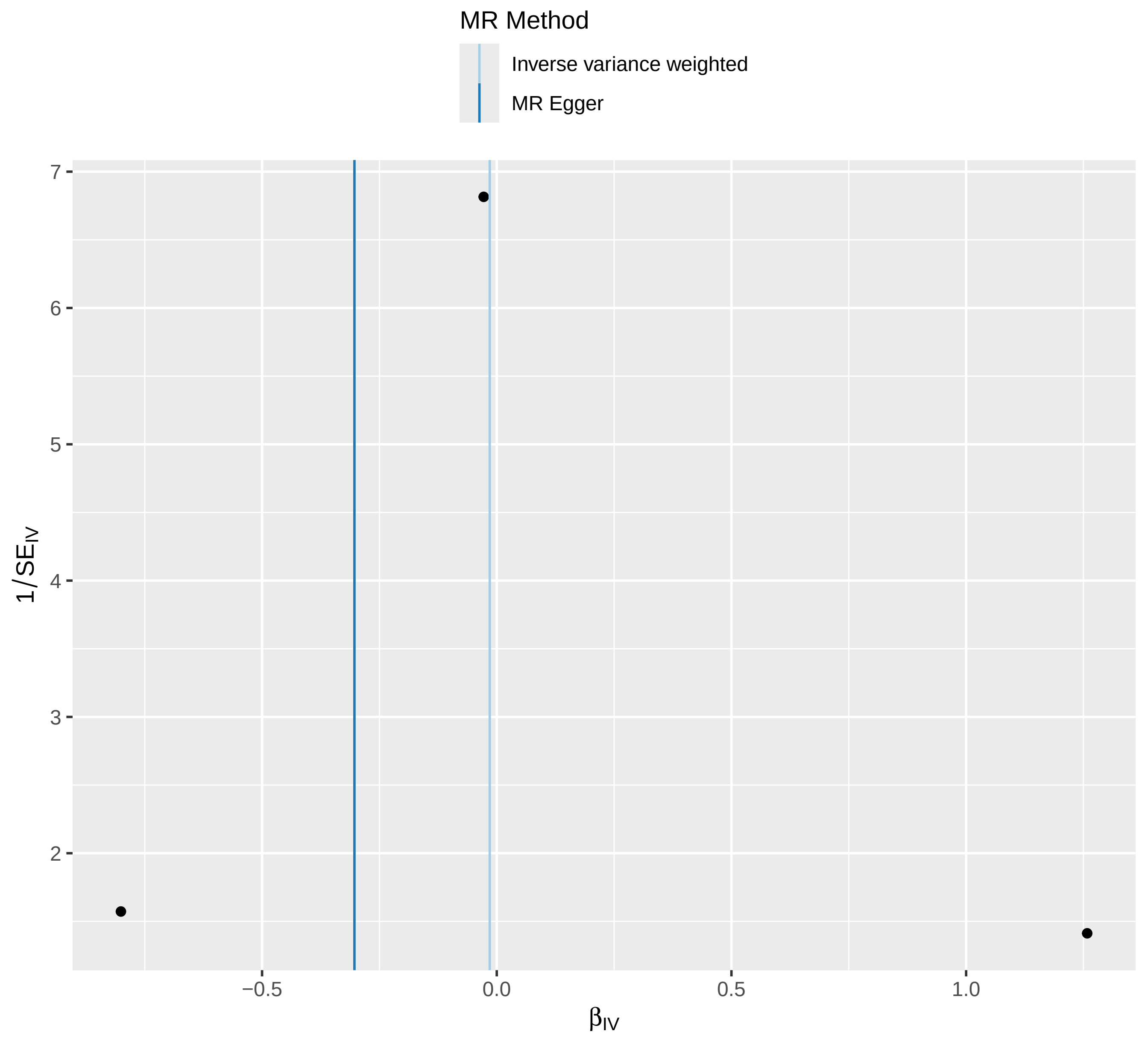

MR Method
Inverse variance weighted
MR Egger
7
6
5
4
3
2
1/SE_IV
−0.5
0.0
0.5
1.0
β_IV

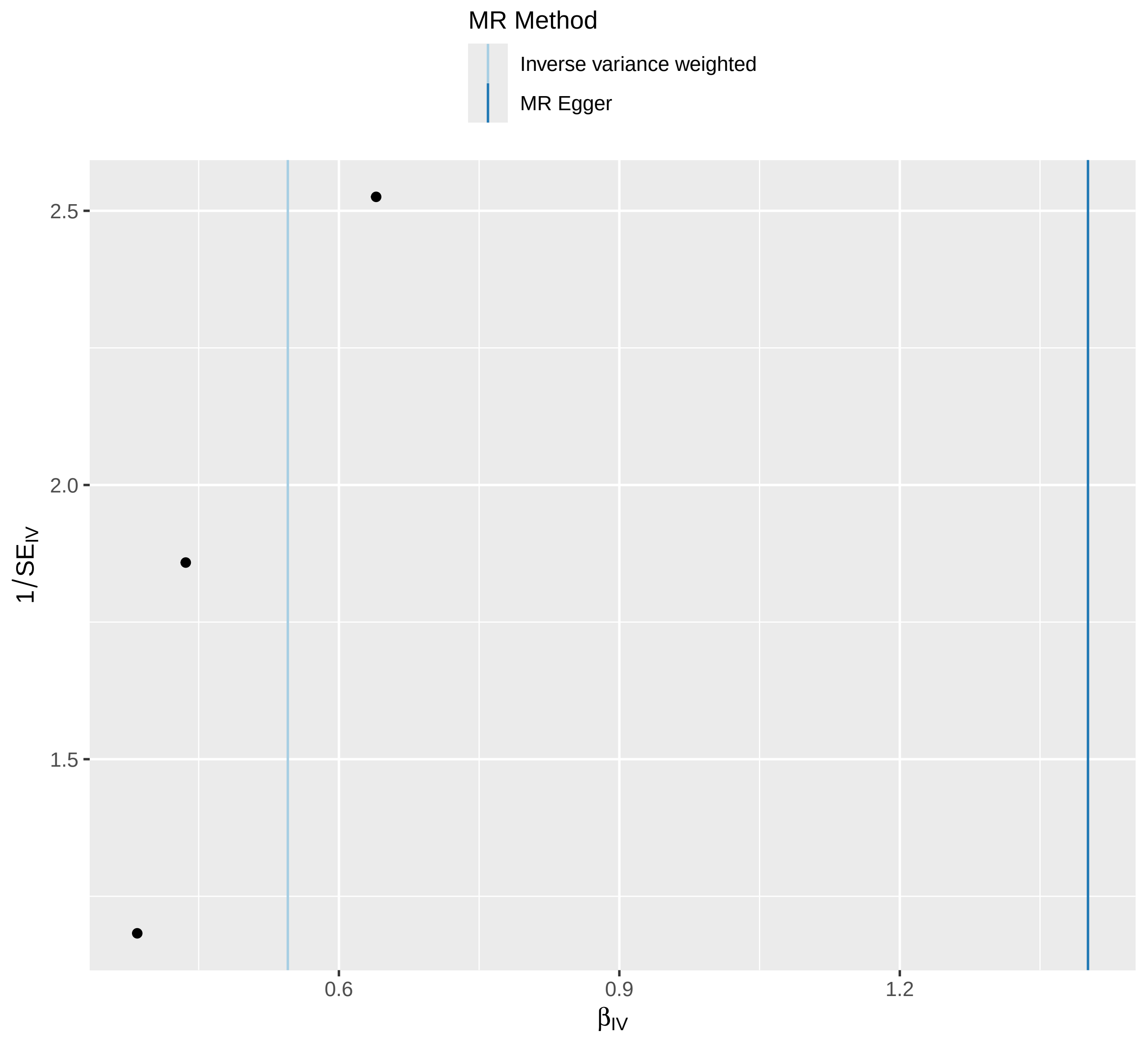

MR Method
Inverse variance weighted
MR Egger
2.5
2.0
1.5
$1/SE_{IV}$
0.6
0.9
1.2
β_{IV}

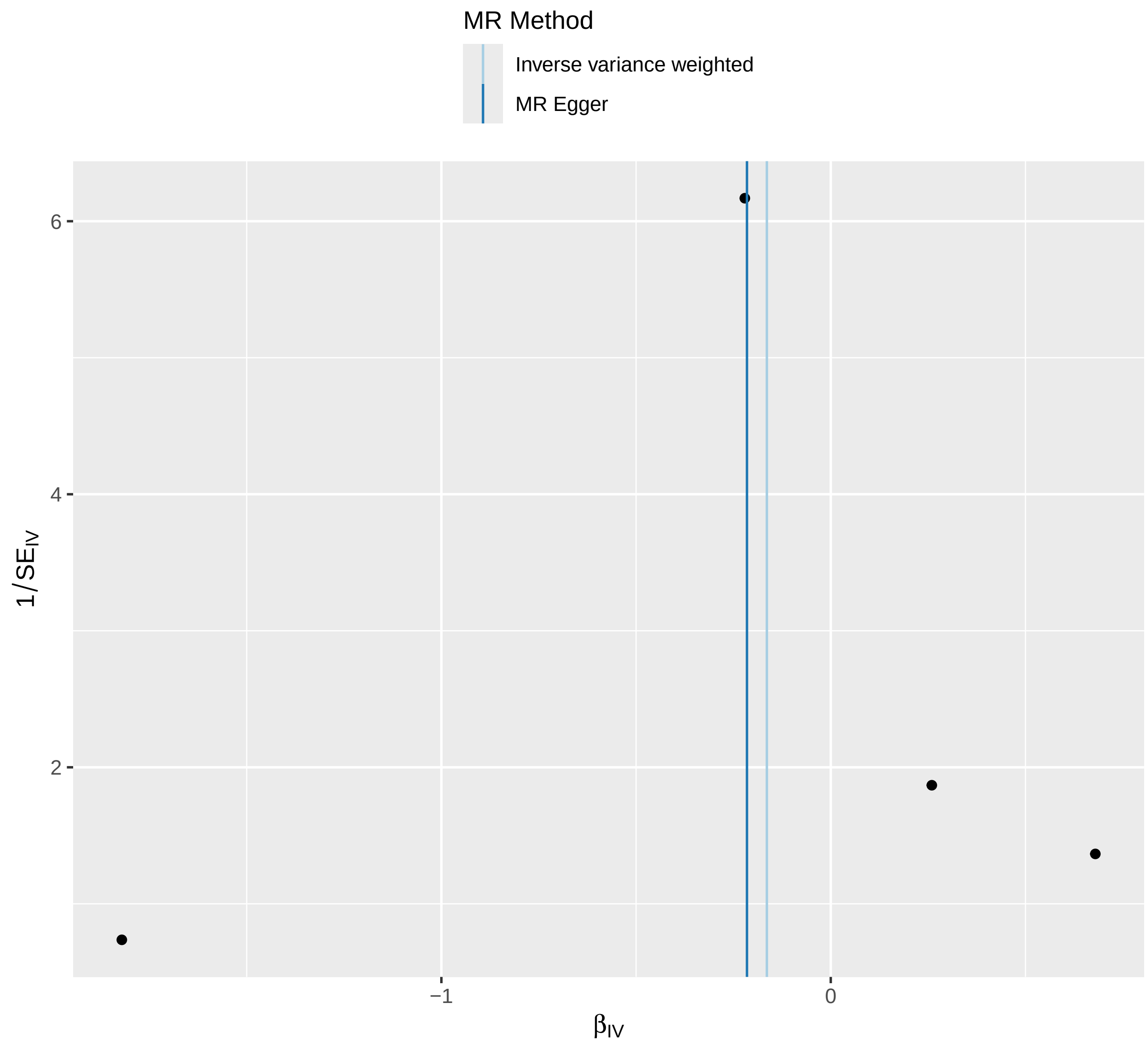
MR Method
Inverse variance weighted
MR Egger
6
4
2
1/SE$_{IV}$
−1
0
β$_{IV}$

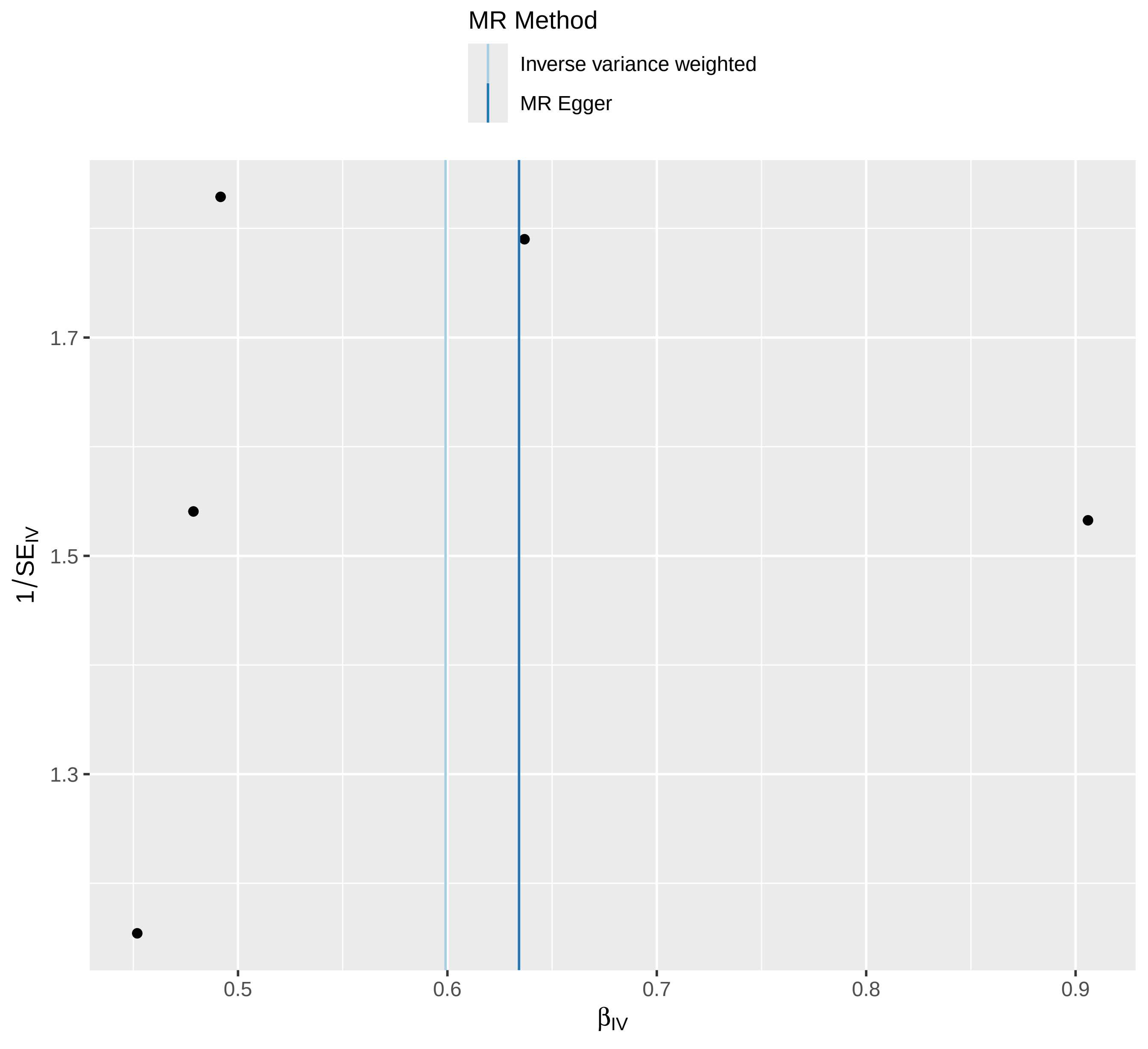
MR Method
Inverse variance weighted
MR Egger
1.7
1.5
1.3
$1/SE_{IV}$
0.5
0.6
0.7
0.8
0.9
β_{IV}

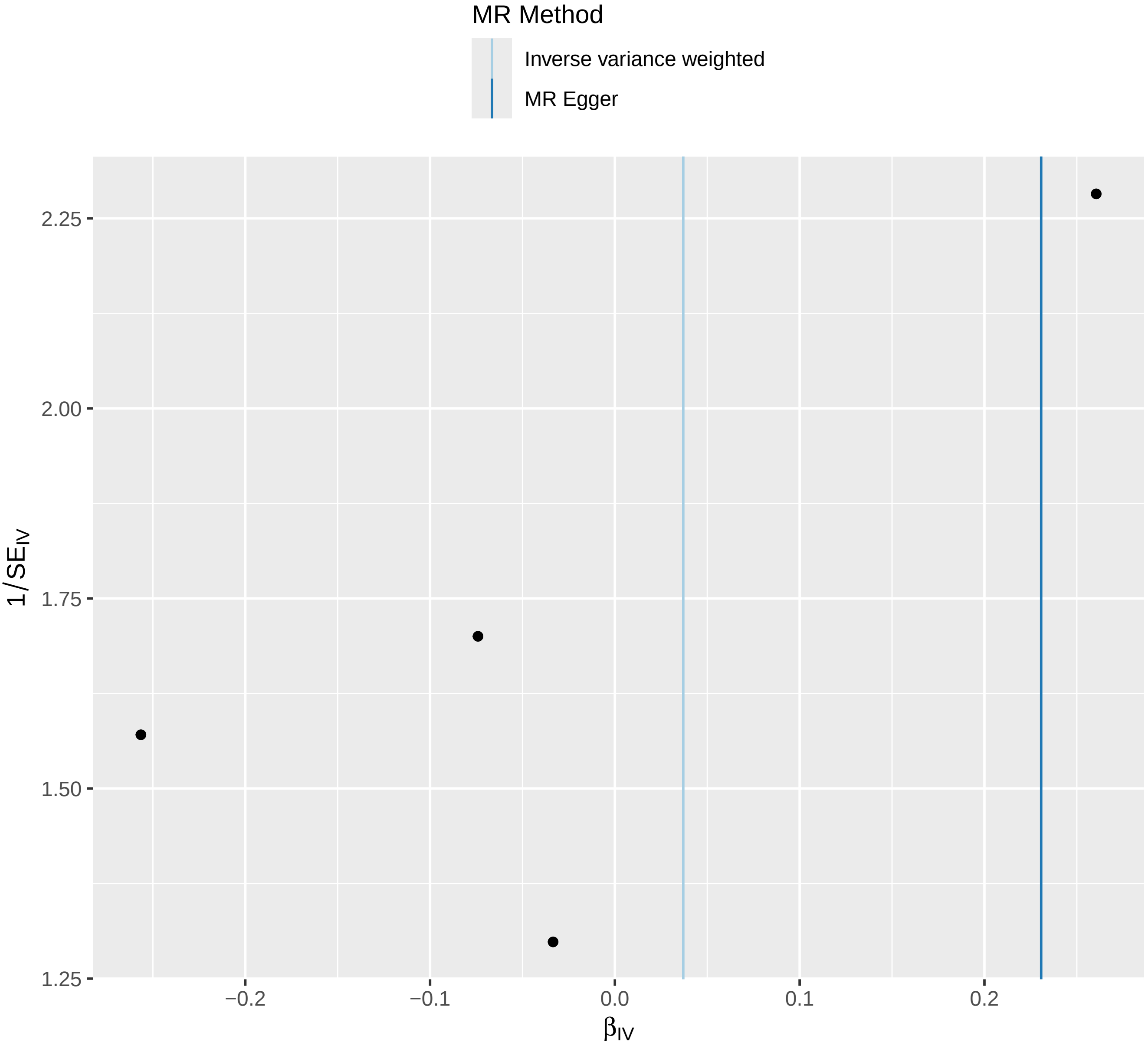
MR Method
Inverse variance weighted
MR Egger
2.25
2.00
1.75
1.50
1.25
$1/SE_{IV}$
−0.2
−0.1
0.0
0.1
0.2
β_{IV}

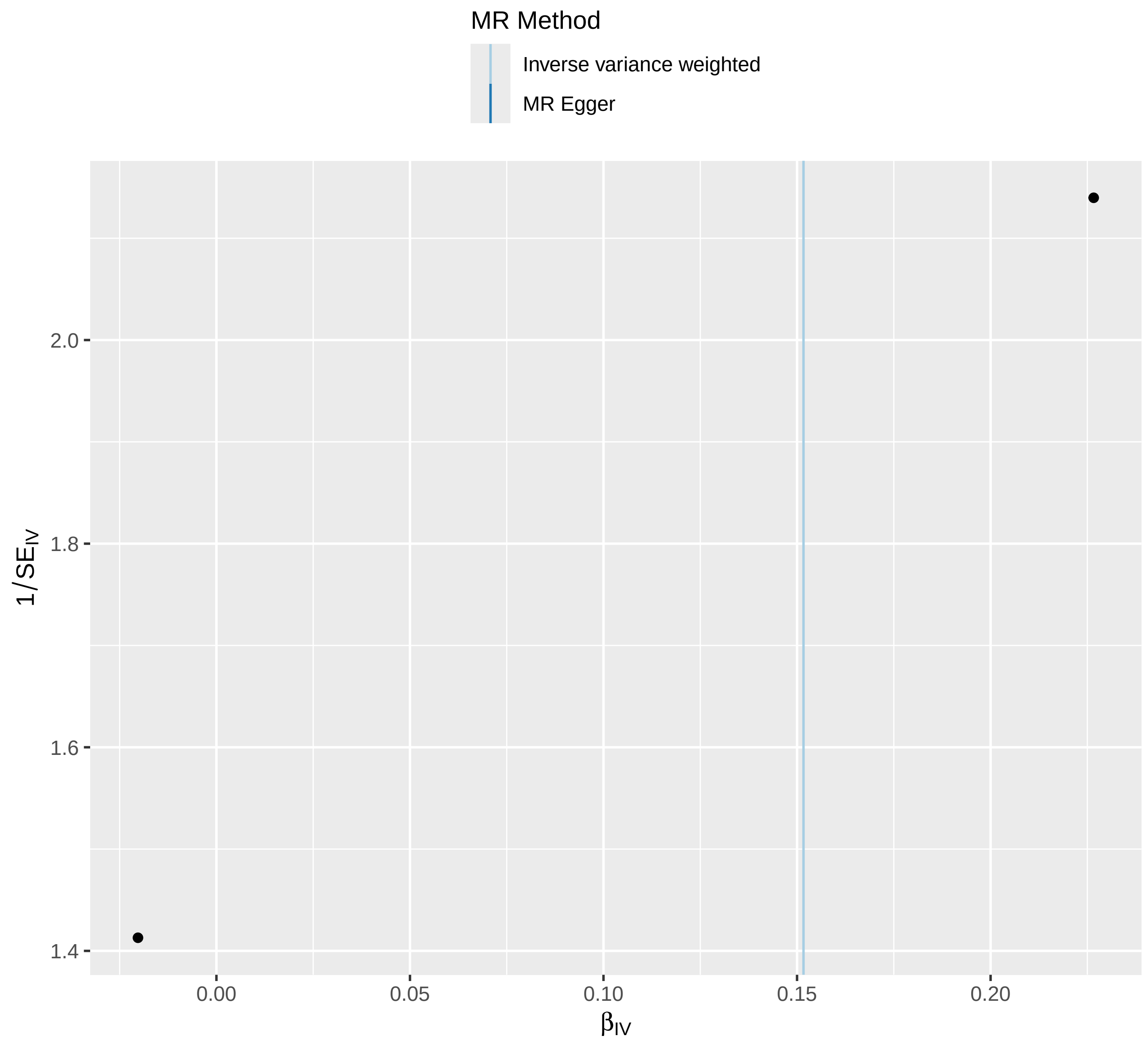
MR Method
Inverse variance weighted
MR Egger
2.0
1.8
1.6
1.4
1/SE$_{IV}$
0.00
0.05
0.10
0.15
0.20
β_{IV}

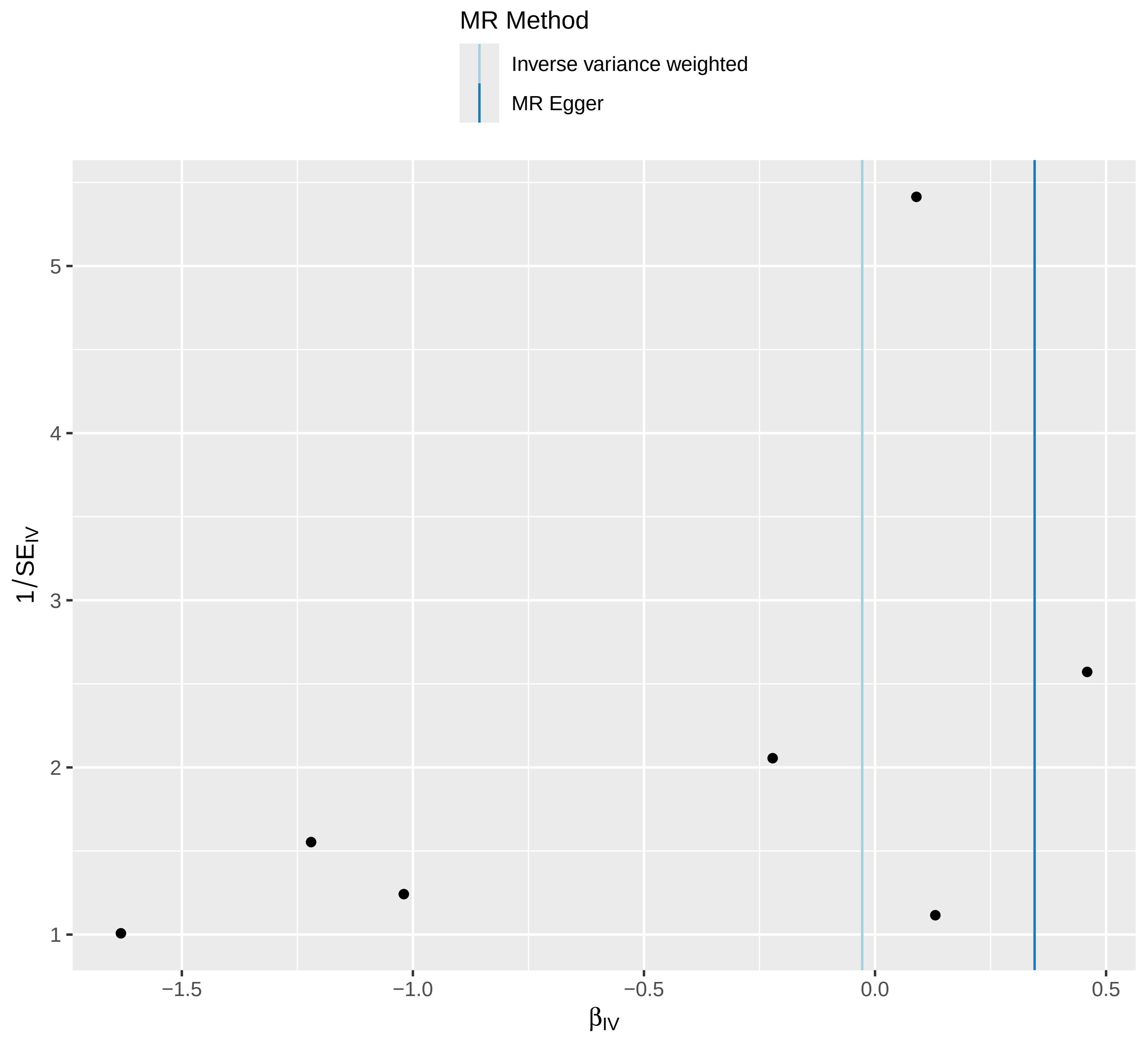
MR Method
Inverse variance weighted
MR Egger
1/SE_IV
β_IV
5
4
3
2
1
−1.5
−1.0
−0.5
0.0
0.5

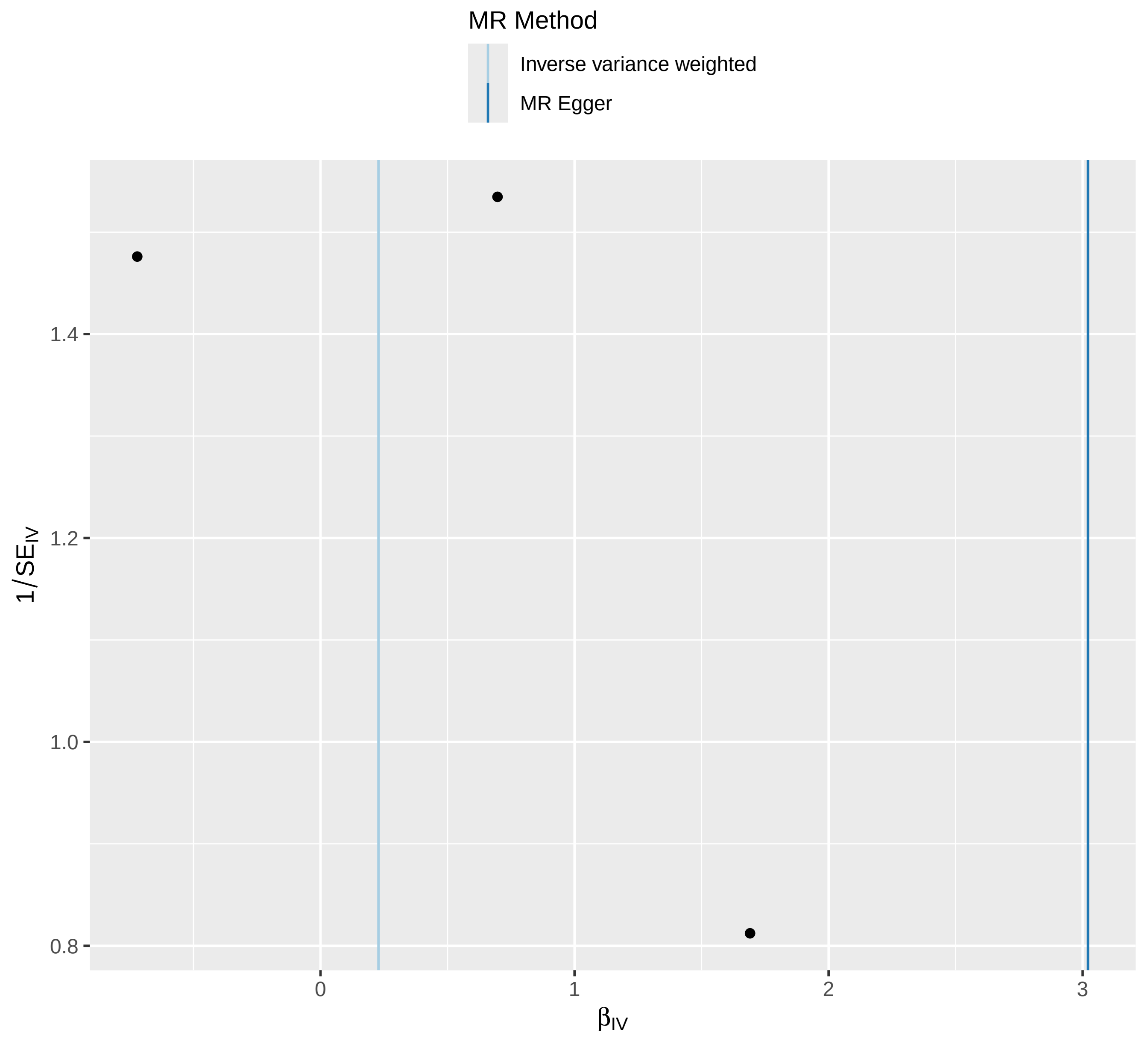

MR Method
Inverse variance weighted
MR Egger
1.4
1.2
1.0
0.8
1/SE_IV
0
1
2
3
β_IV

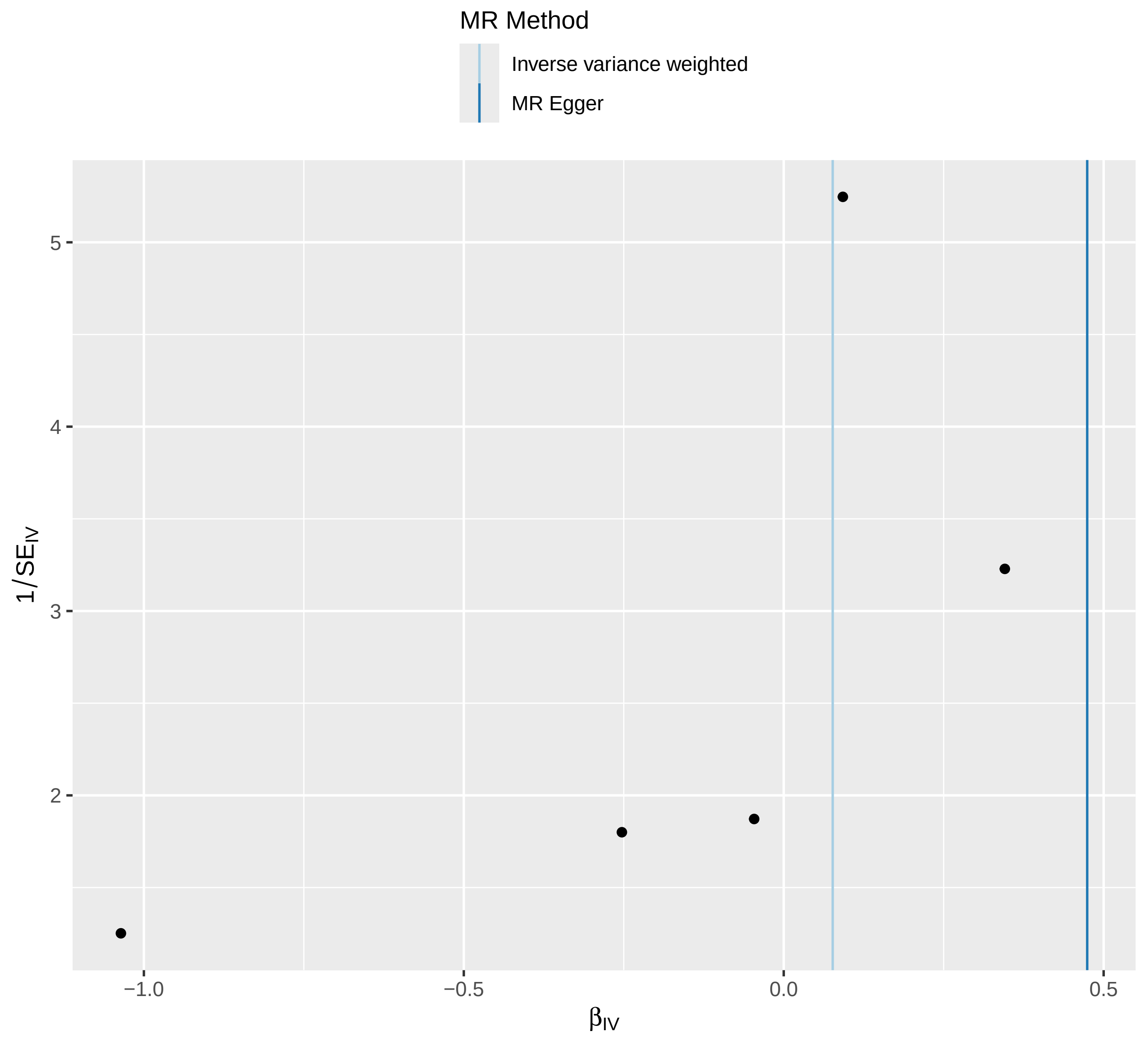

MR Method
Inverse variance weighted
MR Egger
5
4
3
2
1/SE$_{IV}$
−1.0
−0.5
0.0
0.5
β$_{IV}$

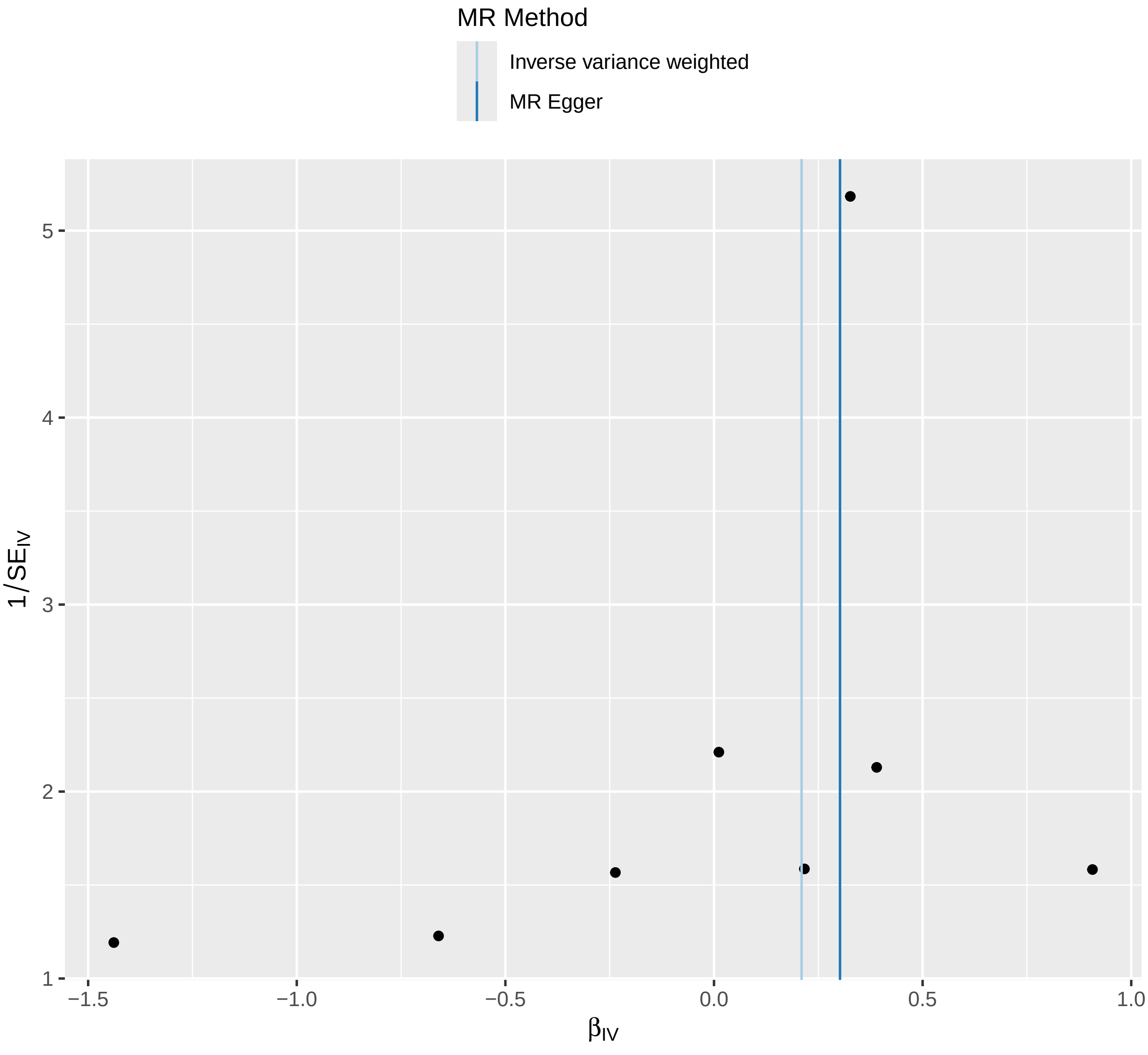

MR Method
Inverse variance weighted
MR Egger
5
4
3
2
1
1/SE$_{IV}$
−1.5
−1.0
−0.5
0.0
0.5
1.0
β$_{IV}$

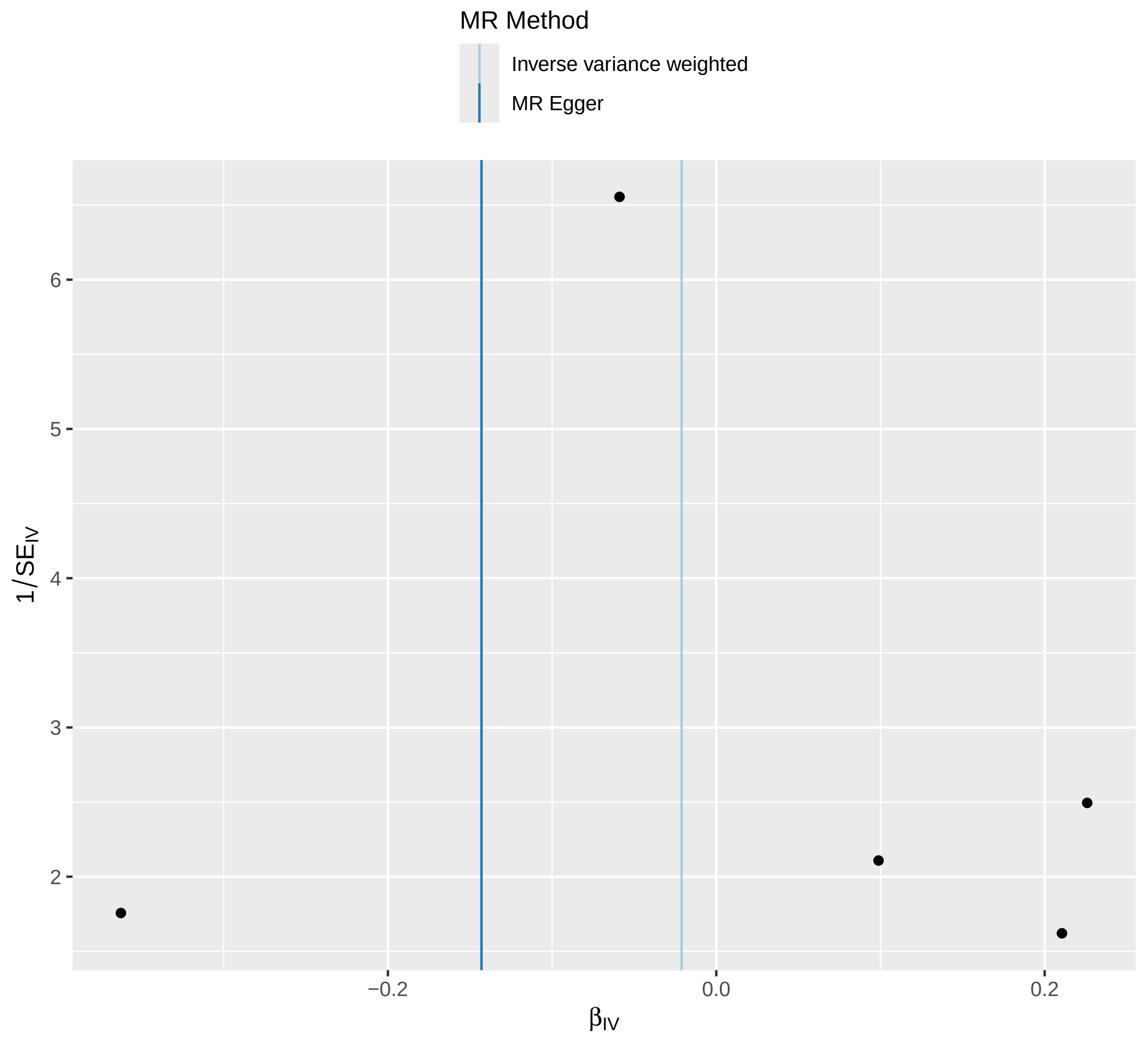
MR Method
Inverse variance weighted
MR Egger
6
5
4
3
2
$1/SE_{IV}$
−0.2
0.0
0.2
β_{IV}

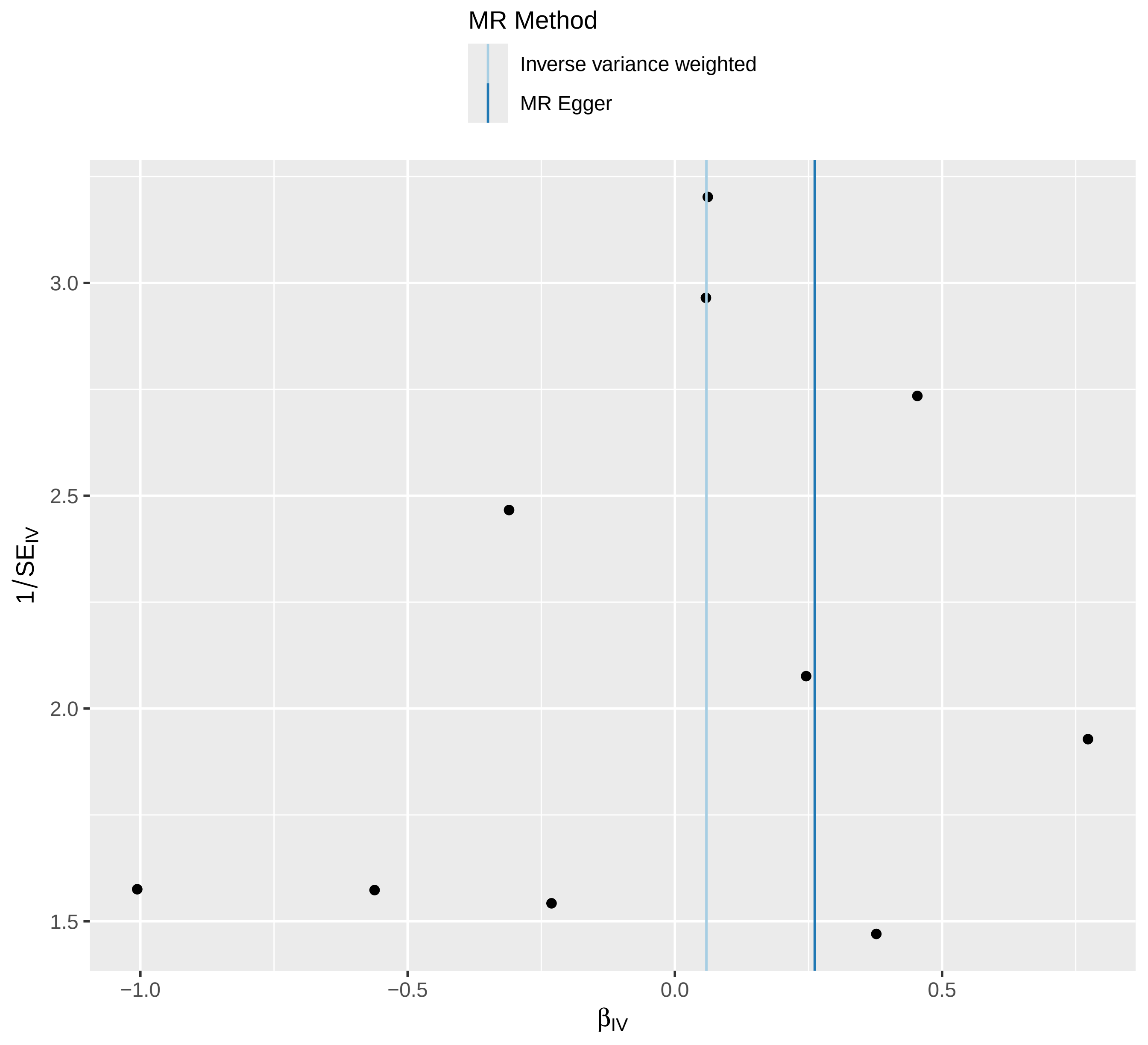

MR Method
Inverse variance weighted
MR Egger
3.0
2.5
2.0
1.5
1/SE_IV
−1.0
−0.5
0.0
0.5
β_IV

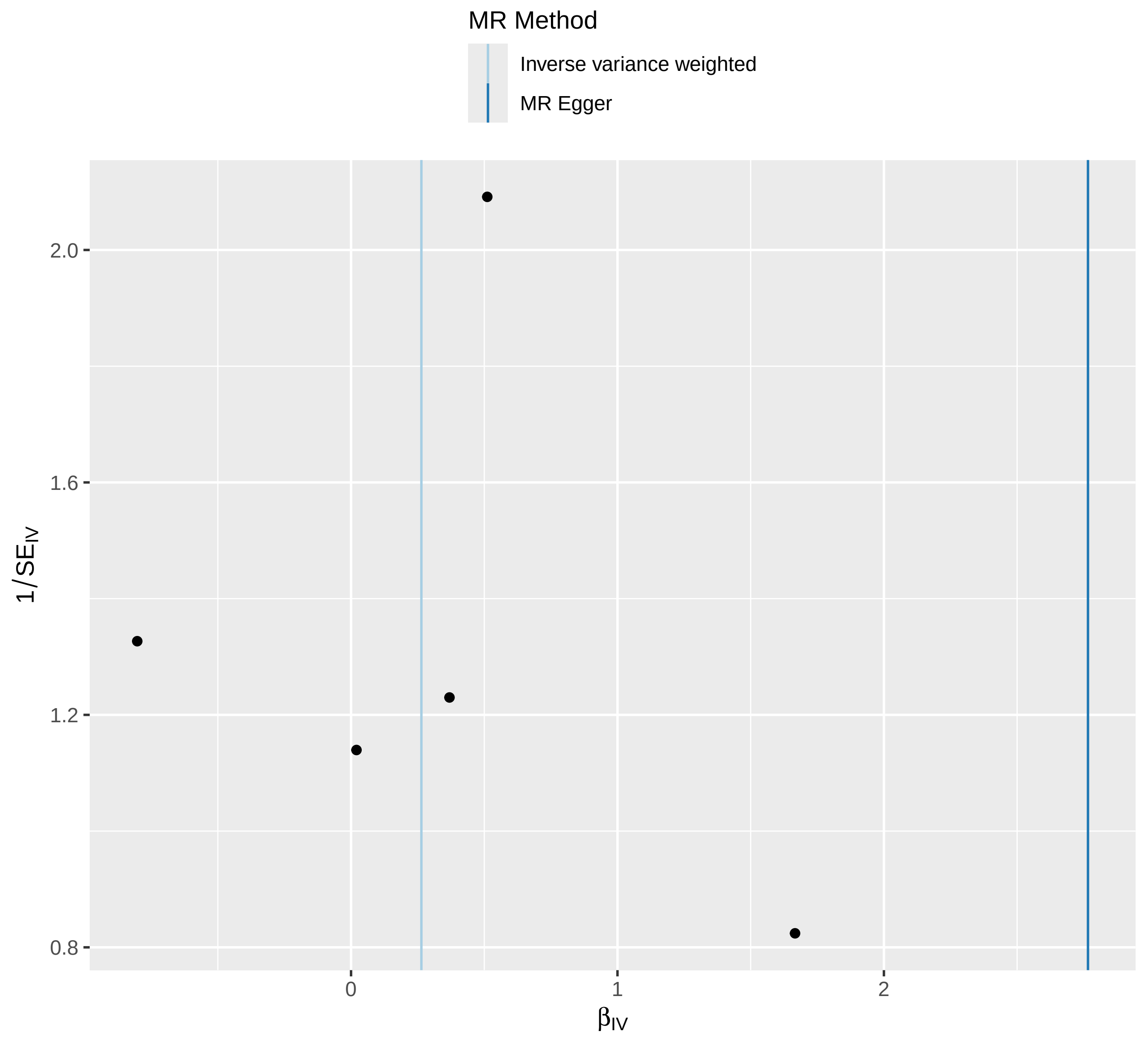
MR Method
Inverse variance weighted
MR Egger
2.0
1.6
1.2
0.8
1/SE_IV
0
1
2
β_IV

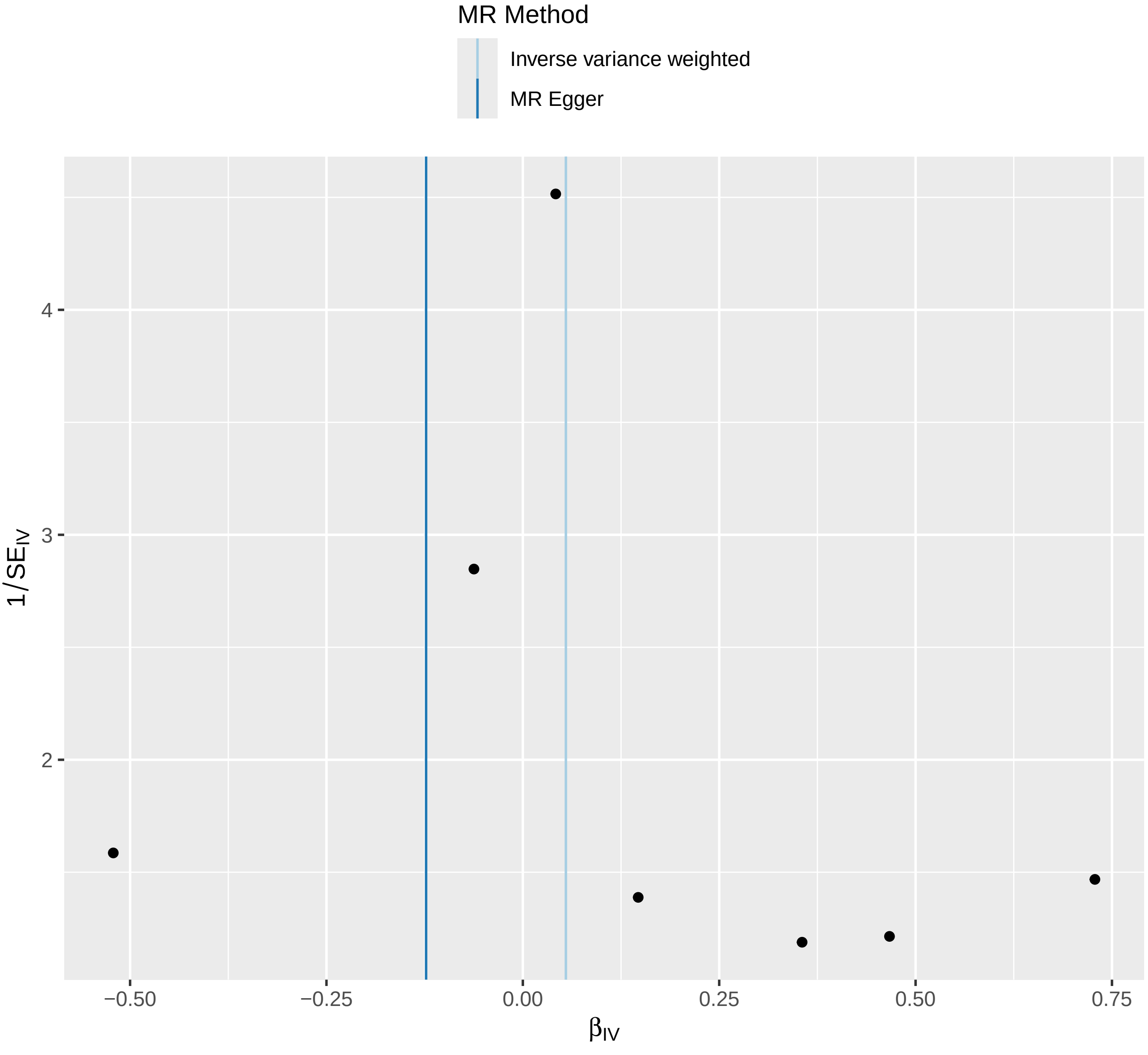
MR Method
Inverse variance weighted
MR Egger
4
3
2
1/SE_IV
−0.50
−0.25
0.00
0.25
0.50
0.75
β_IV

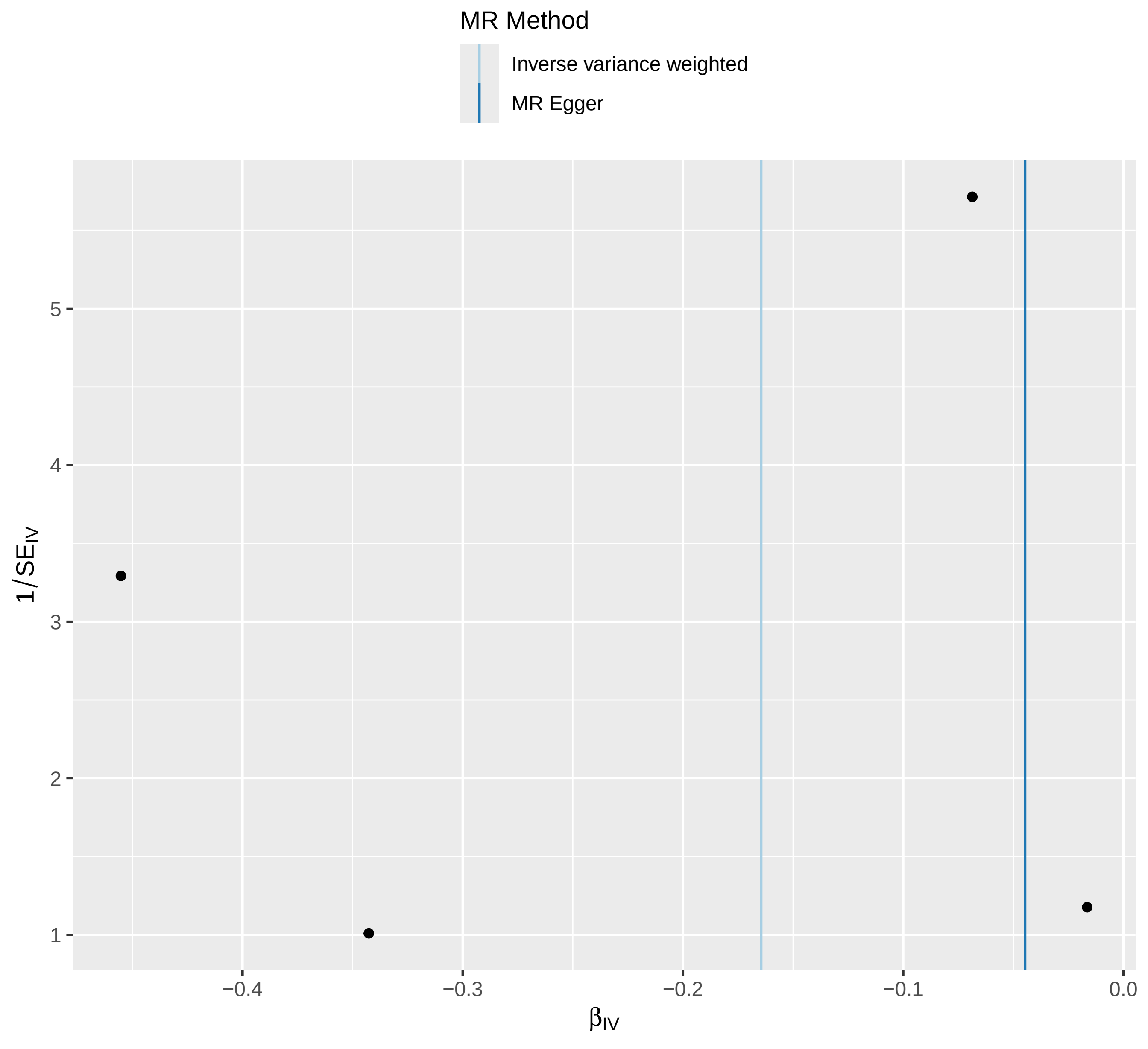
MR Method
Inverse variance weighted
MR Egger
5
4
3
2
1
$1/SE_{IV}$
−0.4
−0.3
−0.2
−0.1
0.0
β_{IV}

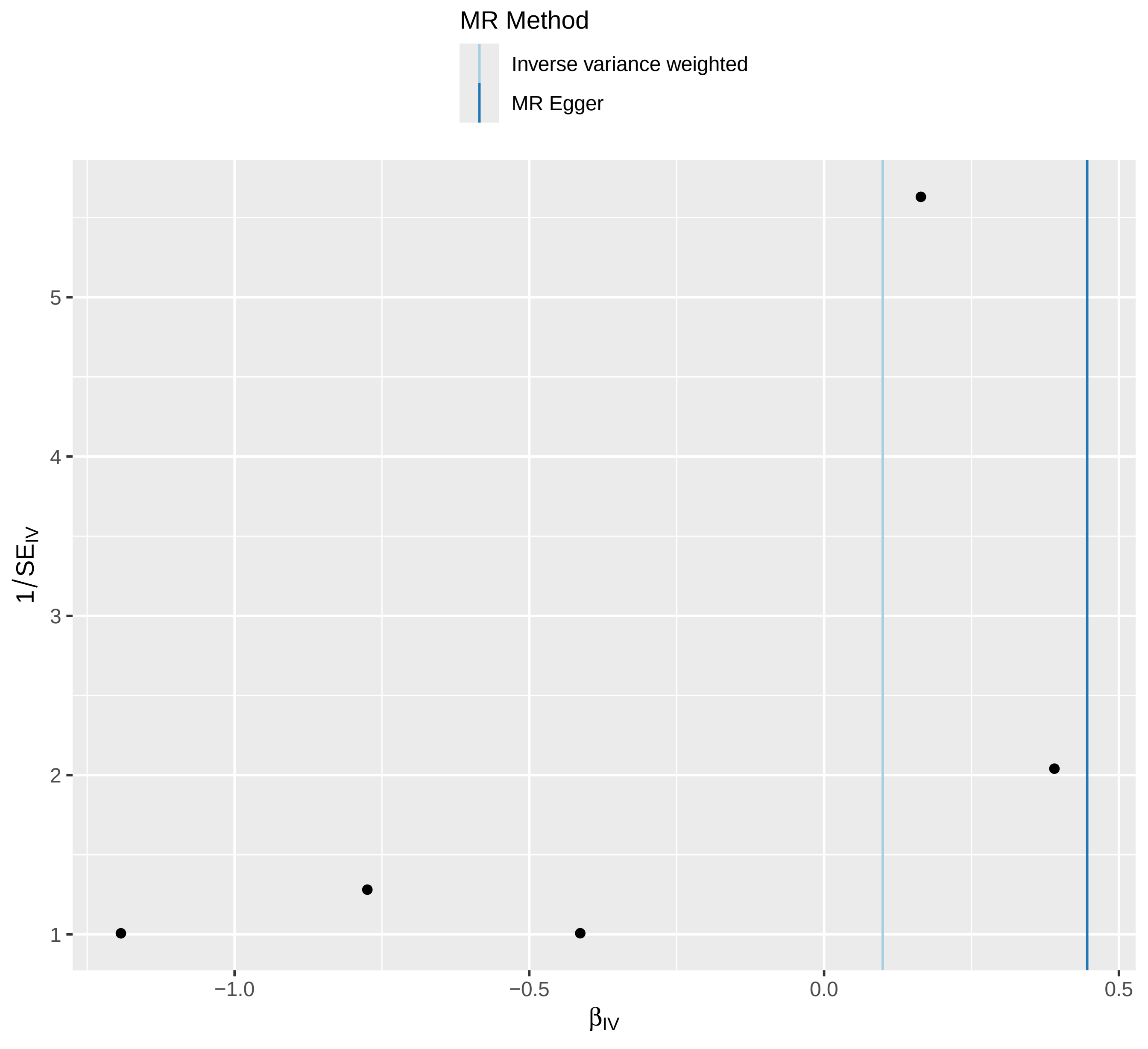
MR Method
Inverse variance weighted
MR Egger
1/SE$_{IV}$
5
4
3
2
1
−1.0
−0.5
0.0
0.5
β_{IV}

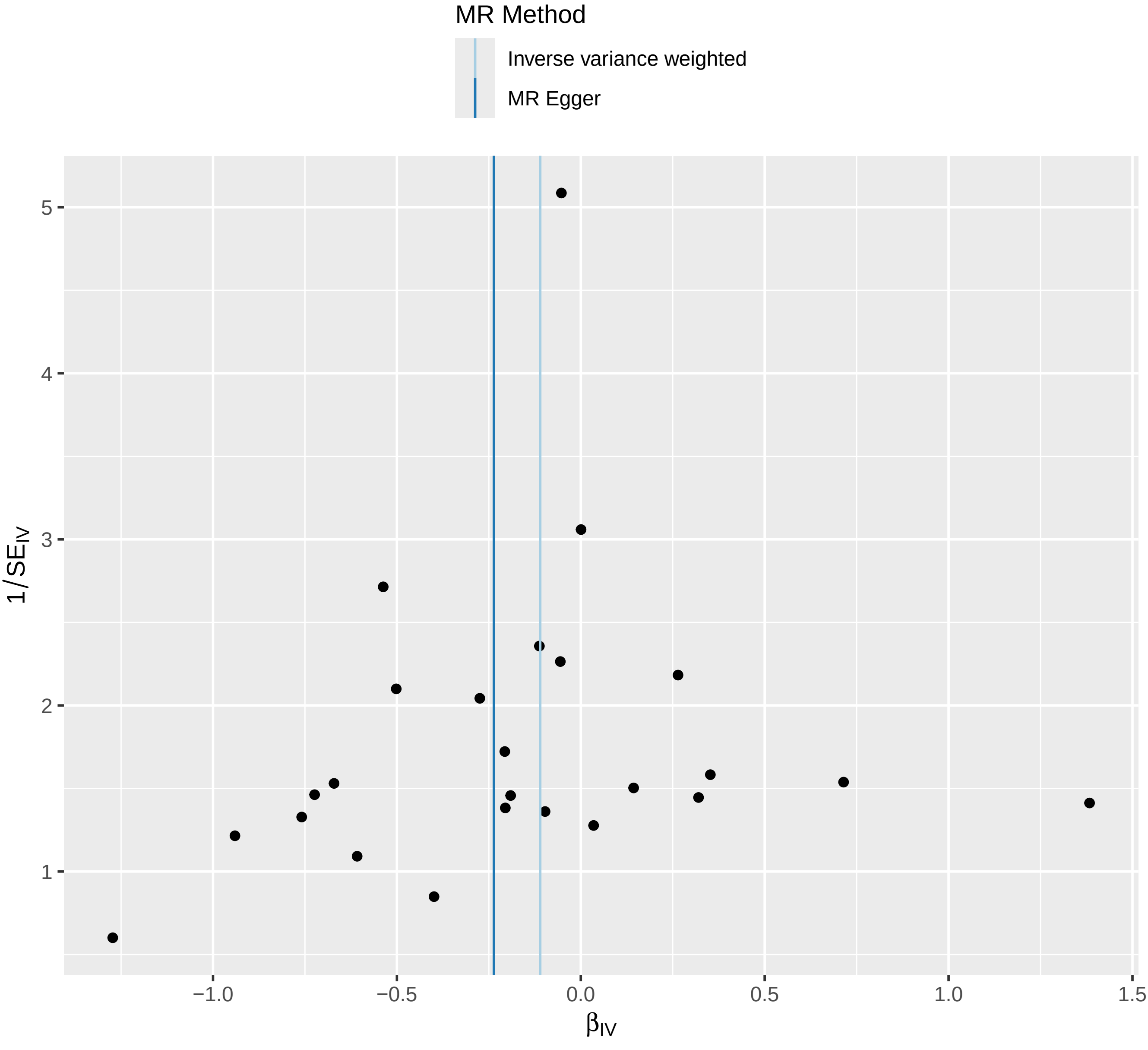

MR Method
Inverse variance weighted
MR Egger
5
4
3
2
1
1/SE$_{IV}$
−1.0
−0.5
0.0
0.5
1.0
1.5
β$_{IV}$

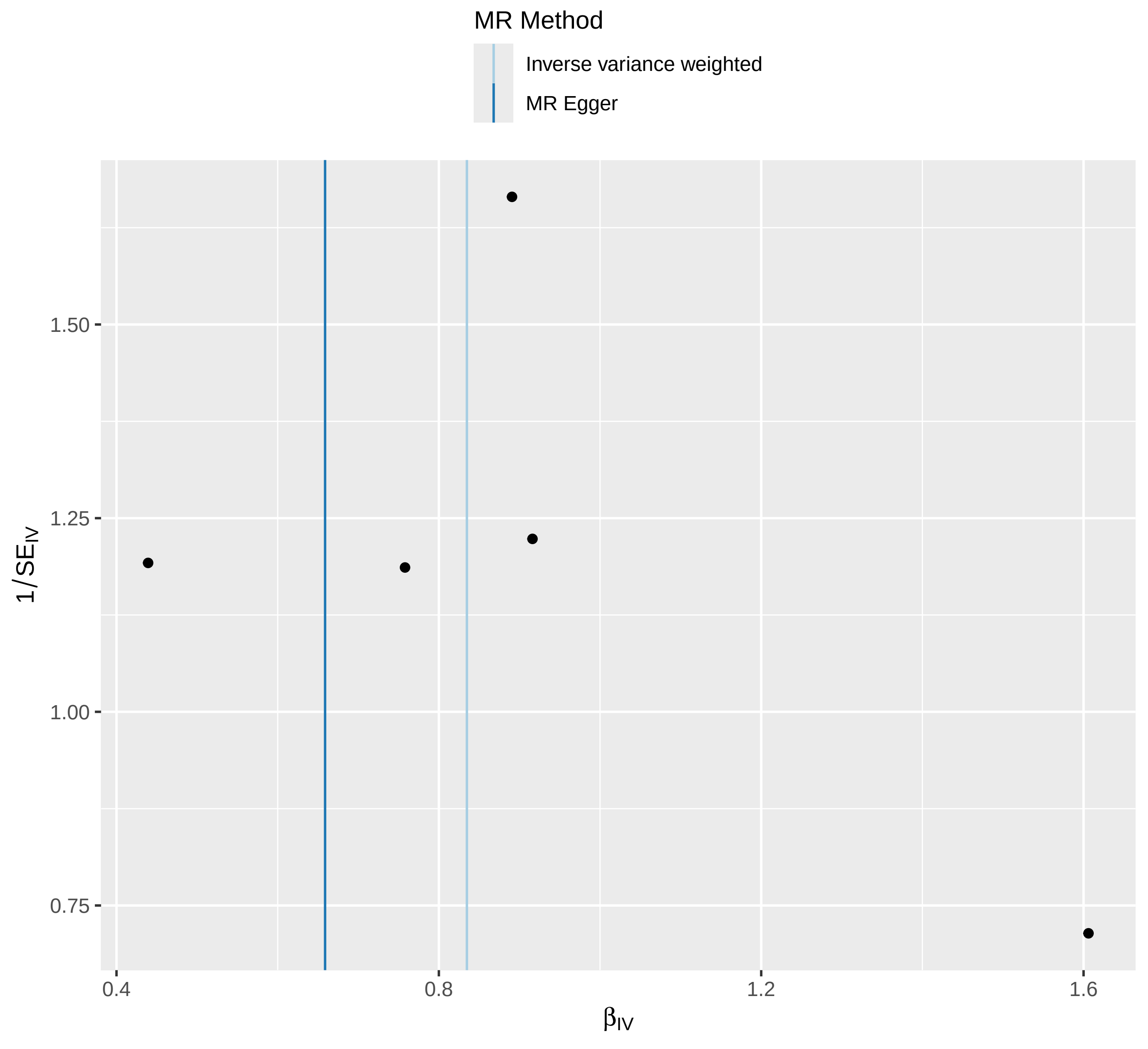

MR Method
Inverse variance weighted
MR Egger
1.50
1.25
1.00
0.75
1/SE_IV
0.4
0.8
1.2
1.6
β_IV

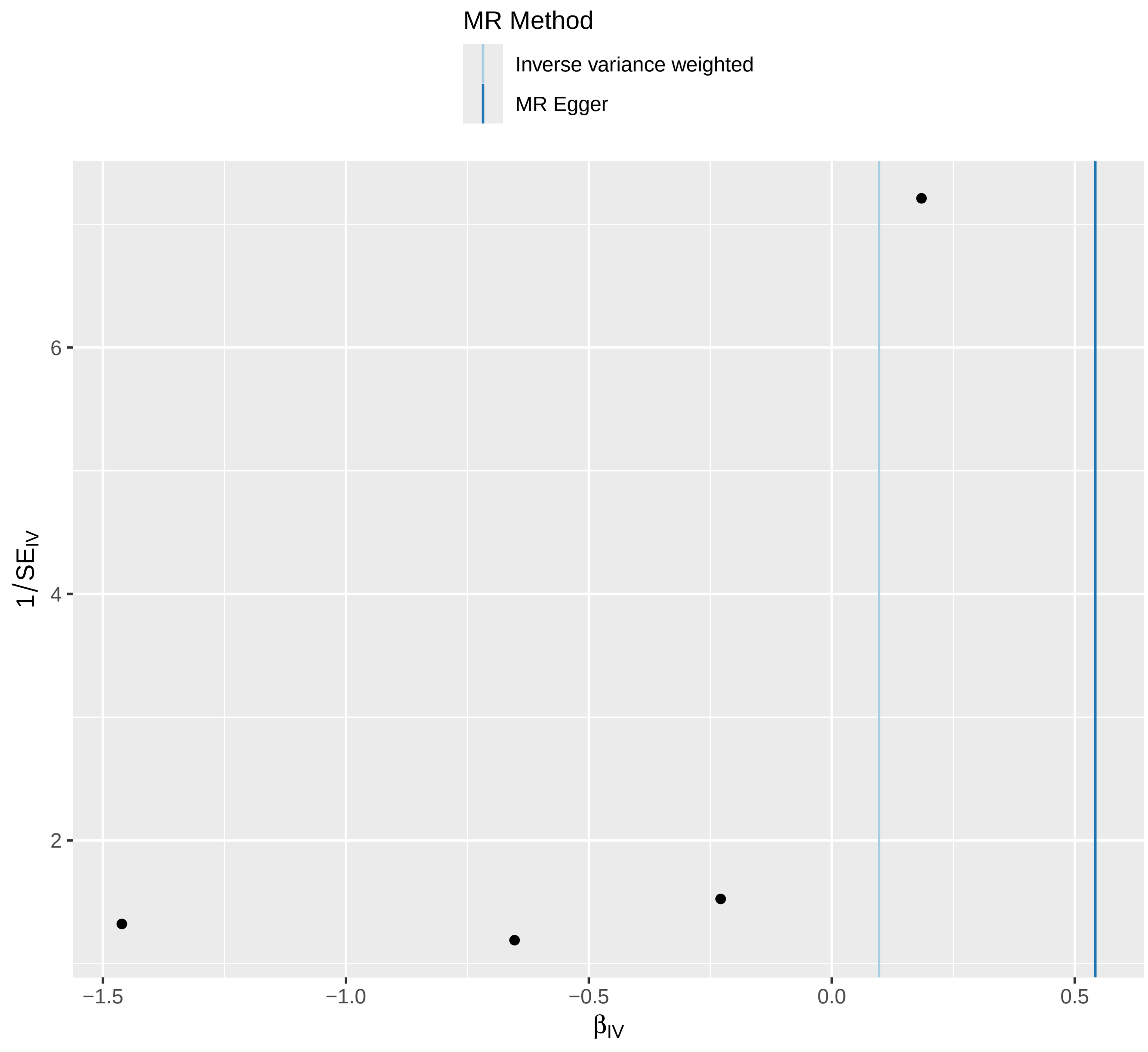

MR Method
Inverse variance weighted
MR Egger
6
4
2
$1/SE_{IV}$
−1.5
−1.0
−0.5
0.0
0.5
β_{IV}

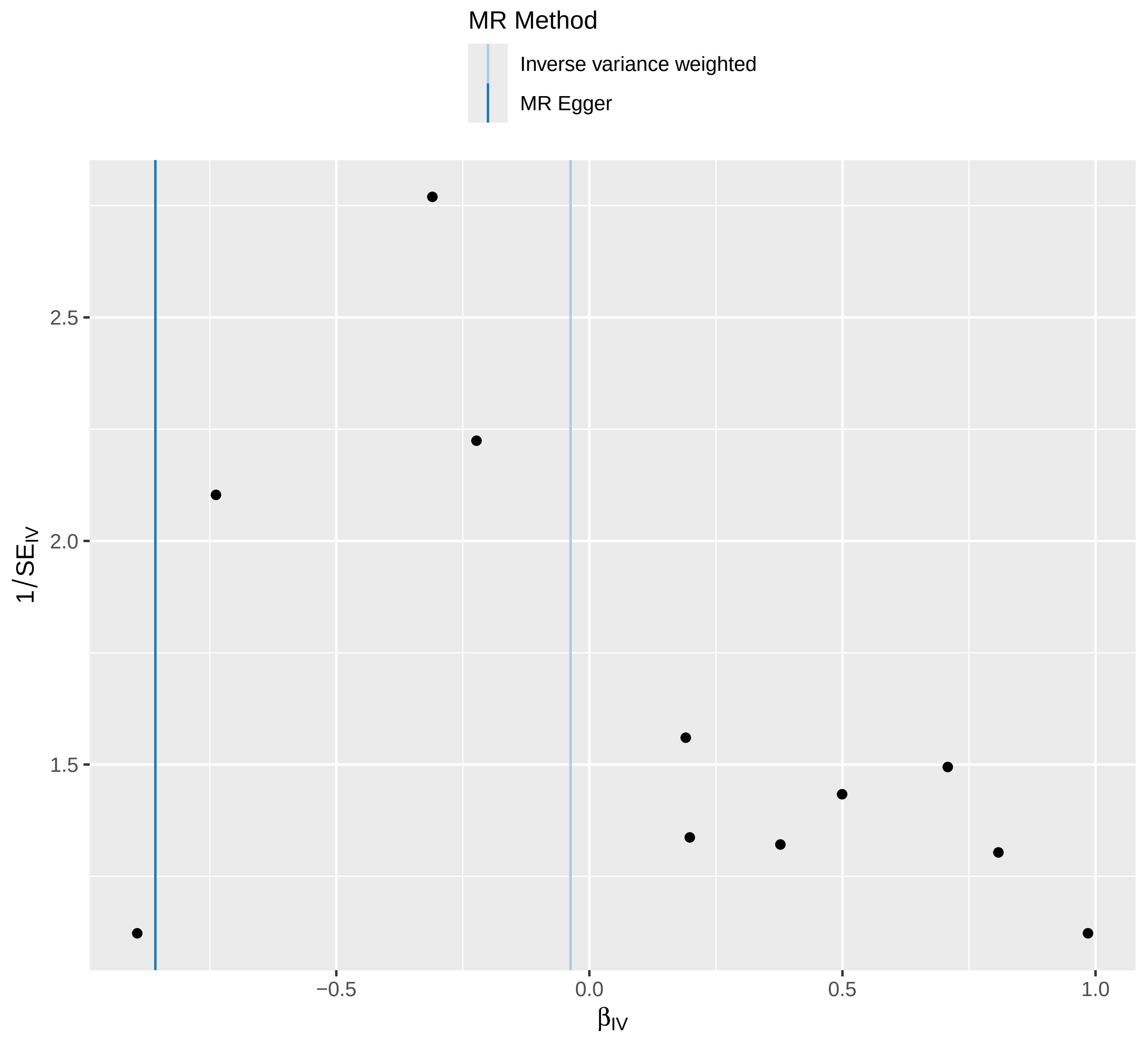
MR Method
Inverse variance weighted
MR Egger
2.5
2.0
1.5
1/SE_IV
−0.5
0.0
0.5
1.0
β_IV

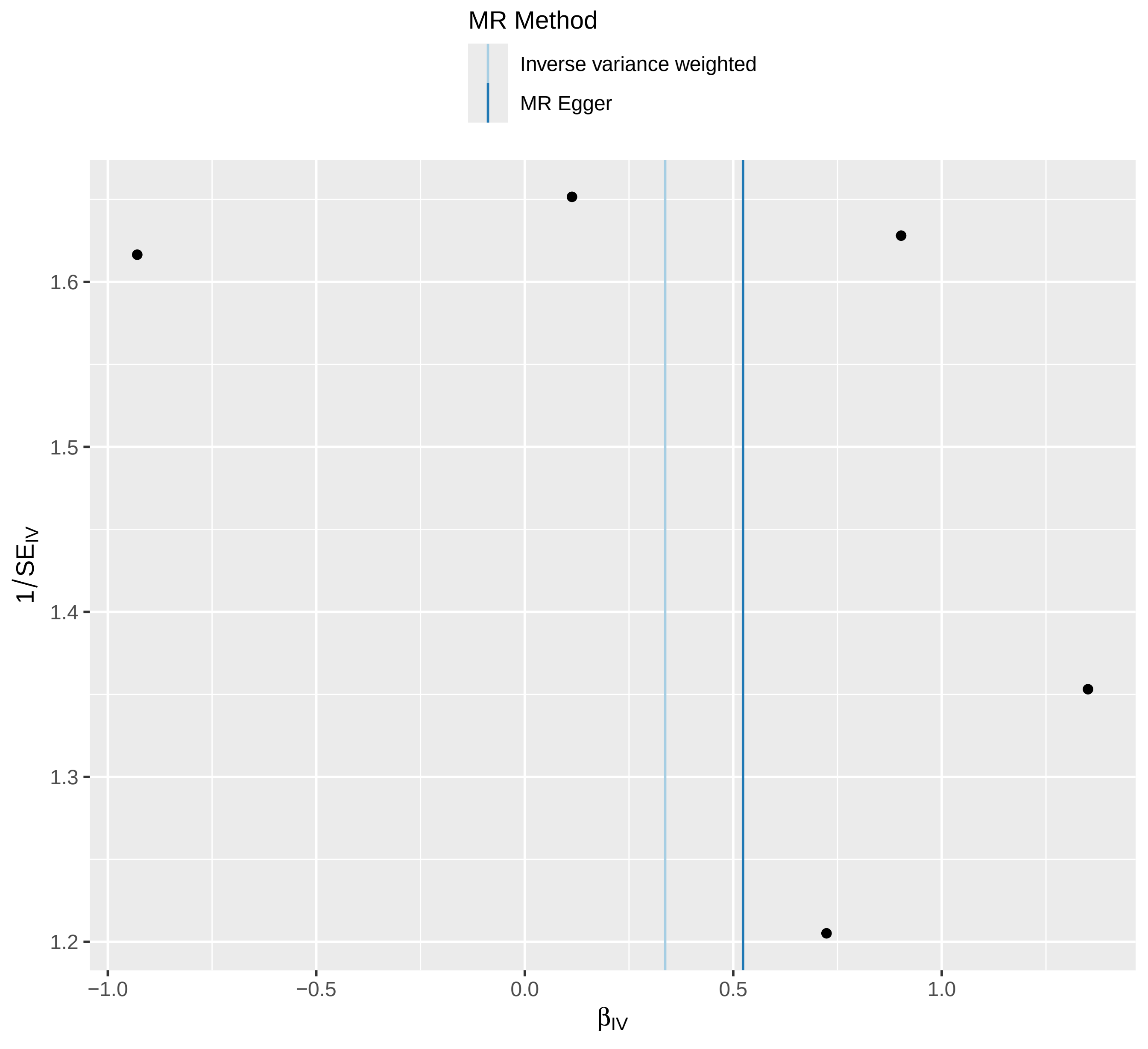
MR Method
Inverse variance weighted
MR Egger
1.6
1.5
1.4
1.3
1.2
1/SE_IV
−1.0
−0.5
0.0
0.5
1.0
β_IV

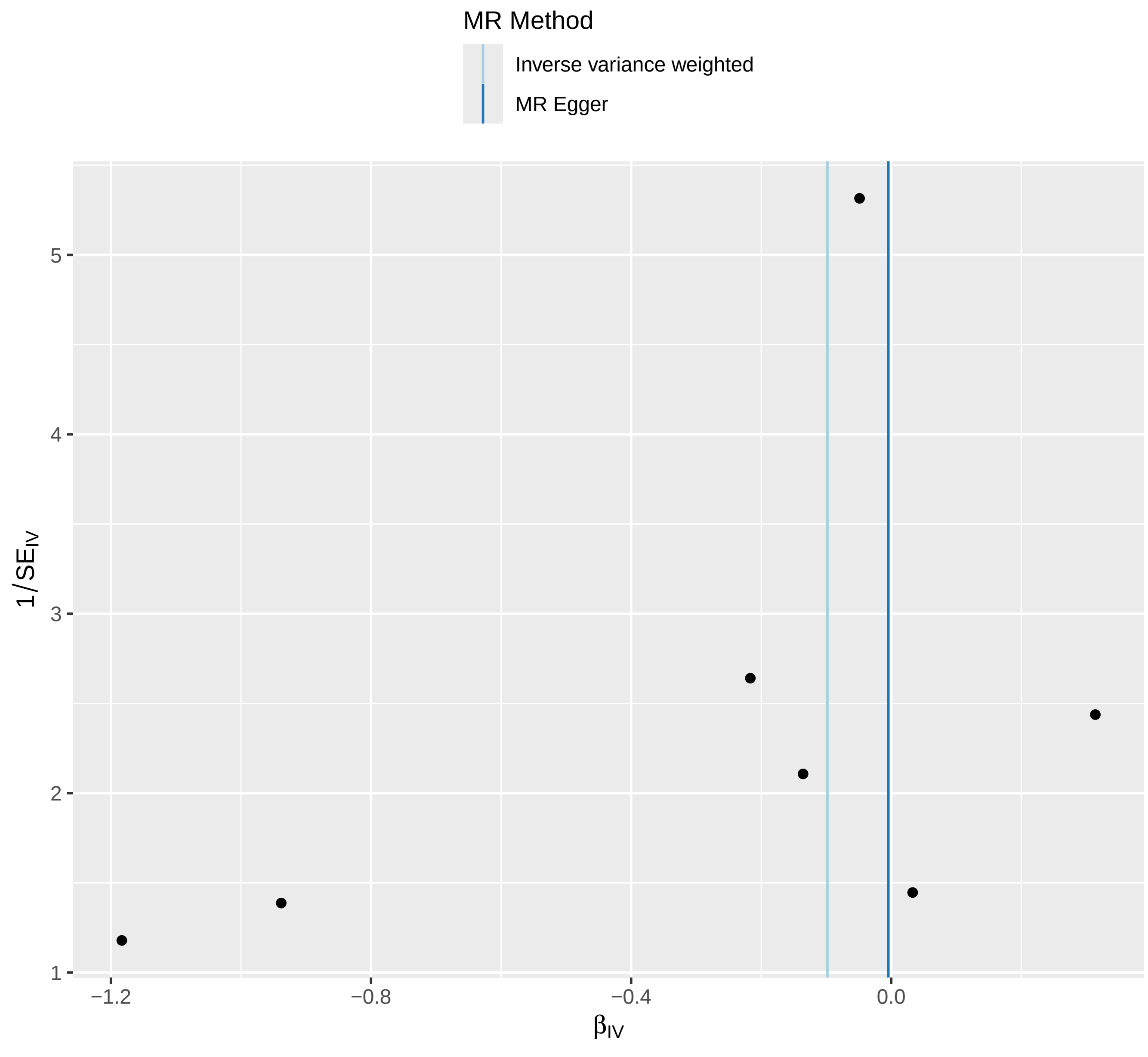

MR Method
Inverse variance weighted
MR Egger
1/SE_IV
5
4
3
2
1
−1.2
−0.8
−0.4
0.0
β_IV

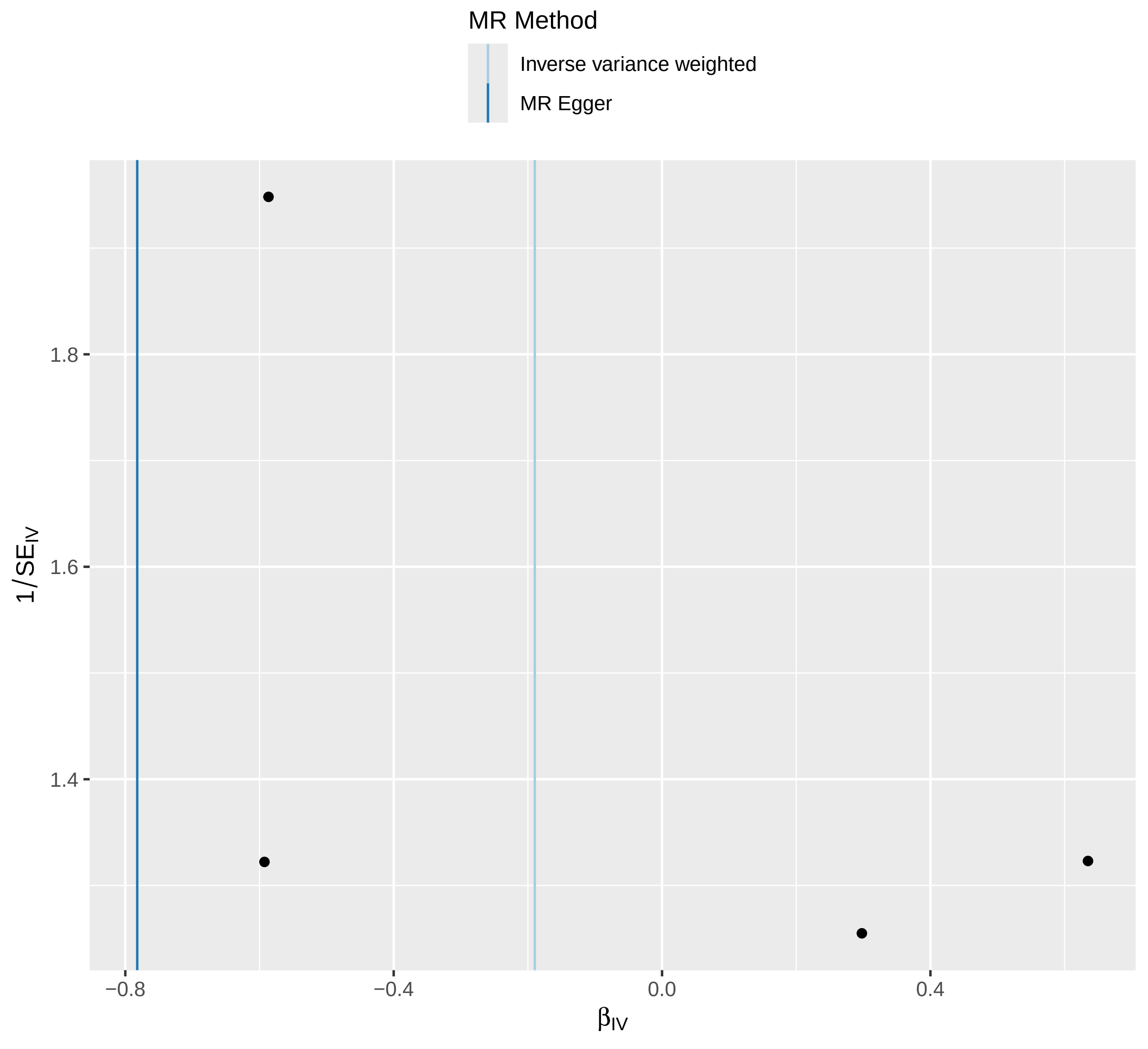
MR Method
Inverse variance weighted
MR Egger
1/SE_IV
1.8
1.6
1.4
−0.8
−0.4
0.0
0.4
β_IV

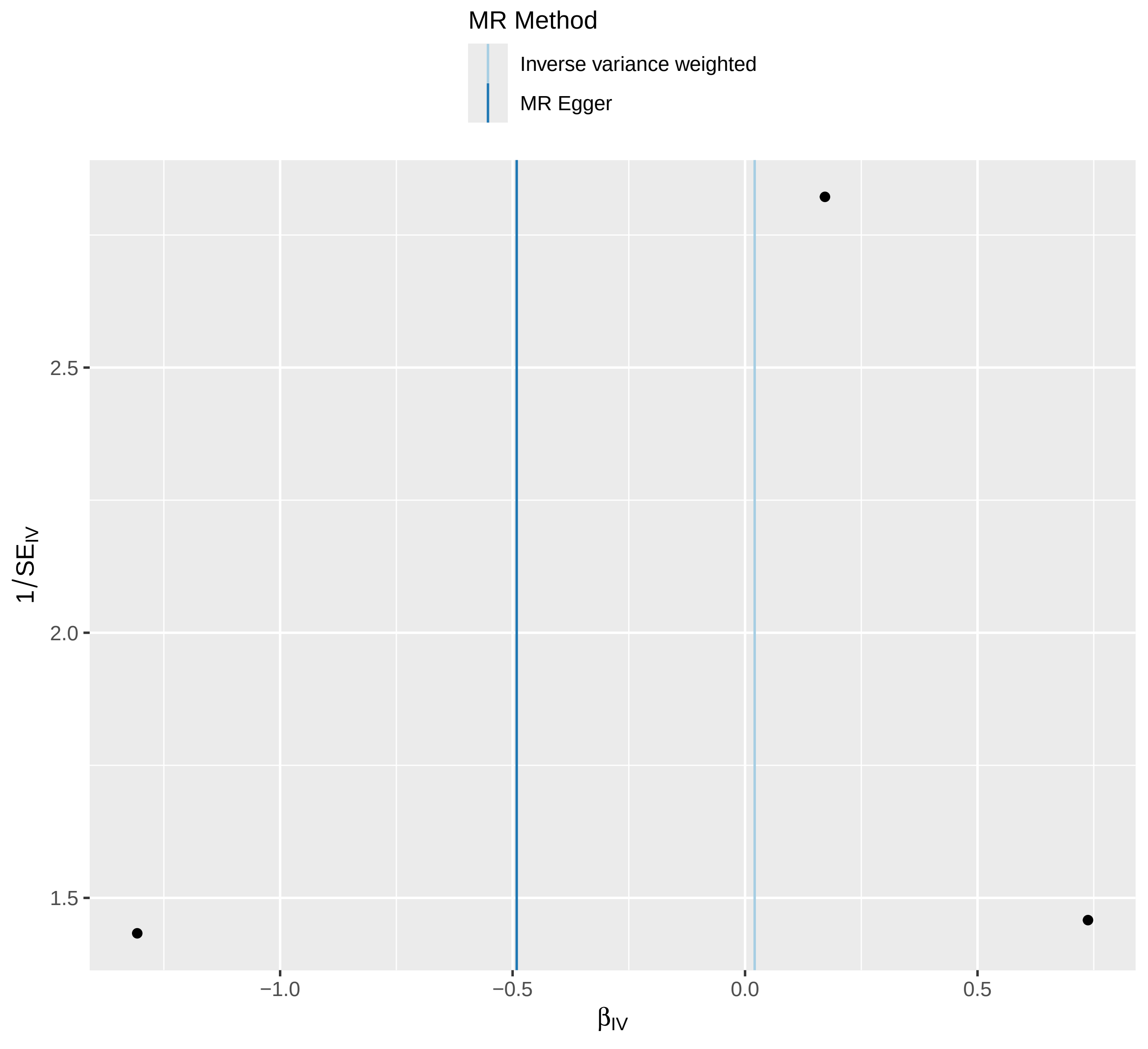

MR Method
Inverse variance weighted
MR Egger
2.5
2.0
1.5
1/SE_IV
−1.0
−0.5
0.0
0.5
β_IV

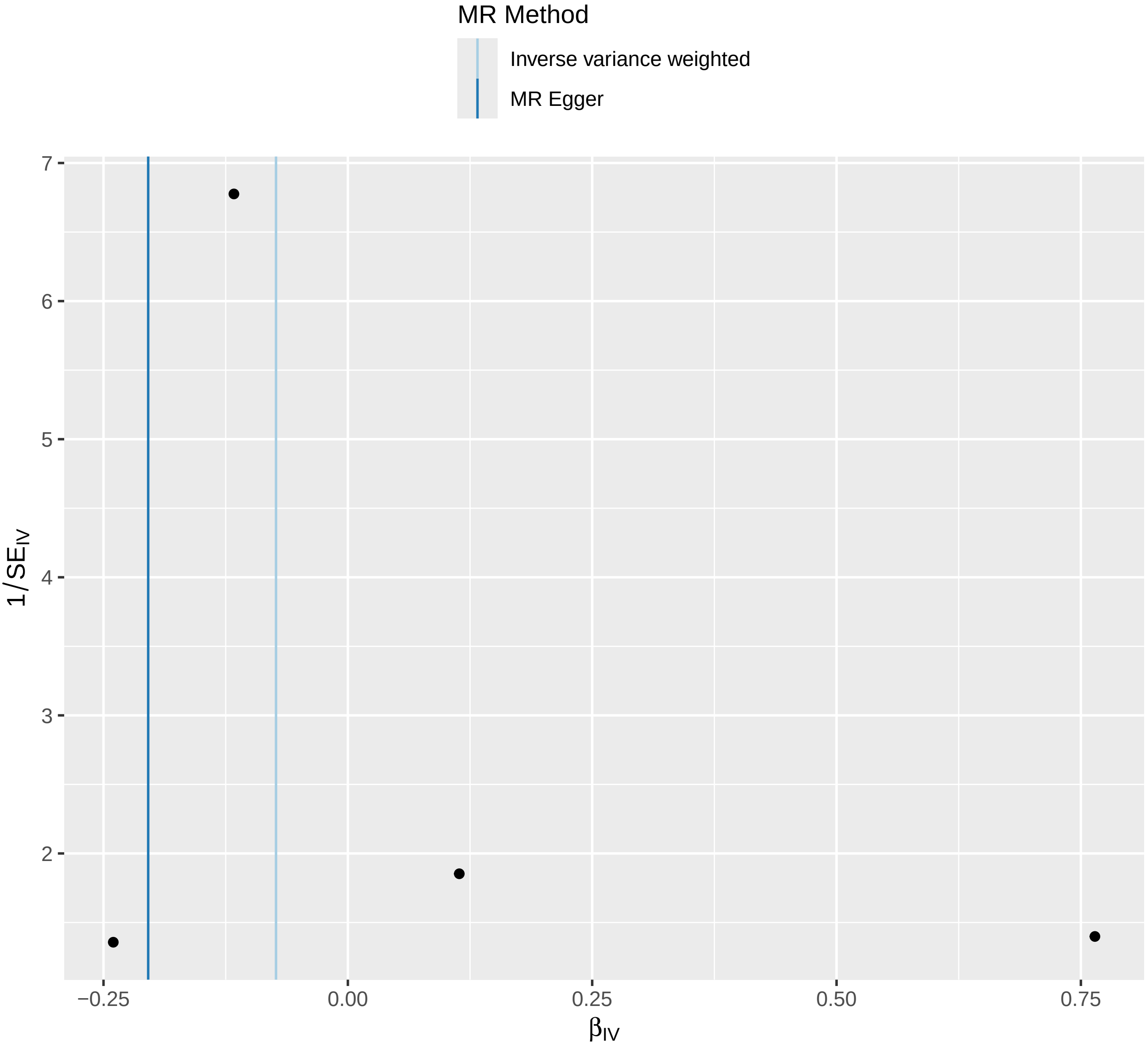

MR Method
Inverse variance weighted
MR Egger
1/SE_IV
7
6
5
4
3
2
β_IV
−0.25
0.00
0.25
0.50
0.75

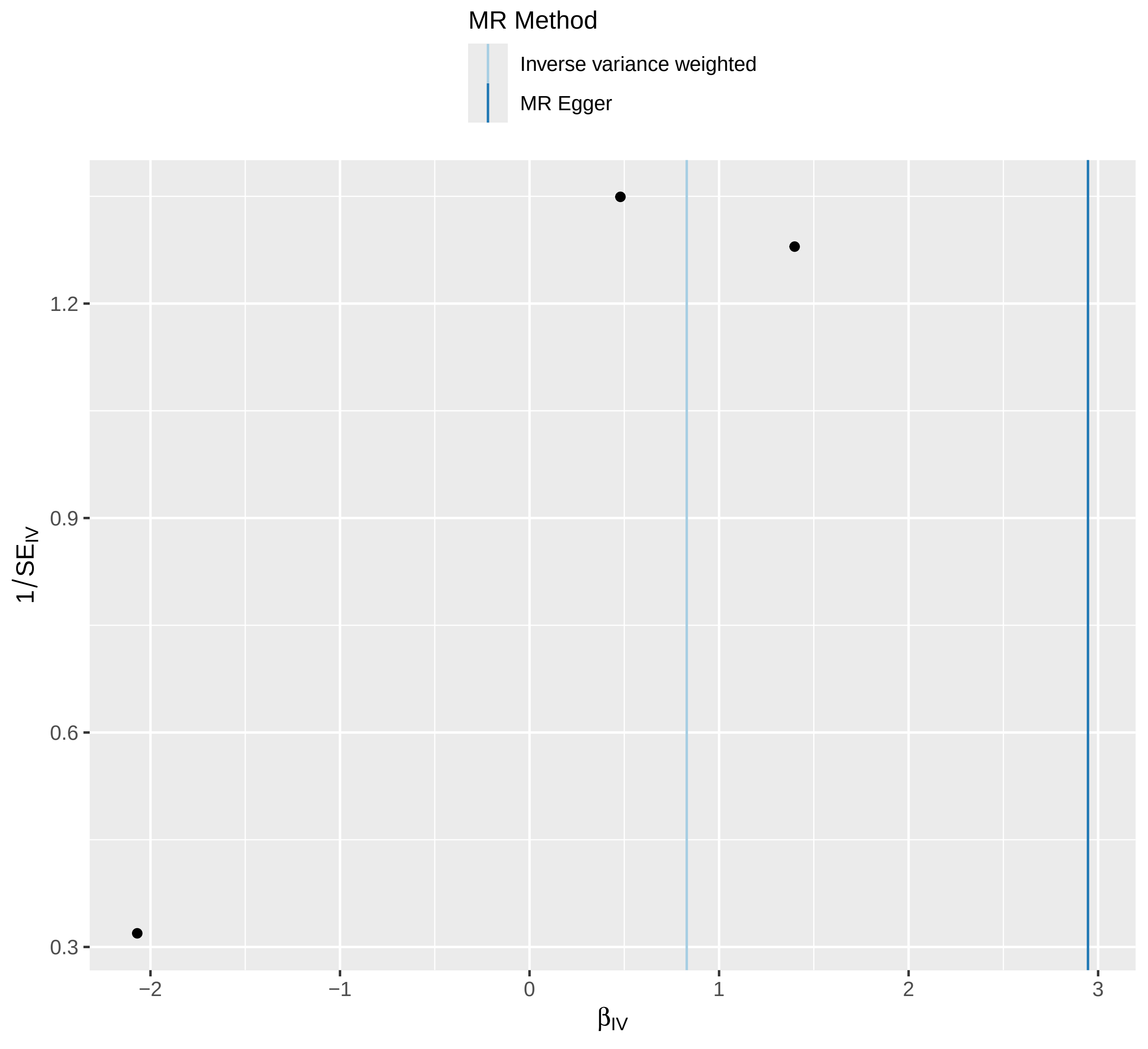
MR Method
Inverse variance weighted
MR Egger
1.2
0.9
0.6
0.3
1/SE_IV
−2
−1
0
1
2
3
β_IV

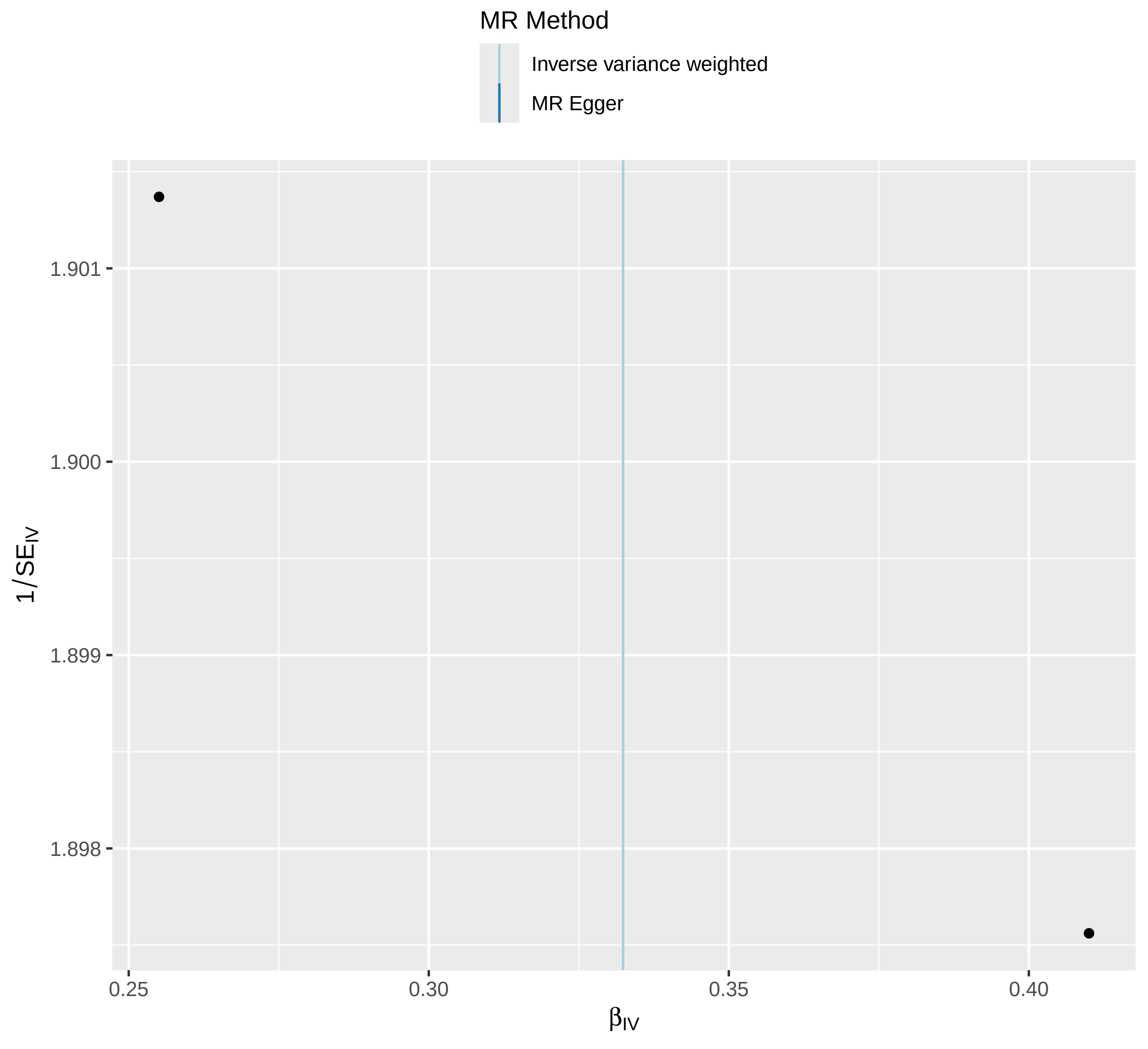
MR Method
Inverse variance weighted
MR Egger
1.901
1.900
1.899
1.898
$1/SE_{IV}$
0.25
0.30
0.35
0.40
β_{IV}

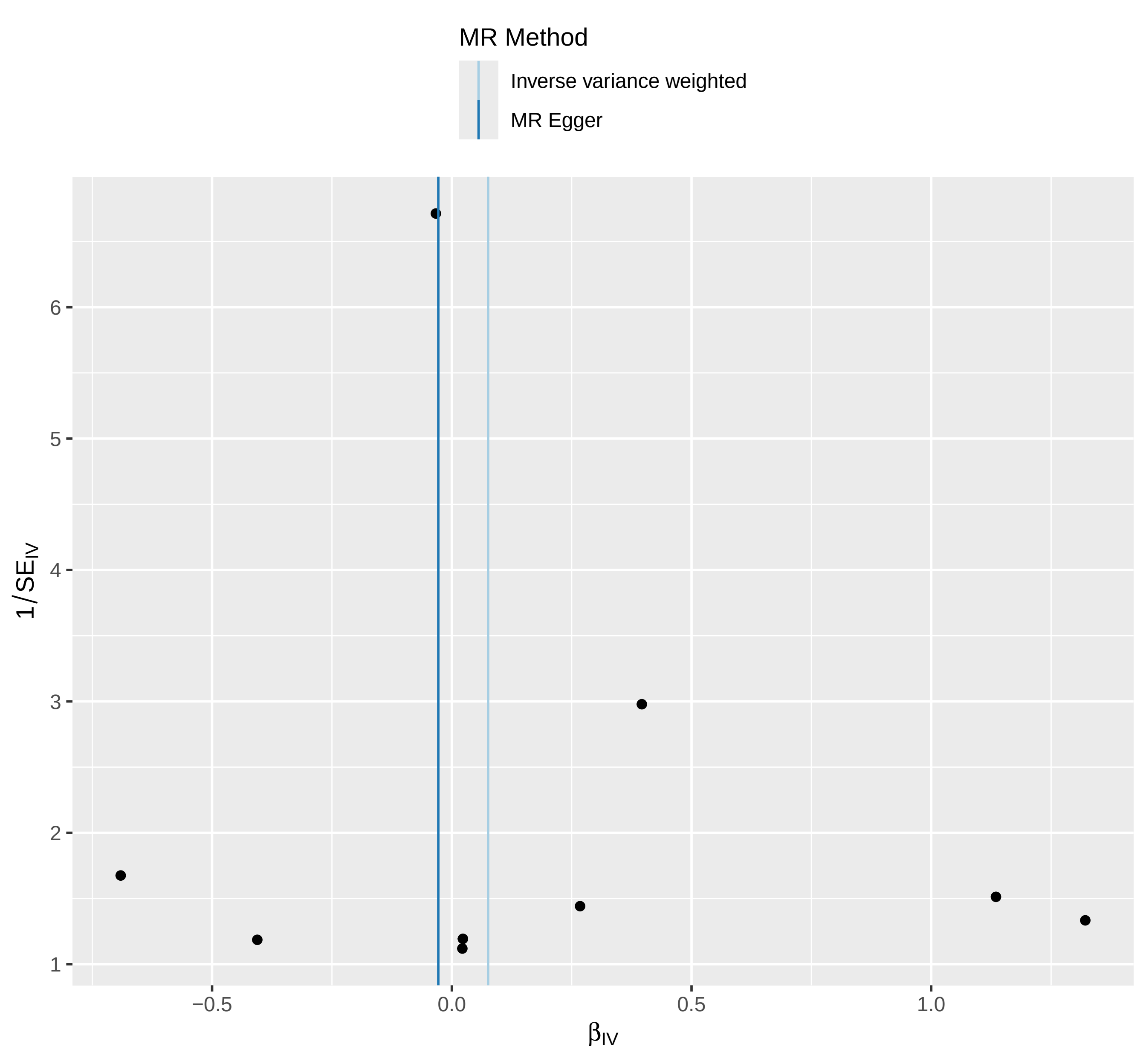
MR Method
Inverse variance weighted
MR Egger
1/SE_IV
β_IV
−0.5
0.0
0.5
1.0
1
2
3
4
5
6

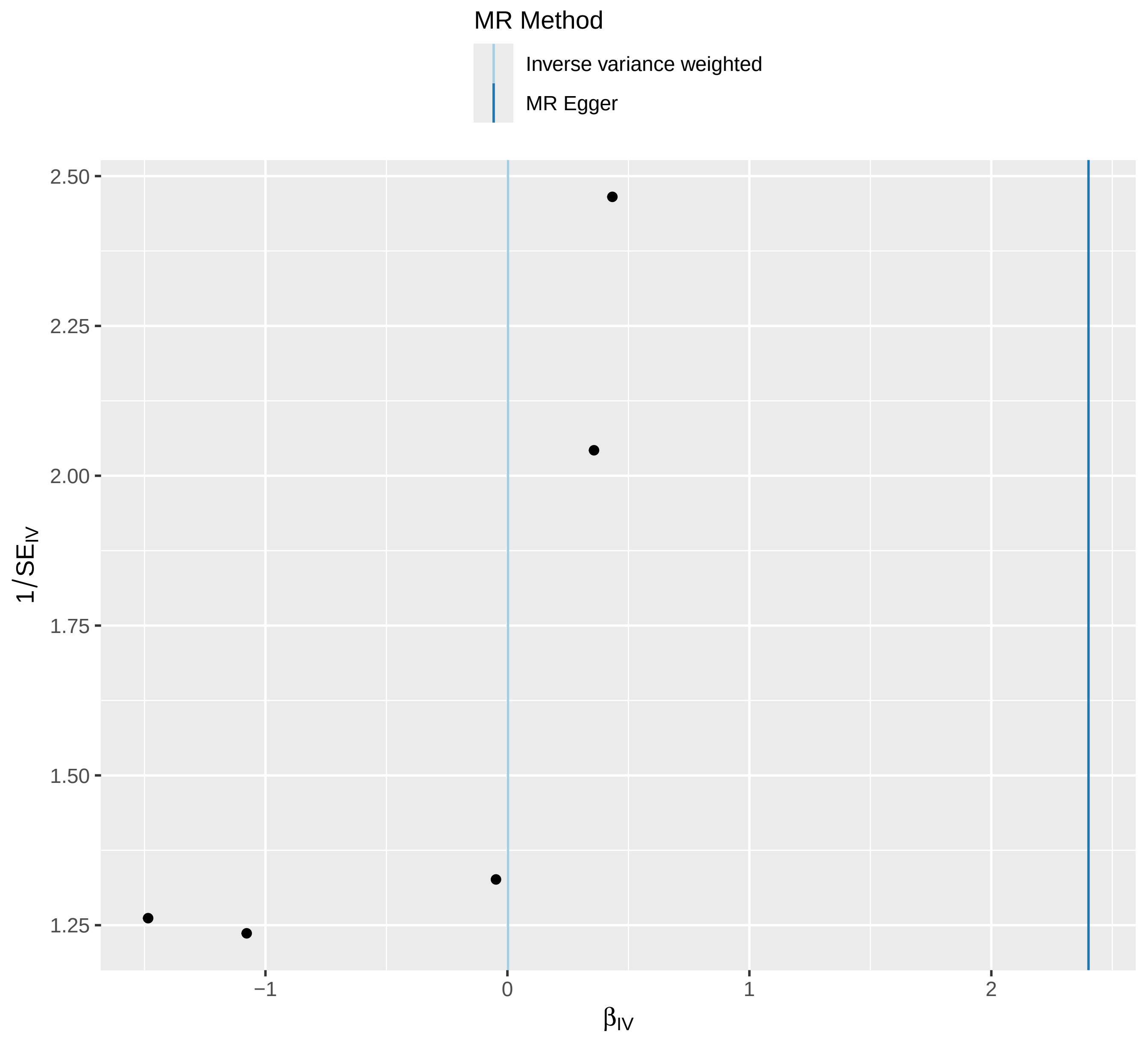
MR Method
Inverse variance weighted
MR Egger
2.50
2.25
2.00
1.75
1.50
1.25
1/SE_IV
−1
0
1
2
β_IV

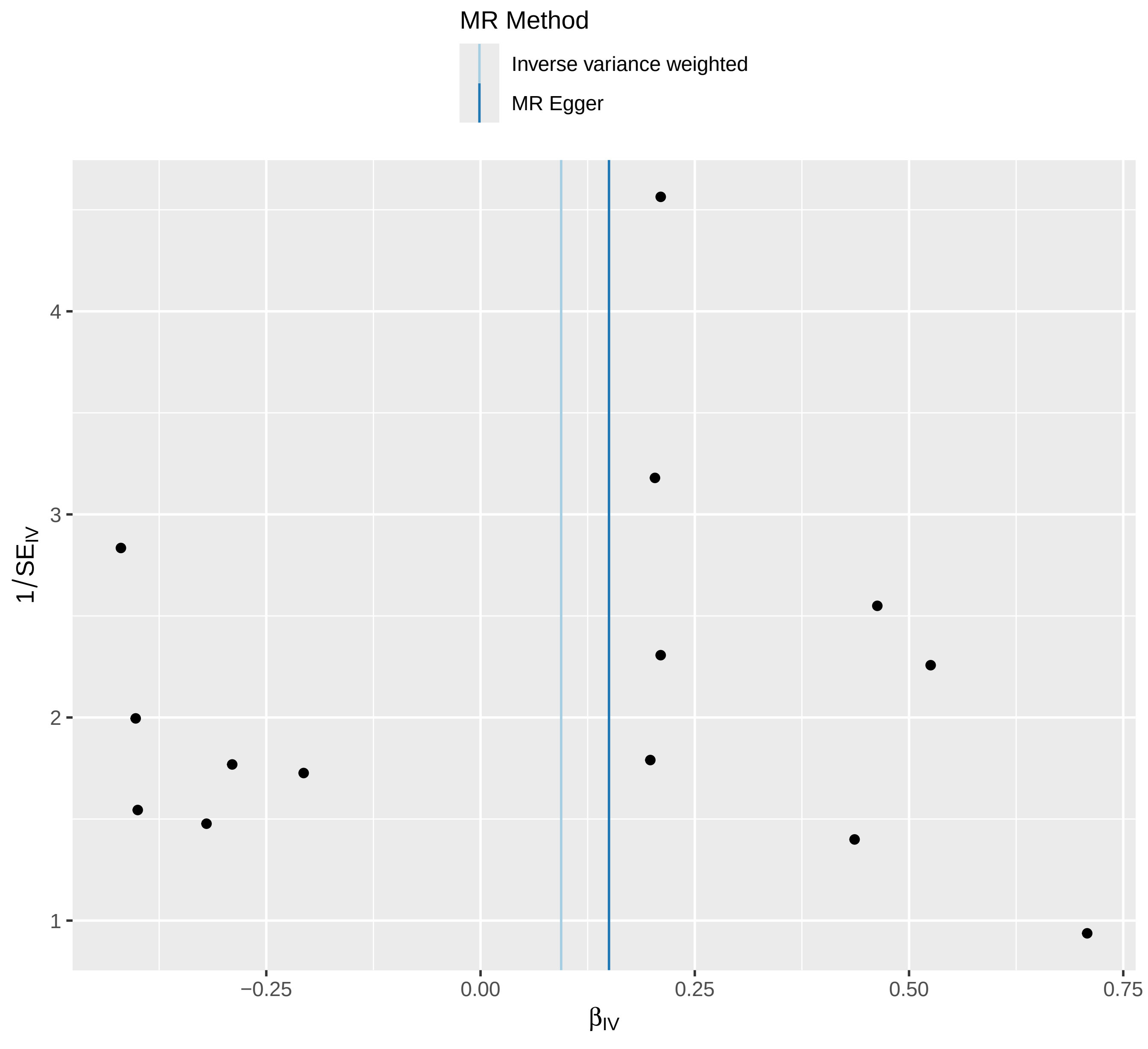
MR Method
Inverse variance weighted
MR Egger
$1/SE_{IV}$
4
3
2
1
−0.25
0.00
0.25
0.50
0.75
β_{IV}

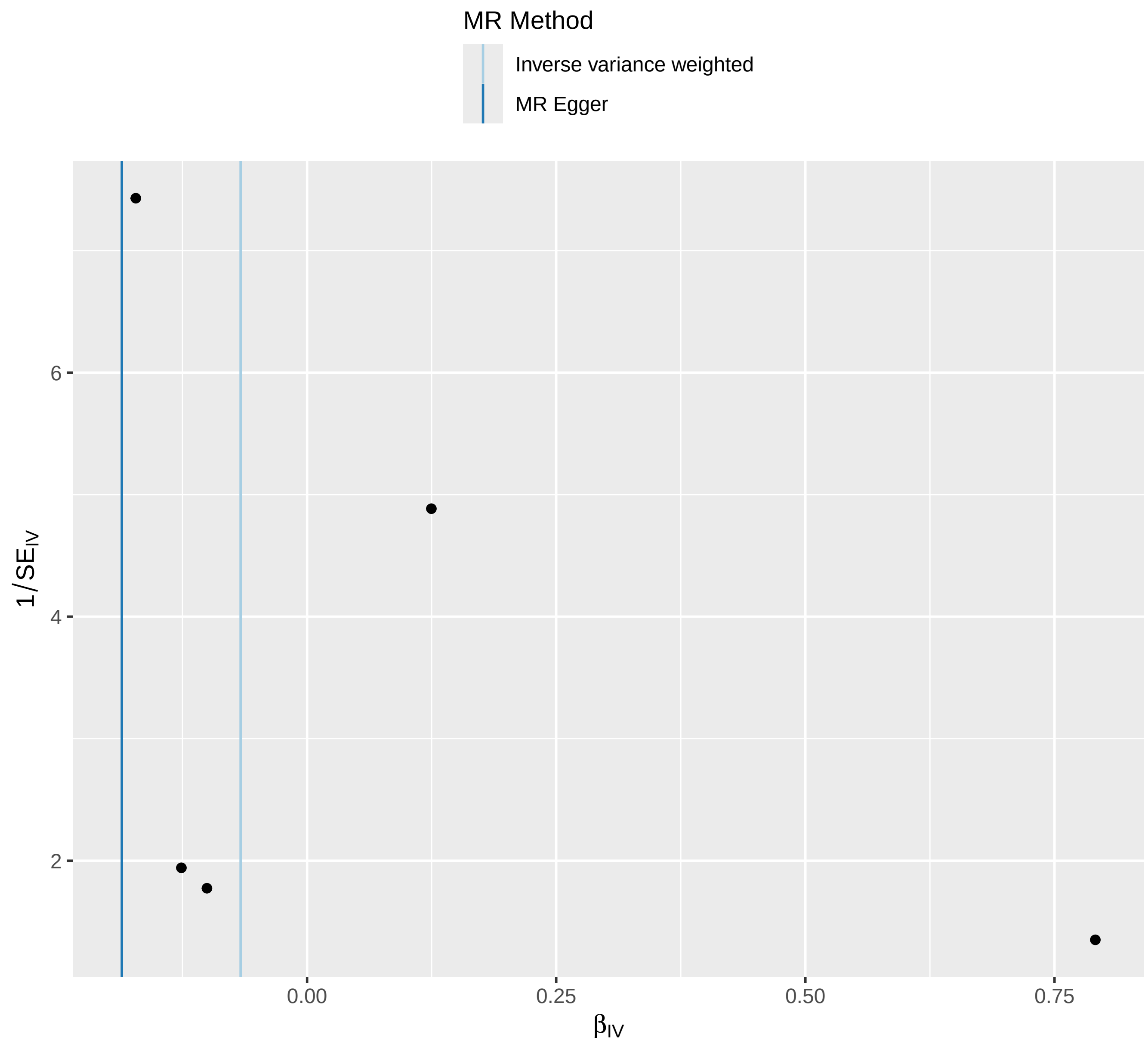

MR Method
Inverse variance weighted
MR Egger
6
4
2
1/SE_IV
0.00
0.25
0.50
0.75
β_IV

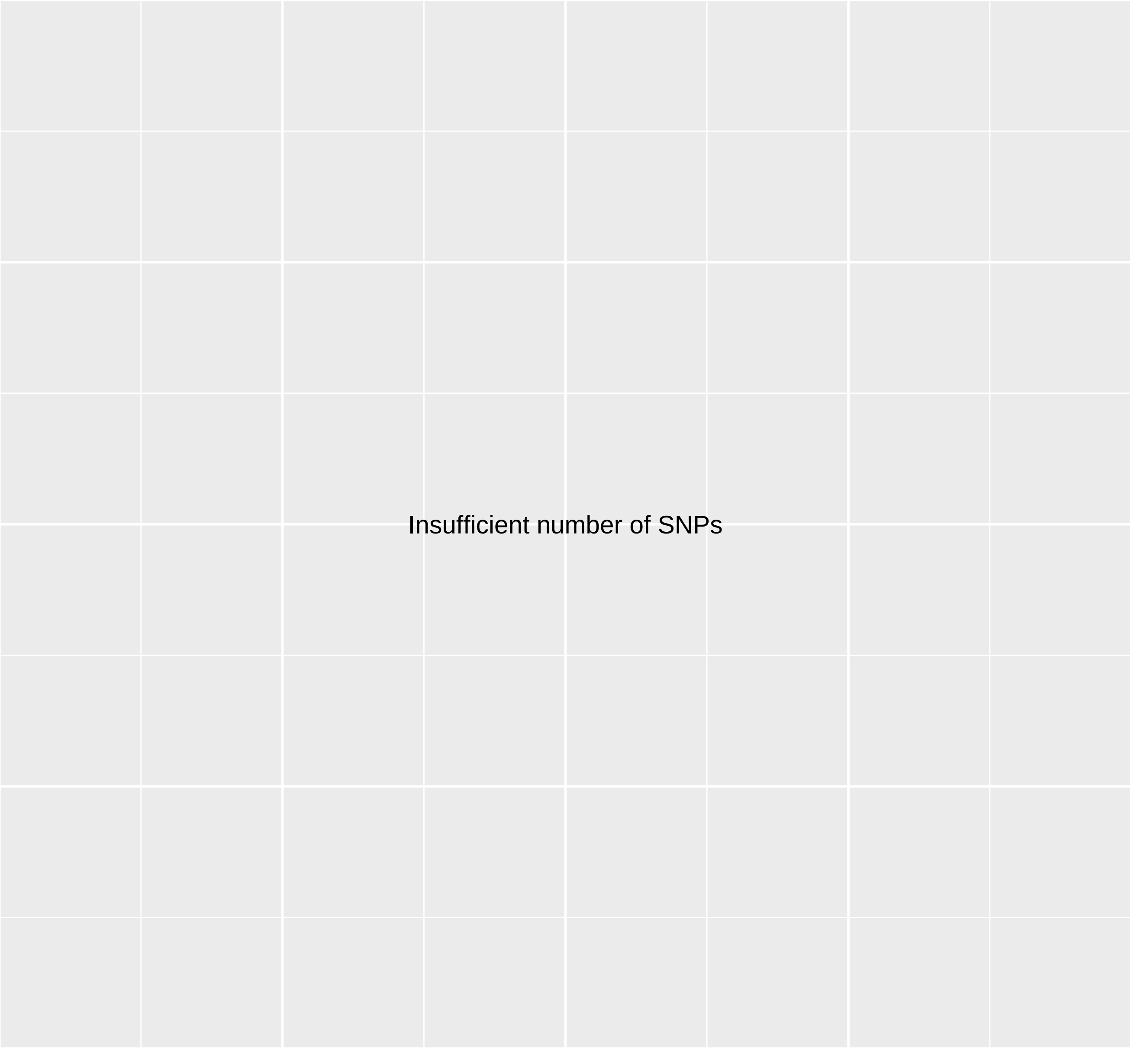
Insufficient number of SNPs

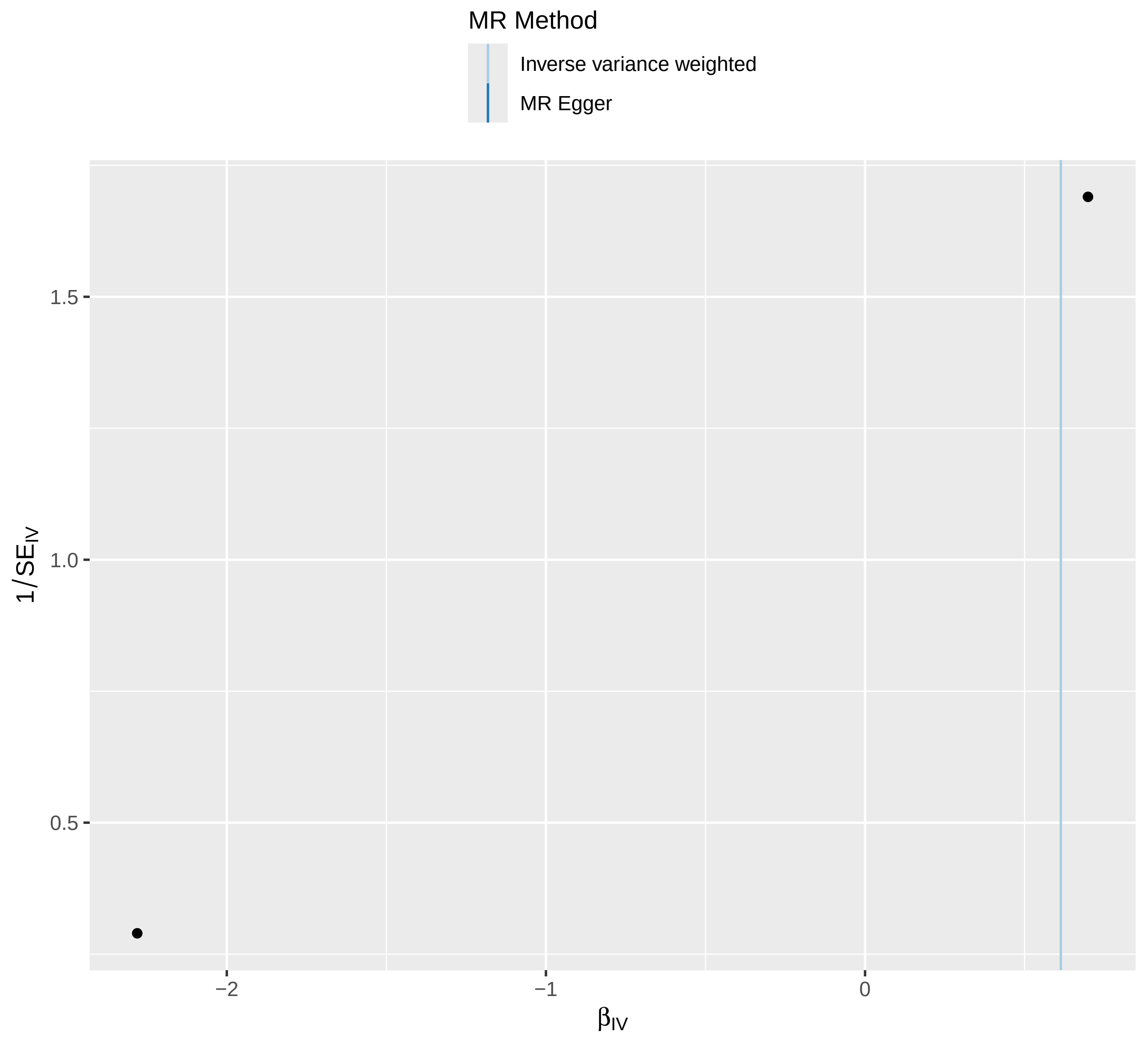
MR Method
Inverse variance weighted
MR Egger
1.5
1.0
0.5
−2
−1
0
$1/SE_{IV}$
β_{IV}

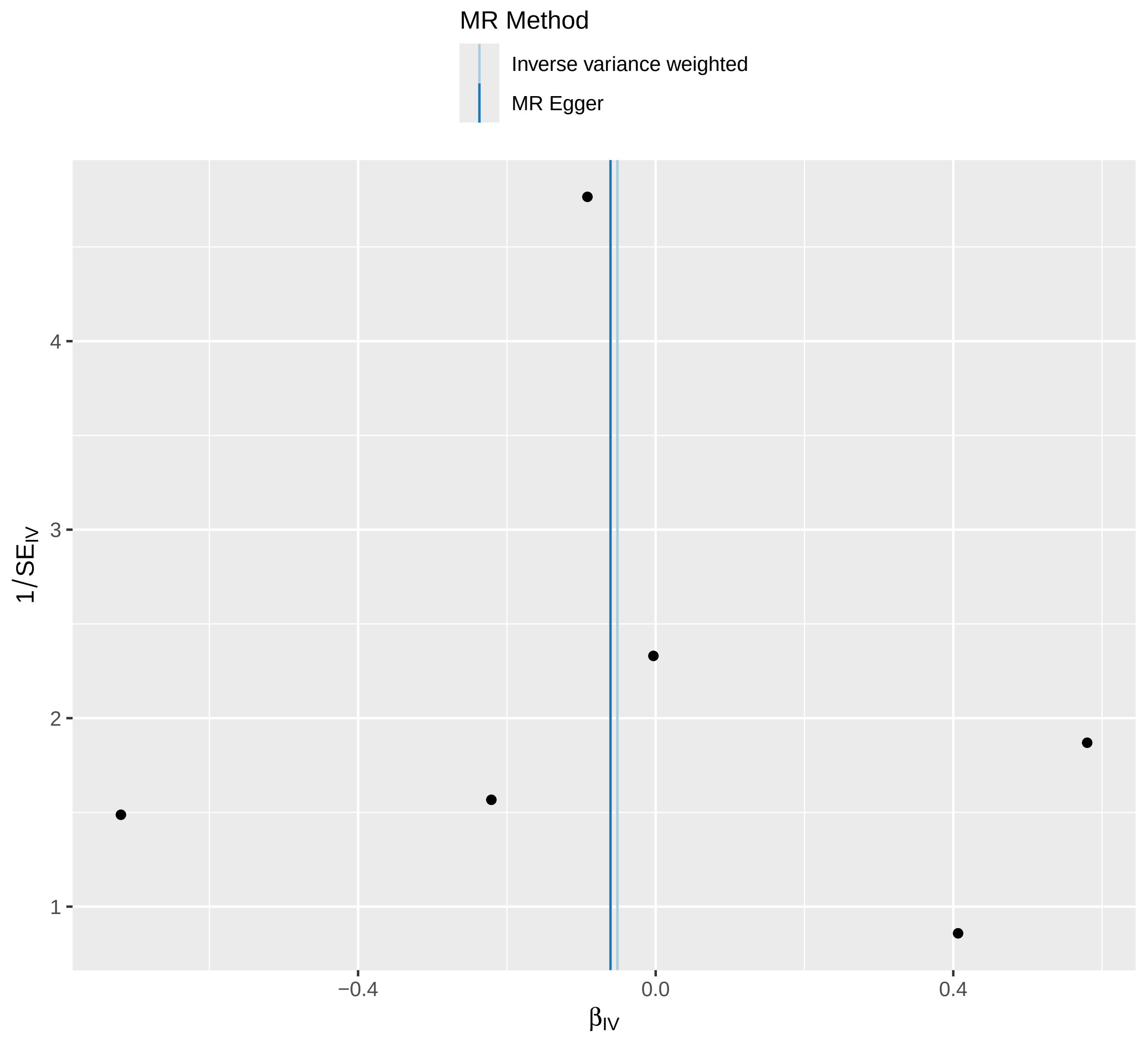

MR Method
Inverse variance weighted
MR Egger
1/SE_IV
4
3
2
1
−0.4
0.0
0.4
β_IV

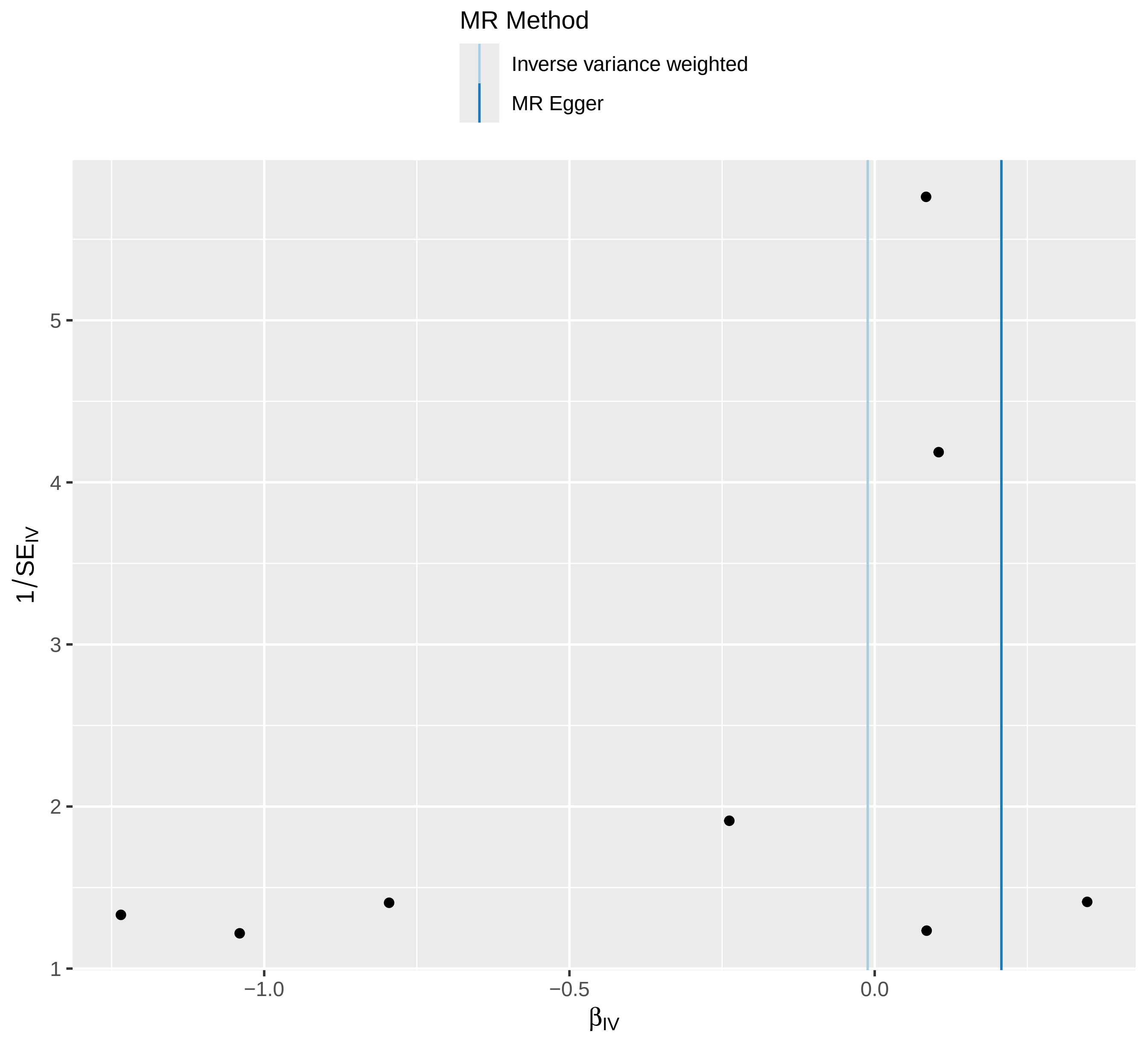

MR Method
Inverse variance weighted
MR Egger
1/SE_IV
5
4
3
2
1
−1.0
−0.5
0.0
β_IV

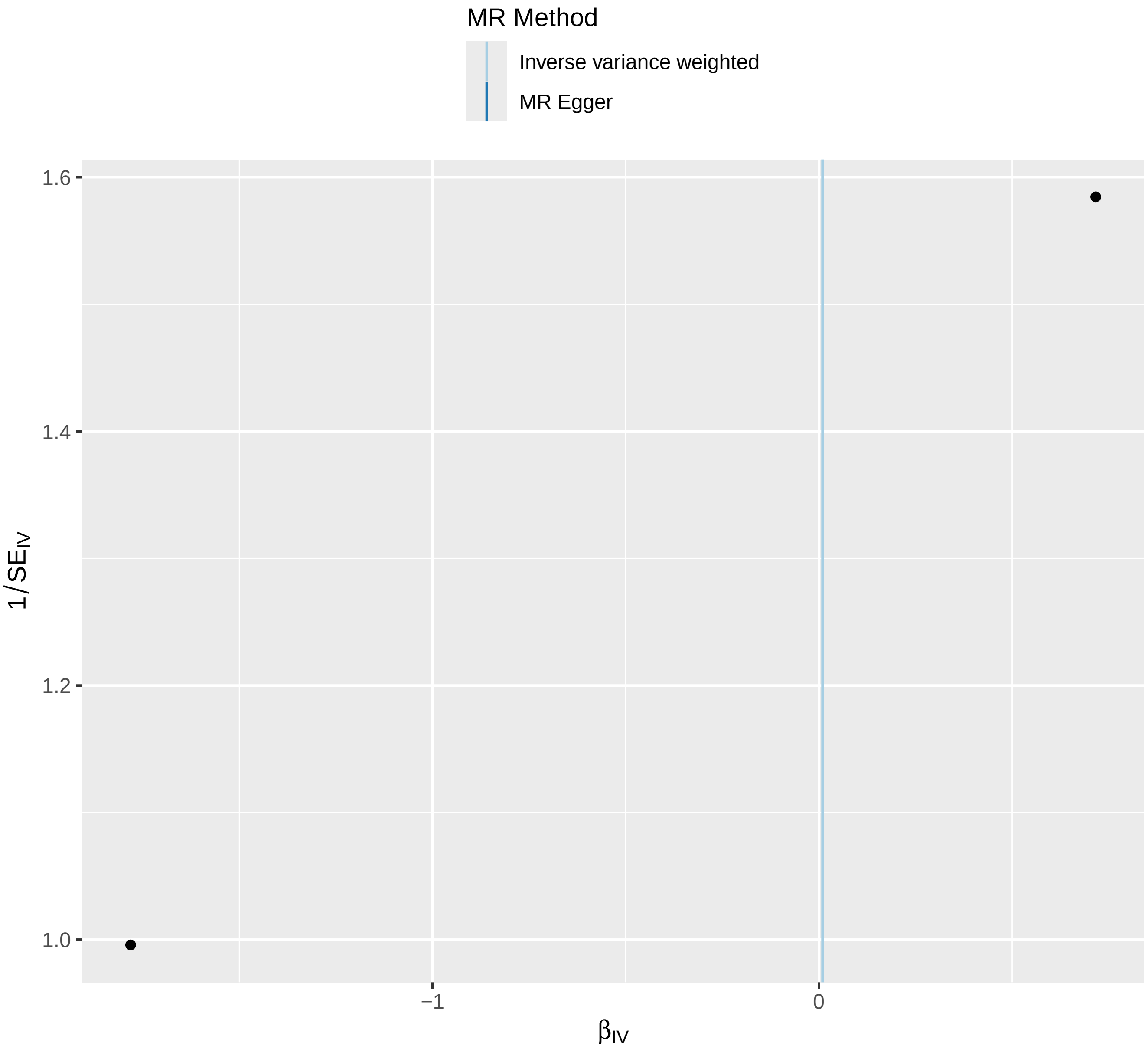
MR Method
Inverse variance weighted
MR Egger
1.6
1.4
1.2
1.0
1/SE$_{IV}$
−1
0
β$_{IV}$

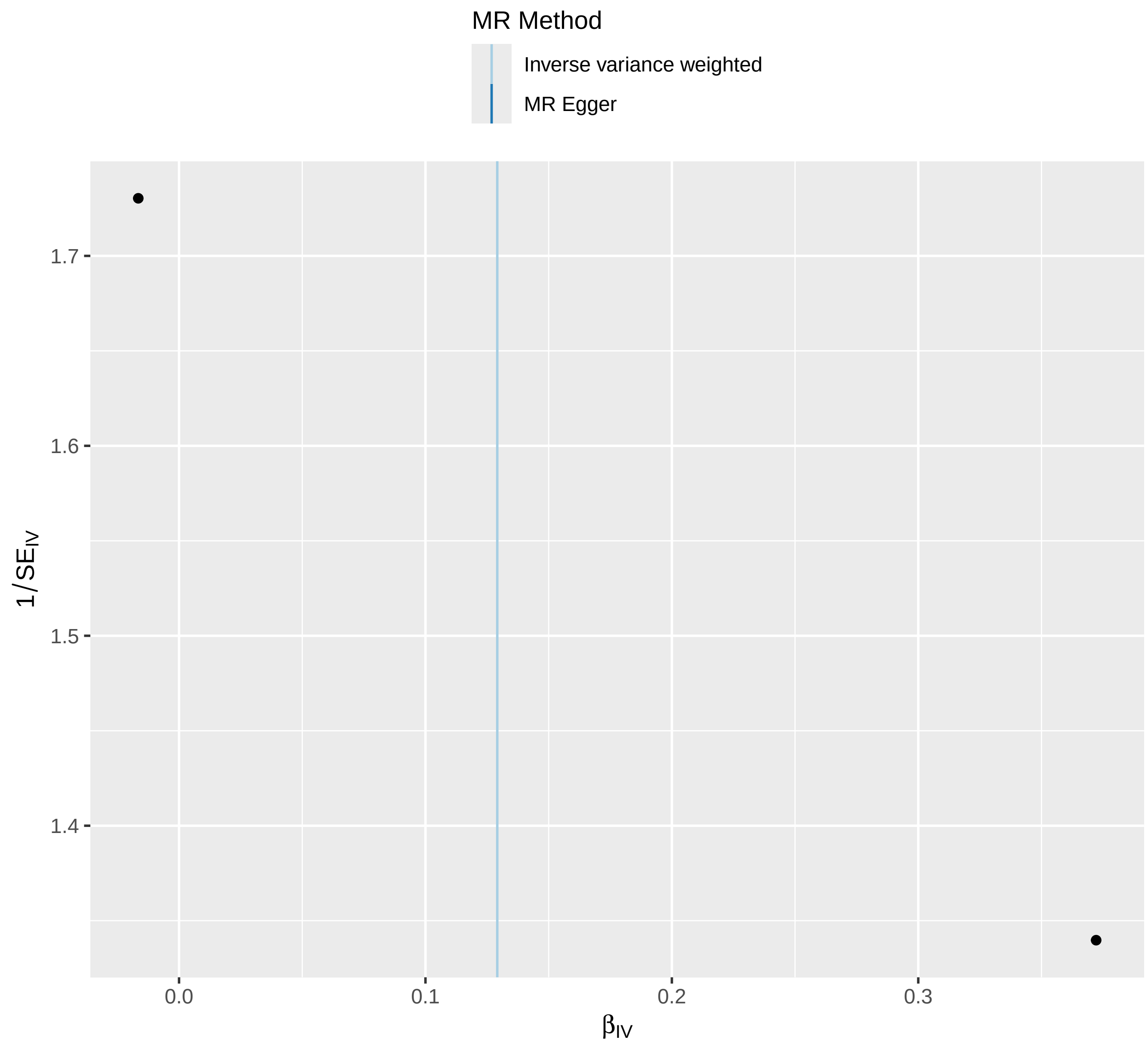

MR Method
Inverse variance weighted
MR Egger
1.7
1.6
1.5
1.4
1/SE_IV
0.0
0.1
0.2
0.3
β_IV

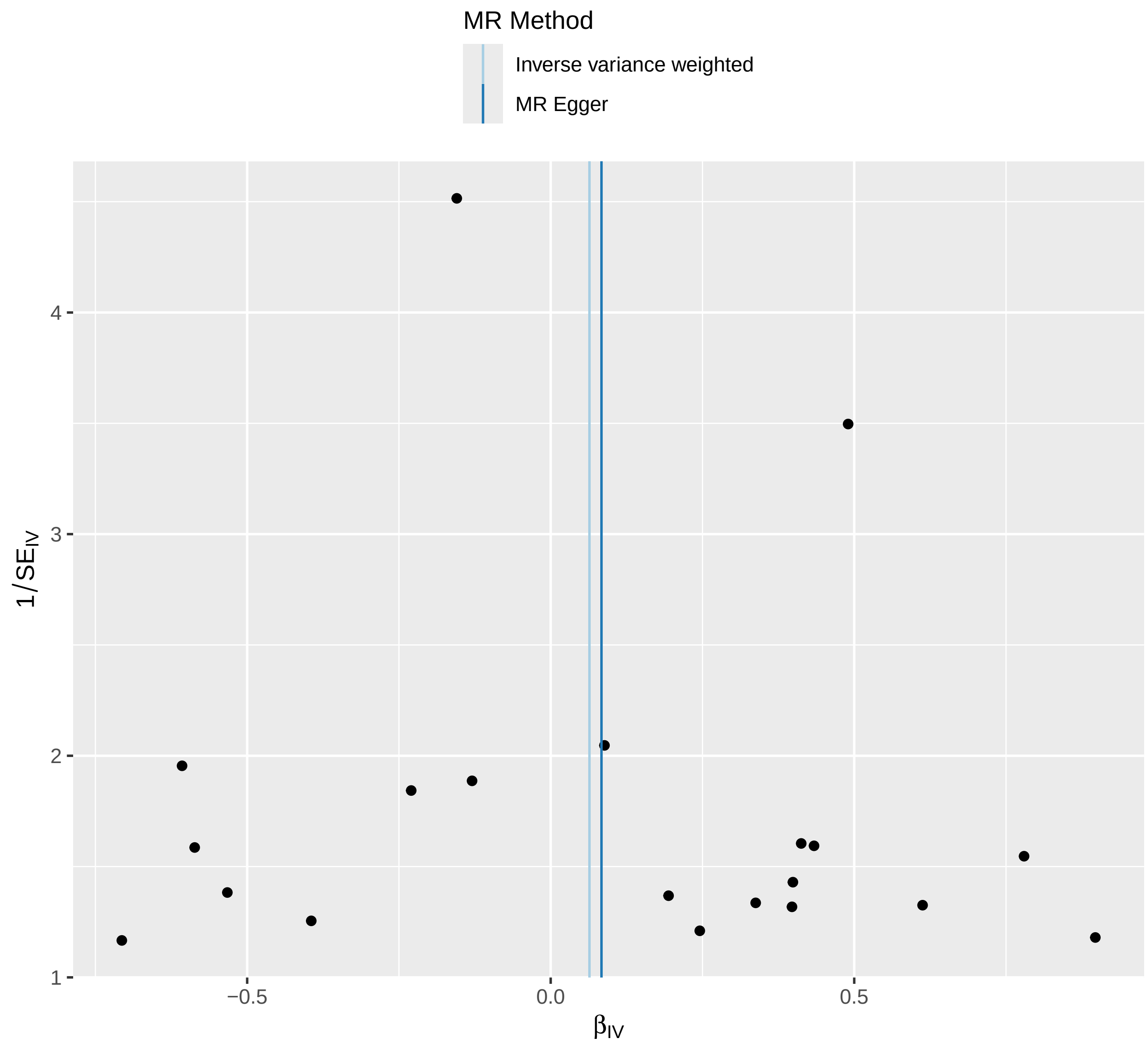
MR Method
Inverse variance weighted
MR Egger
4
3
2
1
1/SE_IV
−0.5
0.0
0.5
β_IV

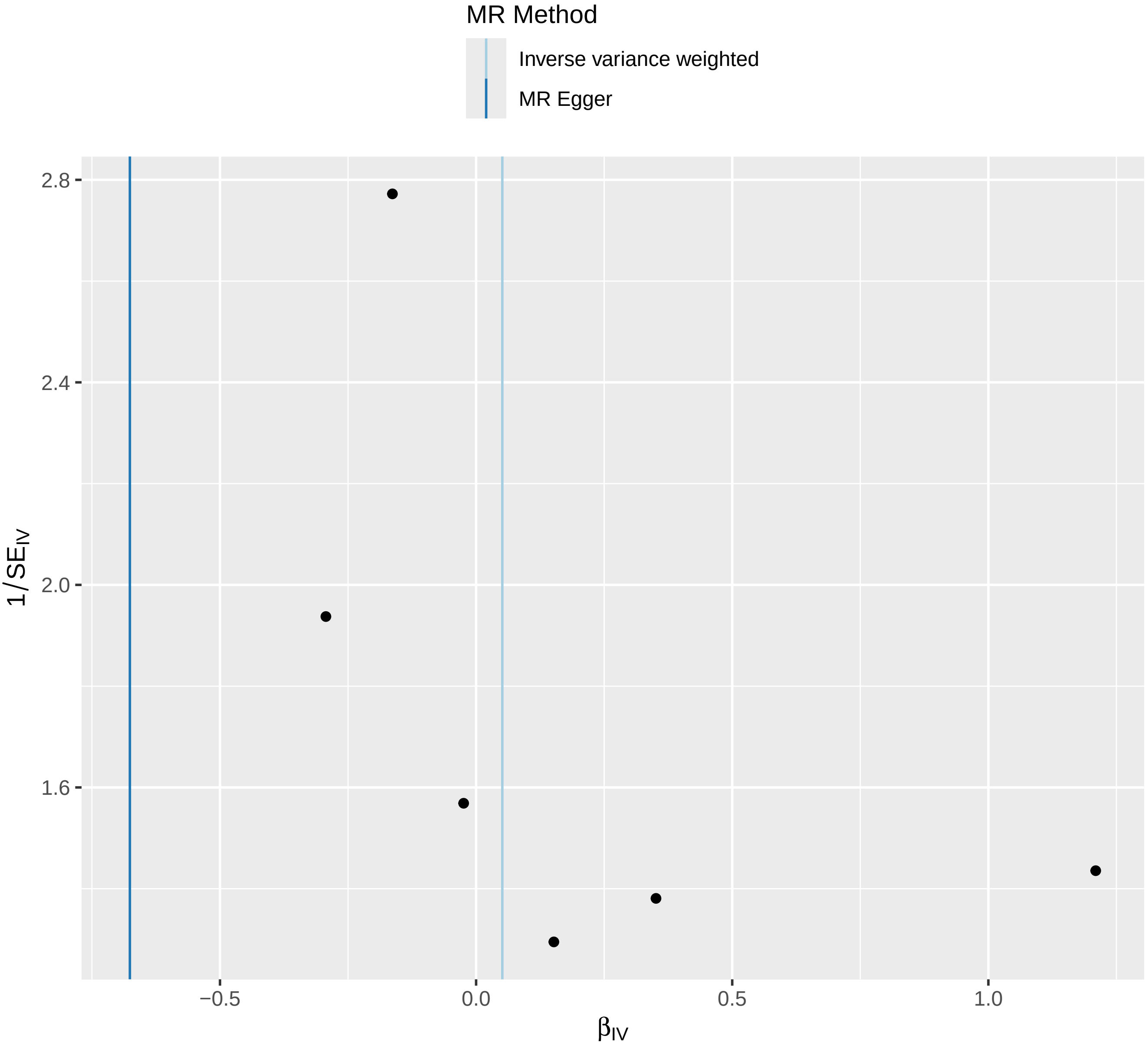
MR Method
Inverse variance weighted
MR Egger
2.8
2.4
2.0
1.6
1/SE_IV
−0.5
0.0
0.5
1.0
β_IV

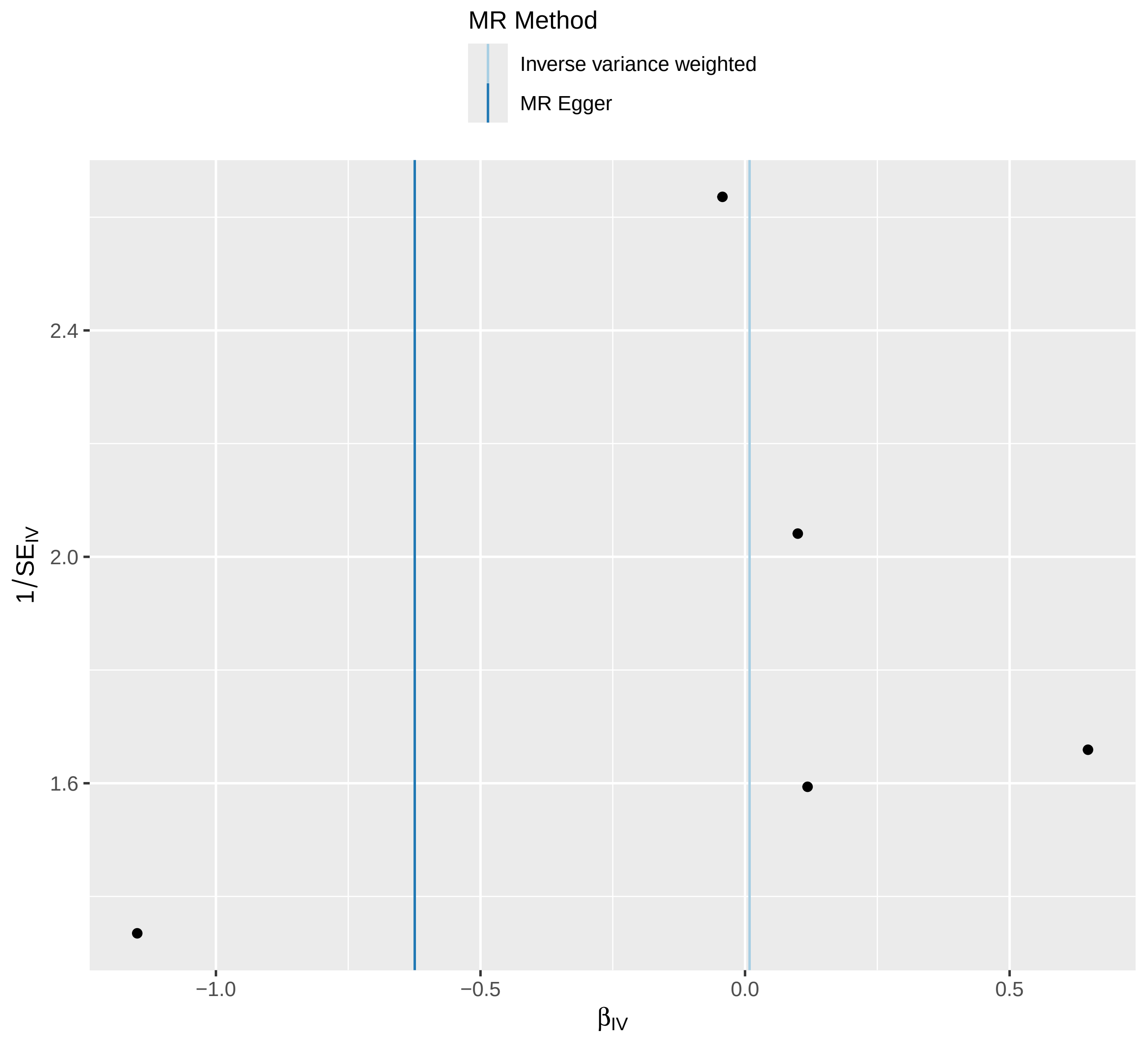
MR Method
Inverse variance weighted
MR Egger
2.4
2.0
1.6
1/SE$_{IV}$
−1.0
−0.5
0.0
0.5
β$_{IV}$

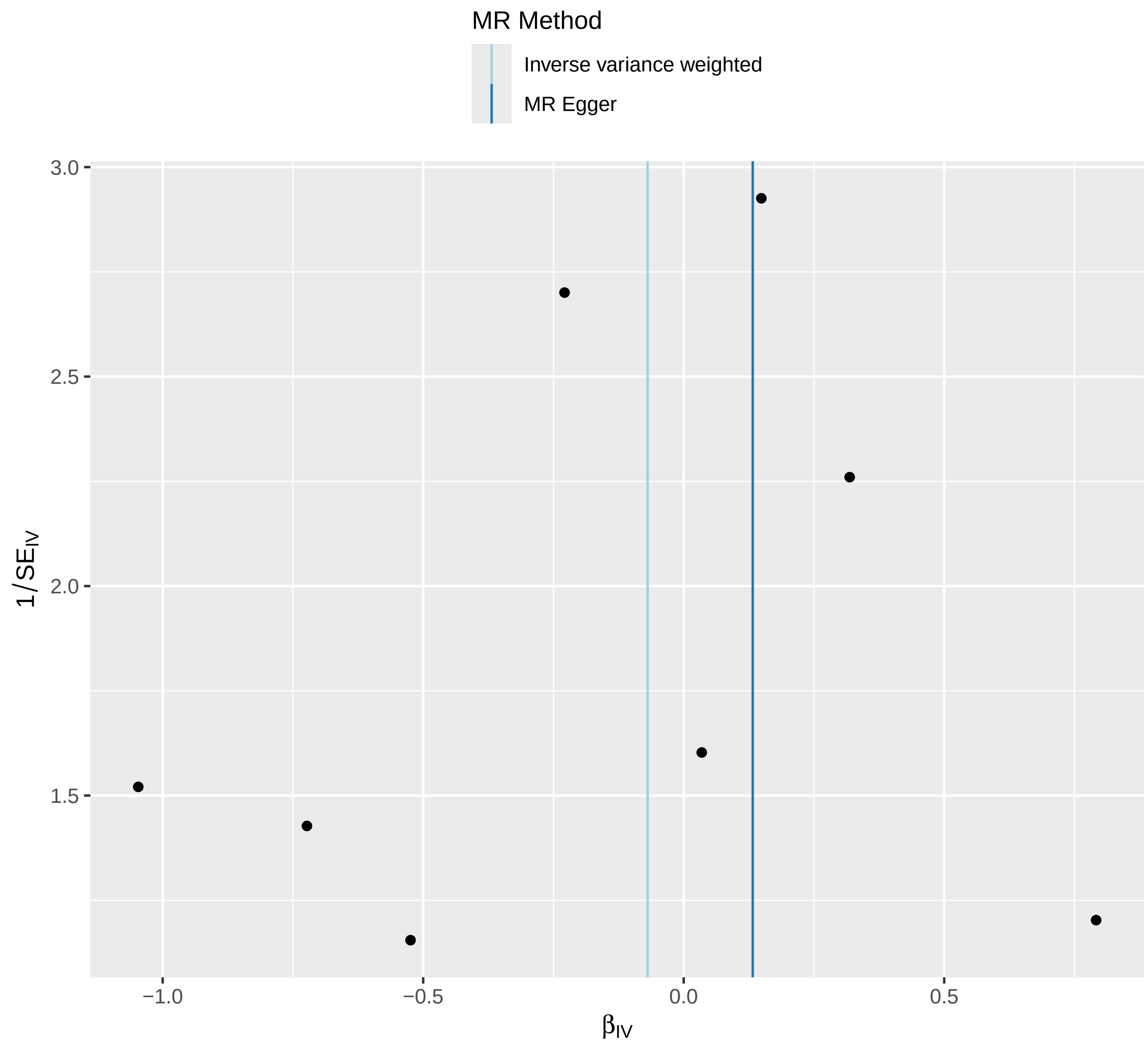

MR Method
Inverse variance weighted
MR Egger
3.0
2.5
2.0
1.5
1/SE_IV
−1.0
−0.5
0.0
0.5
β_IV

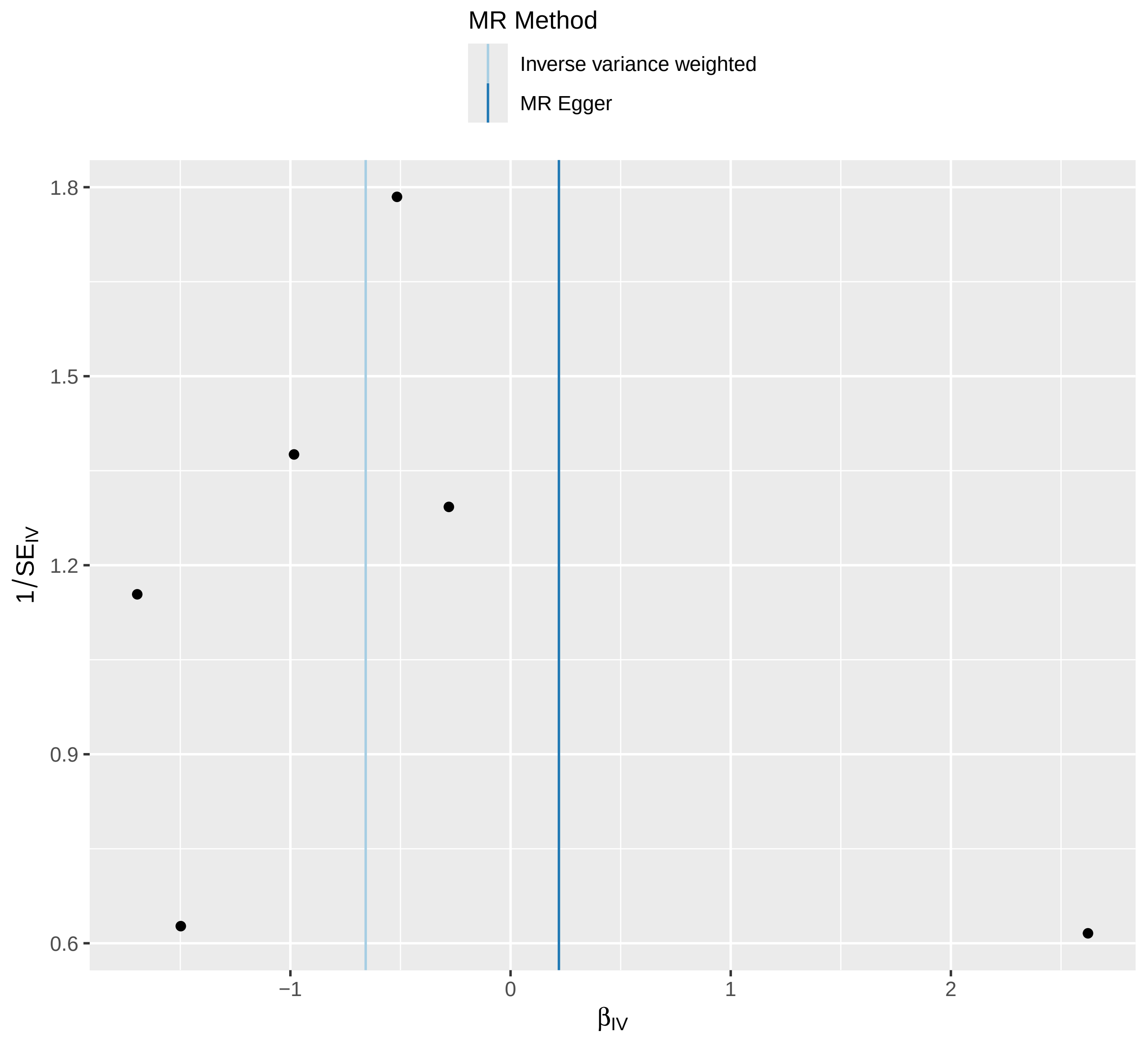

MR Method
Inverse variance weighted
MR Egger
1.8
1.5
1.2
0.9
0.6
1/SE_IV
−1
0
1
2
β_IV

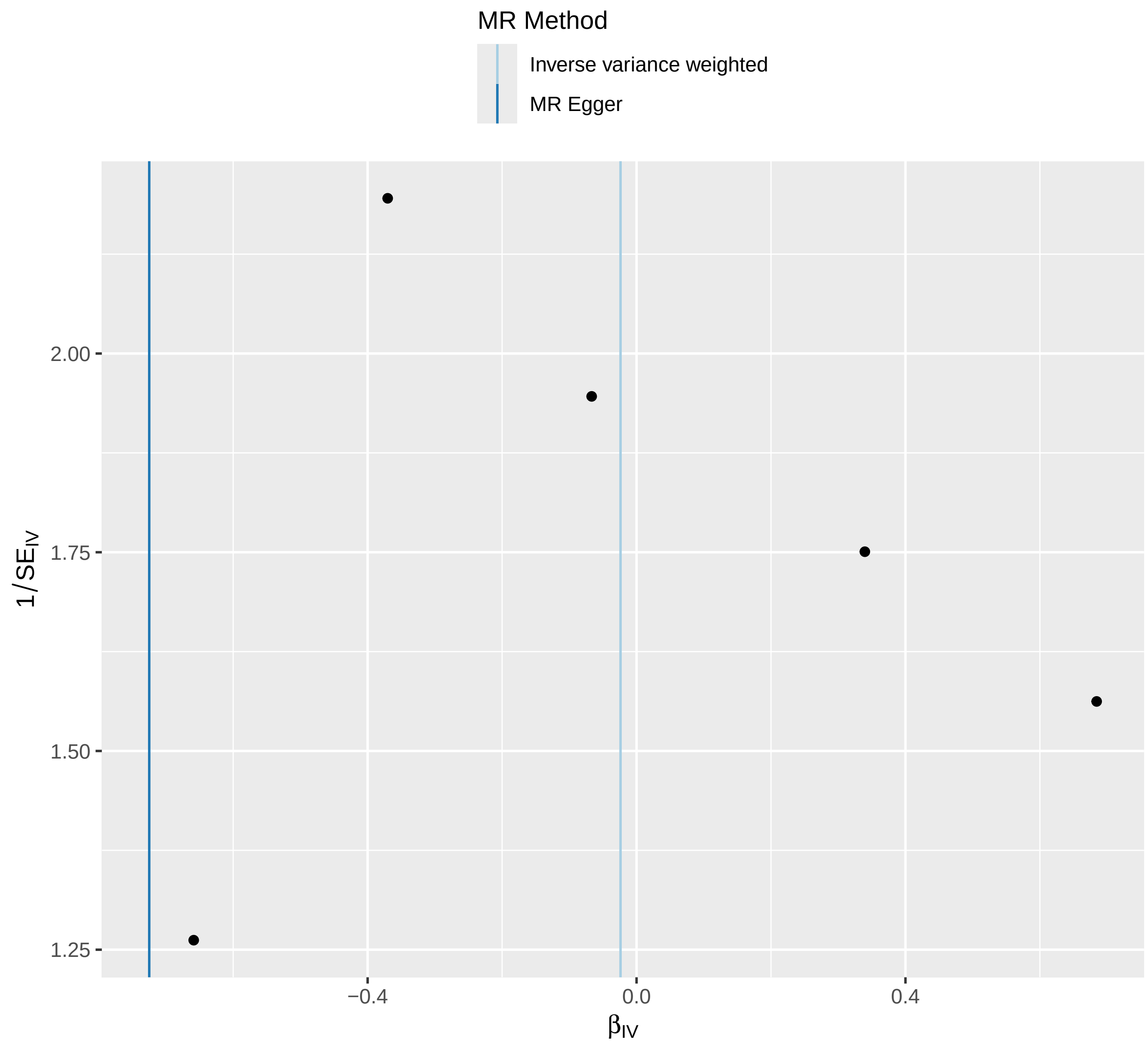
MR Method
Inverse variance weighted
MR Egger
2.00
1.75
1.50
1.25
1/SE_IV
−0.4
0.0
0.4
β_IV

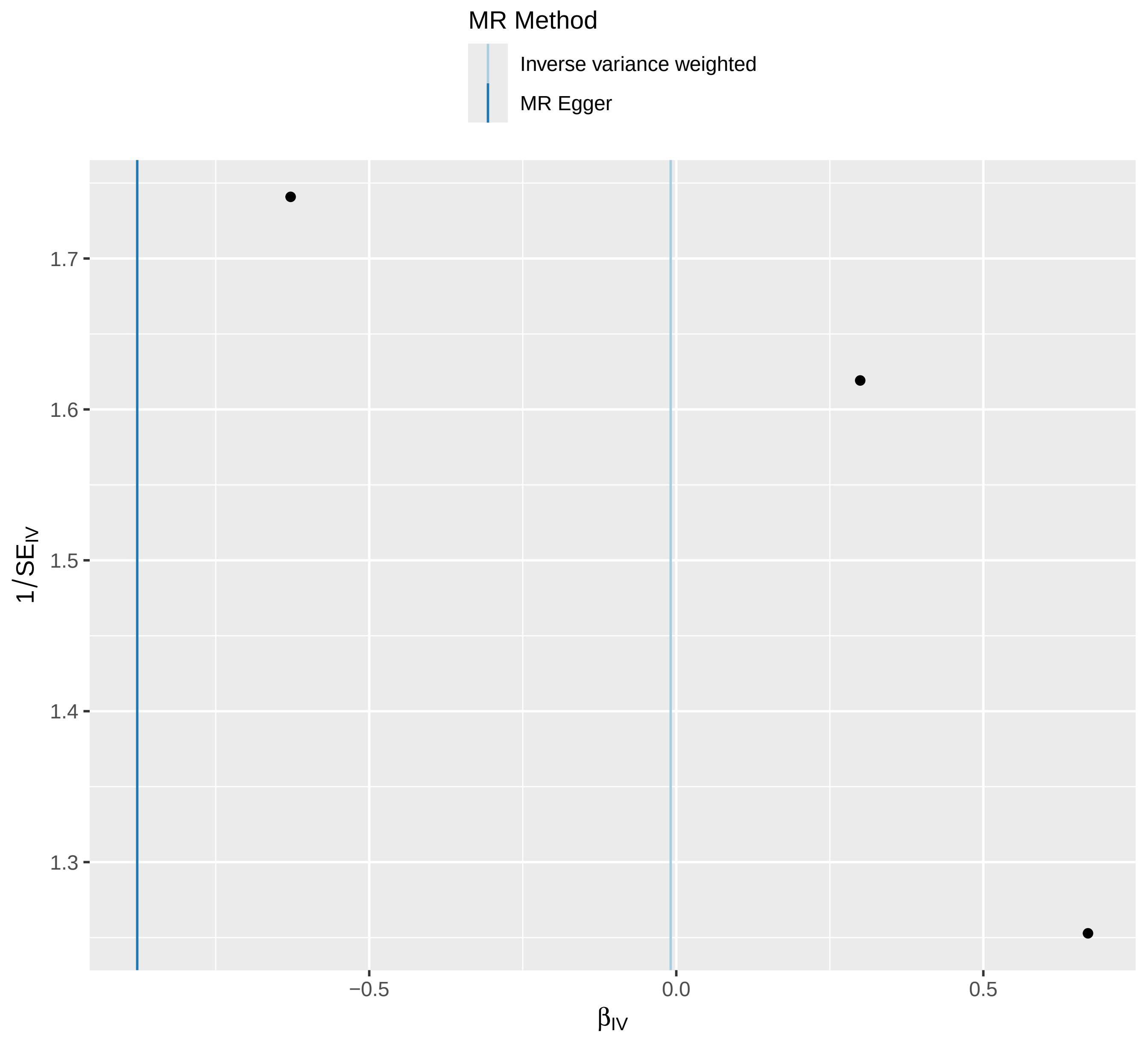
MR Method
Inverse variance weighted
MR Egger
1.7
1.6
1.5
1.4
1.3
$1/SE_{IV}$
−0.5
0.0
0.5
β_{IV}

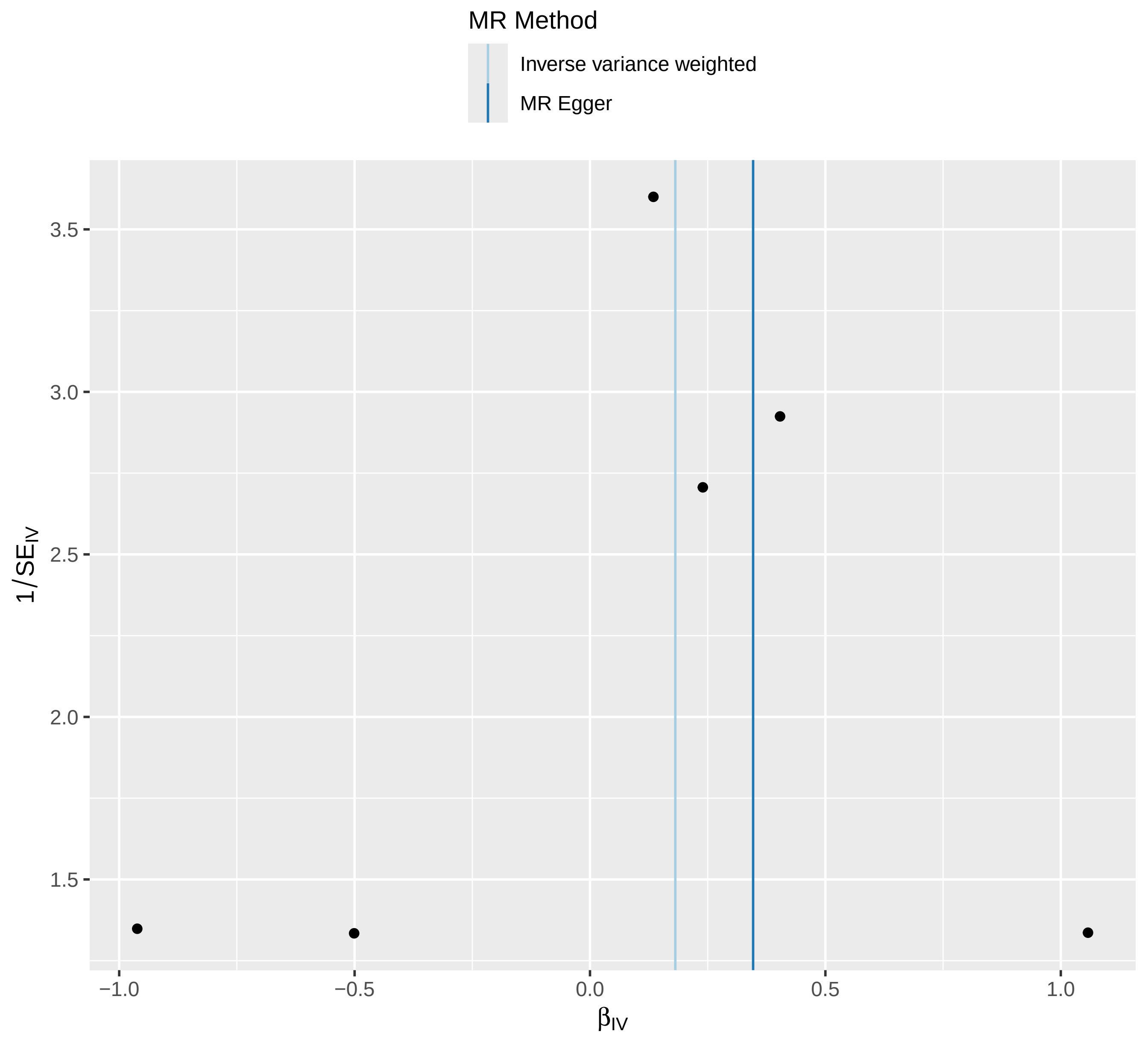
MR Method
Inverse variance weighted
MR Egger
3.5
3.0
2.5
2.0
1.5
$1/SE_{IV}$
−1.0
−0.5
0.0
0.5
1.0
β_{IV}

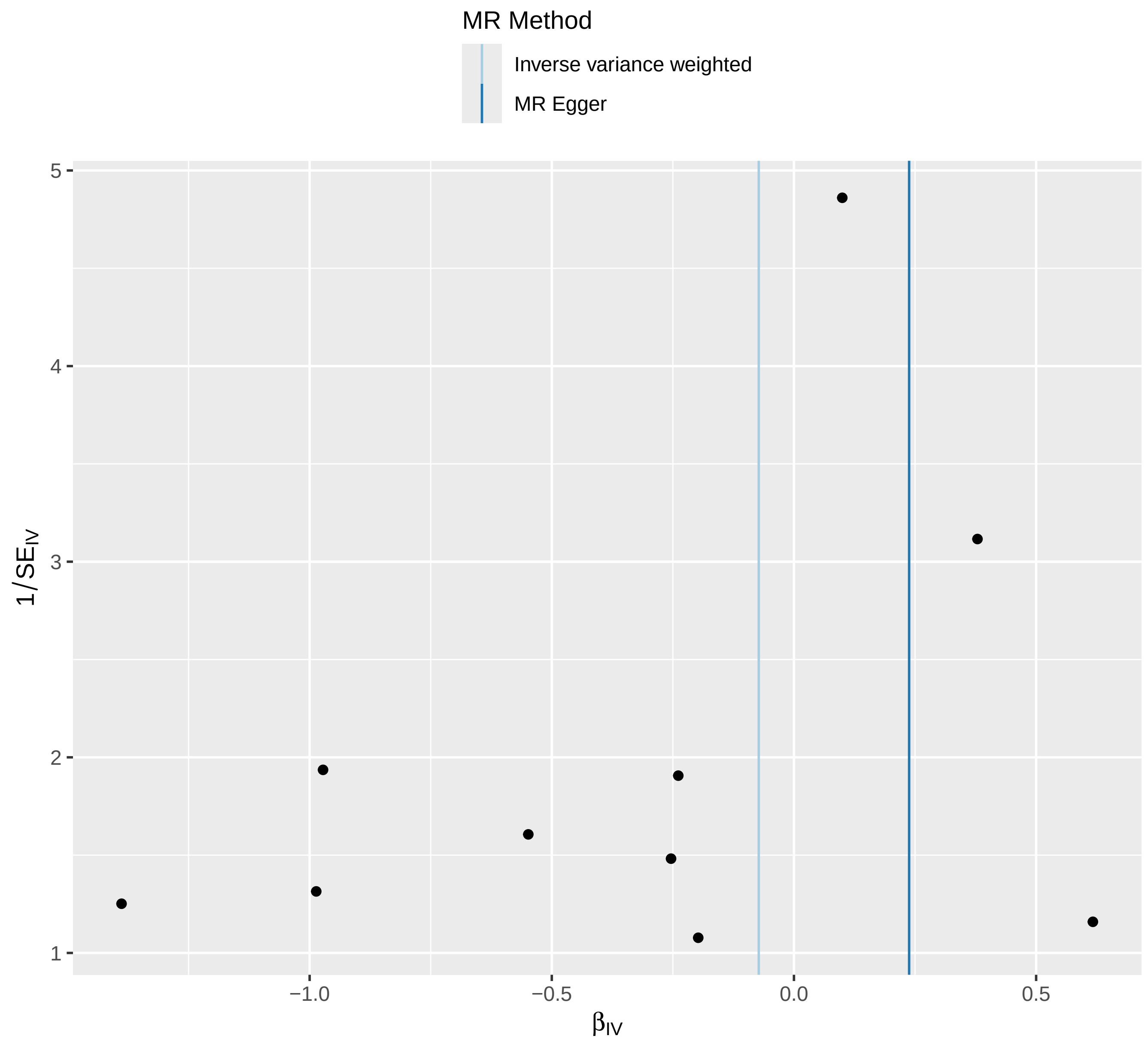
MR Method
Inverse variance weighted
MR Egger
1/SE$_{IV}$
5
4
3
2
1
−1.0
−0.5
0.0
0.5
β$_{IV}$

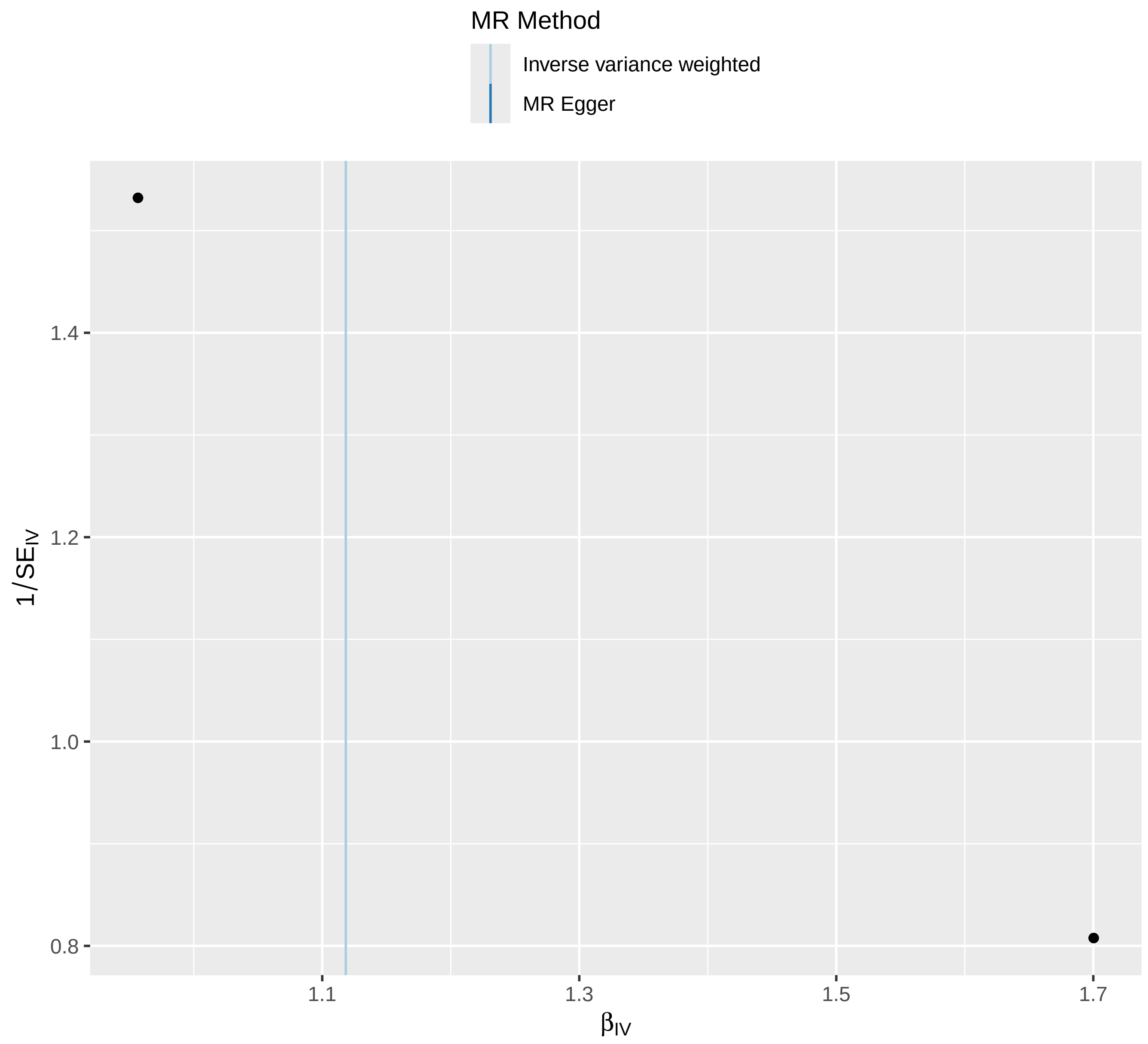
MR Method
Inverse variance weighted
MR Egger
1/SE$_{IV}$
1.4
1.2
1.0
0.8
1.1
1.3
1.5
1.7
β$_{IV}$

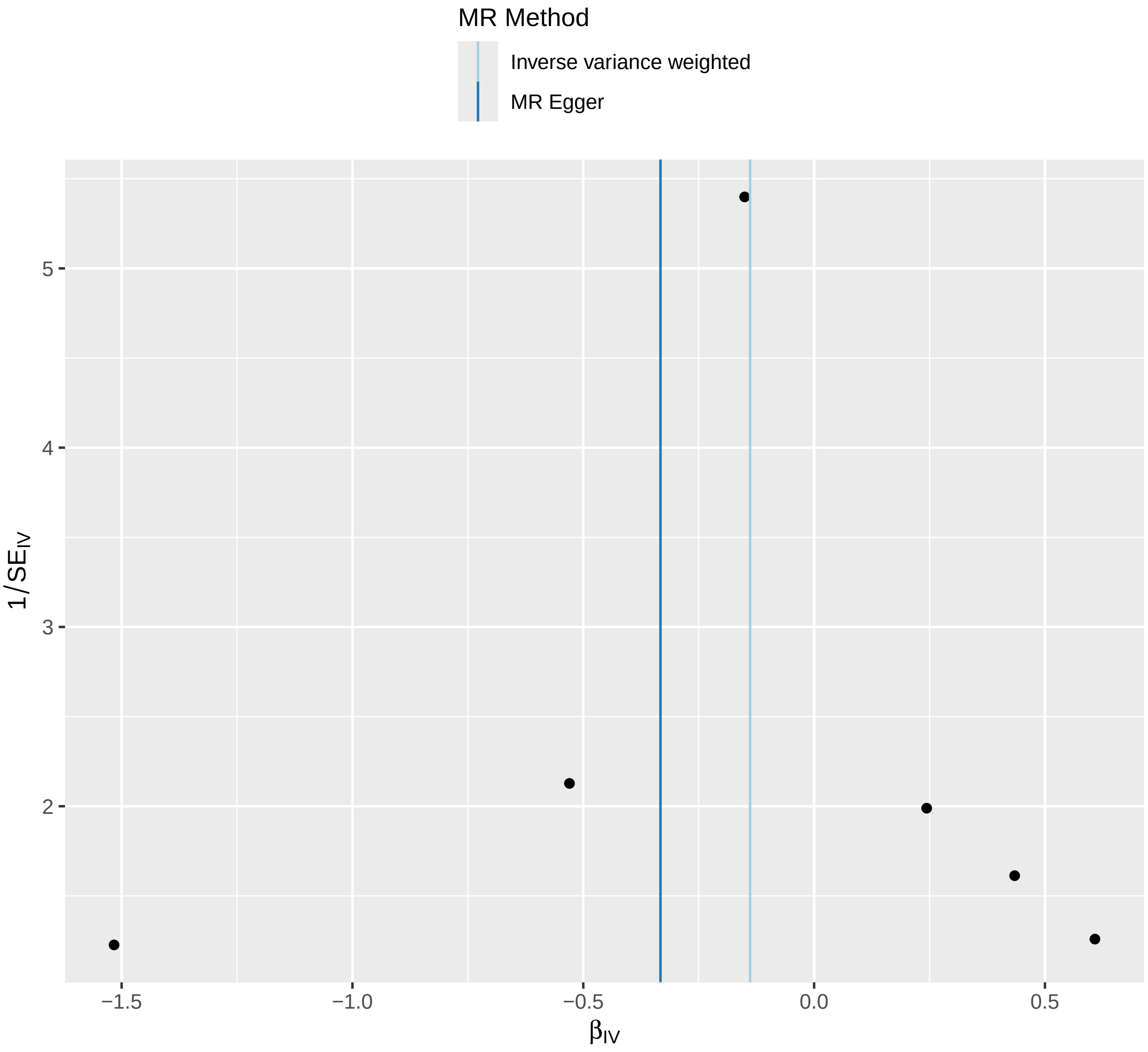

MR Method
Inverse variance weighted
MR Egger
5
4
3
2
1/SE$_{IV}$
−1.5
−1.0
−0.5
0.0
0.5
β$_{IV}$

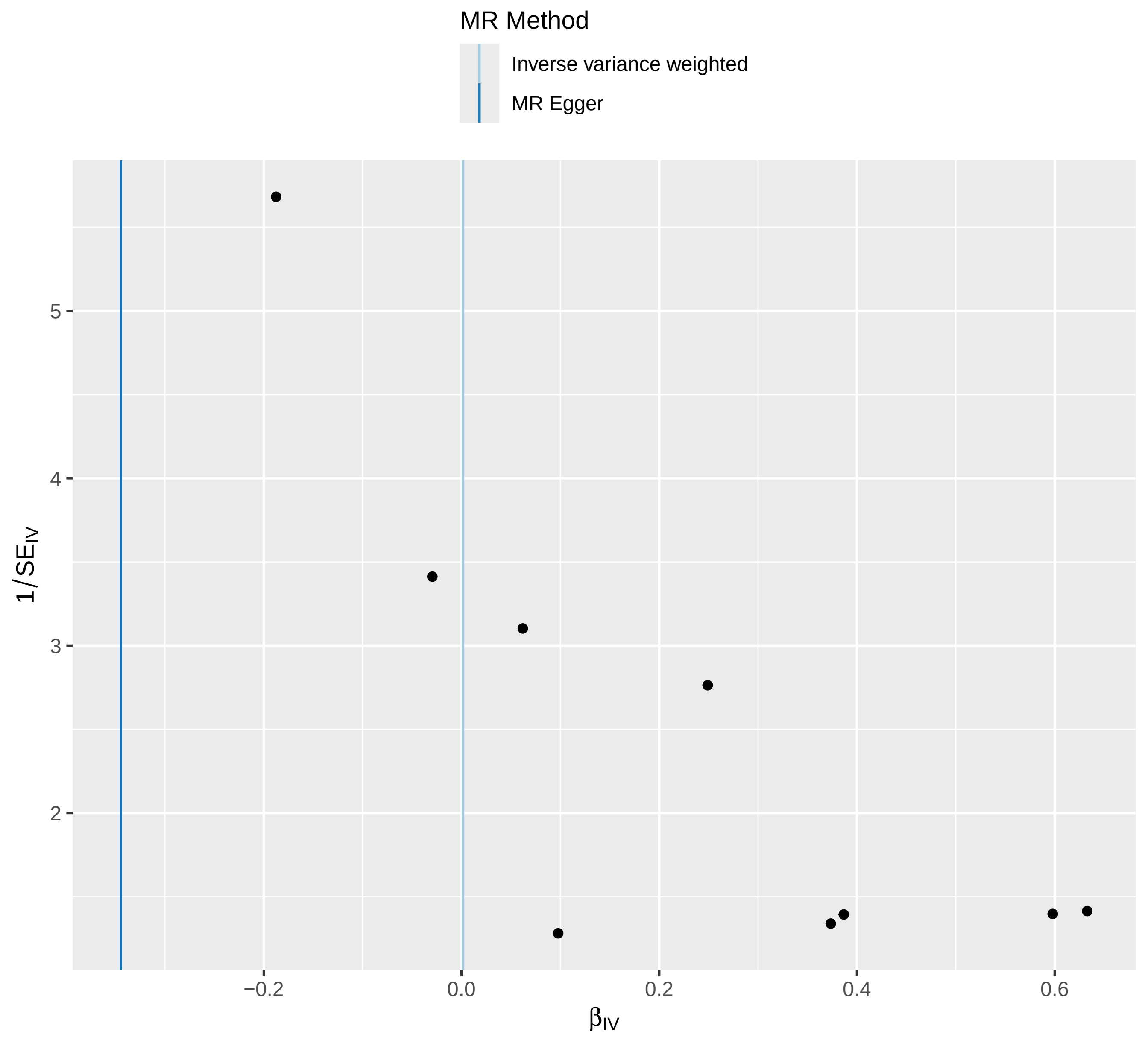

MR Method
Inverse variance weighted
MR Egger
5
4
3
2
1/SE$_{IV}$
−0.2
0.0
0.2
0.4
0.6
β$_{IV}$

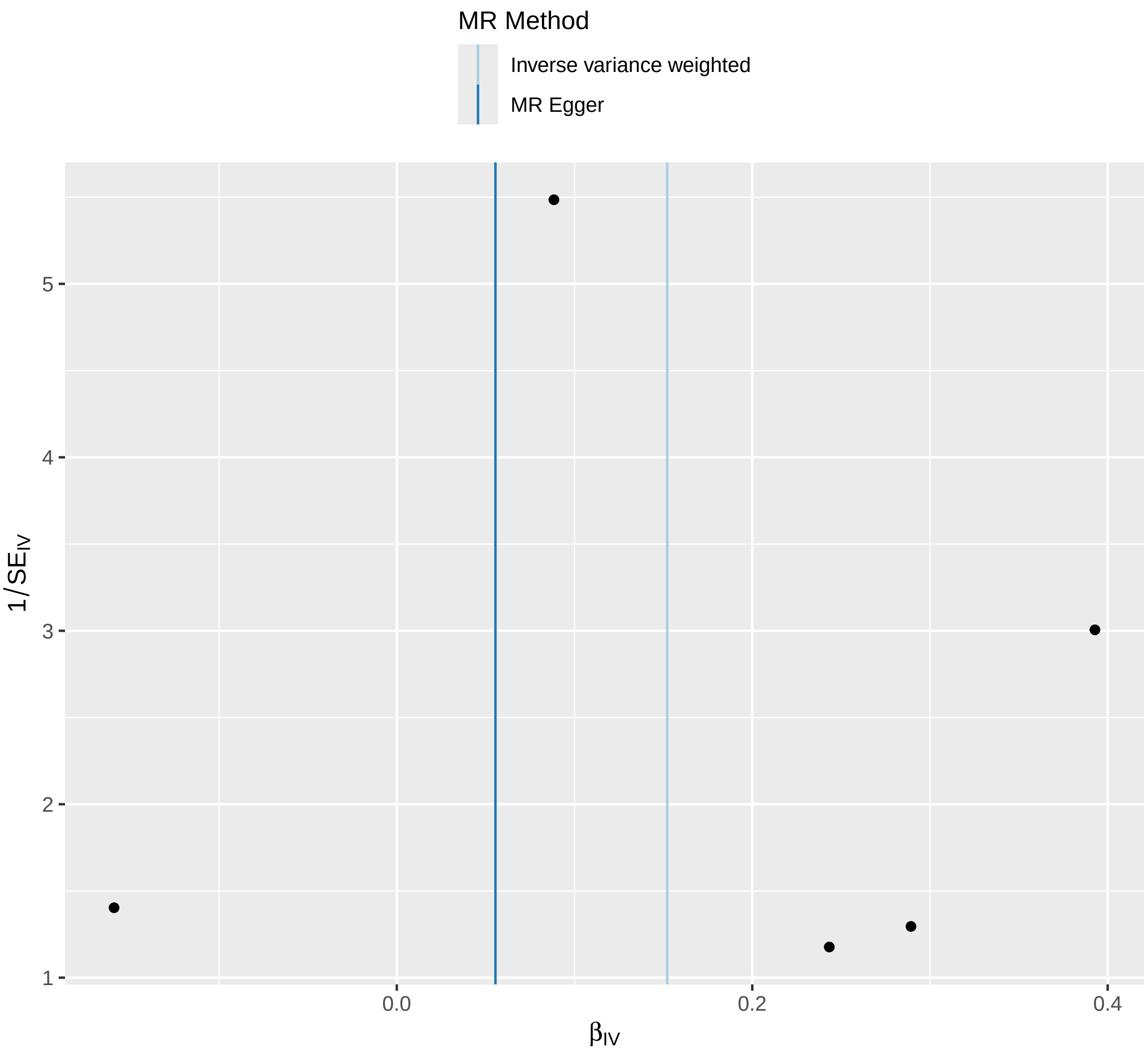
MR Method
Inverse variance weighted
MR Egger
1/SE_{IV}
5
4
3
2
1
0.0
0.2
0.4
β_{IV}

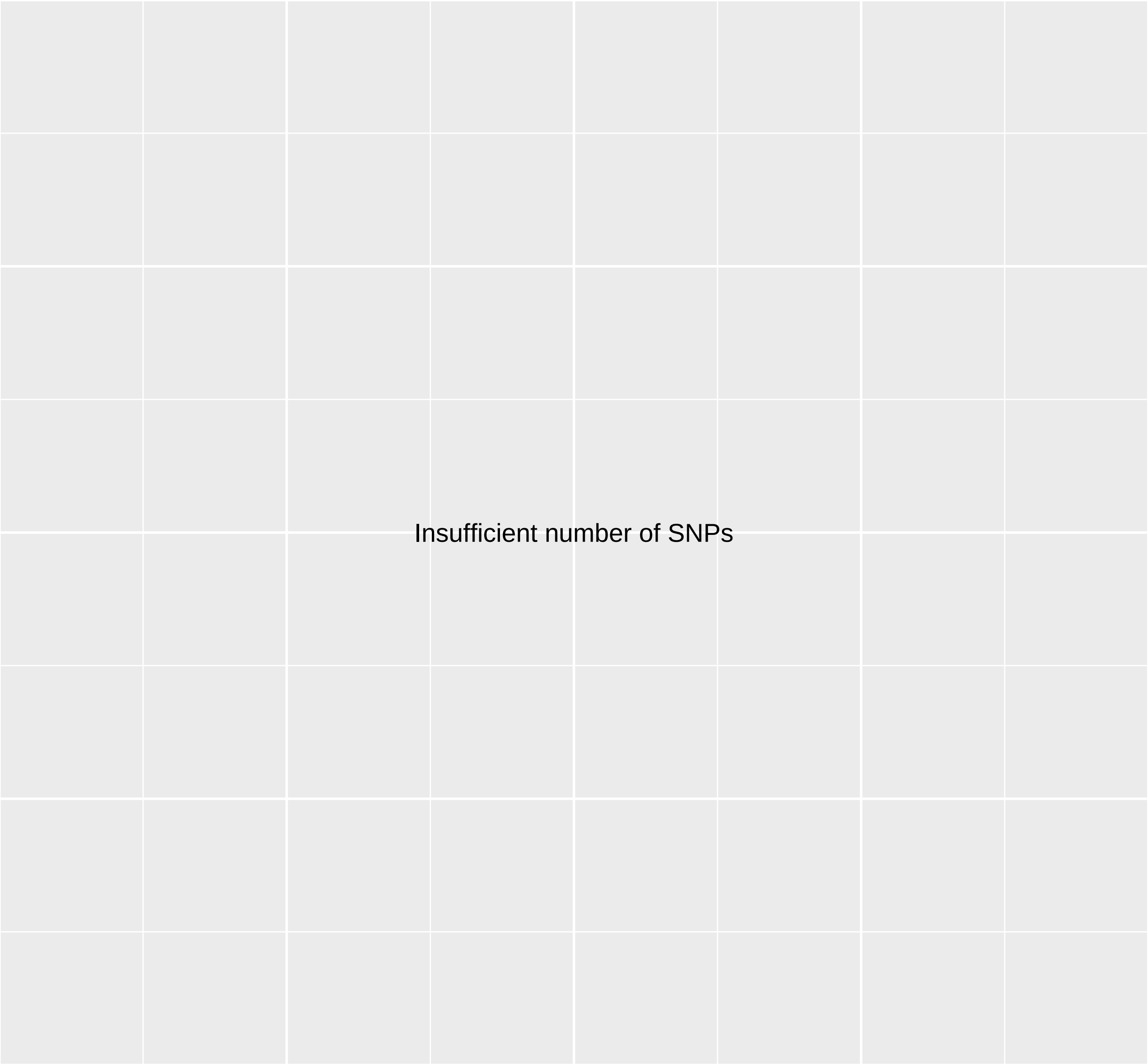
Insufficient number of SNPs

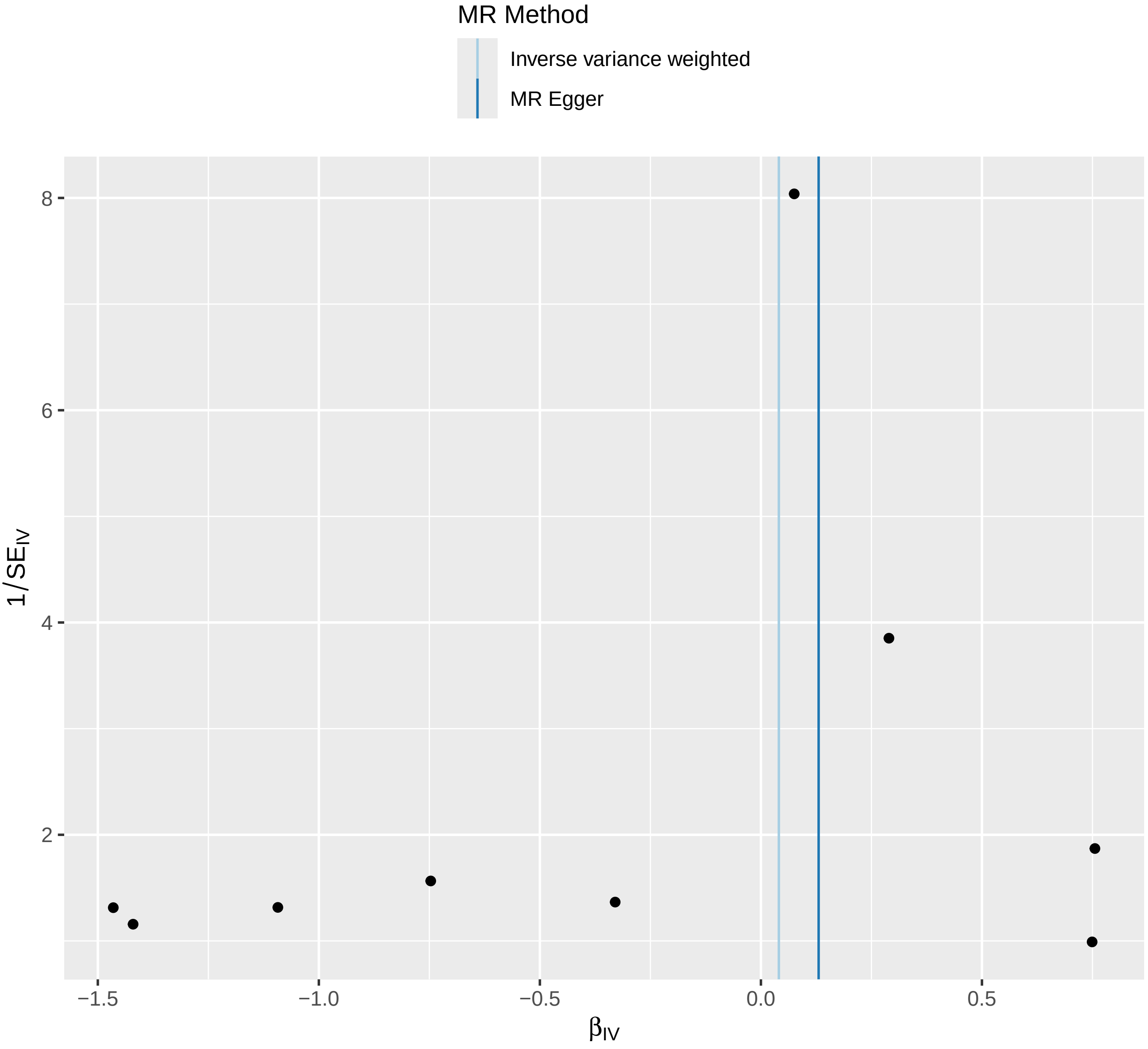
MR Method
Inverse variance weighted
MR Egger
8
6
4
2
$1/SE_{IV}$
−1.5
−1.0
−0.5
0.0
0.5
β_{IV}

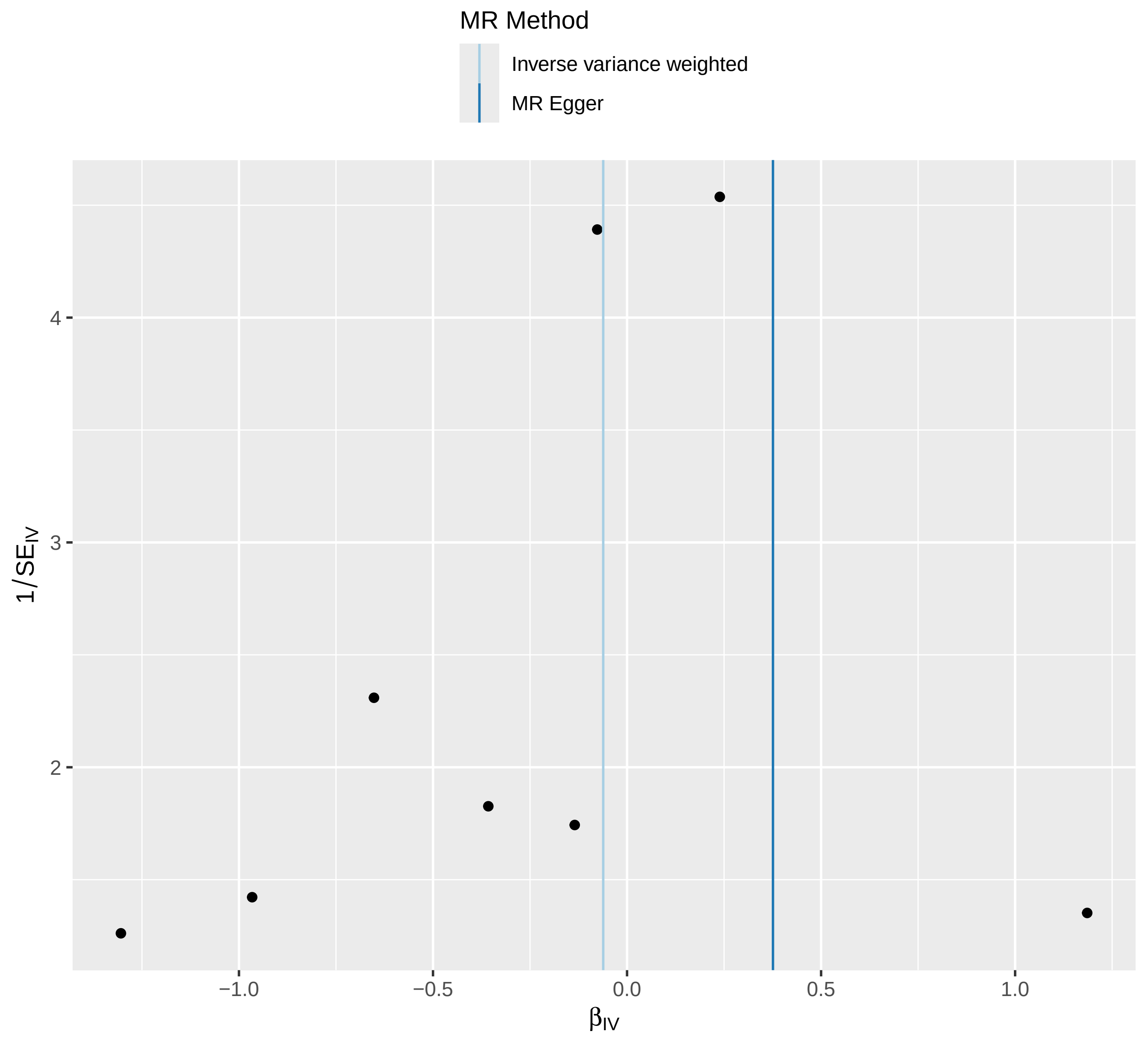
MR Method
Inverse variance weighted
MR Egger
1/SE_IV
β_IV
4
3
2
−1.0
−0.5
0.0
0.5
1.0

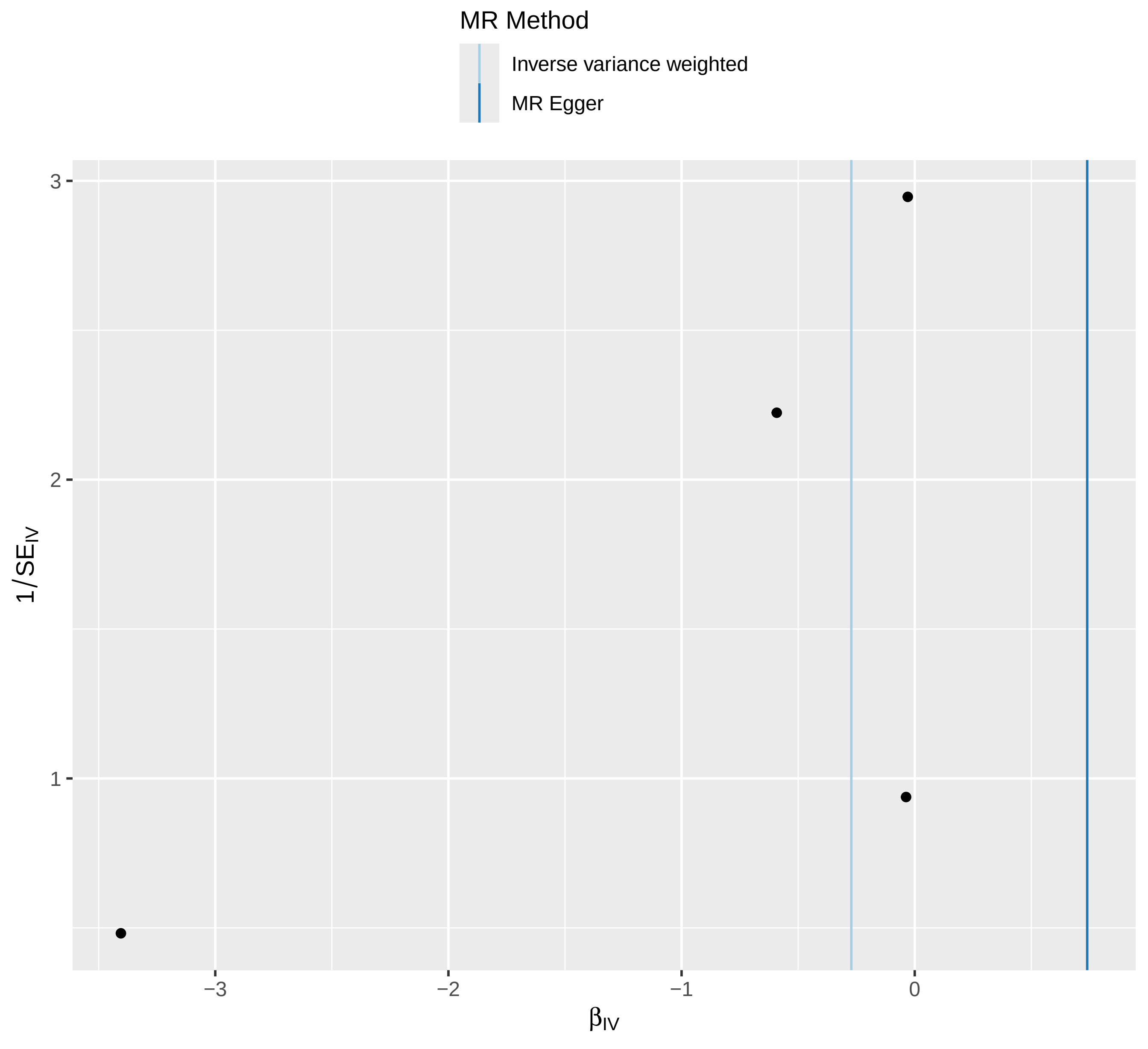

MR Method
Inverse variance weighted
MR Egger
3
2
1
1/SE_IV
−3
−2
−1
0
β_IV

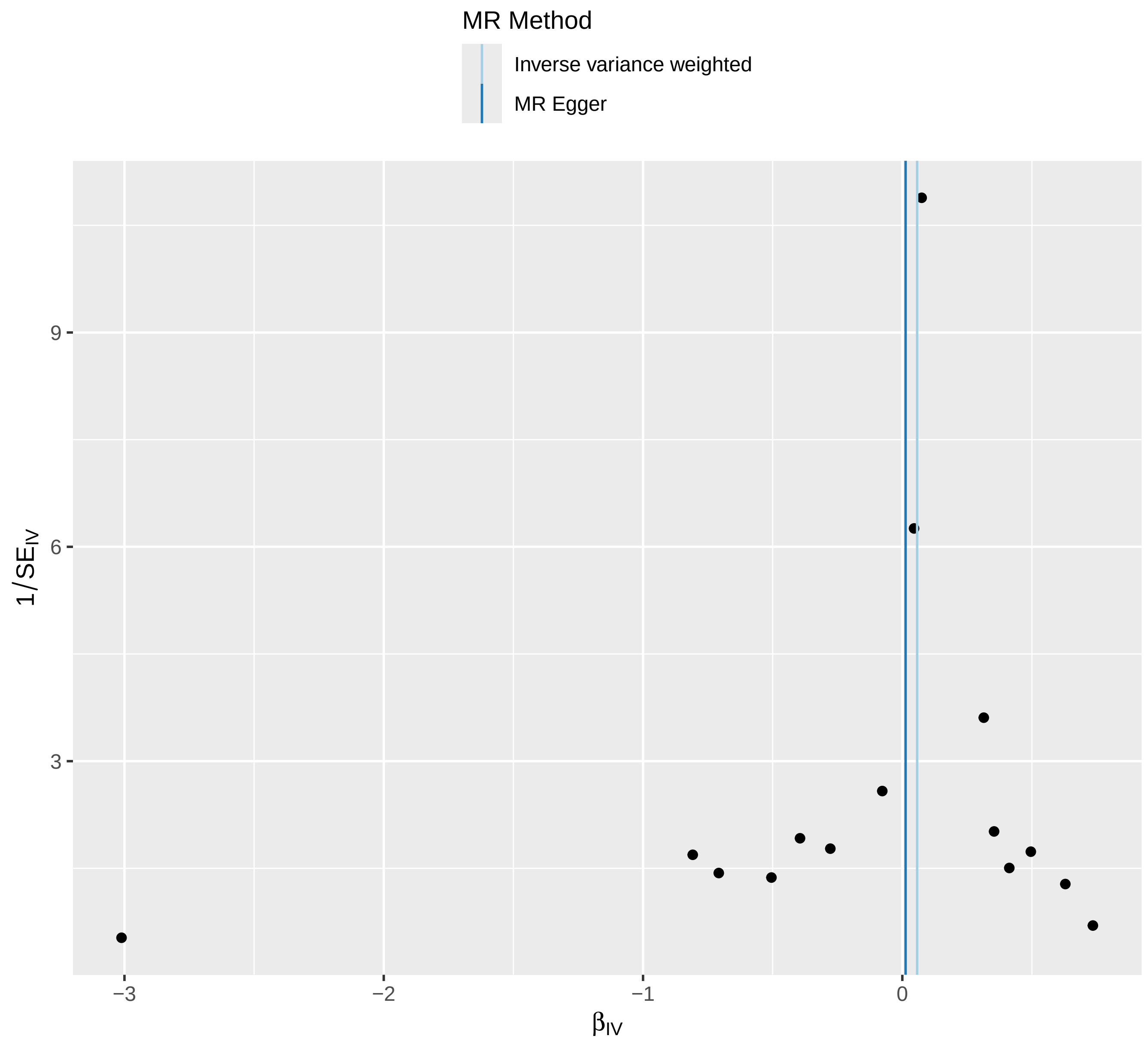
MR Method
Inverse variance weighted
MR Egger
9
6
3
1/SE_IV
−3
−2
−1
0
β_IV

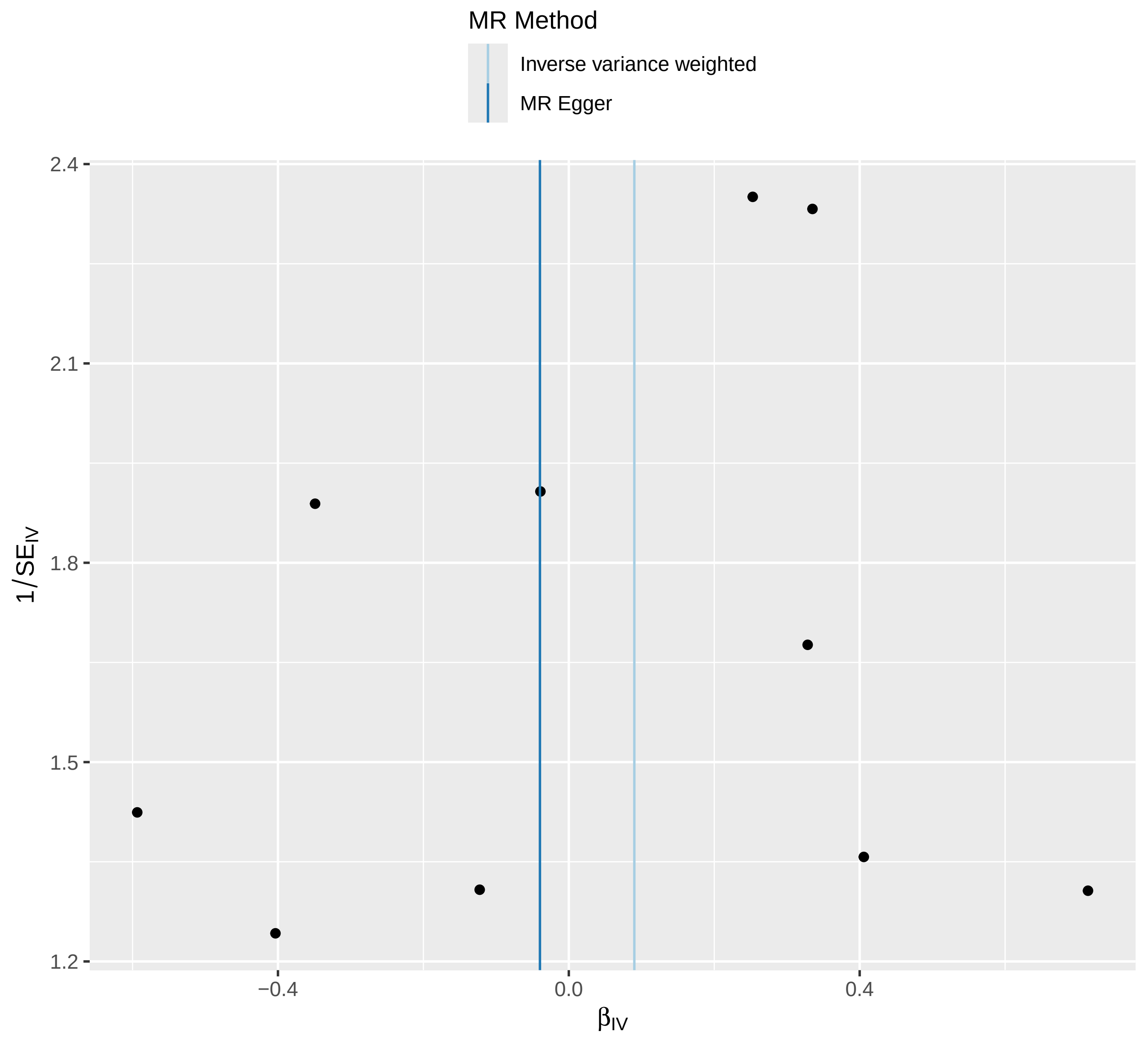

MR Method
Inverse variance weighted
MR Egger
2.4
2.1
1.8
1.5
1.2
1/SE_IV
−0.4
0.0
0.4
β_IV

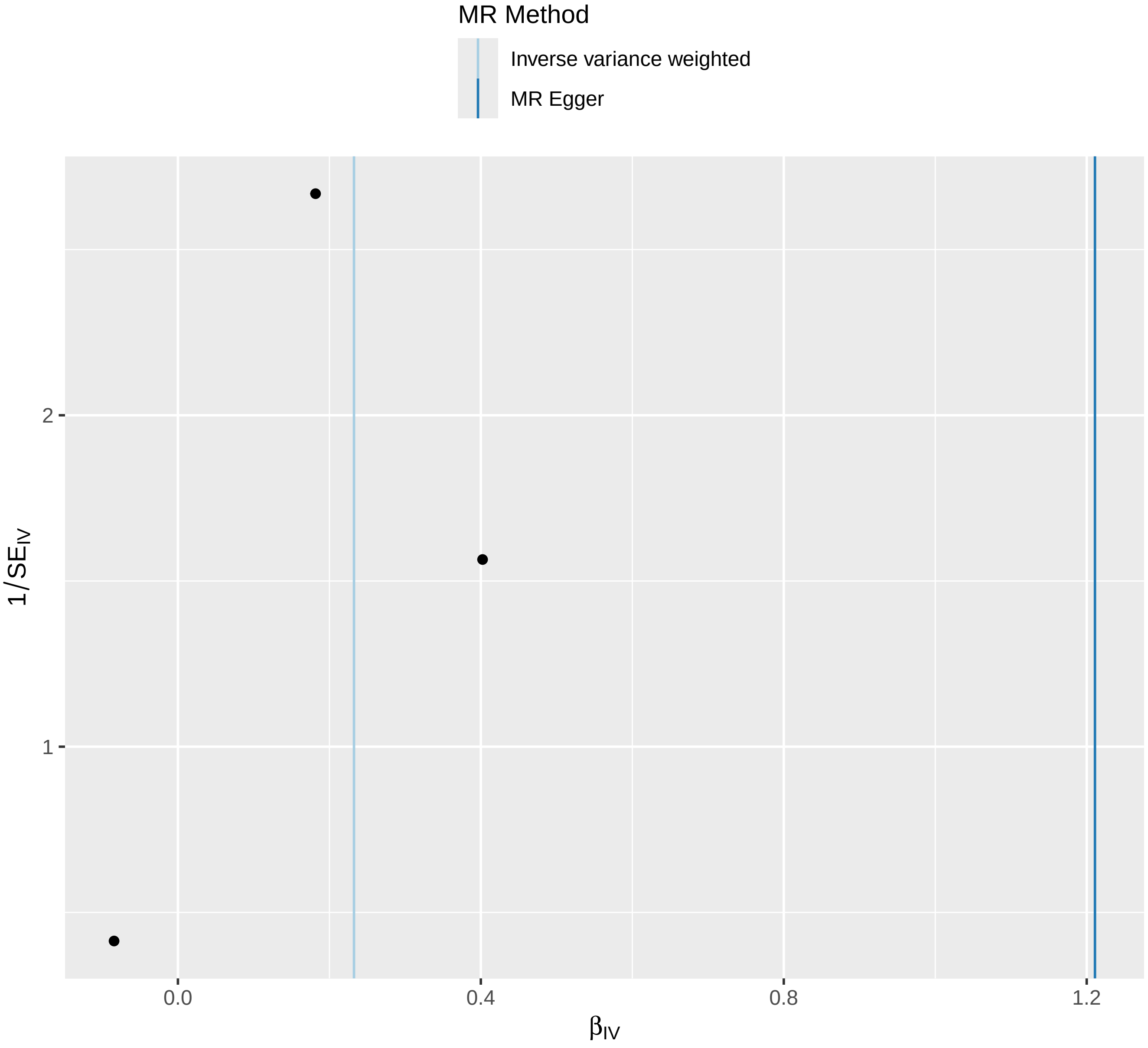
MR Method
Inverse variance weighted
MR Egger
1/SE_IV
2
1
0.0
0.4
0.8
1.2
β_IV

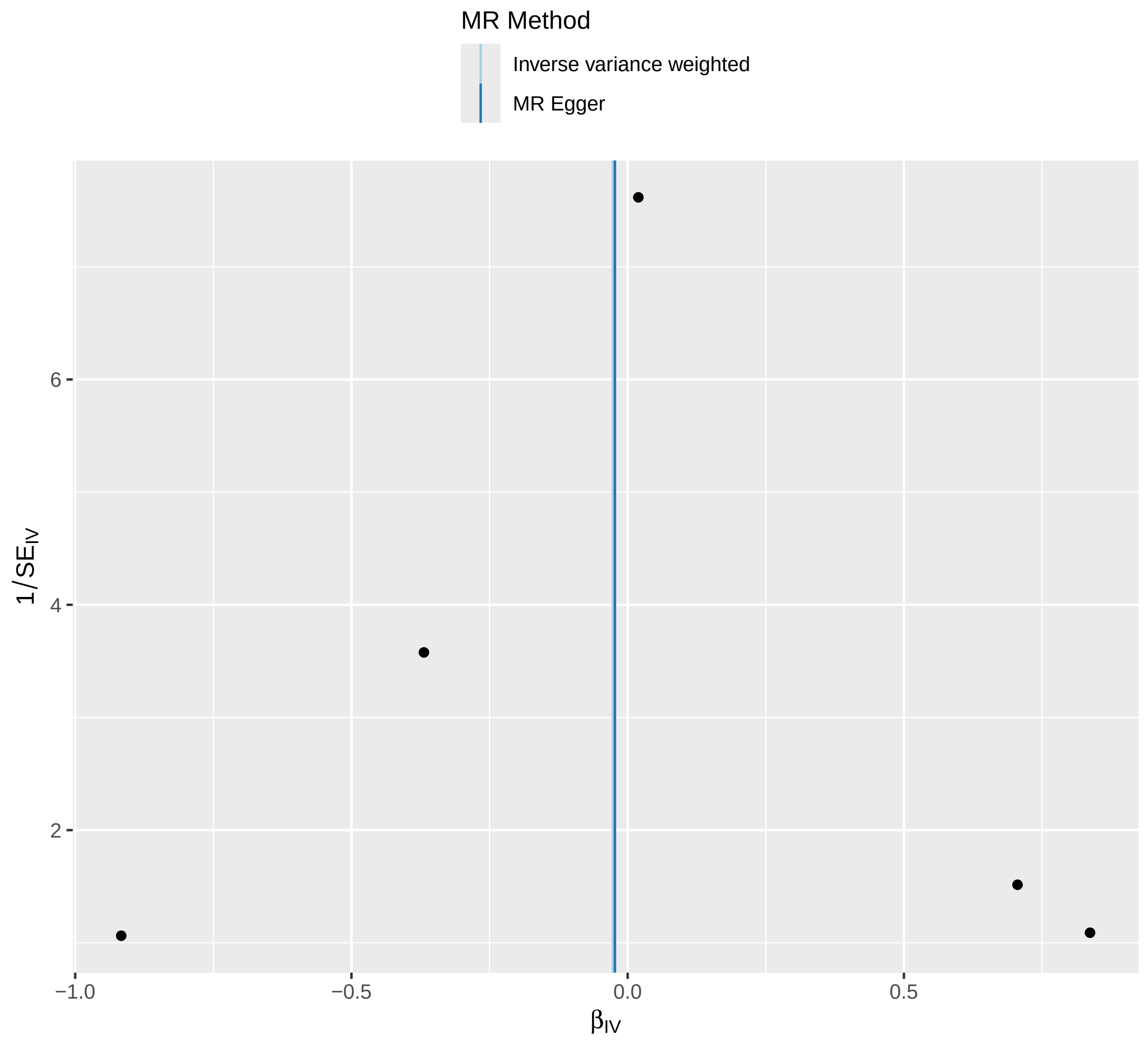

MR Method
Inverse variance weighted
MR Egger
6
4
2
1/SE_IV
−1.0
−0.5
0.0
0.5
β_IV

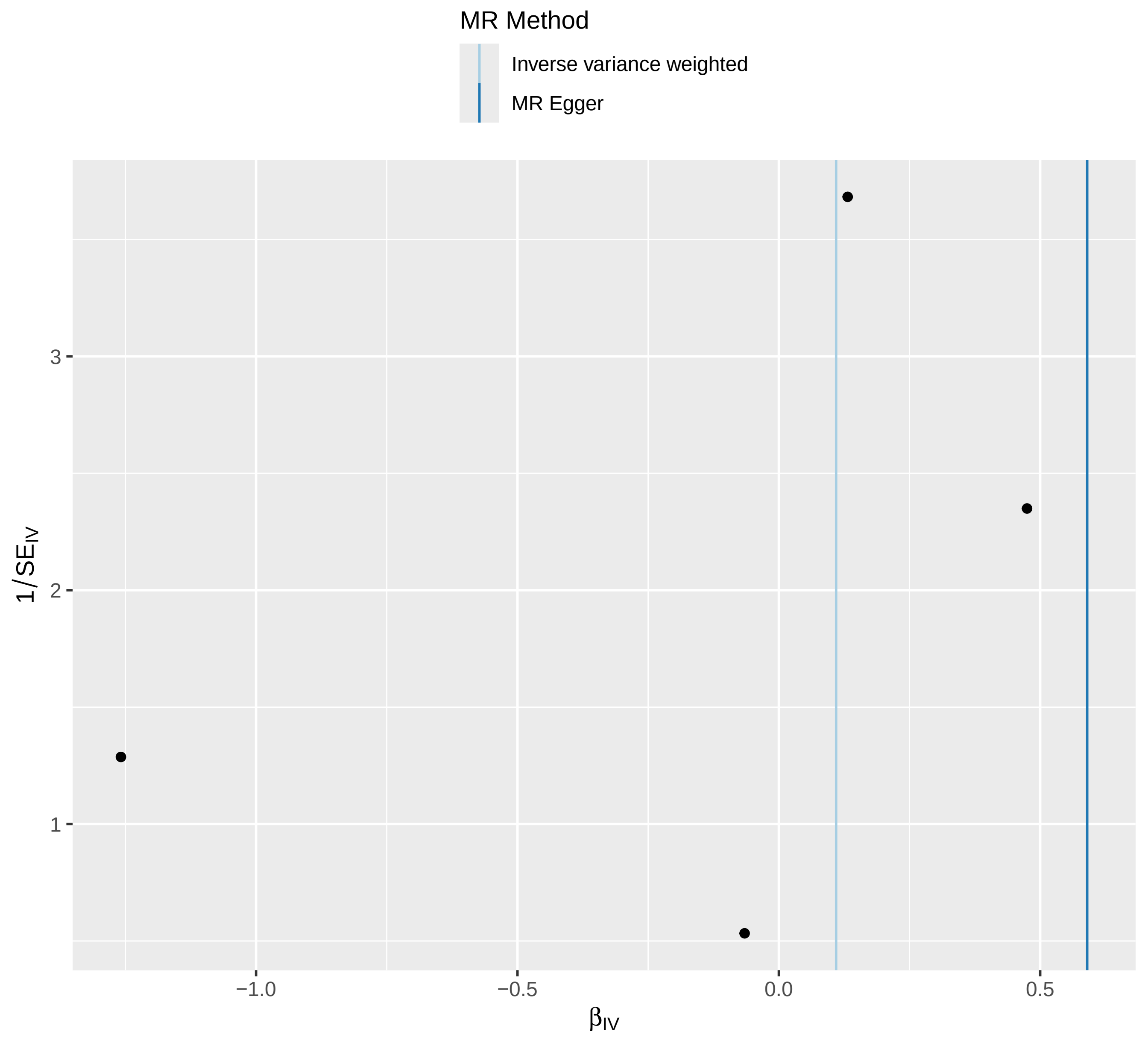

MR Method
Inverse variance weighted
MR Egger
1/SE_IV
3
2
1
−1.0
−0.5
0.0
0.5
β_IV

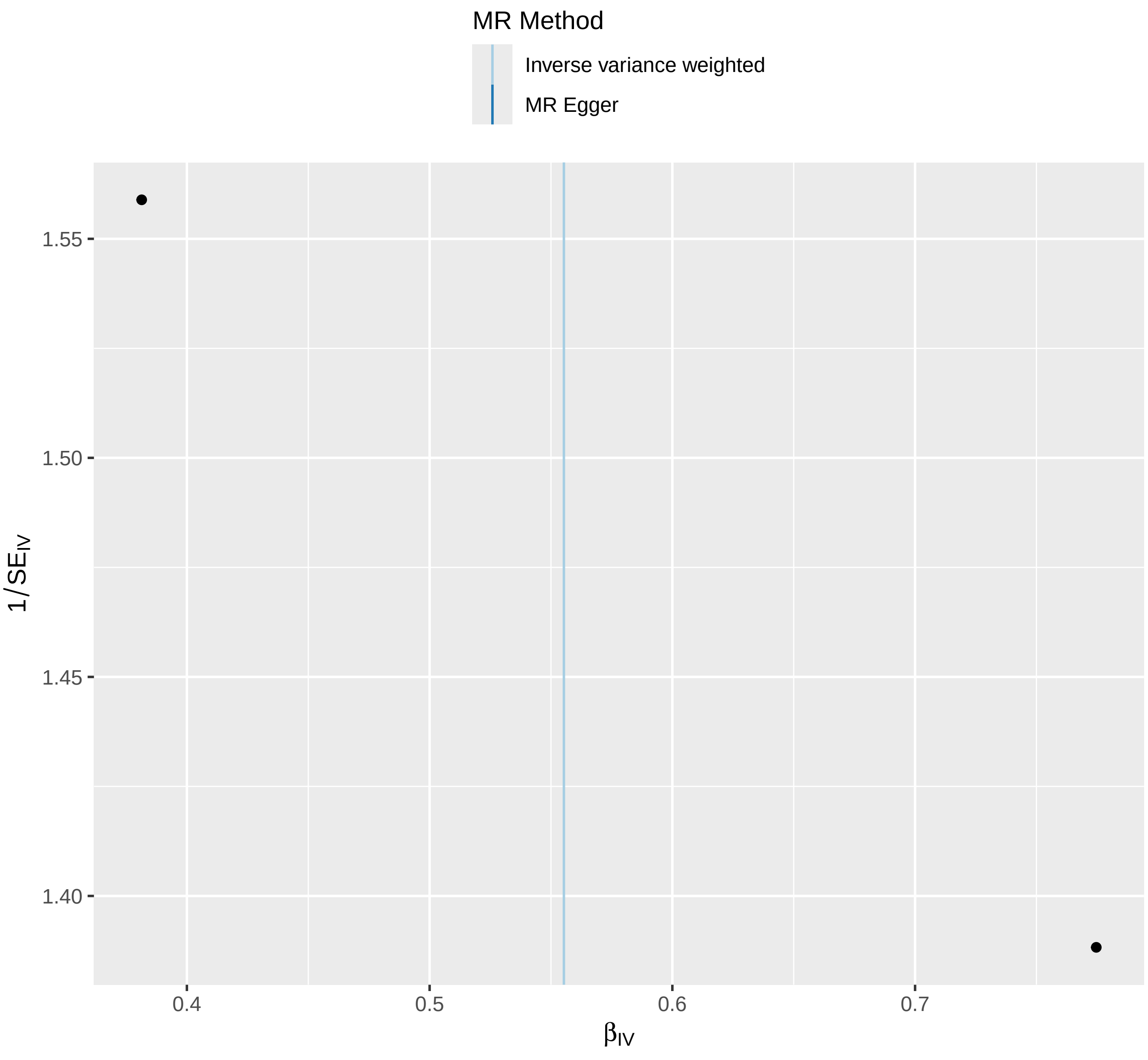
MR Method
Inverse variance weighted
MR Egger
1.55
1.50
1.45
1.40
1/SE_IV
0.4
0.5
0.6
0.7
β_IV

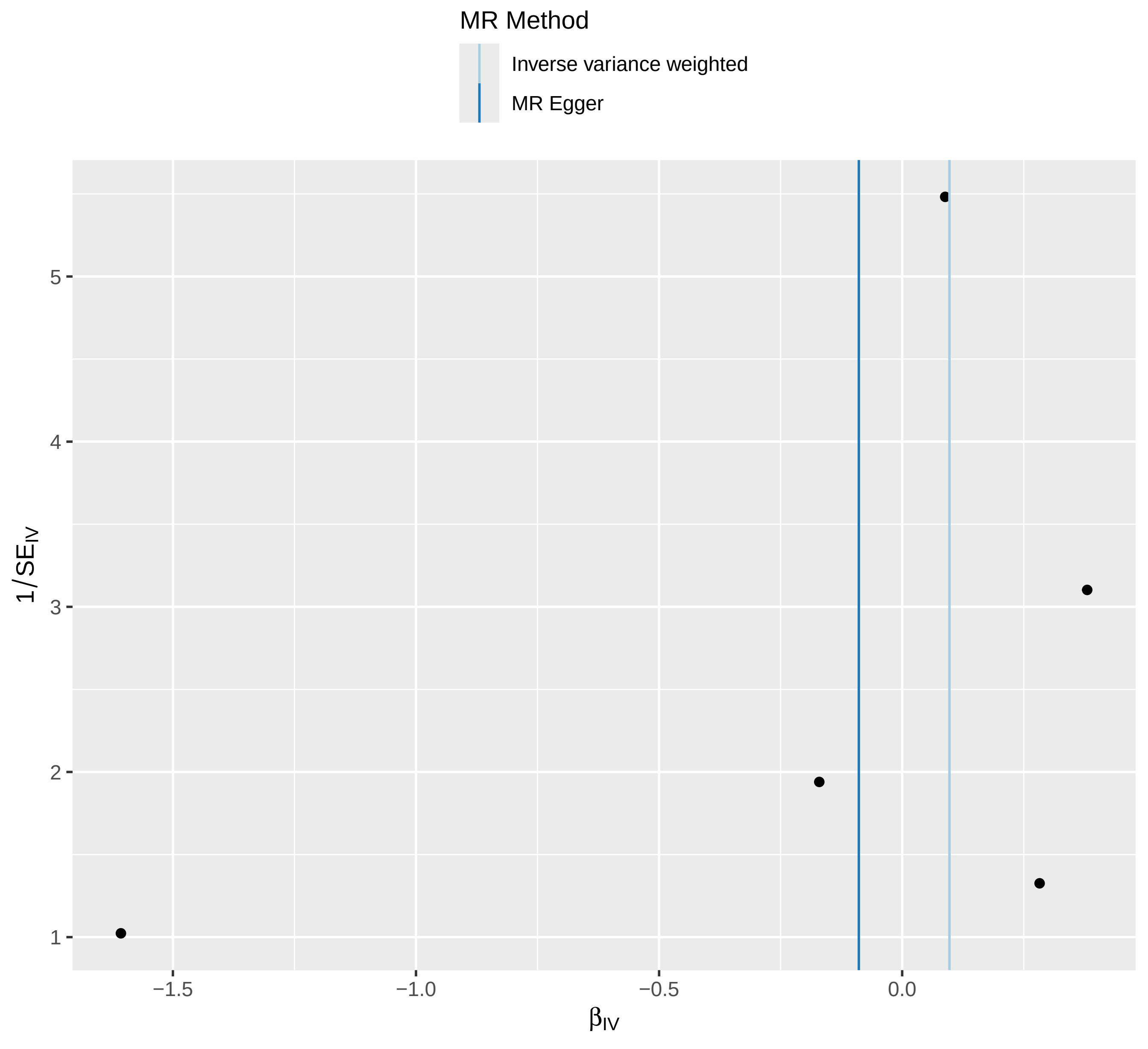
MR Method
Inverse variance weighted
MR Egger
1/SE_IV
5
4
3
2
1
−1.5
−1.0
−0.5
0.0
β_IV

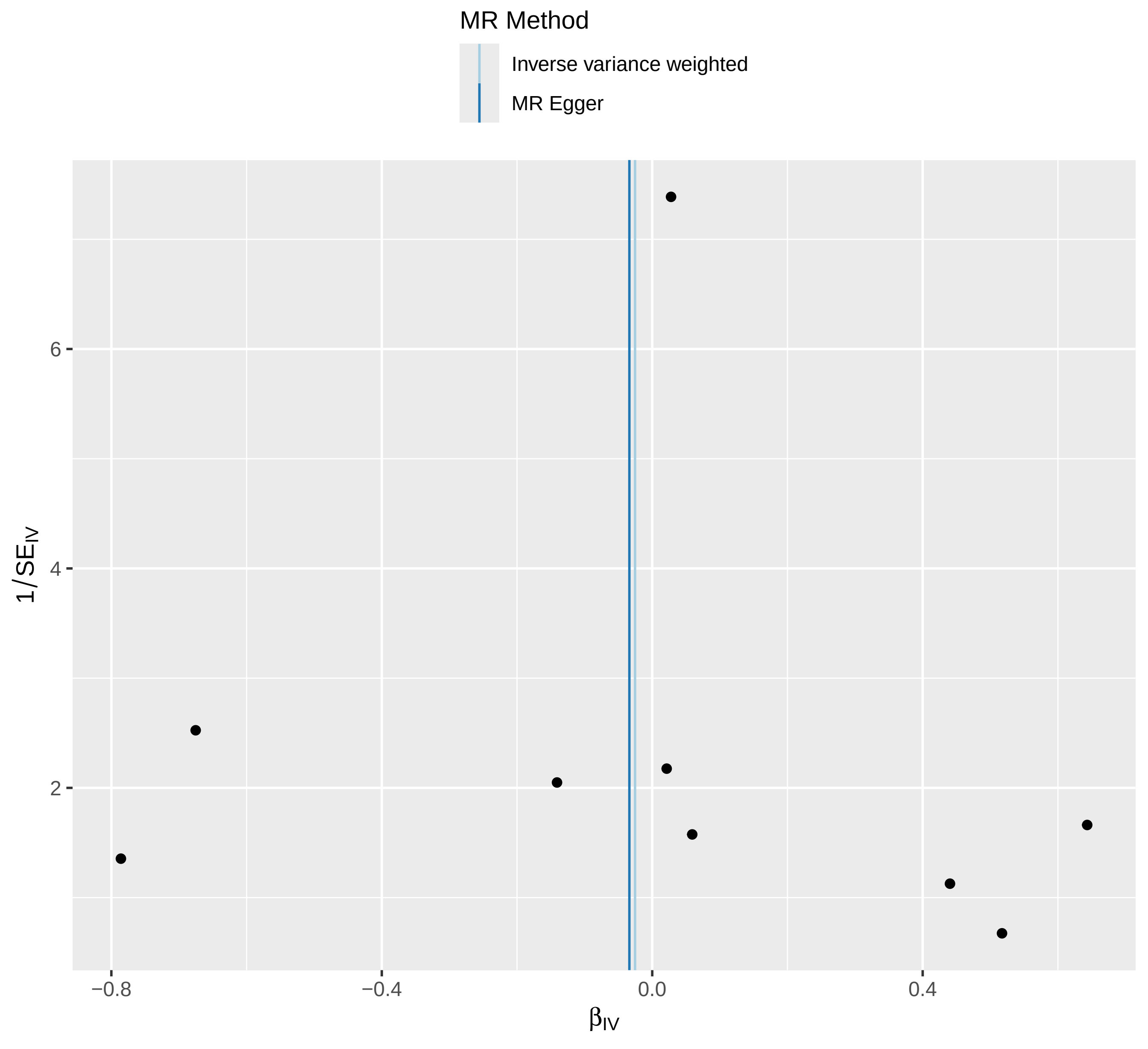

MR Method
Inverse variance weighted
MR Egger
1/SE_IV
6
4
2
−0.8
−0.4
0.0
0.4
β_IV

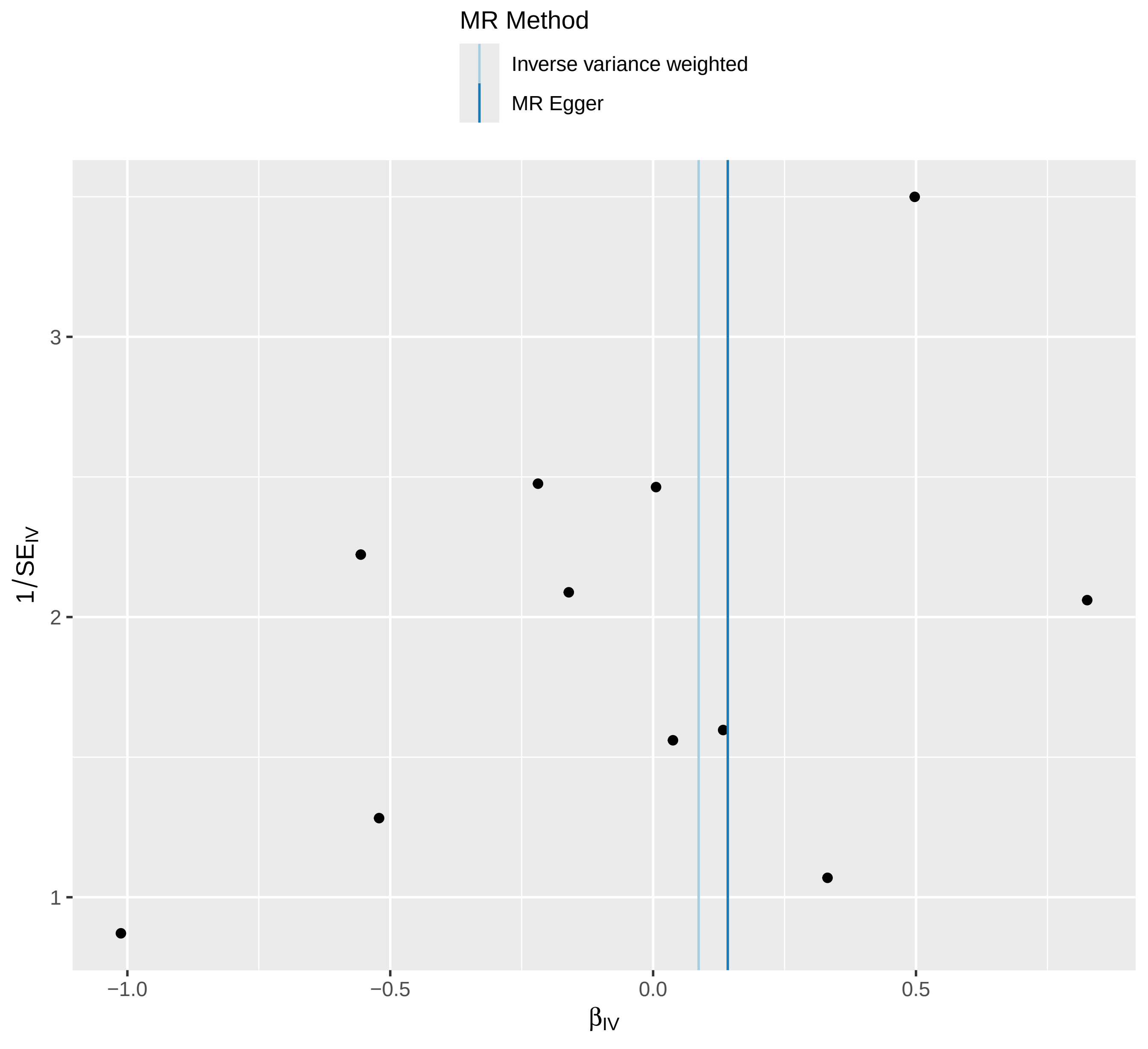

MR Method
Inverse variance weighted
MR Egger
1/SE_IV
3
2
1
−1.0
−0.5
0.0
0.5
β_IV

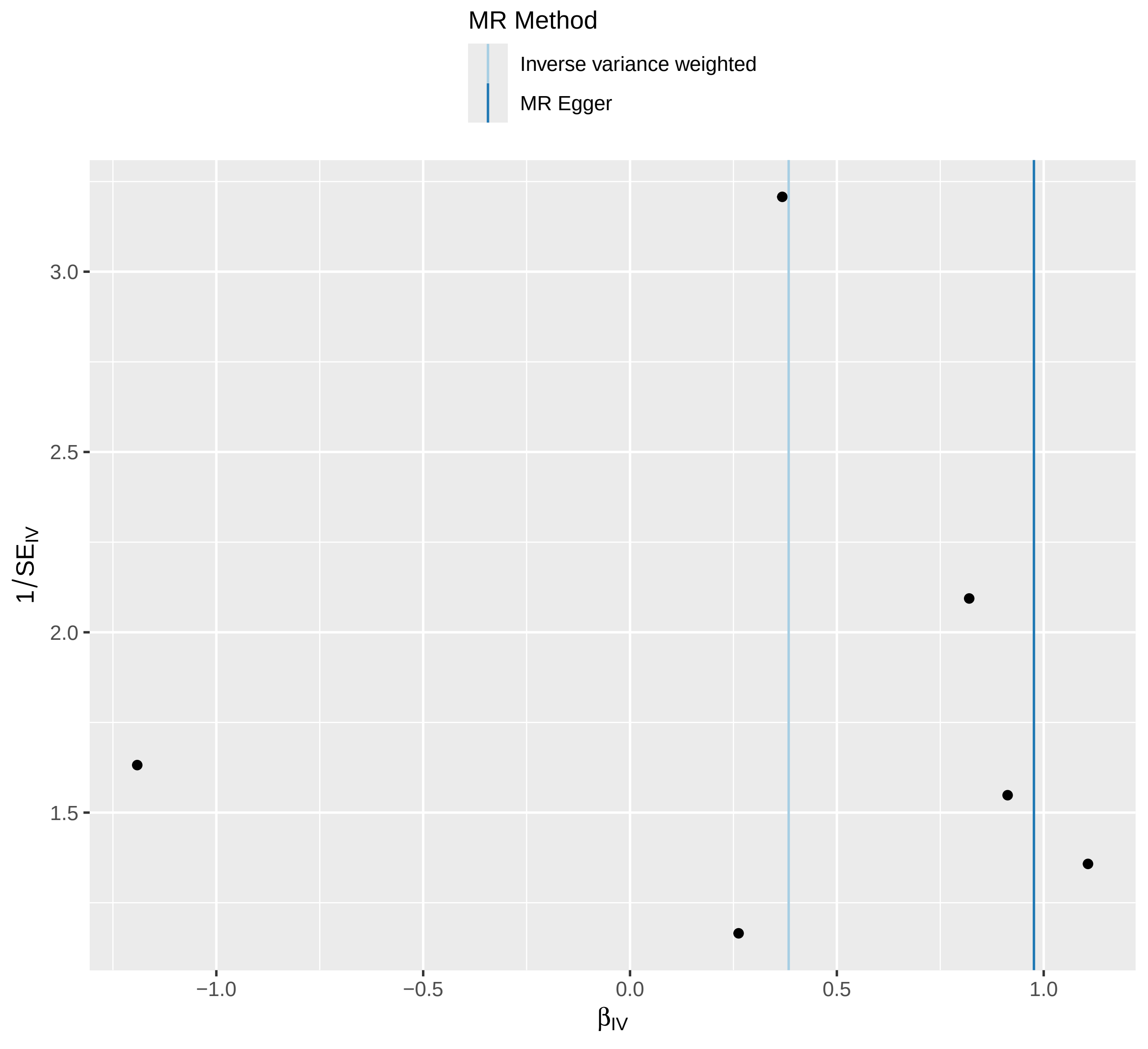

MR Method
Inverse variance weighted
MR Egger
3.0
2.5
2.0
1.5
1/SE_IV
−1.0
−0.5
0.0
0.5
1.0
β_IV

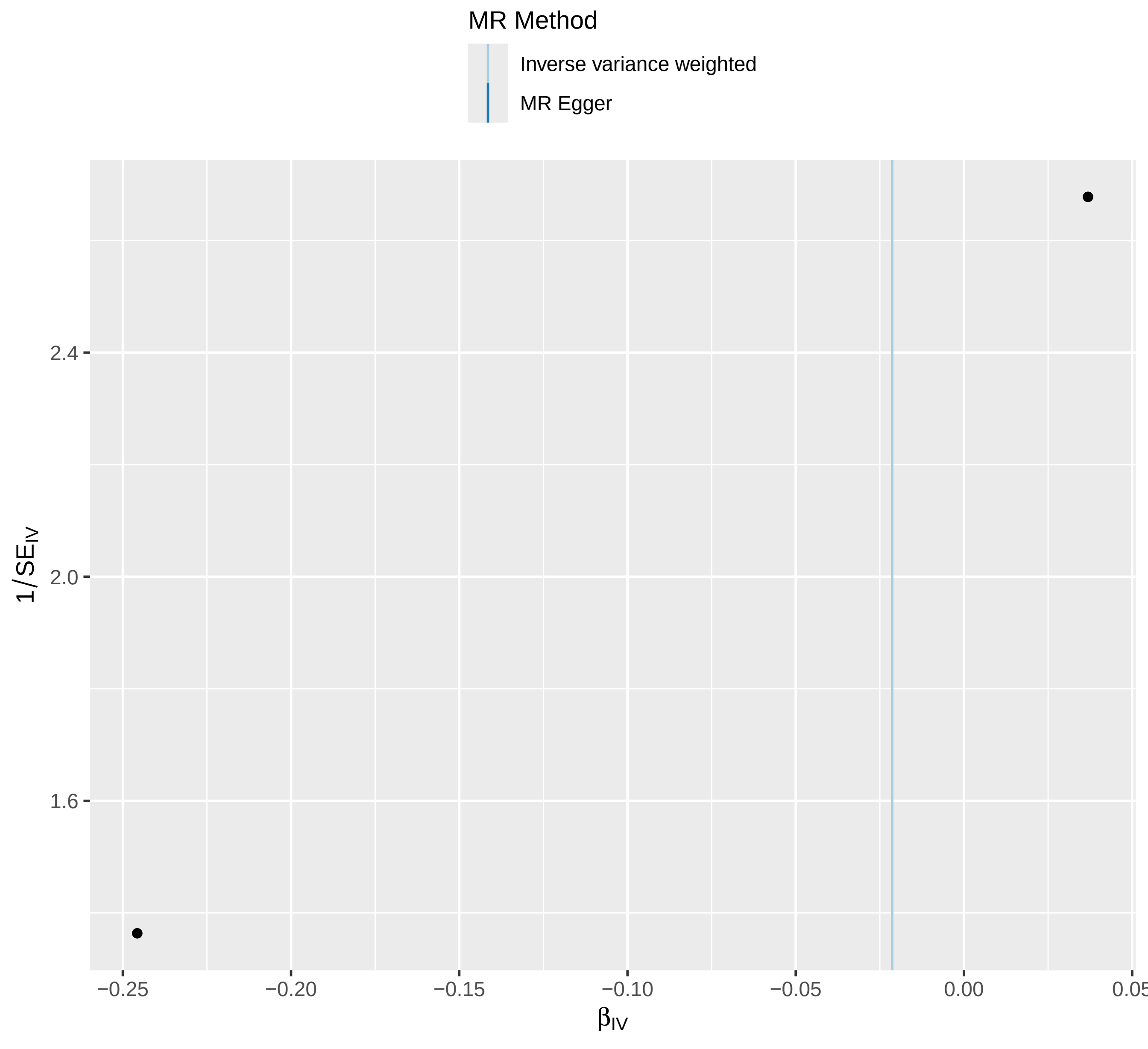

MR Method
Inverse variance weighted
MR Egger
2.4
2.0
1.6
1/SE_IV
−0.25
−0.20
−0.15
−0.10
−0.05
0.00
0.05
β_IV

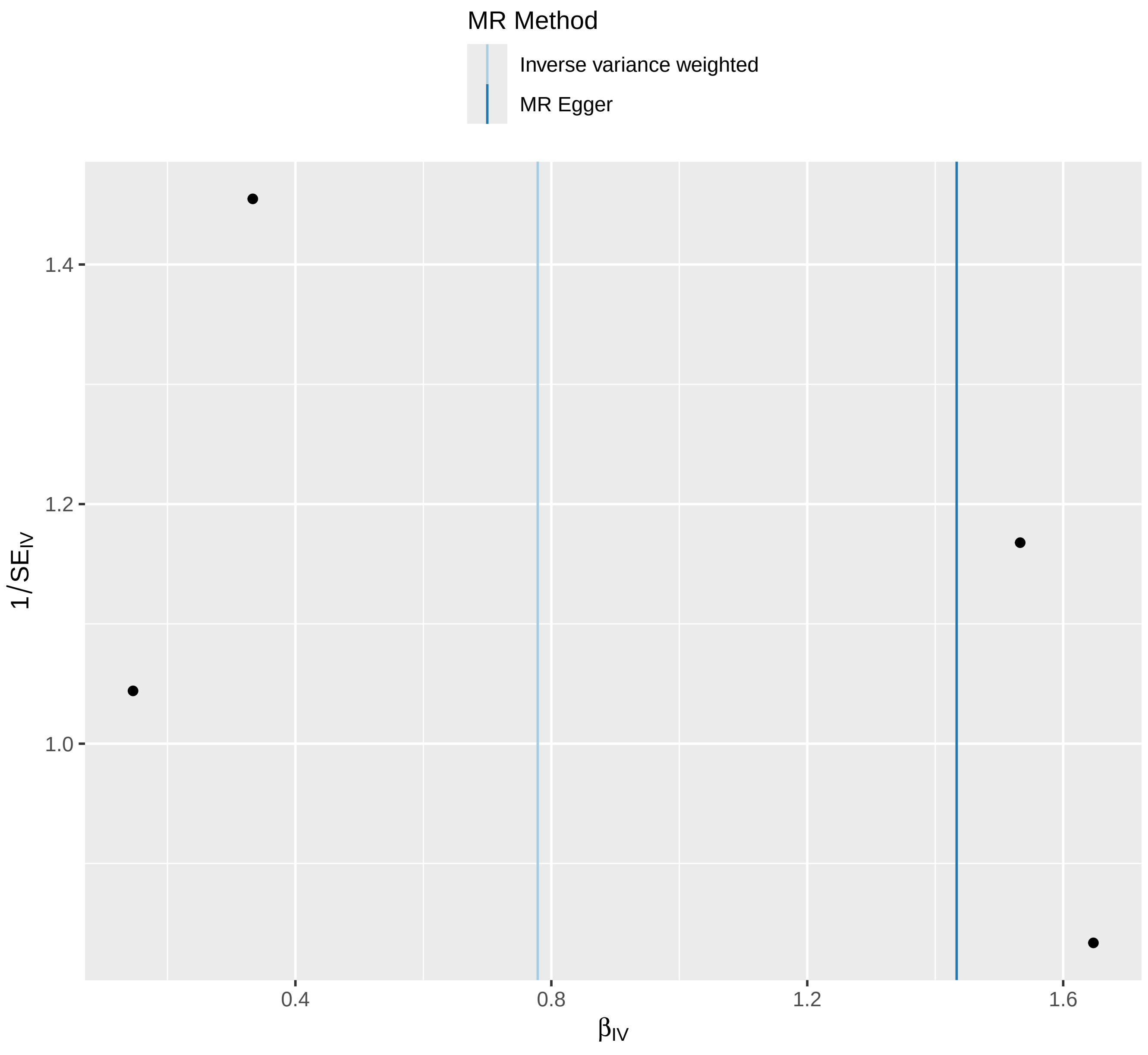
MR Method
Inverse variance weighted
MR Egger
1.4
1.2
1.0
1/SE$_{IV}$
0.4
0.8
1.2
1.6
β_{IV}

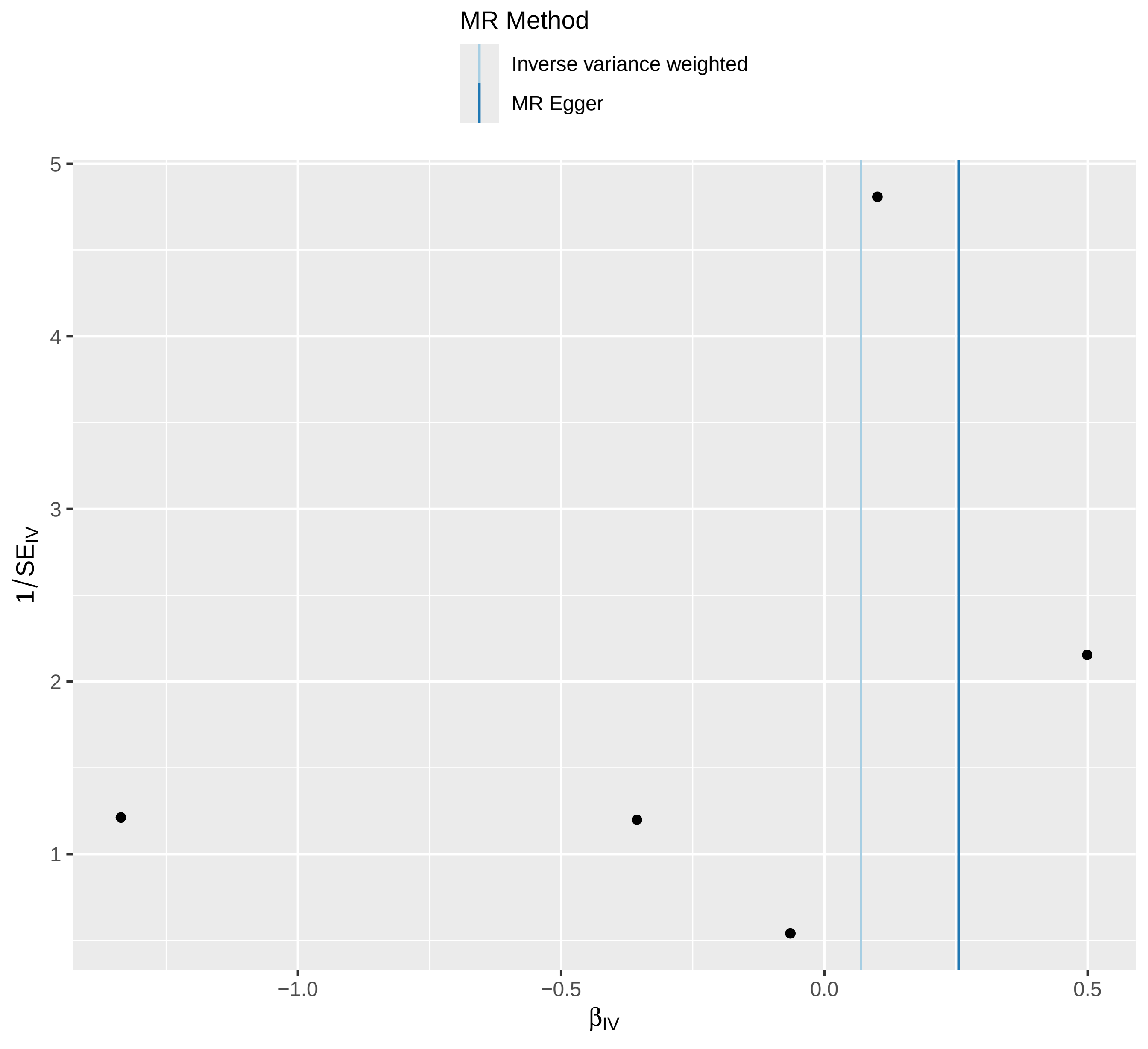
MR Method
Inverse variance weighted
MR Egger
5
4
3
2
1
1/SE_IV
−1.0
−0.5
0.0
0.5
β_IV

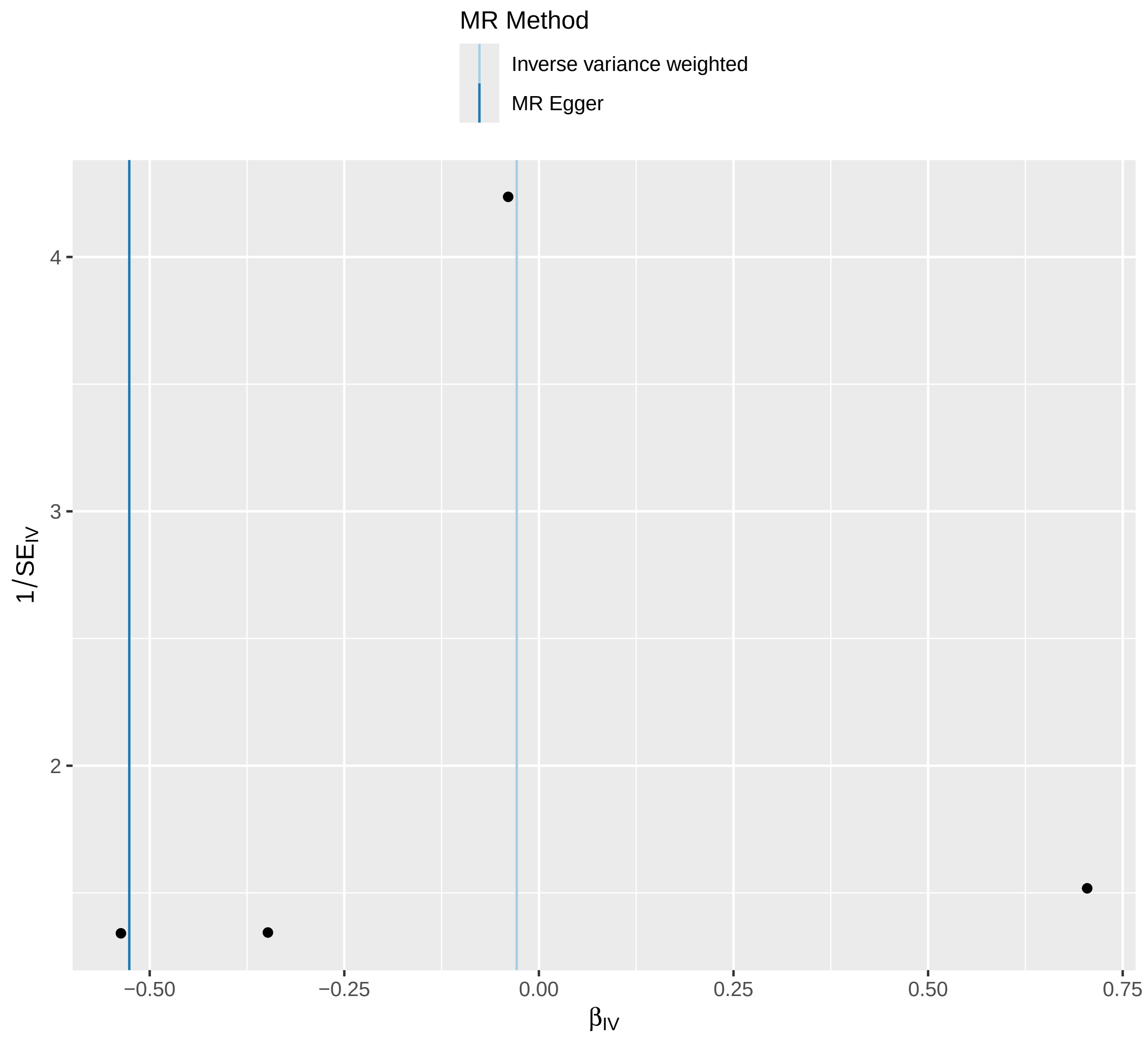
MR Method
Inverse variance weighted
MR Egger
4
3
2
1/SE_IV
−0.50
−0.25
0.00
0.25
0.50
0.75
β_IV

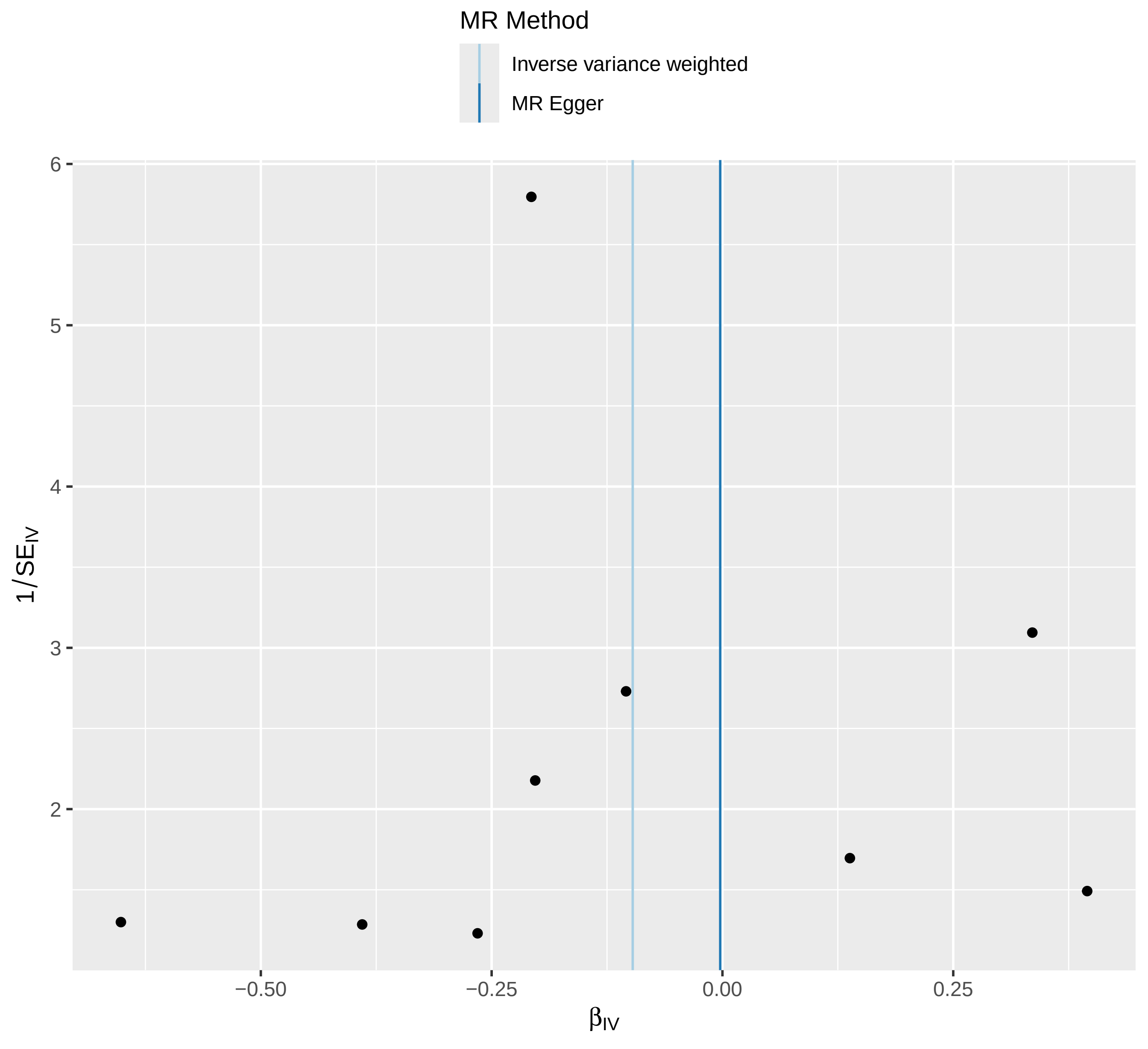

MR Method
Inverse variance weighted
MR Egger
6
5
4
3
2
1/SE_IV
−0.50
−0.25
0.00
0.25
β_IV

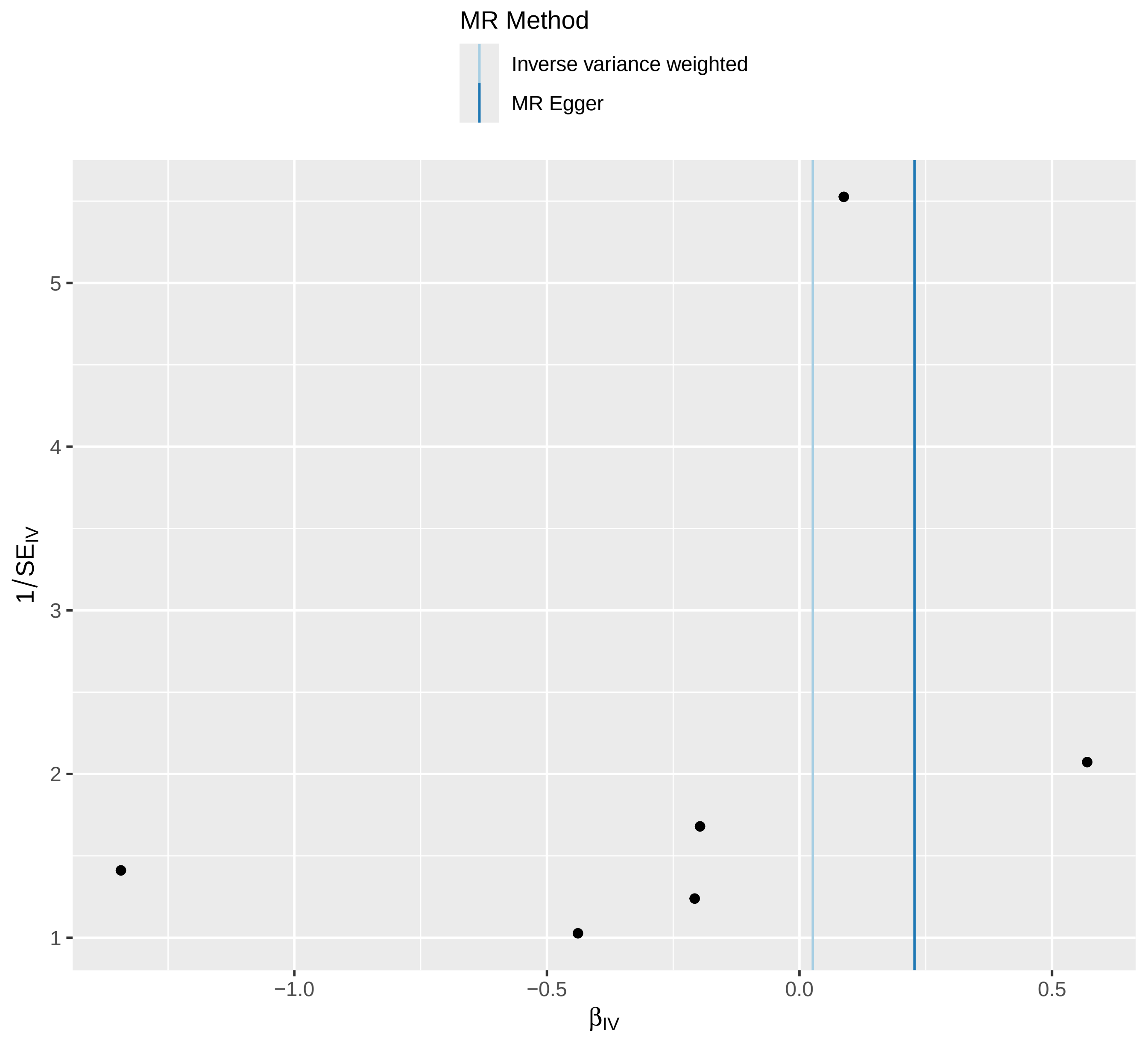
MR Method
Inverse variance weighted
MR Egger
1/SE$_{IV}$
5
4
3
2
1
−1.0
−0.5
0.0
0.5
β$_{IV}$

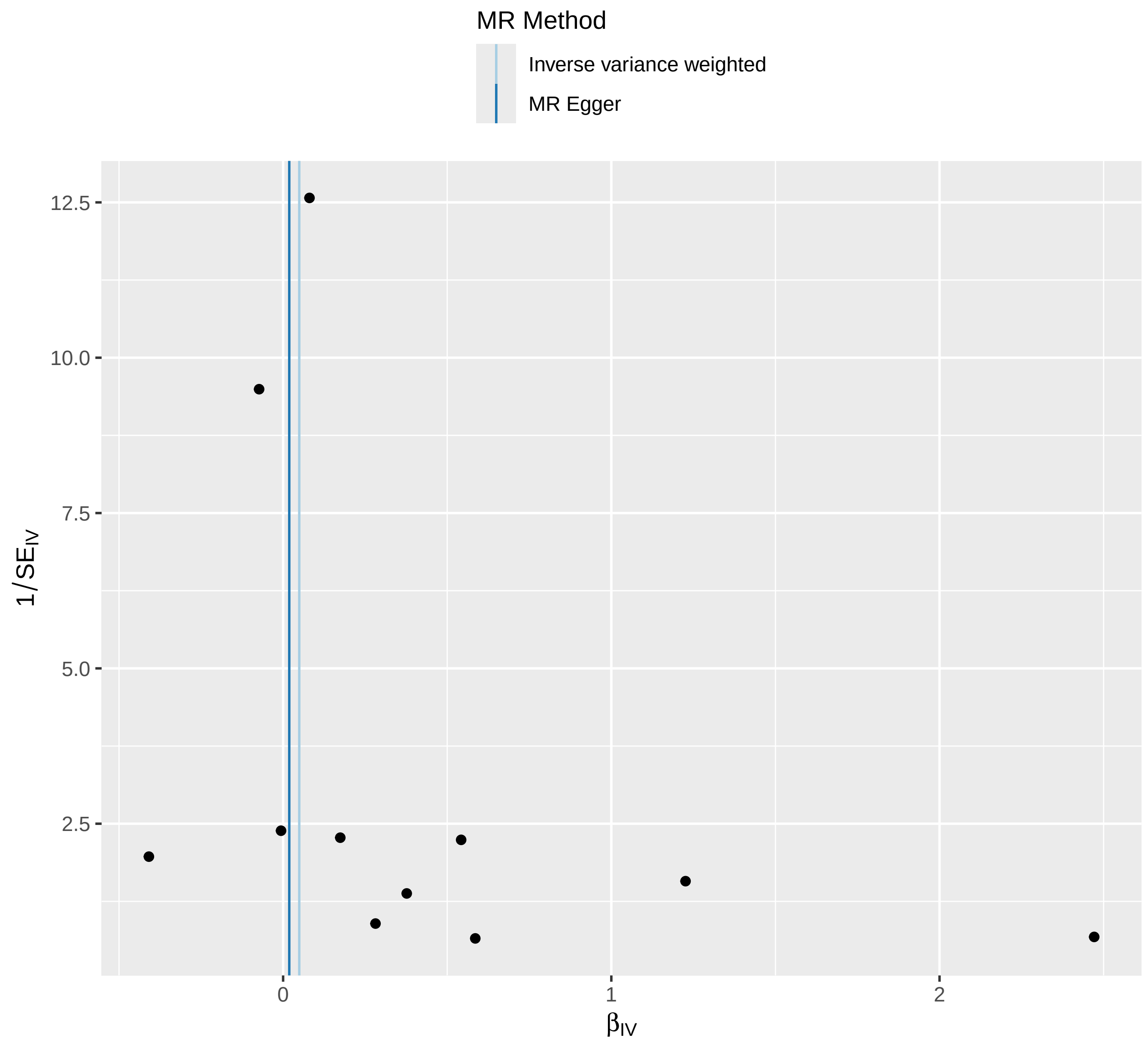
MR Method
Inverse variance weighted
MR Egger
12.5
10.0
7.5
5.0
2.5
$1/SE_{IV}$
0
1
2
β_{IV}

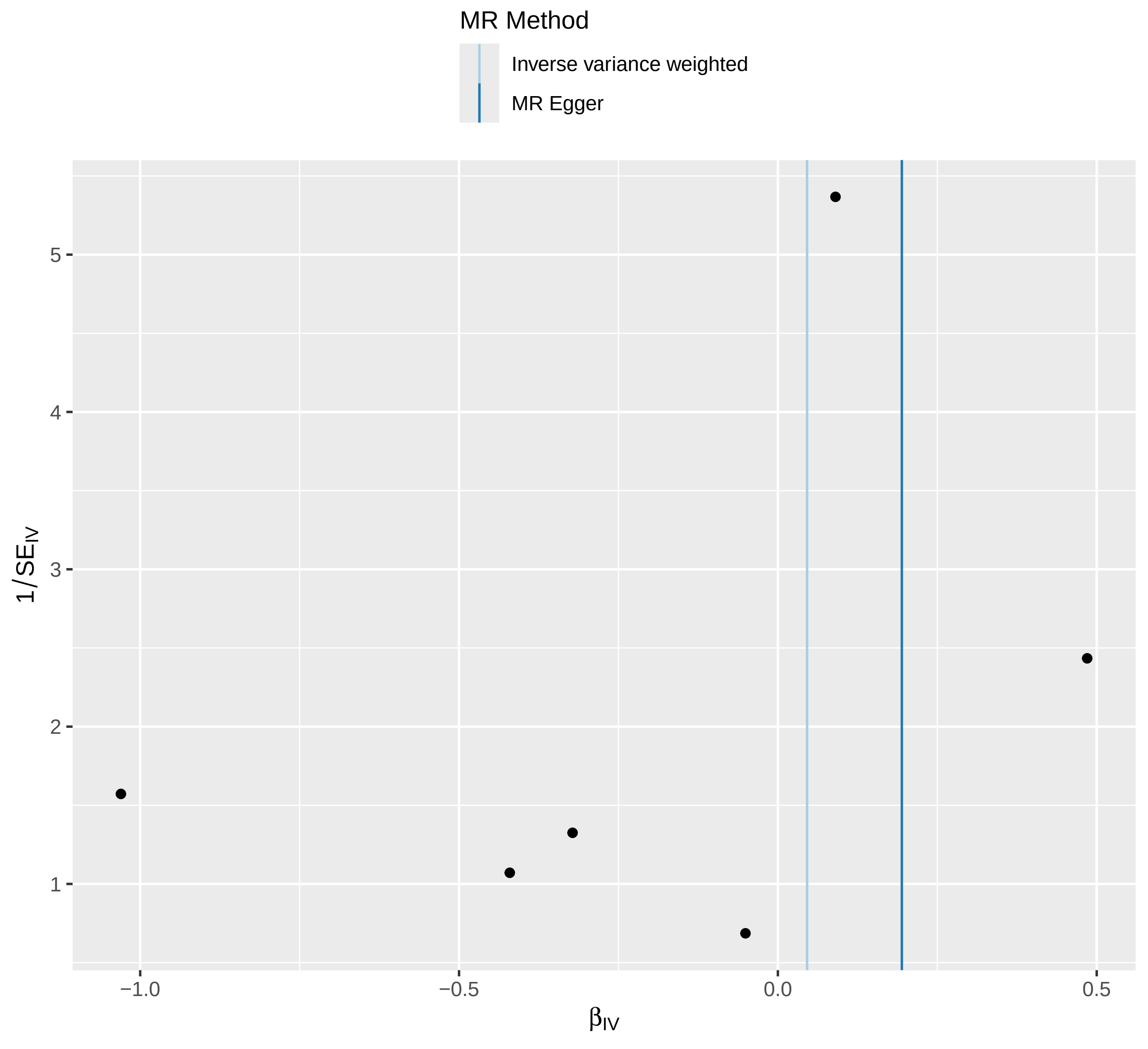
MR Method
Inverse variance weighted
MR Egger
5
4
3
2
1
−1.0
−0.5
0.0
0.5
$1/SE_{IV}$
β_{IV}

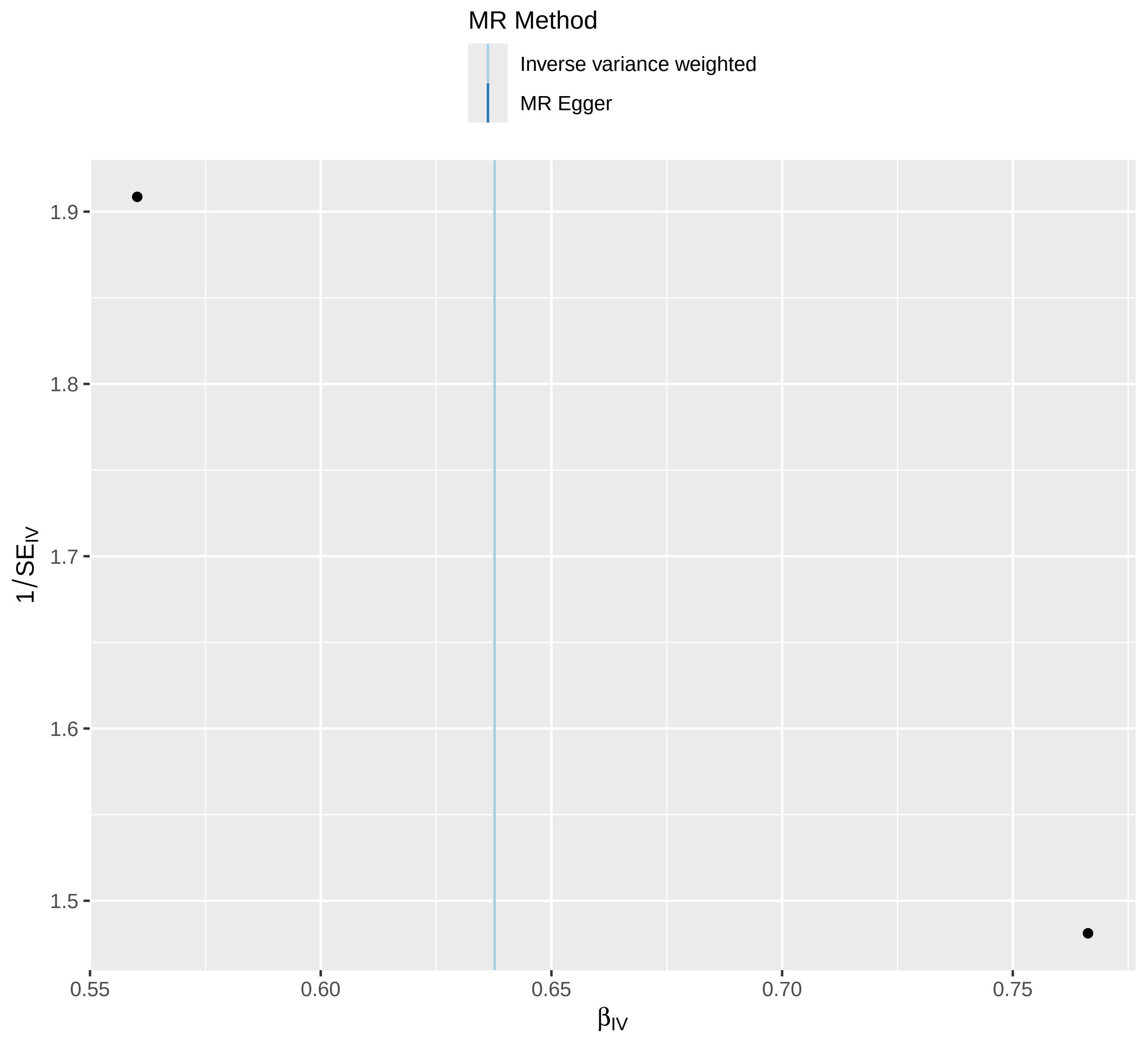
MR Method
Inverse variance weighted
MR Egger
1.9
1.8
1.7
1.6
1.5
$1/SE_{IV}$
0.55
0.60
0.65
0.70
0.75
β_{IV}

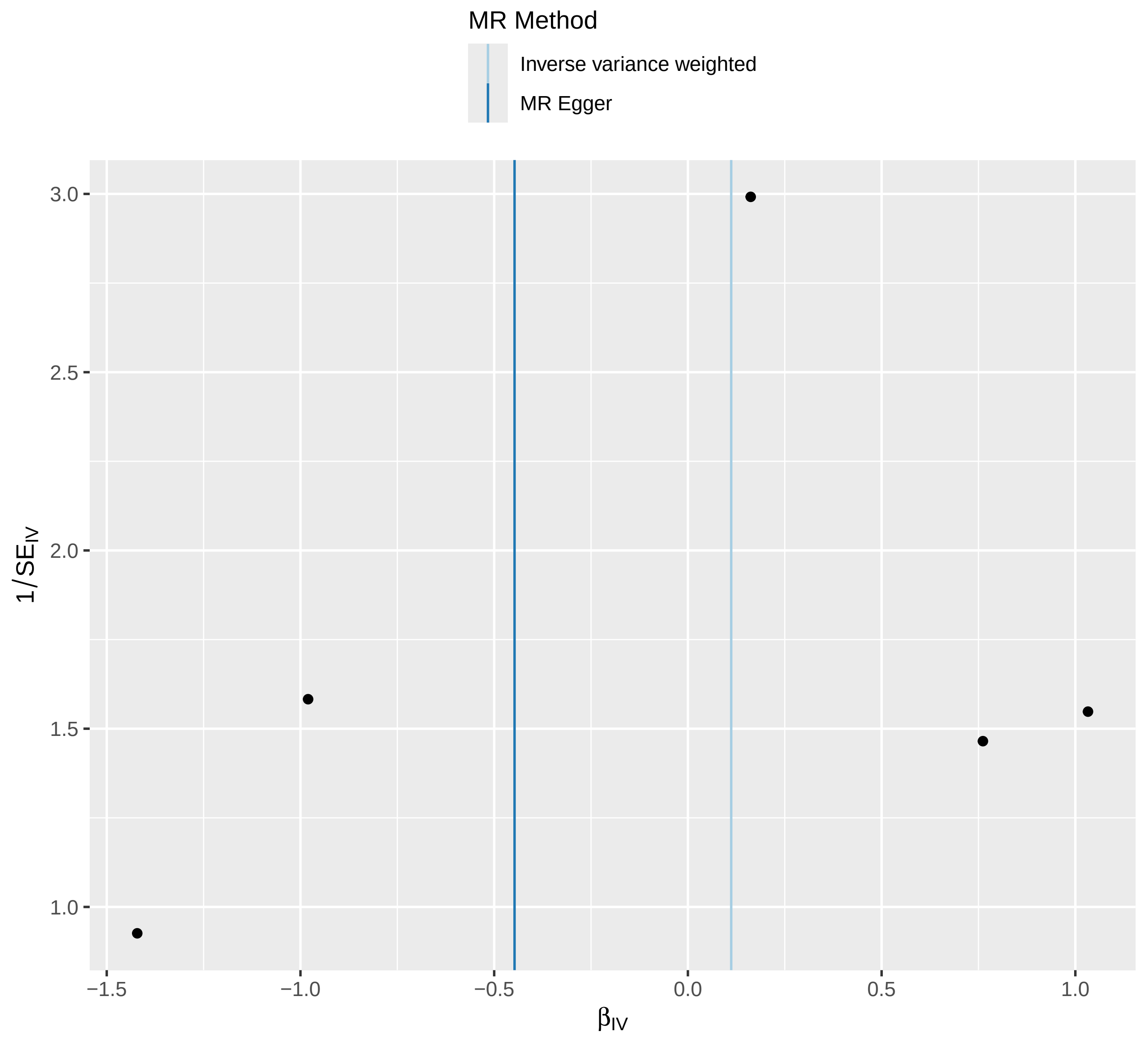
MR Method
Inverse variance weighted
MR Egger
3.0
2.5
2.0
1.5
1.0
1/SE$_{IV}$
−1.5
−1.0
−0.5
0.0
0.5
1.0
β$_{IV}$

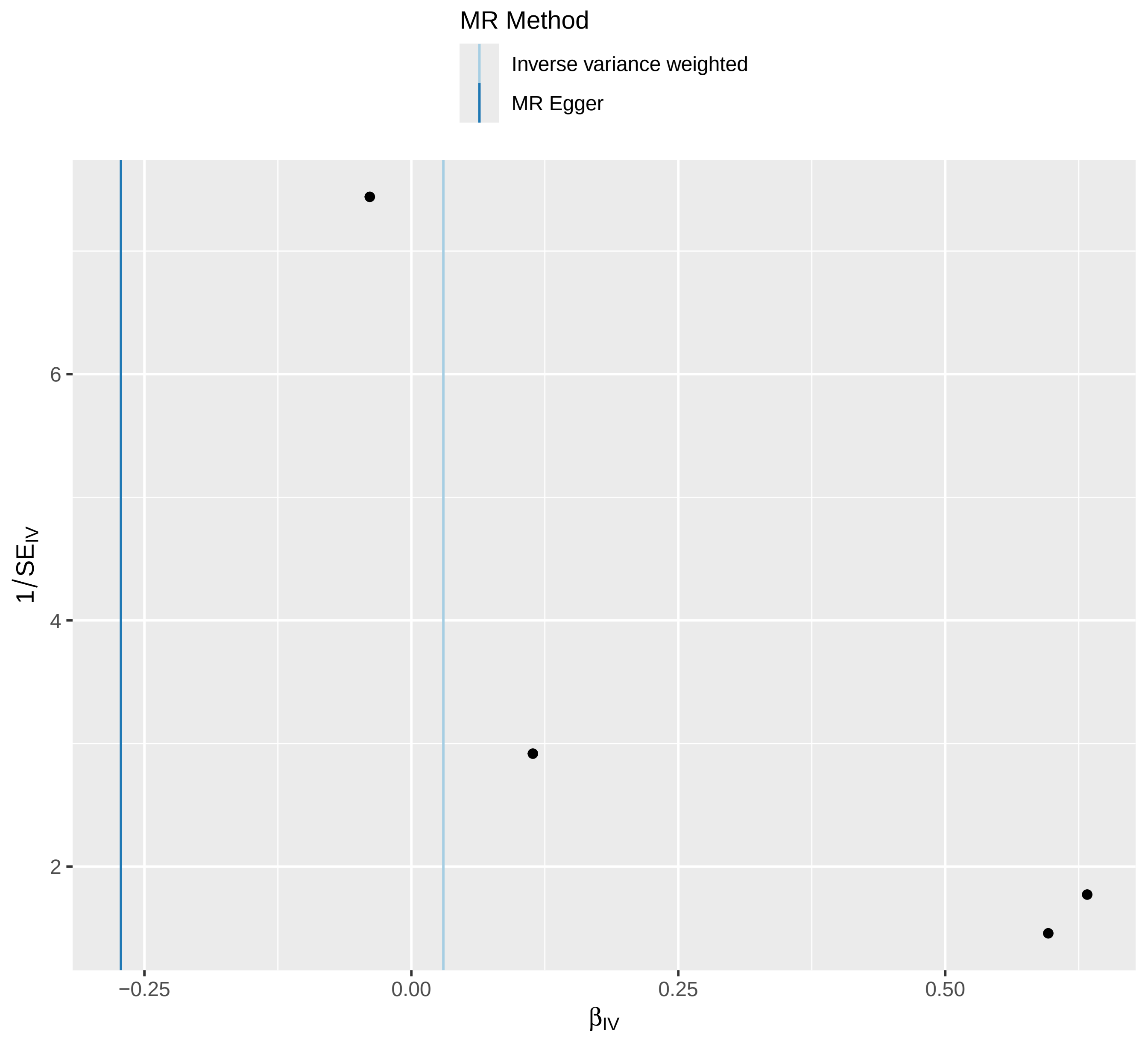
MR Method
Inverse variance weighted
MR Egger
6
4
2
1/SE_IV
−0.25
0.00
0.25
0.50
β_IV

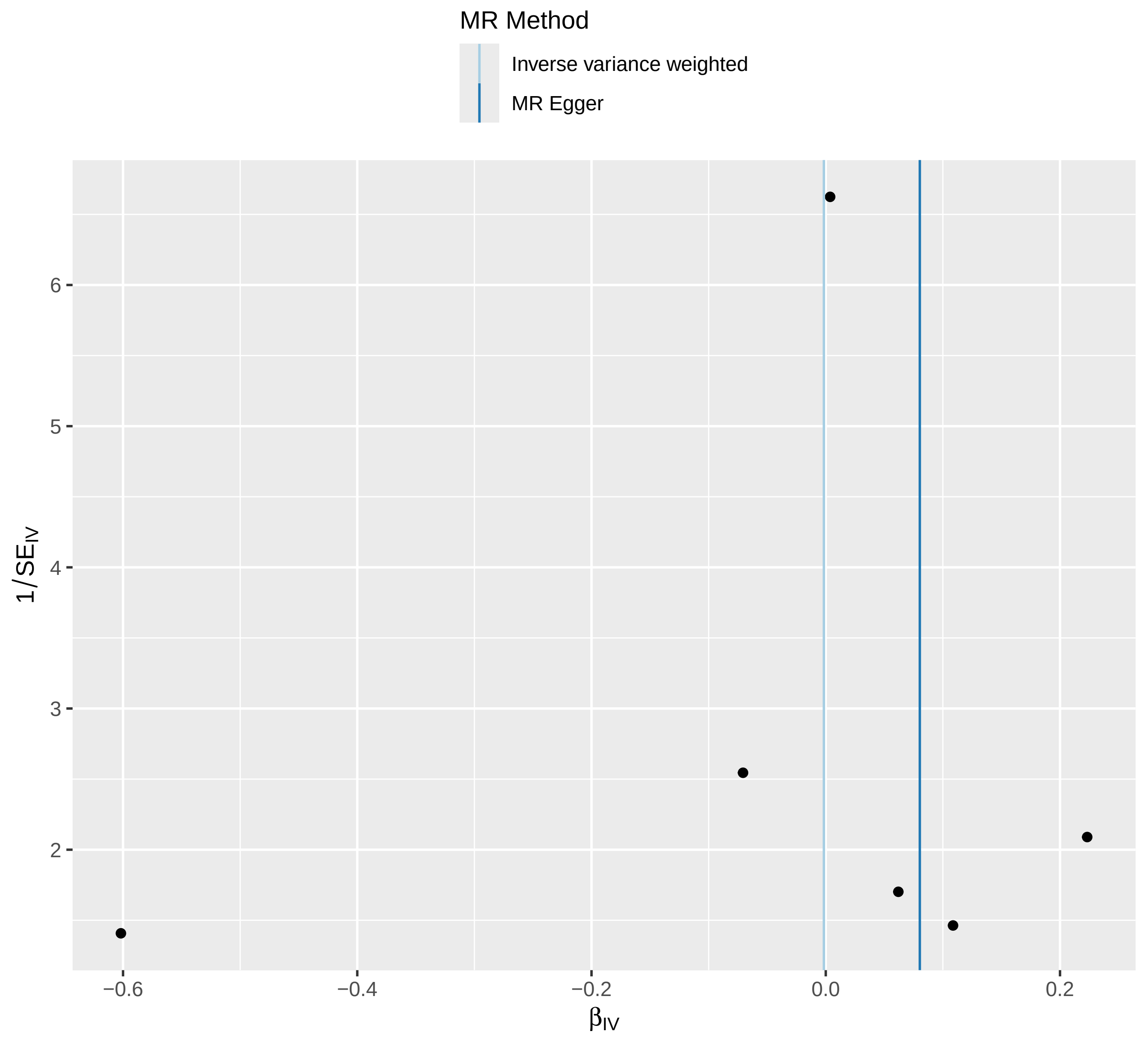
MR Method
Inverse variance weighted
MR Egger
6
5
4
3
2
1/SE_IV
−0.6
−0.4
−0.2
0.0
0.2
β_IV

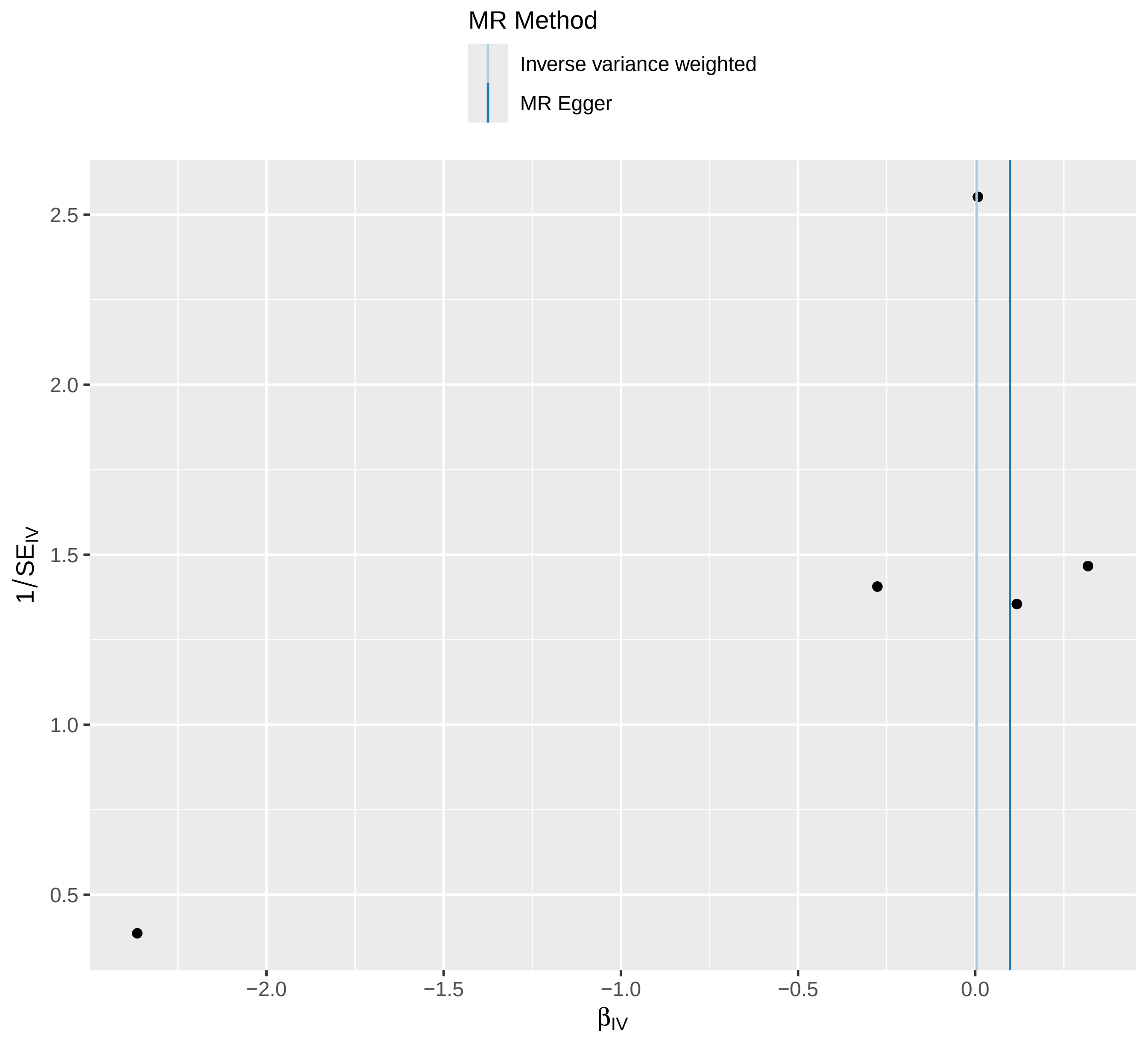
MR Method
Inverse variance weighted
MR Egger
2.5
2.0
1.5
1.0
0.5
1/SE$_{IV}$
−2.0
−1.5
−1.0
−0.5
0.0
β_{IV}

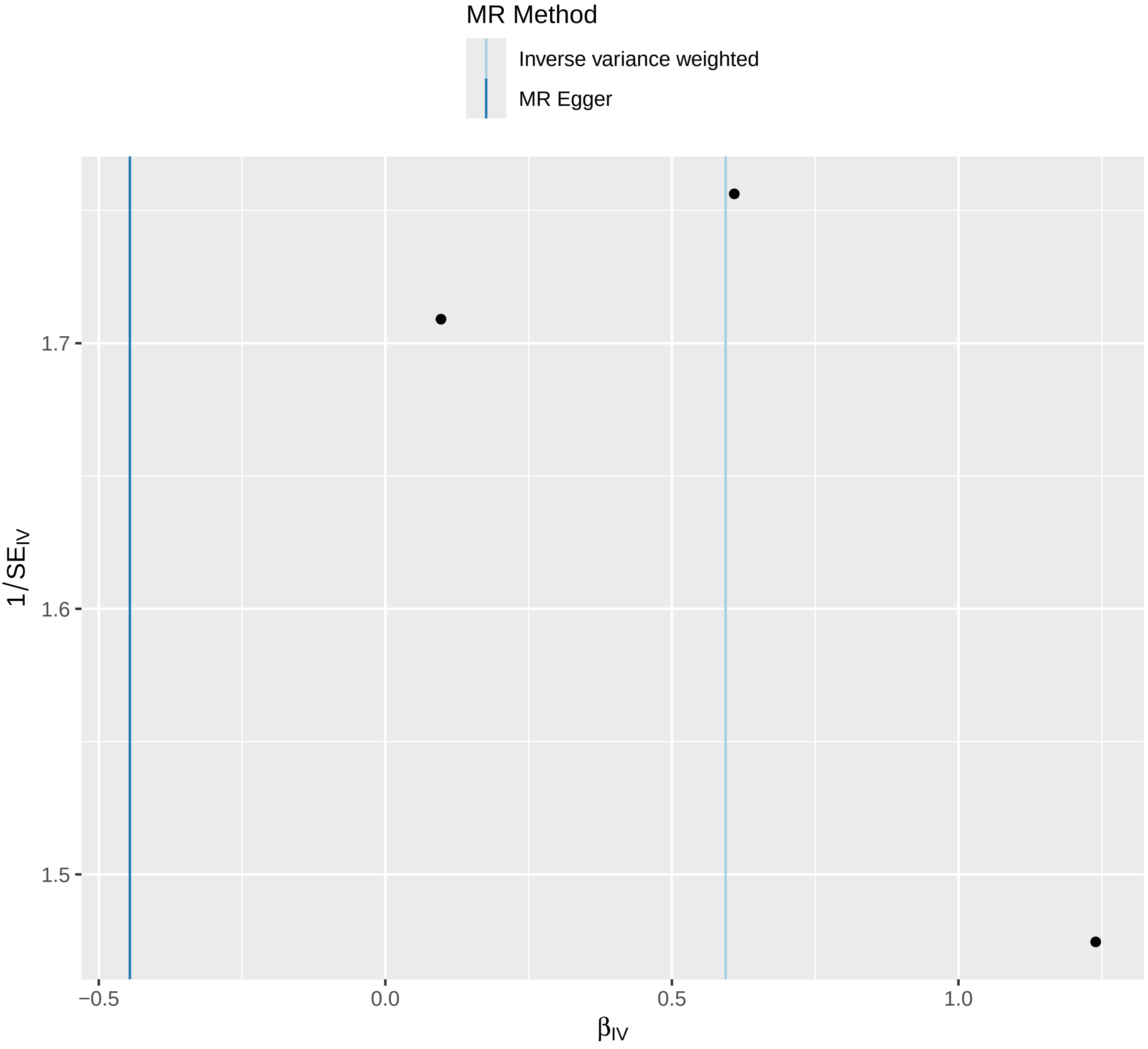

MR Method
Inverse variance weighted
MR Egger
1.7
1.6
1.5
1/SE_IV
−0.5
0.0
0.5
1.0
β_IV

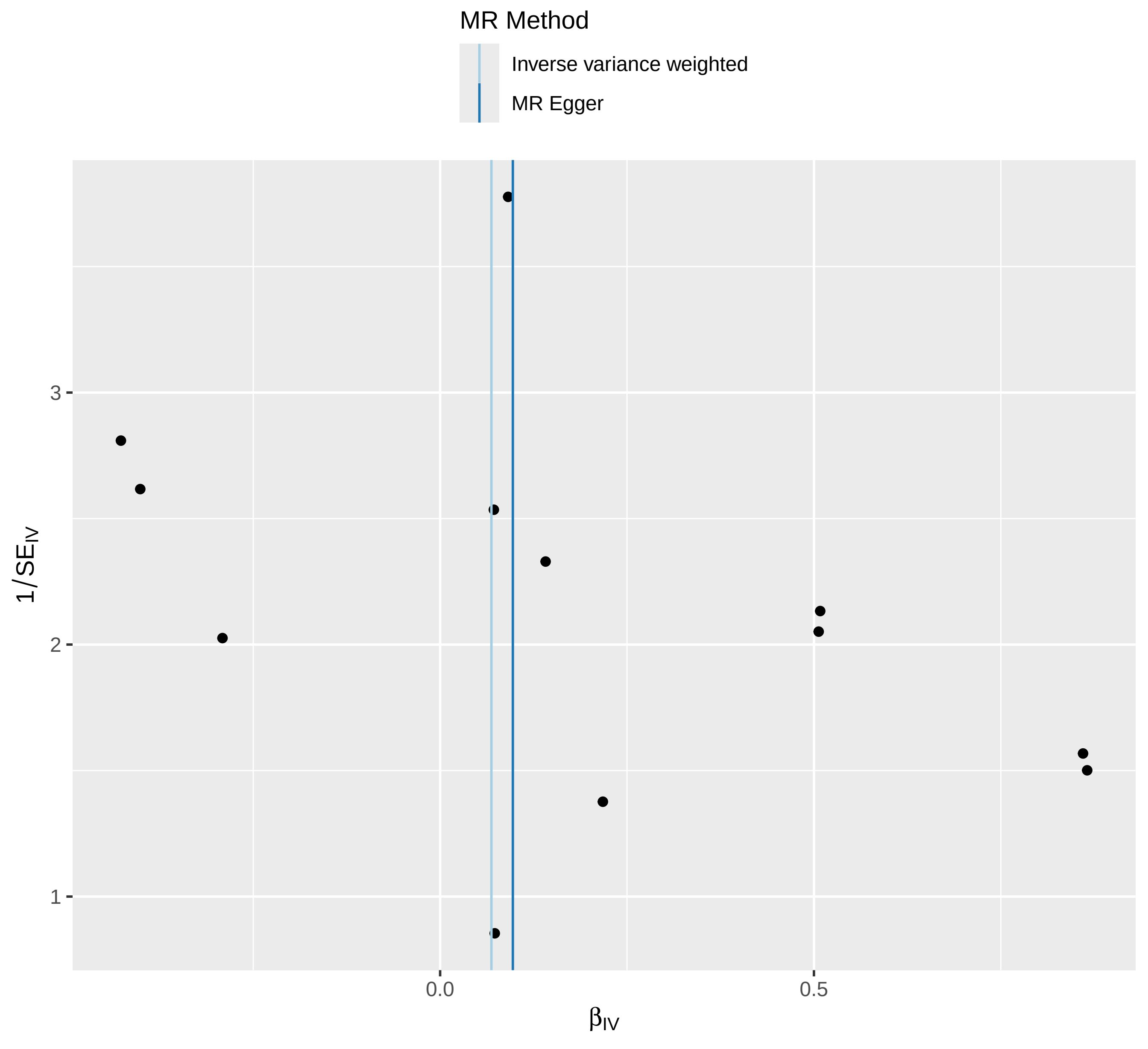
MR Method
Inverse variance weighted
MR Egger
3
2
1
1/SE_IV
0.0
0.5
β_IV

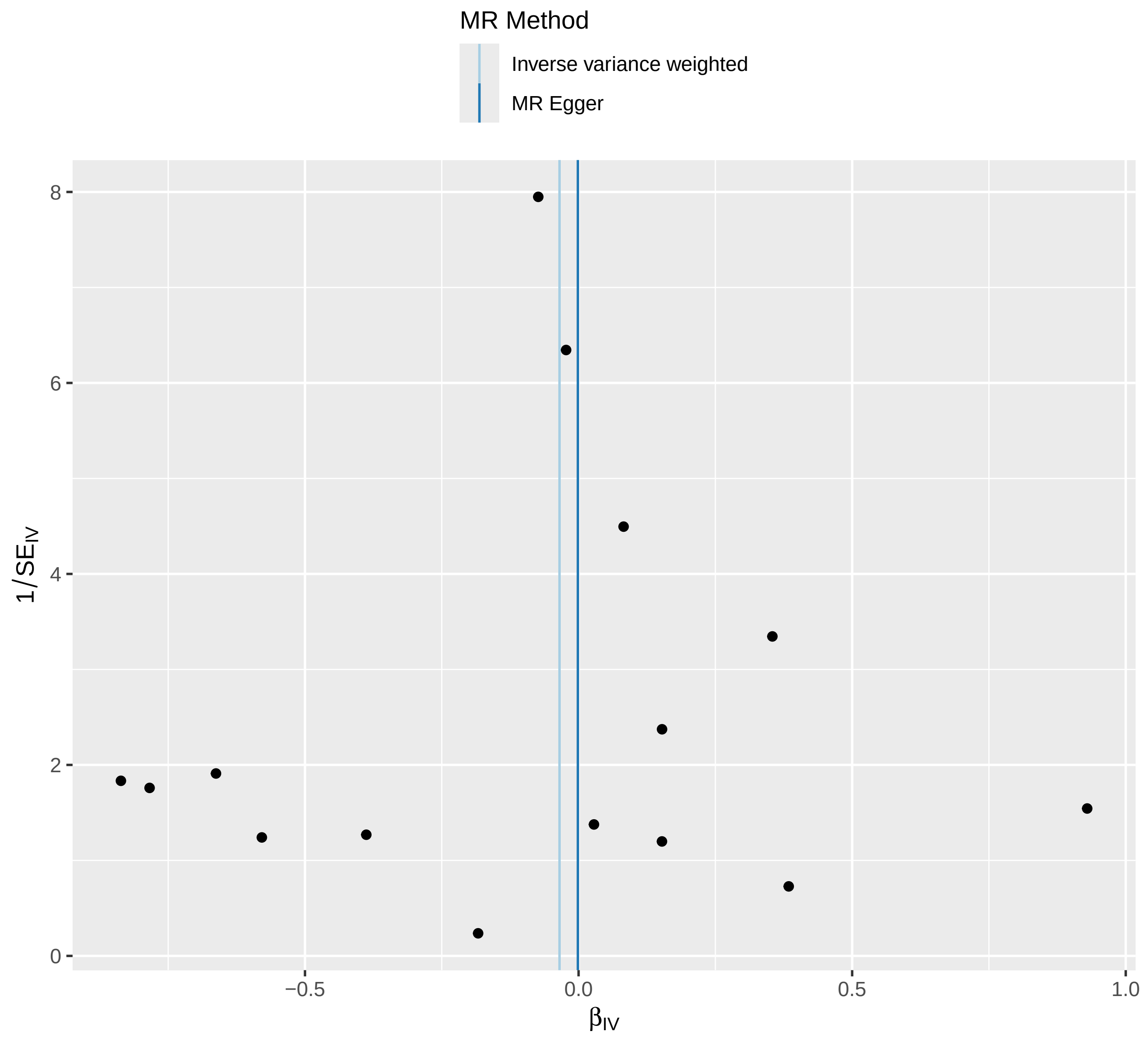

MR Method
Inverse variance weighted
MR Egger
8
6
4
2
0
1/SE_IV
−0.5
0.0
0.5
1.0
β_IV

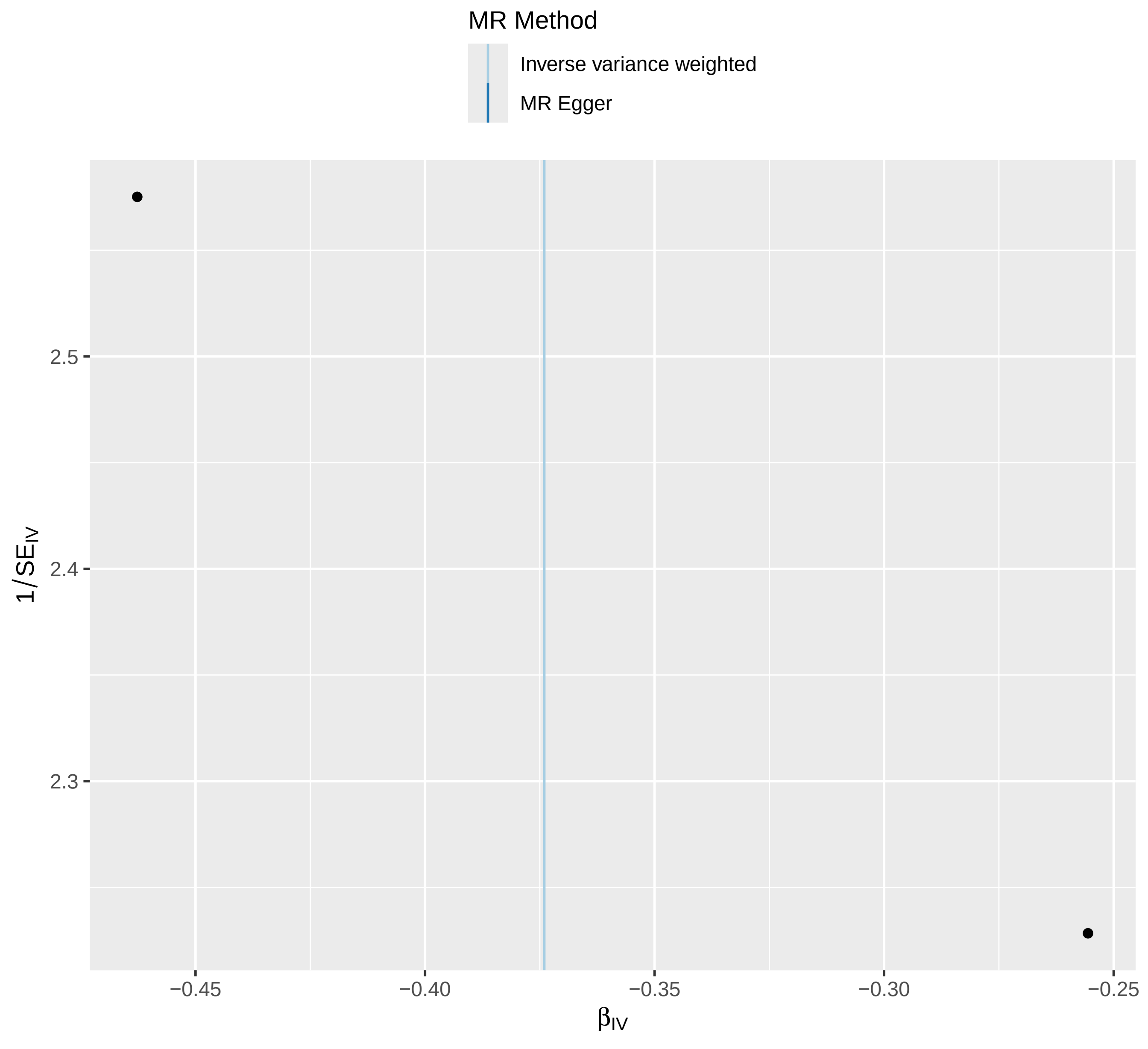
MR Method
Inverse variance weighted
MR Egger
1/SE_IV
2.5
2.4
2.3
β_IV
−0.45
−0.40
−0.35
−0.30
−0.25

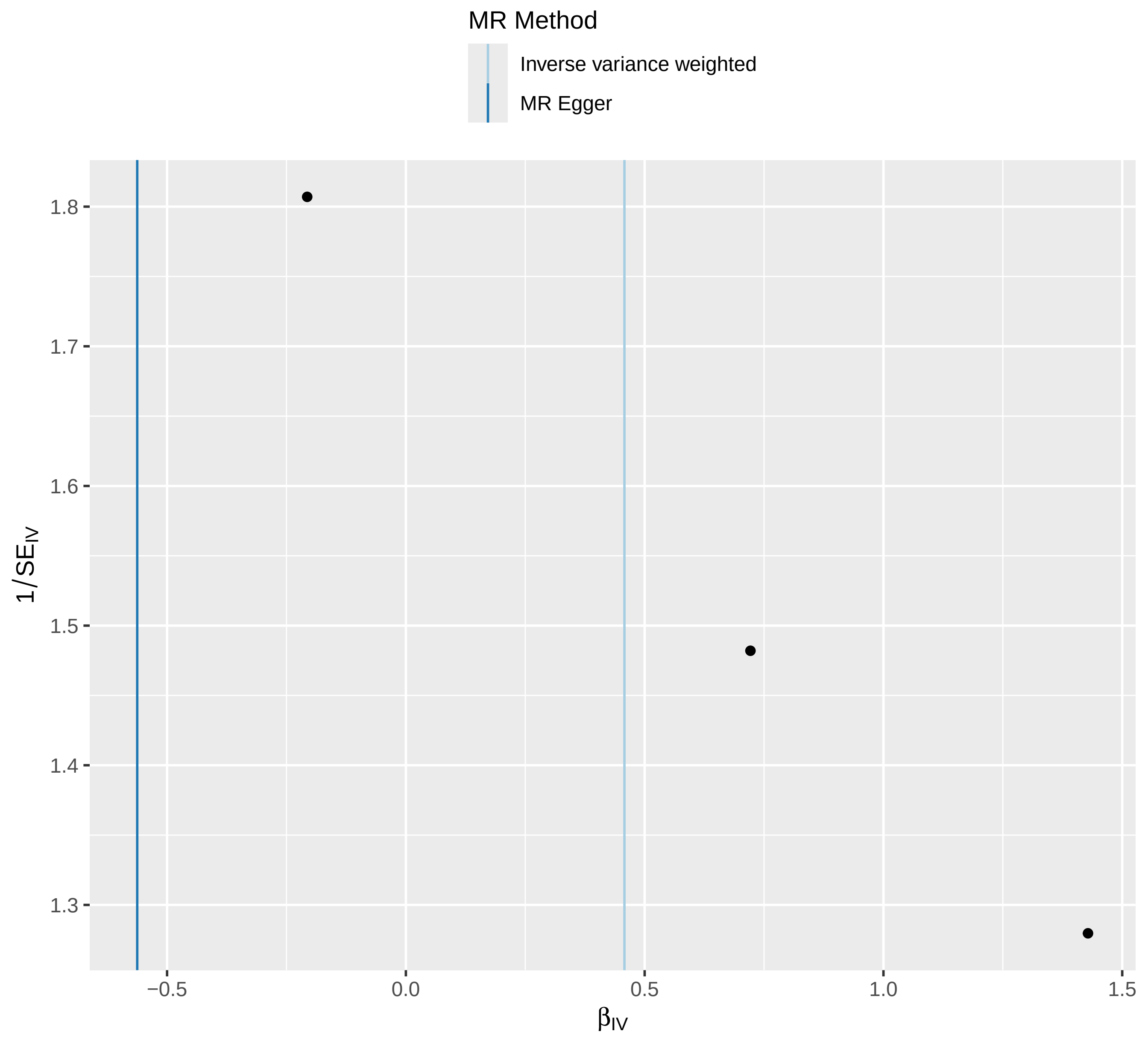
MR Method
Inverse variance weighted
MR Egger
1.8
1.7
1.6
1.5
1.4
1.3
1/SE$_{IV}$
−0.5
0.0
0.5
1.0
1.5
β$_{IV}$

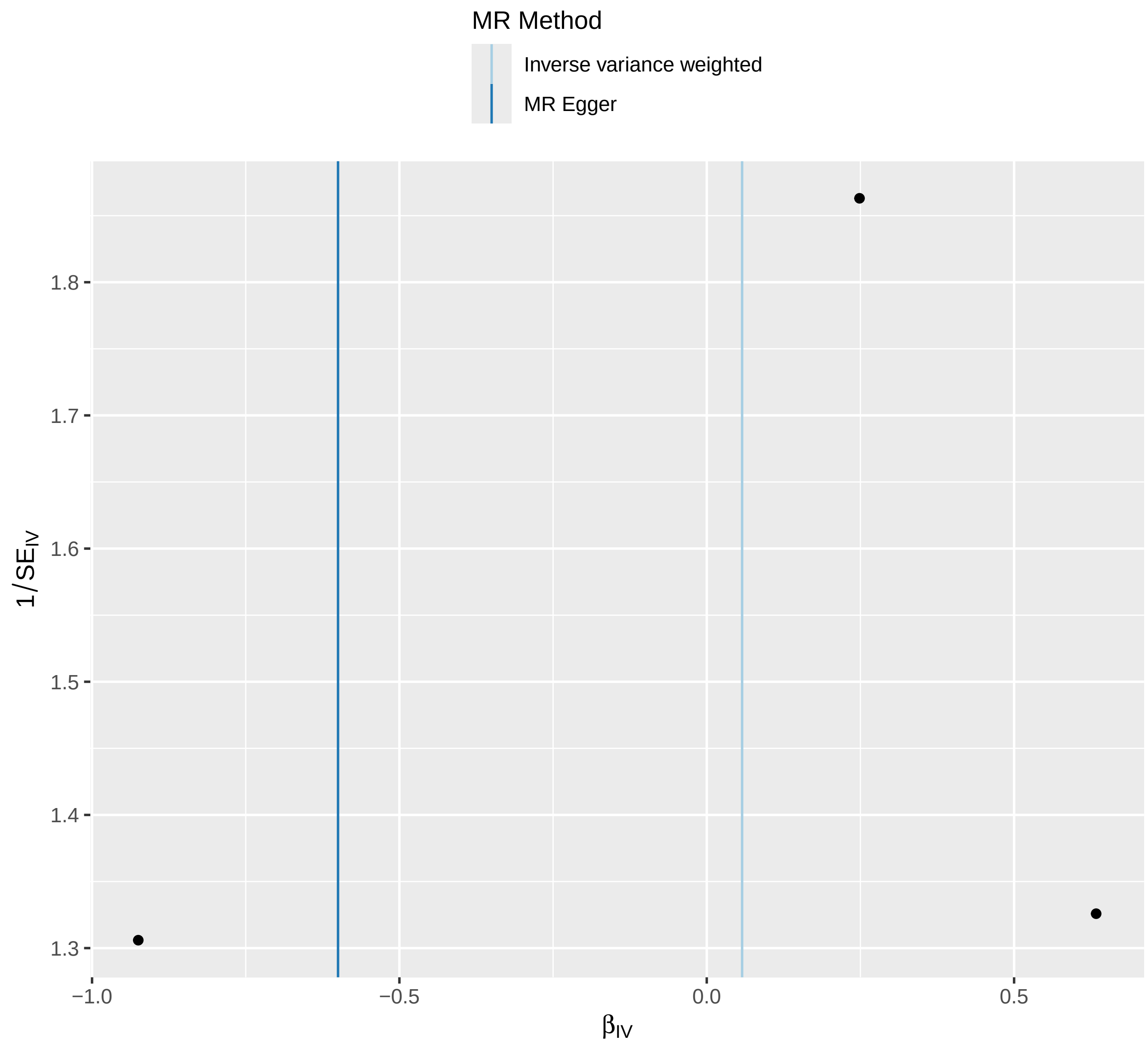
MR Method
Inverse variance weighted
MR Egger
1.8
1.7
1.6
1.5
1.4
1.3
$1/SE_{IV}$
−1.0
−0.5
0.0
0.5
β_{IV}

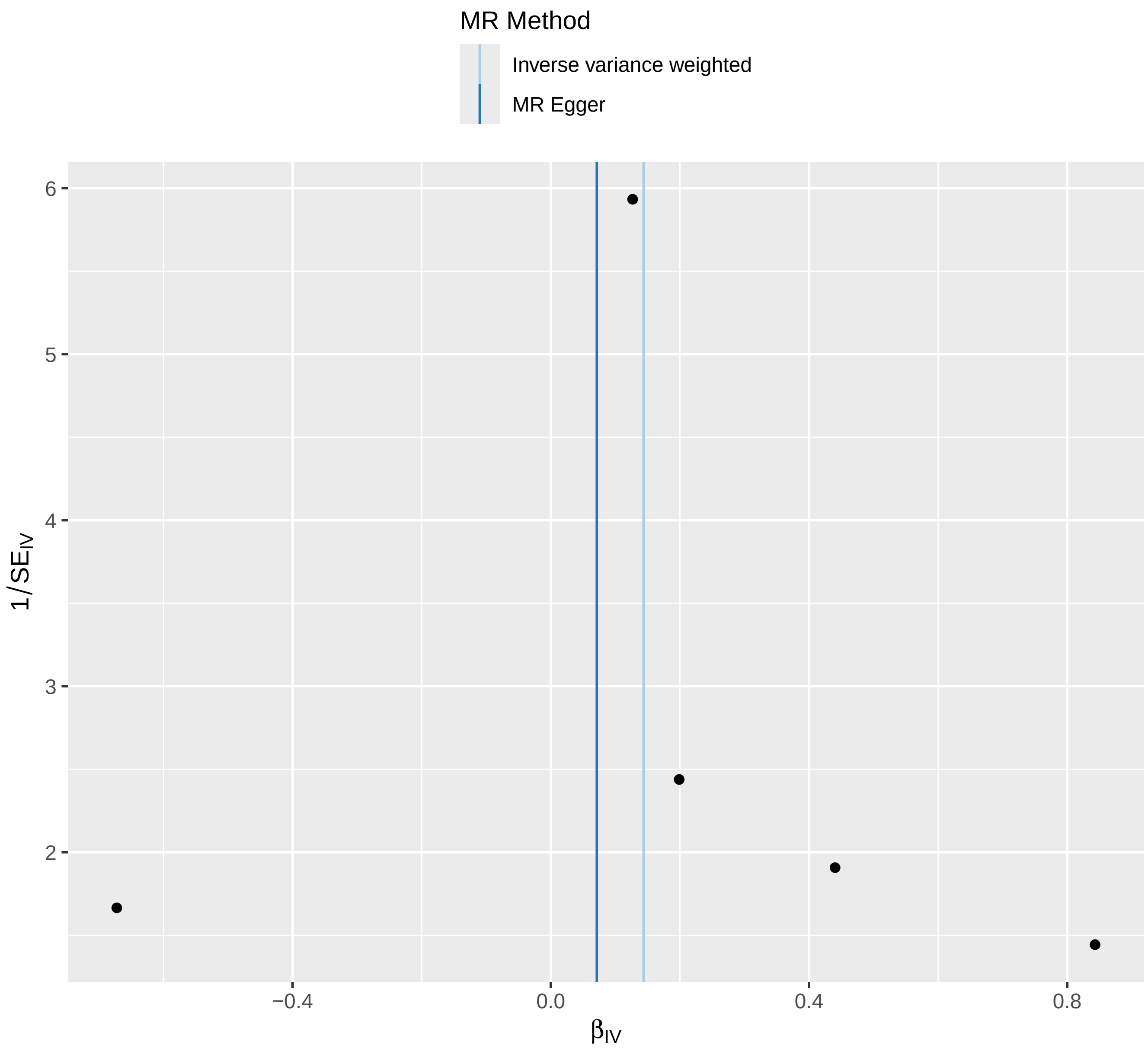
MR Method
Inverse variance weighted
MR Egger
6
5
4
3
2
1/SE_IV
−0.4
0.0
0.4
0.8
β_IV

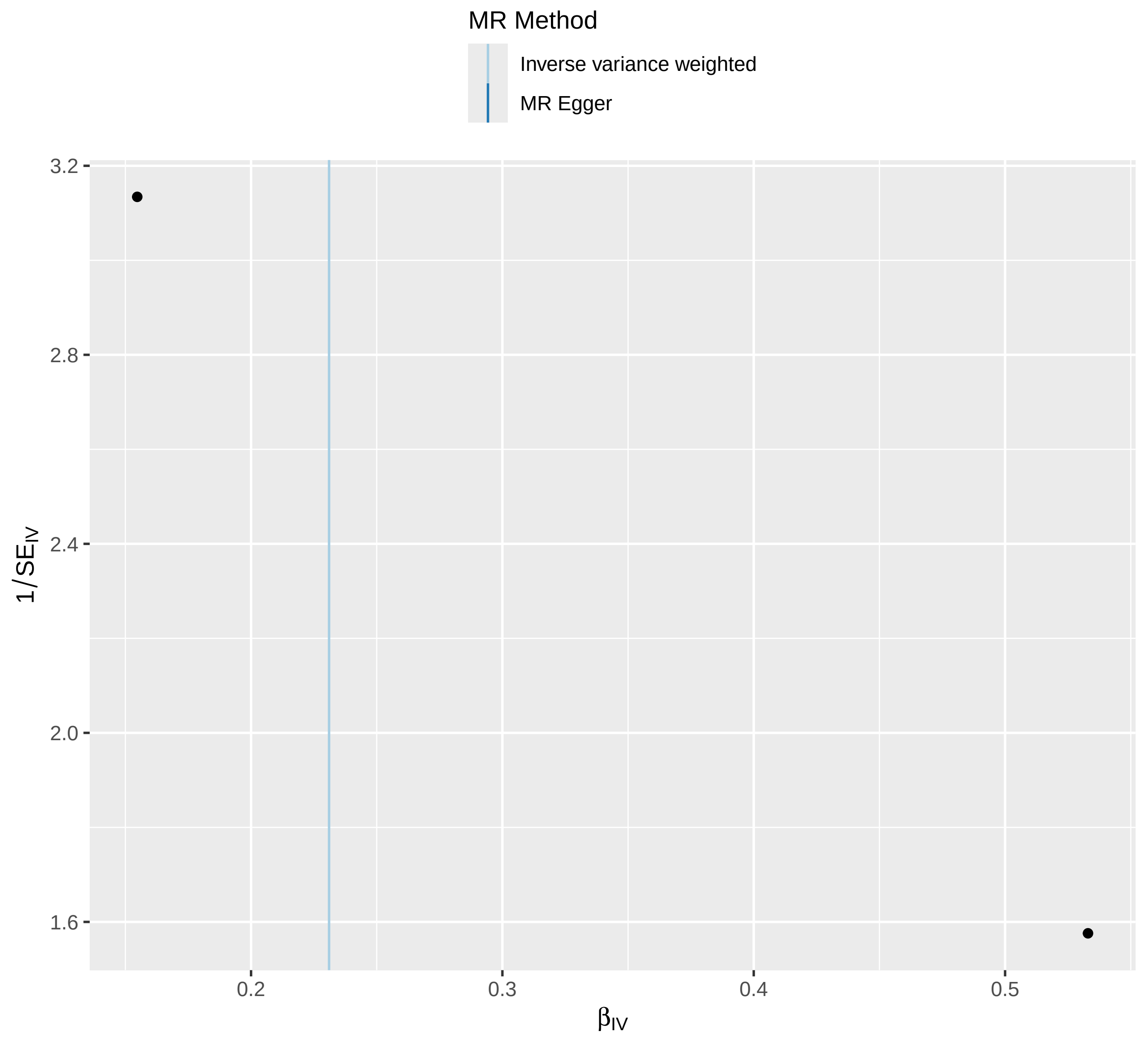

MR Method
Inverse variance weighted
MR Egger
3.2
2.8
2.4
2.0
1.6
1/SE_IV
0.2
0.3
0.4
0.5
β_IV

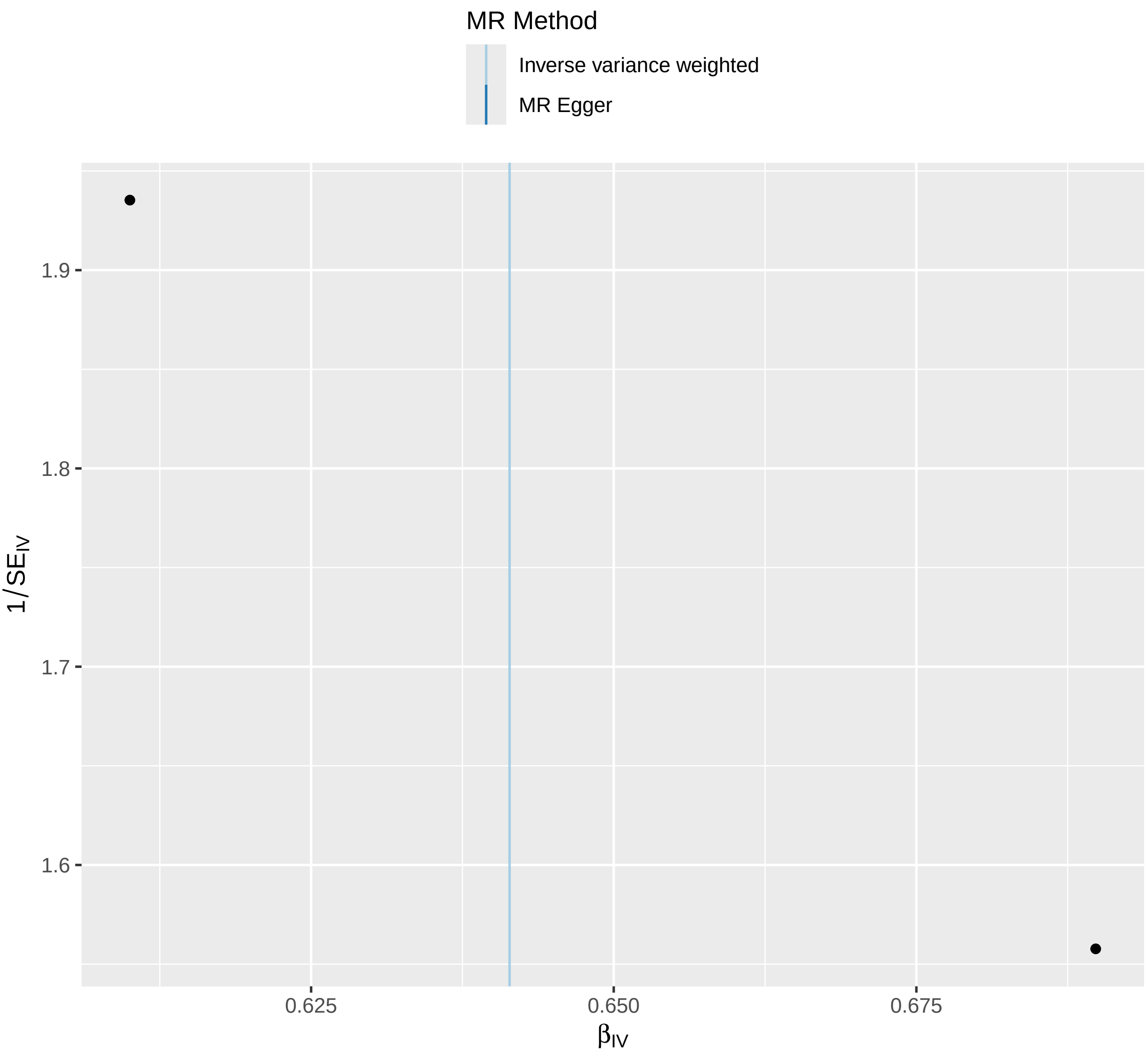
MR Method
Inverse variance weighted
MR Egger
1.9
1.8
1.7
1.6
1/SE_IV
0.625
0.650
0.675
β_IV

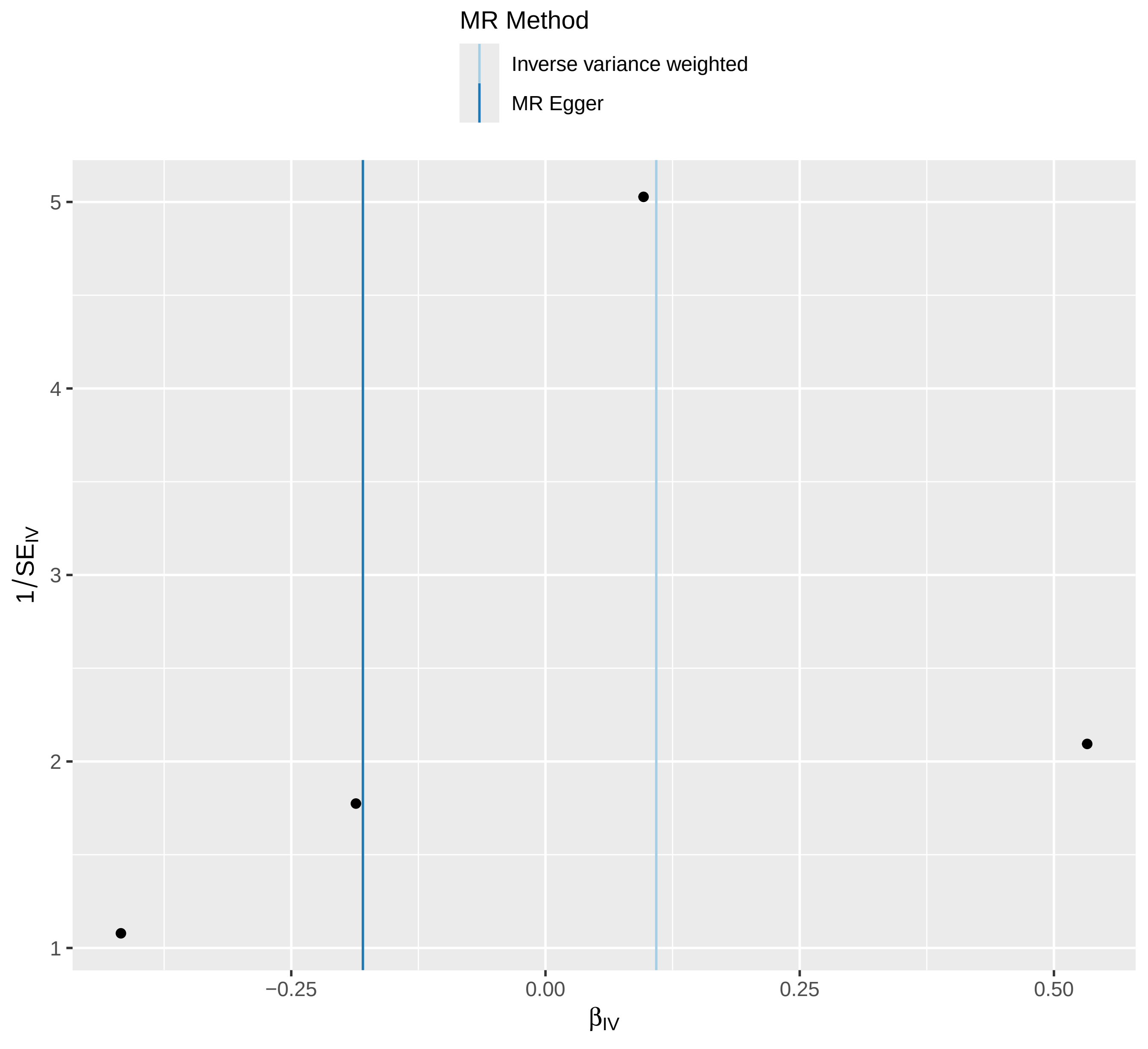

MR Method
Inverse variance weighted
MR Egger
5
4
3
2
1
1/SE_IV
−0.25
0.00
0.25
0.50
β_IV

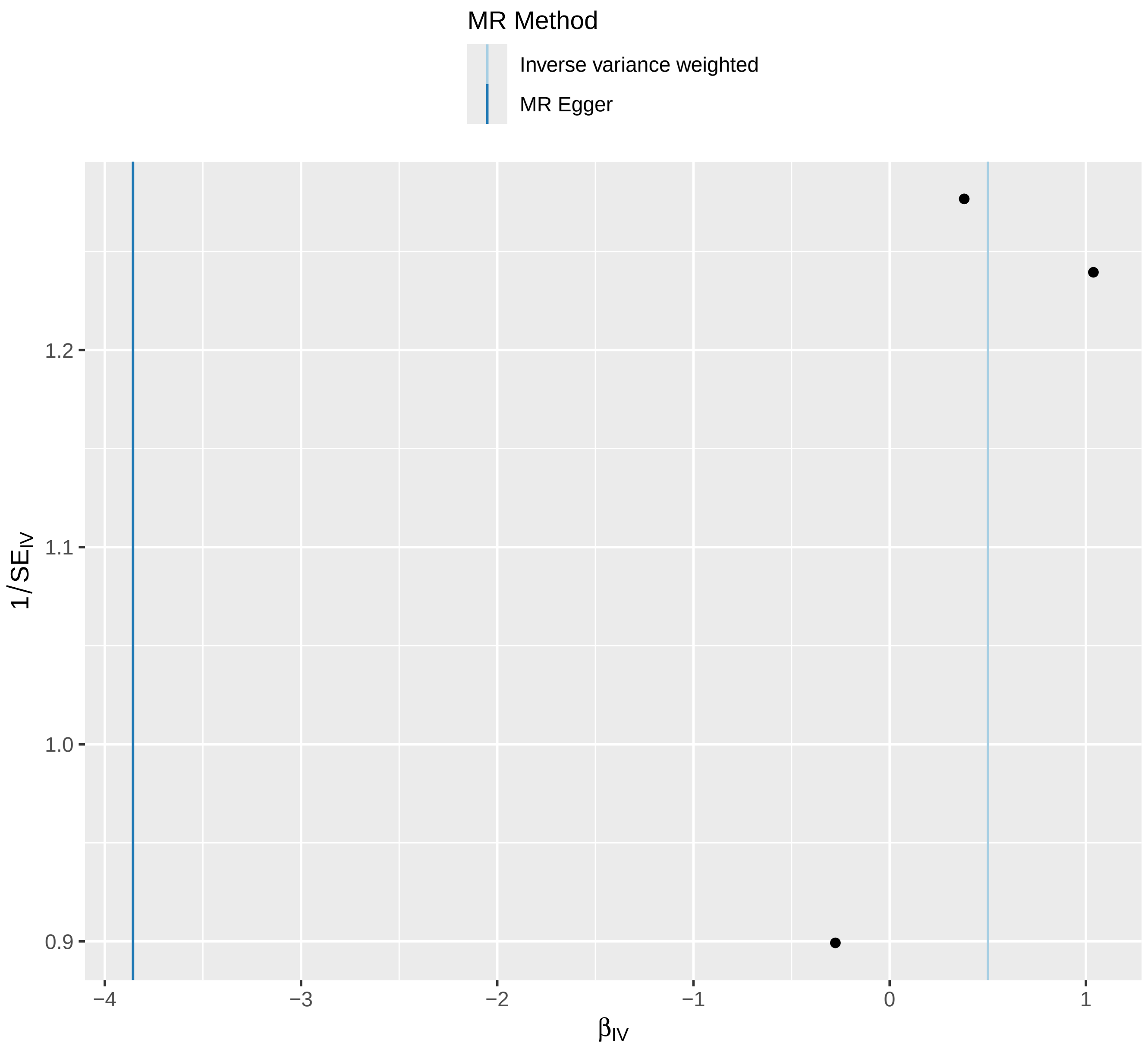
MR Method
Inverse variance weighted
MR Egger
1.2
1.1
1.0
0.9
−4
−3
−2
−1
0
1
$1/SE_{IV}$
β_{IV}

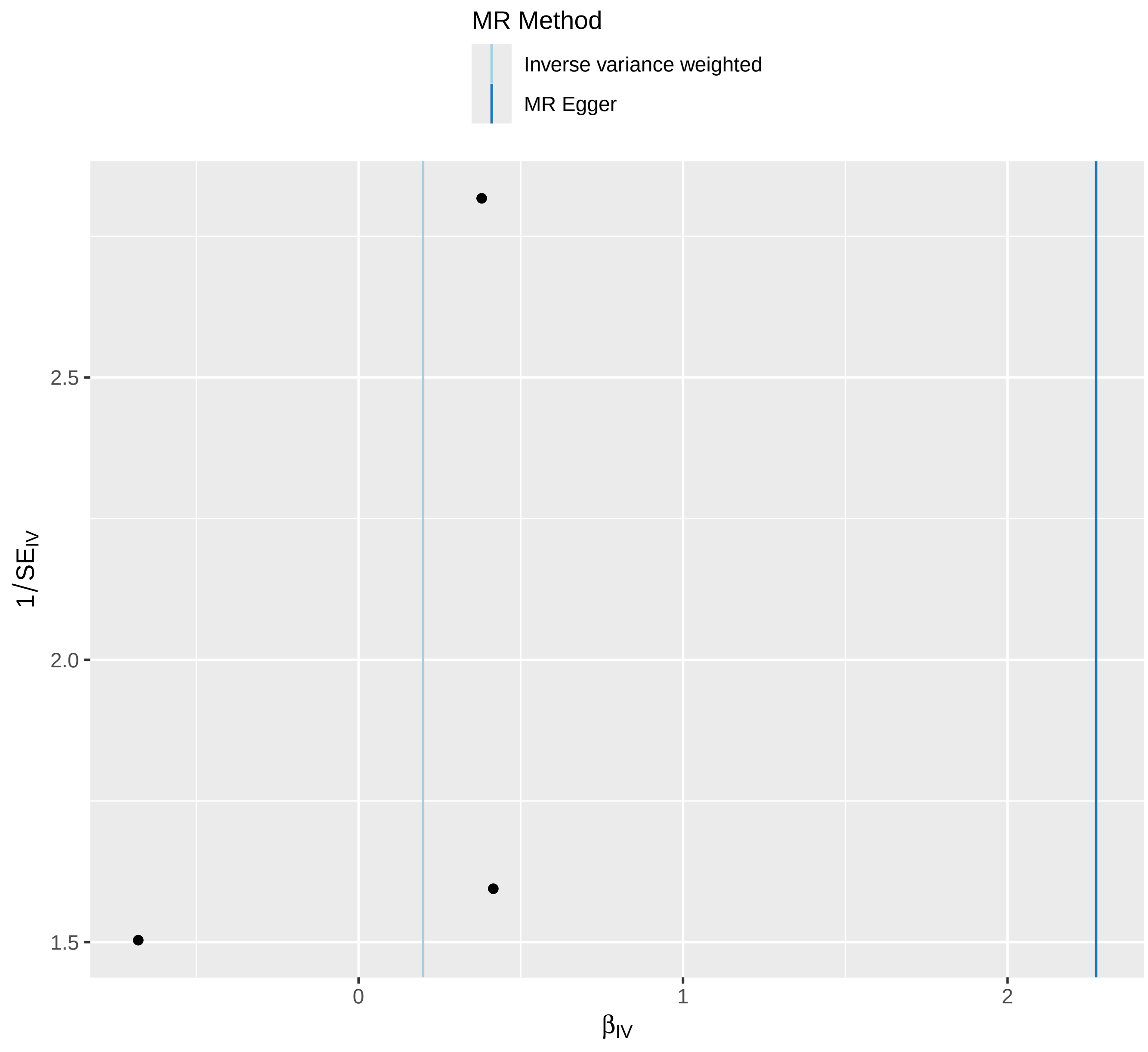
MR Method
Inverse variance weighted
MR Egger
2.5
2.0
1.5
1/SE_IV
0
1
2
β_IV

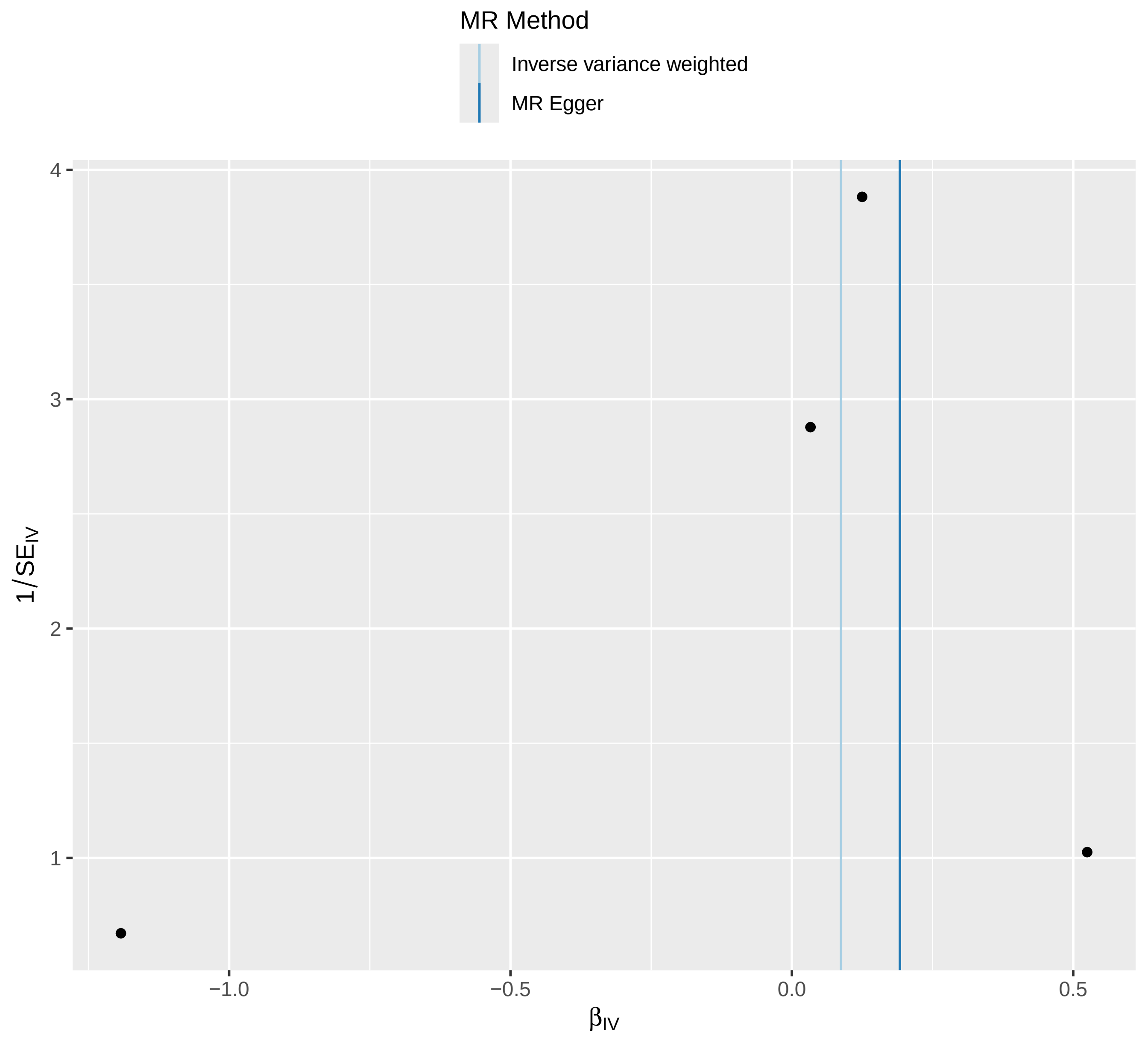
MR Method
Inverse variance weighted
MR Egger
4
3
2
1
1/SE_IV
−1.0
−0.5
0.0
0.5
β_IV

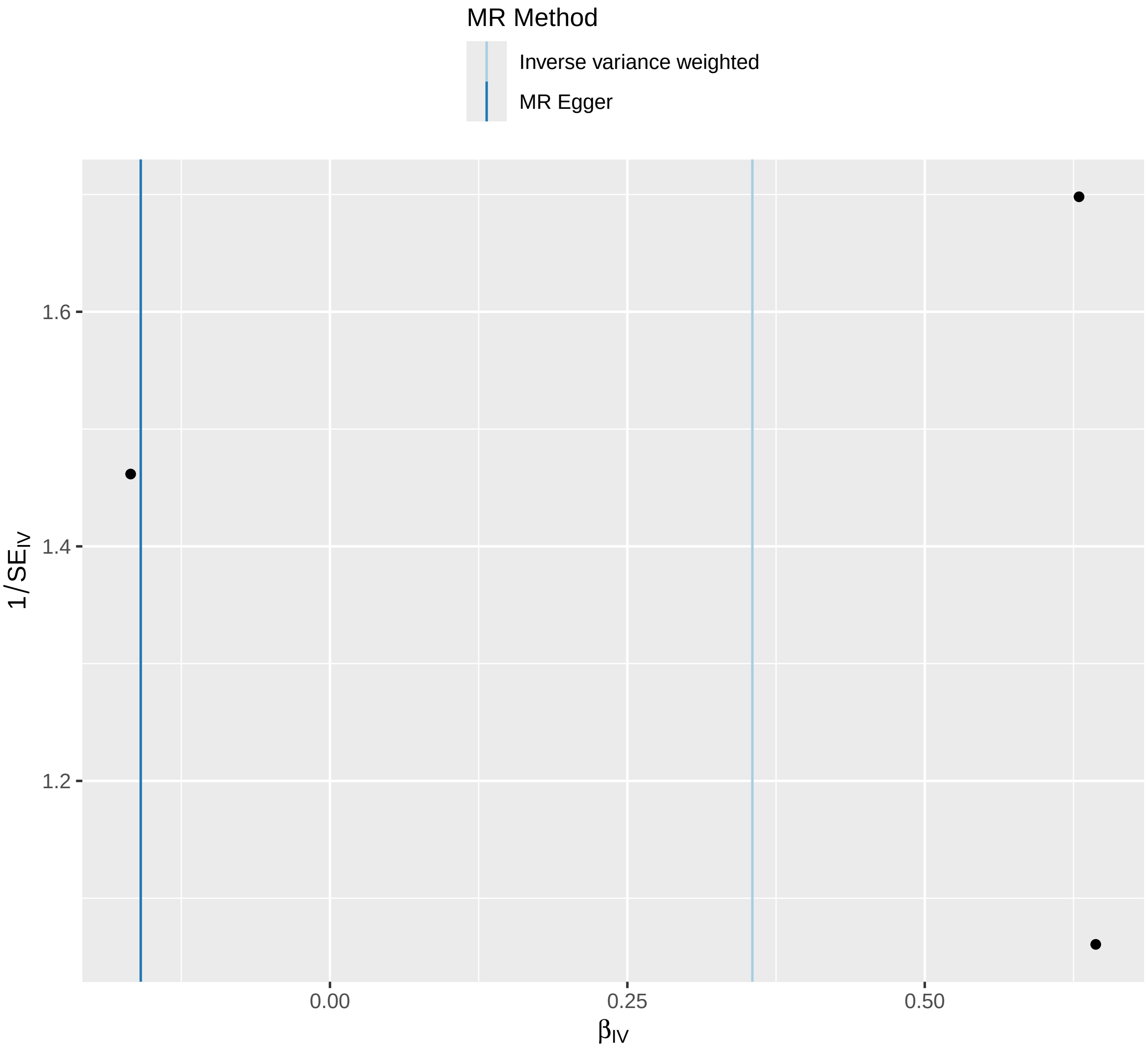

MR Method
Inverse variance weighted
MR Egger
1.6
1.4
1.2
$1/SE_{IV}$
0.00
0.25
0.50
β_{IV}

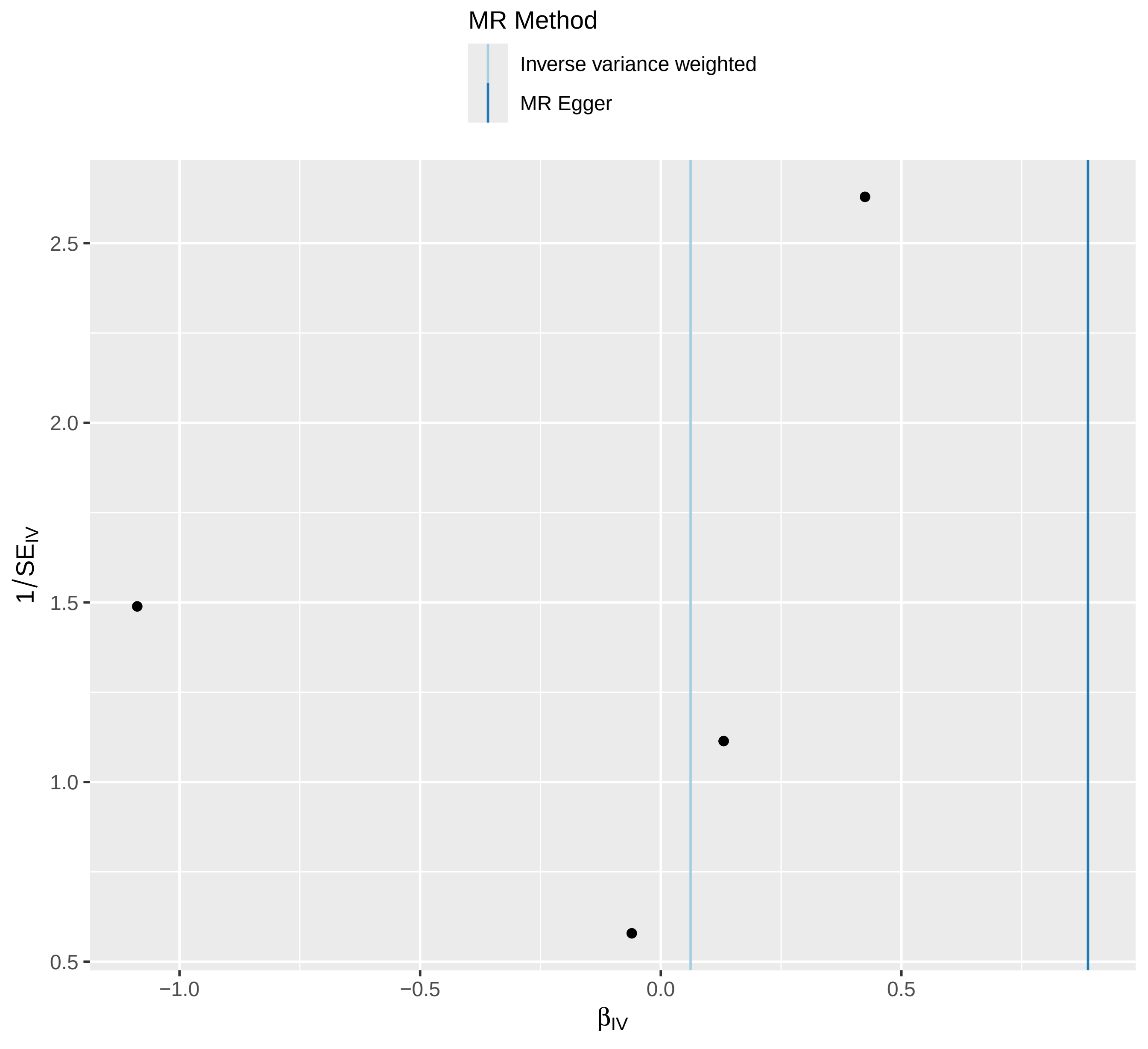

MR Method
Inverse variance weighted
MR Egger
2.5
2.0
1.5
1.0
0.5
1/SE_IV
−1.0
−0.5
0.0
0.5
β_IV

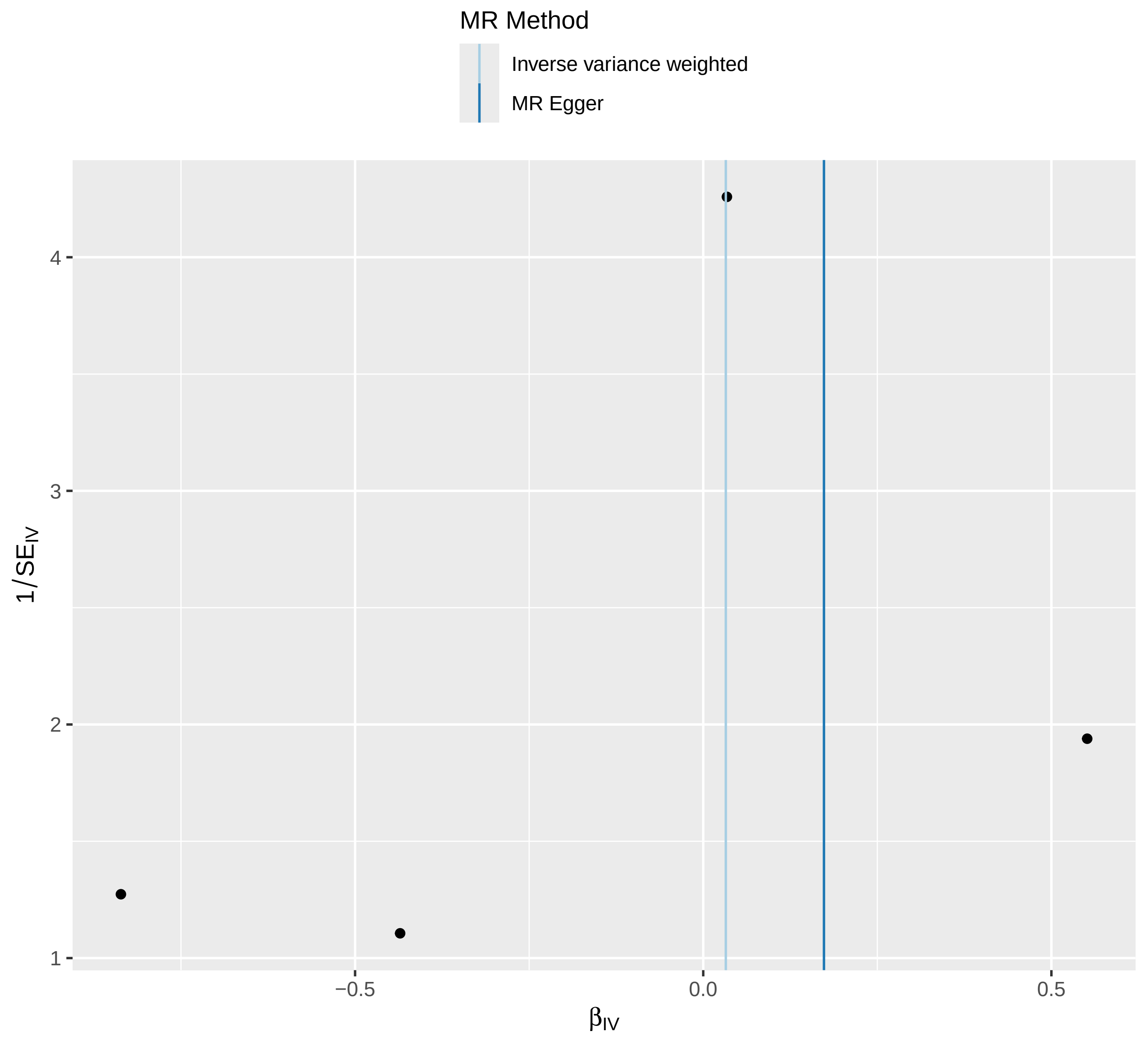

MR Method
Inverse variance weighted
MR Egger
4
3
2
1
1/SE_IV
−0.5
0.0
0.5
β_IV

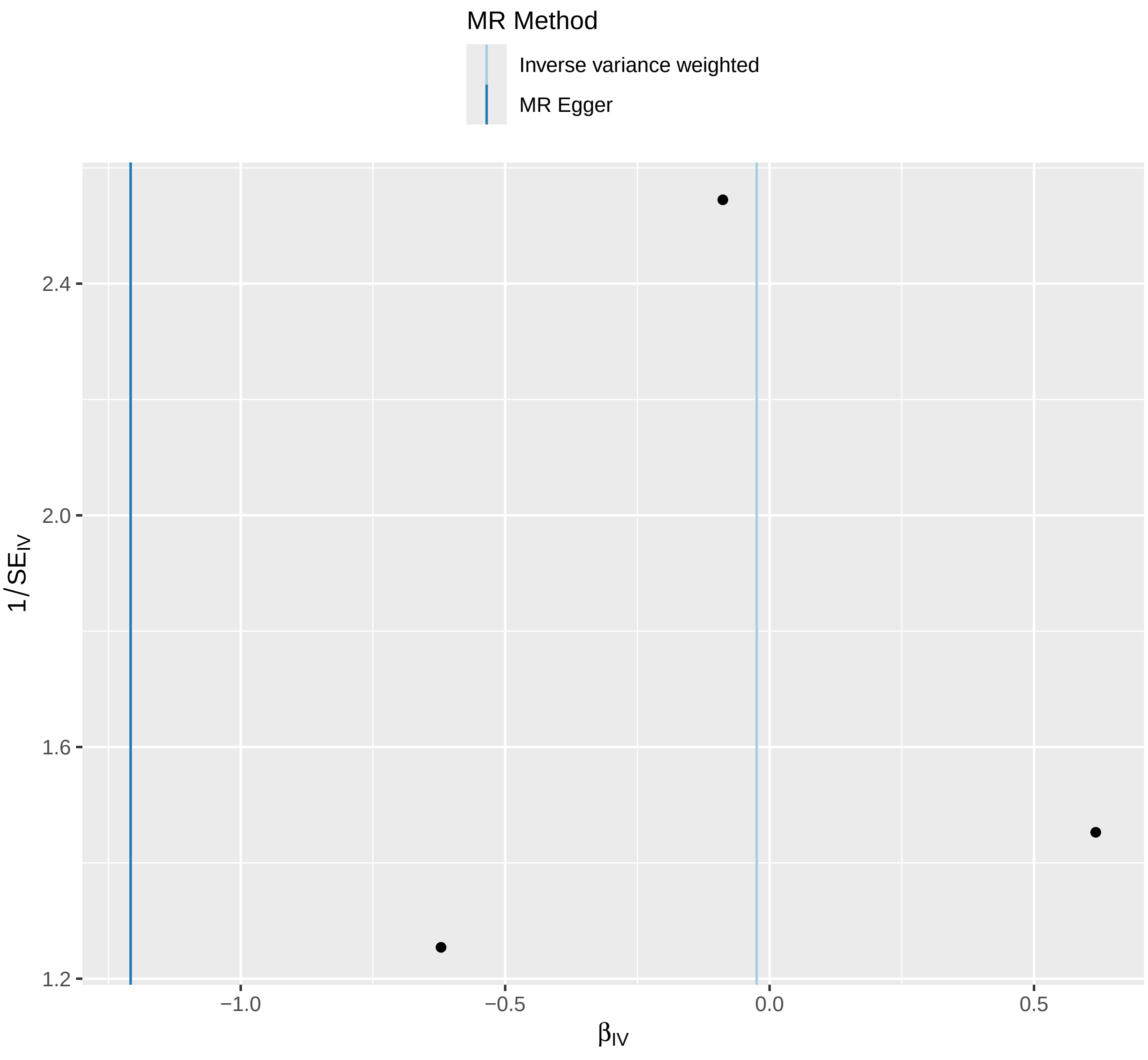
MR Method
Inverse variance weighted
MR Egger
2.4
2.0
1.6
1.2
1/SE_IV
−1.0
−0.5
0.0
0.5
β_IV

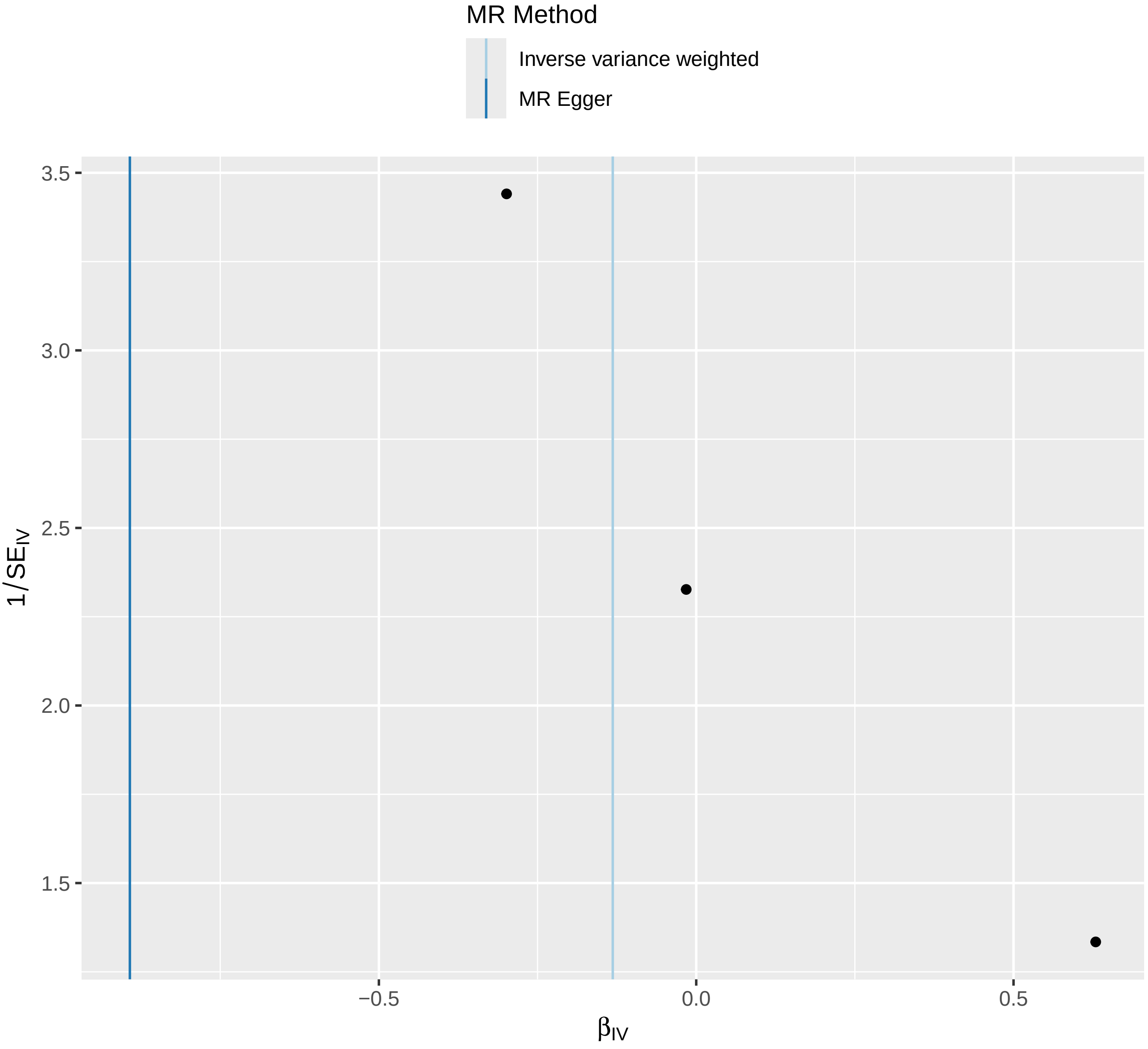
MR Method
Inverse variance weighted
MR Egger
3.5
3.0
2.5
2.0
1.5
$1/SE_{IV}$
−0.5
0.0
0.5
β_{IV}

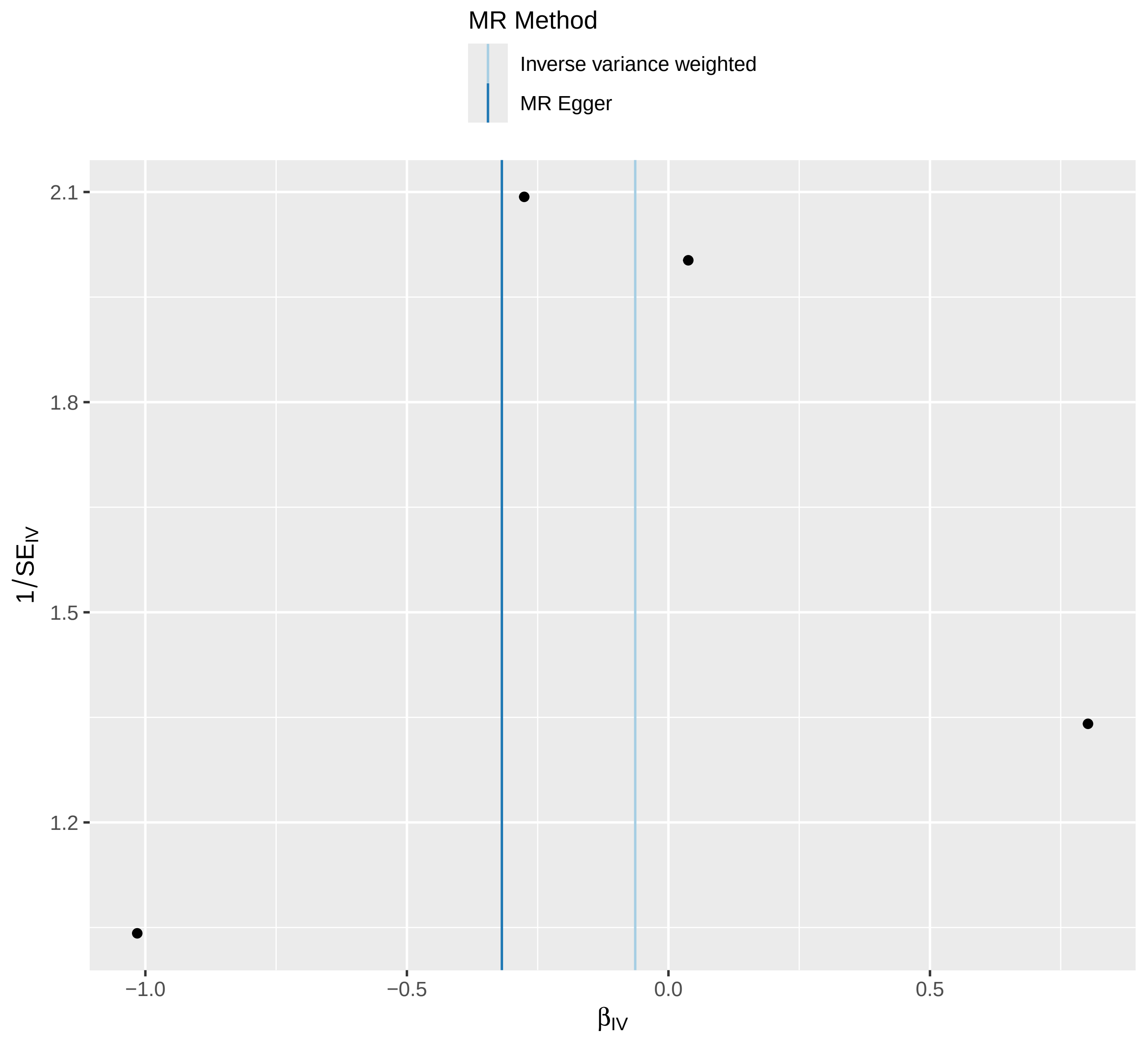

MR Method
Inverse variance weighted
MR Egger
2.1
1.8
1.5
1.2
1/SE$_{IV}$
−1.0
−0.5
0.0
0.5
β$_{IV}$

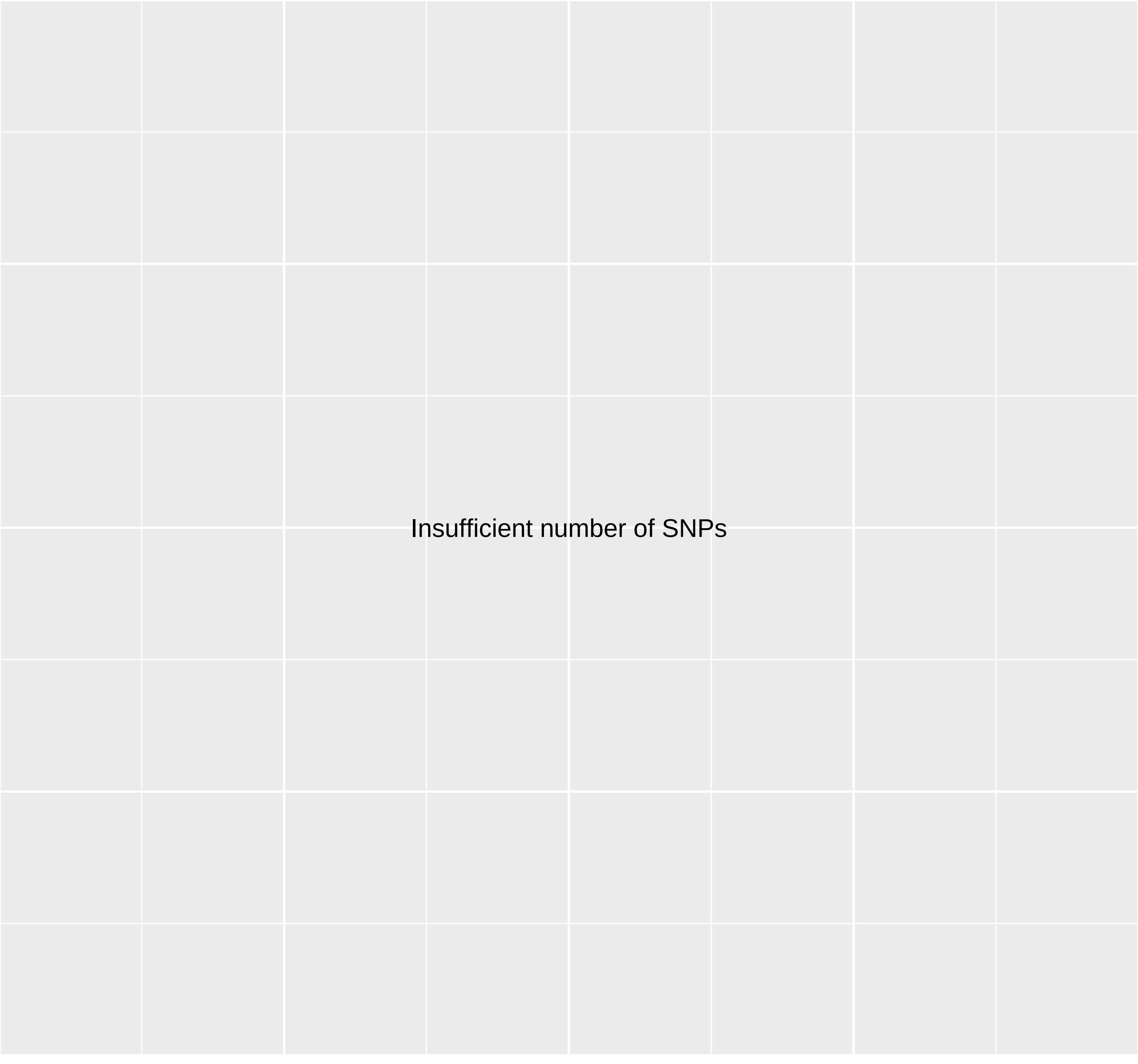
Insufficient number of SNPs

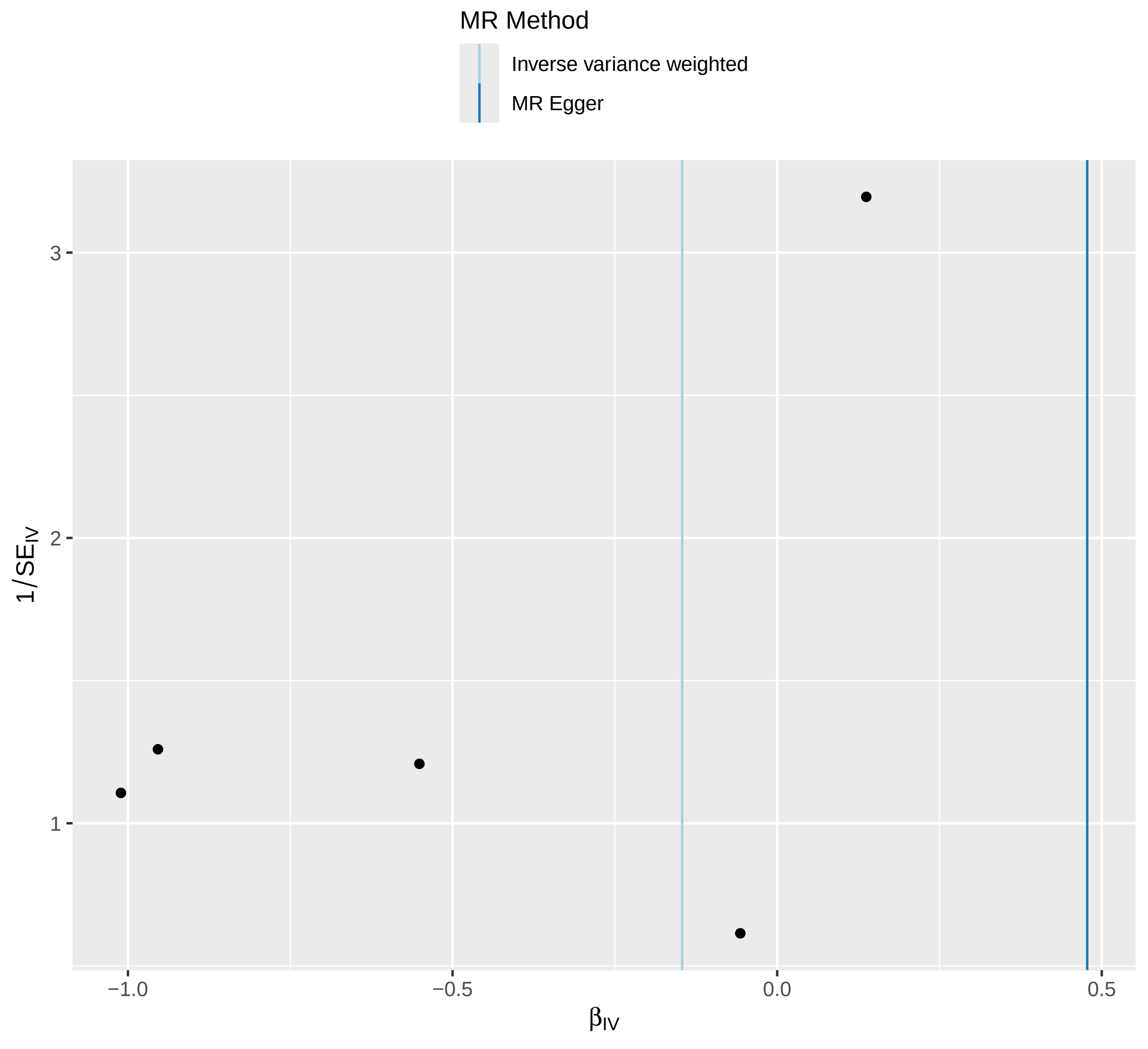
MR Method
Inverse variance weighted
MR Egger
3
2
1
1/SE_IV
−1.0
−0.5
0.0
0.5
β_IV

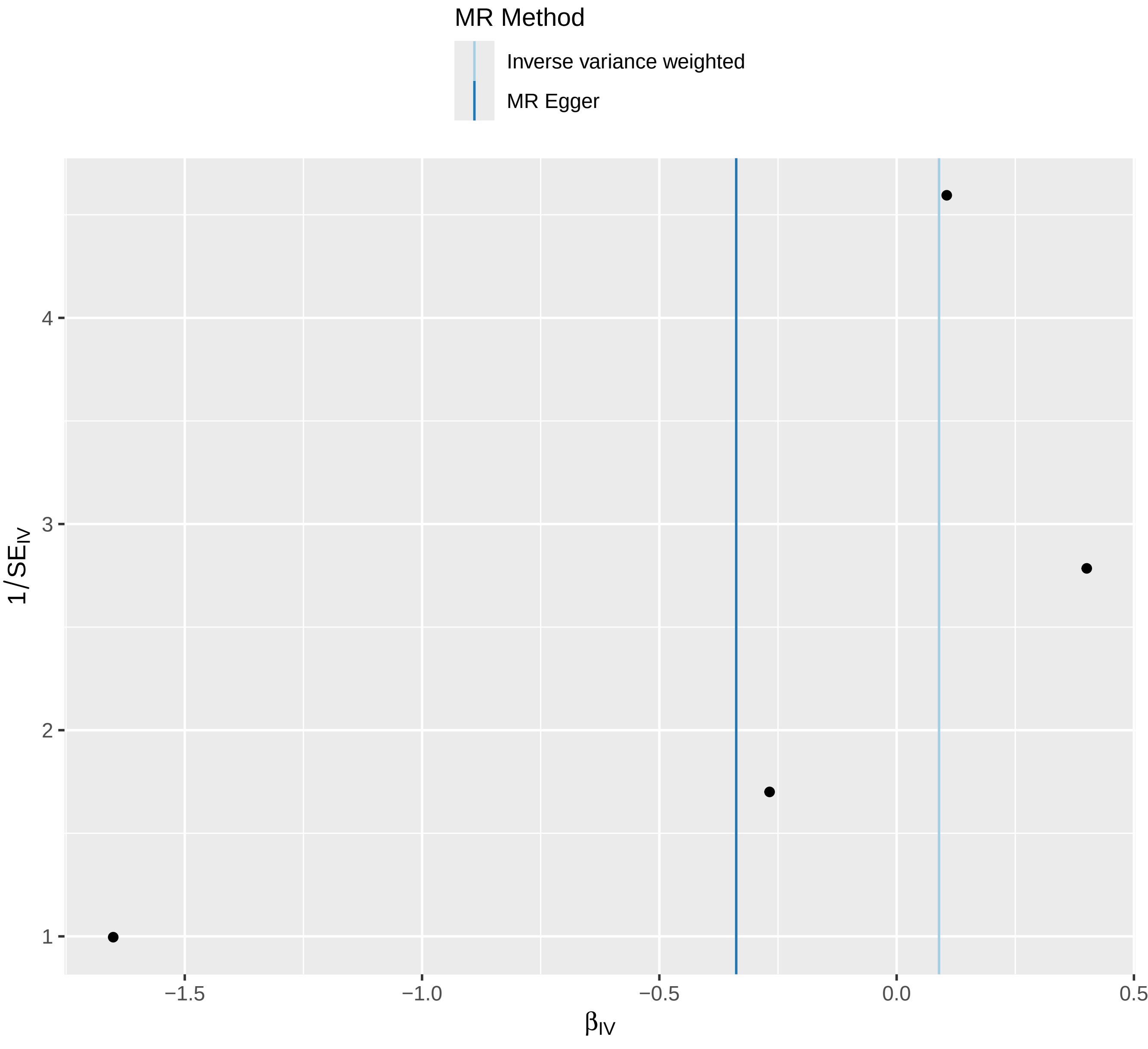

MR Method
Inverse variance weighted
MR Egger
4
3
2
1
$1/SE_{IV}$
−1.5
−1.0
−0.5
0.0
0.5
β_{IV}

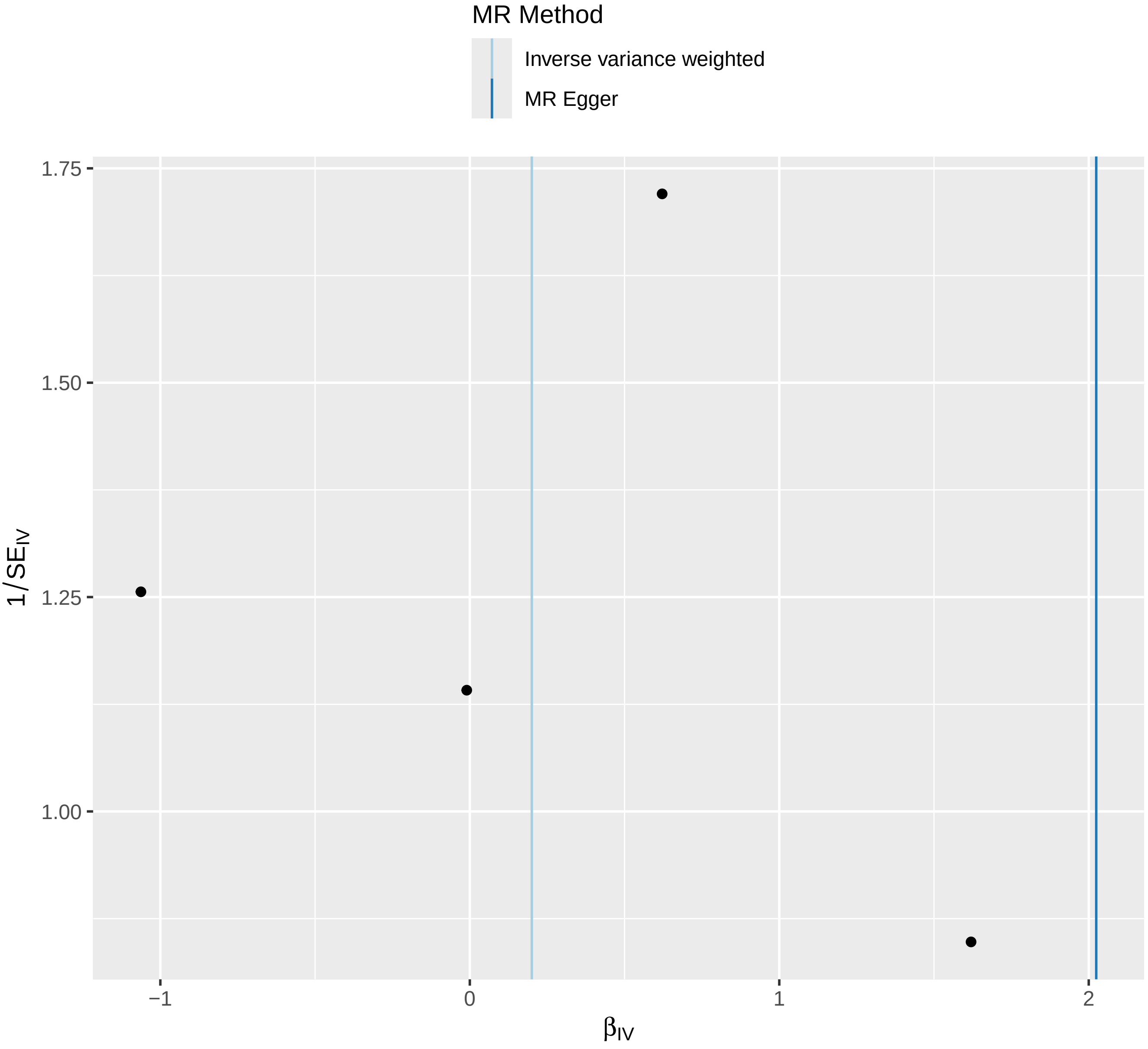
MR Method
Inverse variance weighted
MR Egger
1.75
1.50
1.25
1.00
1/SE$_{IV}$
−1
0
1
2
β$_{IV}$

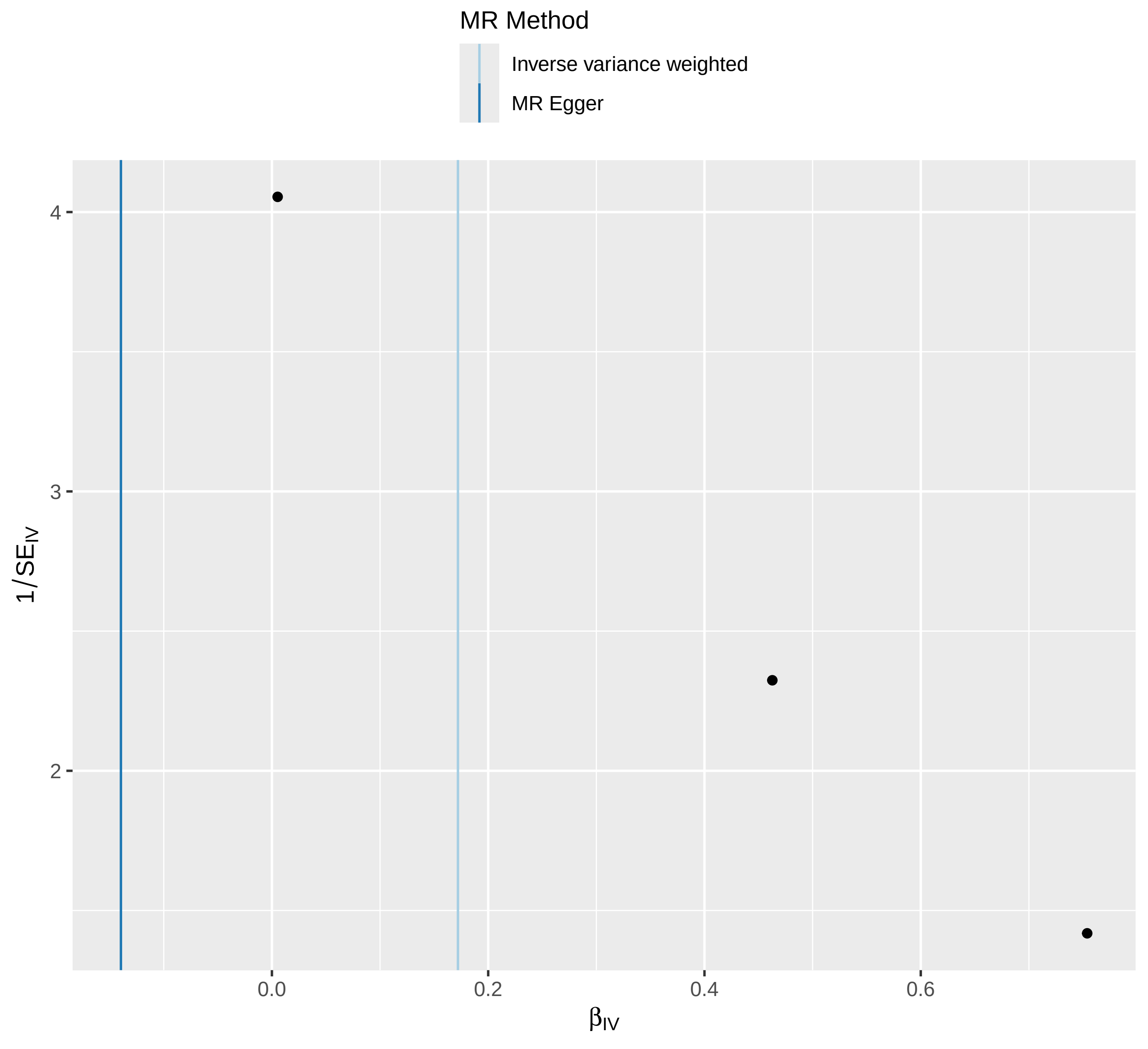
MR Method
Inverse variance weighted
MR Egger
4
3
2
0.0
0.2
0.4
0.6
$1/SE_{IV}$
β_{IV}

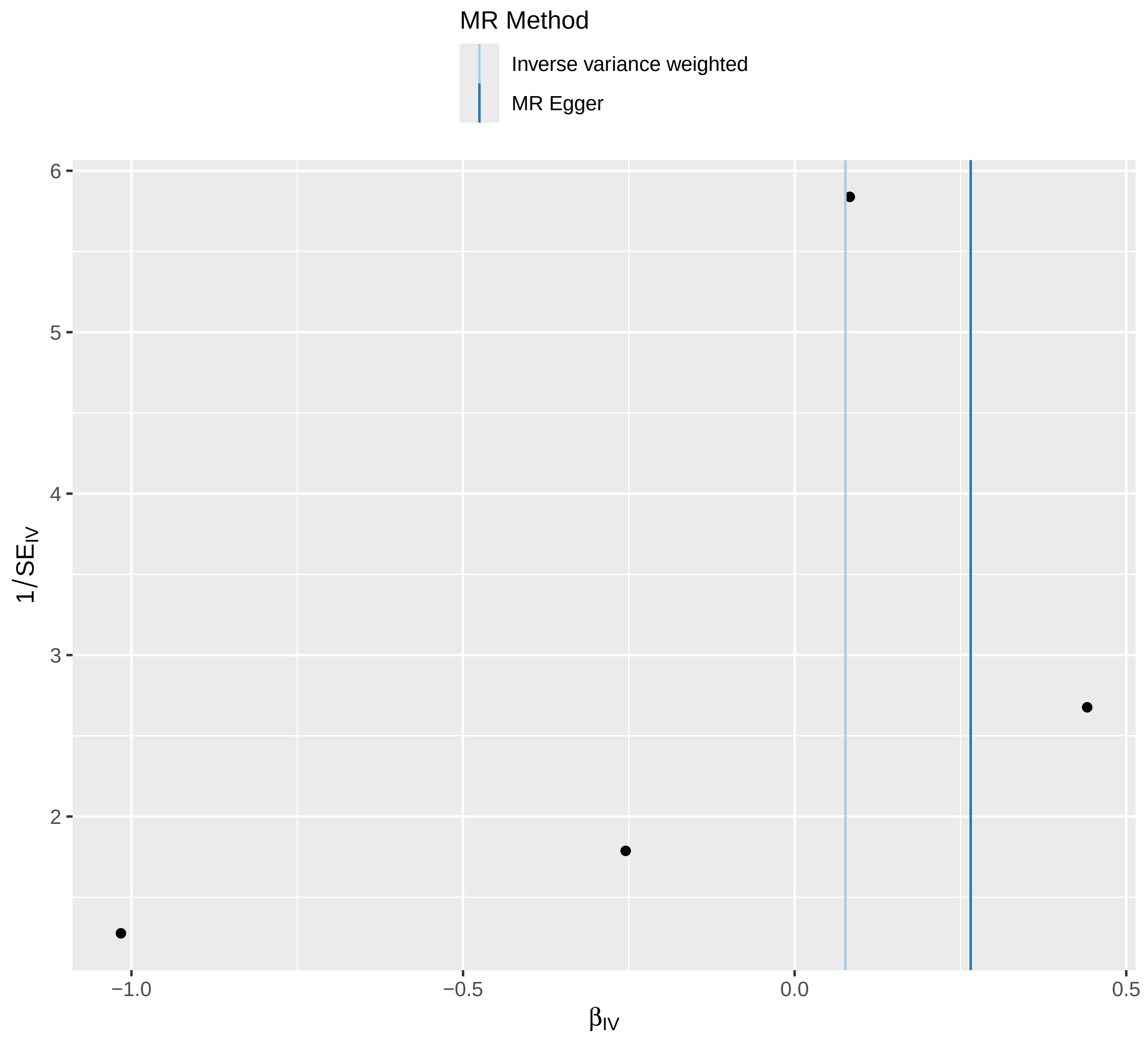

MR Method
Inverse variance weighted
MR Egger
6
5
4
3
2
1/SE_IV
−1.0
−0.5
0.0
0.5
β_IV

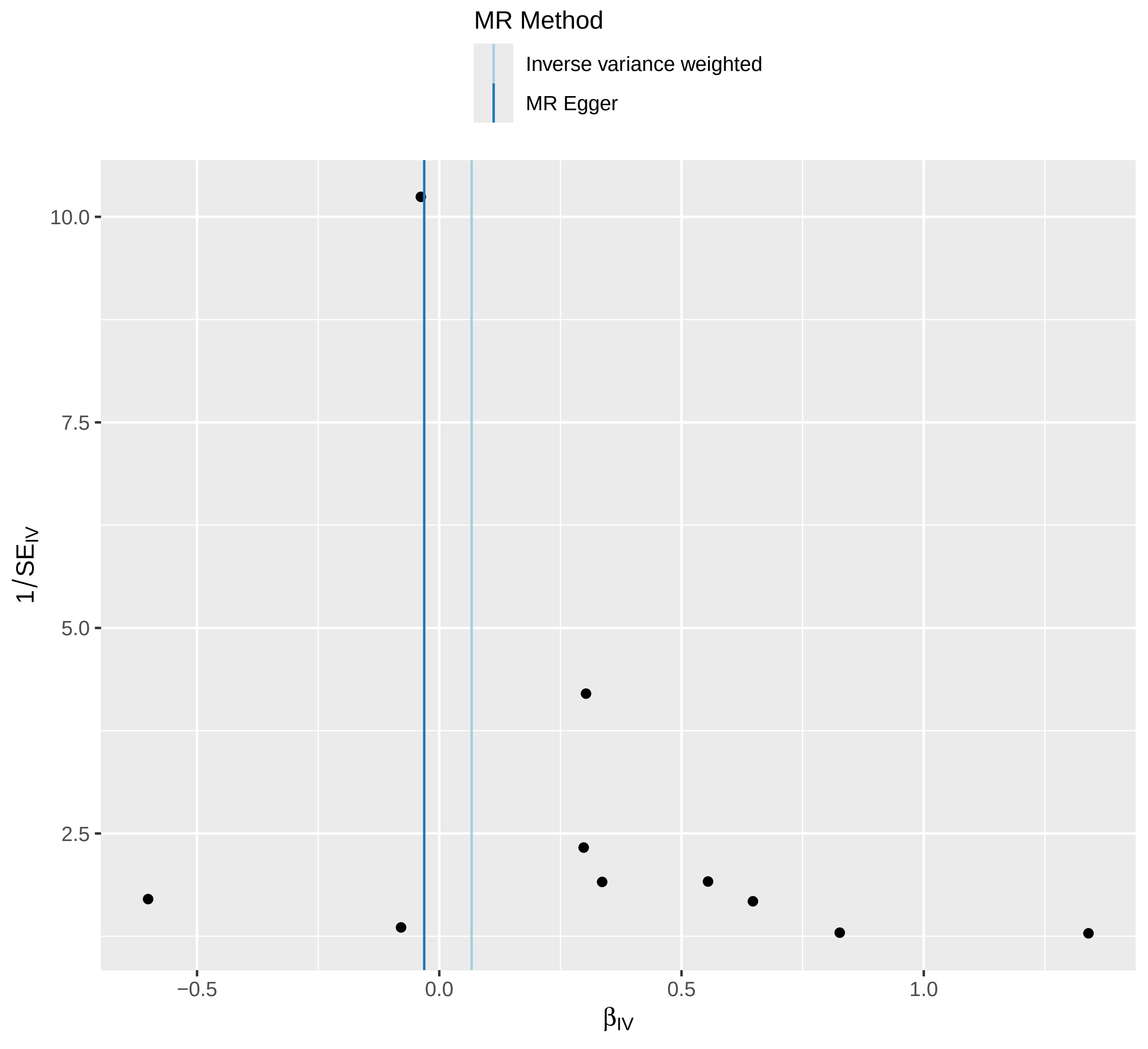
MR Method
Inverse variance weighted
MR Egger
10.0
7.5
5.0
2.5
1/SE_IV
−0.5
0.0
0.5
1.0
β_IV

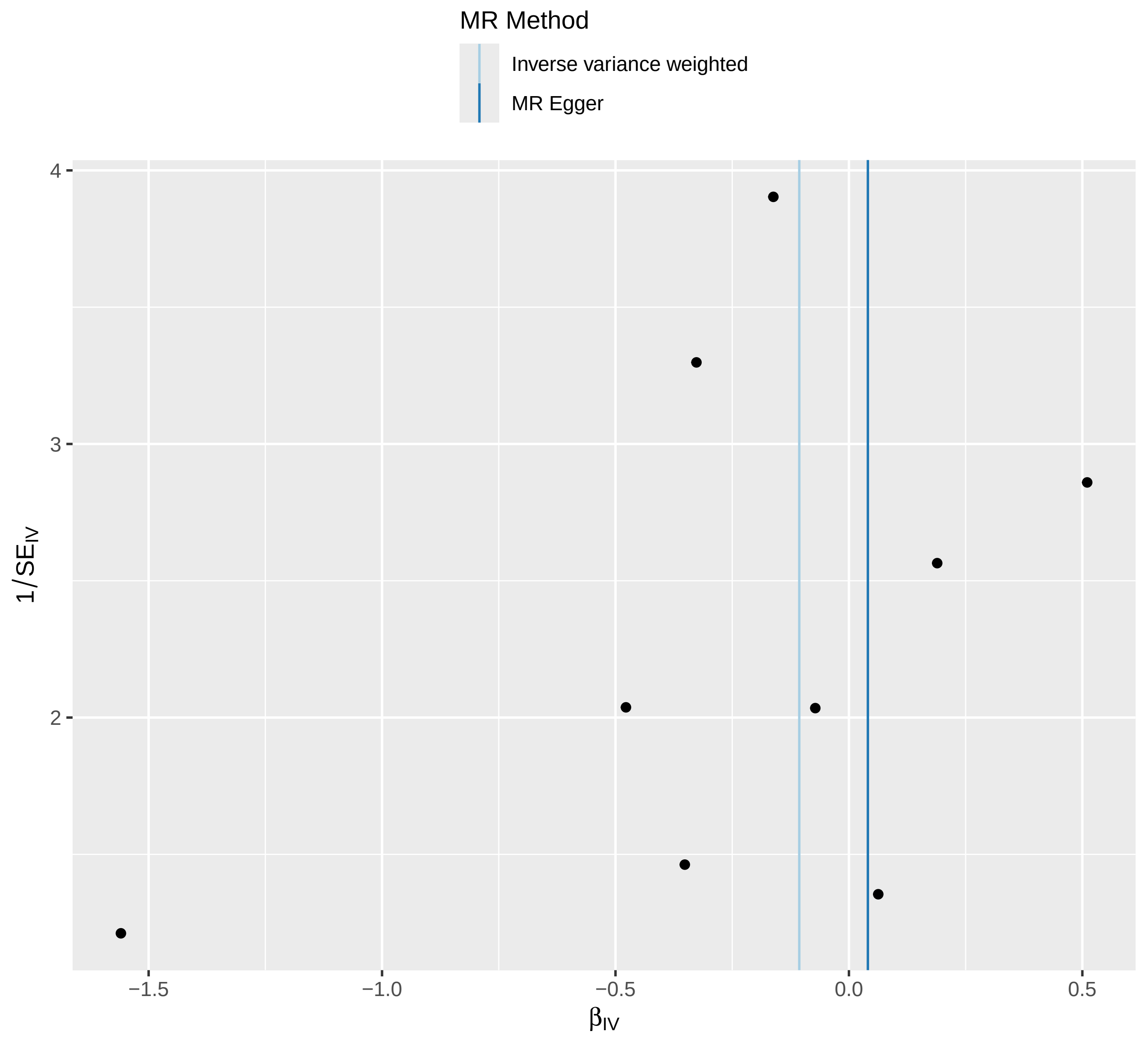
MR Method
Inverse variance weighted
MR Egger
4
3
2
1/SE_IV
−1.5
−1.0
−0.5
0.0
0.5
β_IV

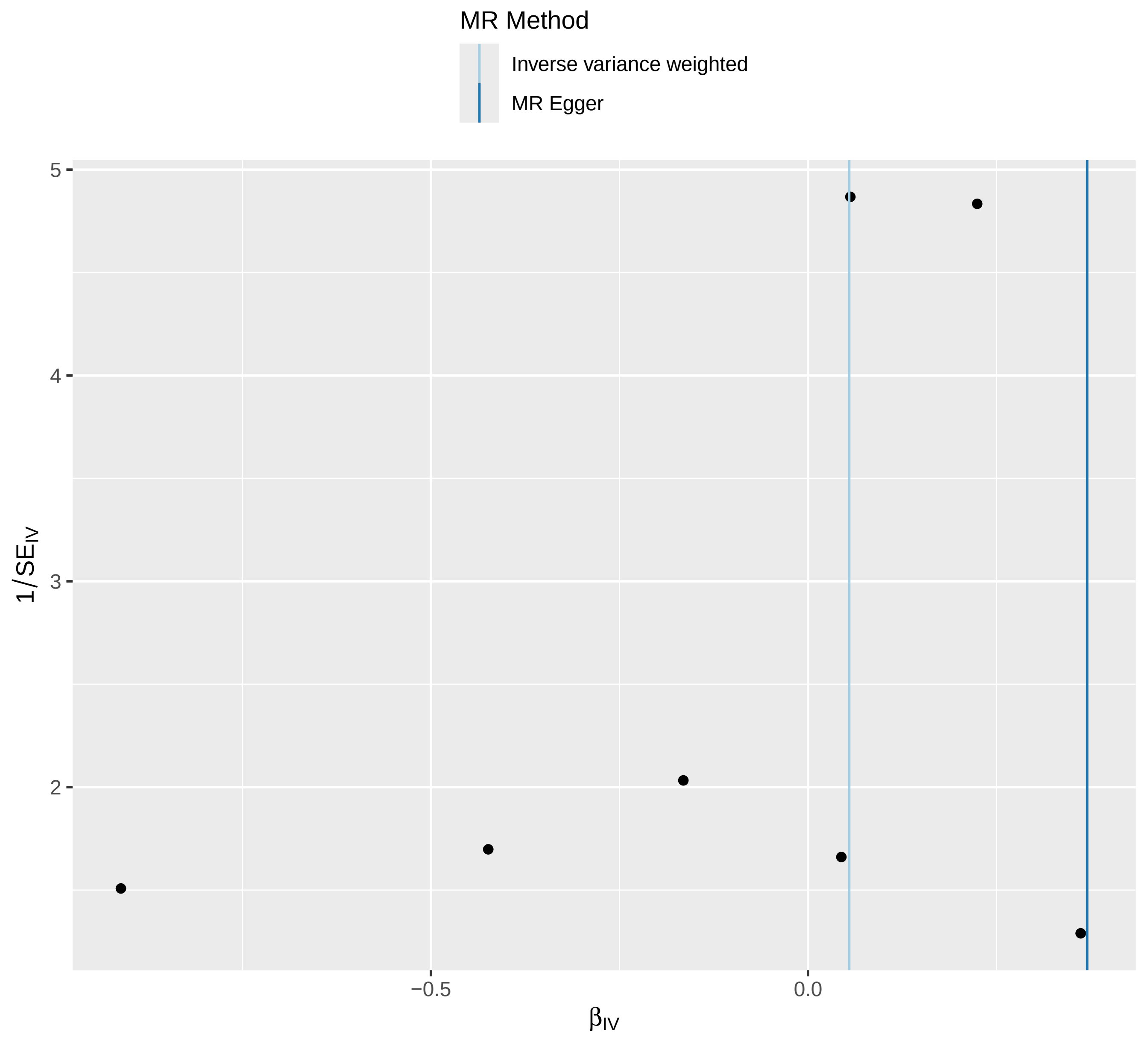

MR Method
Inverse variance weighted
MR Egger
5
4
3
2
1/SE$_{IV}$
−0.5
0.0
β$_{IV}$

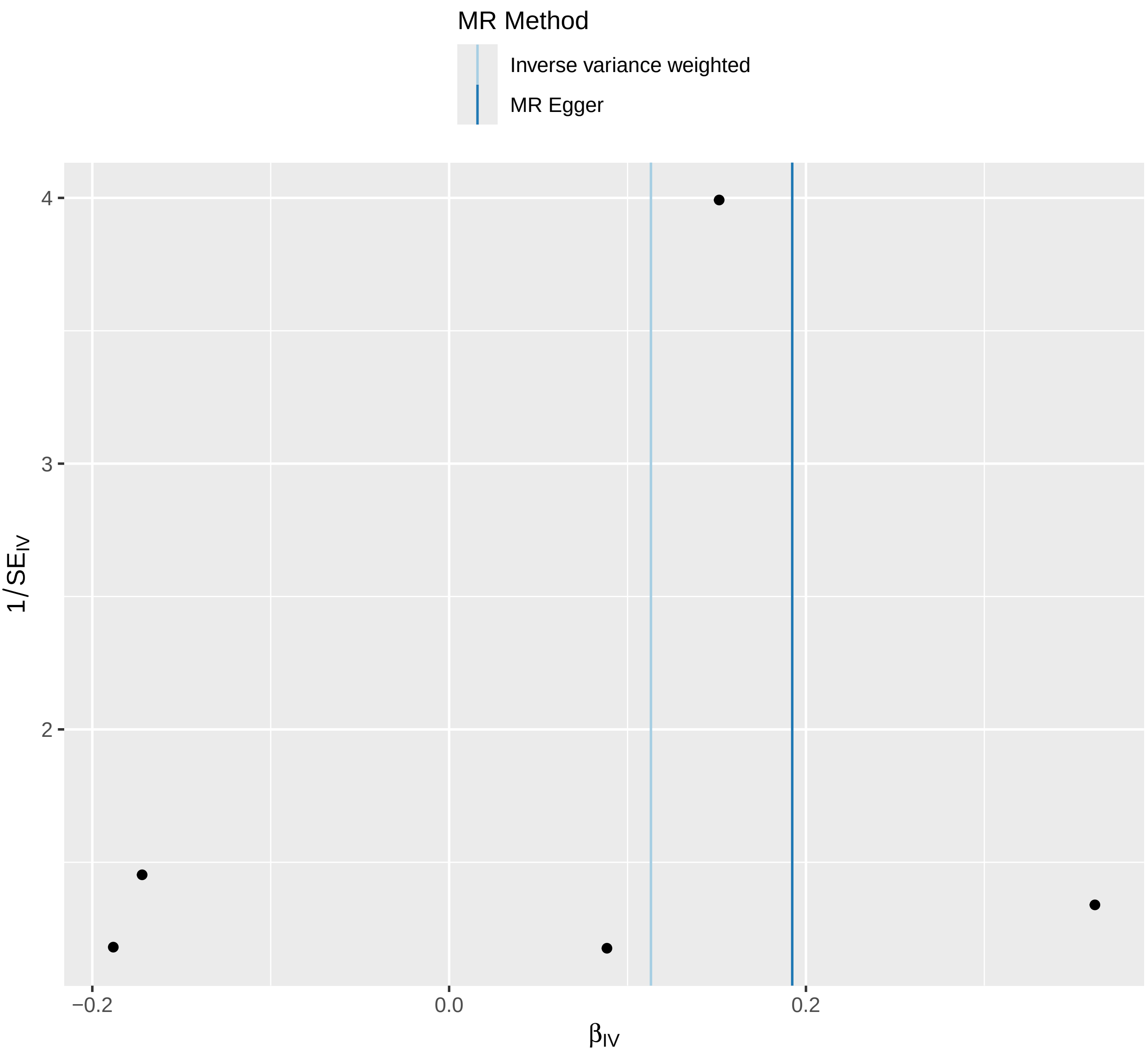
MR Method
Inverse variance weighted
MR Egger
4
3
2
$1/SE_{IV}$
−0.2
0.0
0.2
β_{IV}

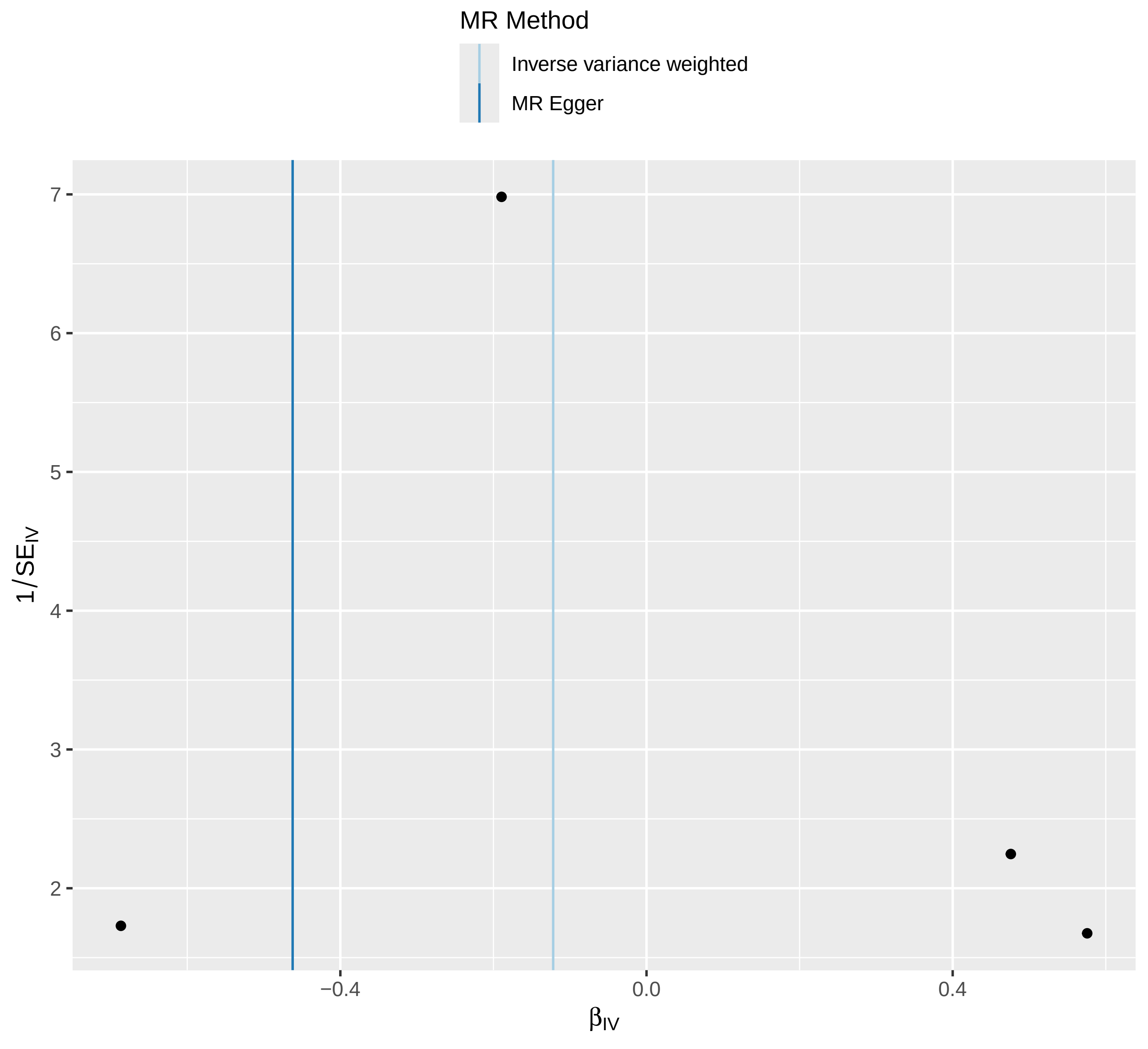
MR Method
Inverse variance weighted
MR Egger
7
6
5
4
3
2
1/SE_IV
−0.4
0.0
0.4
β_IV

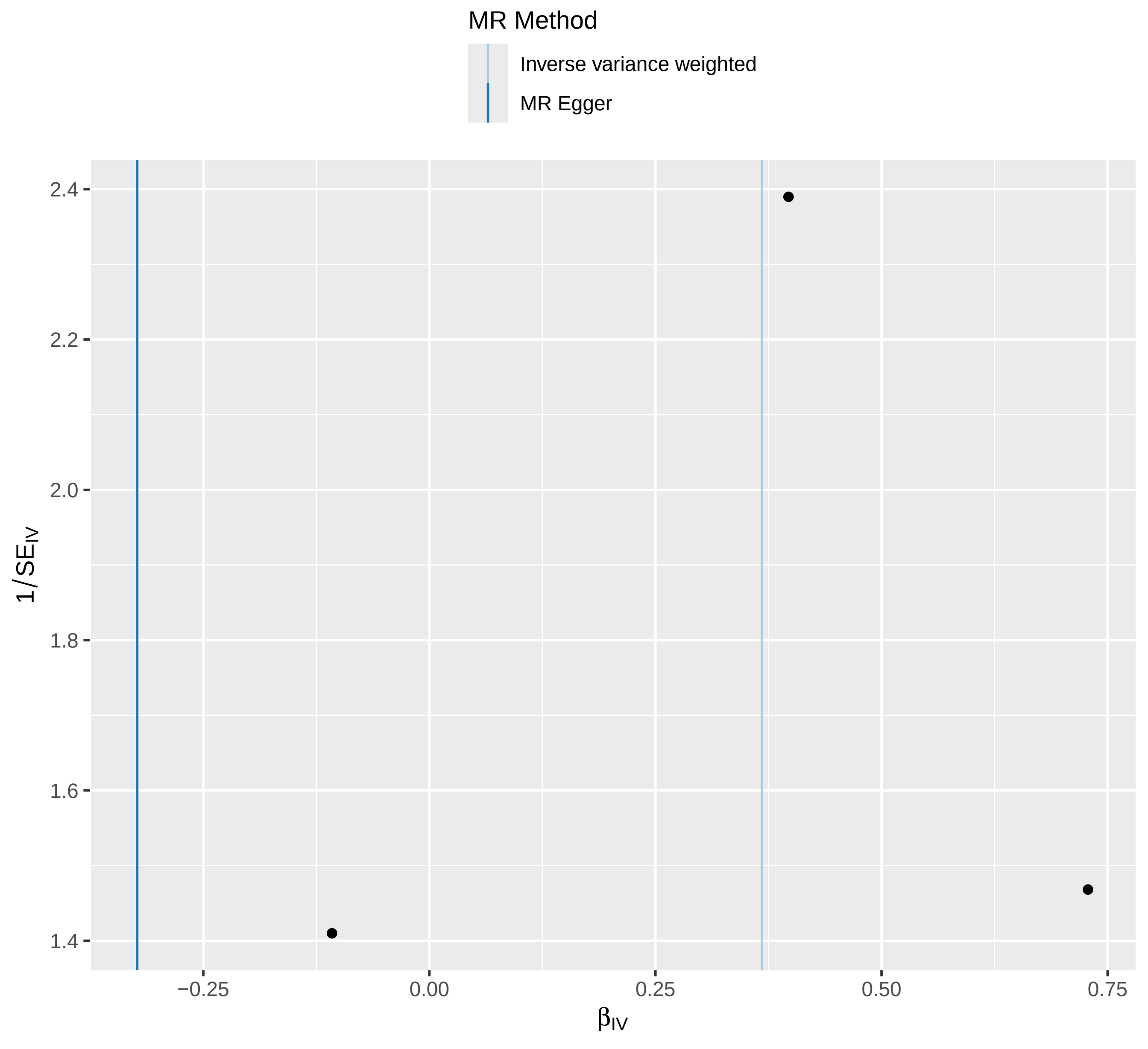
MR Method
Inverse variance weighted
MR Egger
2.4
2.2
2.0
1.8
1.6
1.4
1/SE$_{IV}$
−0.25
0.00
0.25
0.50
0.75
β$_{IV}$

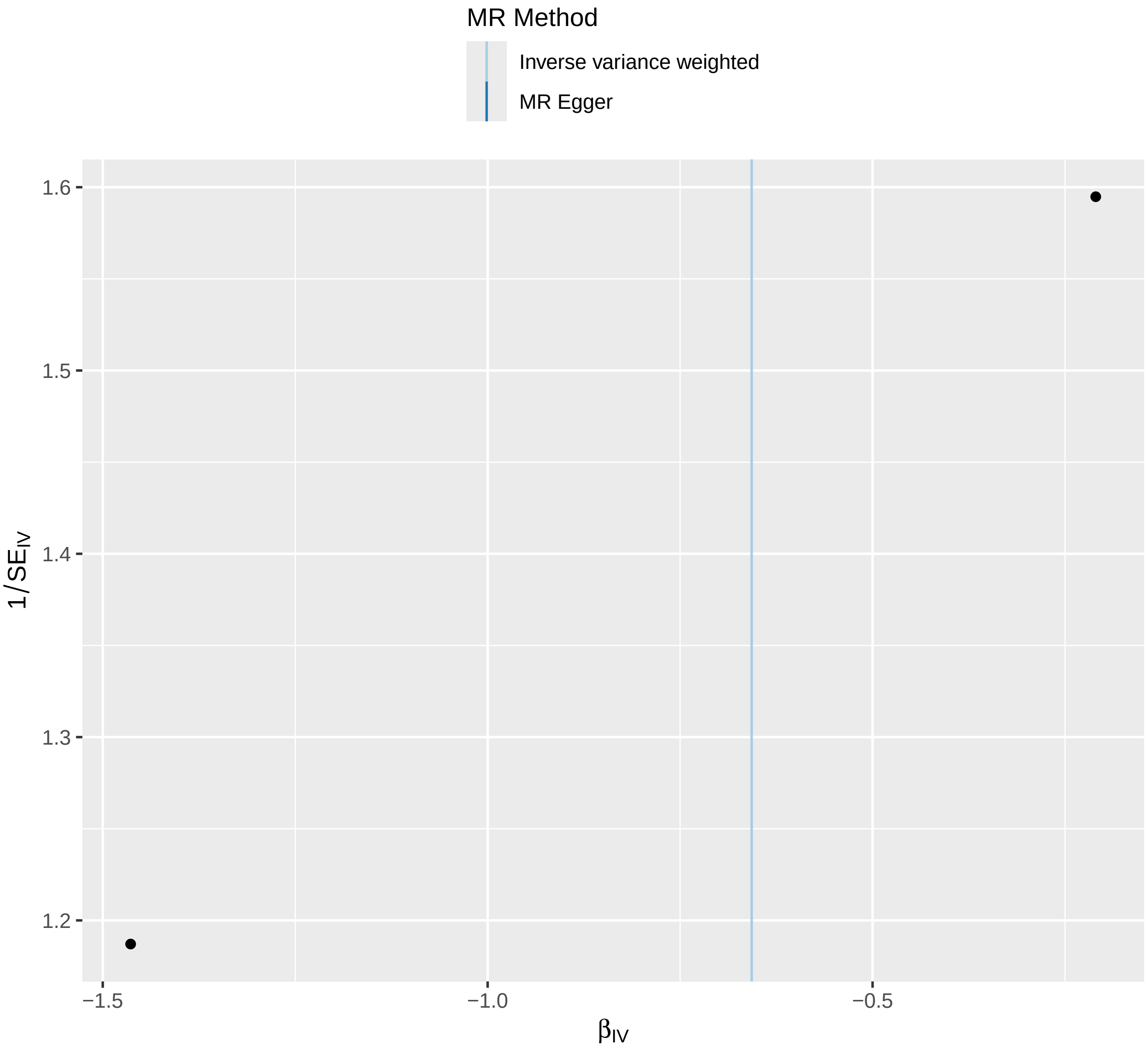

MR Method
Inverse variance weighted
MR Egger
1.6
1.5
1.4
1.3
1.2
1/SE$_{IV}$
−1.5
−1.0
−0.5
β$_{IV}$

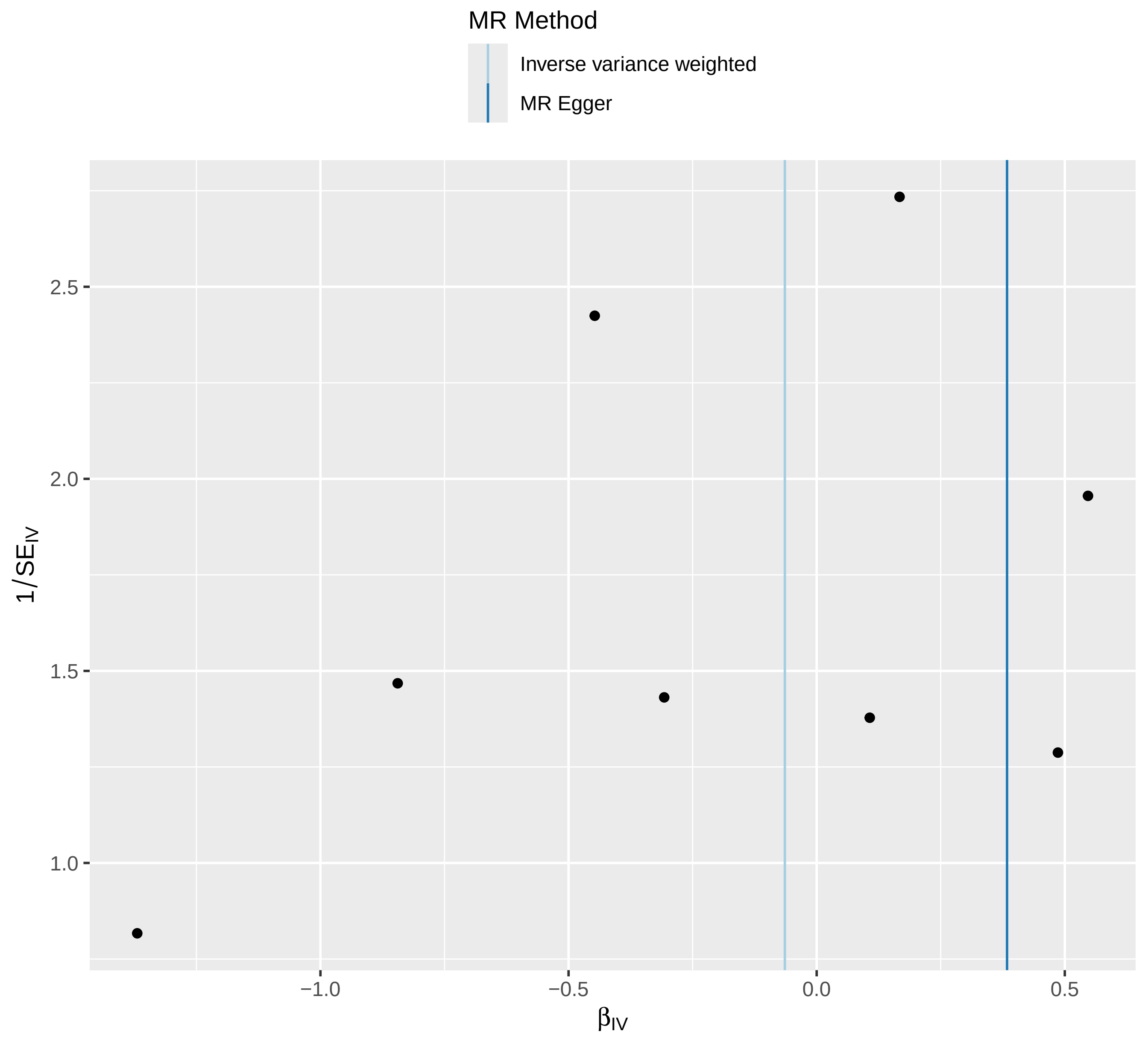
MR Method
Inverse variance weighted
MR Egger
2.5
2.0
1.5
1.0
−1.0
−0.5
0.0
0.5
1/SE_IV
β_IV

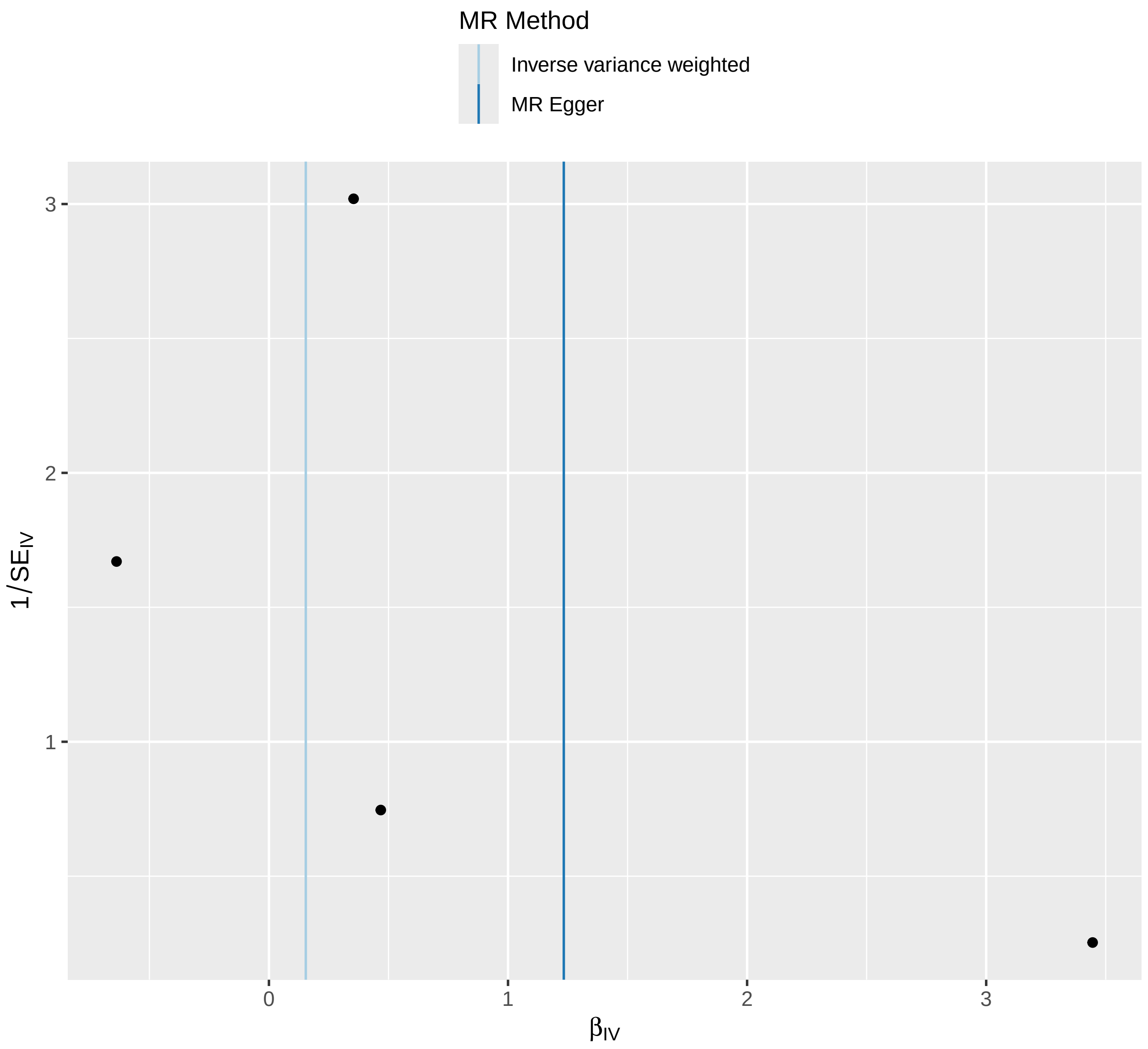

MR Method
Inverse variance weighted
MR Egger
3
2
1
0
1
2
3
1/SE$_{IV}$
β$_{IV}$

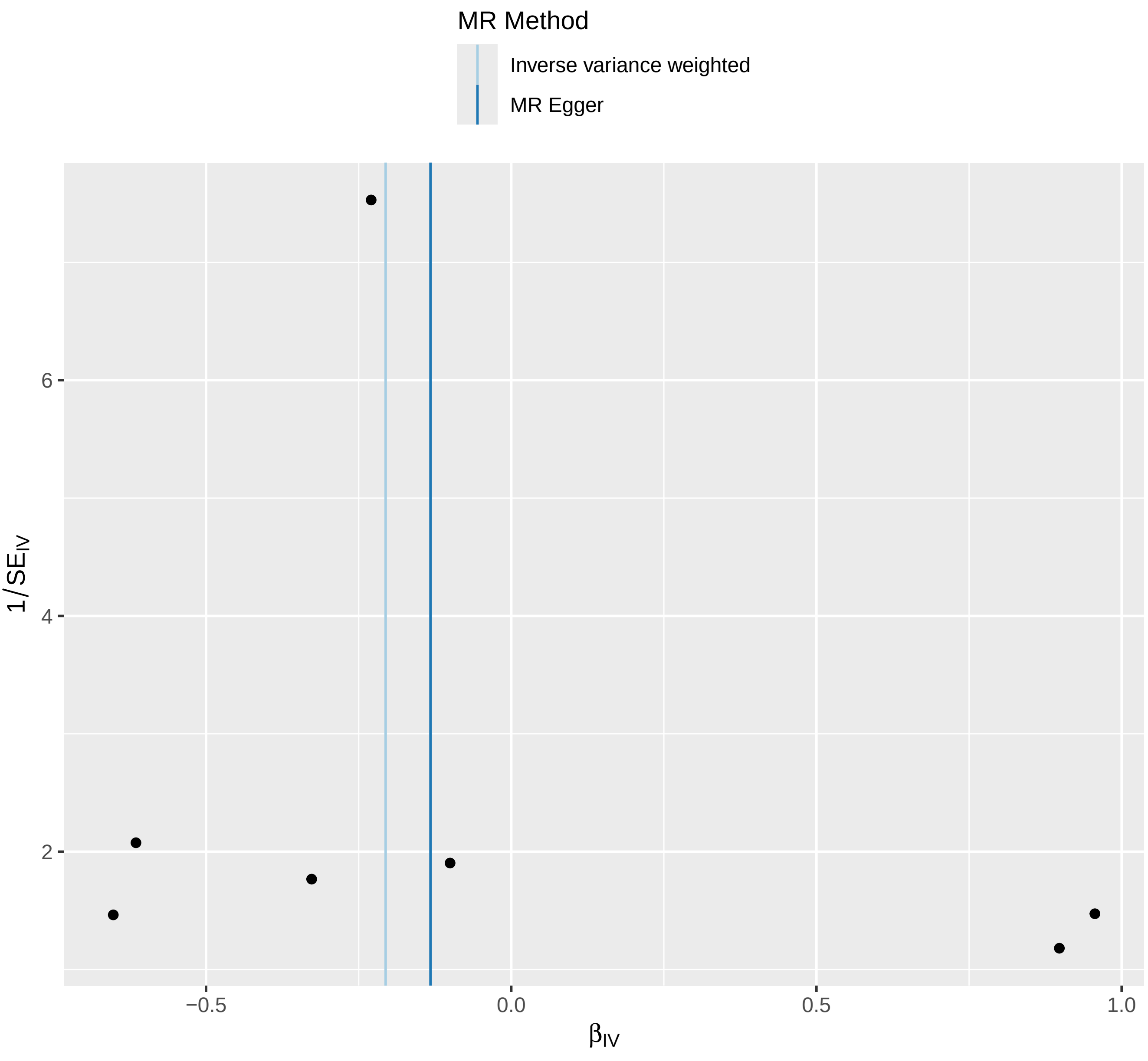
MR Method
Inverse variance weighted
MR Egger
1/SE$_{IV}$
6
4
2
−0.5
0.0
0.5
1.0
β$_{IV}$

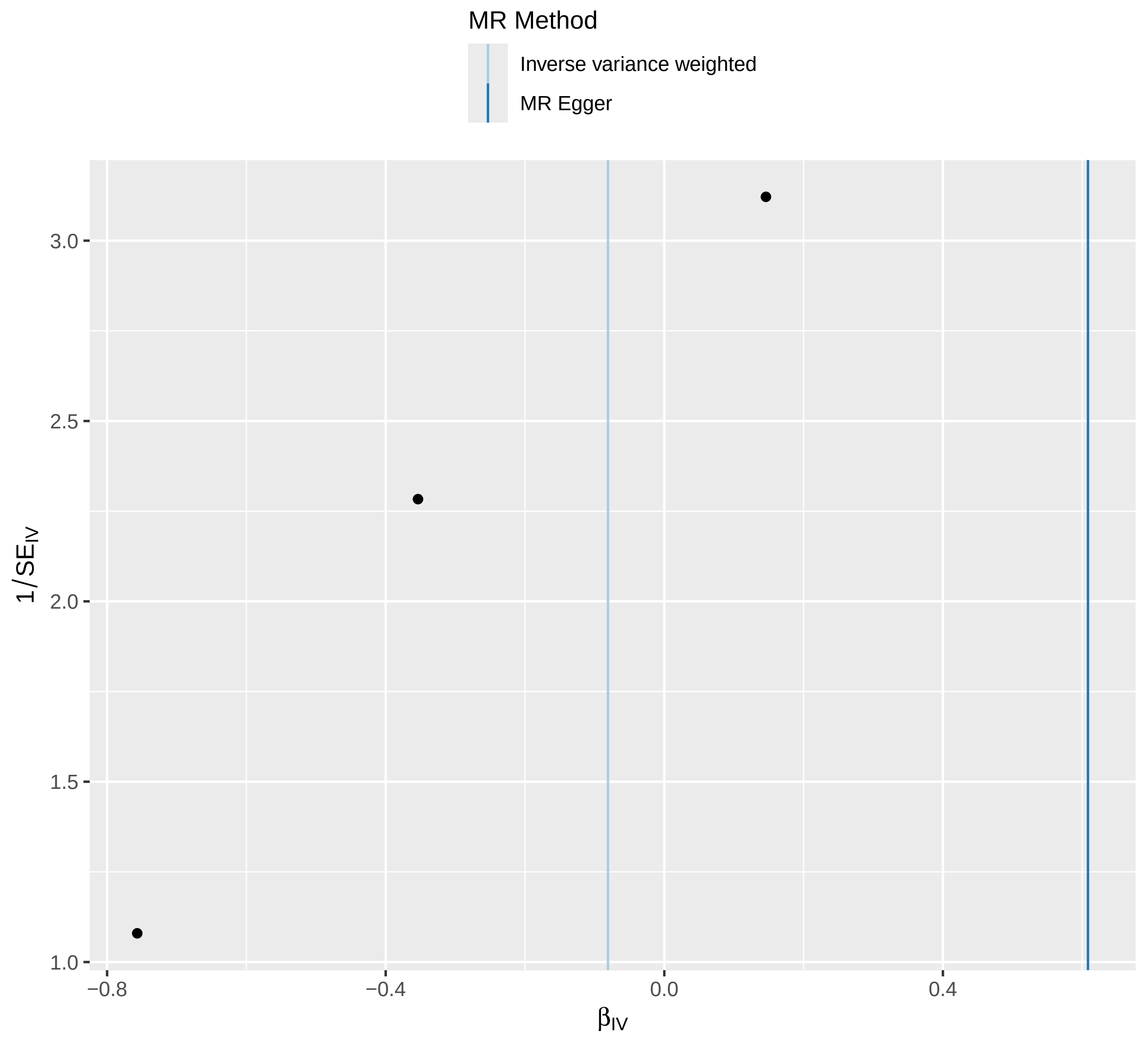

MR Method
Inverse variance weighted
MR Egger
1/SE_IV
3.0
2.5
2.0
1.5
1.0
−0.8
−0.4
0.0
0.4
β_IV

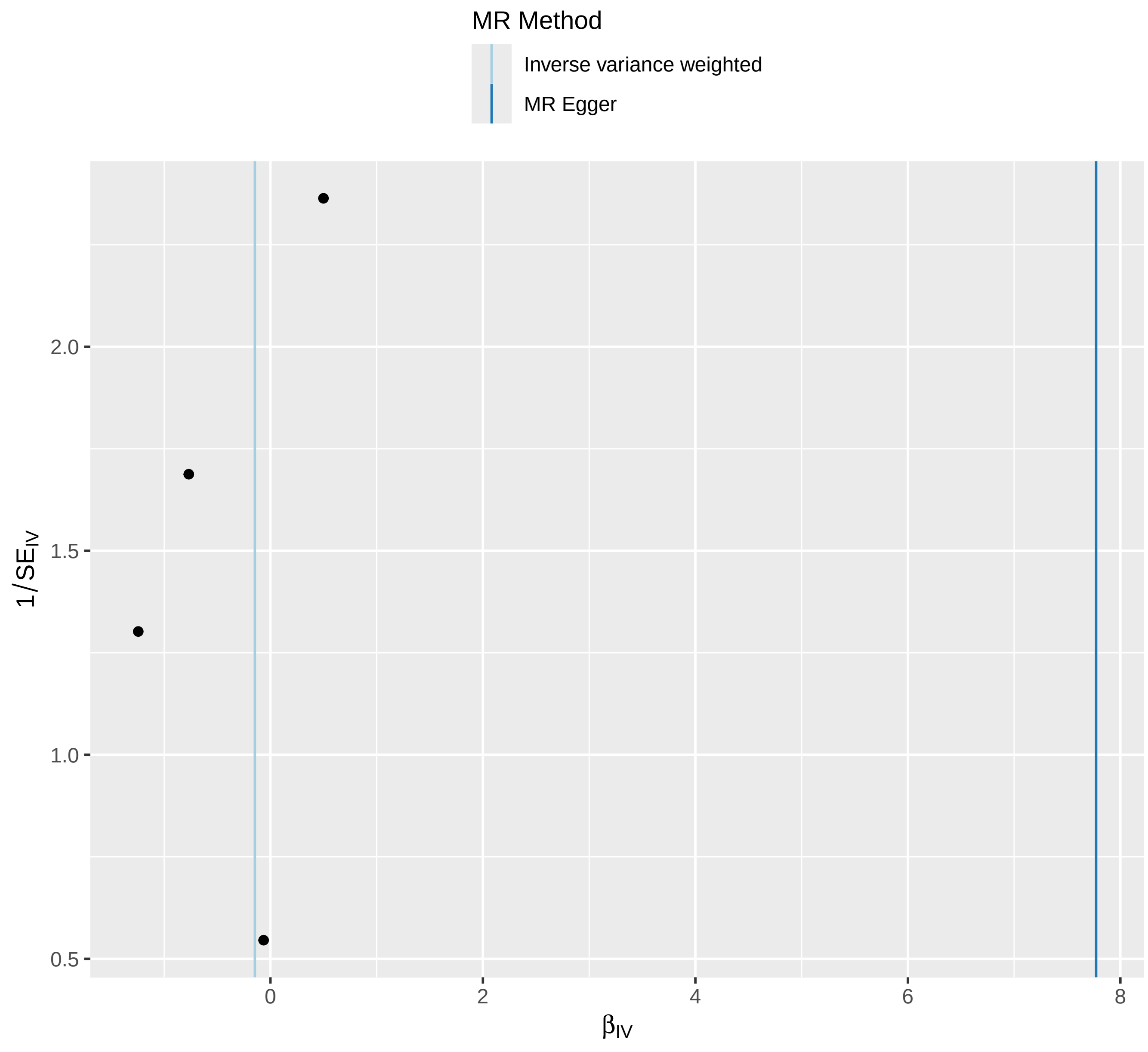

MR Method
Inverse variance weighted
MR Egger
2.0
1.5
1.0
0.5
$1/SE_{IV}$
0
2
4
6
8
β_{IV}

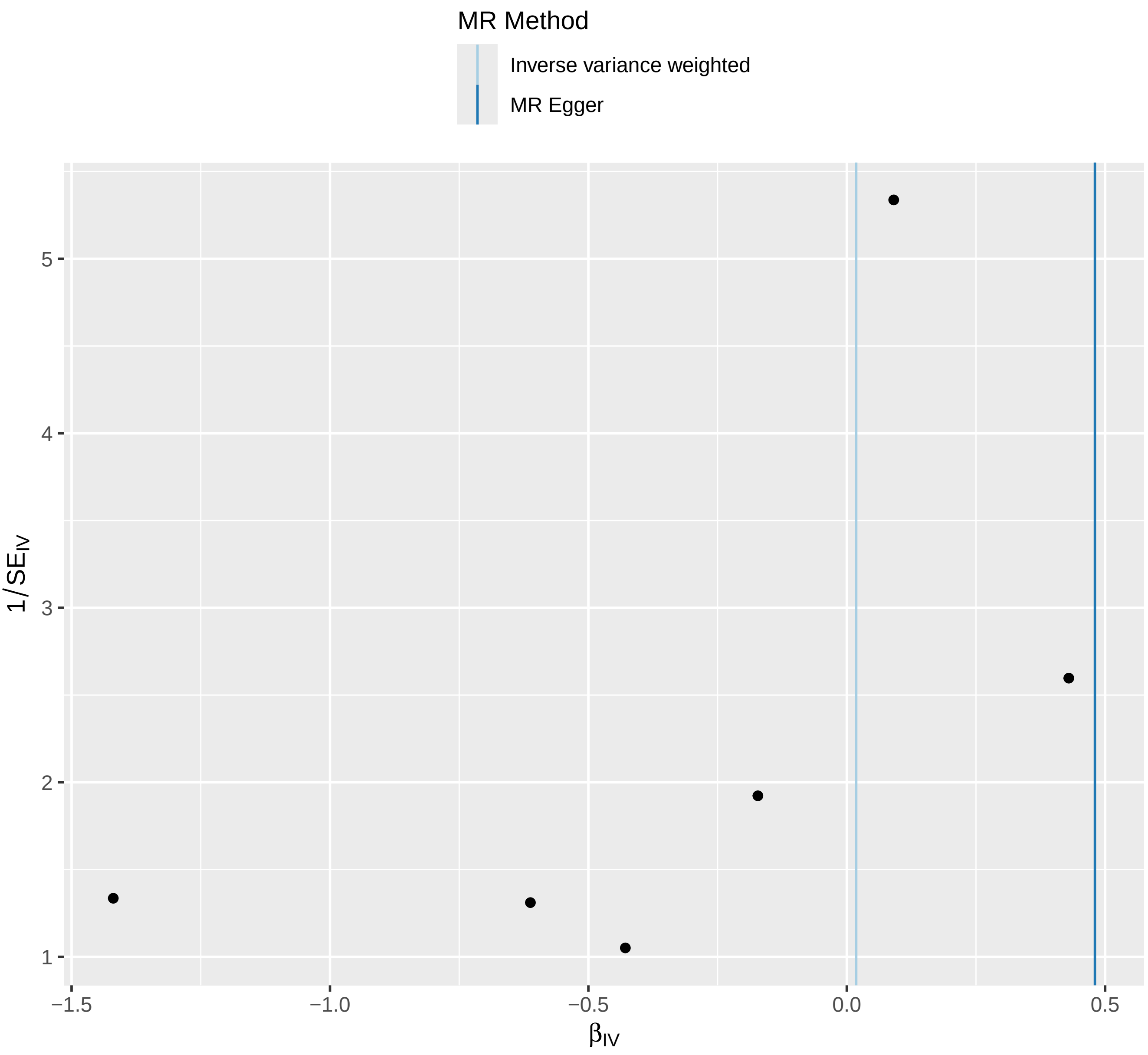

MR Method
Inverse variance weighted
MR Egger
5
4
3
2
1
−1.5
−1.0
−0.5
0.0
0.5
1/SE_IV
β_IV

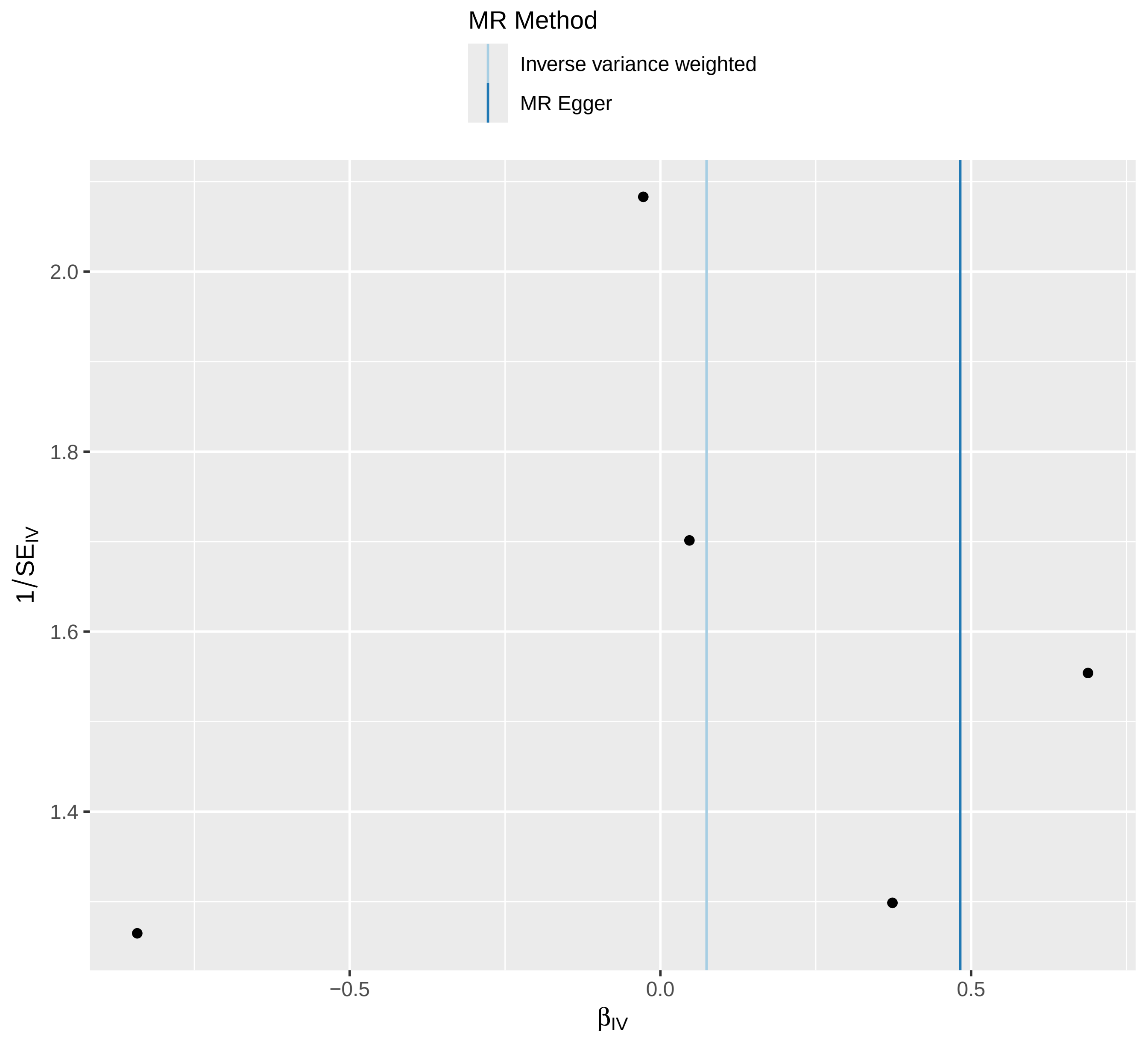
MR Method
Inverse variance weighted
MR Egger
2.0
1.8
1.6
1.4
−0.5
0.0
0.5
$1/SE_{IV}$
β_{IV}

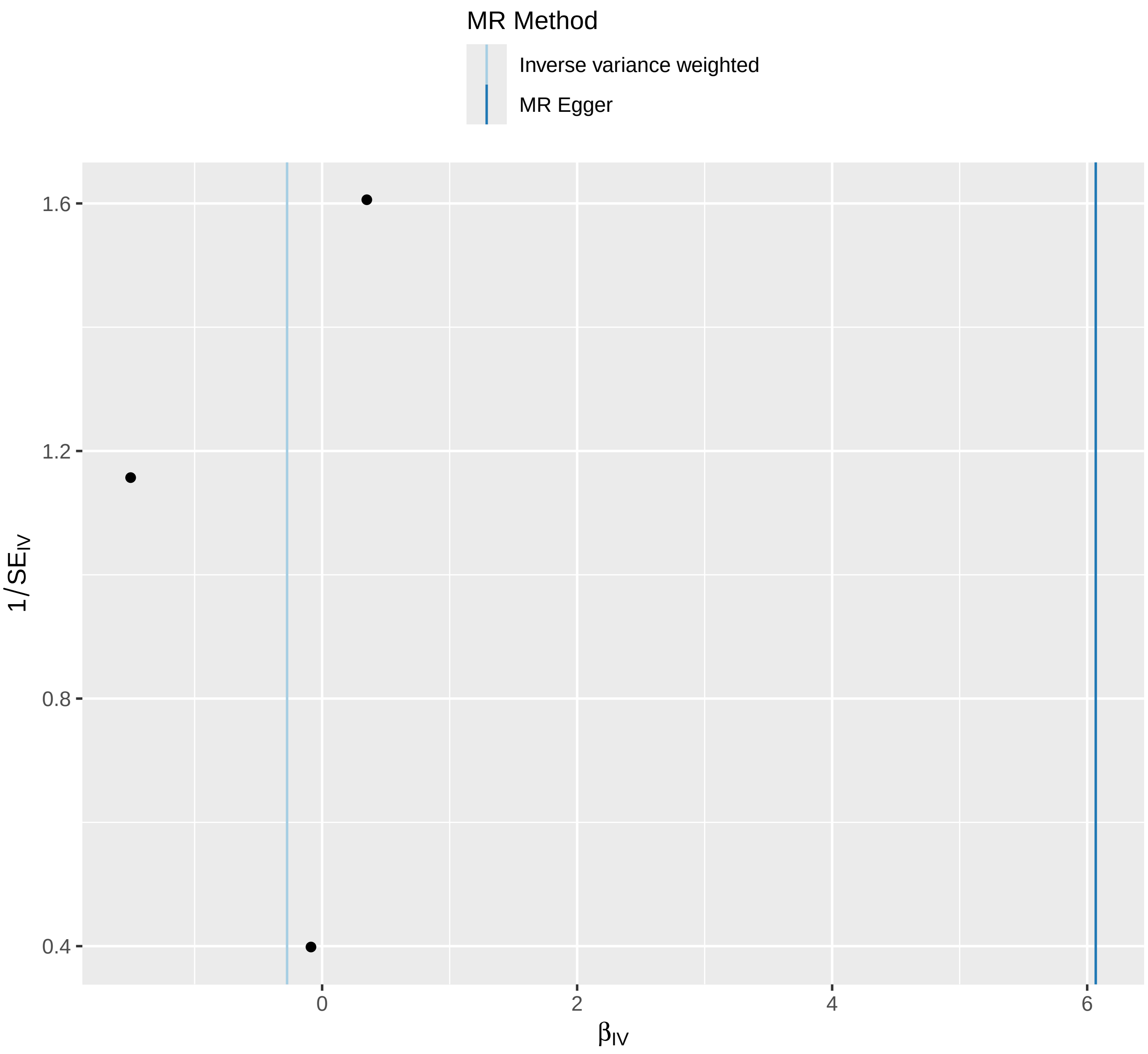
MR Method
Inverse variance weighted
MR Egger
1.6
1.2
0.8
0.4
1/SE$_{IV}$
0
2
4
6
β$_{IV}$

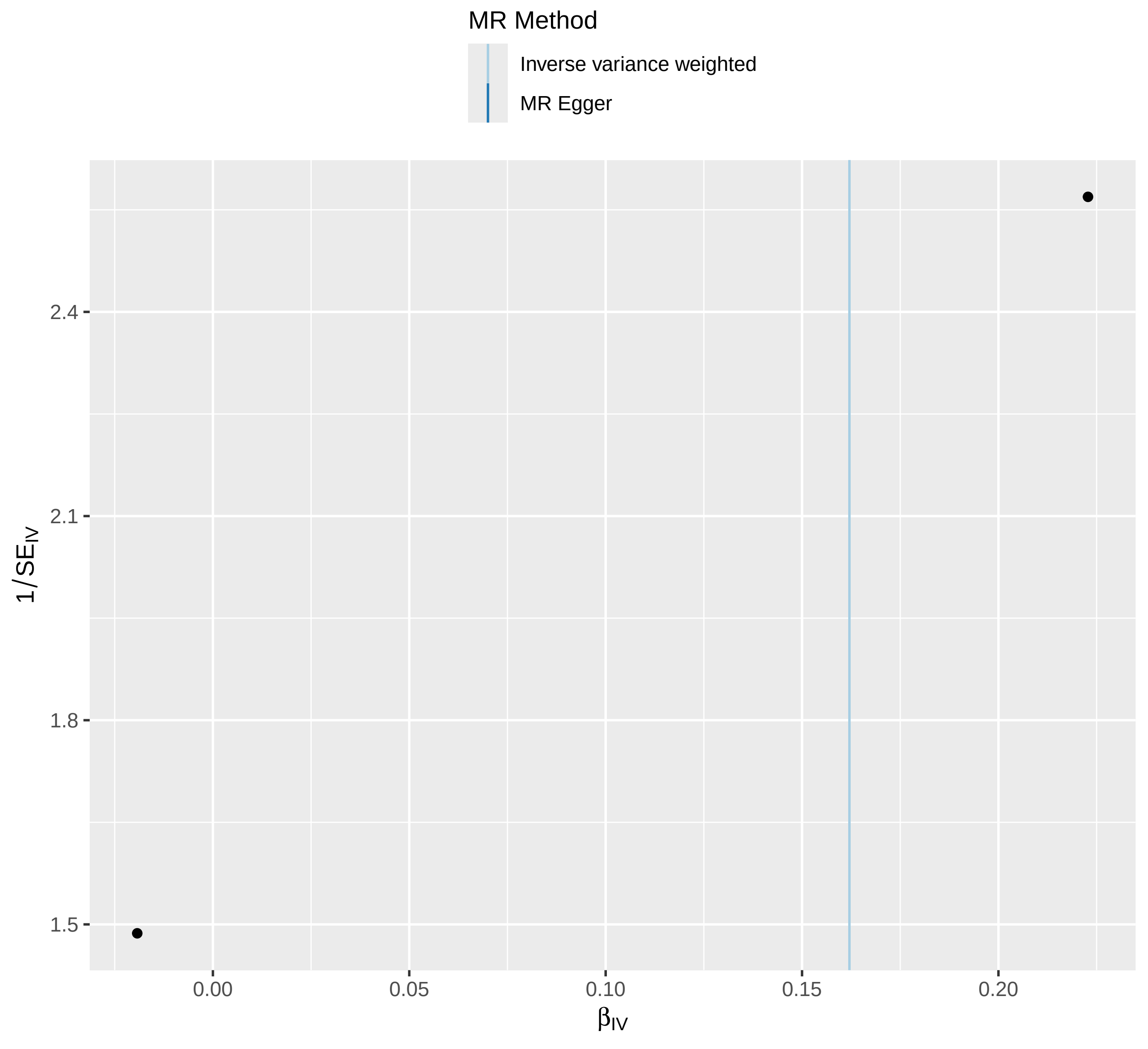

MR Method
Inverse variance weighted
MR Egger
2.4
2.1
1.8
1.5
$1/SE_{IV}$
0.00
0.05
0.10
0.15
0.20
β_{IV}

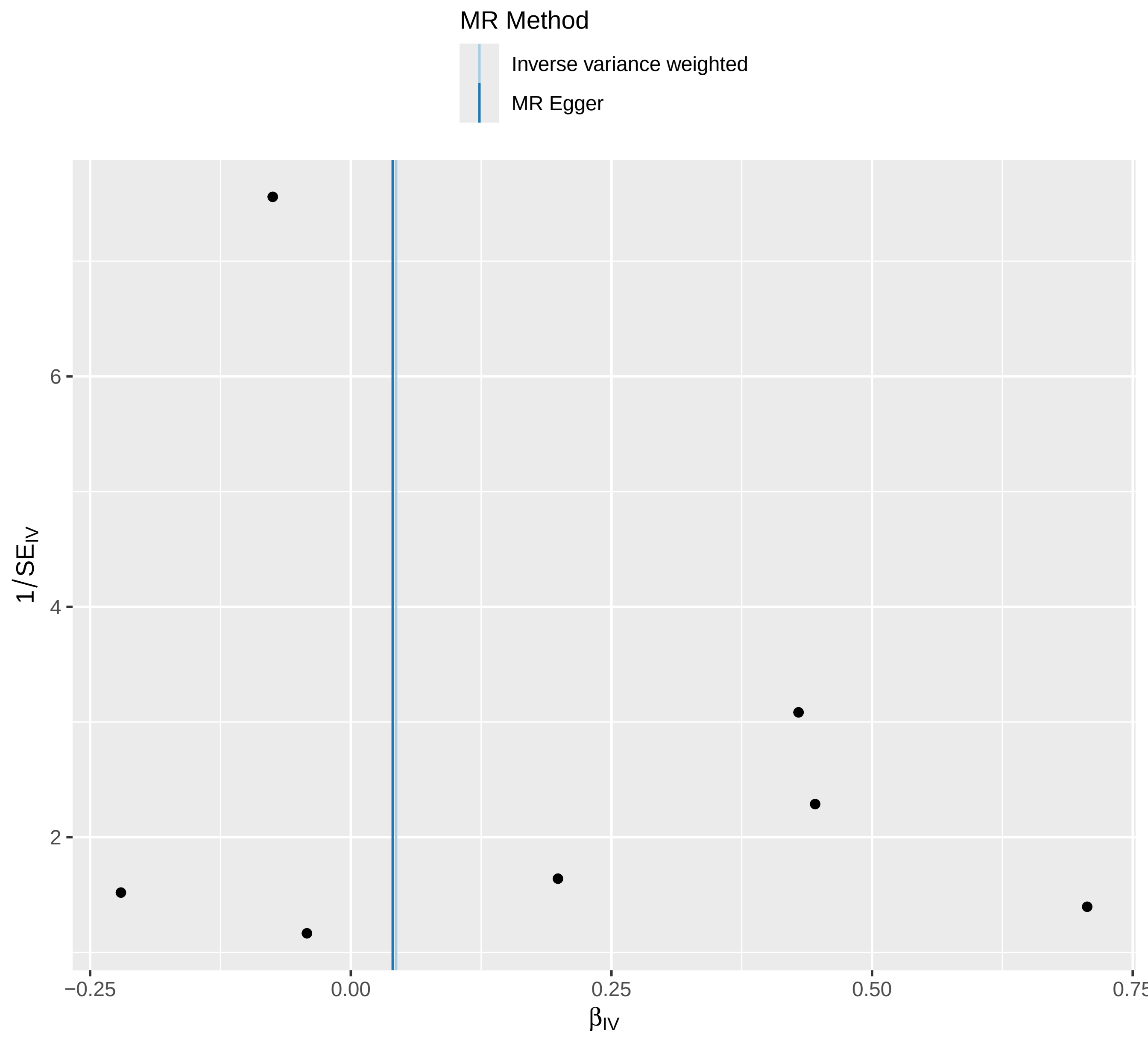

MR Method
Inverse variance weighted
MR Egger
6
4
2
1/SE_IV
−0.25
0.00
0.25
0.50
0.75
β_IV

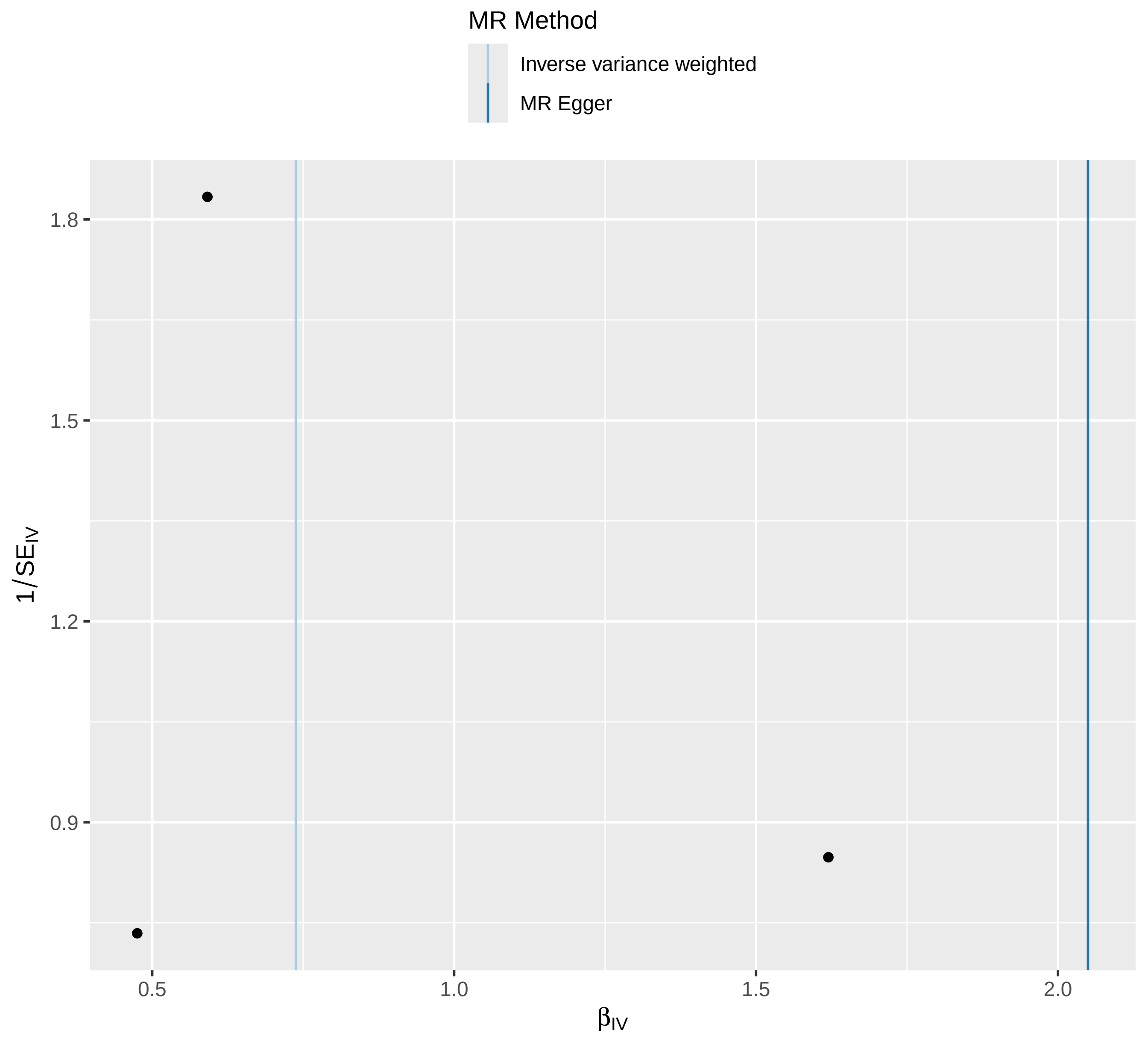
MR Method
Inverse variance weighted
MR Egger
1.8
1.5
1.2
0.9
0.5
1.0
1.5
2.0
$1/SE_{IV}$
β_{IV}

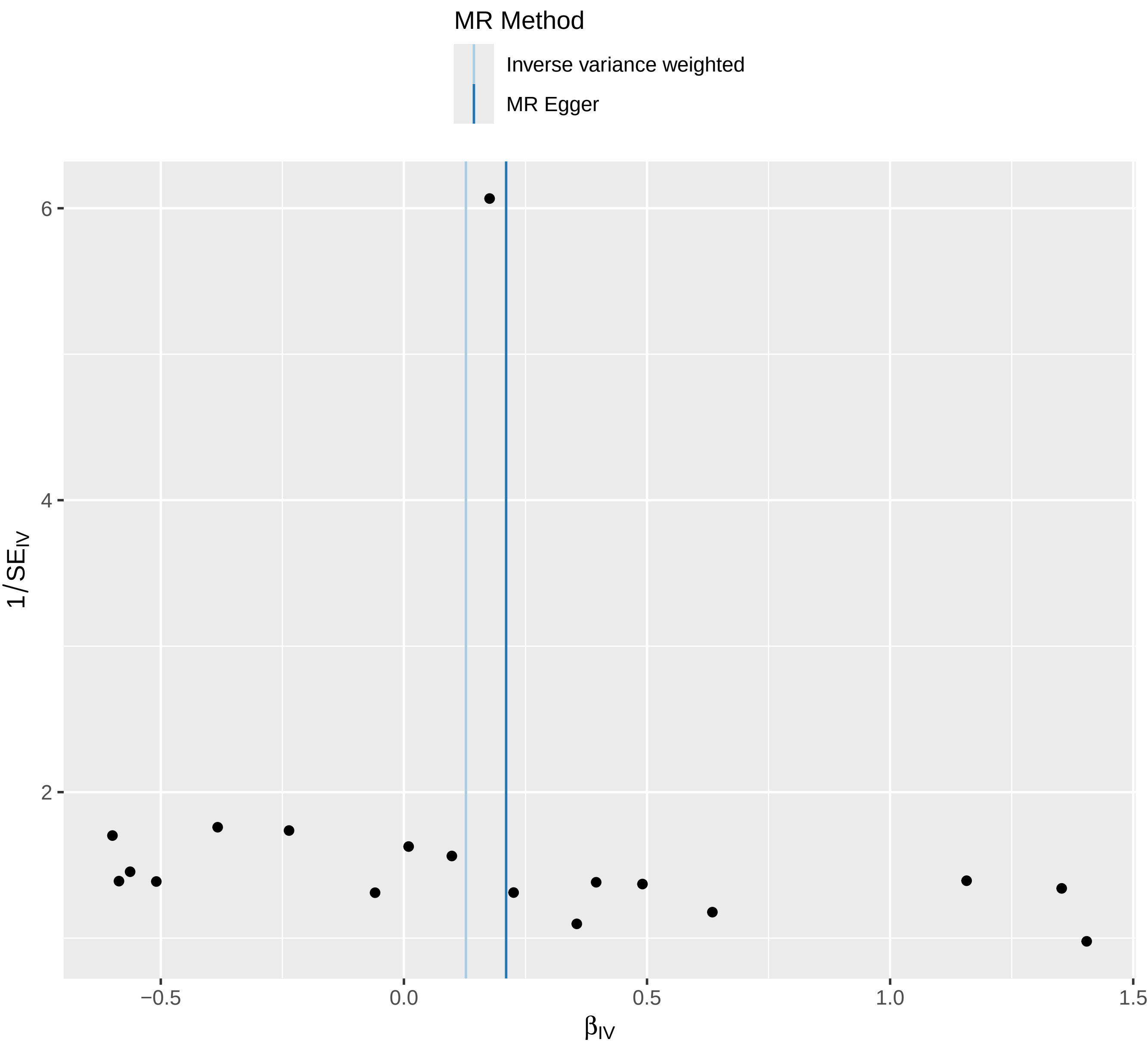

MR Method
Inverse variance weighted
MR Egger
6
4
2
1/SE_IV
−0.5
0.0
0.5
1.0
1.5
β_IV

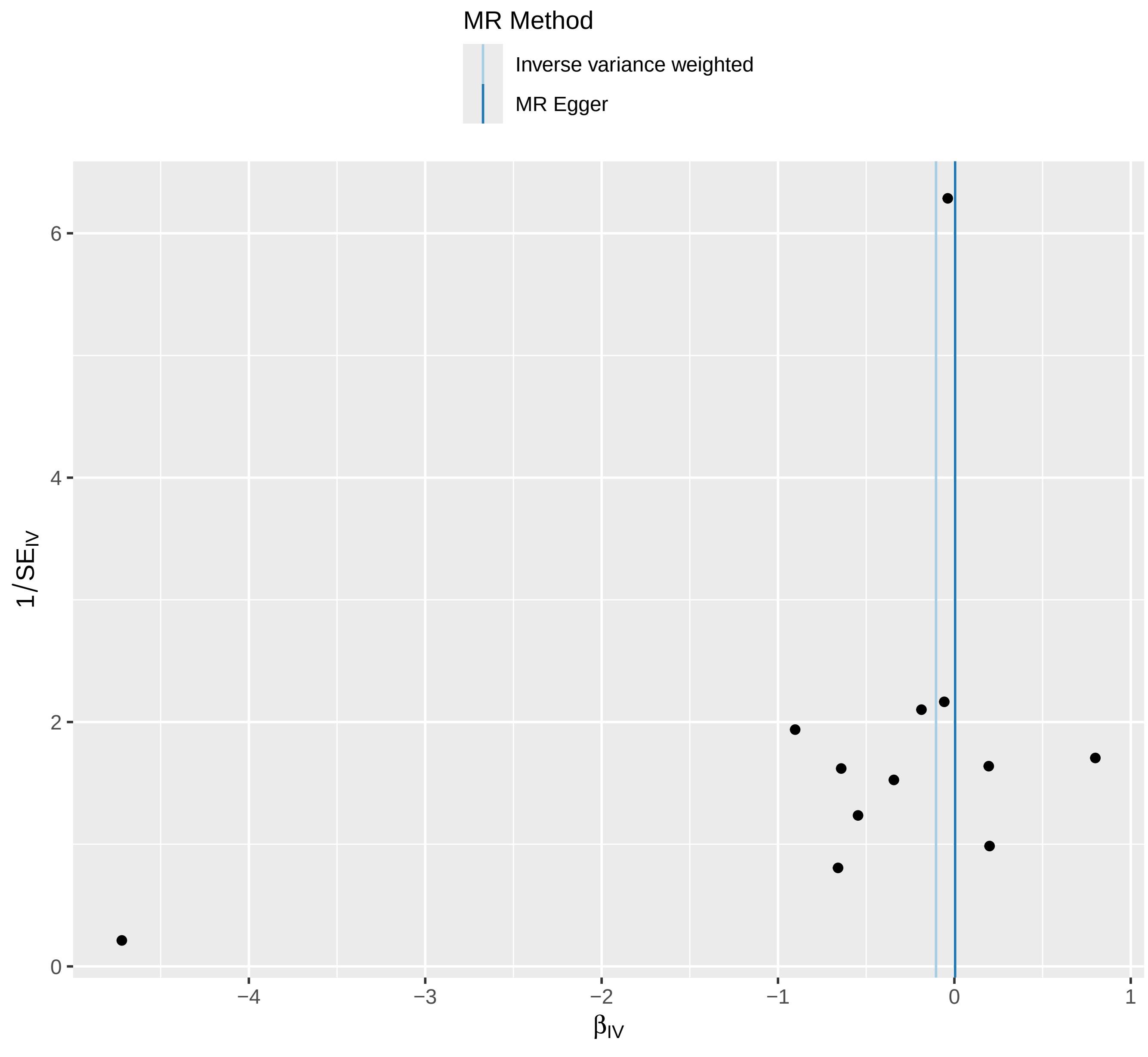

MR Method
Inverse variance weighted
MR Egger
6
4
2
0
1/SE_IV
−4
−3
−2
−1
0
1
β_IV

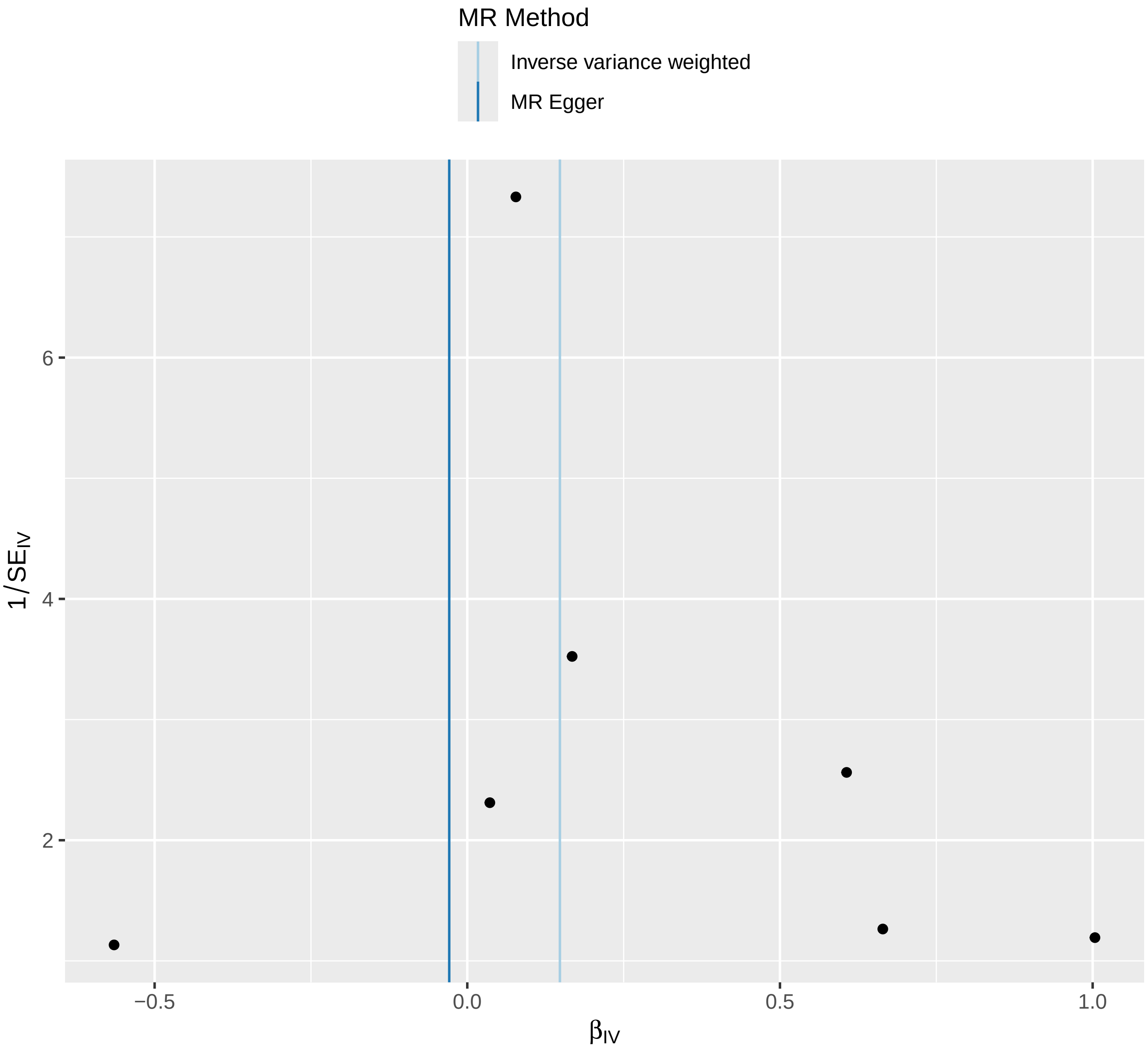
MR Method
Inverse variance weighted
MR Egger
6
4
2
1/SE_IV
−0.5
0.0
0.5
1.0
β_IV

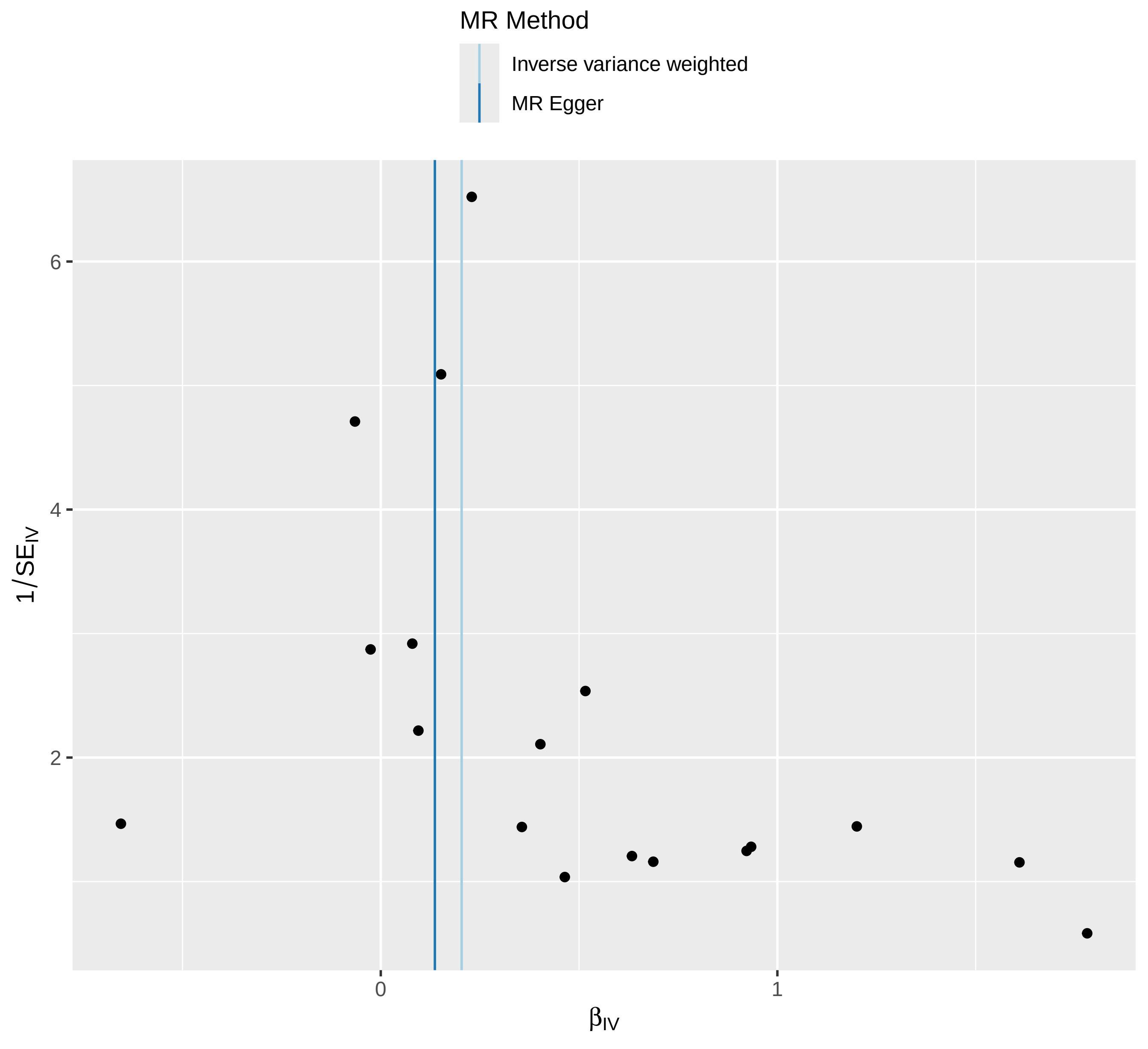
MR Method
Inverse variance weighted
MR Egger
6
4
2
0
1
1/SE_IV
β_IV

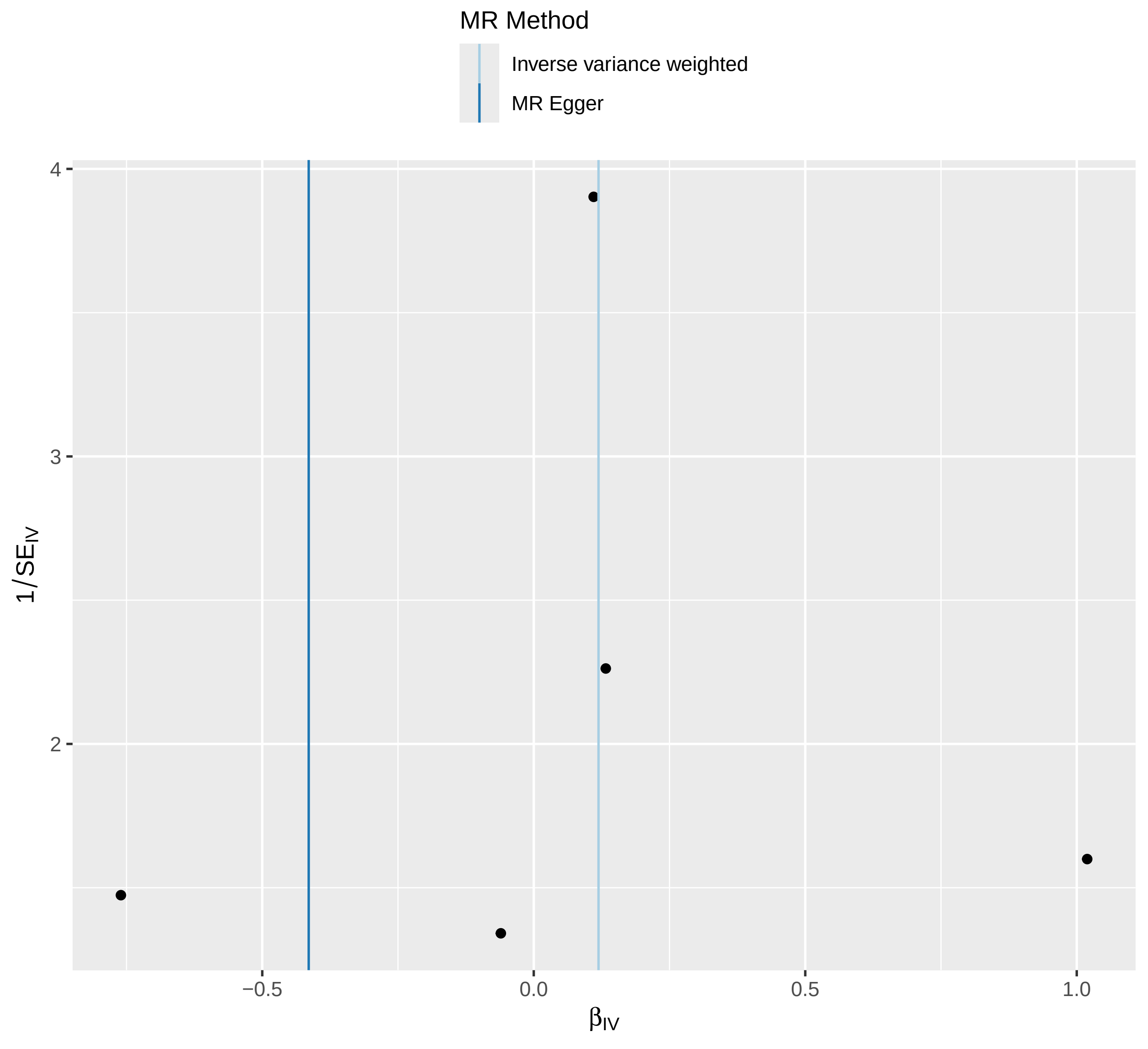
MR Method
Inverse variance weighted
MR Egger
4
3
2
$1/SE_{IV}$
−0.5
0.0
0.5
1.0
β_{IV}

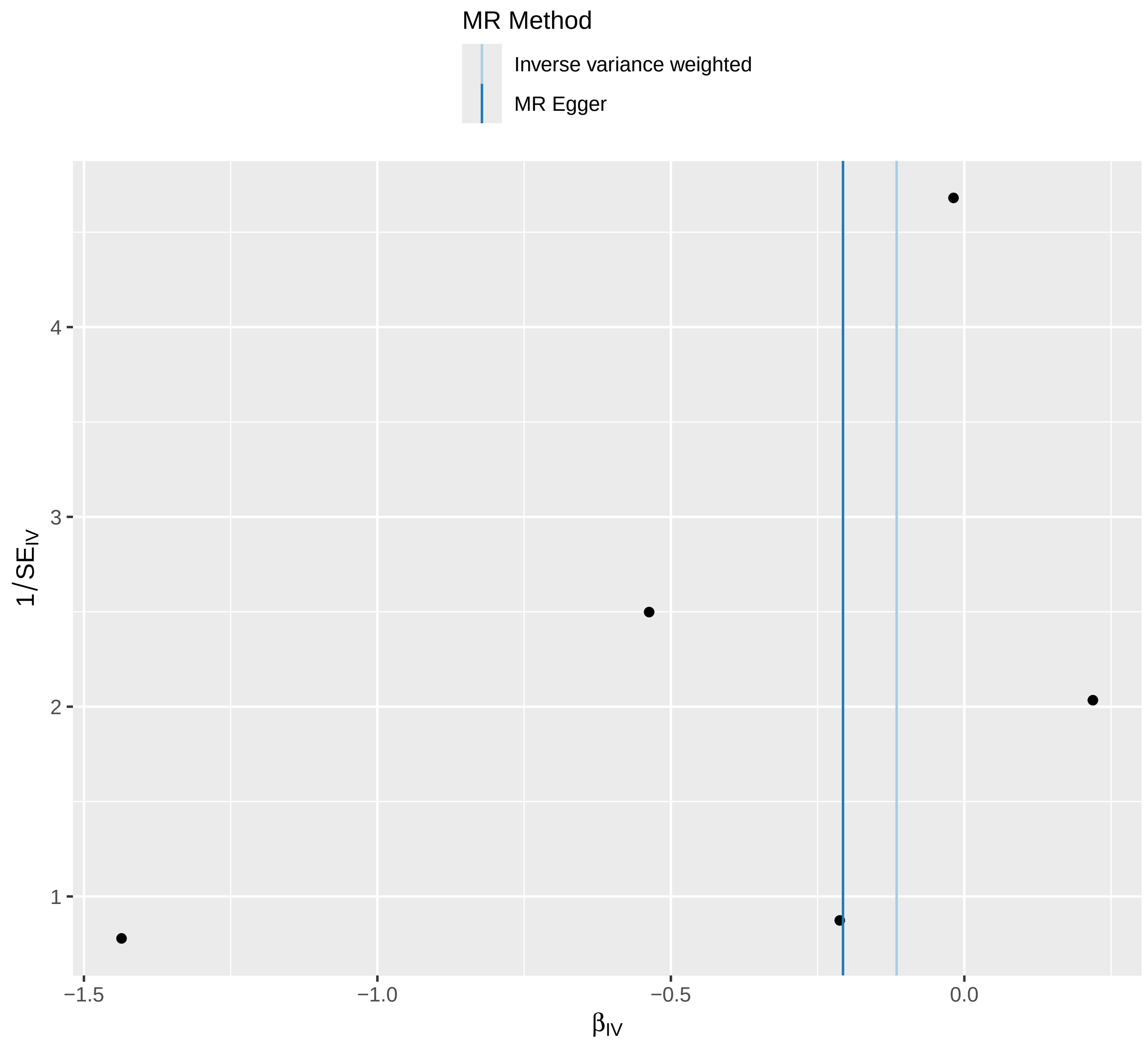

MR Method
Inverse variance weighted
MR Egger
4
3
2
1
1/SE$_{IV}$
−1.5
−1.0
−0.5
0.0
β$_{IV}$

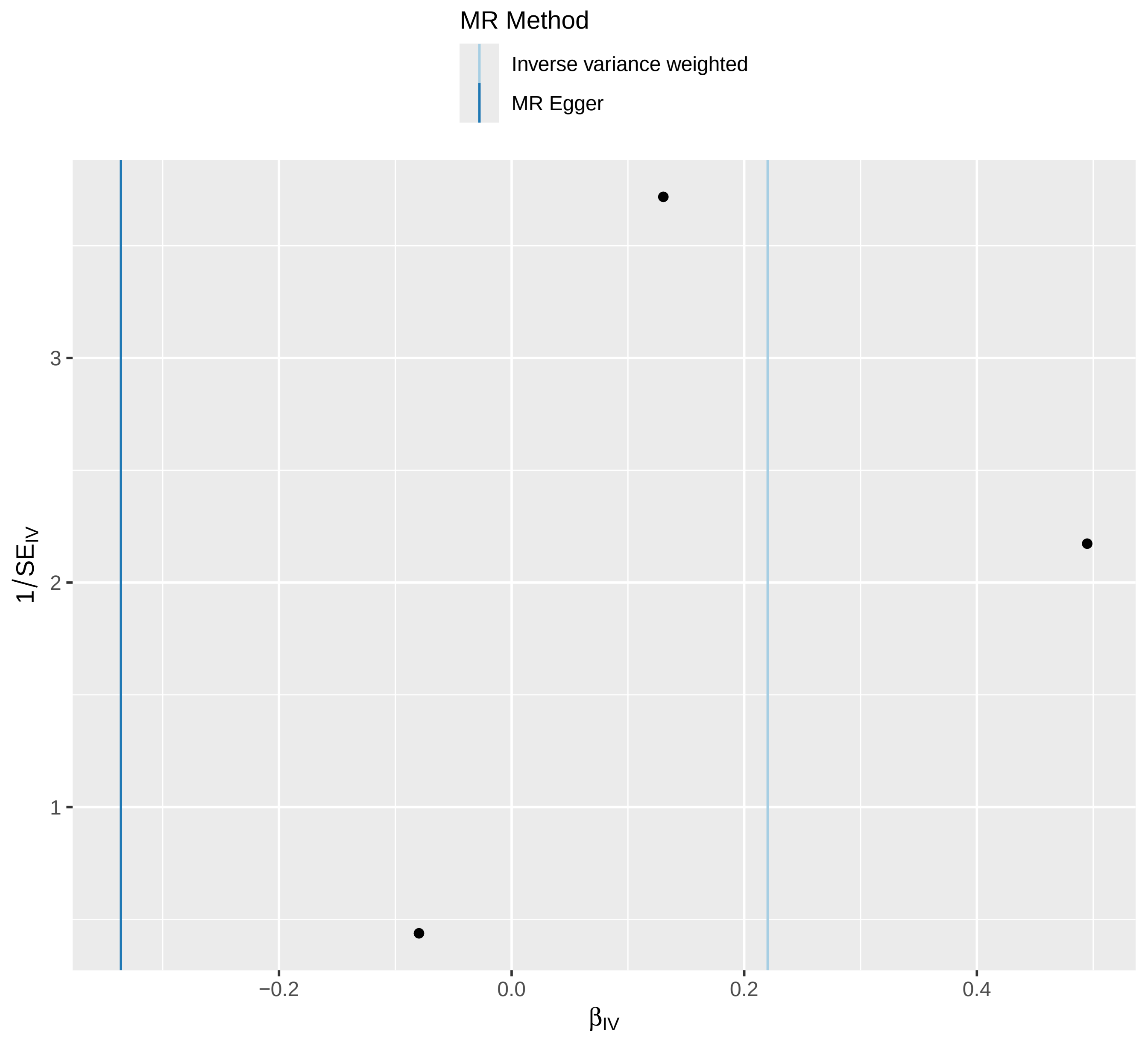
MR Method
Inverse variance weighted
MR Egger
3
2
1
−0.2
0.0
0.2
0.4
$1/SE_{IV}$
β_{IV}

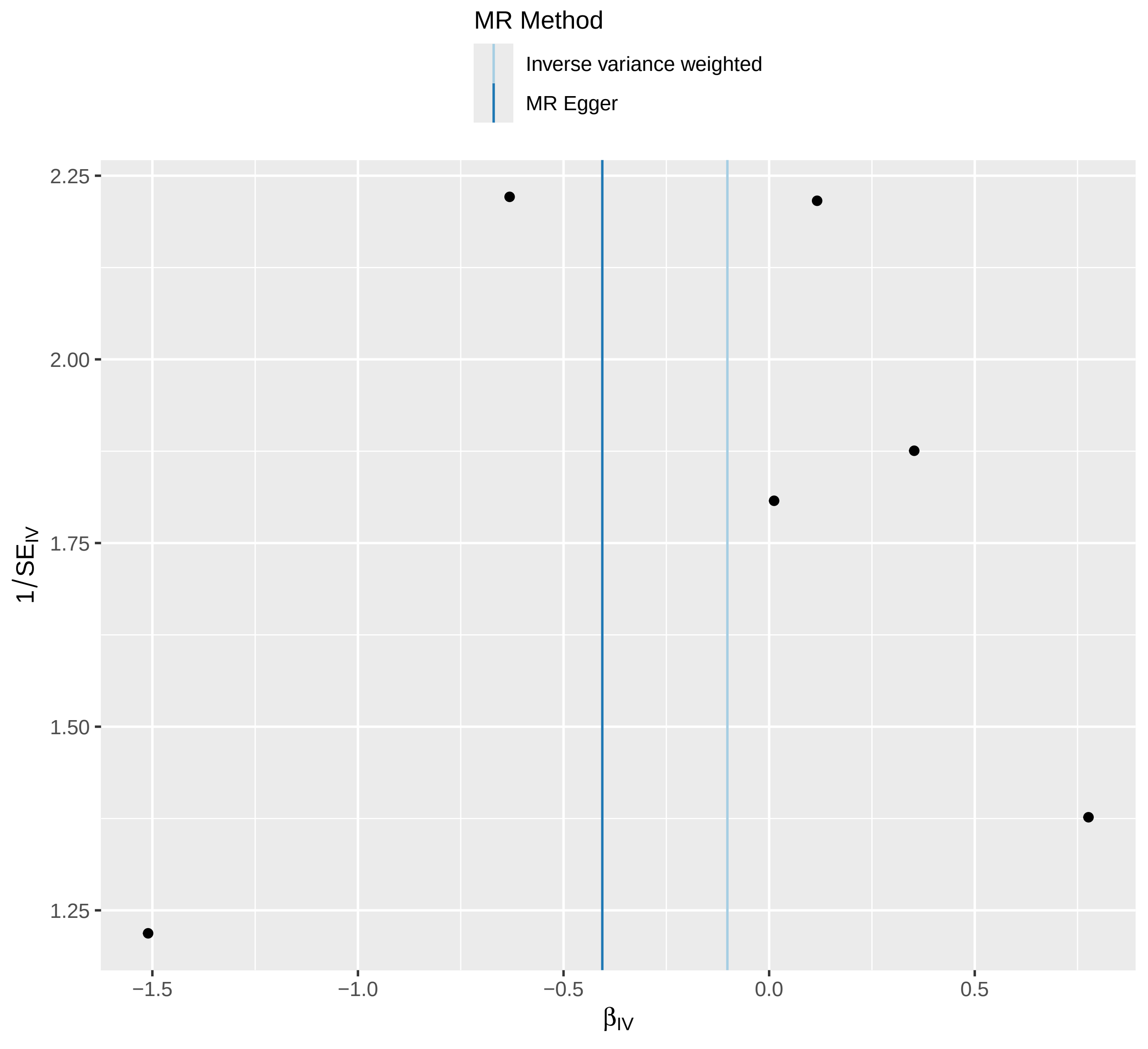

MR Method
Inverse variance weighted
MR Egger
2.25
2.00
1.75
1.50
1.25
1/SE_IV
−1.5
−1.0
−0.5
0.0
0.5
β_IV

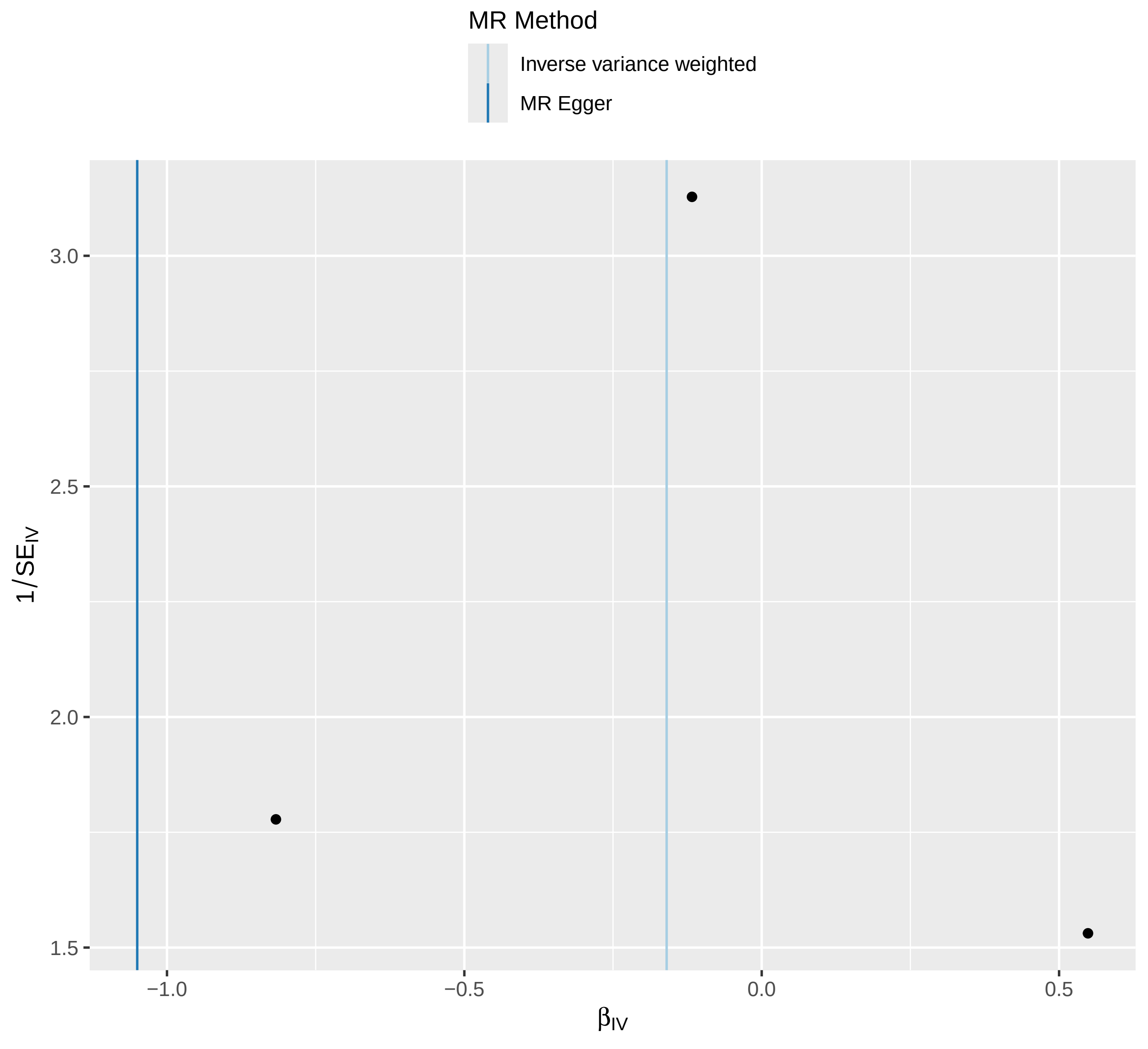
MR Method
Inverse variance weighted
MR Egger
1/SE_IV
3.0
2.5
2.0
1.5
−1.0
−0.5
0.0
0.5
β_IV

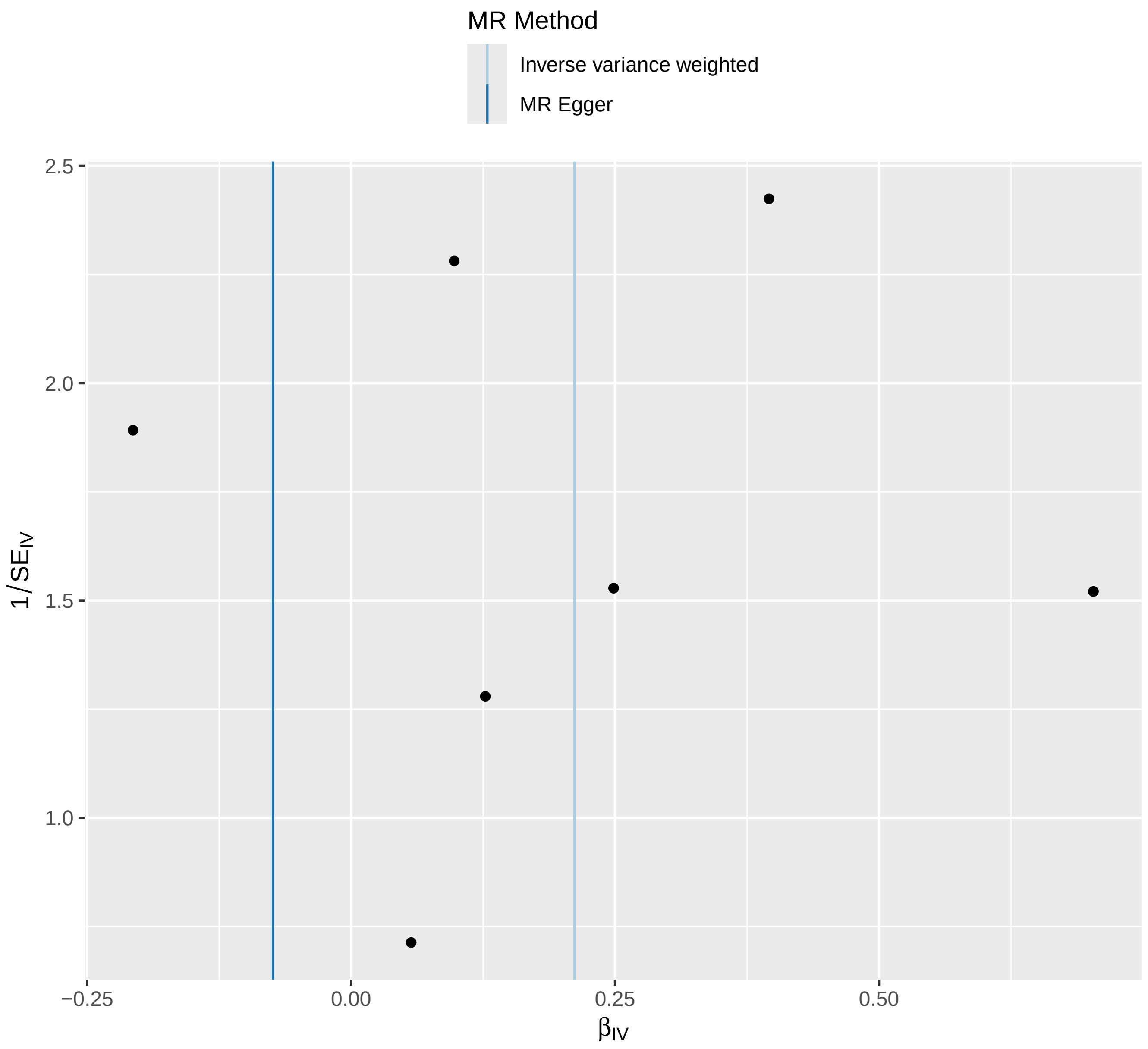

MR Method
Inverse variance weighted
MR Egger
2.5
2.0
1.5
1.0
$1/SE_{IV}$
−0.25
0.00
0.25
0.50
β_{IV}

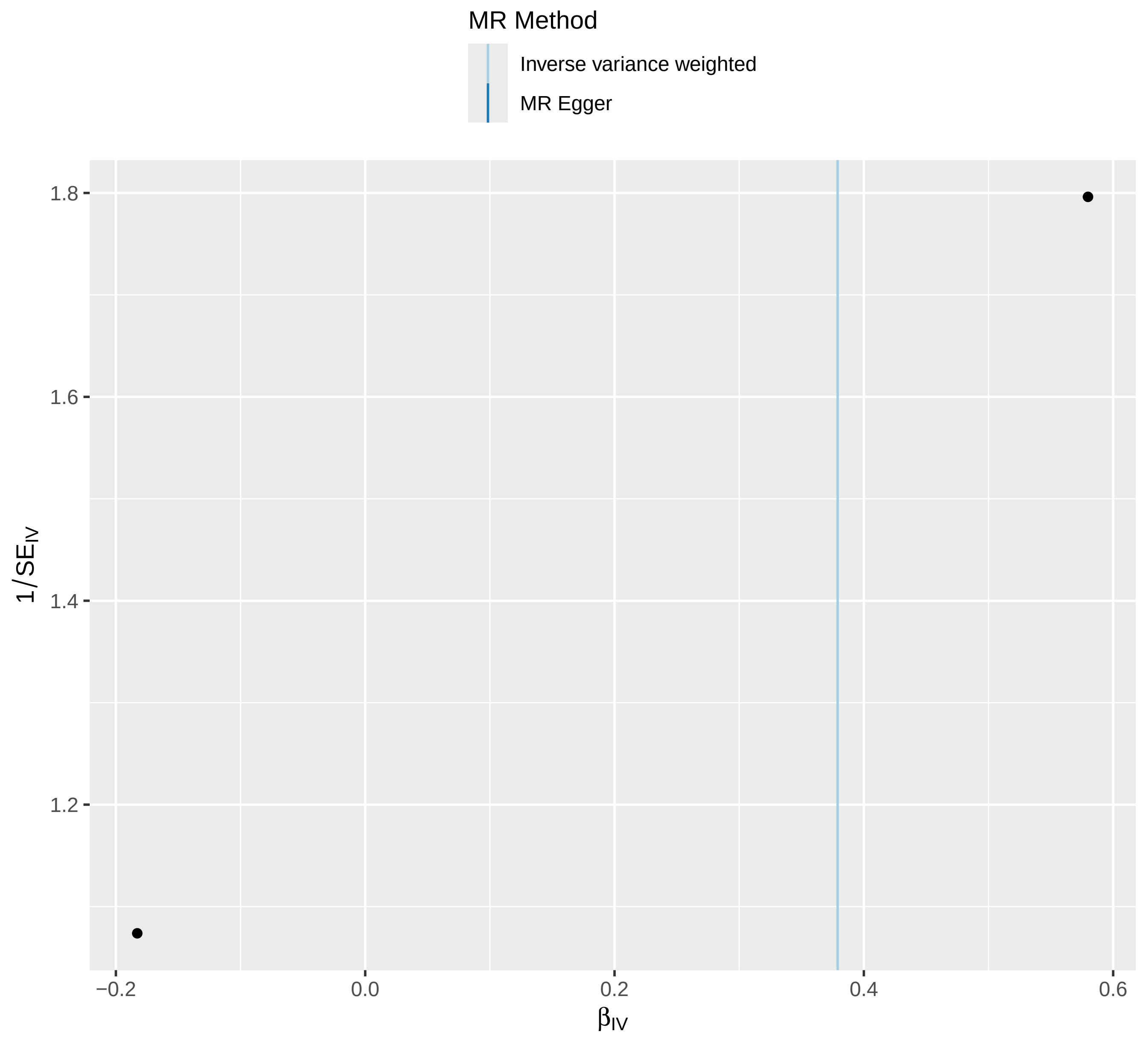
MR Method
Inverse variance weighted
MR Egger
1.8
1.6
1.4
1.2
1/SE_IV
−0.2
0.0
0.2
0.4
0.6
β_IV

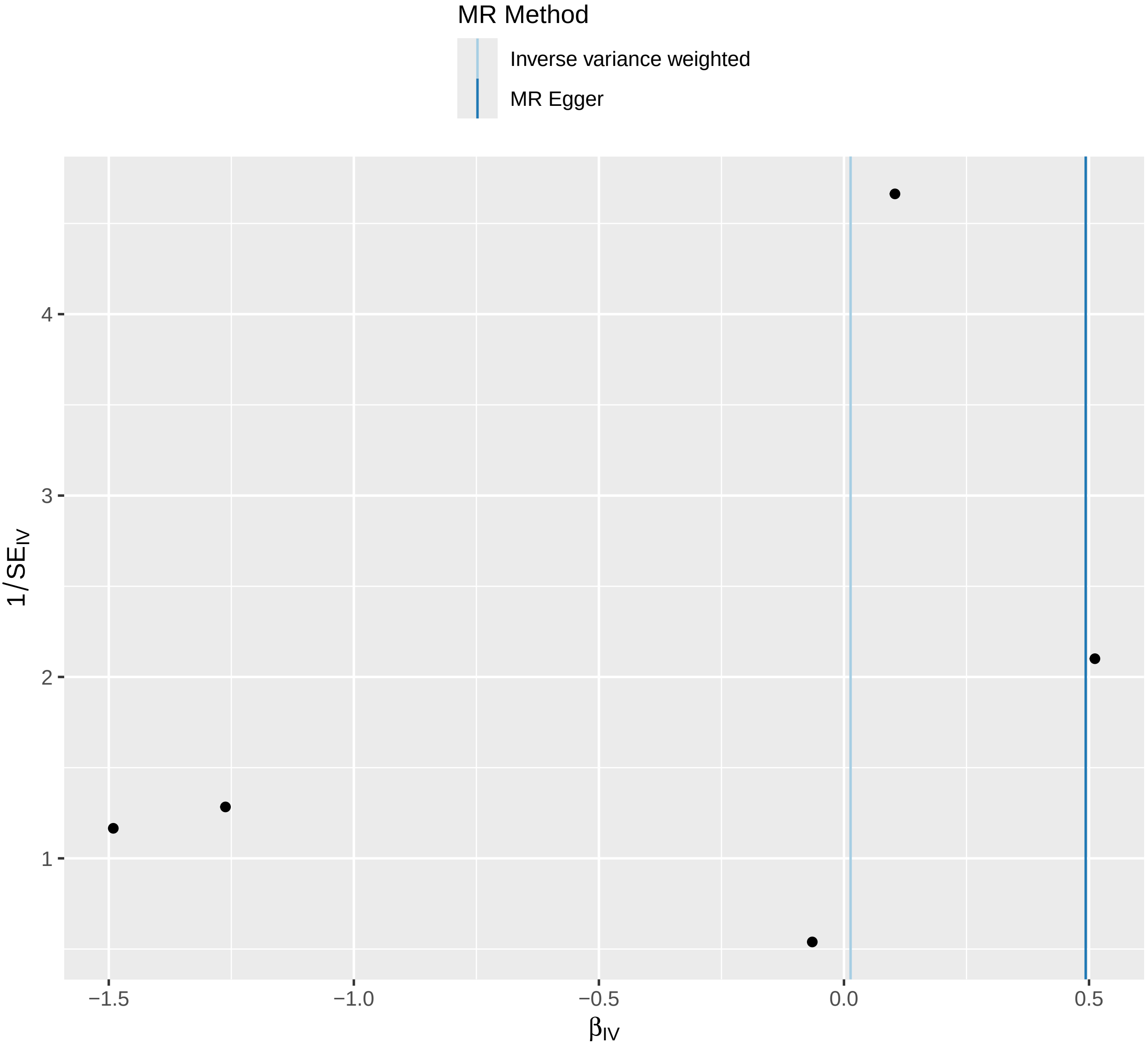
MR Method
Inverse variance weighted
MR Egger
1/SE_IV
β_IV
4
3
2
1
−1.5
−1.0
−0.5
0.0
0.5

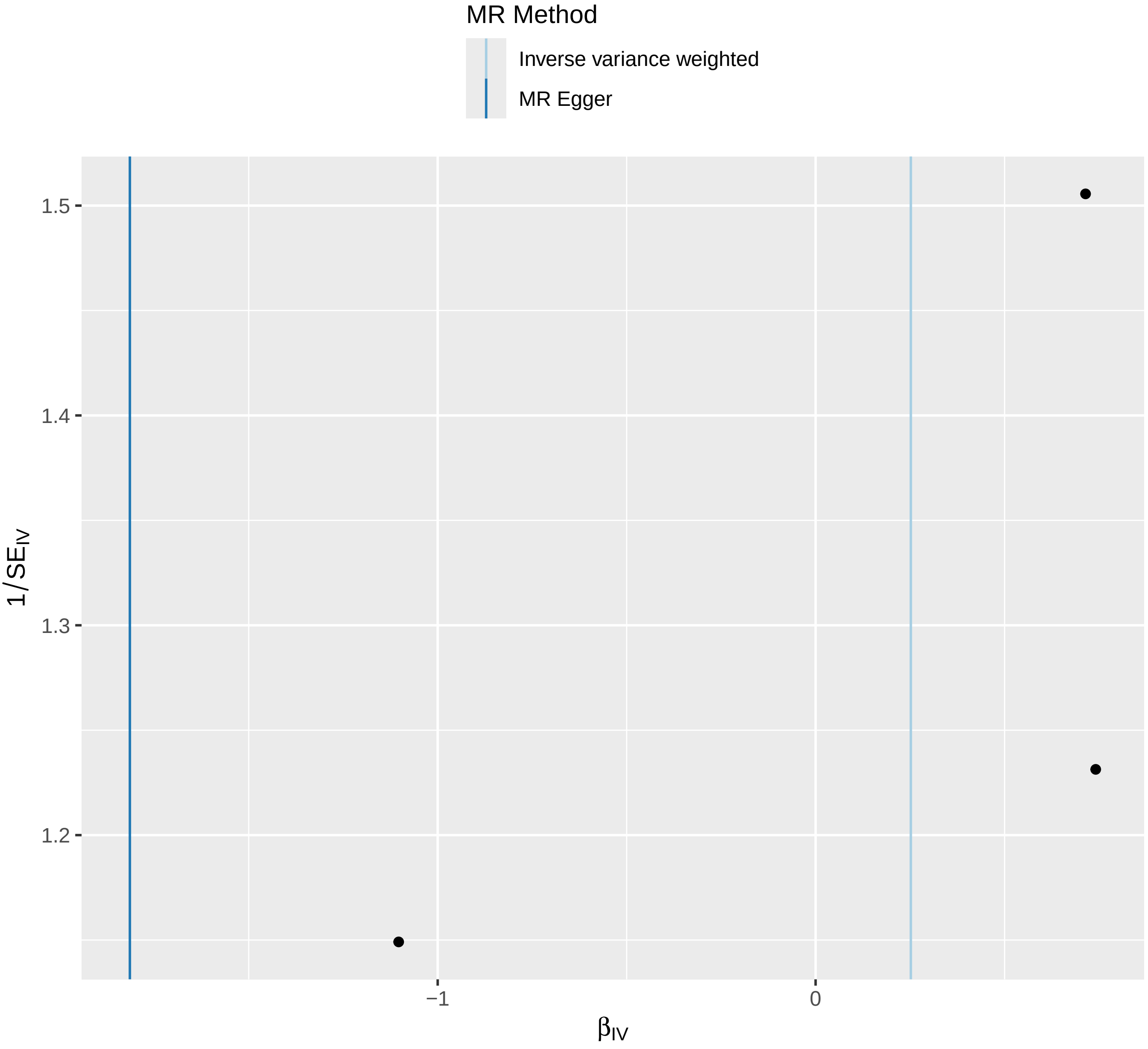
MR Method
Inverse variance weighted
MR Egger
1.5
1.4
1.3
1.2
−1
0
$1/SE_{IV}$
β_{IV}

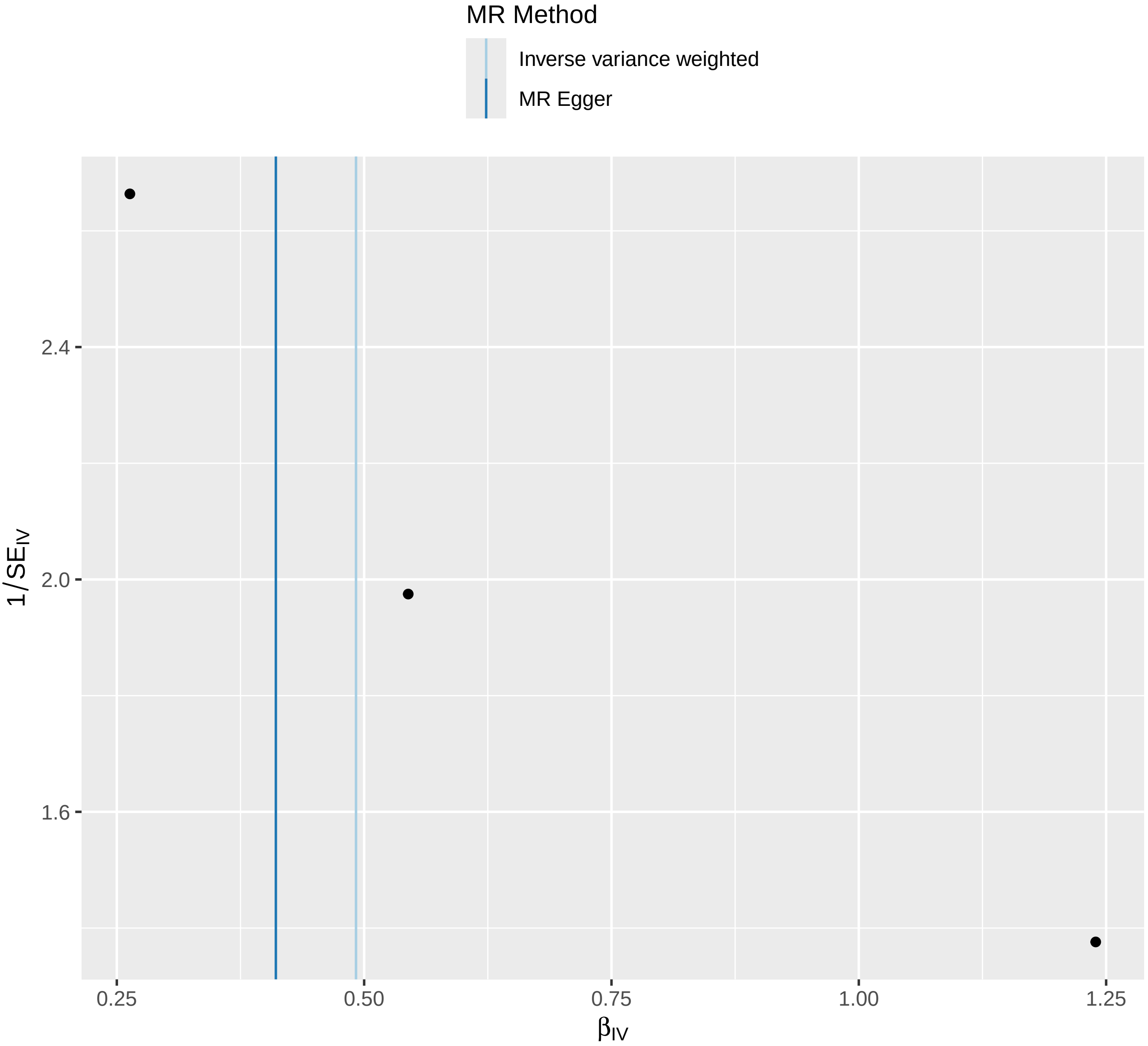
MR Method
Inverse variance weighted
MR Egger
2.4
2.0
1.6
0.25
0.50
0.75
1.00
1.25
$1/SE_{IV}$
β_{IV}

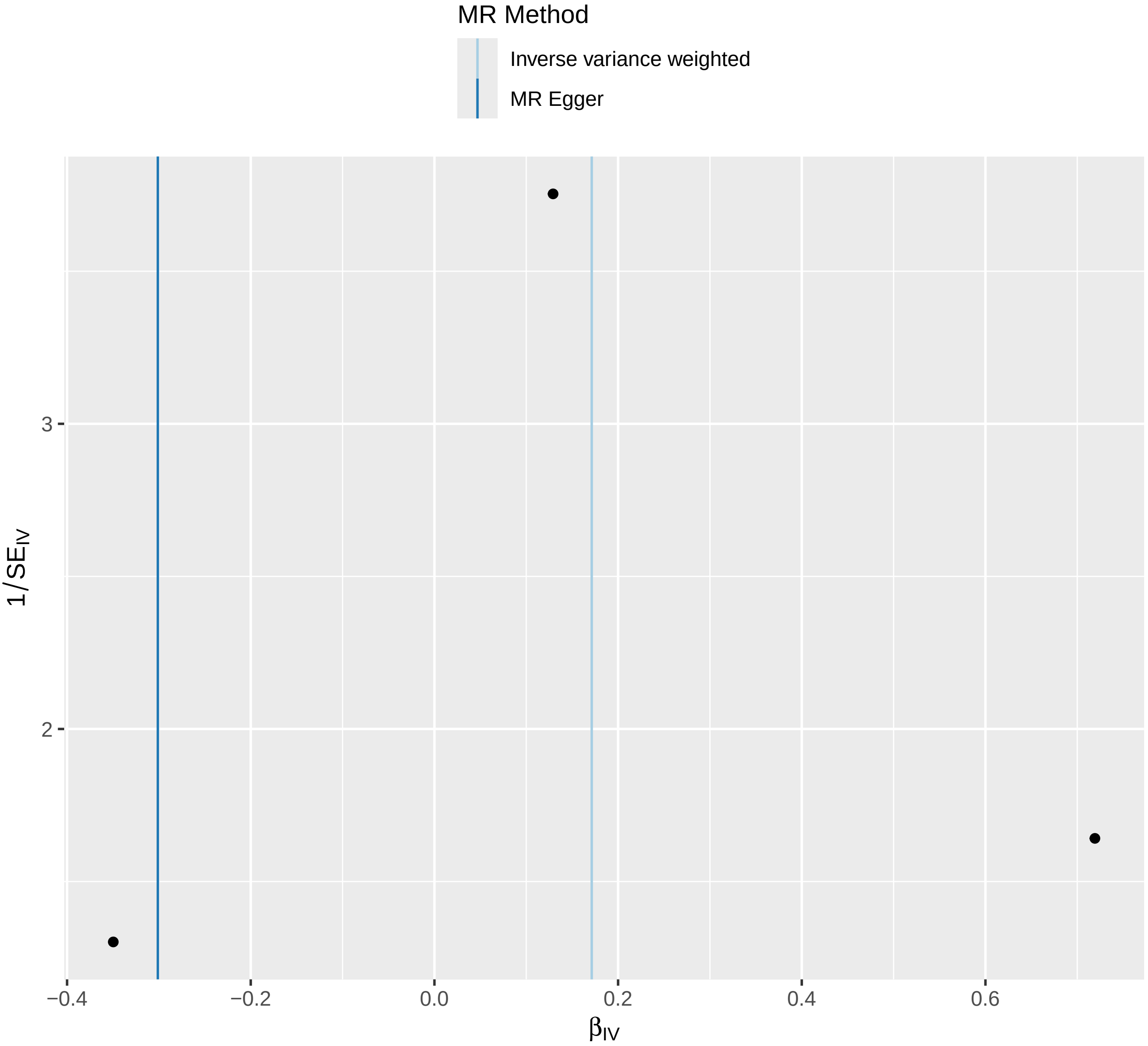
MR Method
Inverse variance weighted
MR Egger
3
2
1/SE_IV
−0.4
−0.2
0.0
0.2
0.4
0.6
β_IV

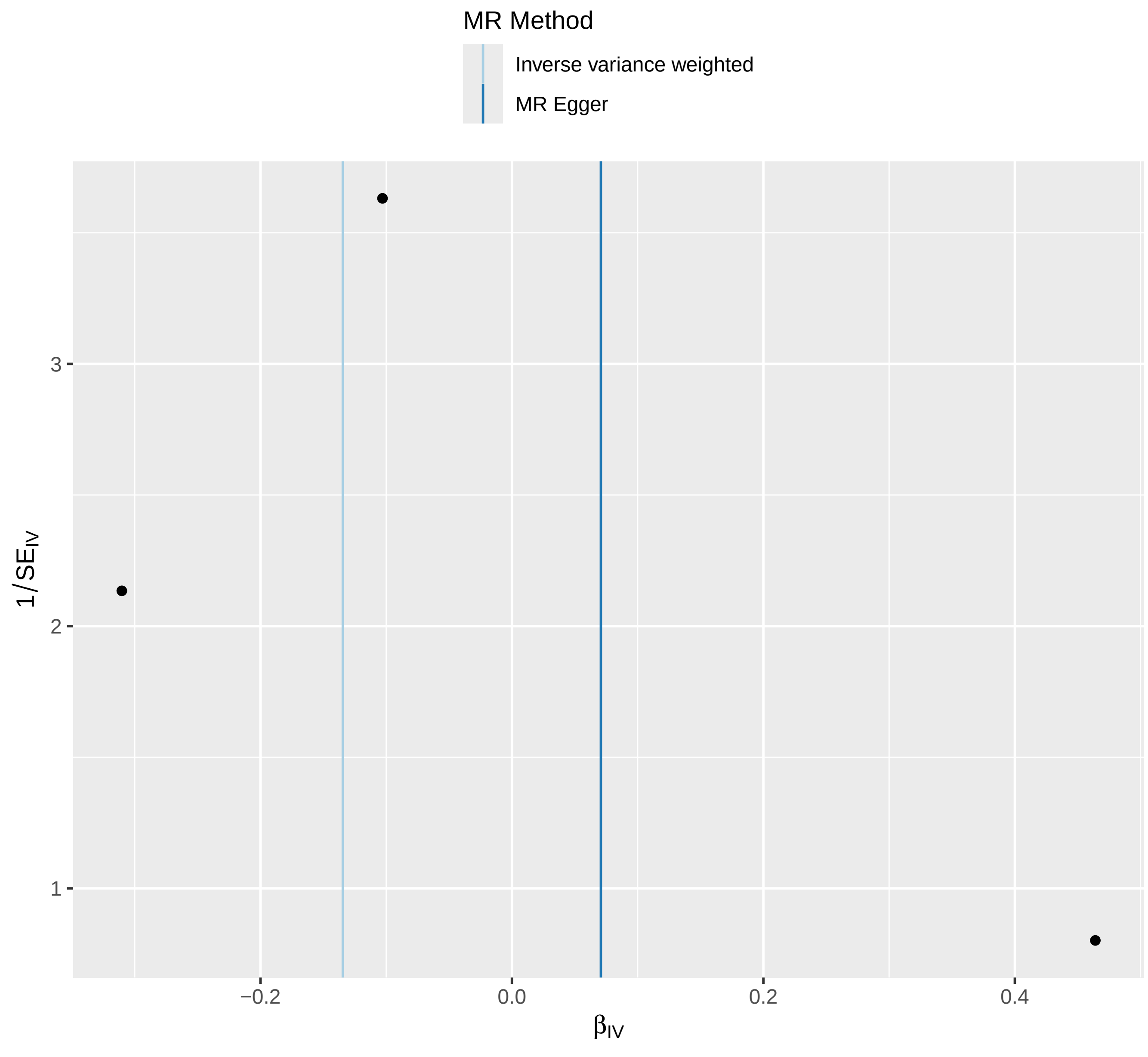
MR Method
Inverse variance weighted
MR Egger
1/SE_IV
3
2
1
−0.2
0.0
0.2
0.4
β_IV

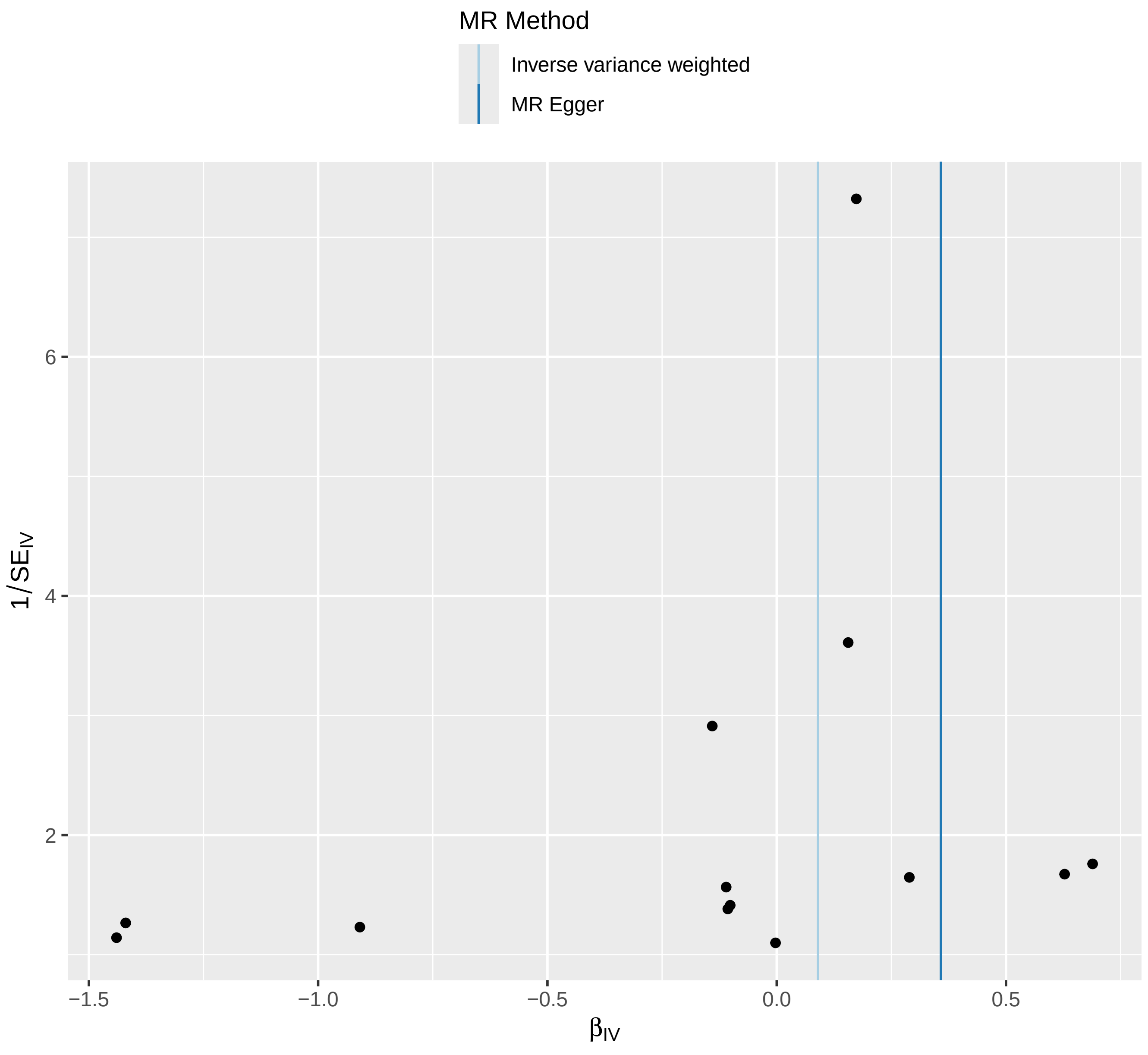
MR Method
Inverse variance weighted
MR Egger
1/SE$_{IV}$
6
4
2
−1.5
−1.0
−0.5
0.0
0.5
β_{IV}

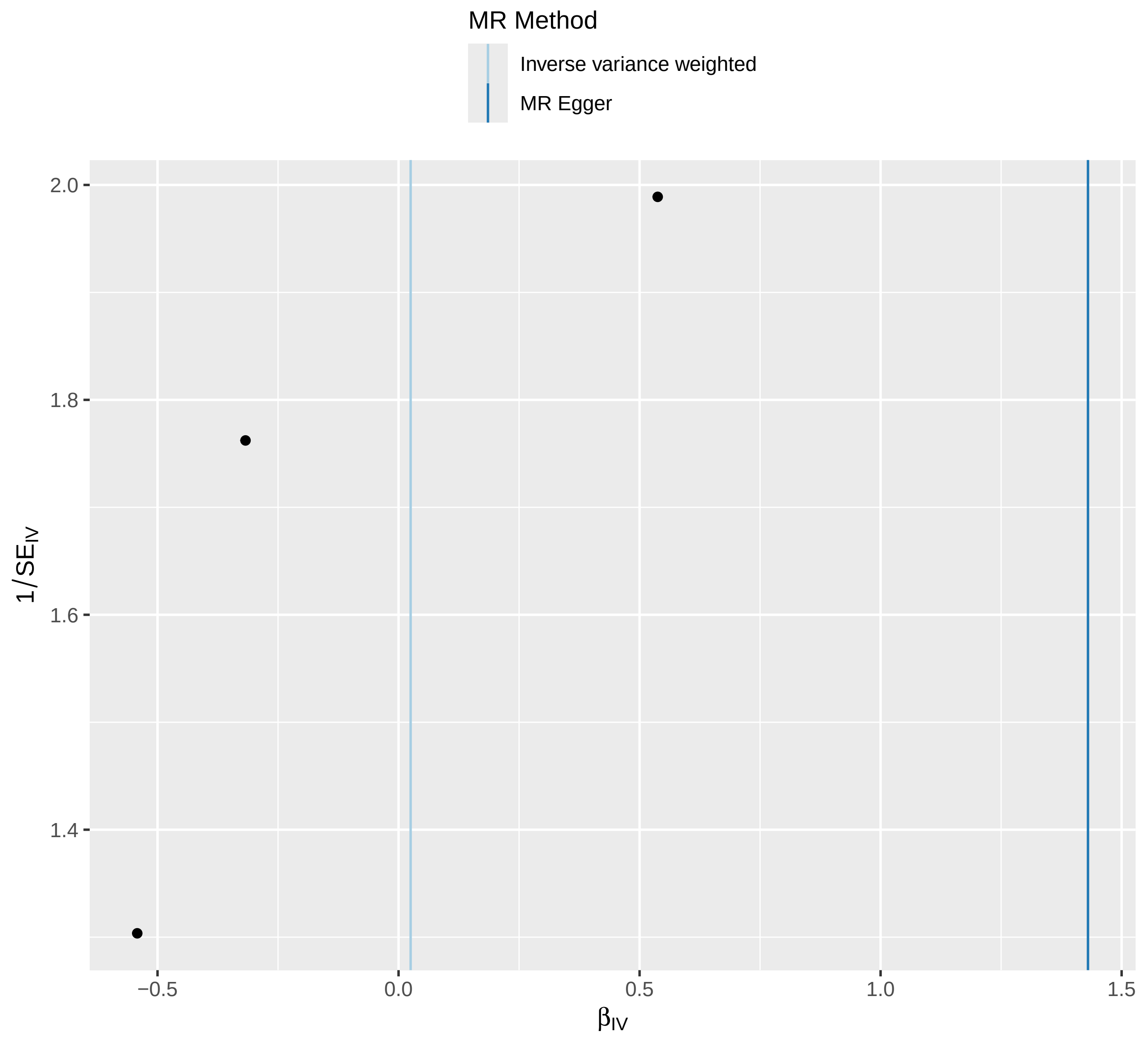

MR Method
Inverse variance weighted
MR Egger
2.0
1.8
1.6
1.4
1/SE_IV
−0.5
0.0
0.5
1.0
1.5
β_IV

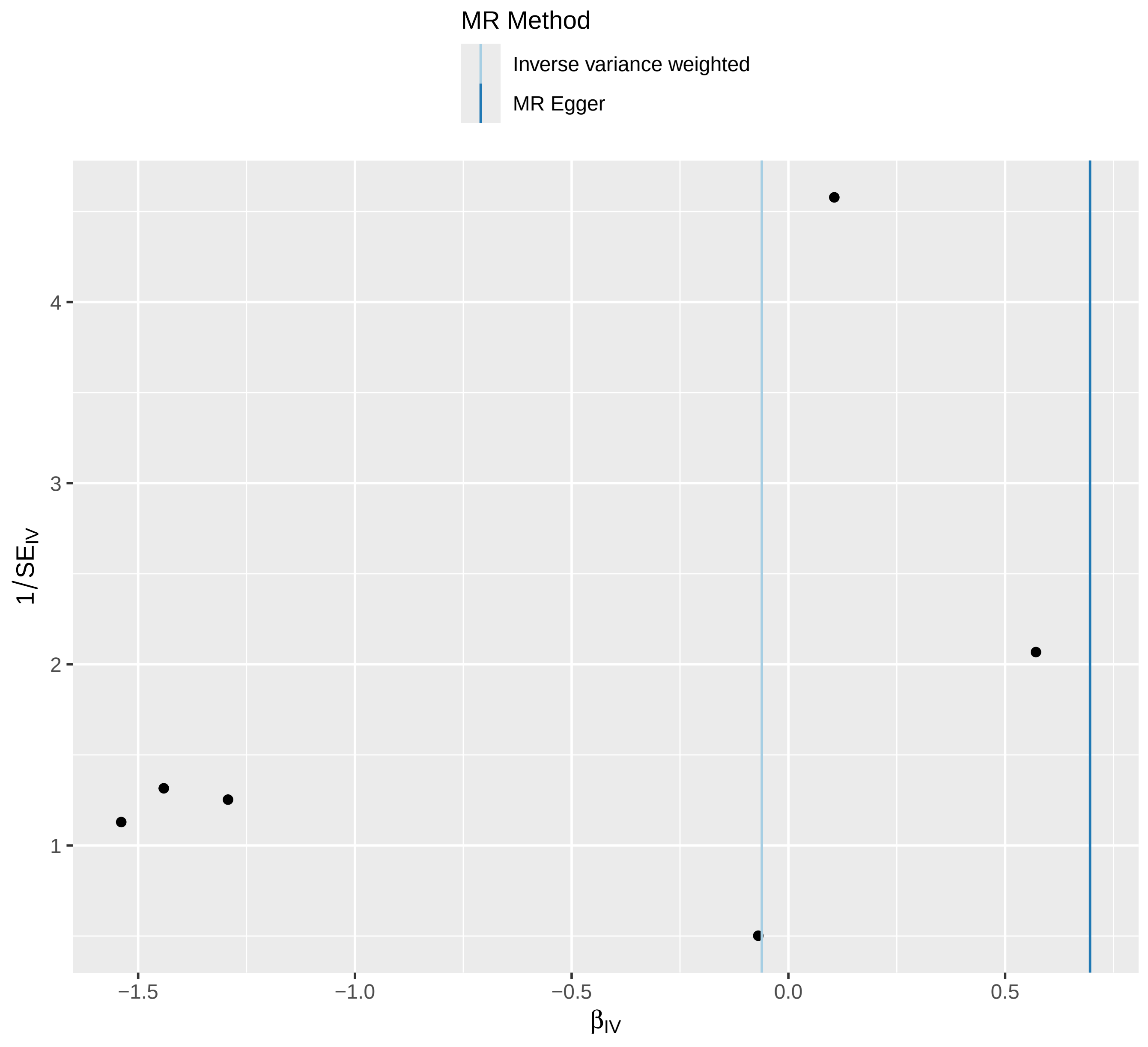

MR Method
Inverse variance weighted
MR Egger
4
3
2
1
1/SE$_{IV}$
−1.5
−1.0
−0.5
0.0
0.5
β$_{IV}$

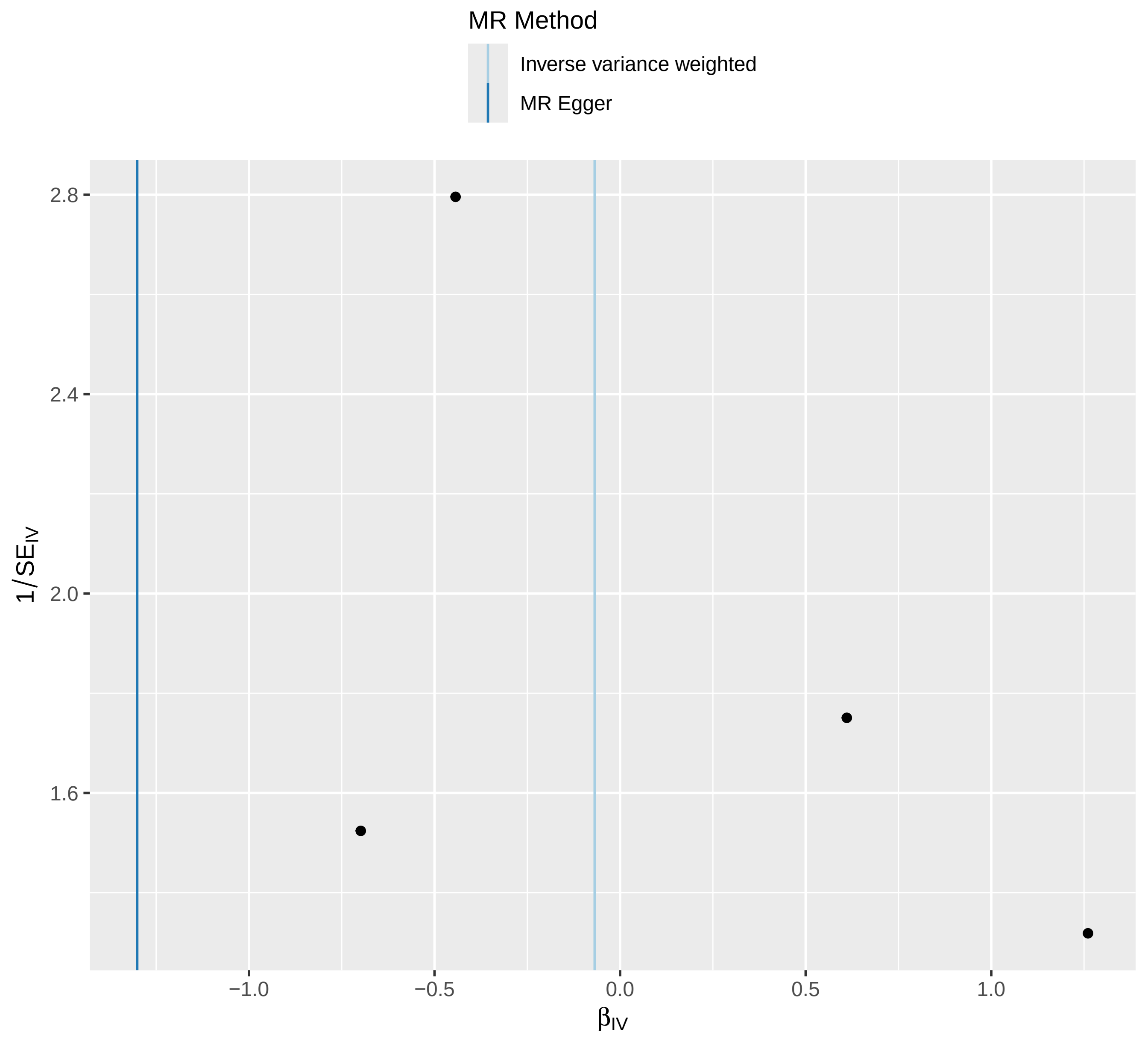
MR Method
Inverse variance weighted
MR Egger
2.8
2.4
2.0
1.6
1/SE_IV
−1.0
−0.5
0.0
0.5
1.0
β_IV

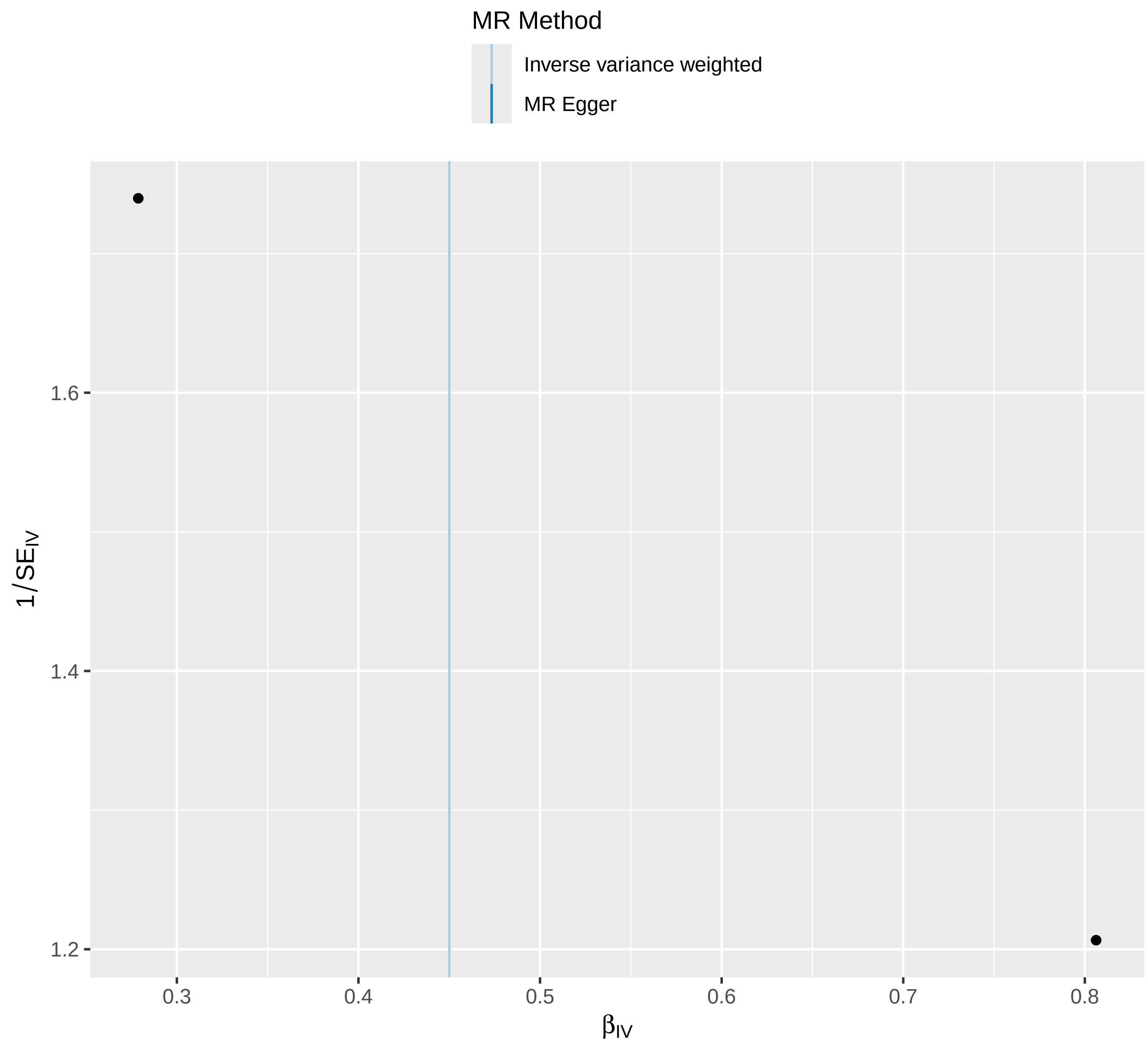
MR Method
Inverse variance weighted
MR Egger
1.6
1.4
1.2
$1/SE_{IV}$
0.3
0.4
0.5
0.6
0.7
0.8
β_{IV}

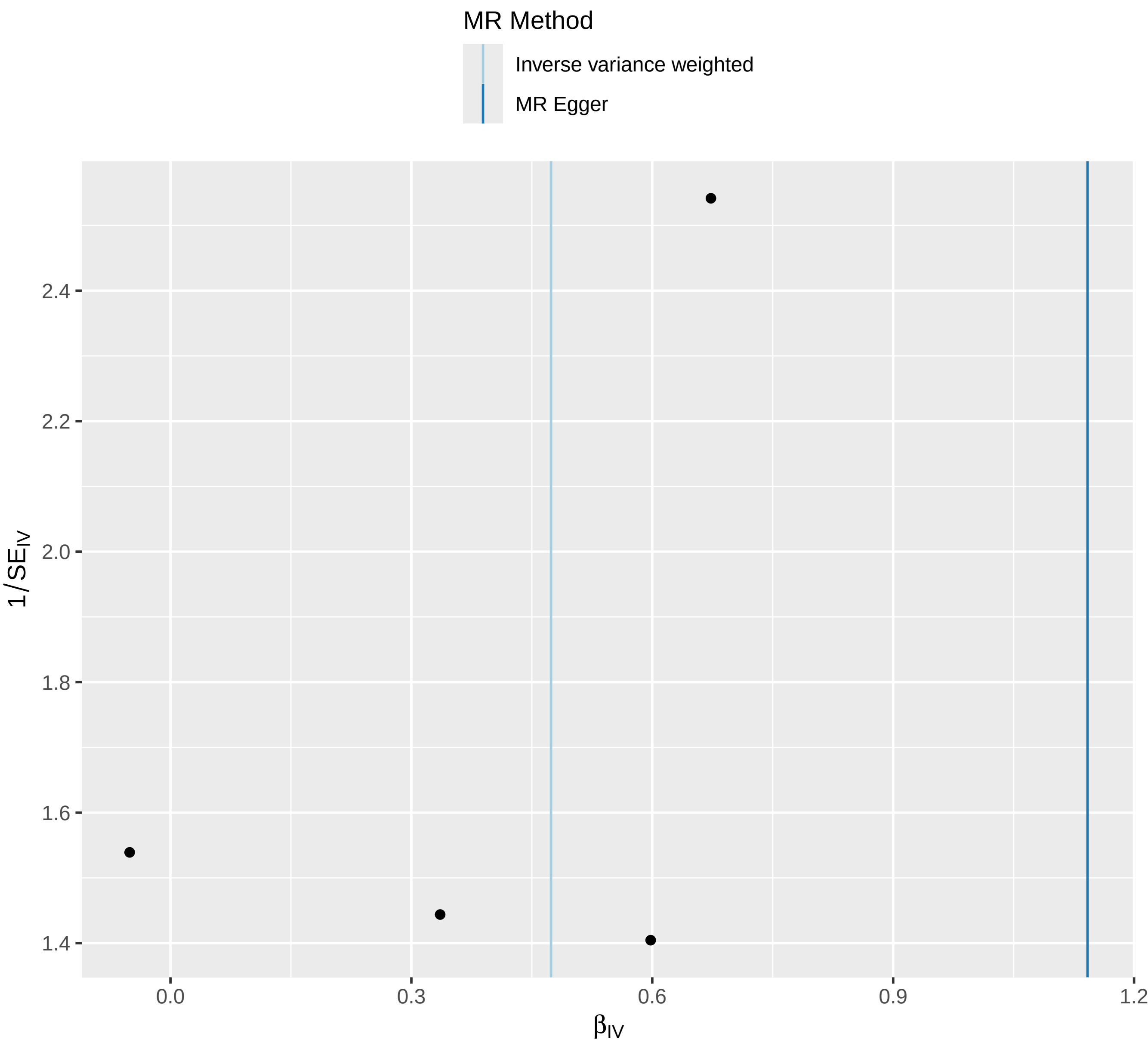
MR Method
Inverse variance weighted
MR Egger
1/SE_IV
2.4
2.2
2.0
1.8
1.6
1.4
0.0
0.3
0.6
0.9
1.2
β_IV

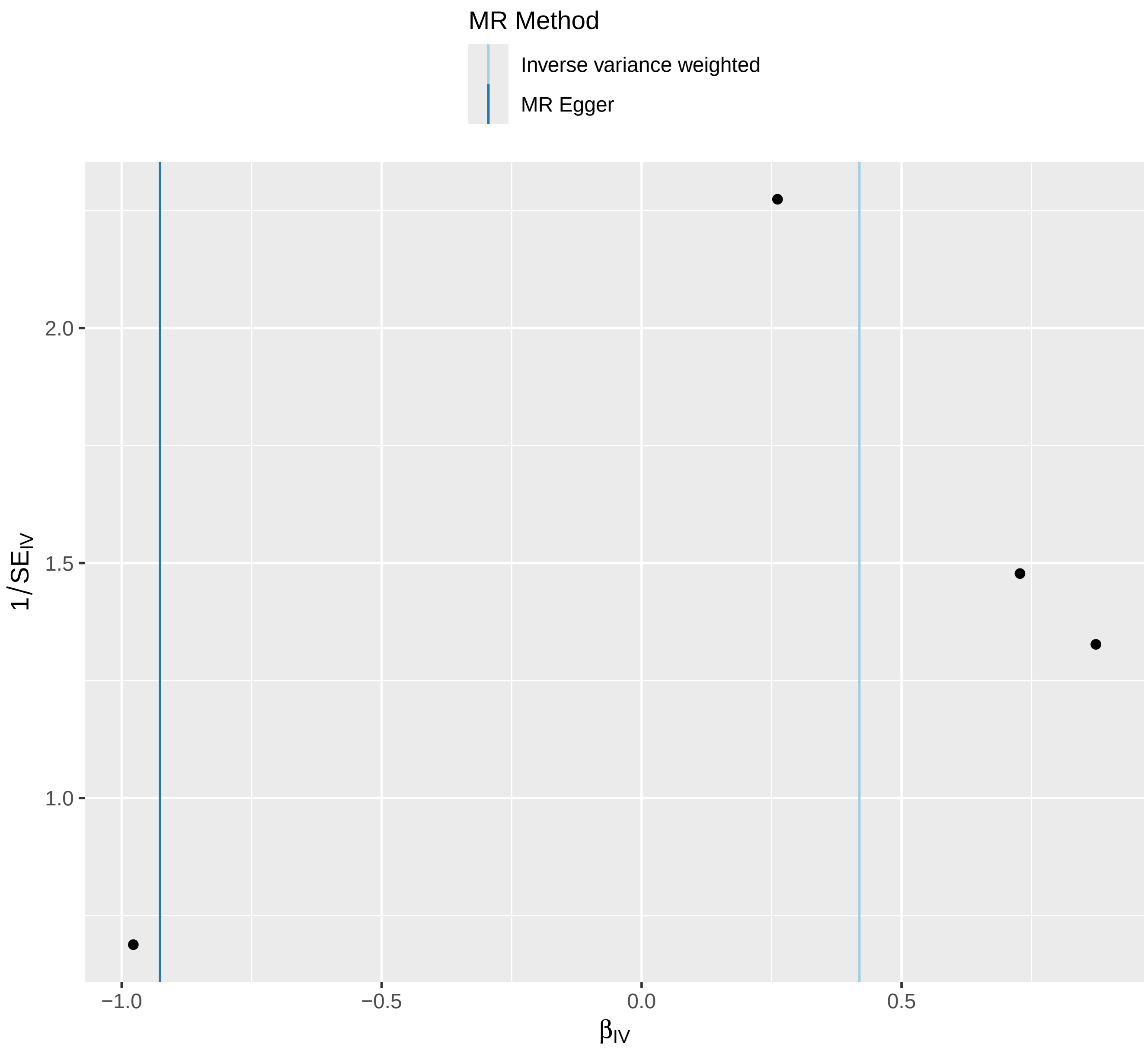
MR Method
Inverse variance weighted
MR Egger
2.0
1.5
1.0
1/SE$_{IV}$
−1.0
−0.5
0.0
0.5
β$_{IV}$

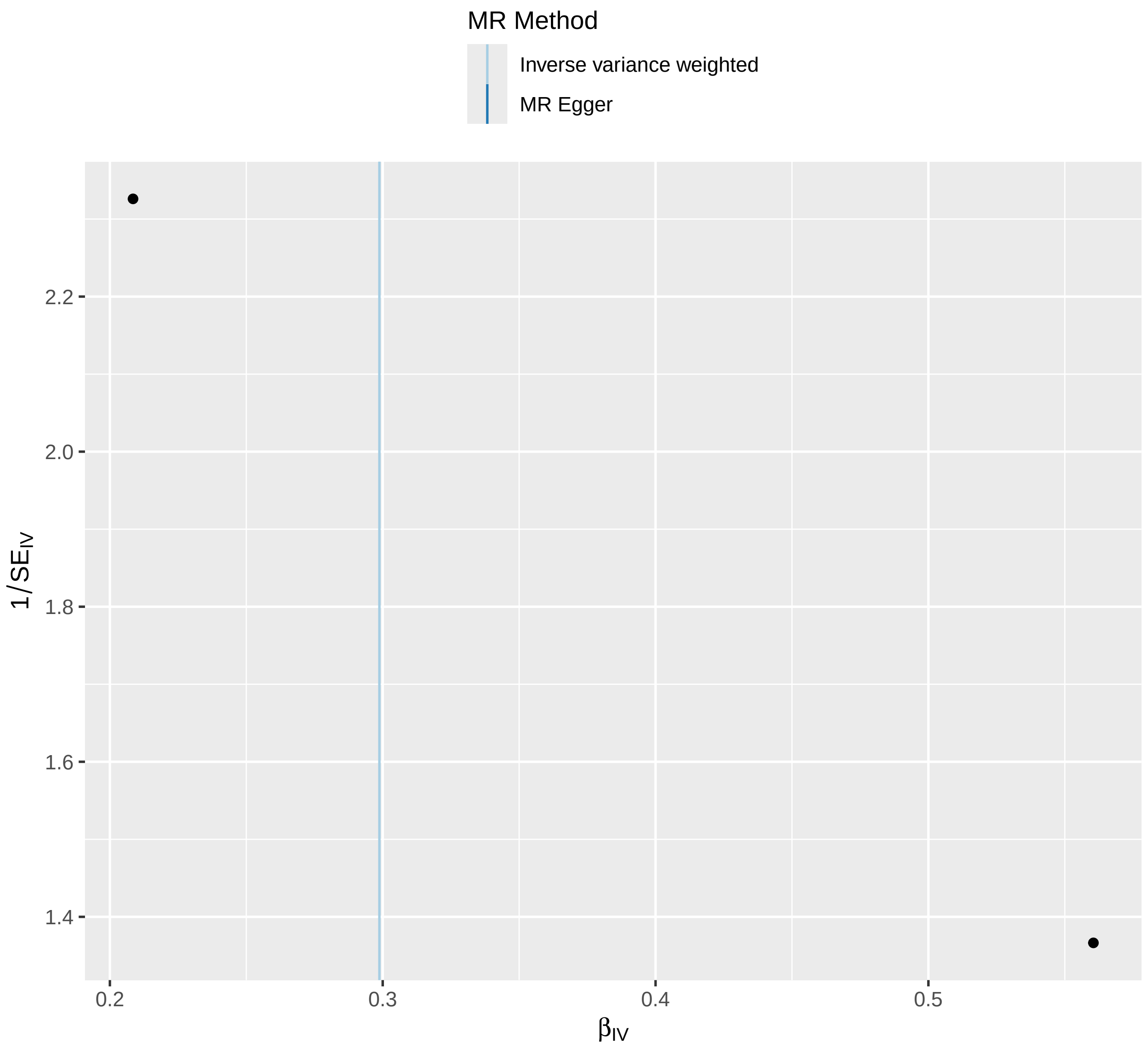

MR Method
Inverse variance weighted
MR Egger
2.2
2.0
1.8
1.6
1.4
1/SE_IV
0.2
0.3
0.4
0.5
β_IV

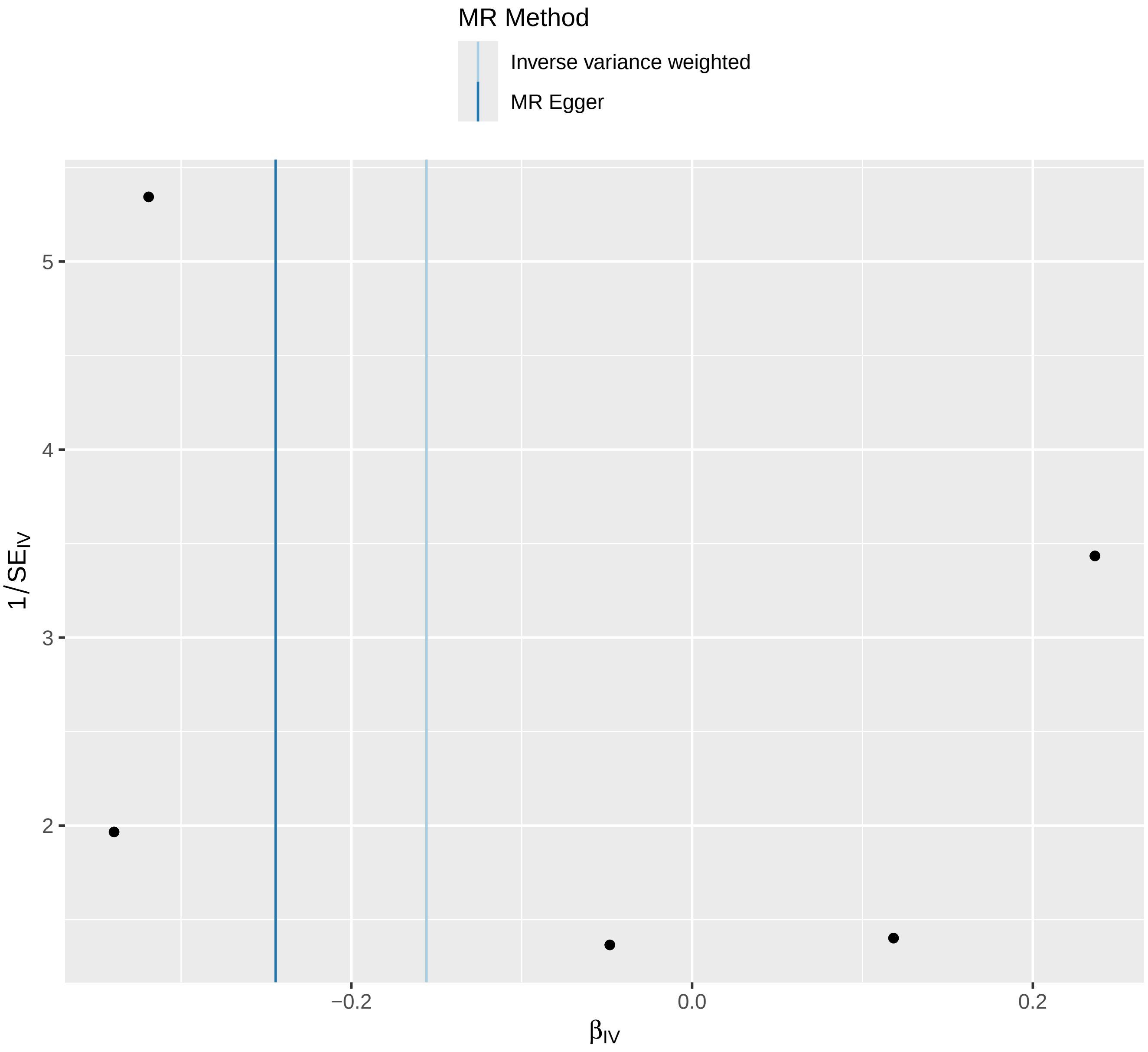

MR Method
Inverse variance weighted
MR Egger
5
4
3
2
−0.2
0.0
0.2
$1/SE_{IV}$
β_{IV}

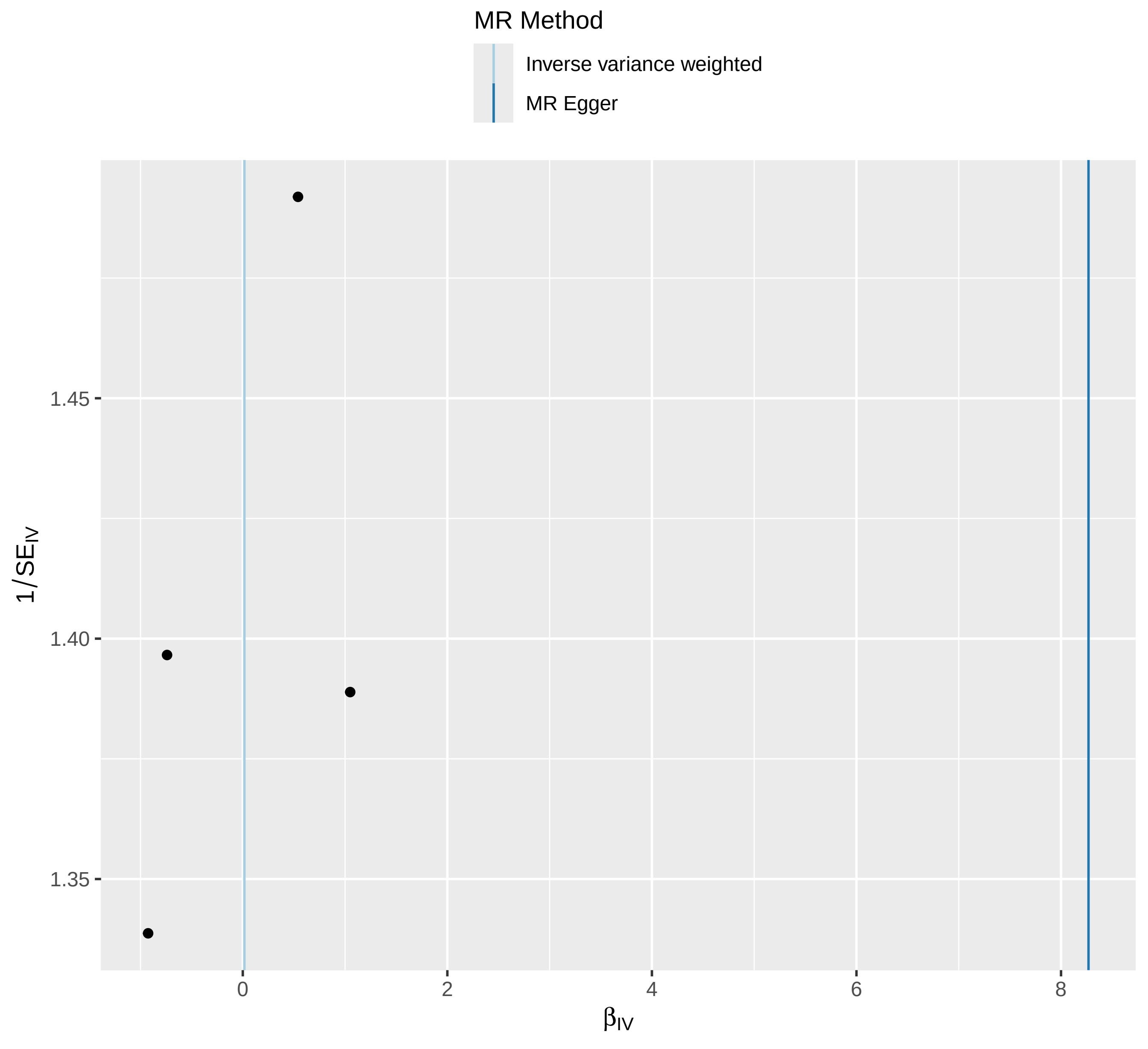

MR Method
Inverse variance weighted
MR Egger
1.45
1.40
1.35
$1/SE_{IV}$
0
2
4
6
8
β_{IV}

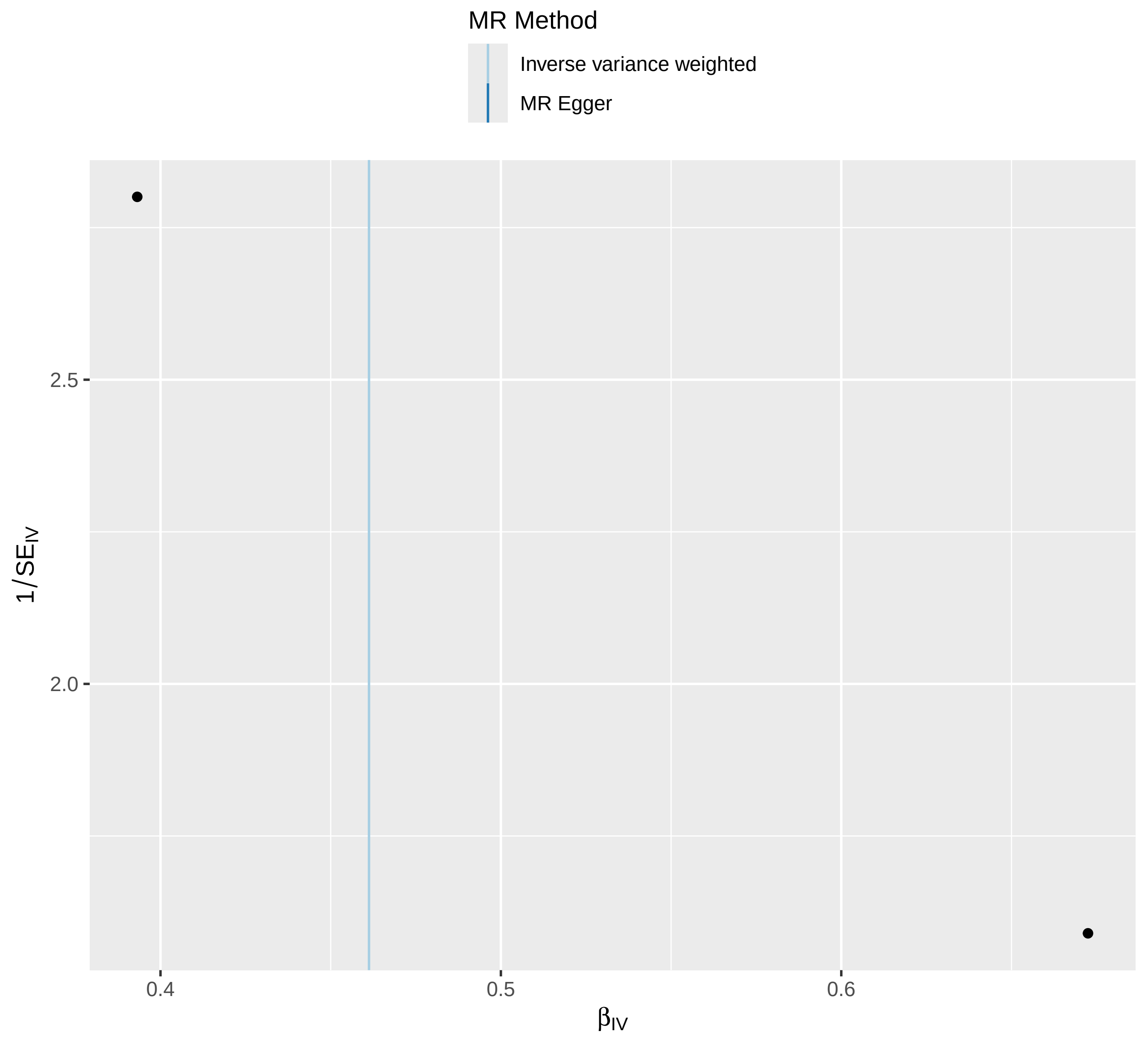

MR Method
Inverse variance weighted
MR Egger
2.5
2.0
1/SE_IV
0.4
0.5
0.6
β_IV

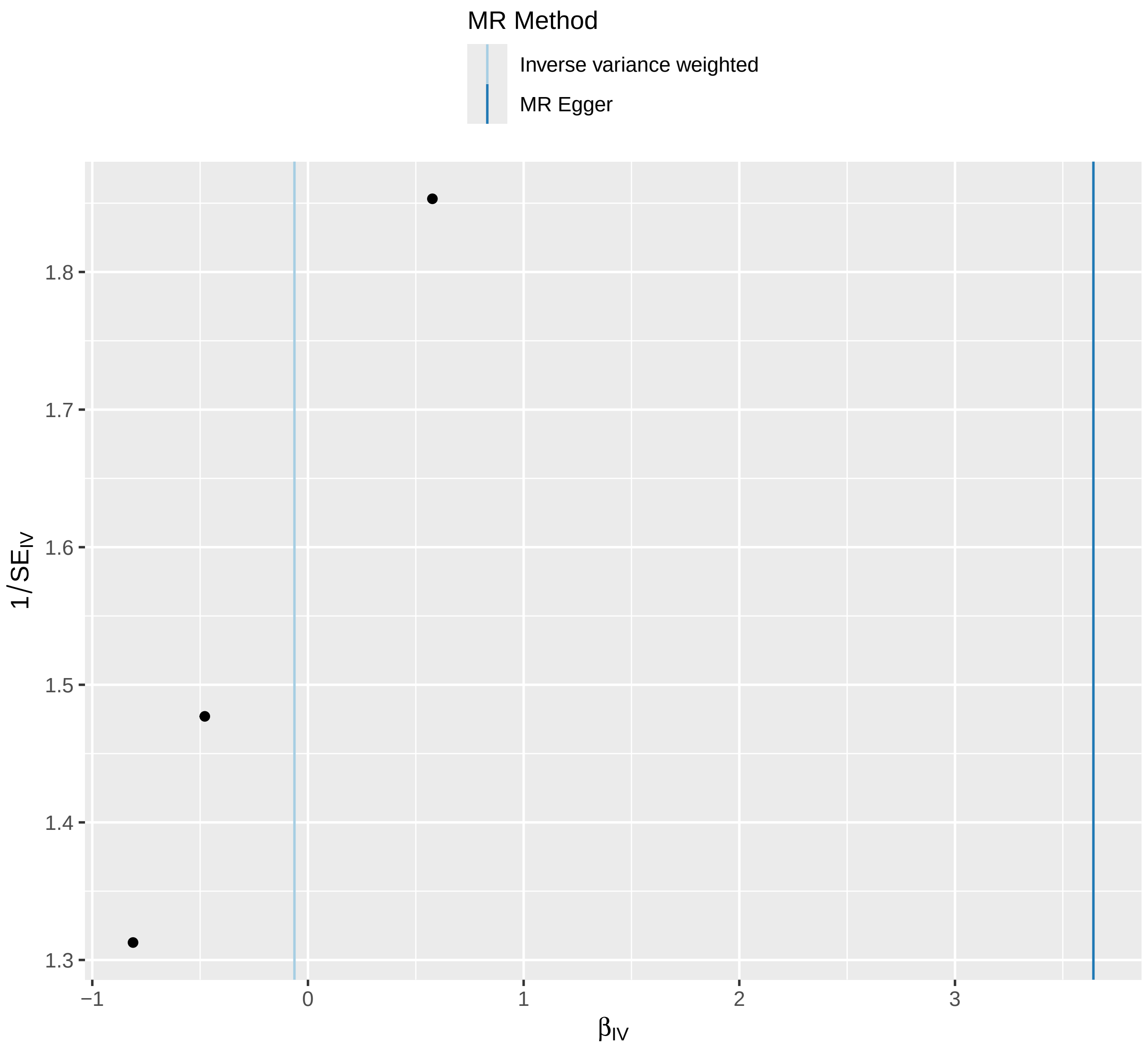

MR Method
Inverse variance weighted
MR Egger
1.8
1.7
1.6
1.5
1.4
1.3
1/SE_IV
−1
0
1
2
3
β_IV

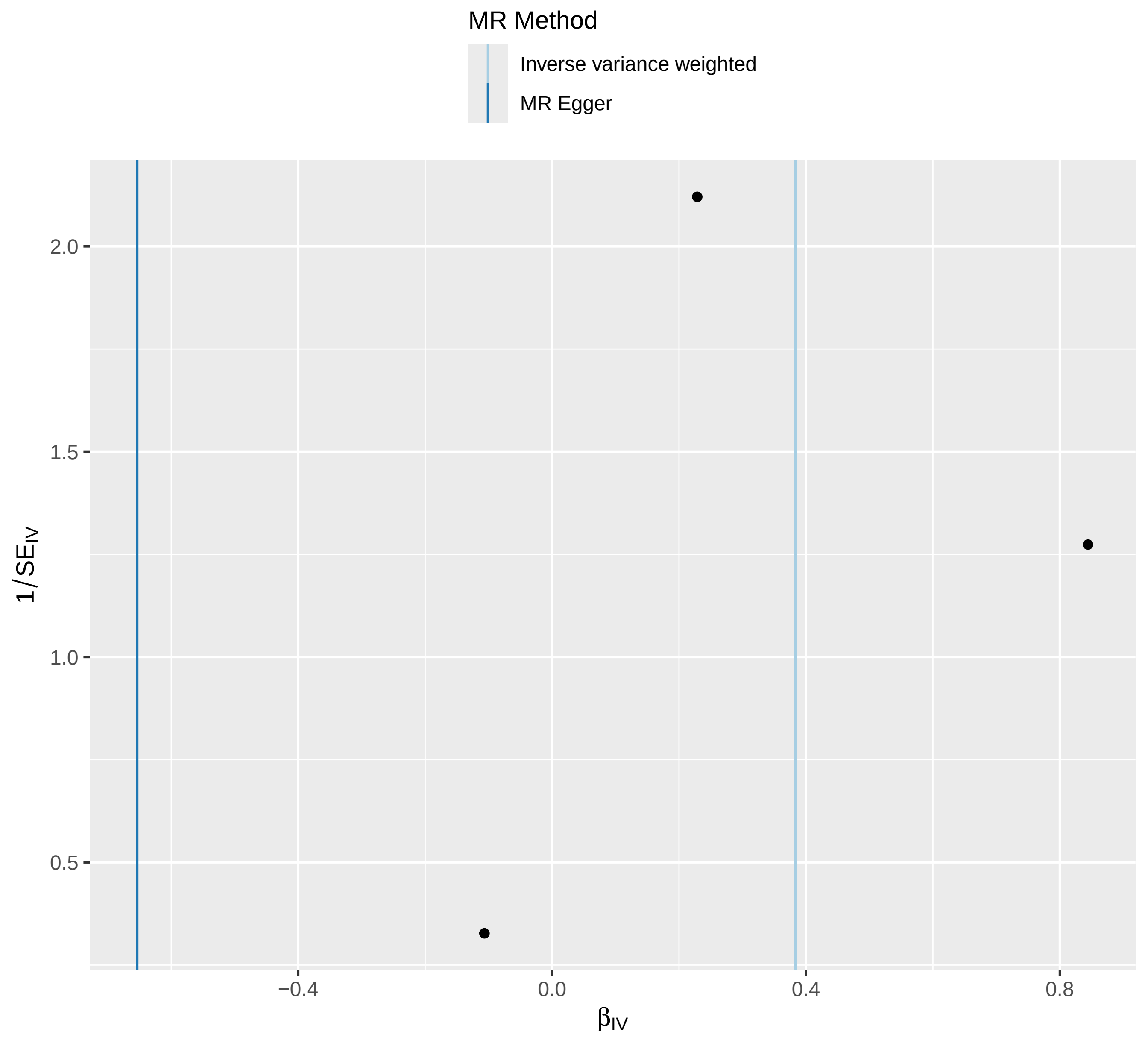

MR Method
Inverse variance weighted
MR Egger
2.0
1.5
1.0
0.5
1/SE$_{IV}$
−0.4
0.0
0.4
0.8
β$_{IV}$

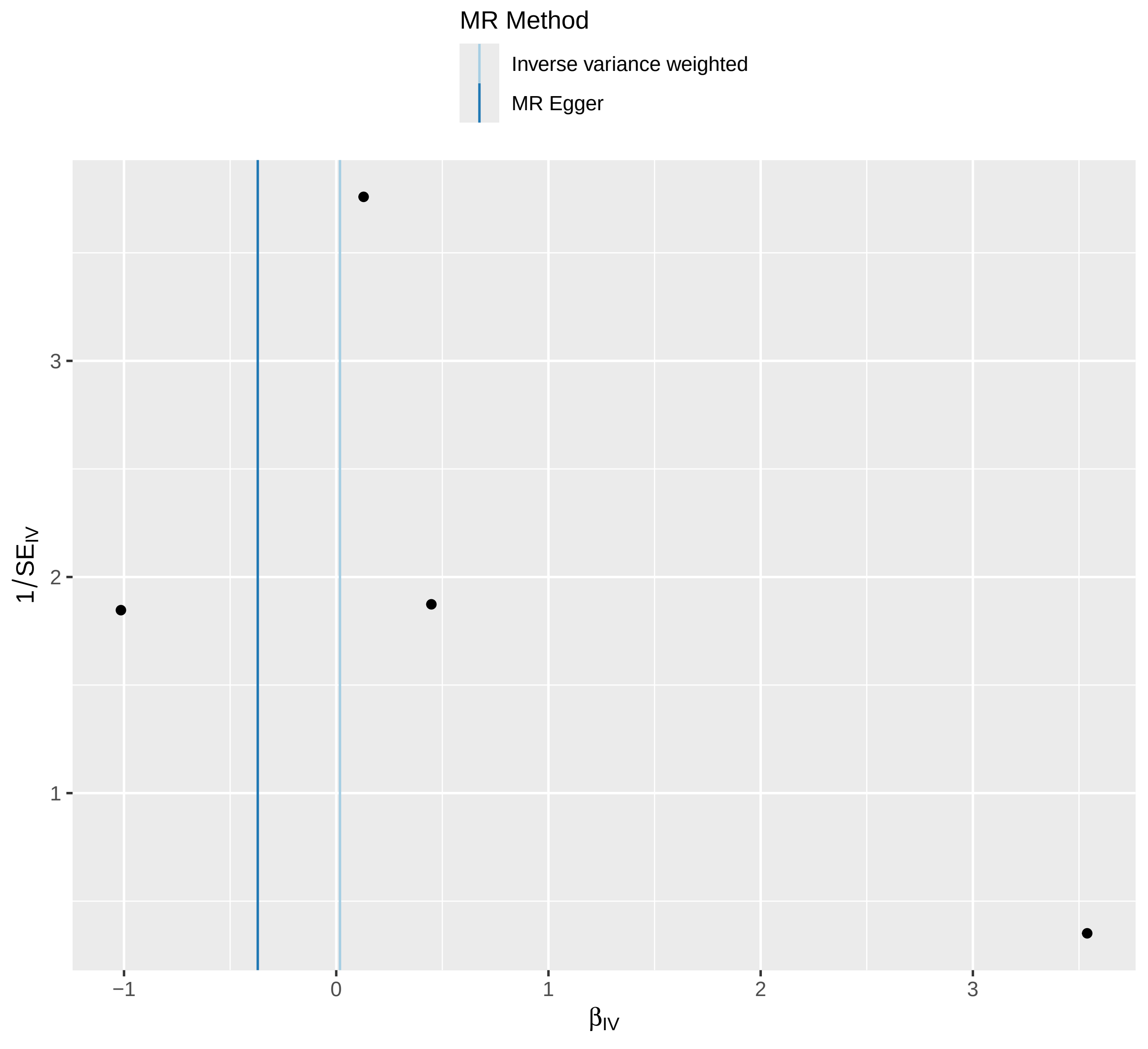
MR Method
Inverse variance weighted
MR Egger
1/SE_IV
3
2
1
−1
0
1
2
3
β_IV

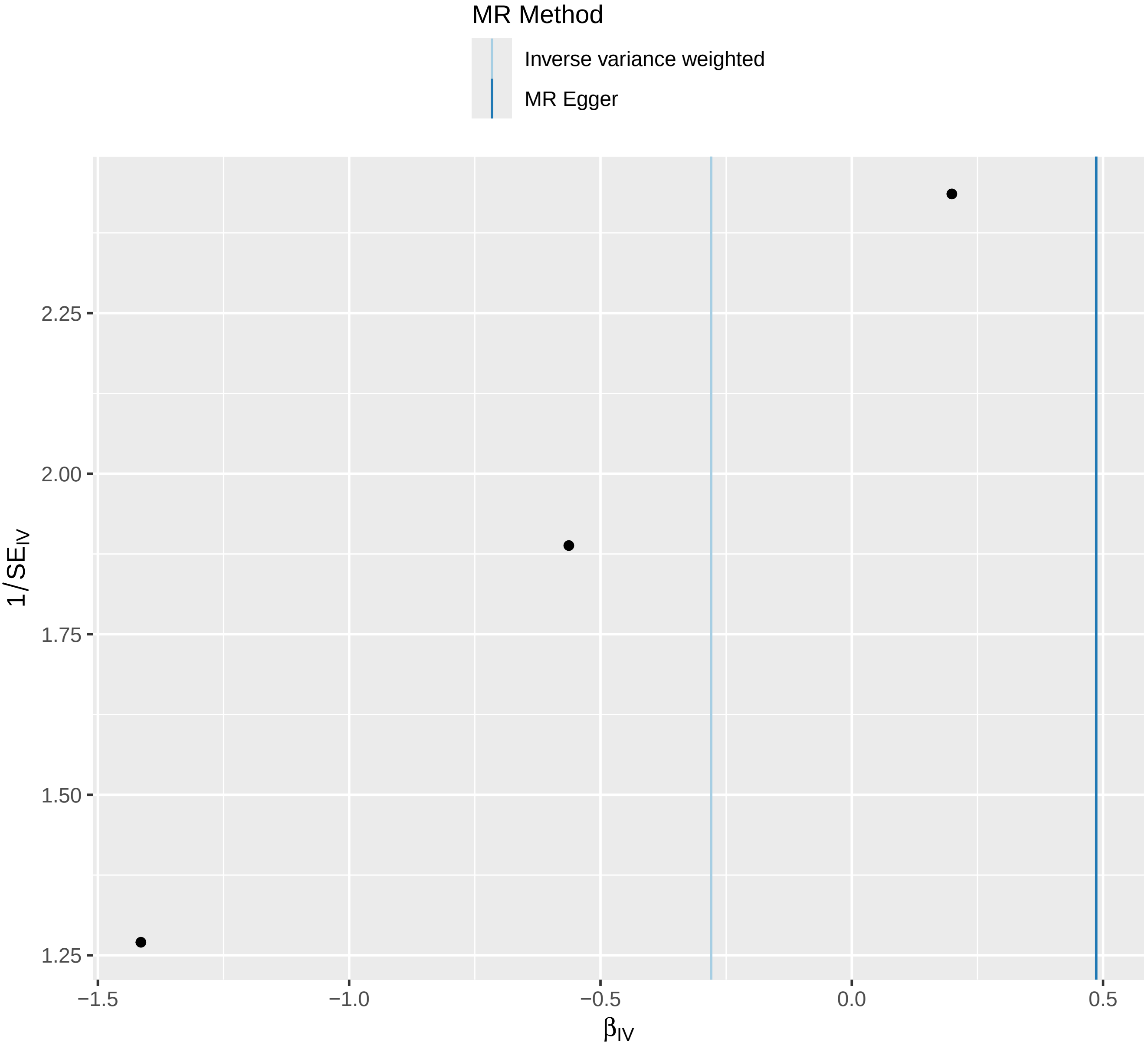
MR Method
Inverse variance weighted
MR Egger
2.25
2.00
1.75
1.50
1.25
$1/SE_{IV}$
−1.5
−1.0
−0.5
0.0
0.5
β_{IV}

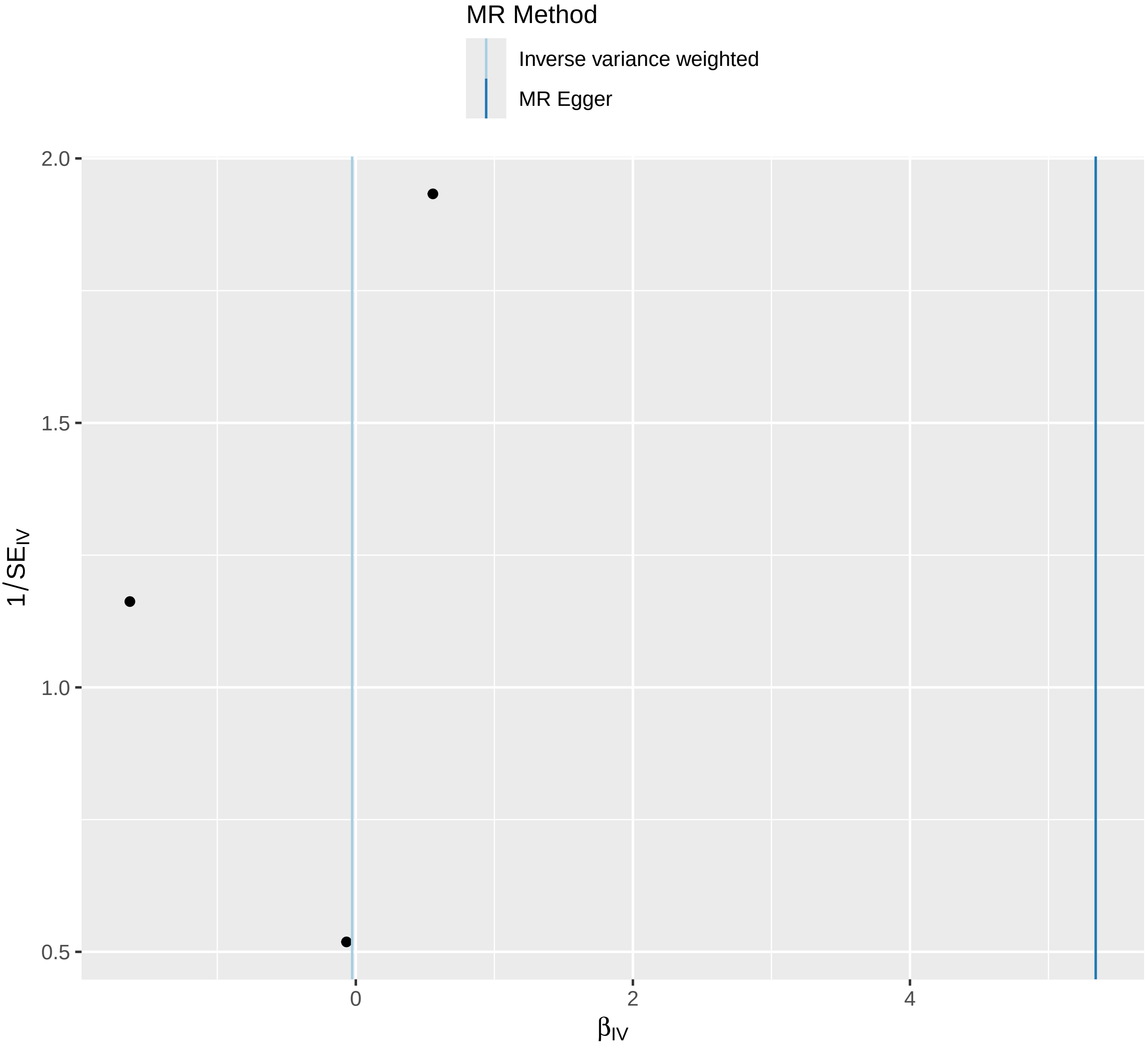
MR Method
Inverse variance weighted
MR Egger
2.0
1.5
1.0
0.5
0
2
4
1/SE_IV
β_IV

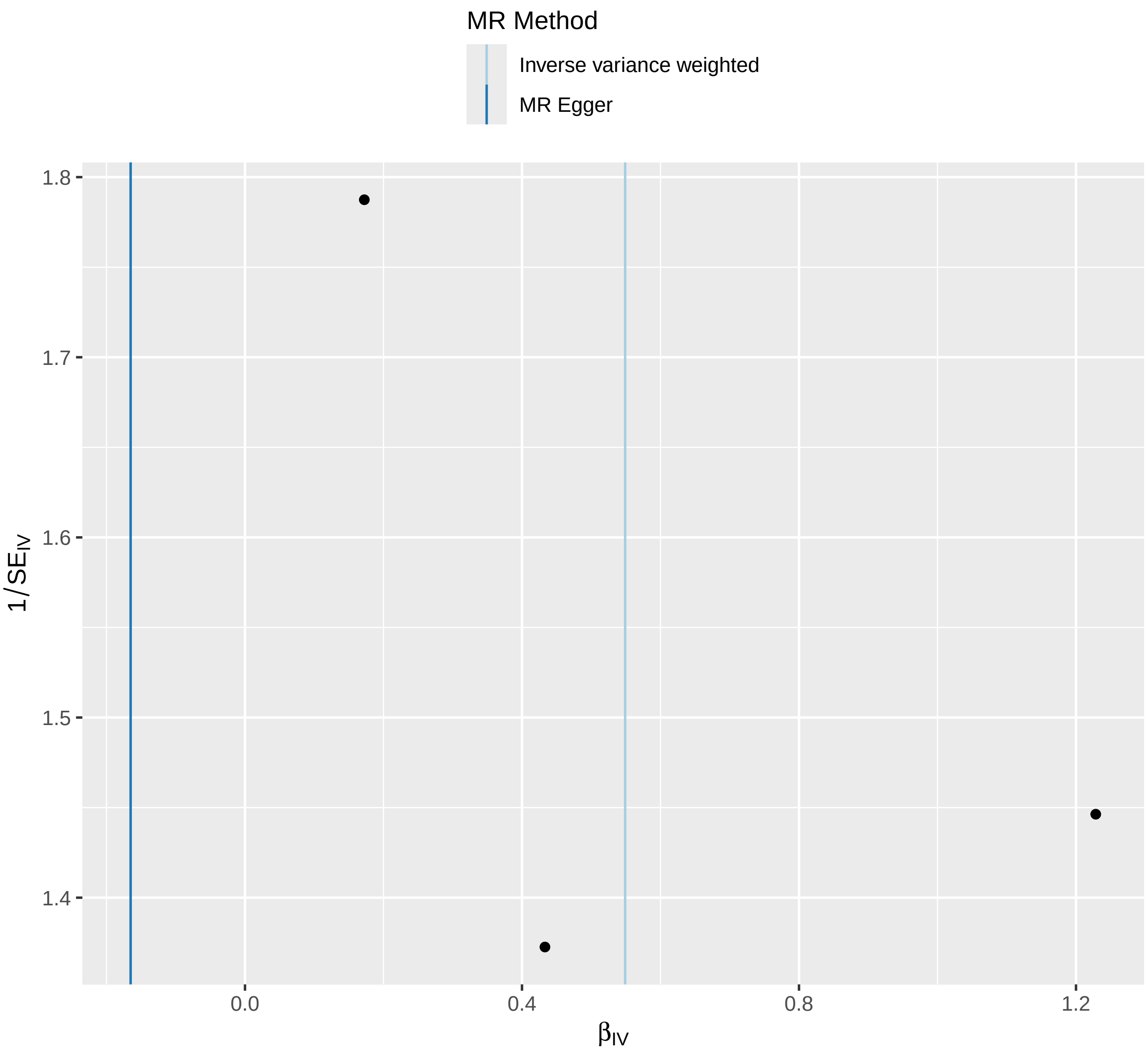

MR Method
Inverse variance weighted
MR Egger
1.8
1.7
1.6
1.5
1.4
1/SE_IV
0.0
0.4
0.8
1.2
β_IV

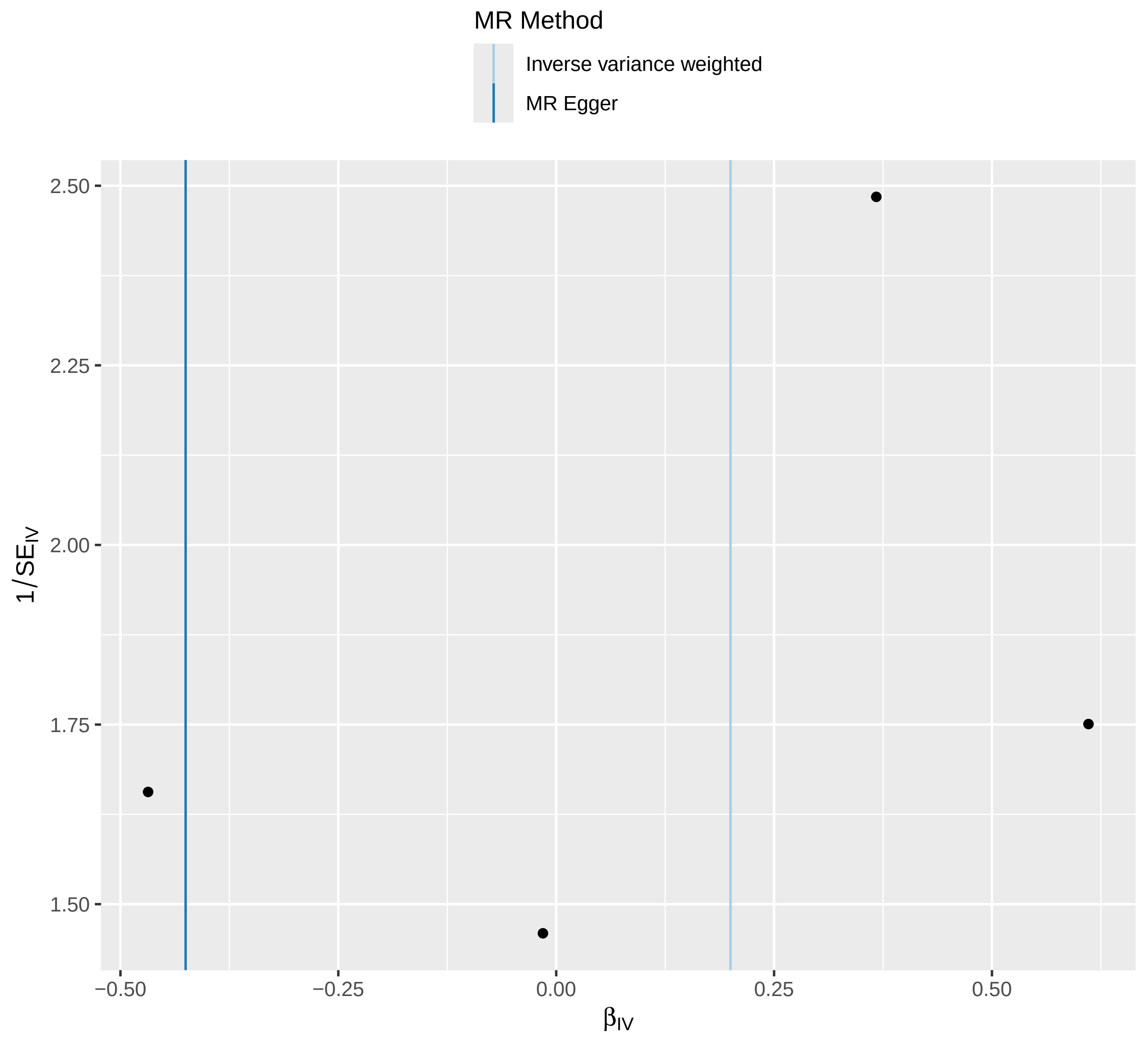

MR Method
Inverse variance weighted
MR Egger
2.50
2.25
2.00
1.75
1.50
1/SE_IV
−0.50
−0.25
0.00
0.25
0.50
β_IV

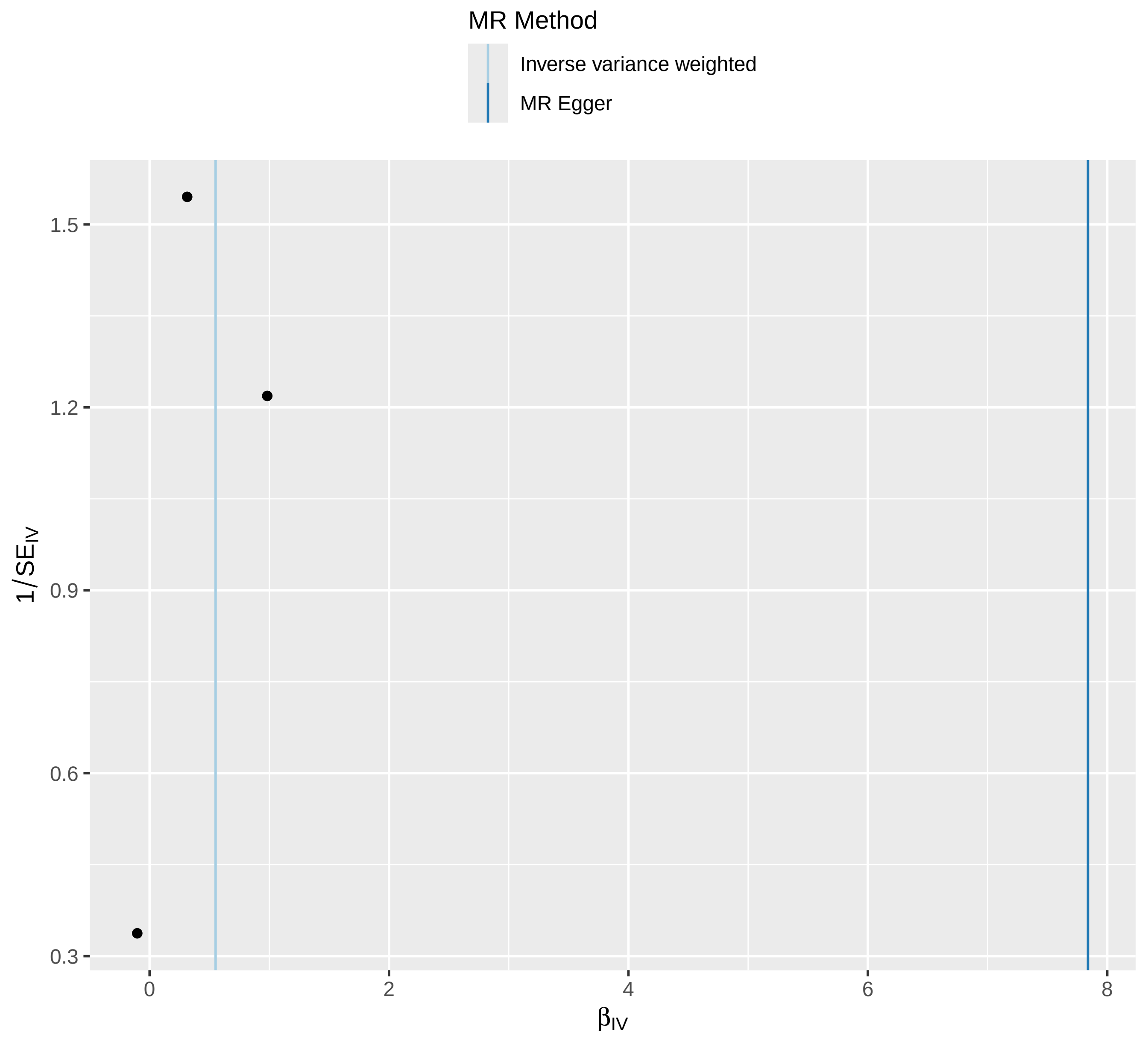

MR Method
Inverse variance weighted
MR Egger
1.5
1.2
0.9
0.6
0.3
0
2
4
6
8
1/SE_IV
β_IV

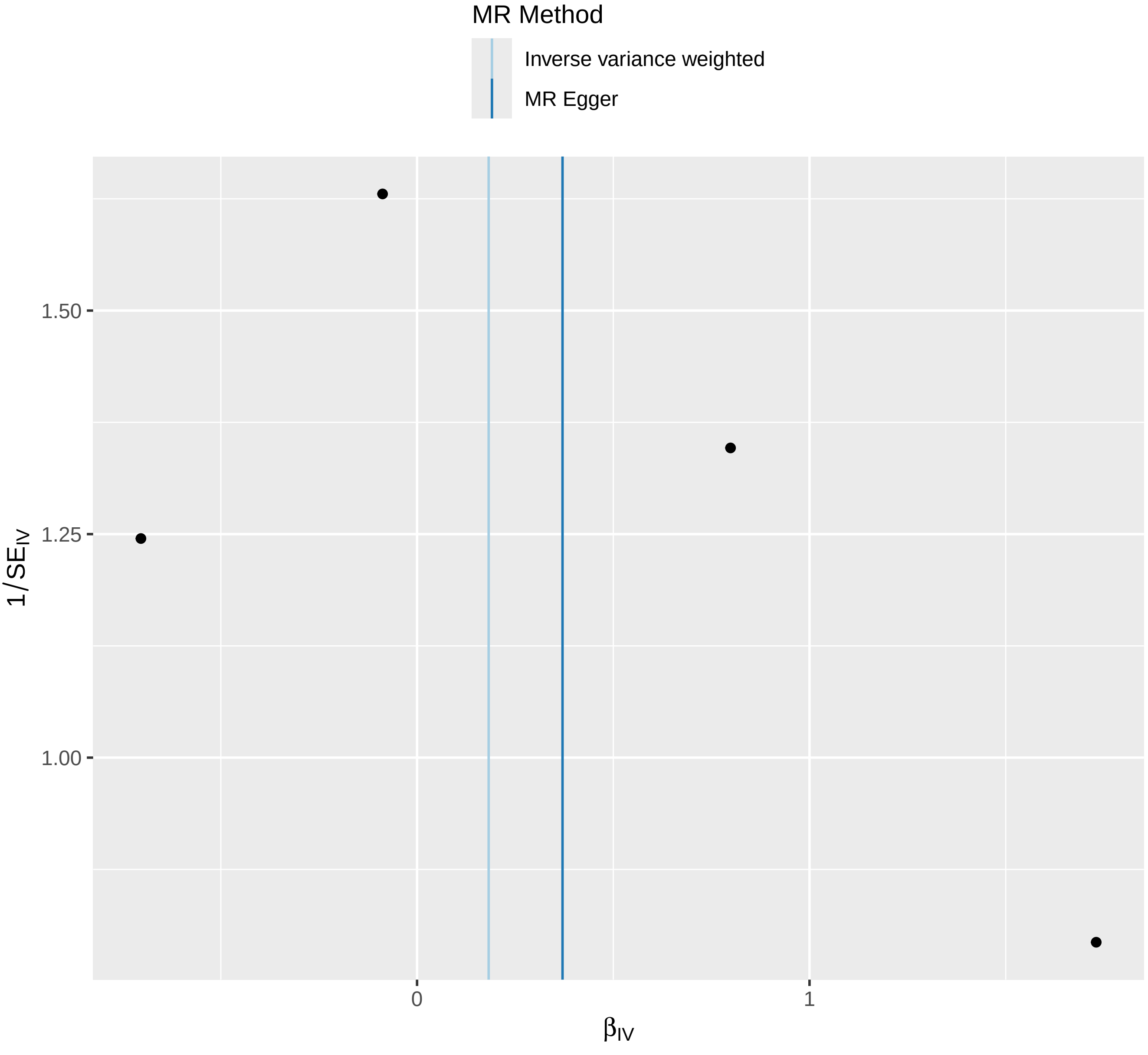
MR Method
Inverse variance weighted
MR Egger
1.50
1.25
1.00
0
1
$1/SE_{IV}$
β_{IV}

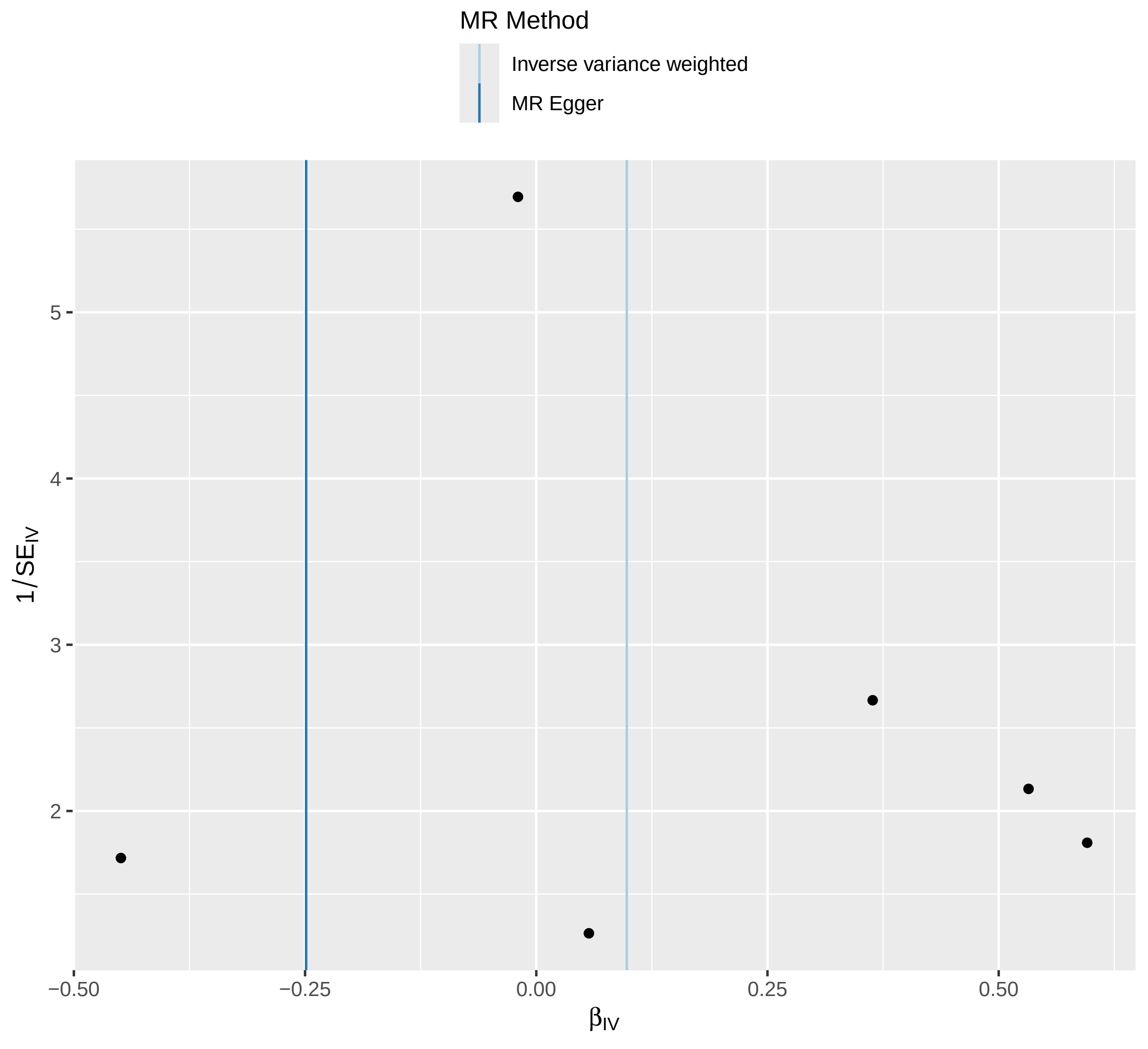
MR Method
Inverse variance weighted
MR Egger
1/SE$_{IV}$
5
4
3
2
−0.50
−0.25
0.00
0.25
0.50
β$_{IV}$

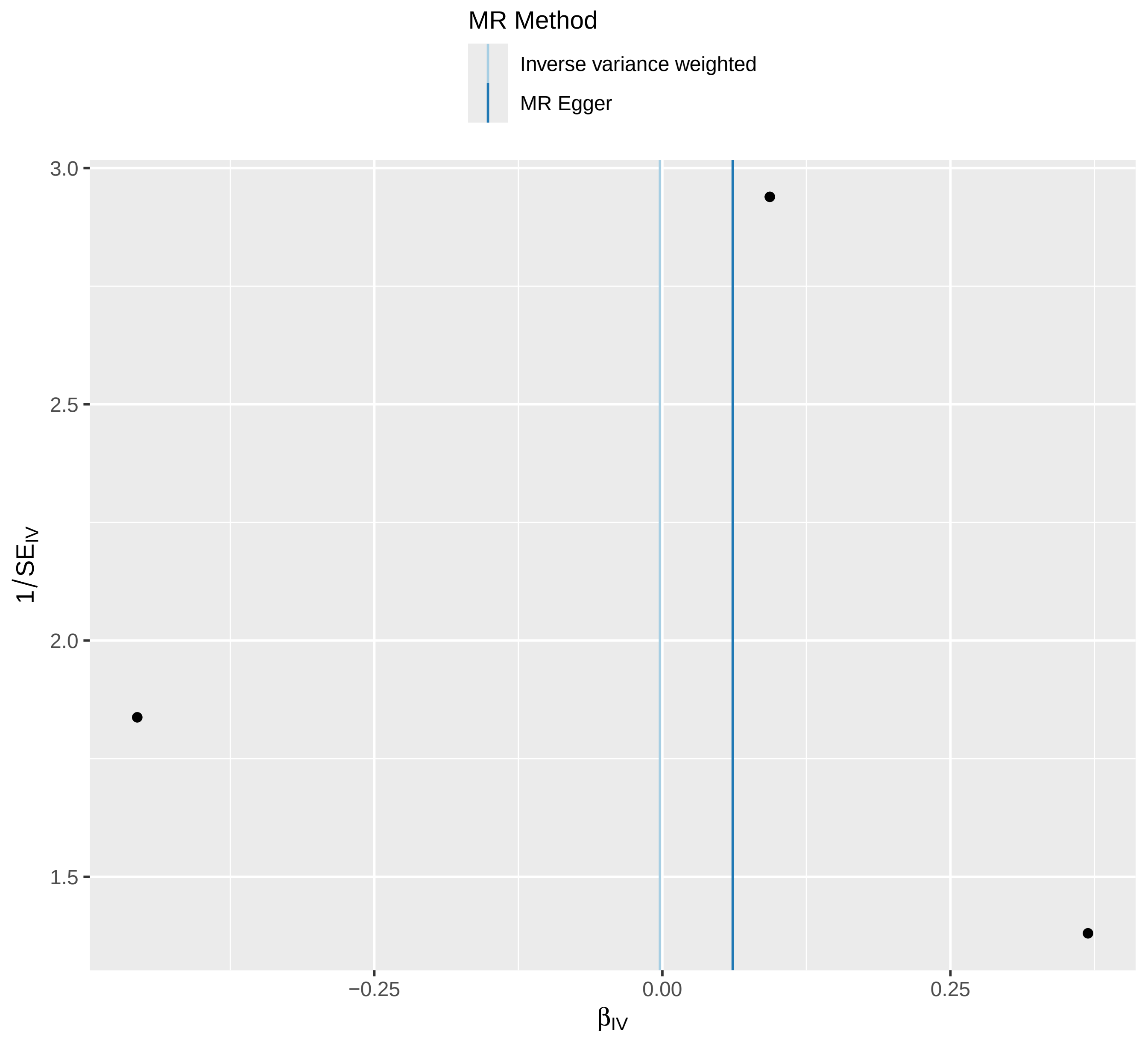
MR Method
Inverse variance weighted
MR Egger
3.0
2.5
2.0
1.5
1/SE_IV
−0.25
0.00
0.25
β_IV

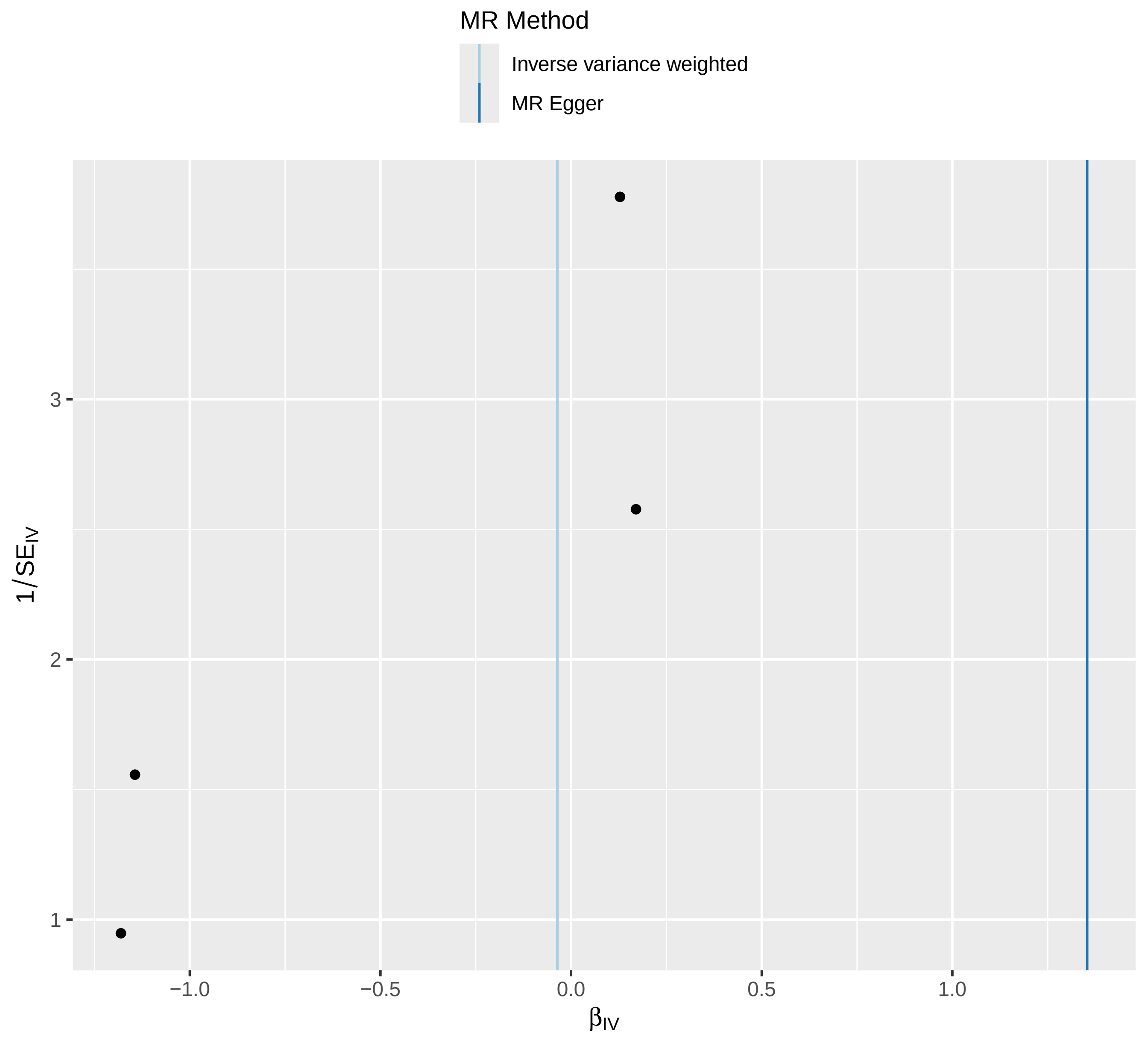
MR Method
Inverse variance weighted
MR Egger
3
2
1
1/SE_IV
−1.0
−0.5
0.0
0.5
1.0
β_IV

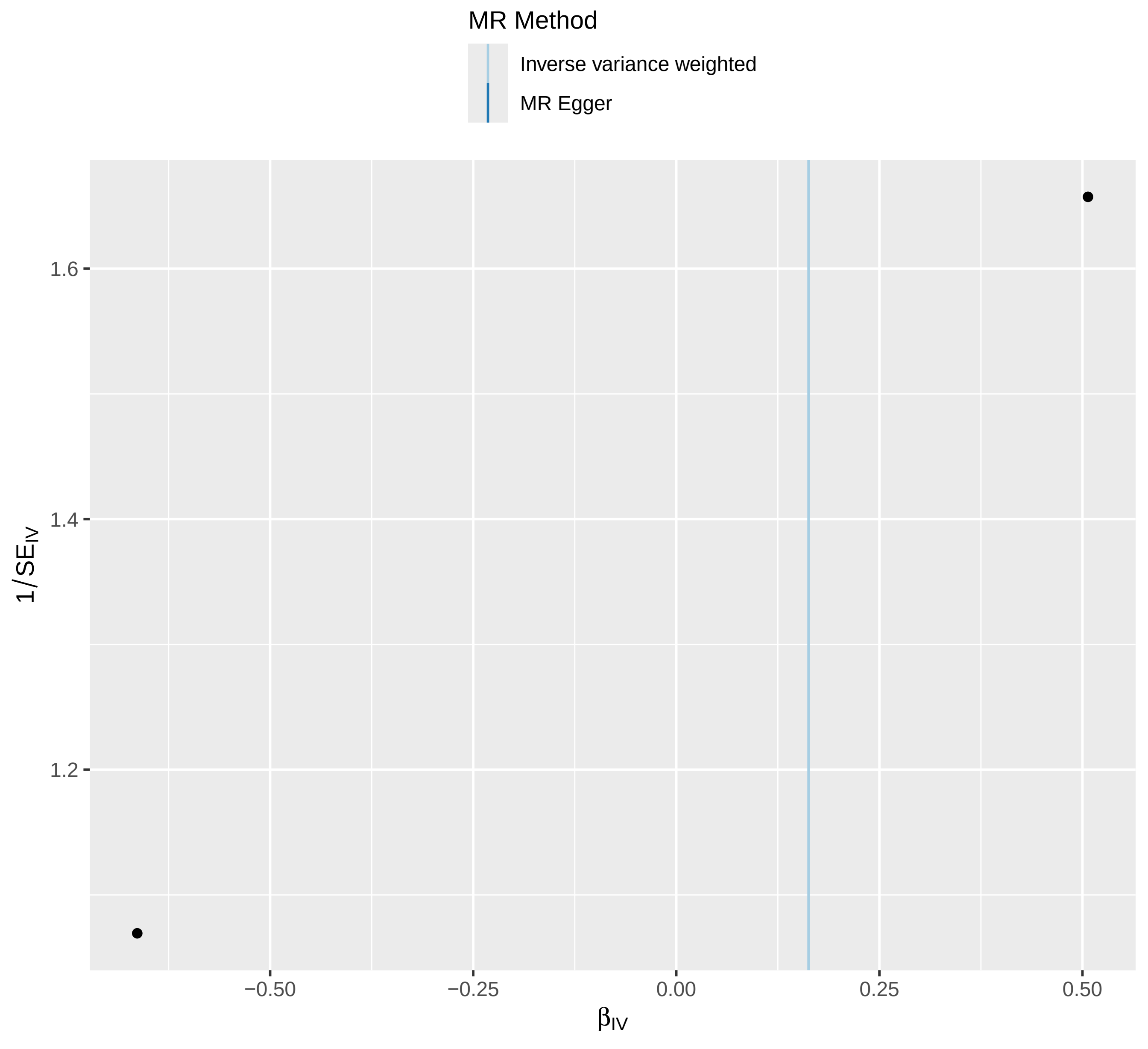

MR Method
Inverse variance weighted
MR Egger
1.6
1.4
1.2
1/SE_IV
−0.50
−0.25
0.00
0.25
0.50
β_IV

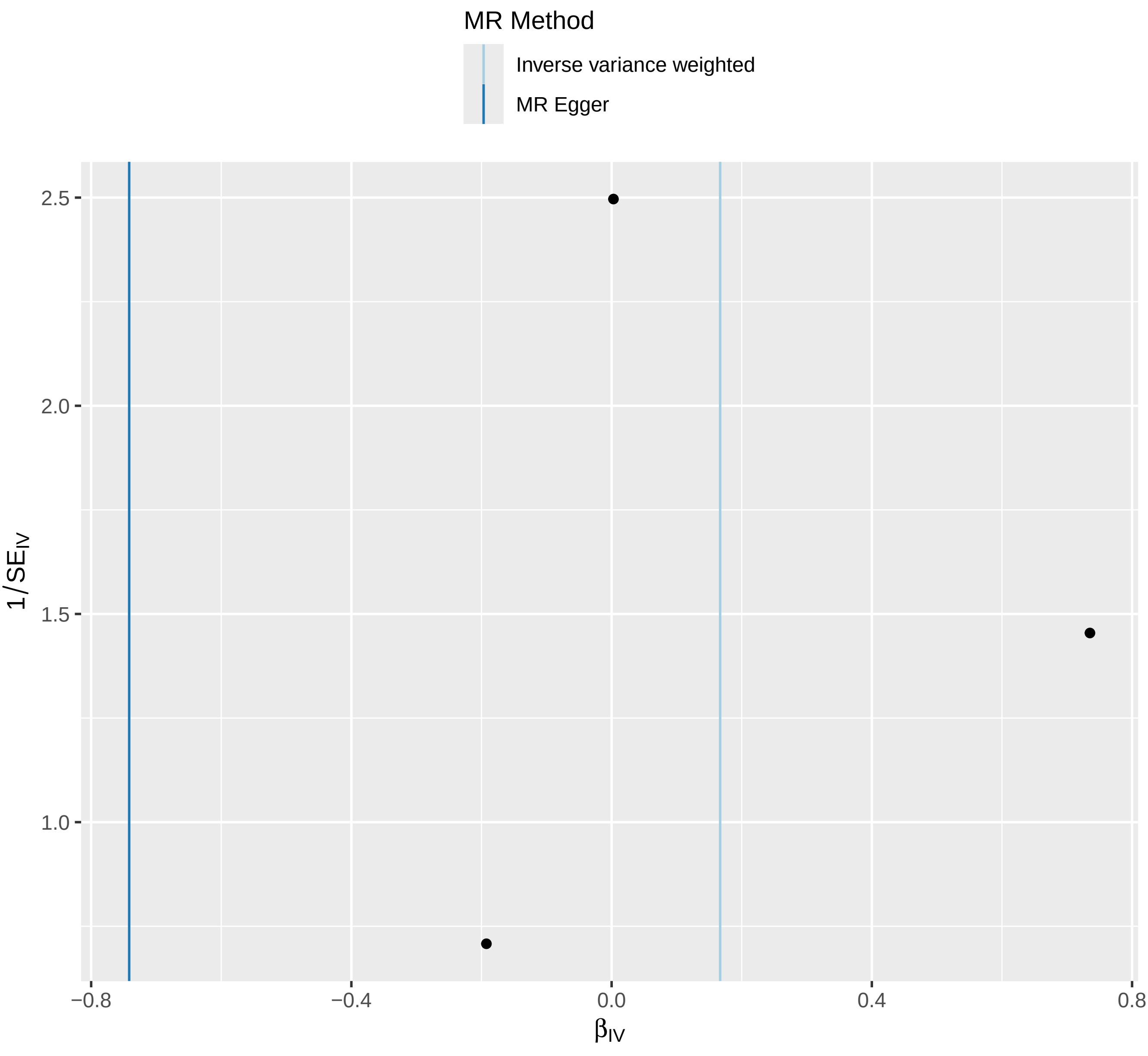

MR Method
Inverse variance weighted
MR Egger
2.5
2.0
1.5
1.0
1/SE_IV
−0.8
−0.4
0.0
0.4
0.8
β_IV

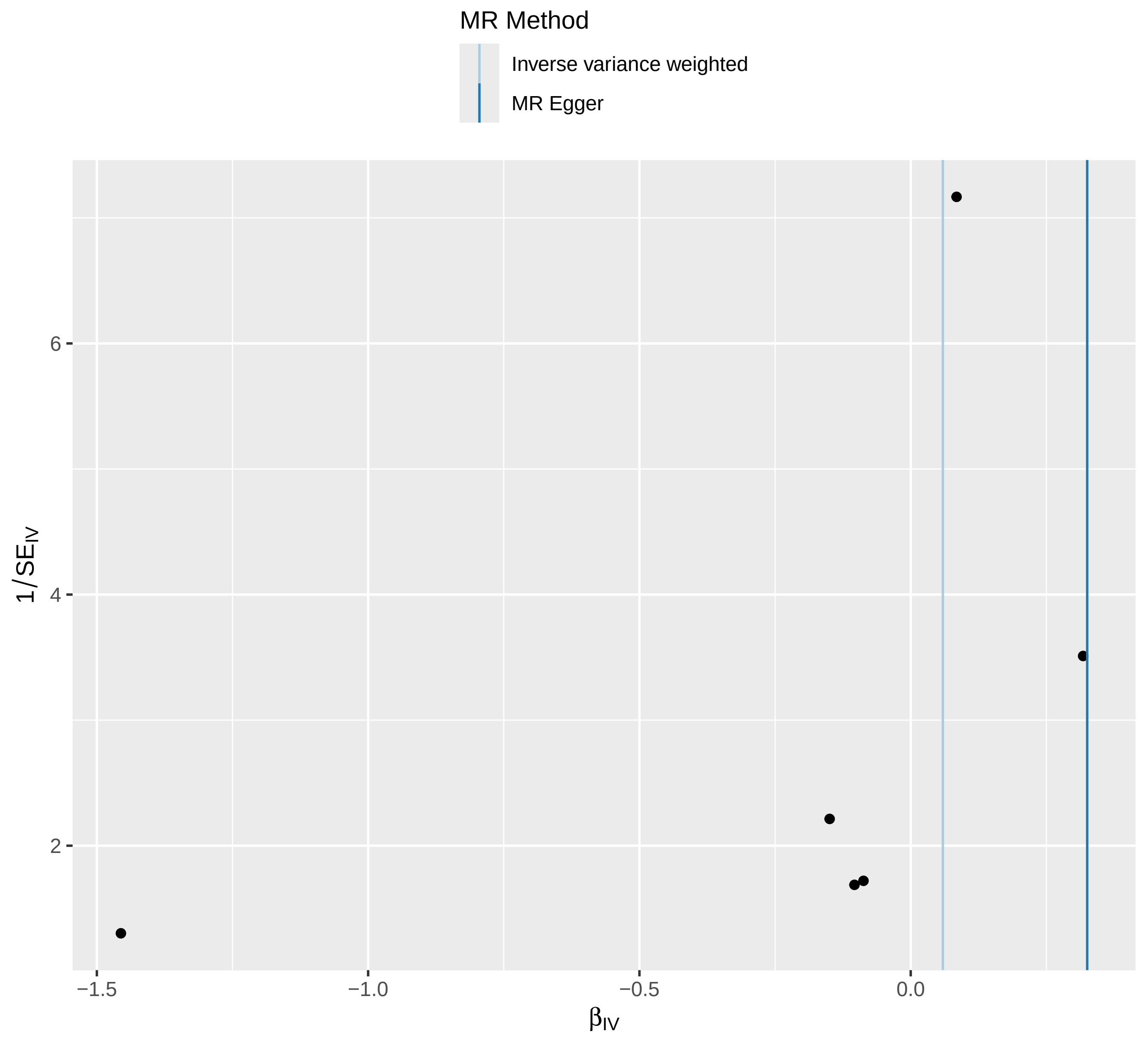
MR Method
Inverse variance weighted
MR Egger
6
4
2
1/SE$_{IV}$
−1.5
−1.0
−0.5
0.0
β$_{IV}$

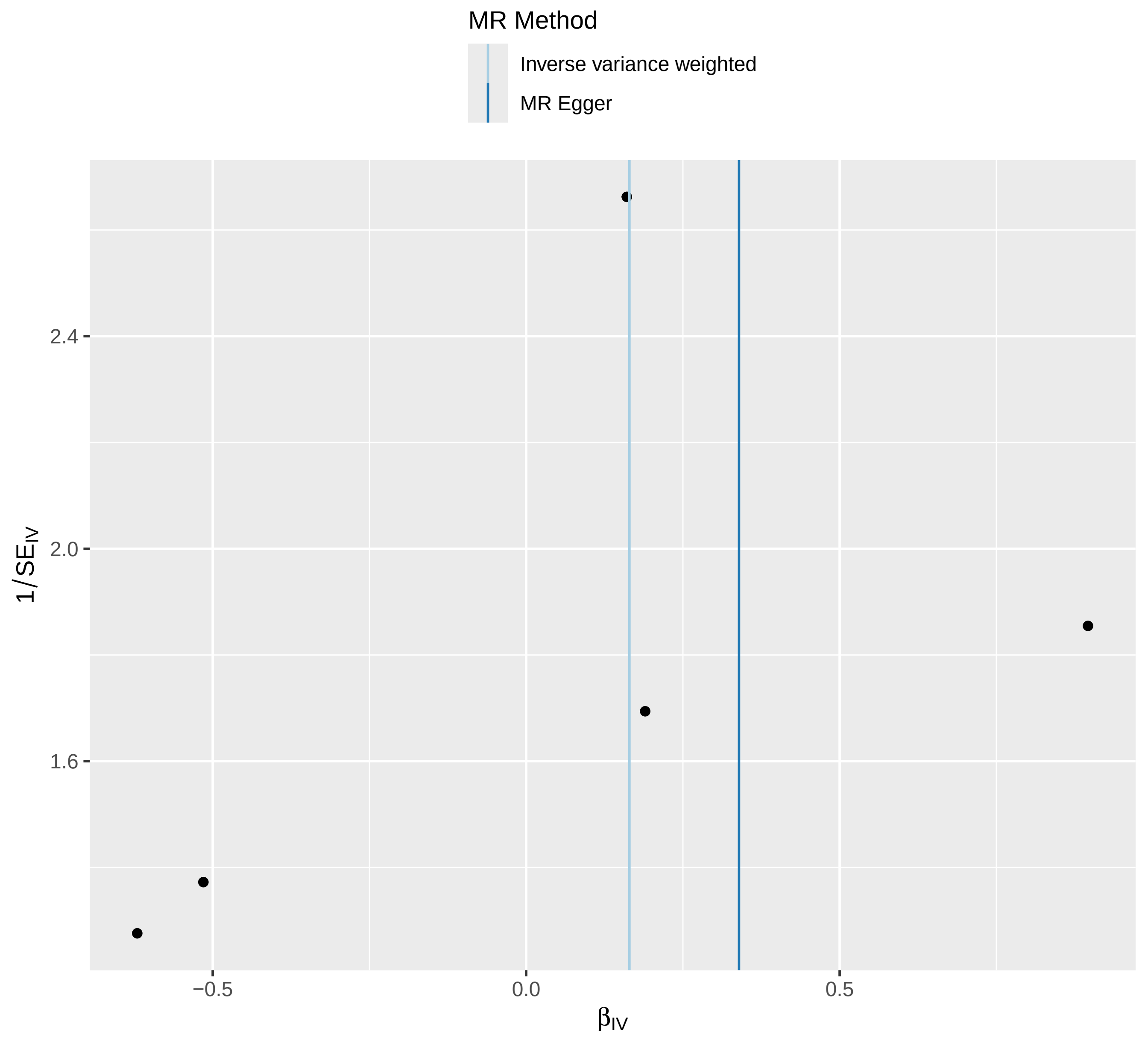
MR Method
Inverse variance weighted
MR Egger
2.4
2.0
1.6
1/SE$_{IV}$
−0.5
0.0
0.5
β$_{IV}$

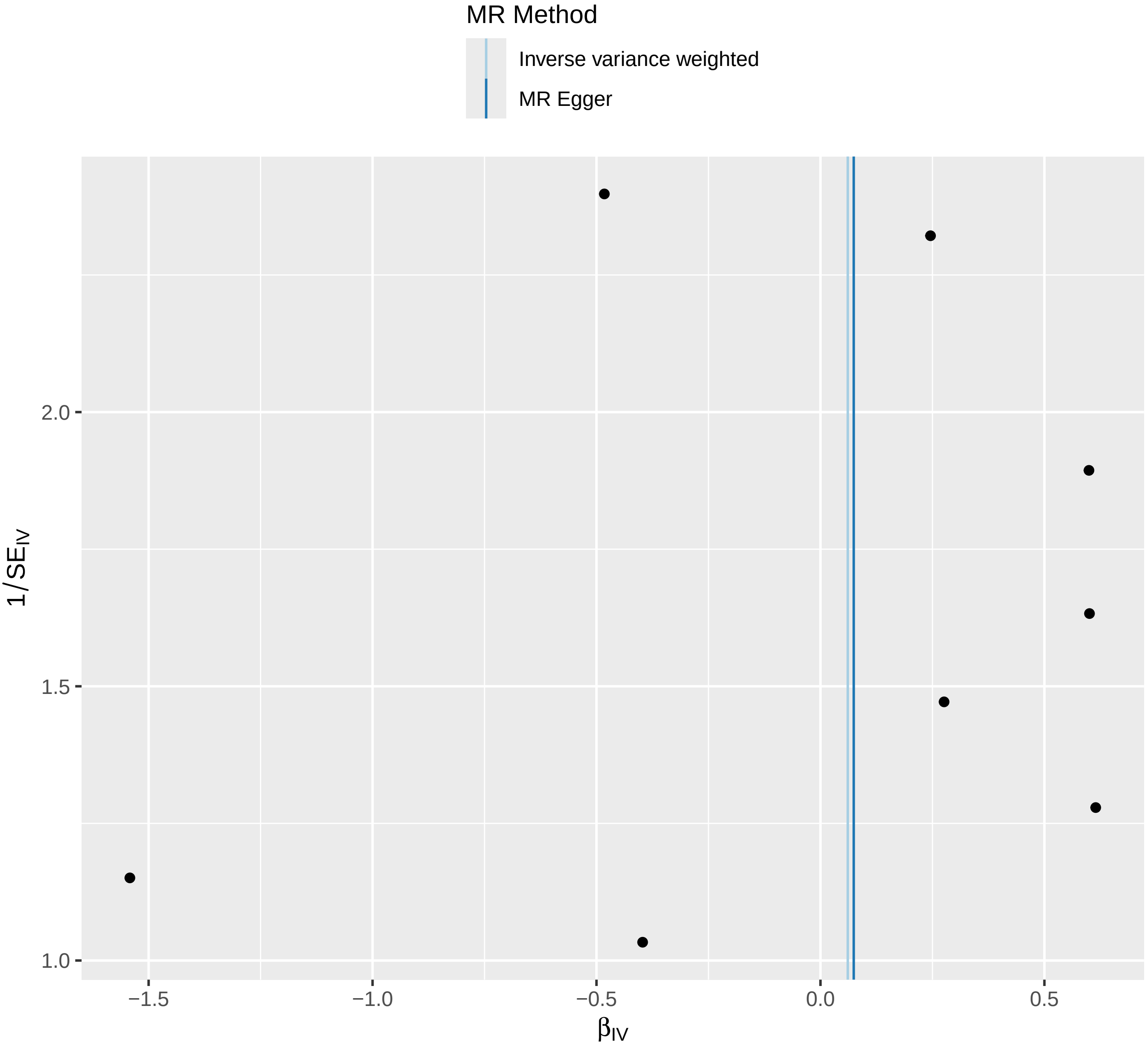

MR Method
Inverse variance weighted
MR Egger
1/SE_IV
2.0
1.5
1.0
−1.5
−1.0
−0.5
0.0
0.5
β_IV

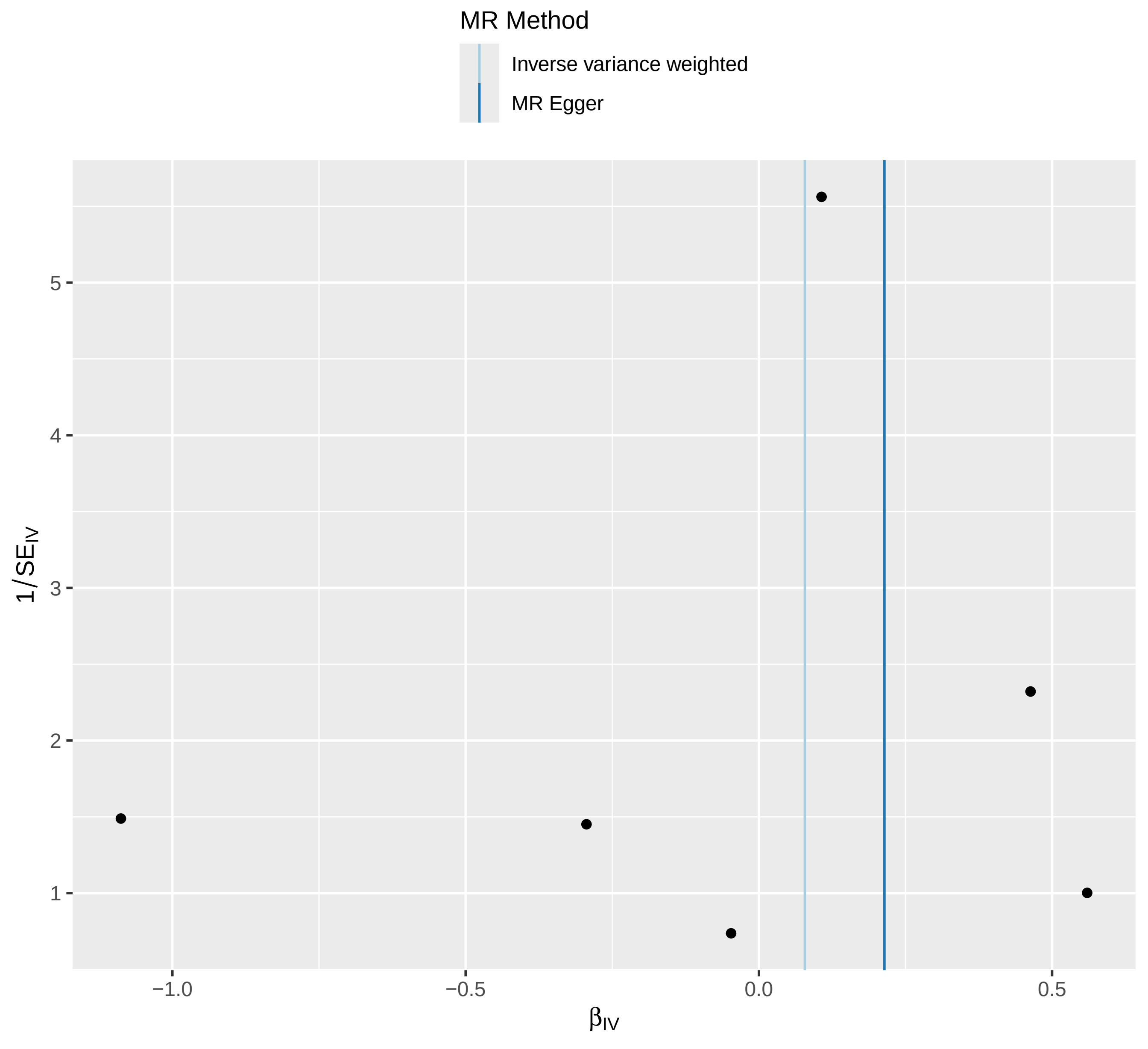
MR Method
Inverse variance weighted
MR Egger
1/SE_IV
5
4
3
2
1
−1.0
−0.5
0.0
0.5
β_IV

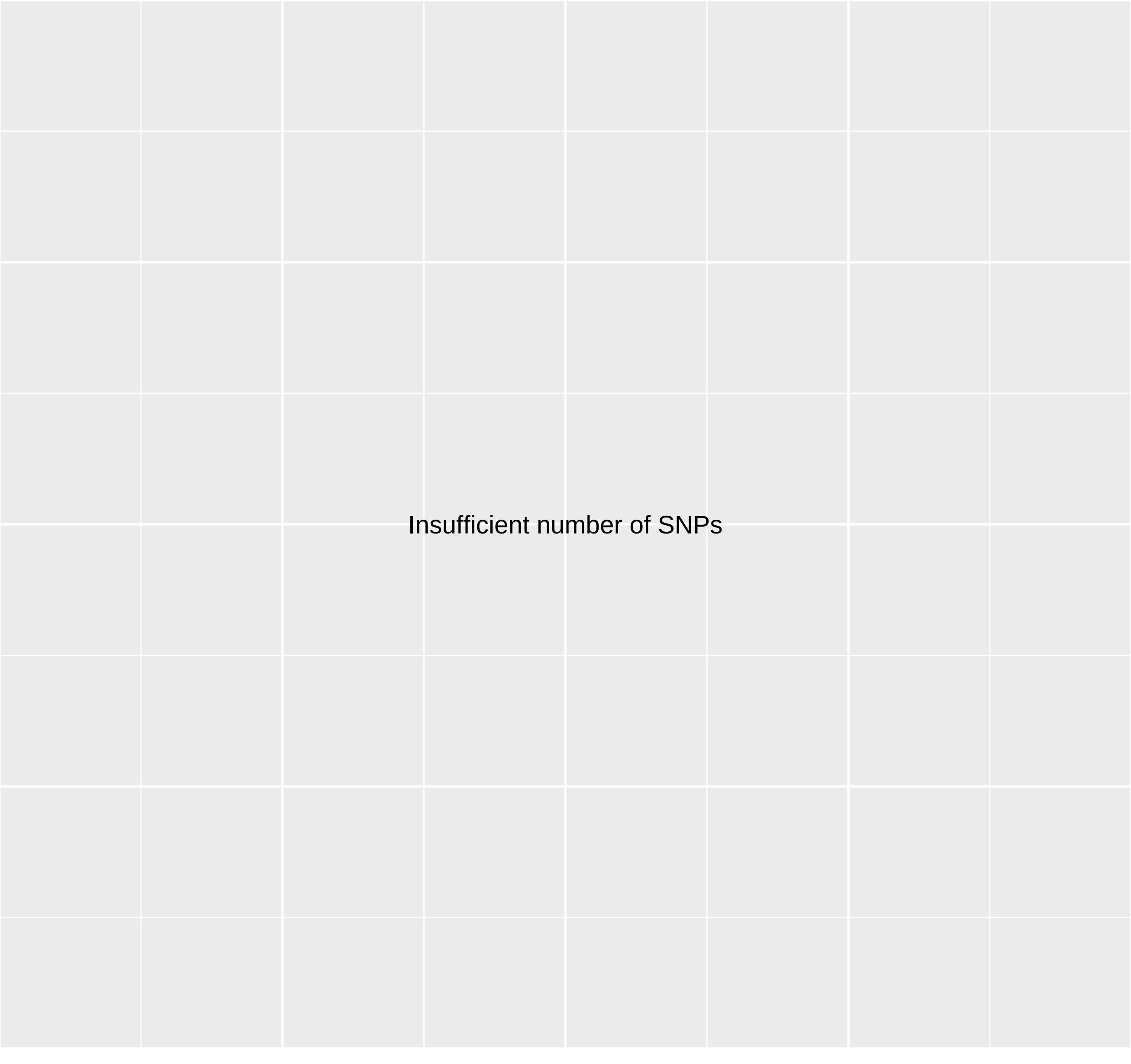
Insufficient number of SNPs

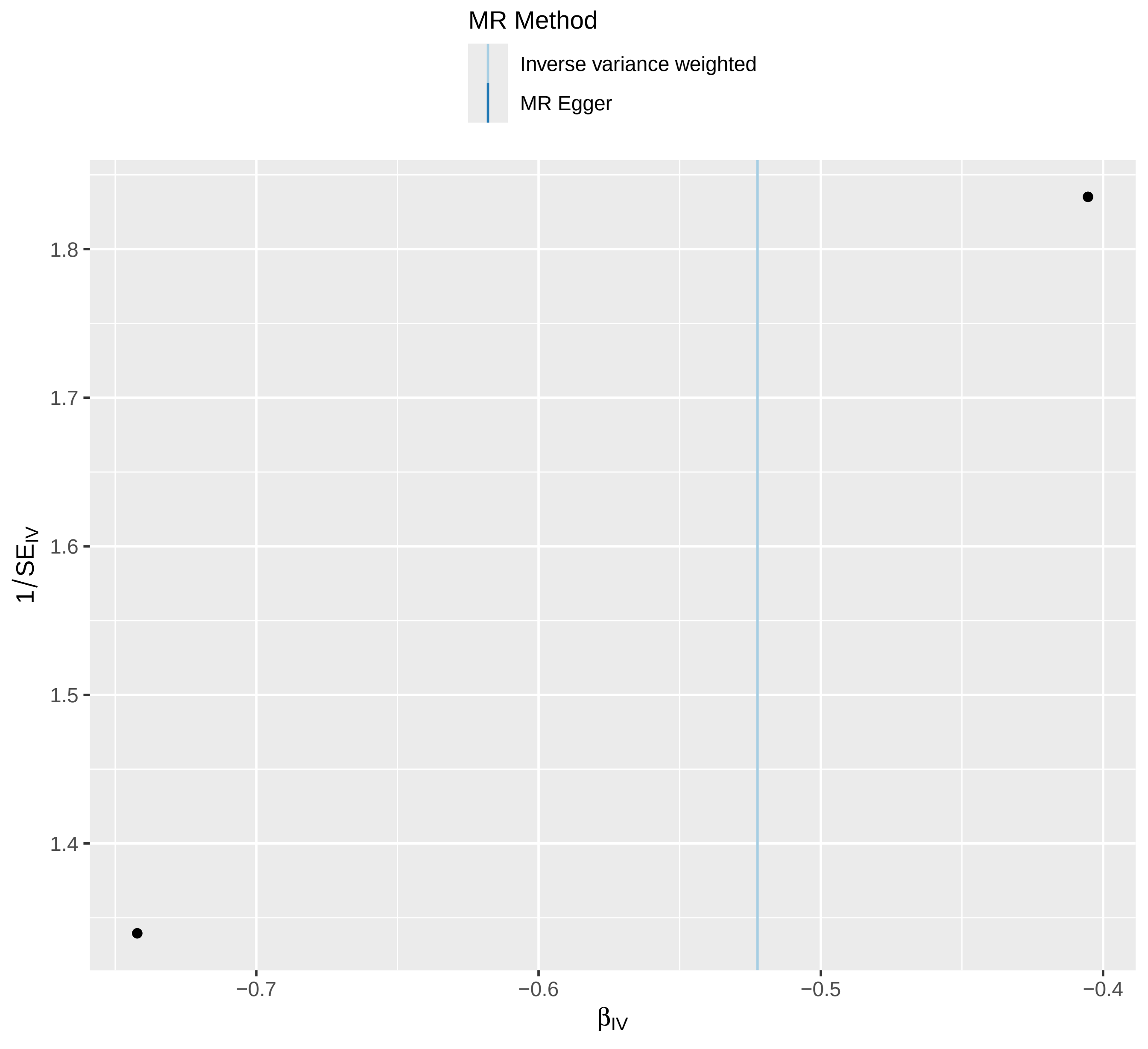
MR Method
Inverse variance weighted
MR Egger
1.8
1.7
1.6
1.5
1.4
1/SE_IV
−0.7
−0.6
−0.5
−0.4
β_IV

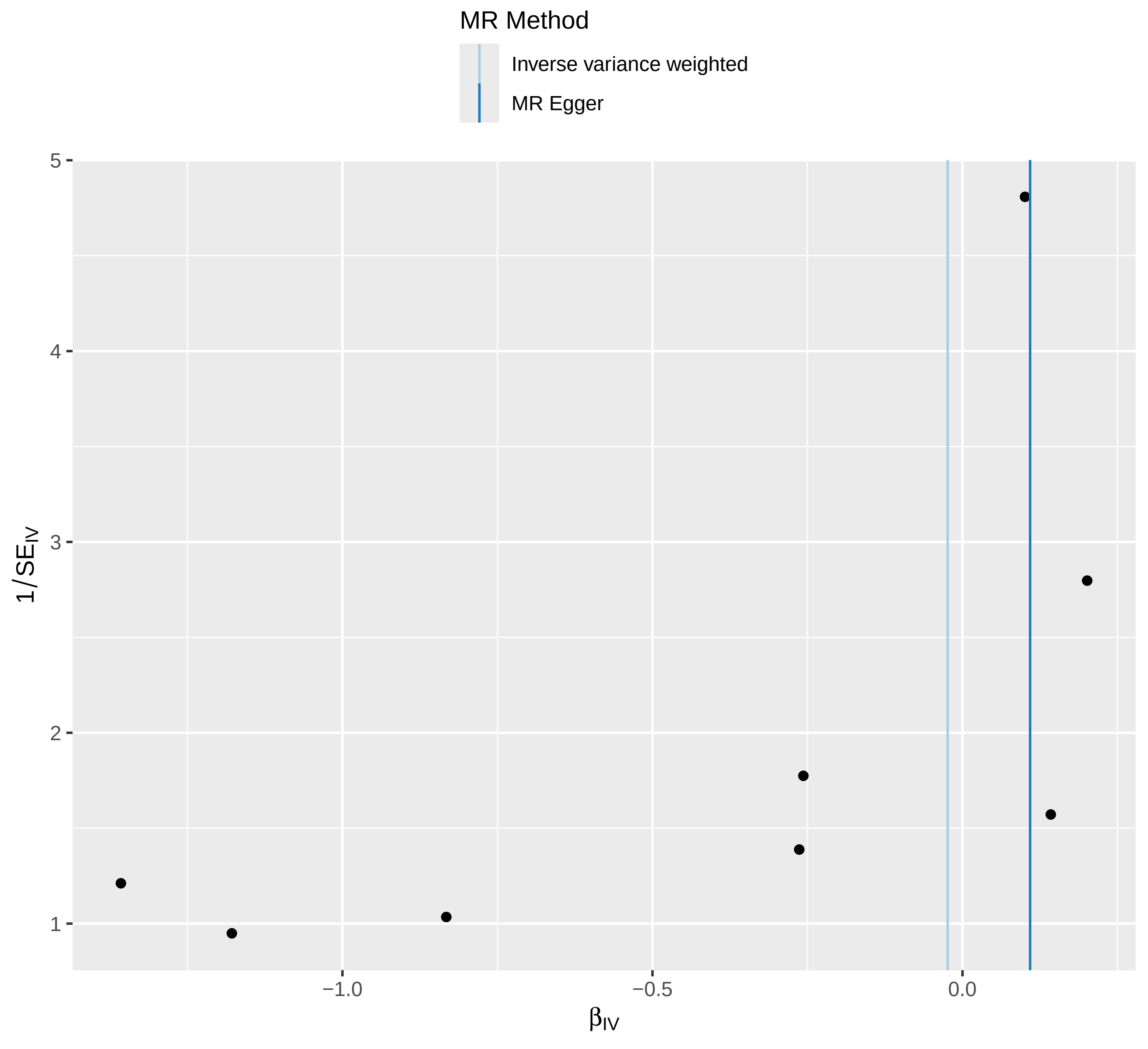
MR Method
Inverse variance weighted
MR Egger
5
4
3
2
1
1/SE_IV
−1.0
−0.5
0.0
β_IV

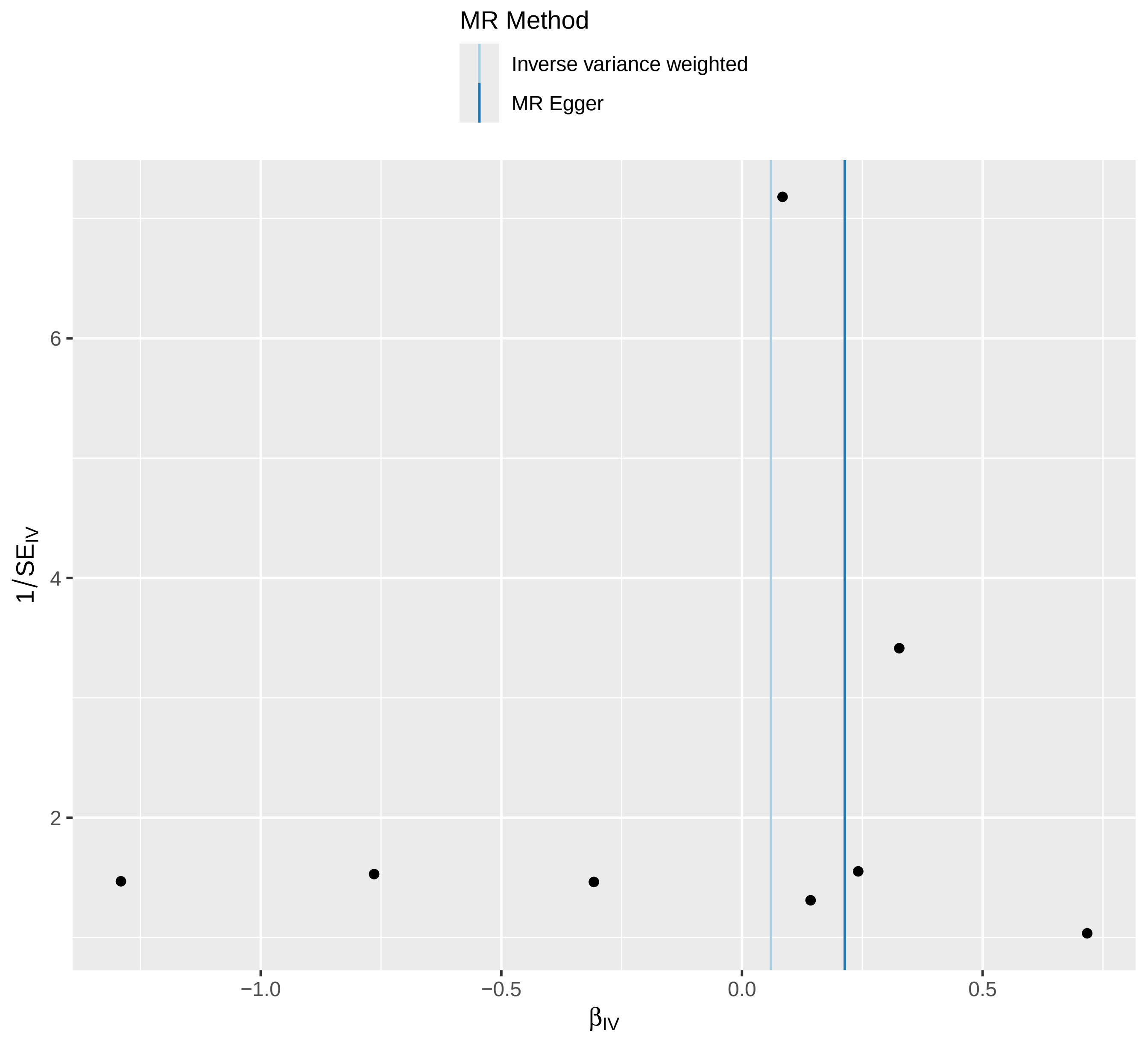
MR Method
Inverse variance weighted
MR Egger
1/SE$_{IV}$
6
4
2
−1.0
−0.5
0.0
0.5
β_{IV}

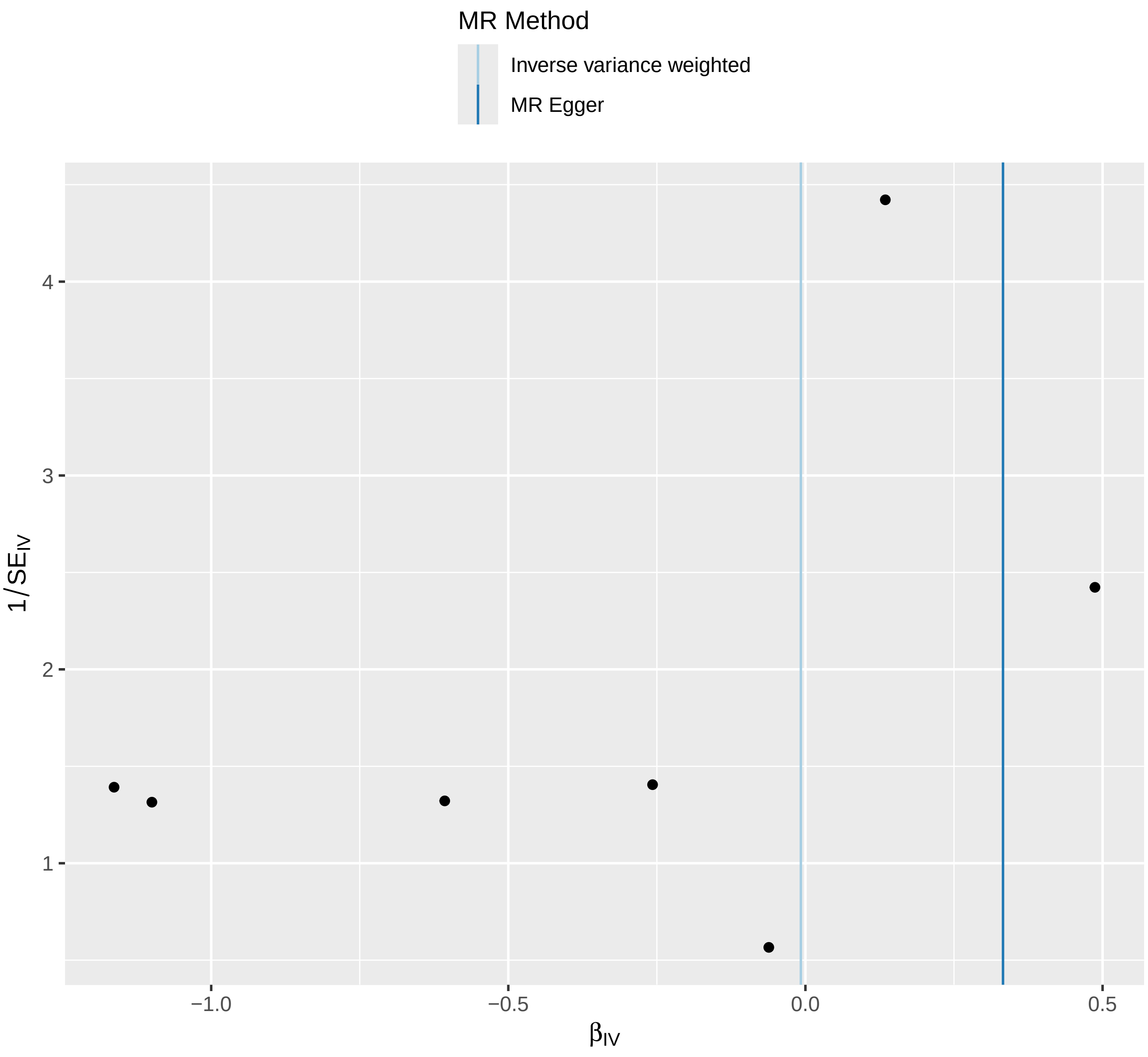

MR Method
Inverse variance weighted
MR Egger
1/SE_IV
4
3
2
1
−1.0
−0.5
0.0
0.5
β_IV

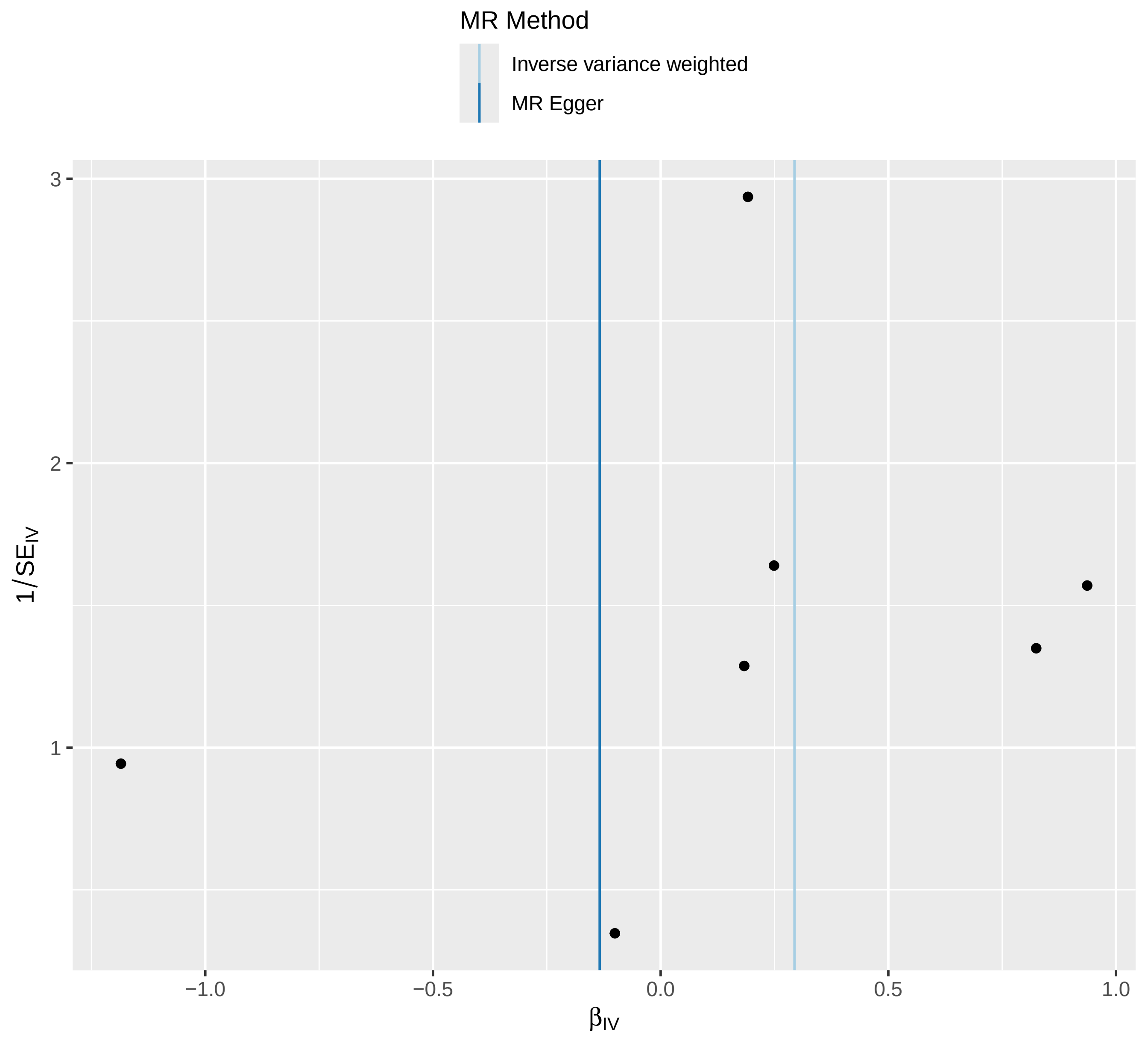
MR Method
Inverse variance weighted
MR Egger
3
2
1
1/SE$_{IV}$
−1.0
−0.5
0.0
0.5
1.0
β$_{IV}$

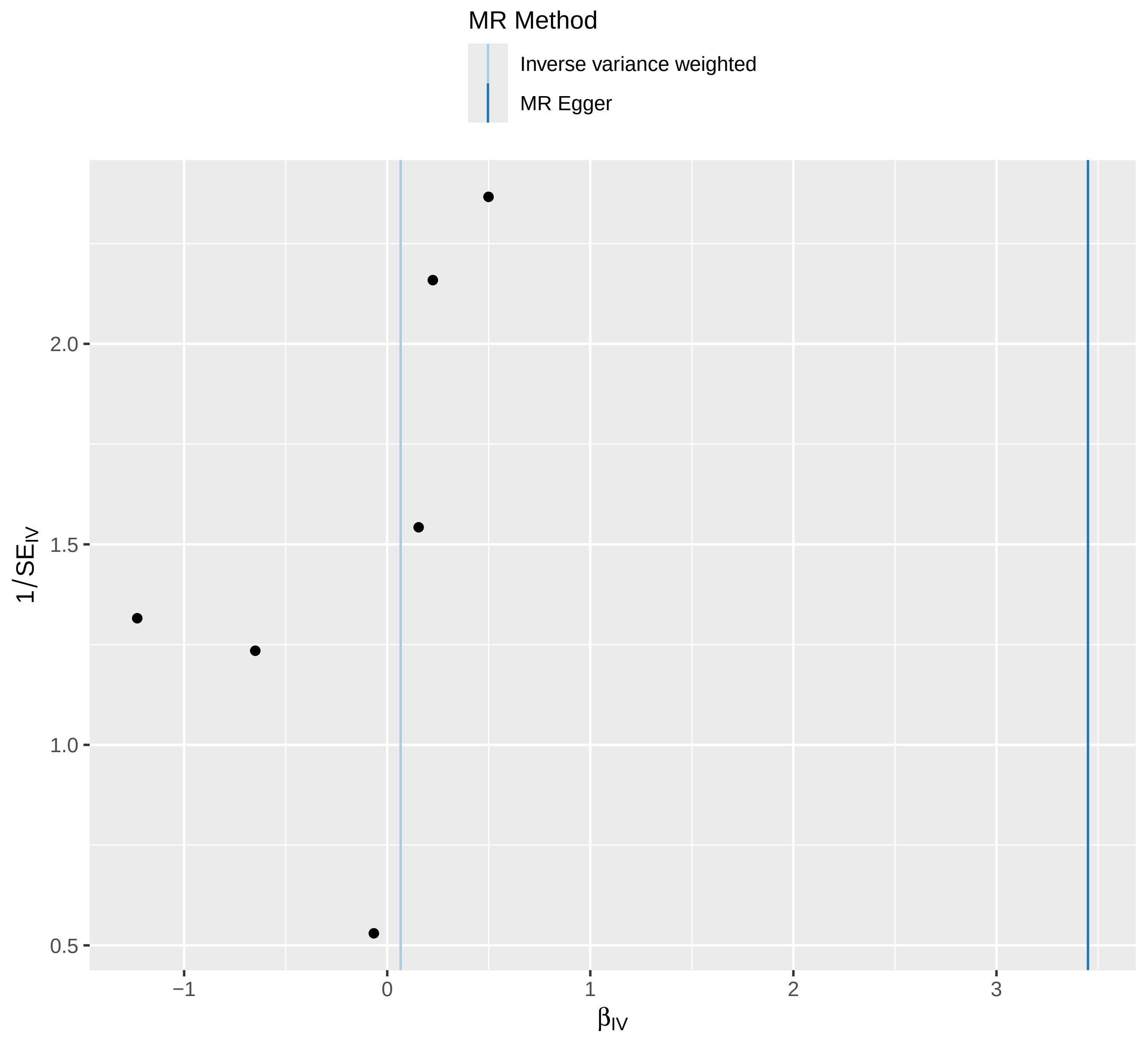

MR Method
Inverse variance weighted
MR Egger
2.0
1.5
1.0
0.5
−1
0
1
2
3
$1/SE_{IV}$
β_{IV}

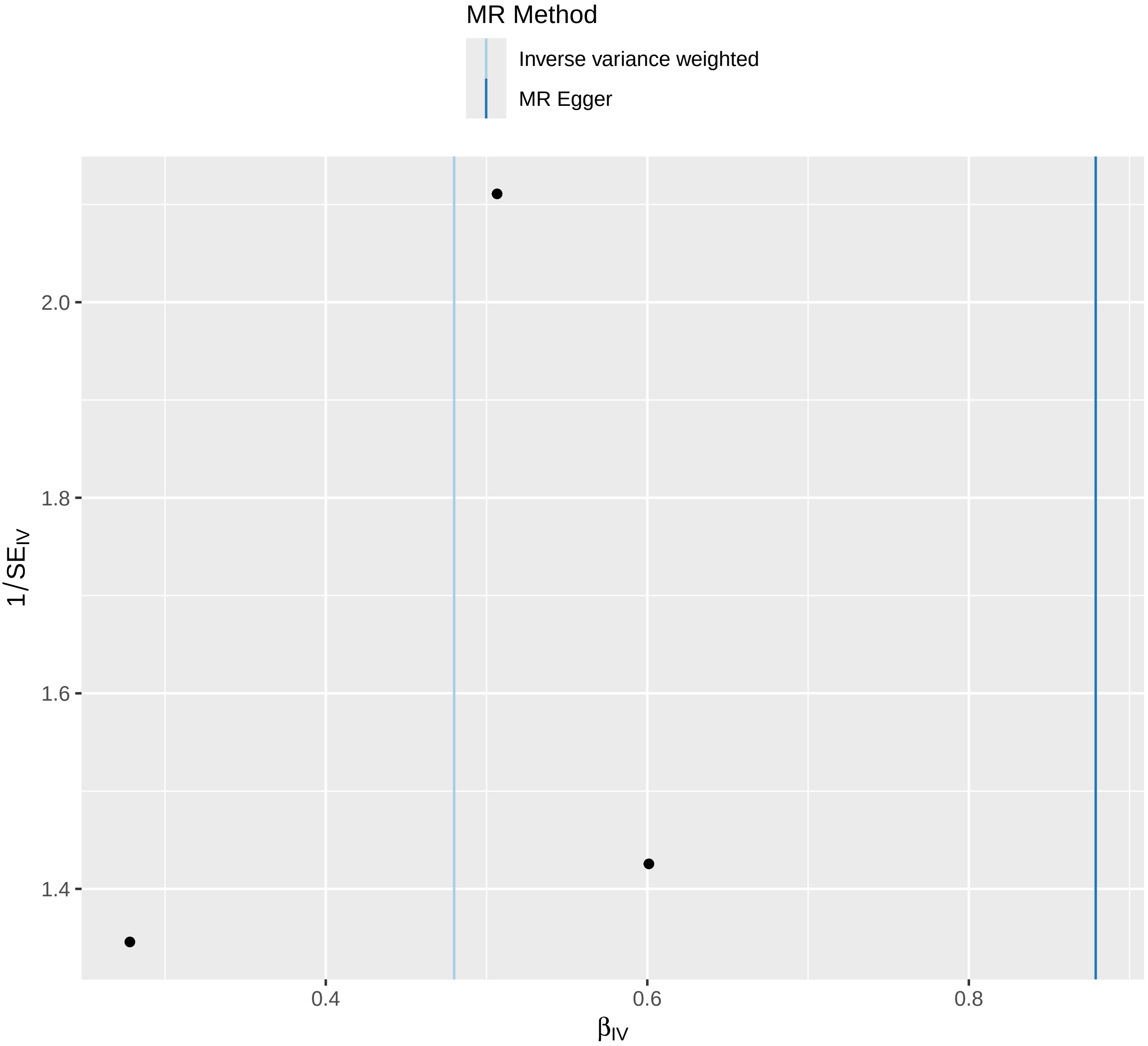
MR Method
Inverse variance weighted
MR Egger
2.0
1.8
1.6
1.4
1/SE_IV
0.4
0.6
0.8
β_IV

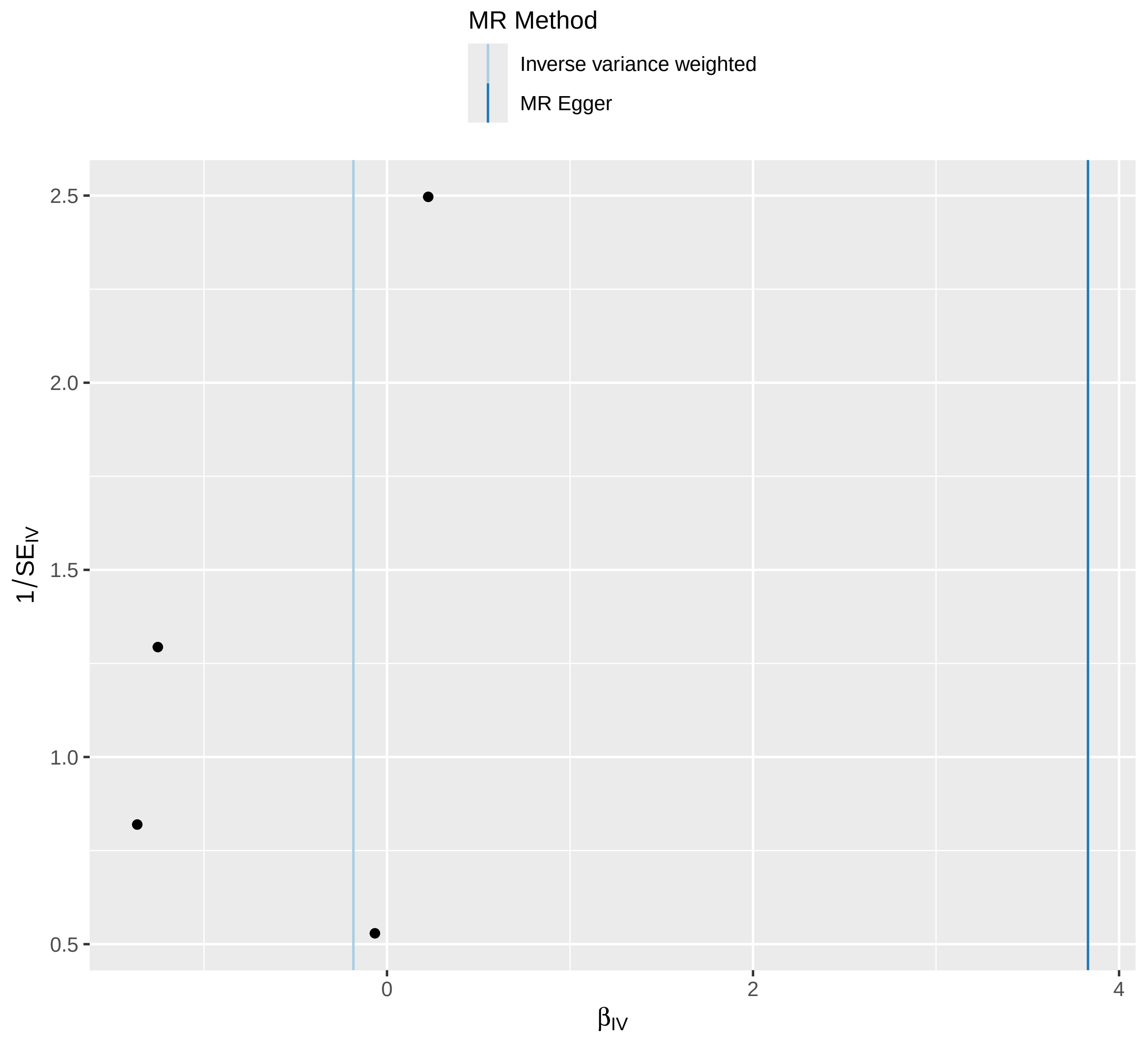

MR Method
Inverse variance weighted
MR Egger
2.5
2.0
1.5
1.0
0.5
1/SE$_{IV}$
0
2
4
β$_{IV}$

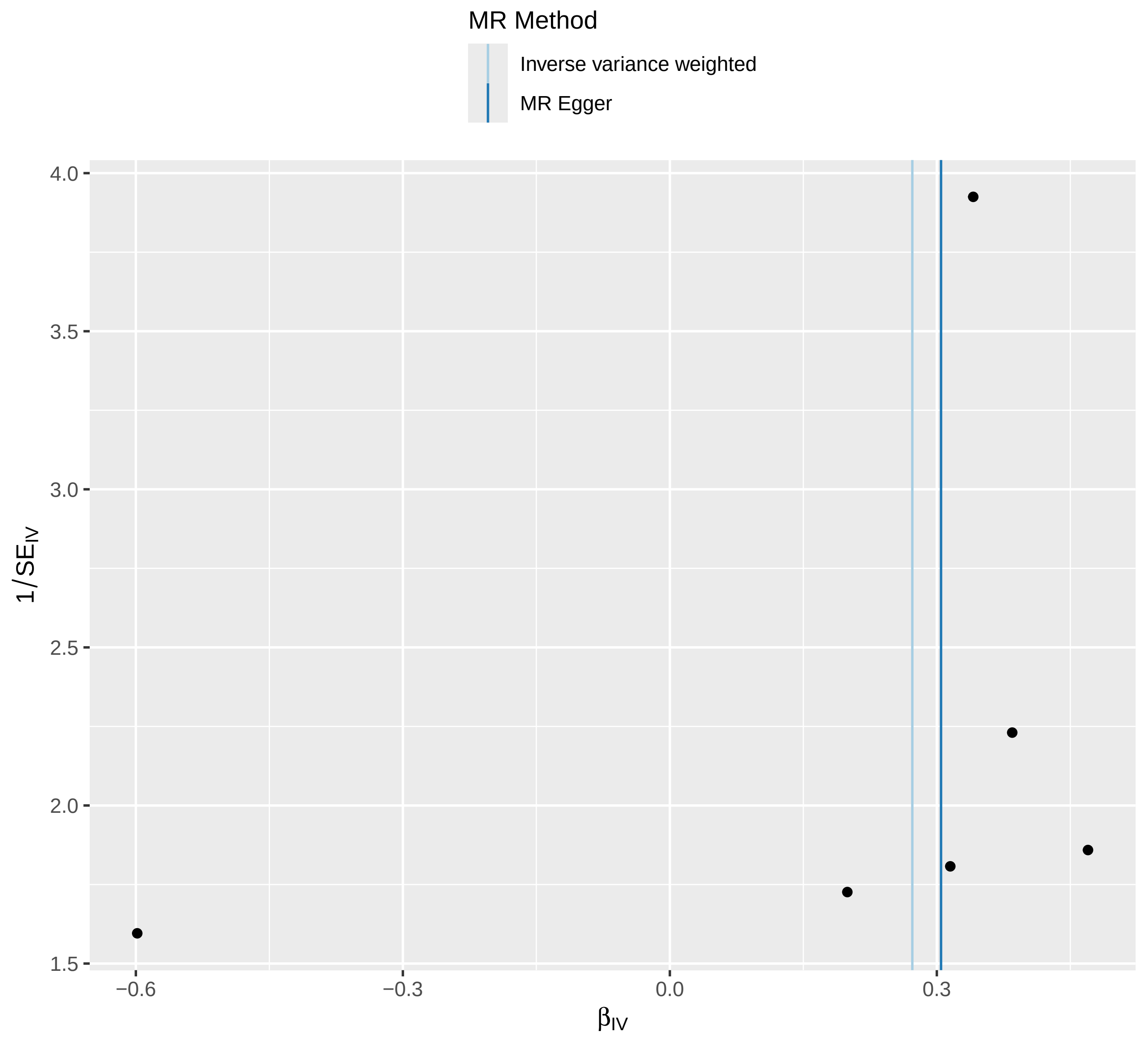
MR Method
Inverse variance weighted
MR Egger
1/SE_IV
4.0
3.5
3.0
2.5
2.0
1.5
−0.6
−0.3
0.0
0.3
β_IV

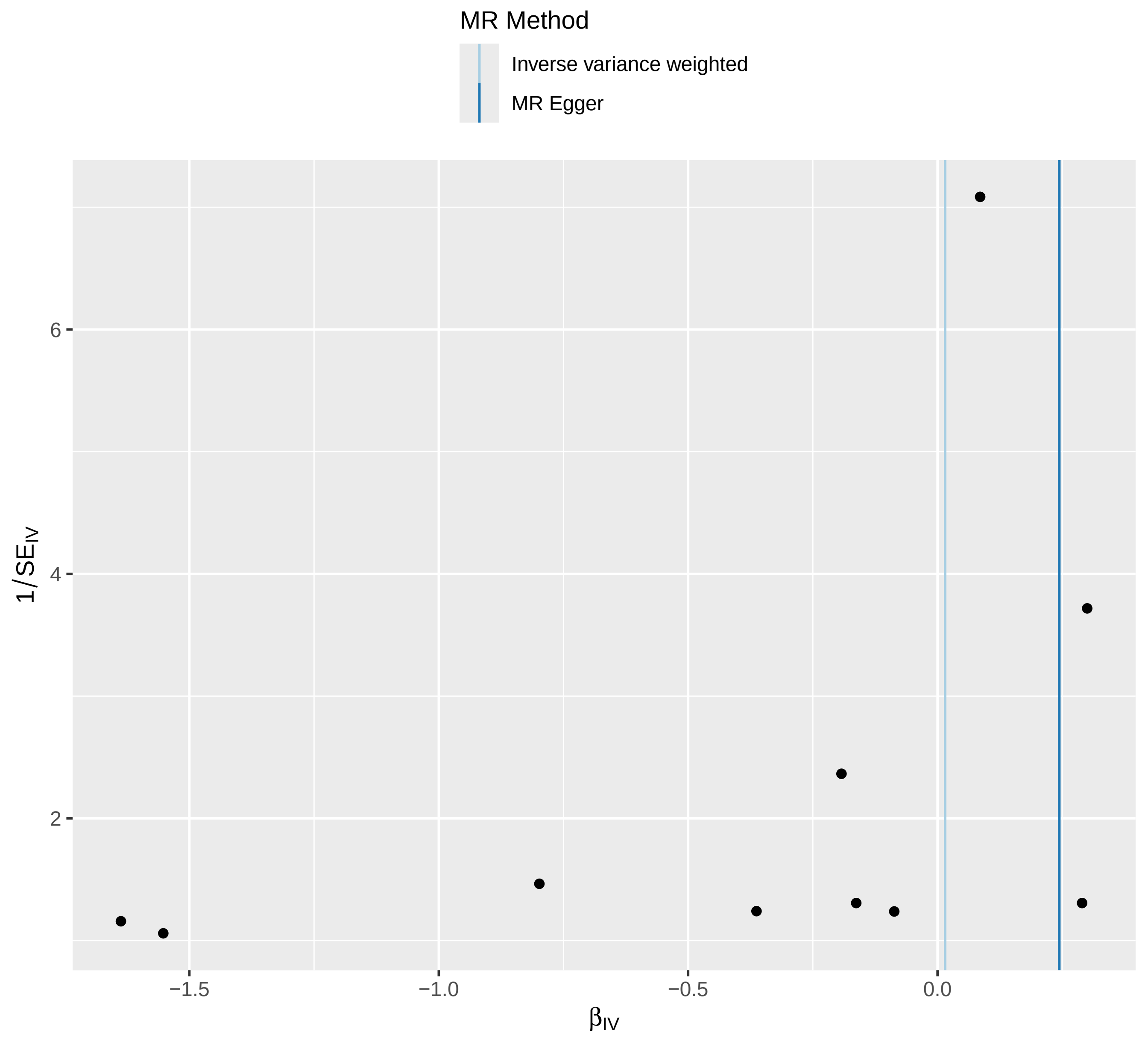

MR Method
Inverse variance weighted
MR Egger
6
4
2
1/SE_IV
−1.5
−1.0
−0.5
0.0
β_IV

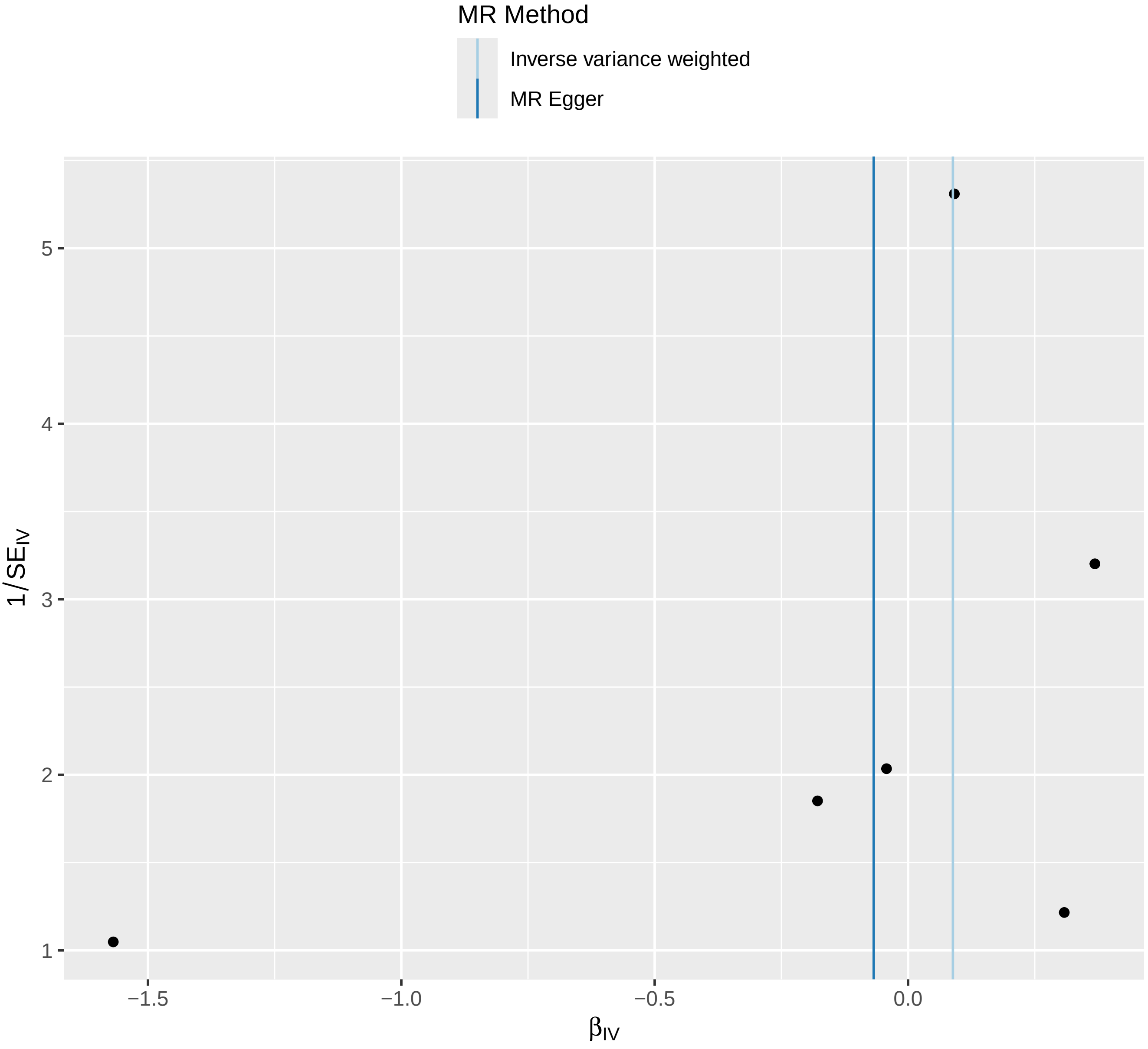

MR Method
Inverse variance weighted
MR Egger
5
4
3
2
1
1/SE_IV
−1.5
−1.0
−0.5
0.0
β_IV

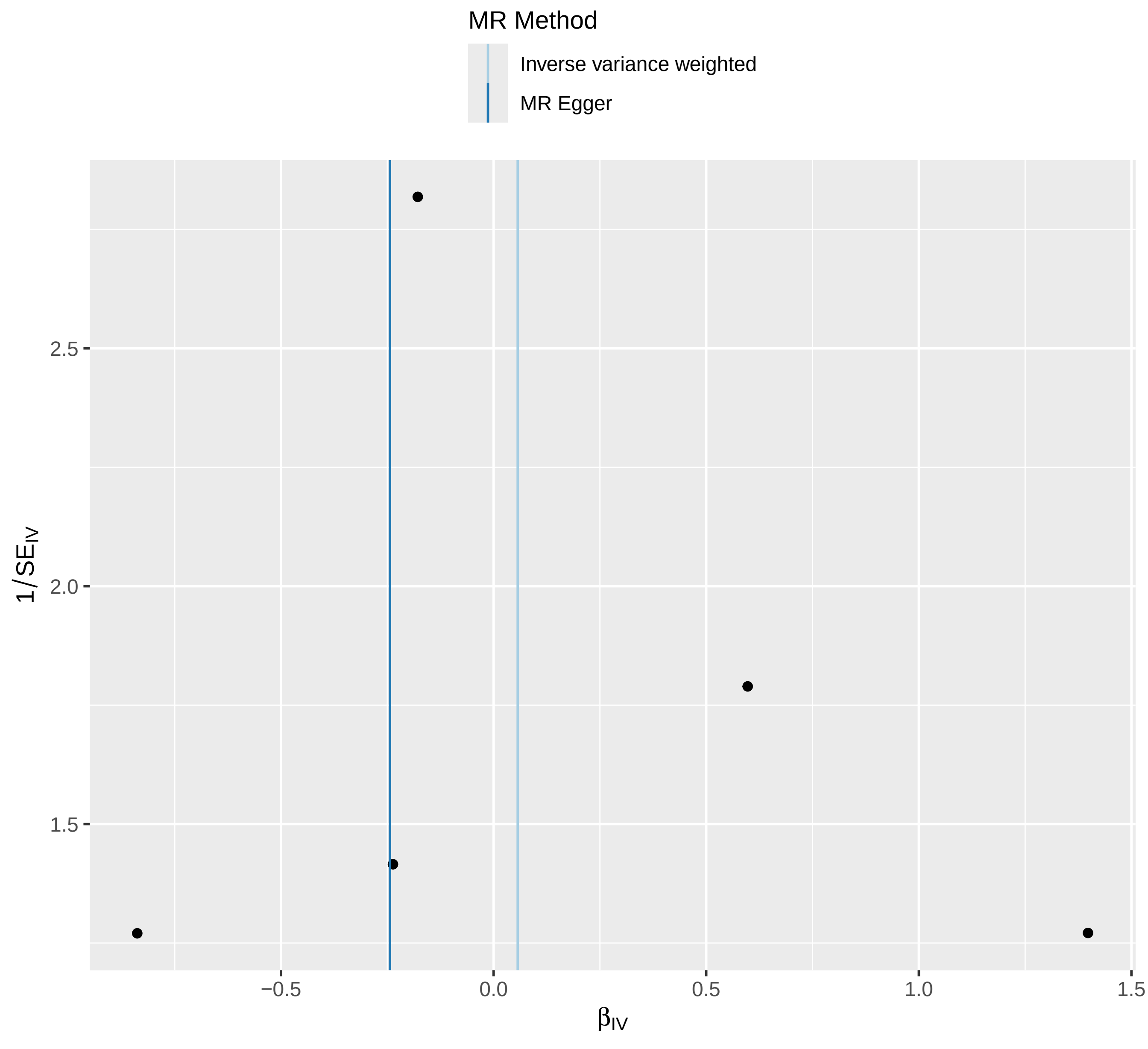

MR Method
Inverse variance weighted
MR Egger
2.5
2.0
1.5
1/SE_IV
−0.5
0.0
0.5
1.0
1.5
β_IV

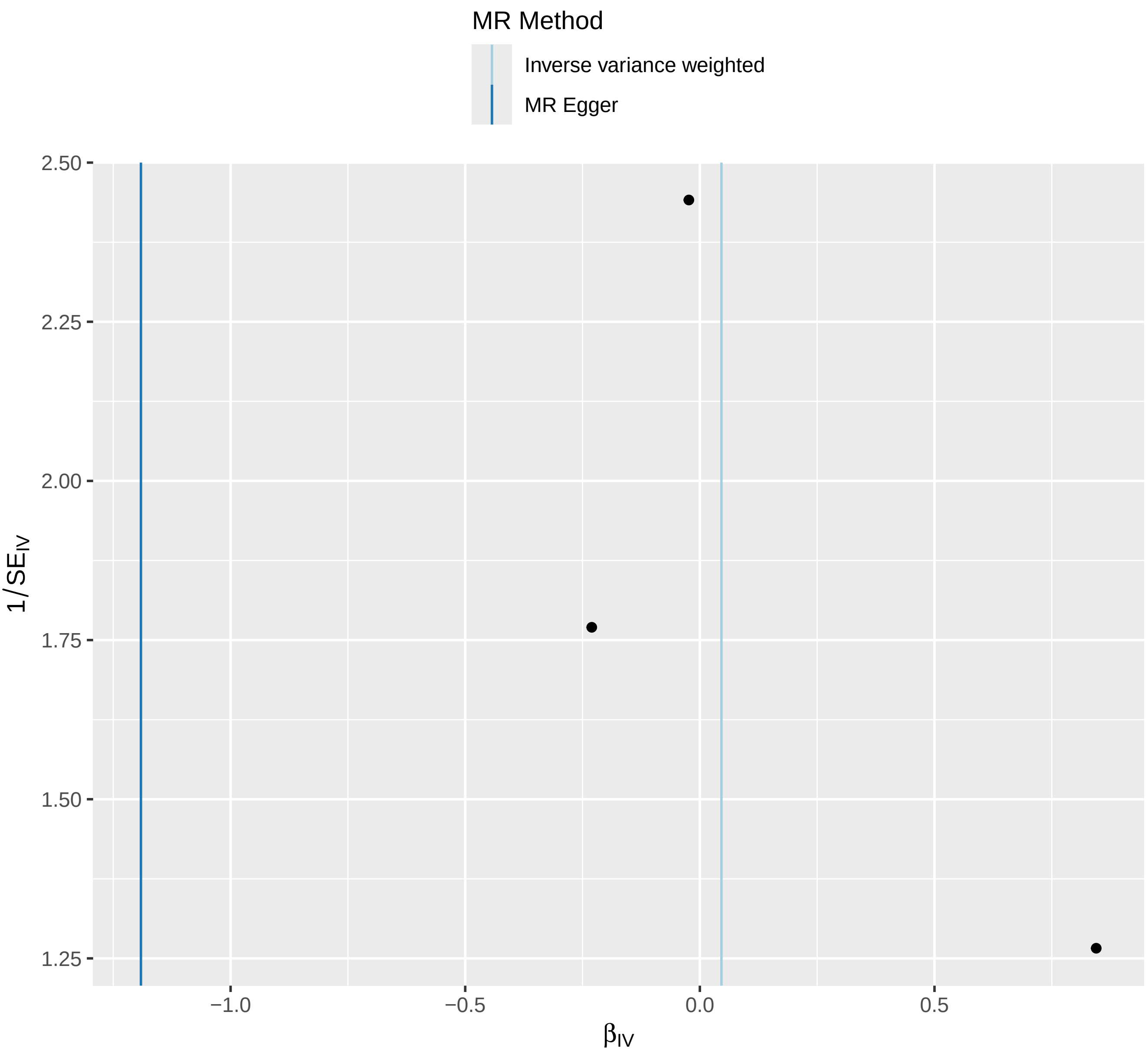
MR Method
Inverse variance weighted
MR Egger
2.50
2.25
2.00
1.75
1.50
1.25
1/SE_IV
−1.0
−0.5
0.0
0.5
β_IV

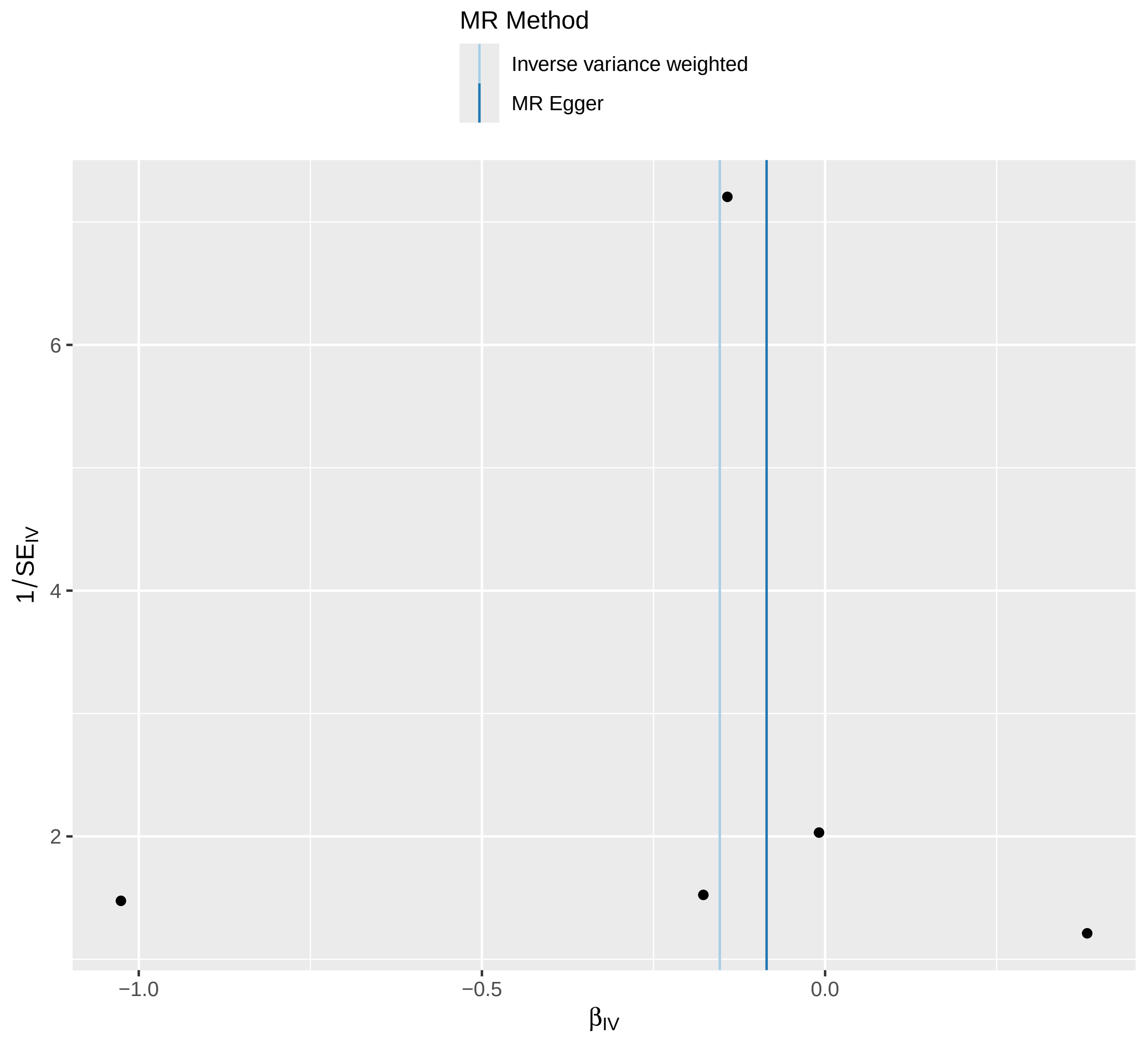

MR Method
Inverse variance weighted
MR Egger
6
4
2
1/SE$_{IV}$
−1.0
−0.5
0.0
β$_{IV}$

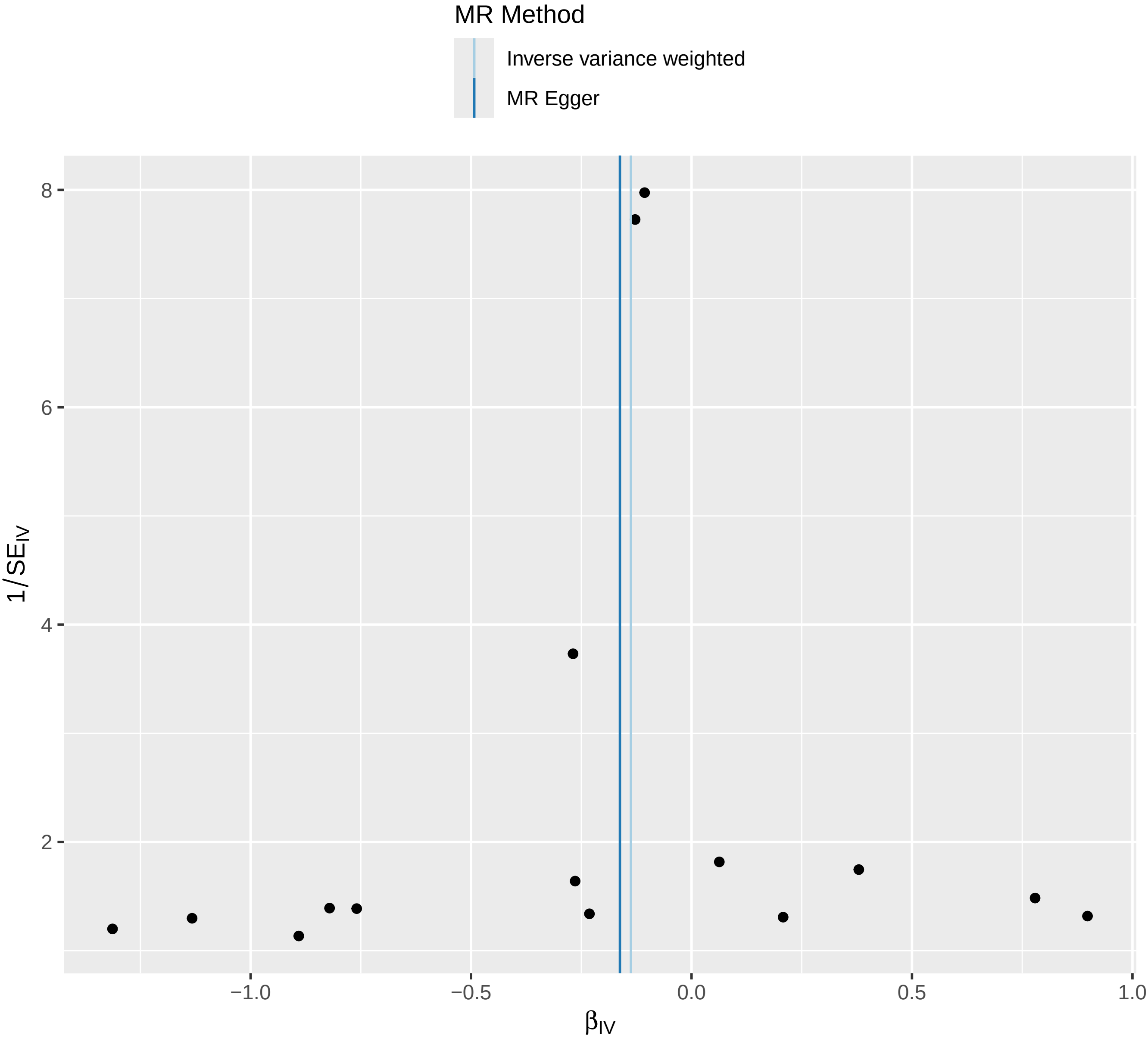

MR Method
Inverse variance weighted
MR Egger
8
6
4
2
1/SE$_{IV}$
−1.0
−0.5
0.0
0.5
1.0
β$_{IV}$

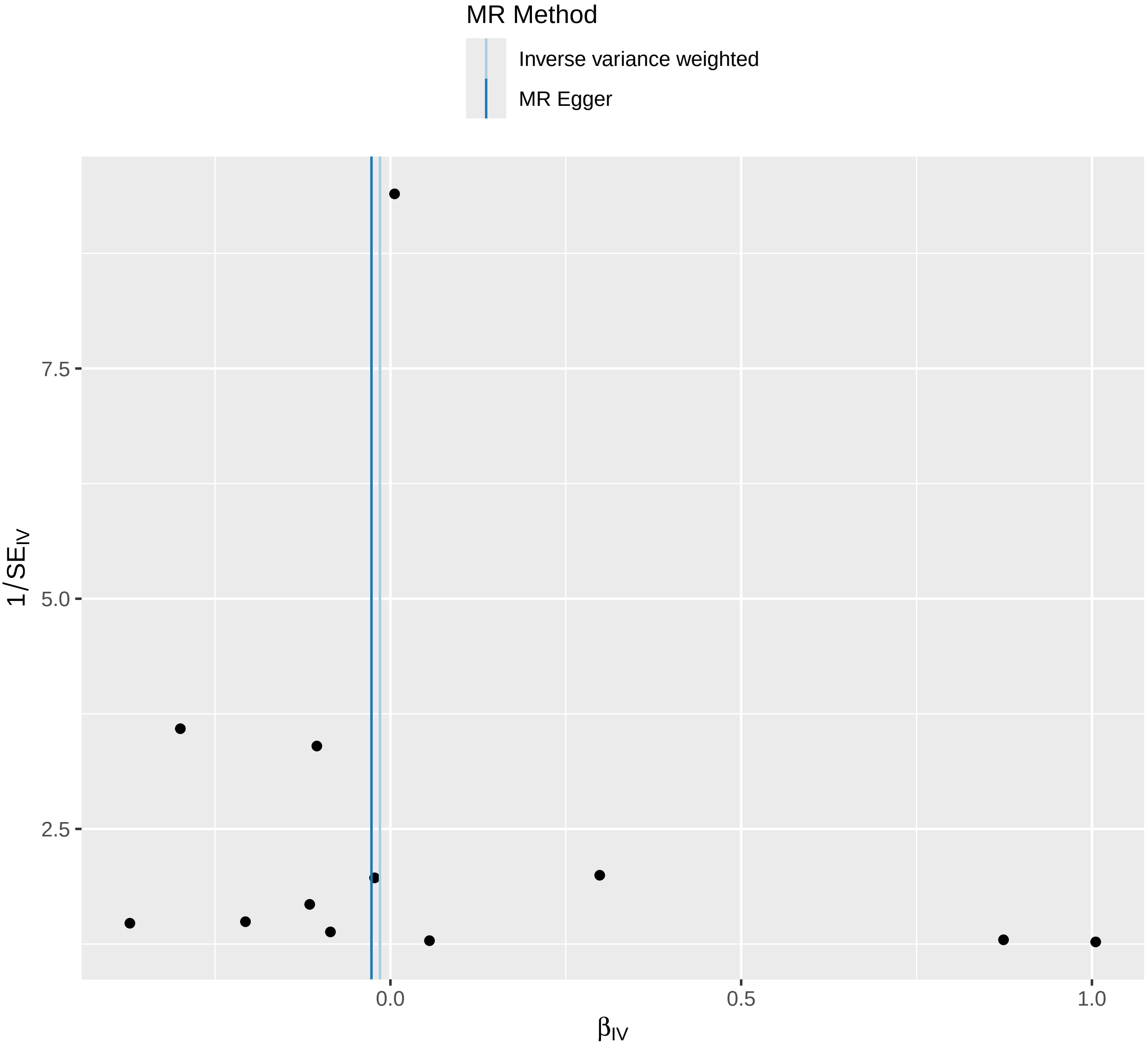

MR Method
Inverse variance weighted
MR Egger
7.5
5.0
2.5
1/SE_IV
0.0
0.5
1.0
β_IV

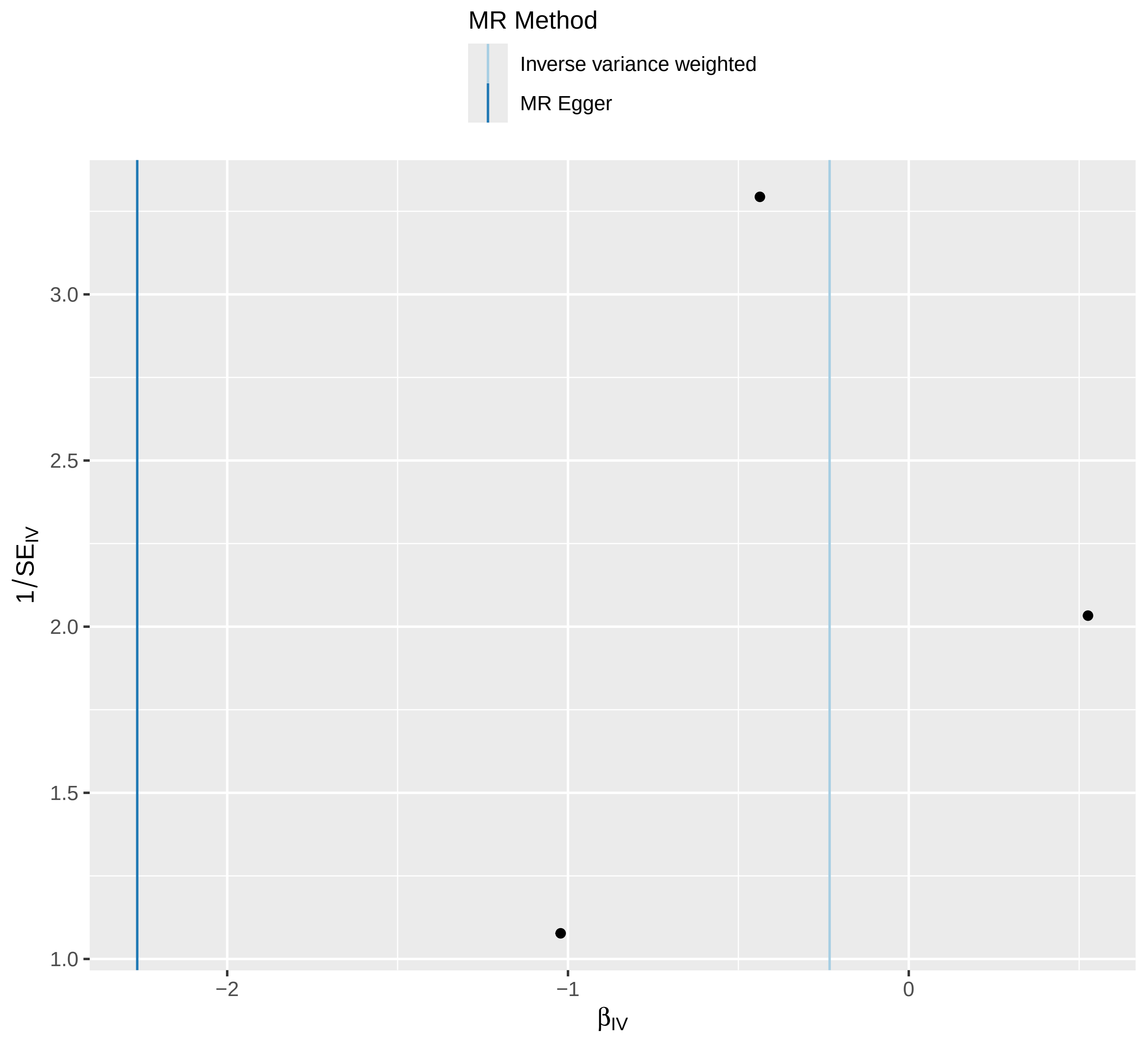

MR Method
Inverse variance weighted
MR Egger
3.0
2.5
2.0
1.5
1.0
1/SE_IV
−2
−1
0
β_IV

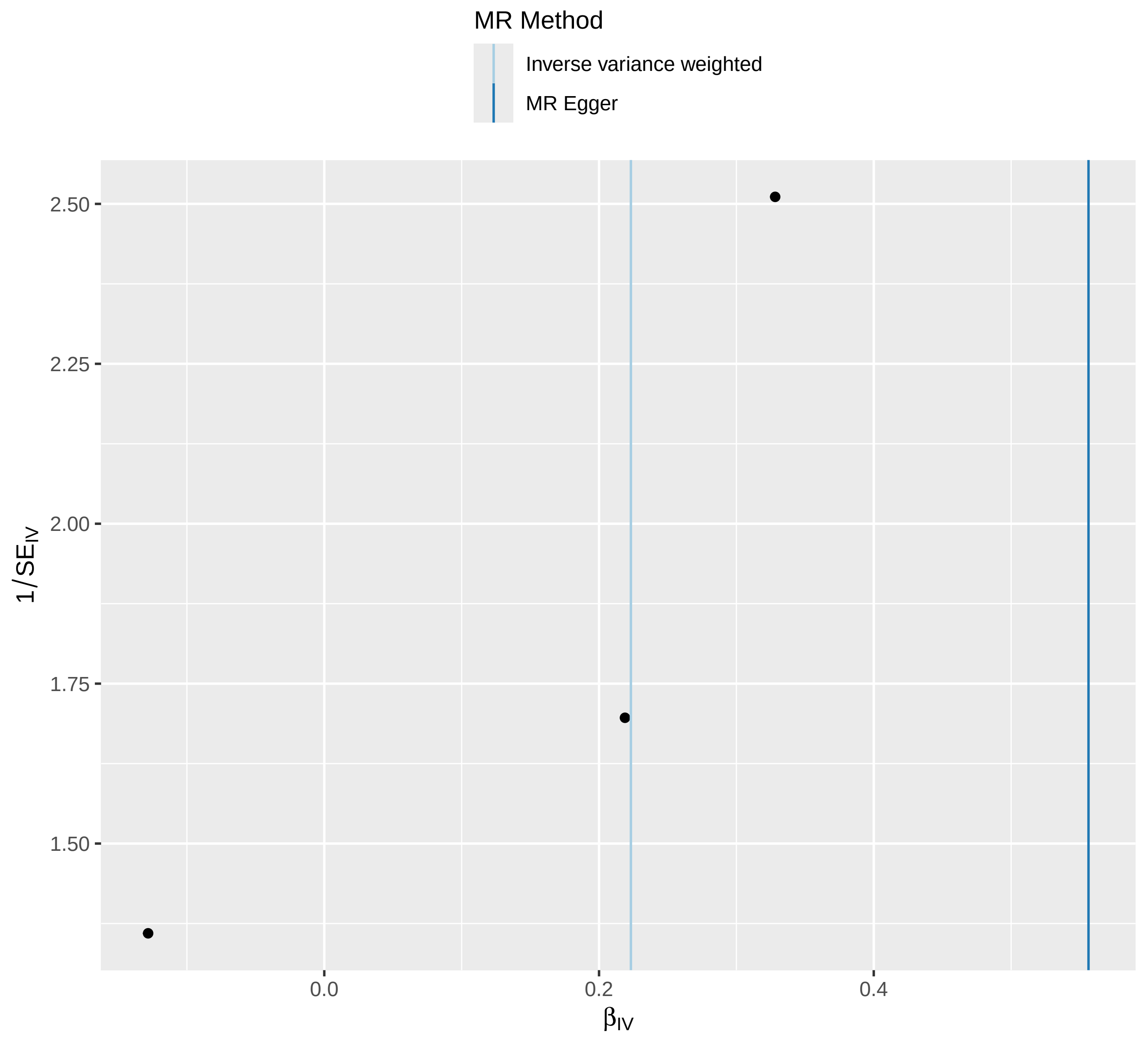
MR Method
Inverse variance weighted
MR Egger
2.50
2.25
2.00
1.75
1.50
1/SE_IV
0.0
0.2
0.4
β_IV

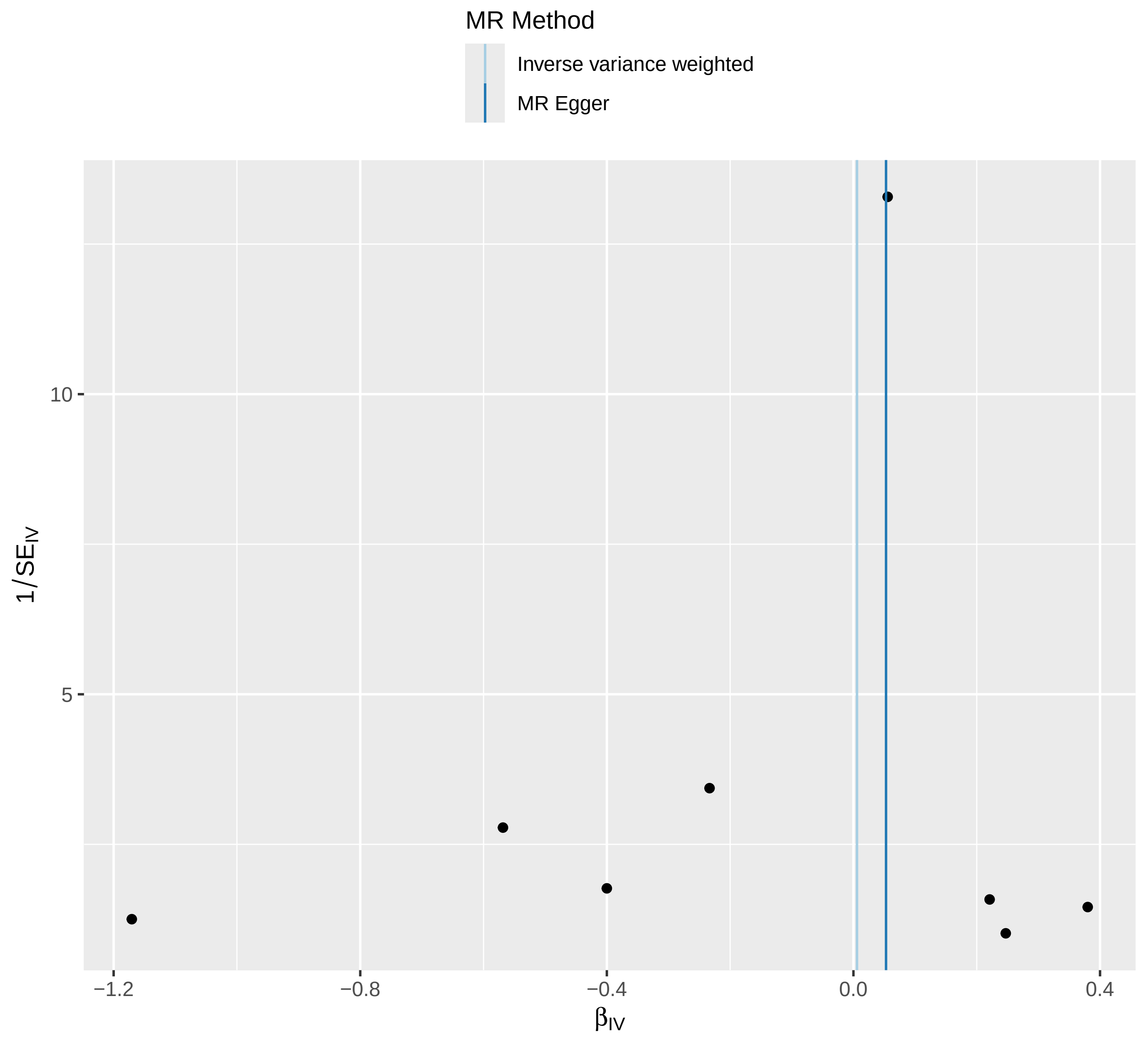

MR Method
Inverse variance weighted
MR Egger
10
5
1/SE_IV
−1.2
−0.8
−0.4
0.0
0.4
β_IV

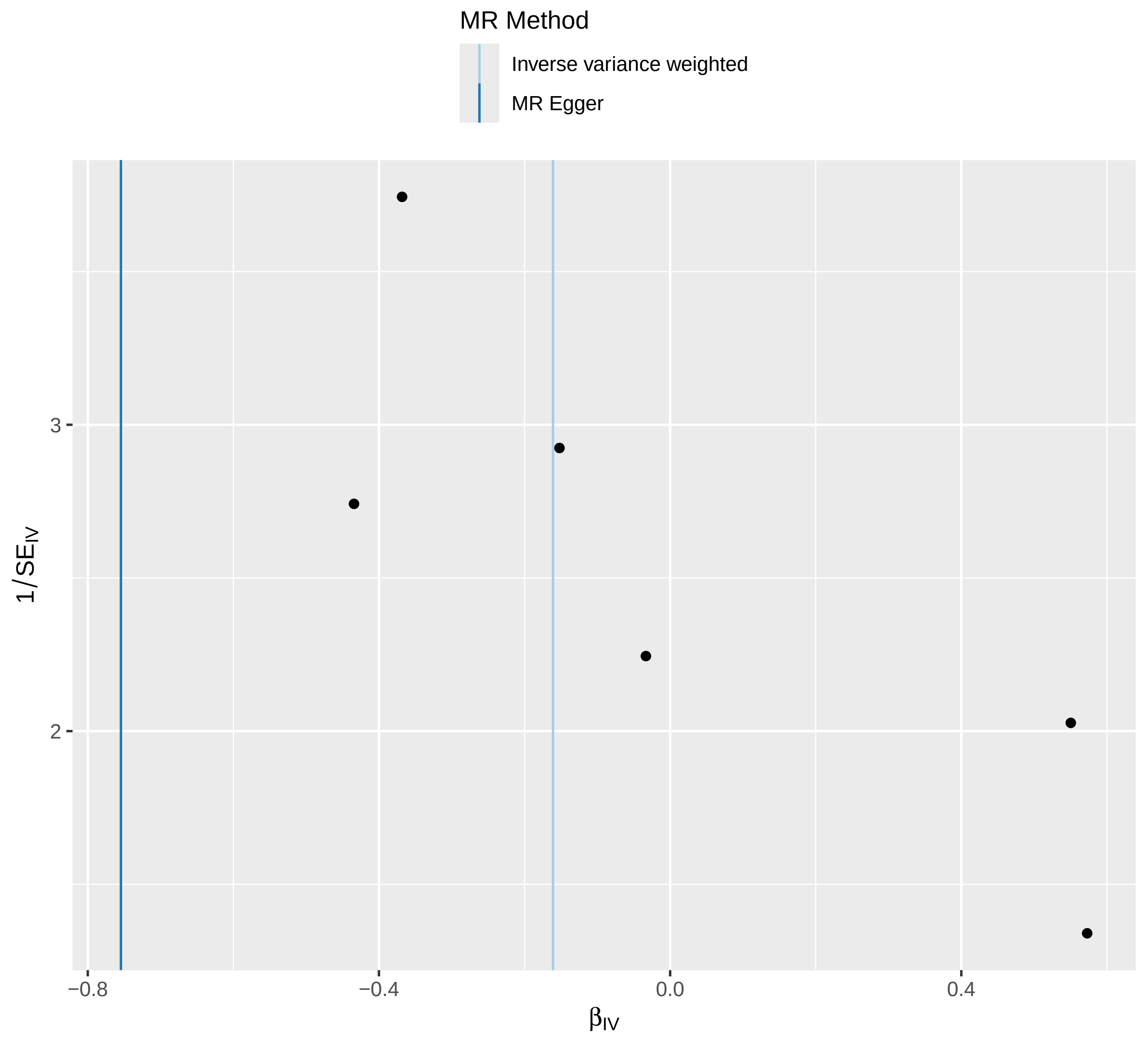

MR Method
Inverse variance weighted
MR Egger
3
2
1/SE$_{IV}$
−0.8
−0.4
0.0
0.4
β$_{IV}$

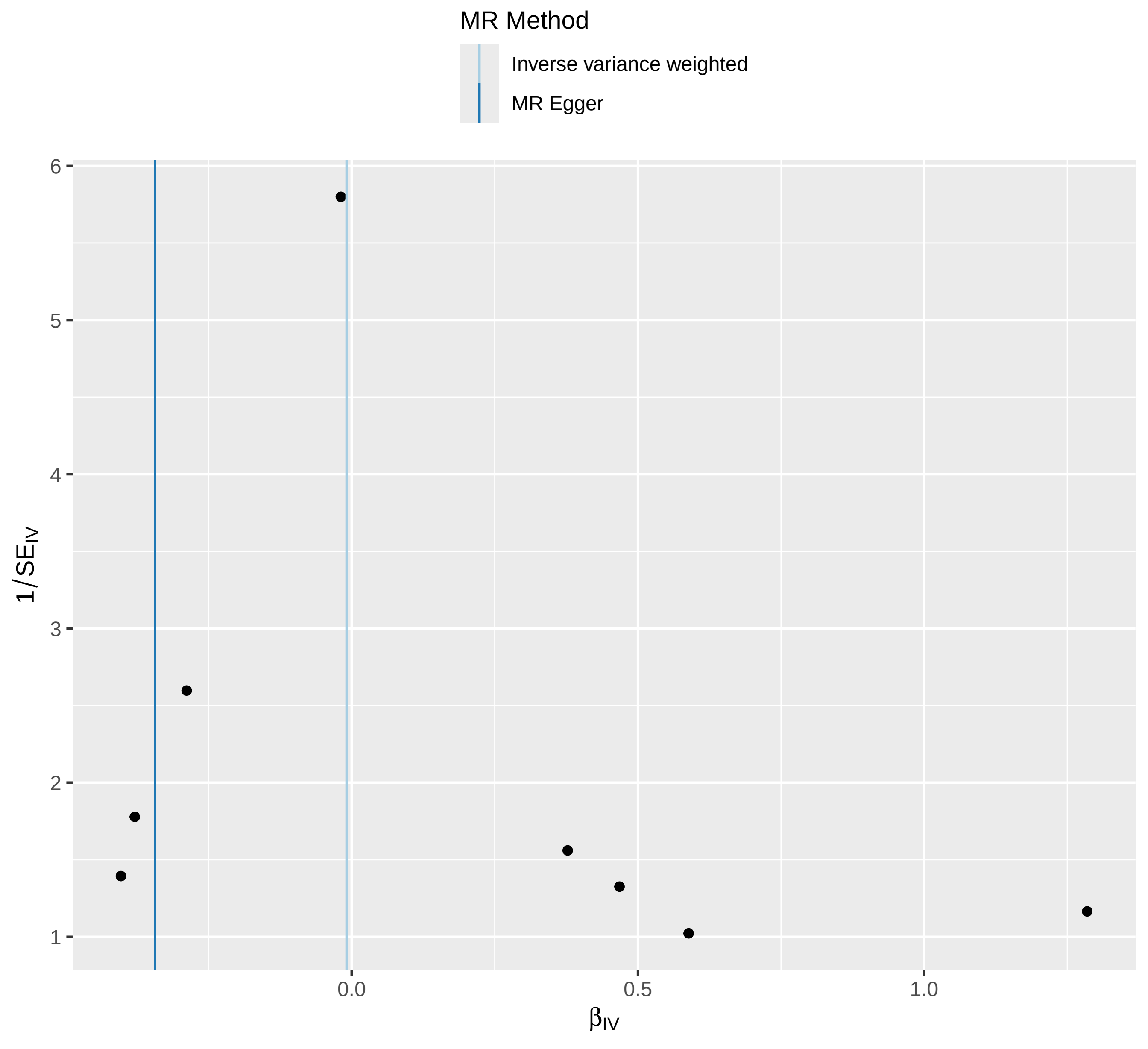
MR Method
Inverse variance weighted
MR Egger
1/SE_IV
6
5
4
3
2
1
0.0
0.5
1.0
β_IV

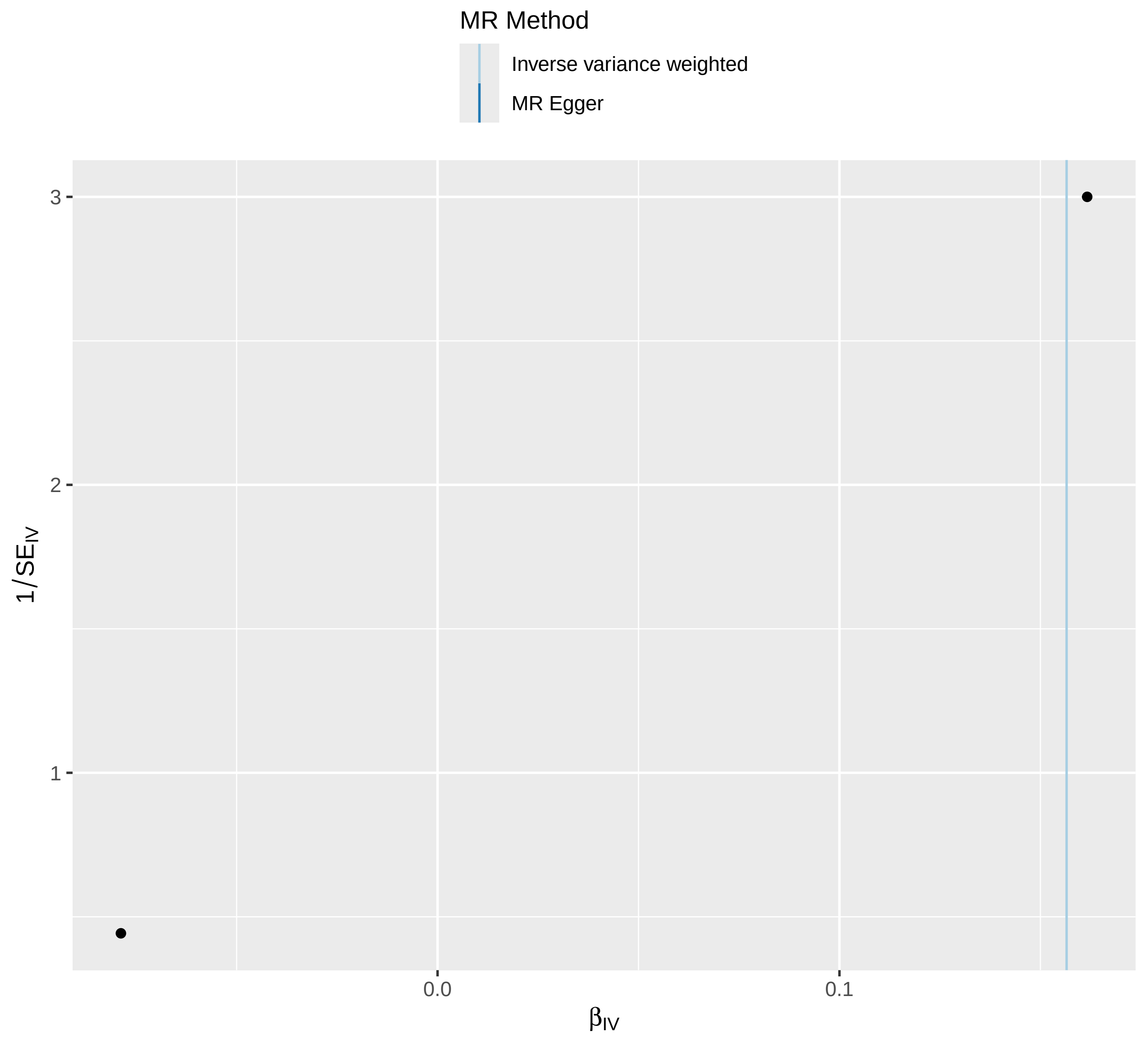

MR Method
Inverse variance weighted
MR Egger
3
2
1
1/SE_IV
0.0
0.1
β_IV

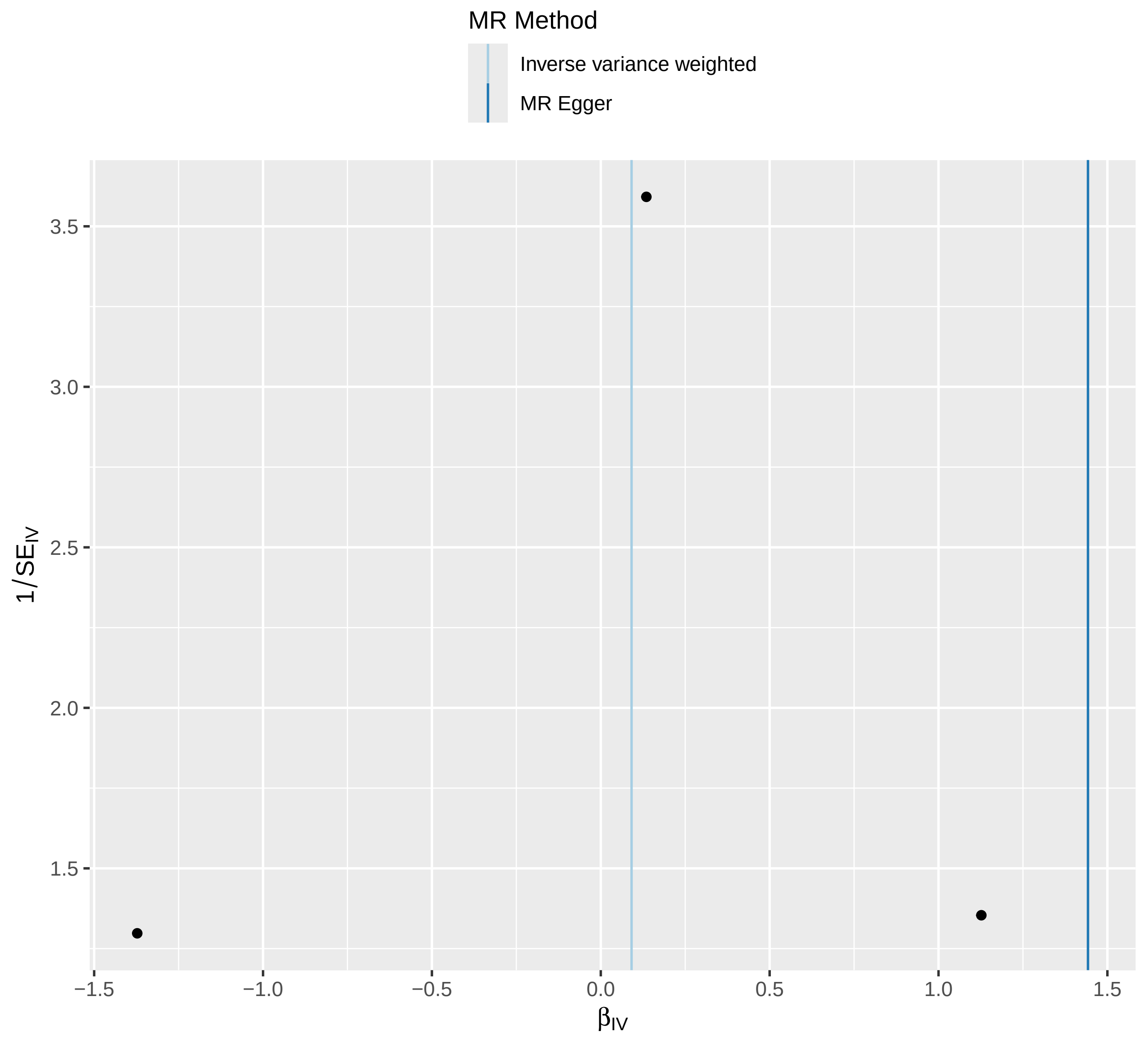

MR Method
Inverse variance weighted
MR Egger
3.5
3.0
2.5
2.0
1.5
1/SE_IV
−1.5
−1.0
−0.5
0.0
0.5
1.0
1.5
β_IV

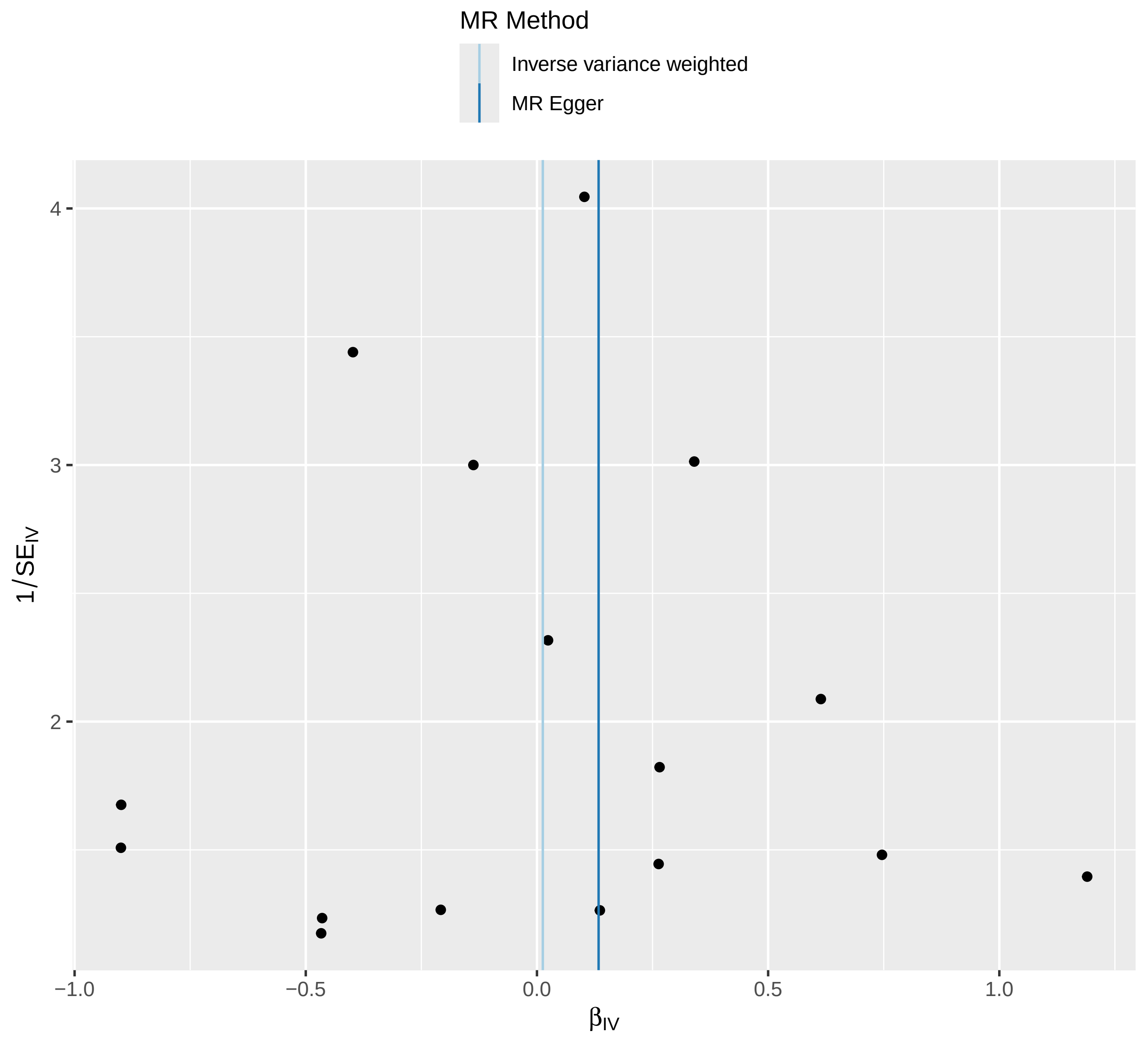
MR Method
Inverse variance weighted
MR Egger
4
3
2
−1.0
−0.5
0.0
0.5
1.0
$1/SE_{IV}$
β_{IV}

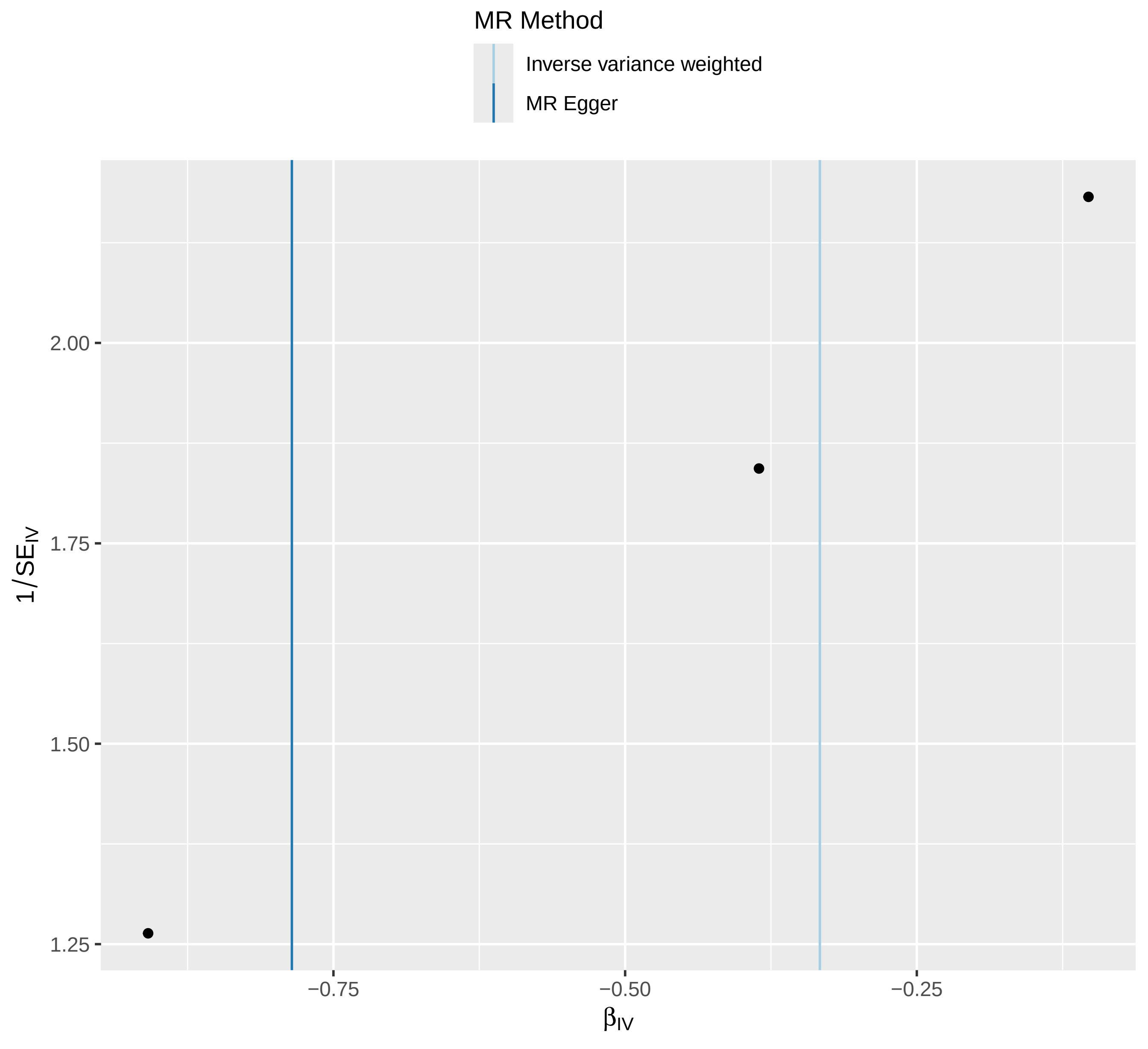
MR Method
Inverse variance weighted
MR Egger
1/SE_IV
2.00
1.75
1.50
1.25
−0.75
−0.50
−0.25
β_IV

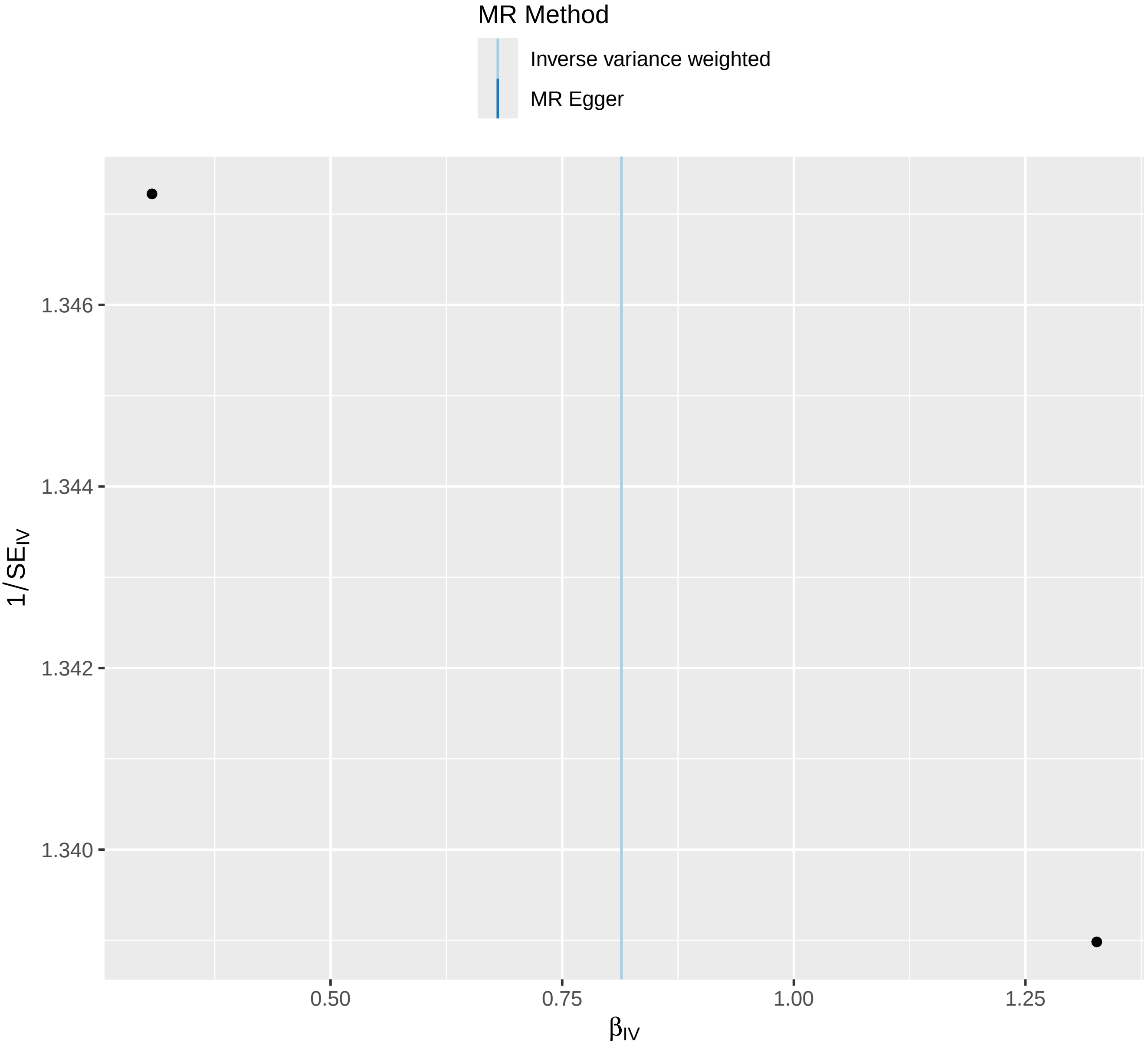
MR Method
Inverse variance weighted
MR Egger
1.346
1.344
1.342
1.340
$1/SE_{IV}$
0.50
0.75
1.00
1.25
β_{IV}

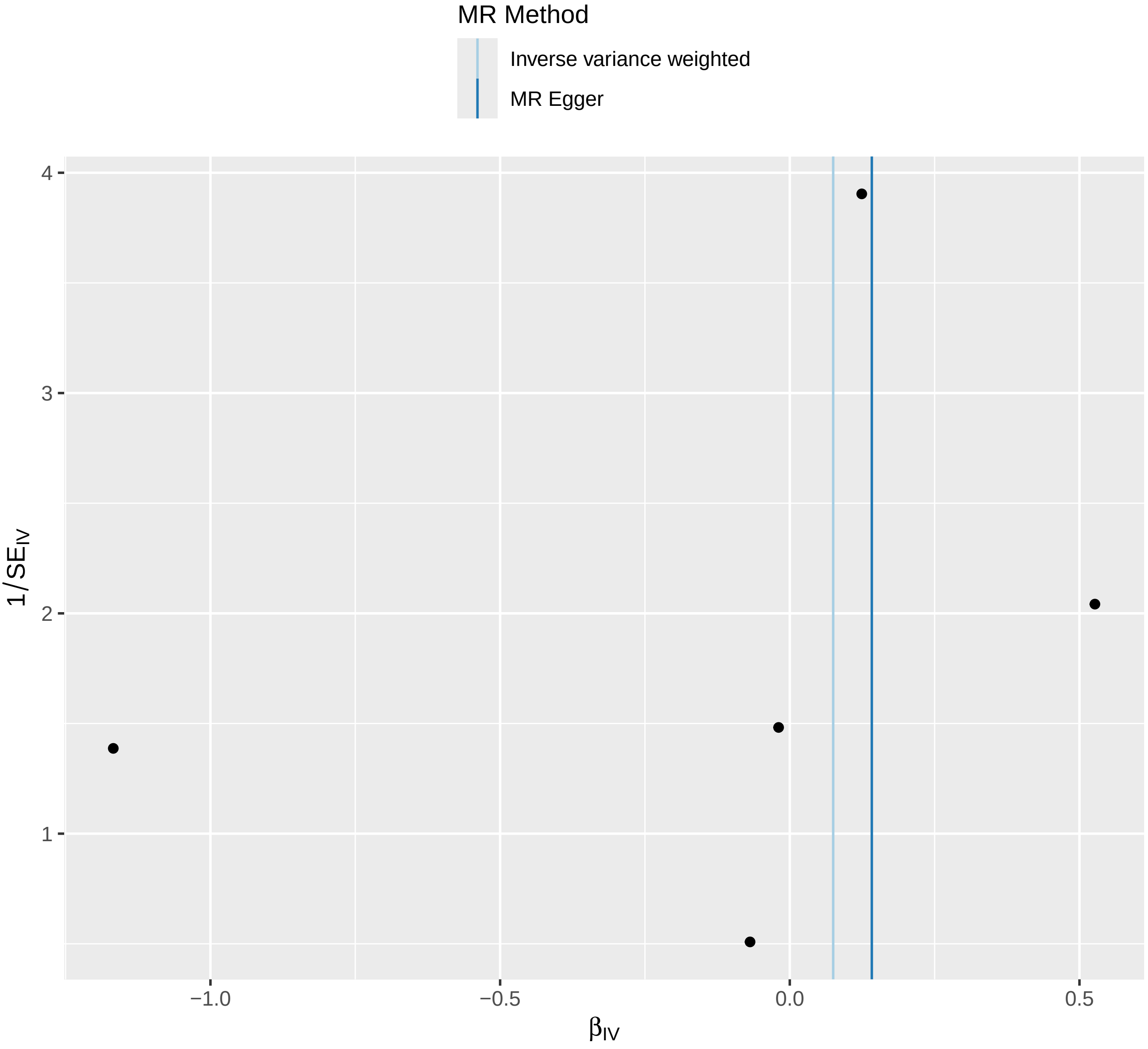
MR Method
Inverse variance weighted
MR Egger
4
3
2
1
1/SE_{IV}
−1.0
−0.5
0.0
0.5
β_{IV}

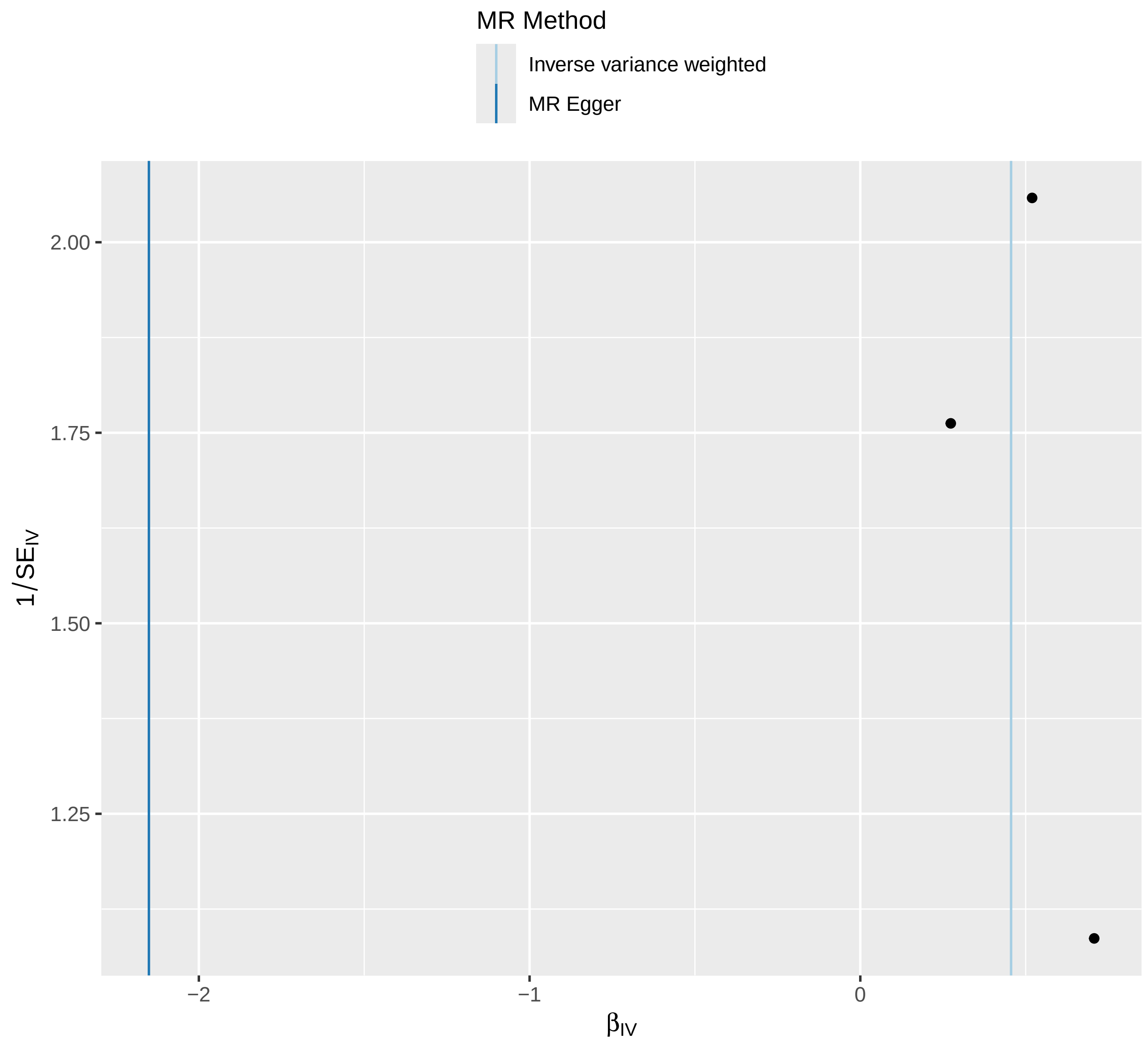
MR Method
Inverse variance weighted
MR Egger
2.00
1.75
1.50
1.25
1/SE_IV
−2
−1
0
β_IV

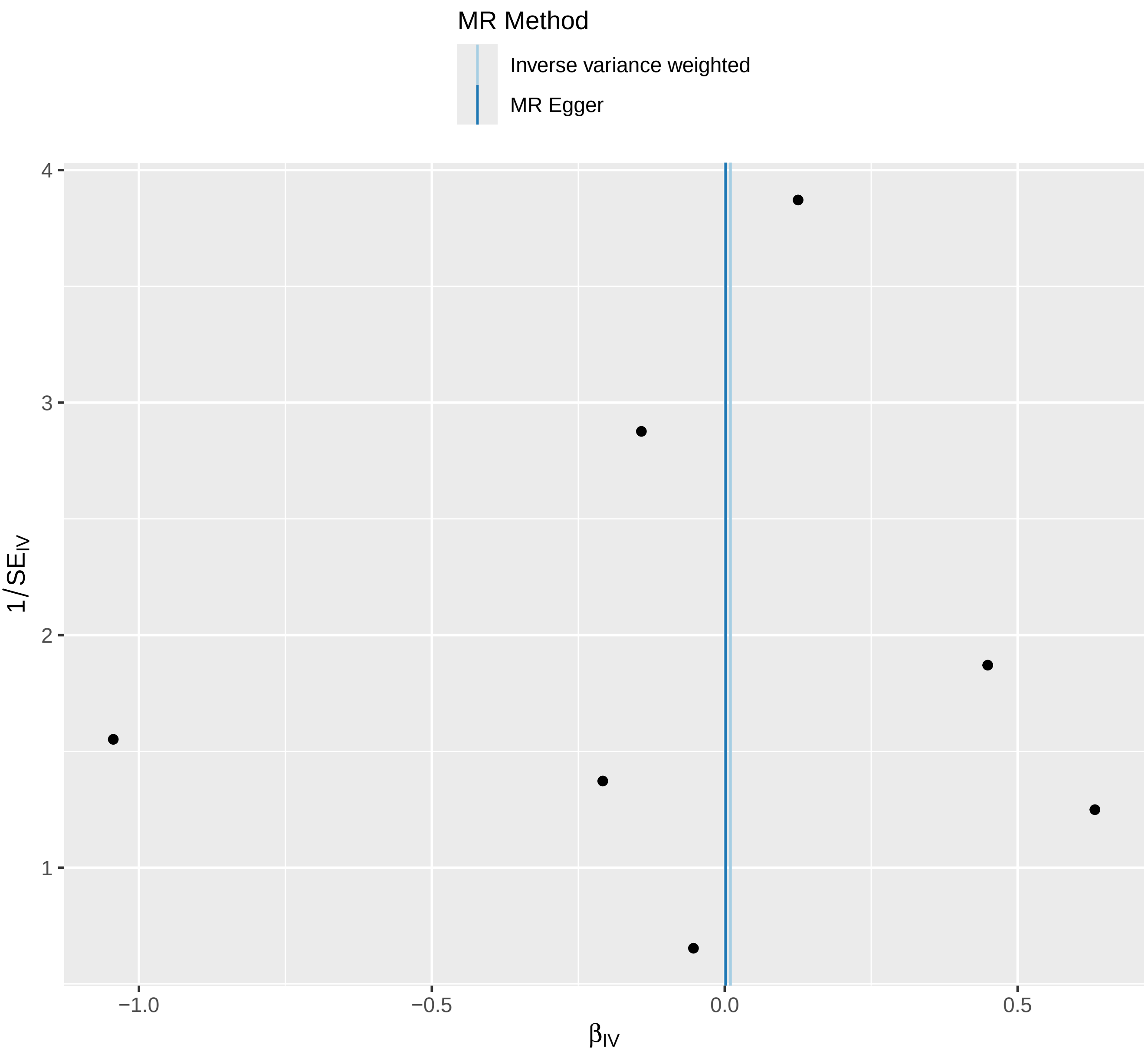

MR Method
Inverse variance weighted
MR Egger
4
3
2
1
1/SE$_{IV}$
−1.0
−0.5
0.0
0.5
β$_{IV}$

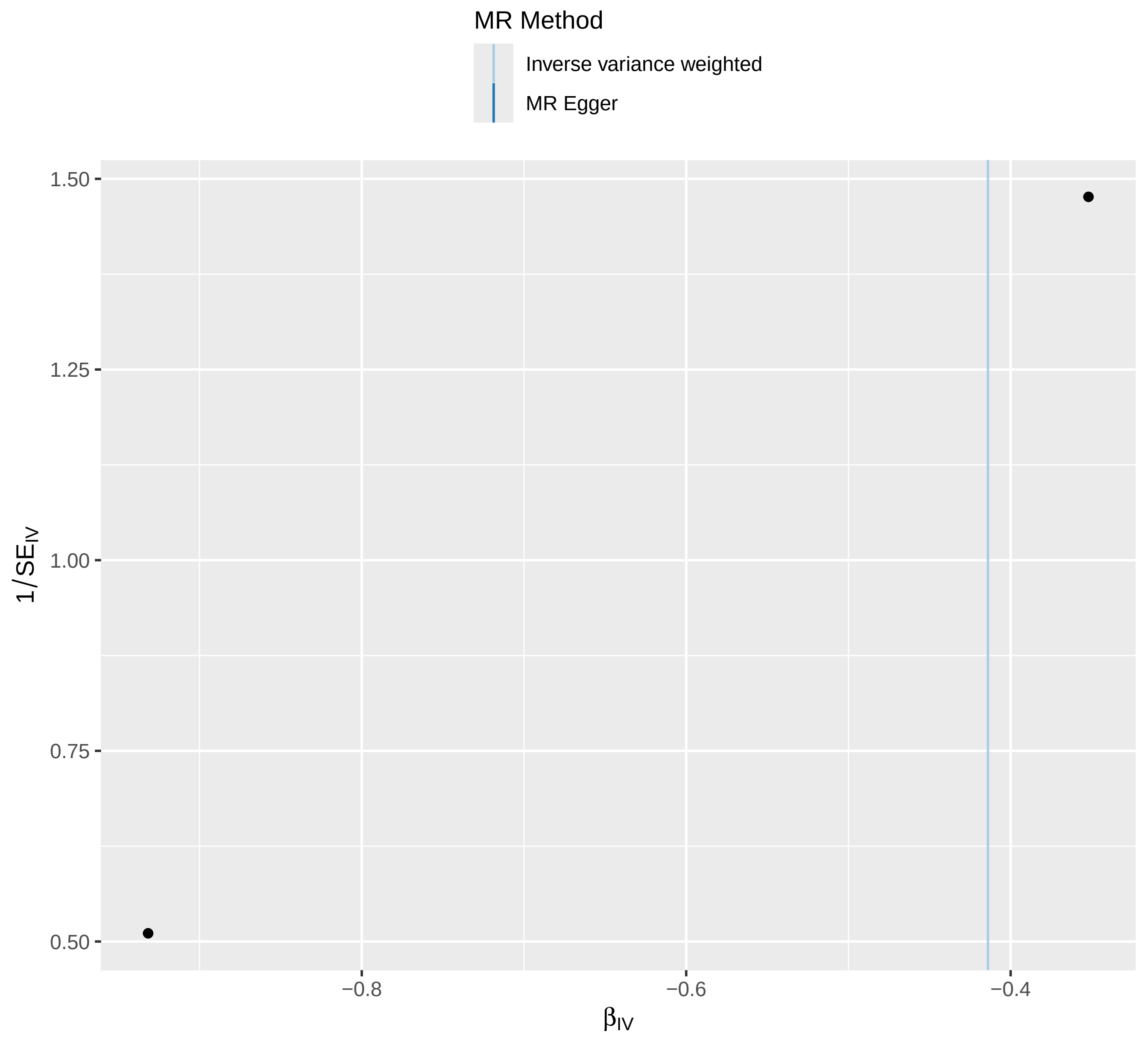
MR Method
Inverse variance weighted
MR Egger
1.50
1.25
1.00
0.75
0.50
$1/SE_{IV}$
−0.8
−0.6
−0.4
β_{IV}

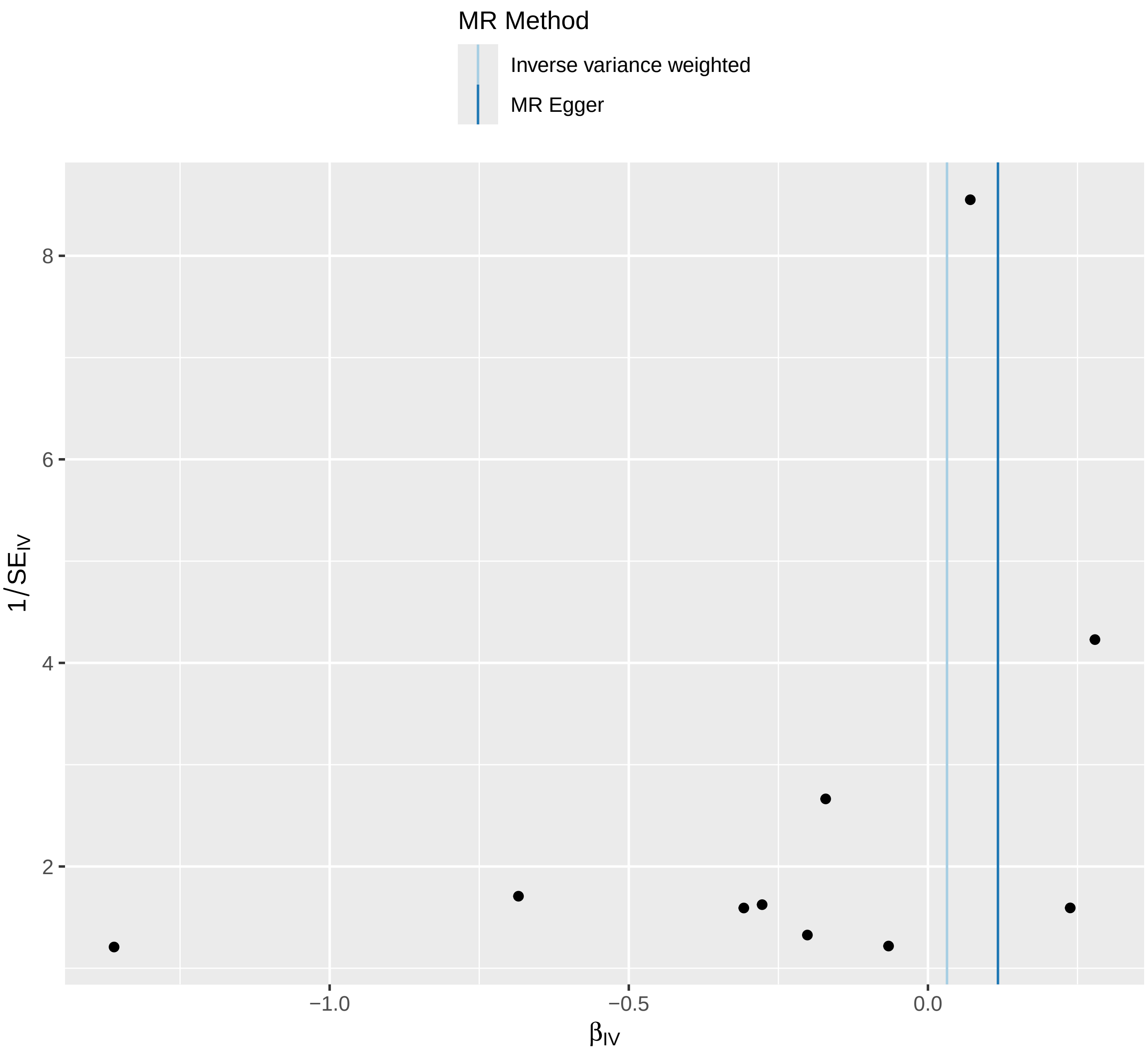

MR Method
Inverse variance weighted
MR Egger
8
6
4
2
1/SE$_{IV}$
−1.0
−0.5
0.0
β$_{IV}$

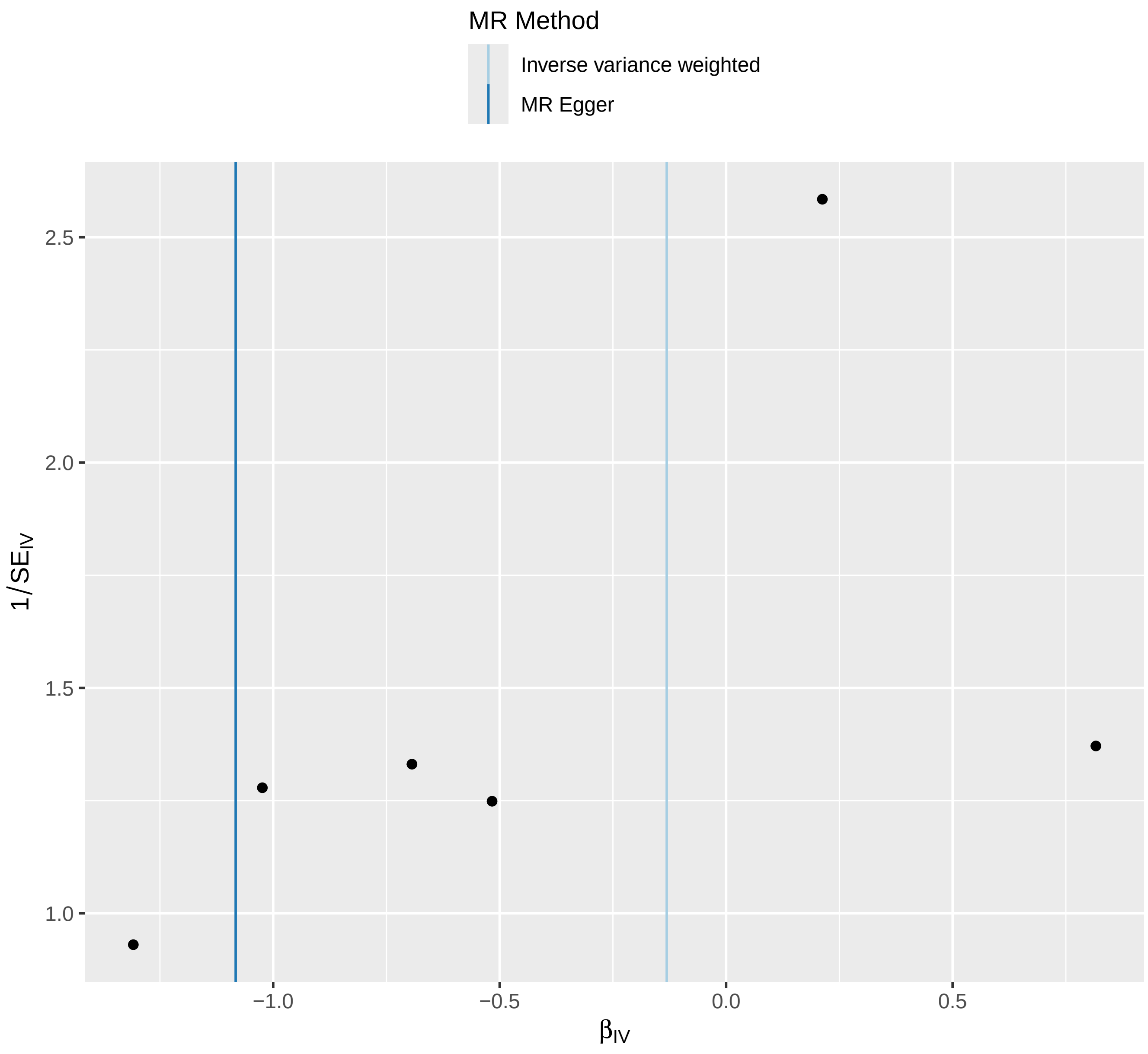
MR Method
Inverse variance weighted
MR Egger
2.5
2.0
1.5
1.0
1/SE_IV
−1.0
−0.5
0.0
0.5
β_IV

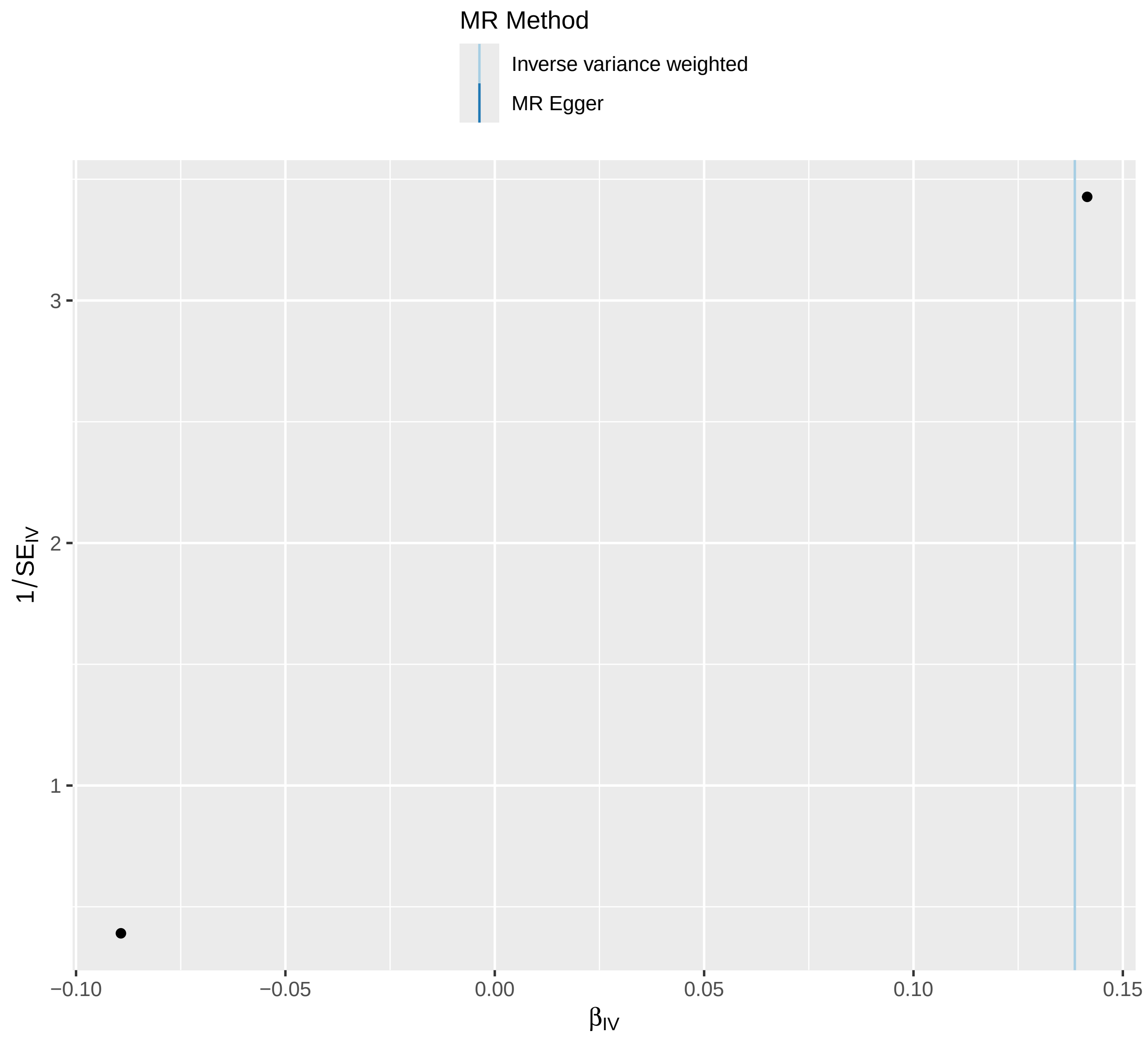

MR Method
Inverse variance weighted
MR Egger
1/SE_{IV}
3
2
1
−0.10
−0.05
0.00
0.05
0.10
0.15
β_{IV}

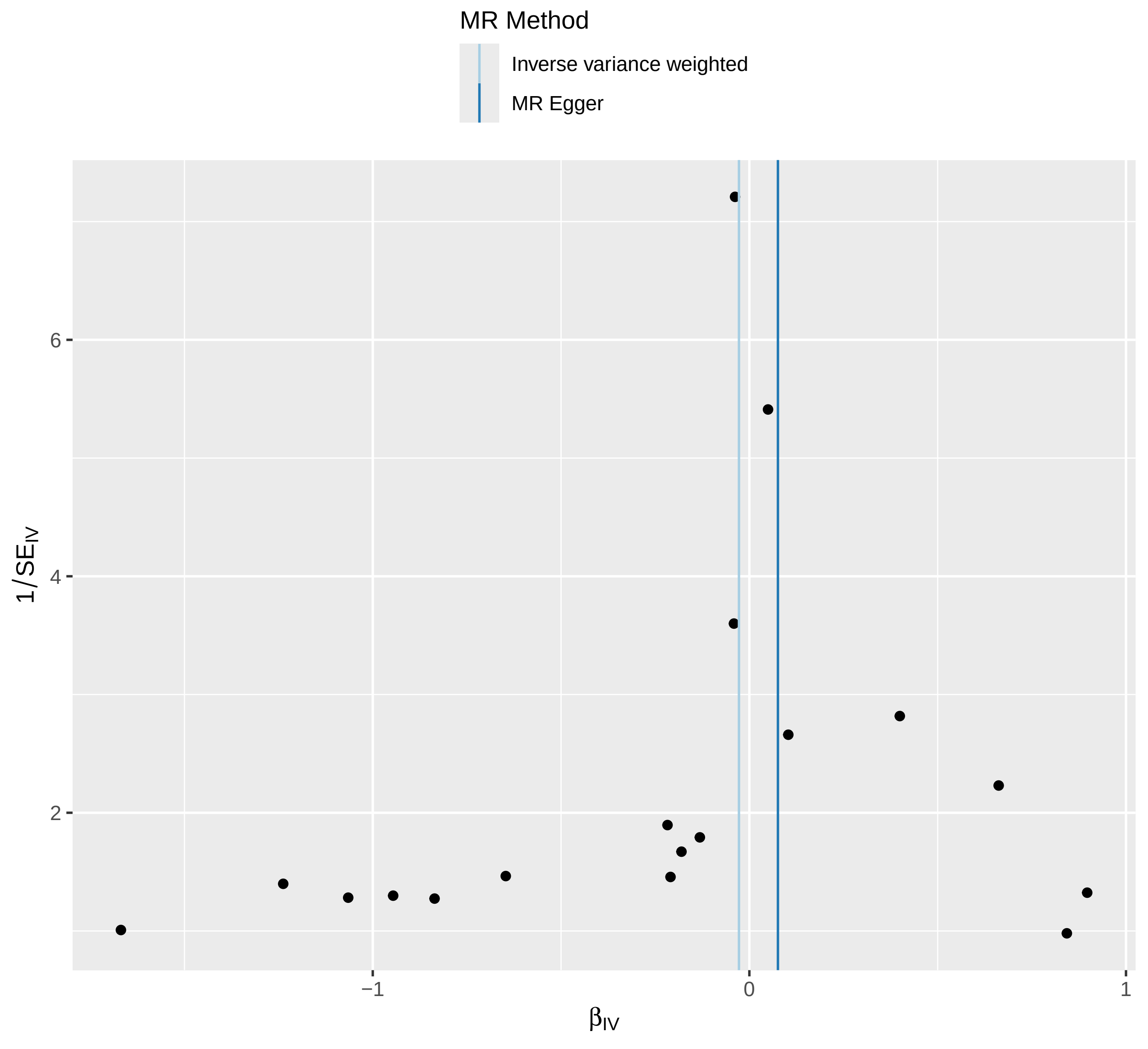

MR Method
Inverse variance weighted
MR Egger
1/SE_IV
6
4
2
−1
0
1
β_IV

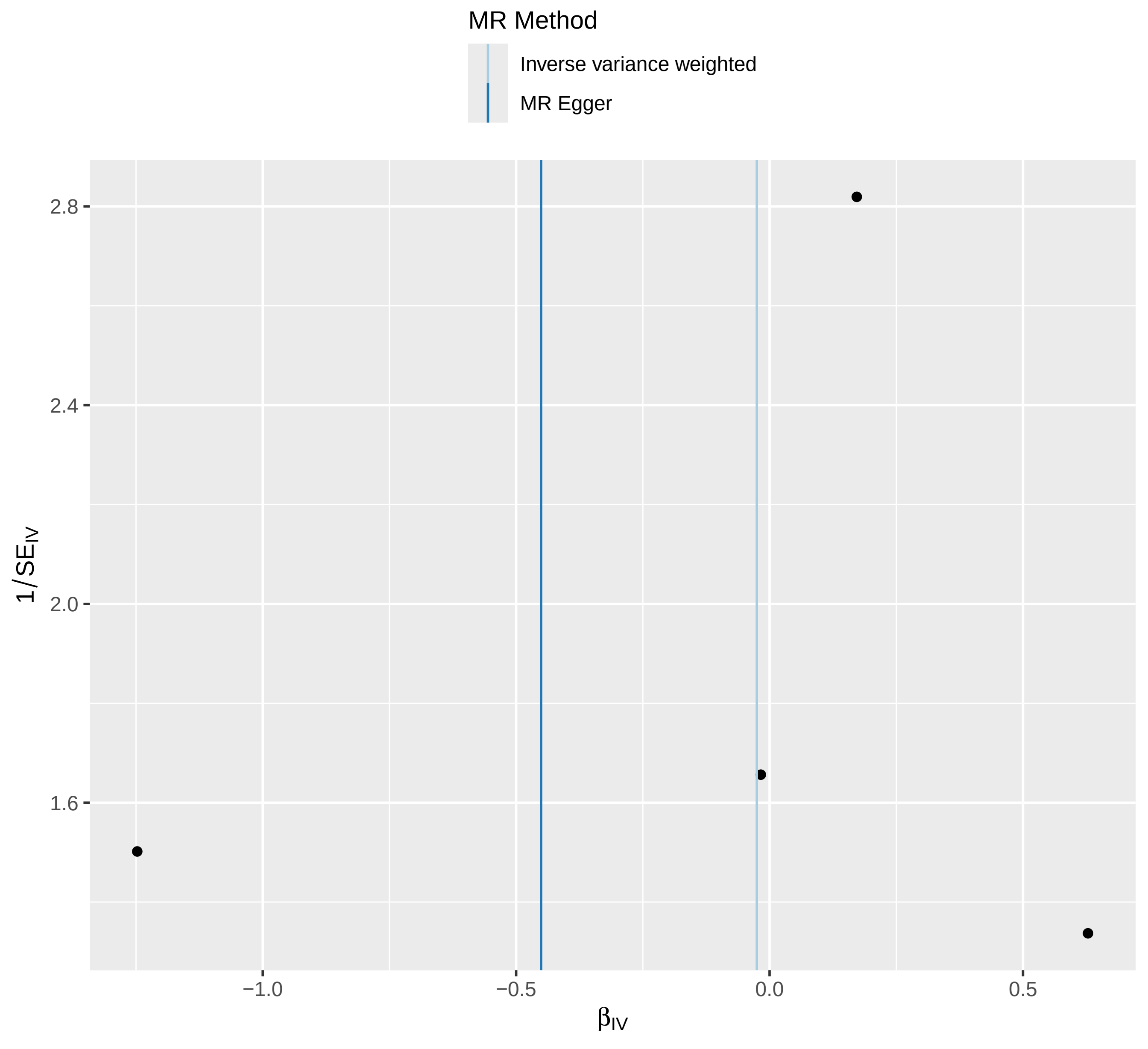
MR Method
Inverse variance weighted
MR Egger
2.8
2.4
2.0
1.6
1/SE_IV
−1.0
−0.5
0.0
0.5
β_IV

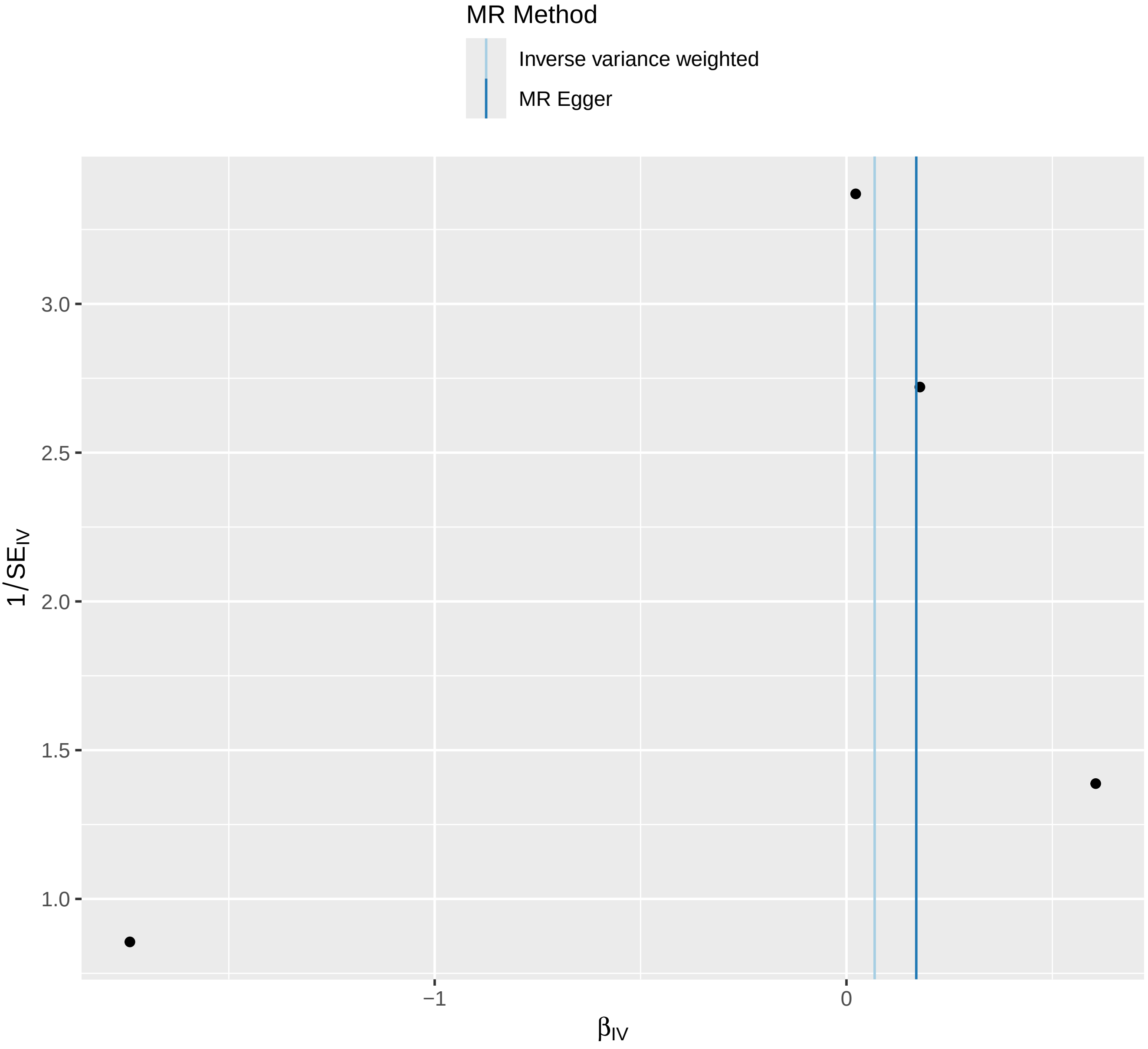

MR Method
Inverse variance weighted
MR Egger
3.0
2.5
2.0
1.5
1.0
1/SE_IV
−1
0
β_IV

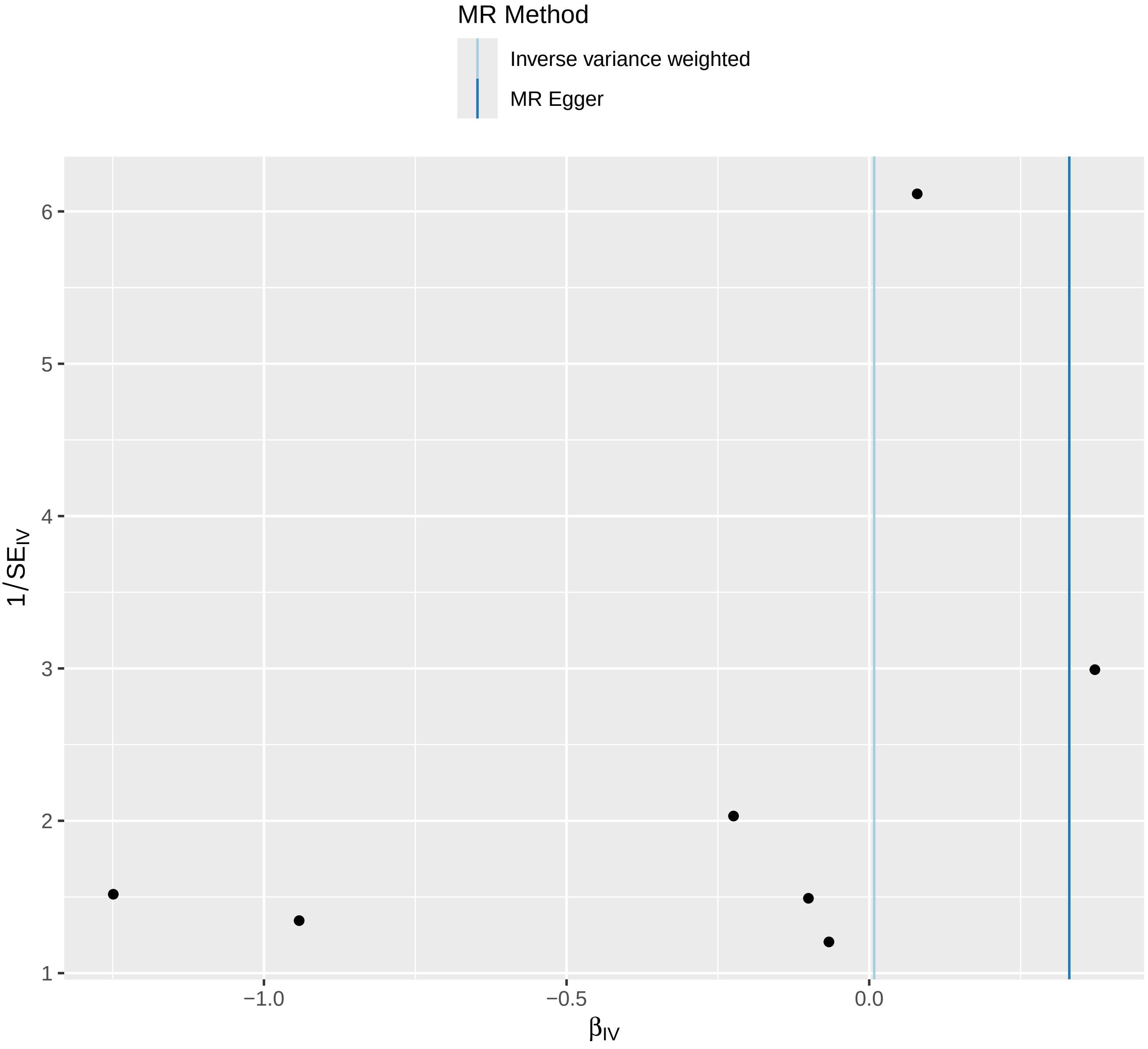

MR Method
Inverse variance weighted
MR Egger
6
5
4
3
2
1
1/SE_IV
−1.0
−0.5
0.0
β_IV

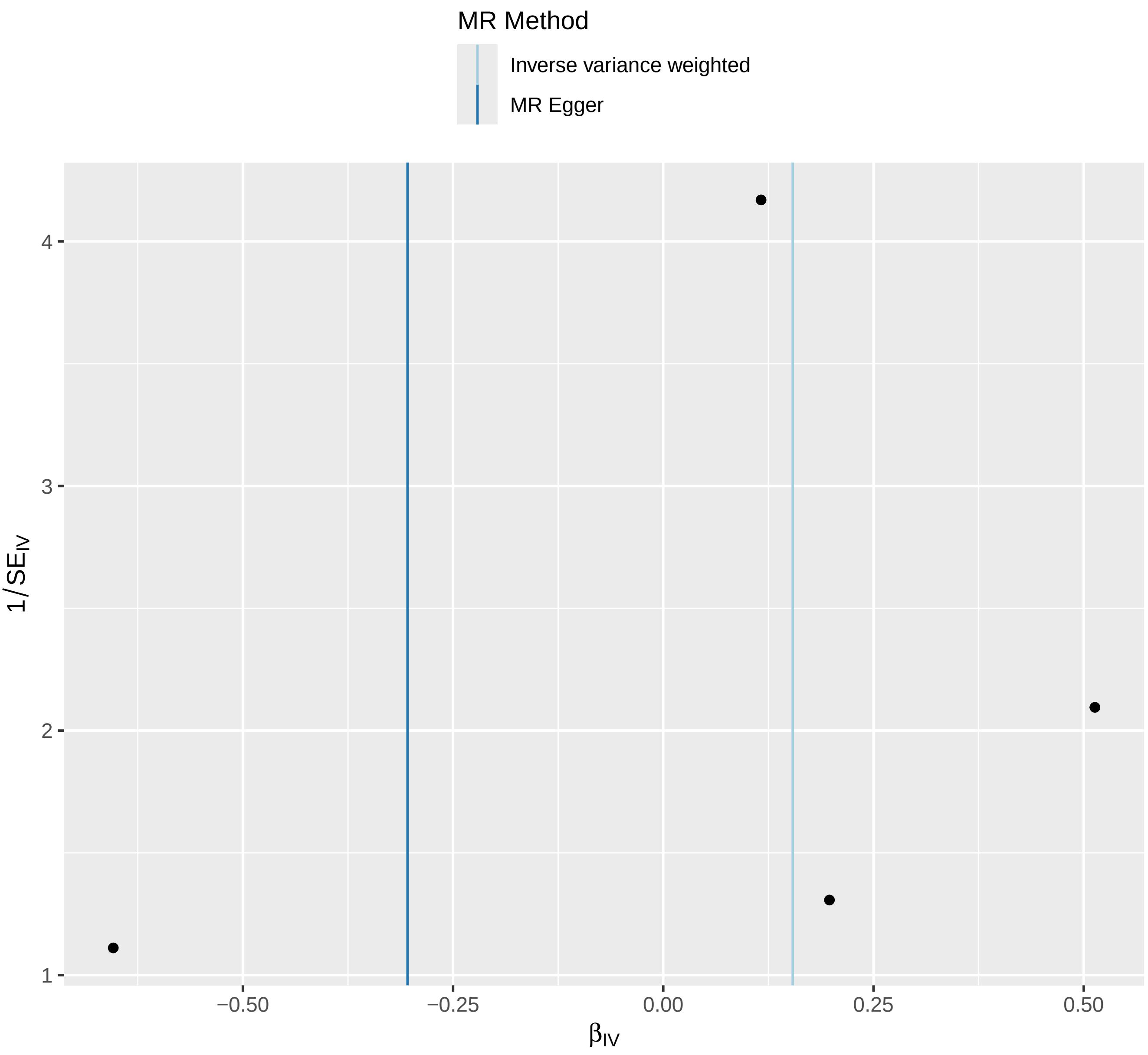
MR Method
Inverse variance weighted
MR Egger
1/SE_IV
4
3
2
1
−0.50
−0.25
0.00
0.25
0.50
β_IV

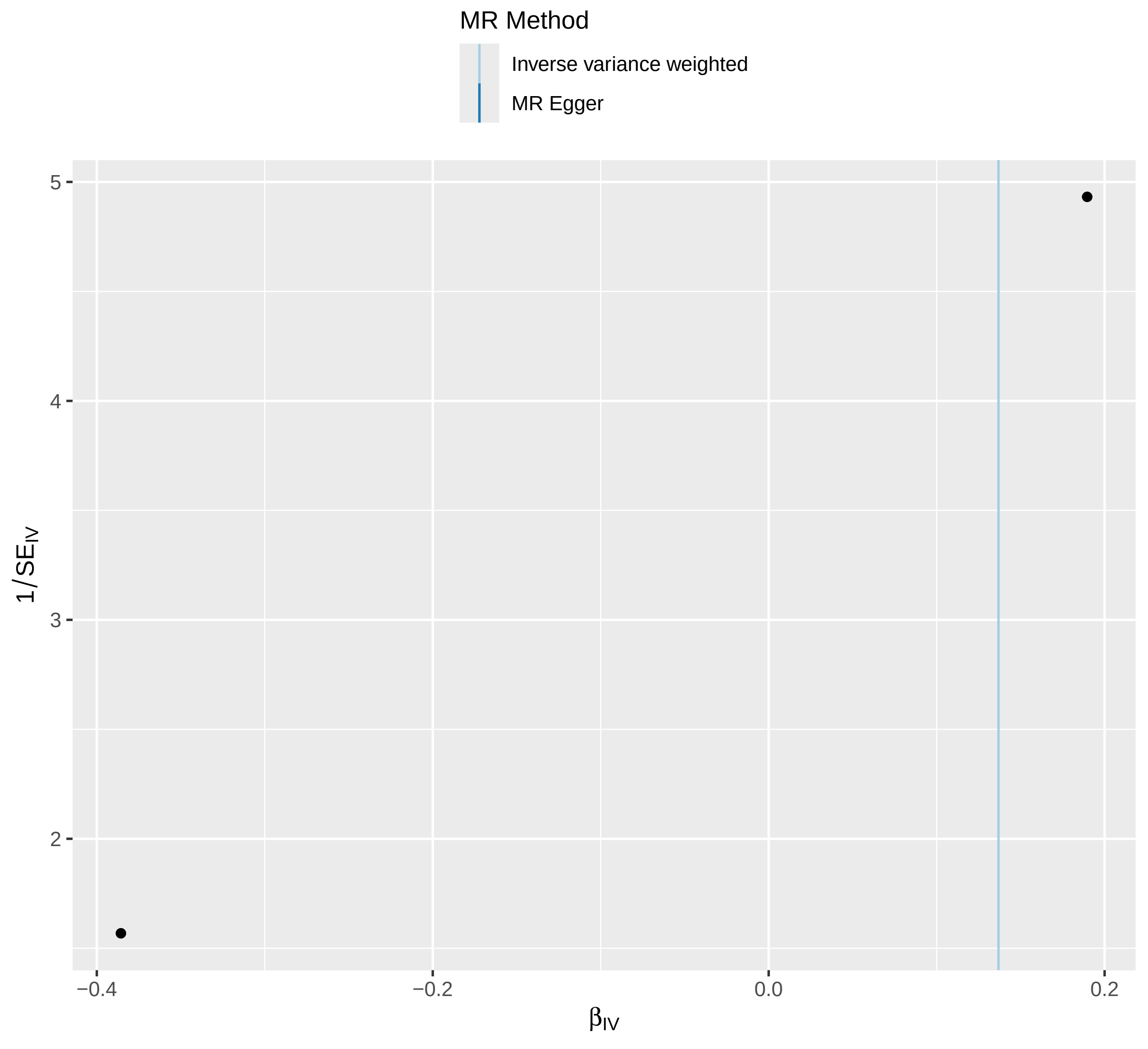
MR Method
Inverse variance weighted
MR Egger
1/SE_IV
5
4
3
2
−0.4
−0.2
0.0
0.2
β_IV

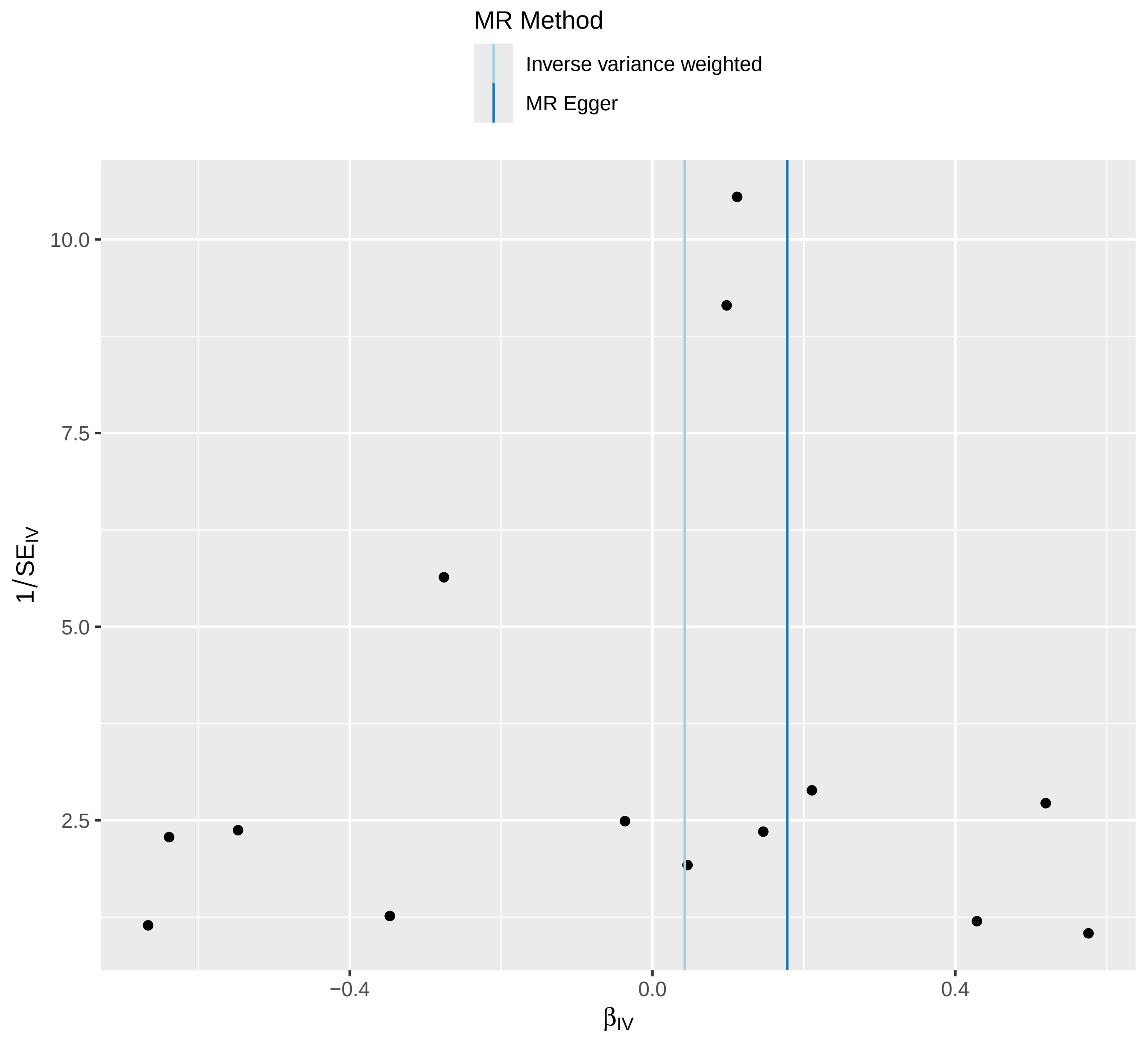

MR Method
Inverse variance weighted
MR Egger
10.0
7.5
5.0
2.5
1/SE_IV
−0.4
0.0
0.4
β_IV

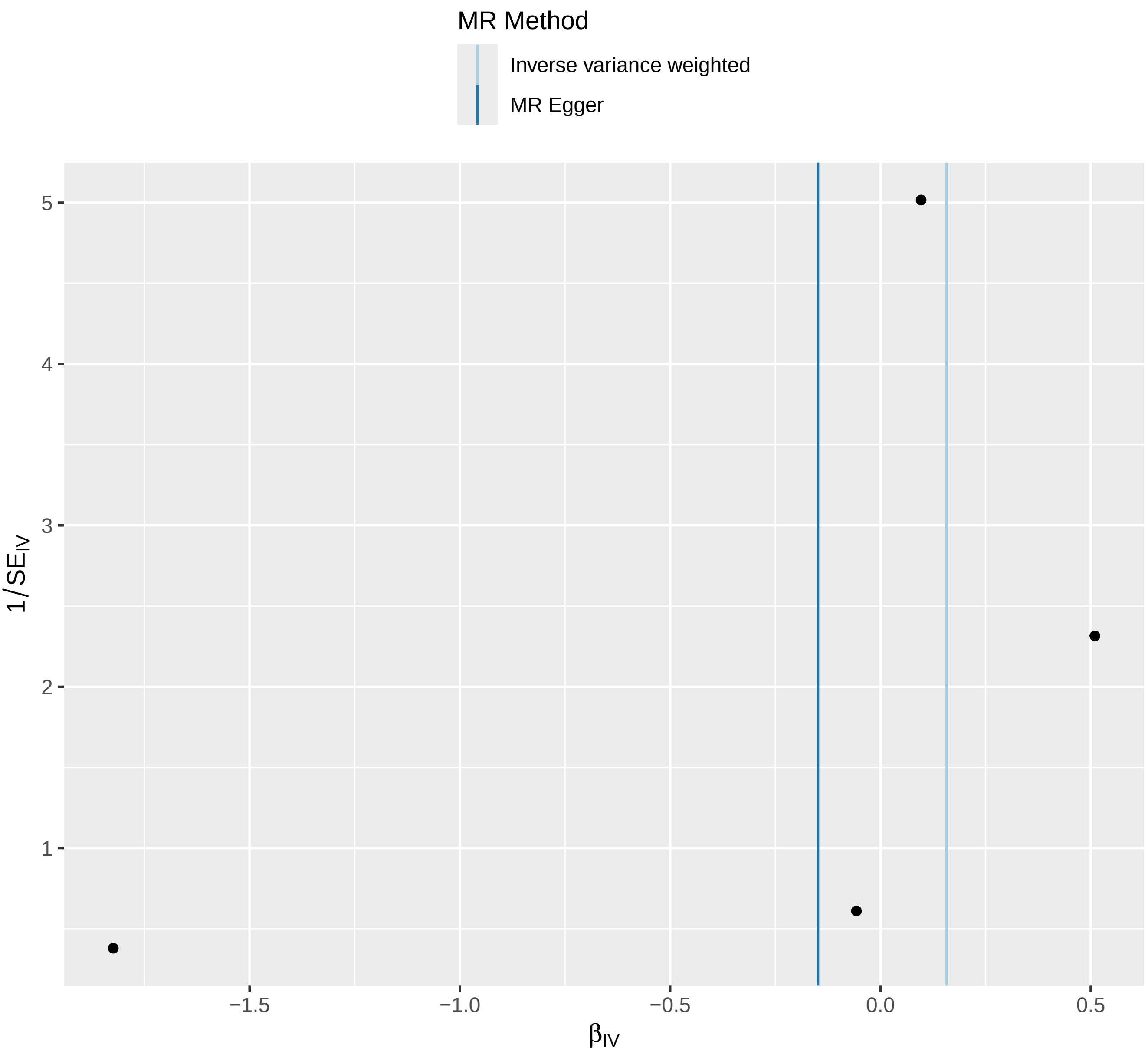

MR Method
Inverse variance weighted
MR Egger
1/SE$_{IV}$
5
4
3
2
1
−1.5
−1.0
−0.5
0.0
0.5
β_{IV}

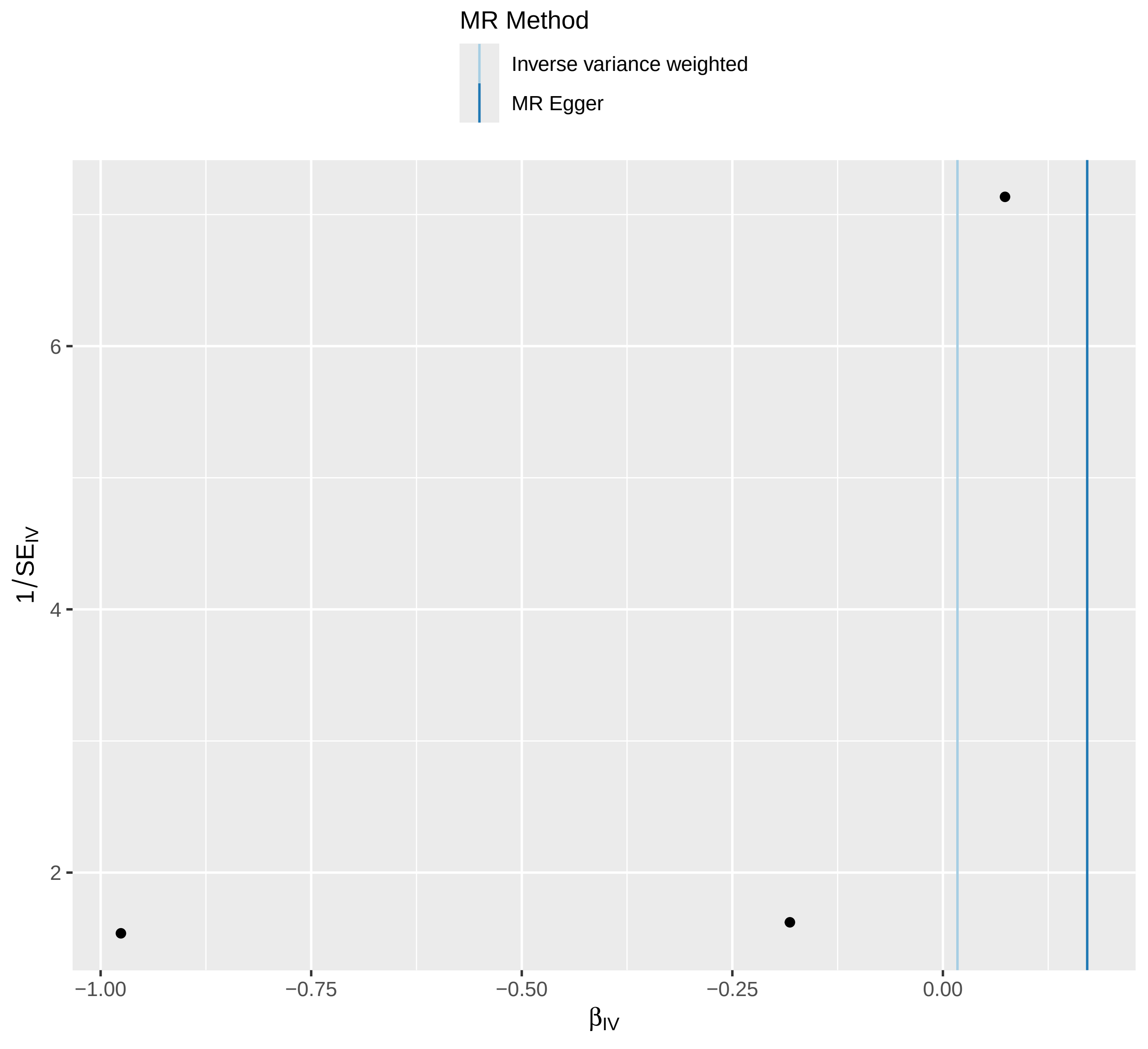
MR Method
Inverse variance weighted
MR Egger
1/SE_IV
6
4
2
−1.00
−0.75
−0.50
−0.25
0.00
β_IV

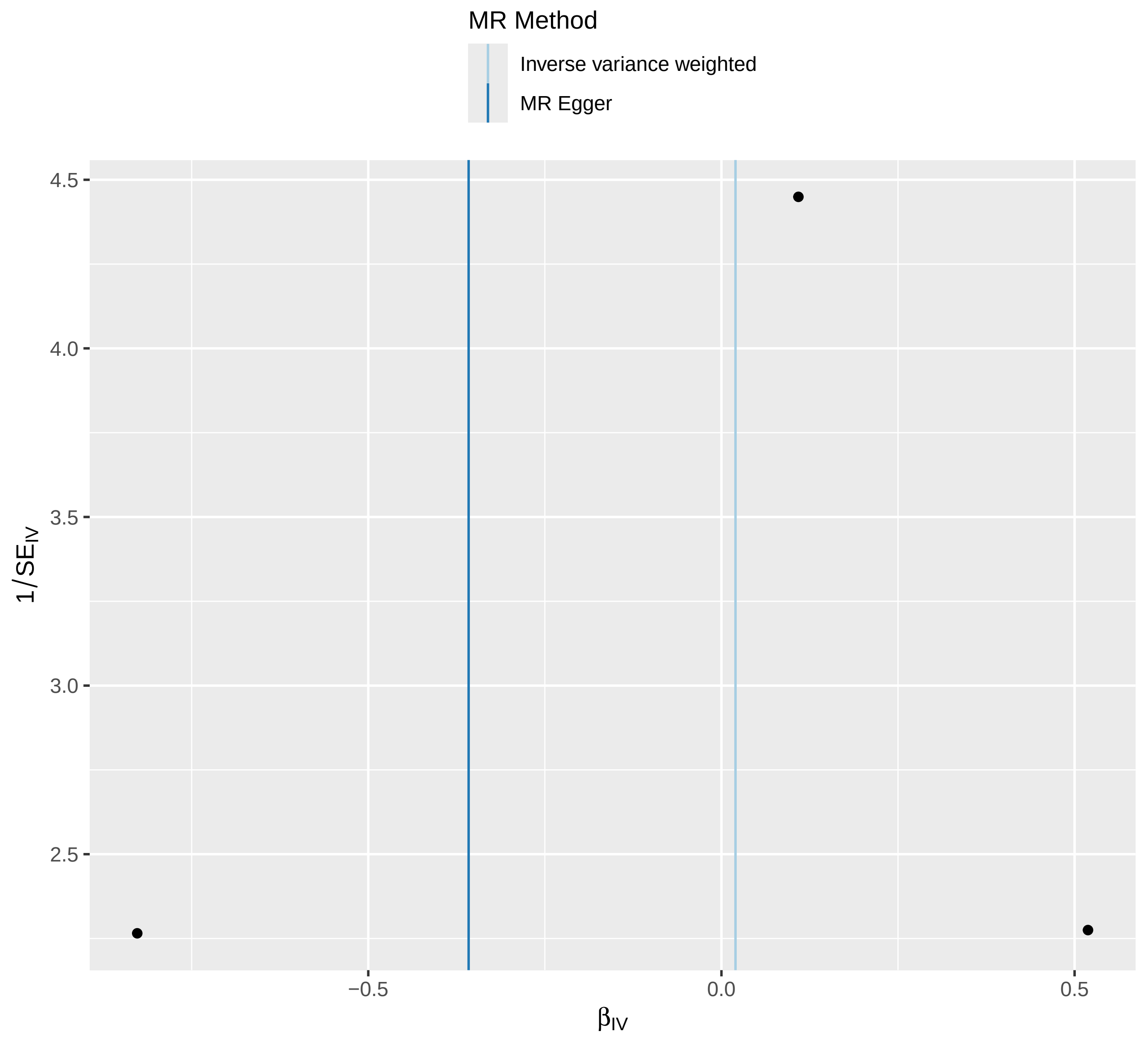
MR Method
Inverse variance weighted
MR Egger
4.5
4.0
3.5
3.0
2.5
1/SE_IV
−0.5
0.0
0.5
β_IV

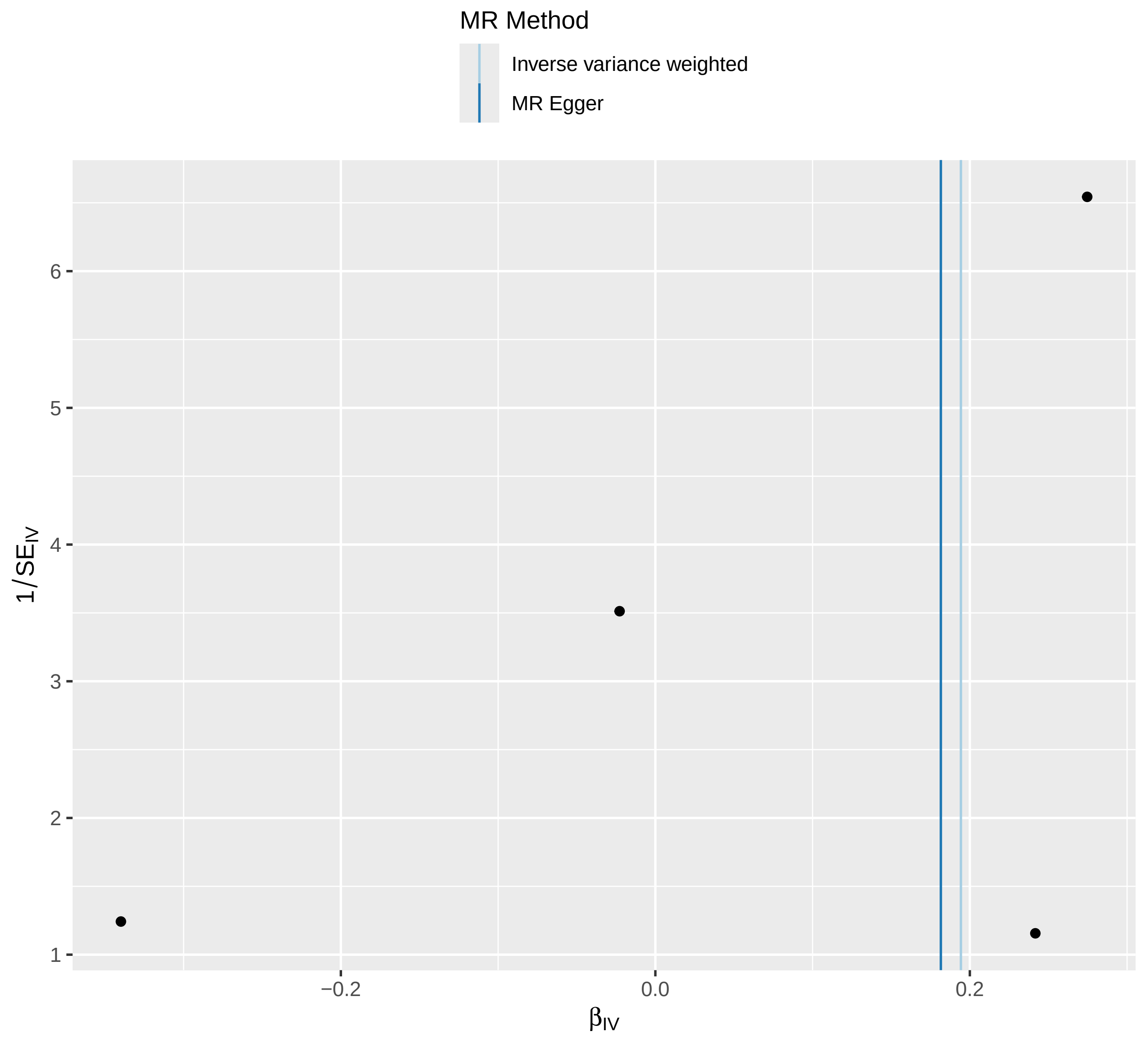
MR Method
Inverse variance weighted
MR Egger
6
5
4
3
2
1
1/SE_IV
−0.2
0.0
0.2
β_IV

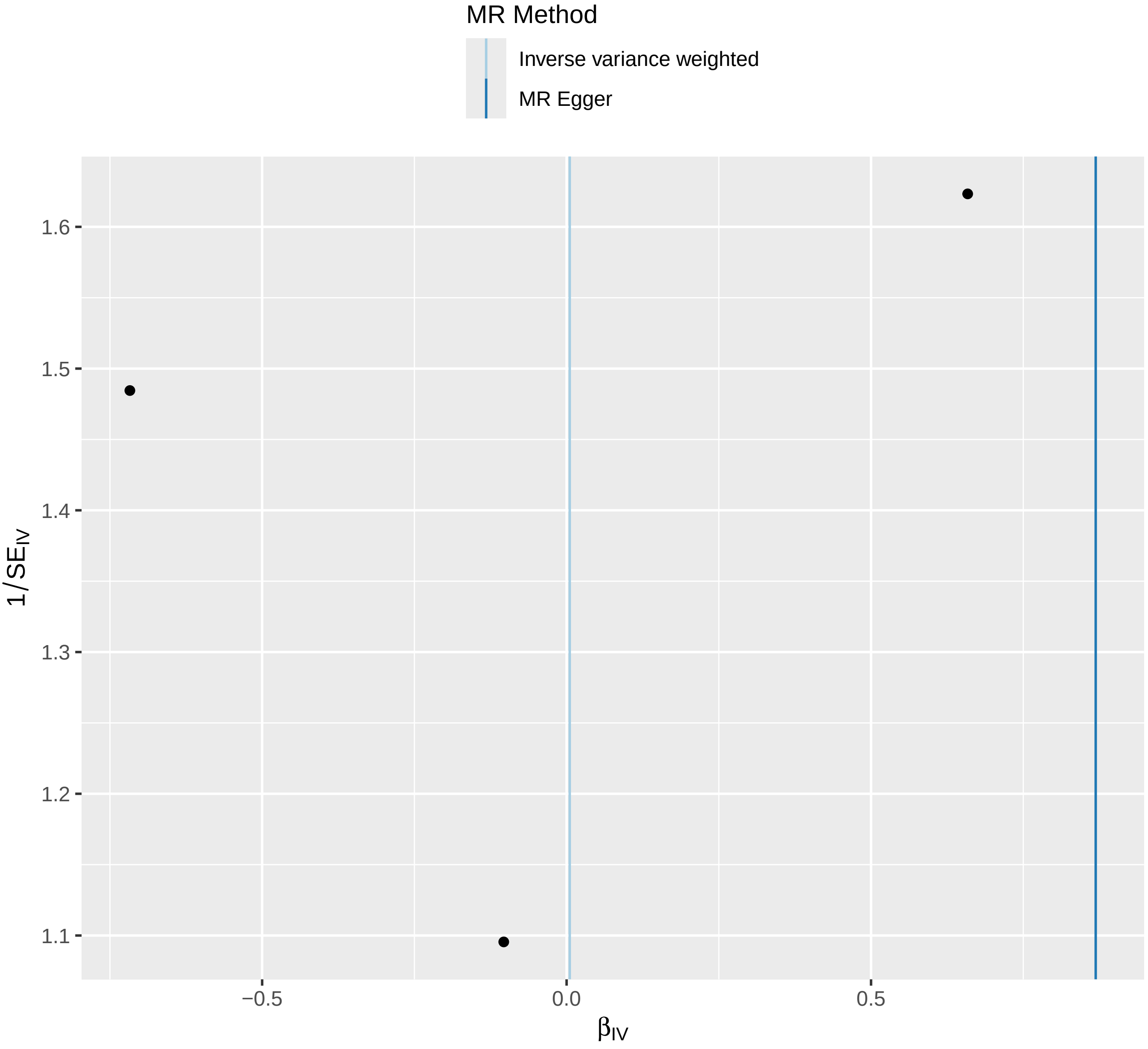

MR Method
Inverse variance weighted
MR Egger
1.6
1.5
1.4
1.3
1.2
1.1
$1/SE_{IV}$
−0.5
0.0
0.5
β_{IV}

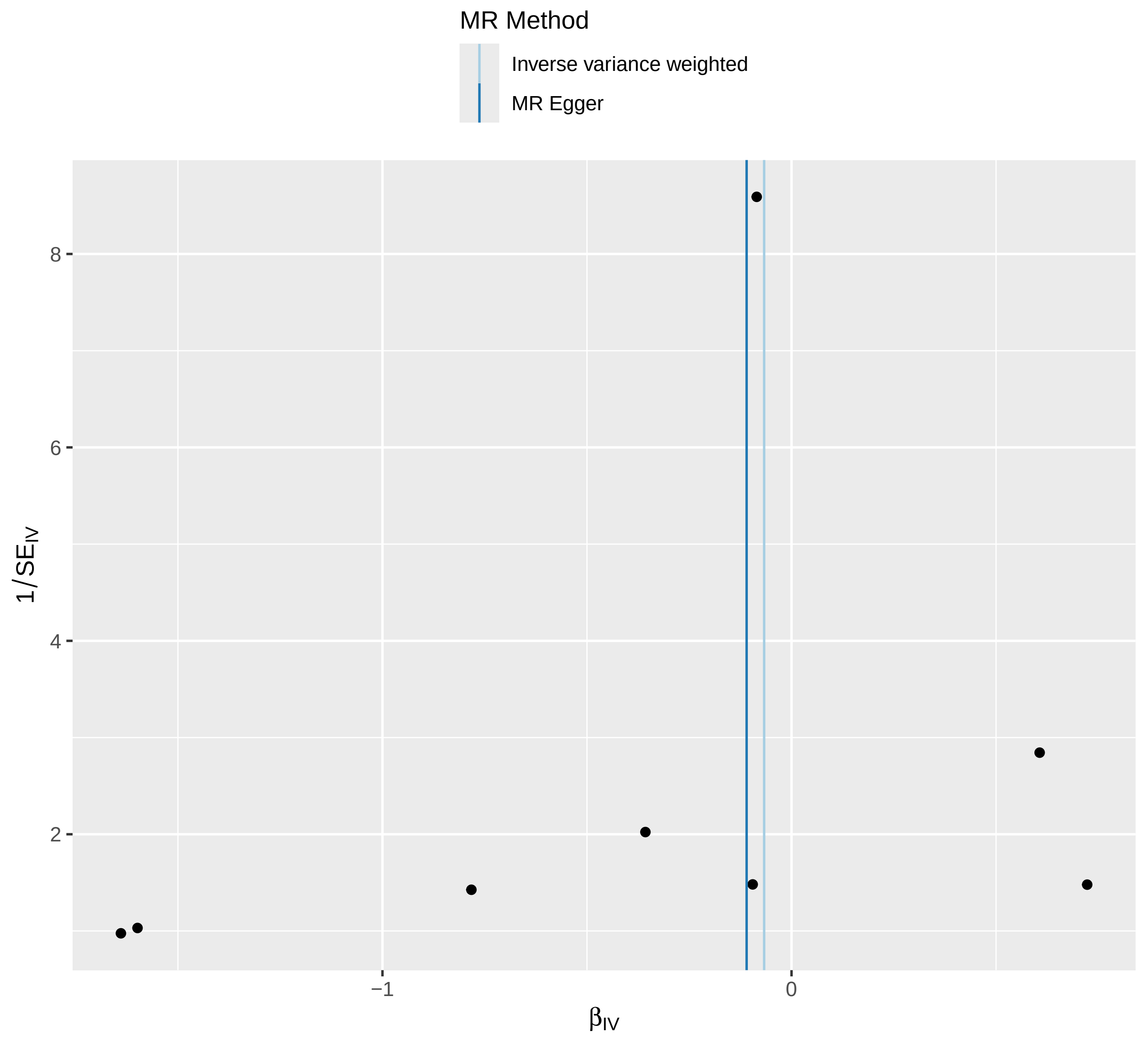
MR Method
Inverse variance weighted
MR Egger
8
6
4
2
1/SE_IV
−1
0
β_IV

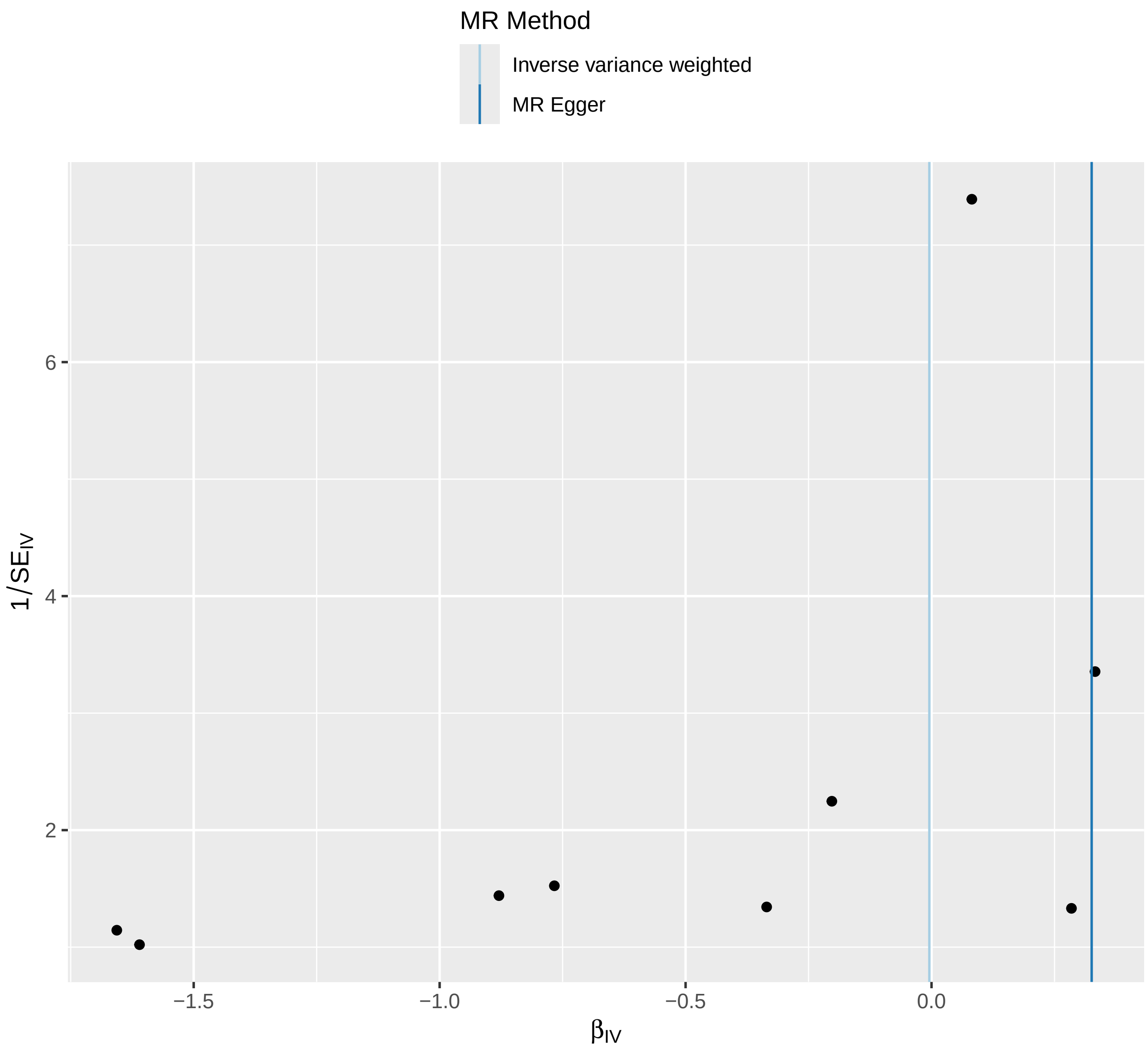
MR Method
Inverse variance weighted
MR Egger
6
4
2
$1/SE_{IV}$
−1.5
−1.0
−0.5
0.0
β_{IV}

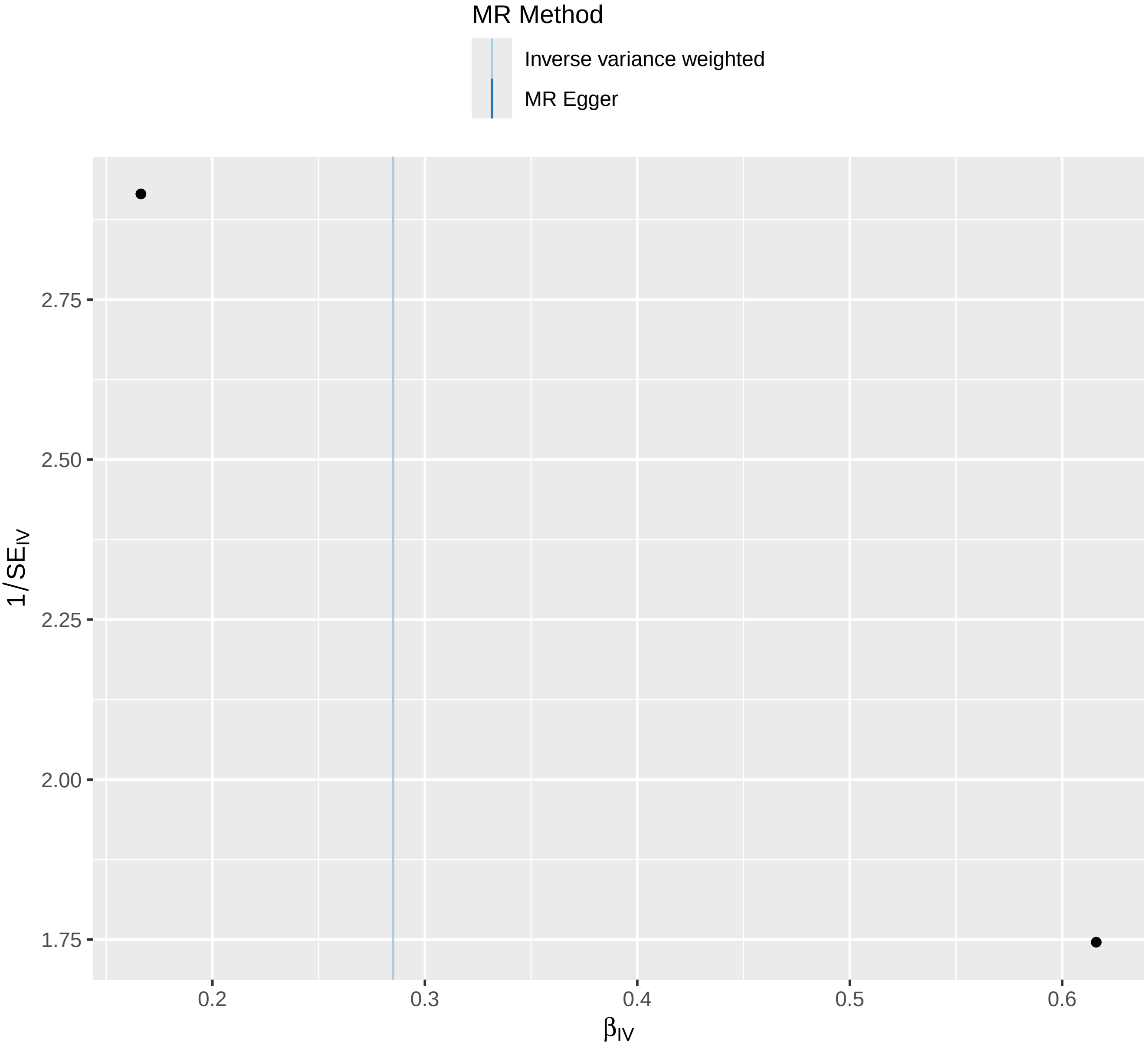
MR Method
Inverse variance weighted
MR Egger
2.75
2.50
2.25
2.00
1.75
1/SE_IV
0.2
0.3
0.4
0.5
0.6
β_IV

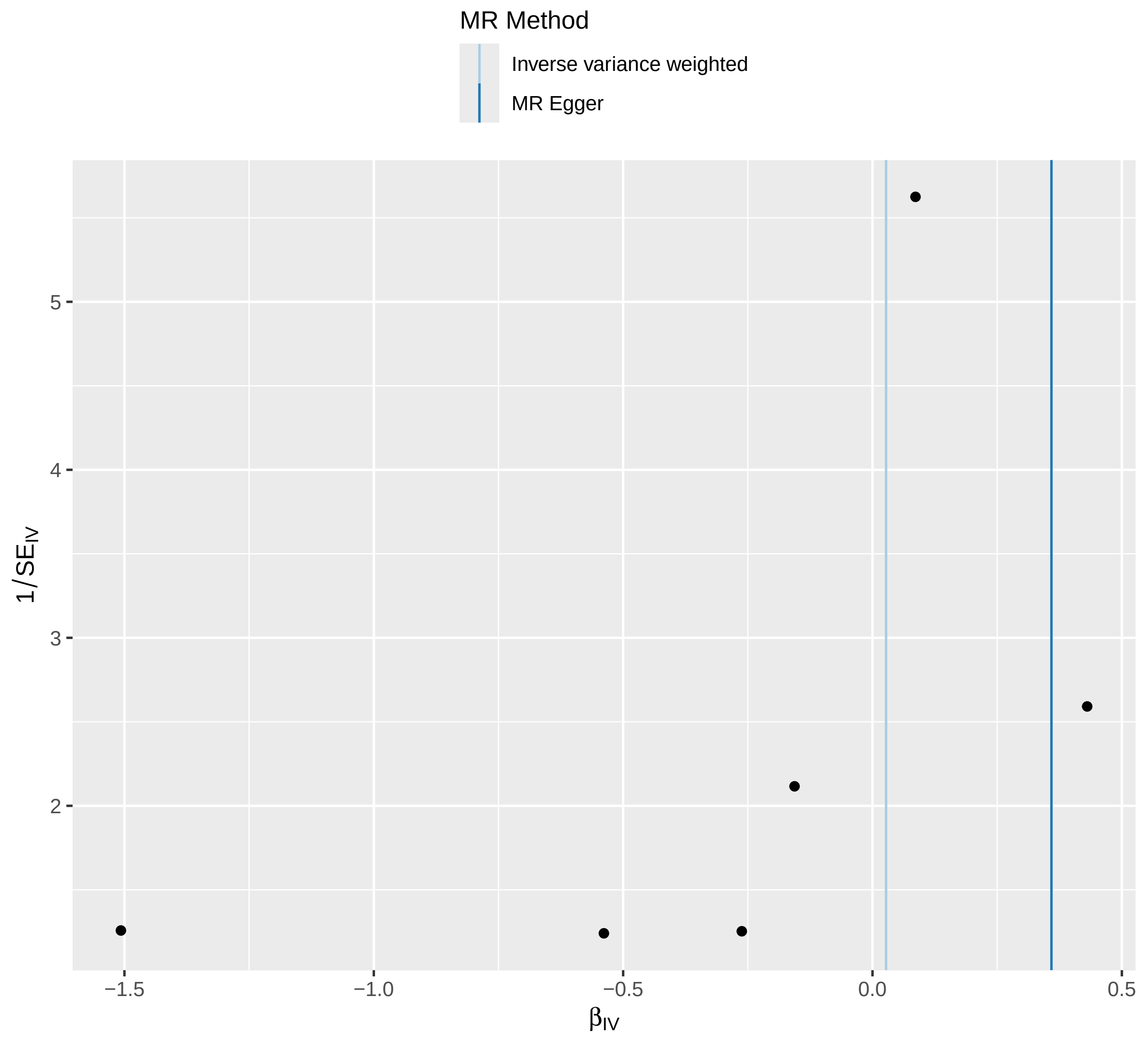
MR Method
Inverse variance weighted
MR Egger
1/SE_IV
β_IV
5
4
3
2
−1.5
−1.0
−0.5
0.0
0.5

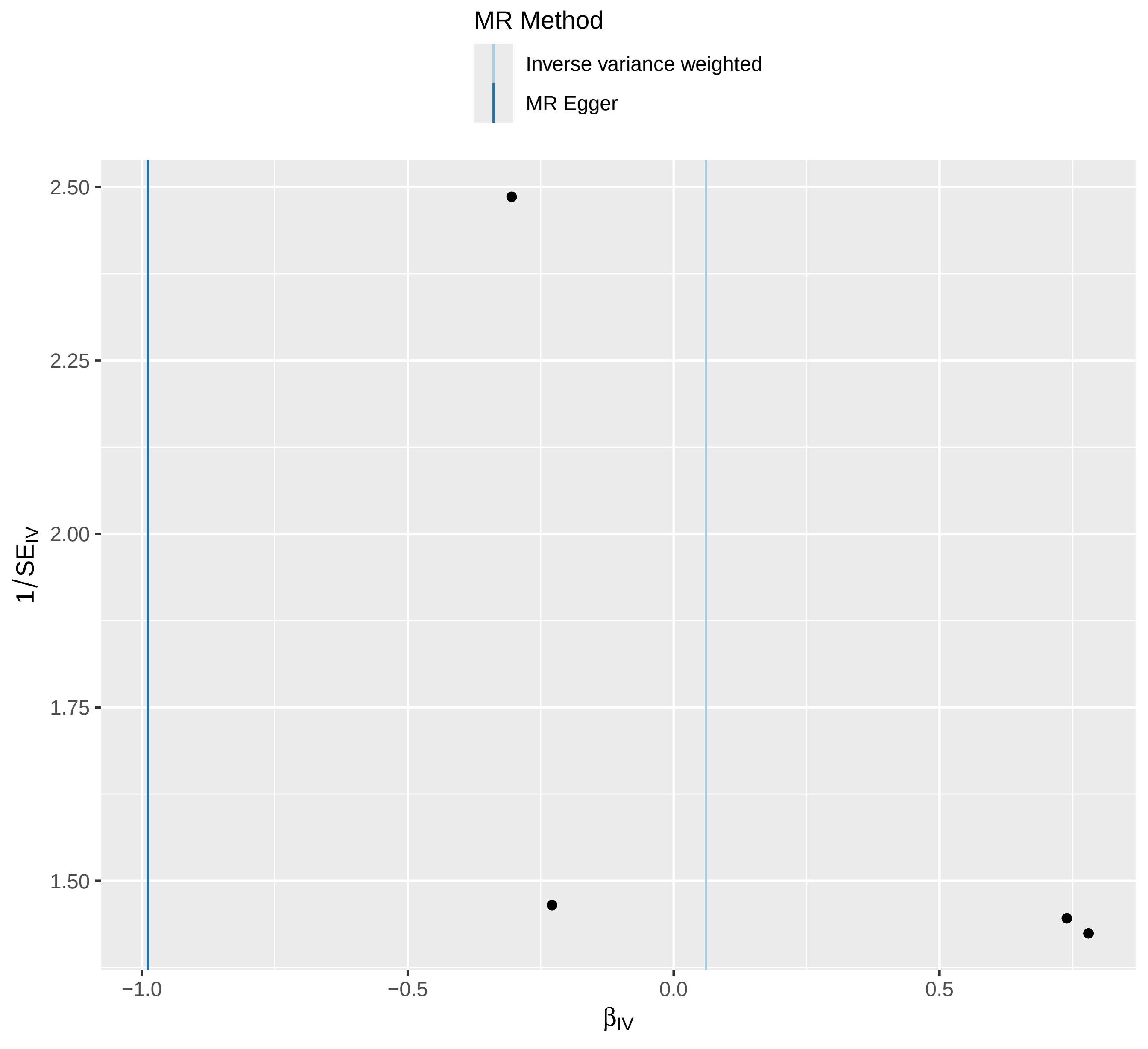
MR Method
Inverse variance weighted
MR Egger
2.50
2.25
2.00
1.75
1.50
$1/SE_{IV}$
−1.0
−0.5
0.0
0.5
β_{IV}

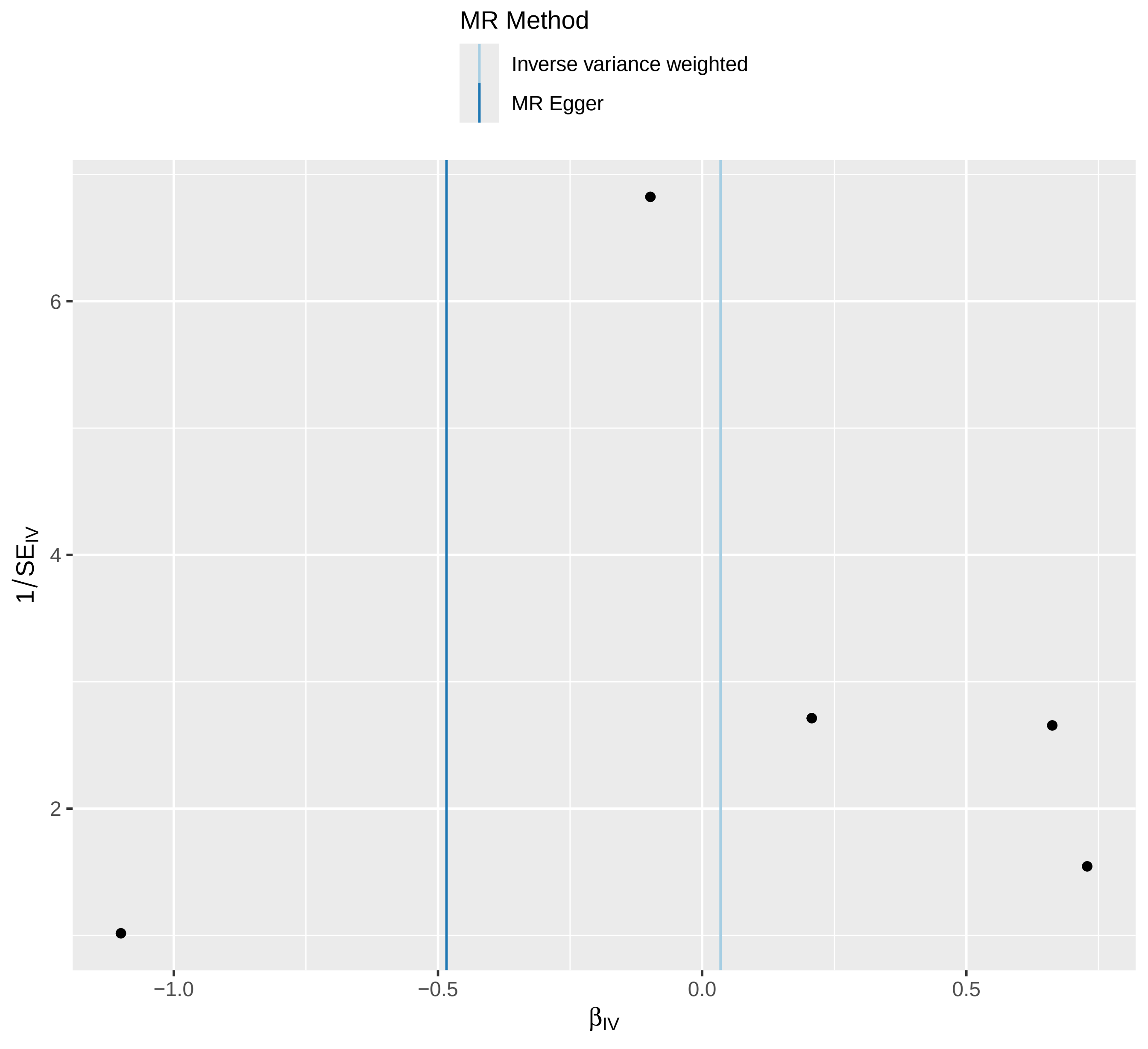

MR Method
Inverse variance weighted
MR Egger
6
4
2
1/SE_{IV}
−1.0
−0.5
0.0
0.5
β_{IV}

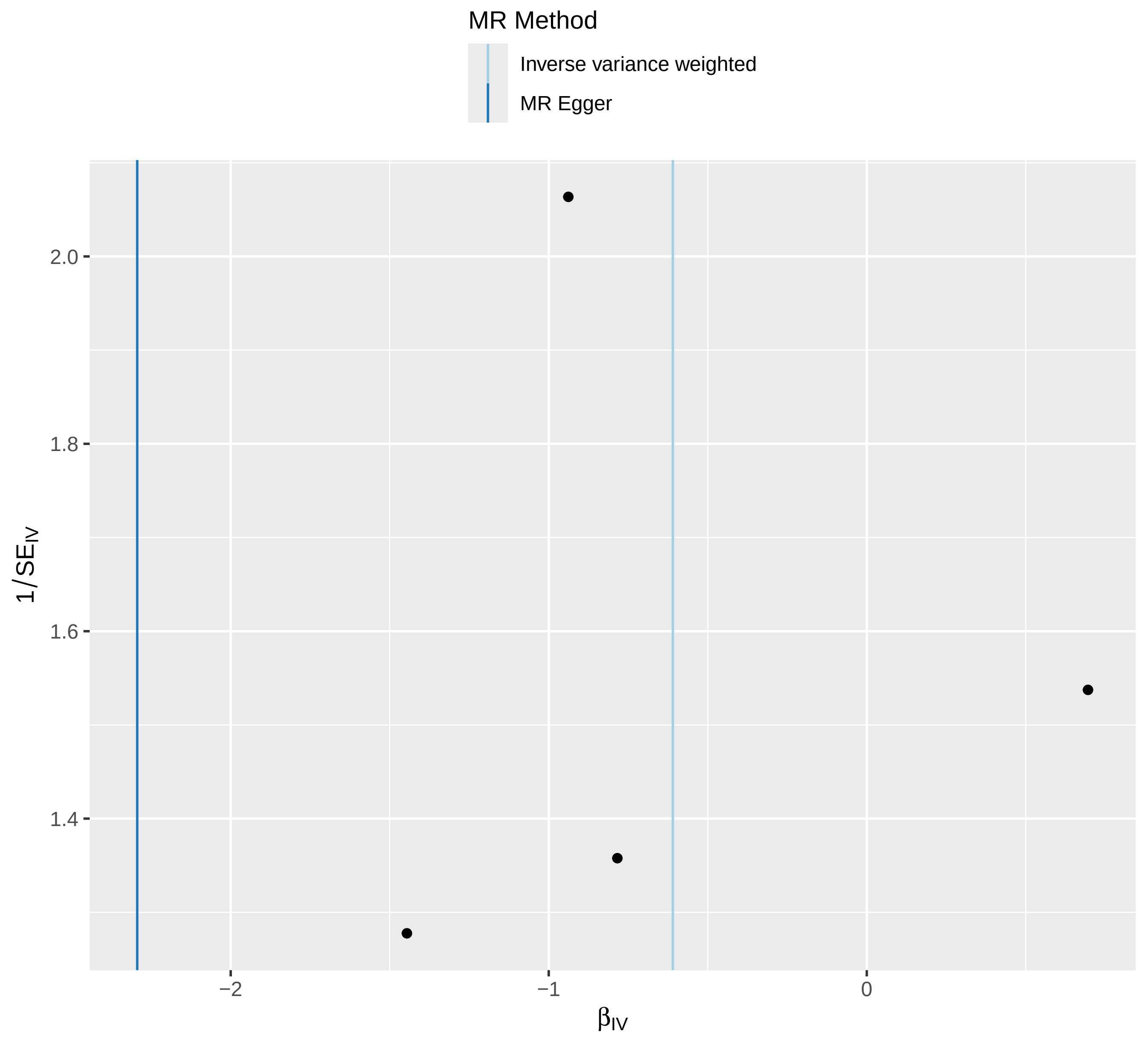

MR Method
Inverse variance weighted
MR Egger
2.0
1.8
1.6
1.4
−2
−1
0
1/SE_IV
β_IV

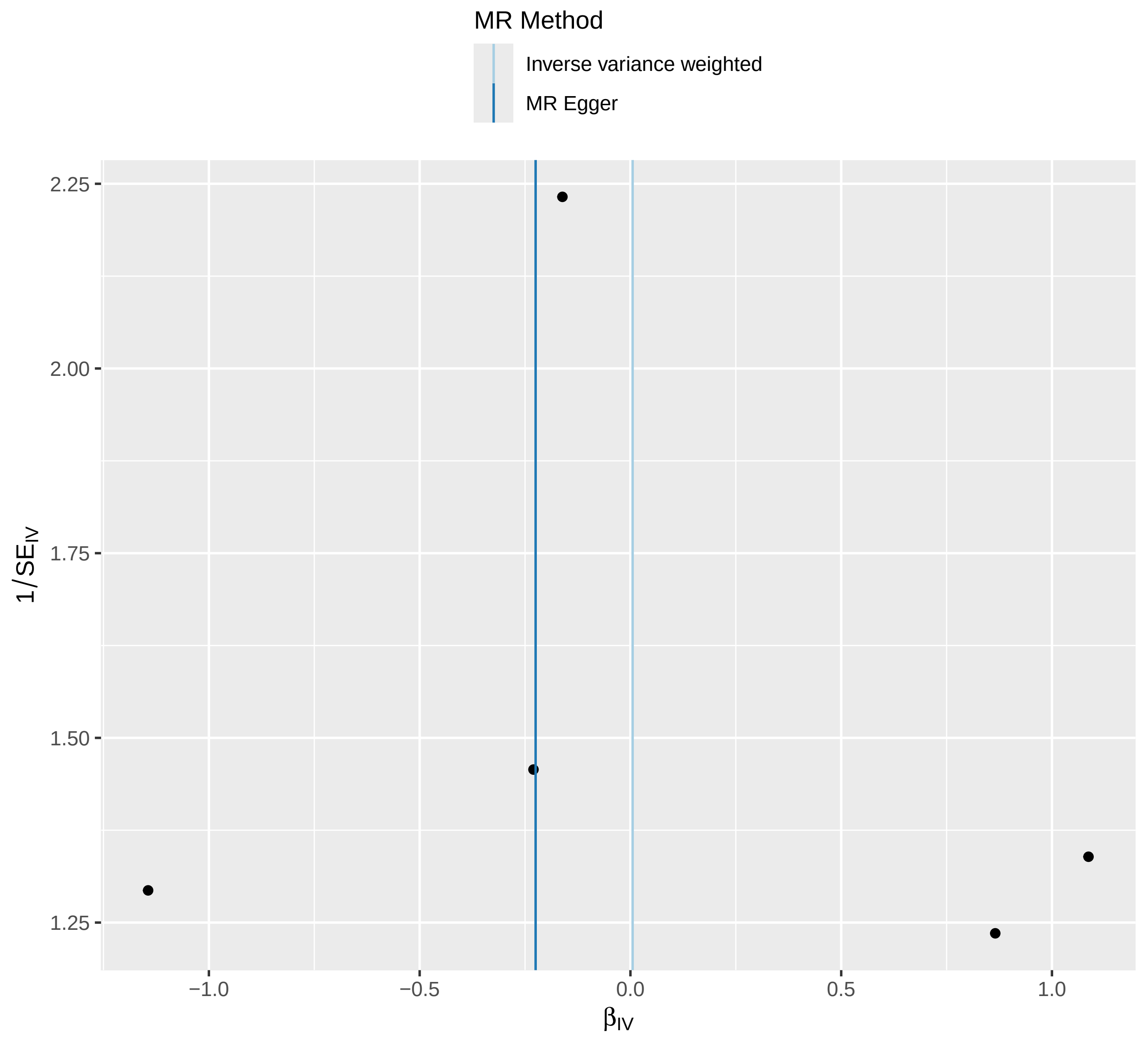
MR Method
Inverse variance weighted
MR Egger
2.25
2.00
1.75
1.50
1.25
$1/SE_{IV}$
−1.0
−0.5
0.0
0.5
1.0
β_{IV}

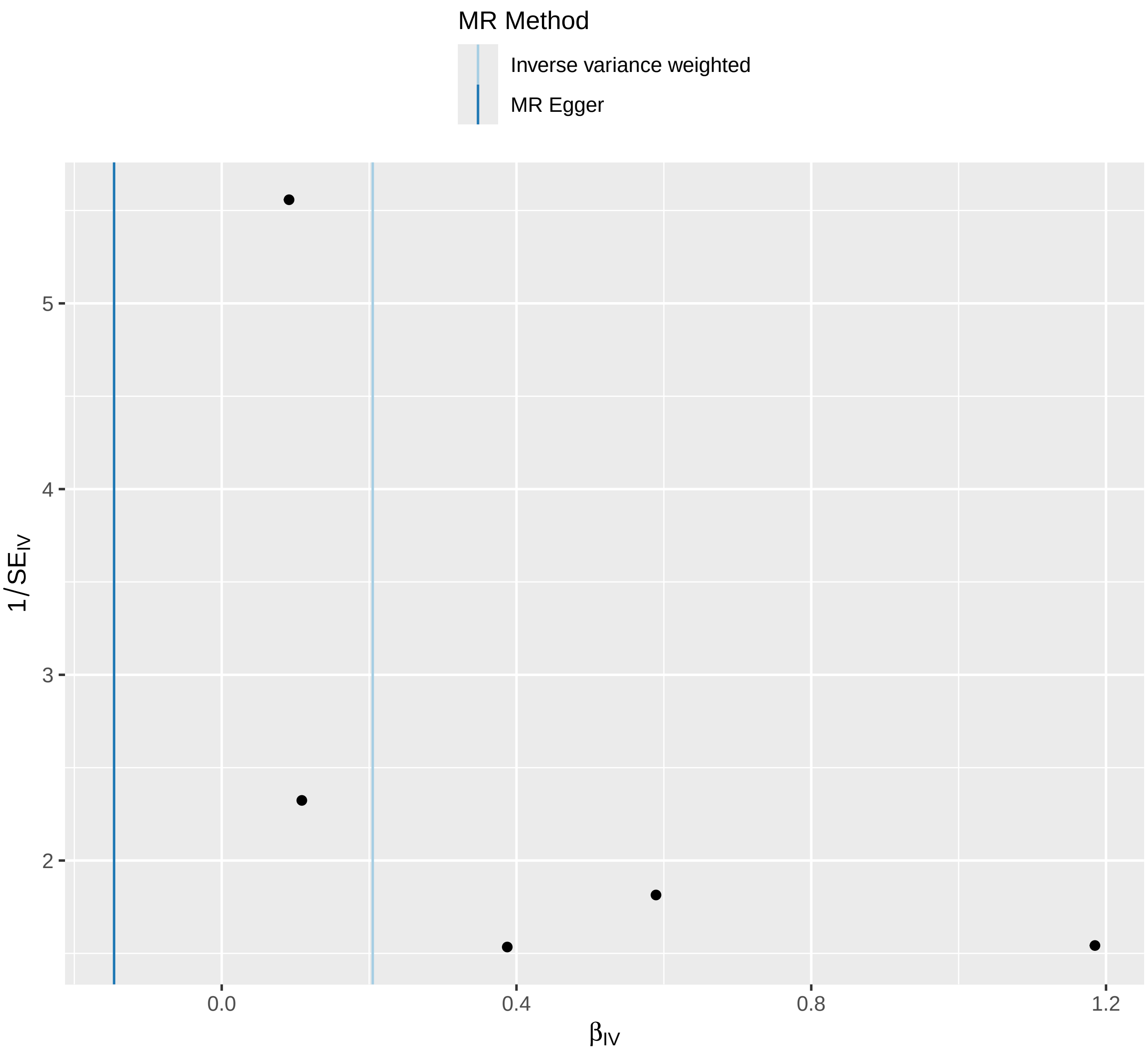
MR Method
Inverse variance weighted
MR Egger
1/SE_{IV}
5
4
3
2
0.0
0.4
0.8
1.2
β_{IV}

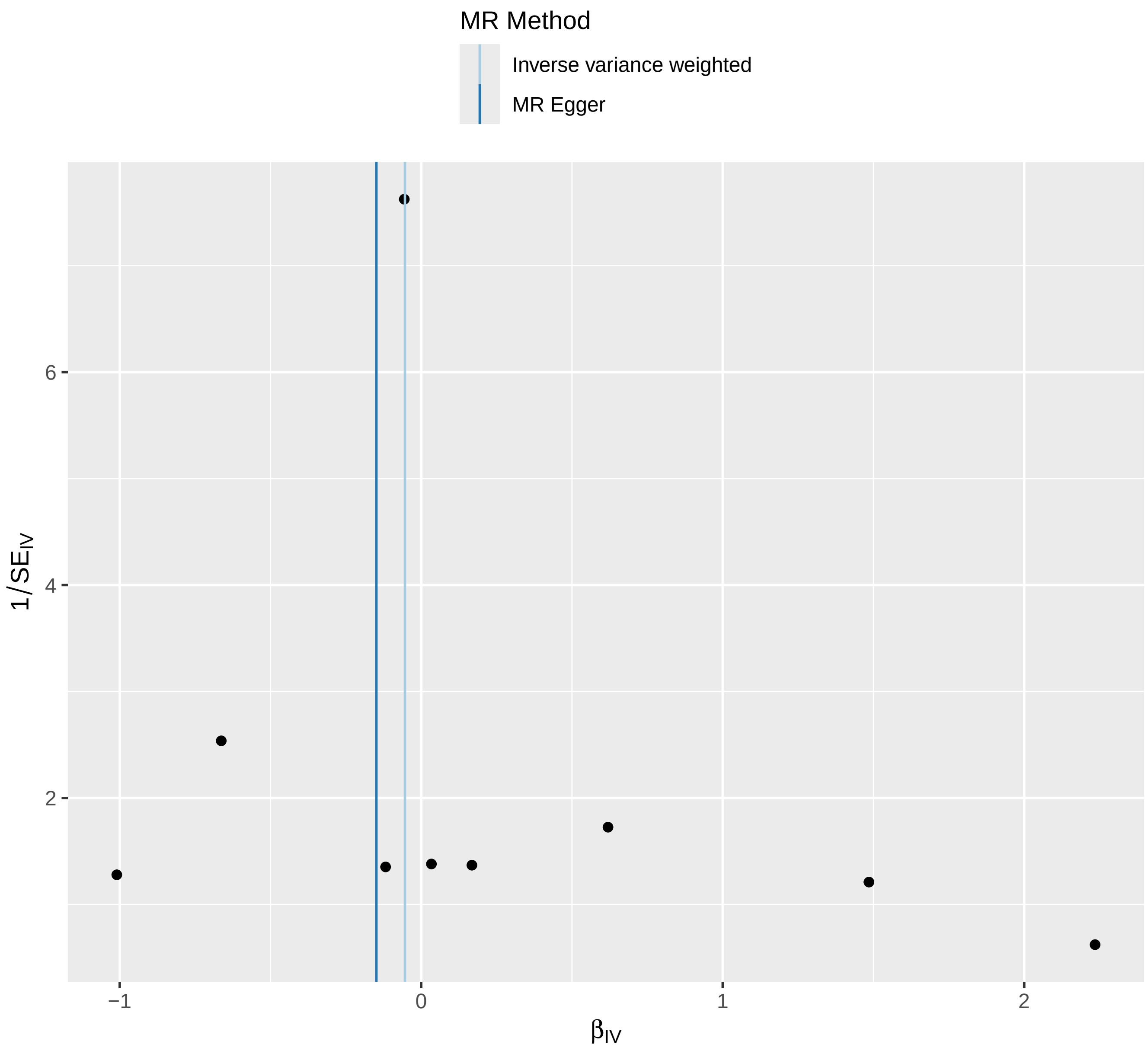
MR Method
Inverse variance weighted
MR Egger
6
4
2
1/SE$_{IV}$
−1
0
1
2
β_{IV}

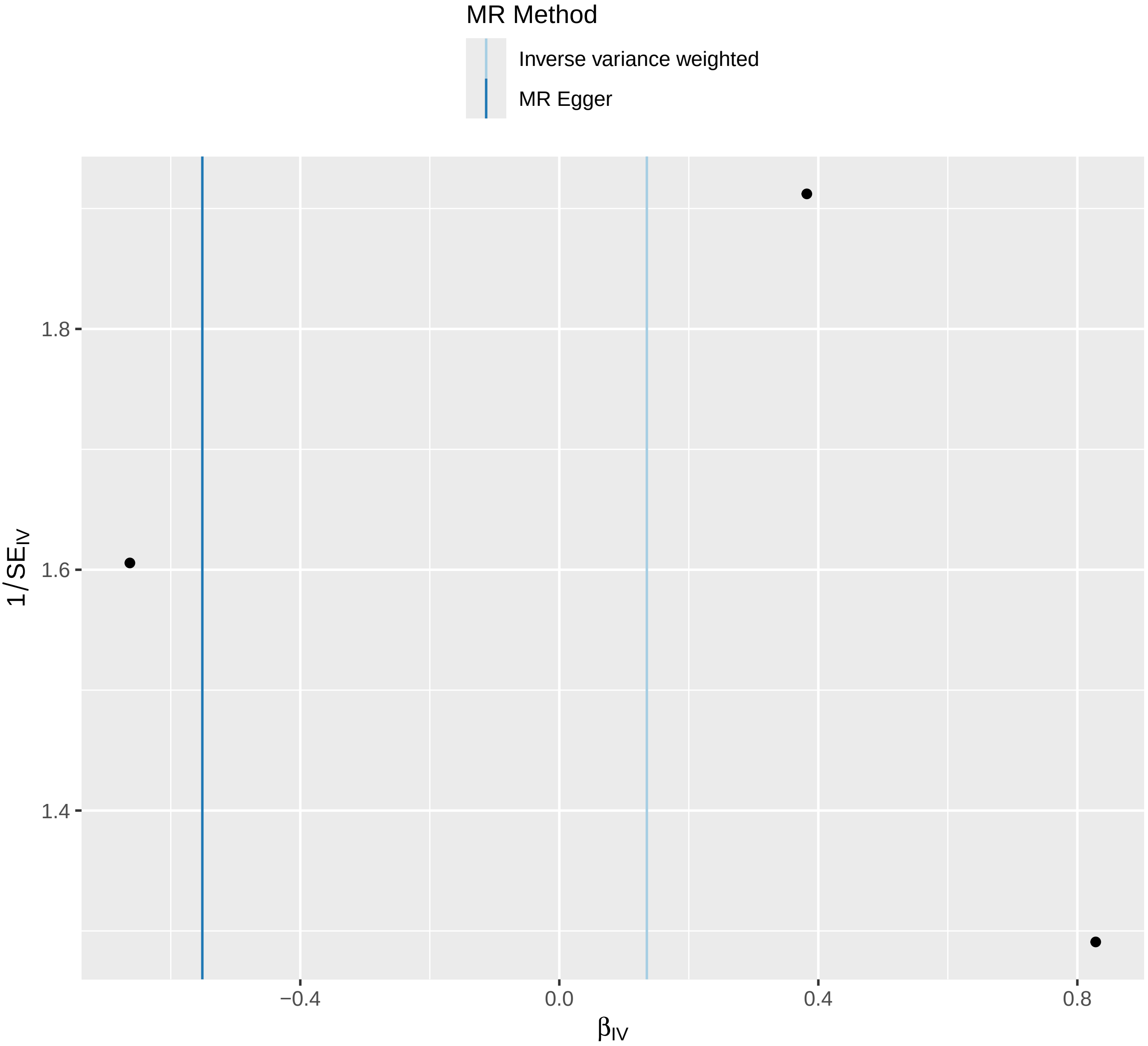
MR Method
Inverse variance weighted
MR Egger
1.8
1.6
1.4
$1/SE_{IV}$
−0.4
0.0
0.4
0.8
β_{IV}

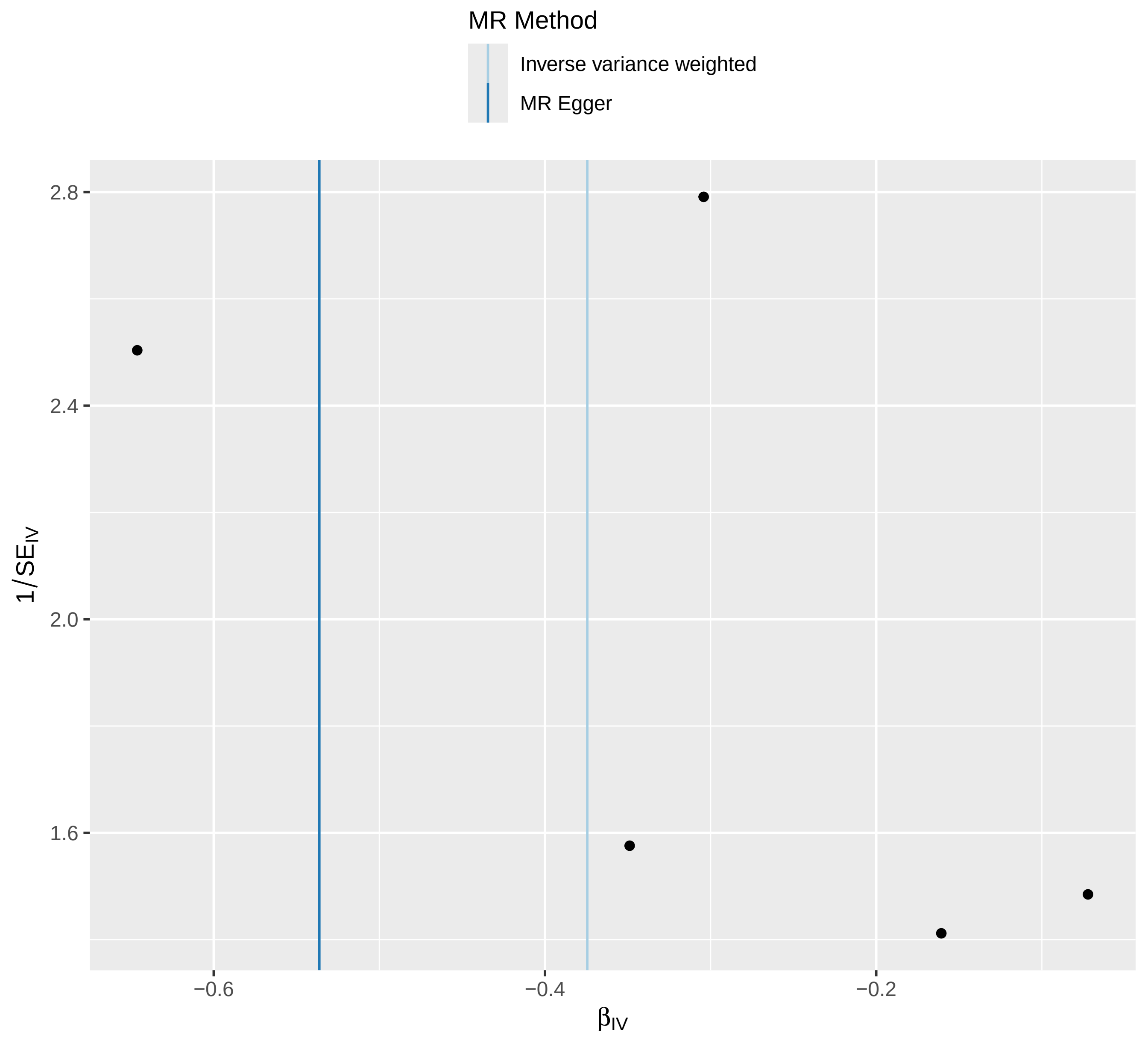
MR Method
Inverse variance weighted
MR Egger
2.8
2.4
2.0
1.6
1/SE$_{IV}$
−0.6
−0.4
−0.2
β$_{IV}$

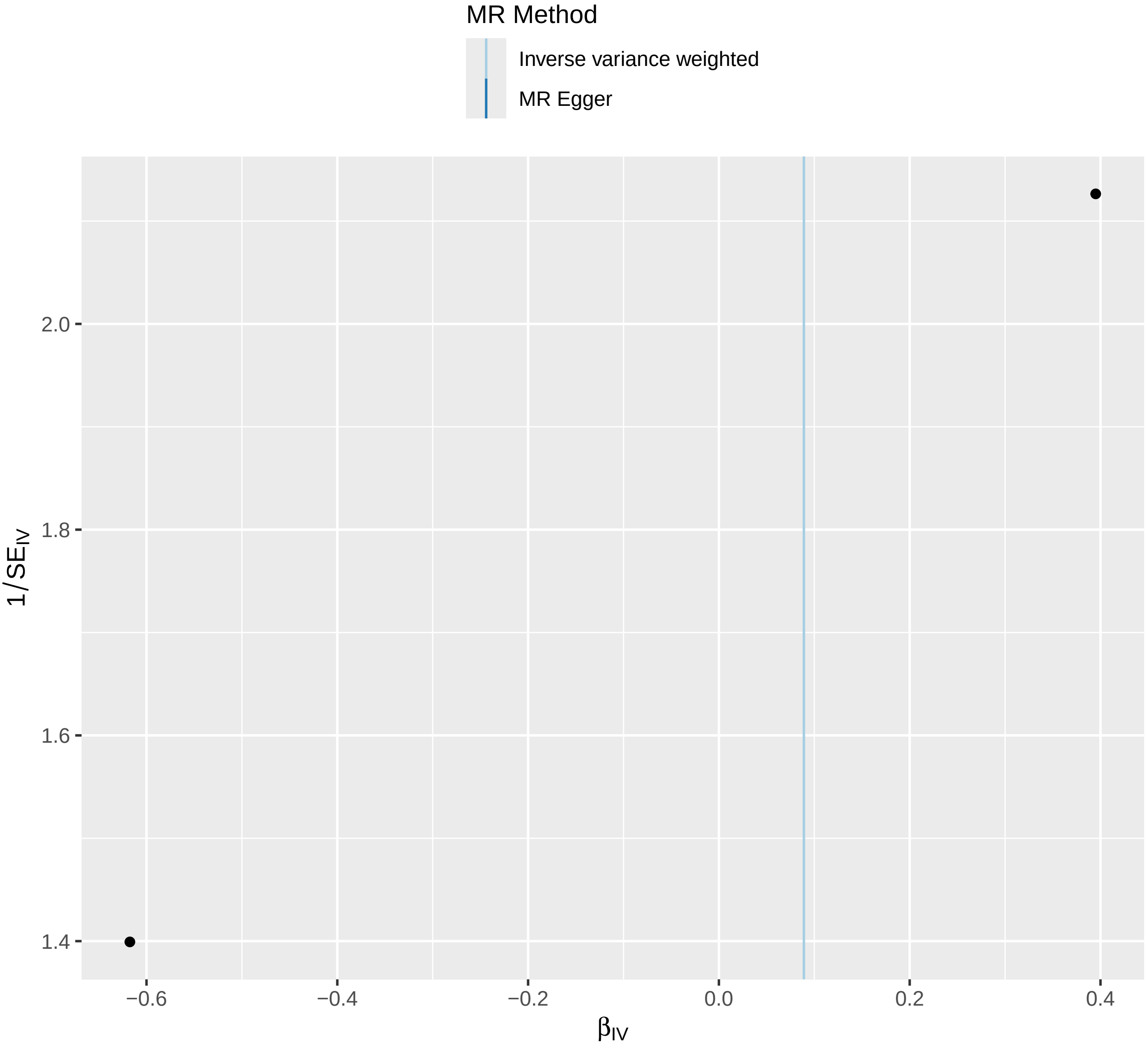
MR Method
Inverse variance weighted
MR Egger
2.0
1.8
1.6
1.4
1/SE$_{IV}$
−0.6
−0.4
−0.2
0.0
0.2
0.4
β_{IV}

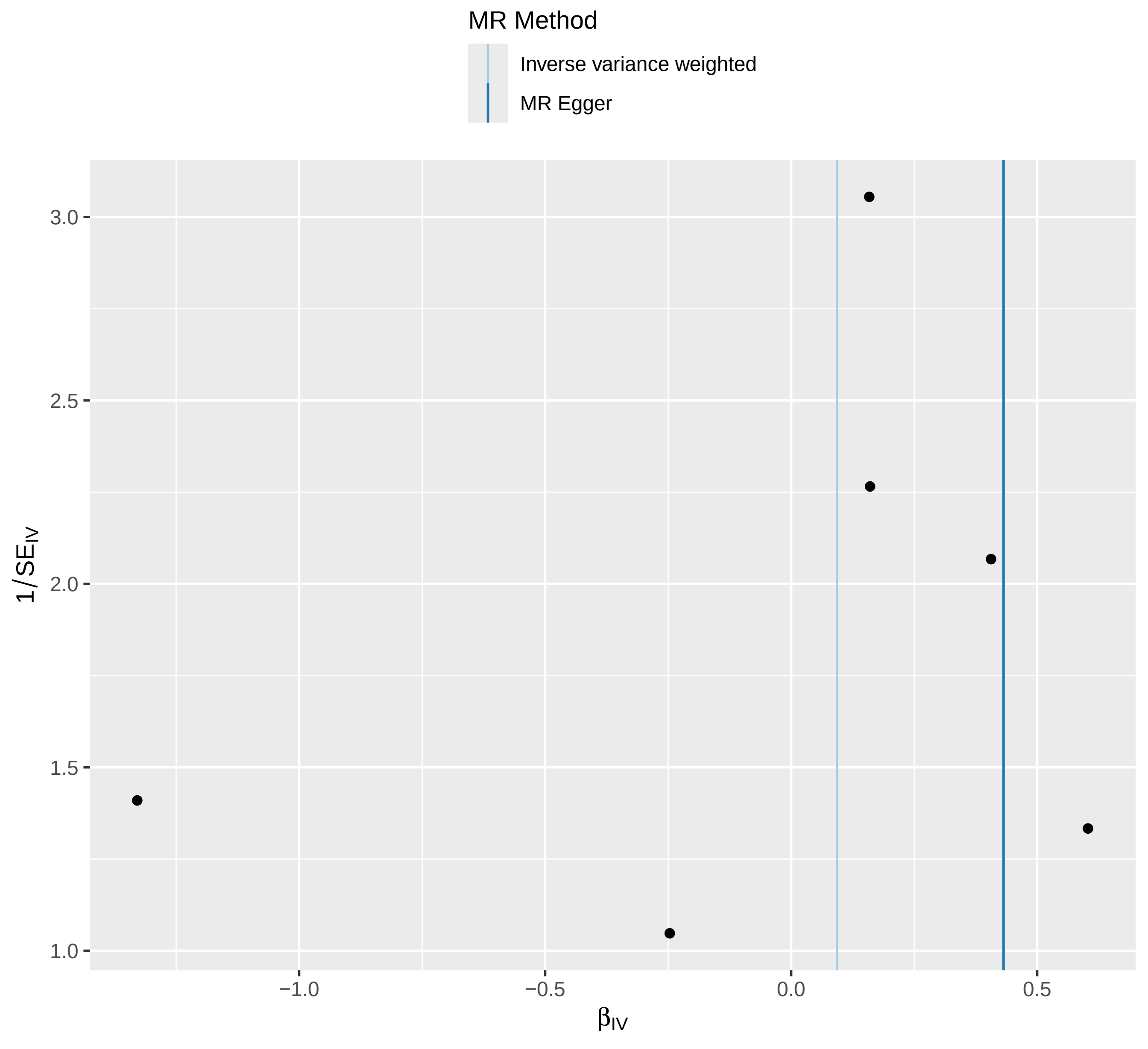

MR Method
Inverse variance weighted
MR Egger
3.0
2.5
2.0
1.5
1.0
1/SE_IV
−1.0
−0.5
0.0
0.5
β_IV

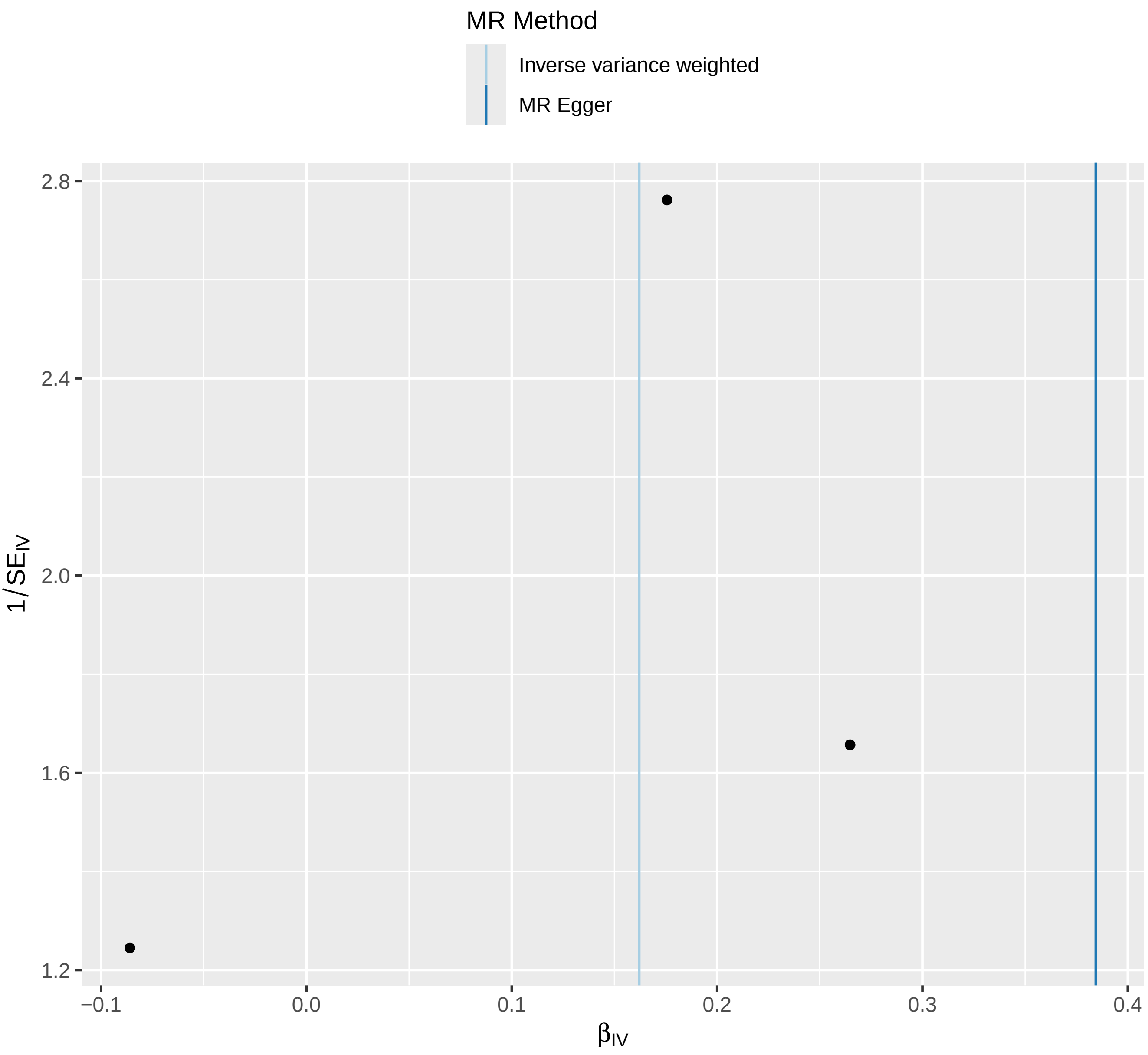
MR Method
Inverse variance weighted
MR Egger
2.8
2.4
2.0
1.6
1.2
$1/SE_{IV}$
−0.1
0.0
0.1
0.2
0.3
0.4
β_{IV}

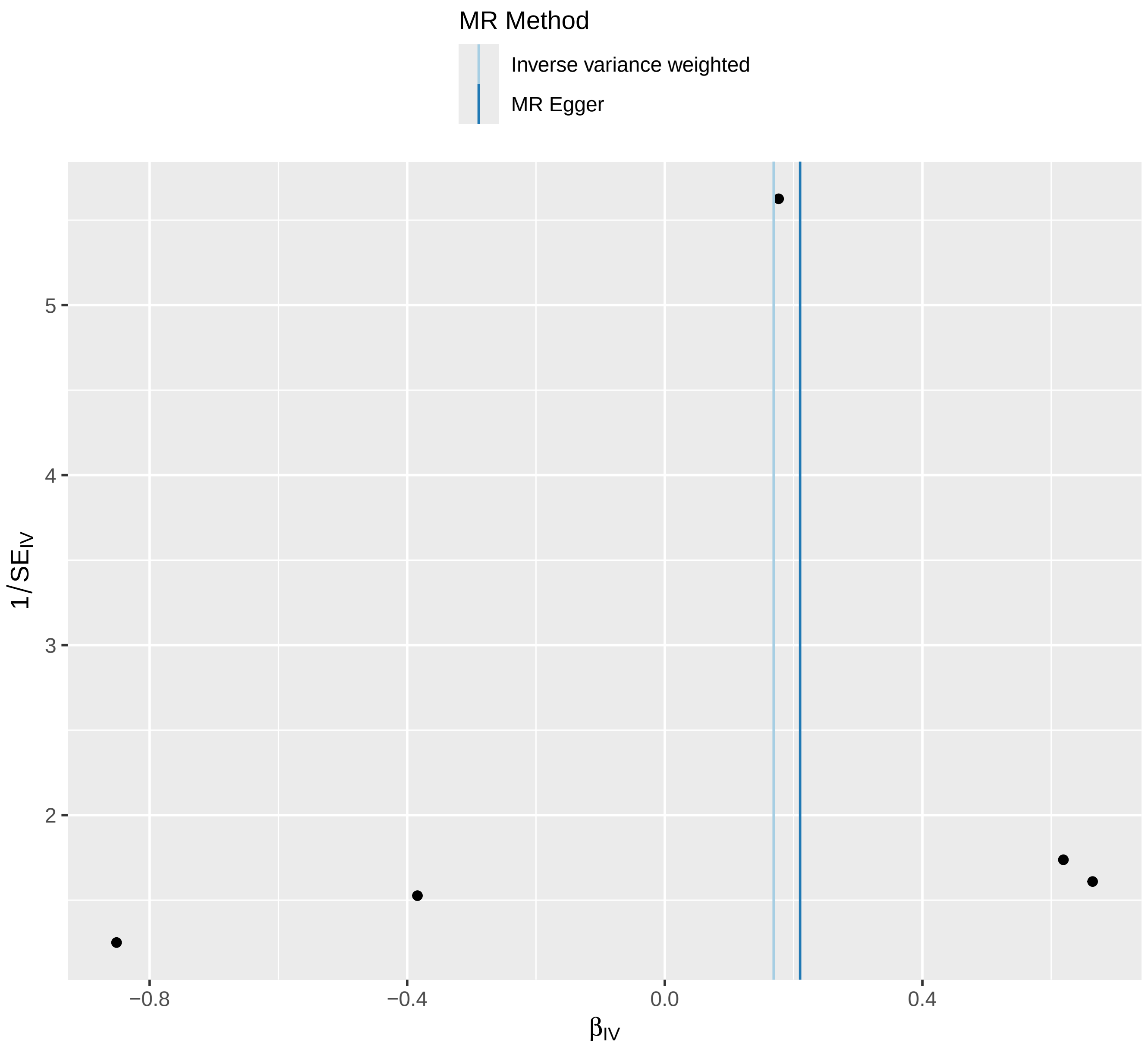
MR Method
Inverse variance weighted
MR Egger
5
4
3
2
1/SE$_{IV}$
−0.8
−0.4
0.0
0.4
β$_{IV}$

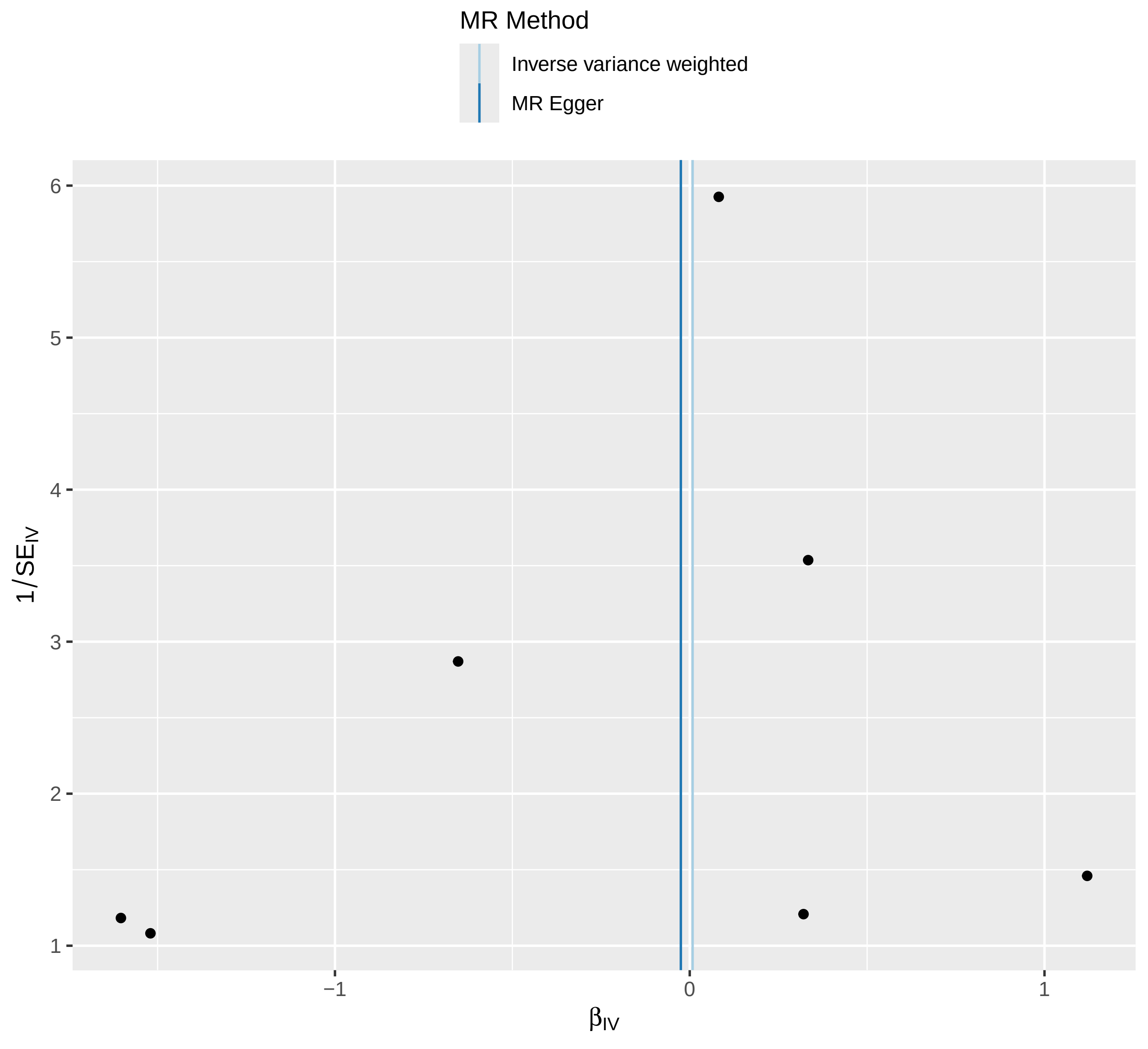
MR Method
Inverse variance weighted
MR Egger
6
5
4
3
2
1
1/SE_IV
−1
0
1
β_IV

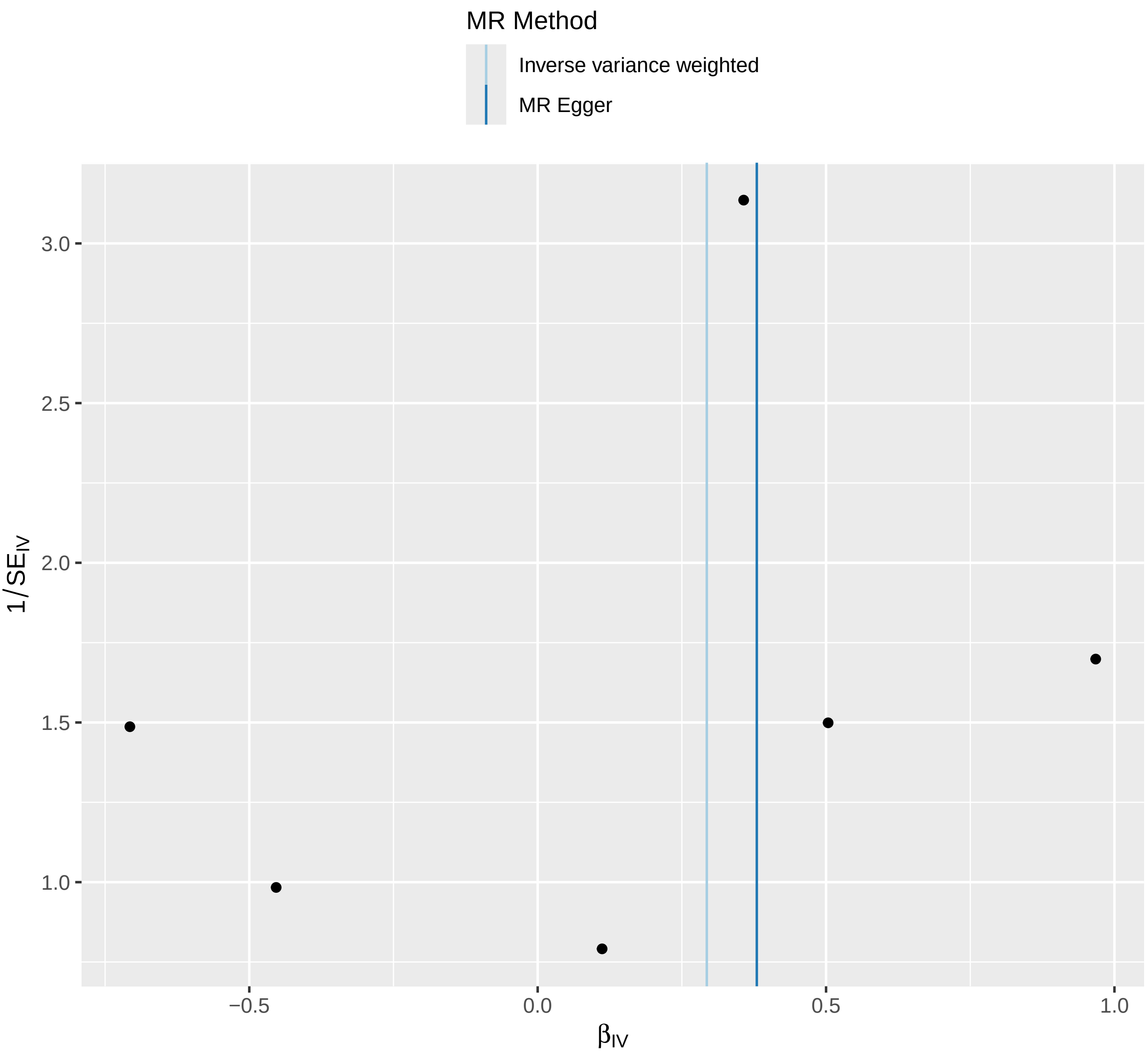
MR Method
Inverse variance weighted
MR Egger
3.0
2.5
2.0
1.5
1.0
1/SE_IV
−0.5
0.0
0.5
1.0
β_IV

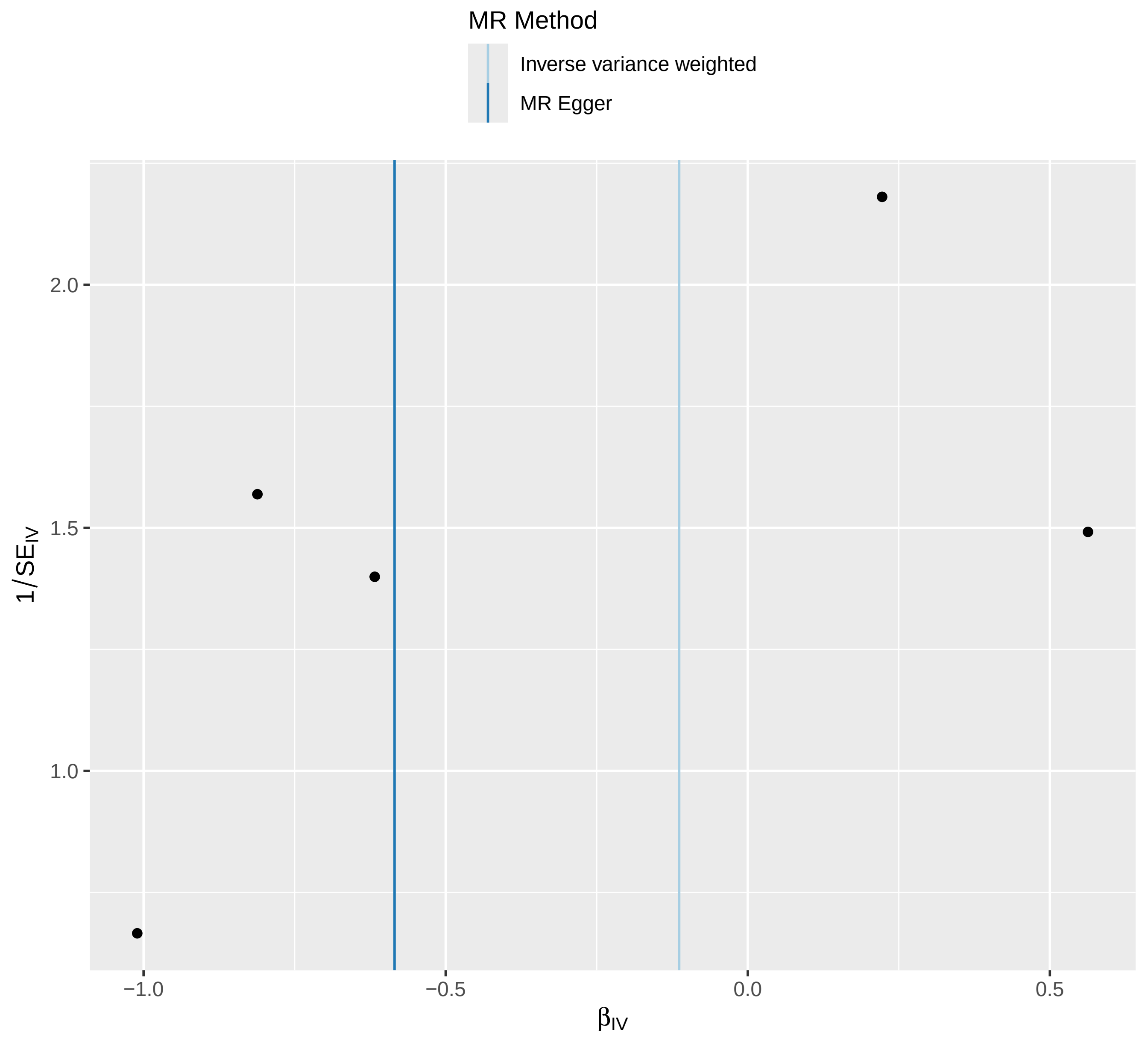
MR Method
Inverse variance weighted
MR Egger
2.0
1.5
1.0
1/SE_IV
−1.0
−0.5
0.0
0.5
β_IV

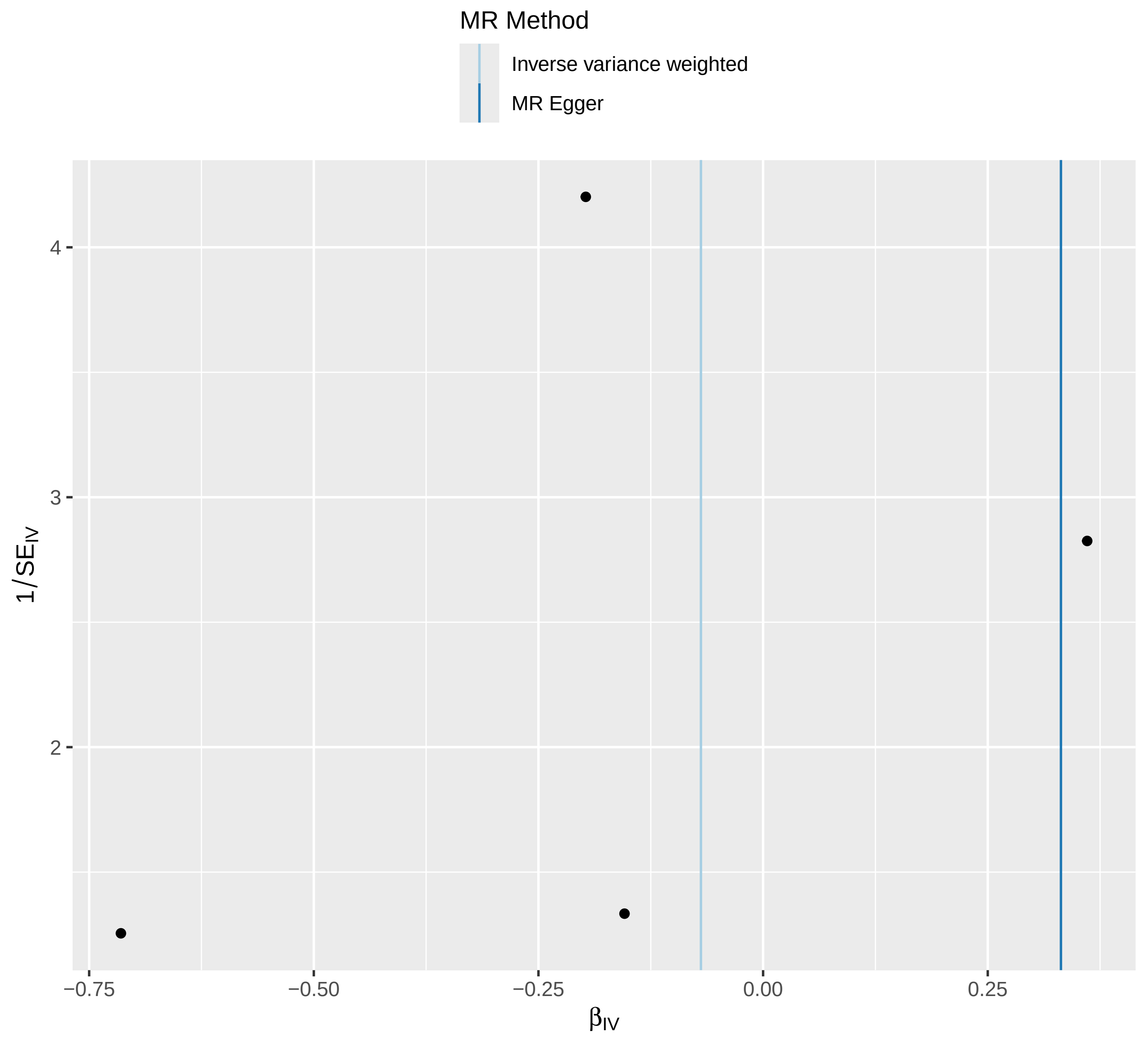
MR Method
Inverse variance weighted
MR Egger
4
3
2
1/SE$_{IV}$
−0.75
−0.50
−0.25
0.00
0.25
β$_{IV}$

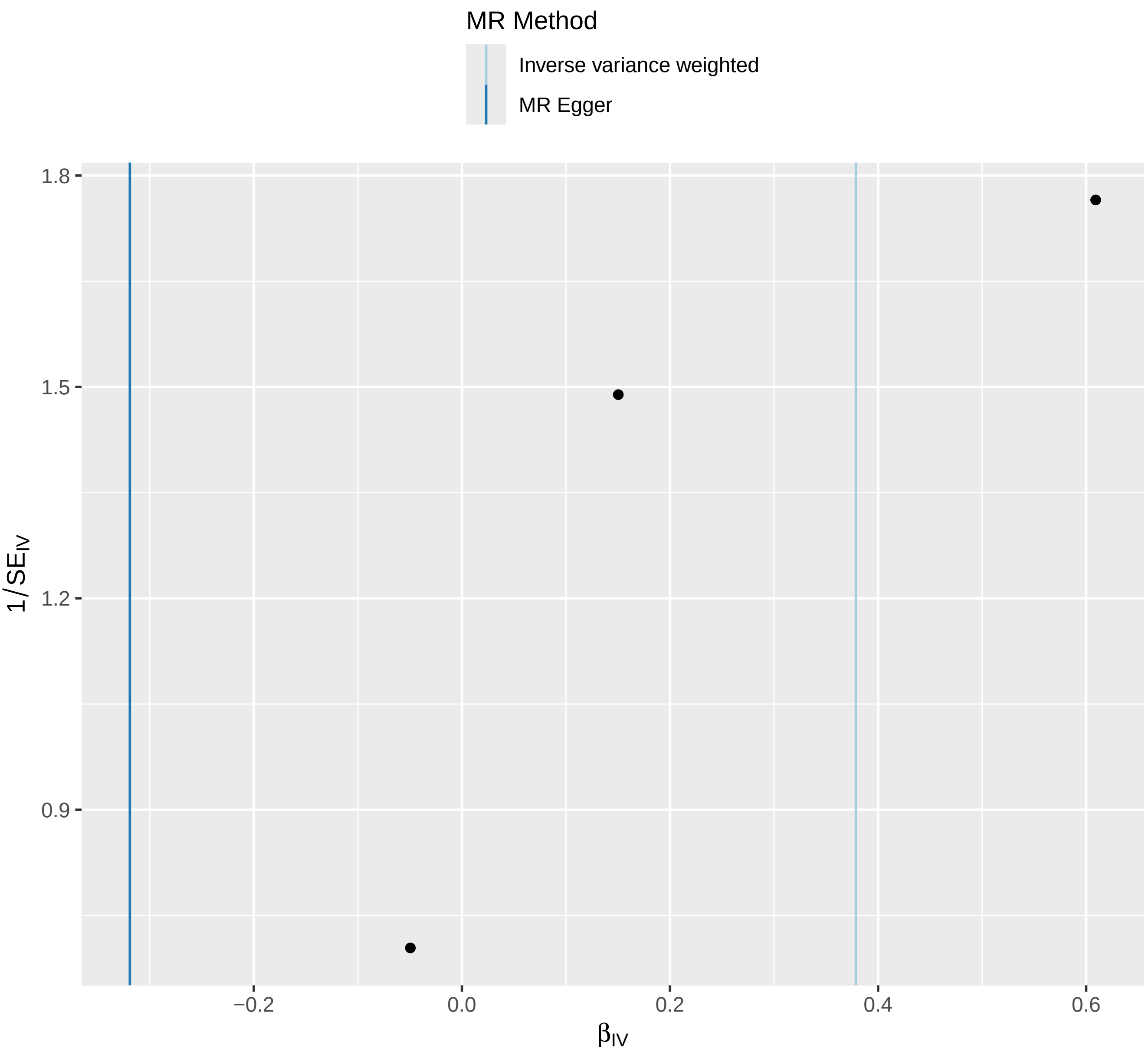

MR Method
Inverse variance weighted
MR Egger
1.8
1.5
1.2
0.9
1/SE_IV
−0.2
0.0
0.2
0.4
0.6
β_IV

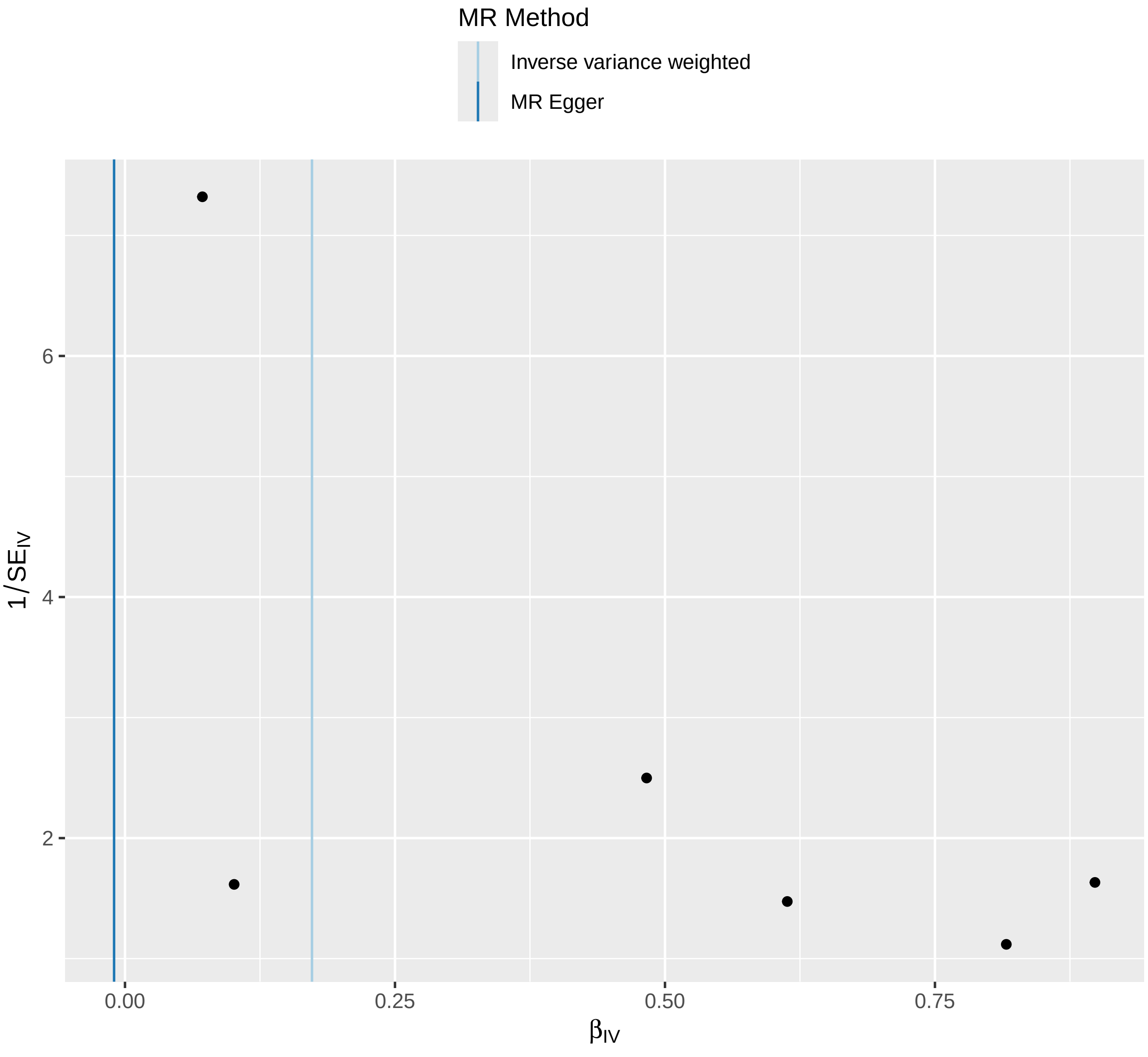
MR Method
Inverse variance weighted
MR Egger
6
4
2
$1/SE_{IV}$
0.00
0.25
0.50
0.75
β_{IV}

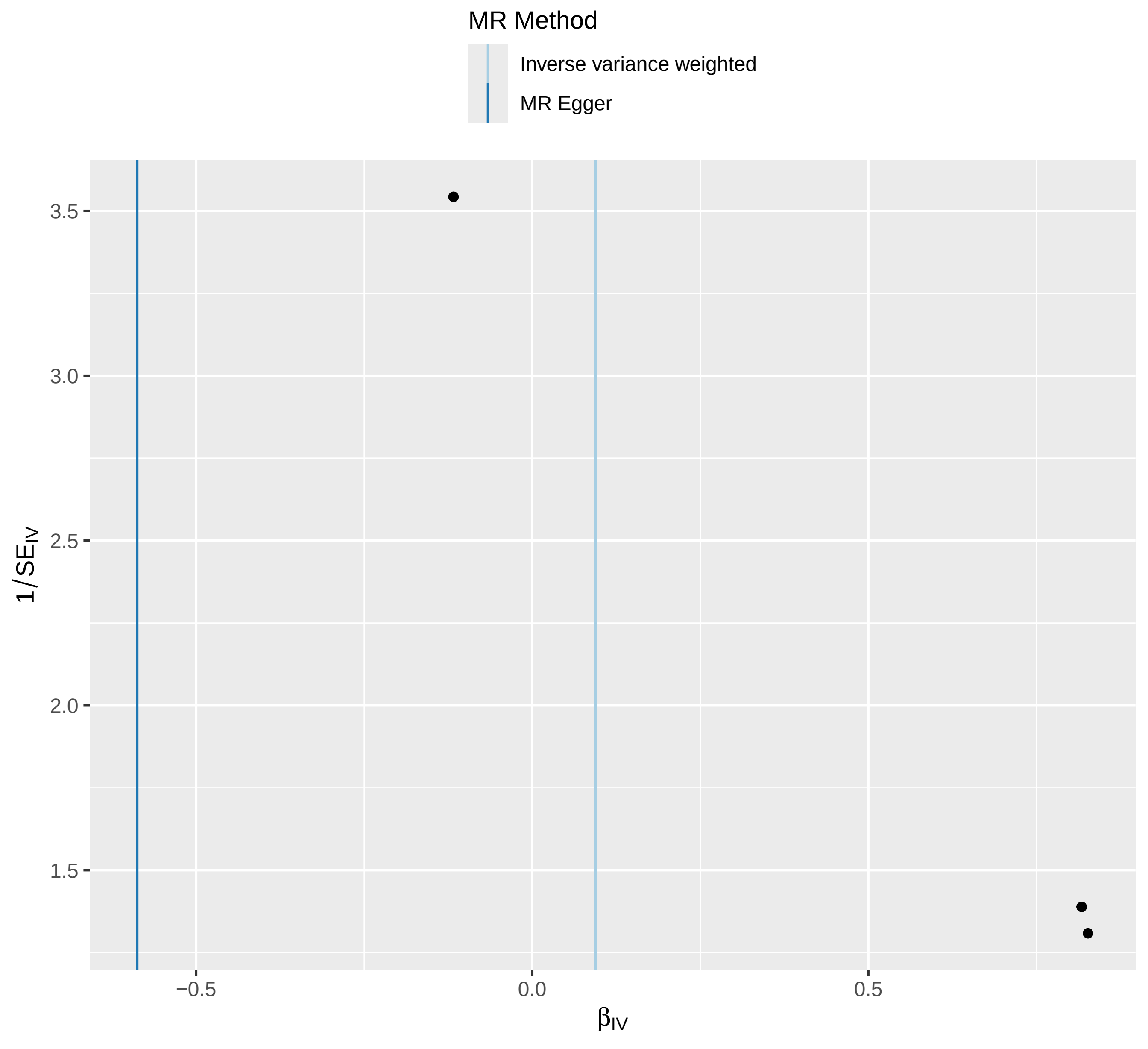
MR Method
Inverse variance weighted
MR Egger
3.5
3.0
2.5
2.0
1.5
1/SE_IV
−0.5
0.0
0.5
β_IV

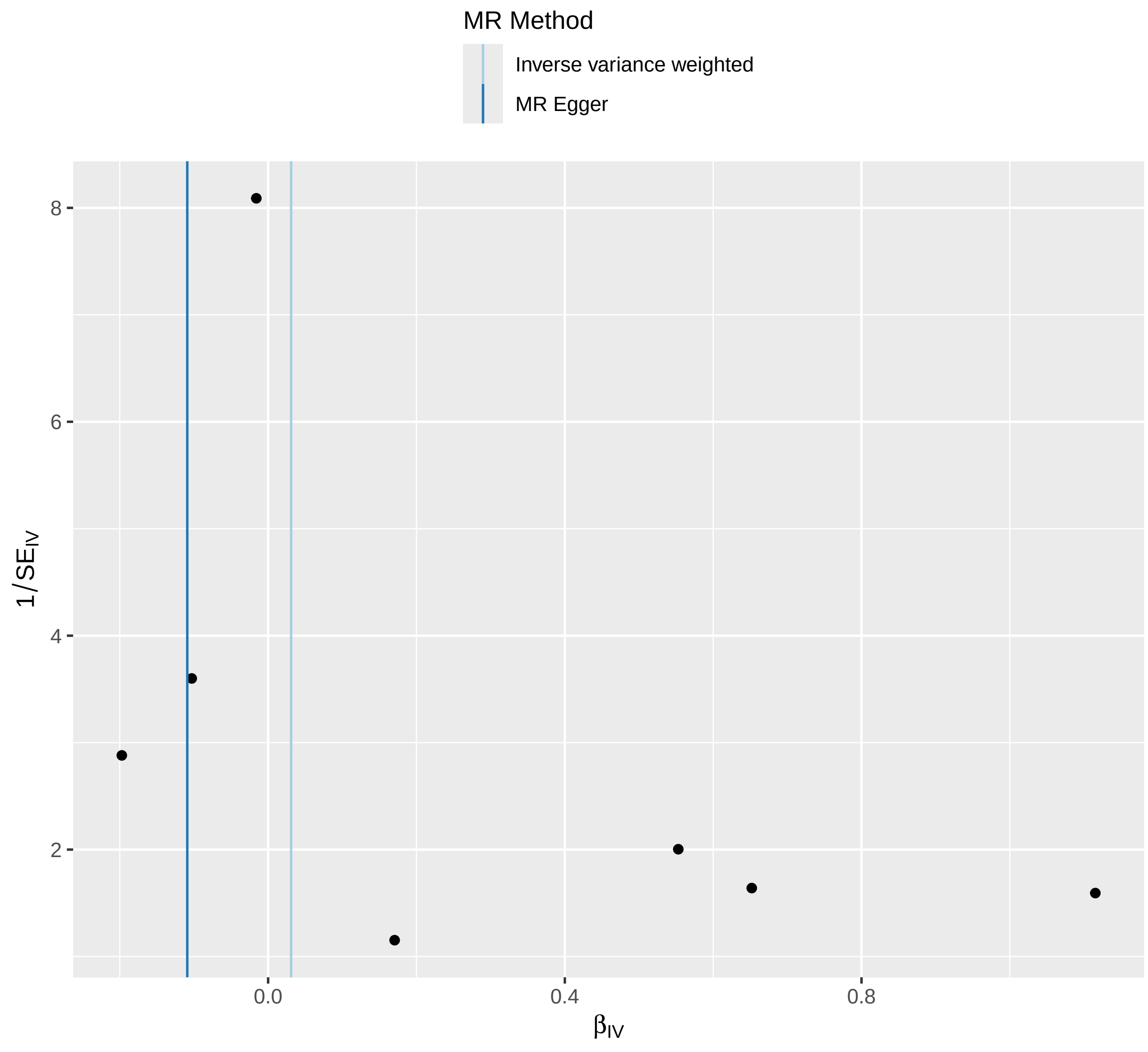
MR Method
Inverse variance weighted
MR Egger
1/SE_IV
8
6
4
2
0.0
0.4
0.8
β_IV

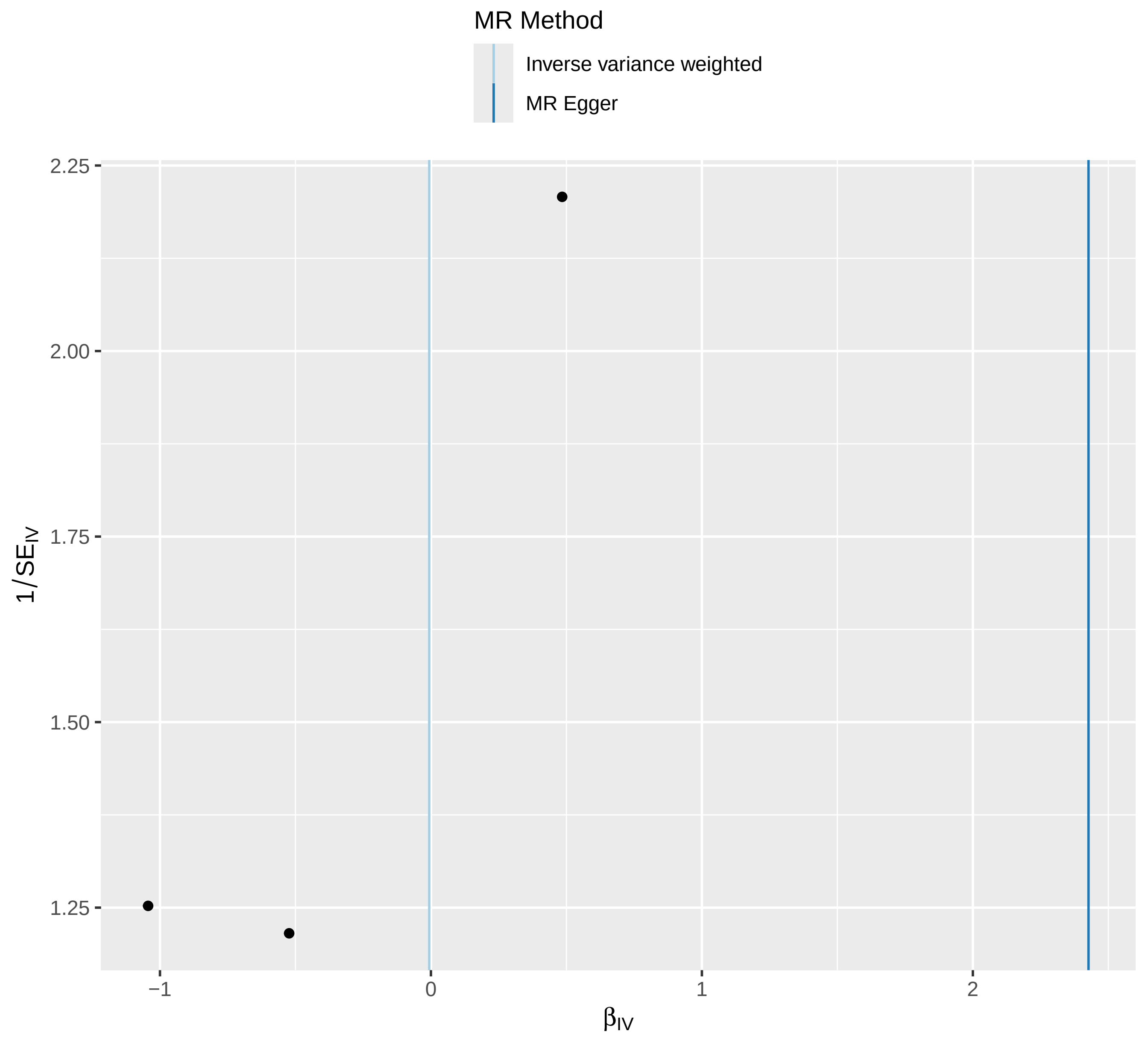
MR Method
Inverse variance weighted
MR Egger
2.25
2.00
1.75
1.50
1.25
1/SE_IV
−1
0
1
2
β_IV

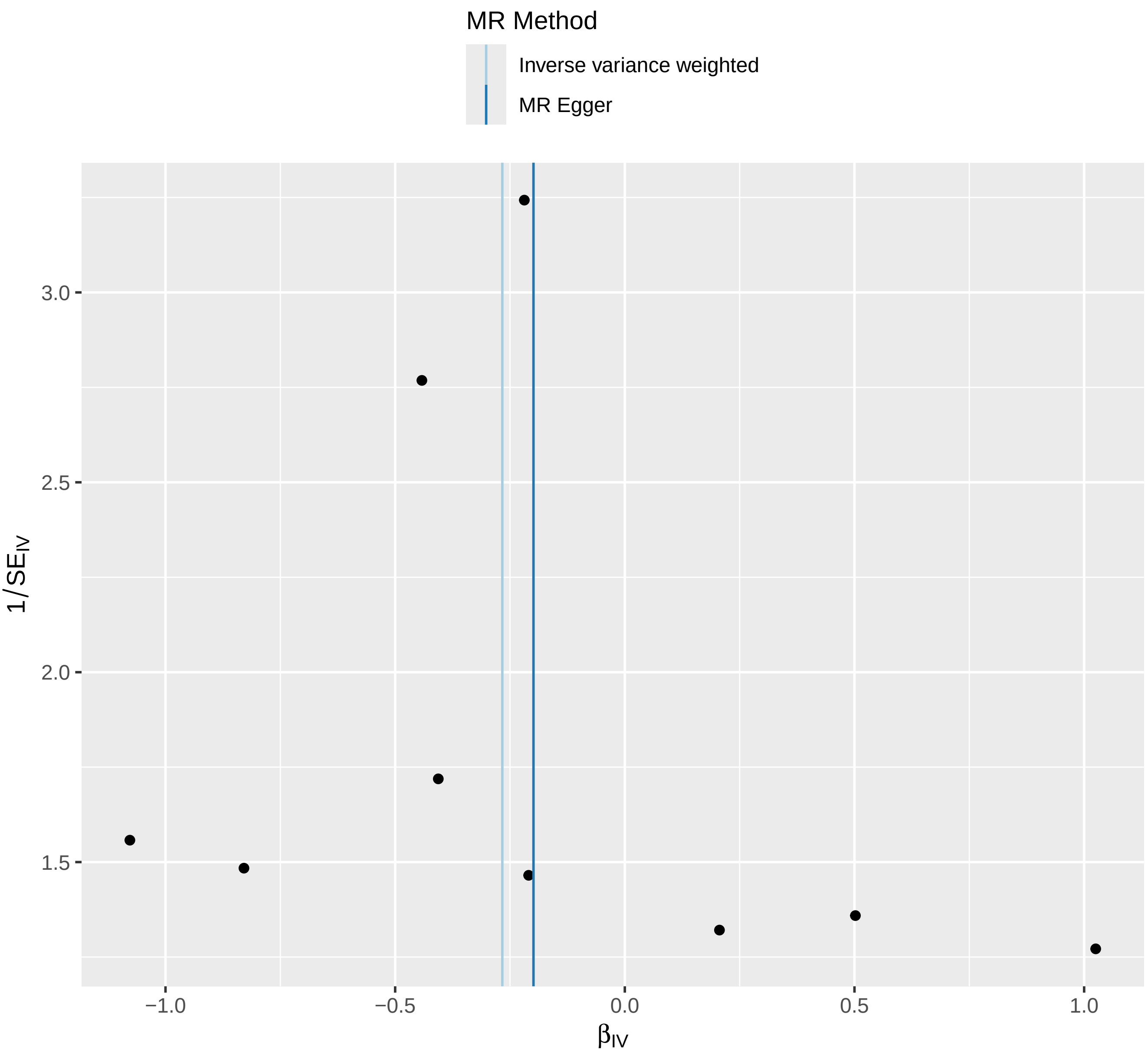
MR Method
Inverse variance weighted
MR Egger
3.0
2.5
2.0
1.5
1/SE$_{IV}$
−1.0
−0.5
0.0
0.5
1.0
β$_{IV}$

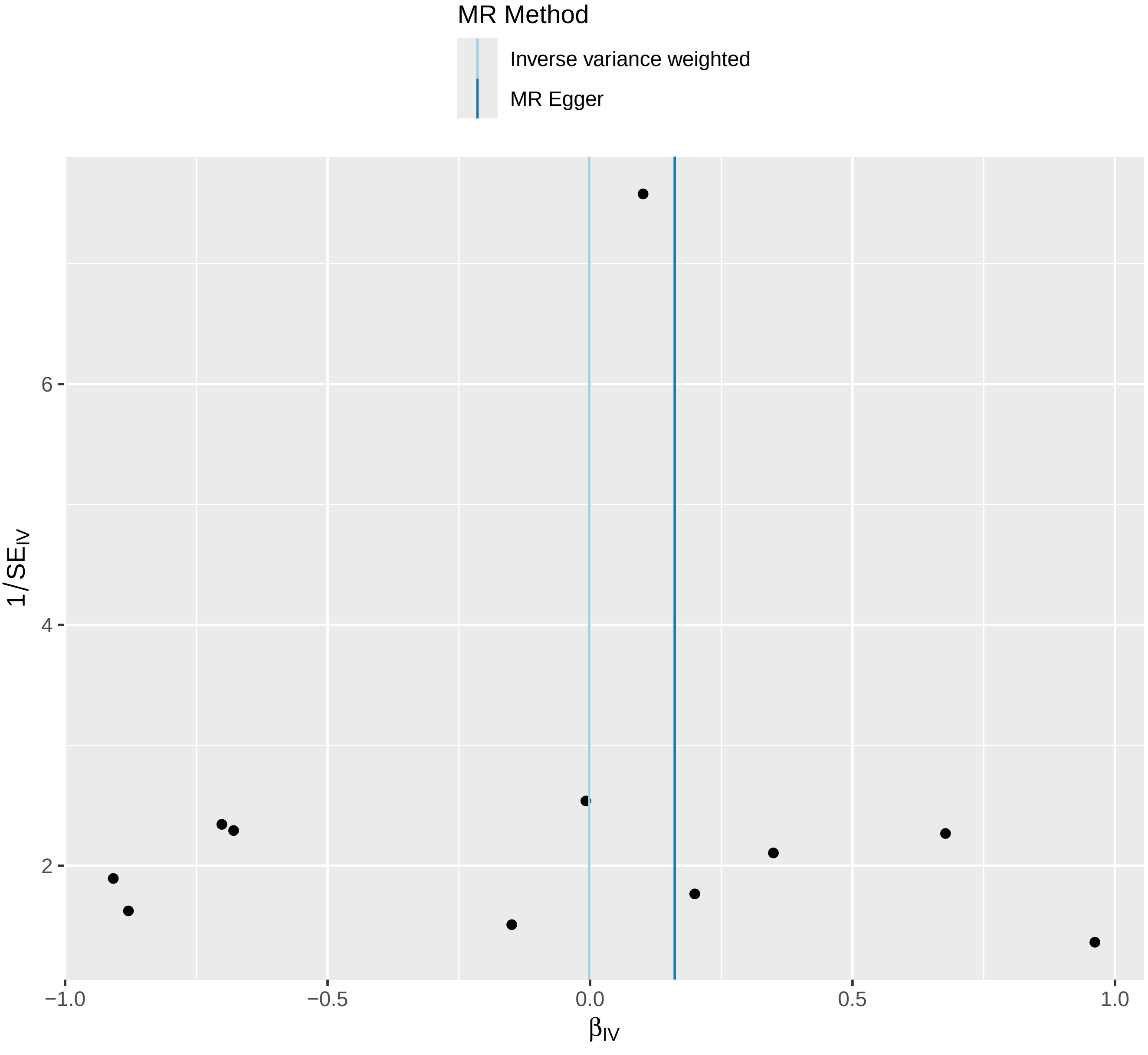

MR Method
Inverse variance weighted
MR Egger
1/SE_IV
6
4
2
−1.0
−0.5
0.0
0.5
1.0
β_IV

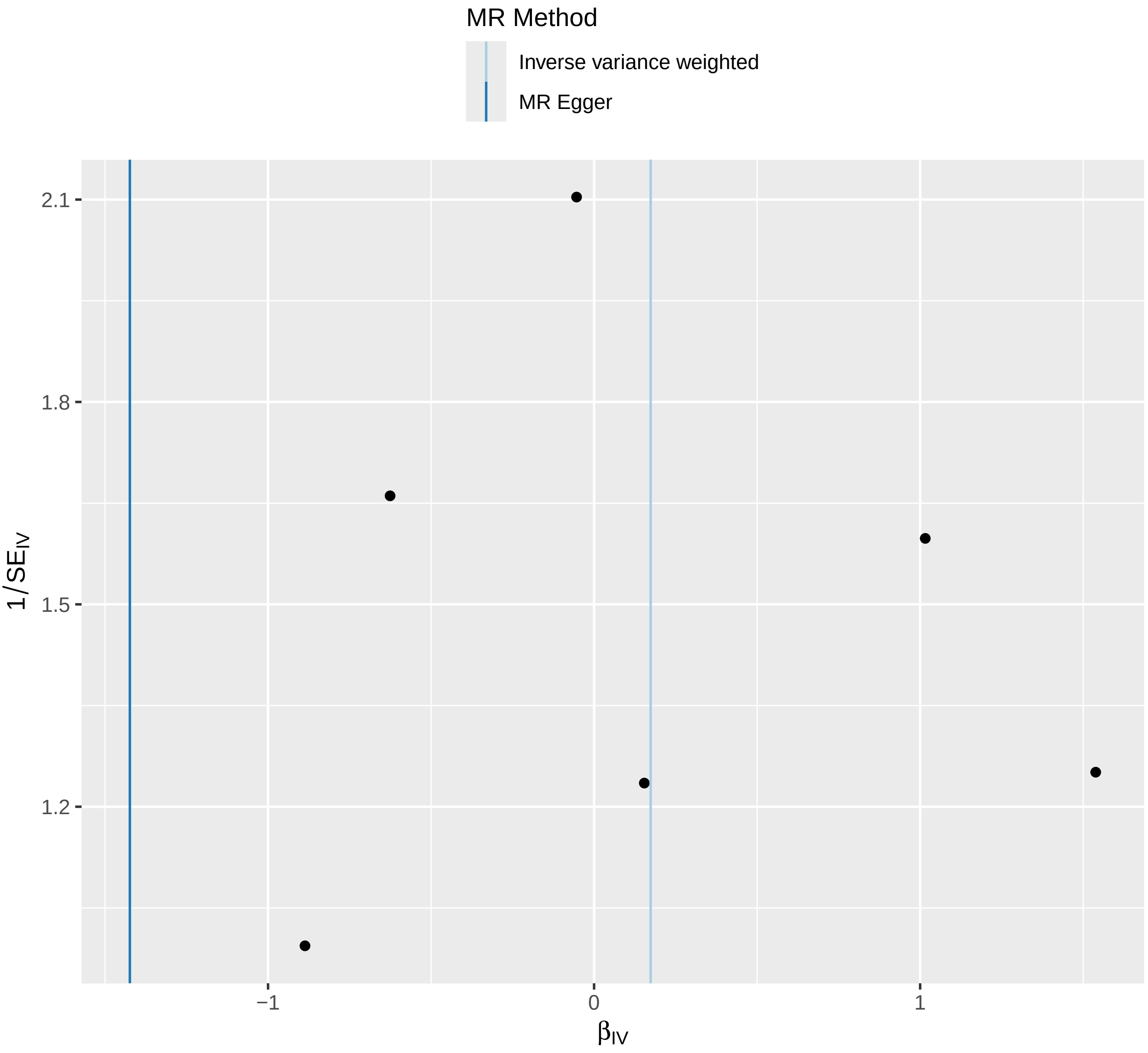

MR Method
Inverse variance weighted
MR Egger
2.1
1.8
1.5
1.2
1/SE_IV
−1
0
1
β_IV

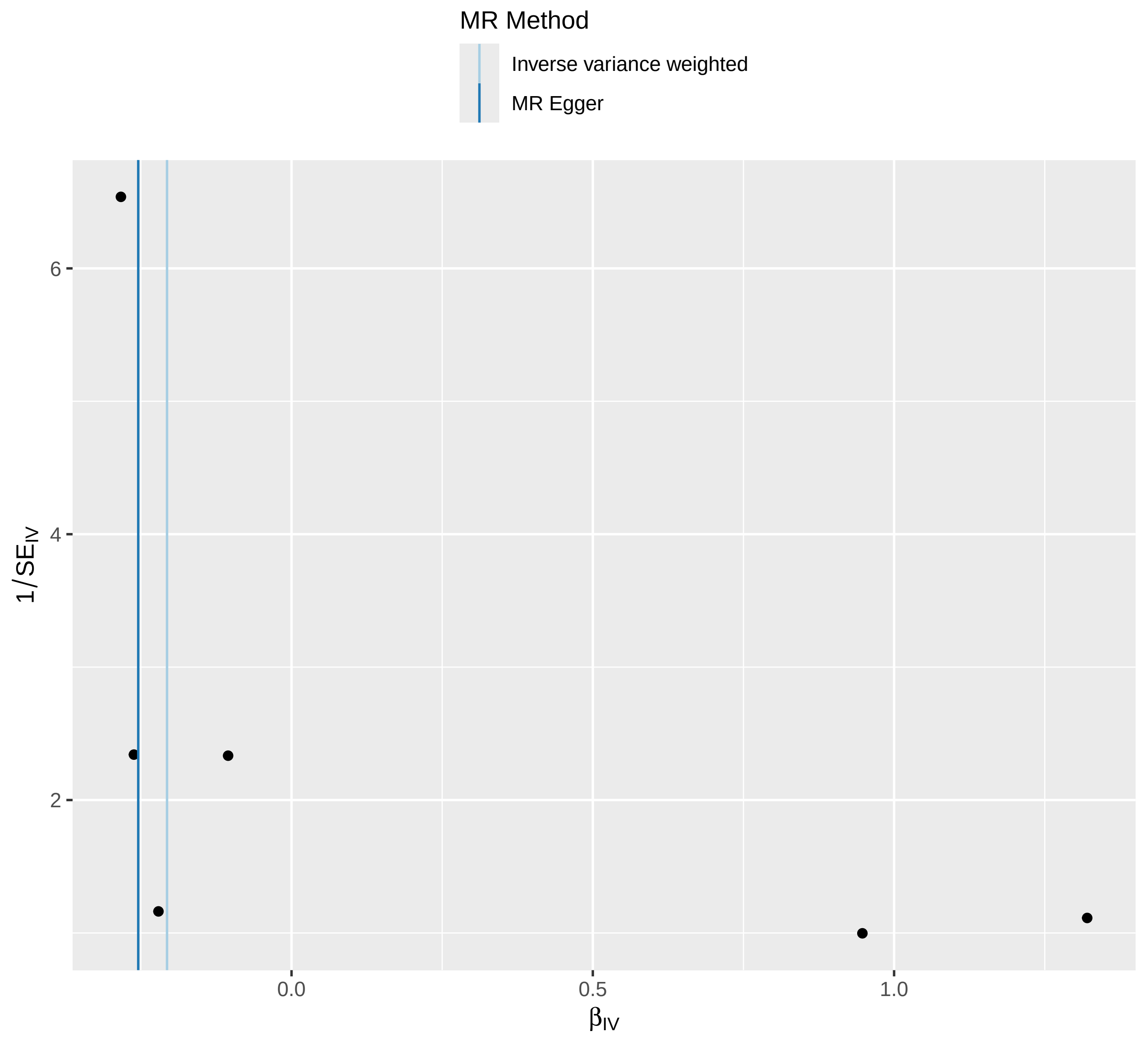

MR Method
Inverse variance weighted
MR Egger
6
4
2
$1/SE_{IV}$
0.0
0.5
1.0
β_{IV}

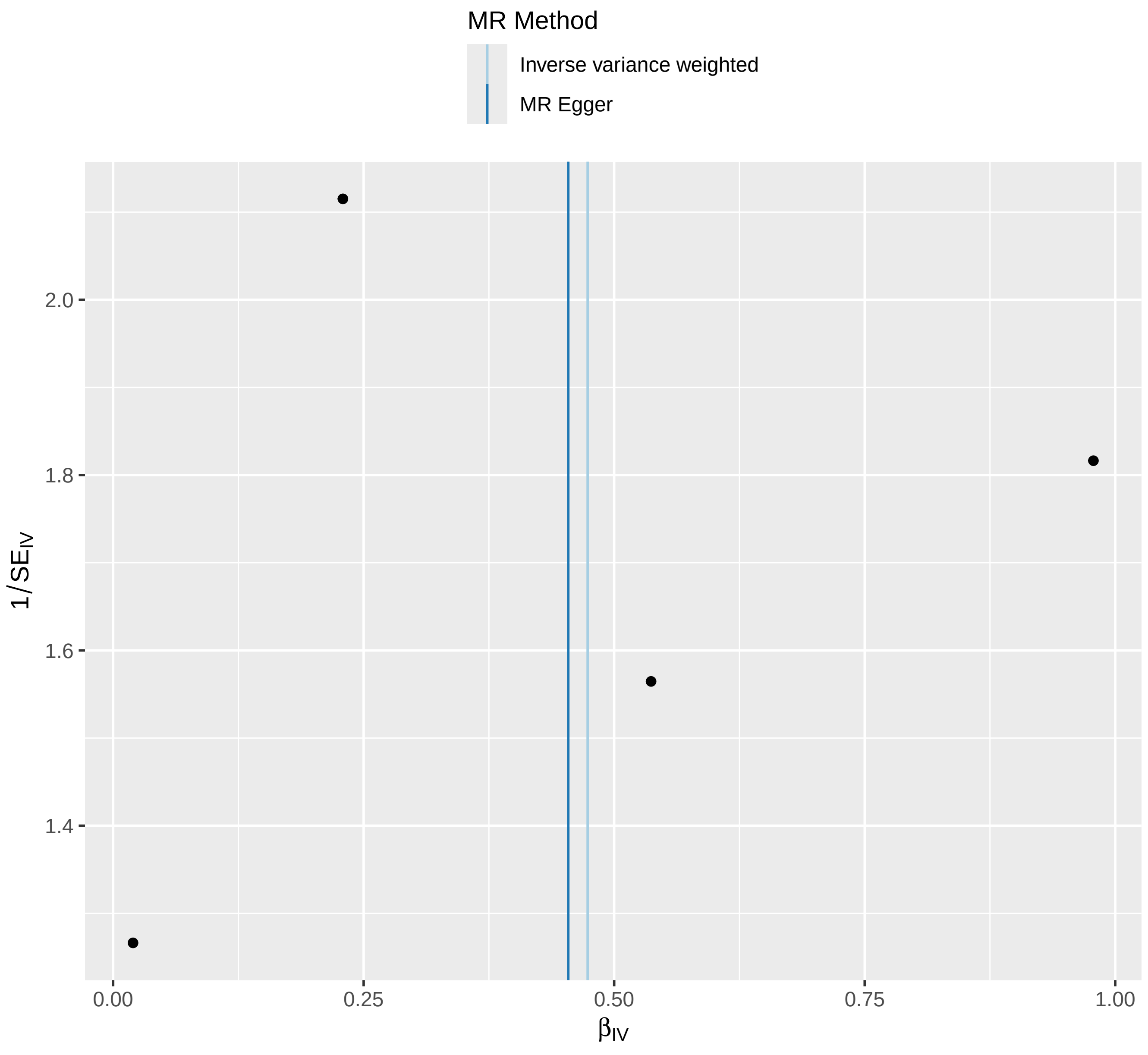

MR Method
Inverse variance weighted
MR Egger
2.0
1.8
1.6
1.4
1/SE_IV
0.00
0.25
0.50
0.75
1.00
β_IV

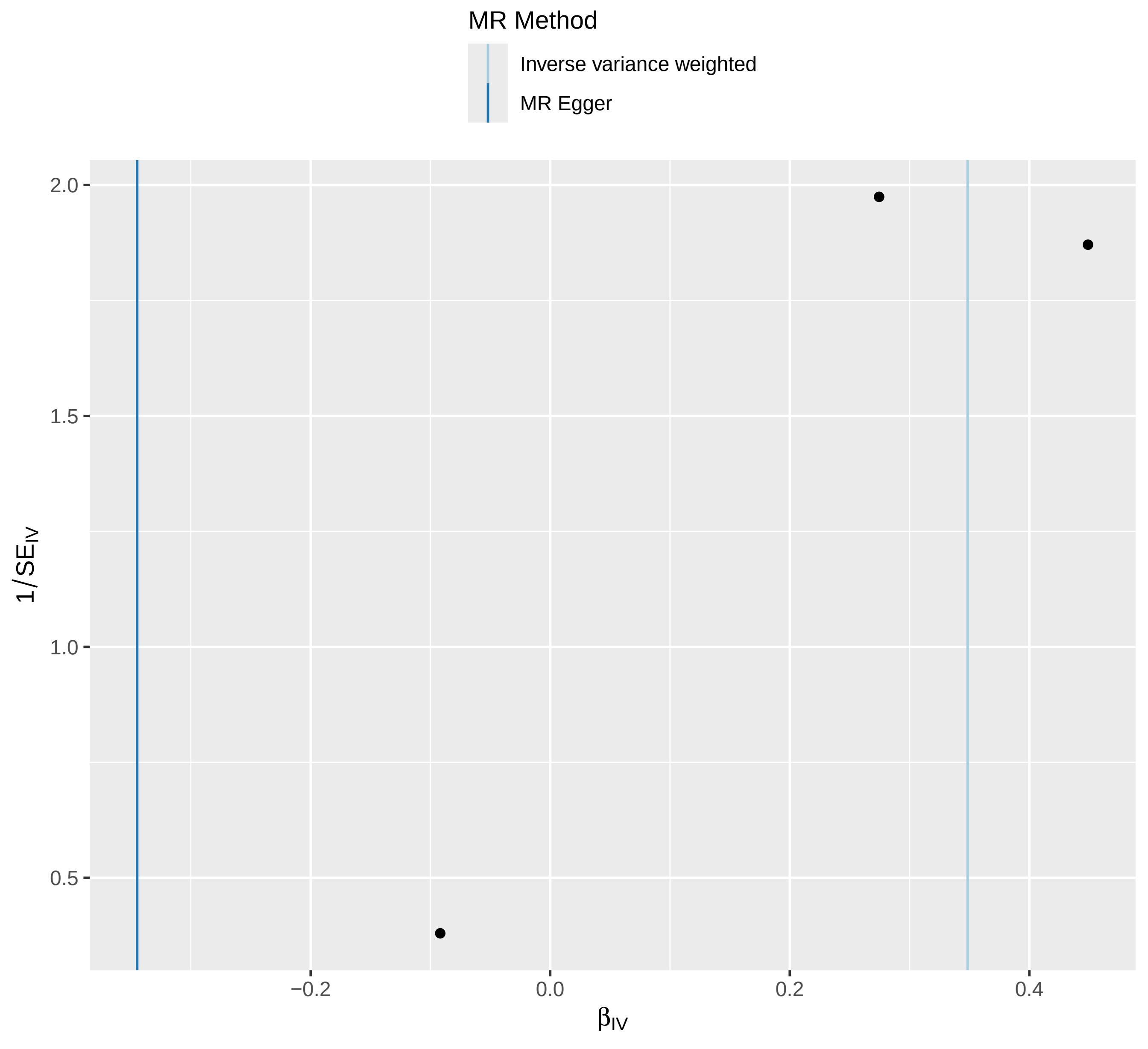
MR Method
Inverse variance weighted
MR Egger
2.0
1.5
1.0
0.5
1/SE_IV
−0.2
0.0
0.2
0.4
β_IV

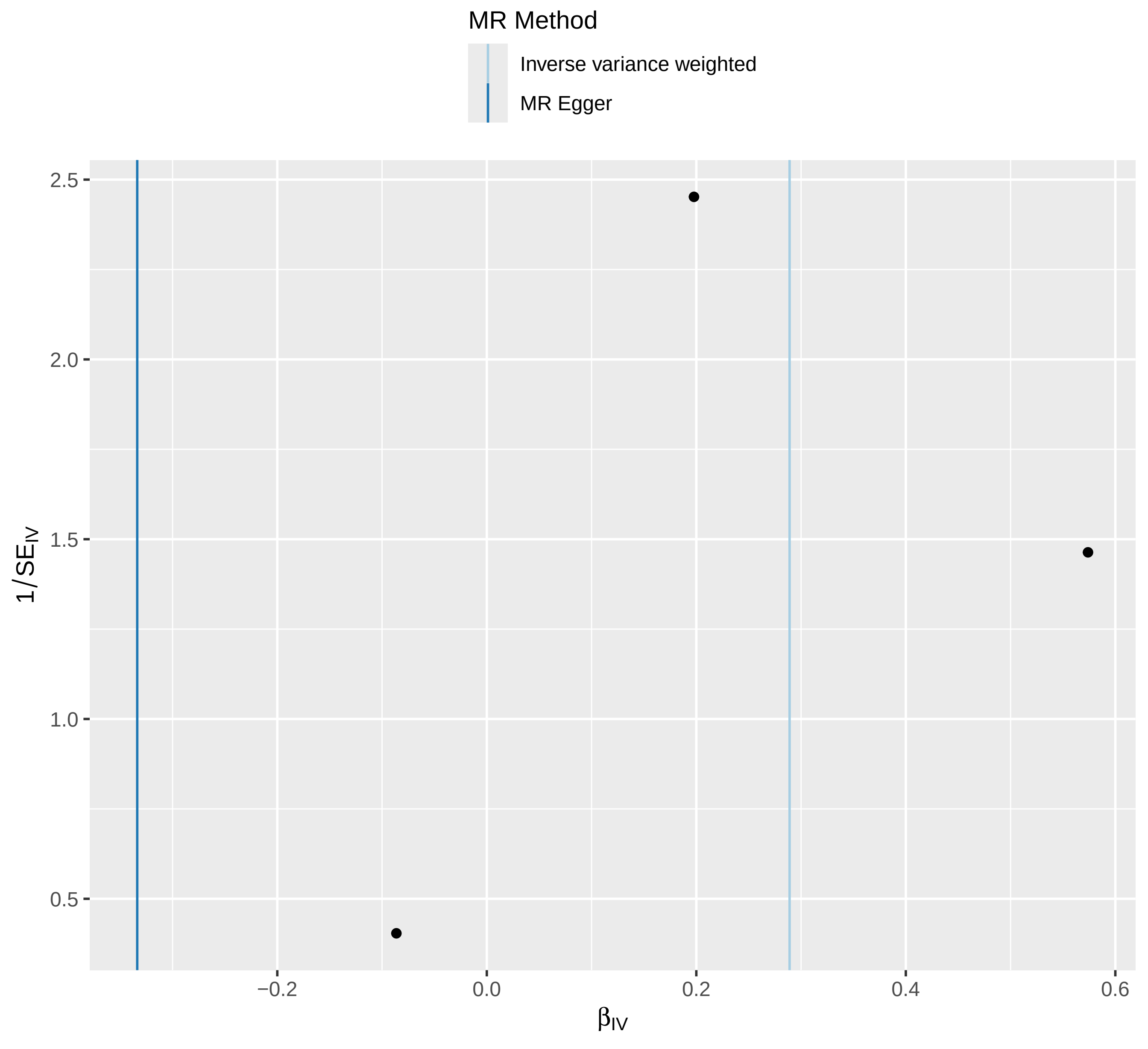

MR Method
Inverse variance weighted
MR Egger
2.5
2.0
1.5
1.0
0.5
1/SE_IV
−0.2
0.0
0.2
0.4
0.6
β_IV

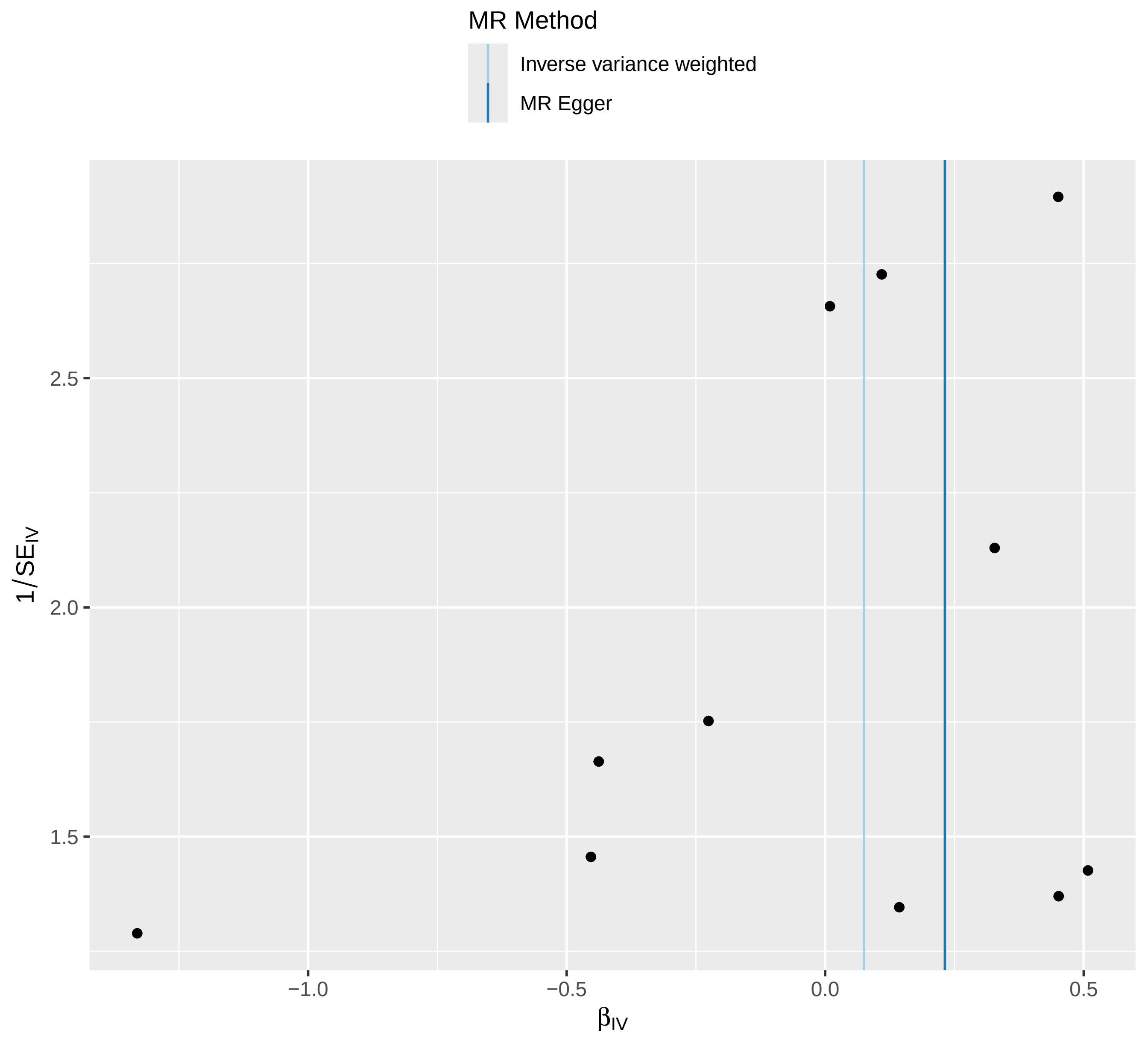
MR Method
Inverse variance weighted
MR Egger
2.5
2.0
1.5
1/SE_IV
−1.0
−0.5
0.0
0.5
β_IV

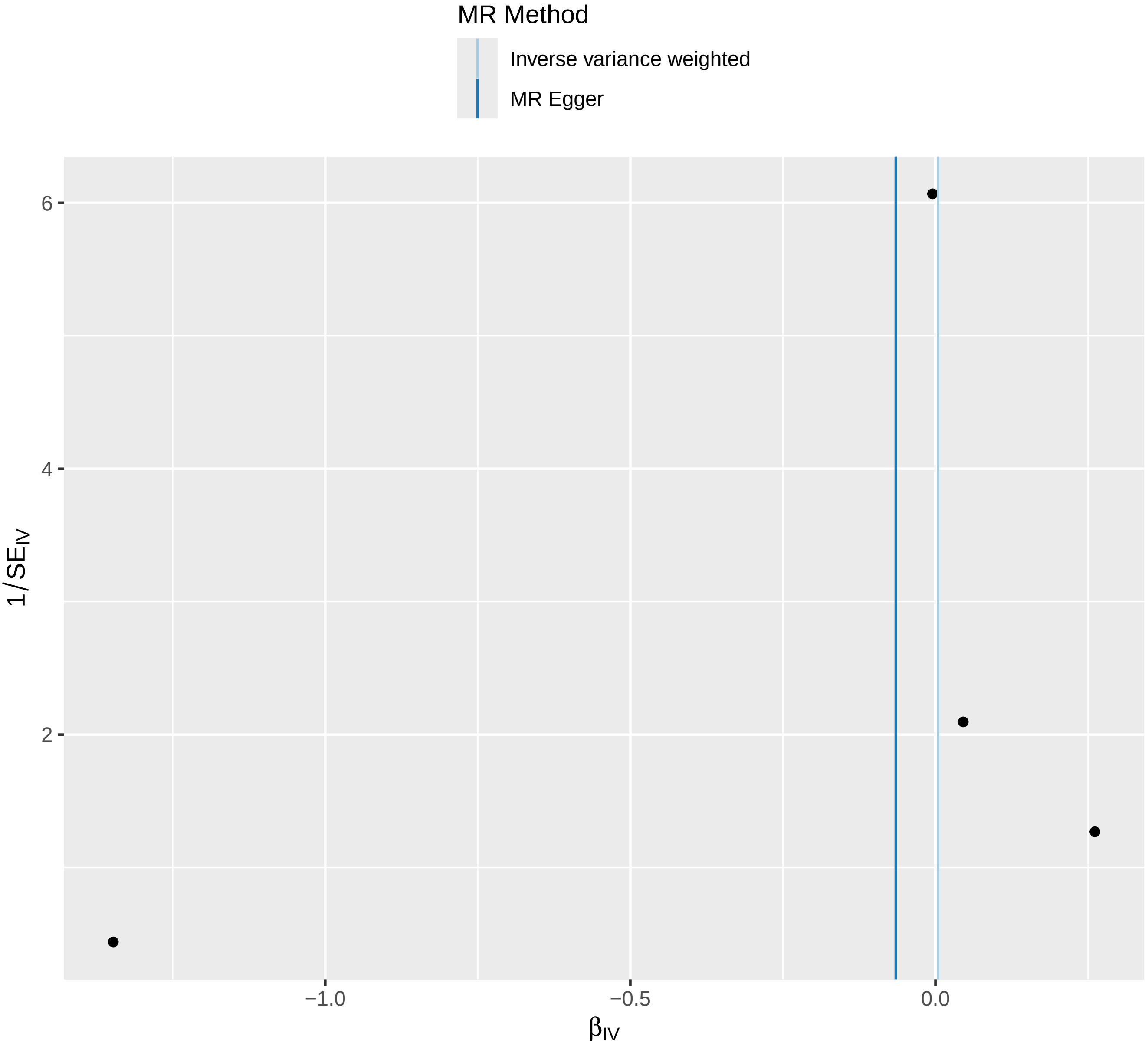
MR Method
Inverse variance weighted
MR Egger
6
4
2
1/SE$_{IV}$
−1.0
−0.5
0.0
β_{IV}

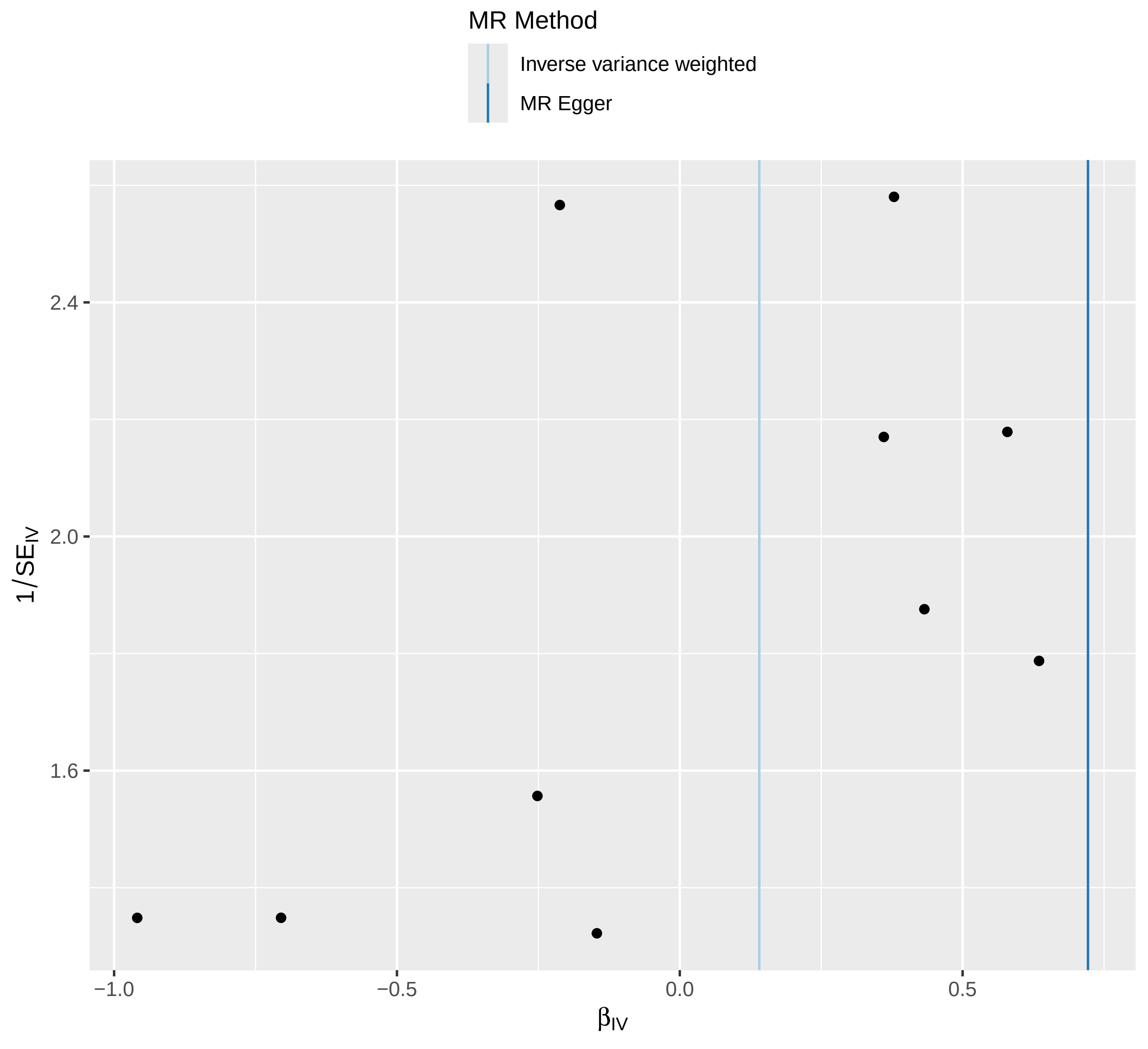

MR Method
Inverse variance weighted
MR Egger
2.4
2.0
1.6
1/SE$_{IV}$
−1.0
−0.5
0.0
0.5
β$_{IV}$

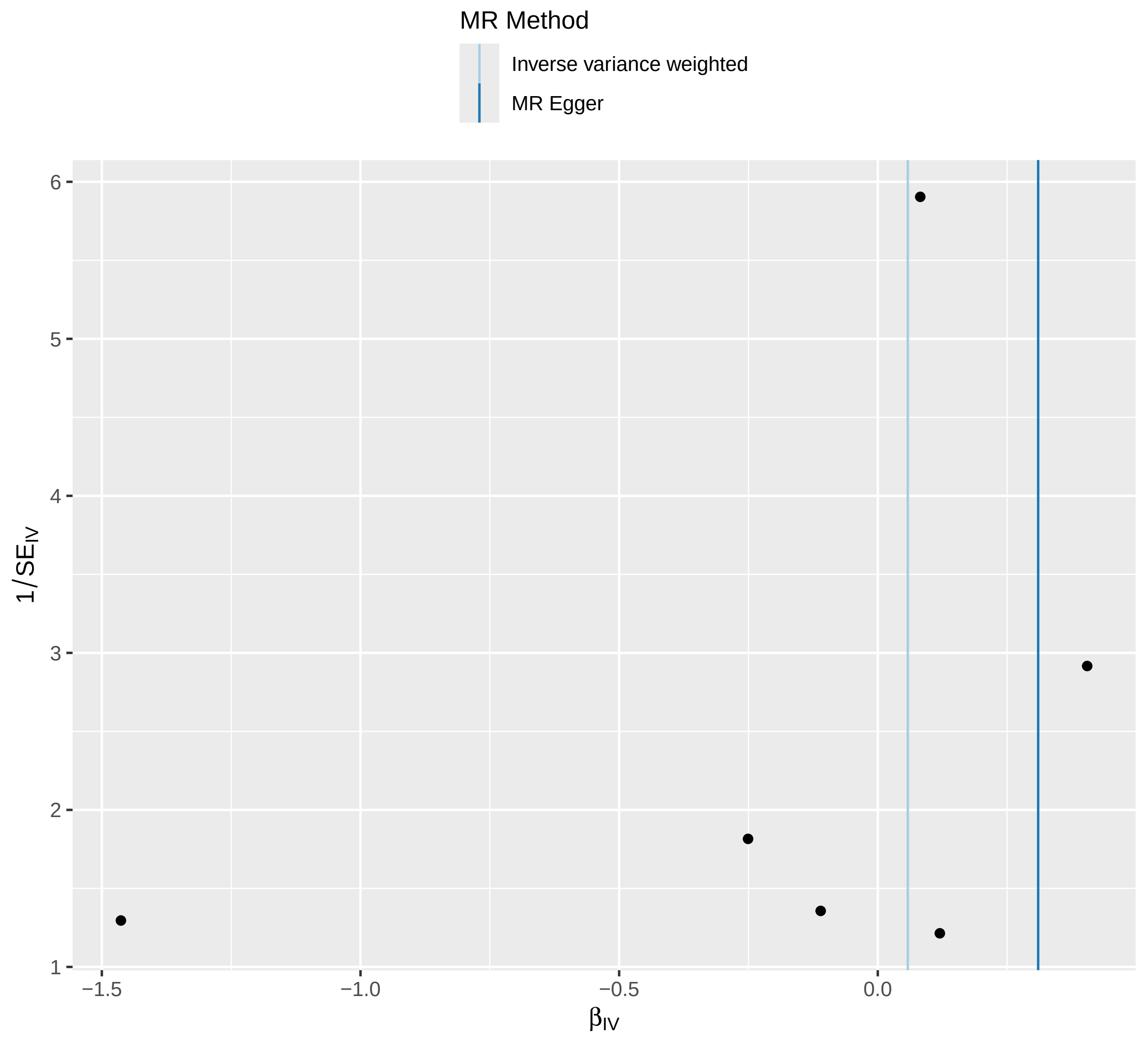
MR Method
Inverse variance weighted
MR Egger
$1/SE_{IV}$
6
5
4
3
2
1
−1.5
−1.0
−0.5
0.0
β_{IV}

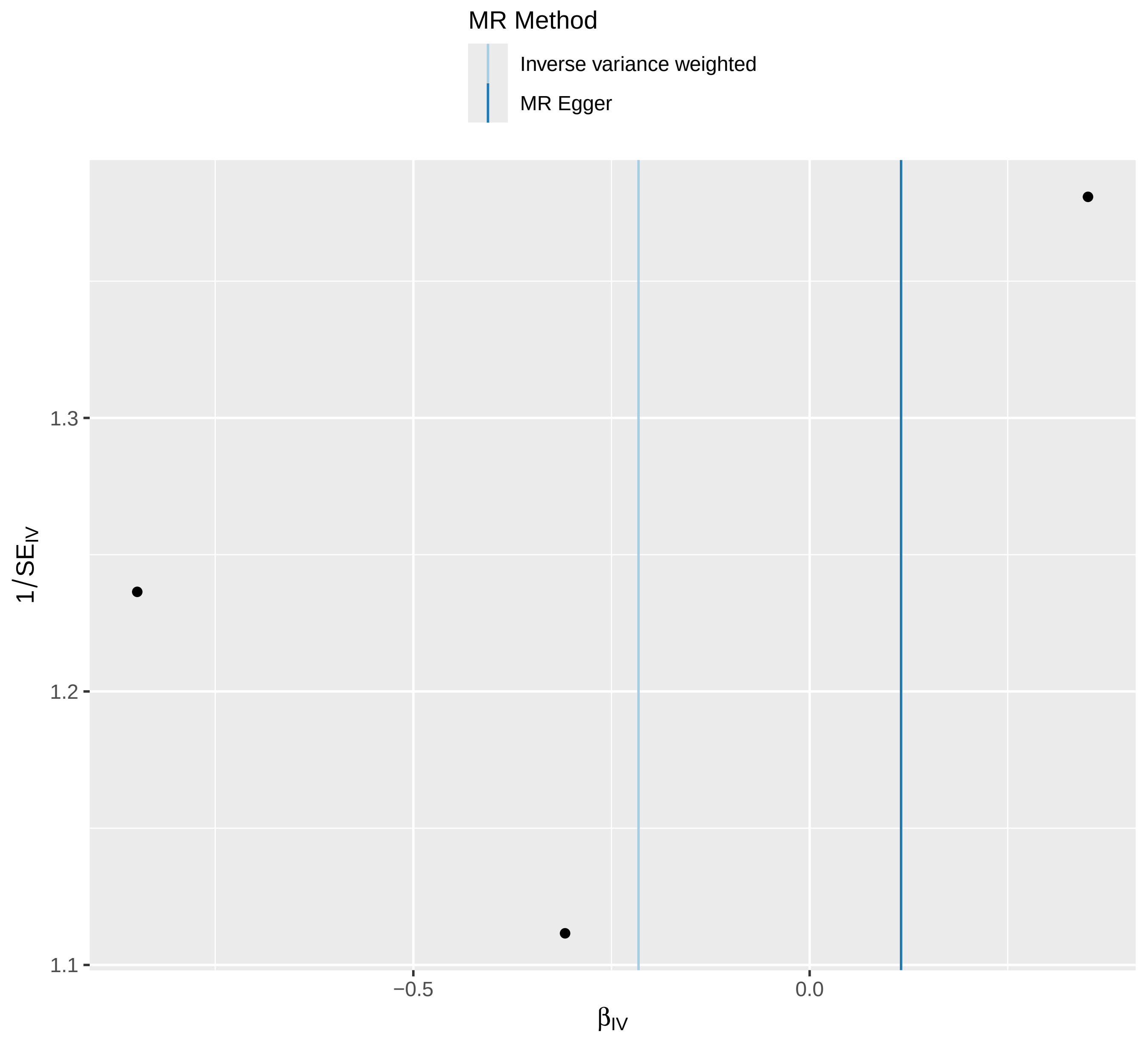
MR Method
Inverse variance weighted
MR Egger
1.3
1.2
1.1
$1/SE_{IV}$
−0.5
0.0
β_{IV}

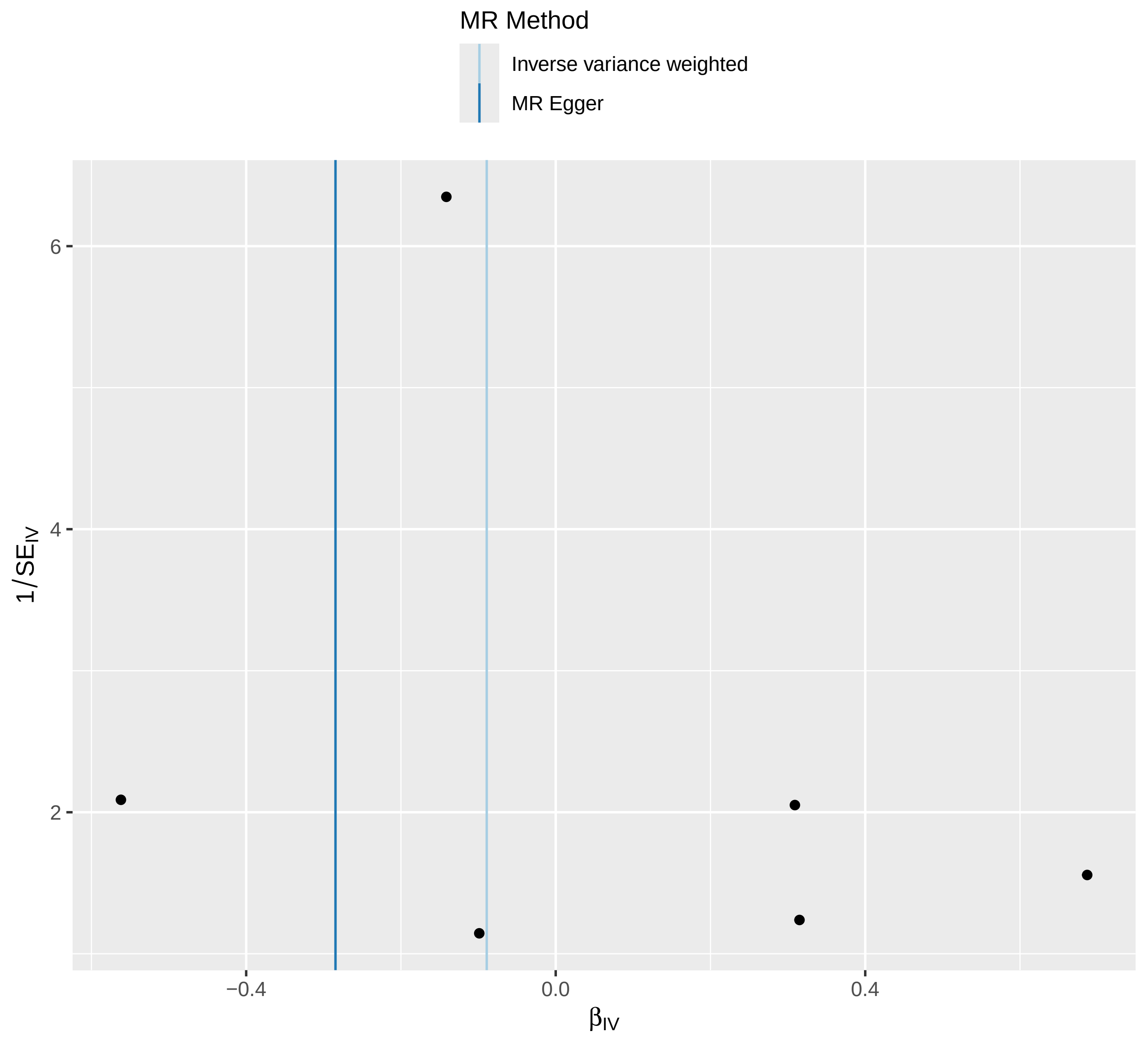

MR Method
Inverse variance weighted
MR Egger
6
4
2
1/SE$_{IV}$
−0.4
0.0
0.4
β_{IV}

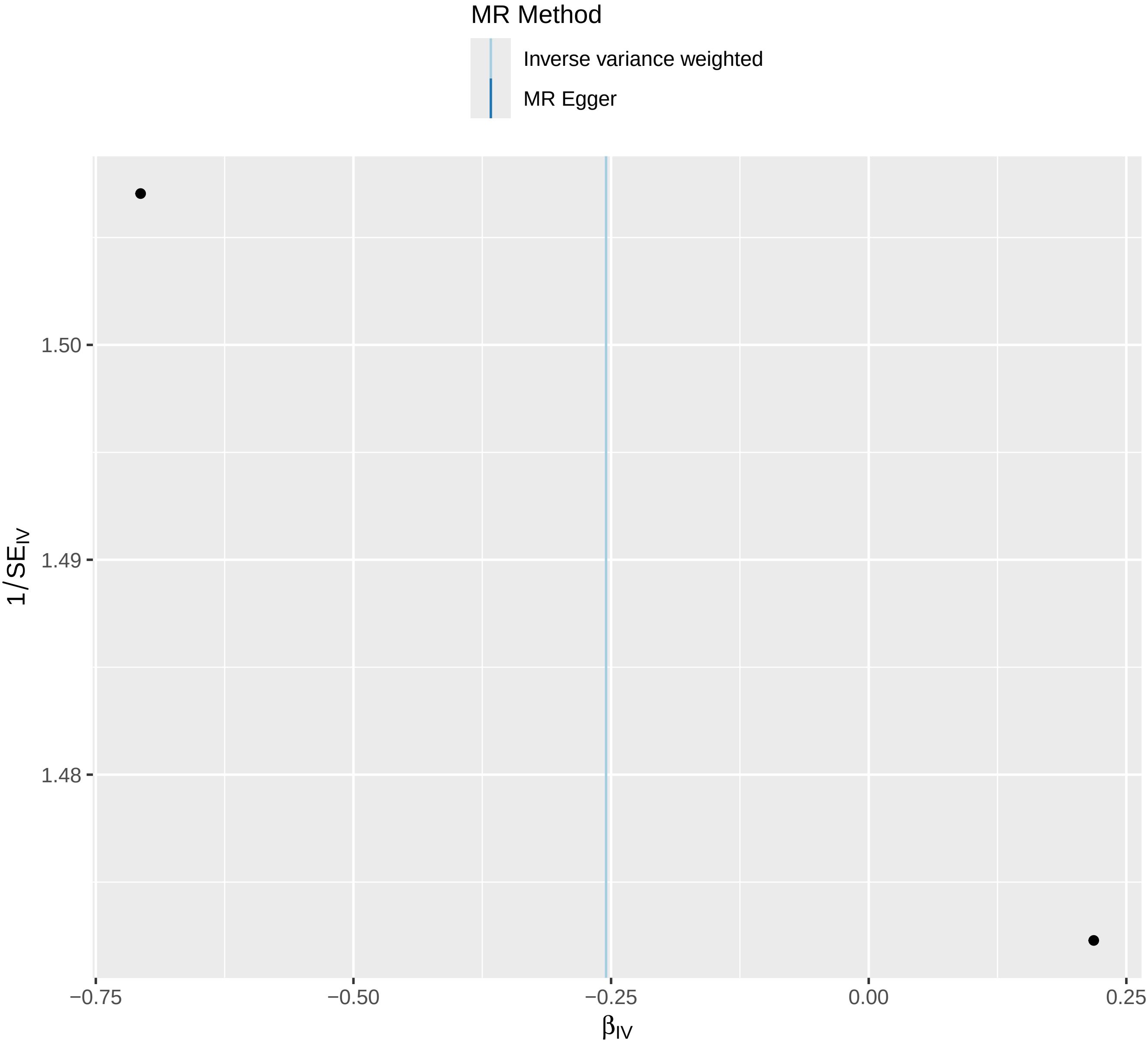

MR Method
Inverse variance weighted
MR Egger
1.50
1.49
1.48
1/SE$_{IV}$
−0.75
−0.50
−0.25
0.00
0.25
β$_{IV}$

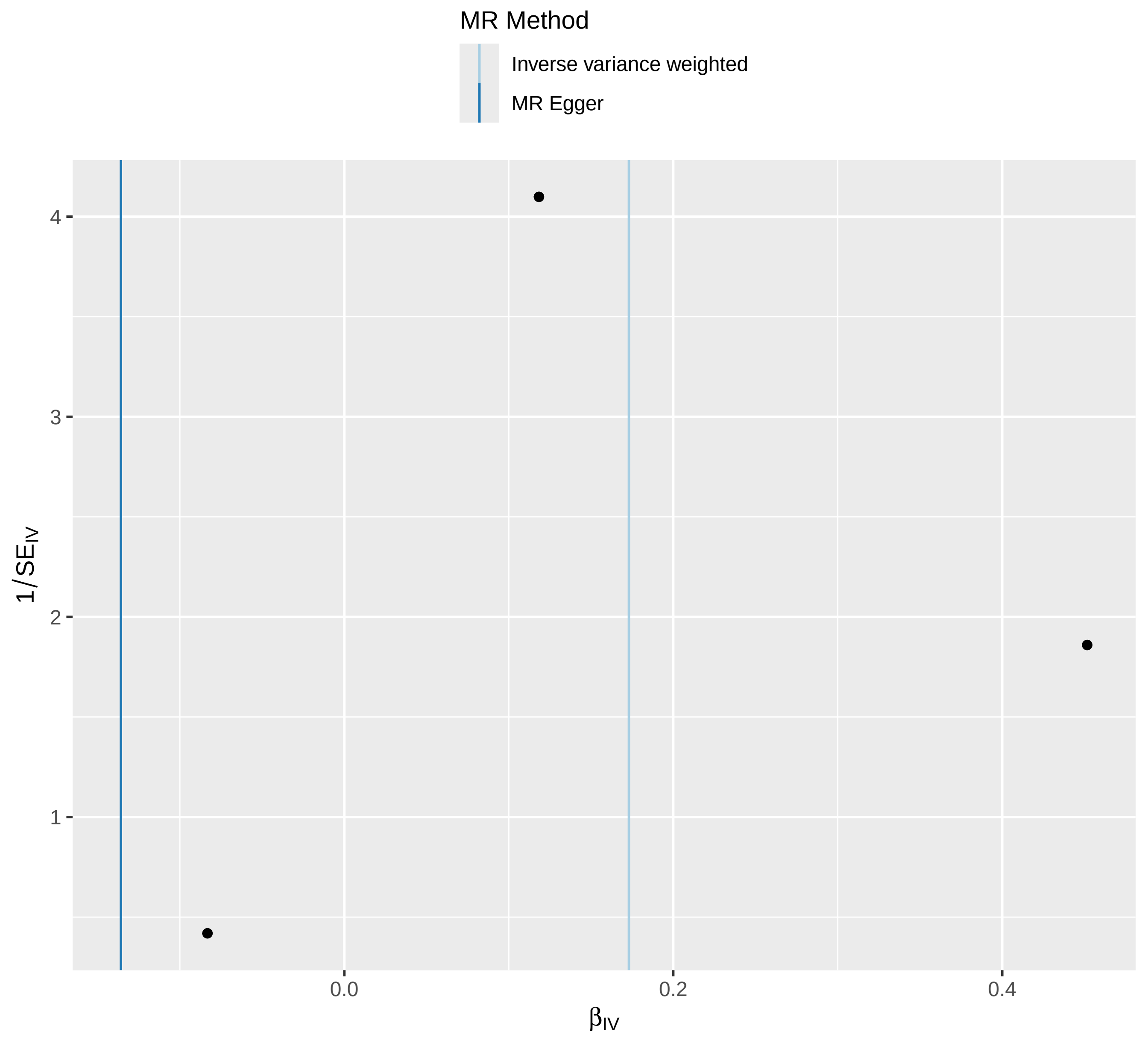
MR Method
Inverse variance weighted
MR Egger
1/SE_IV
4
3
2
1
0.0
0.2
0.4
β_IV

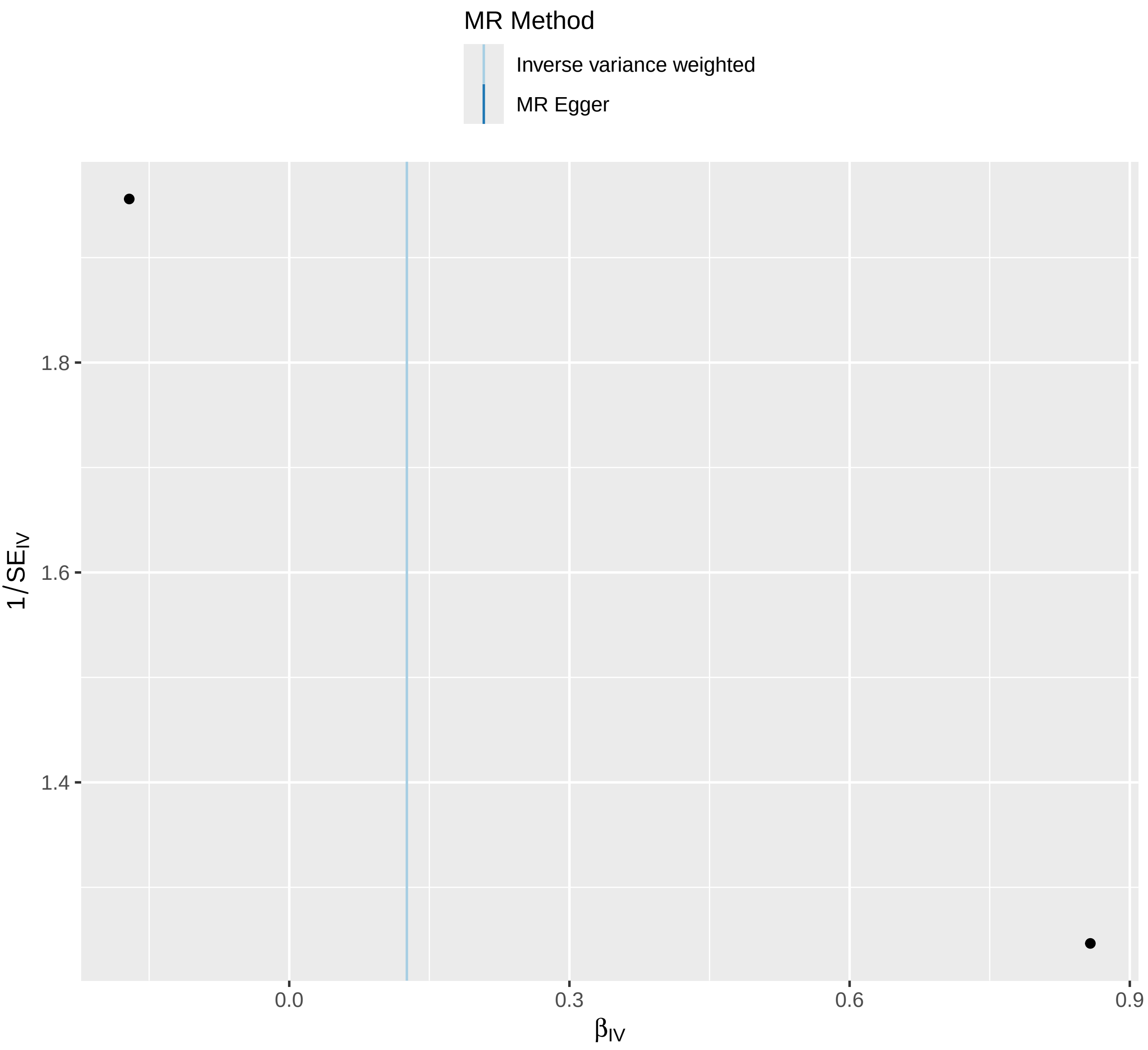
MR Method
Inverse variance weighted
MR Egger
1.8
1.6
1.4
1/SE$_{IV}$
0.0
0.3
0.6
0.9
β$_{IV}$

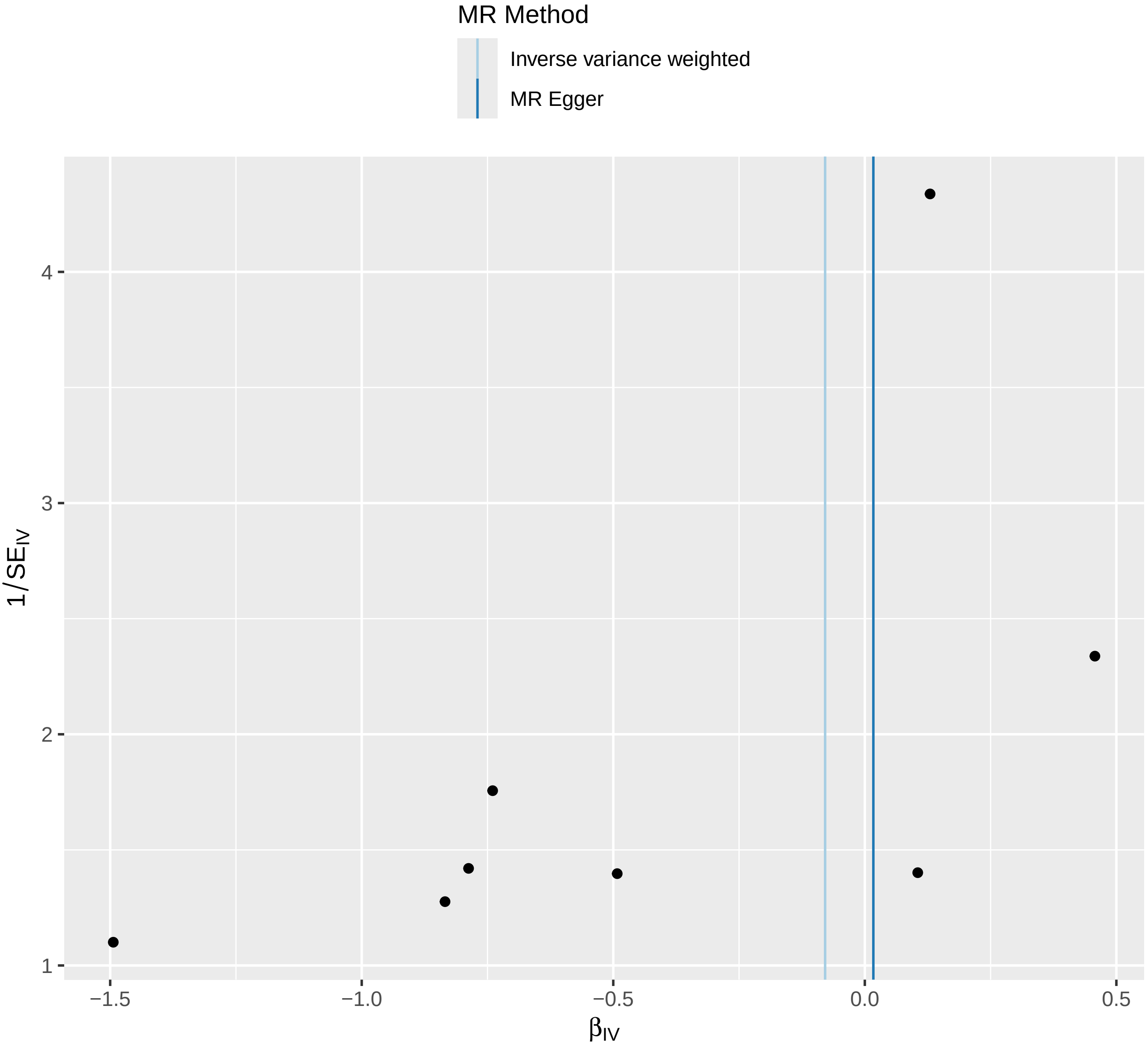
MR Method
Inverse variance weighted
MR Egger
1/SE$_{IV}$
4
3
2
1
−1.5
−1.0
−0.5
0.0
0.5
β$_{IV}$

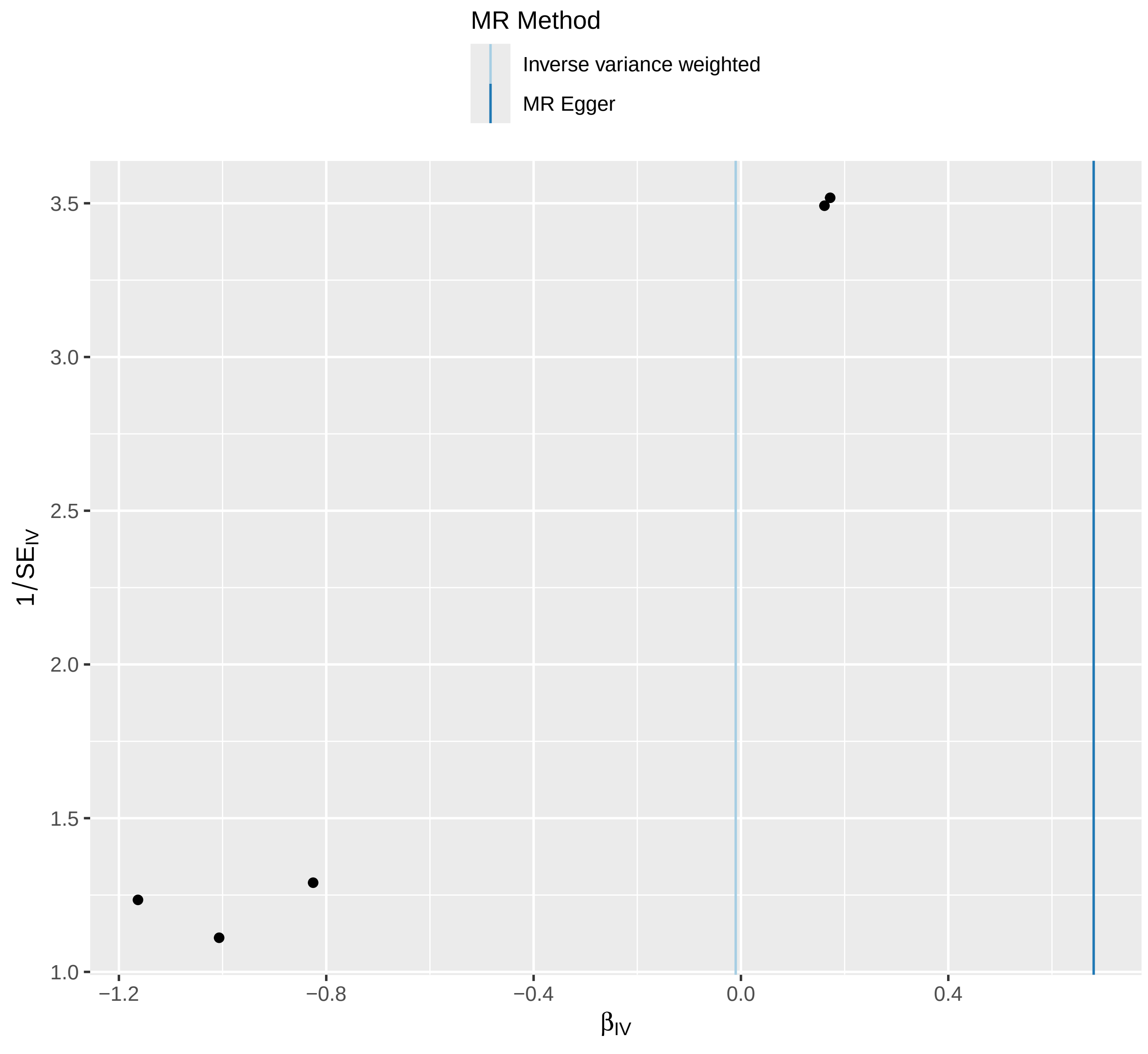
MR Method
Inverse variance weighted
MR Egger
1/SE_IV
3.5
3.0
2.5
2.0
1.5
1.0
−1.2
−0.8
−0.4
0.0
0.4
β_IV

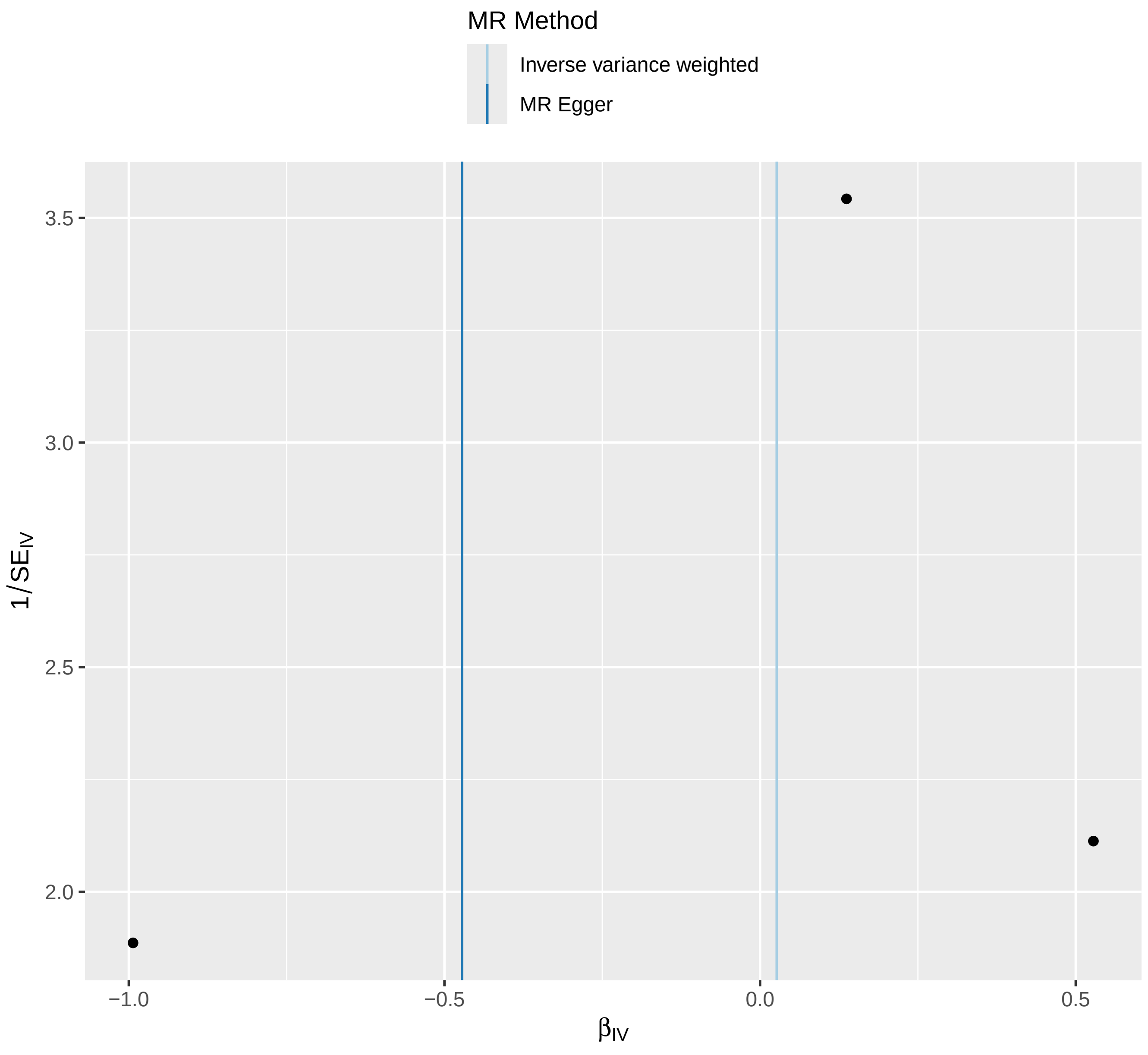
MR Method
Inverse variance weighted
MR Egger
3.5
3.0
2.5
2.0
−1.0
−0.5
0.0
0.5
$1/SE_{IV}$
β_{IV}

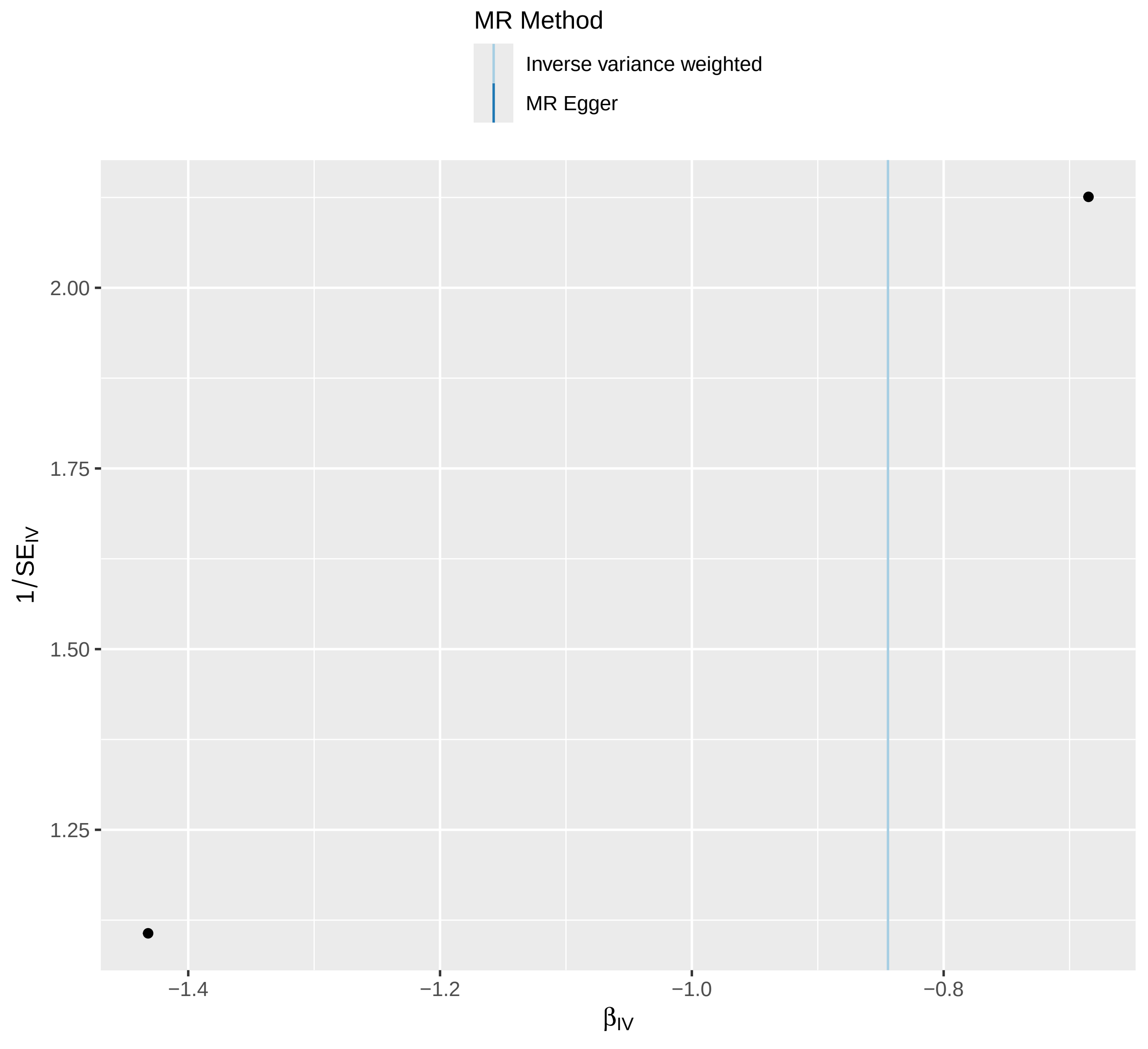
MR Method
Inverse variance weighted
MR Egger
2.00
1.75
1.50
1.25
$1/SE_{IV}$
−1.4
−1.2
−1.0
−0.8
β_{IV}

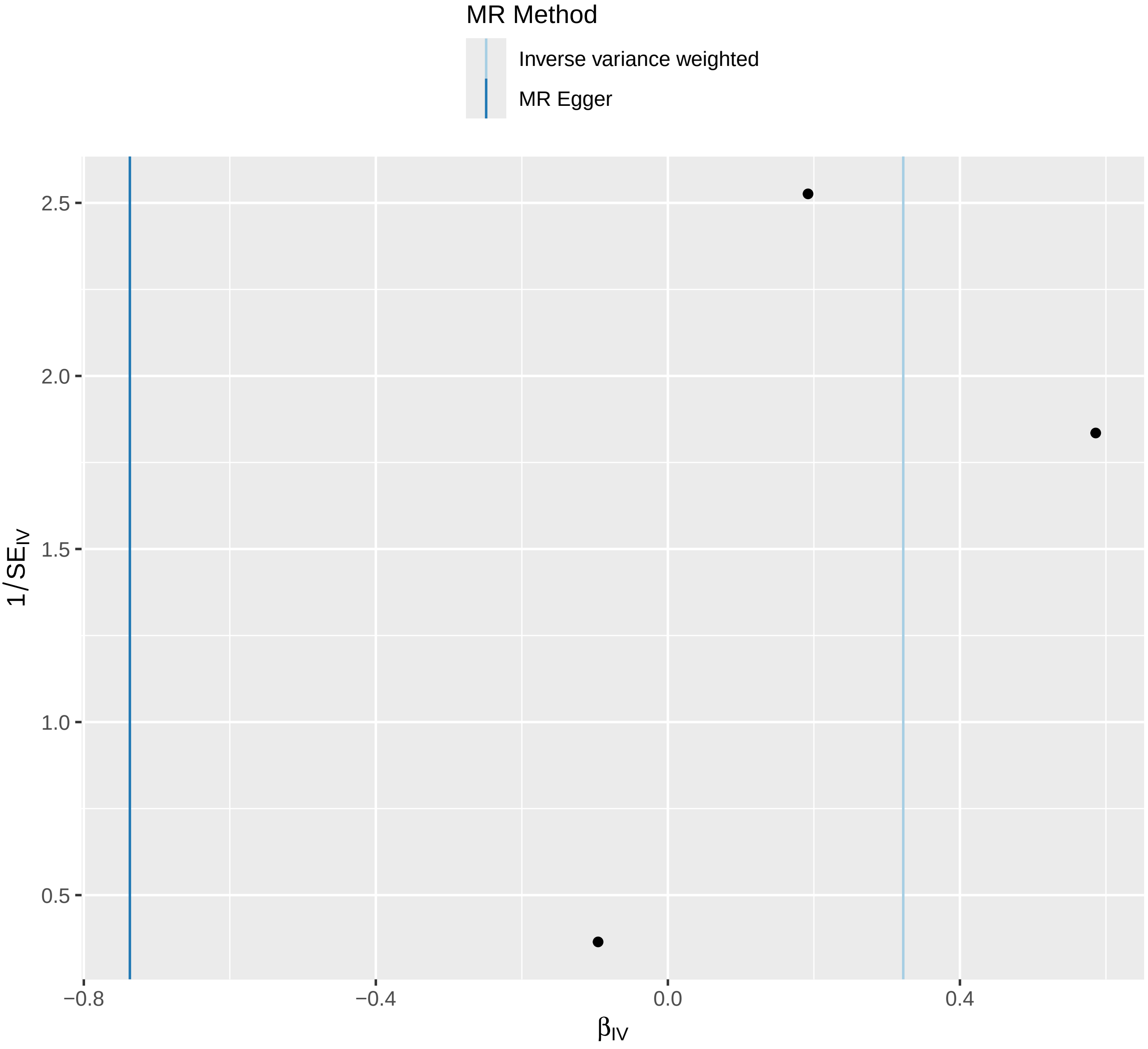
MR Method
Inverse variance weighted
MR Egger
2.5
2.0
1.5
1.0
0.5
1/SE$_{IV}$
−0.8
−0.4
0.0
0.4
β$_{IV}$

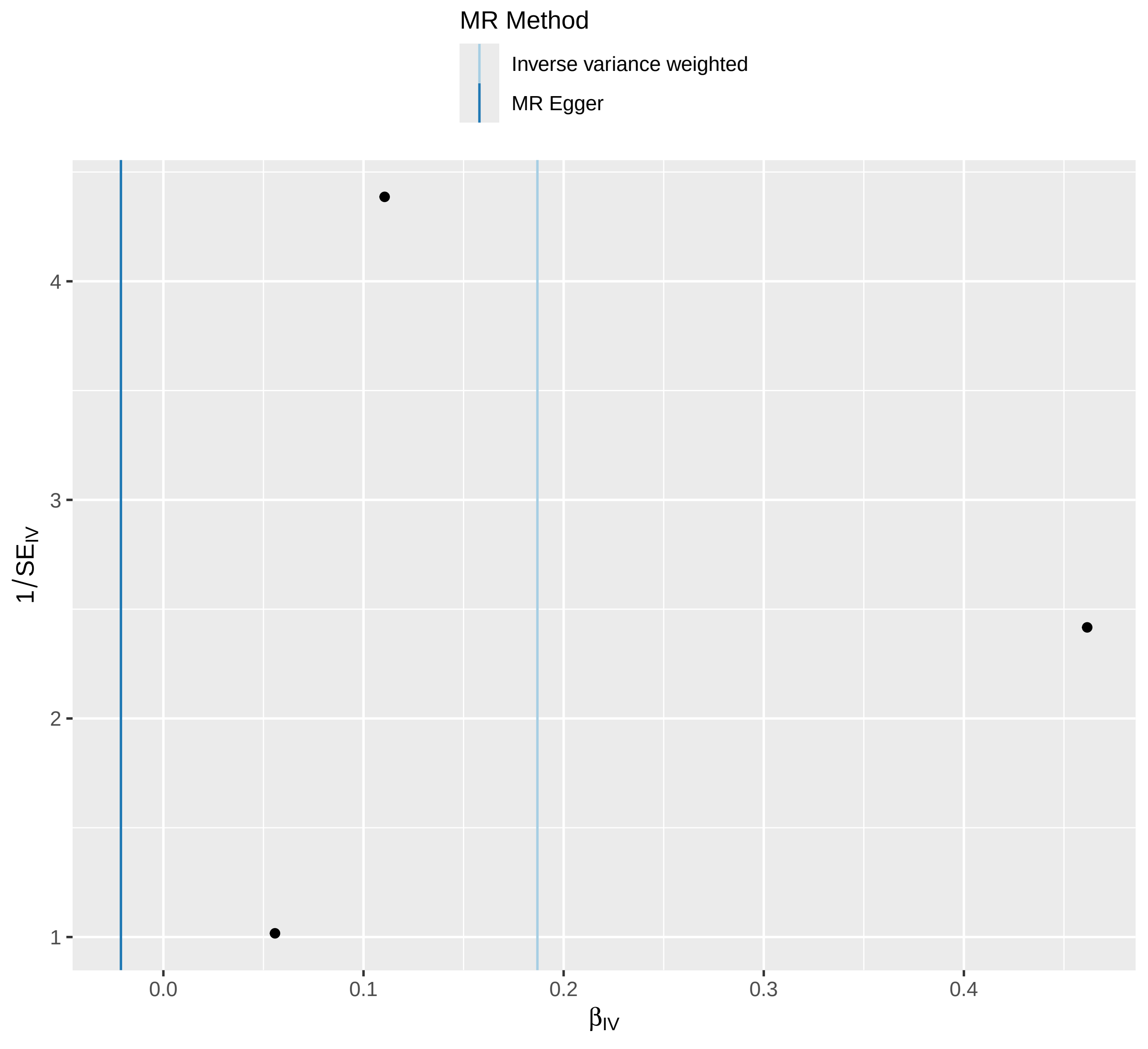

MR Method
Inverse variance weighted
MR Egger
1/SE_IV
4
3
2
1
0.0
0.1
0.2
0.3
0.4
β_IV

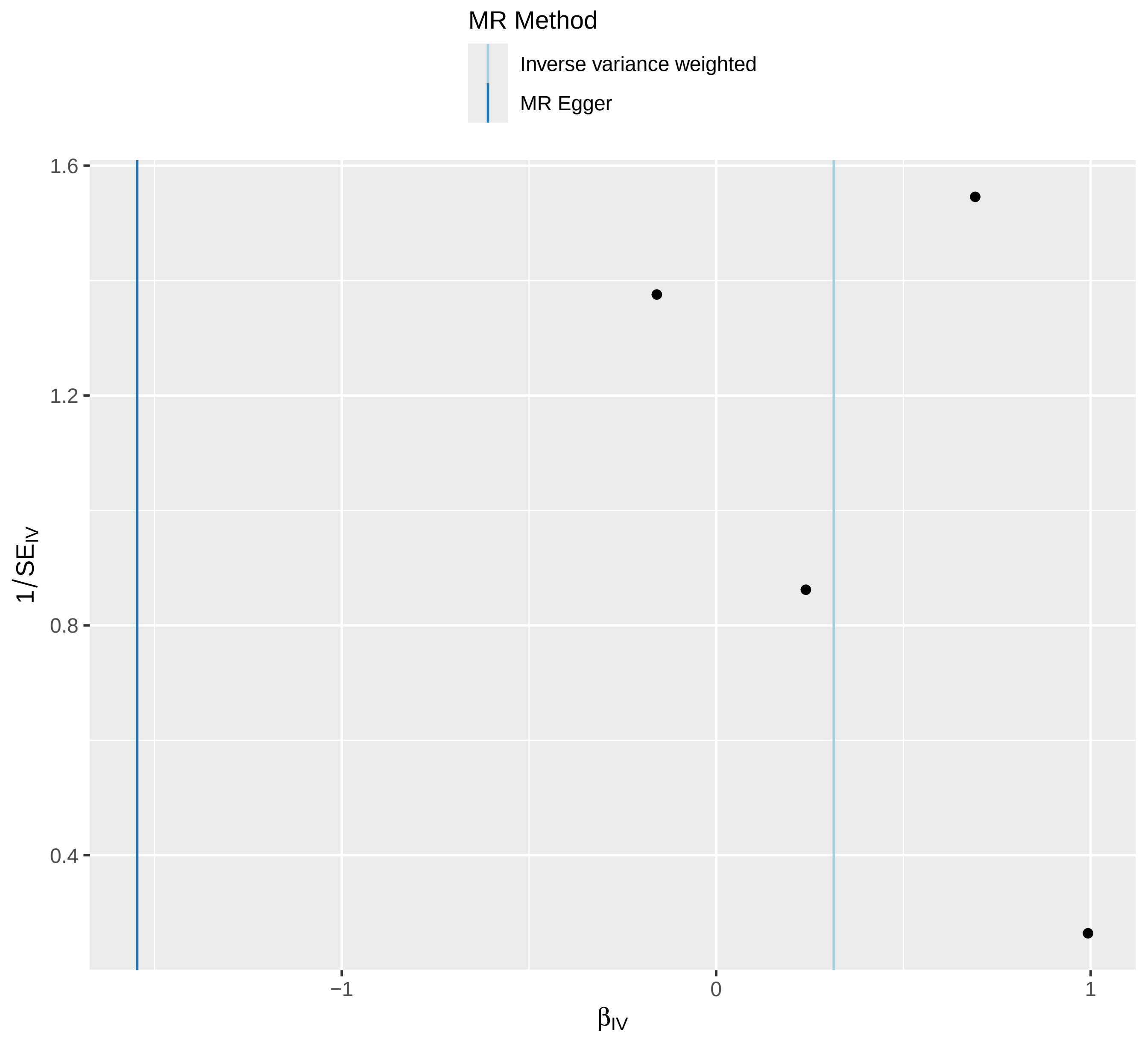

MR Method
Inverse variance weighted
MR Egger
1.6
1.2
0.8
0.4
$1/SE_{IV}$
−1
0
1
β_{IV}

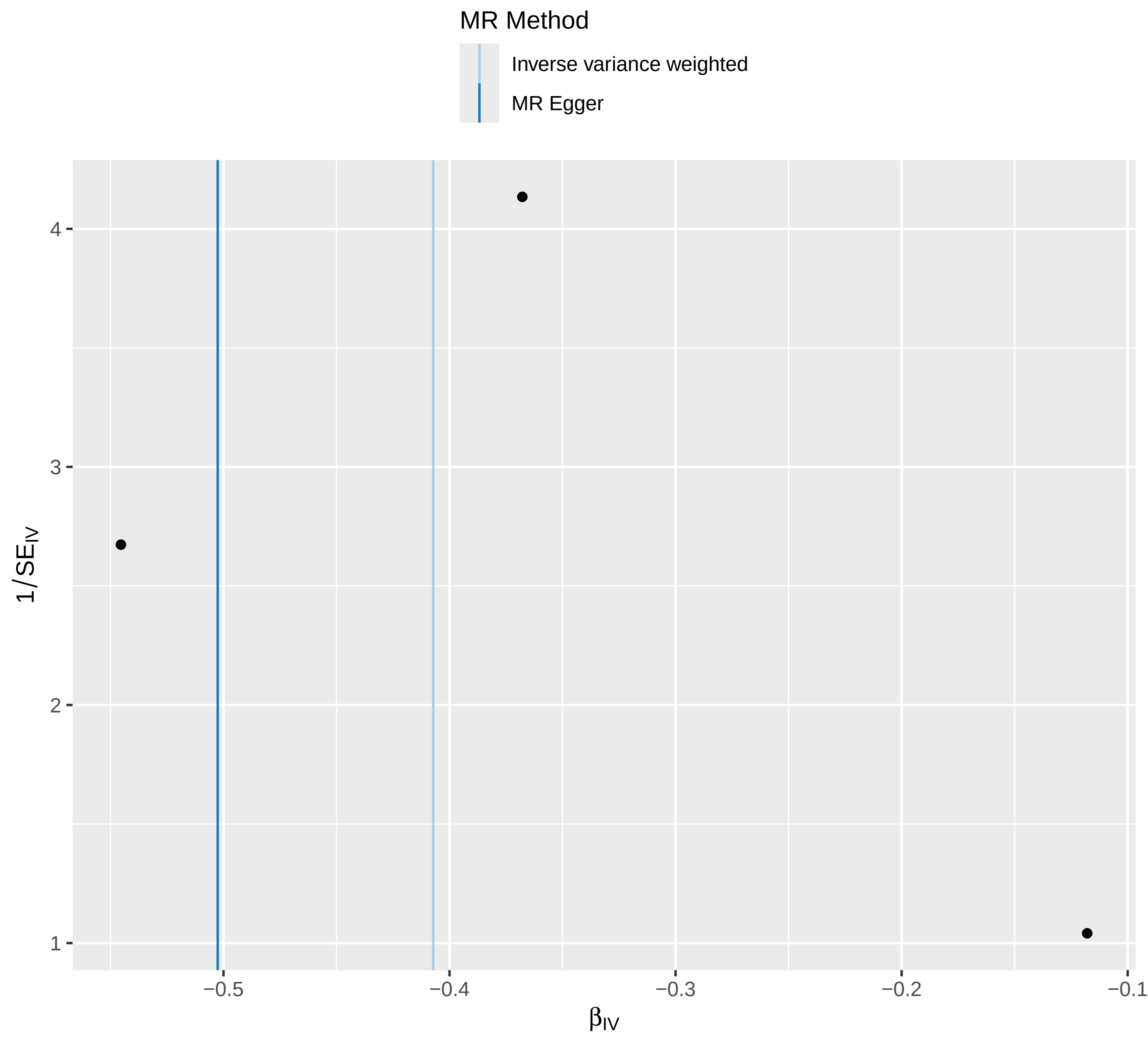
MR Method
Inverse variance weighted
MR Egger
1/SE_IV
4
3
2
1
−0.5
−0.4
−0.3
−0.2
−0.1
β_IV

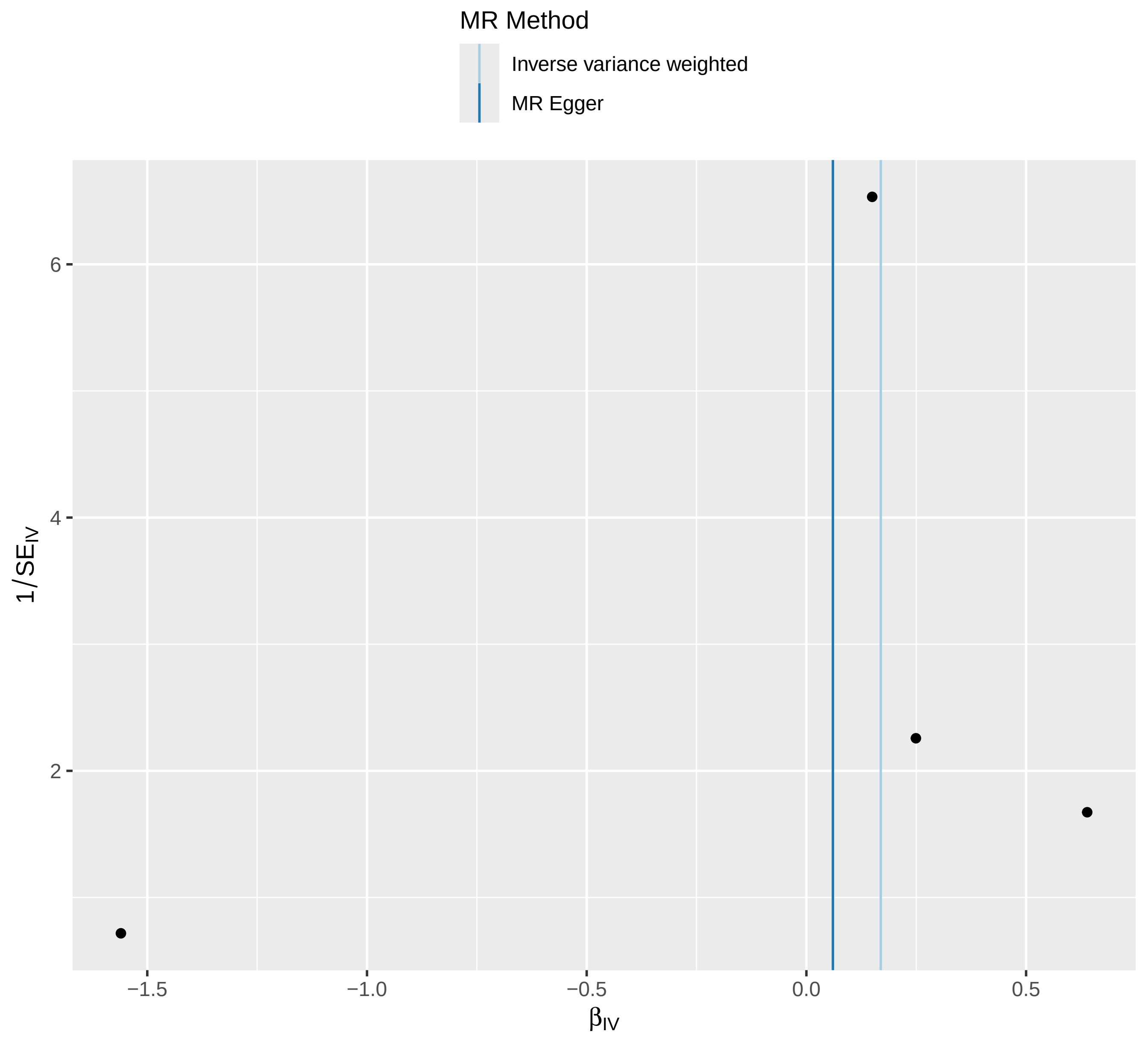
MR Method
Inverse variance weighted
MR Egger
6
4
2
1/SE_IV
−1.5
−1.0
−0.5
0.0
0.5
β_IV

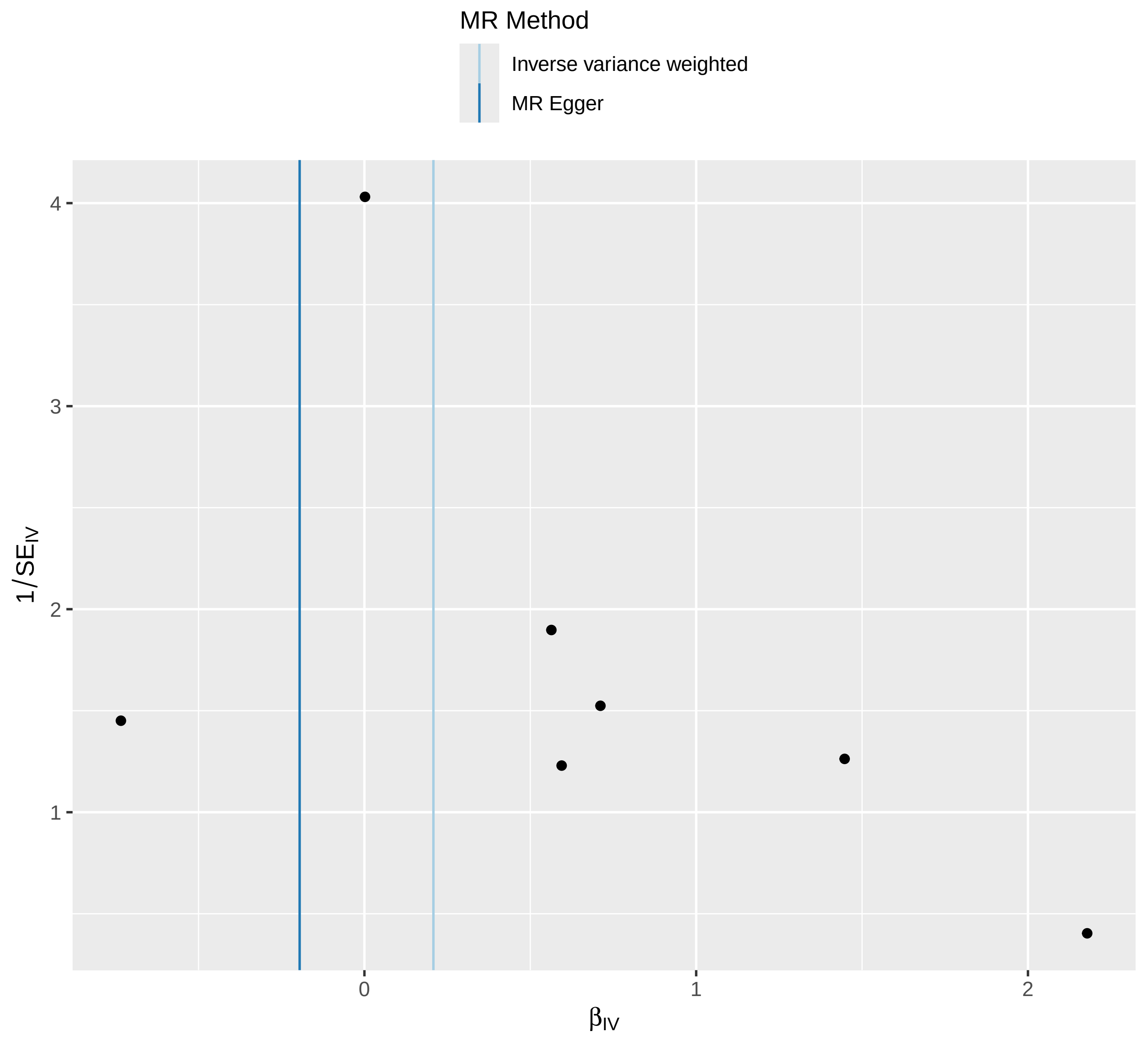

MR Method
Inverse variance weighted
MR Egger
4
3
2
1
1/SE_IV
0
1
2
β_IV

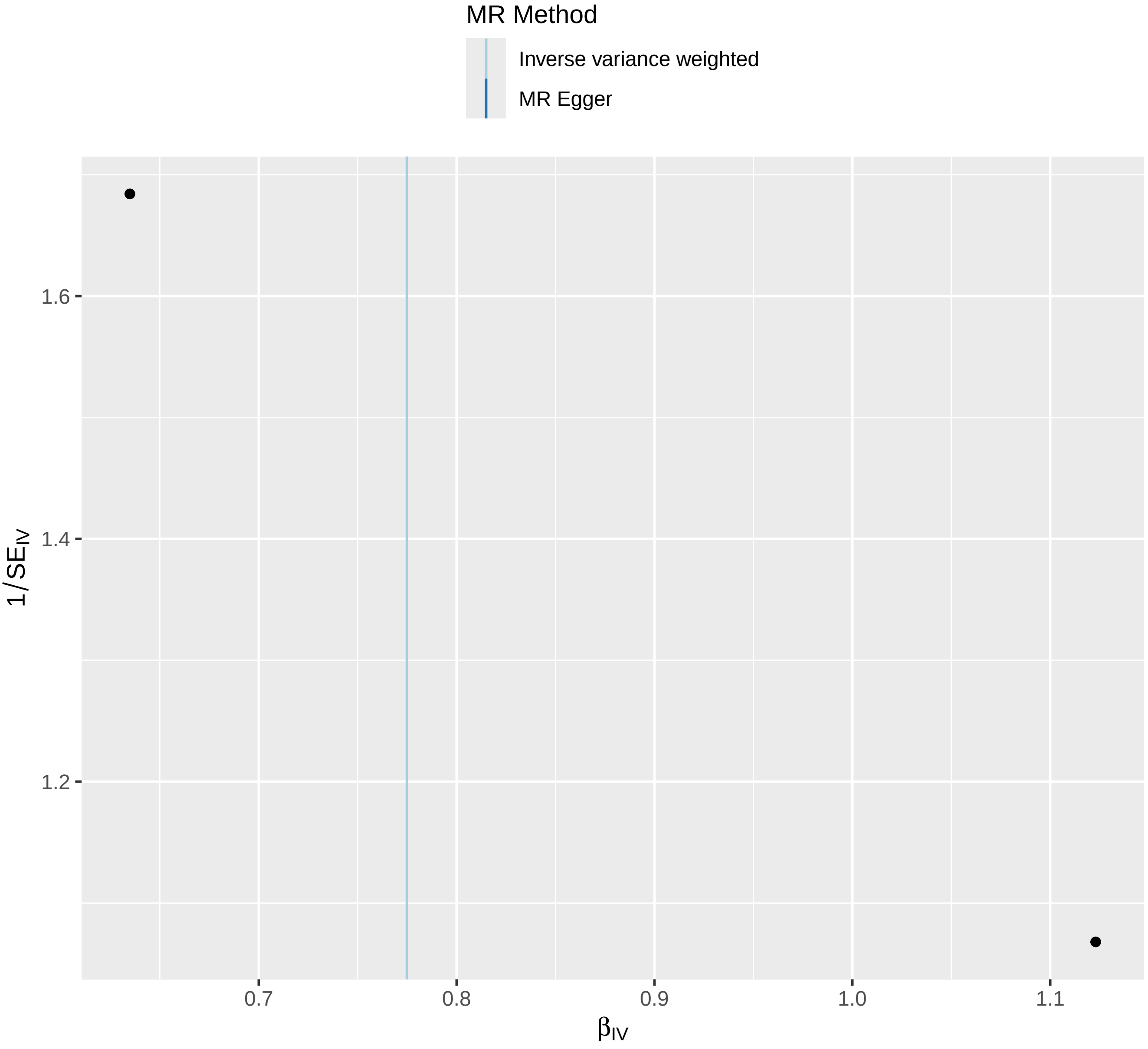

MR Method
Inverse variance weighted
MR Egger
1.6
1.4
1.2
0.7
0.8
0.9
1.0
1.1
1/SE_IV
β_IV

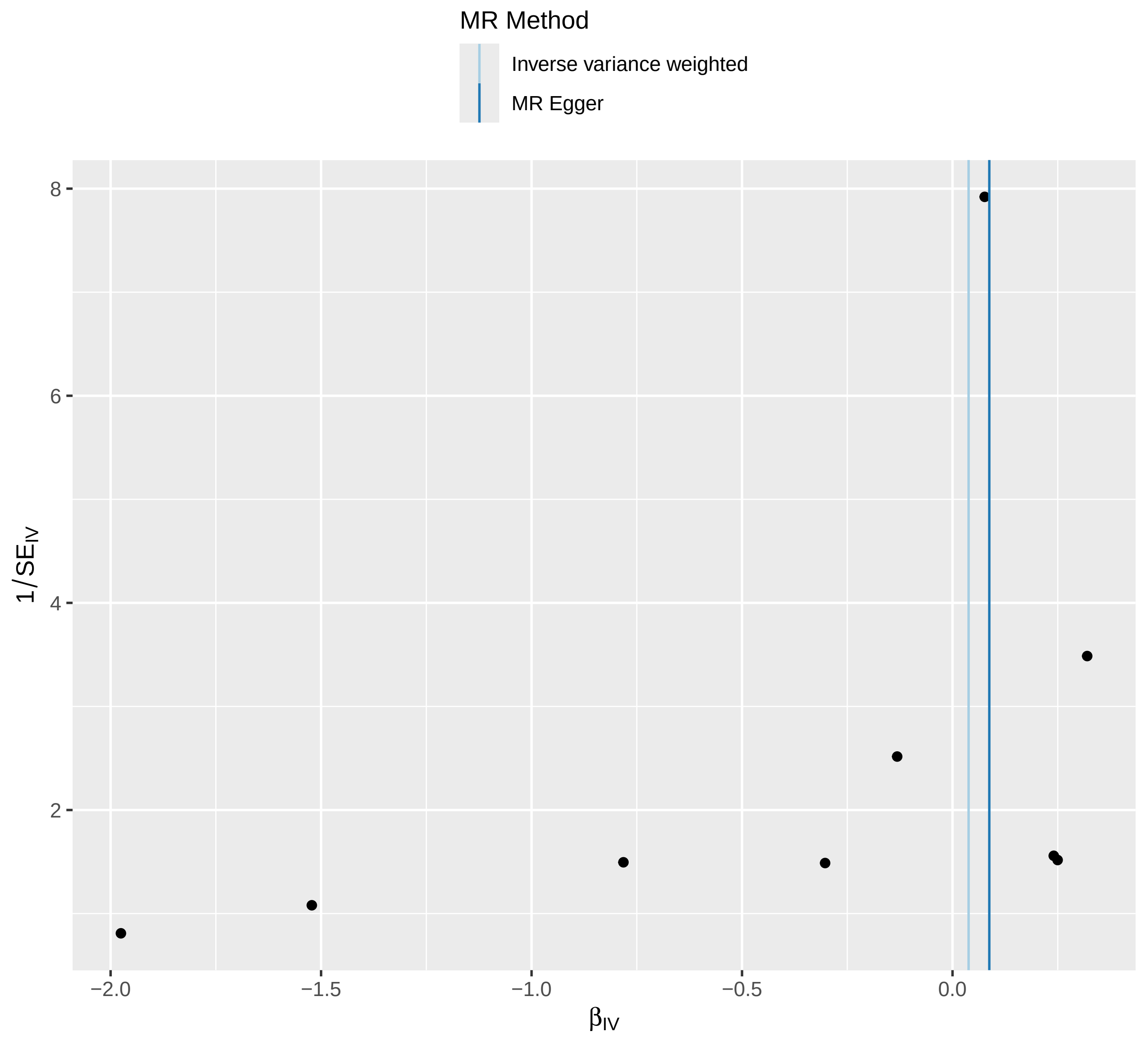
MR Method
Inverse variance weighted
MR Egger
8
6
4
2
1/SE_IV
−2.0
−1.5
−1.0
−0.5
0.0
β_IV

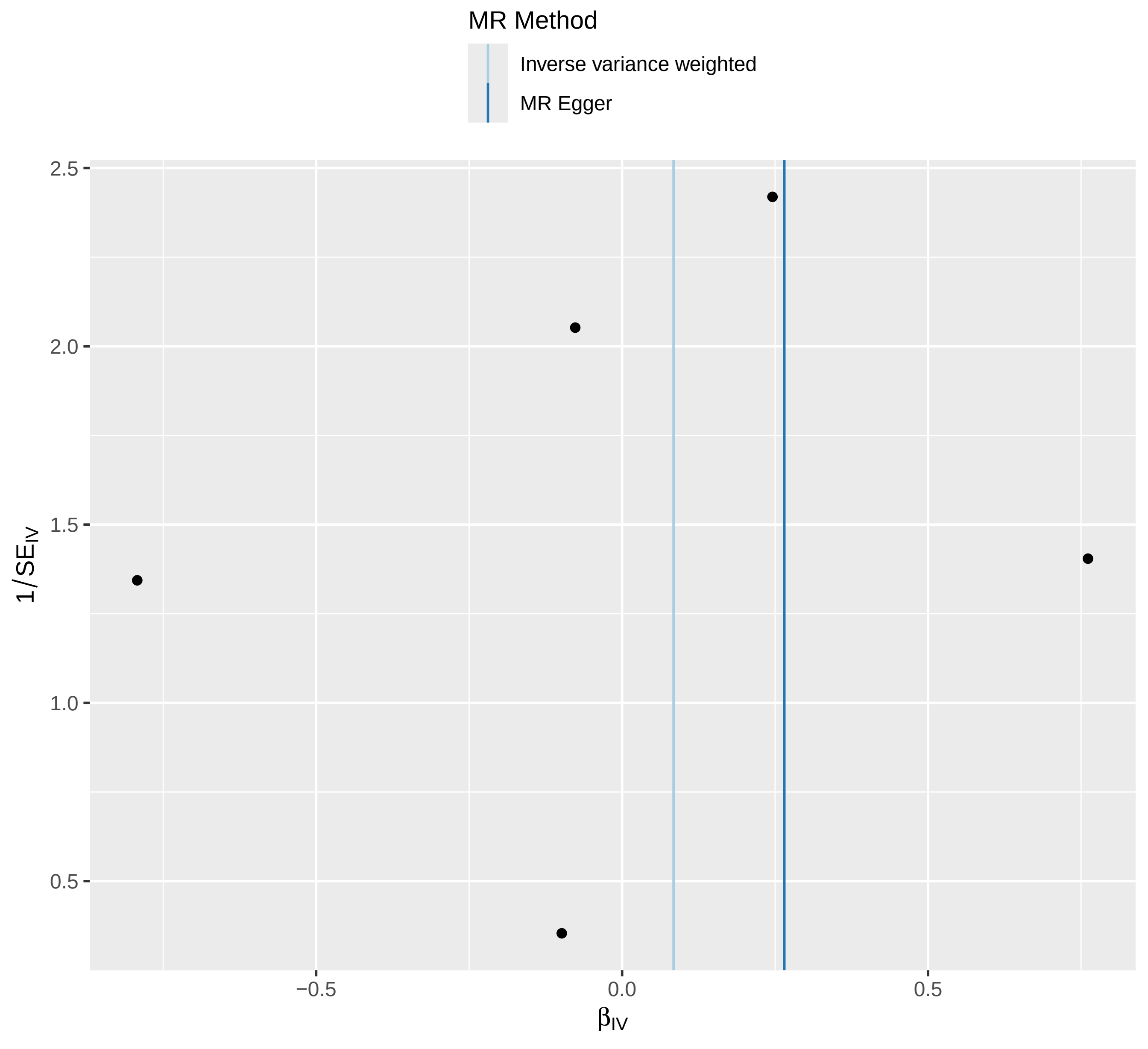
MR Method
Inverse variance weighted
MR Egger
2.5
2.0
1.5
1.0
0.5
1/SE_IV
−0.5
0.0
0.5
β_IV

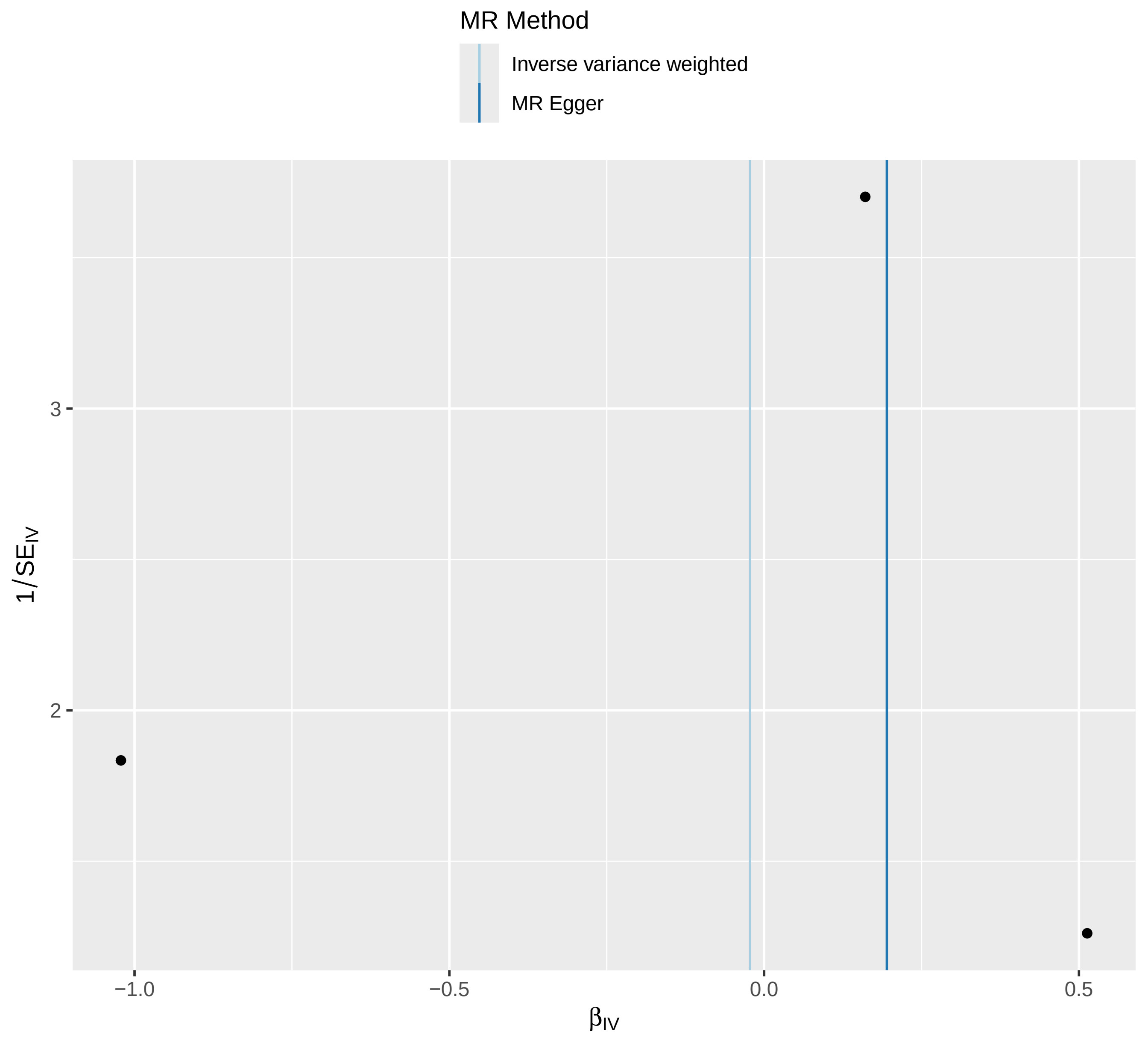

MR Method
Inverse variance weighted
MR Egger
3
2
1/SE_IV
−1.0
−0.5
0.0
0.5
β_IV

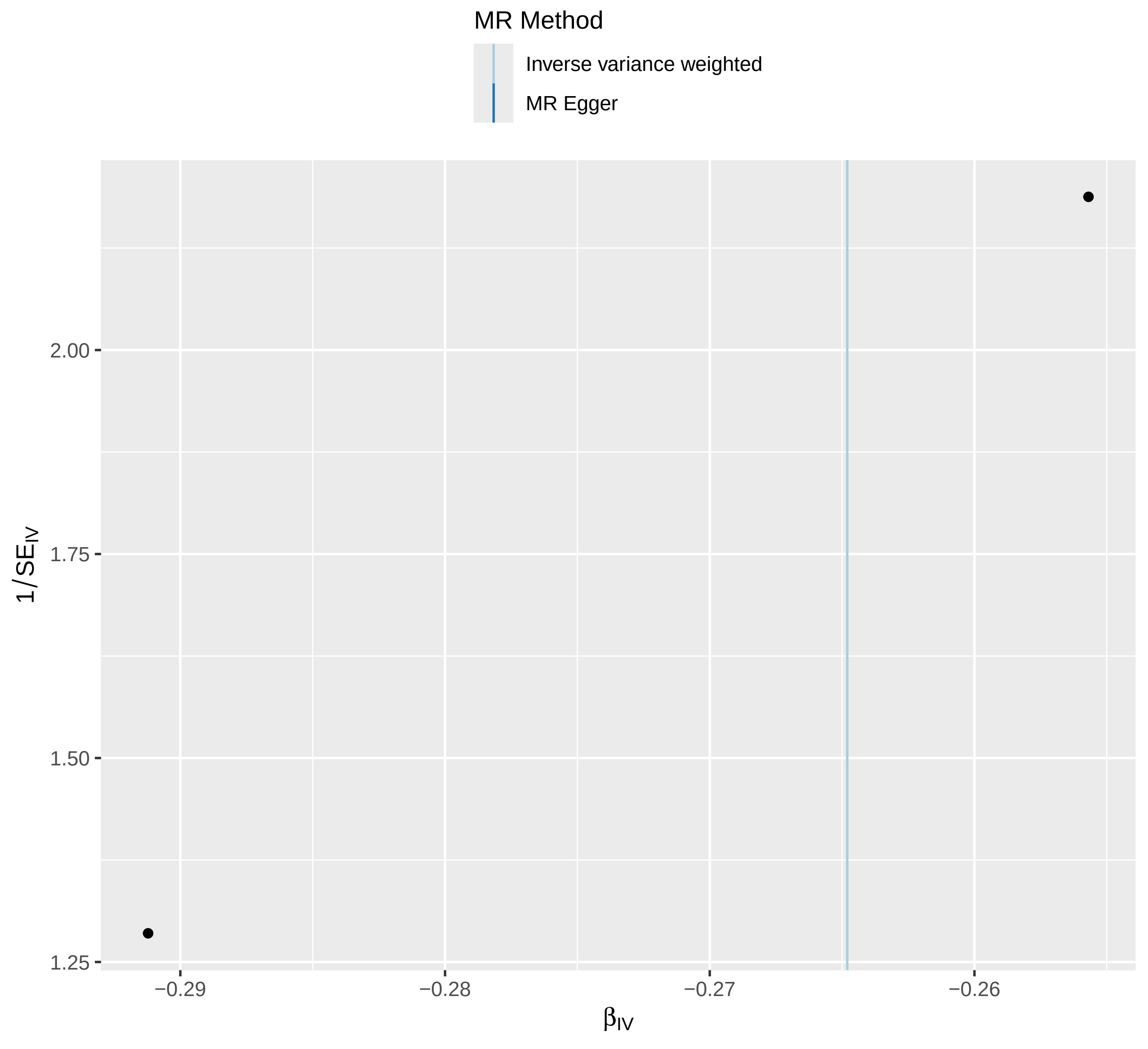
MR Method
Inverse variance weighted
MR Egger
2.00
1.75
1.50
1.25
−0.29
−0.28
−0.27
−0.26
$1/SE_{IV}$
β_{IV}

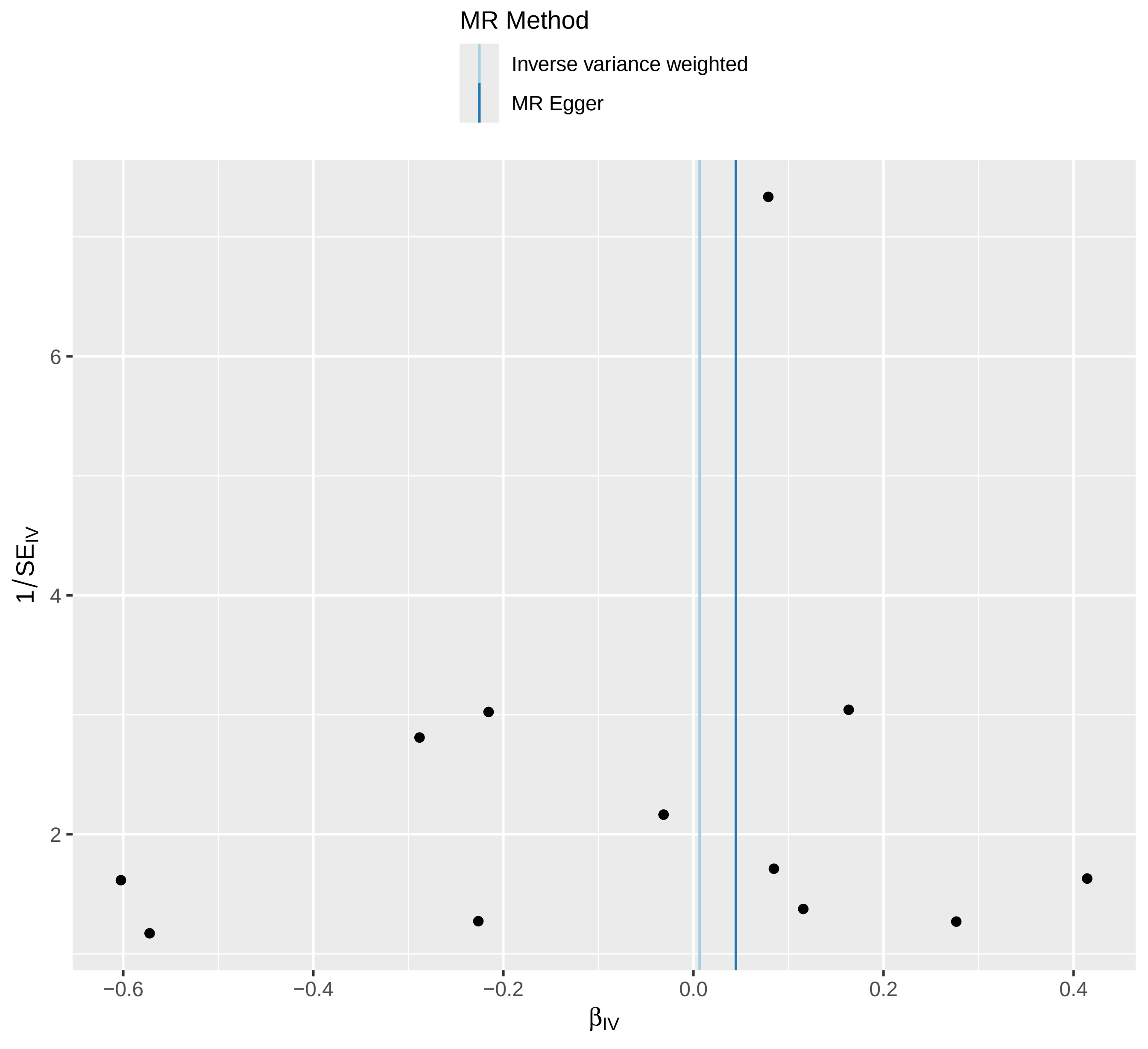

MR Method
Inverse variance weighted
MR Egger
6
4
2
−0.6
−0.4
−0.2
0.0
0.2
0.4
$1/SE_{IV}$
β_{IV}

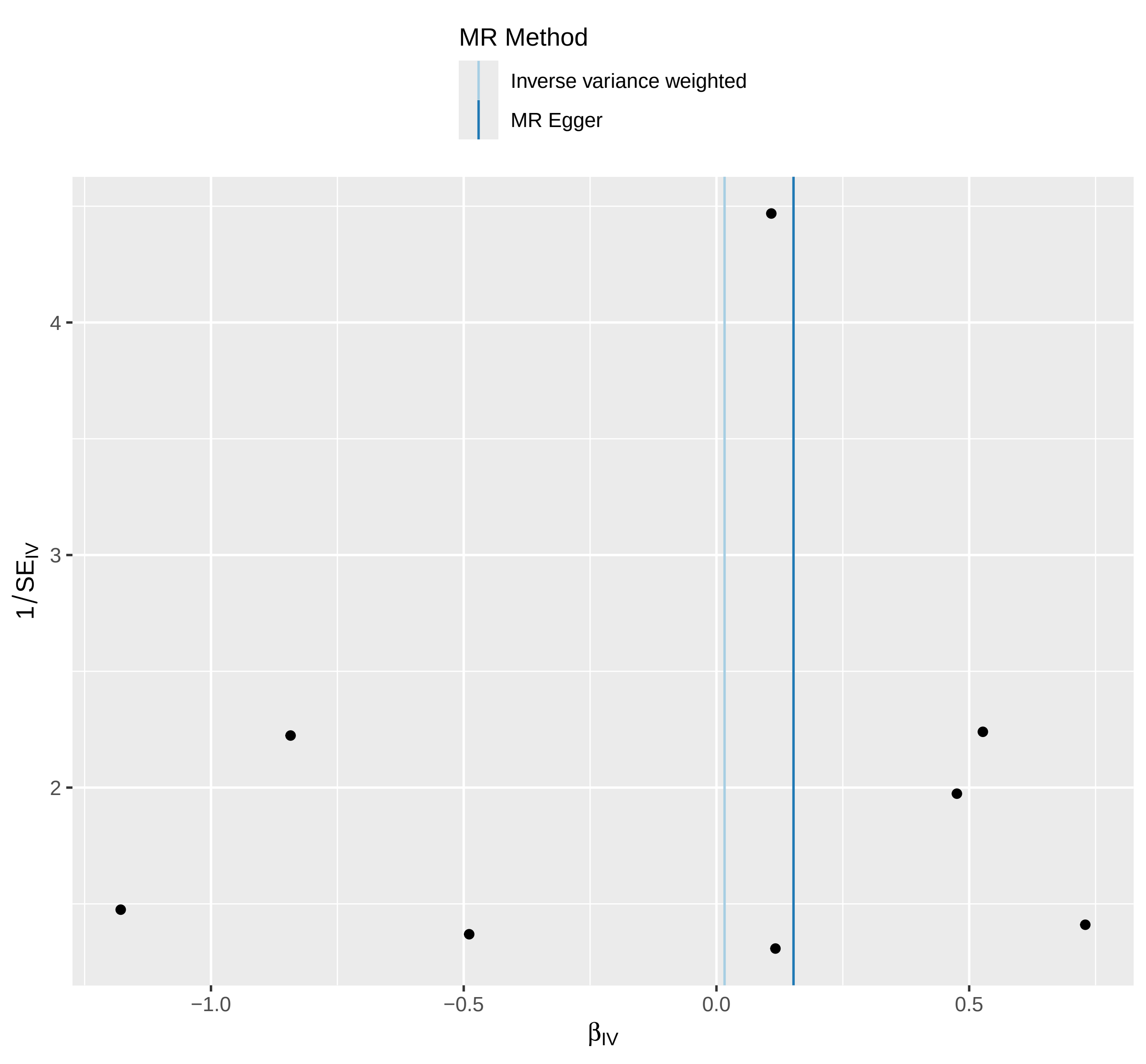
MR Method
Inverse variance weighted
MR Egger
4
3
2
1/SE$_{IV}$
−1.0
−0.5
0.0
0.5
β$_{IV}$

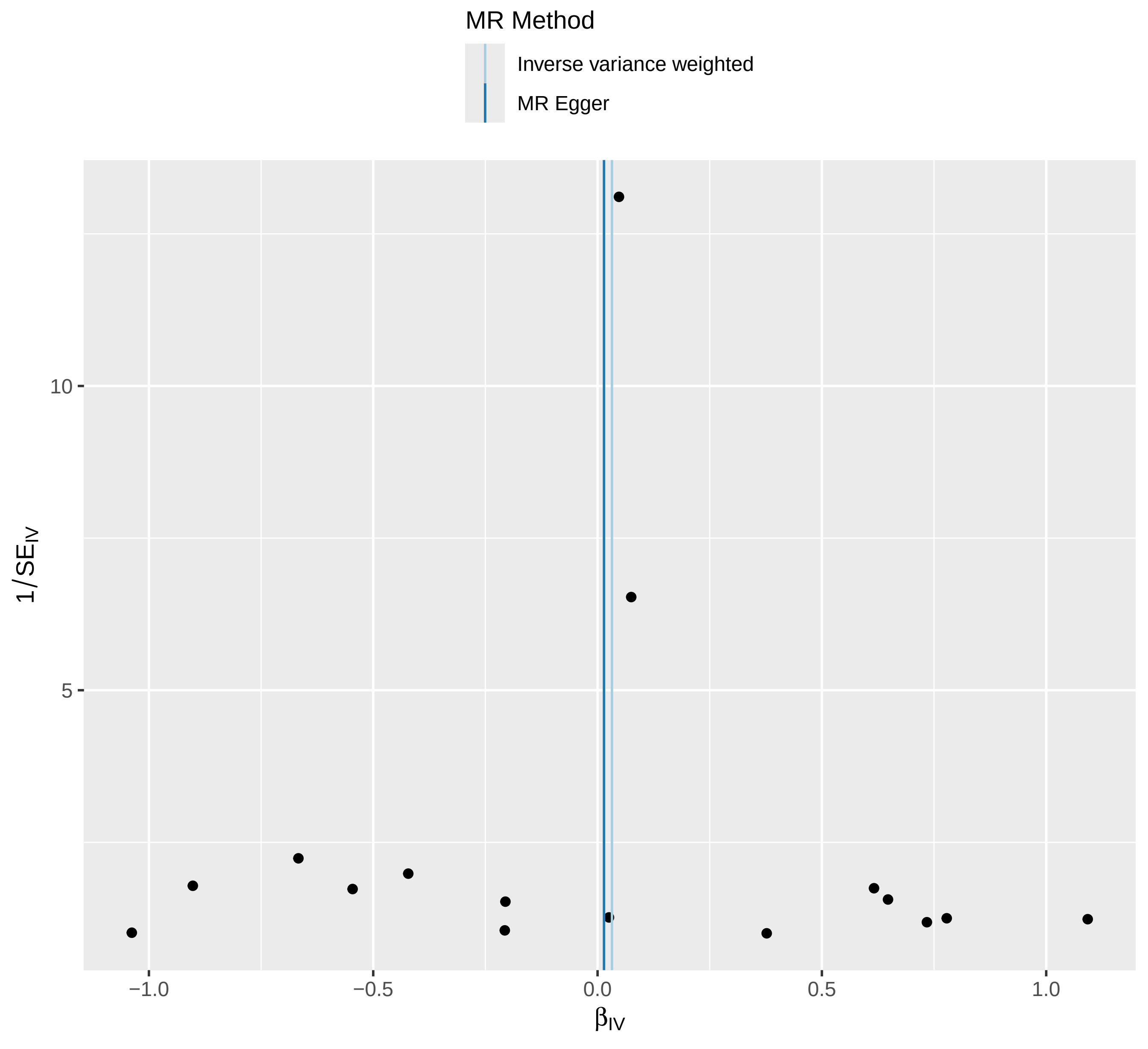

MR Method
Inverse variance weighted
MR Egger
10
5
1/SE$_{IV}$
−1.0
−0.5
0.0
0.5
1.0
β$_{IV}$

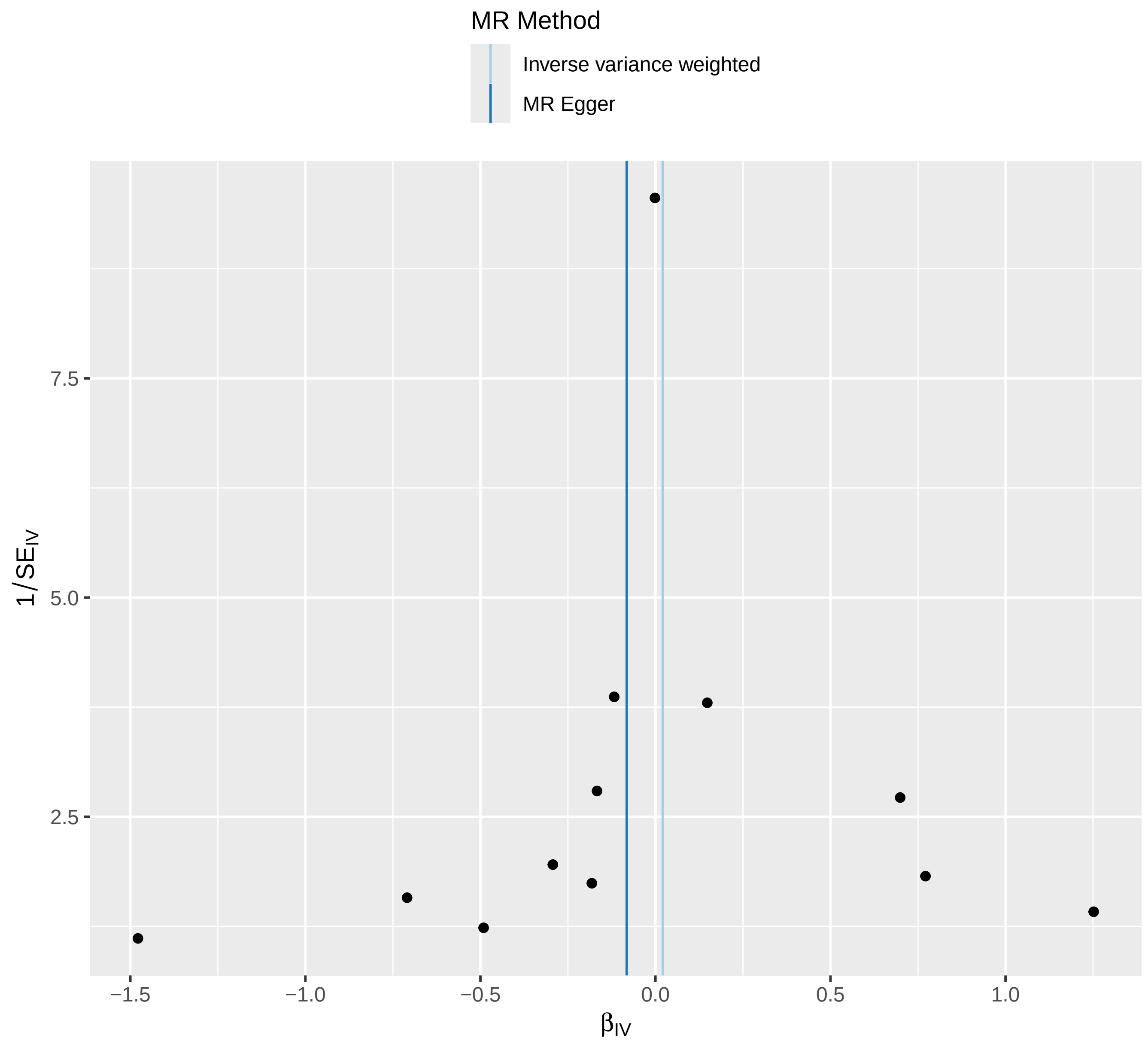
MR Method
Inverse variance weighted
MR Egger
7.5
5.0
2.5
1/SE_IV
−1.5
−1.0
−0.5
0.0
0.5
1.0
β_IV

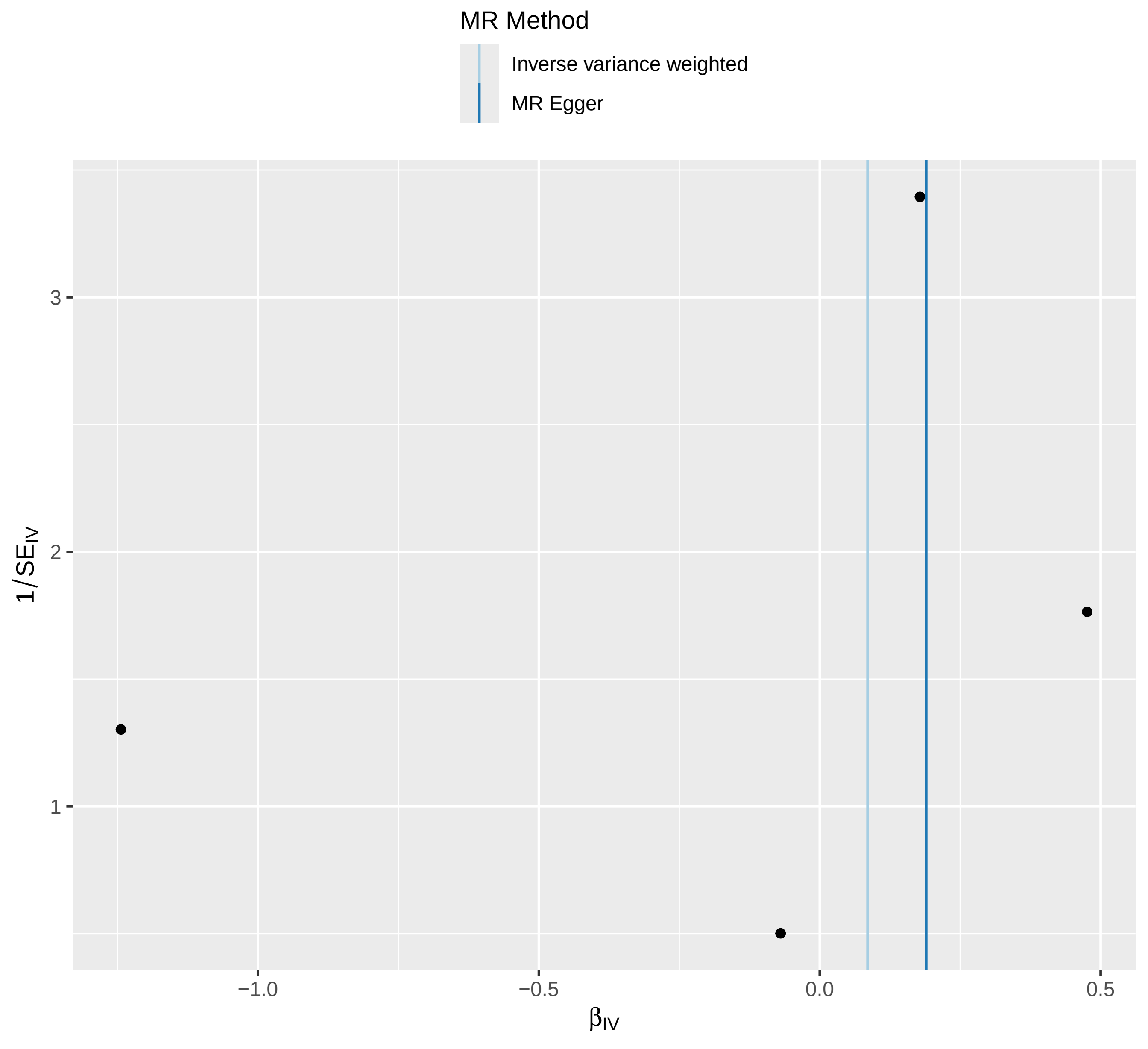

MR Method
Inverse variance weighted
MR Egger
1/SE_IV
3
2
1
−1.0
−0.5
0.0
0.5
β_IV

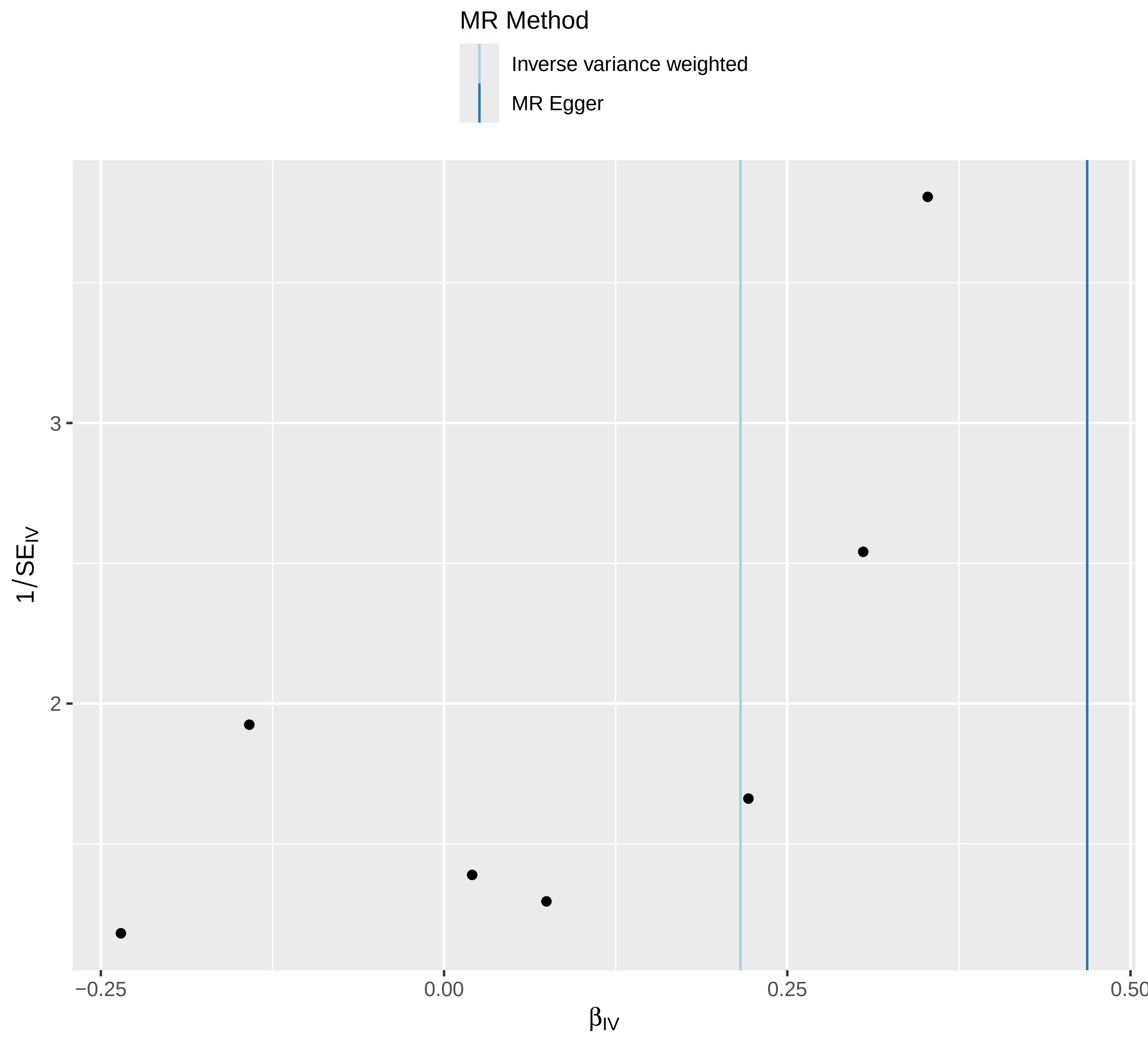

MR Method
Inverse variance weighted
MR Egger
1/SE_IV
3
2
−0.25
0.00
0.25
0.50
β_IV

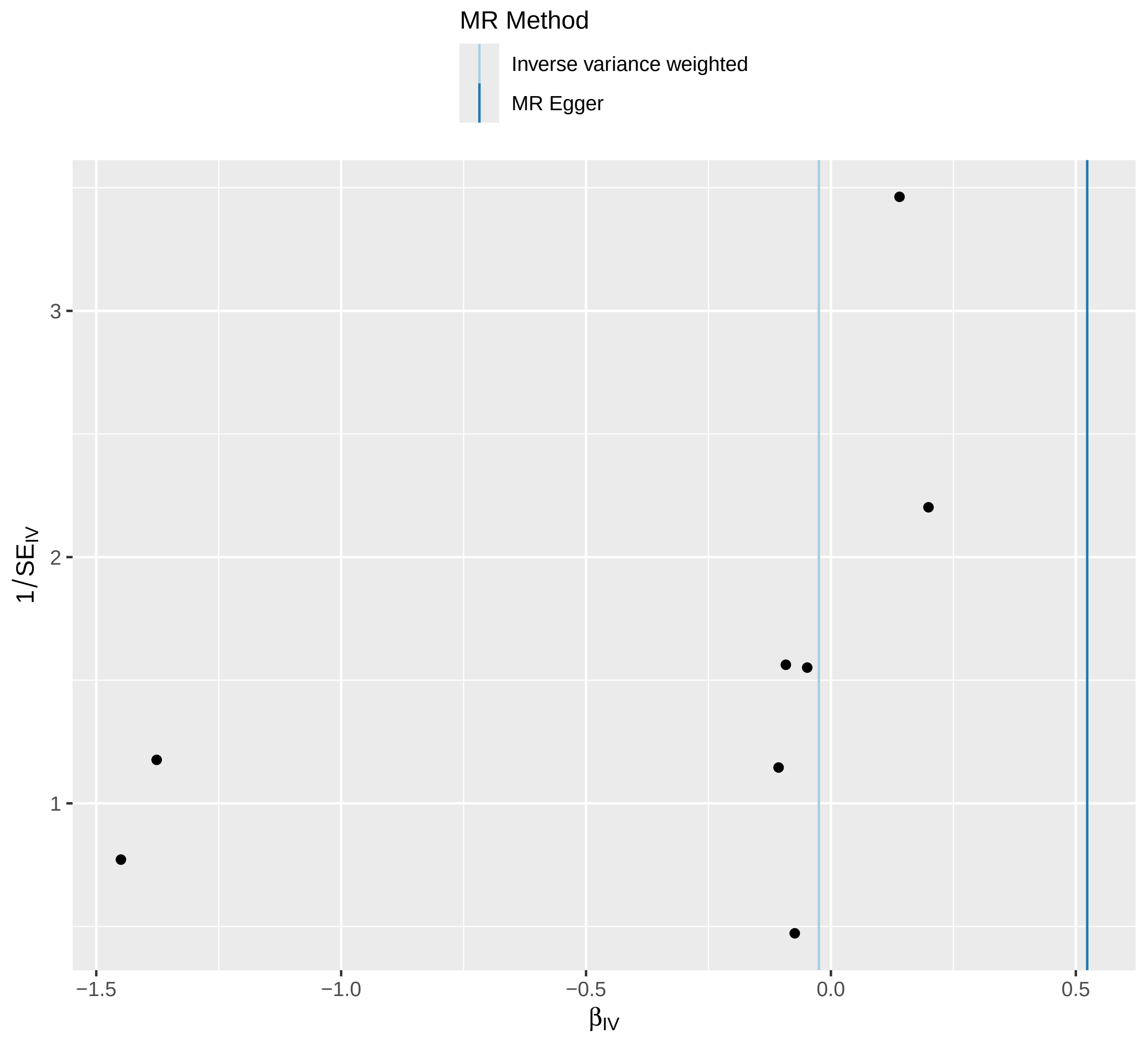

MR Method
Inverse variance weighted
MR Egger
1/SE_IV
3
2
1
−1.5
−1.0
−0.5
0.0
0.5
β_IV

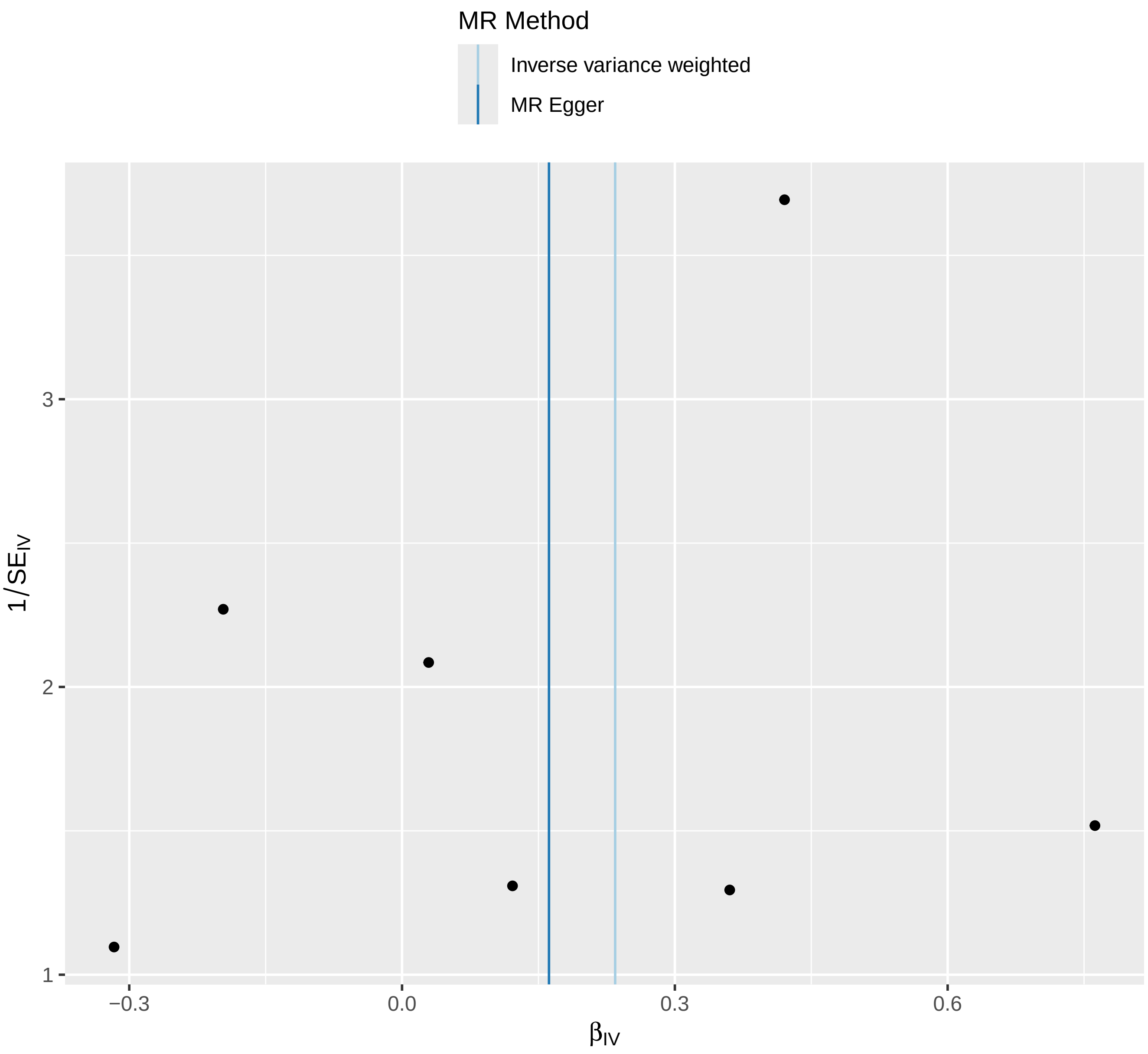
MR Method
Inverse variance weighted
MR Egger
3
2
1
$1/SE_{IV}$
−0.3
0.0
0.3
0.6
β_{IV}

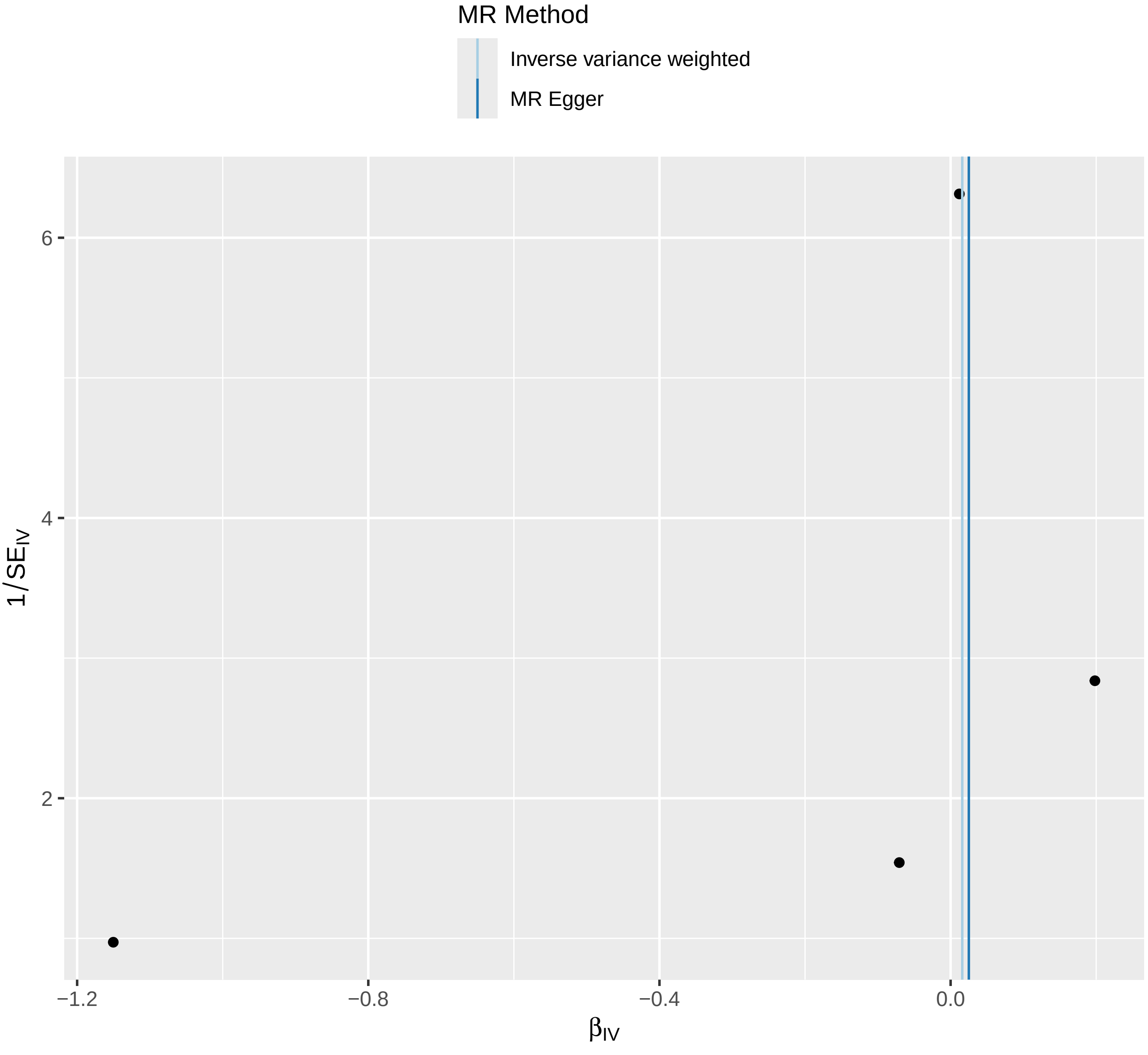

MR Method
Inverse variance weighted
MR Egger
1/SE_IV
6
4
2
−1.2
−0.8
−0.4
0.0
β_IV

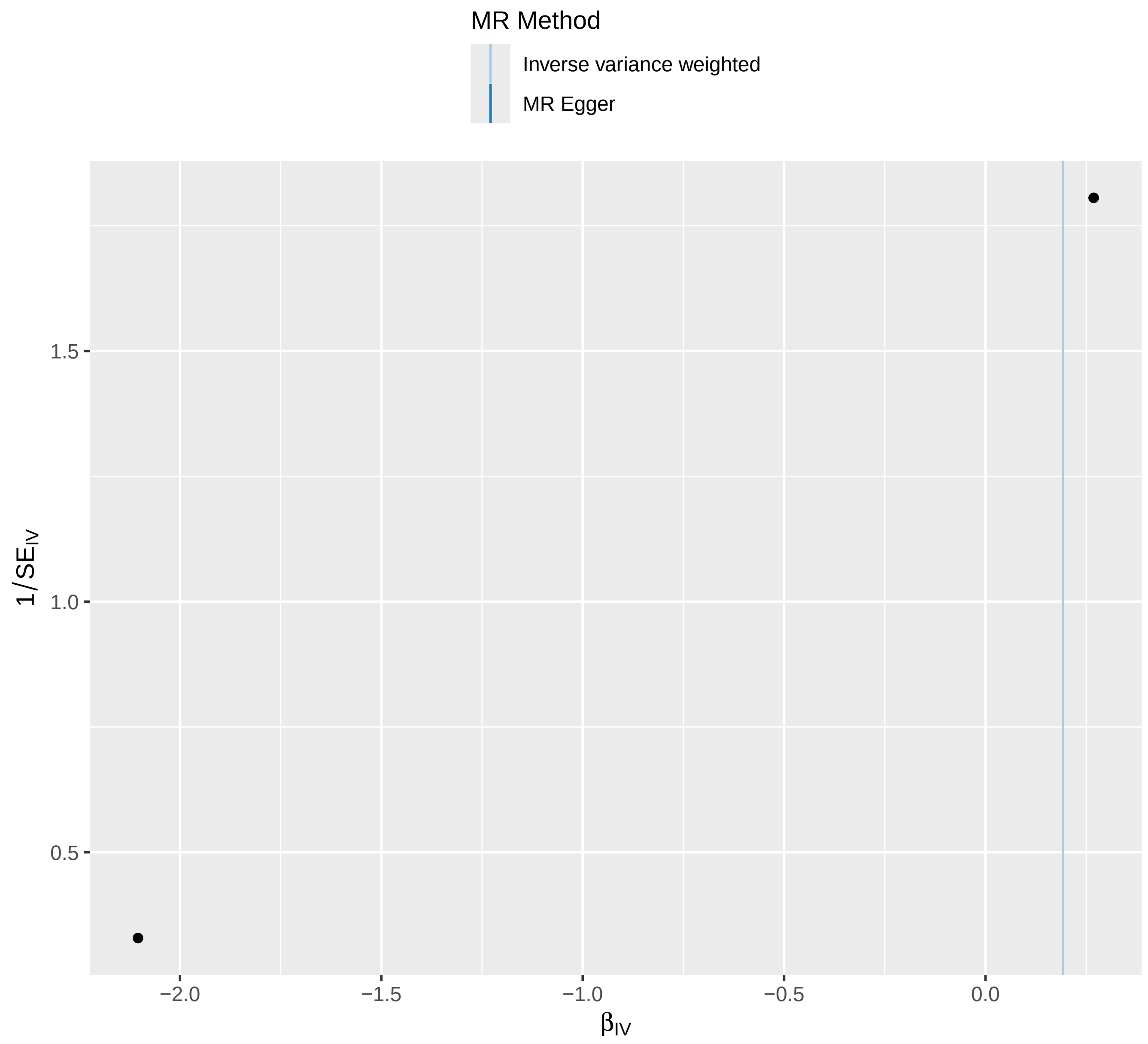

MR Method
Inverse variance weighted
MR Egger
1.5
1.0
0.5
$1/SE_{IV}$
−2.0
−1.5
−1.0
−0.5
0.0
β_{IV}

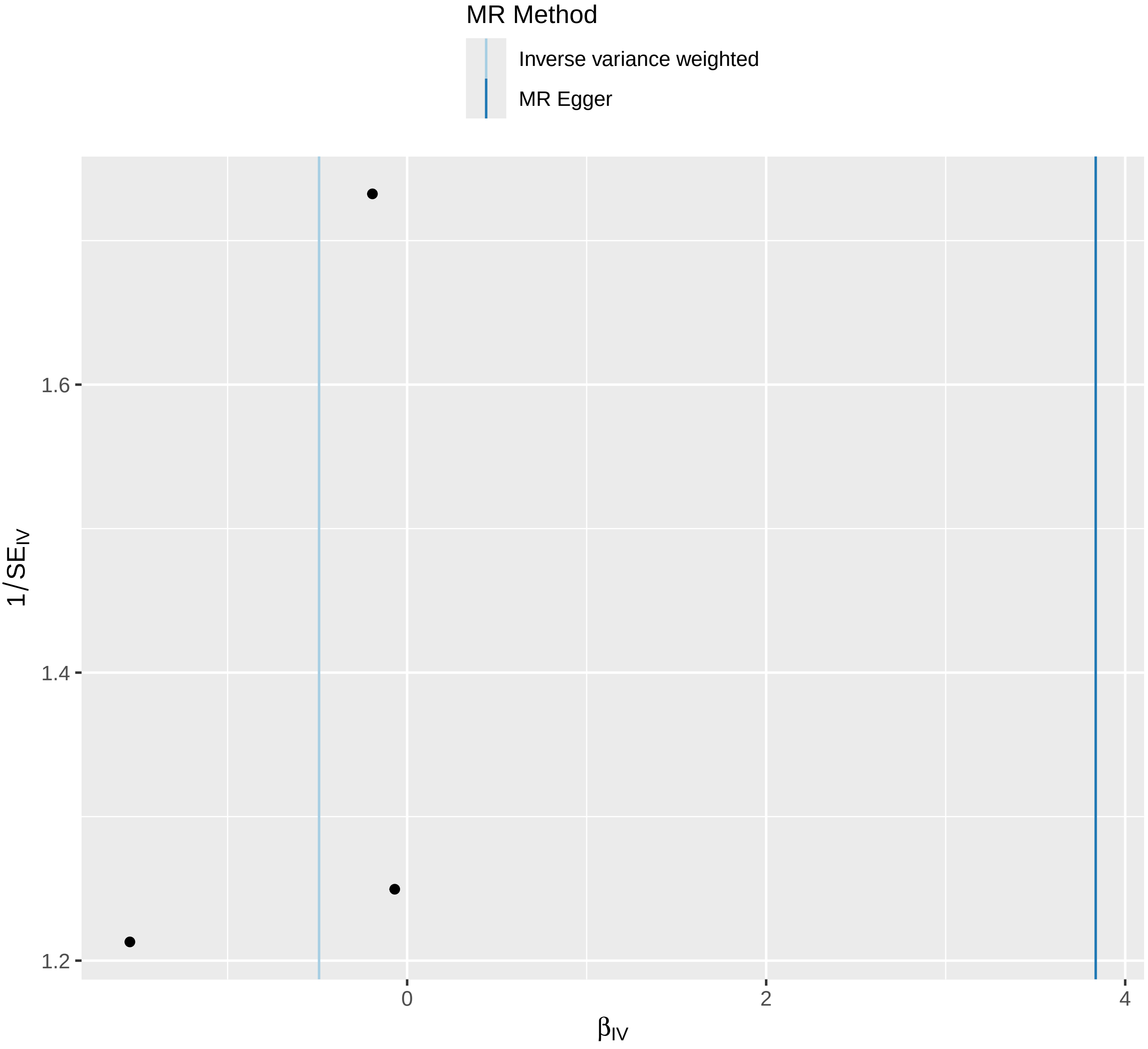
MR Method
Inverse variance weighted
MR Egger
1.6
1.4
1.2
1/SE$_{IV}$
0
2
4
β$_{IV}$

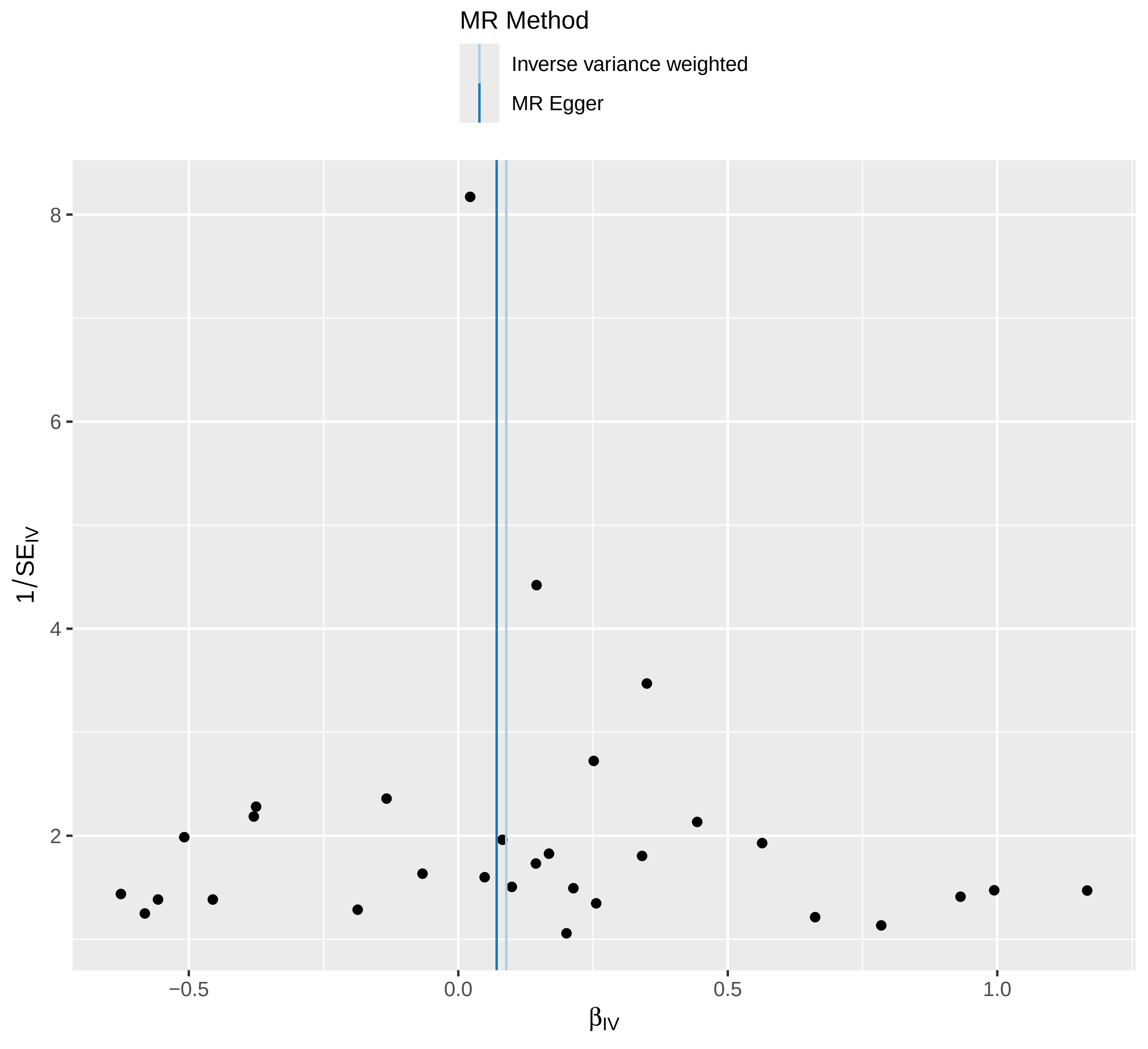
MR Method
Inverse variance weighted
MR Egger
1/SE$_{IV}$
8
6
4
2
−0.5
0.0
0.5
1.0
β$_{IV}$

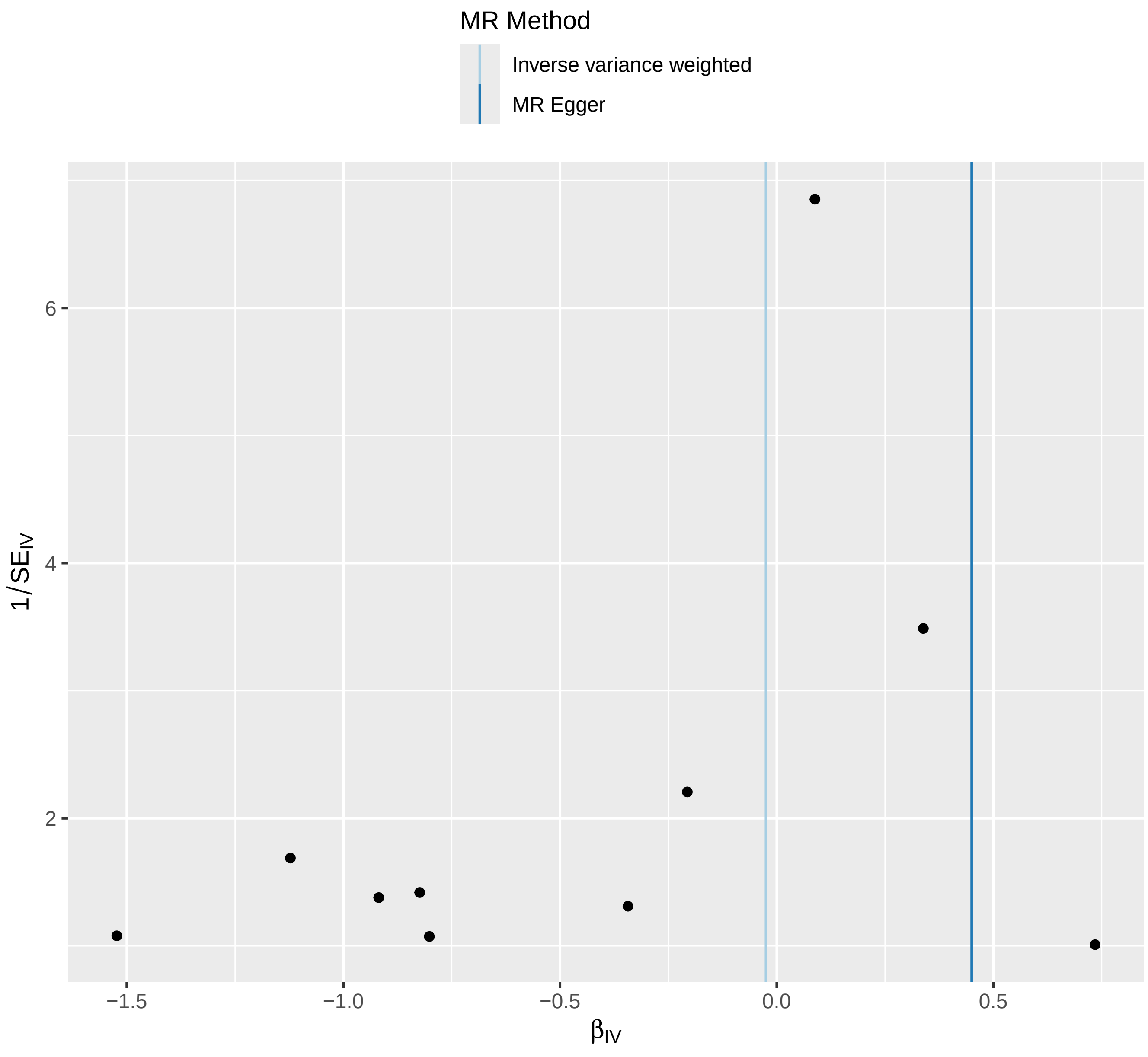

MR Method
Inverse variance weighted
MR Egger
6
4
2
$1/SE_{IV}$
−1.5
−1.0
−0.5
0.0
0.5
β_{IV}

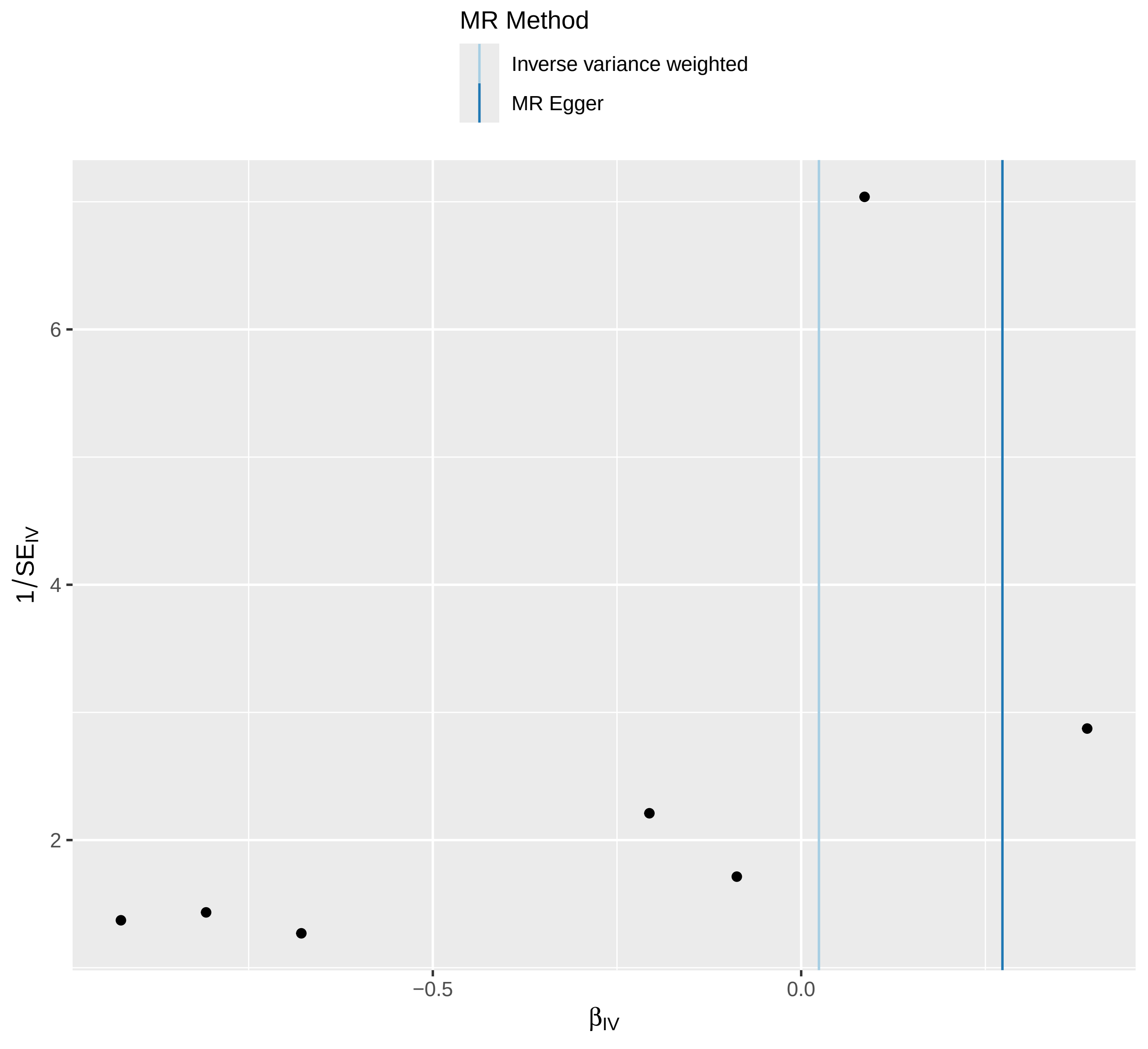
MR Method
Inverse variance weighted
MR Egger
1/SE_IV
6
4
2
−0.5
0.0
β_IV

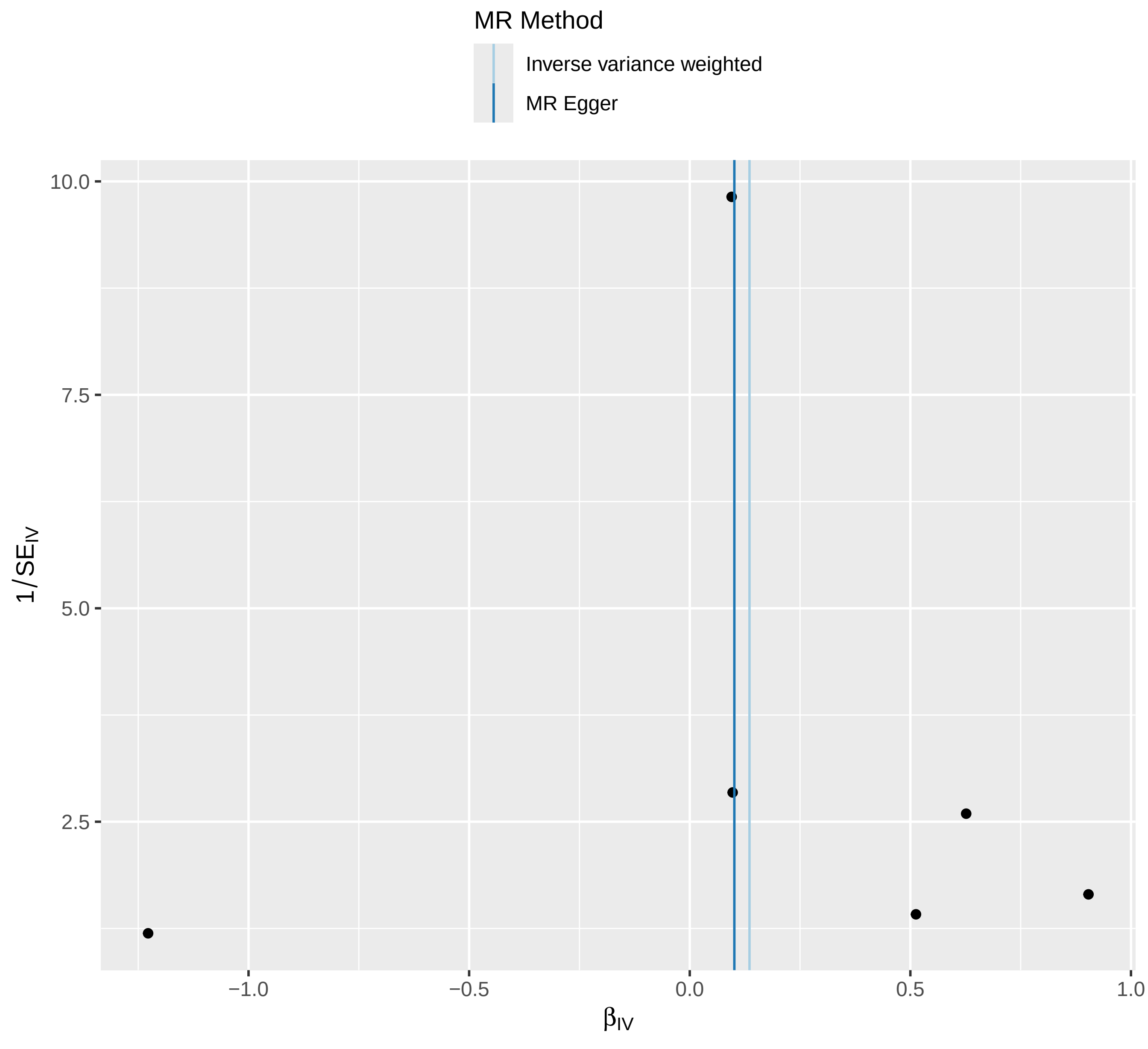

MR Method
Inverse variance weighted
MR Egger
10.0
7.5
5.0
2.5
1/SE_IV
−1.0
−0.5
0.0
0.5
1.0
β_IV

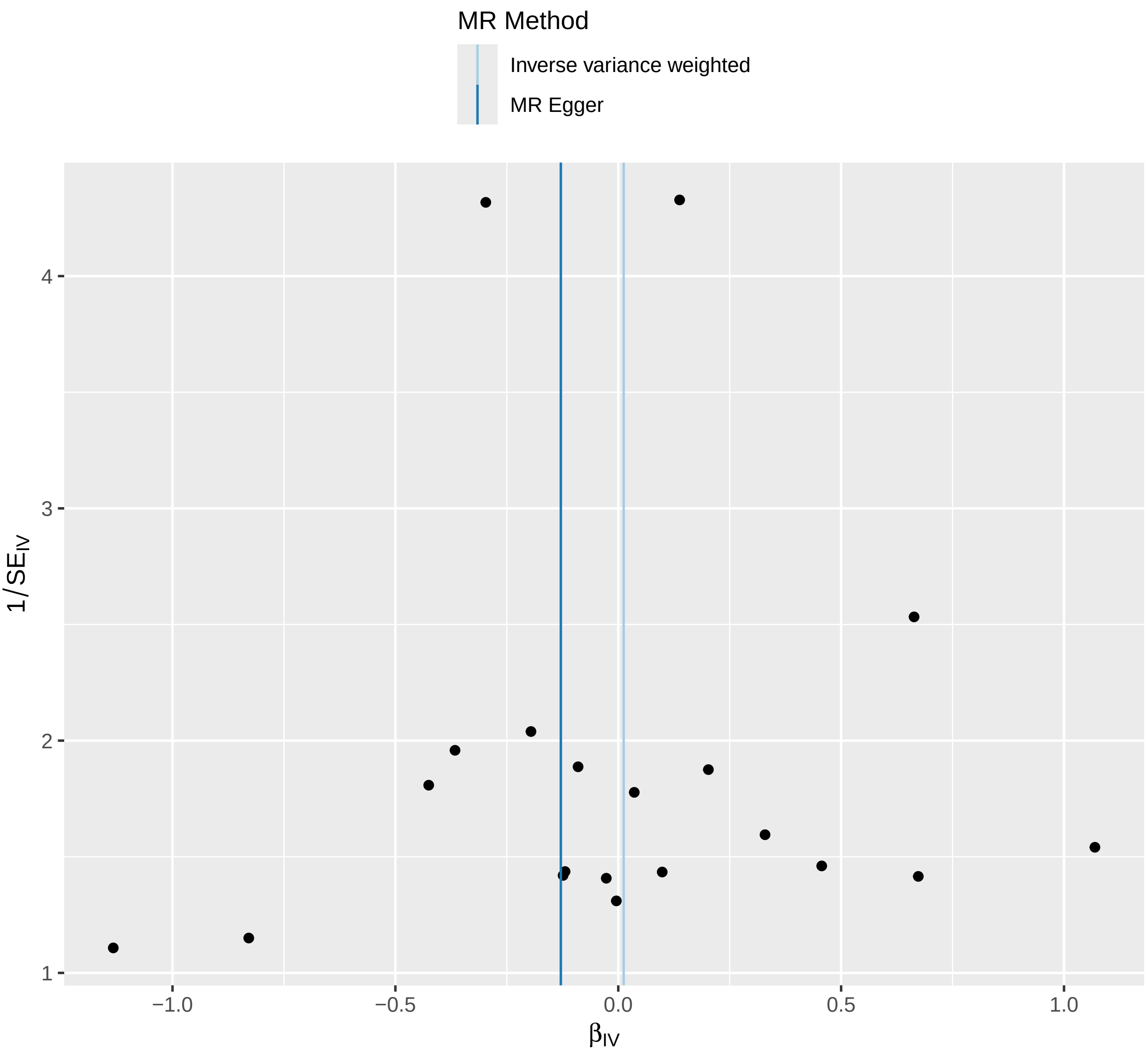
MR Method
Inverse variance weighted
MR Egger
4
3
2
1
1/SE_{IV}
−1.0
−0.5
0.0
0.5
1.0
β_{IV}

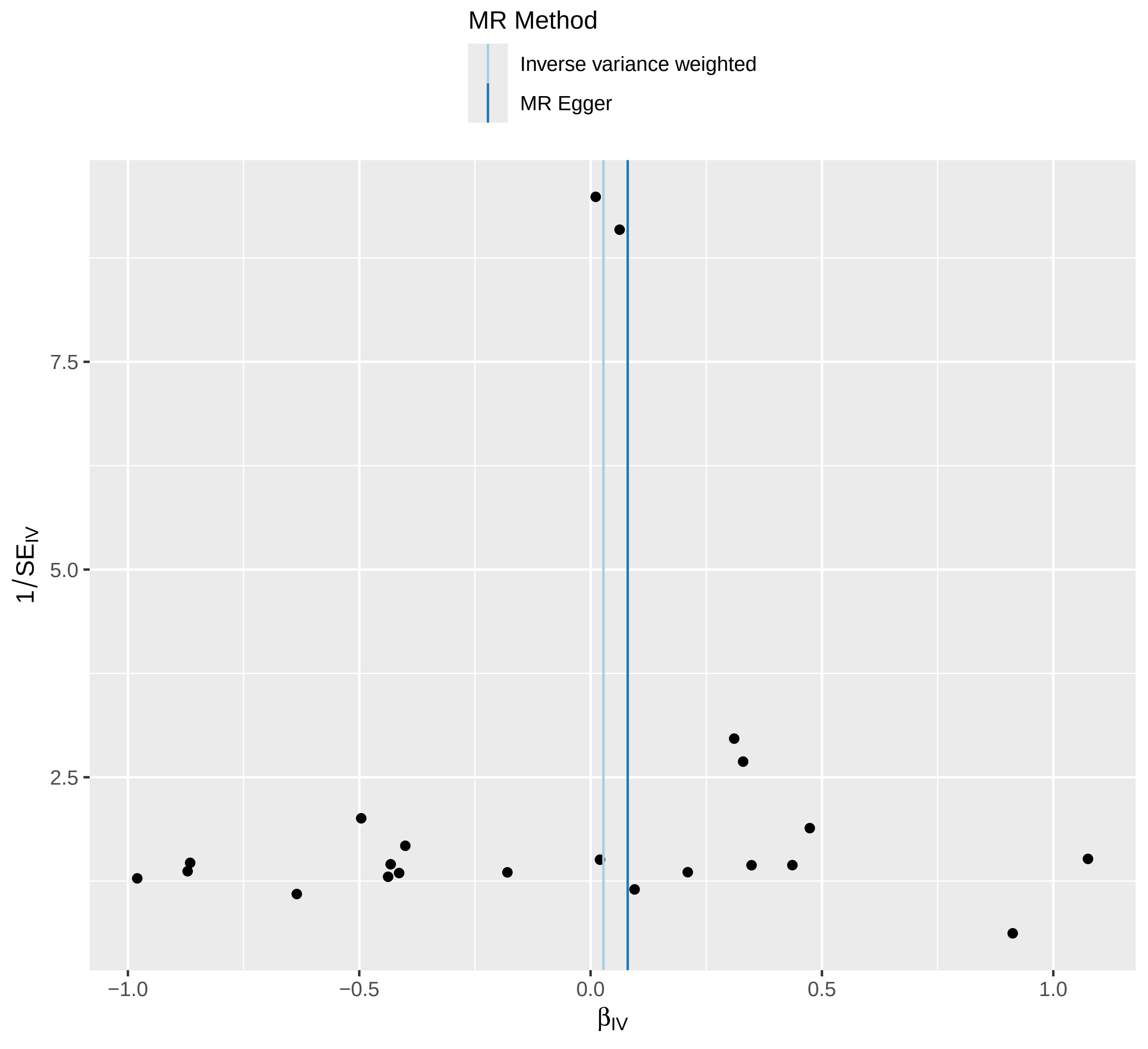
MR Method
Inverse variance weighted
MR Egger
1/SE_IV
7.5
5.0
2.5
−1.0
−0.5
0.0
0.5
1.0
β_IV

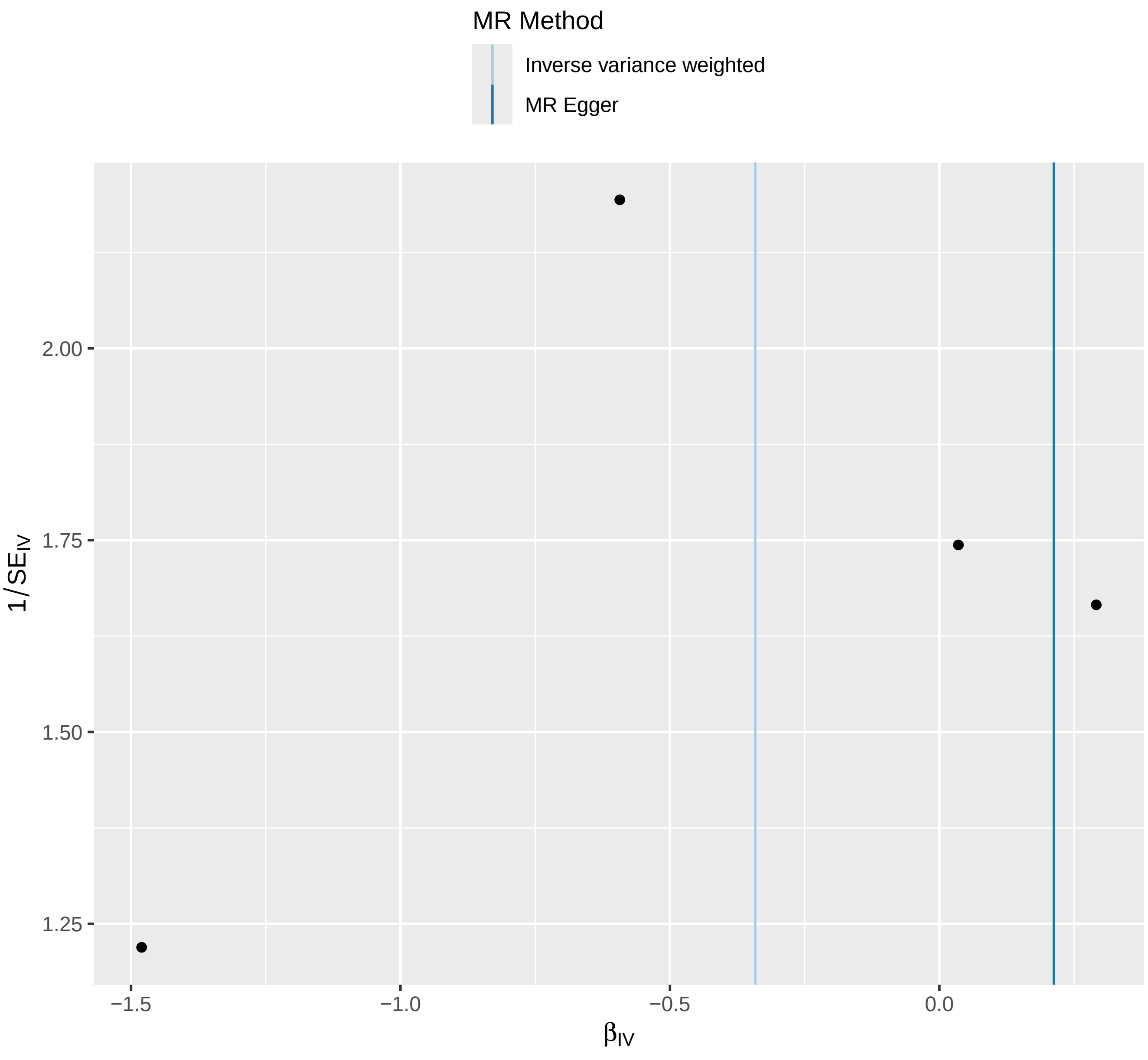

MR Method
Inverse variance weighted
MR Egger
2.00
1.75
1.50
1.25
1/SE_IV
−1.5
−1.0
−0.5
0.0
β_IV

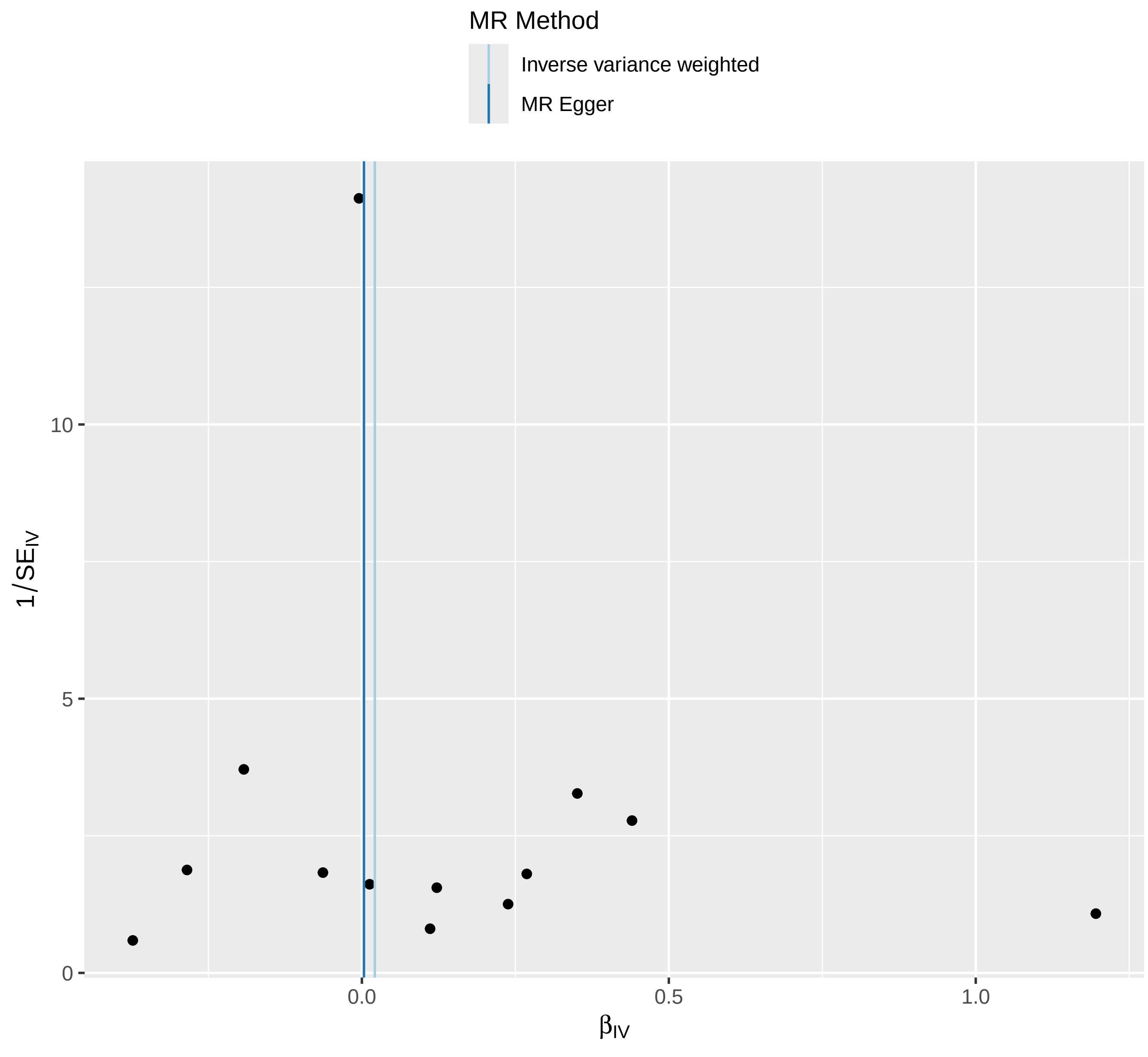
MR Method
Inverse variance weighted
MR Egger
1/SE_IV
10
5
0
0.0
0.5
1.0
β_IV

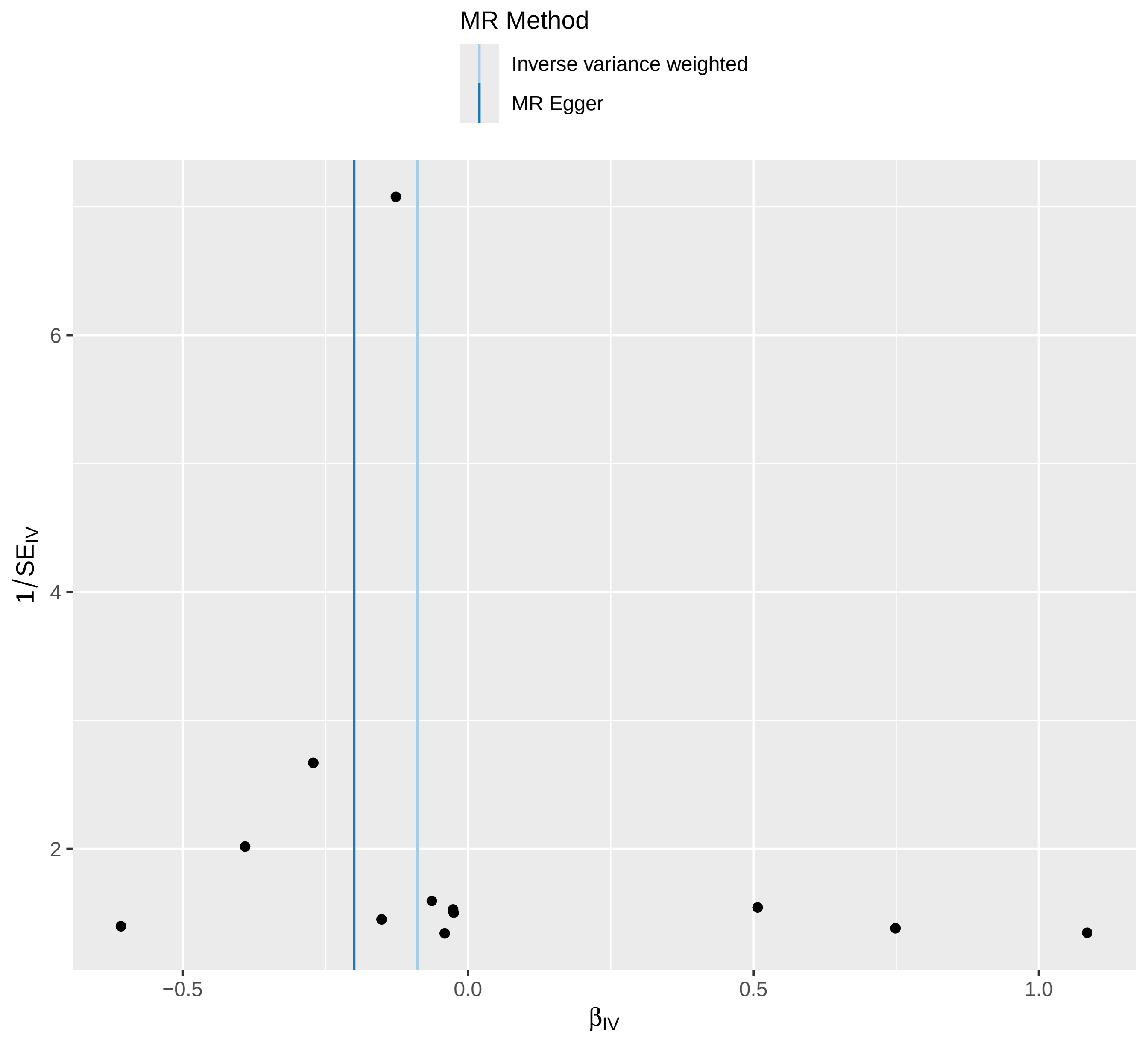

MR Method
Inverse variance weighted
MR Egger
6
4
2
1/SE_IV
−0.5
0.0
0.5
1.0
β_IV

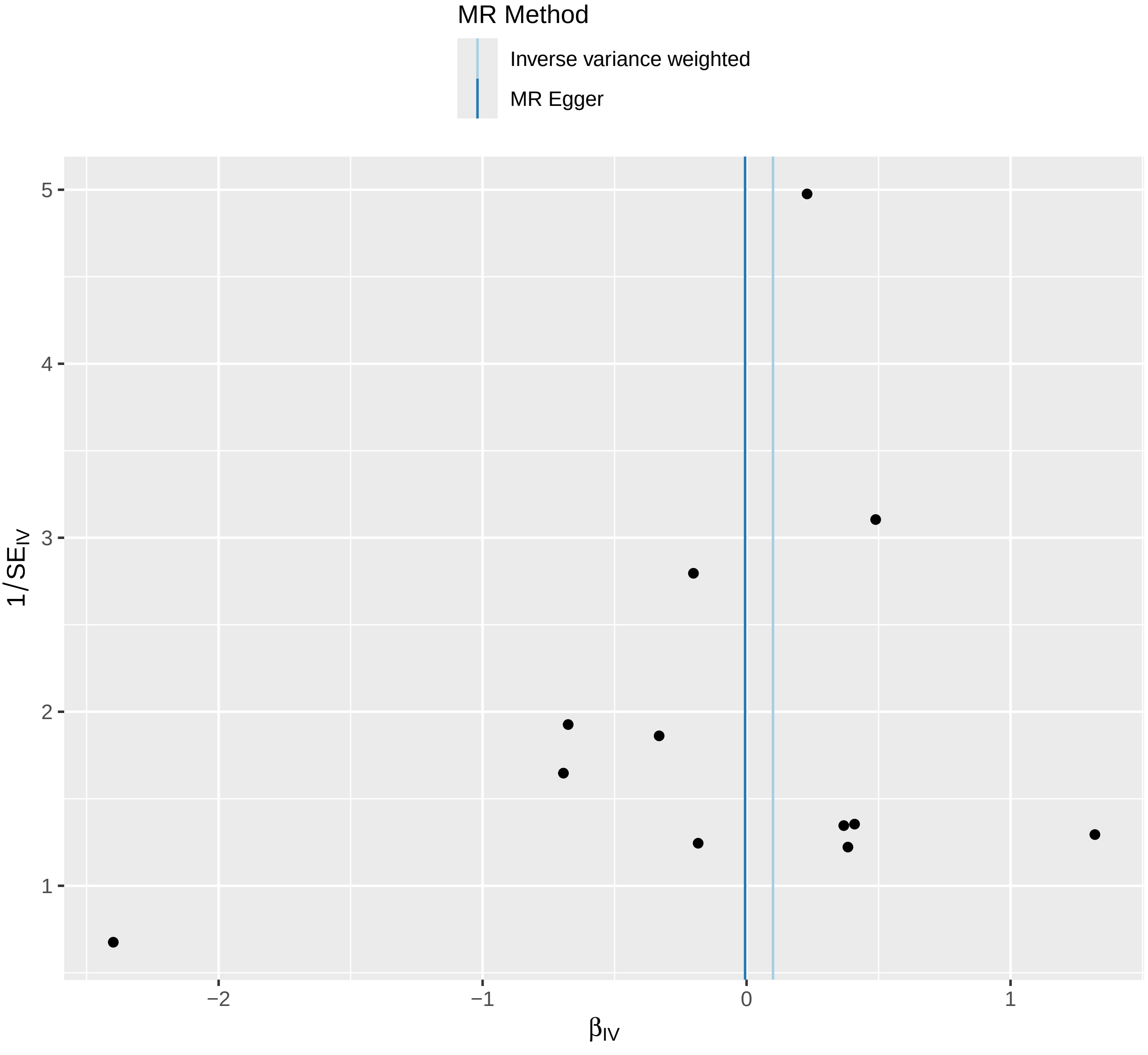

MR Method
Inverse variance weighted
MR Egger
5
4
3
2
1
1/SE_IV
−2
−1
0
1
β_IV

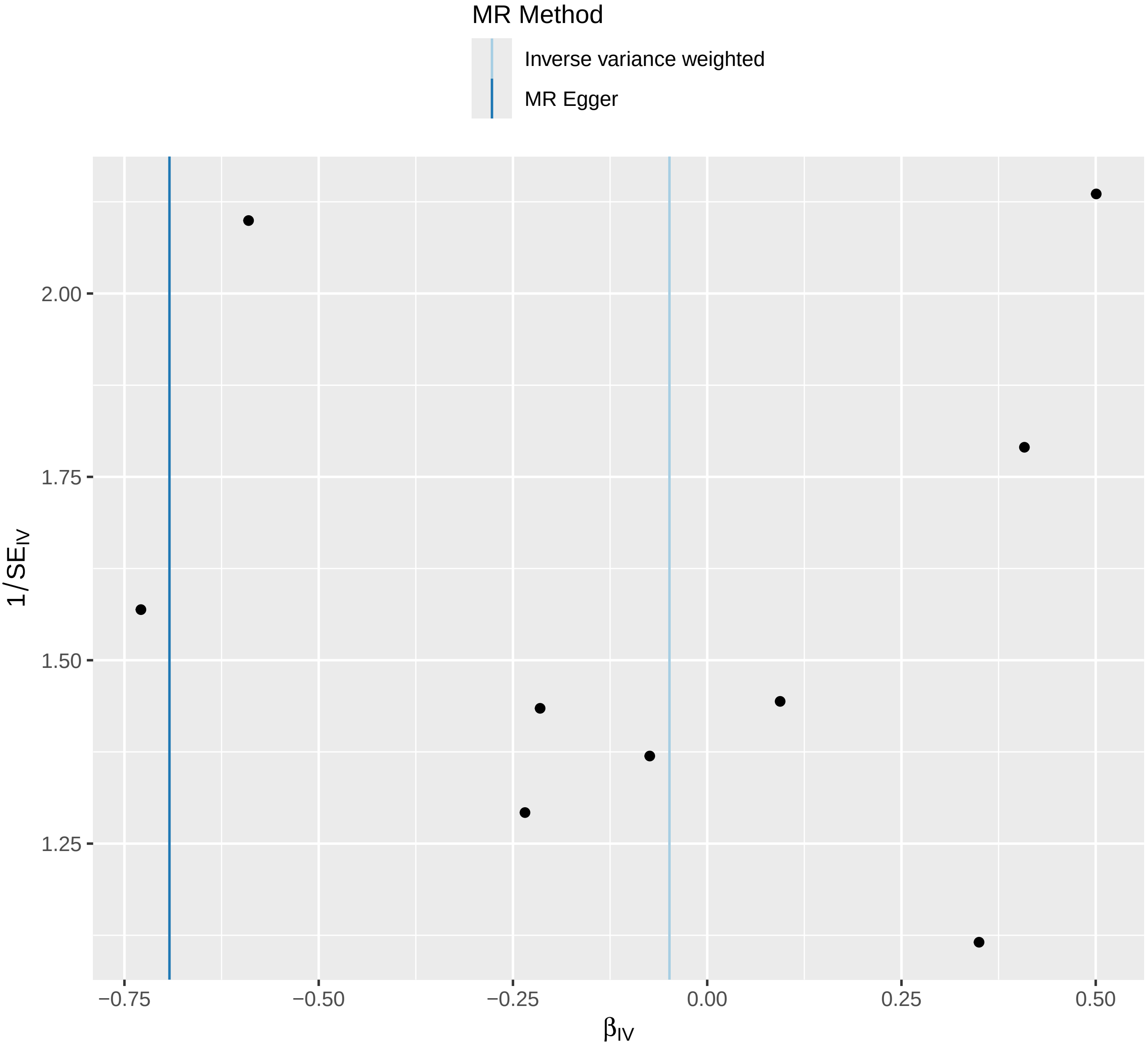
MR Method
Inverse variance weighted
MR Egger
2.00
1.75
1.50
1.25
1/SE_IV
−0.75
−0.50
−0.25
0.00
0.25
0.50
β_IV

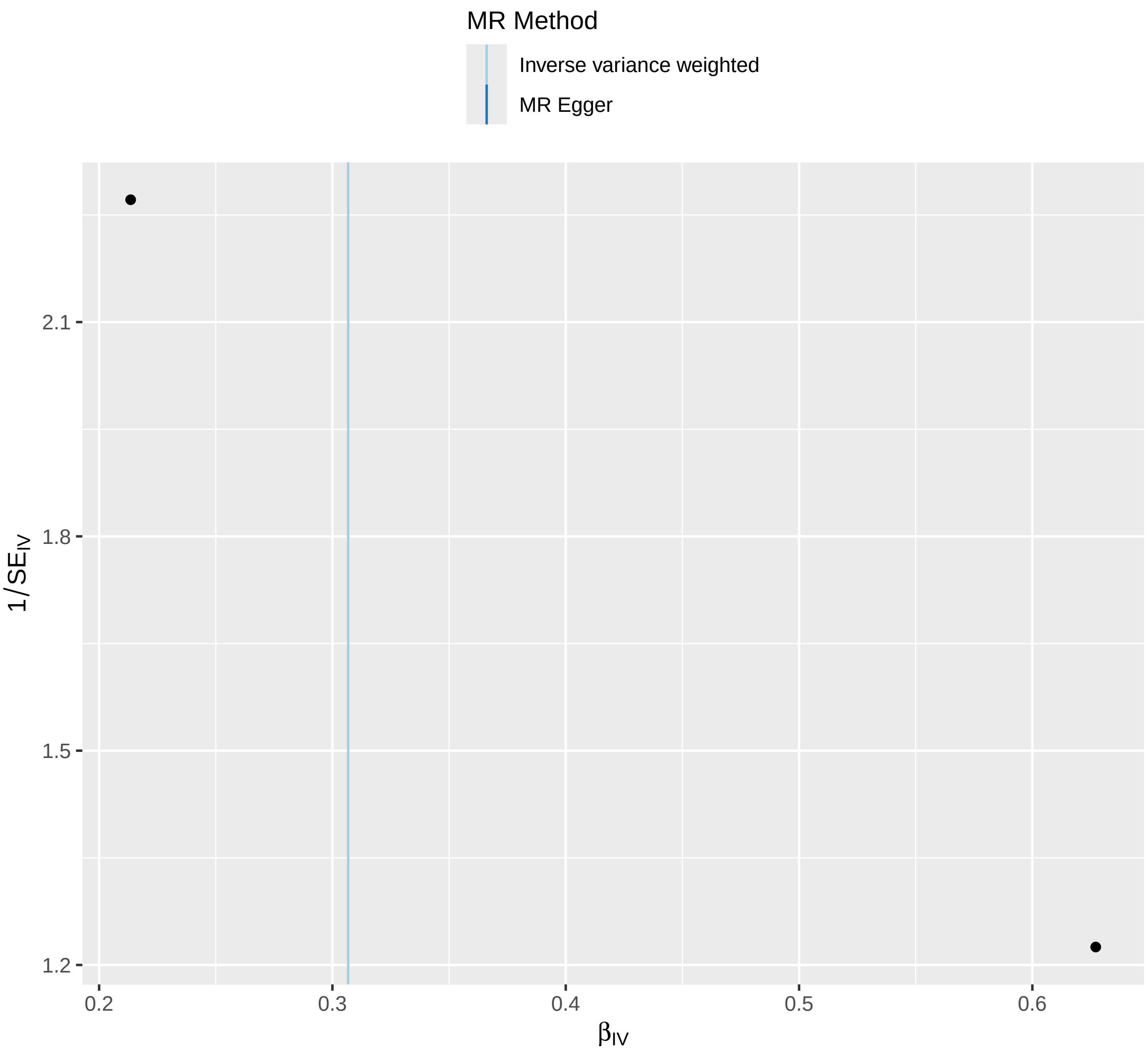
MR Method
Inverse variance weighted
MR Egger
2.1
1.8
1.5
1.2
$1/SE_{IV}$
0.2
0.3
0.4
0.5
0.6
β_{IV}

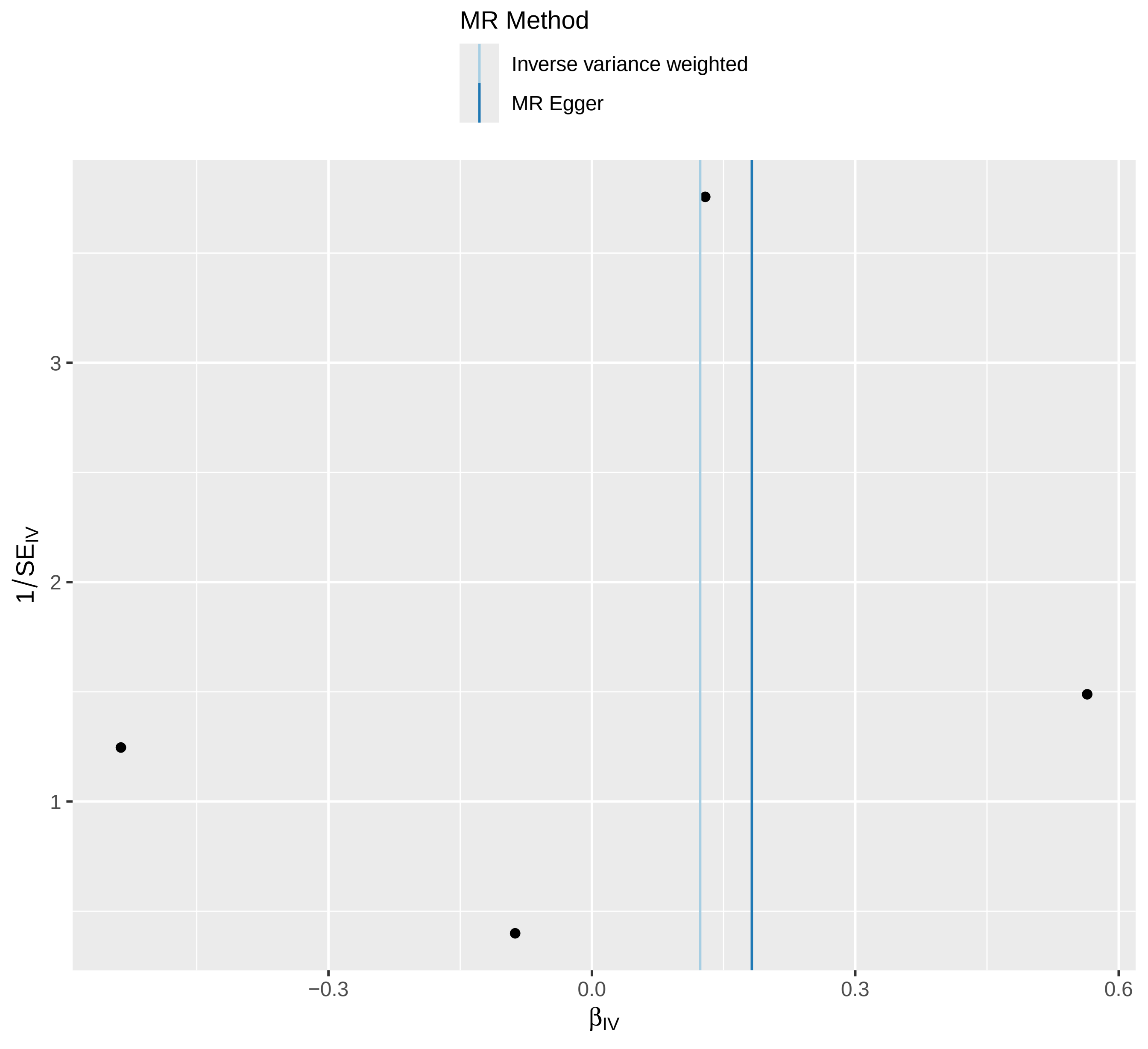

MR Method
Inverse variance weighted
MR Egger
3
2
1
$1/SE_{IV}$
−0.3
0.0
0.3
0.6
β_{IV}

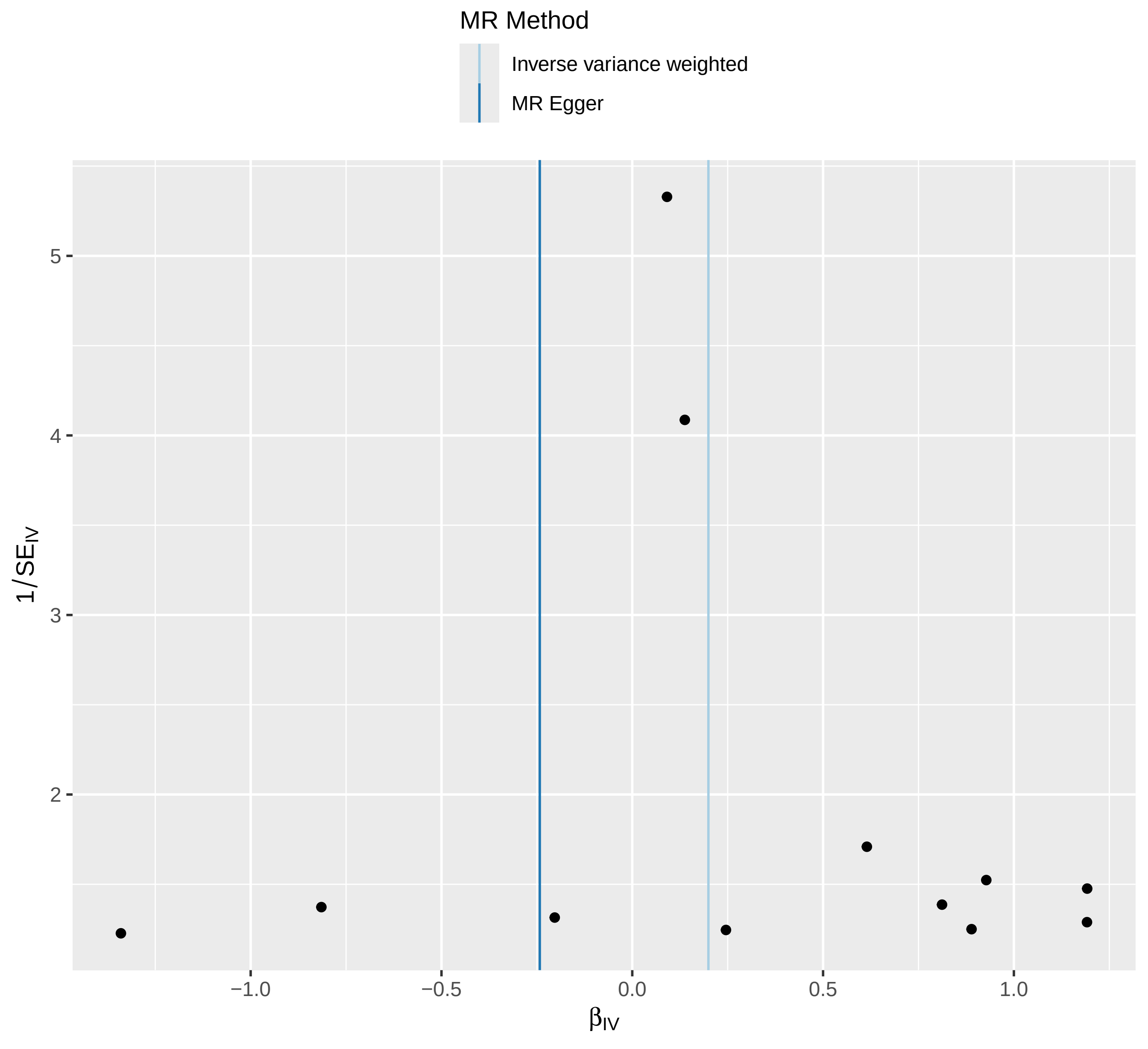
MR Method
Inverse variance weighted
MR Egger
1/SE_IV
5
4
3
2
−1.0
−0.5
0.0
0.5
1.0
β_IV

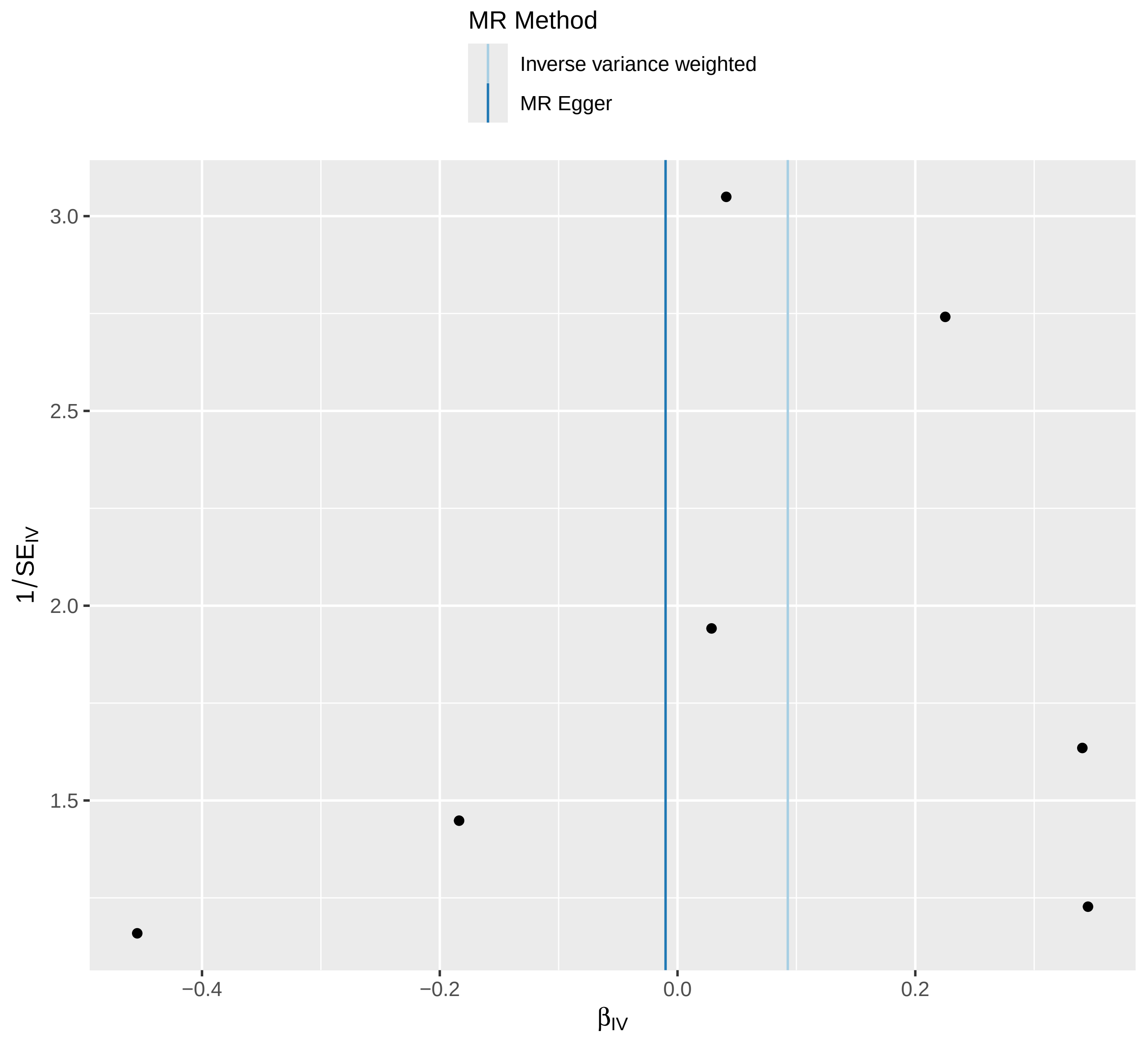

MR Method
Inverse variance weighted
MR Egger
3.0
2.5
2.0
1.5
$1/SE_{IV}$
−0.4
−0.2
0.0
0.2
β_{IV}

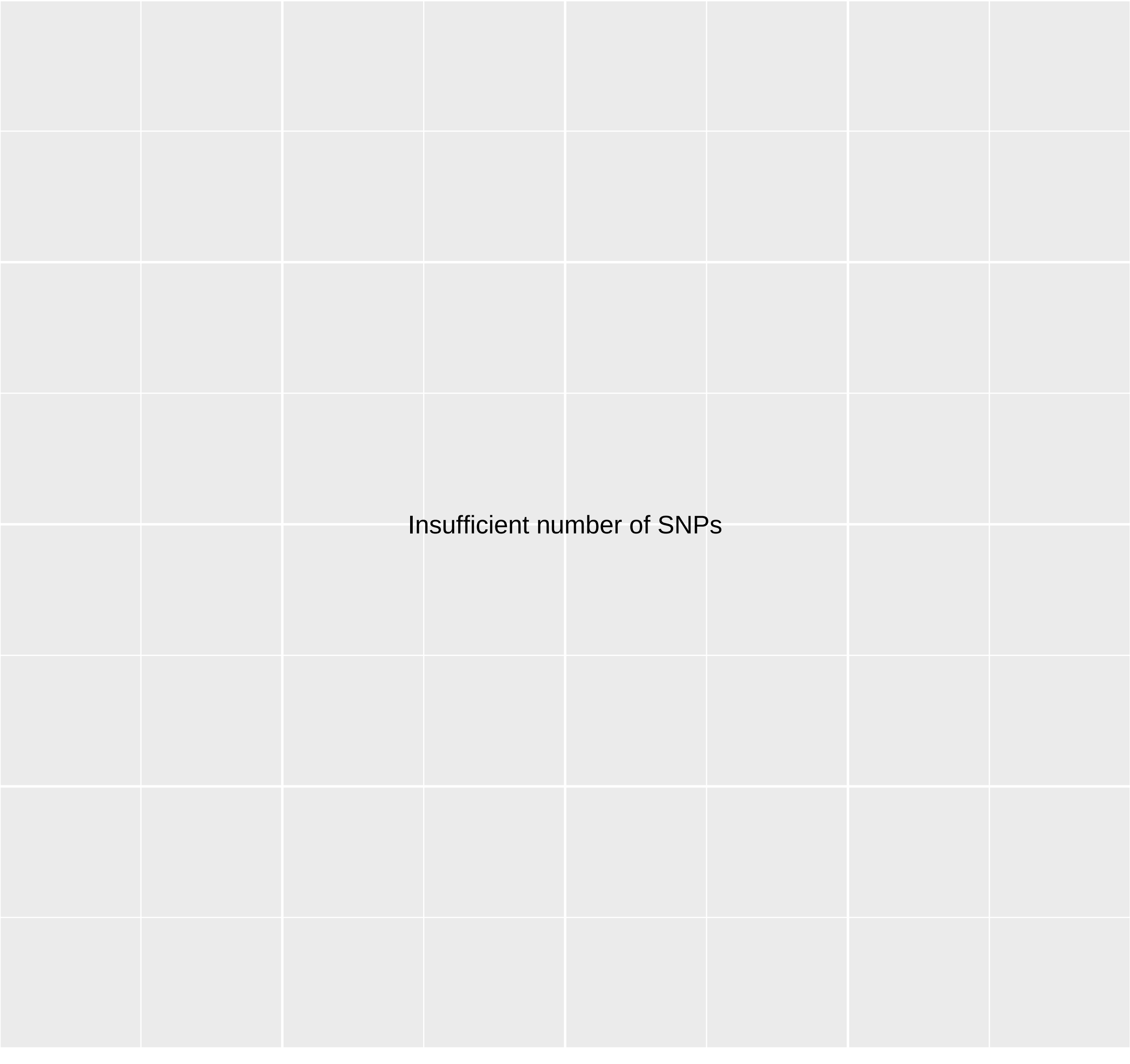
Insufficient number of SNPs

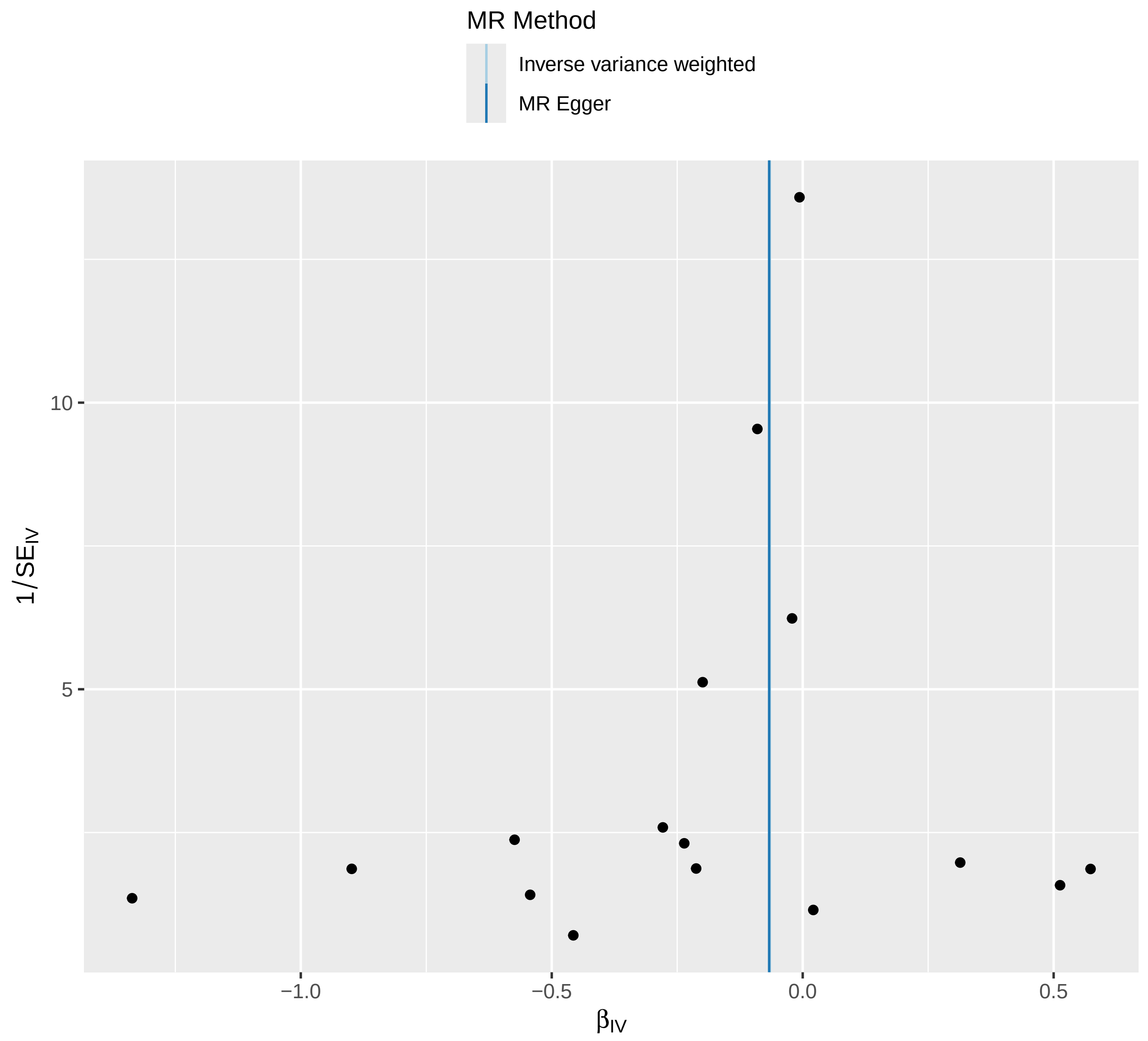

MR Method
Inverse variance weighted
MR Egger
1/SE$_{IV}$
10
5
−1.0
−0.5
0.0
0.5
β$_{IV}$

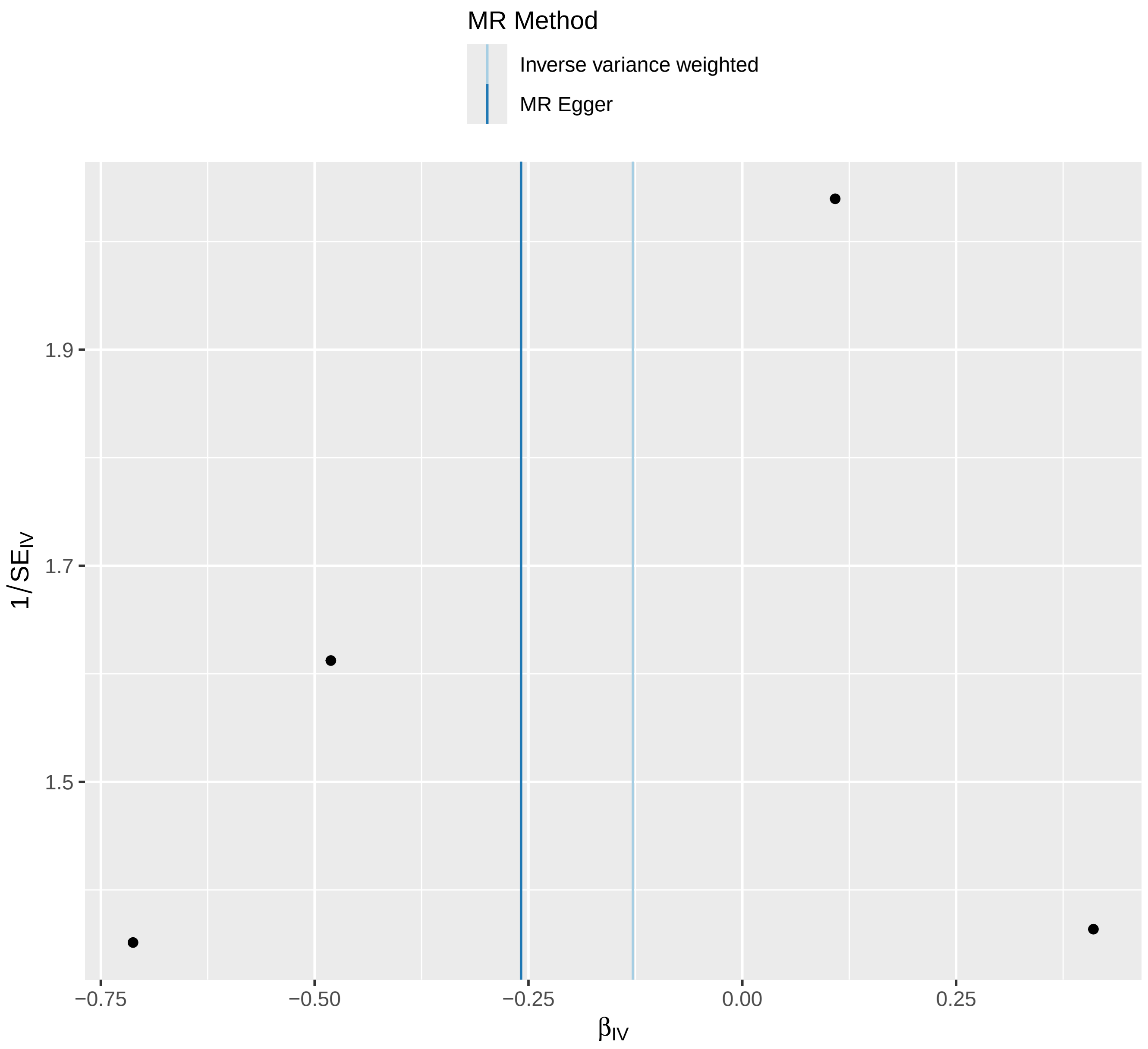
MR Method
Inverse variance weighted
MR Egger
1.9
1.7
1.5
1/SE$_{IV}$
−0.75
−0.50
−0.25
0.00
0.25
β$_{IV}$

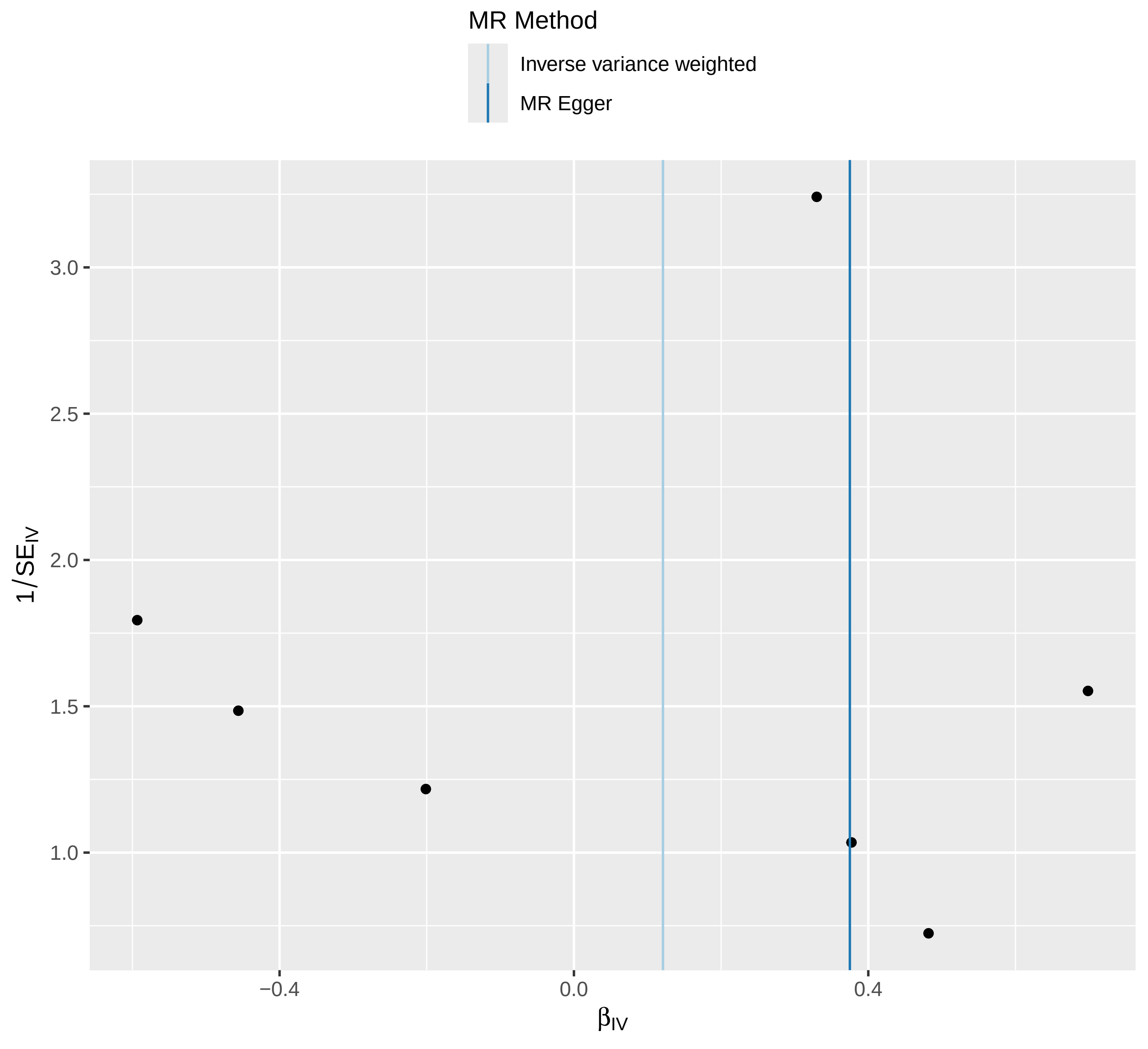

MR Method
Inverse variance weighted
MR Egger
3.0
2.5
2.0
1.5
1.0
1/SE$_{IV}$
−0.4
0.0
0.4
β$_{IV}$

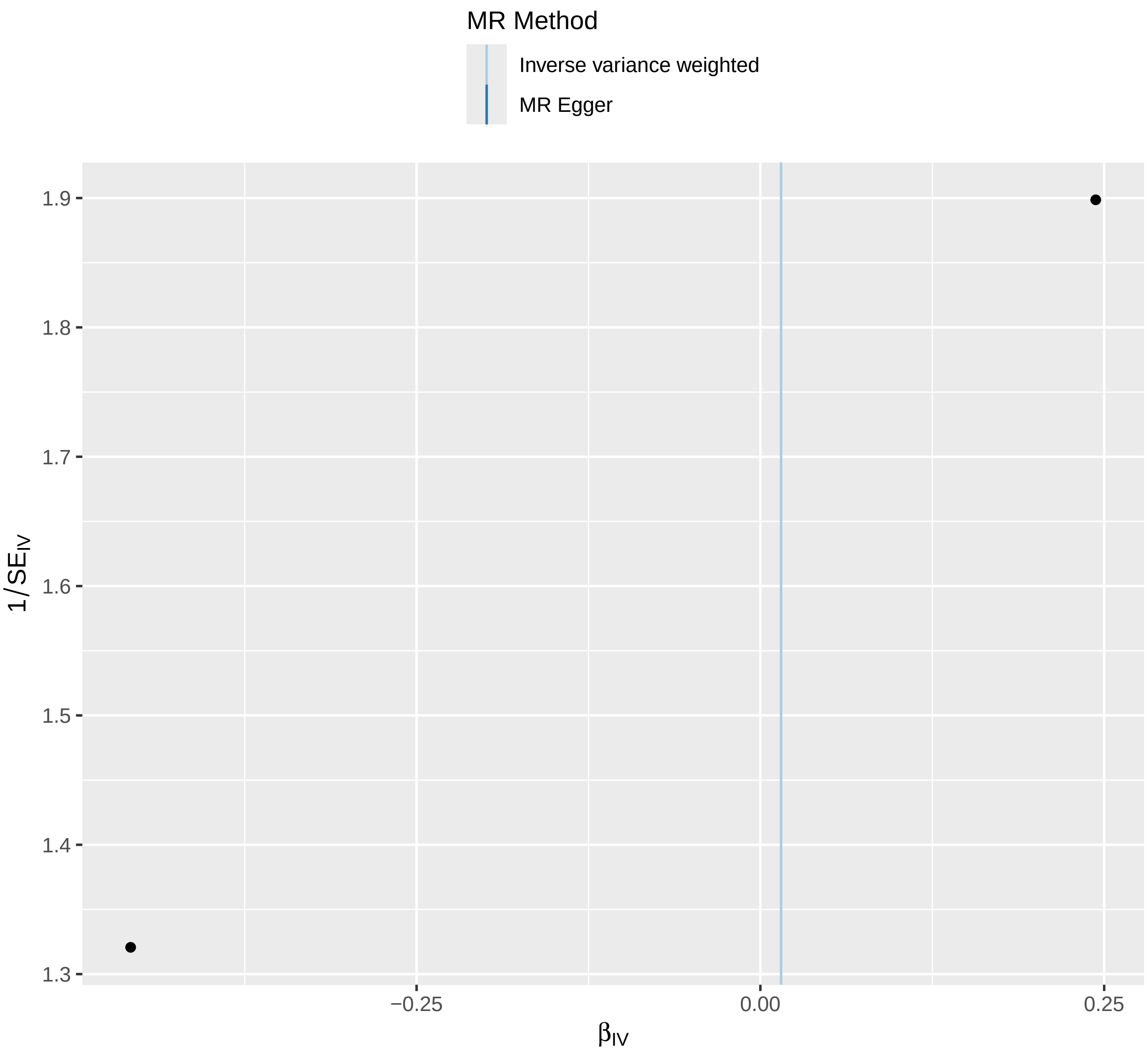

MR Method
Inverse variance weighted
MR Egger
1.9
1.8
1.7
1.6
1.5
1.4
1.3
1/SE_IV
−0.25
0.00
0.25
β_IV

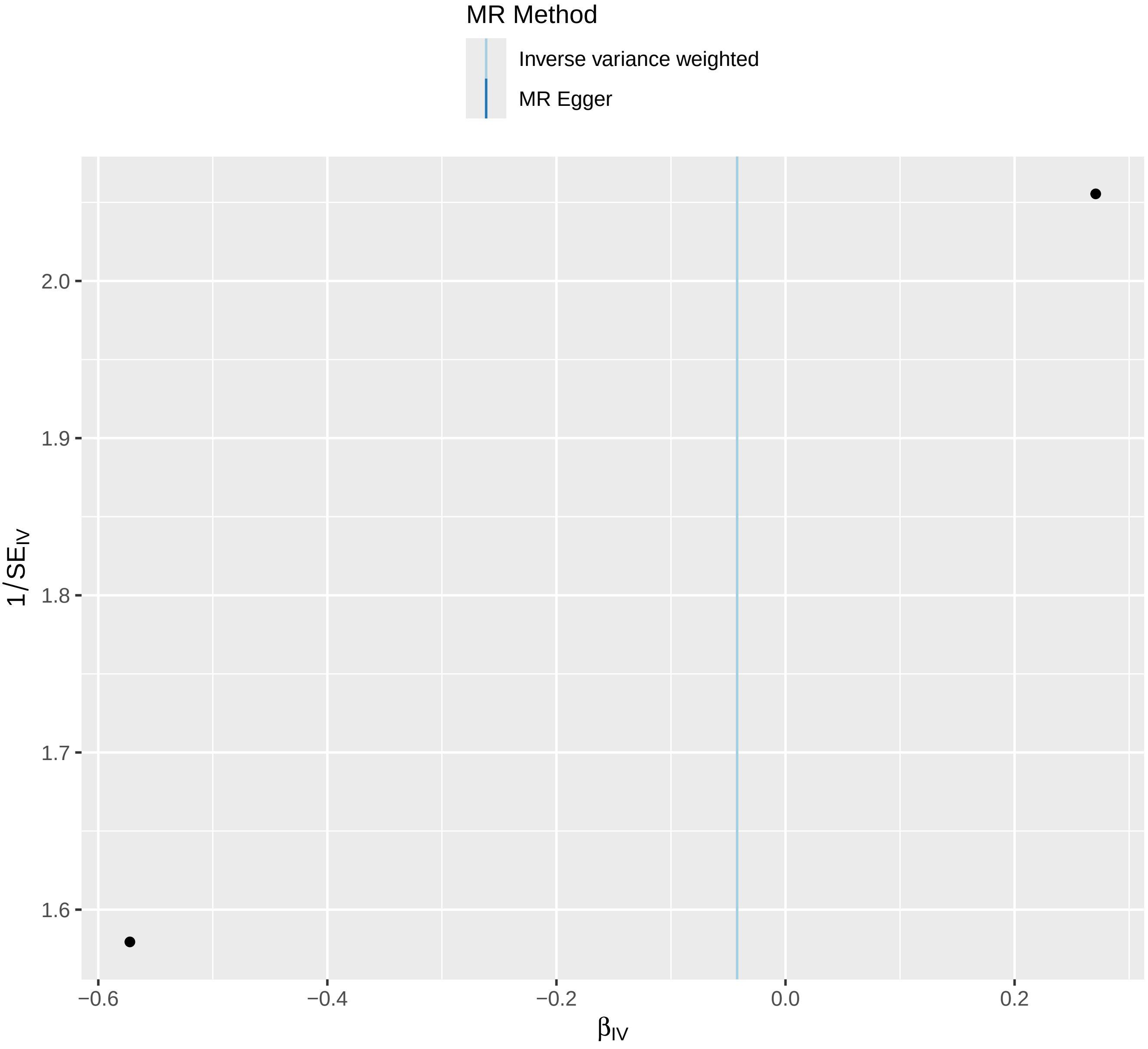

MR Method
Inverse variance weighted
MR Egger
2.0
1.9
1.8
1.7
1.6
$1/SE_{IV}$
−0.6
−0.4
−0.2
0.0
0.2
β_{IV}

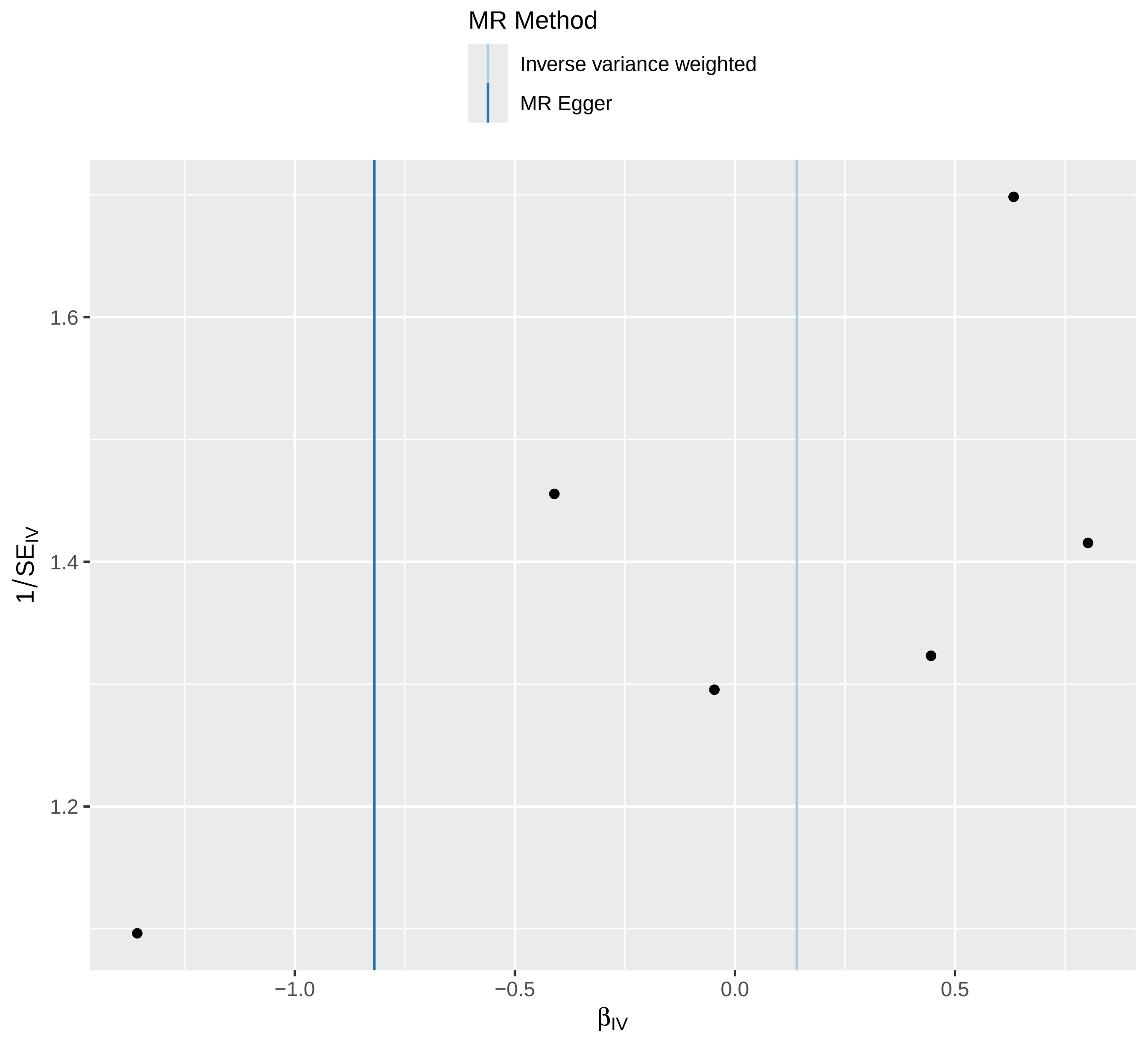
MR Method
Inverse variance weighted
MR Egger
1.6
1.4
1.2
1/SE_IV
−1.0
−0.5
0.0
0.5
β_IV

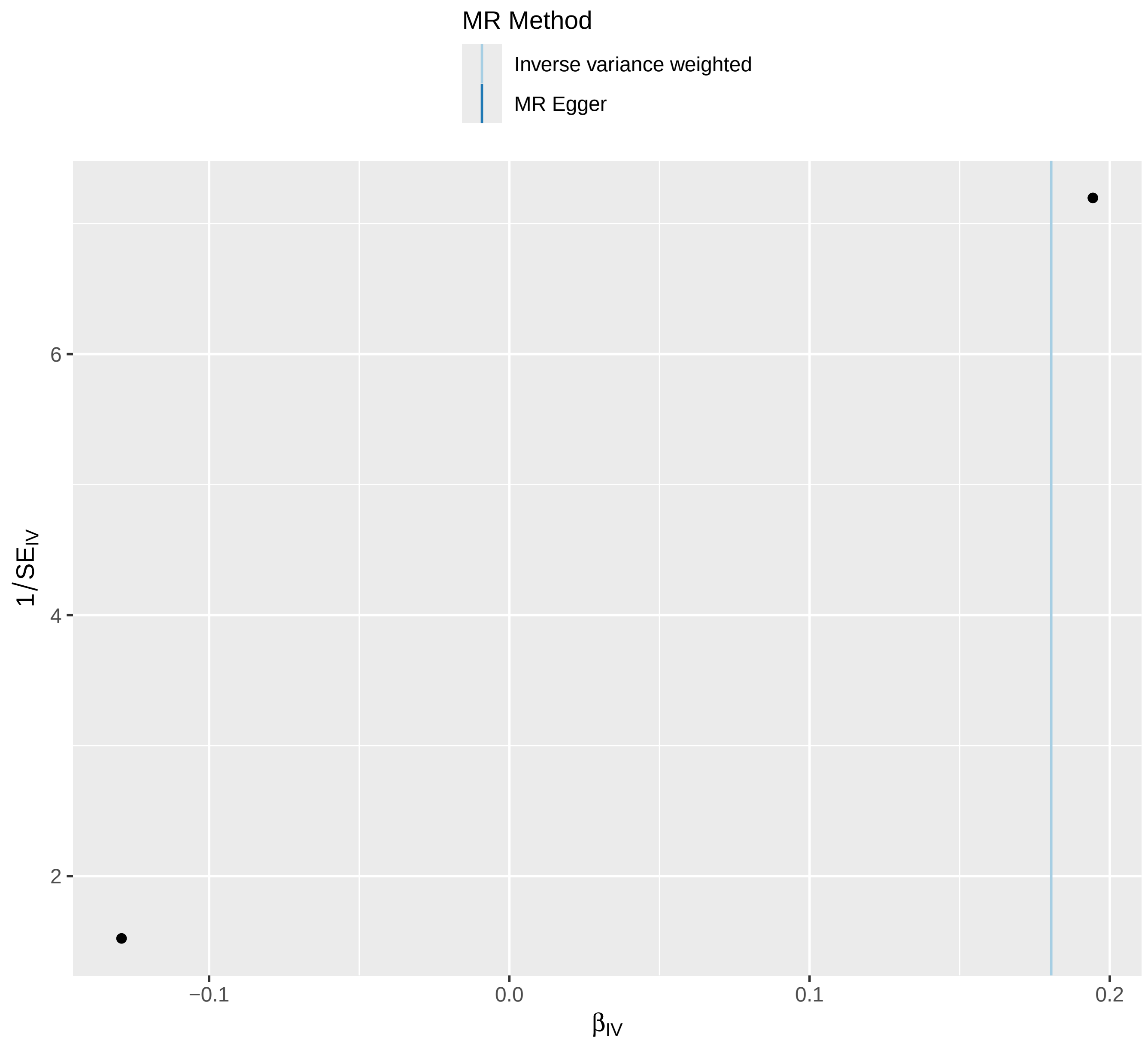

MR Method
Inverse variance weighted
MR Egger
6
4
2
1/SE$_{IV}$
−0.1
0.0
0.1
0.2
β$_{IV}$

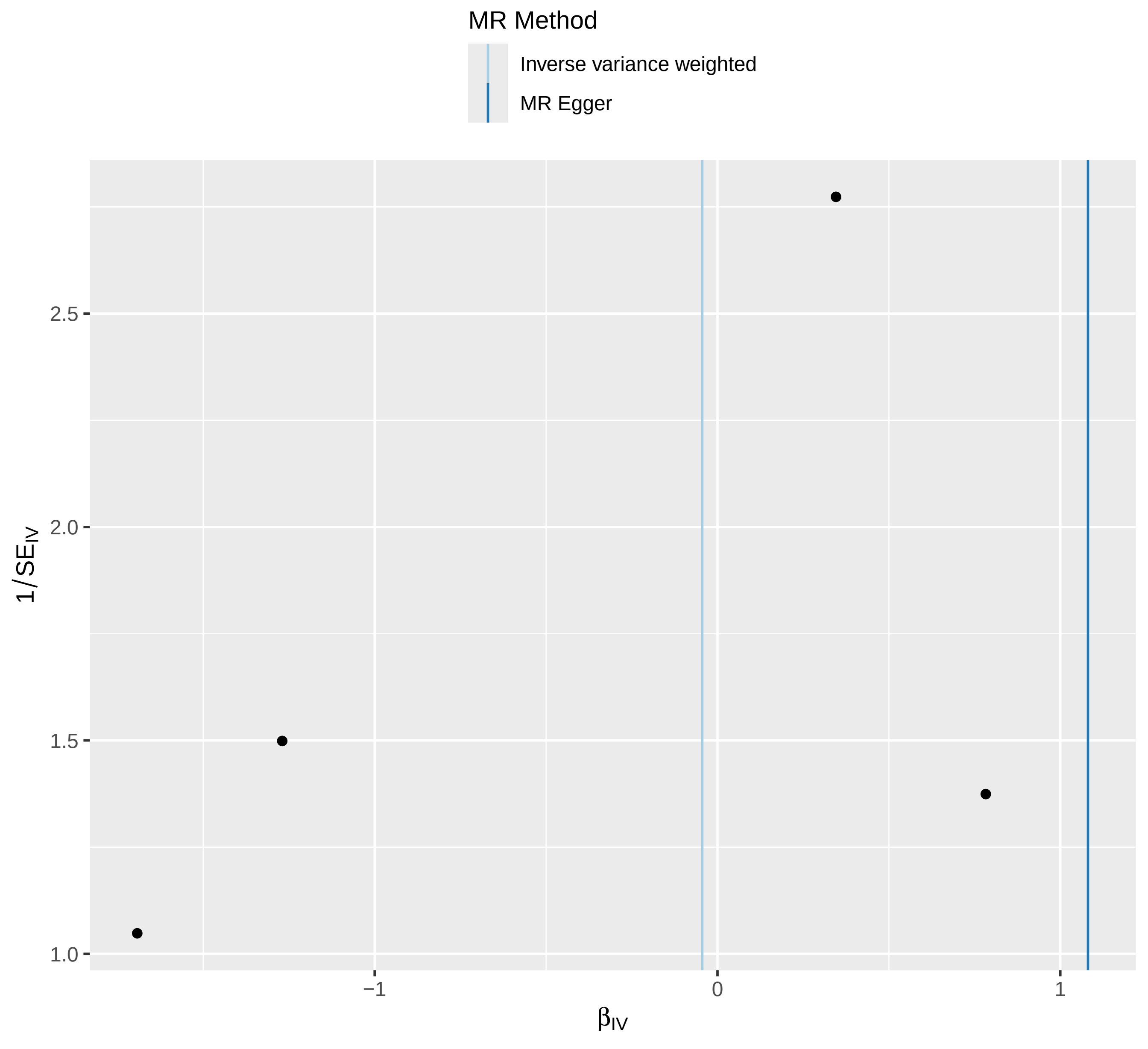
MR Method
Inverse variance weighted
MR Egger
2.5
2.0
1.5
1.0
1/SE_IV
−1
0
1
β_IV

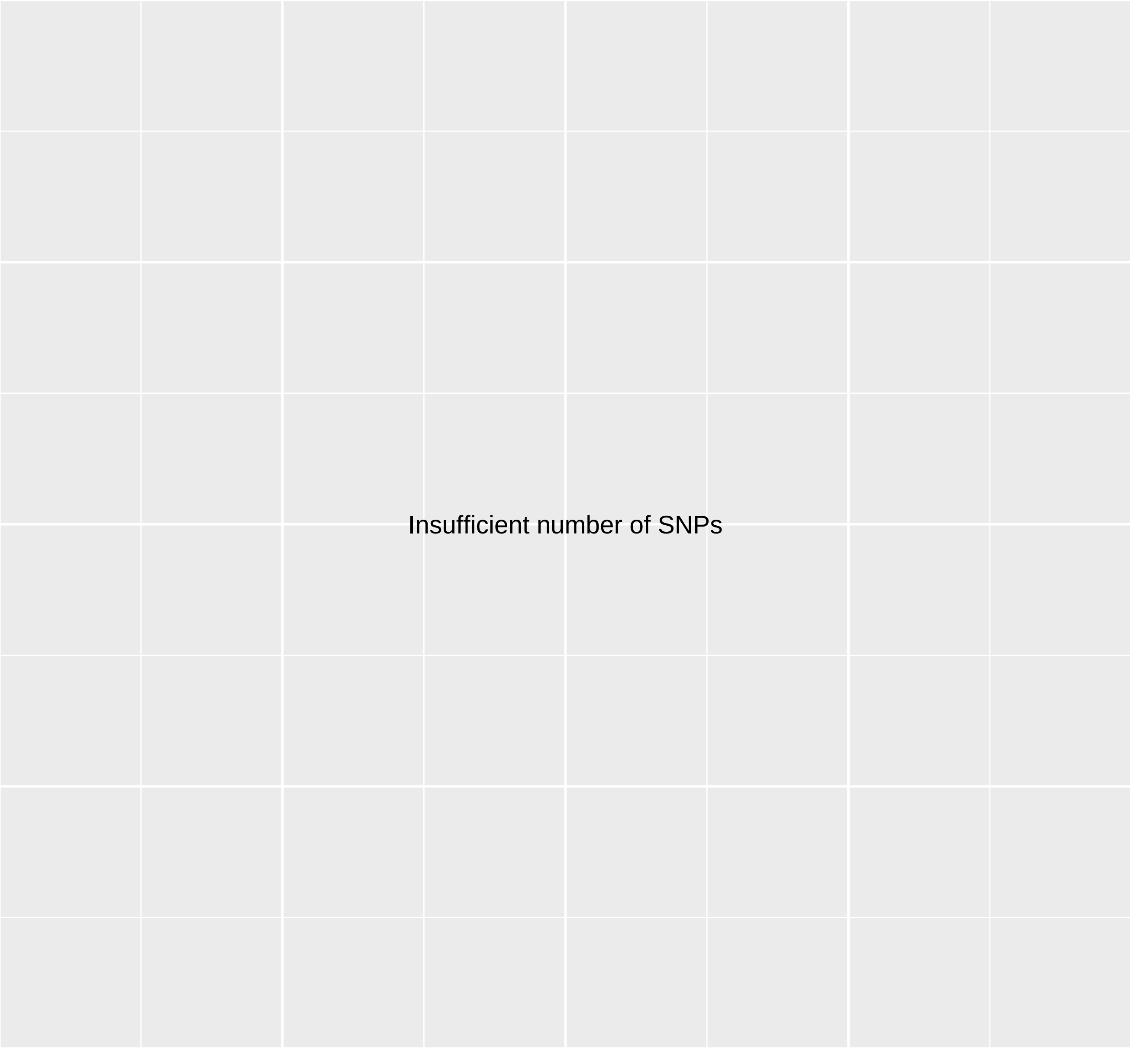
Insufficient number of SNPs

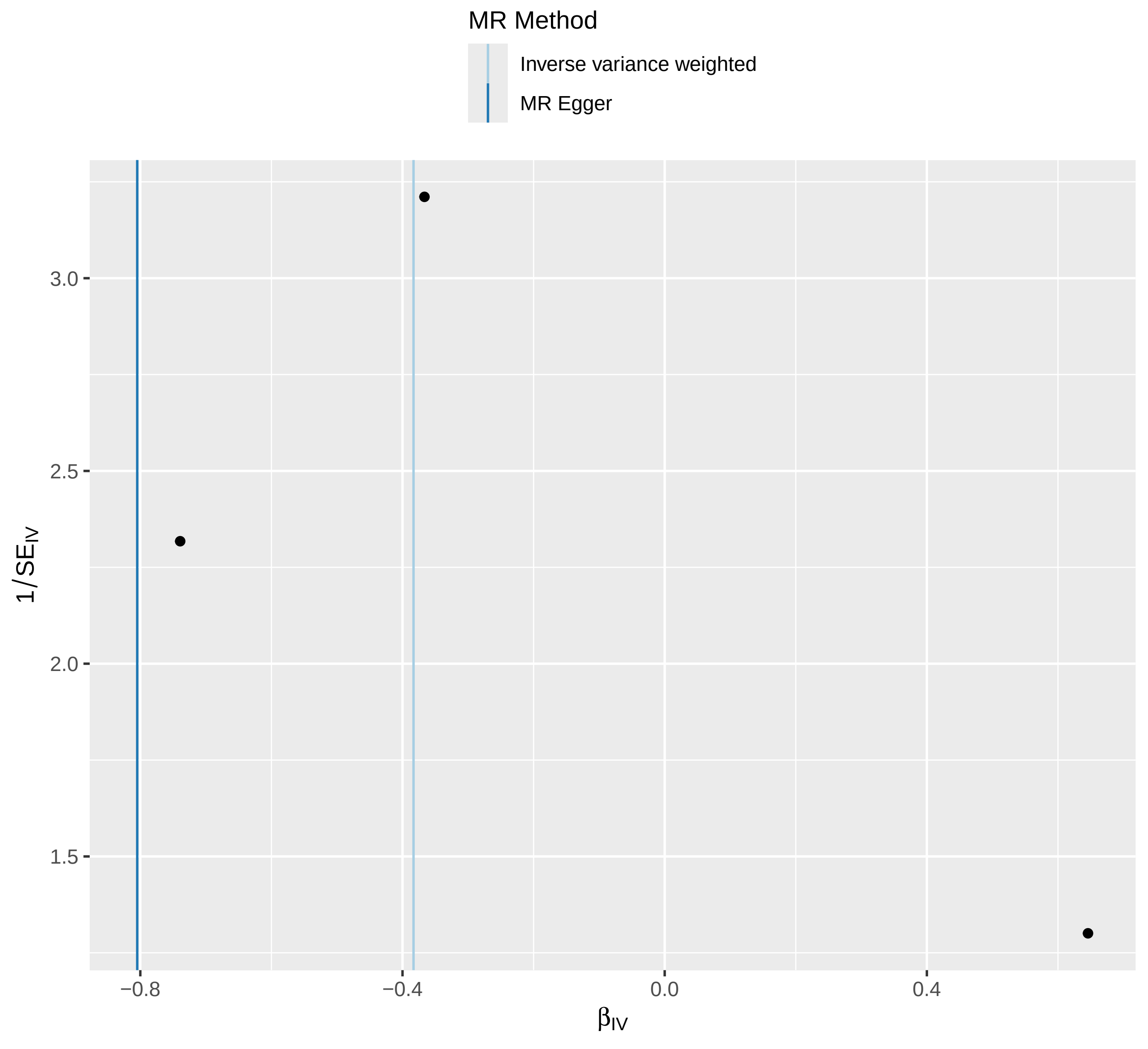
MR Method
Inverse variance weighted
MR Egger
3.0
2.5
2.0
1.5
1/SE_IV
−0.8
−0.4
0.0
0.4
β_IV

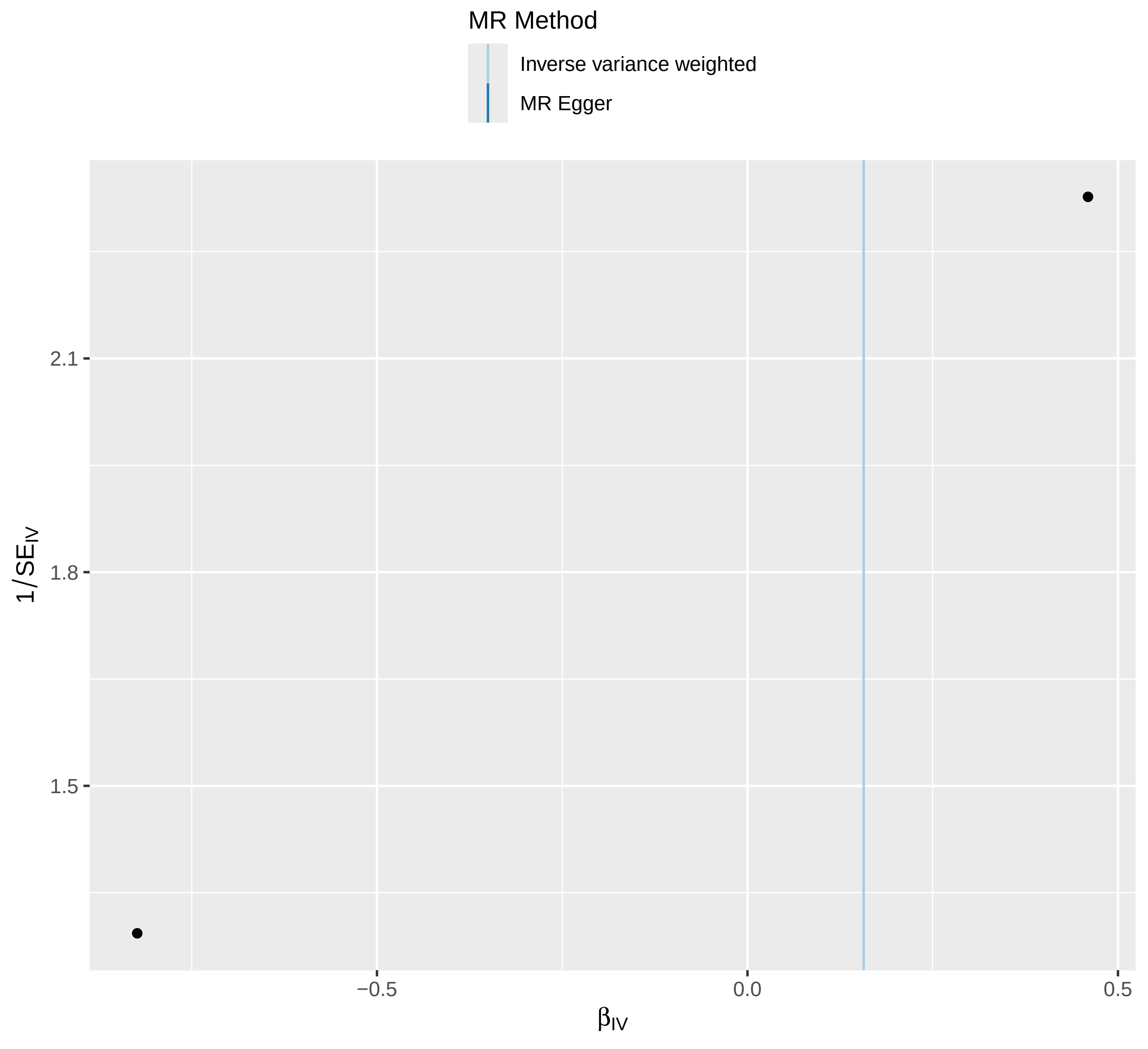
MR Method
Inverse variance weighted
MR Egger
2.1
1.8
1.5
1/SE_IV
−0.5
0.0
0.5
β_IV

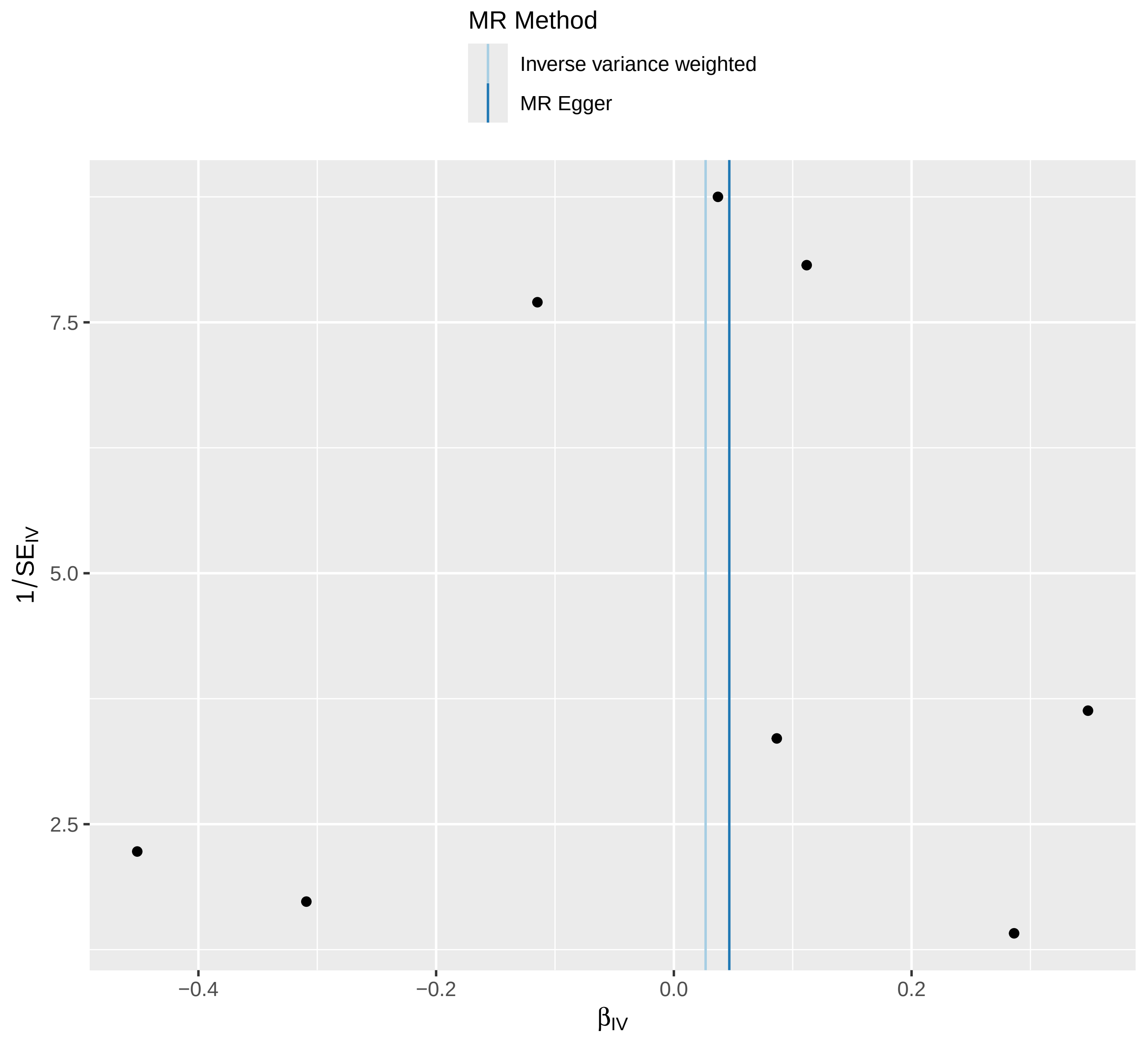
MR Method
Inverse variance weighted
MR Egger
7.5
5.0
2.5
$1/SE_{IV}$
−0.4
−0.2
0.0
0.2
β_{IV}

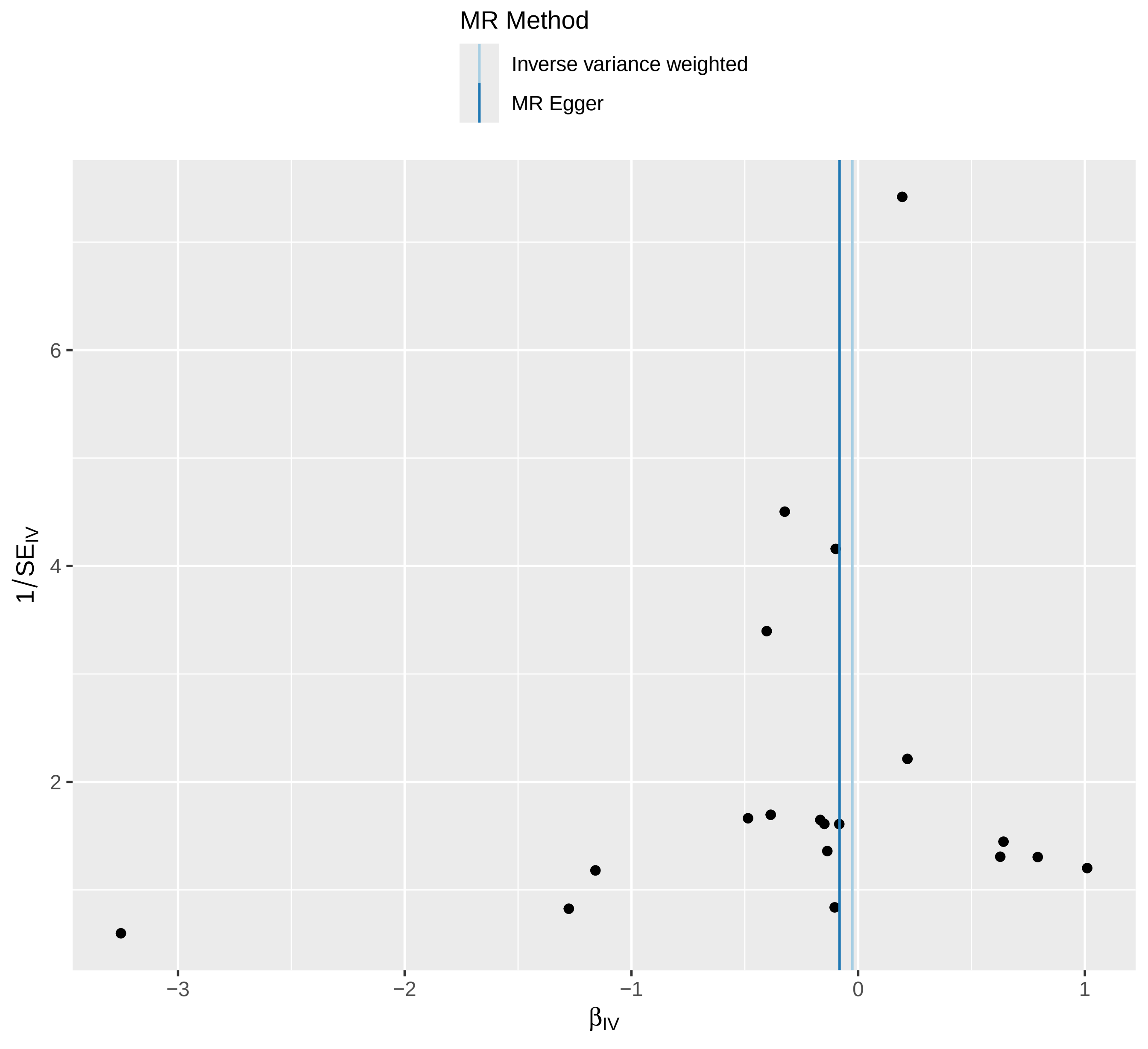
MR Method
Inverse variance weighted
MR Egger
6
4
2
1/SE_IV
−3
−2
−1
0
1
β_IV

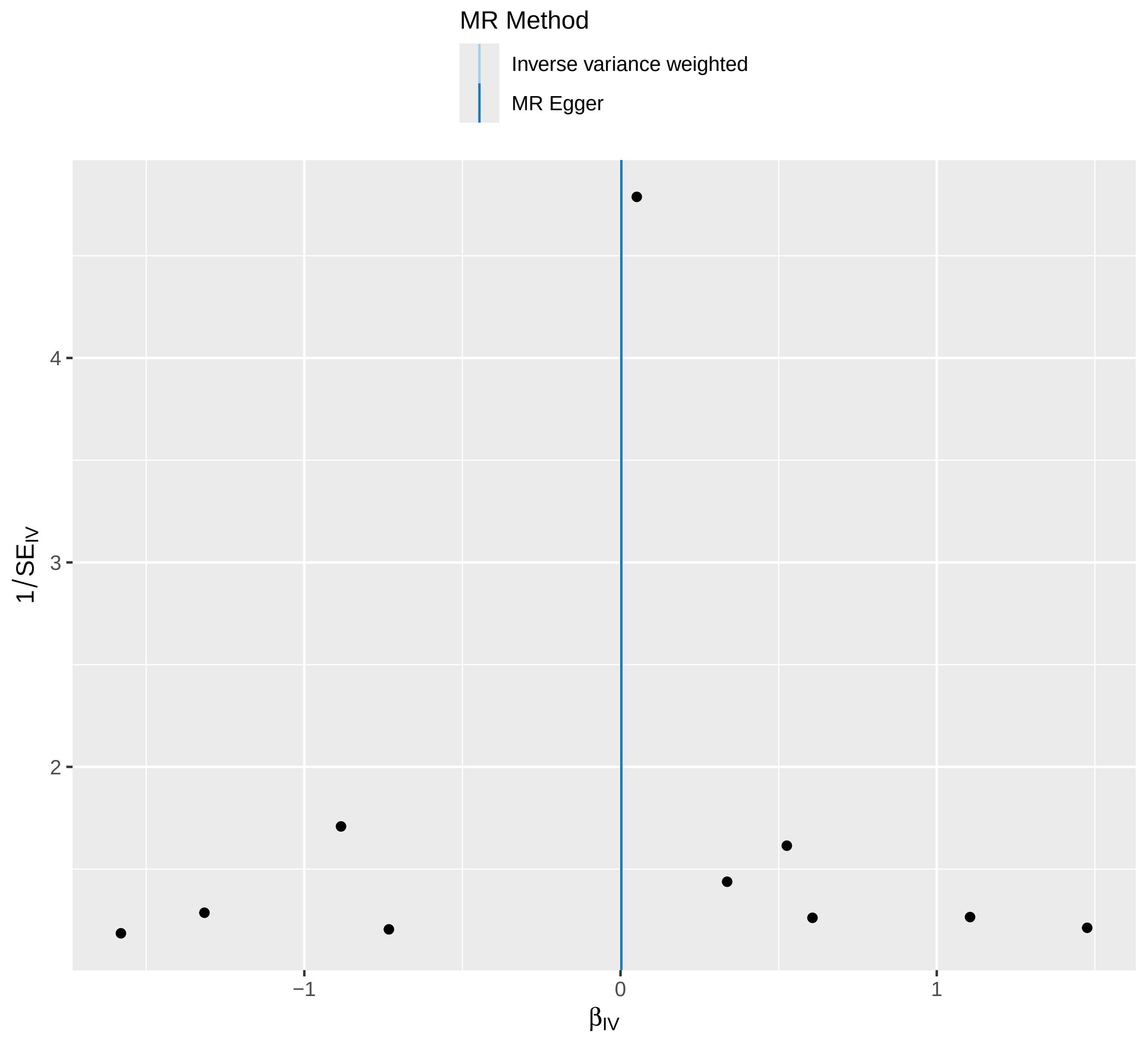

MR Method
Inverse variance weighted
MR Egger
4
3
2
−1
0
1
$1/SE_{IV}$
β_{IV}

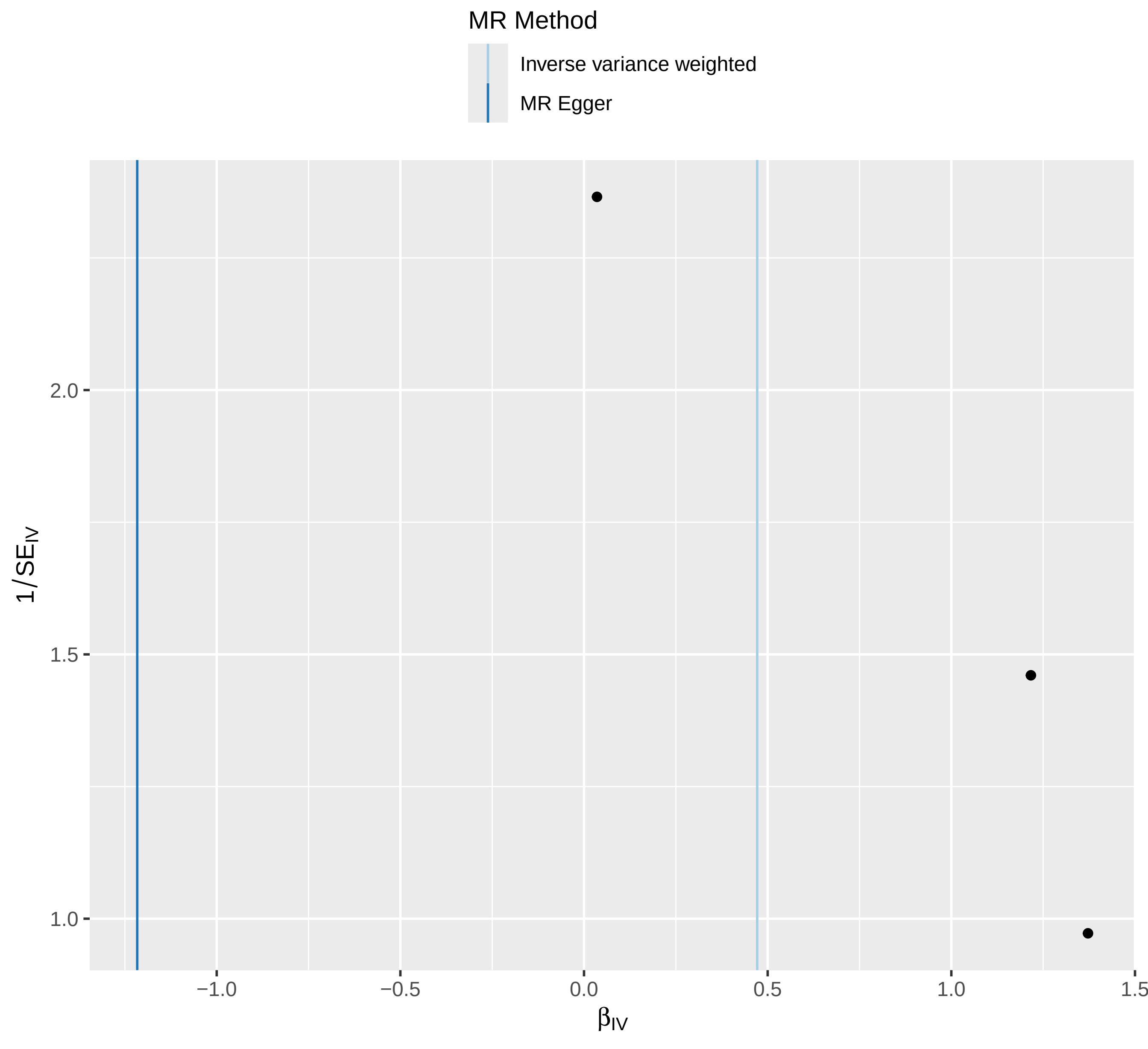
MR Method
Inverse variance weighted
MR Egger
2.0
1.5
1.0
1/SE_IV
−1.0
−0.5
0.0
0.5
1.0
1.5
β_IV

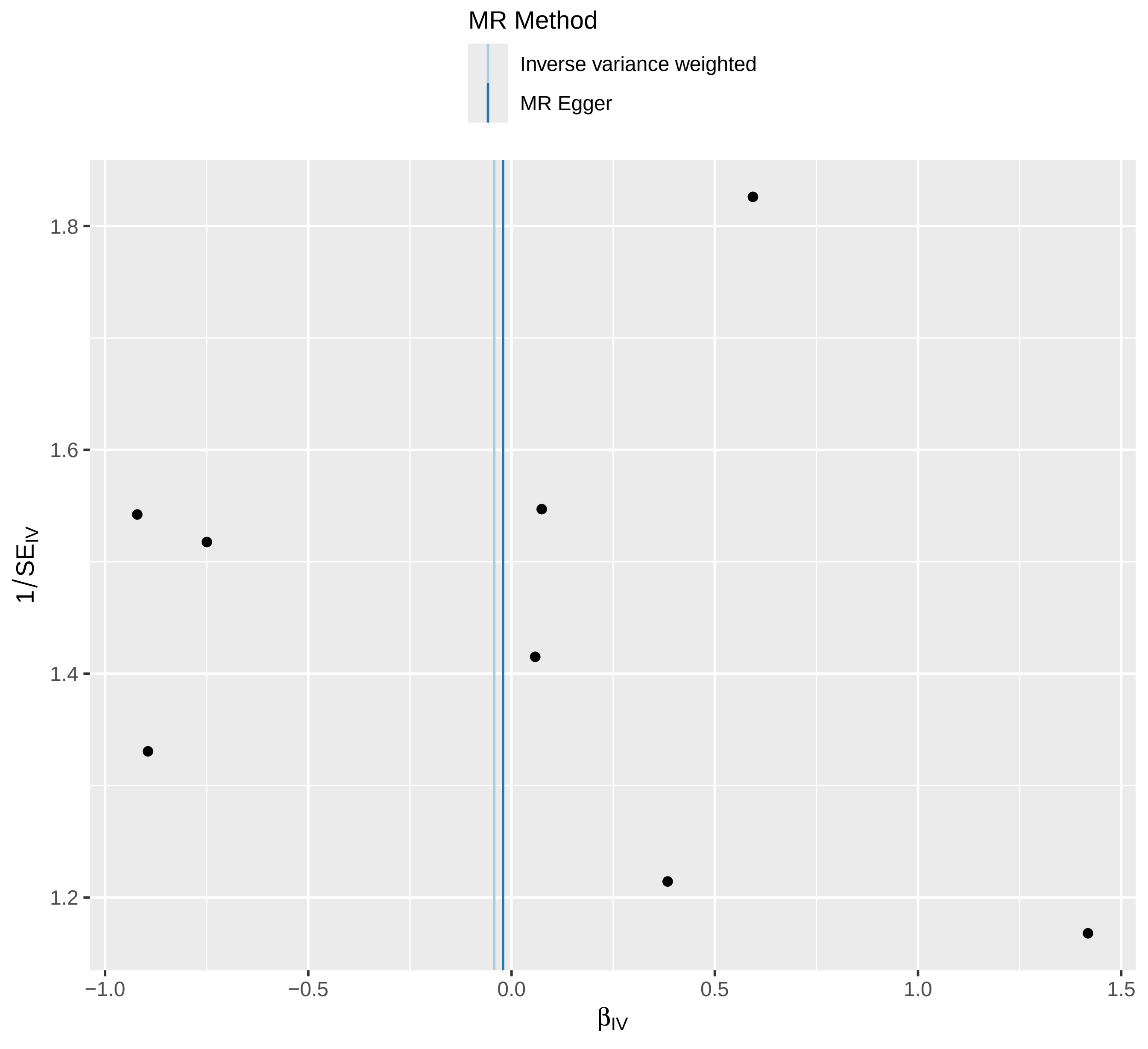

MR Method
Inverse variance weighted
MR Egger
1.8
1.6
1.4
1.2
1/SE_IV
−1.0
−0.5
0.0
0.5
1.0
1.5
β_IV

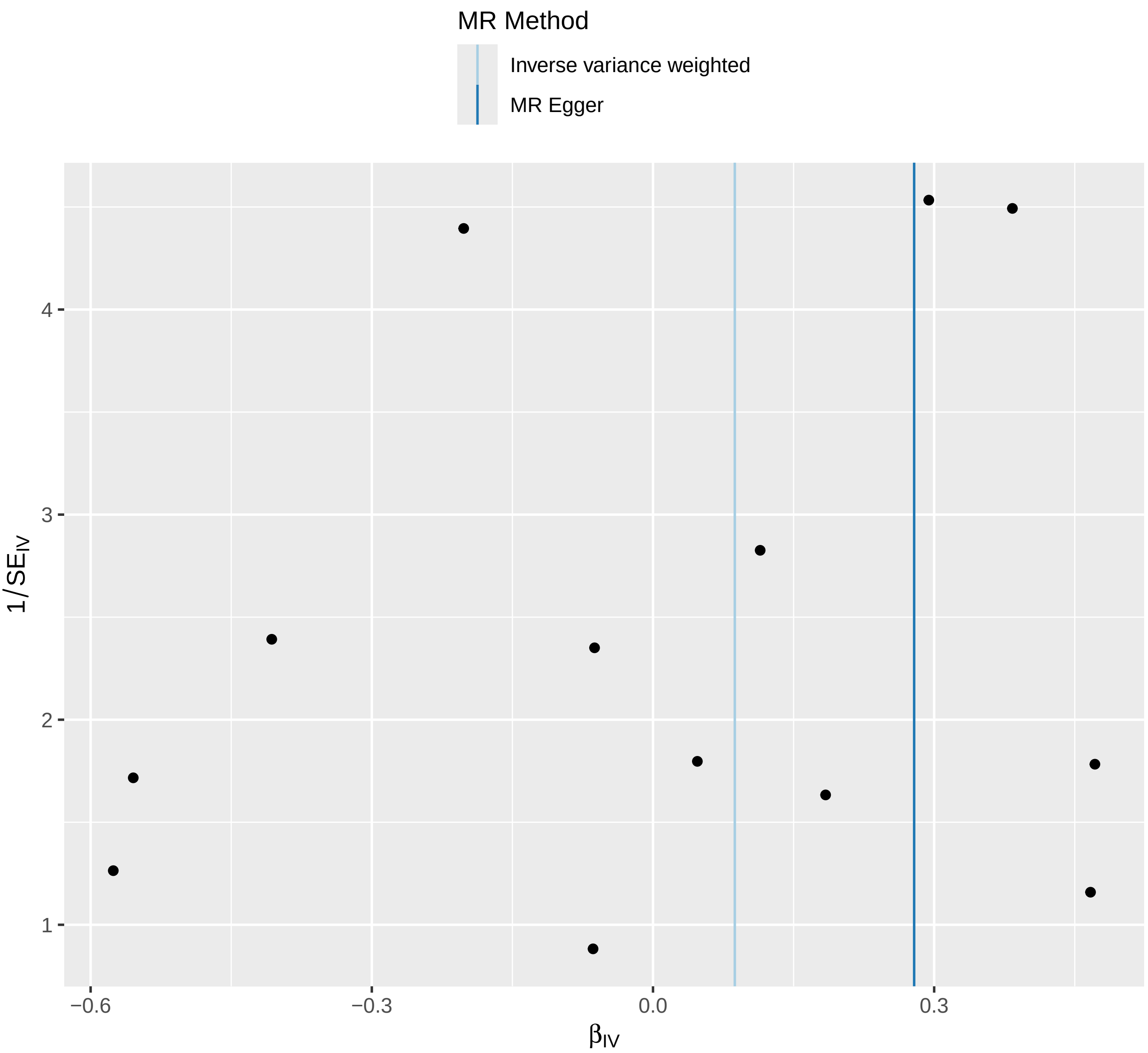

MR Method
Inverse variance weighted
MR Egger
4
3
2
1
$1/SE_{IV}$
−0.6
−0.3
0.0
0.3
β_{IV}

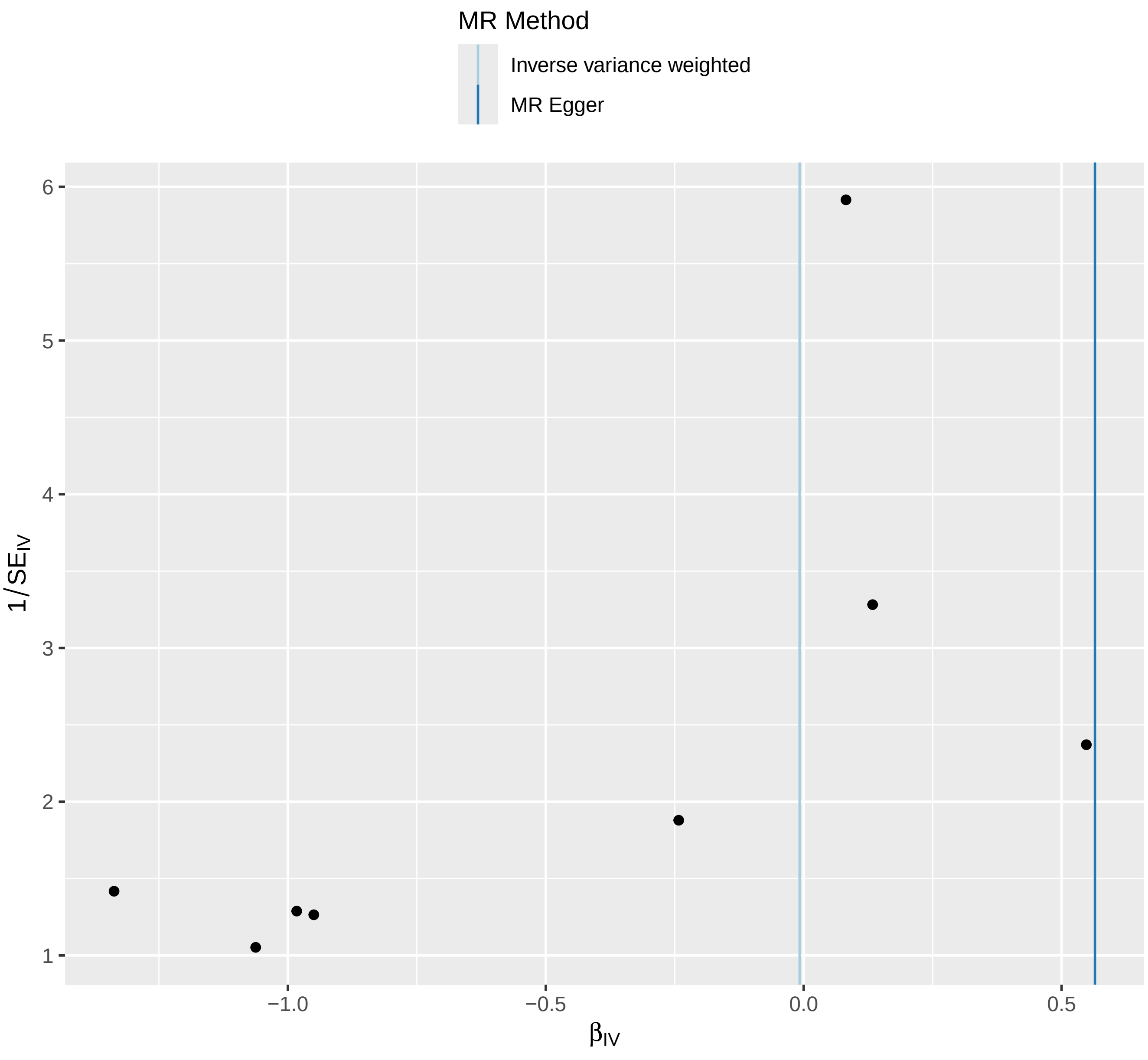
MR Method
Inverse variance weighted
MR Egger
1/SE_{IV}
β_{IV}
6
5
4
3
2
1
−1.0
−0.5
0.0
0.5

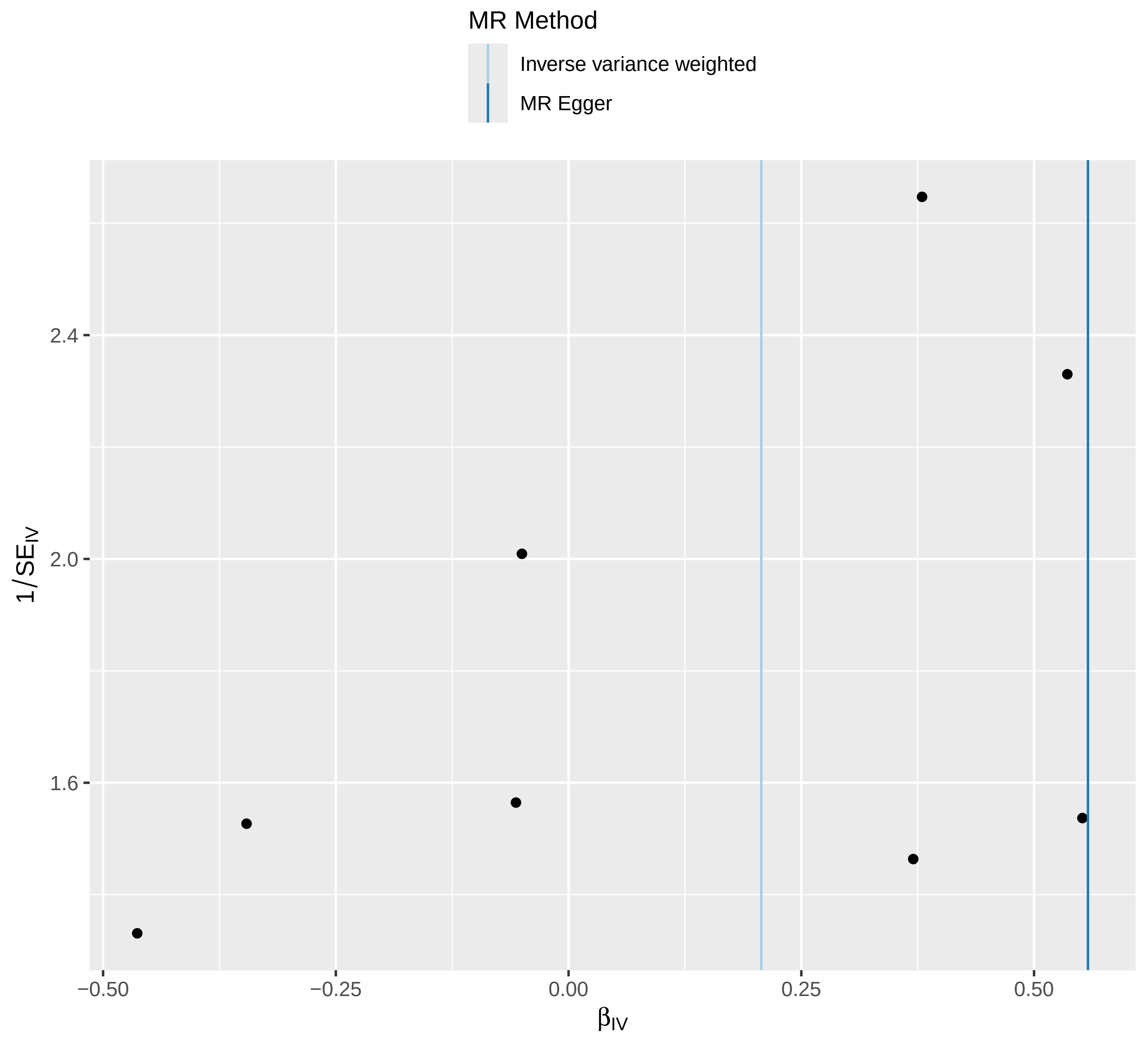
MR Method
Inverse variance weighted
MR Egger
2.4
2.0
1.6
−0.50
−0.25
0.00
0.25
0.50
$1/SE_{IV}$
β_{IV}

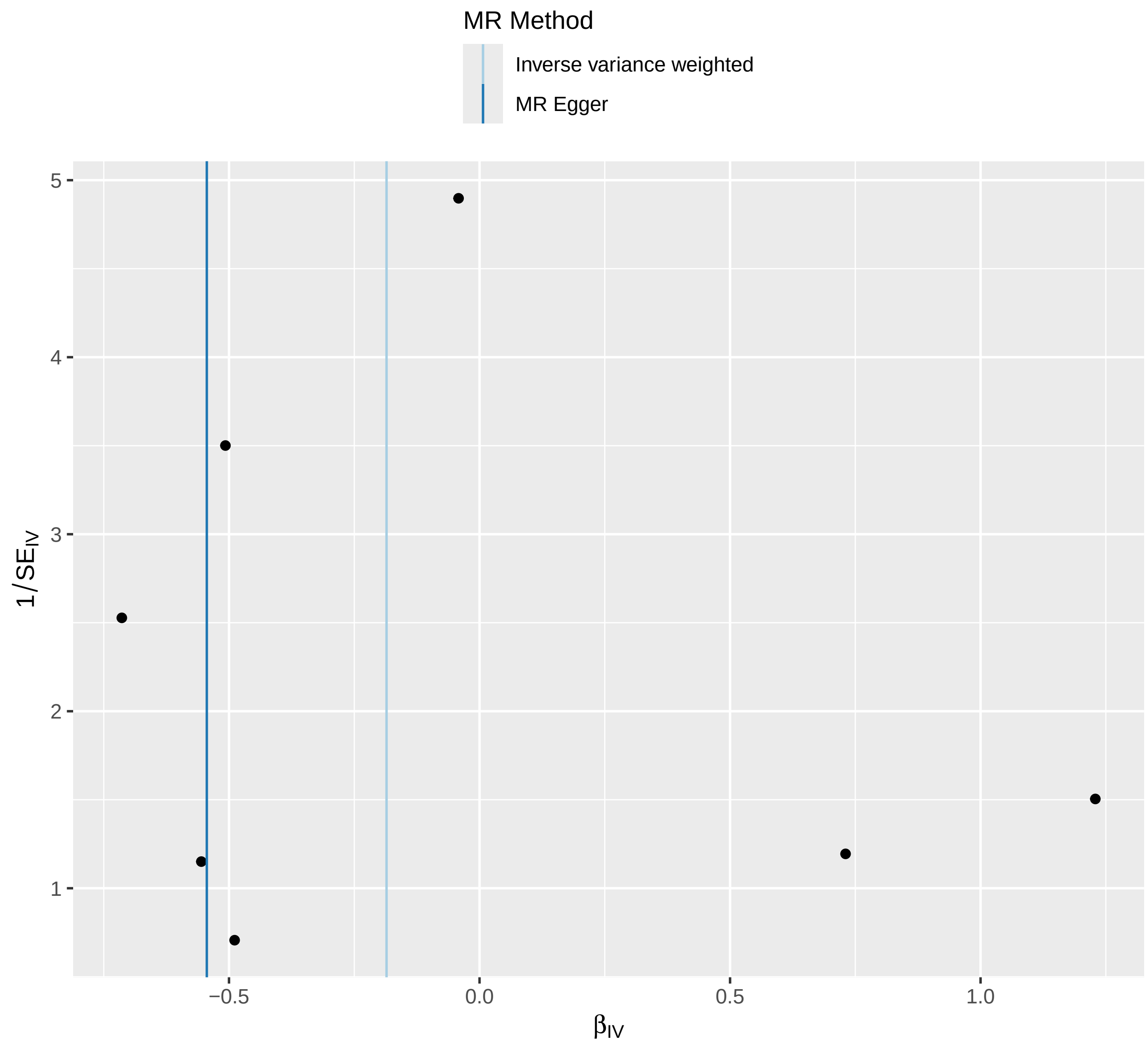

MR Method
Inverse variance weighted
MR Egger
5
4
3
2
1
1/SE$_{IV}$
−0.5
0.0
0.5
1.0
β$_{IV}$

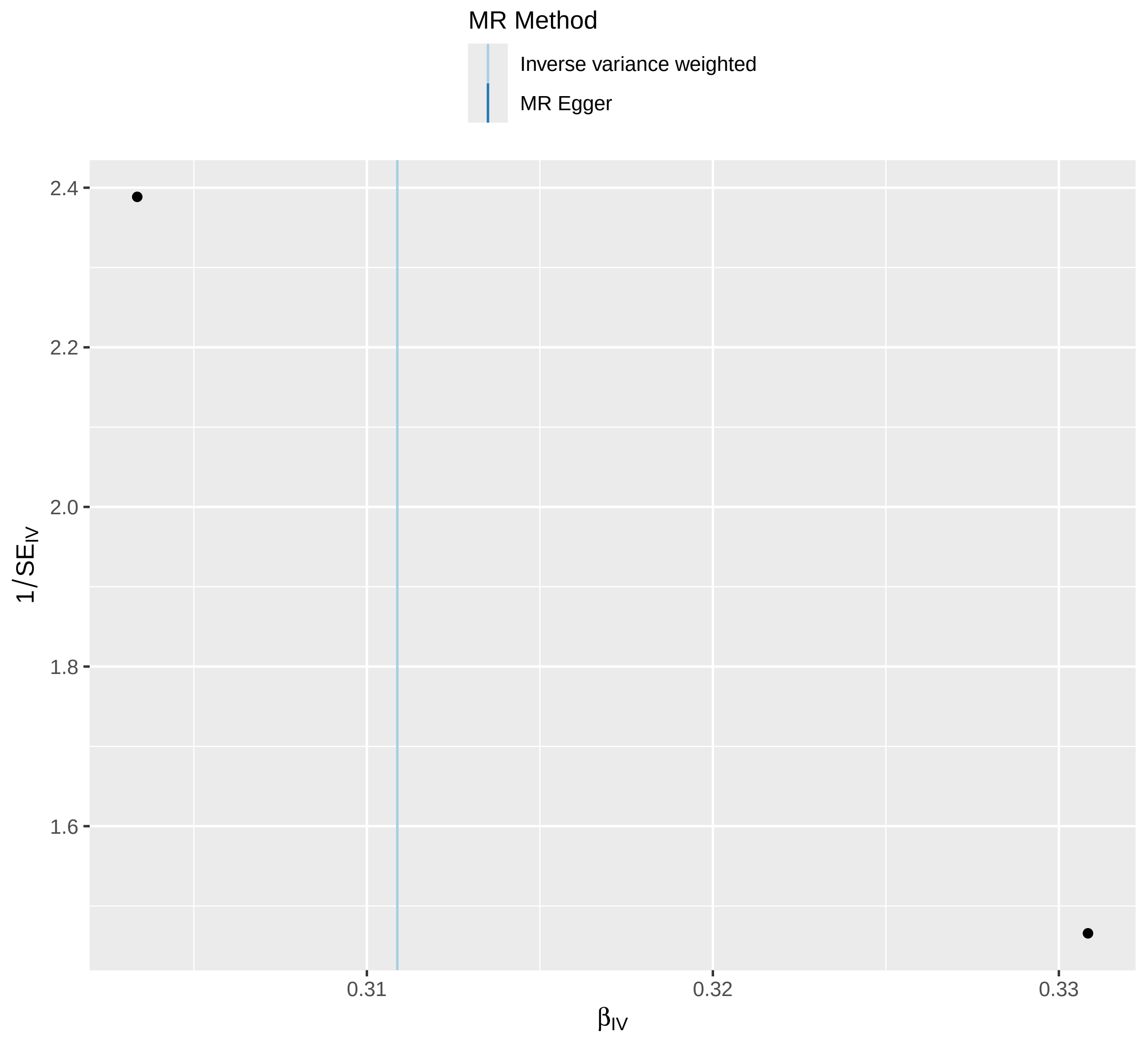

MR Method
Inverse variance weighted
MR Egger
2.4
2.2
2.0
1.8
1.6
1/SE_{IV}
0.31
0.32
0.33
β_{IV}

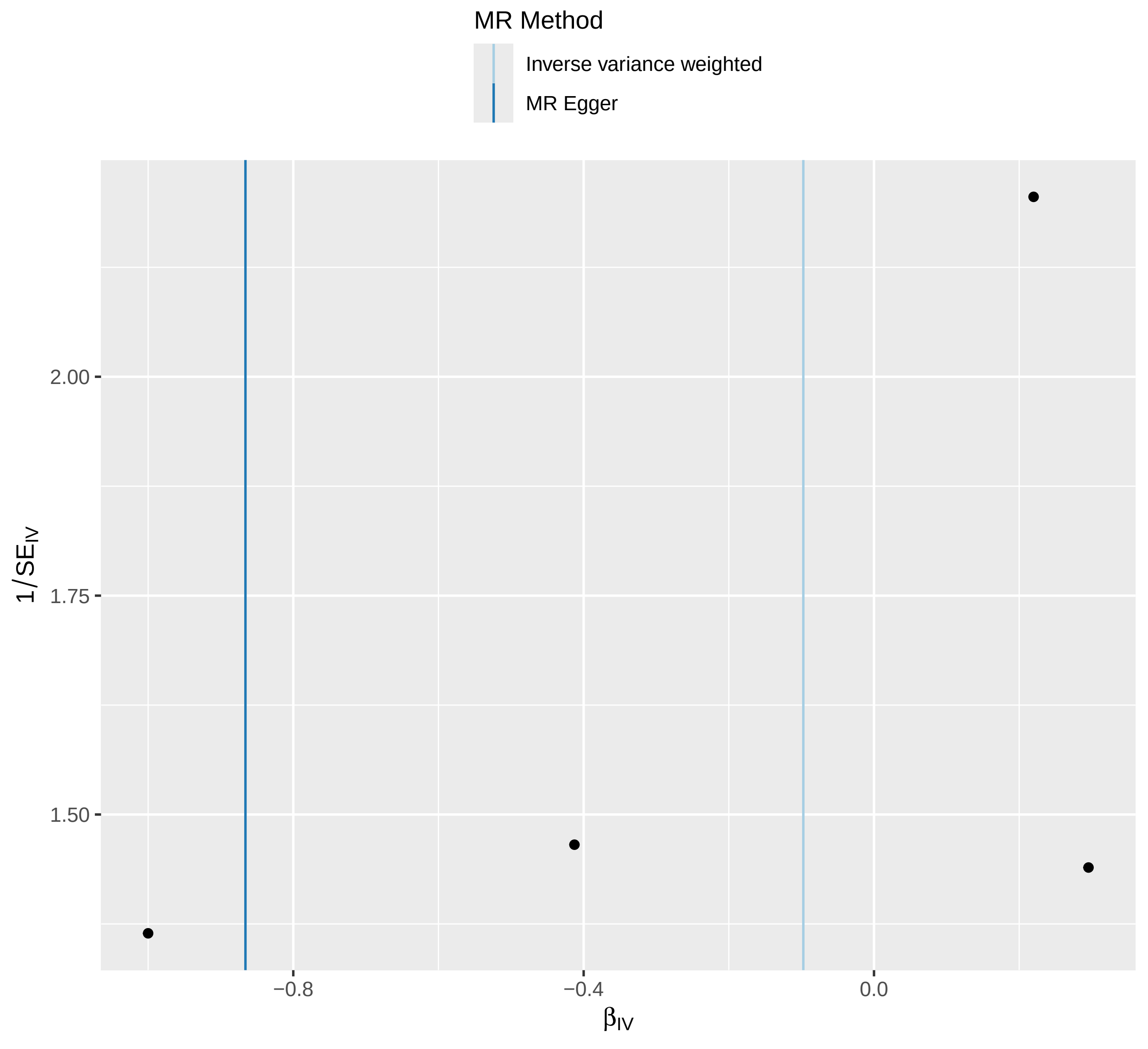

MR Method
Inverse variance weighted
MR Egger
2.00
1.75
1.50
1/SE_IV
−0.8
−0.4
0.0
β_IV

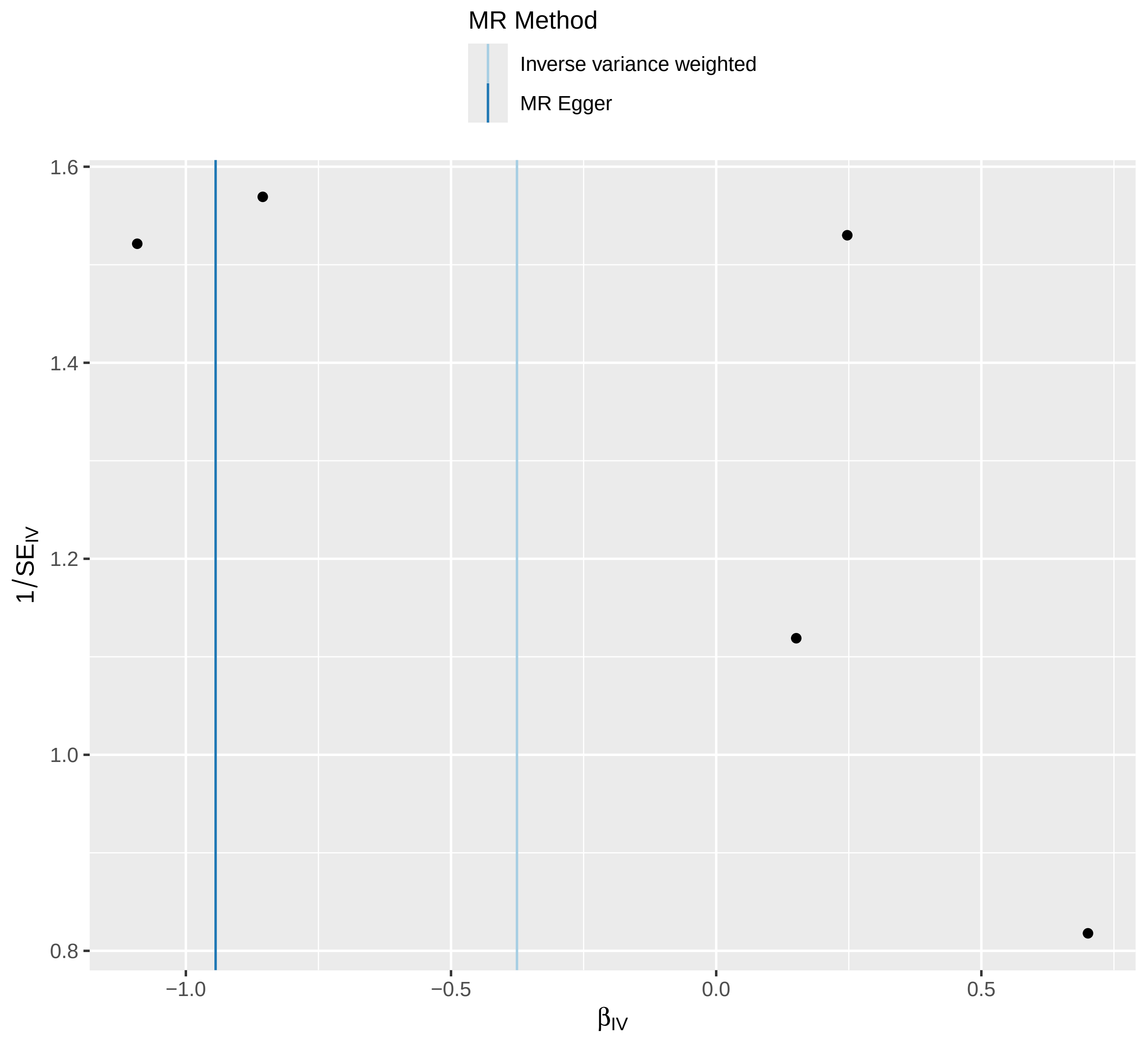

MR Method
Inverse variance weighted
MR Egger
1.6
1.4
1.2
1.0
0.8
1/SE$_{IV}$
−1.0
−0.5
0.0
0.5
β$_{IV}$

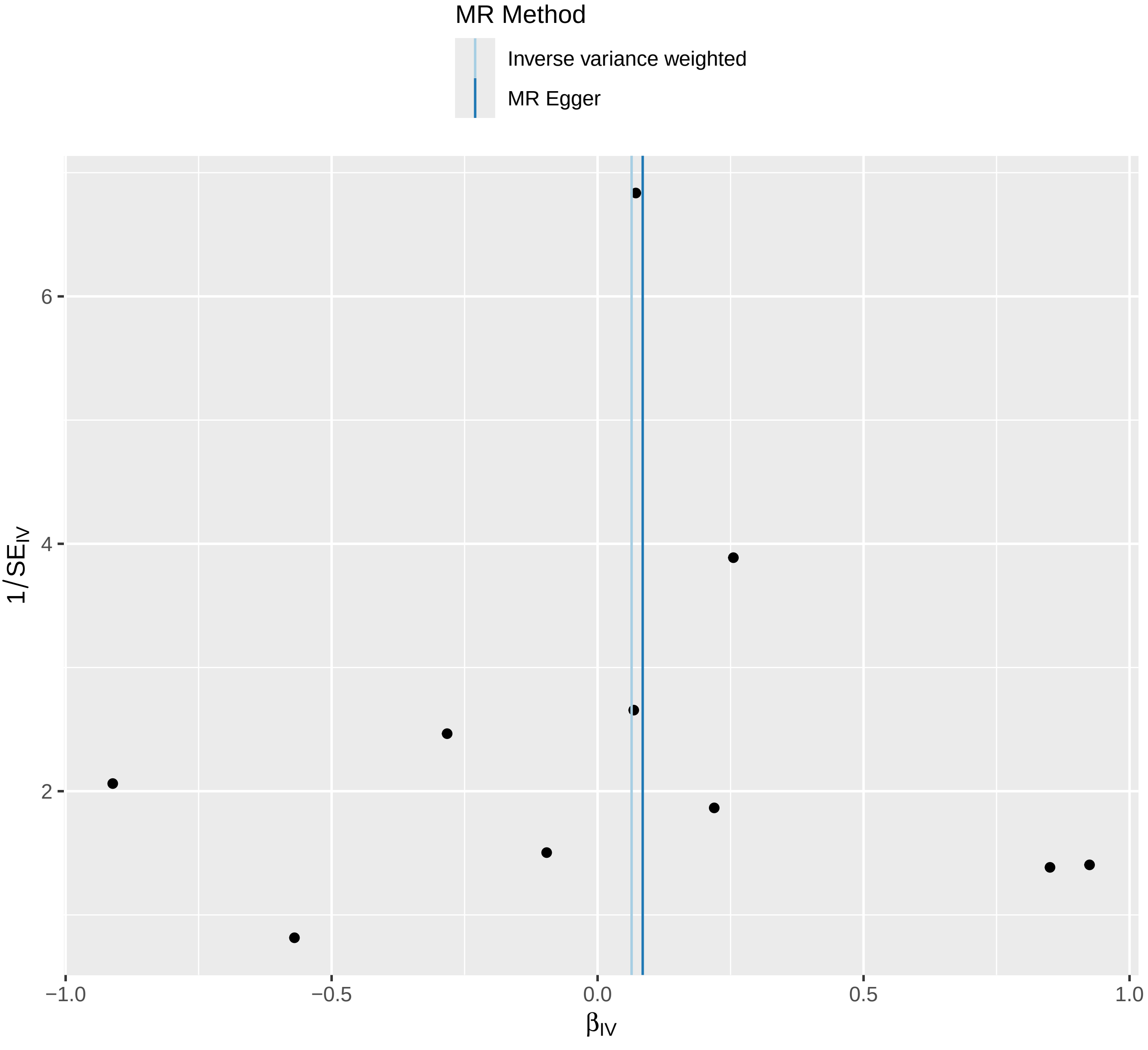

MR Method
Inverse variance weighted
MR Egger
6
4
2
1/SE_IV
−1.0
−0.5
0.0
0.5
1.0
β_IV

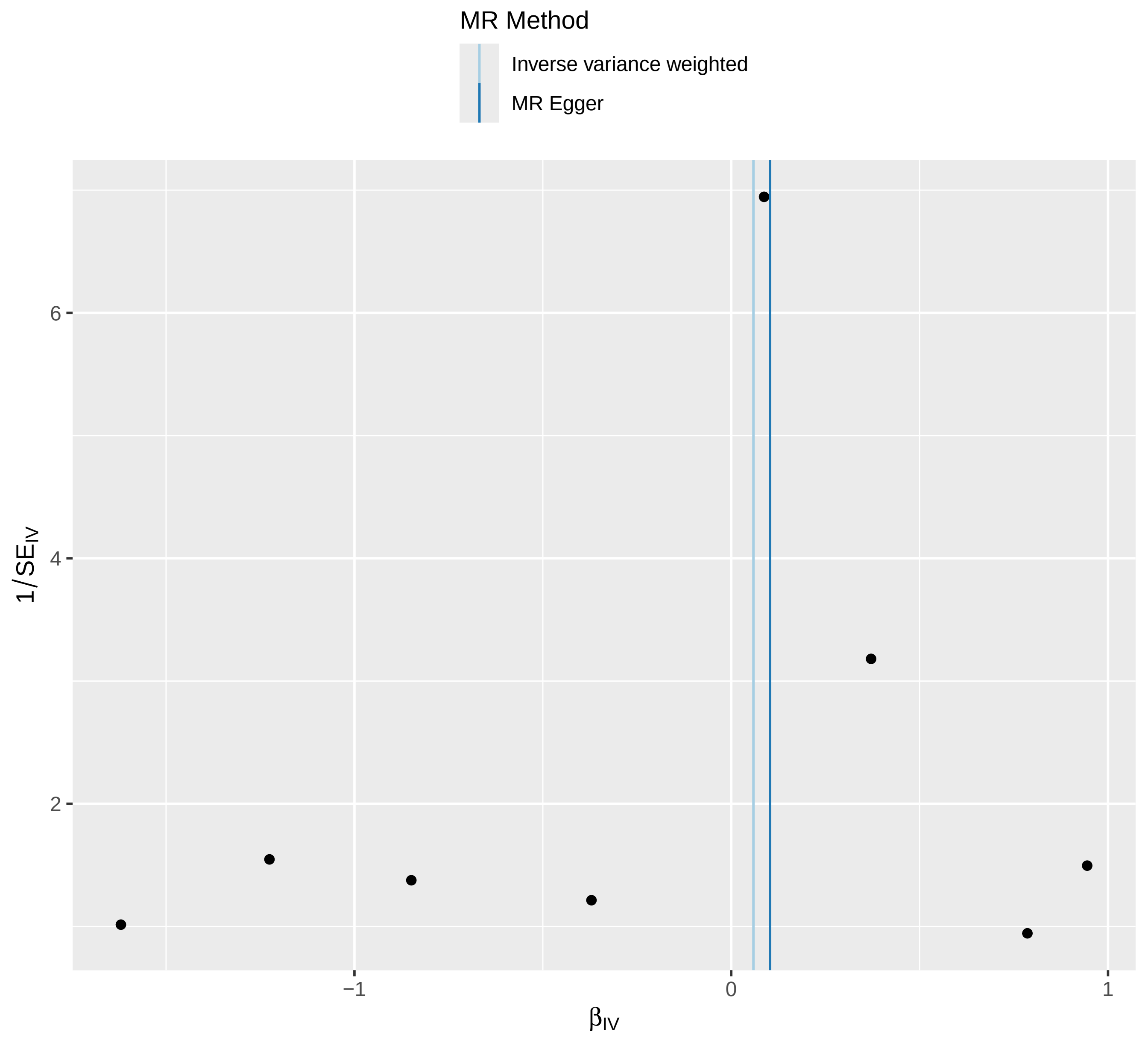
MR Method
Inverse variance weighted
MR Egger
6
4
2
1/SE_IV
−1
0
1
β_IV

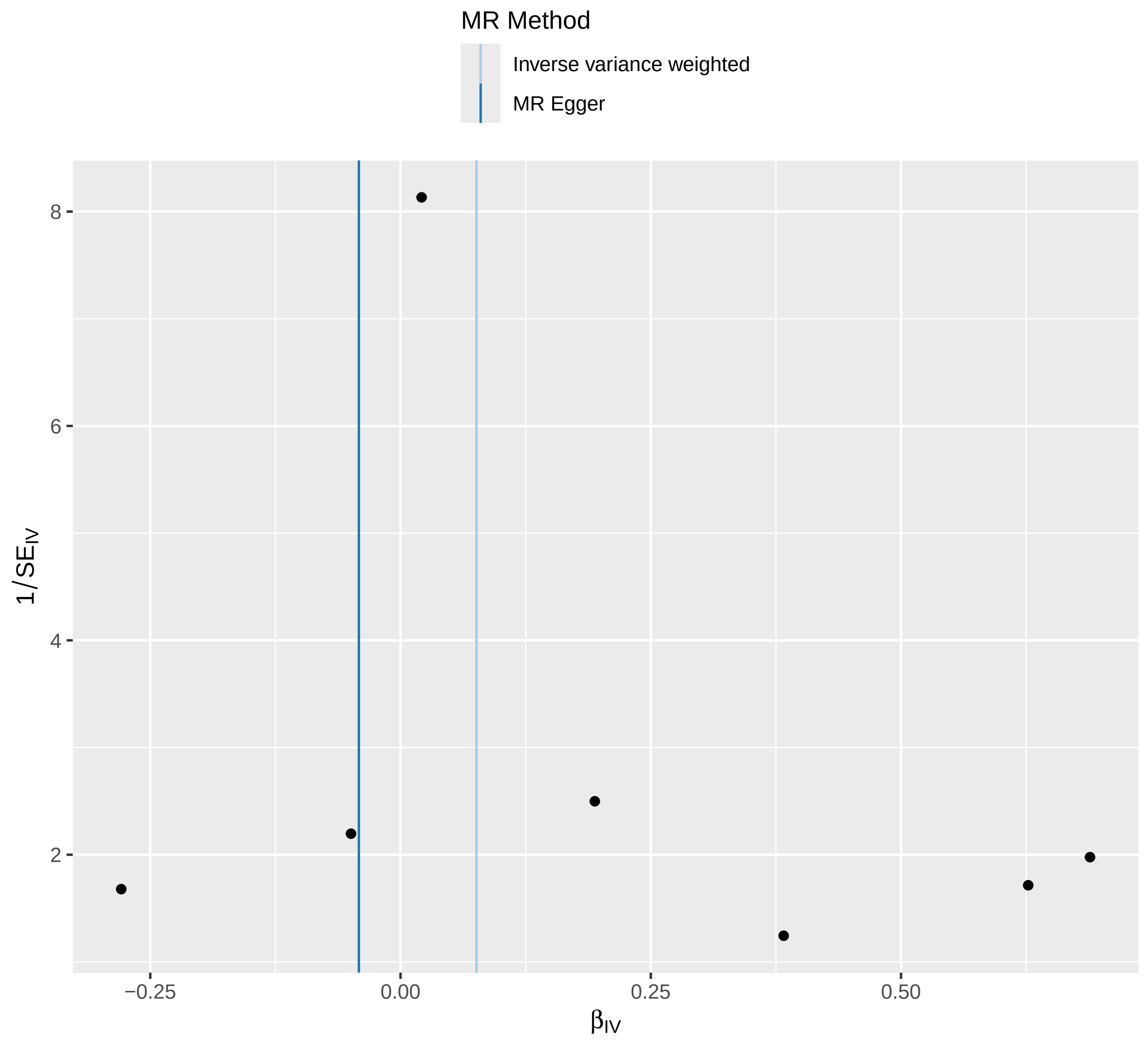
MR Method
Inverse variance weighted
MR Egger
8
6
4
2
1/SE$_{IV}$
−0.25
0.00
0.25
0.50
β$_{IV}$

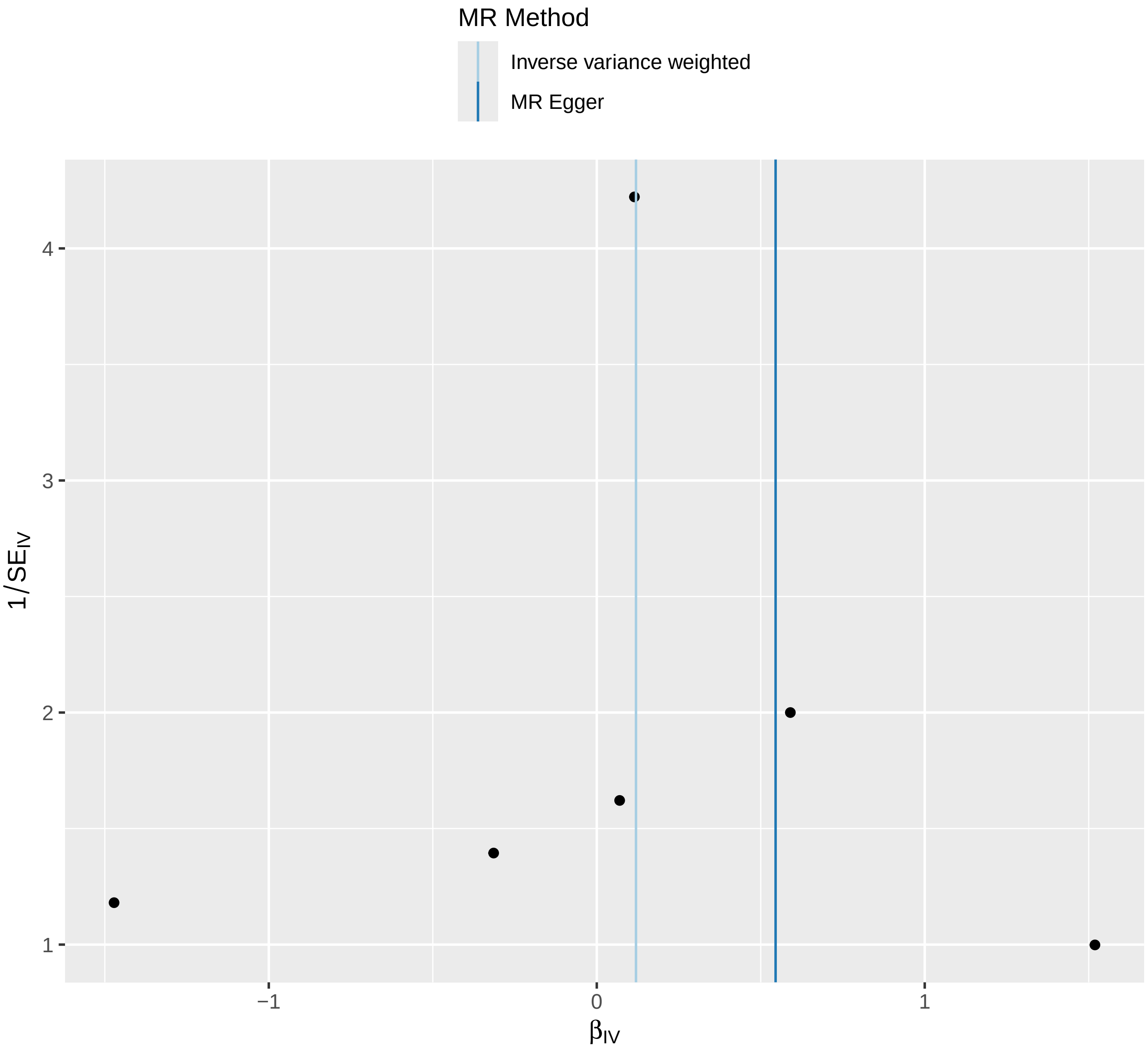
MR Method
Inverse variance weighted
MR Egger
1/SE_{IV}
4
3
2
1
−1
0
1
β_{IV}

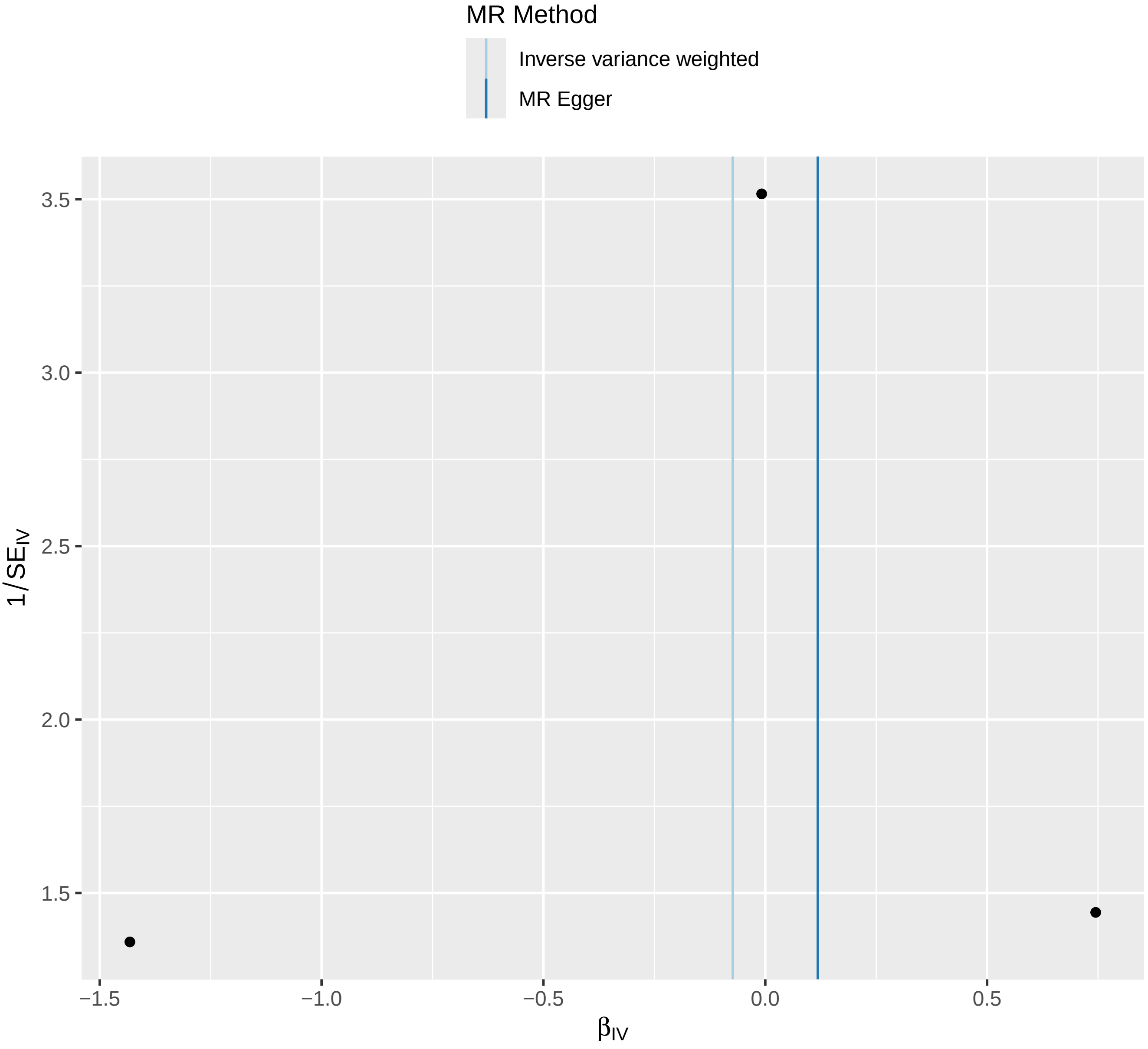
MR Method
Inverse variance weighted
MR Egger
3.5
3.0
2.5
2.0
1.5
1/SE_IV
−1.5
−1.0
−0.5
0.0
0.5
β_IV

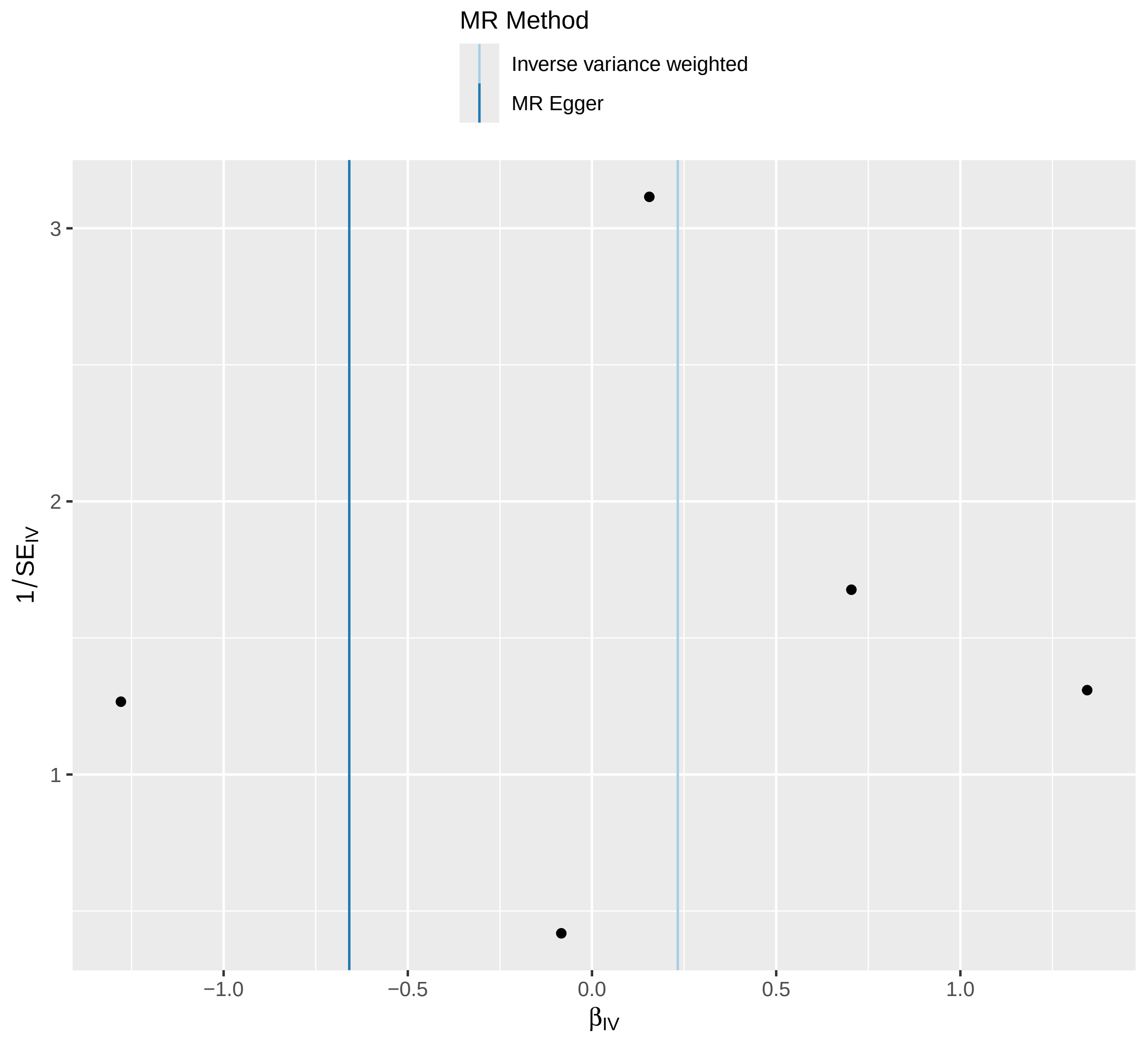
MR Method
Inverse variance weighted
MR Egger
3
2
1
1/SE_IV
−1.0
−0.5
0.0
0.5
1.0
β_IV

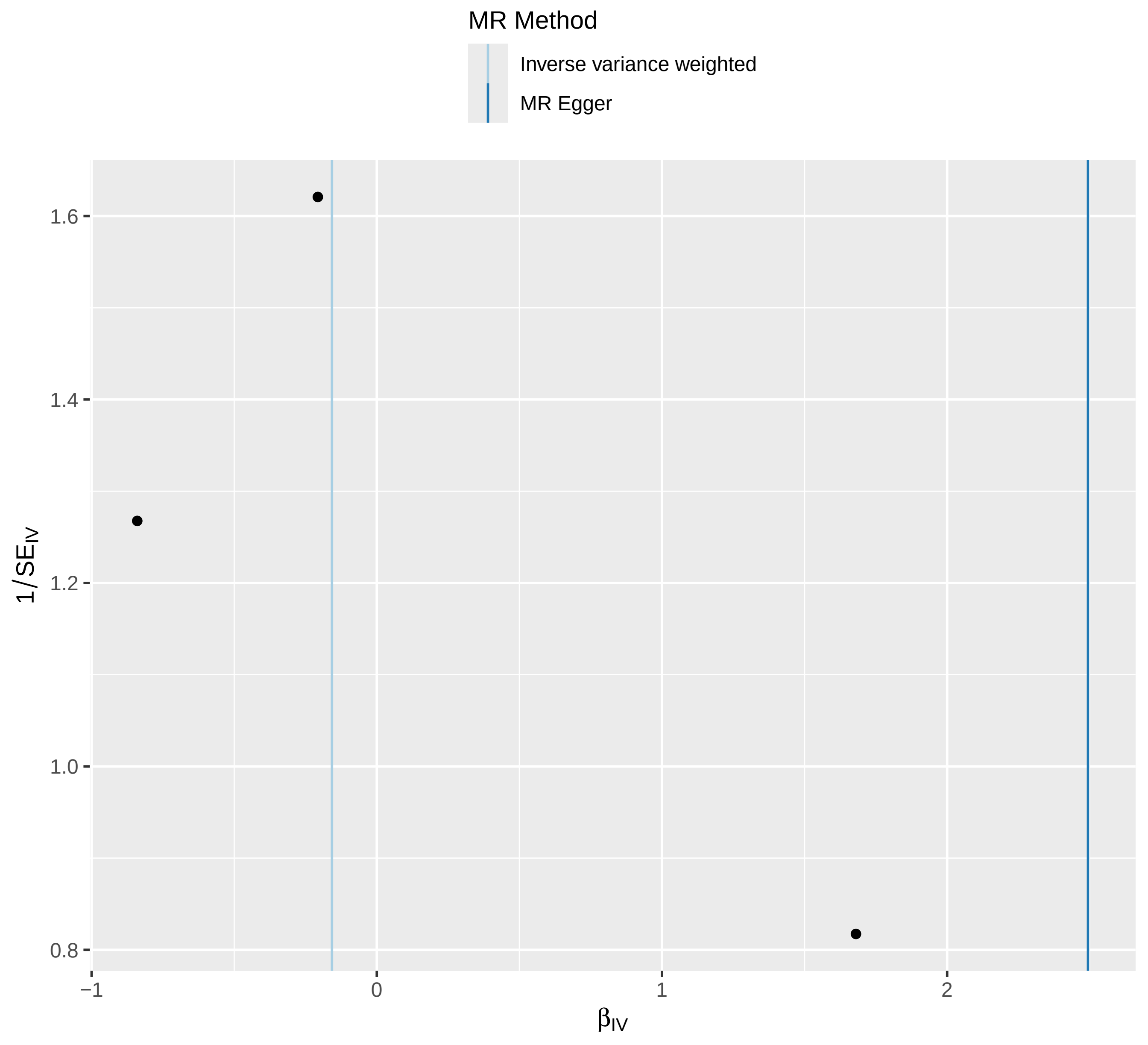

MR Method
Inverse variance weighted
MR Egger
1.6
1.4
1.2
1.0
0.8
1/SE_IV
−1
0
1
2
β_IV

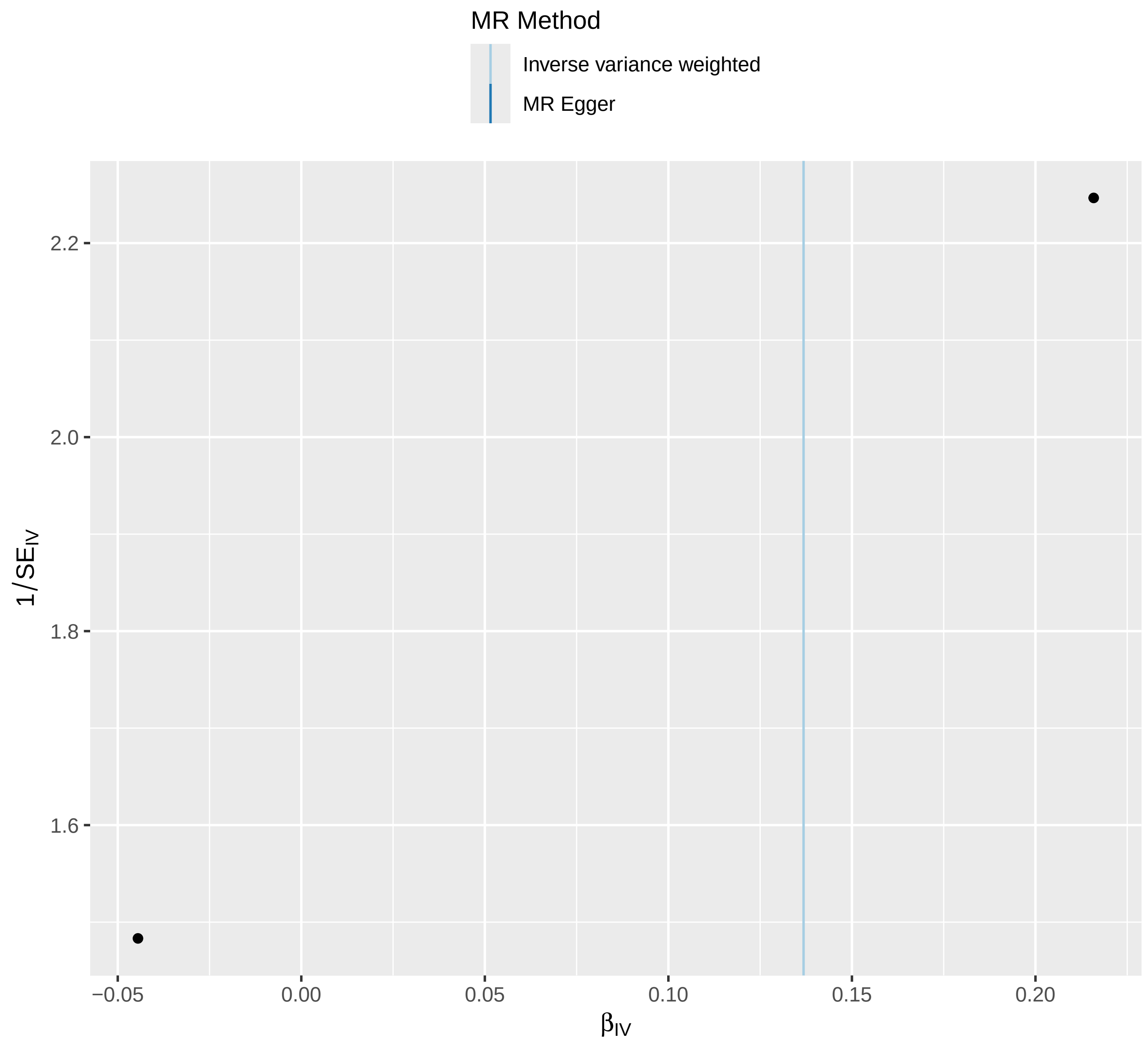

MR Method
Inverse variance weighted
MR Egger
2.2
2.0
1.8
1.6
1/SE_IV
−0.05
0.00
0.05
0.10
0.15
0.20
β_IV

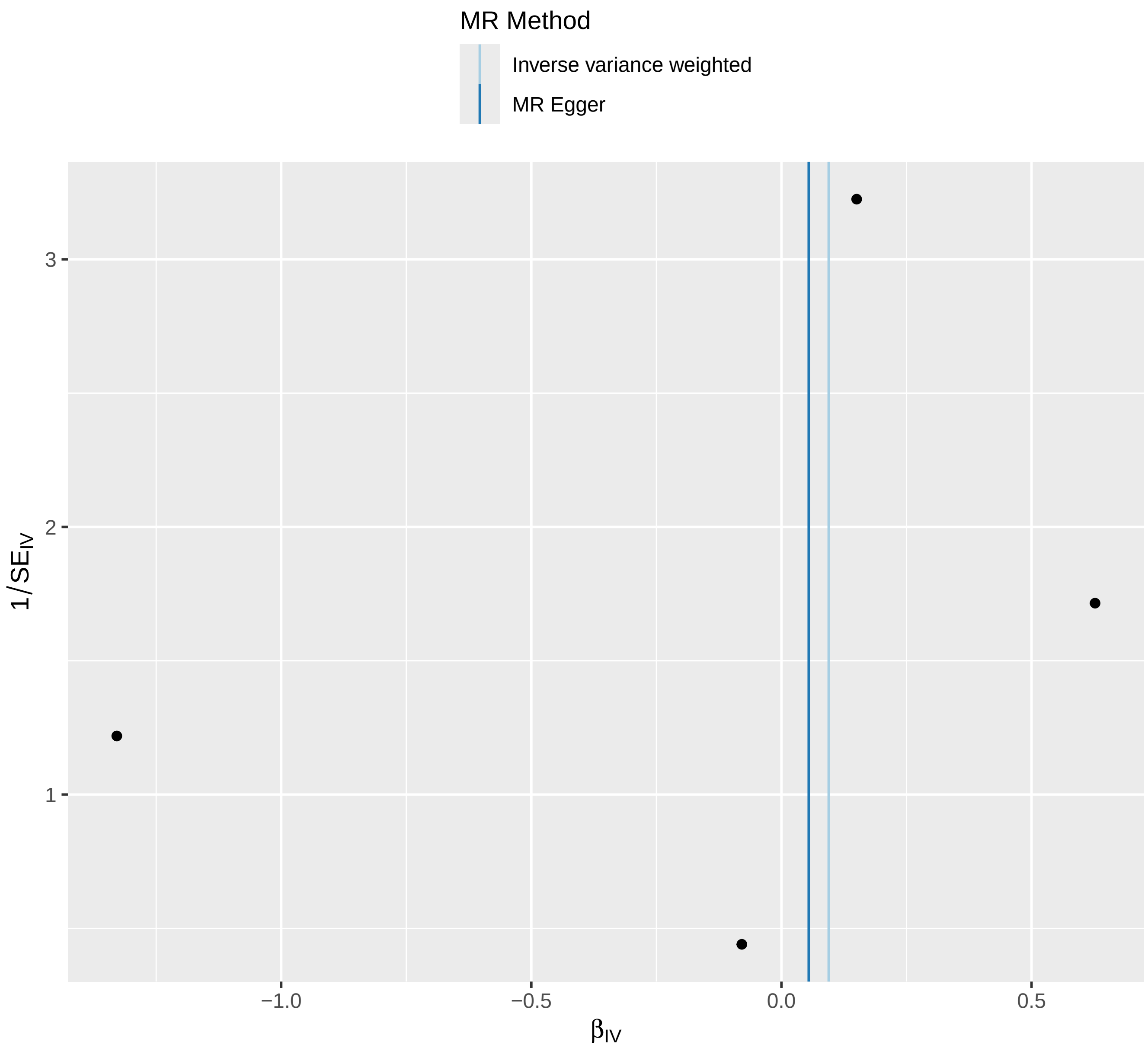
MR Method
Inverse variance weighted
MR Egger
3
2
1
1/SE$_{IV}$
−1.0
−0.5
0.0
0.5
β$_{IV}$

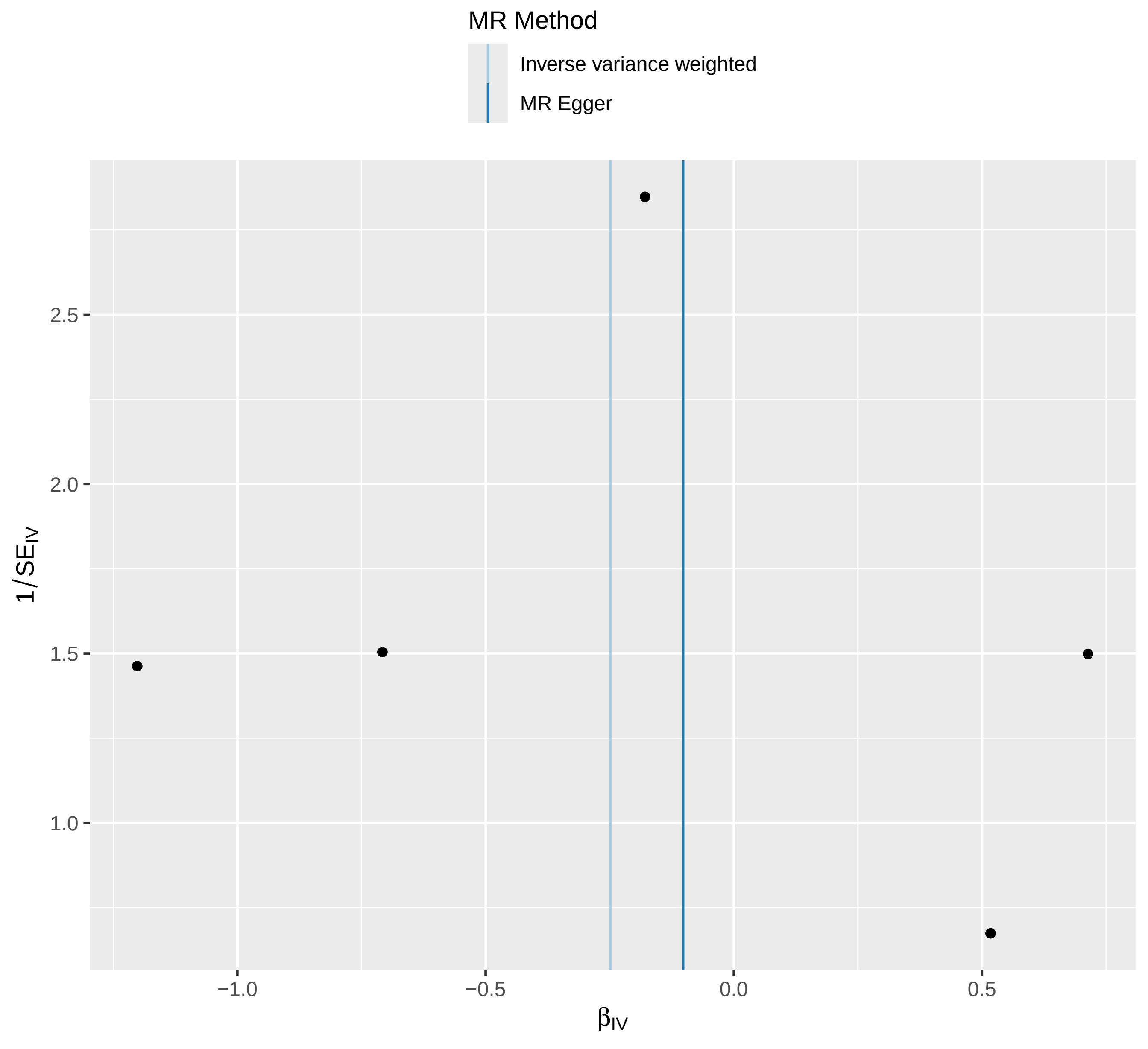
MR Method
Inverse variance weighted
MR Egger
2.5
2.0
1.5
1.0
1/SE_IV
−1.0
−0.5
0.0
0.5
β_IV

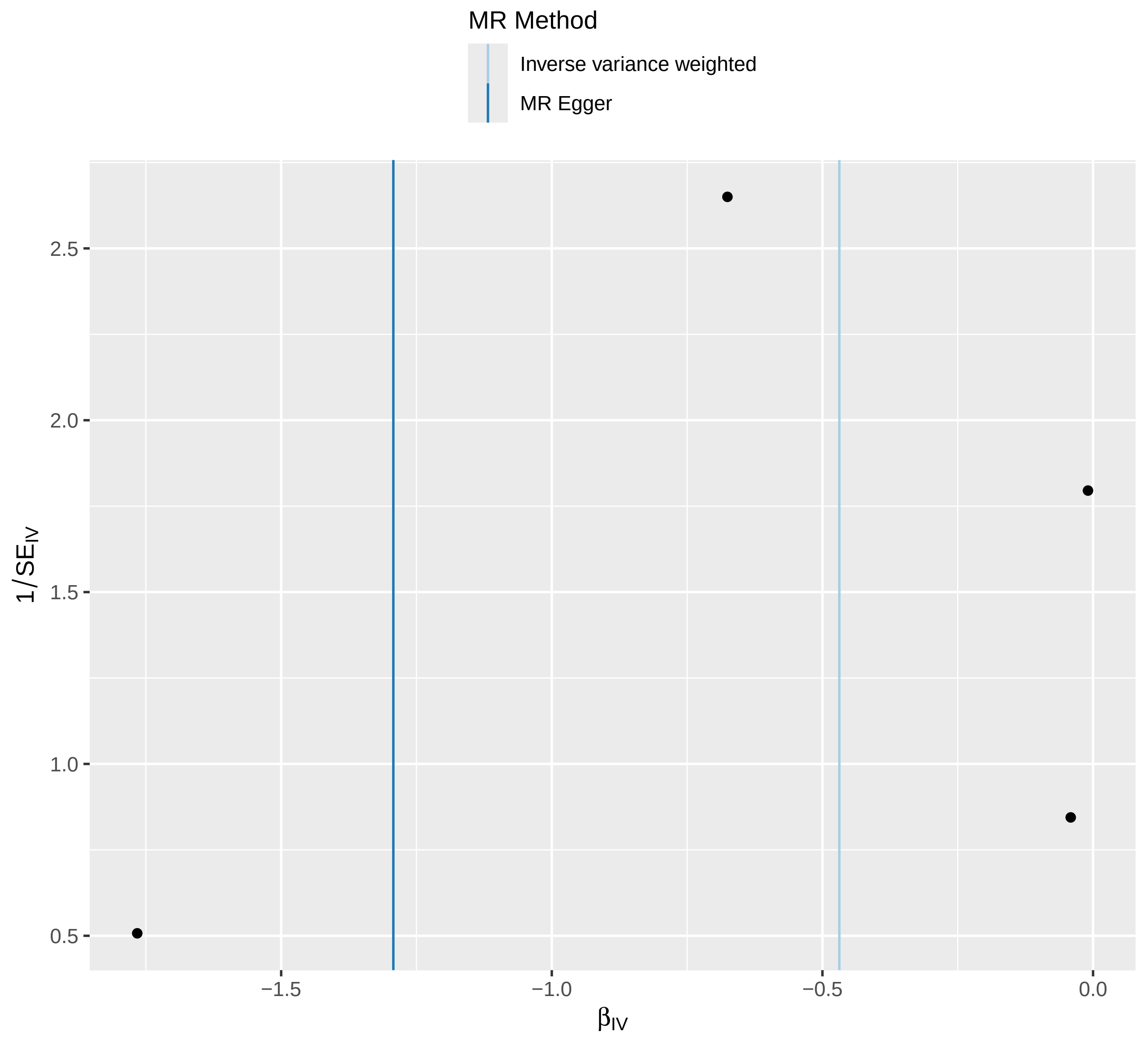

MR Method
Inverse variance weighted
MR Egger
2.5
2.0
1.5
1.0
0.5
1/SE_IV
−1.5
−1.0
−0.5
0.0
β_IV

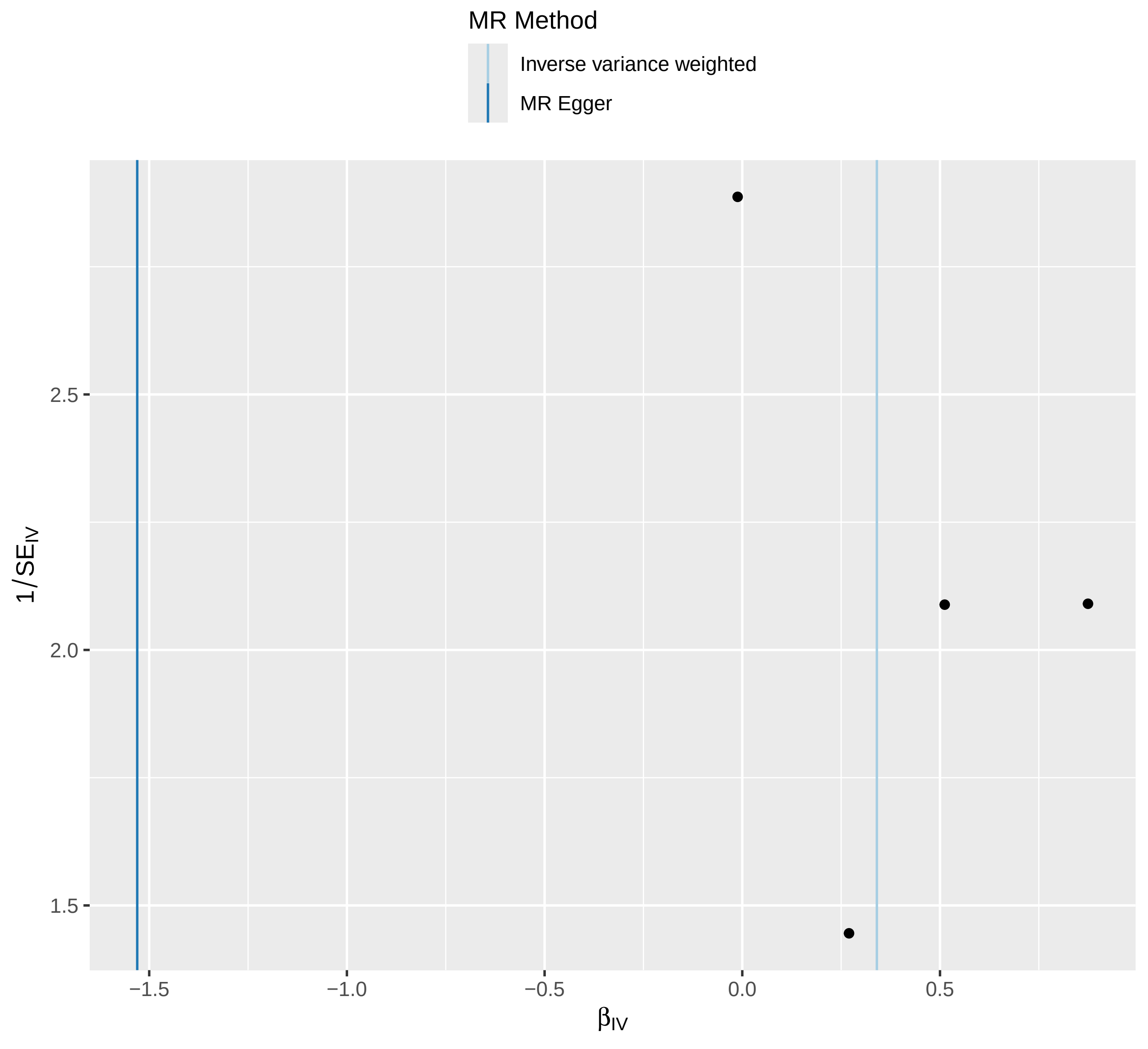

MR Method
Inverse variance weighted
MR Egger
1/SE_IV
2.5
2.0
1.5
−1.5
−1.0
−0.5
0.0
0.5
β_IV

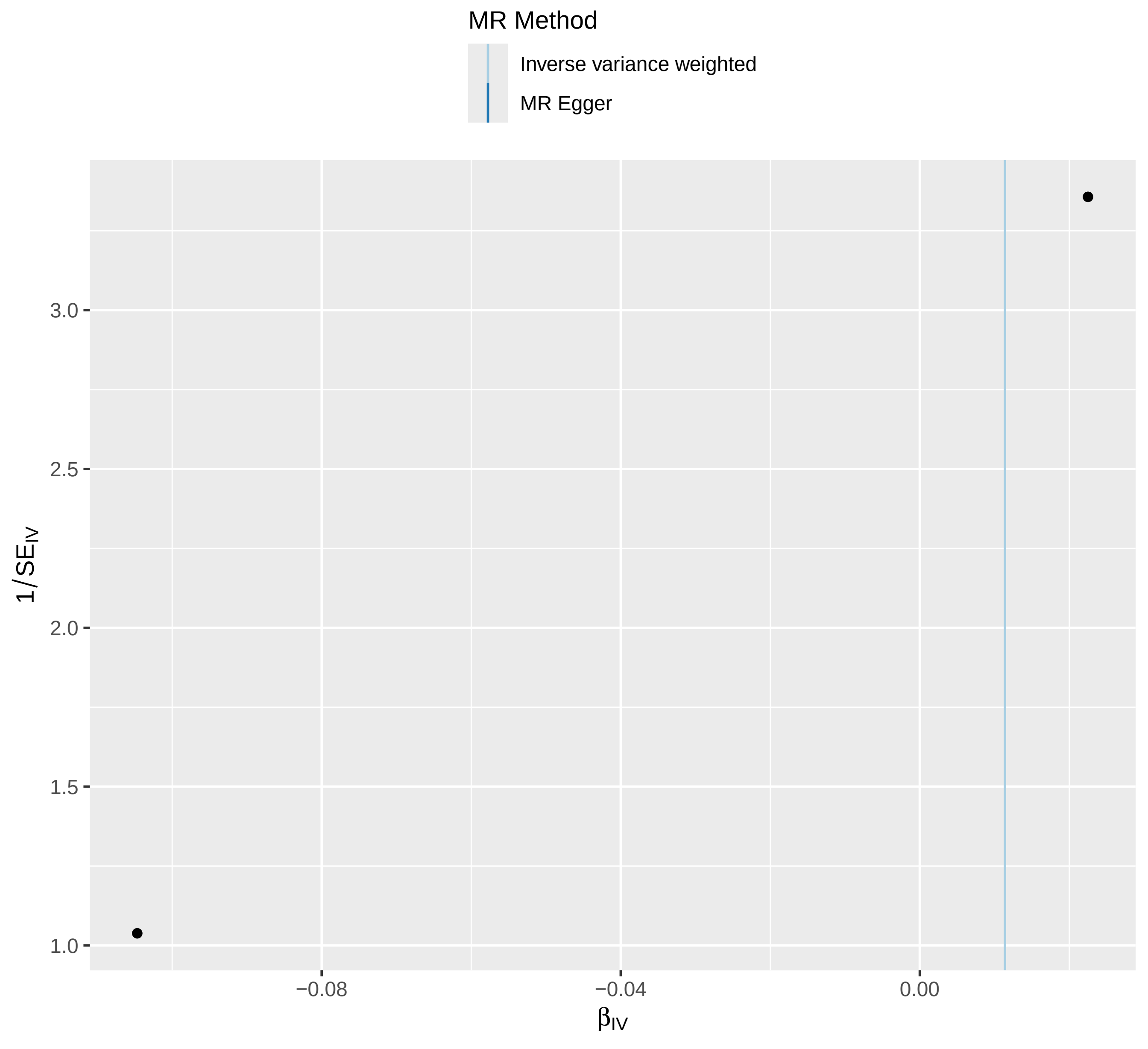

MR Method
Inverse variance weighted
MR Egger
3.0
2.5
2.0
1.5
1.0
1/SE_IV
−0.08
−0.04
0.00
β_IV

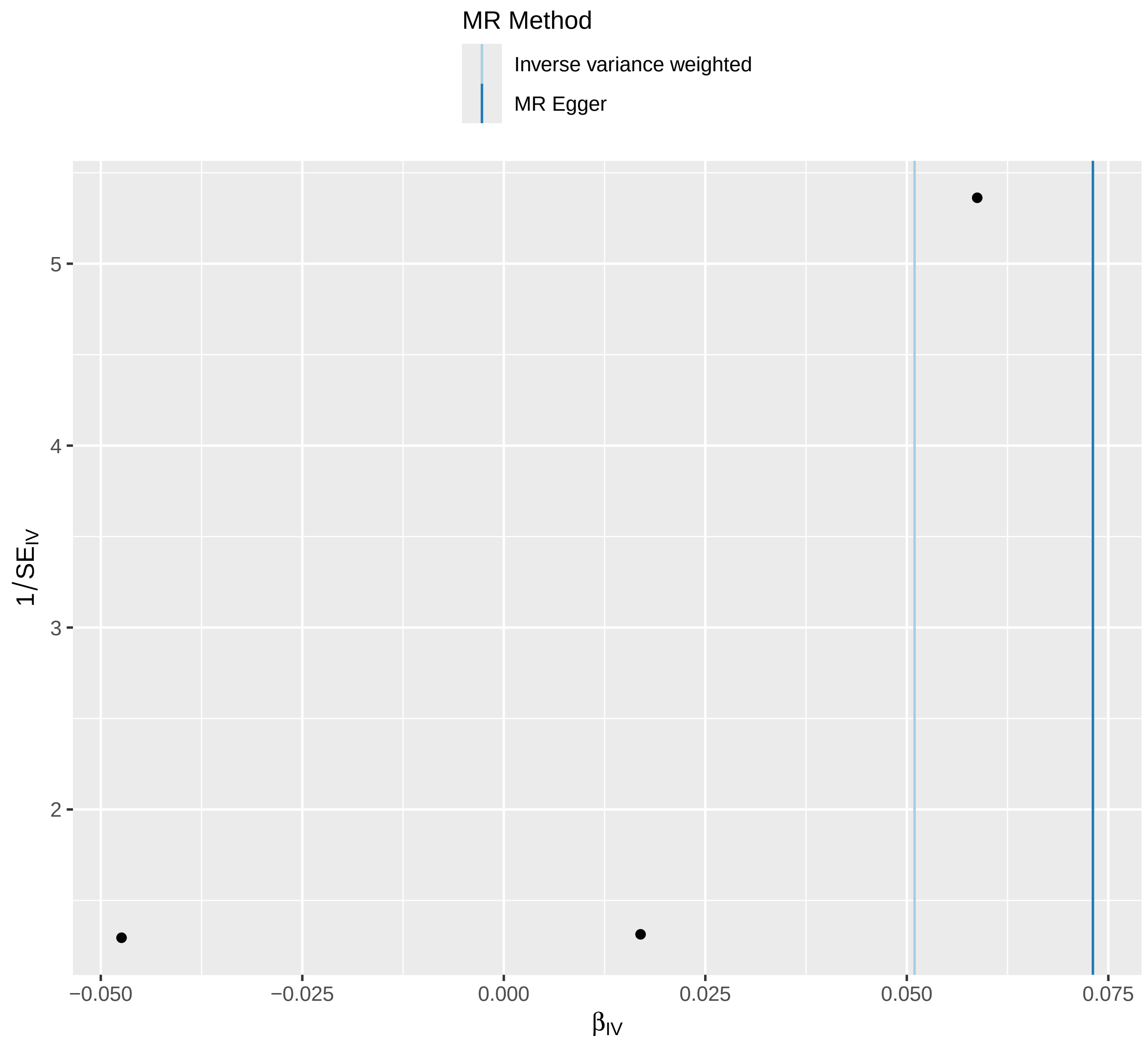
MR Method
Inverse variance weighted
MR Egger
5
4
3
2
1/SE$_{IV}$
−0.050
−0.025
0.000
0.025
0.050
0.075
β$_{IV}$

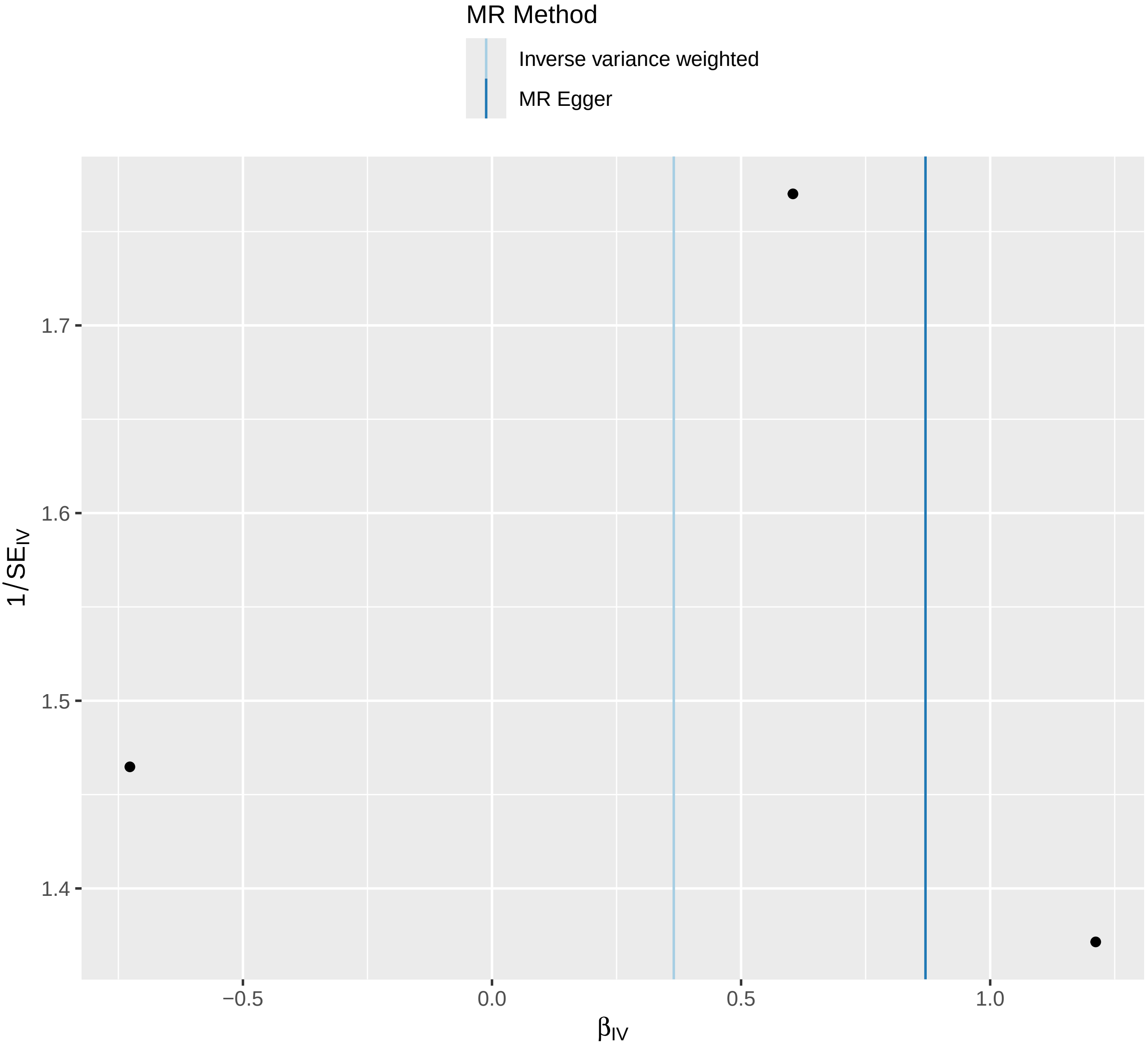
MR Method
Inverse variance weighted
MR Egger
1.7
1.6
1.5
1.4
1/SE$_{IV}$
−0.5
0.0
0.5
1.0
β$_{IV}$

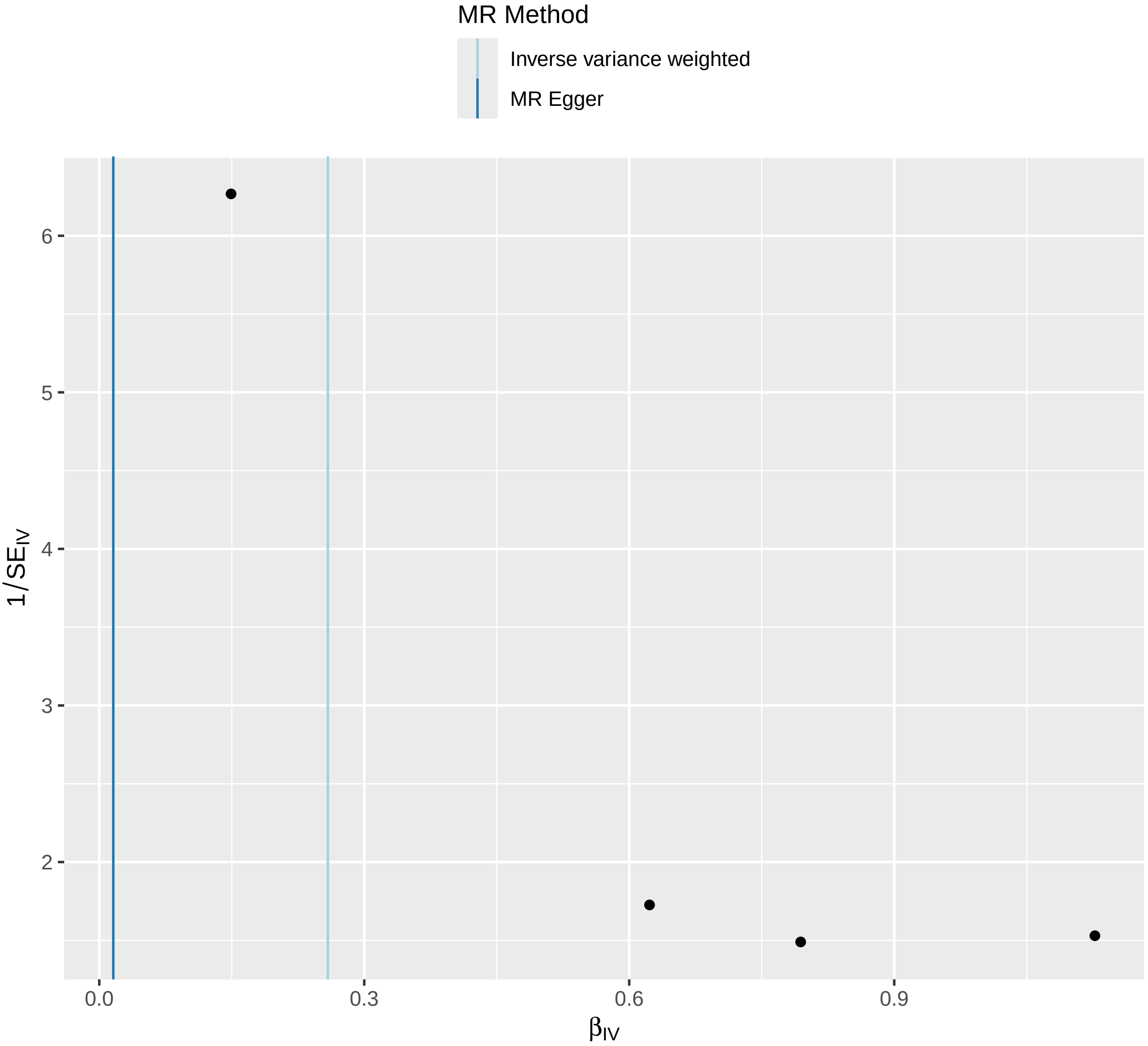
MR Method
Inverse variance weighted
MR Egger
6
5
4
3
2
$1/SE_{IV}$
0.0
0.3
0.6
0.9
β_{IV}

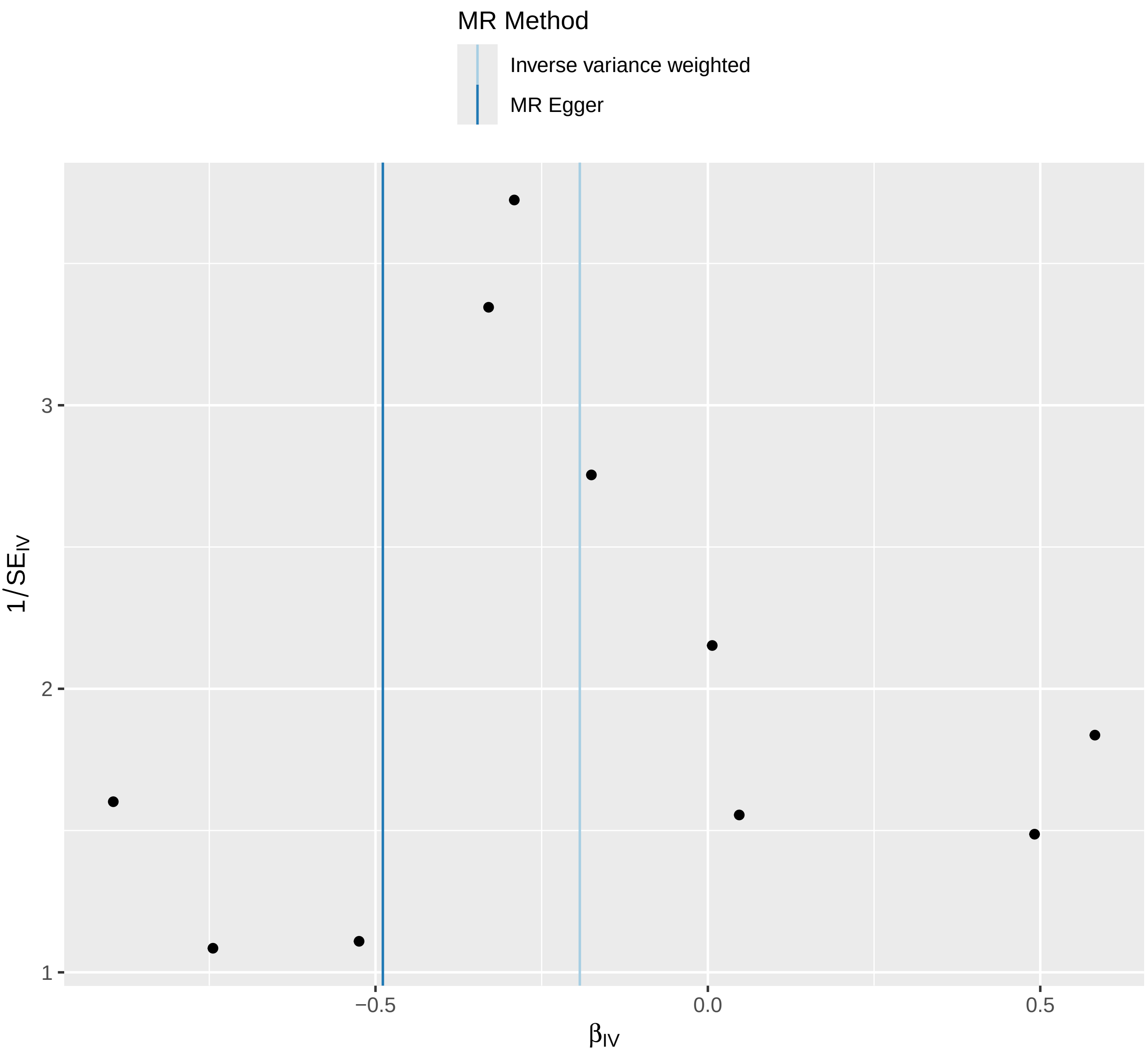
MR Method
Inverse variance weighted
MR Egger
1/SE_IV
3
2
1
−0.5
0.0
0.5
β_IV

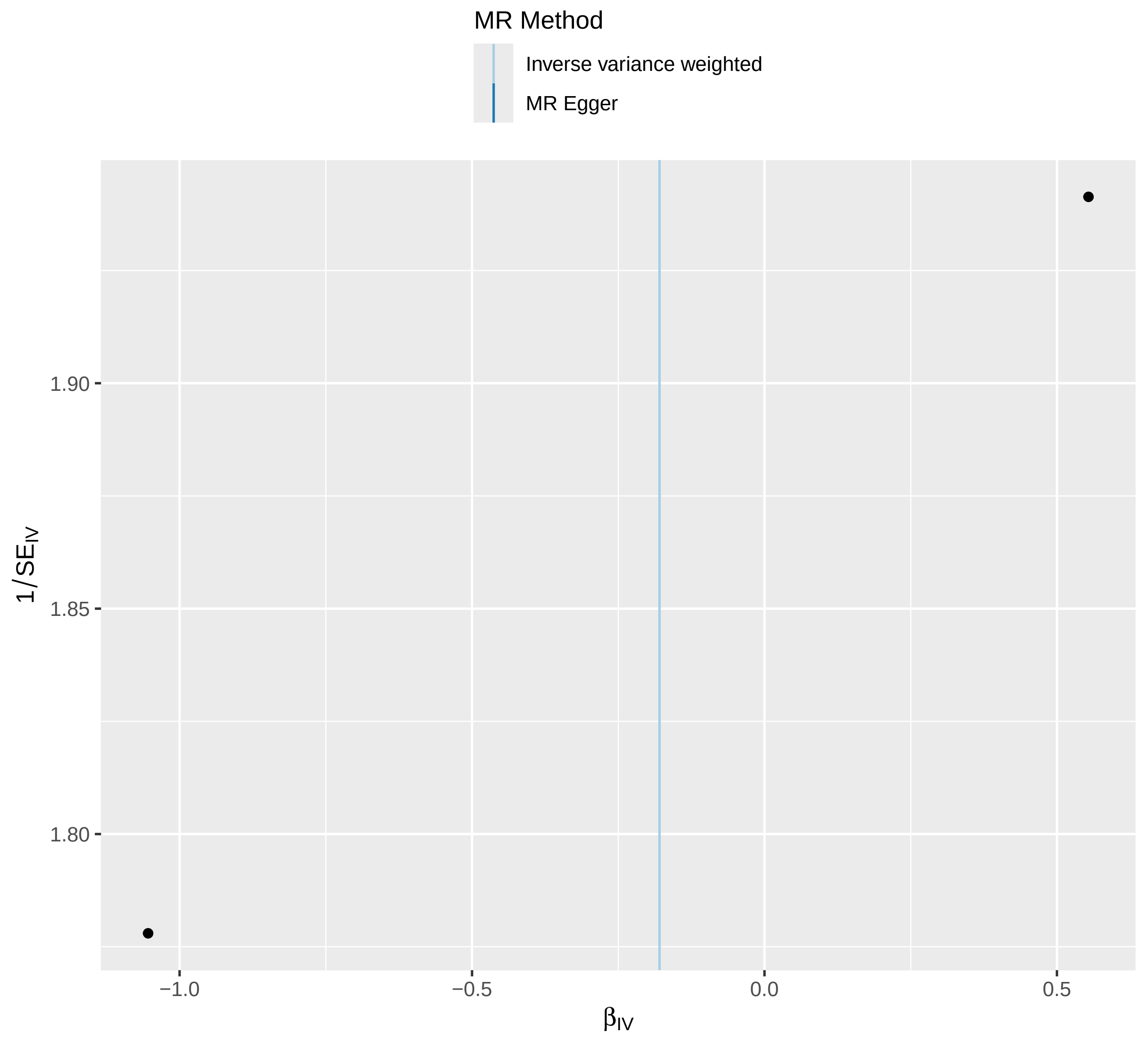

MR Method
Inverse variance weighted
MR Egger
1.90
1.85
1.80
1/SE_IV
−1.0
−0.5
0.0
0.5
β_IV

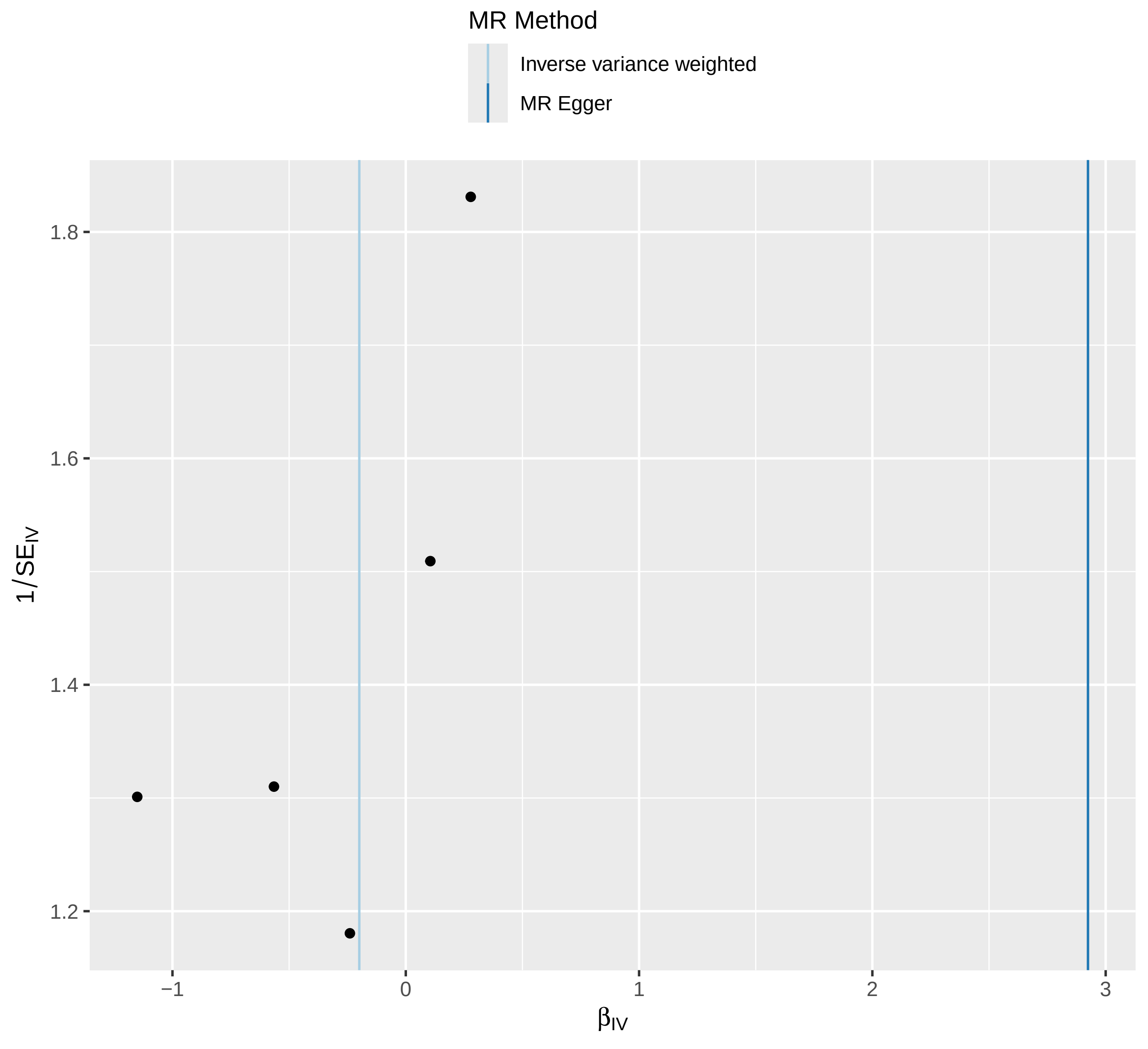

MR Method
Inverse variance weighted
MR Egger
1.8
1.6
1.4
1.2
$1/SE_{IV}$
−1
0
1
2
3
β_{IV}

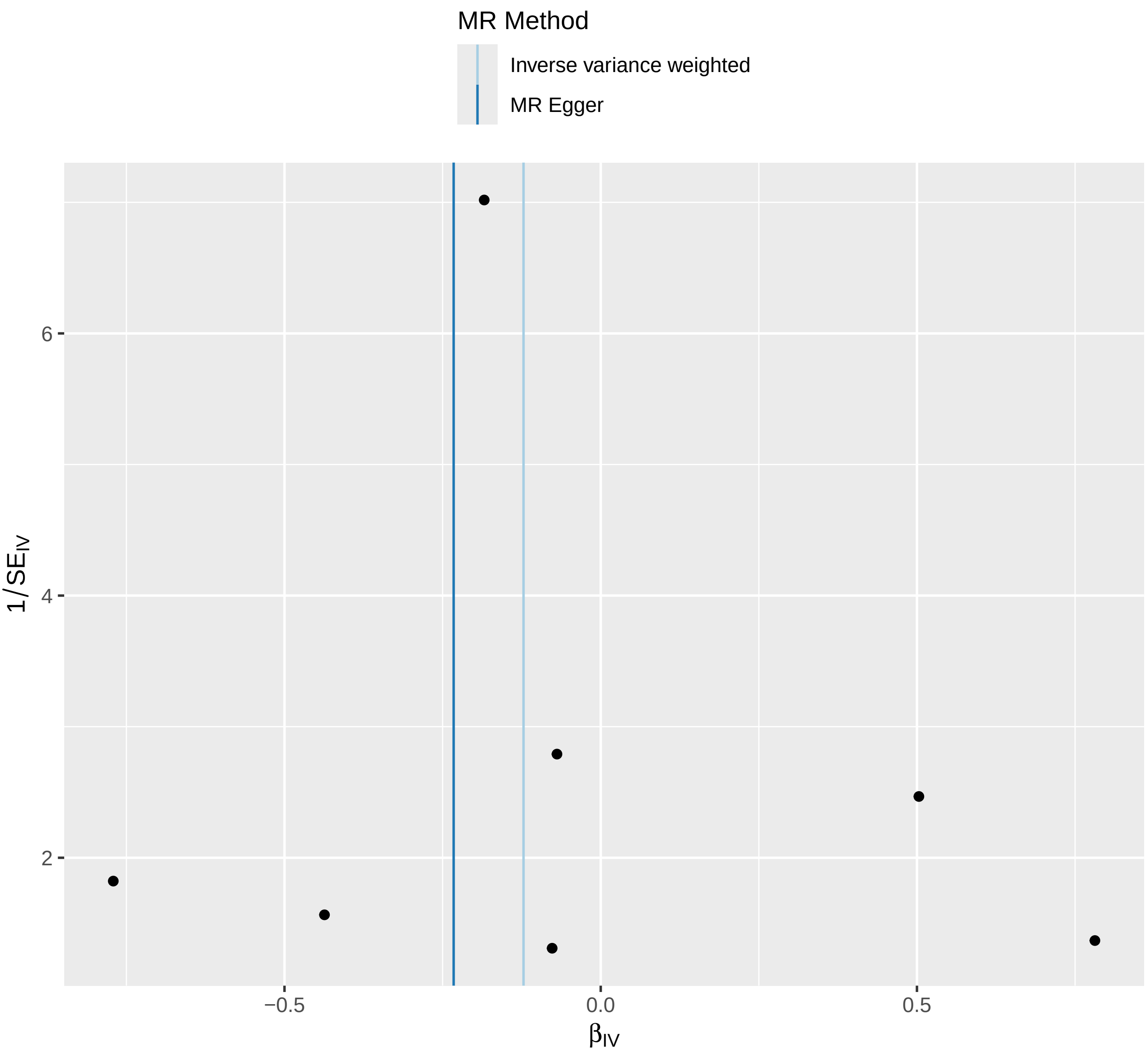
MR Method
Inverse variance weighted
MR Egger
6
4
2
1/SE$_{IV}$
−0.5
0.0
0.5
β$_{IV}$

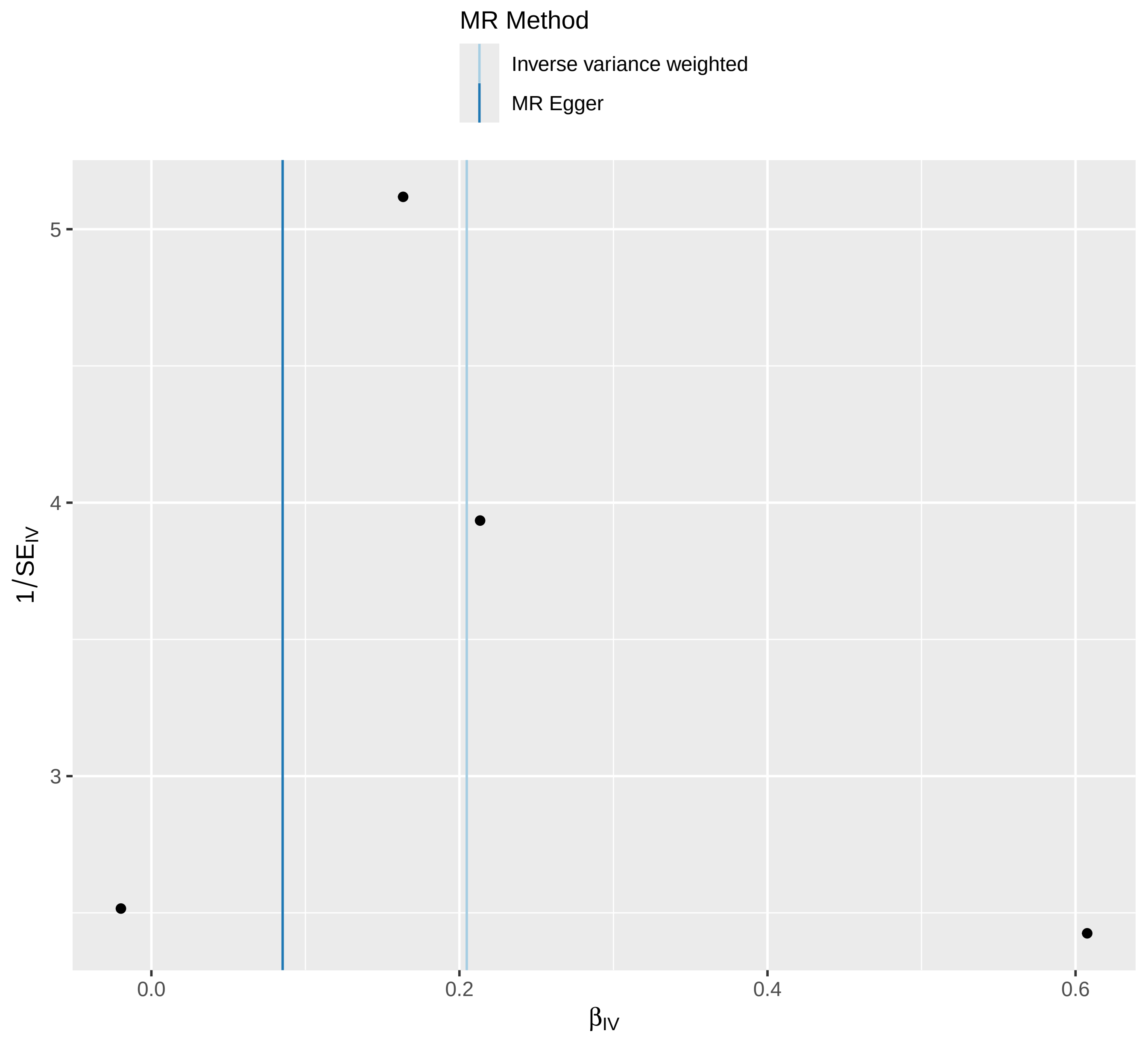

MR Method
Inverse variance weighted
MR Egger
5
4
3
1/SE_IV
0.0
0.2
0.4
0.6
β_IV

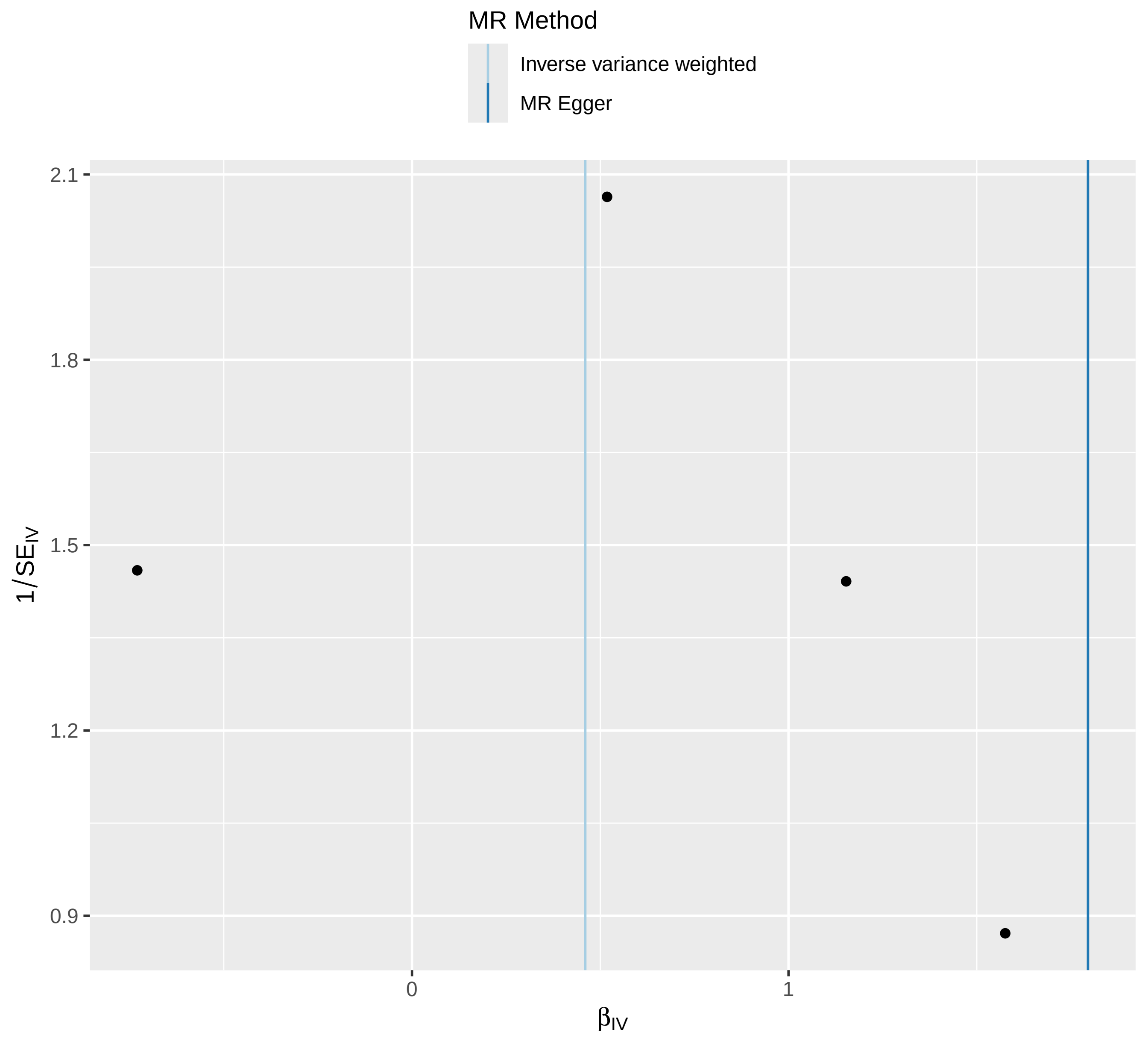
MR Method
Inverse variance weighted
MR Egger
2.1
1.8
1.5
1.2
0.9
$1/SE_{IV}$
0
1
β_{IV}

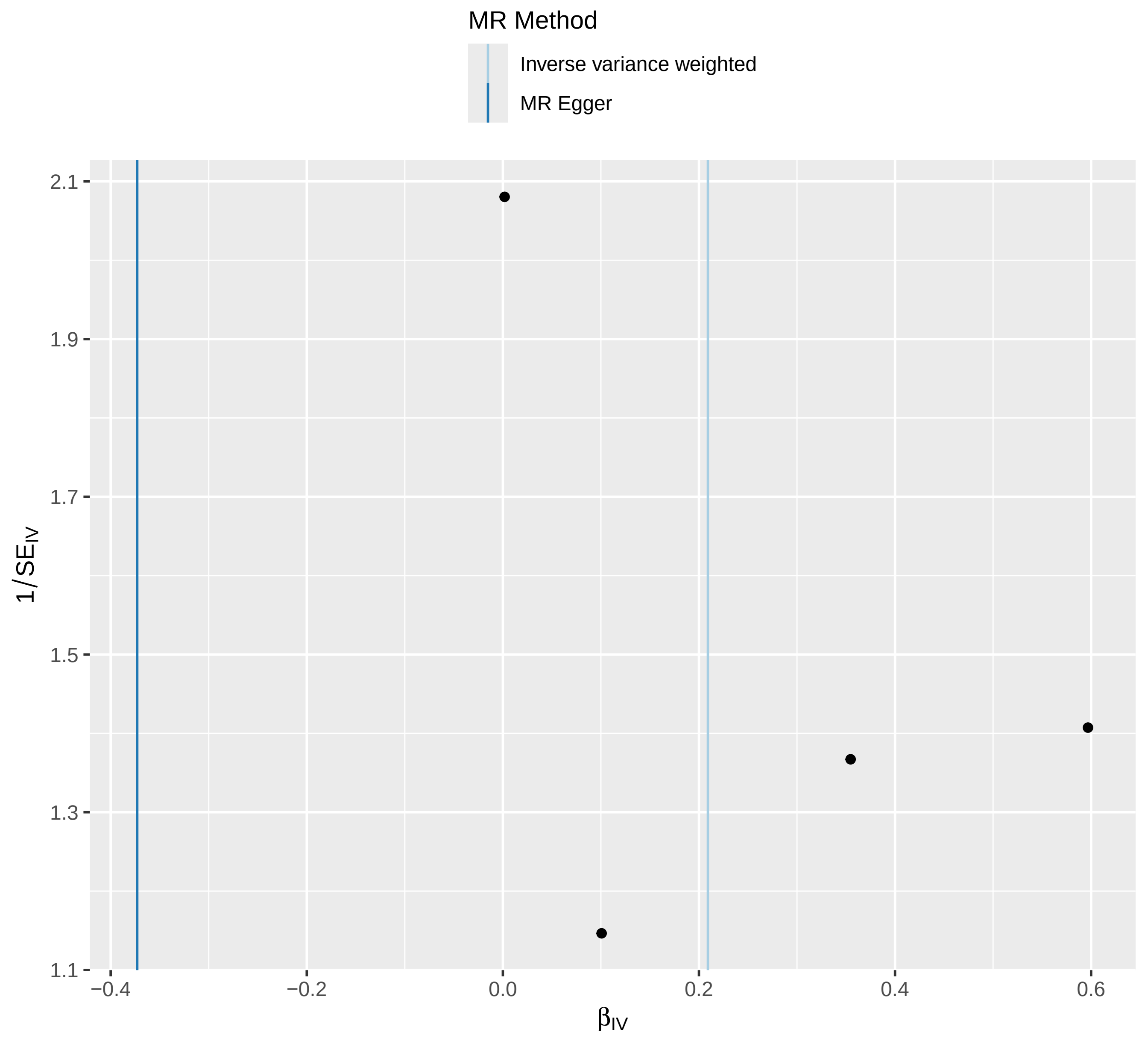

MR Method
Inverse variance weighted
MR Egger
2.1
1.9
1.7
1.5
1.3
1.1
1/SE_IV
−0.4
−0.2
0.0
0.2
0.4
0.6
β_IV

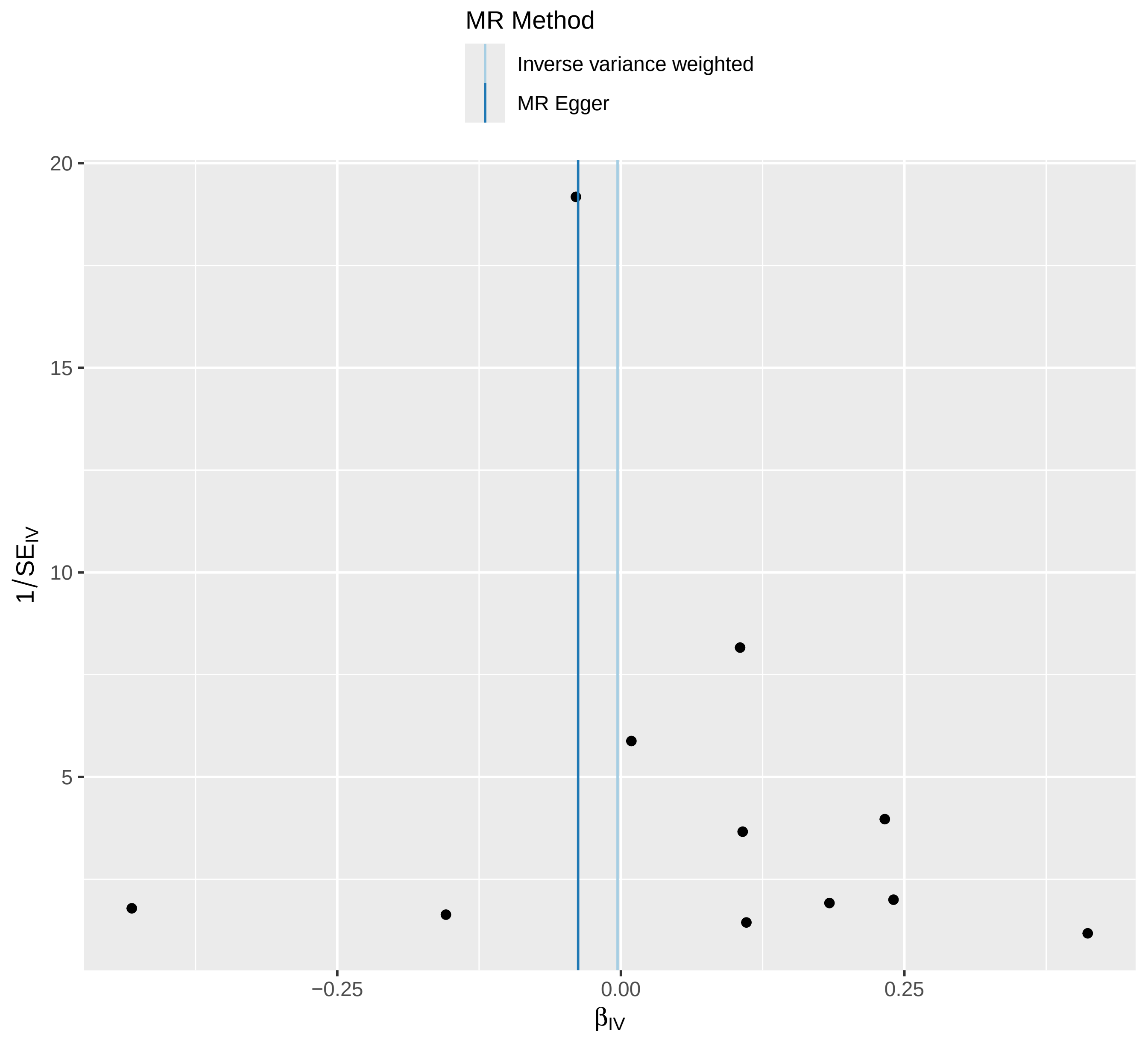
MR Method
Inverse variance weighted
MR Egger
20
15
10
5
1/SE_IV
−0.25
0.00
0.25
β_IV

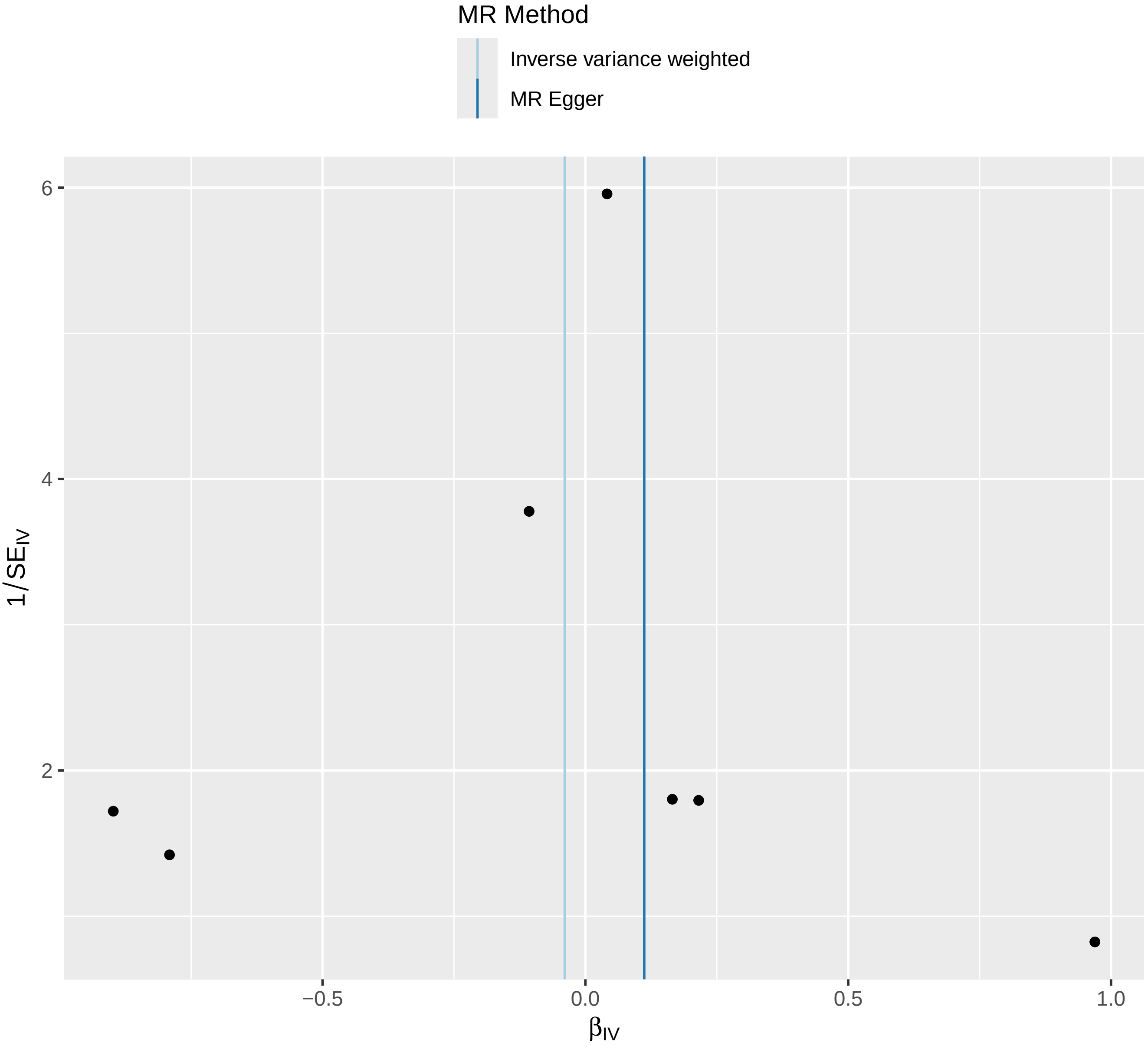
MR Method
Inverse variance weighted
MR Egger
6
4
2
1/SE$_{IV}$
−0.5
0.0
0.5
1.0
β$_{IV}$

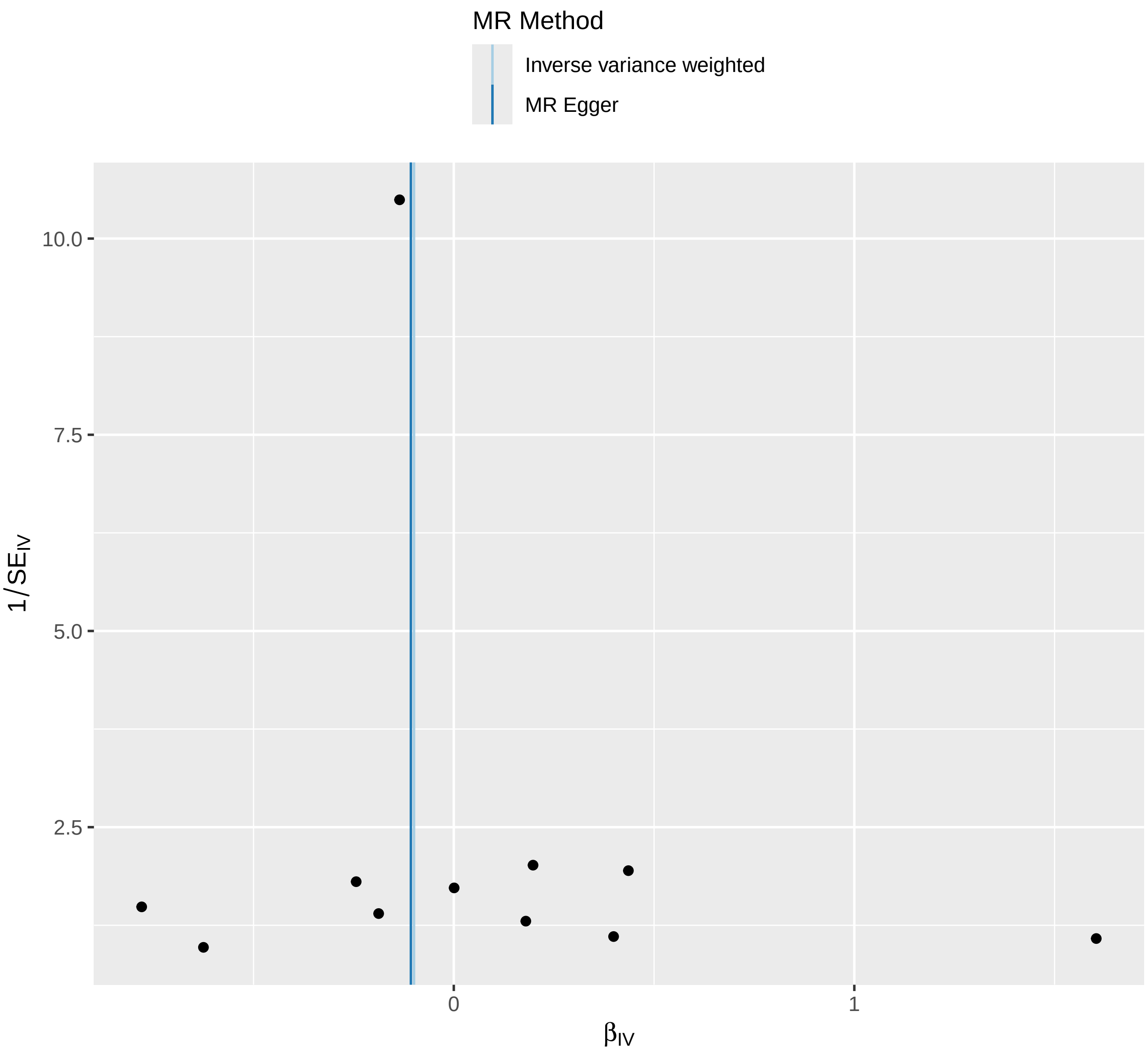

MR Method
Inverse variance weighted
MR Egger
10.0
7.5
5.0
2.5
1/SE_IV
0
1
β_IV

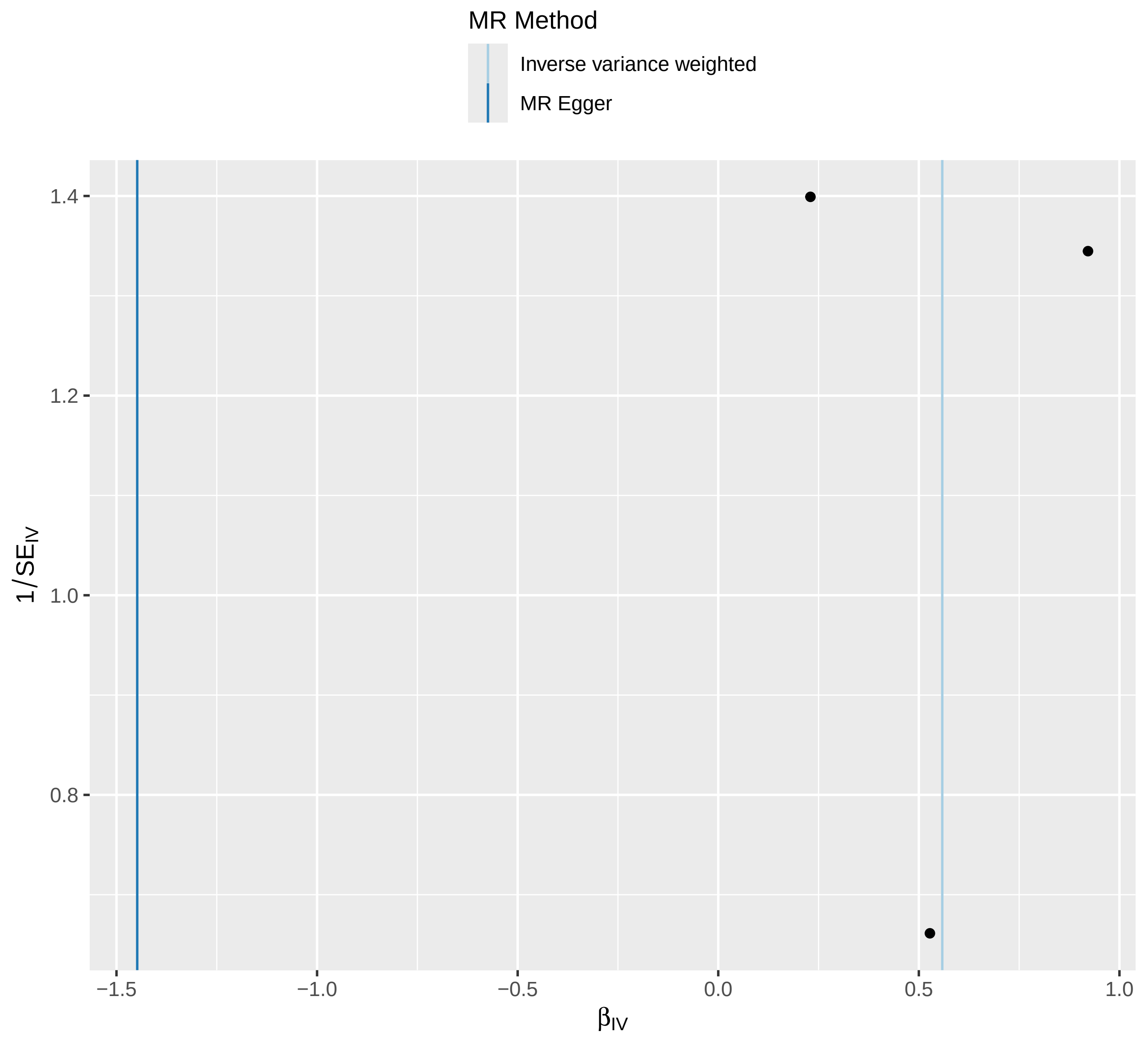
MR Method
Inverse variance weighted
MR Egger
1.4
1.2
1.0
0.8
1/SE_{IV}
−1.5
−1.0
−0.5
0.0
0.5
1.0
β_{IV}

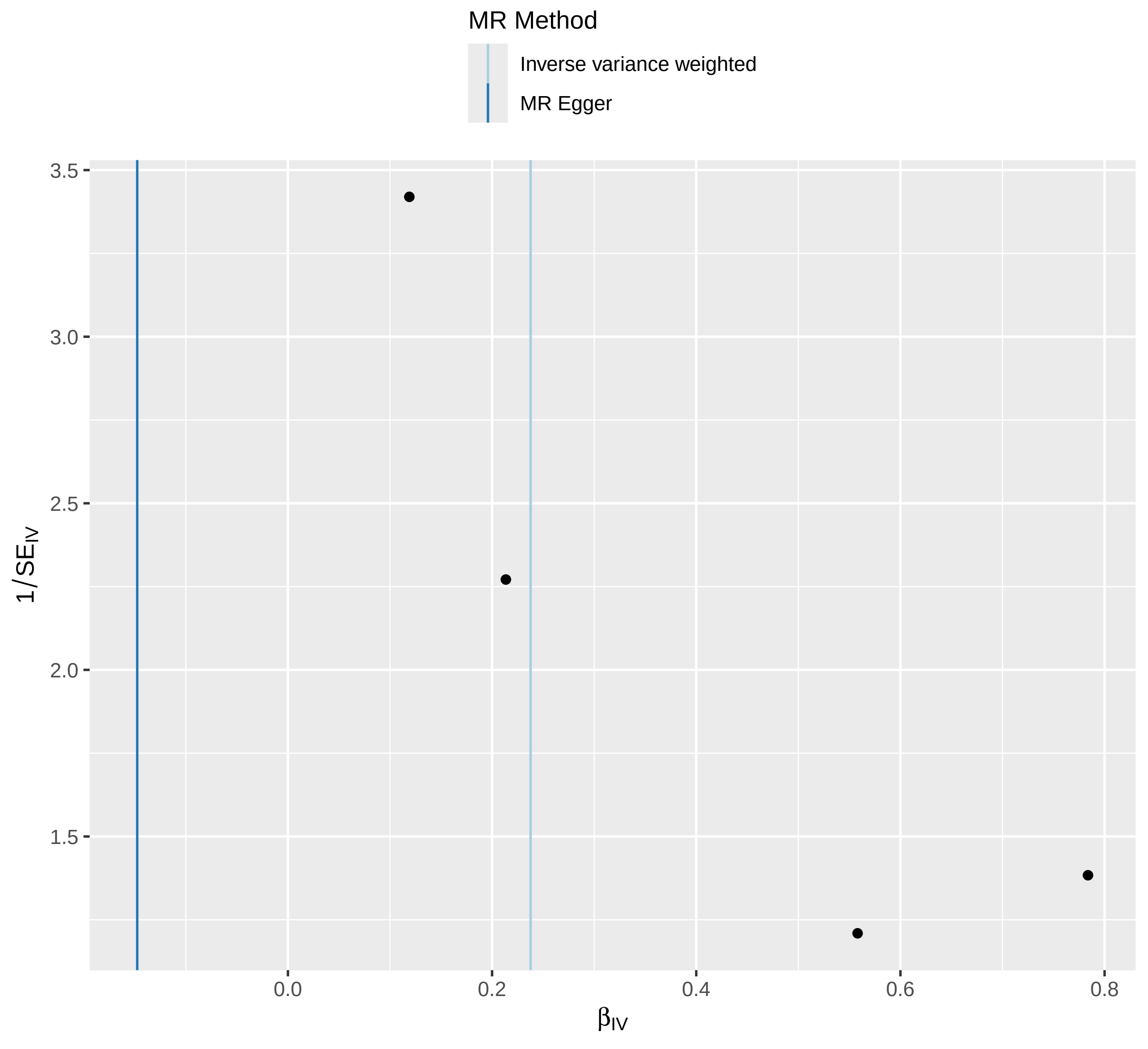

MR Method
Inverse variance weighted
MR Egger
3.5
3.0
2.5
2.0
1.5
1/SE_IV
0.0
0.2
0.4
0.6
0.8
β_IV

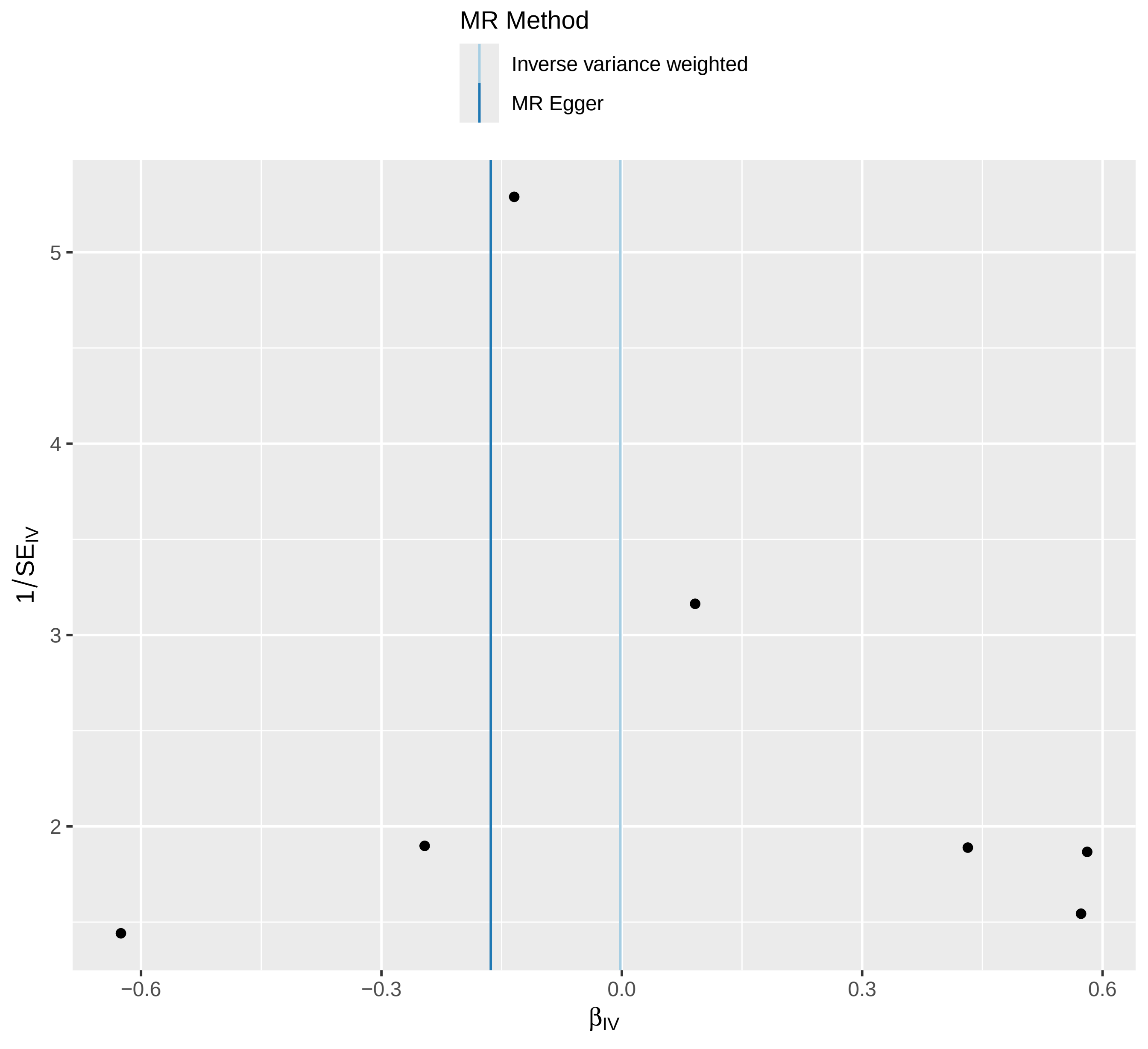

MR Method
Inverse variance weighted
MR Egger
5
4
3
2
1/SE_IV
−0.6
−0.3
0.0
0.3
0.6
β_IV

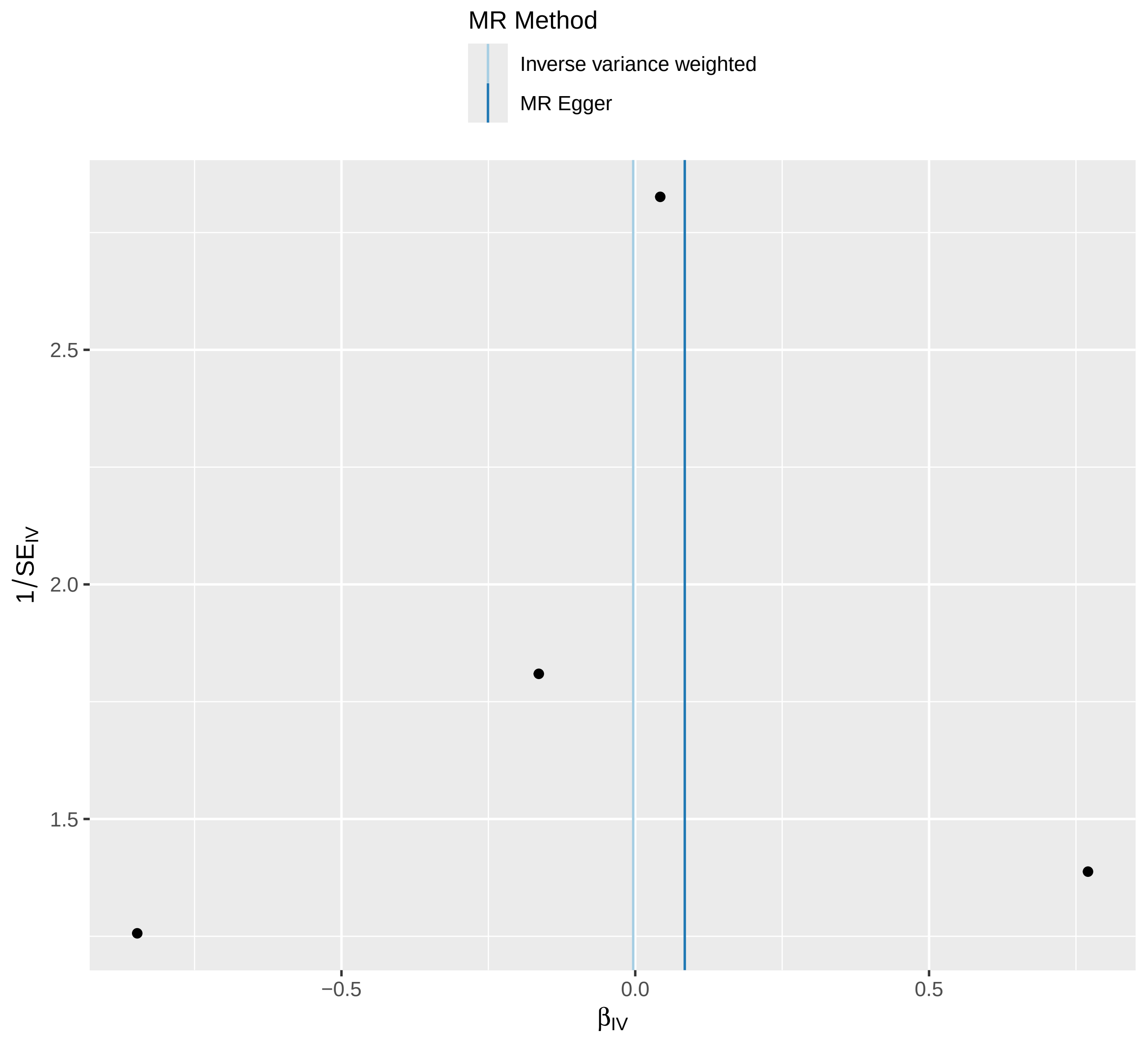
MR Method
Inverse variance weighted
MR Egger
2.5
2.0
1.5
1/SE$_{IV}$
−0.5
0.0
0.5
β$_{IV}$

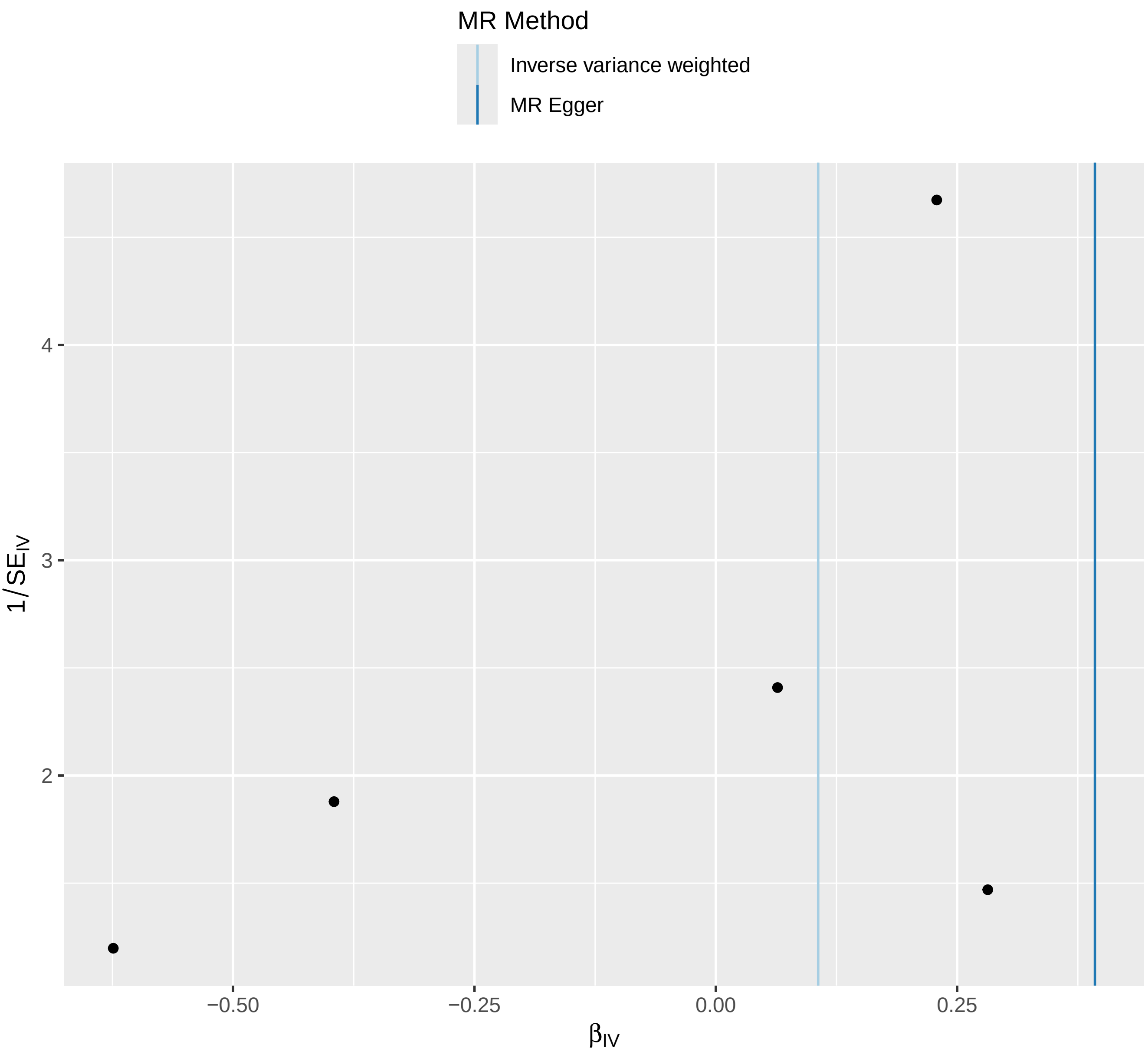
MR Method
Inverse variance weighted
MR Egger
1/SE_IV
4
3
2
−0.50
−0.25
0.00
0.25
β_IV

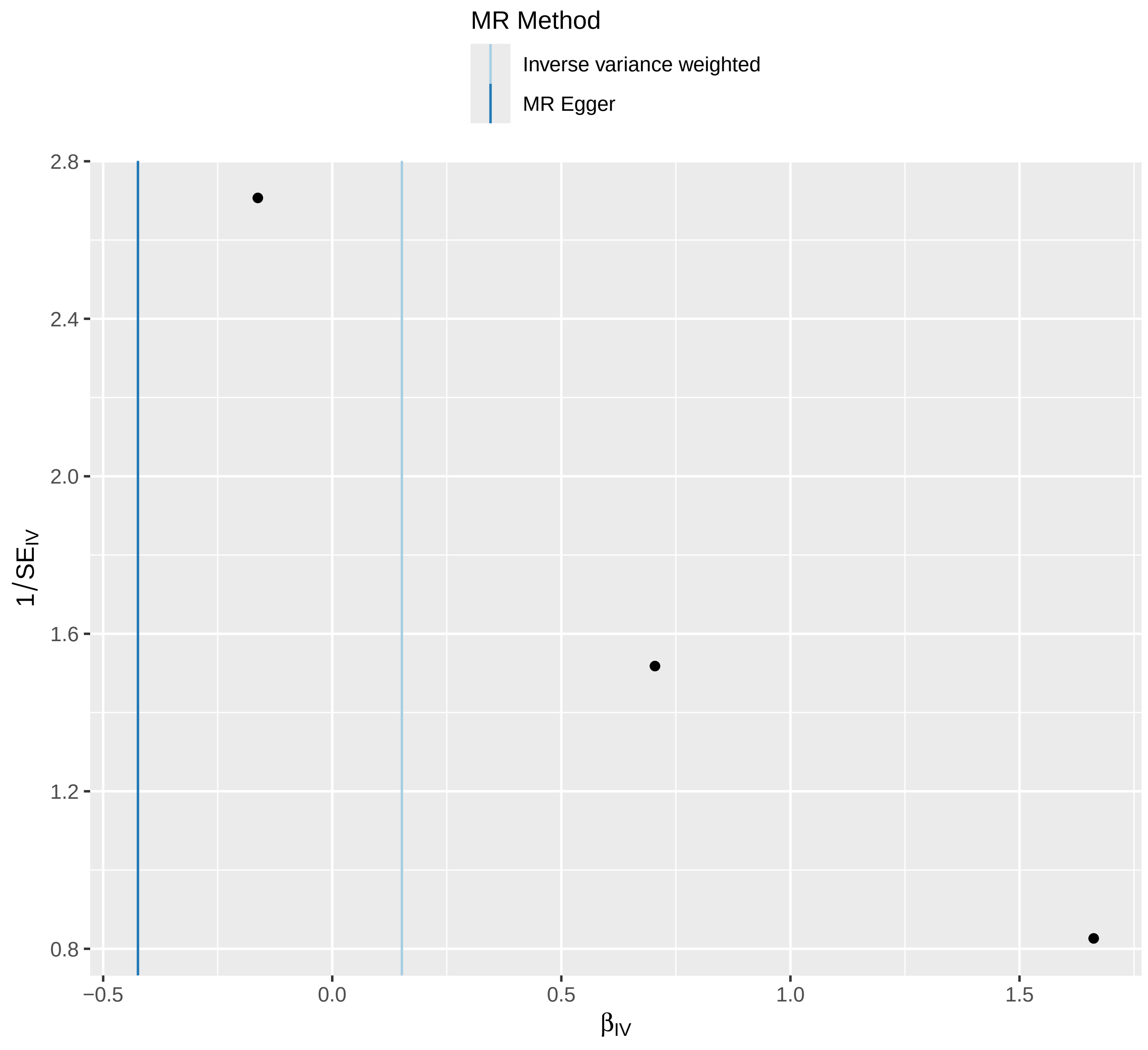

MR Method
Inverse variance weighted
MR Egger
2.8
2.4
2.0
1.6
1.2
0.8
1/SE_IV
−0.5
0.0
0.5
1.0
1.5
β_IV

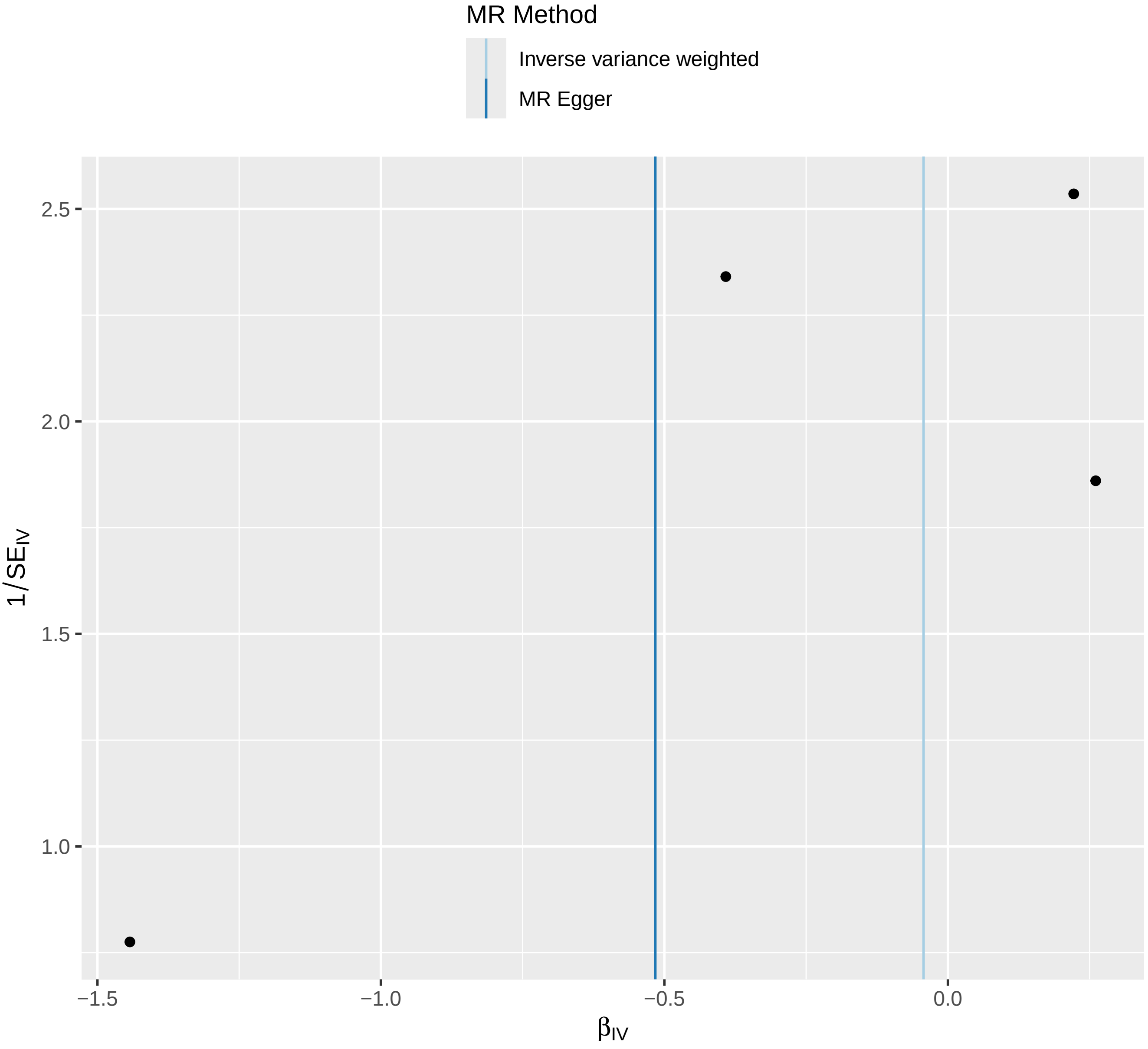
MR Method
Inverse variance weighted
MR Egger
2.5
2.0
1.5
1.0
1/SE_IV
−1.5
−1.0
−0.5
0.0
β_IV

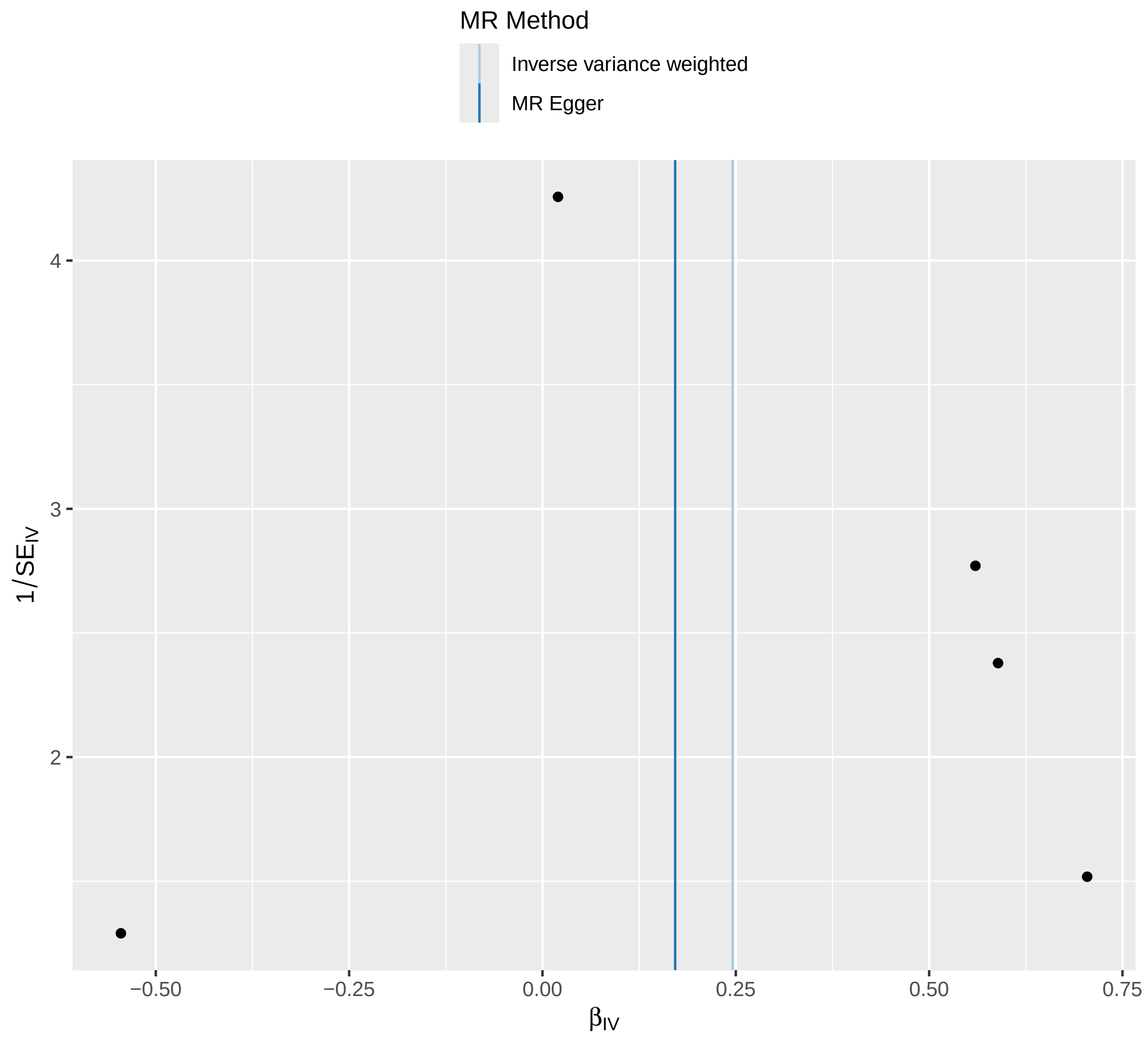
MR Method
Inverse variance weighted
MR Egger
1/SE_IV
4
3
2
−0.50
−0.25
0.00
0.25
0.50
0.75
β_IV

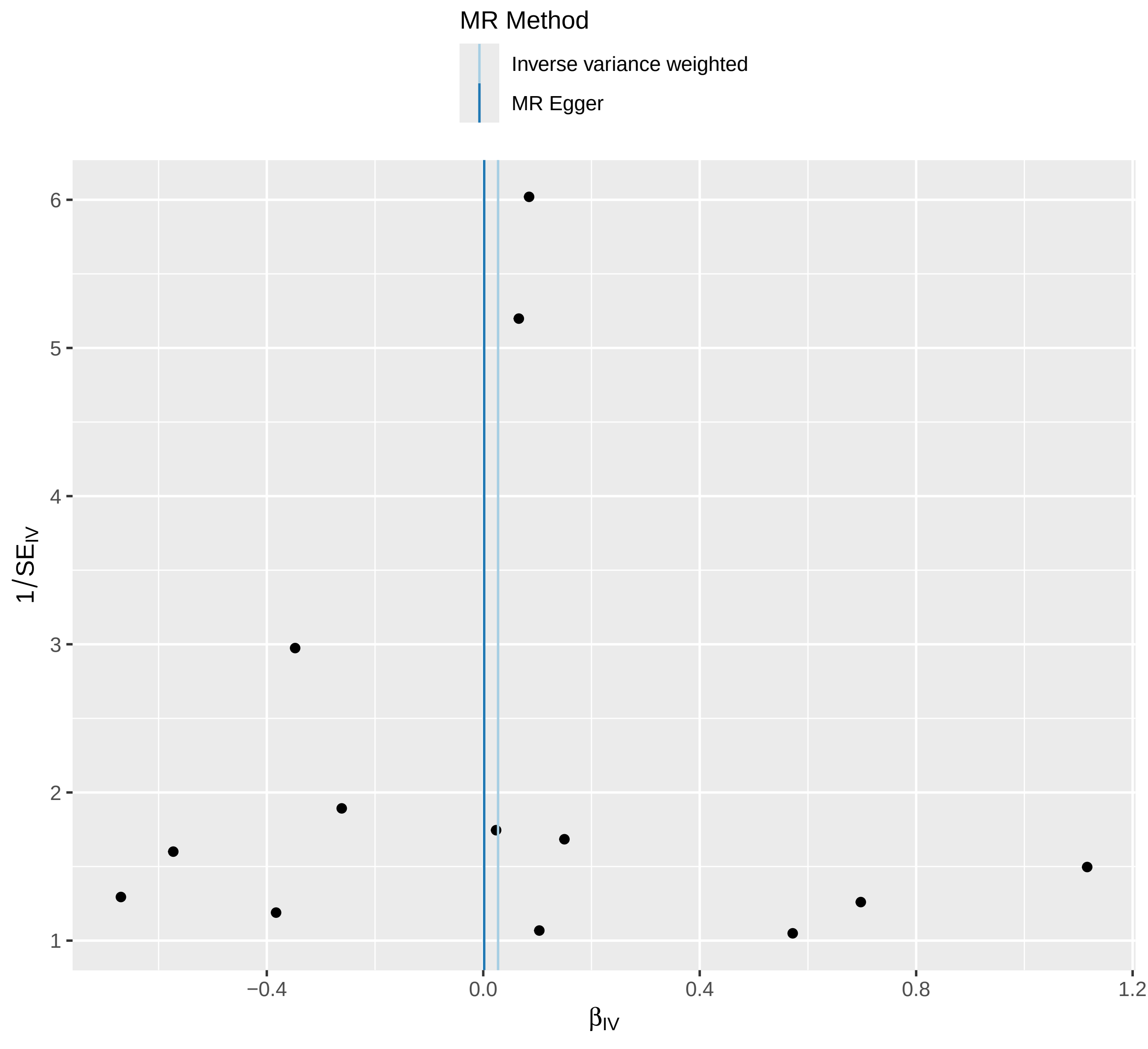

MR Method
Inverse variance weighted
MR Egger
6
5
4
3
2
1
$1/SE_{IV}$
−0.4
0.0
0.4
0.8
1.2
β_{IV}

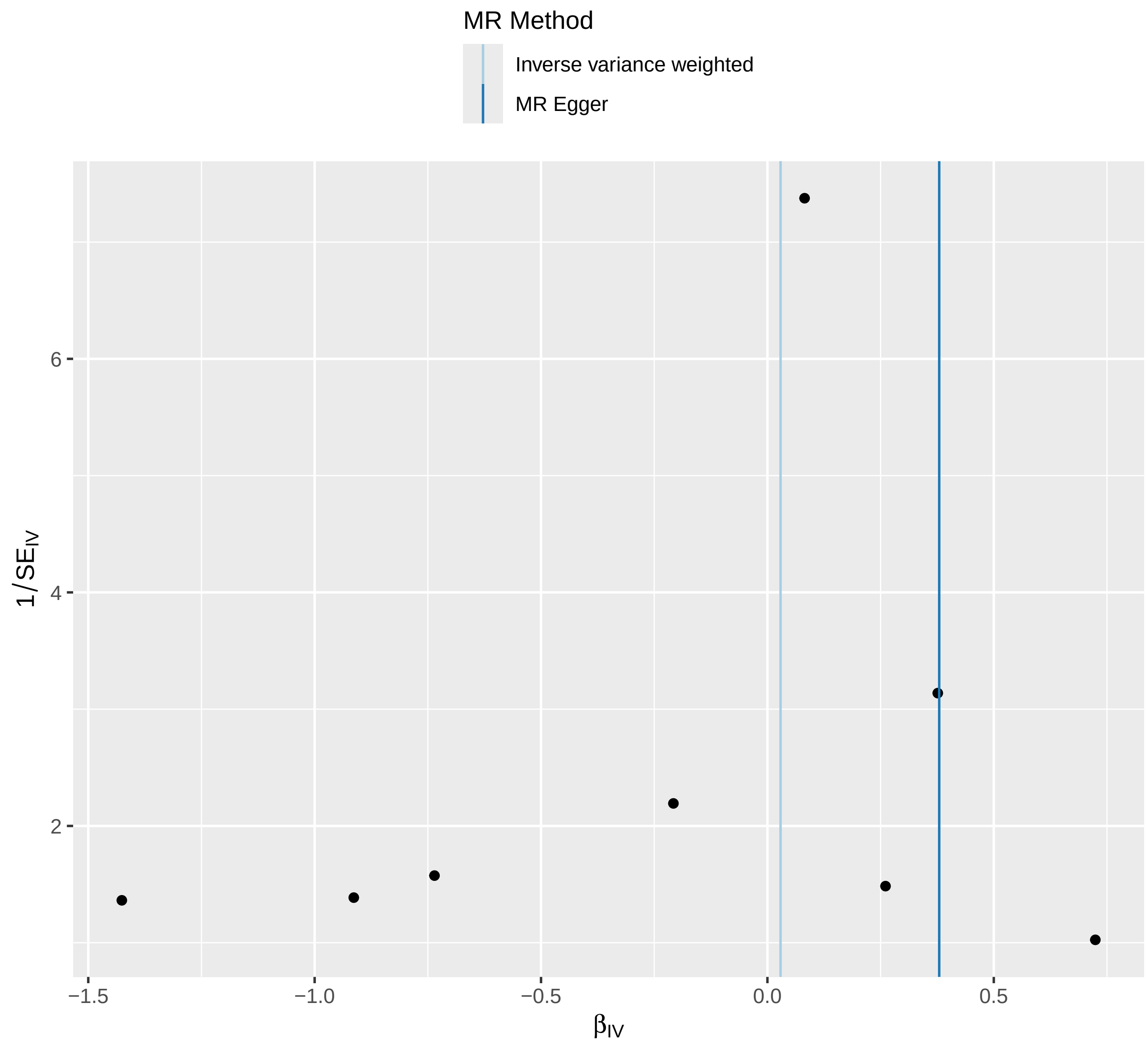

MR Method
Inverse variance weighted
MR Egger
1/SE_IV
6
4
2
−1.5
−1.0
−0.5
0.0
0.5
β_IV

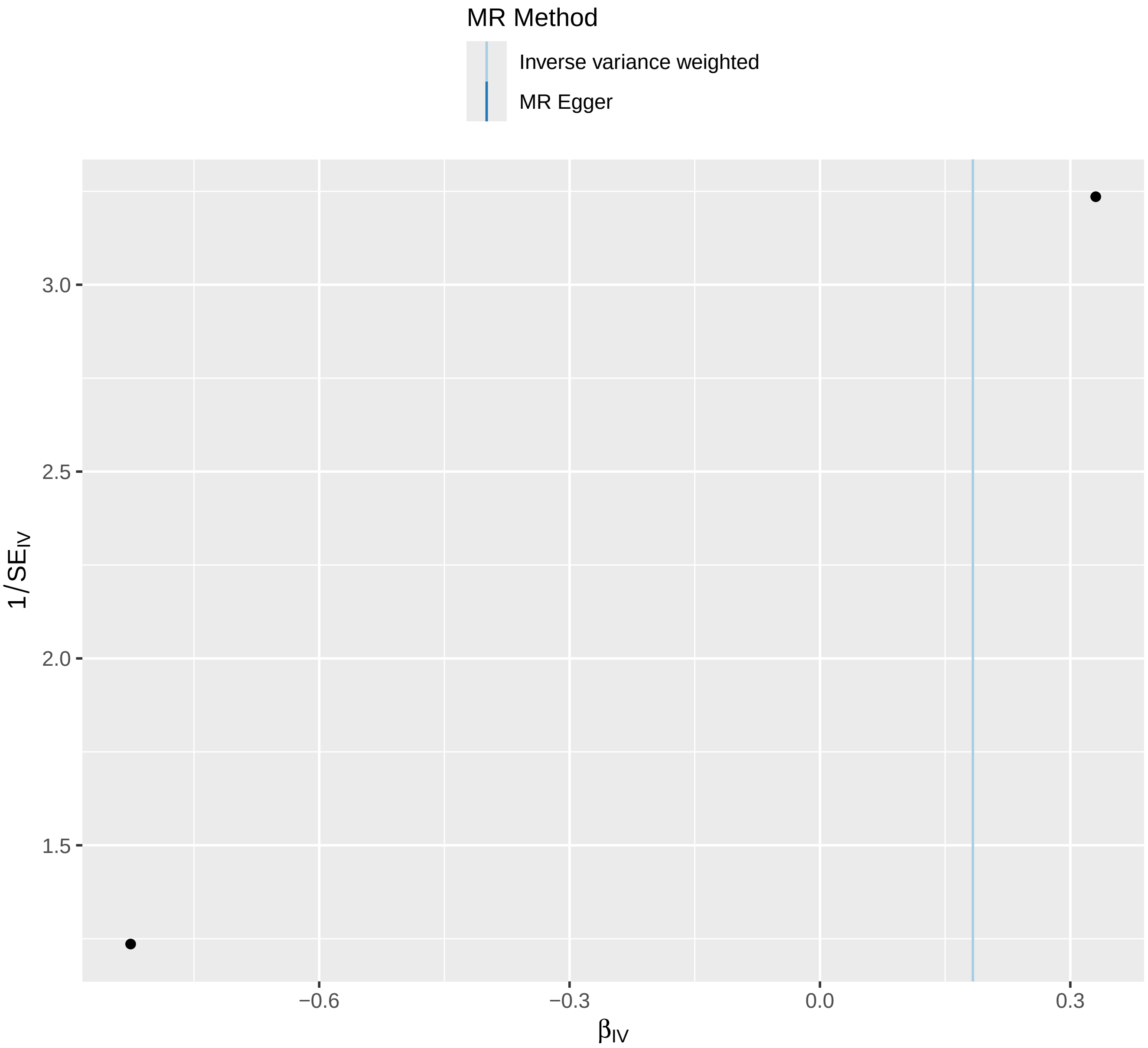
MR Method
Inverse variance weighted
MR Egger
3.0
2.5
2.0
1.5
1/SE_IV
−0.6
−0.3
0.0
0.3
β_IV

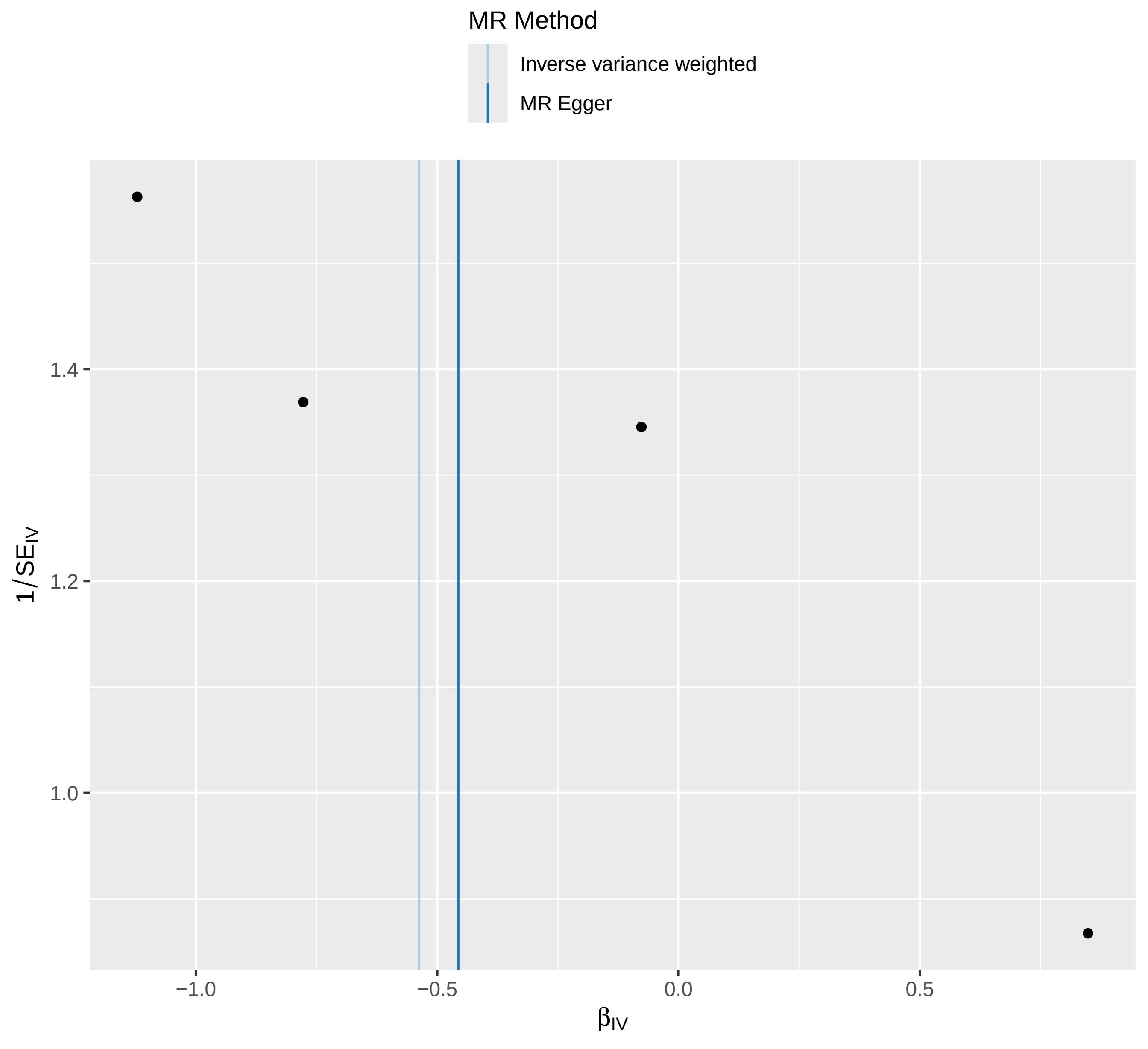

MR Method
Inverse variance weighted
MR Egger
1.4
1.2
1.0
1/SE_IV
−1.0
−0.5
0.0
0.5
β_IV

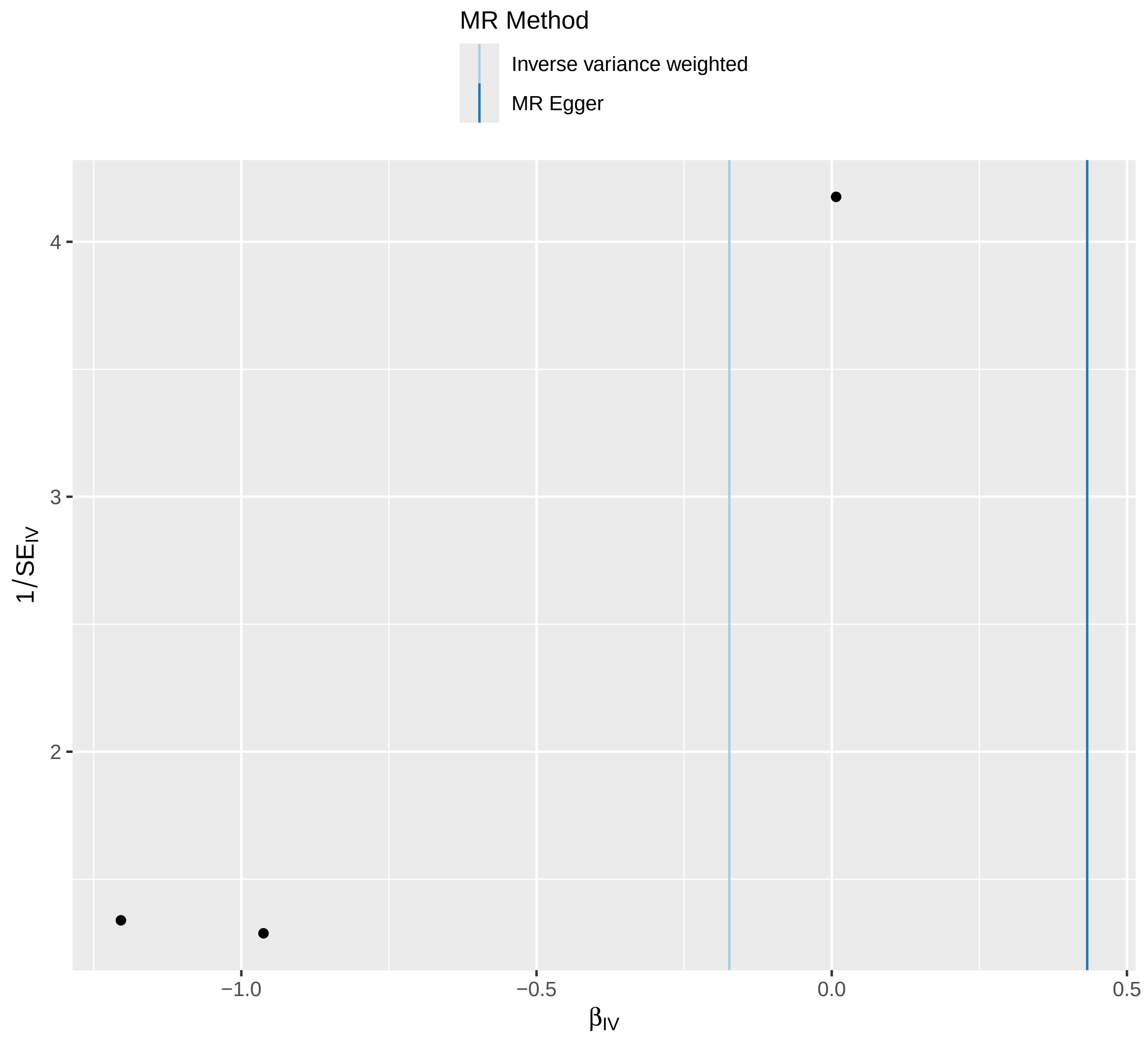

MR Method
Inverse variance weighted
MR Egger
4
3
2
1/SE$_{IV}$
−1.0
−0.5
0.0
0.5
β$_{IV}$

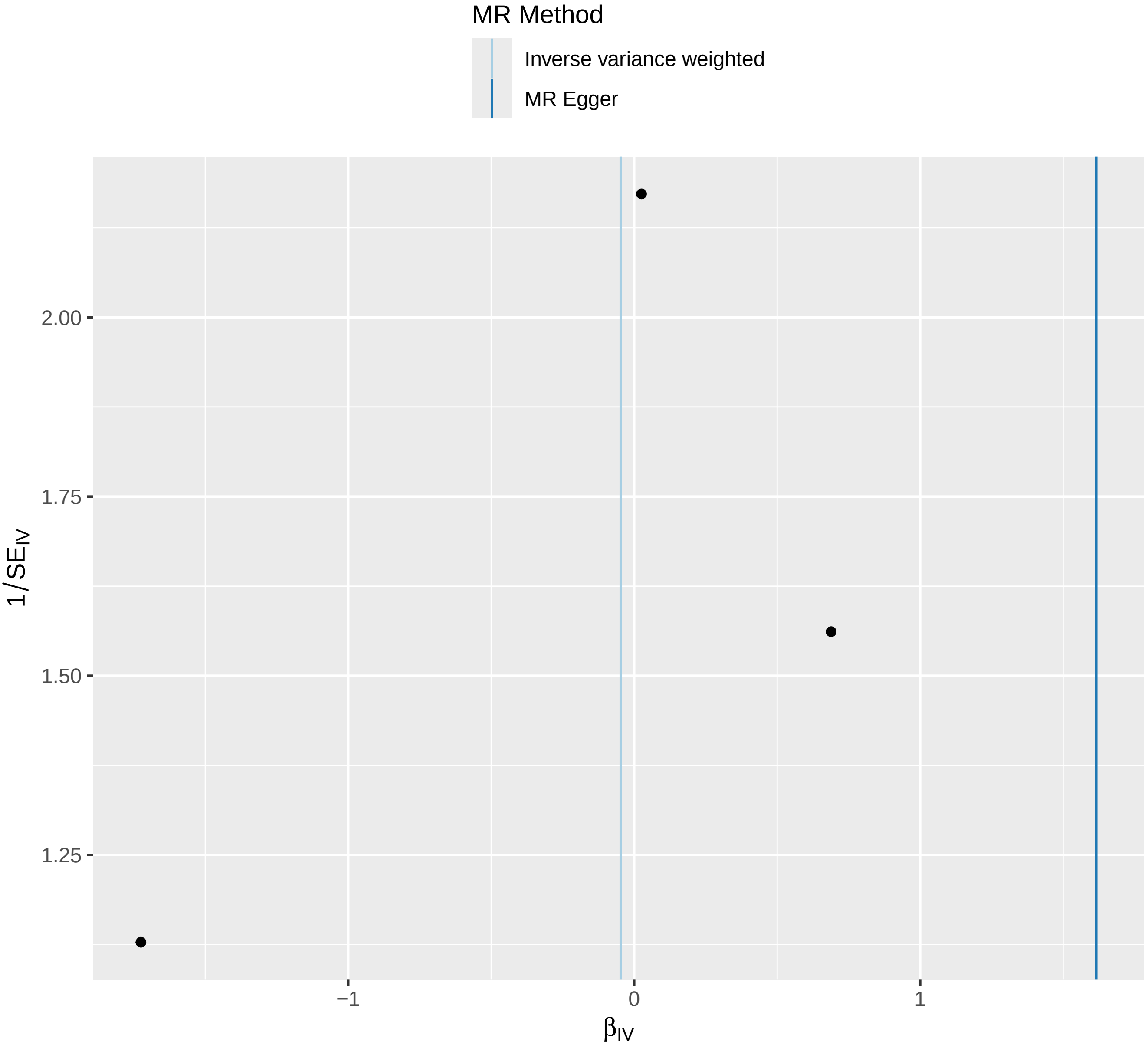
MR Method
Inverse variance weighted
MR Egger
1/SE_IV
2.00
1.75
1.50
1.25
−1
0
1
β_IV

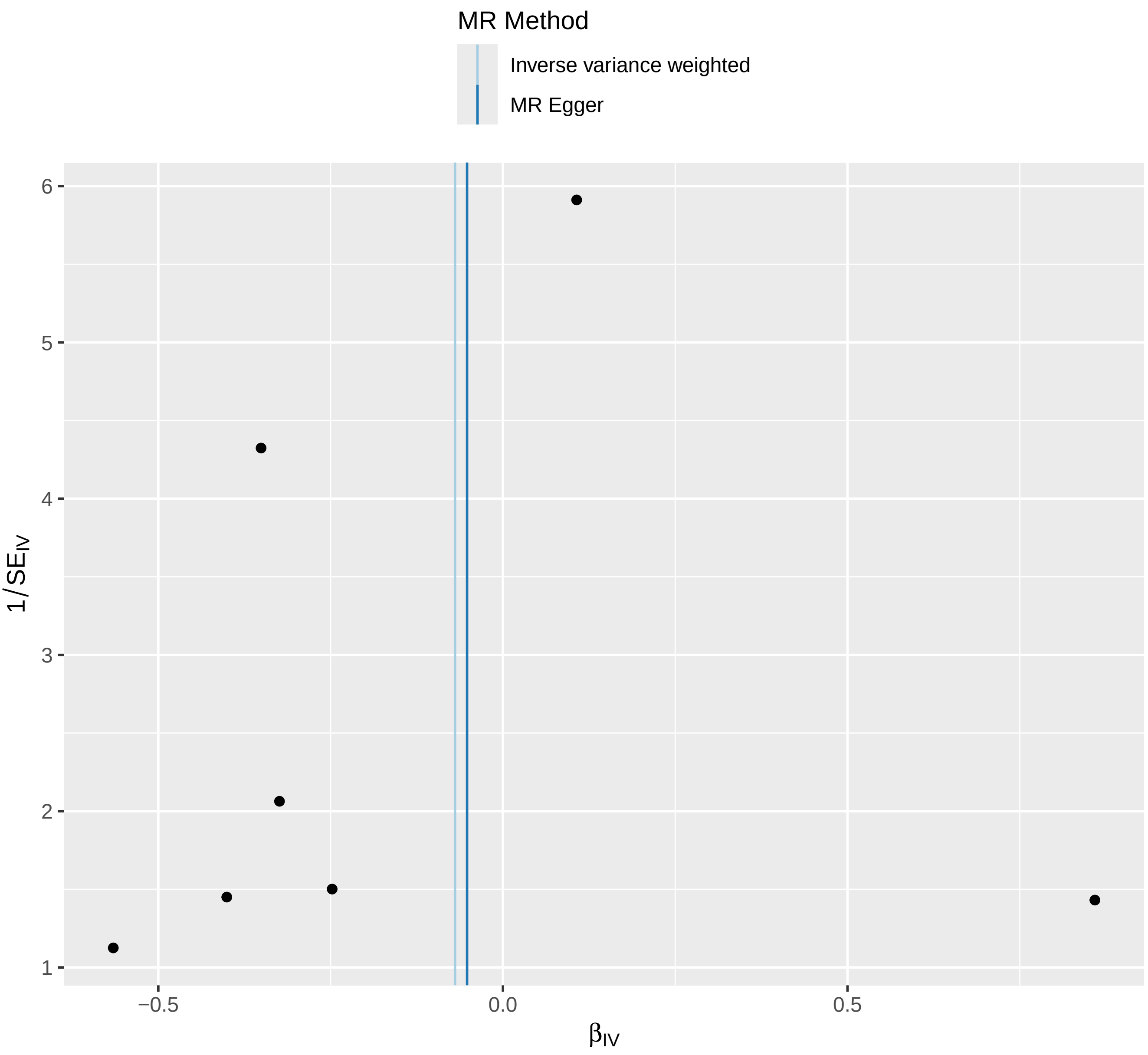

MR Method
Inverse variance weighted
MR Egger
6
5
4
3
2
1
1/SE$_{IV}$
−0.5
0.0
0.5
β$_{IV}$

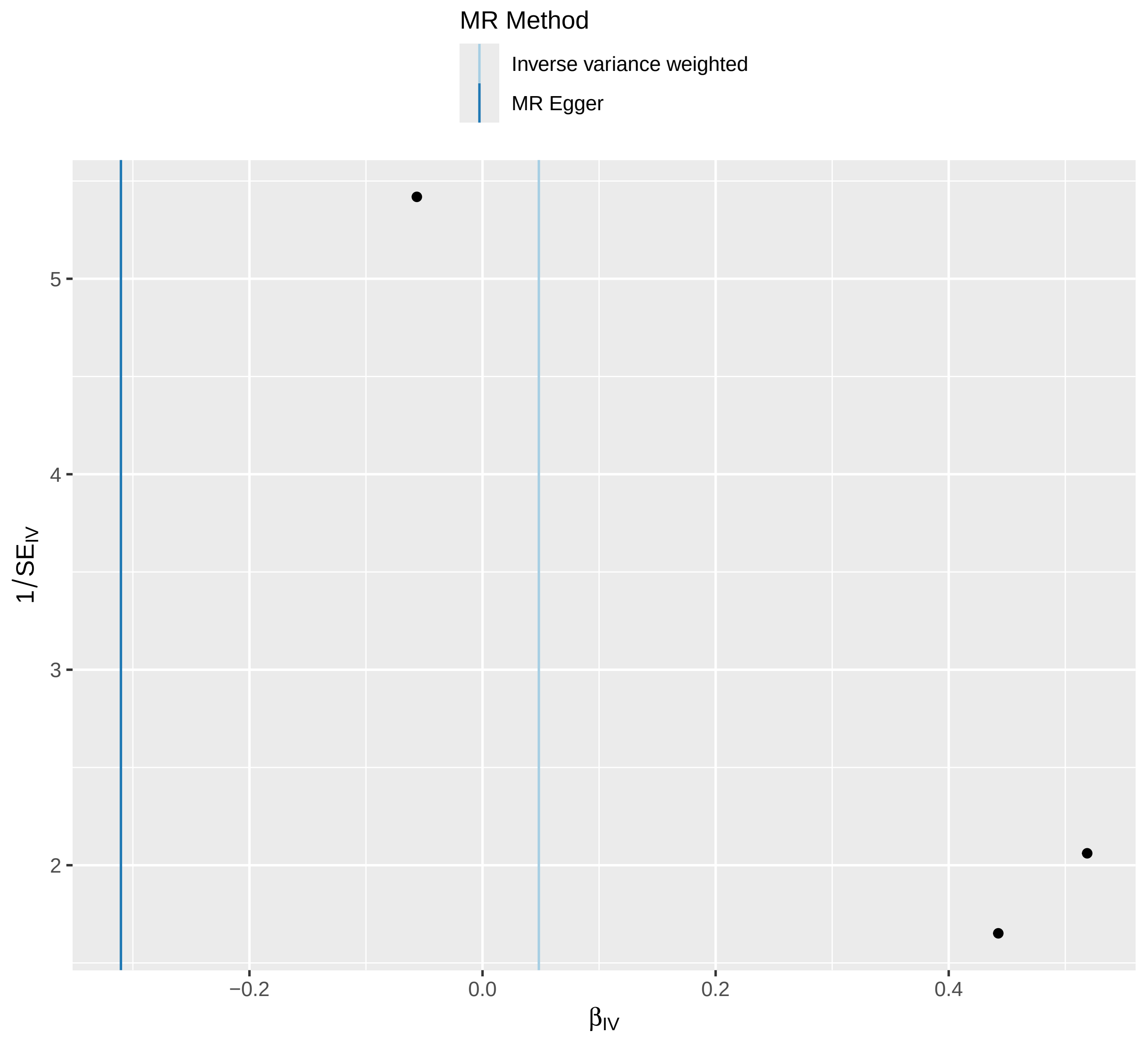
MR Method
Inverse variance weighted
MR Egger
5
4
3
2
1/SE_IV
−0.2
0.0
0.2
0.4
β_IV

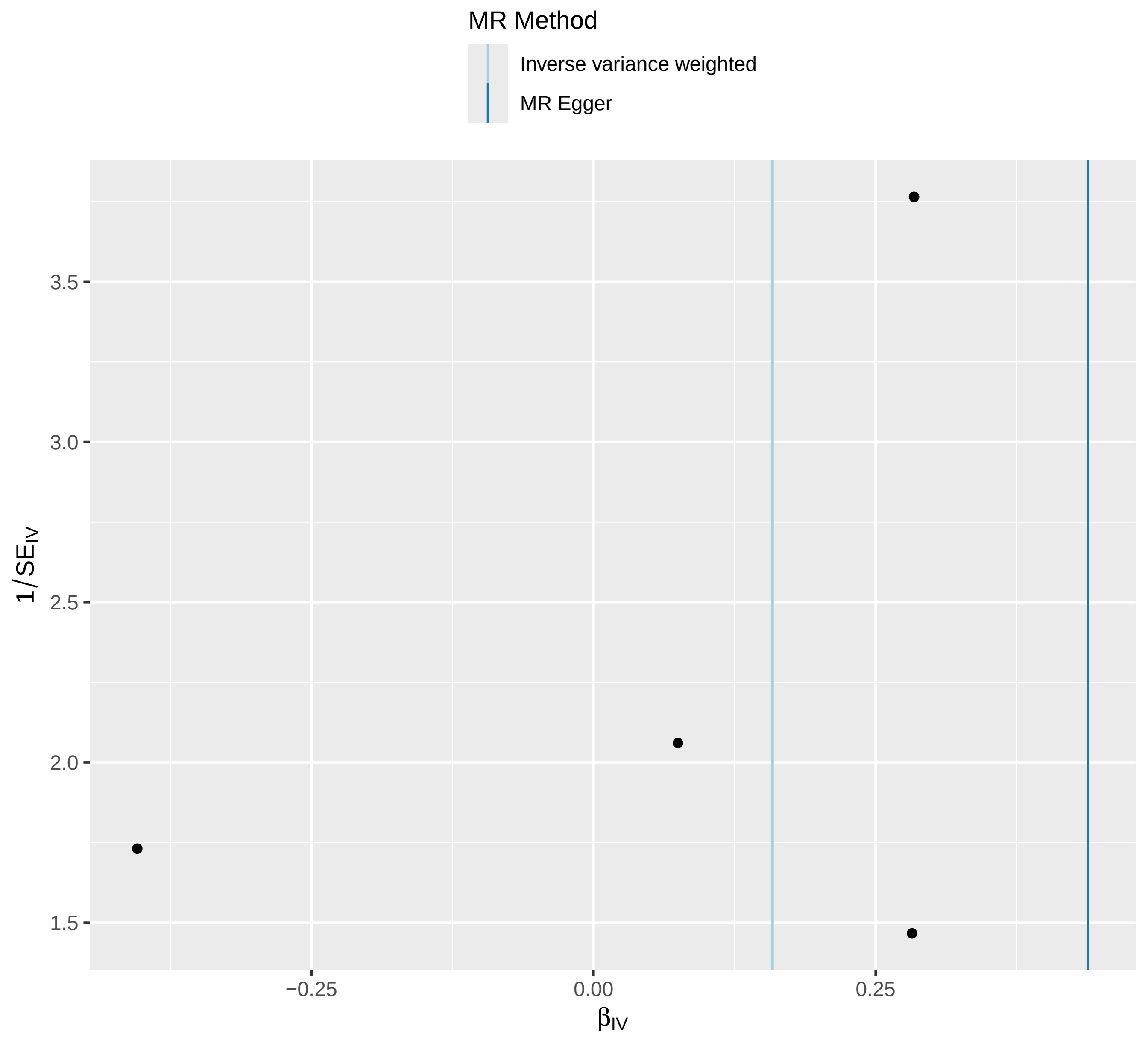
MR Method
Inverse variance weighted
MR Egger
3.5
3.0
2.5
2.0
1.5
1/SE_IV
−0.25
0.00
0.25
β_IV

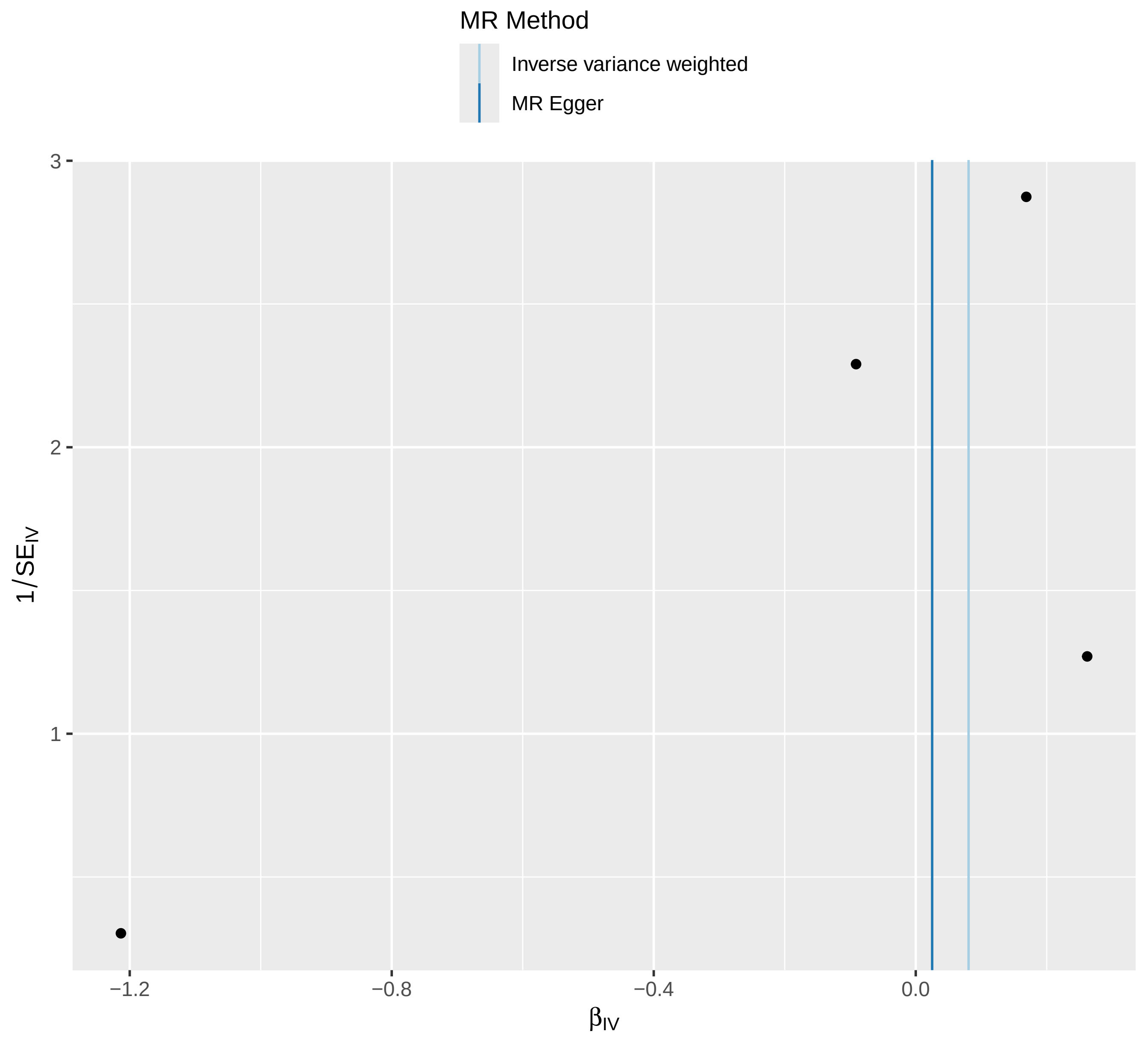

MR Method
Inverse variance weighted
MR Egger
3
2
1
1/SE$_{IV}$
−1.2
−0.8
−0.4
0.0
β_{IV}

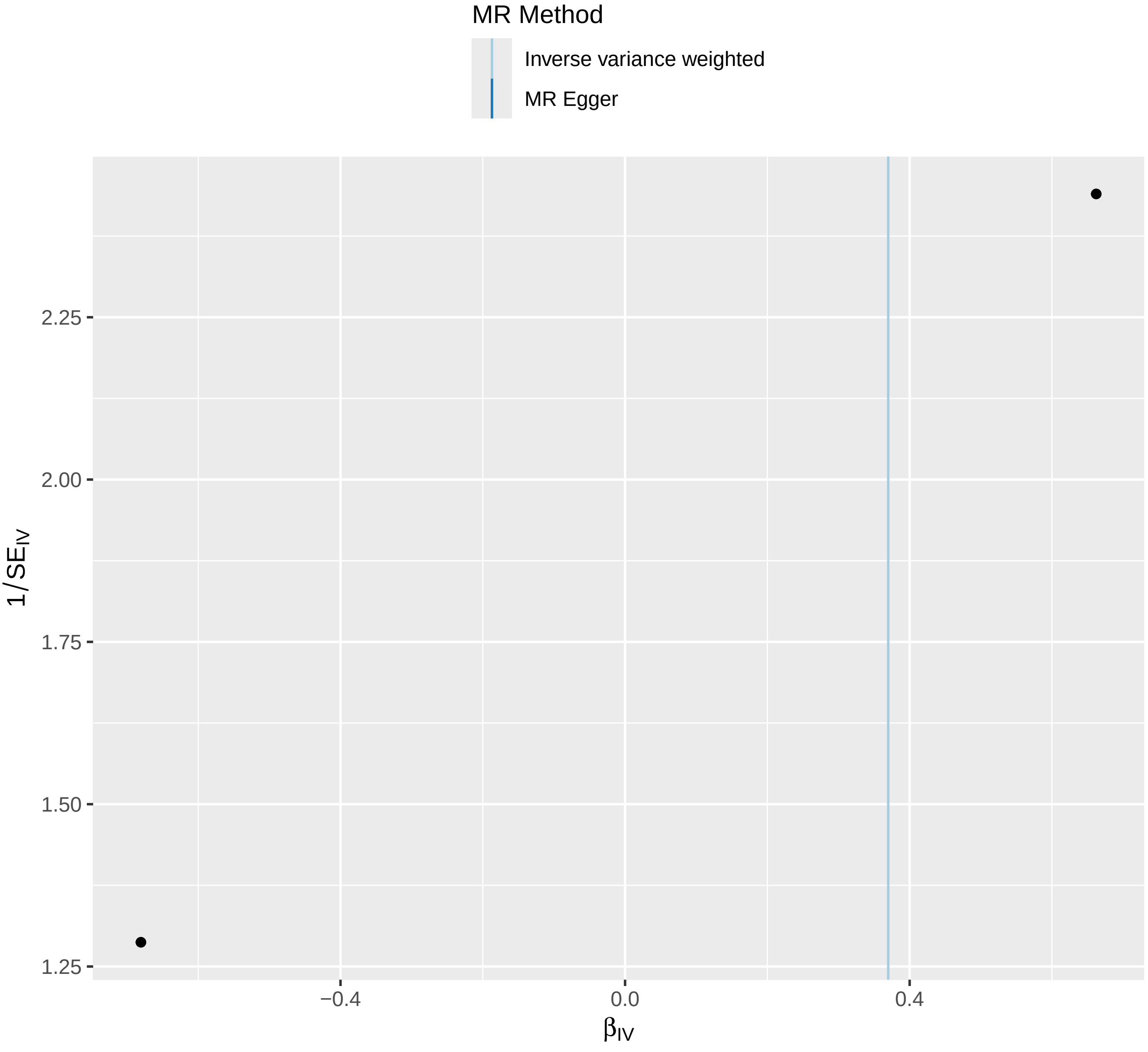

MR Method
Inverse variance weighted
MR Egger
2.25
2.00
1.75
1.50
1.25
1/SE_IV
−0.4
0.0
0.4
β_IV

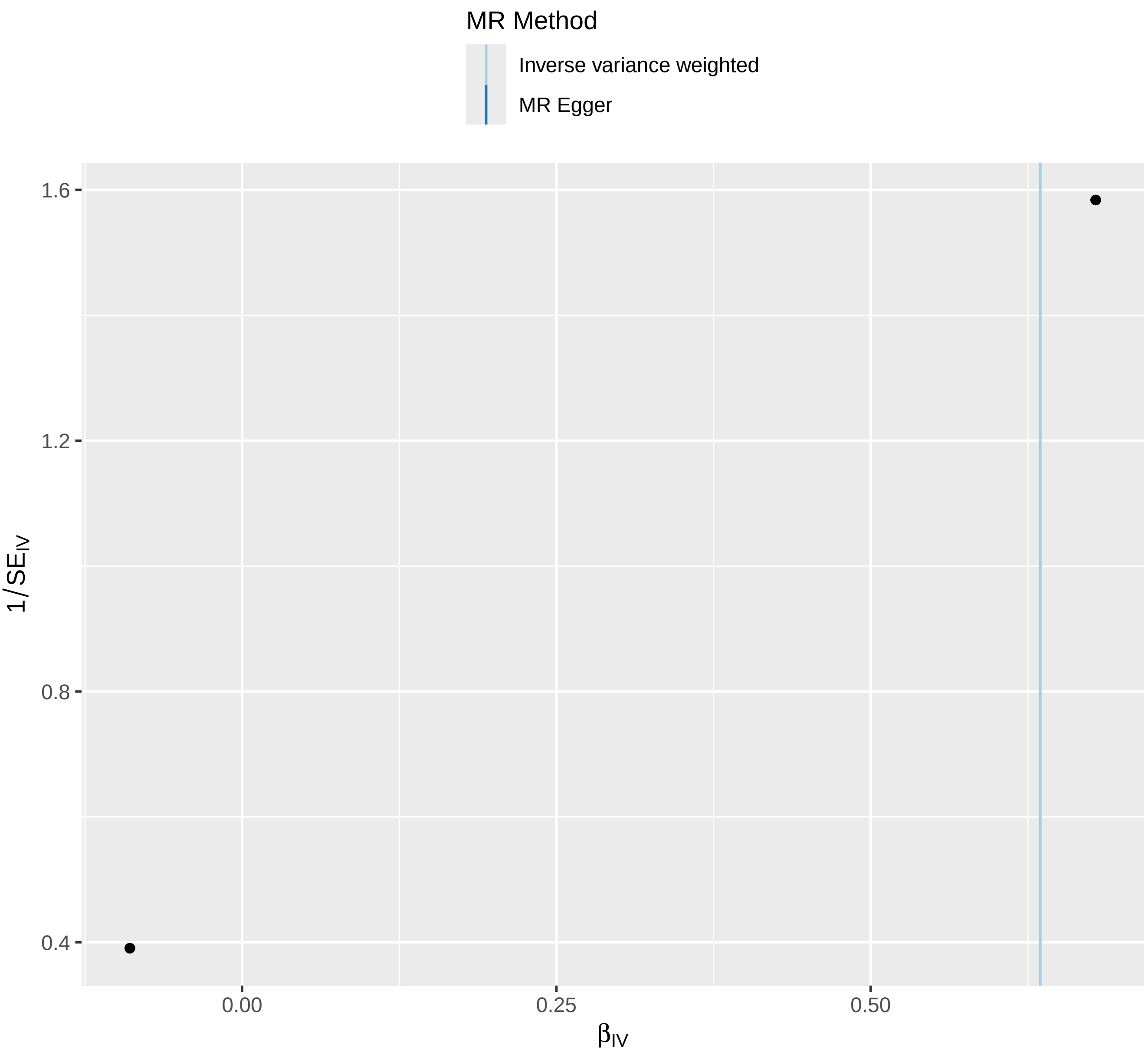
MR Method
Inverse variance weighted
MR Egger
1.6
1.2
0.8
0.4
1/SE_{IV}
0.00
0.25
0.50
β_{IV}

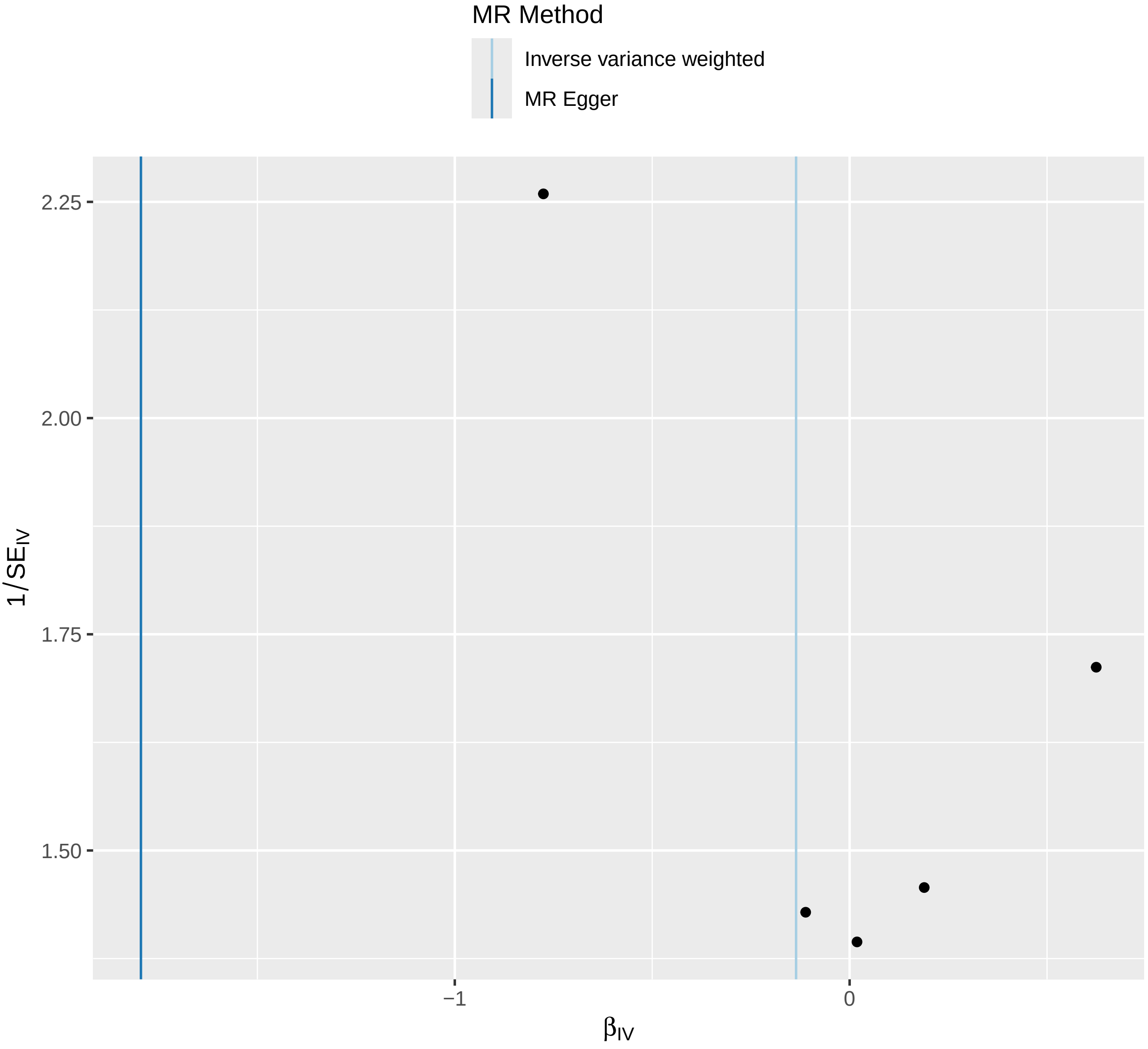
MR Method
Inverse variance weighted
MR Egger
2.25
2.00
1.75
1.50
1/SE_IV
−1
0
β_IV

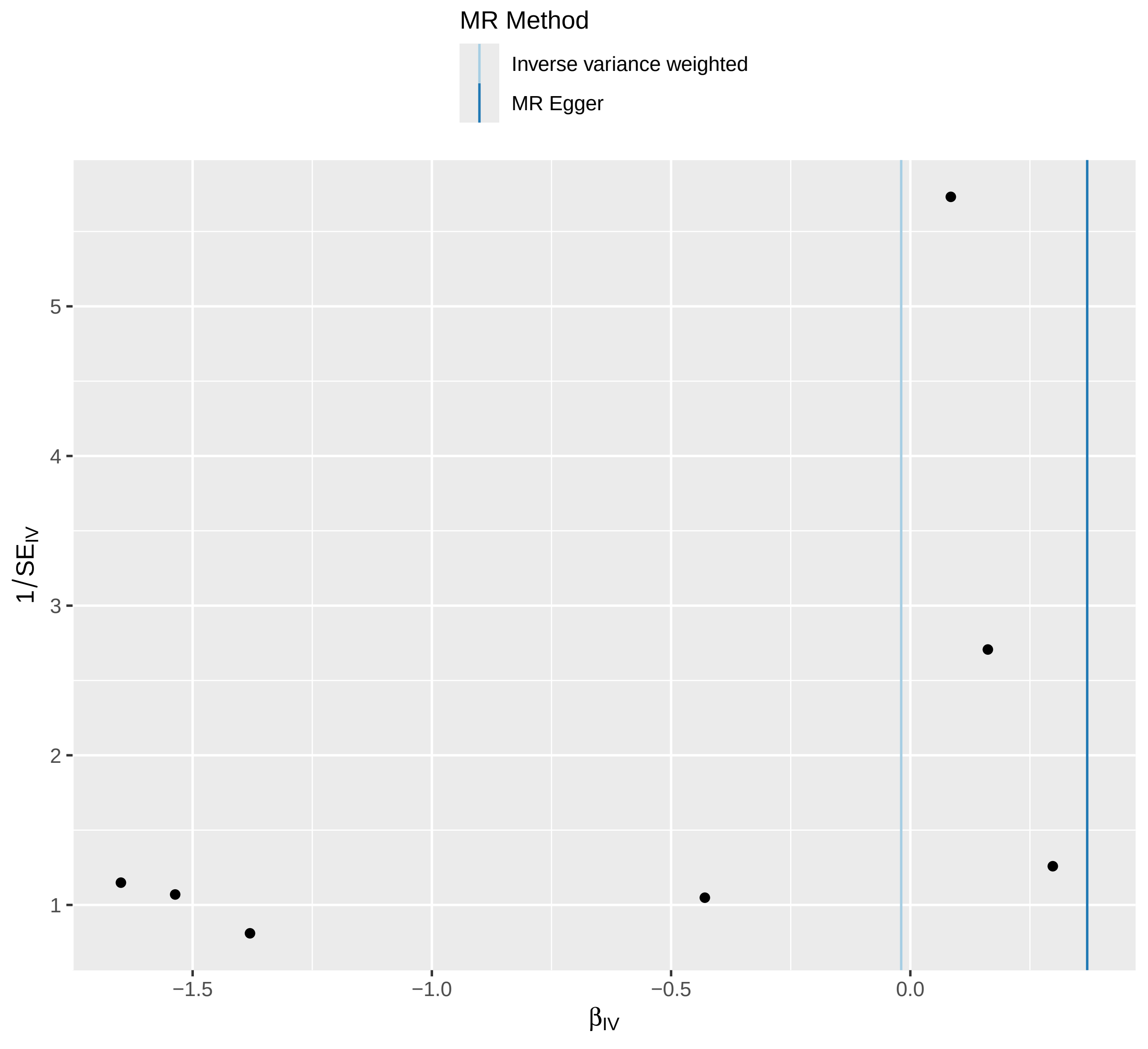
MR Method
Inverse variance weighted
MR Egger
1/SE_IV
β_IV
5
4
3
2
1
−1.5
−1.0
−0.5
0.0

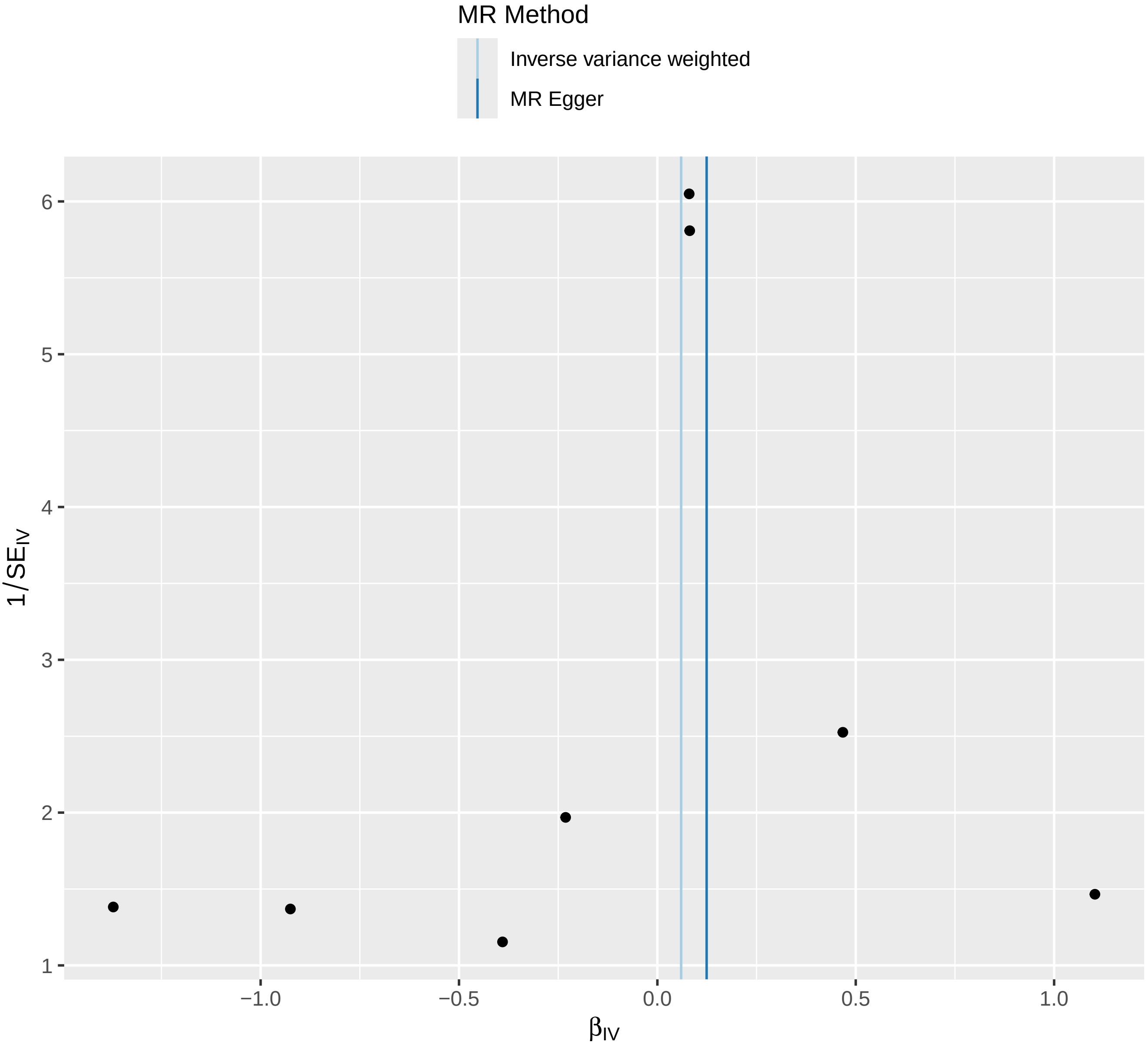

MR Method
Inverse variance weighted
MR Egger
6
5
4
3
2
1
1/SE_IV
−1.0
−0.5
0.0
0.5
1.0
β_IV

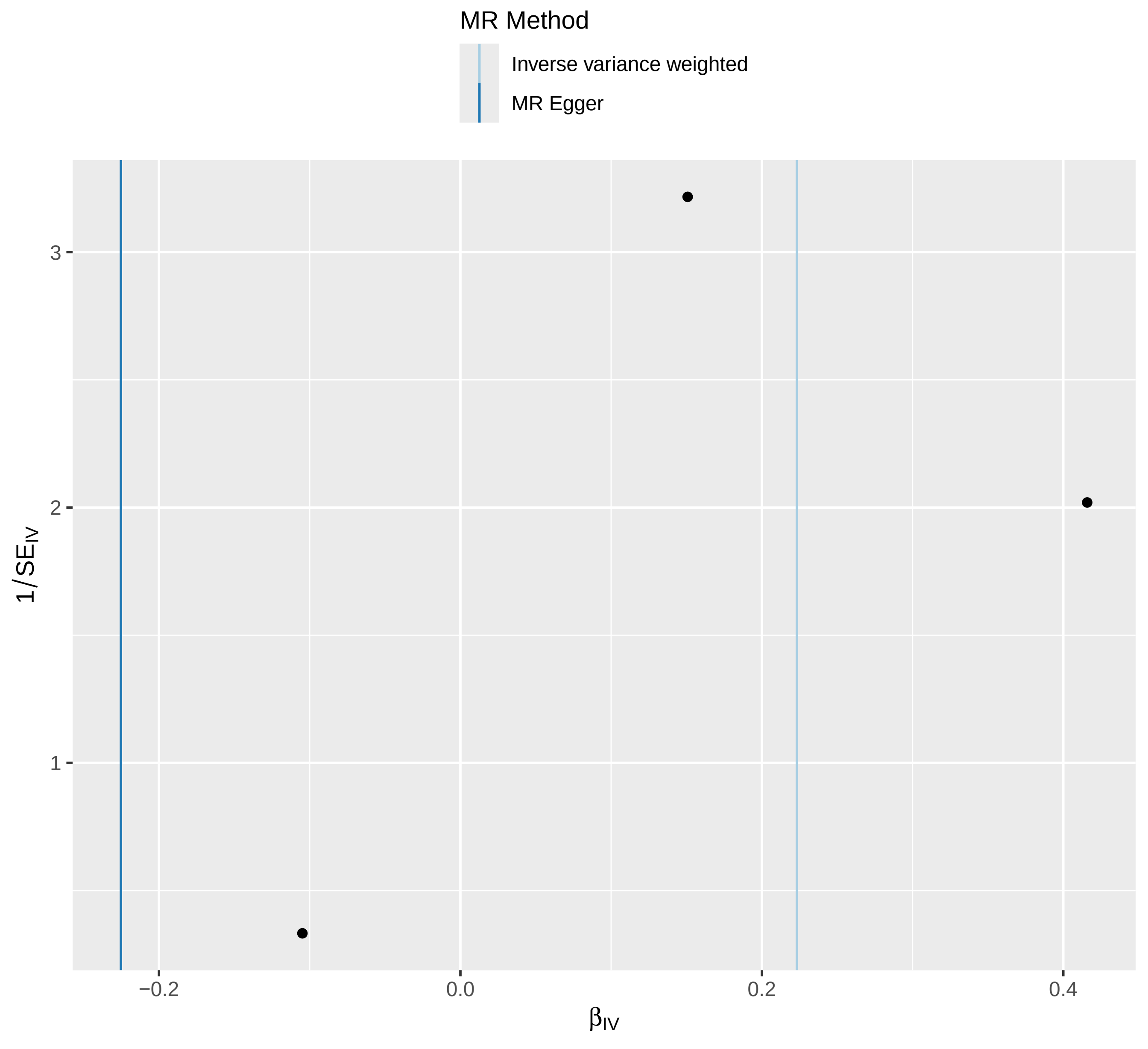
MR Method
Inverse variance weighted
MR Egger
3
2
1
1/SE_IV
−0.2
0.0
0.2
0.4
β_IV

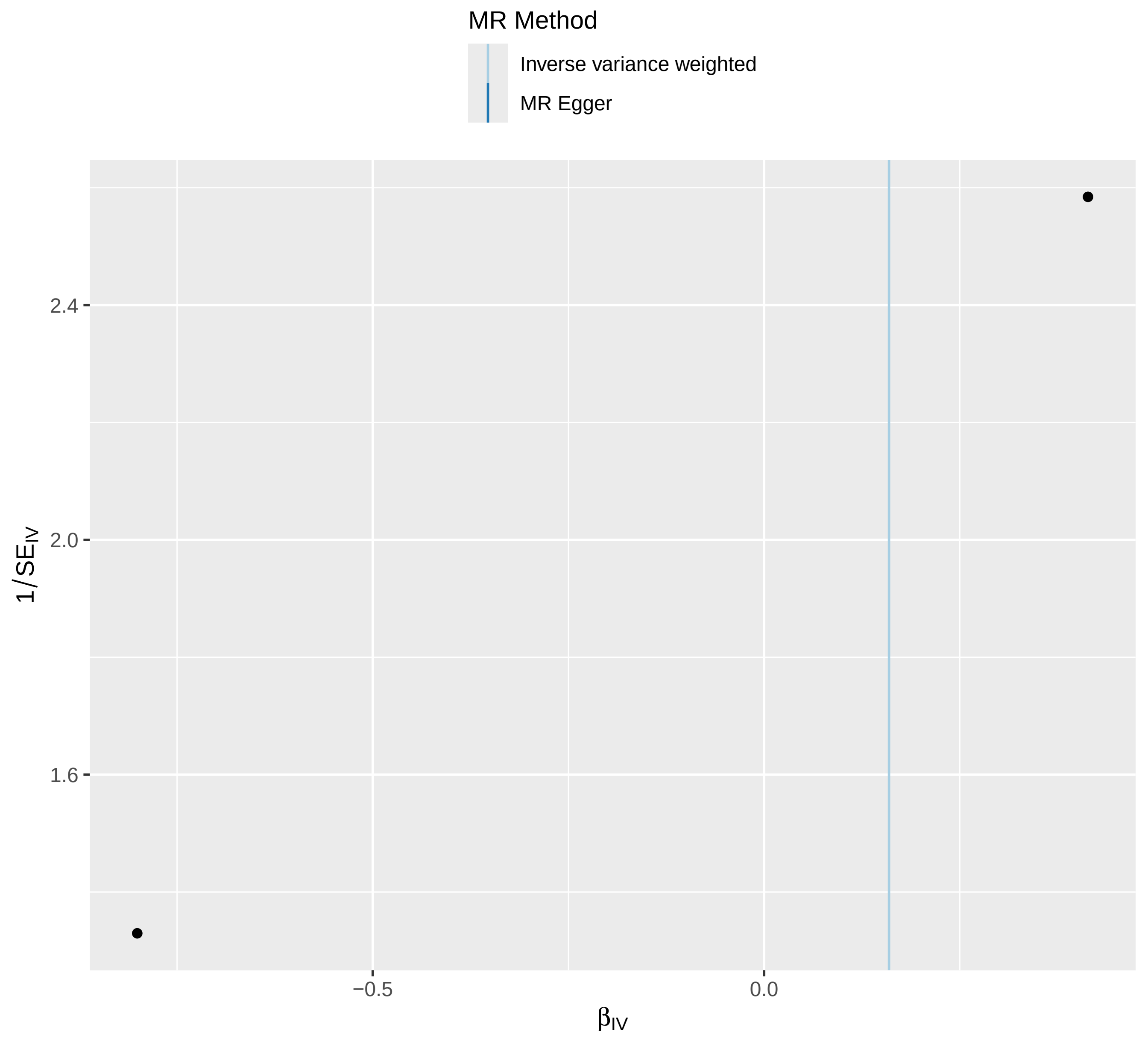

MR Method
Inverse variance weighted
MR Egger
2.4
2.0
1.6
1/SE_IV
−0.5
0.0
β_IV

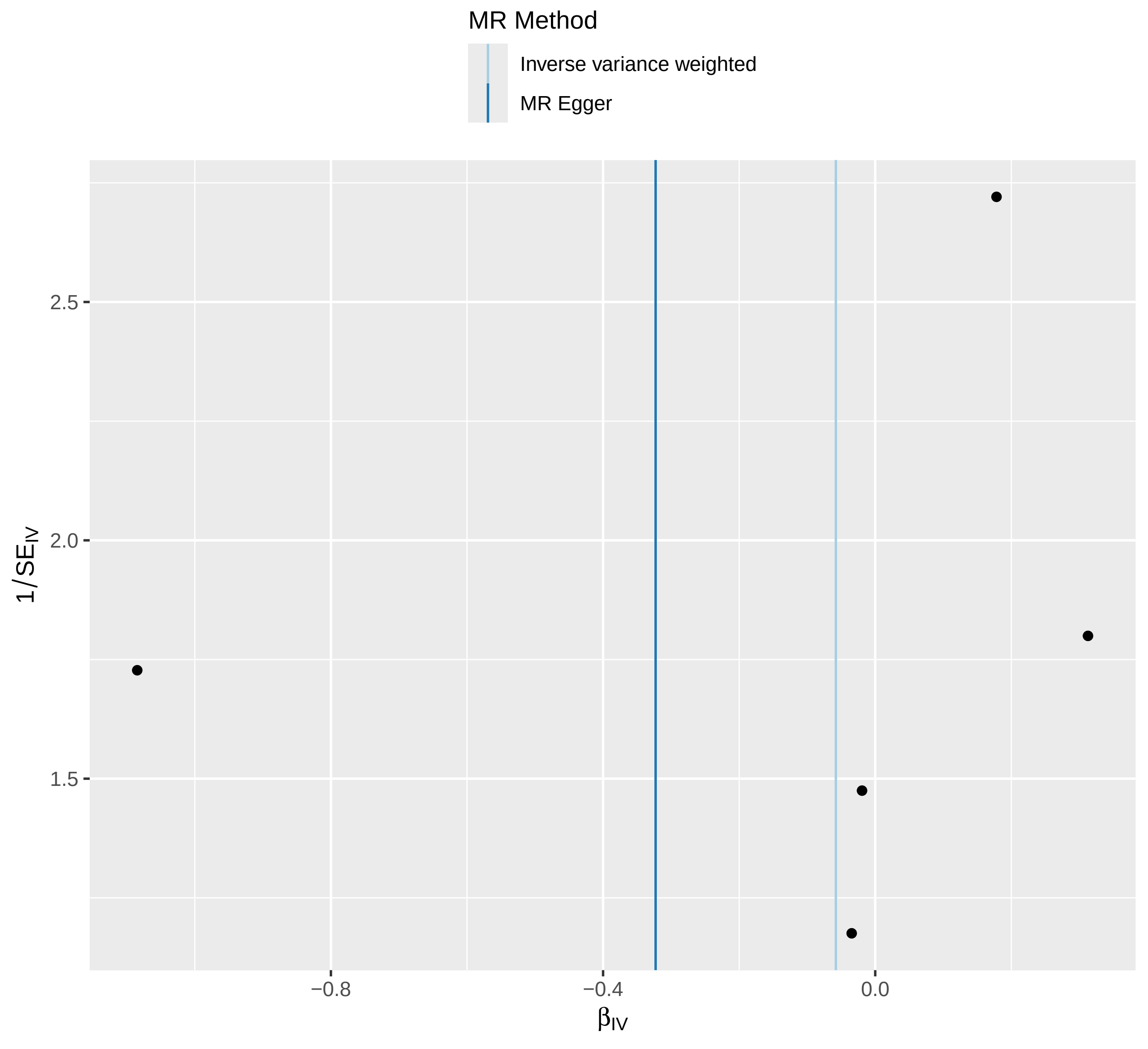

MR Method
Inverse variance weighted
MR Egger
2.5
2.0
1.5
1/SE_IV
−0.8
−0.4
0.0
β_IV

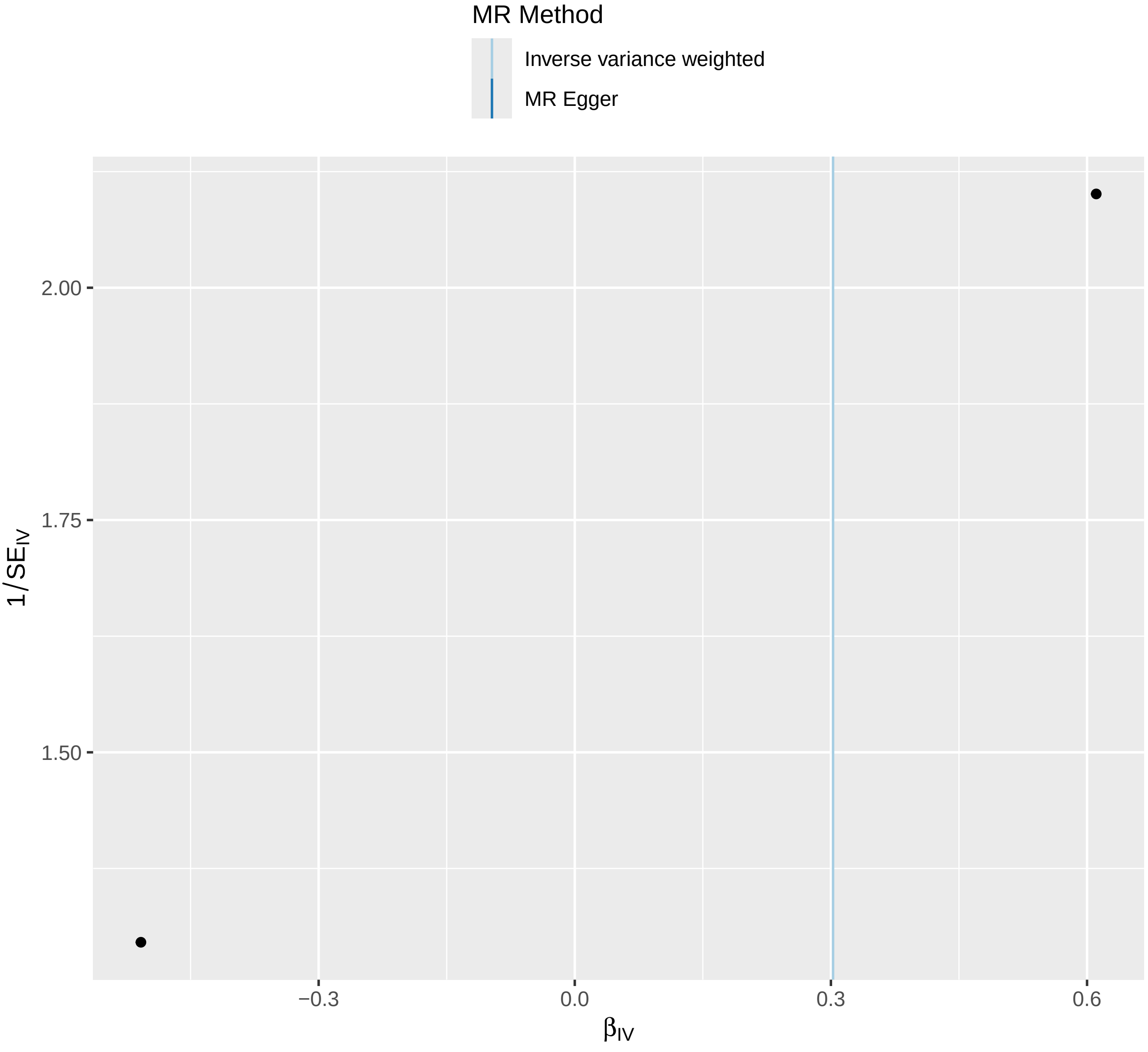
MR Method
Inverse variance weighted
MR Egger
2.00
1.75
1.50
−0.3
0.0
0.3
0.6
$1/SE_{IV}$
β_{IV}

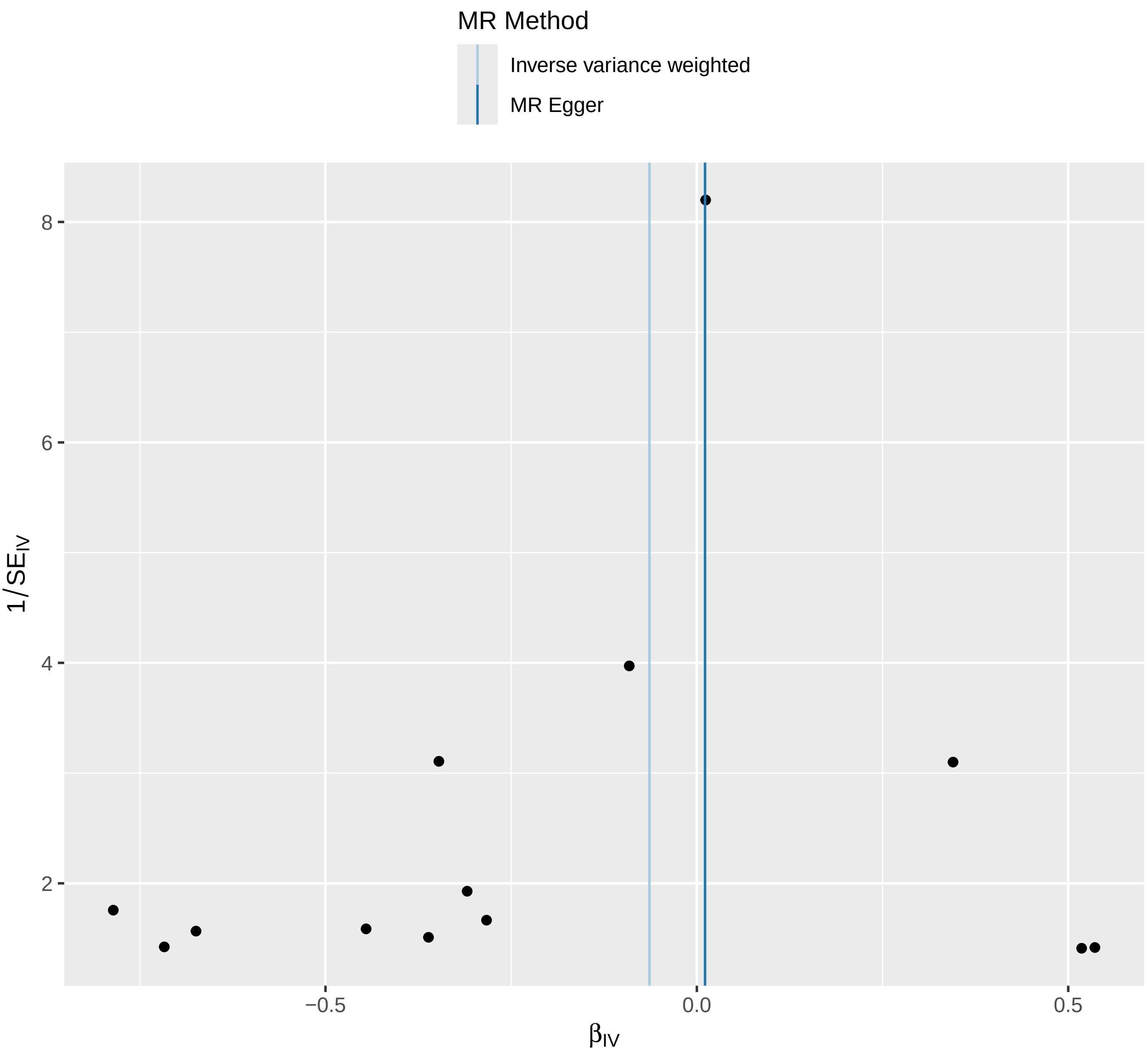
MR Method
Inverse variance weighted
MR Egger
8
6
4
2
1/SE$_{IV}$
−0.5
0.0
0.5
β$_{IV}$

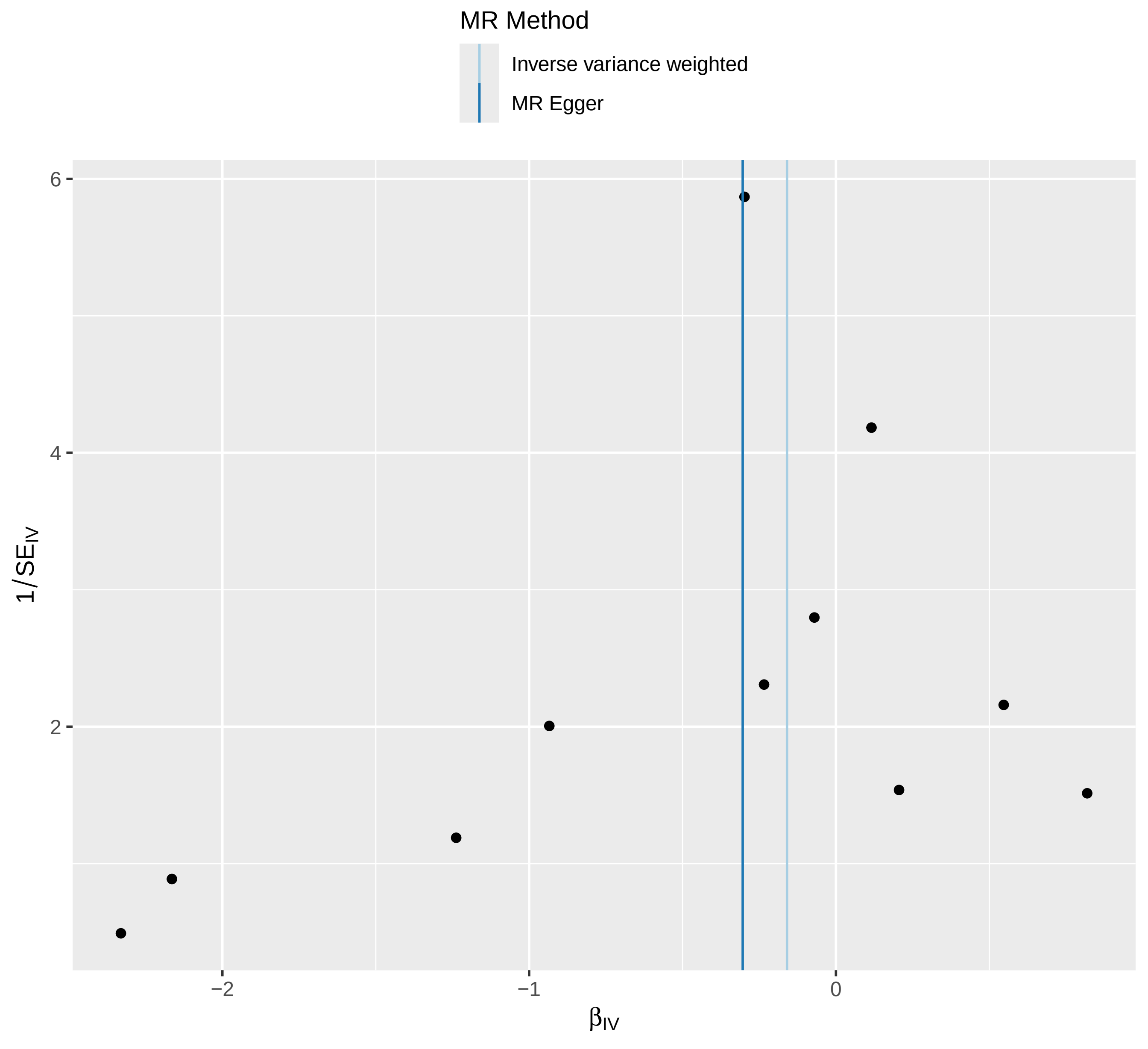
MR Method
Inverse variance weighted
MR Egger
6
4
2
$1/SE_{IV}$
−2
−1
0
β_{IV}

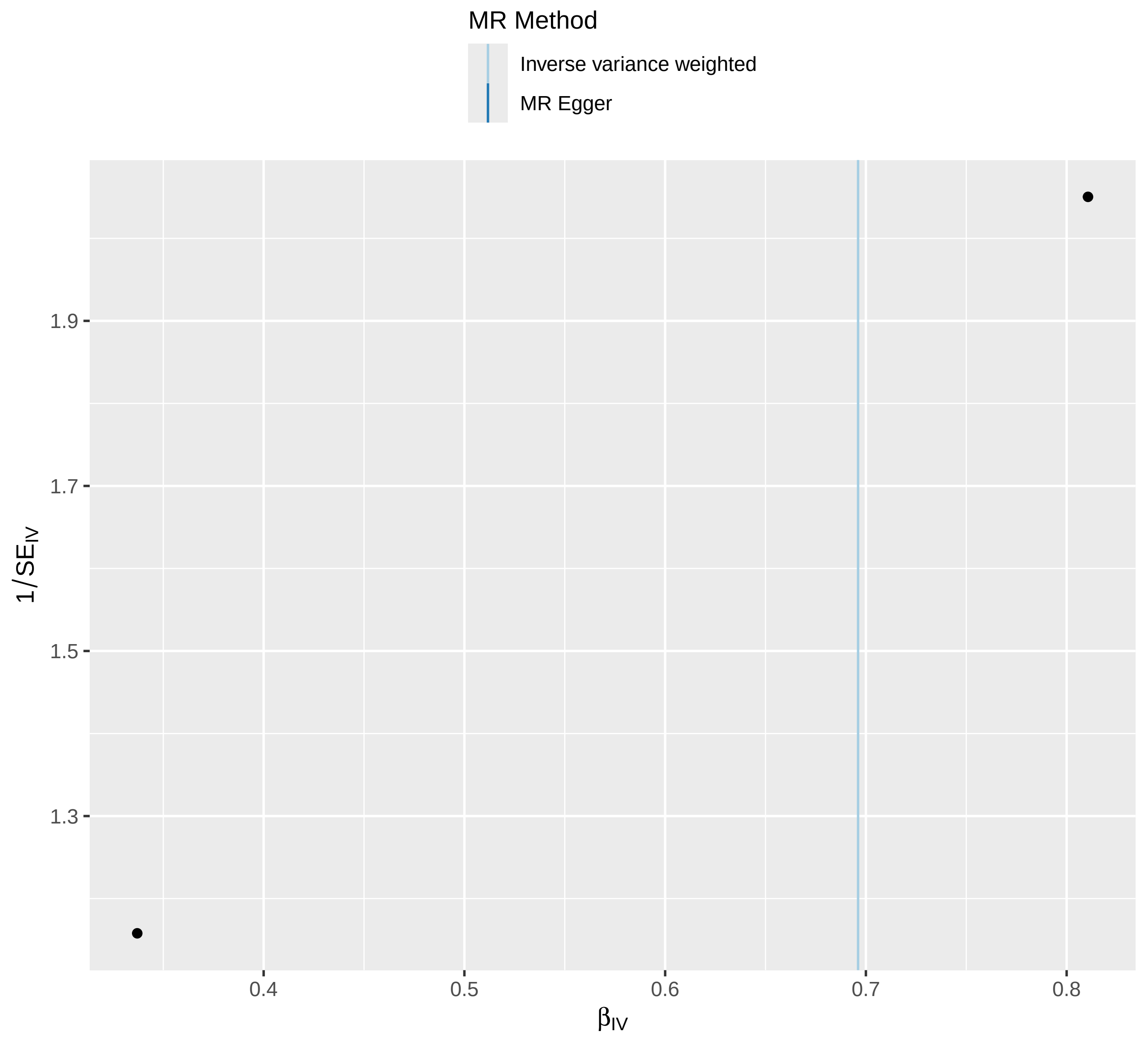
MR Method
Inverse variance weighted
MR Egger
1.9
1.7
1.5
1.3
0.4
0.5
0.6
0.7
0.8
1/SE_IV
β_IV

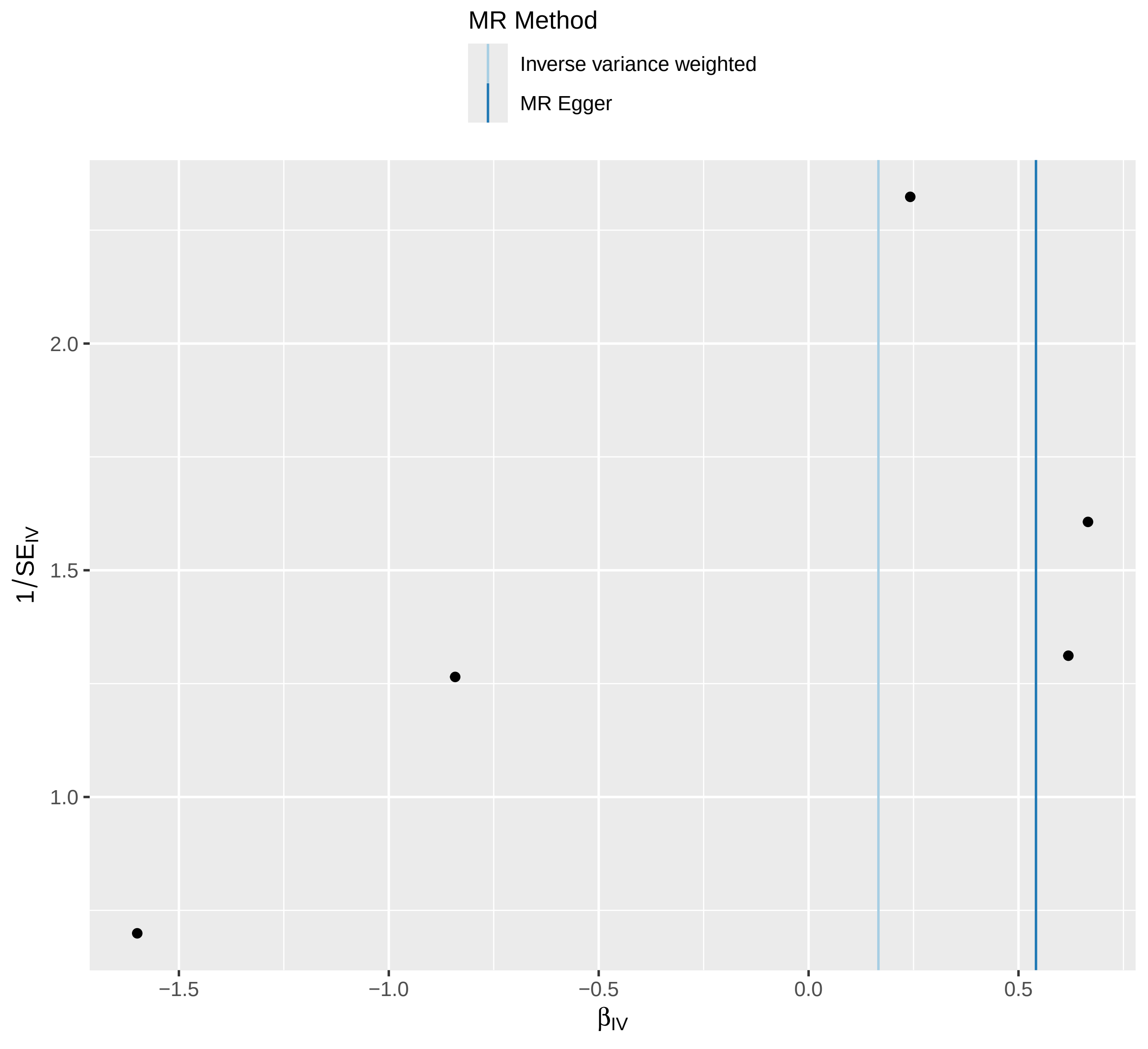

MR Method
Inverse variance weighted
MR Egger
2.0
1.5
1.0
1/SE_IV
−1.5
−1.0
−0.5
0.0
0.5
β_IV

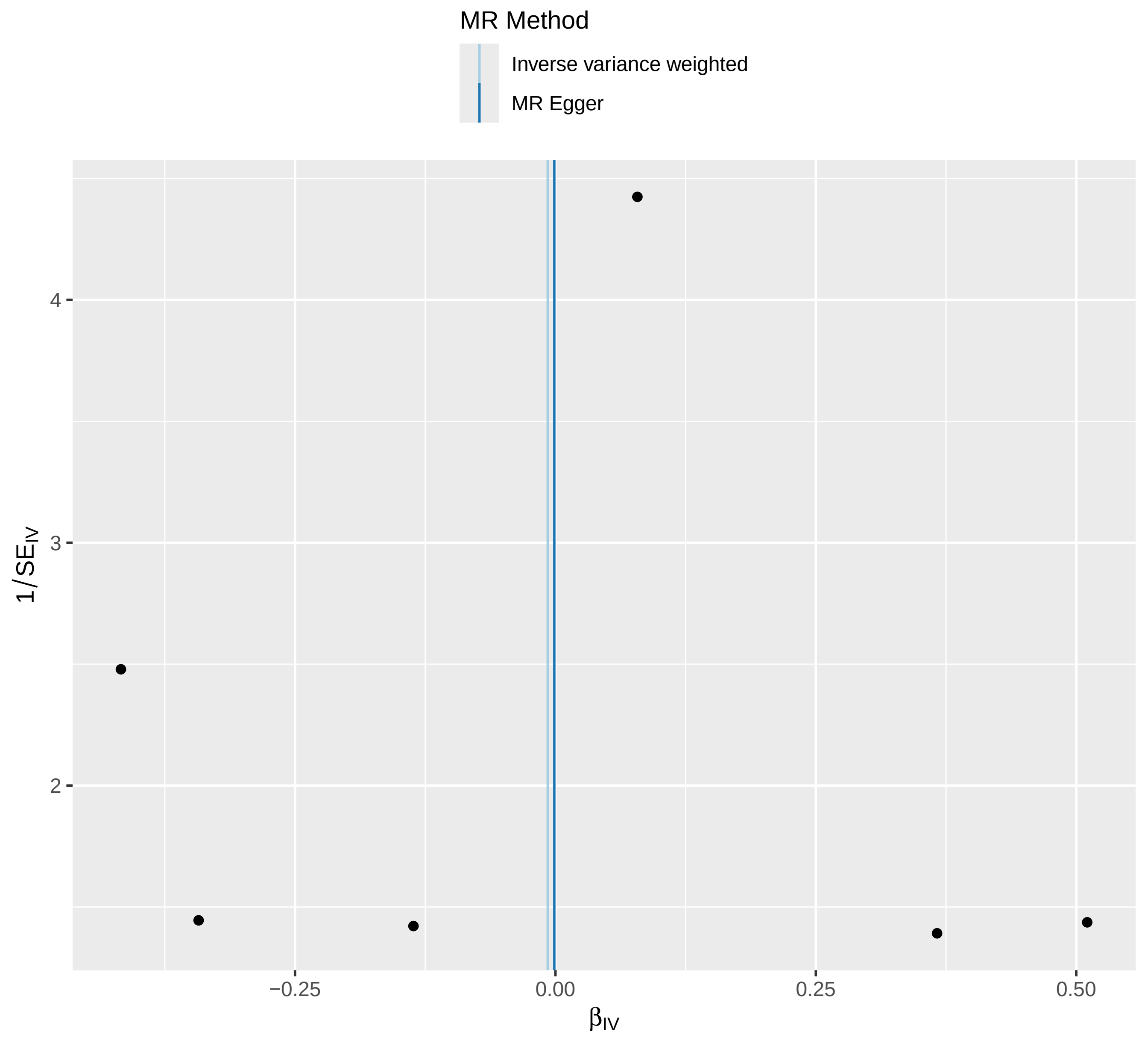

MR Method
Inverse variance weighted
MR Egger
1/SE$_{IV}$
4
3
2
−0.25
0.00
0.25
0.50
β$_{IV}$

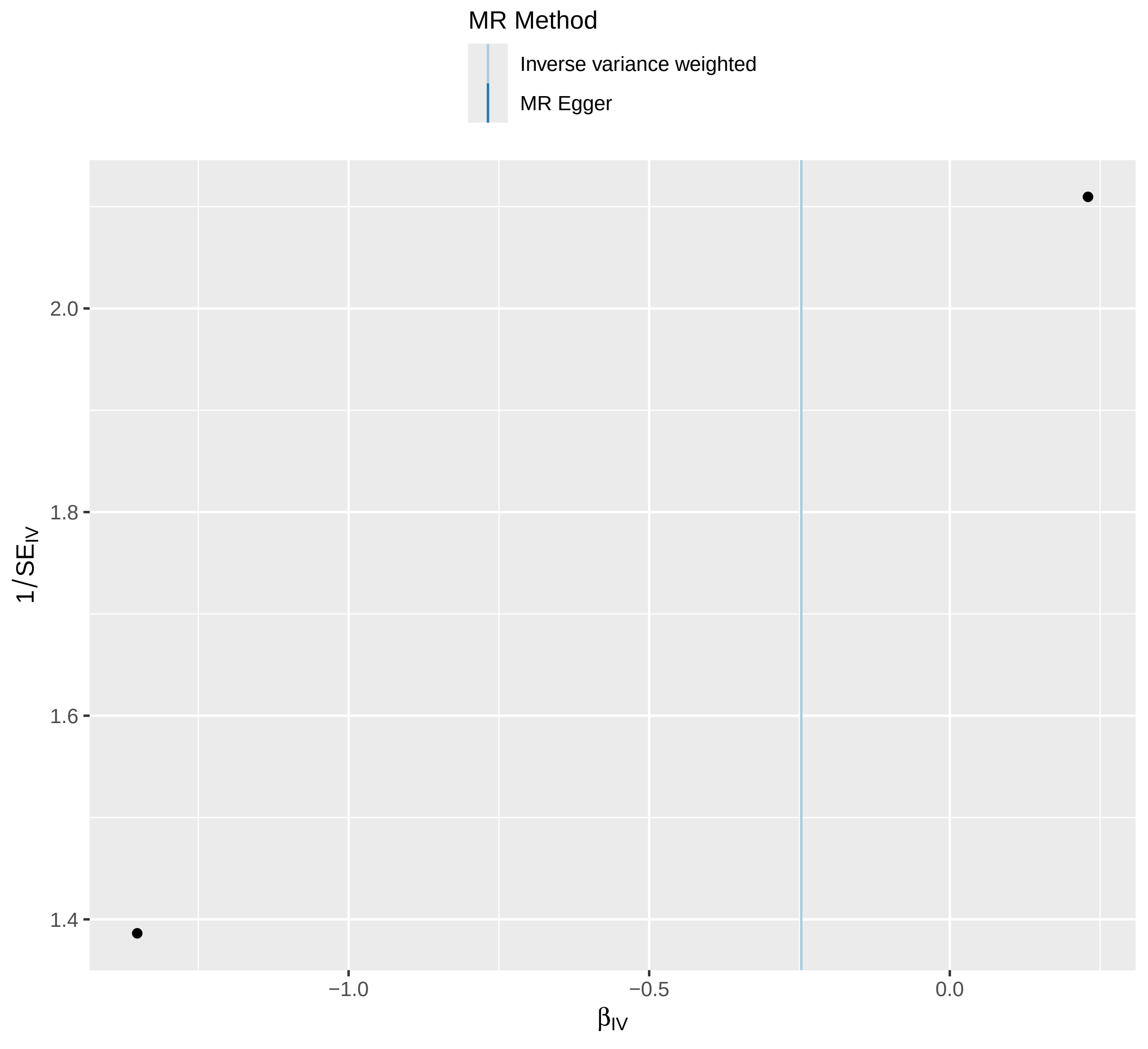
MR Method
Inverse variance weighted
MR Egger
2.0
1.8
1.6
1.4
1/SE_IV
−1.0
−0.5
0.0
β_IV

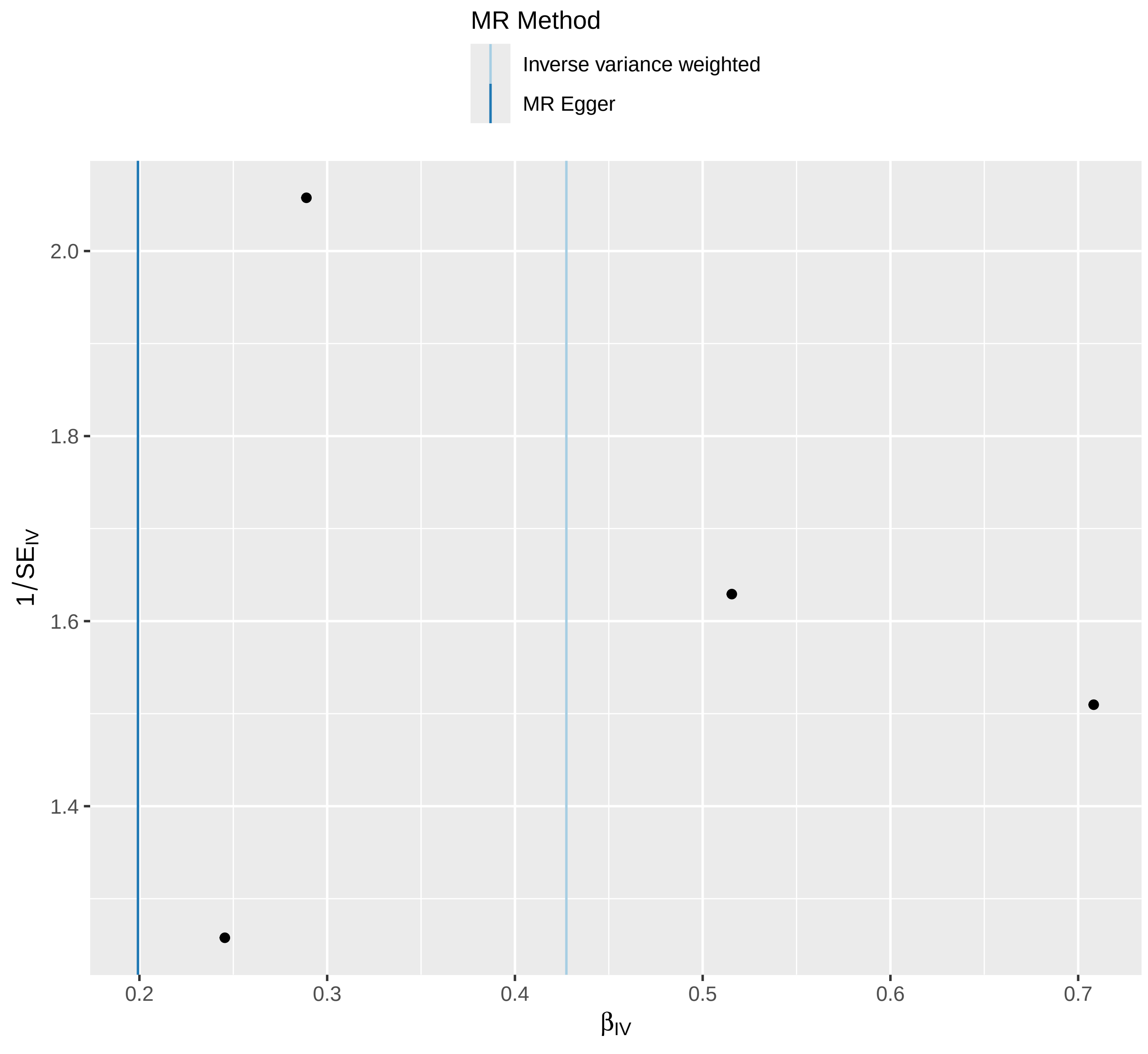
MR Method
Inverse variance weighted
MR Egger
2.0
1.8
1.6
1.4
1/SE$_{IV}$
0.2
0.3
0.4
0.5
0.6
0.7
β$_{IV}$

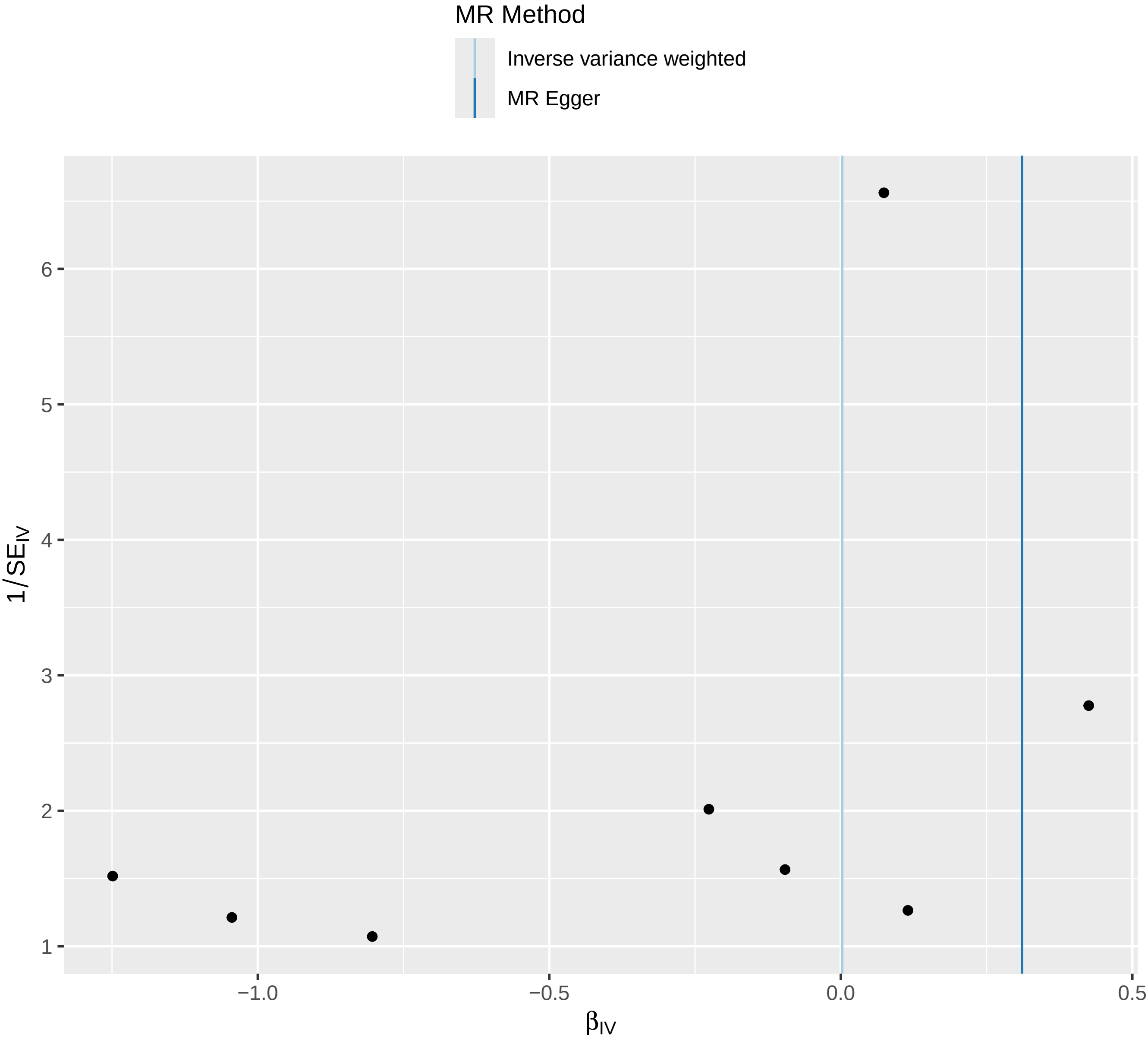

MR Method
Inverse variance weighted
MR Egger
6
5
4
3
2
1
1/SE$_{IV}$
−1.0
−0.5
0.0
0.5
β$_{IV}$

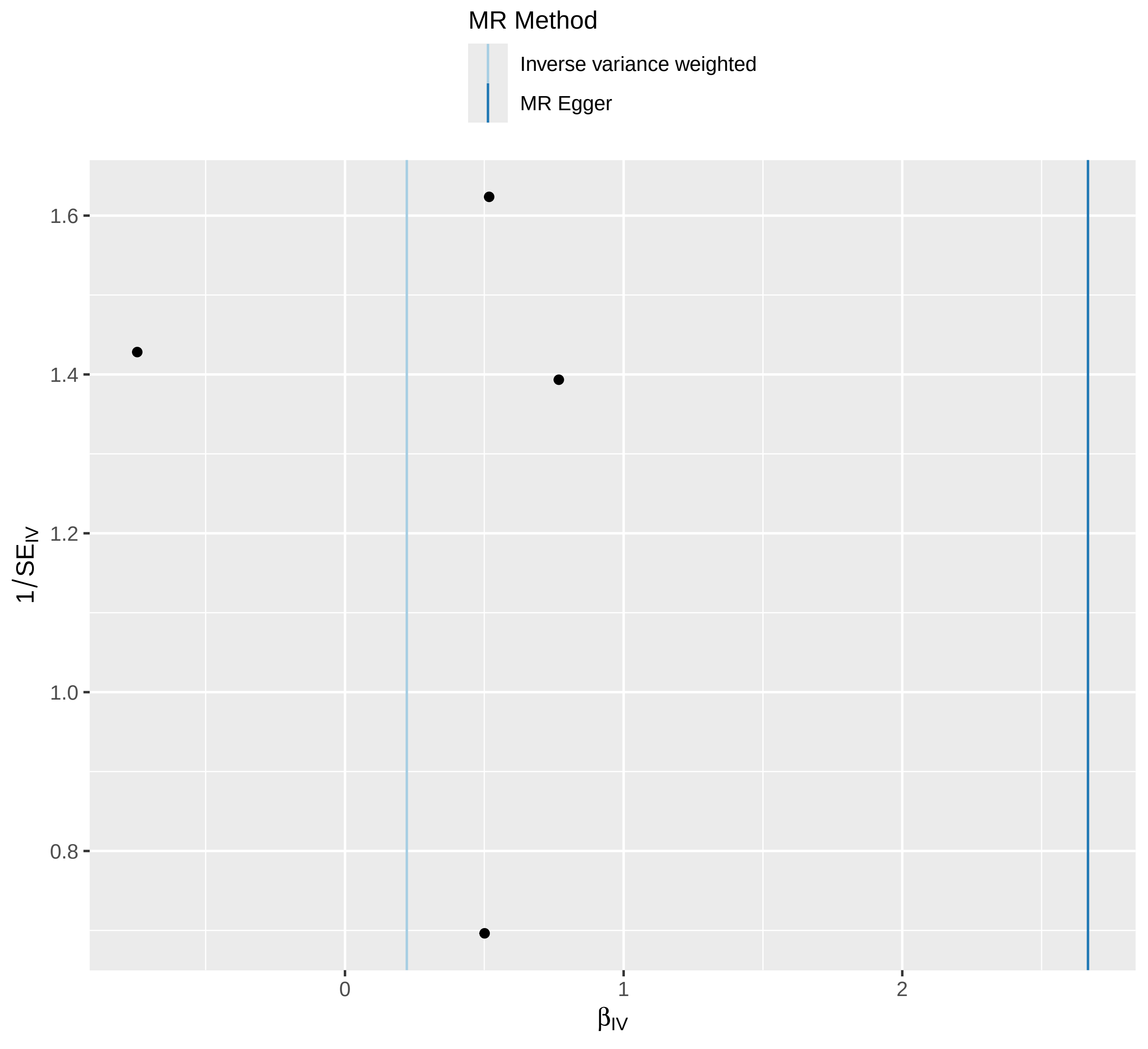

MR Method
Inverse variance weighted
MR Egger
1.6
1.4
1.2
1.0
0.8
1/SE_IV
0
1
2
β_IV

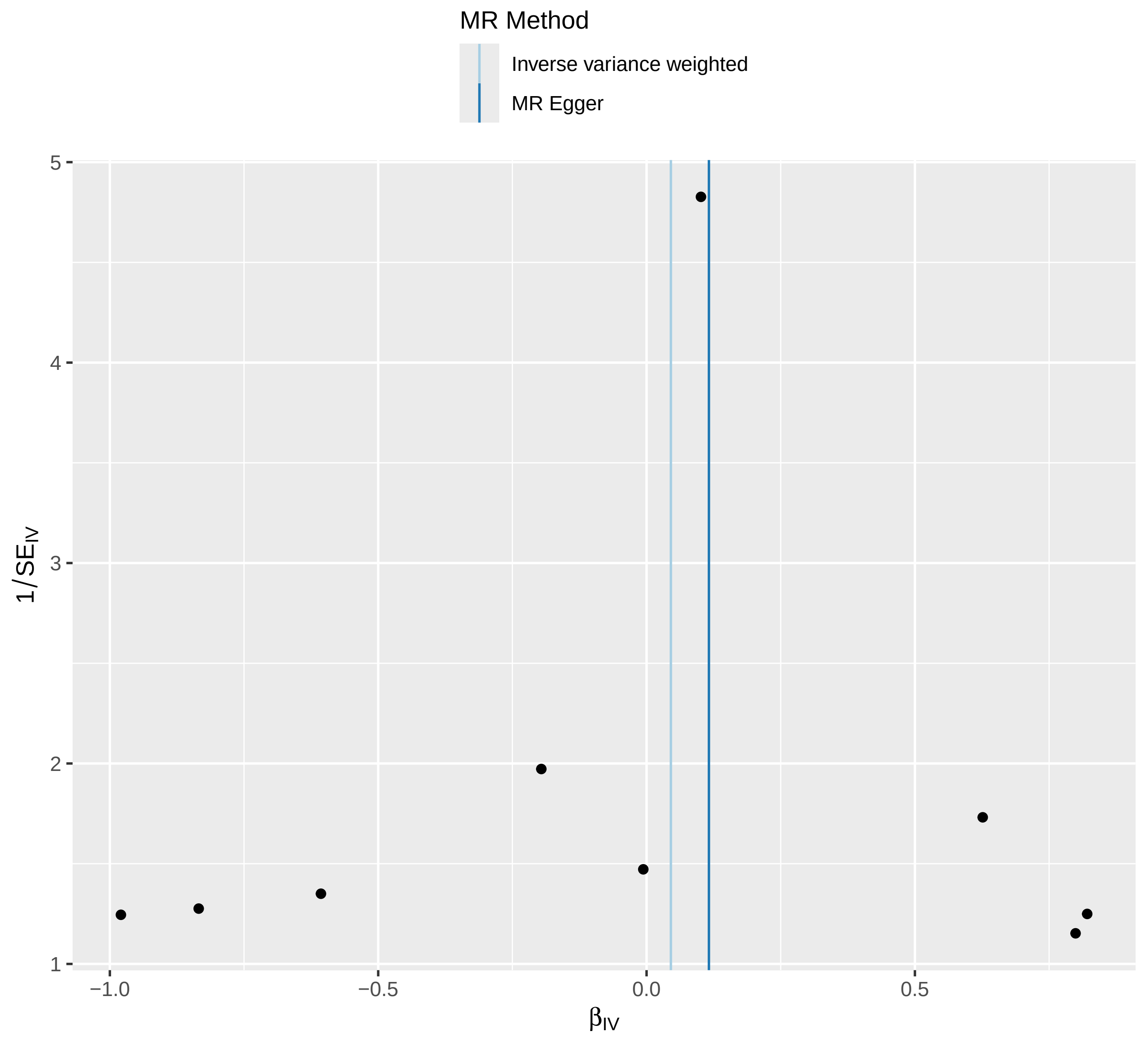
MR Method
Inverse variance weighted
MR Egger
5
4
3
2
1
$1/SE_{IV}$
−1.0
−0.5
0.0
0.5
β_{IV}

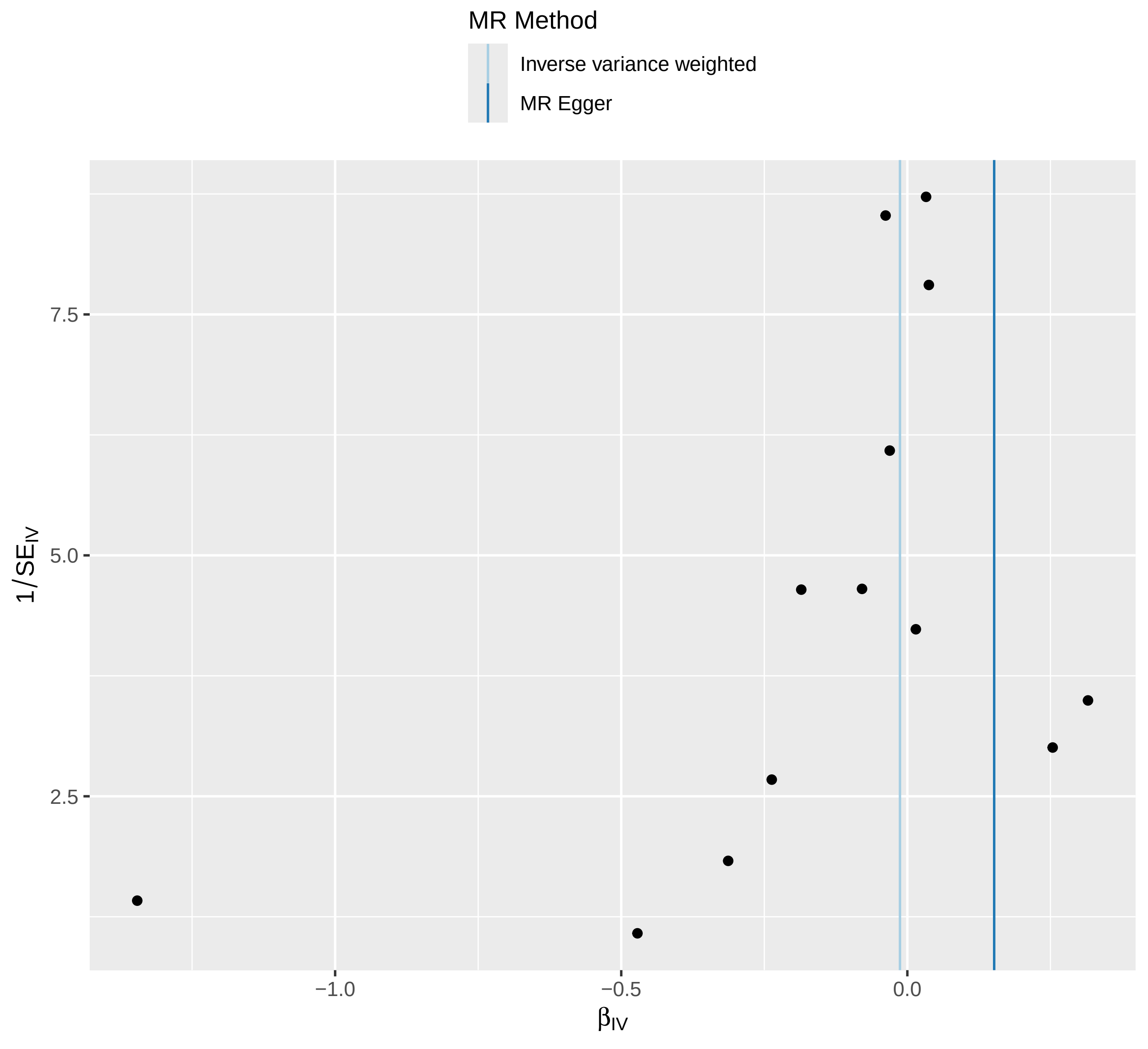

MR Method
Inverse variance weighted
MR Egger
7.5
5.0
2.5
1/SE_IV
−1.0
−0.5
0.0
β_IV

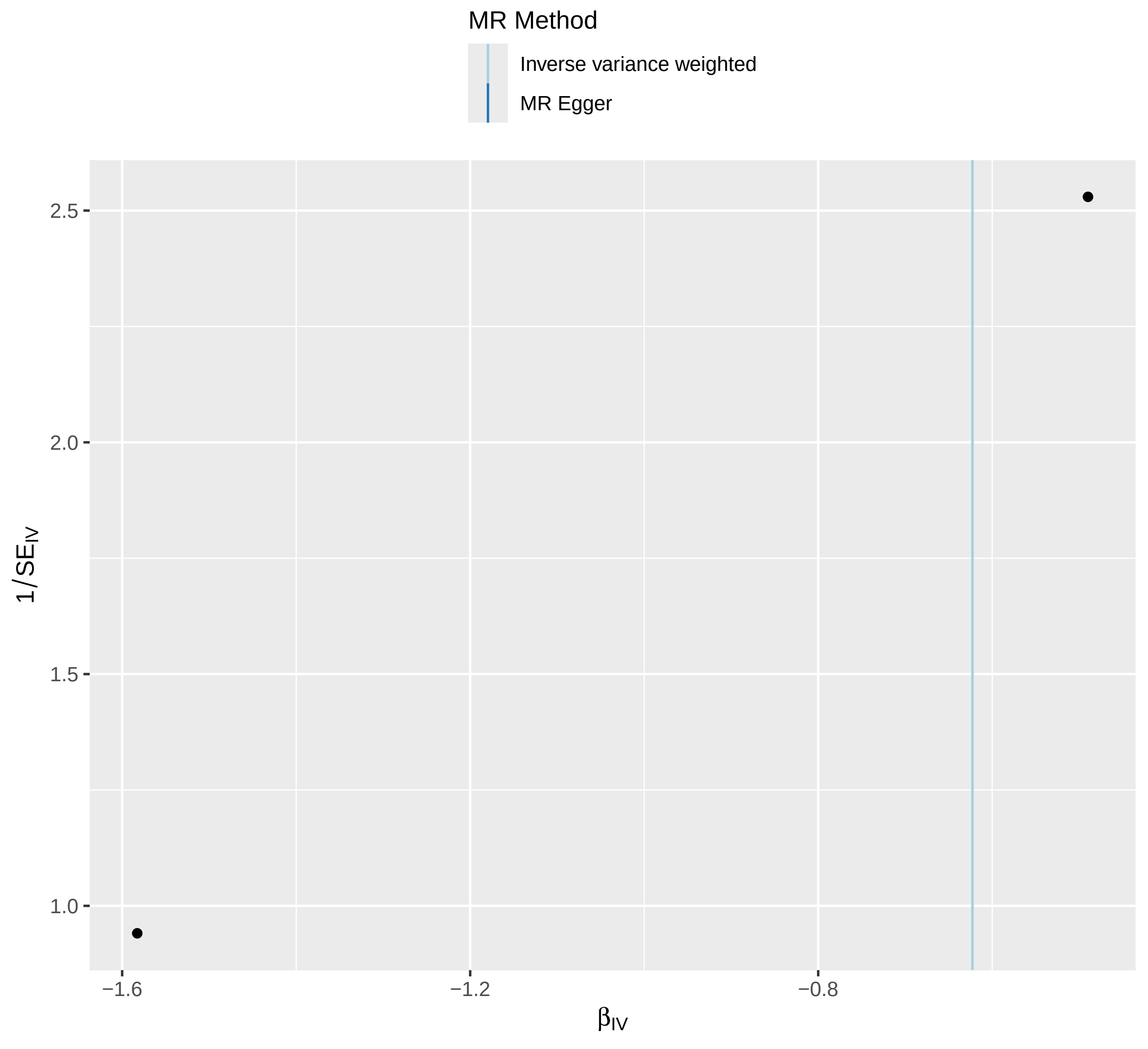
MR Method
Inverse variance weighted
MR Egger
2.5
2.0
1.5
1.0
−1.6
−1.2
−0.8
$1/SE_{IV}$
β_{IV}

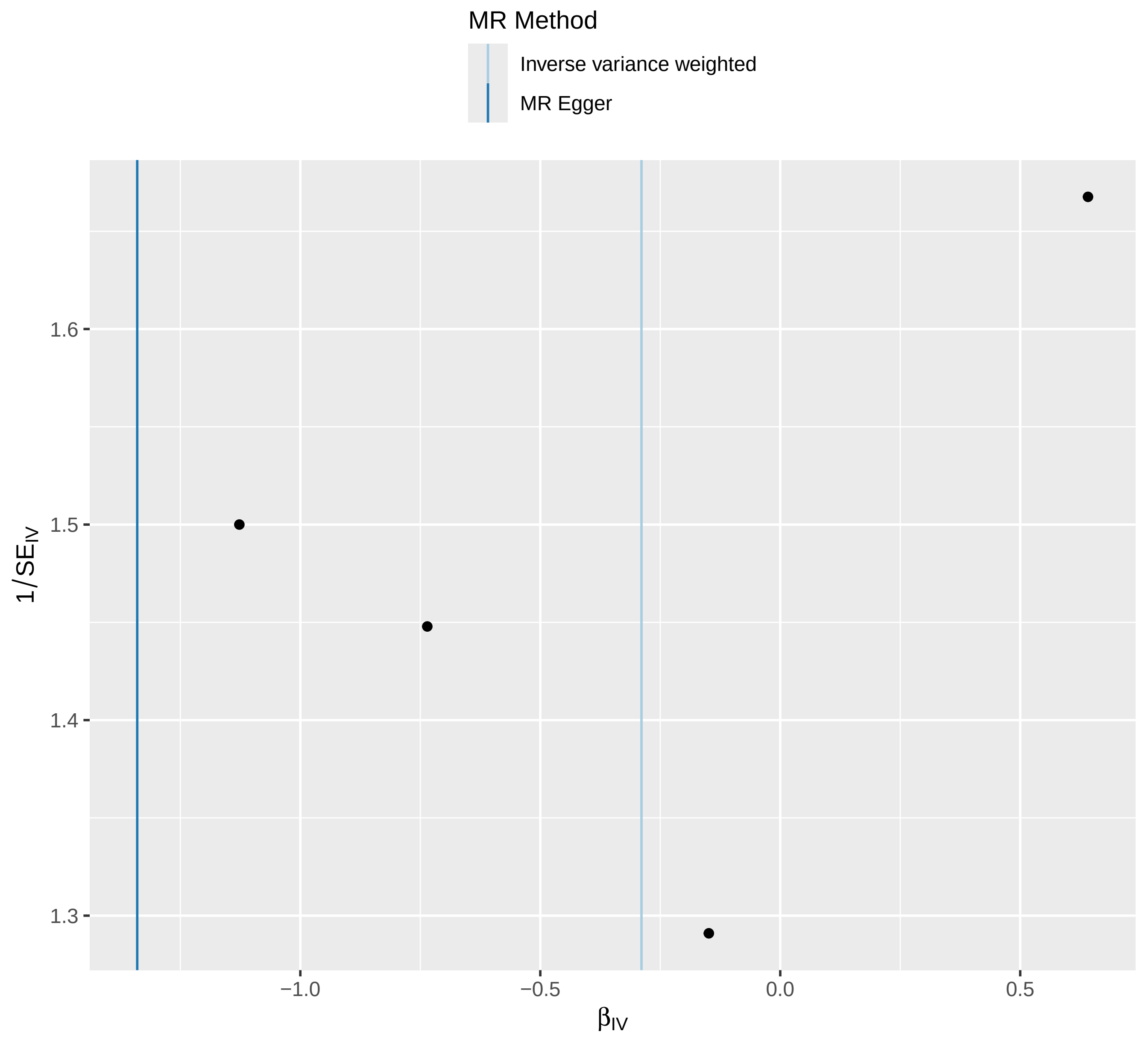

MR Method
Inverse variance weighted
MR Egger
1.6
1.5
1.4
1.3
1/SE$_{IV}$
−1.0
−0.5
0.0
0.5
β$_{IV}$

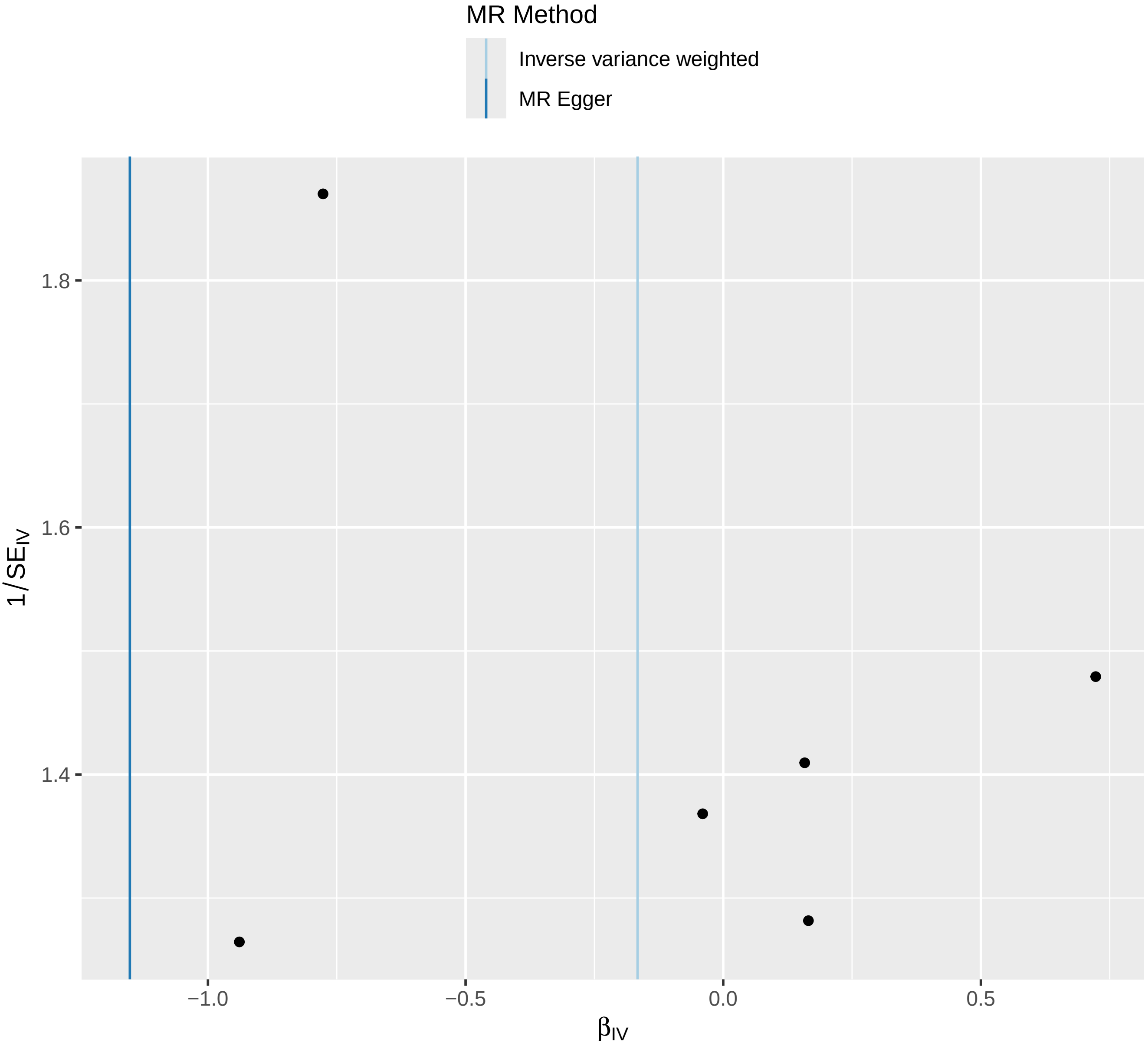

MR Method
Inverse variance weighted
MR Egger
1.8
1.6
1.4
1/SE$_{IV}$
−1.0
−0.5
0.0
0.5
β$_{IV}$

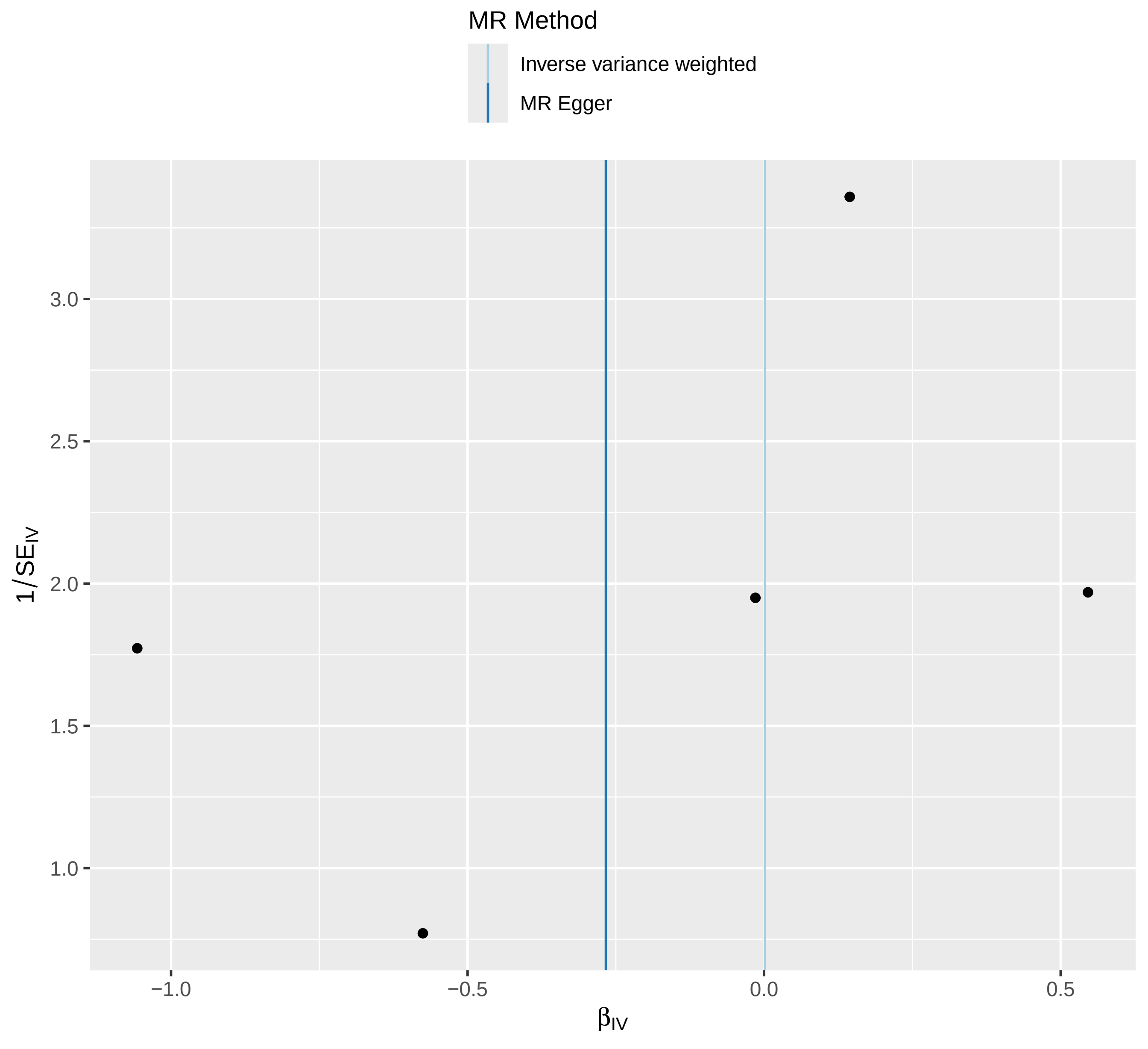
MR Method
Inverse variance weighted
MR Egger
3.0
2.5
2.0
1.5
1.0
1/SE_IV
−1.0
−0.5
0.0
0.5
β_IV

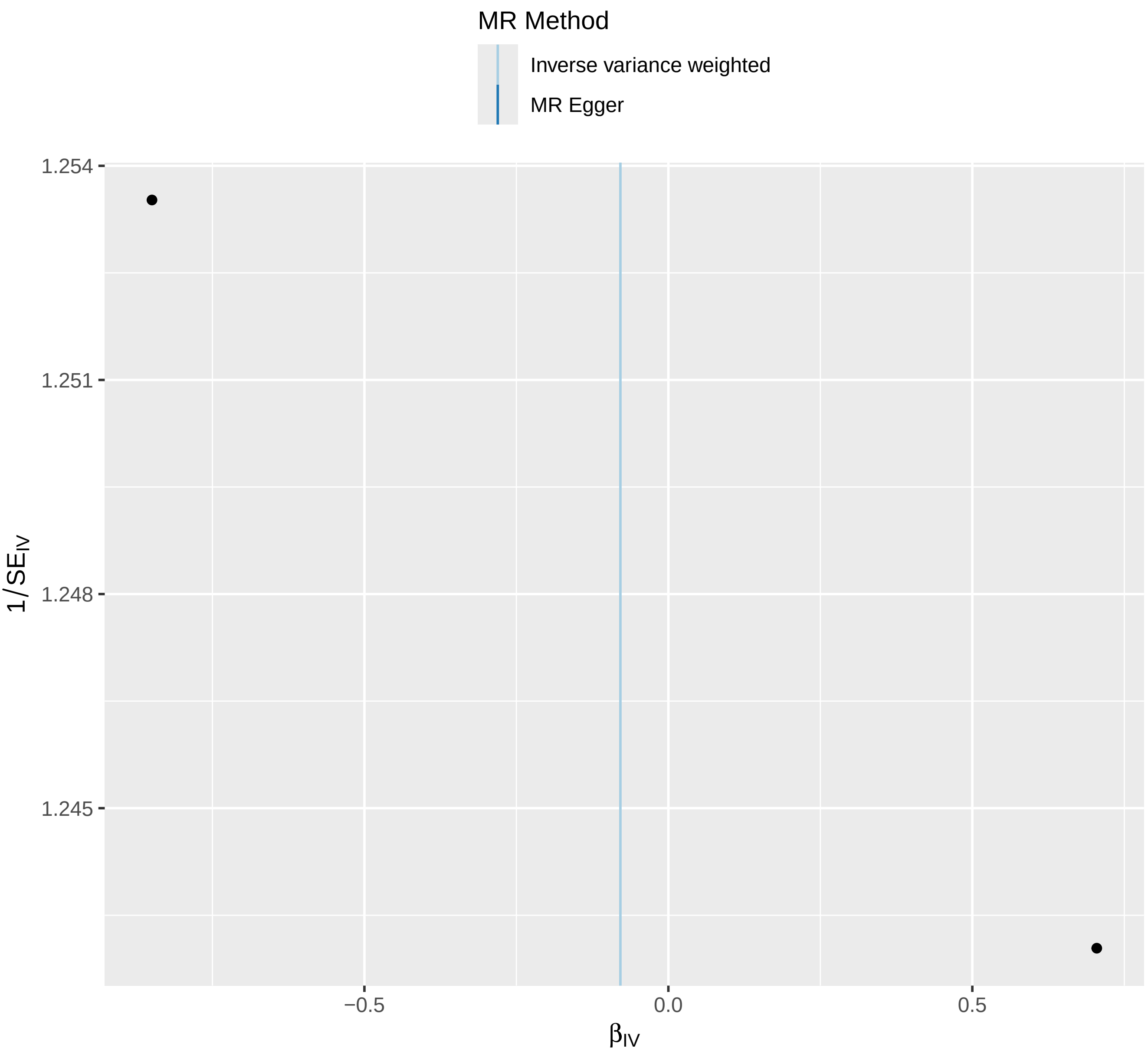

MR Method
Inverse variance weighted
MR Egger
1.254
1.251
1.248
1.245
1/SE_IV
−0.5
0.0
0.5
β_IV

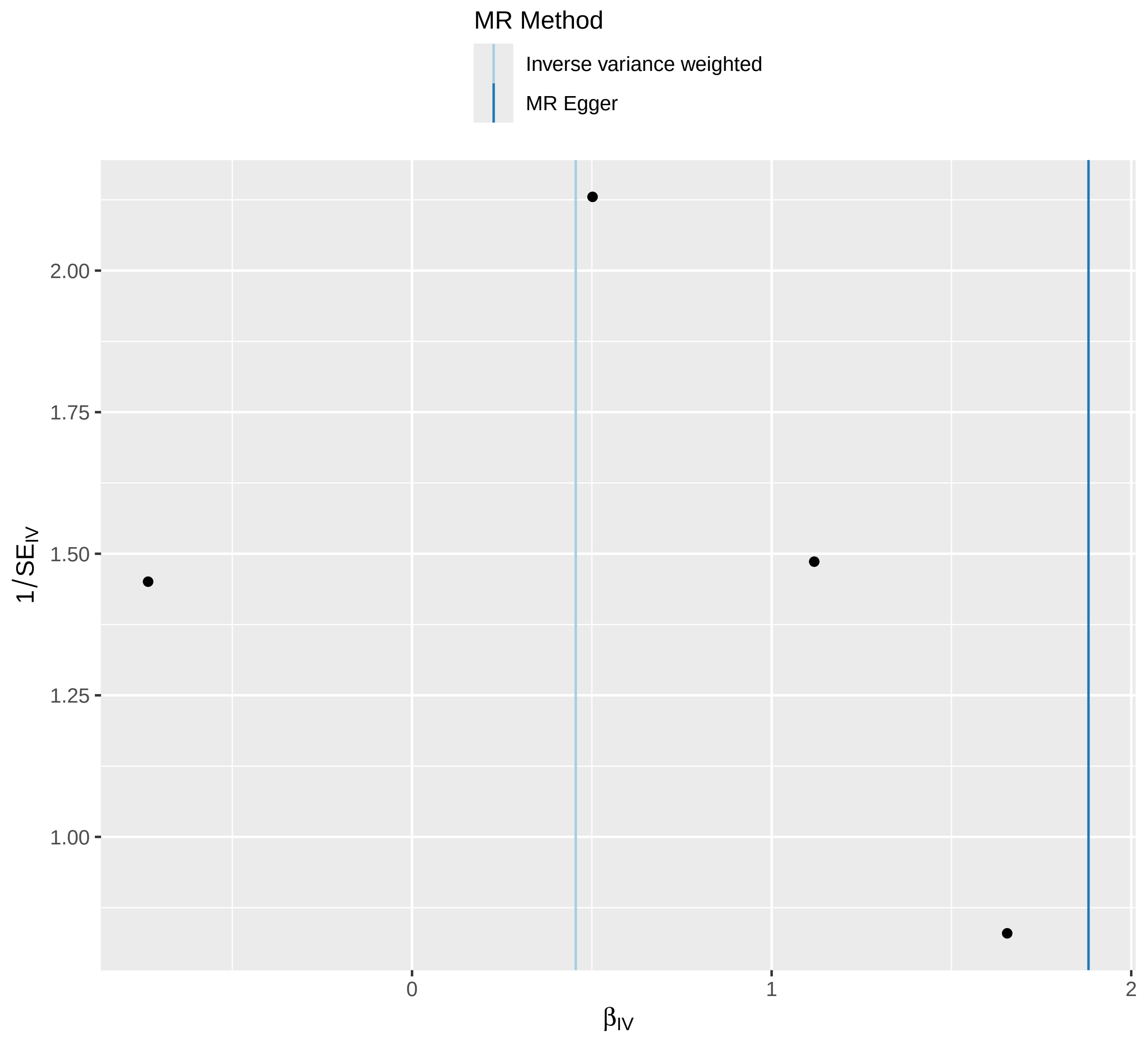

MR Method
Inverse variance weighted
MR Egger
2.00
1.75
1.50
1.25
1.00
0
1
2
$1/SE_{IV}$
β_{IV}

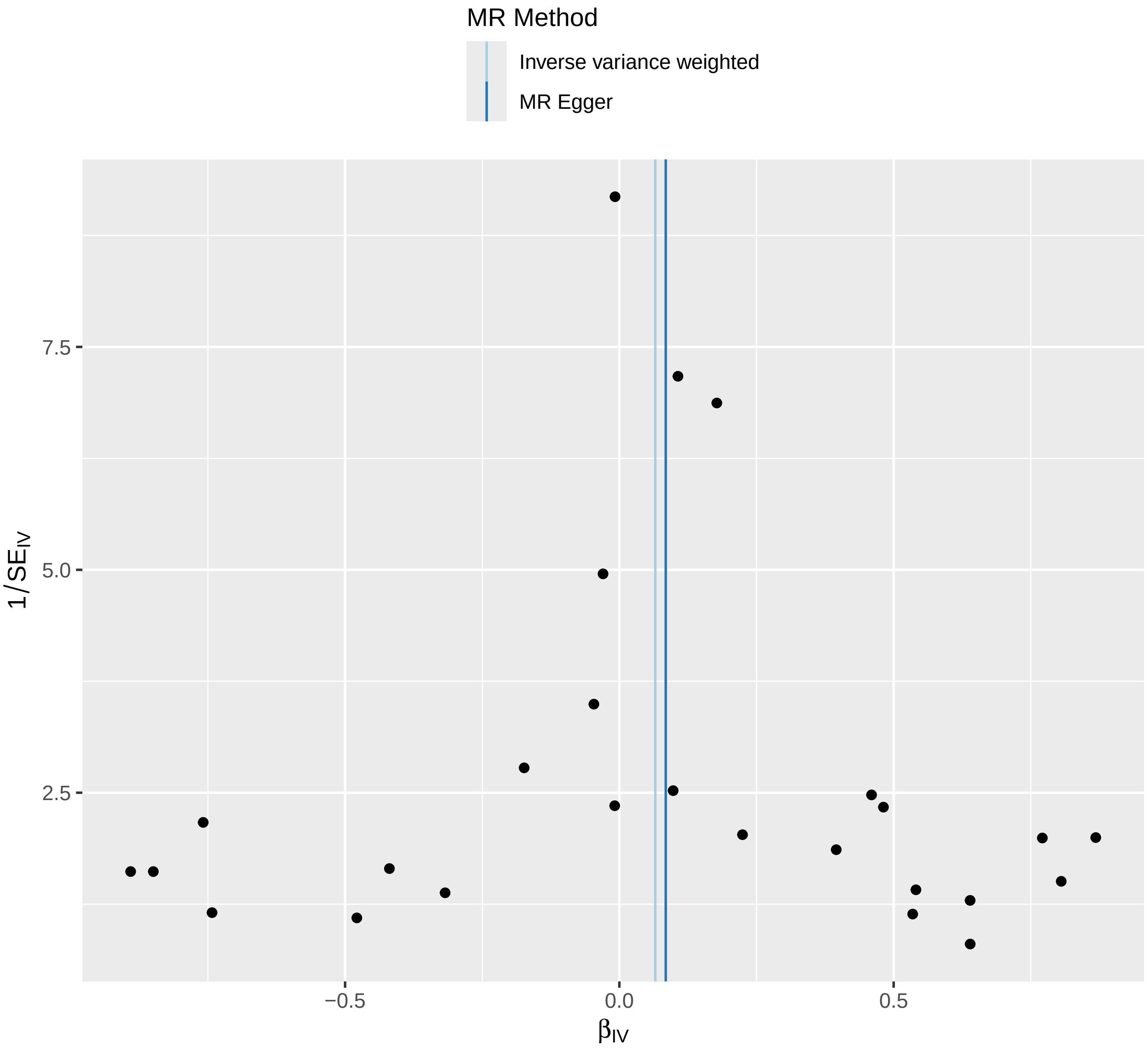
MR Method
Inverse variance weighted
MR Egger
1/SE_IV
7.5
5.0
2.5
−0.5
0.0
0.5
β_IV

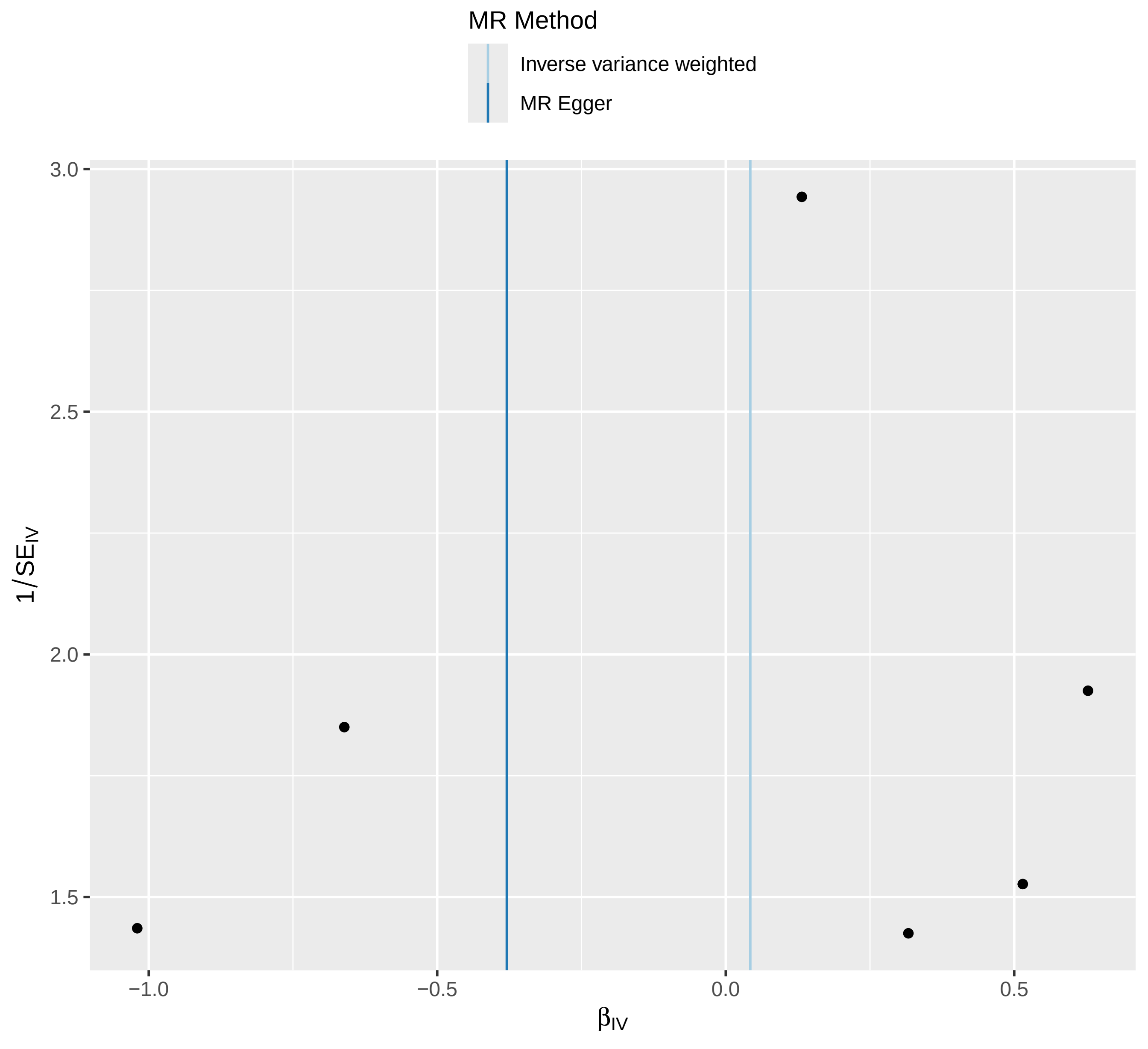
MR Method
Inverse variance weighted
MR Egger
3.0
2.5
2.0
1.5
1/SE$_{IV}$
−1.0
−0.5
0.0
0.5
β$_{IV}$

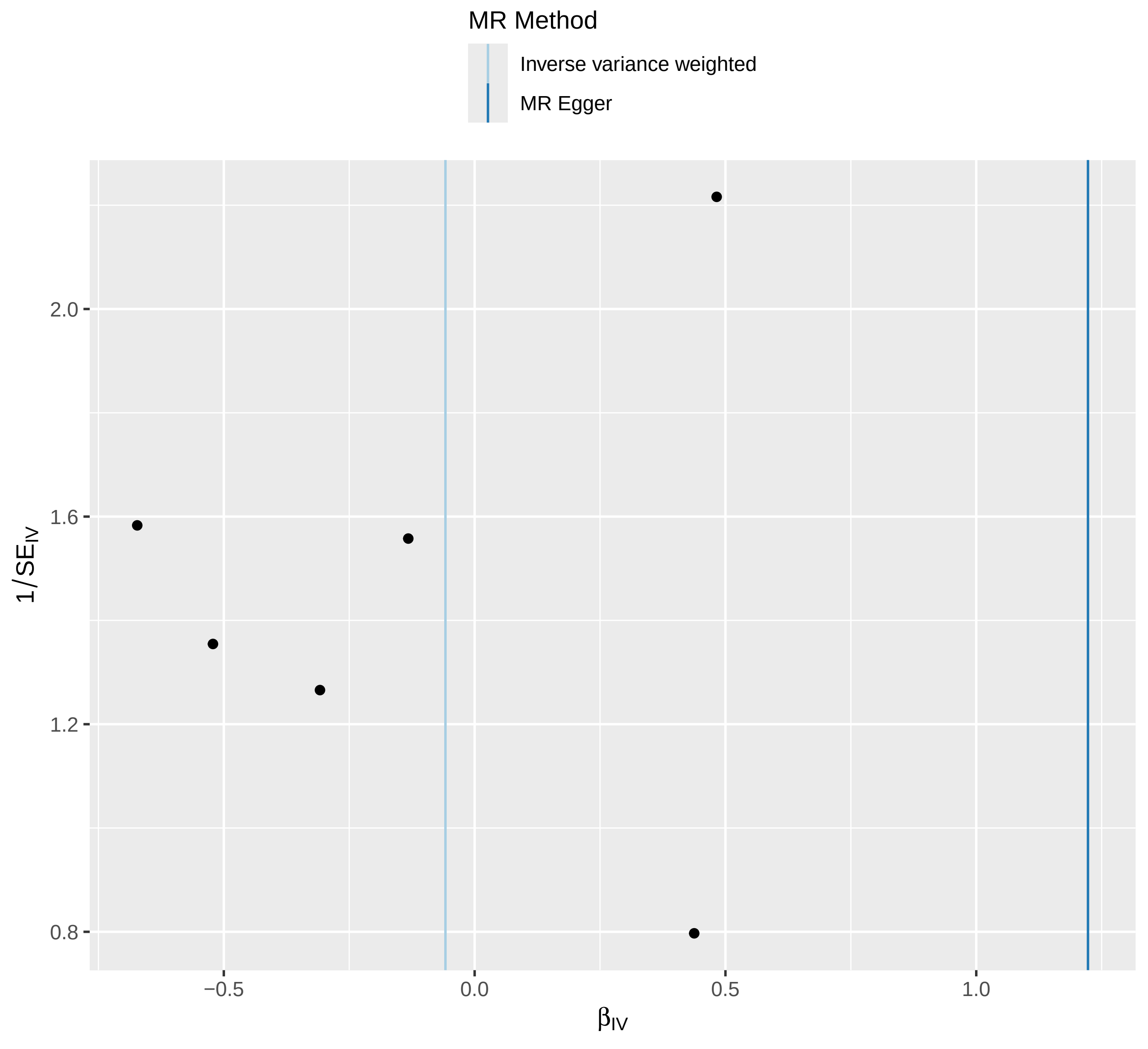

MR Method
Inverse variance weighted
MR Egger
2.0
1.6
1.2
0.8
1/SE_IV
−0.5
0.0
0.5
1.0
β_IV

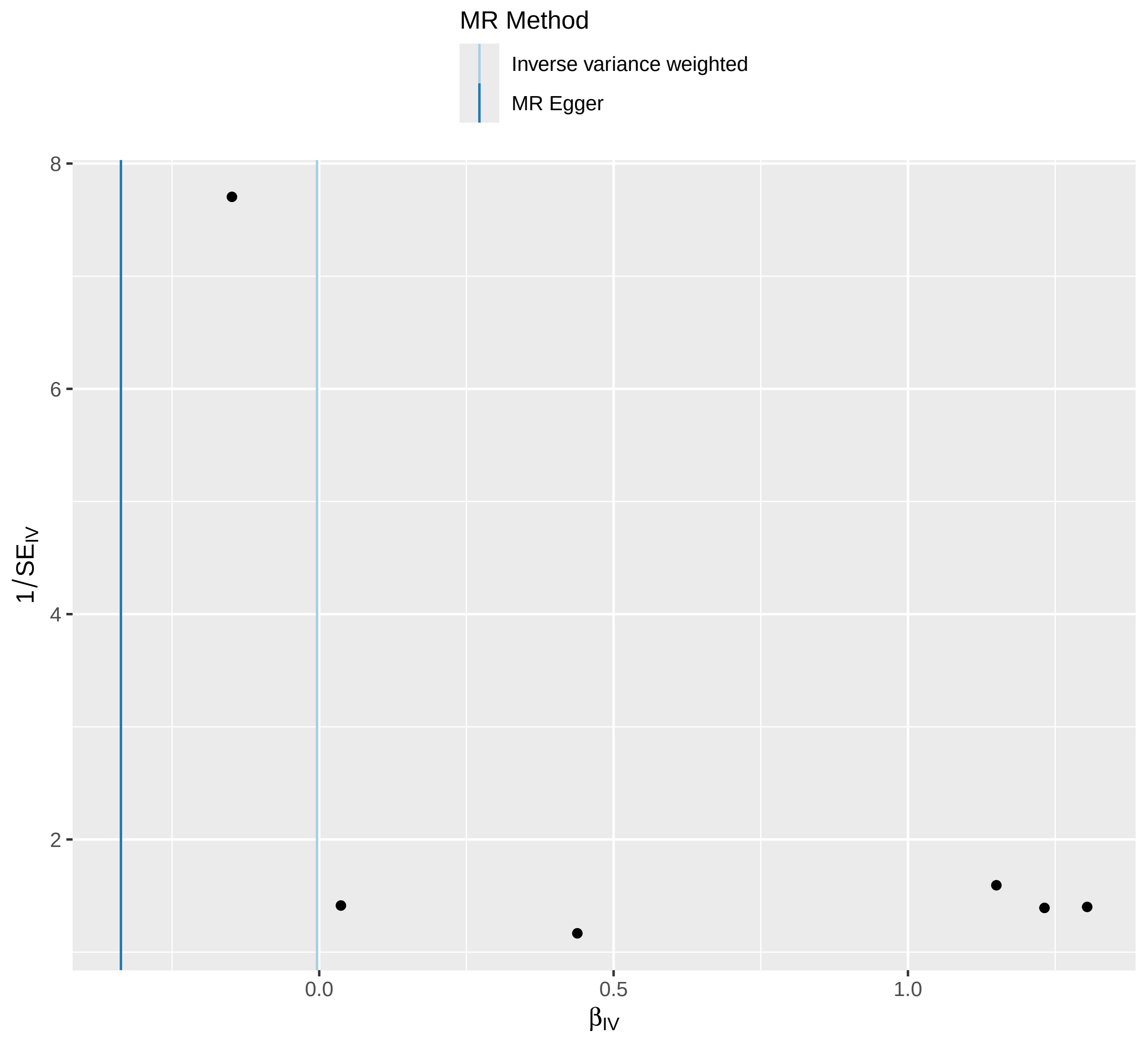
MR Method
Inverse variance weighted
MR Egger
8
6
4
2
0.0
0.5
1.0
$1/SE_{IV}$
β_{IV}

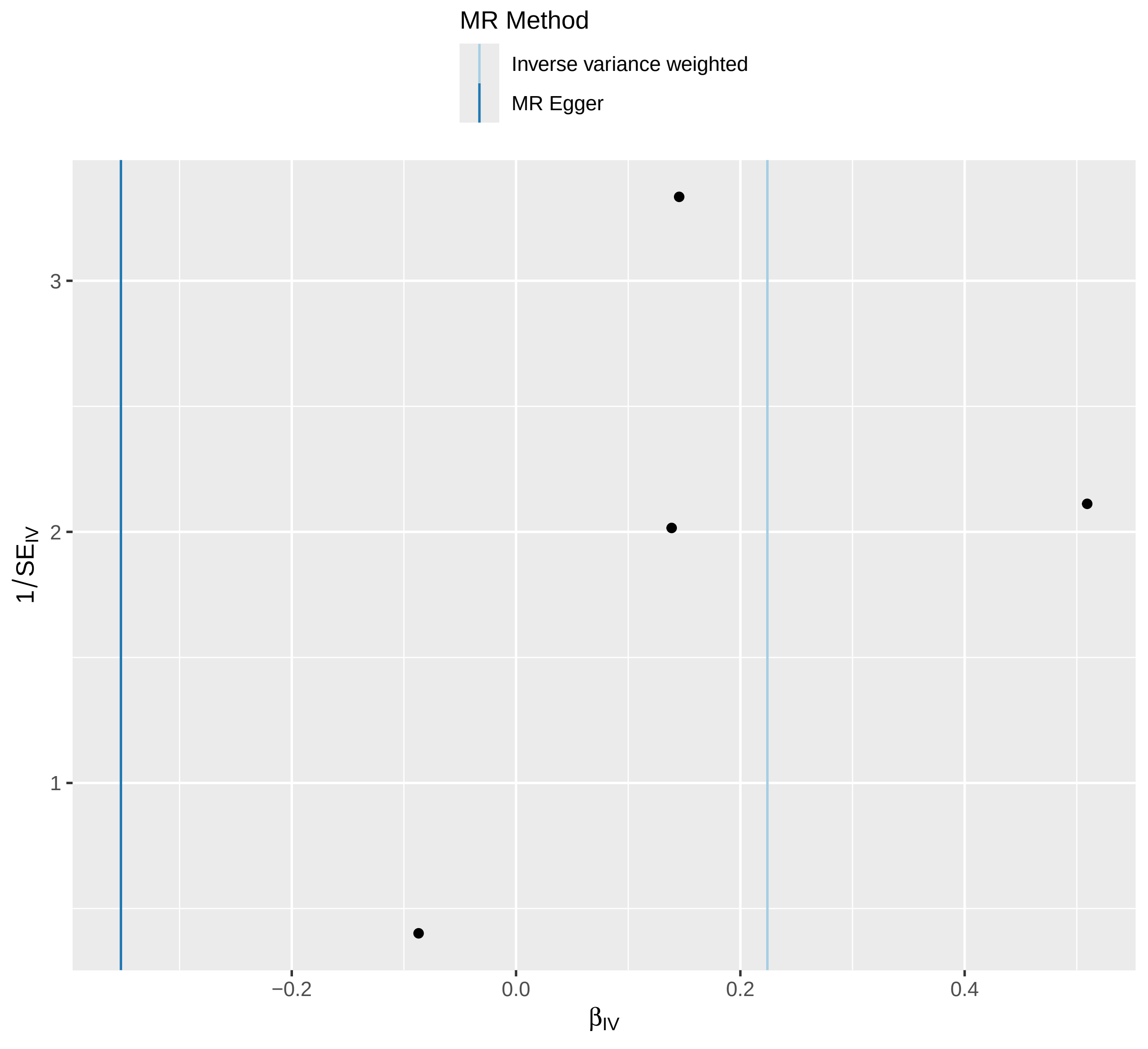

MR Method
Inverse variance weighted
MR Egger
3
2
1
1/SE_IV
−0.2
0.0
0.2
0.4
β_IV

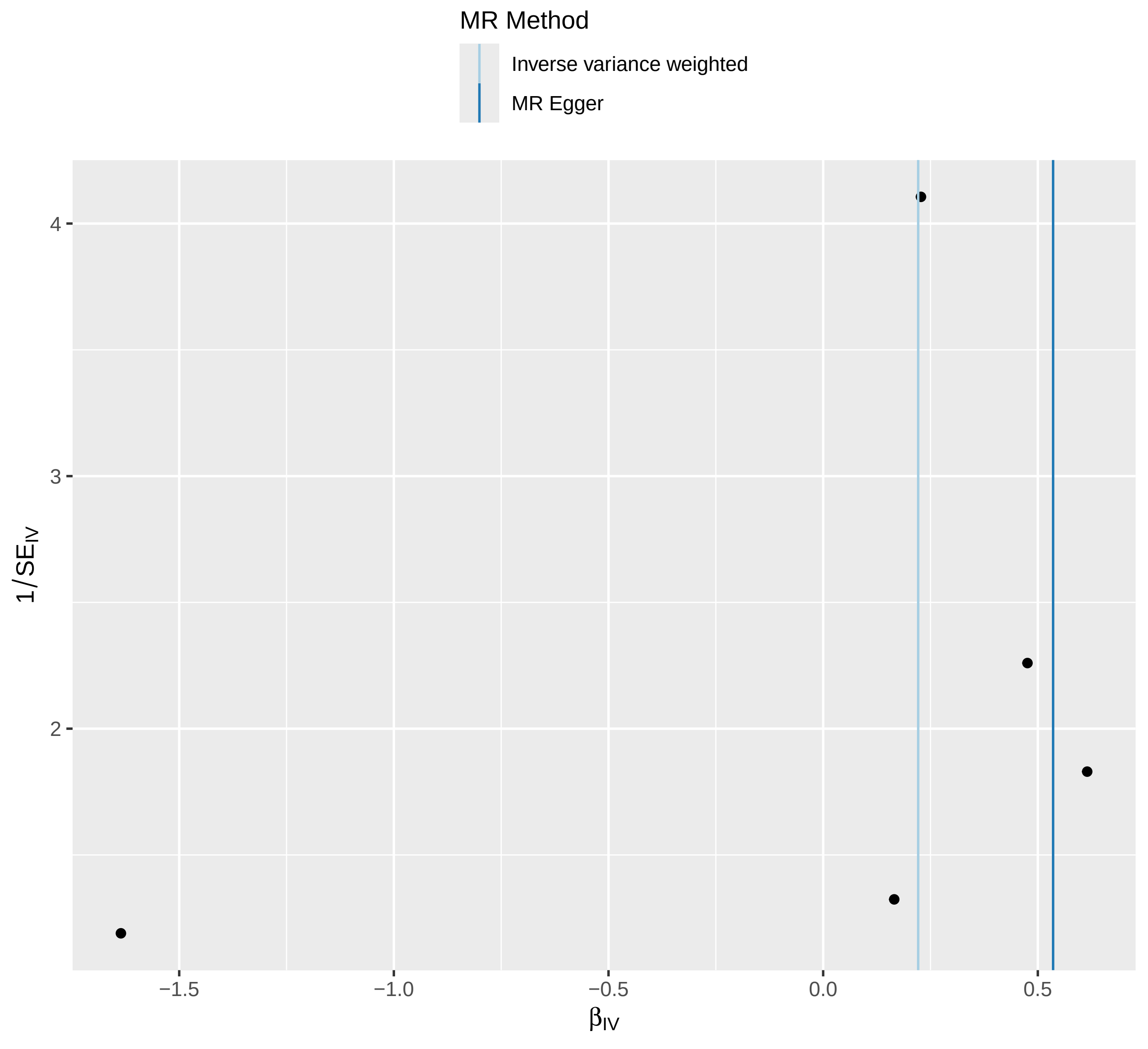

MR Method
Inverse variance weighted
MR Egger
4
3
2
1/SE_IV
−1.5
−1.0
−0.5
0.0
0.5
β_IV

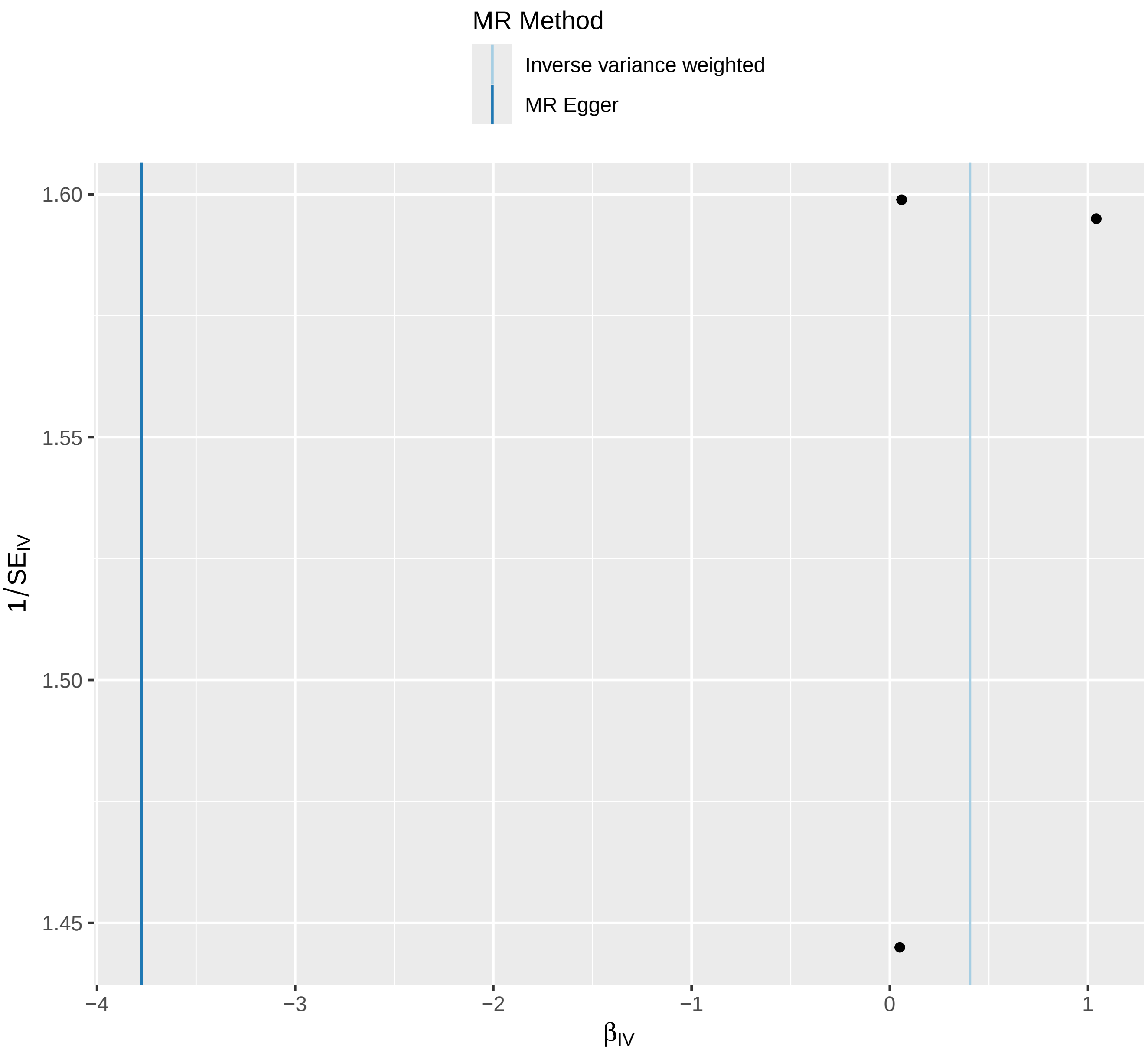

MR Method
Inverse variance weighted
MR Egger
1.60
1.55
1.50
1.45
1/SE$_{IV}$
−4
−3
−2
−1
0
1
β$_{IV}$

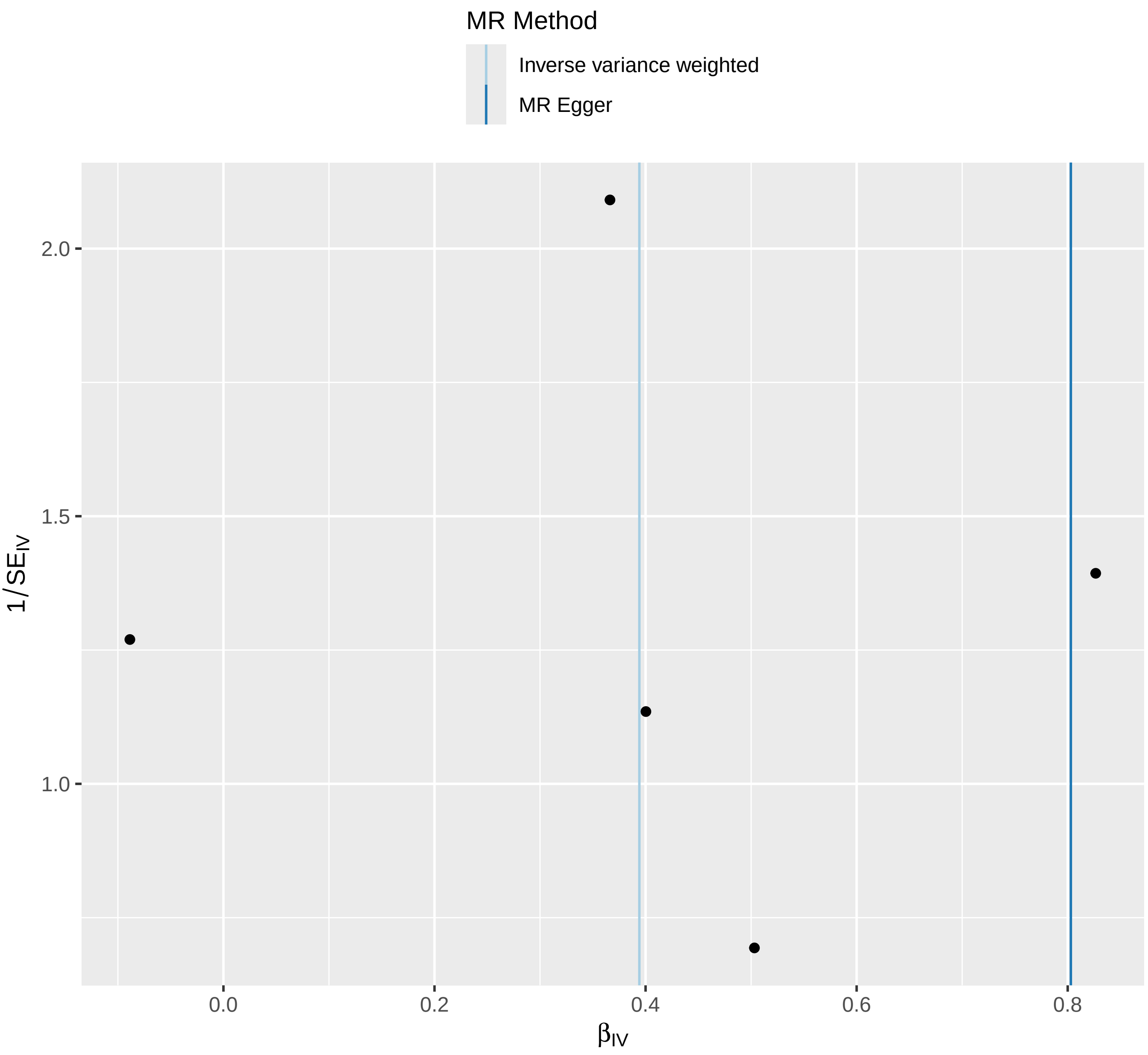
MR Method
Inverse variance weighted
MR Egger
2.0
1.5
1.0
1/SE$_{IV}$
0.0
0.2
0.4
0.6
0.8
β_{IV}

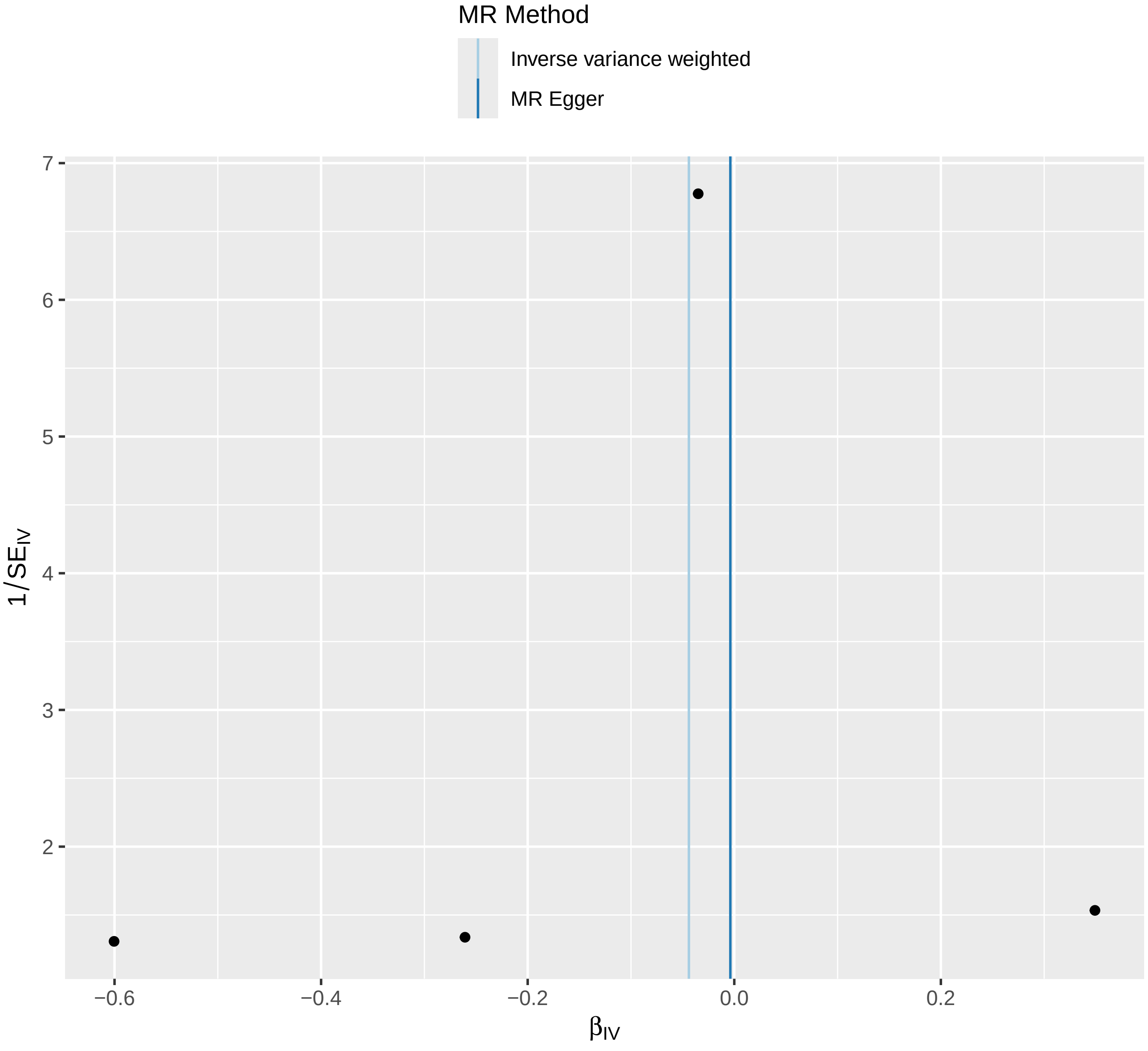

MR Method
Inverse variance weighted
MR Egger
1/SE$_{IV}$
7
6
5
4
3
2
−0.6
−0.4
−0.2
0.0
0.2
β$_{IV}$

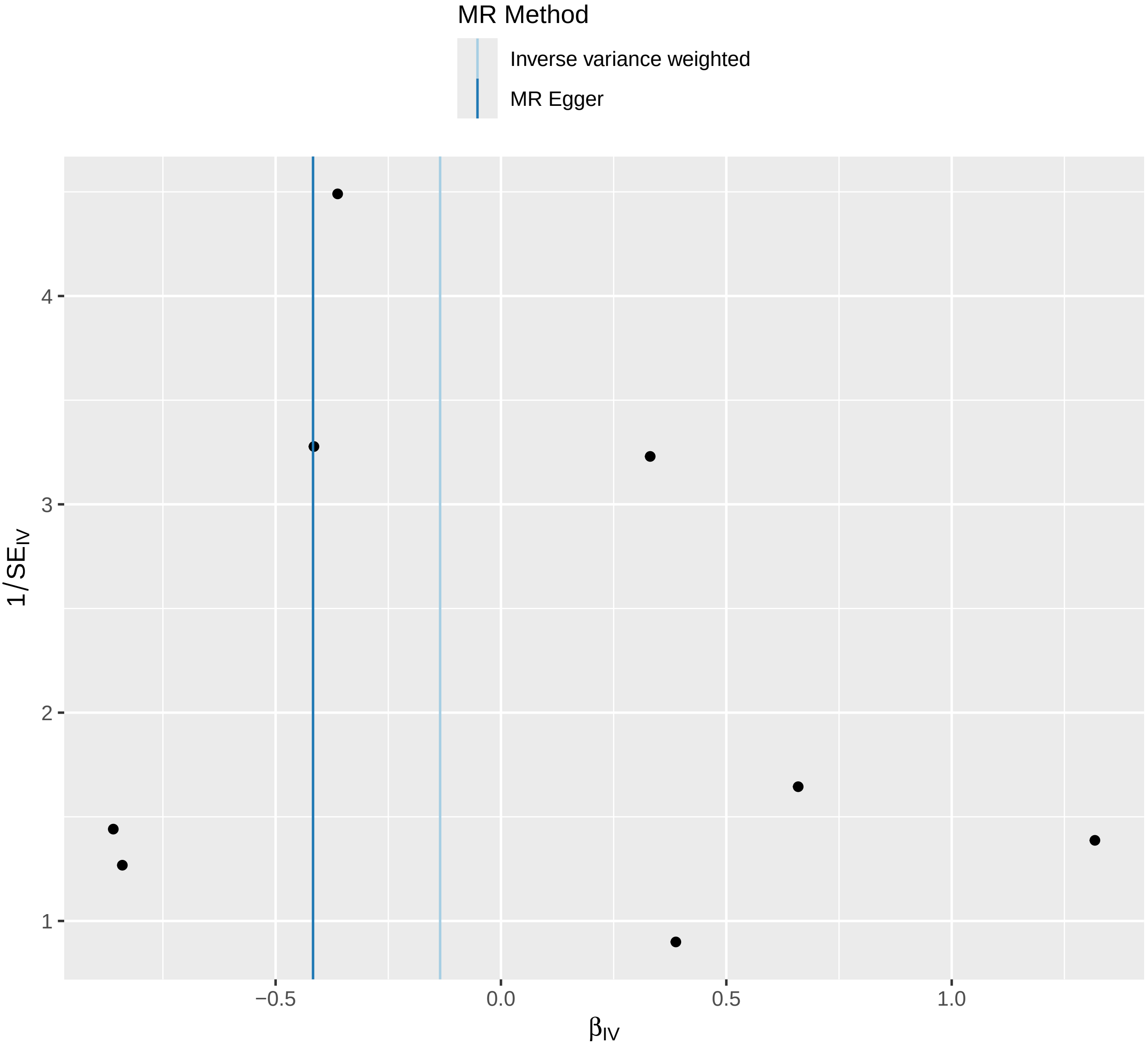

MR Method
Inverse variance weighted
MR Egger
4
3
2
1
1/SE_IV
−0.5
0.0
0.5
1.0
β_IV

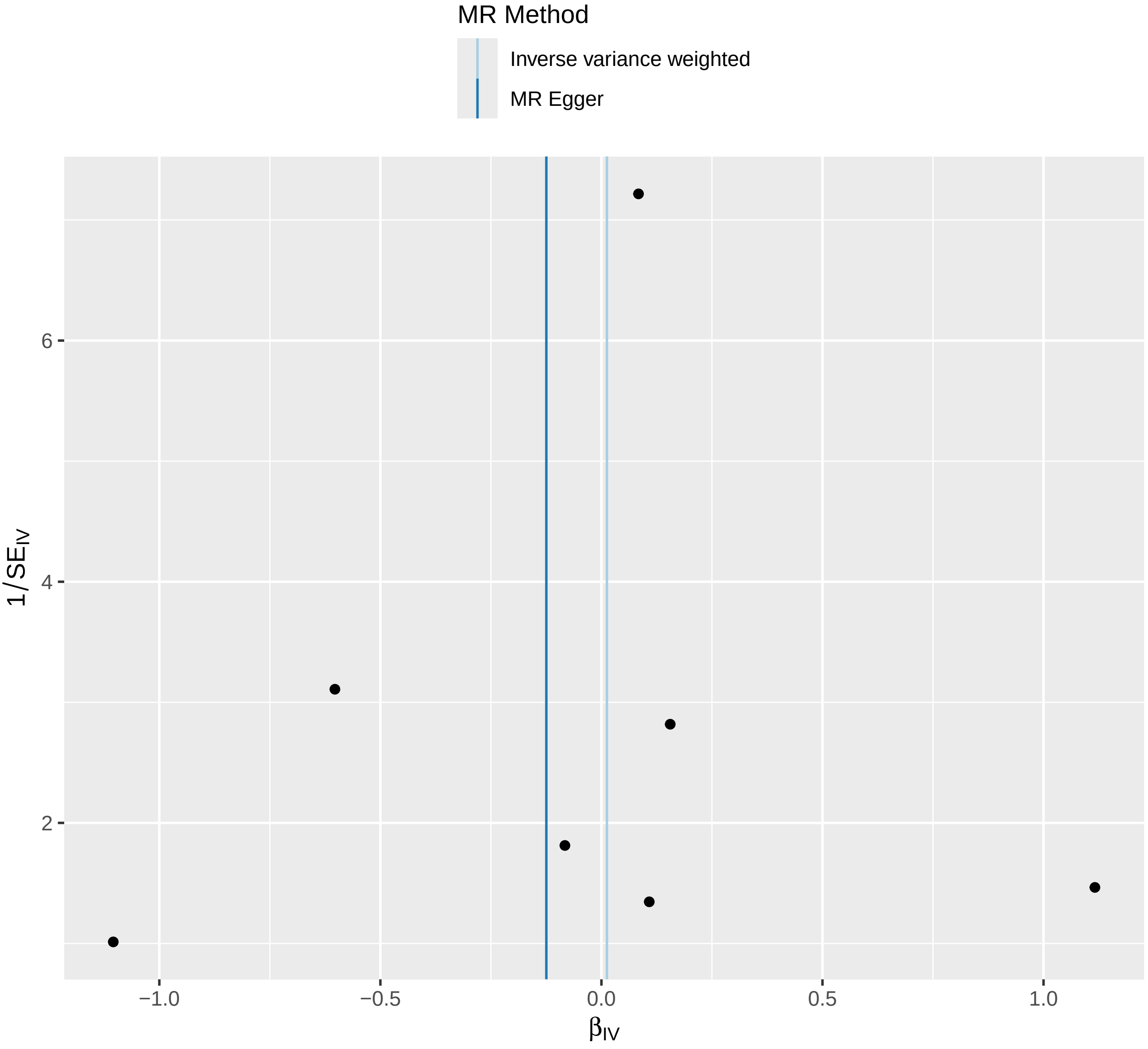

MR Method
Inverse variance weighted
MR Egger
6
4
2
1/SE$_{IV}$
−1.0
−0.5
0.0
0.5
1.0
β$_{IV}$

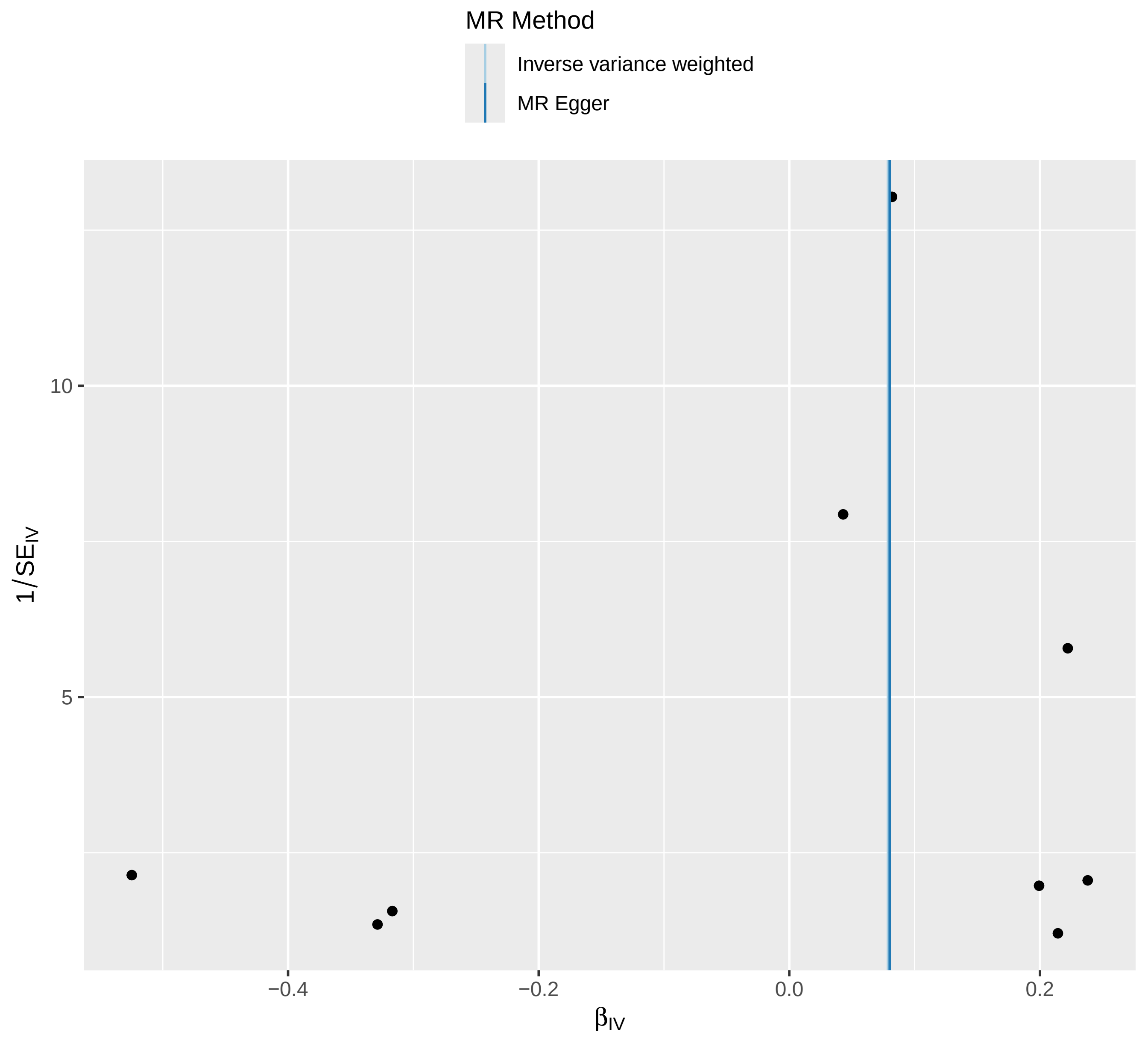
MR Method
Inverse variance weighted
MR Egger
10
5
1/SE_IV
−0.4
−0.2
0.0
0.2
β_IV

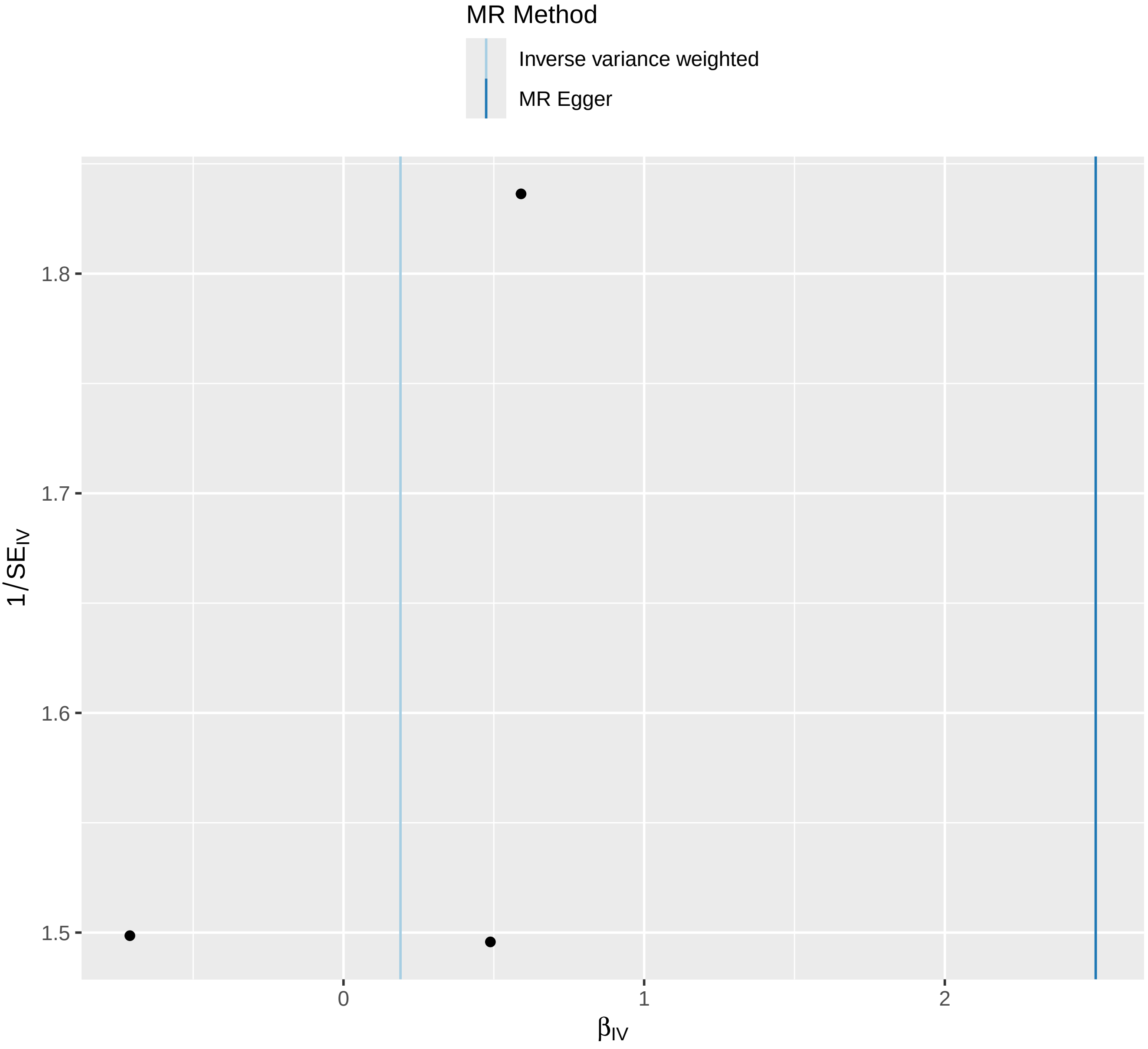

MR Method
Inverse variance weighted
MR Egger
1.8
1.7
1.6
1.5
1/SE$_{IV}$
0
1
2
β$_{IV}$

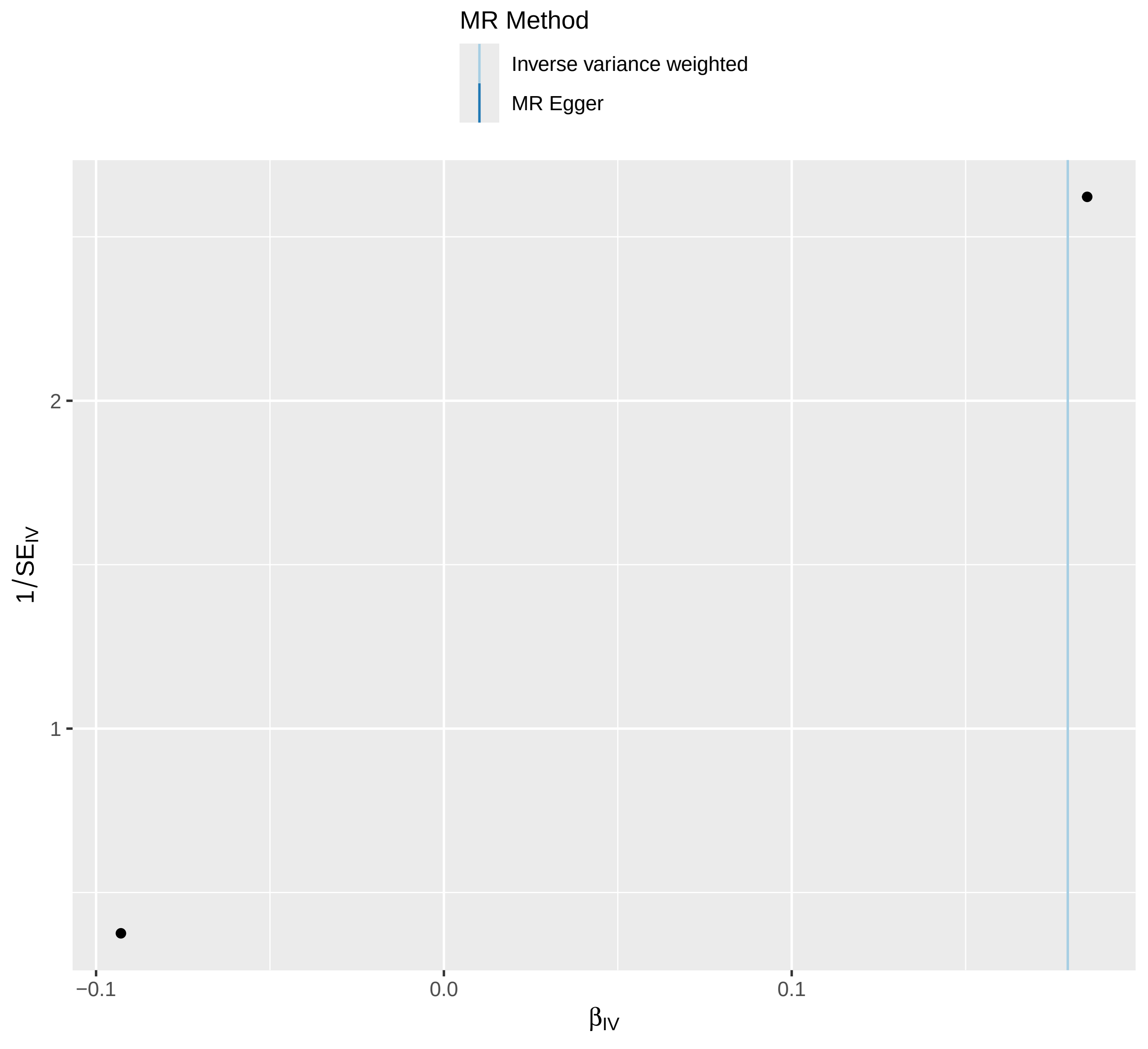

MR Method
Inverse variance weighted
MR Egger
2
1
1/SE_IV
−0.1
0.0
0.1
β_IV

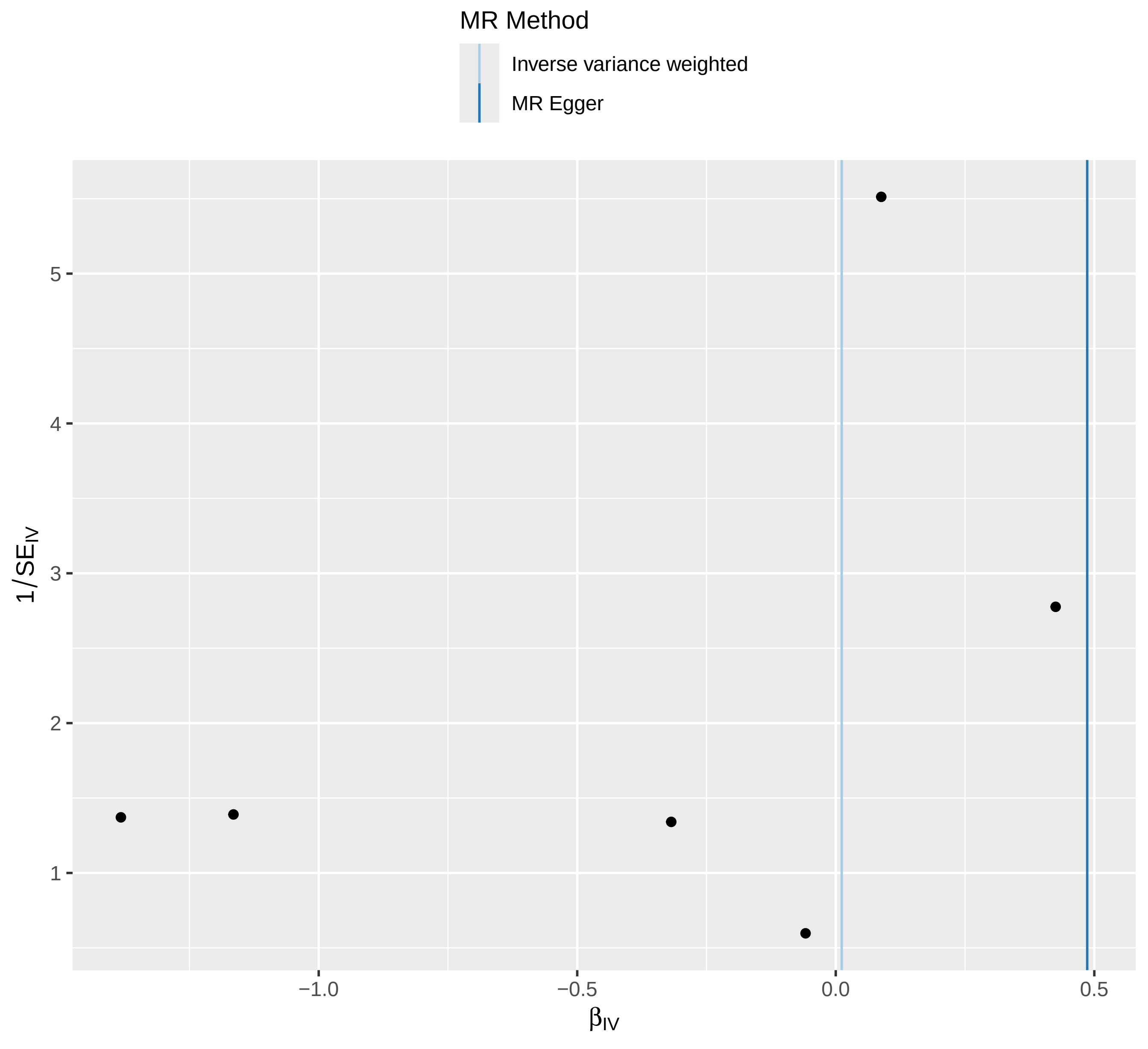
MR Method
Inverse variance weighted
MR Egger
1/SE_IV
5
4
3
2
1
−1.0
−0.5
0.0
0.5
β_IV

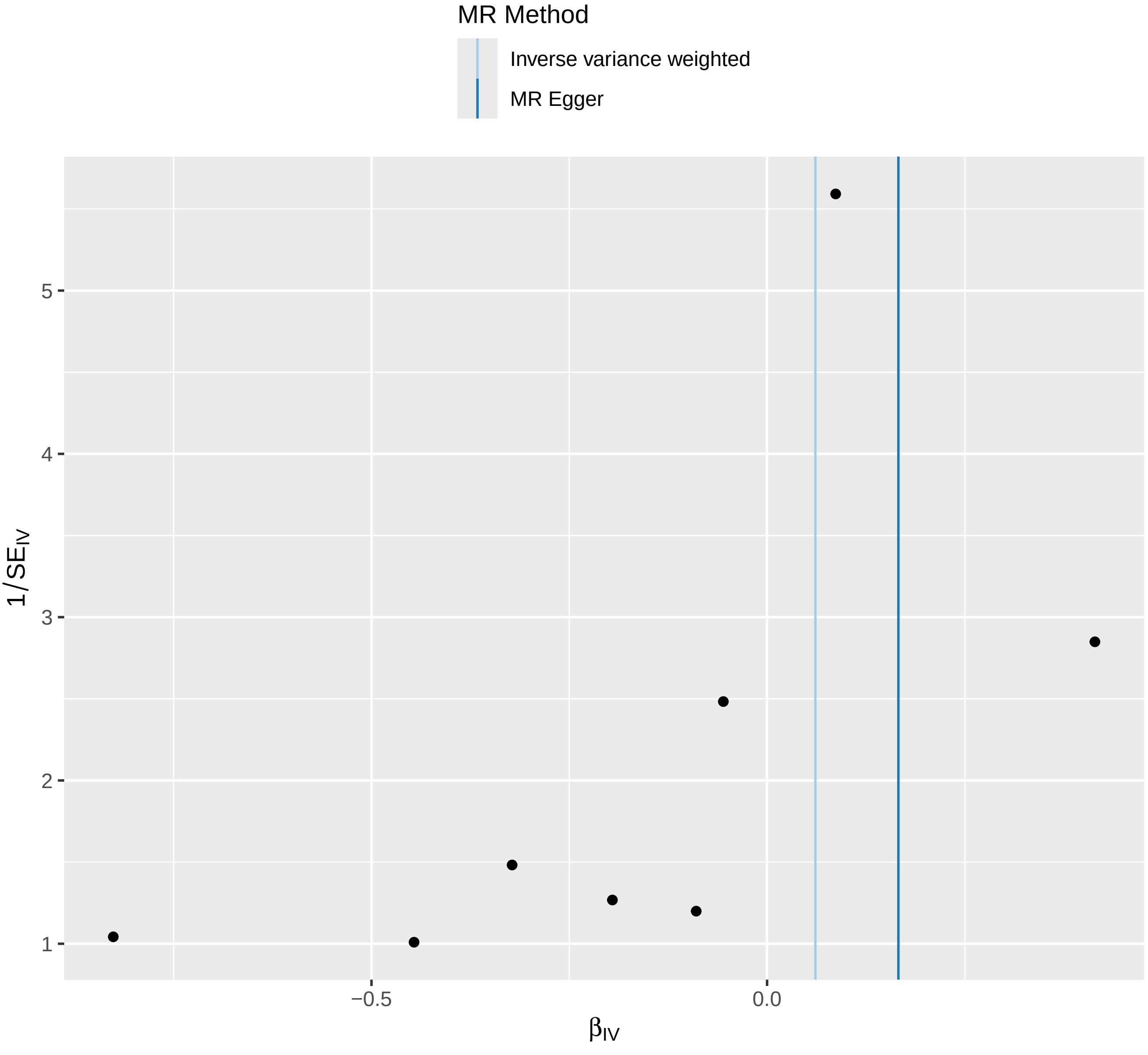
MR Method
Inverse variance weighted
MR Egger
5
4
3
2
1
1/SE_IV
−0.5
0.0
β_IV

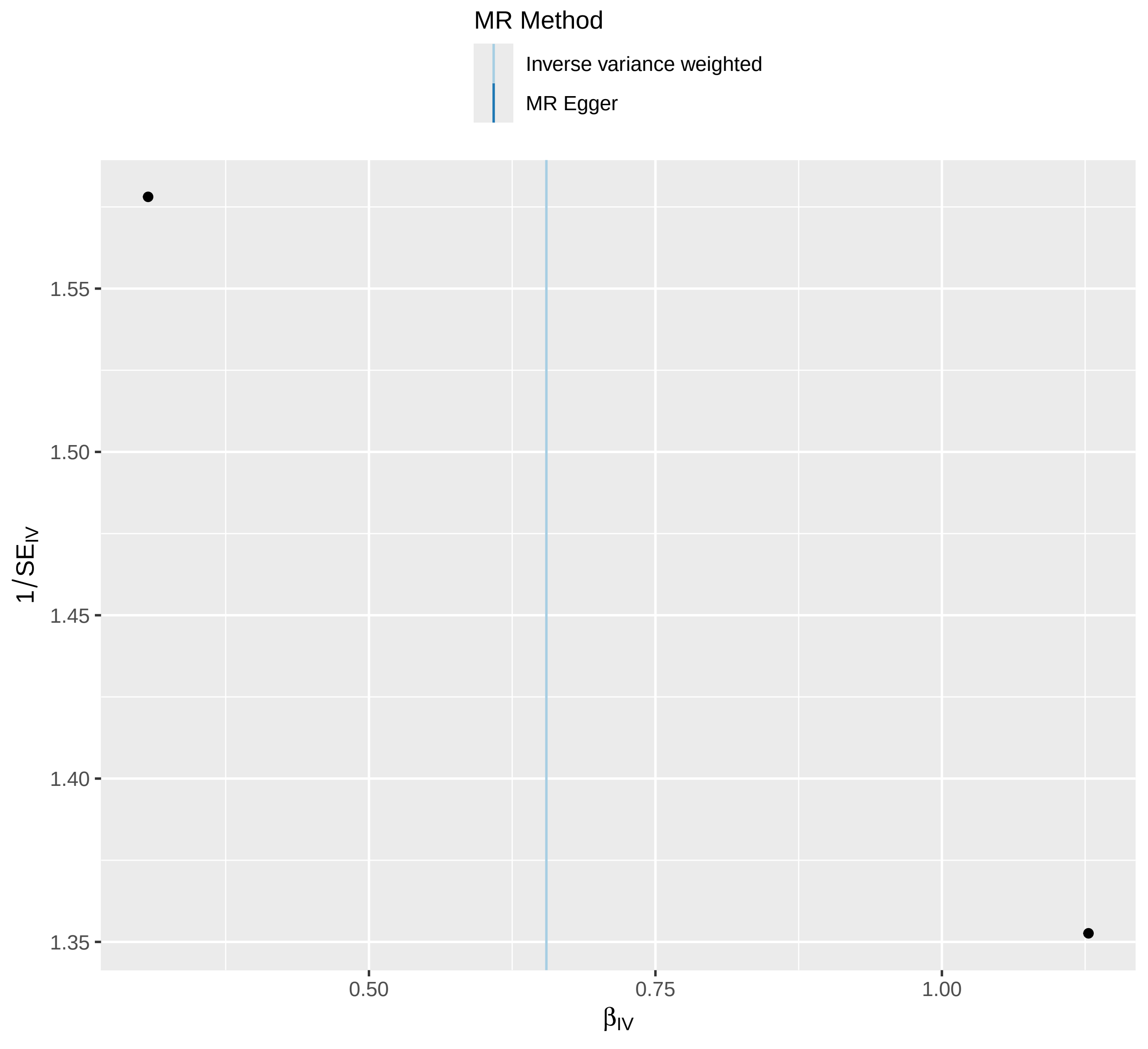
MR Method
Inverse variance weighted
MR Egger
1.55
1.50
1.45
1.40
1.35
1/SE_{IV}
0.50
0.75
1.00
β_{IV}

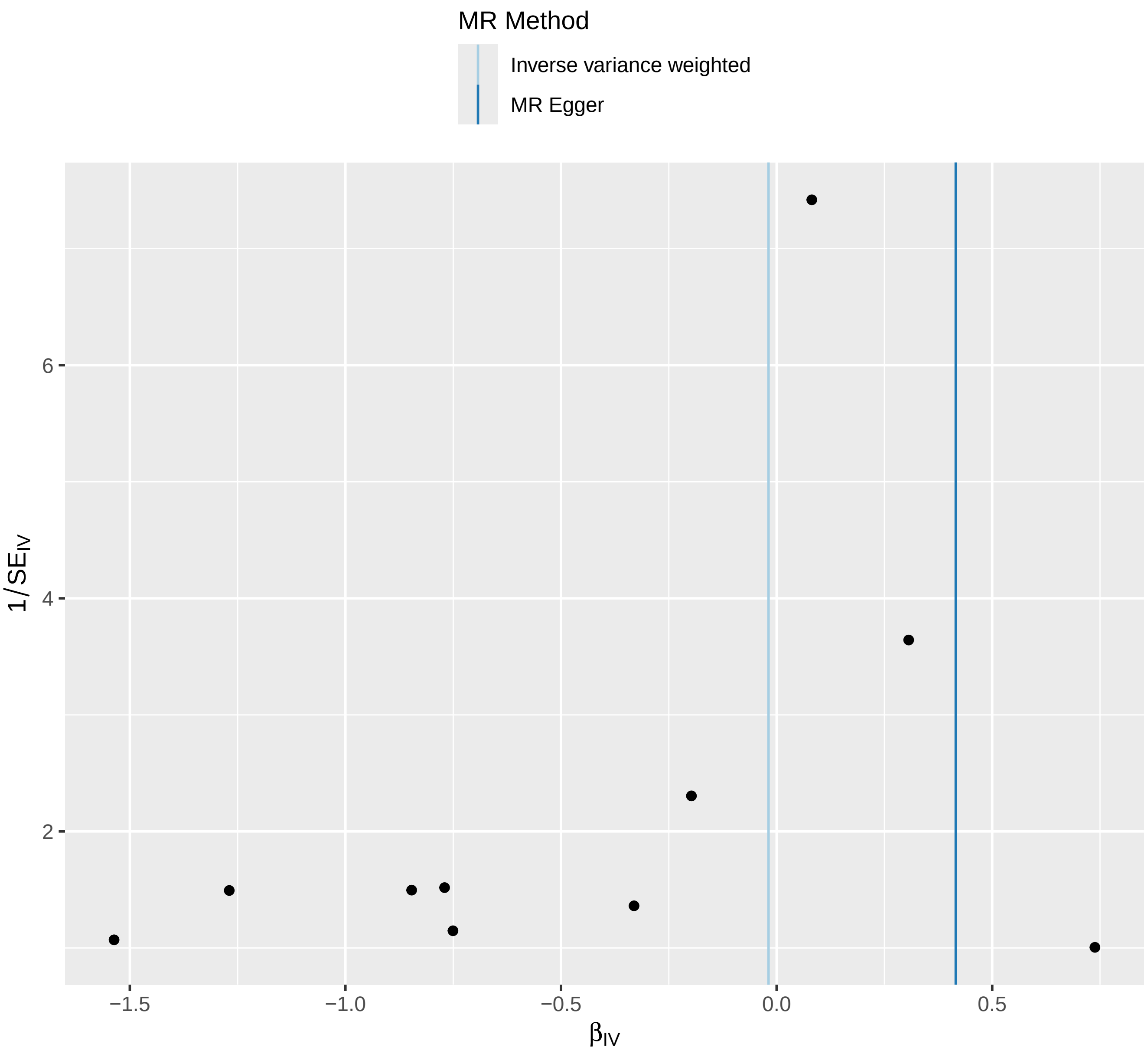
MR Method
Inverse variance weighted
MR Egger
1/SE_IV
6
4
2
−1.5
−1.0
−0.5
0.0
0.5
β_IV

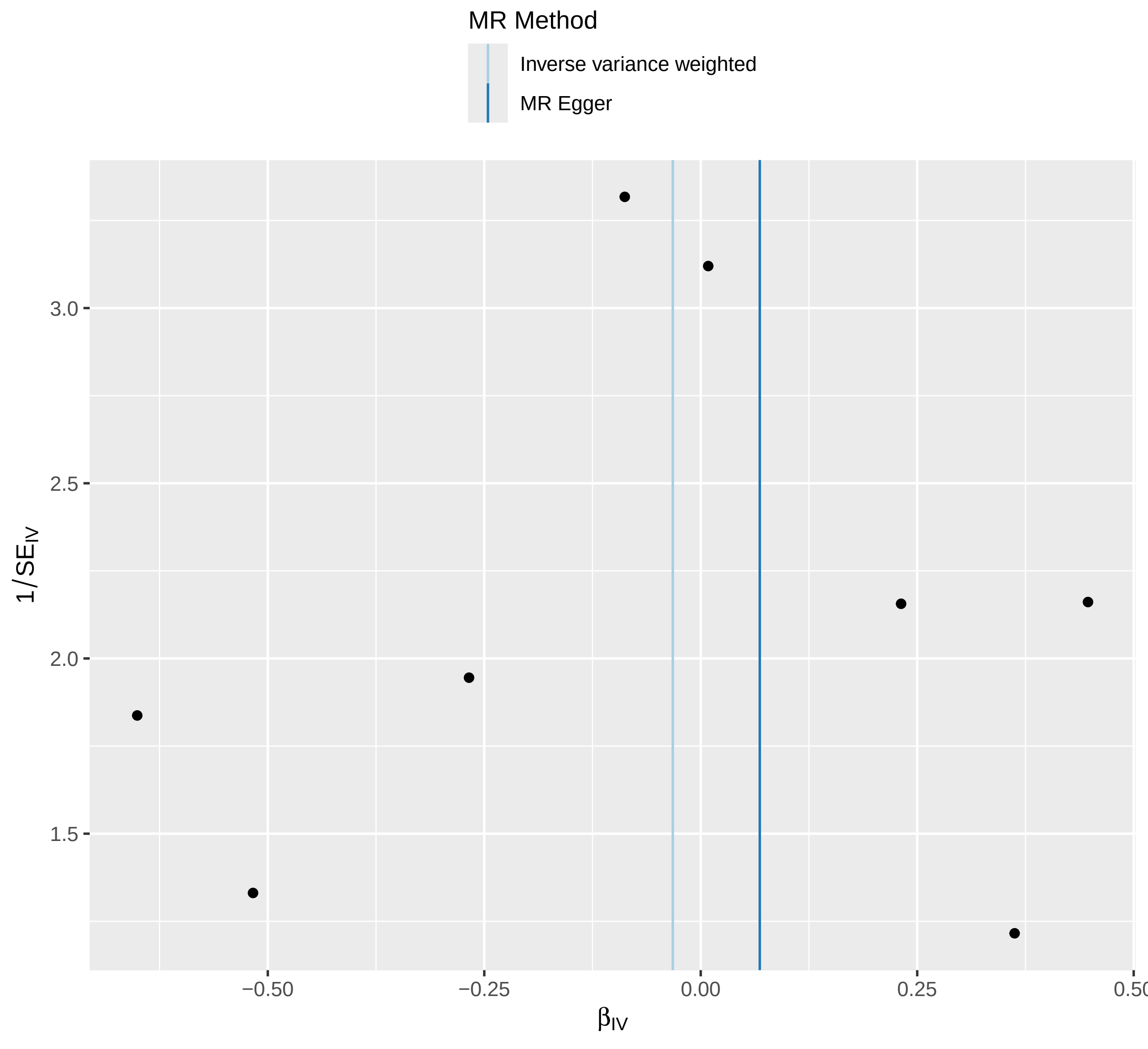
MR Method
Inverse variance weighted
MR Egger
3.0
2.5
2.0
1.5
1/SE_IV
−0.50
−0.25
0.00
0.25
0.50
β_IV

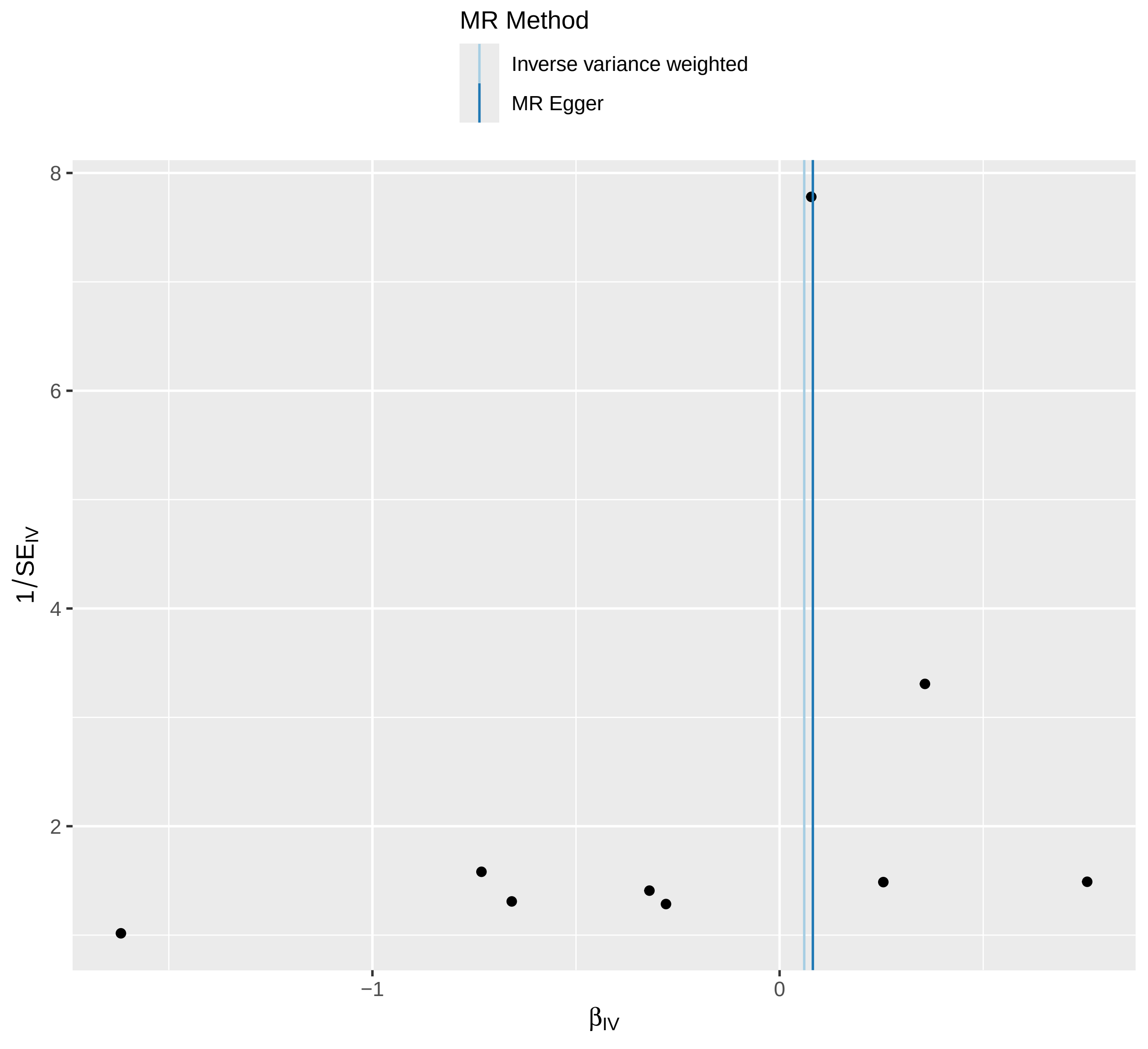
MR Method
Inverse variance weighted
MR Egger
8
6
4
2
−1
0
1/SE$_{IV}$
β$_{IV}$

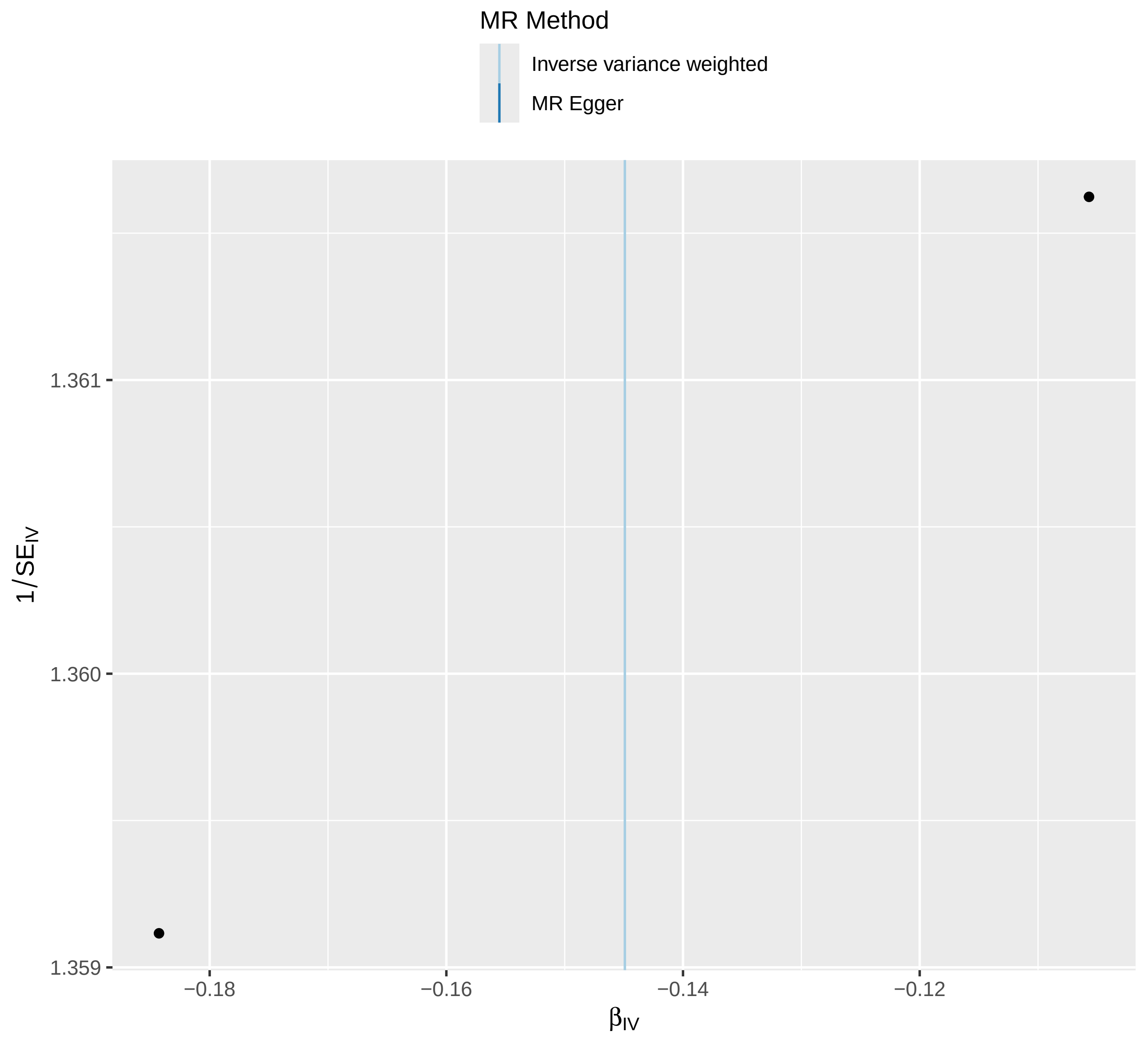
MR Method
Inverse variance weighted
MR Egger
1.361
1.360
1.359
$1/SE_{IV}$
−0.18
−0.16
−0.14
−0.12
β_{IV}

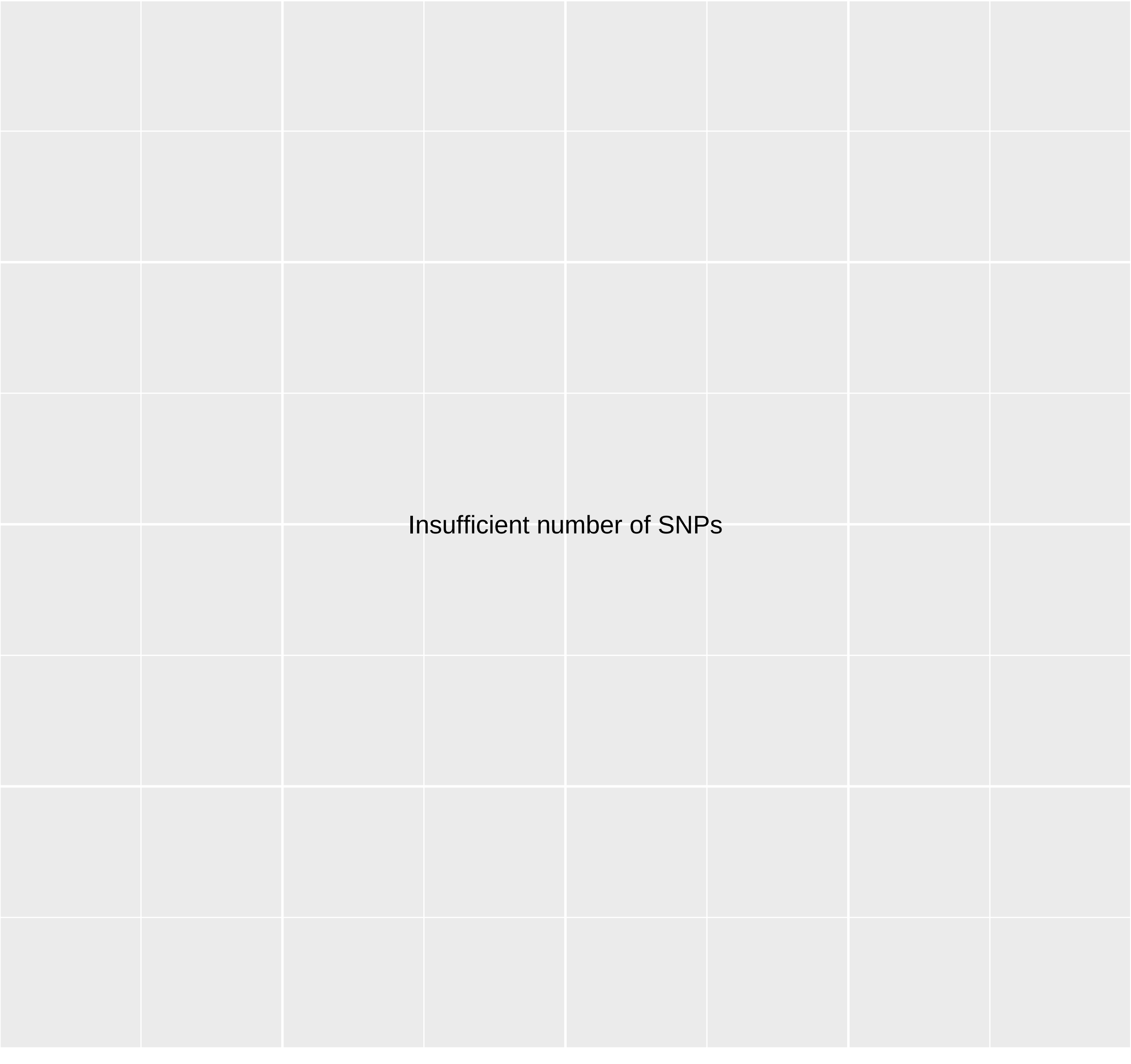
Insufficient number of SNPs

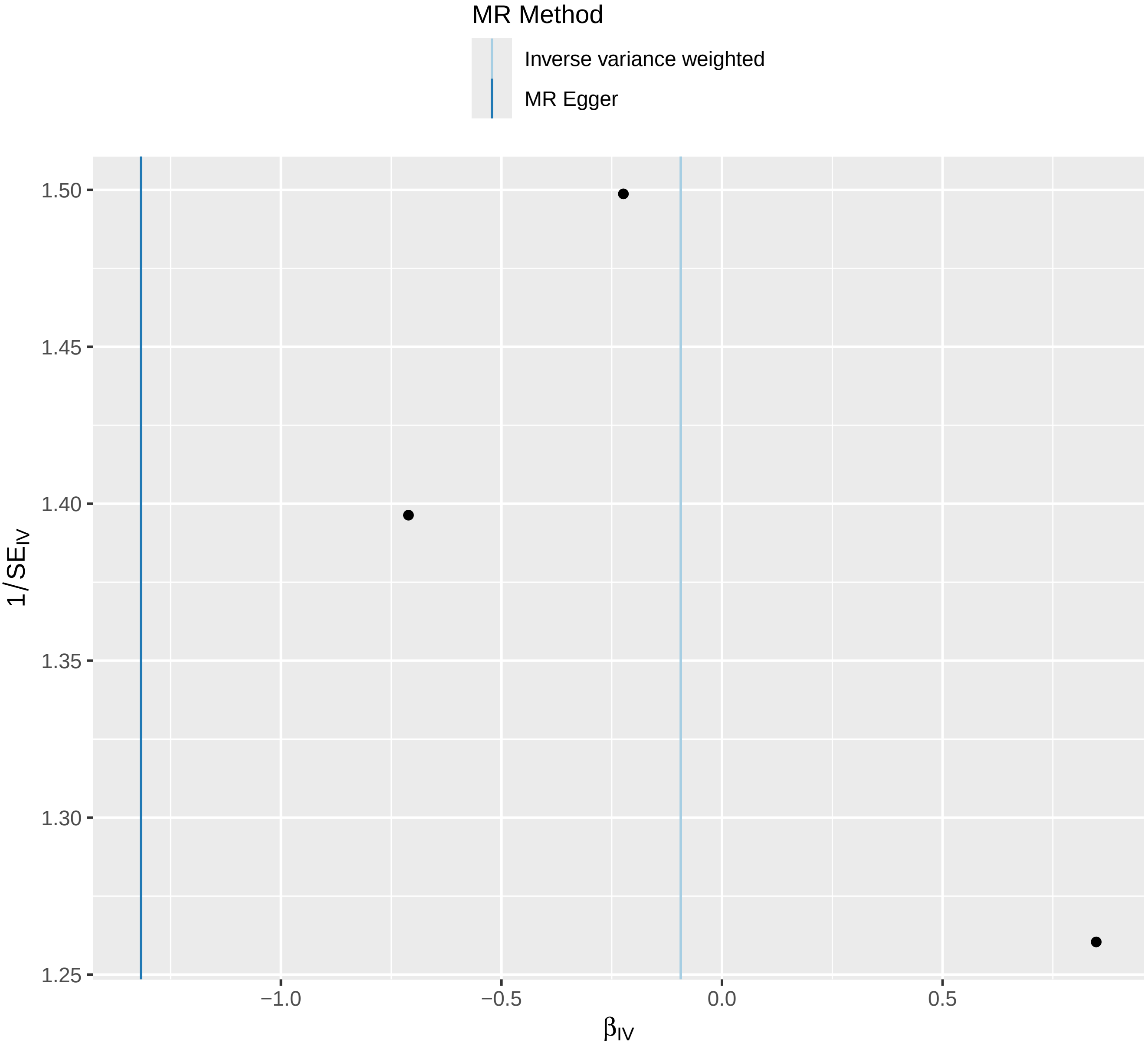
MR Method
Inverse variance weighted
MR Egger
1.50
1.45
1.40
1.35
1.30
1.25
1/SE$_{IV}$
−1.0
−0.5
0.0
0.5
β$_{IV}$

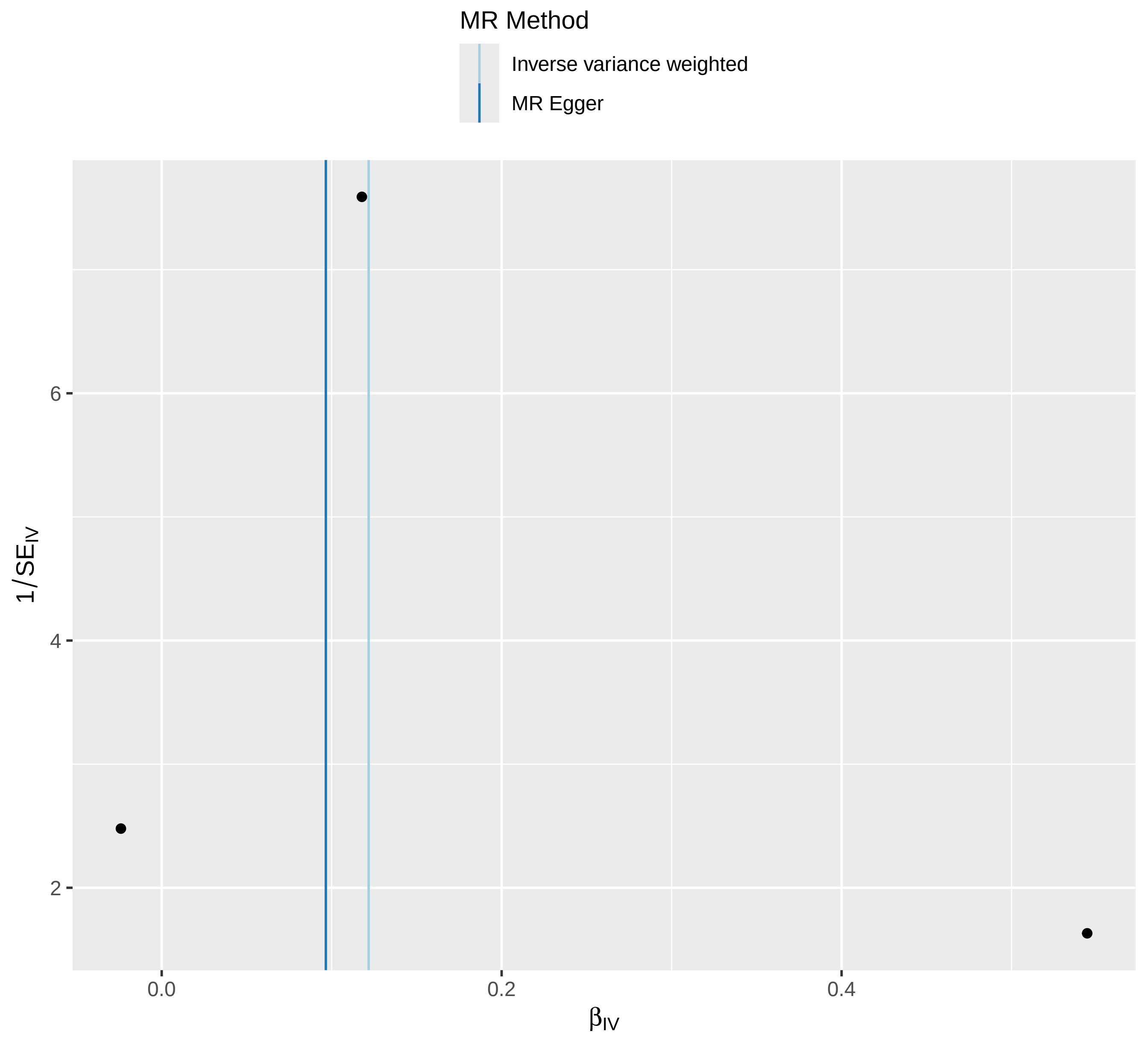

MR Method
Inverse variance weighted
MR Egger
6
4
2
1/SE$_{IV}$
0.0
0.2
0.4
β$_{IV}$

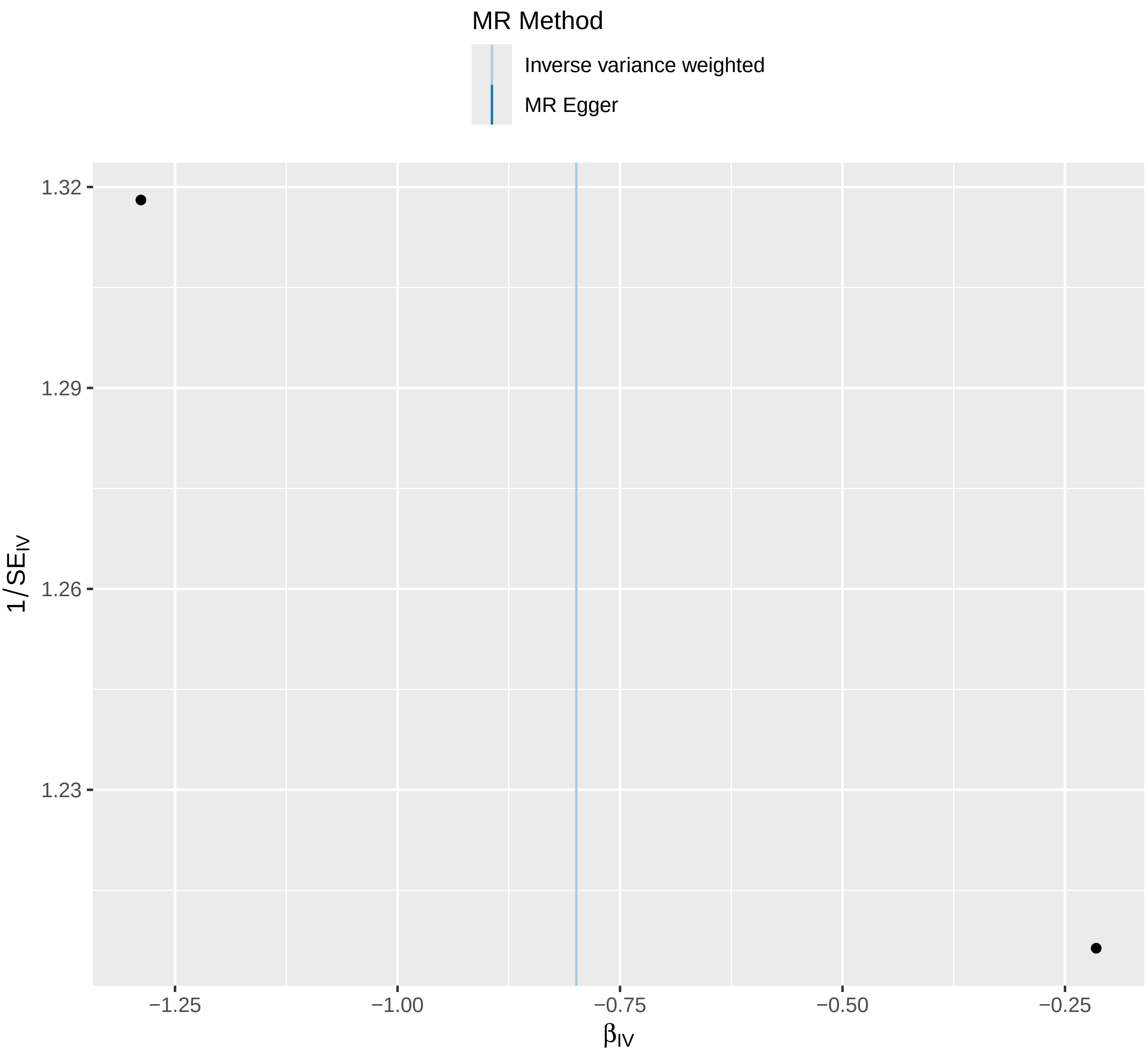
MR Method
Inverse variance weighted
MR Egger
1.32
1.29
1.26
1.23
1/SE_IV
−1.25
−1.00
−0.75
−0.50
−0.25
β_IV

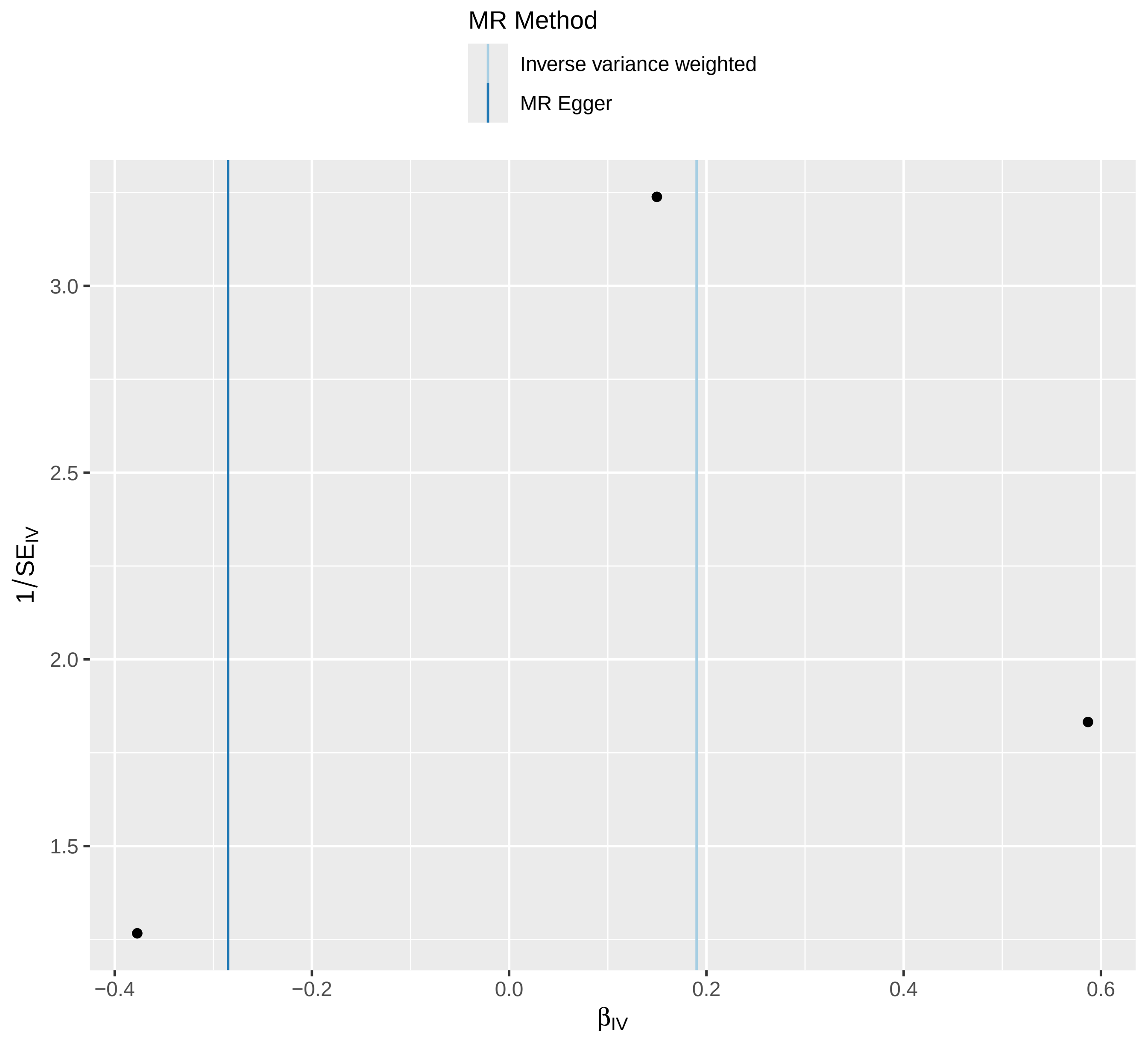

MR Method
Inverse variance weighted
MR Egger
3.0
2.5
2.0
1.5
$1/SE_{IV}$
−0.4
−0.2
0.0
0.2
0.4
0.6
β_{IV}

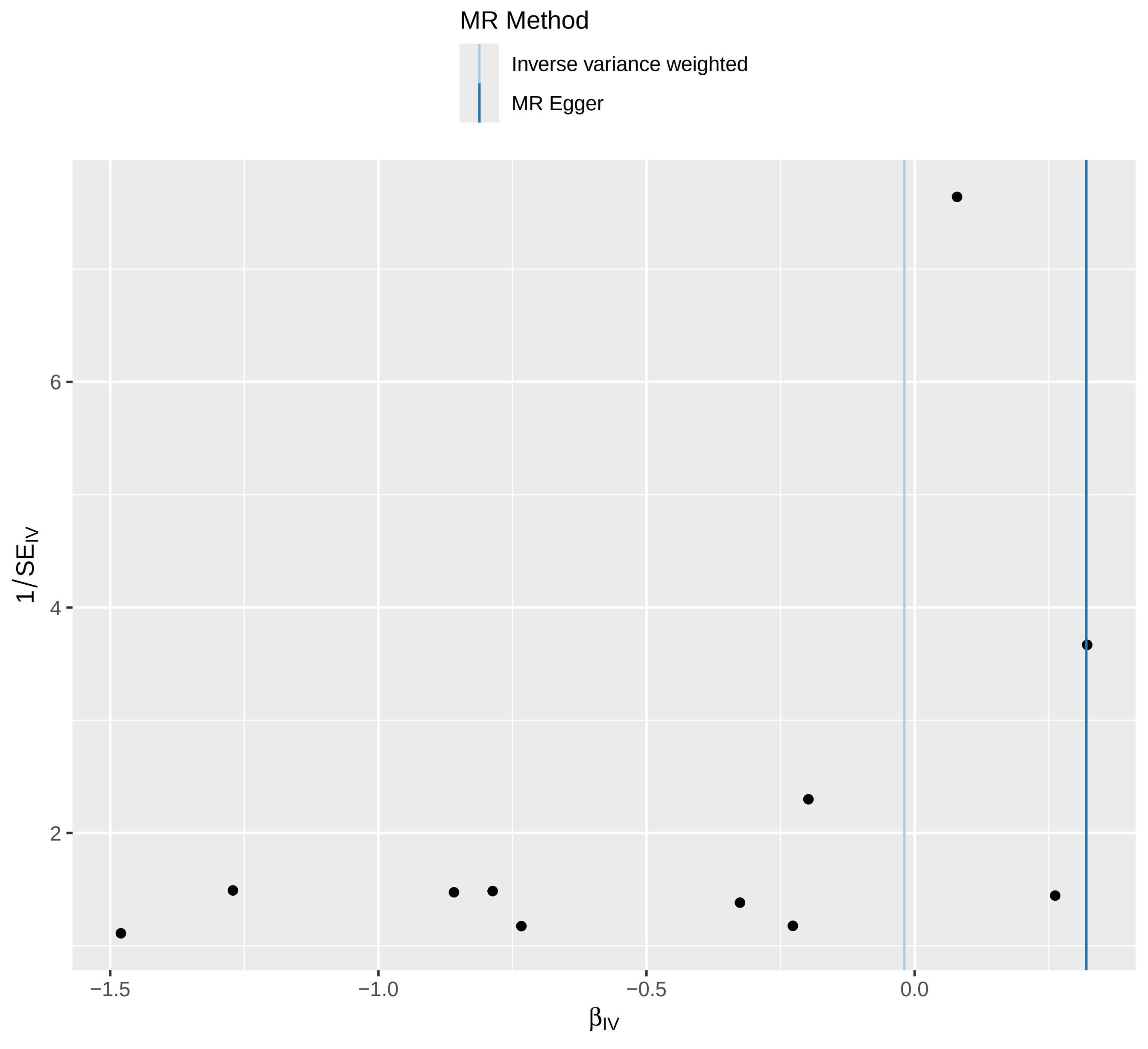

MR Method
Inverse variance weighted
MR Egger
1/SE$_{IV}$
6
4
2
−1.5
−1.0
−0.5
0.0
β$_{IV}$

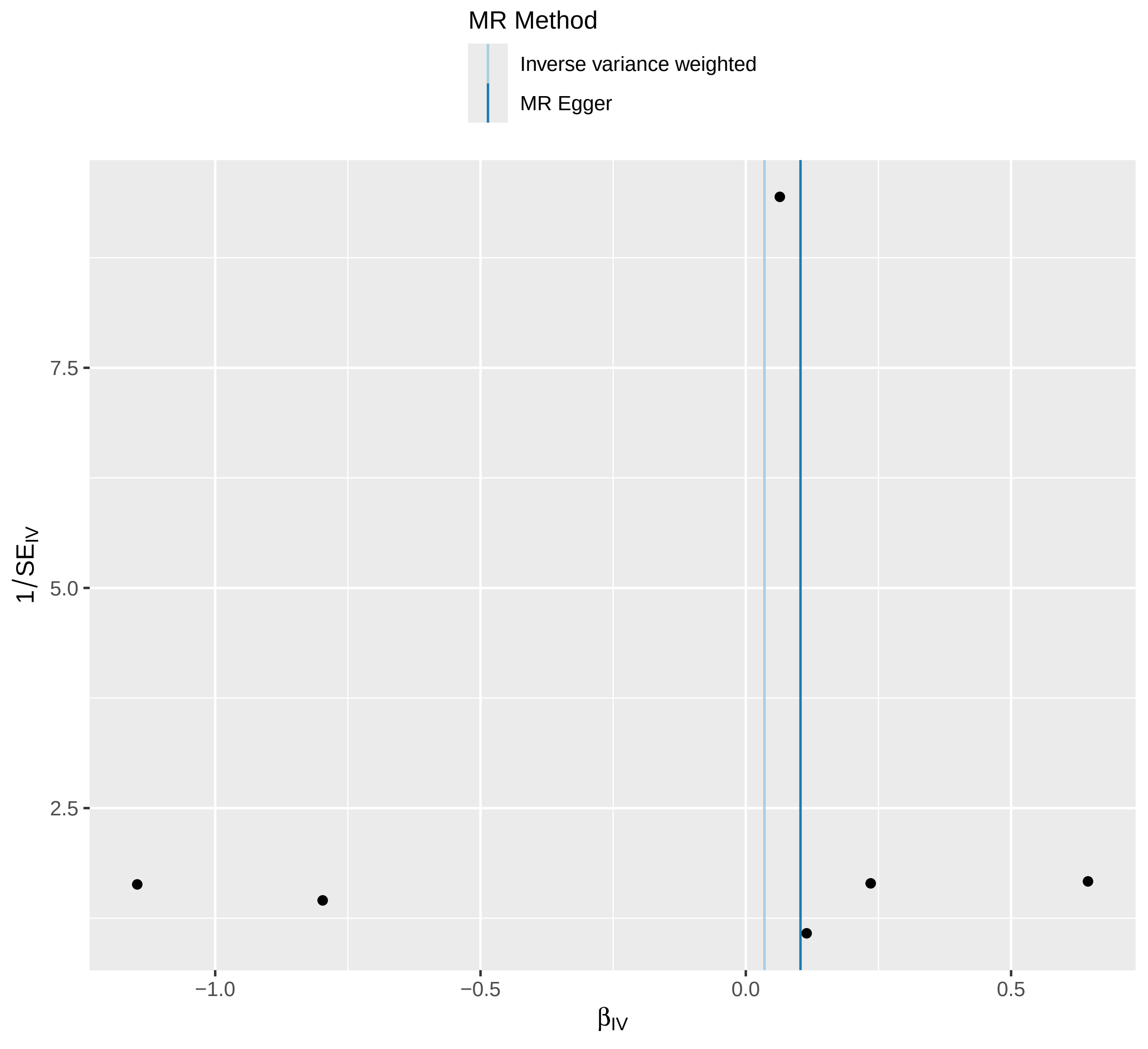

MR Method
Inverse variance weighted
MR Egger
7.5
5.0
2.5
1/SE_IV
−1.0
−0.5
0.0
0.5
β_IV

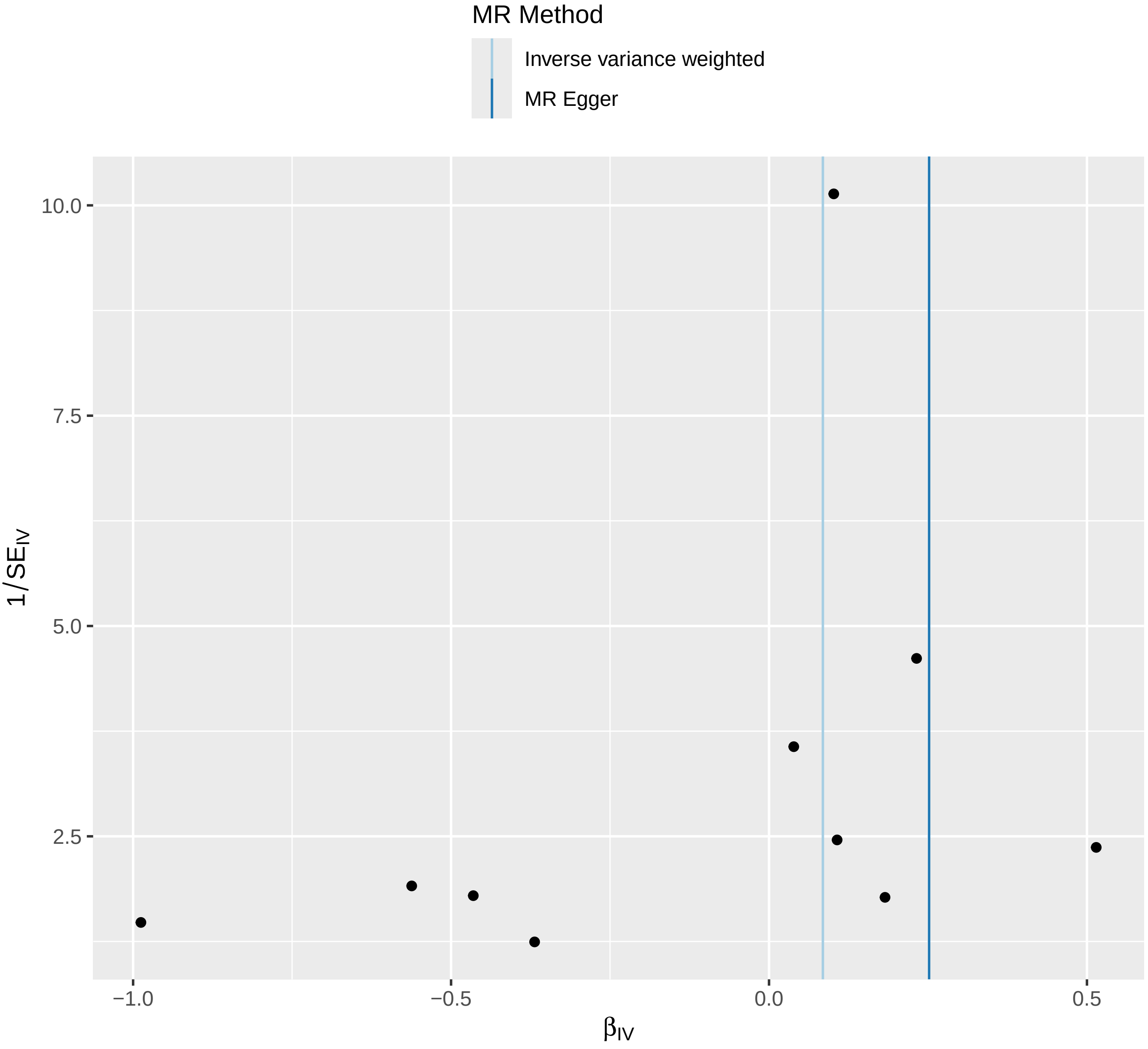
MR Method
Inverse variance weighted
MR Egger
10.0
7.5
5.0
2.5
1/SE_IV
−1.0
−0.5
0.0
0.5
β_IV

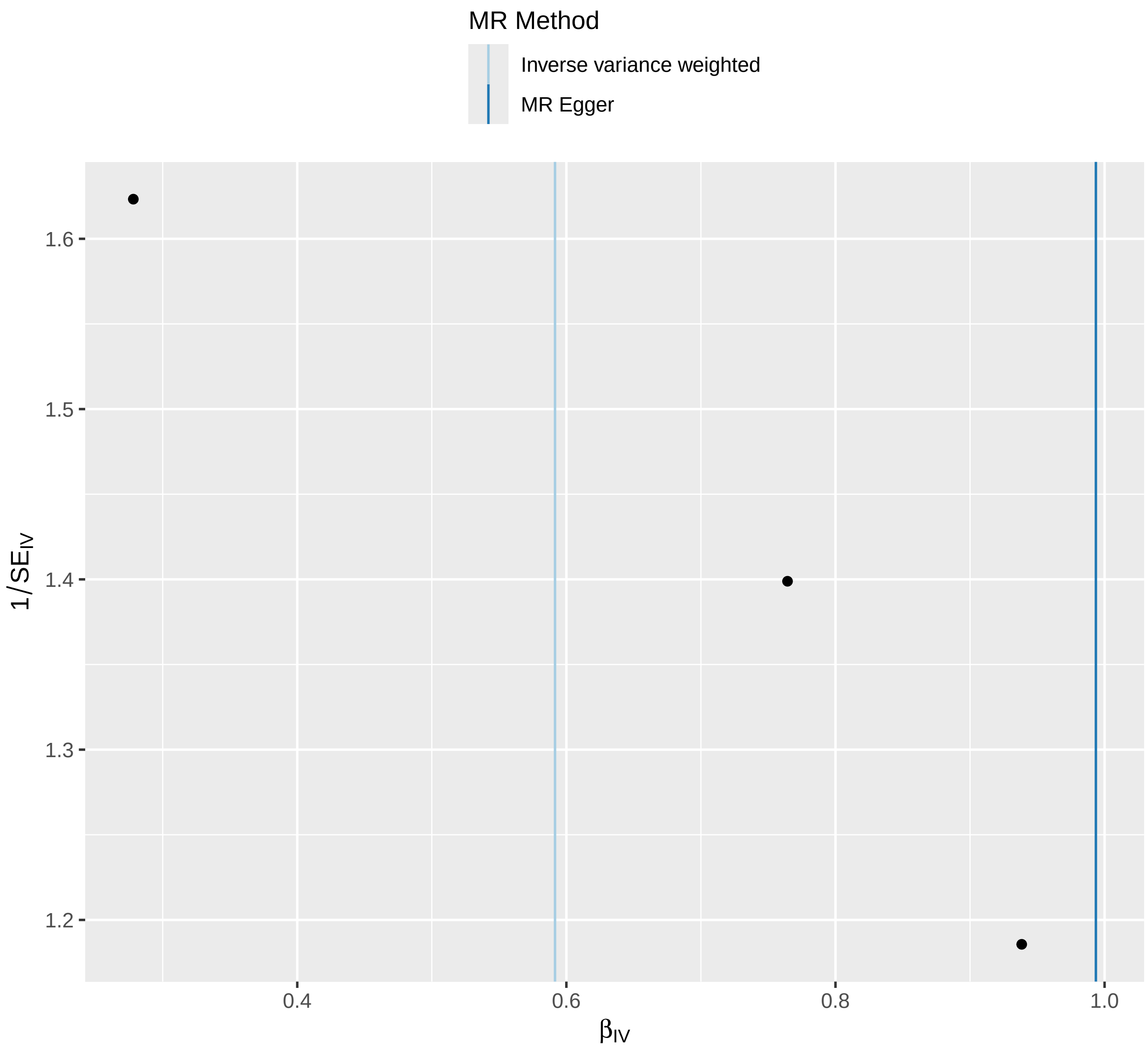
MR Method
Inverse variance weighted
MR Egger
1.6
1.5
1.4
1.3
1.2
1/SE$_{IV}$
0.4
0.6
0.8
1.0
β$_{IV}$

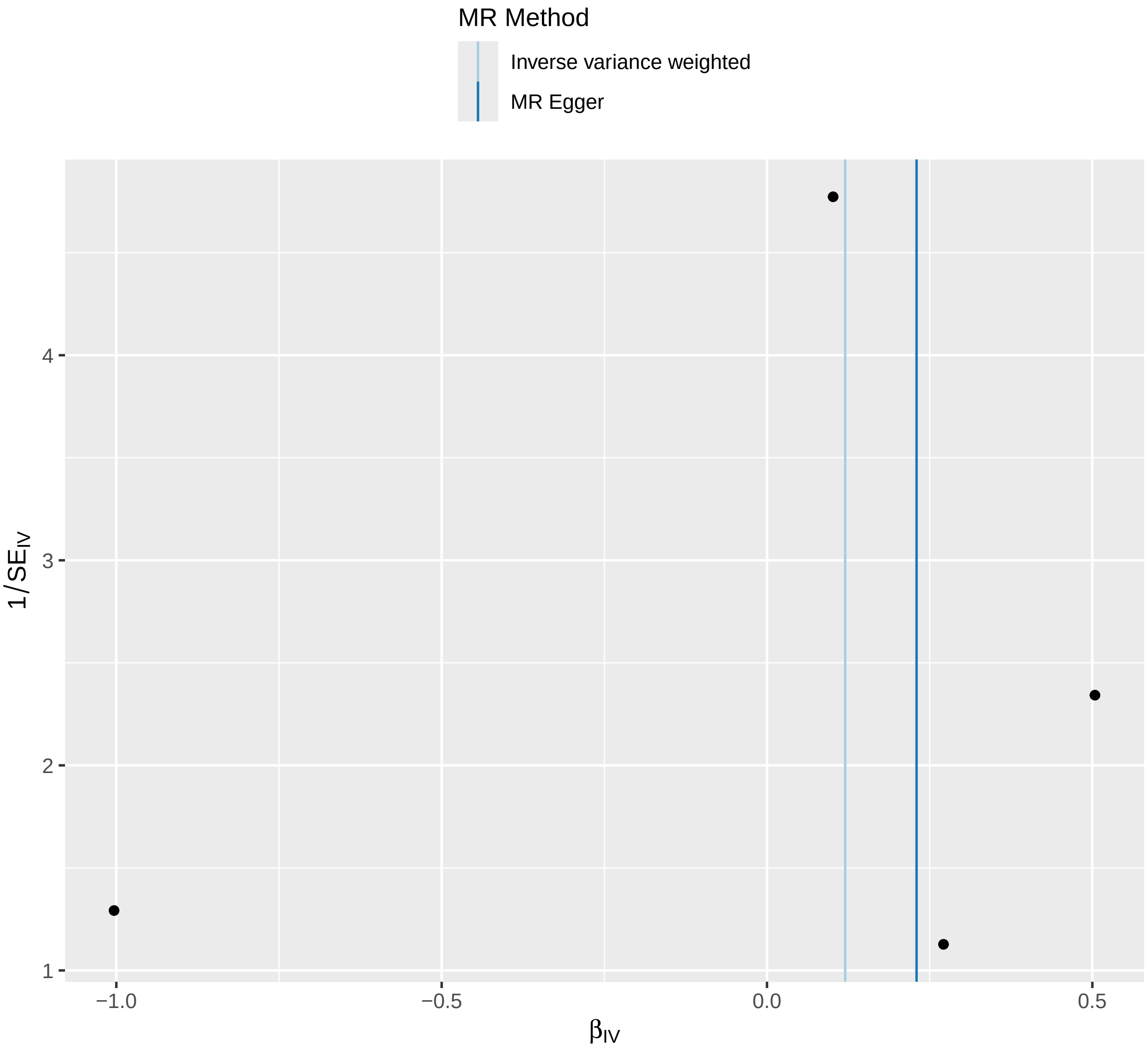

MR Method
Inverse variance weighted
MR Egger
4
3
2
1
−1.0
−0.5
0.0
0.5
$1/SE_{IV}$
β_{IV}

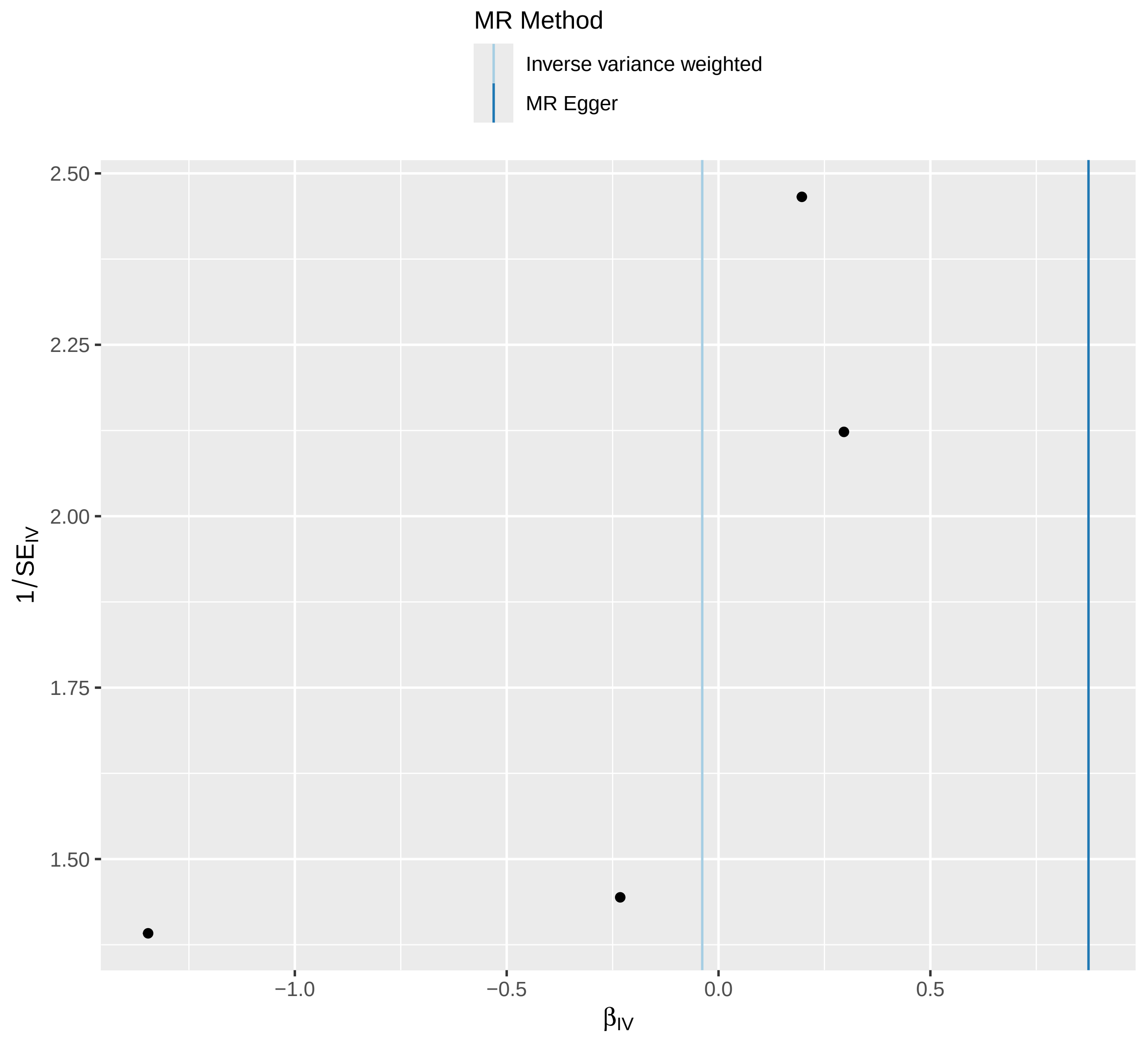

MR Method
Inverse variance weighted
MR Egger
2.50
2.25
2.00
1.75
1.50
1/SE_IV
−1.0
−0.5
0.0
0.5
β_IV

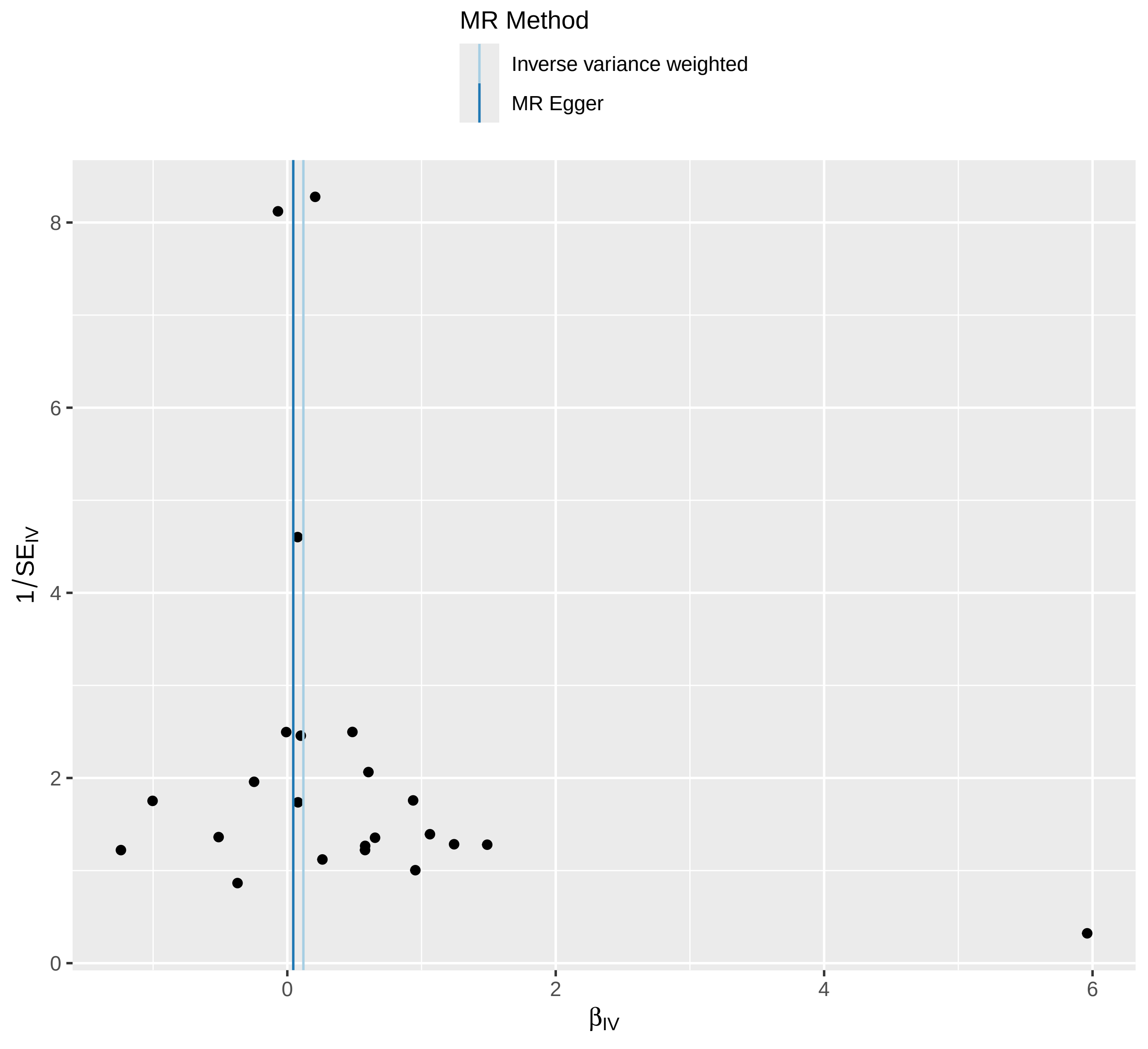

MR Method
Inverse variance weighted
MR Egger
8
6
4
2
0
$1/SE_{IV}$
0
2
4
6
β_{IV}

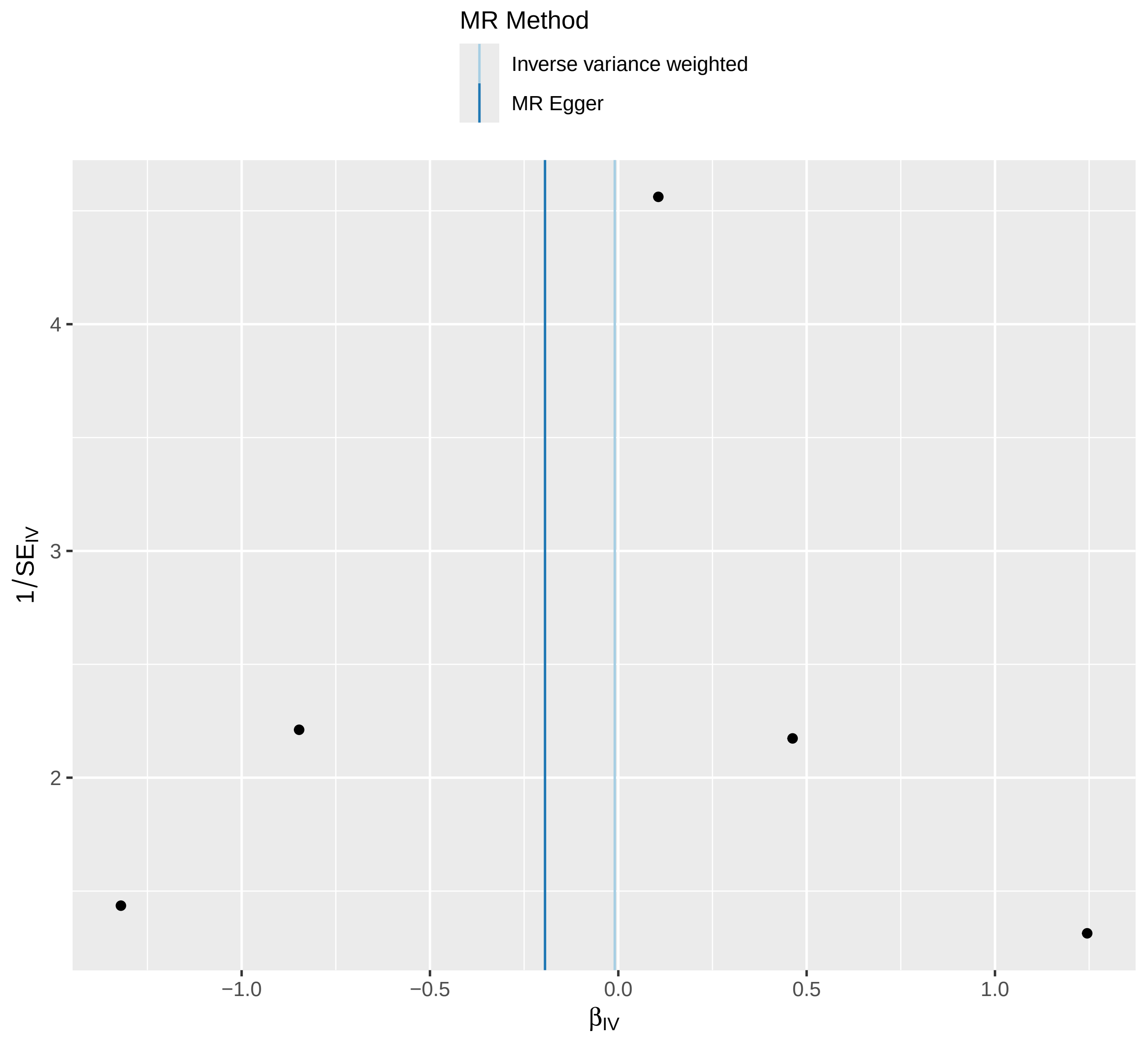

MR Method
Inverse variance weighted
MR Egger
4
3
2
$1/SE_{IV}$
−1.0
−0.5
0.0
0.5
1.0
β_{IV}

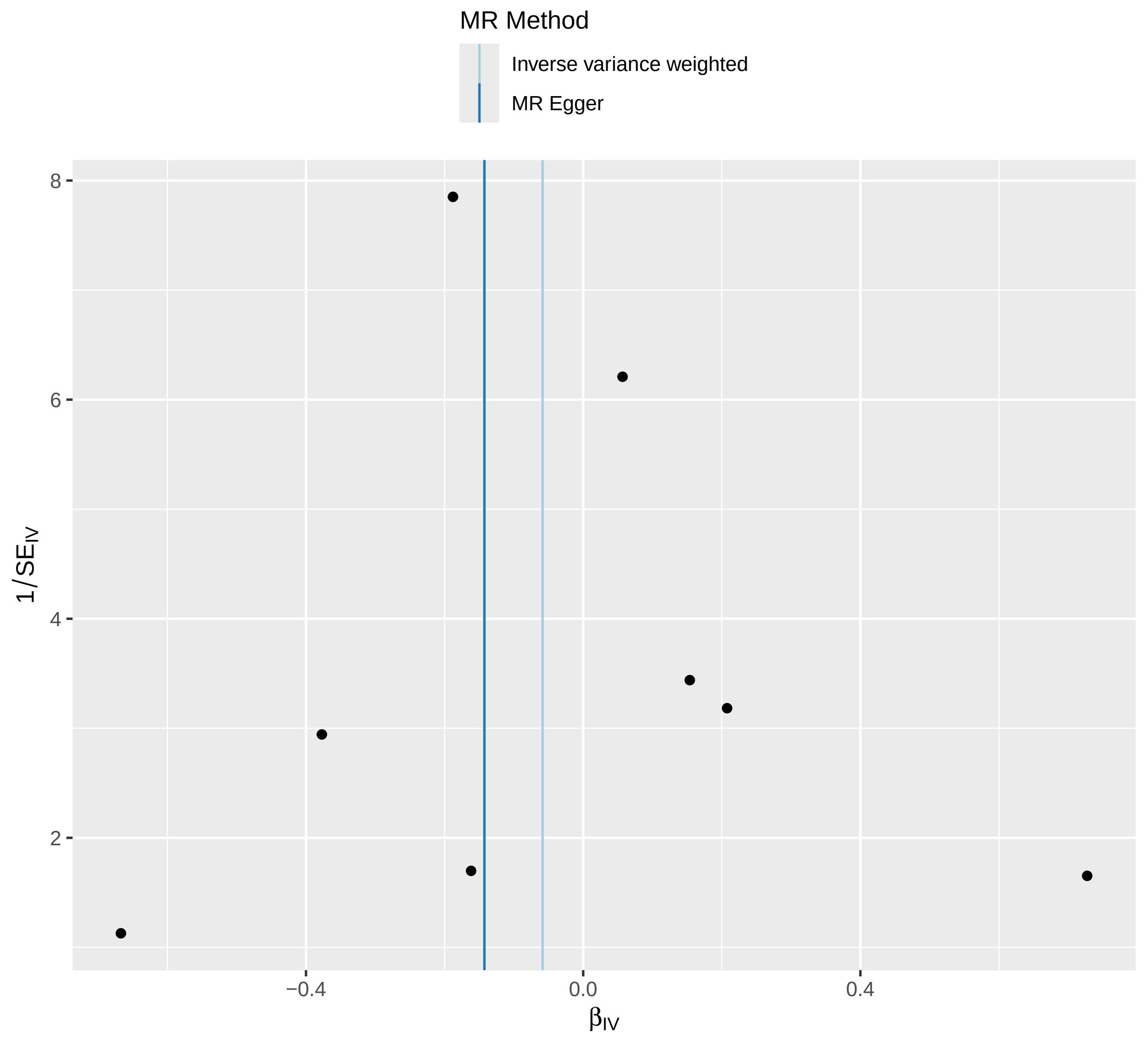
MR Method
Inverse variance weighted
MR Egger
8
6
4
2
1/SE_{IV}
−0.4
0.0
0.4
$β_{IV}$

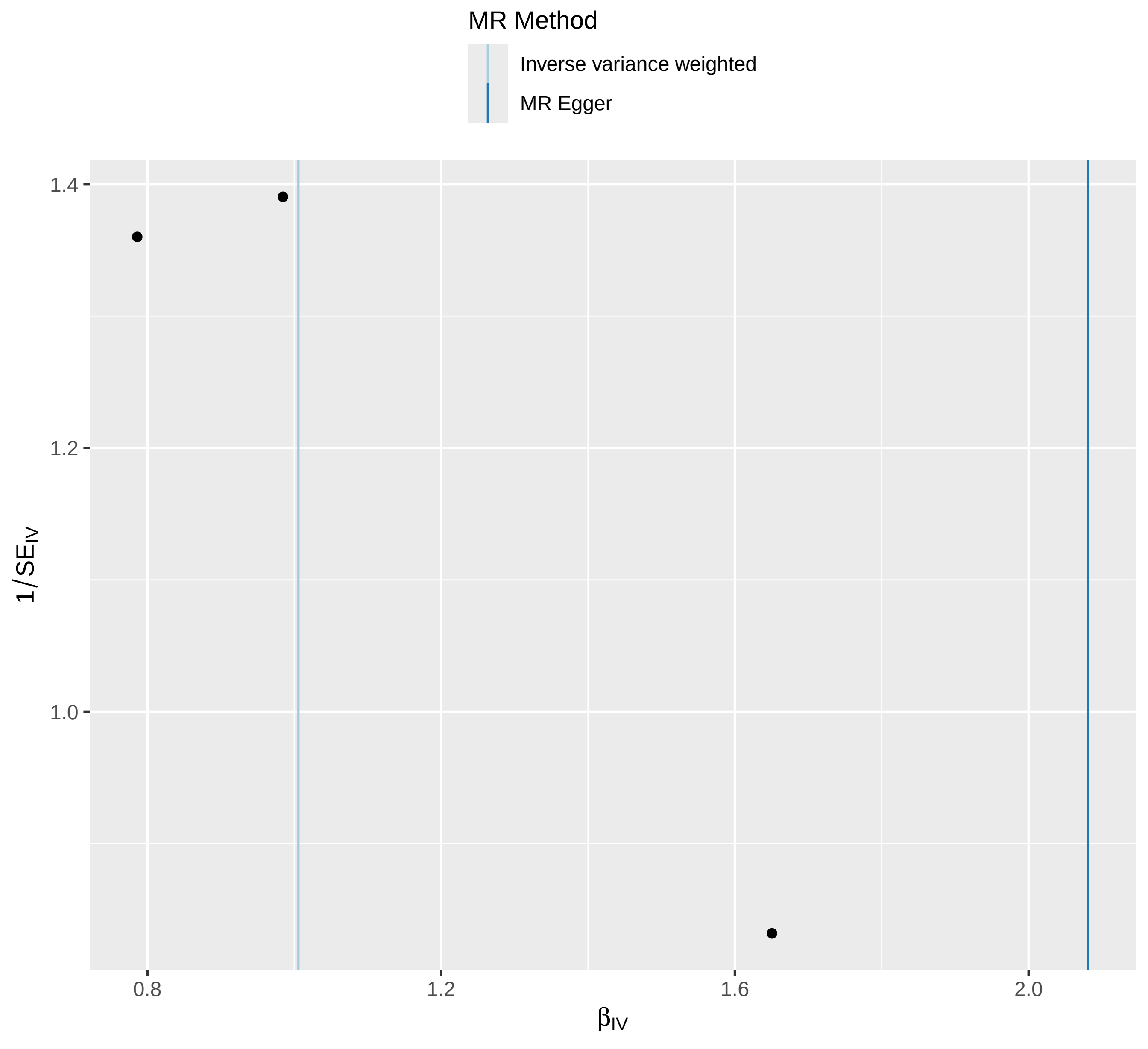
MR Method
Inverse variance weighted
MR Egger
1.4
1.2
1.0
1/SE_IV
0.8
1.2
1.6
2.0
β_IV

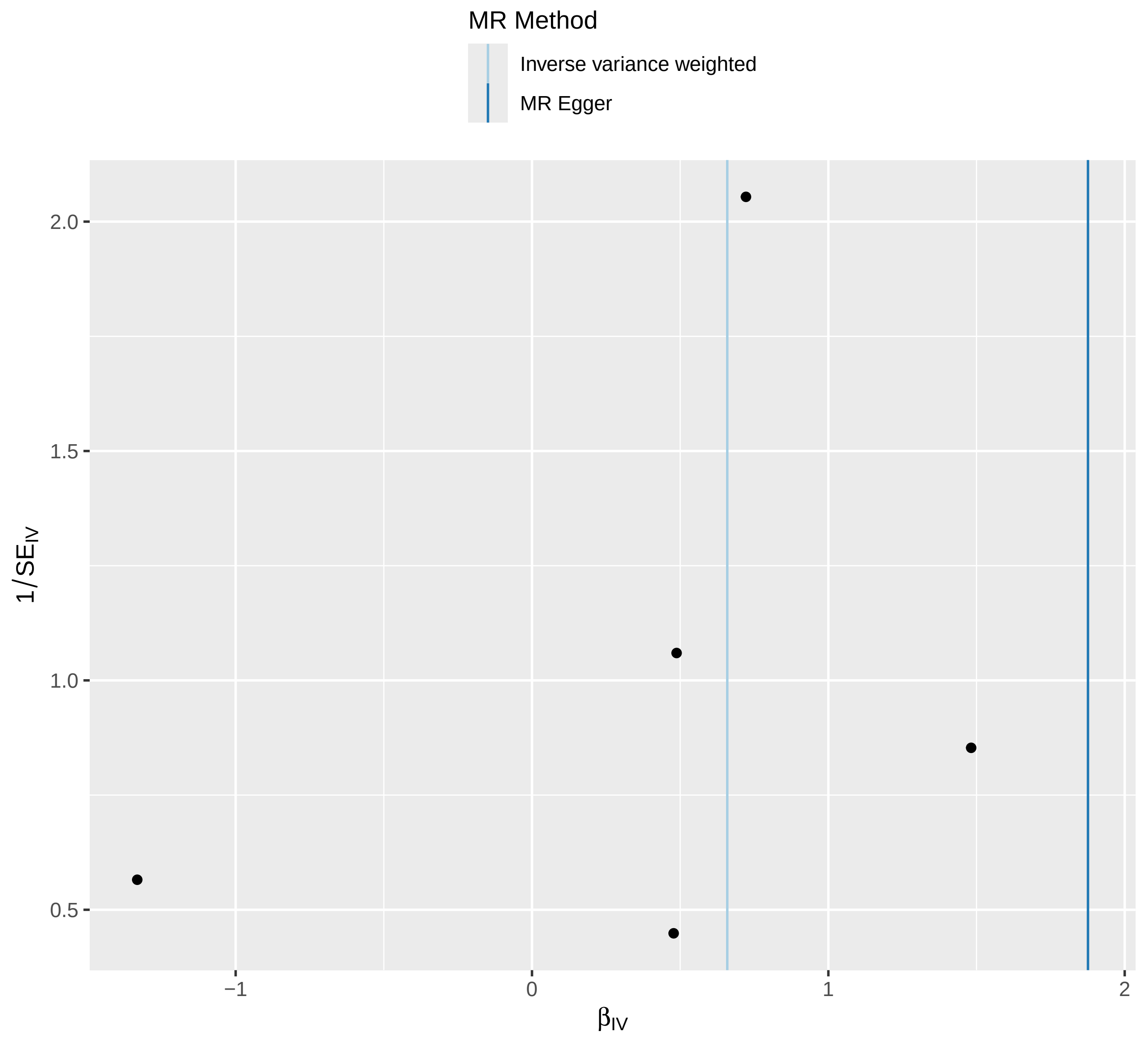
MR Method
Inverse variance weighted
MR Egger
2.0
1.5
1.0
0.5
1/SE$_{IV}$
−1
0
1
2
β$_{IV}$

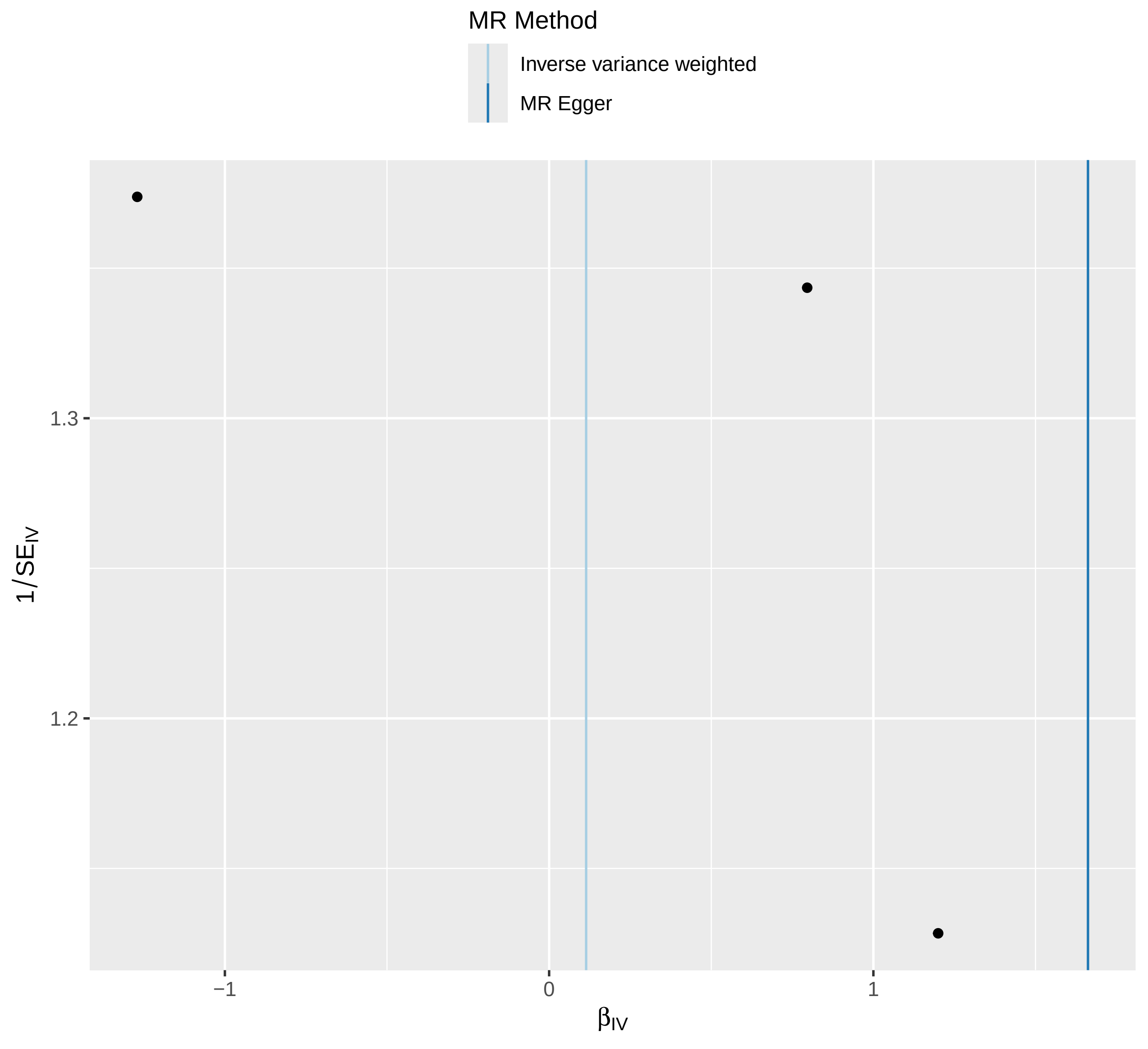

MR Method
Inverse variance weighted
MR Egger
1.3
1.2
1/SE_IV
−1
0
1
β_IV

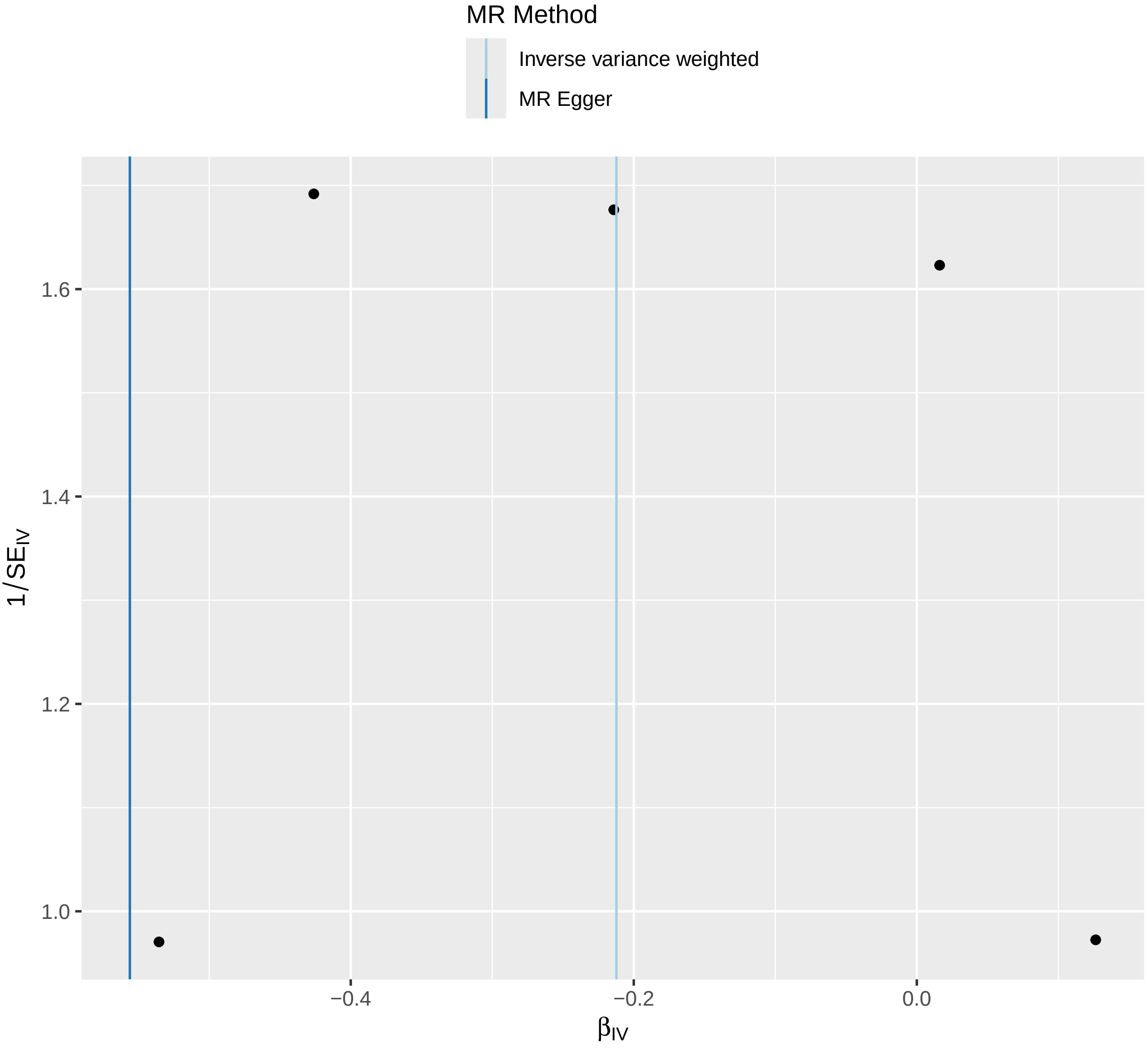
MR Method
Inverse variance weighted
MR Egger
1.6
1.4
1.2
1.0
$1/SE_{IV}$
−0.4
−0.2
0.0
β_{IV}

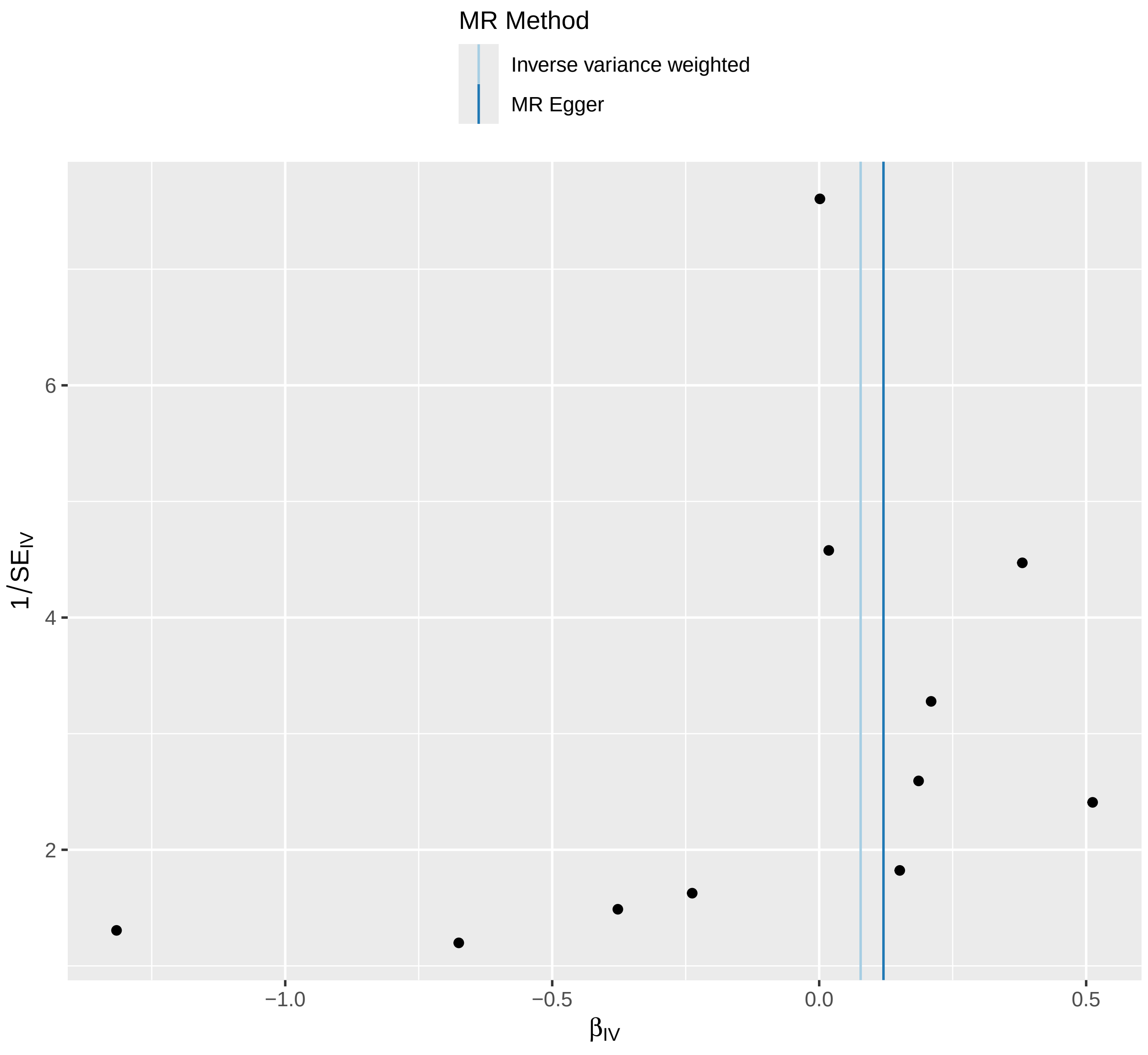

MR Method
Inverse variance weighted
MR Egger
6
4
2
1/SE_IV
−1.0
−0.5
0.0
0.5
β_IV

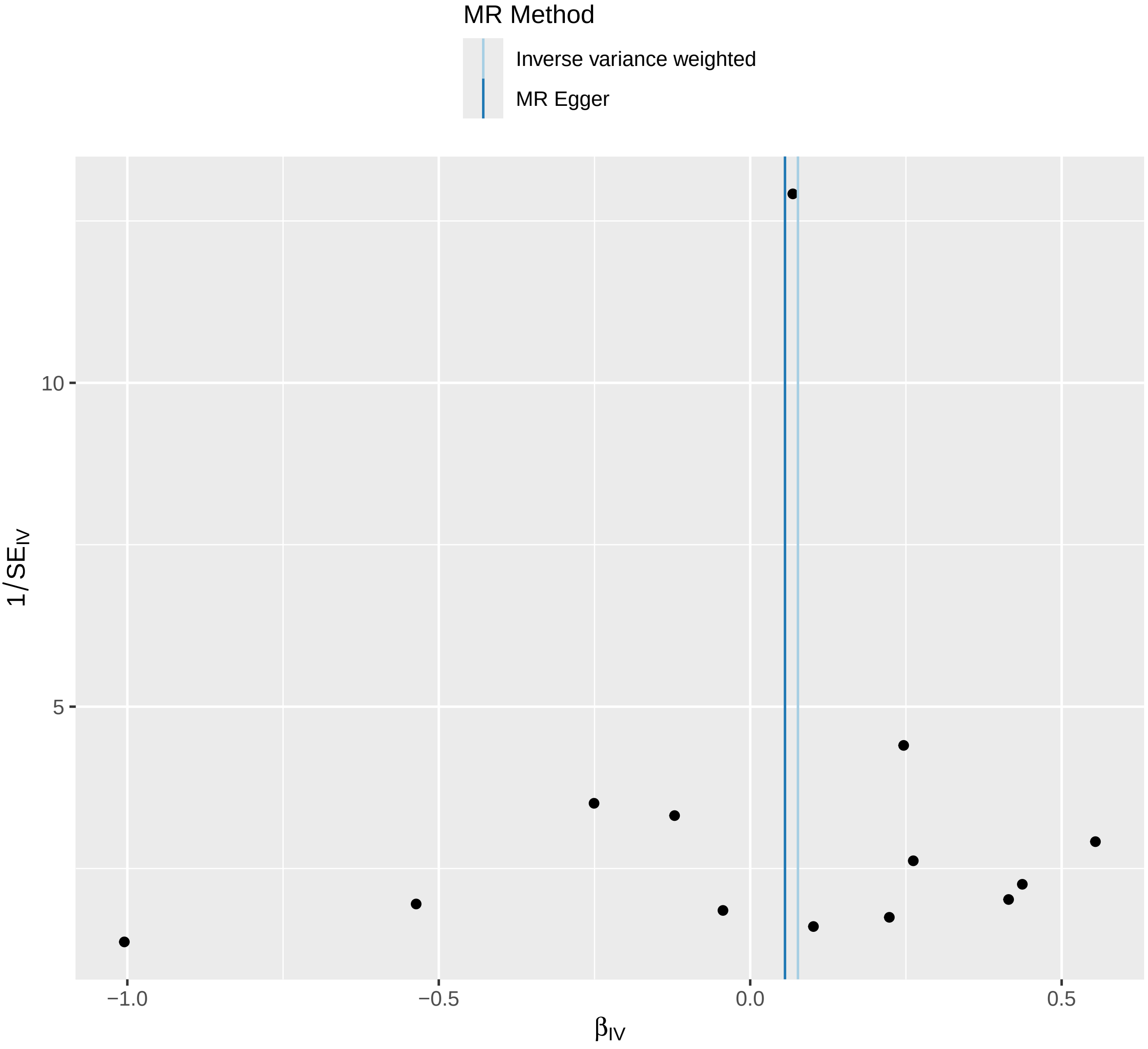

MR Method
Inverse variance weighted
MR Egger
10
5
1/SE_IV
−1.0
−0.5
0.0
0.5
β_IV

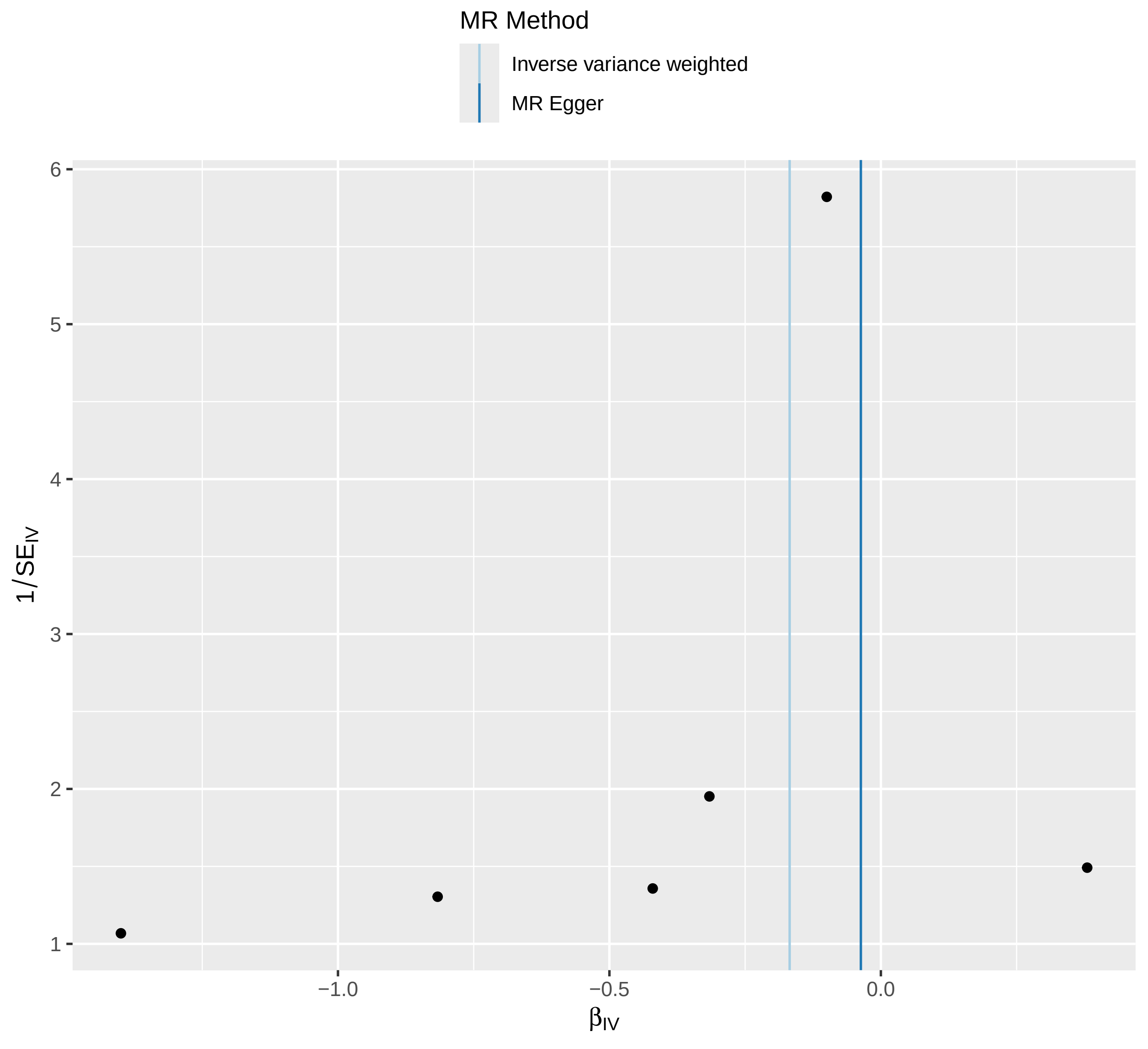
MR Method
Inverse variance weighted
MR Egger
1/SE$_{IV}$
6
5
4
3
2
1
−1.0
−0.5
0.0
β$_{IV}$

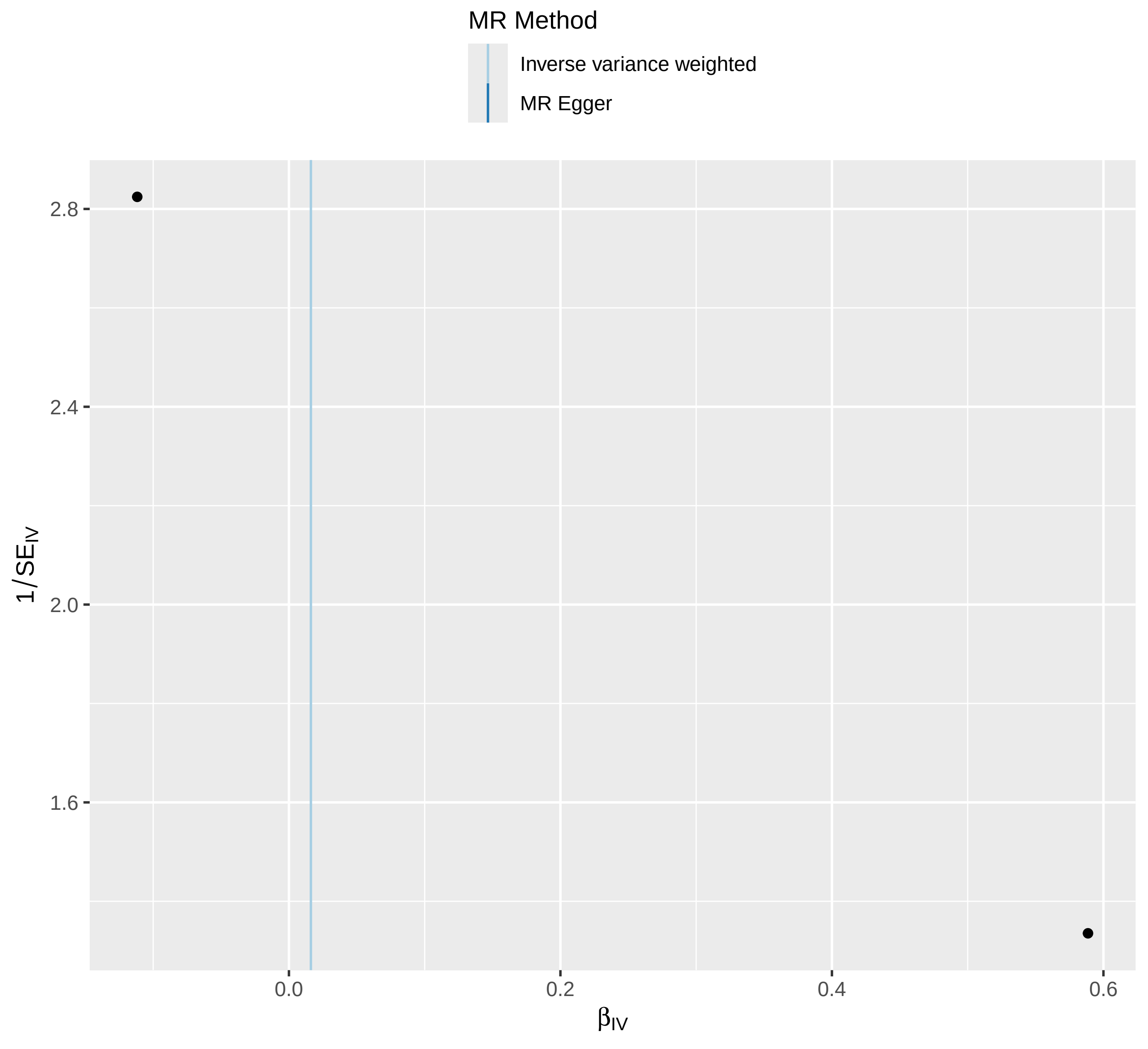

MR Method
Inverse variance weighted
MR Egger
2.8
2.4
2.0
1.6
1/SE_IV
0.0
0.2
0.4
0.6
β_IV

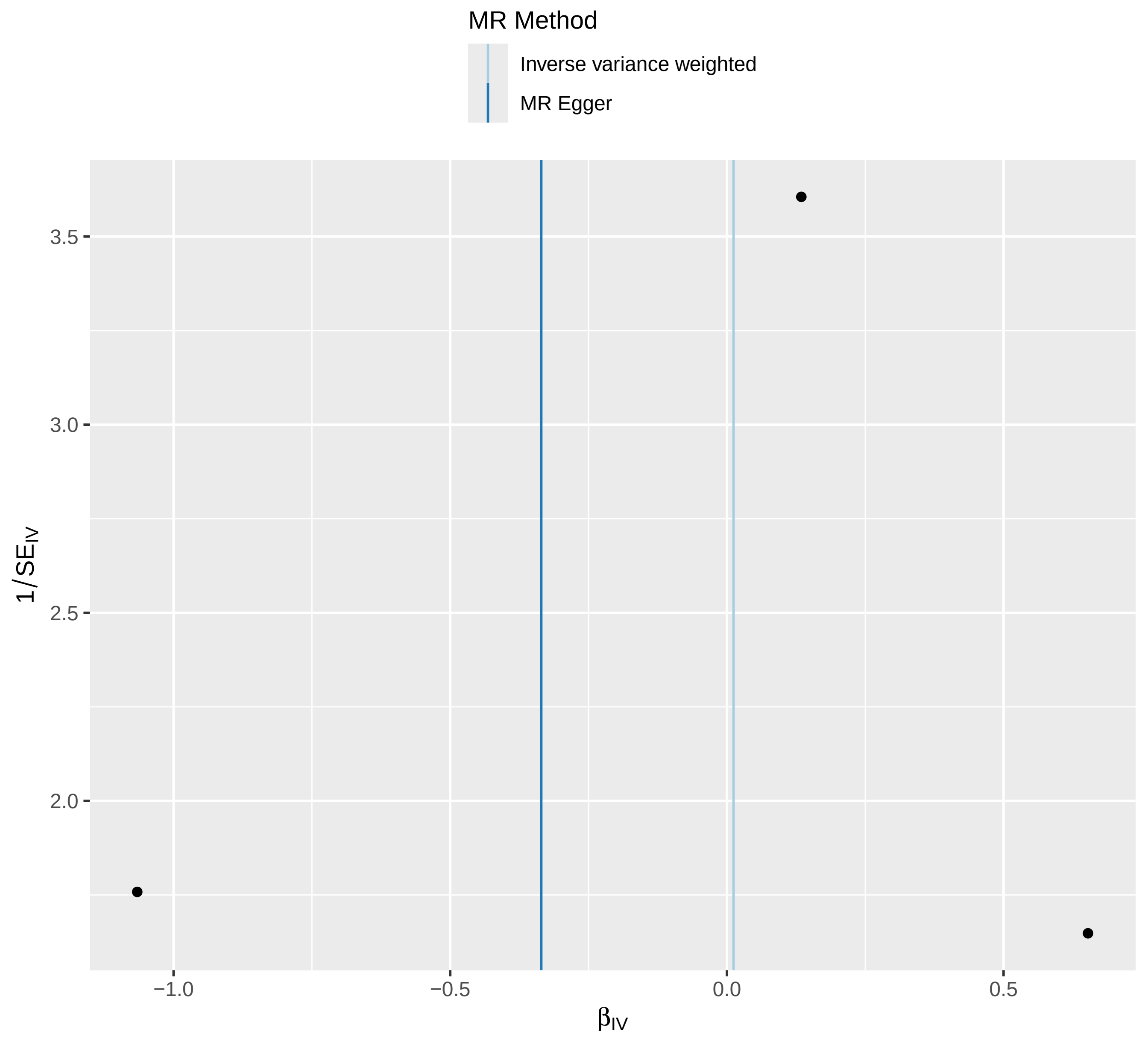
MR Method
Inverse variance weighted
MR Egger
3.5
3.0
2.5
2.0
1/SE_IV
−1.0
−0.5
0.0
0.5
β_IV

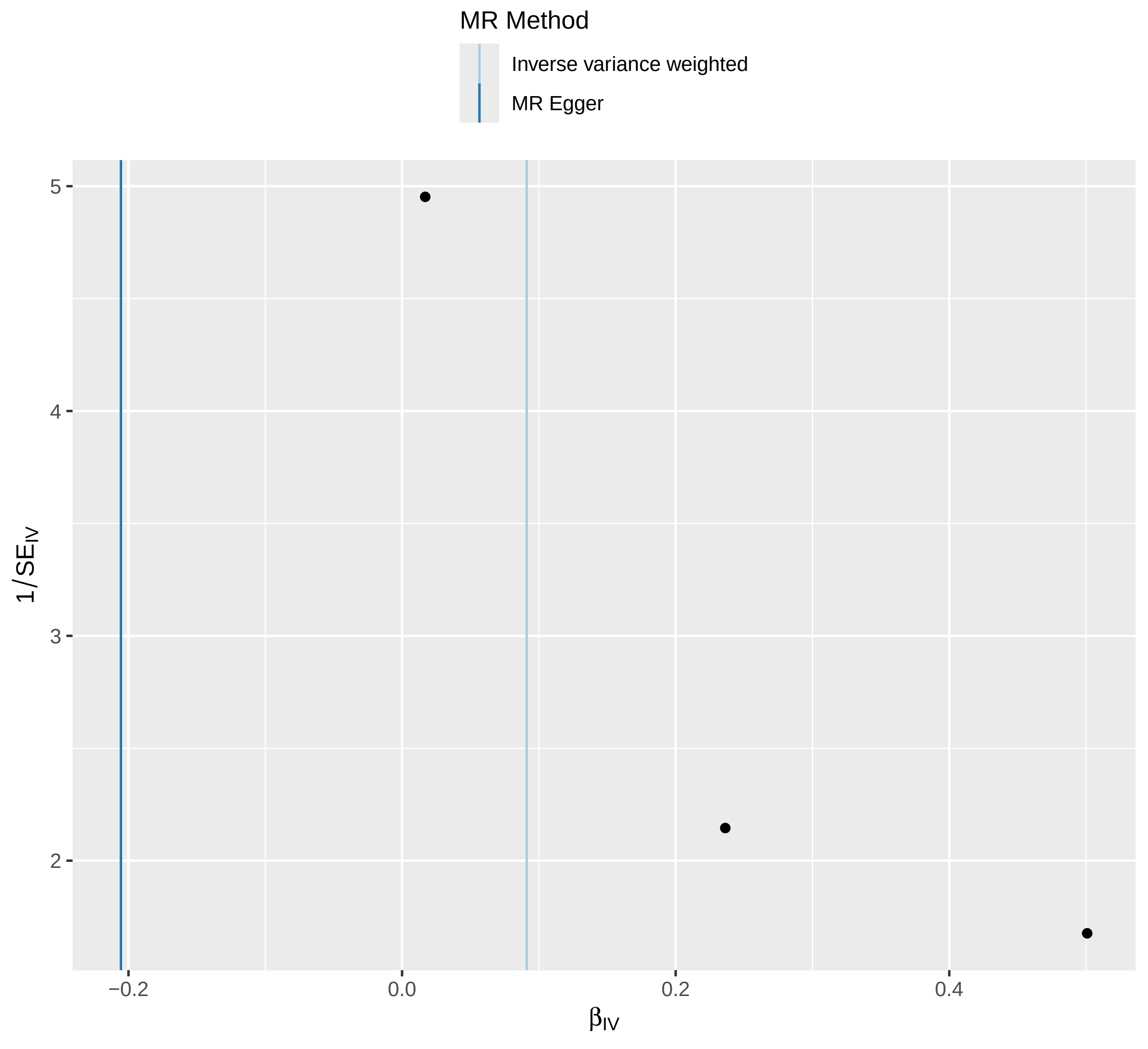

MR Method
Inverse variance weighted
MR Egger
5
4
3
2
1/SE_IV
−0.2
0.0
0.2
0.4
β_IV

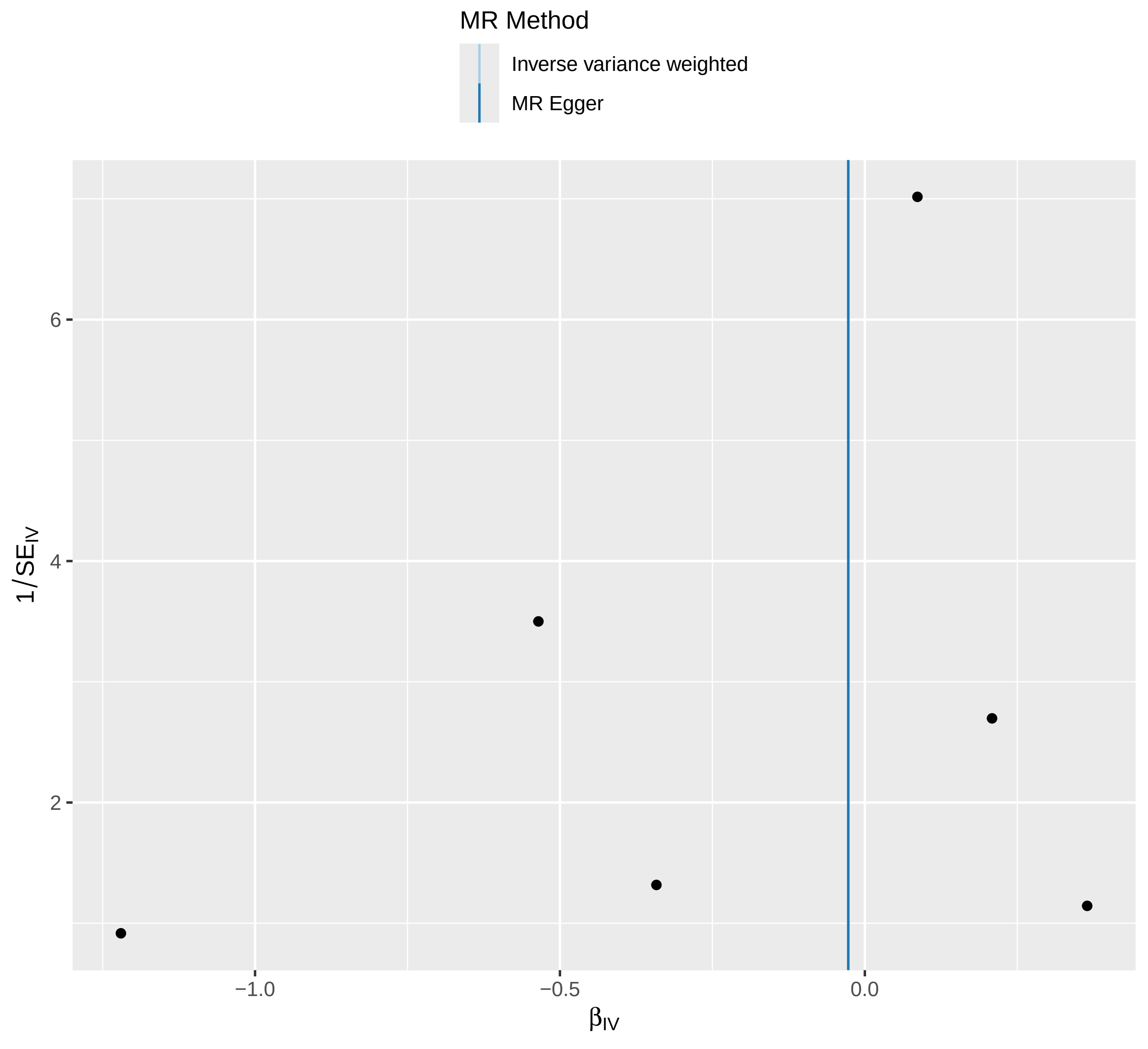
MR Method
Inverse variance weighted
MR Egger
6
4
2
1/SE$_{IV}$
−1.0
−0.5
0.0
β$_{IV}$

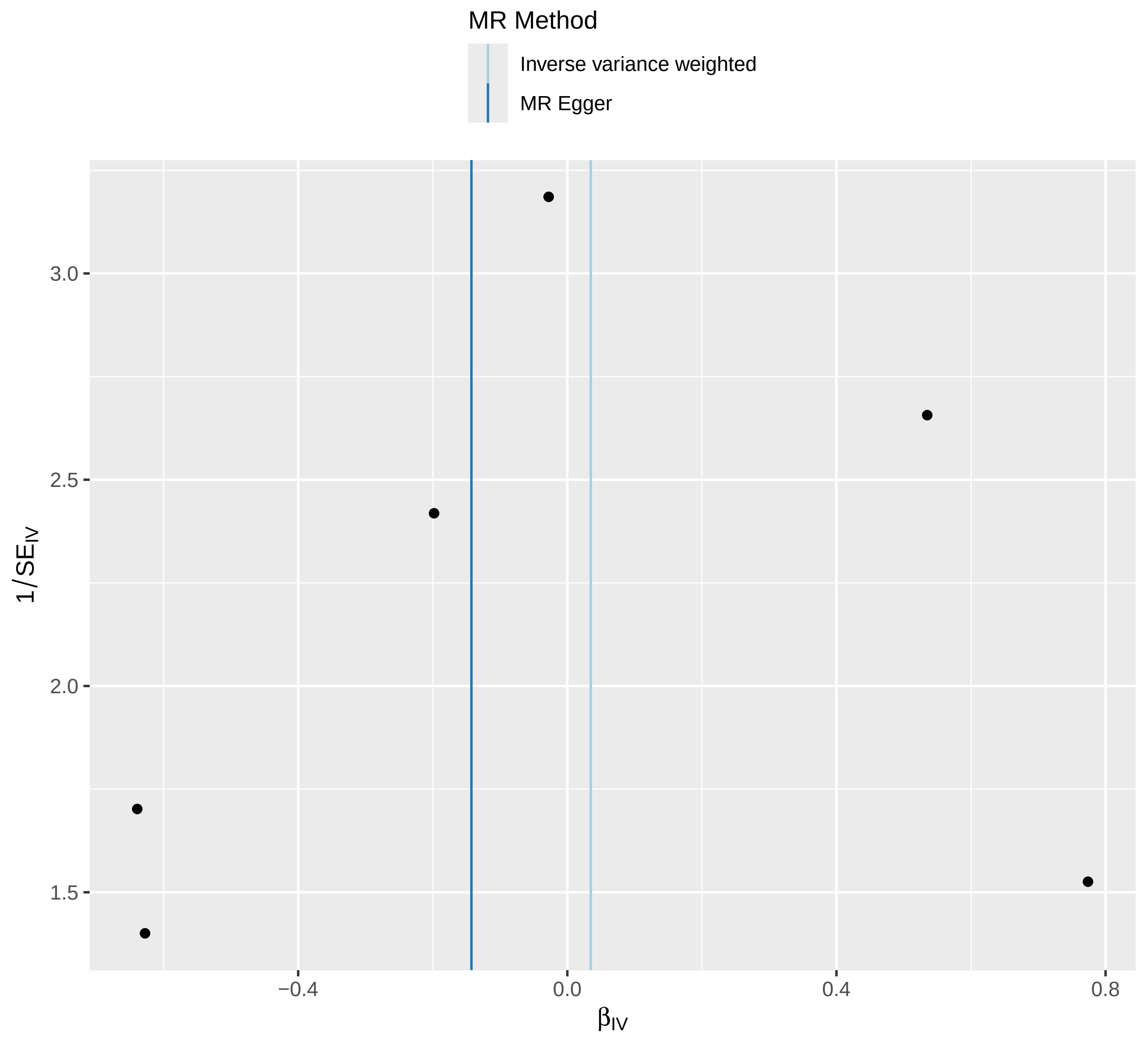

MR Method
Inverse variance weighted
MR Egger
3.0
2.5
2.0
1.5
1/SE_IV
−0.4
0.0
0.4
0.8
β_IV

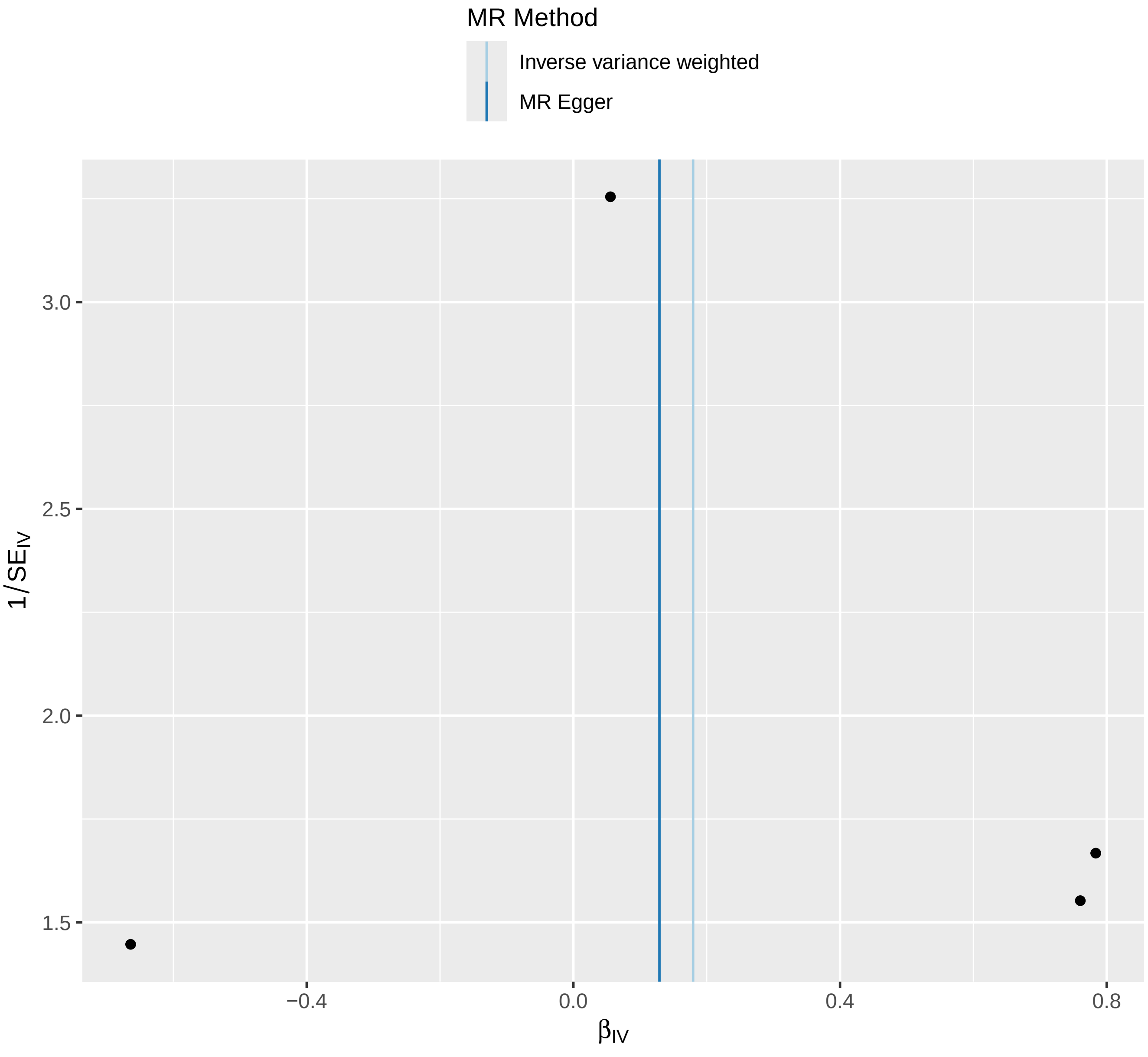

MR Method
Inverse variance weighted
MR Egger
3.0
2.5
2.0
1.5
1/SE_{IV}
−0.4
0.0
0.4
0.8
β_{IV}

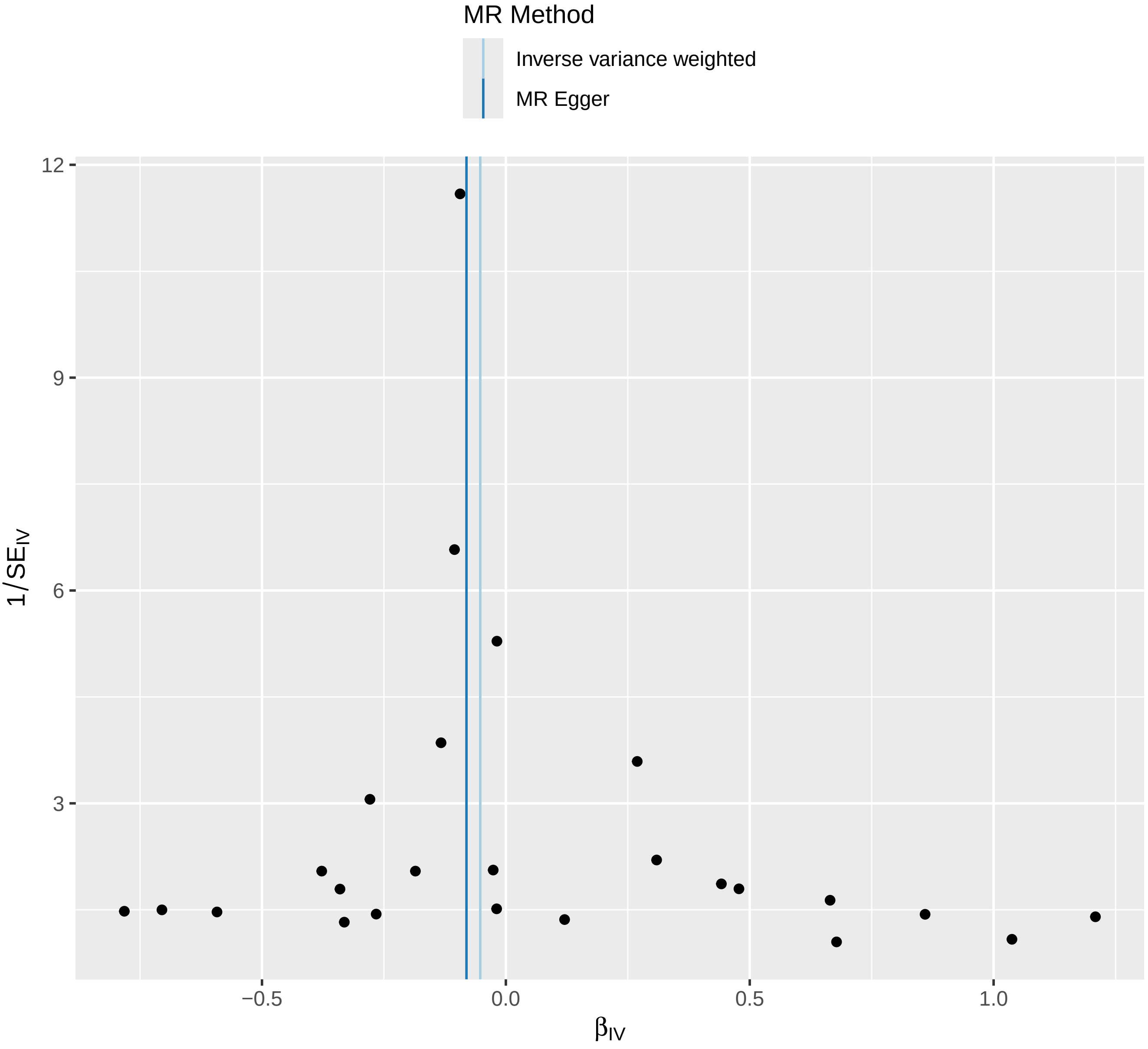

MR Method
Inverse variance weighted
MR Egger
12
9
6
3
1/SE_IV
−0.5
0.0
0.5
1.0
β_IV

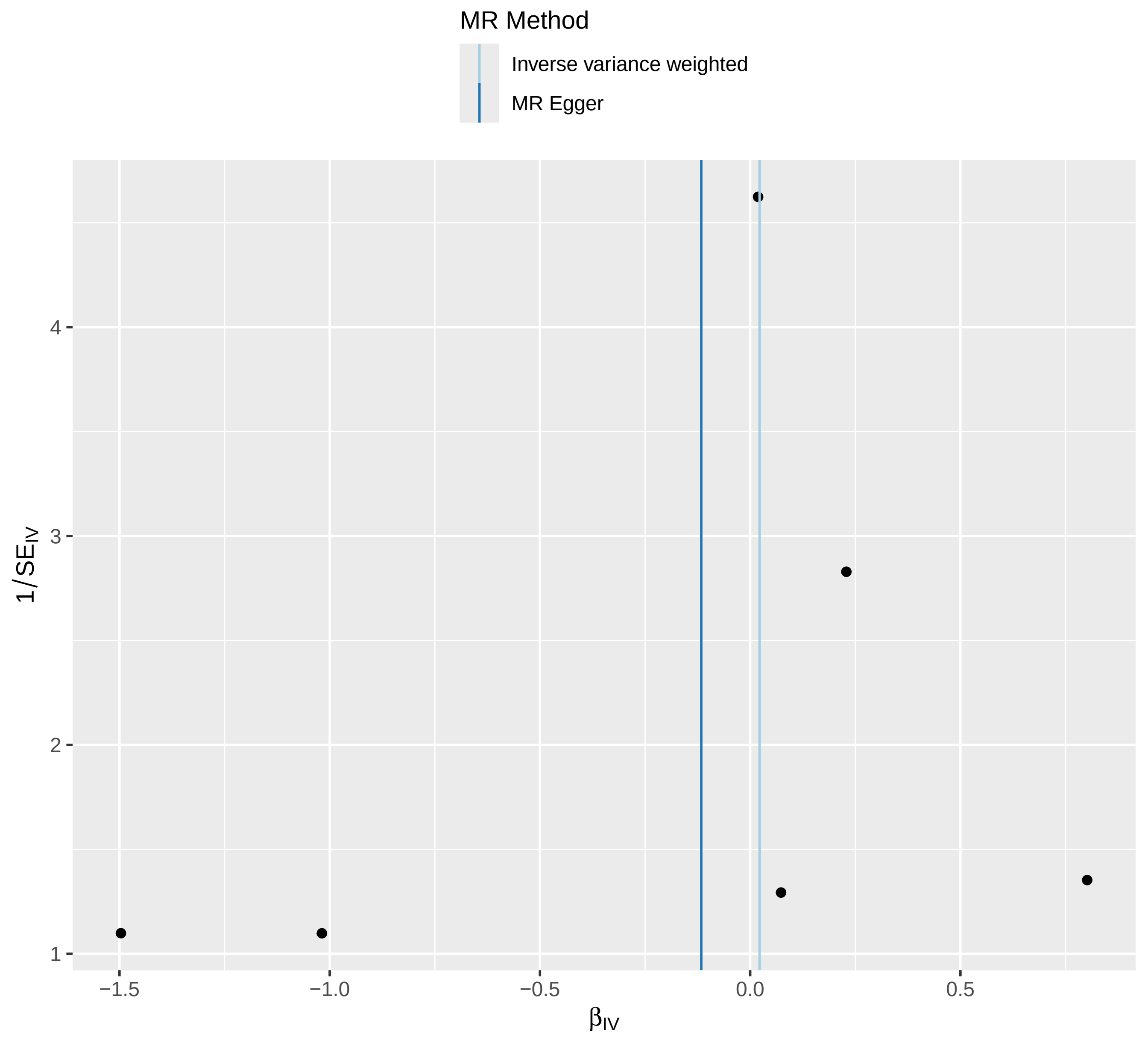

MR Method
Inverse variance weighted
MR Egger
4
3
2
1
1/SE$_{IV}$
−1.5
−1.0
−0.5
0.0
0.5
β$_{IV}$

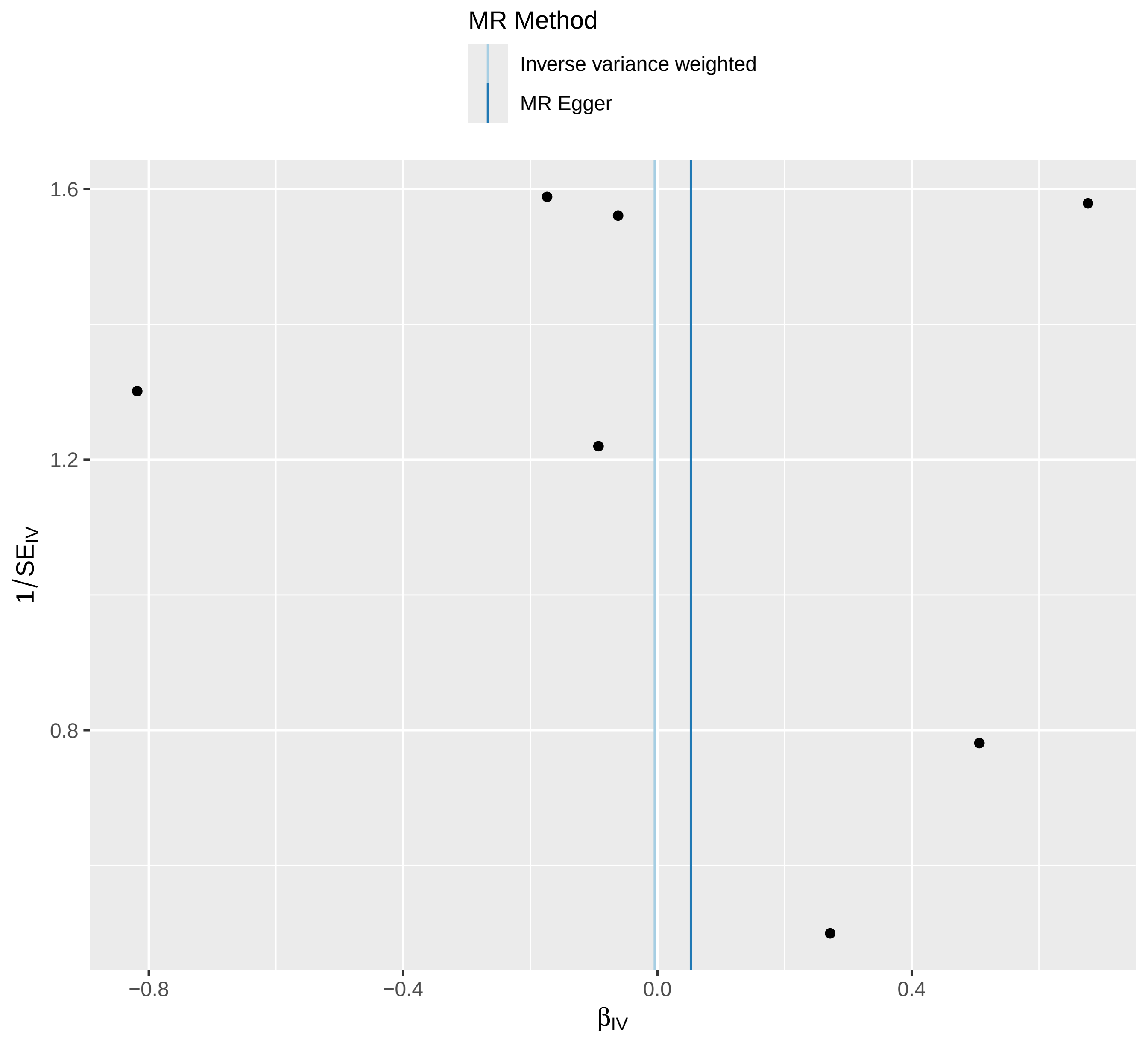
MR Method
Inverse variance weighted
MR Egger
1.6
1.2
0.8
1/SE_IV
−0.8
−0.4
0.0
0.4
β_IV

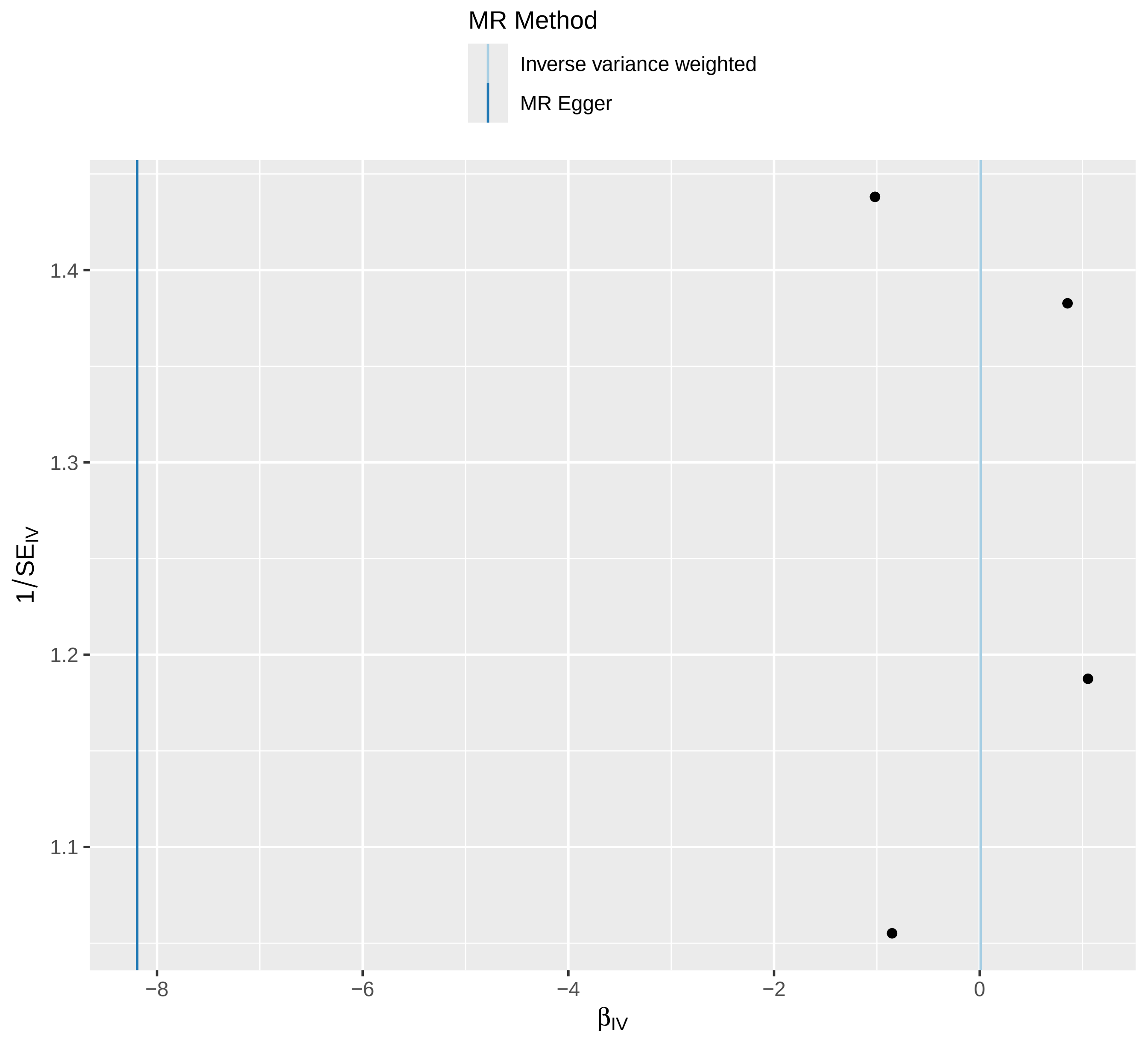
MR Method
Inverse variance weighted
MR Egger
1.4
1.3
1.2
1.1
1/SE_IV
−8
−6
−4
−2
0
β_IV

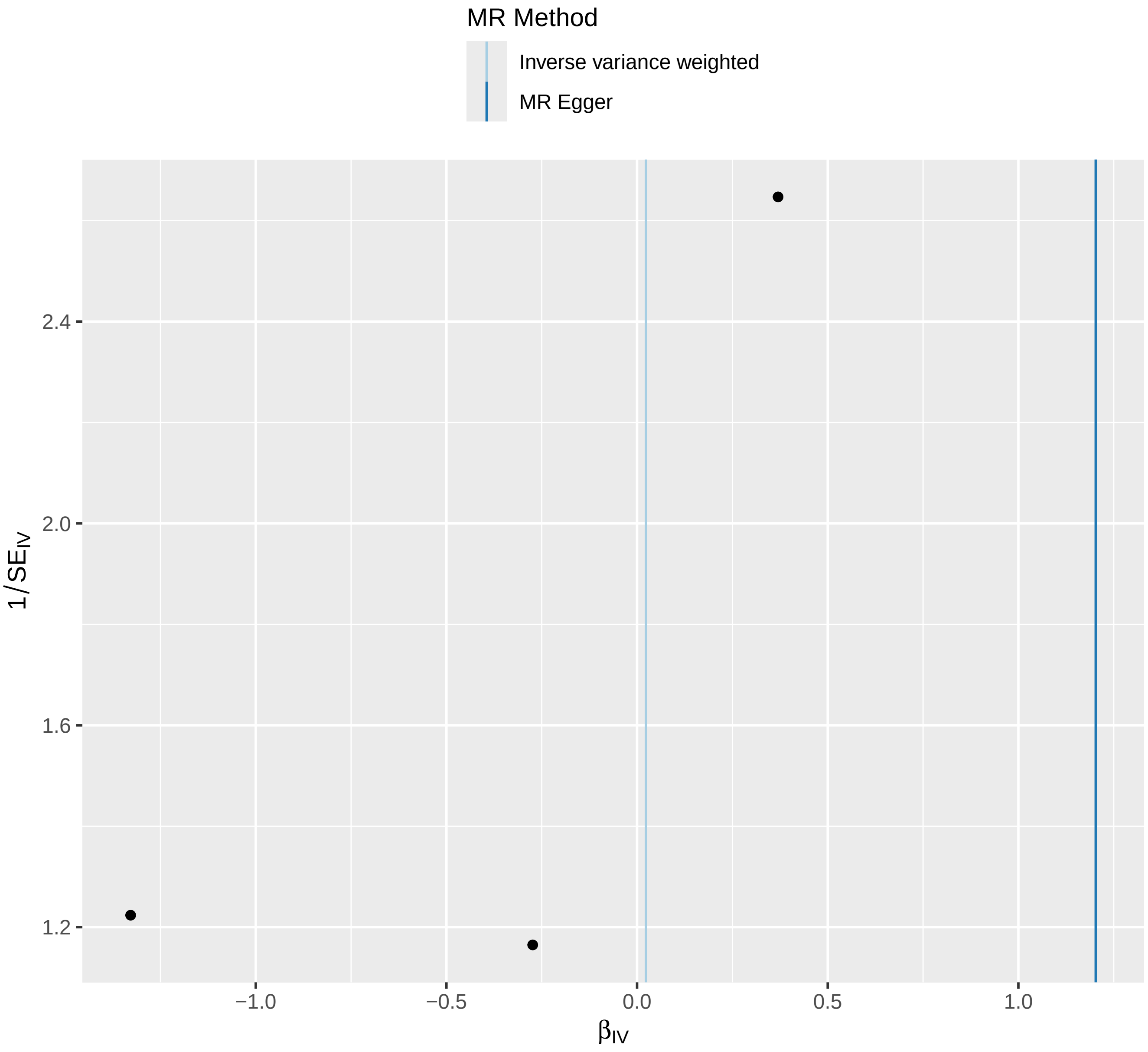

MR Method
Inverse variance weighted
MR Egger
2.4
2.0
1.6
1.2
1/SE$_{IV}$
−1.0
−0.5
0.0
0.5
1.0
β$_{IV}$

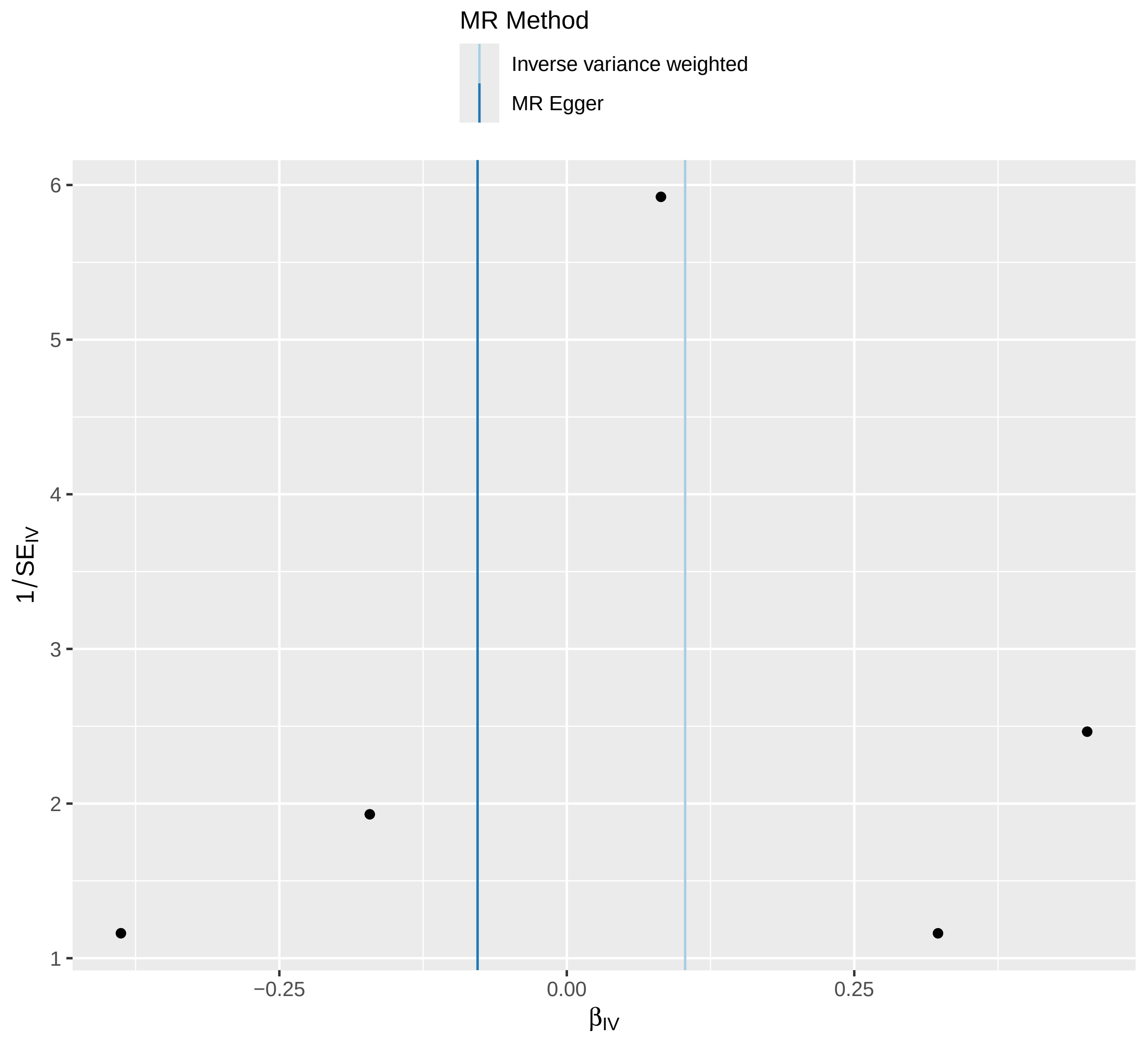

MR Method
Inverse variance weighted
MR Egger
6
5
4
3
2
1
1/SE_IV
−0.25
0.00
0.25
β_IV

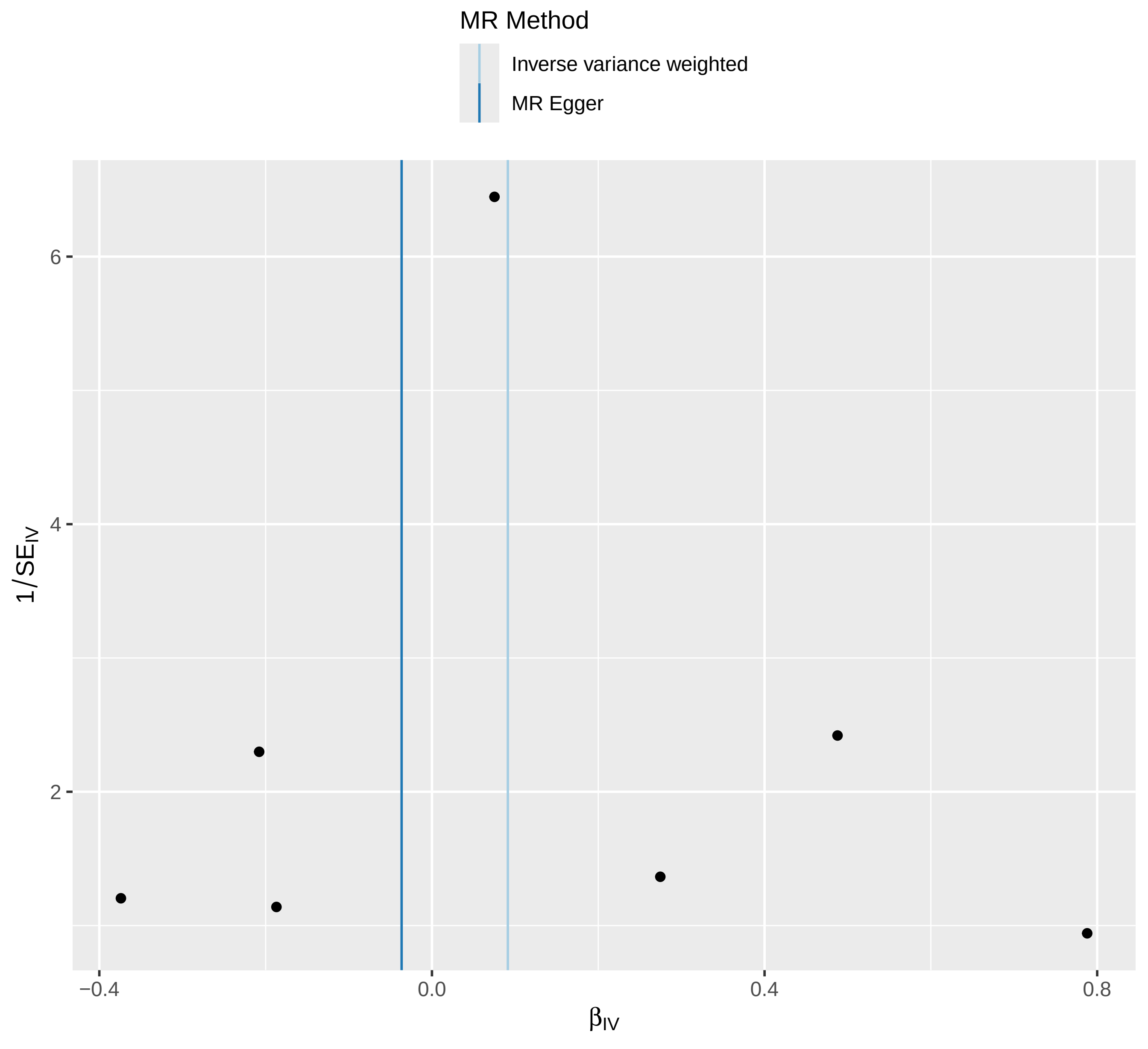

MR Method
Inverse variance weighted
MR Egger
6
4
2
1/SE$_{IV}$
−0.4
0.0
0.4
0.8
β$_{IV}$

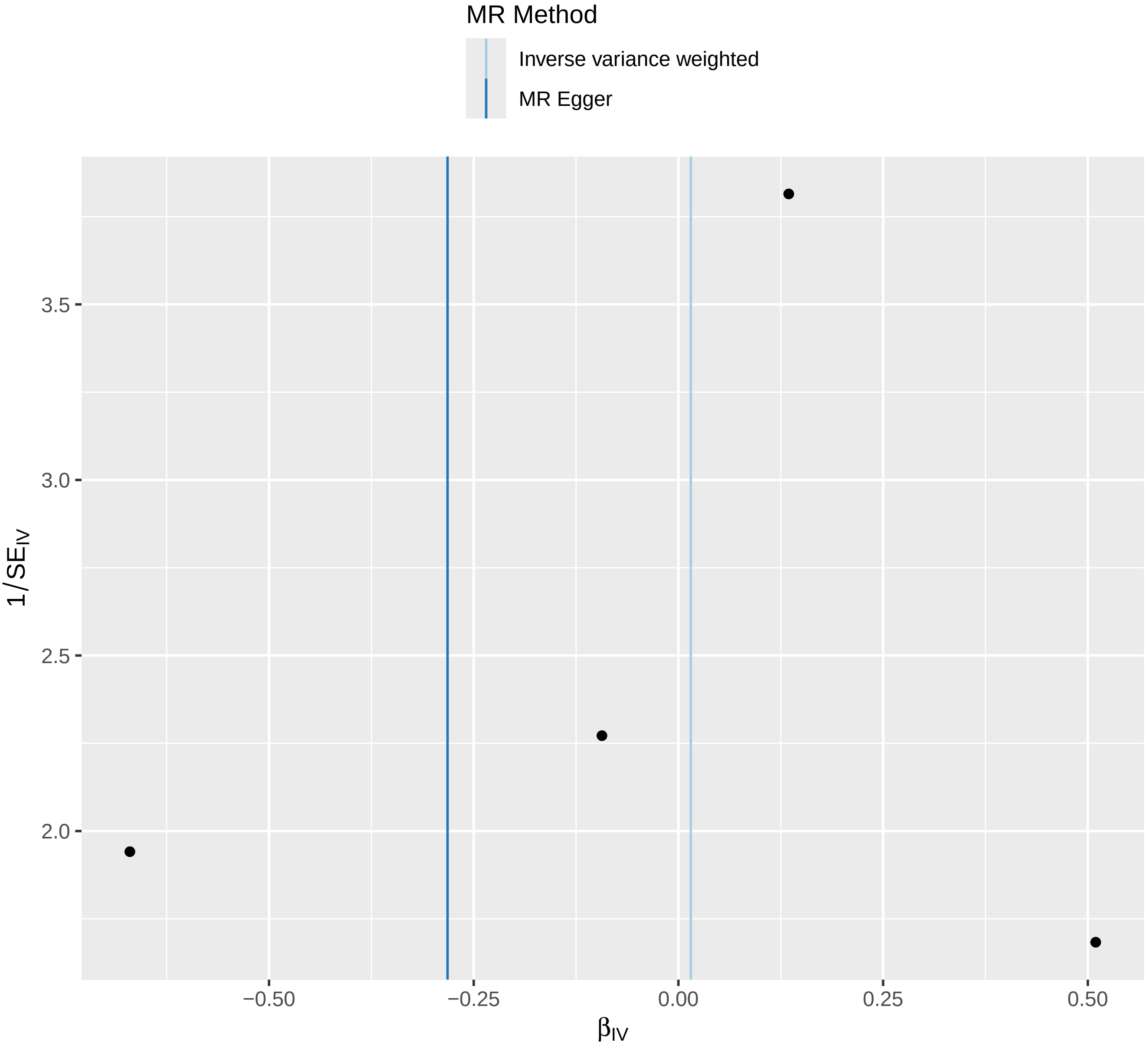

MR Method
Inverse variance weighted
MR Egger
3.5
3.0
2.5
2.0
1/SE$_{IV}$
−0.50
−0.25
0.00
0.25
0.50
β$_{IV}$

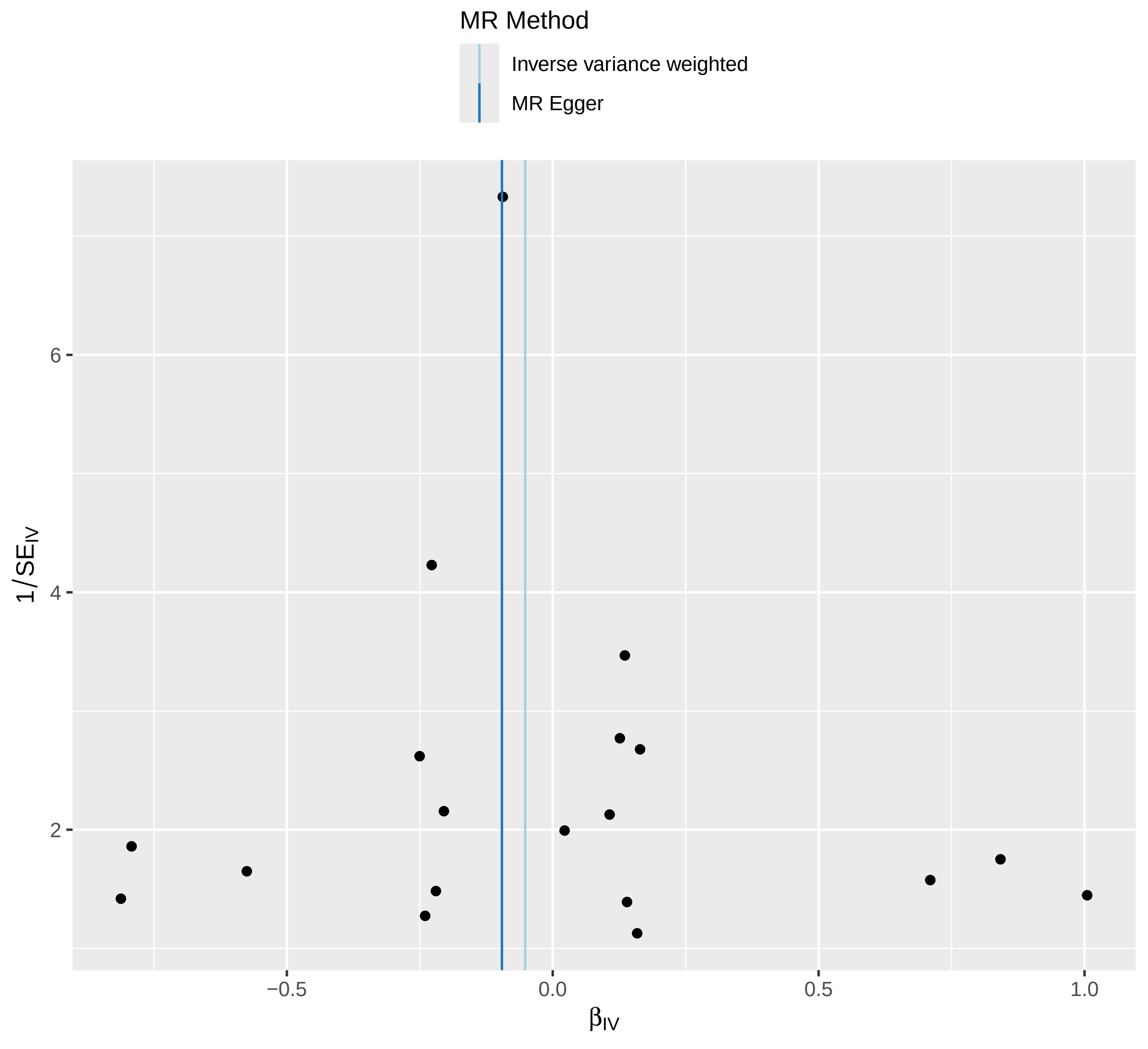

MR Method
Inverse variance weighted
MR Egger
6
4
2
1/SE$_{IV}$
−0.5
0.0
0.5
1.0
β_{IV}

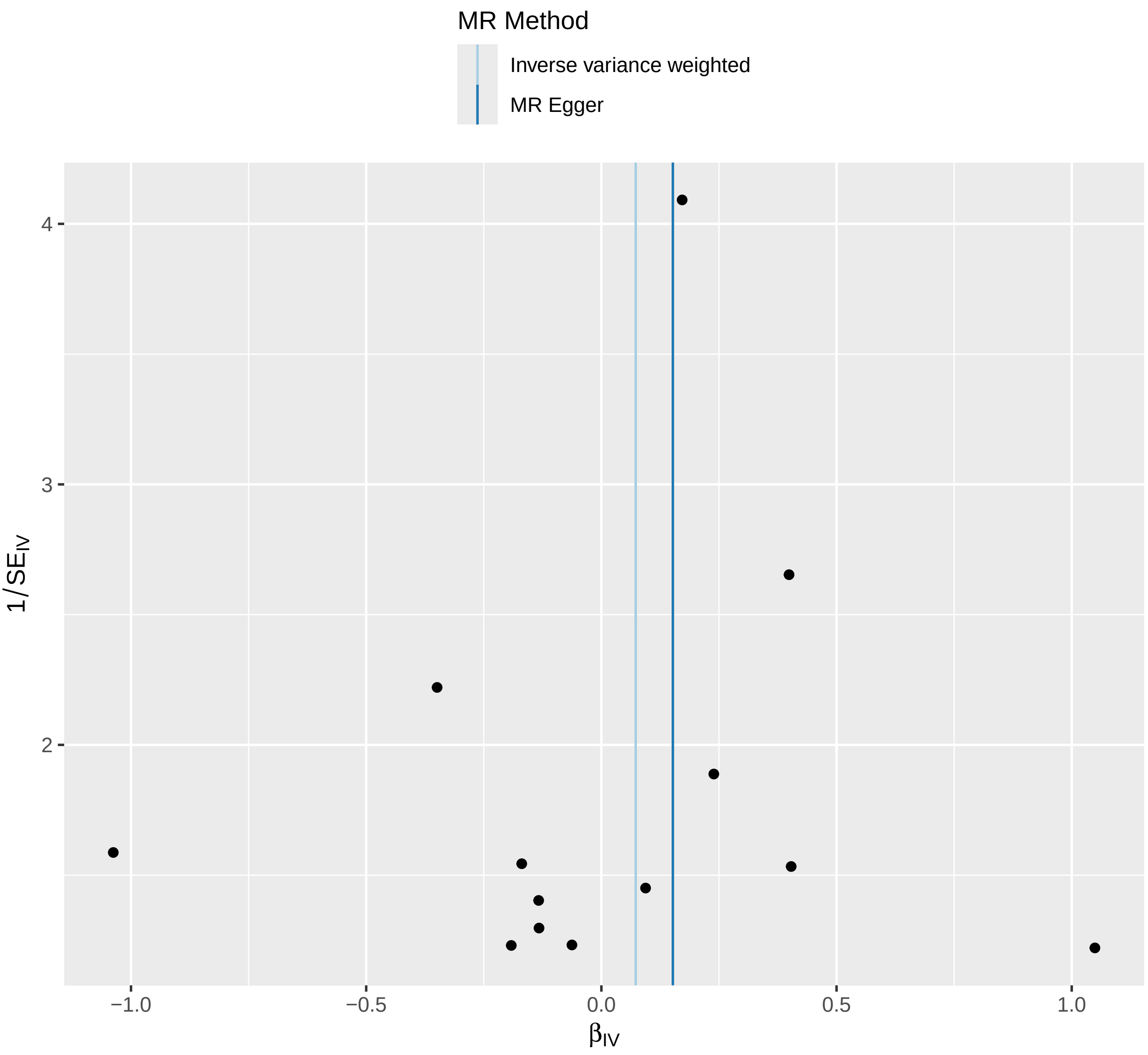
MR Method
Inverse variance weighted
MR Egger
4
3
2
$1/SE_{IV}$
−1.0
−0.5
0.0
0.5
1.0
β_{IV}

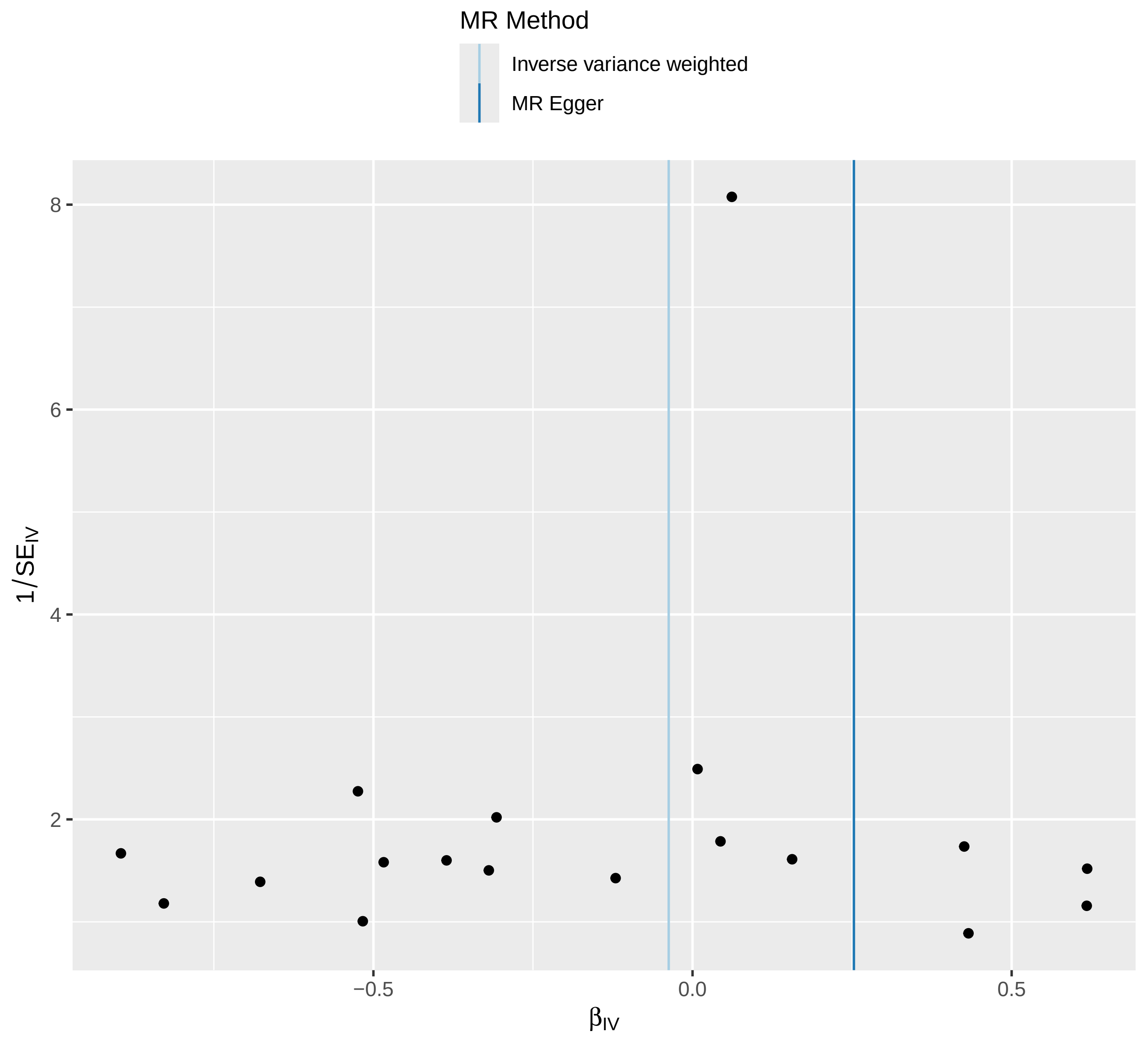

MR Method
Inverse variance weighted
MR Egger
8
6
4
2
1/SE_IV
−0.5
0.0
0.5
β_IV

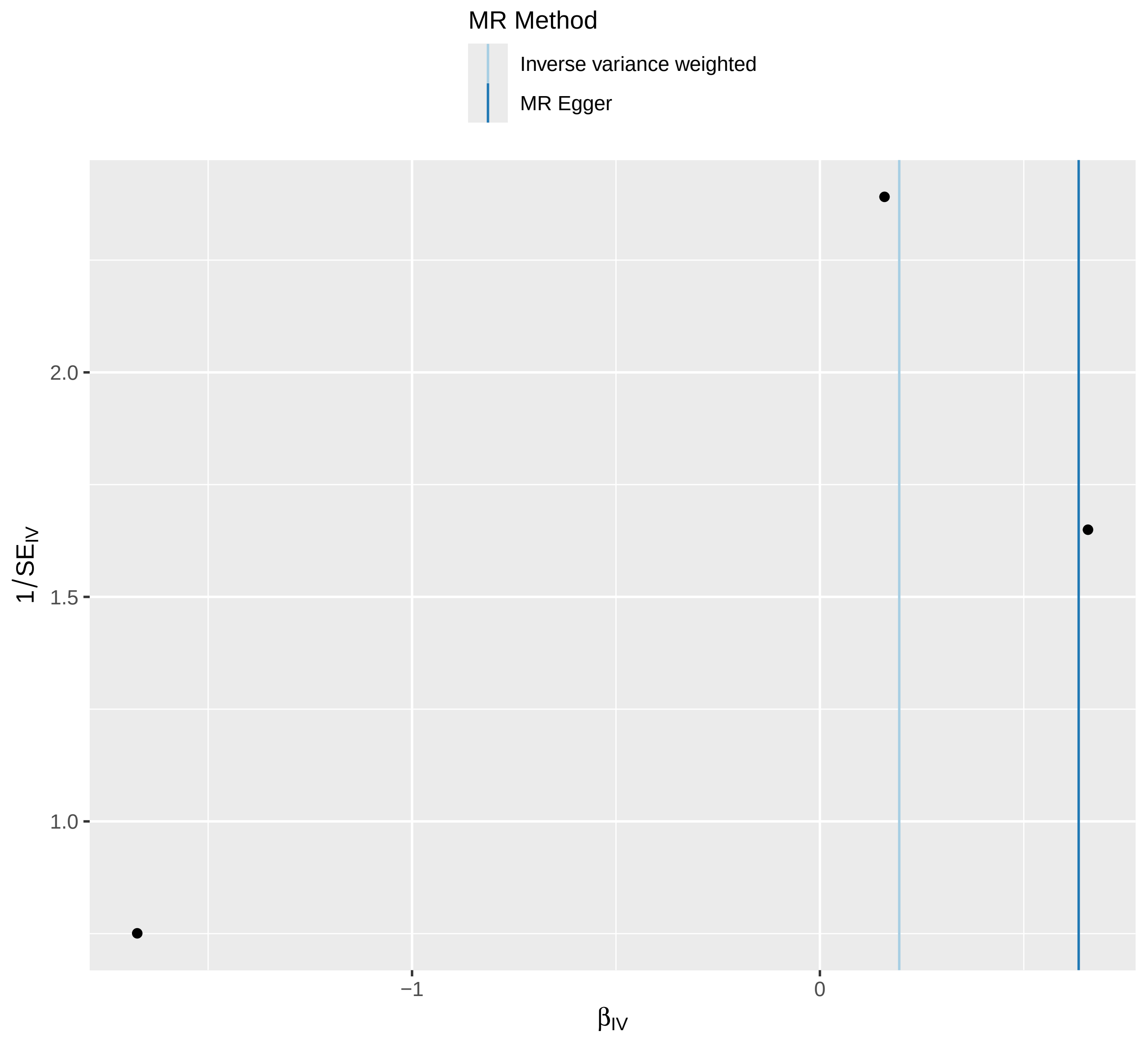
MR Method
Inverse variance weighted
MR Egger
2.0
1.5
1.0
1/SE_IV
−1
0
β_IV

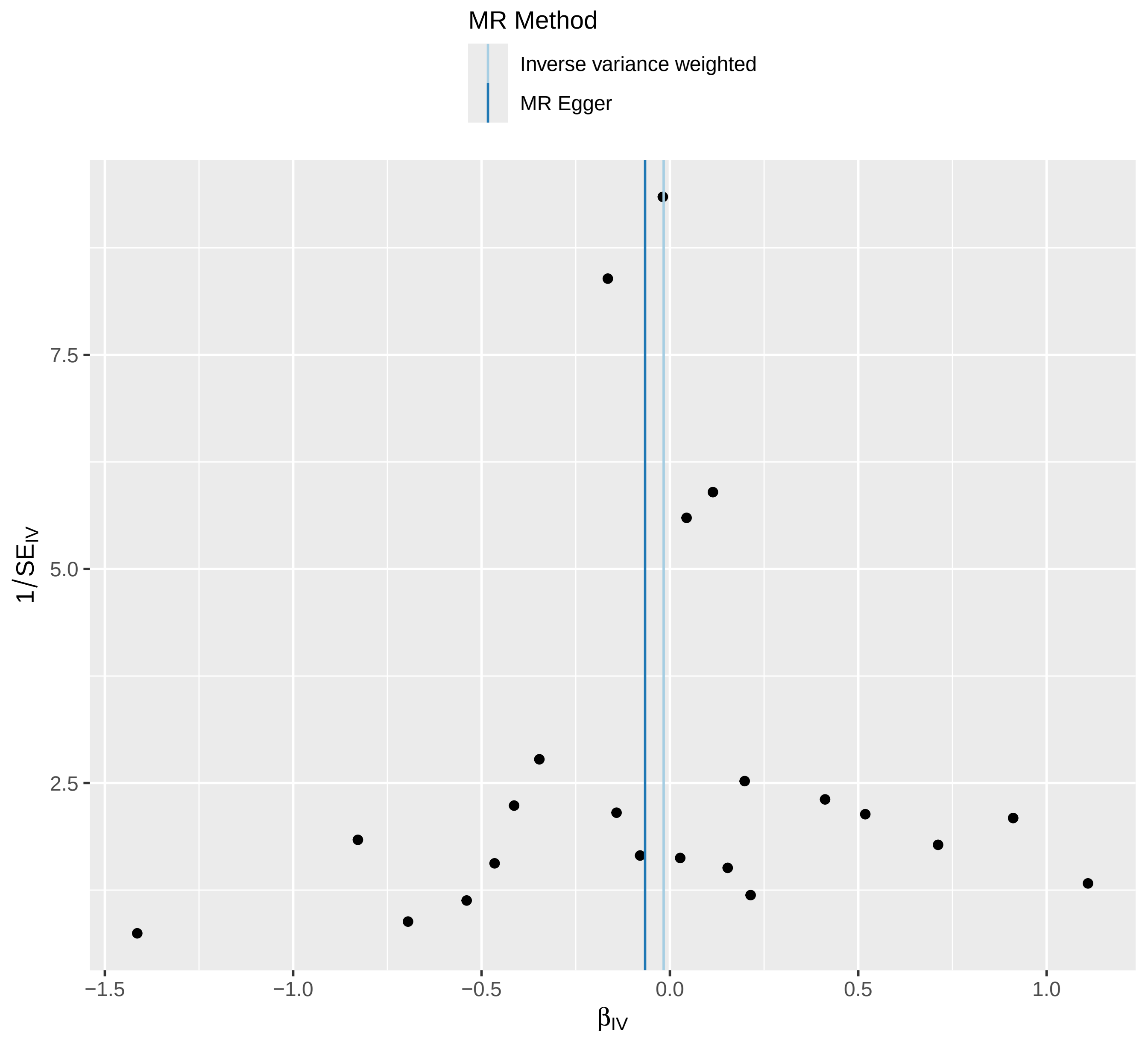

MR Method
Inverse variance weighted
MR Egger
7.5
5.0
2.5
$1/SE_{IV}$
−1.5
−1.0
−0.5
0.0
0.5
1.0
β_{IV}

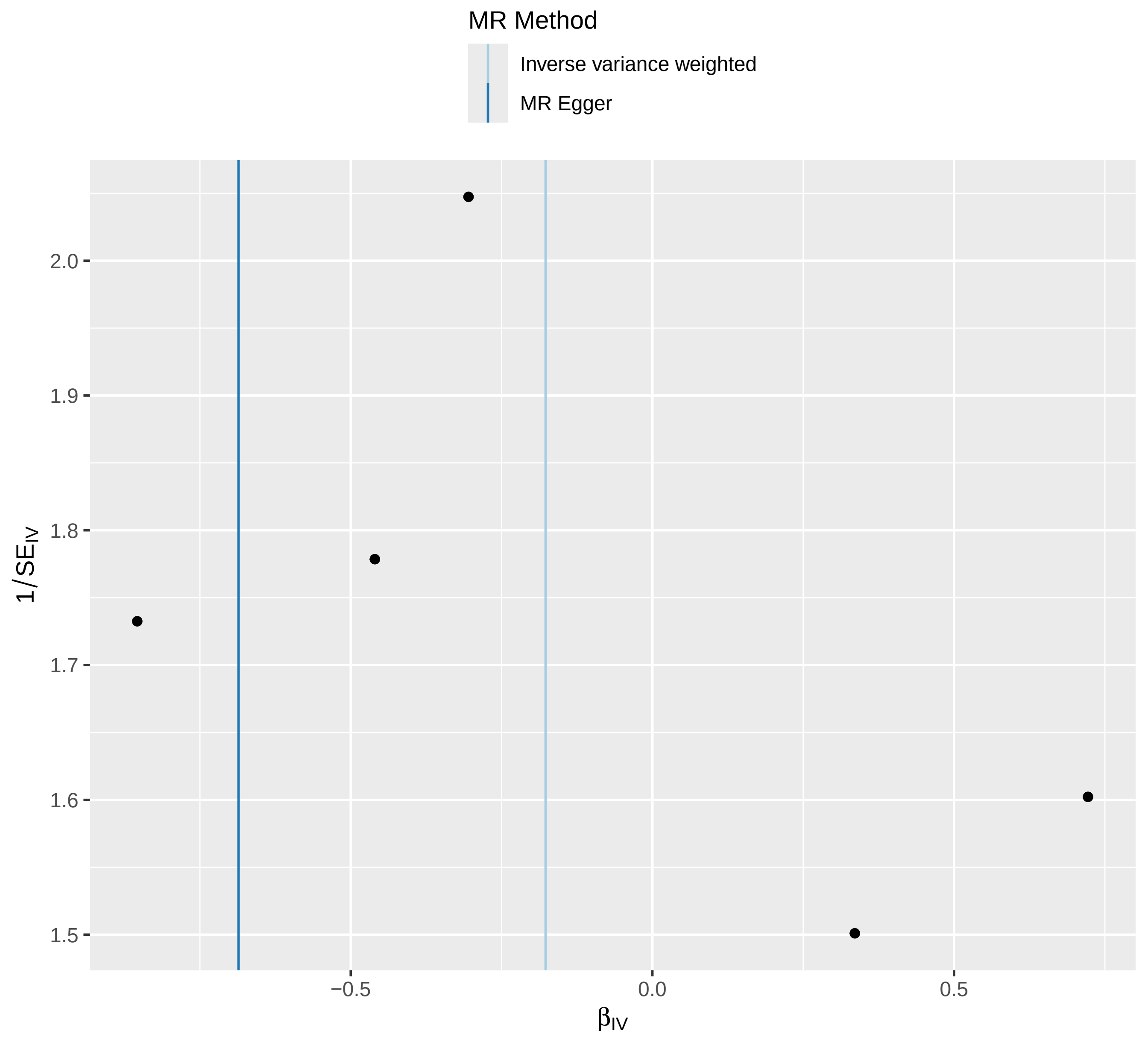

MR Method
Inverse variance weighted
MR Egger
2.0
1.9
1.8
1.7
1.6
1.5
1/SE_IV
−0.5
0.0
0.5
β_IV

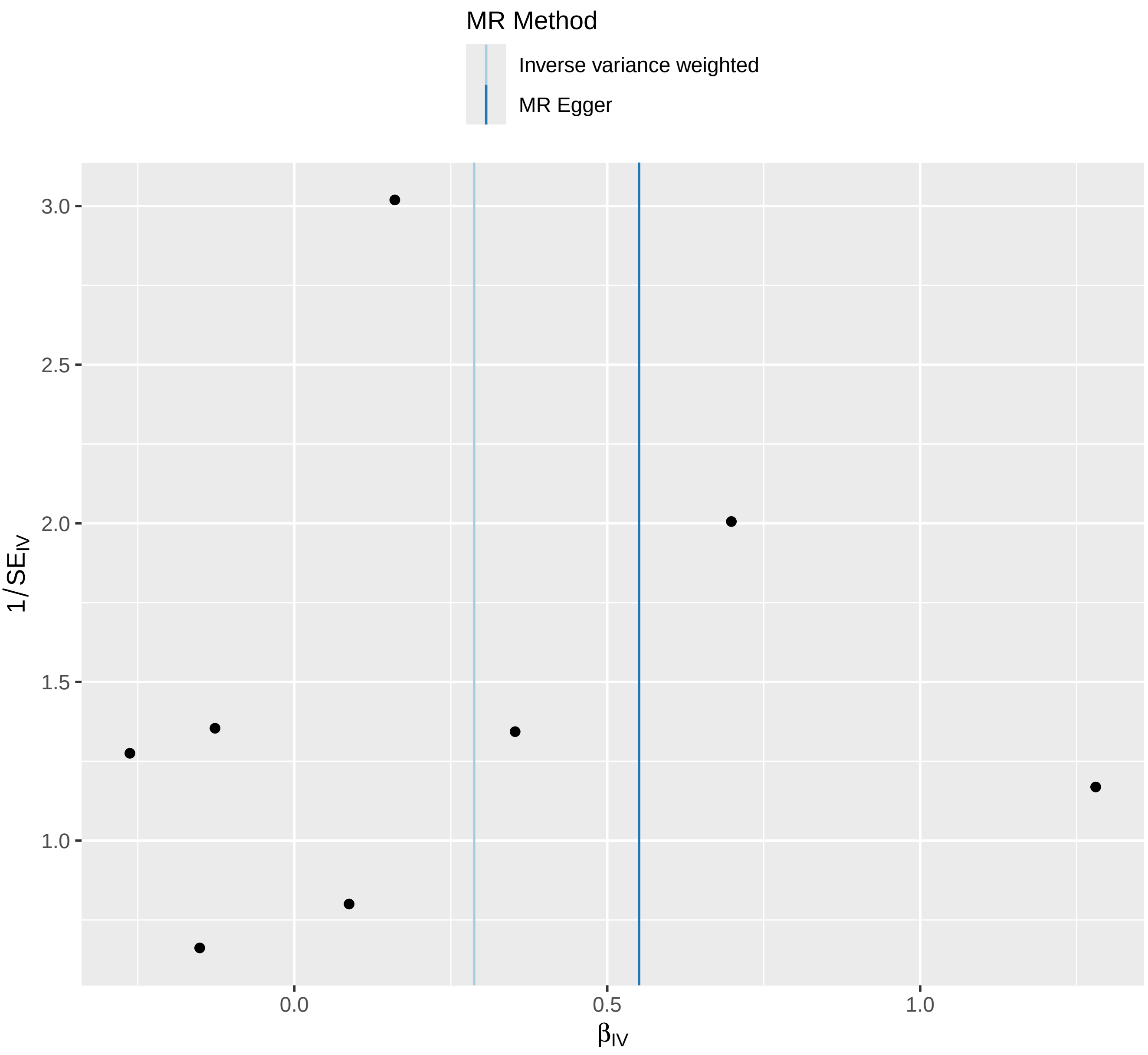

MR Method
Inverse variance weighted
MR Egger
3.0
2.5
2.0
1.5
1.0
$1/SE_{IV}$
0.0
0.5
1.0
β_{IV}

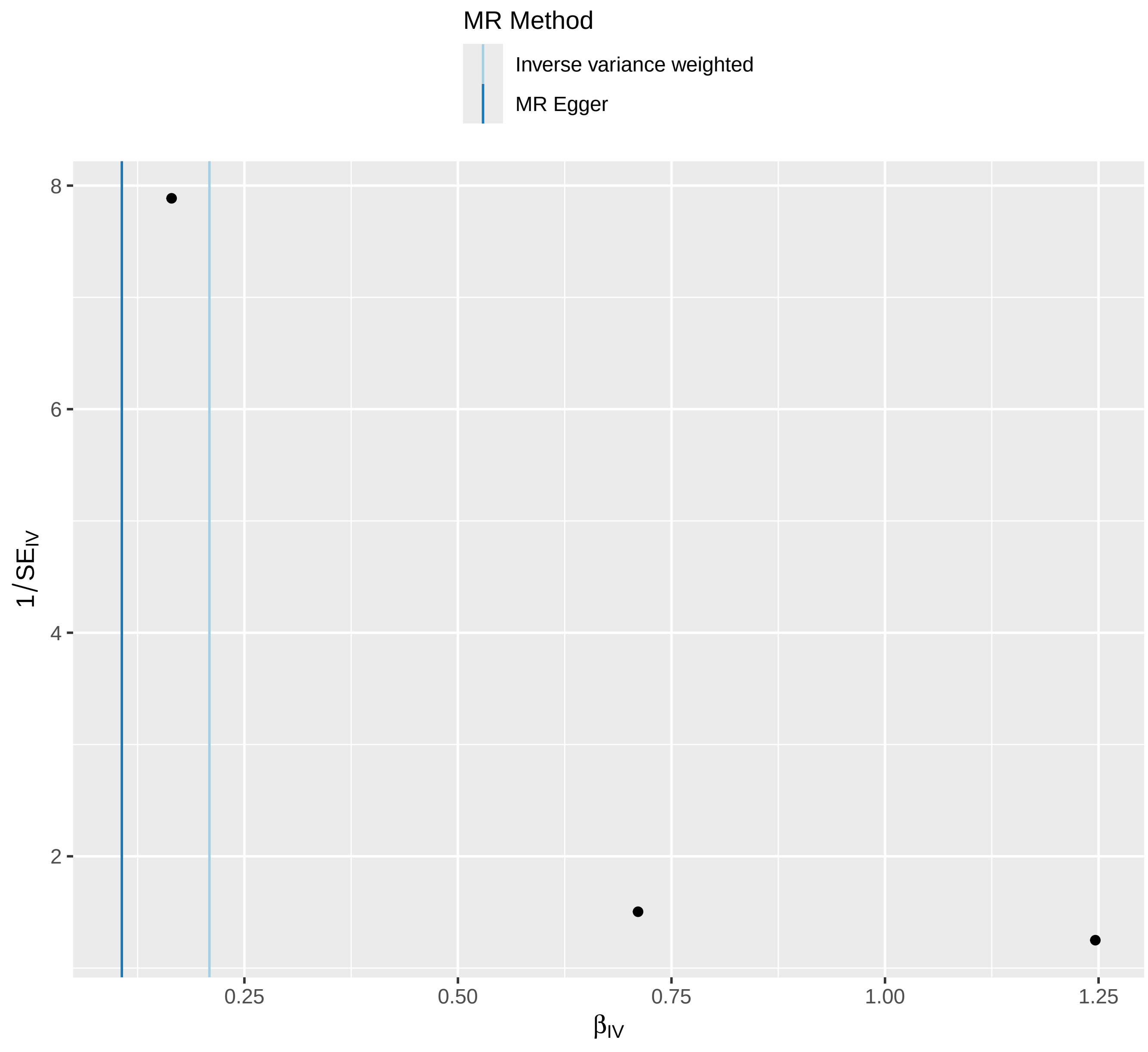

MR Method
Inverse variance weighted
MR Egger
8
6
4
2
0.25
0.50
0.75
1.00
1.25
β_{IV}
$1/SE_{IV}$

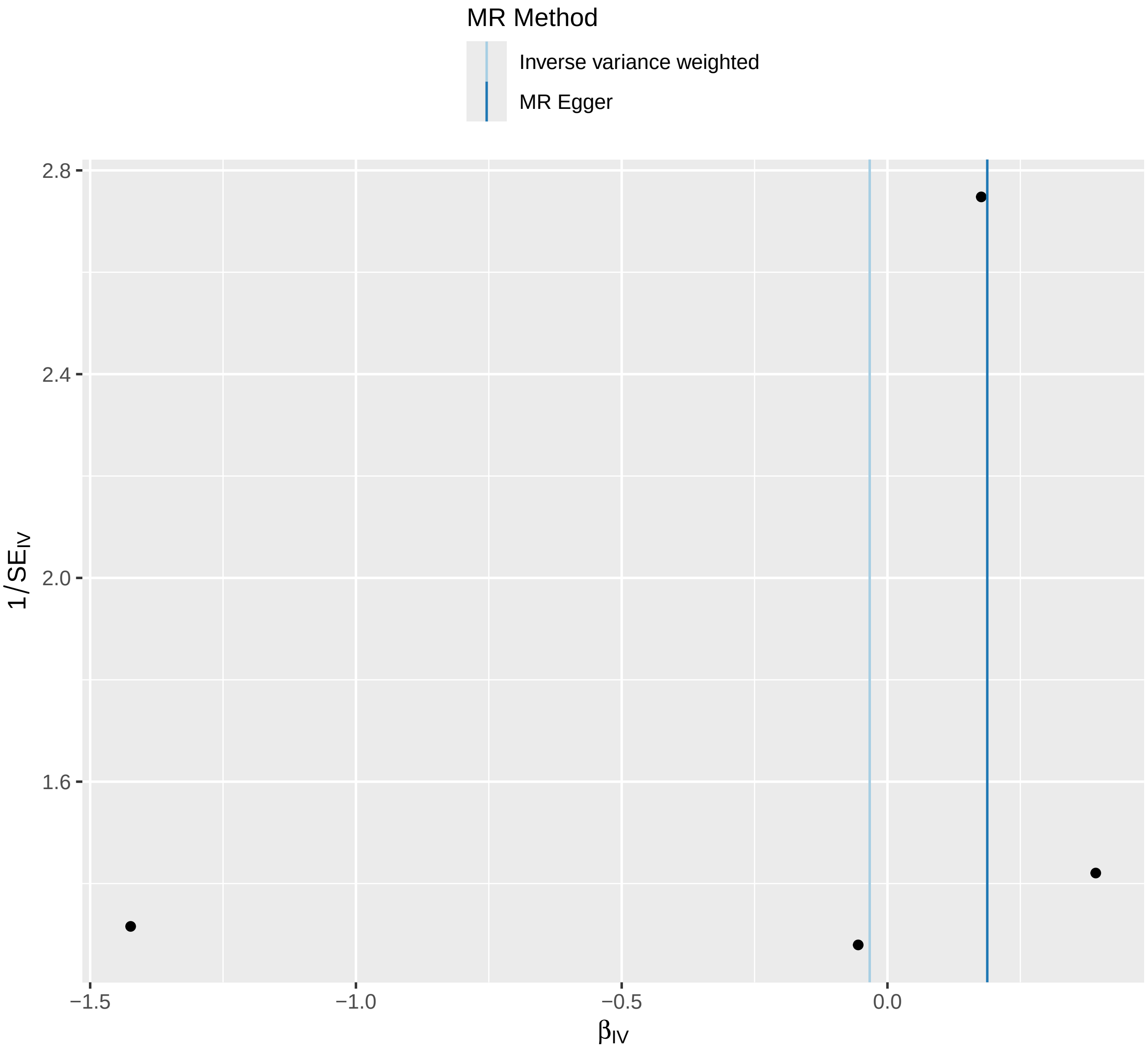
MR Method
Inverse variance weighted
MR Egger
2.8
2.4
2.0
1.6
1/SE_IV
−1.5
−1.0
−0.5
0.0
β_IV

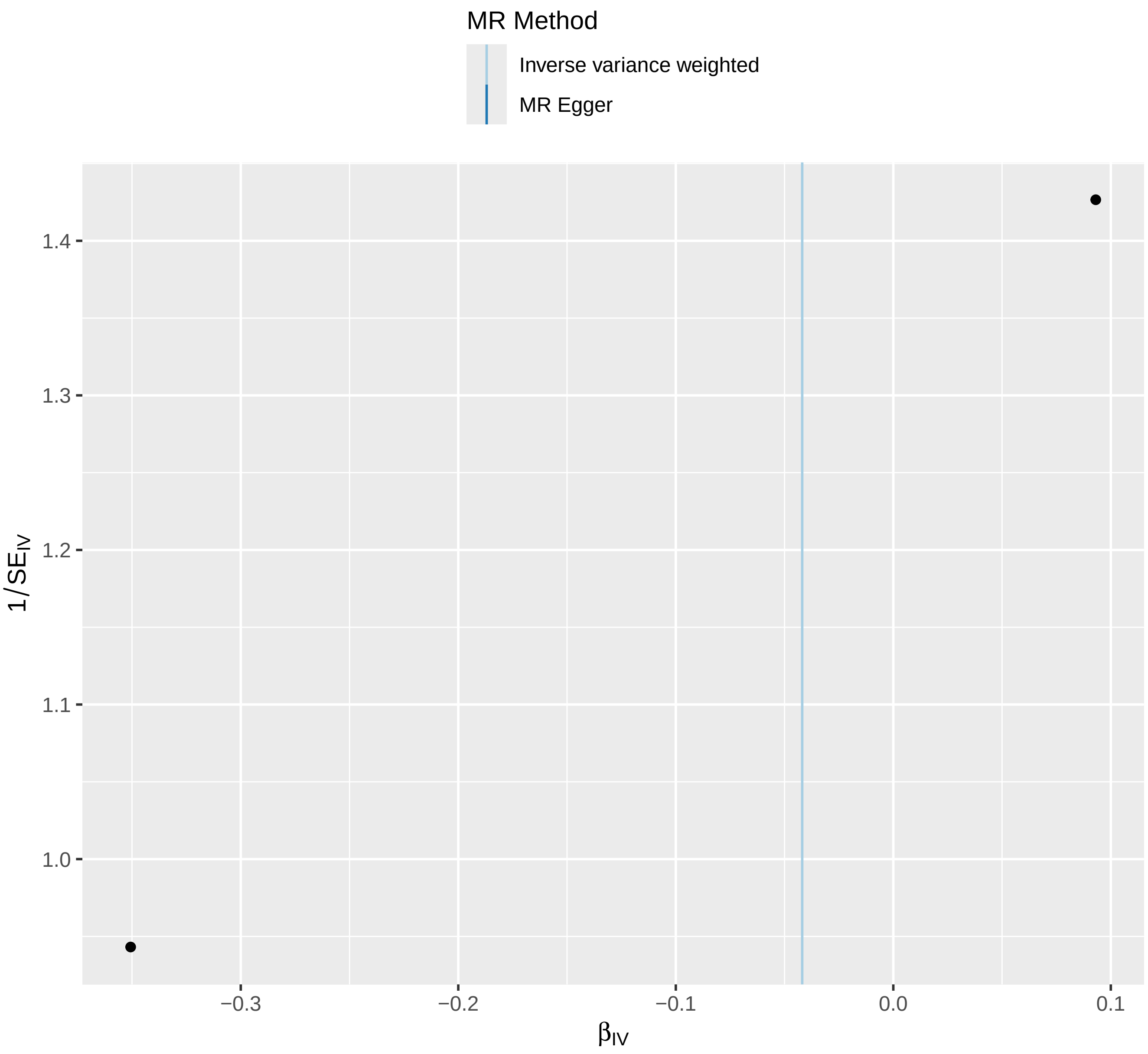

MR Method
Inverse variance weighted
MR Egger
1.4
1.3
1.2
1.1
1.0
$1/SE_{IV}$
−0.3
−0.2
−0.1
0.0
0.1
β_{IV}

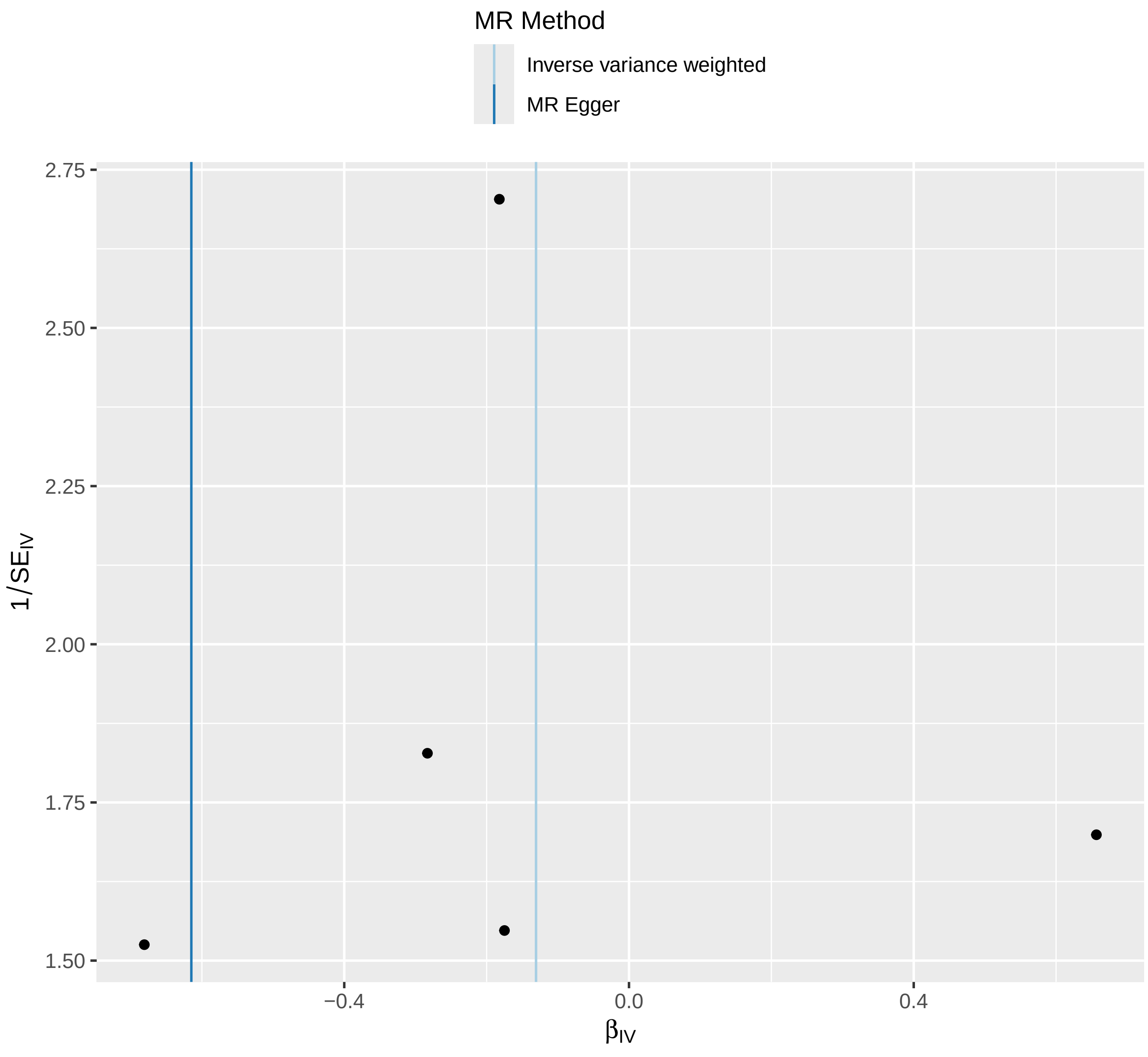
MR Method
Inverse variance weighted
MR Egger
2.75
2.50
2.25
2.00
1.75
1.50
1/SE_IV
−0.4
0.0
0.4
β_IV

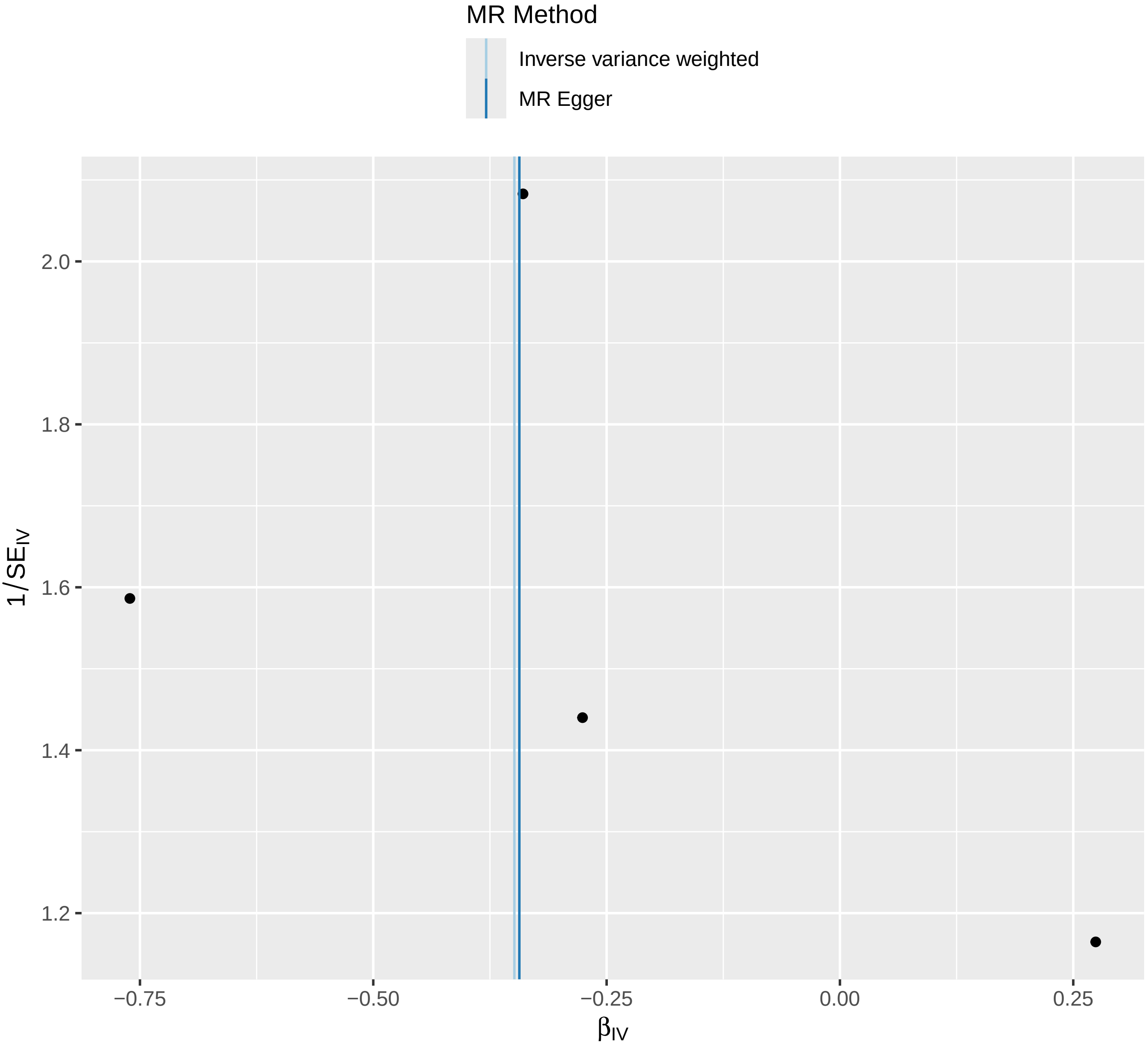

MR Method
Inverse variance weighted
MR Egger
2.0
1.8
1.6
1.4
1.2
1/SE_IV
−0.75
−0.50
−0.25
0.00
0.25
β_IV

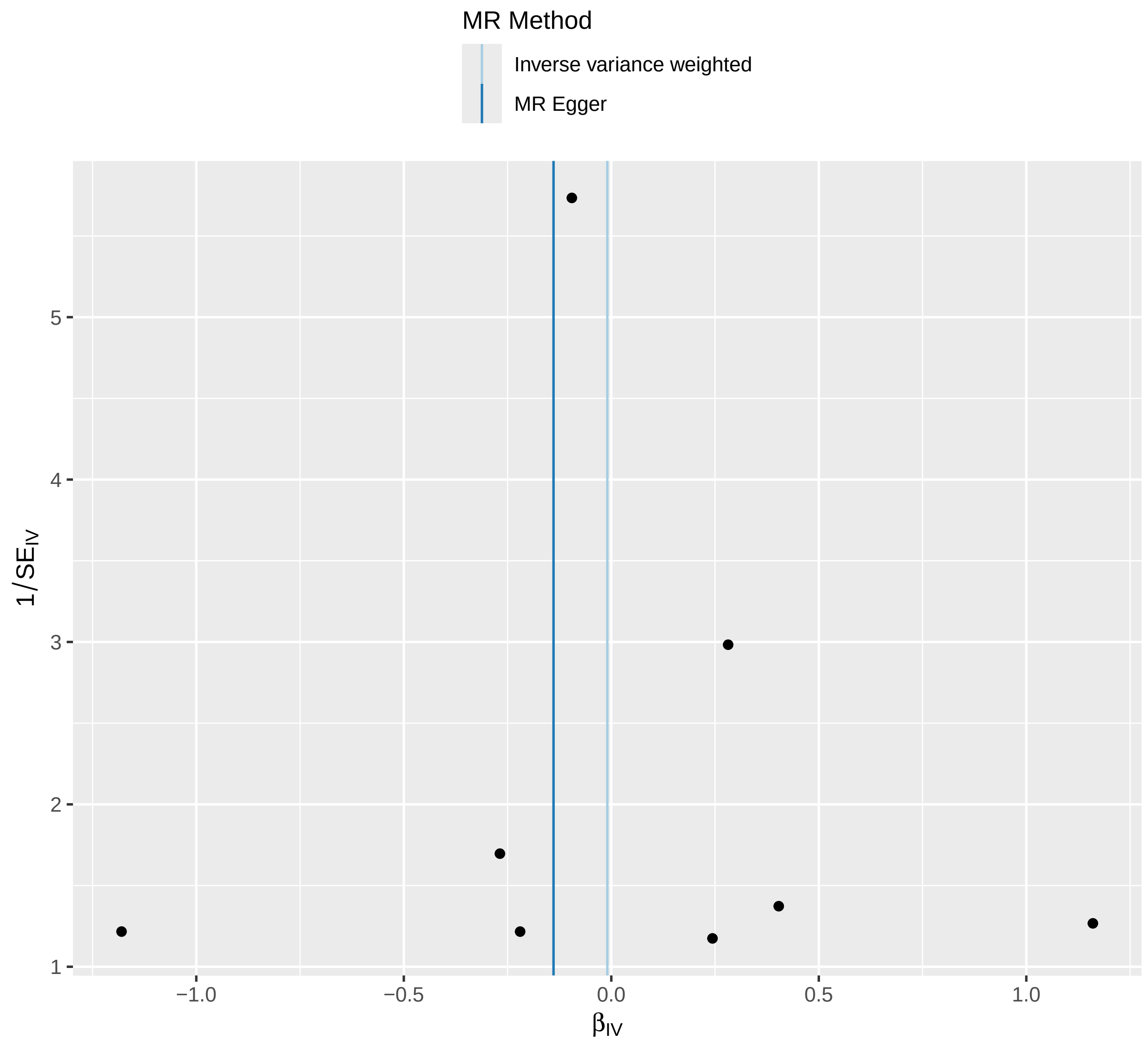

MR Method
Inverse variance weighted
MR Egger
1/SE$_{IV}$
5
4
3
2
1
−1.0
−0.5
0.0
0.5
1.0
β$_{IV}$

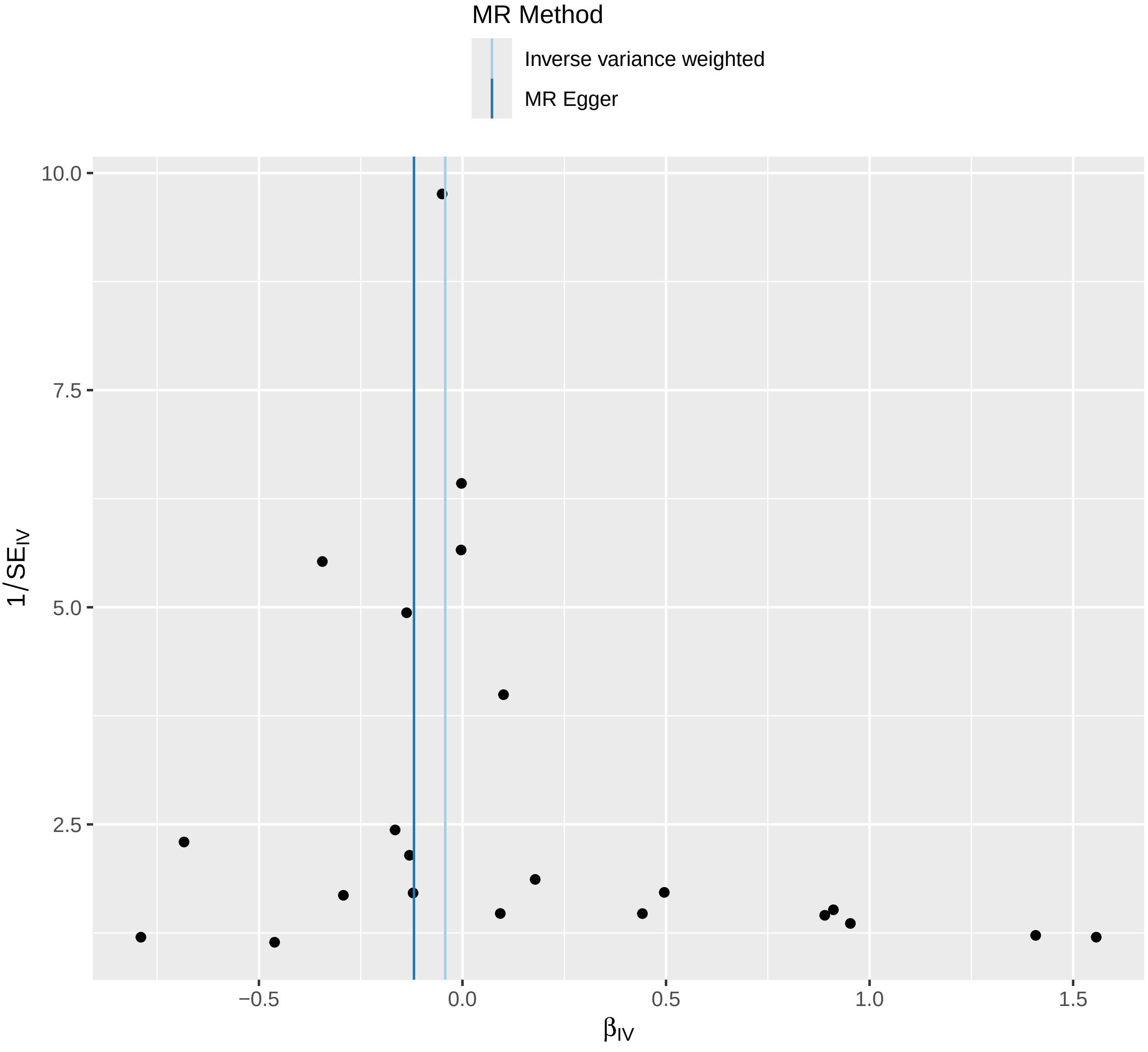

MR Method
Inverse variance weighted
MR Egger
10.0
7.5
5.0
2.5
1/SE$_{IV}$
−0.5
0.0
0.5
1.0
1.5
β$_{IV}$

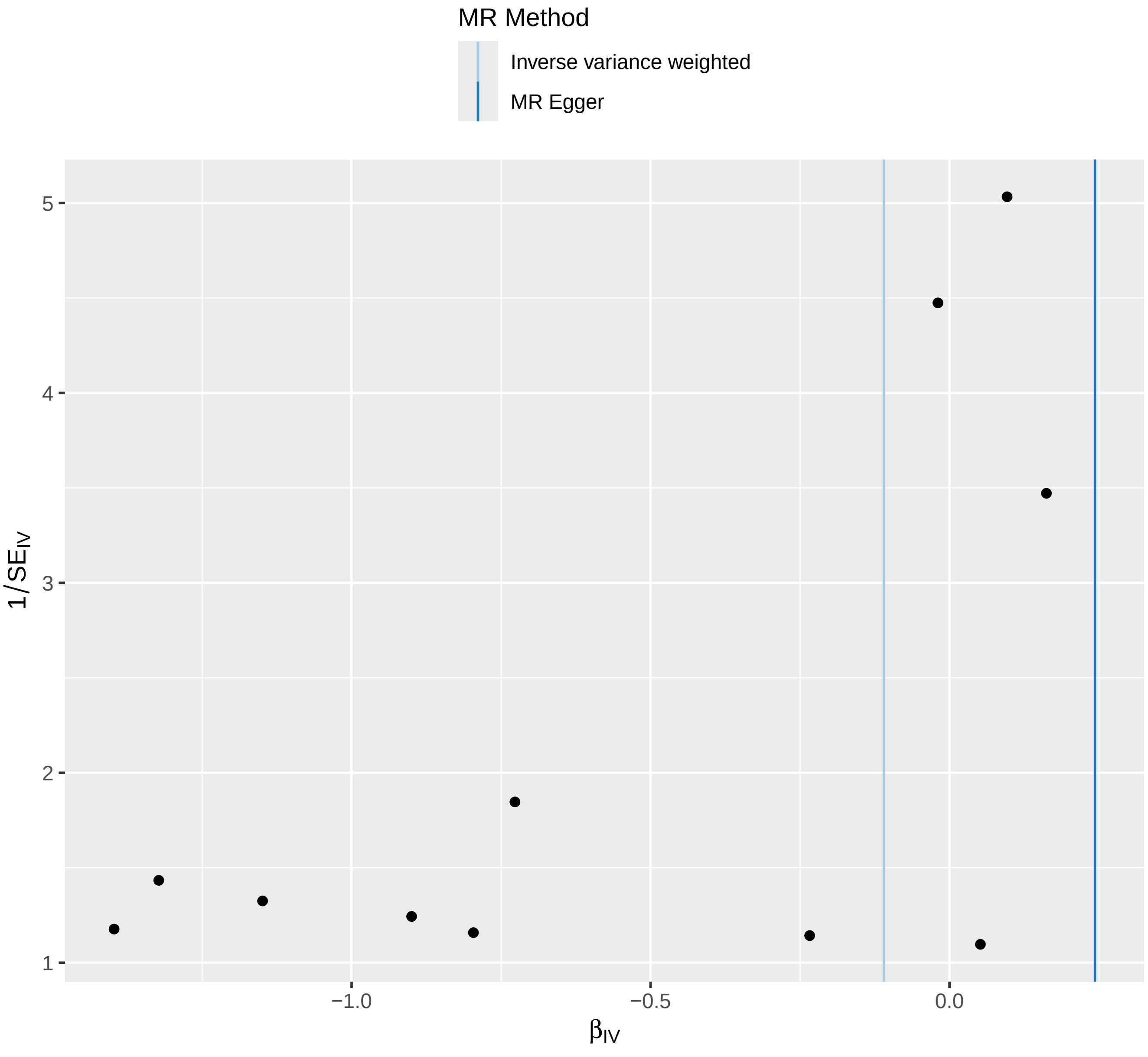

MR Method
Inverse variance weighted
MR Egger
5
4
3
2
1
1/SE_IV
−1.0
−0.5
0.0
β_IV

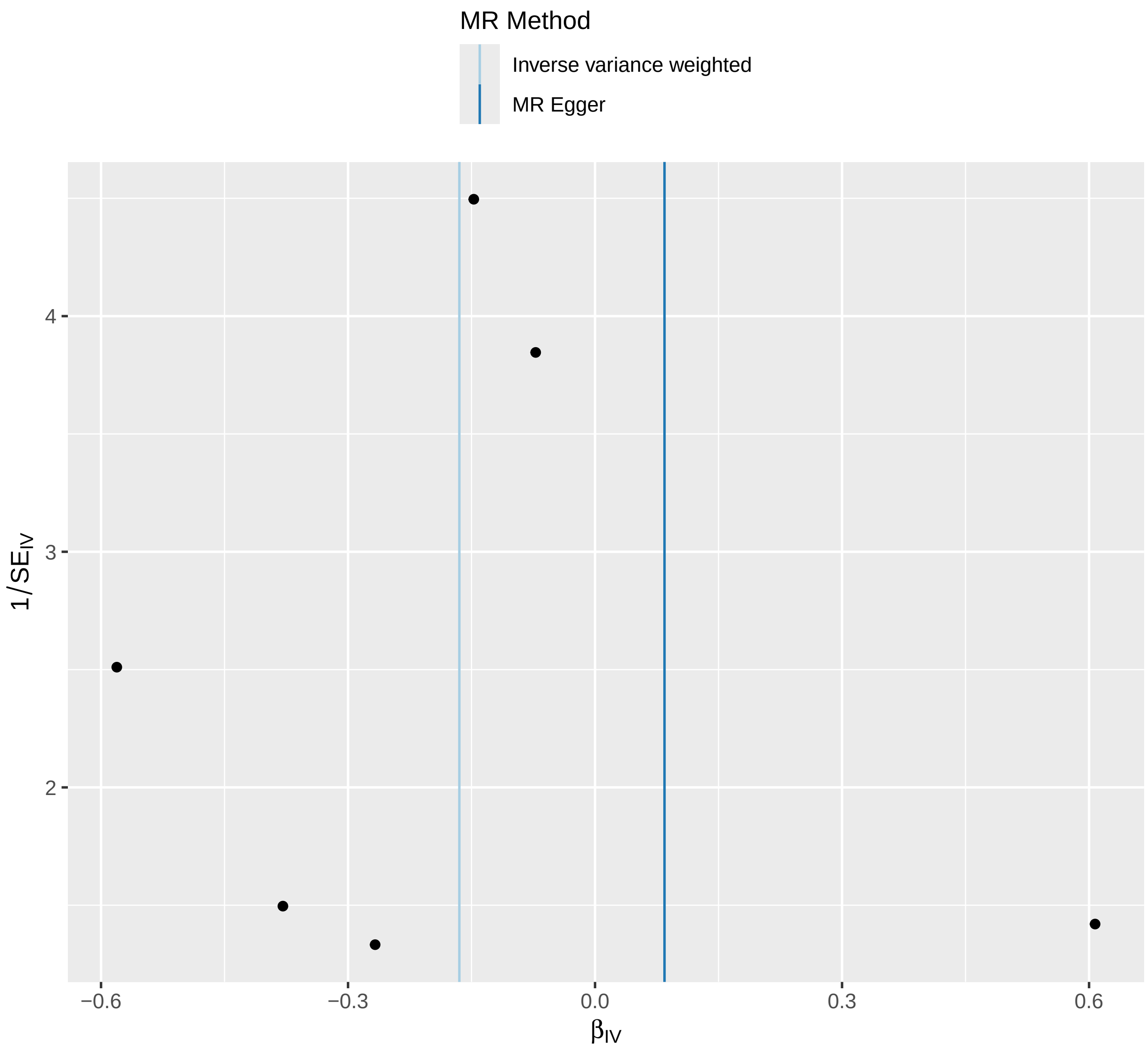

MR Method
Inverse variance weighted
MR Egger
4
3
2
1/SE_IV
−0.6
−0.3
0.0
0.3
0.6
β_IV

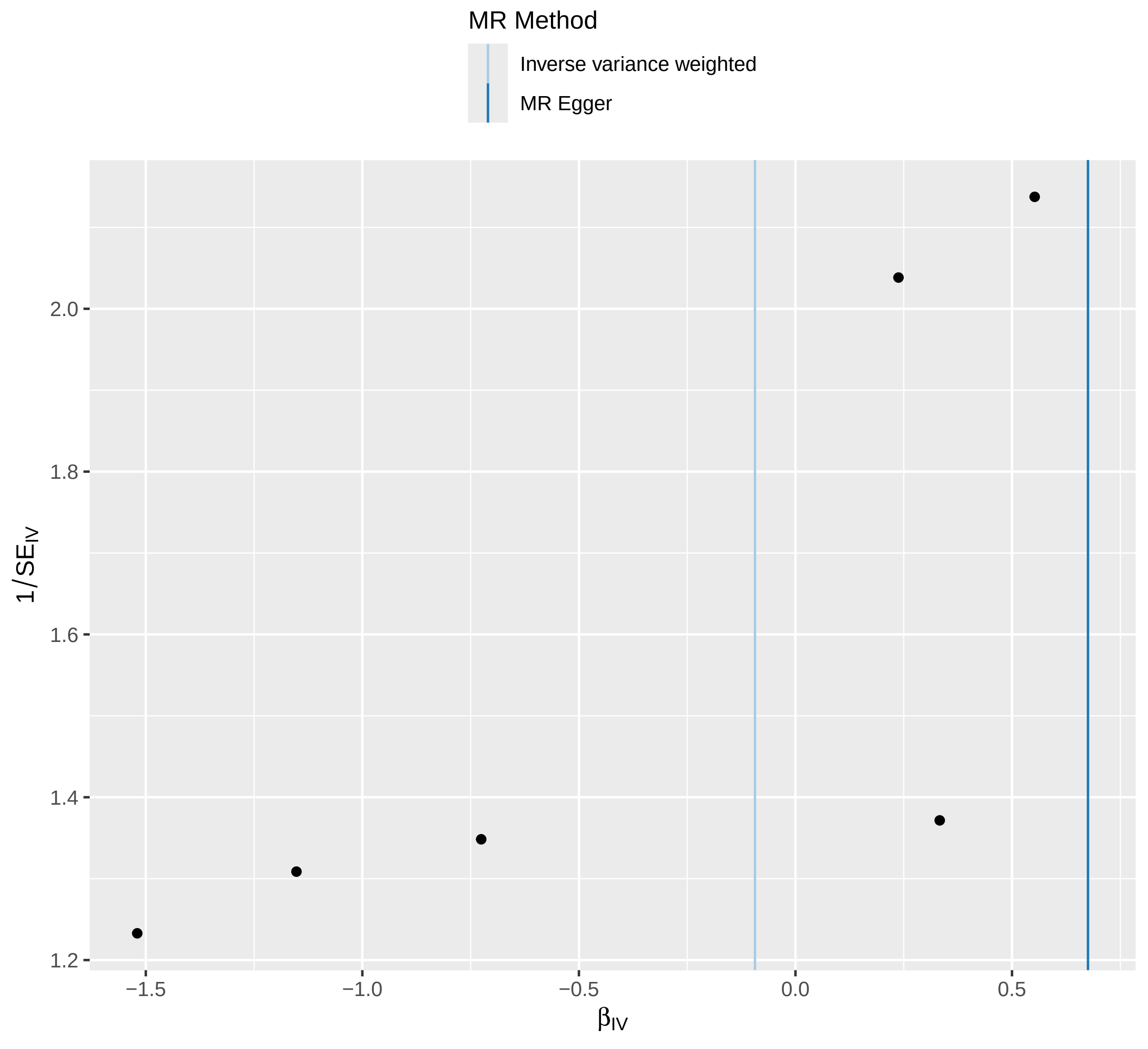
MR Method
Inverse variance weighted
MR Egger
2.0
1.8
1.6
1.4
1.2
1/SE_IV
−1.5
−1.0
−0.5
0.0
0.5
β_IV

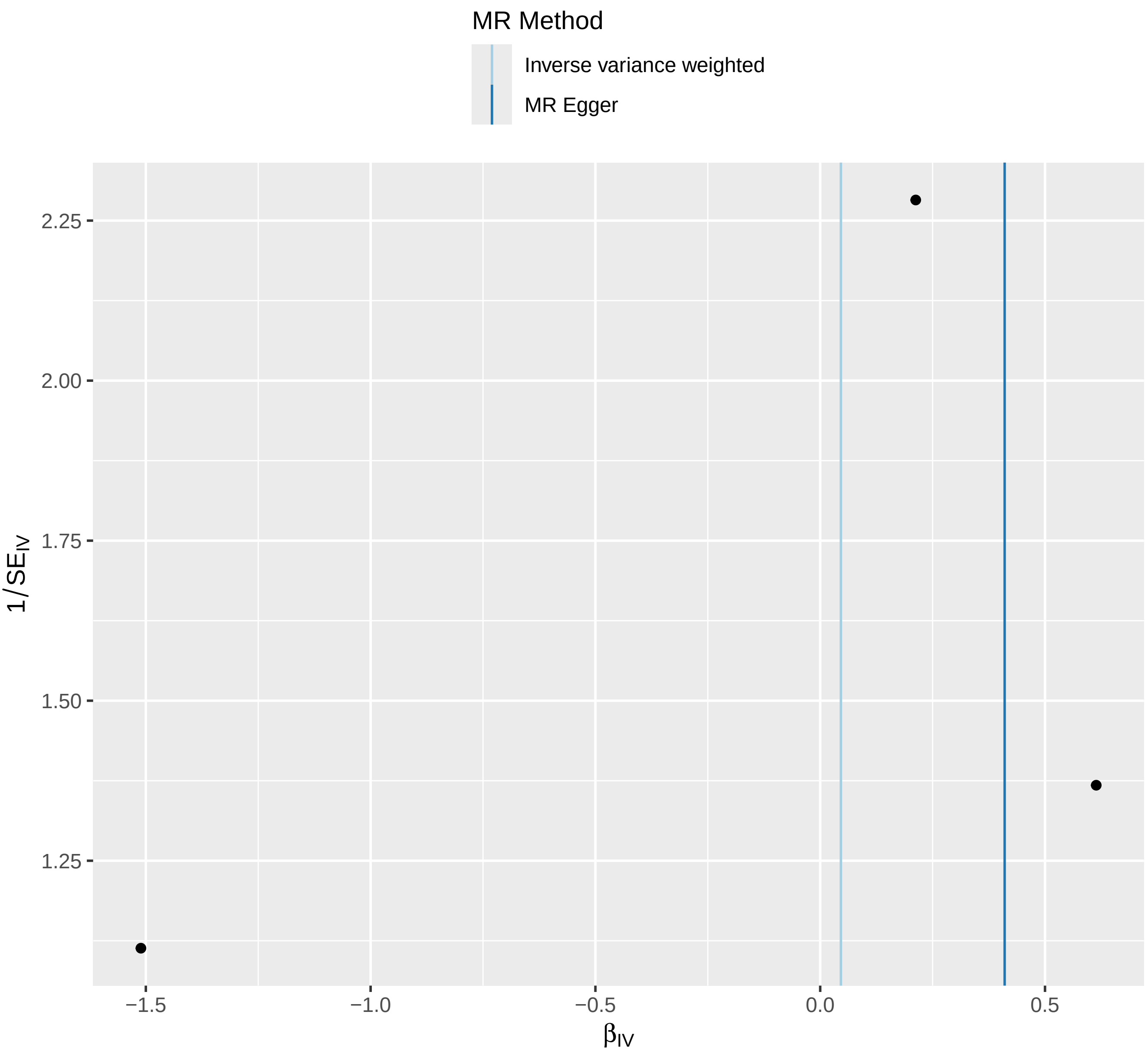
MR Method
Inverse variance weighted
MR Egger
2.25
2.00
1.75
1.50
1.25
1/SE_IV
−1.5
−1.0
−0.5
0.0
0.5
β_IV

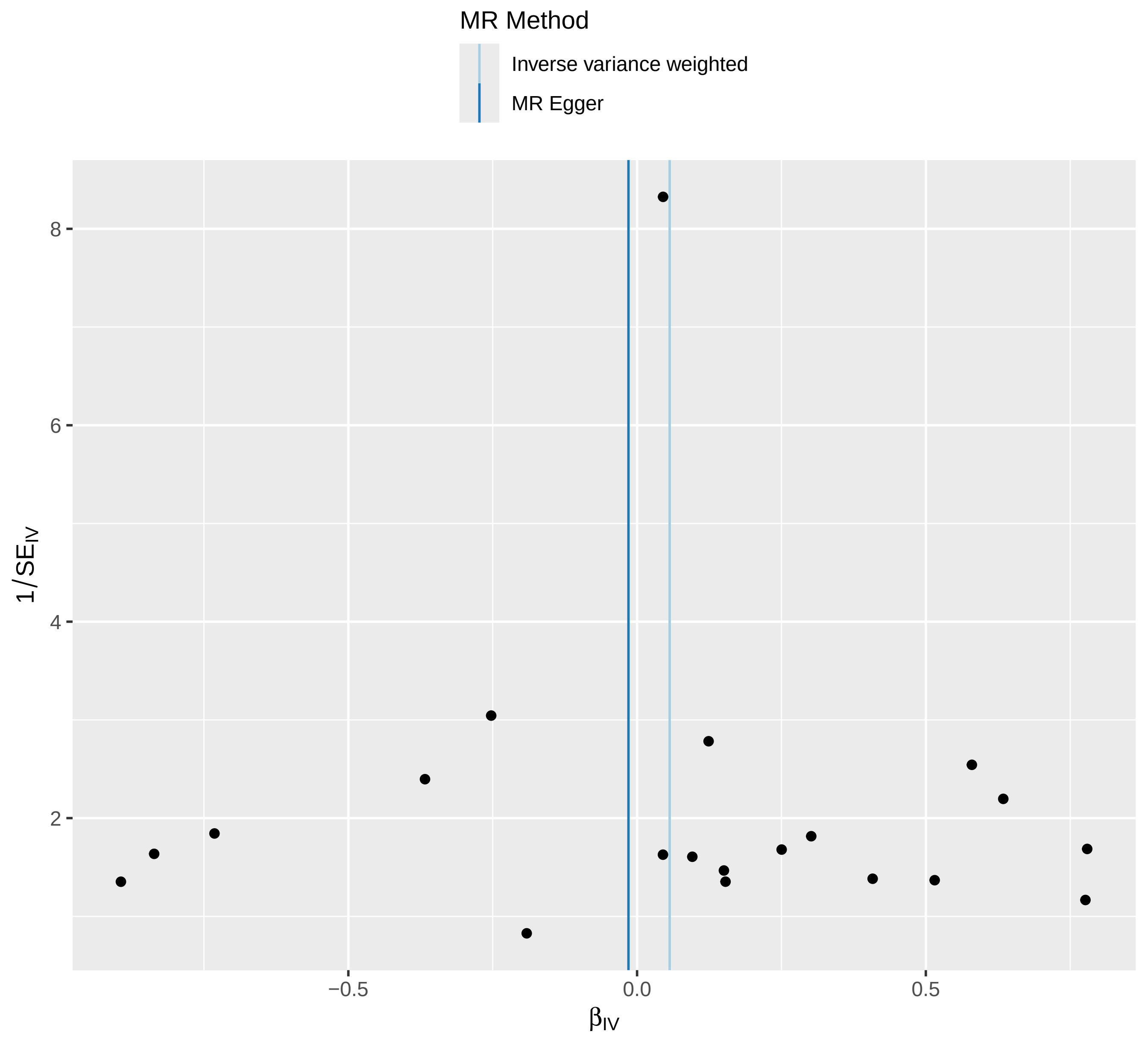
MR Method
Inverse variance weighted
MR Egger
1/SE_IV
8
6
4
2
−0.5
0.0
0.5
β_IV

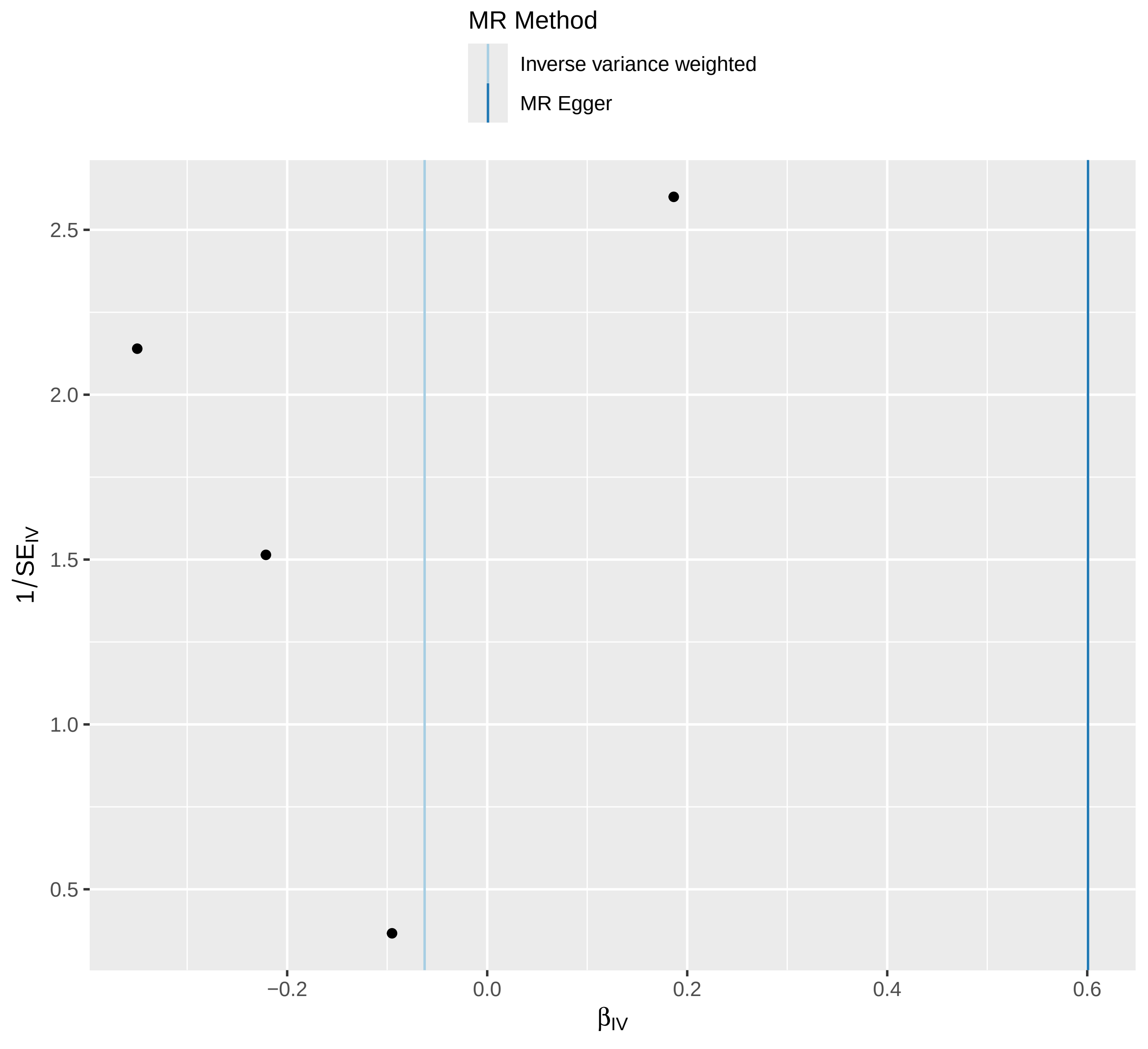
MR Method
Inverse variance weighted
MR Egger
1/SE_{IV}
2.5
2.0
1.5
1.0
0.5
−0.2
0.0
0.2
0.4
0.6
β_{IV}

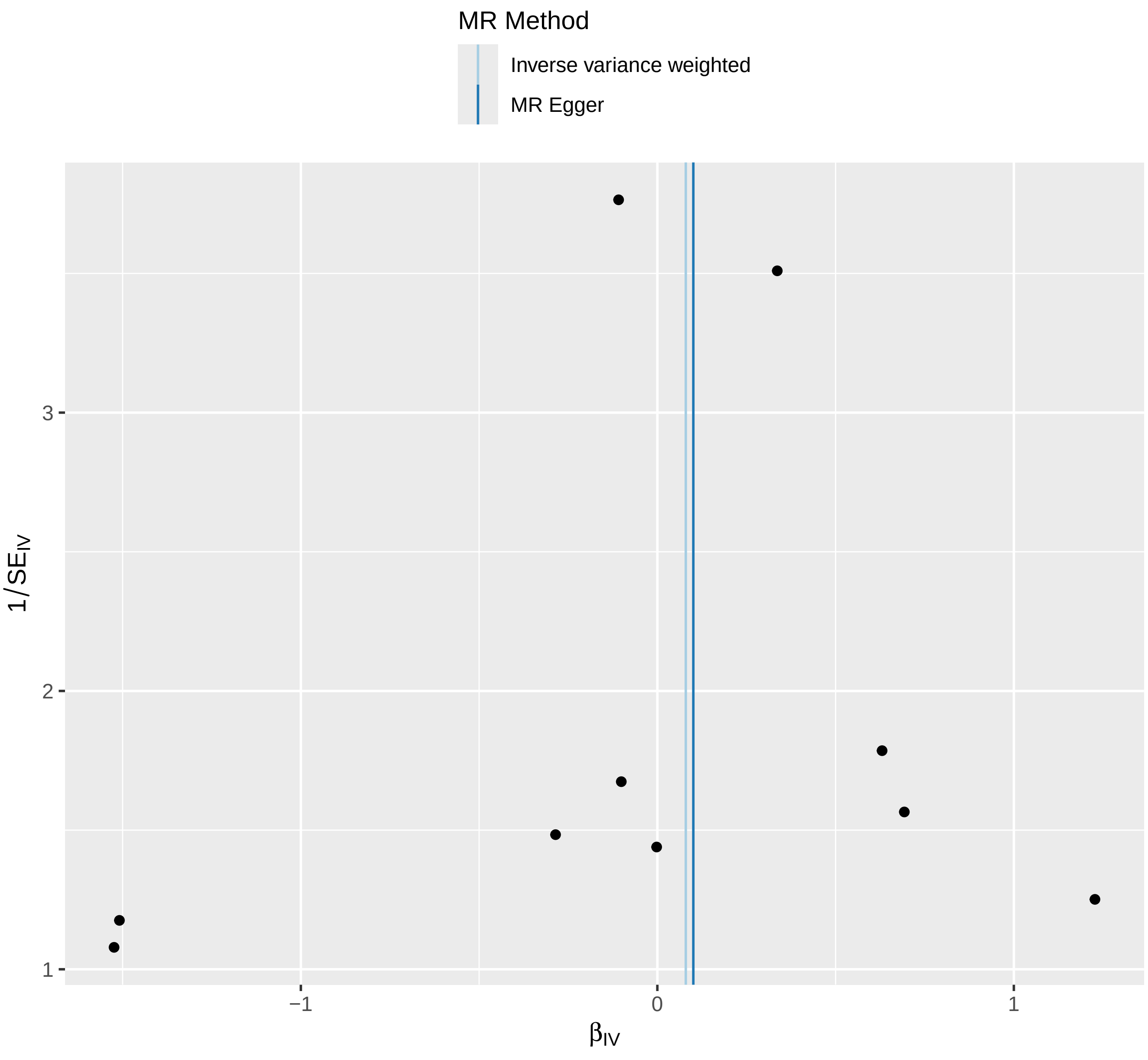

MR Method
Inverse variance weighted
MR Egger
1/SE_IV
3
2
1
−1
0
1
β_IV

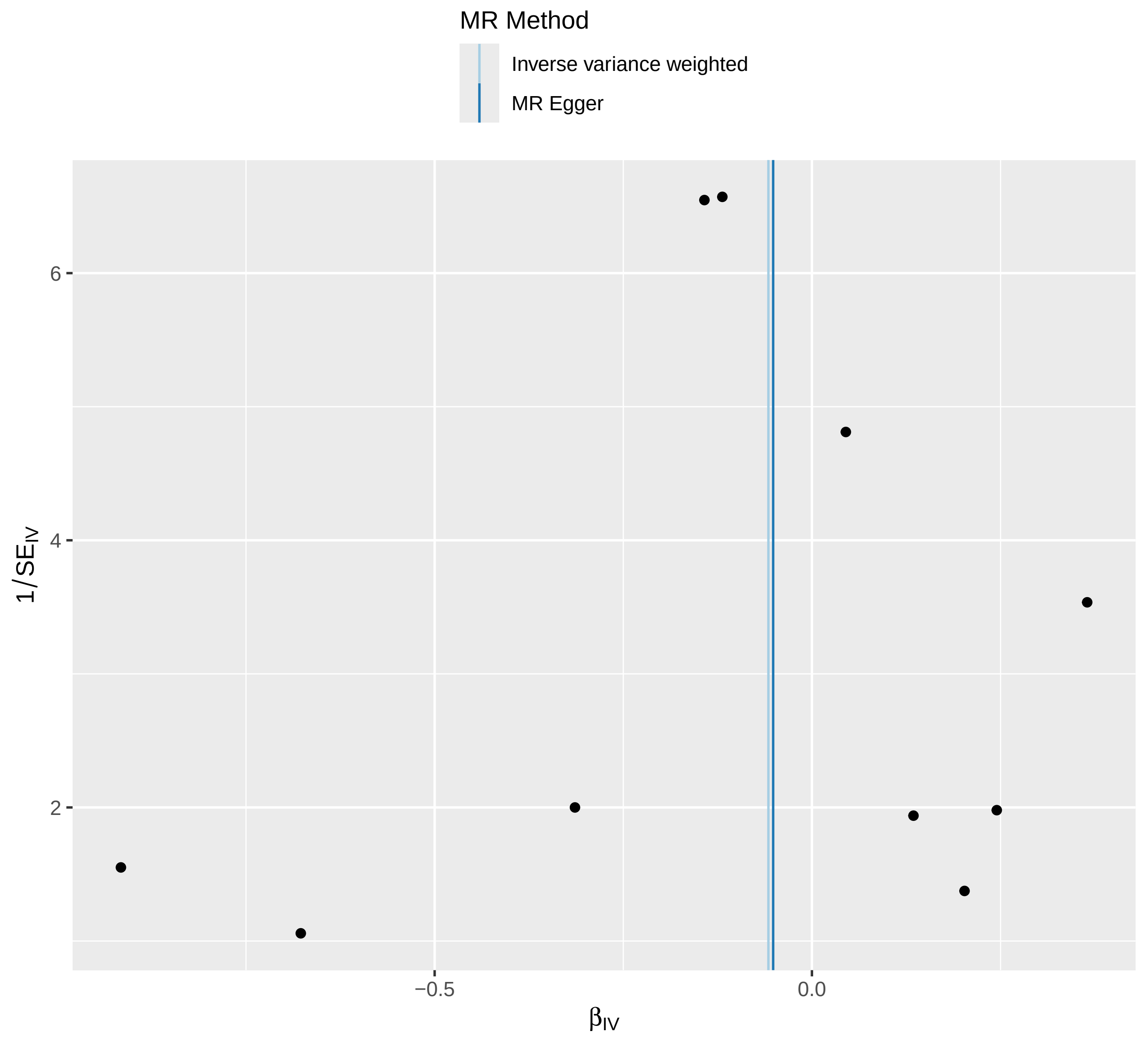
MR Method
Inverse variance weighted
MR Egger
6
4
2
1/SE_IV
−0.5
0.0
β_IV

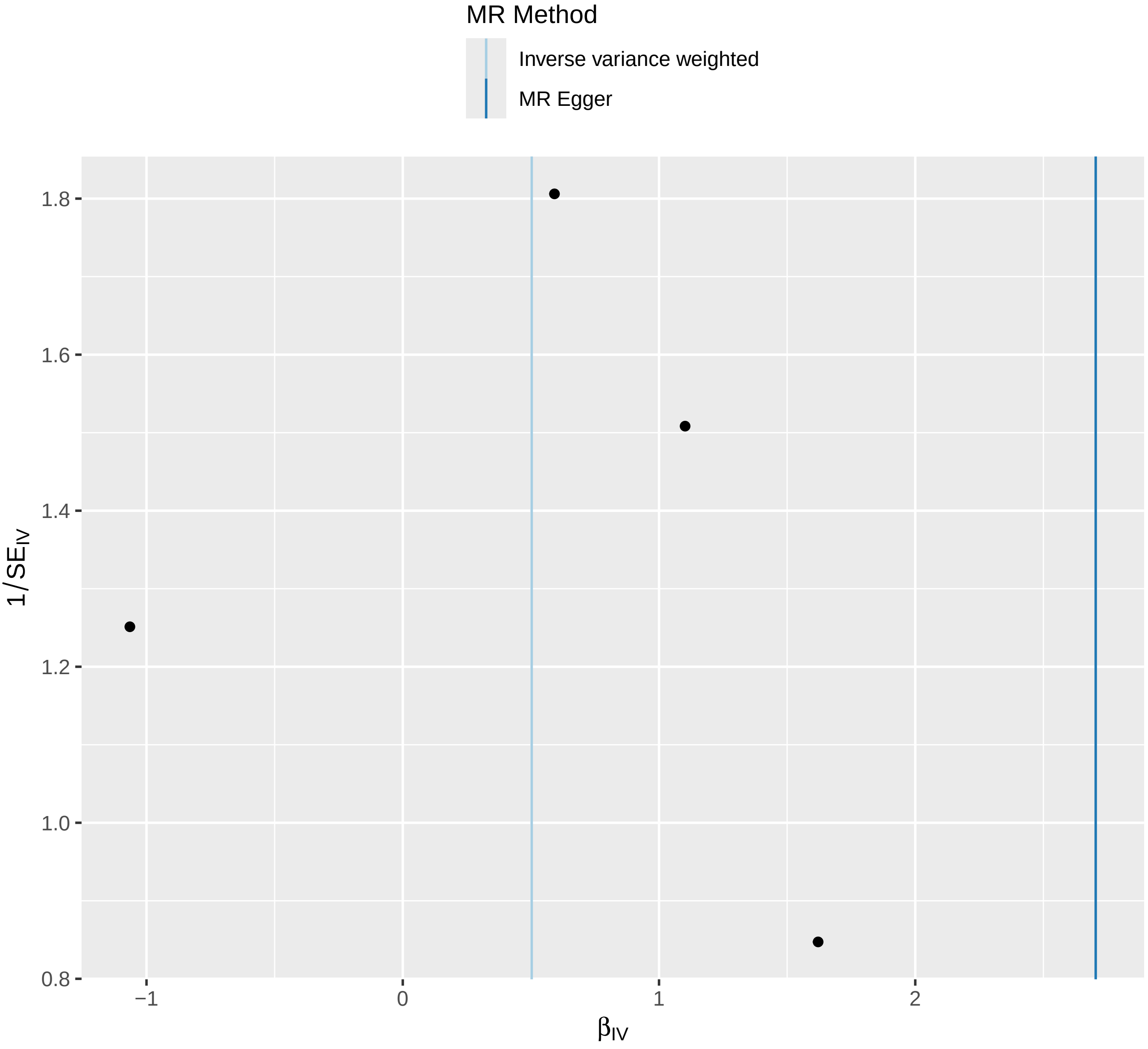
MR Method
Inverse variance weighted
MR Egger
1.8
1.6
1.4
1.2
1.0
0.8
1/SE$_{IV}$
−1
0
1
2
β$_{IV}$

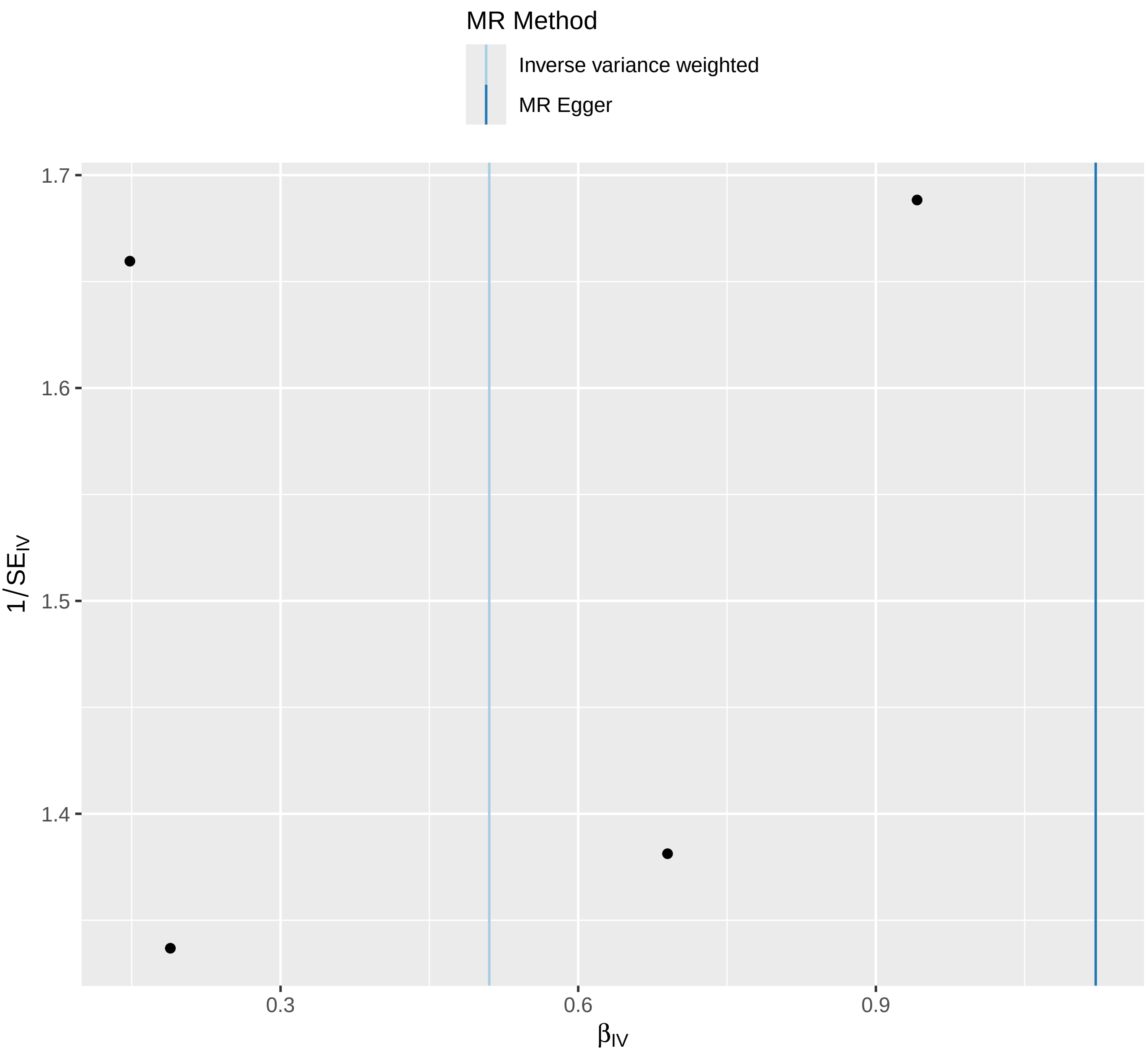
MR Method
Inverse variance weighted
MR Egger
1.7
1.6
1.5
1.4
1/SE_IV
0.3
0.6
0.9
β_IV

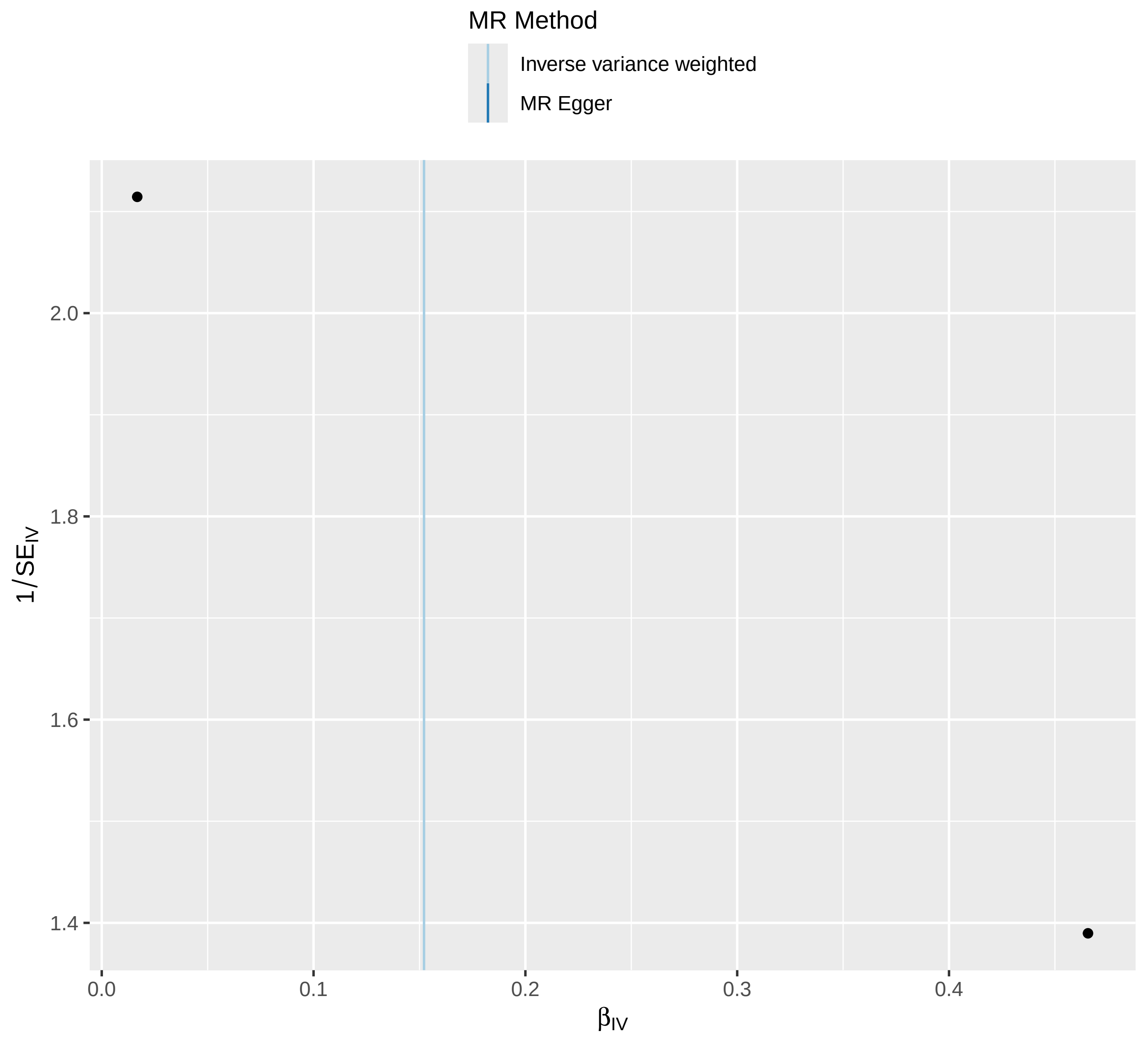
MR Method
Inverse variance weighted
MR Egger
2.0
1.8
1.6
1.4
1/SE_IV
0.0
0.1
0.2
0.3
0.4
β_IV

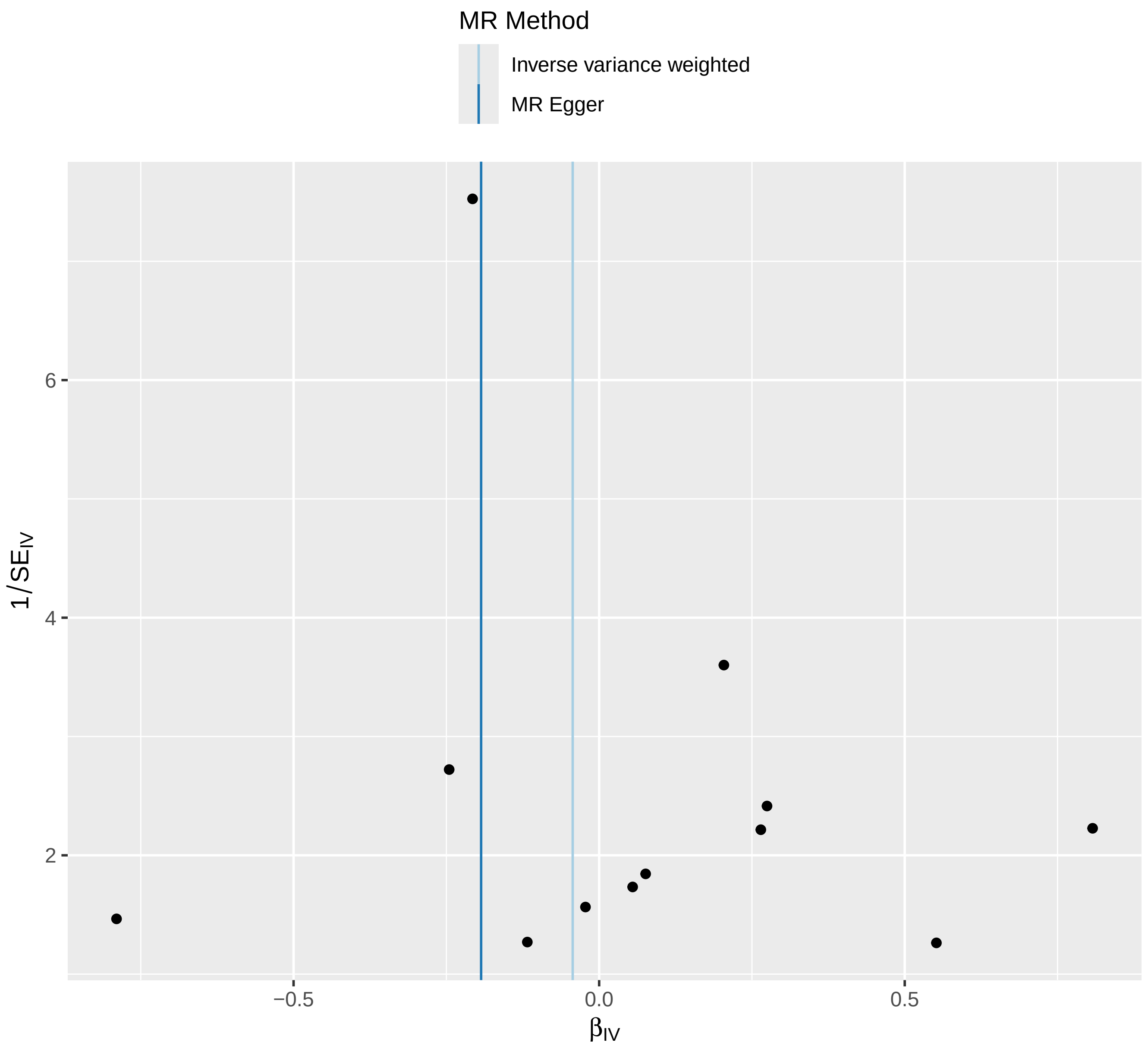

MR Method
Inverse variance weighted
MR Egger
6
4
2
1/SE_IV
−0.5
0.0
0.5
β_IV

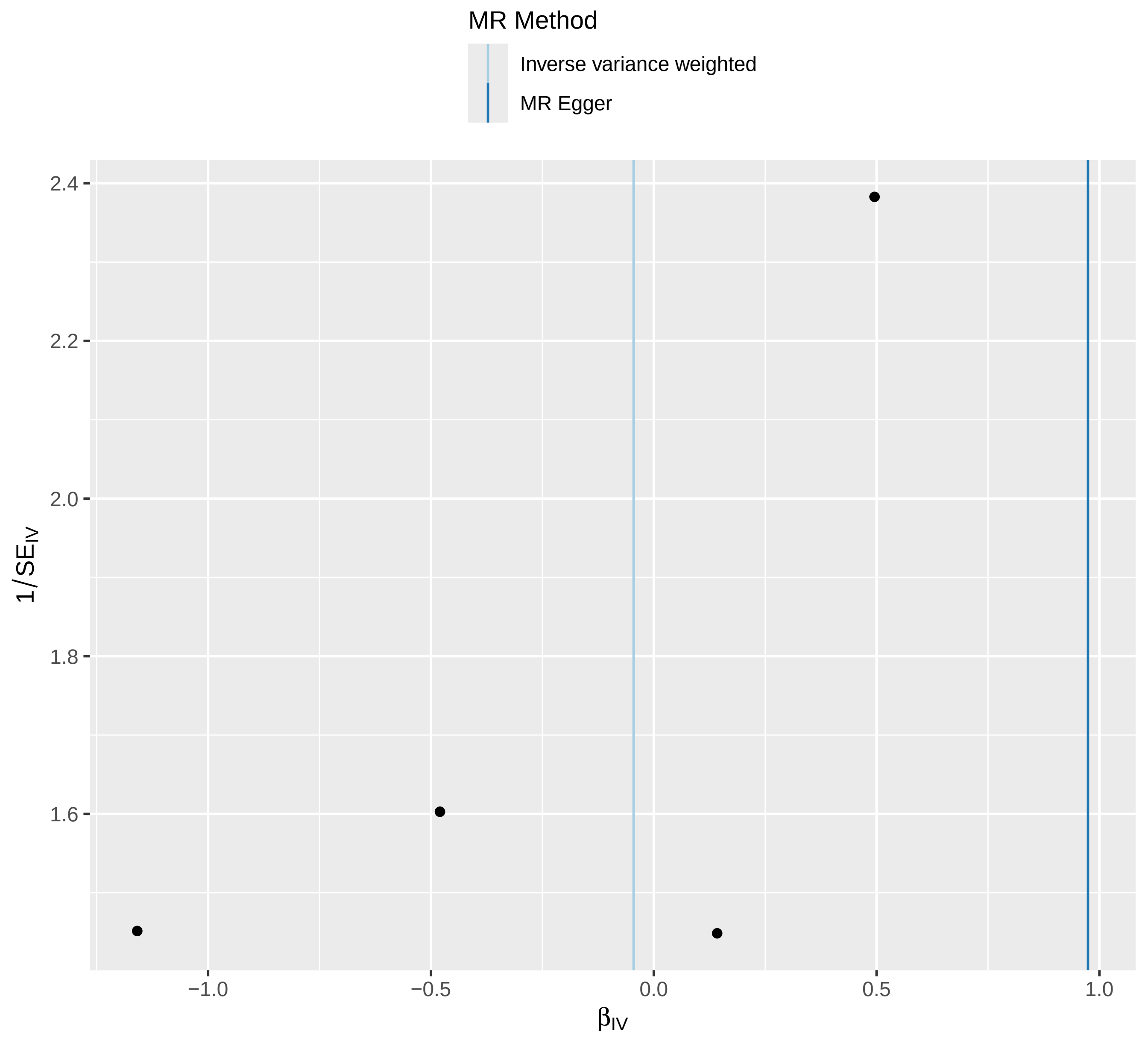
MR Method
Inverse variance weighted
MR Egger
2.4
2.2
2.0
1.8
1.6
$1/SE_{IV}$
−1.0
−0.5
0.0
0.5
1.0
β_{IV}

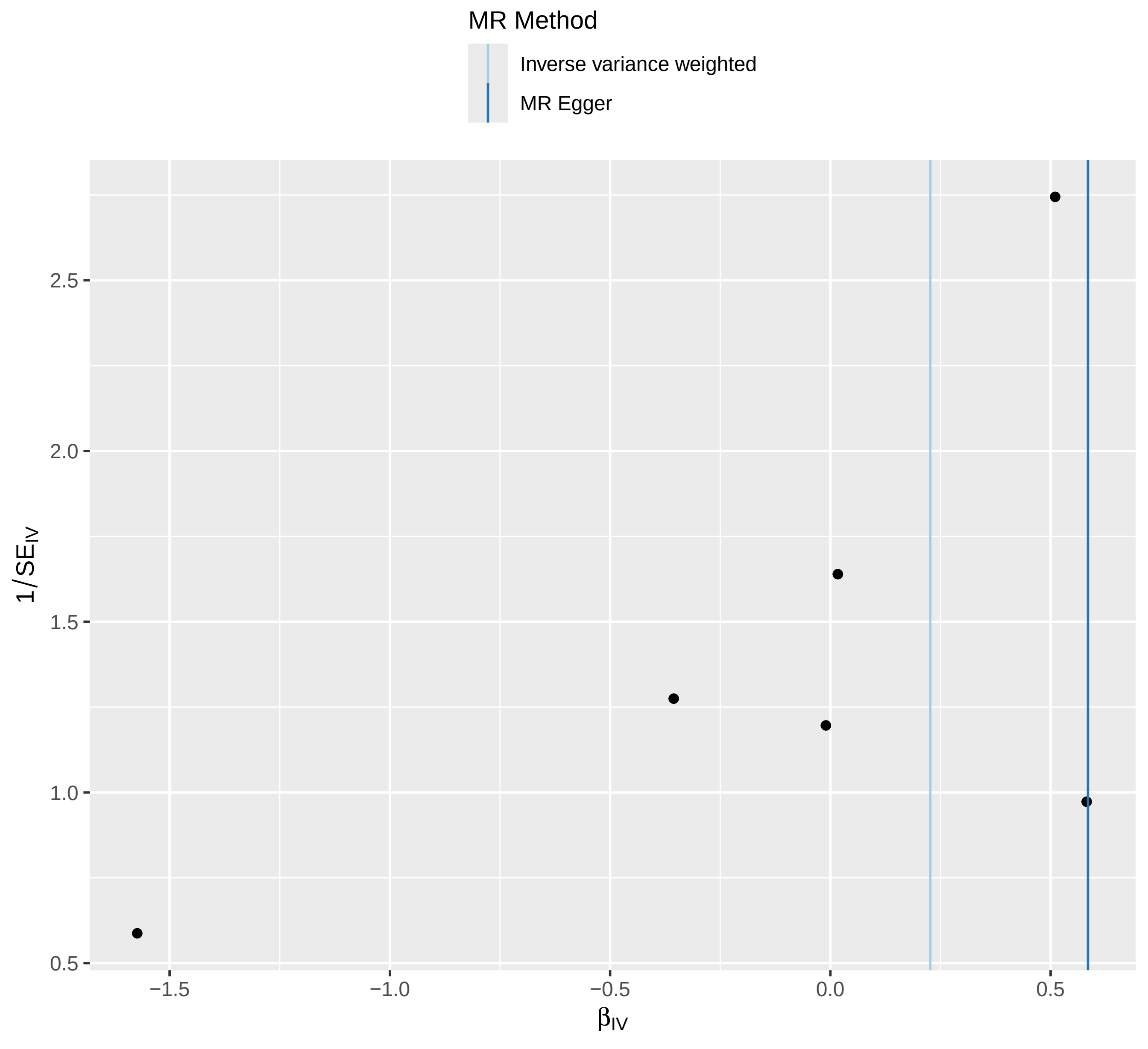

MR Method
Inverse variance weighted
MR Egger
2.5
2.0
1.5
1.0
0.5
1/SE$_{IV}$
−1.5
−1.0
−0.5
0.0
0.5
β$_{IV}$

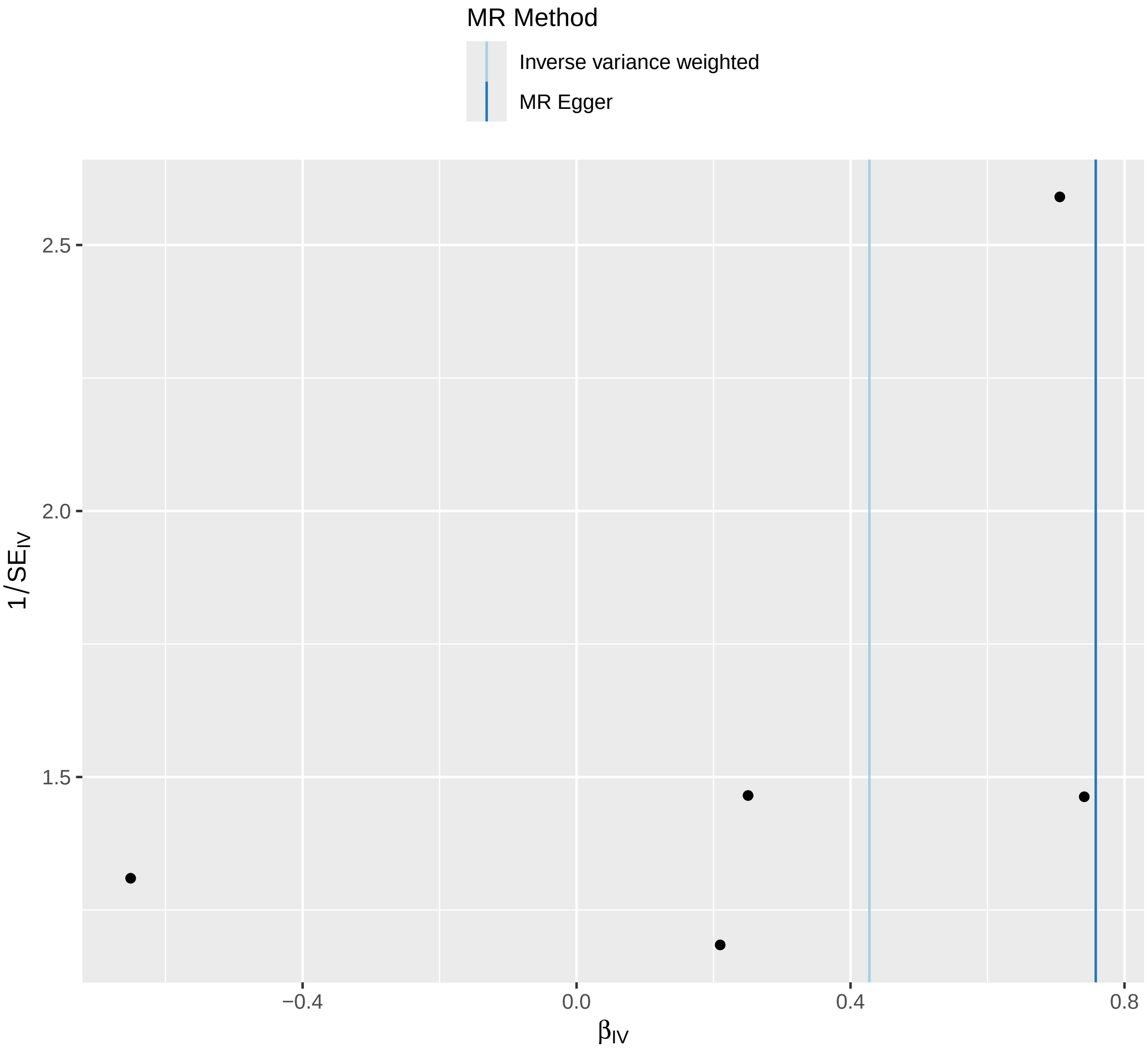

MR Method
Inverse variance weighted
MR Egger
2.5
2.0
1.5
1/SE$_{IV}$
−0.4
0.0
0.4
0.8
β$_{IV}$

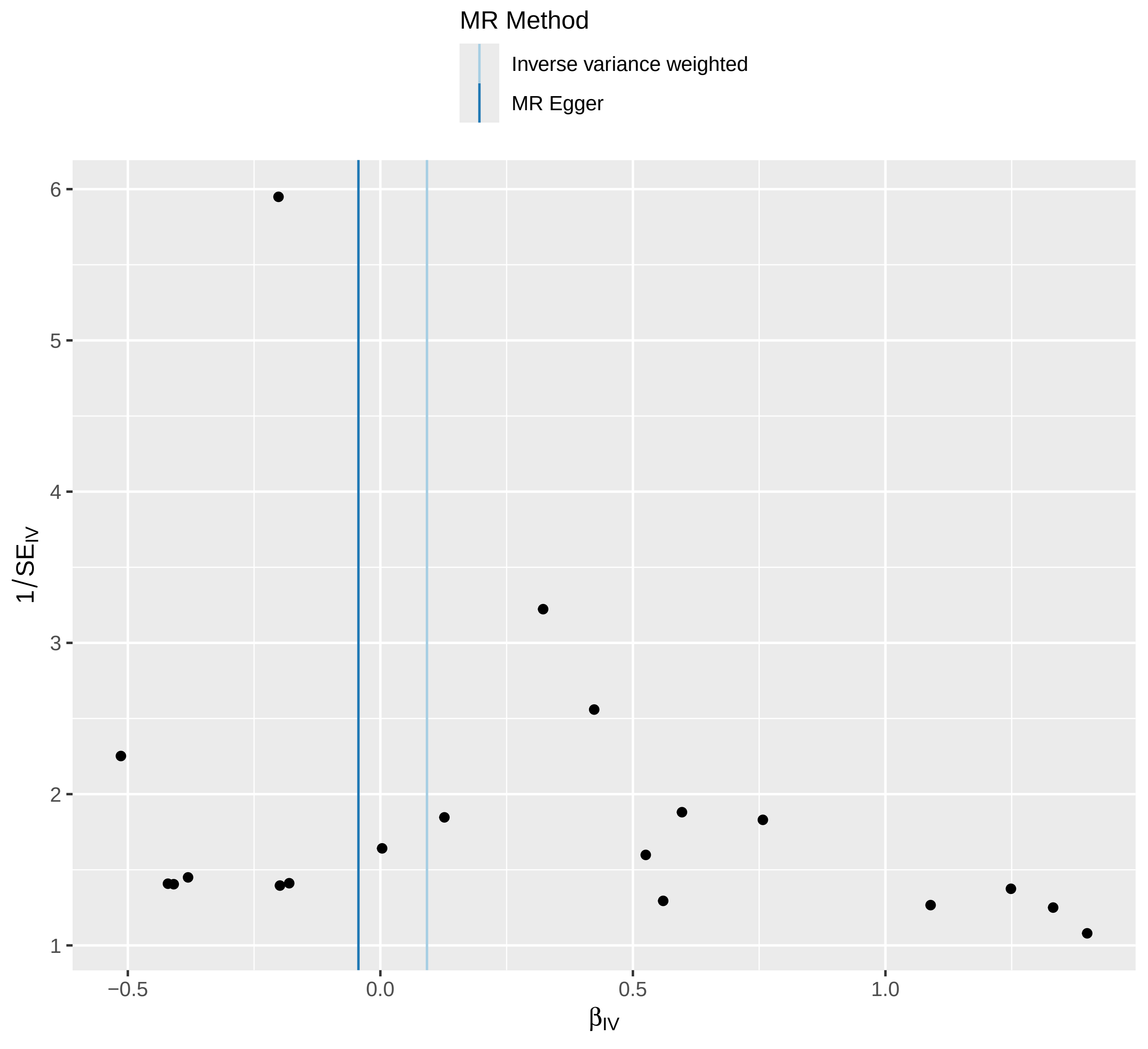

MR Method
Inverse variance weighted
MR Egger
6
5
4
3
2
1
1/SE_{IV}
−0.5
0.0
0.5
1.0
β_{IV}

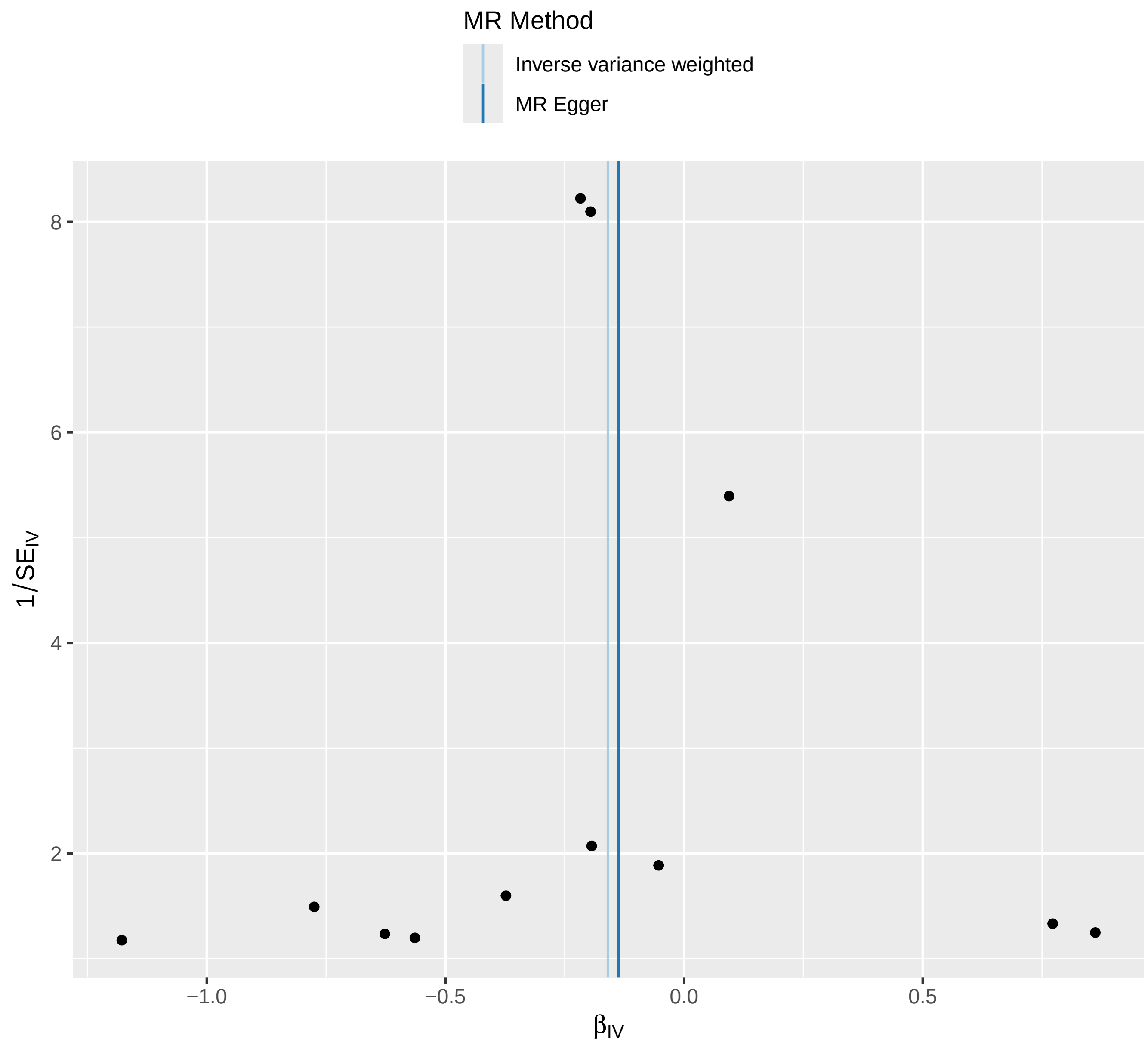
MR Method
Inverse variance weighted
MR Egger
8
6
4
2
$1/SE_{IV}$
−1.0
−0.5
0.0
0.5
β_{IV}

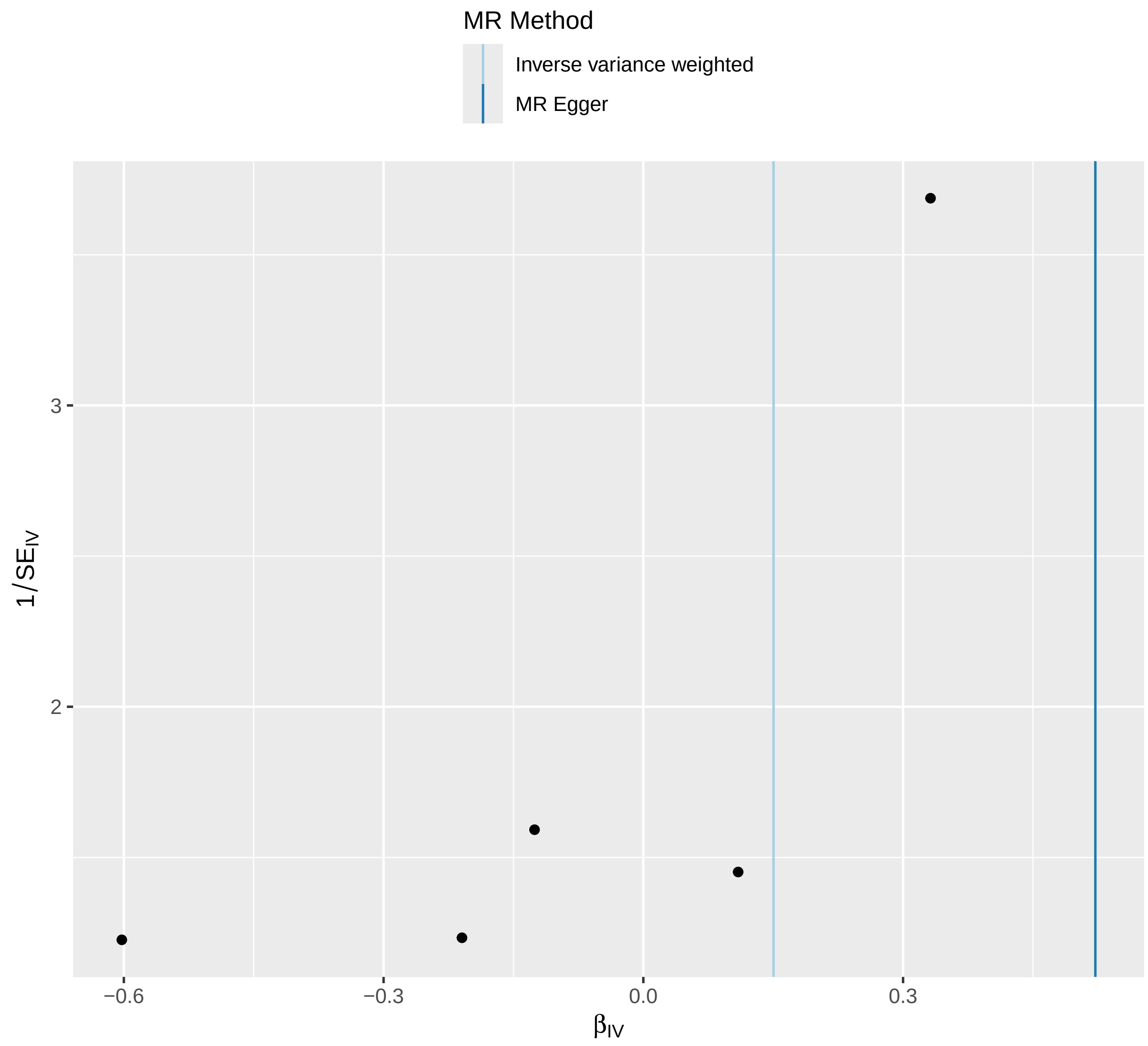

MR Method
Inverse variance weighted
MR Egger
3
2
1/SE_IV
−0.6
−0.3
0.0
0.3
β_IV

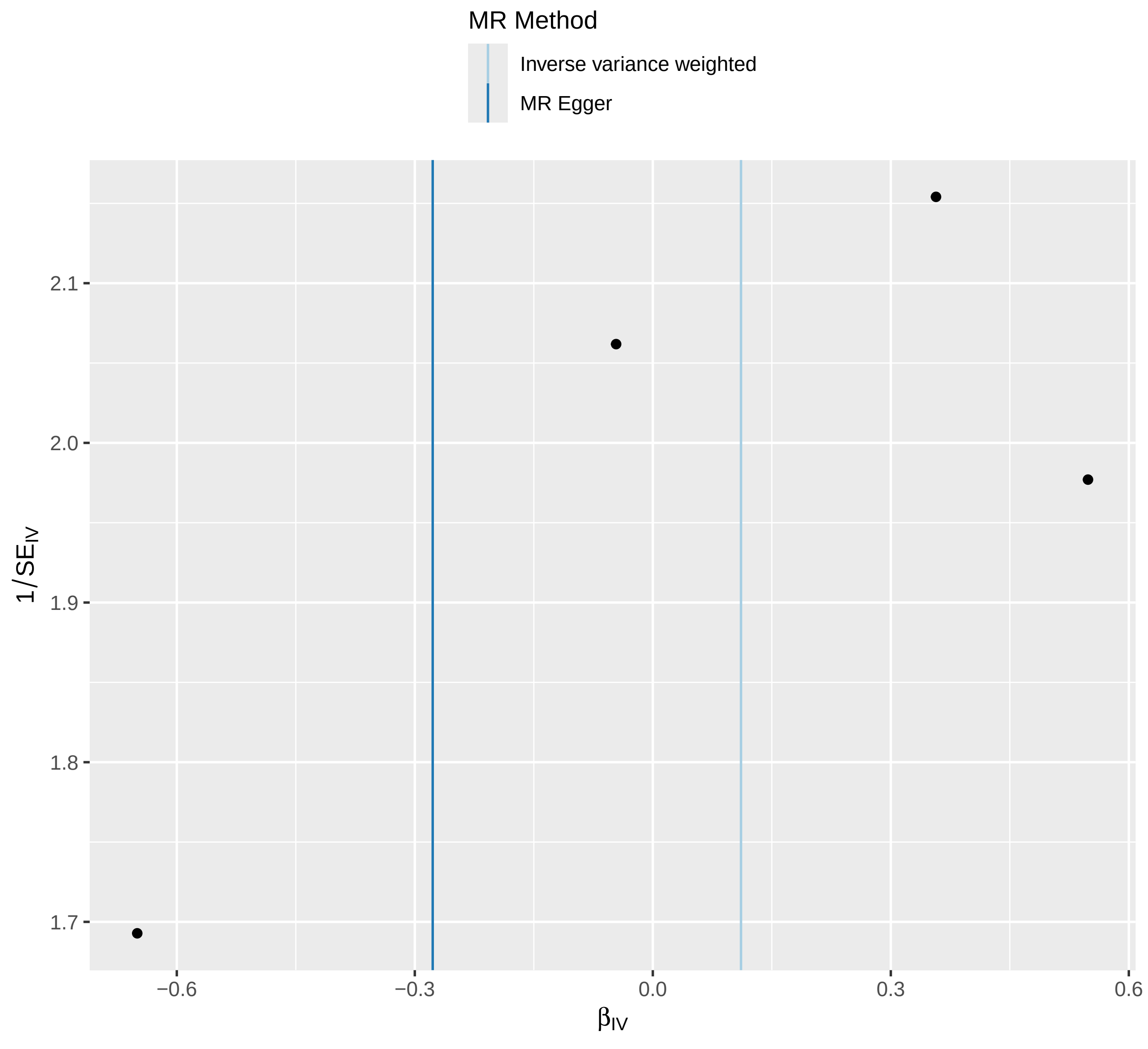
MR Method
Inverse variance weighted
MR Egger
2.1
2.0
1.9
1.8
1.7
1/SE_IV
−0.6
−0.3
0.0
0.3
0.6
β_IV

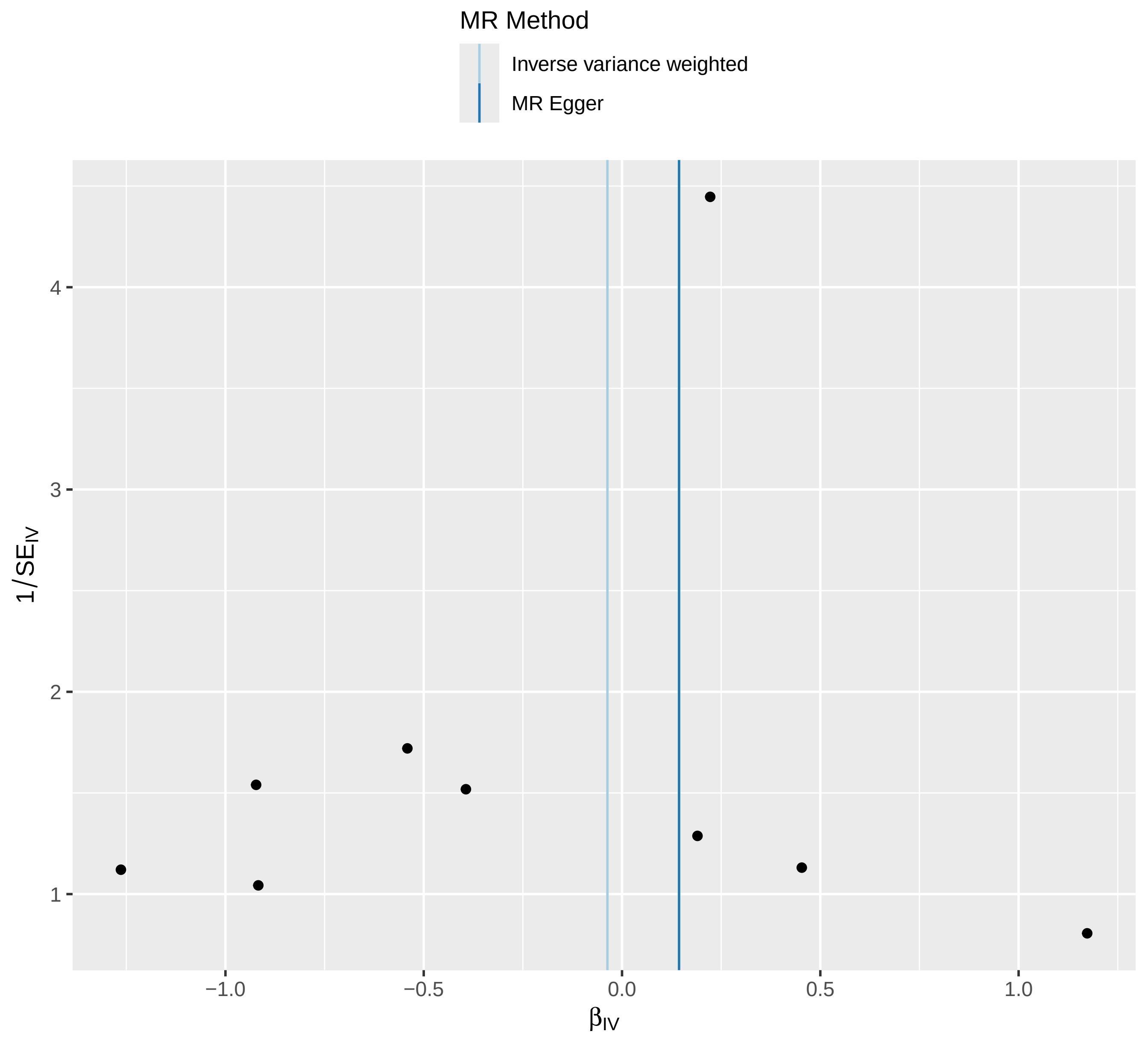

MR Method
Inverse variance weighted
MR Egger
4
3
2
1
1/SE_IV
−1.0
−0.5
0.0
0.5
1.0
β_IV

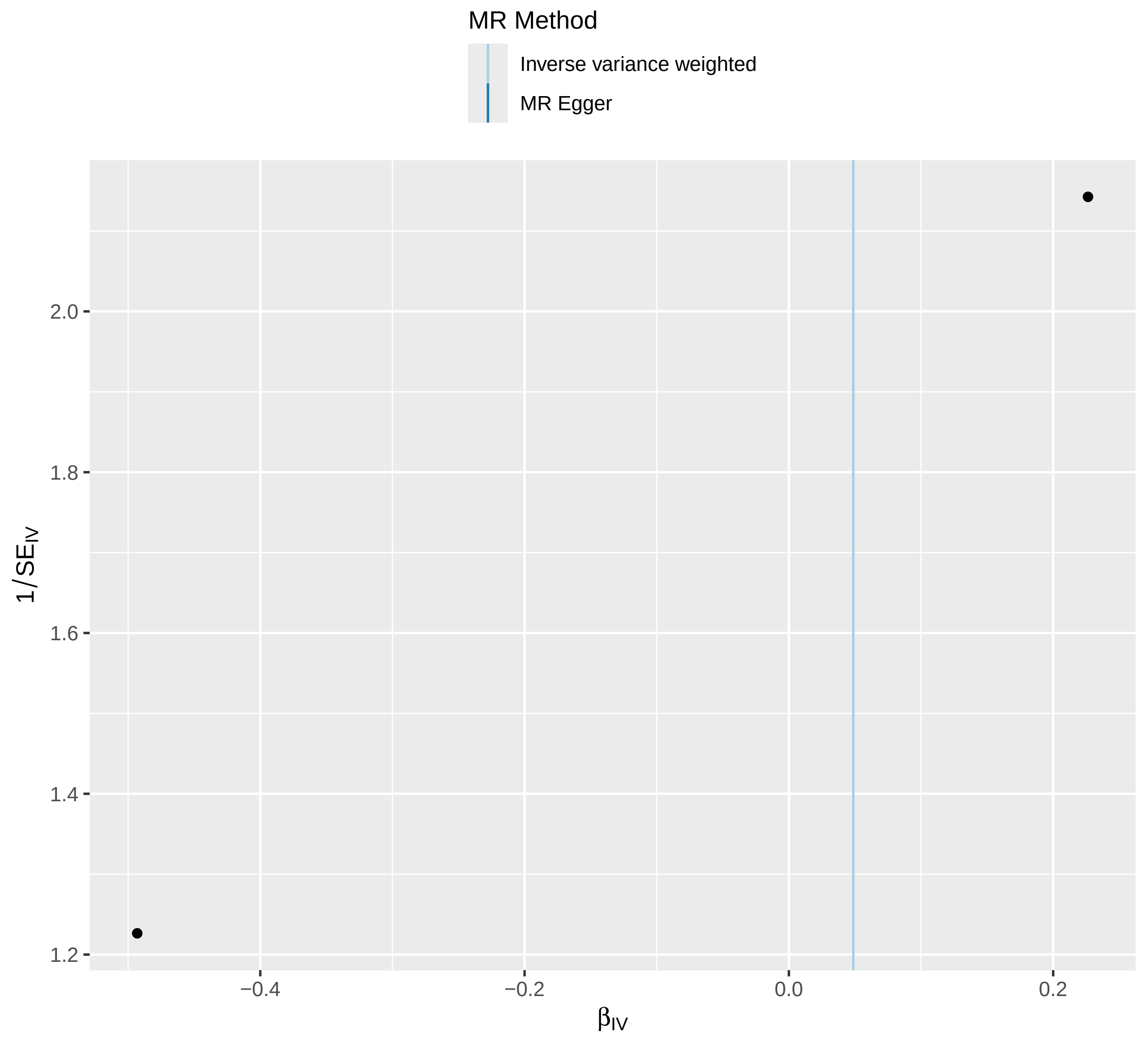
MR Method
Inverse variance weighted
MR Egger
2.0
1.8
1.6
1.4
1.2
1/SE$_{IV}$
−0.4
−0.2
0.0
0.2
β$_{IV}$

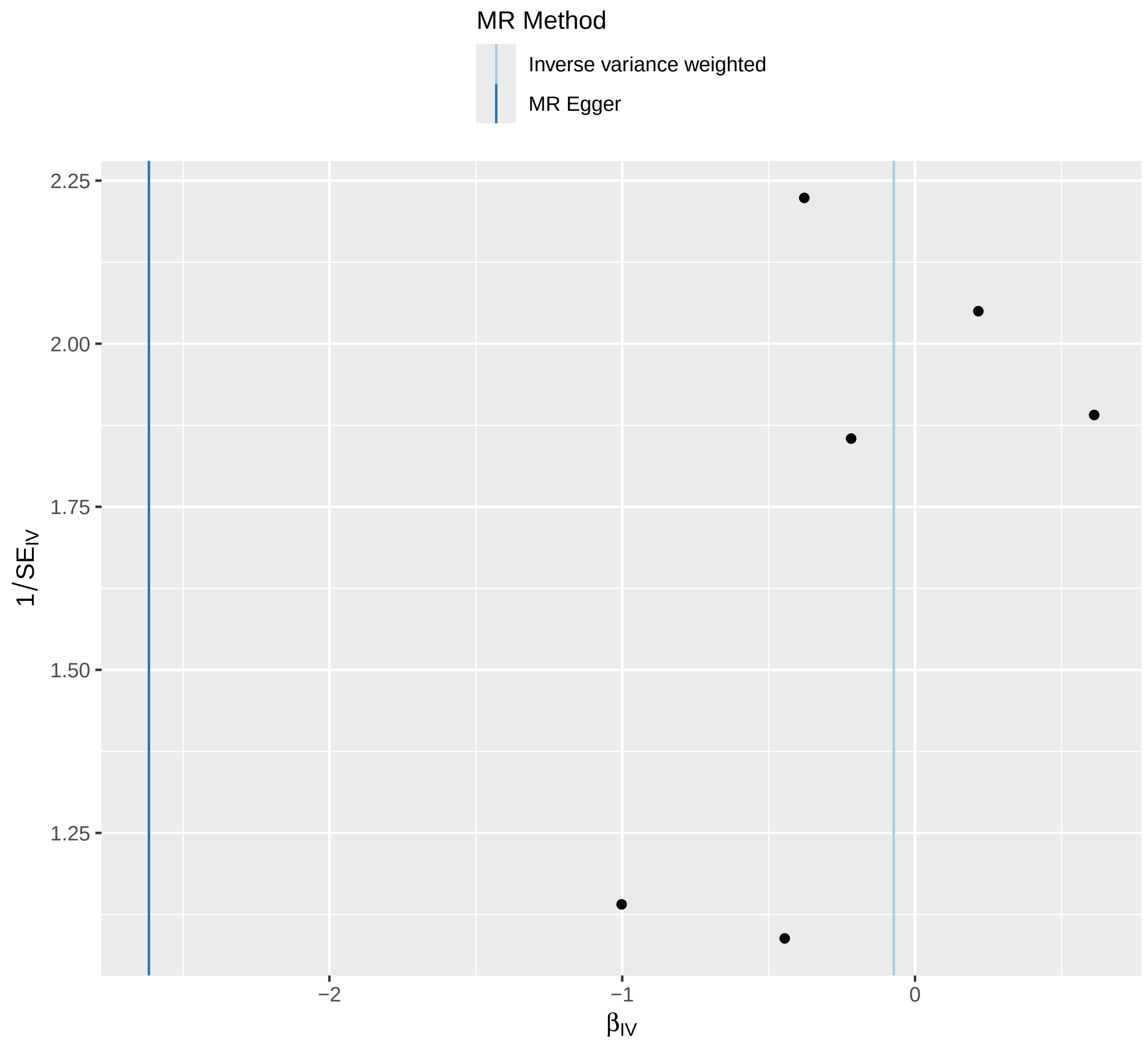

MR Method
Inverse variance weighted
MR Egger
2.25
2.00
1.75
1.50
1.25
1/SE_IV
−2
−1
0
β_IV

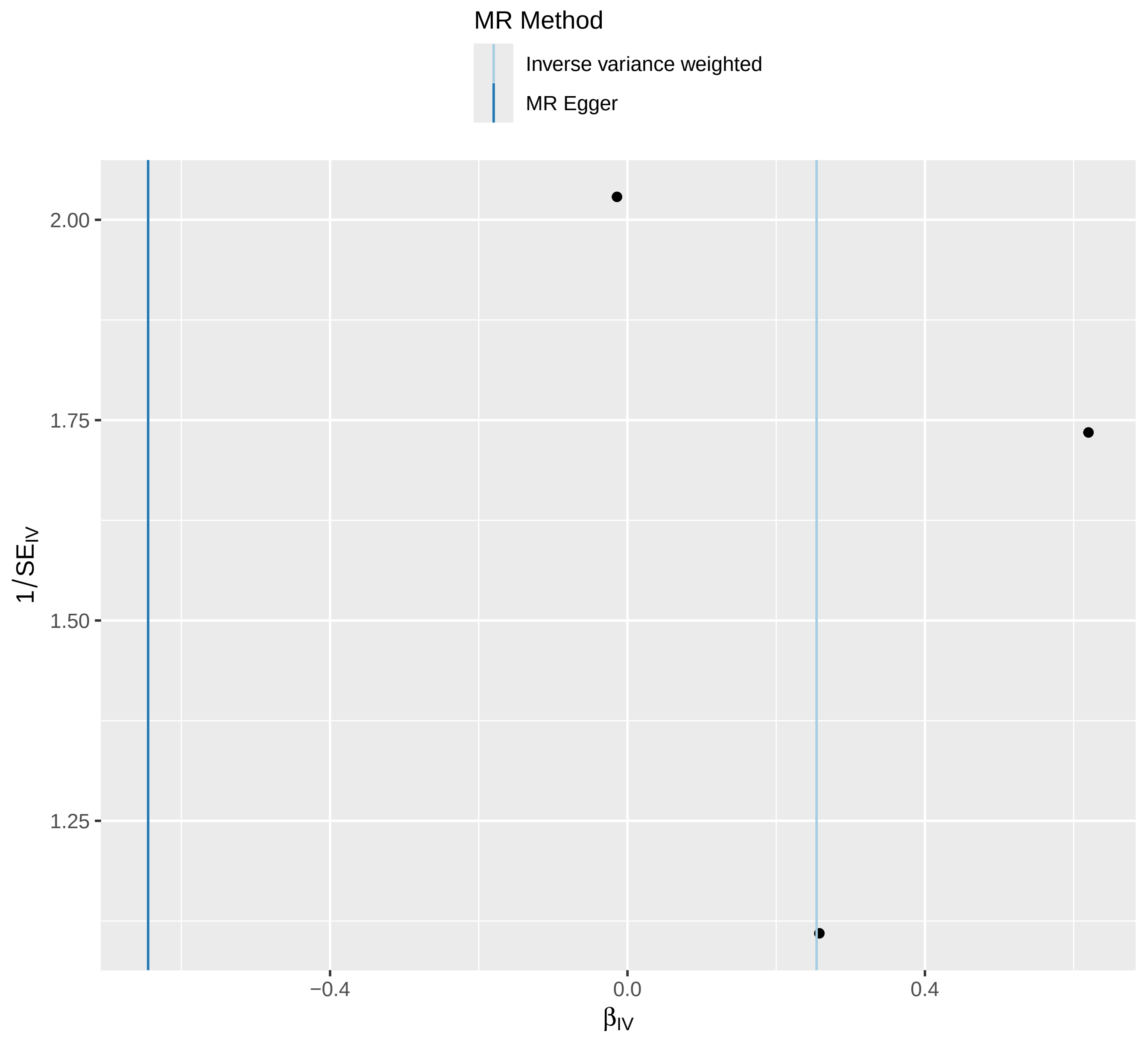

MR Method
Inverse variance weighted
MR Egger
2.00
1.75
1.50
1.25
1/SE$_{IV}$
−0.4
0.0
0.4
β$_{IV}$

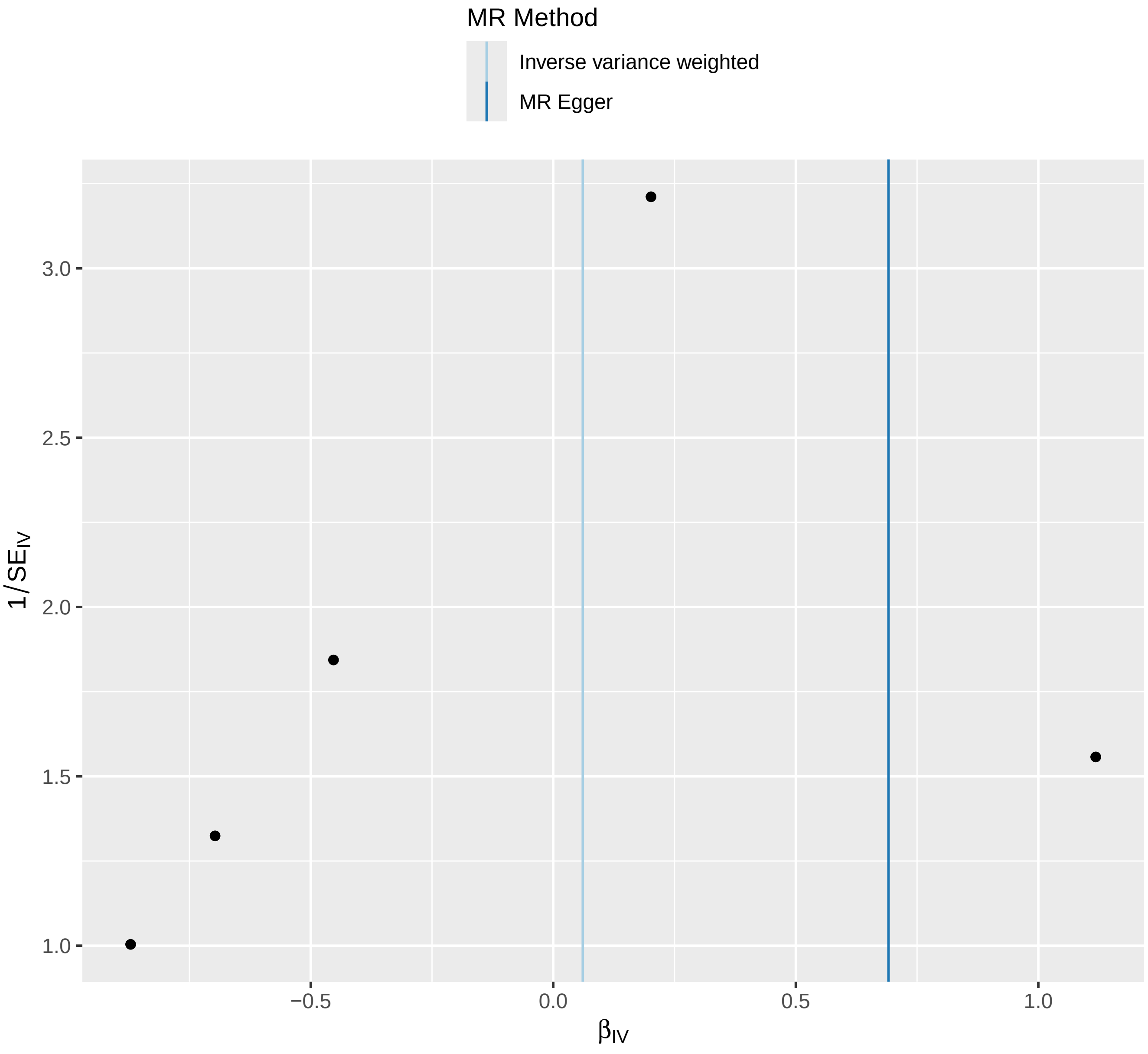

MR Method
Inverse variance weighted
MR Egger
3.0
2.5
2.0
1.5
1.0
1/SE$_{IV}$
−0.5
0.0
0.5
1.0
β$_{IV}$

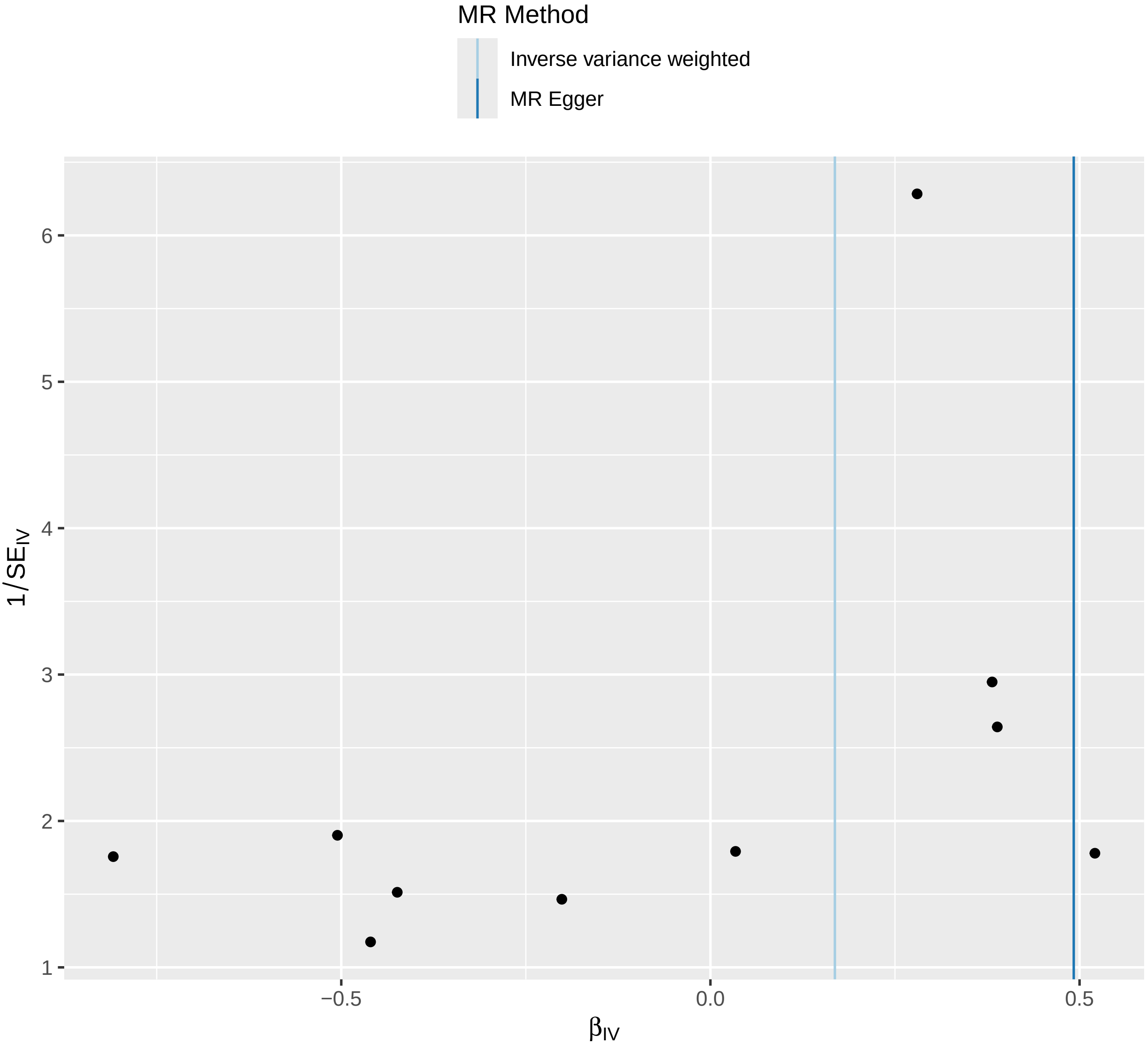

MR Method
Inverse variance weighted
MR Egger
1/SE$_{IV}$
6
5
4
3
2
1
−0.5
0.0
0.5
β$_{IV}$

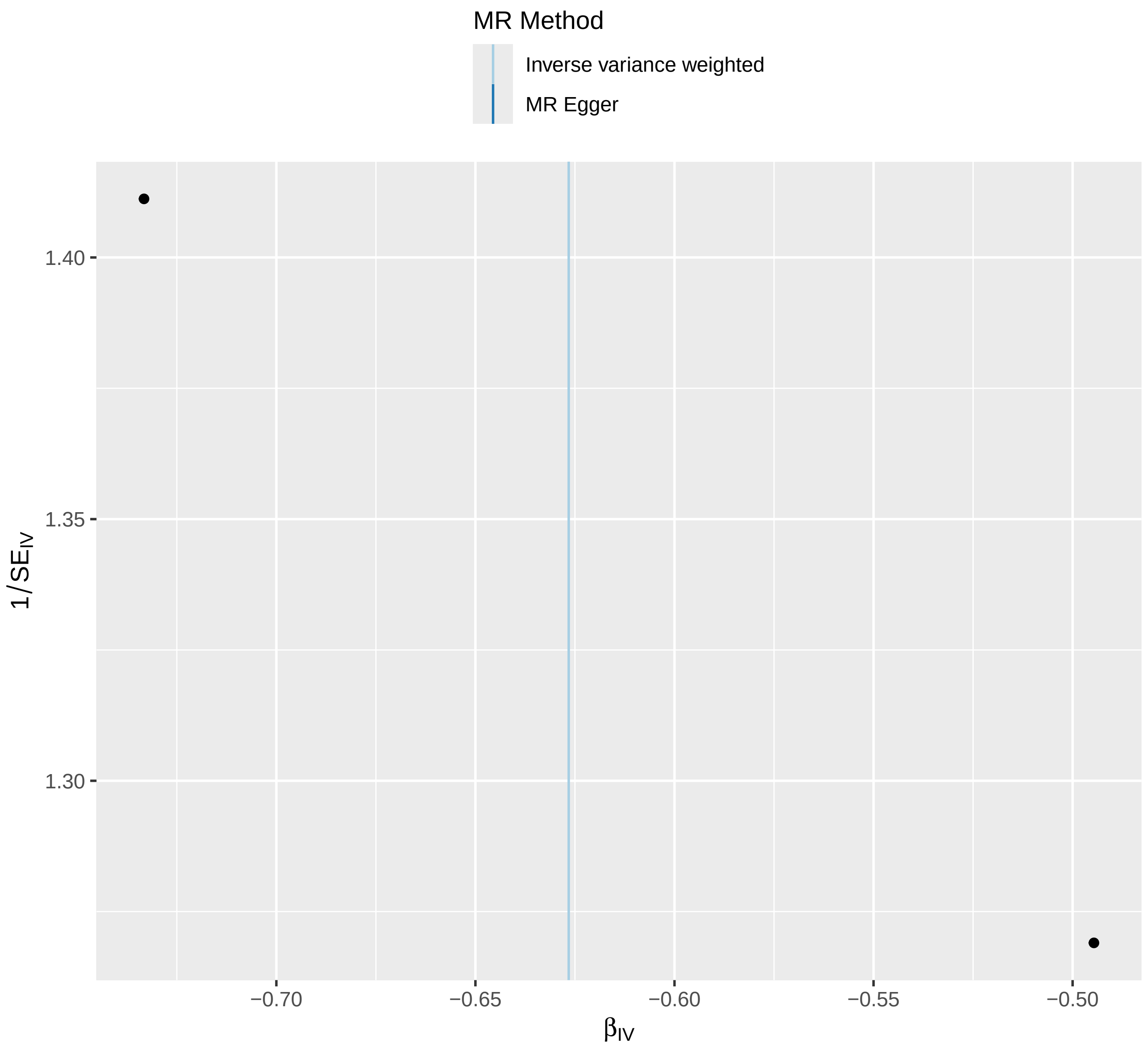

MR Method
Inverse variance weighted
MR Egger
1.40
1.35
1.30
1/SE$_{IV}$
−0.70
−0.65
−0.60
−0.55
−0.50
β$_{IV}$

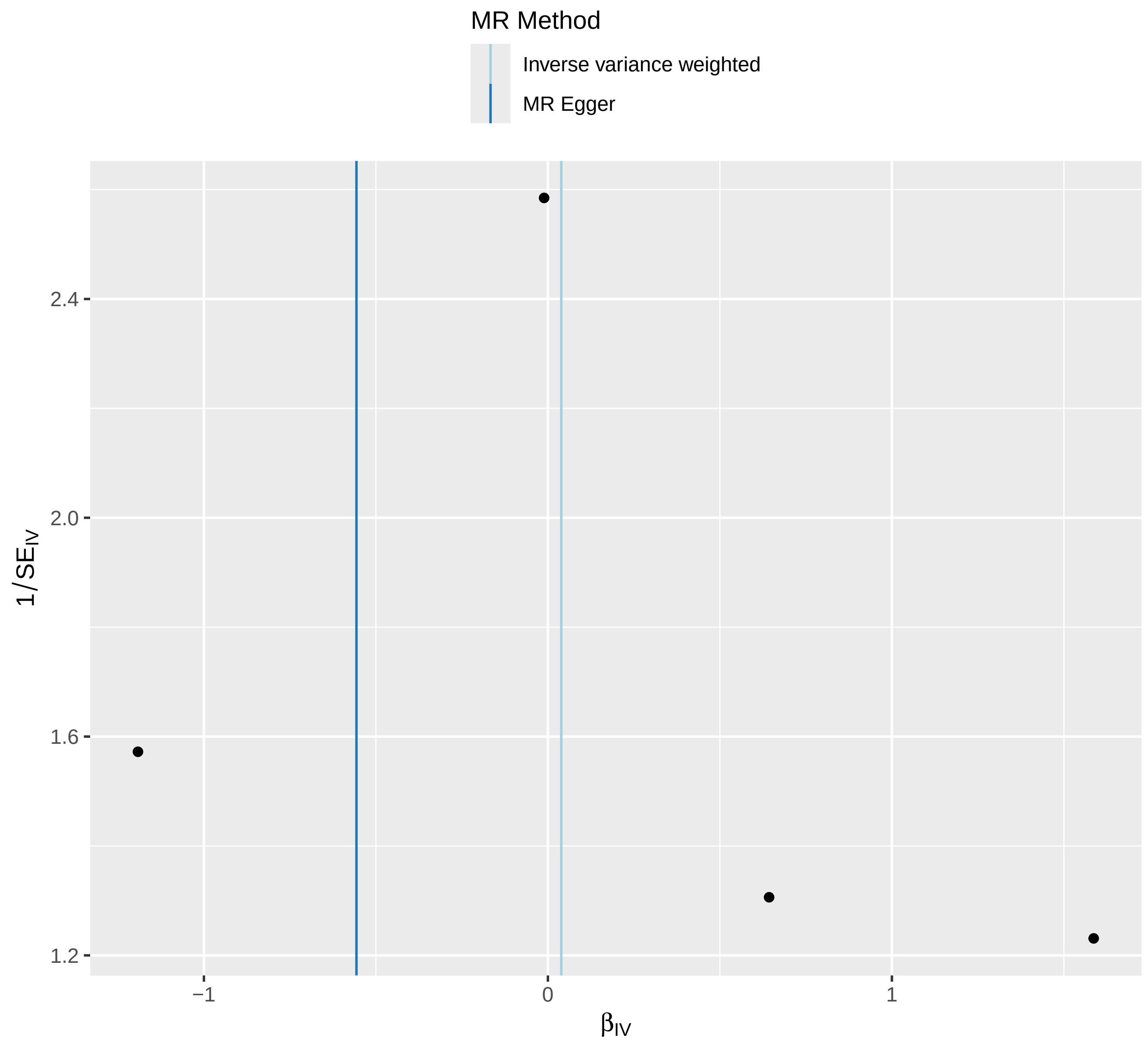
MR Method
Inverse variance weighted
MR Egger
2.4
2.0
1.6
1.2
1/SE_IV
−1
0
1
β_IV

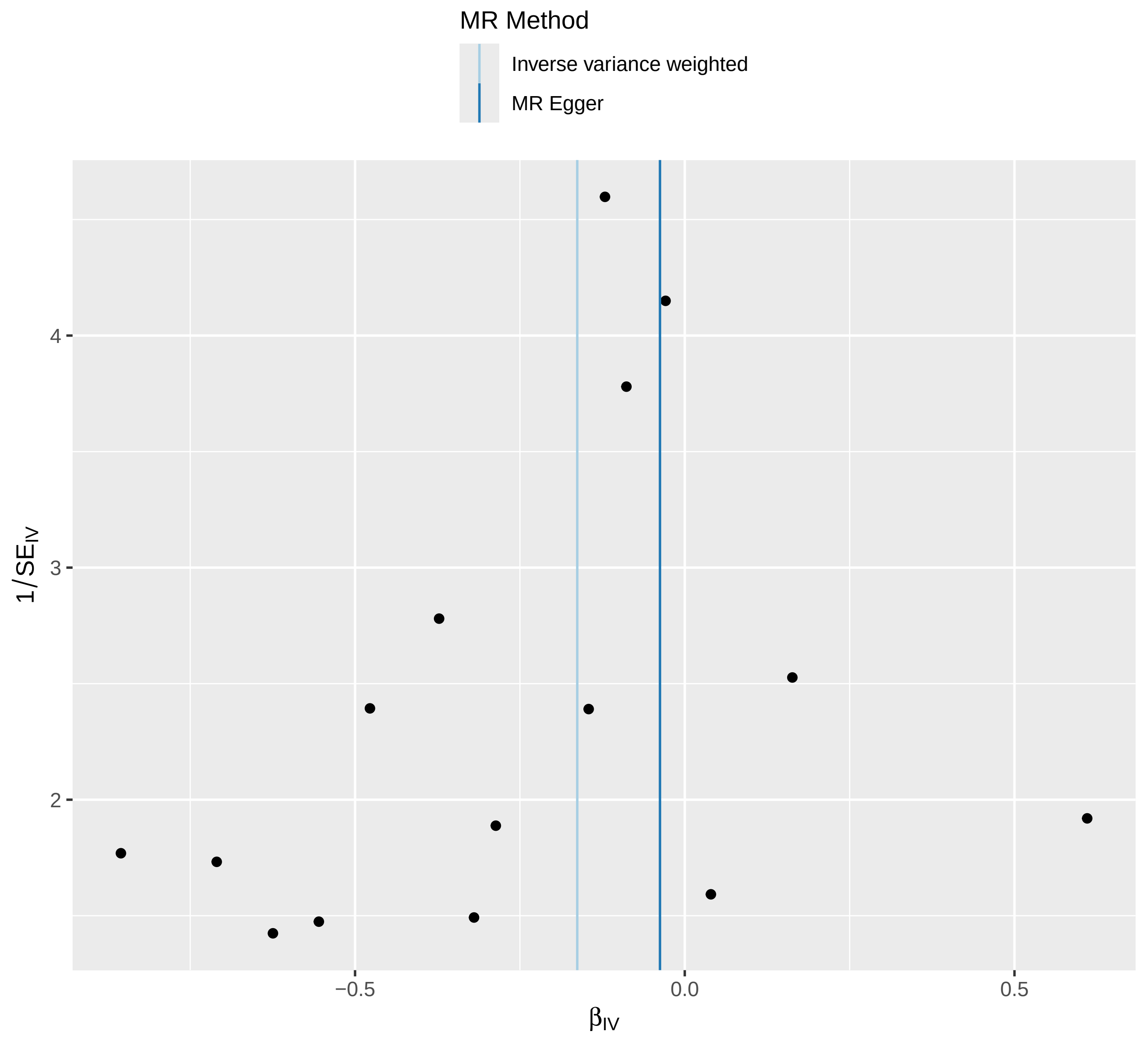
MR Method
Inverse variance weighted
MR Egger
$1/SE_{IV}$
4
3
2
−0.5
0.0
0.5
β_{IV}

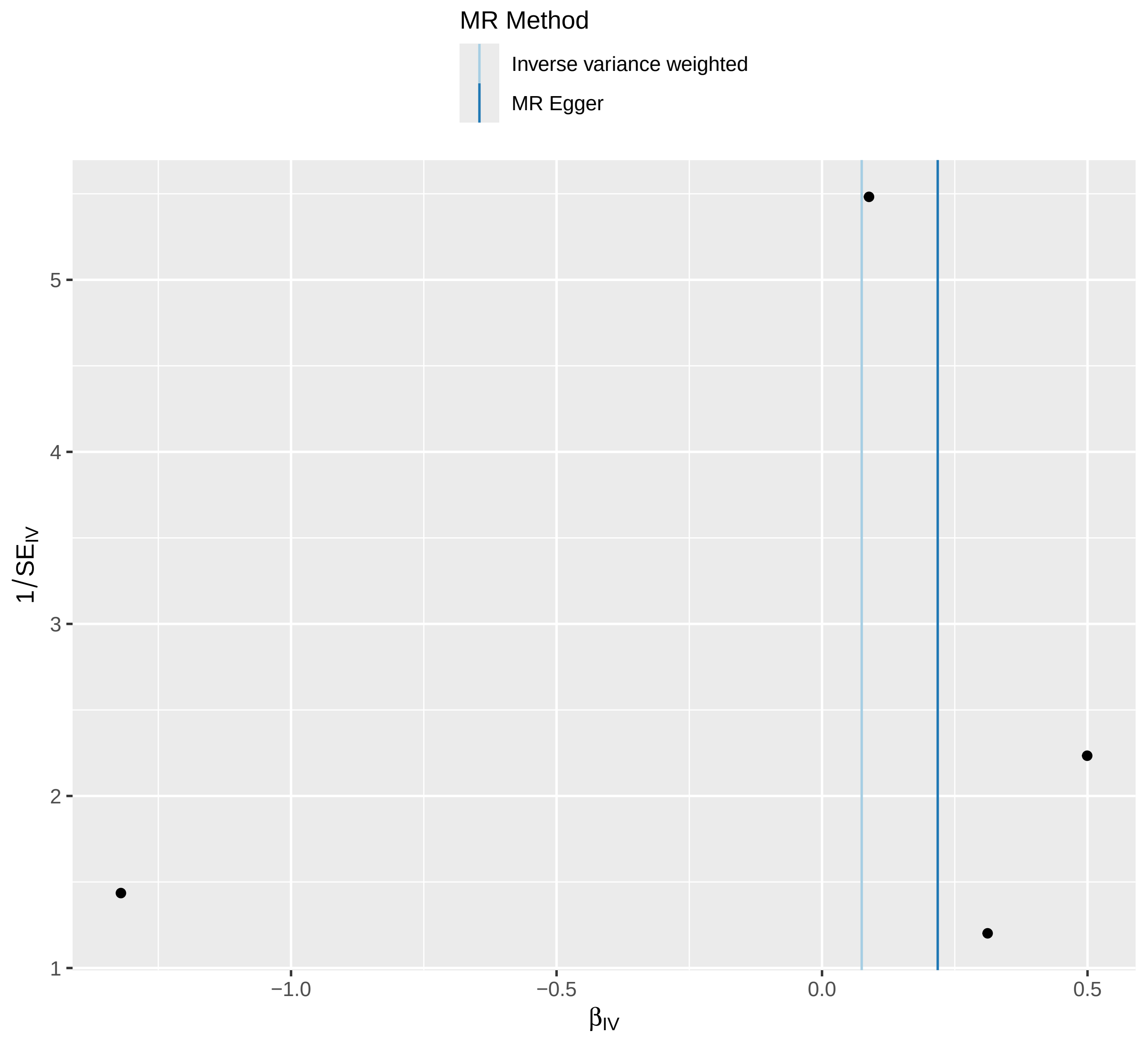
MR Method
Inverse variance weighted
MR Egger
1/SE_{IV}
5
4
3
2
1
−1.0
−0.5
0.0
0.5
β_{IV}

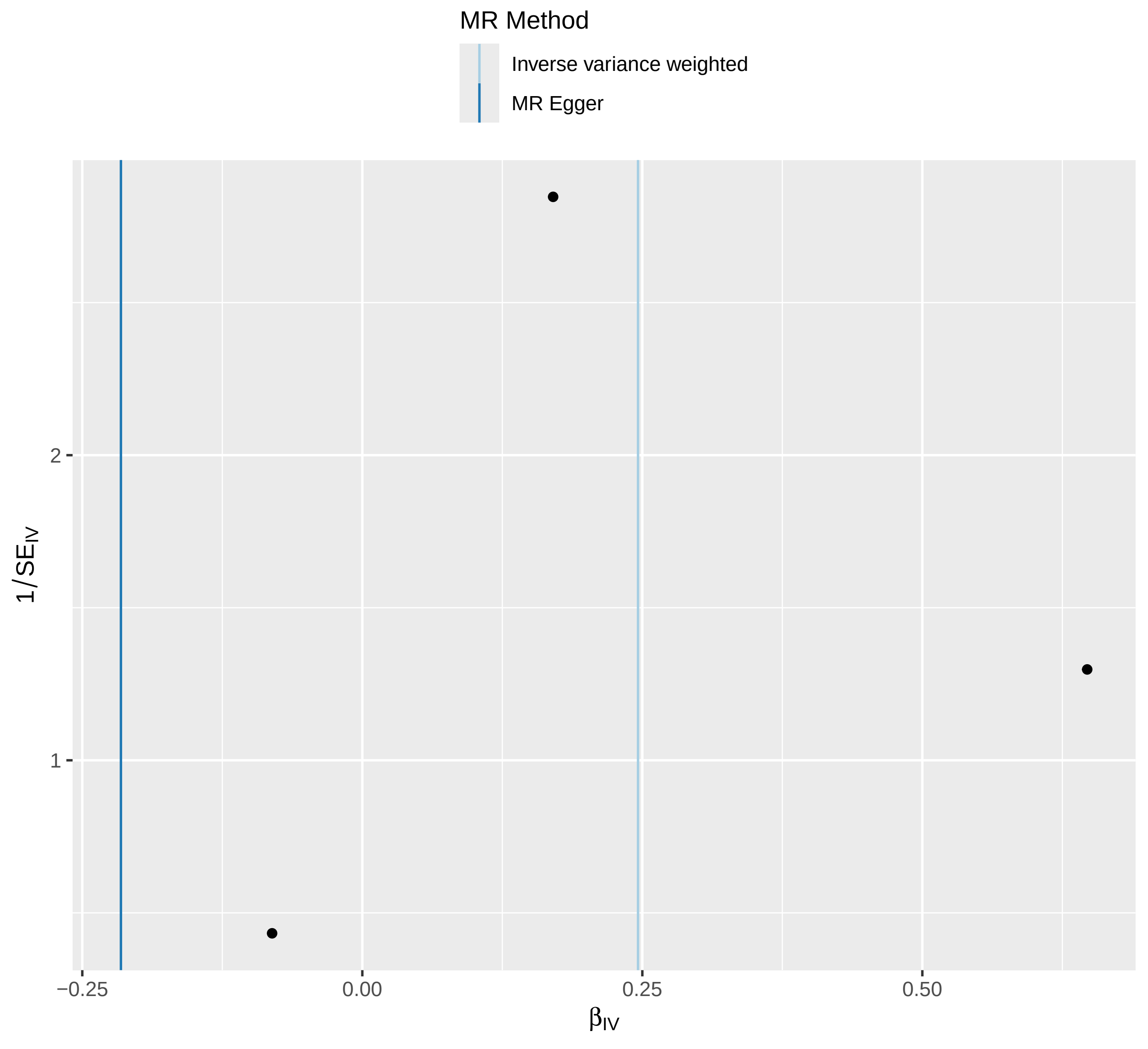

MR Method
Inverse variance weighted
MR Egger
2
1
1/SE$_{IV}$
−0.25
0.00
0.25
0.50
β$_{IV}$

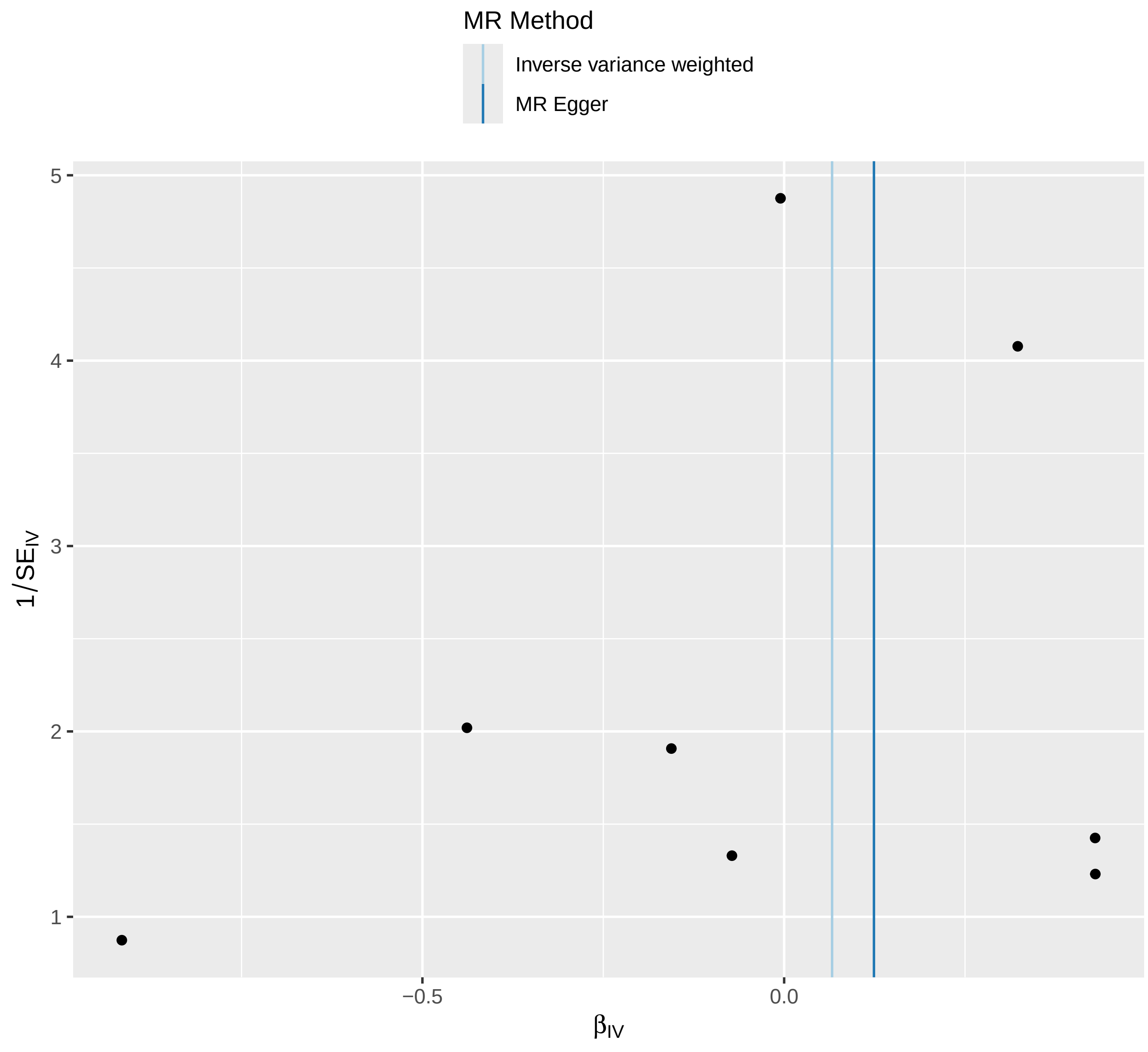
MR Method
Inverse variance weighted
MR Egger
5
4
3
2
1
1/SE_IV
−0.5
0.0
β_IV

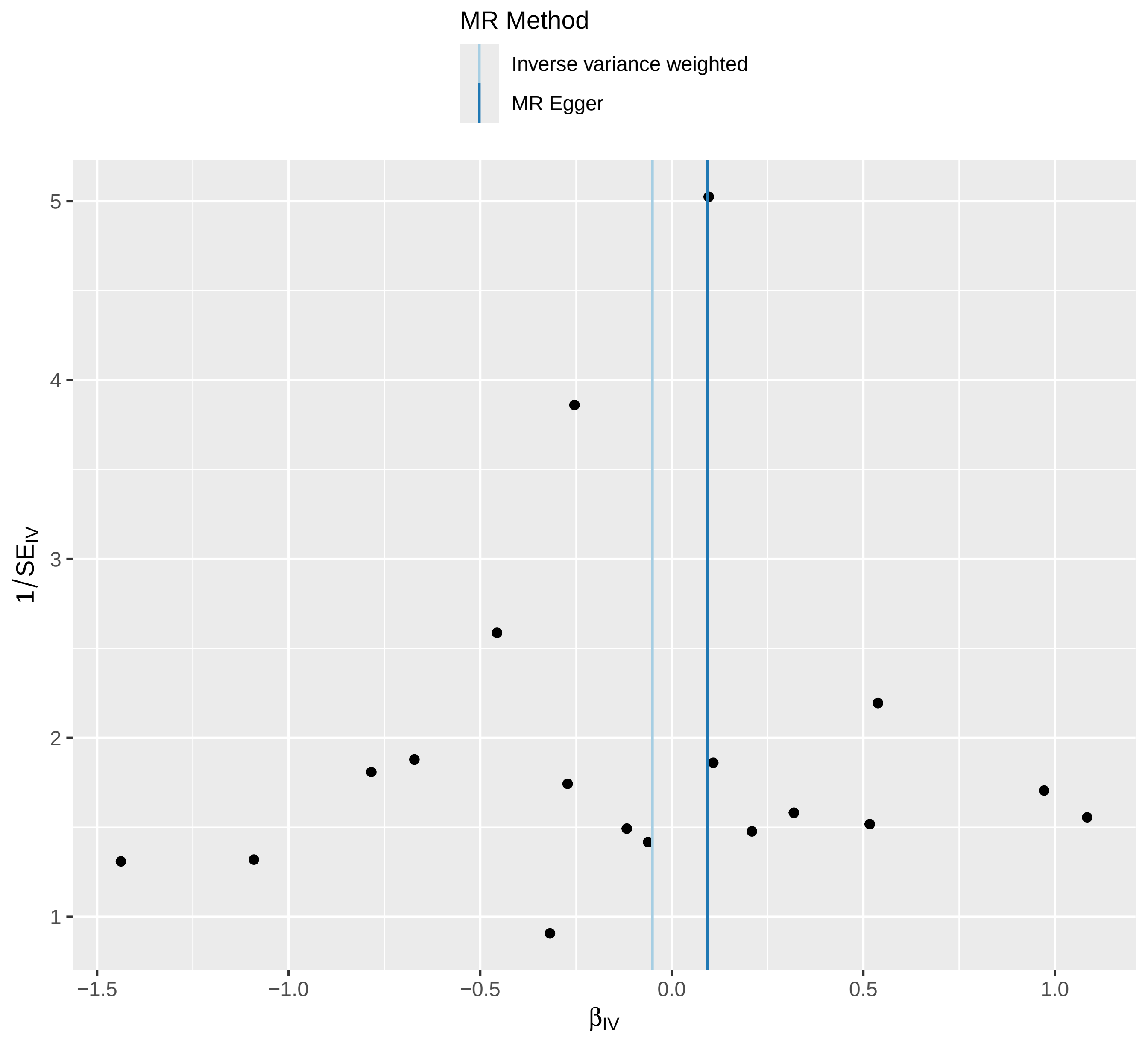
MR Method
Inverse variance weighted
MR Egger
5
4
3
2
1
1/SE_IV
−1.5
−1.0
−0.5
0.0
0.5
1.0
β_IV

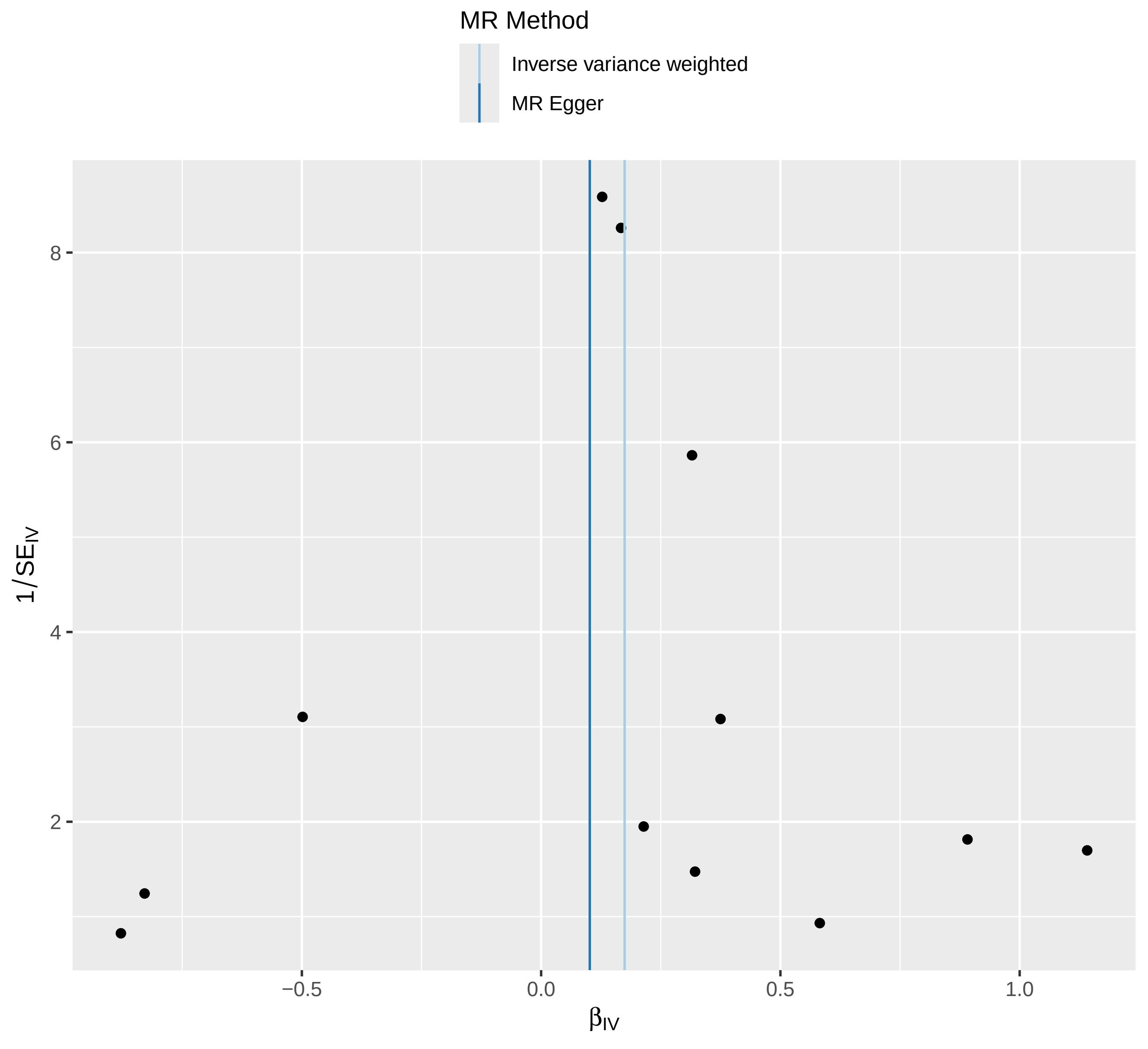
MR Method
Inverse variance weighted
MR Egger
8
6
4
2
1/SE$_{IV}$
−0.5
0.0
0.5
1.0
β_{IV}

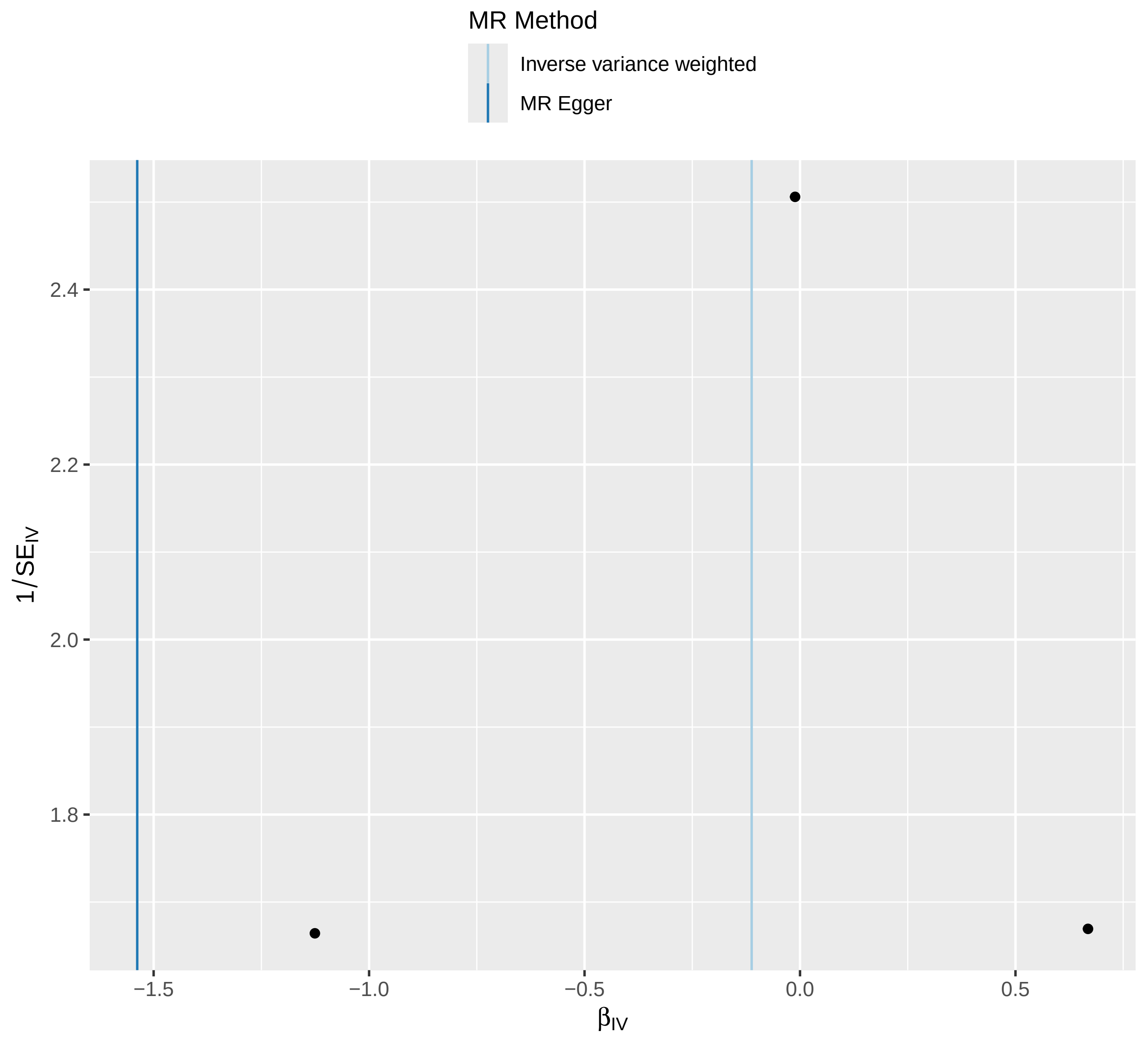

MR Method
Inverse variance weighted
MR Egger
2.4
2.2
2.0
1.8
$1/SE_{IV}$
−1.5
−1.0
−0.5
0.0
0.5
β_{IV}

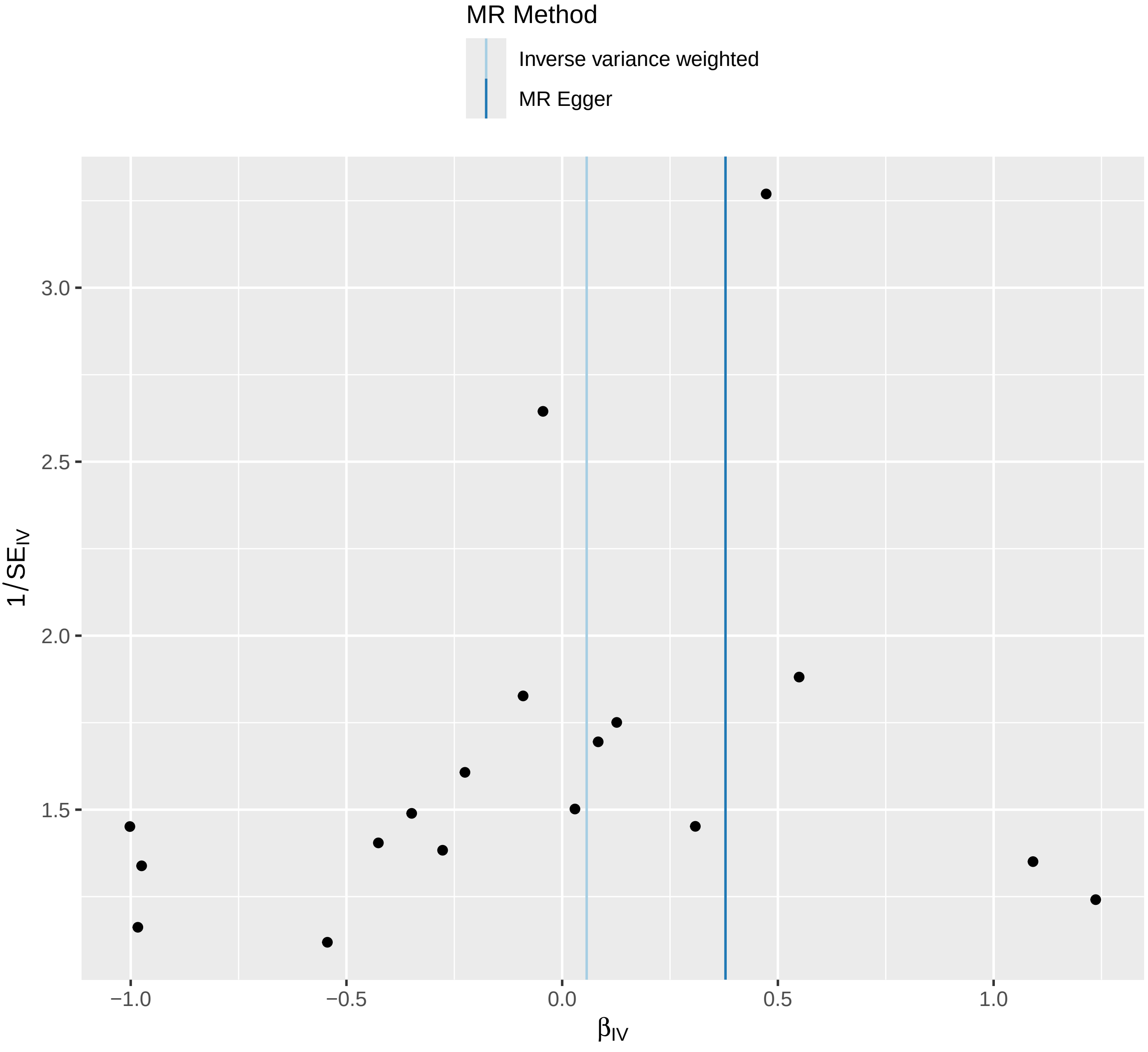

MR Method
Inverse variance weighted
MR Egger
3.0
2.5
2.0
1.5
1/SE$_{IV}$
−1.0
−0.5
0.0
0.5
1.0
β$_{IV}$

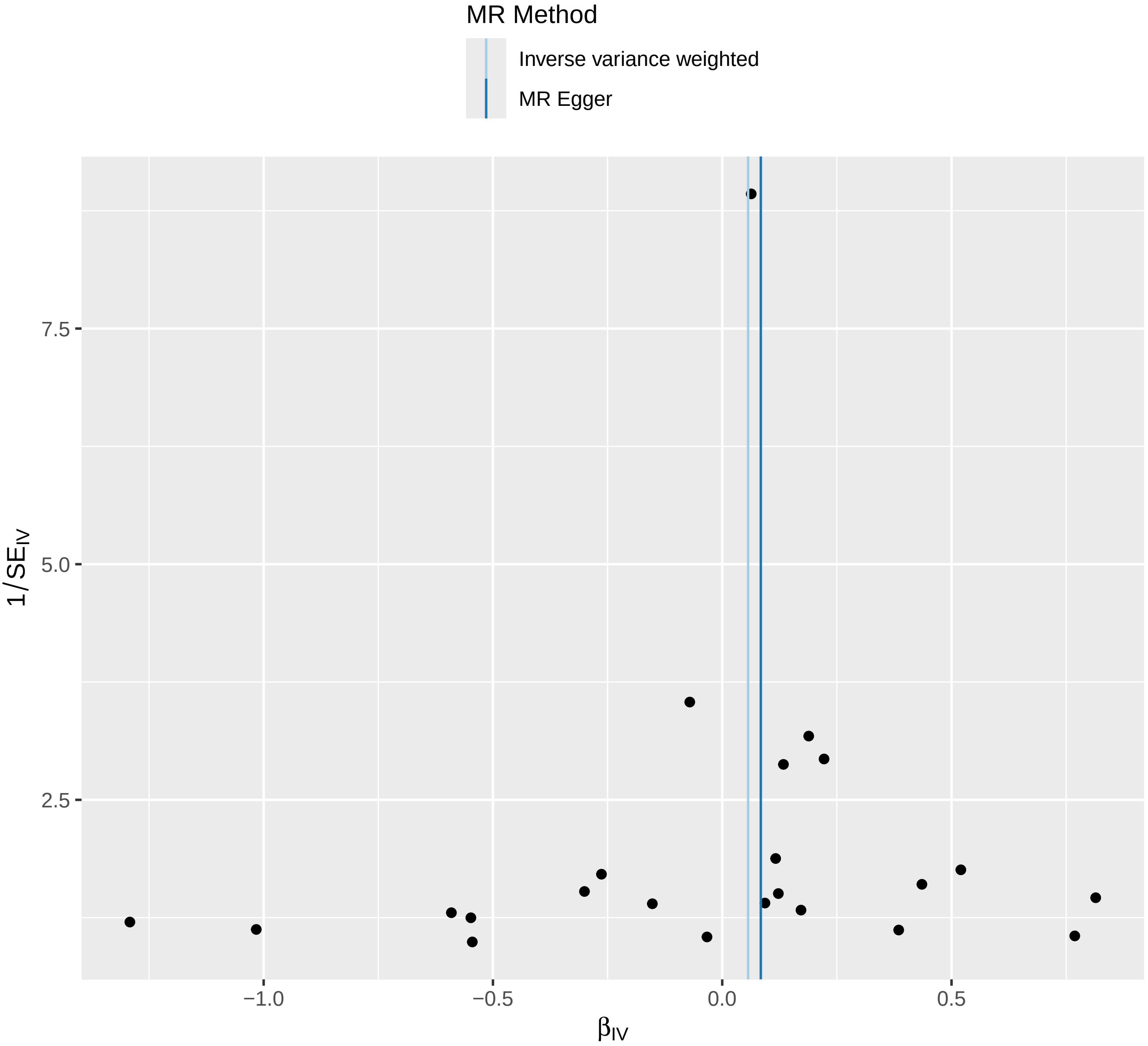

MR Method
Inverse variance weighted
MR Egger
7.5
5.0
2.5
1/SE_IV
−1.0
−0.5
0.0
0.5
β_IV

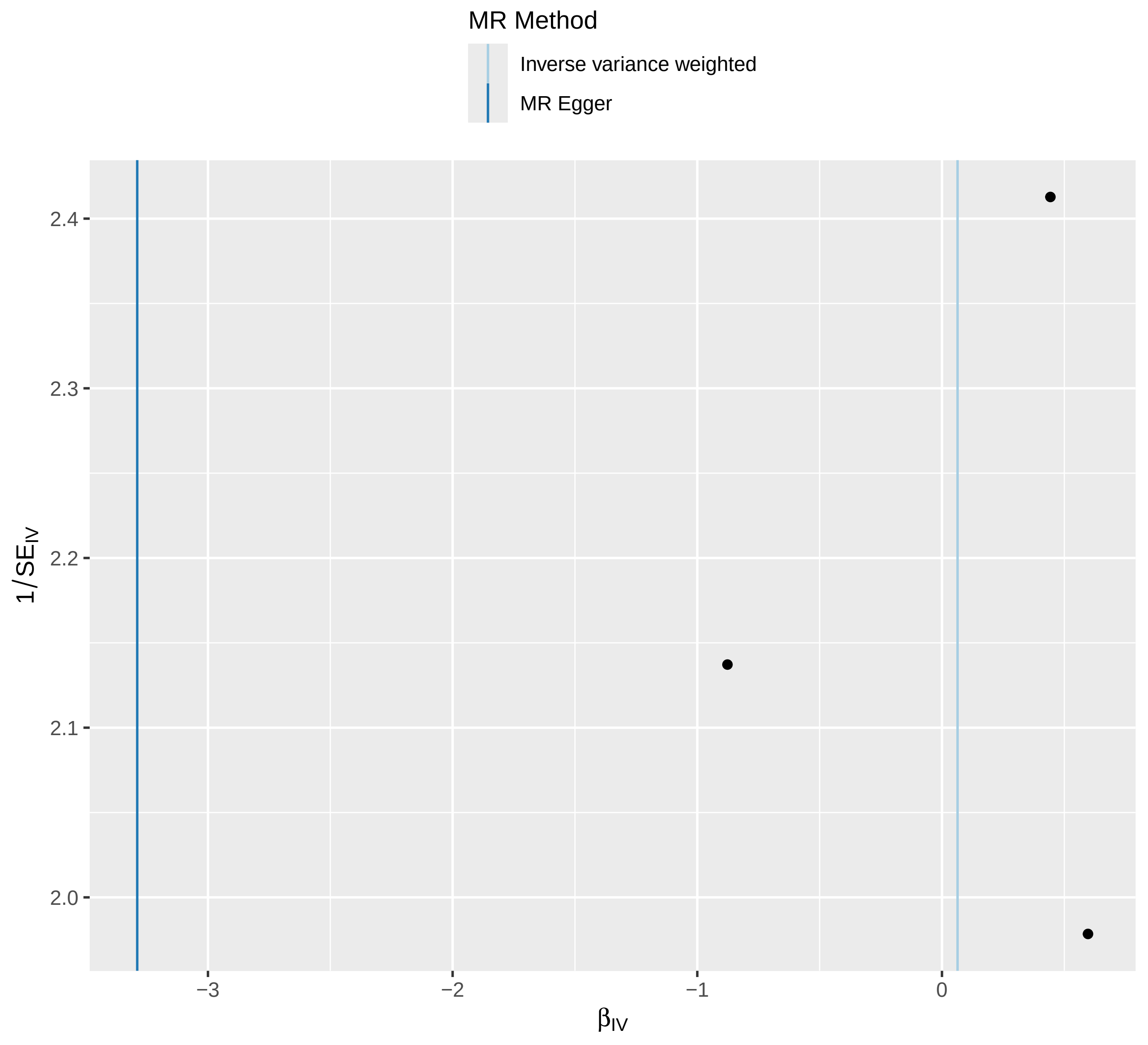
MR Method
Inverse variance weighted
MR Egger
2.4
2.3
2.2
2.1
2.0
1/SE$_{IV}$
−3
−2
−1
0
β$_{IV}$

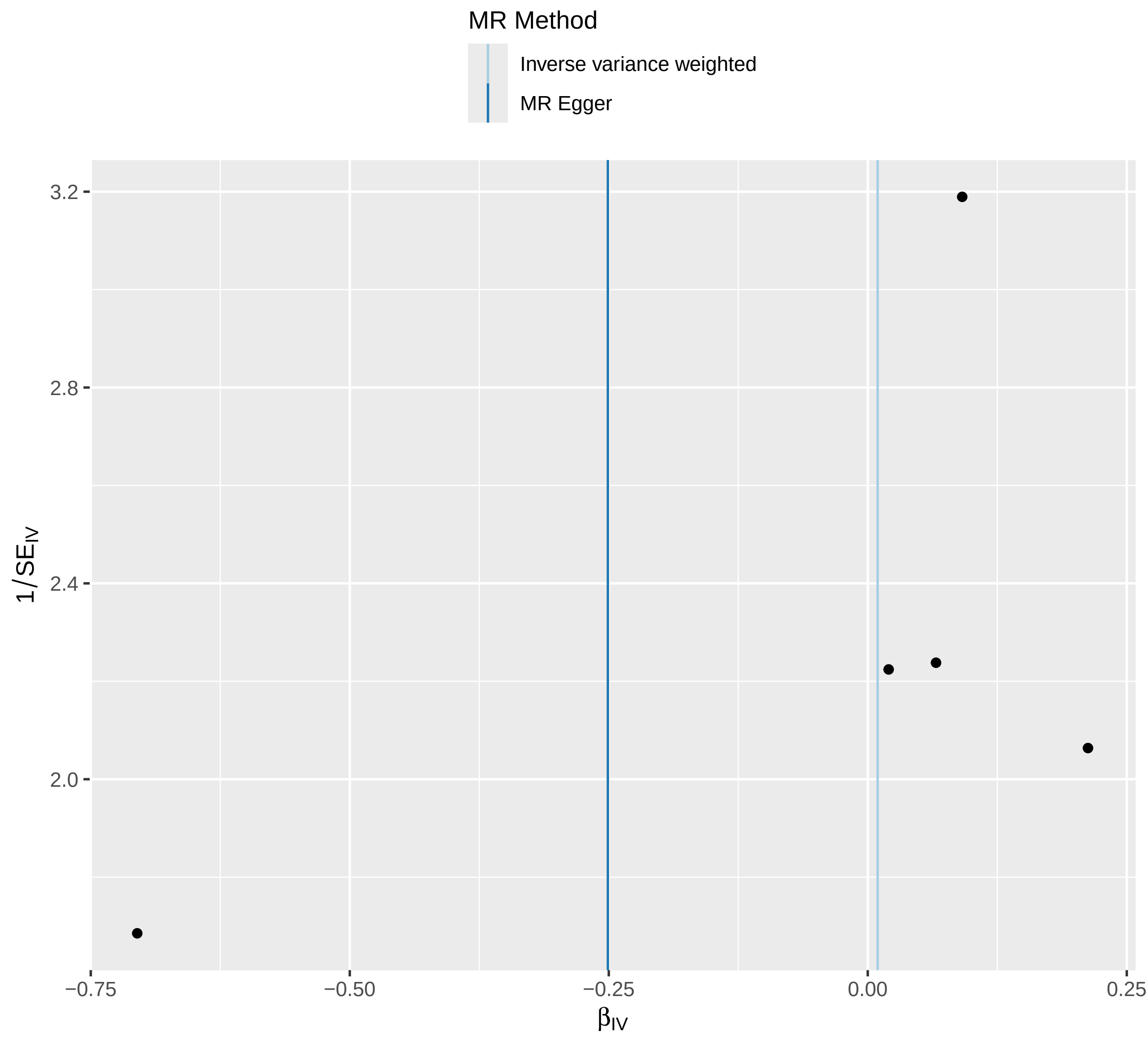
MR Method
Inverse variance weighted
MR Egger
3.2
2.8
2.4
2.0
$1/SE_{IV}$
−0.75
−0.50
−0.25
0.00
0.25
β_{IV}

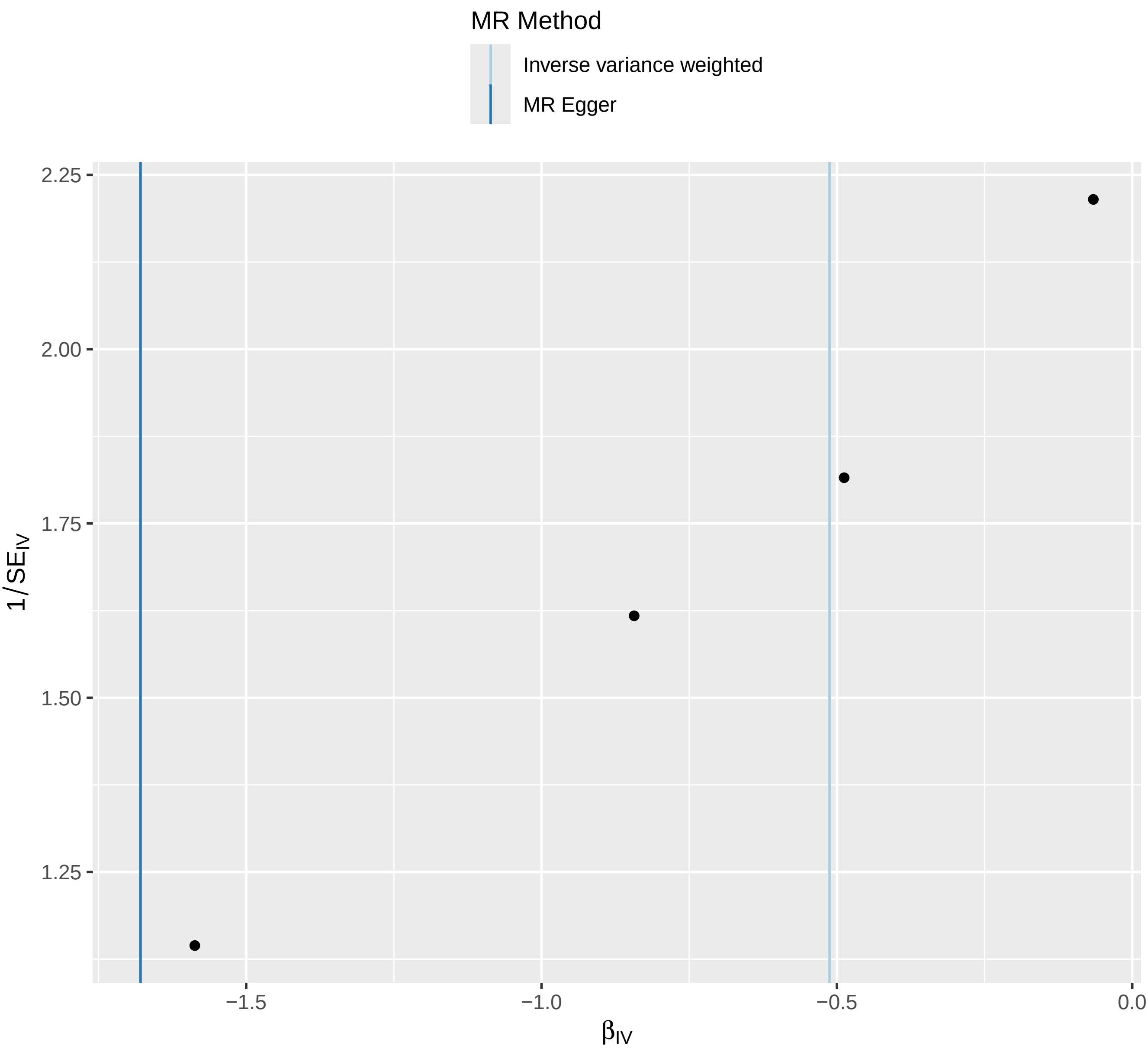
MR Method
Inverse variance weighted
MR Egger
2.25
2.00
1.75
1.50
1.25
$1/SE_{IV}$
−1.5
−1.0
−0.5
0.0
β_{IV}

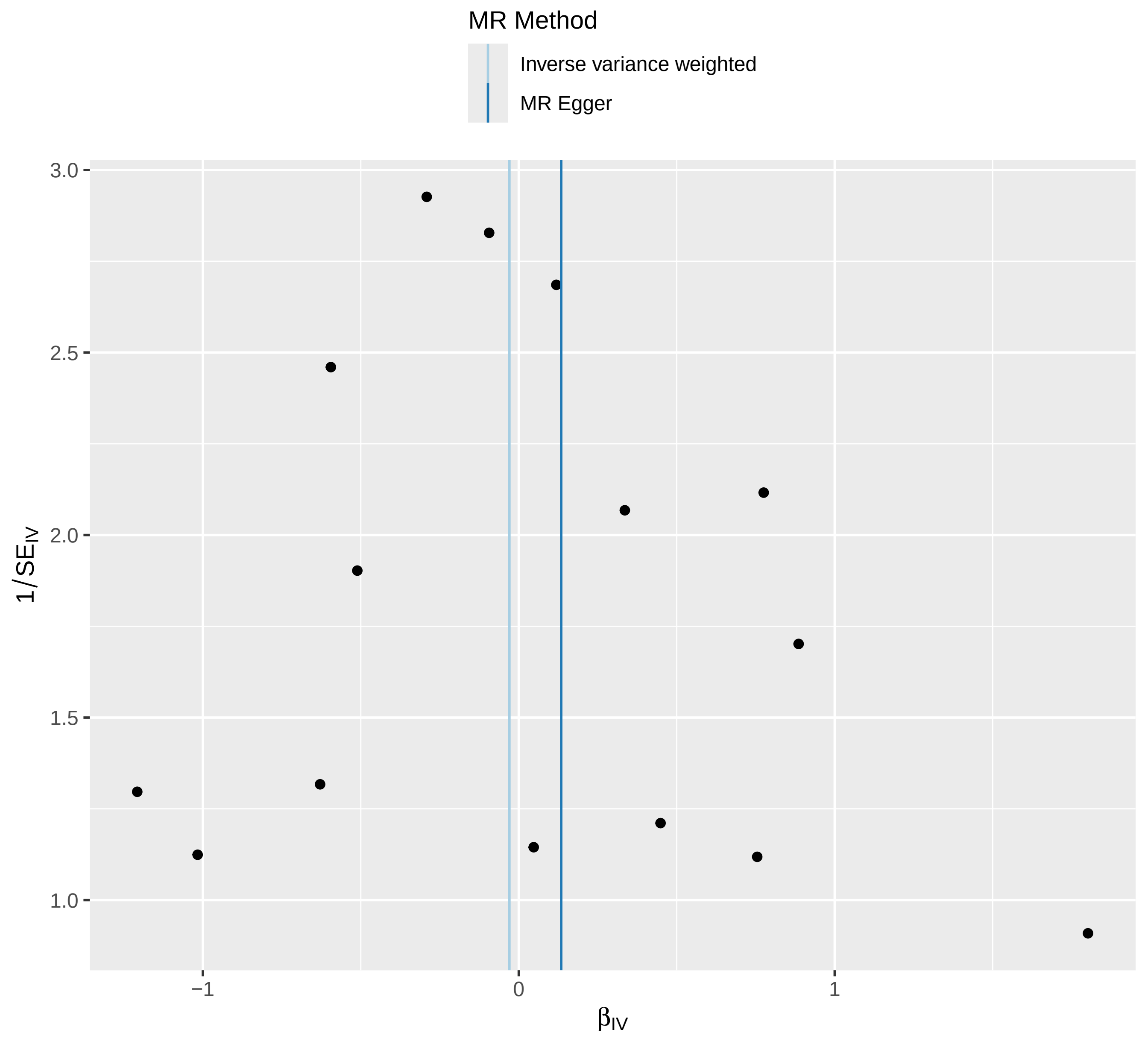
MR Method
Inverse variance weighted
MR Egger
3.0
2.5
2.0
1.5
1.0
1/SE_IV
−1
0
1
β_IV

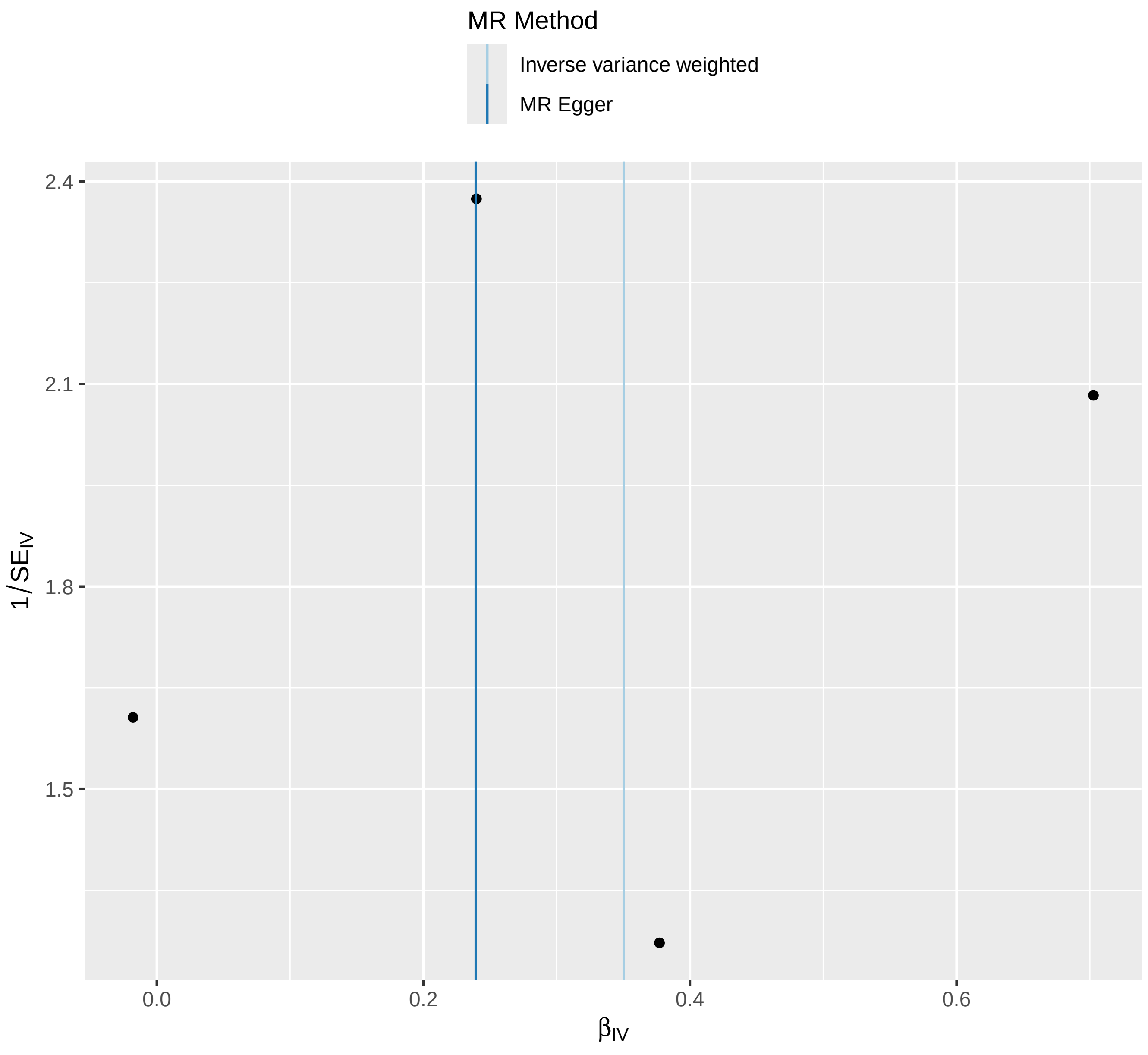

MR Method
Inverse variance weighted
MR Egger
2.4
2.1
1.8
1.5
1/SE_IV
0.0
0.2
0.4
0.6
β_IV

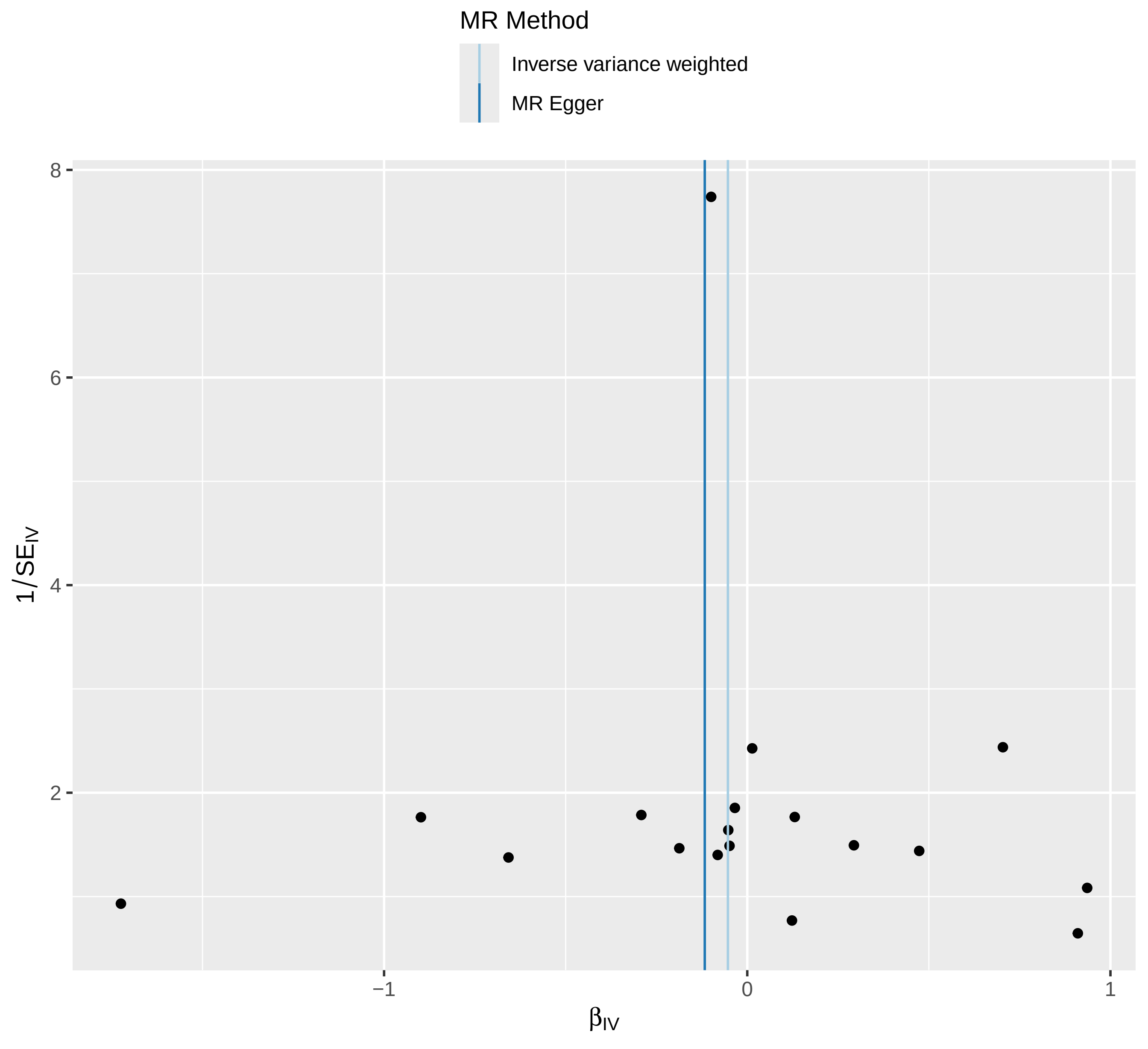
MR Method
Inverse variance weighted
MR Egger
1/SE_IV
β_IV
8
6
4
2
−1
0
1

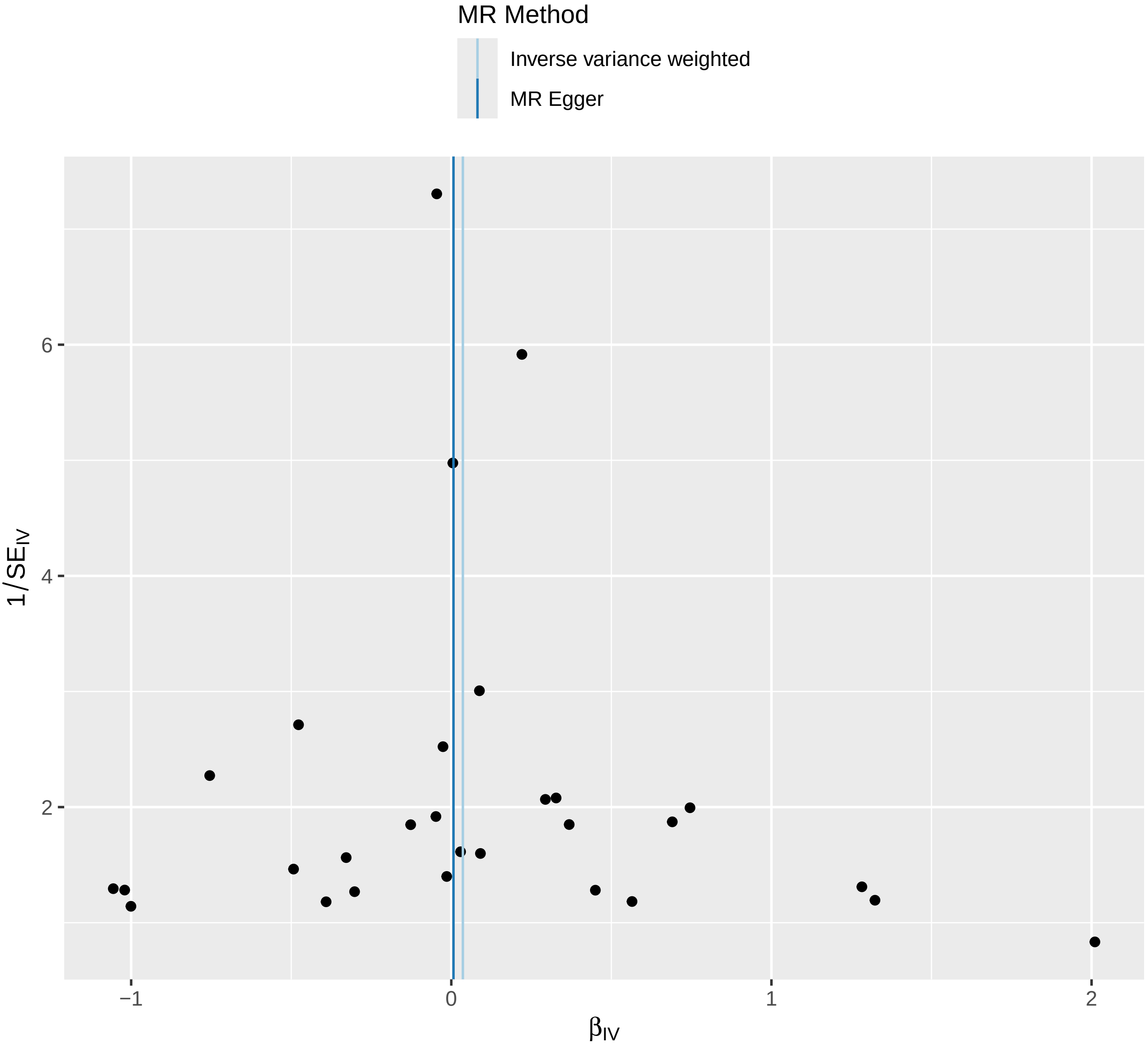

MR Method
Inverse variance weighted
MR Egger
6
4
2
1/SE$_{IV}$
−1
0
1
2
β$_{IV}$

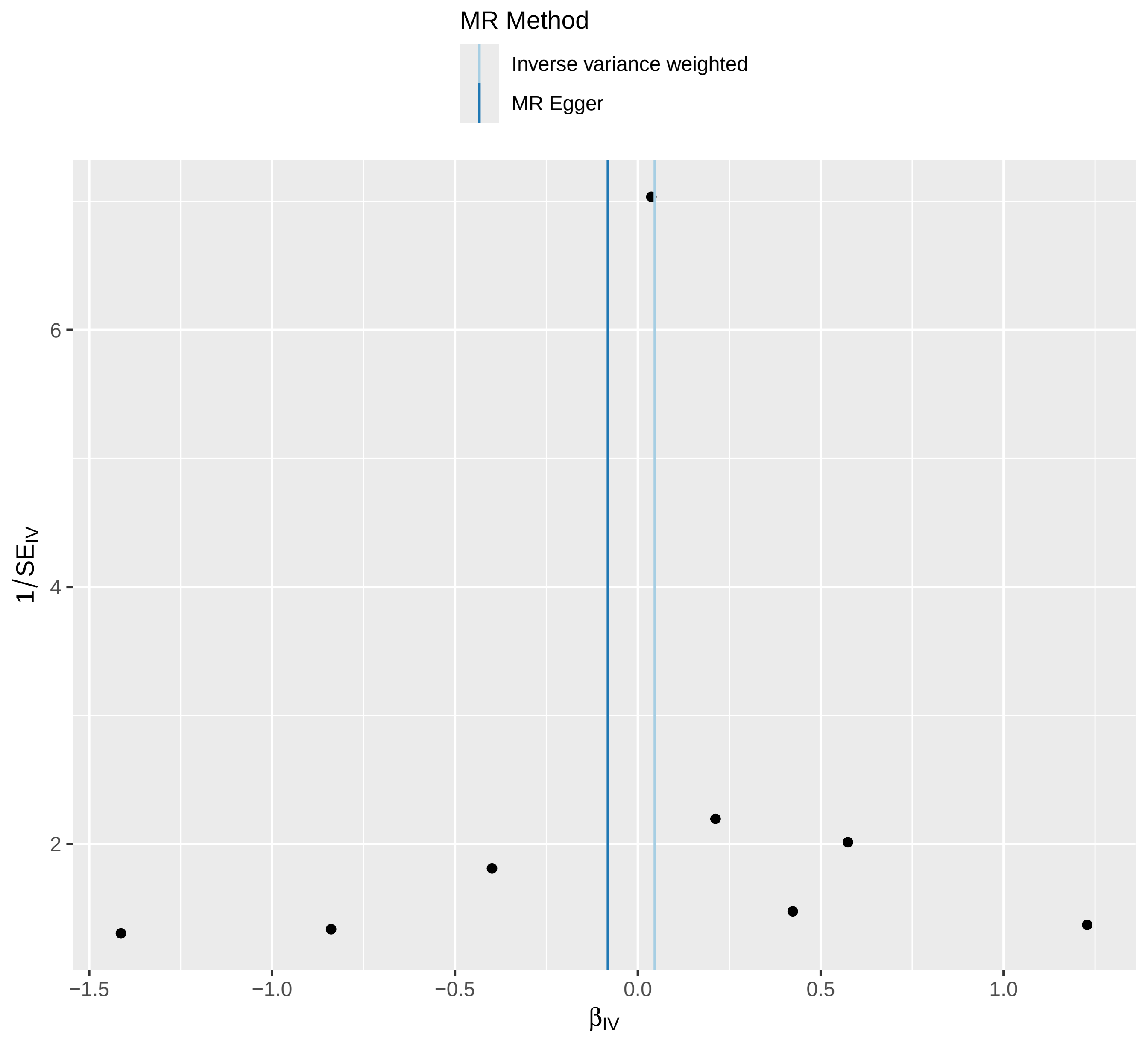
MR Method
Inverse variance weighted
MR Egger
1/SE$_{IV}$
6
4
2
−1.5
−1.0
−0.5
0.0
0.5
1.0
β$_{IV}$

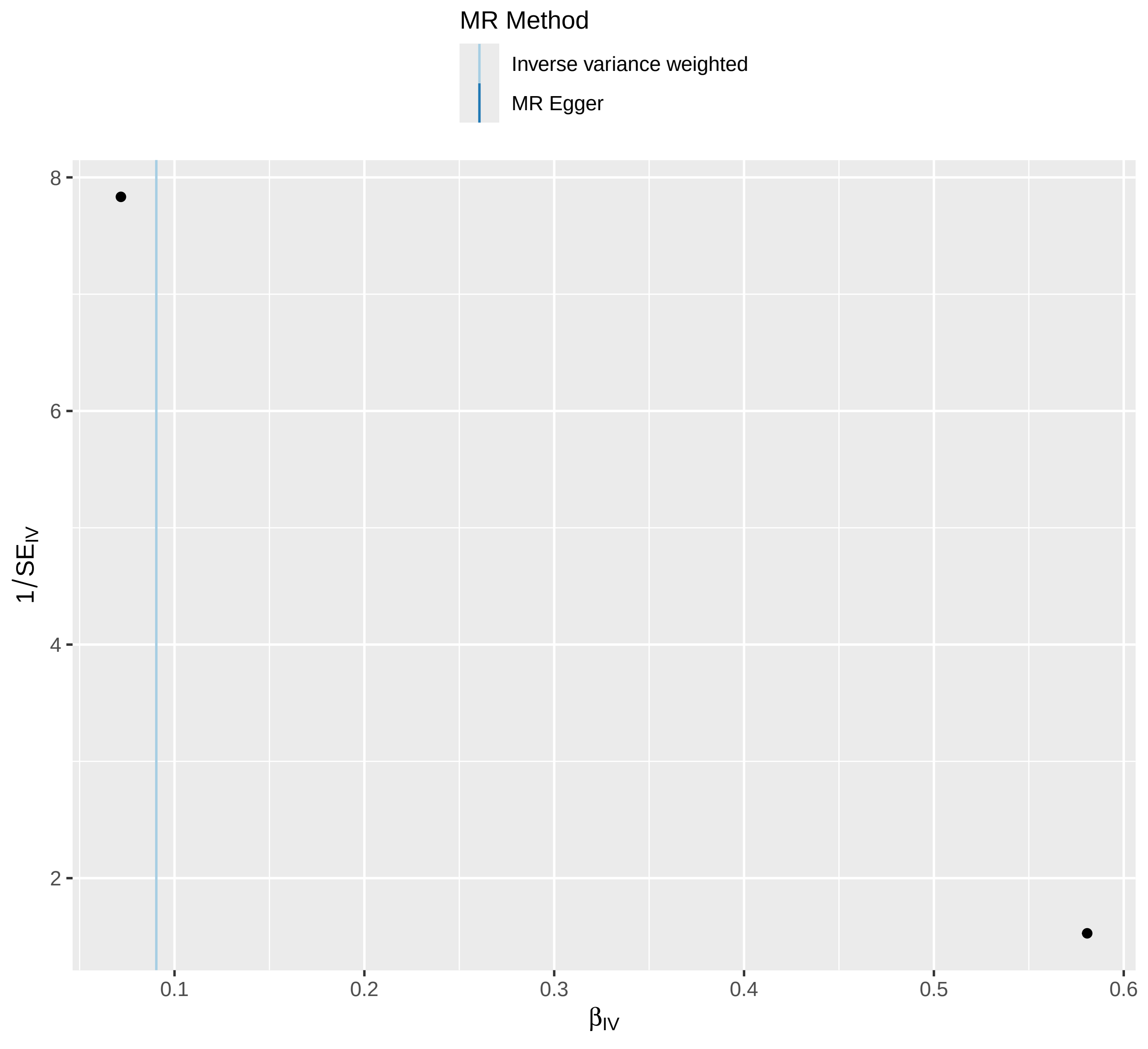

MR Method
Inverse variance weighted
MR Egger
1/SE_IV
β_IV
8
6
4
2
0.1
0.2
0.3
0.4
0.5
0.6

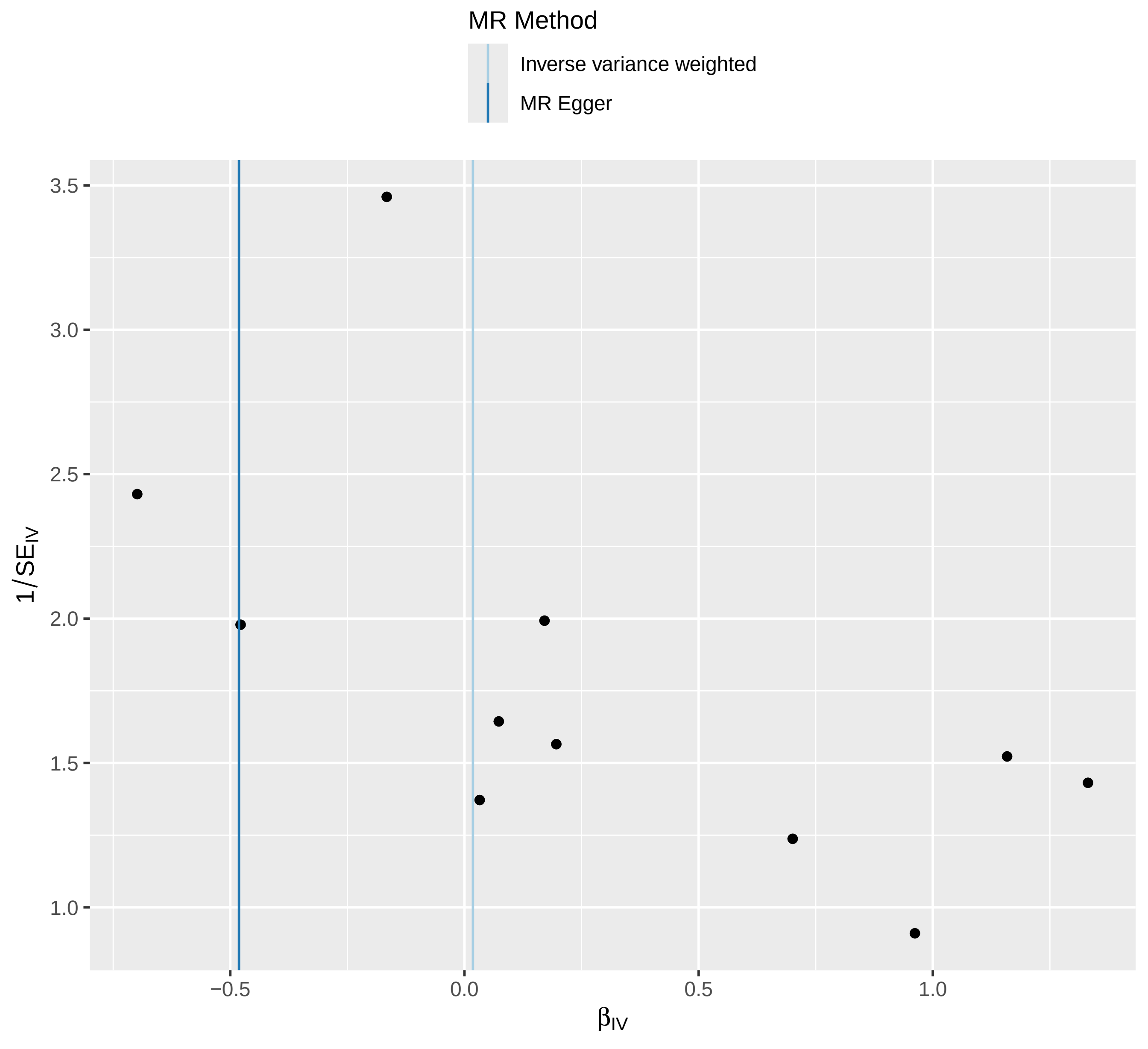

MR Method
Inverse variance weighted
MR Egger
3.5
3.0
2.5
2.0
1.5
1.0
$1/SE_{IV}$
−0.5
0.0
0.5
1.0
β_{IV}

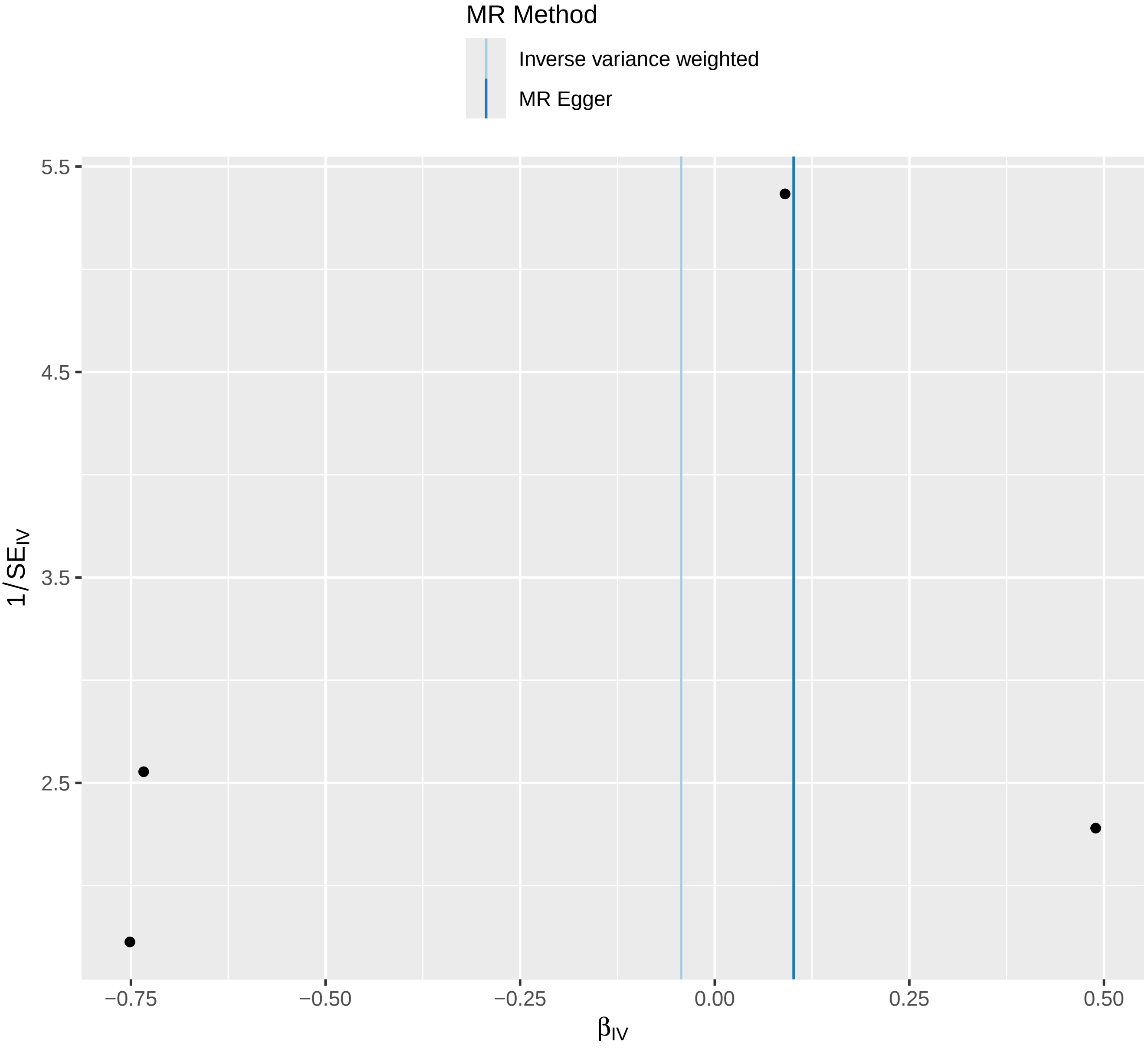

MR Method
Inverse variance weighted
MR Egger
5.5
4.5
3.5
2.5
1/SE$_{IV}$
−0.75
−0.50
−0.25
0.00
0.25
0.50
β$_{IV}$

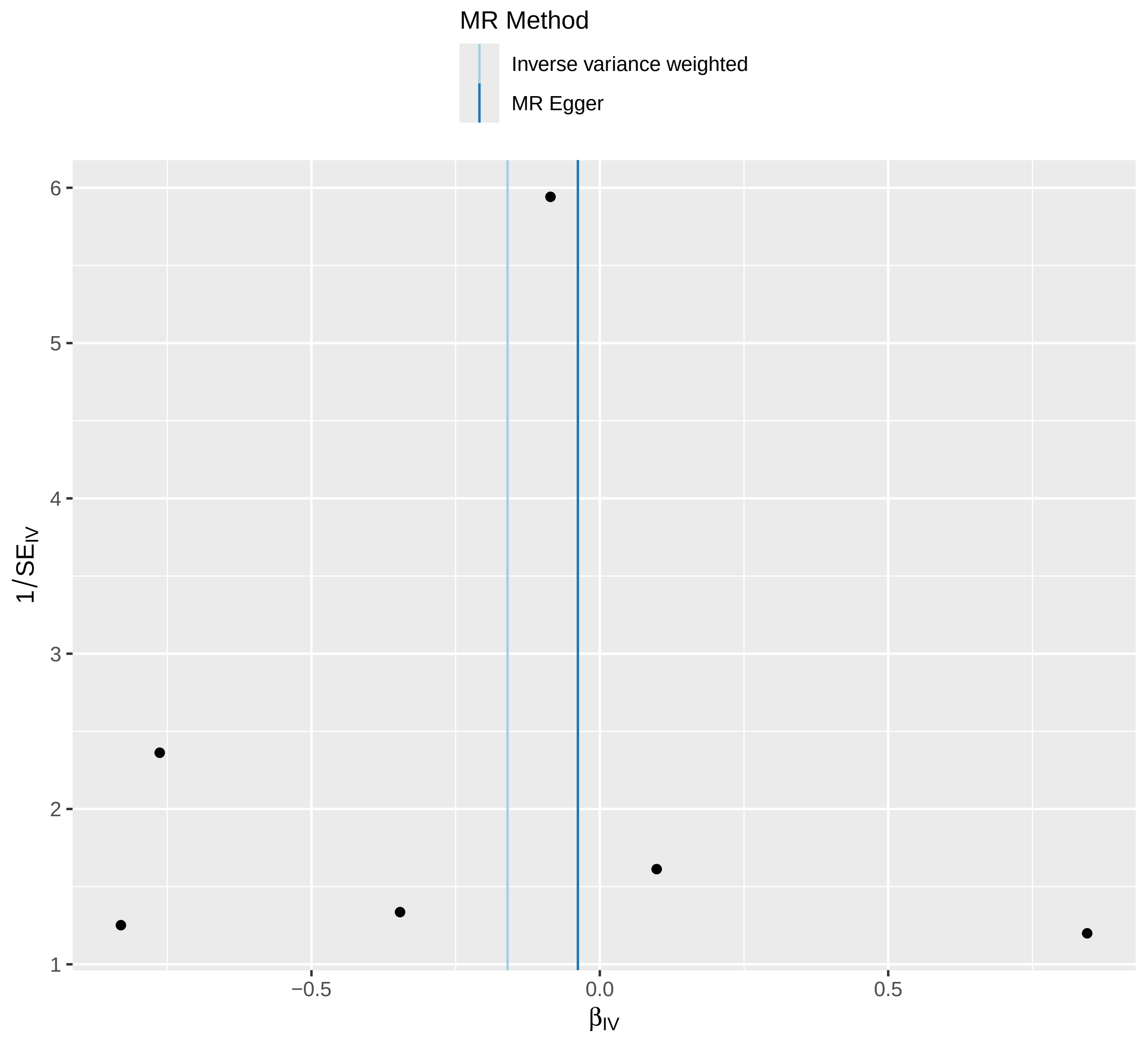
MR Method
Inverse variance weighted
MR Egger
6
5
4
3
2
1
1/SE_IV
−0.5
0.0
0.5
β_IV

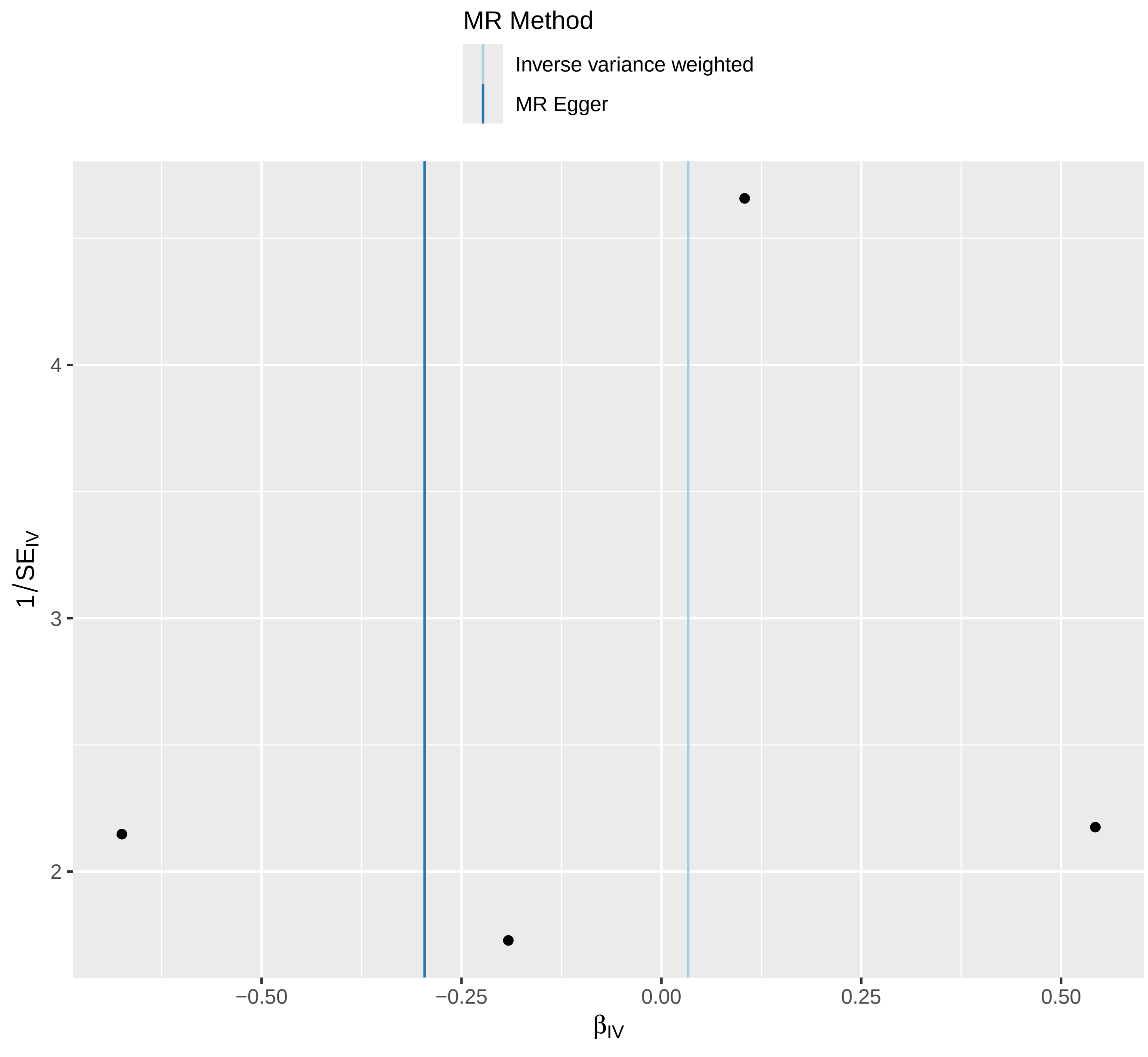
MR Method
Inverse variance weighted
MR Egger
4
3
2
1/SE_IV
−0.50
−0.25
0.00
0.25
0.50
β_IV

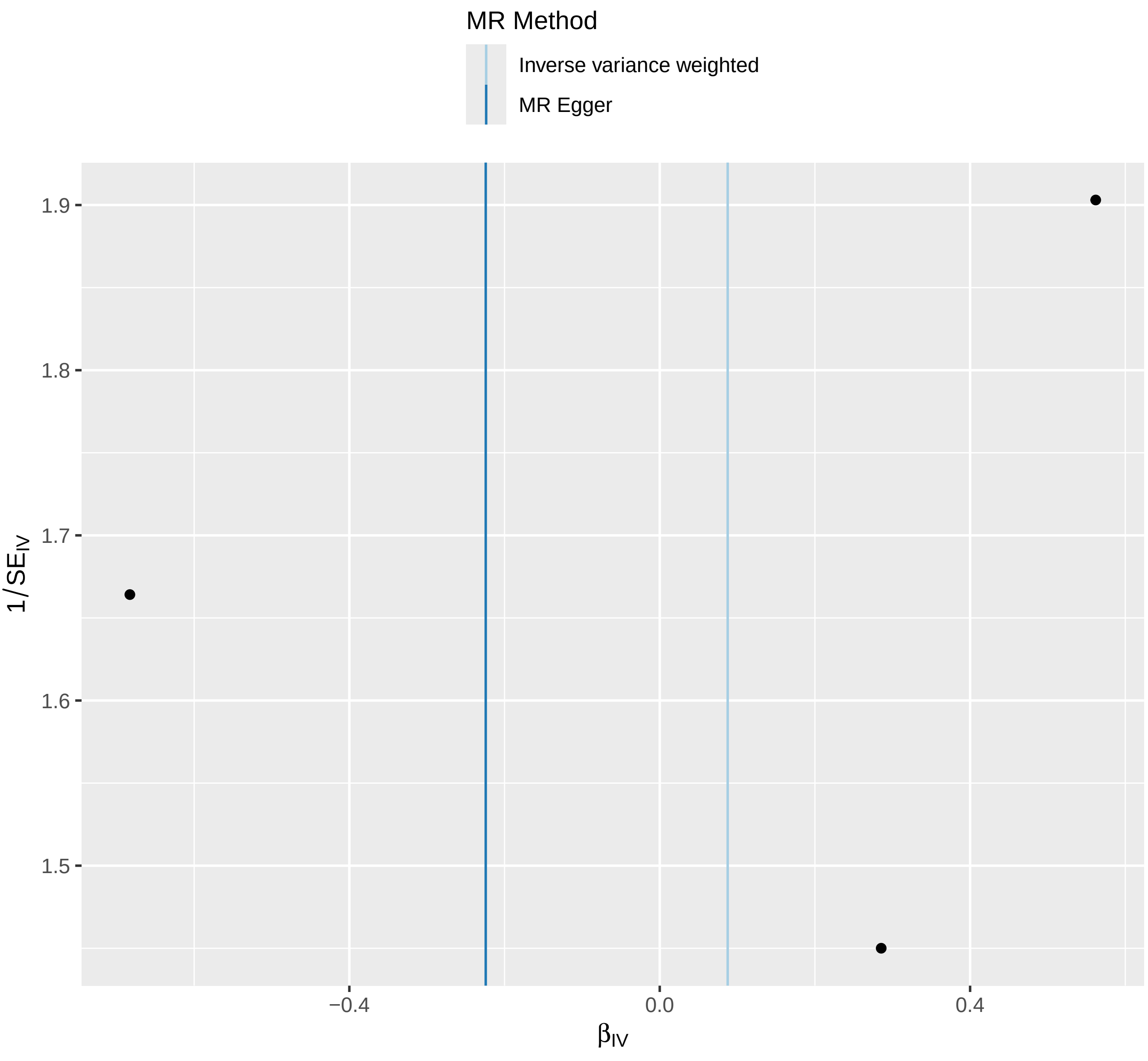
MR Method
Inverse variance weighted
MR Egger
1.9
1.8
1.7
1.6
1.5
1/SE_IV
−0.4
0.0
0.4
β_IV

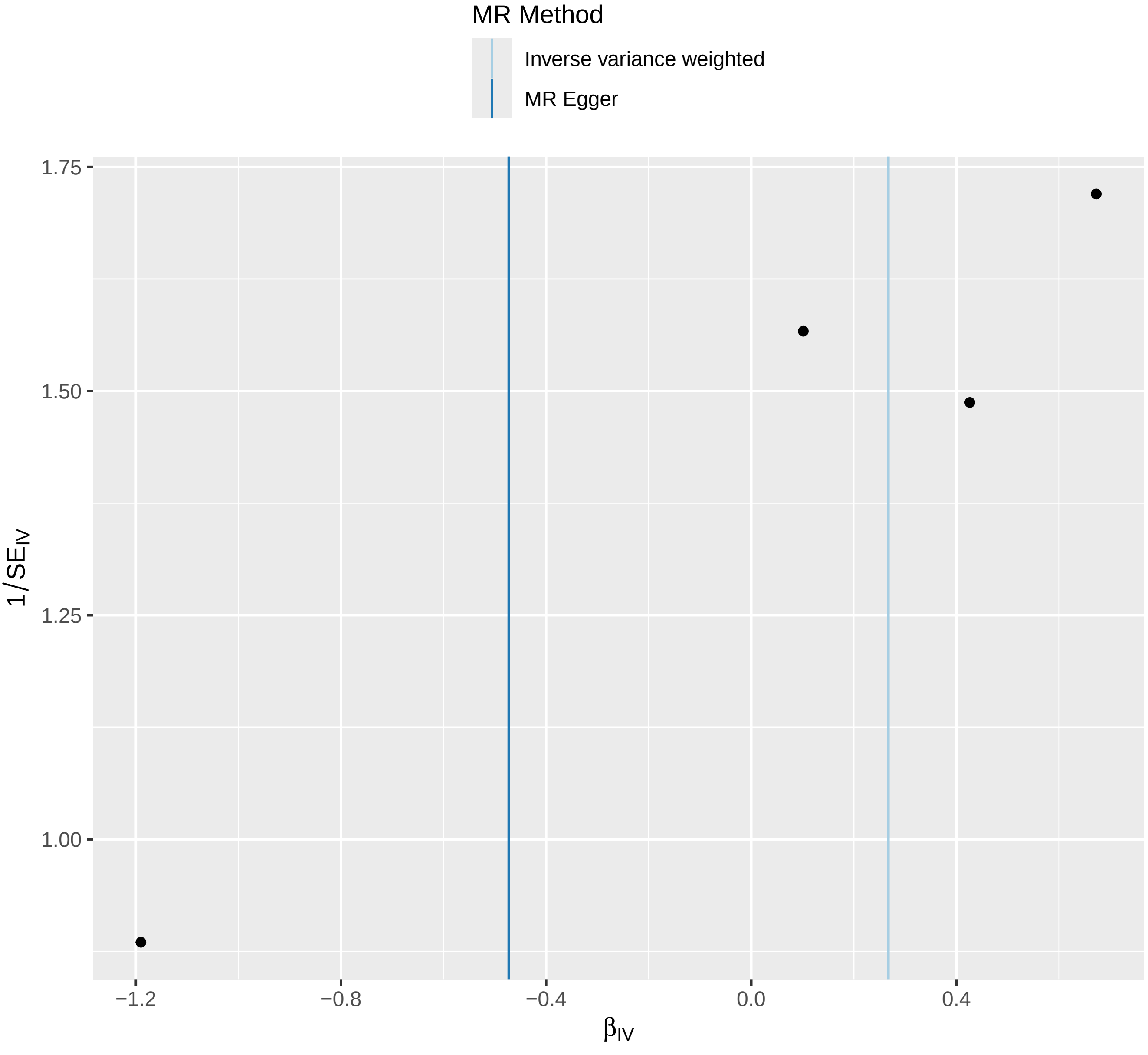
MR Method
Inverse variance weighted
MR Egger
1.75
1.50
1.25
1.00
$1/SE_{IV}$
−1.2
−0.8
−0.4
0.0
0.4
β_{IV}

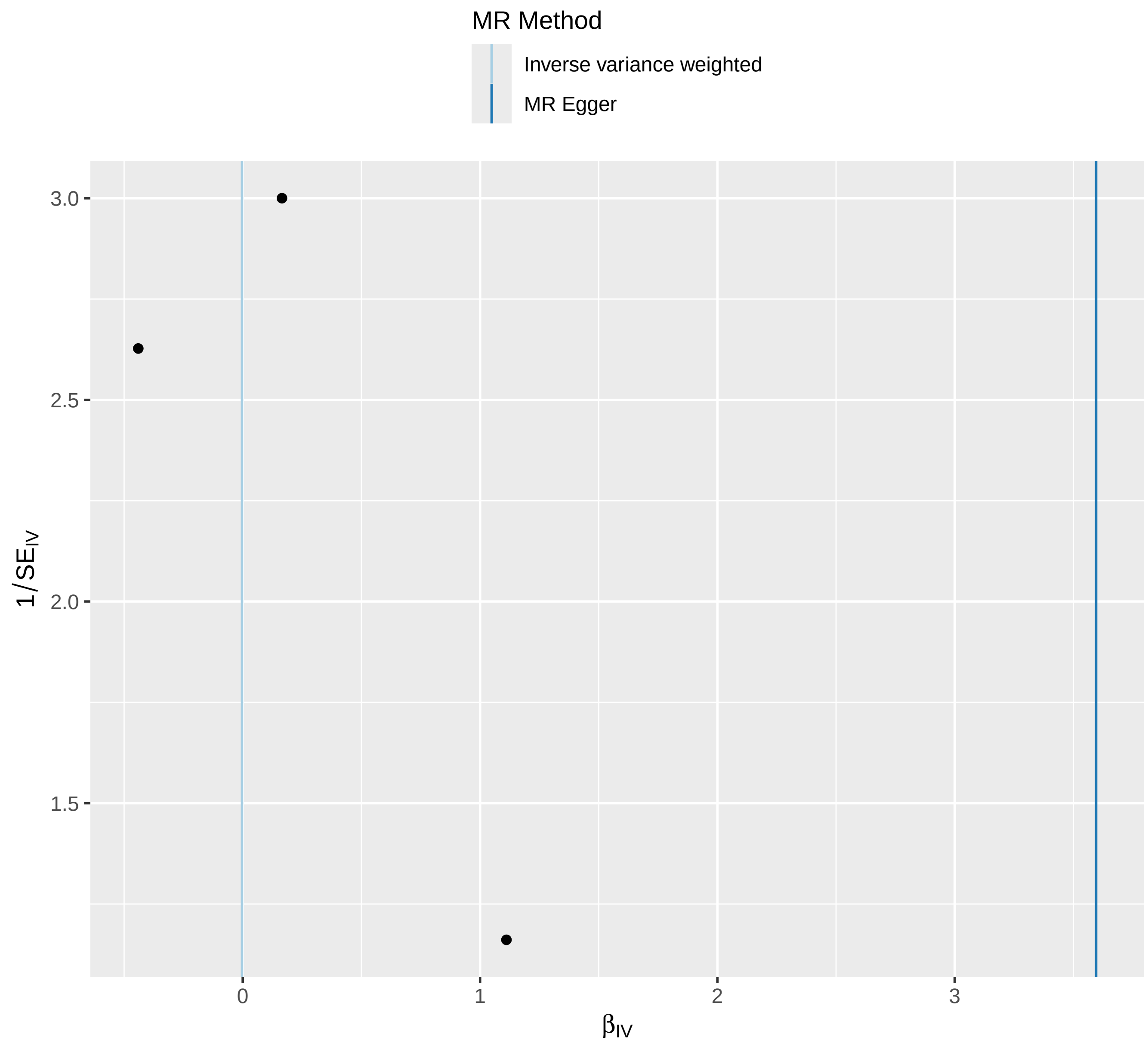
MR Method
Inverse variance weighted
MR Egger
1/SE_IV
3.0
2.5
2.0
1.5
0
1
2
3
β_IV

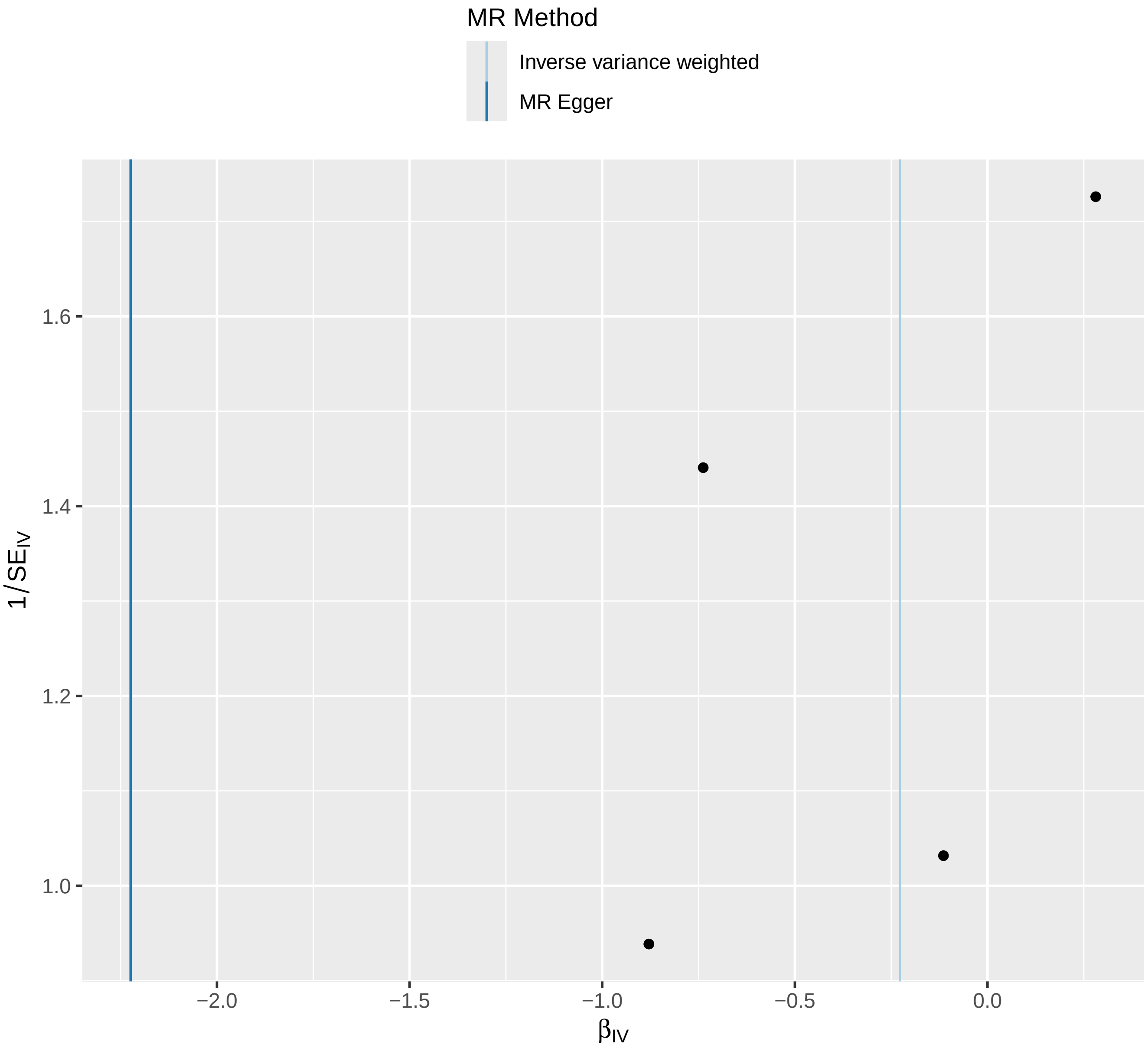
MR Method
Inverse variance weighted
MR Egger
1.6
1.4
1.2
1.0
1/SE$_{IV}$
−2.0
−1.5
−1.0
−0.5
0.0
β$_{IV}$

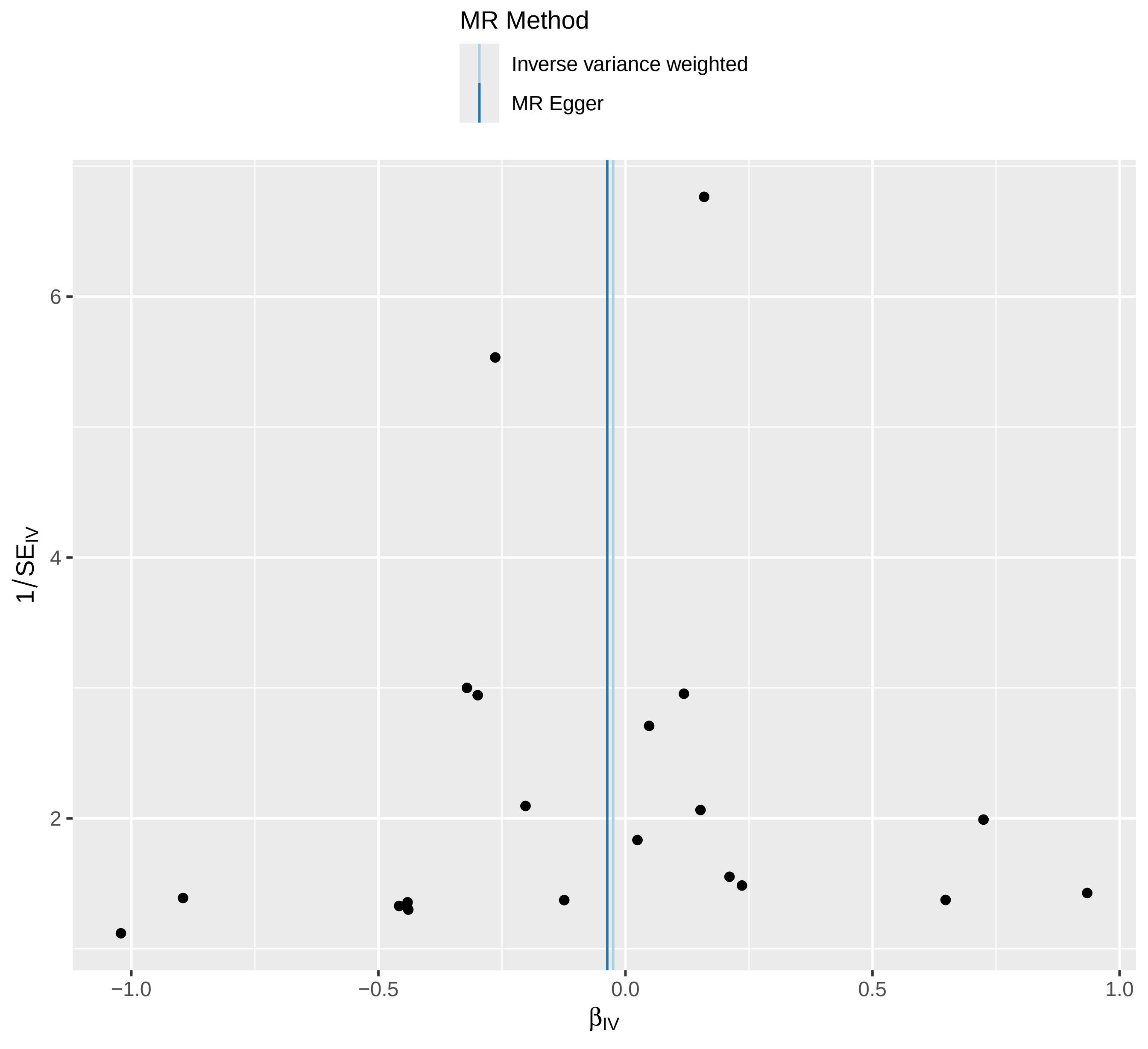
MR Method
Inverse variance weighted
MR Egger
6
4
2
1/SE_IV
−1.0
−0.5
0.0
0.5
1.0
β_IV

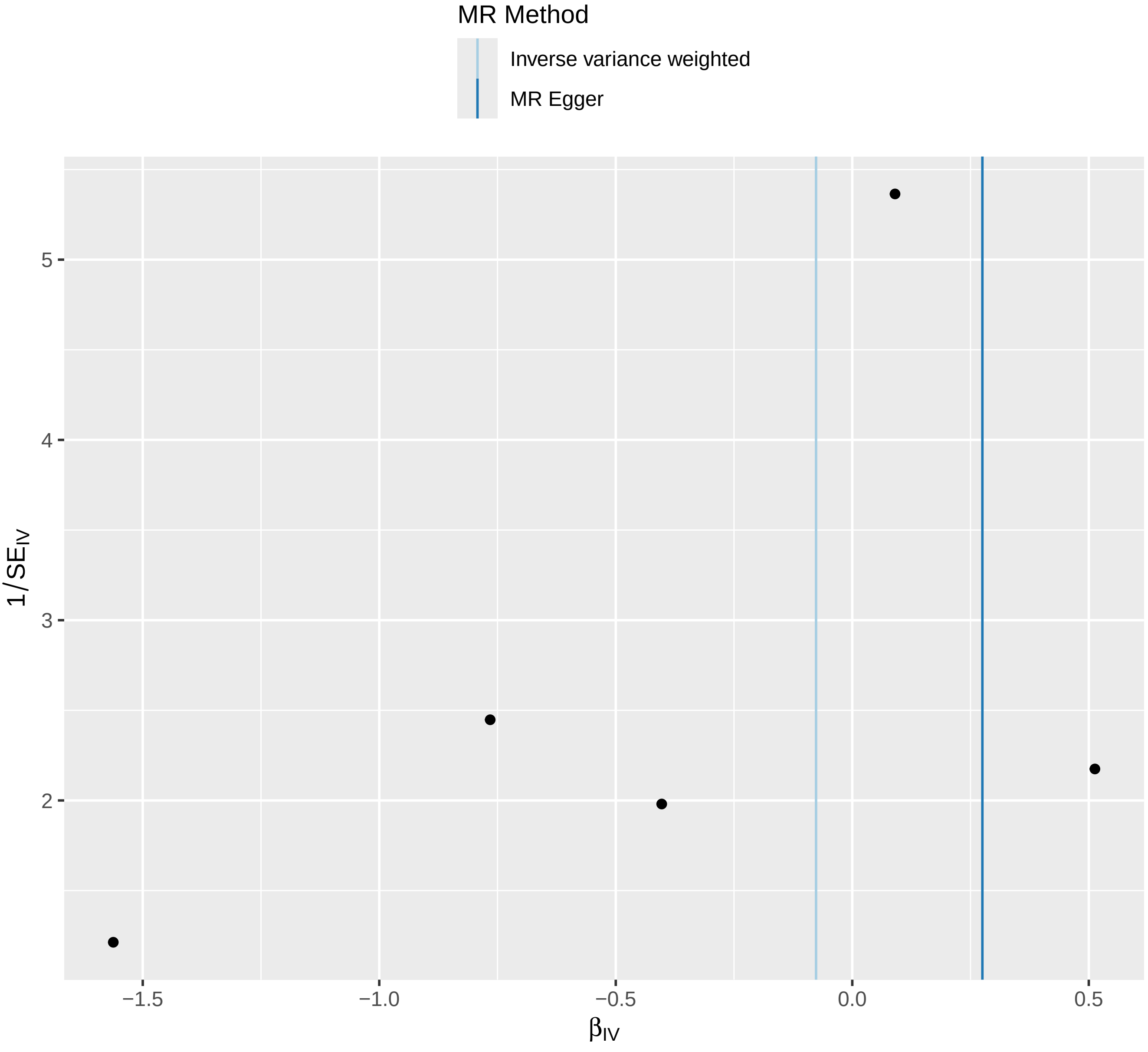
MR Method
Inverse variance weighted
MR Egger
5
4
3
2
1/SE$_{IV}$
−1.5
−1.0
−0.5
0.0
0.5
β$_{IV}$

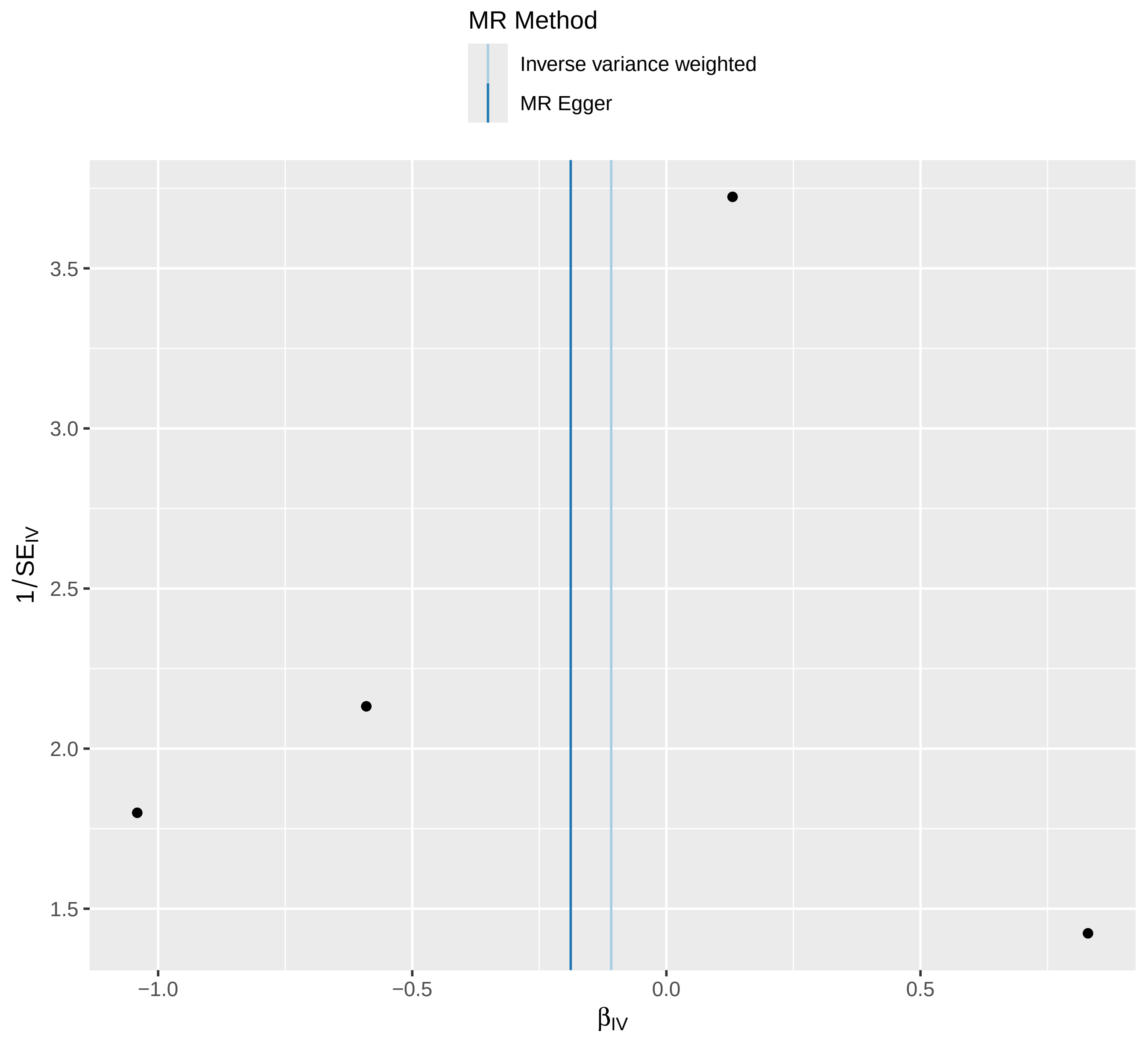
MR Method
Inverse variance weighted
MR Egger
3.5
3.0
2.5
2.0
1.5
1/SE$_{IV}$
−1.0
−0.5
0.0
0.5
β$_{IV}$

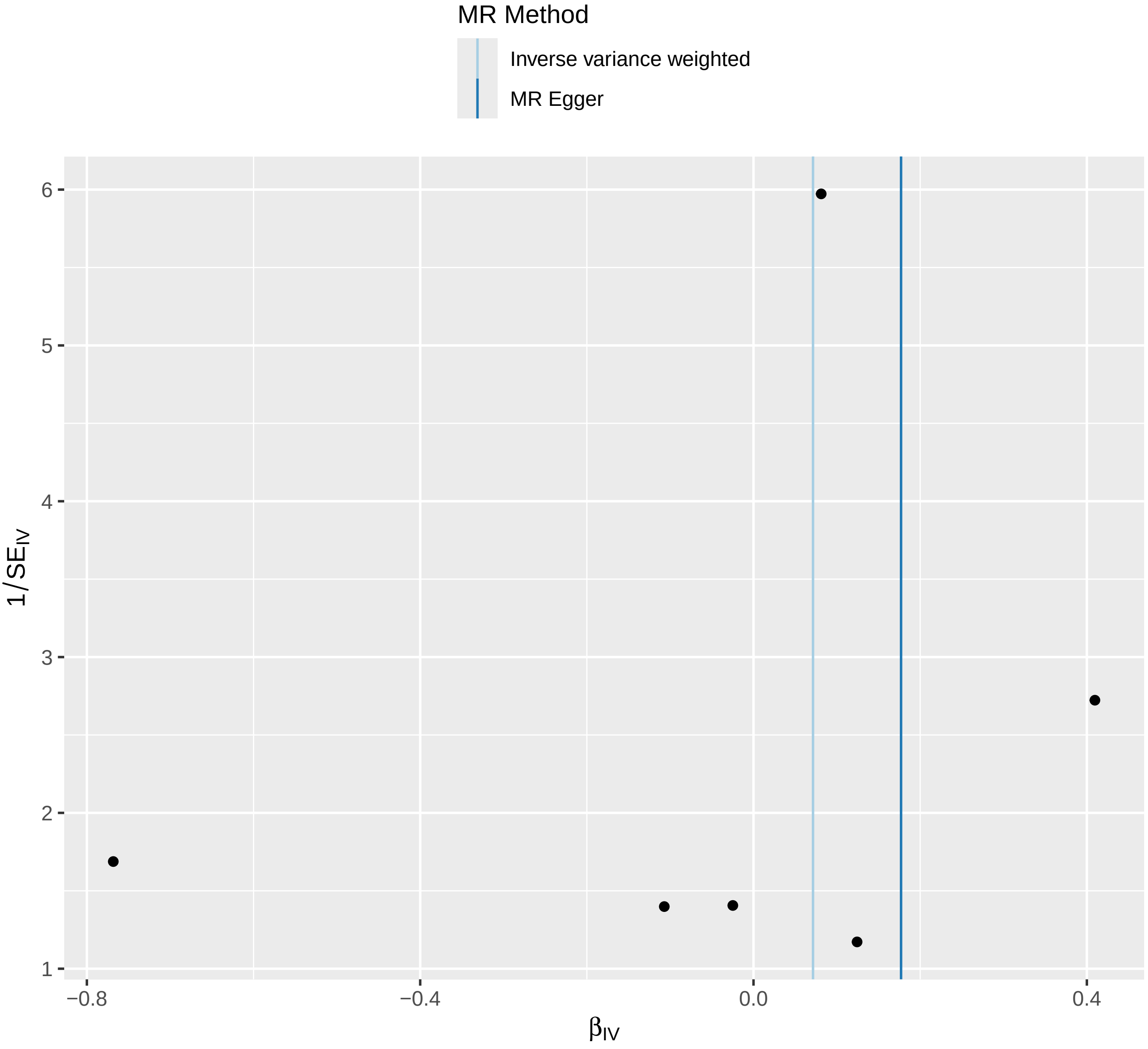

MR Method
Inverse variance weighted
MR Egger
6
5
4
3
2
1
1/SE$_{IV}$
−0.8
−0.4
0.0
0.4
β$_{IV}$

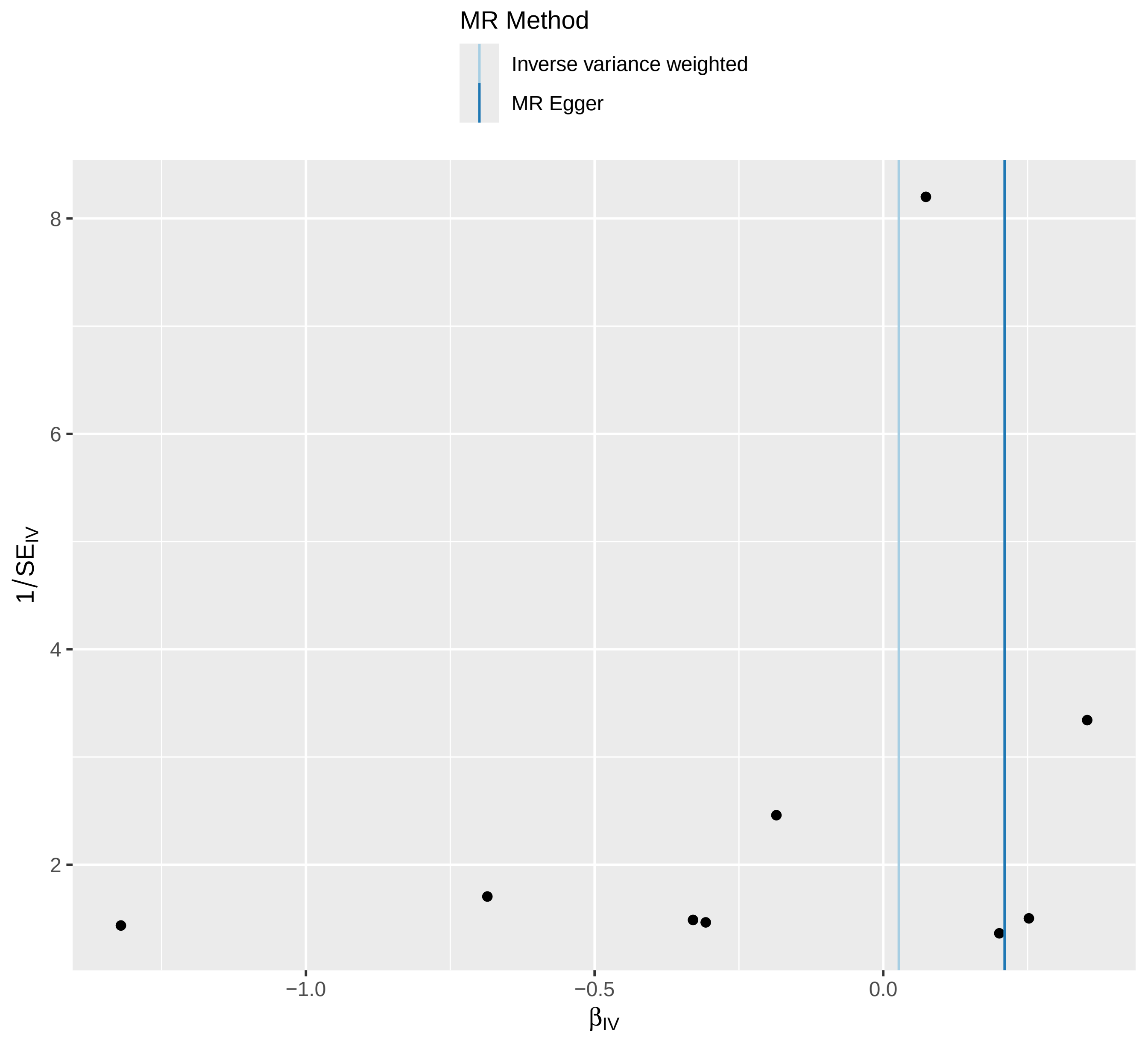
MR Method
Inverse variance weighted
MR Egger
8
6
4
2
1/SE_IV
−1.0
−0.5
0.0
β_IV

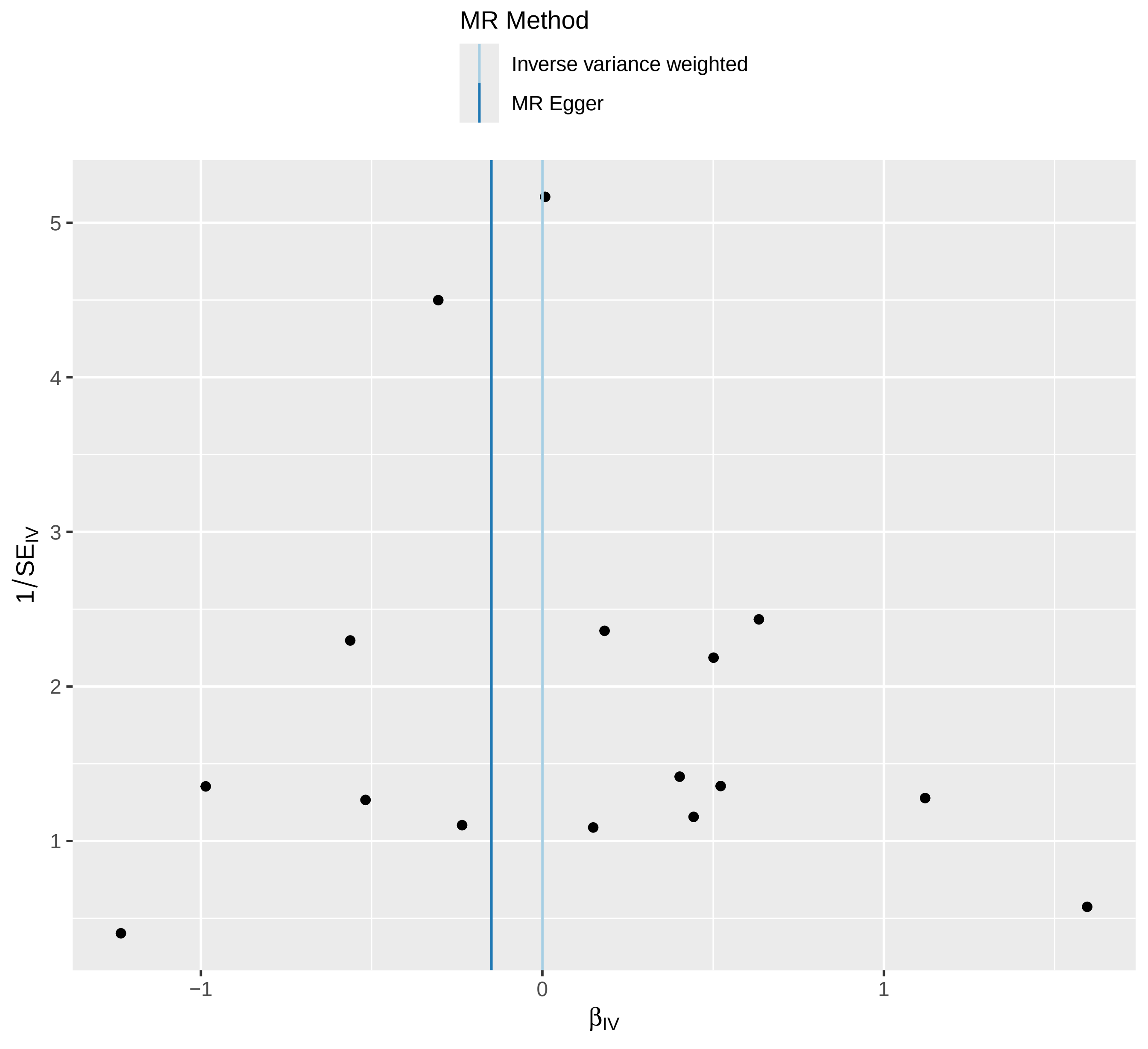

MR Method
Inverse variance weighted
MR Egger
5
4
3
2
1
$1/SE_{IV}$
−1
0
1
β_{IV}

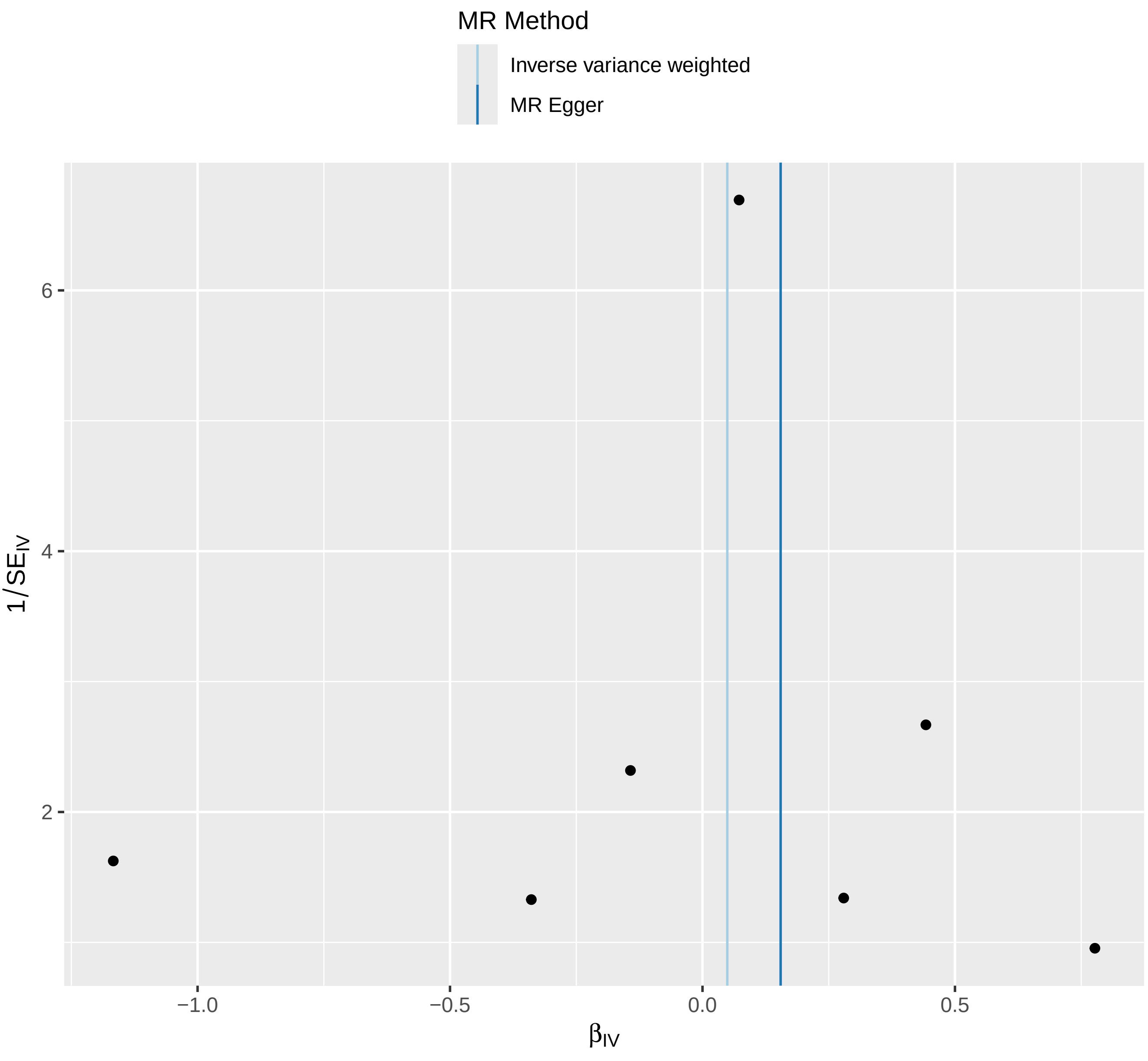
MR Method
Inverse variance weighted
MR Egger
6
4
2
1/SE_IV
−1.0
−0.5
0.0
0.5
β_IV

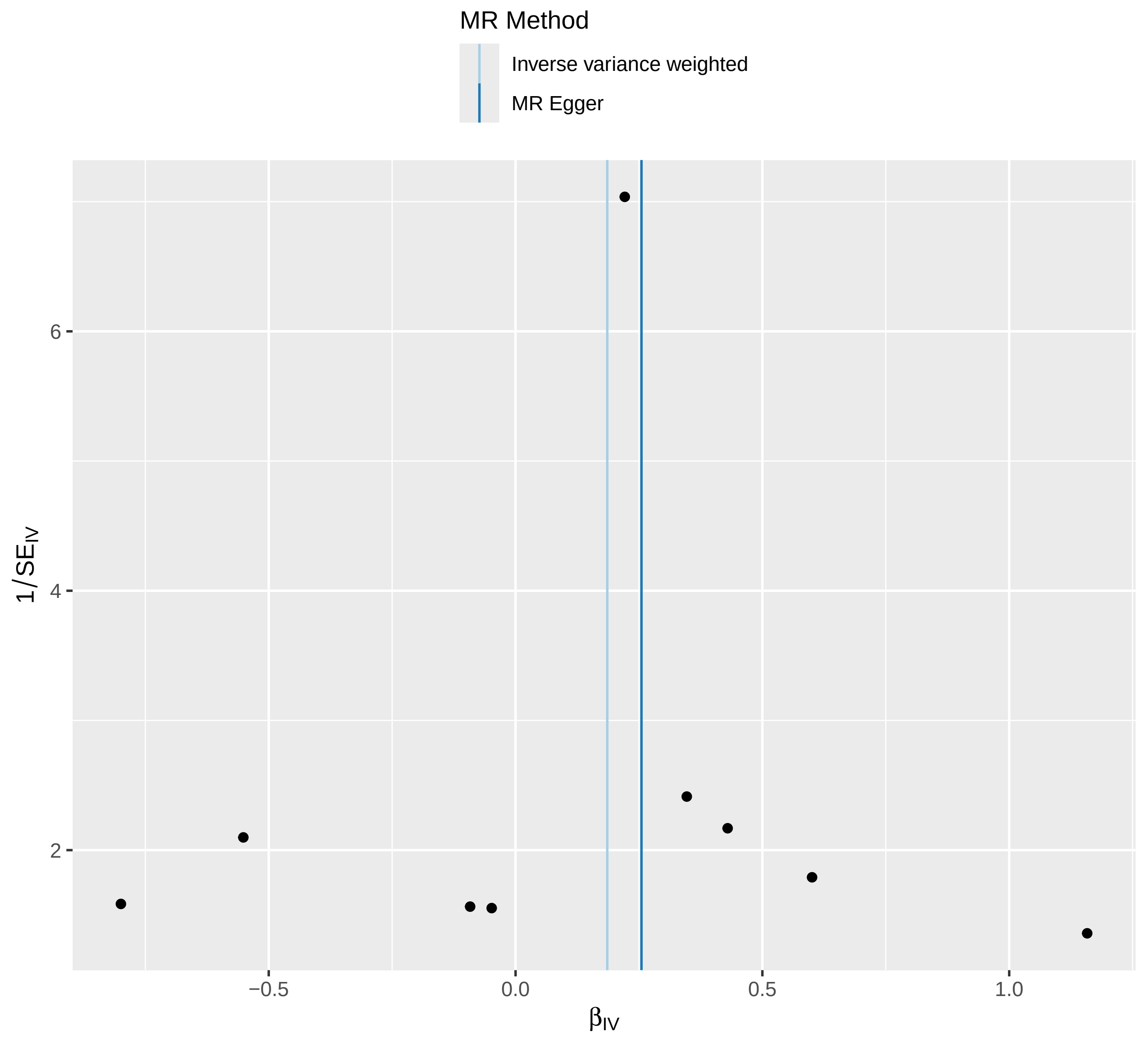

MR Method
Inverse variance weighted
MR Egger
6
4
2
1/SE$_{IV}$
−0.5
0.0
0.5
1.0
β_{IV}

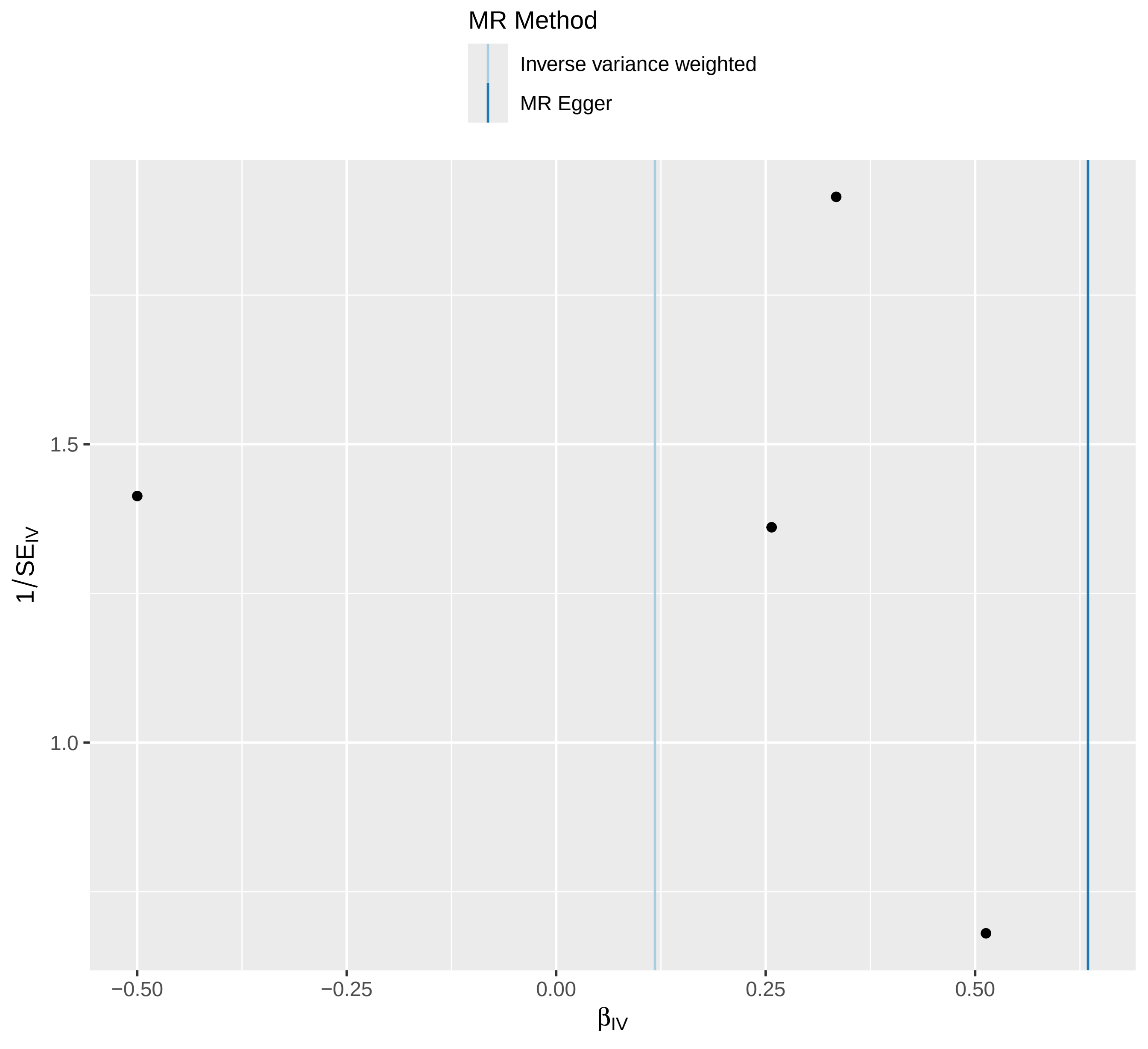

MR Method
Inverse variance weighted
MR Egger
1.5
1.0
−0.50
−0.25
0.00
0.25
0.50
$1/SE_{IV}$
β_{IV}

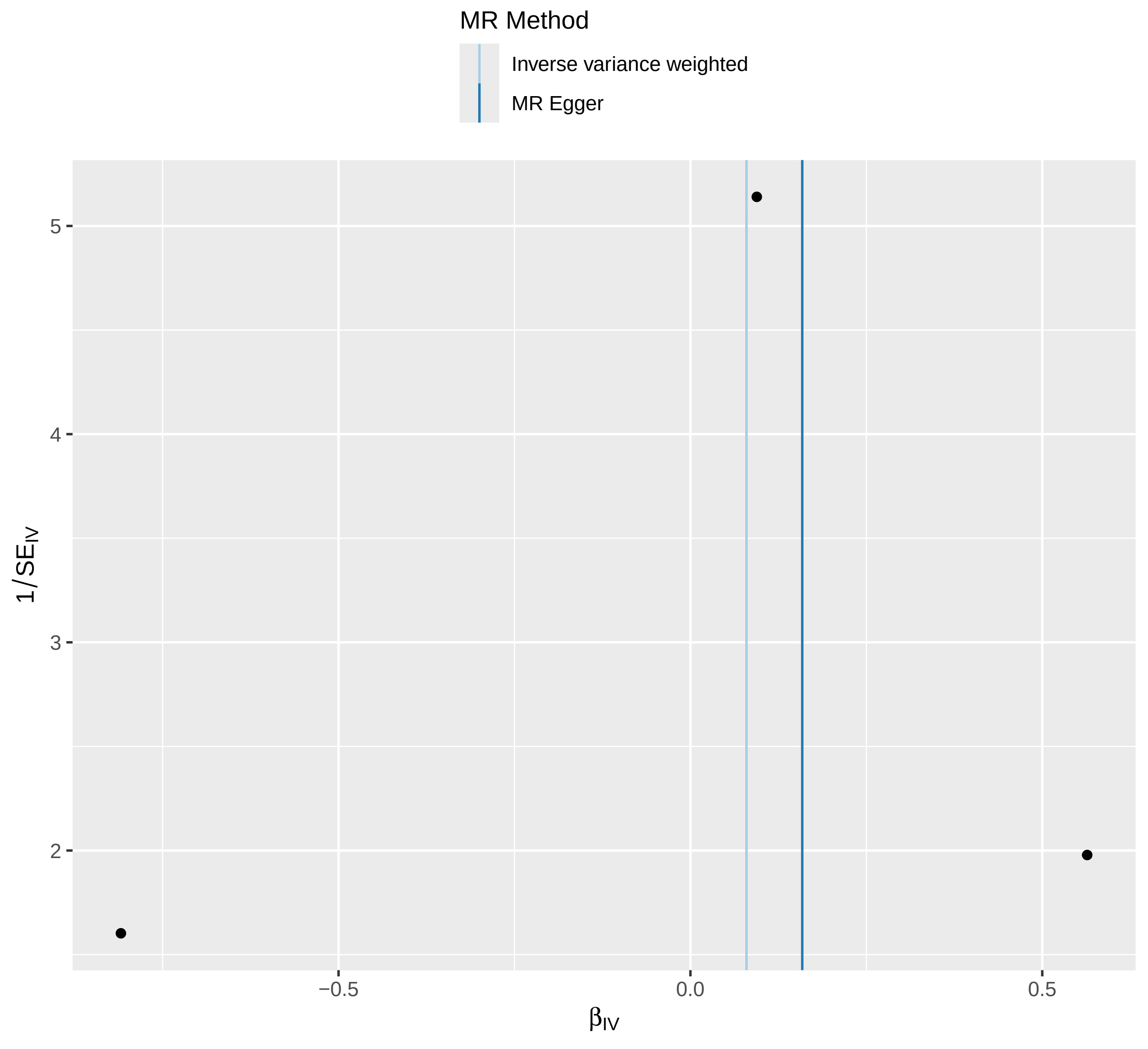
MR Method
Inverse variance weighted
MR Egger
5
4
3
2
1/SE_IV
−0.5
0.0
0.5
β_IV

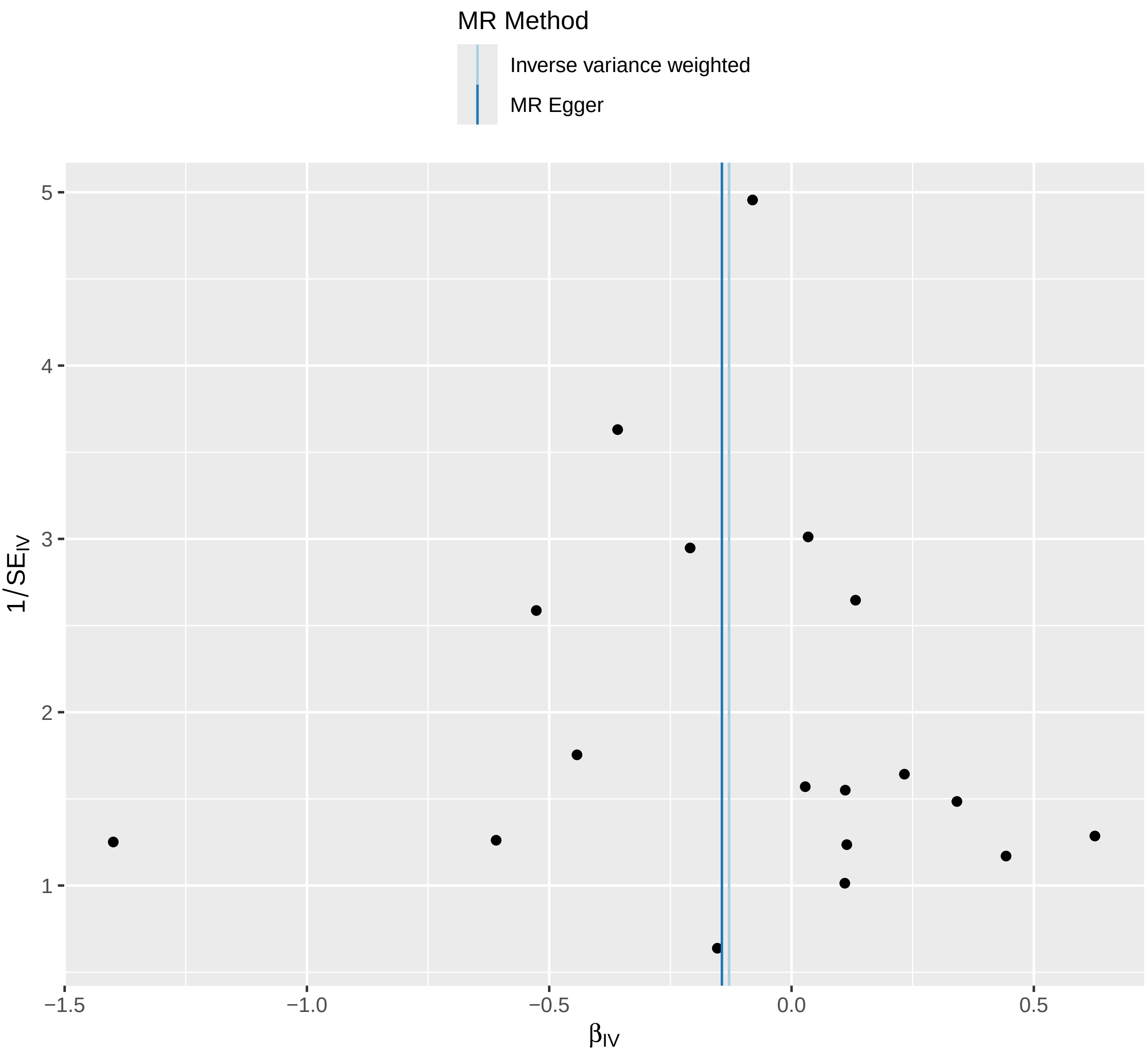

MR Method
Inverse variance weighted
MR Egger
5
4
3
2
1
$1/SE_{IV}$
−1.5
−1.0
−0.5
0.0
0.5
β_{IV}

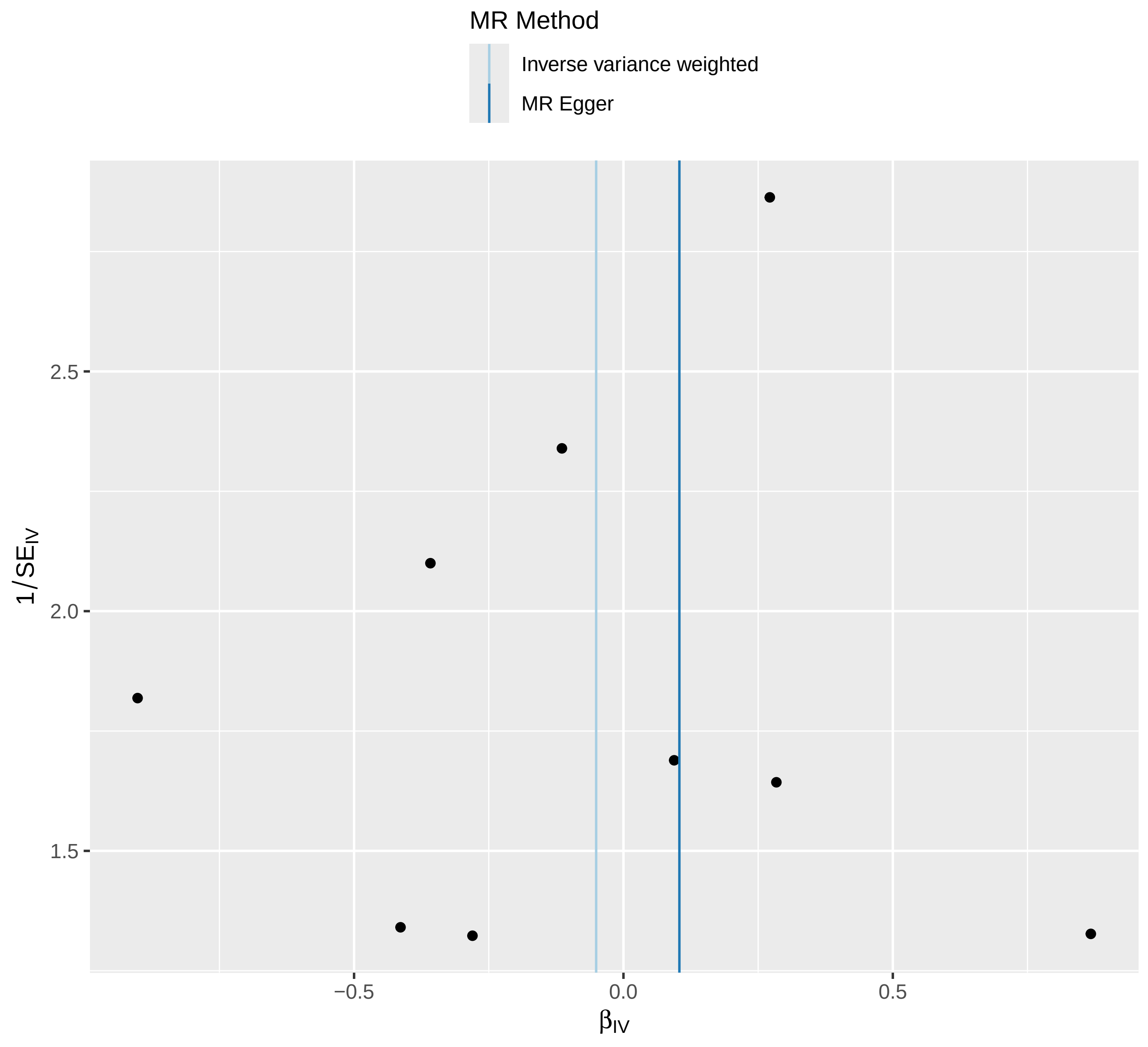

MR Method
Inverse variance weighted
MR Egger
2.5
2.0
1.5
1/SE$_{IV}$
−0.5
0.0
0.5
β$_{IV}$

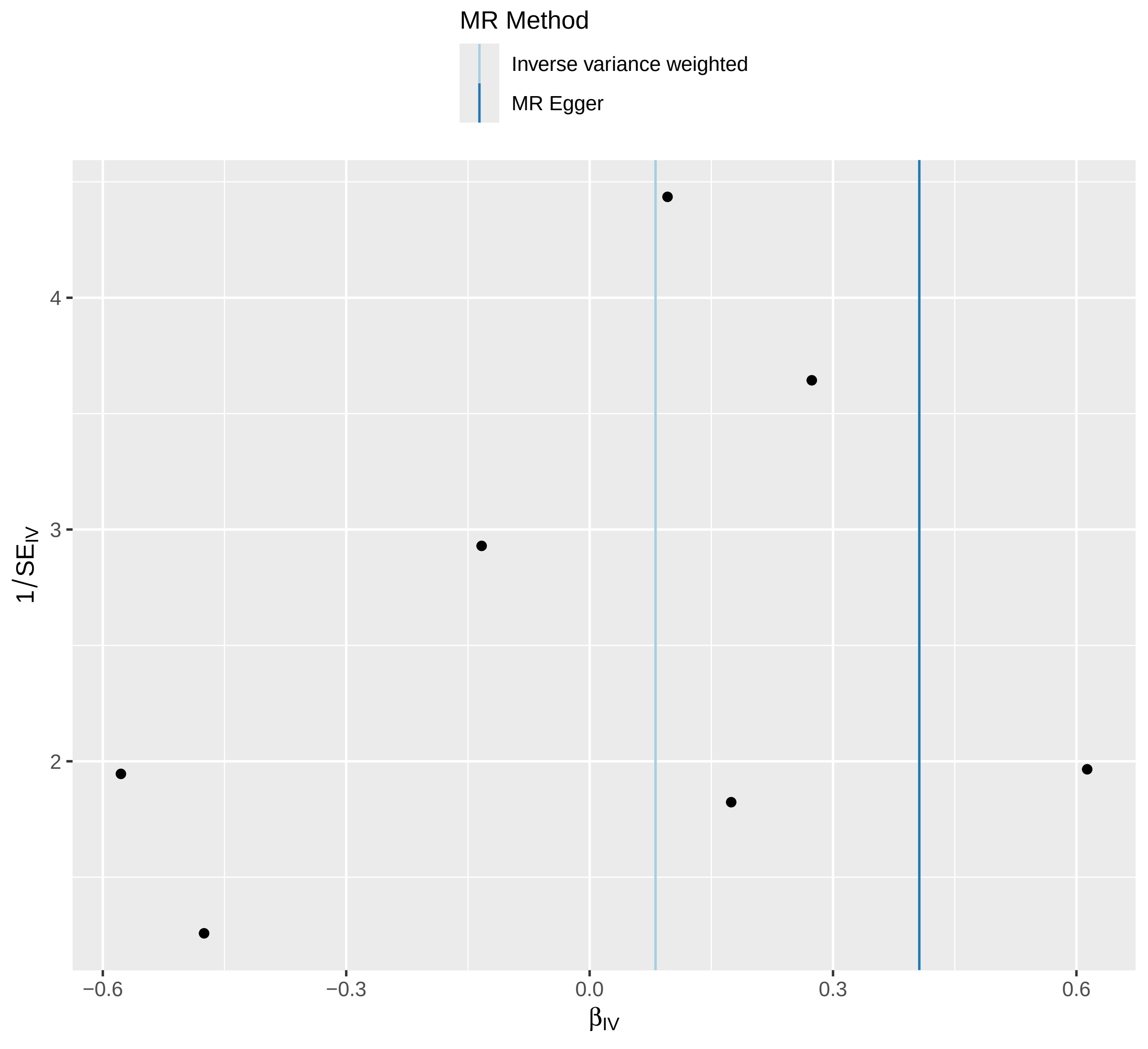
MR Method
Inverse variance weighted
MR Egger
4
3
2
$1/SE_{IV}$
−0.6
−0.3
0.0
0.3
0.6
β_{IV}

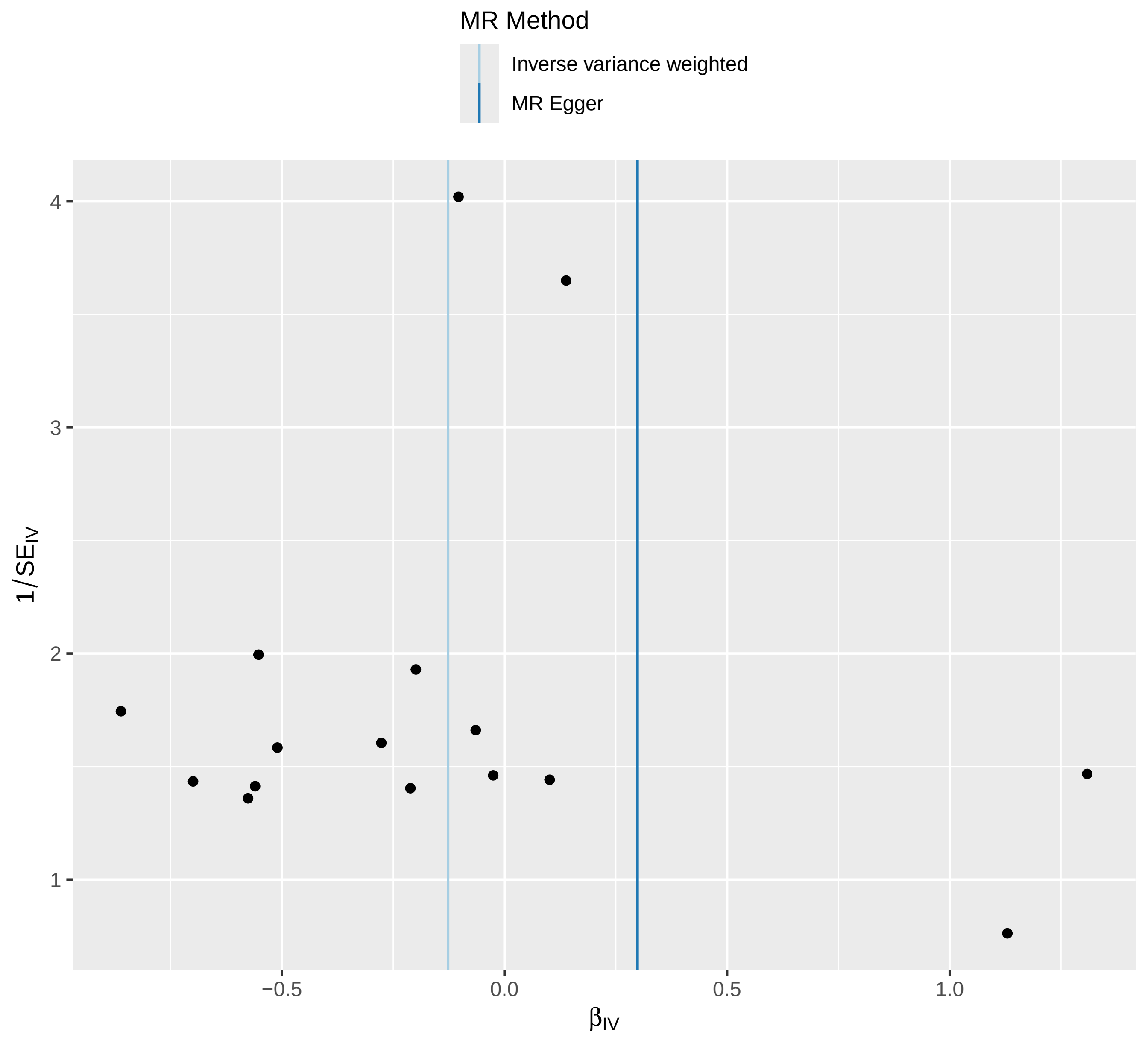

MR Method
Inverse variance weighted
MR Egger
4
3
2
1
1/SE_IV
−0.5
0.0
0.5
1.0
β_IV

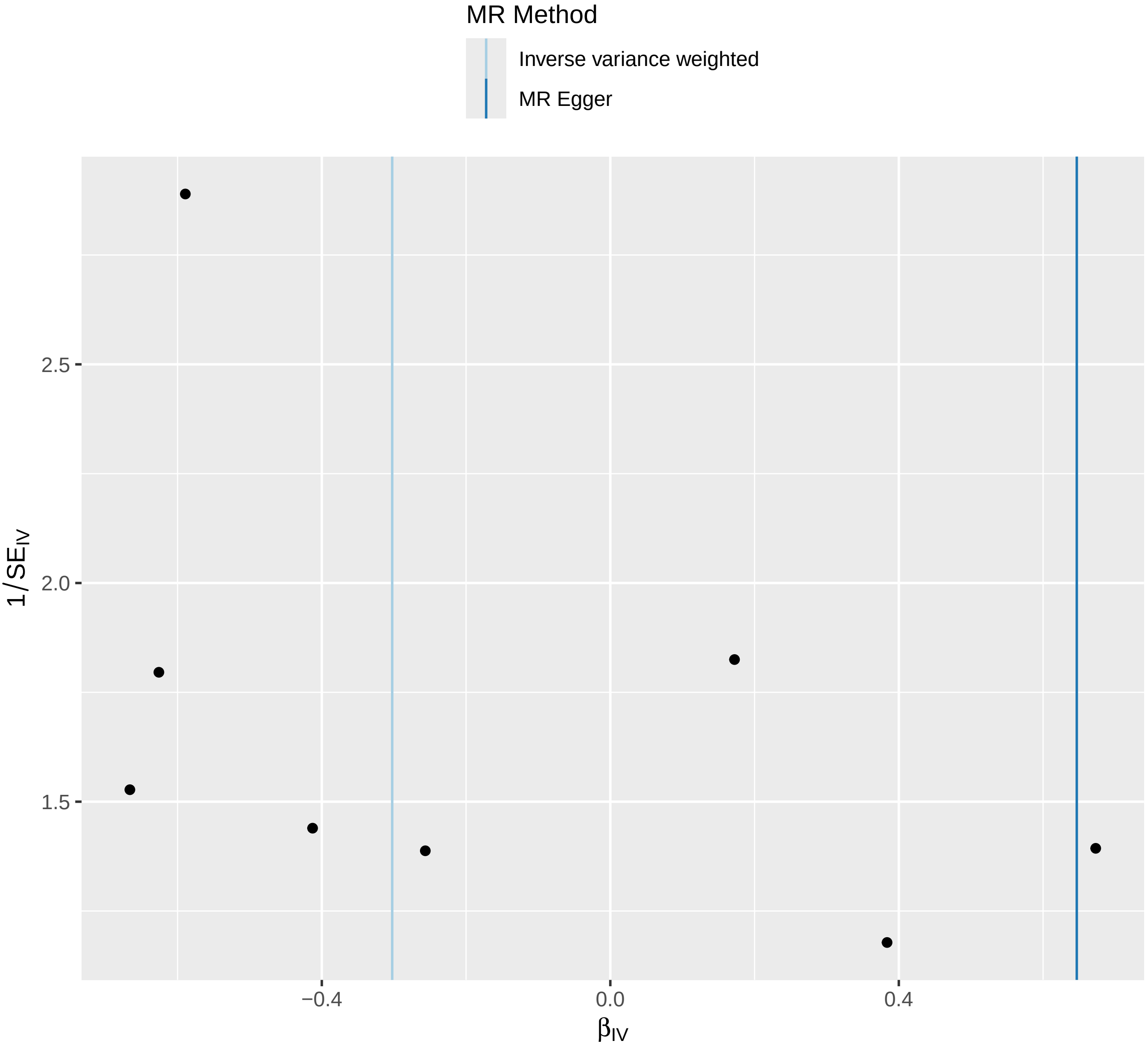
MR Method
Inverse variance weighted
MR Egger
2.5
2.0
1.5
1/SE_IV
−0.4
0.0
0.4
β_IV

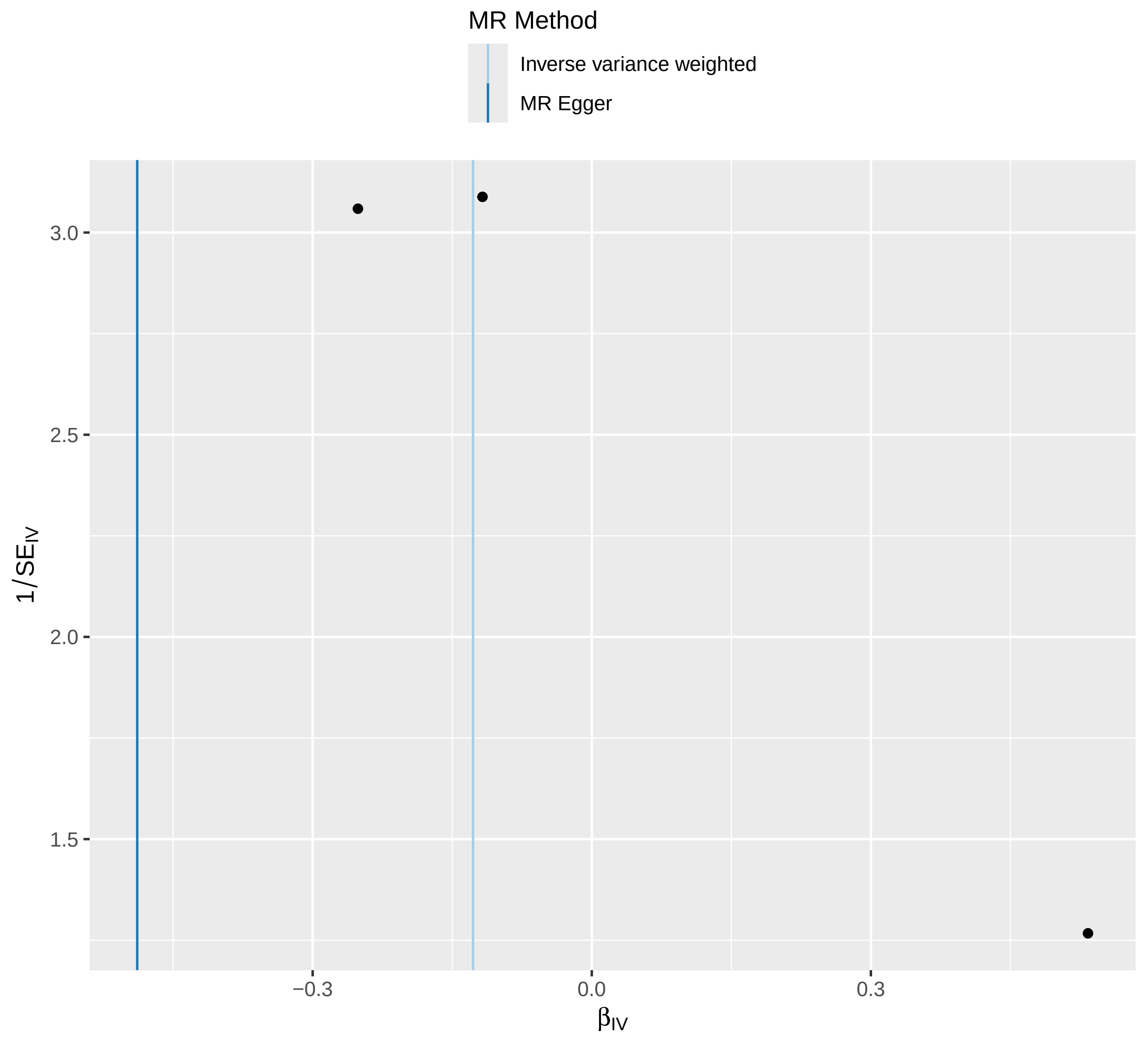

MR Method
Inverse variance weighted
MR Egger
3.0
2.5
2.0
1.5
1/SE_IV
−0.3
0.0
0.3
β_IV

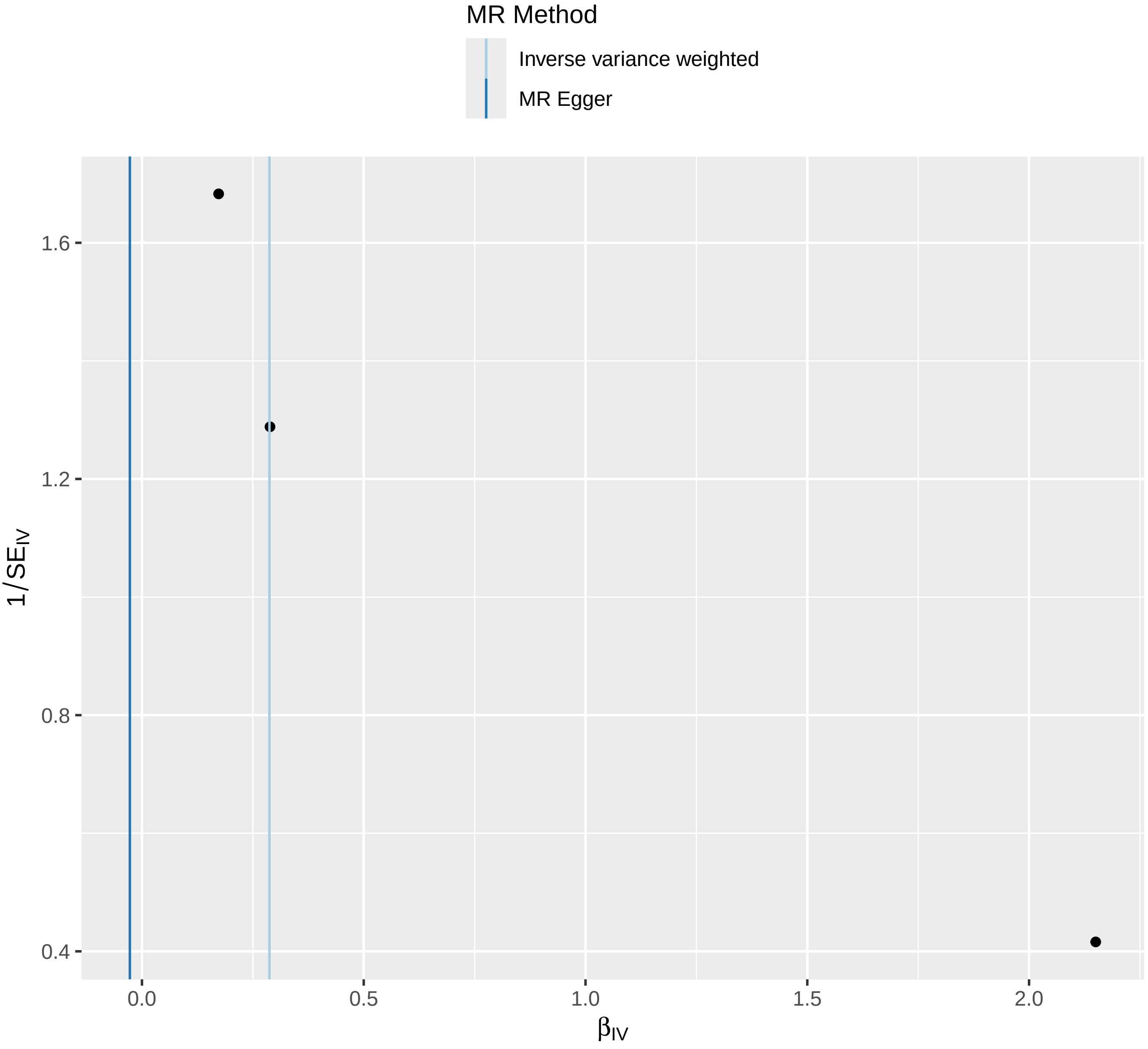

MR Method
Inverse variance weighted
MR Egger
1.6
1.2
0.8
0.4
1/SE_IV
0.0
0.5
1.0
1.5
2.0
β_IV

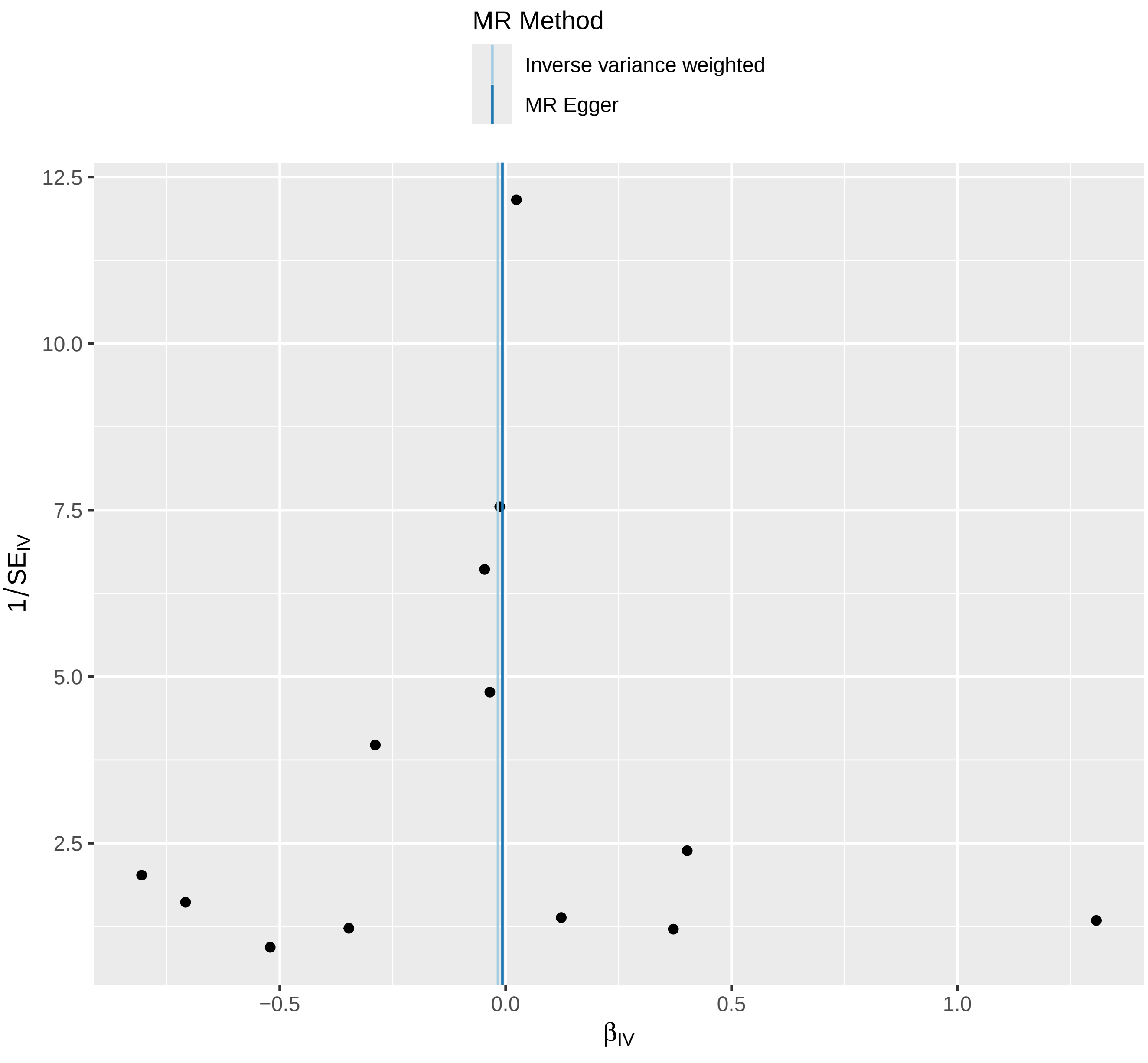
MR Method
Inverse variance weighted
MR Egger
12.5
10.0
7.5
5.0
2.5
$1/SE_{IV}$
−0.5
0.0
0.5
1.0
β_{IV}

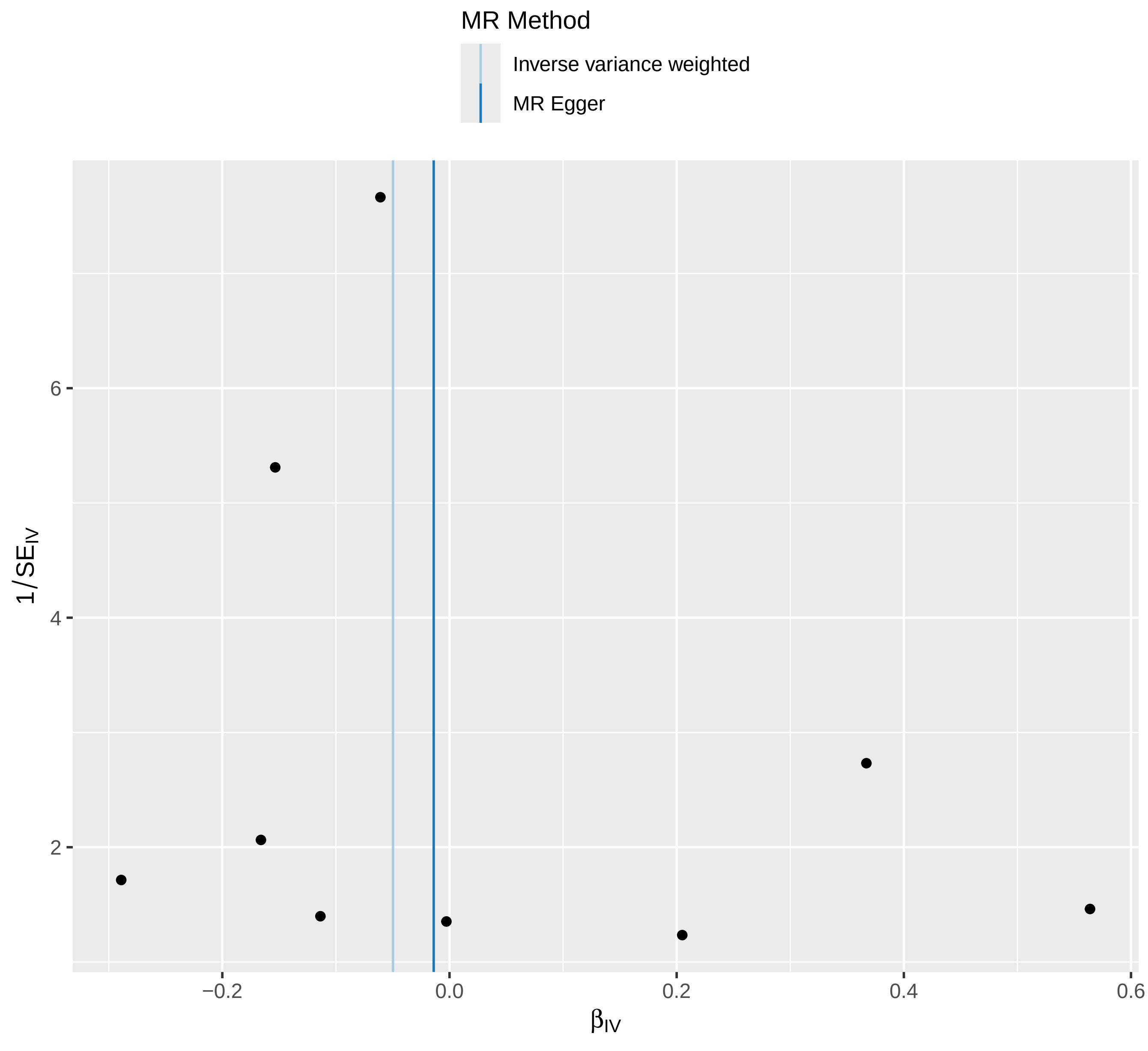

MR Method
Inverse variance weighted
MR Egger
6
4
2
1/SE_IV
−0.2
0.0
0.2
0.4
0.6
β_IV

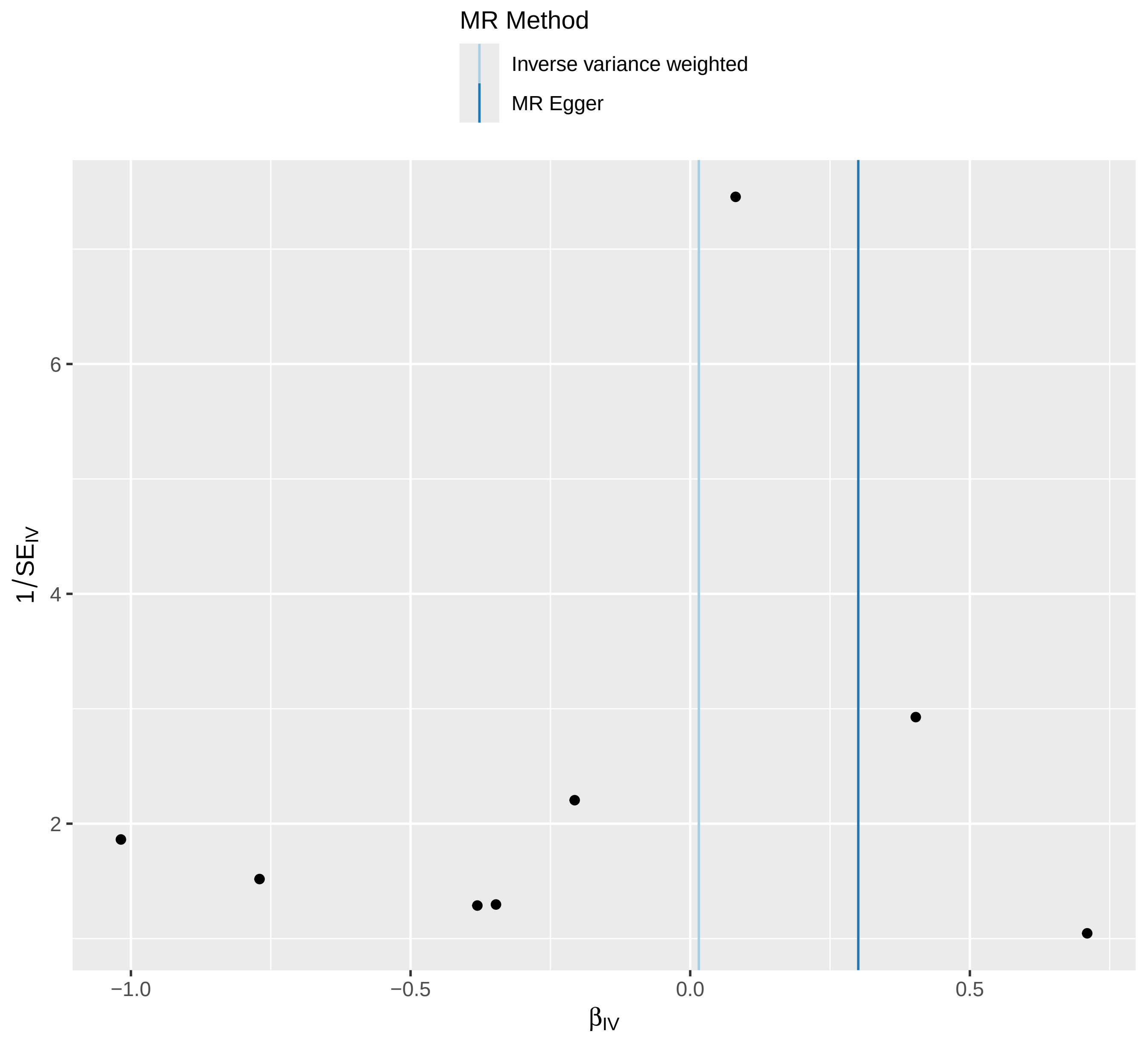

MR Method
Inverse variance weighted
MR Egger
6
4
2
1/SE$_{IV}$
−1.0
−0.5
0.0
0.5
β$_{IV}$

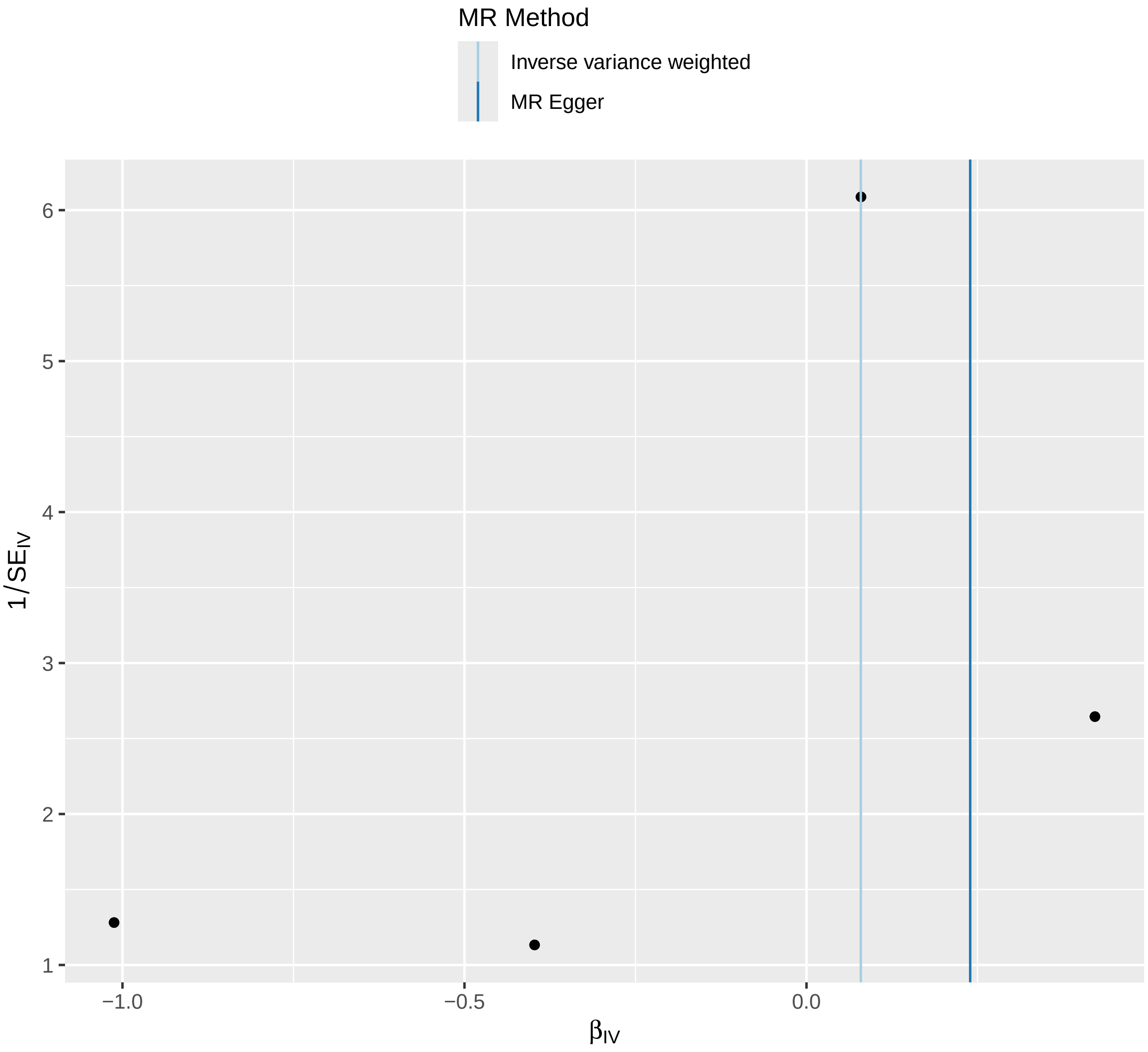
MR Method
Inverse variance weighted
MR Egger
1/SE$_{IV}$
6
5
4
3
2
1
−1.0
−0.5
0.0
β$_{IV}$

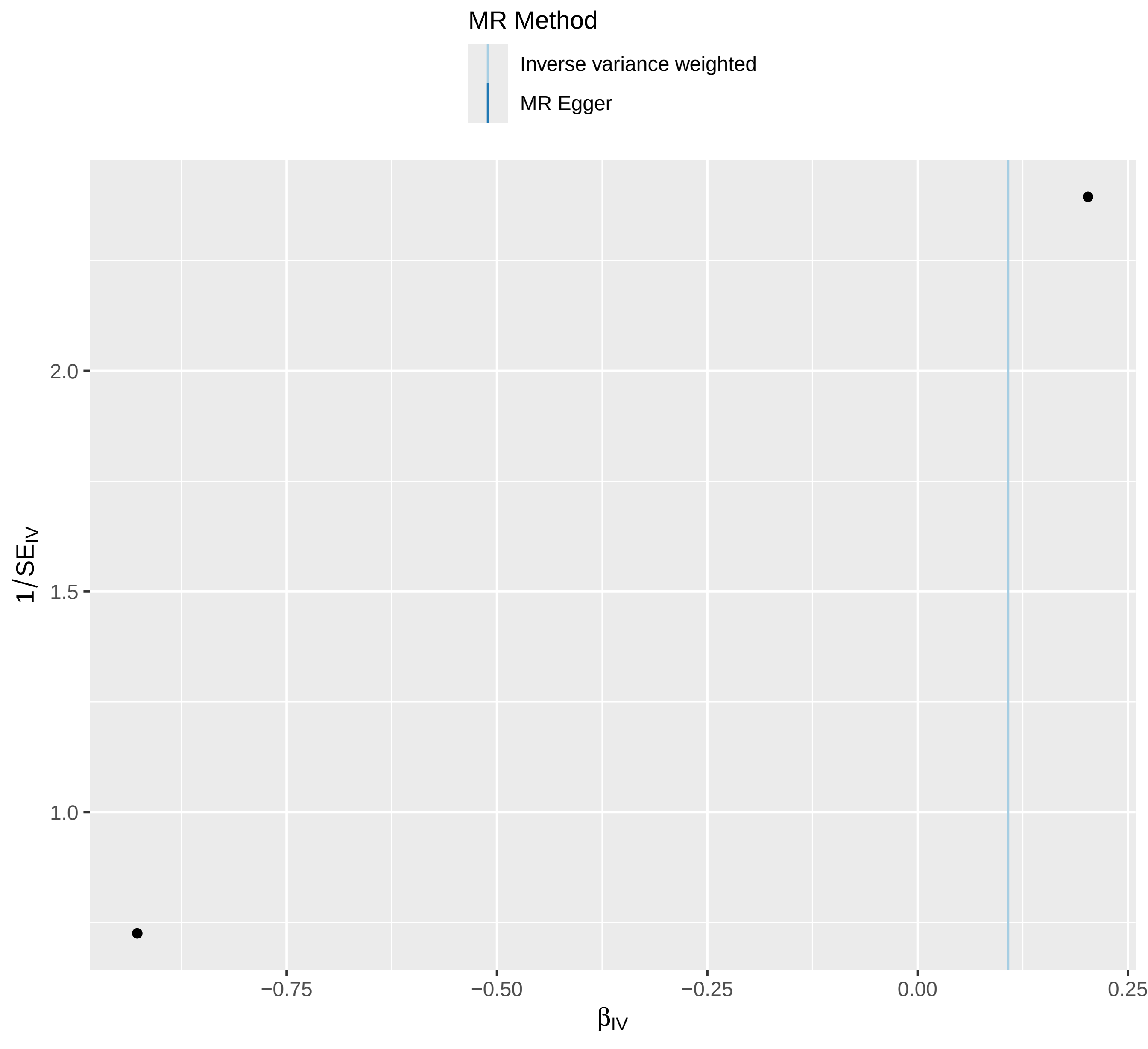

MR Method
Inverse variance weighted
MR Egger
2.0
1.5
1.0
1/SE_IV
−0.75
−0.50
−0.25
0.00
0.25
β_IV

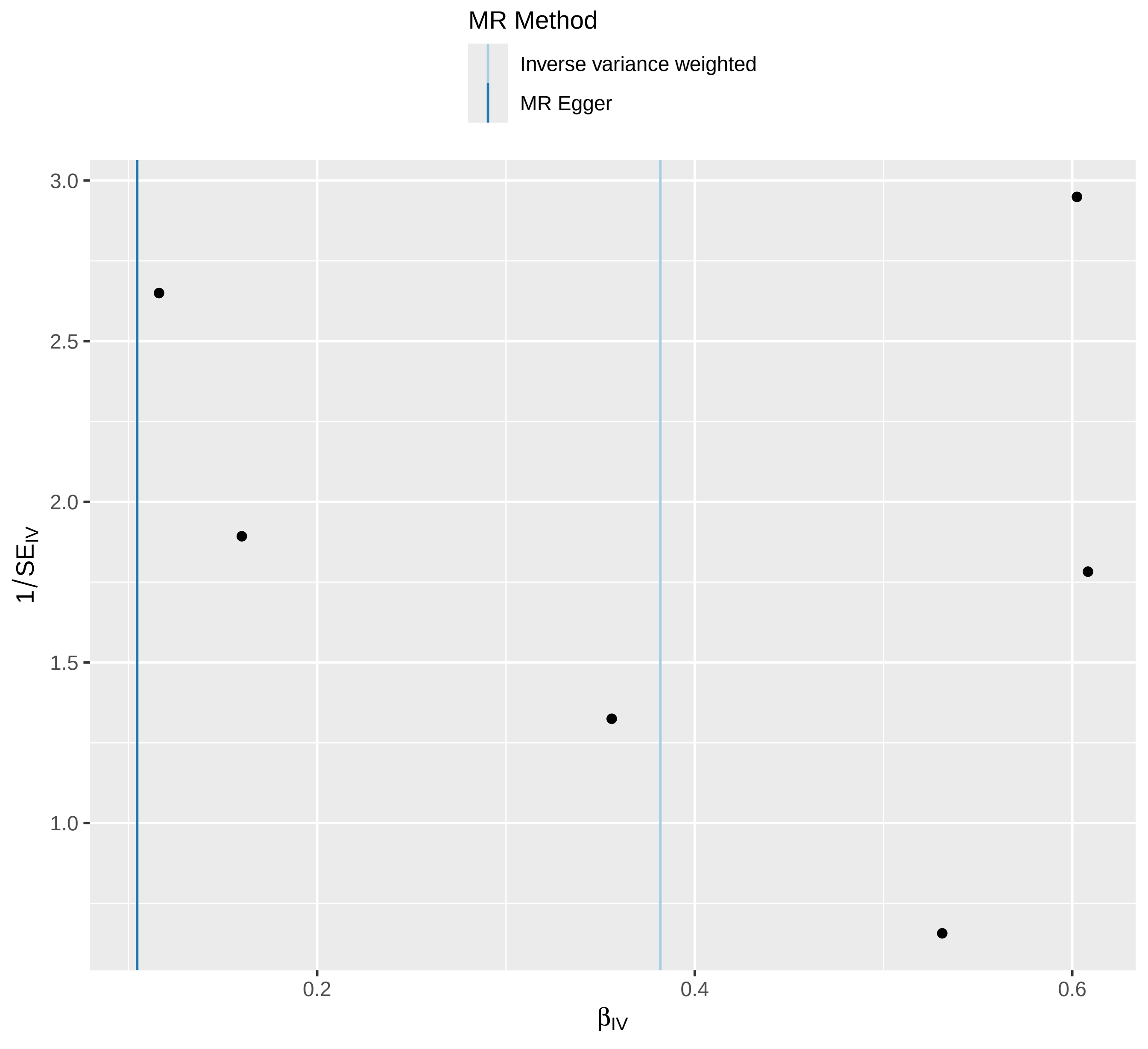

MR Method
Inverse variance weighted
MR Egger
3.0
2.5
2.0
1.5
1.0
1/SE_IV
0.2
0.4
0.6
β_IV

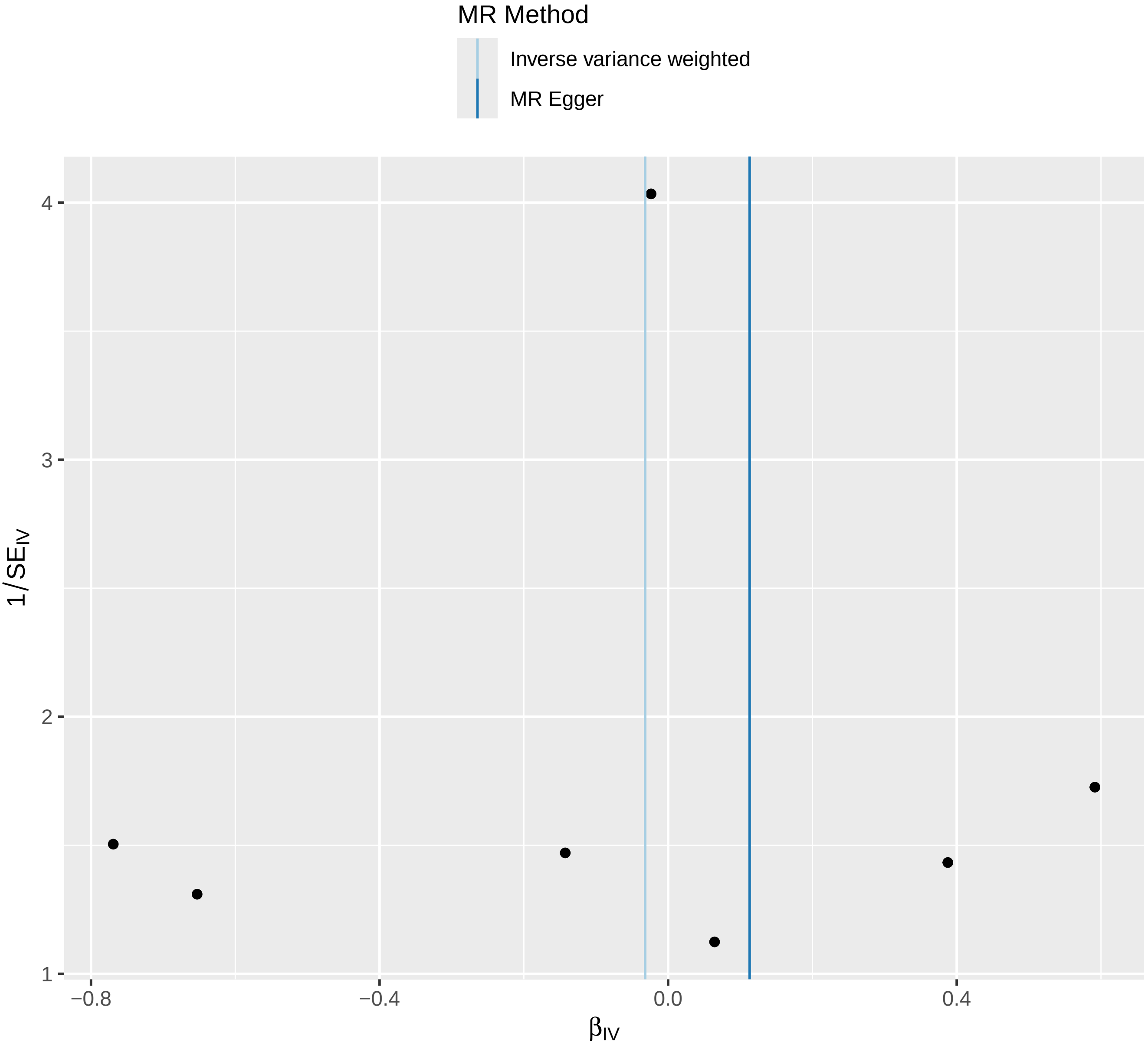
MR Method
Inverse variance weighted
MR Egger
4
3
2
1
1/SE_IV
−0.8
−0.4
0.0
0.4
β_IV

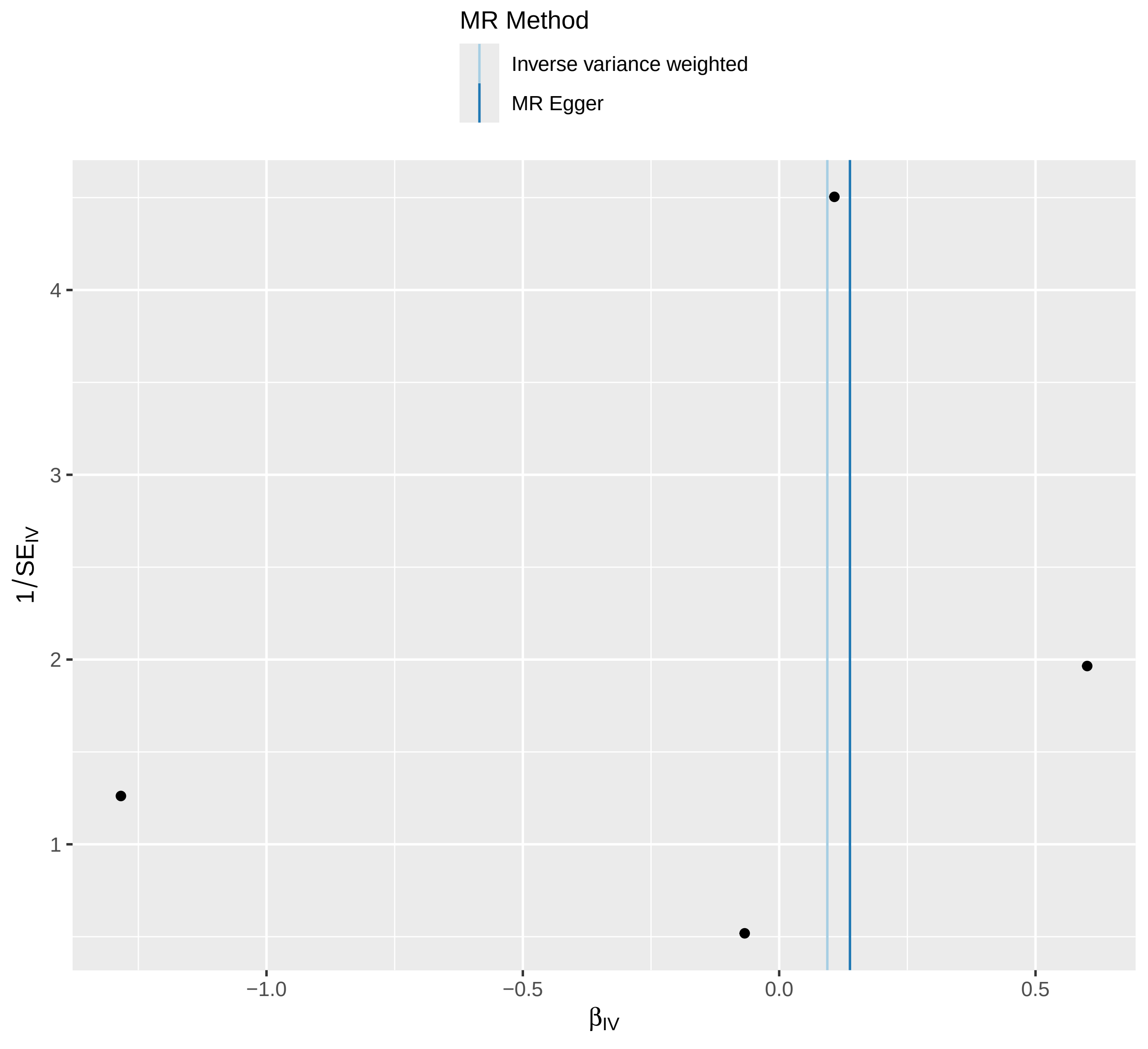

MR Method
Inverse variance weighted
MR Egger
4
3
2
1
1/SE$_{IV}$
−1.0
−0.5
0.0
0.5
β$_{IV}$

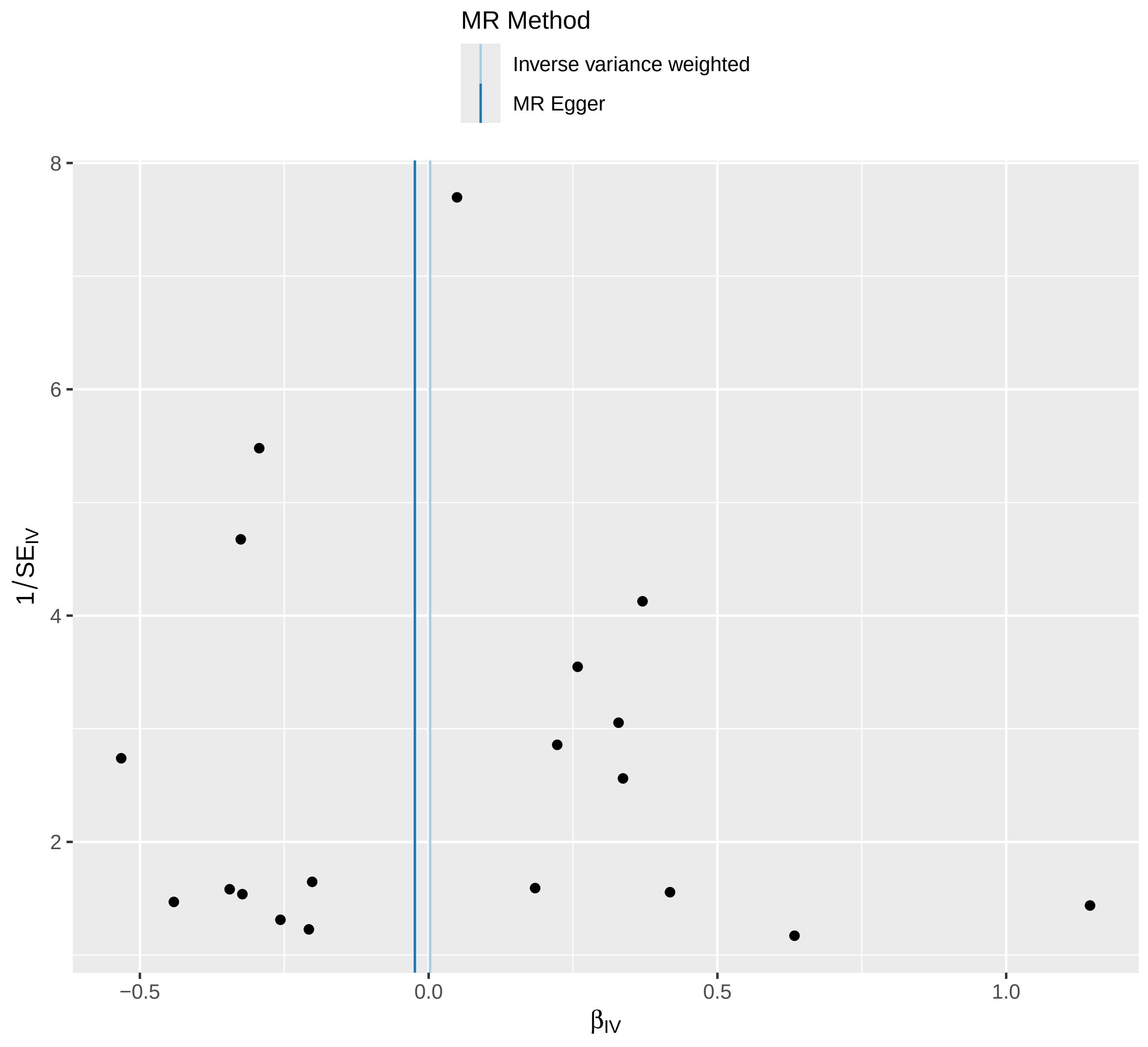

MR Method
Inverse variance weighted
MR Egger
8
6
4
2
1/SE$_{IV}$
−0.5
0.0
0.5
1.0
β$_{IV}$

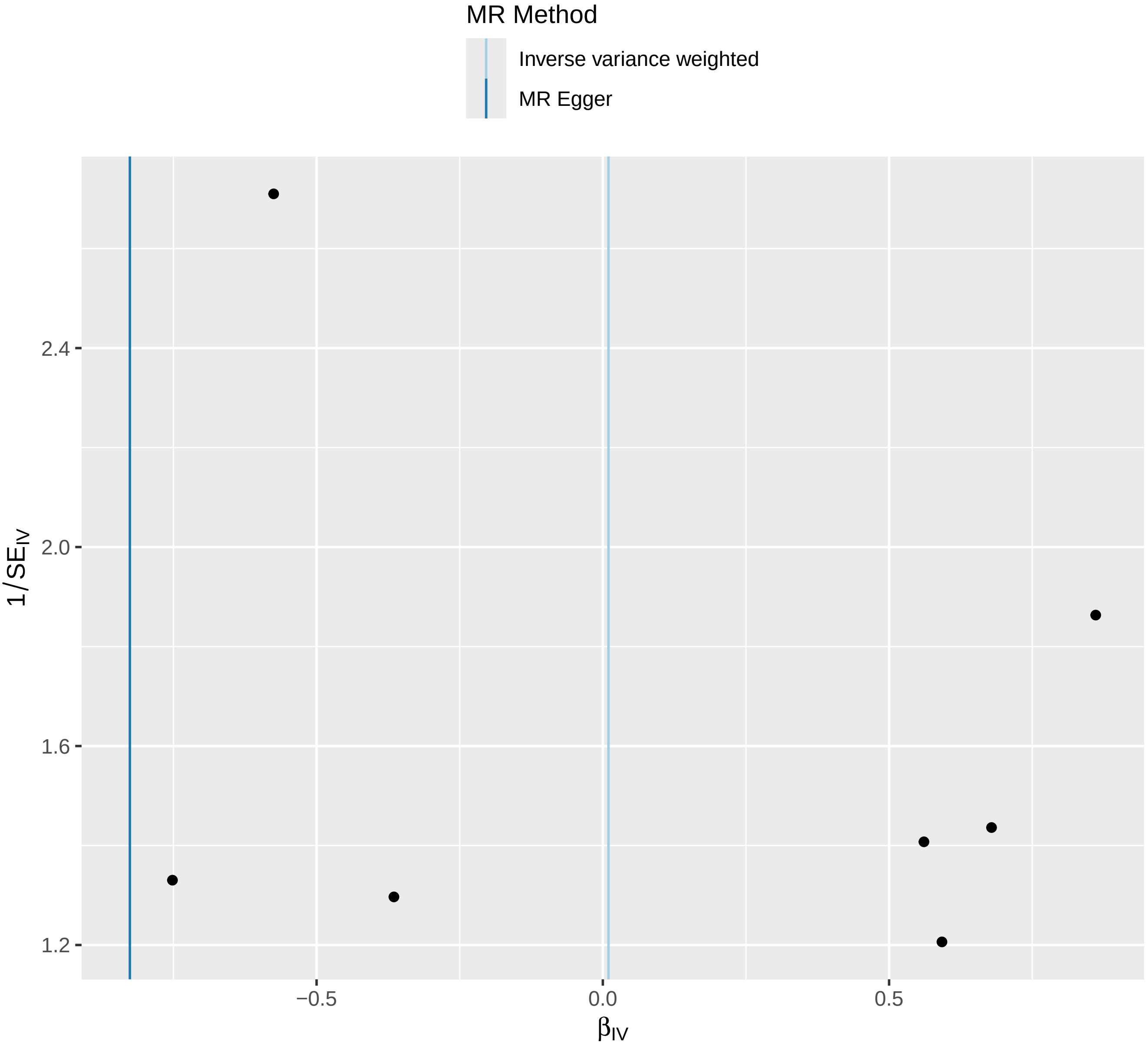
MR Method
Inverse variance weighted
MR Egger
2.4
2.0
1.6
1.2
1/SE$_{IV}$
−0.5
0.0
0.5
β$_{IV}$

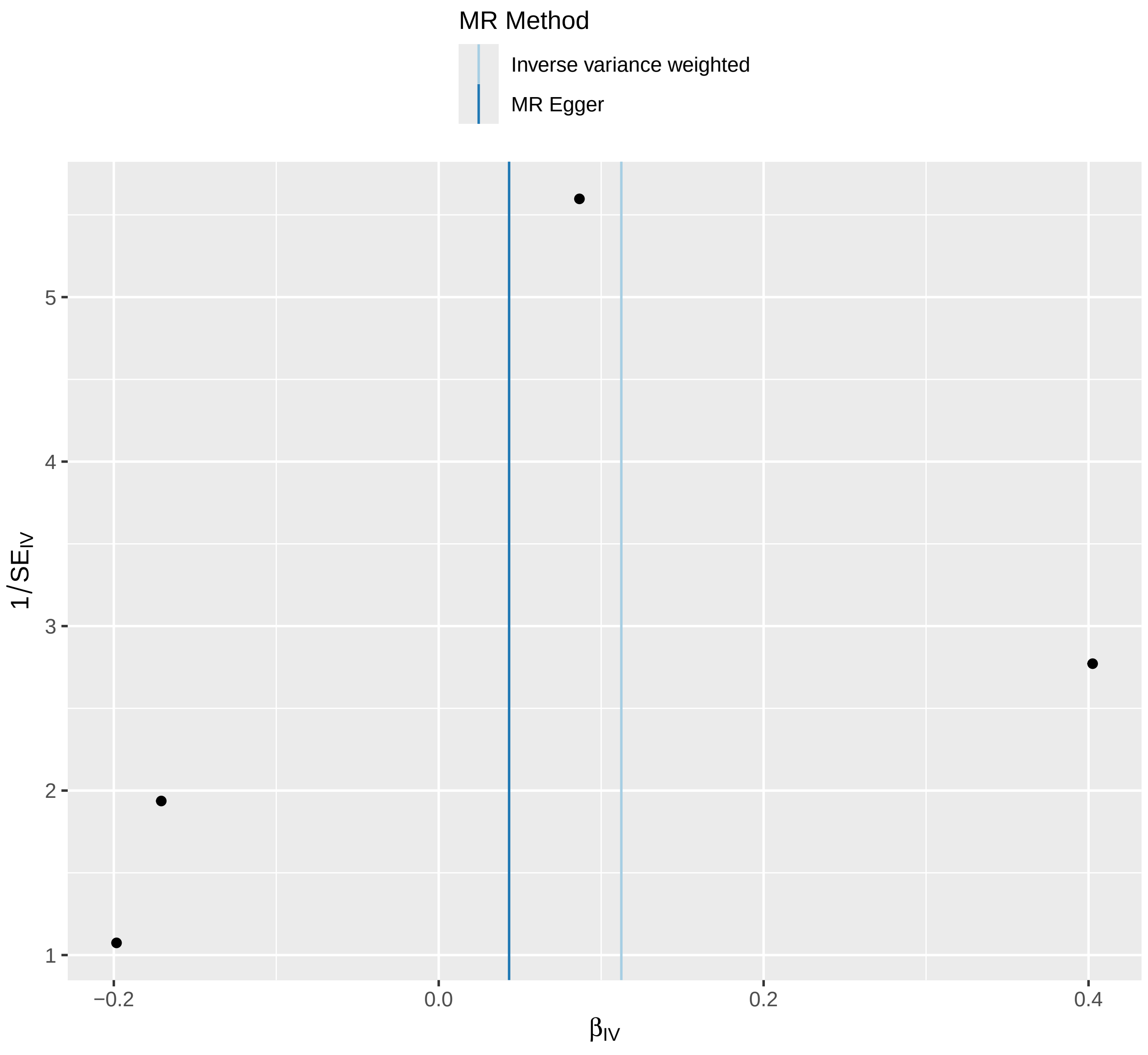

MR Method
Inverse variance weighted
MR Egger
1/SE_IV
5
4
3
2
1
−0.2
0.0
0.2
0.4
β_IV

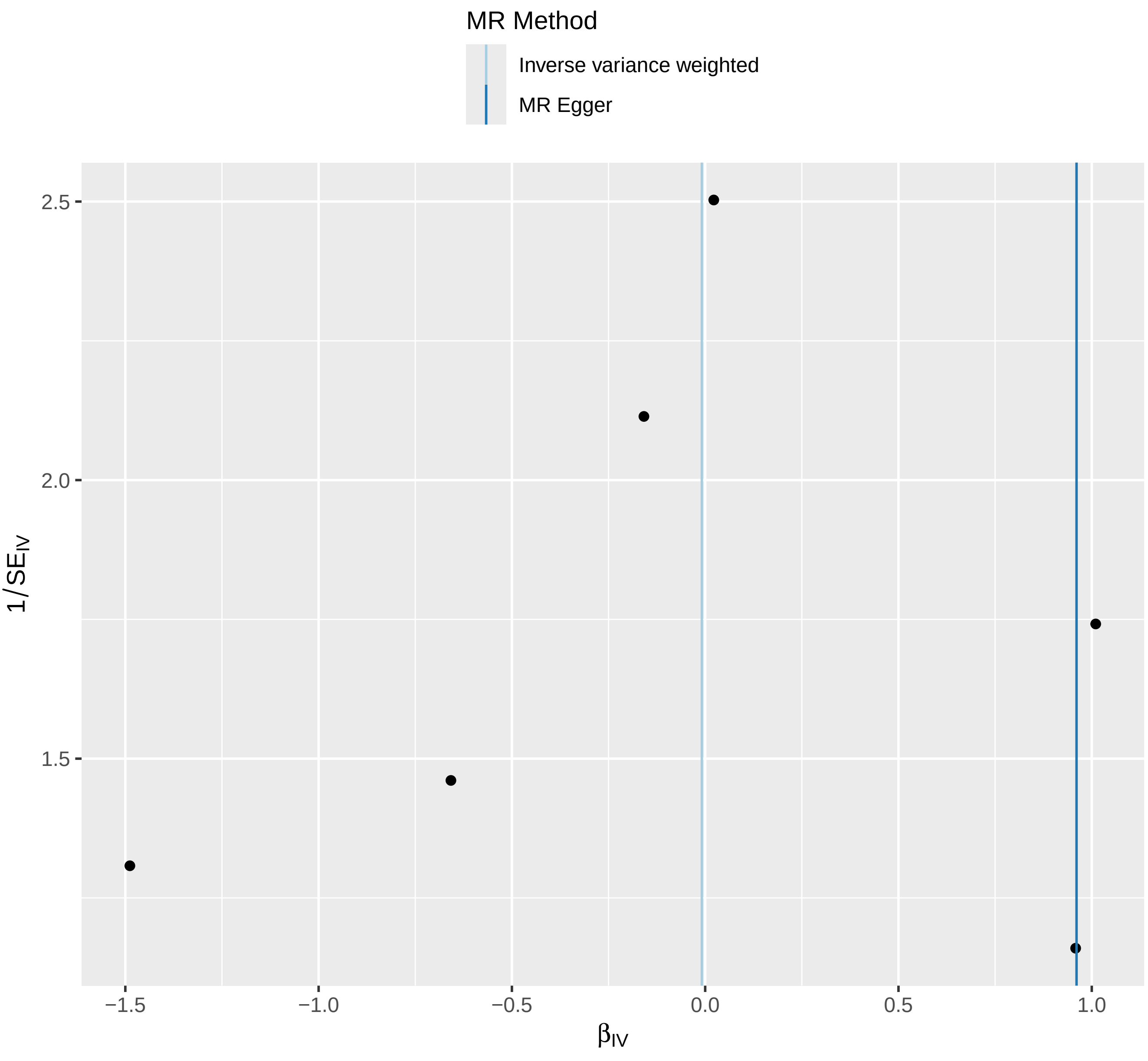
MR Method
Inverse variance weighted
MR Egger
2.5
2.0
1.5
1/SE$_{IV}$
−1.5
−1.0
−0.5
0.0
0.5
1.0
β$_{IV}$

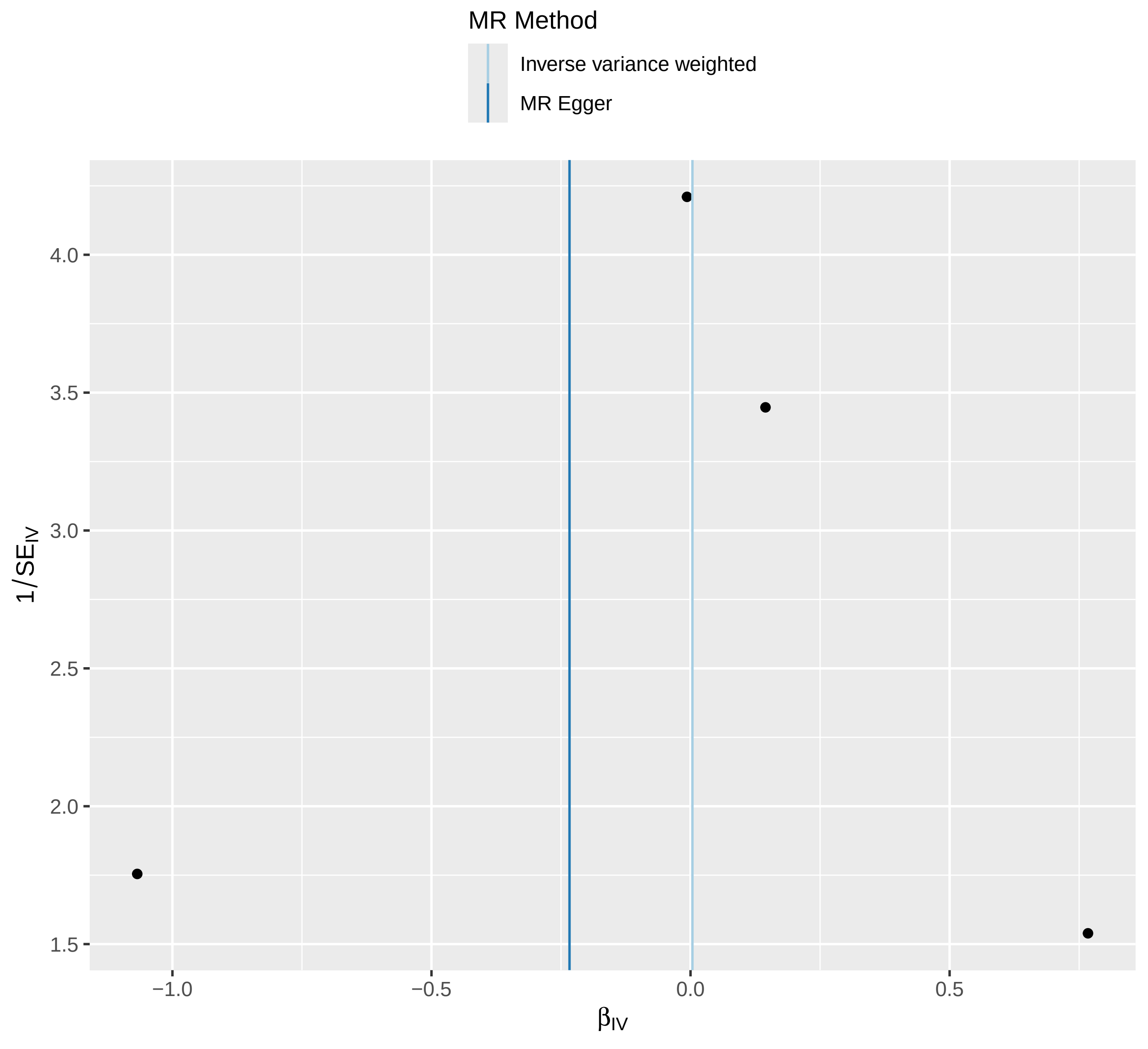

MR Method
Inverse variance weighted
MR Egger
4.0
3.5
3.0
2.5
2.0
1.5
1/SE_IV
−1.0
−0.5
0.0
0.5
β_IV

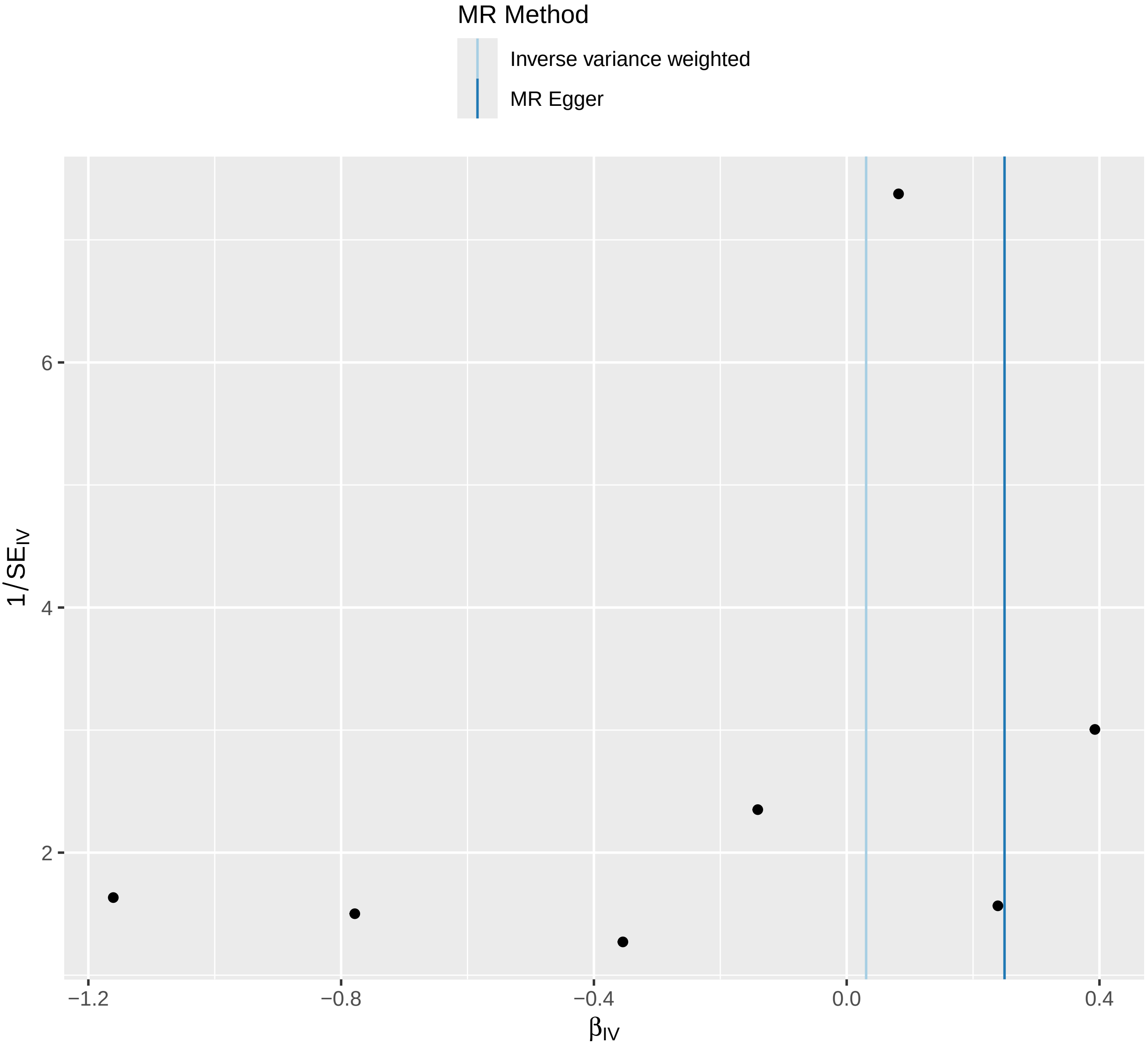

MR Method
Inverse variance weighted
MR Egger
1/SE$_{IV}$
6
4
2
−1.2
−0.8
−0.4
0.0
0.4
β$_{IV}$

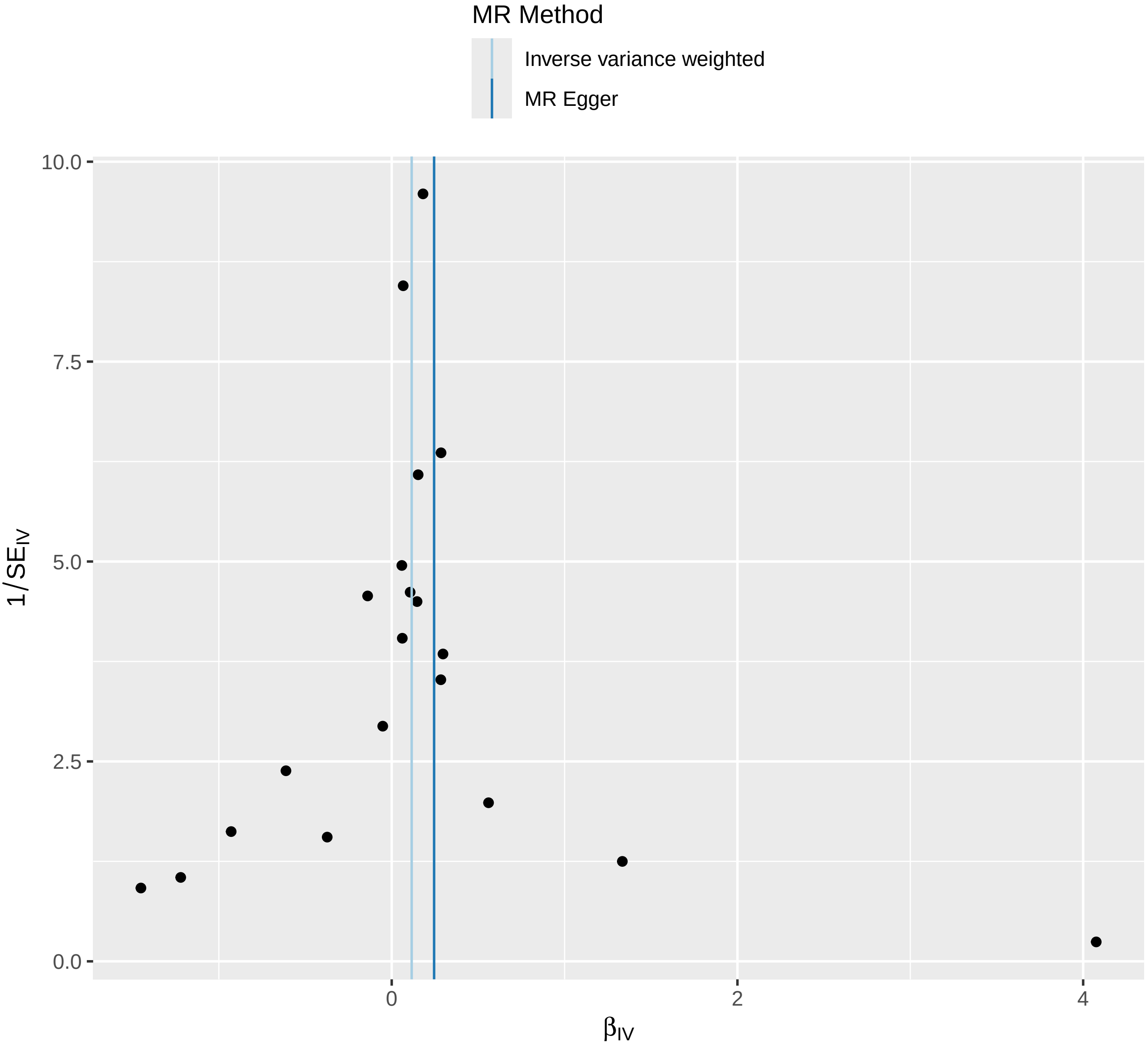

MR Method
Inverse variance weighted
MR Egger
10.0
7.5
5.0
2.5
0.0
$1/SE_{IV}$
0
2
4
β_{IV}

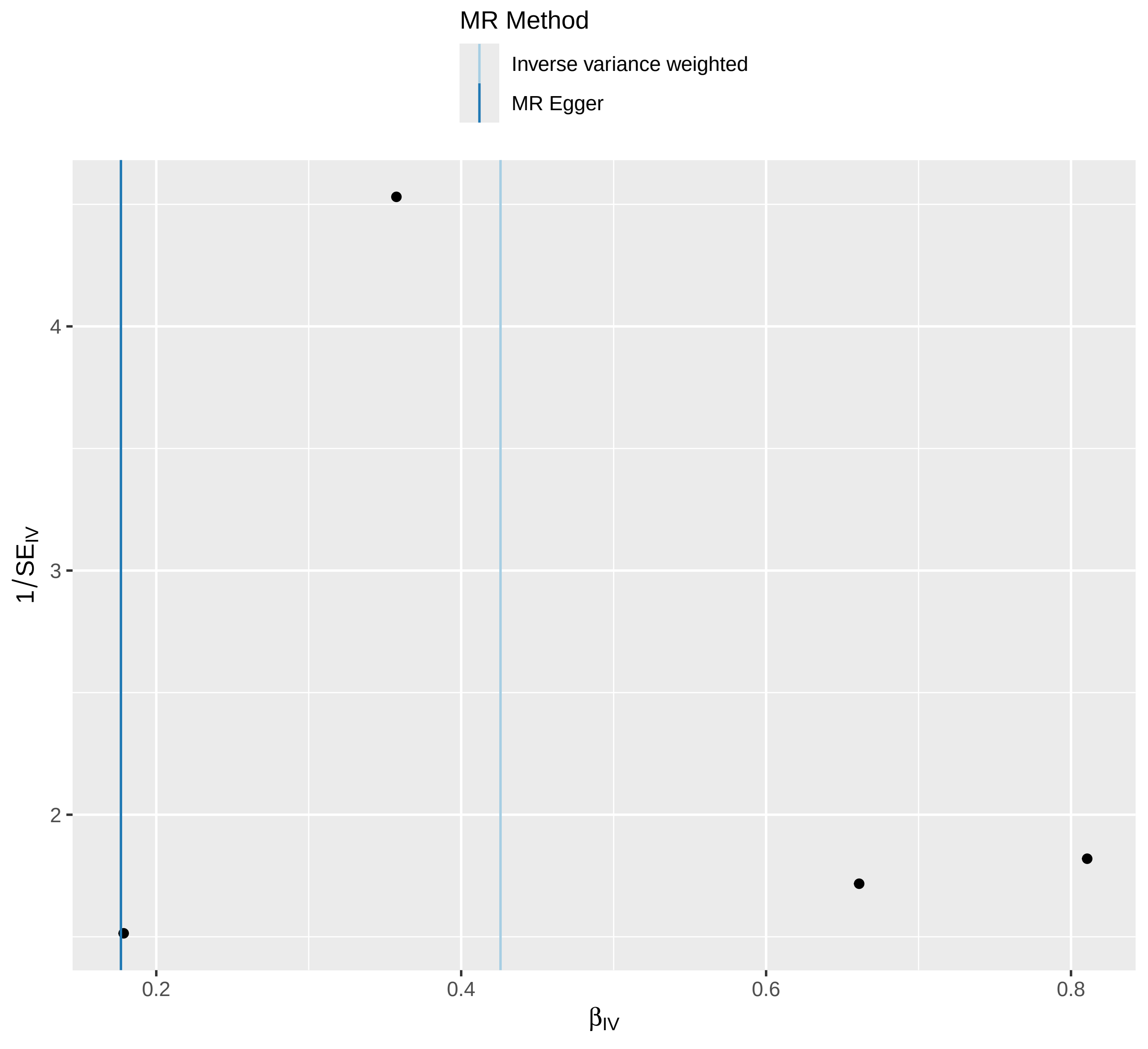
MR Method
Inverse variance weighted
MR Egger
4
3
2
0.2
0.4
0.6
0.8
1/SE_IV
β_IV

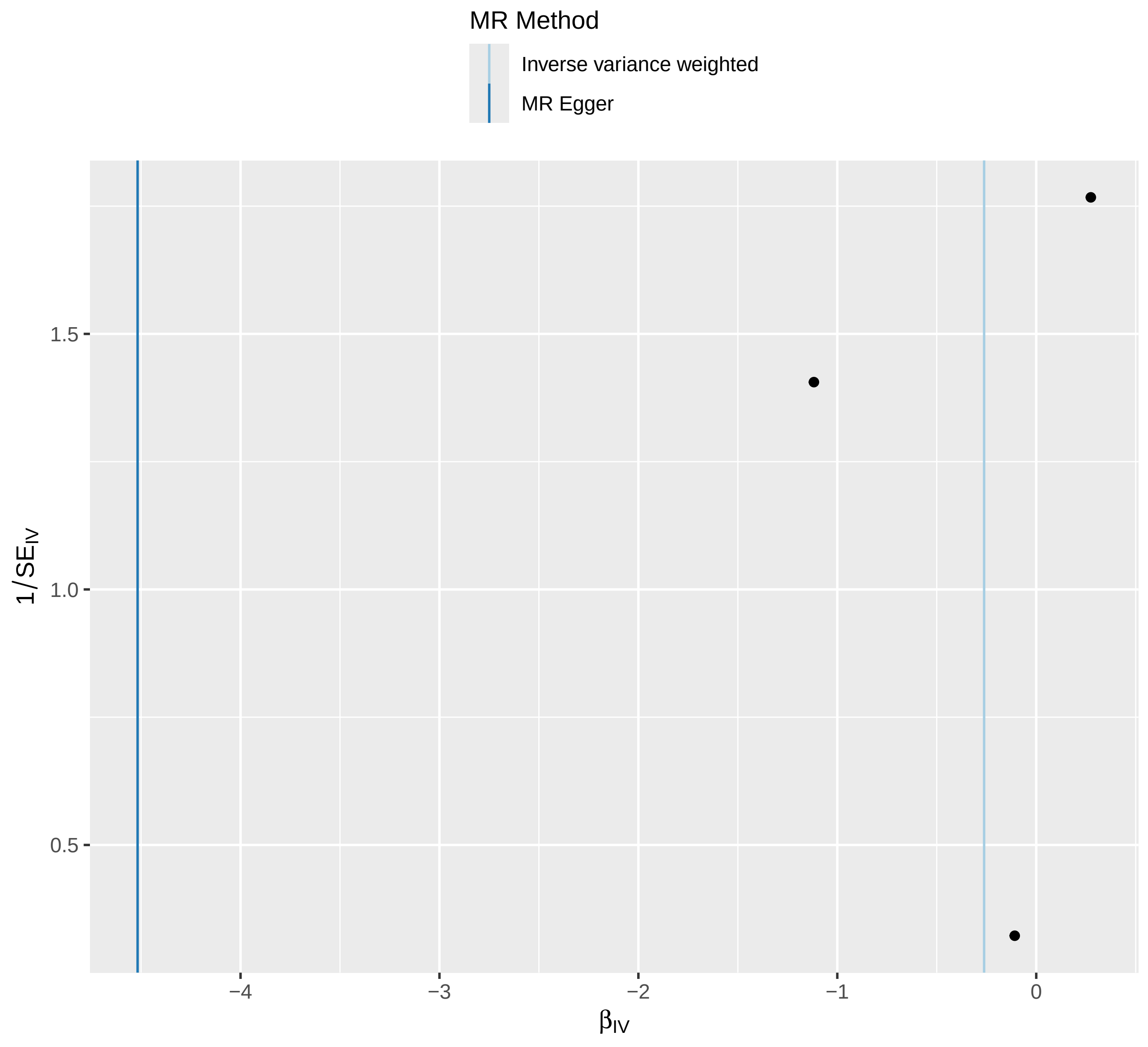
MR Method
Inverse variance weighted
MR Egger
1.5
1.0
0.5
1/SE_IV
−4
−3
−2
−1
0
β_IV

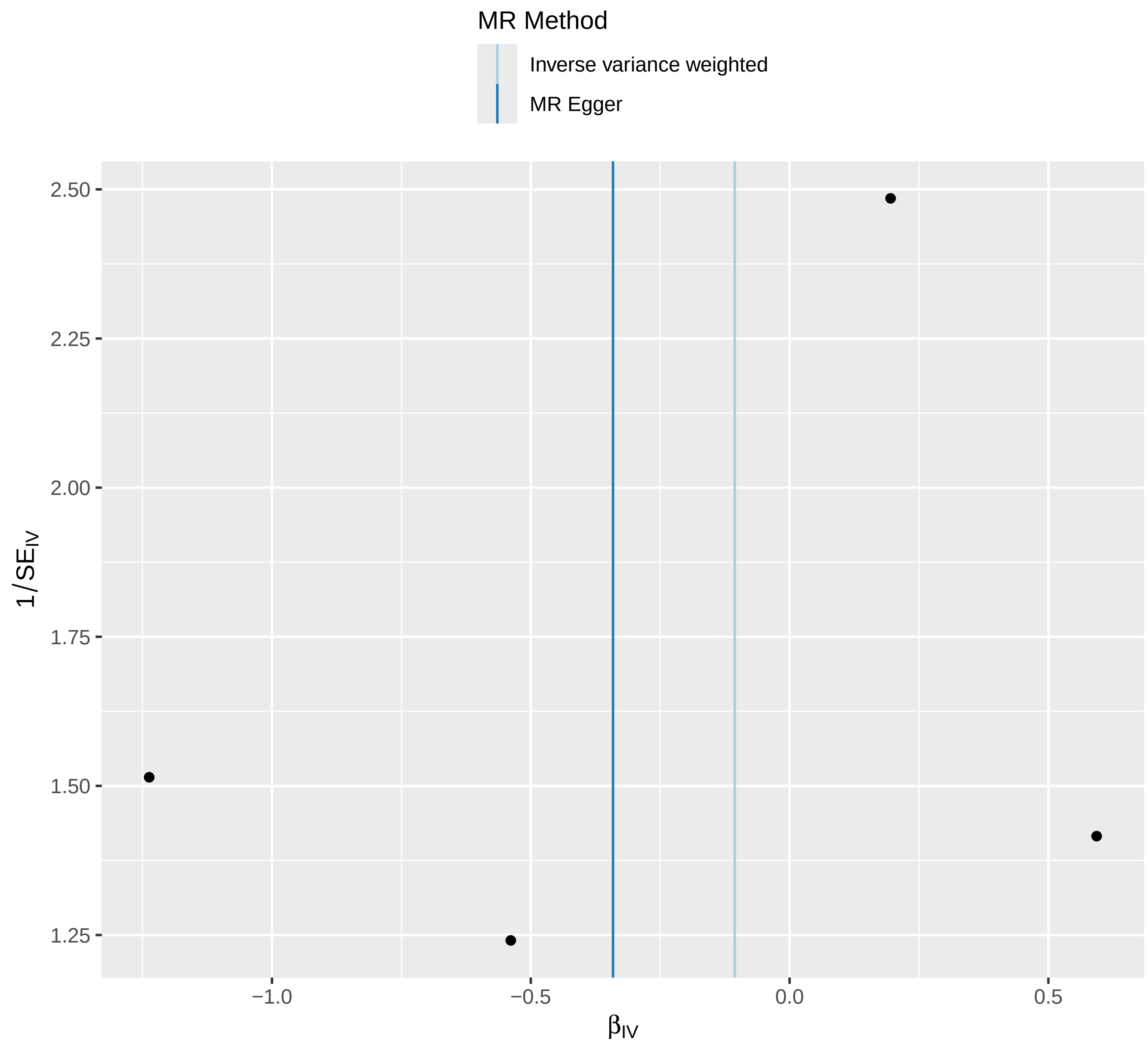

MR Method
Inverse variance weighted
MR Egger
2.50
2.25
2.00
1.75
1.50
1.25
1/SE_IV
−1.0
−0.5
0.0
0.5
β_IV

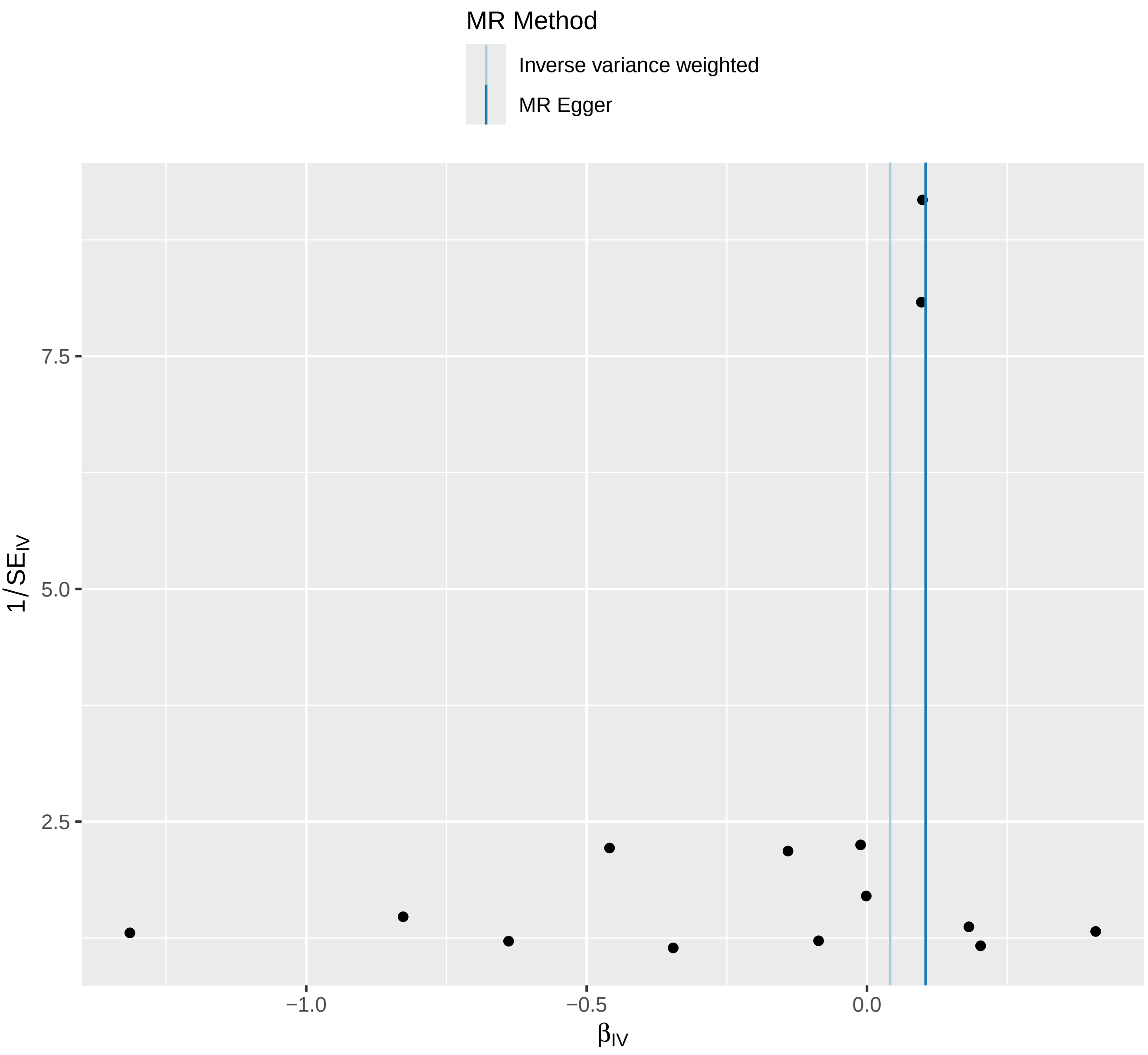
MR Method
Inverse variance weighted
MR Egger
7.5
5.0
2.5
1/SE$_{IV}$
−1.0
−0.5
0.0
β_{IV}

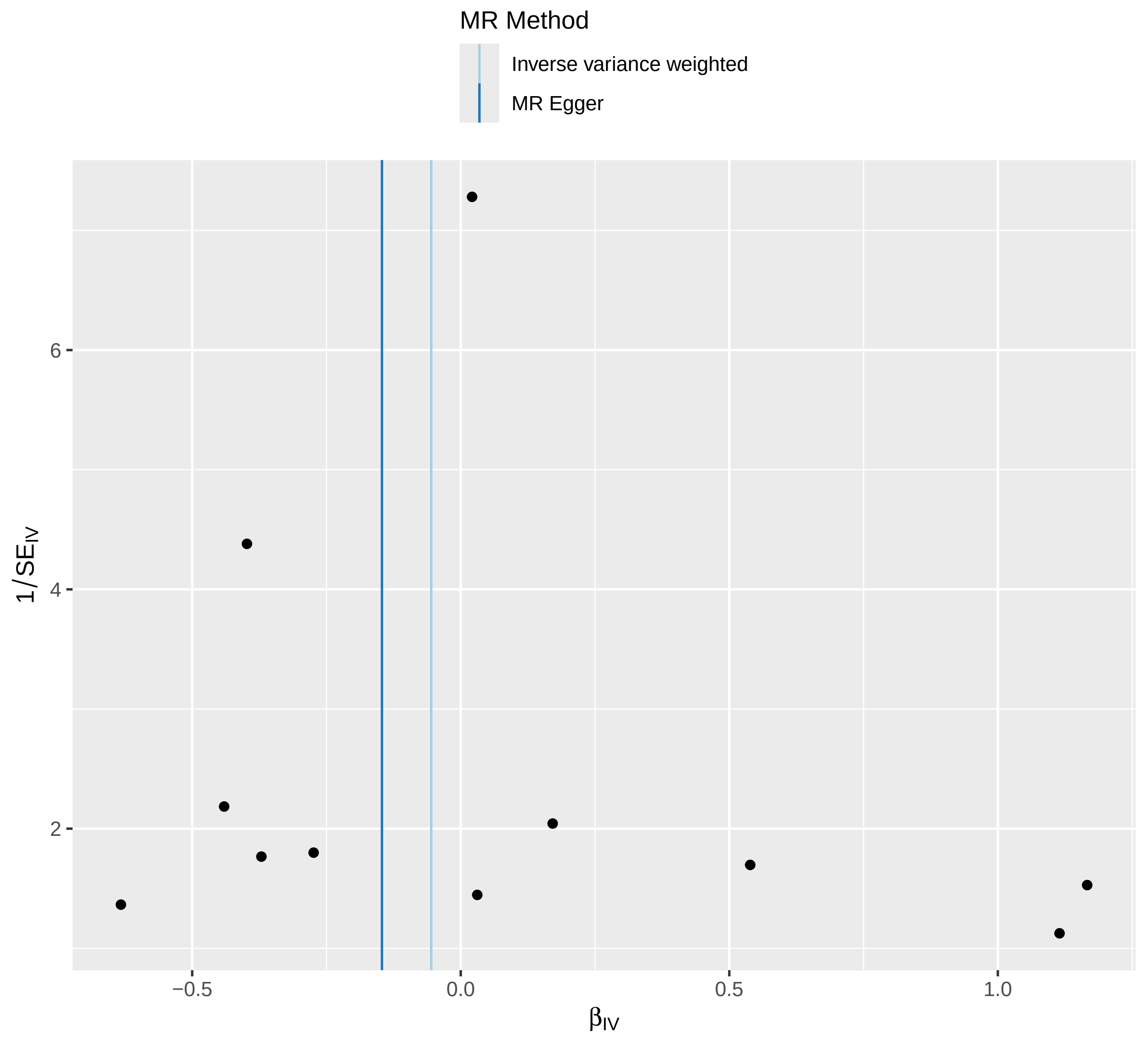
MR Method
Inverse variance weighted
MR Egger
6
4
2
$1/SE_{IV}$
−0.5
0.0
0.5
1.0
β_{IV}

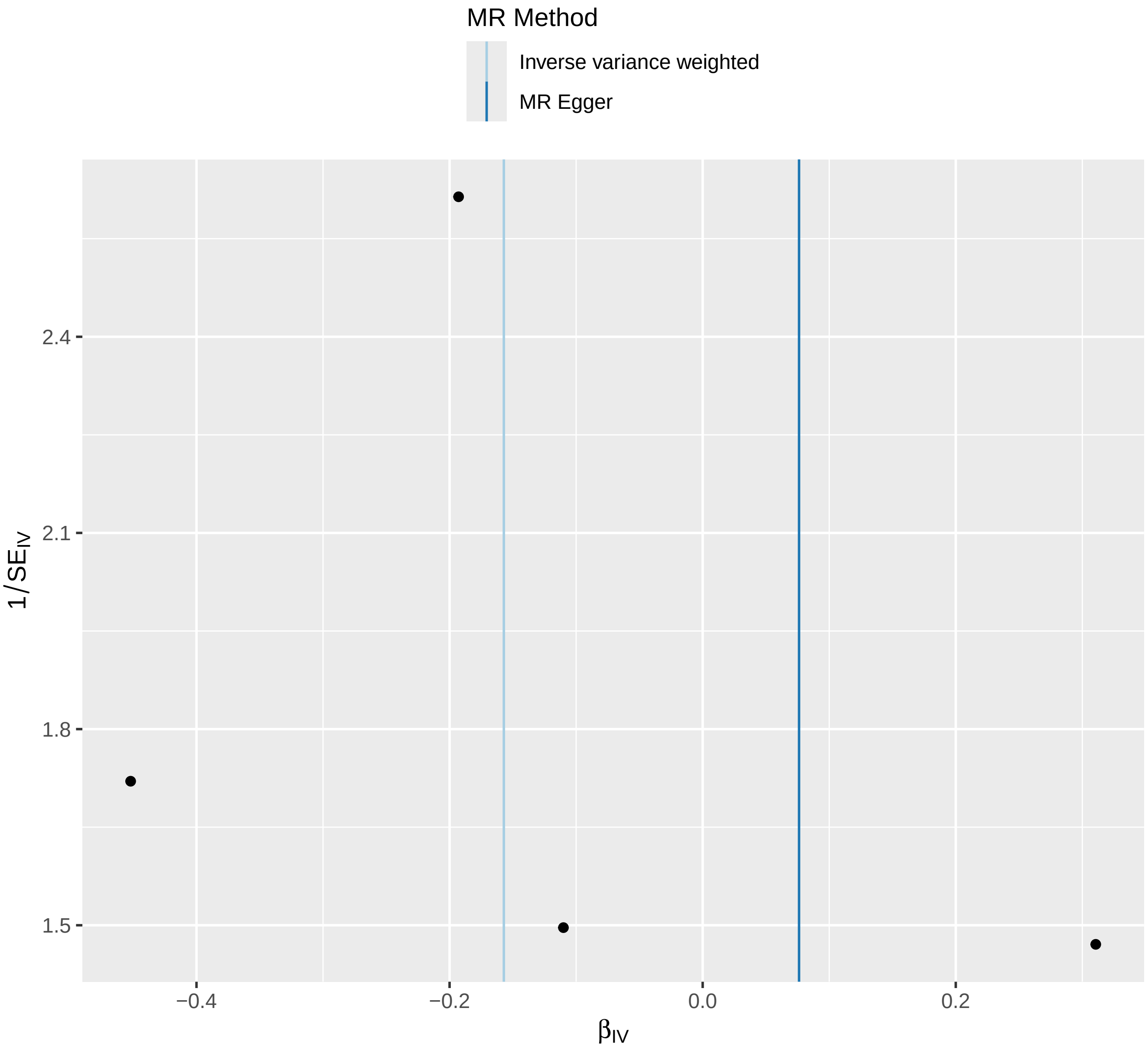
MR Method
Inverse variance weighted
MR Egger
2.4
2.1
1.8
1.5
1/SE$_{IV}$
−0.4
−0.2
0.0
0.2
β$_{IV}$

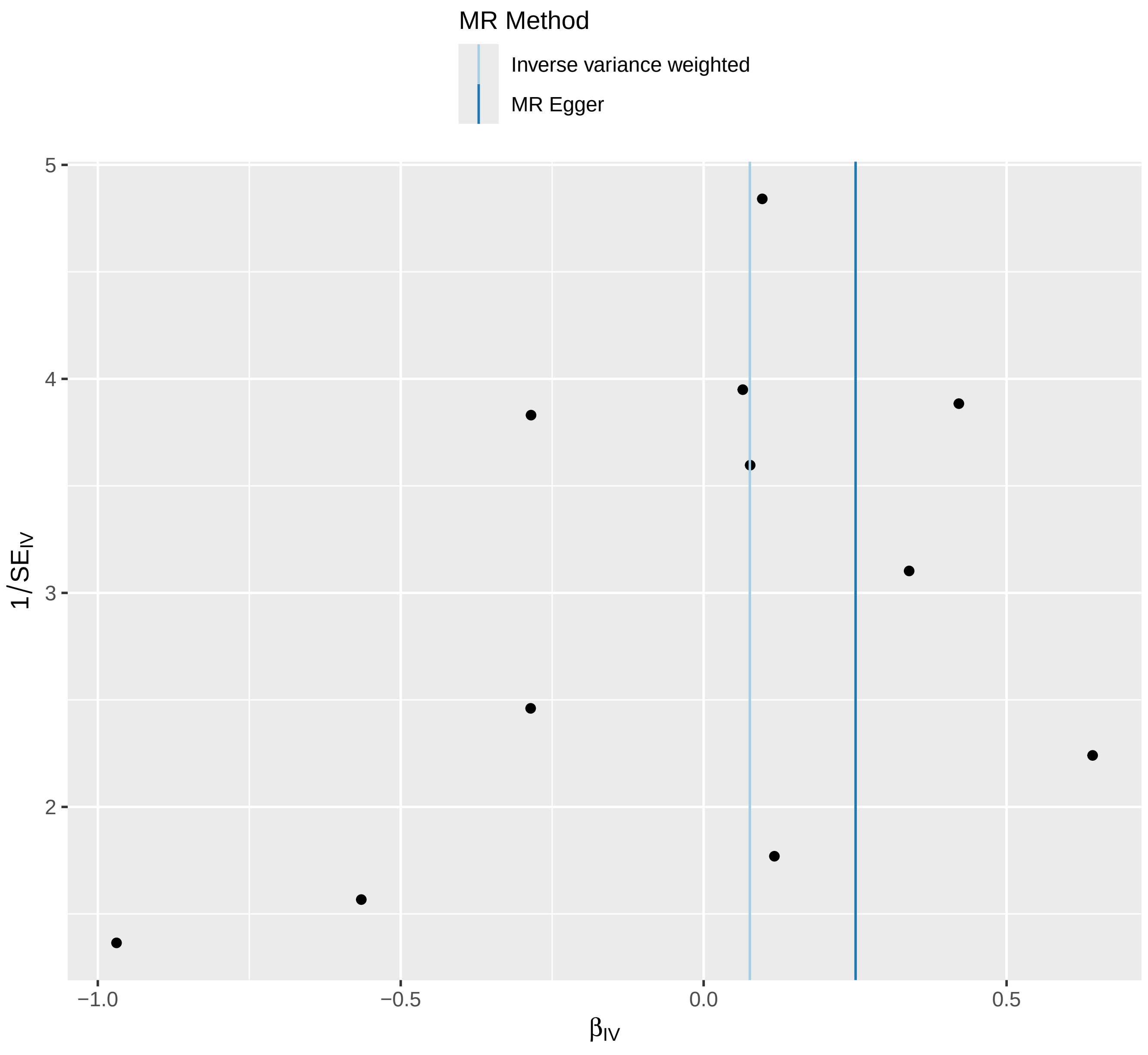

MR Method
Inverse variance weighted
MR Egger
5
4
3
2
1/SE$_{IV}$
−1.0
−0.5
0.0
0.5
β$_{IV}$

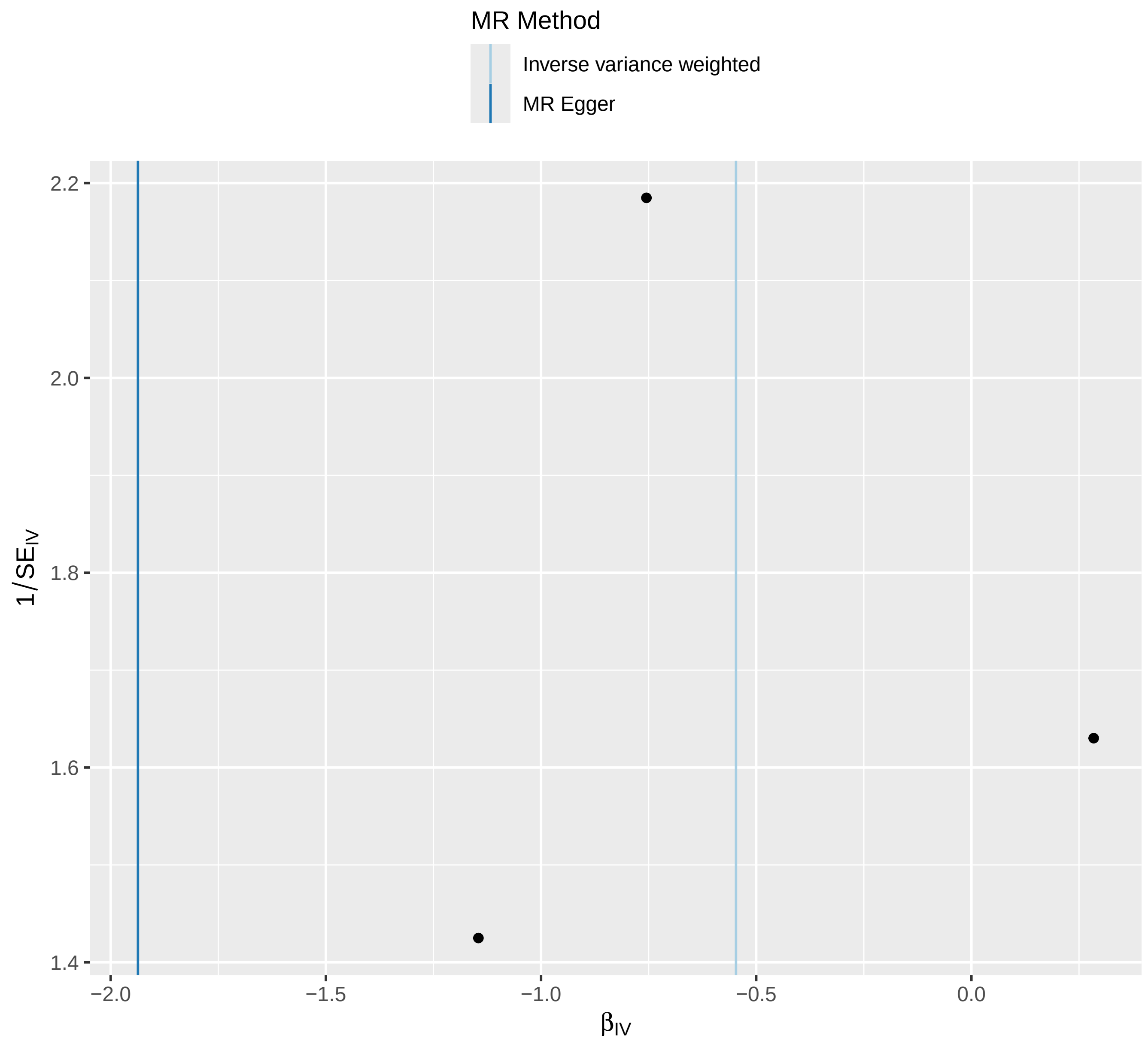
MR Method
Inverse variance weighted
MR Egger
2.2
2.0
1.8
1.6
1.4
1/SE$_{IV}$
−2.0
−1.5
−1.0
−0.5
0.0
β$_{IV}$

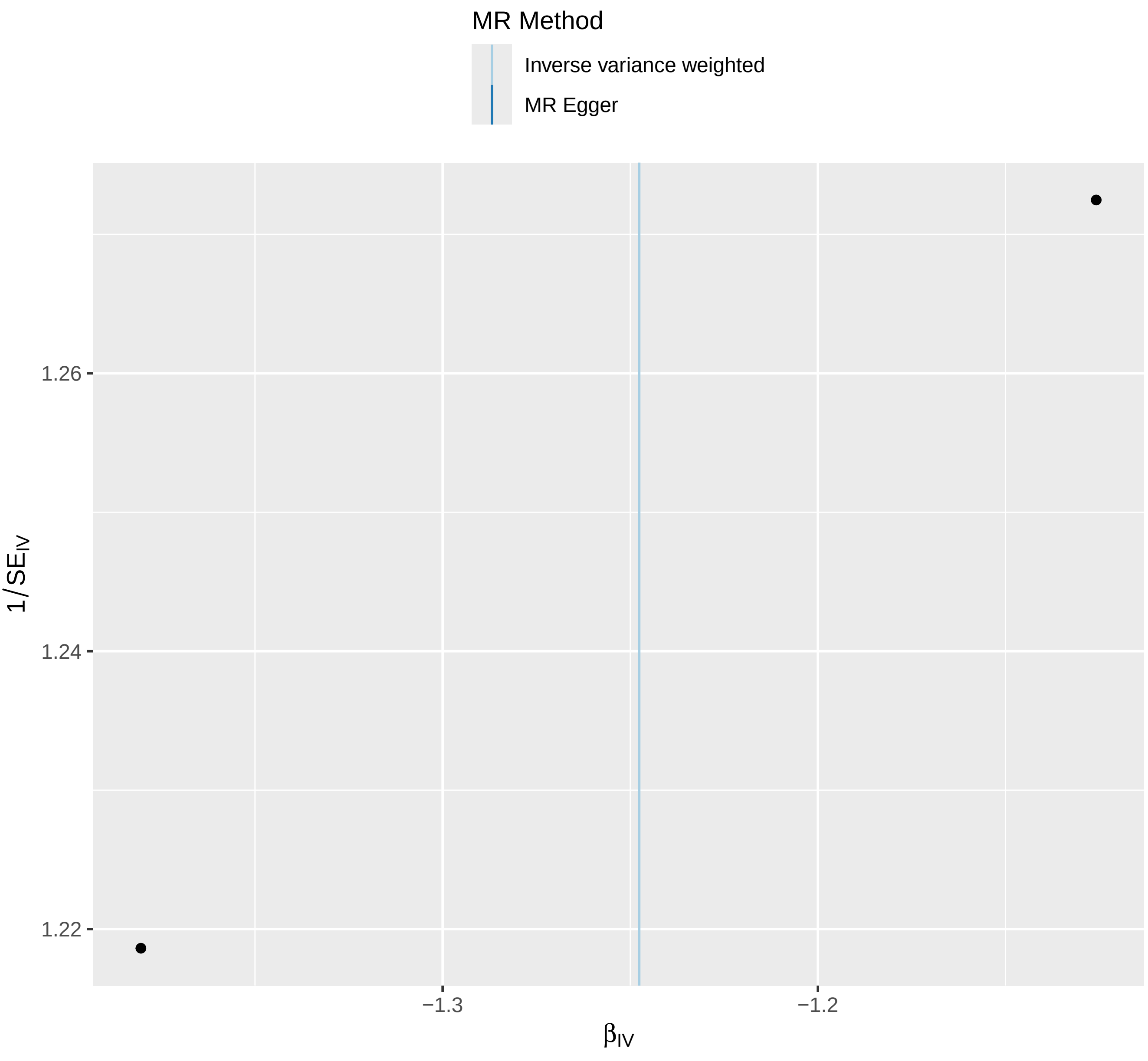

MR Method
Inverse variance weighted
MR Egger
1.26
1.24
1.22
1/SE_IV
−1.3
−1.2
β_IV

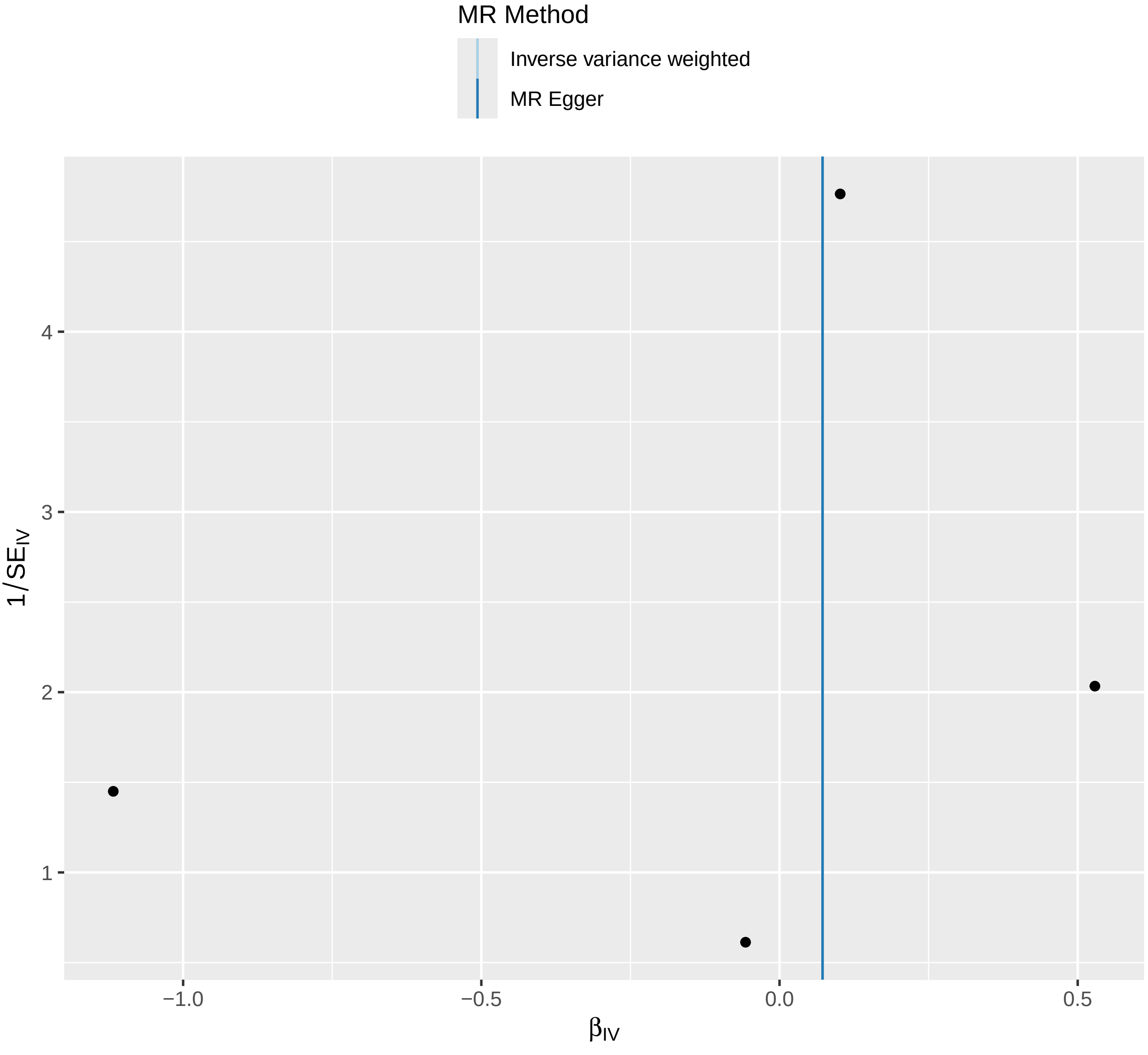
MR Method
Inverse variance weighted
MR Egger
4
3
2
1
1/SE_IV
−1.0
−0.5
0.0
0.5
β_IV

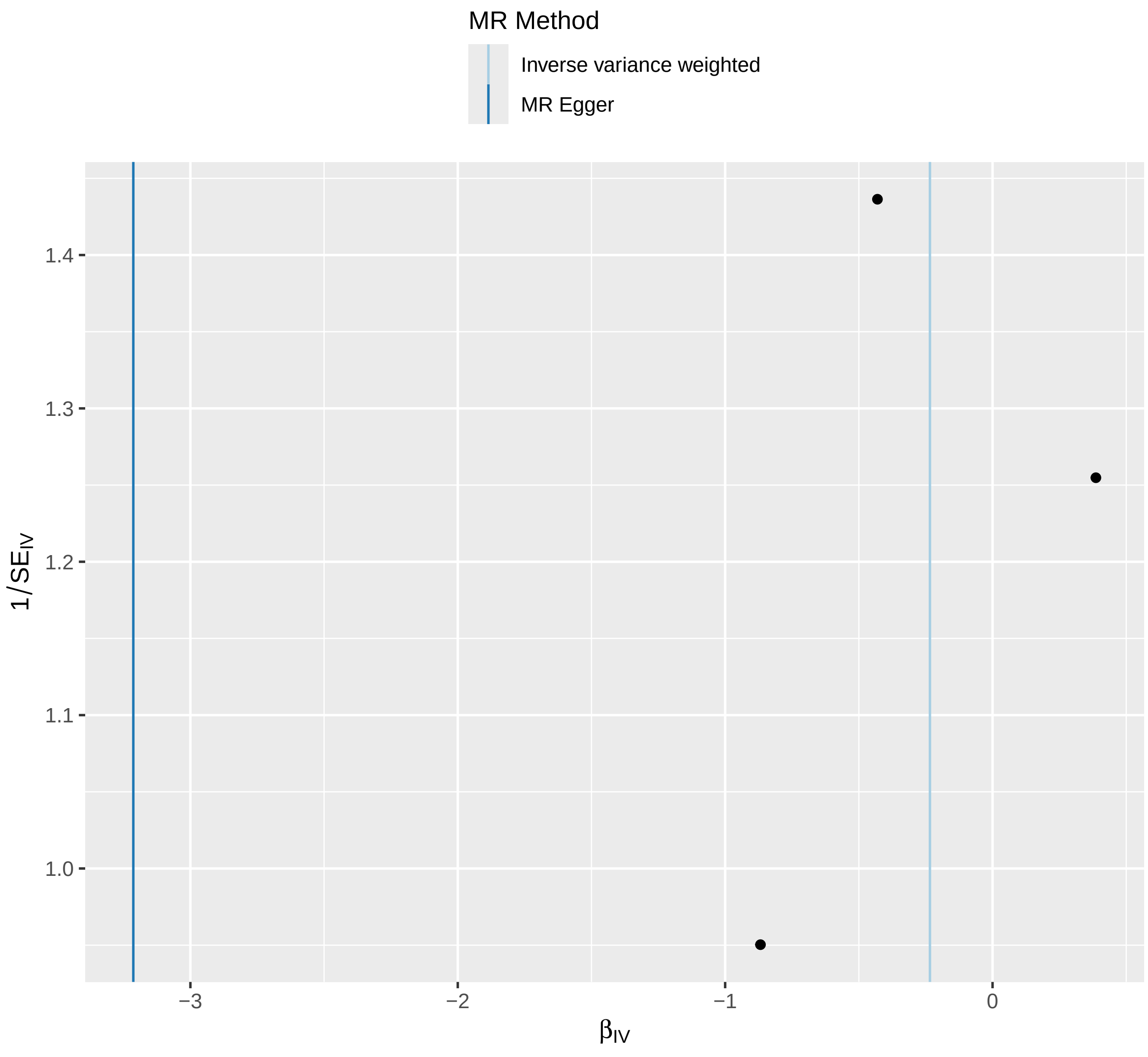
MR Method
Inverse variance weighted
MR Egger
1.4
1.3
1.2
1.1
1.0
1/SE_IV
−3
−2
−1
0
β_IV

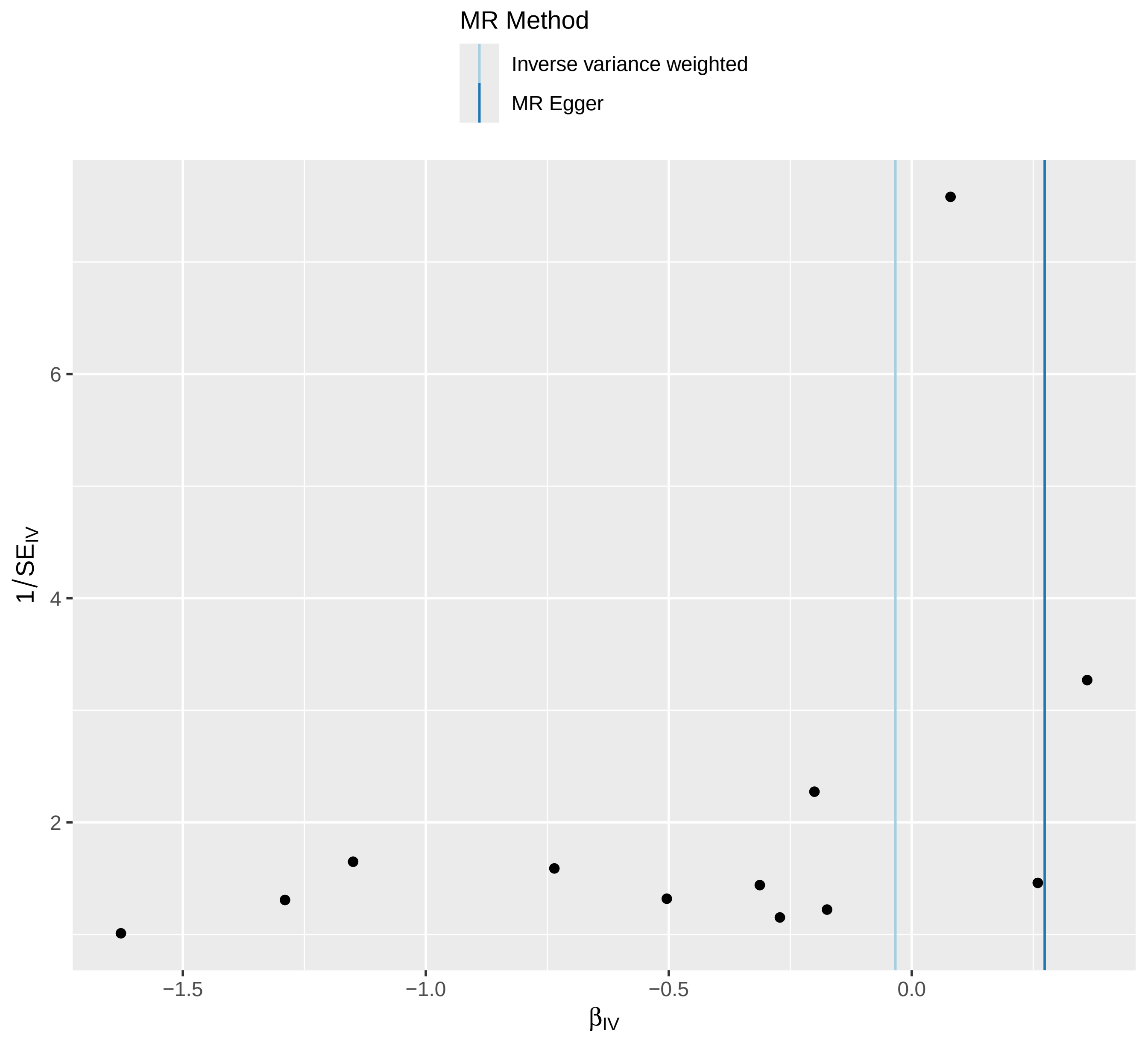
MR Method
Inverse variance weighted
MR Egger
6
4
2
1/SE$_{IV}$
−1.5
−1.0
−0.5
0.0
β$_{IV}$

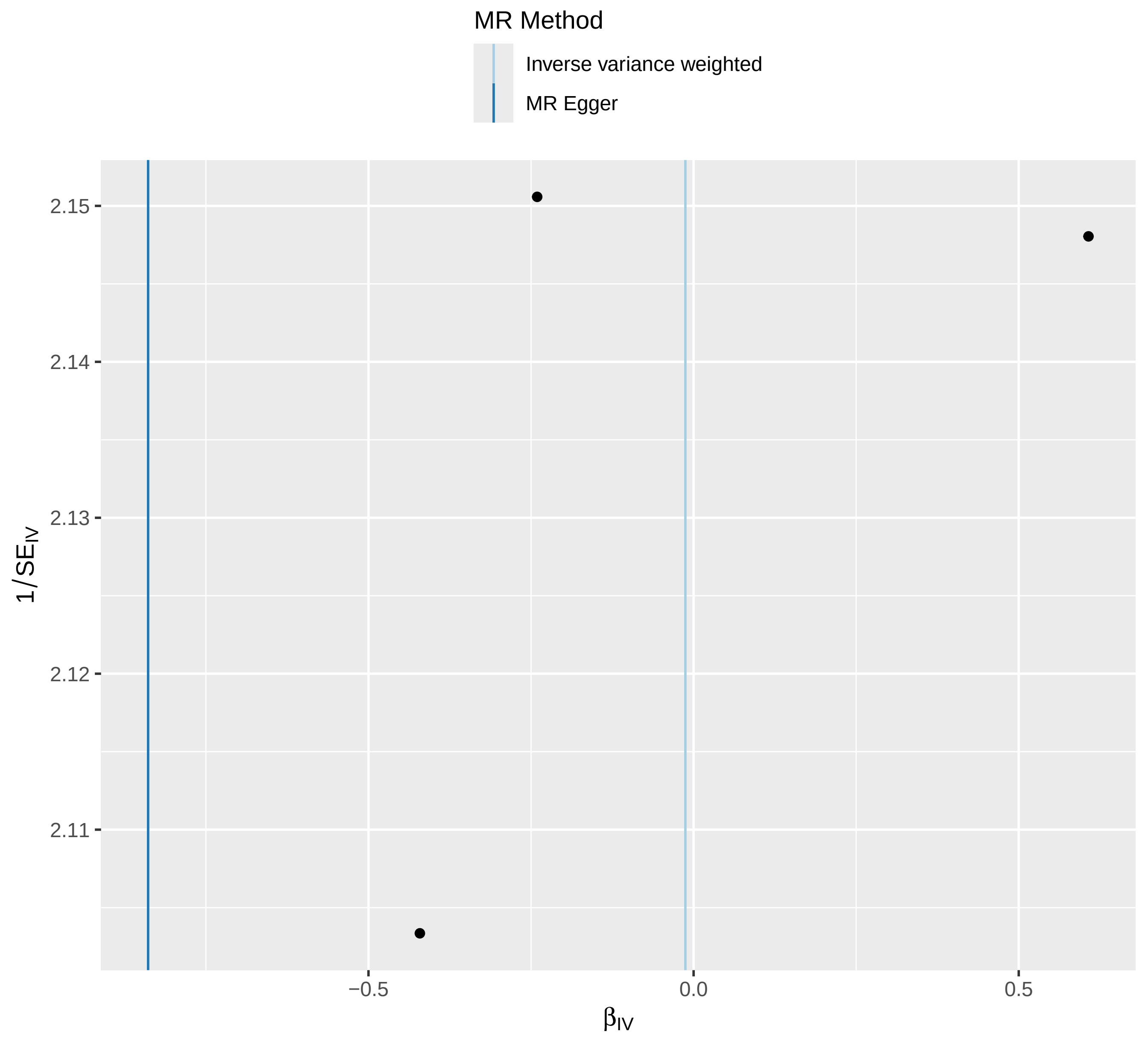
MR Method
Inverse variance weighted
MR Egger
2.15
2.14
2.13
2.12
2.11
1/SE$_{IV}$
−0.5
0.0
0.5
β$_{IV}$

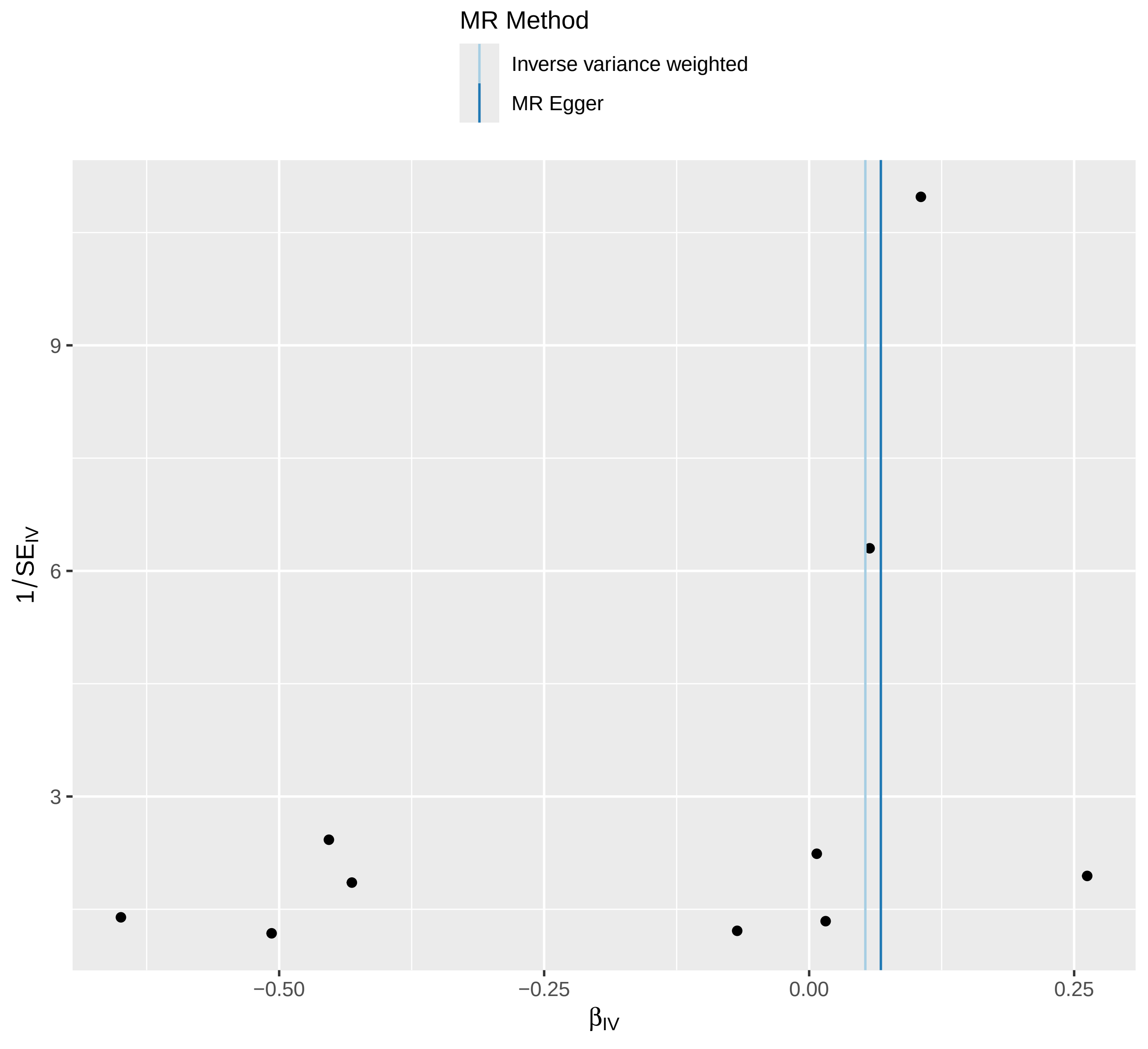

MR Method
Inverse variance weighted
MR Egger
1/SE_IV
9
6
3
−0.50
−0.25
0.00
0.25
β_IV

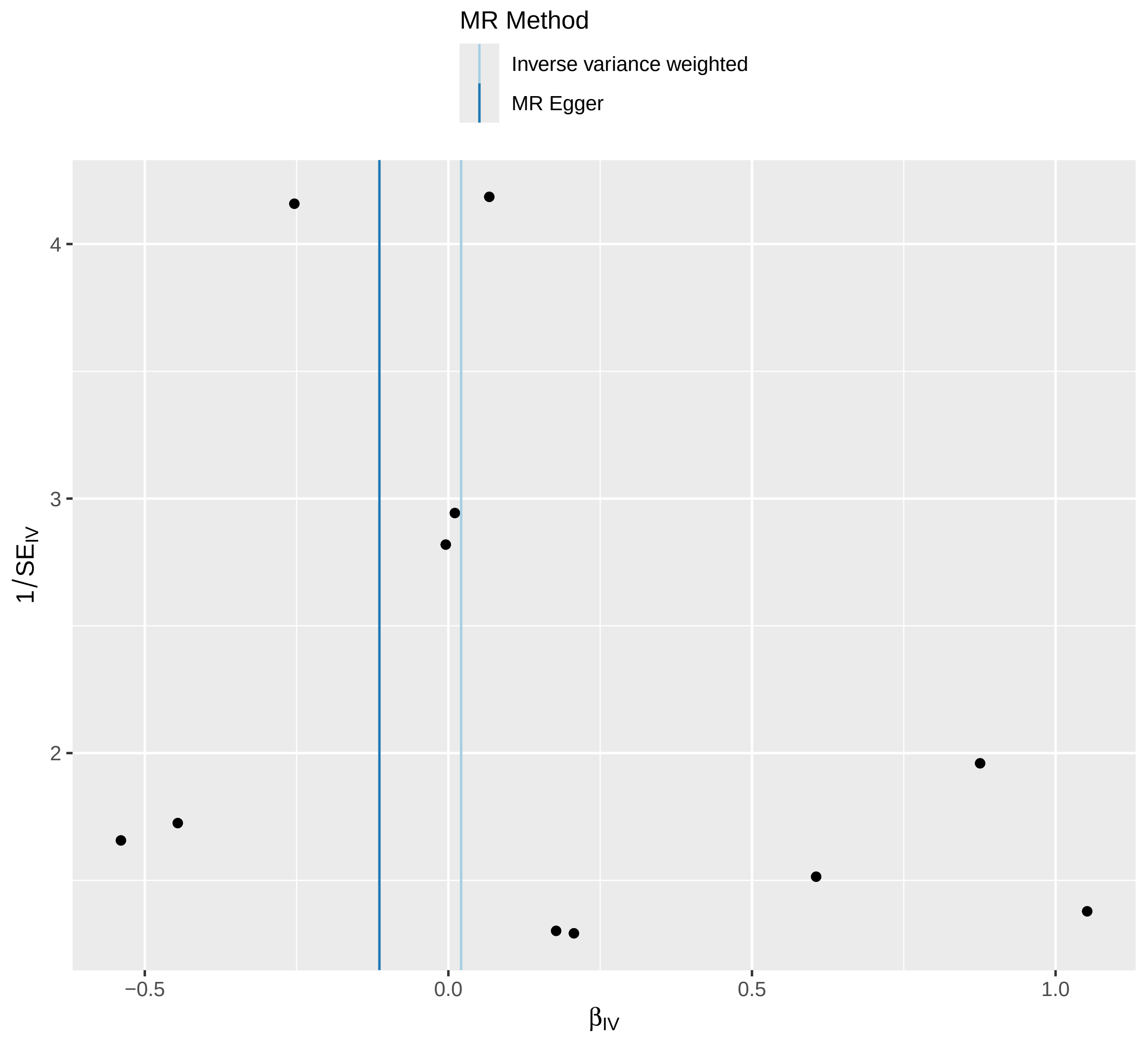
MR Method
Inverse variance weighted
MR Egger
4
3
2
1/SE$_{IV}$
−0.5
0.0
0.5
1.0
β$_{IV}$

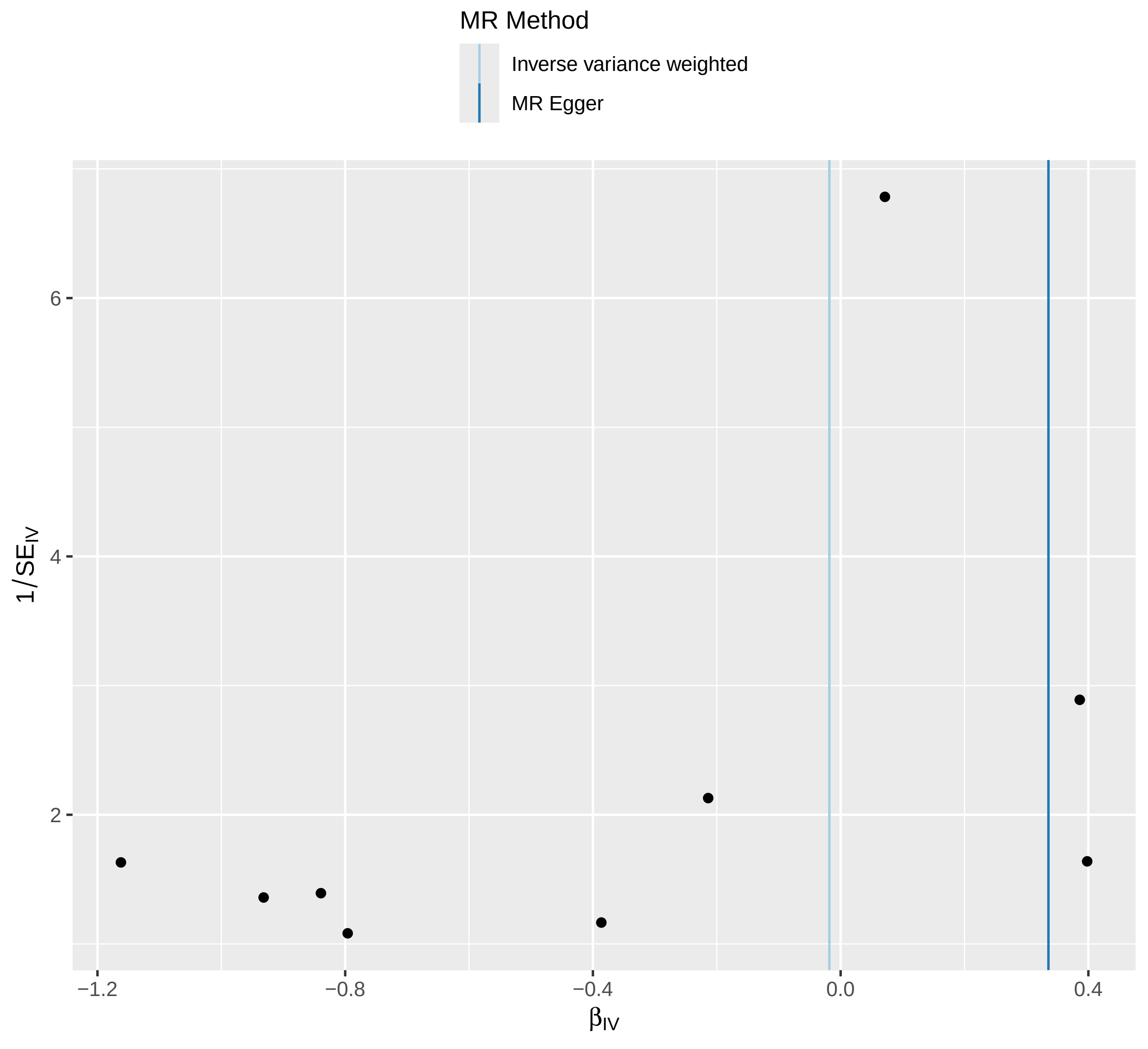

MR Method
Inverse variance weighted
MR Egger
6
4
2
1/SE$_{IV}$
−1.2
−0.8
−0.4
0.0
0.4
β$_{IV}$

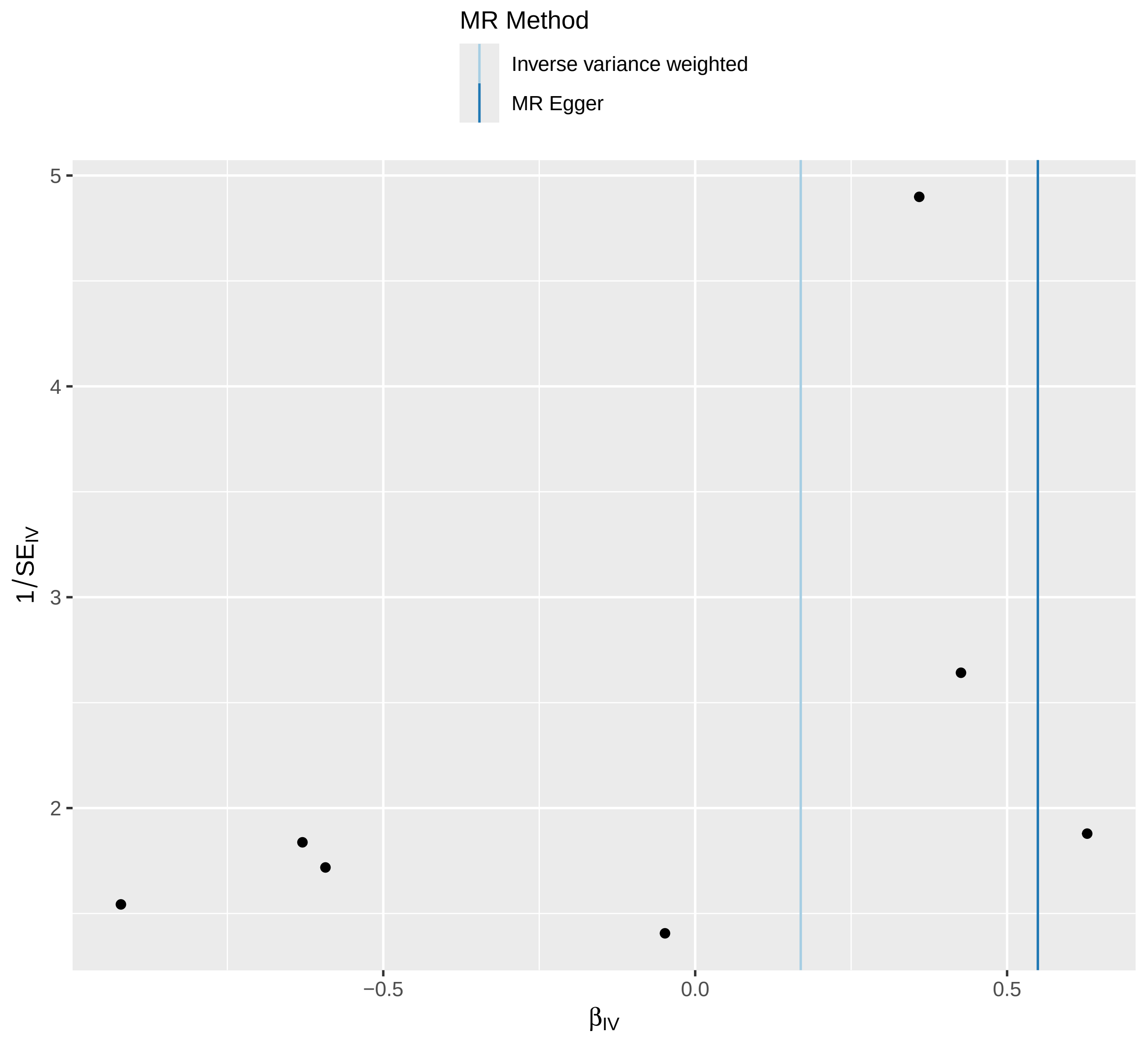

MR Method
Inverse variance weighted
MR Egger
5
4
3
2
1/SE$_{IV}$
−0.5
0.0
0.5
β_{IV}

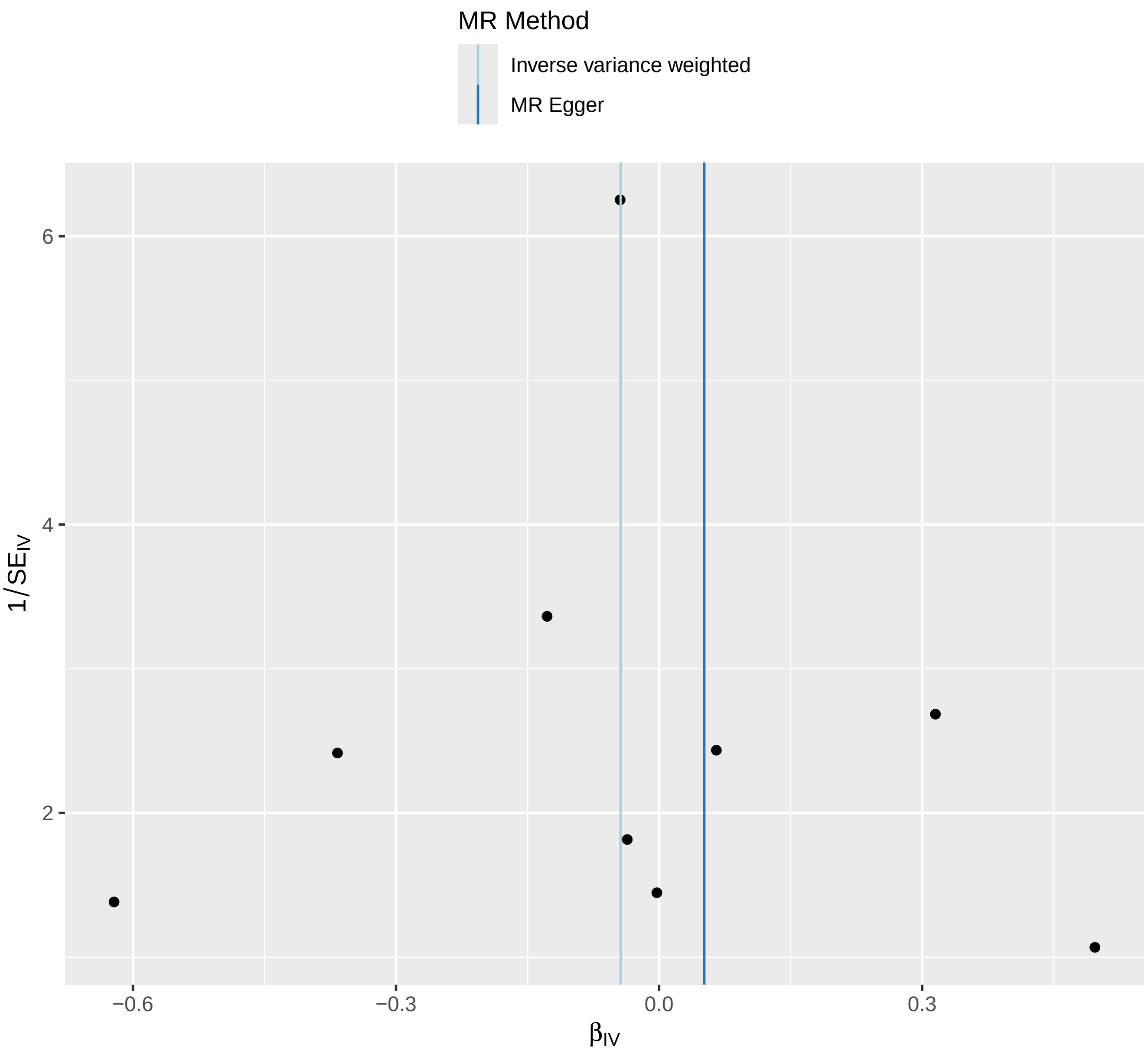
MR Method
Inverse variance weighted
MR Egger
6
4
2
$1/SE_{IV}$
−0.6
−0.3
0.0
0.3
β_{IV}

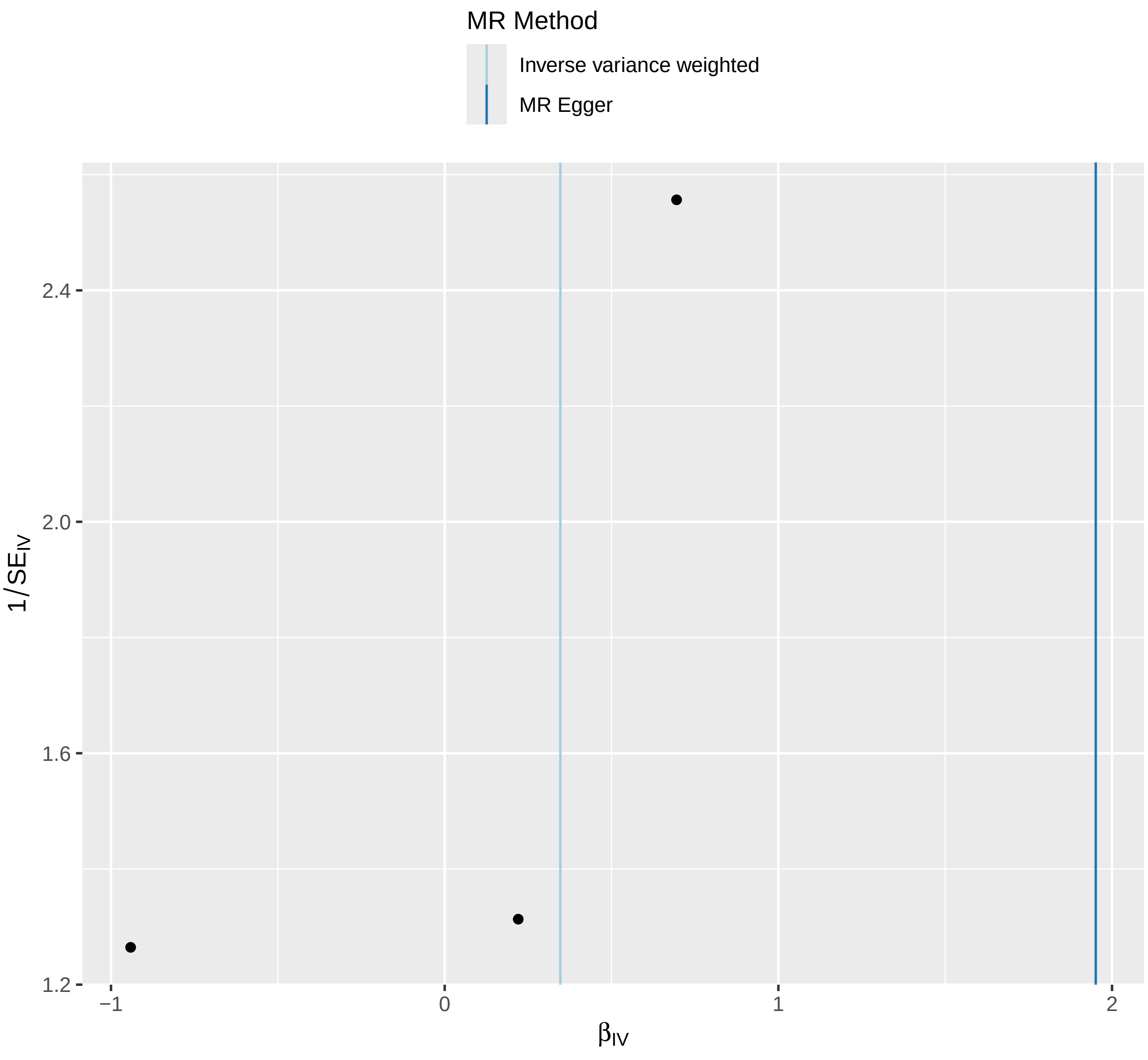
MR Method
Inverse variance weighted
MR Egger
2.4
2.0
1.6
1.2
−1
0
1
2
1/SE_IV
β_IV

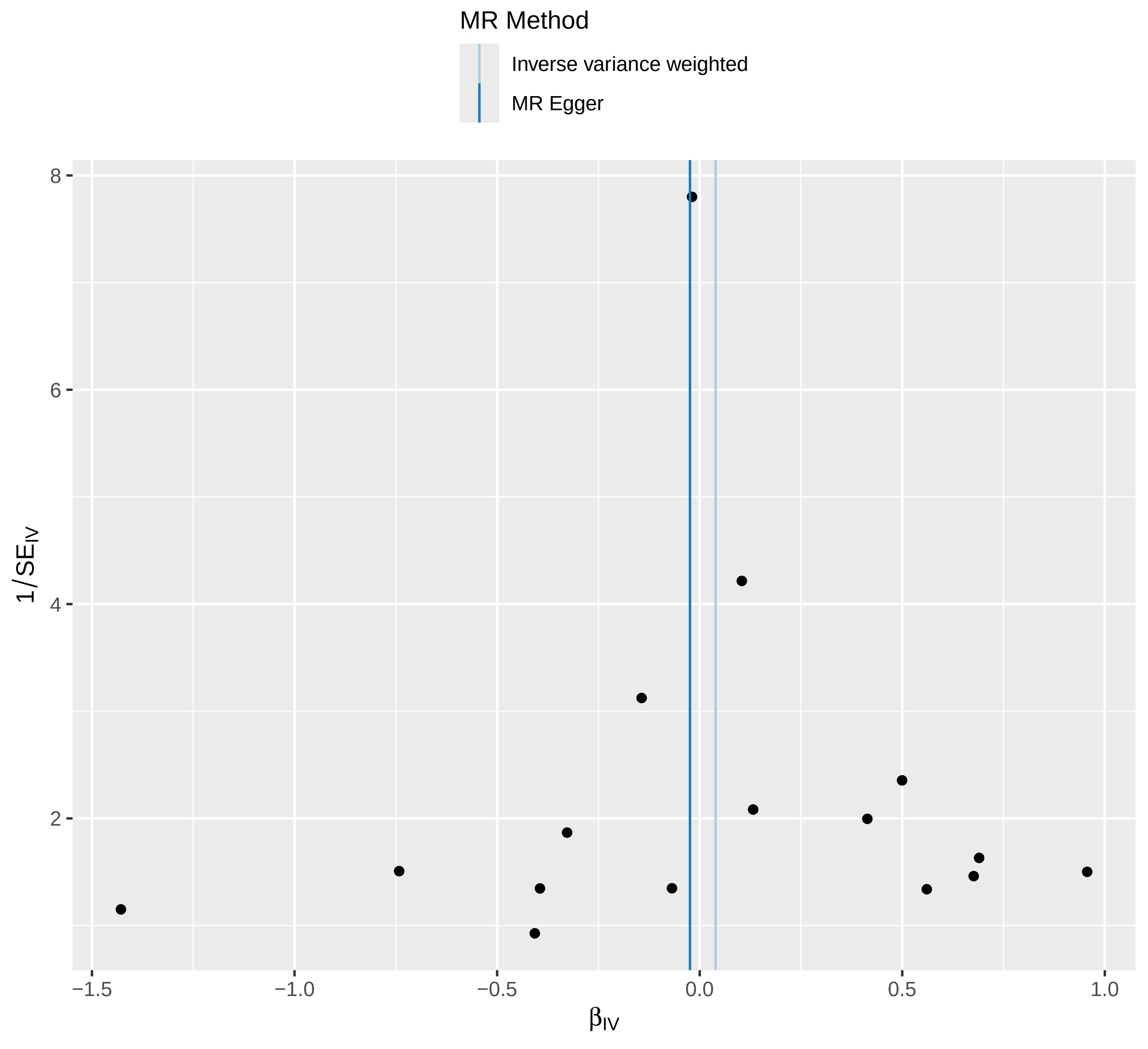
MR Method
Inverse variance weighted
MR Egger
8
6
4
2
1/SE_IV
−1.5
−1.0
−0.5
0.0
0.5
1.0
β_IV

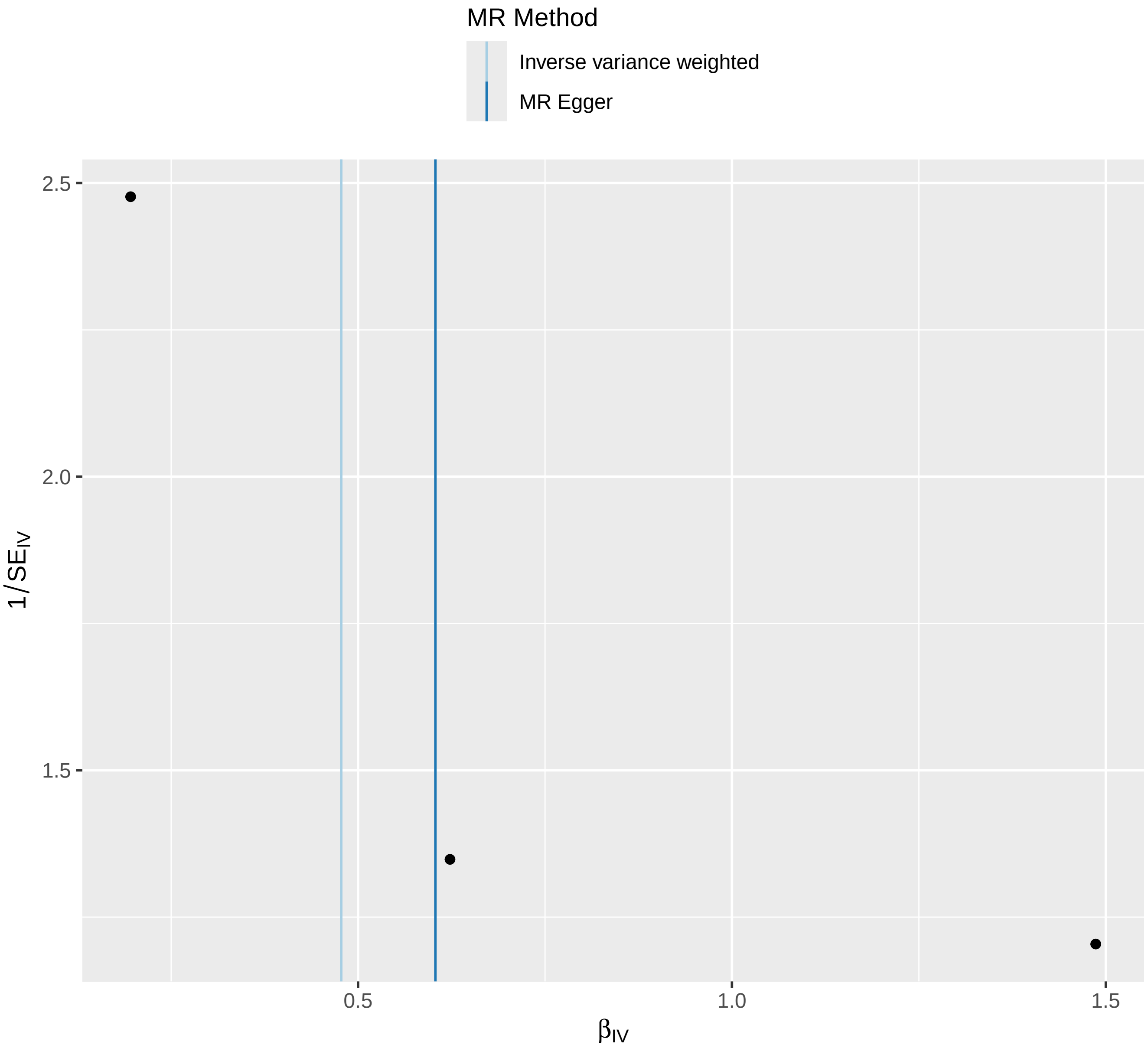
MR Method
Inverse variance weighted
MR Egger
2.5
2.0
1.5
0.5
1.0
1.5
$1/SE_{IV}$
β_{IV}

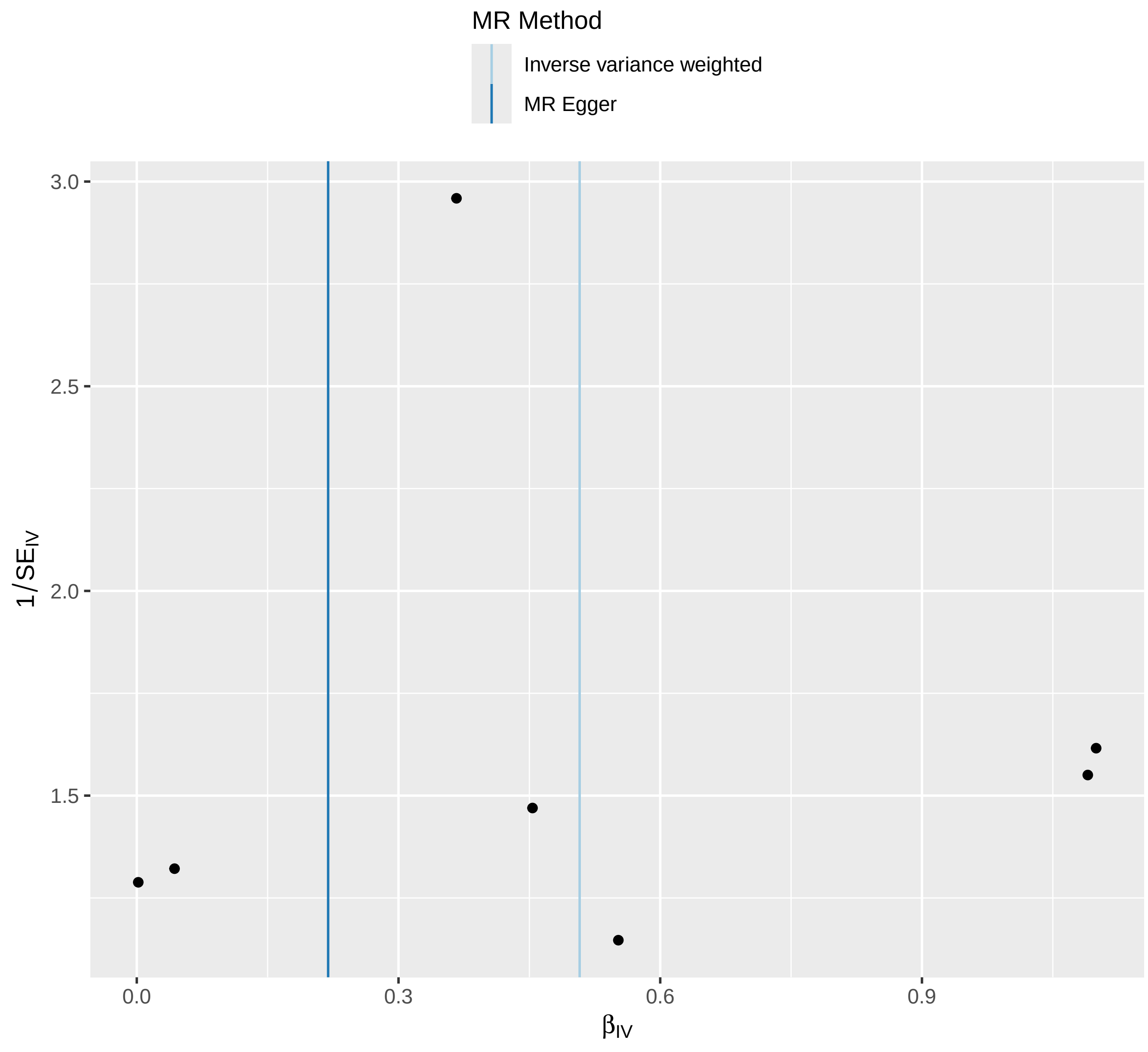
MR Method
Inverse variance weighted
MR Egger
3.0
2.5
2.0
1.5
$1/SE_{IV}$
0.0
0.3
0.6
0.9
β_{IV}

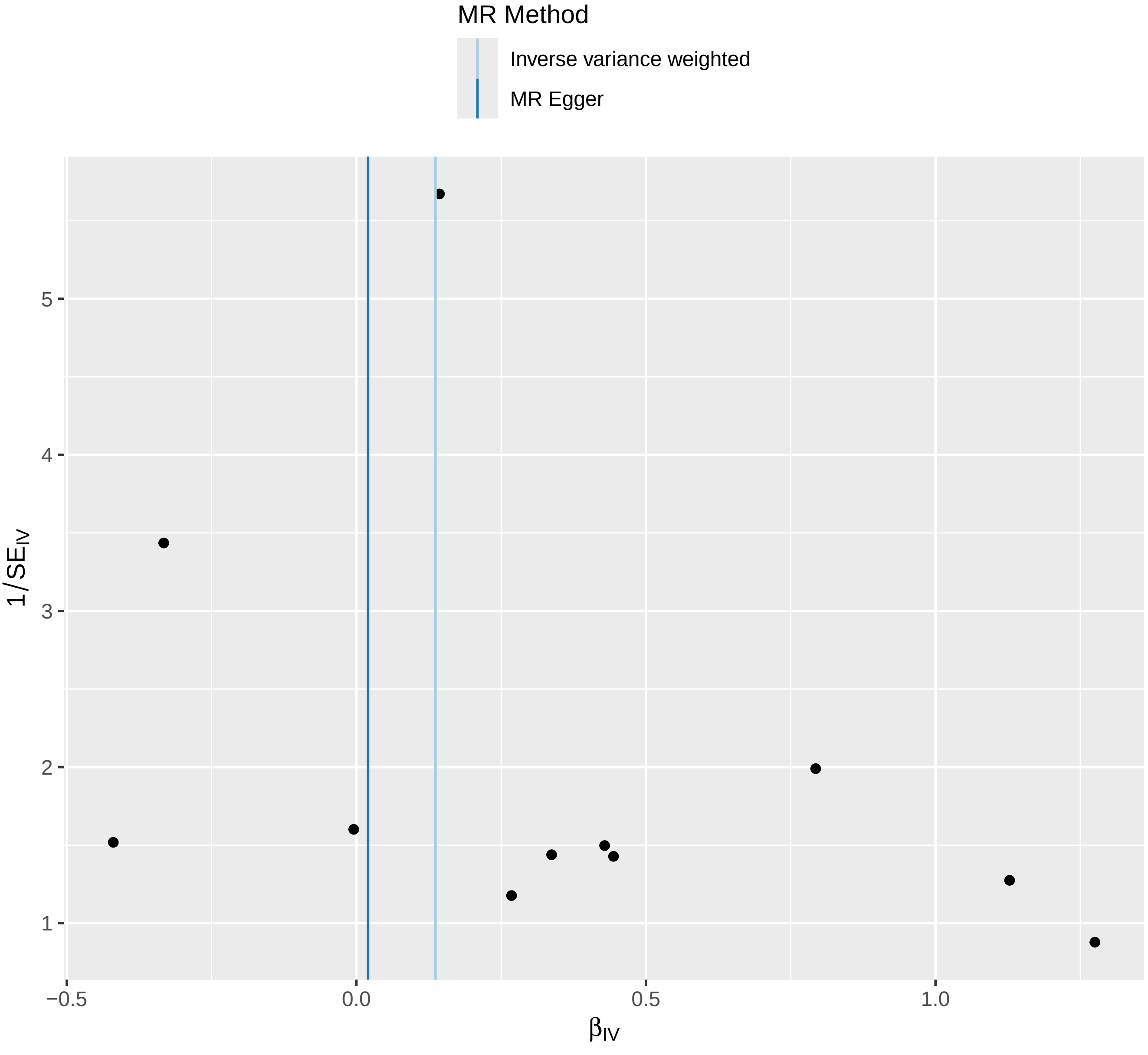

MR Method
Inverse variance weighted
MR Egger
1/SE_IV
5
4
3
2
1
−0.5
0.0
0.5
1.0
β_IV

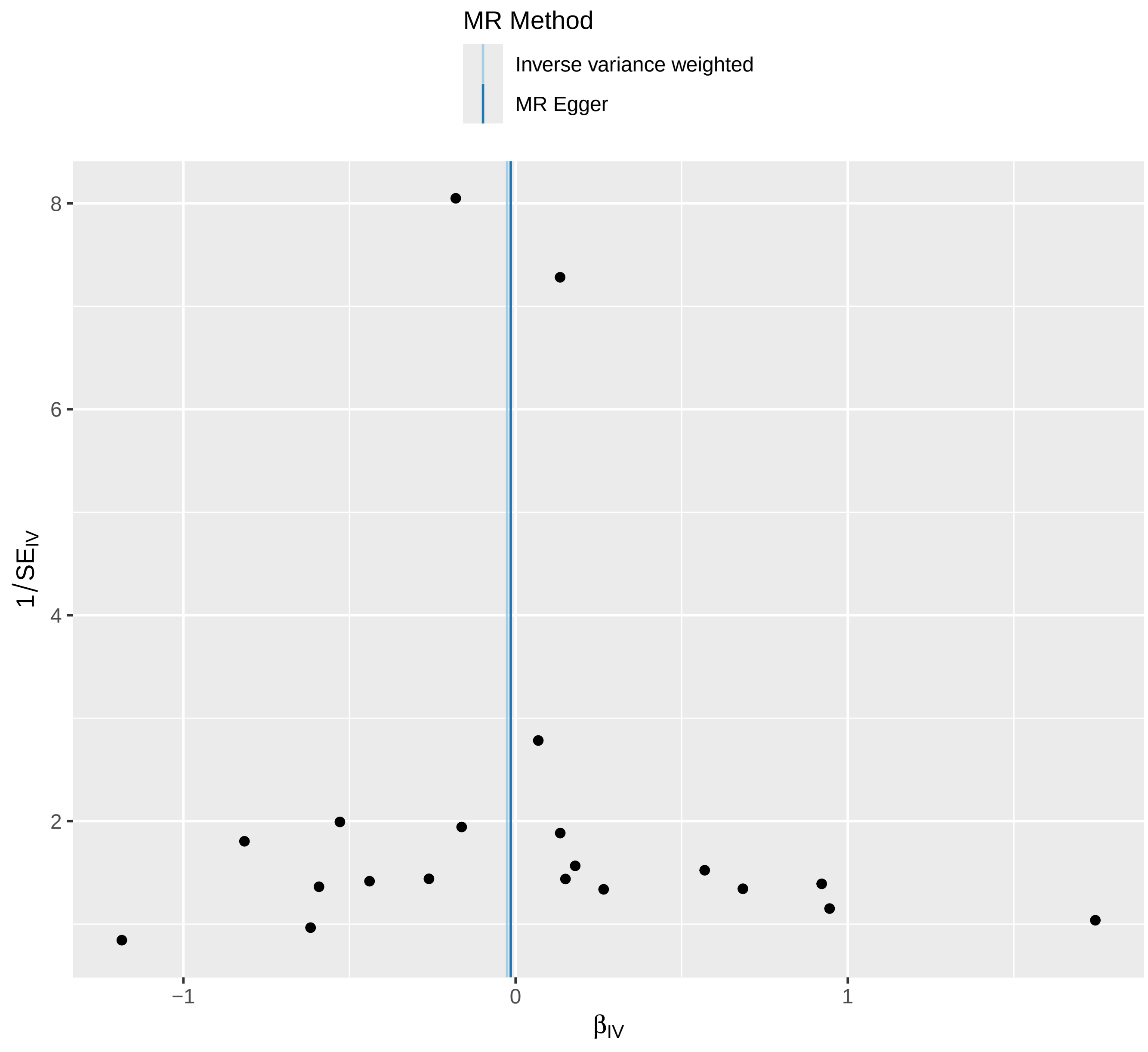
MR Method
Inverse variance weighted
MR Egger
8
6
4
2
1/SE$_{IV}$
−1
0
1
β$_{IV}$

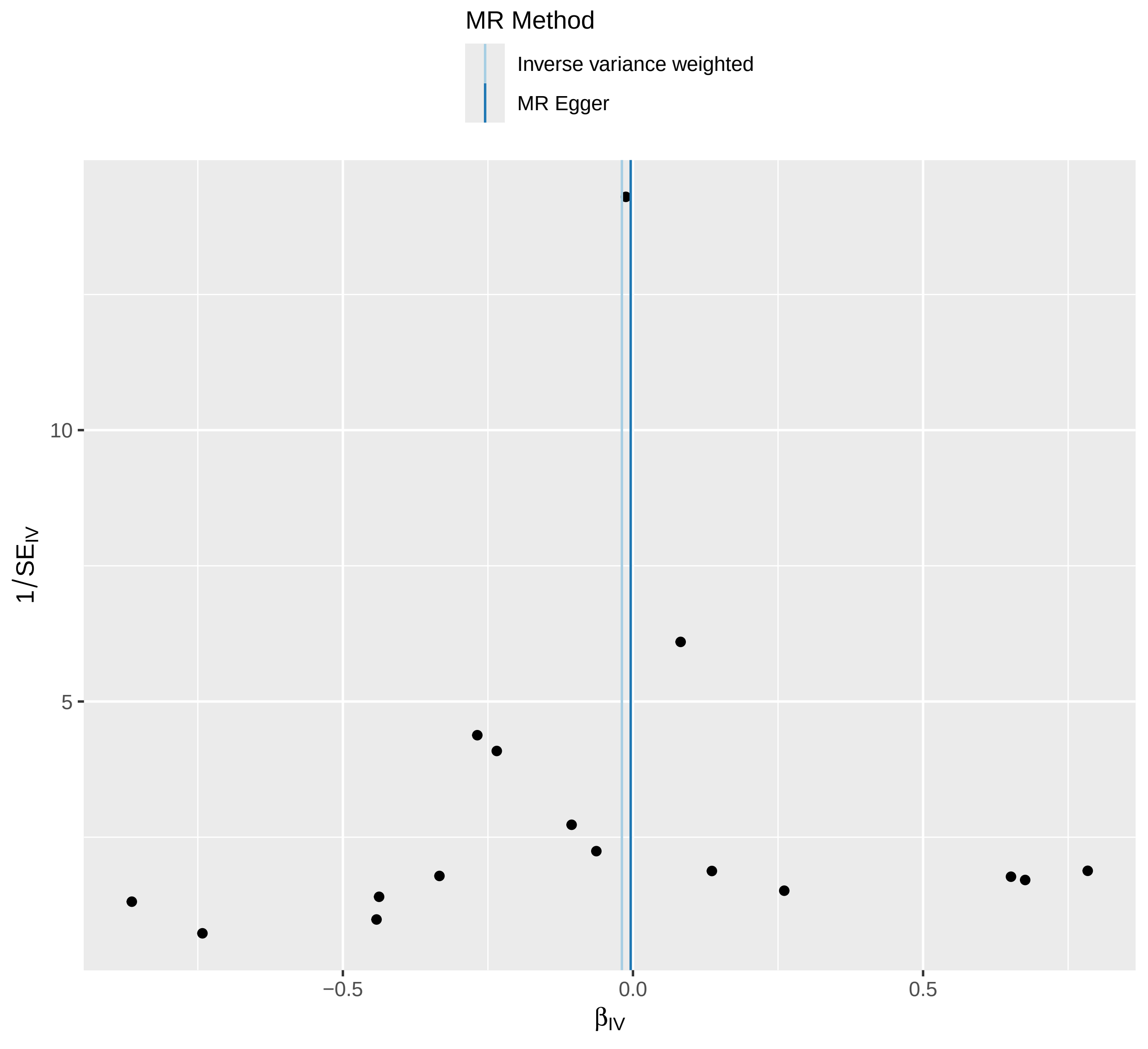

MR Method
Inverse variance weighted
MR Egger
10
5
1/SE_IV
−0.5
0.0
0.5
β_IV

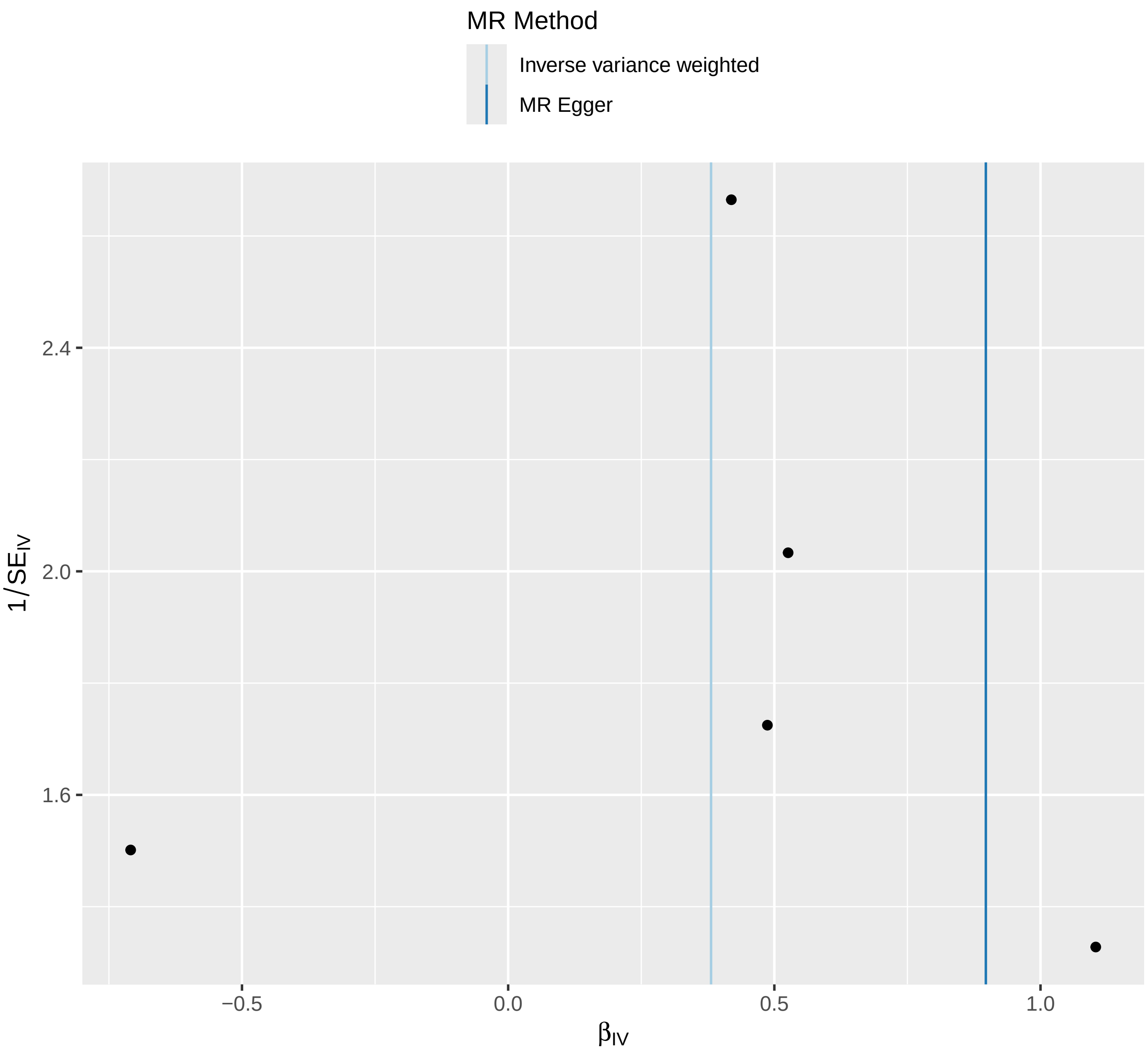

MR Method
Inverse variance weighted
MR Egger
2.4
2.0
1.6
1/SE_IV
−0.5
0.0
0.5
1.0
β_IV

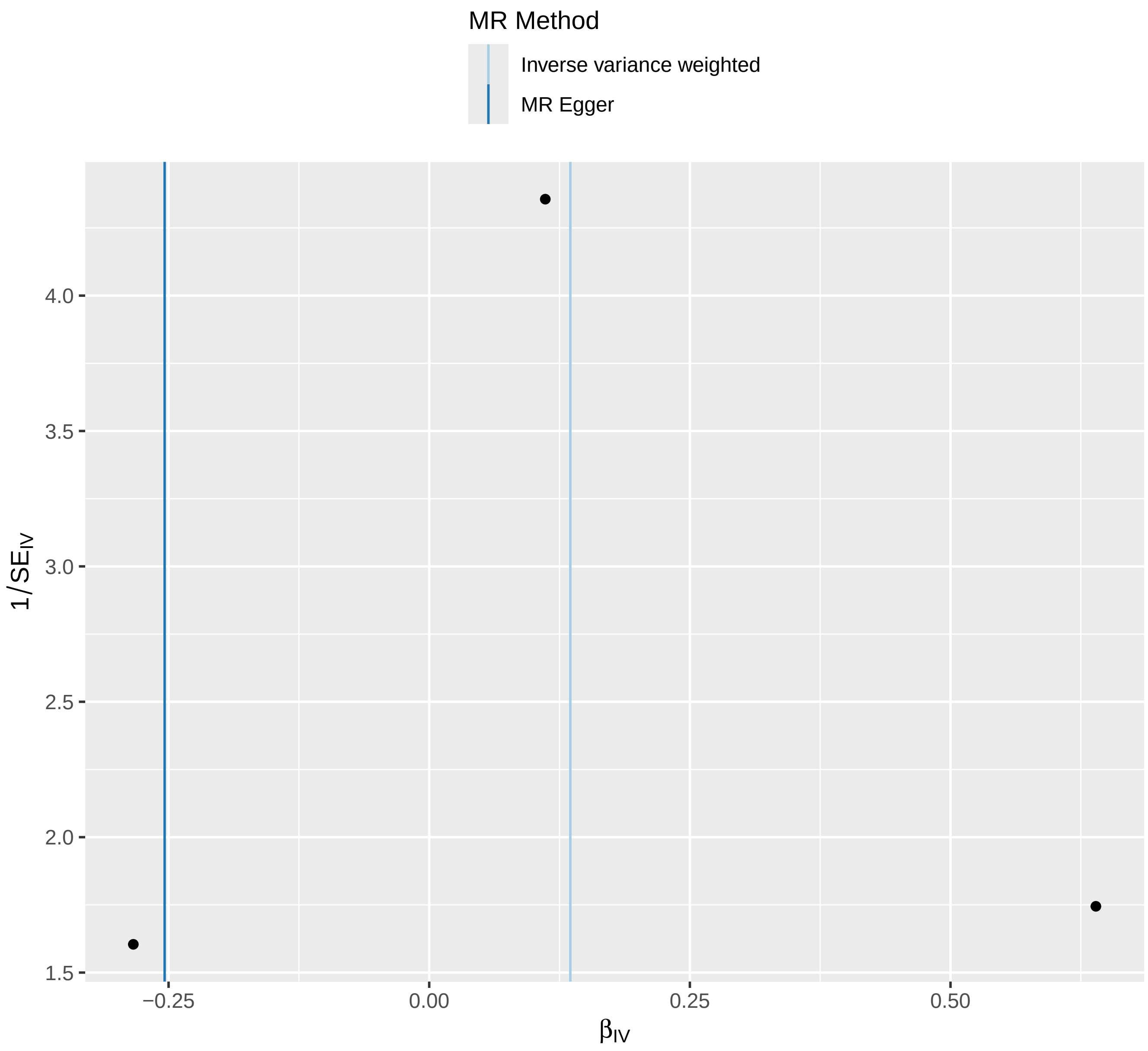
MR Method
Inverse variance weighted
MR Egger
4.0
3.5
3.0
2.5
2.0
1.5
1/SE_IV
−0.25
0.00
0.25
0.50
β_IV

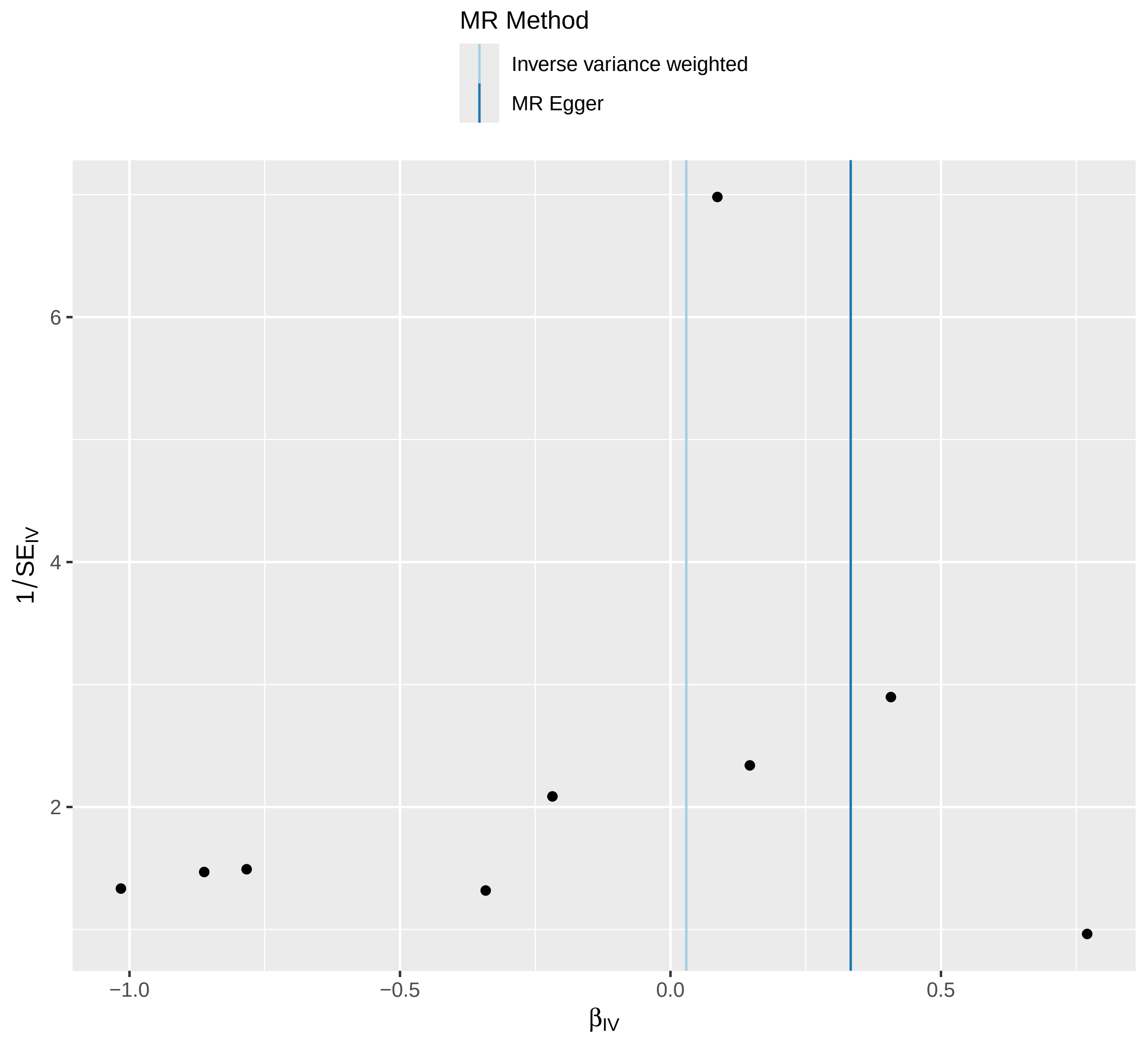

MR Method
Inverse variance weighted
MR Egger
6
4
2
1/SE_IV
−1.0
−0.5
0.0
0.5
β_IV

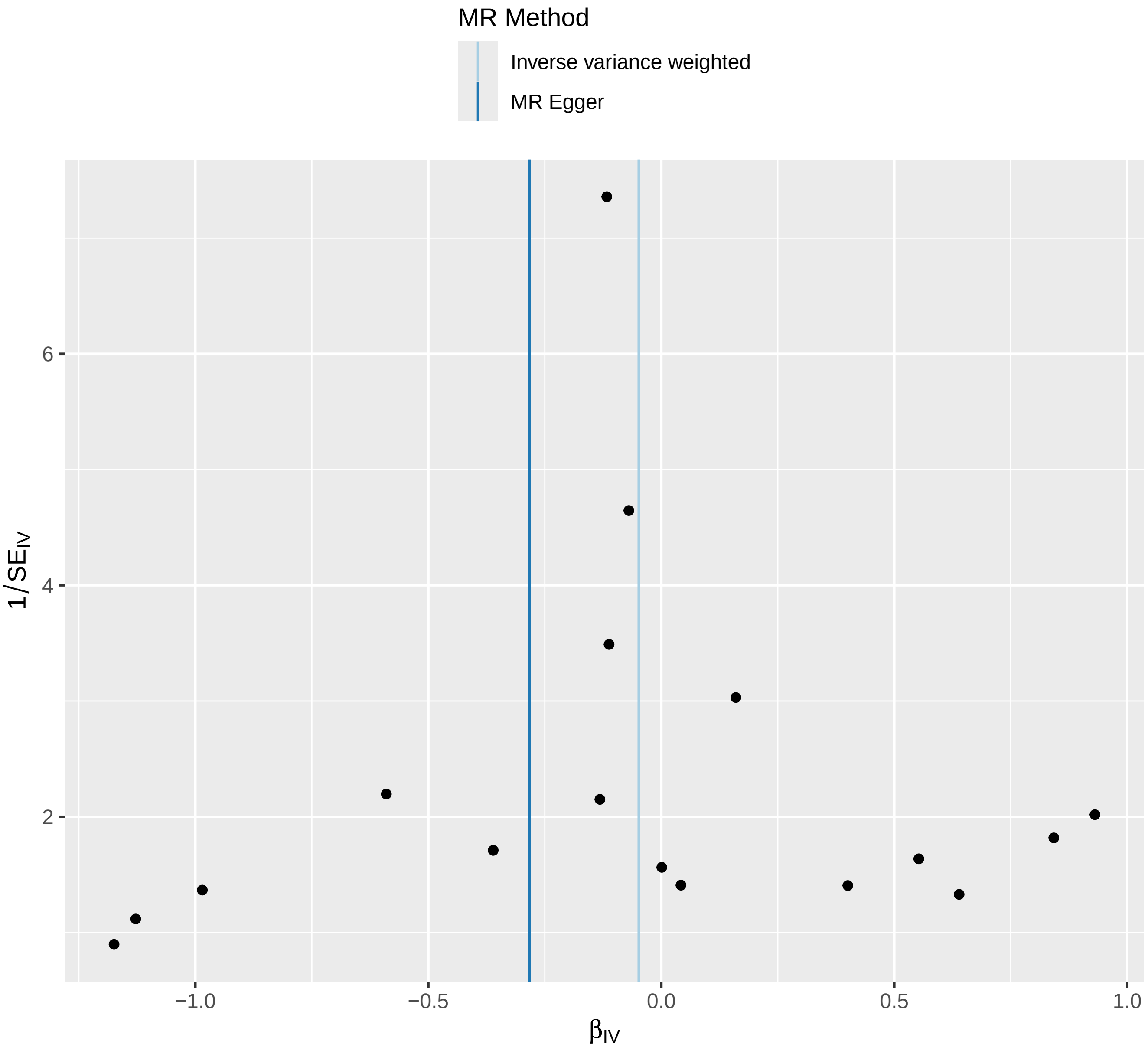

MR Method
Inverse variance weighted
MR Egger
6
4
2
1/SE$_{IV}$
−1.0
−0.5
0.0
0.5
1.0
β$_{IV}$

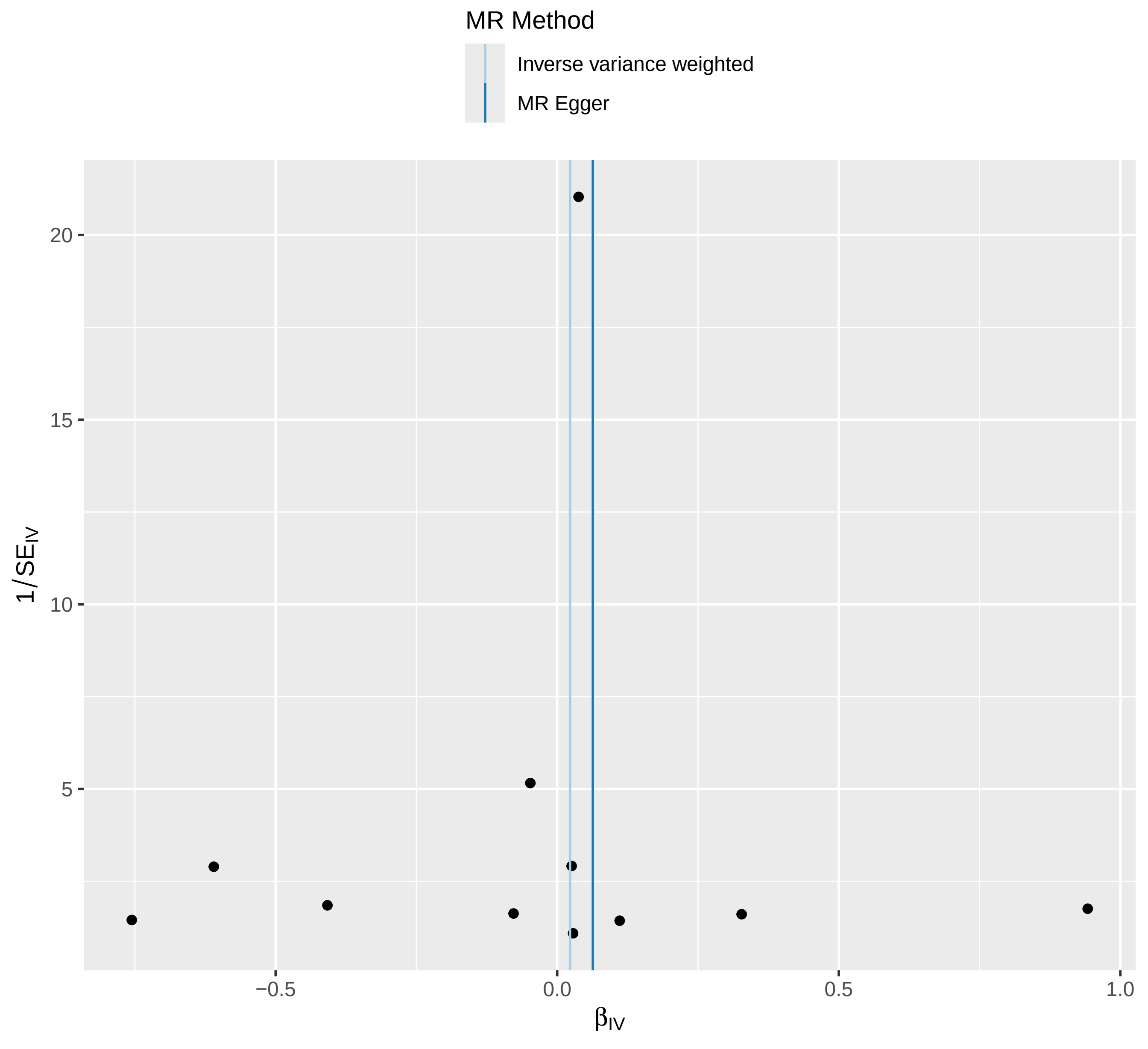

MR Method
Inverse variance weighted
MR Egger
20
15
10
5
1/SE$_{IV}$
−0.5
0.0
0.5
1.0
β$_{IV}$

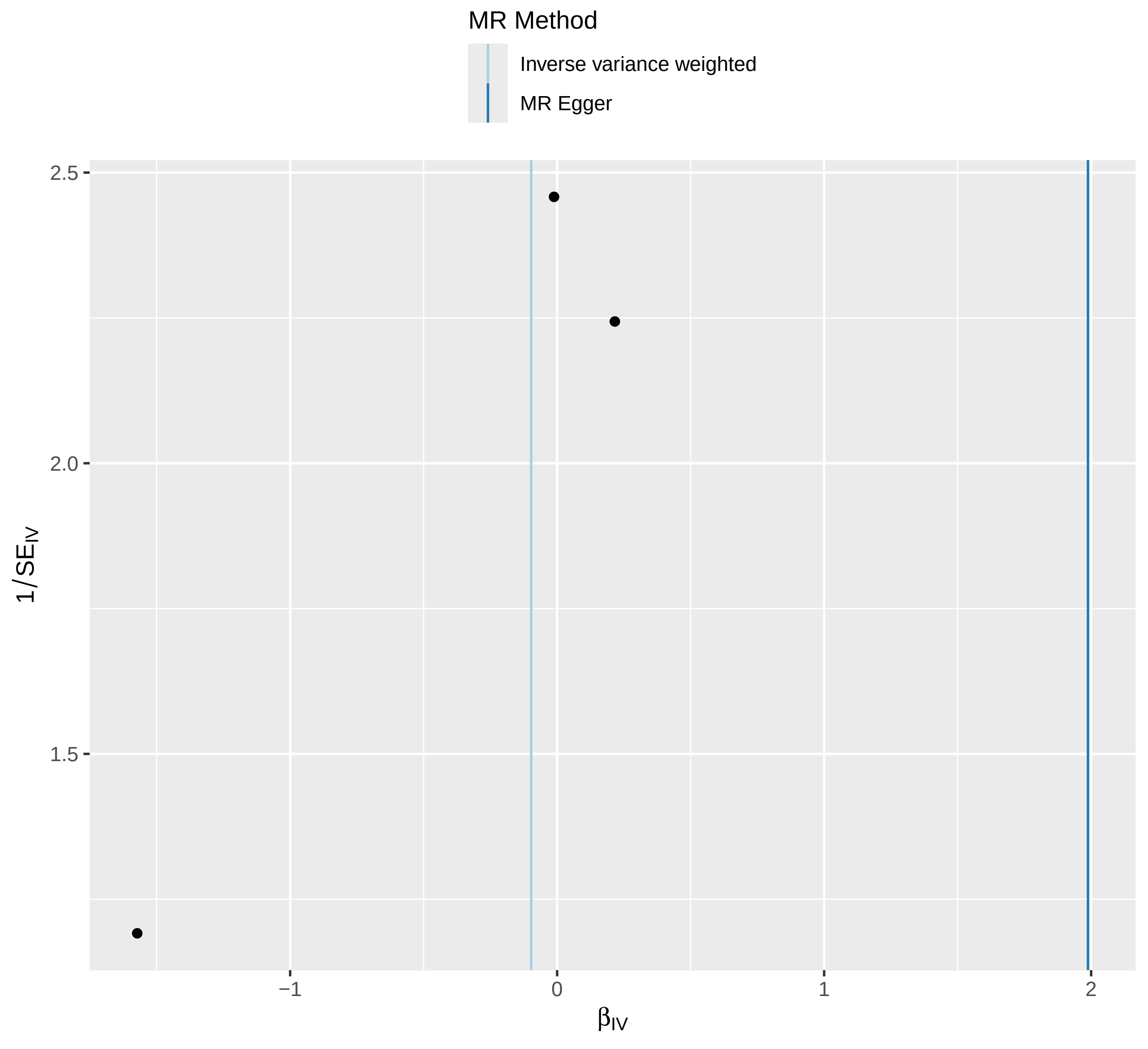
MR Method
Inverse variance weighted
MR Egger
2.5
2.0
1.5
1/SE$_{IV}$
−1
0
1
2
β_{IV}

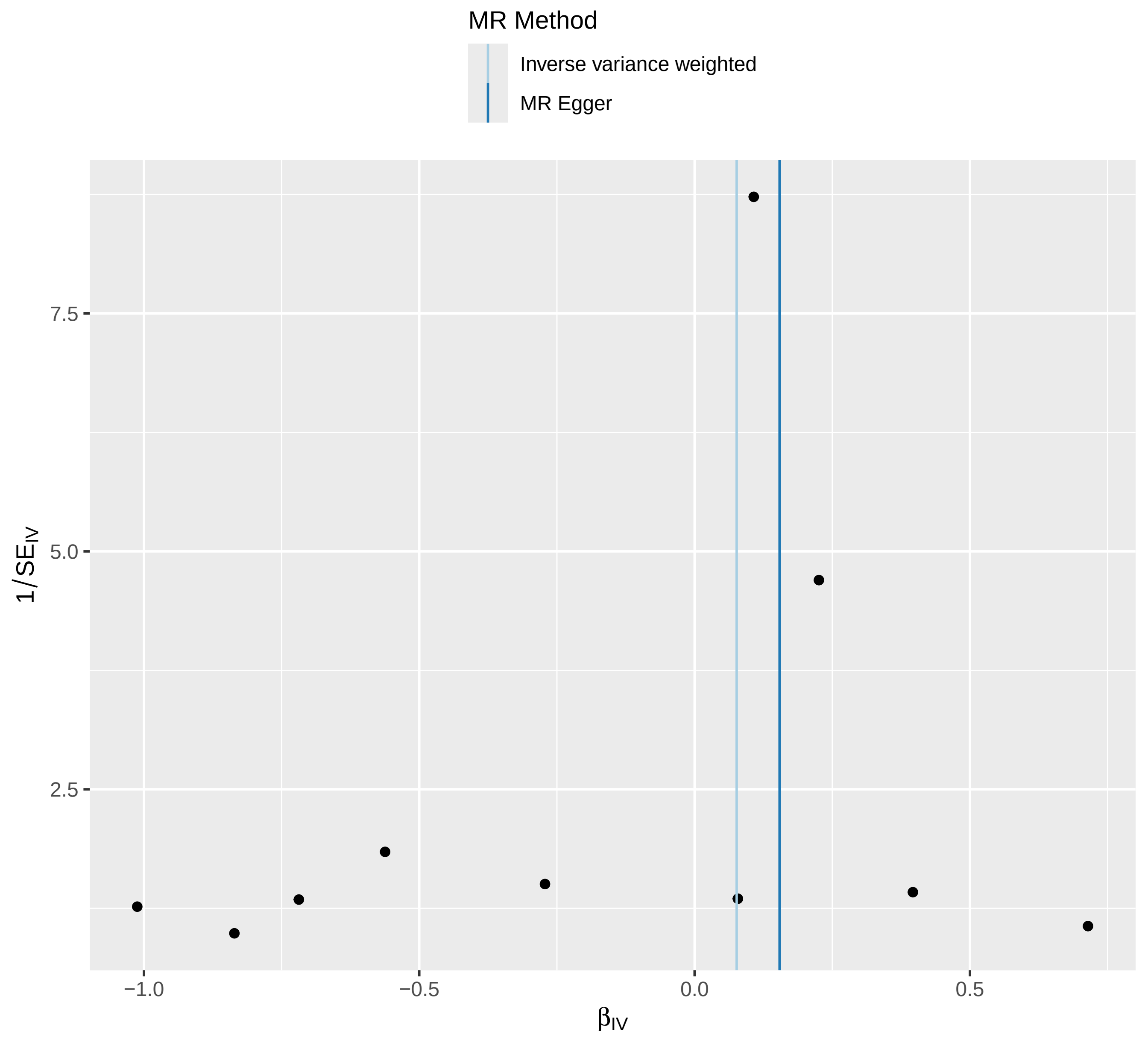

MR Method
Inverse variance weighted
MR Egger
7.5
5.0
2.5
1/SE_IV
−1.0
−0.5
0.0
0.5
β_IV

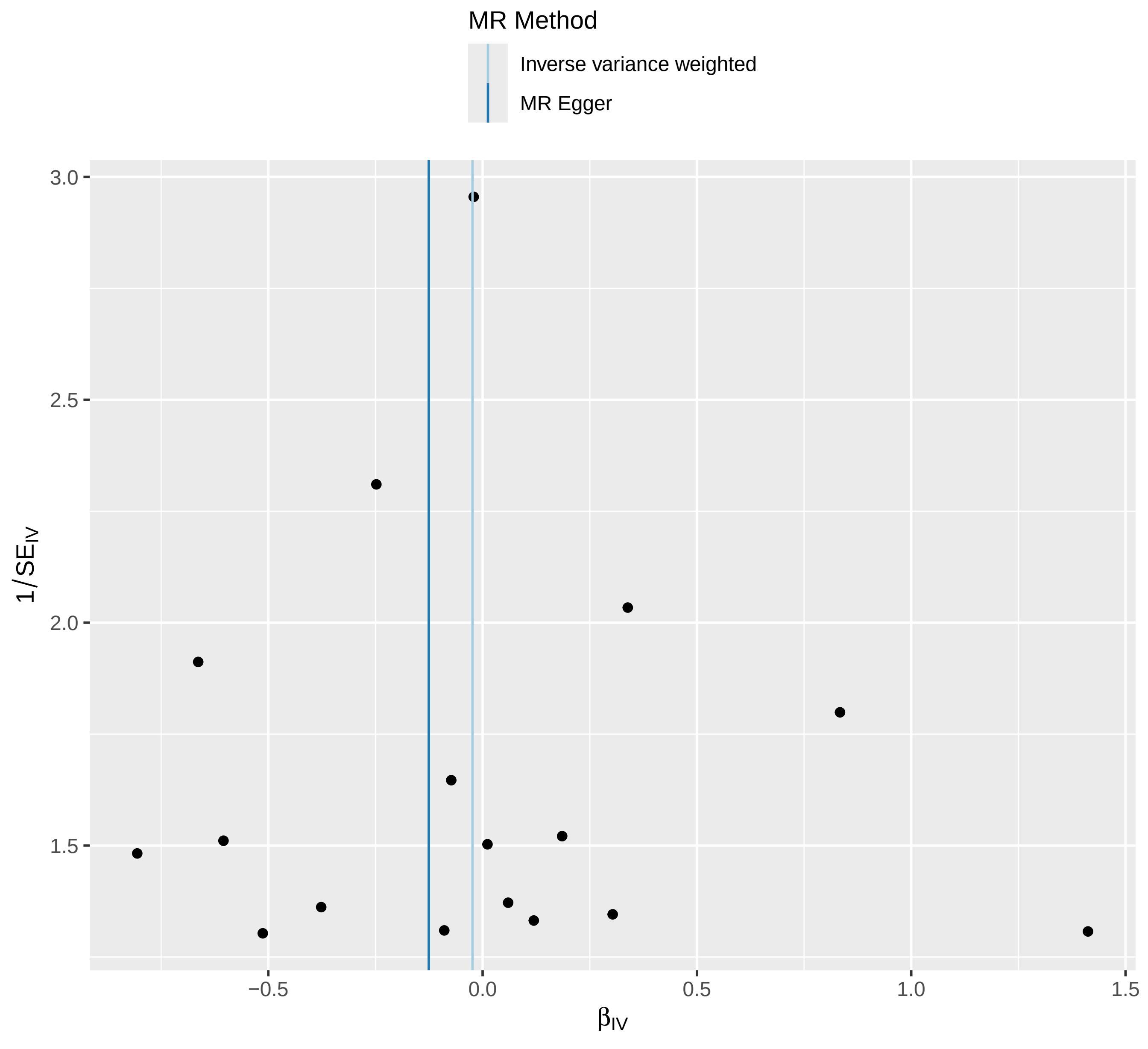
MR Method
Inverse variance weighted
MR Egger
3.0
2.5
2.0
1.5
$1/SE_{IV}$
−0.5
0.0
0.5
1.0
1.5
β_{IV}

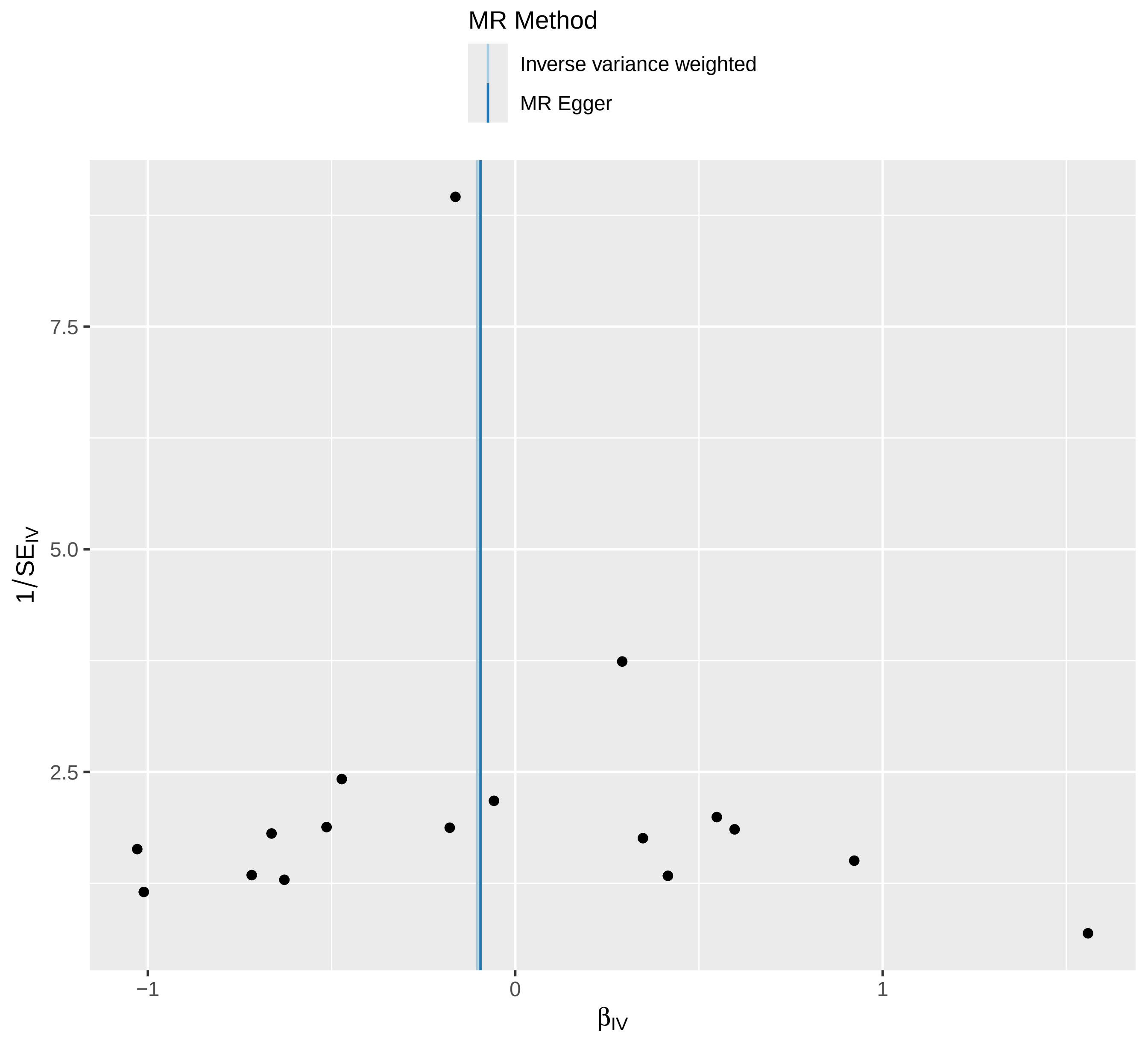

MR Method
Inverse variance weighted
MR Egger
7.5
5.0
2.5
1/SE$_{IV}$
−1
0
1
β$_{IV}$

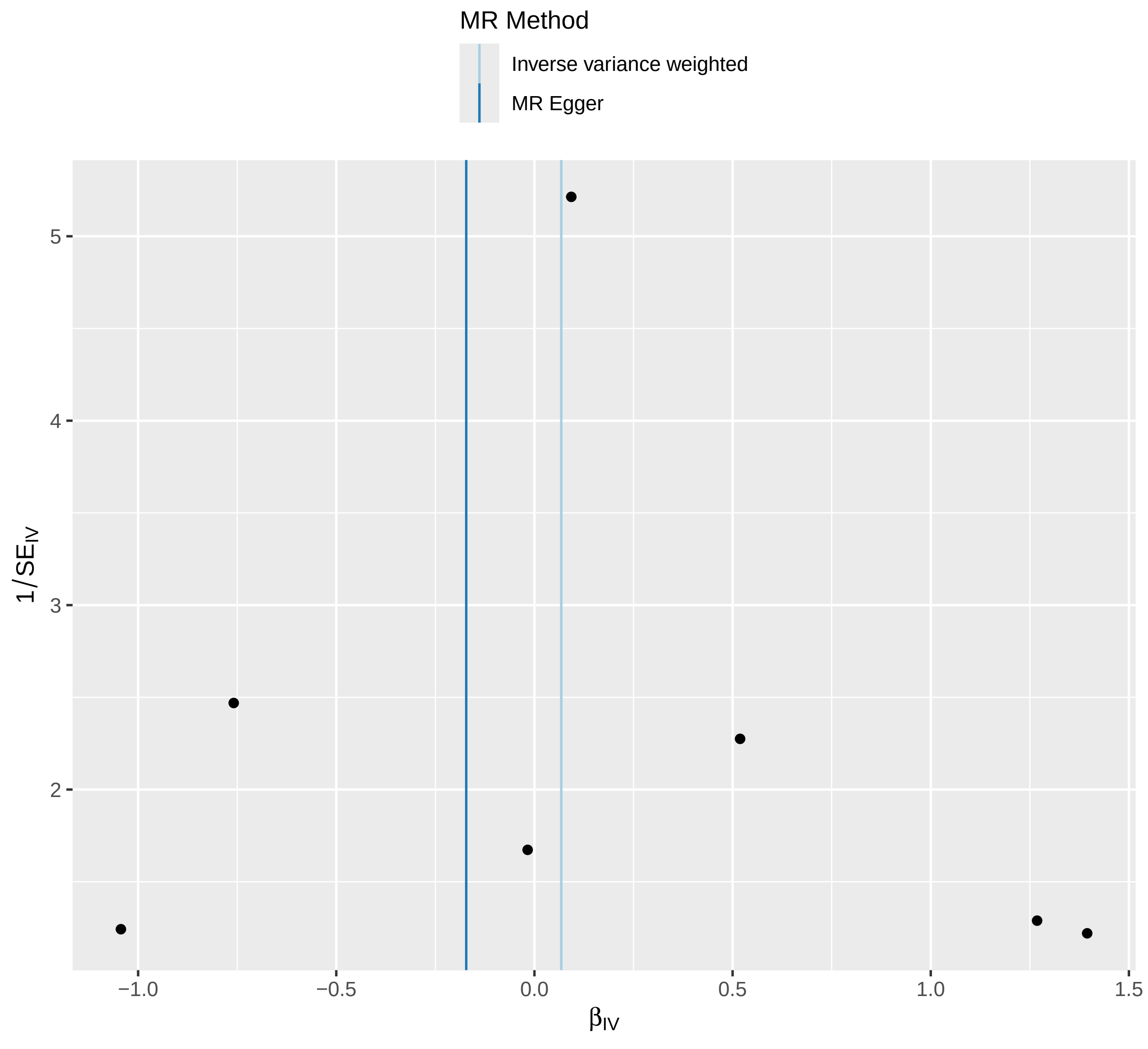
MR Method
Inverse variance weighted
MR Egger
5
4
3
2
1/SE_IV
−1.0
−0.5
0.0
0.5
1.0
1.5
β_IV

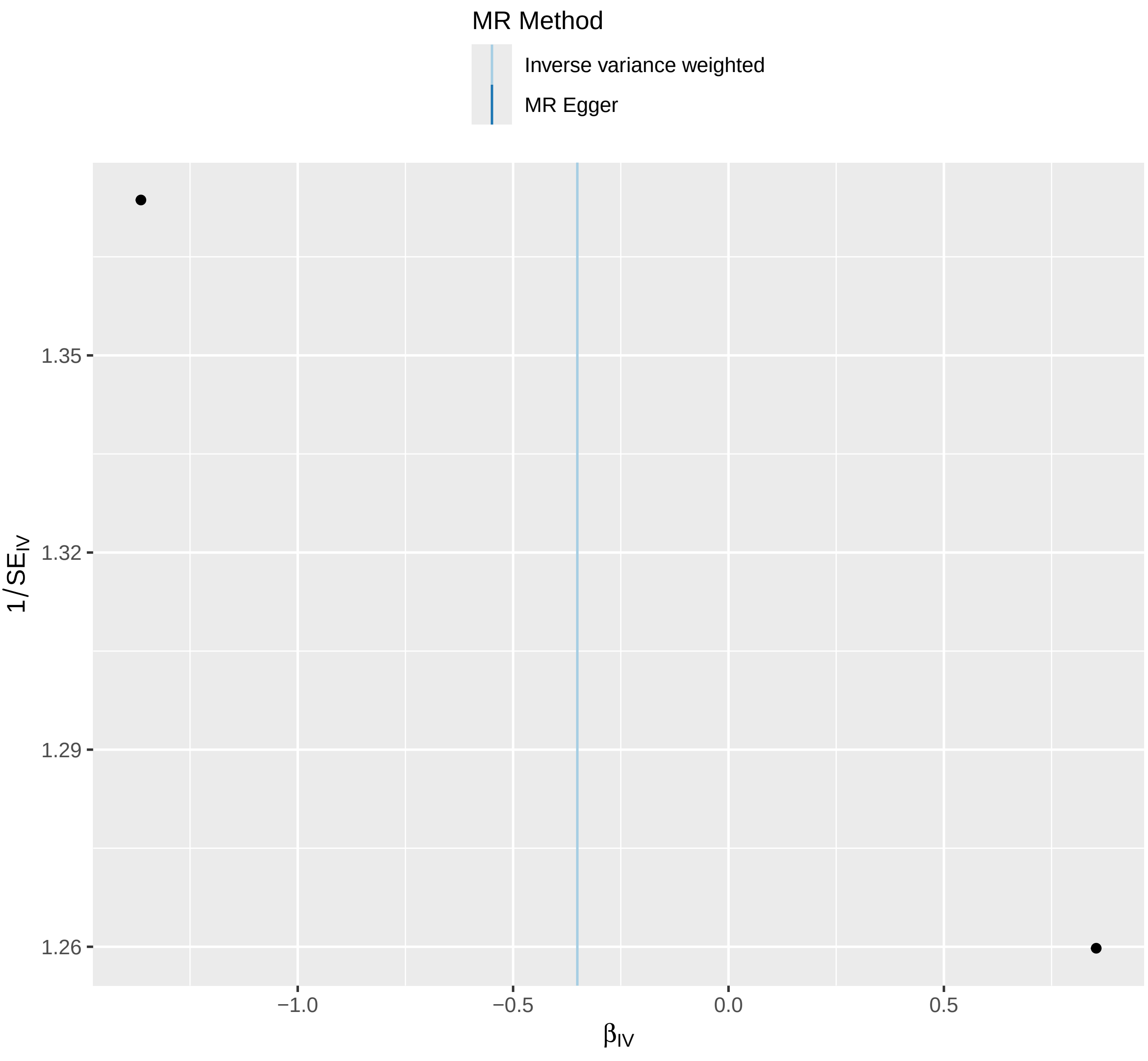
MR Method
Inverse variance weighted
MR Egger
1.35
1.32
1.29
1.26
1/SE_IV
−1.0
−0.5
0.0
0.5
β_IV

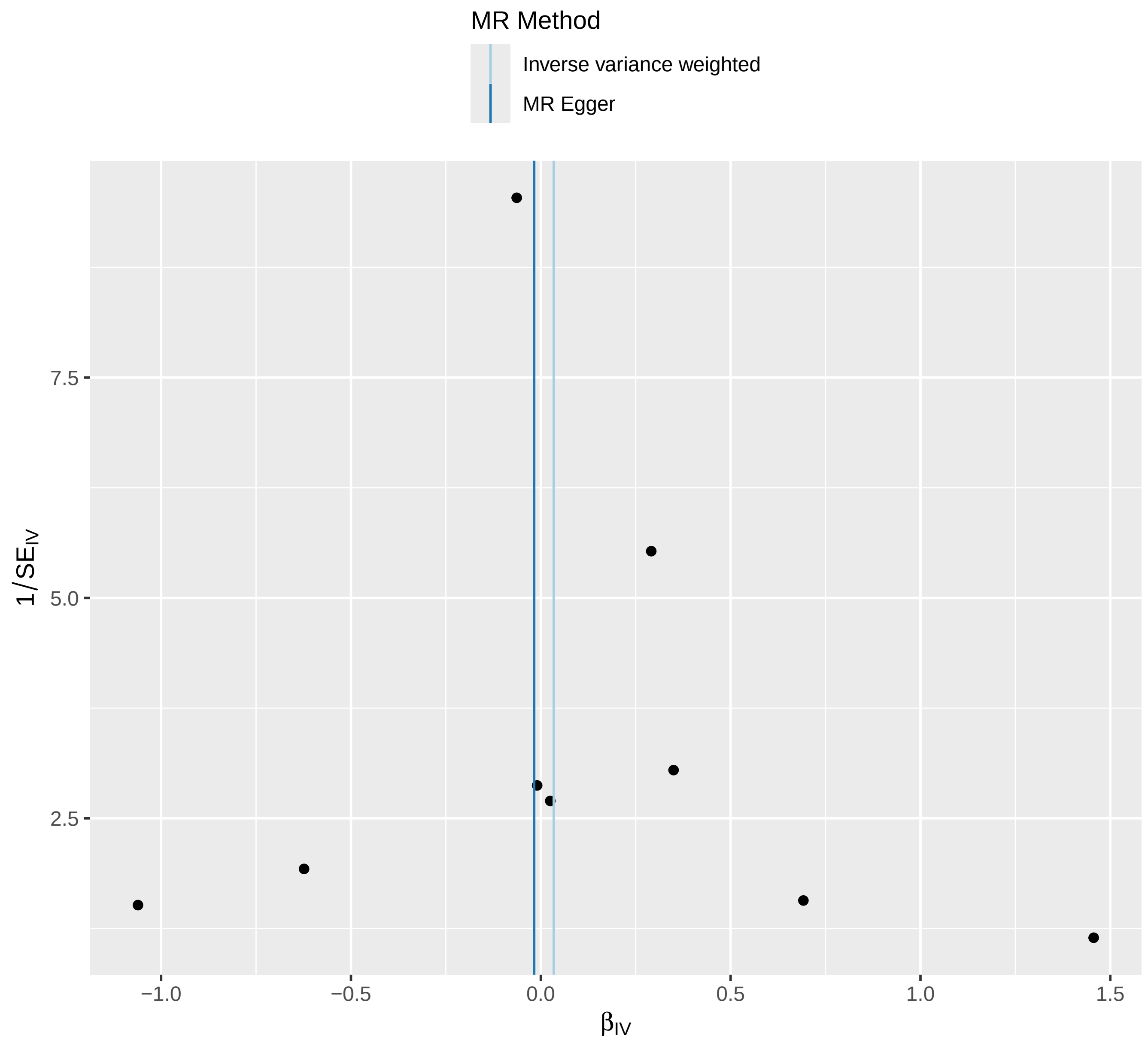
MR Method
Inverse variance weighted
MR Egger
7.5
5.0
2.5
1/SE$_{IV}$
−1.0
−0.5
0.0
0.5
1.0
1.5
β$_{IV}$

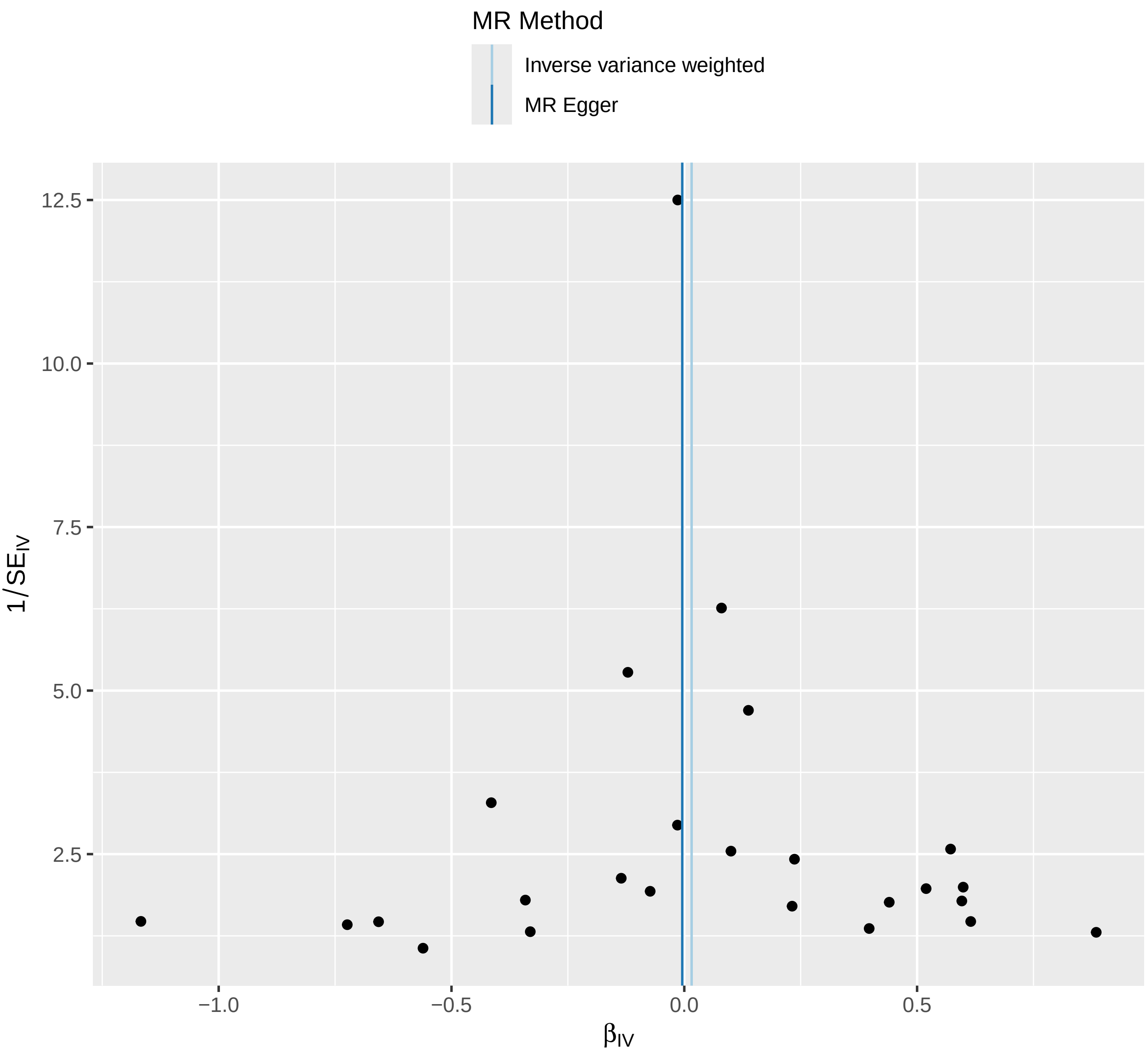

MR Method
Inverse variance weighted
MR Egger
12.5
10.0
7.5
5.0
2.5
$1/SE_{IV}$
−1.0
−0.5
0.0
0.5
β_{IV}

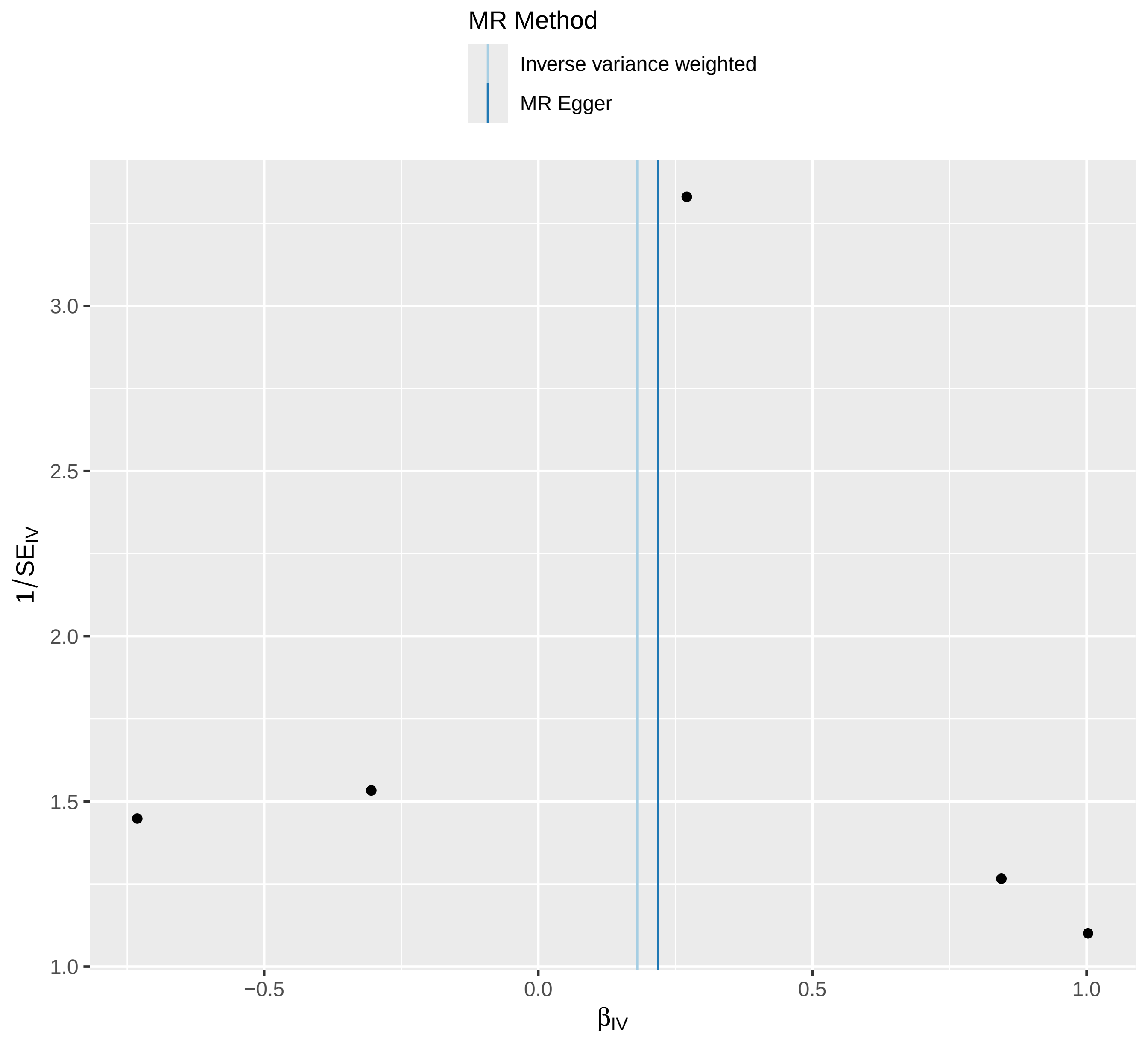
MR Method
Inverse variance weighted
MR Egger
1/SE_IV
3.0
2.5
2.0
1.5
1.0
−0.5
0.0
0.5
1.0
β_IV

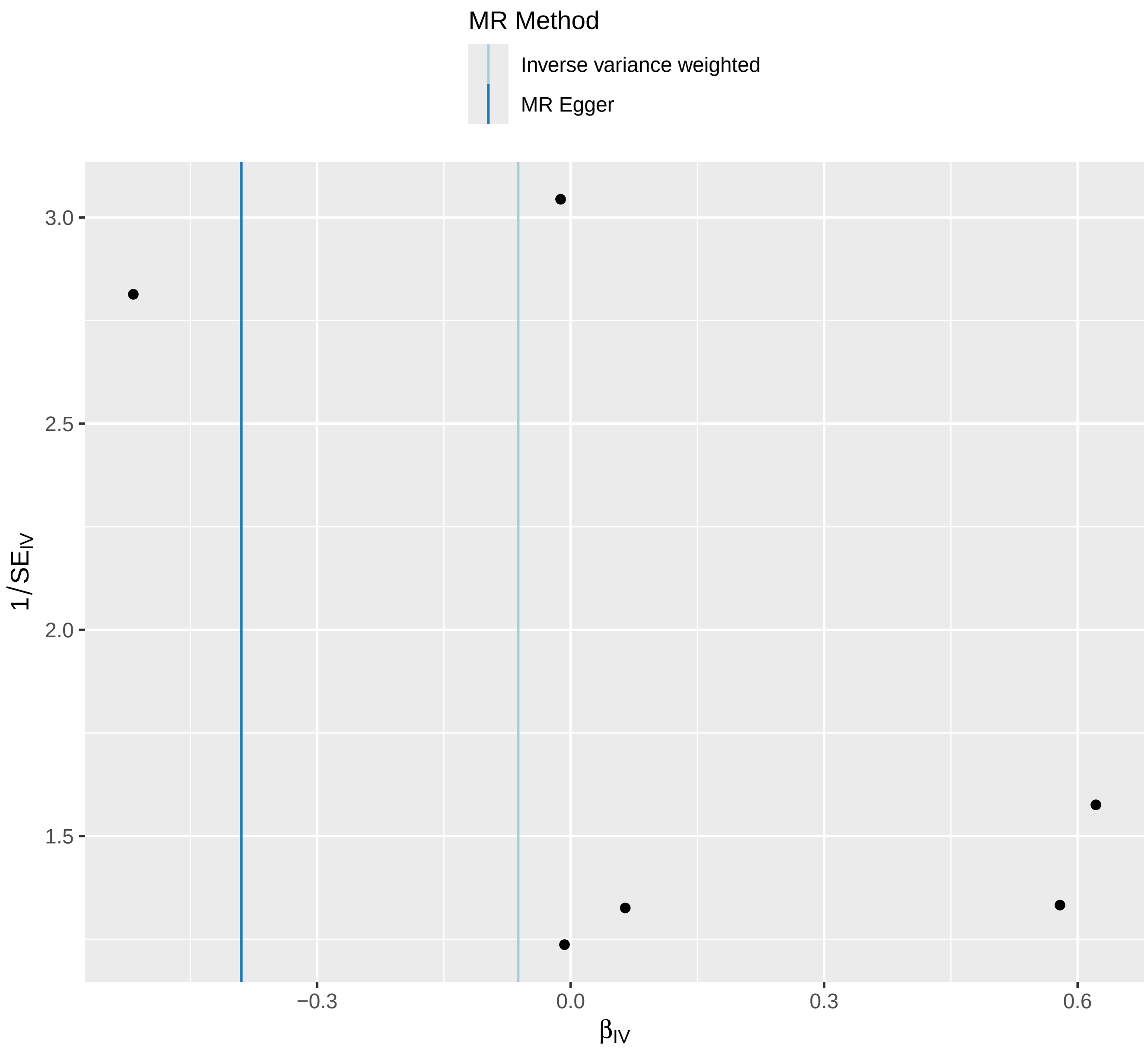

MR Method
Inverse variance weighted
MR Egger
3.0
2.5
2.0
1.5
$1/SE_{IV}$
−0.3
0.0
0.3
0.6
β_{IV}

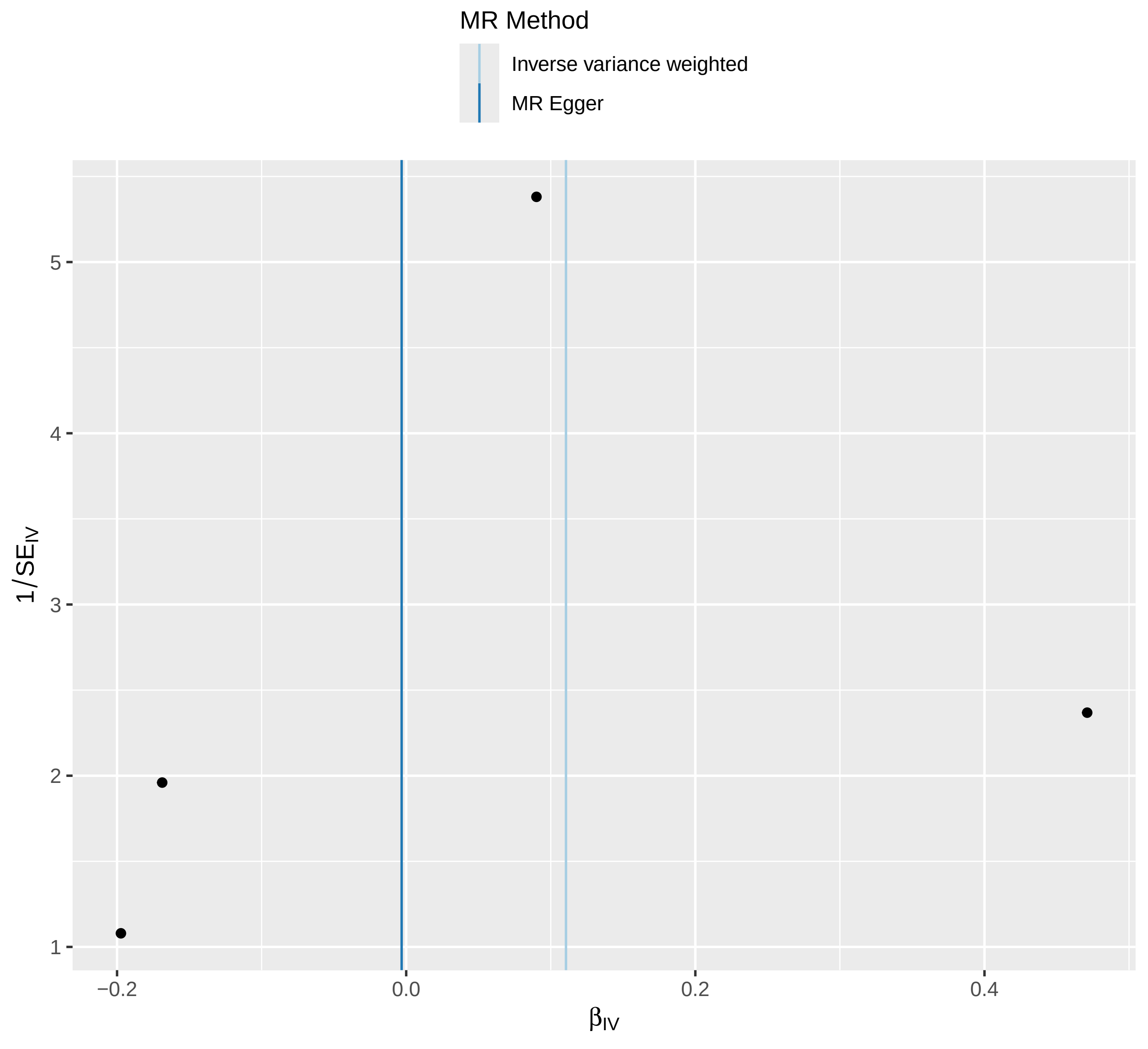

MR Method
Inverse variance weighted
MR Egger
5
4
3
2
1
$1/SE_{IV}$
−0.2
0.0
0.2
0.4
β_{IV}

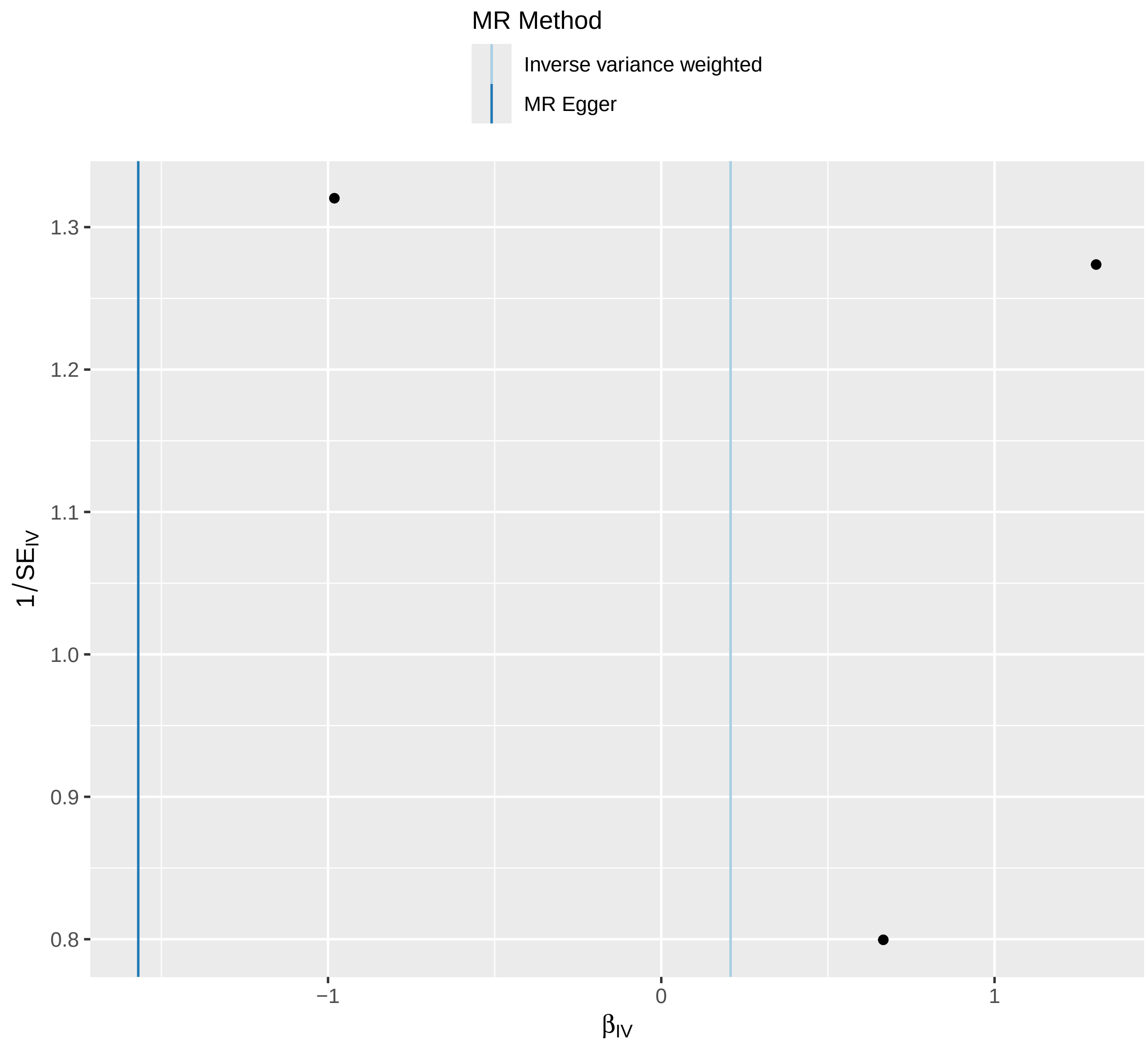
MR Method
Inverse variance weighted
MR Egger
1.3
1.2
1.1
1.0
0.9
0.8
1/SE_IV
−1
0
1
β_IV

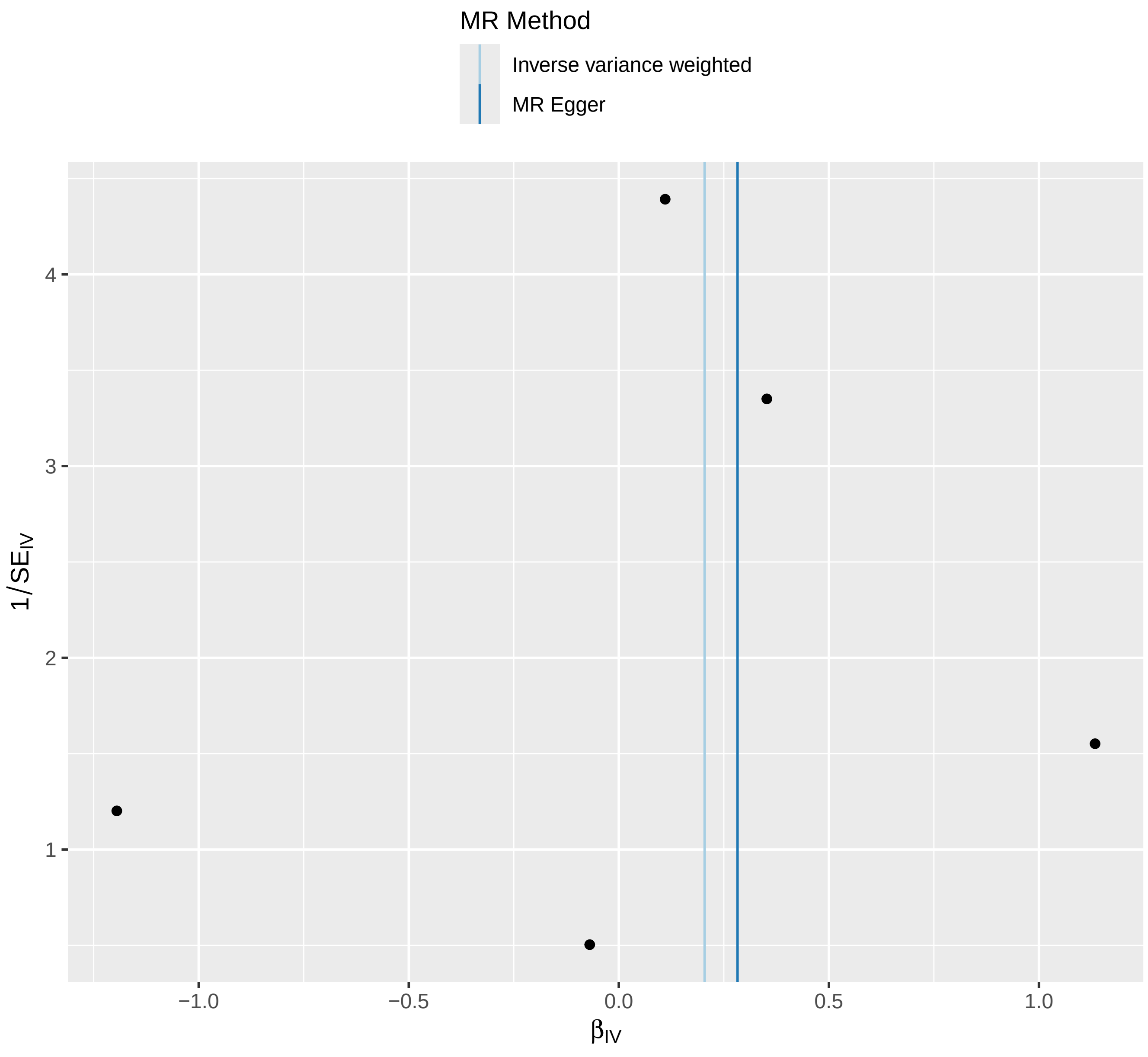

MR Method
Inverse variance weighted
MR Egger
4
3
2
1
1/SE_IV
−1.0
−0.5
0.0
0.5
1.0
β_IV

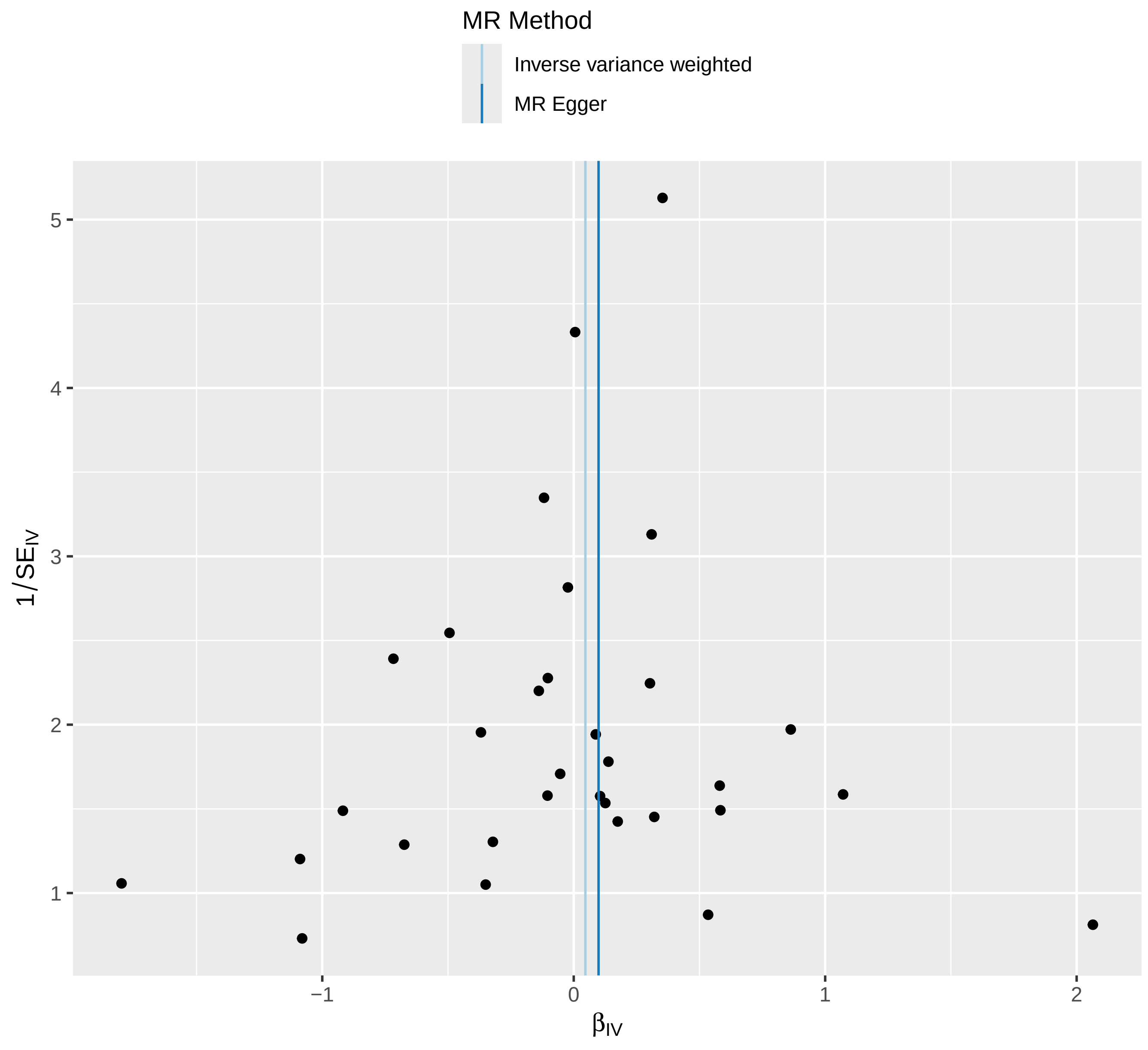

MR Method
Inverse variance weighted
MR Egger
5
4
3
2
1
1/SE_IV
−1
0
1
2
β_IV

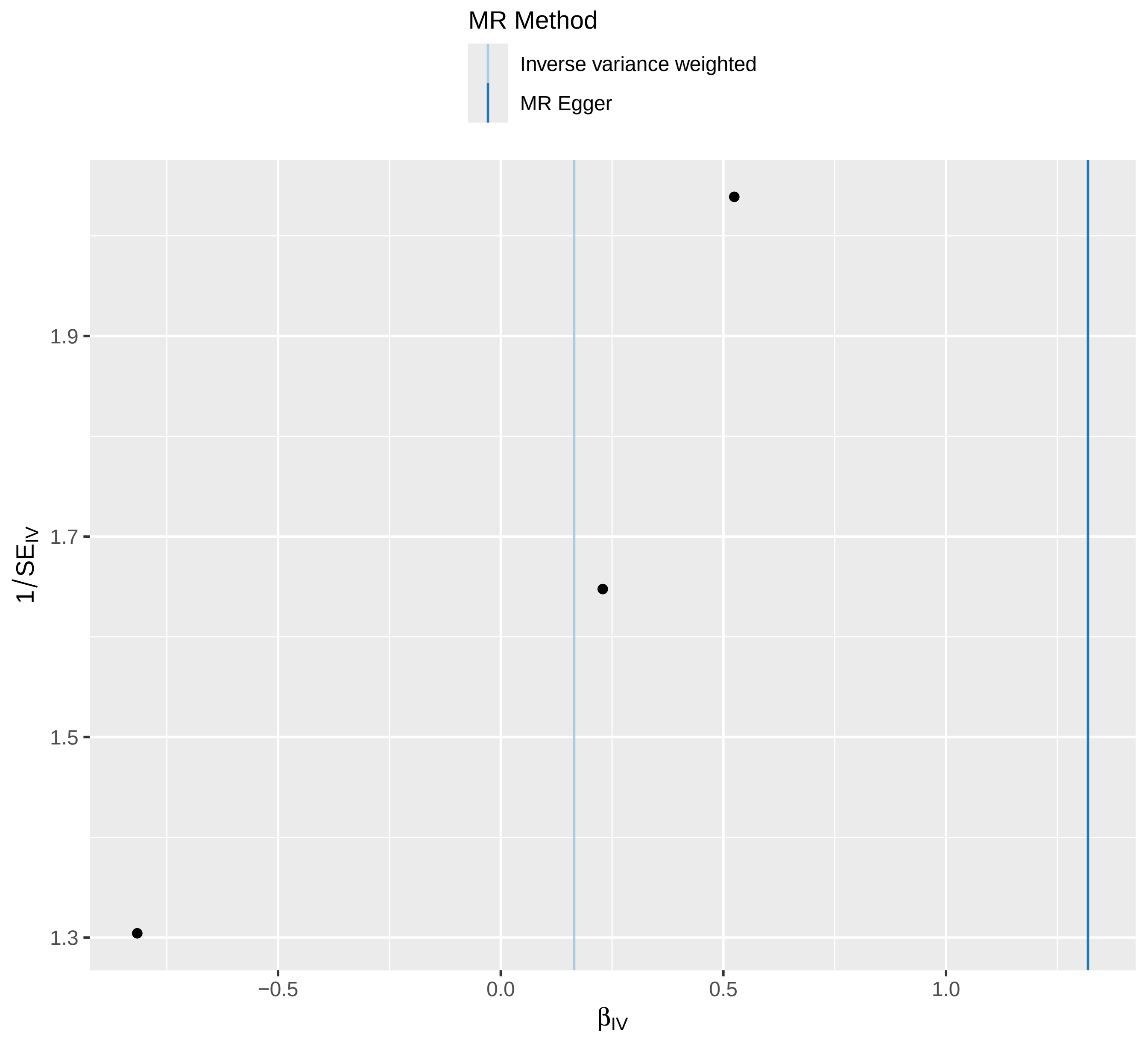
MR Method
Inverse variance weighted
MR Egger
1.9
1.7
1.5
1.3
$1/SE_{IV}$
−0.5
0.0
0.5
1.0
β_{IV}

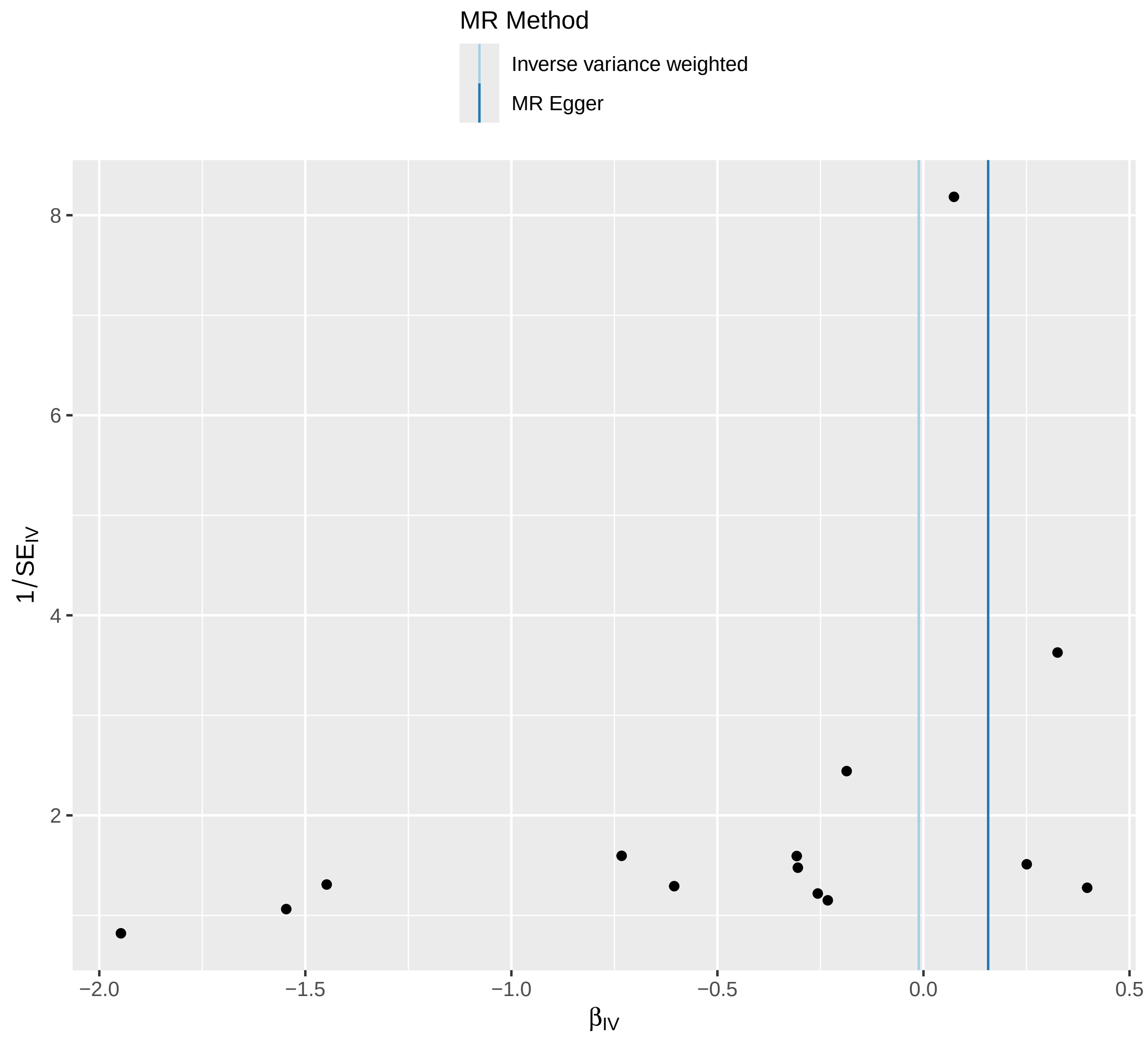
MR Method
Inverse variance weighted
MR Egger
8
6
4
2
1/SE$_{IV}$
−2.0
−1.5
−1.0
−0.5
0.0
0.5
β$_{IV}$

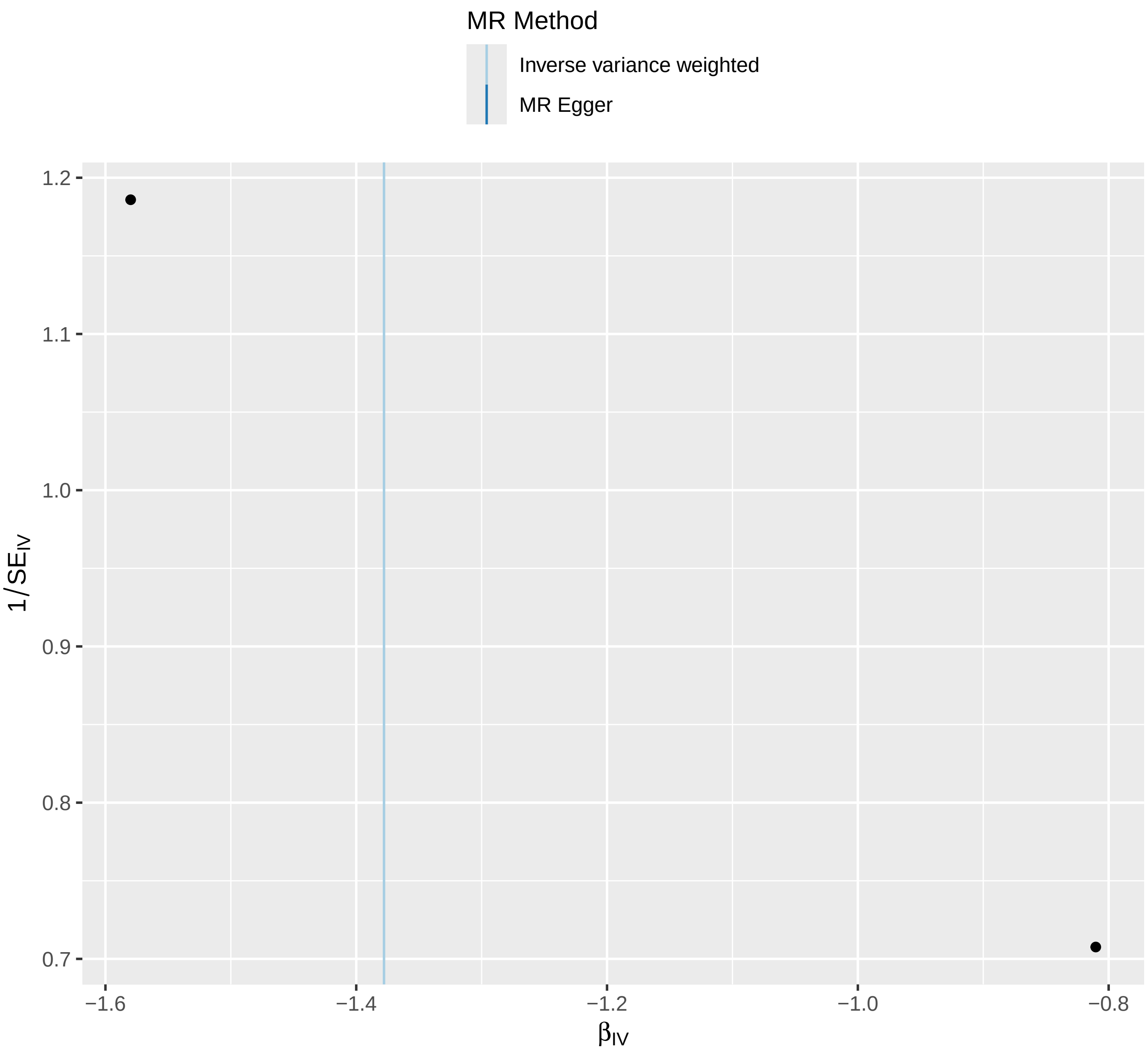

MR Method
Inverse variance weighted
MR Egger
1.2
1.1
1.0
0.9
0.8
0.7
1/SE_IV
−1.6
−1.4
−1.2
−1.0
−0.8
β_IV

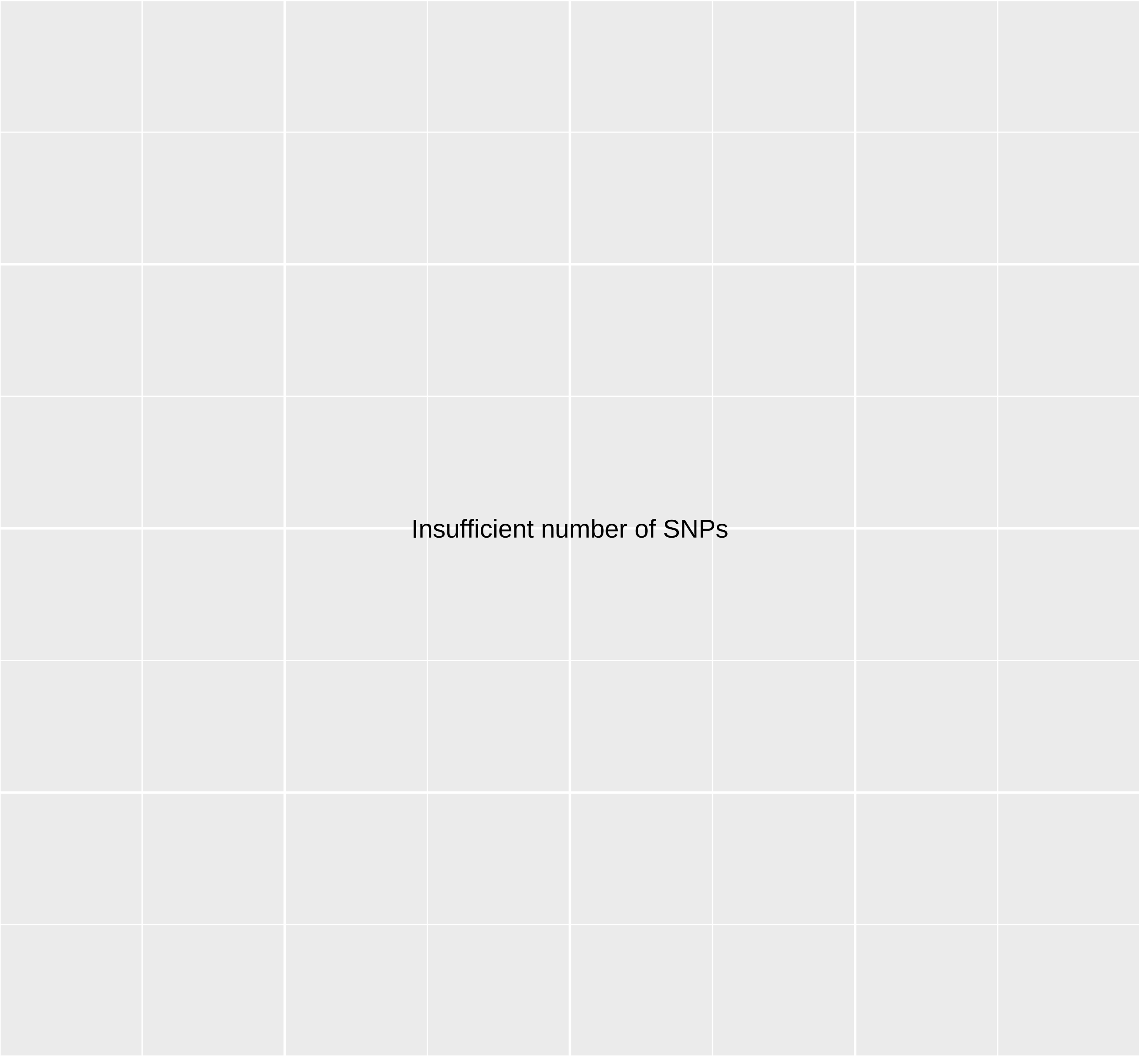
Insufficient number of SNPs

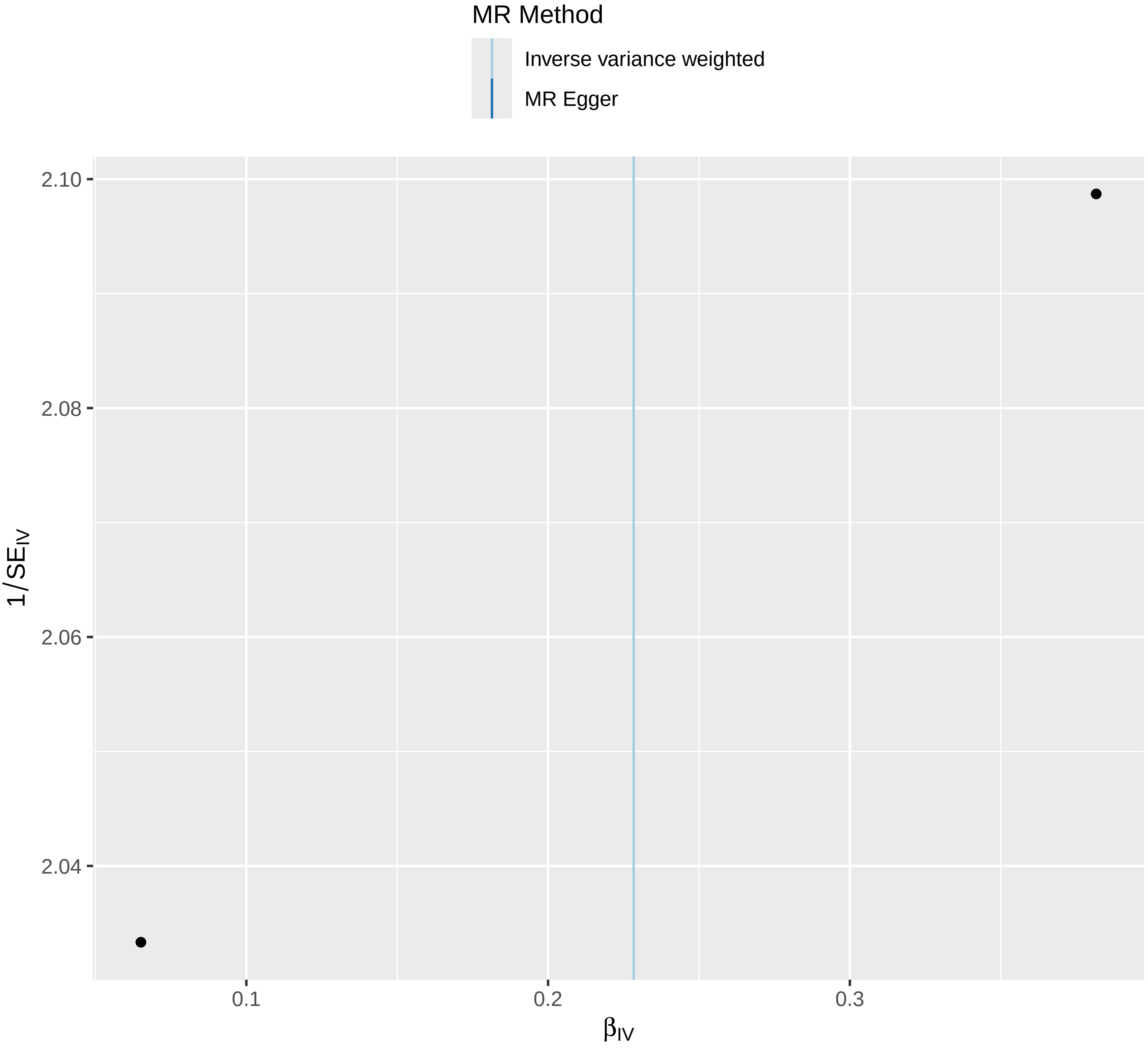
MR Method
Inverse variance weighted
MR Egger
2.10
2.08
2.06
2.04
0.1
0.2
0.3
$1/SE_{IV}$
β_{IV}

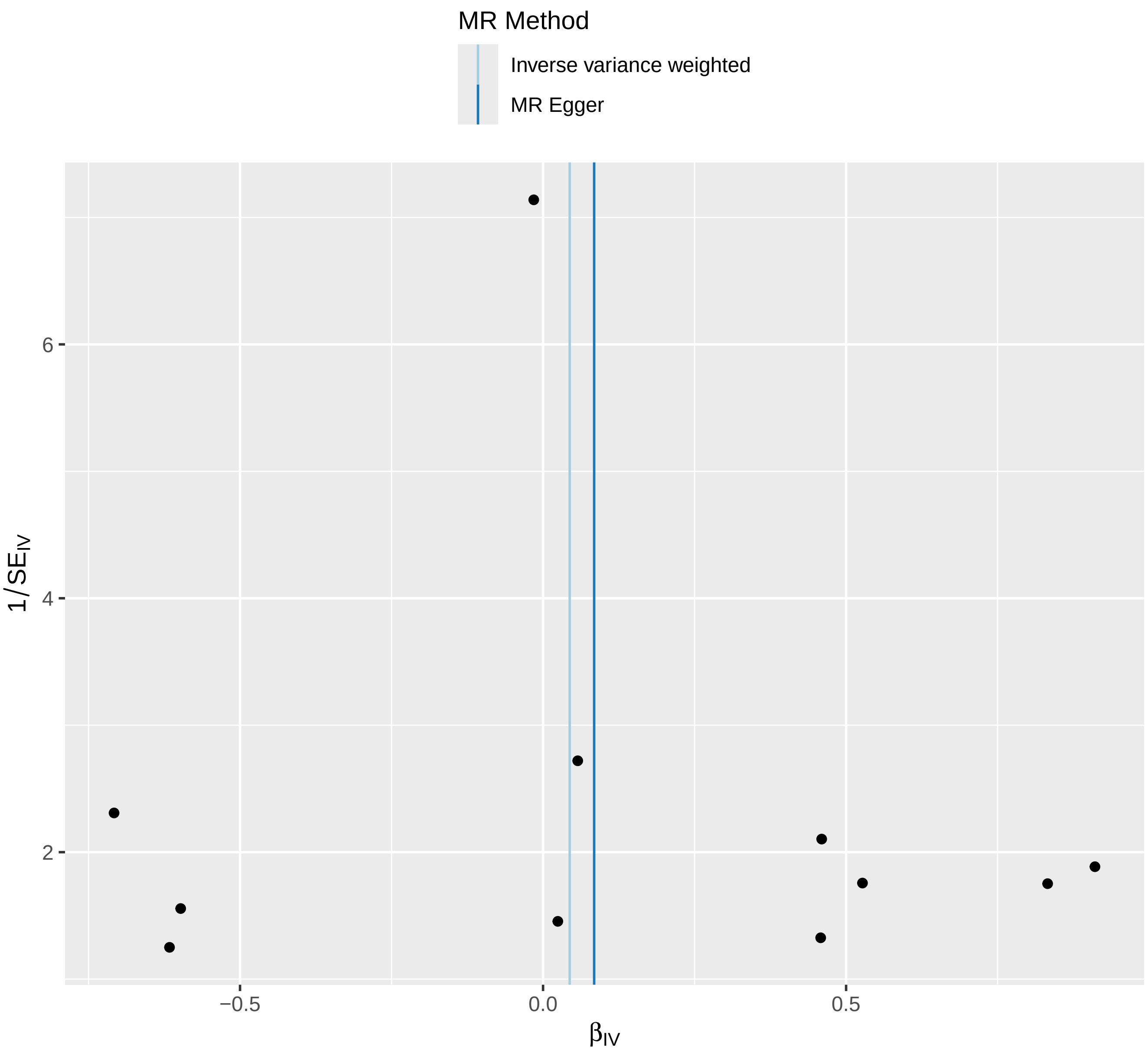
MR Method
Inverse variance weighted
MR Egger
6
4
2
1/SE$_{IV}$
−0.5
0.0
0.5
β$_{IV}$

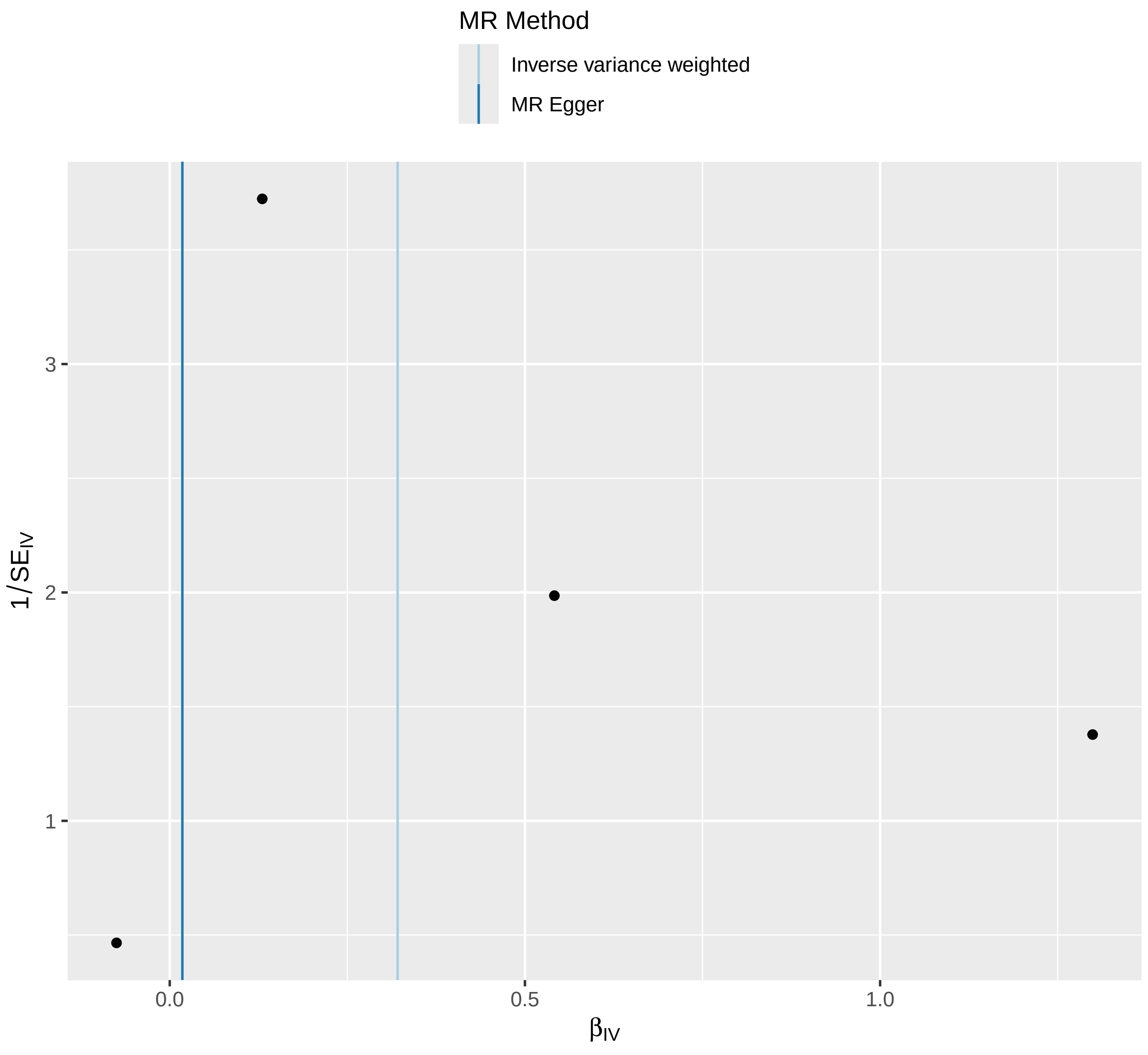

MR Method
Inverse variance weighted
MR Egger
3
2
1
1/SE_IV
0.0
0.5
1.0
β_IV

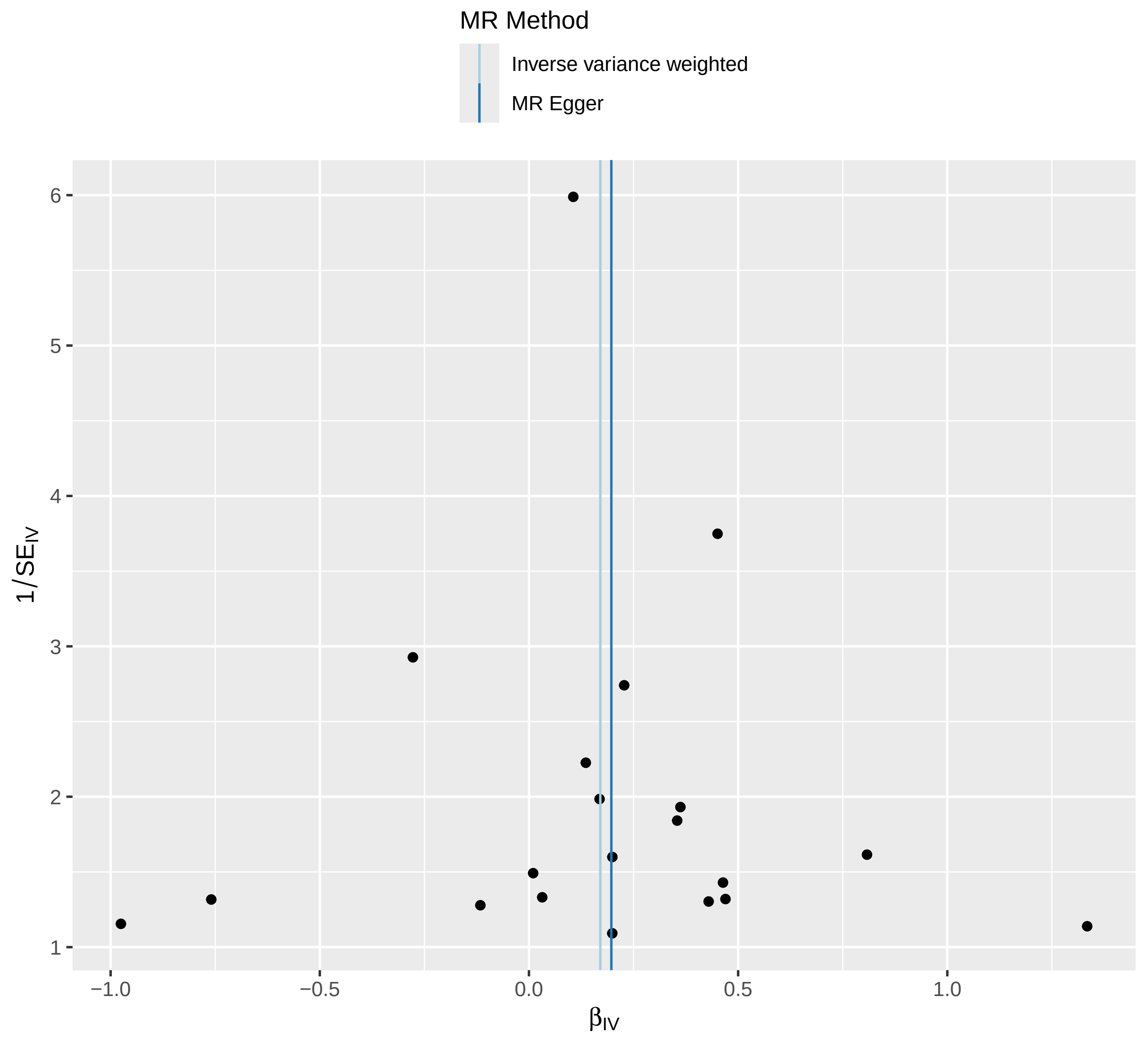
MR Method
Inverse variance weighted
MR Egger
6
5
4
3
2
1
$1/SE_{IV}$
−1.0
−0.5
0.0
0.5
1.0
β_{IV}

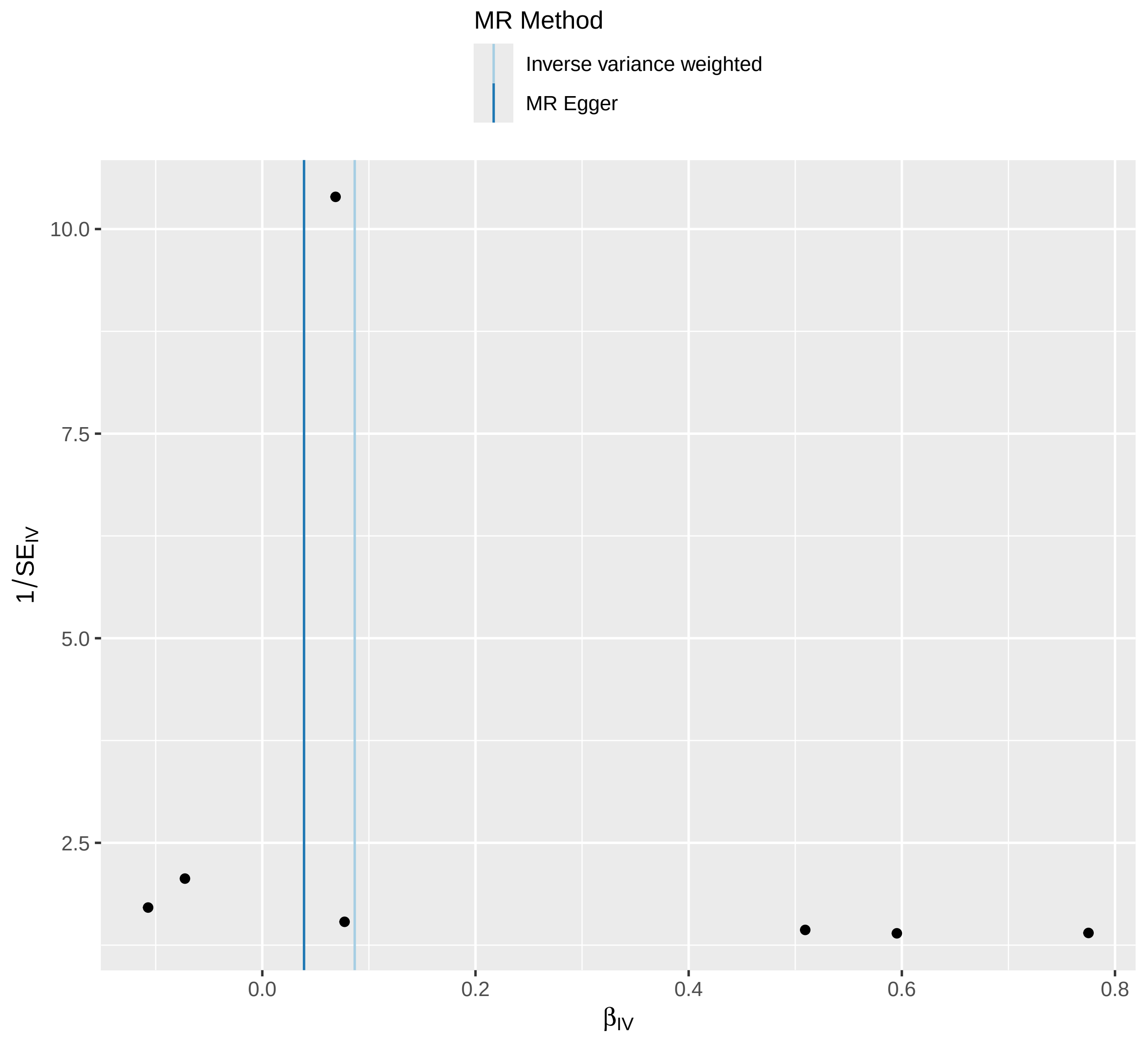
MR Method
Inverse variance weighted
MR Egger
10.0
7.5
5.0
2.5
1/SE_IV
0.0
0.2
0.4
0.6
0.8
β_IV

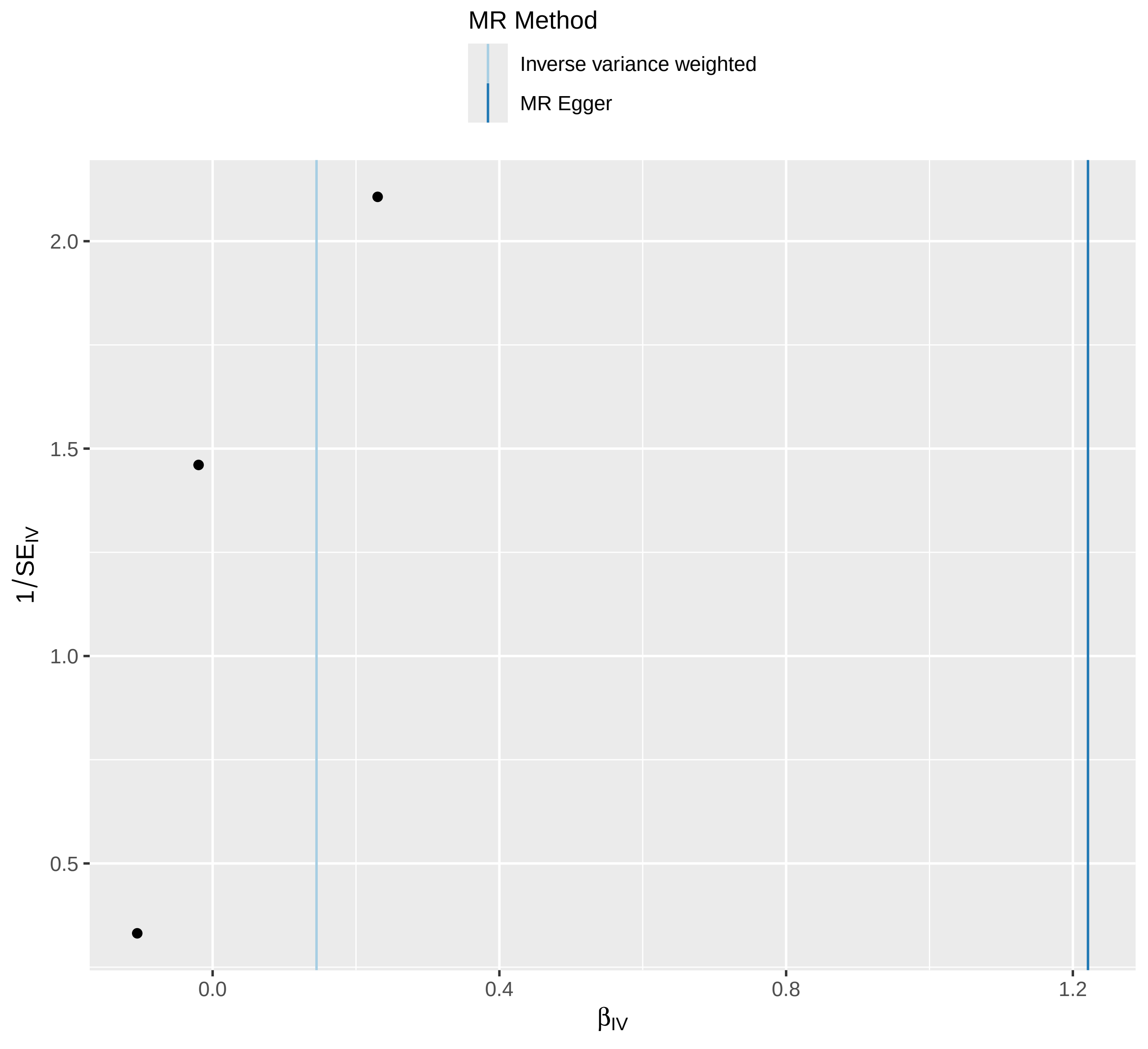
MR Method
Inverse variance weighted
MR Egger
2.0
1.5
1.0
0.5
0.0
0.4
0.8
1.2
$1/SE_{IV}$
β_{IV}

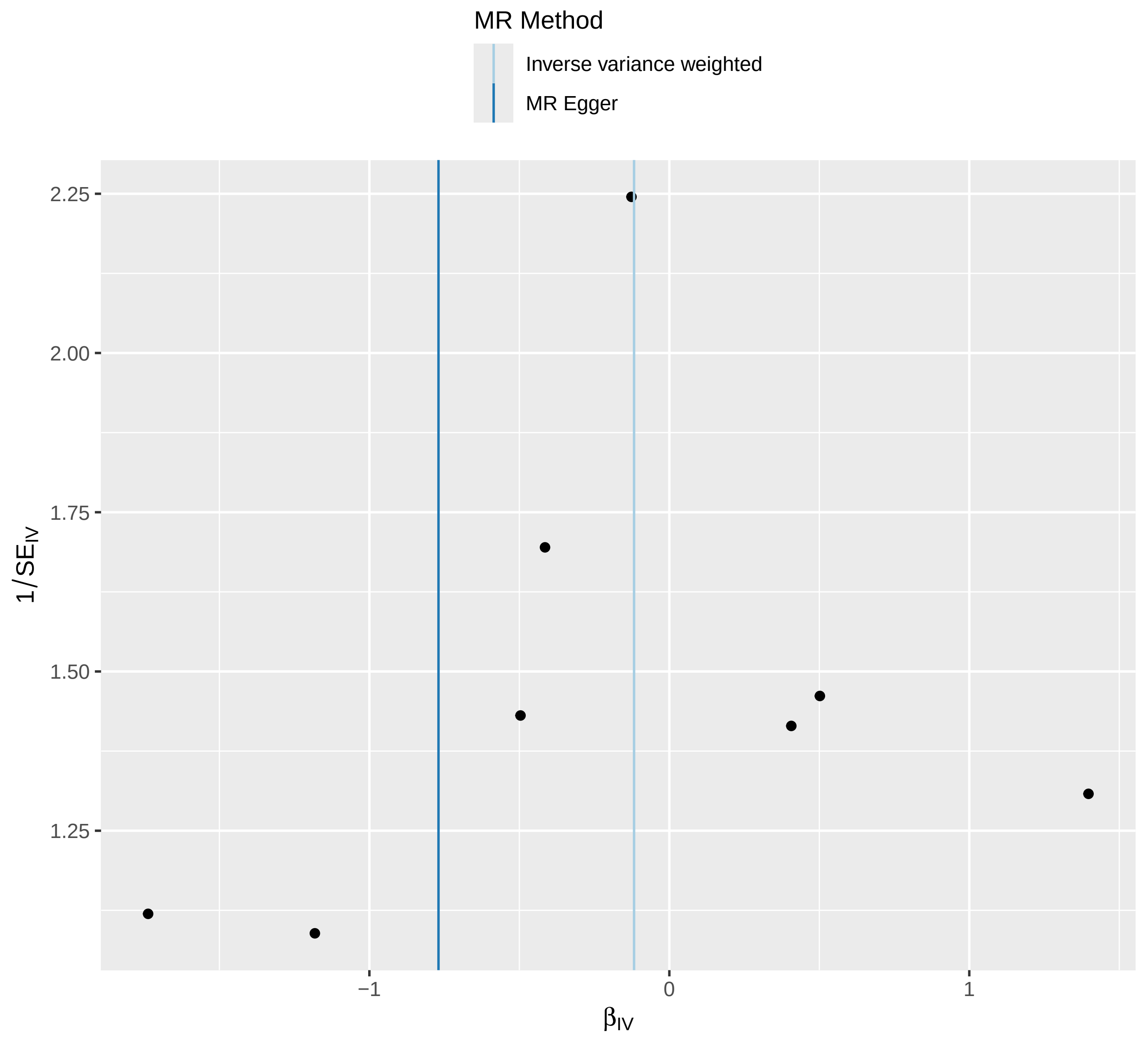
MR Method
Inverse variance weighted
MR Egger
2.25
2.00
1.75
1.50
1.25
1/SE_IV
−1
0
1
β_IV

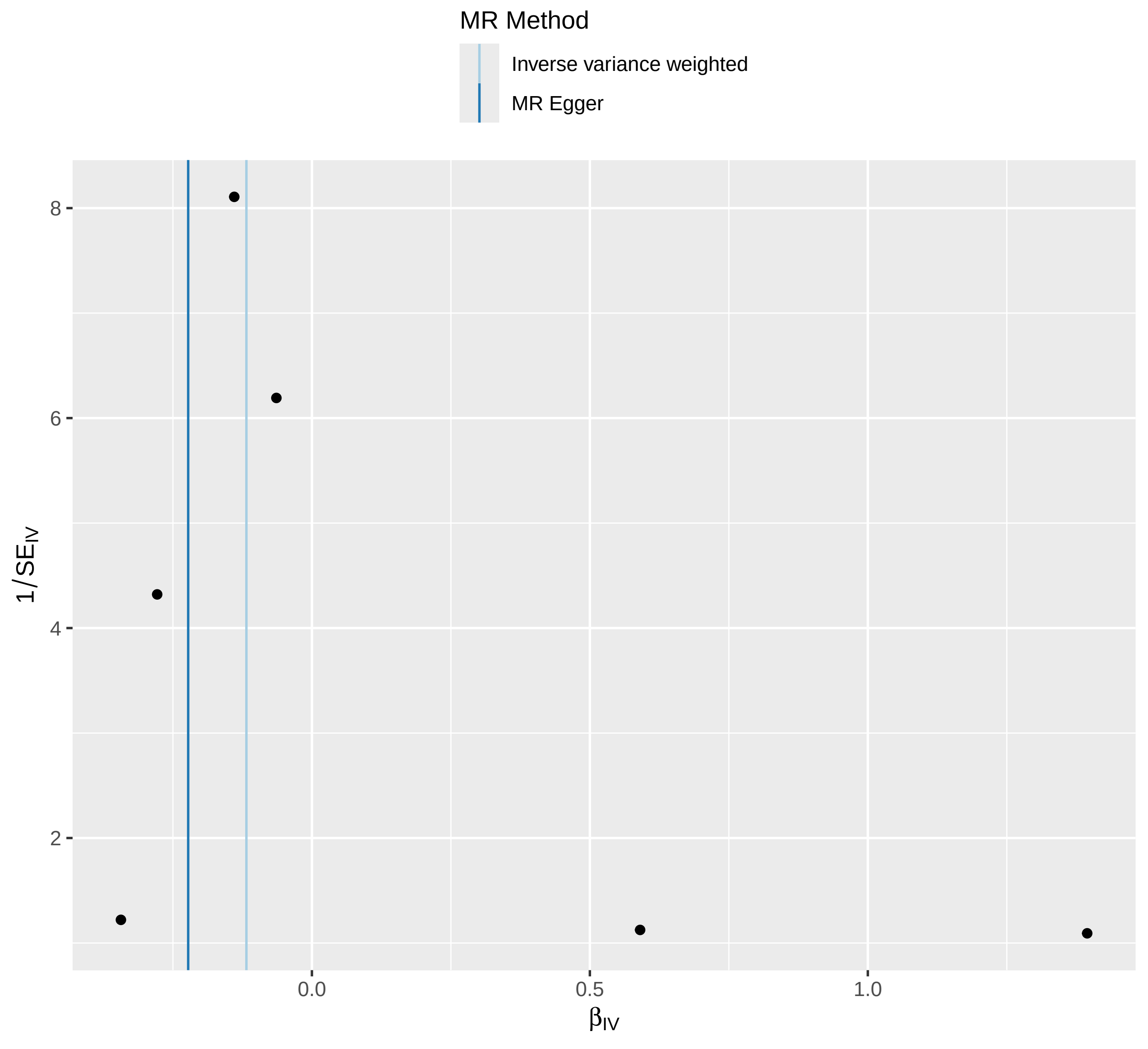

MR Method
Inverse variance weighted
MR Egger
8
6
4
2
1/SE_IV
0.0
0.5
1.0
β_IV

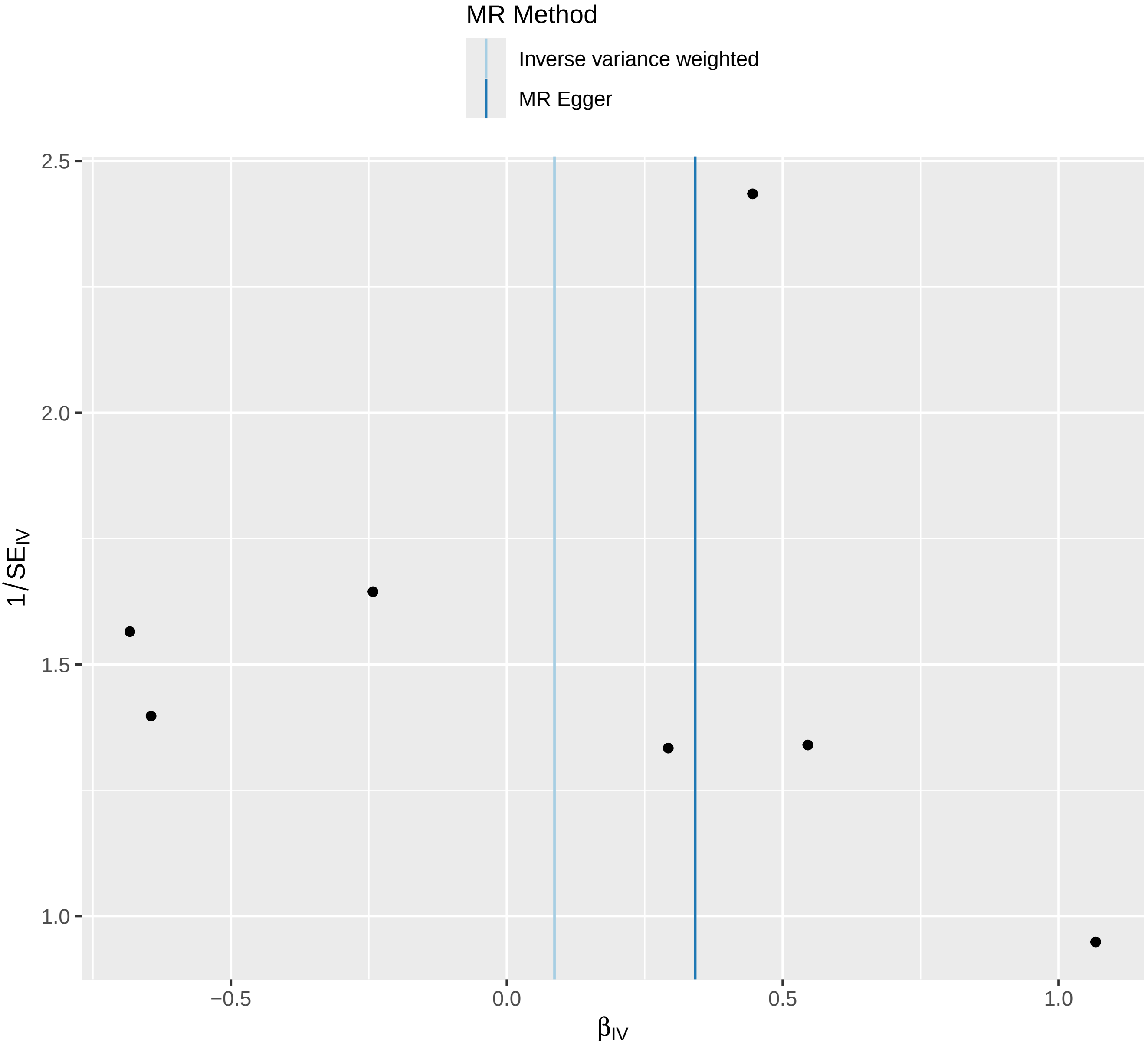
MR Method
Inverse variance weighted
MR Egger
2.5
2.0
1.5
1.0
$1/SE_{IV}$
−0.5
0.0
0.5
1.0
β_{IV}

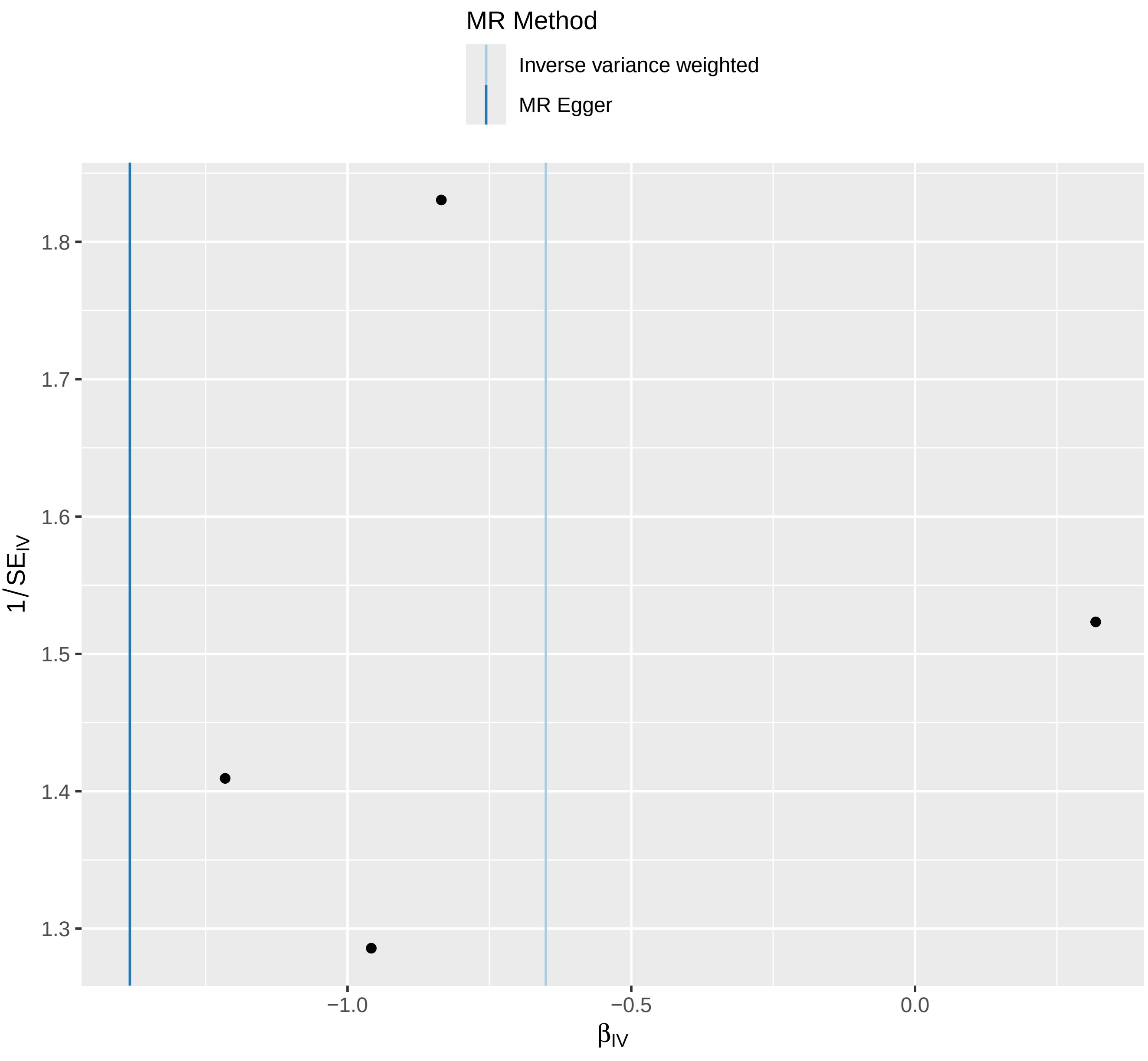
MR Method
Inverse variance weighted
MR Egger
1.8
1.7
1.6
1.5
1.4
1.3
−1.0
−0.5
0.0
$1/SE_{IV}$
β_{IV}

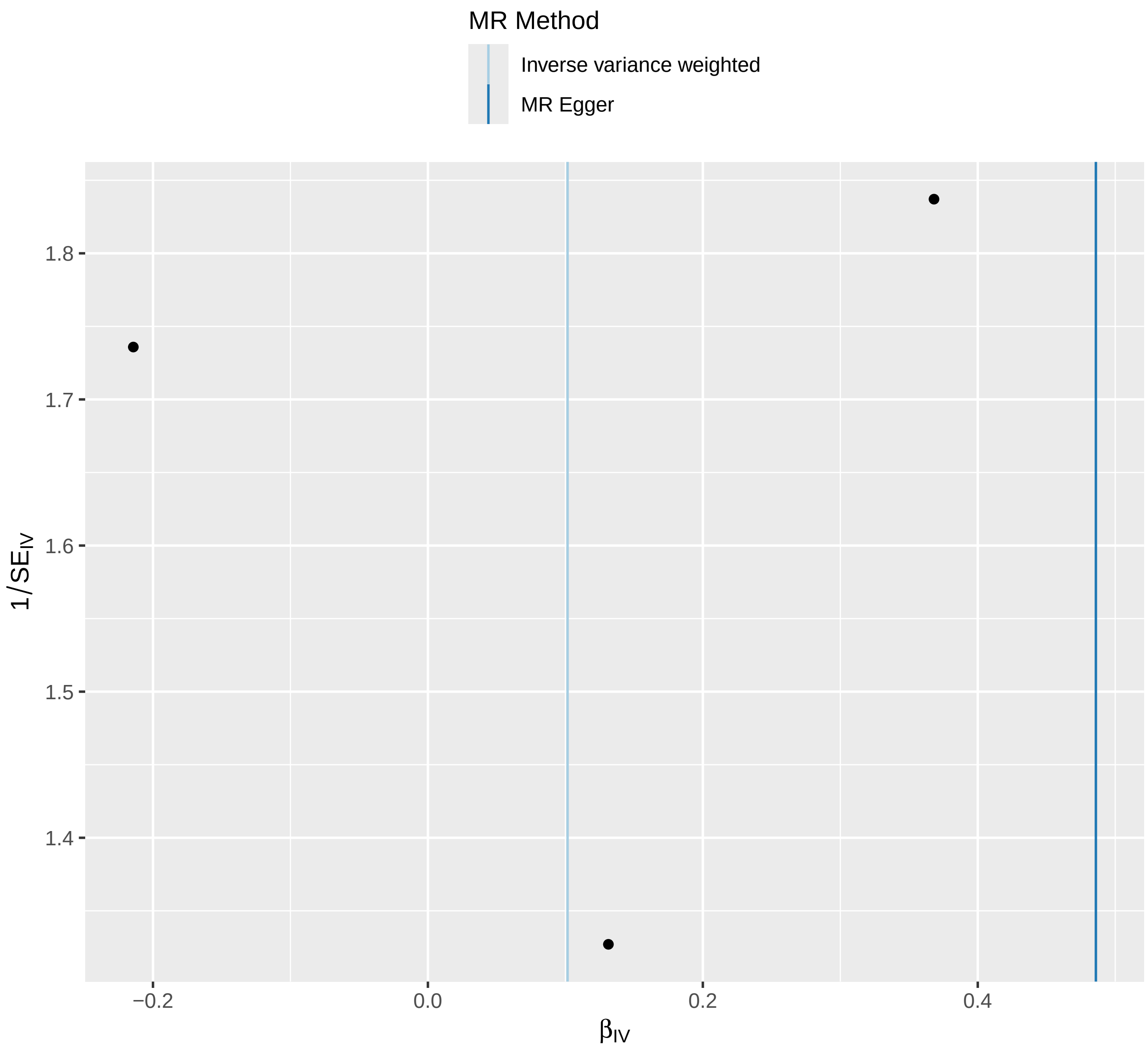
MR Method
Inverse variance weighted
MR Egger
1.8
1.7
1.6
1.5
1.4
−0.2
0.0
0.2
0.4
$1/SE_{IV}$
β_{IV}

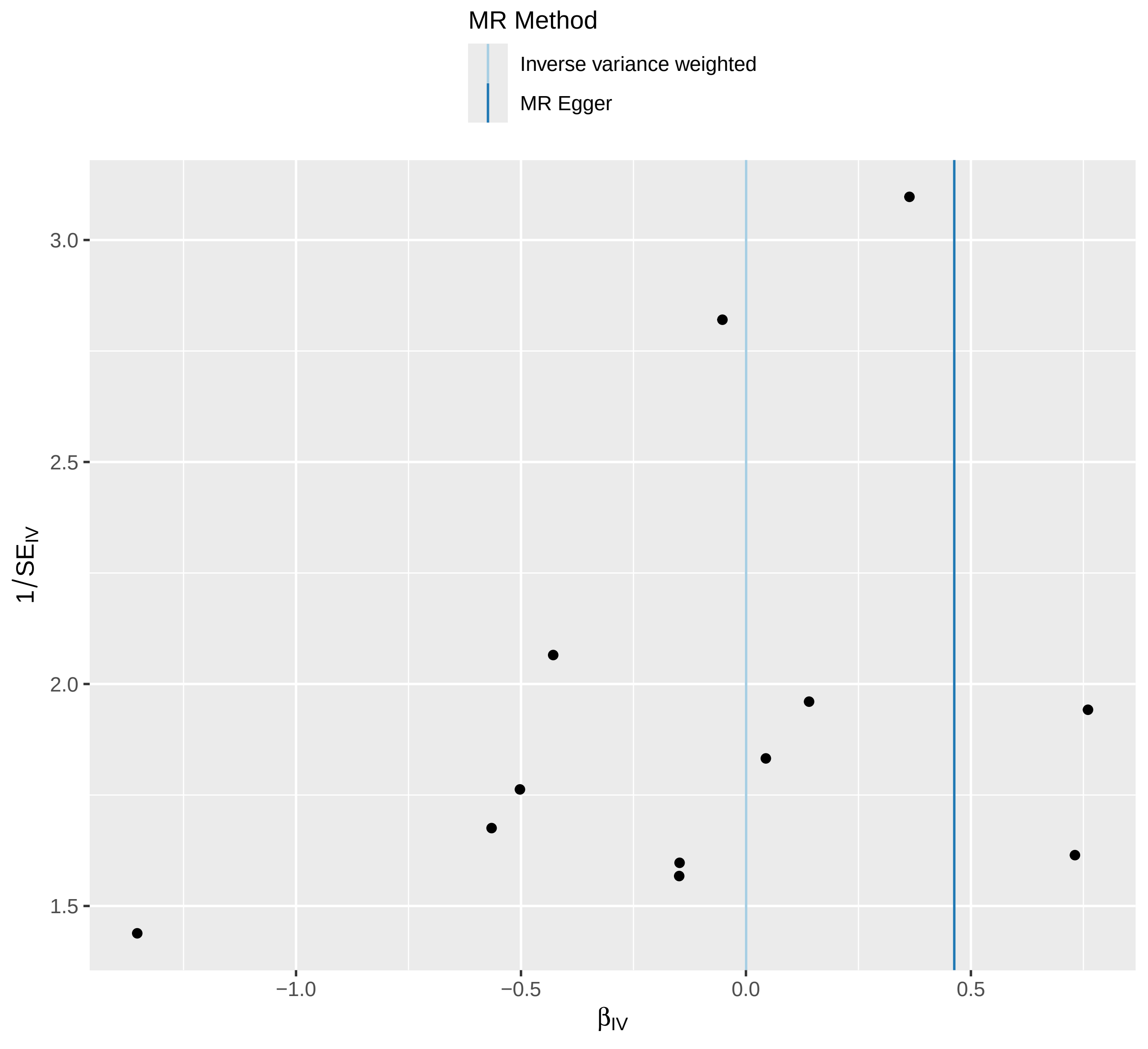
MR Method
Inverse variance weighted
MR Egger
3.0
2.5
2.0
1.5
−1.0
−0.5
0.0
0.5
$1/SE_{IV}$
β_{IV}

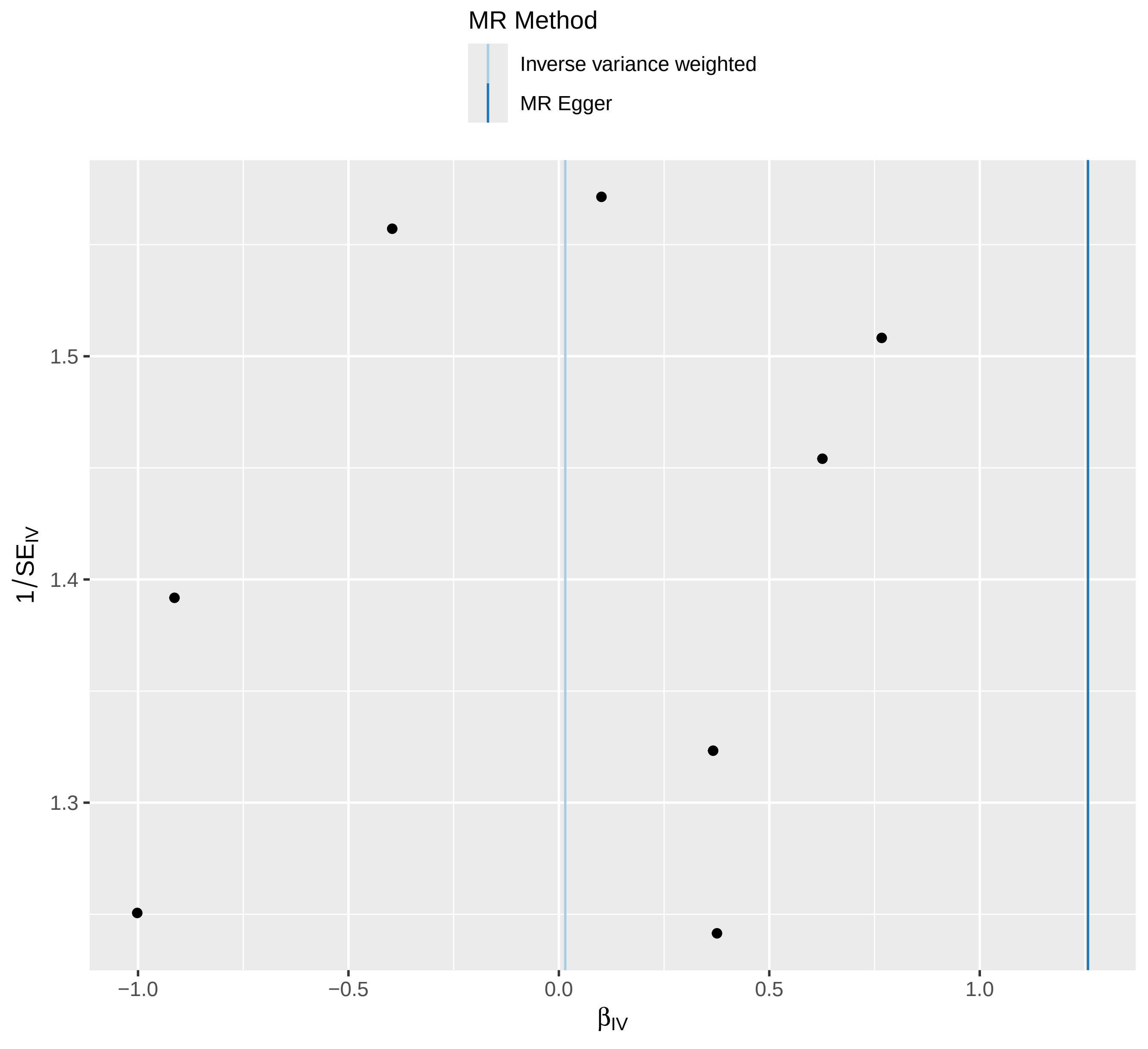

MR Method
Inverse variance weighted
MR Egger
1.5
1.4
1.3
−1.0
−0.5
0.0
0.5
1.0
1/SE$_{IV}$
β$_{IV}$

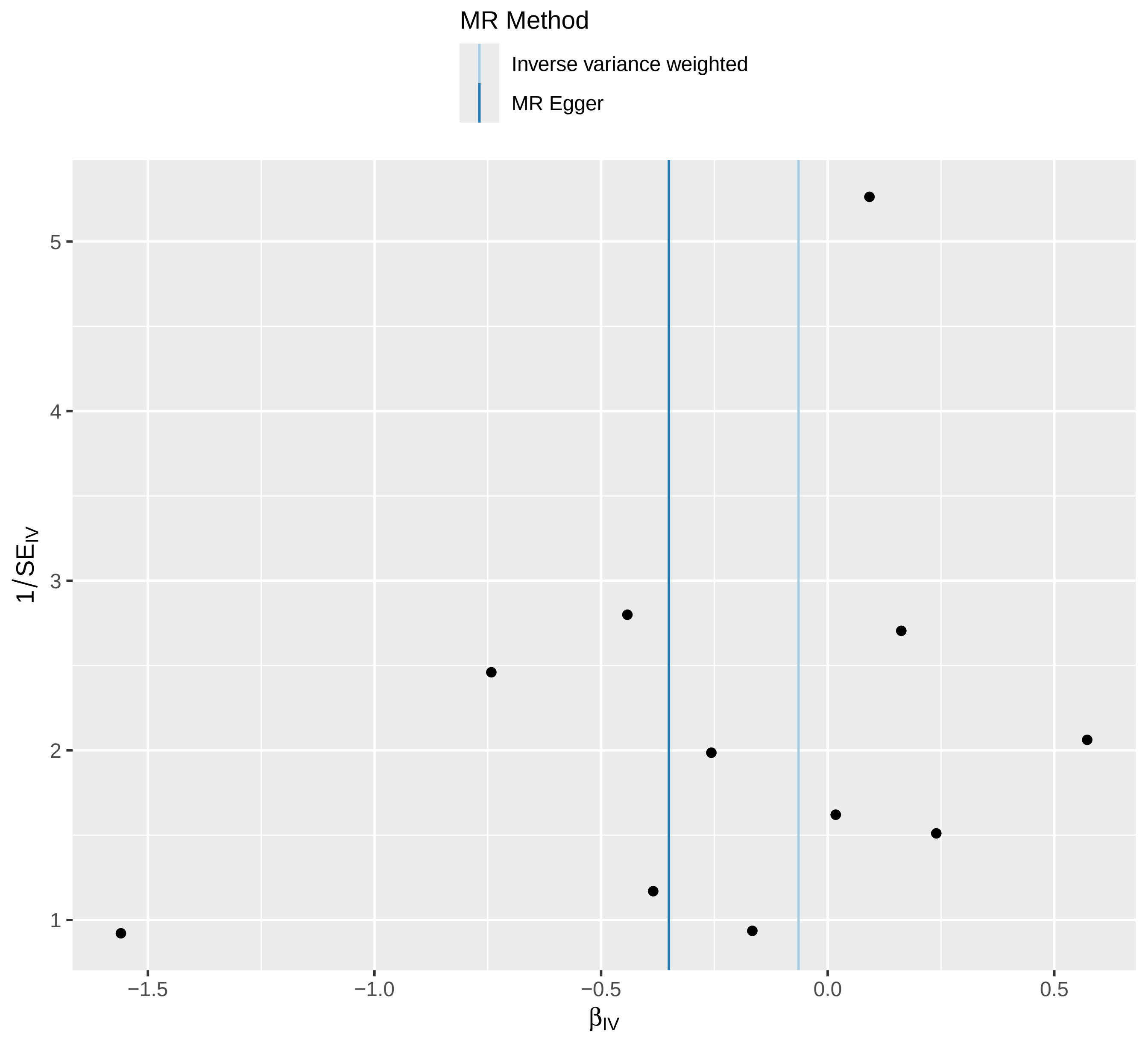

MR Method
Inverse variance weighted
MR Egger
5
4
3
2
1
1/SE_IV
−1.5
−1.0
−0.5
0.0
0.5
β_IV

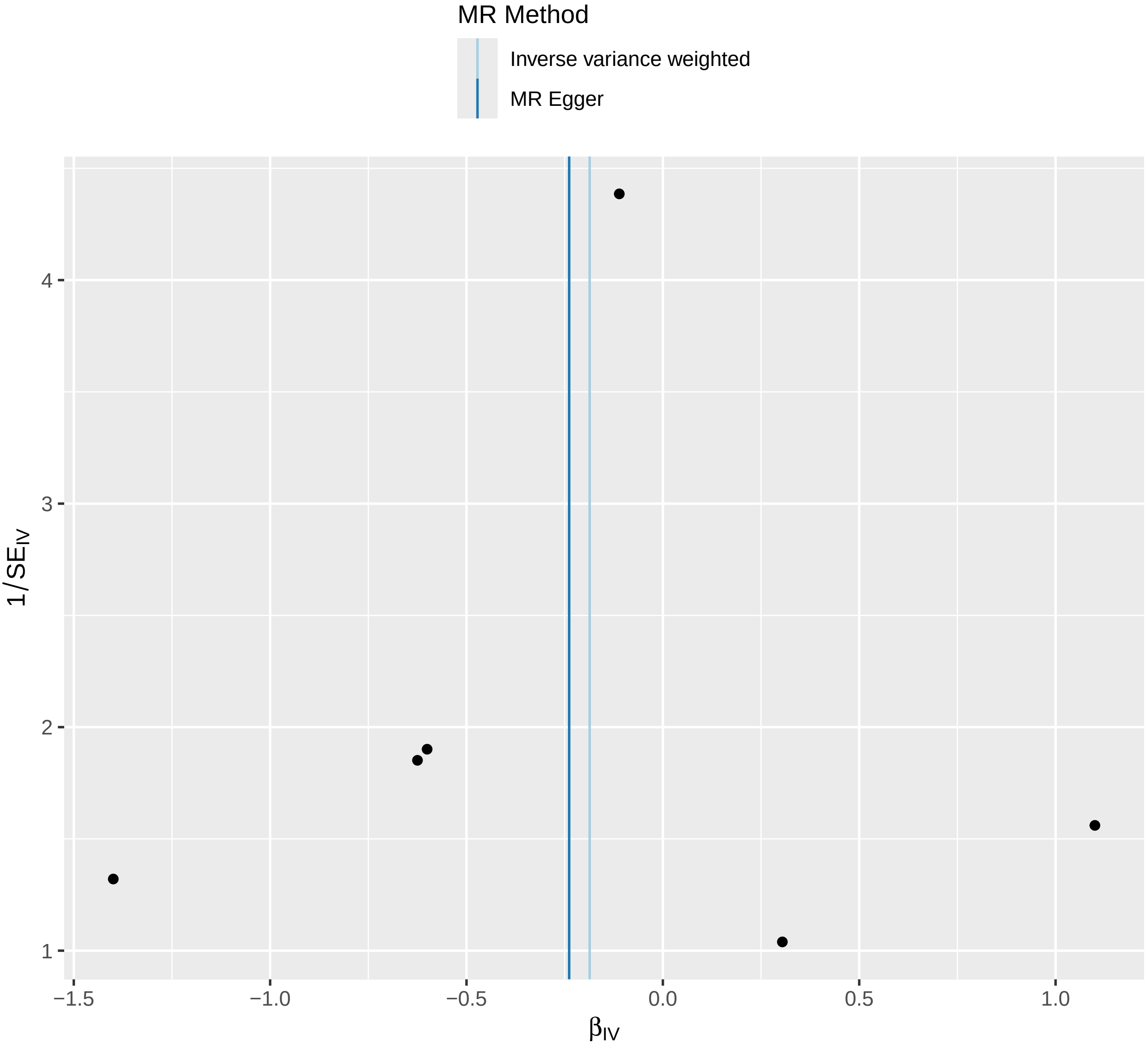

MR Method
Inverse variance weighted
MR Egger
1/SE_IV
β_IV
4
3
2
1
−1.5
−1.0
−0.5
0.0
0.5
1.0

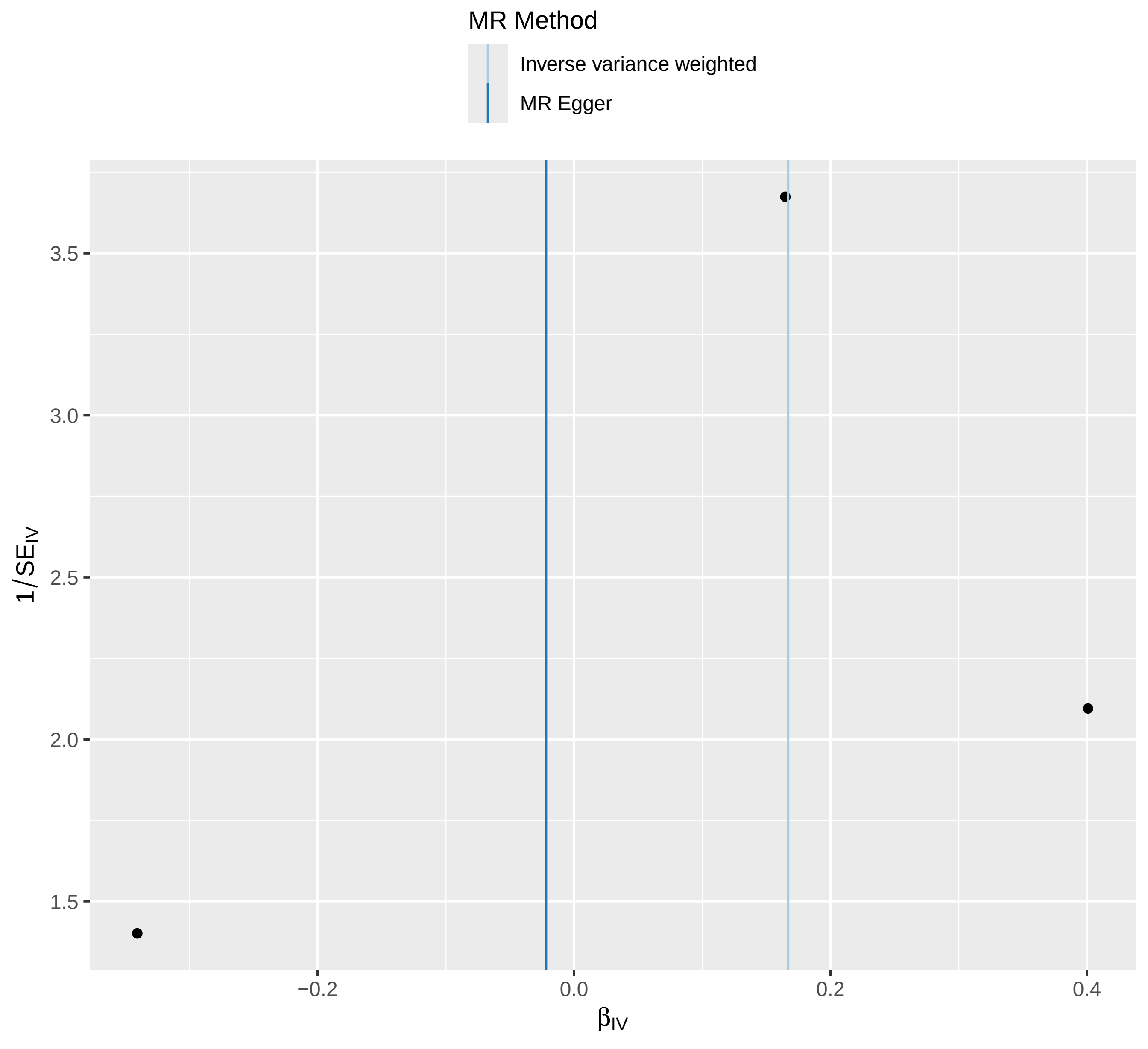

MR Method
Inverse variance weighted
MR Egger
3.5
3.0
2.5
2.0
1.5
$1/SE_{IV}$
−0.2
0.0
0.2
0.4
β_{IV}

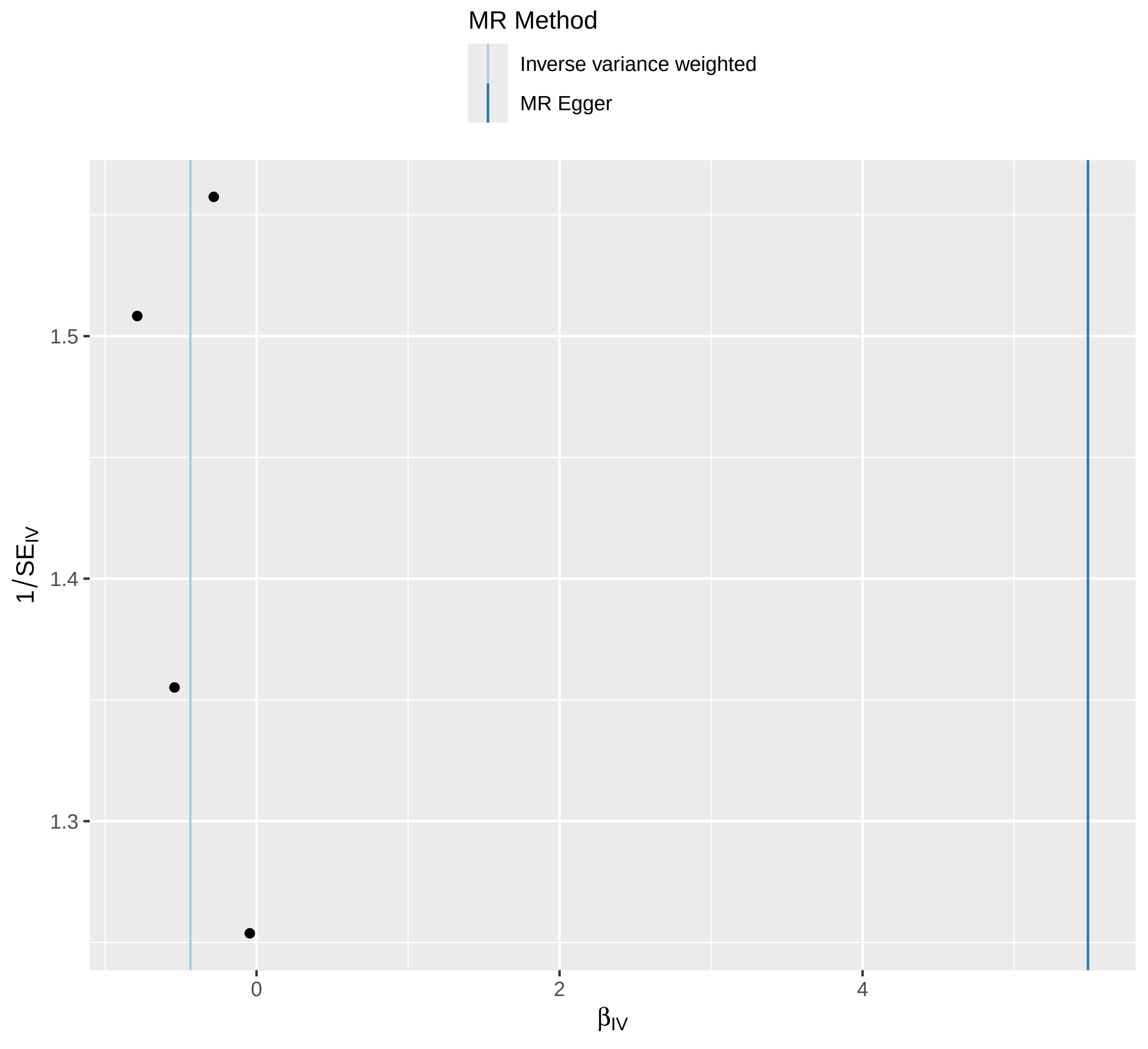

MR Method
Inverse variance weighted
MR Egger
1.5
1.4
1.3
1/SE$_{IV}$
0
2
4
β_{IV}

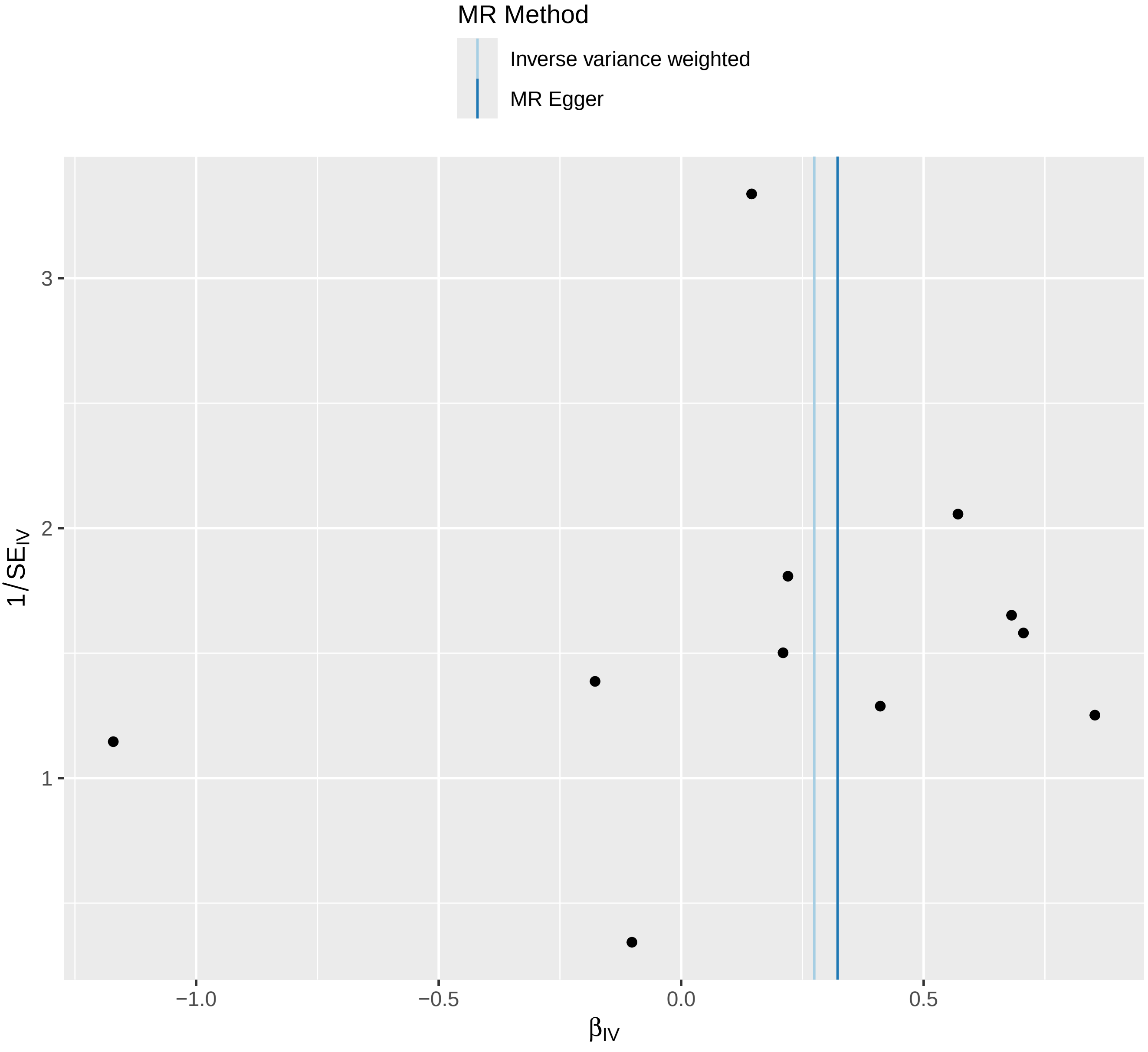
MR Method
Inverse variance weighted
MR Egger
3
2
1
1/SE_IV
−1.0
−0.5
0.0
0.5
β_IV

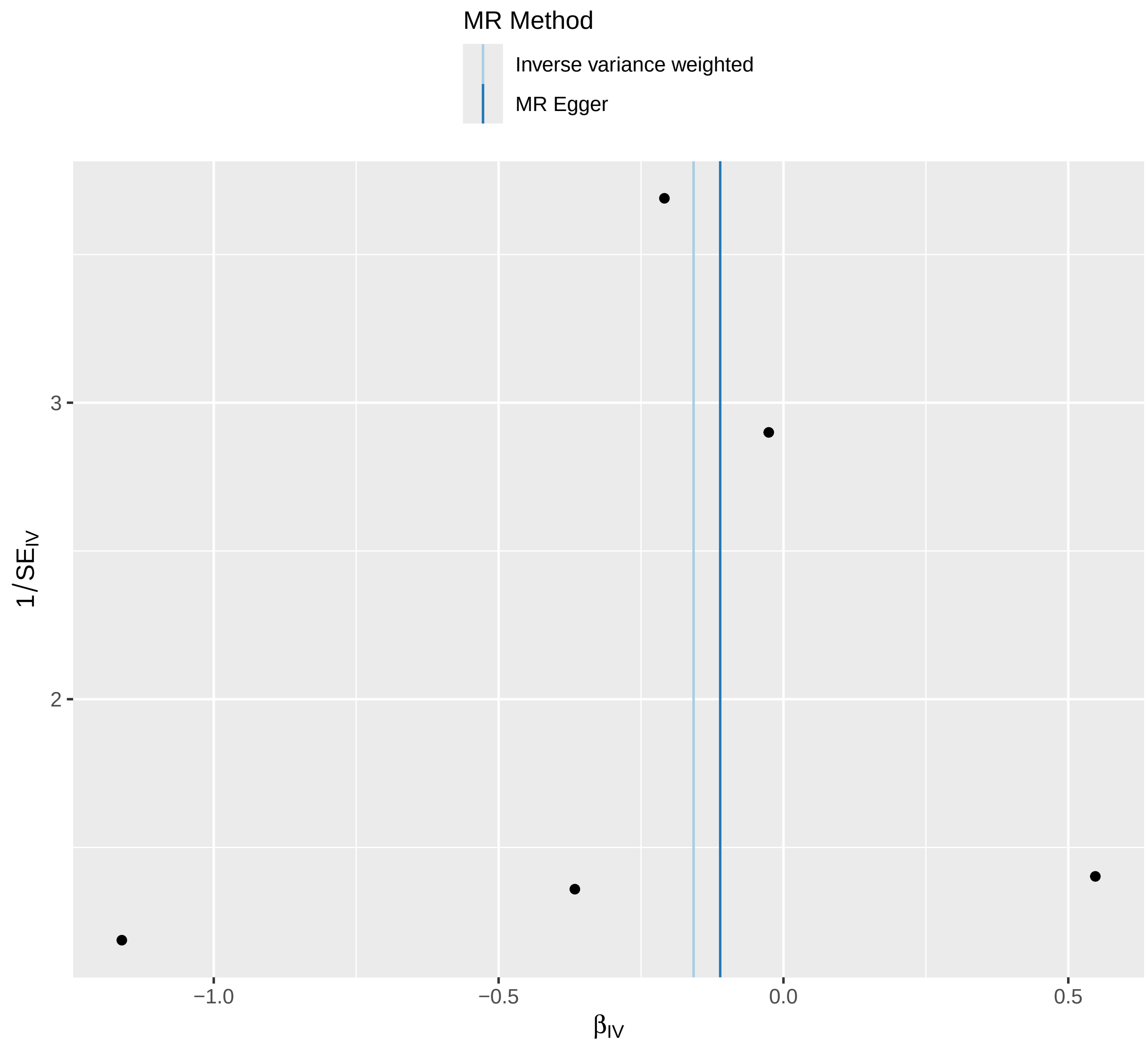

MR Method
Inverse variance weighted
MR Egger
3
2
1/SE_{IV}
−1.0
−0.5
0.0
0.5
β_{IV}

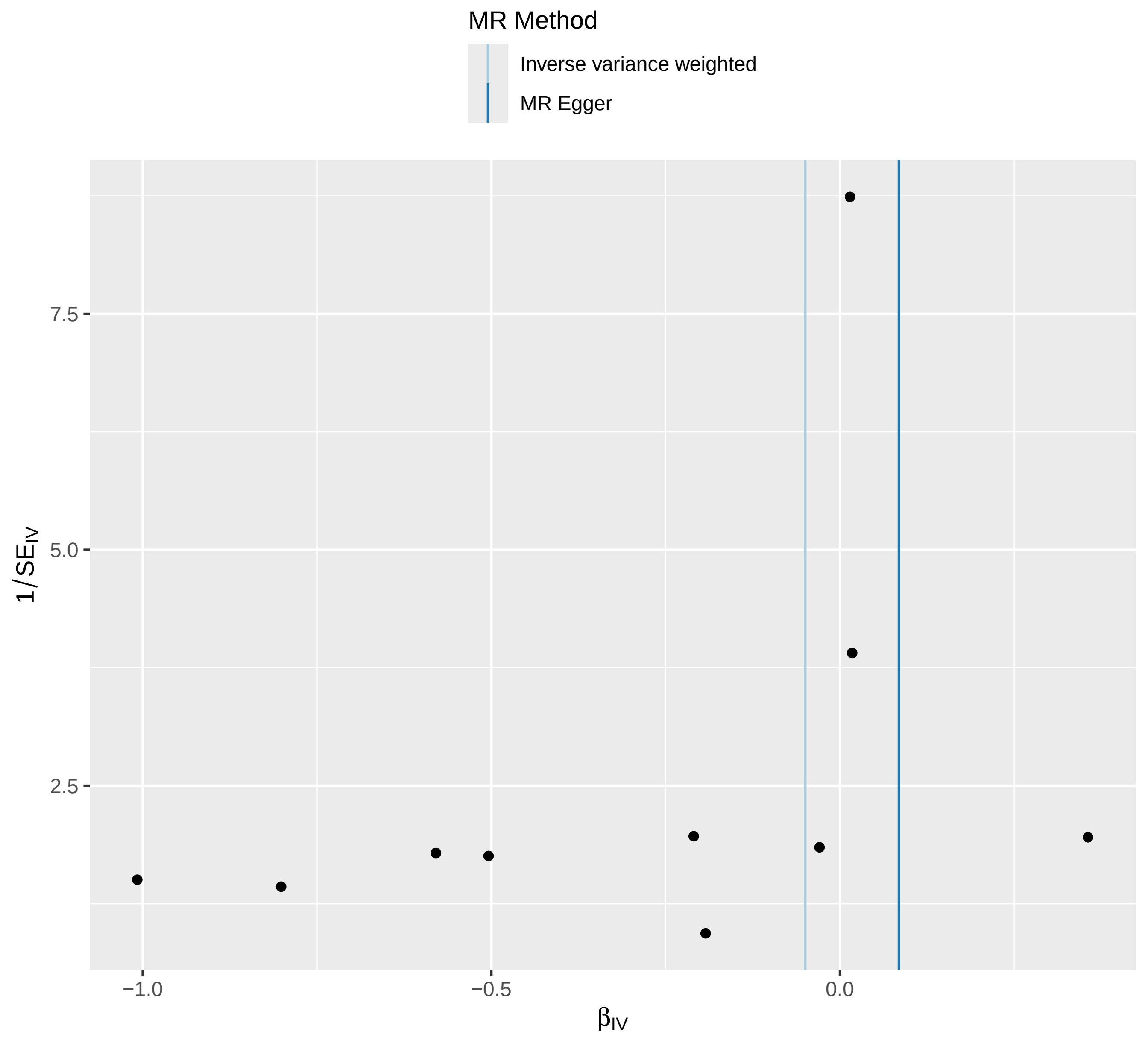
MR Method
Inverse variance weighted
MR Egger
7.5
5.0
2.5
1/SE$_{IV}$
−1.0
−0.5
0.0
β$_{IV}$

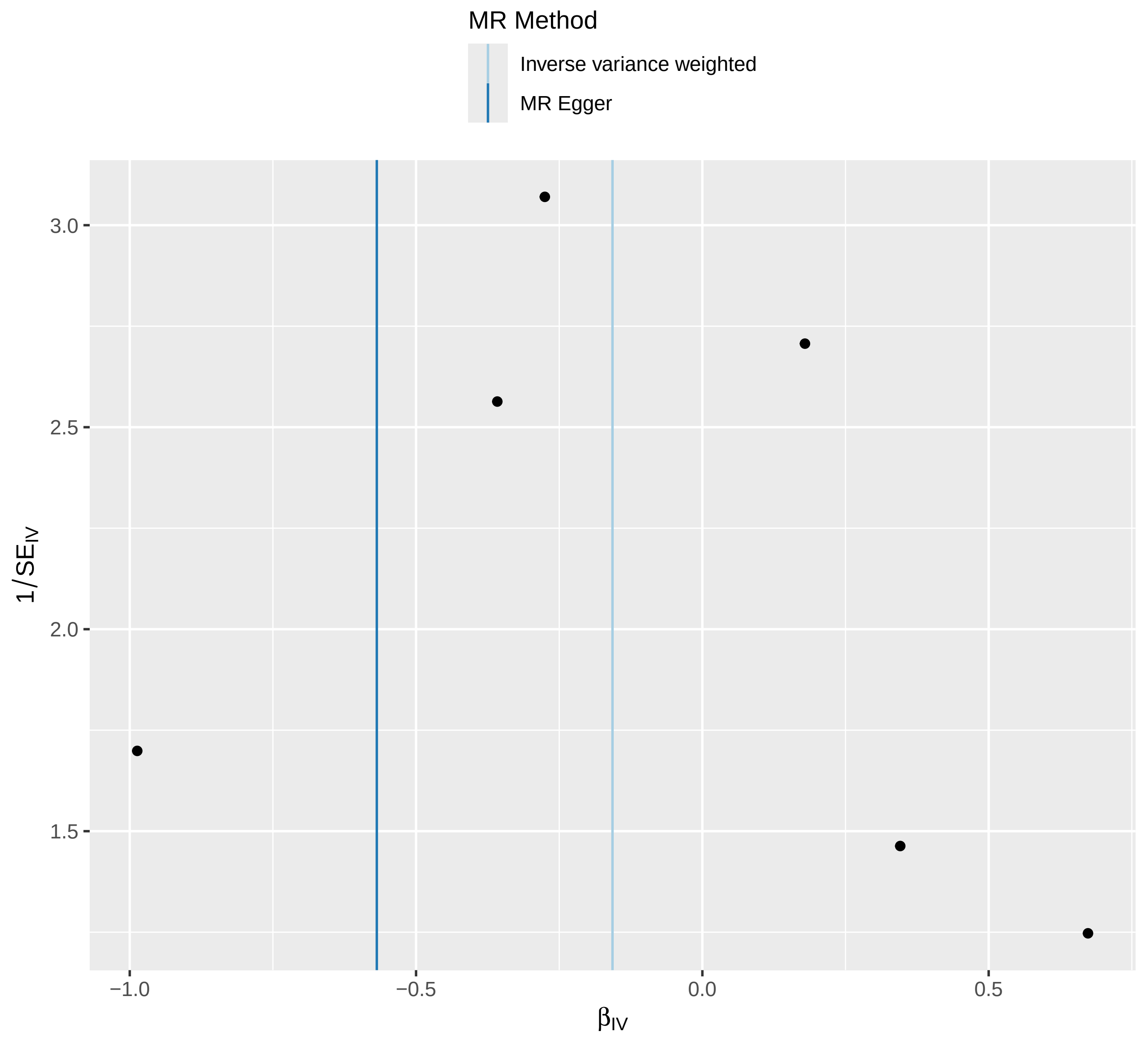

MR Method
Inverse variance weighted
MR Egger
3.0
2.5
2.0
1.5
1/SE_IV
−1.0
−0.5
0.0
0.5
β_IV

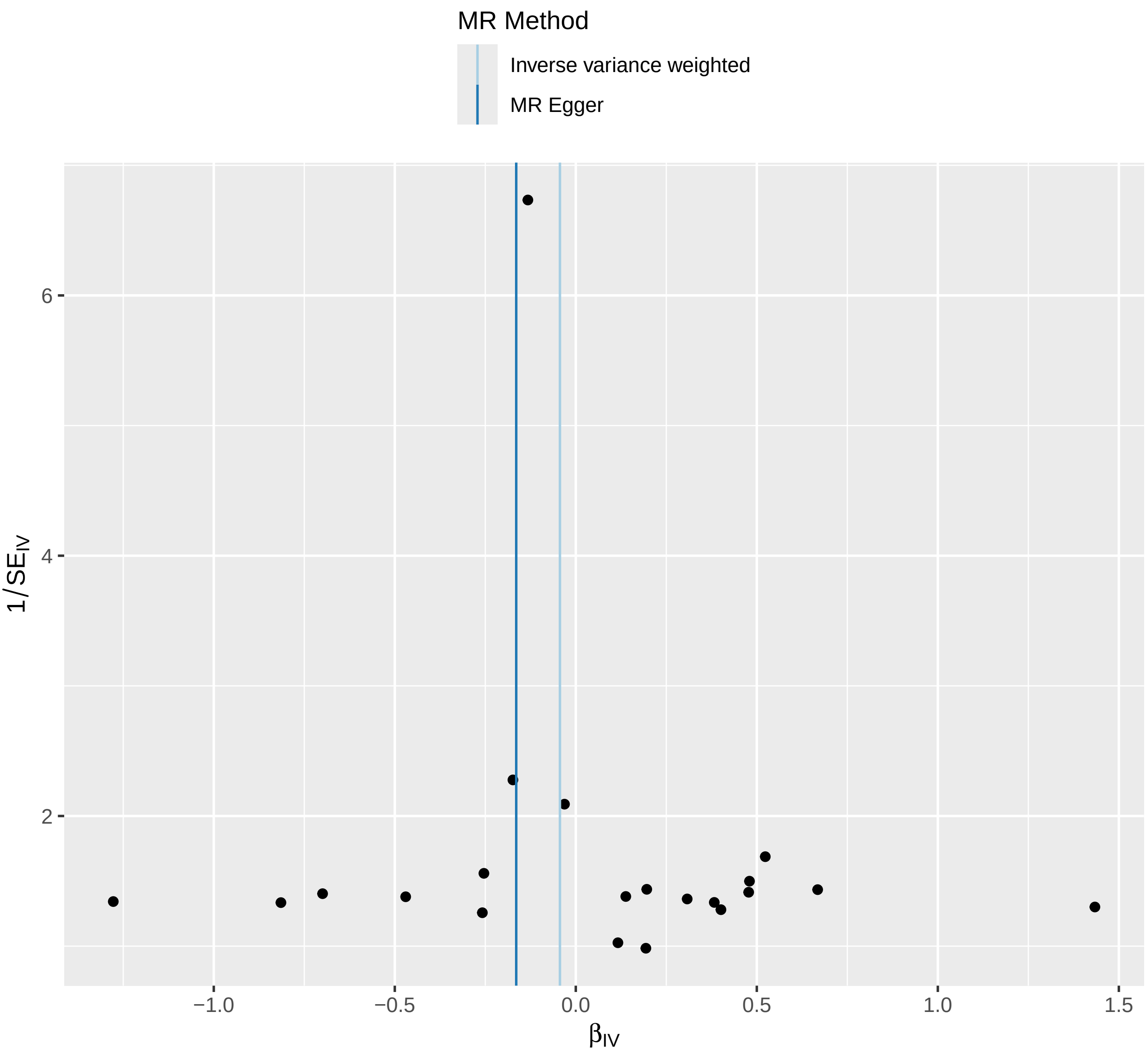

MR Method
Inverse variance weighted
MR Egger
6
4
2
1/SE_IV
−1.0
−0.5
0.0
0.5
1.0
1.5
β_IV

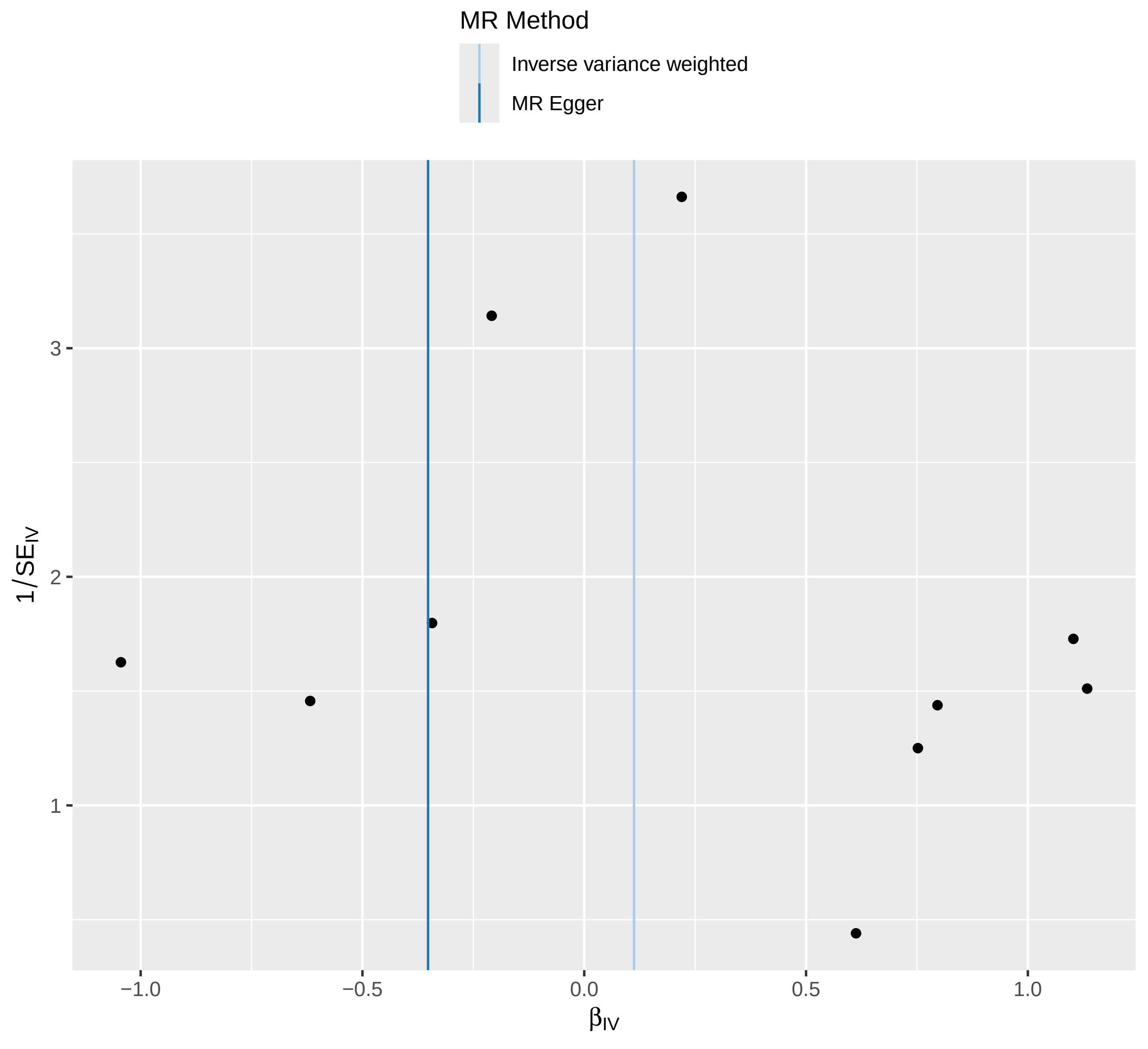
MR Method
Inverse variance weighted
MR Egger
1/SE_{IV}
3
2
1
−1.0
−0.5
0.0
0.5
1.0
β_{IV}

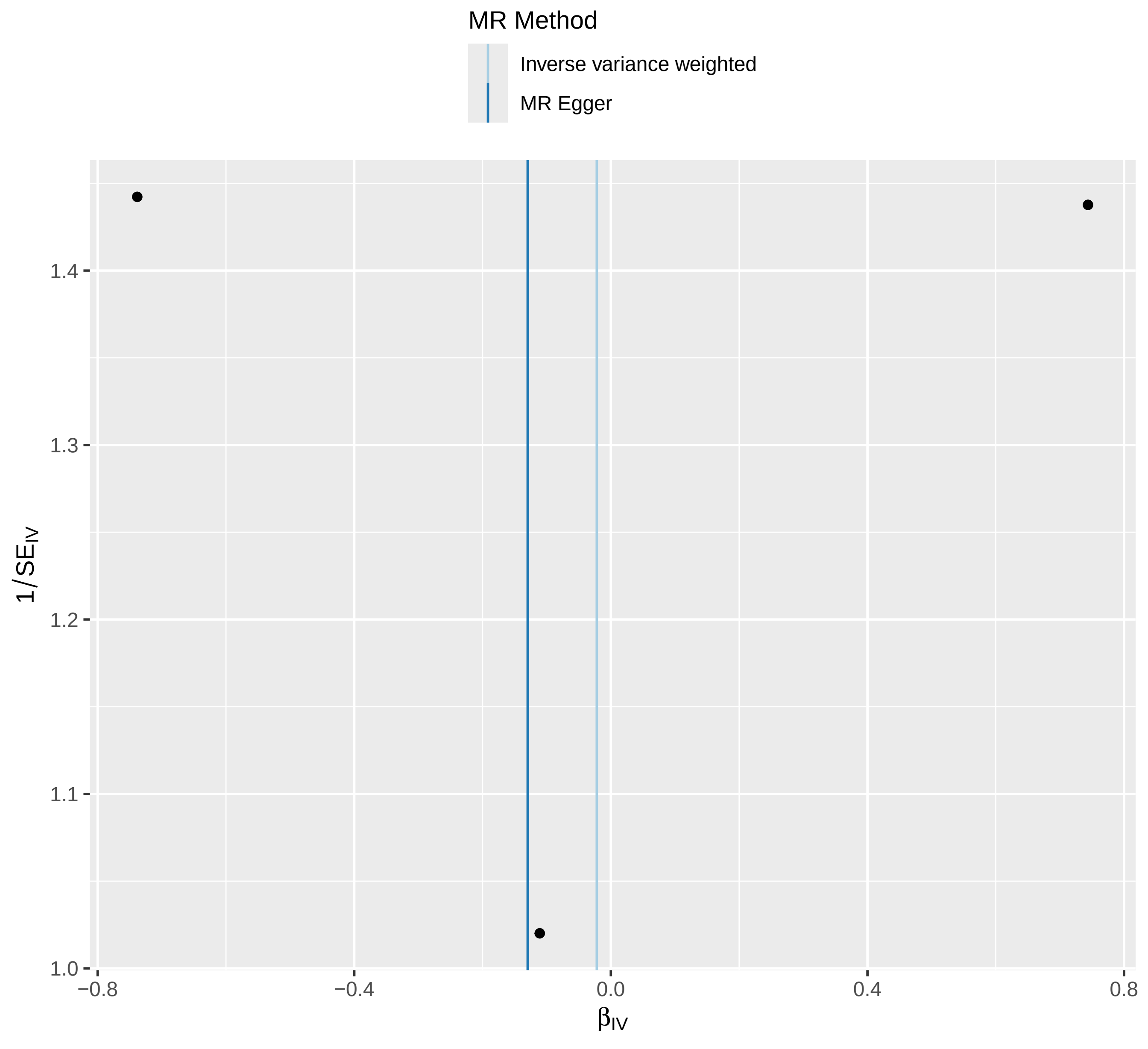
MR Method
Inverse variance weighted
MR Egger
1.4
1.3
1.2
1.1
1.0
−0.8
−0.4
0.0
0.4
0.8
1/SE_IV
β_IV

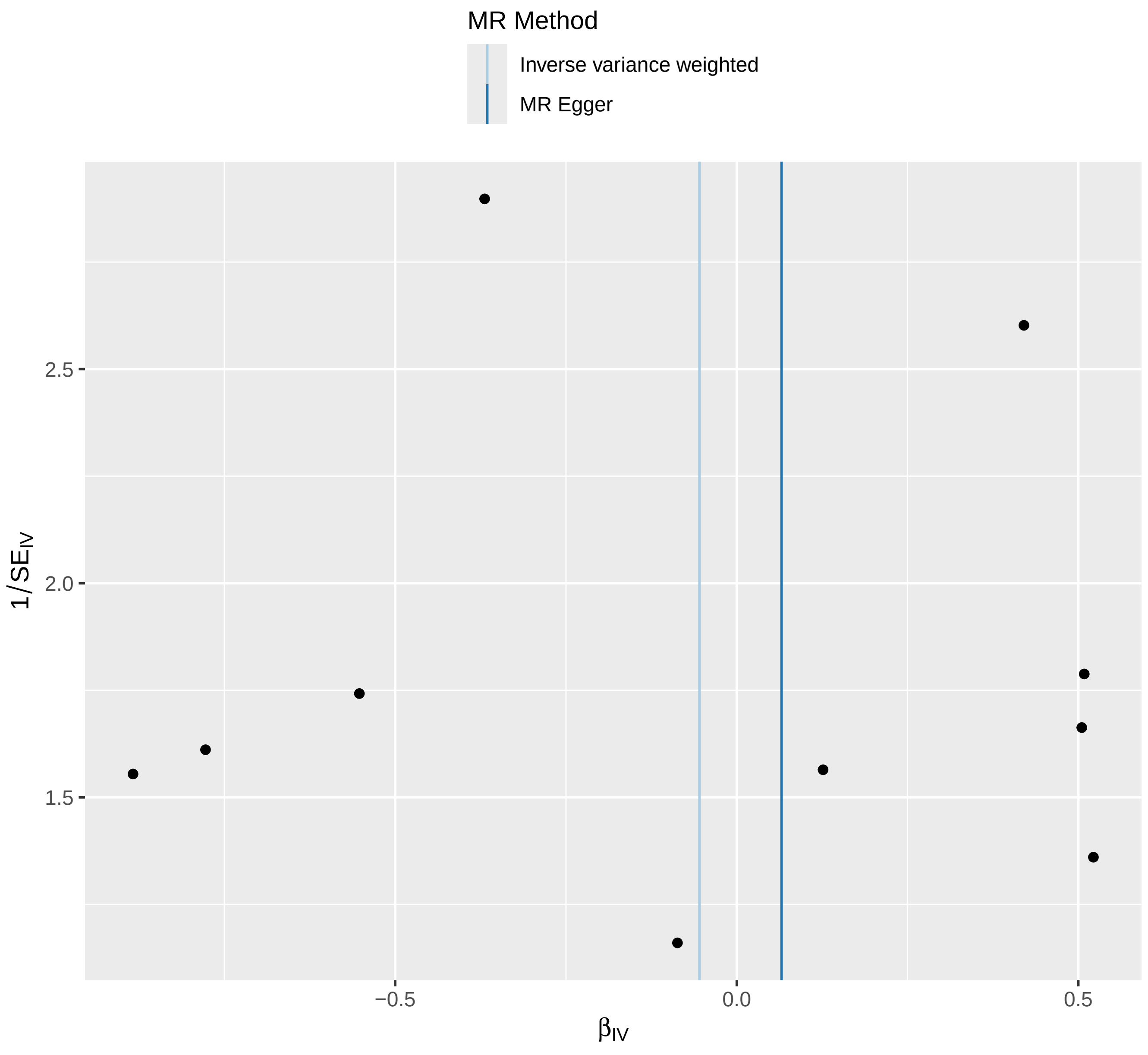

MR Method
Inverse variance weighted
MR Egger
2.5
2.0
1.5
1/SE$_{IV}$
−0.5
0.0
0.5
β$_{IV}$

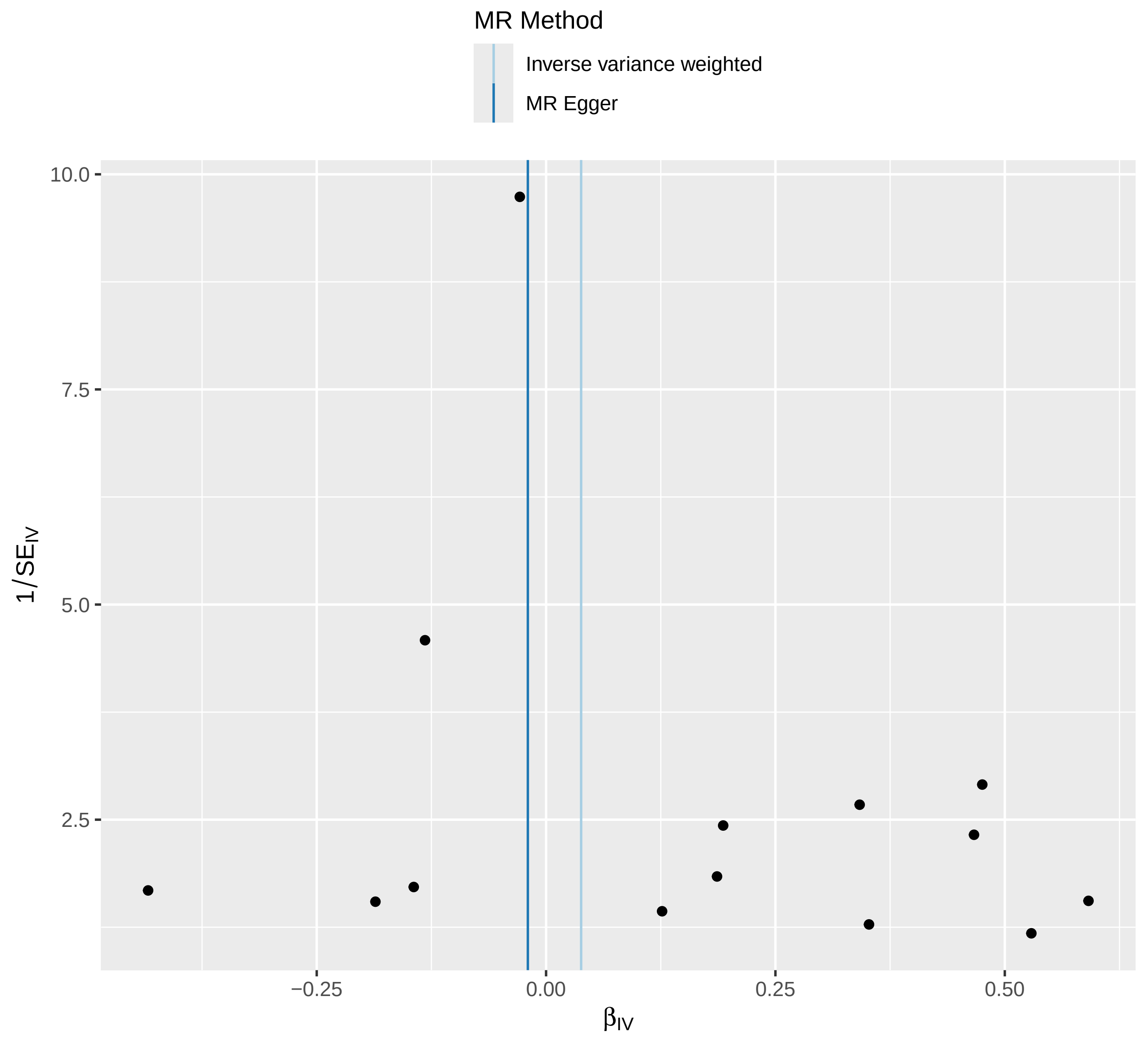
MR Method
Inverse variance weighted
MR Egger
10.0
7.5
5.0
2.5
1/SE_IV
−0.25
0.00
0.25
0.50
β_IV

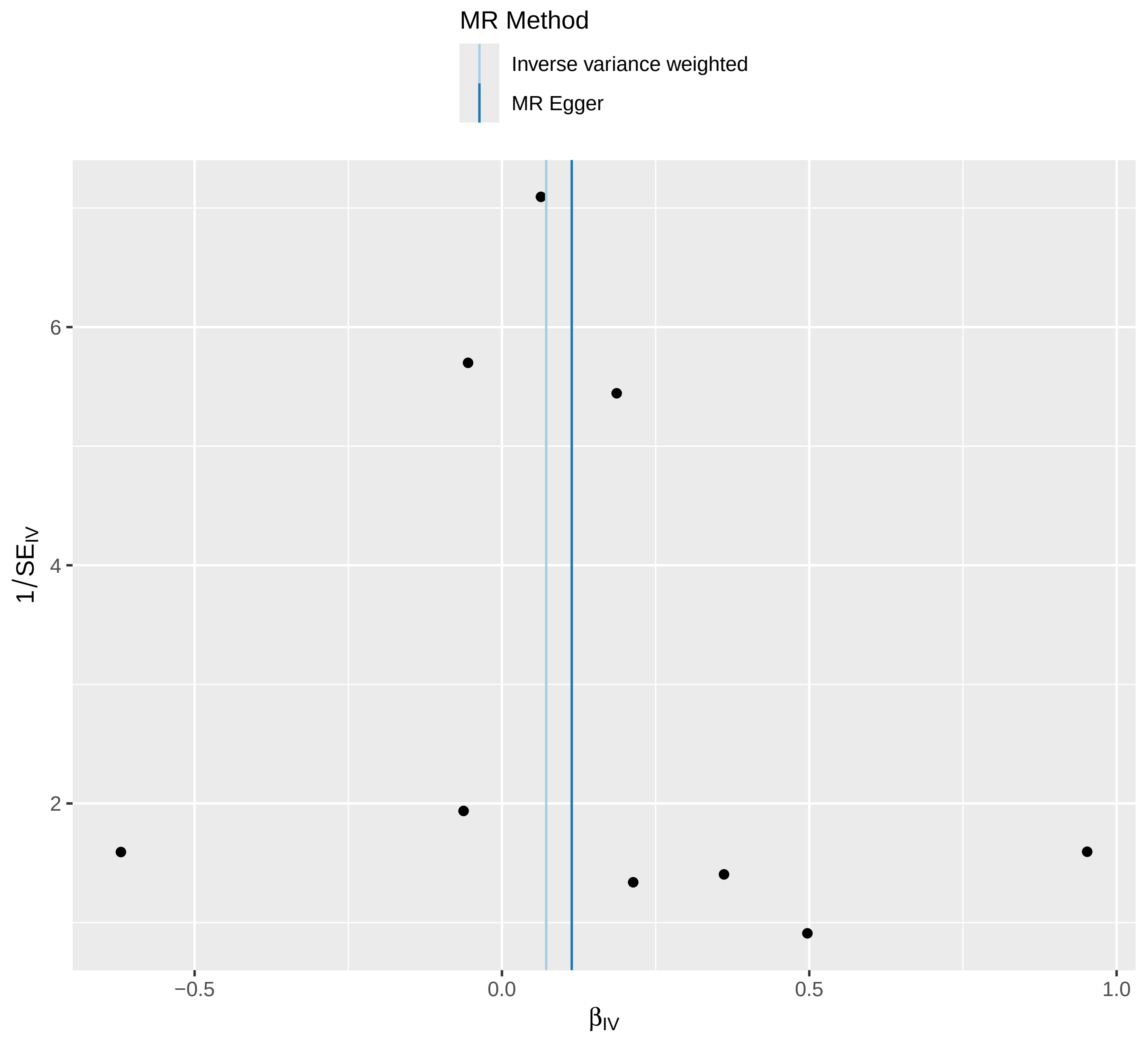
MR Method
Inverse variance weighted
MR Egger
1/SE_IV
6
4
2
−0.5
0.0
0.5
1.0
β_IV

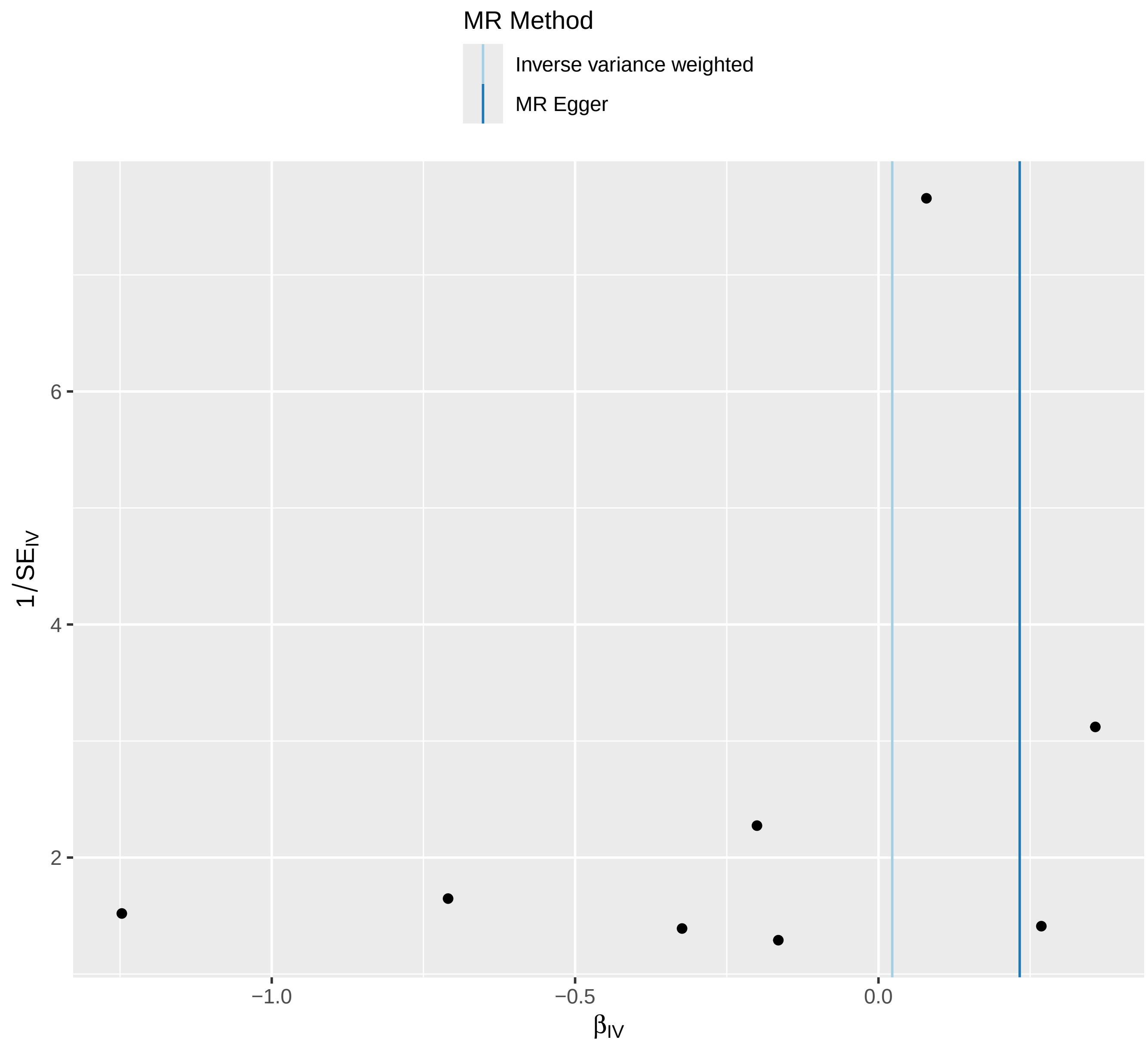

MR Method
Inverse variance weighted
MR Egger
6
4
2
1/SE$_{IV}$
−1.0
−0.5
0.0
β$_{IV}$

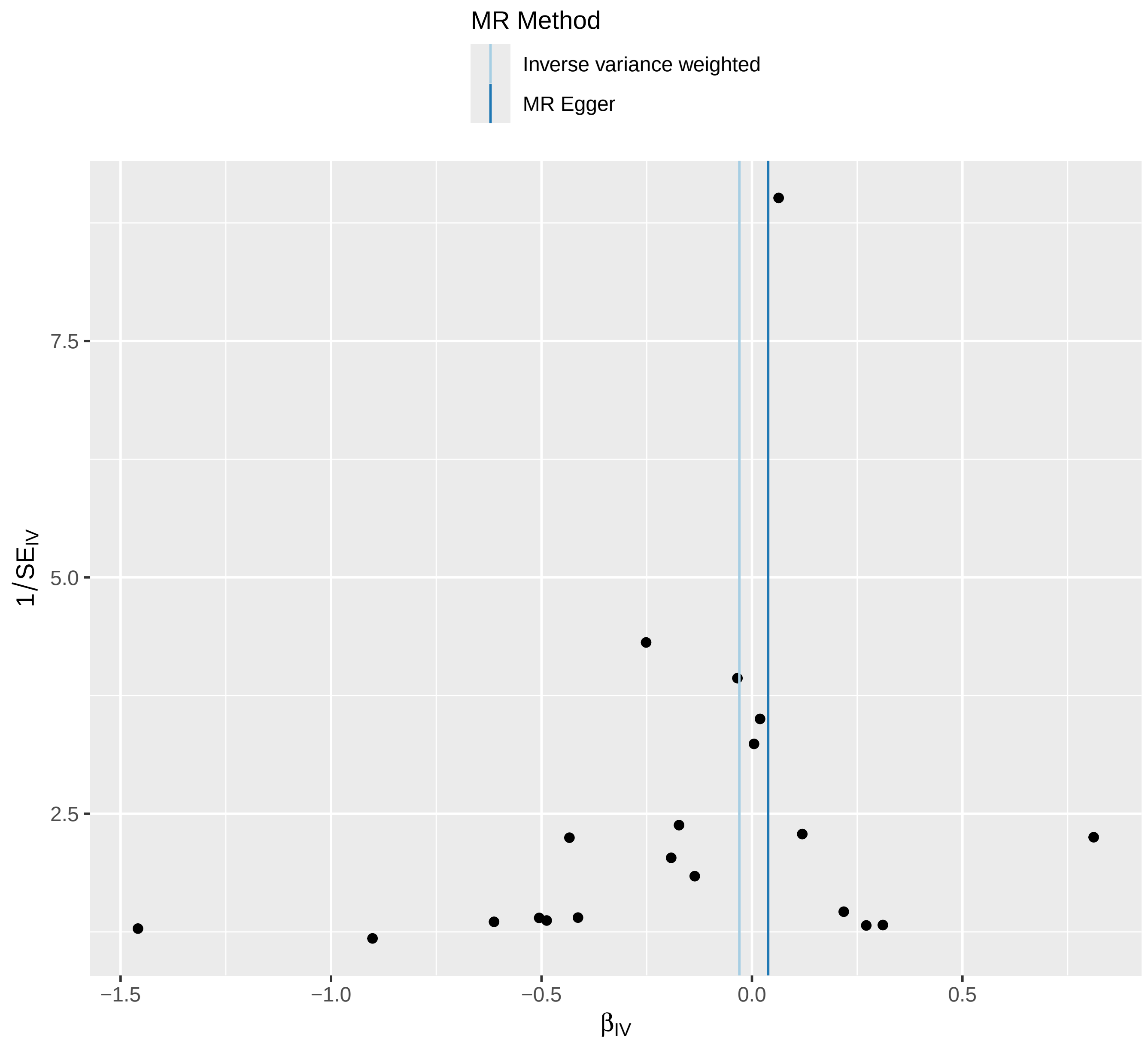

MR Method
Inverse variance weighted
MR Egger
7.5
5.0
2.5
1/SE$_{IV}$
−1.5
−1.0
−0.5
0.0
0.5
β$_{IV}$

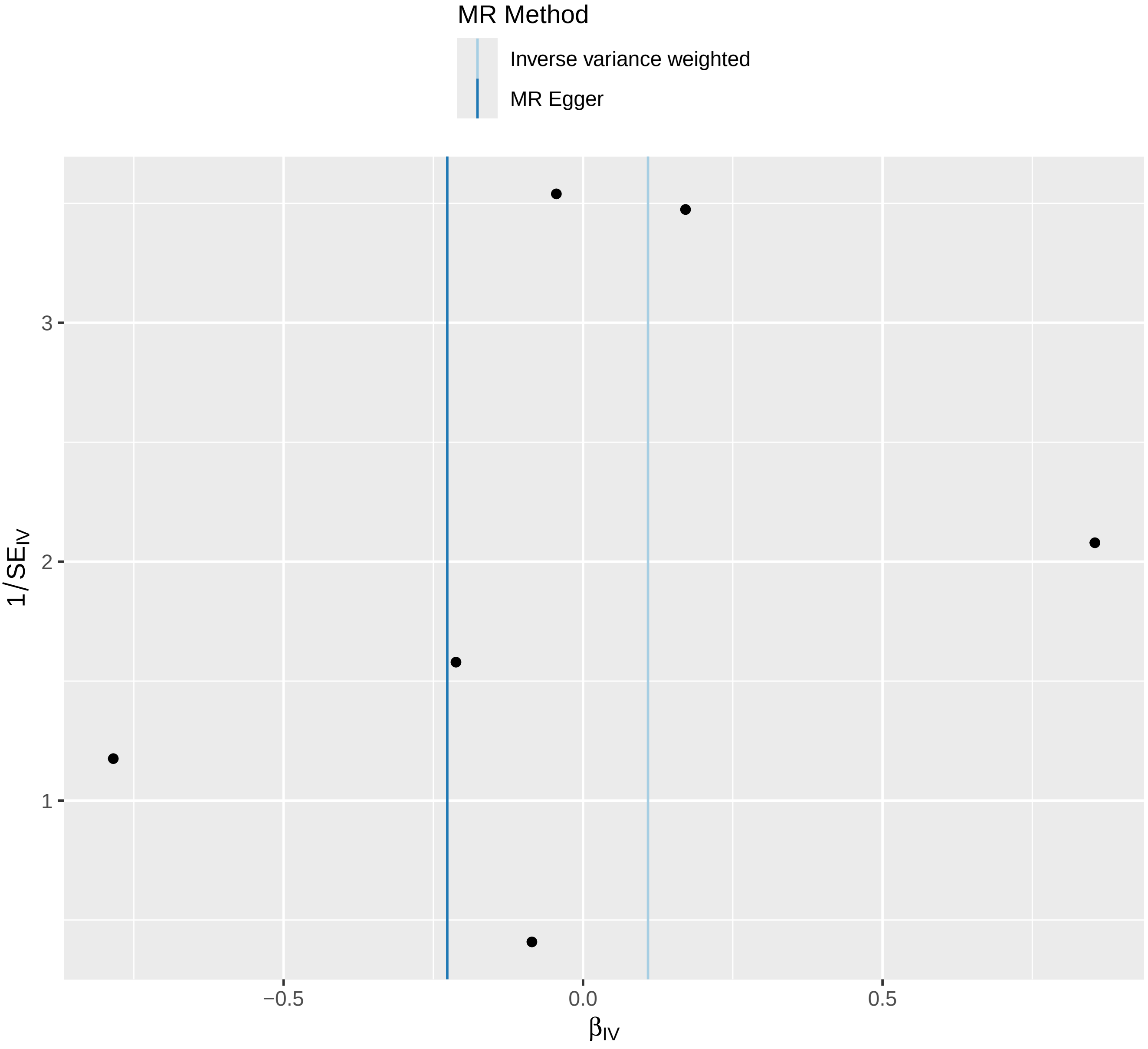

MR Method
Inverse variance weighted
MR Egger
3
2
1
1/SE_IV
−0.5
0.0
0.5
β_IV

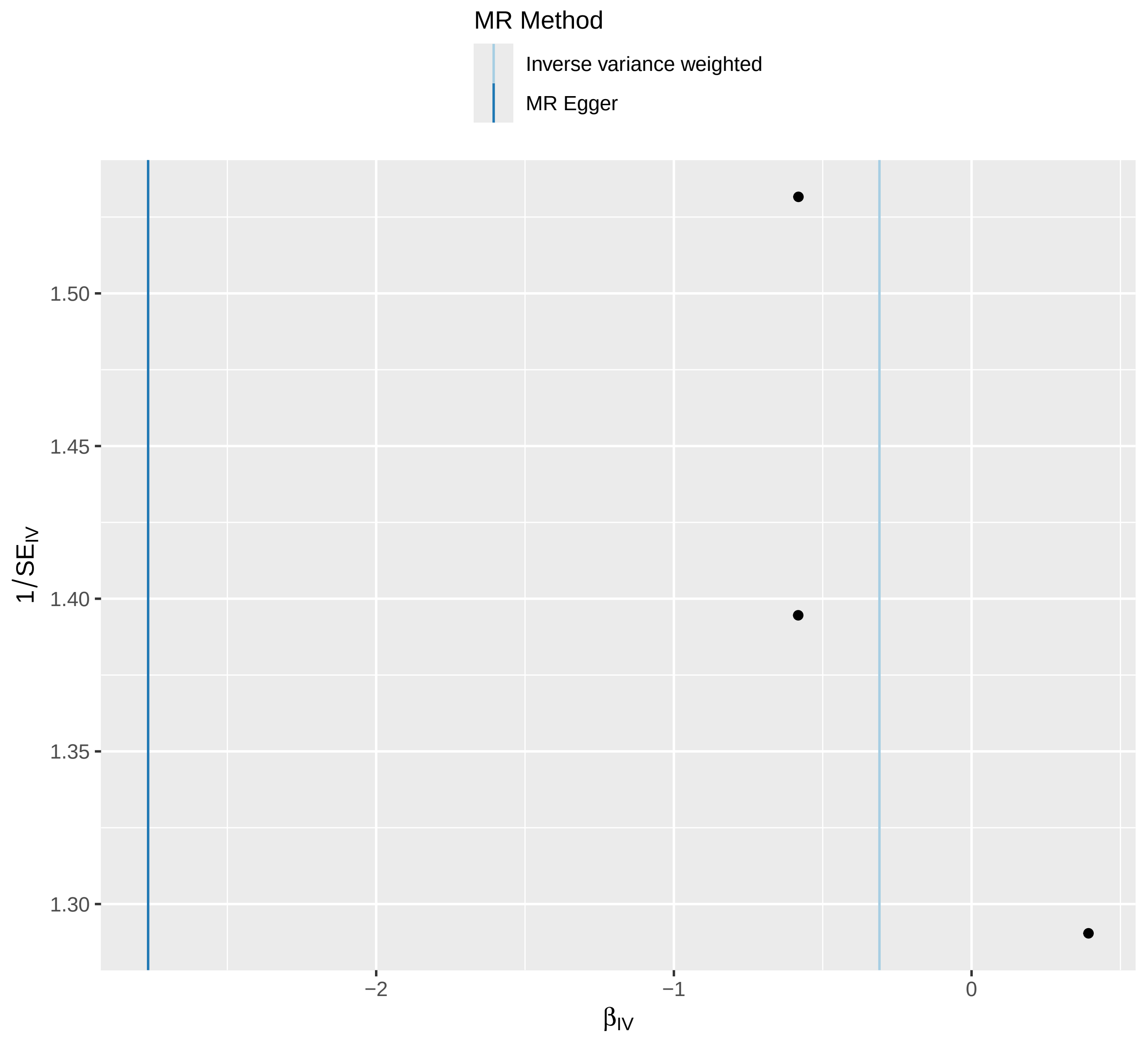
MR Method
Inverse variance weighted
MR Egger
1.50
1.45
1.40
1.35
1.30
1/SE_IV
−2
−1
0
β_IV

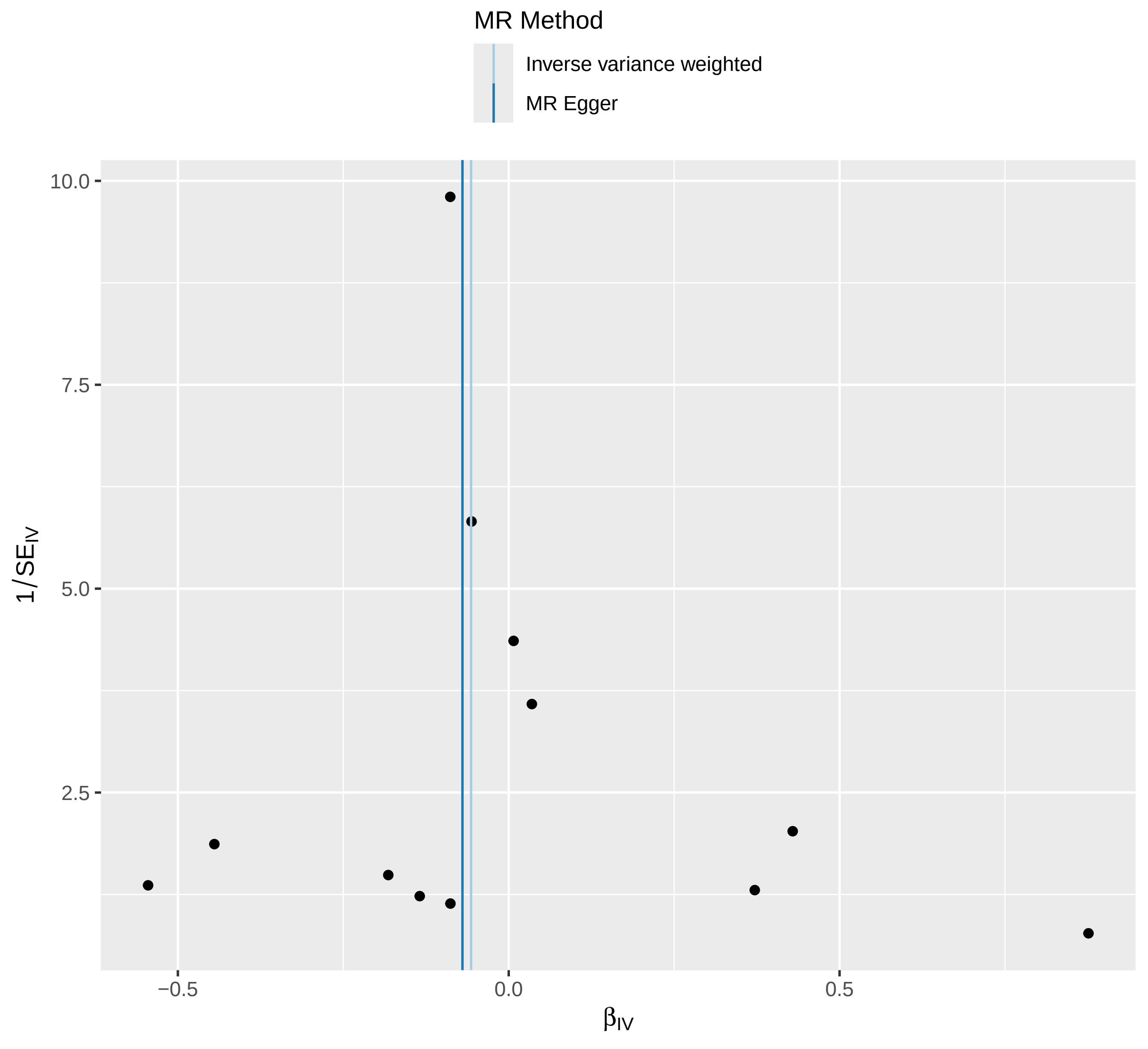

MR Method
Inverse variance weighted
MR Egger
10.0
7.5
5.0
2.5
$1/SE_{IV}$
−0.5
0.0
0.5
β_{IV}

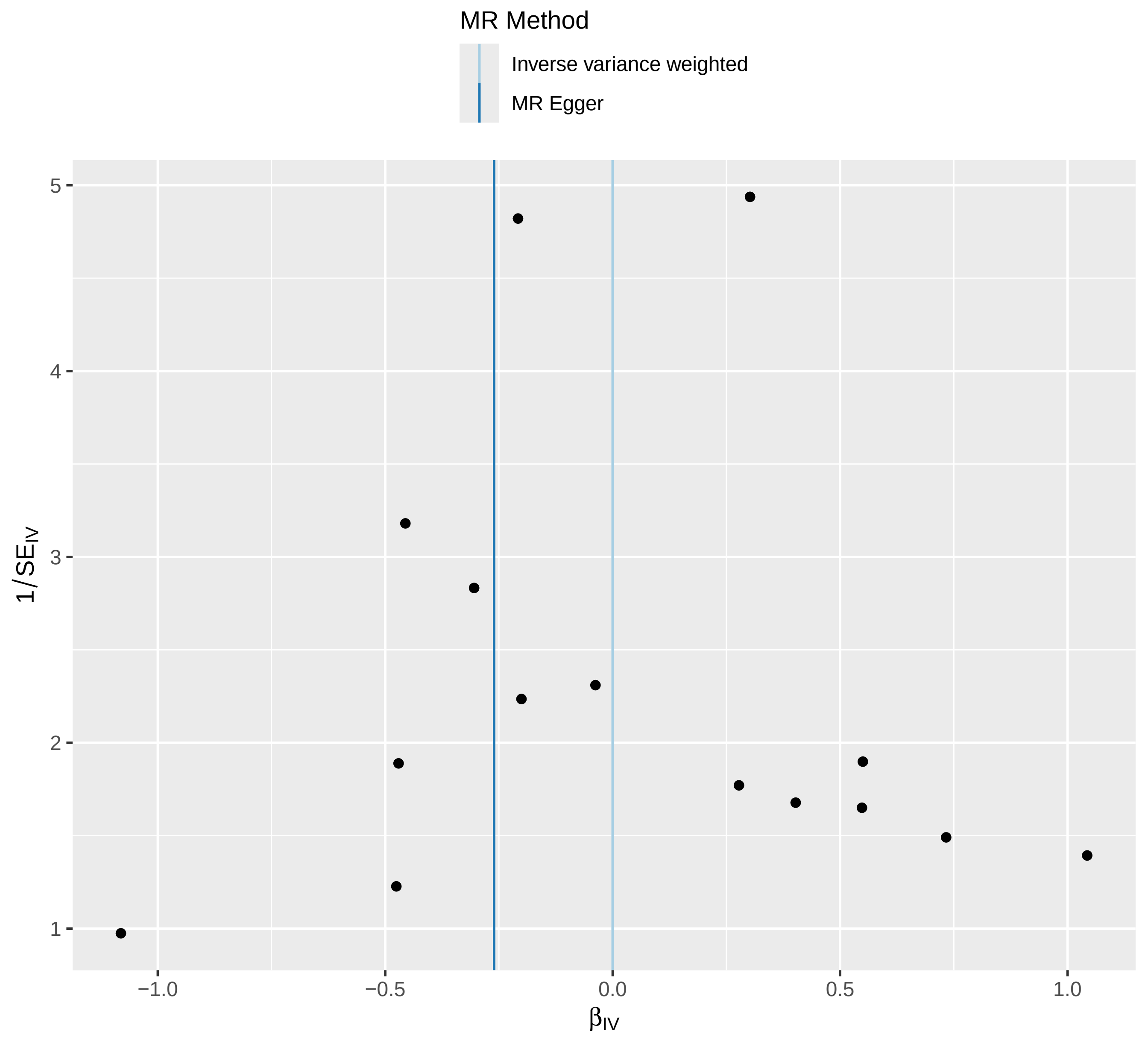
MR Method
Inverse variance weighted
MR Egger
1/SE_IV
β_IV
5
4
3
2
1
−1.0
−0.5
0.0
0.5
1.0

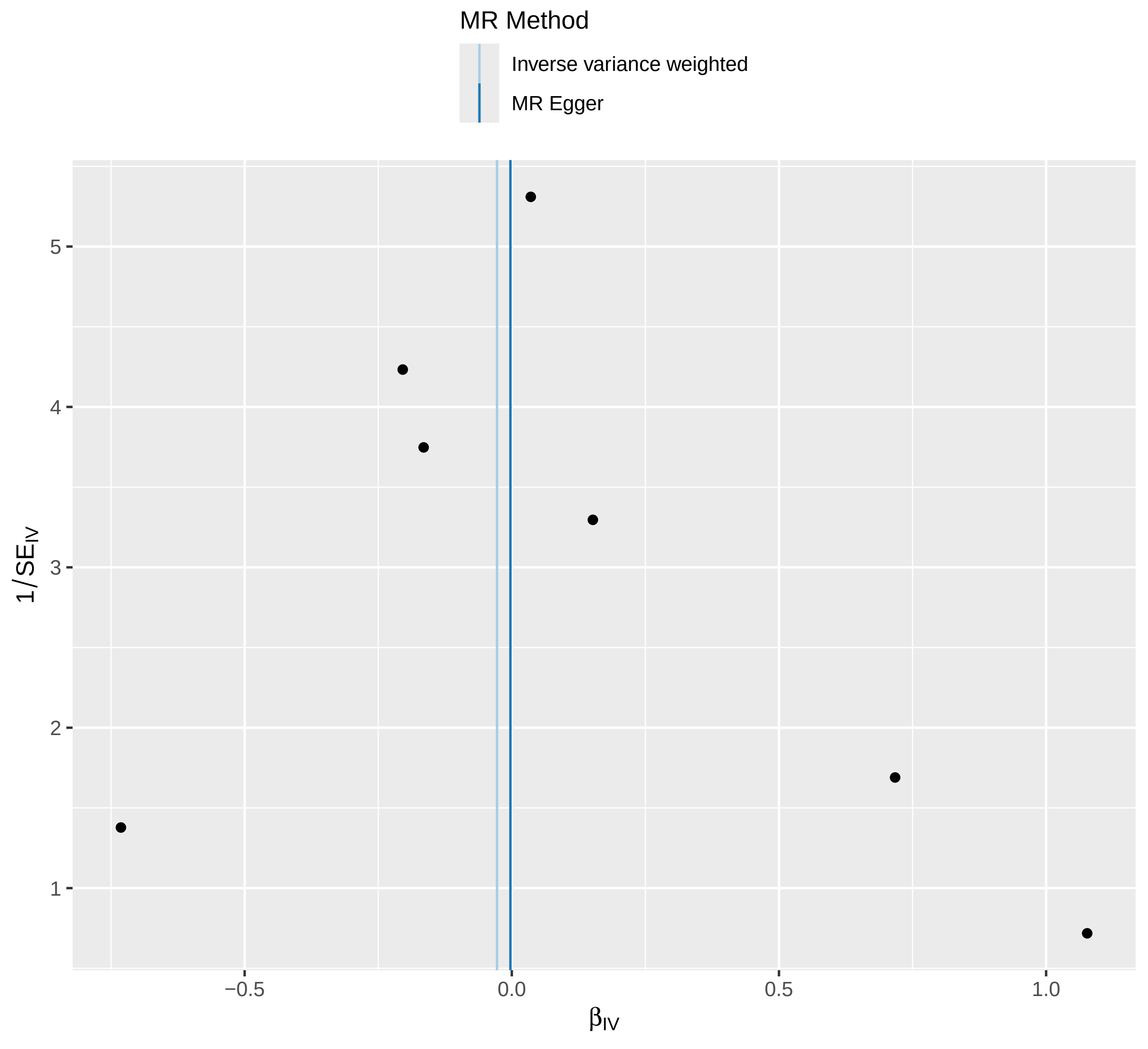
MR Method
Inverse variance weighted
MR Egger
5
4
3
2
1
$1/SE_{IV}$
−0.5
0.0
0.5
1.0
β_{IV}

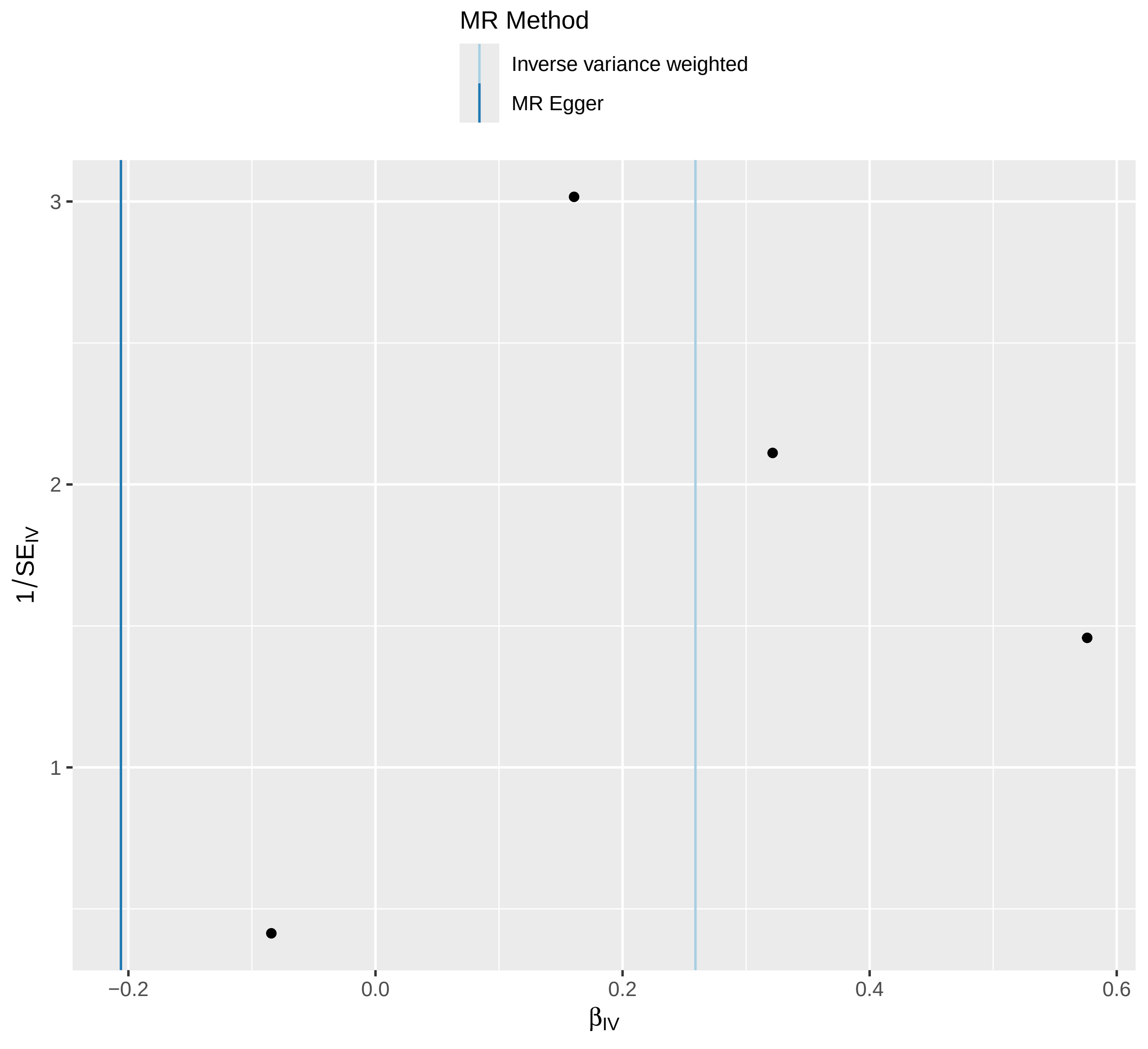

MR Method
Inverse variance weighted
MR Egger
3
2
1
1/SE_IV
−0.2
0.0
0.2
0.4
0.6
β_IV

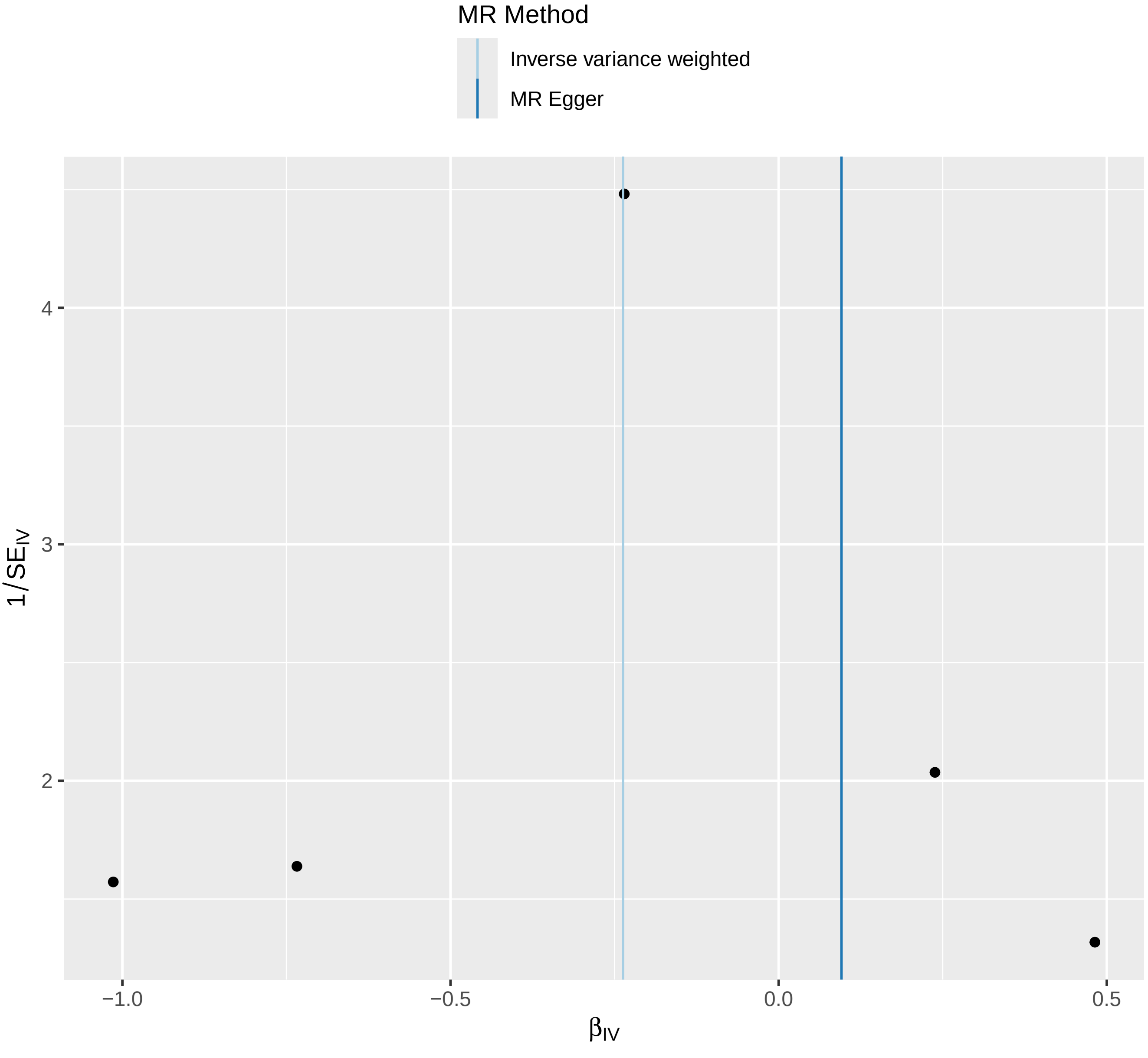

MR Method
Inverse variance weighted
MR Egger
4
3
2
1/SE$_{IV}$
−1.0
−0.5
0.0
0.5
β$_{IV}$

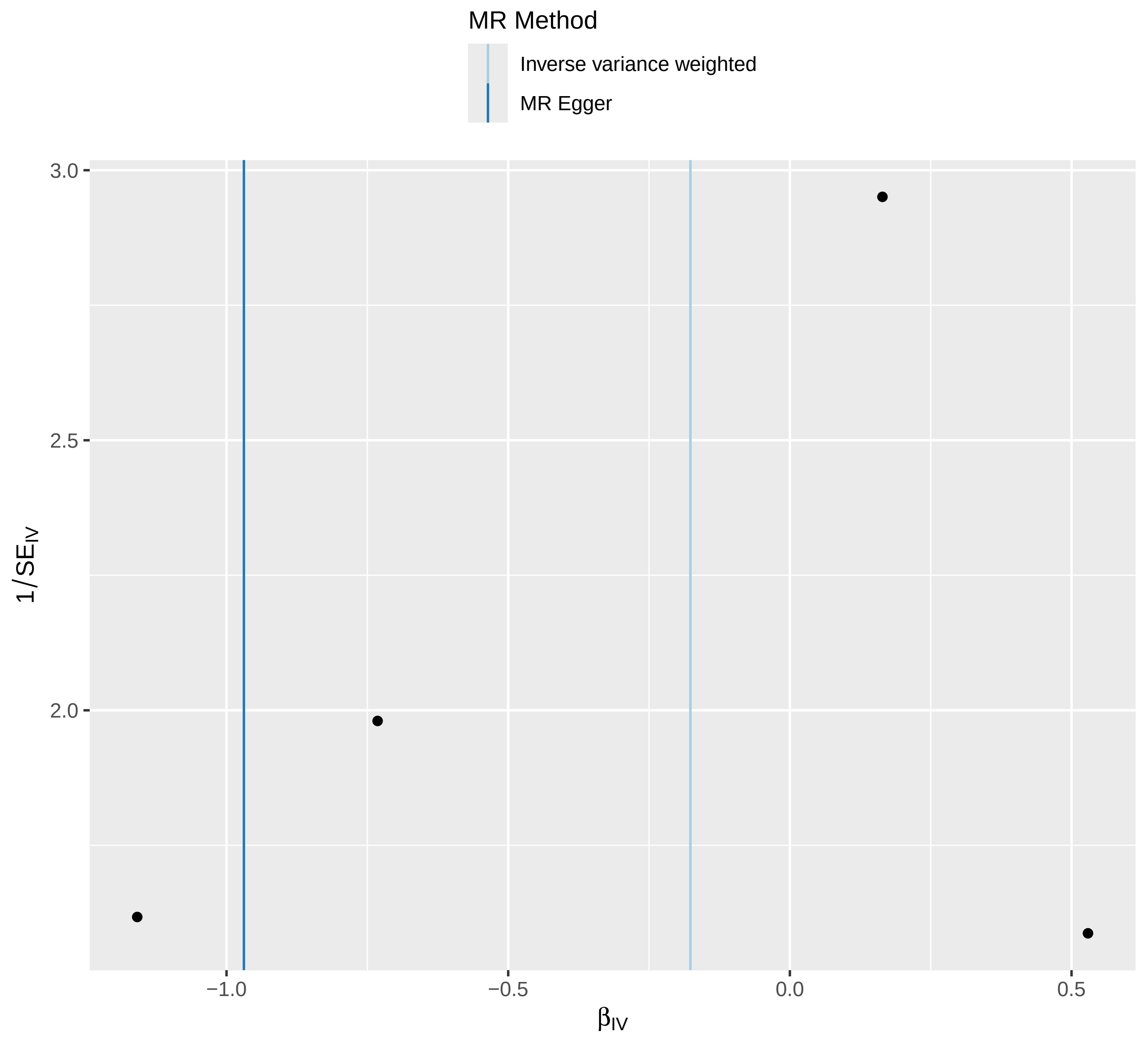
MR Method
Inverse variance weighted
MR Egger
3.0
2.5
2.0
1/SE$_{IV}$
−1.0
−0.5
0.0
0.5
β$_{IV}$

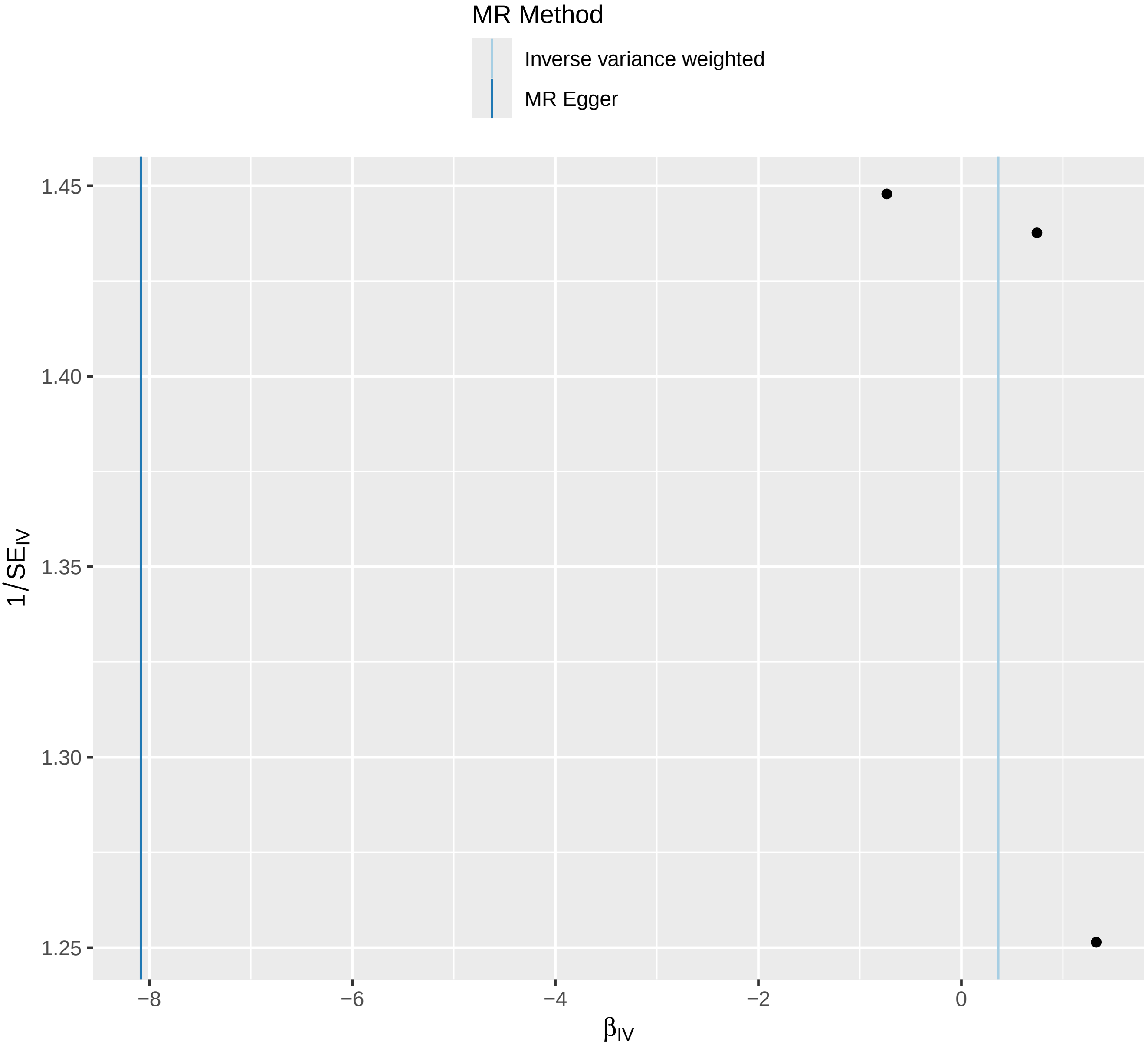

MR Method
Inverse variance weighted
MR Egger
1.45
1.40
1.35
1.30
1.25
$1/SE_{IV}$
−8
−6
−4
−2
0
β_{IV}

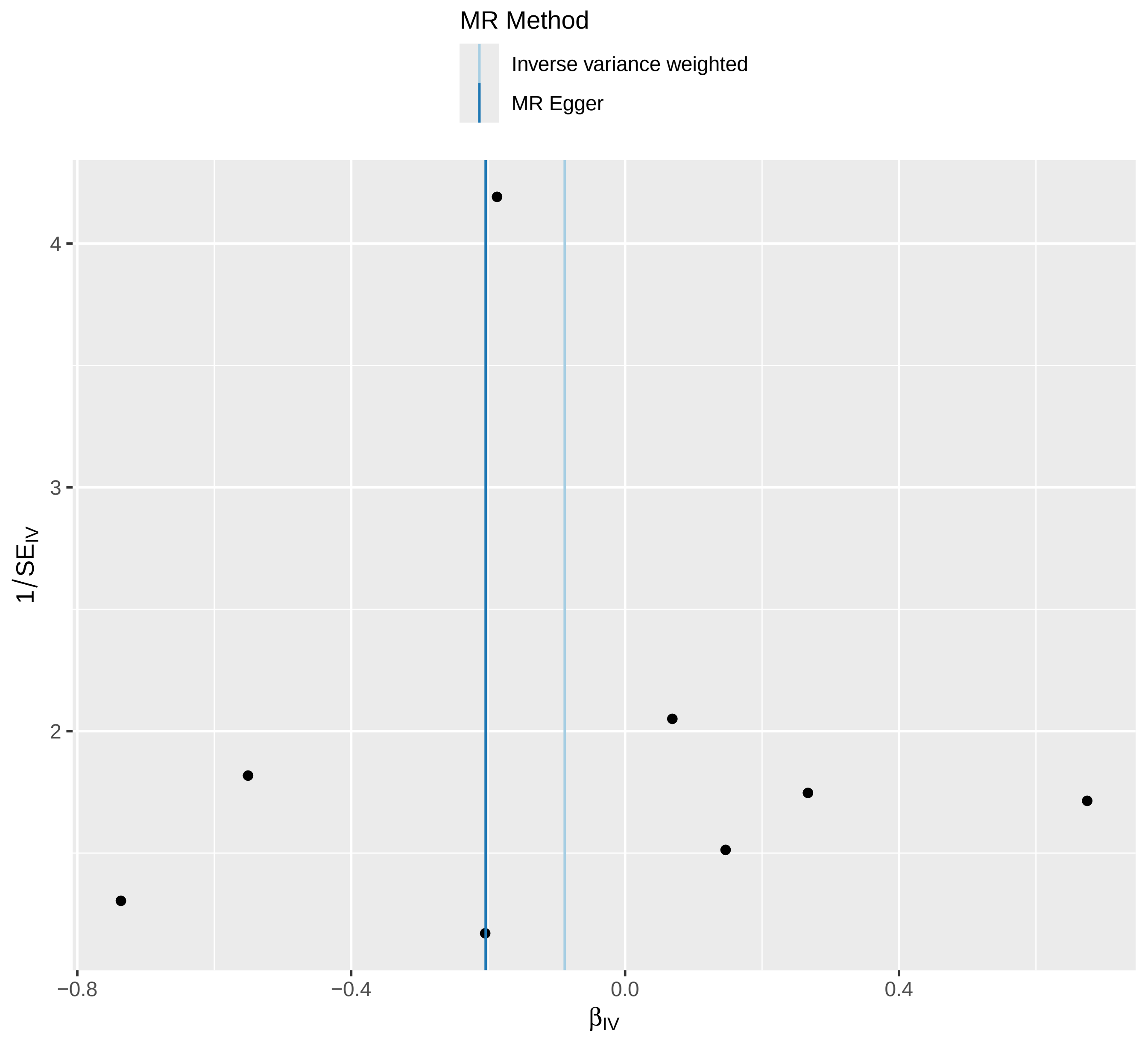

MR Method
Inverse variance weighted
MR Egger
4
3
2
1/SE_IV
−0.8
−0.4
0.0
0.4
β_IV

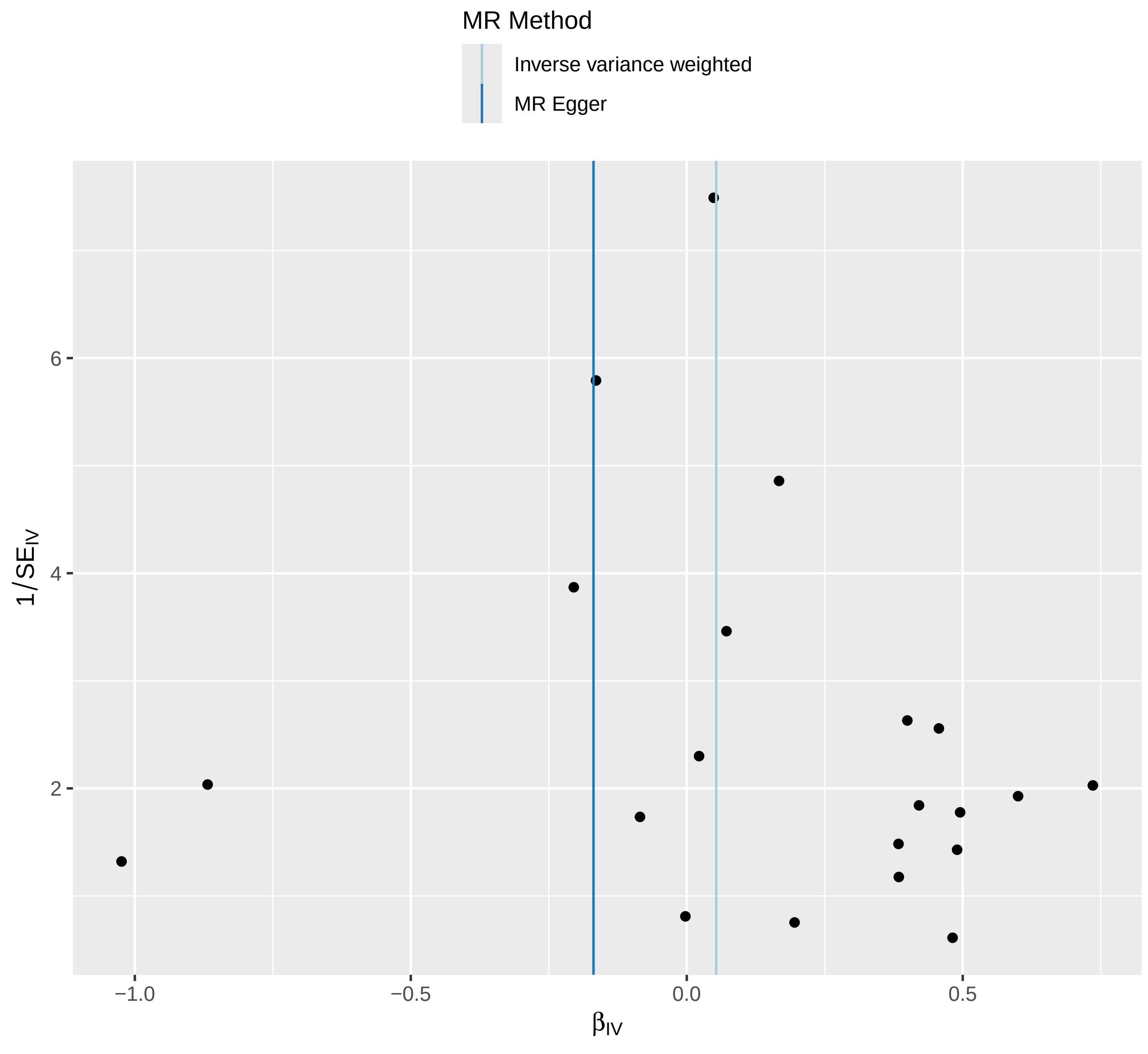
MR Method
Inverse variance weighted
MR Egger
6
4
2
1/SE_IV
−1.0
−0.5
0.0
0.5
β_IV

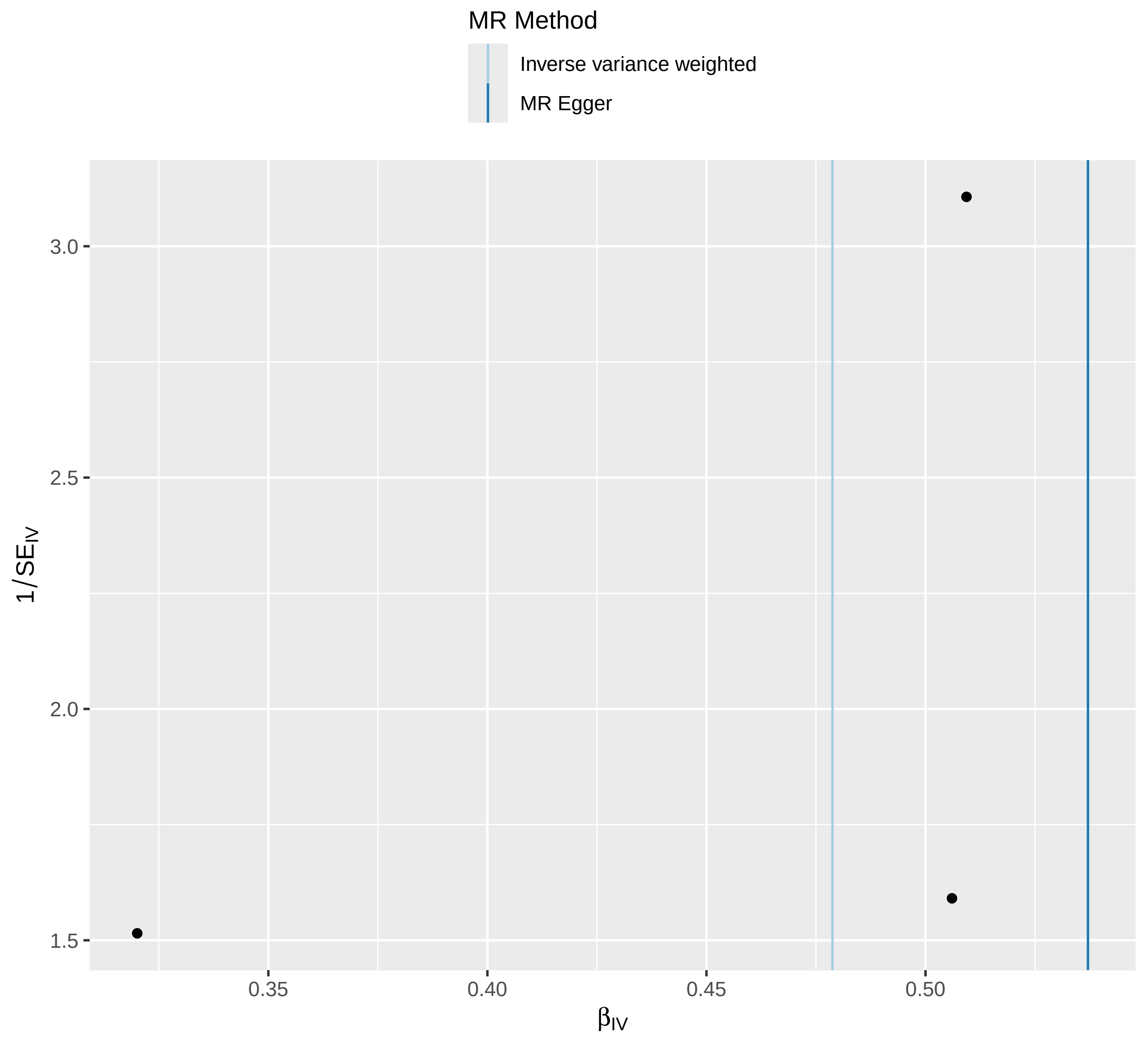
MR Method
Inverse variance weighted
MR Egger
3.0
2.5
2.0
1.5
1/SE_IV
0.35
0.40
0.45
0.50
β_IV

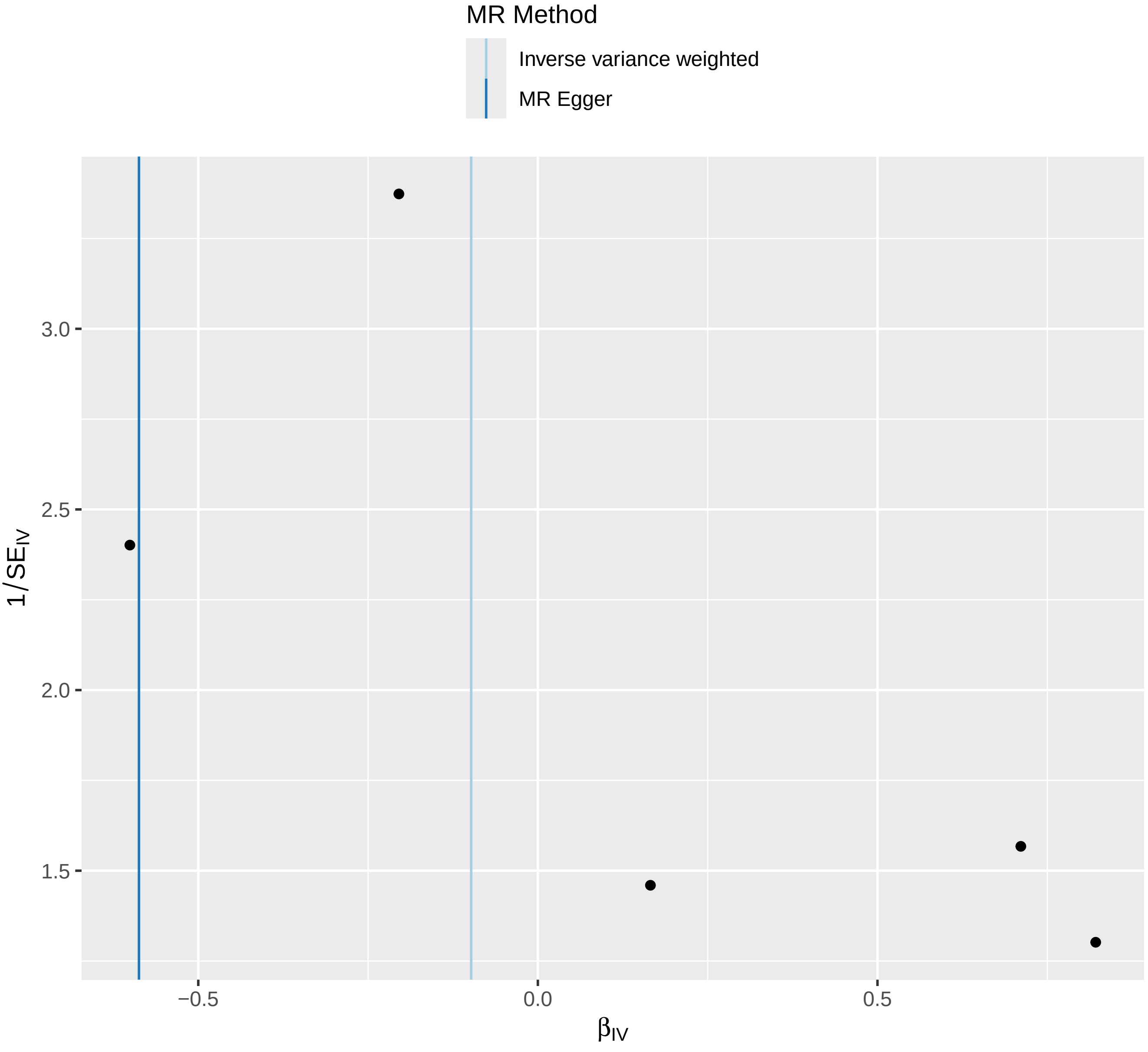

MR Method
Inverse variance weighted
MR Egger
3.0
2.5
2.0
1.5
1/SE_IV
−0.5
0.0
0.5
β_IV

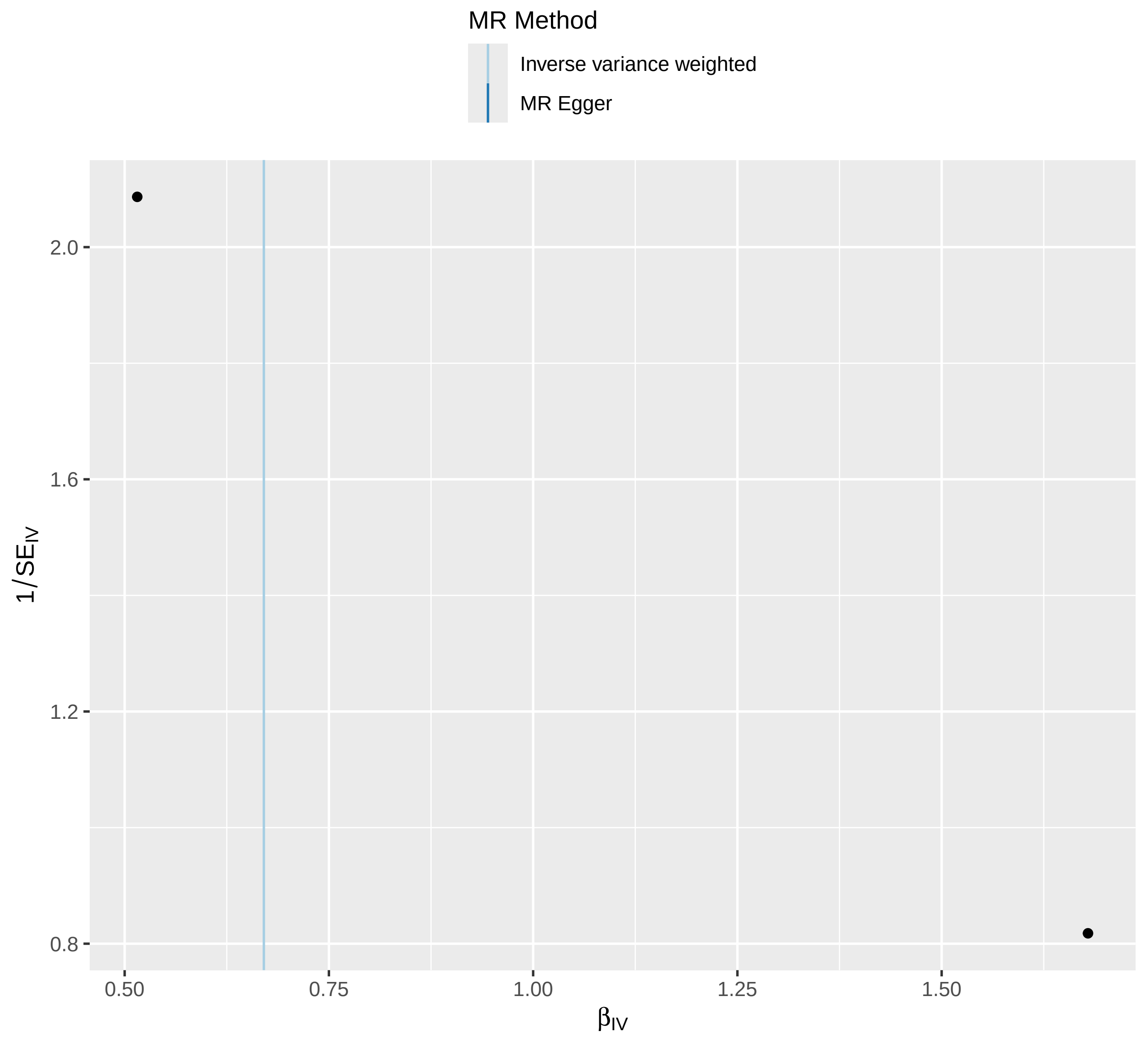

MR Method
Inverse variance weighted
MR Egger
2.0
1.6
1.2
0.8
$1/SE_{IV}$
0.50
0.75
1.00
1.25
1.50
β_{IV}

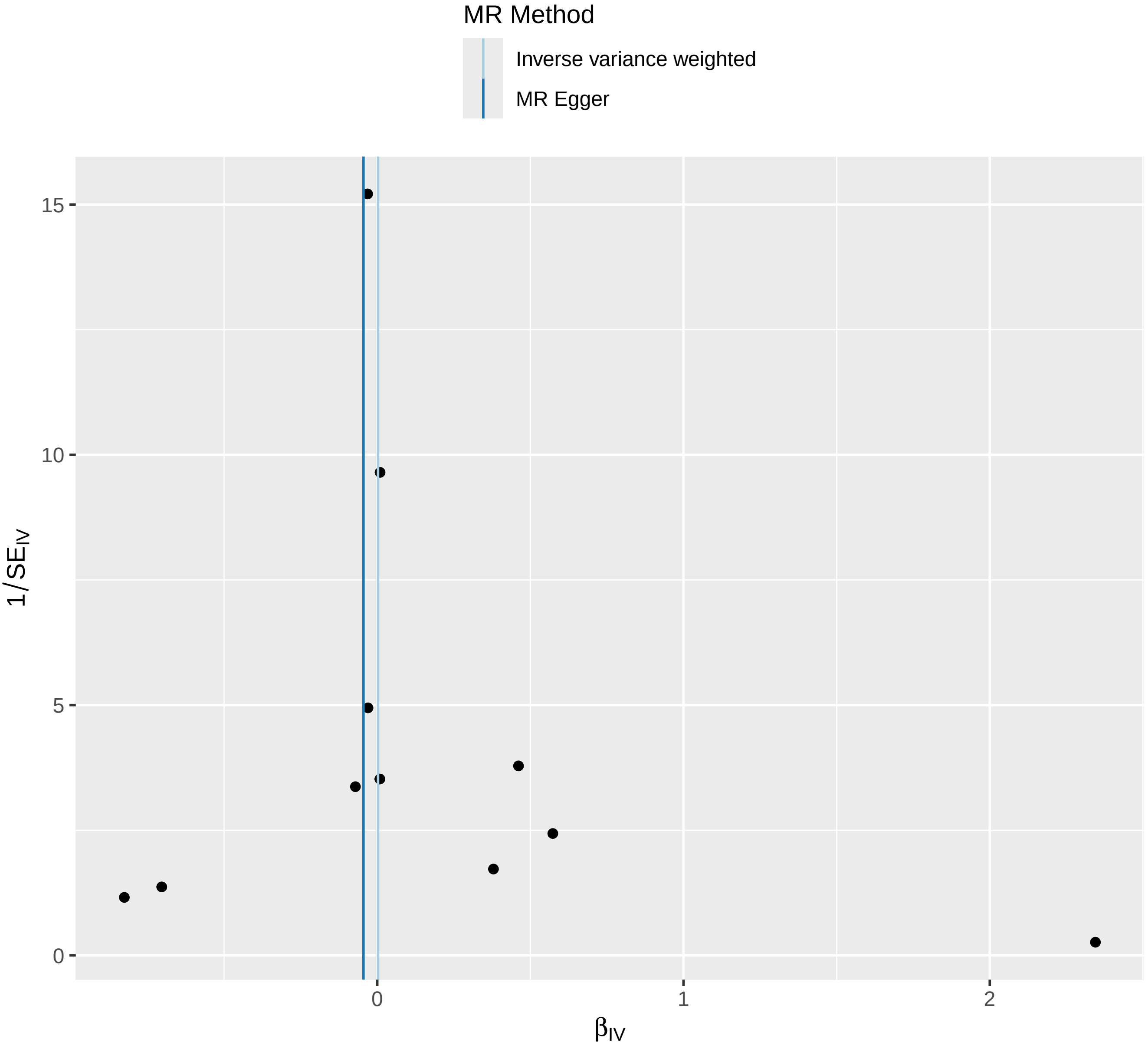
MR Method
Inverse variance weighted
MR Egger
15
10
5
0
1/SE$_{IV}$
0
1
2
β$_{IV}$

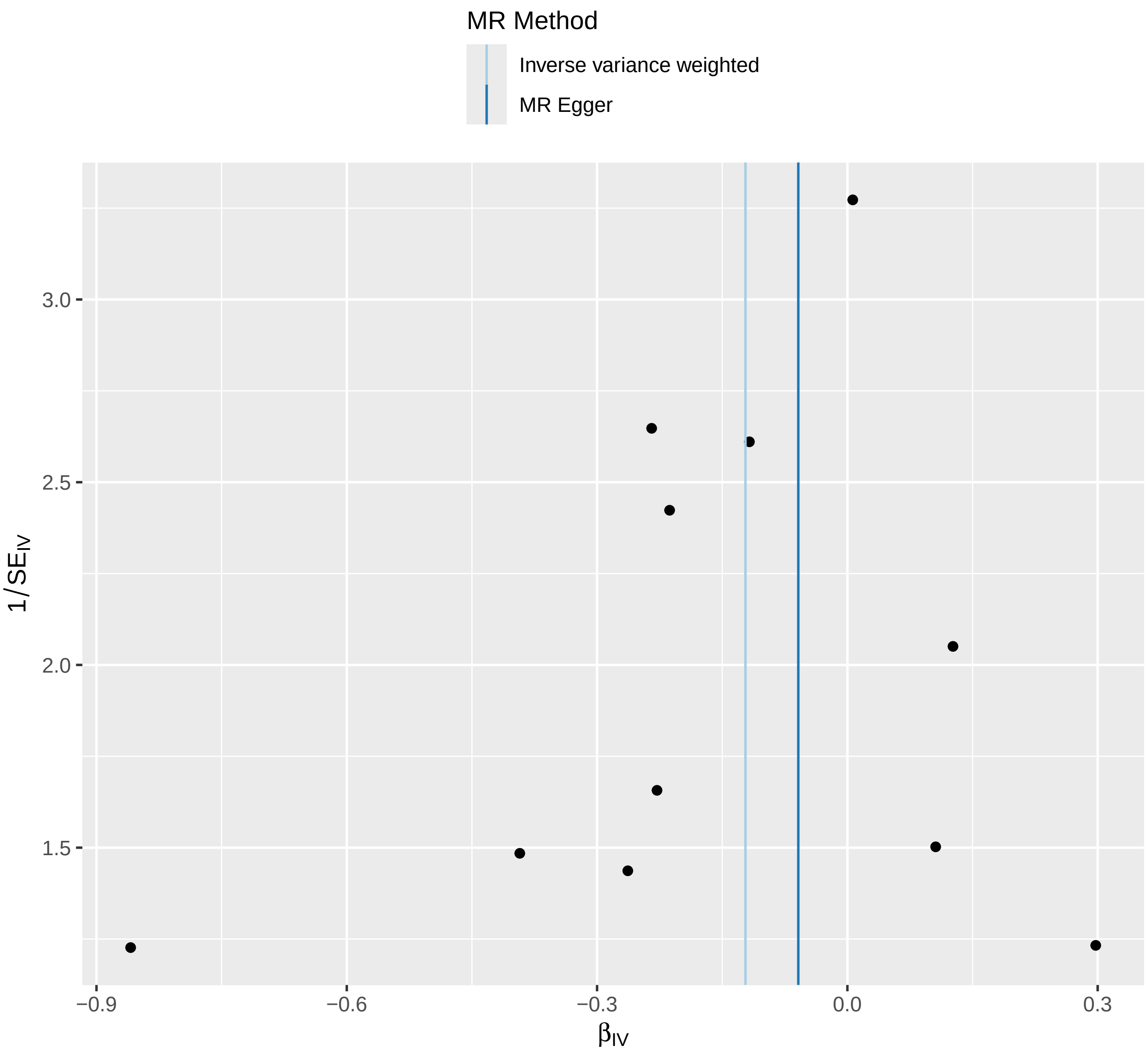
MR Method
Inverse variance weighted
MR Egger
1/SE_IV
3.0
2.5
2.0
1.5
−0.9
−0.6
−0.3
0.0
0.3
β_IV

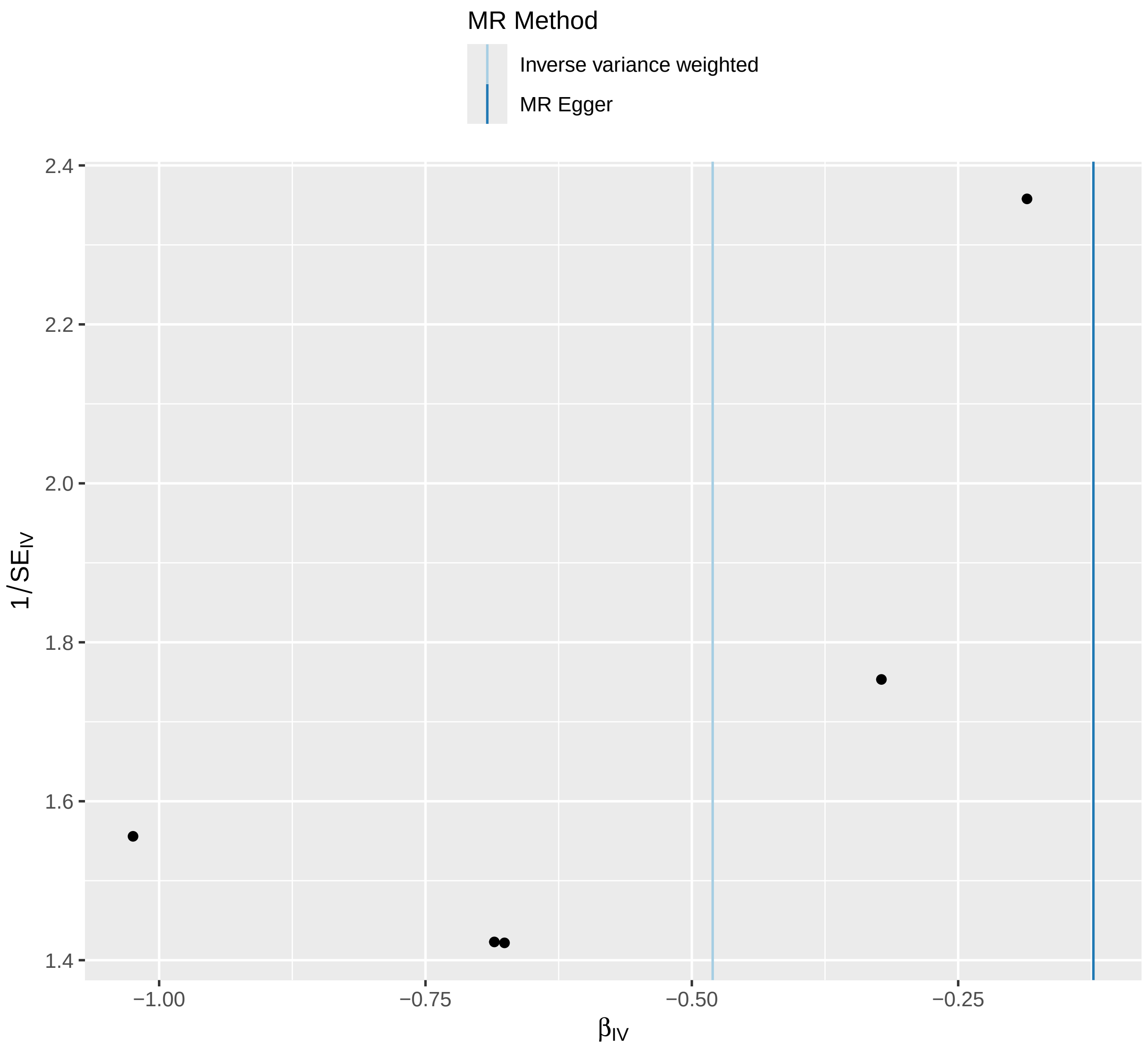
MR Method
Inverse variance weighted
MR Egger
2.4
2.2
2.0
1.8
1.6
1.4
1/SE_IV
−1.00
−0.75
−0.50
−0.25
β_IV

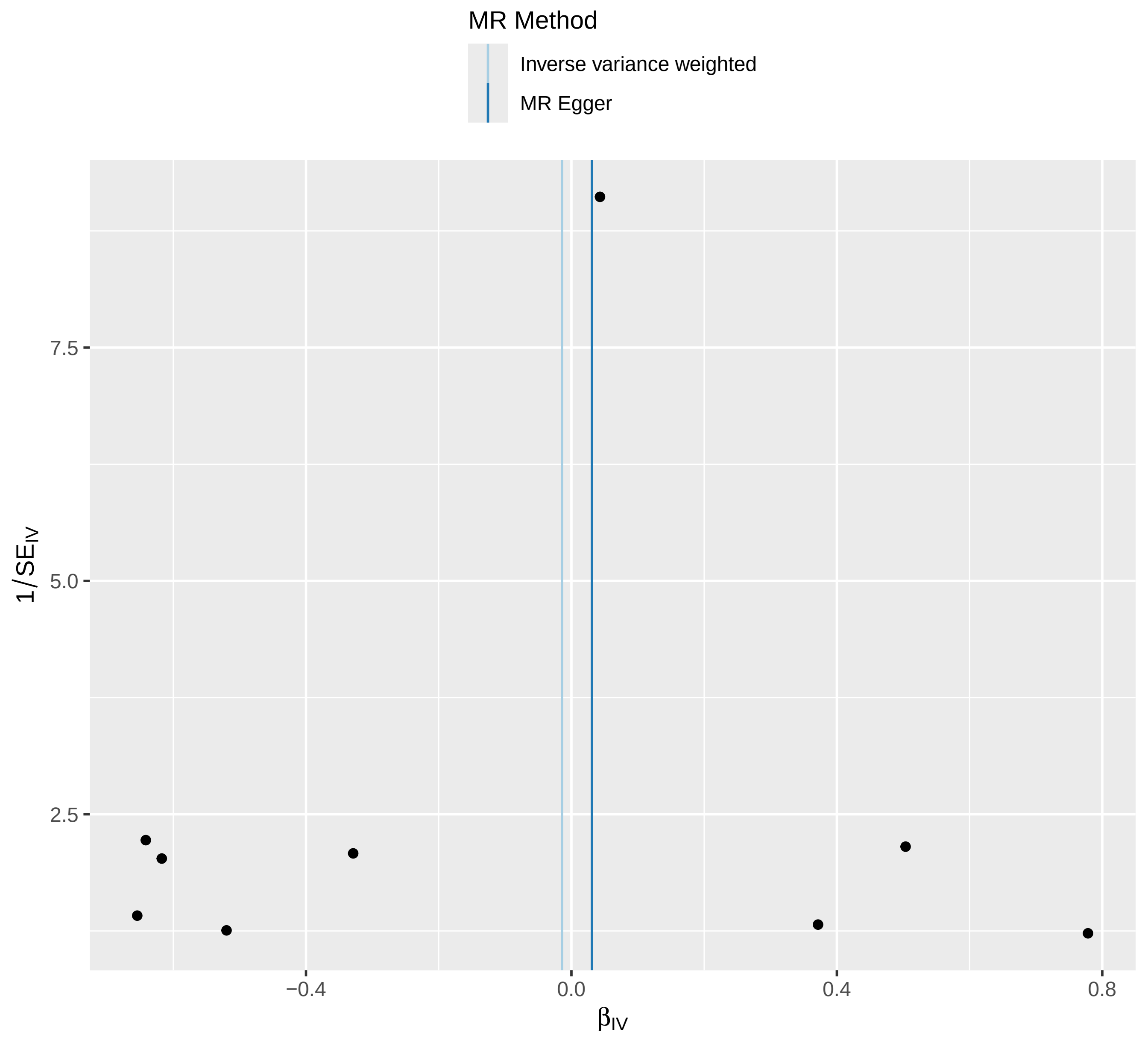

MR Method
Inverse variance weighted
MR Egger
7.5
5.0
2.5
$1/SE_{IV}$
−0.4
0.0
0.4
0.8
β_{IV}

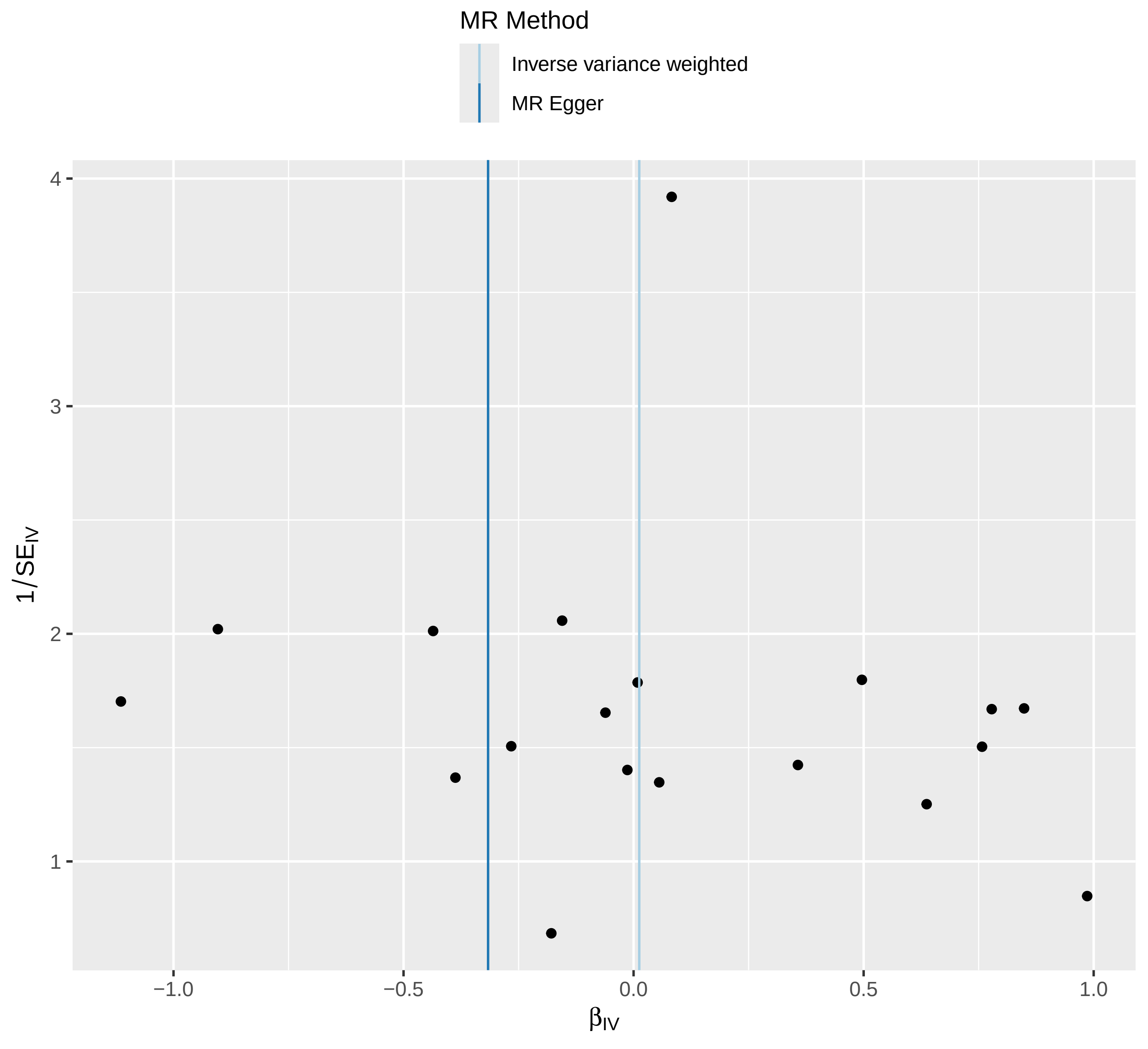
MR Method
Inverse variance weighted
MR Egger
$1/SE_{IV}$
4
3
2
1
−1.0
−0.5
0.0
0.5
1.0
β_{IV}

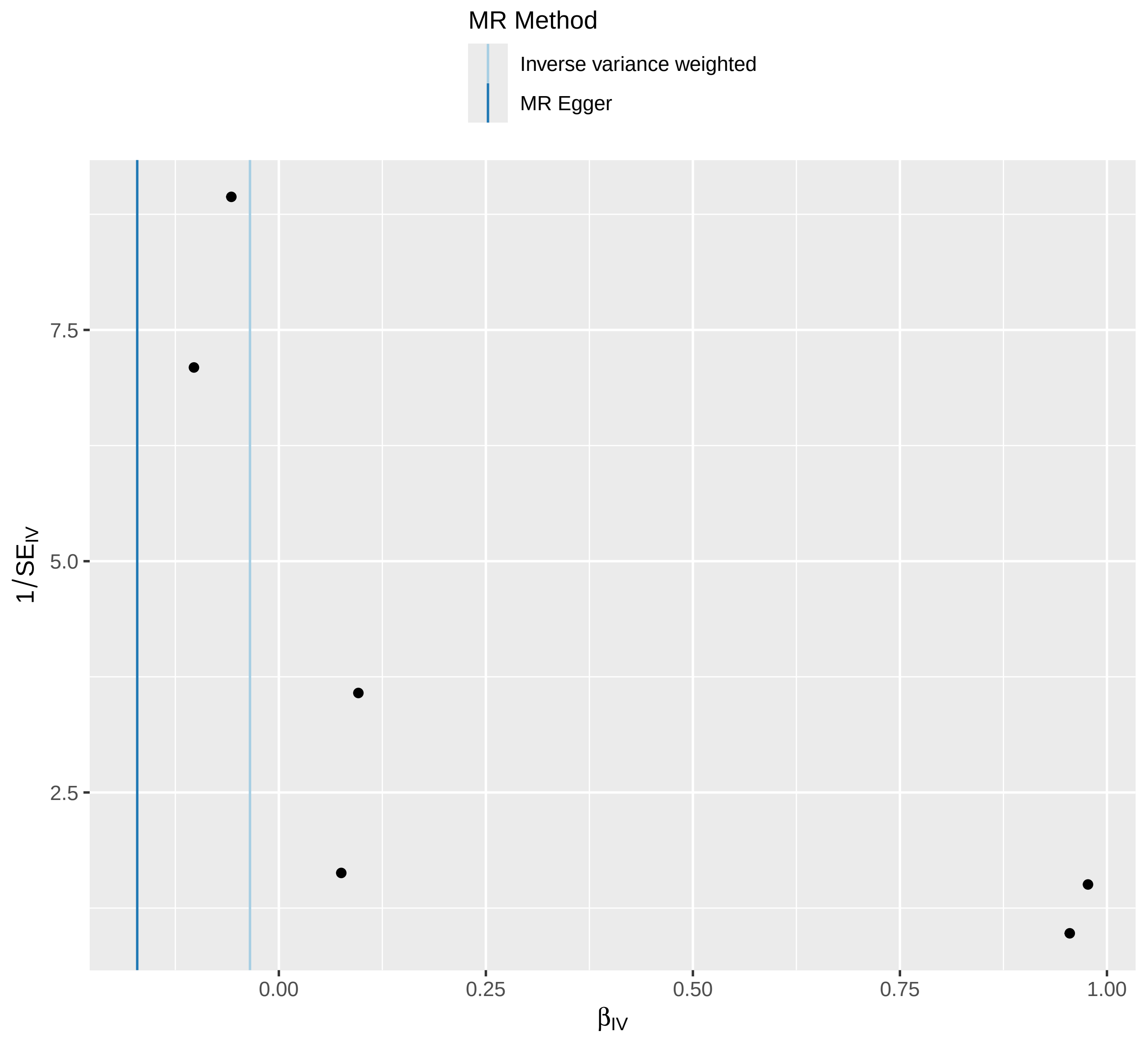
MR Method
Inverse variance weighted
MR Egger
7.5
5.0
2.5
$1/SE_{IV}$
0.00
0.25
0.50
0.75
1.00
β_{IV}

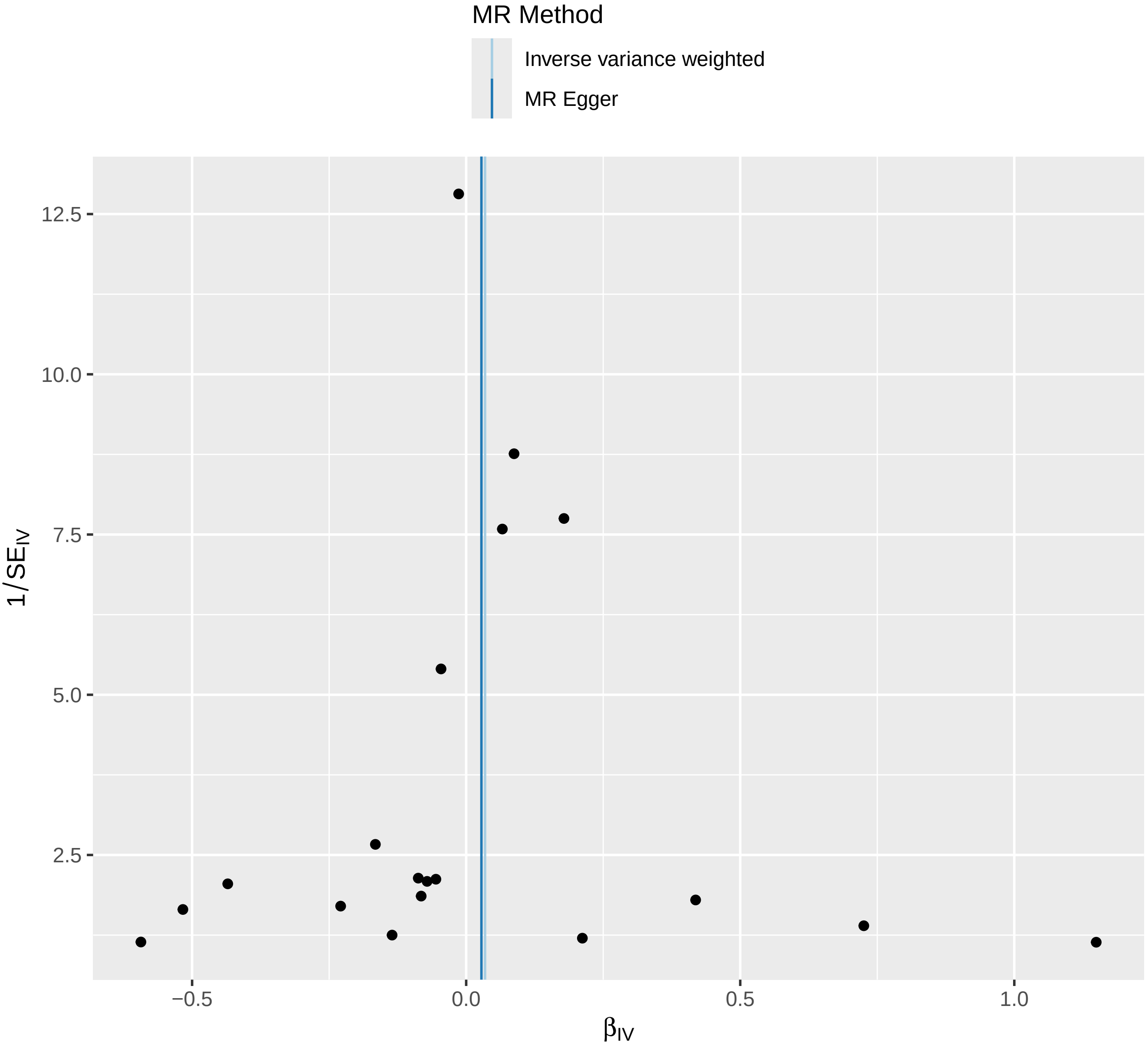
MR Method
Inverse variance weighted
MR Egger
12.5
10.0
7.5
5.0
2.5
1/SE_IV
−0.5
0.0
0.5
1.0
β_IV

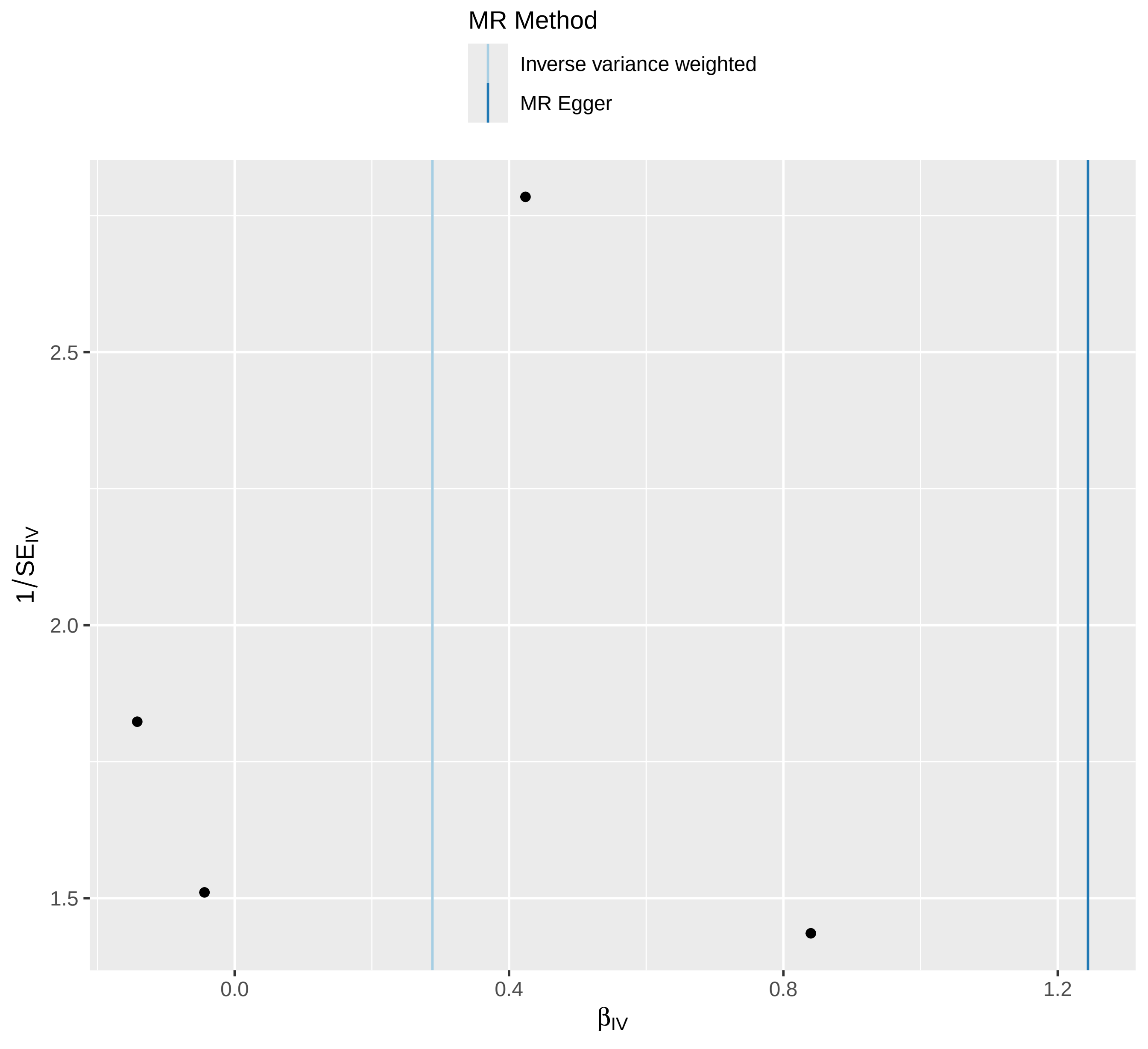
MR Method
Inverse variance weighted
MR Egger
1/SE$_{IV}$
2.5
2.0
1.5
0.0
0.4
0.8
1.2
β$_{IV}$

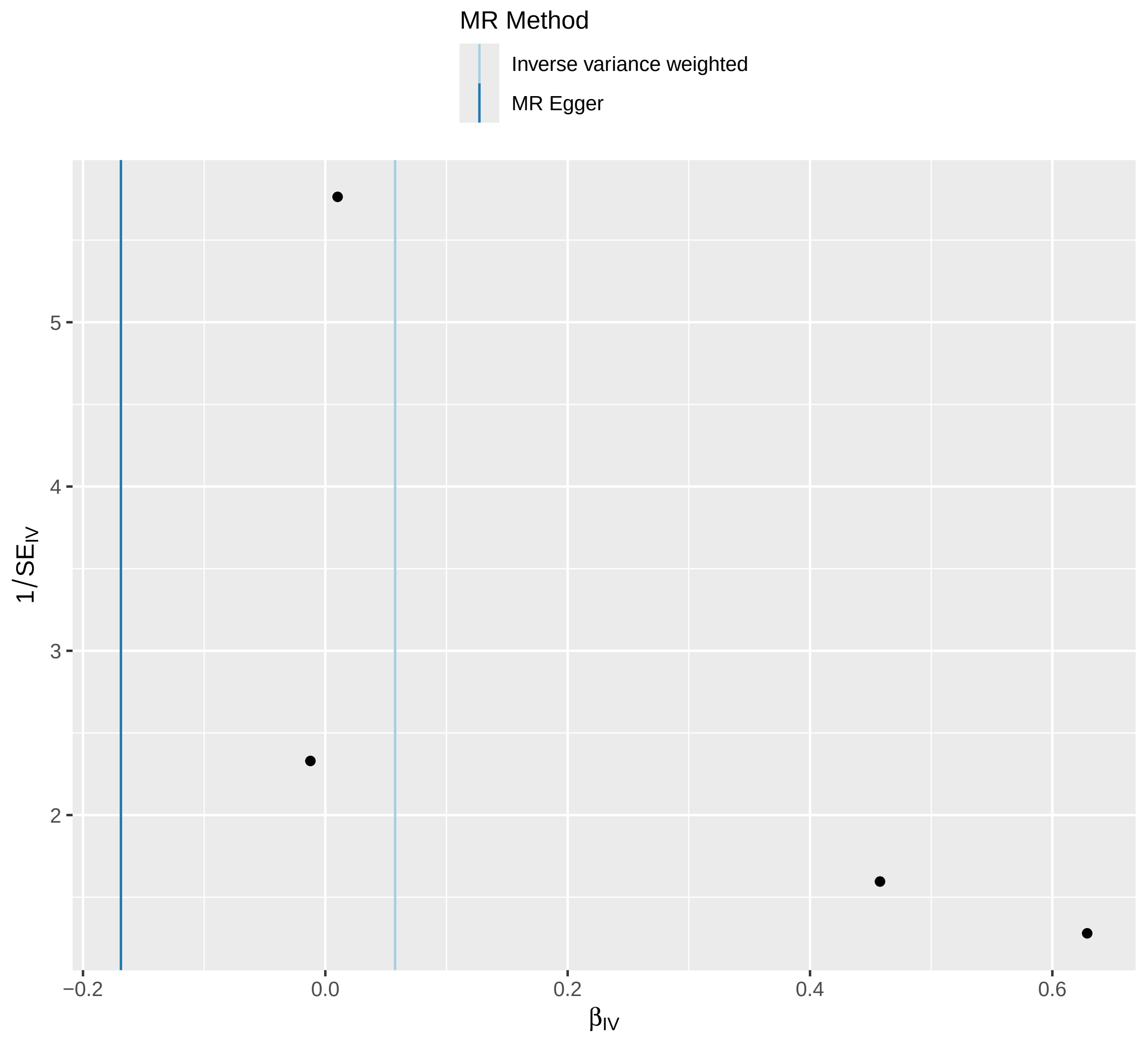

MR Method
Inverse variance weighted
MR Egger
5
4
3
2
1/SE_IV
−0.2
0.0
0.2
0.4
0.6
β_IV

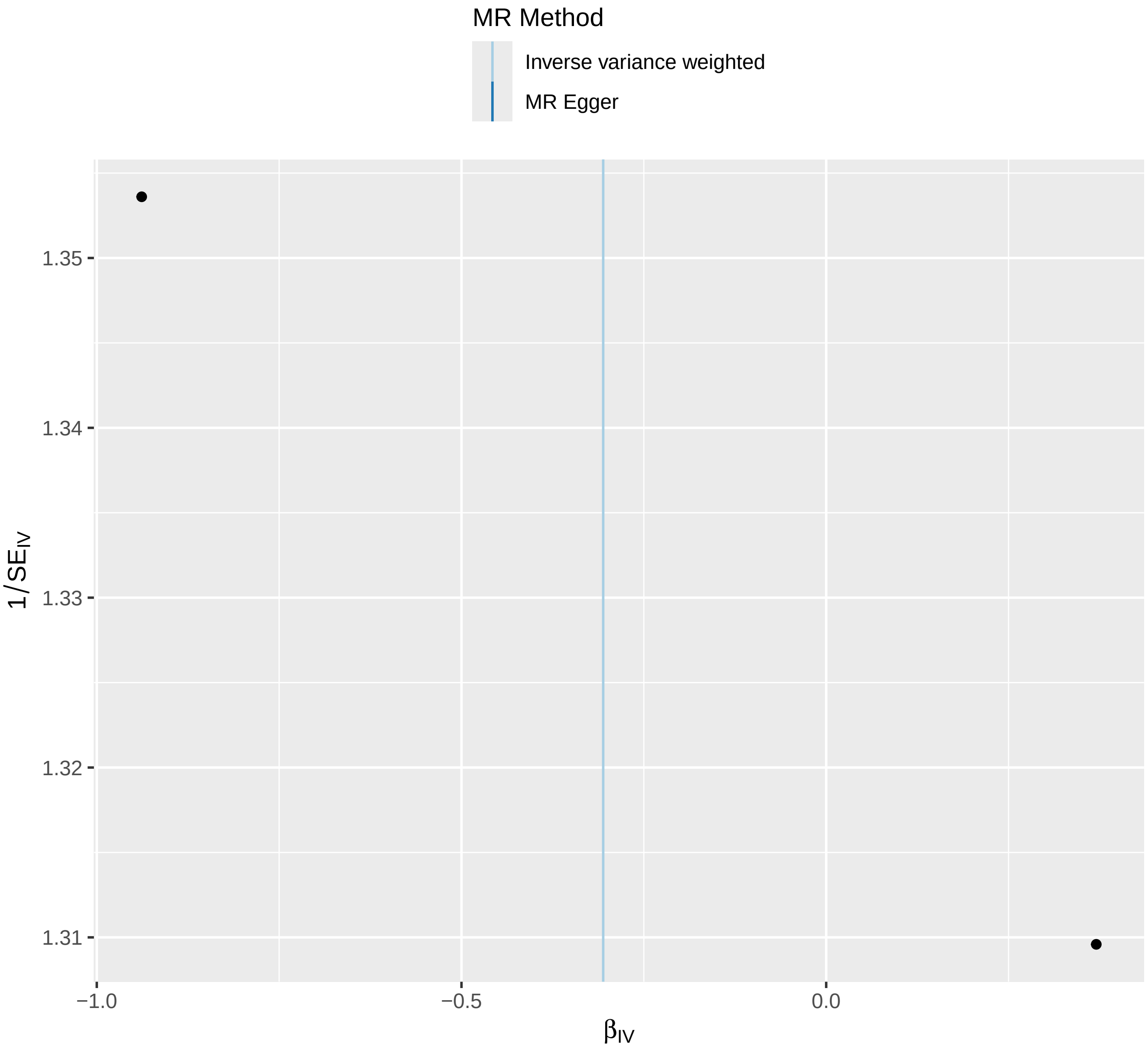

MR Method
Inverse variance weighted
MR Egger
1.35
1.34
1.33
1.32
1.31
$1/SE_{IV}$
−1.0
−0.5
0.0
β_{IV}

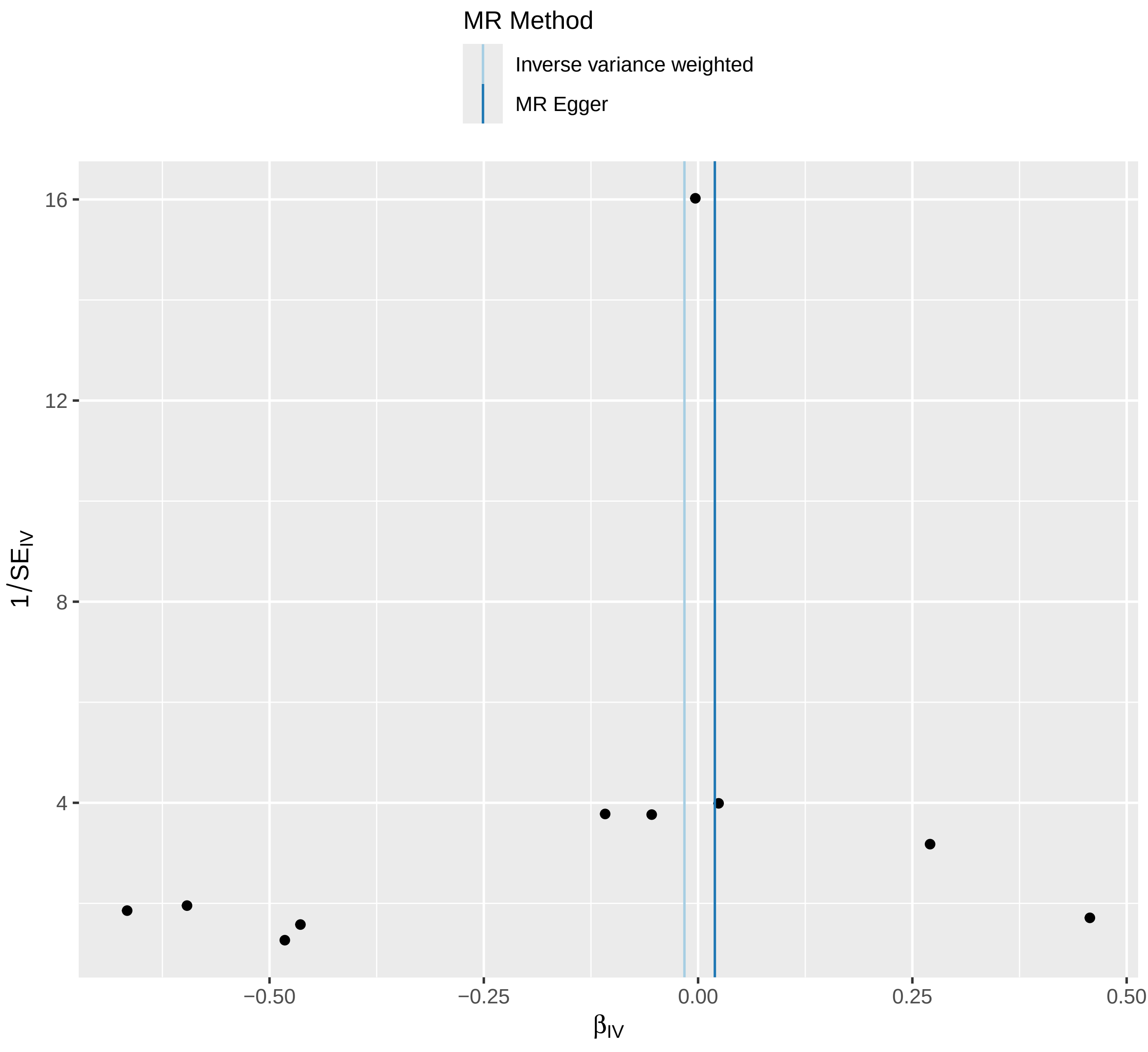
MR Method
Inverse variance weighted
MR Egger
16
12
8
4
1/SE$_{IV}$
−0.50
−0.25
0.00
0.25
0.50
β$_{IV}$

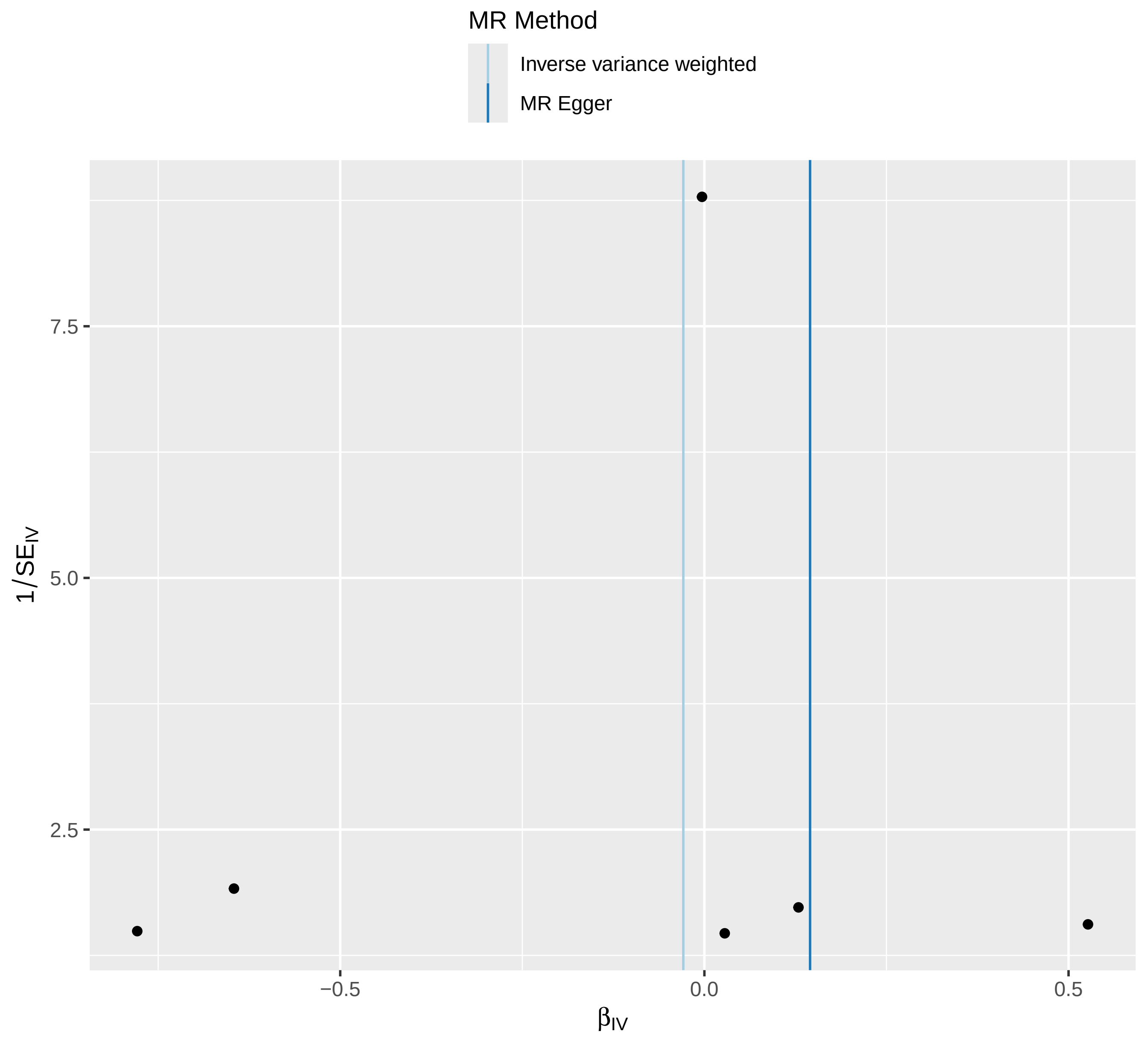
MR Method
Inverse variance weighted
MR Egger
7.5
5.0
2.5
1/SE$_{IV}$
−0.5
0.0
0.5
β$_{IV}$

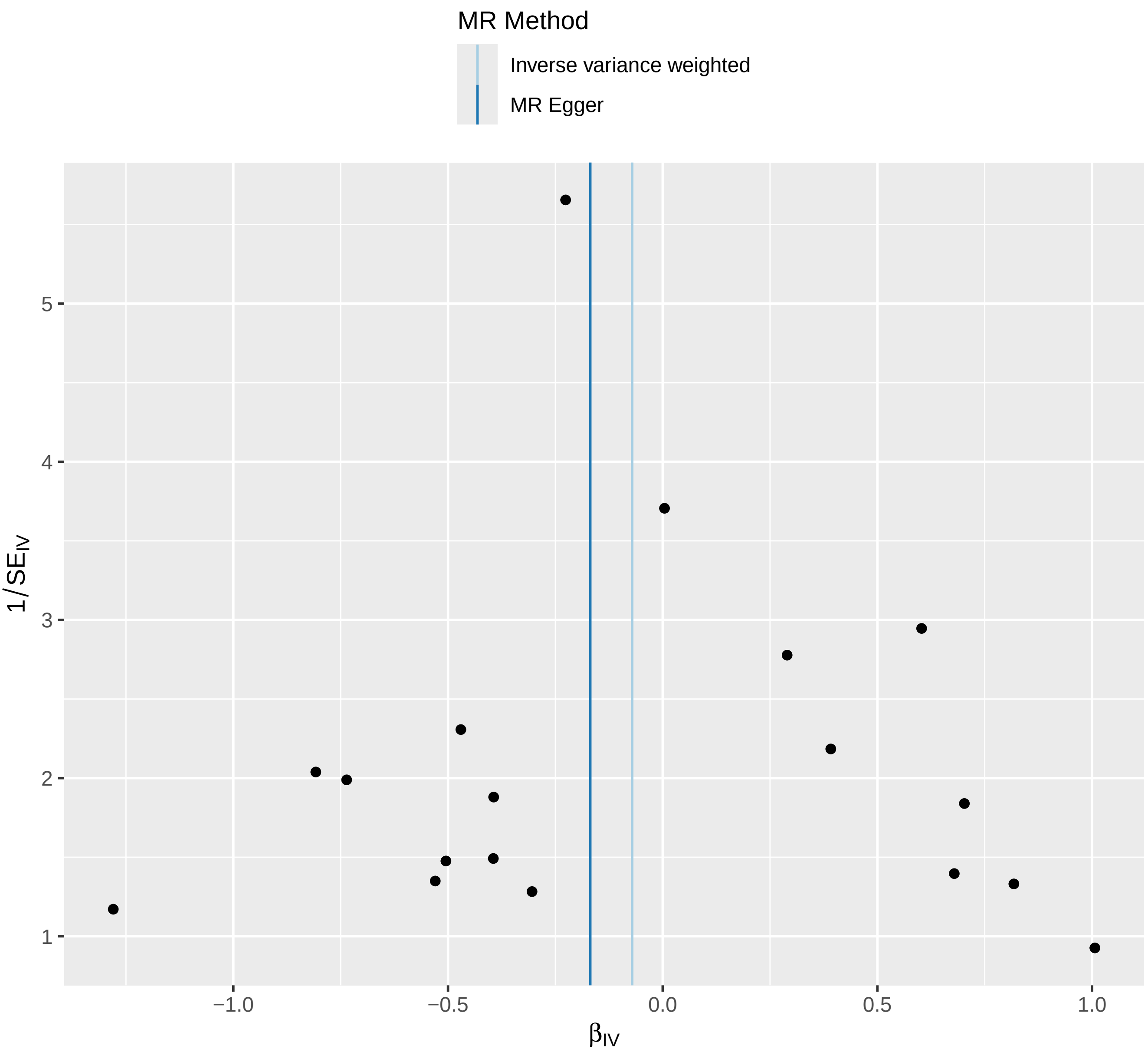

MR Method
Inverse variance weighted
MR Egger
1/SE$_{IV}$
5
4
3
2
1
−1.0
−0.5
0.0
0.5
1.0
β$_{IV}$

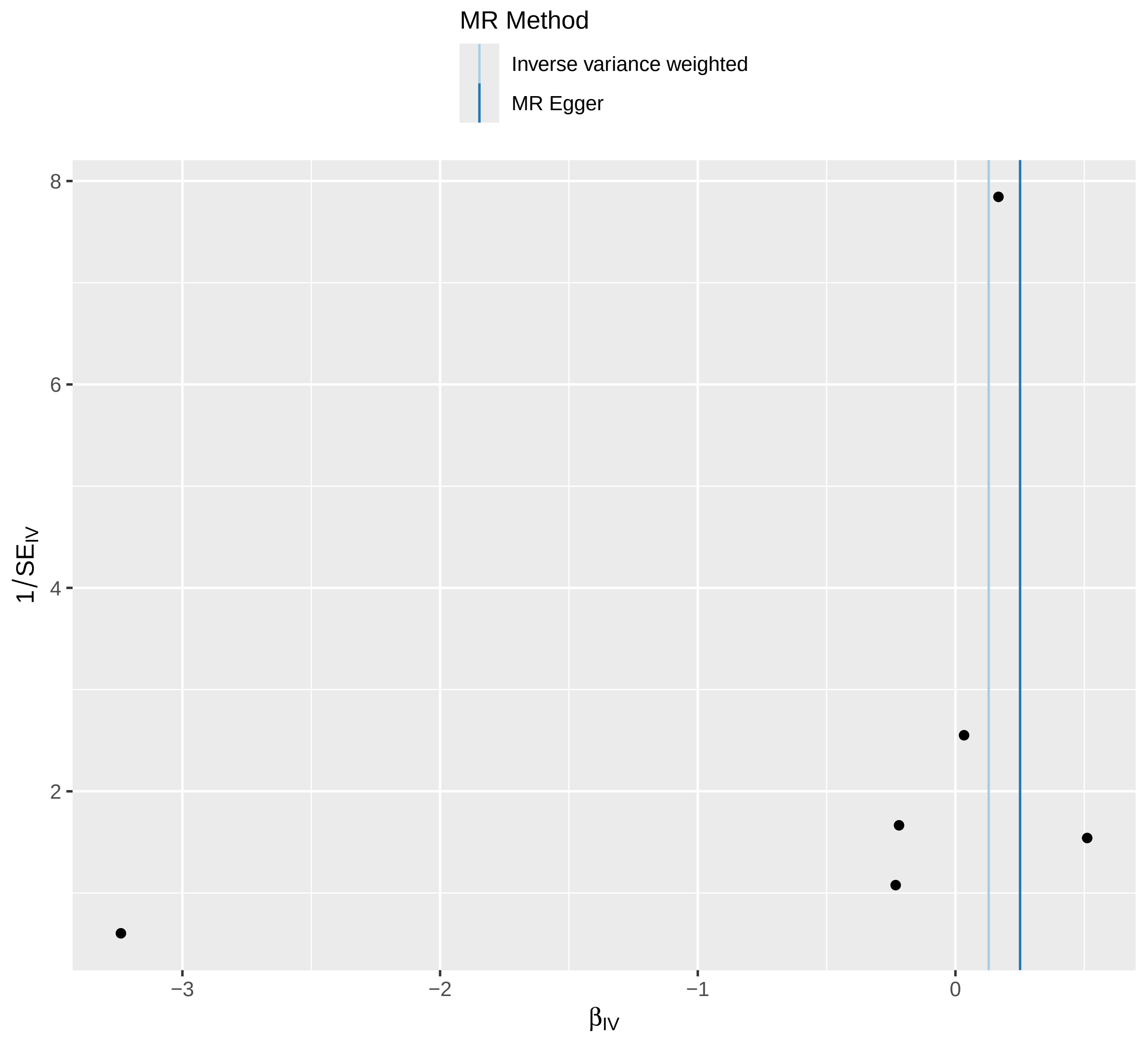

MR Method
Inverse variance weighted
MR Egger
8
6
4
2
1/SE_IV
−3
−2
−1
0
β_IV

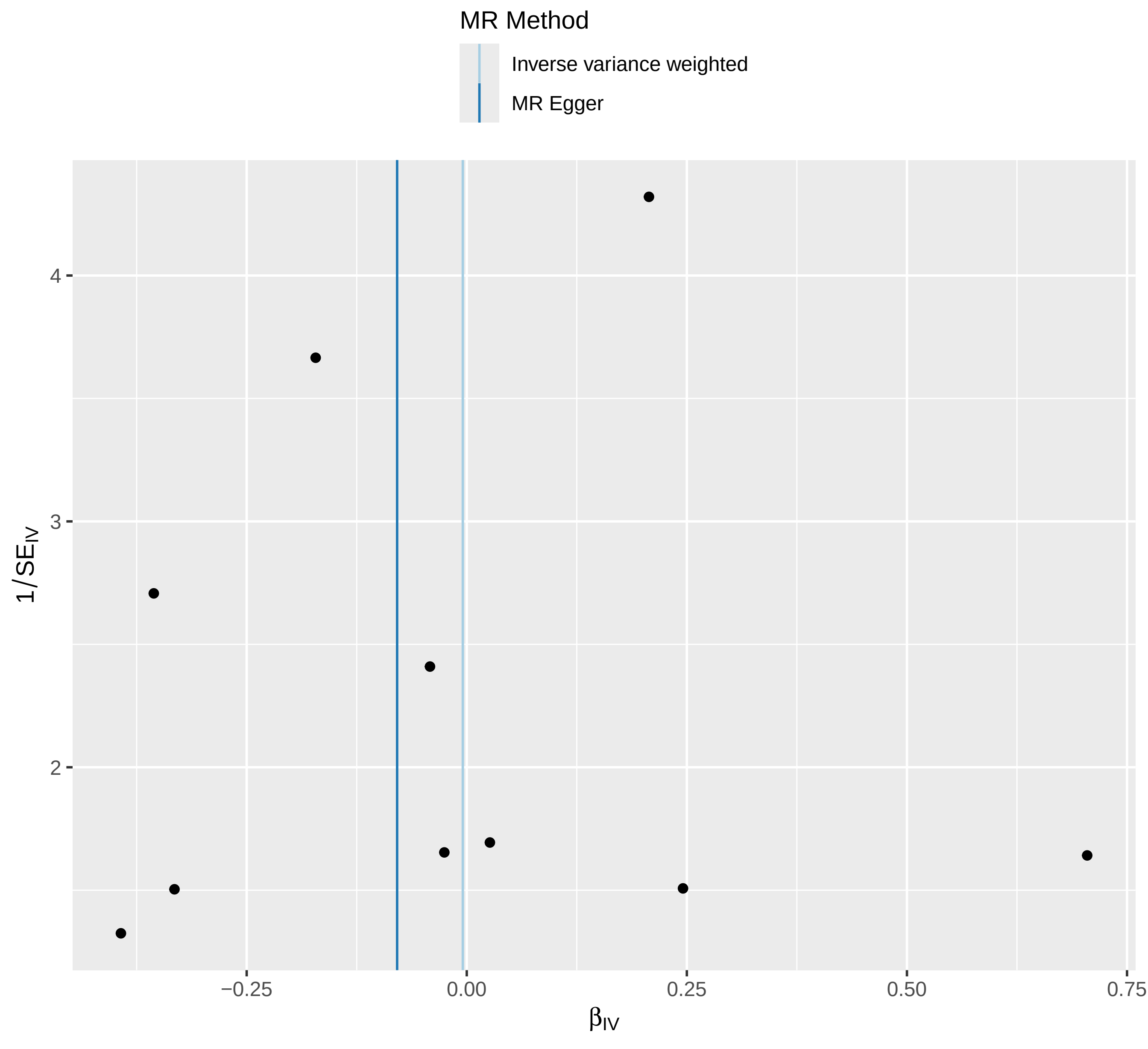
MR Method
Inverse variance weighted
MR Egger
4
3
2
1/SE_IV
−0.25
0.00
0.25
0.50
0.75
β_IV

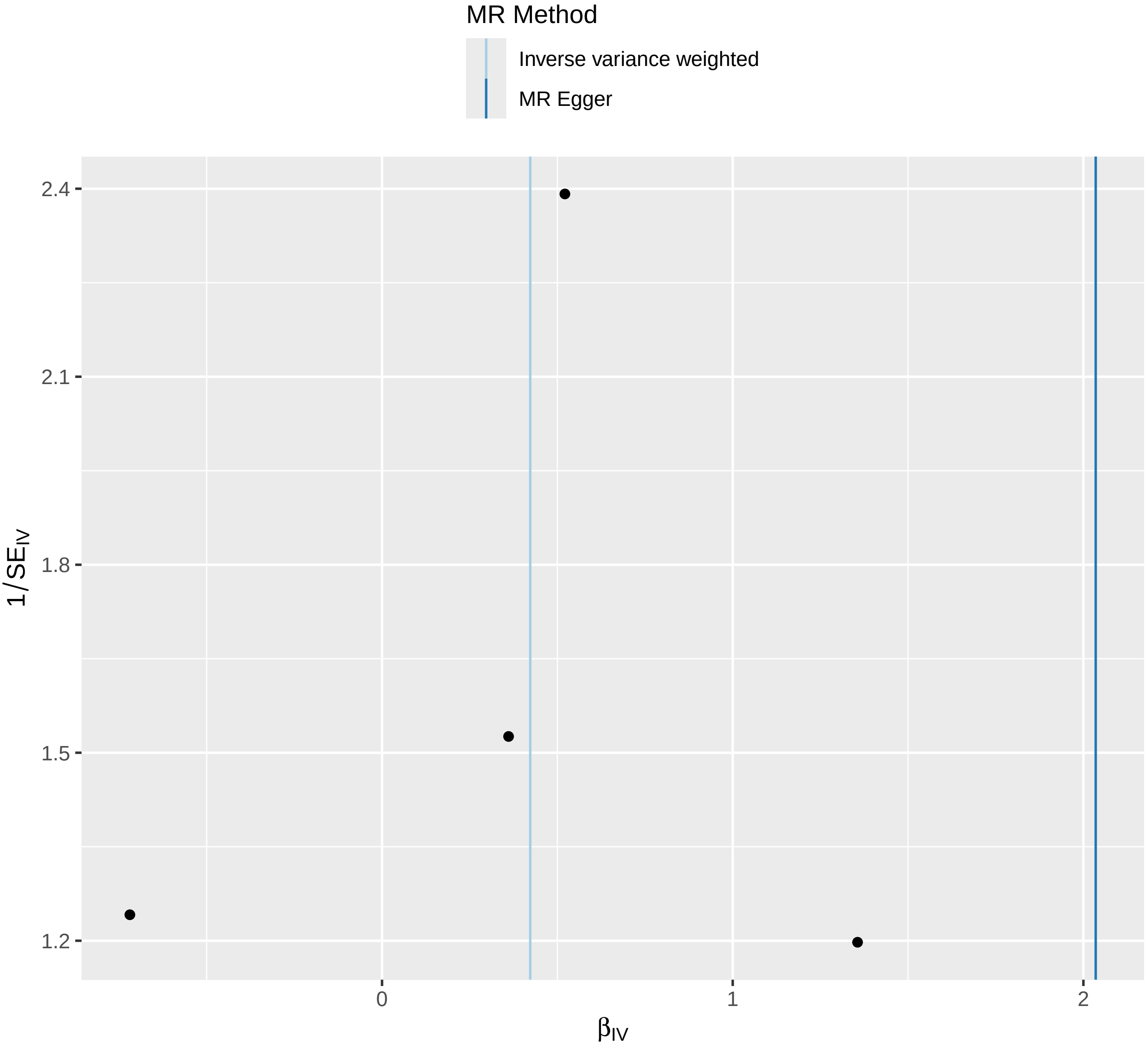

MR Method
Inverse variance weighted
MR Egger
2.4
2.1
1.8
1.5
1.2
1/SE_IV
0
1
2
β_IV

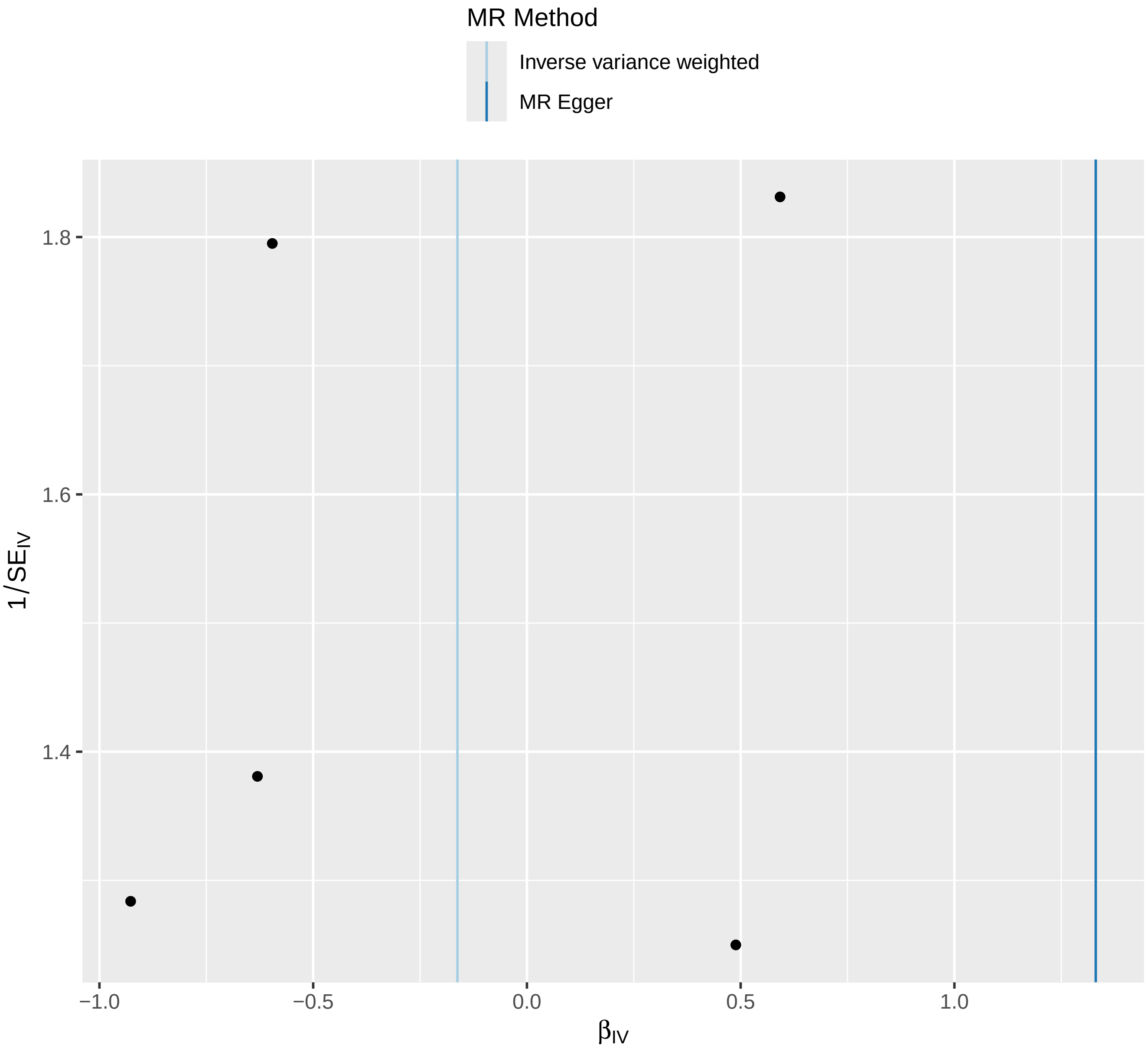
MR Method
Inverse variance weighted
MR Egger
1.8
1.6
1.4
1/SE_{IV}
−1.0
−0.5
0.0
0.5
1.0
$β_{IV}$

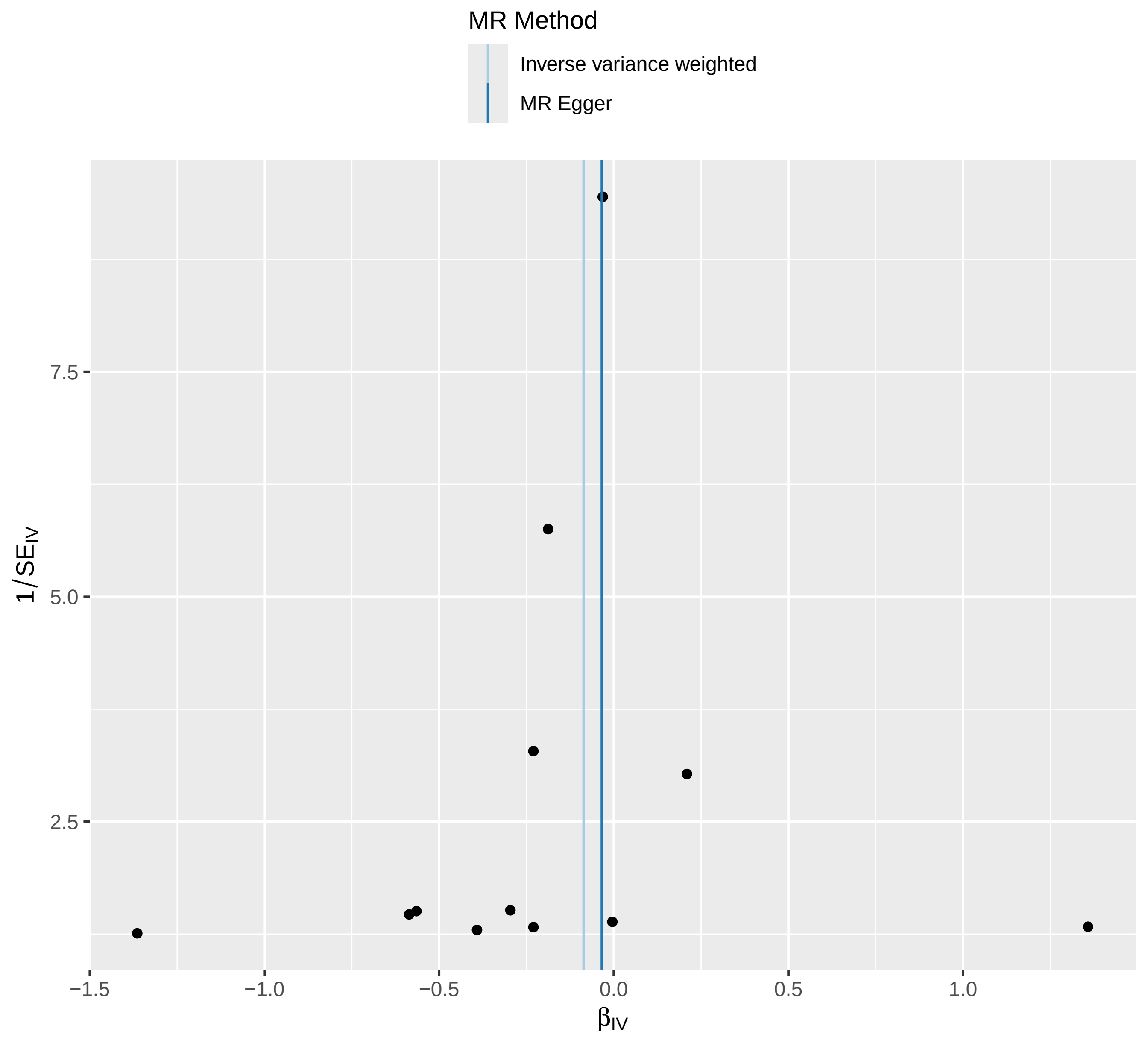
MR Method
Inverse variance weighted
MR Egger
7.5
5.0
2.5
$1/SE_{IV}$
−1.5
−1.0
−0.5
0.0
0.5
1.0
β_{IV}

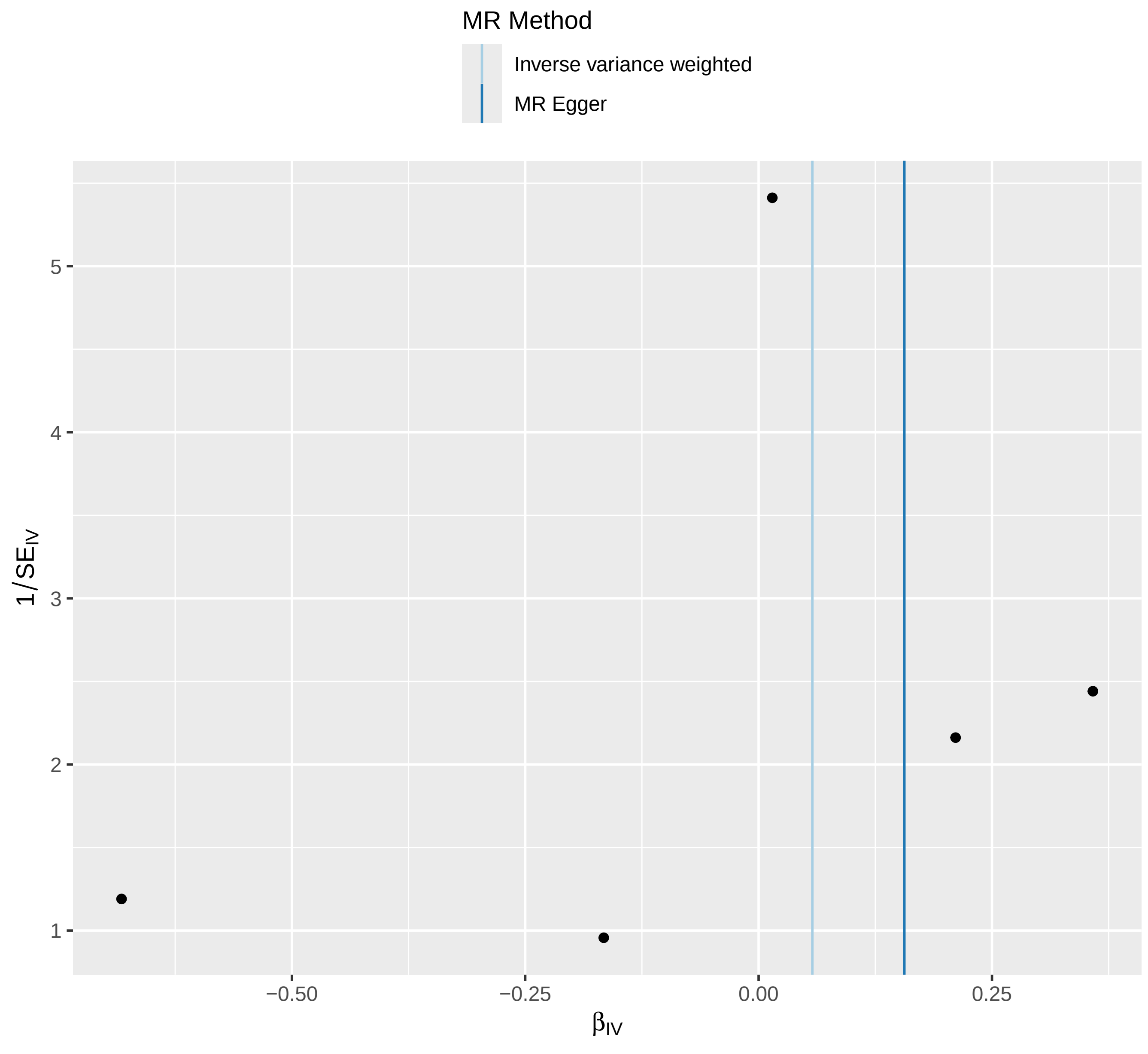

MR Method
Inverse variance weighted
MR Egger
5
4
3
2
1
1/SE_IV
−0.50
−0.25
0.00
0.25
β_IV

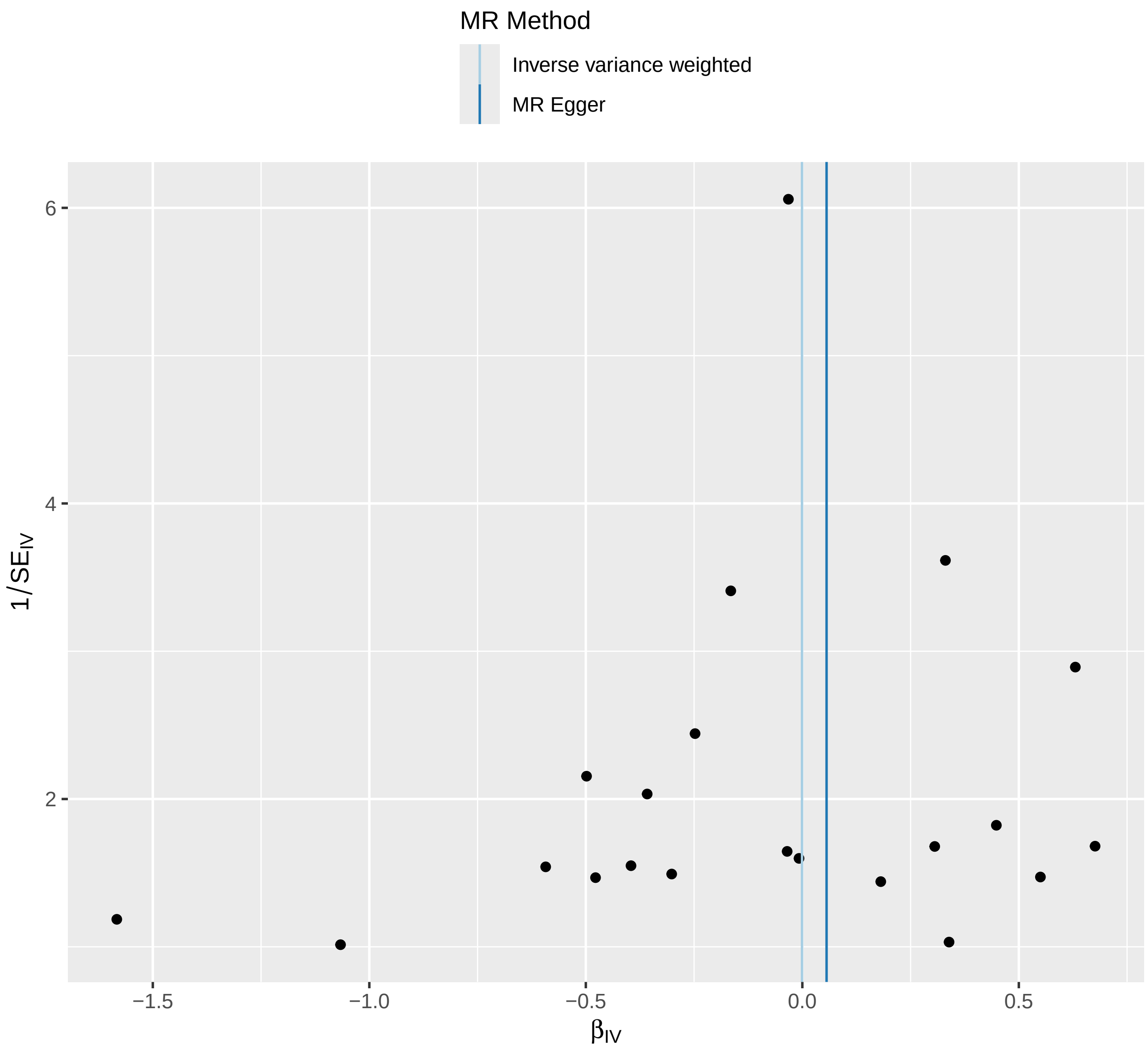

MR Method
Inverse variance weighted
MR Egger
6
4
2
1/SE$_{IV}$
−1.5
−1.0
−0.5
0.0
0.5
β$_{IV}$

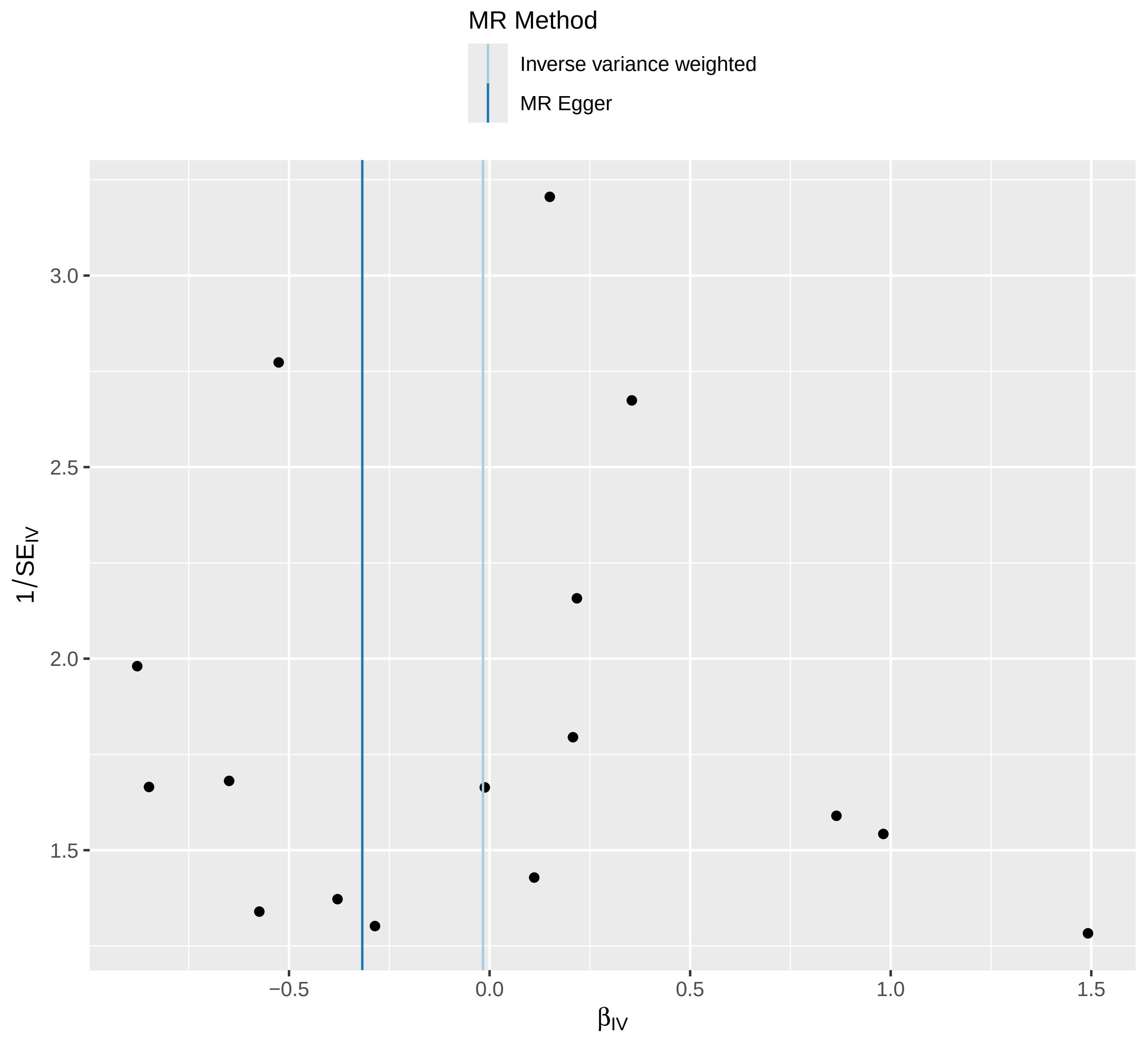
MR Method
Inverse variance weighted
MR Egger
1/SE_IV
3.0
2.5
2.0
1.5
−0.5
0.0
0.5
1.0
1.5
β_IV

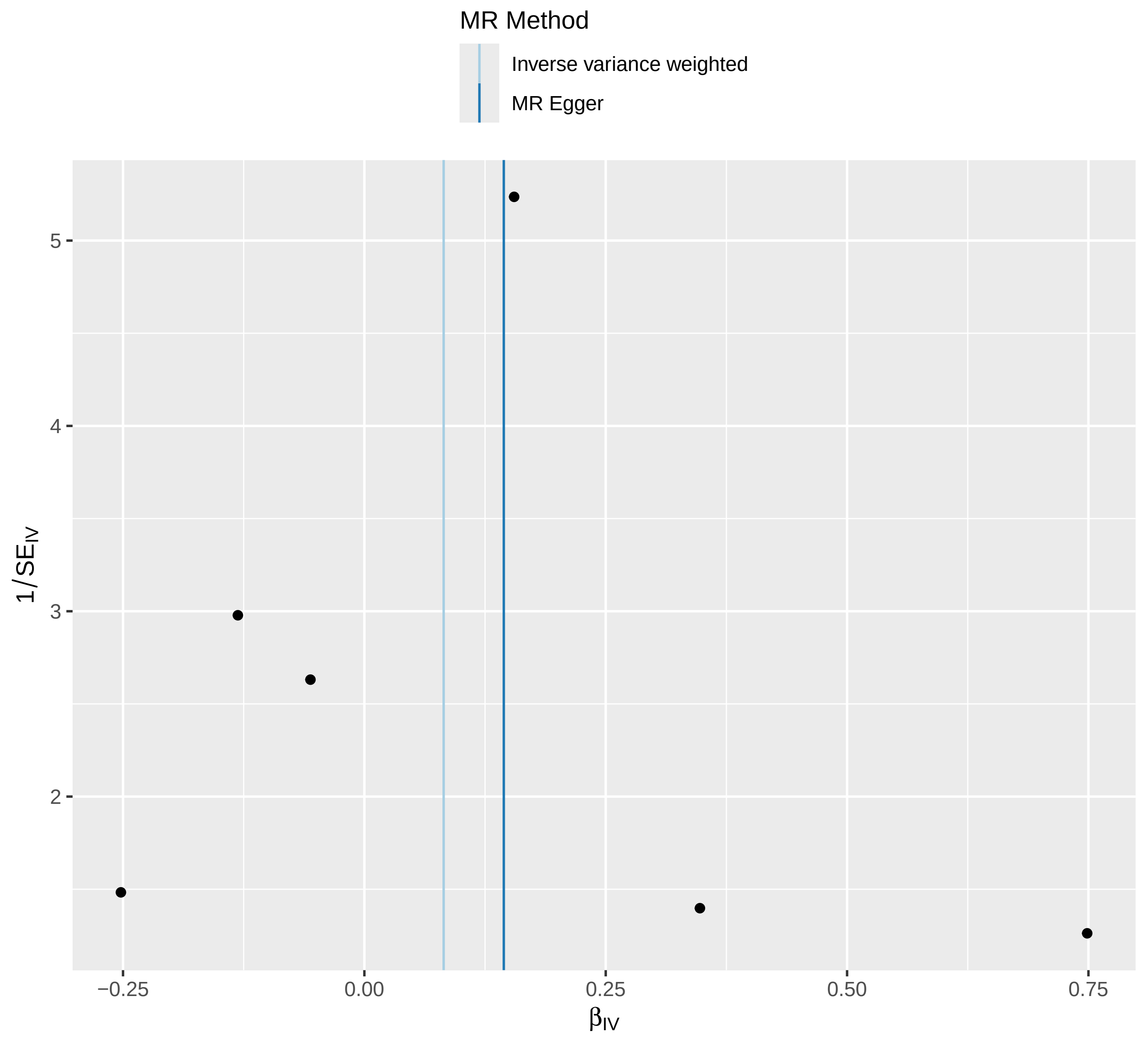

MR Method
Inverse variance weighted
MR Egger
5
4
3
2
1/SE_IV
−0.25
0.00
0.25
0.50
0.75
β_IV

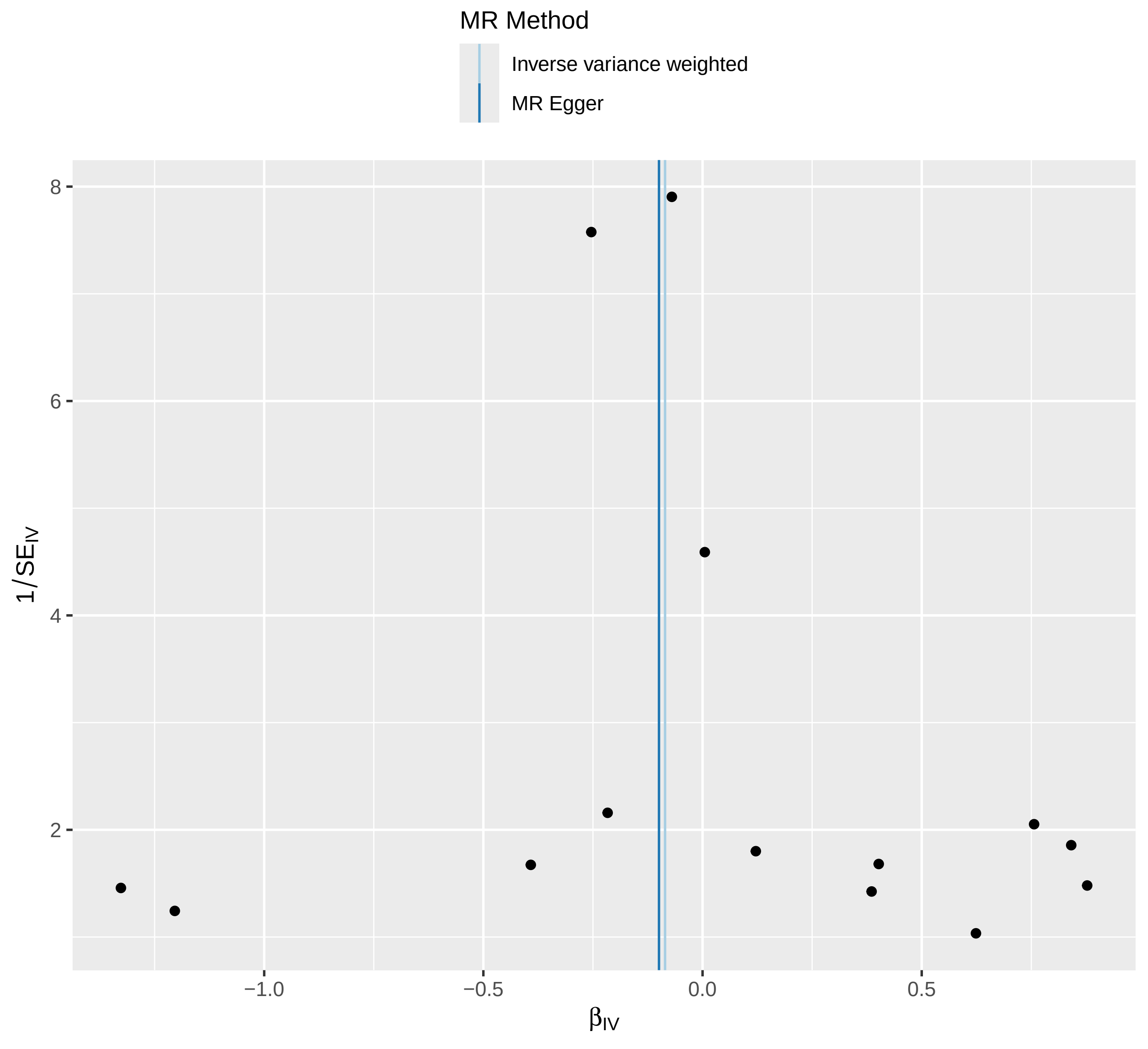
MR Method
Inverse variance weighted
MR Egger
8
6
4
2
1/SE_IV
−1.0
−0.5
0.0
0.5
β_IV

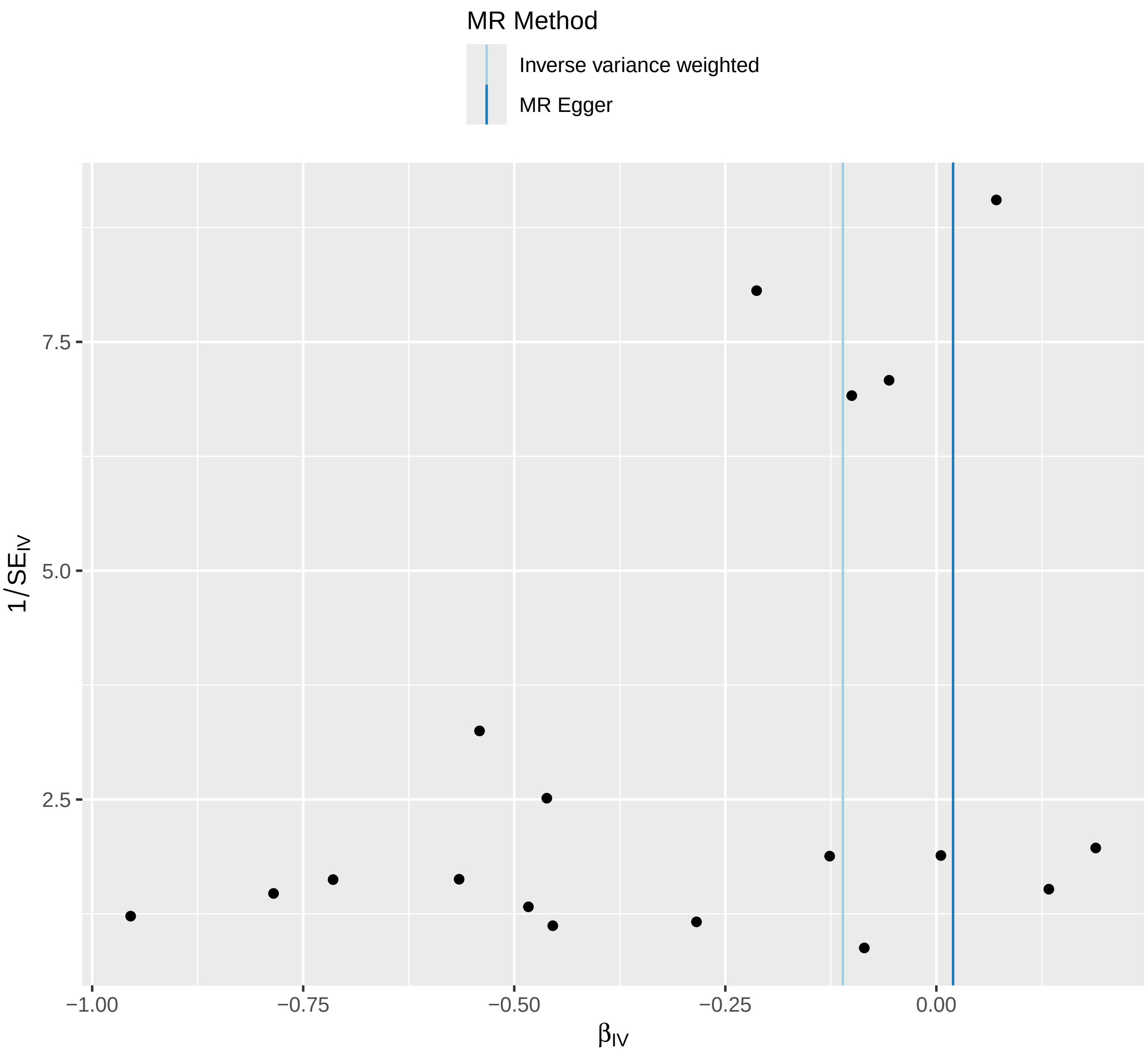
MR Method
Inverse variance weighted
MR Egger
7.5
5.0
2.5
1/SE_IV
−1.00
−0.75
−0.50
−0.25
0.00
β_IV

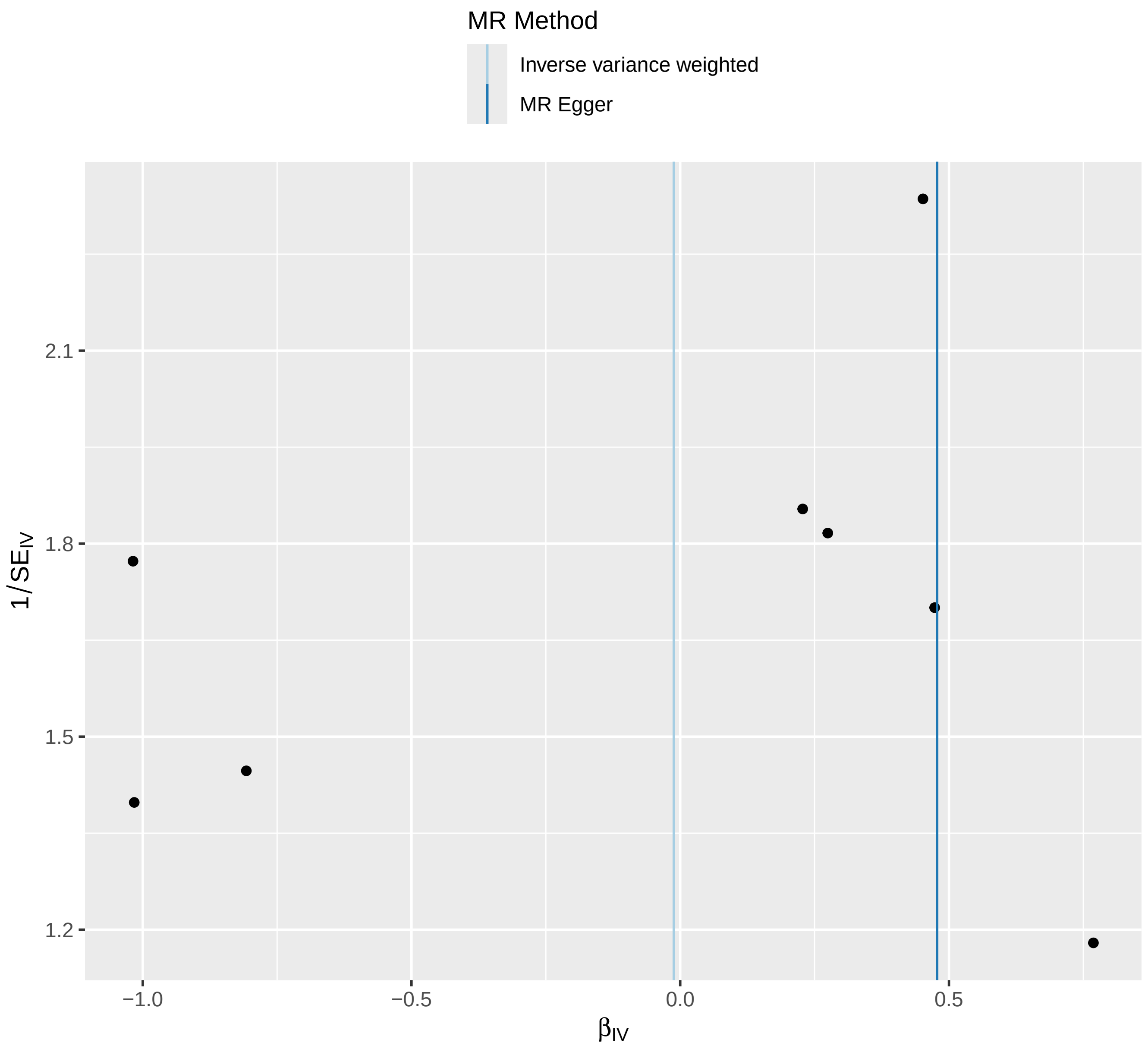
MR Method
Inverse variance weighted
MR Egger
2.1
1.8
1.5
1.2
1/SE_IV
−1.0
−0.5
0.0
0.5
β_IV

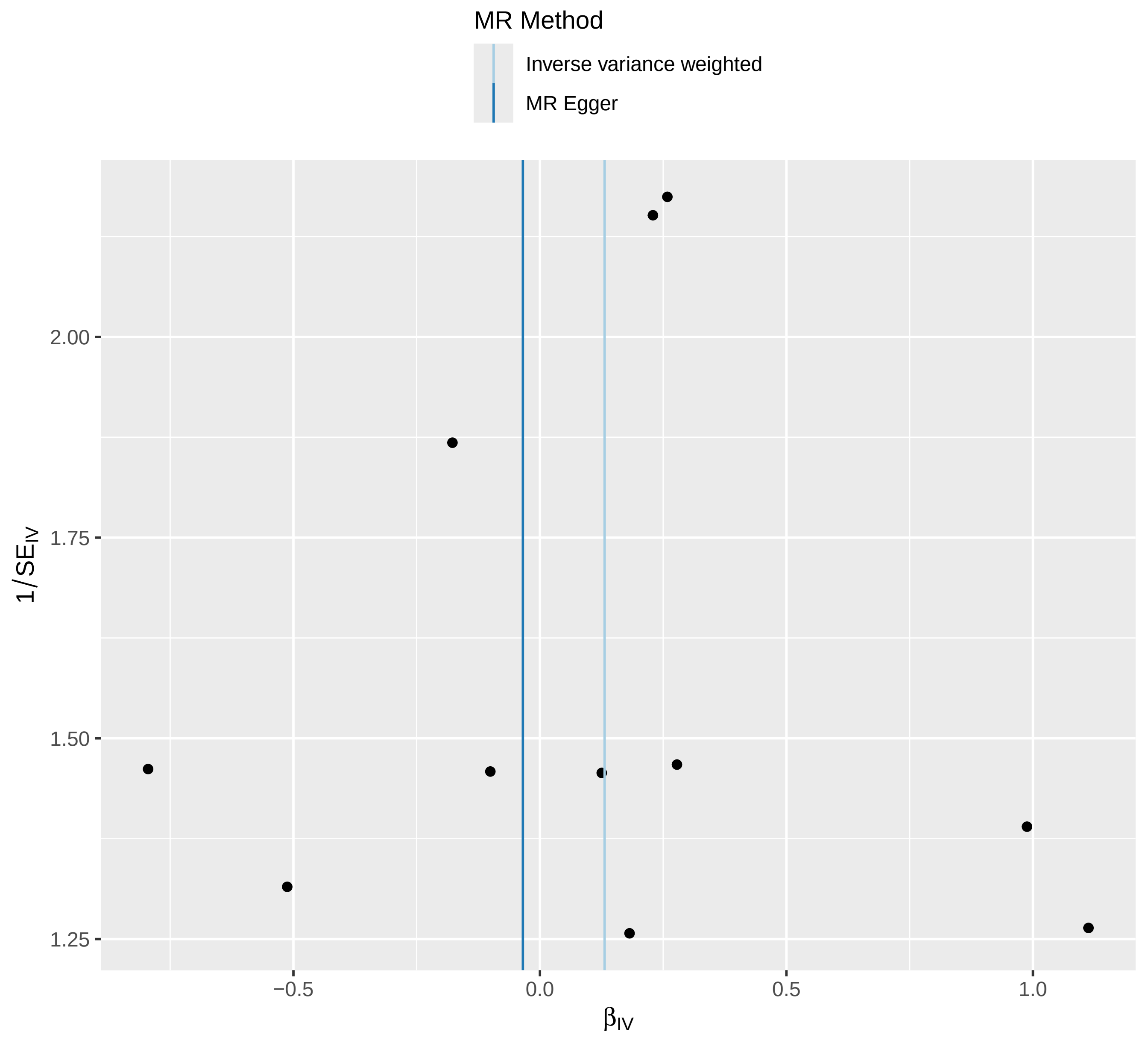

MR Method
Inverse variance weighted
MR Egger
1/SE_IV
2.00
1.75
1.50
1.25
−0.5
0.0
0.5
1.0
β_IV

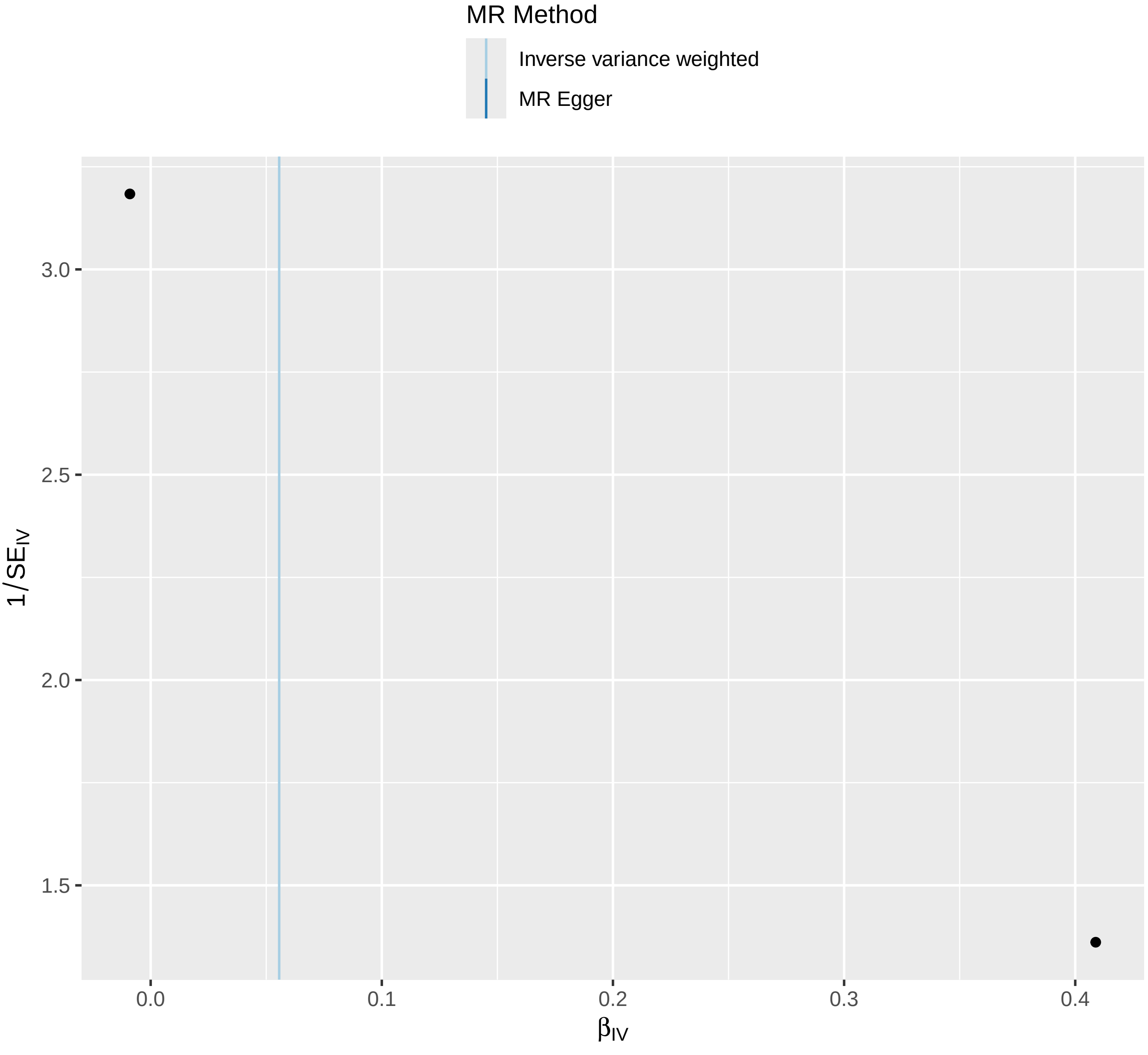

MR Method
Inverse variance weighted
MR Egger
1/SE$_{IV}$
3.0
2.5
2.0
1.5
0.0
0.1
0.2
0.3
0.4
β$_{IV}$

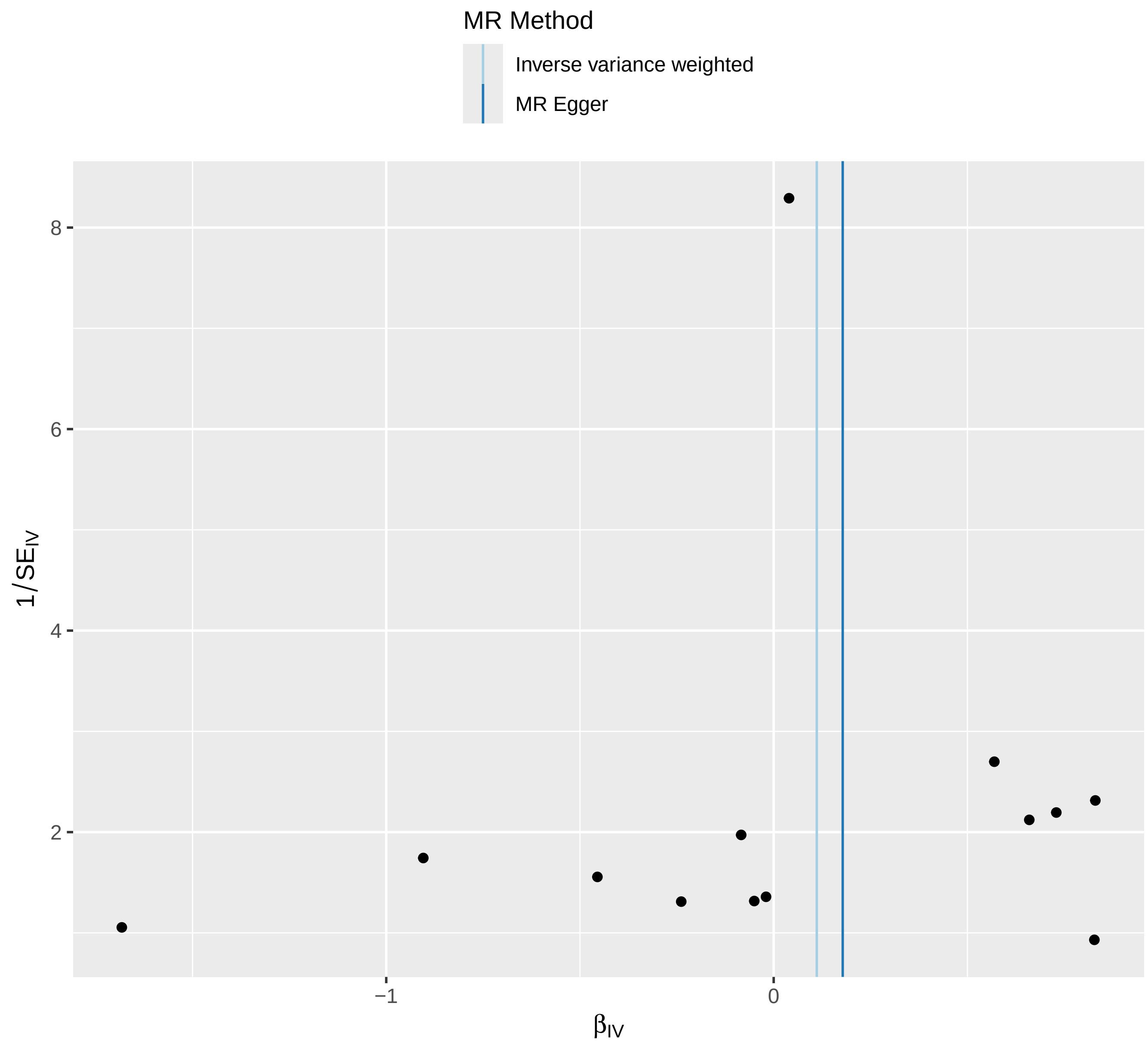
MR Method
Inverse variance weighted
MR Egger
$1/SE_{IV}$
8
6
4
2
−1
0
β_{IV}

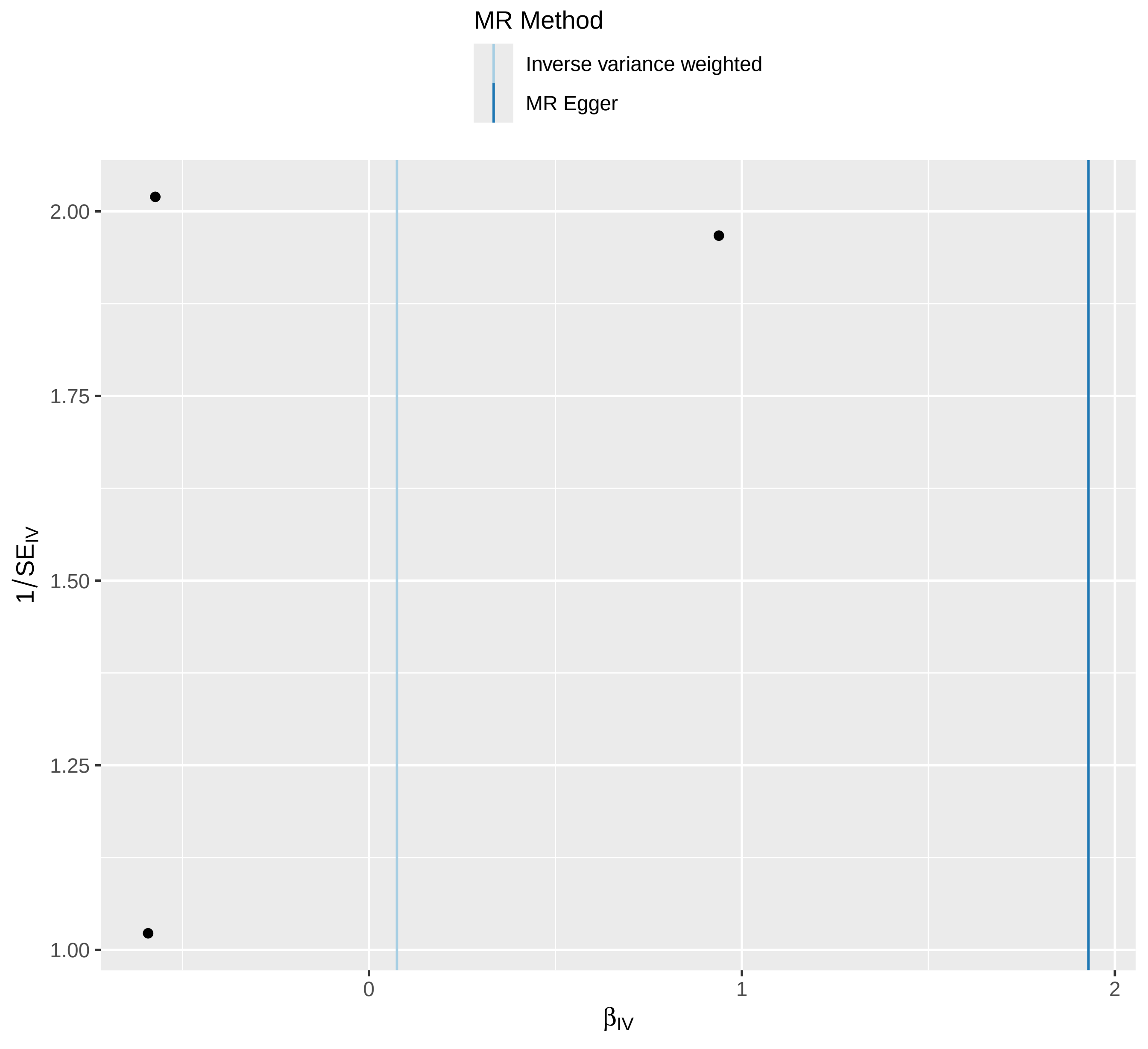
MR Method
Inverse variance weighted
MR Egger
2.00
1.75
1.50
1.25
1.00
$1/SE_{IV}$
0
1
2
β_{IV}

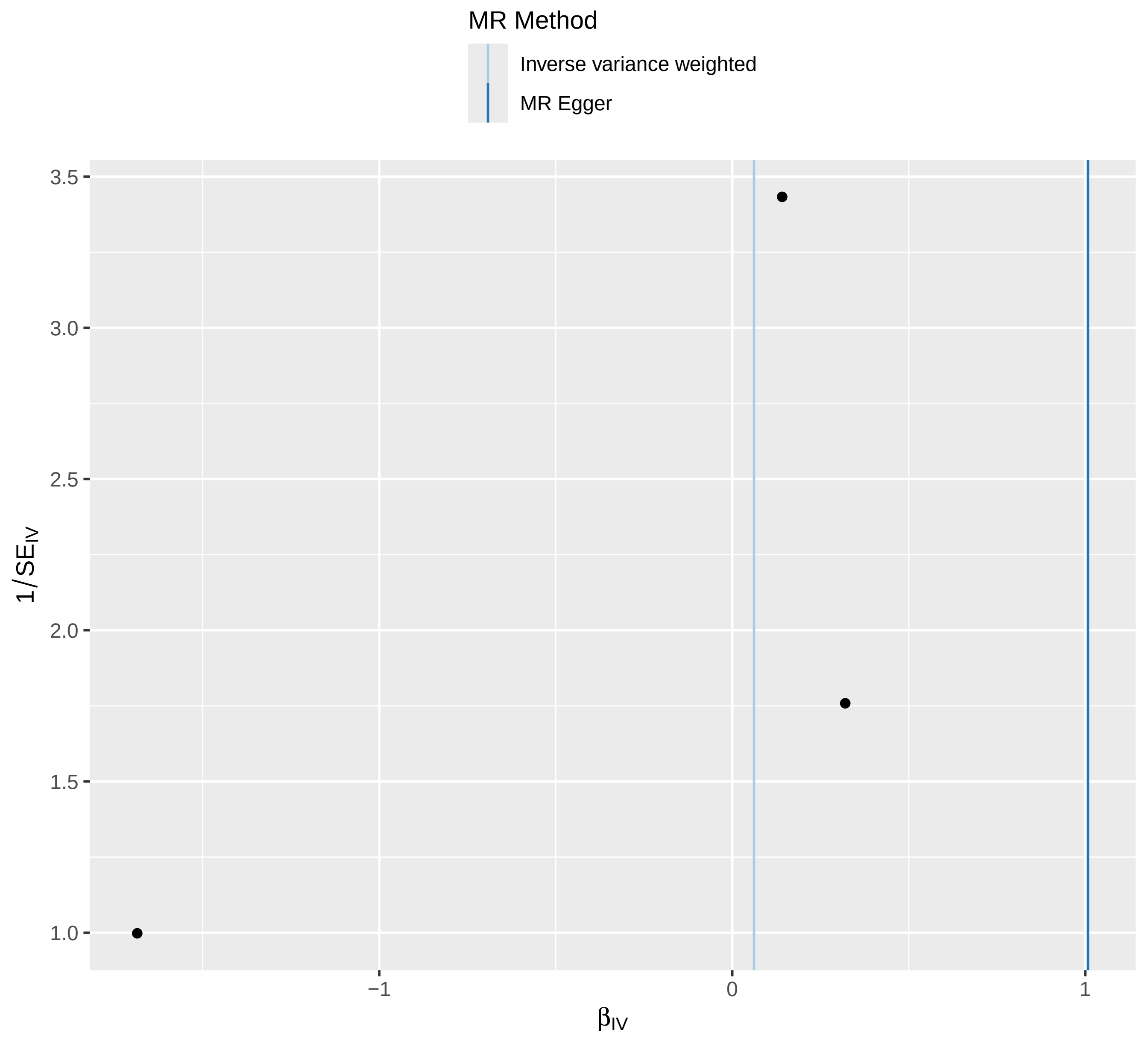
MR Method
Inverse variance weighted
MR Egger
3.5
3.0
2.5
2.0
1.5
1.0
1/SE_IV
−1
0
1
β_IV

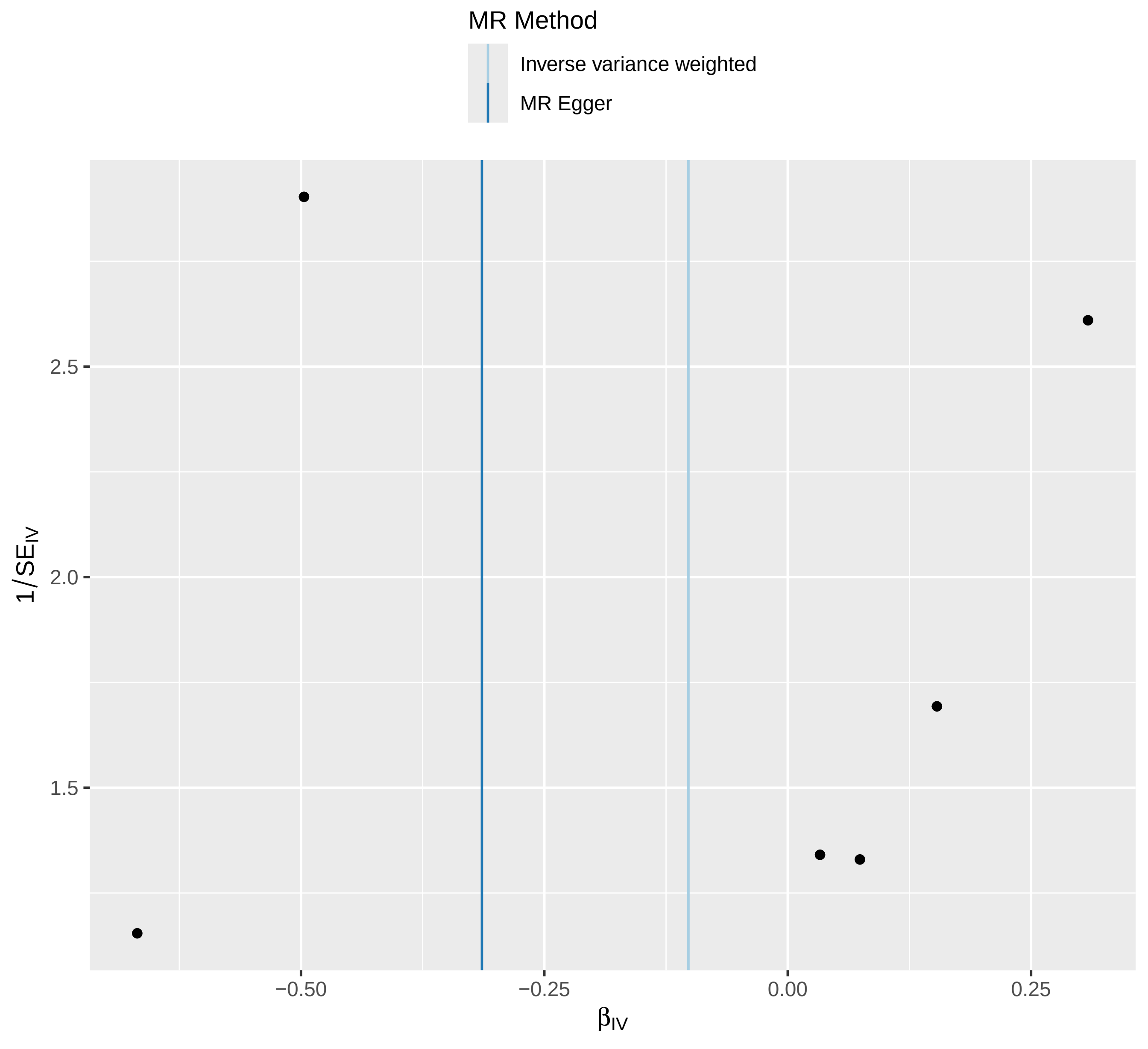
MR Method
Inverse variance weighted
MR Egger
2.5
2.0
1.5
−0.50
−0.25
0.00
0.25
$1/SE_{IV}$
β_{IV}

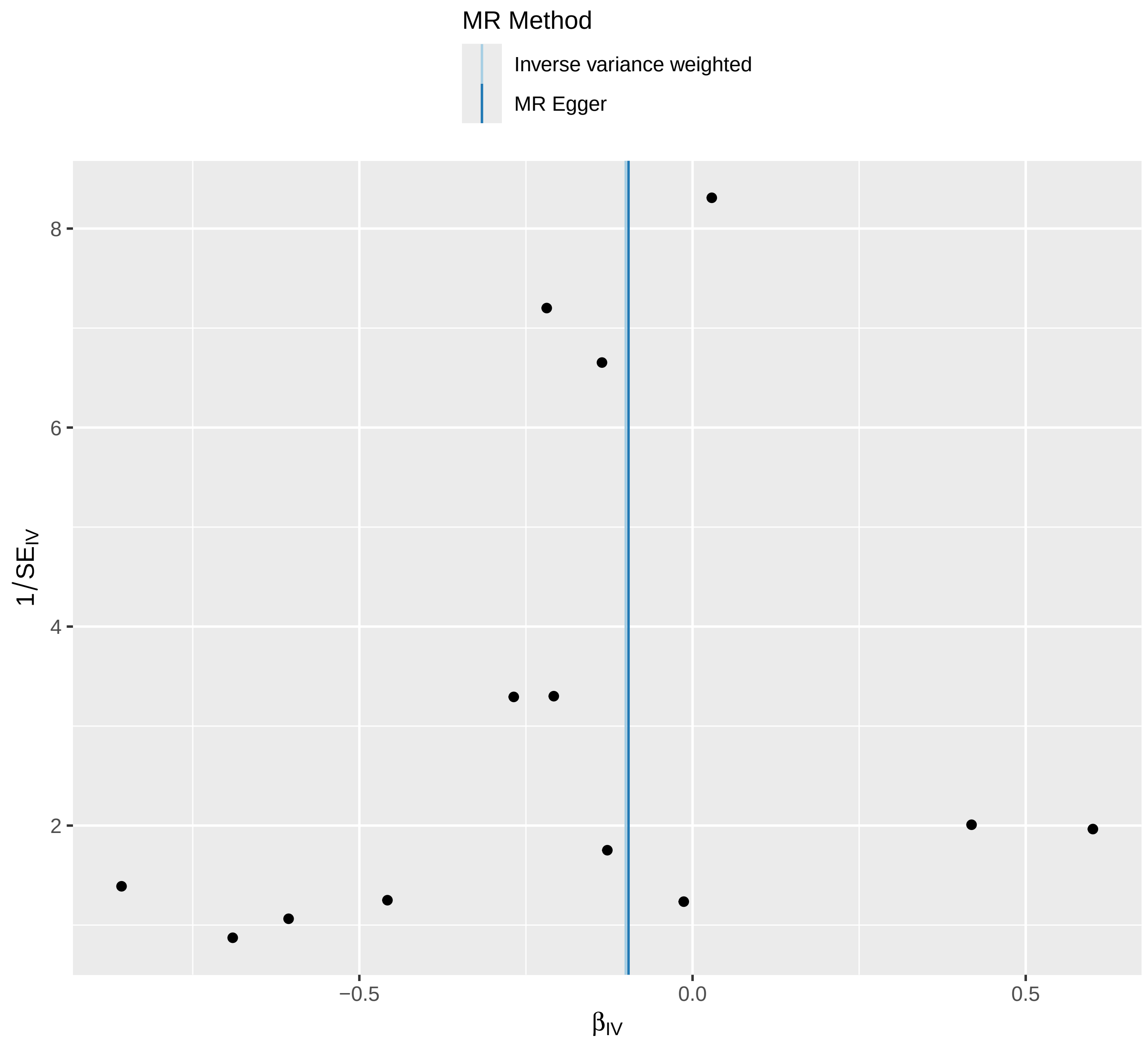

MR Method
Inverse variance weighted
MR Egger
1/SE$_{IV}$
8
6
4
2
−0.5
0.0
0.5
β_{IV}

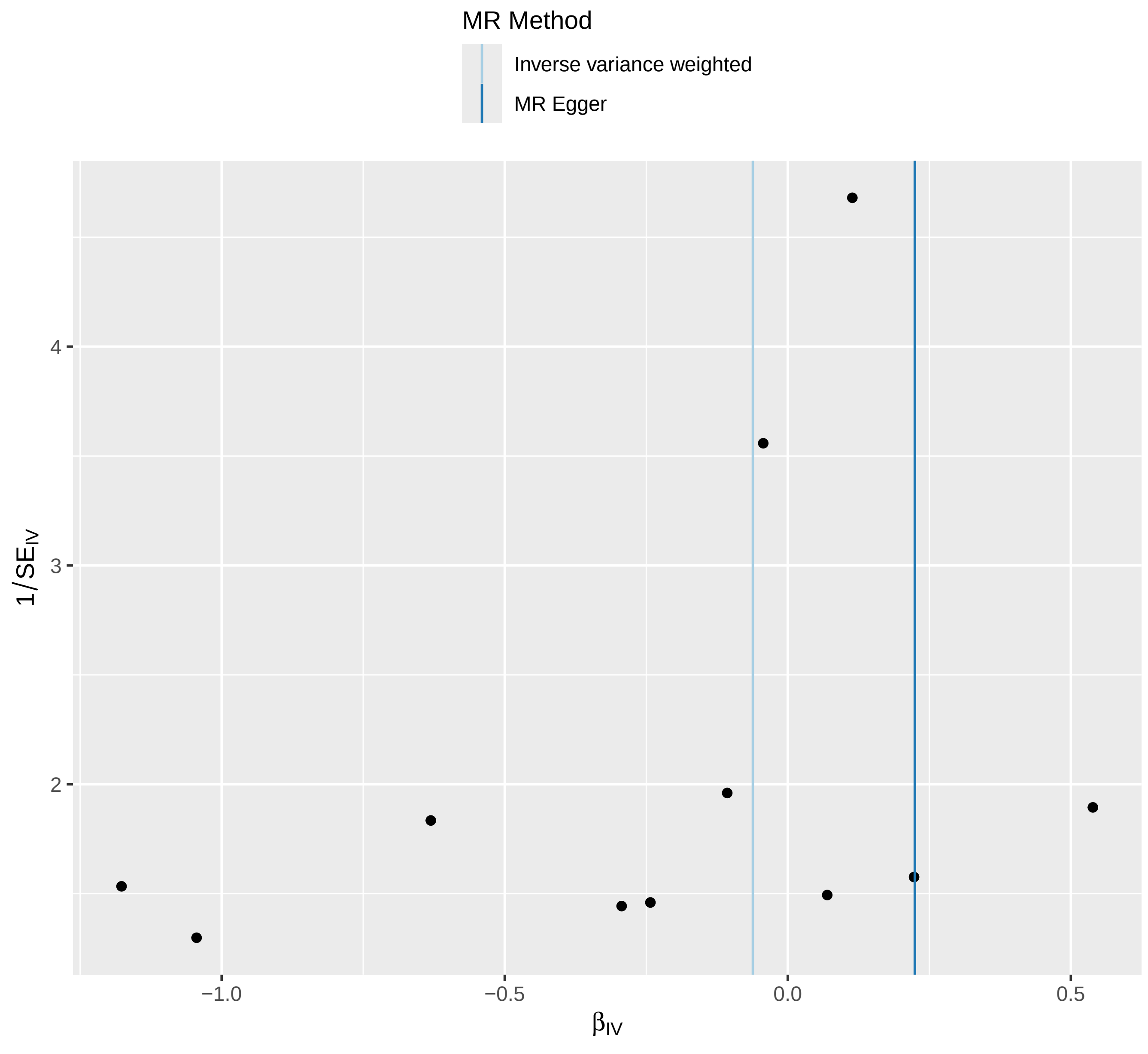

MR Method
Inverse variance weighted
MR Egger
4
3
2
1/SE_IV
−1.0
−0.5
0.0
0.5
β_IV

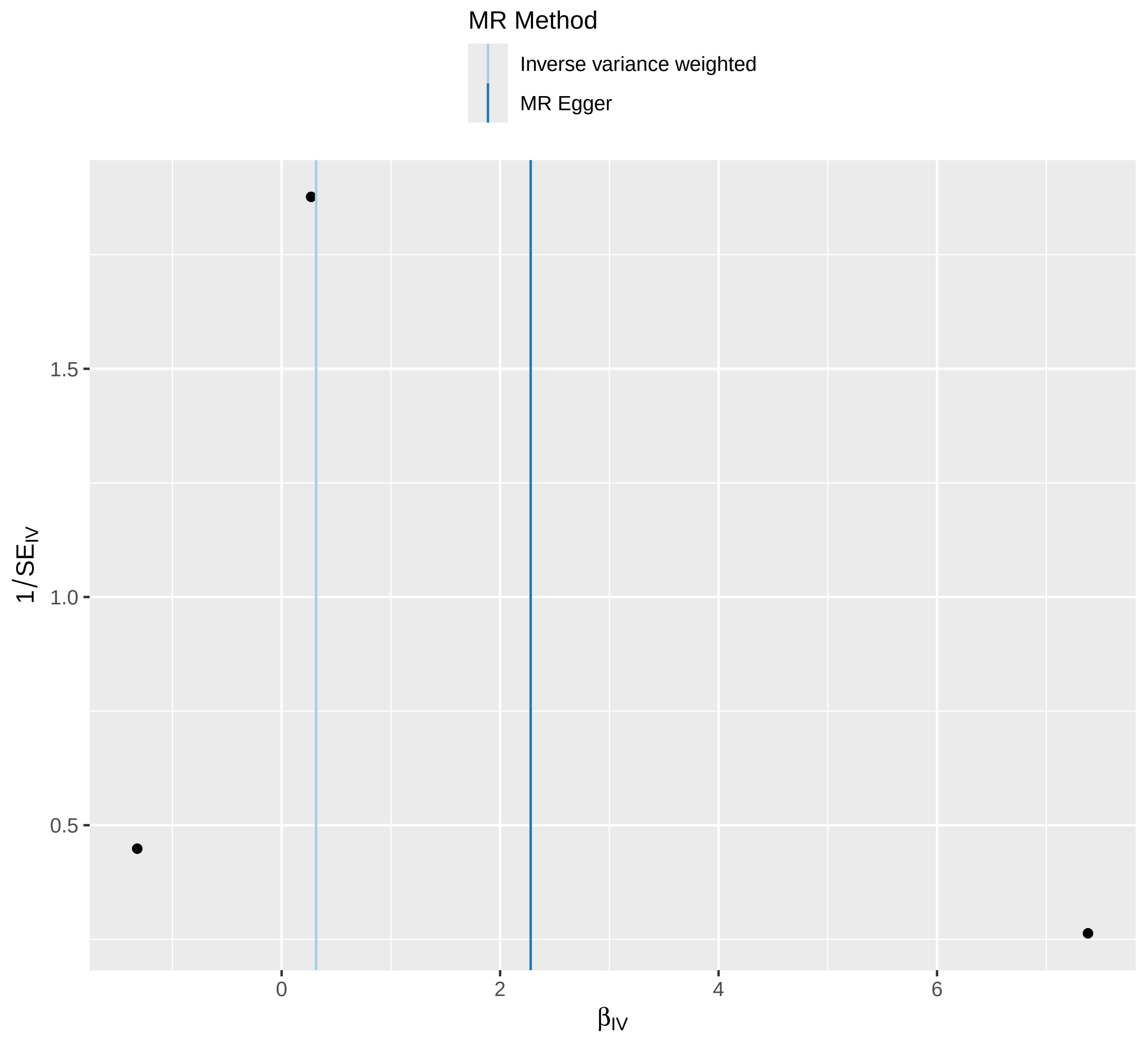
MR Method
Inverse variance weighted
MR Egger
1/SE_IV
1.5
1.0
0.5
0
2
4
6
β_IV

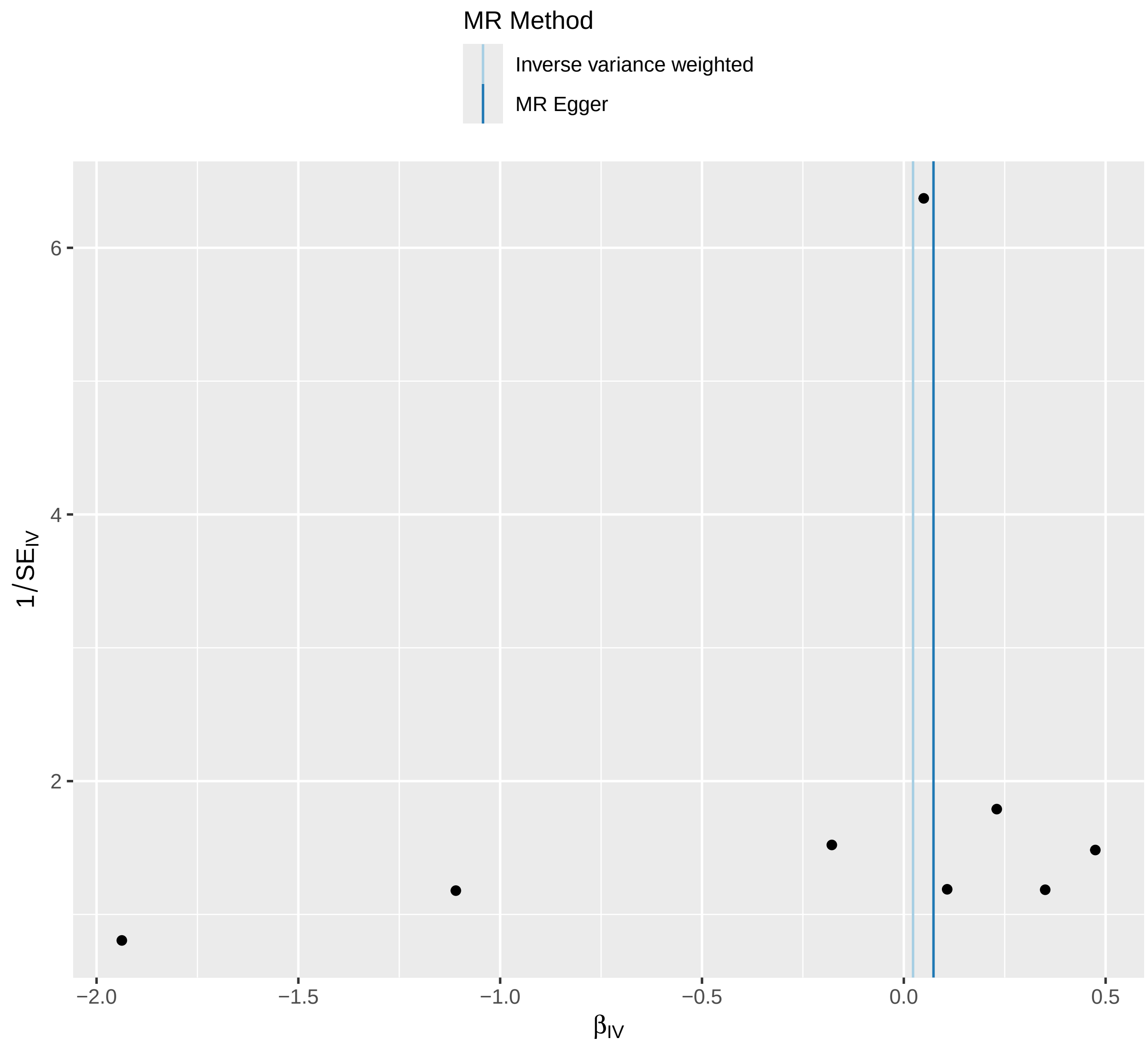
MR Method
Inverse variance weighted
MR Egger
6
4
2
1/SE$_{IV}$
−2.0
−1.5
−1.0
−0.5
0.0
0.5
β$_{IV}$

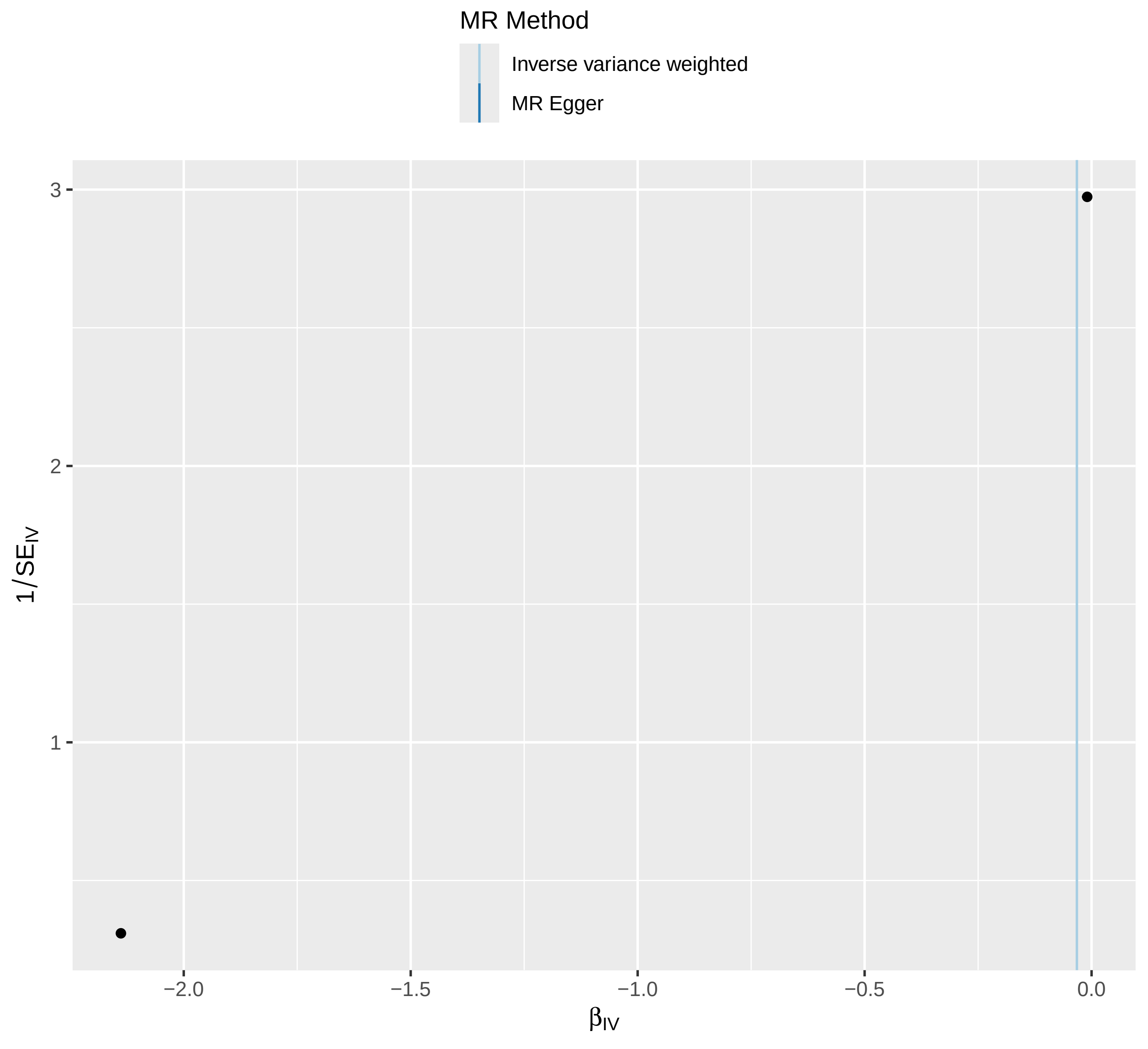
MR Method
Inverse variance weighted
MR Egger
3
2
1
1/SE$_{IV}$
−2.0
−1.5
−1.0
−0.5
0.0
β$_{IV}$

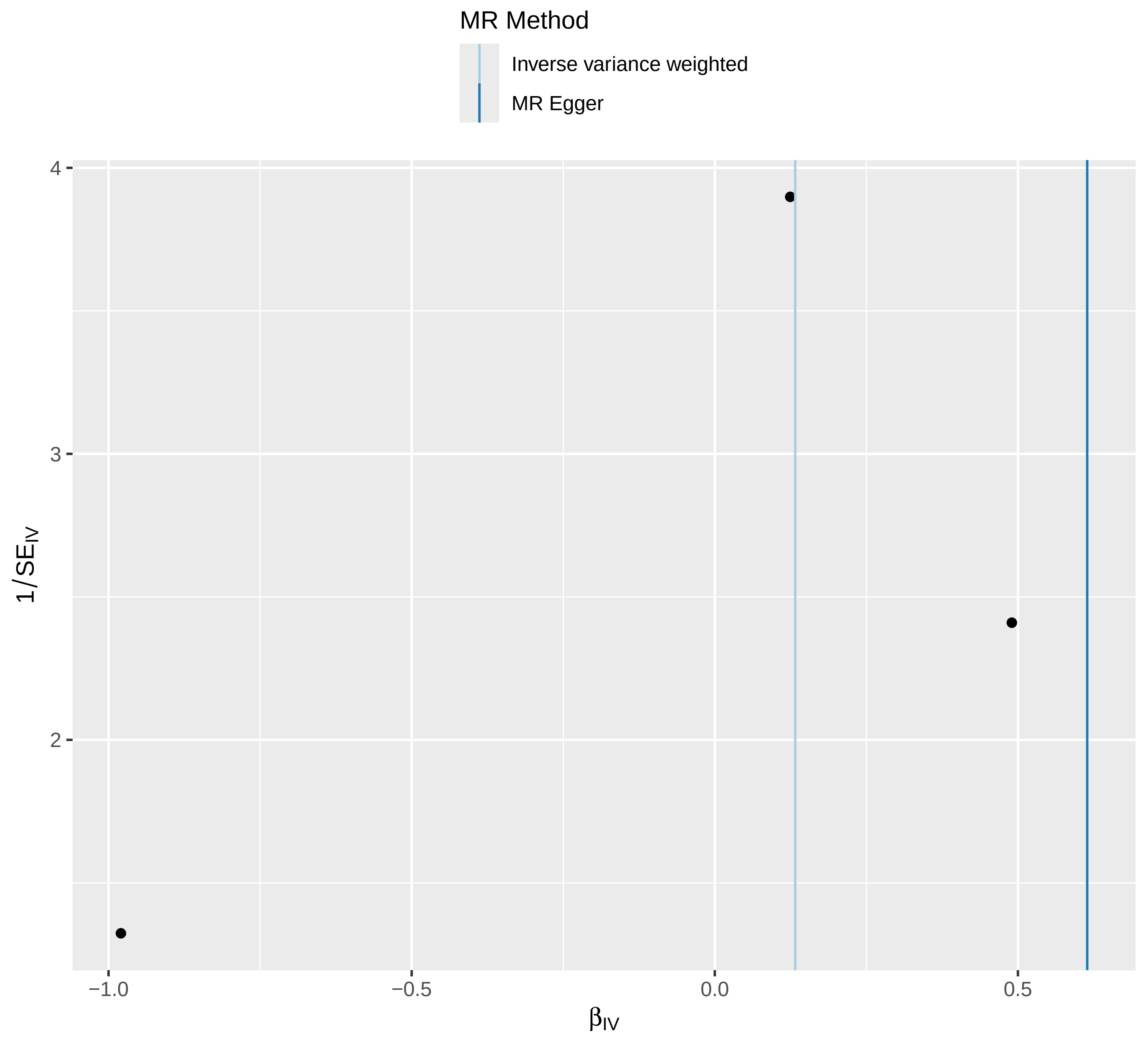
MR Method
Inverse variance weighted
MR Egger
4
3
2
1/SE_IV
−1.0
−0.5
0.0
0.5
β_IV

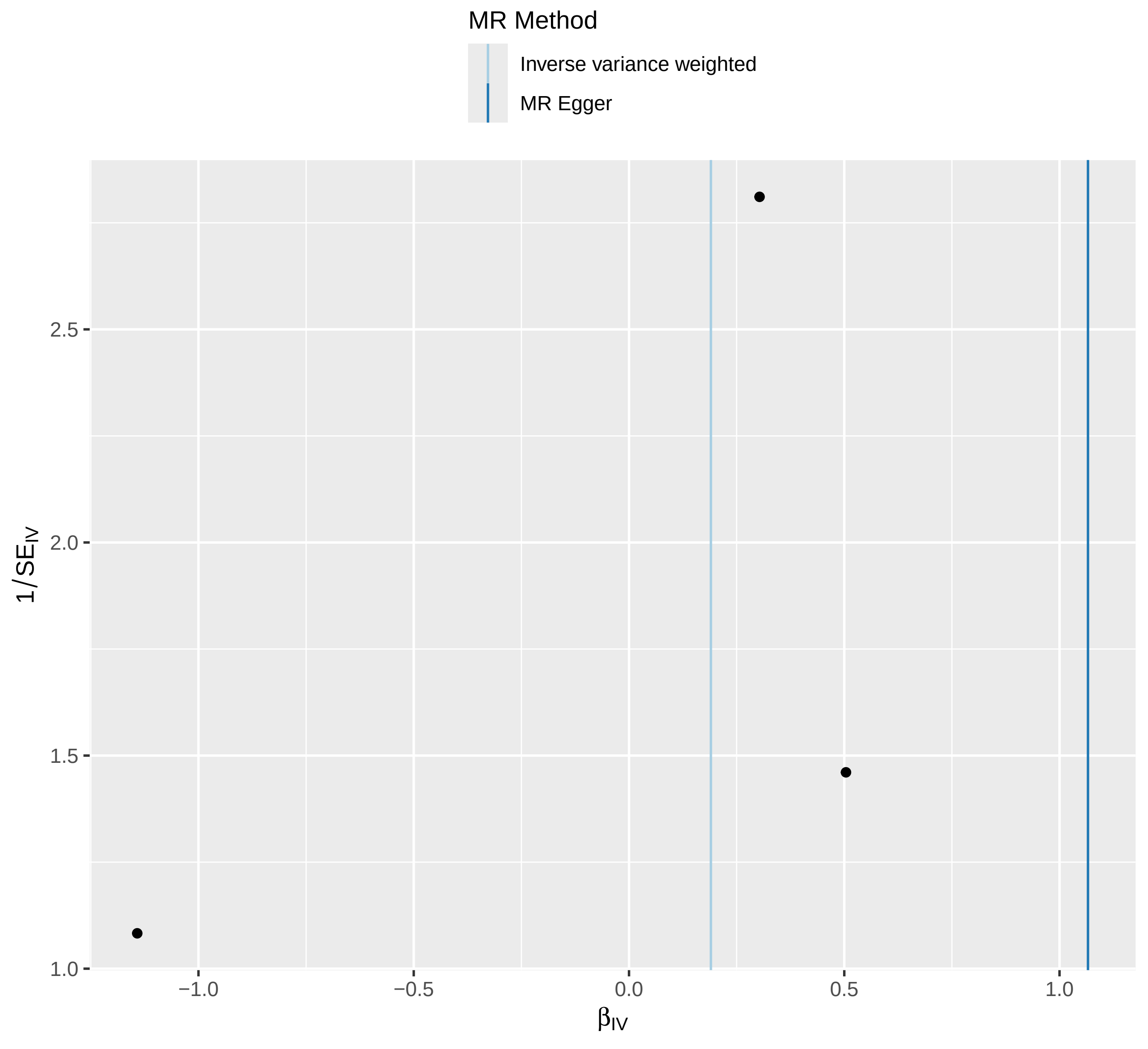

MR Method
Inverse variance weighted
MR Egger
1/SE_IV
2.5
2.0
1.5
1.0
−1.0
−0.5
0.0
0.5
1.0
β_IV

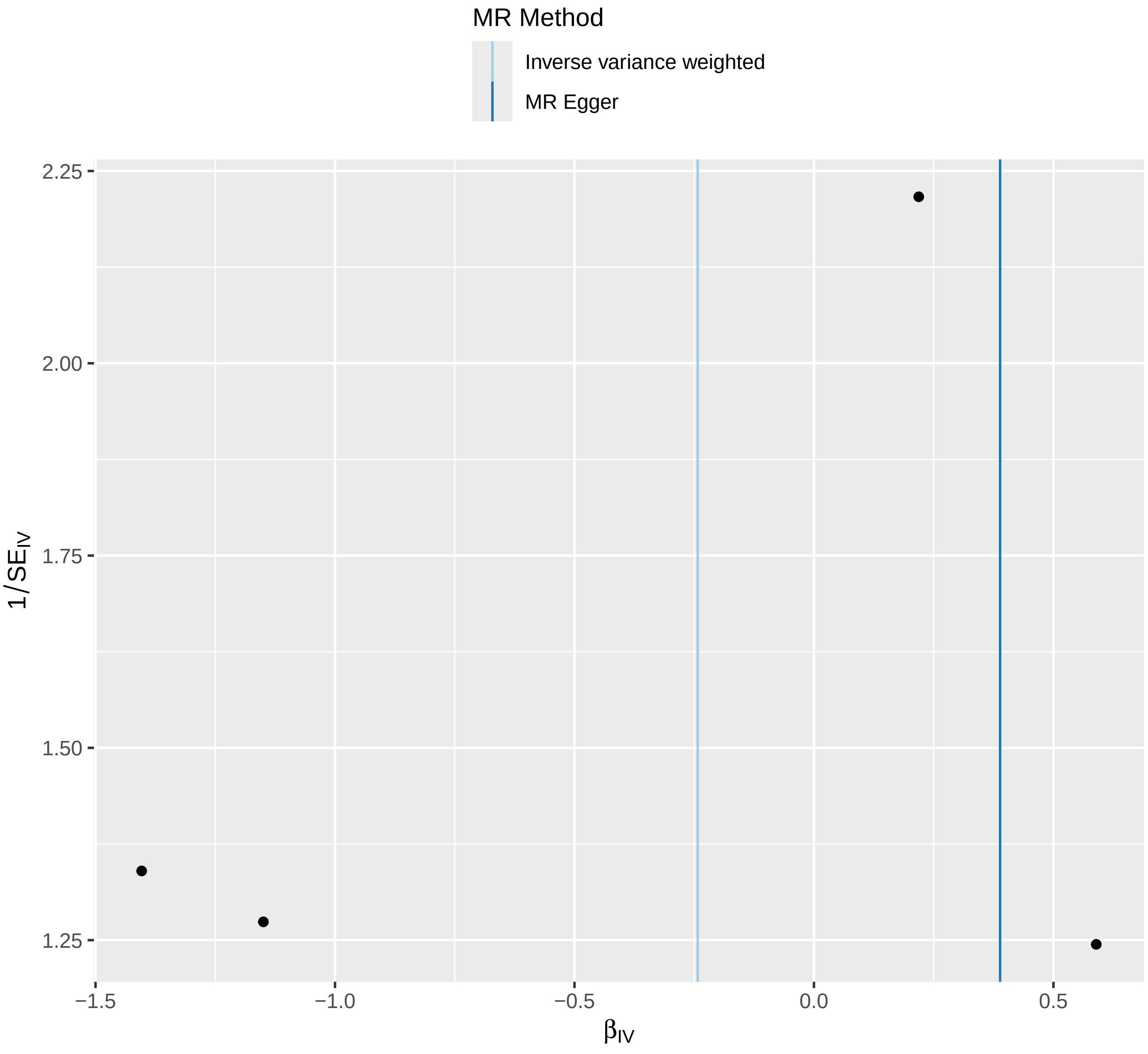
MR Method
Inverse variance weighted
MR Egger
2.25
2.00
1.75
1.50
1.25
$1/SE_{IV}$
−1.5
−1.0
−0.5
0.0
0.5
β_{IV}

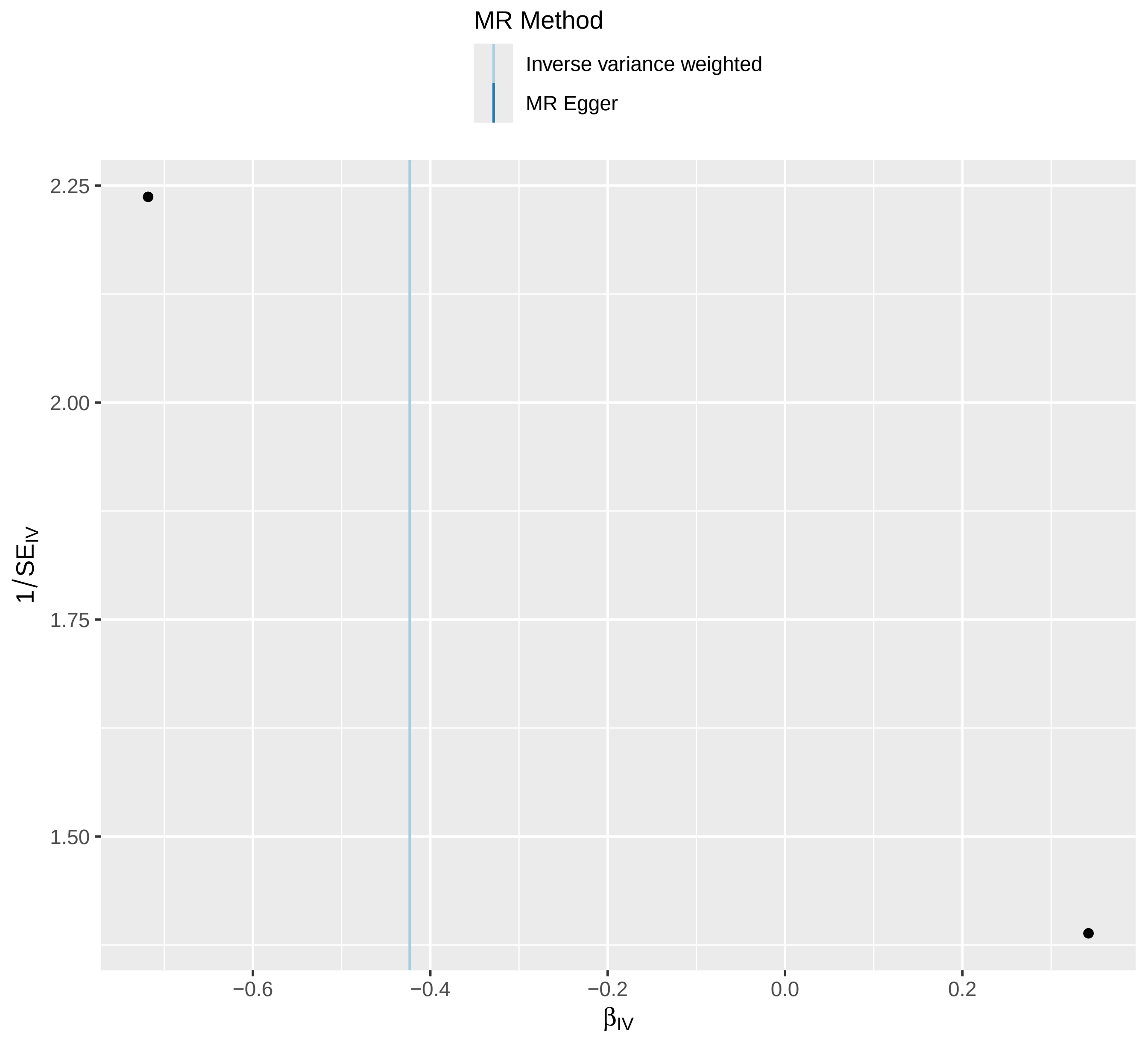
MR Method
Inverse variance weighted
MR Egger
2.25
2.00
1.75
1.50
1/SE_IV
−0.6
−0.4
−0.2
0.0
0.2
β_IV

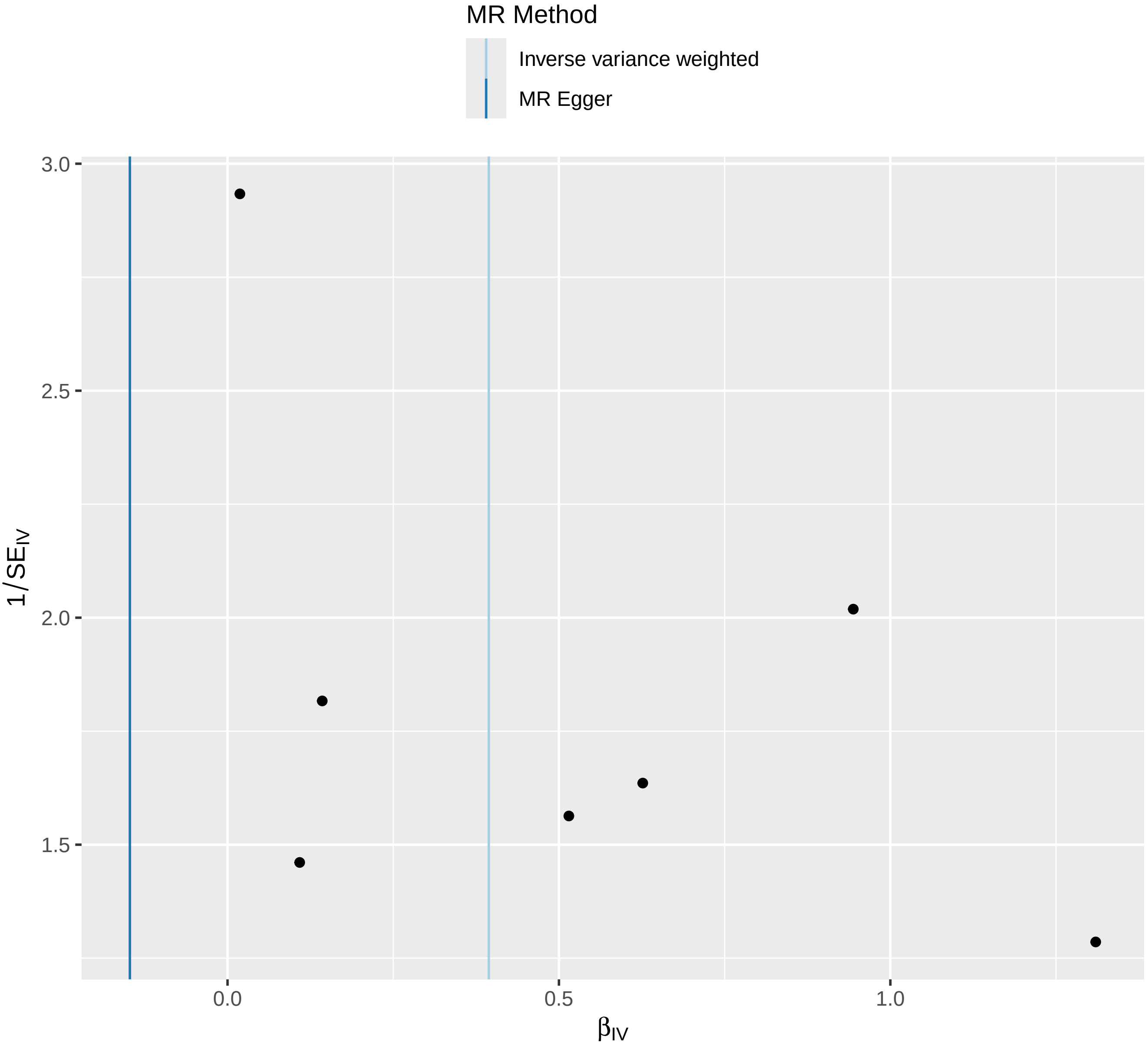

MR Method
Inverse variance weighted
MR Egger
3.0
2.5
2.0
1.5
$1/SE_{IV}$
0.0
0.5
1.0
β_{IV}

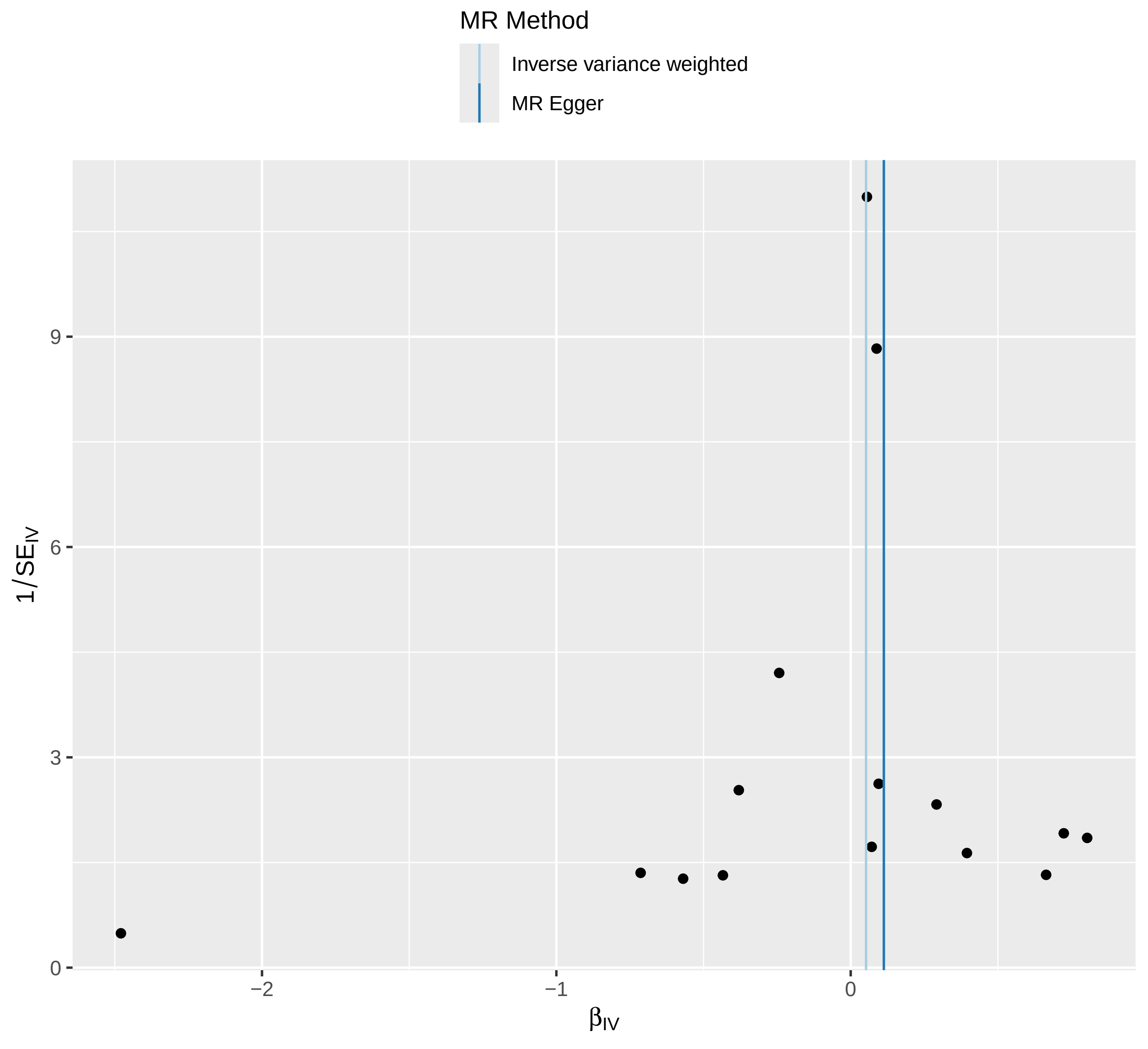

MR Method
Inverse variance weighted
MR Egger
1/SE_IV
9
6
3
0
−2
−1
0
β_IV

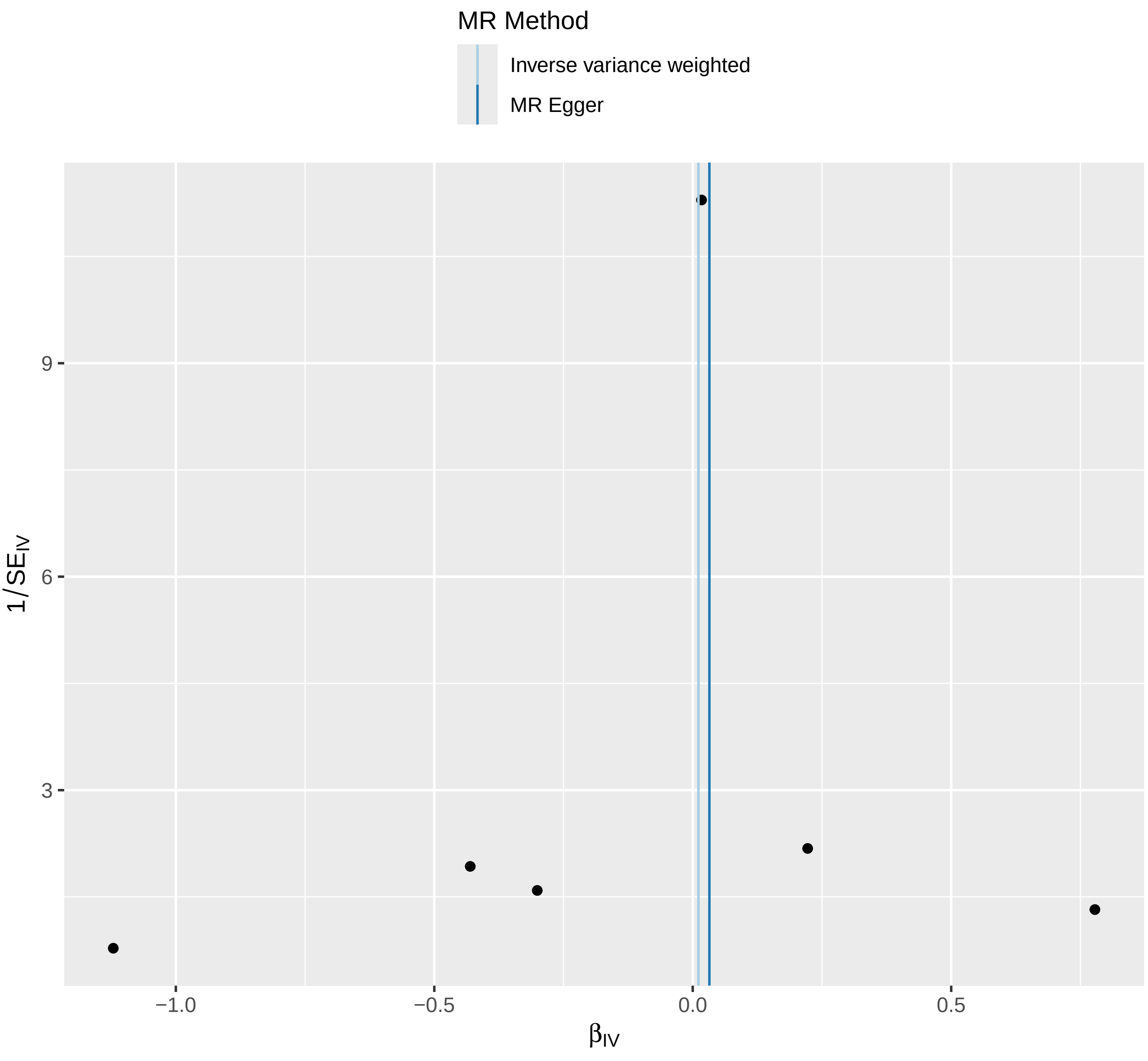

MR Method
Inverse variance weighted
MR Egger
9
6
3
$1/SE_{IV}$
−1.0
−0.5
0.0
0.5
β_{IV}

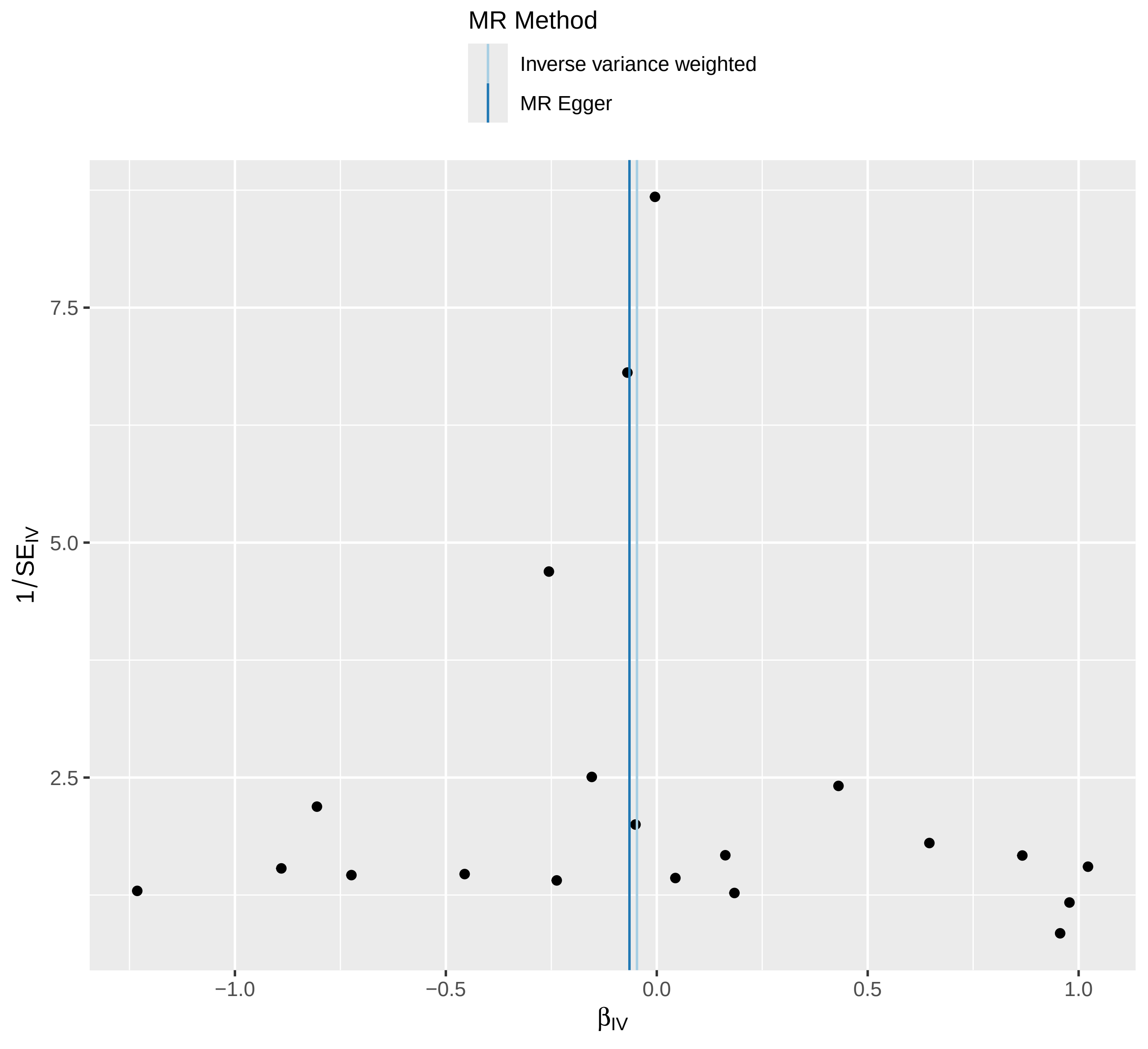
MR Method
Inverse variance weighted
MR Egger
7.5
5.0
2.5
1/SE_IV
−1.0
−0.5
0.0
0.5
1.0
β_IV

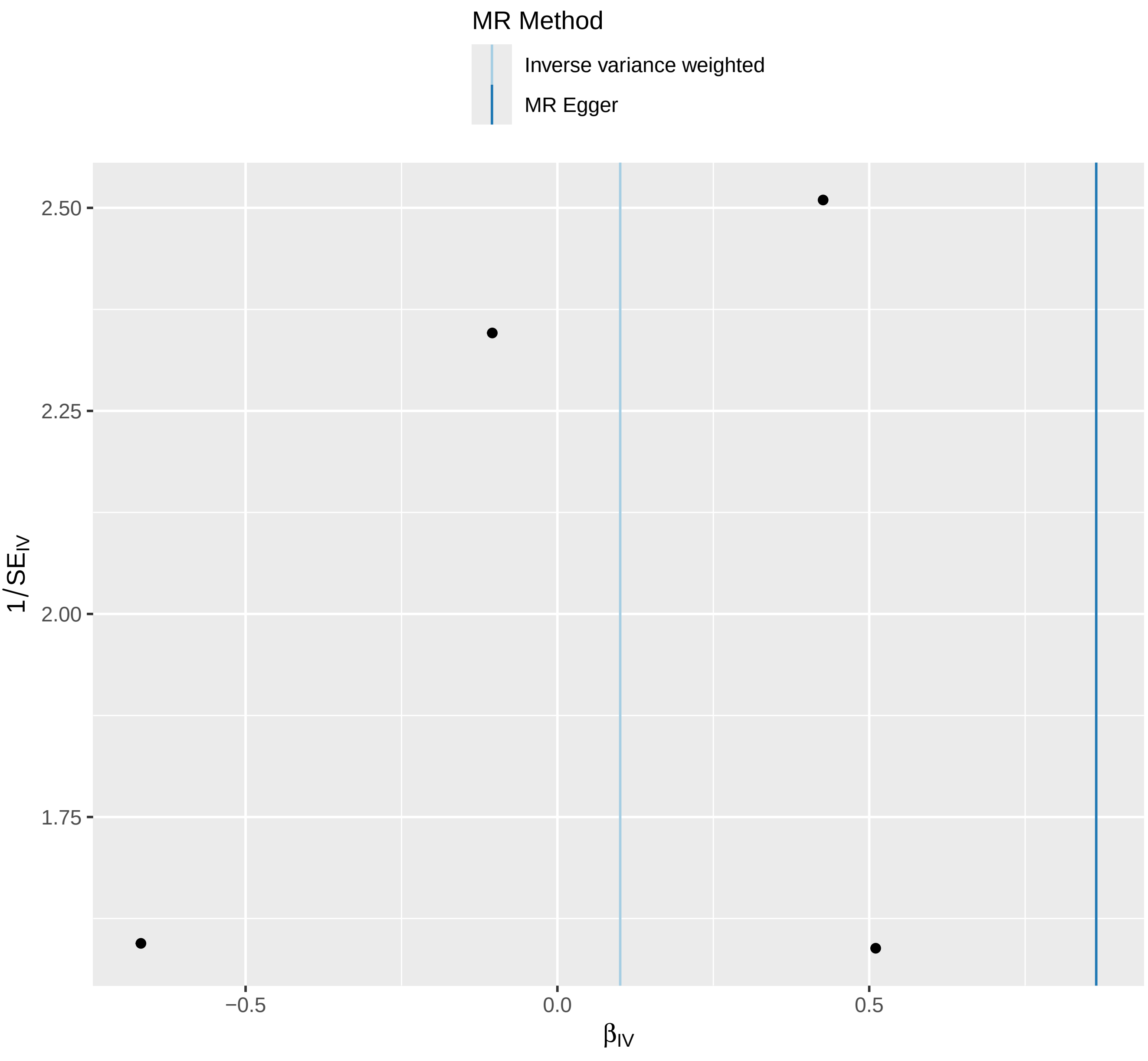
MR Method
Inverse variance weighted
MR Egger
2.50
2.25
2.00
1.75
1/SE_IV
−0.5
0.0
0.5
β_IV

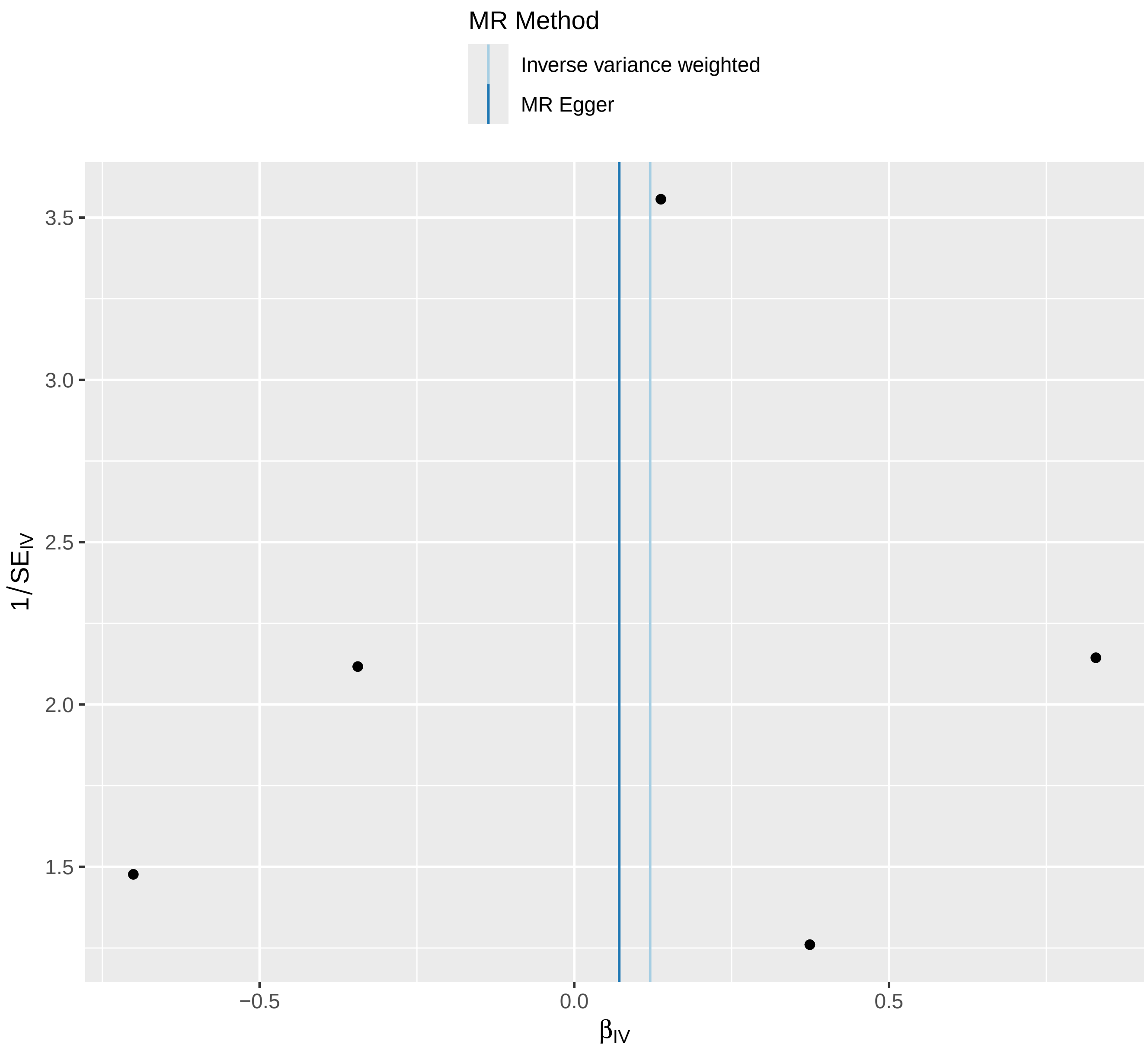
MR Method
Inverse variance weighted
MR Egger
3.5
3.0
2.5
2.0
1.5
1/SE_IV
−0.5
0.0
0.5
β_IV

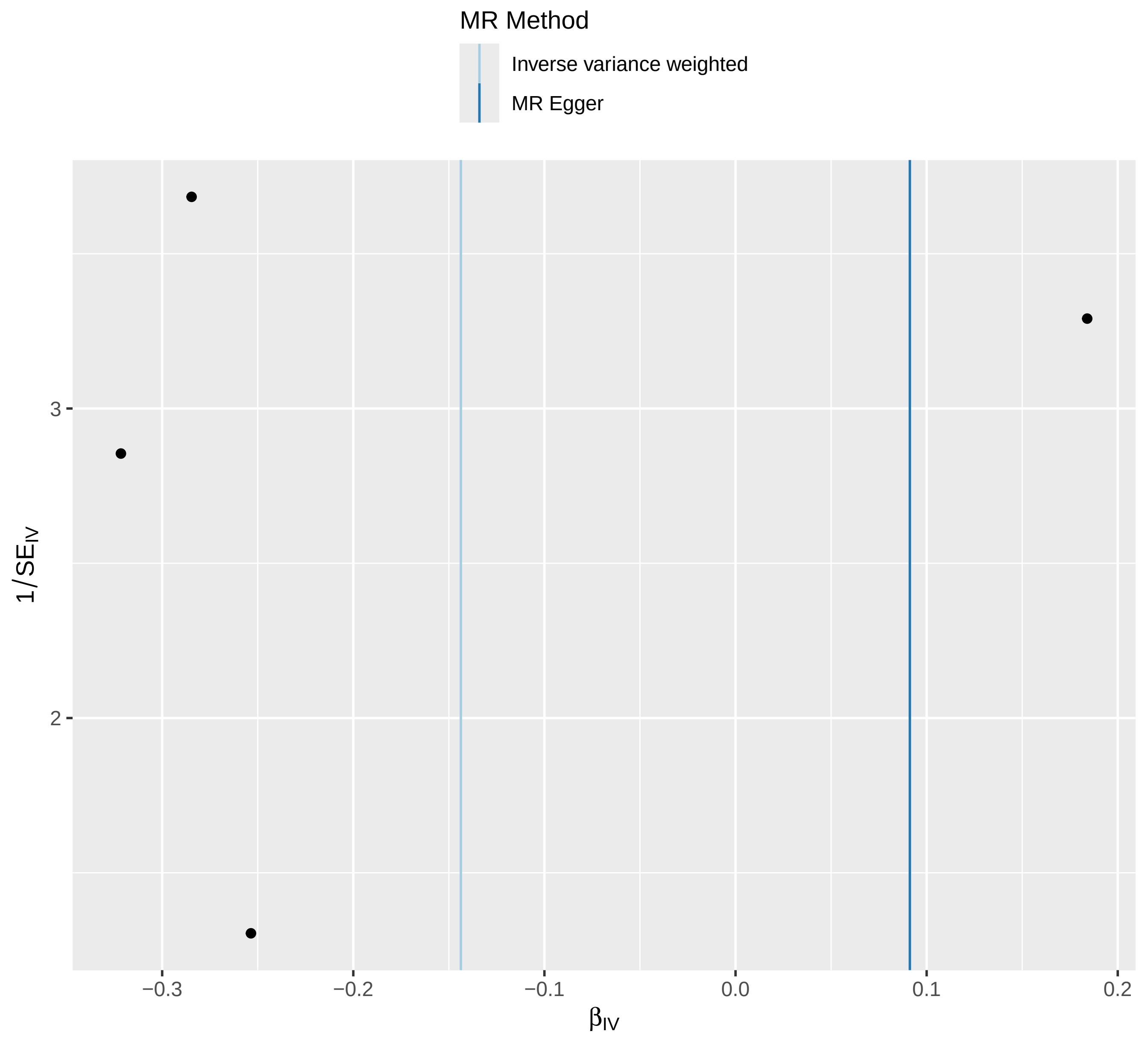
MR Method
Inverse variance weighted
MR Egger
3
2
1/SE_IV
−0.3
−0.2
−0.1
0.0
0.1
0.2
β_IV

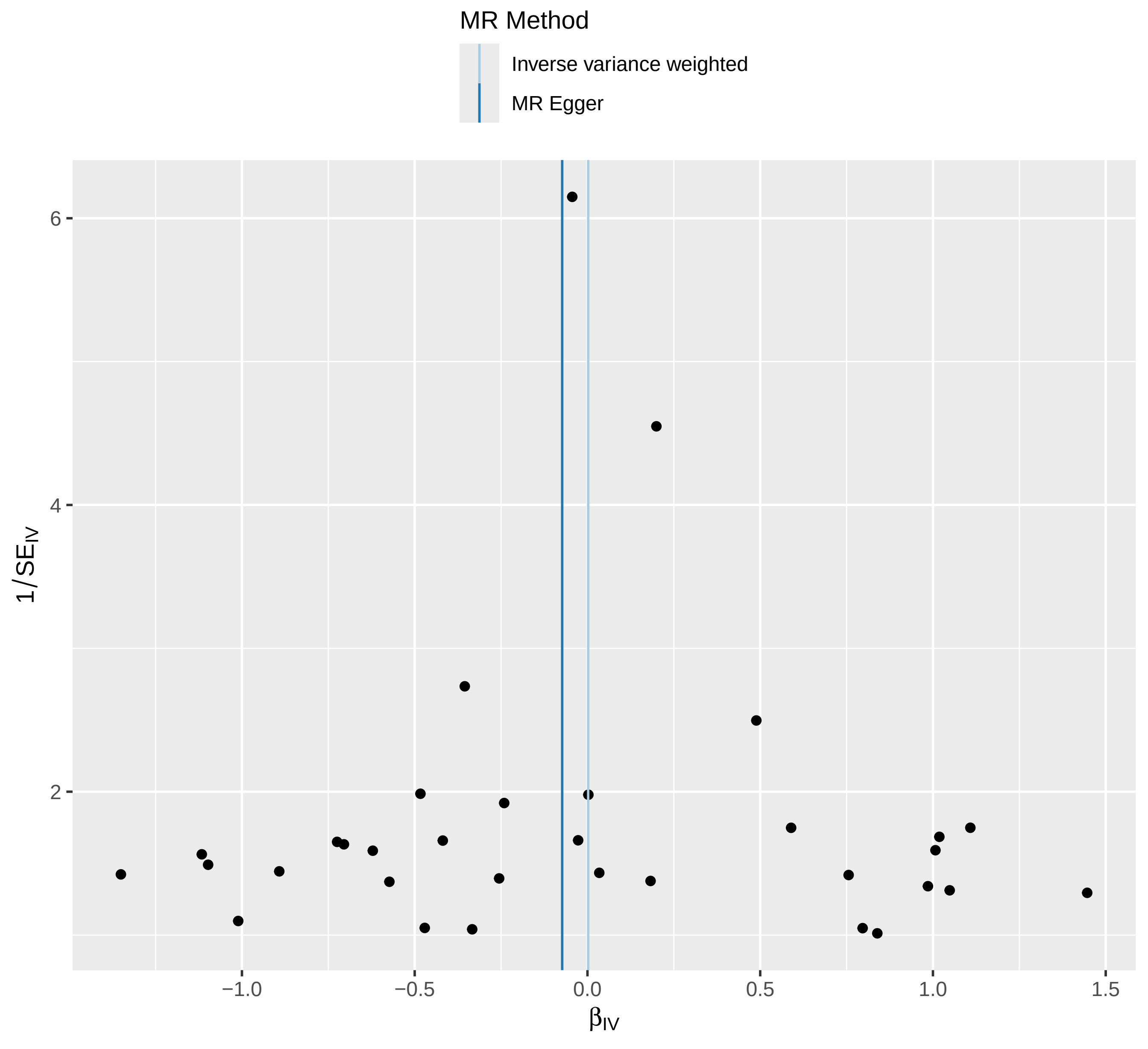
MR Method
Inverse variance weighted
MR Egger
1/SE$_{IV}$
6
4
2
−1.0
−0.5
0.0
0.5
1.0
1.5
β$_{IV}$

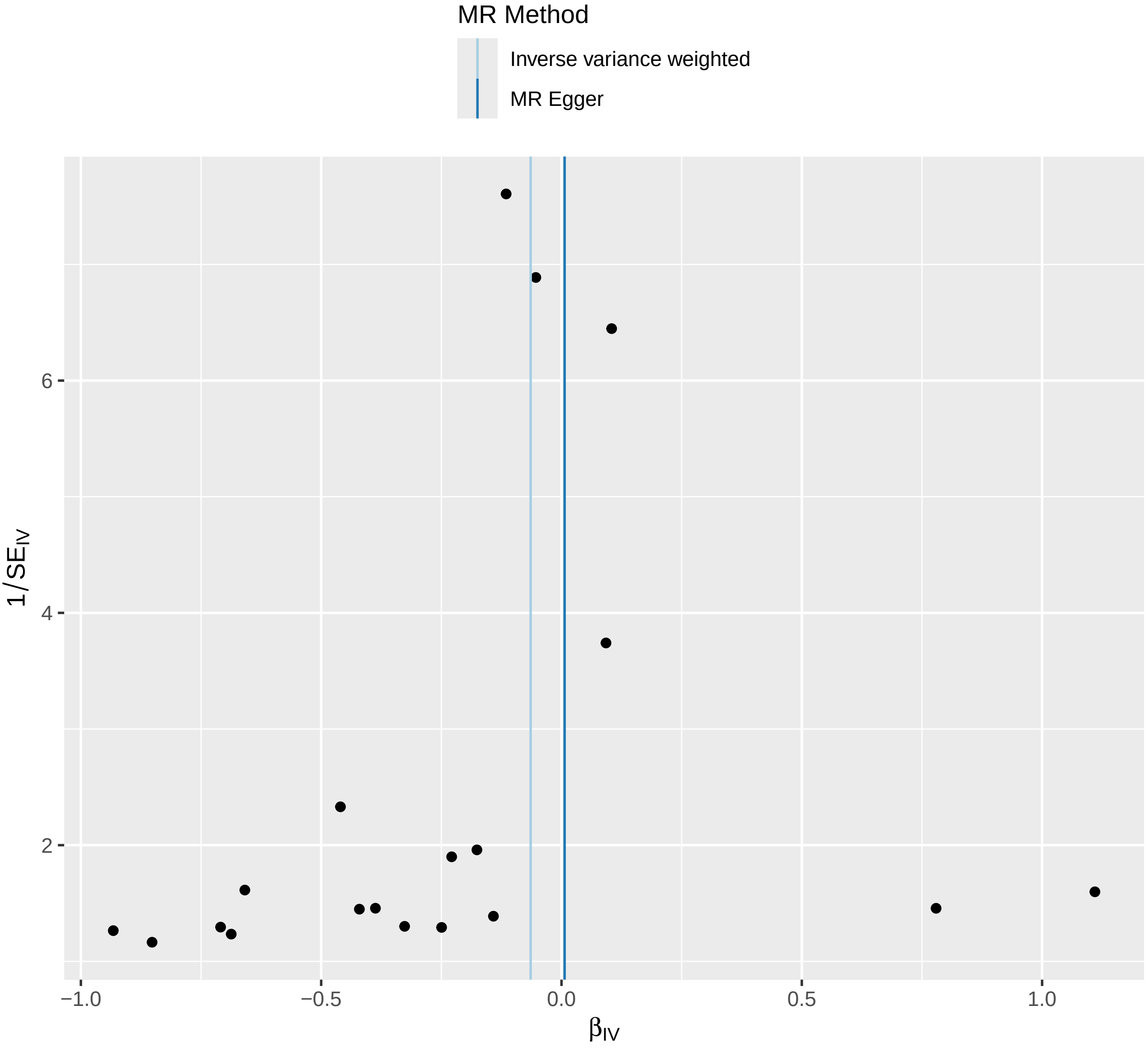
MR Method
Inverse variance weighted
MR Egger
1/SE$_{IV}$
6
4
2
−1.0
−0.5
0.0
0.5
1.0
β$_{IV}$

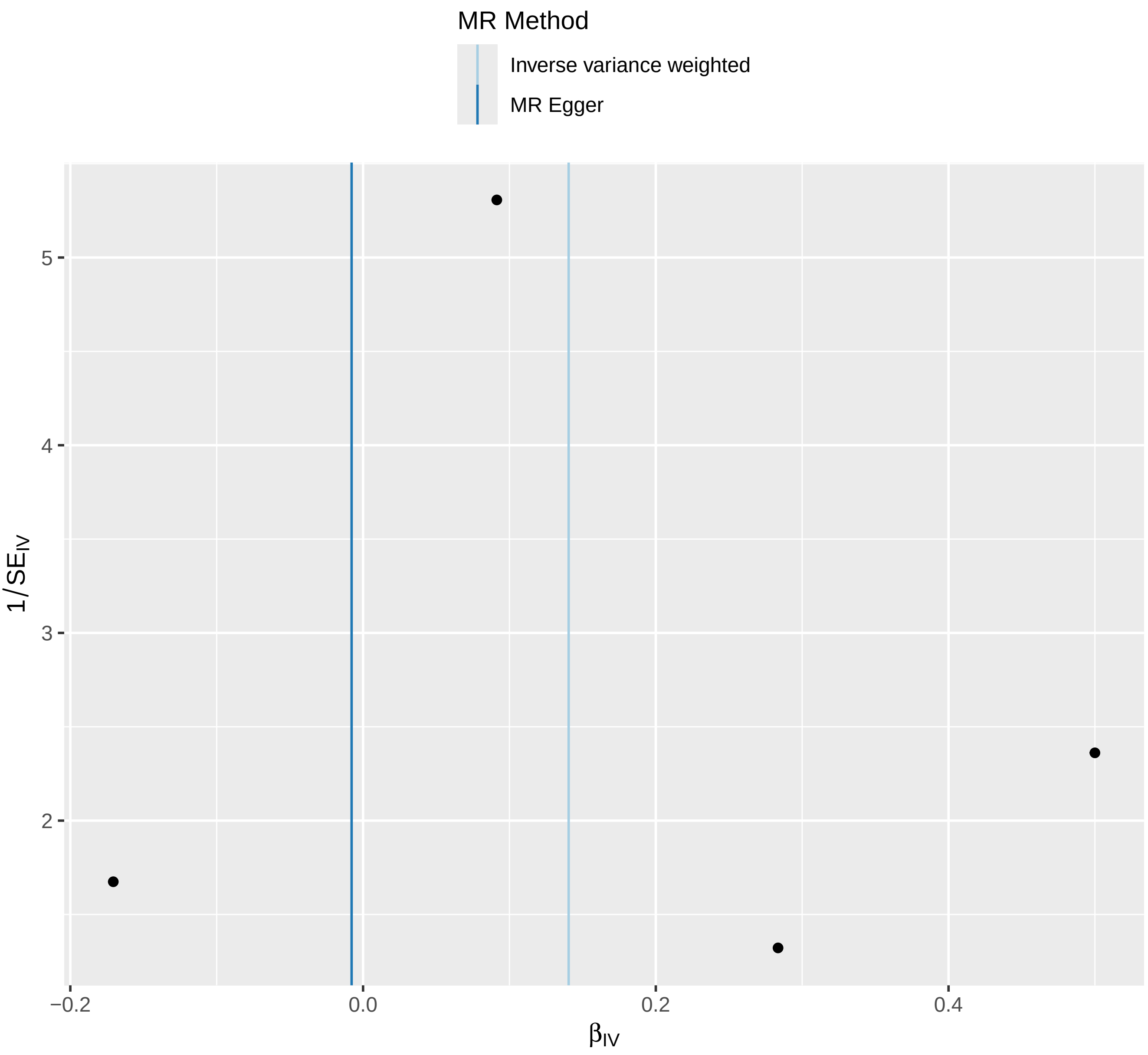

MR Method
Inverse variance weighted
MR Egger
1/SE$_{IV}$
5
4
3
2
−0.2
0.0
0.2
0.4
β$_{IV}$

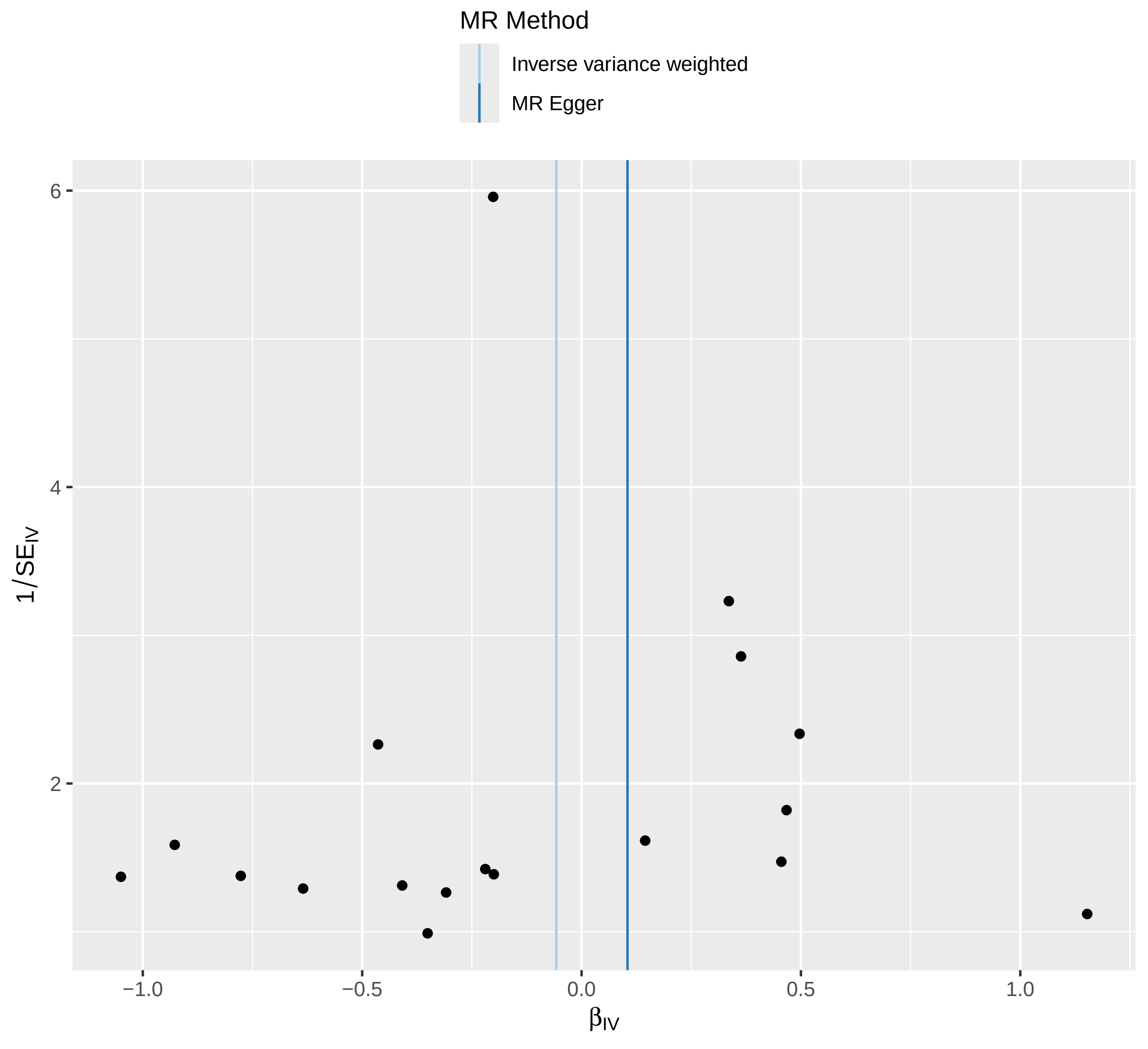

MR Method
Inverse variance weighted
MR Egger
6
4
2
1/SE$_{IV}$
−1.0
−0.5
0.0
0.5
1.0
β$_{IV}$

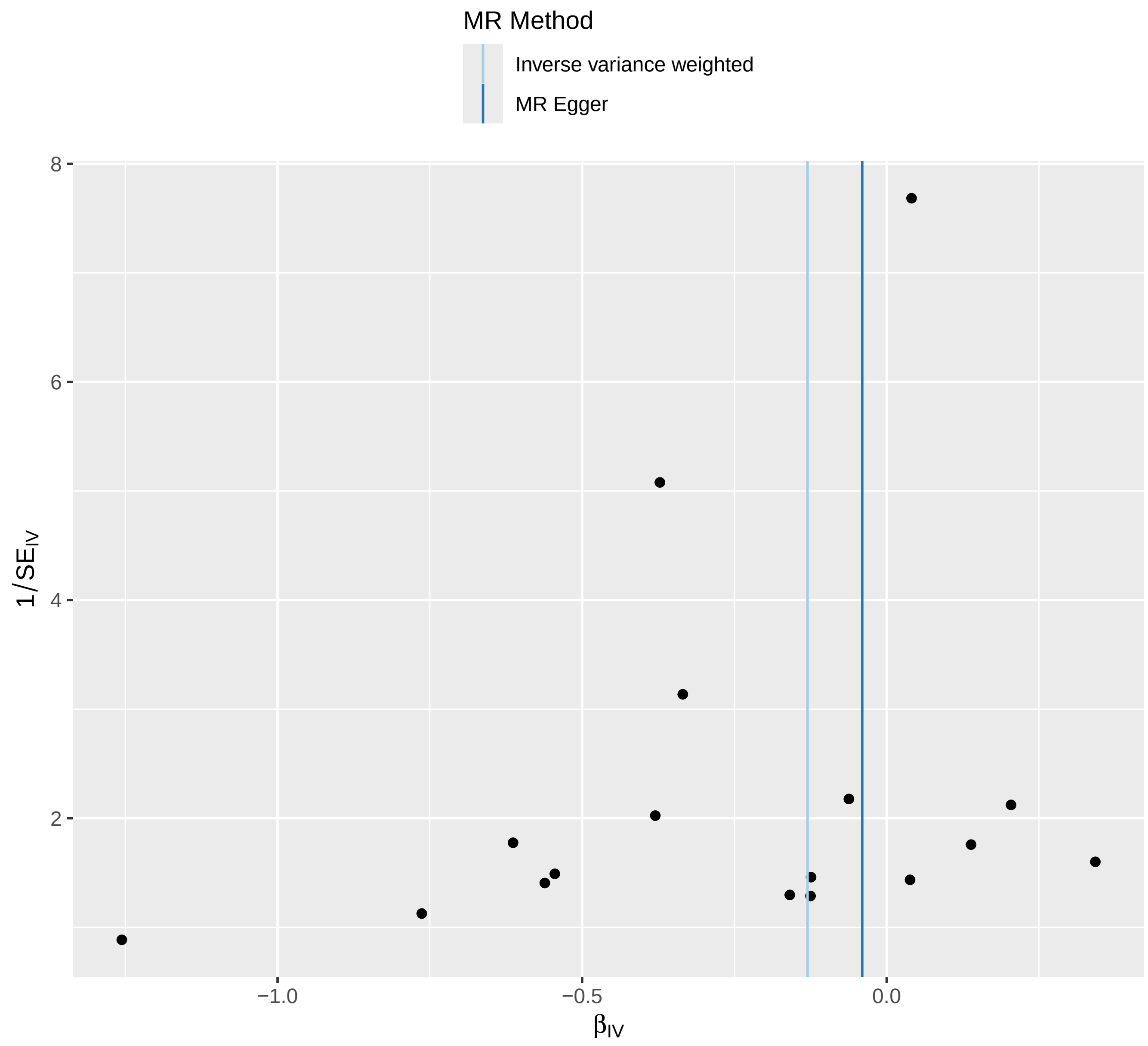
MR Method
Inverse variance weighted
MR Egger
8
6
4
2
1/SE_IV
−1.0
−0.5
0.0
β_IV

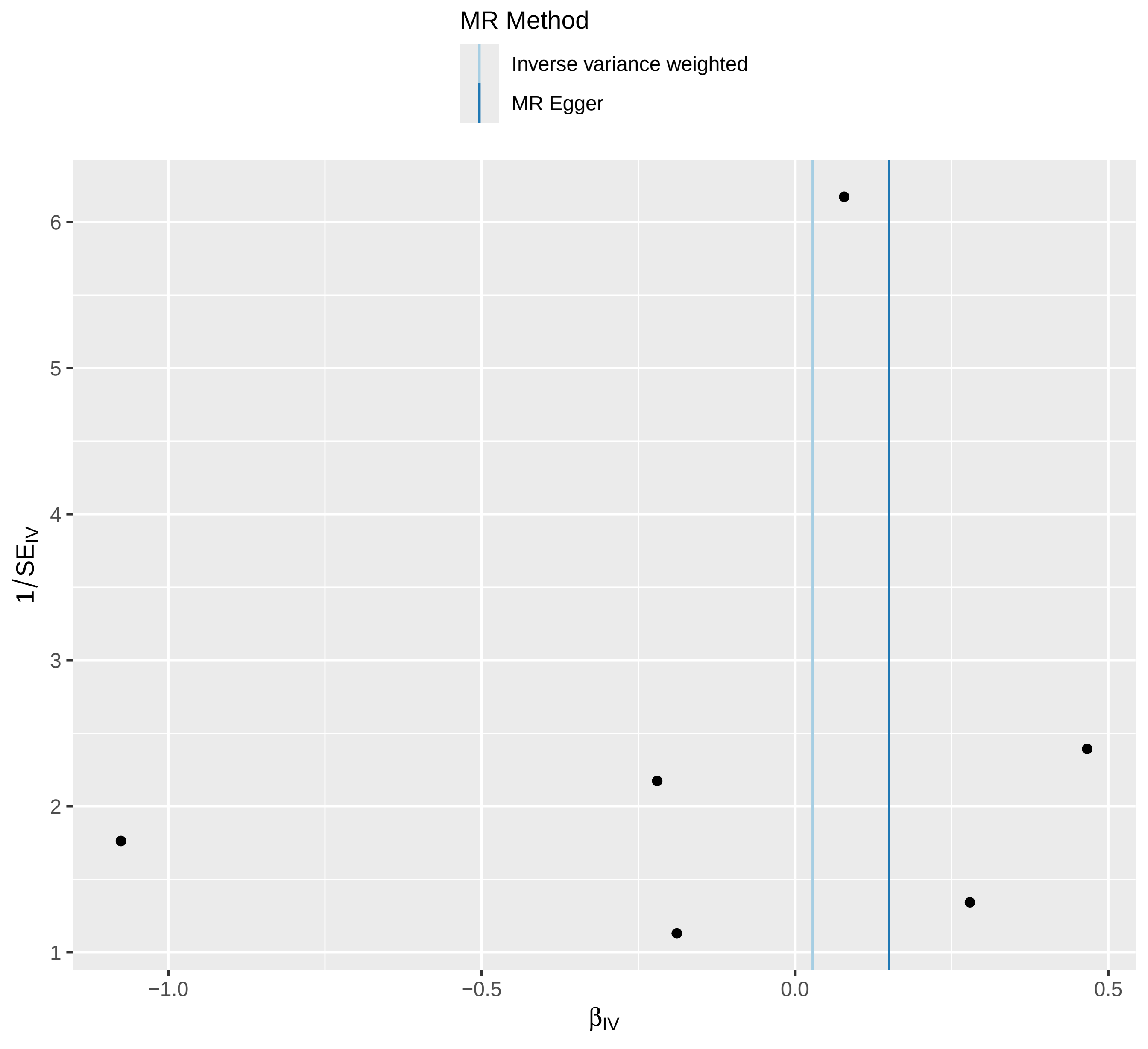

MR Method
Inverse variance weighted
MR Egger
6
5
4
3
2
1
1/SE_IV
−1.0
−0.5
0.0
0.5
β_IV

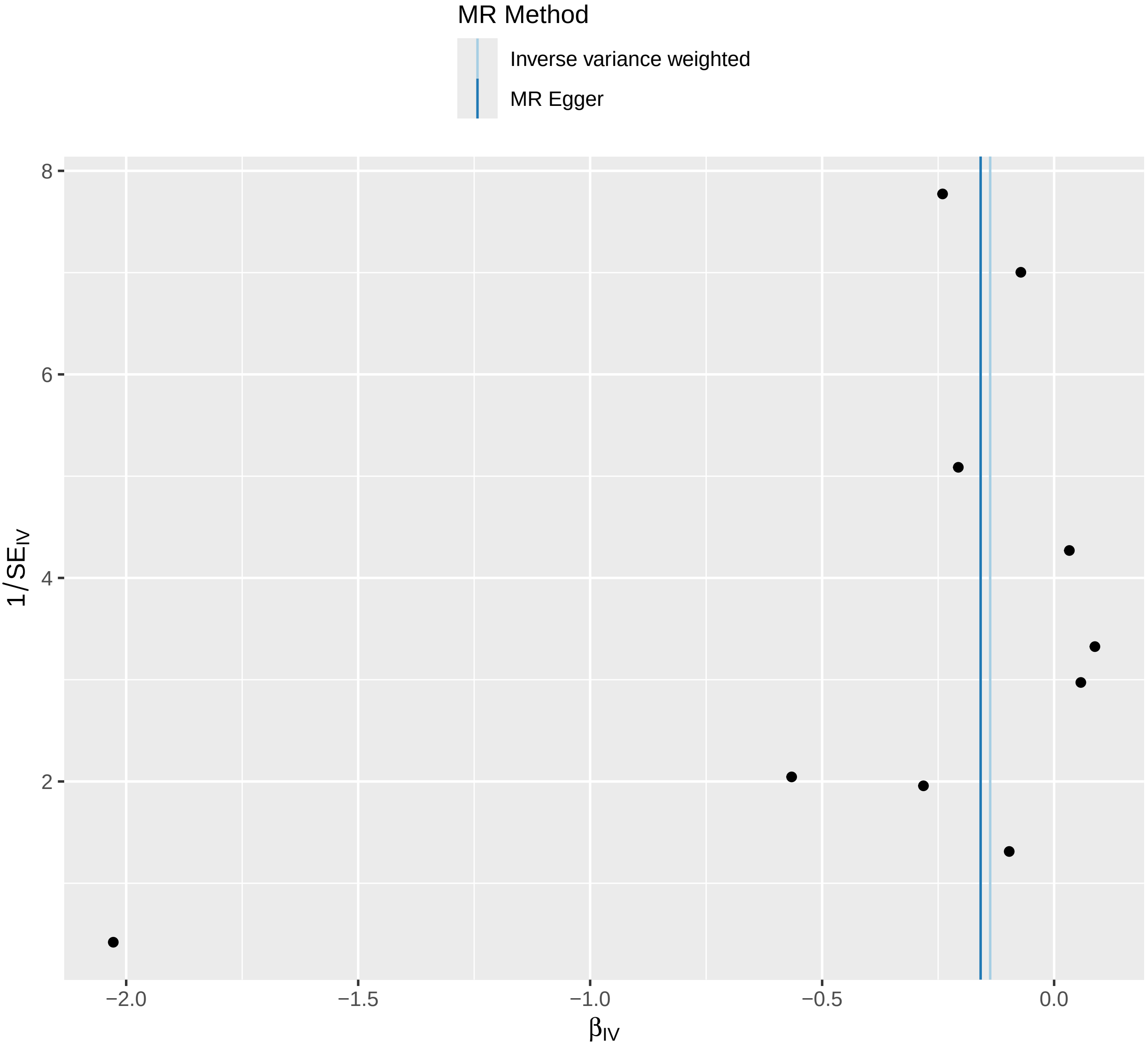

MR Method
Inverse variance weighted
MR Egger
8
6
4
2
$1/SE_{IV}$
−2.0
−1.5
−1.0
−0.5
0.0
β_{IV}

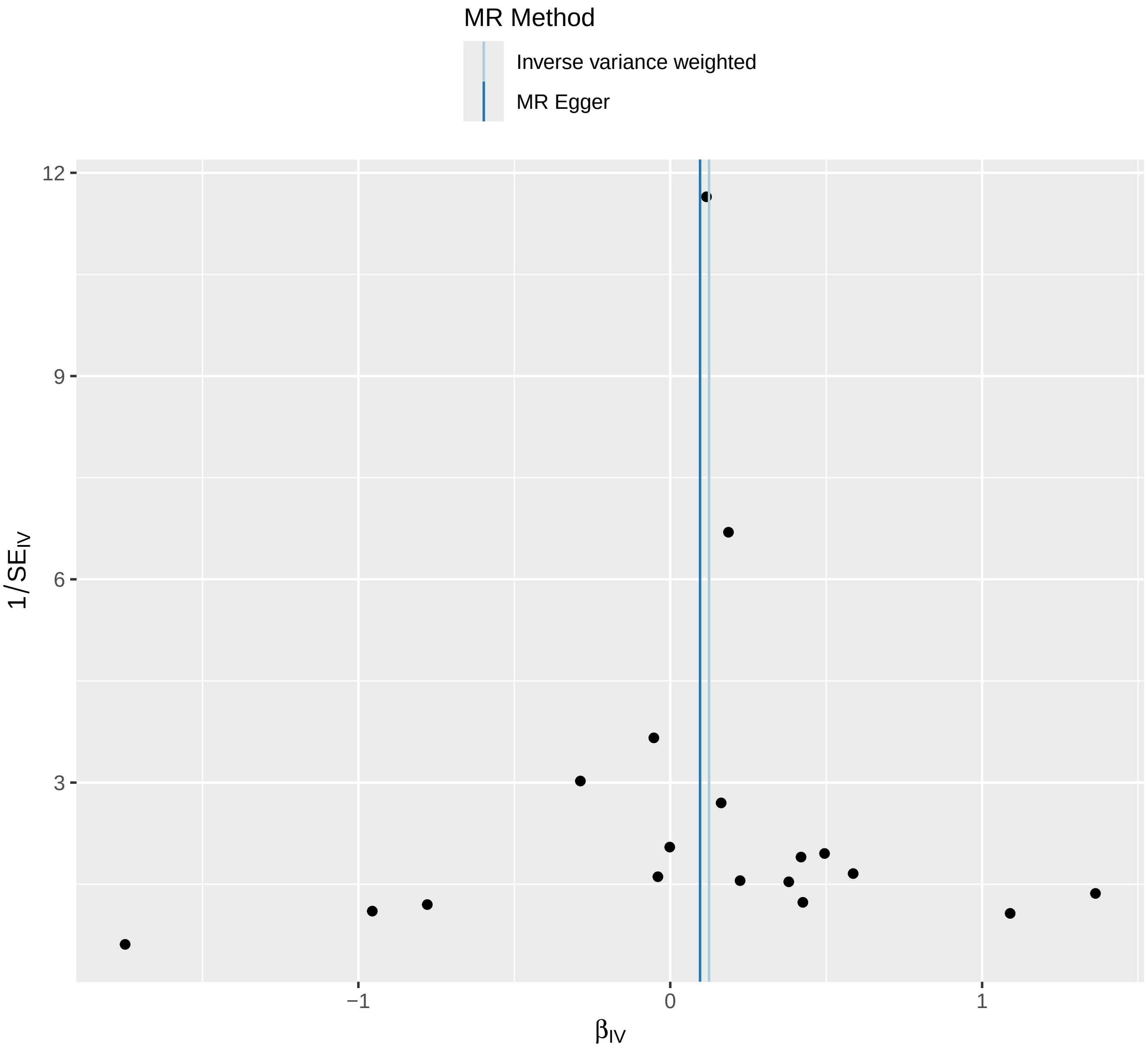
MR Method
Inverse variance weighted
MR Egger
12
9
6
3
−1
0
1
$1/SE_{IV}$
β_{IV}

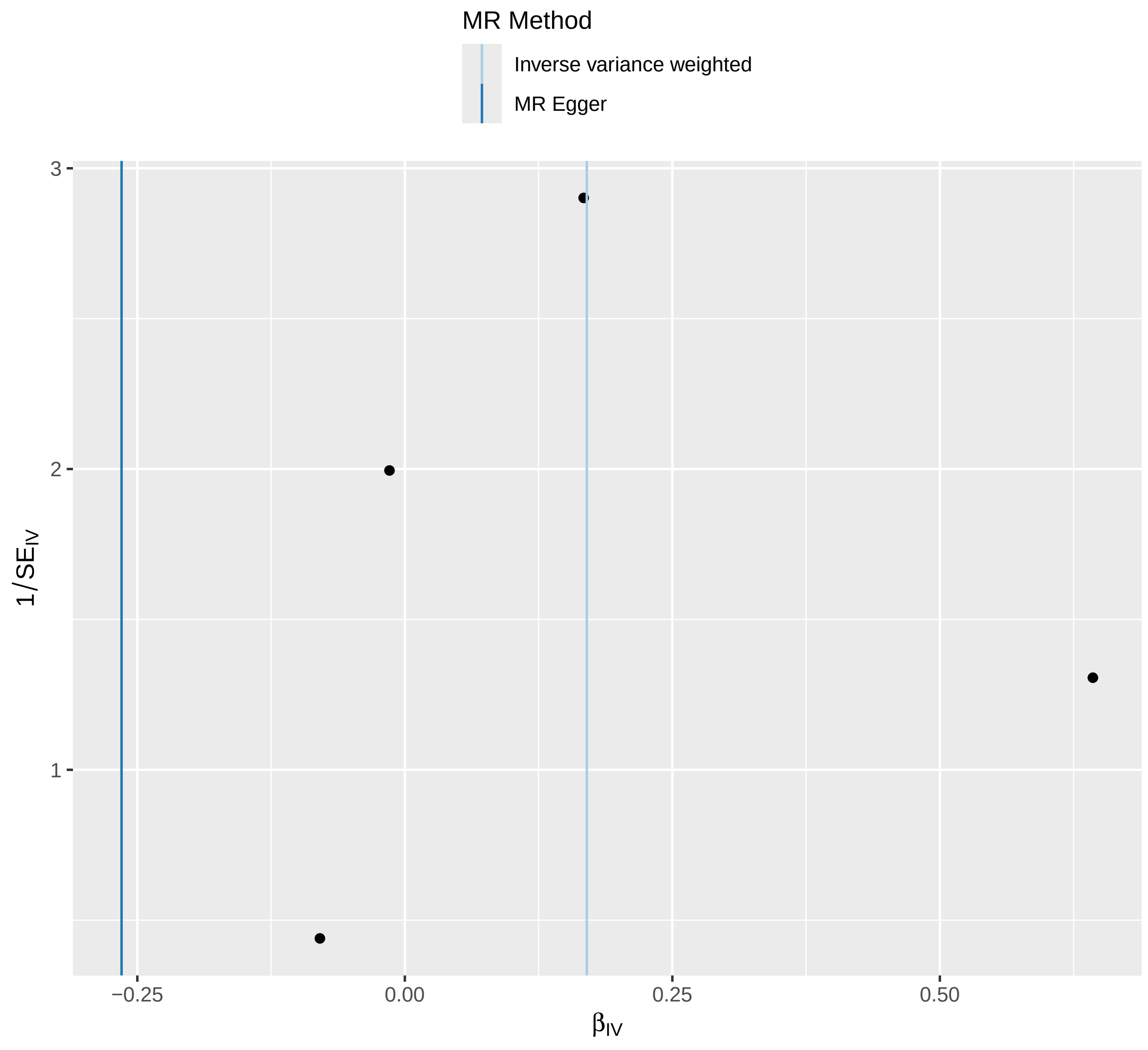
MR Method
Inverse variance weighted
MR Egger
3
2
1
1/SE$_{IV}$
−0.25
0.00
0.25
0.50
β$_{IV}$

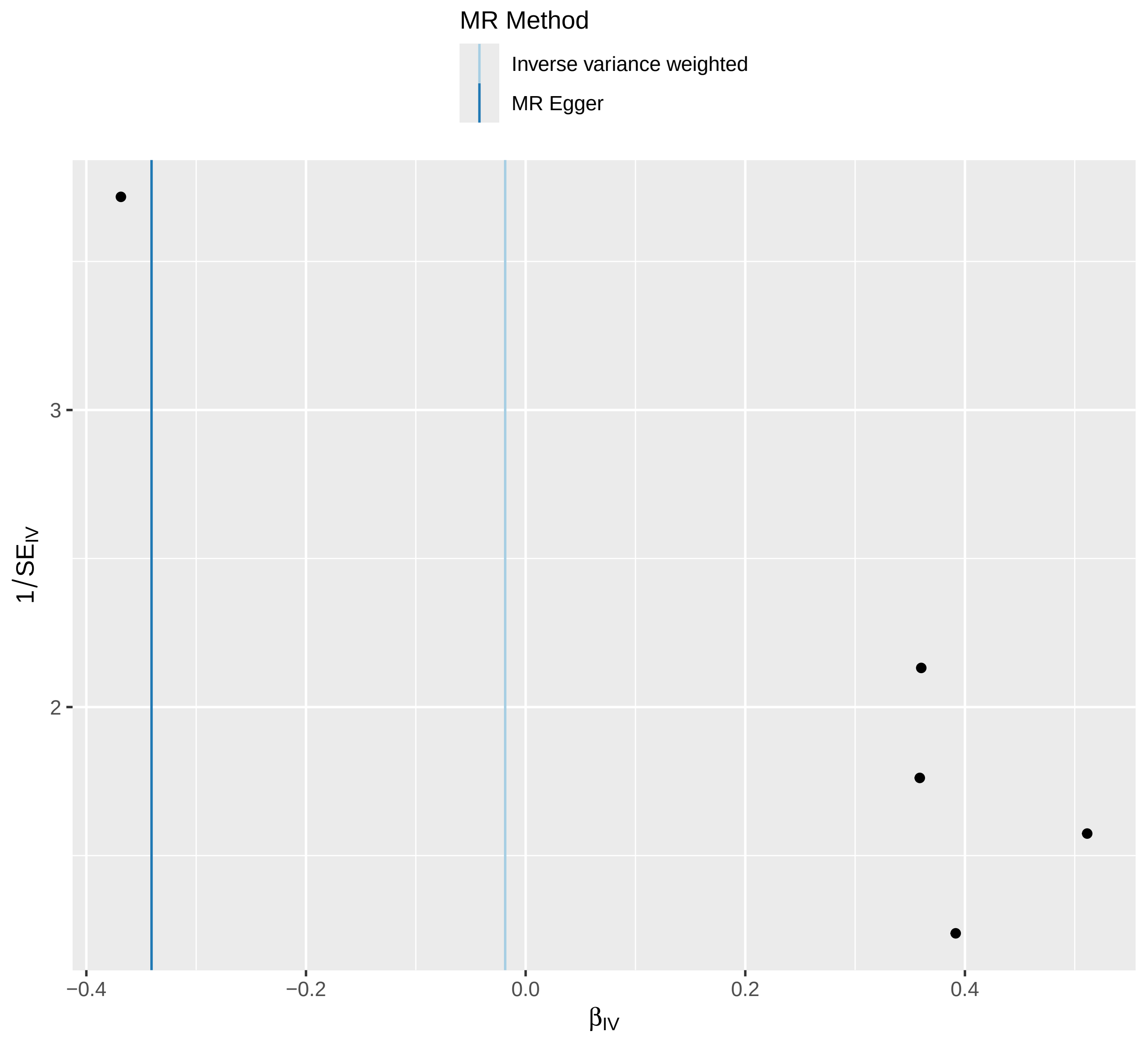
MR Method
Inverse variance weighted
MR Egger
3
2
1/SE_IV
−0.4
−0.2
0.0
0.2
0.4
β_IV

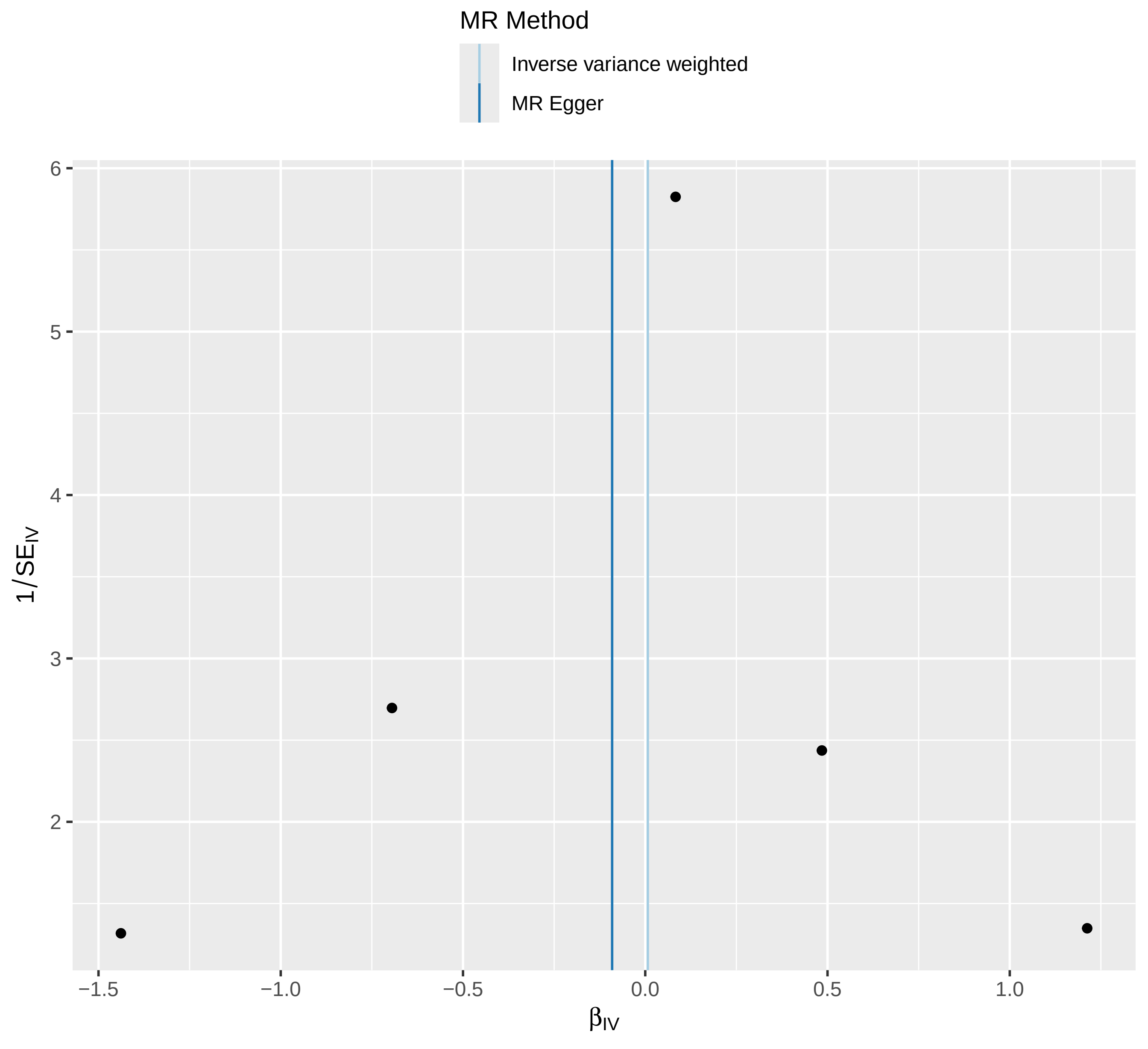
MR Method
Inverse variance weighted
MR Egger
6
5
4
3
2
1/SE_IV
−1.5
−1.0
−0.5
0.0
0.5
1.0
β_IV

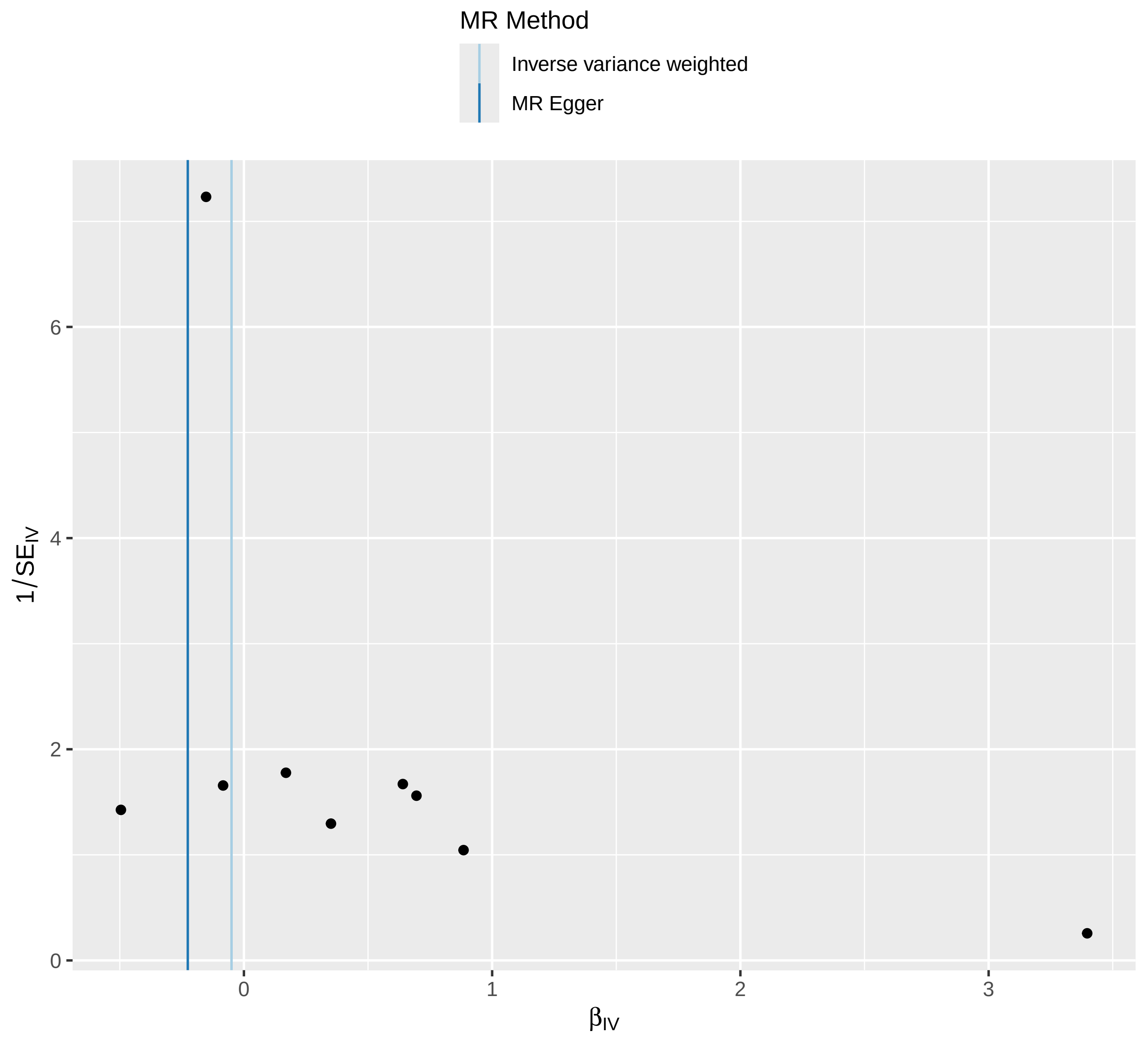

MR Method
Inverse variance weighted
MR Egger
$1/SE_{IV}$
β_{IV}
0
2
4
6
0
1
2
3

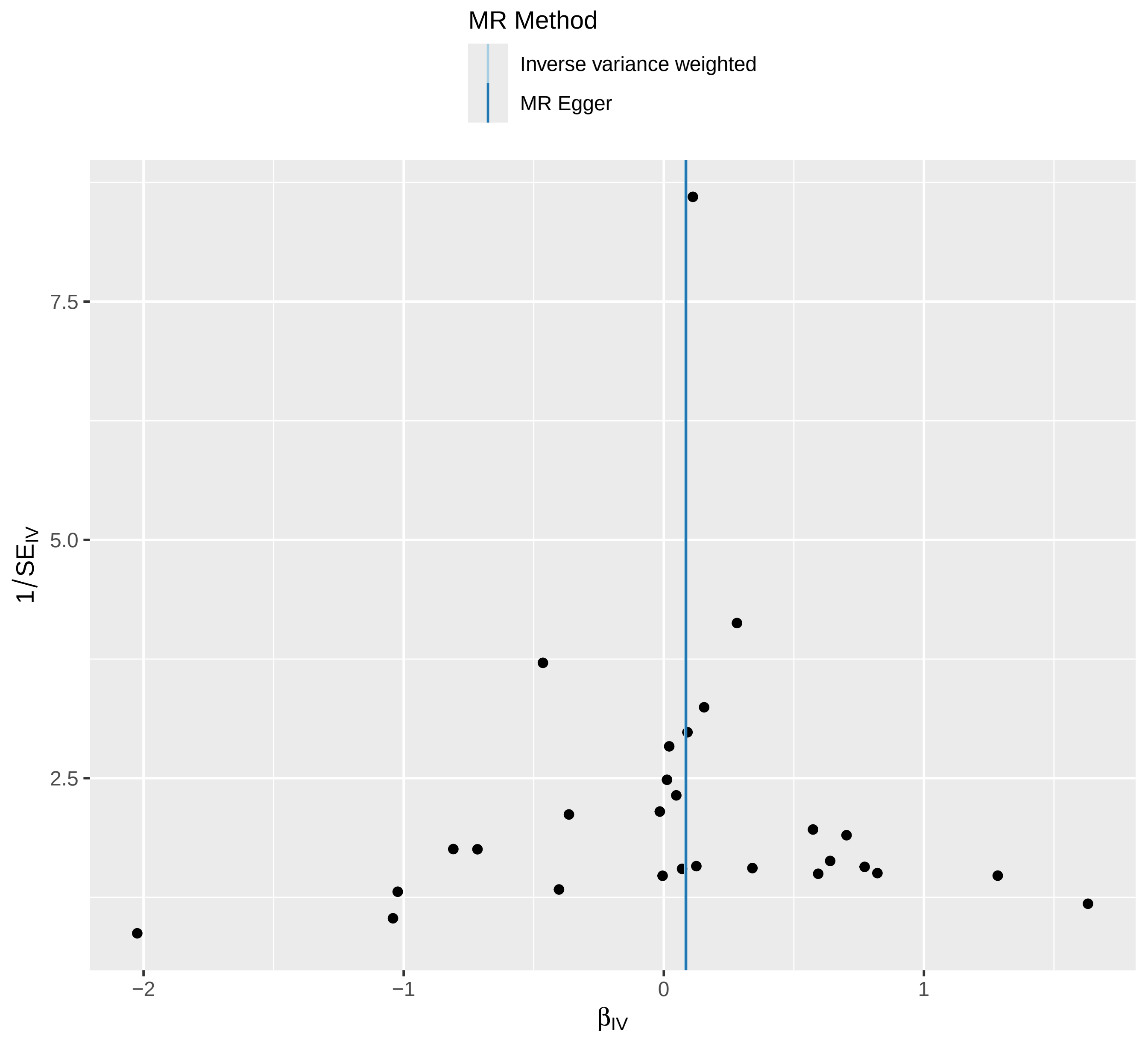
MR Method
Inverse variance weighted
MR Egger
7.5
5.0
2.5
1/SE_IV
−2
−1
0
1
β_IV

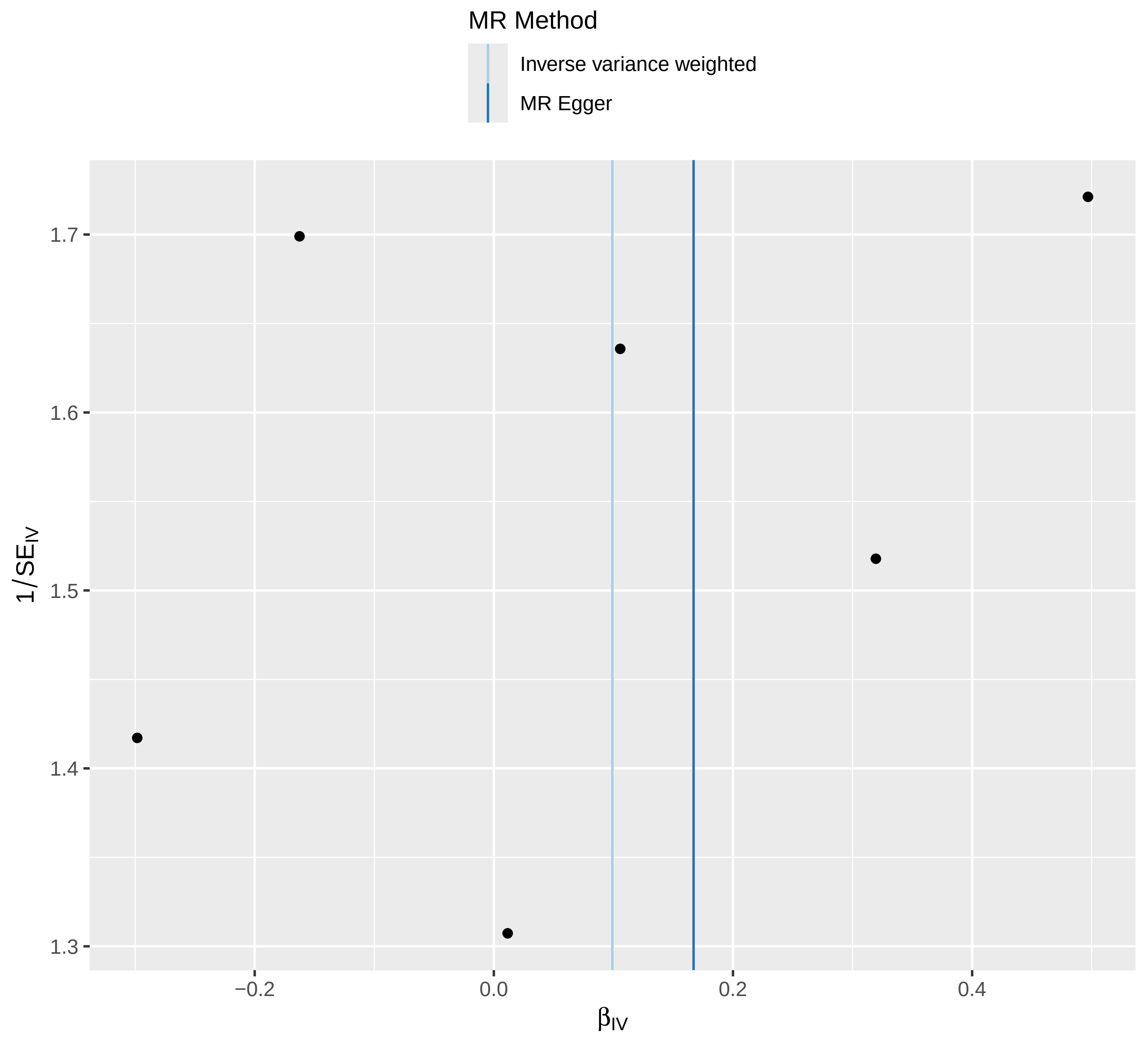

MR Method
Inverse variance weighted
MR Egger
1.7
1.6
1.5
1.4
1.3
1/SE$_{IV}$
−0.2
0.0
0.2
0.4
β$_{IV}$

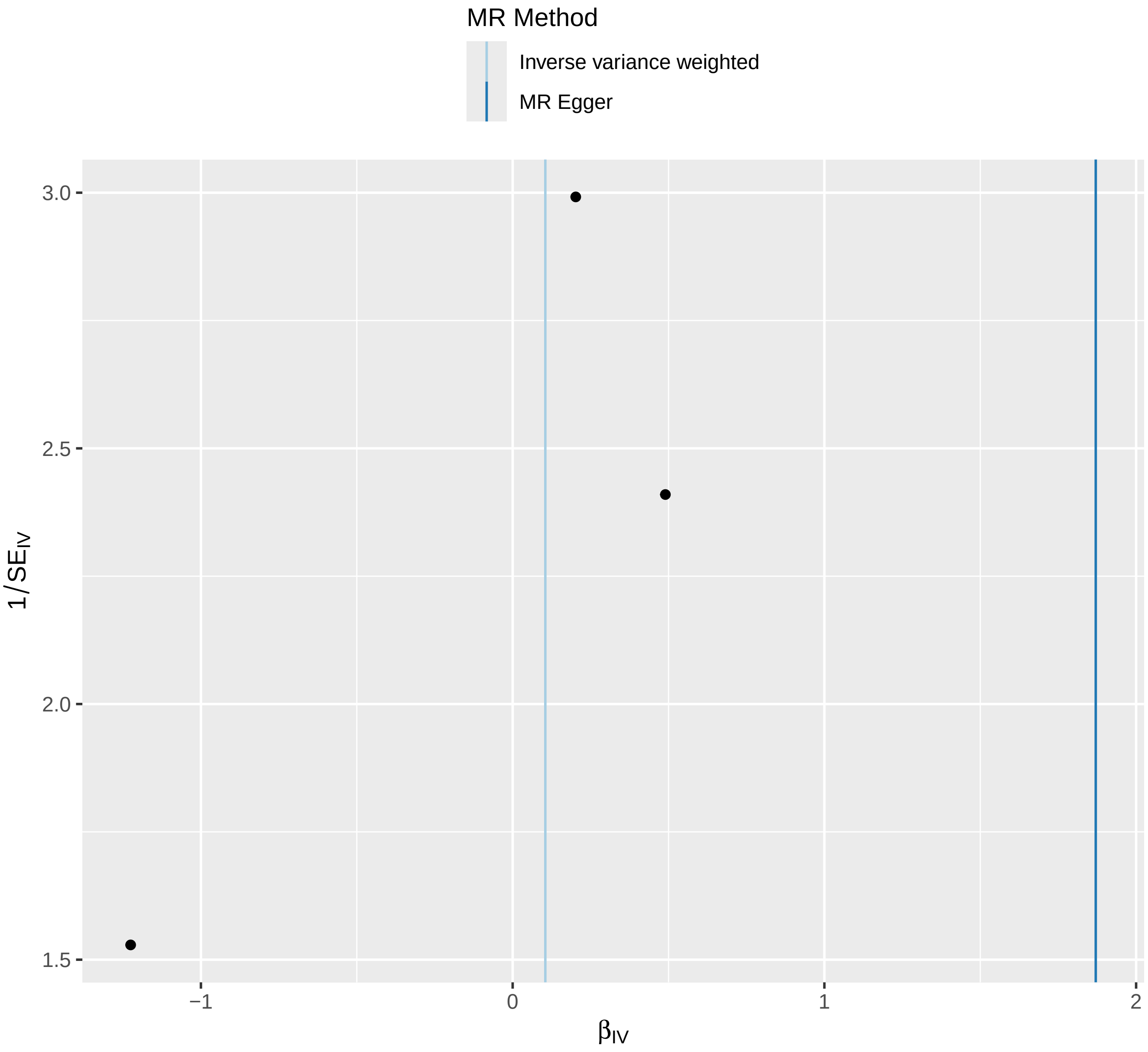

MR Method
Inverse variance weighted
MR Egger
3.0
2.5
2.0
1.5
$1/SE_{IV}$
−1
0
1
2
β_{IV}

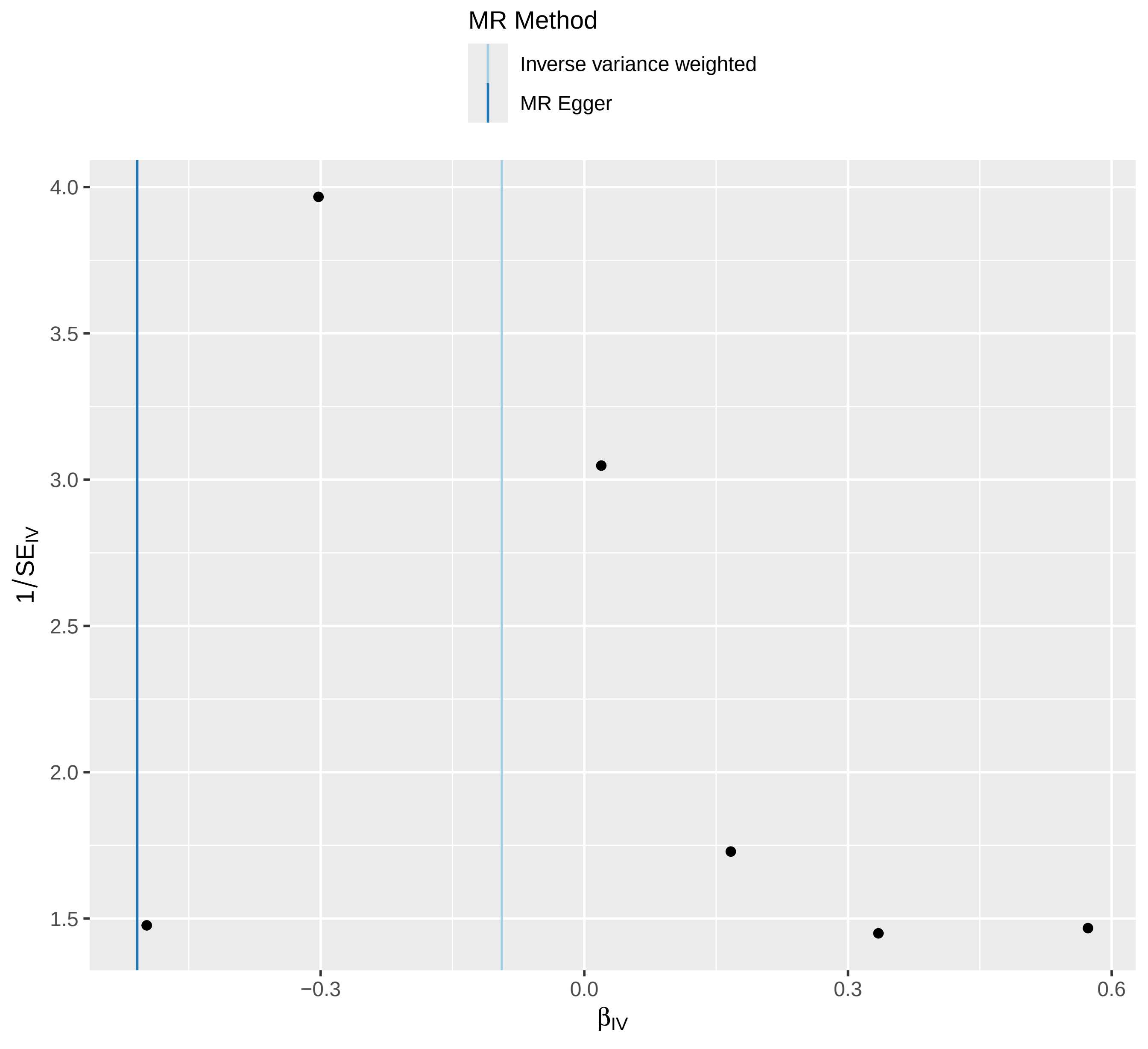

MR Method
Inverse variance weighted
MR Egger
4.0
3.5
3.0
2.5
2.0
1.5
1/SE$_{IV}$
−0.3
0.0
0.3
0.6
β$_{IV}$

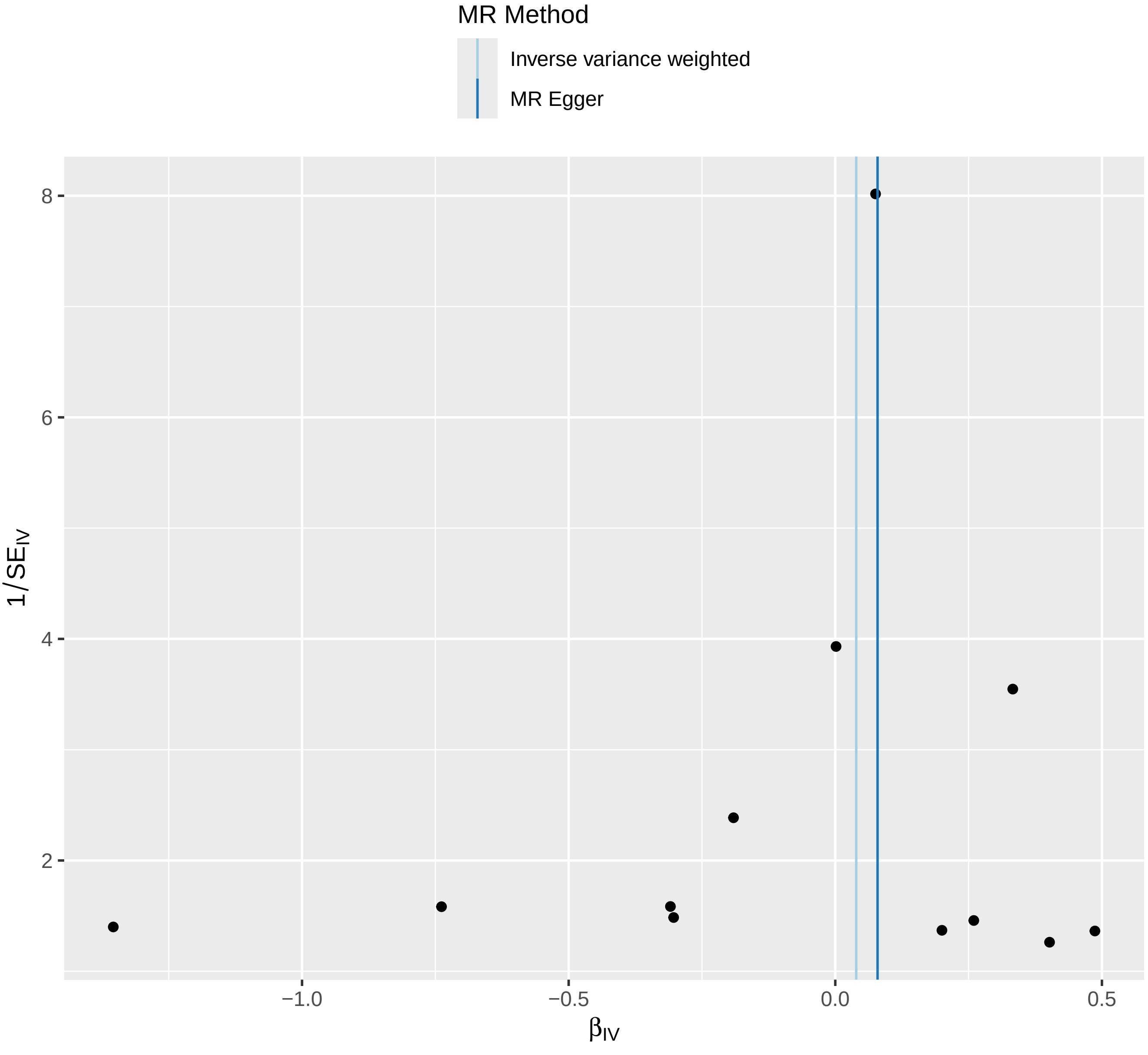

MR Method
Inverse variance weighted
MR Egger
1/SE_IV
8
6
4
2
−1.0
−0.5
0.0
0.5
β_IV

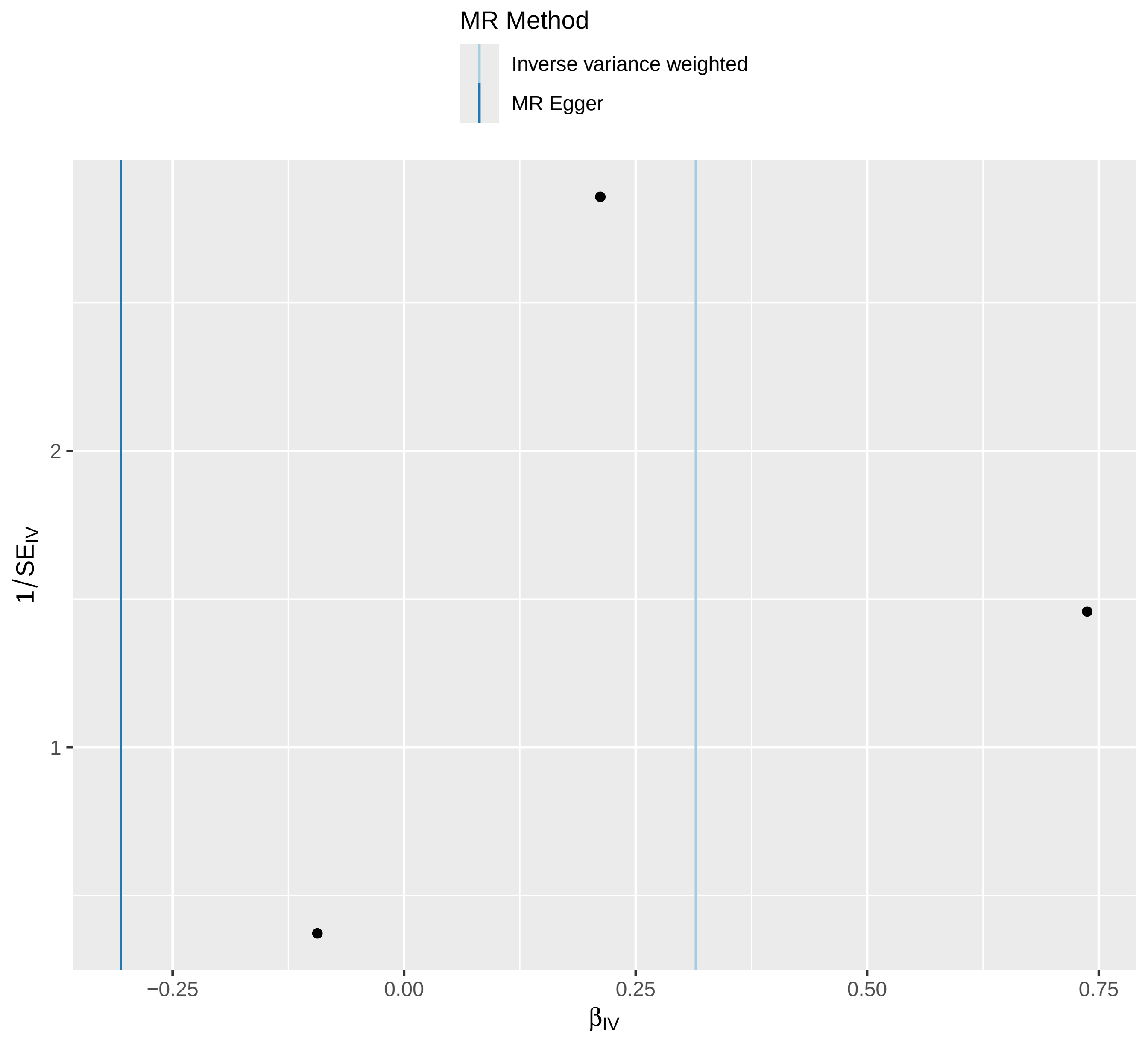

MR Method
Inverse variance weighted
MR Egger
2
1
1/SE_IV
−0.25
0.00
0.25
0.50
0.75
β_IV

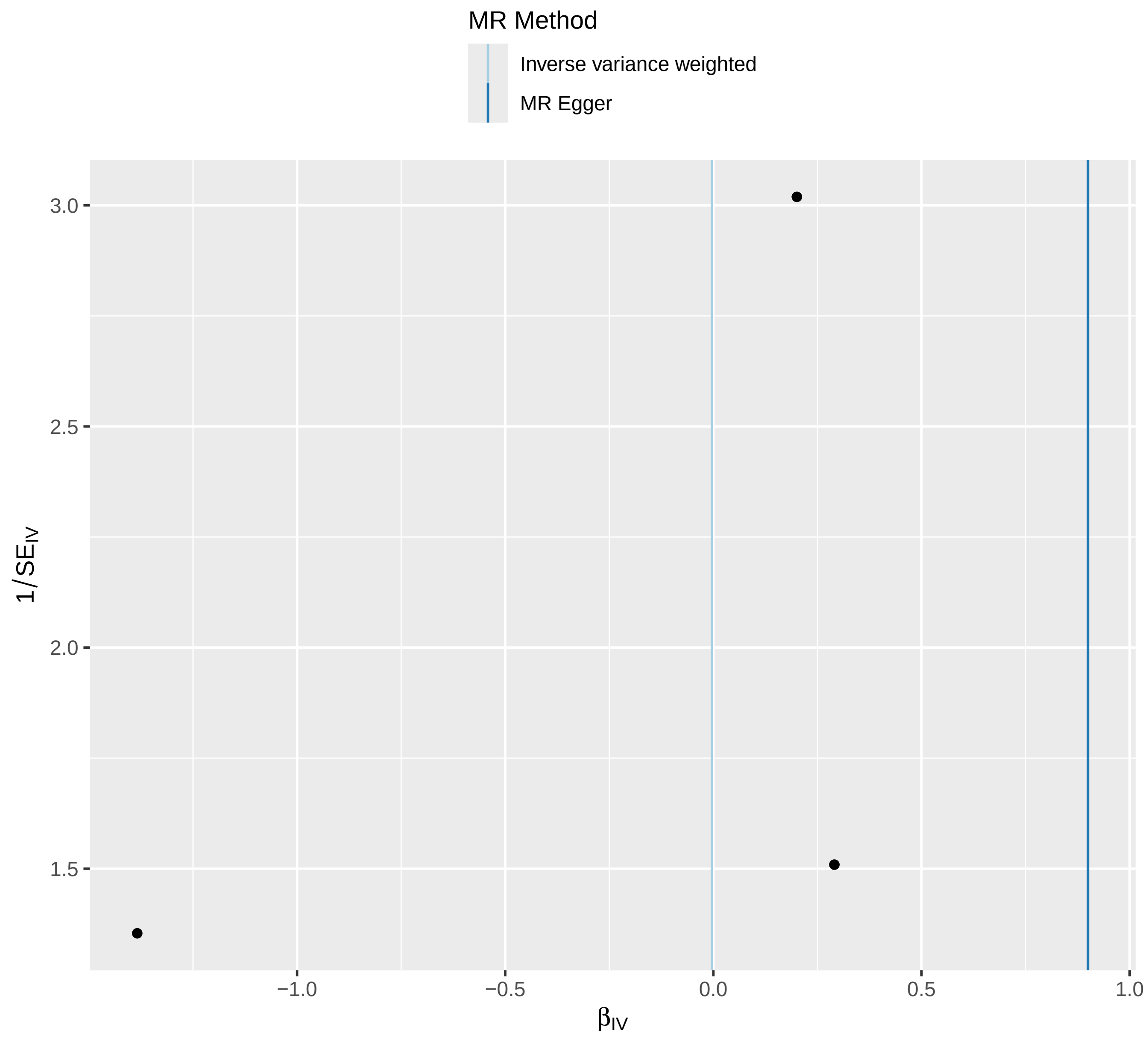
MR Method
Inverse variance weighted
MR Egger
3.0
2.5
2.0
1.5
$1/SE_{IV}$
−1.0
−0.5
0.0
0.5
1.0
β_{IV}

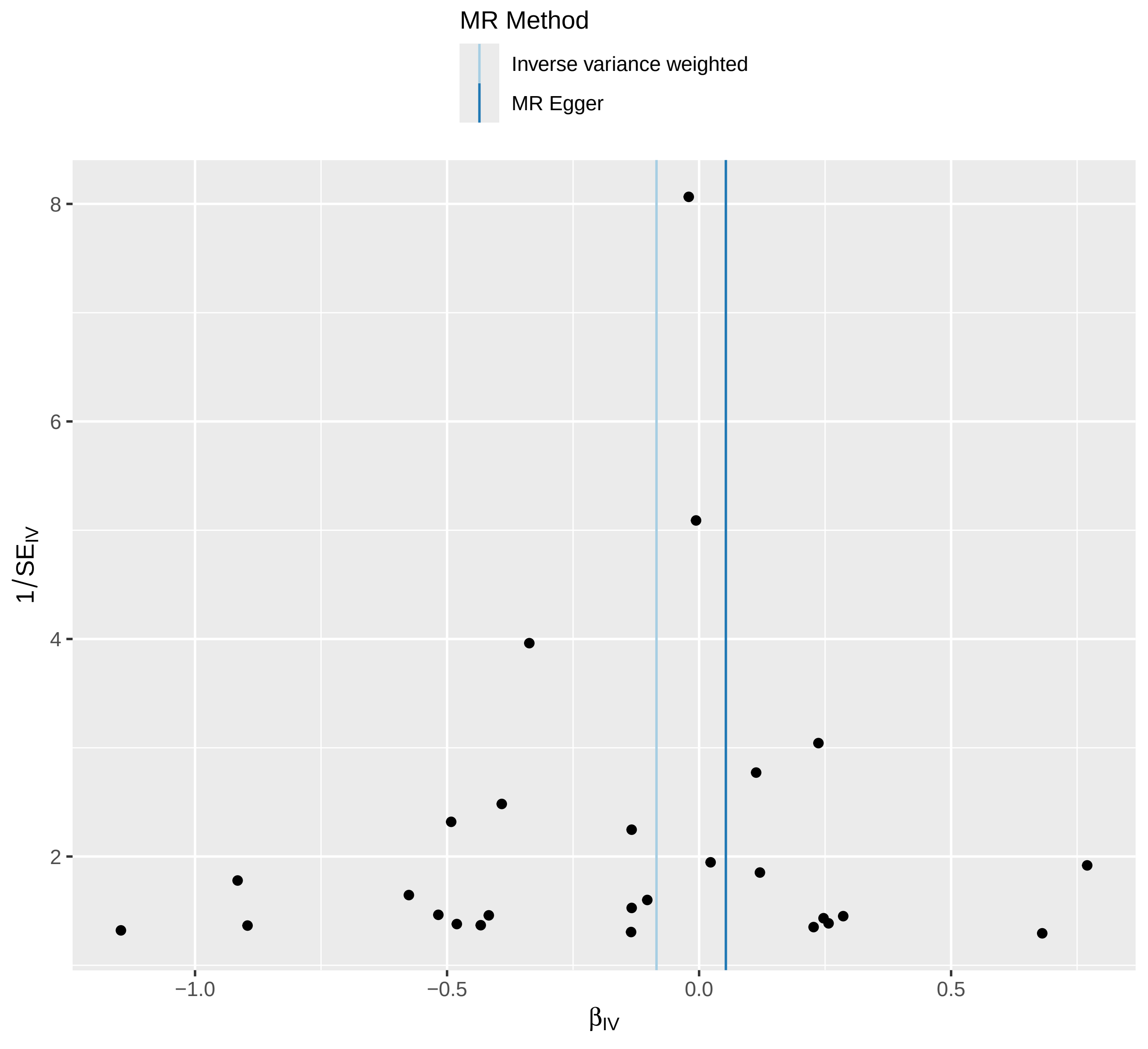
MR Method
Inverse variance weighted
MR Egger
8
6
4
2
1/SE_IV
−1.0
−0.5
0.0
0.5
β_IV

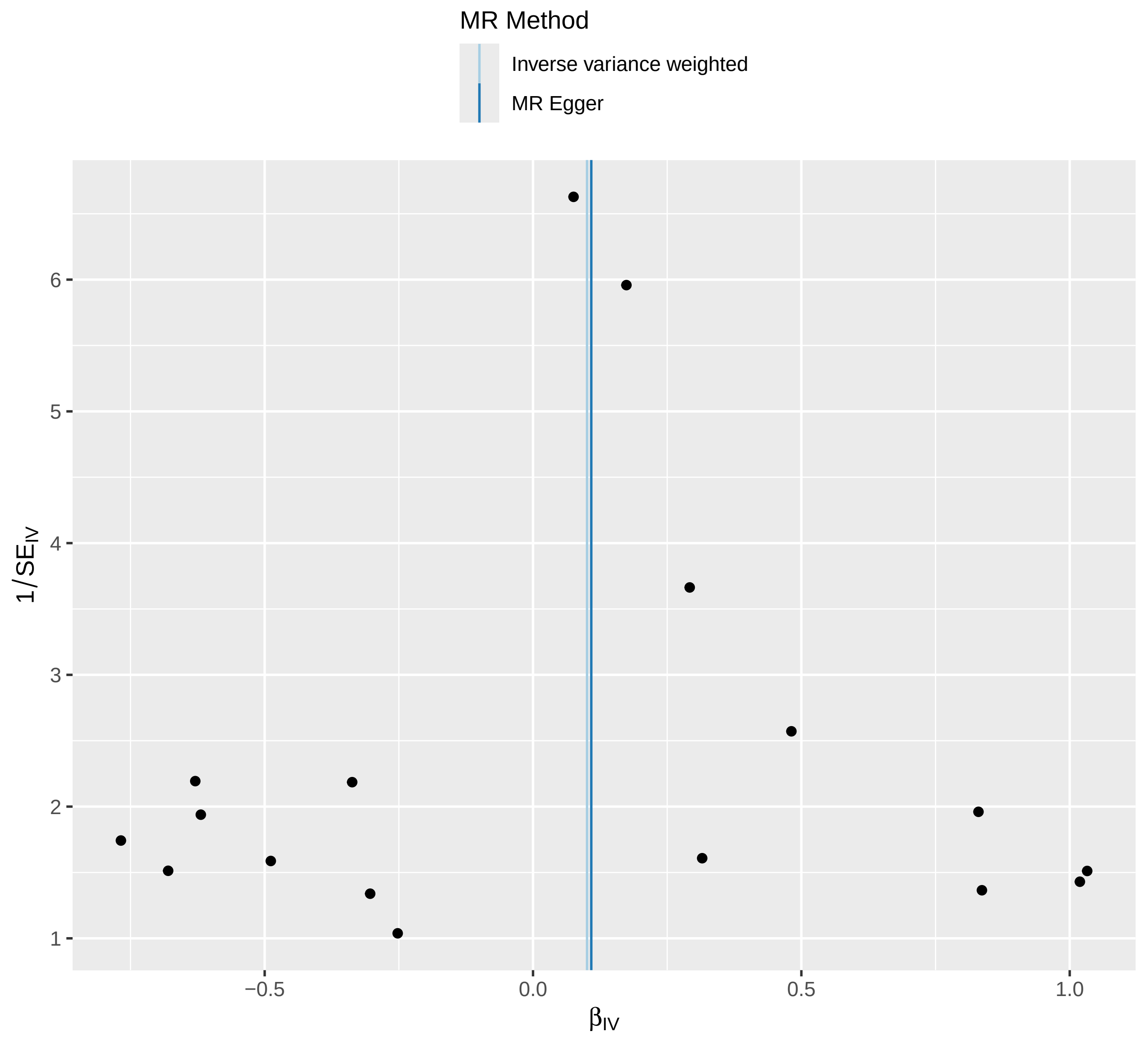
MR Method
Inverse variance weighted
MR Egger
1/SE_IV
6
5
4
3
2
1
−0.5
0.0
0.5
1.0
β_IV

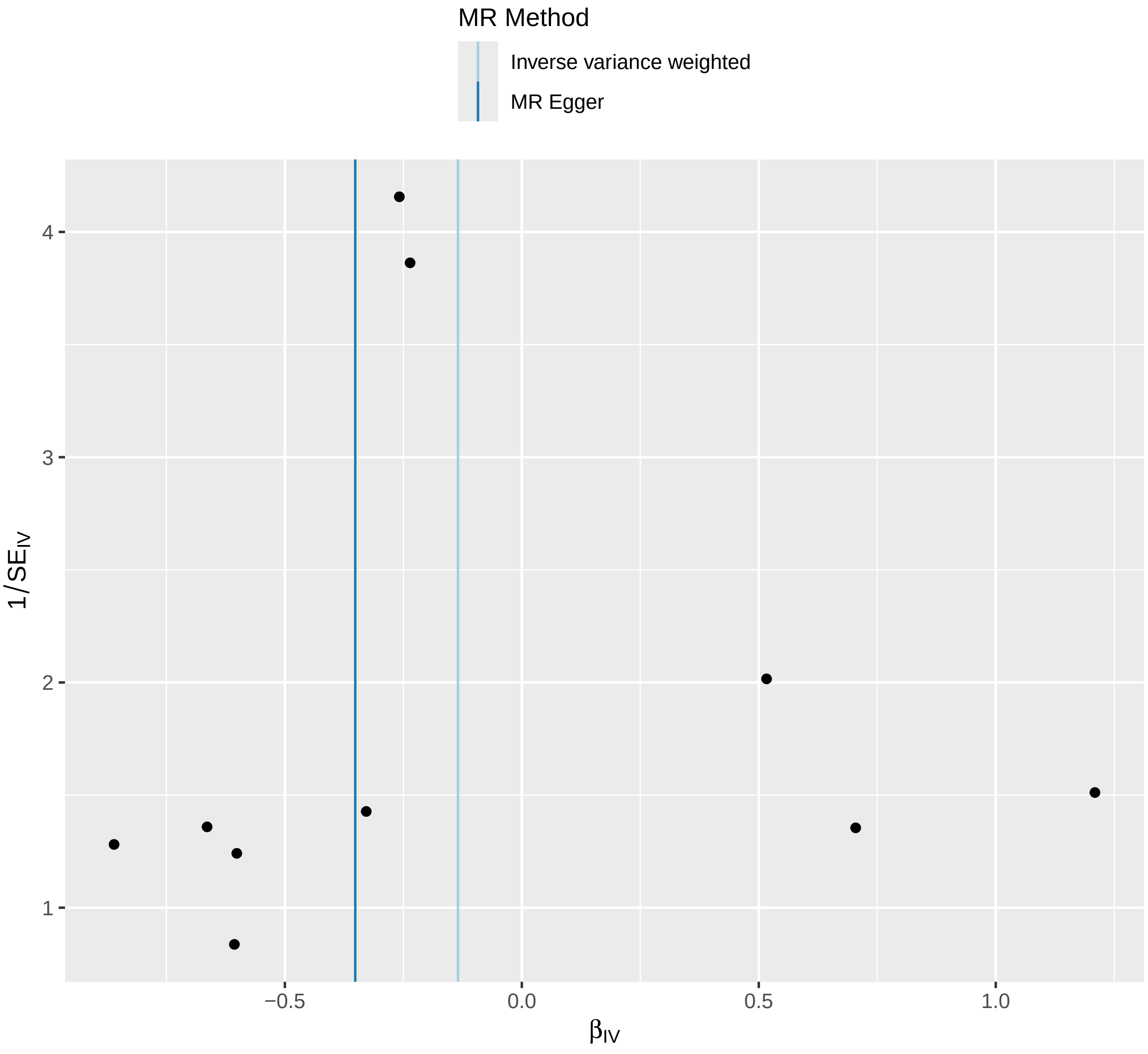

MR Method
Inverse variance weighted
MR Egger
4
3
2
1
$1/SE_{IV}$
−0.5
0.0
0.5
1.0
β_{IV}

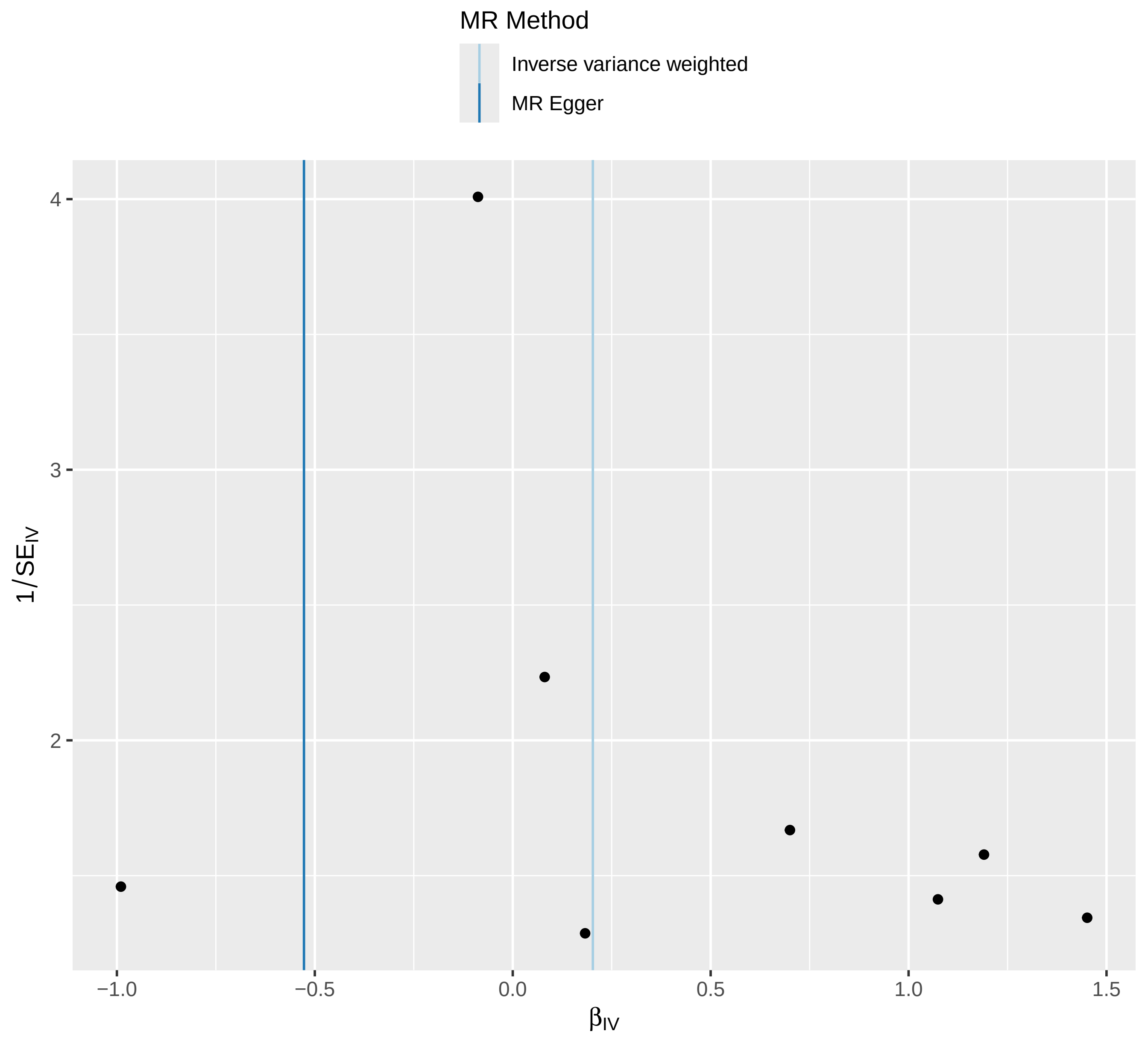
MR Method
Inverse variance weighted
MR Egger
4
3
2
1/SE_IV
−1.0
−0.5
0.0
0.5
1.0
1.5
β_IV

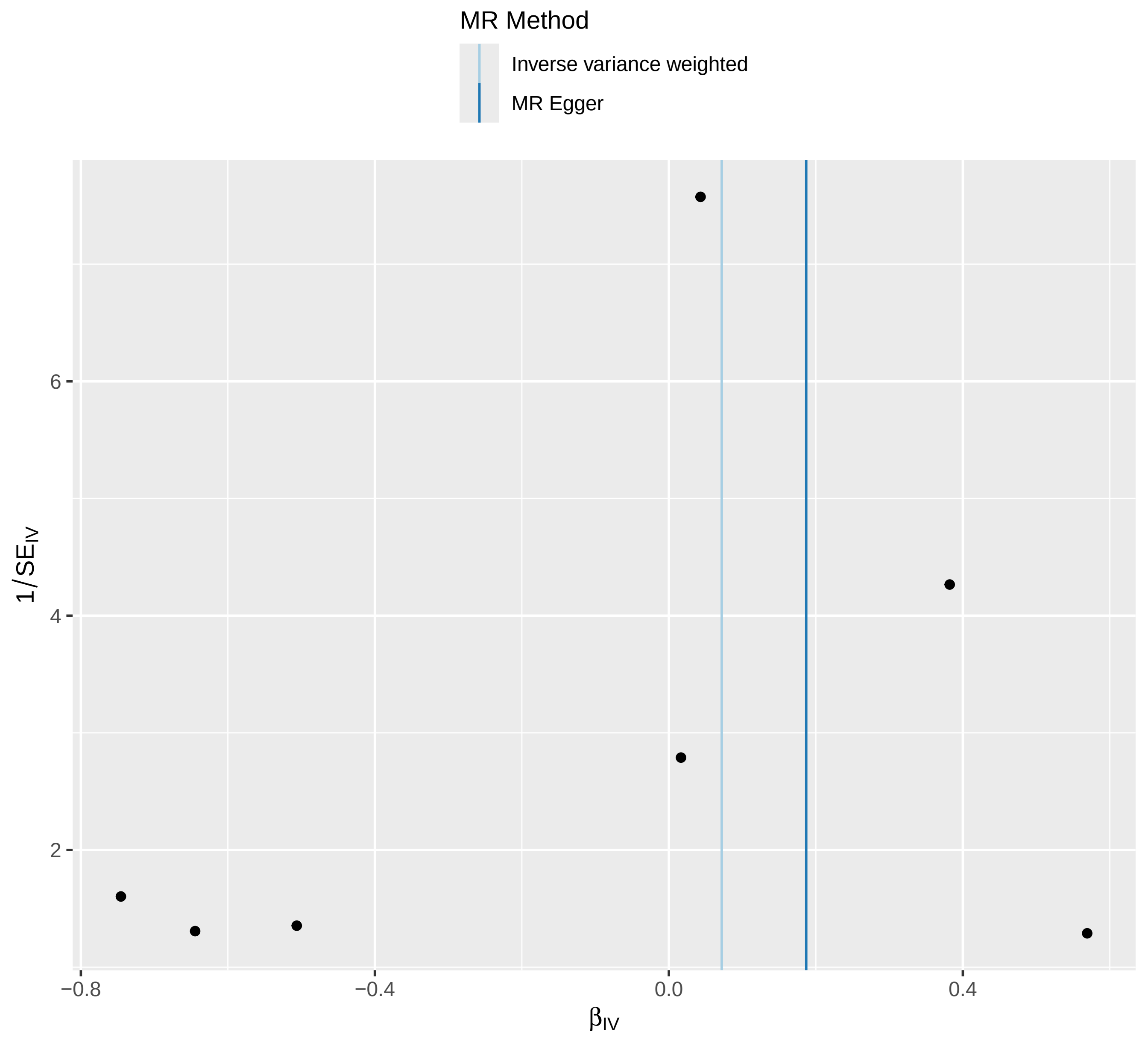
MR Method
Inverse variance weighted
MR Egger
6
4
2
1/SE_IV
−0.8
−0.4
0.0
0.4
β_IV

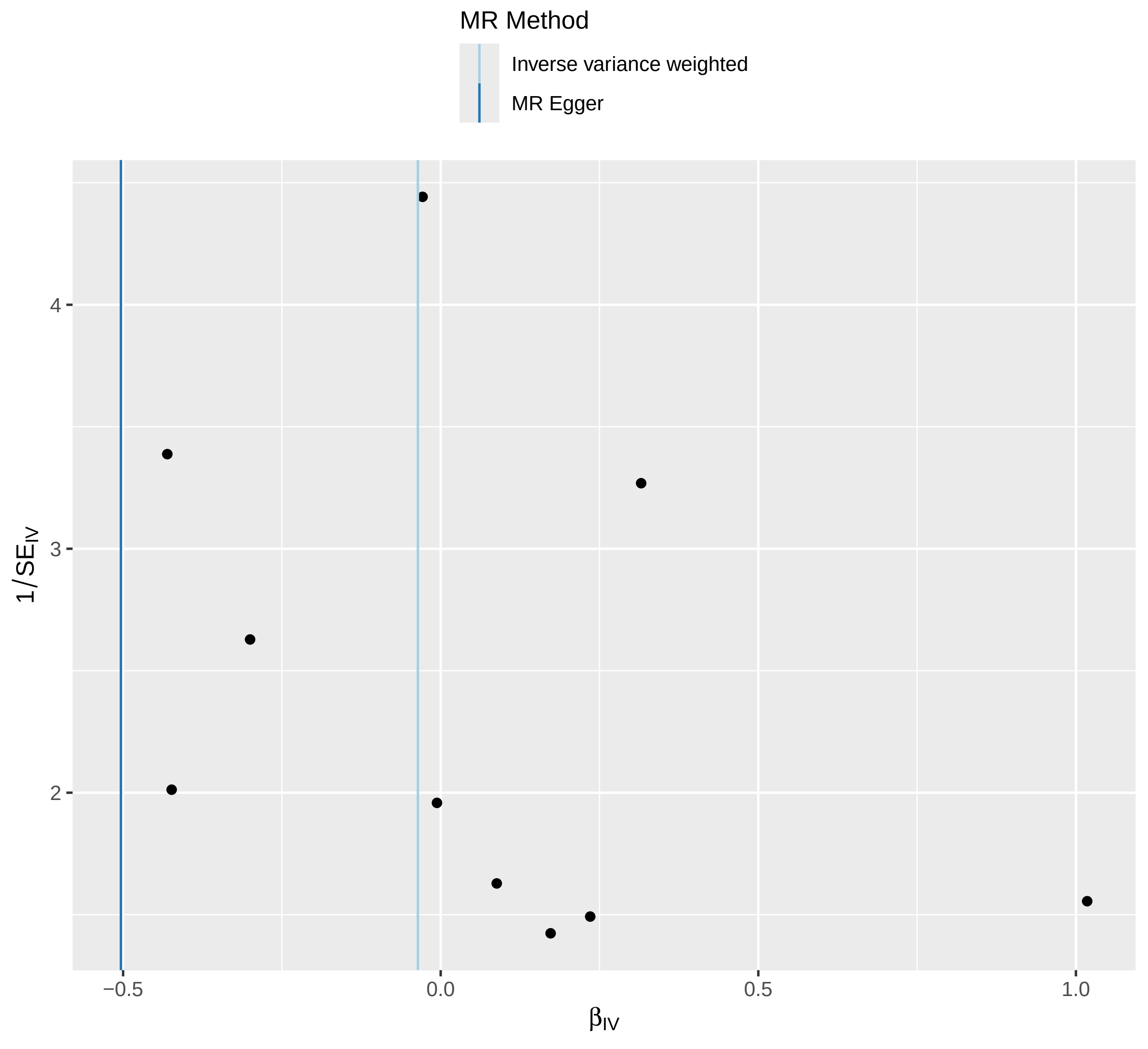

MR Method
Inverse variance weighted
MR Egger
4
3
2
1/SE_IV
−0.5
0.0
0.5
1.0
β_IV

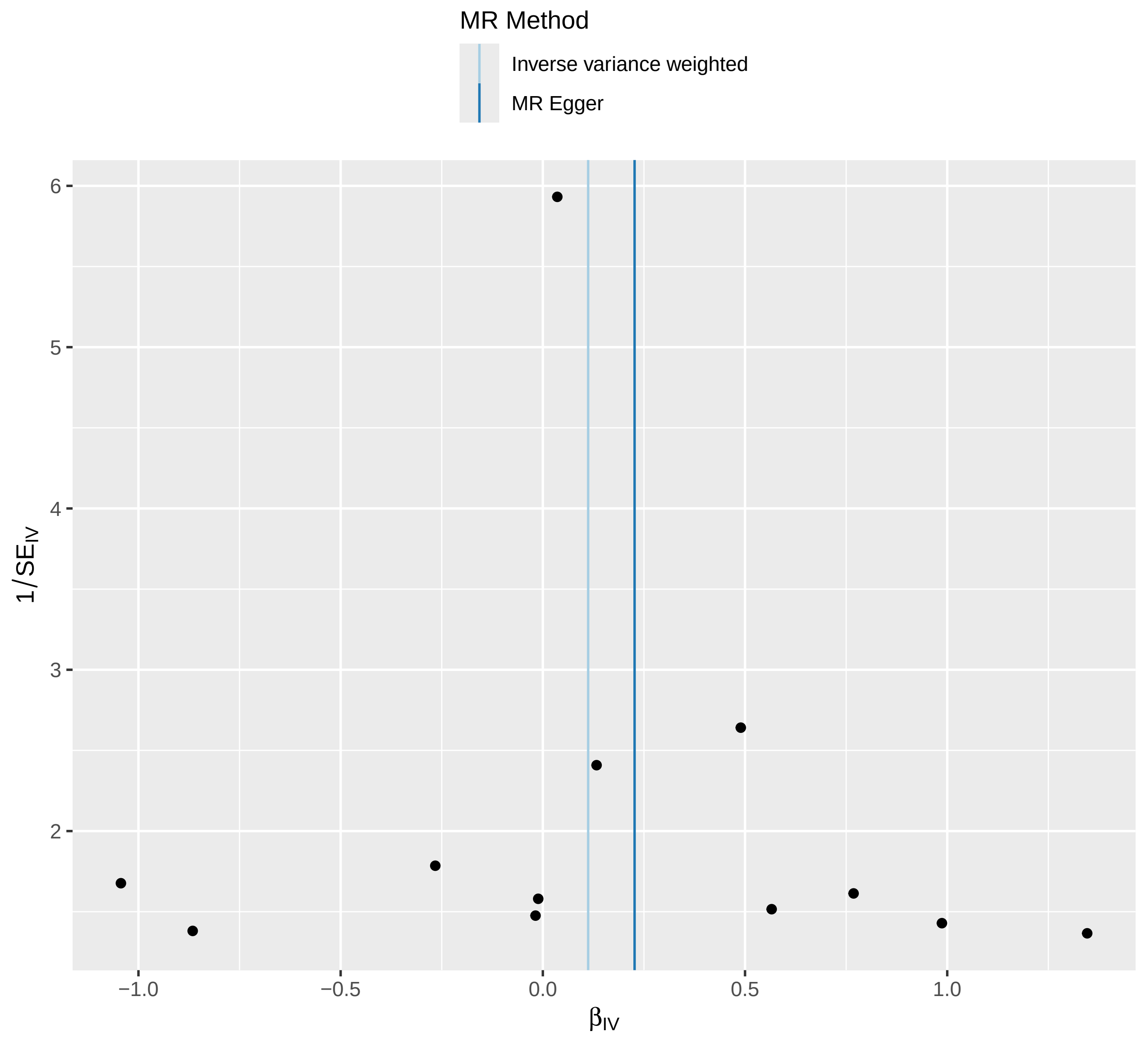

MR Method
Inverse variance weighted
MR Egger
6
5
4
3
2
1/SE$_{IV}$
−1.0
−0.5
0.0
0.5
1.0
β$_{IV}$

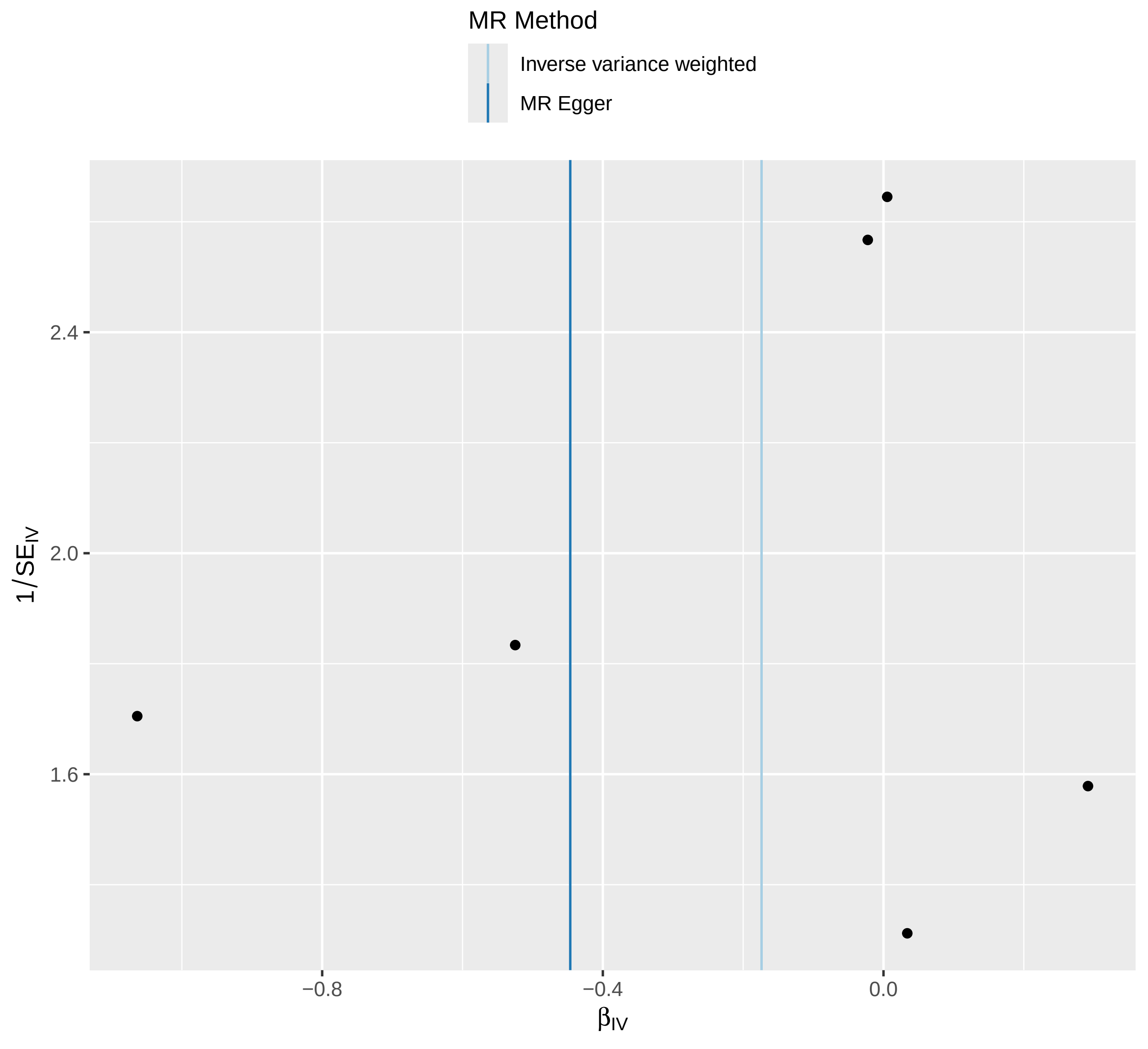

MR Method
Inverse variance weighted
MR Egger
2.4
2.0
1.6
1/SE_IV
−0.8
−0.4
0.0
β_IV

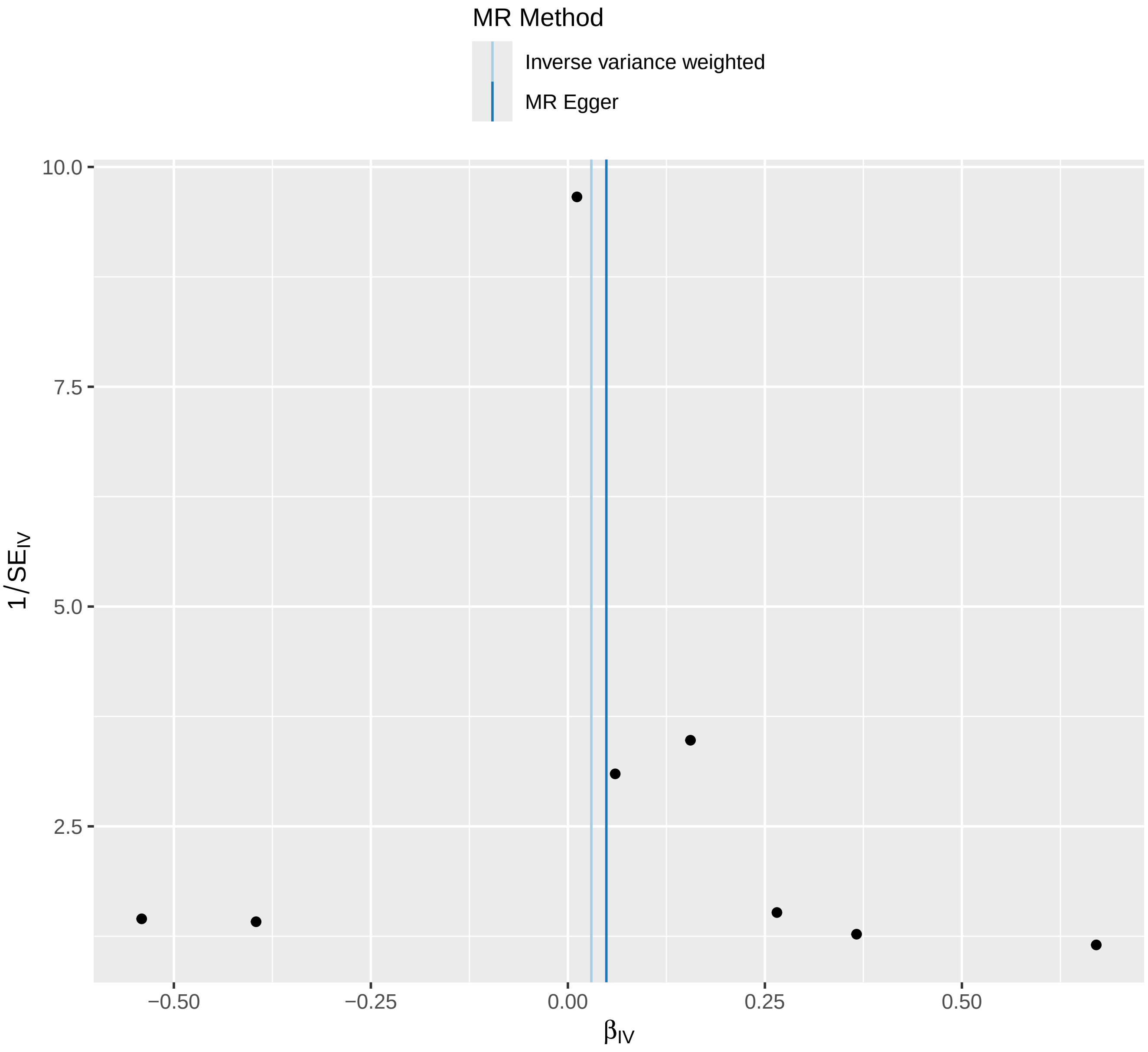
MR Method
Inverse variance weighted
MR Egger
10.0
7.5
5.0
2.5
1/SE$_{IV}$
−0.50
−0.25
0.00
0.25
0.50
β$_{IV}$

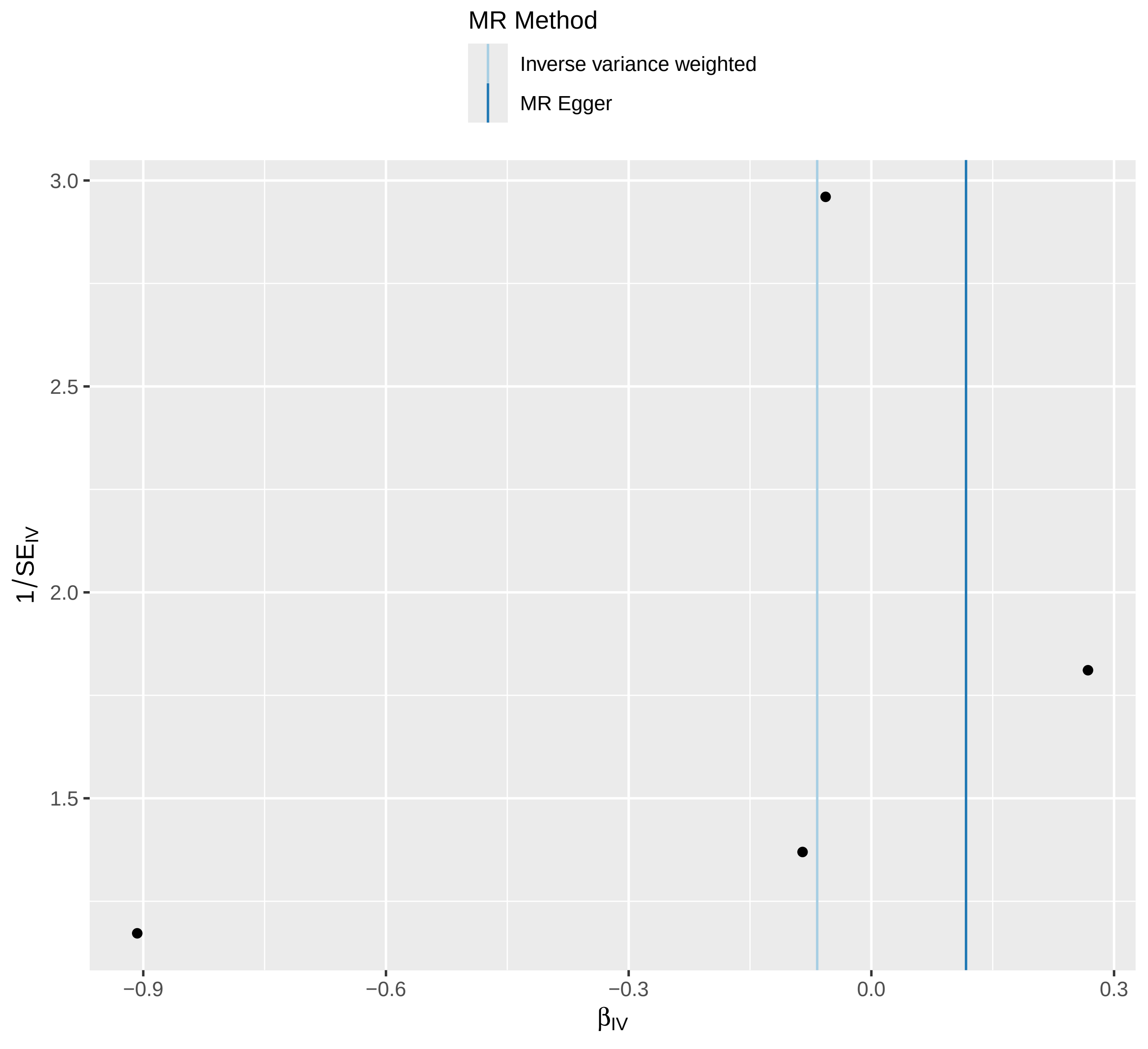
MR Method
Inverse variance weighted
MR Egger
3.0
2.5
2.0
1.5
1/SE_IV
−0.9
−0.6
−0.3
0.0
0.3
β_IV

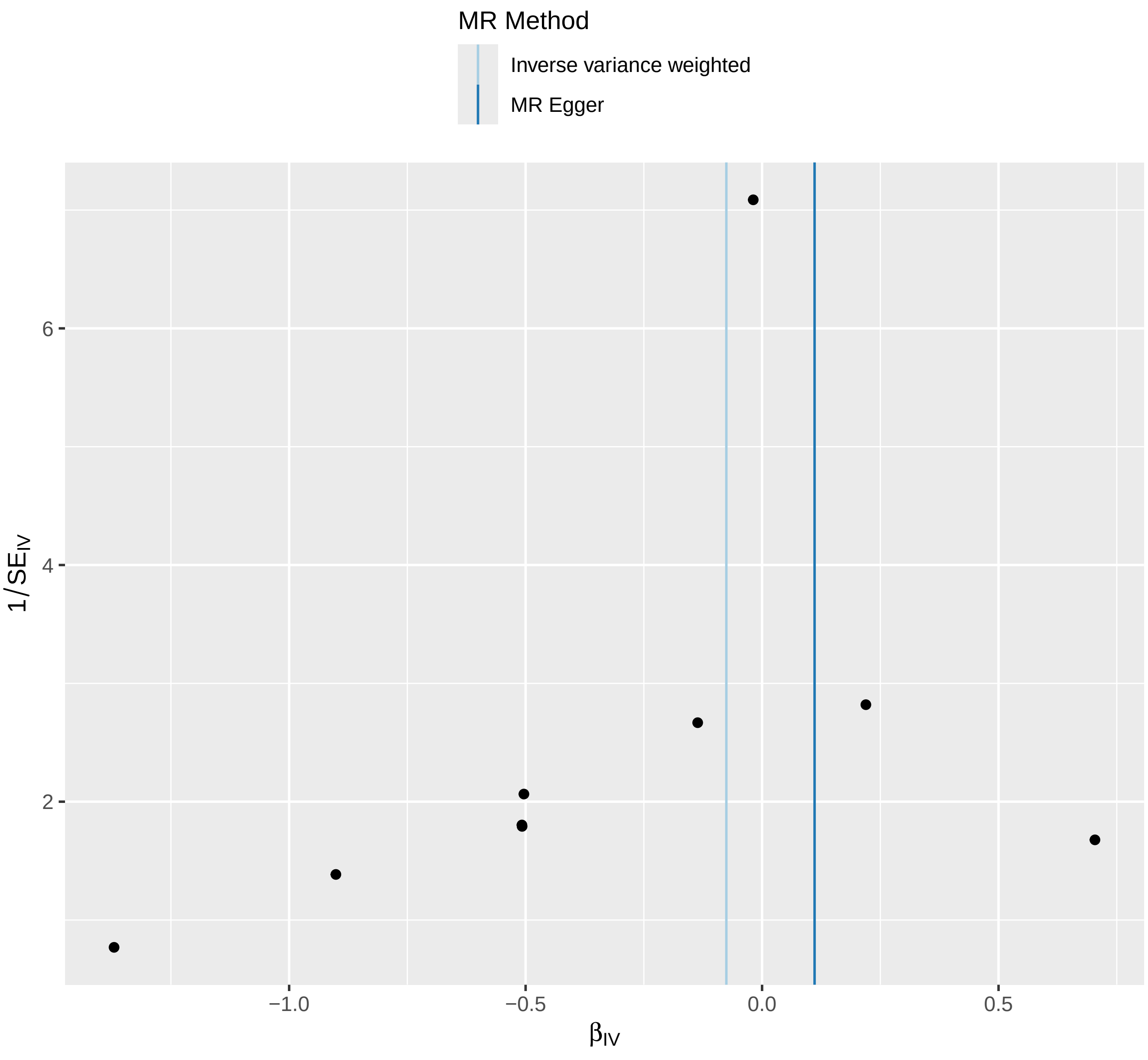
MR Method
Inverse variance weighted
MR Egger
6
4
2
1/SE$_{IV}$
−1.0
−0.5
0.0
0.5
β$_{IV}$

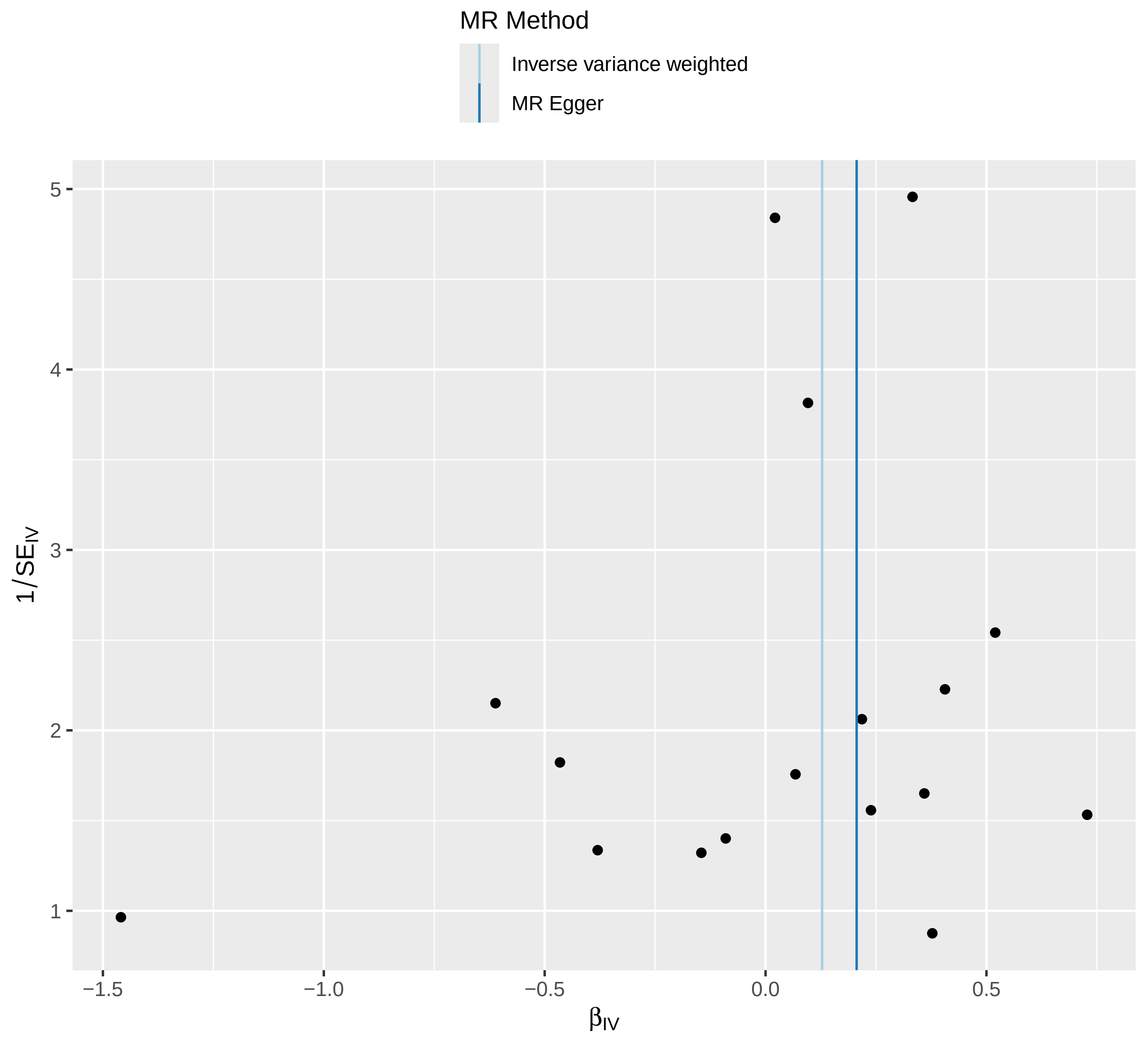

MR Method
Inverse variance weighted
MR Egger
1/SE$_{IV}$
5
4
3
2
1
−1.5
−1.0
−0.5
0.0
0.5
β$_{IV}$

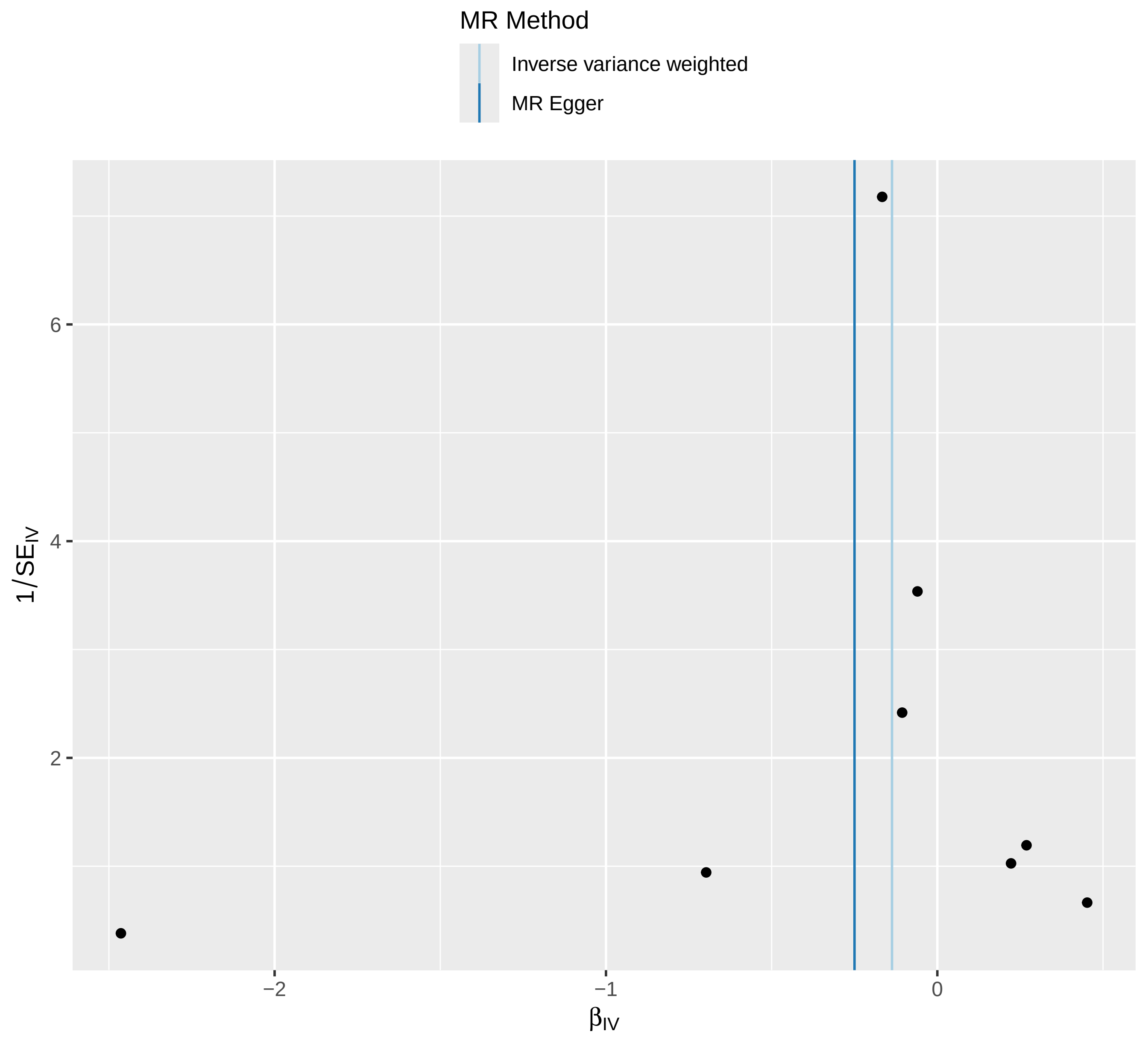

MR Method
Inverse variance weighted
MR Egger
6
4
2
1/SE_IV
−2
−1
0
β_IV

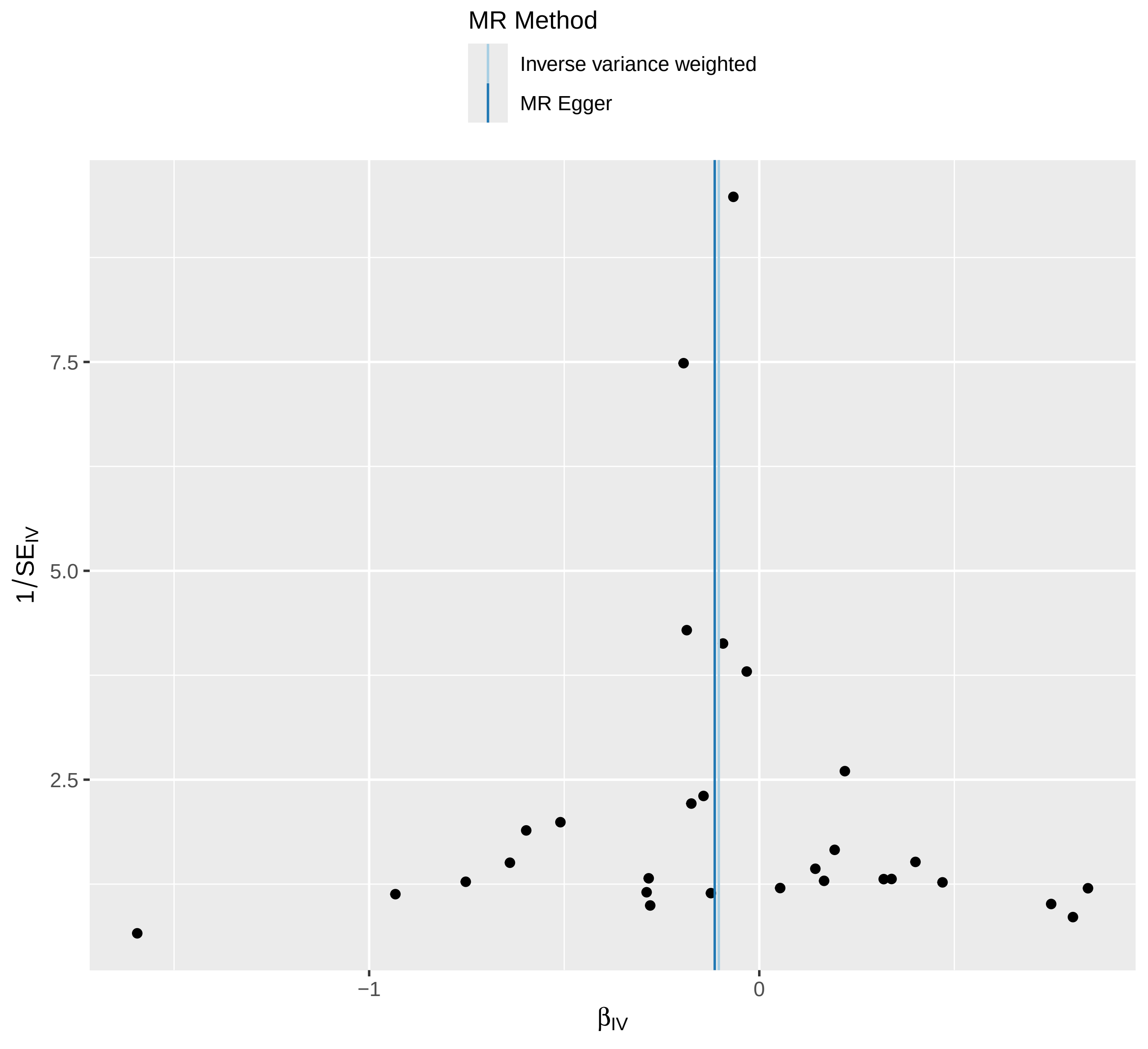
MR Method
Inverse variance weighted
MR Egger
7.5
5.0
2.5
1/SE$_{IV}$
−1
0
β$_{IV}$

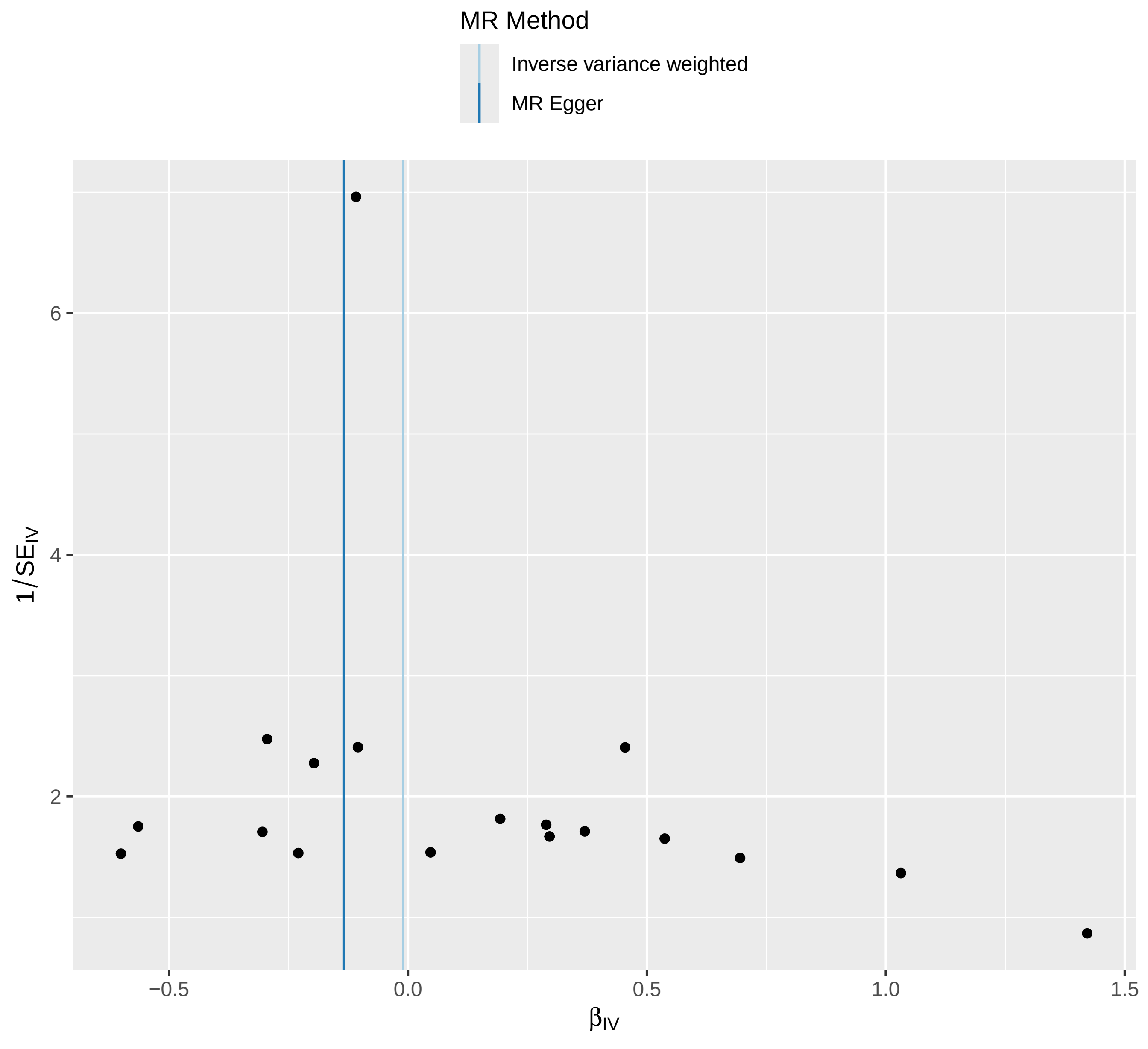
MR Method
Inverse variance weighted
MR Egger
6
4
2
1/SE_IV
−0.5
0.0
0.5
1.0
1.5
β_IV

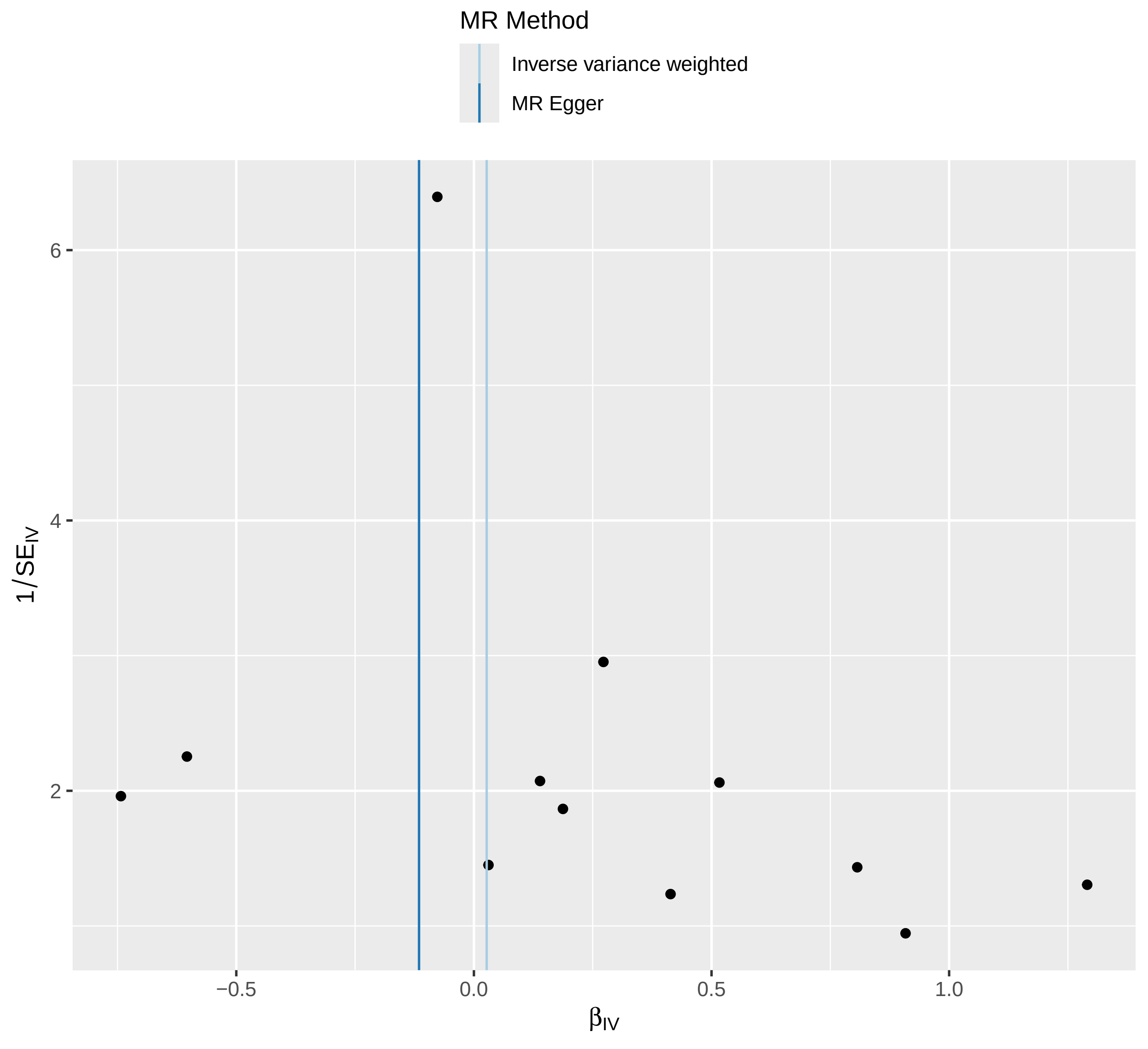

MR Method
Inverse variance weighted
MR Egger
6
4
2
1/SE_IV
−0.5
0.0
0.5
1.0
β_IV

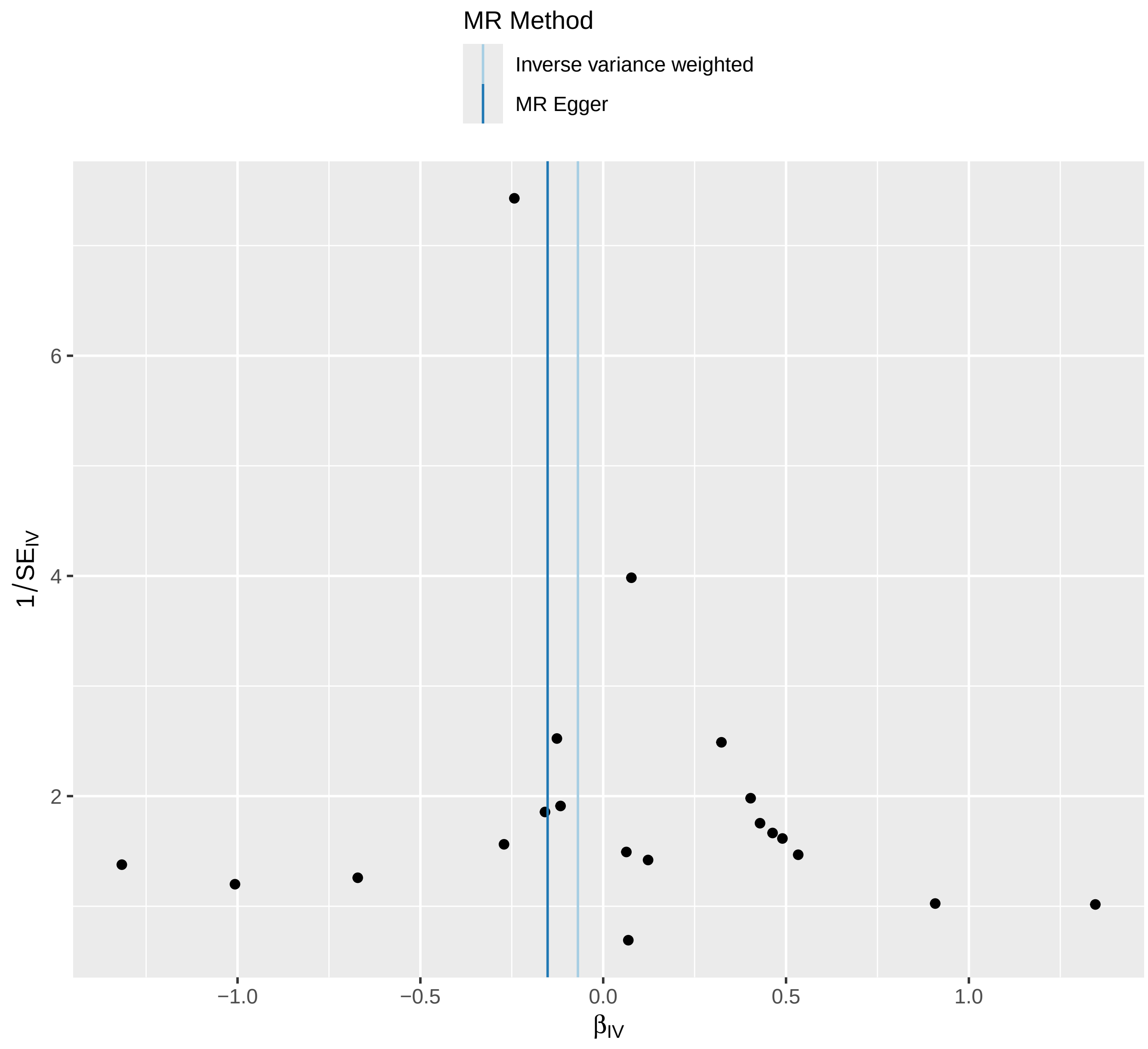
MR Method
Inverse variance weighted
MR Egger
1/SE$_{IV}$
6
4
2
−1.0
−0.5
0.0
0.5
1.0
β$_{IV}$

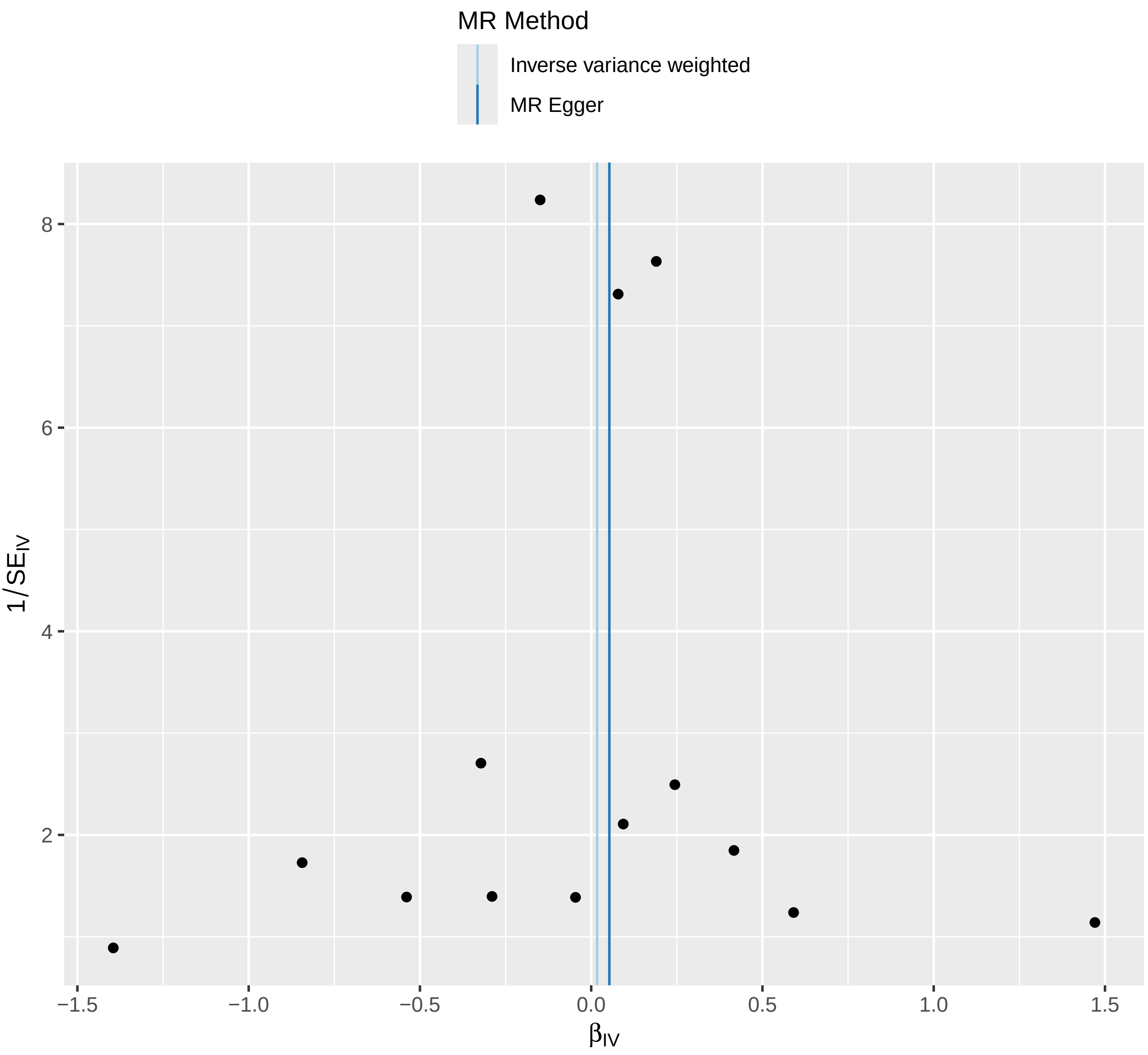
MR Method
Inverse variance weighted
MR Egger
1/SE$_{IV}$
8
6
4
2
−1.5
−1.0
−0.5
0.0
0.5
1.0
1.5
β$_{IV}$

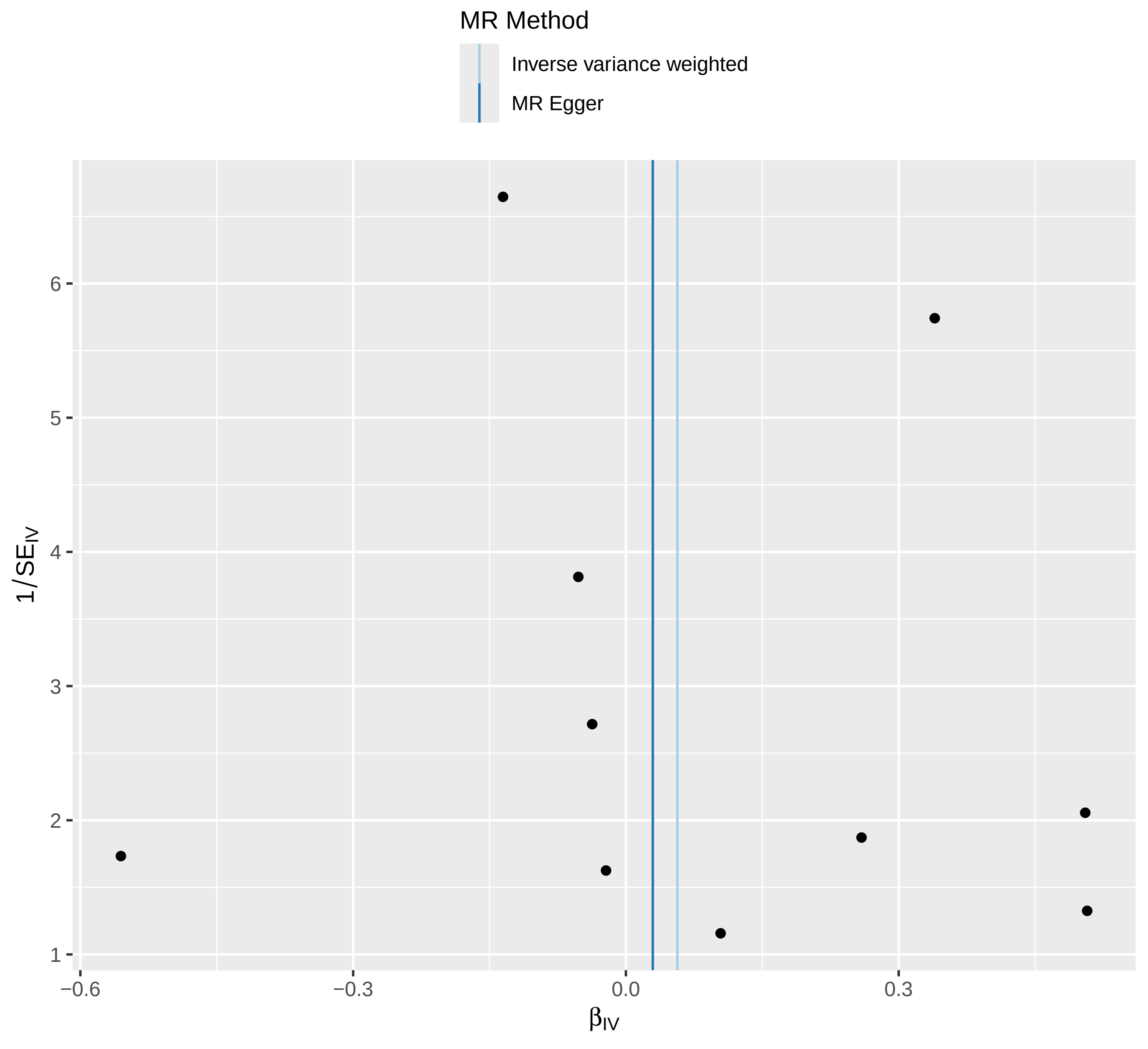

MR Method
Inverse variance weighted
MR Egger
6
5
4
3
2
1
1/SE_IV
−0.6
−0.3
0.0
0.3
β_IV

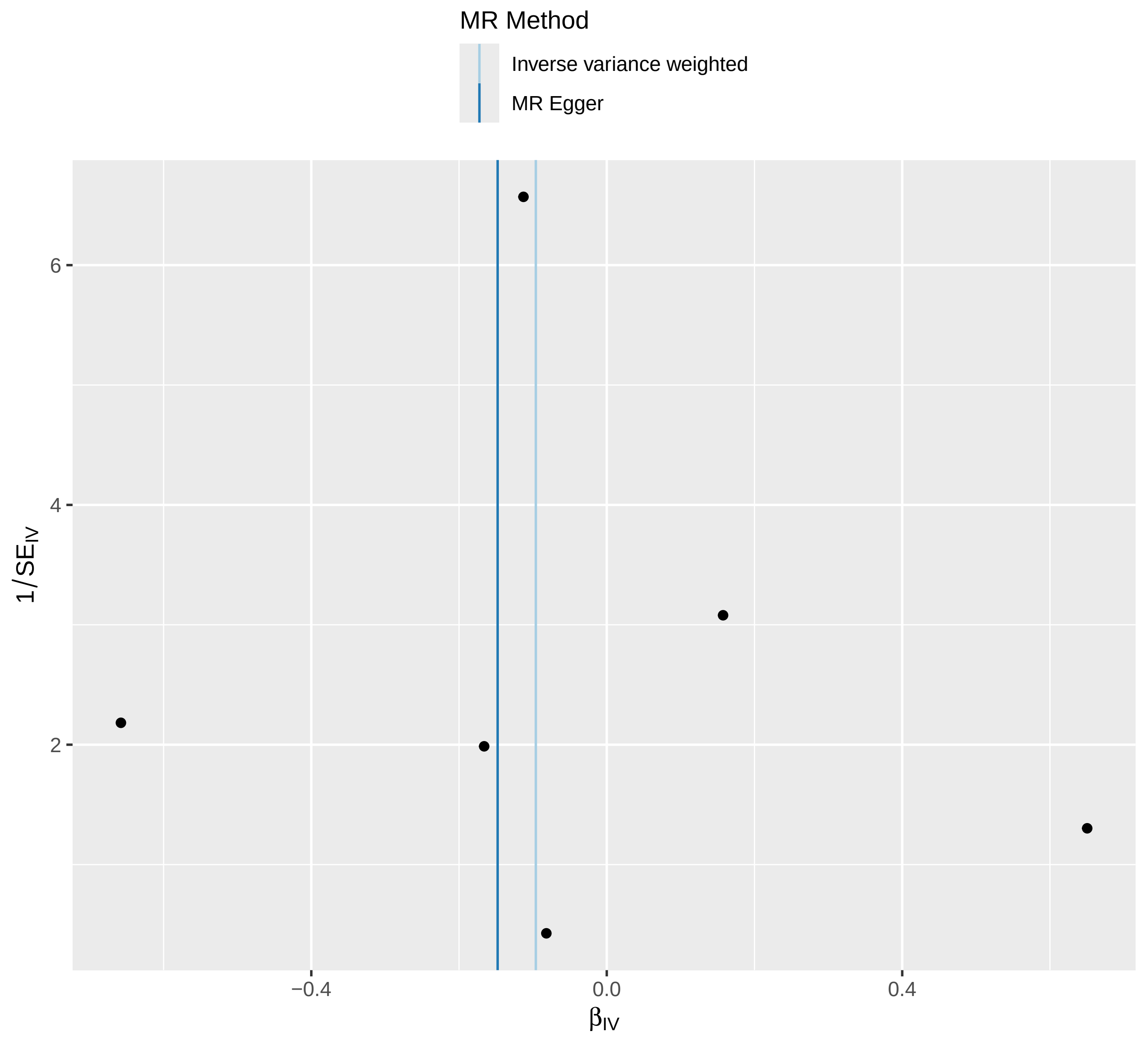

MR Method
Inverse variance weighted
MR Egger
6
4
2
$1/SE_{IV}$
−0.4
0.0
0.4
β_{IV}

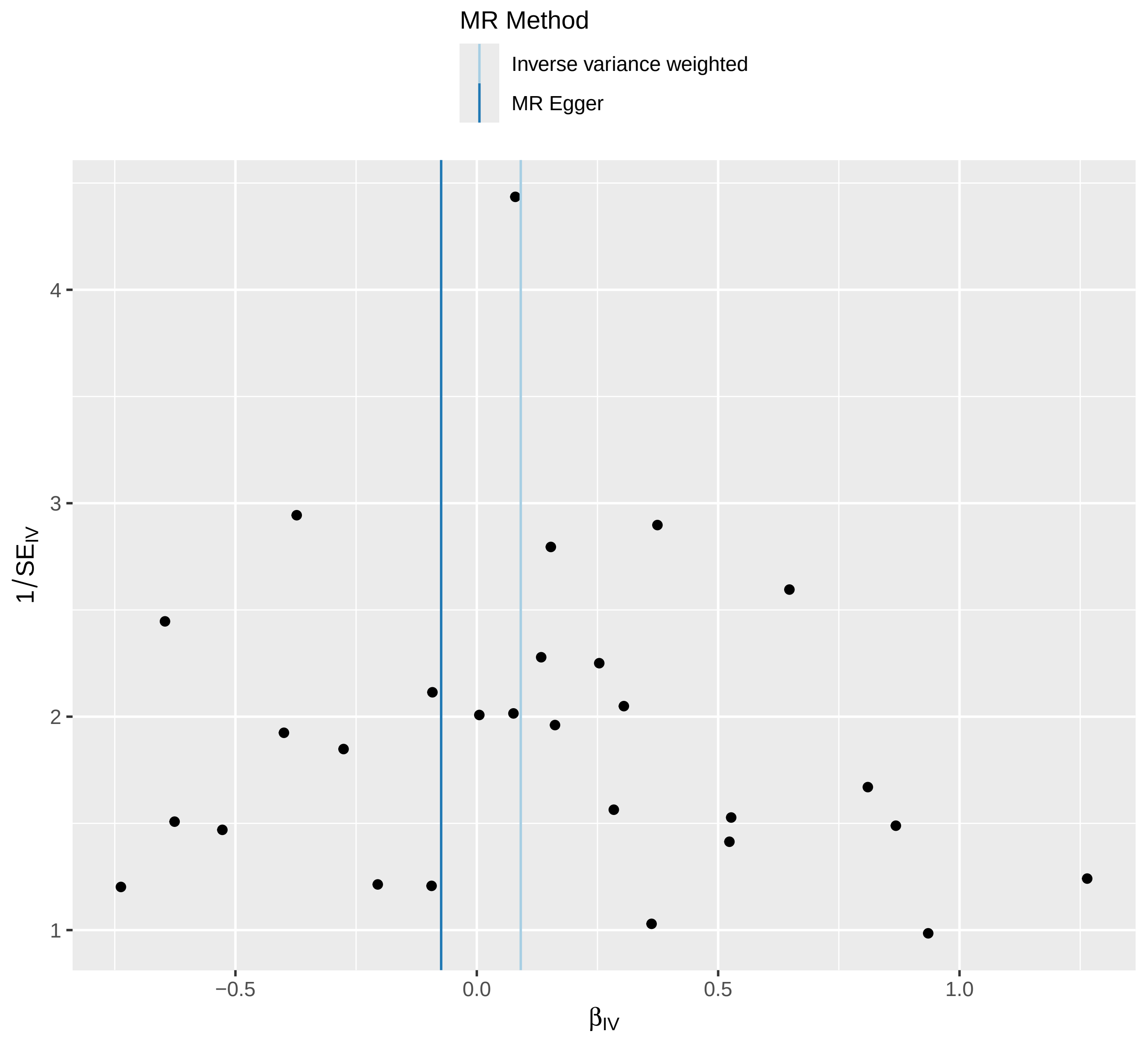

MR Method
Inverse variance weighted
MR Egger
1/SE$_{IV}$
4
3
2
1
−0.5
0.0
0.5
1.0
β$_{IV}$

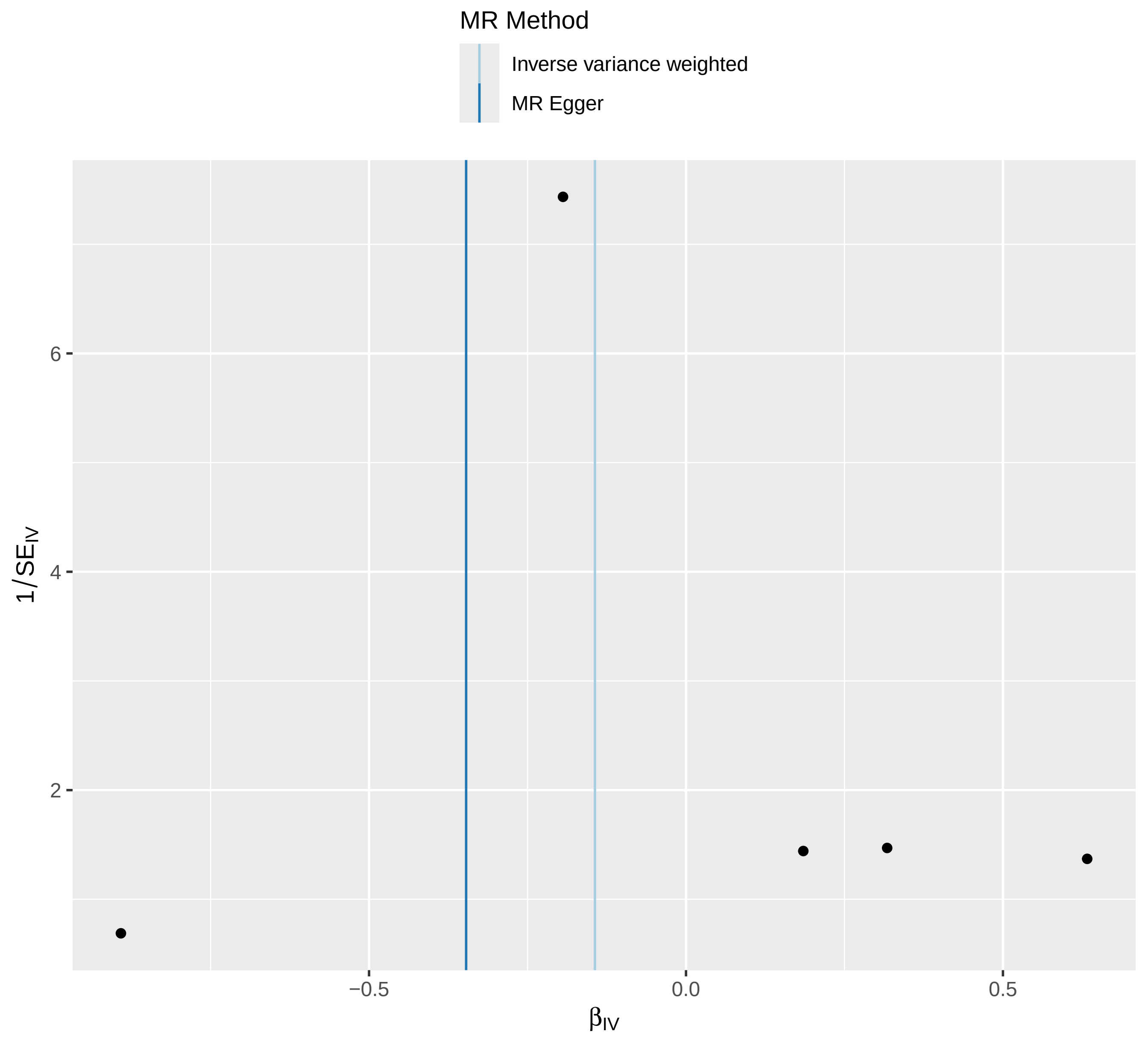

MR Method
Inverse variance weighted
MR Egger
6
4
2
1/SE_IV
−0.5
0.0
0.5
β_IV

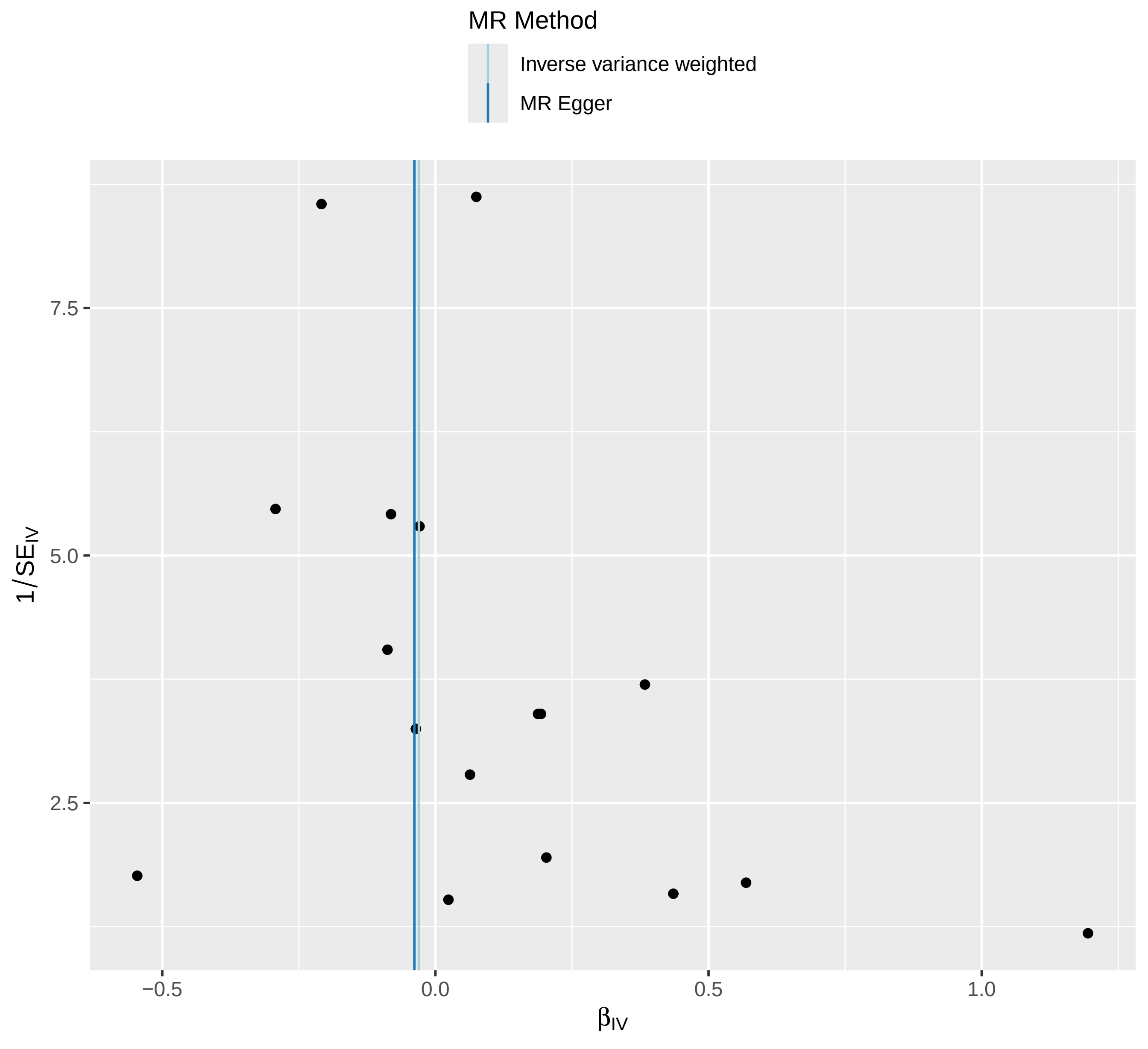

MR Method
Inverse variance weighted
MR Egger
7.5
5.0
2.5
1/SE_IV
−0.5
0.0
0.5
1.0
β_IV

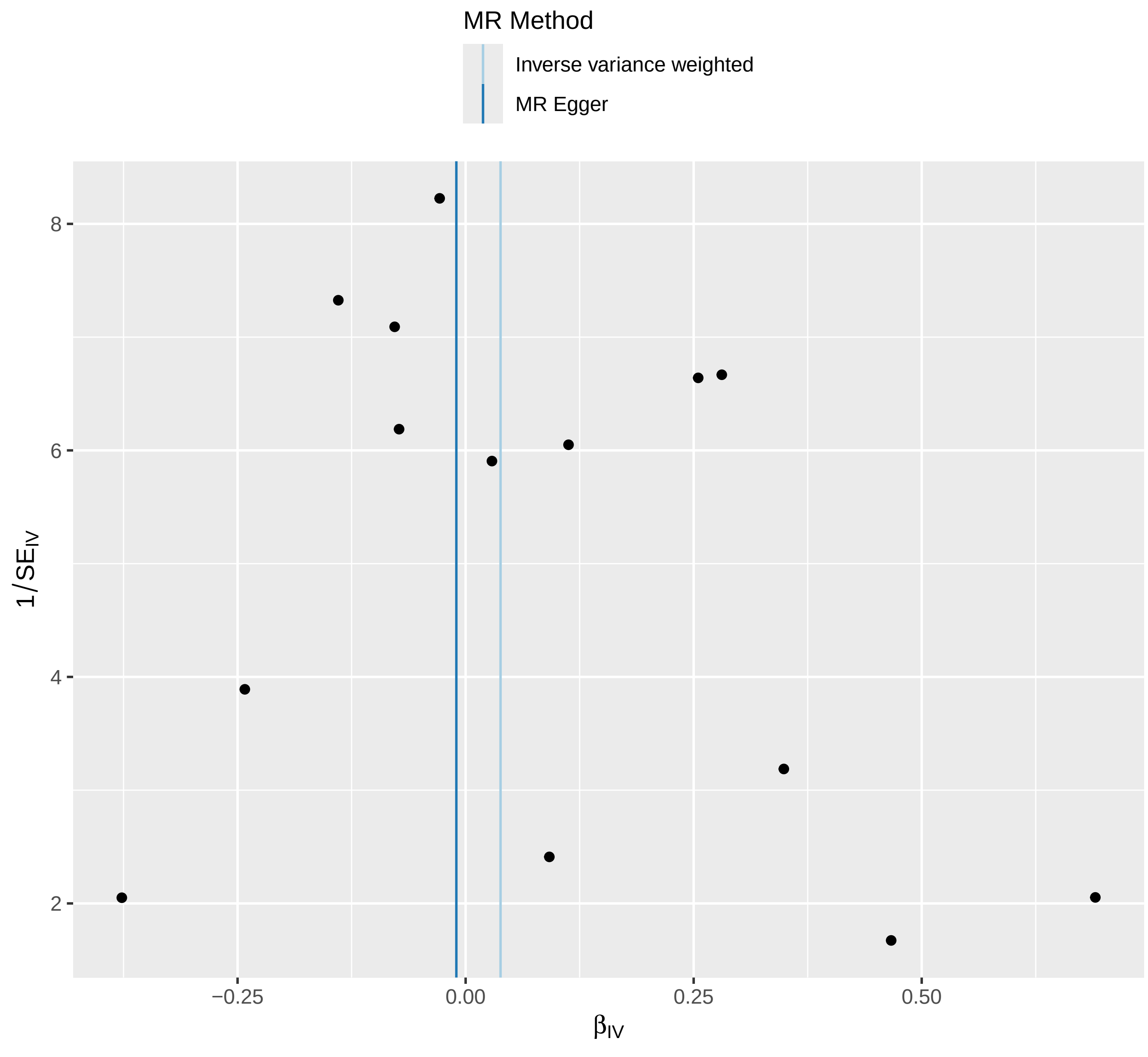

MR Method
Inverse variance weighted
MR Egger
8
6
4
2
1/SE_{IV}
−0.25
0.00
0.25
0.50
β_{IV}

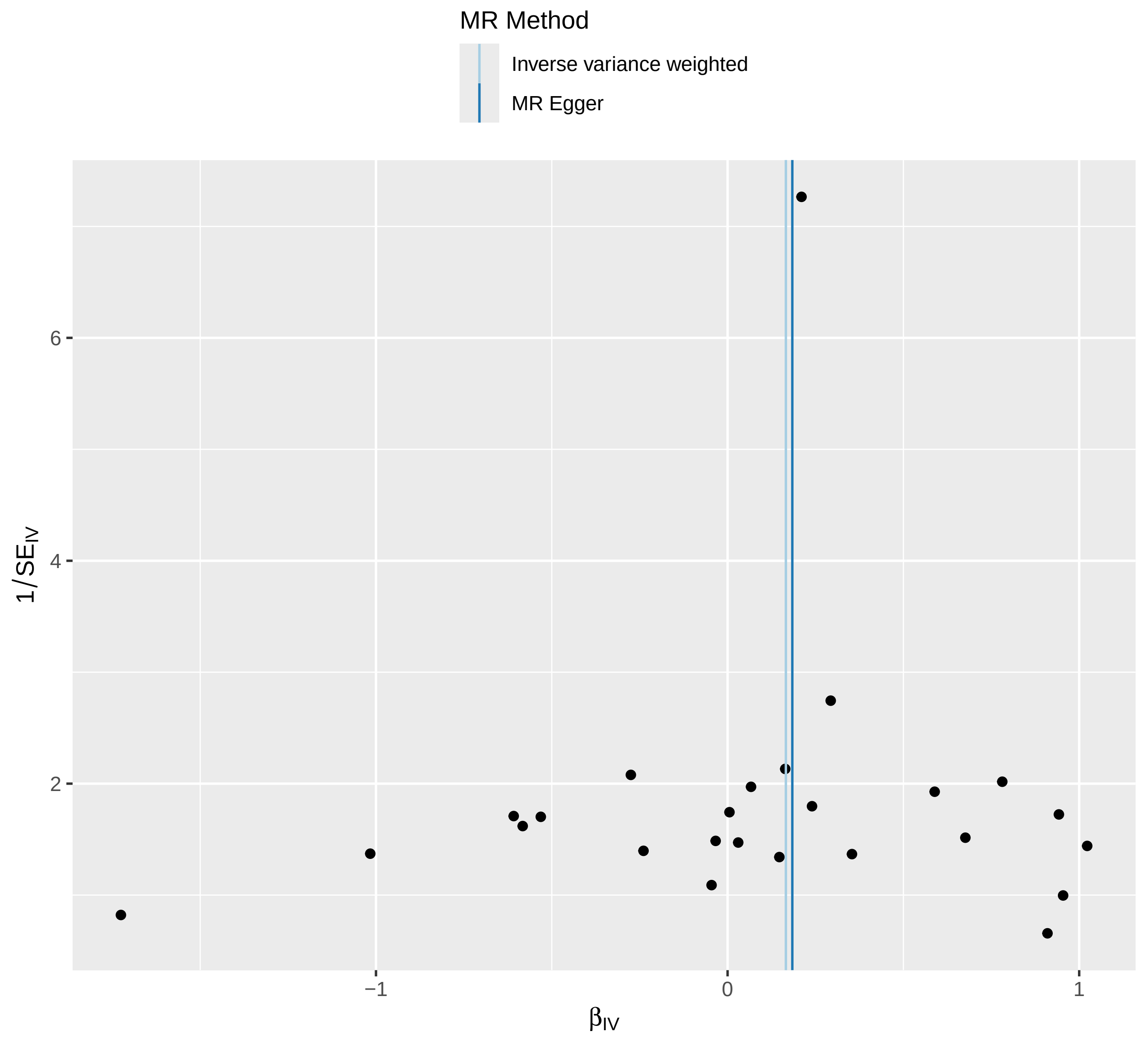
MR Method
Inverse variance weighted
MR Egger
6
4
2
1/SE_IV
−1
0
1
β_IV

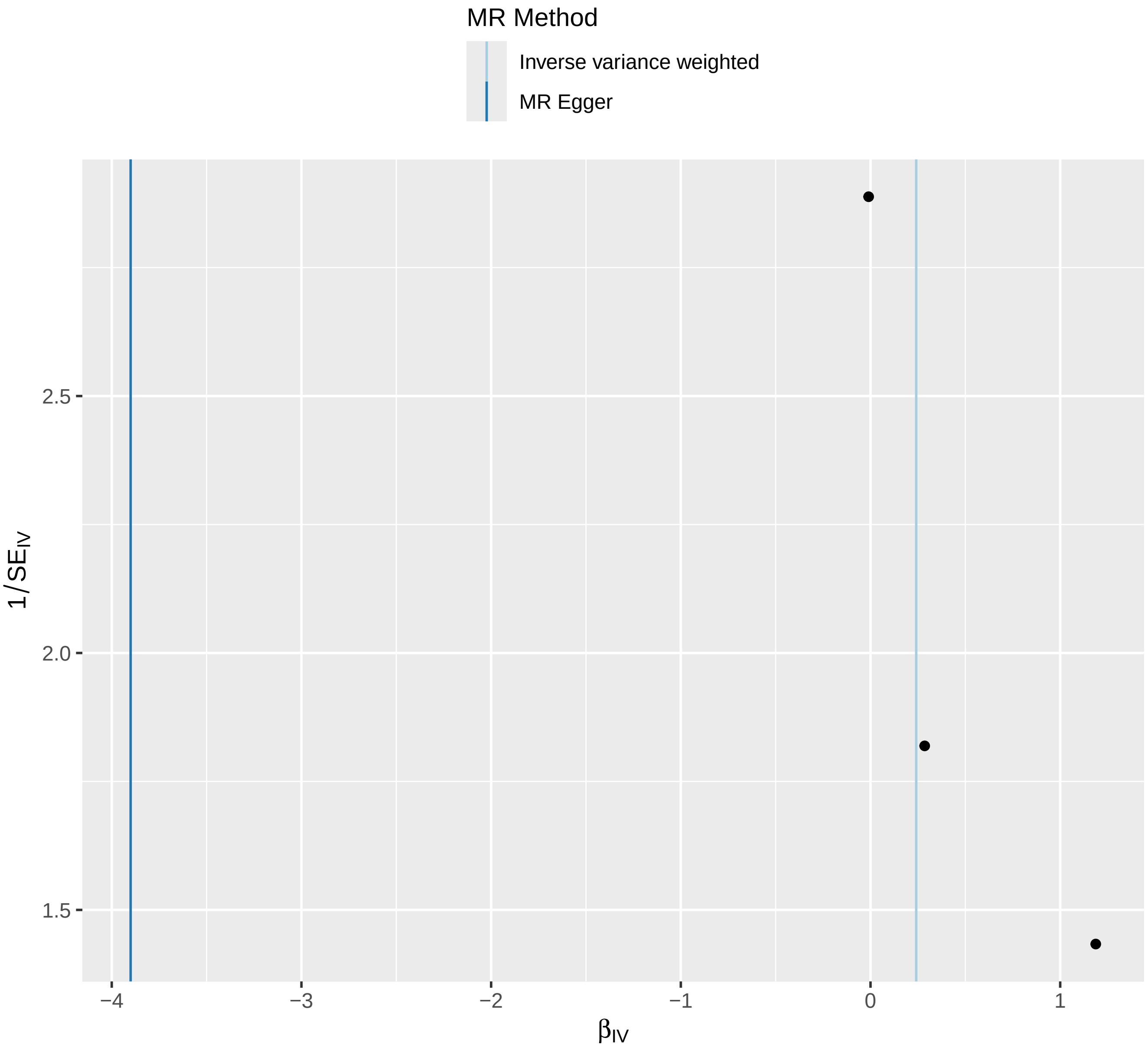
MR Method
Inverse variance weighted
MR Egger
1/SE_IV
2.5
2.0
1.5
−4
−3
−2
−1
0
1
β_IV

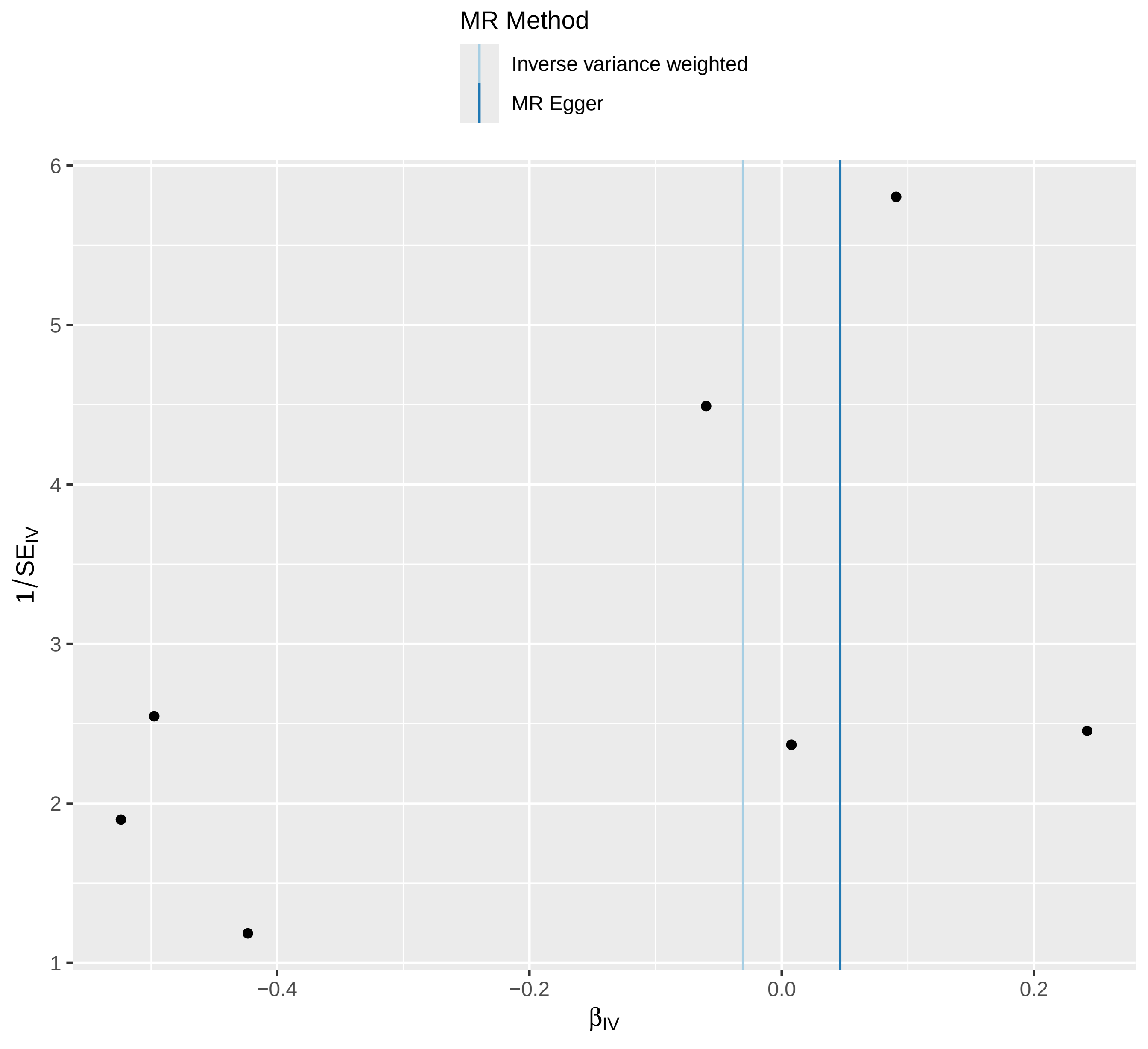

MR Method
Inverse variance weighted
MR Egger
1/SE_IV
β_IV
6
5
4
3
2
1
−0.4
−0.2
0.0
0.2

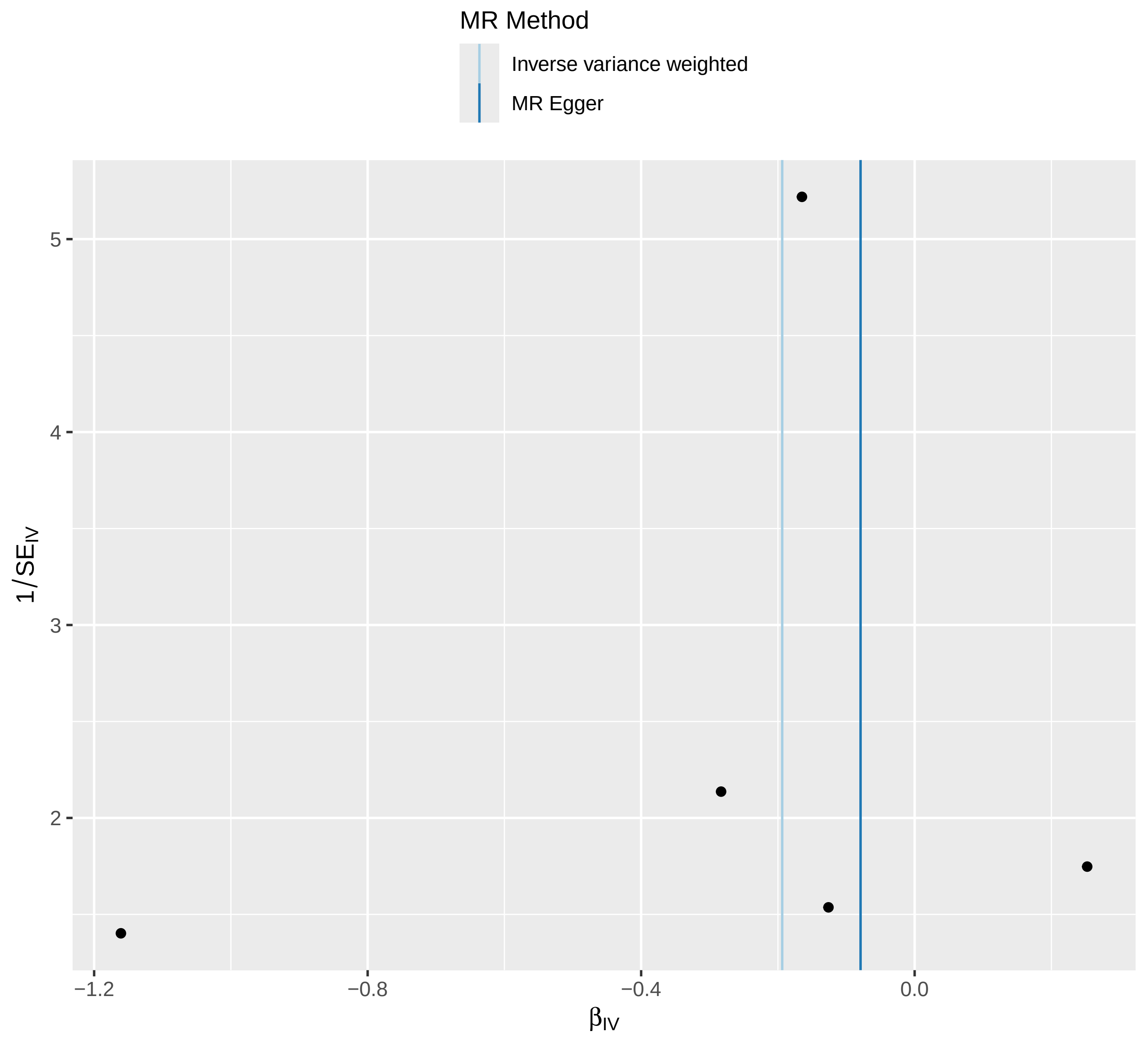
MR Method
Inverse variance weighted
MR Egger
5
4
3
2
1/SE_IV
−1.2
−0.8
−0.4
0.0
β_IV

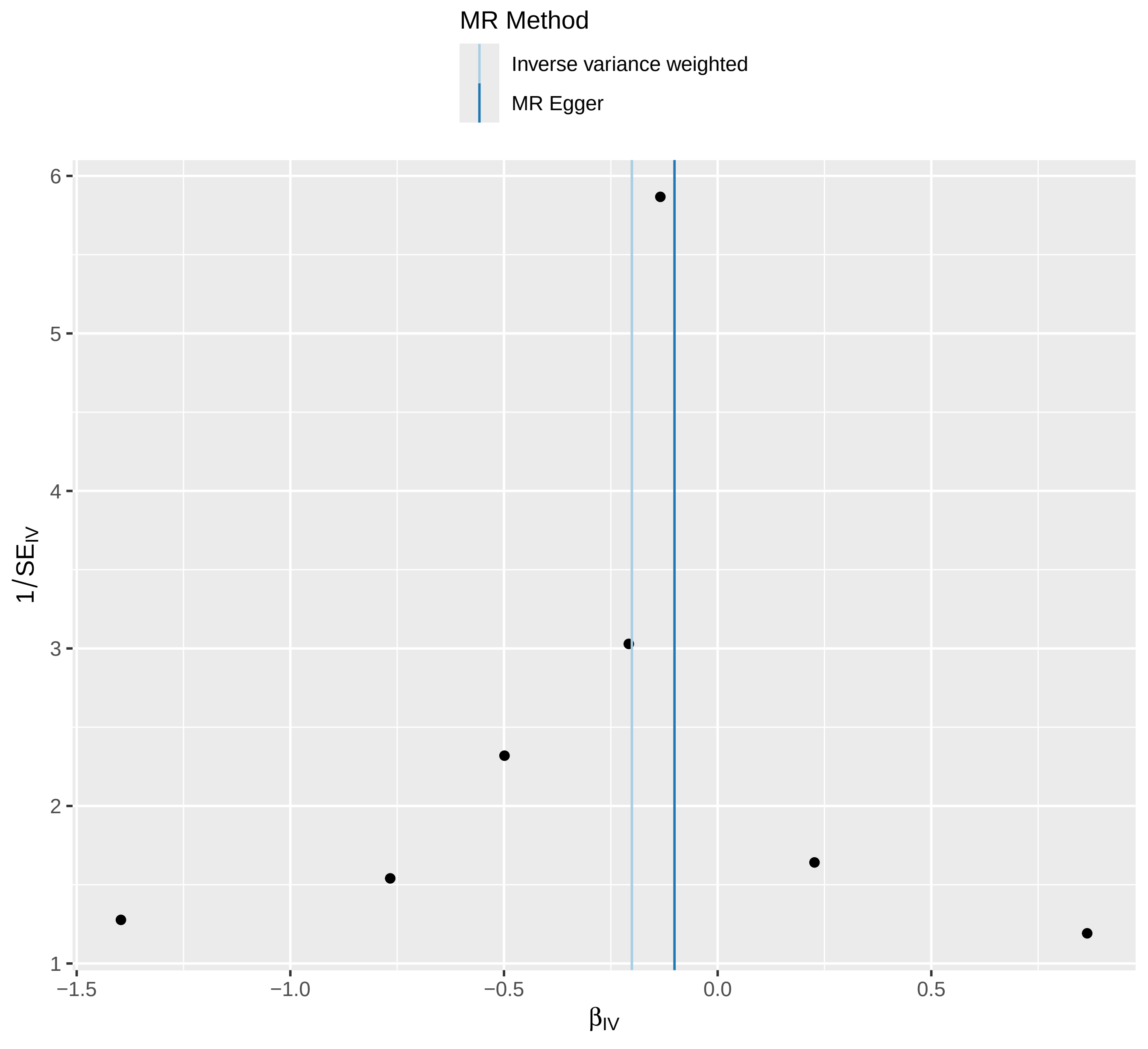
MR Method
Inverse variance weighted
MR Egger
6
5
4
3
2
1
1/SE$_{IV}$
−1.5
−1.0
−0.5
0.0
0.5
β$_{IV}$

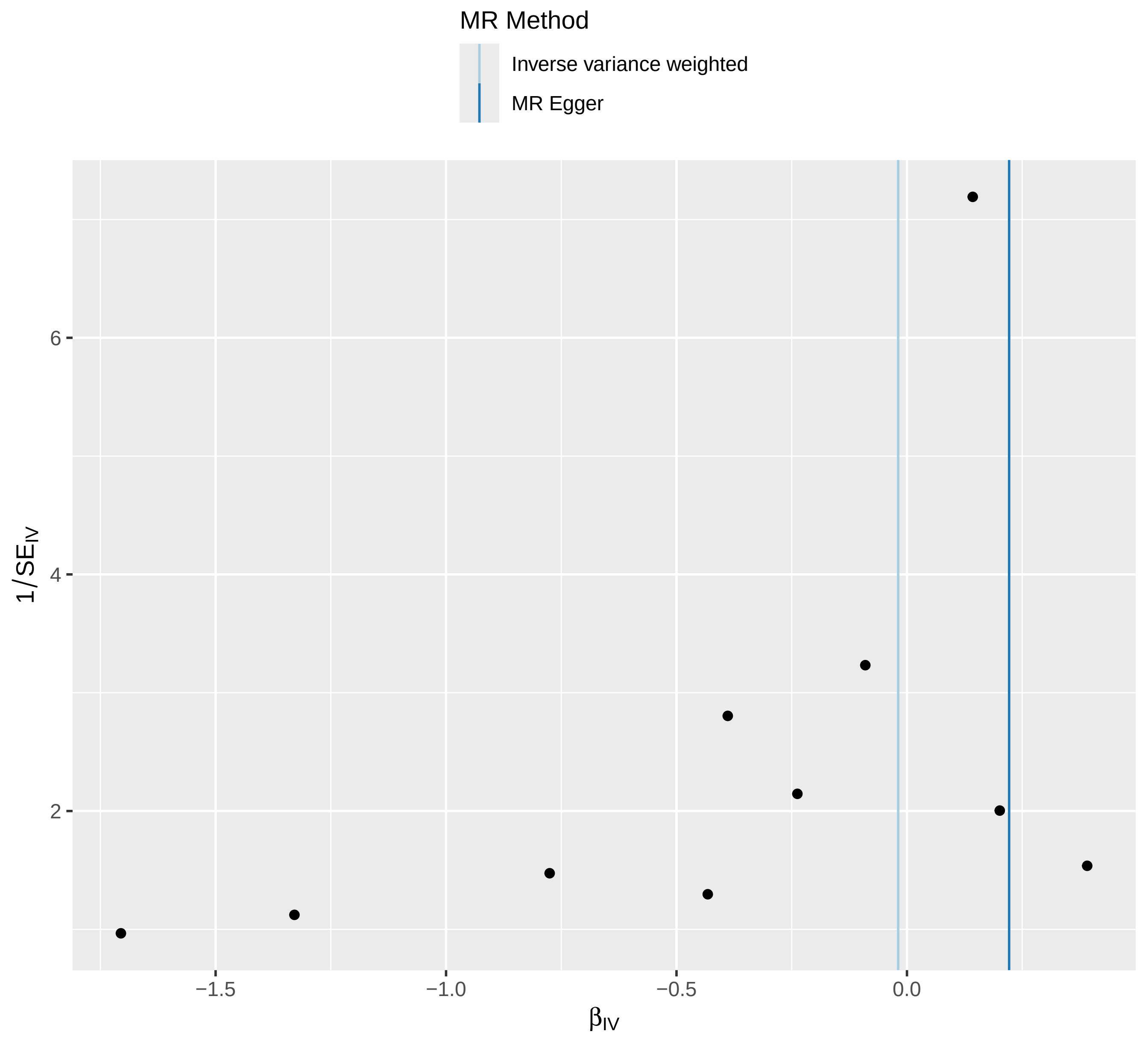
MR Method
Inverse variance weighted
MR Egger
1/SE$_{IV}$
6
4
2
−1.5
−1.0
−0.5
0.0
β$_{IV}$

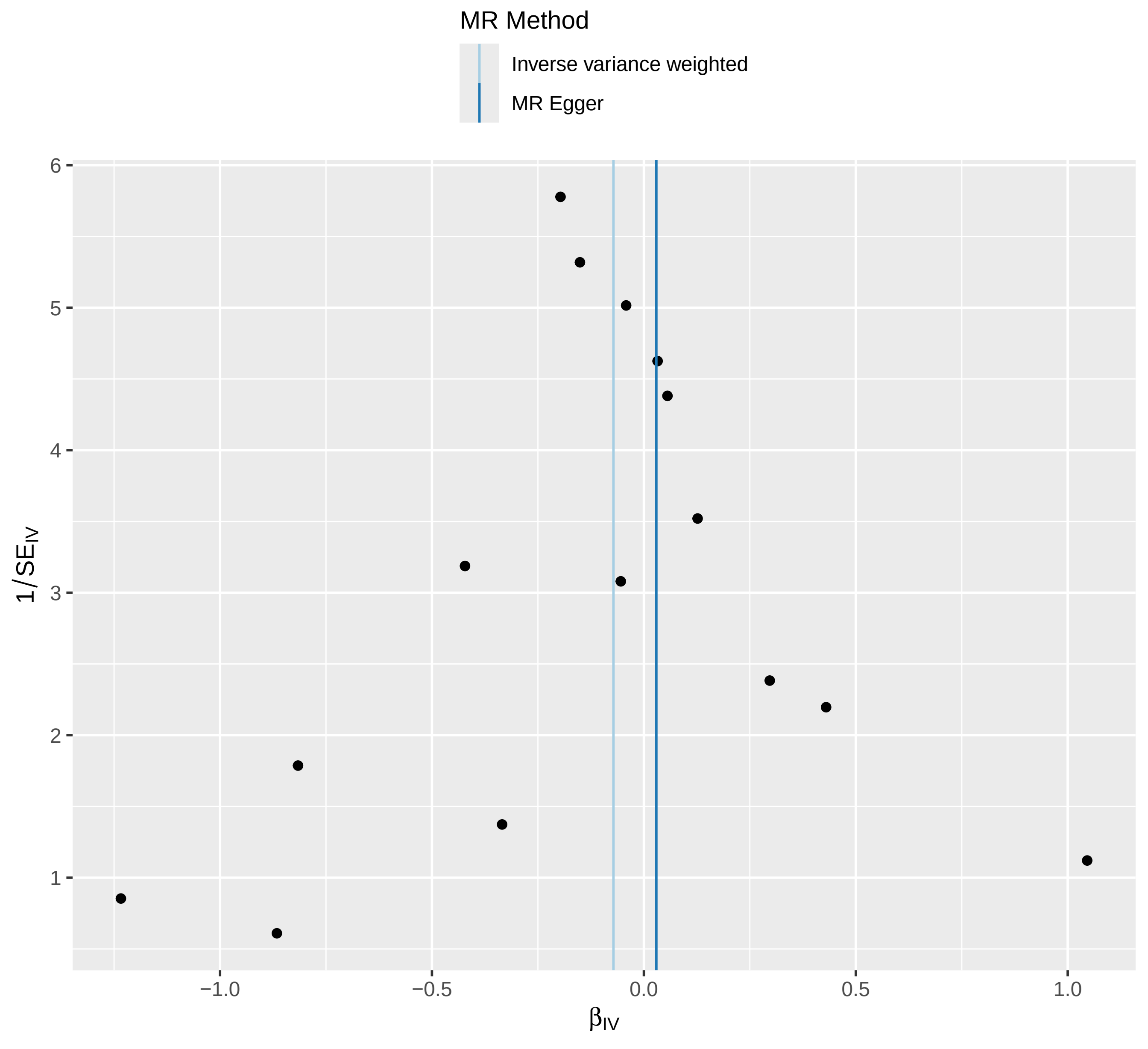
MR Method
Inverse variance weighted
MR Egger
6
5
4
3
2
1
$1/SE_{IV}$
−1.0
−0.5
0.0
0.5
1.0
β_{IV}

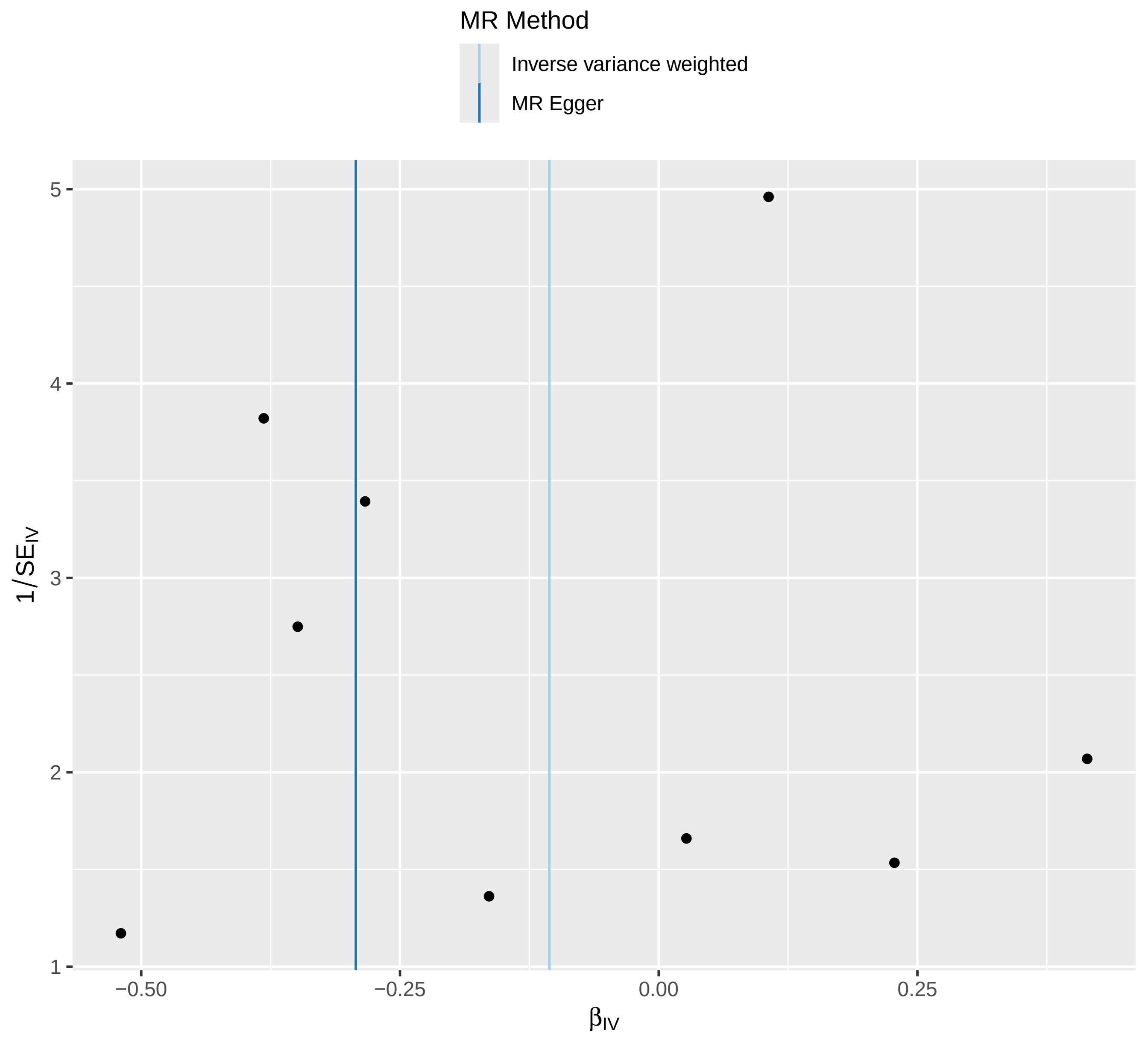

MR Method
Inverse variance weighted
MR Egger
5
4
3
2
1
1/SE_IV
−0.50
−0.25
0.00
0.25
β_IV

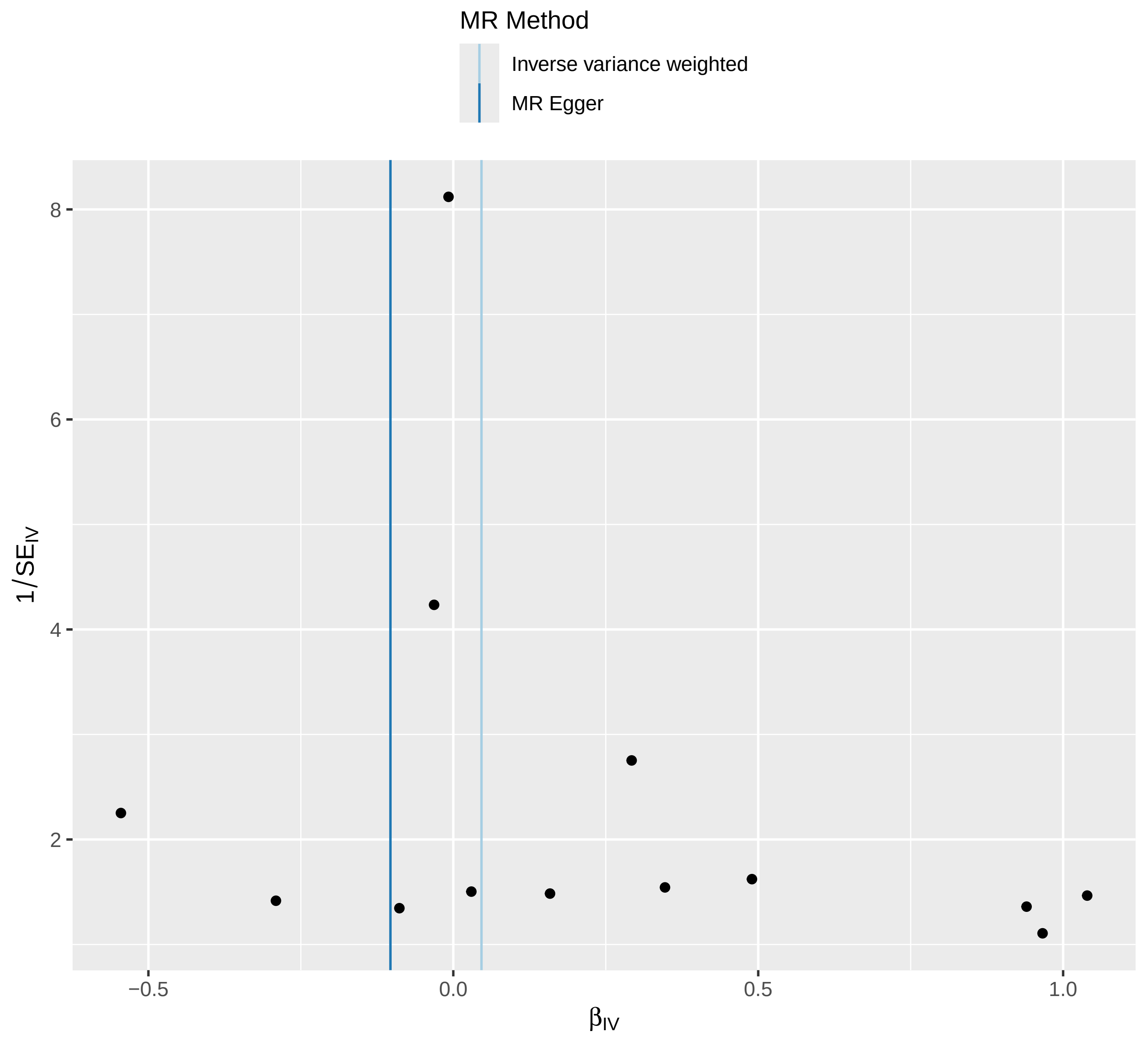

MR Method
Inverse variance weighted
MR Egger
8
6
4
2
1/SE_IV
−0.5
0.0
0.5
1.0
β_IV

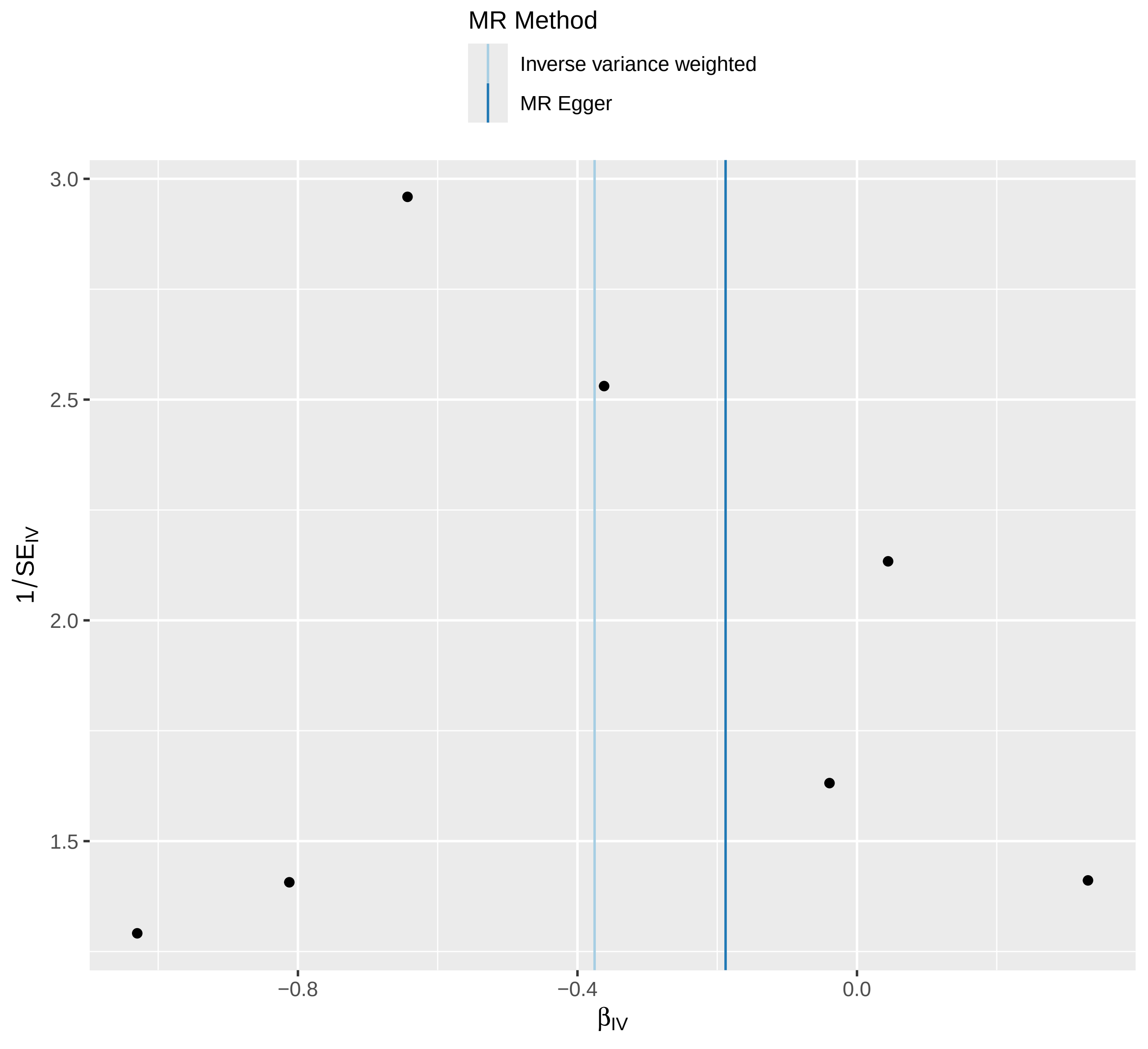
MR Method
Inverse variance weighted
MR Egger
3.0
2.5
2.0
1.5
1/SE_IV
−0.8
−0.4
0.0
β_IV

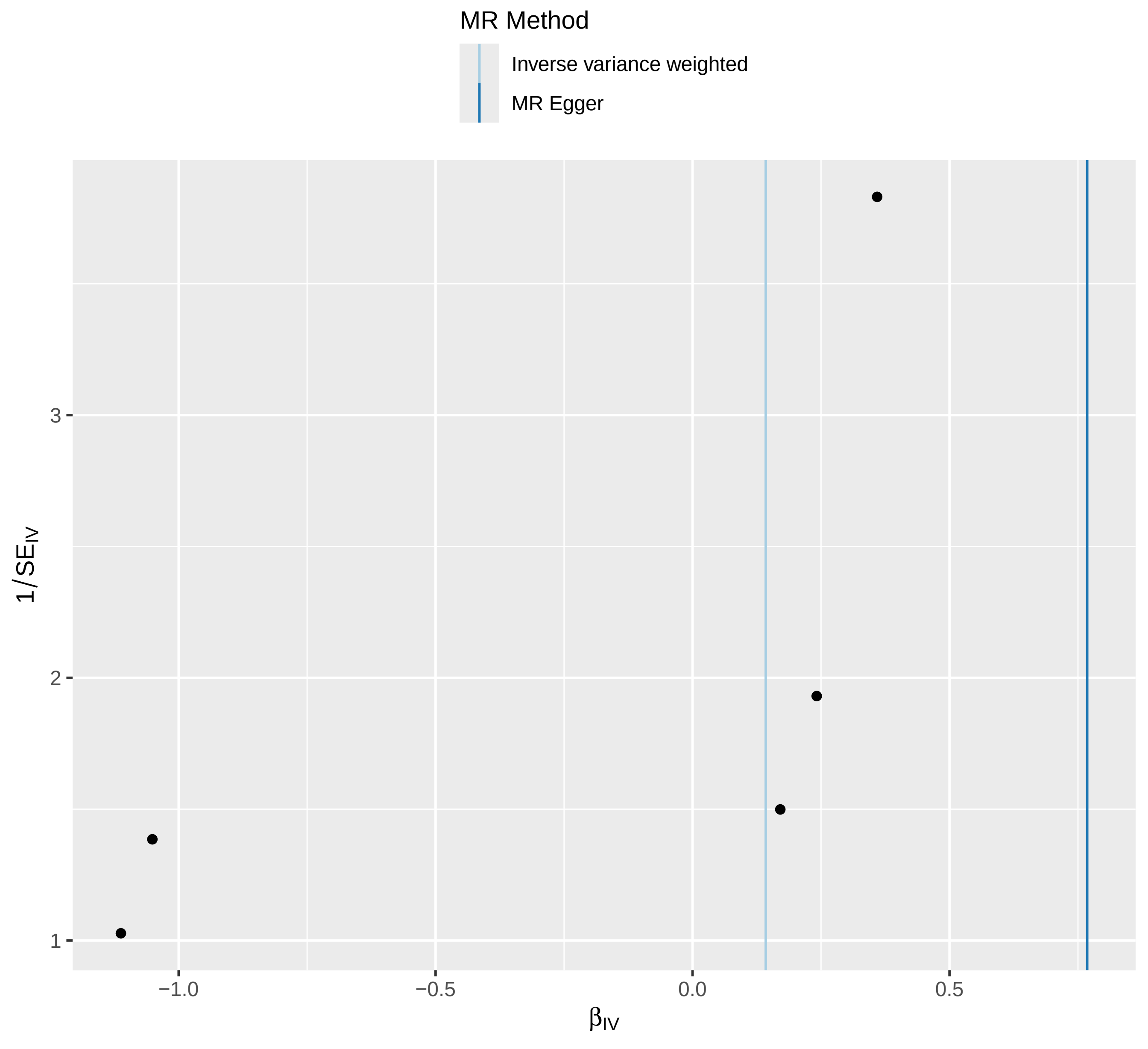

MR Method
Inverse variance weighted
MR Egger
3
2
1
$1/SE_{IV}$
−1.0
−0.5
0.0
0.5
β_{IV}

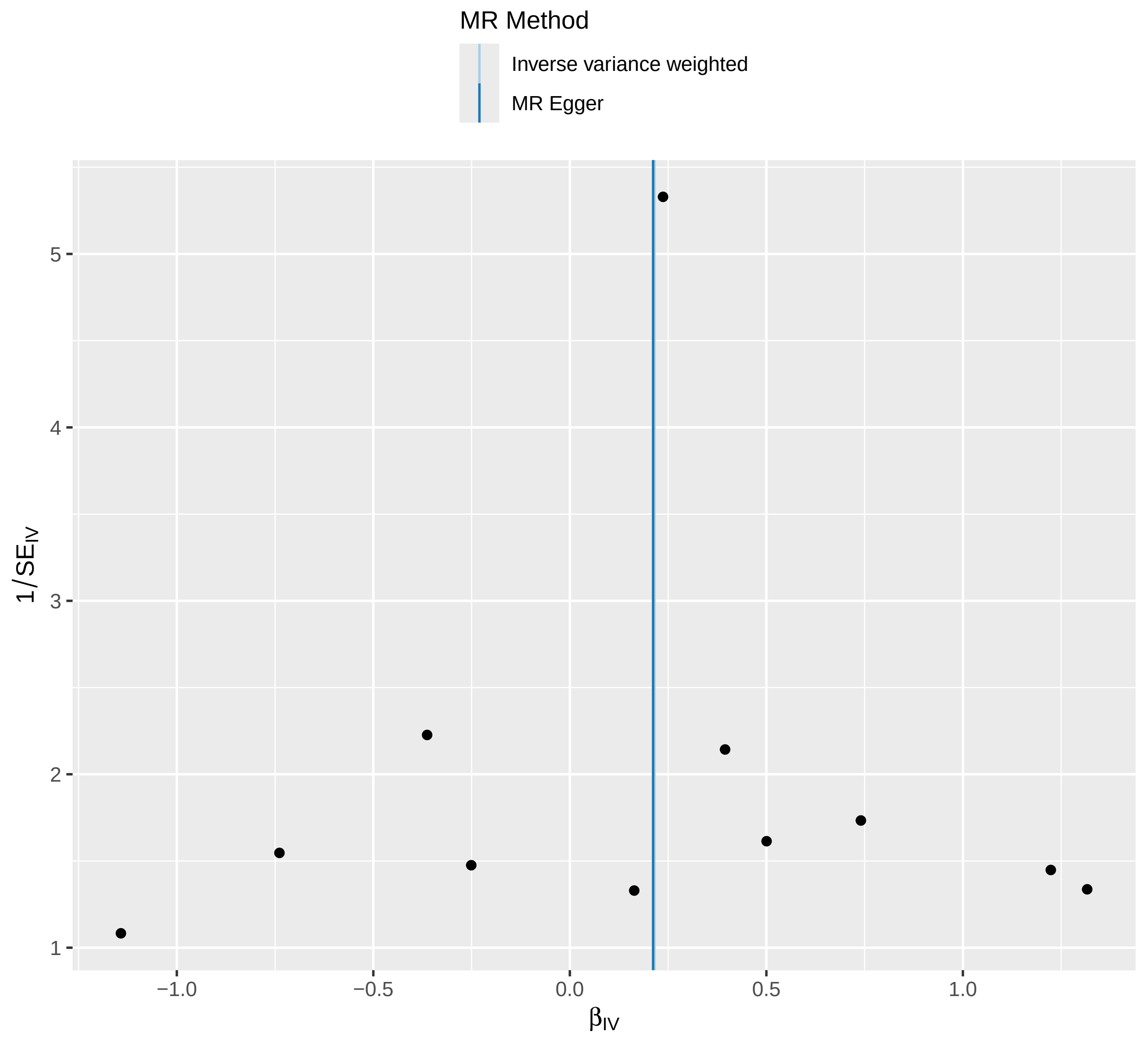
MR Method
Inverse variance weighted
MR Egger
1/SE_IV
5
4
3
2
1
−1.0
−0.5
0.0
0.5
1.0
β_IV

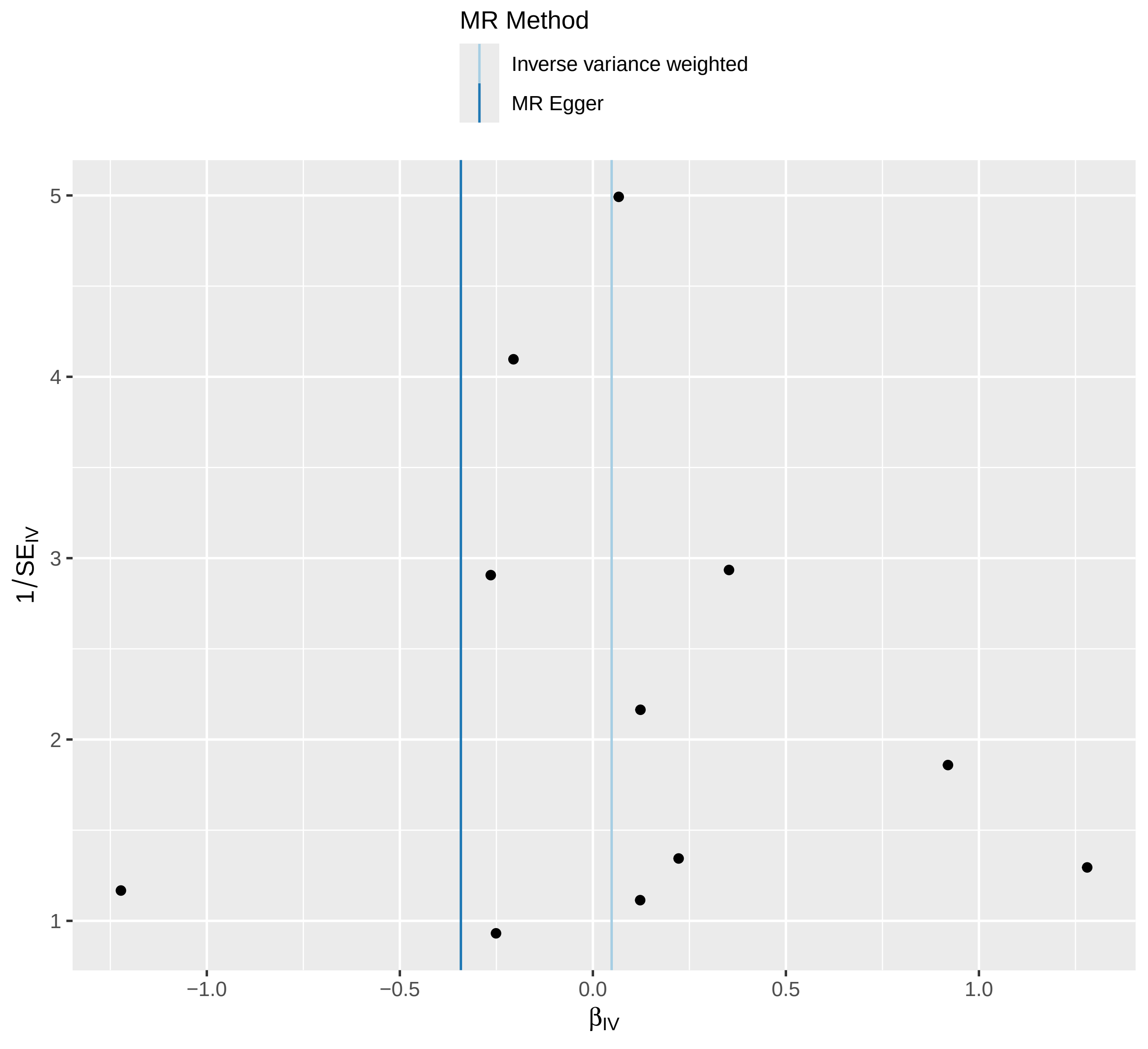
MR Method
Inverse variance weighted
MR Egger
1/SE_IV
5
4
3
2
1
−1.0
−0.5
0.0
0.5
1.0
β_IV

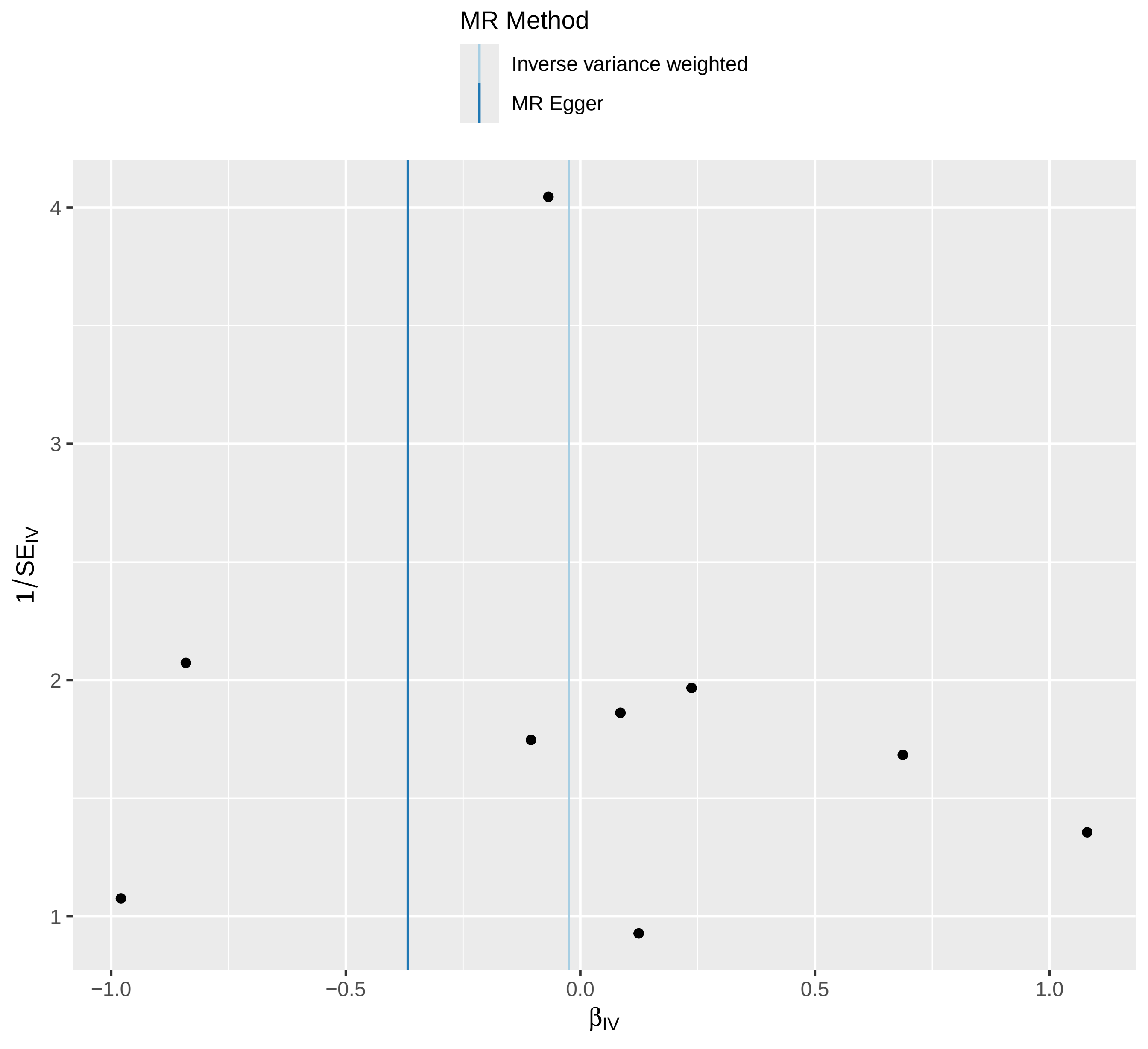

MR Method
Inverse variance weighted
MR Egger
4
3
2
1
1/SE$_{IV}$
−1.0
−0.5
0.0
0.5
1.0
β$_{IV}$

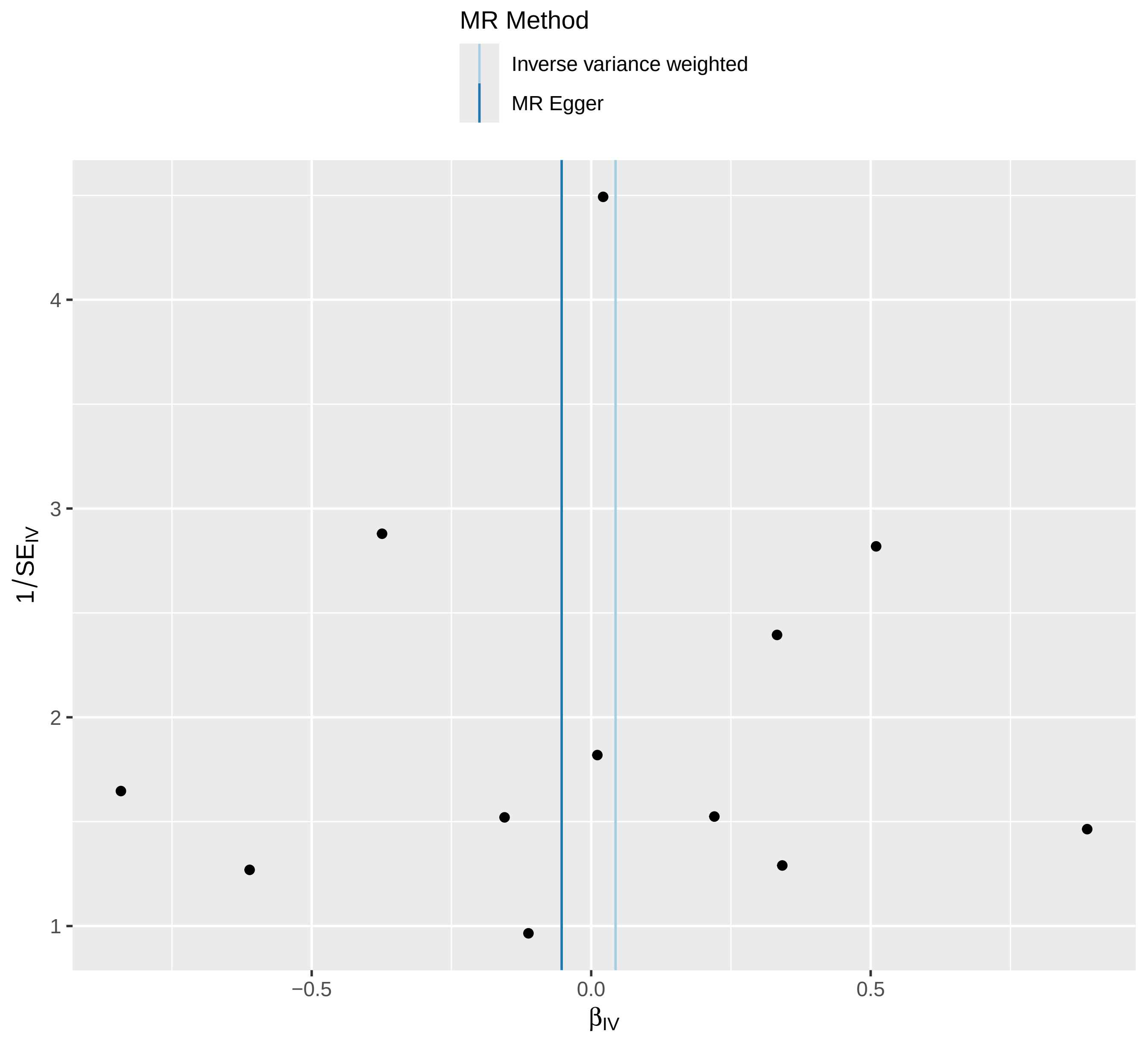
MR Method
Inverse variance weighted
MR Egger
1/SE_IV
4
3
2
1
−0.5
0.0
0.5
β_IV

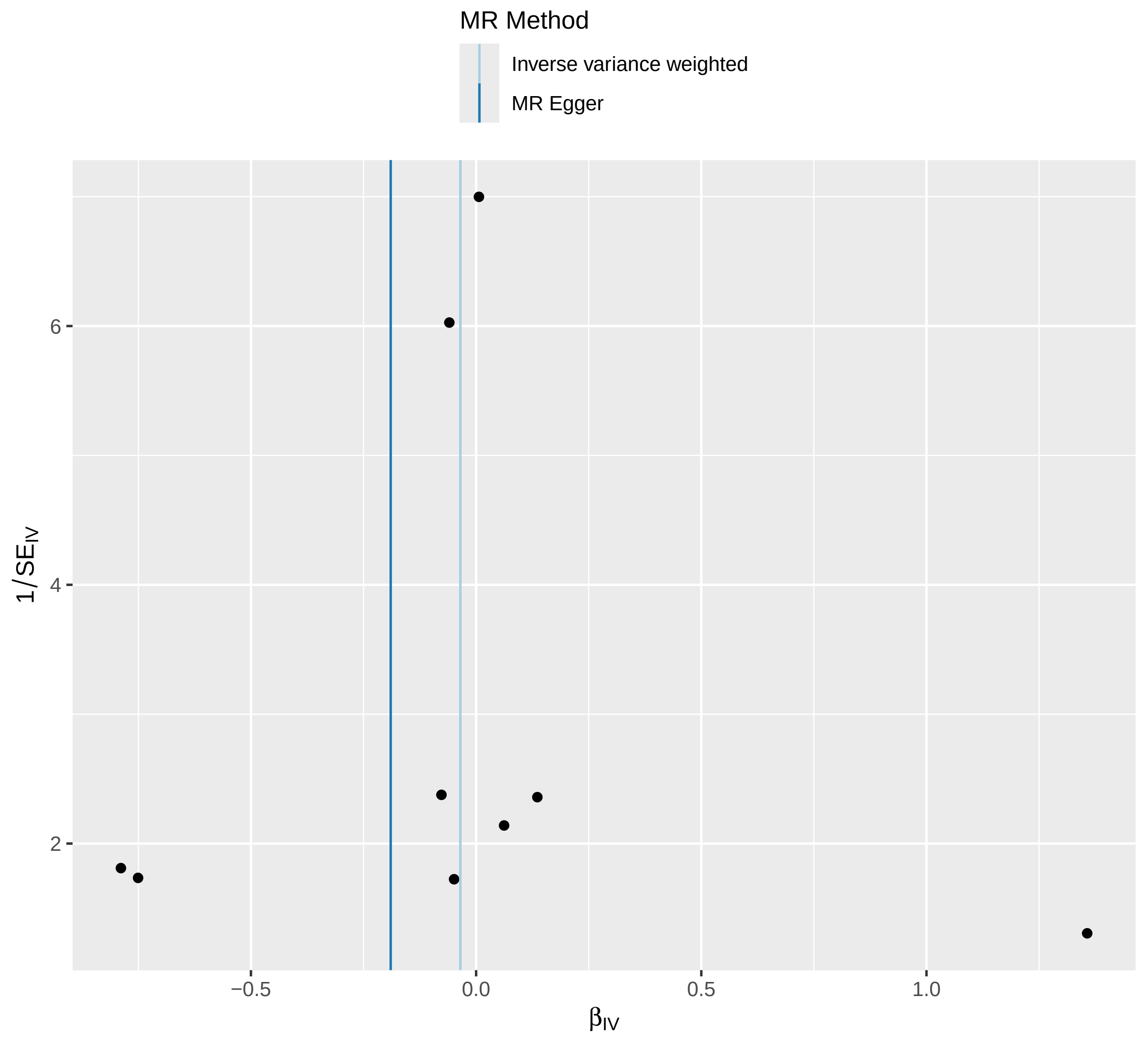

MR Method
Inverse variance weighted
MR Egger
1/SE$_{IV}$
6
4
2
−0.5
0.0
0.5
1.0
β$_{IV}$

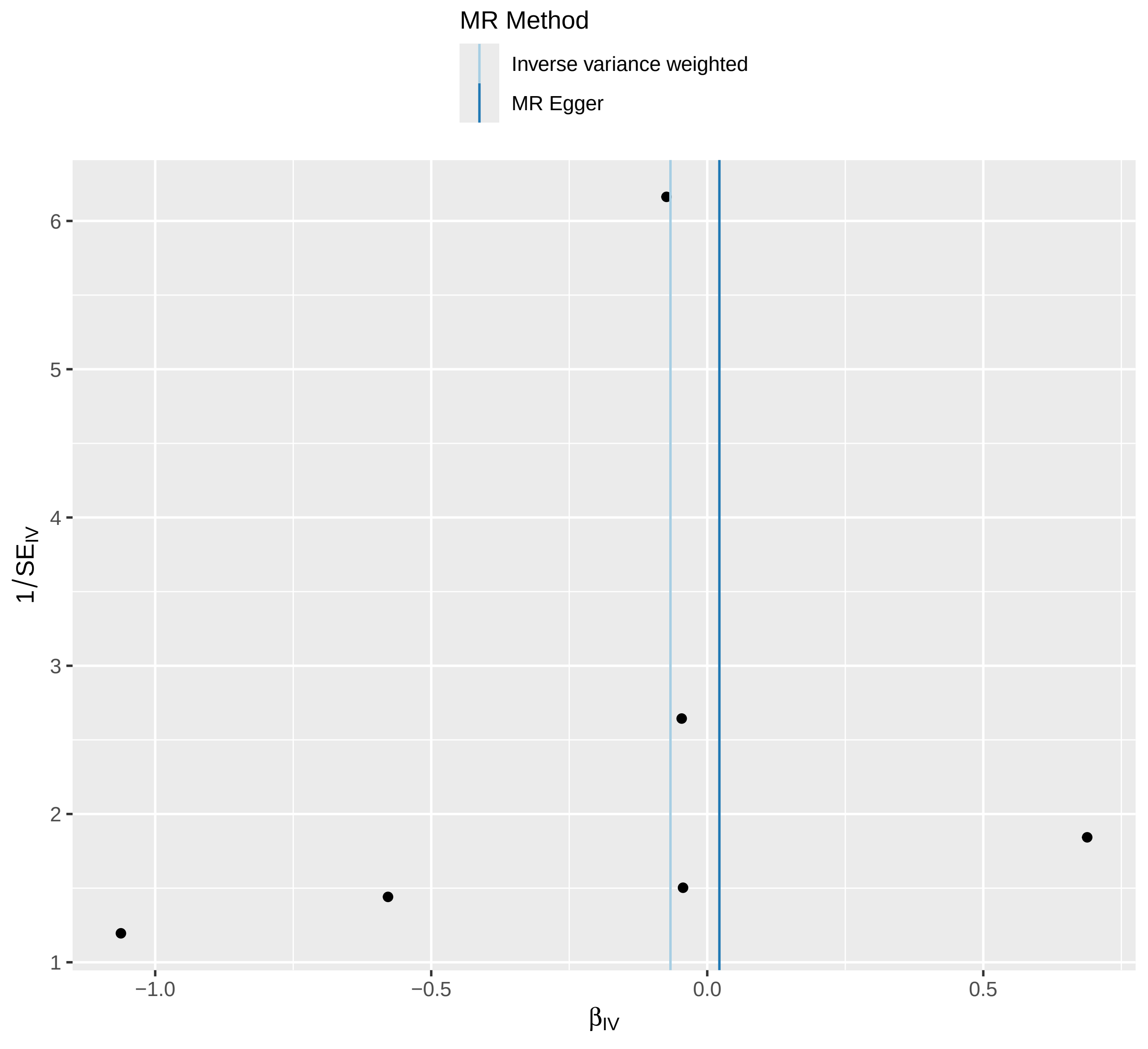

MR Method
Inverse variance weighted
MR Egger
6
5
4
3
2
1
1/SE_IV
−1.0
−0.5
0.0
0.5
β_IV

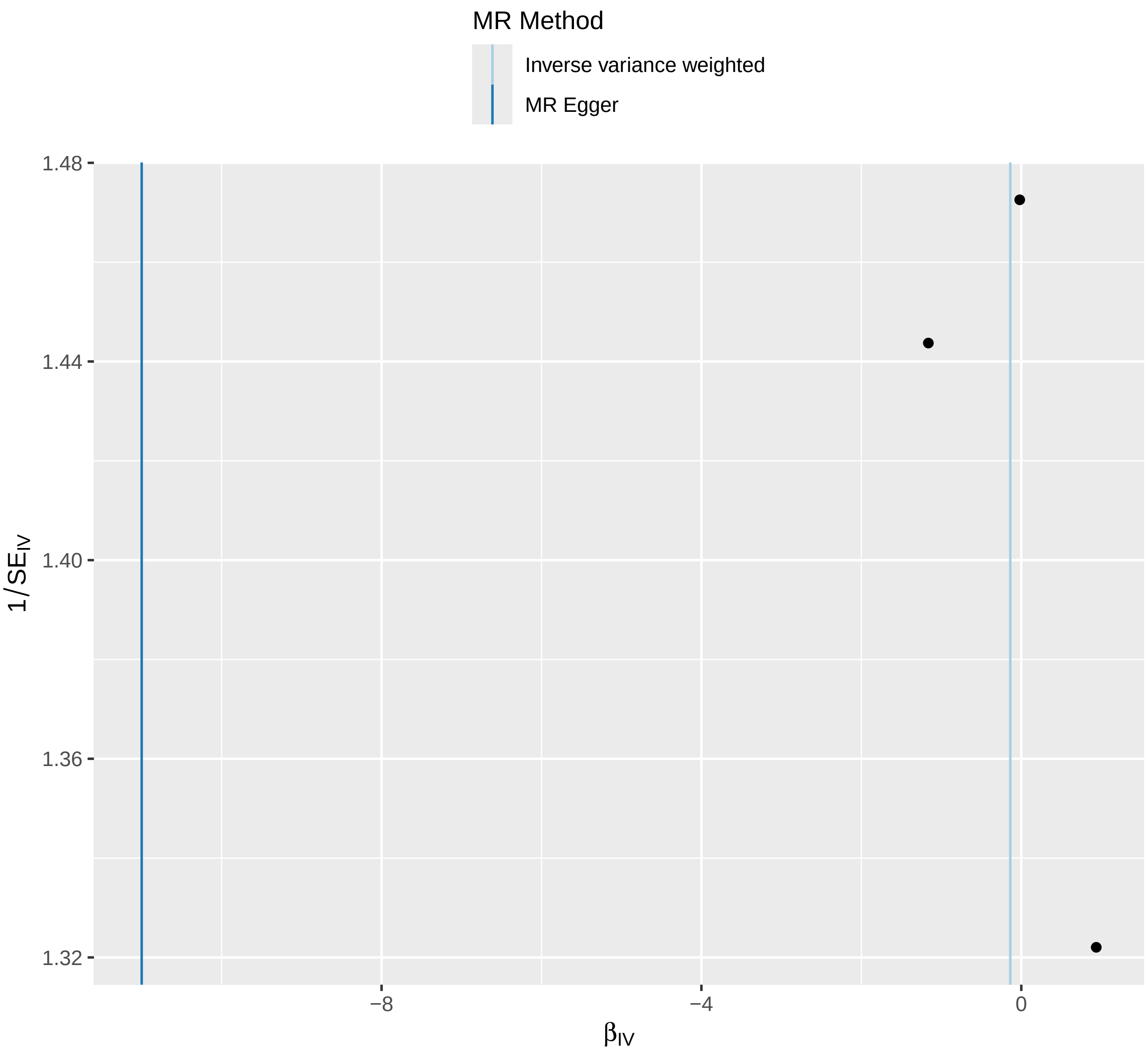
MR Method
Inverse variance weighted
MR Egger
1.48
1.44
1.40
1.36
1.32
1/SE_IV
−8
−4
0
β_IV

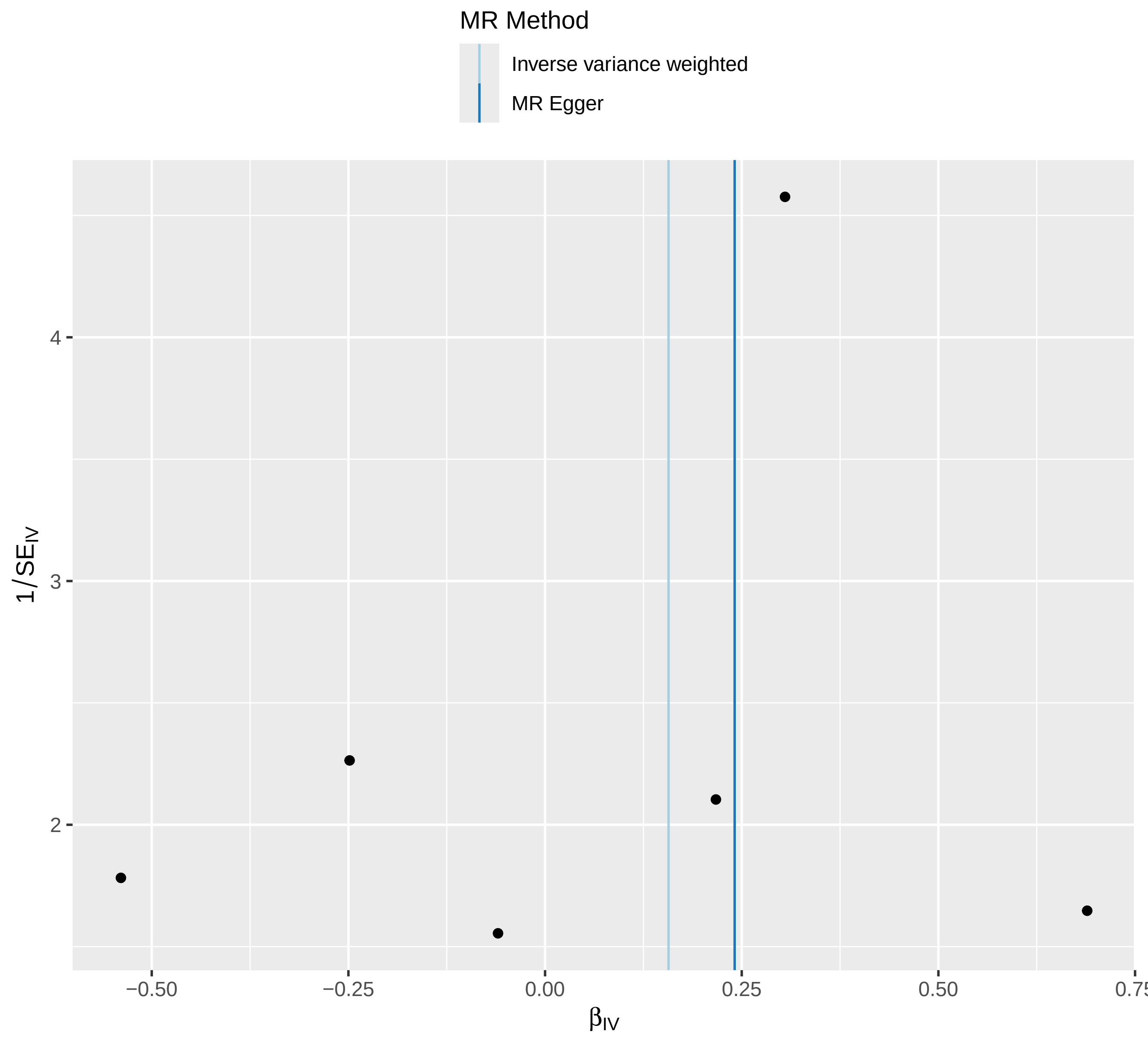
MR Method
Inverse variance weighted
MR Egger
1/SE$_{IV}$
4
3
2
−0.50
−0.25
0.00
0.25
0.50
0.75
β$_{IV}$

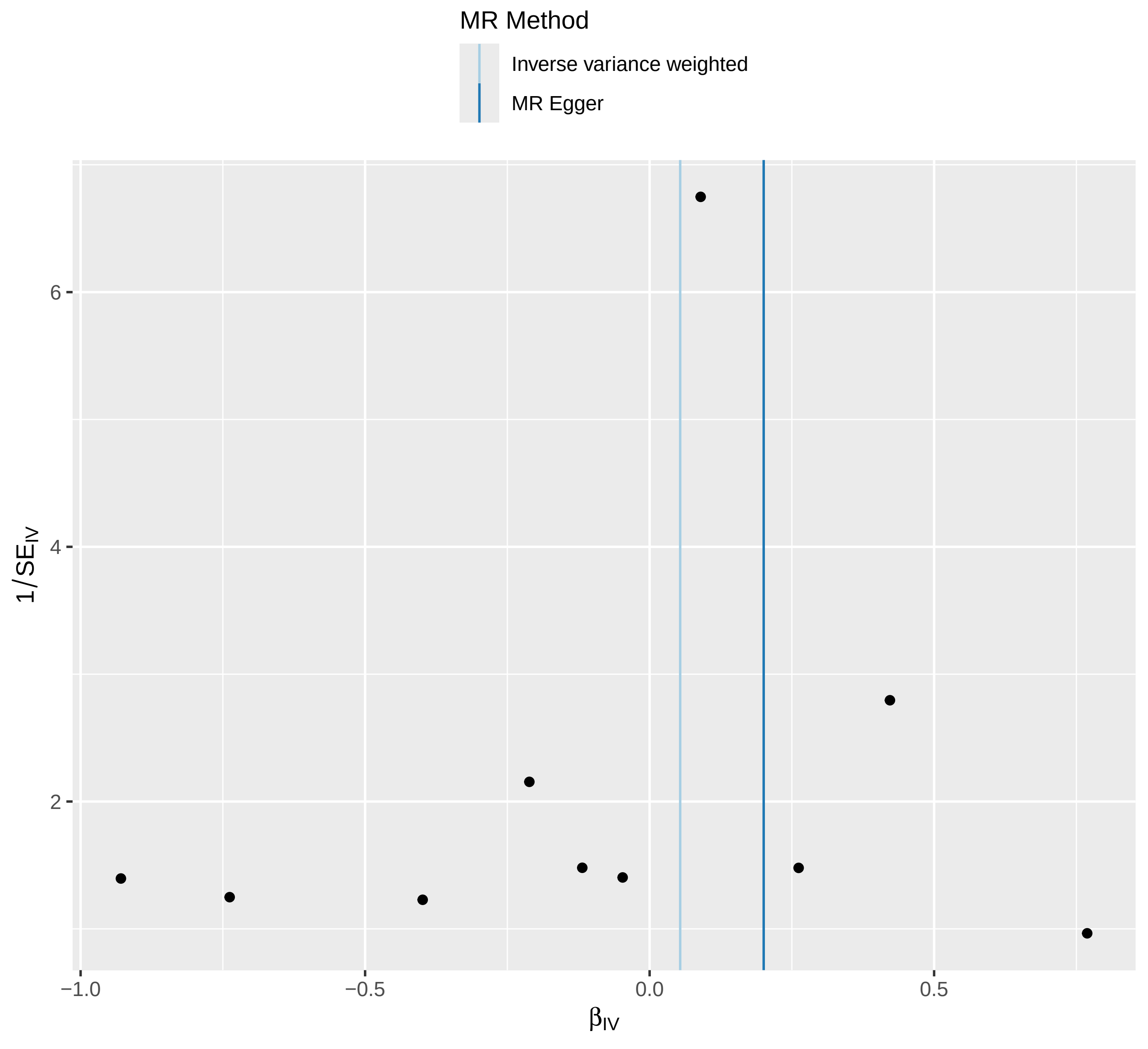

MR Method
Inverse variance weighted
MR Egger
6
4
2
1/SE_IV
−1.0
−0.5
0.0
0.5
β_IV

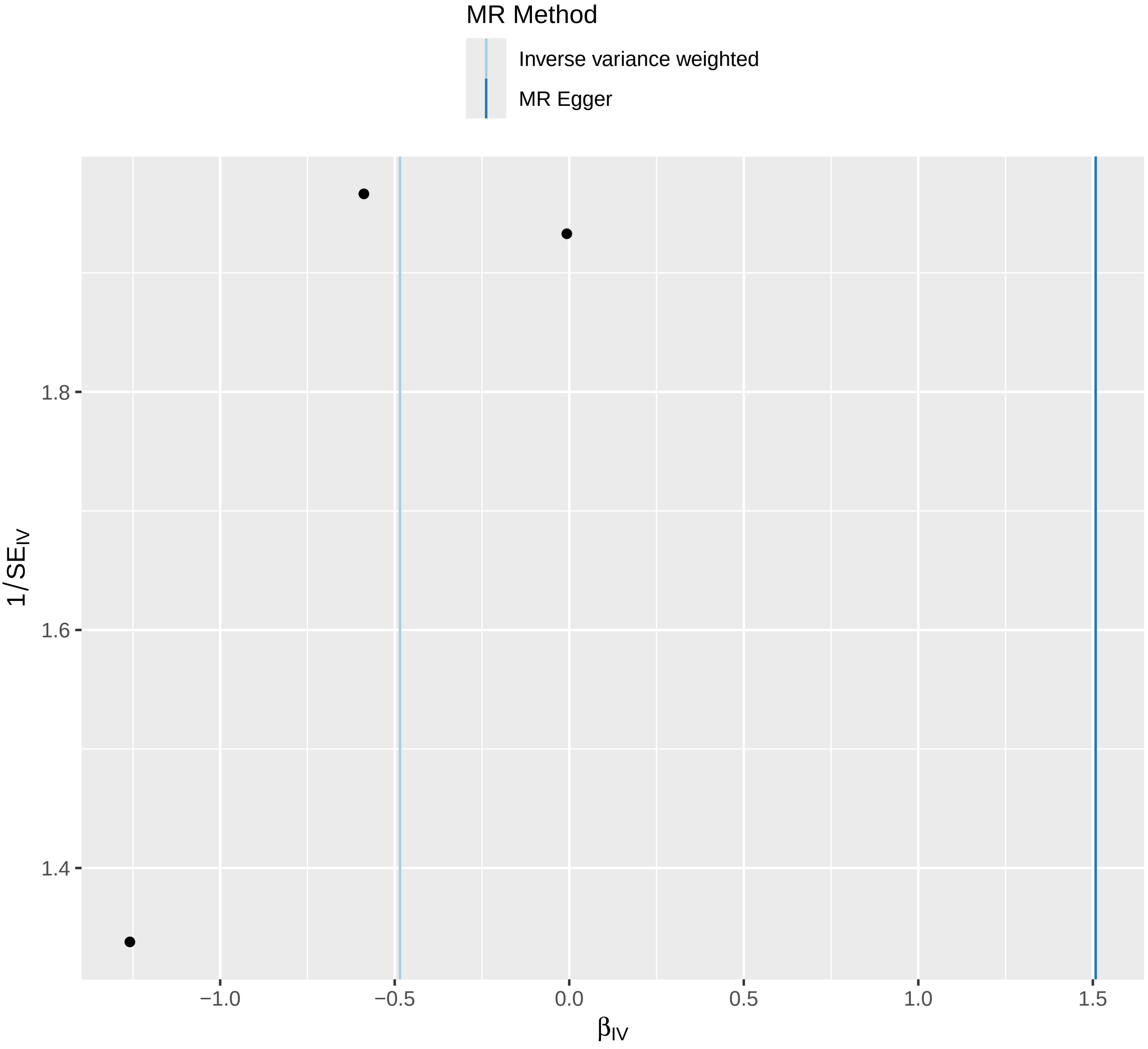
MR Method
Inverse variance weighted
MR Egger
1.8
1.6
1.4
$1/SE_{IV}$
−1.0
−0.5
0.0
0.5
1.0
1.5
β_{IV}

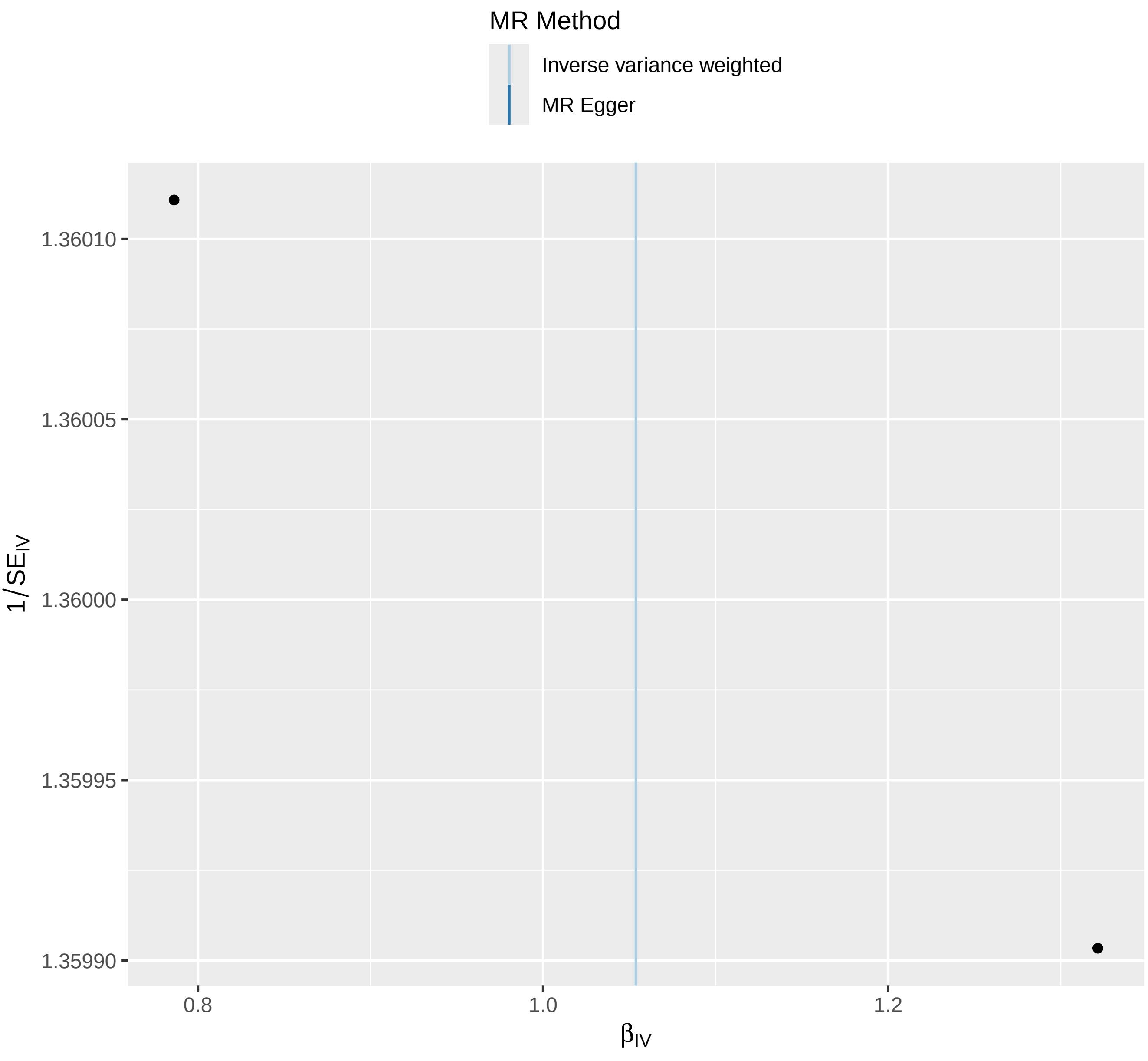
MR Method
Inverse variance weighted
MR Egger
1.36010
1.36005
1.36000
1.35995
1.35990
0.8
1.0
1.2
$1/SE_{IV}$
β_{IV}

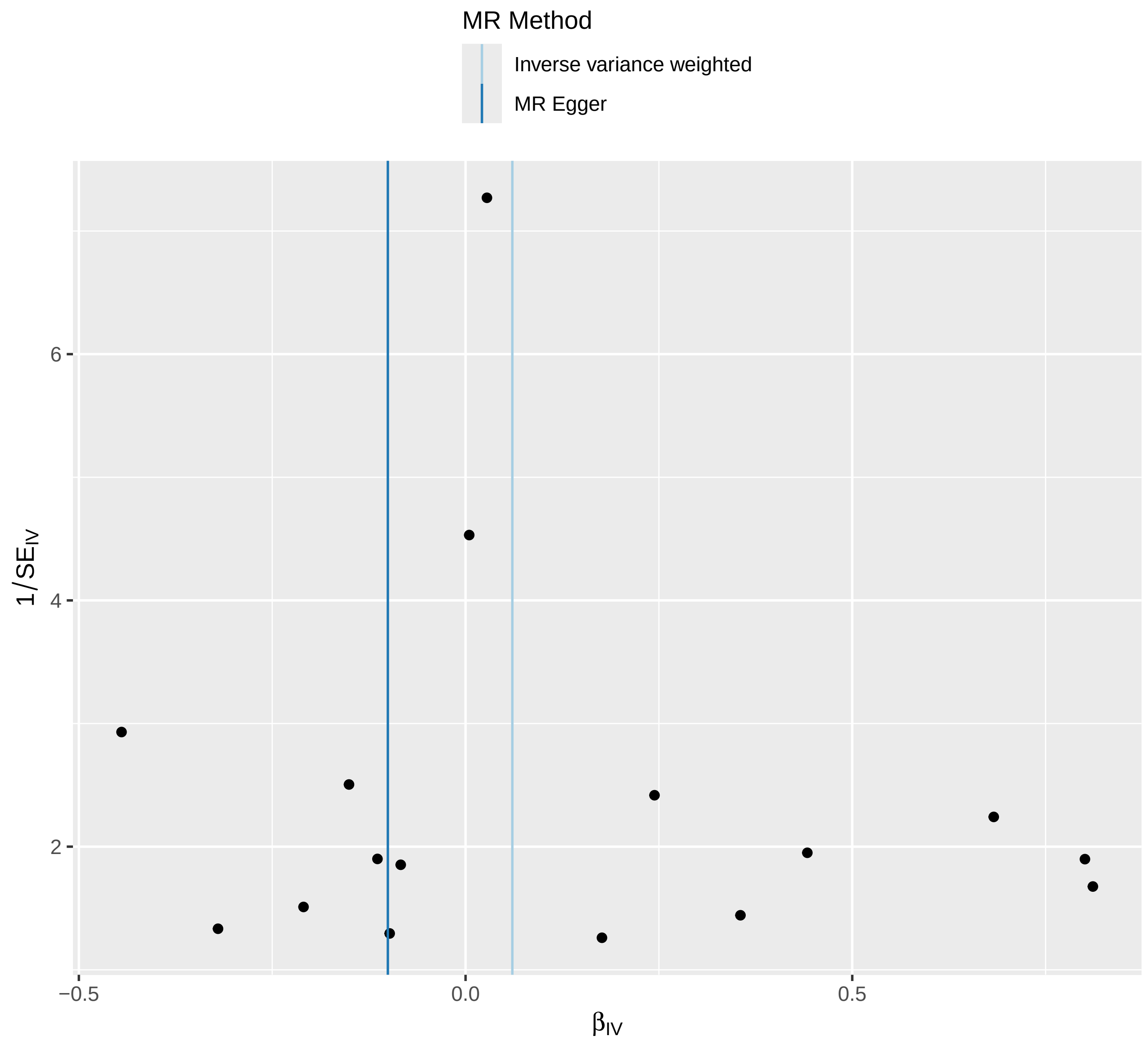

MR Method
Inverse variance weighted
MR Egger
6
4
2
1/SE_IV
−0.5
0.0
0.5
β_IV

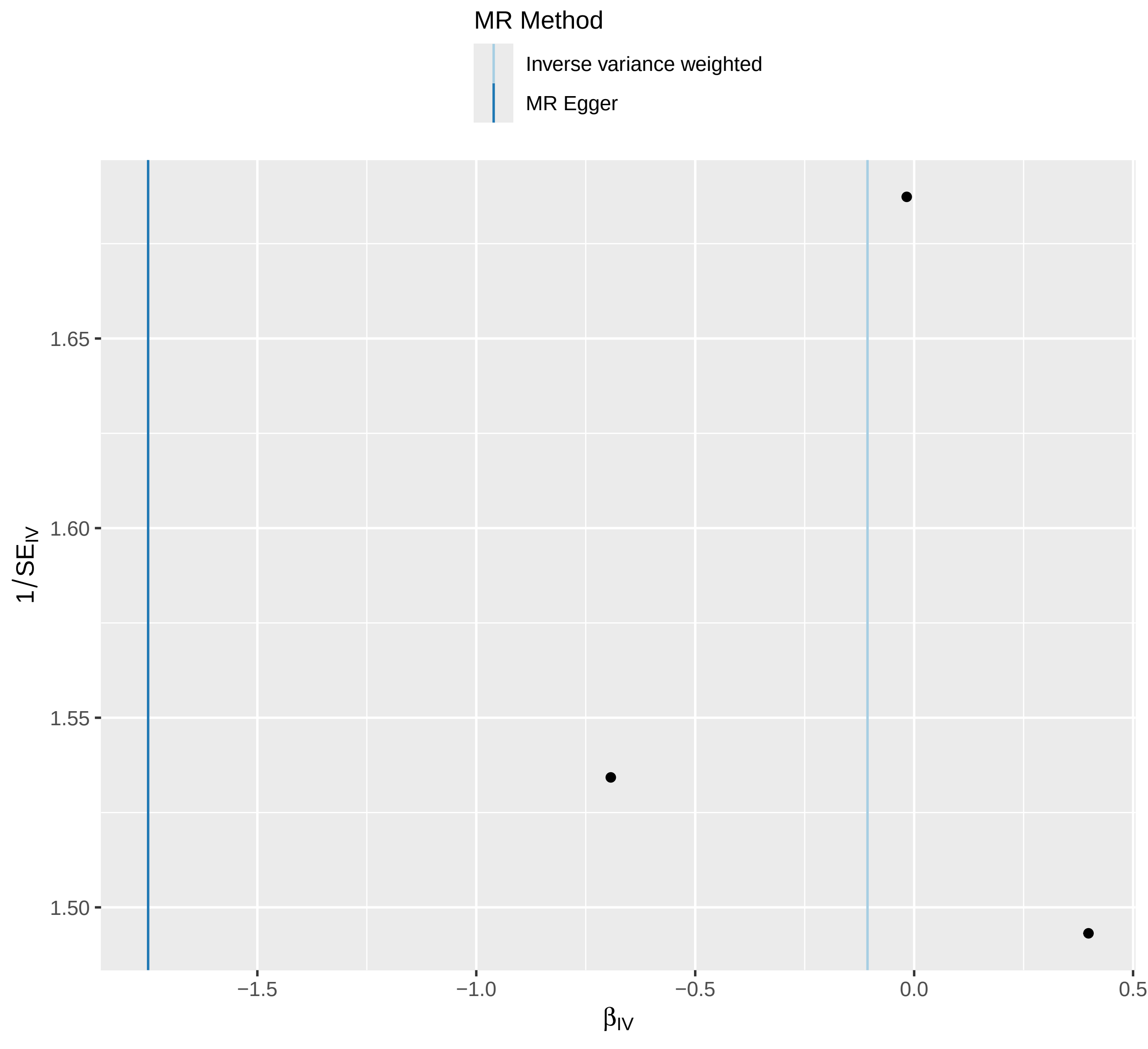

MR Method
Inverse variance weighted
MR Egger
1.65
1.60
1.55
1.50
1/SE$_{IV}$
−1.5
−1.0
−0.5
0.0
0.5
β_{IV}

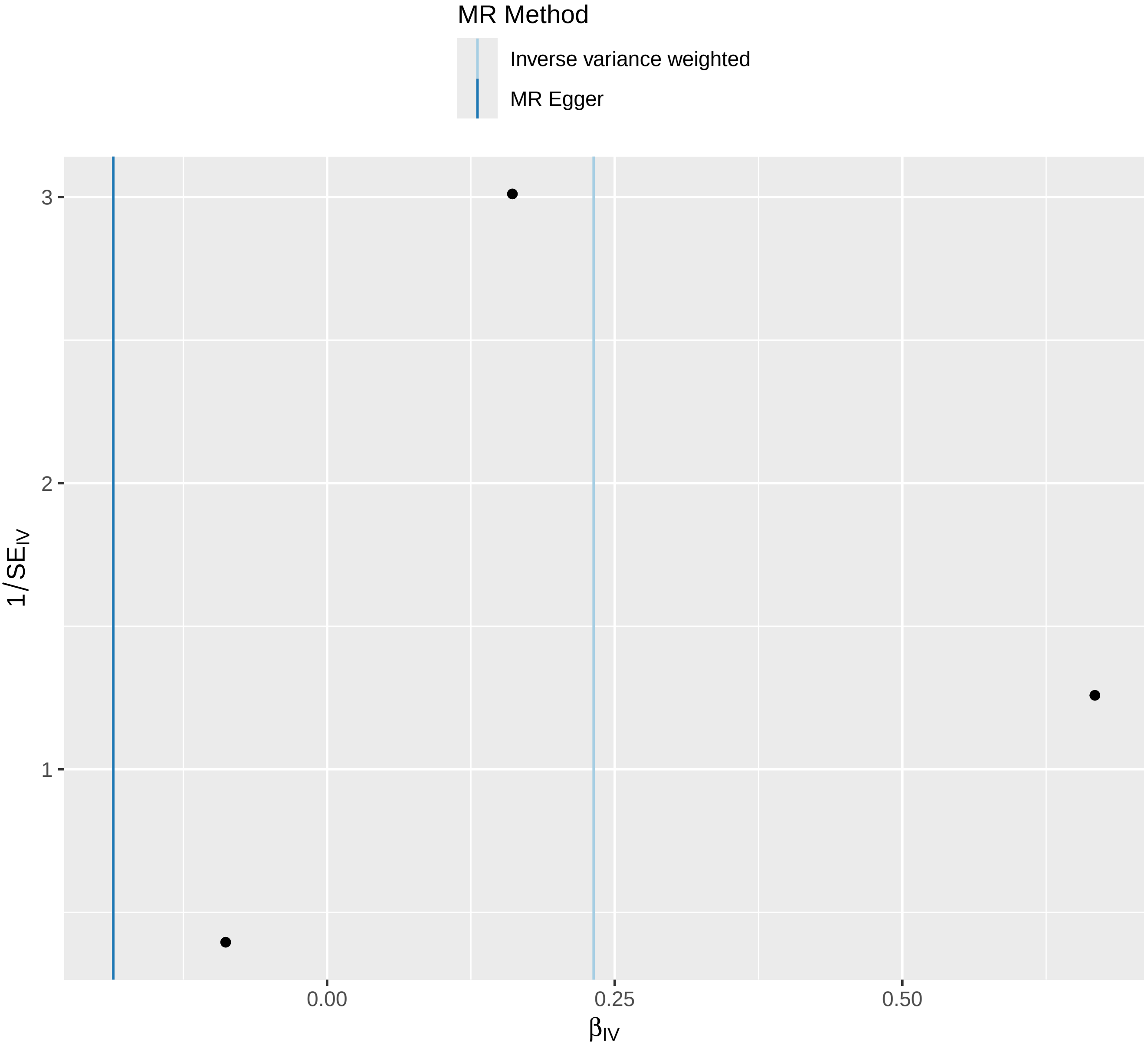
MR Method
Inverse variance weighted
MR Egger
1/SE$_{IV}$
3
2
1
0.00
0.25
0.50
β$_{IV}$

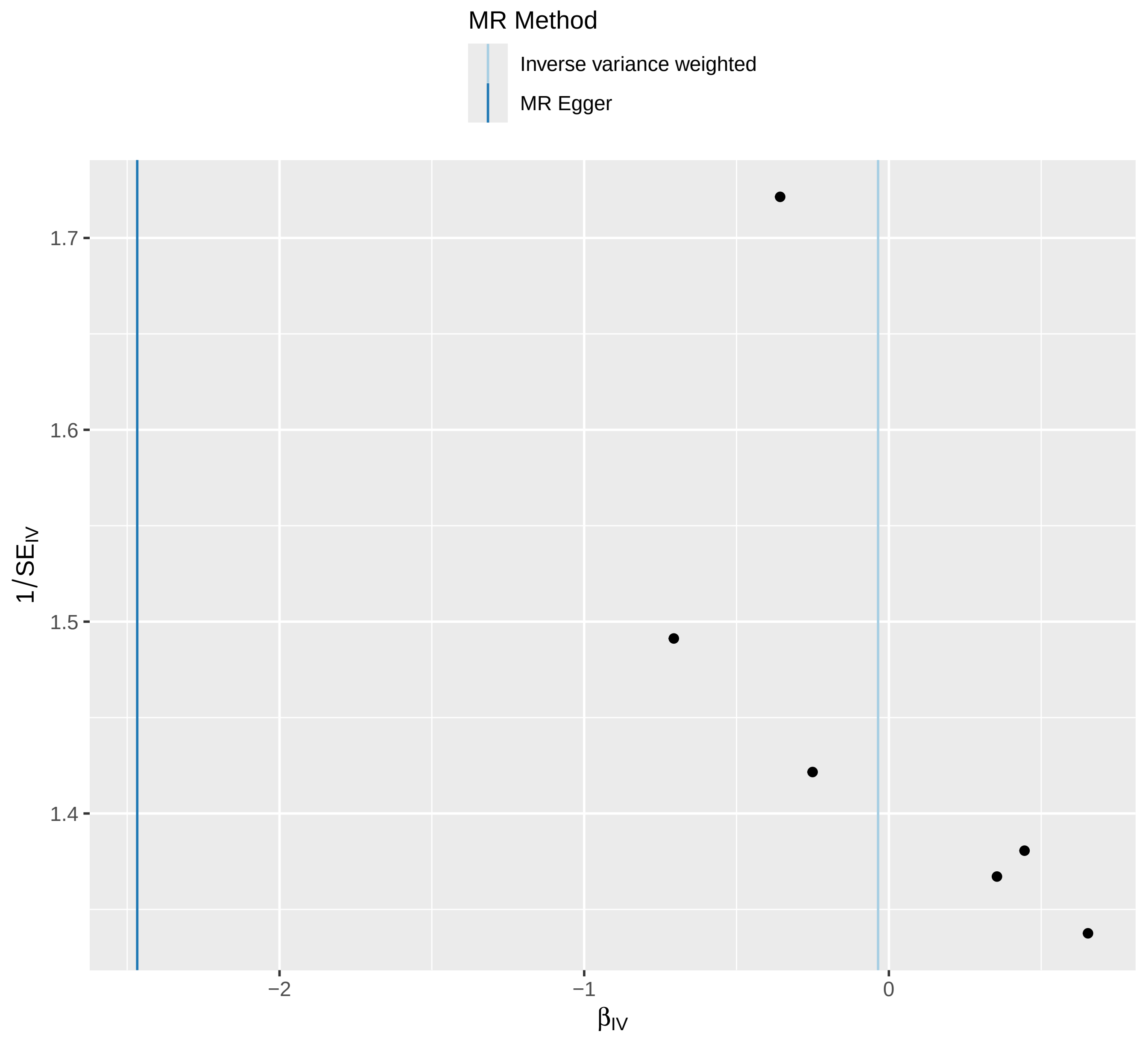
MR Method
Inverse variance weighted
MR Egger
1.7
1.6
1.5
1.4
1/SE$_{IV}$
−2
−1
0
β$_{IV}$

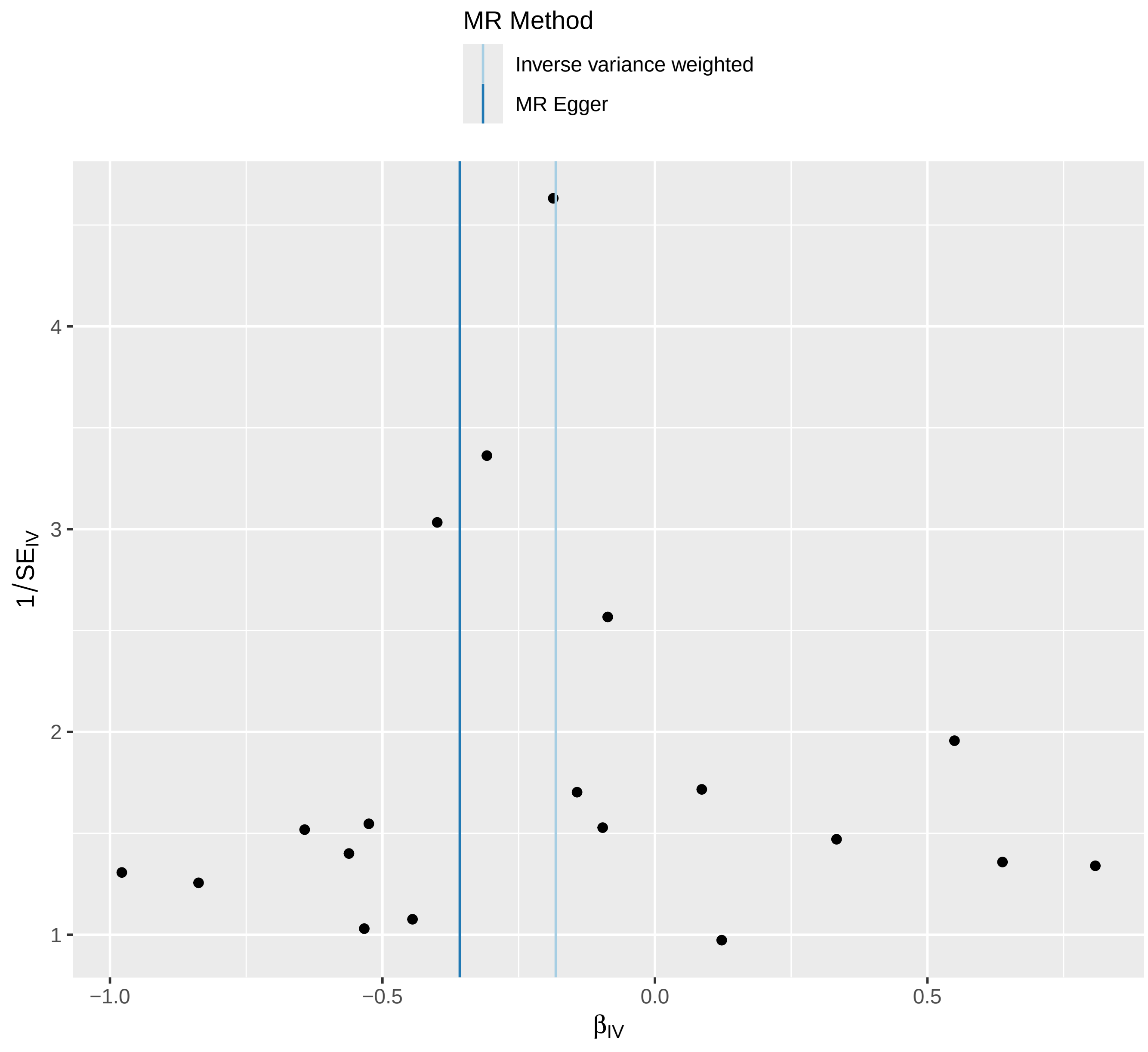
MR Method
Inverse variance weighted
MR Egger
1/SE$_{IV}$
4
3
2
1
−1.0
−0.5
0.0
0.5
β$_{IV}$

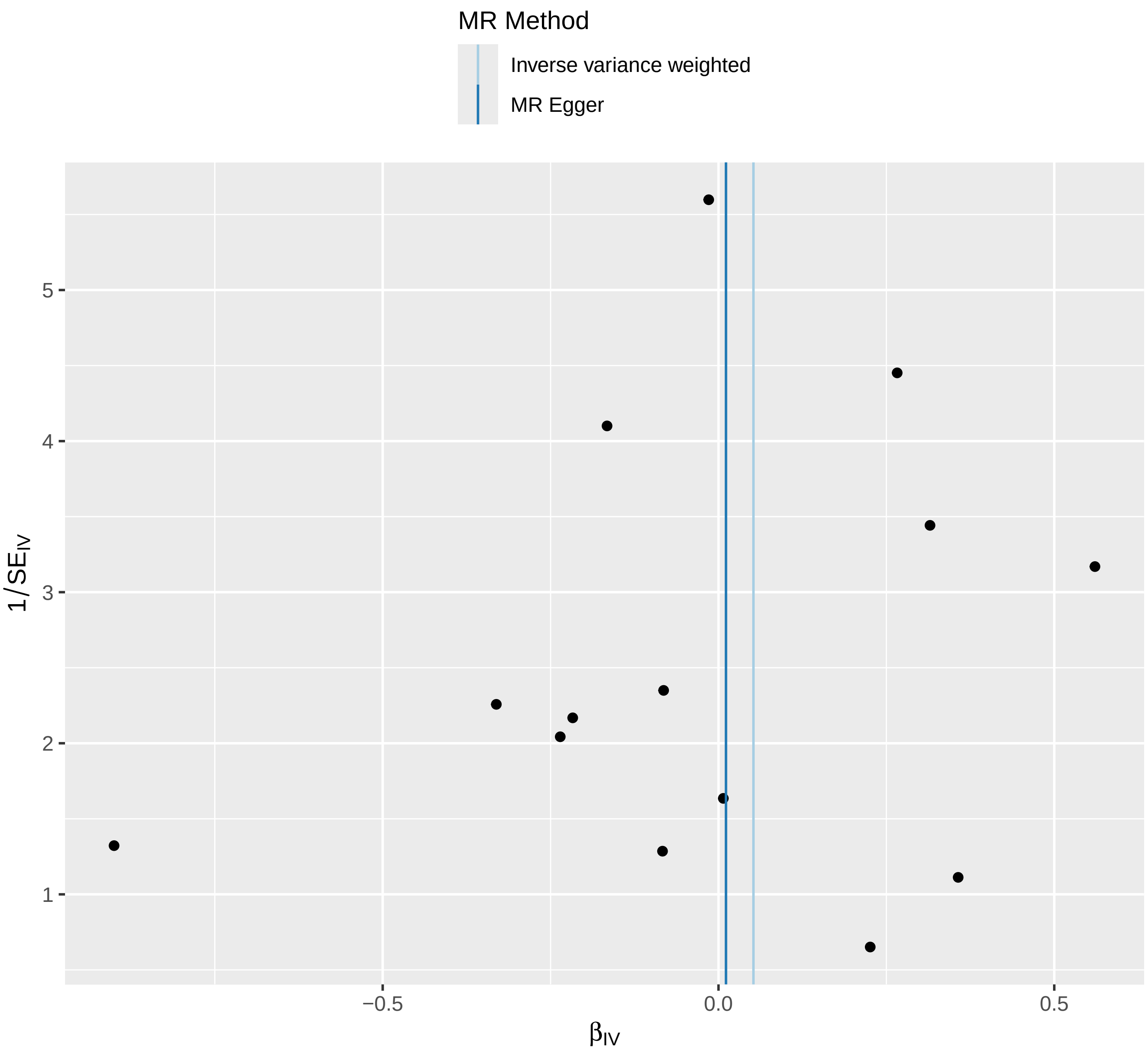

MR Method
Inverse variance weighted
MR Egger
5
4
3
2
1
1/SE_IV
−0.5
0.0
0.5
β_IV

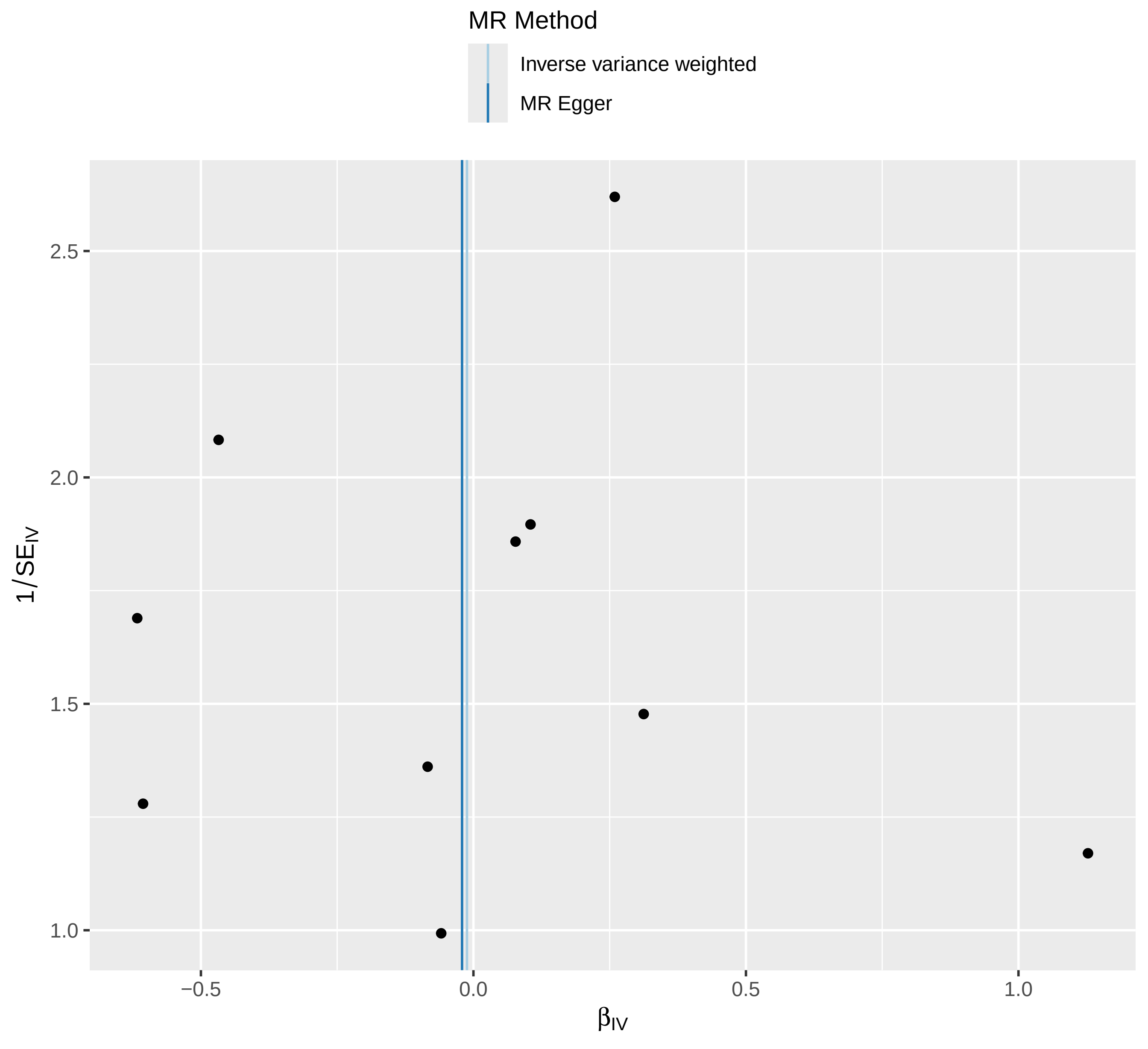

MR Method
Inverse variance weighted
MR Egger
2.5
2.0
1.5
1.0
1/SE_IV
−0.5
0.0
0.5
1.0
β_IV

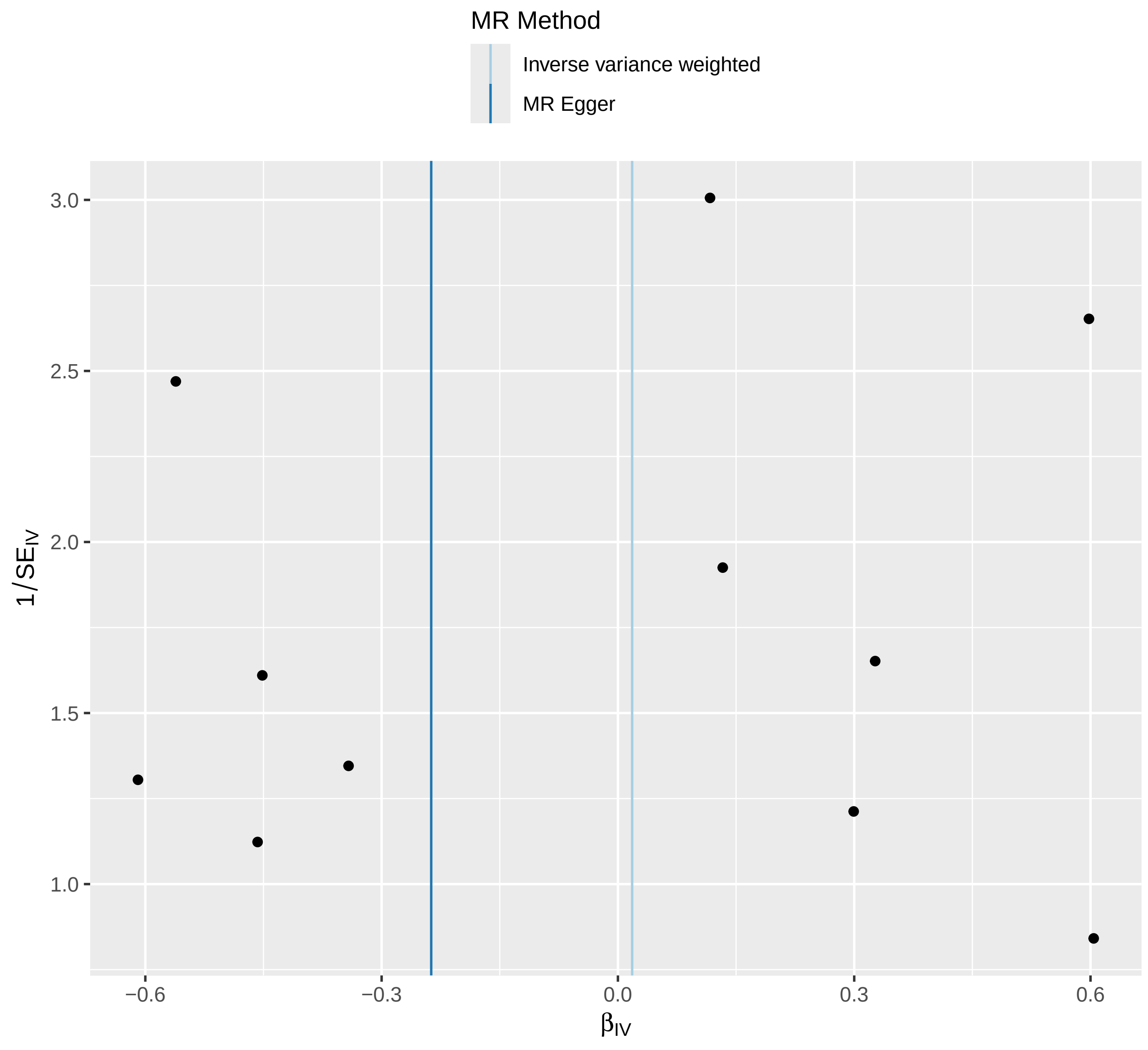

MR Method
Inverse variance weighted
MR Egger
3.0
2.5
2.0
1.5
1.0
1/SE_IV
−0.6
−0.3
0.0
0.3
0.6
β_IV

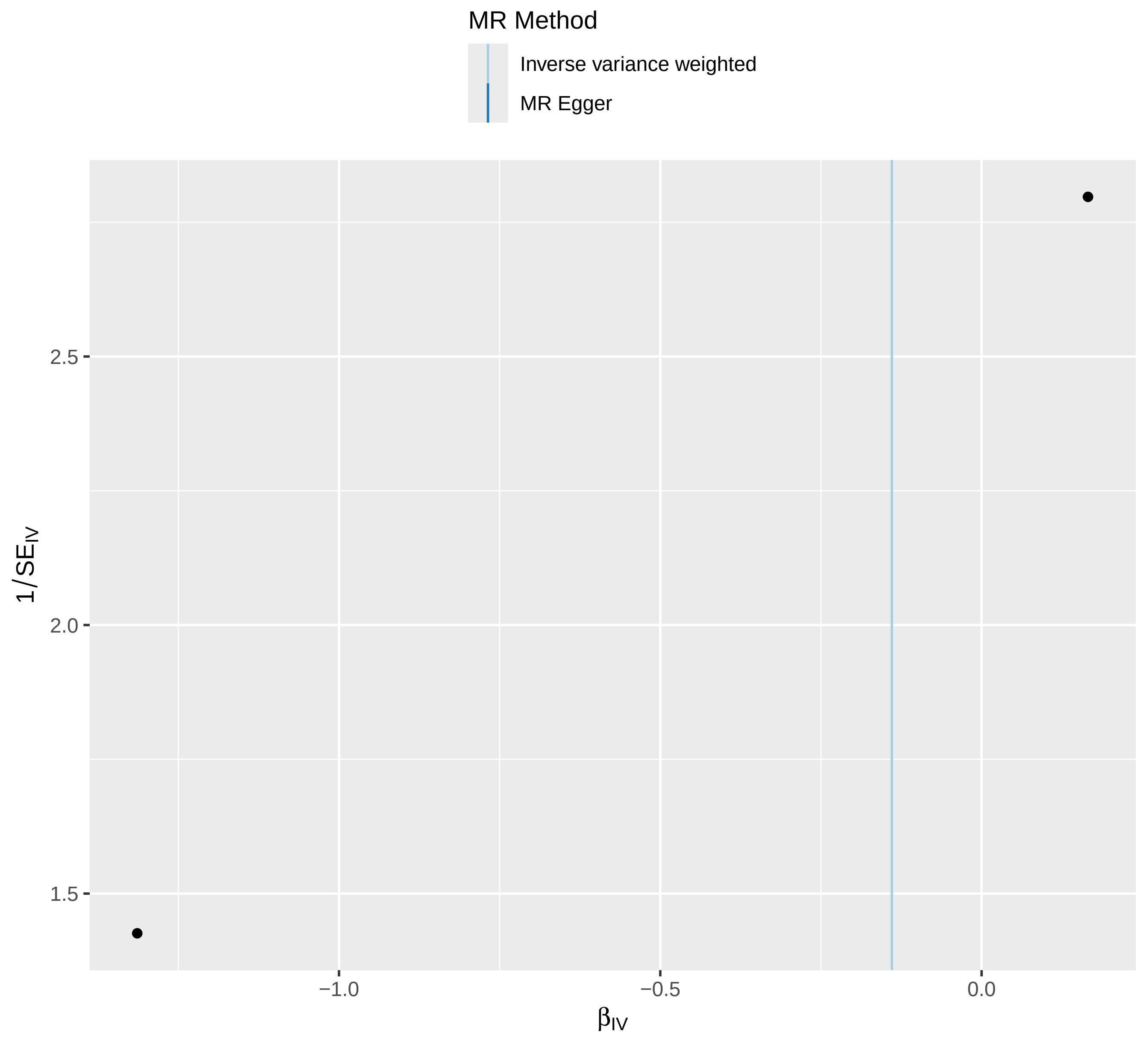
MR Method
Inverse variance weighted
MR Egger
2.5
2.0
1.5
1/SE_IV
−1.0
−0.5
0.0
β_IV

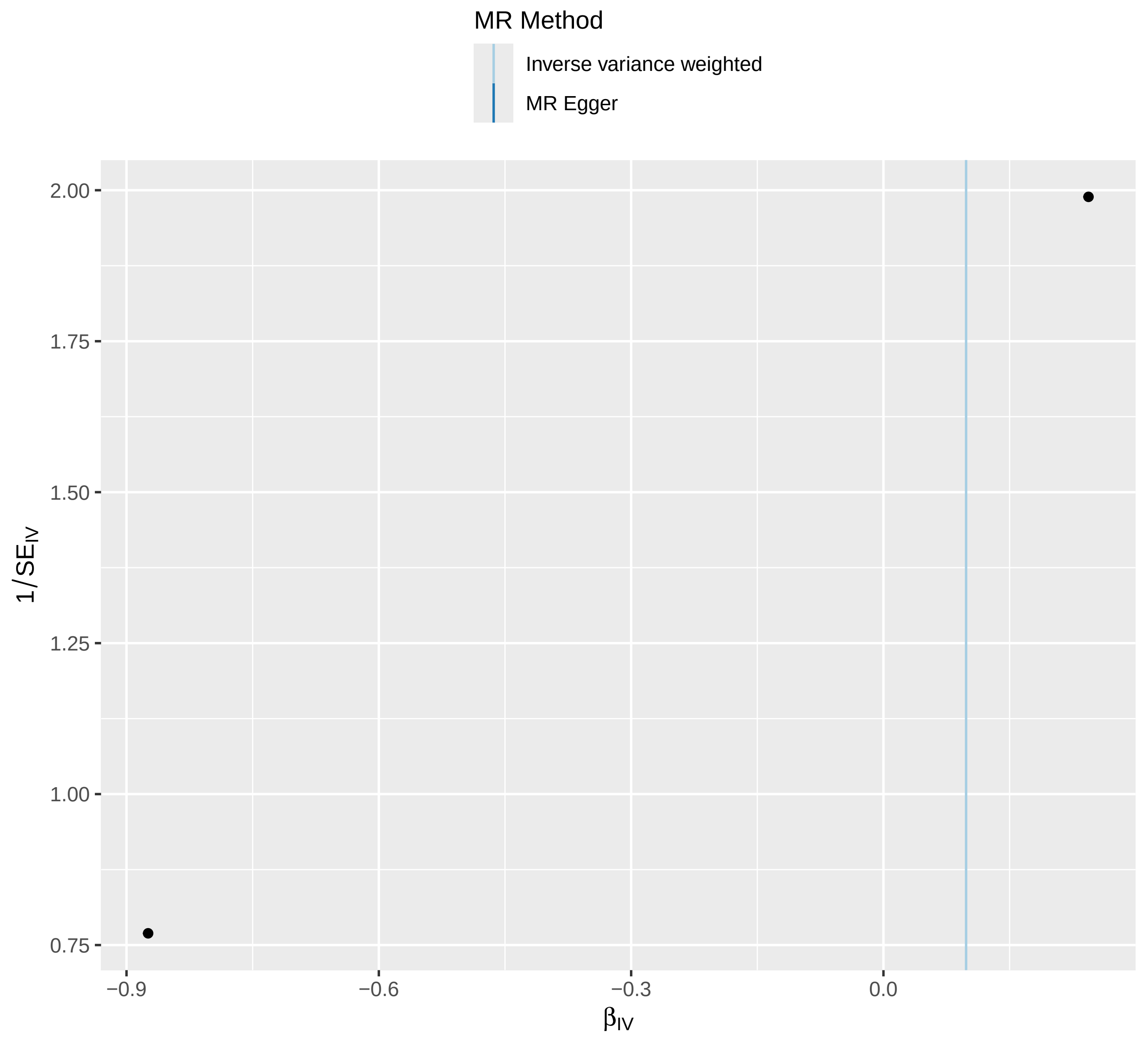

MR Method
Inverse variance weighted
MR Egger
2.00
1.75
1.50
1.25
1.00
0.75
$1/SE_{IV}$
−0.9
−0.6
−0.3
0.0
β_{IV}

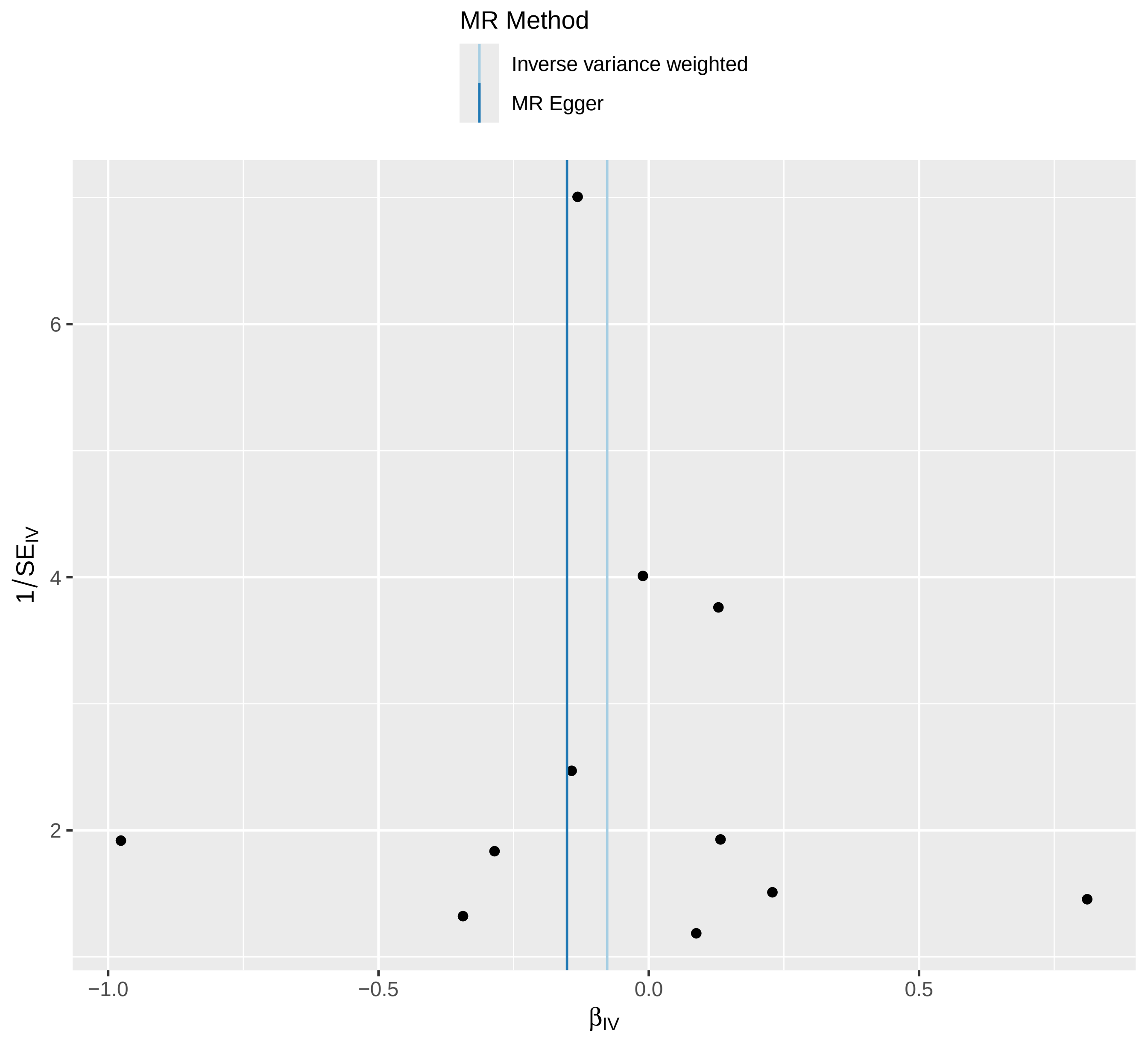
MR Method
Inverse variance weighted
MR Egger
6
4
2
1/SE_IV
−1.0
−0.5
0.0
0.5
β_IV

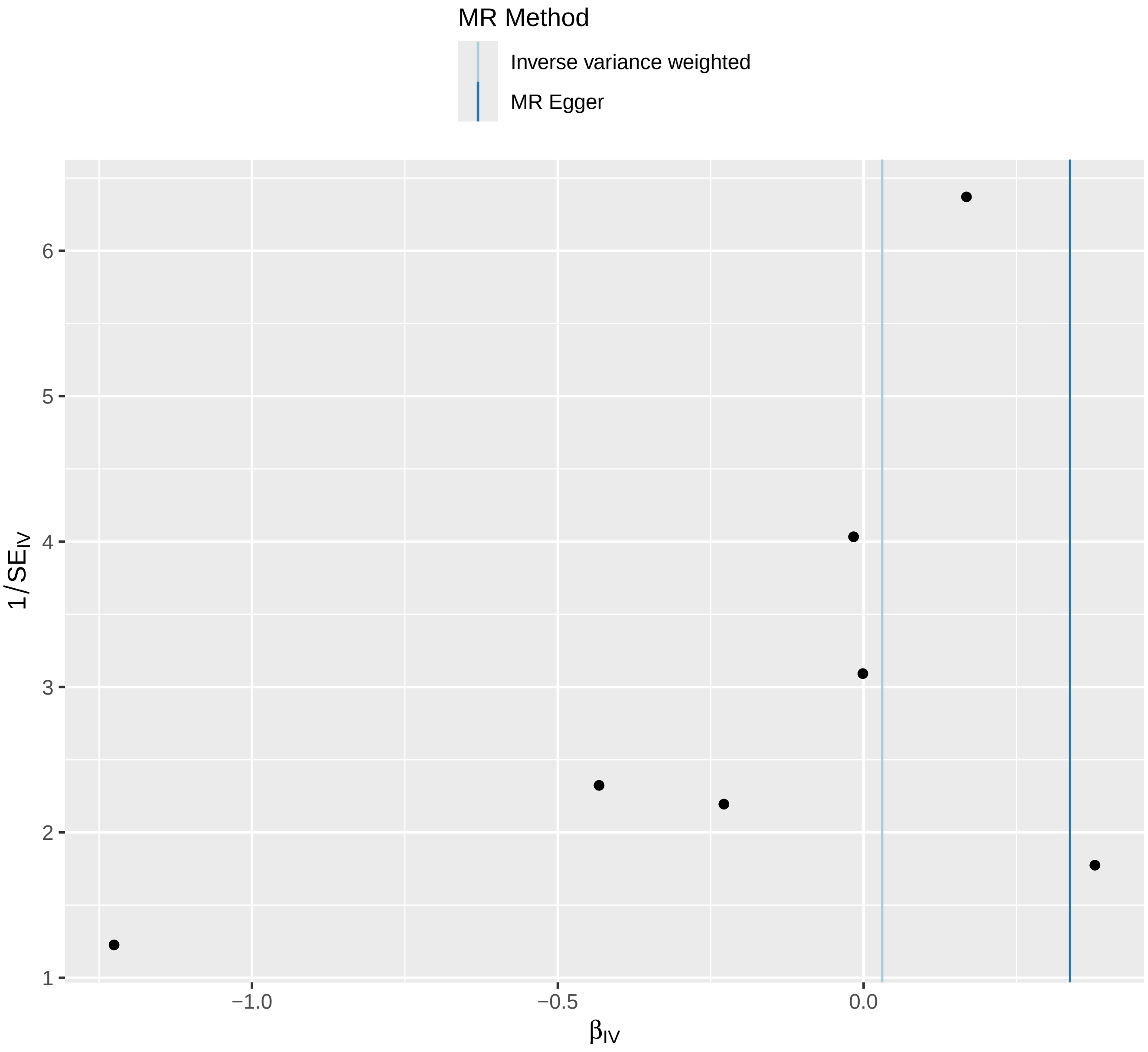

MR Method
Inverse variance weighted
MR Egger
1/SE_IV
6
5
4
3
2
1
−1.0
−0.5
0.0
β_IV

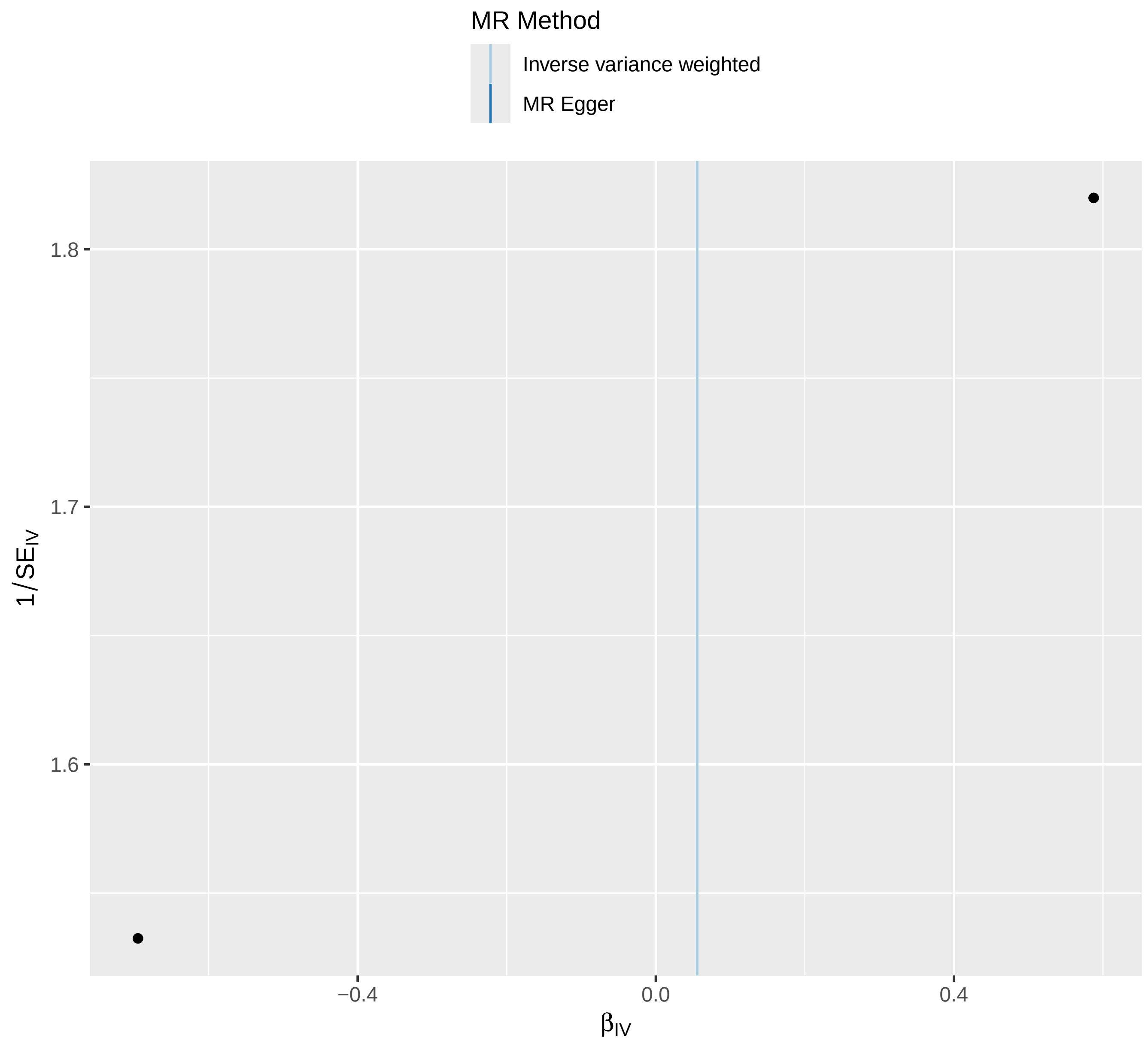

MR Method
Inverse variance weighted
MR Egger
1.8
1.7
1.6
1/SE_IV
−0.4
0.0
0.4
β_IV

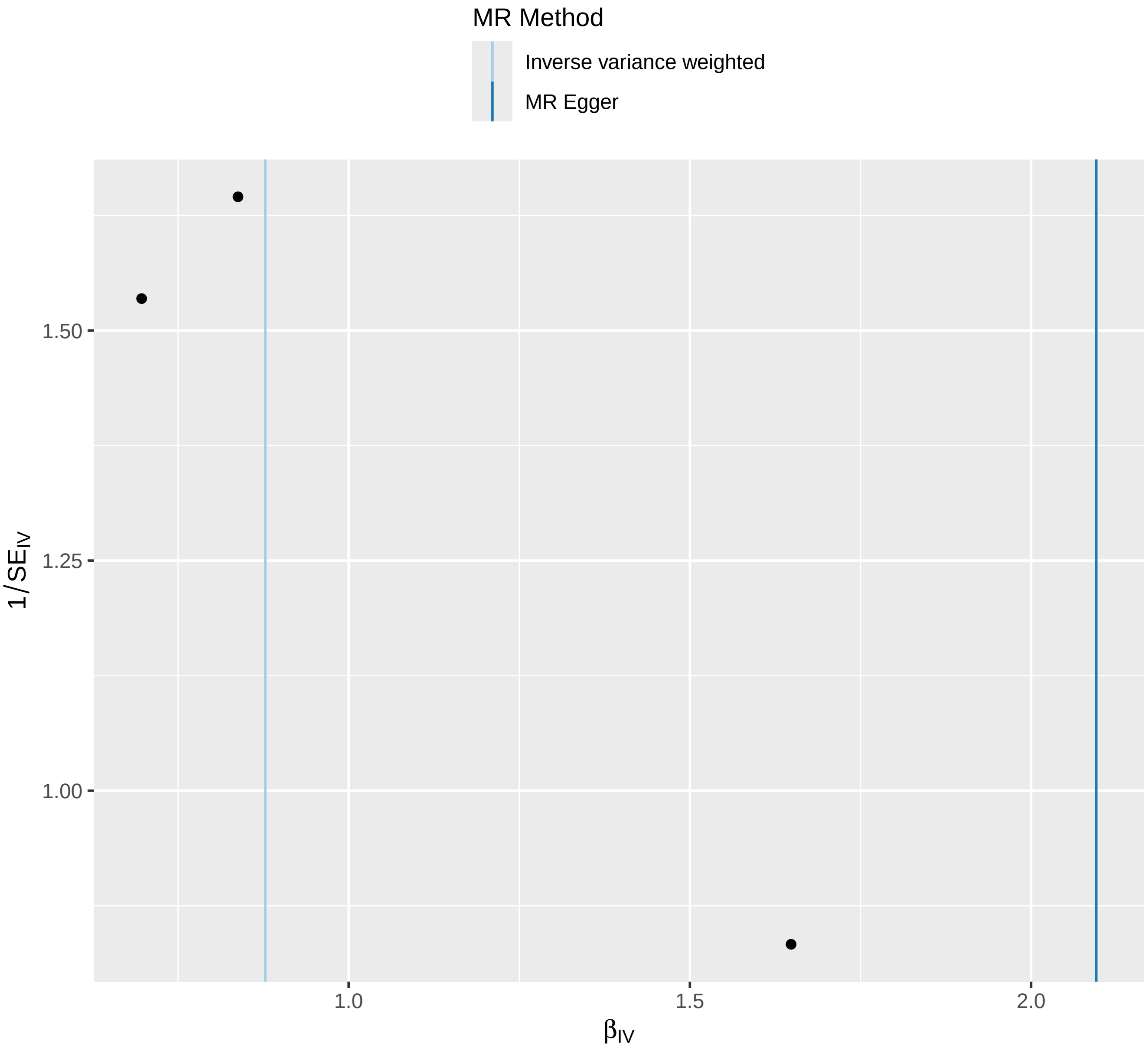

MR Method
Inverse variance weighted
MR Egger
1.50
1.25
1.00
$1/SE_{IV}$
1.0
1.5
2.0
β_{IV}

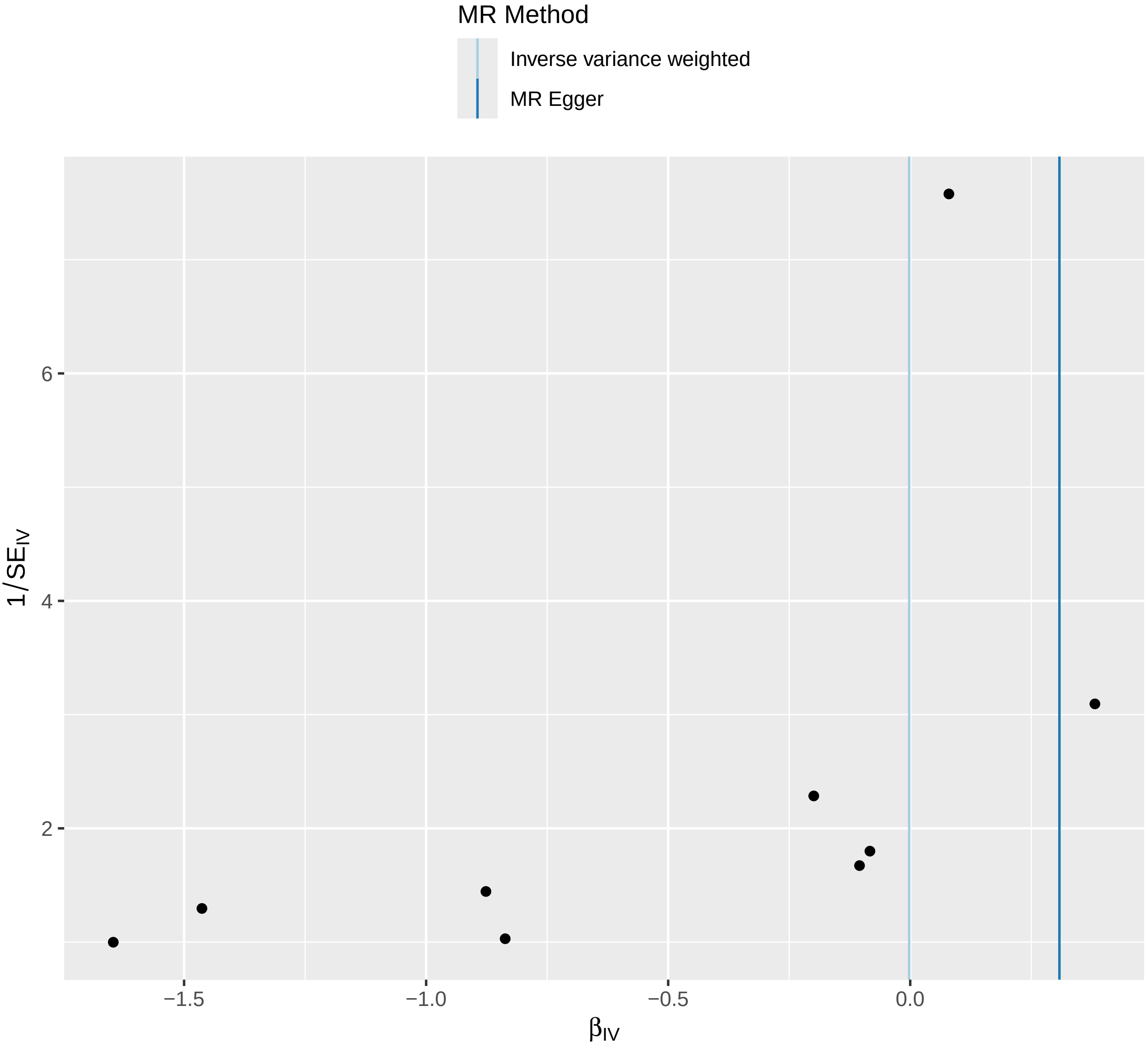
MR Method
Inverse variance weighted
MR Egger
1/SE_IV
6
4
2
−1.5
−1.0
−0.5
0.0
β_IV

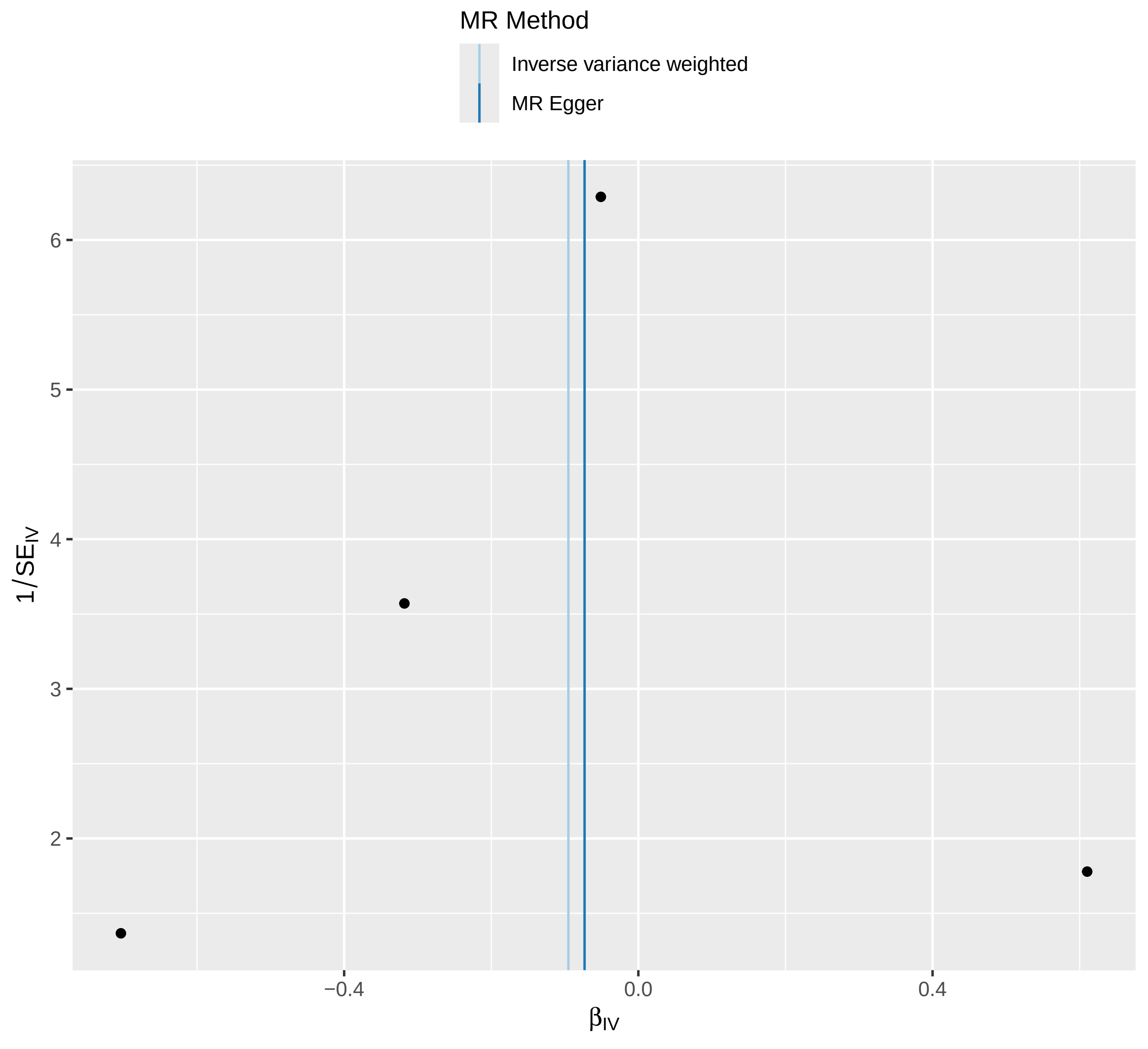
MR Method
Inverse variance weighted
MR Egger
6
5
4
3
2
1/SE_IV
−0.4
0.0
0.4
β_IV

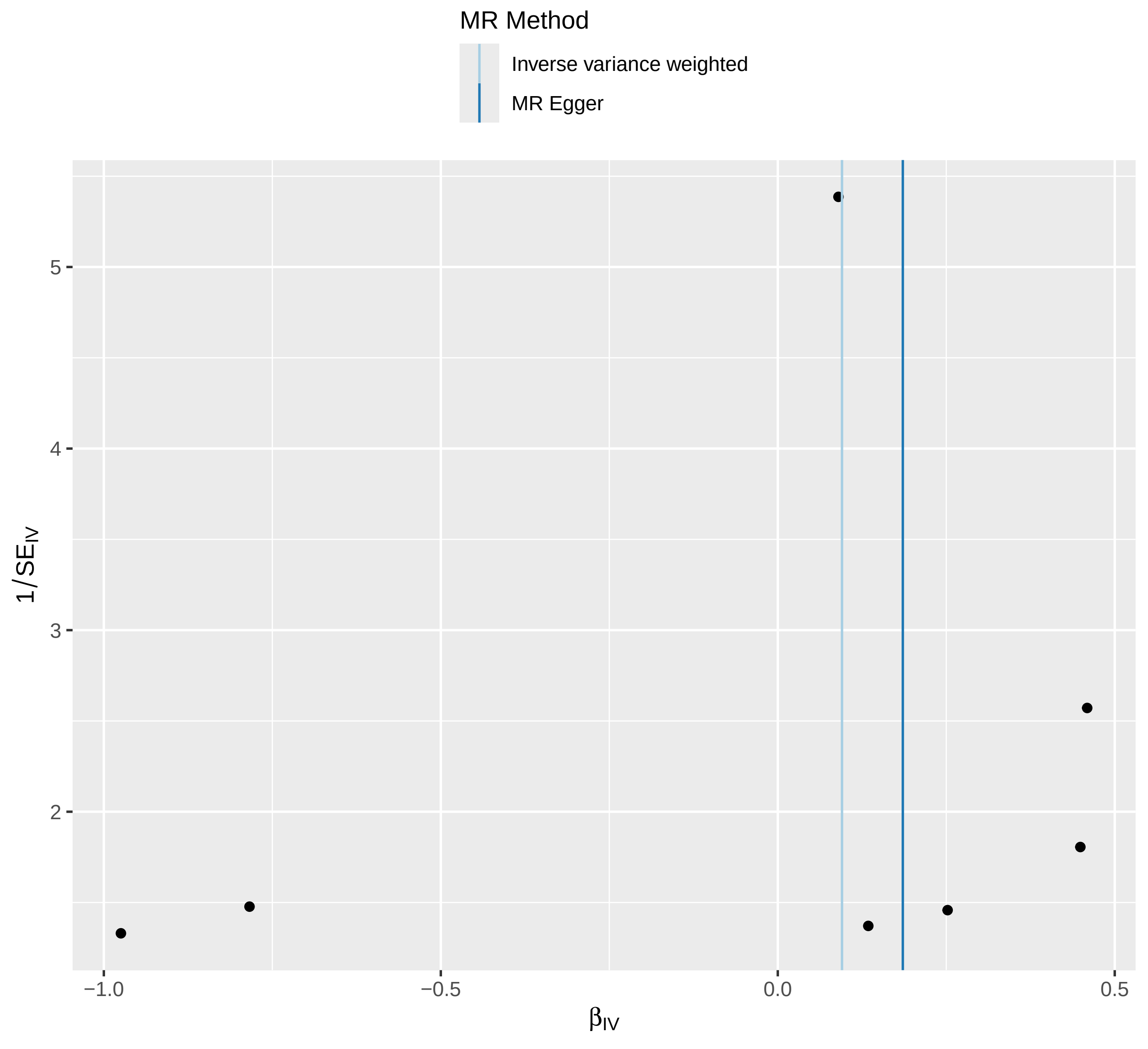
MR Method
Inverse variance weighted
MR Egger
1/SE_IV
β_IV
5
4
3
2
−1.0
−0.5
0.0
0.5

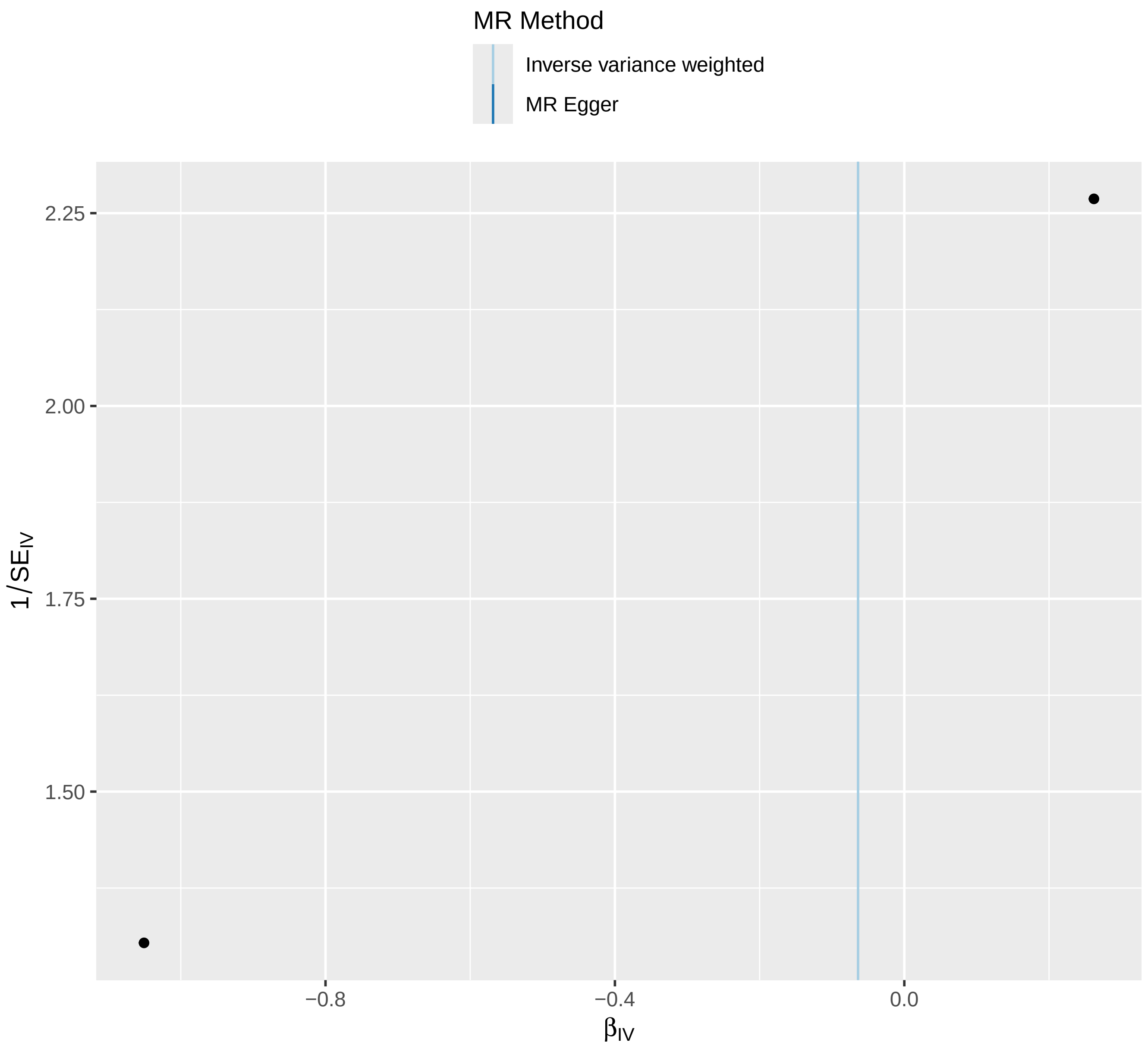
MR Method
Inverse variance weighted
MR Egger
2.25
2.00
1.75
1.50
1/SE_IV
−0.8
−0.4
0.0
β_IV

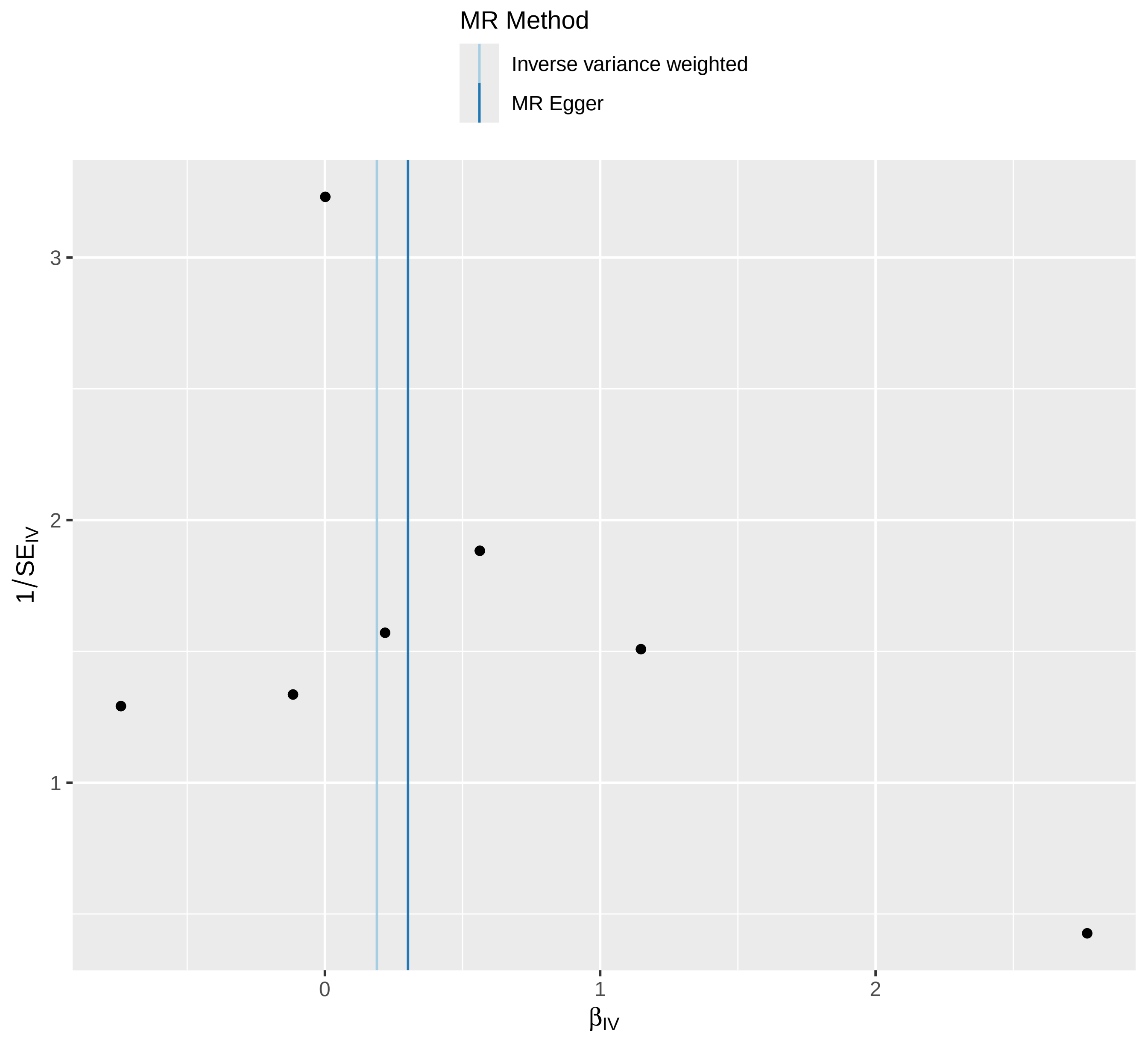

MR Method
Inverse variance weighted
MR Egger
3
2
1
$1/SE_{IV}$
0
1
2
β_{IV}

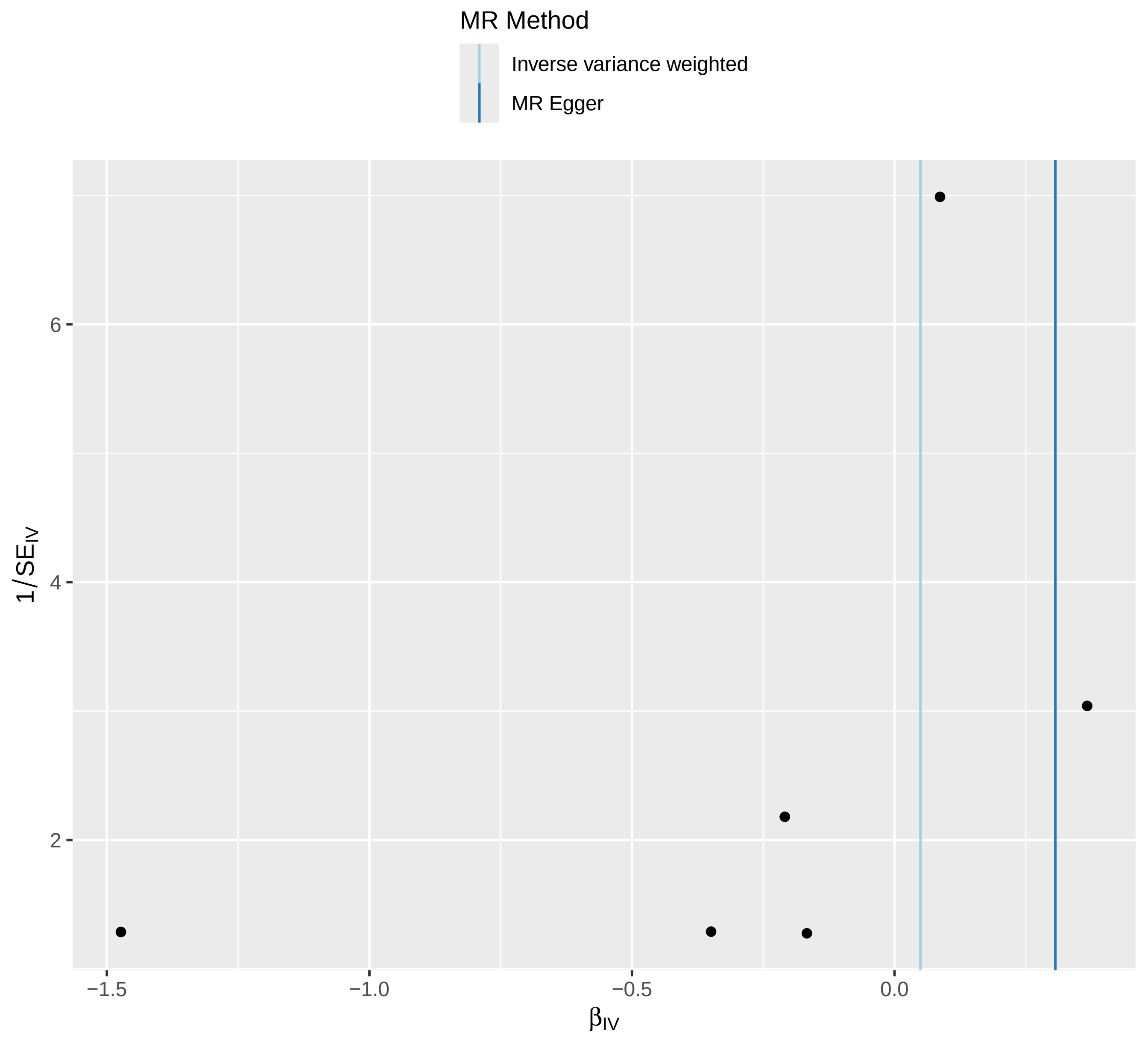

MR Method
Inverse variance weighted
MR Egger
6
4
2
1/SE$_{IV}$
−1.5
−1.0
−0.5
0.0
β$_{IV}$

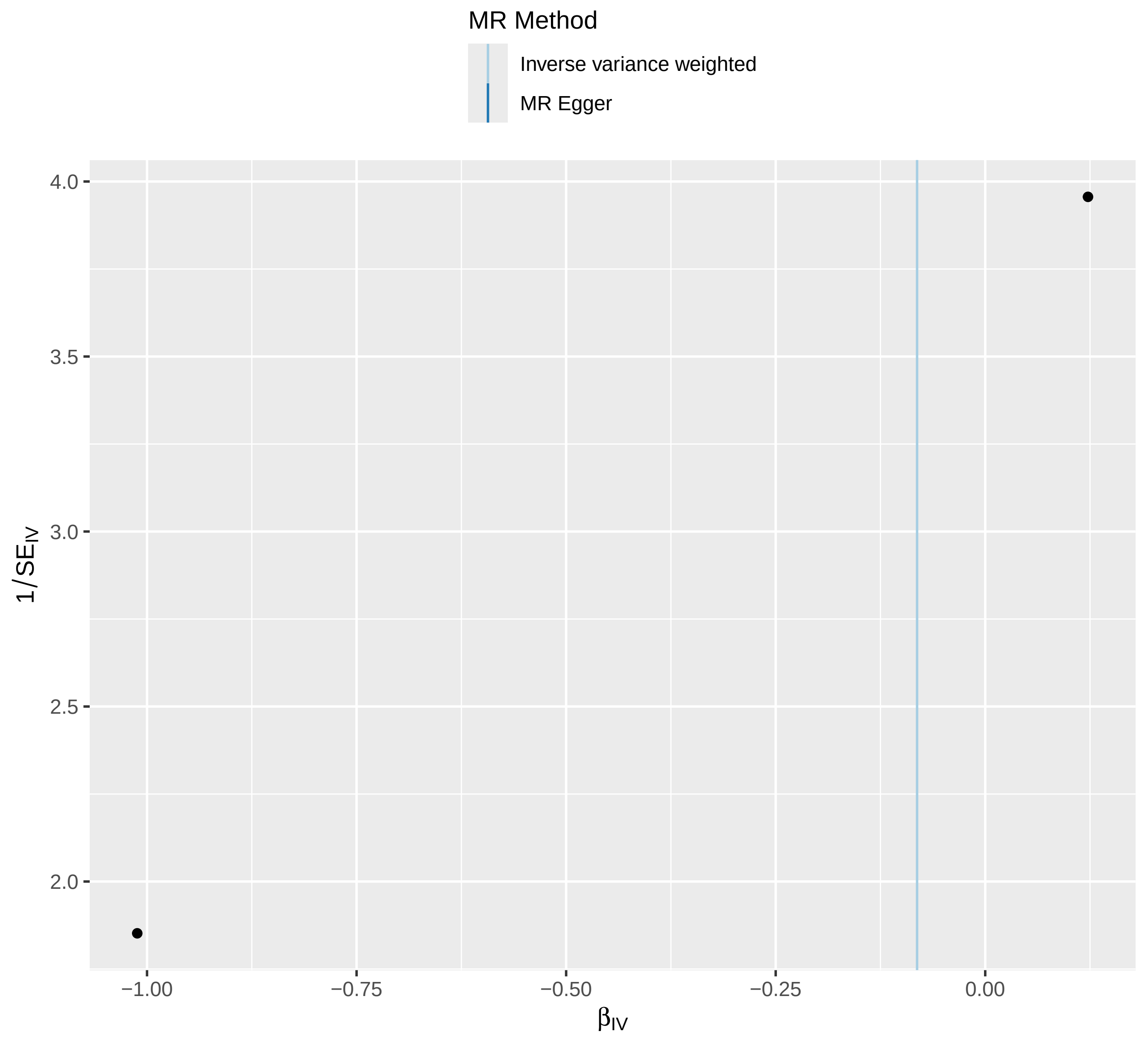
MR Method
Inverse variance weighted
MR Egger
4.0
3.5
3.0
2.5
2.0
$1/SE_{IV}$
−1.00
−0.75
−0.50
−0.25
0.00
β_{IV}

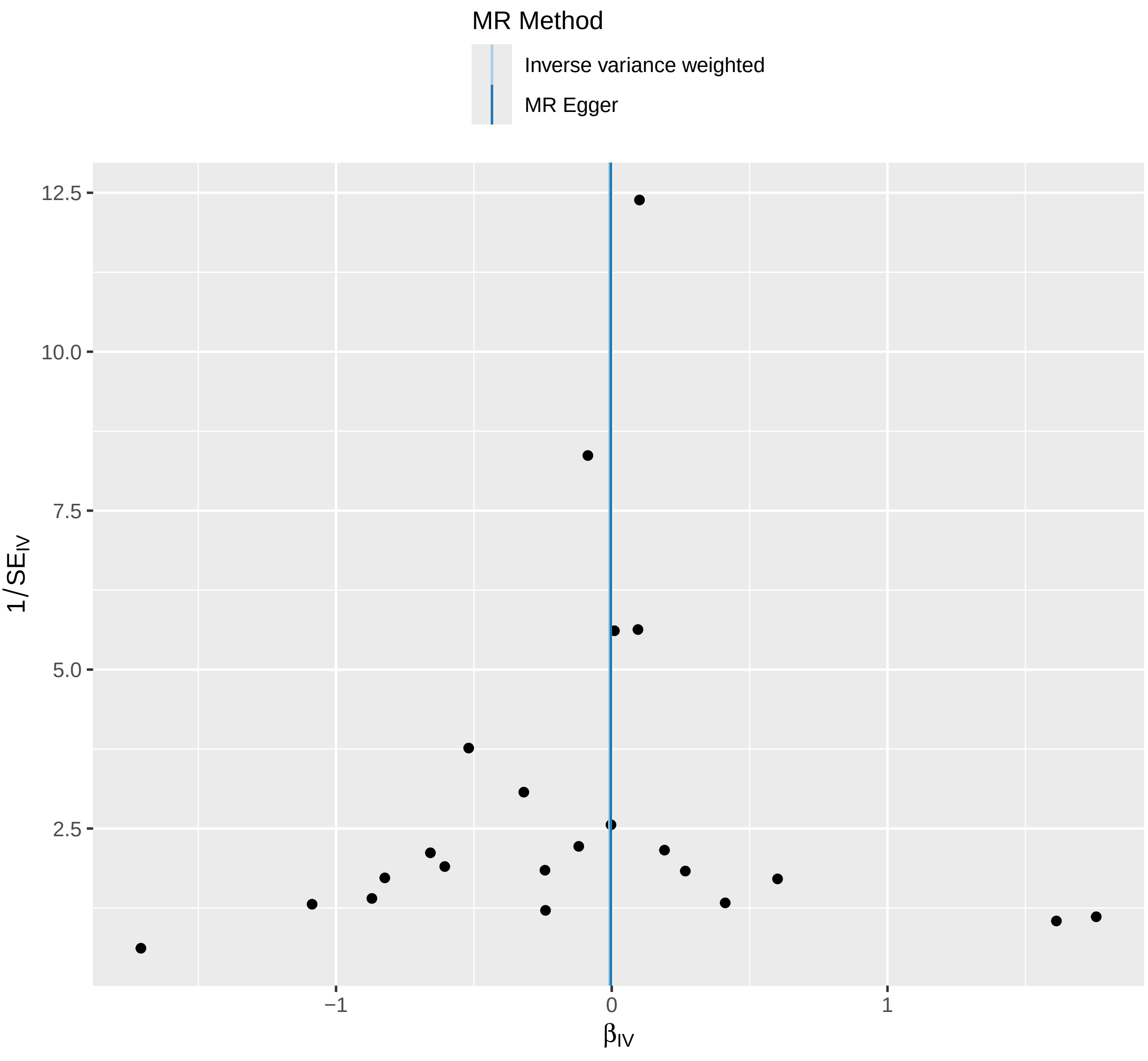
MR Method
Inverse variance weighted
MR Egger
12.5
10.0
7.5
5.0
2.5
$1/SE_{IV}$
−1
0
1
β_{IV}

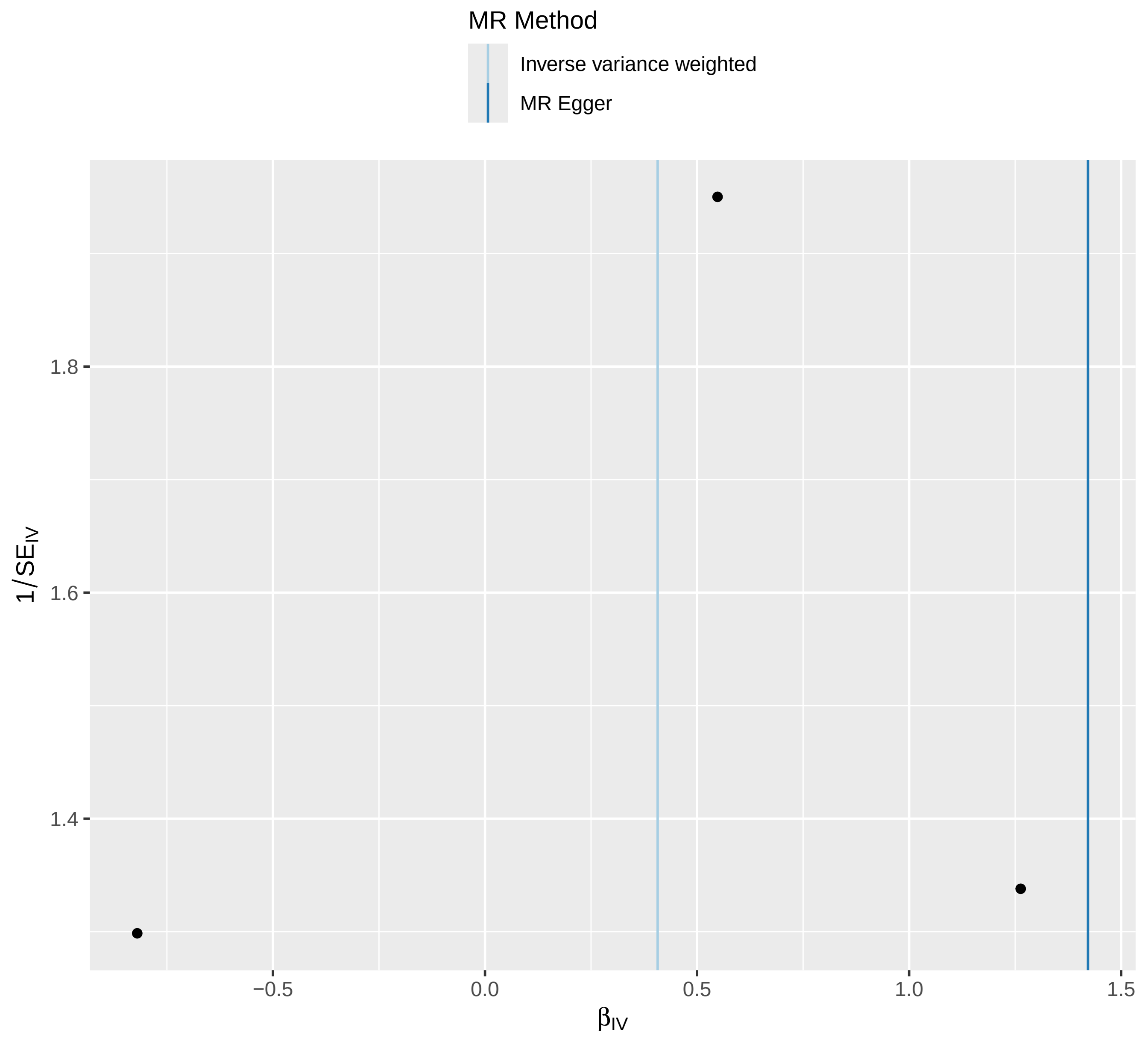

MR Method
Inverse variance weighted
MR Egger
1.8
1.6
1.4
1/SE$_{IV}$
−0.5
0.0
0.5
1.0
1.5
β$_{IV}$

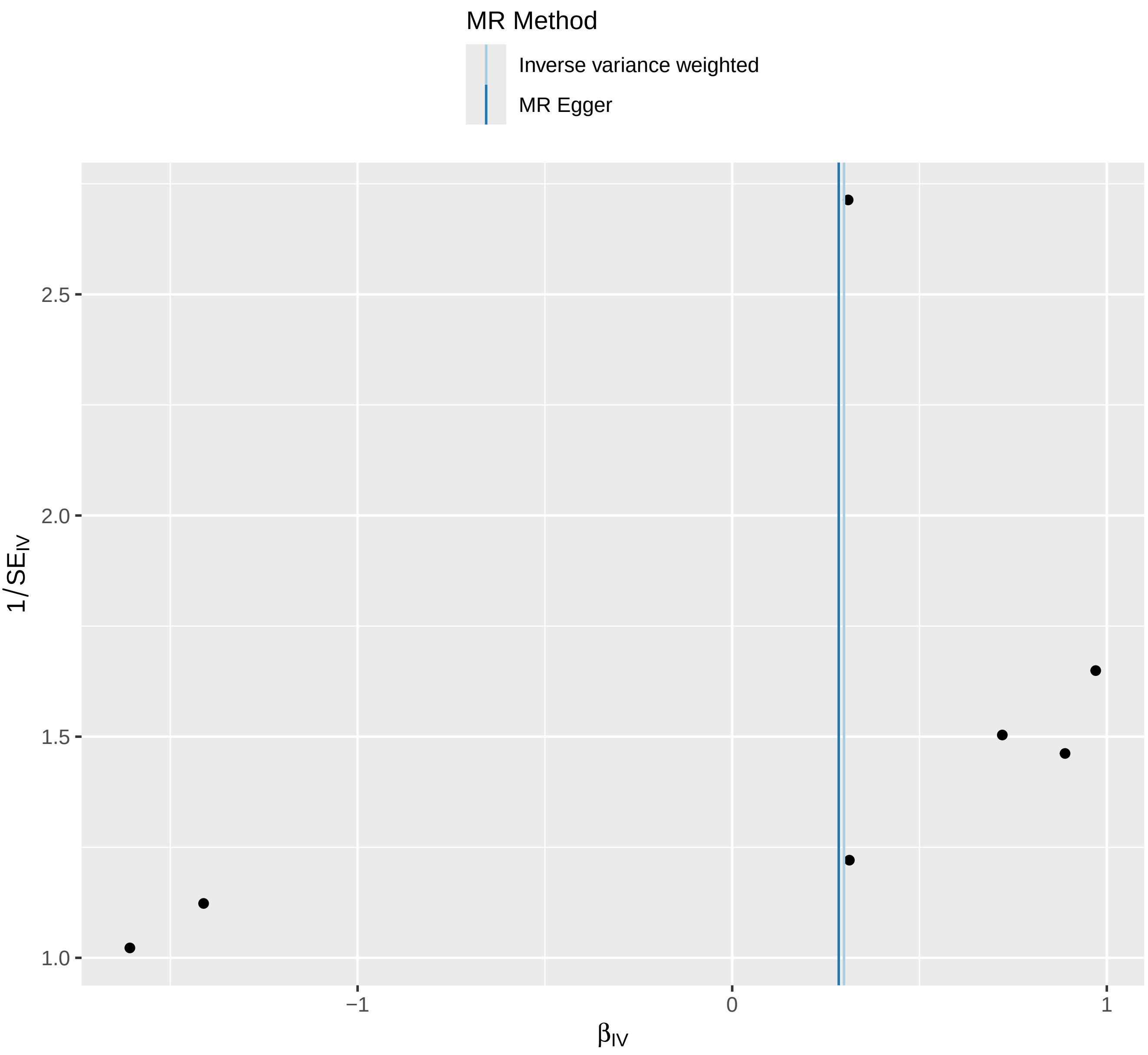
MR Method
Inverse variance weighted
MR Egger
1/SE_IV
2.5
2.0
1.5
1.0
−1
0
1
β_IV

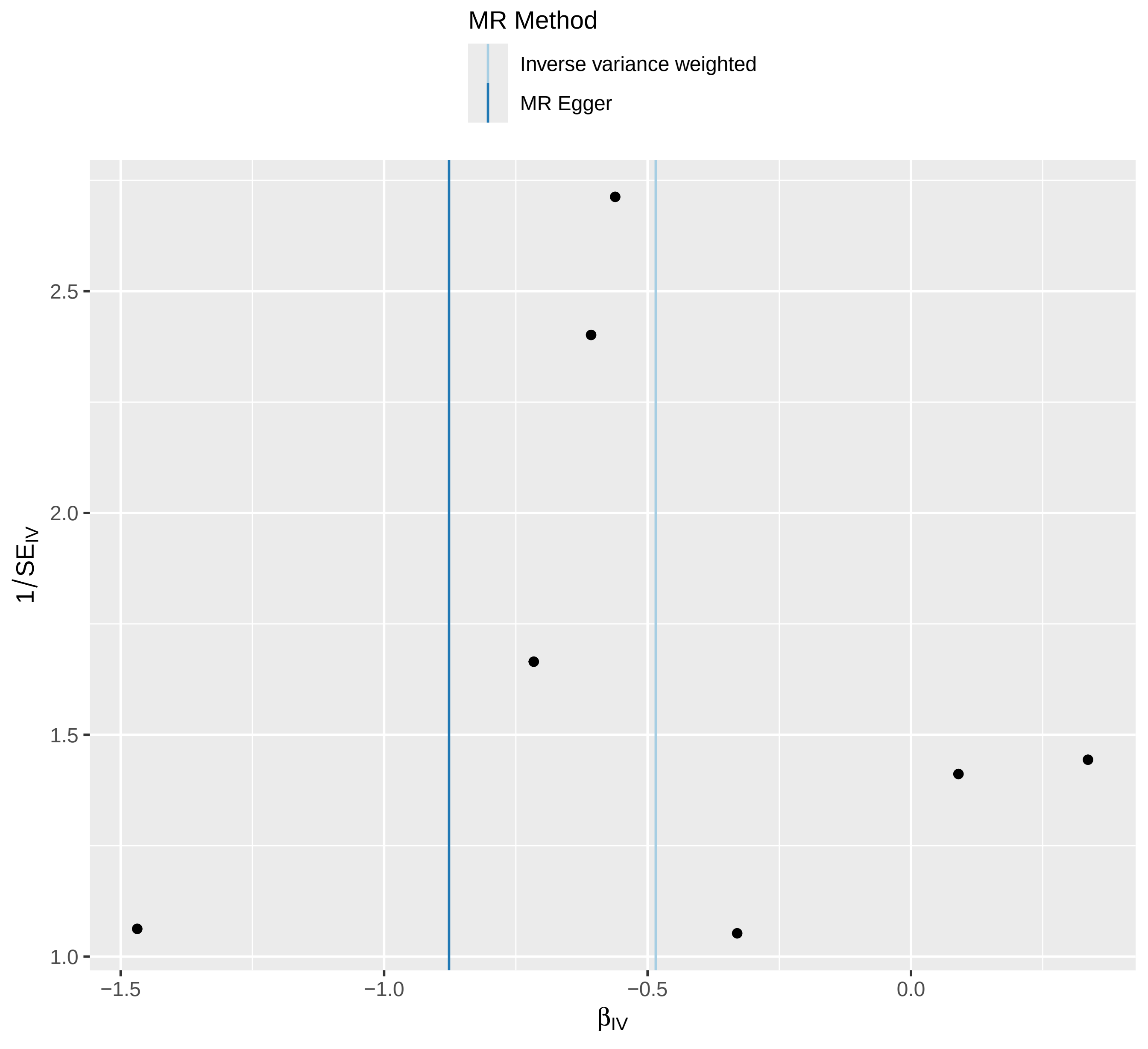
MR Method
Inverse variance weighted
MR Egger
1/SE_IV
β_IV
2.5
2.0
1.5
1.0
−1.5
−1.0
−0.5
0.0

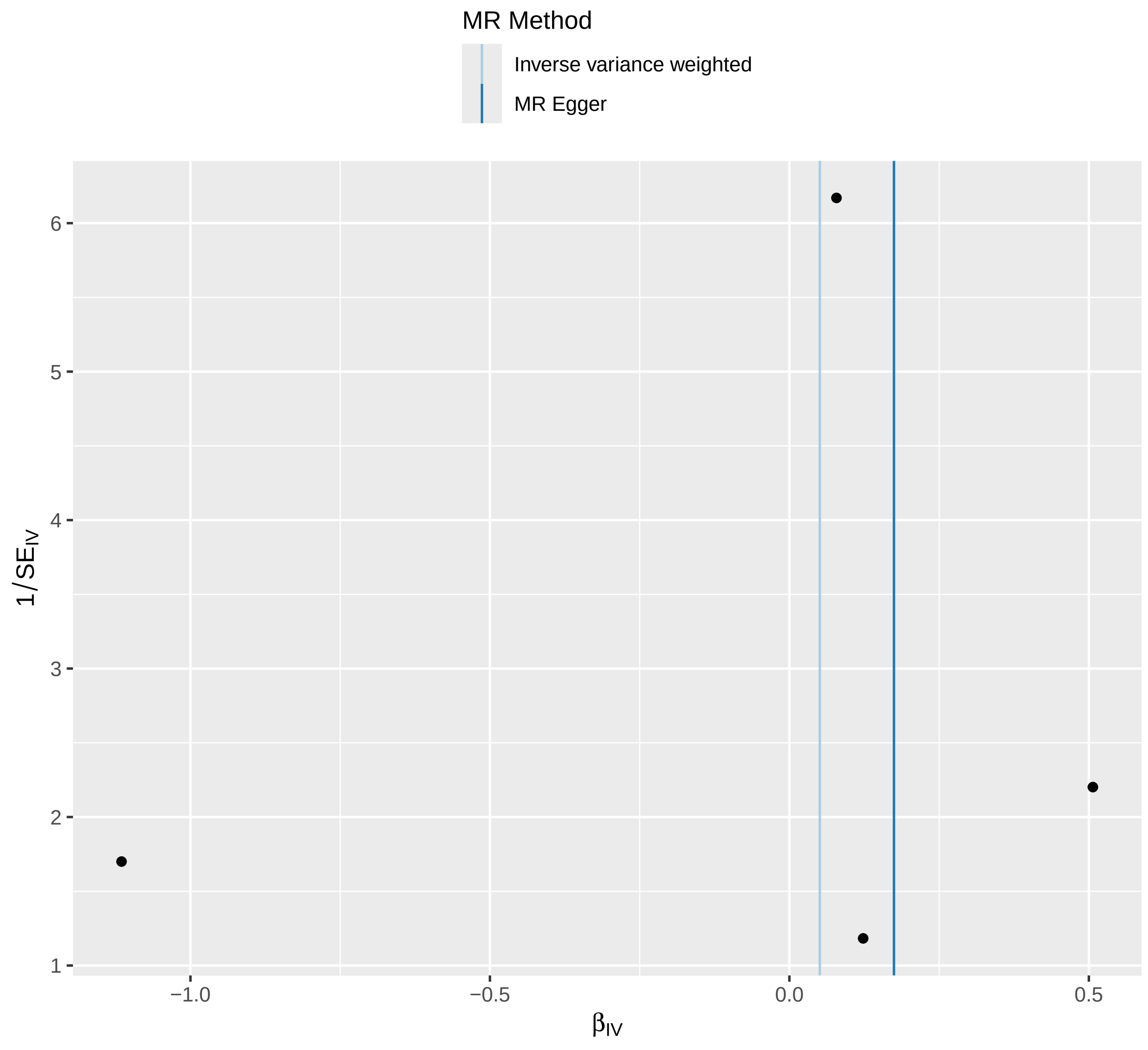
MR Method
Inverse variance weighted
MR Egger
1/SE_IV
β_IV
6
5
4
3
2
1
−1.0
−0.5
0.0
0.5

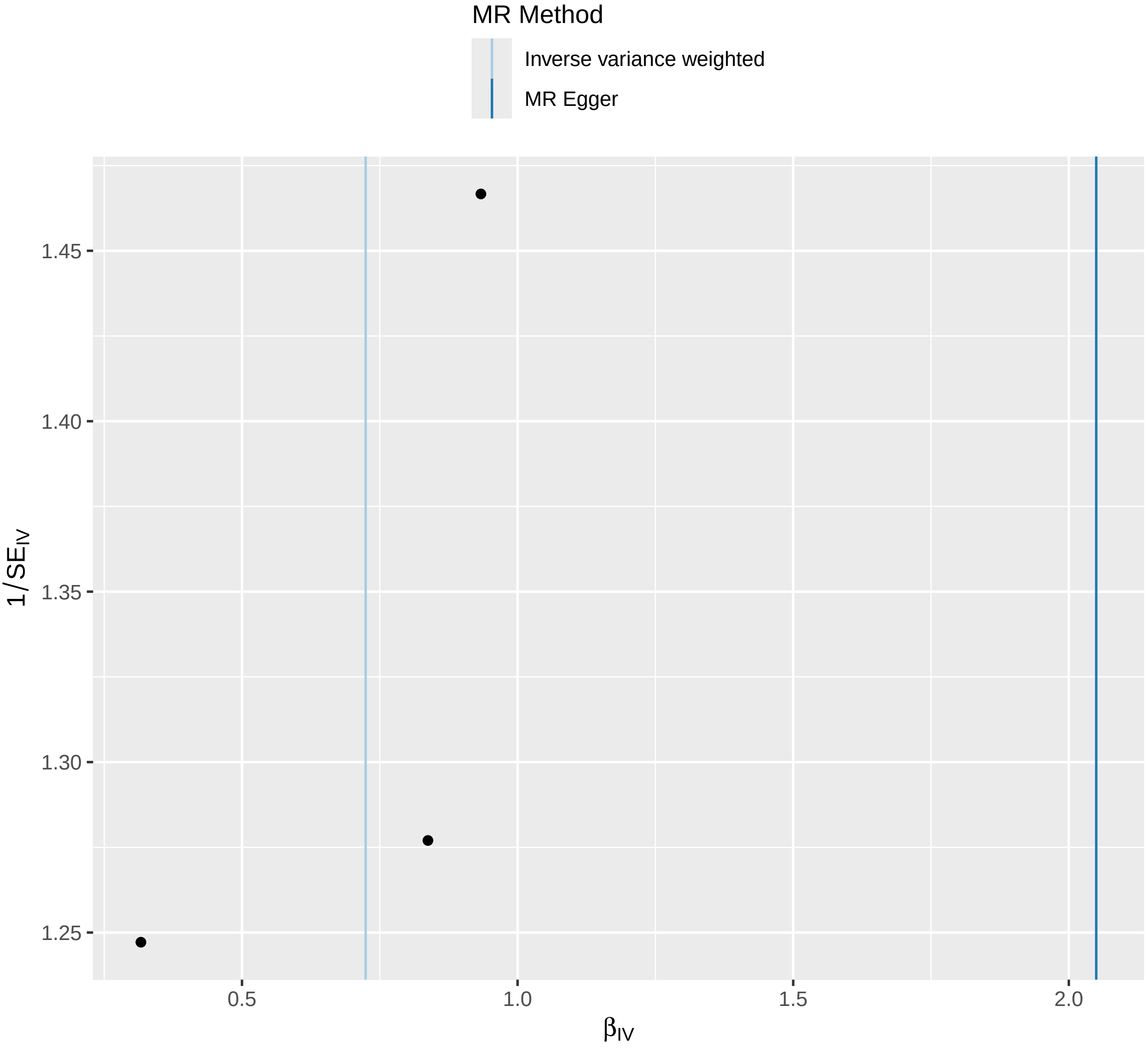
MR Method
Inverse variance weighted
MR Egger
1/SE_IV
1.45
1.40
1.35
1.30
1.25
0.5
1.0
1.5
2.0
β_IV

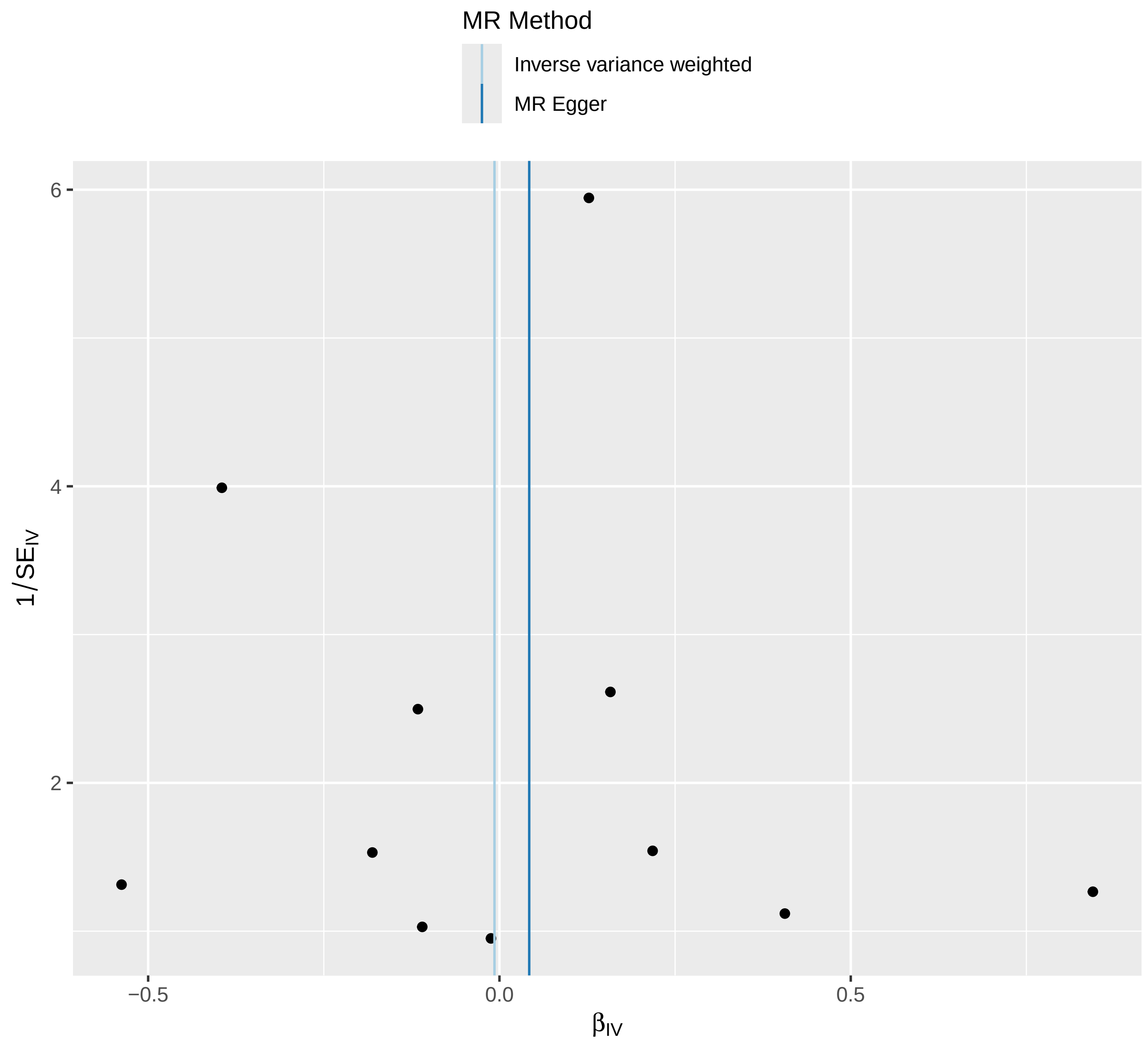

MR Method
Inverse variance weighted
MR Egger
6
4
2
1/SE$_{IV}$
−0.5
0.0
0.5
β$_{IV}$

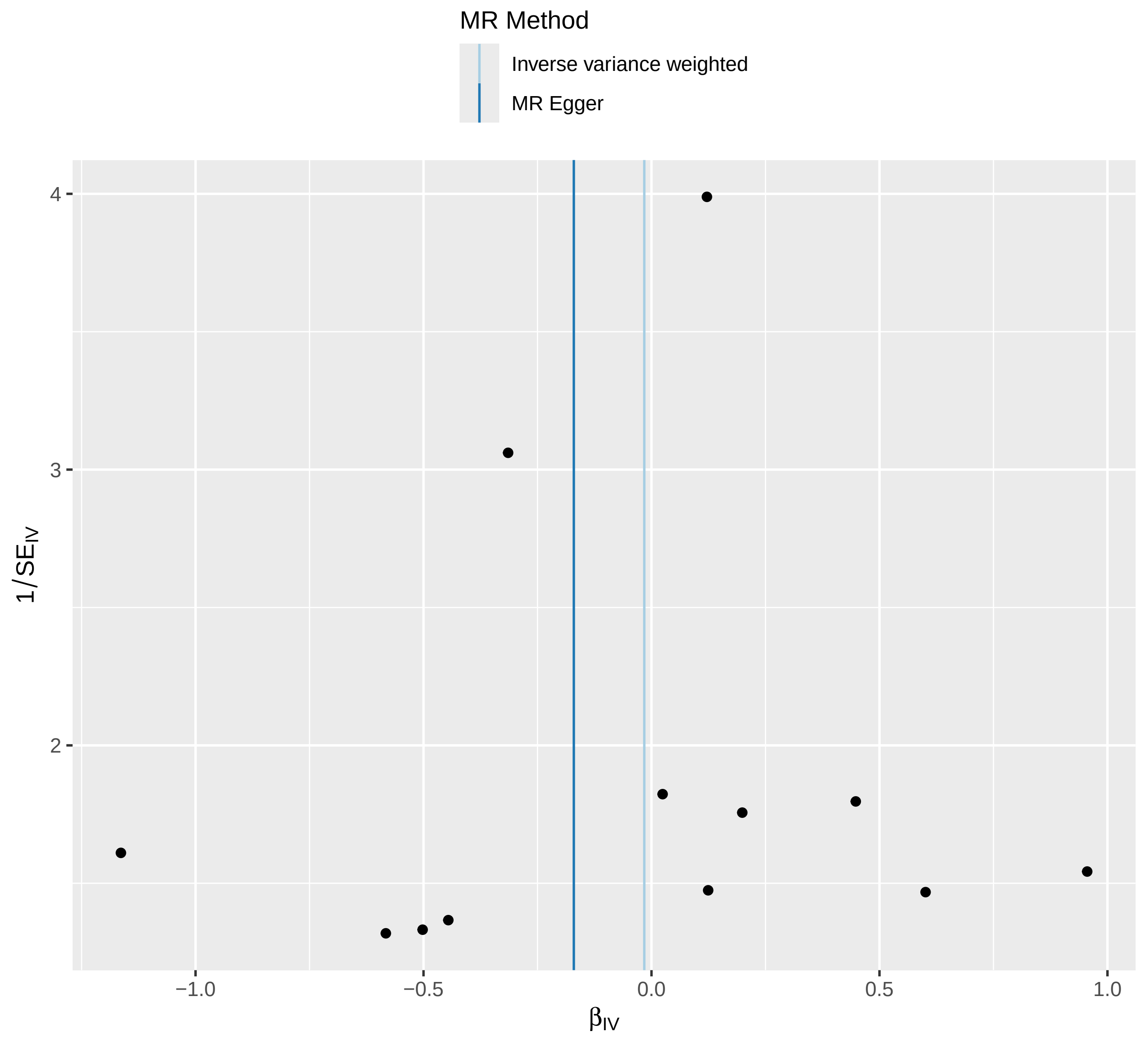

MR Method
Inverse variance weighted
MR Egger
4
3
2
1/SE$_{IV}$
−1.0
−0.5
0.0
0.5
1.0
β$_{IV}$

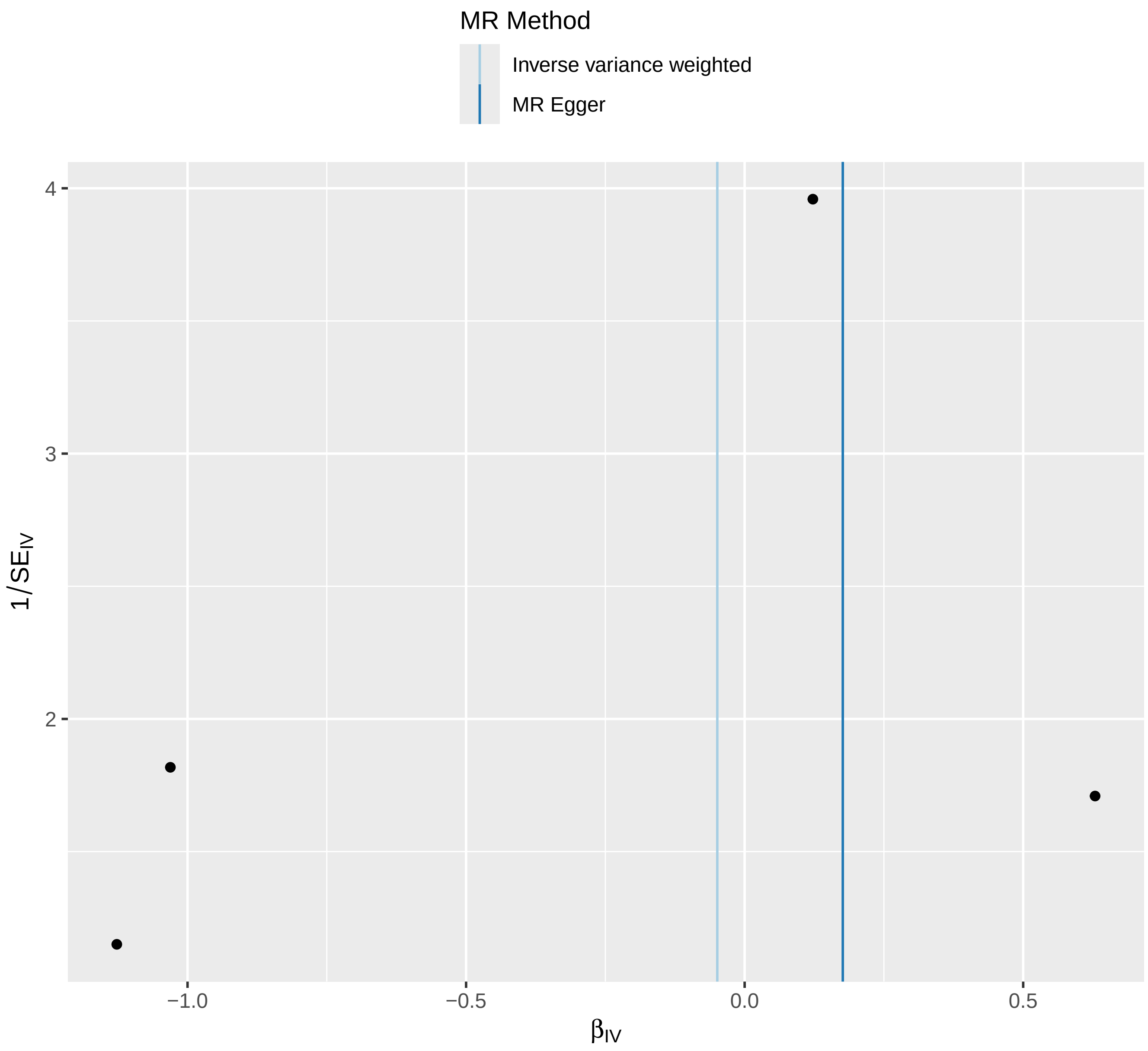
MR Method
Inverse variance weighted
MR Egger
4
3
2
1/SE$_{IV}$
−1.0
−0.5
0.0
0.5
β$_{IV}$

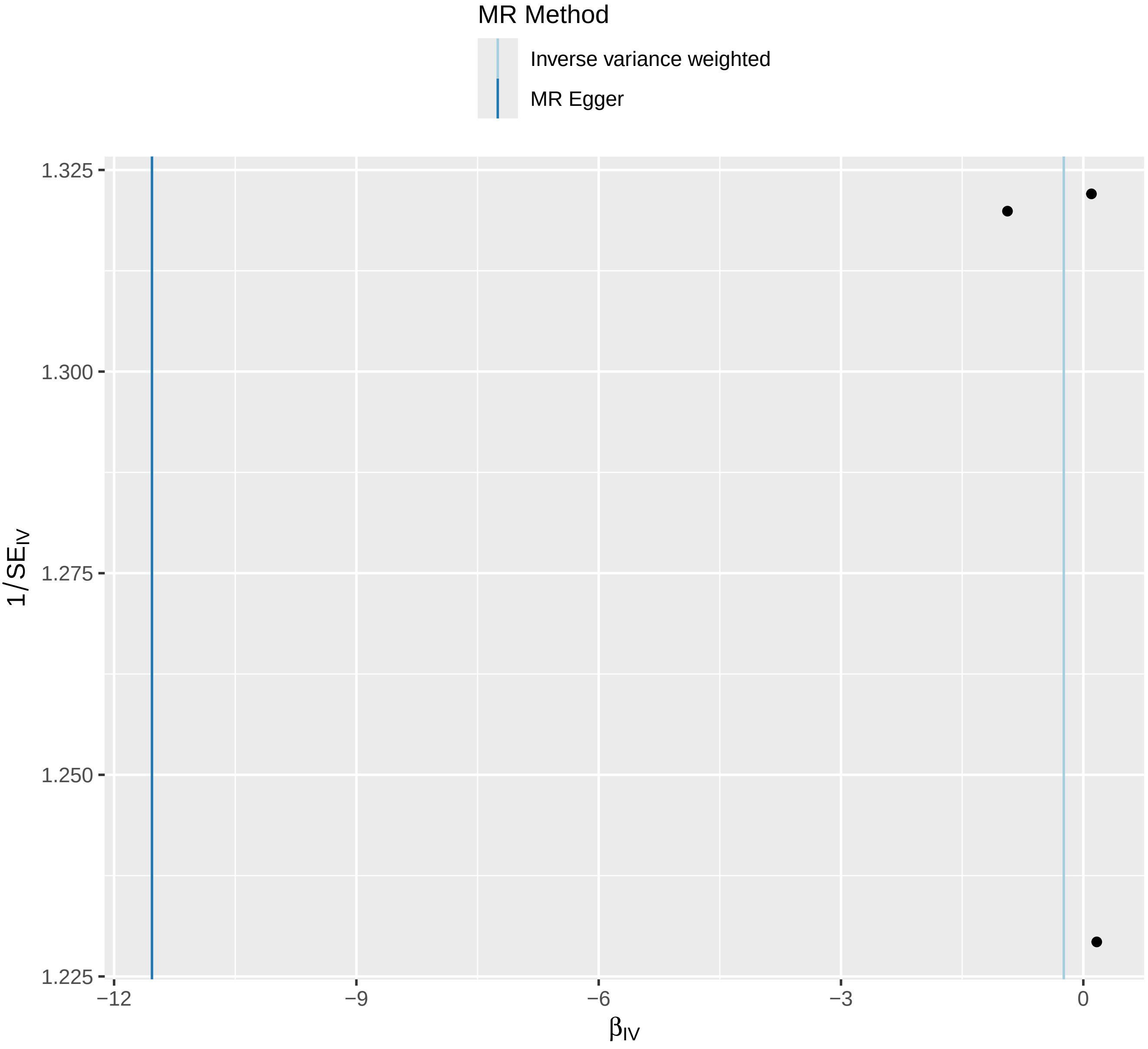

MR Method
Inverse variance weighted
MR Egger
1.325
1.300
1.275
1.250
1.225
1/SE_IV
−12
−9
−6
−3
0
β_IV

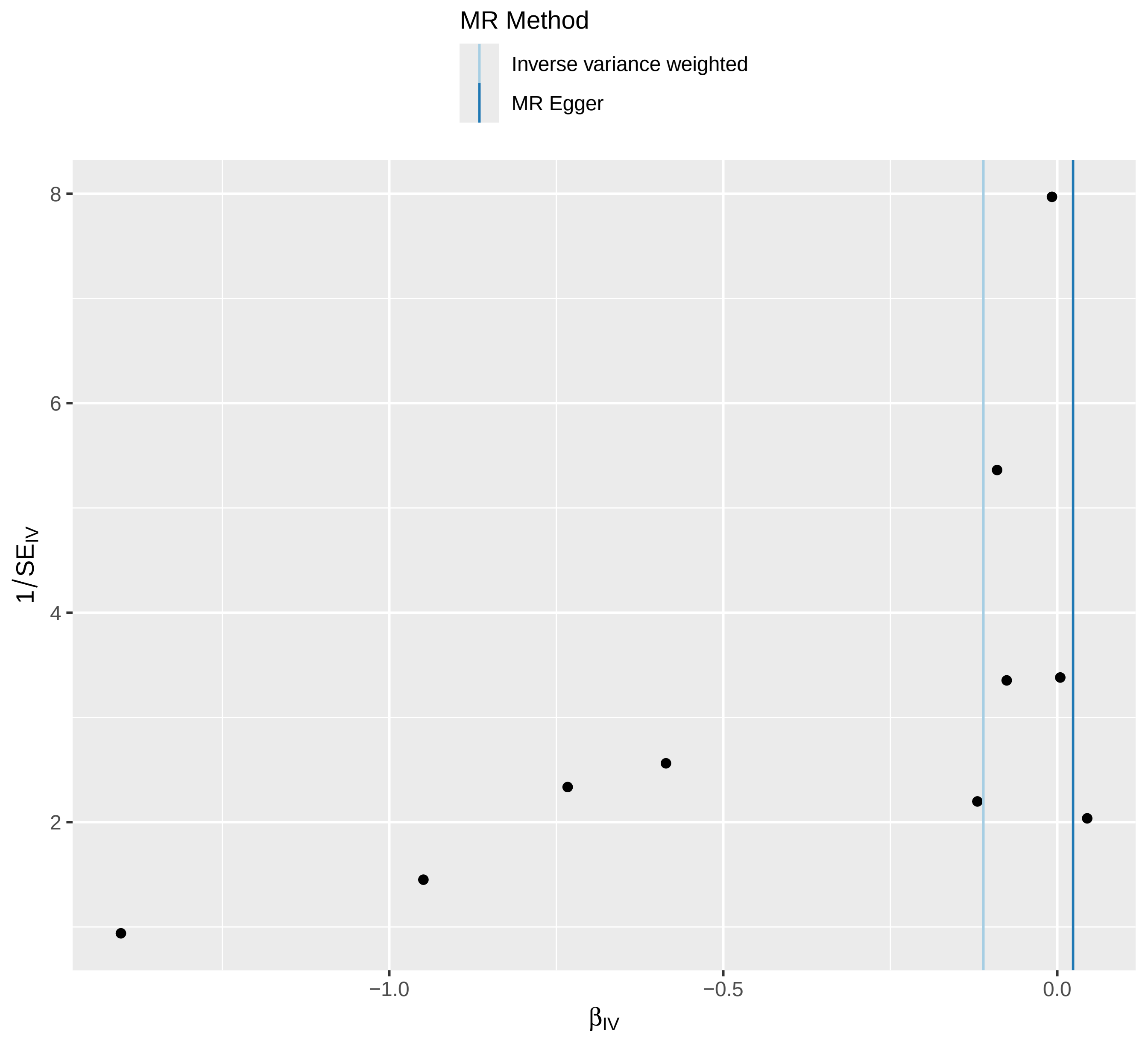

MR Method
Inverse variance weighted
MR Egger
8
6
4
2
1/SE_IV
−1.0
−0.5
0.0
β_IV

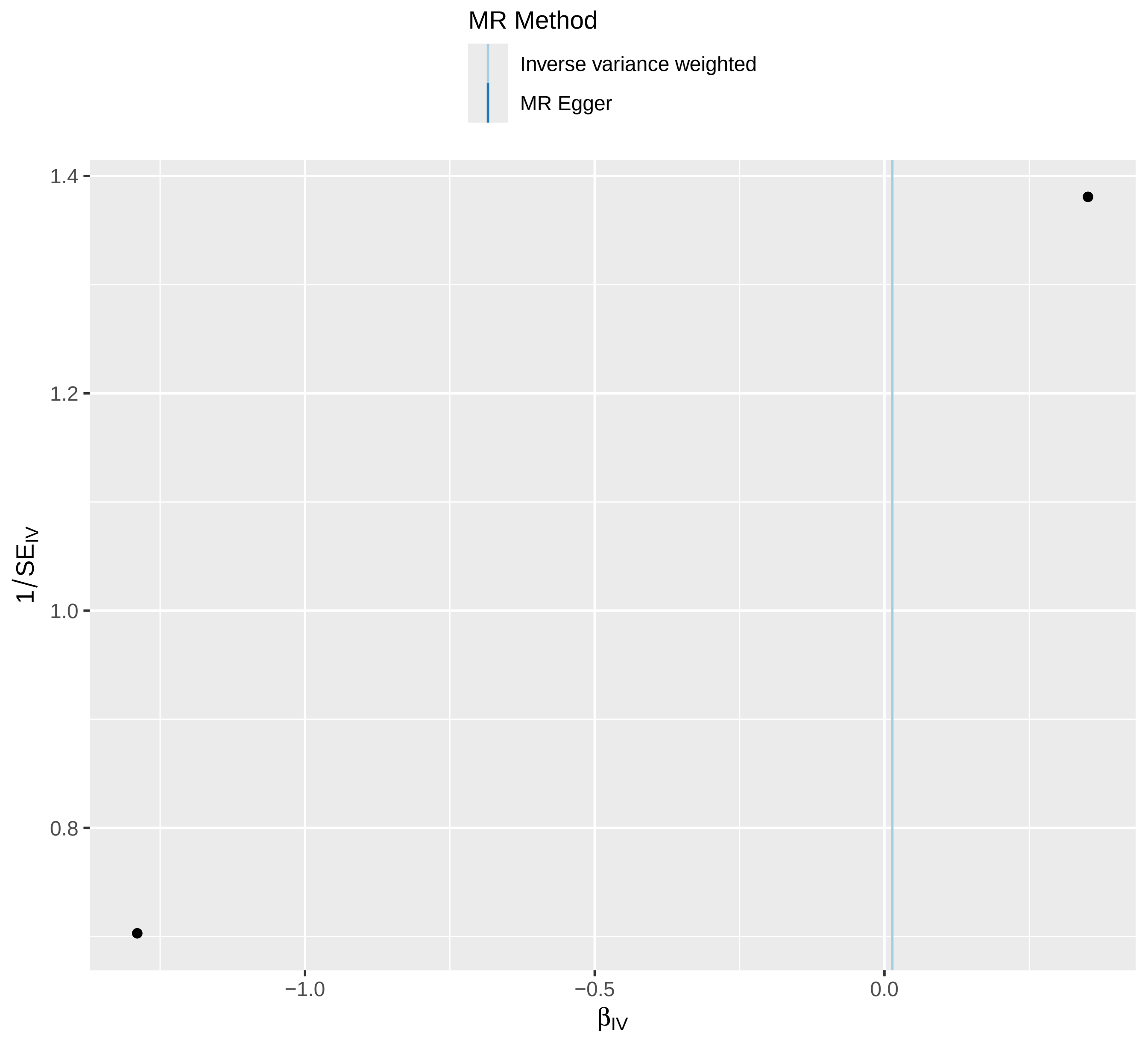
MR Method
Inverse variance weighted
MR Egger
1.4
1.2
1.0
0.8
1/SE_IV
−1.0
−0.5
0.0
β_IV

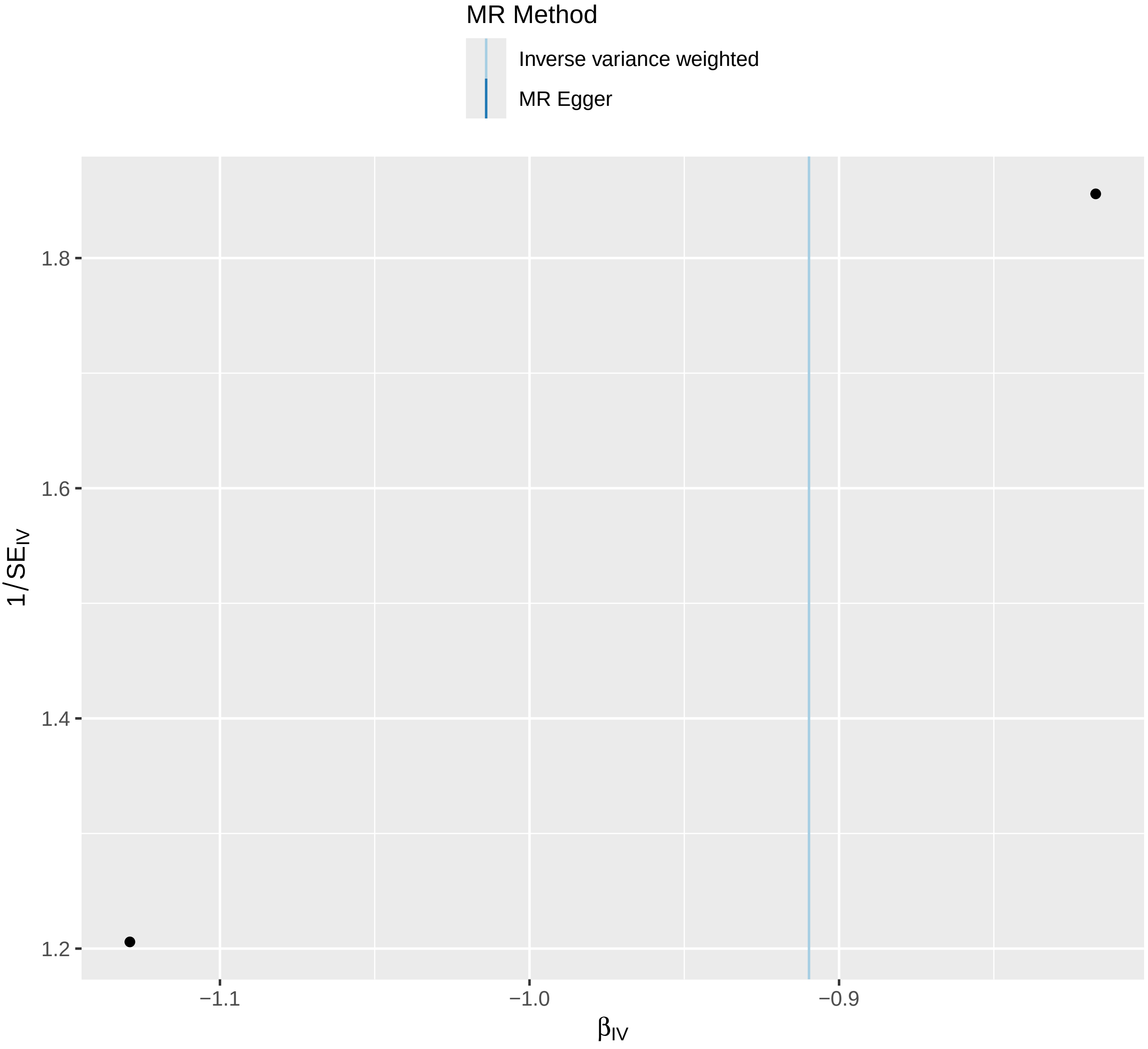

MR Method
Inverse variance weighted
MR Egger
1.8
1.6
1.4
1.2
$1/SE_{IV}$
−1.1
−1.0
−0.9
β_{IV}

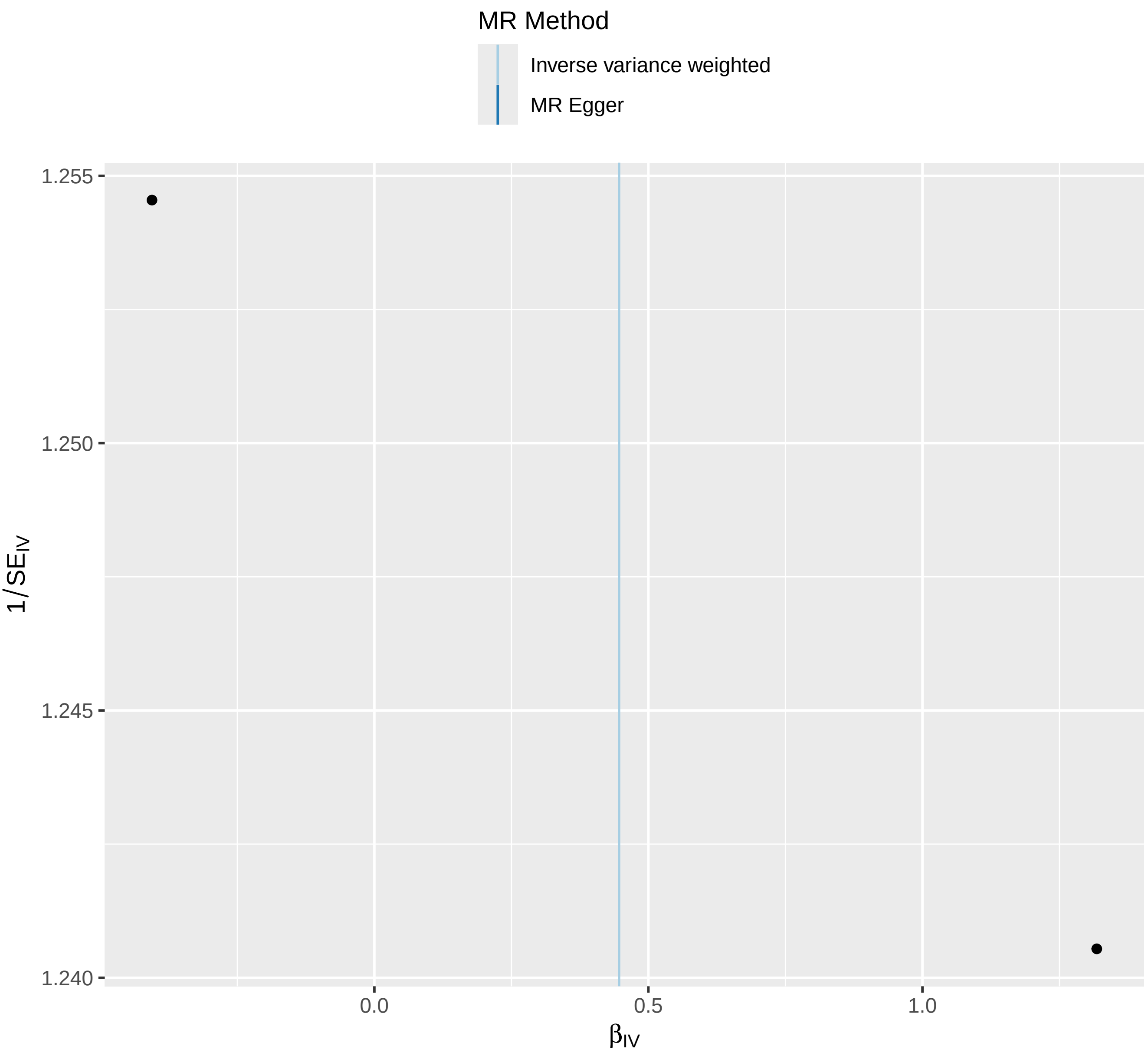

MR Method
Inverse variance weighted
MR Egger
1.255
1.250
1.245
1.240
1/SE$_{IV}$
0.0
0.5
1.0
β$_{IV}$

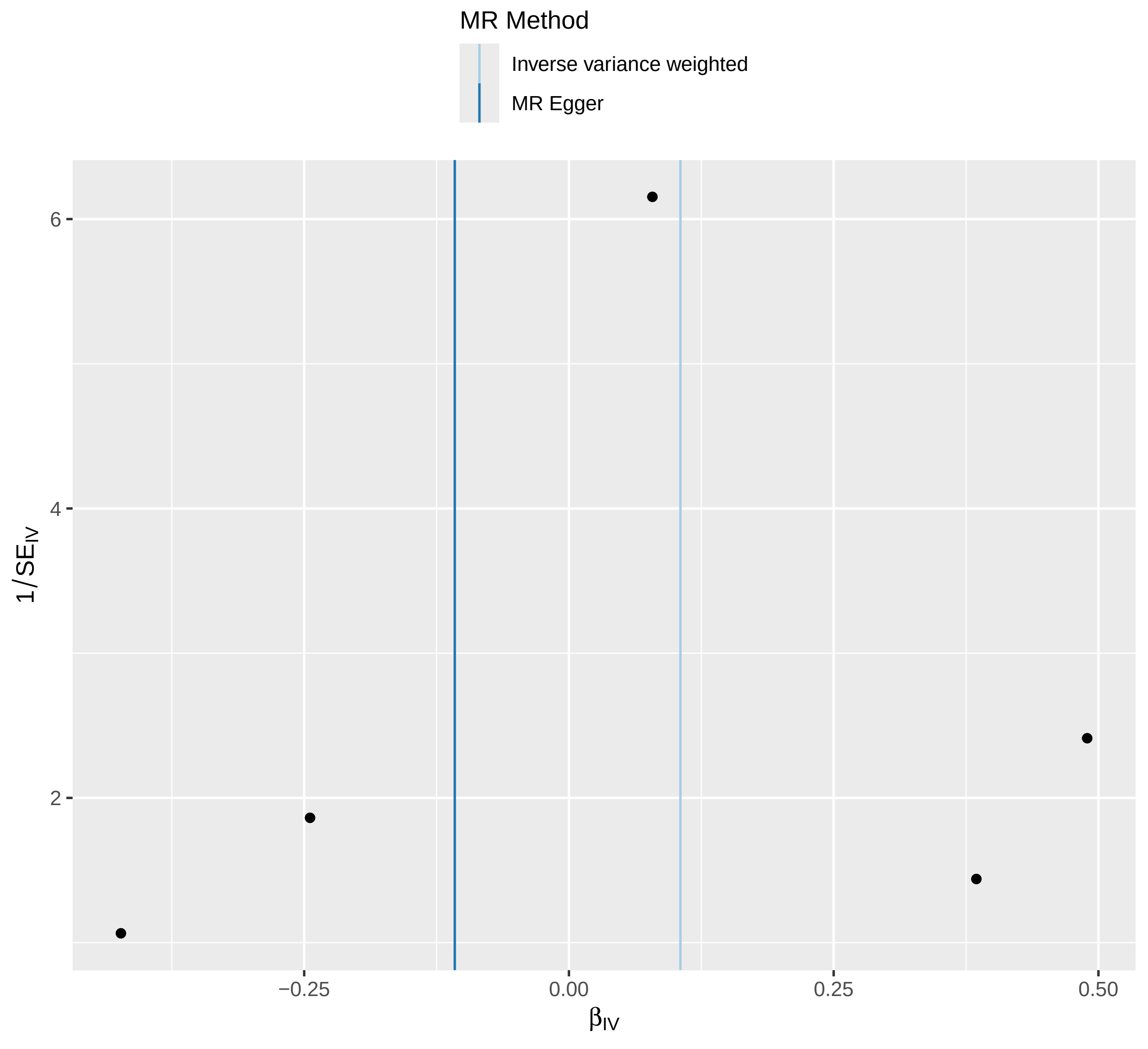
MR Method
Inverse variance weighted
MR Egger
6
4
2
1/SE_IV
−0.25
0.00
0.25
0.50
β_IV

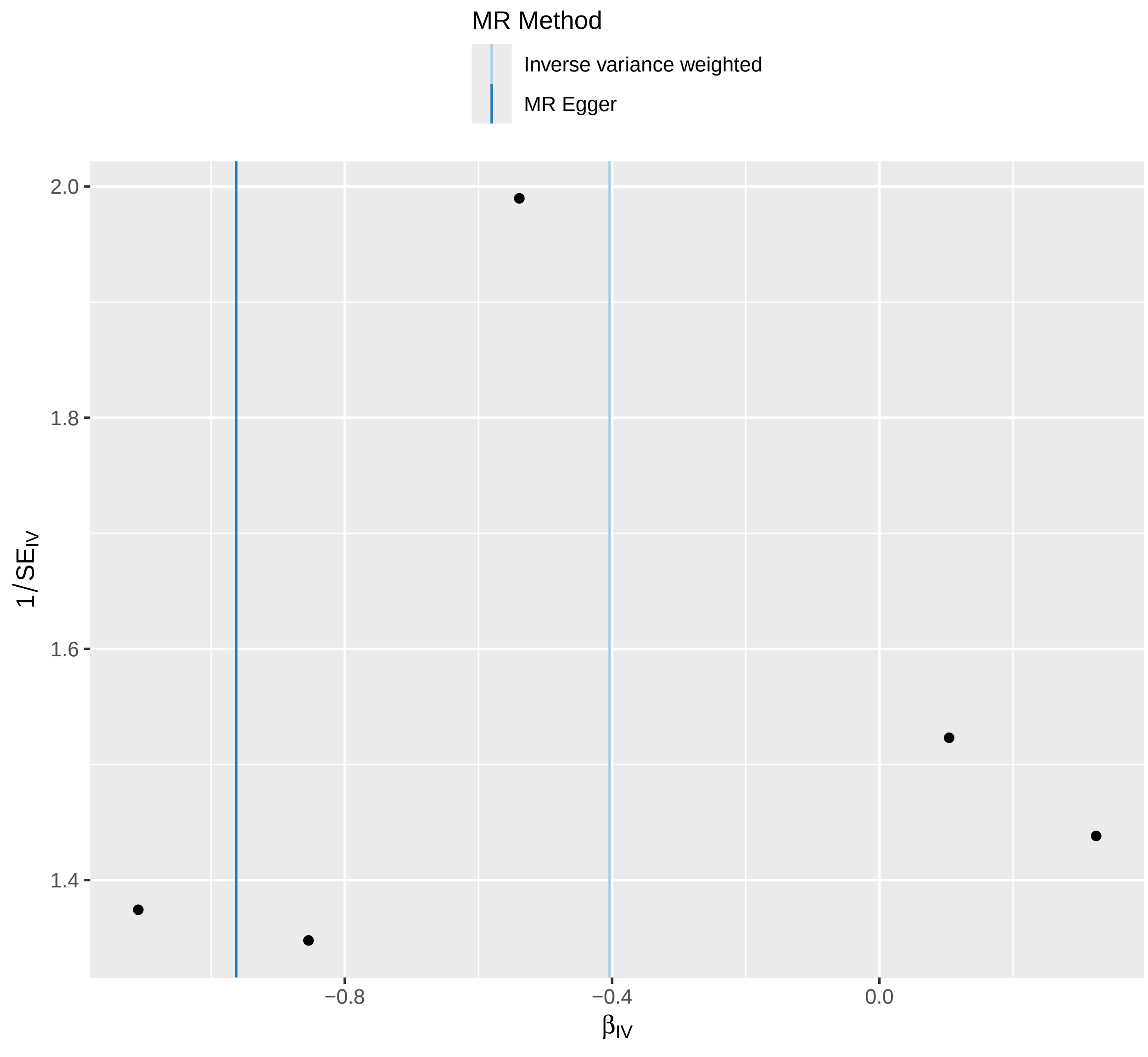
MR Method
Inverse variance weighted
MR Egger
2.0
1.8
1.6
1.4
1/SE_IV
−0.8
−0.4
0.0
β_IV

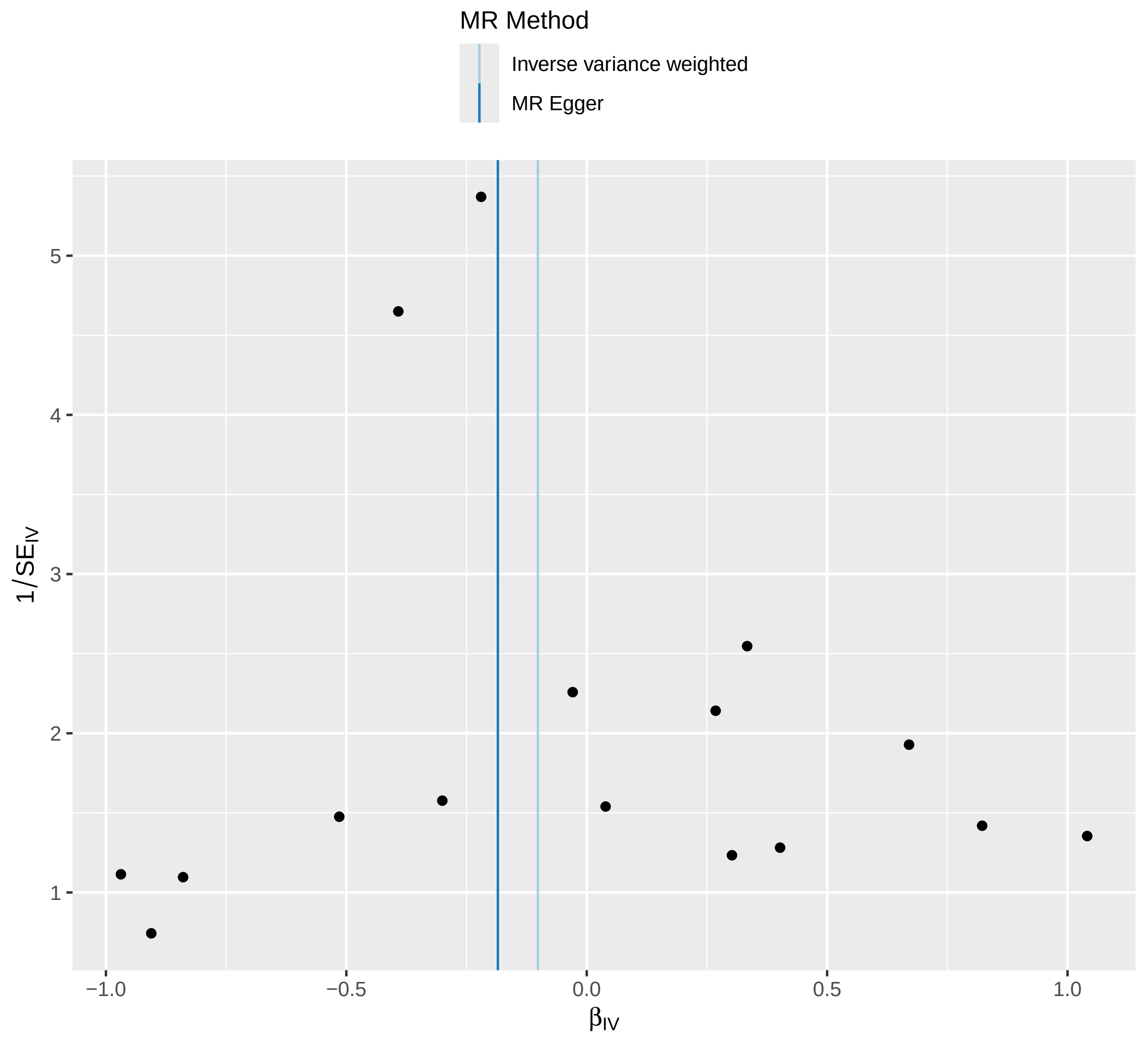
MR Method
Inverse variance weighted
MR Egger
1/SE_IV
5
4
3
2
1
−1.0
−0.5
0.0
0.5
1.0
β_IV

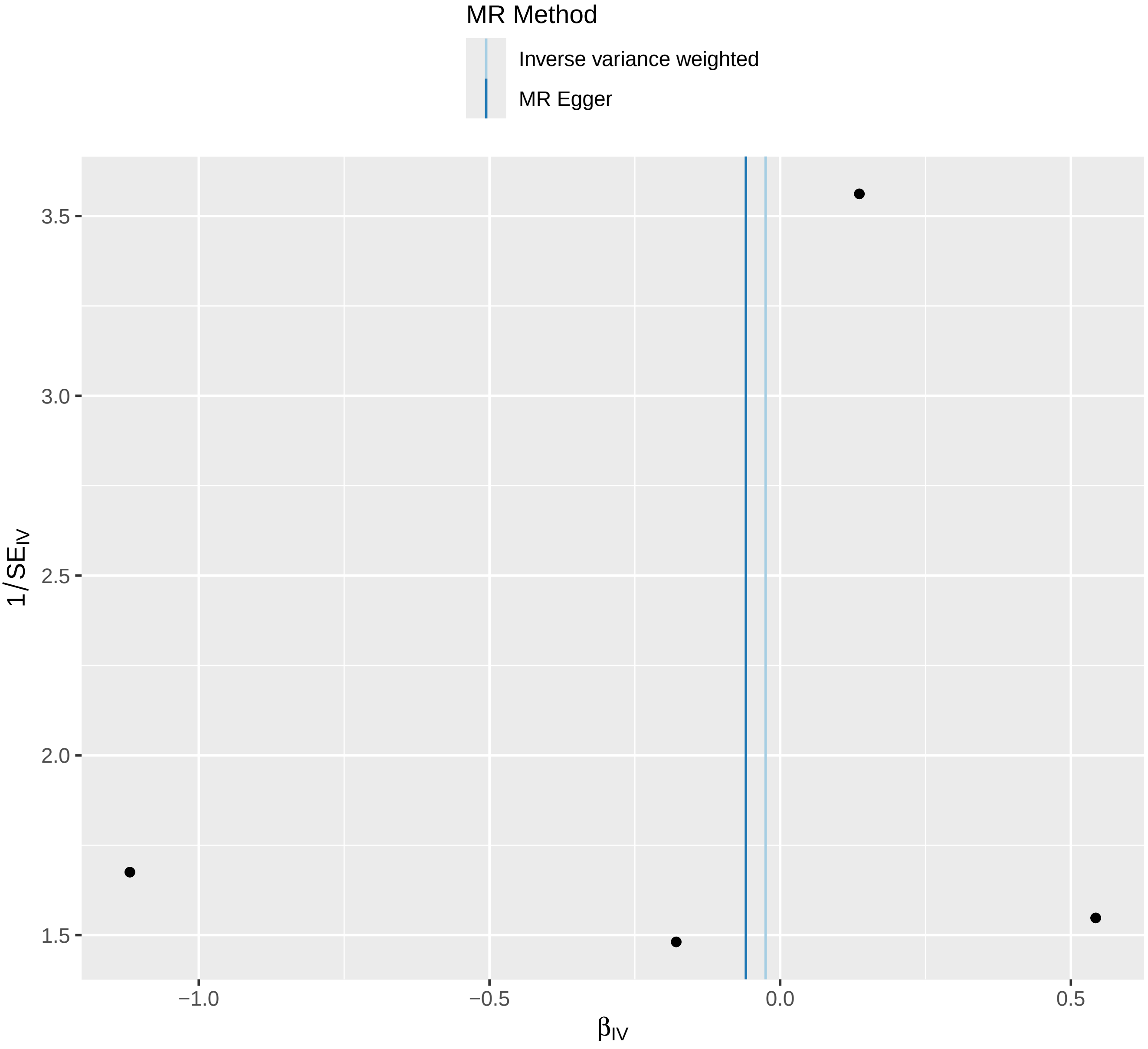

MR Method
Inverse variance weighted
MR Egger
3.5
3.0
2.5
2.0
1.5
1/SE_IV
−1.0
−0.5
0.0
0.5
β_IV

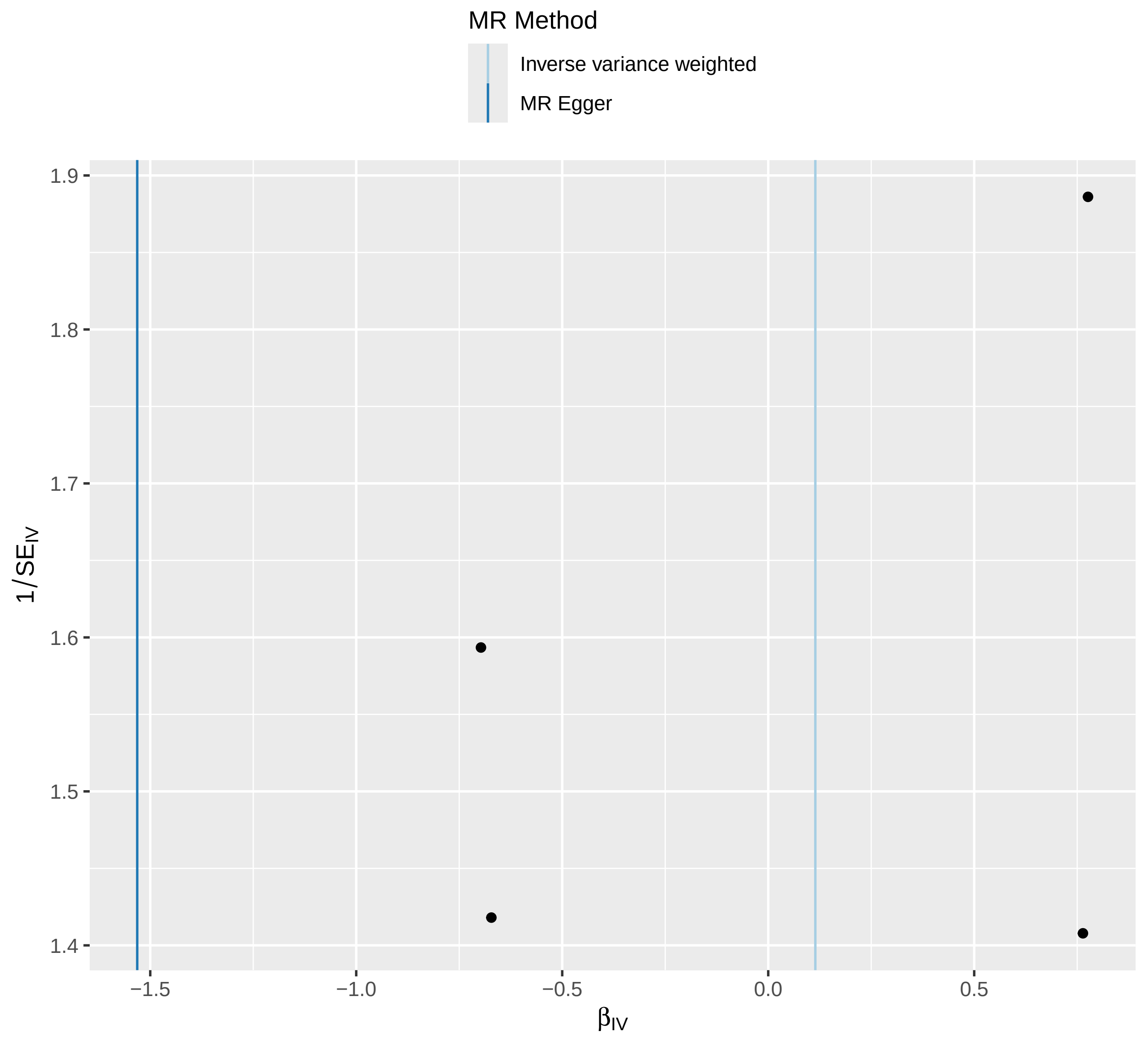

MR Method
Inverse variance weighted
MR Egger
1.9
1.8
1.7
1.6
1.5
1.4
1/SE_IV
−1.5
−1.0
−0.5
0.0
0.5
β_IV

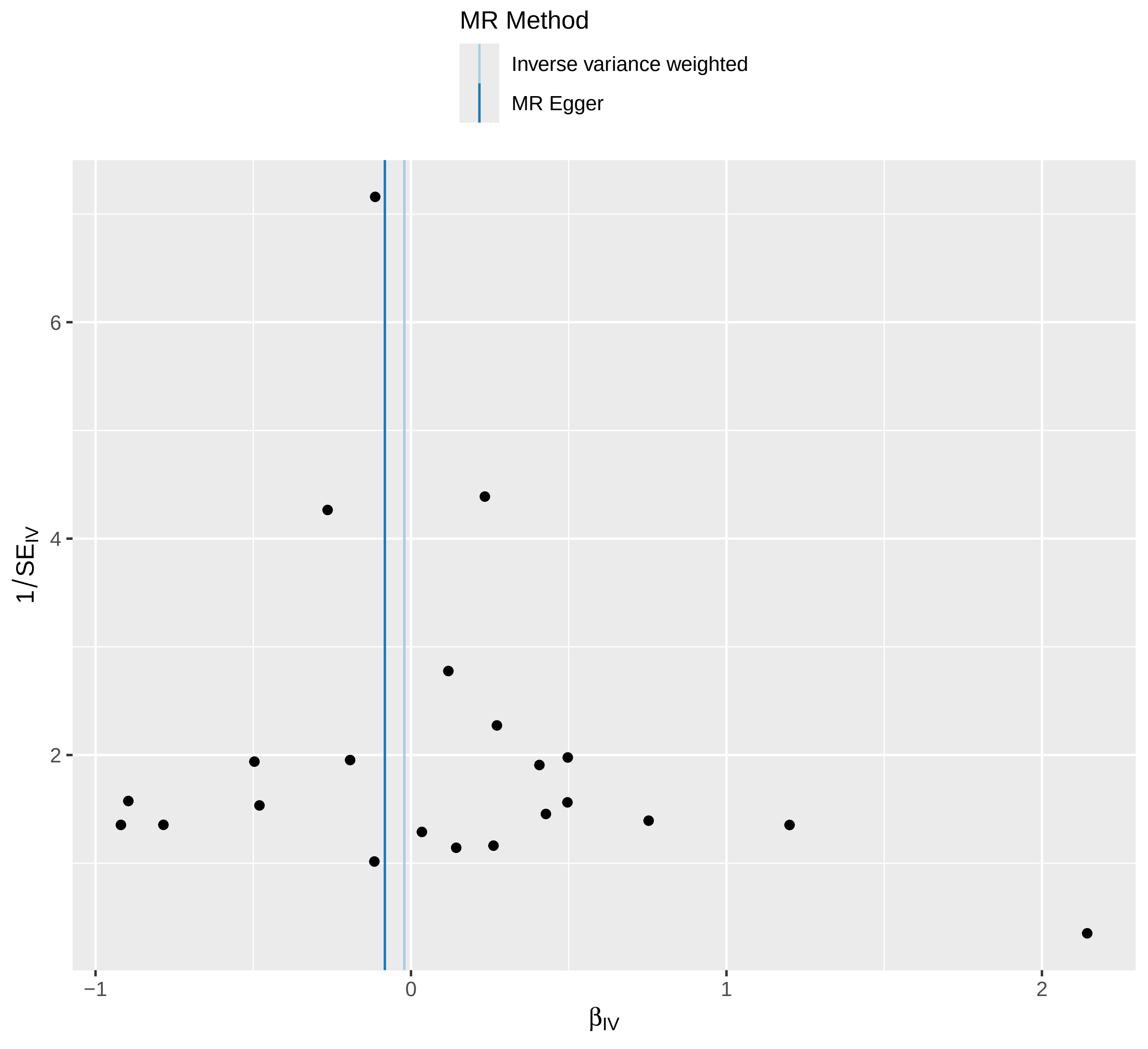

MR Method
Inverse variance weighted
MR Egger
6
4
2
1/SE$_{IV}$
−1
0
1
2
β$_{IV}$

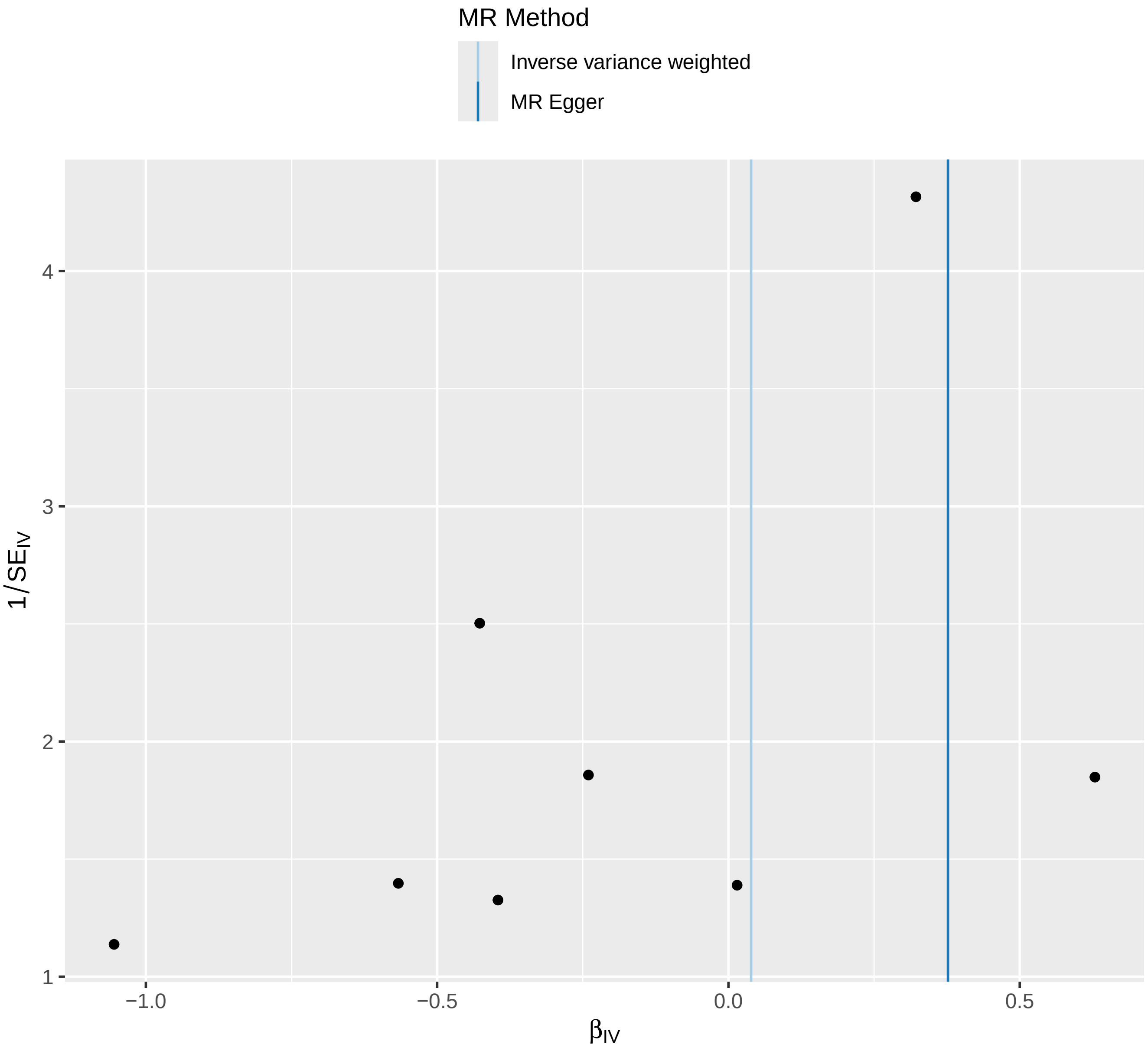

MR Method
Inverse variance weighted
MR Egger
4
3
2
1
1/SE_IV
−1.0
−0.5
0.0
0.5
β_IV

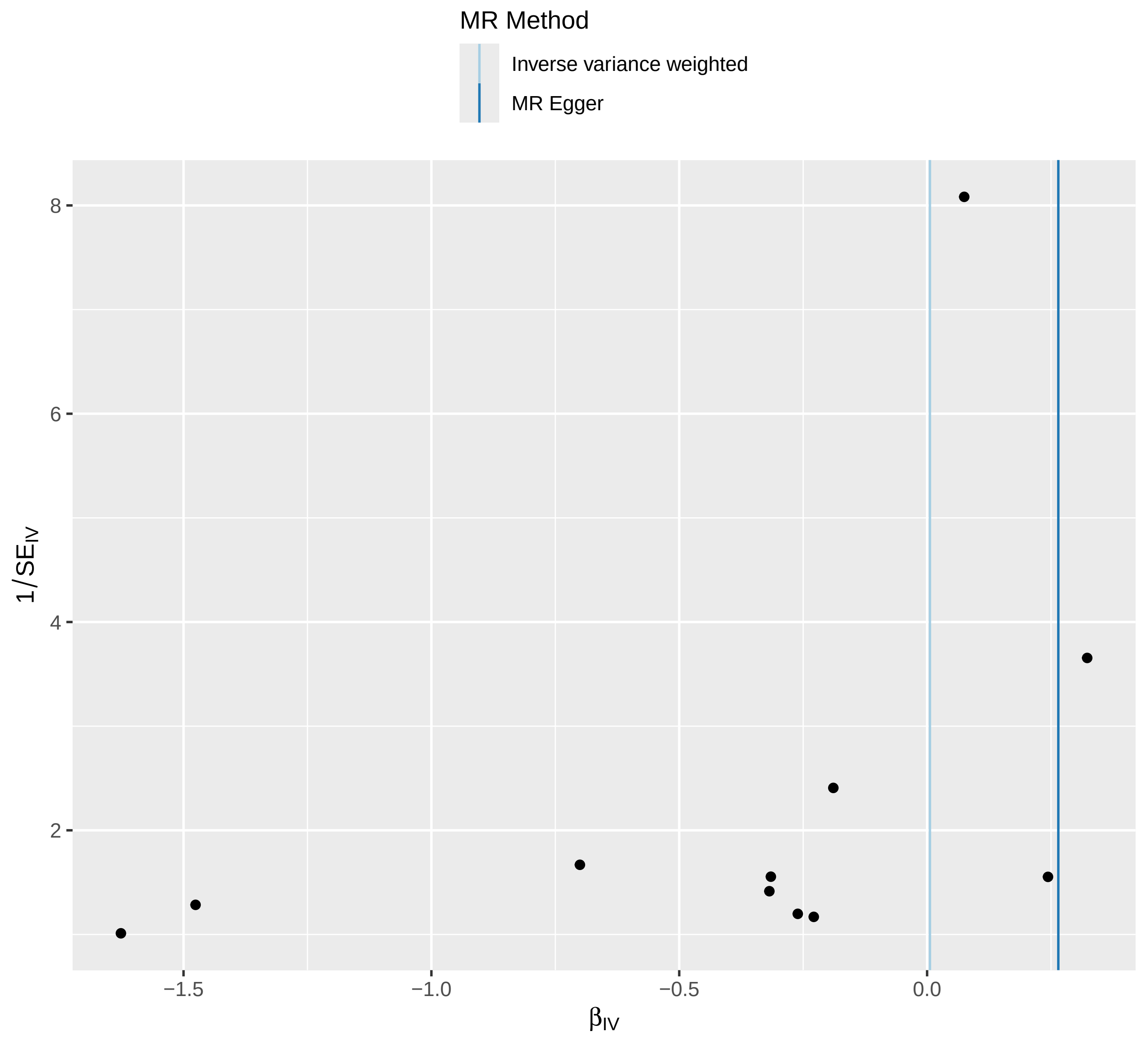
MR Method
Inverse variance weighted
MR Egger
8
6
4
2
1/SE_IV
−1.5
−1.0
−0.5
0.0
β_IV

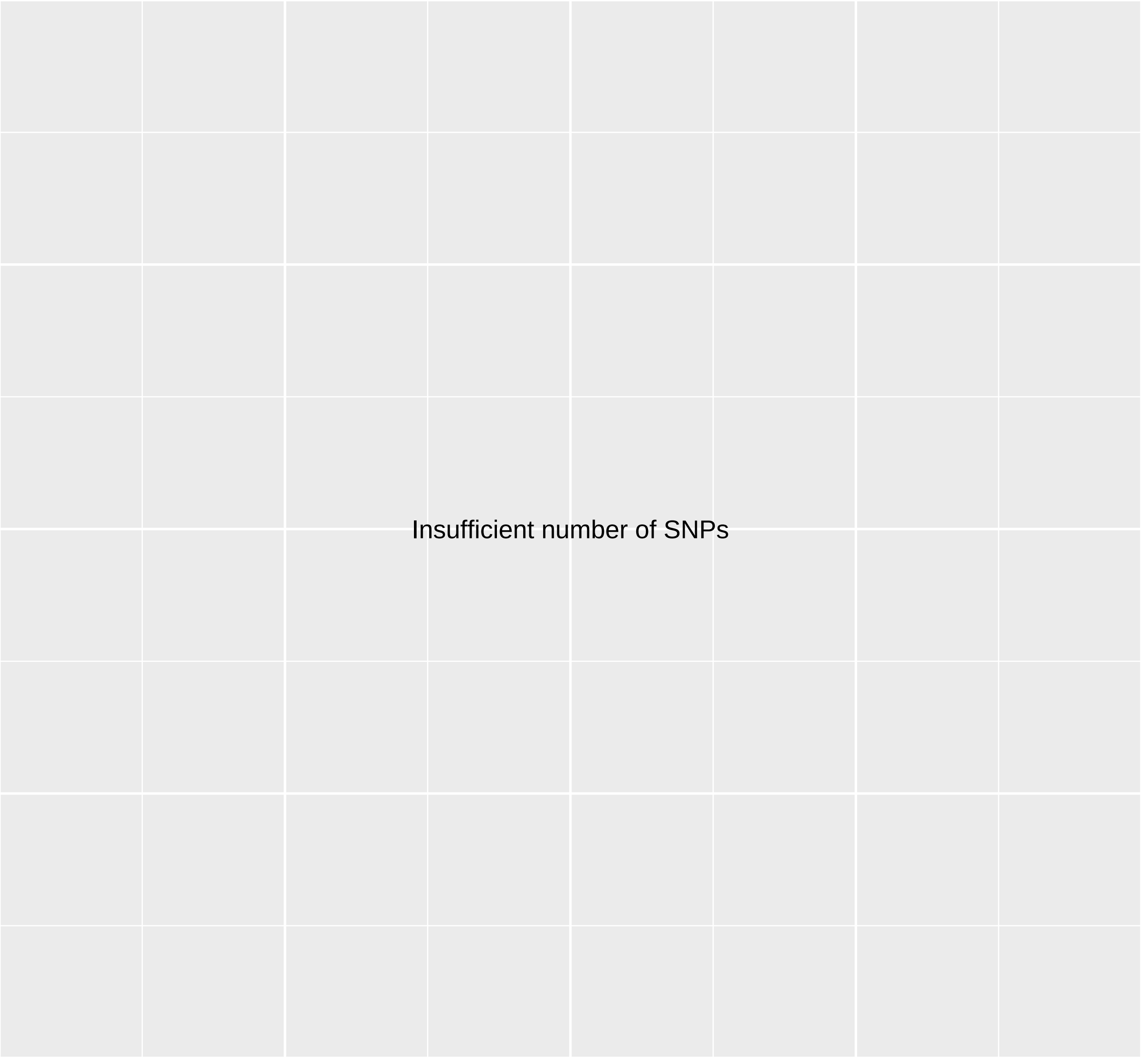
Insufficient number of SNPs

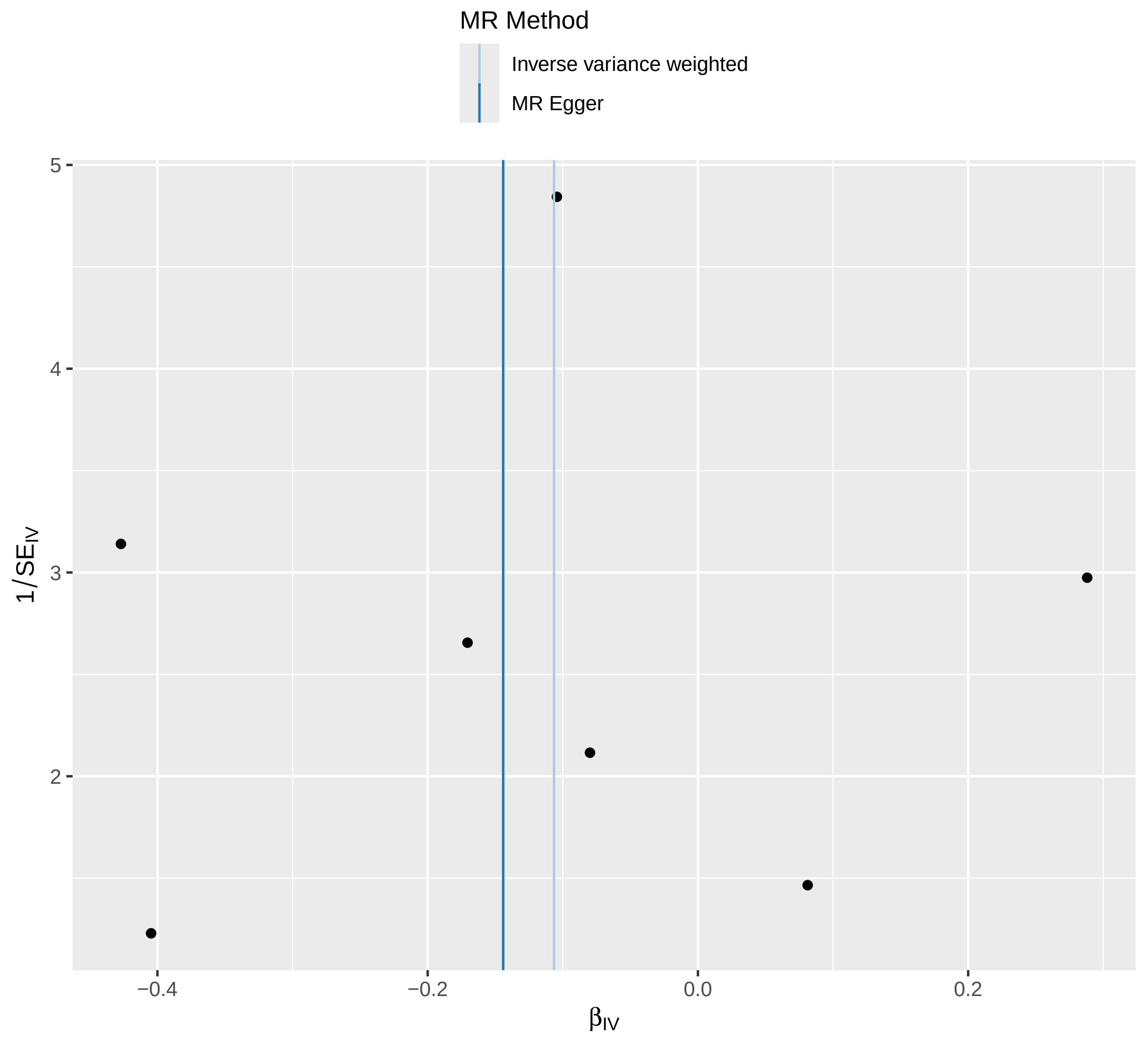
MR Method
Inverse variance weighted
MR Egger
5
4
3
2
1/SE_IV
−0.4
−0.2
0.0
0.2
β_IV

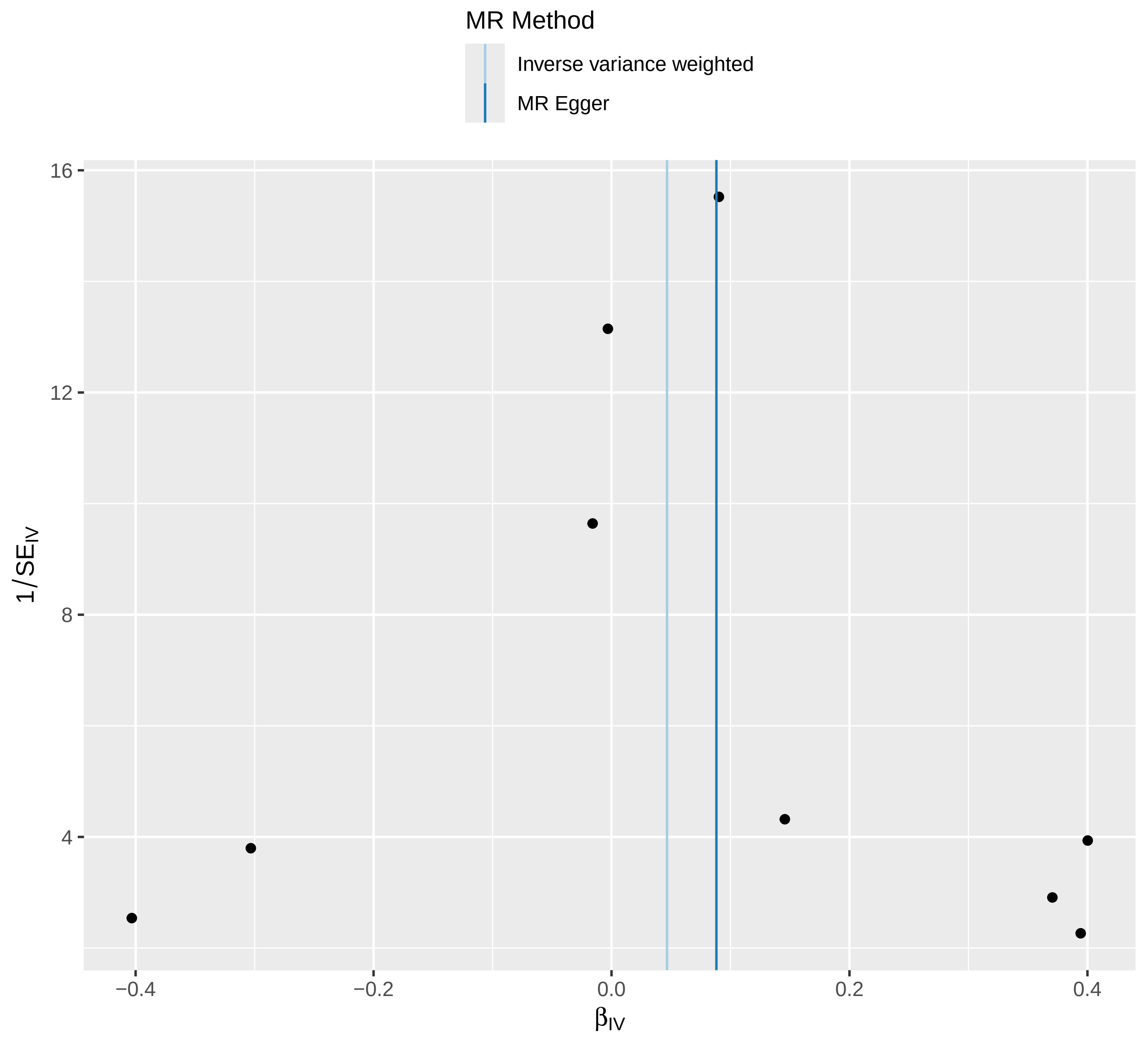

MR Method
Inverse variance weighted
MR Egger
16
12
8
4
1/SE_IV
−0.4
−0.2
0.0
0.2
0.4
β_IV

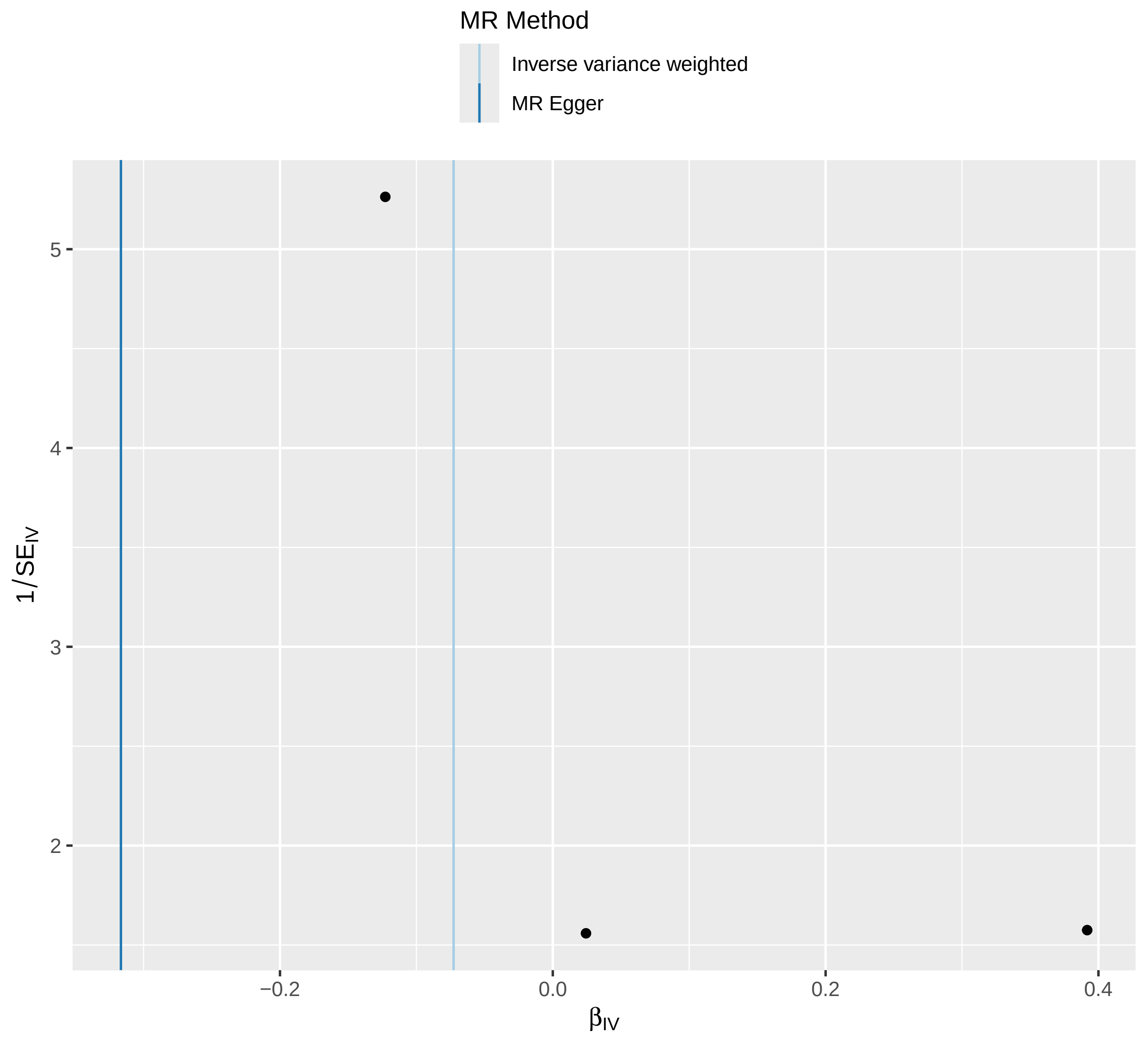
MR Method
Inverse variance weighted
MR Egger
5
4
3
2
1/SE_IV
−0.2
0.0
0.2
0.4
β_IV

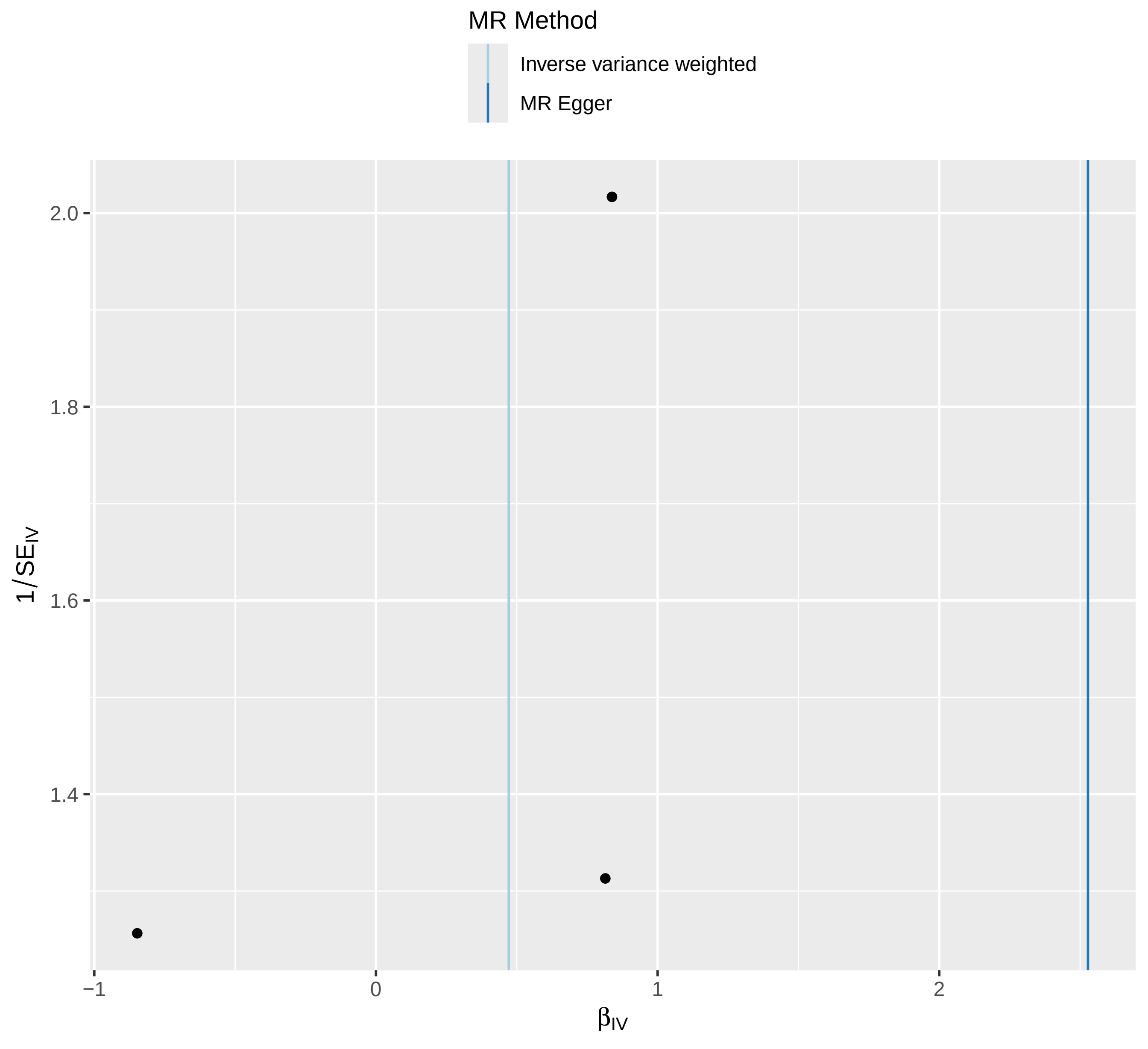

MR Method
Inverse variance weighted
MR Egger
2.0
1.8
1.6
1.4
1/SE_IV
−1
0
1
2
β_IV

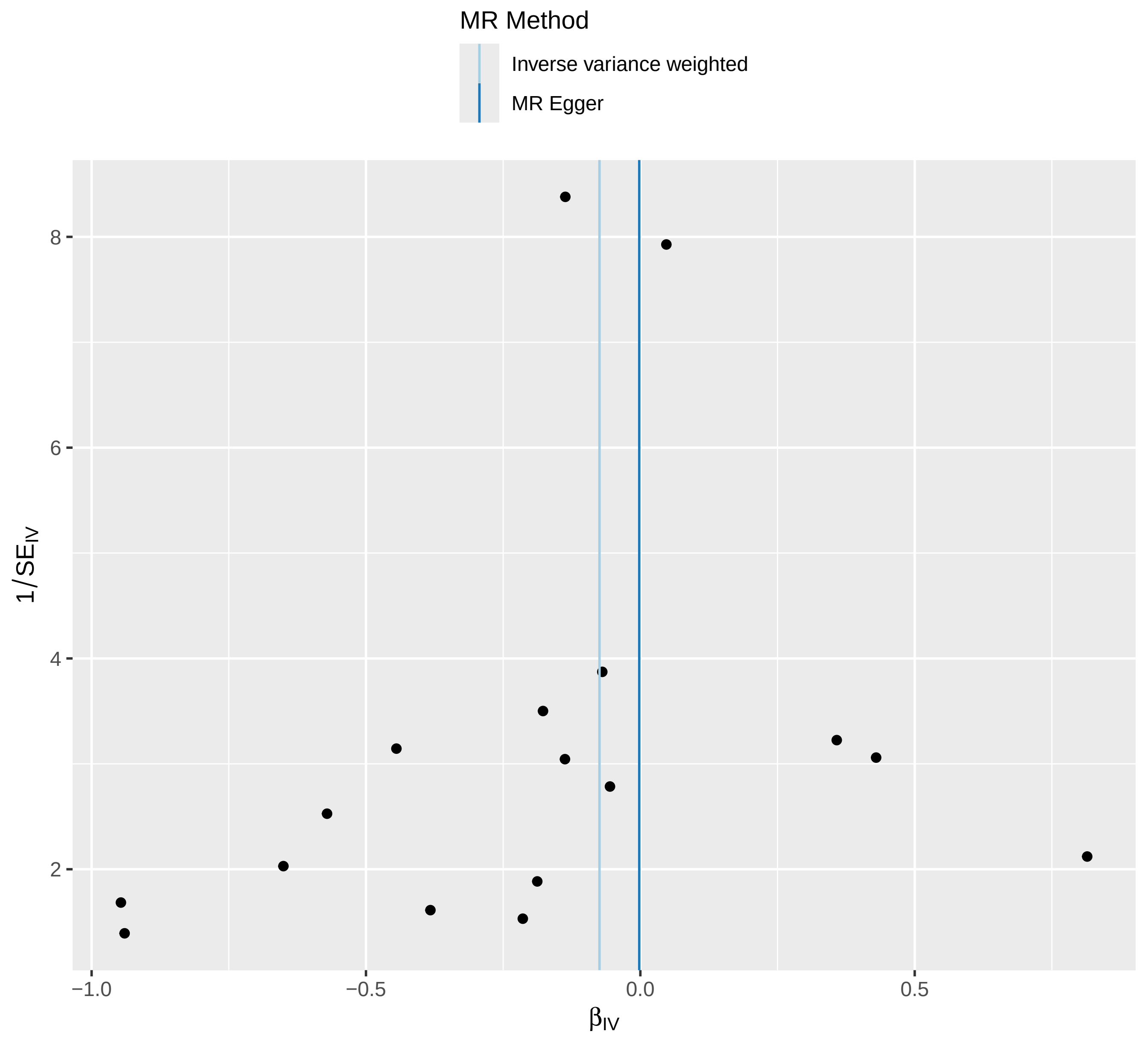

MR Method
Inverse variance weighted
MR Egger
1/SE$_{IV}$
8
6
4
2
−1.0
−0.5
0.0
0.5
β$_{IV}$

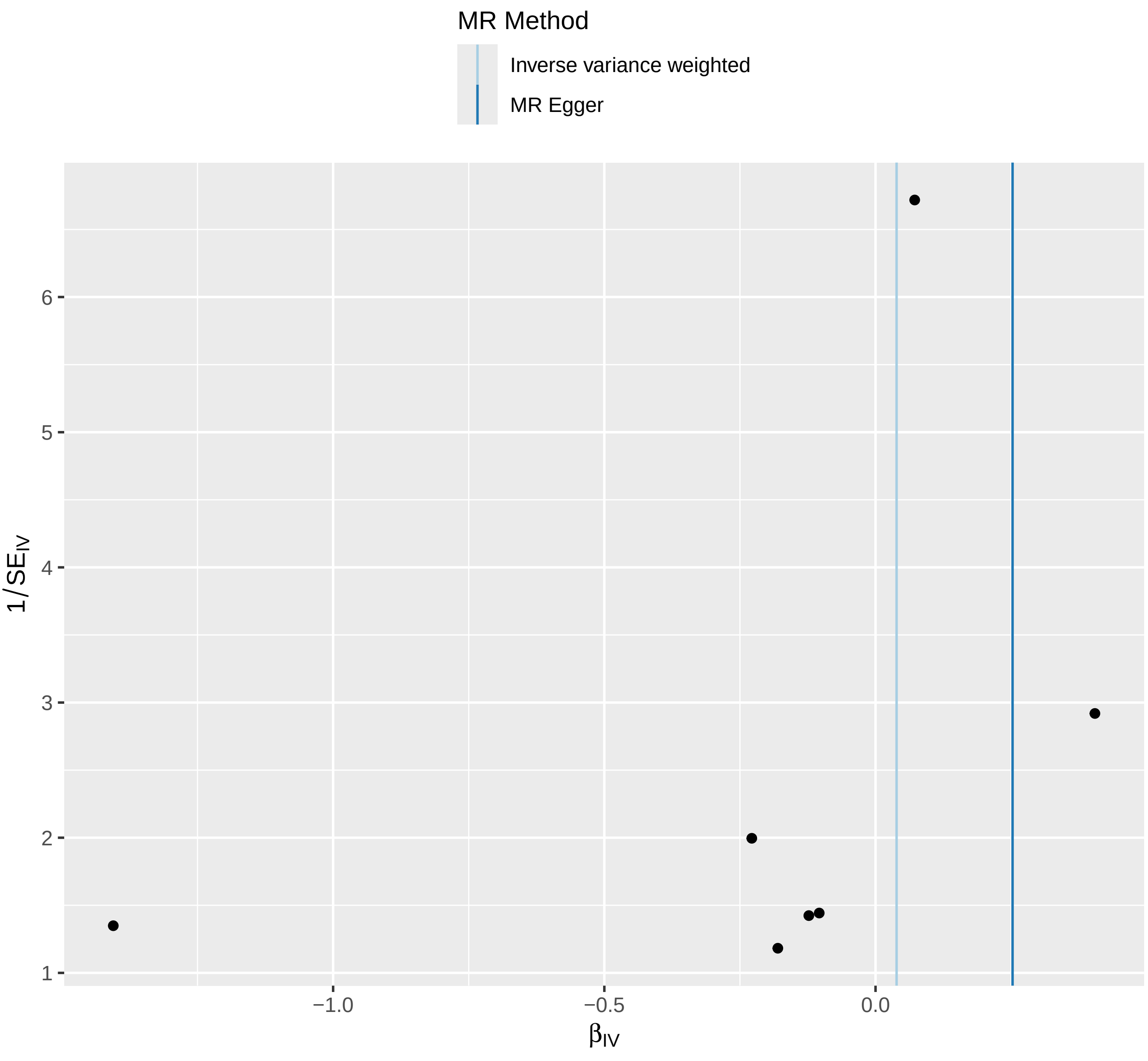
MR Method
Inverse variance weighted
MR Egger
1/SE_IV
β_IV
6
5
4
3
2
1
−1.0
−0.5
0.0

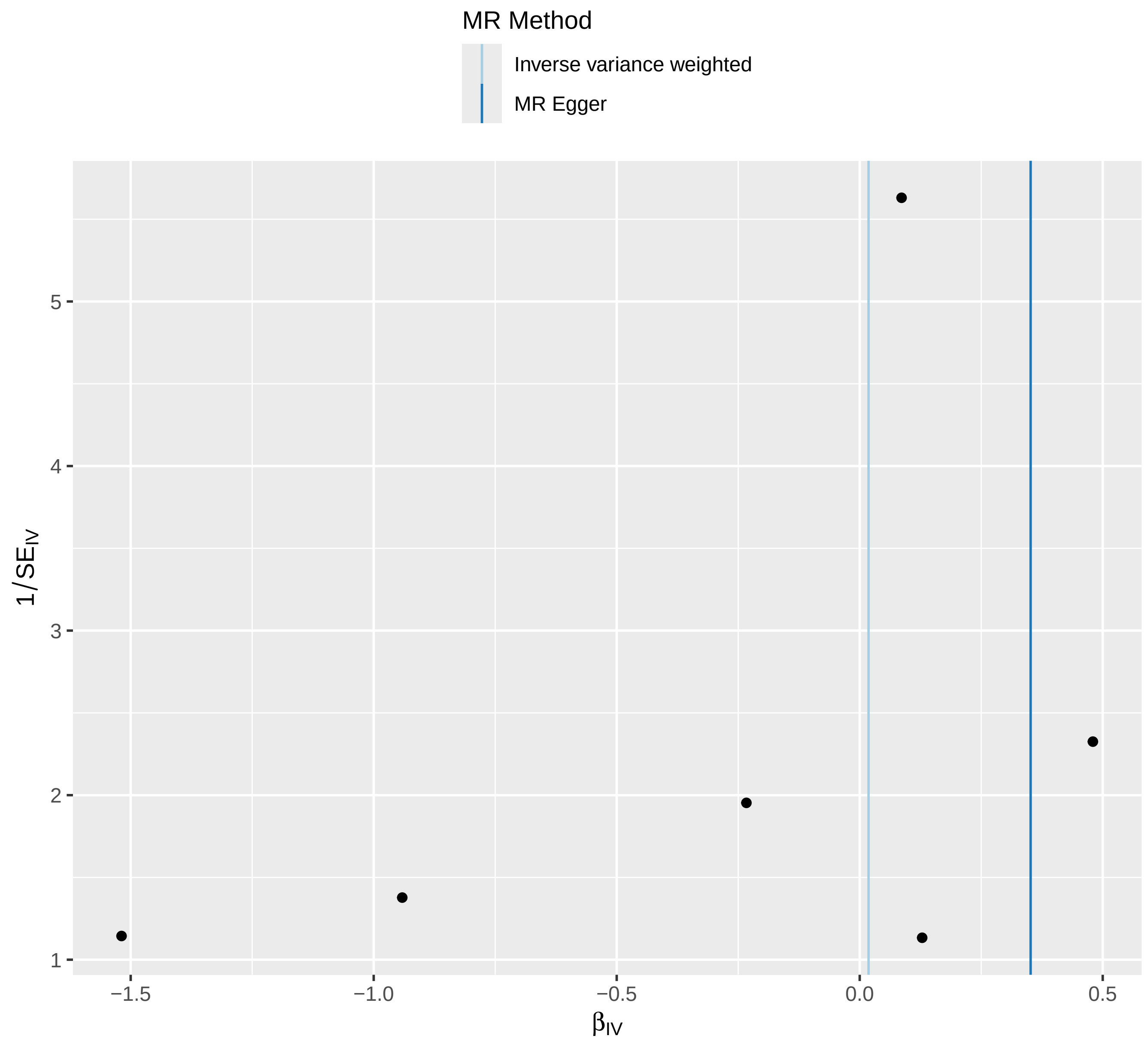

MR Method
Inverse variance weighted
MR Egger
1/SE$_{IV}$
5
4
3
2
1
−1.5
−1.0
−0.5
0.0
0.5
β$_{IV}$

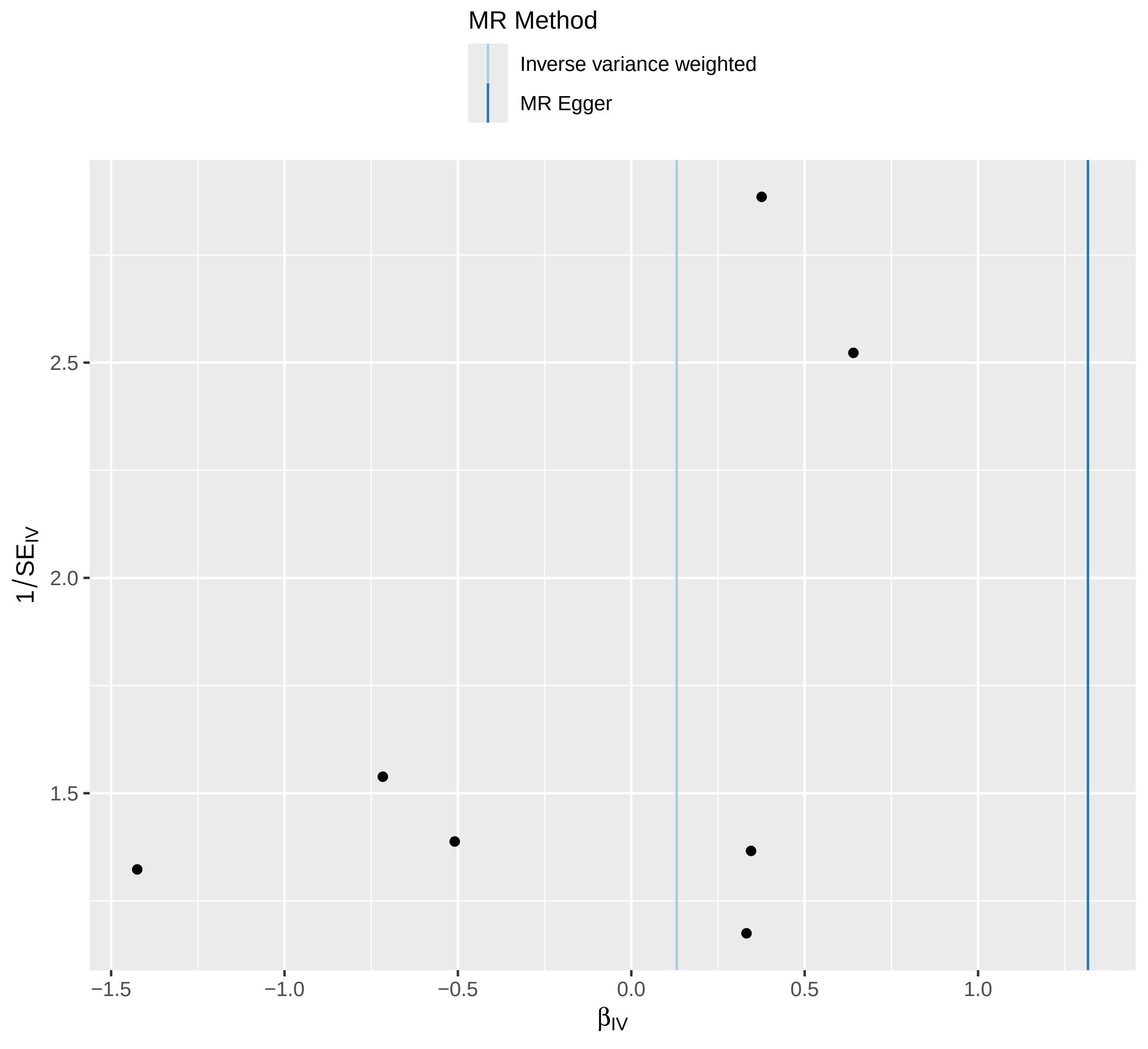
MR Method
Inverse variance weighted
MR Egger
2.5
2.0
1.5
1/SE$_{IV}$
−1.5
−1.0
−0.5
0.0
0.5
1.0
β$_{IV}$

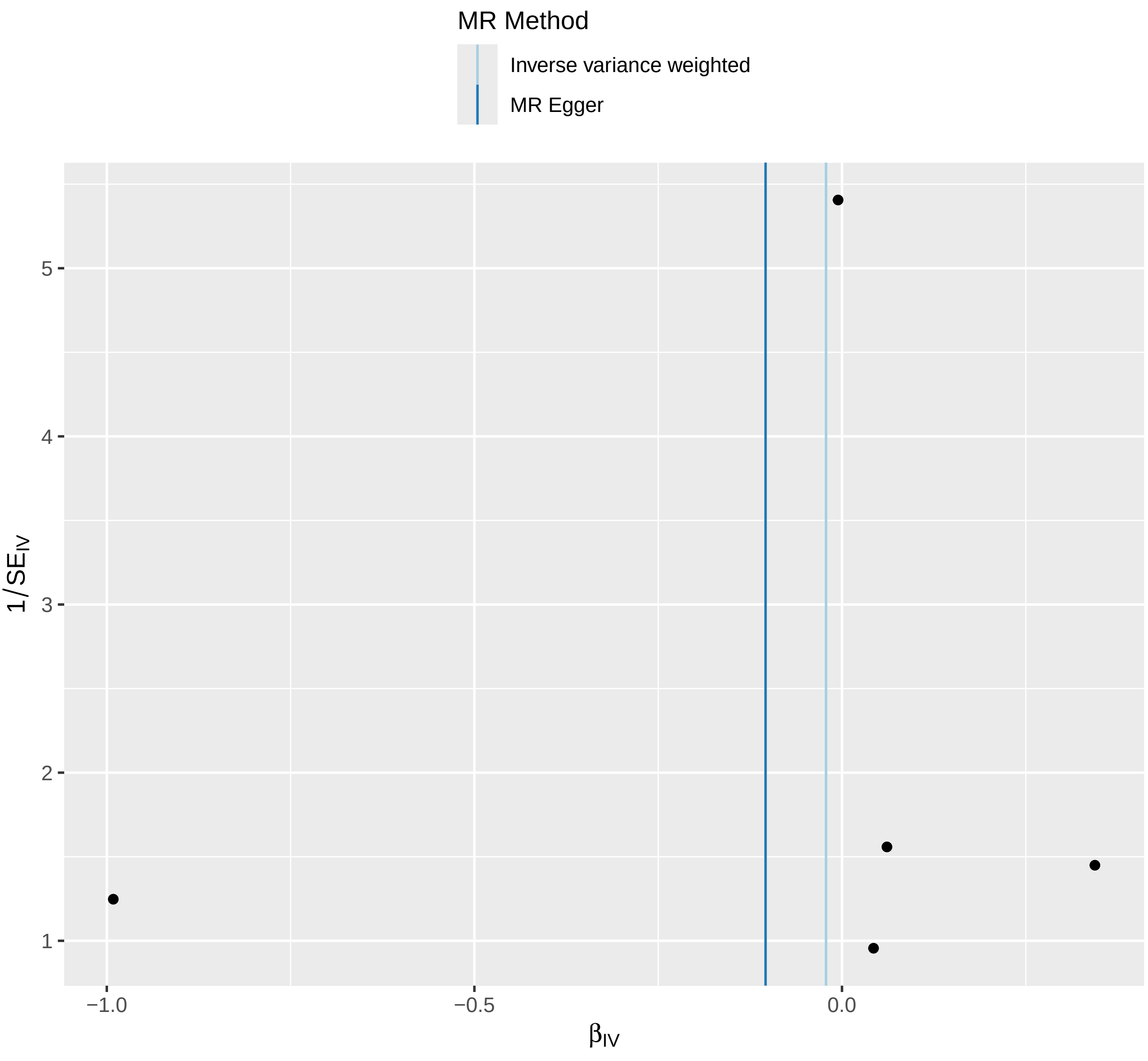

MR Method
Inverse variance weighted
MR Egger
5
4
3
2
1
1/SE_IV
−1.0
−0.5
0.0
β_IV

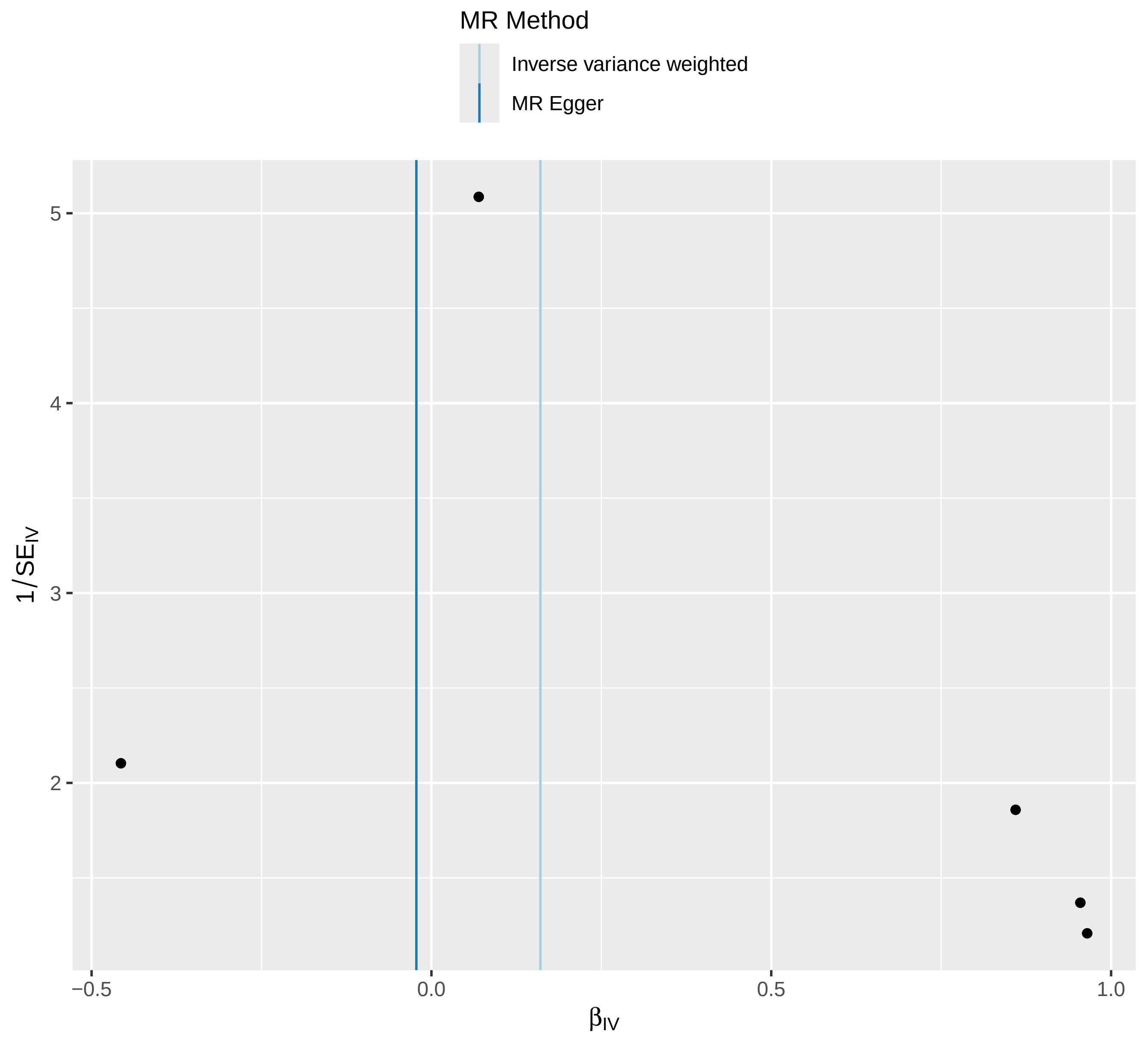
MR Method
Inverse variance weighted
MR Egger
5
4
3
2
1/SE_IV
−0.5
0.0
0.5
1.0
β_IV

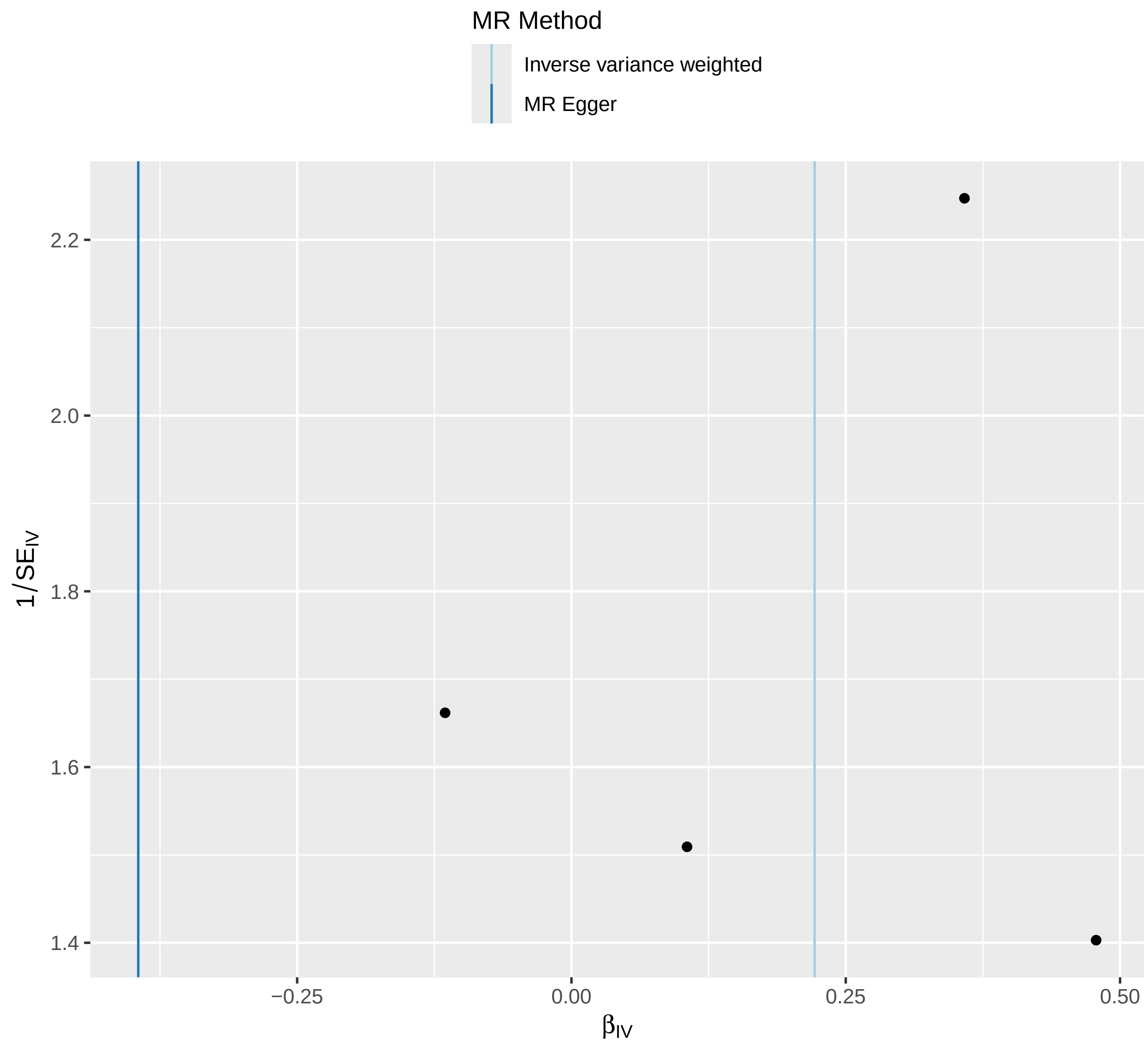
MR Method
Inverse variance weighted
MR Egger
2.2
2.0
1.8
1.6
1.4
$1/SE_{IV}$
−0.25
0.00
0.25
0.50
β_{IV}

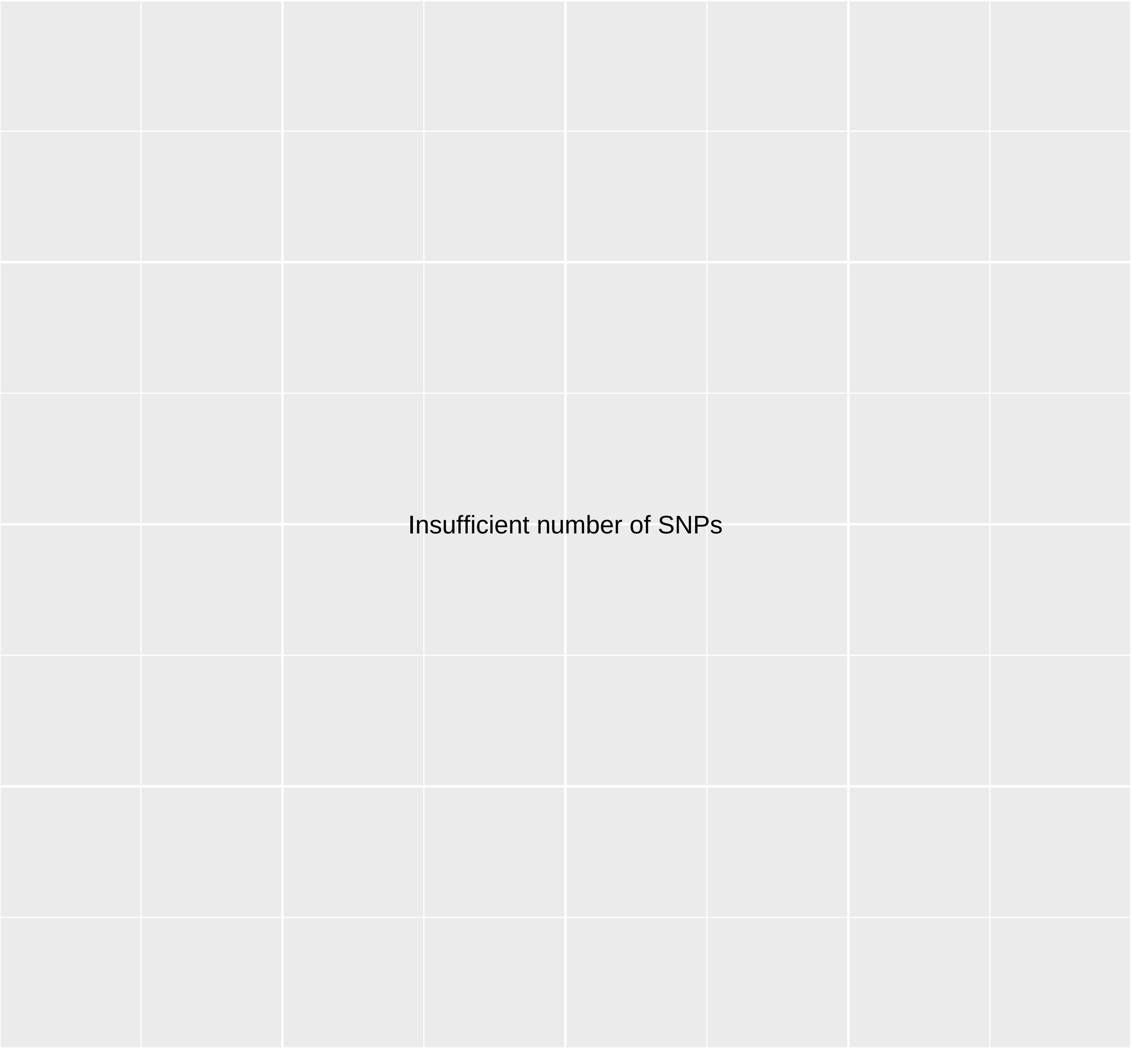
Insufficient number of SNPs

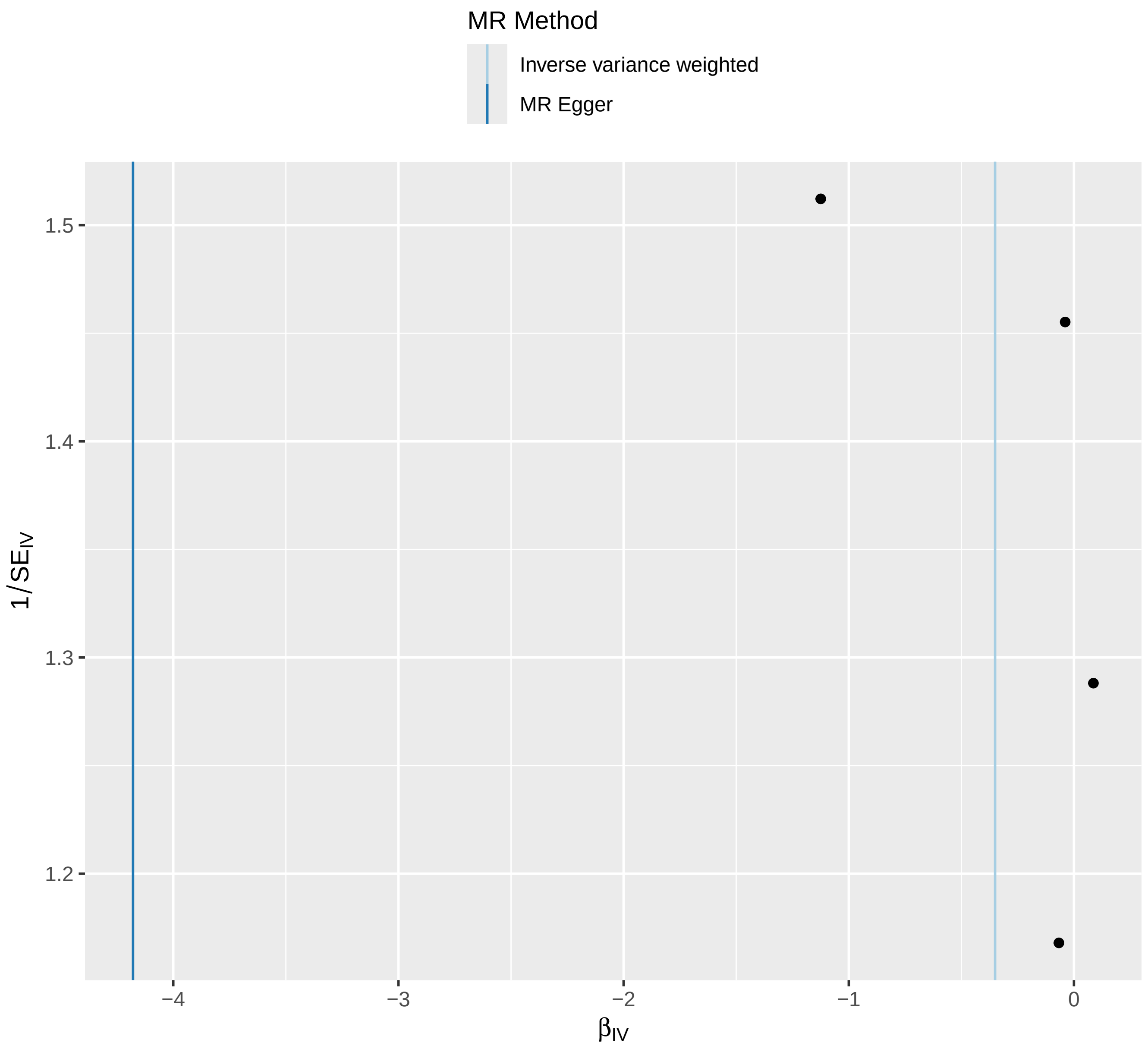
MR Method
Inverse variance weighted
MR Egger
1.5
1.4
1.3
1.2
1/SE_IV
−4
−3
−2
−1
0
β_IV

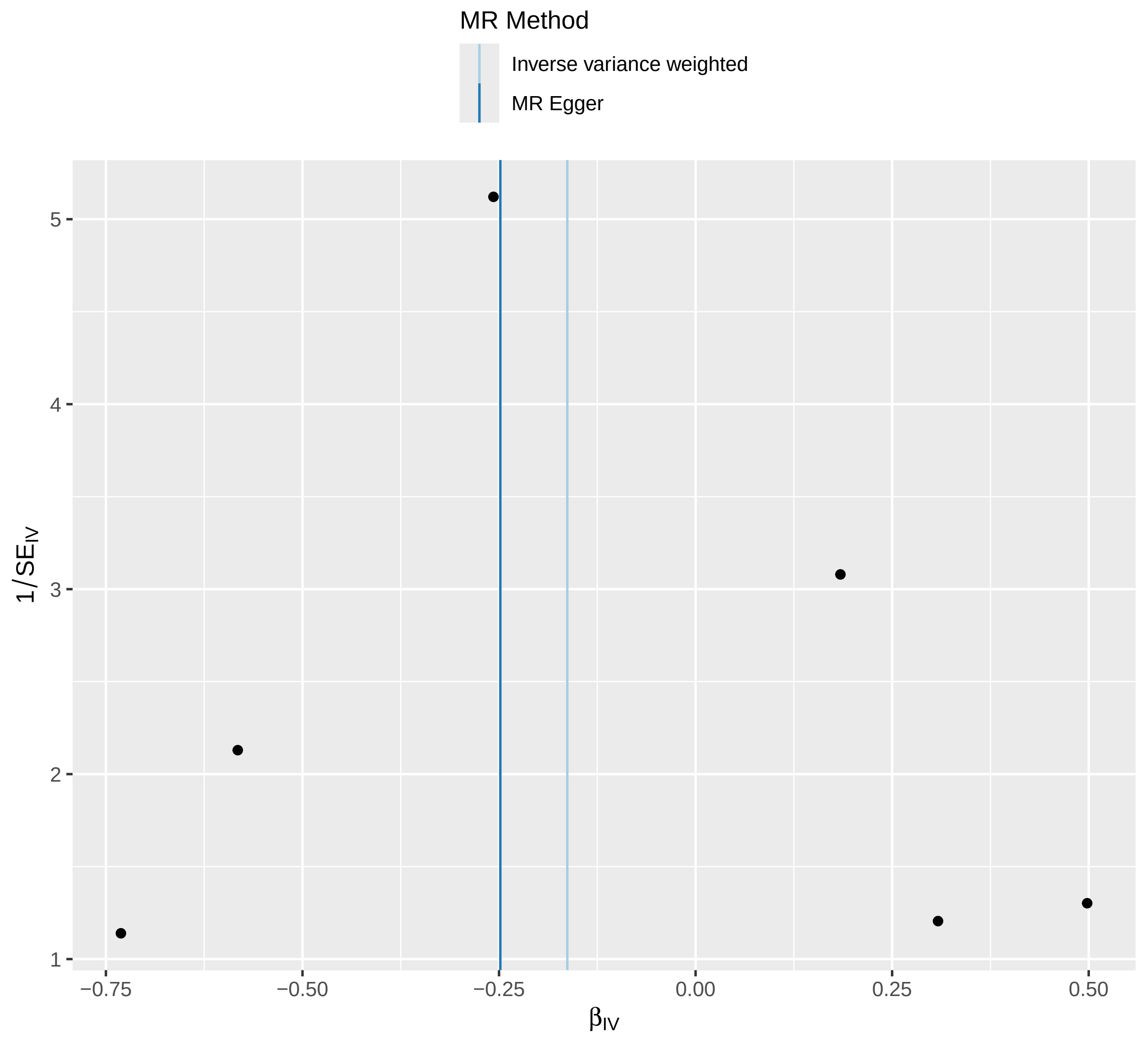

MR Method
Inverse variance weighted
MR Egger
5
4
3
2
1
1/SE$_{IV}$
−0.75
−0.50
−0.25
0.00
0.25
0.50
β$_{IV}$

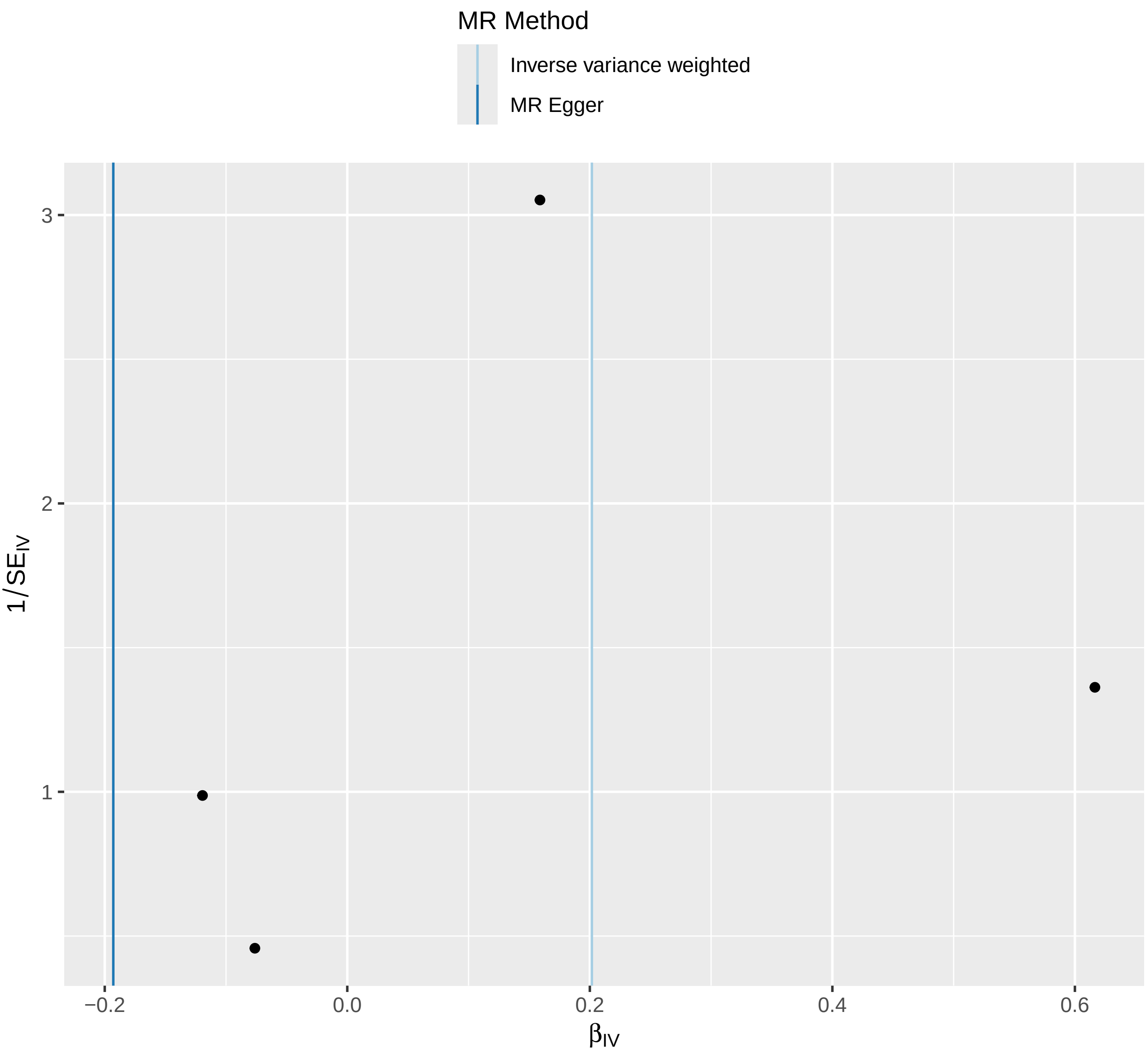

MR Method
Inverse variance weighted
MR Egger
3
2
1
1/SE$_{IV}$
−0.2
0.0
0.2
0.4
0.6
β$_{IV}$

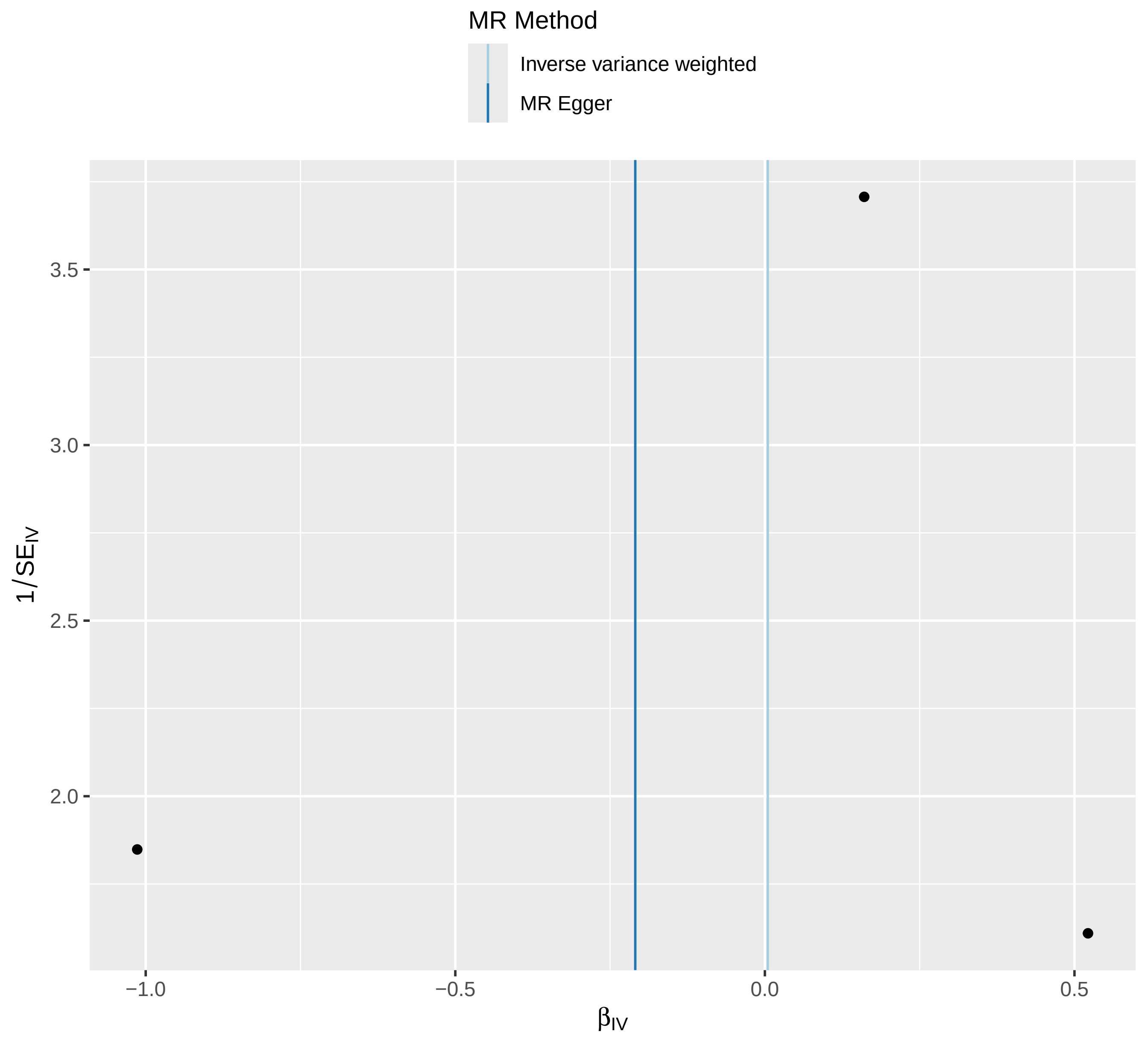

MR Method
Inverse variance weighted
MR Egger
3.5
3.0
2.5
2.0
$1/SE_{IV}$
−1.0
−0.5
0.0
0.5
β_{IV}

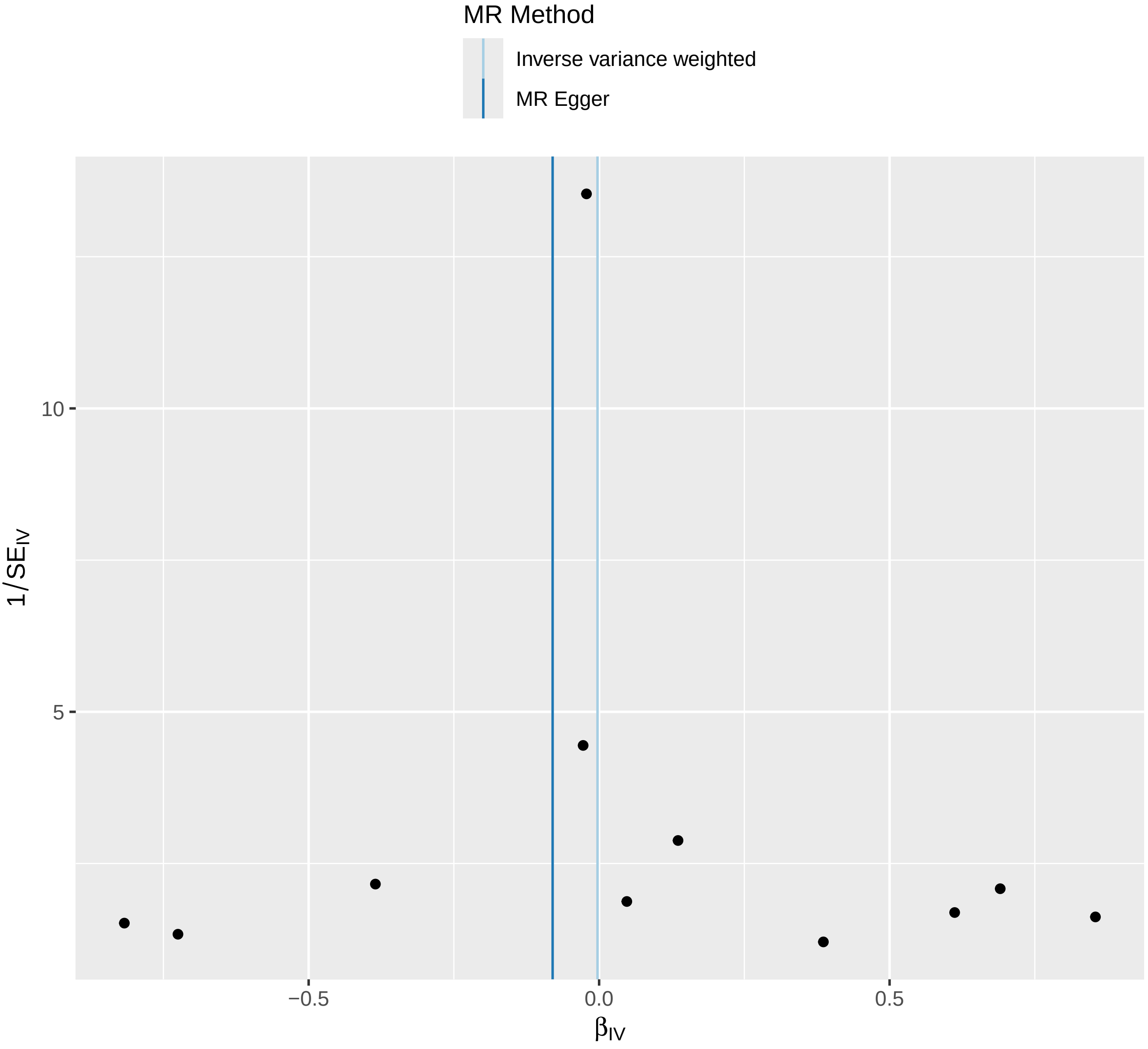
MR Method
Inverse variance weighted
MR Egger
10
5
$1/SE_{IV}$
−0.5
0.0
0.5
β_{IV}

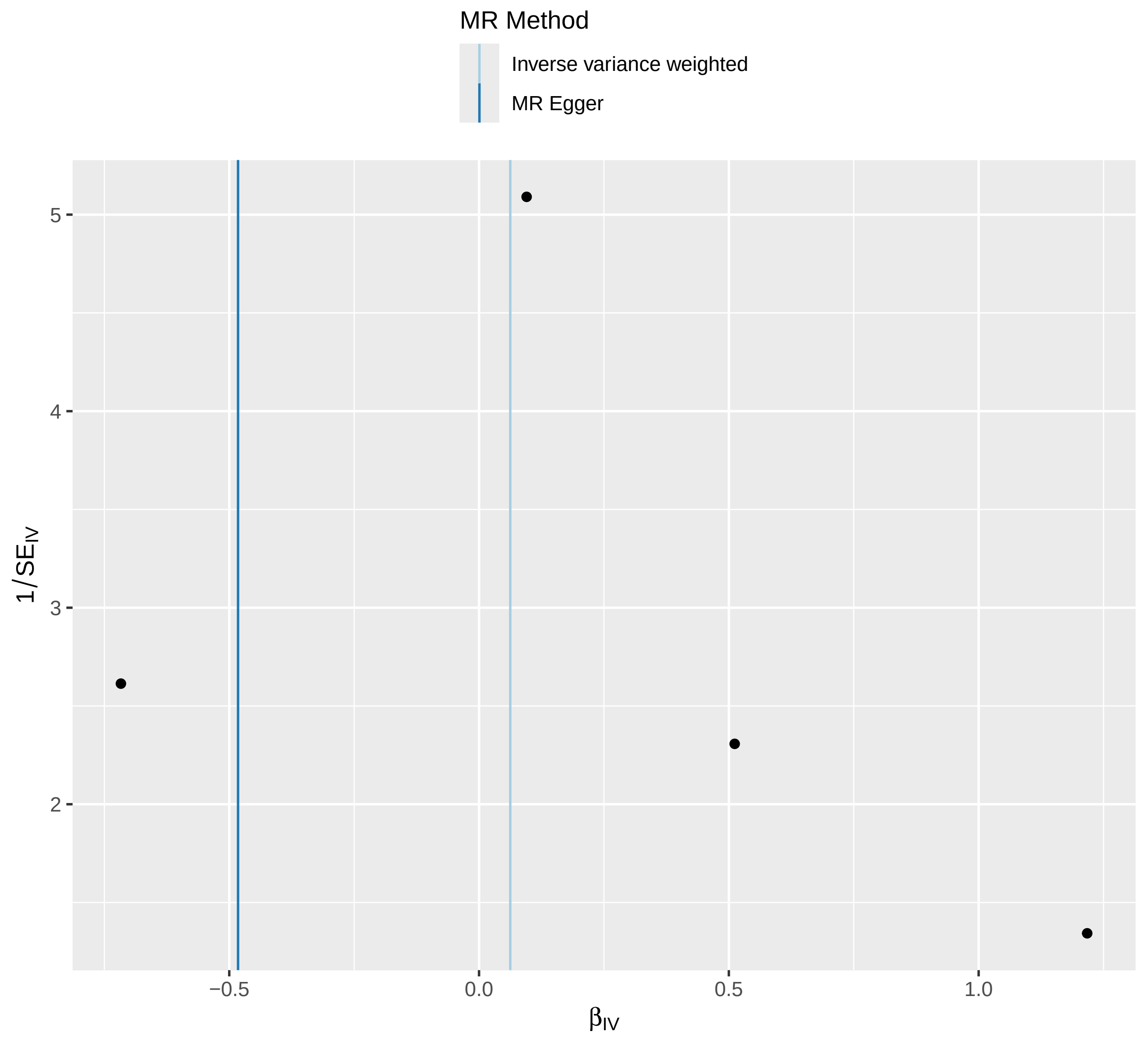
MR Method
Inverse variance weighted
MR Egger
1/SE_IV
5
4
3
2
−0.5
0.0
0.5
1.0
β_IV

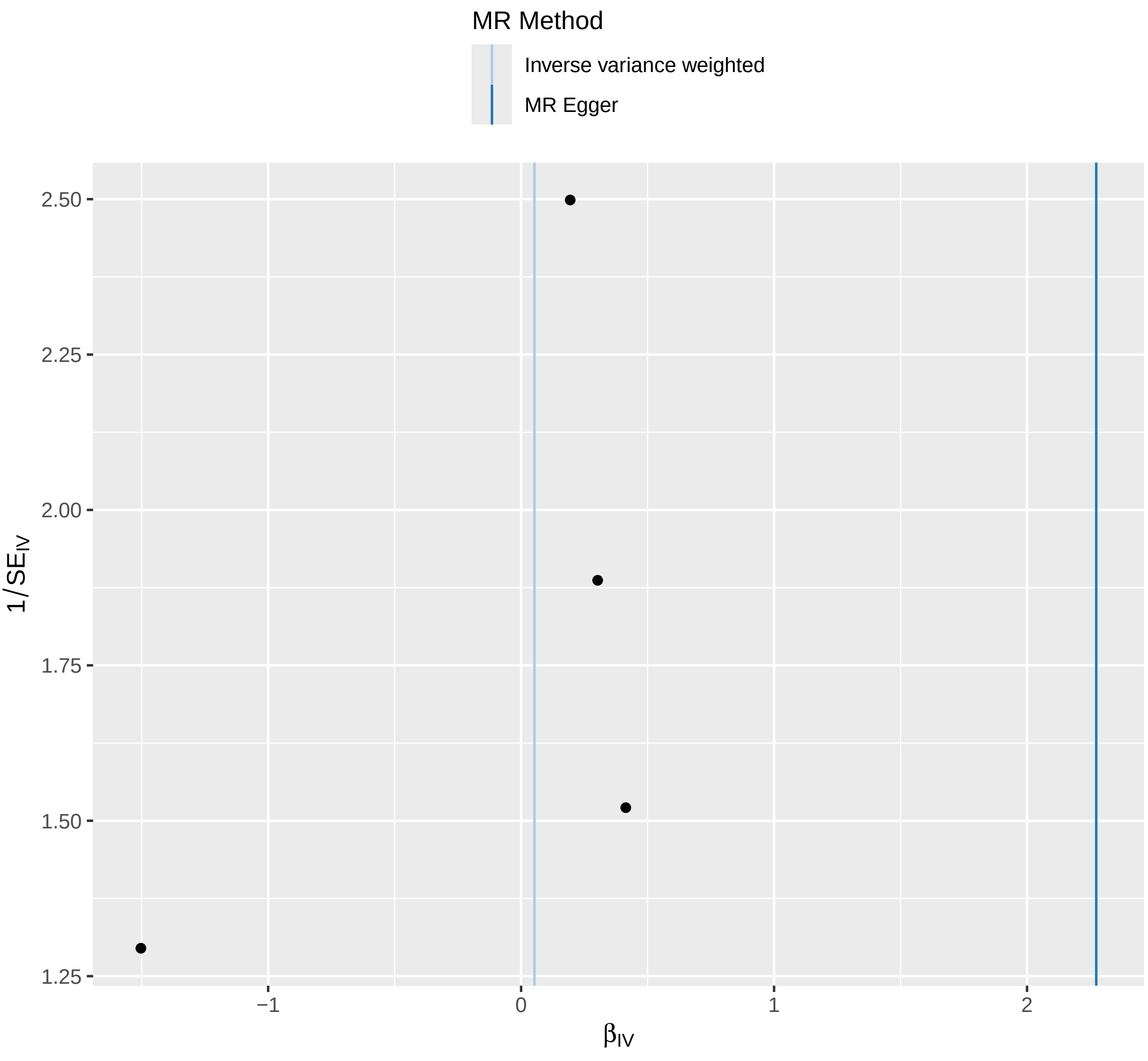
MR Method
Inverse variance weighted
MR Egger
2.50
2.25
2.00
1.75
1.50
1.25
1/SE_IV
−1
0
1
2
β_IV

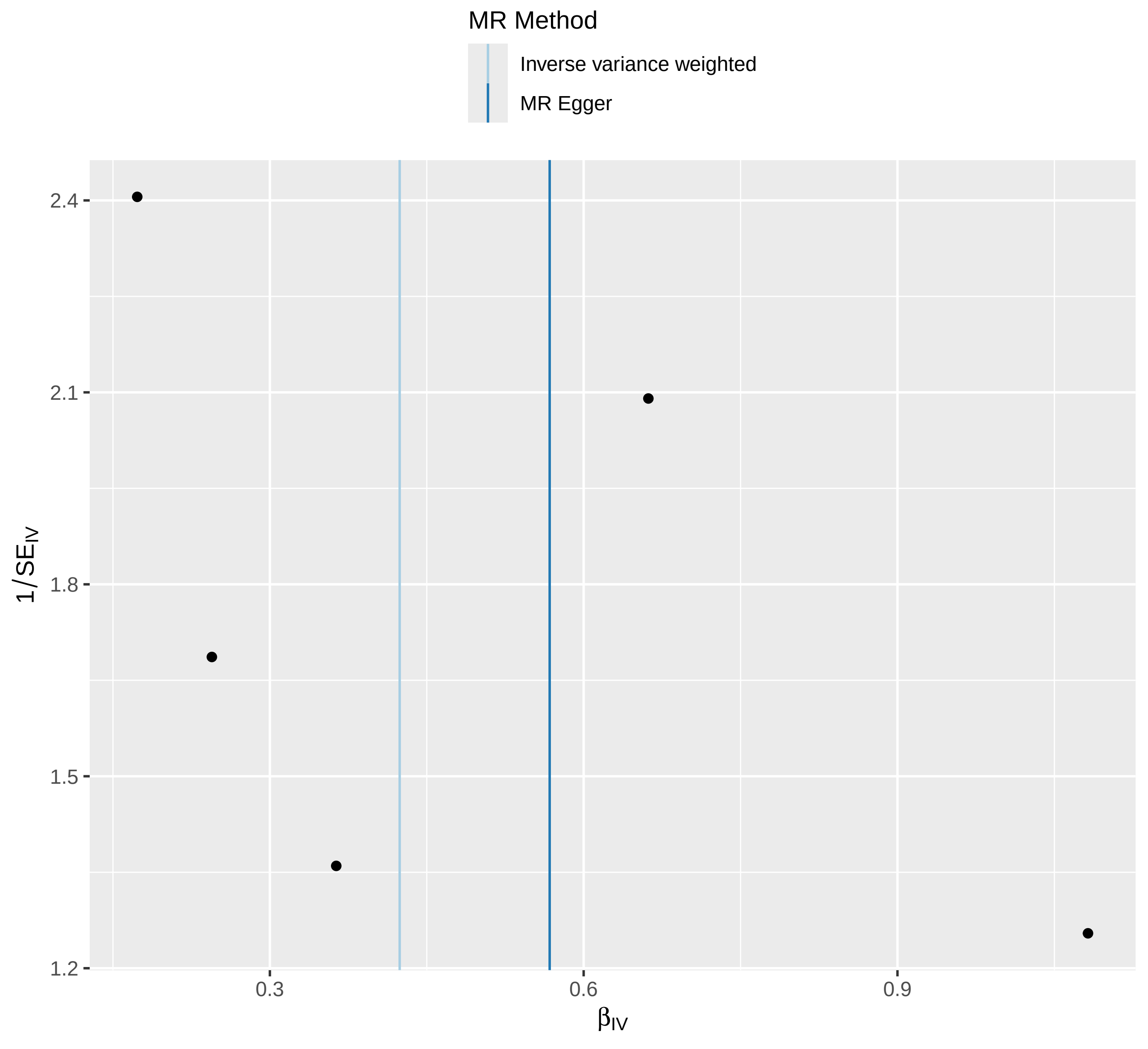

MR Method
Inverse variance weighted
MR Egger
2.4
2.1
1.8
1.5
1.2
$1/SE_{IV}$
0.3
0.6
0.9
β_{IV}

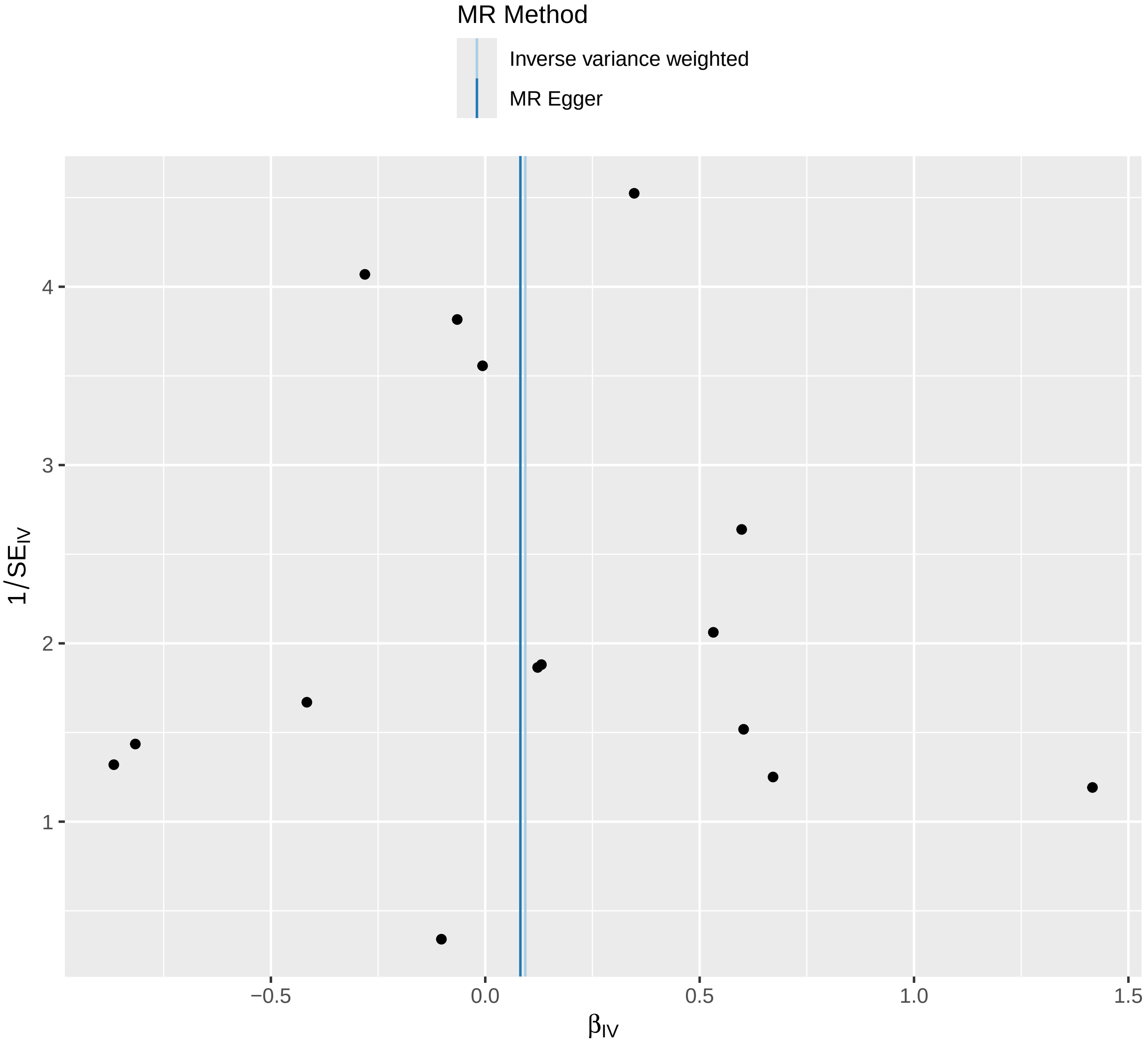
MR Method
Inverse variance weighted
MR Egger
1/SE_IV
4
3
2
1
−0.5
0.0
0.5
1.0
1.5
β_IV

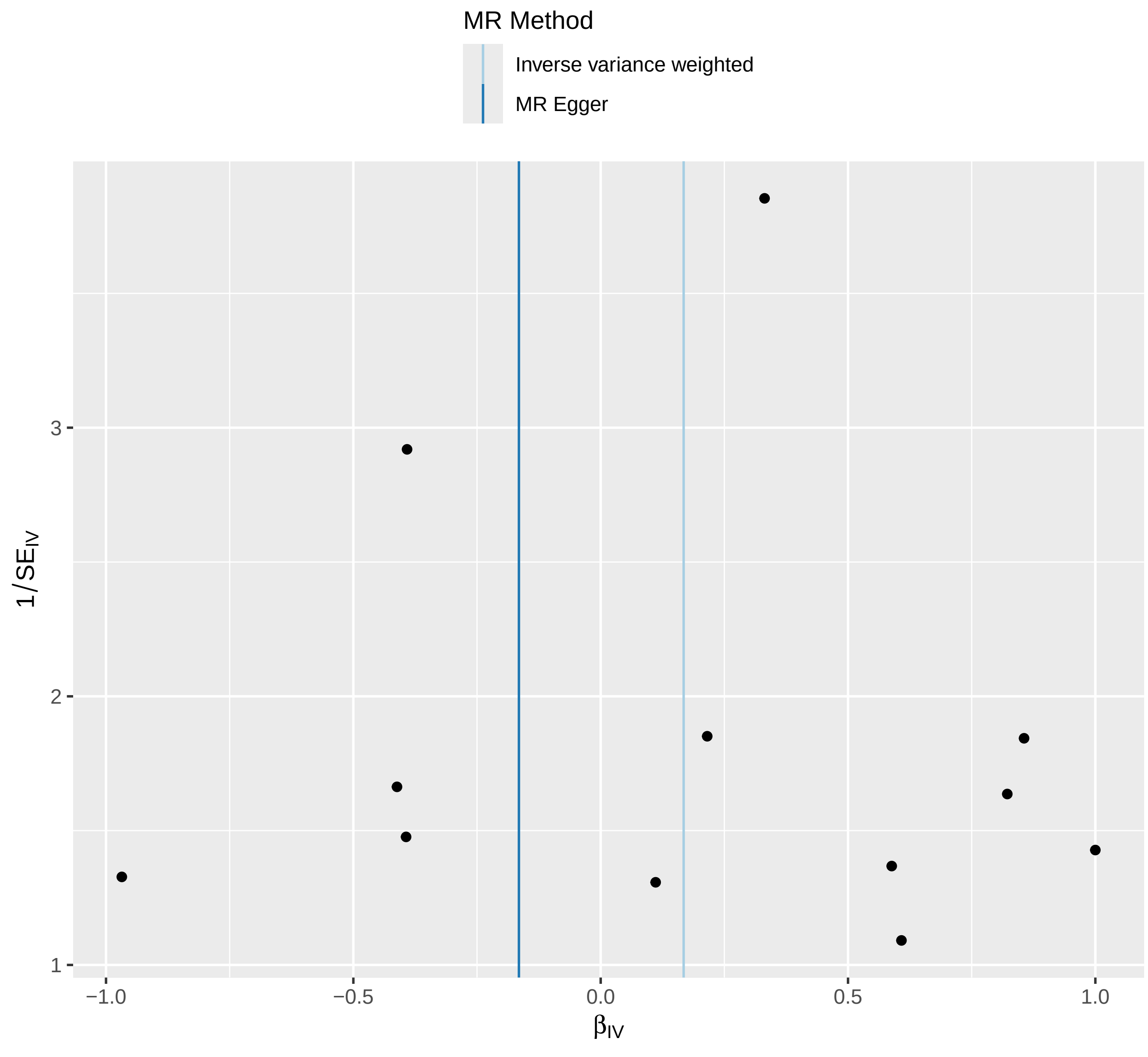
MR Method
Inverse variance weighted
MR Egger
1/SE_IV
3
2
1
−1.0
−0.5
0.0
0.5
1.0
β_IV

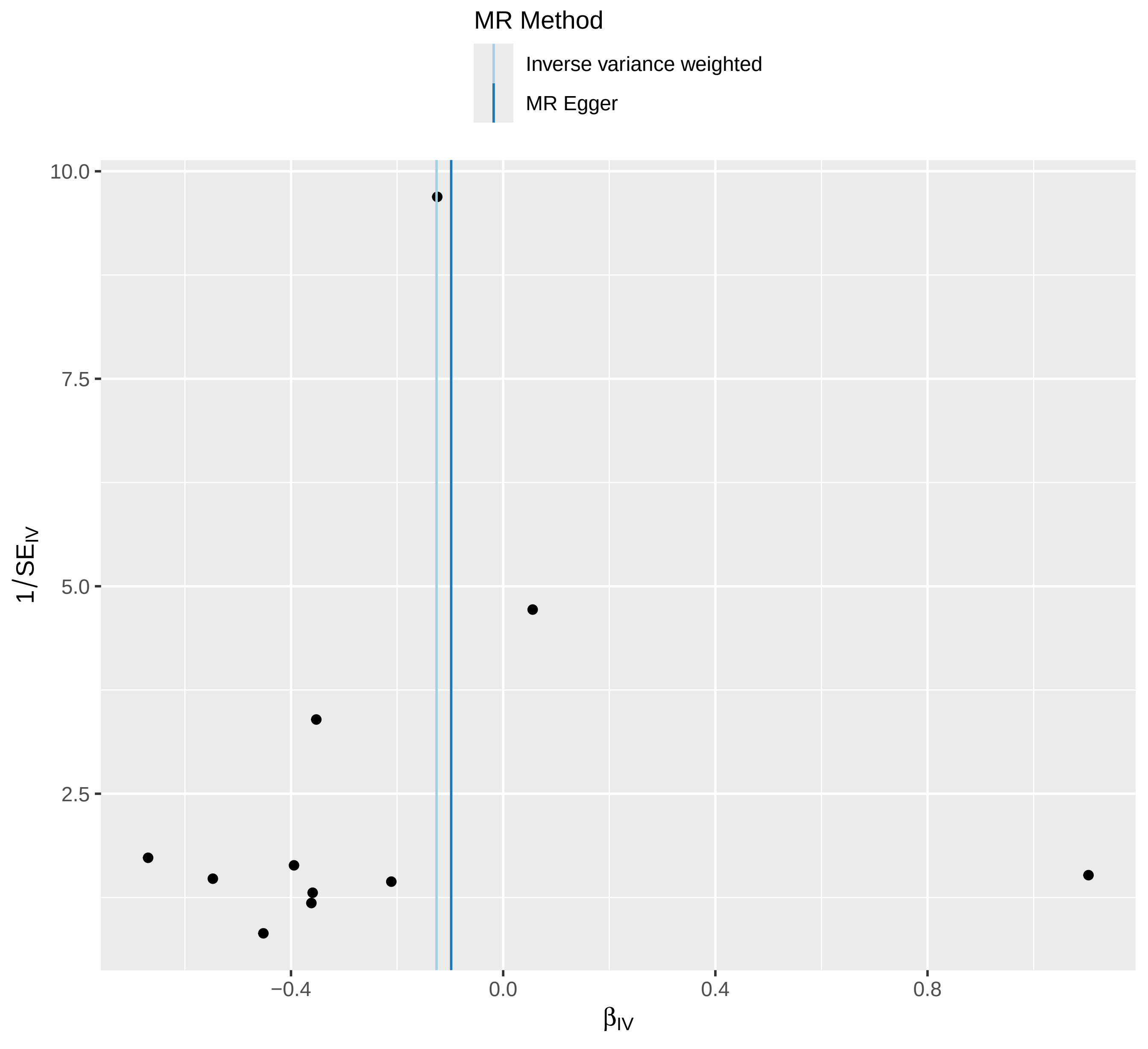
MR Method
Inverse variance weighted
MR Egger
10.0
7.5
5.0
2.5
1/SE_IV
−0.4
0.0
0.4
0.8
β_IV

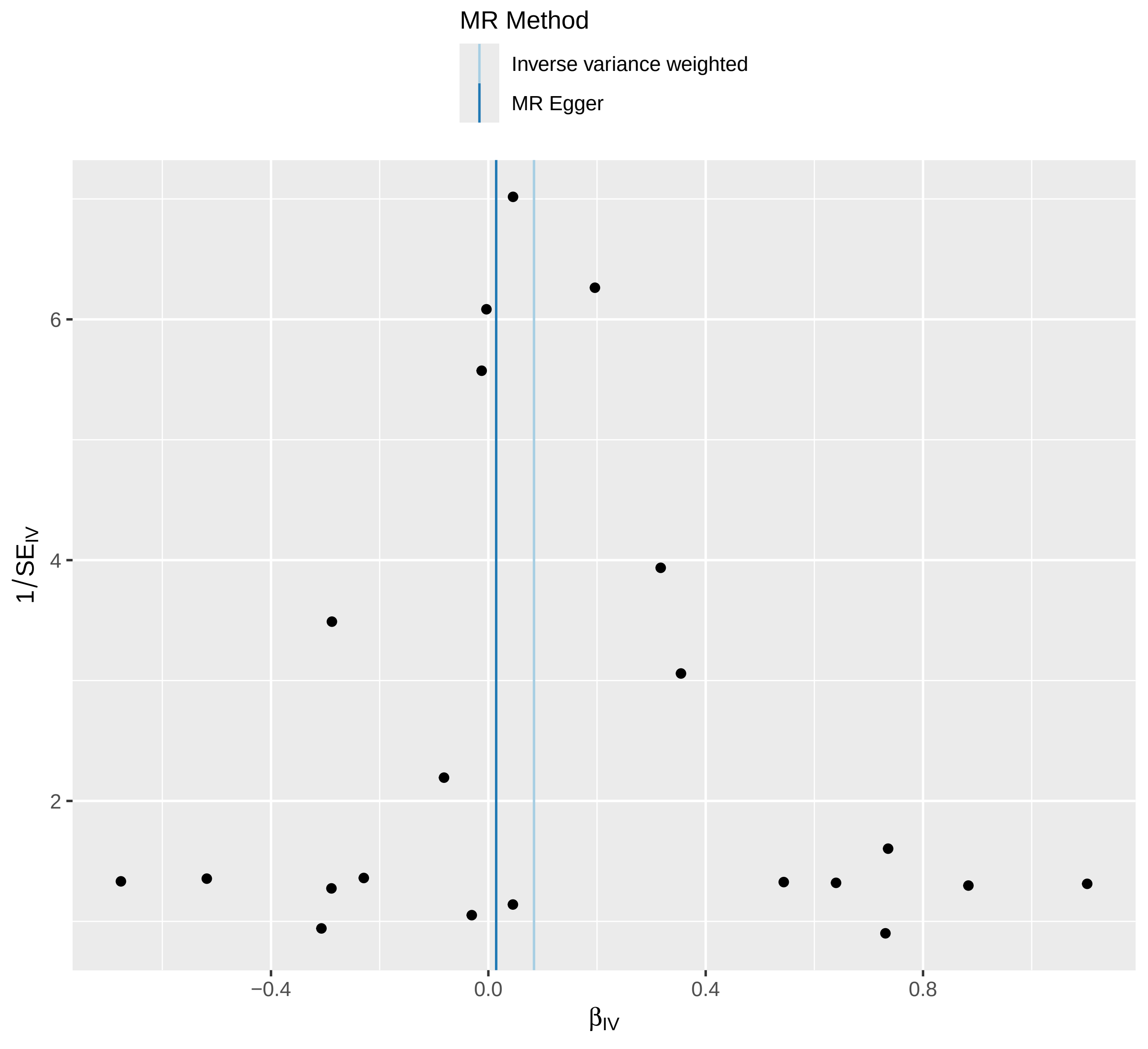

MR Method
Inverse variance weighted
MR Egger
6
4
2
1/SE$_{IV}$
−0.4
0.0
0.4
0.8
β$_{IV}$

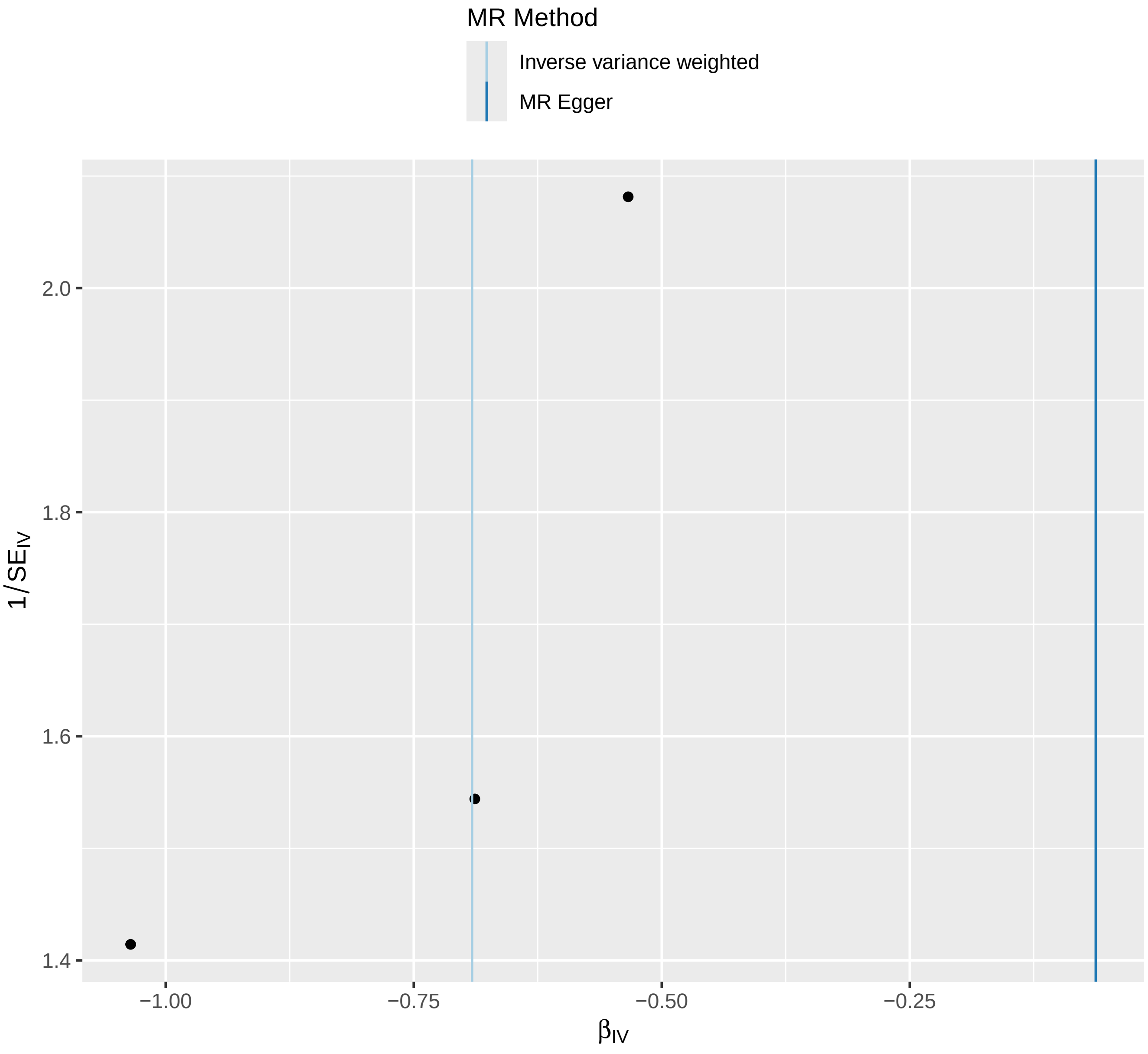

MR Method
Inverse variance weighted
MR Egger
2.0
1.8
1.6
1.4
$1/SE_{IV}$
−1.00
−0.75
−0.50
−0.25
β_{IV}

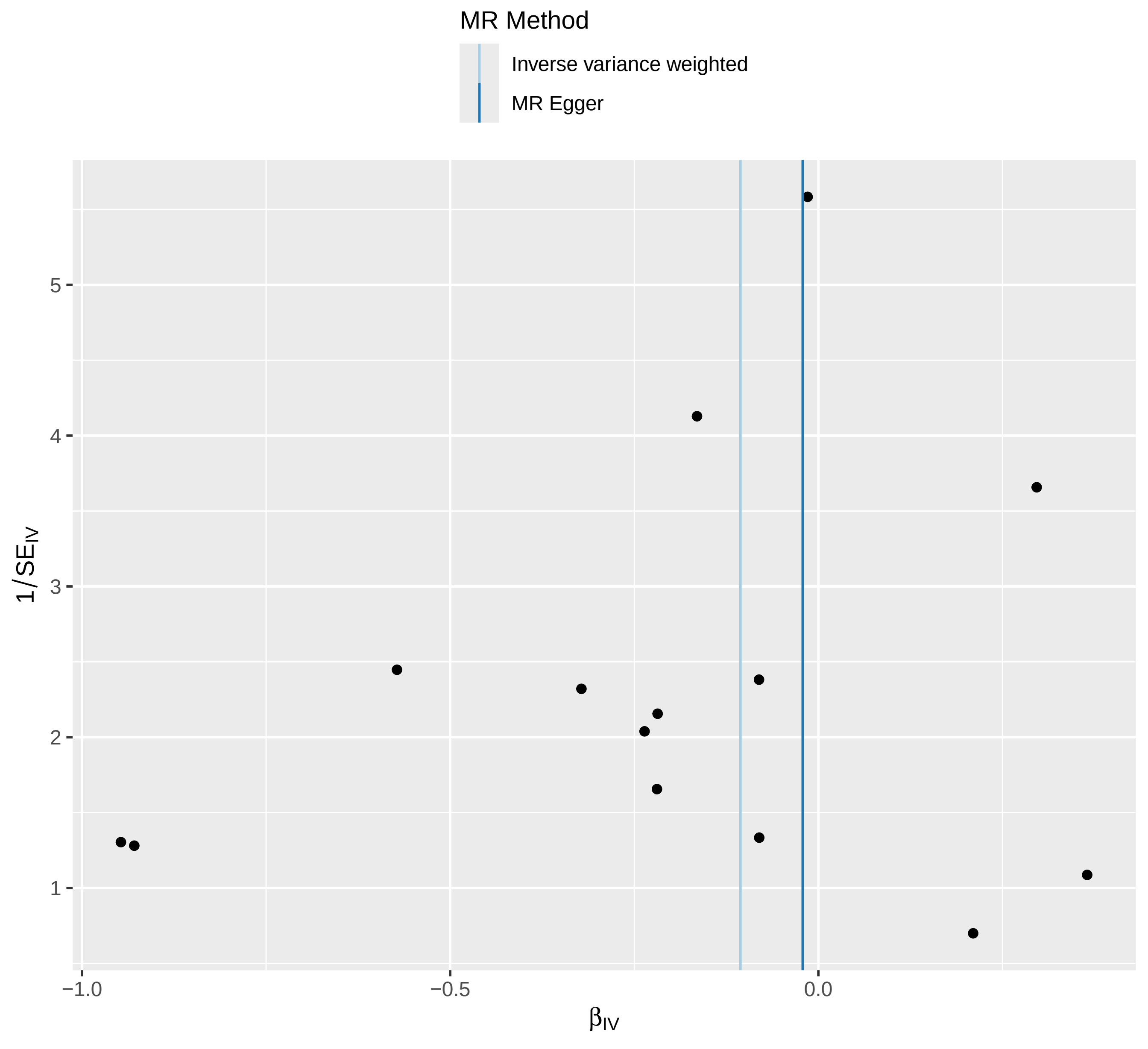

MR Method
Inverse variance weighted
MR Egger
1/SE_IV
5
4
3
2
1
−1.0
−0.5
0.0
β_IV

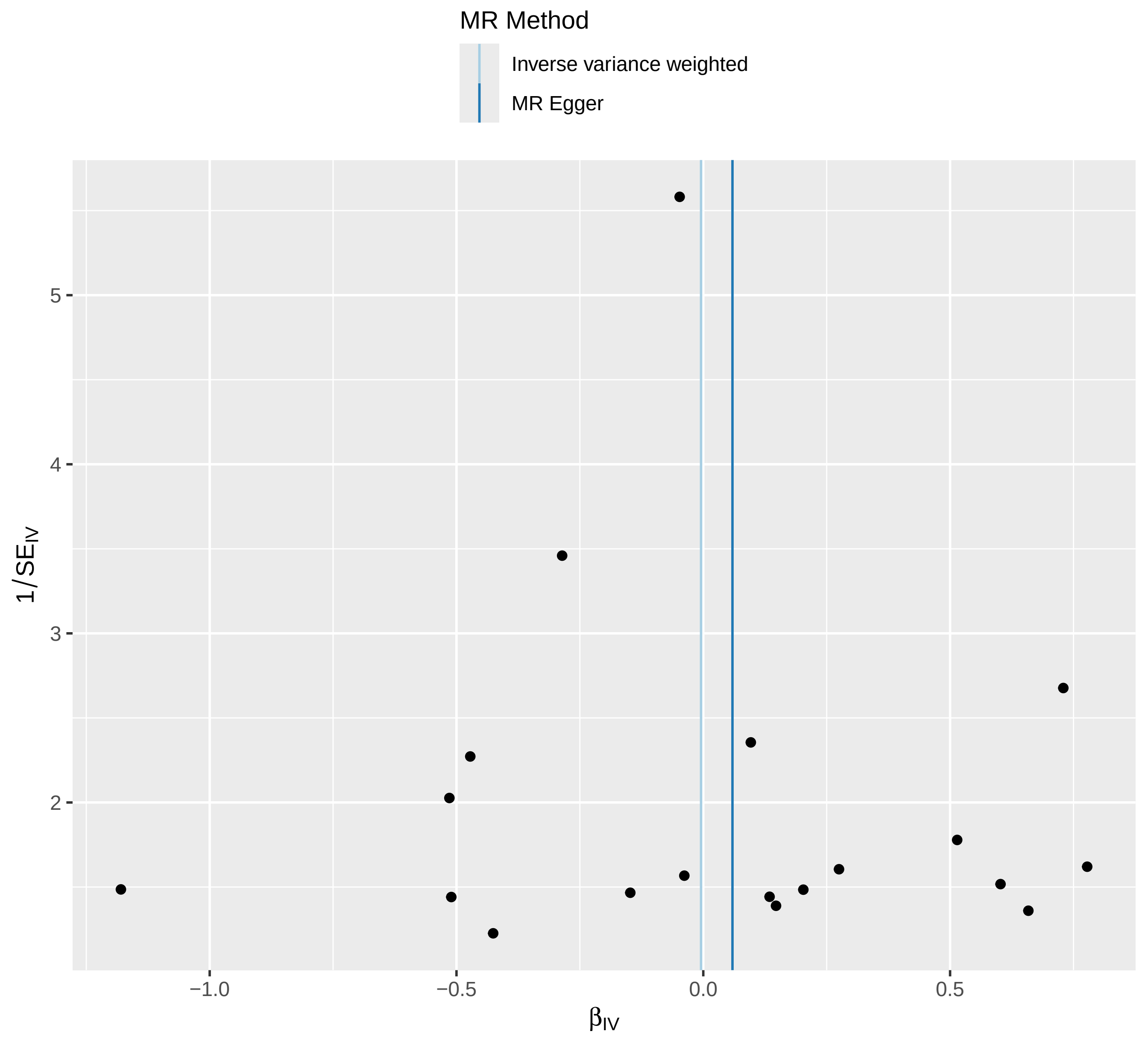
MR Method
Inverse variance weighted
MR Egger
5
4
3
2
1/SE$_{IV}$
−1.0
−0.5
0.0
0.5
β$_{IV}$

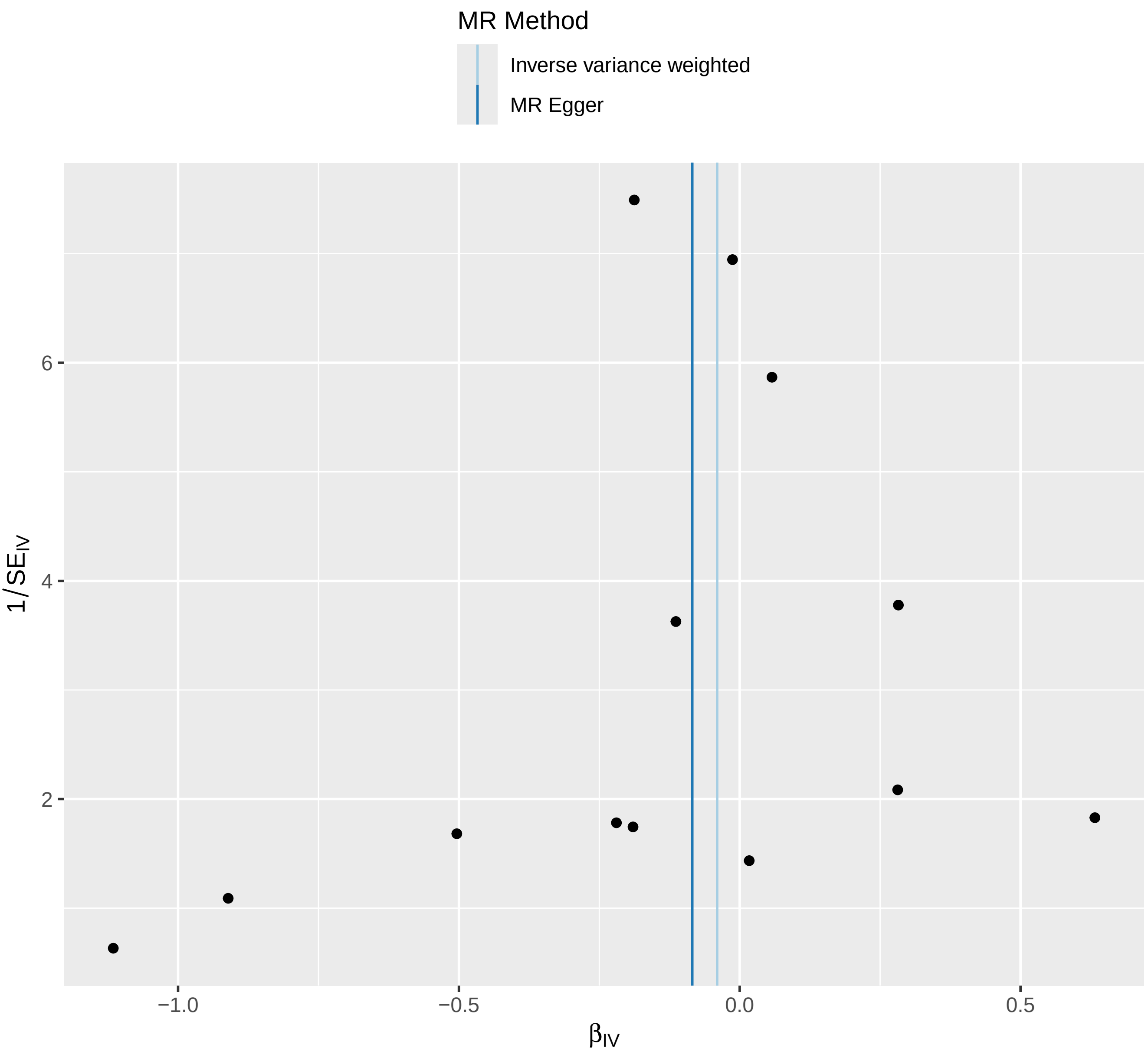
MR Method
Inverse variance weighted
MR Egger
6
4
2
1/SE$_{IV}$
−1.0
−0.5
0.0
0.5
β$_{IV}$

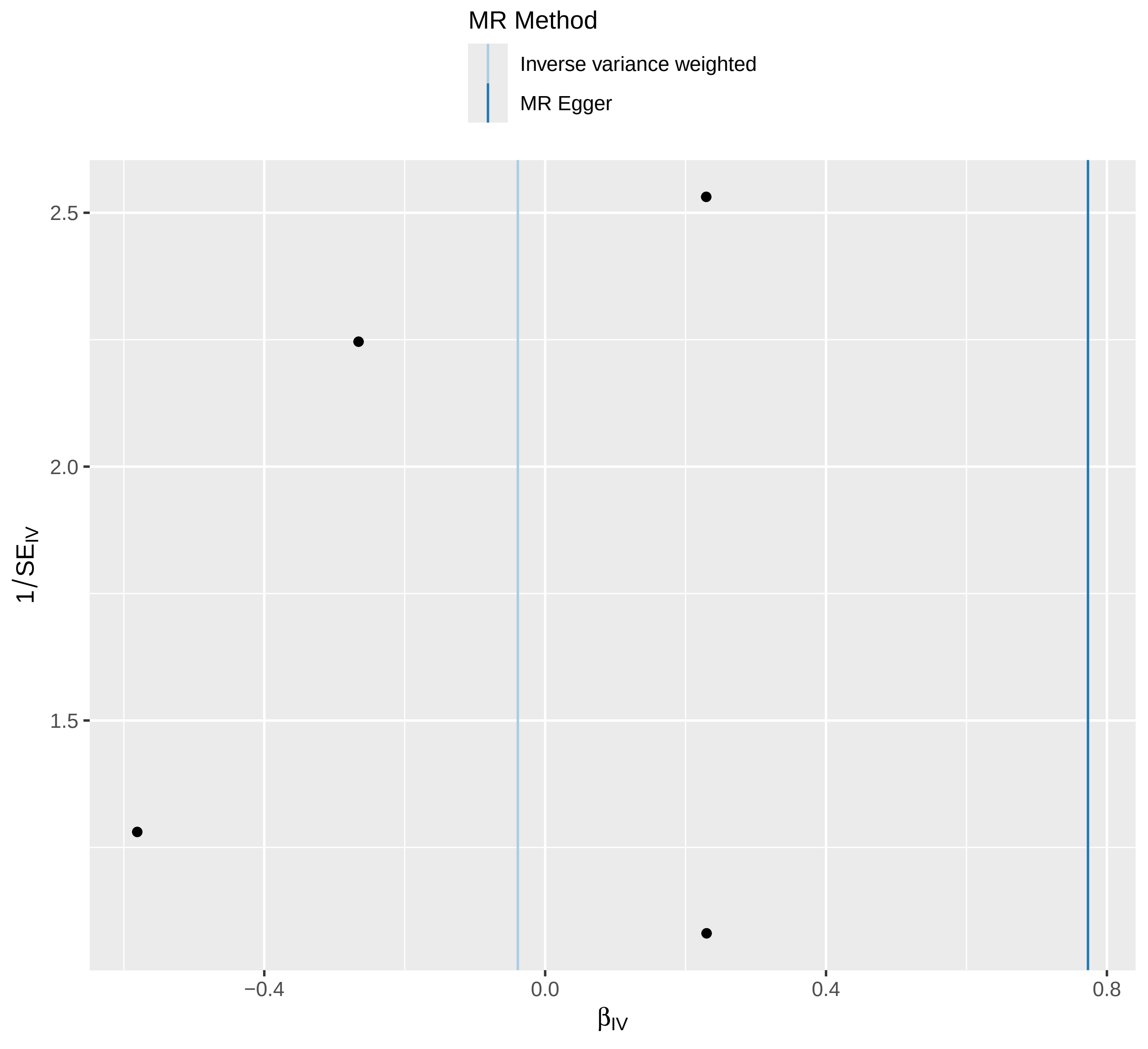

MR Method
Inverse variance weighted
MR Egger
2.5
2.0
1.5
$1/SE_{IV}$
−0.4
0.0
0.4
0.8
β_{IV}

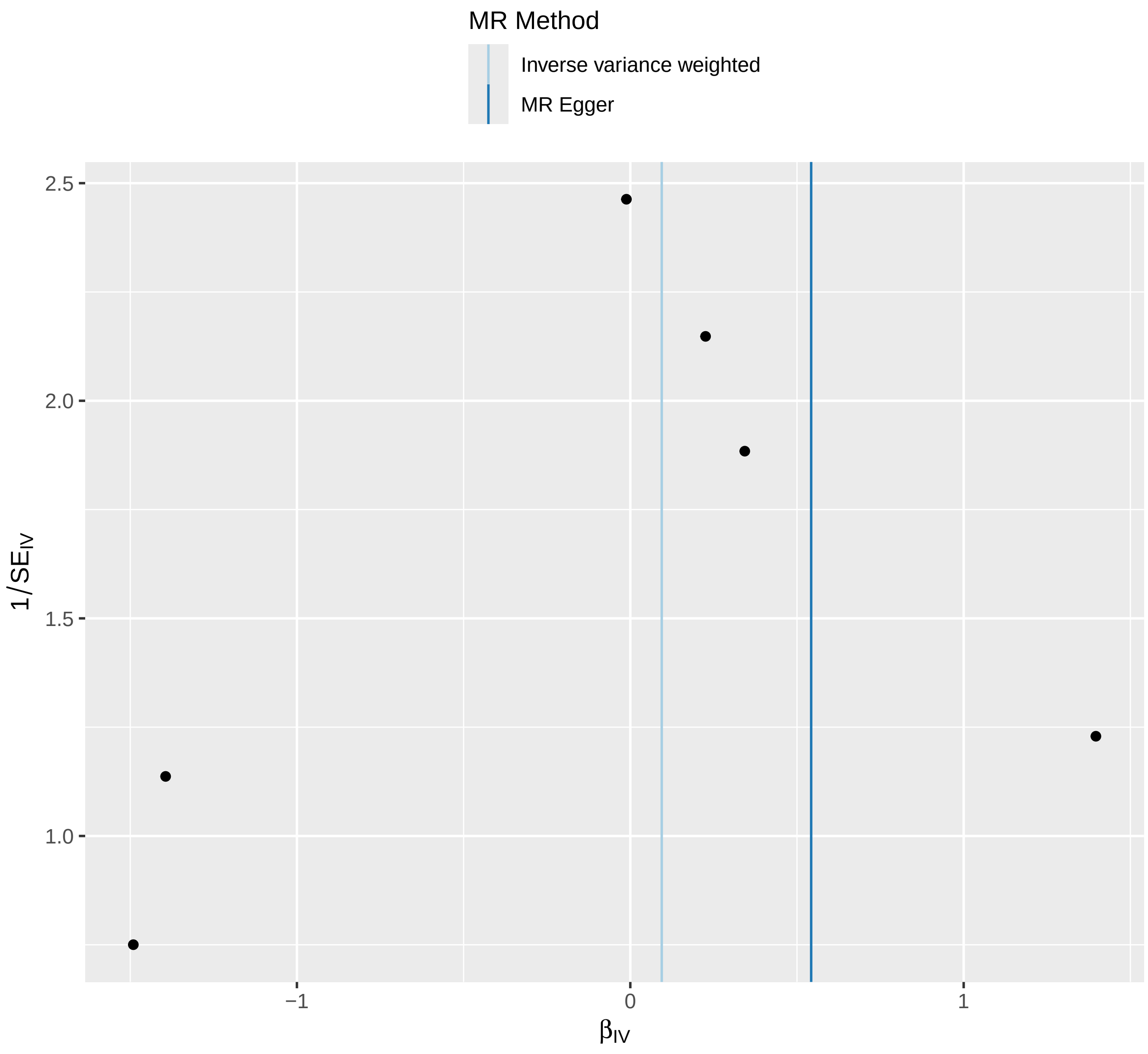
MR Method
Inverse variance weighted
MR Egger
2.5
2.0
1.5
1.0
−1
0
1
$1/SE_{IV}$
β_{IV}

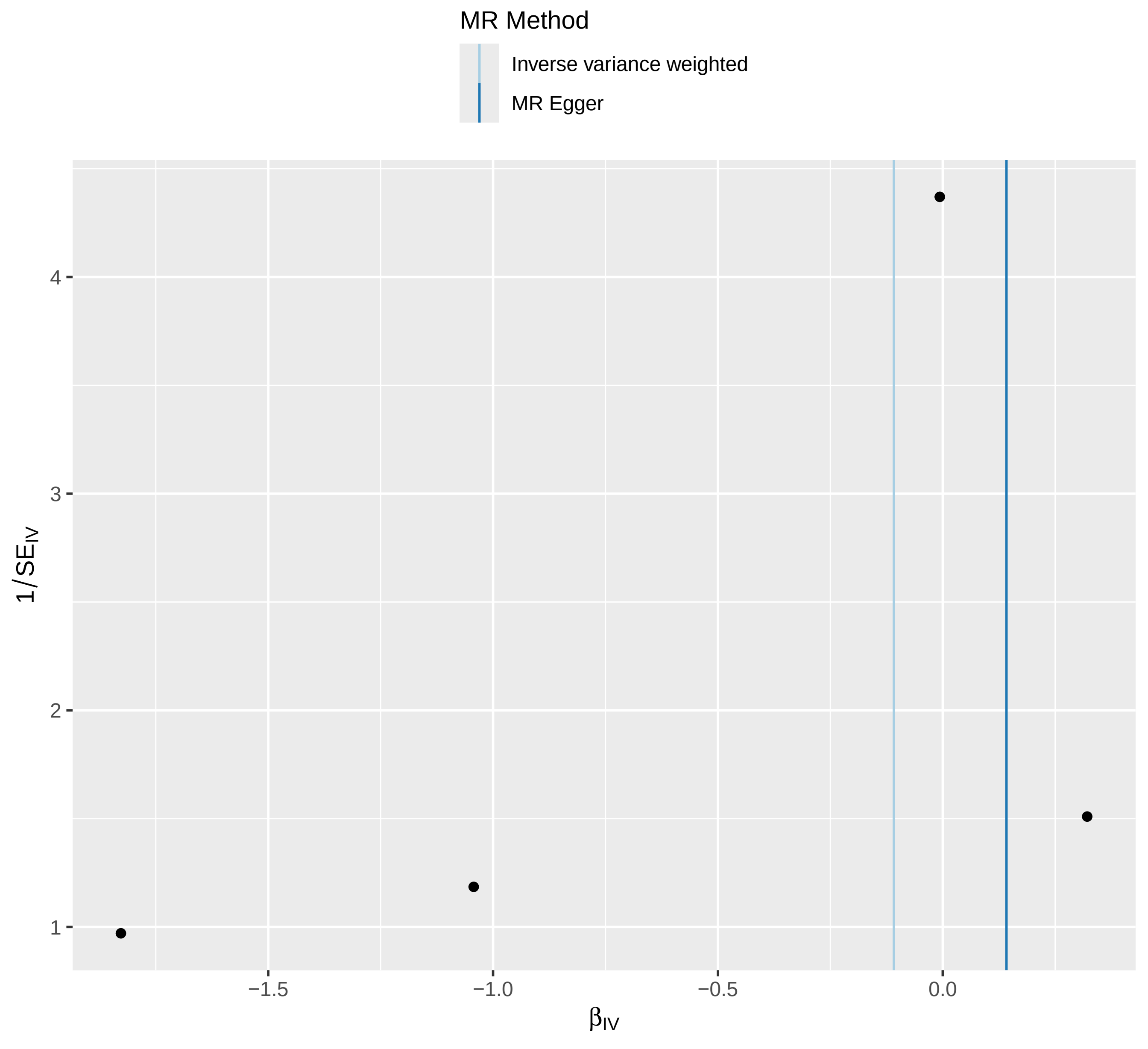

MR Method
Inverse variance weighted
MR Egger
4
3
2
1
1/SE_IV
−1.5
−1.0
−0.5
0.0
β_IV

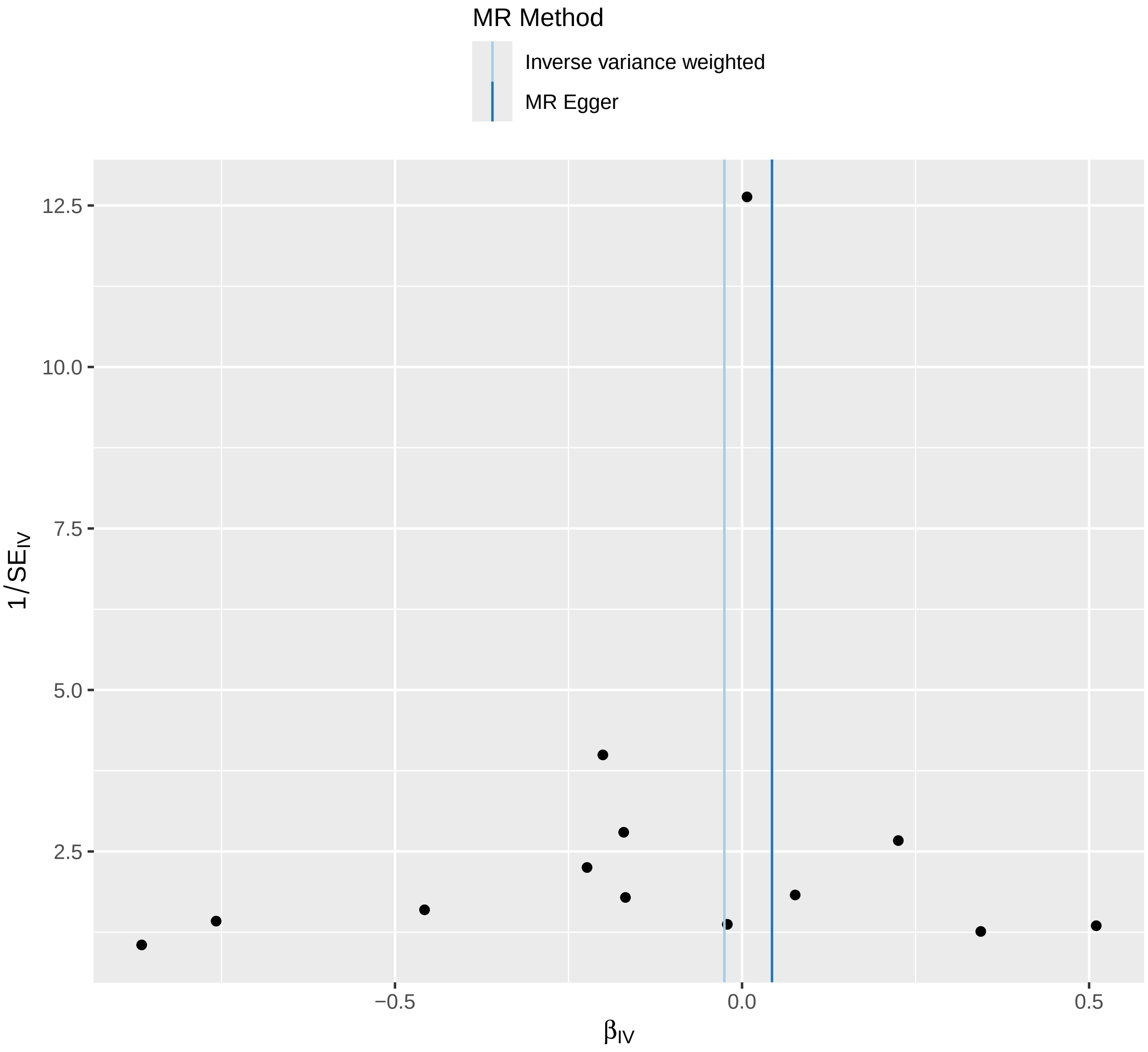
MR Method
Inverse variance weighted
MR Egger
12.5
10.0
7.5
5.0
2.5
$1/SE_{IV}$
−0.5
0.0
0.5
β_{IV}

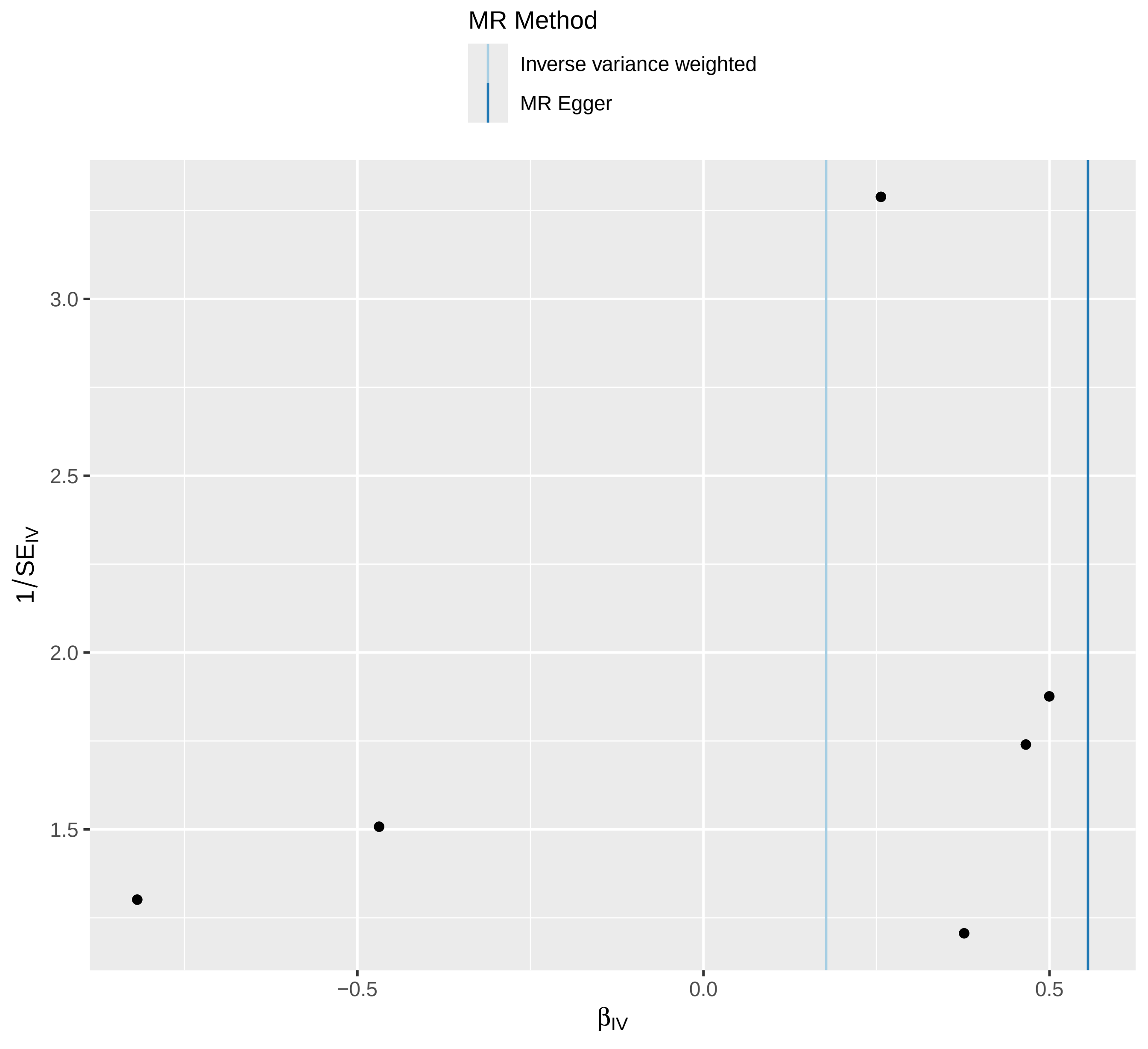

MR Method
Inverse variance weighted
MR Egger
3.0
2.5
2.0
1.5
$1/SE_{IV}$
−0.5
0.0
0.5
β_{IV}

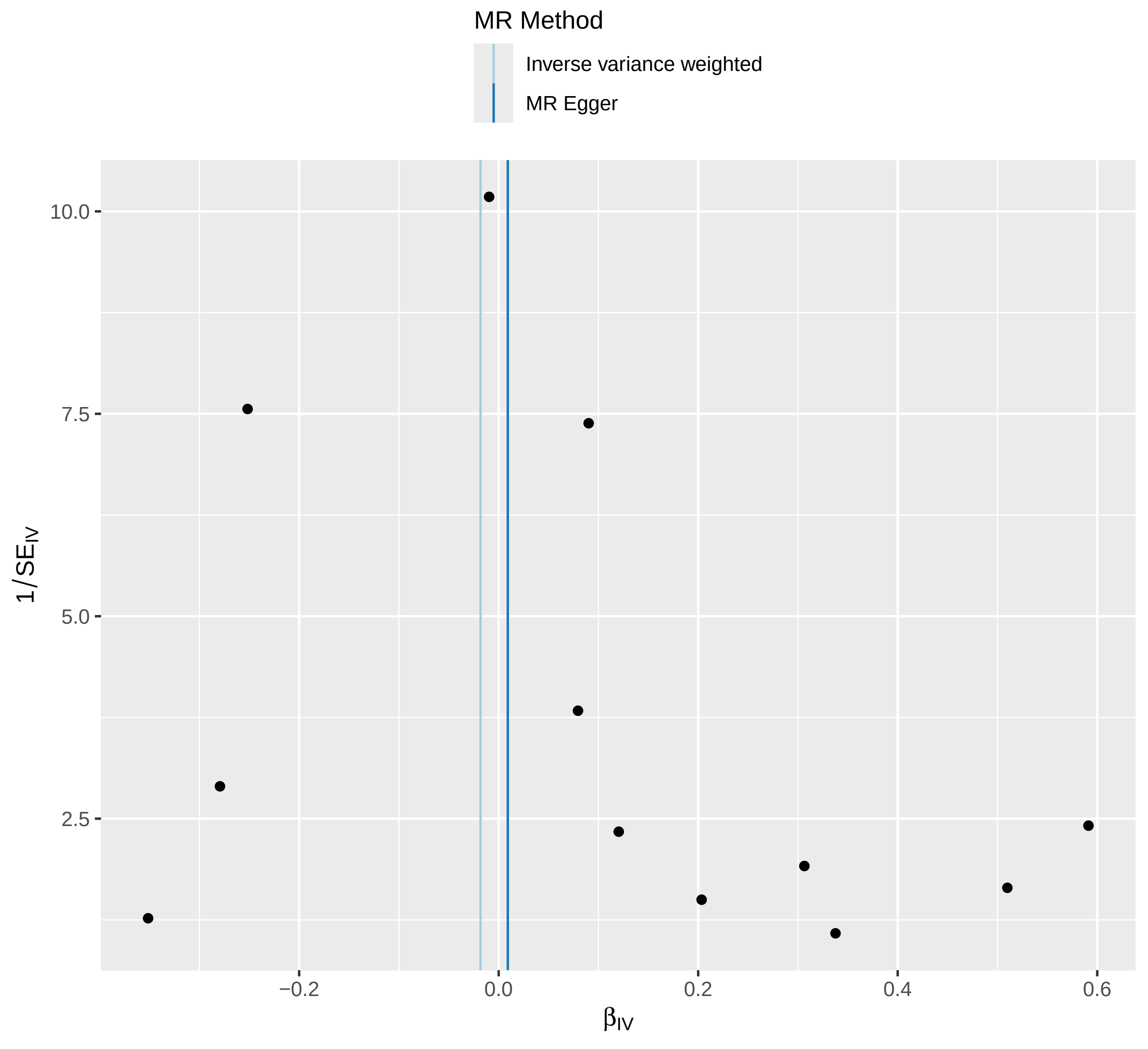

MR Method
Inverse variance weighted
MR Egger
10.0
7.5
5.0
2.5
$1/SE_{IV}$
−0.2
0.0
0.2
0.4
0.6
β_{IV}

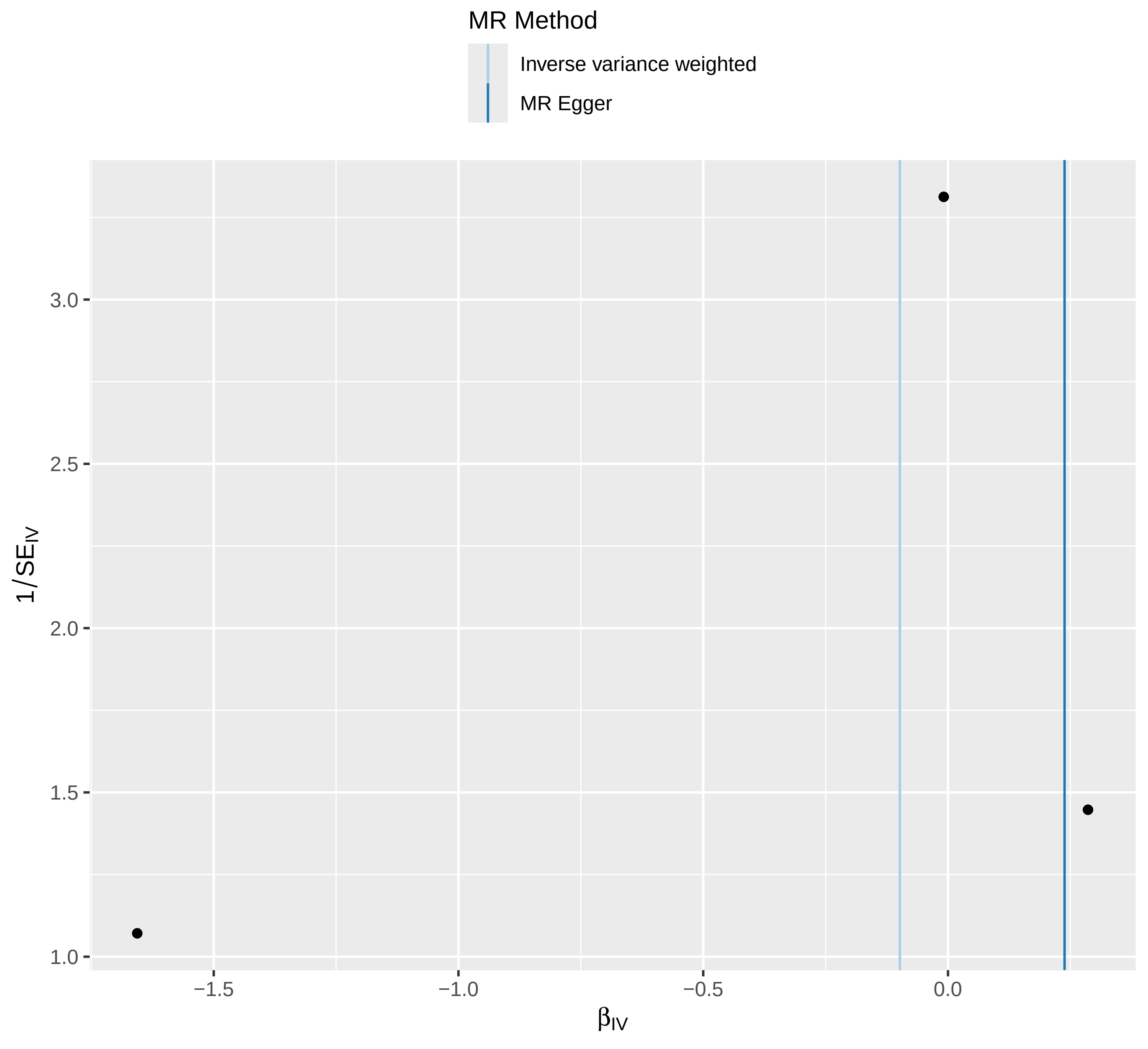

MR Method
Inverse variance weighted
MR Egger
1/SE$_{IV}$
3.0
2.5
2.0
1.5
1.0
−1.5
−1.0
−0.5
0.0
β$_{IV}$

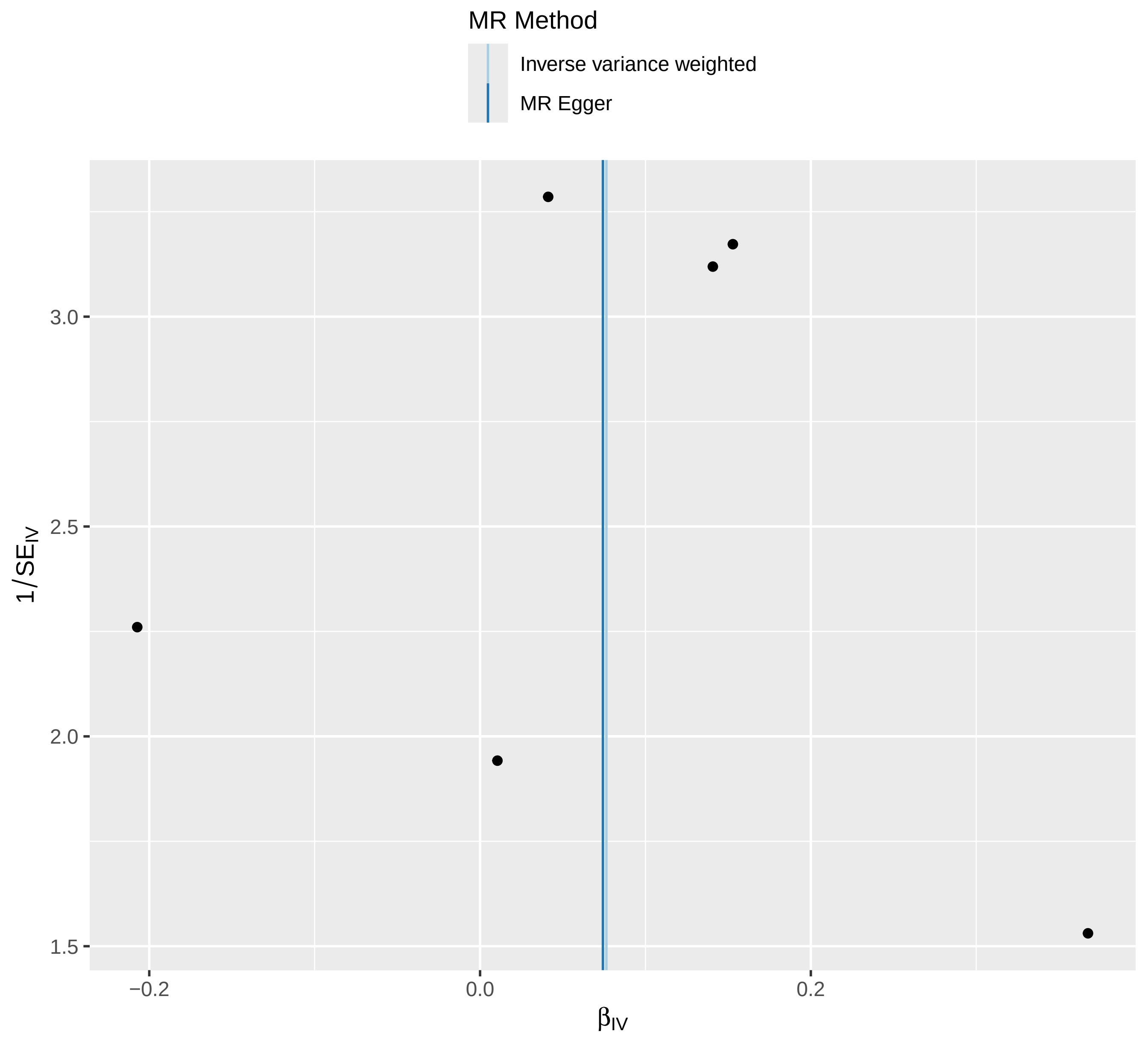
MR Method
Inverse variance weighted
MR Egger
1/SE_IV
3.0
2.5
2.0
1.5
−0.2
0.0
0.2
β_IV

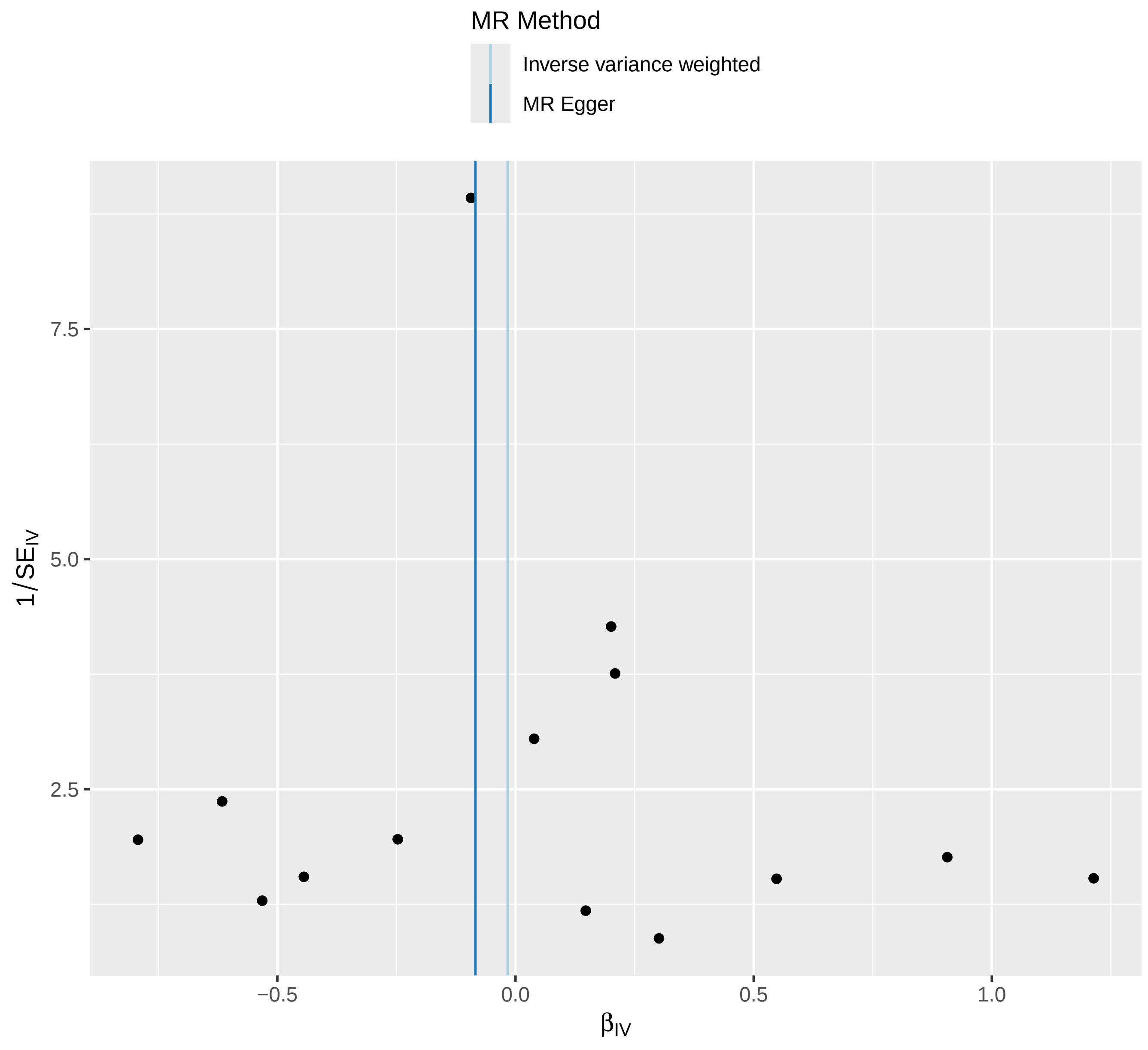

MR Method
Inverse variance weighted
MR Egger
7.5
5.0
2.5
1/SE_IV
−0.5
0.0
0.5
1.0
β_IV

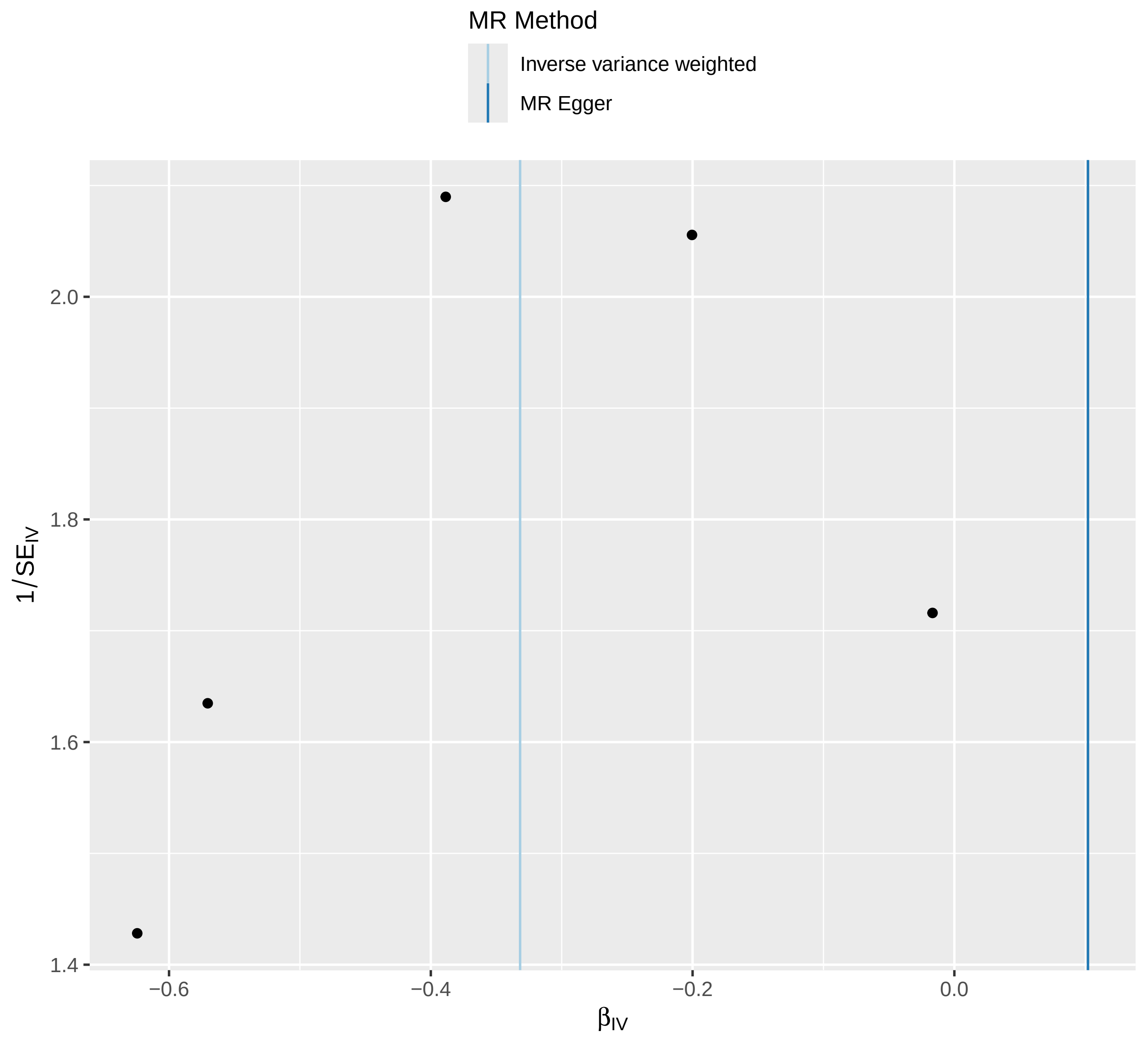

MR Method
Inverse variance weighted
MR Egger
2.0
1.8
1.6
1.4
1/SE_IV
−0.6
−0.4
−0.2
0.0
β_IV

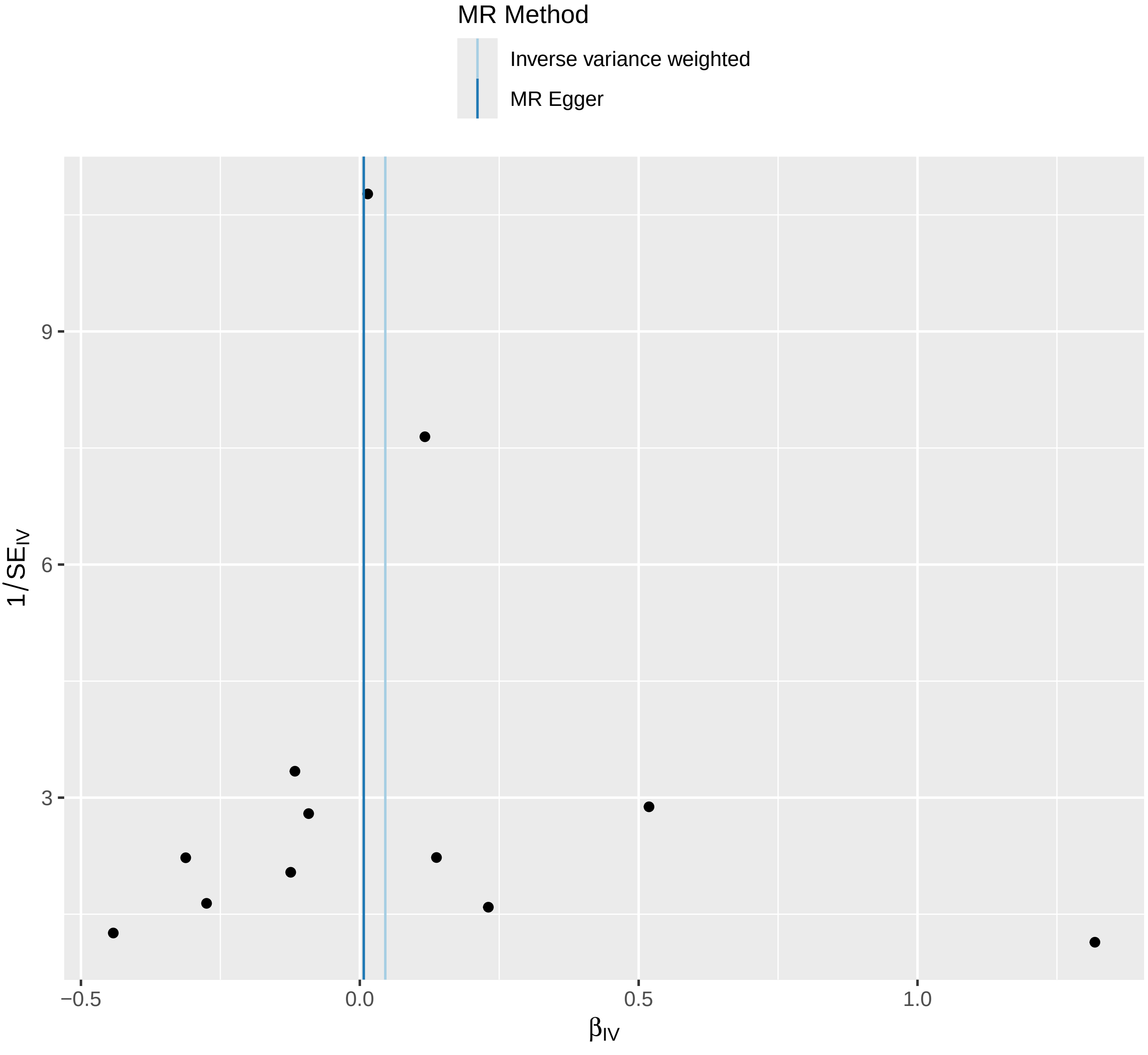

MR Method
Inverse variance weighted
MR Egger
9
6
3
1/SE$_{IV}$
−0.5
0.0
0.5
1.0
β$_{IV}$

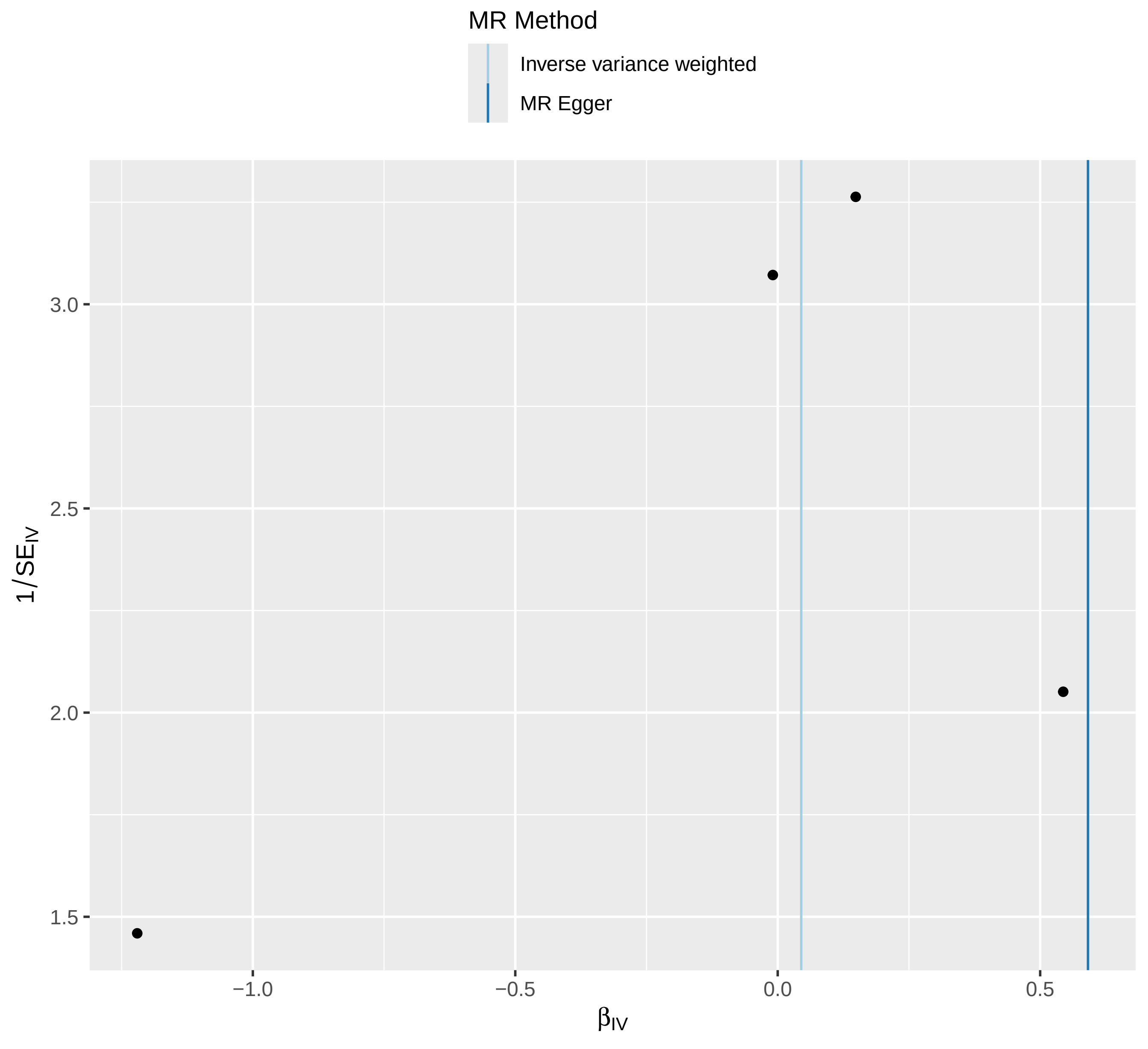
MR Method
Inverse variance weighted
MR Egger
3.0
2.5
2.0
1.5
−1.0
−0.5
0.0
0.5
$1/SE_{IV}$
β_{IV}

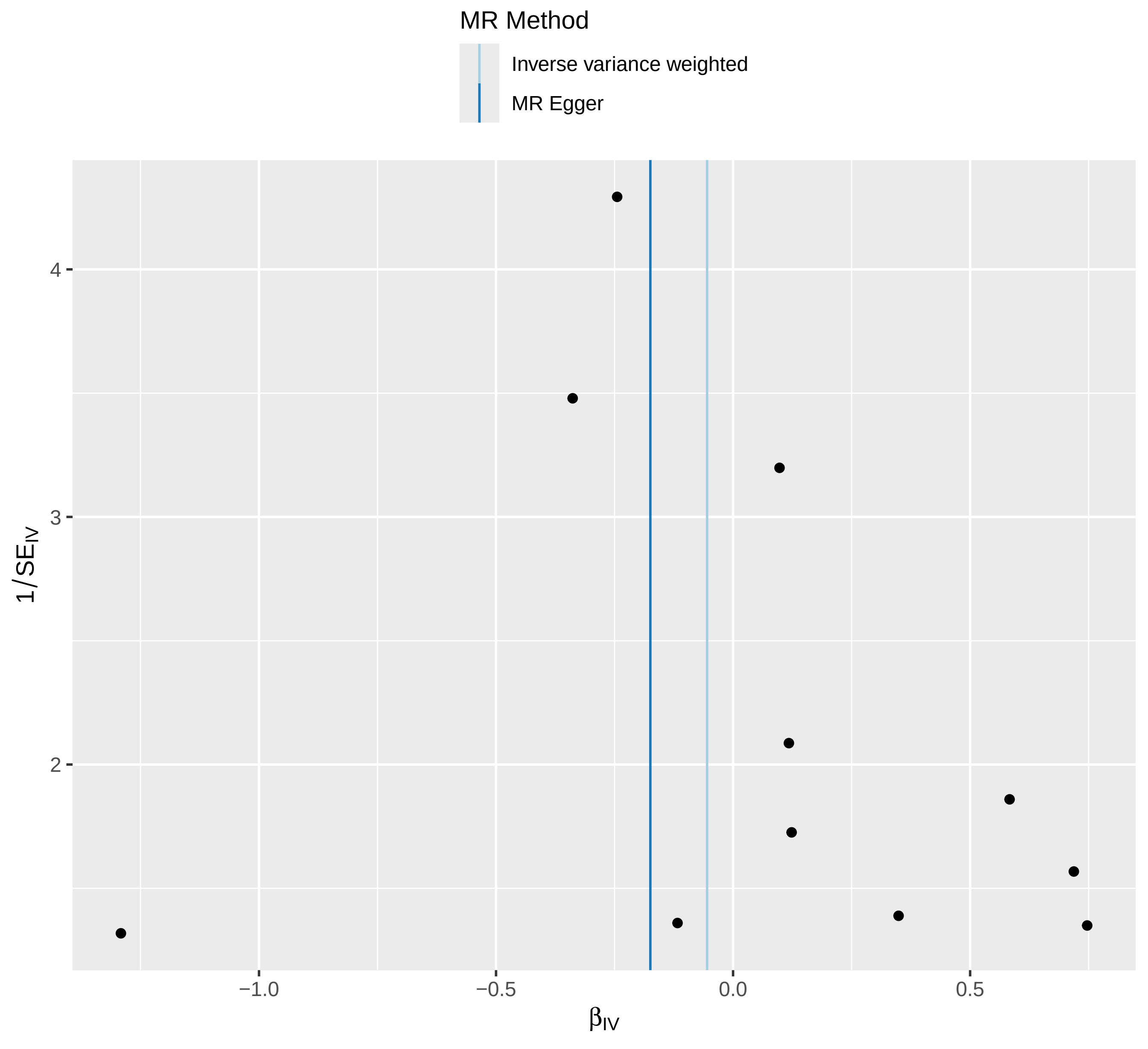

MR Method
Inverse variance weighted
MR Egger
4
3
2
1/SE$_{IV}$
−1.0
−0.5
0.0
0.5
β$_{IV}$

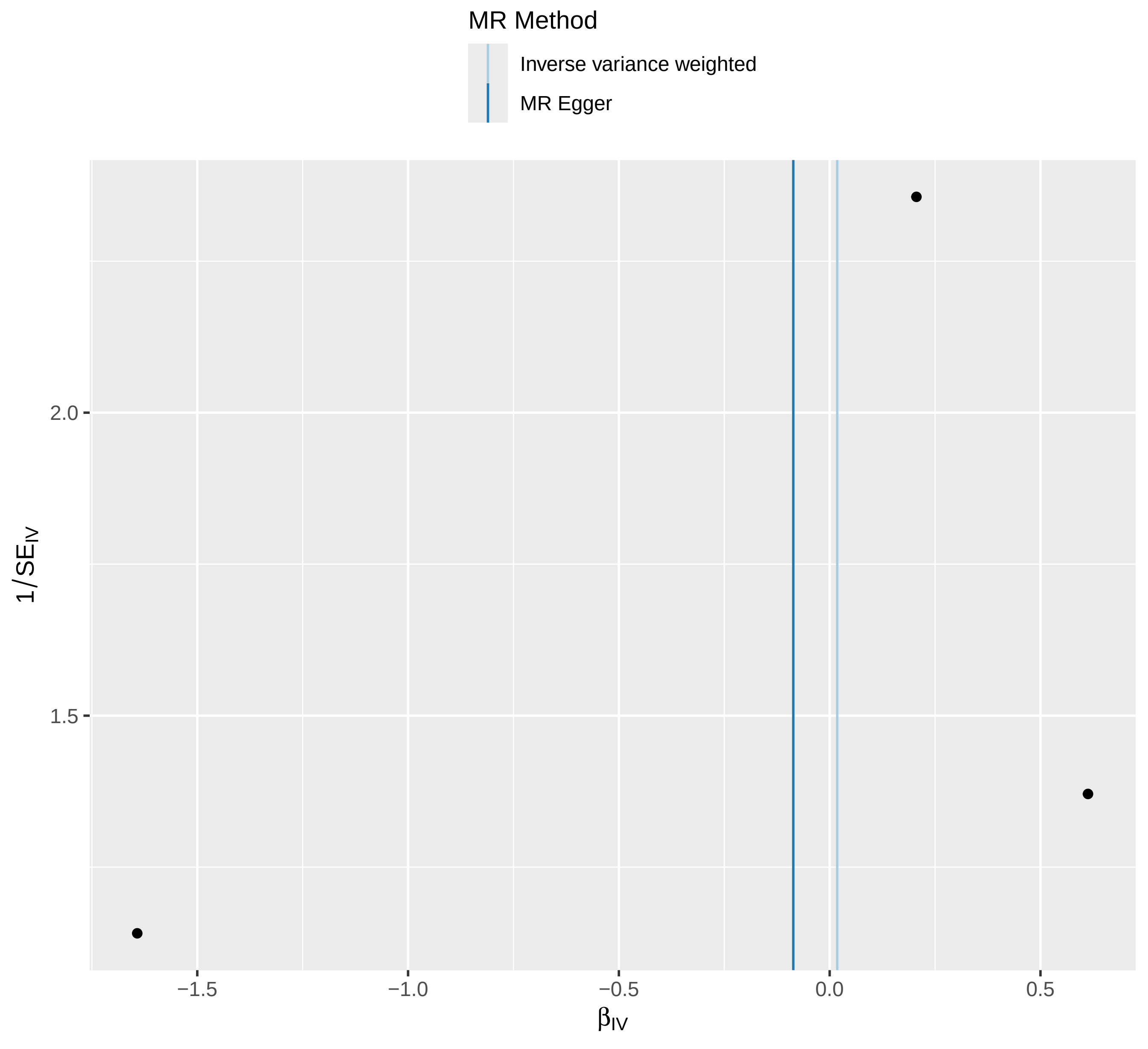

MR Method
Inverse variance weighted
MR Egger
2.0
1.5
$1/SE_{IV}$
−1.5
−1.0
−0.5
0.0
0.5
β_{IV}

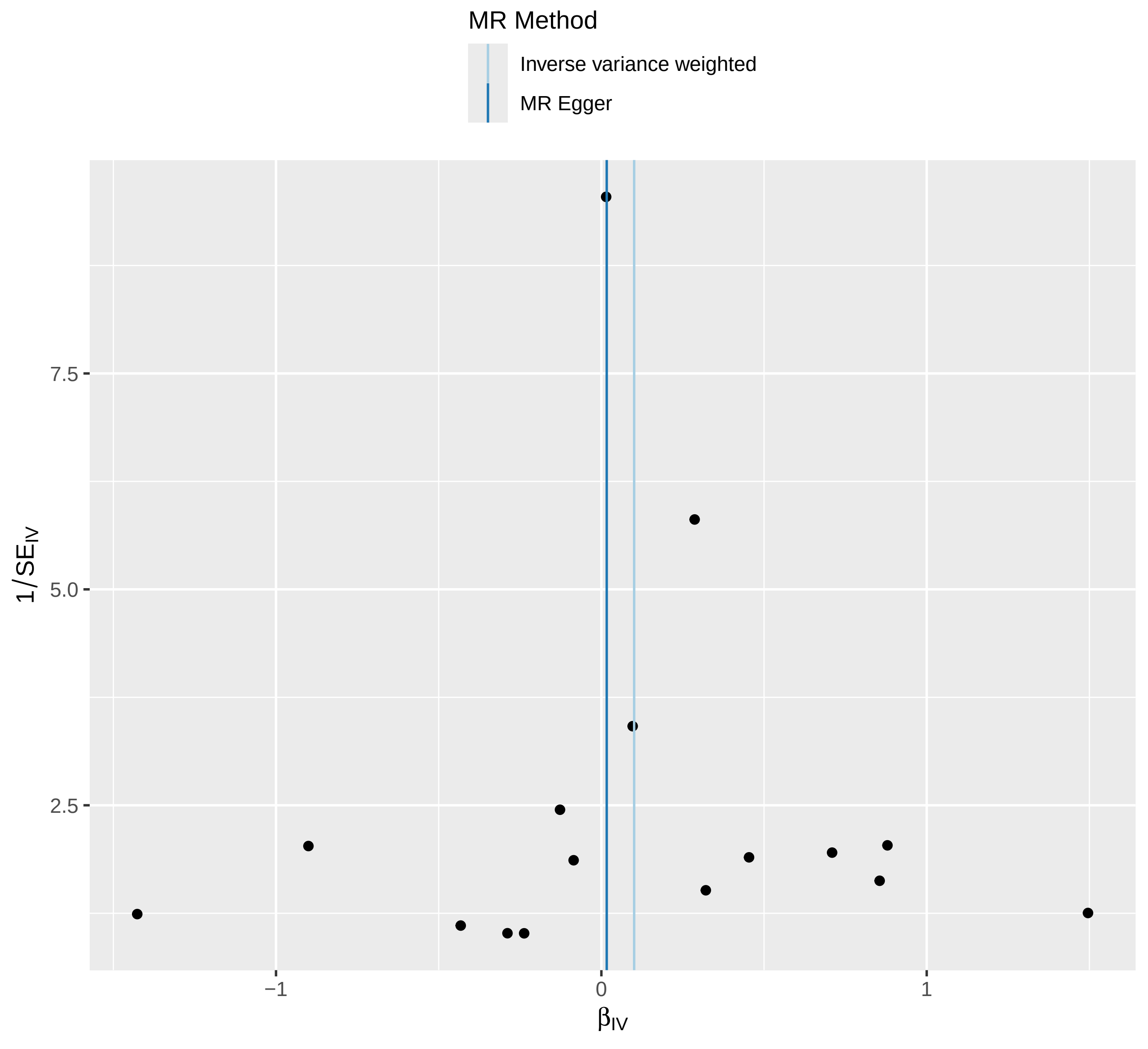

MR Method
Inverse variance weighted
MR Egger
7.5
5.0
2.5
1/SE$_{IV}$
−1
0
1
β$_{IV}$

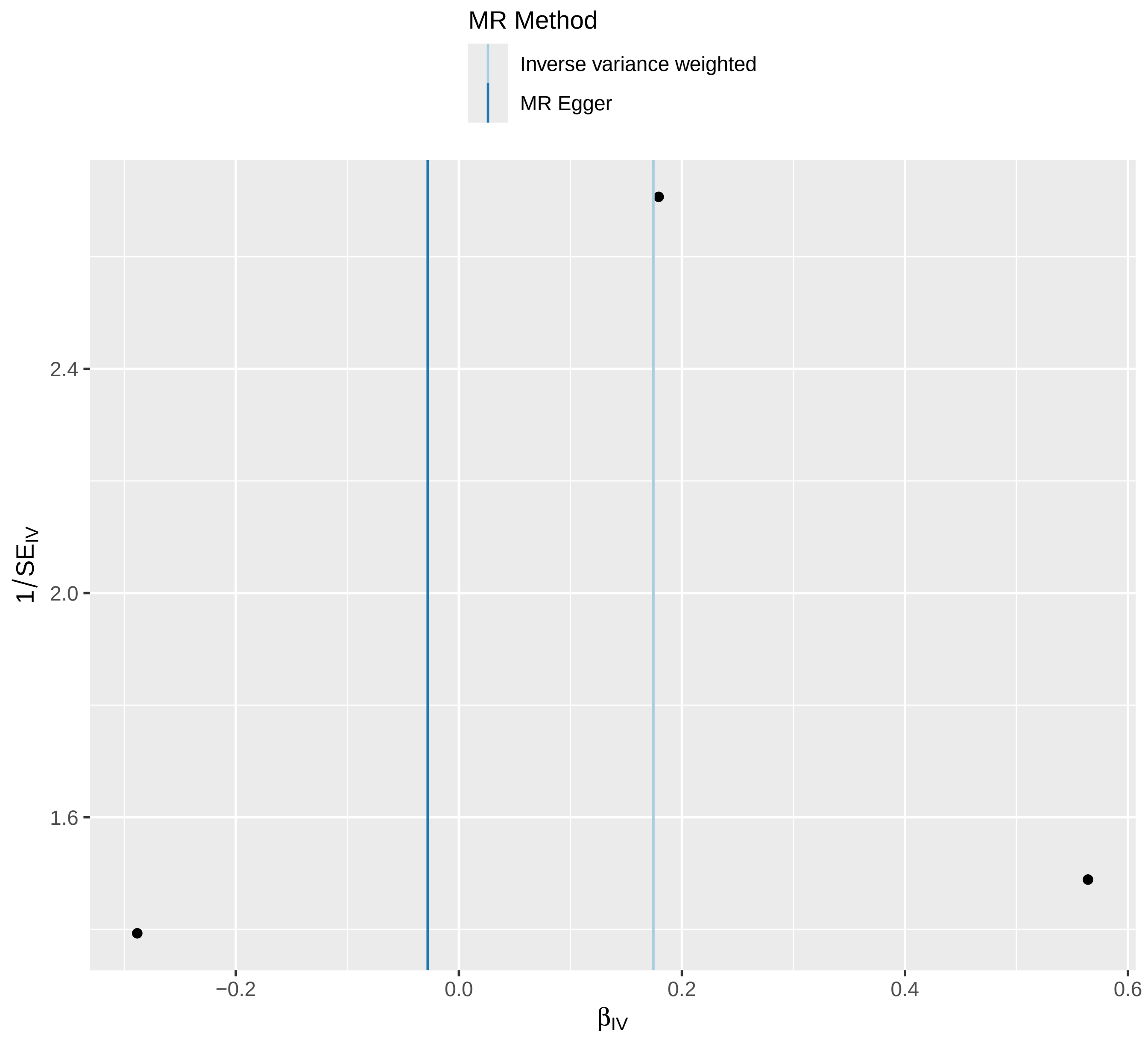
MR Method
Inverse variance weighted
MR Egger
2.4
2.0
1.6
1/SE_IV
−0.2
0.0
0.2
0.4
0.6
β_IV

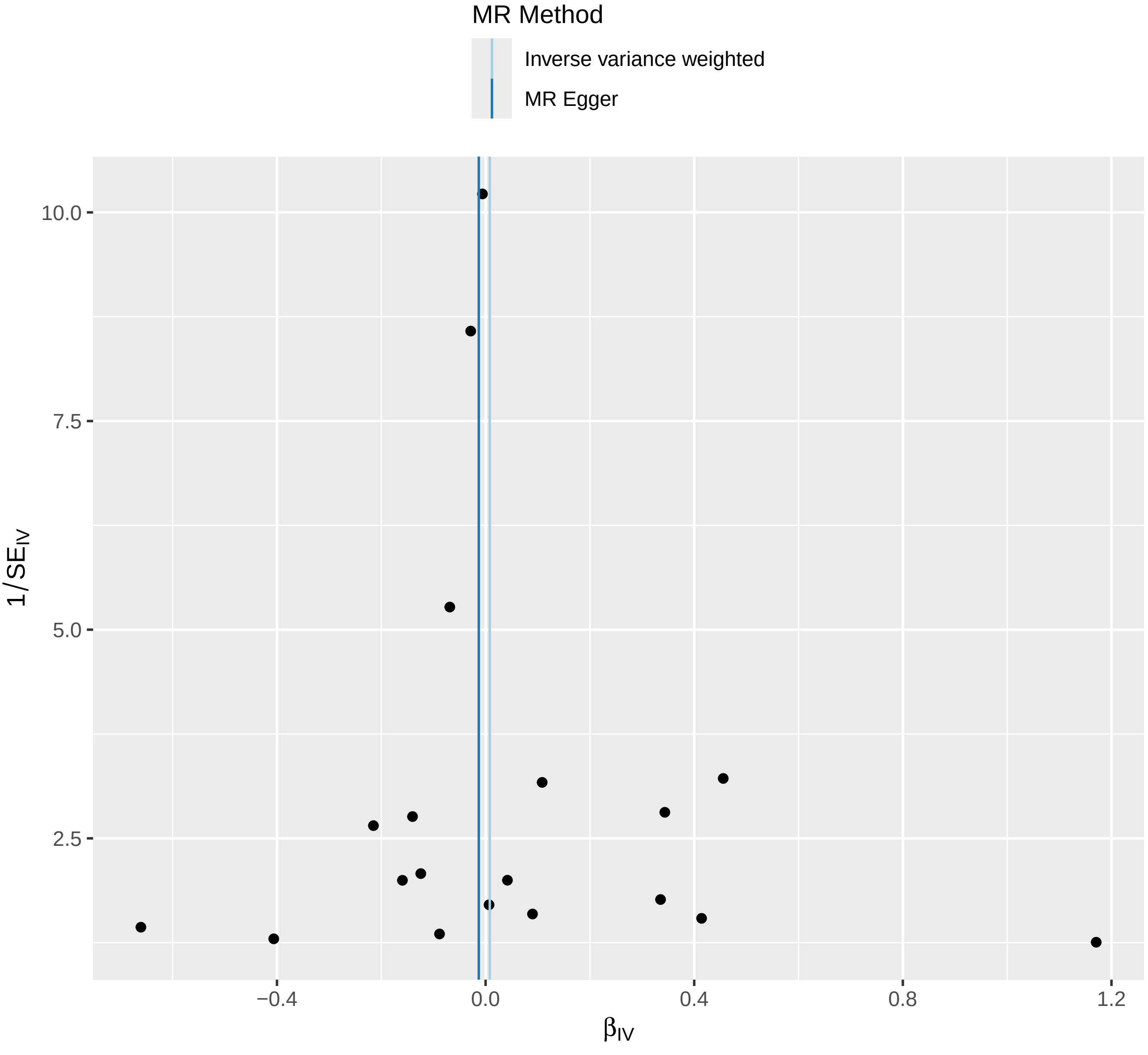

MR Method
Inverse variance weighted
MR Egger
10.0
7.5
5.0
2.5
1/SE_IV
−0.4
0.0
0.4
0.8
1.2
β_IV

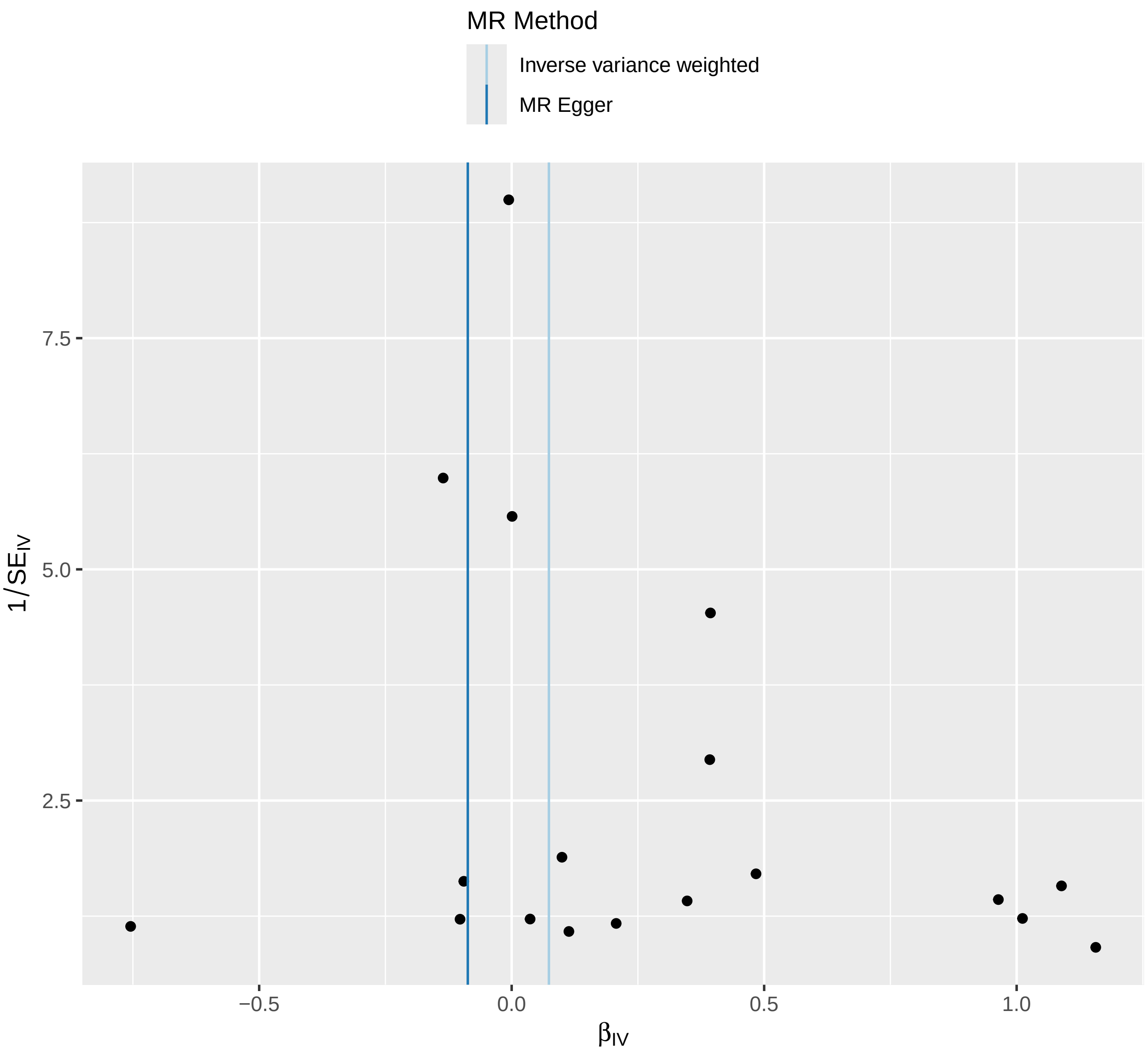
MR Method
Inverse variance weighted
MR Egger
7.5
5.0
2.5
1/SE$_{IV}$
−0.5
0.0
0.5
1.0
β$_{IV}$

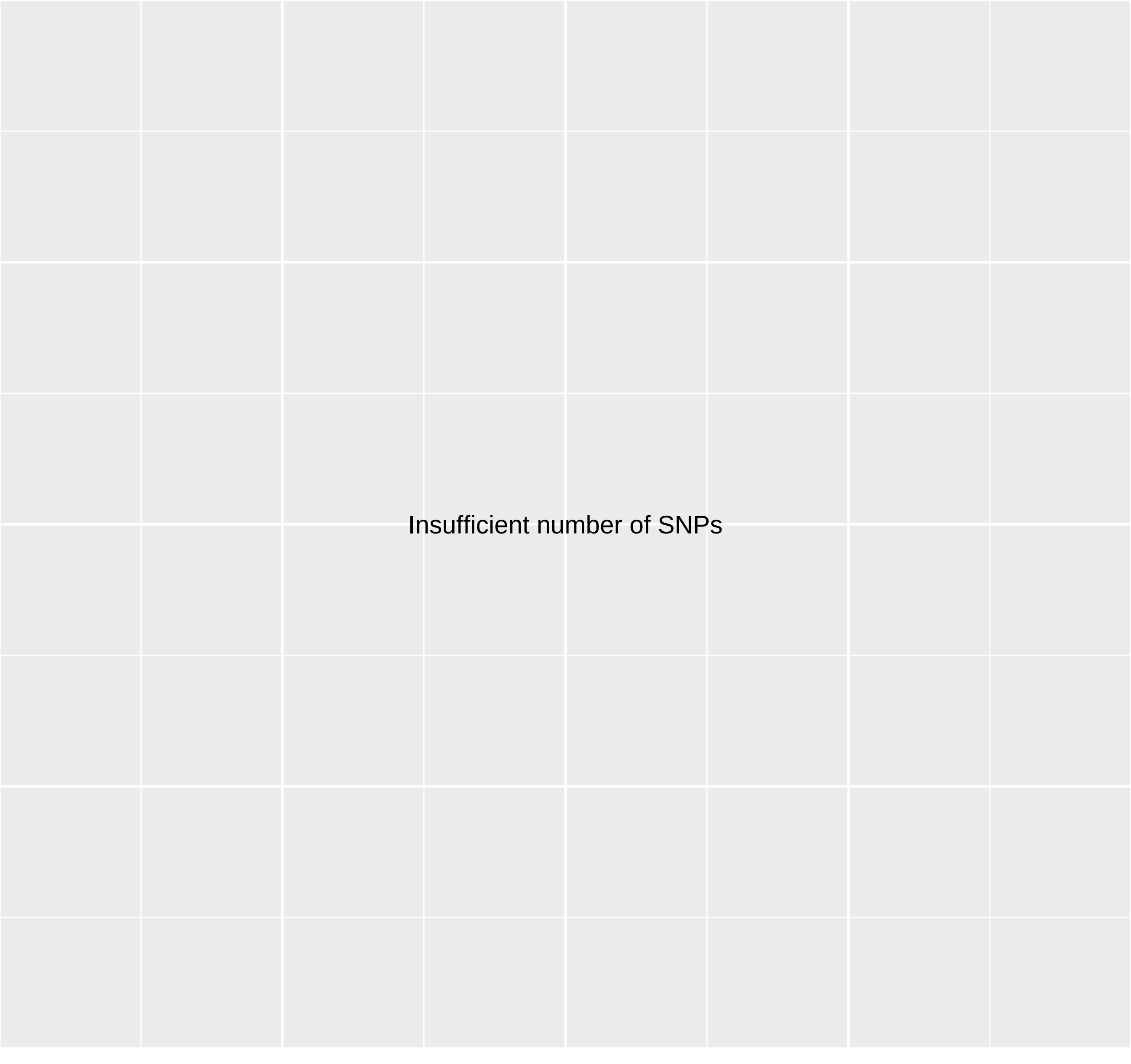
Insufficient number of SNPs

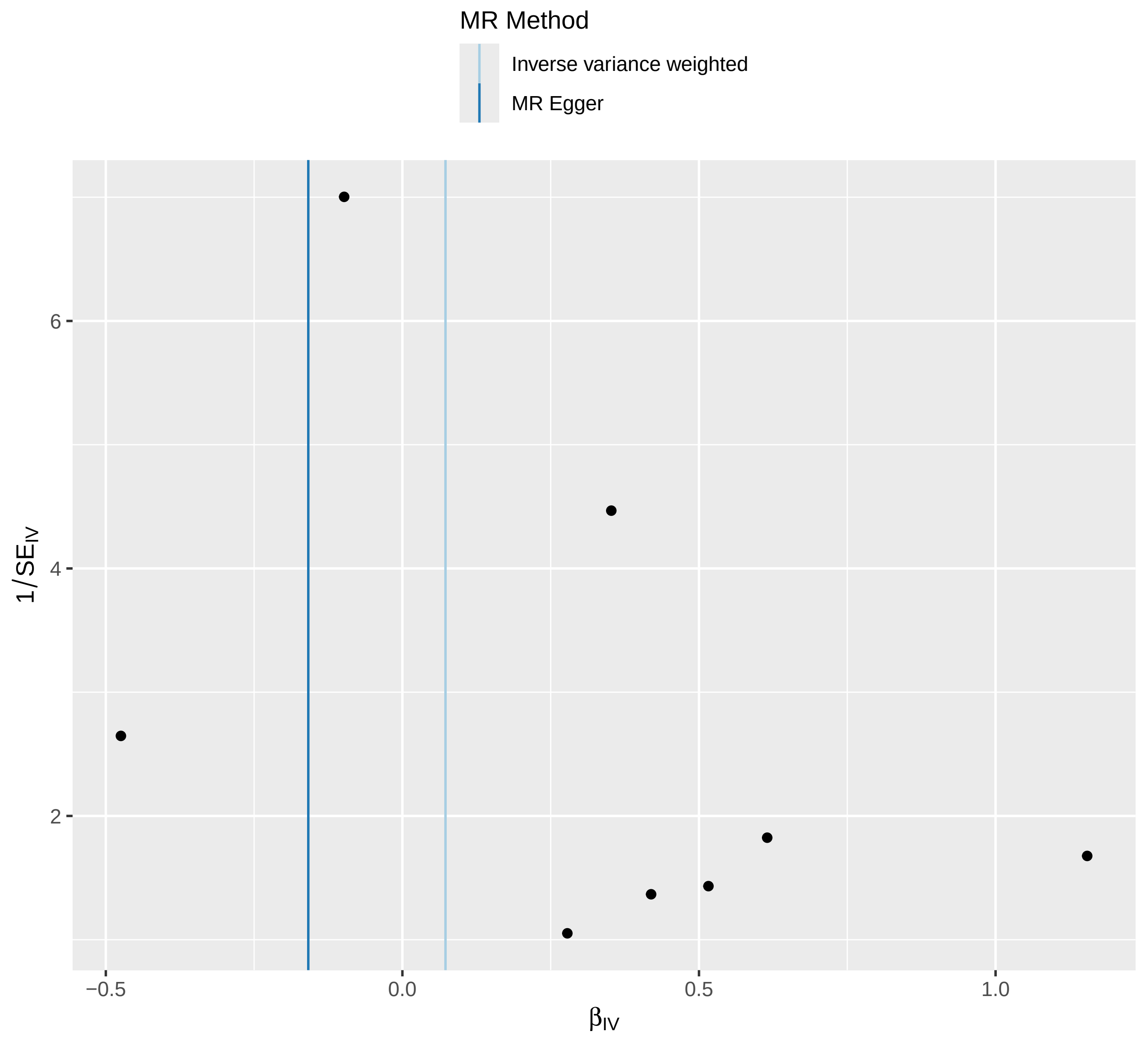
MR Method
Inverse variance weighted
MR Egger
1/SE_IV
6
4
2
−0.5
0.0
0.5
1.0
β_IV

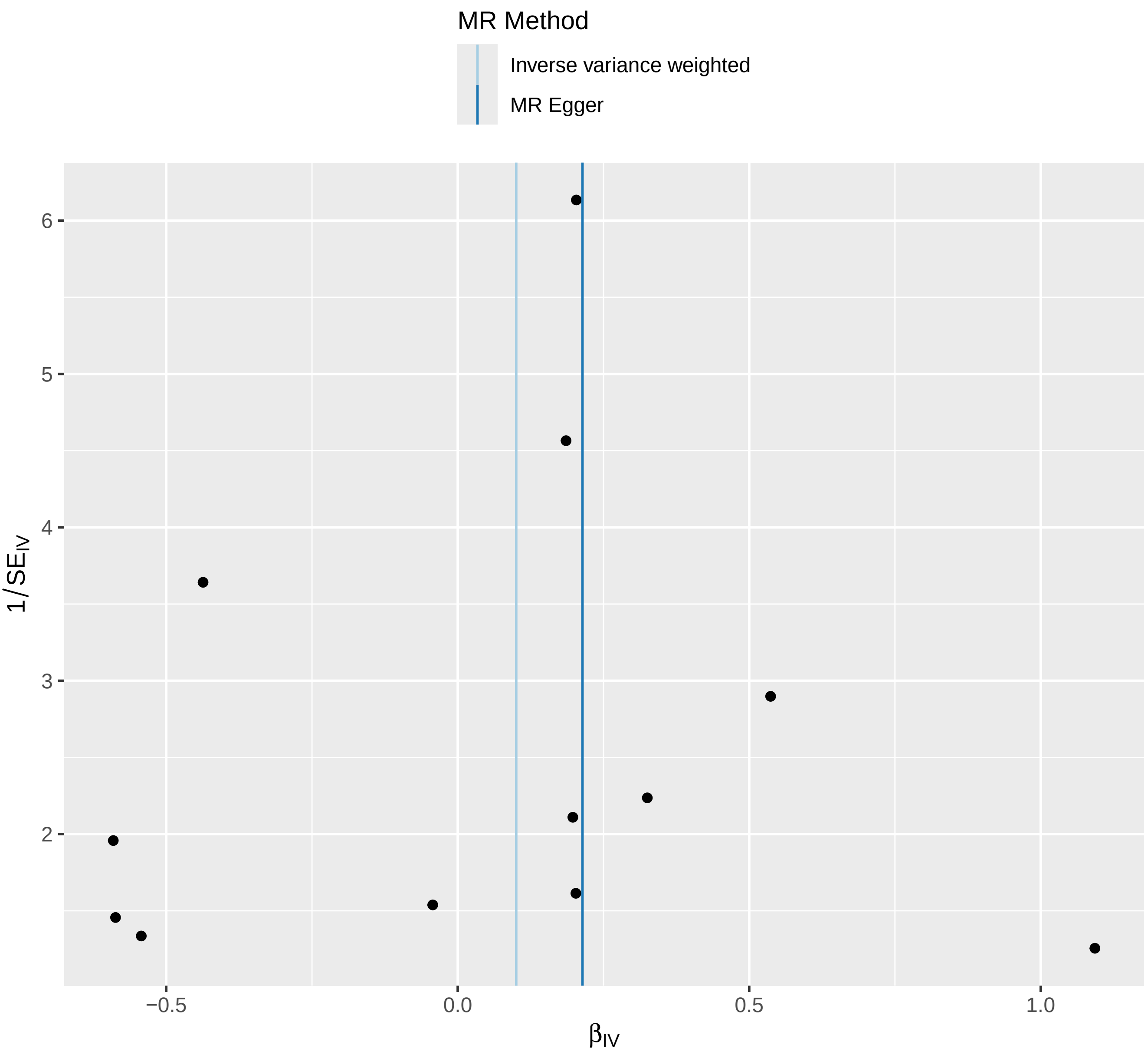
MR Method
Inverse variance weighted
MR Egger
6
5
4
3
2
1/SE_IV
−0.5
0.0
0.5
1.0
β_IV

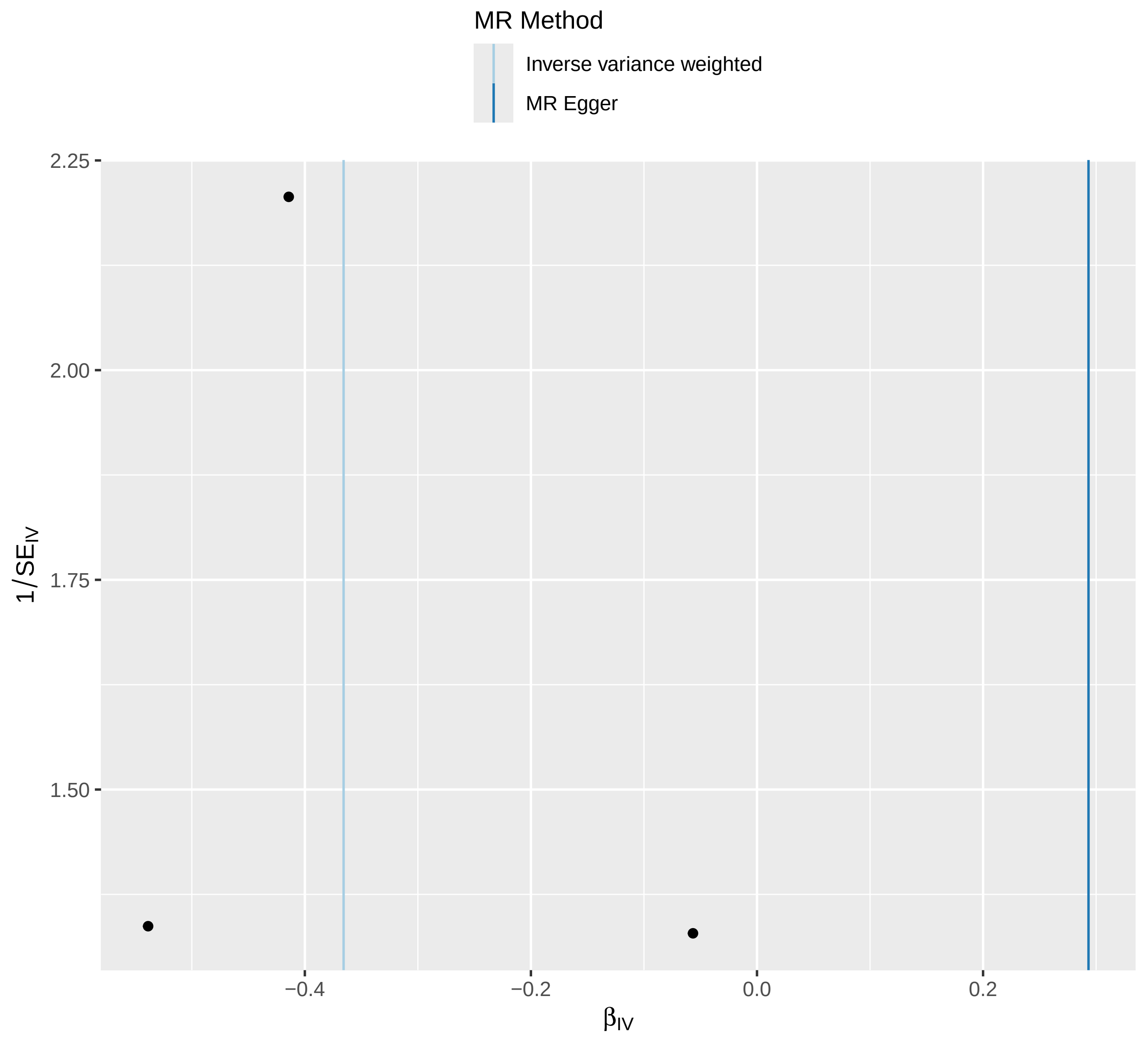
MR Method
Inverse variance weighted
MR Egger
2.25
2.00
1.75
1.50
1/SE_IV
−0.4
−0.2
0.0
0.2
β_IV

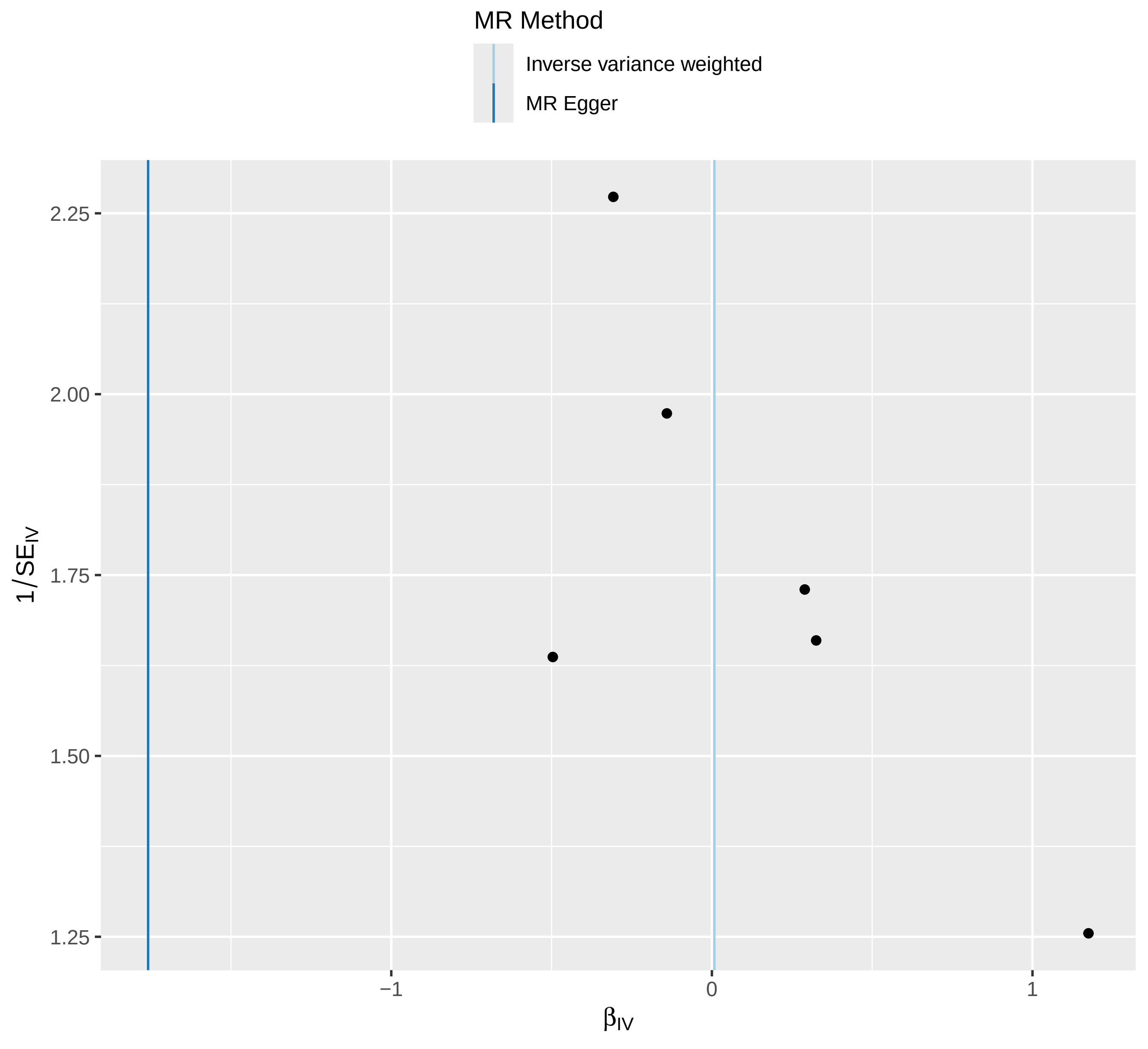

MR Method
Inverse variance weighted
MR Egger
2.25
2.00
1.75
1.50
1.25
1/SE_IV
−1
0
1
β_IV

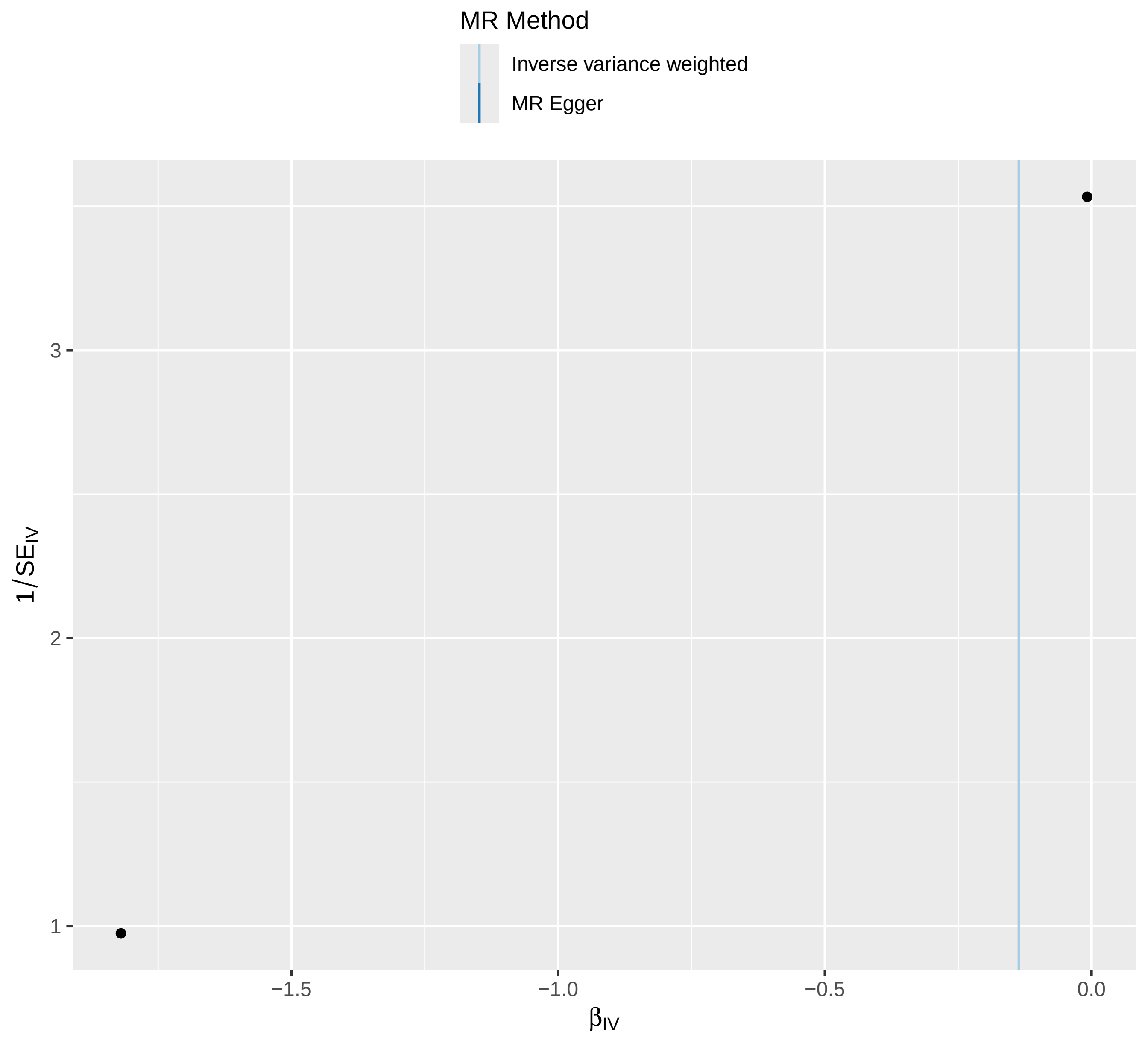

MR Method
Inverse variance weighted
MR Egger
3
2
1
1/SE$_{IV}$
−1.5
−1.0
−0.5
0.0
β$_{IV}$

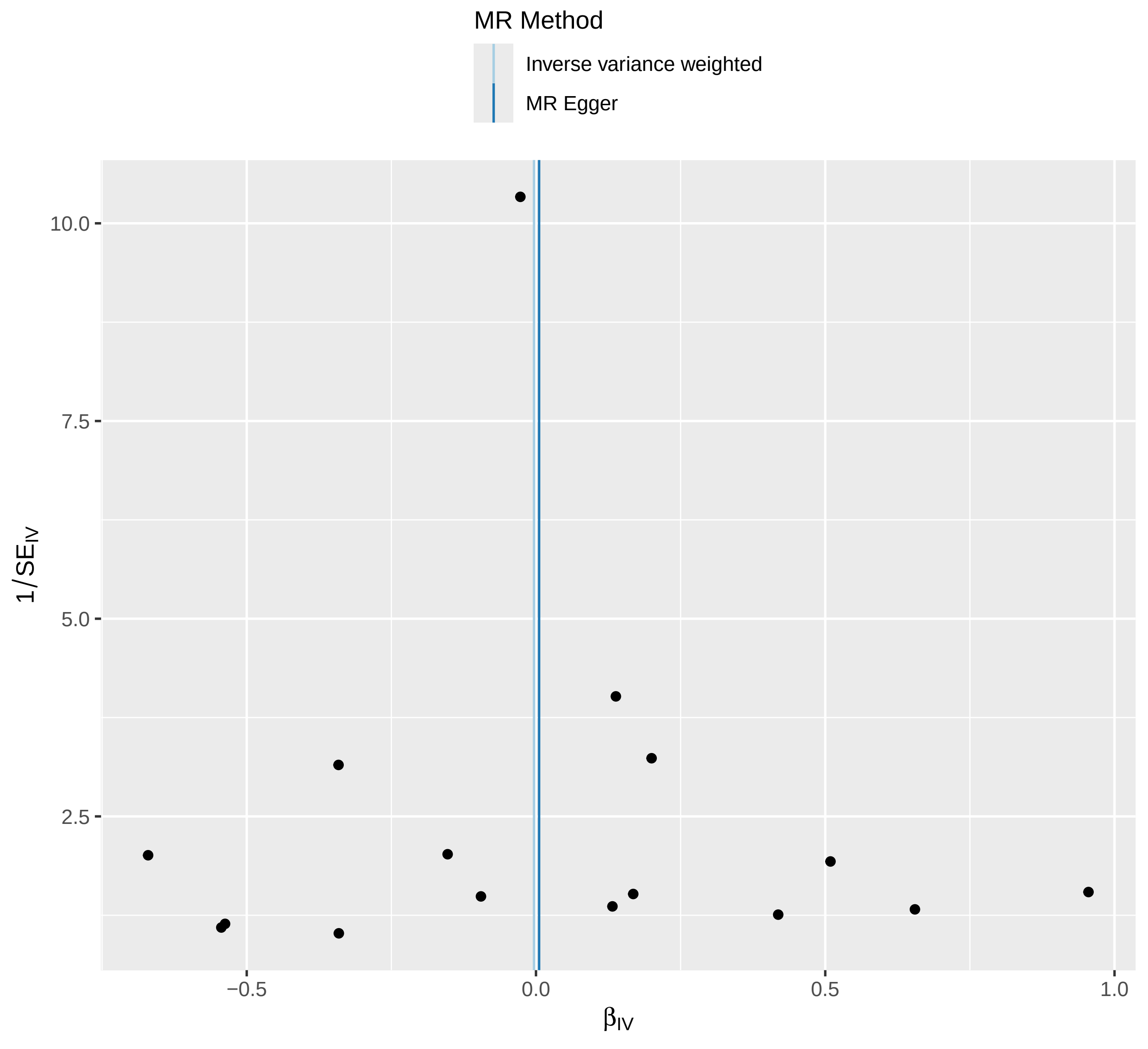
MR Method
Inverse variance weighted
MR Egger
10.0
7.5
5.0
2.5
1/SE_IV
−0.5
0.0
0.5
1.0
β_IV

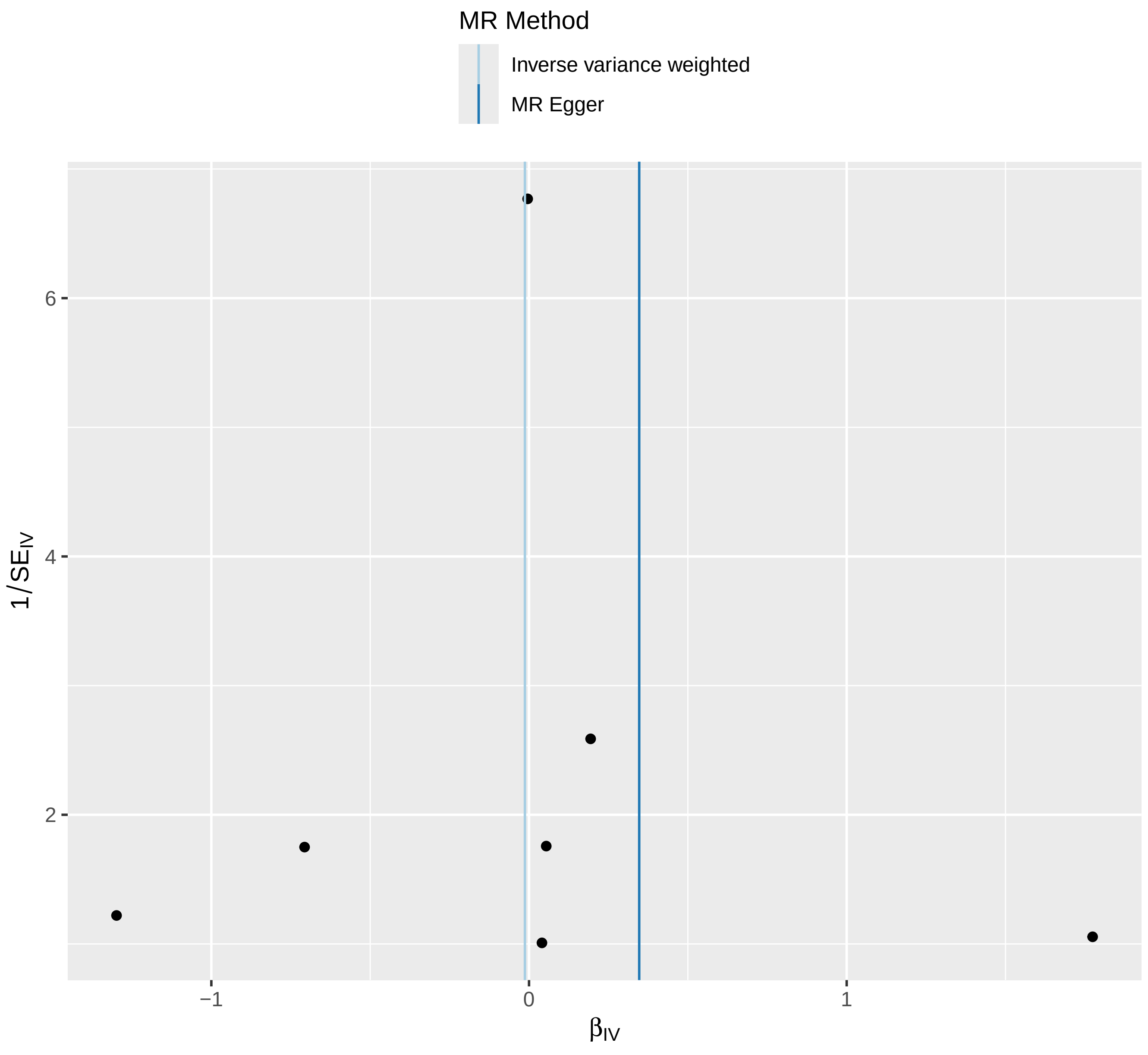

MR Method
Inverse variance weighted
MR Egger
6
4
2
1/SE$_{IV}$
−1
0
1
β$_{IV}$

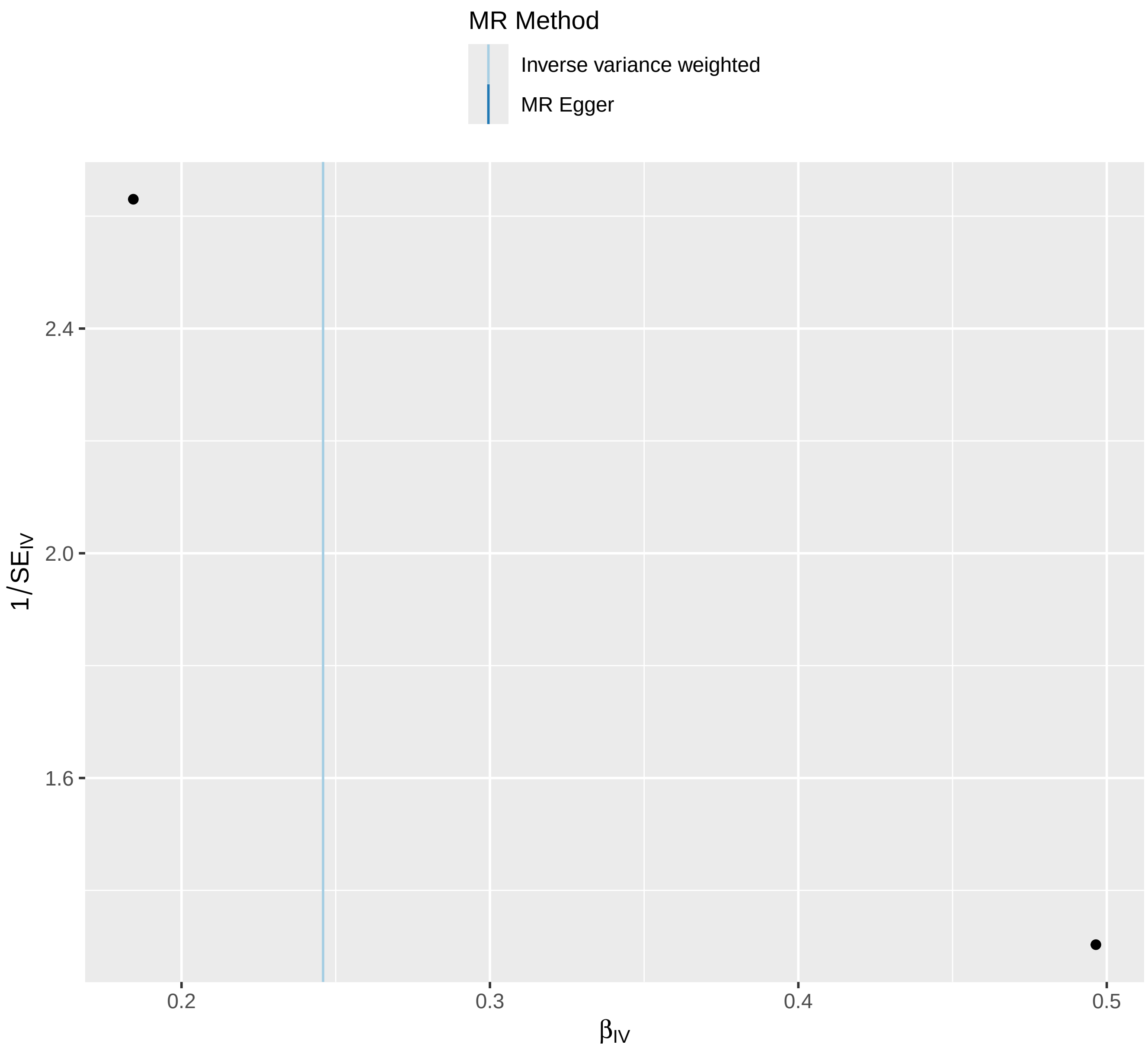

MR Method
Inverse variance weighted
MR Egger
2.4
2.0
1.6
1/SE$_{IV}$
0.2
0.3
0.4
0.5
β$_{IV}$

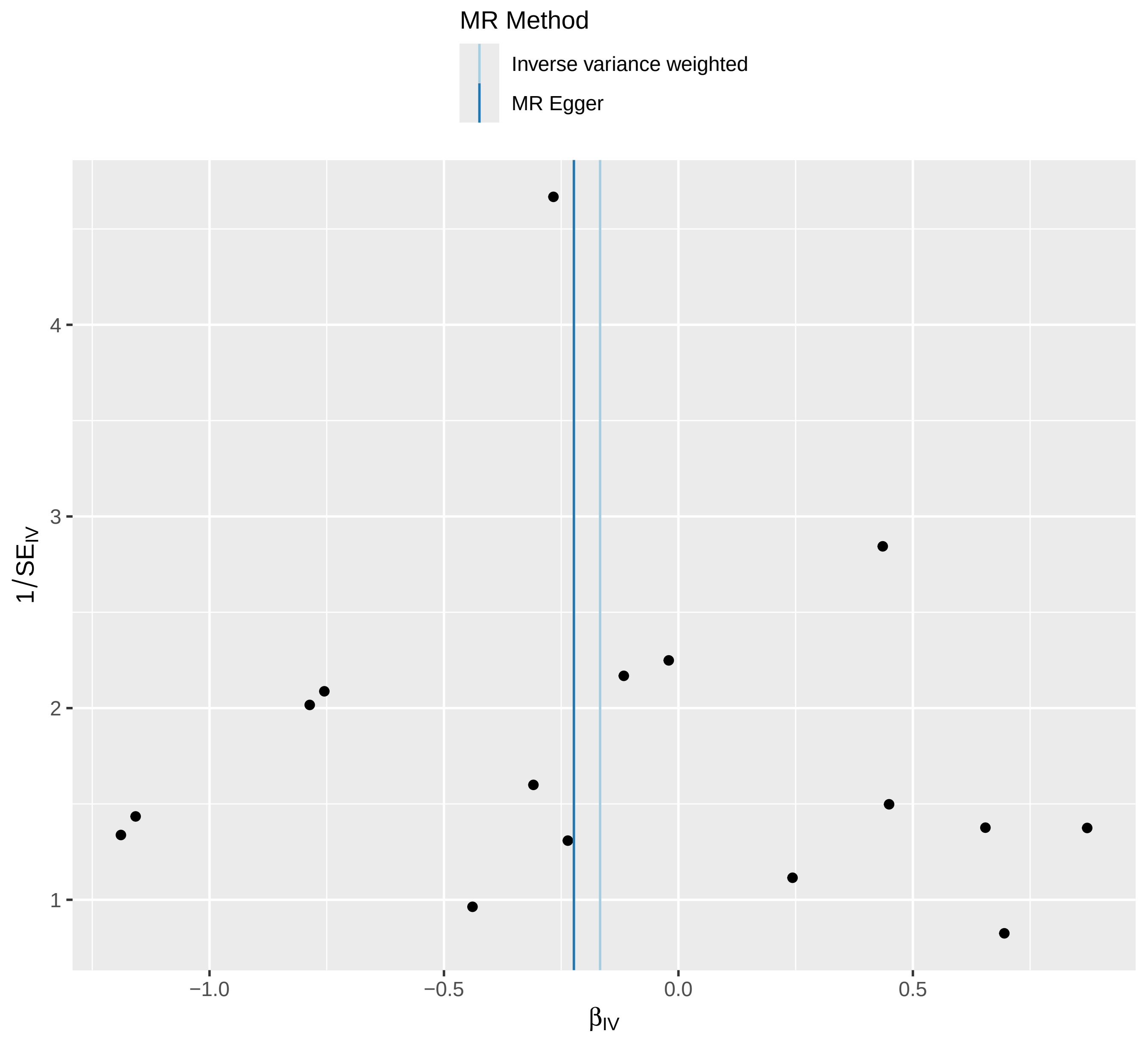

MR Method
Inverse variance weighted
MR Egger
1/SE_IV
4
3
2
1
−1.0
−0.5
0.0
0.5
β_IV

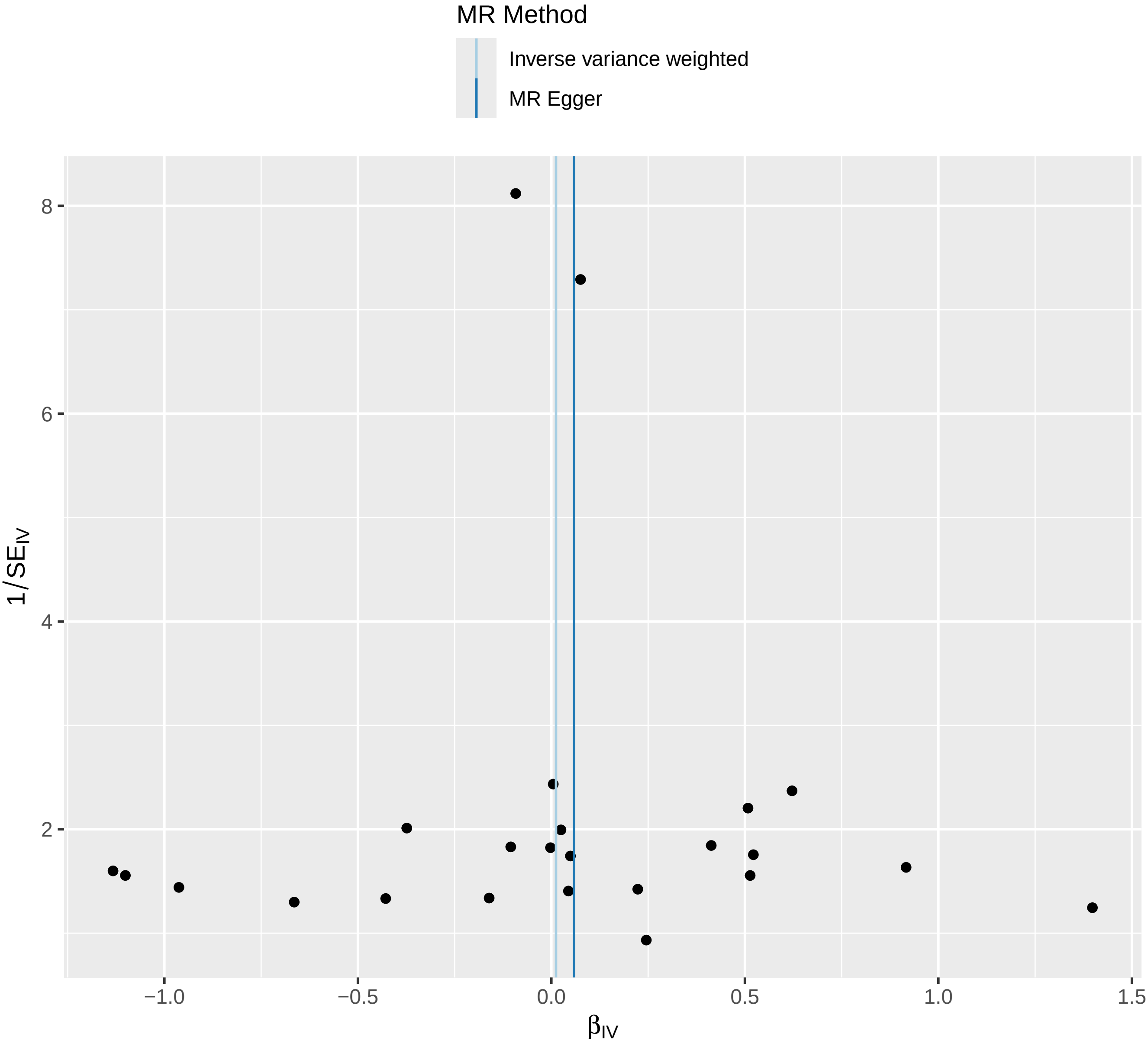
MR Method
Inverse variance weighted
MR Egger
1/SE_IV
8
6
4
2
−1.0
−0.5
0.0
0.5
1.0
1.5
β_IV

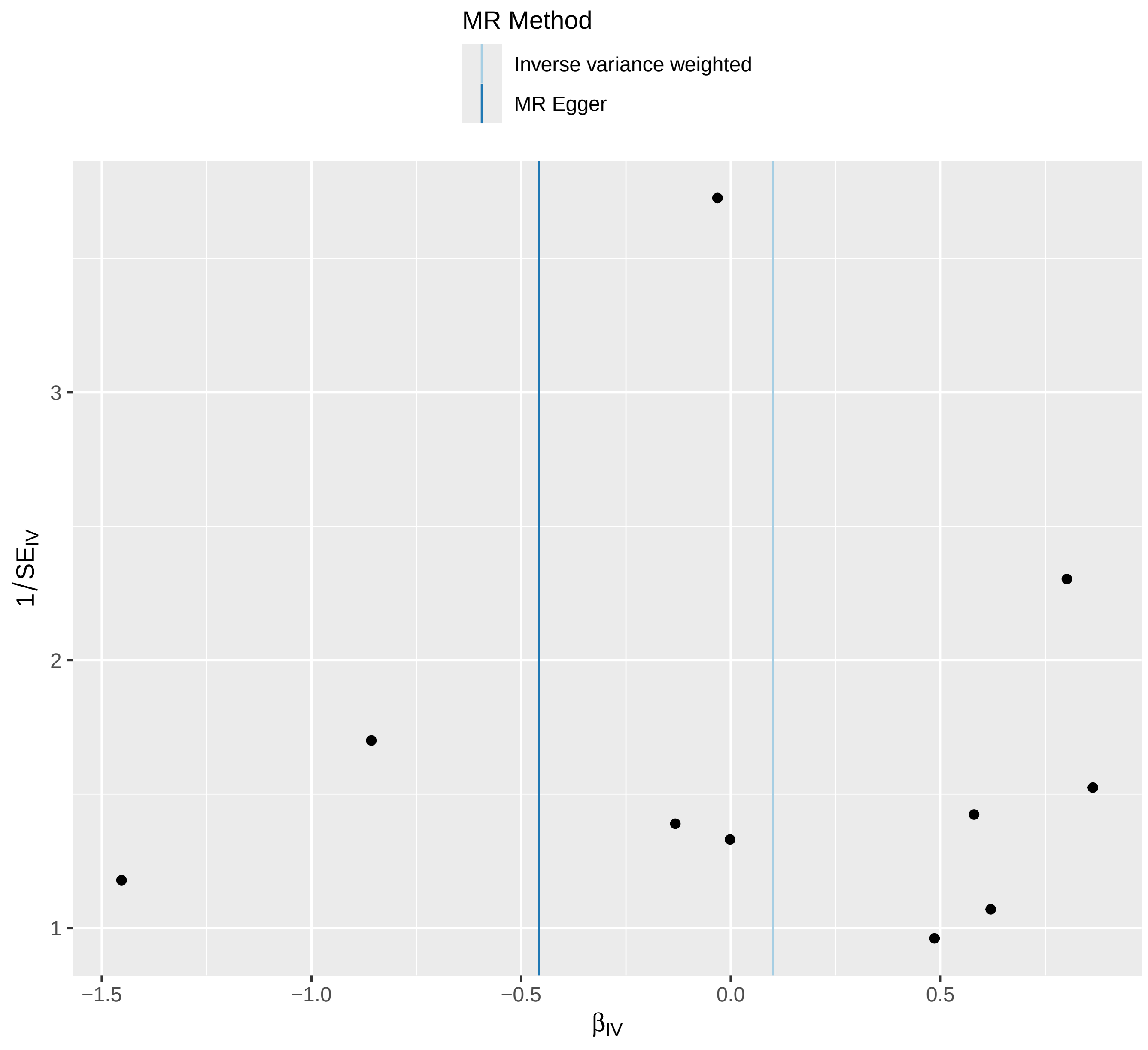

MR Method
Inverse variance weighted
MR Egger
1/SE_IV
3
2
1
−1.5
−1.0
−0.5
0.0
0.5
β_IV

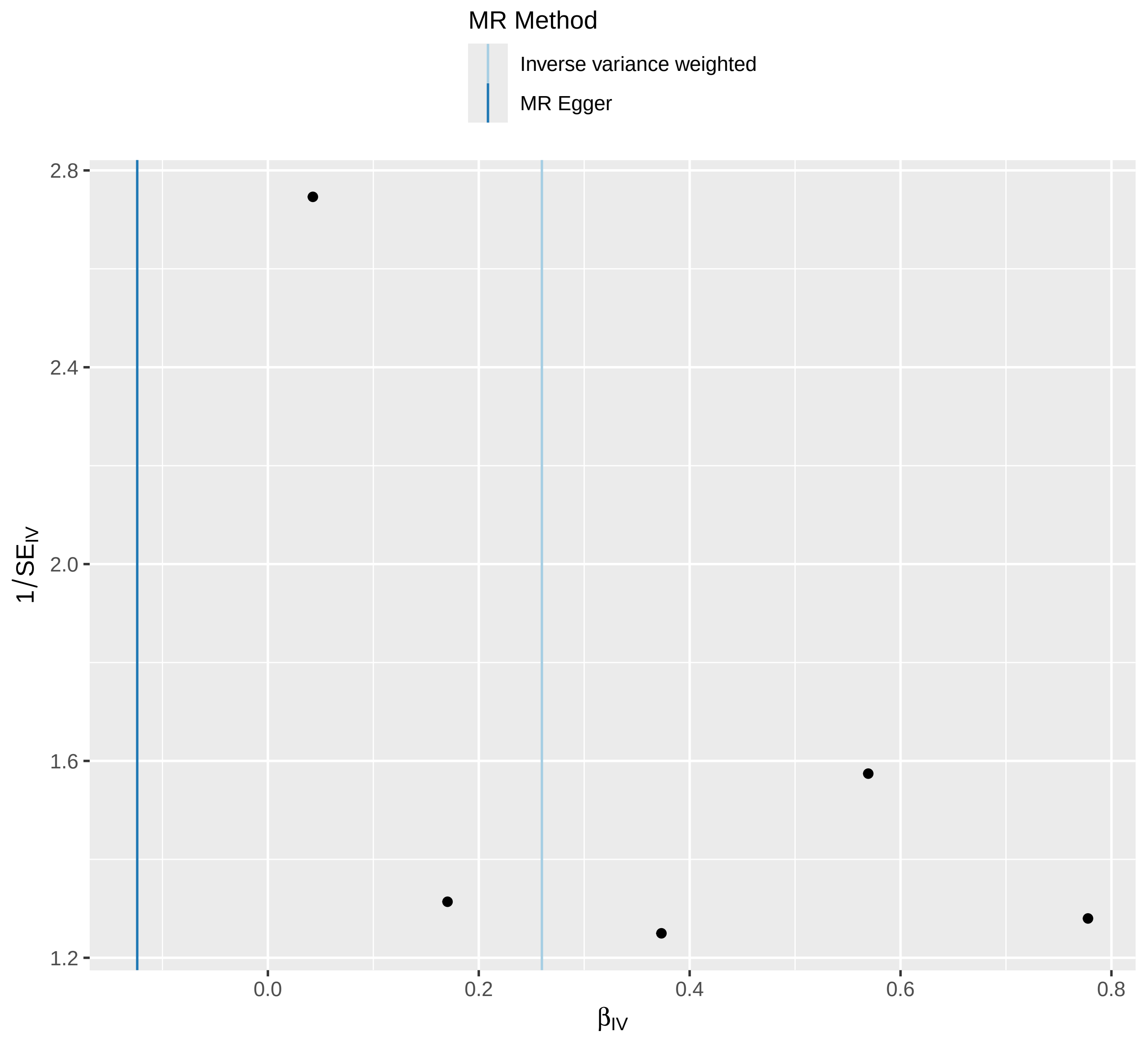
MR Method
Inverse variance weighted
MR Egger
2.8
2.4
2.0
1.6
1.2
1/SE_IV
0.0
0.2
0.4
0.6
0.8
β_IV

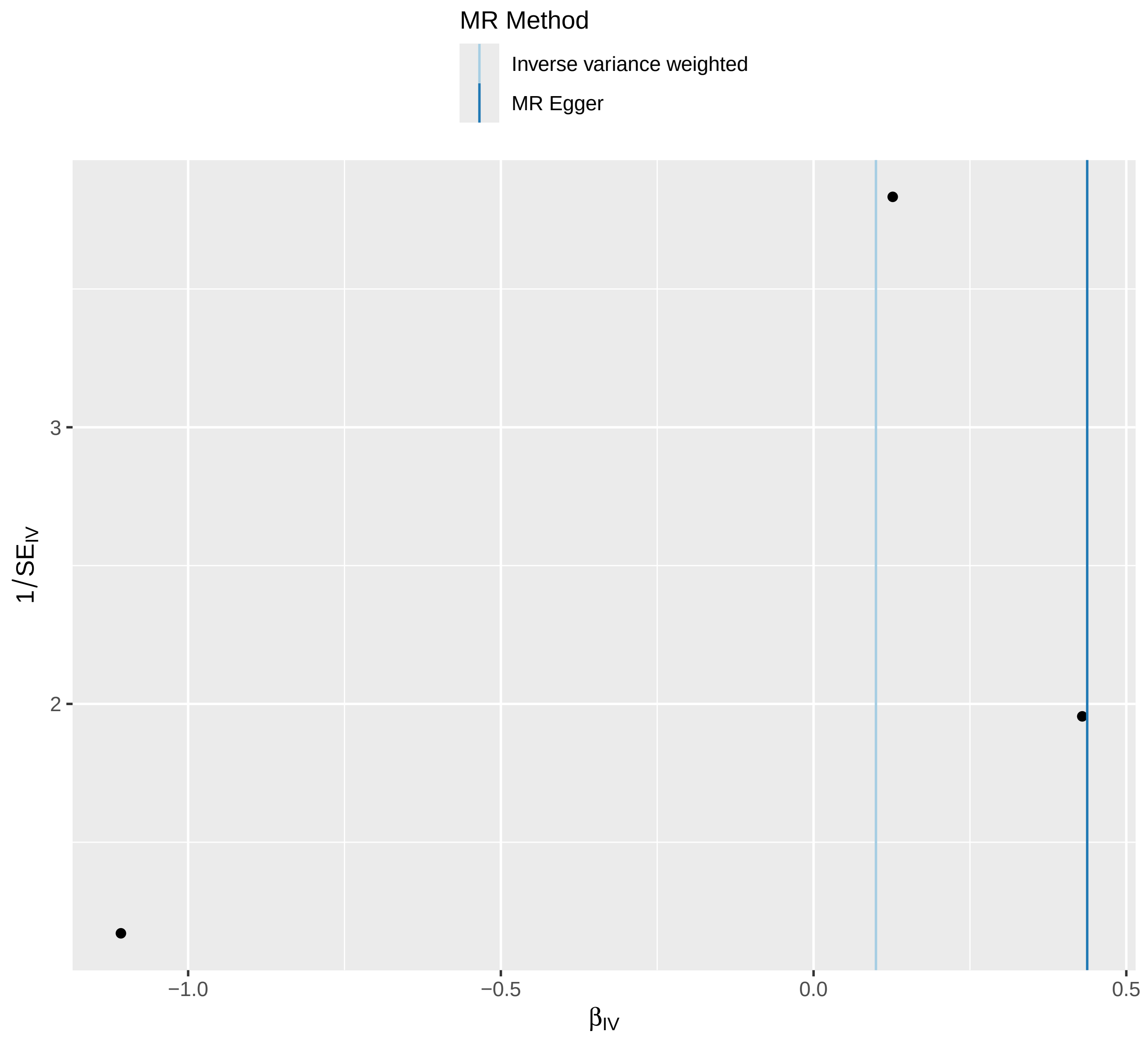
MR Method
Inverse variance weighted
MR Egger
1/SE_IV
3
2
β_IV
−1.0
−0.5
0.0
0.5

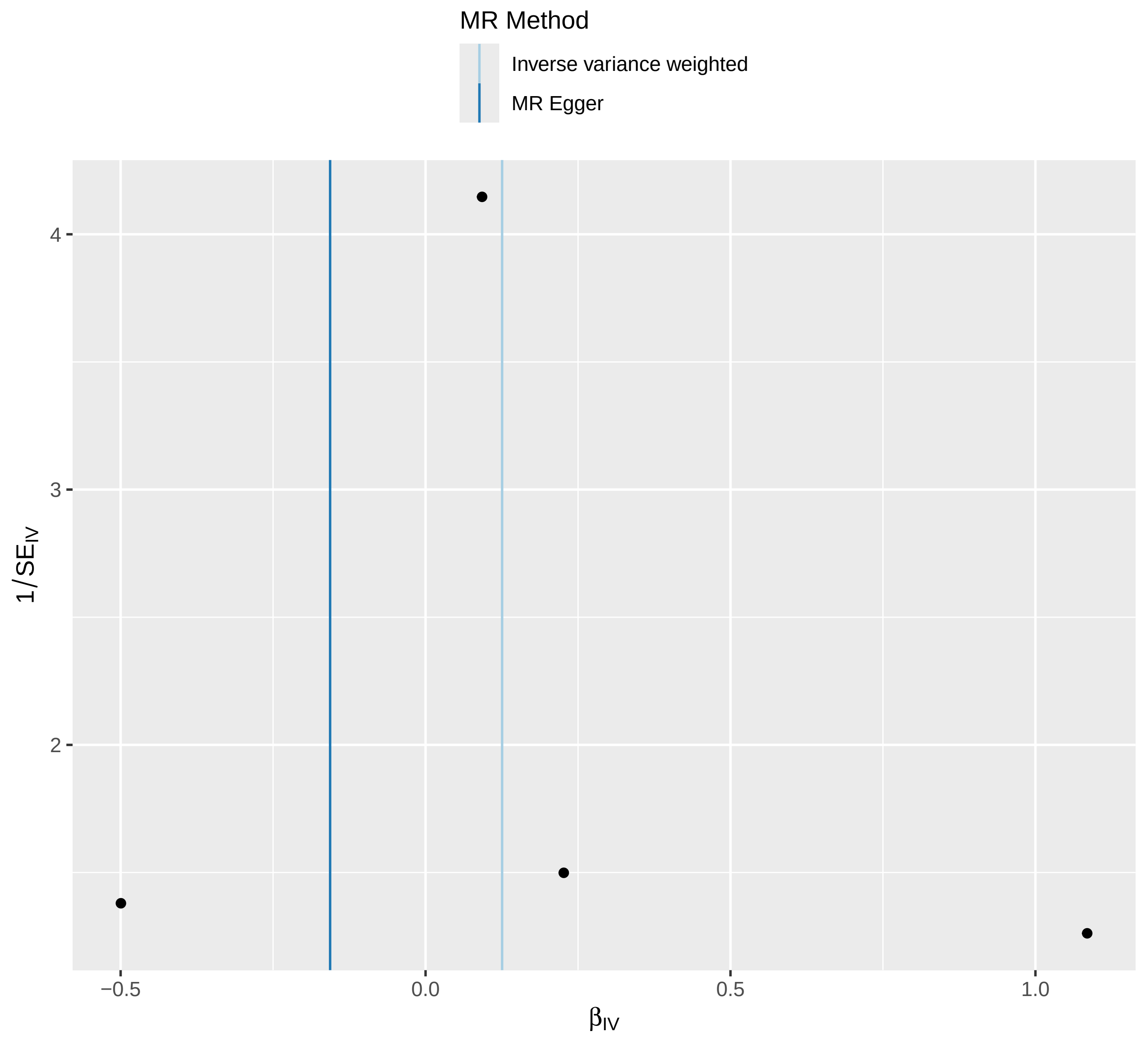
MR Method
Inverse variance weighted
MR Egger
4
3
2
$1/SE_{IV}$
−0.5
0.0
0.5
1.0
β_{IV}

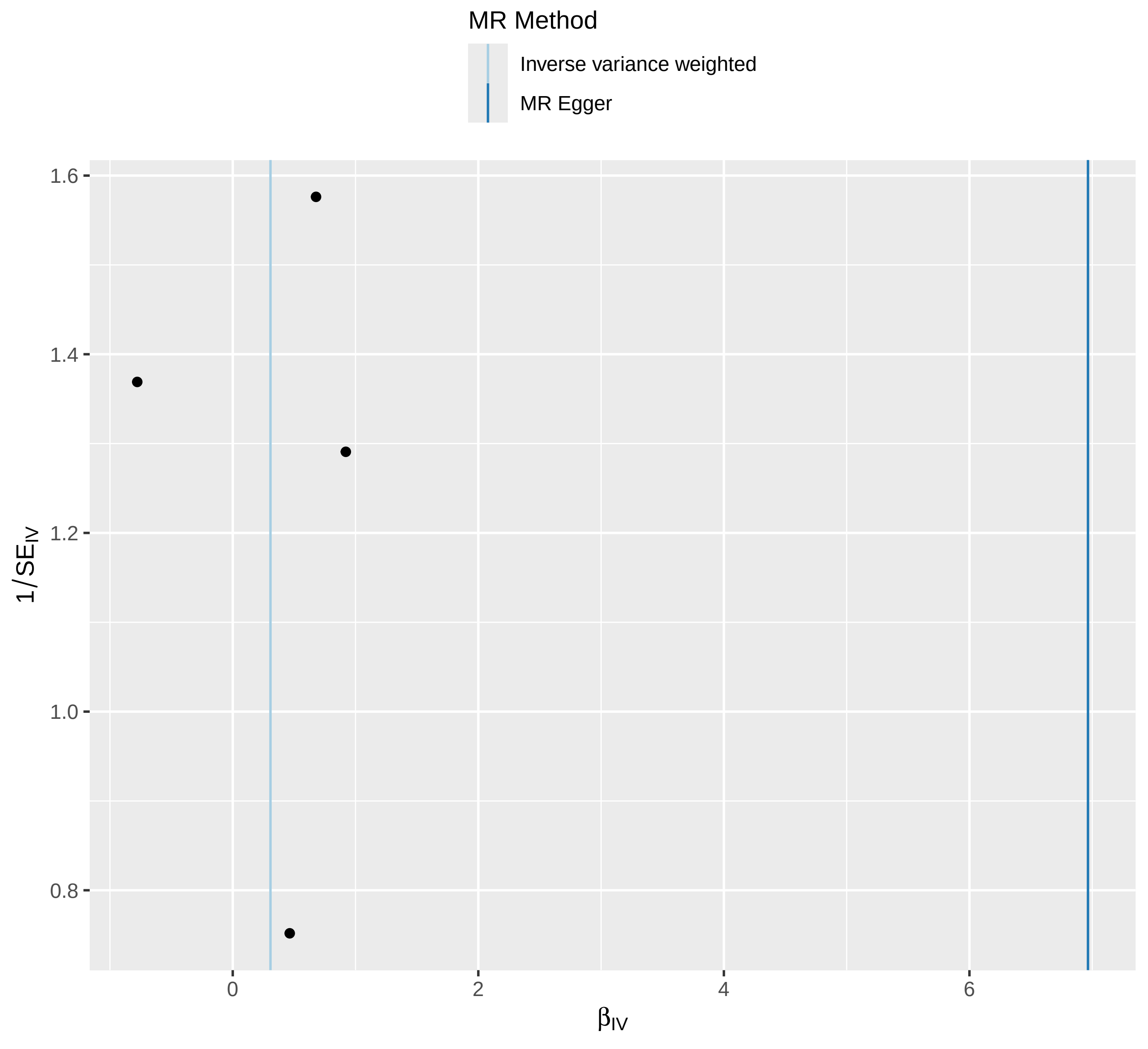
MR Method
Inverse variance weighted
MR Egger
1.6
1.4
1.2
1.0
0.8
$1/SE_{IV}$
0
2
4
6
β_{IV}

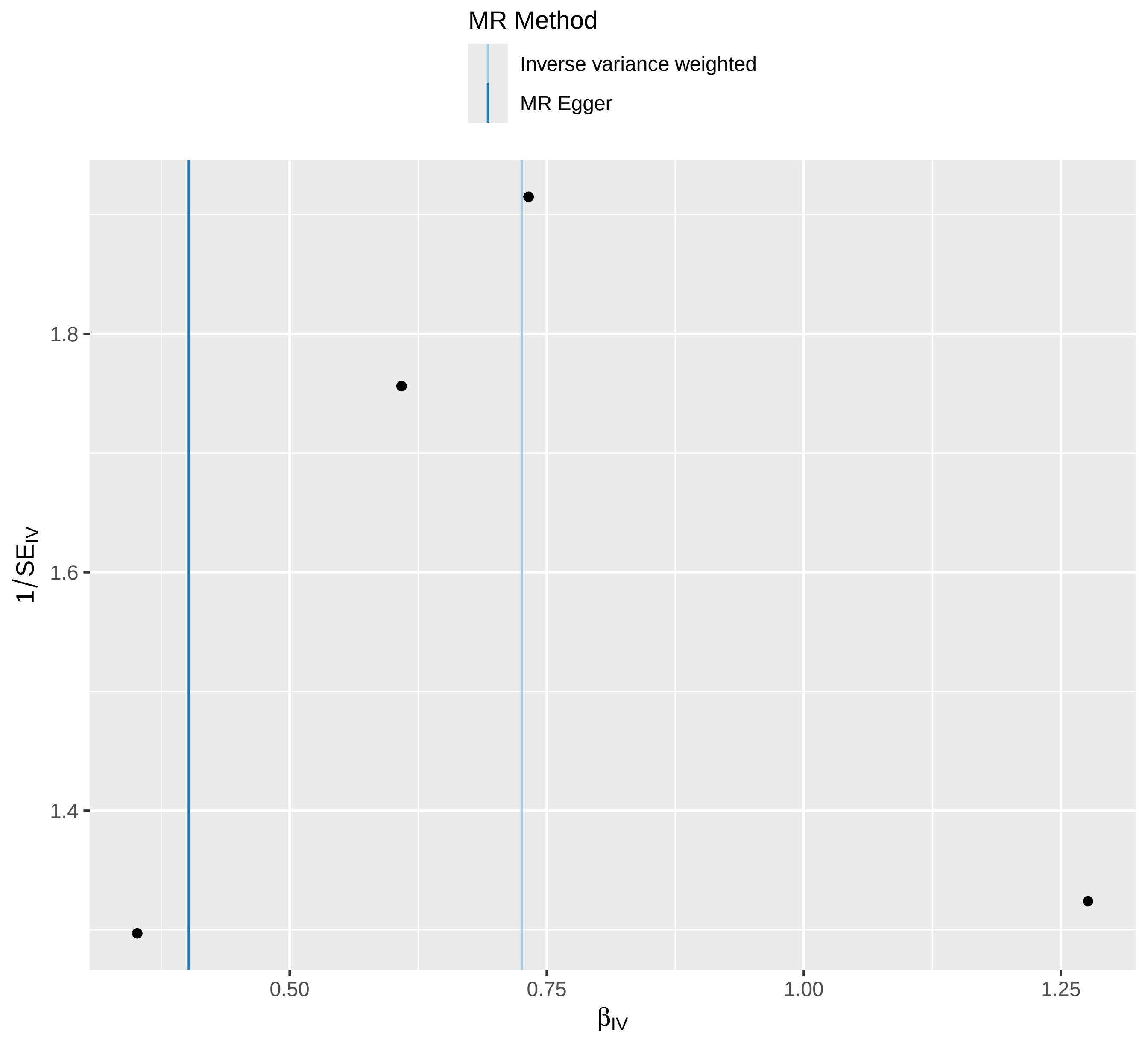
MR Method
Inverse variance weighted
MR Egger
1.8
1.6
1.4
1/SE_IV
0.50
0.75
1.00
1.25
β_IV

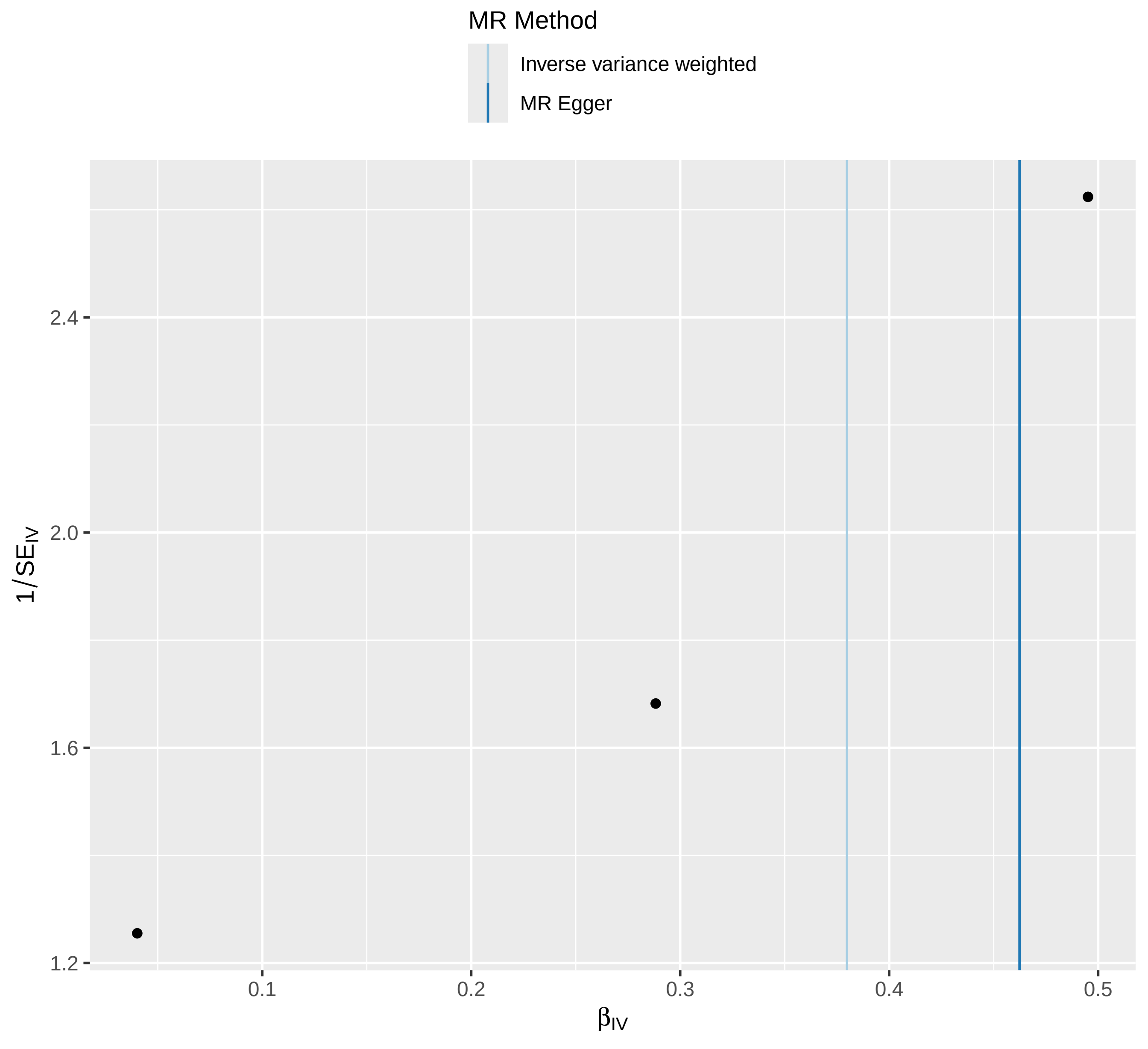
MR Method
Inverse variance weighted
MR Egger
2.4
2.0
1.6
1.2
1/SE_IV
0.1
0.2
0.3
0.4
0.5
β_IV

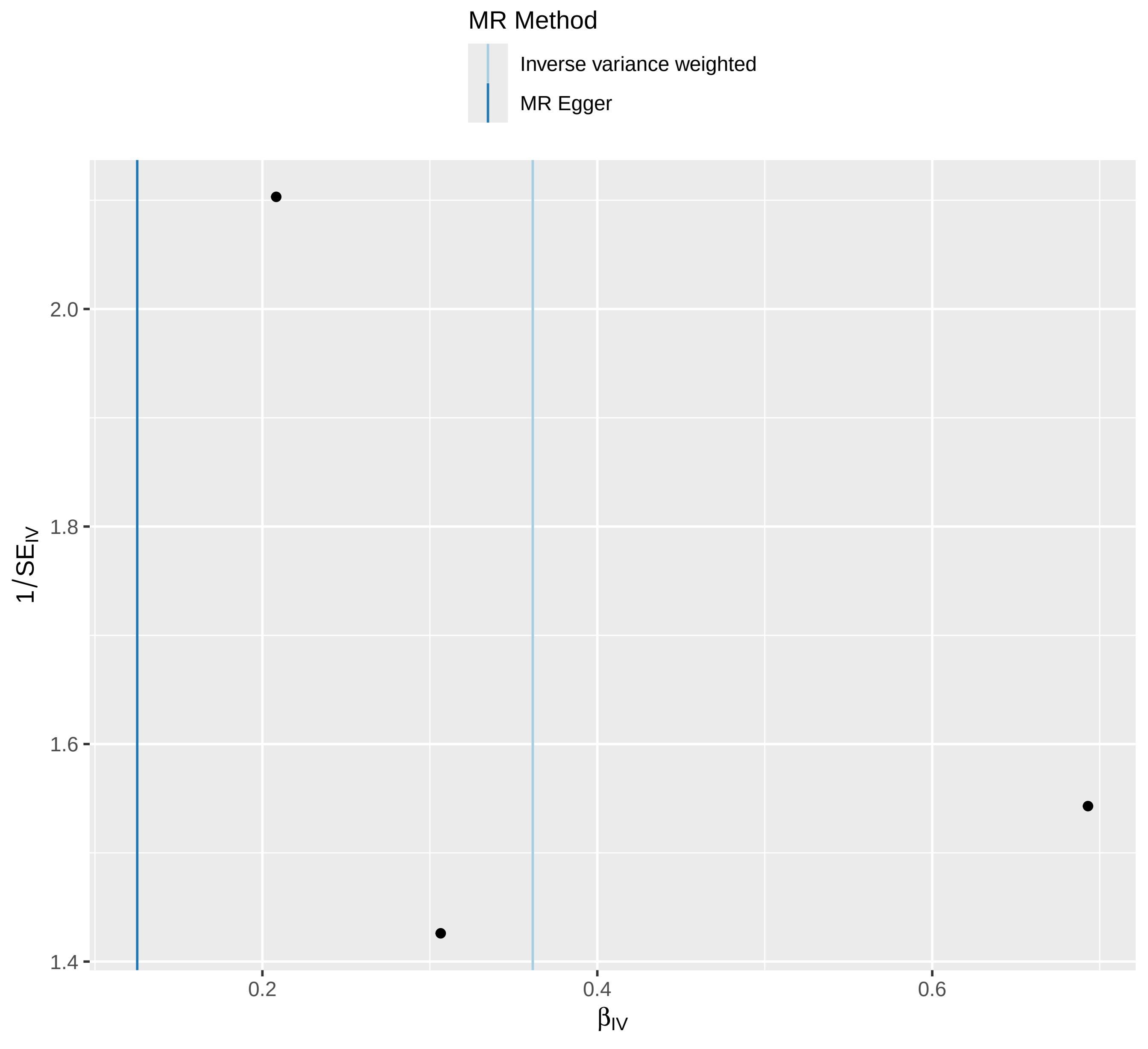
MR Method
Inverse variance weighted
MR Egger
2.0
1.8
1.6
1.4
1/SE_IV
0.2
0.4
0.6
β_IV

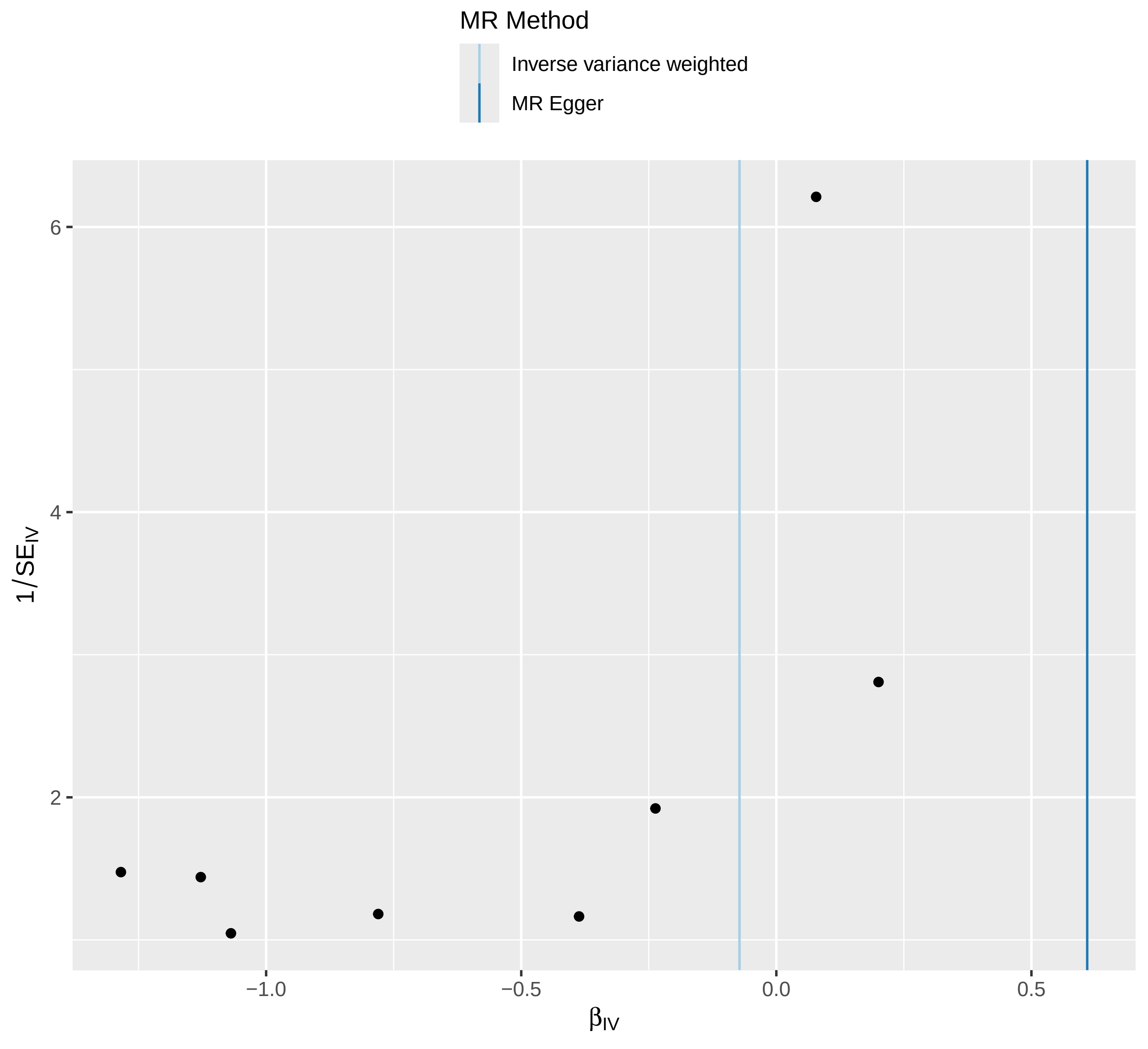

MR Method
Inverse variance weighted
MR Egger
6
4
2
1/SE$_{IV}$
−1.0
−0.5
0.0
0.5
β$_{IV}$

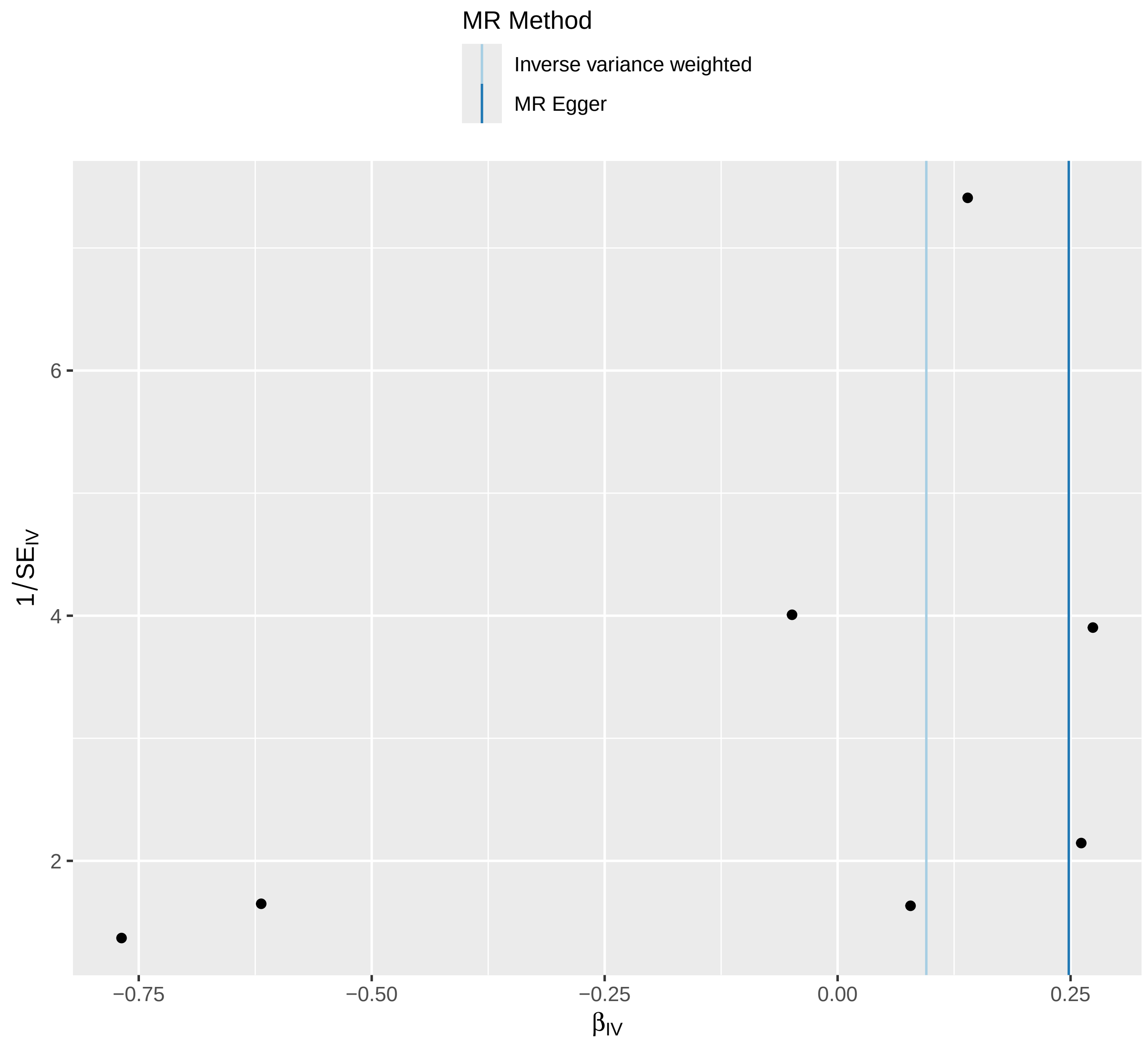
MR Method
Inverse variance weighted
MR Egger
6
4
2
1/SE_IV
−0.75
−0.50
−0.25
0.00
0.25
β_IV

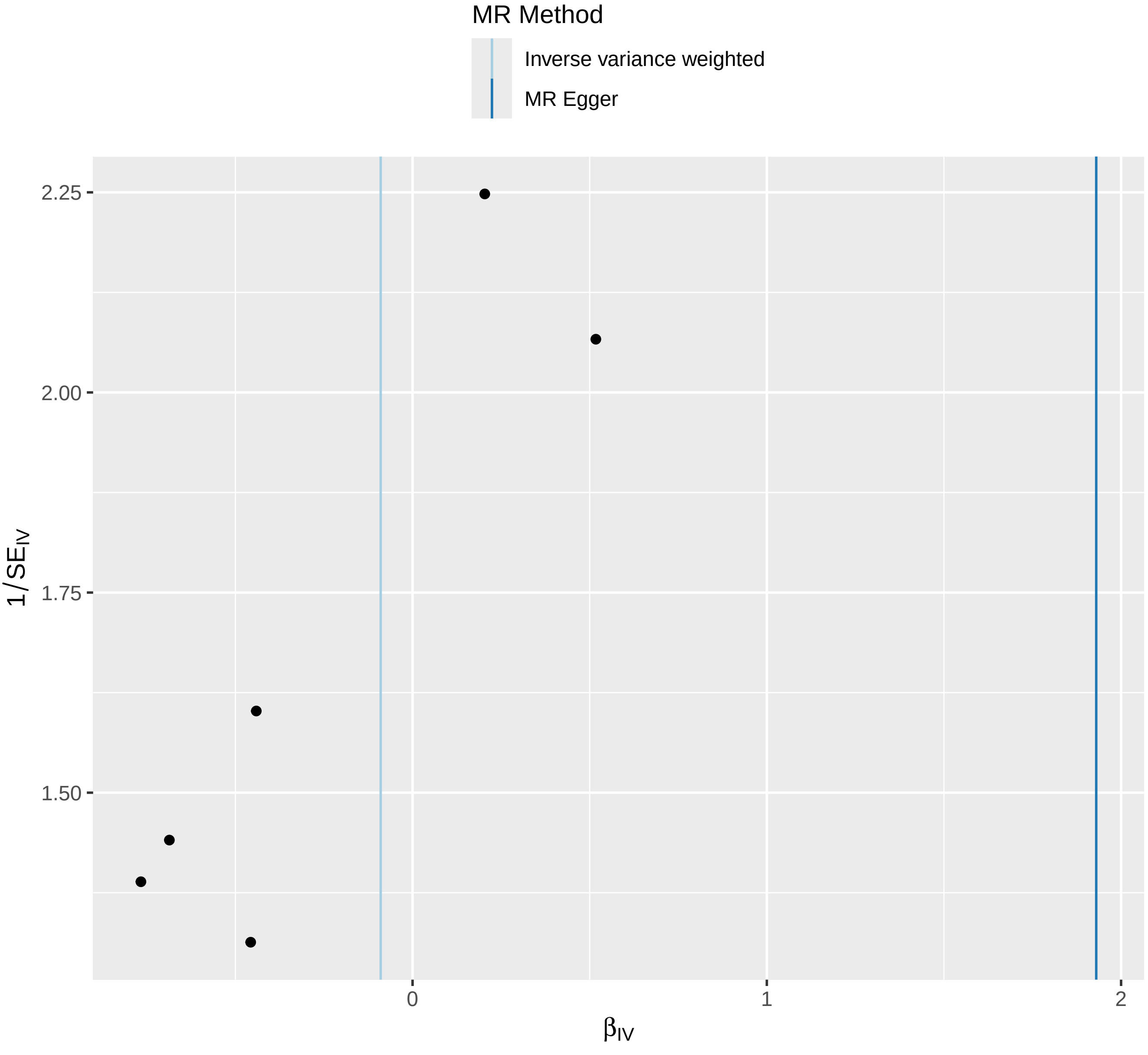
MR Method
Inverse variance weighted
MR Egger
2.25
2.00
1.75
1.50
1/SE_IV
0
1
2
β_IV

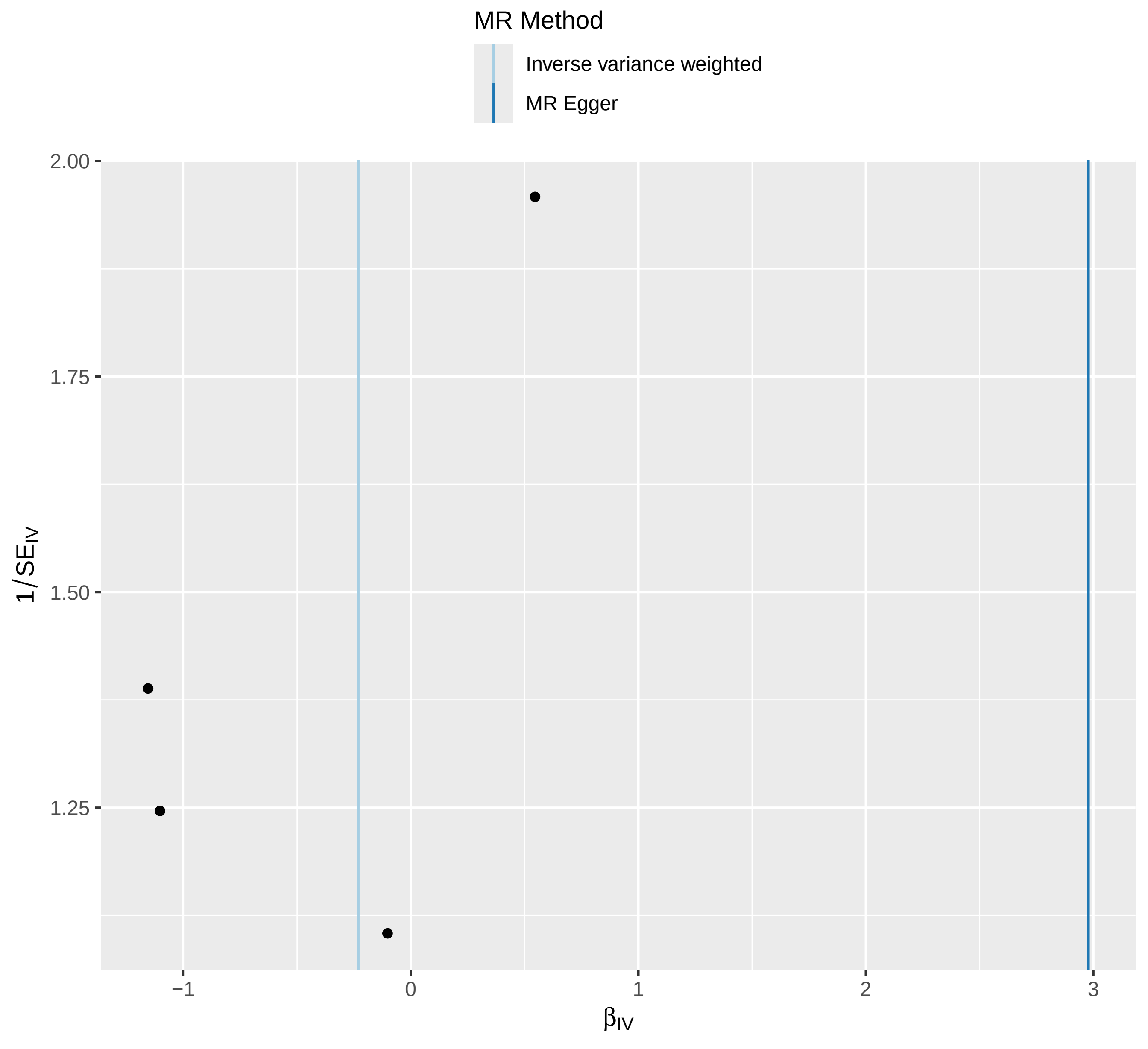
MR Method
Inverse variance weighted
MR Egger
2.00
1.75
1.50
1.25
1/SE_IV
−1
0
1
2
3
β_IV

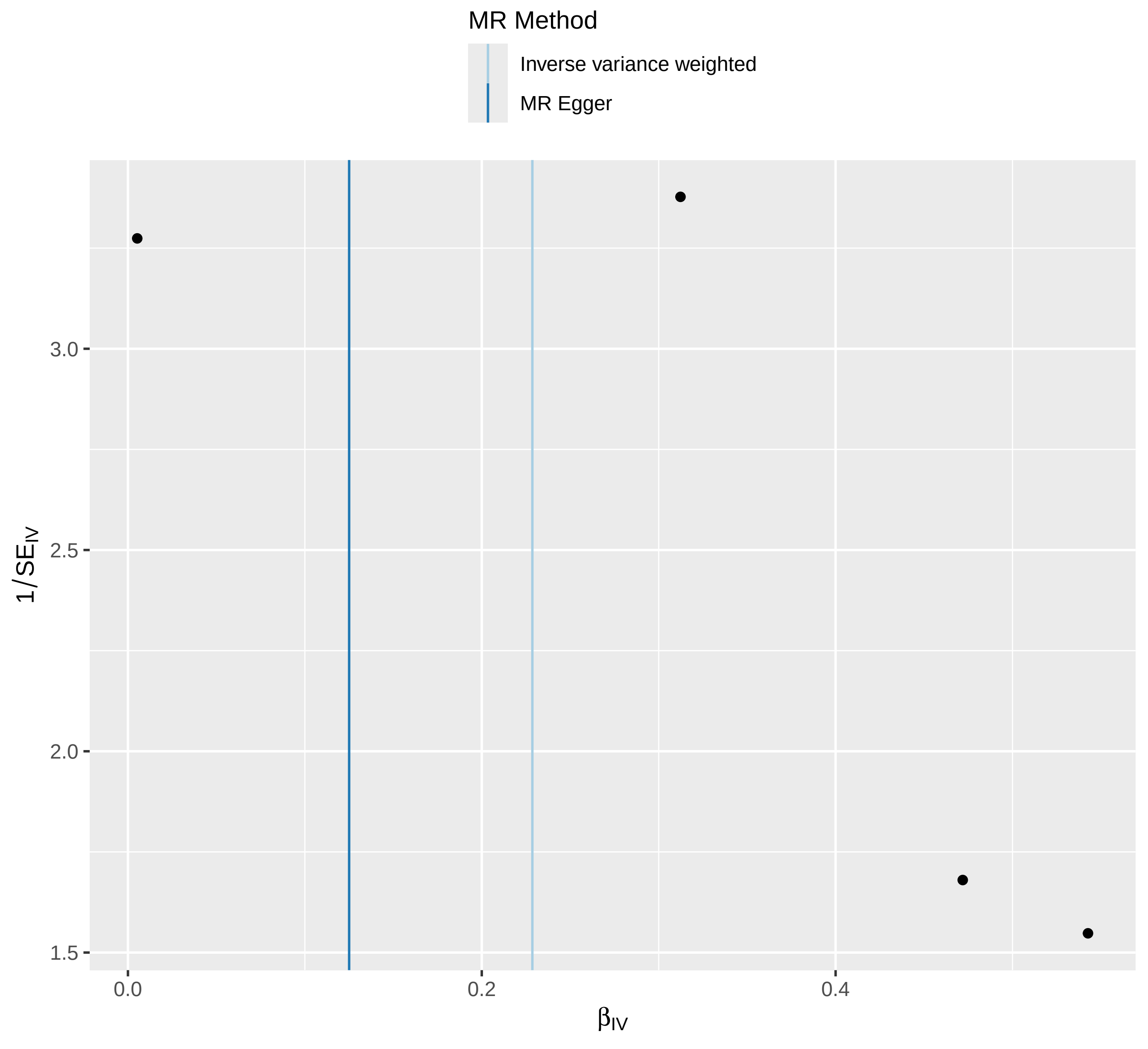

MR Method
Inverse variance weighted
MR Egger
1/SE$_{IV}$
3.0
2.5
2.0
1.5
0.0
0.2
0.4
β$_{IV}$

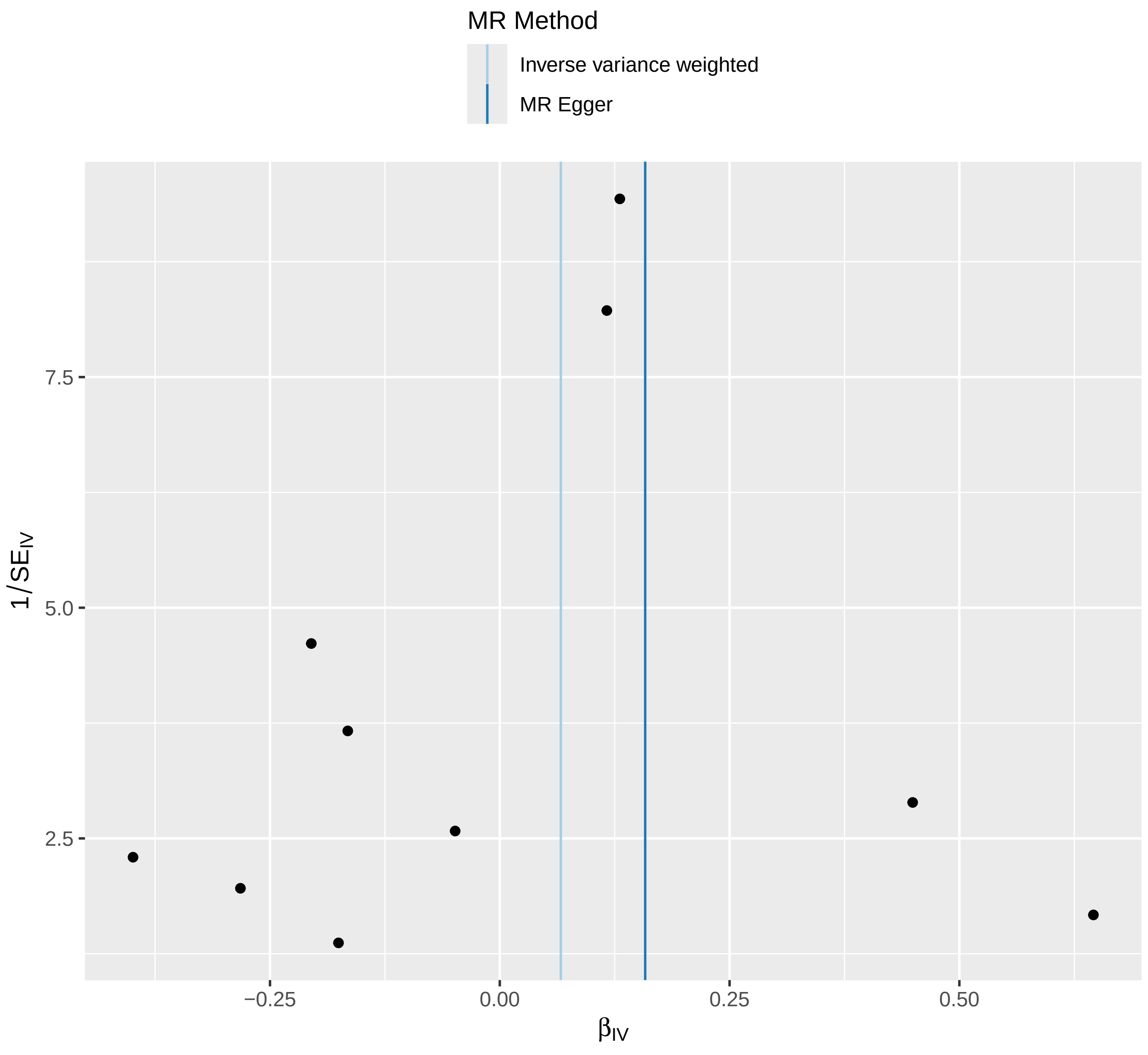

MR Method
Inverse variance weighted
MR Egger
1/SE$_{IV}$
7.5
5.0
2.5
−0.25
0.00
0.25
0.50
β$_{IV}$

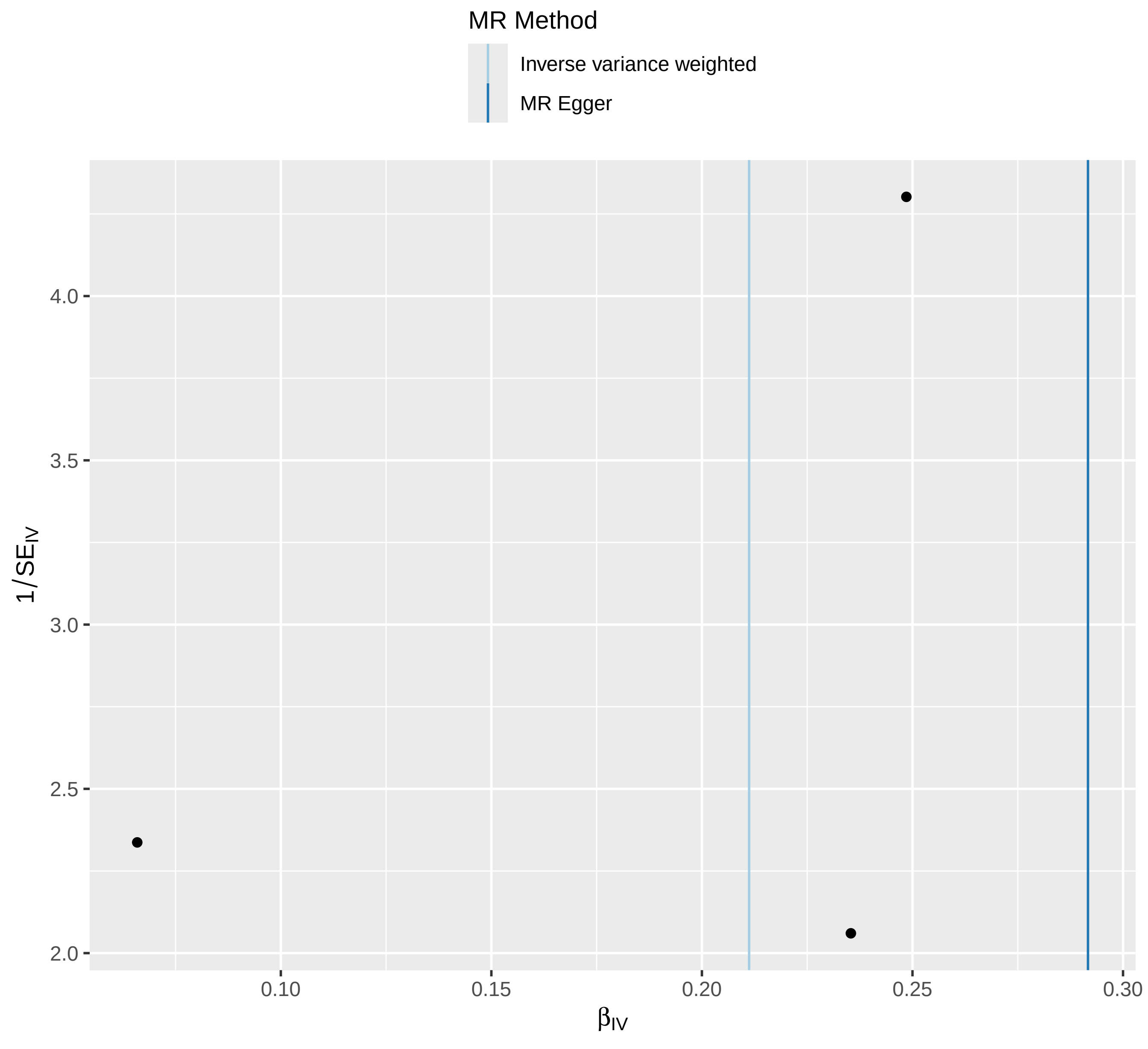
MR Method
Inverse variance weighted
MR Egger
4.0
3.5
3.0
2.5
2.0
1/SE_IV
0.10
0.15
0.20
0.25
0.30
β_IV

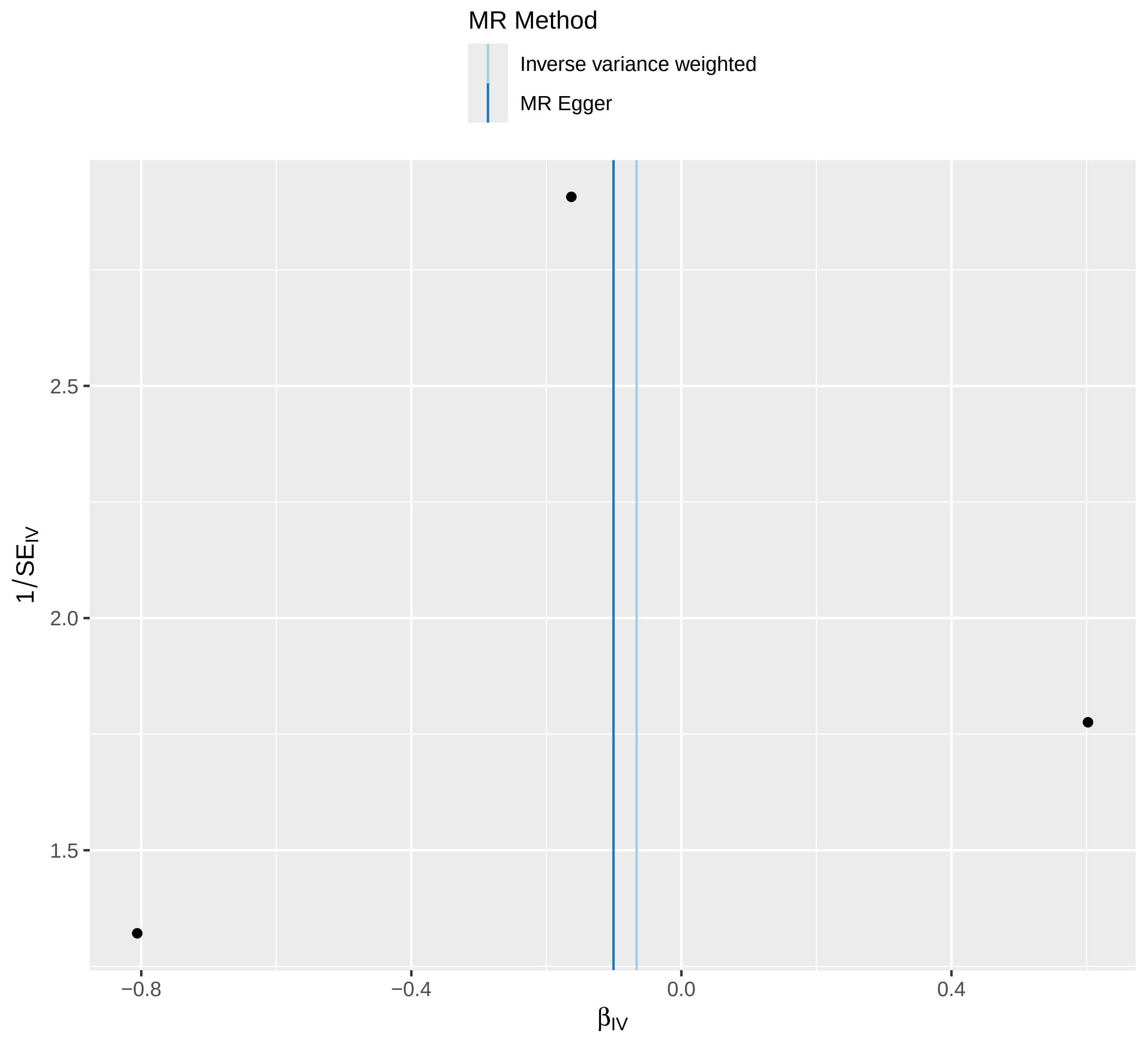

MR Method
Inverse variance weighted
MR Egger
2.5
2.0
1.5
1/SE_IV
−0.8
−0.4
0.0
0.4
β_IV

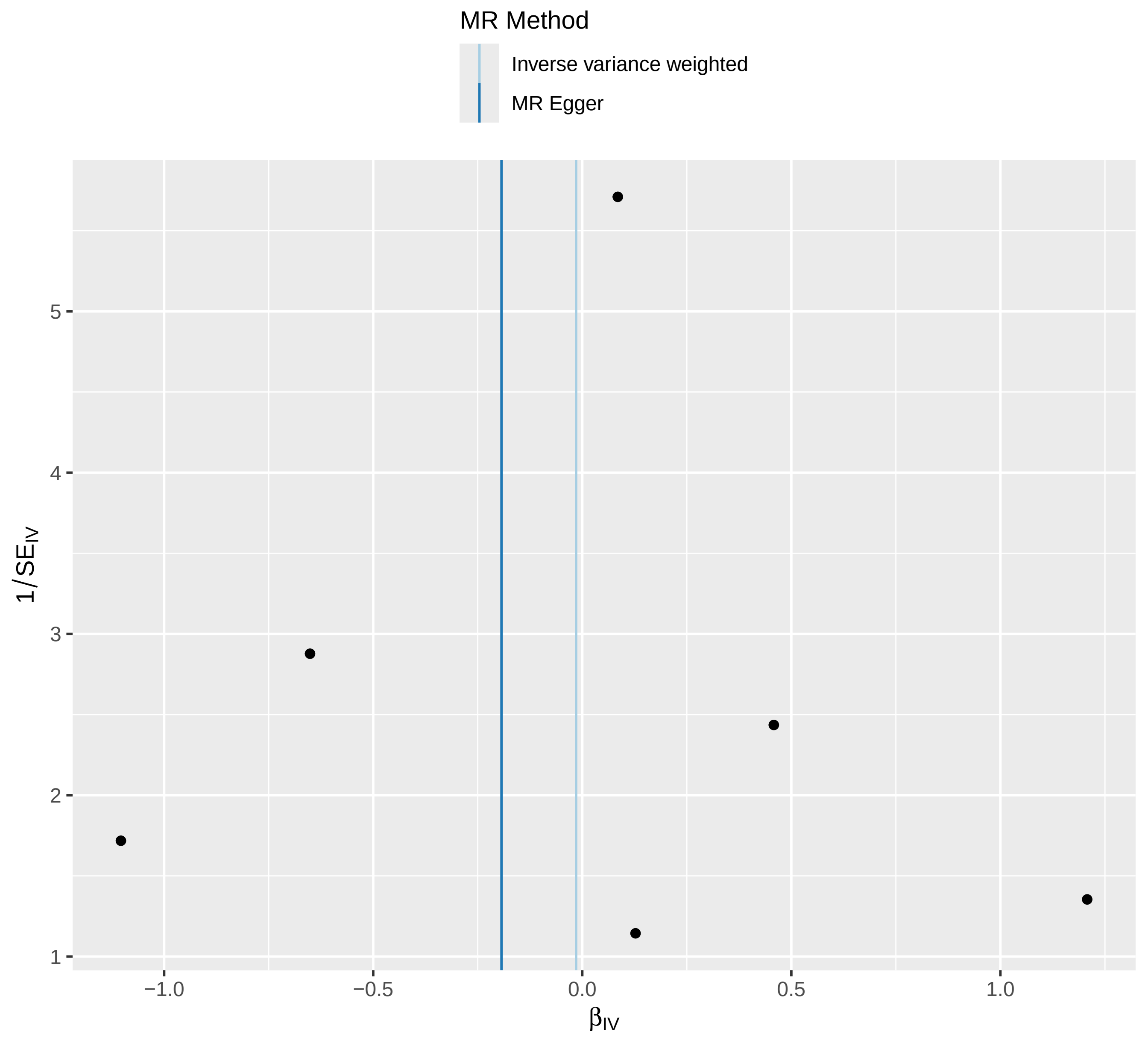

MR Method
Inverse variance weighted
MR Egger
1/SE_IV
5
4
3
2
1
−1.0
−0.5
0.0
0.5
1.0
β_IV

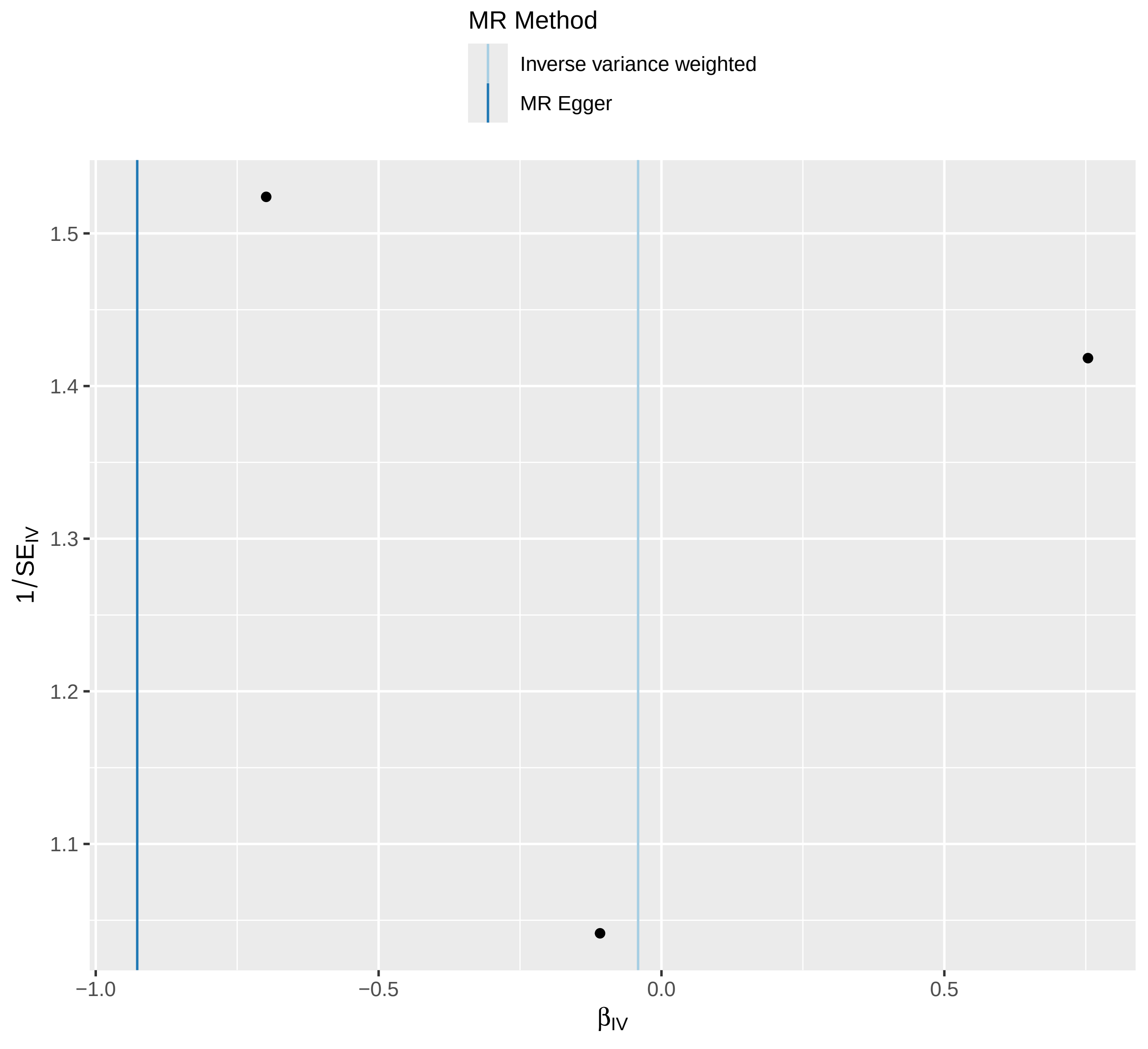

MR Method
Inverse variance weighted
MR Egger
1.5
1.4
1.3
1.2
1.1
1/SE$_{IV}$
−1.0
−0.5
0.0
0.5
β$_{IV}$

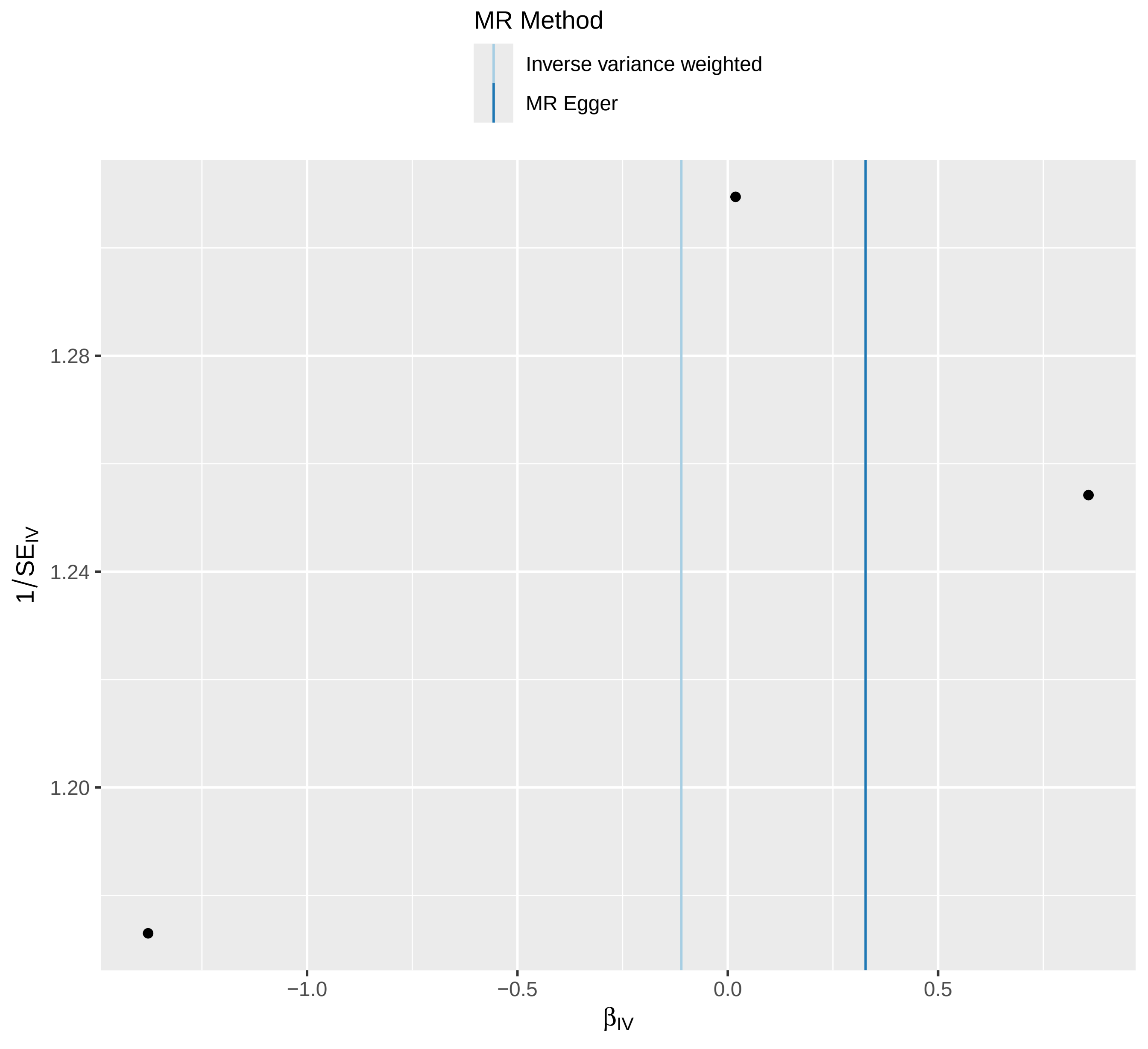

MR Method
Inverse variance weighted
MR Egger
1.28
1.24
1.20
1/SE$_{IV}$
−1.0
−0.5
0.0
0.5
β$_{IV}$

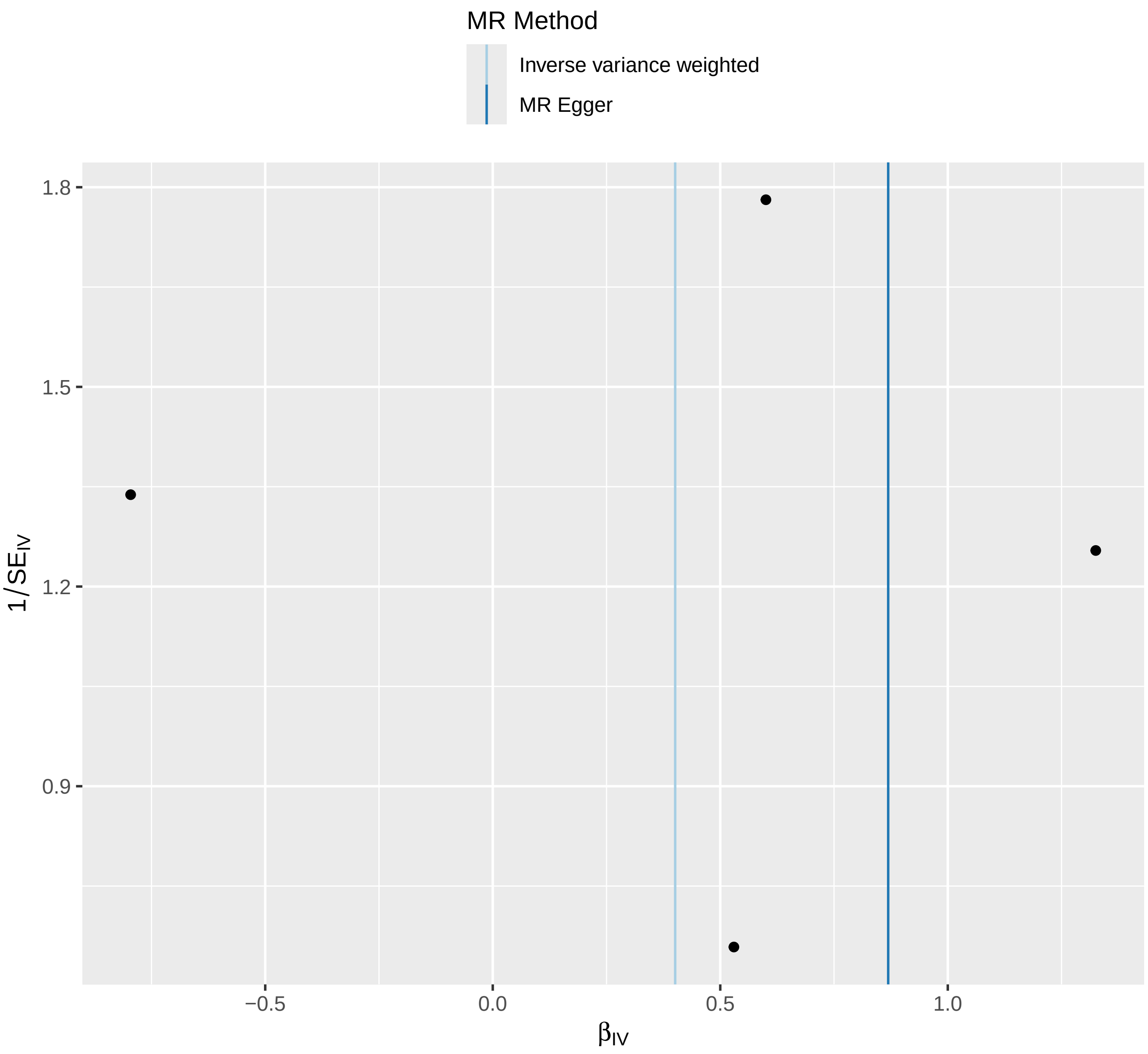
MR Method
Inverse variance weighted
MR Egger
1.8
1.5
1.2
0.9
1/SE_IV
−0.5
0.0
0.5
1.0
β_IV

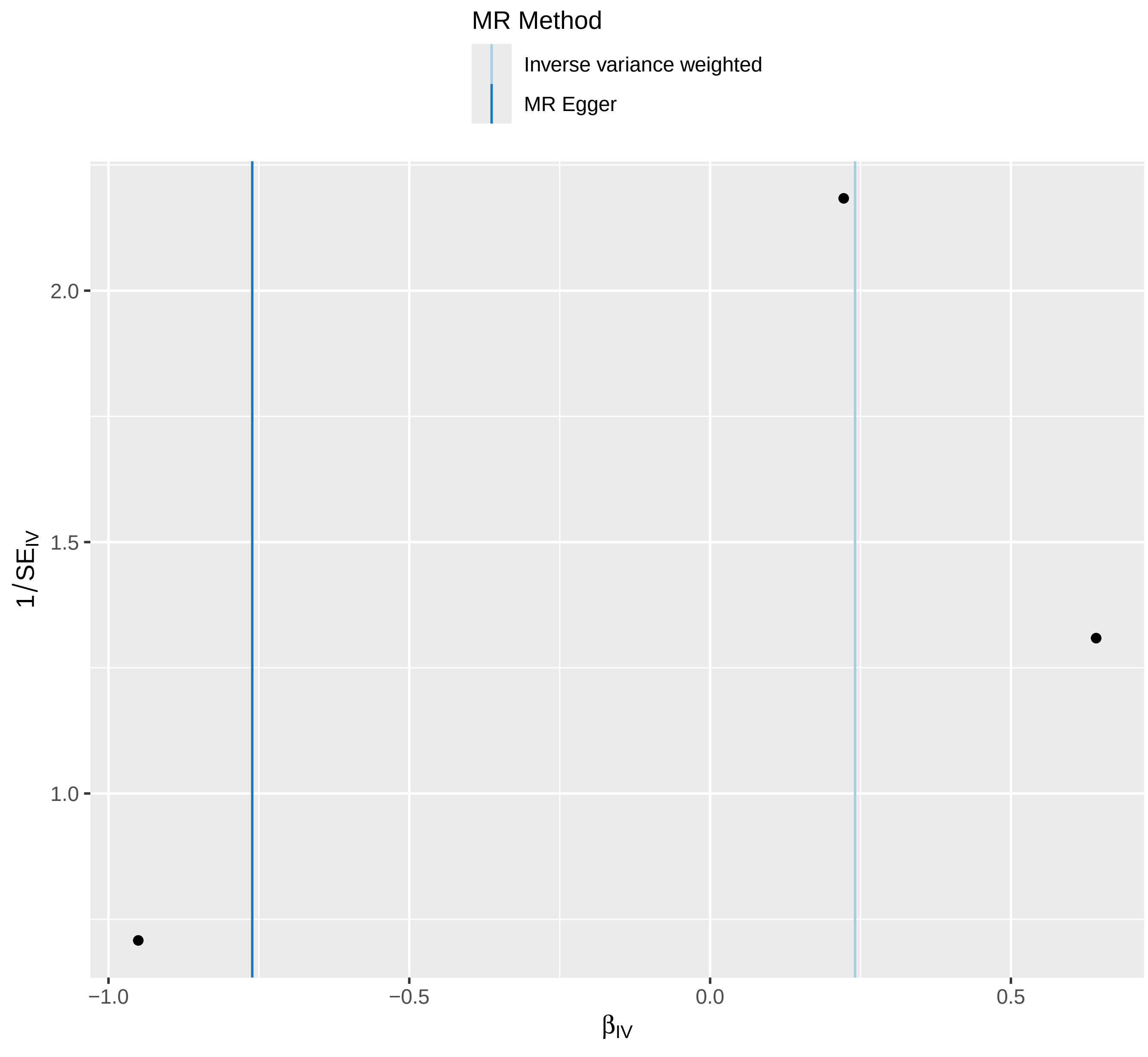
MR Method
Inverse variance weighted
MR Egger
2.0
1.5
1.0
1/SE_IV
−1.0
−0.5
0.0
0.5
β_IV

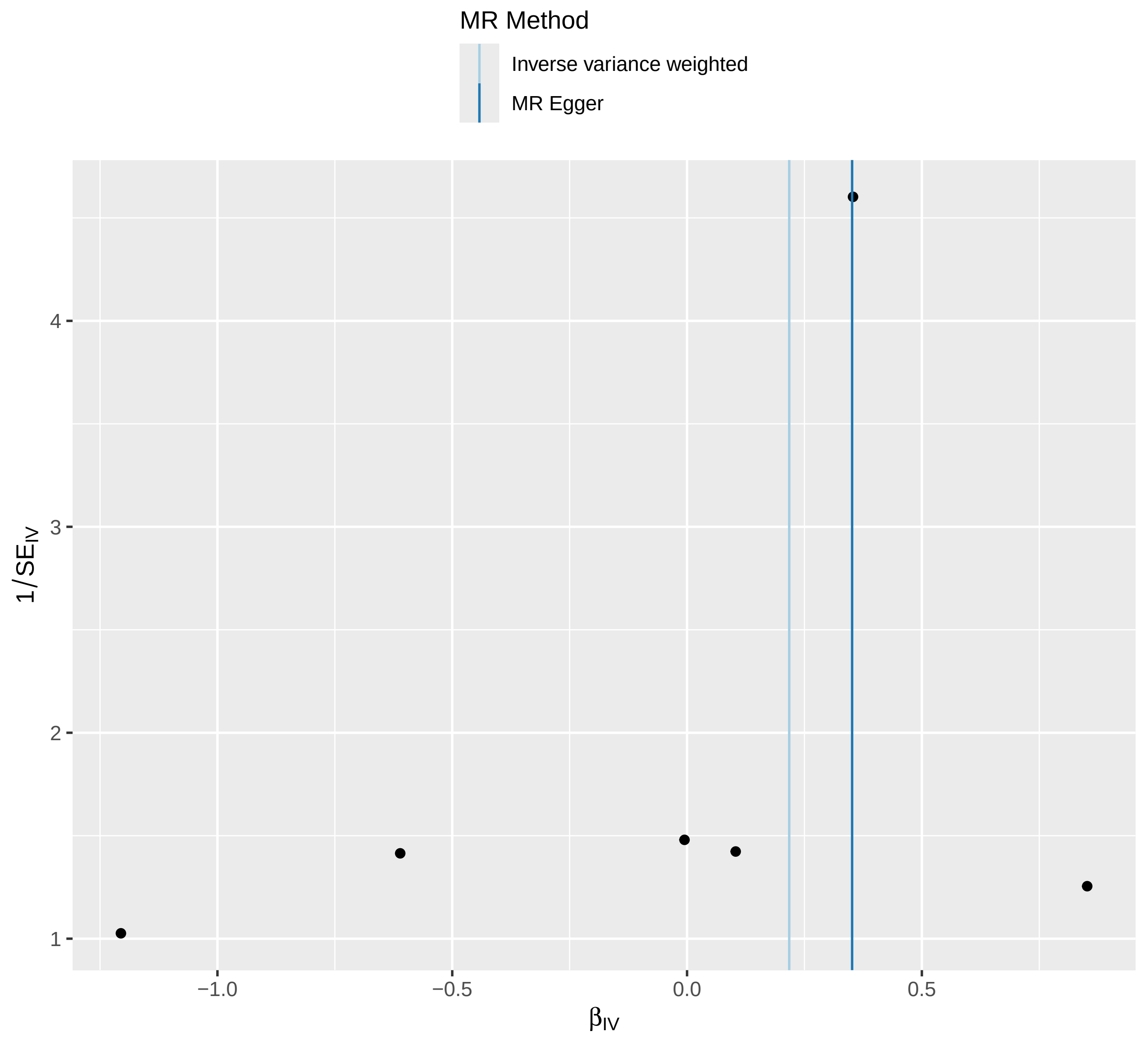
MR Method
Inverse variance weighted
MR Egger
4
3
2
1
$1/SE_{IV}$
−1.0
−0.5
0.0
0.5
β_{IV}

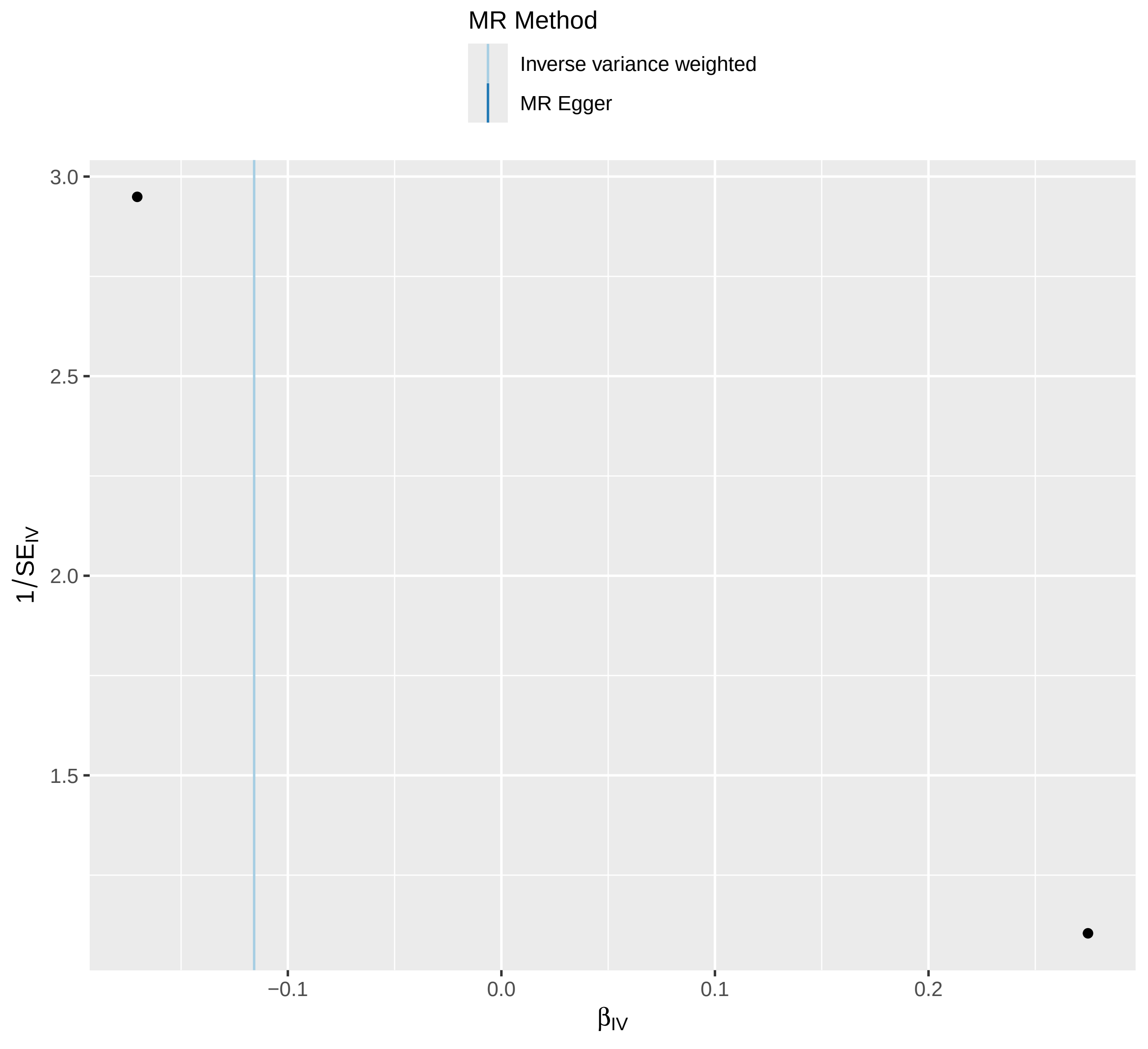
MR Method
Inverse variance weighted
MR Egger
3.0
2.5
2.0
1.5
1/SE_IV
−0.1
0.0
0.1
0.2
β_IV

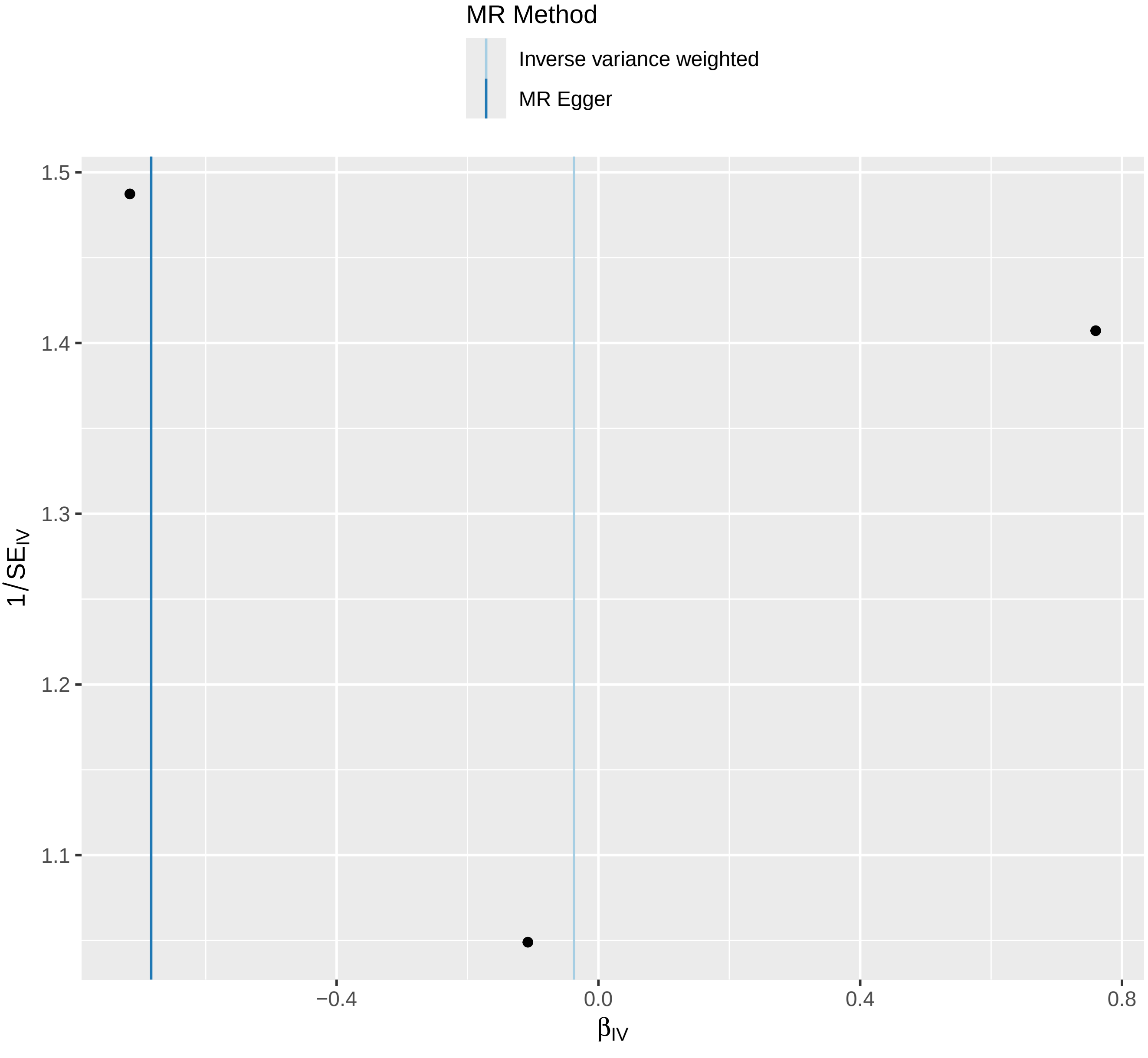
MR Method
Inverse variance weighted
MR Egger
1.5
1.4
1.3
1.2
1.1
1/SE$_{IV}$
−0.4
0.0
0.4
0.8
β$_{IV}$

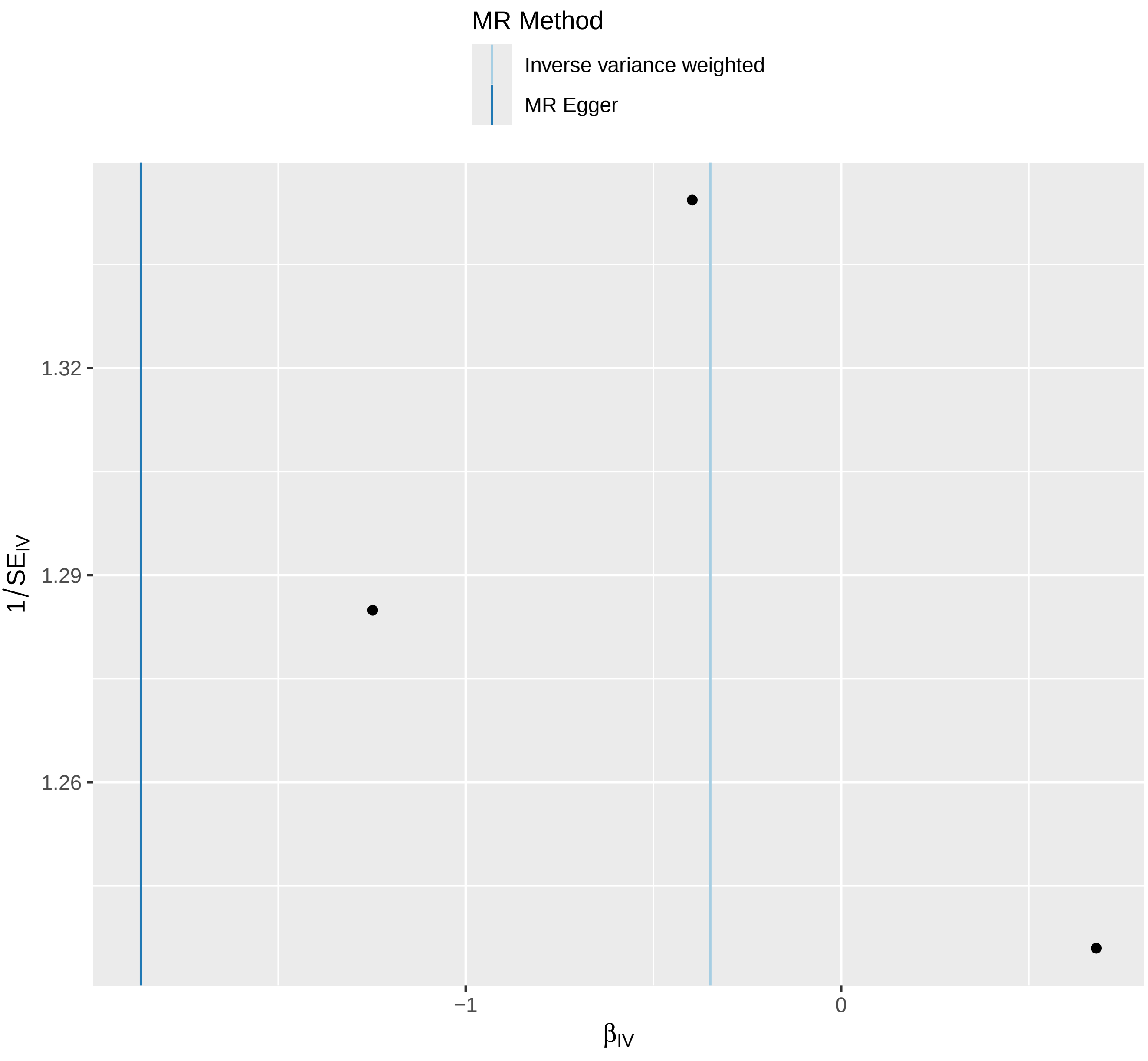
MR Method
Inverse variance weighted
MR Egger
1.32
1.29
1.26
1/SE_IV
−1
0
β_IV

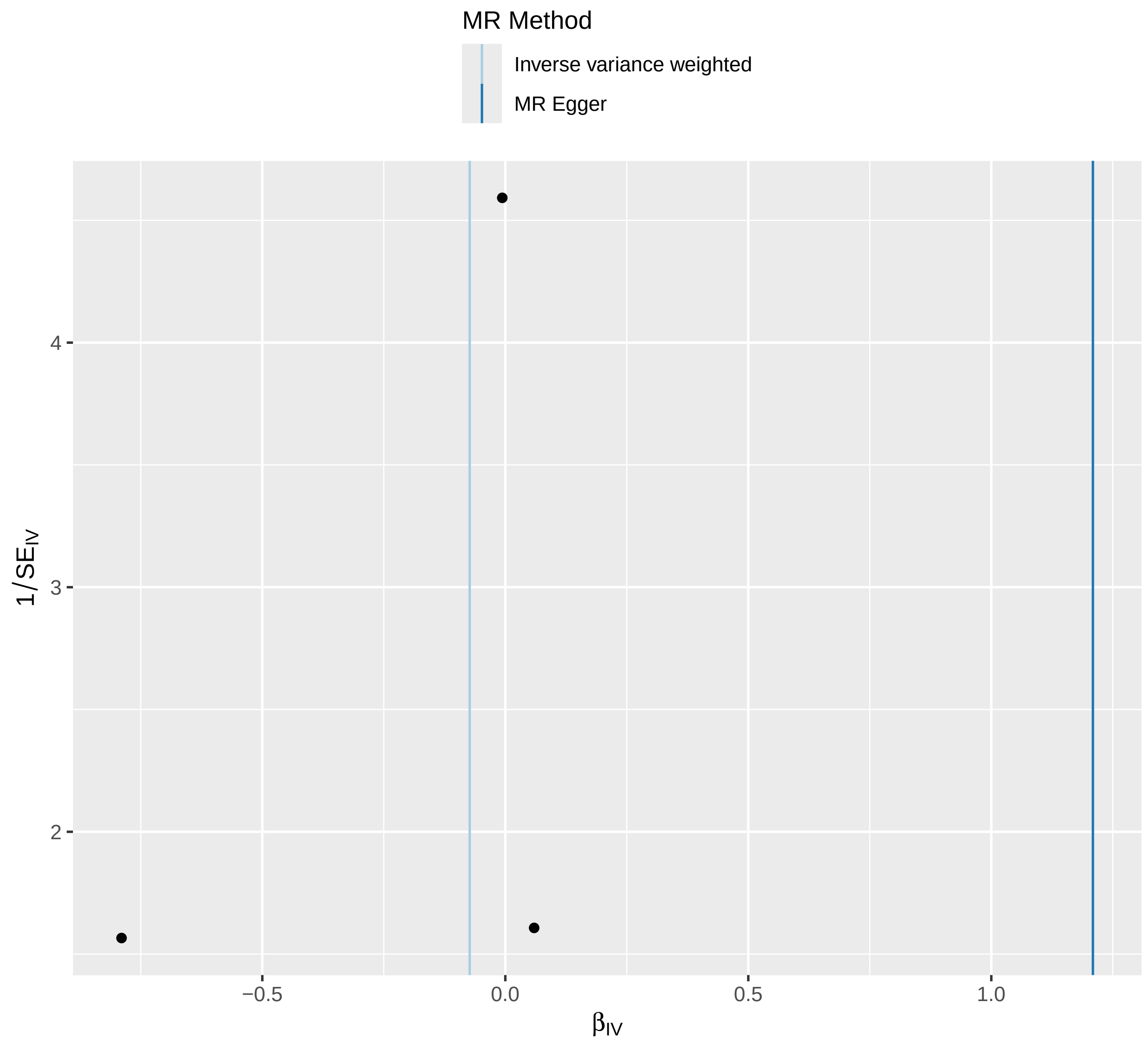

MR Method
Inverse variance weighted
MR Egger
1/SE_IV
4
3
2
−0.5
0.0
0.5
1.0
β_IV

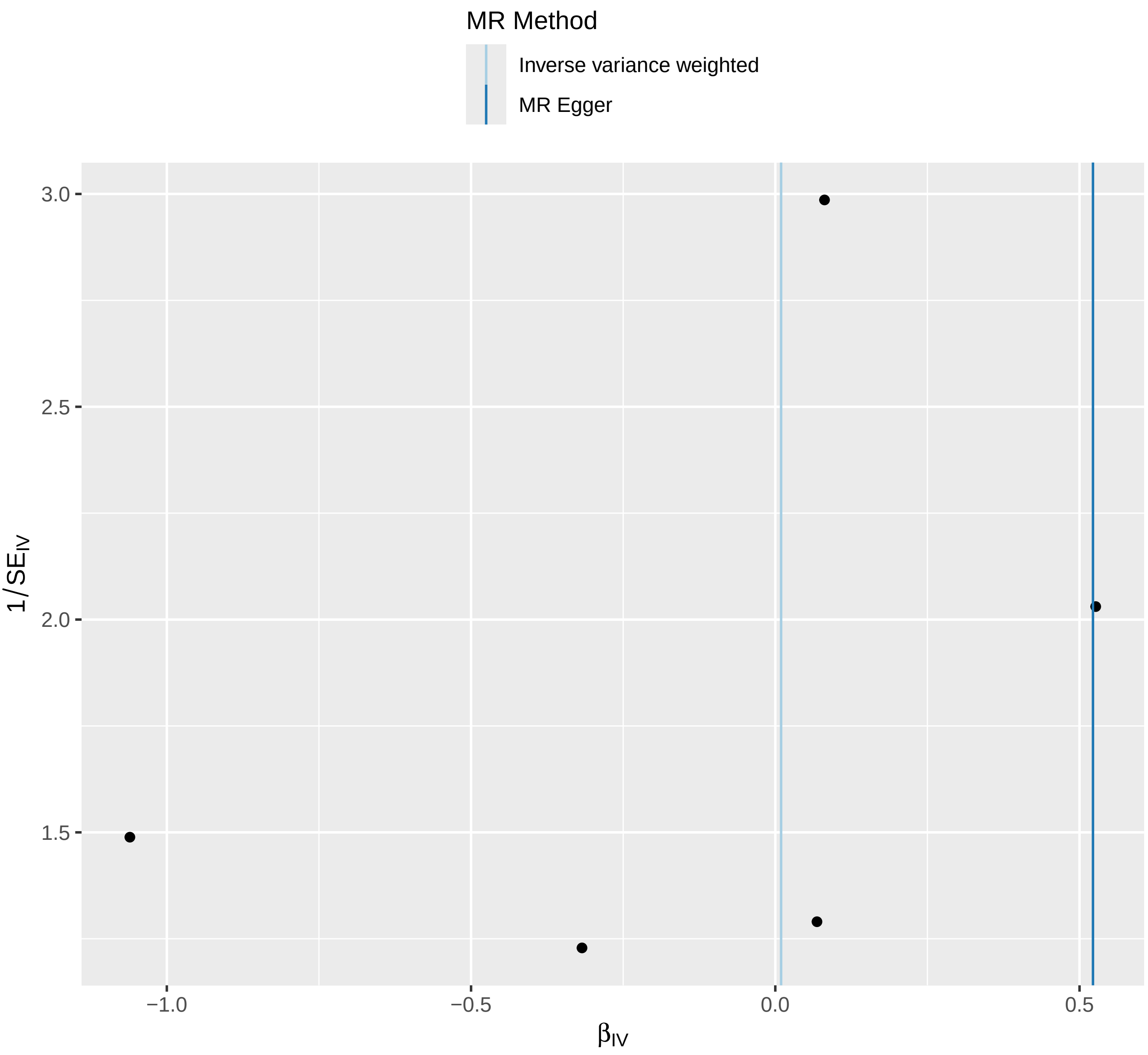
MR Method
Inverse variance weighted
MR Egger
3.0
2.5
2.0
1.5
1/SE$_{IV}$
−1.0
−0.5
0.0
0.5
β$_{IV}$

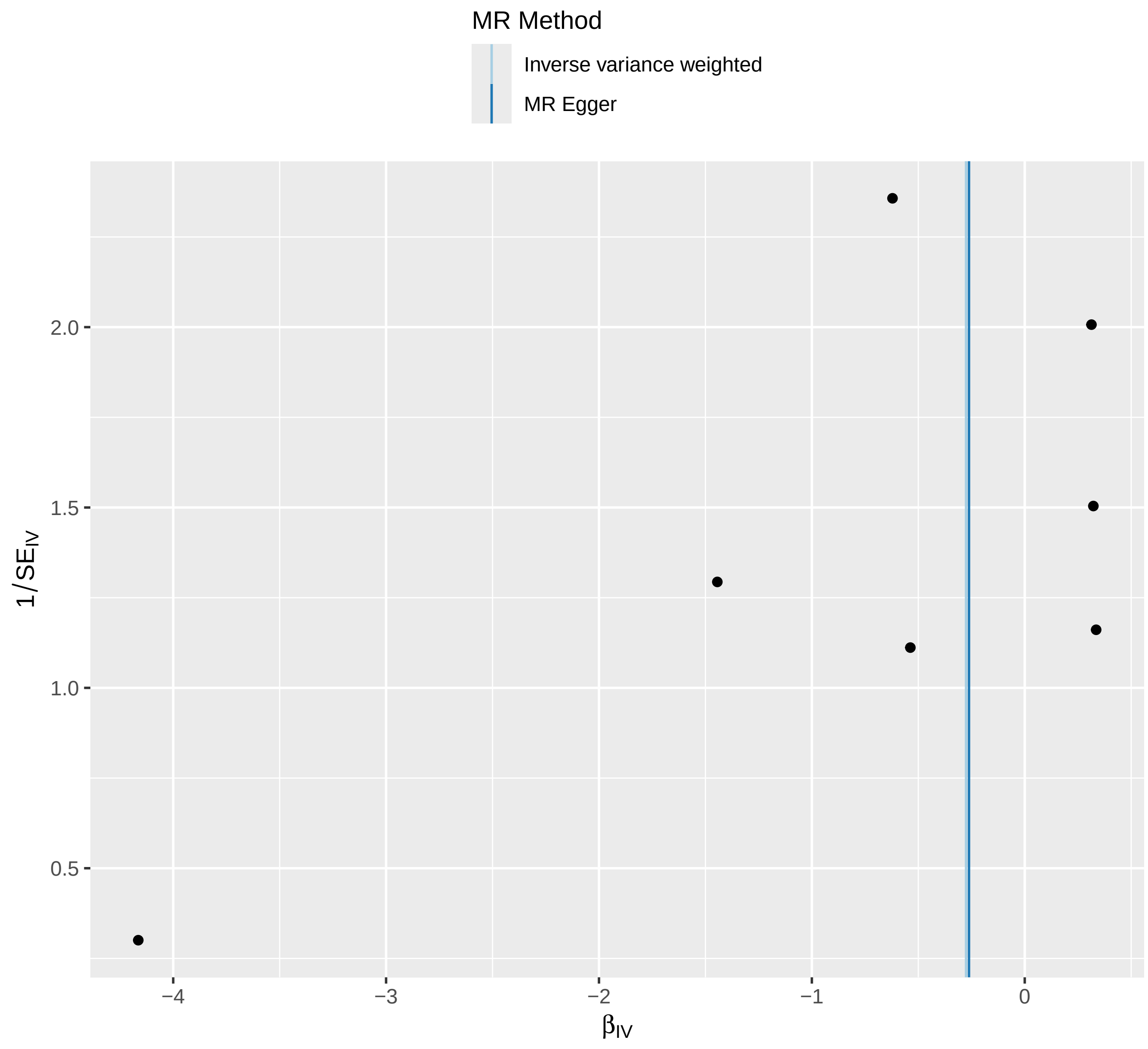
MR Method
Inverse variance weighted
MR Egger
2.0
1.5
1.0
0.5
1/SE_IV
−4
−3
−2
−1
0
β_IV

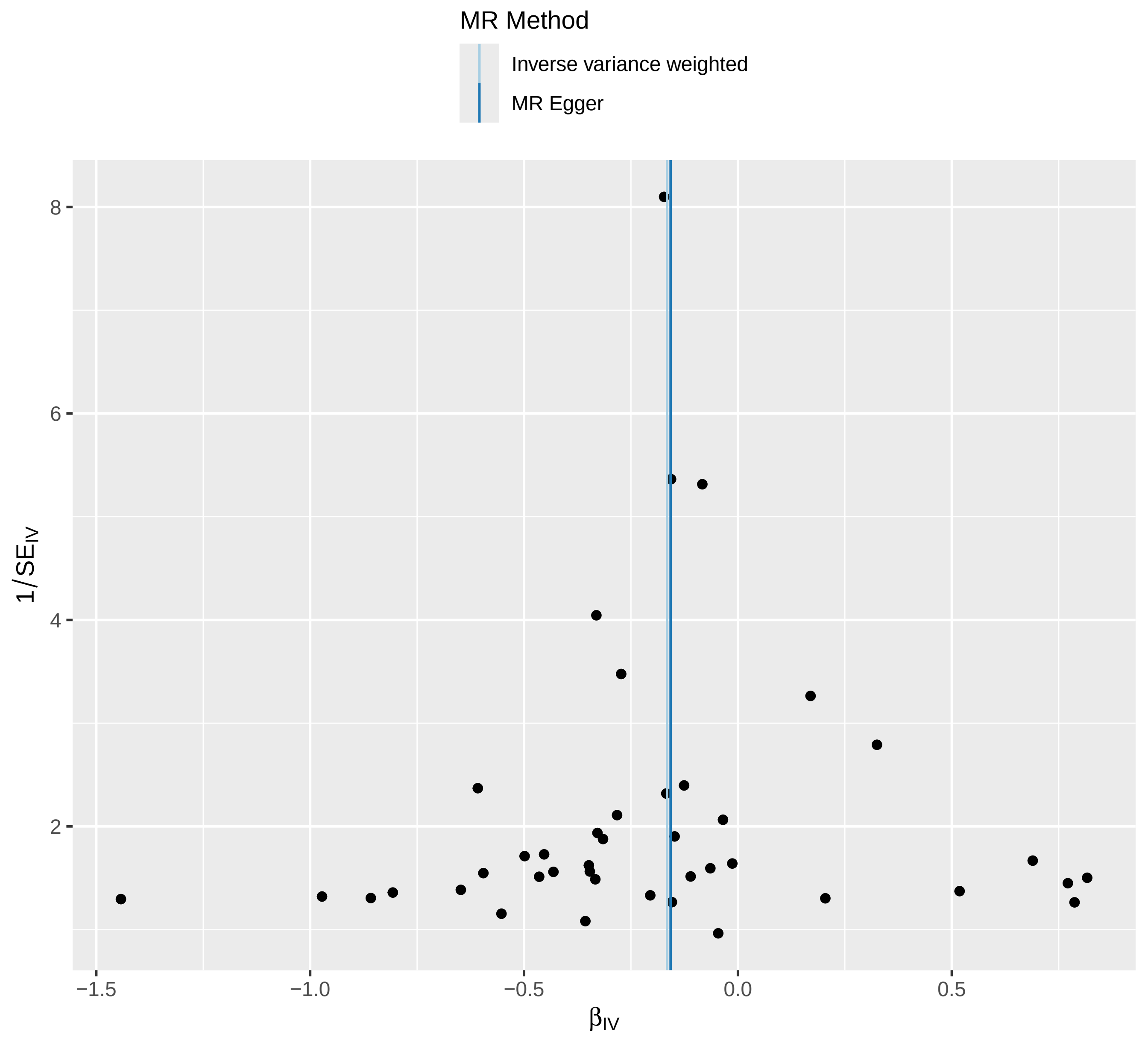
MR Method
Inverse variance weighted
MR Egger
1/SE$_{IV}$
8
6
4
2
−1.5
−1.0
−0.5
0.0
0.5
β_{IV}

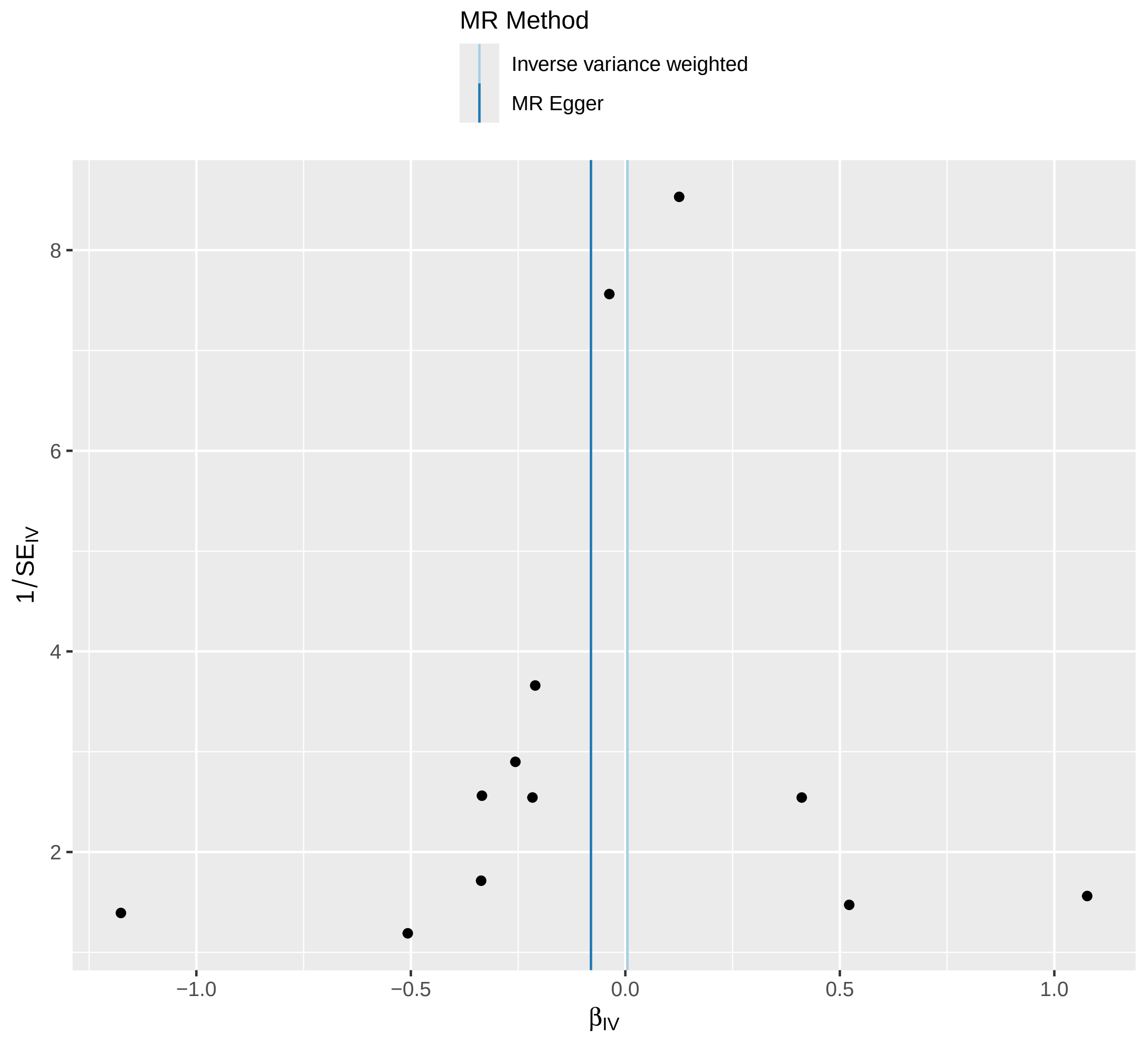

MR Method
Inverse variance weighted
MR Egger
8
6
4
2
$1/SE_{IV}$
−1.0
−0.5
0.0
0.5
1.0
β_{IV}

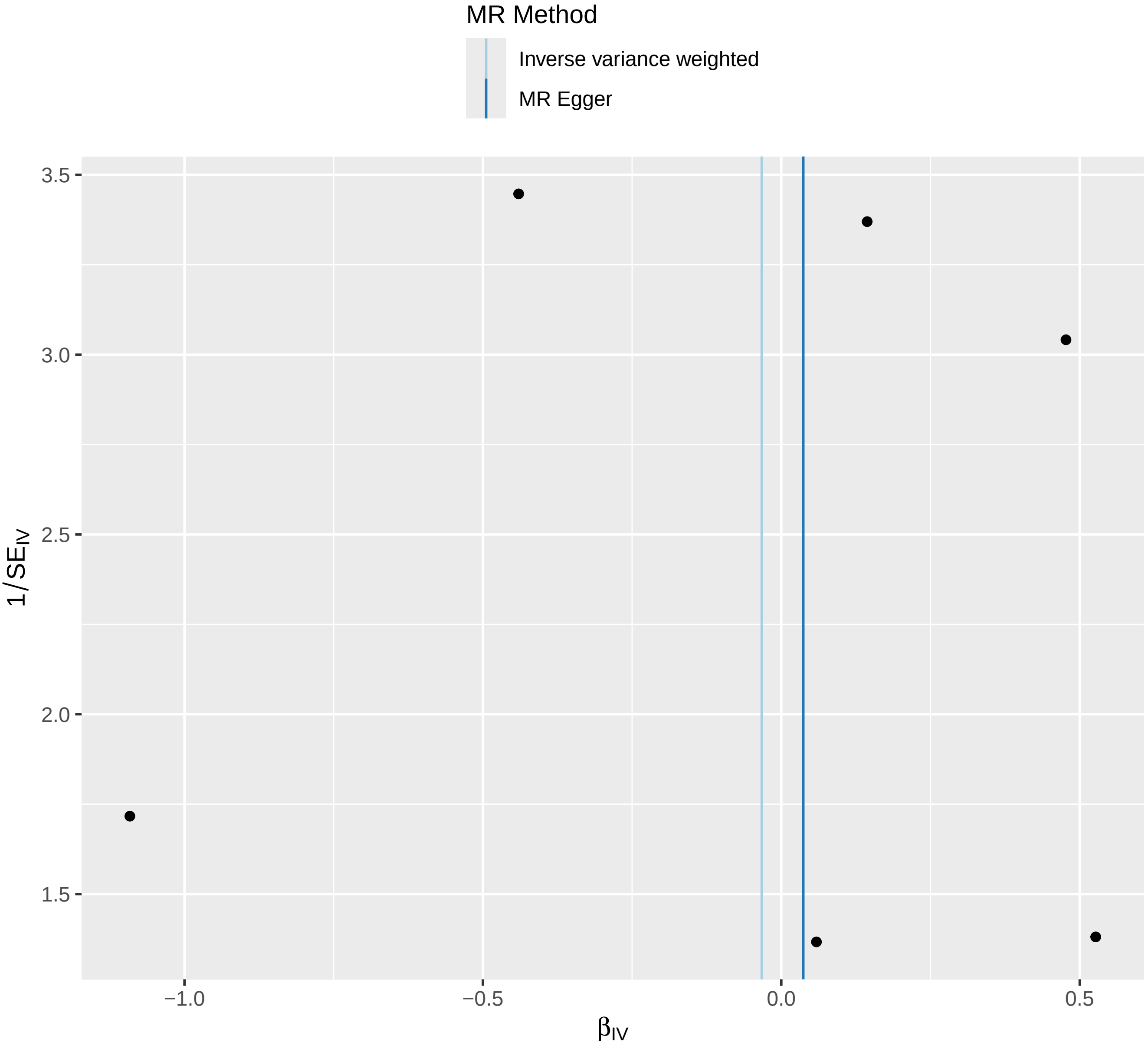

MR Method
Inverse variance weighted
MR Egger
3.5
3.0
2.5
2.0
1.5
$1/SE_{IV}$
−1.0
−0.5
0.0
0.5
β_{IV}

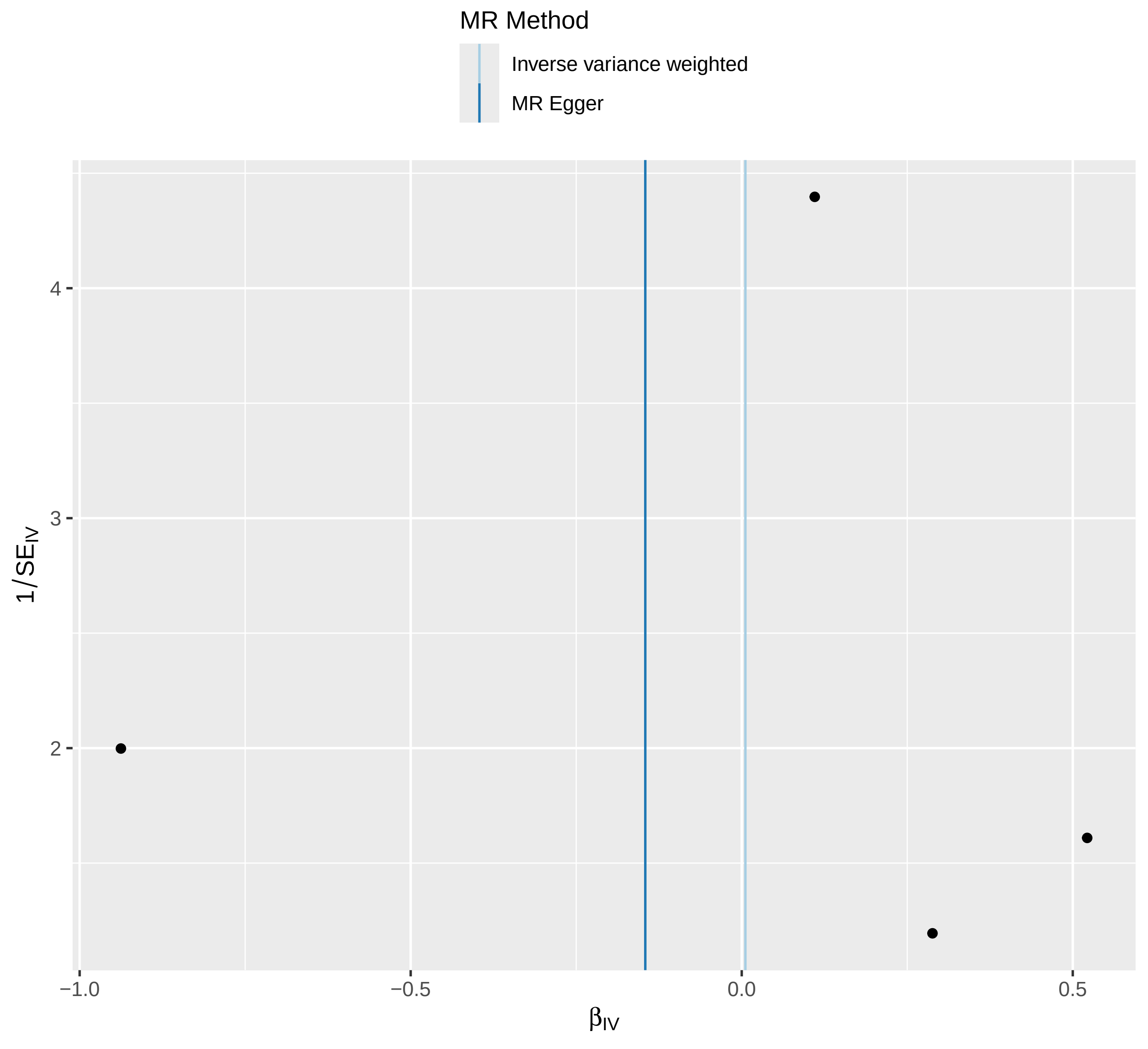
MR Method
Inverse variance weighted
MR Egger
4
3
2
1/SE_IV
−1.0
−0.5
0.0
0.5
β_IV

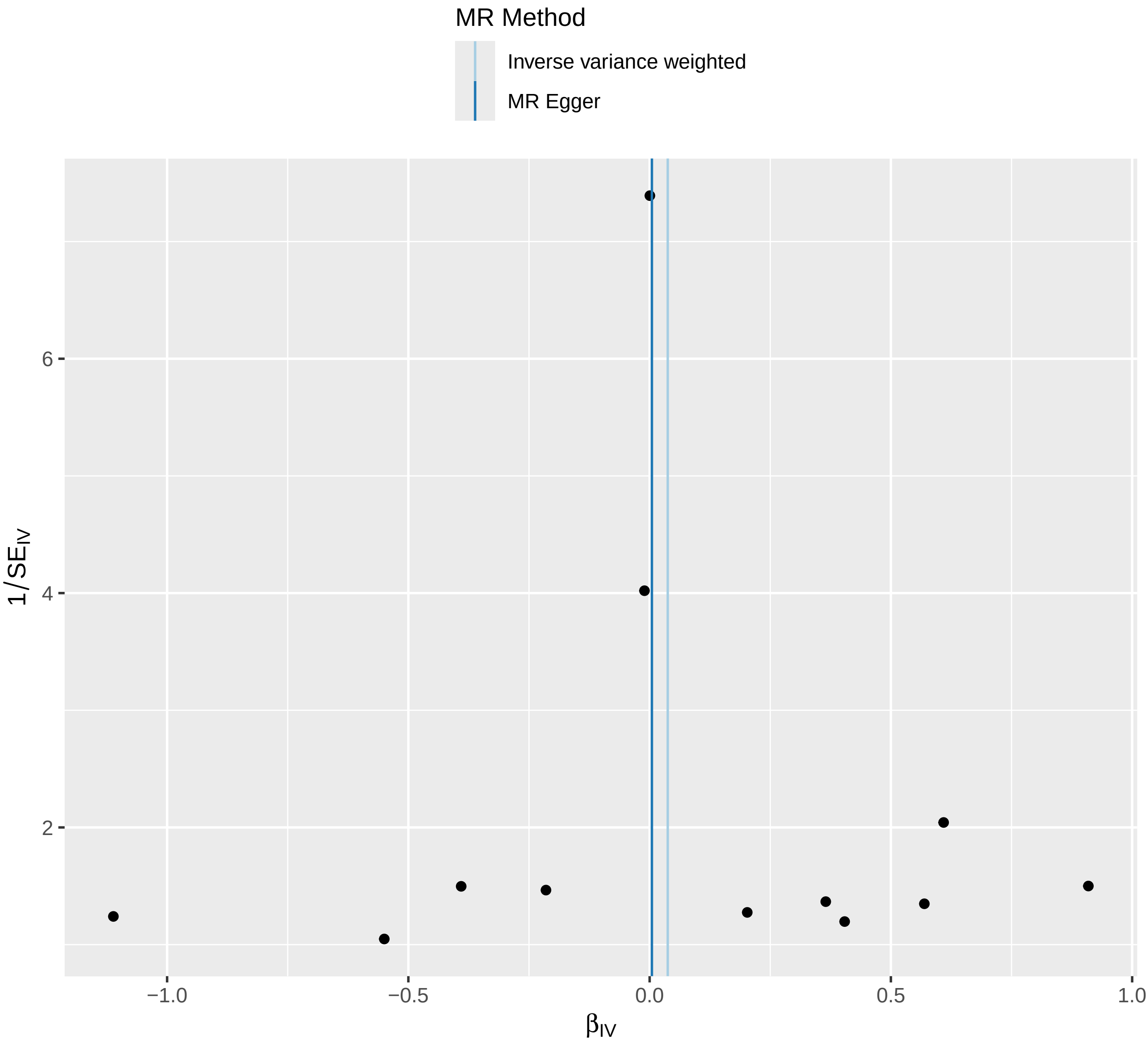
MR Method
Inverse variance weighted
MR Egger
6
4
2
1/SE_IV
−1.0
−0.5
0.0
0.5
1.0
β_IV

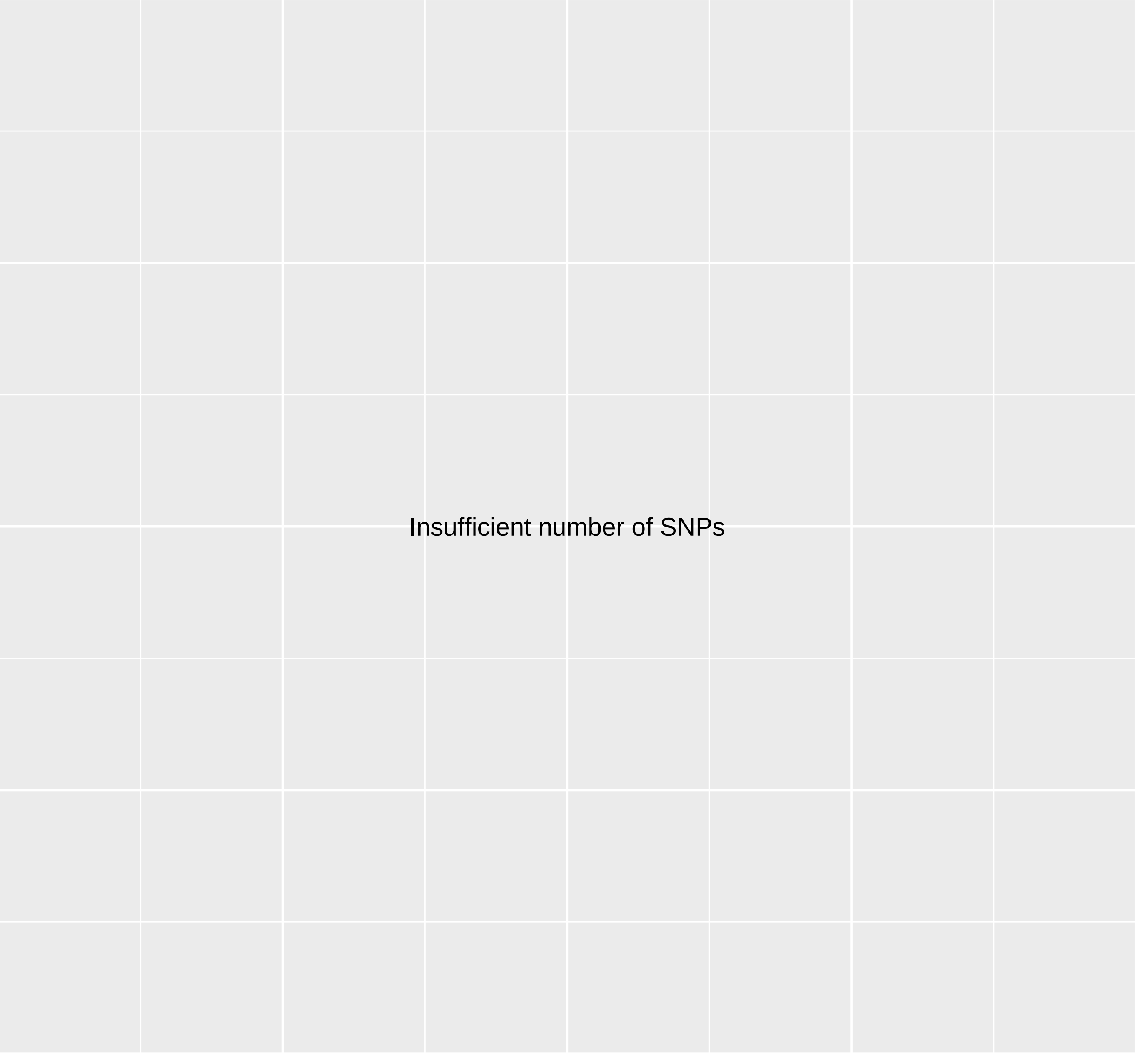
Insufficient number of SNPs

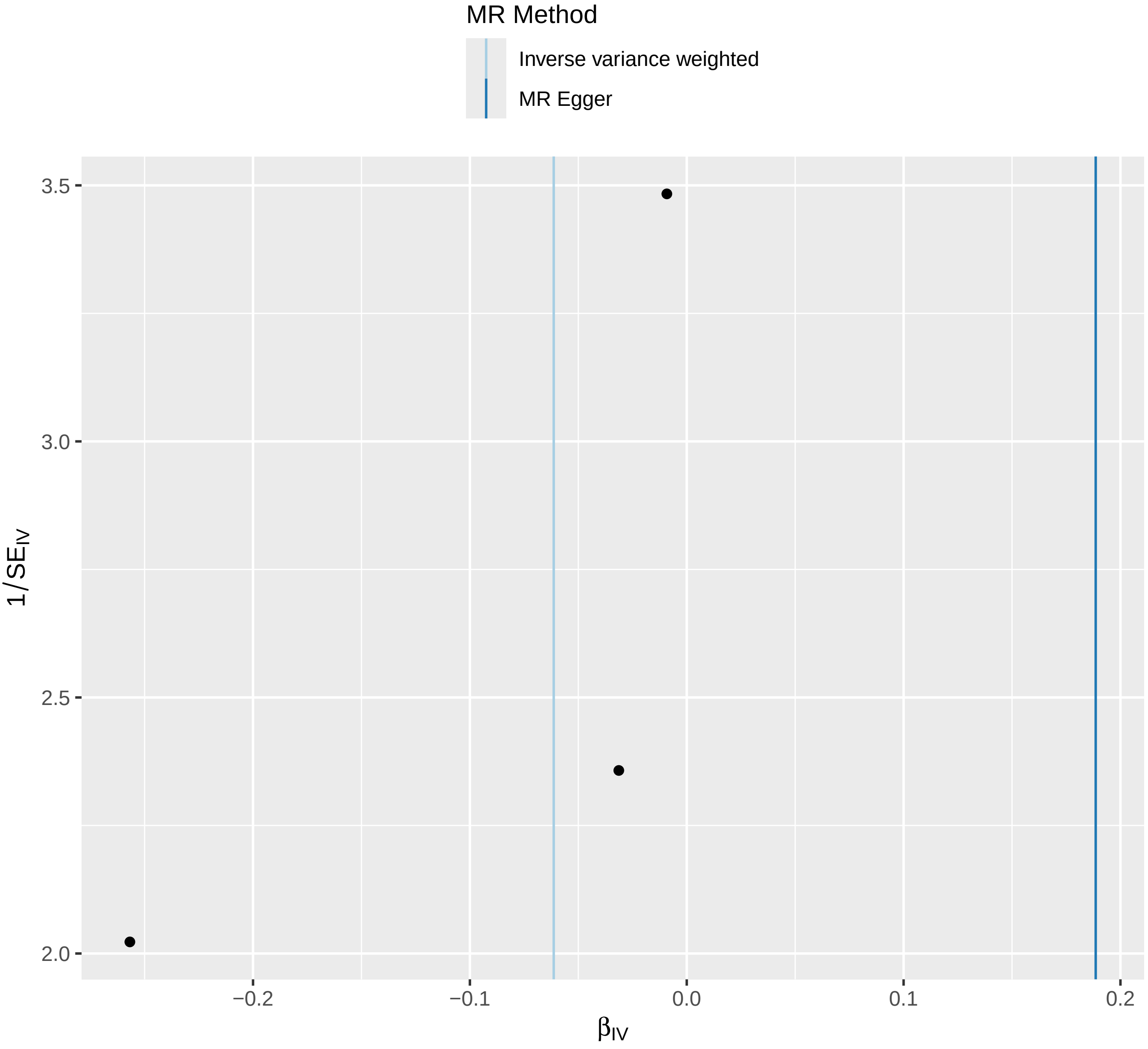
MR Method
Inverse variance weighted
MR Egger
3.5
3.0
2.5
2.0
1/SE_IV
−0.2
−0.1
0.0
0.1
0.2
β_IV

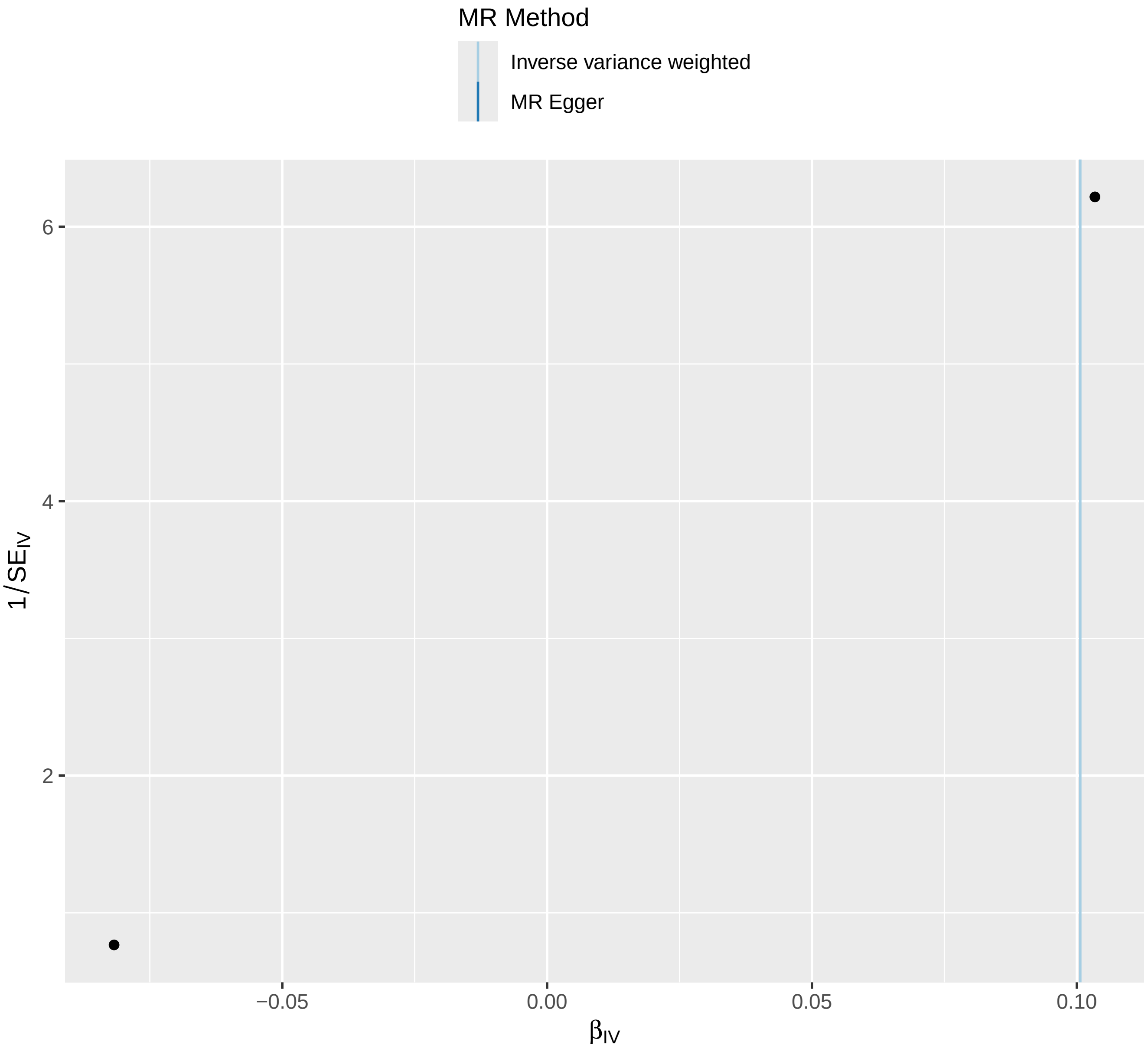

MR Method
Inverse variance weighted
MR Egger
6
4
2
1/SE_IV
−0.05
0.00
0.05
0.10
β_IV

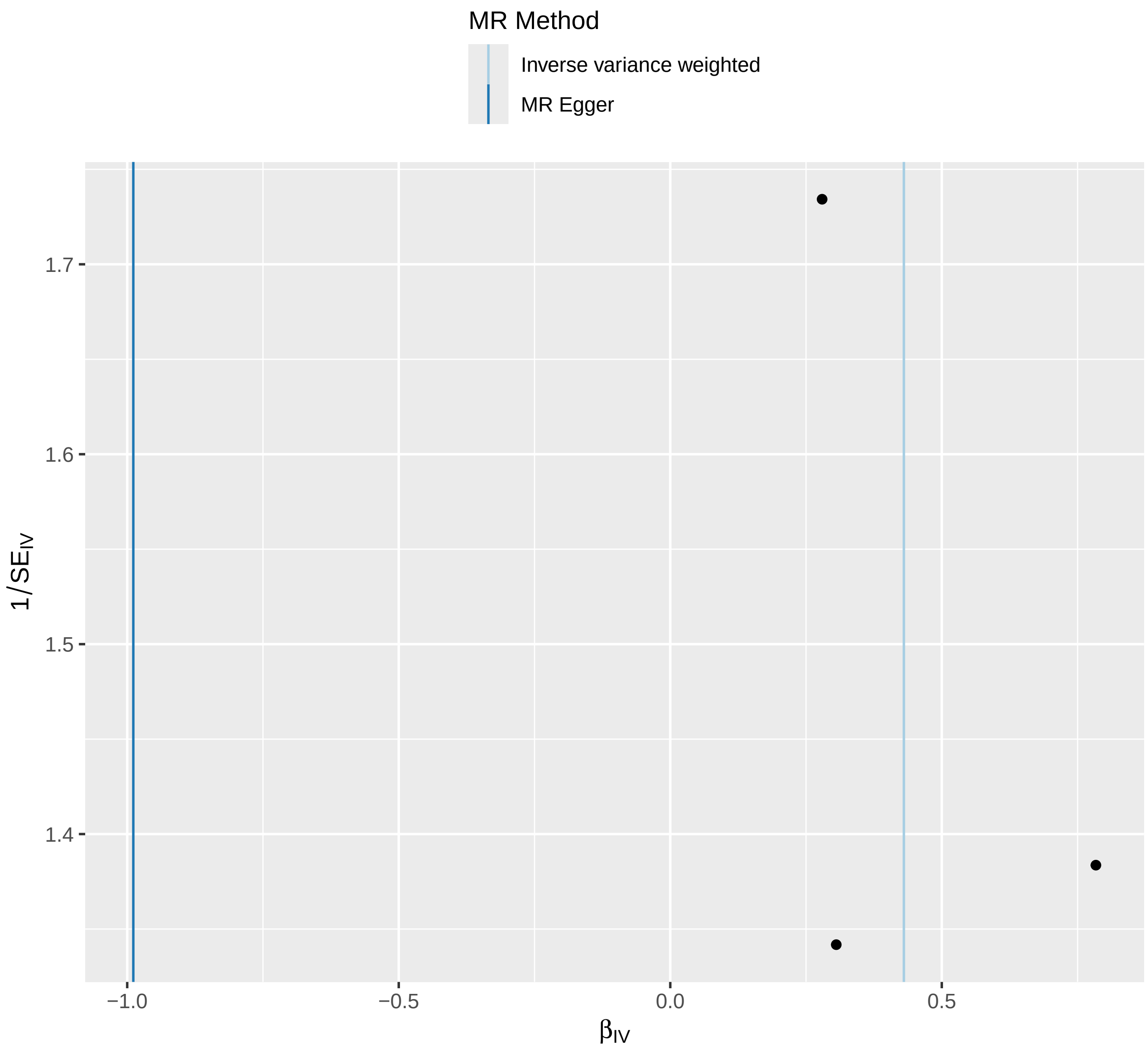
MR Method
Inverse variance weighted
MR Egger
1.7
1.6
1.5
1.4
1/SE$_{IV}$
−1.0
−0.5
0.0
0.5
β$_{IV}$

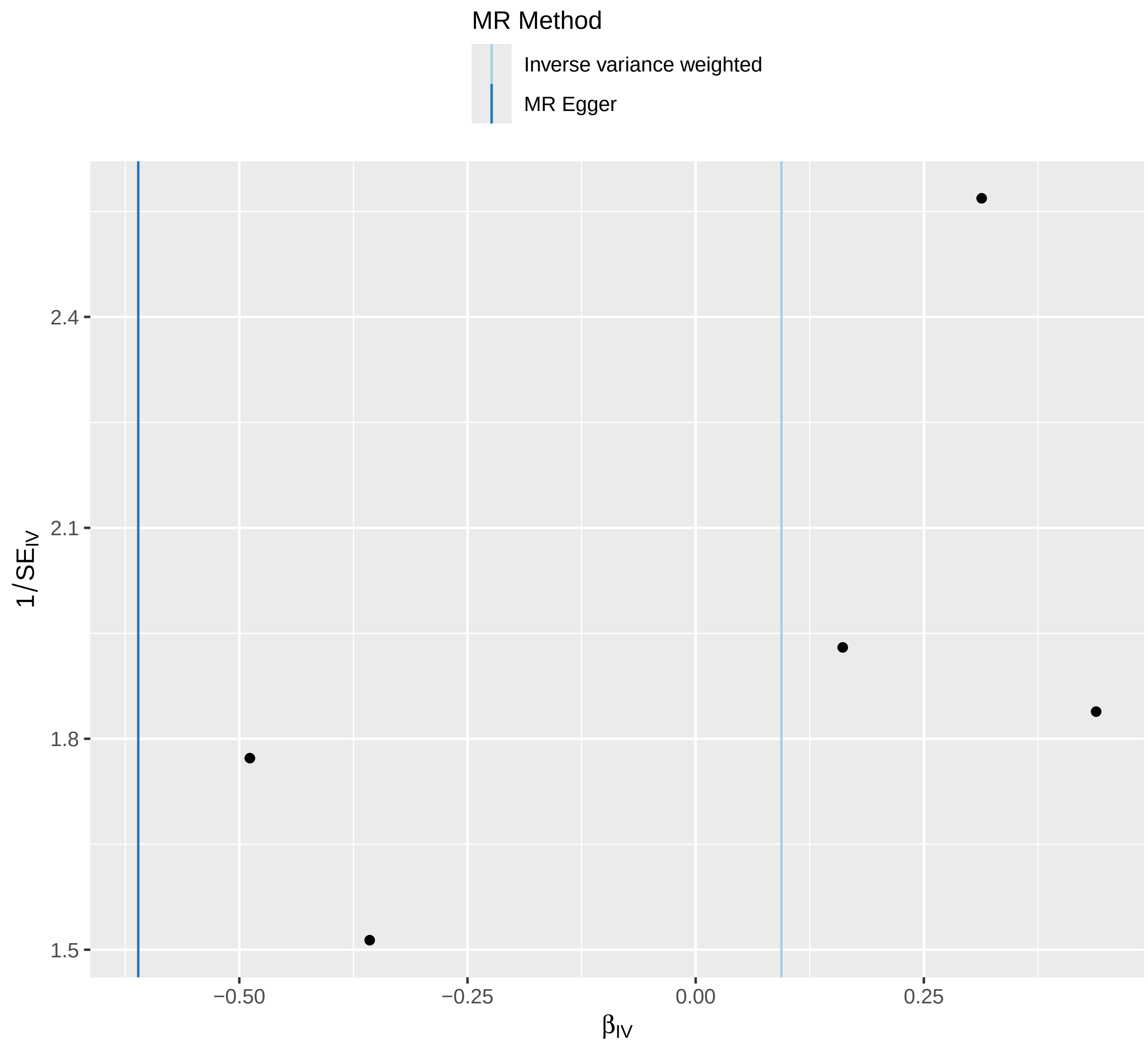

MR Method
Inverse variance weighted
MR Egger
2.4
2.1
1.8
1.5
$1/SE_{IV}$
−0.50
−0.25
0.00
0.25
β_{IV}

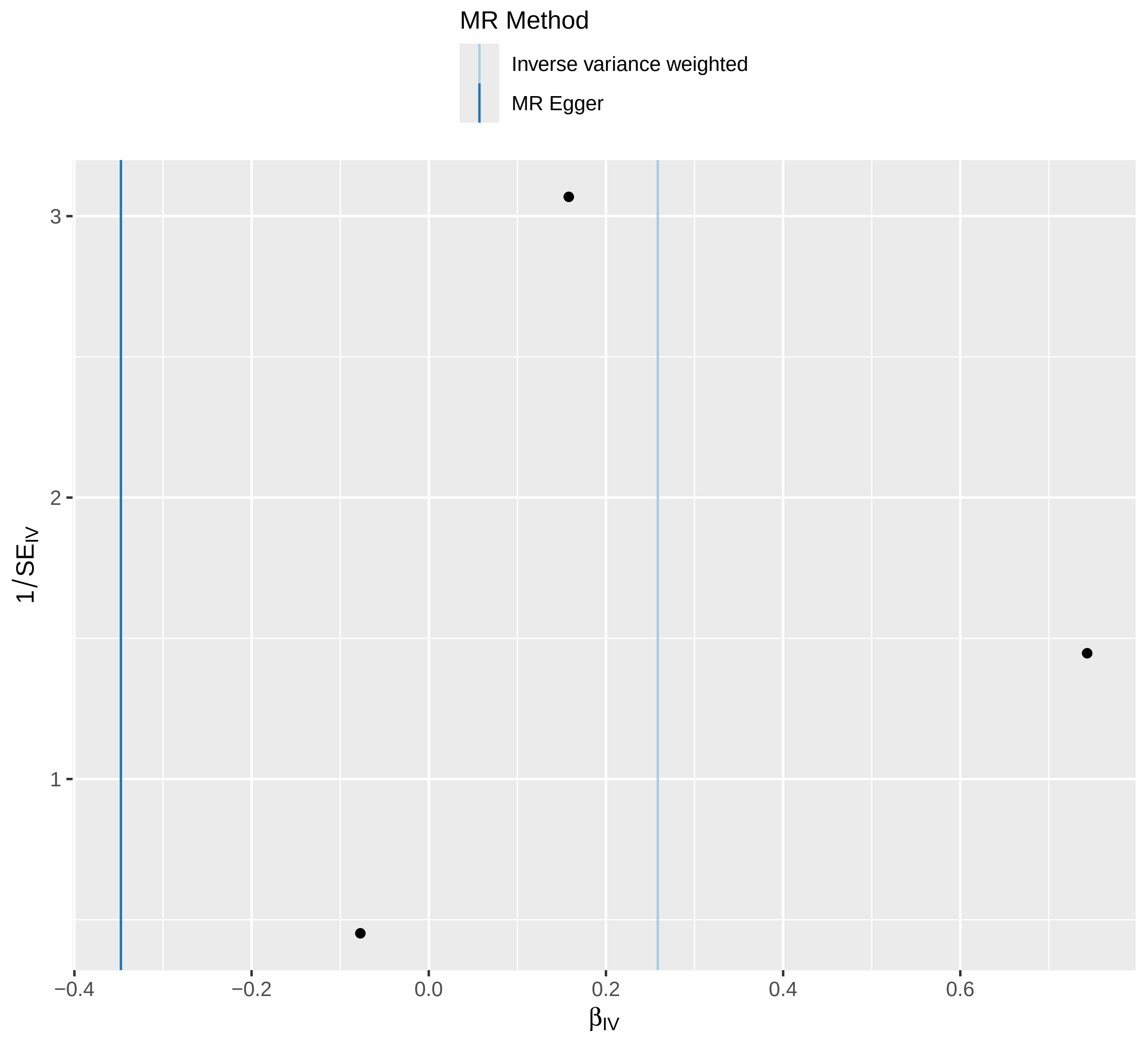
MR Method
Inverse variance weighted
MR Egger
3
2
1
1/SE_IV
−0.4
−0.2
0.0
0.2
0.4
0.6
β_IV

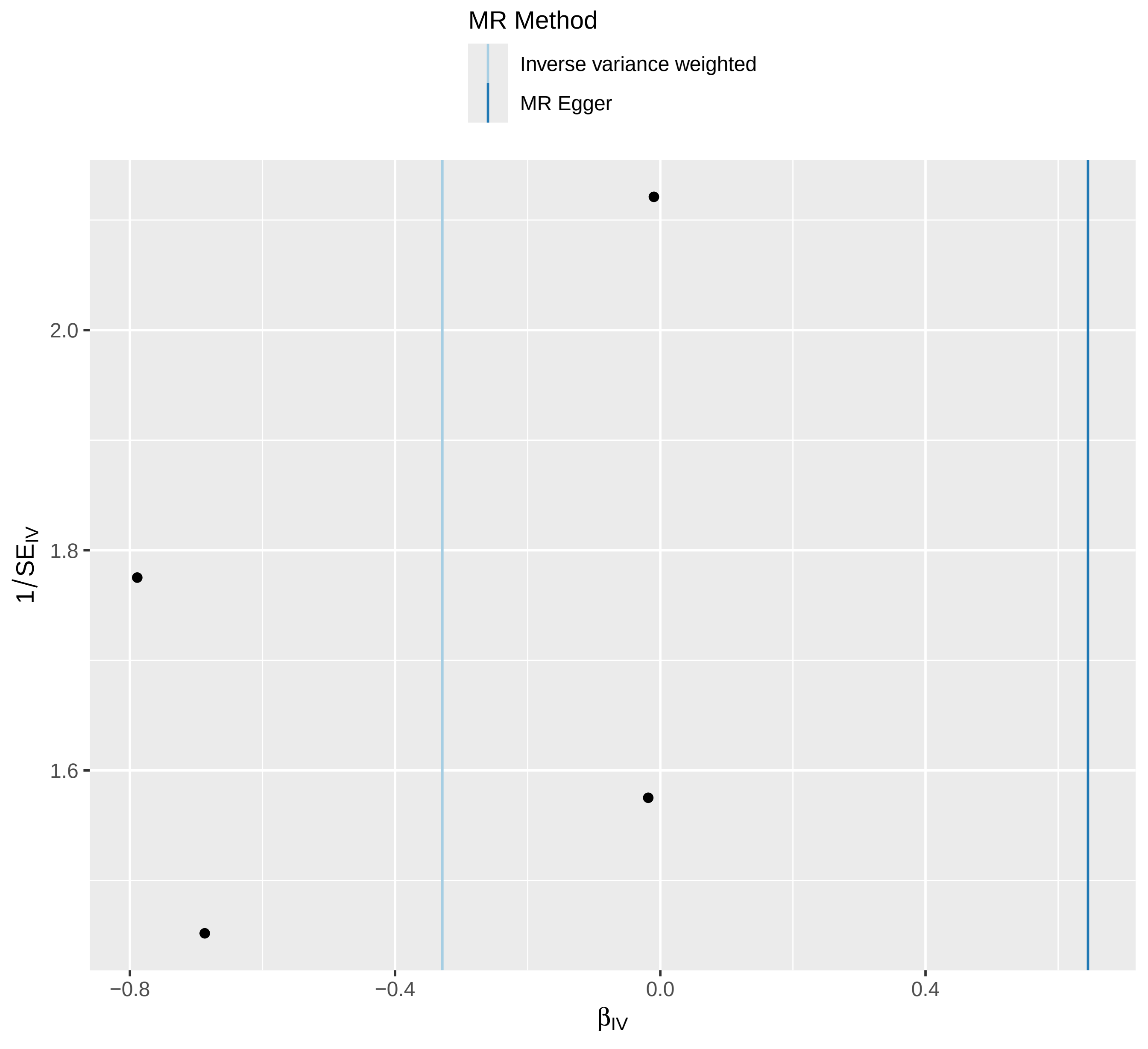
MR Method
Inverse variance weighted
MR Egger
2.0
1.8
1.6
1/SE$_{IV}$
−0.8
−0.4
0.0
0.4
β$_{IV}$

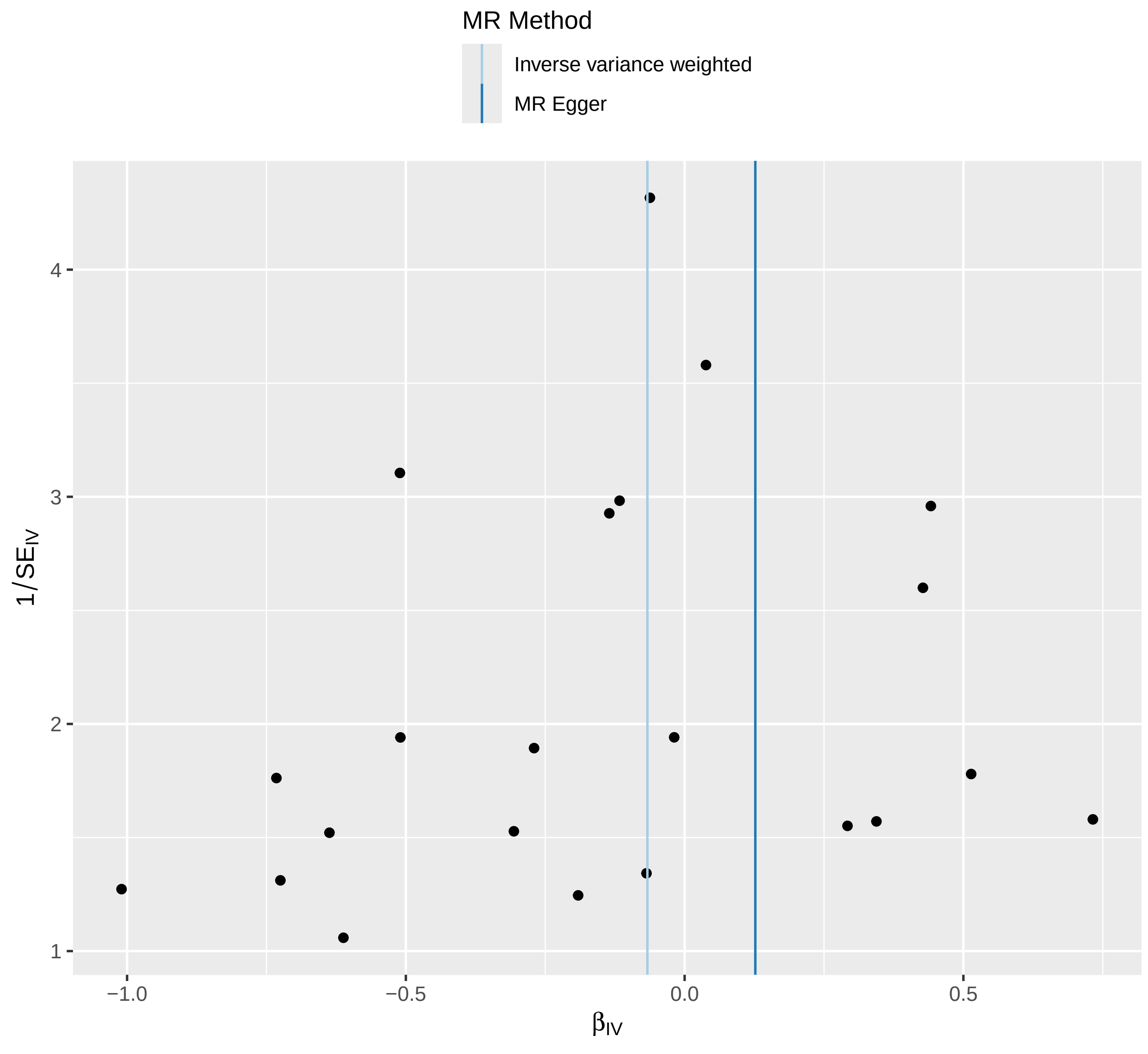
MR Method
Inverse variance weighted
MR Egger
1/SE$_{IV}$
β$_{IV}$
4
3
2
1
−1.0
−0.5
0.0
0.5

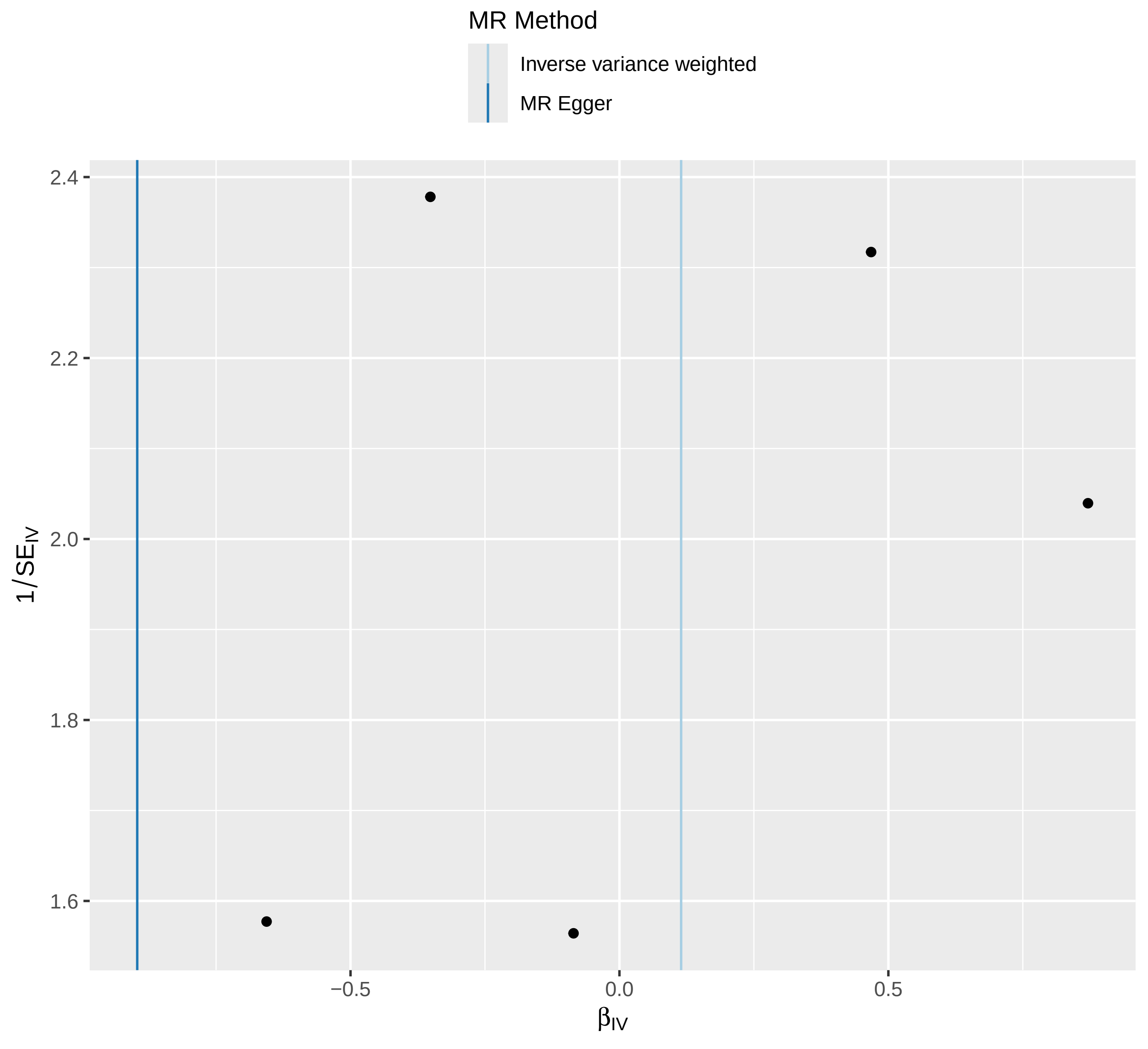
MR Method
Inverse variance weighted
MR Egger
2.4
2.2
2.0
1.8
1.6
1/SE_IV
−0.5
0.0
0.5
β_IV

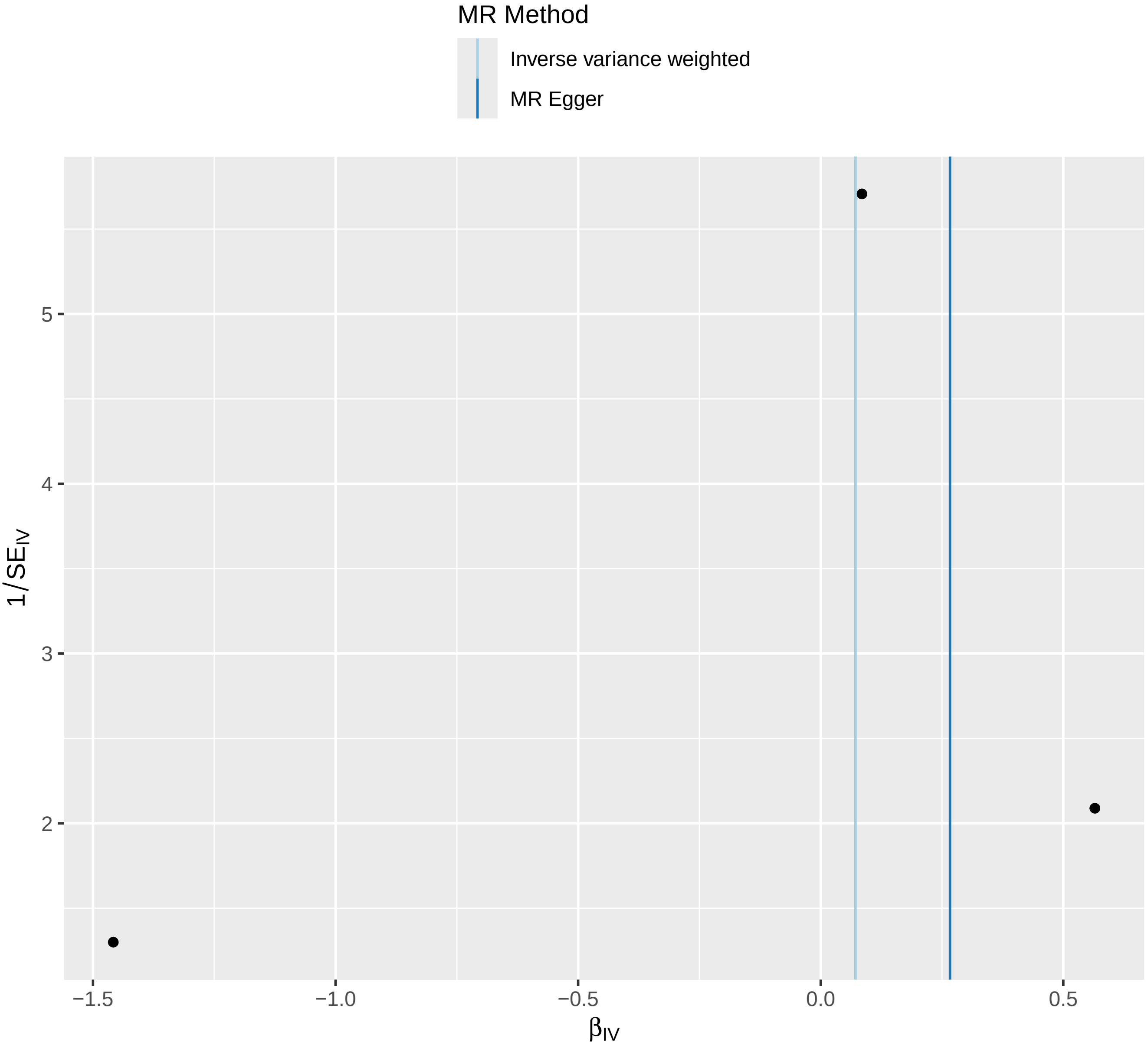

MR Method
Inverse variance weighted
MR Egger
5
4
3
2
1/SE_IV
−1.5
−1.0
−0.5
0.0
0.5
β_IV

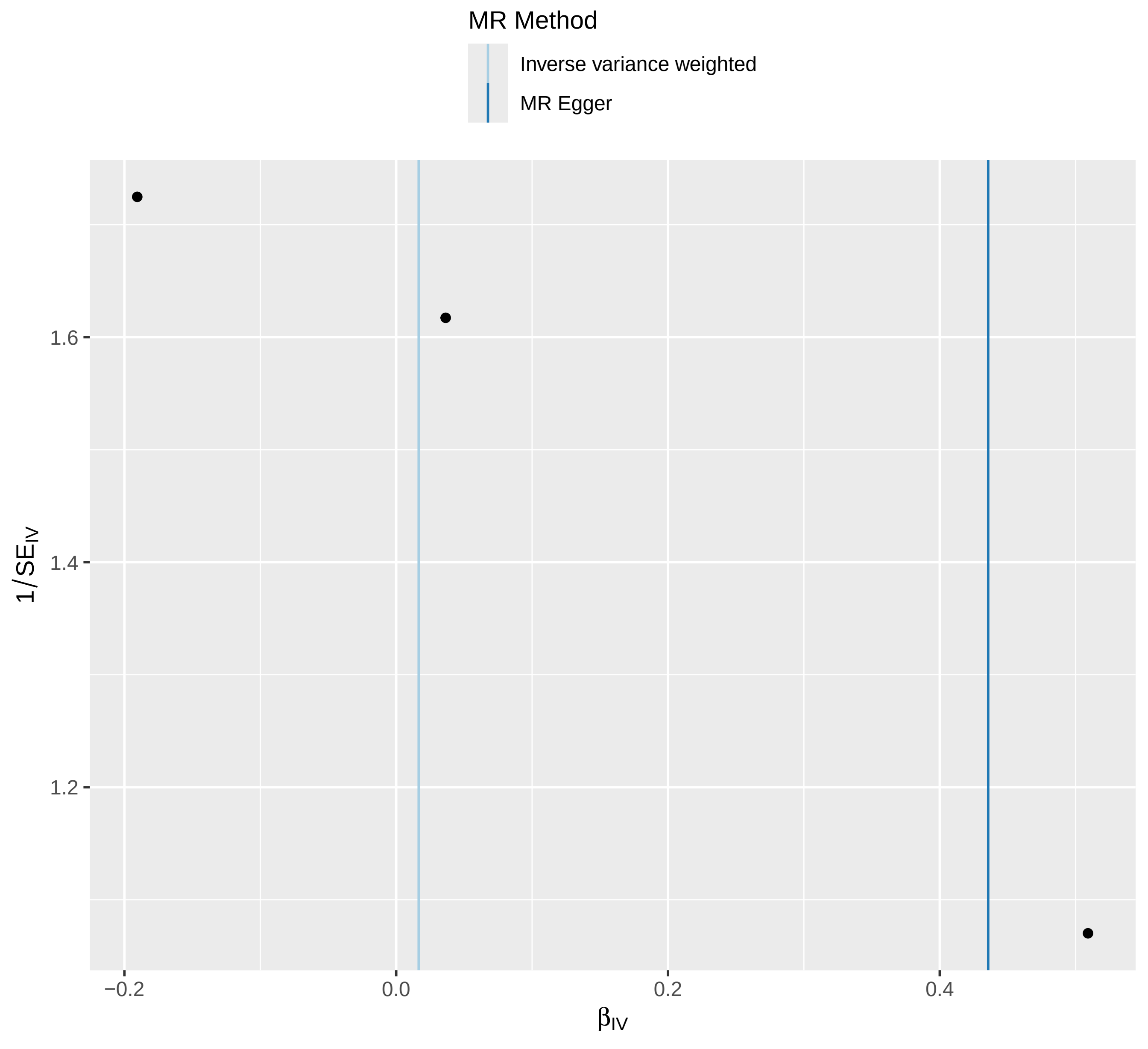

MR Method
Inverse variance weighted
MR Egger
1.6
1.4
1.2
1/SE_IV
−0.2
0.0
0.2
0.4
β_IV

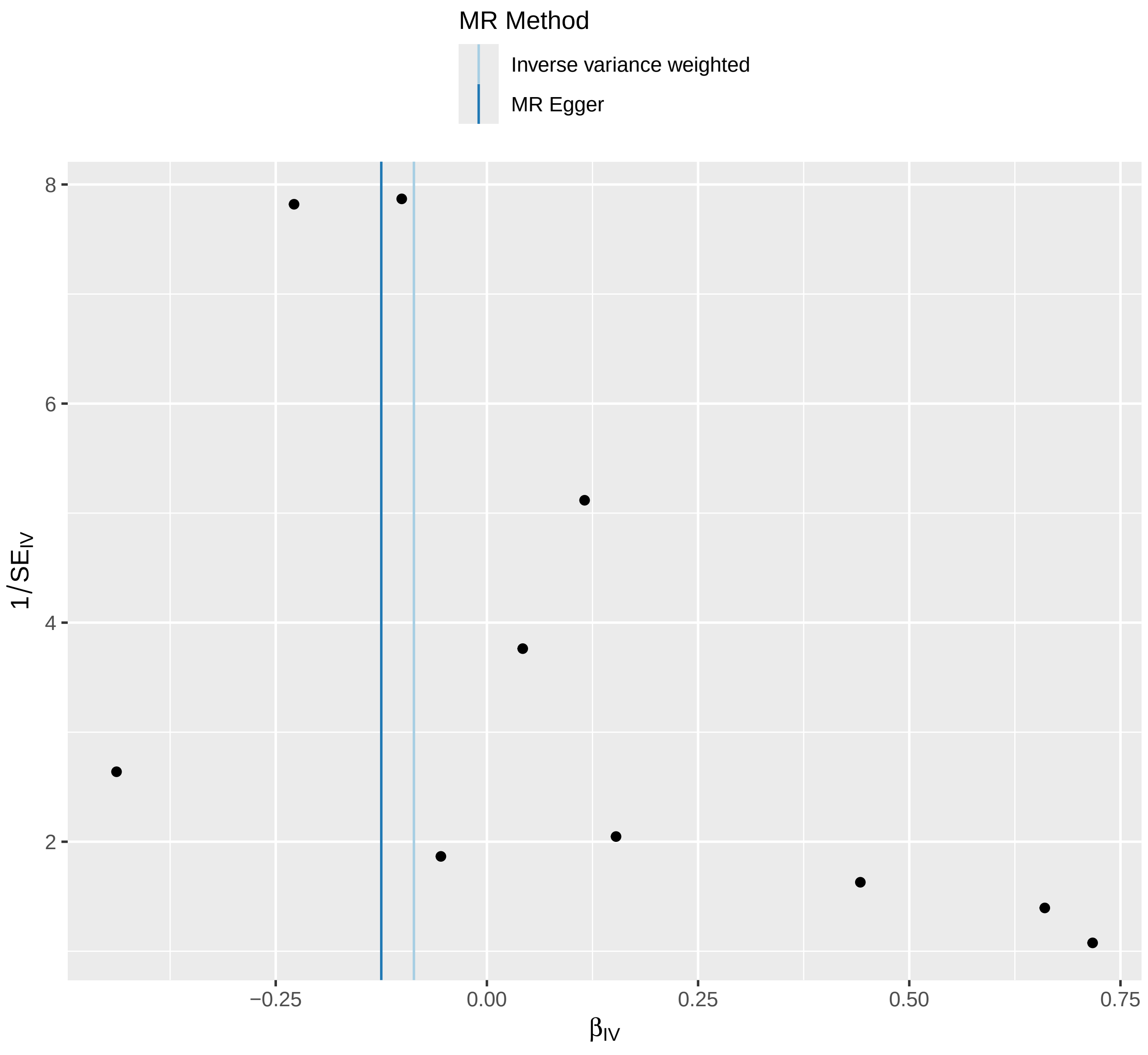
MR Method
Inverse variance weighted
MR Egger
8
6
4
2
1/SE_IV
−0.25
0.00
0.25
0.50
0.75
β_IV

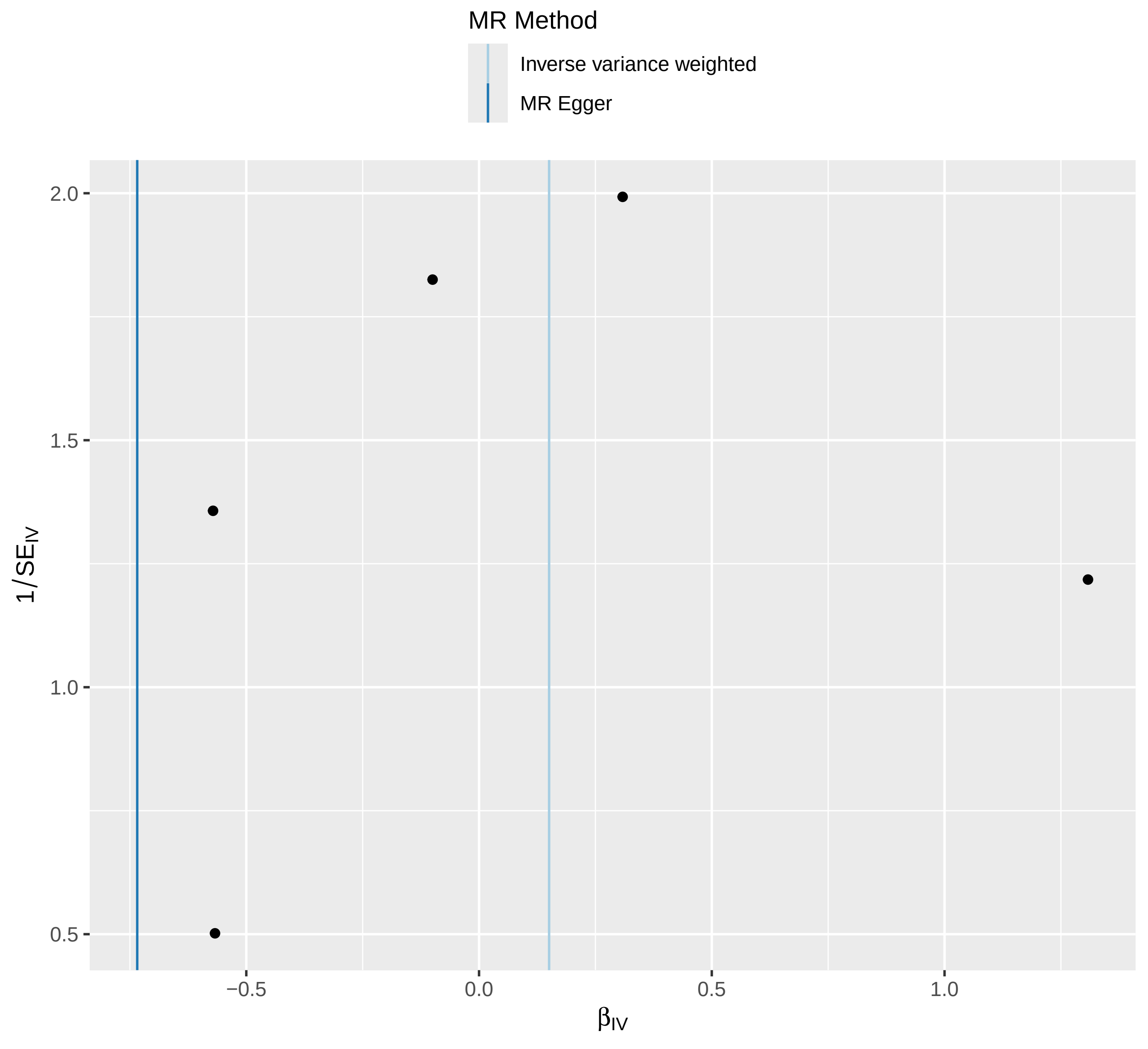
MR Method
Inverse variance weighted
MR Egger
2.0
1.5
1.0
0.5
1/SE_IV
−0.5
0.0
0.5
1.0
β_IV

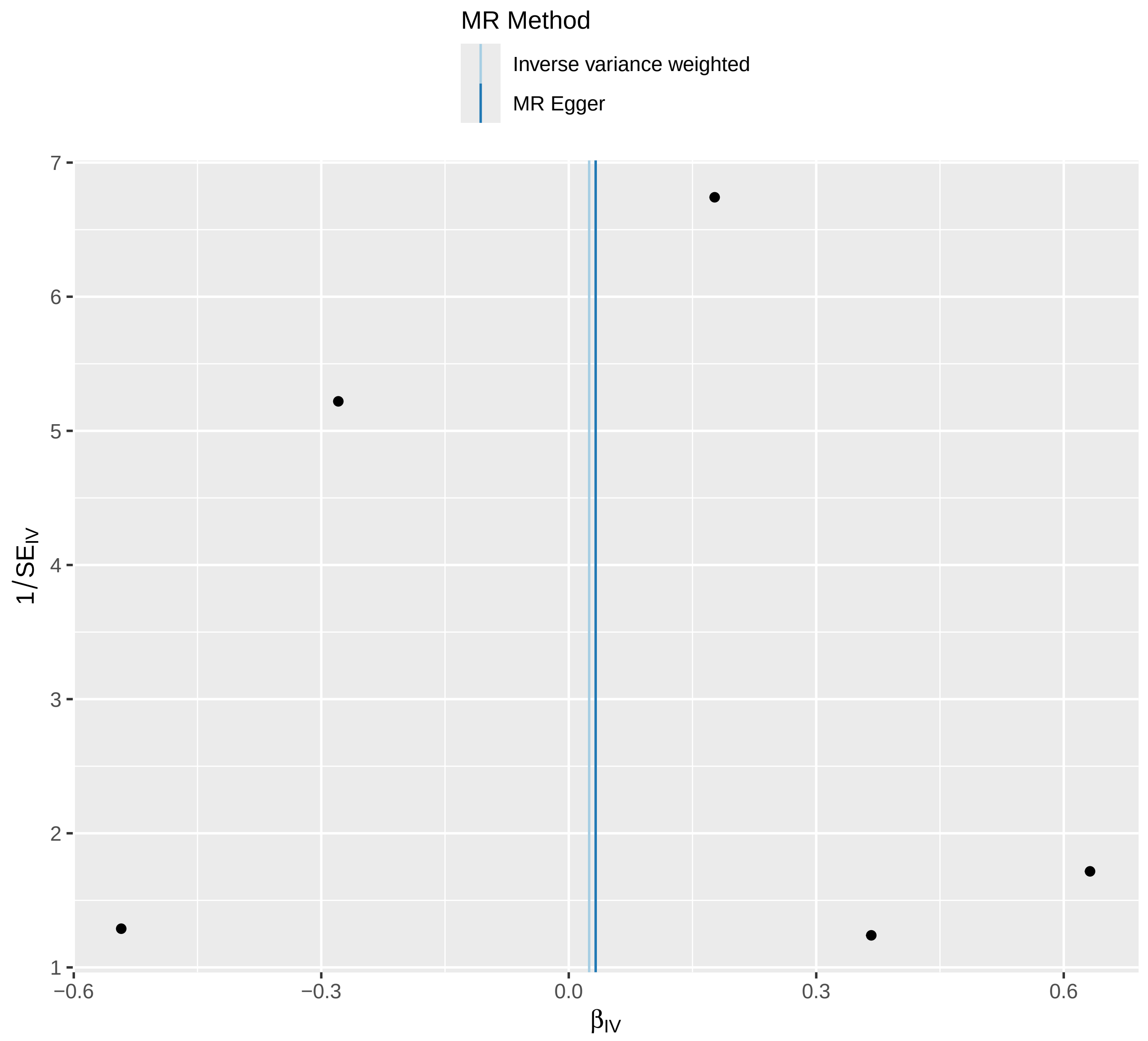
MR Method
Inverse variance weighted
MR Egger
1/SE$_{IV}$
7
6
5
4
3
2
1
−0.6
−0.3
0.0
0.3
0.6
β$_{IV}$

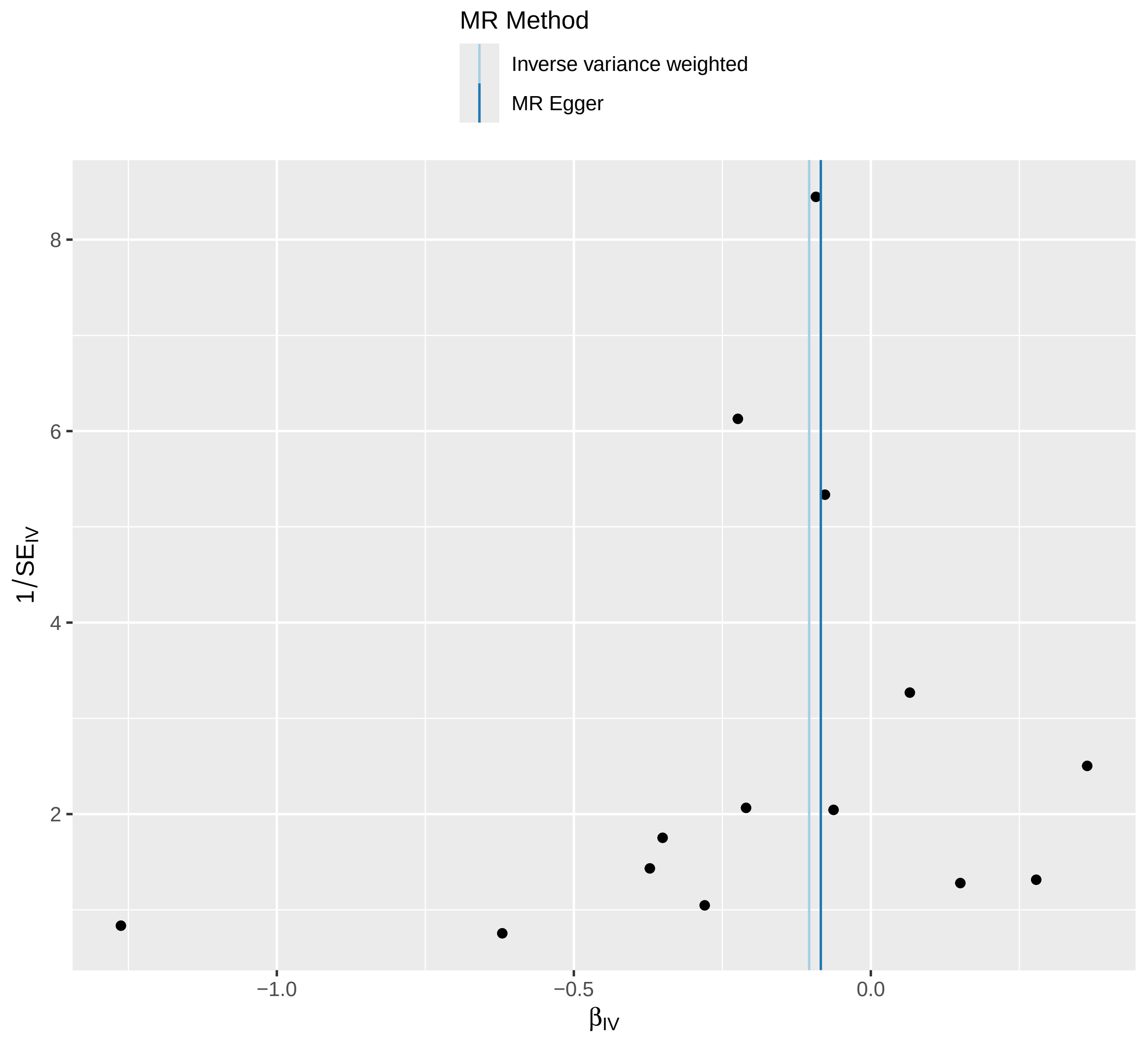
MR Method
Inverse variance weighted
MR Egger
8
6
4
2
−1.0
−0.5
0.0
$1/SE_{IV}$
β_{IV}

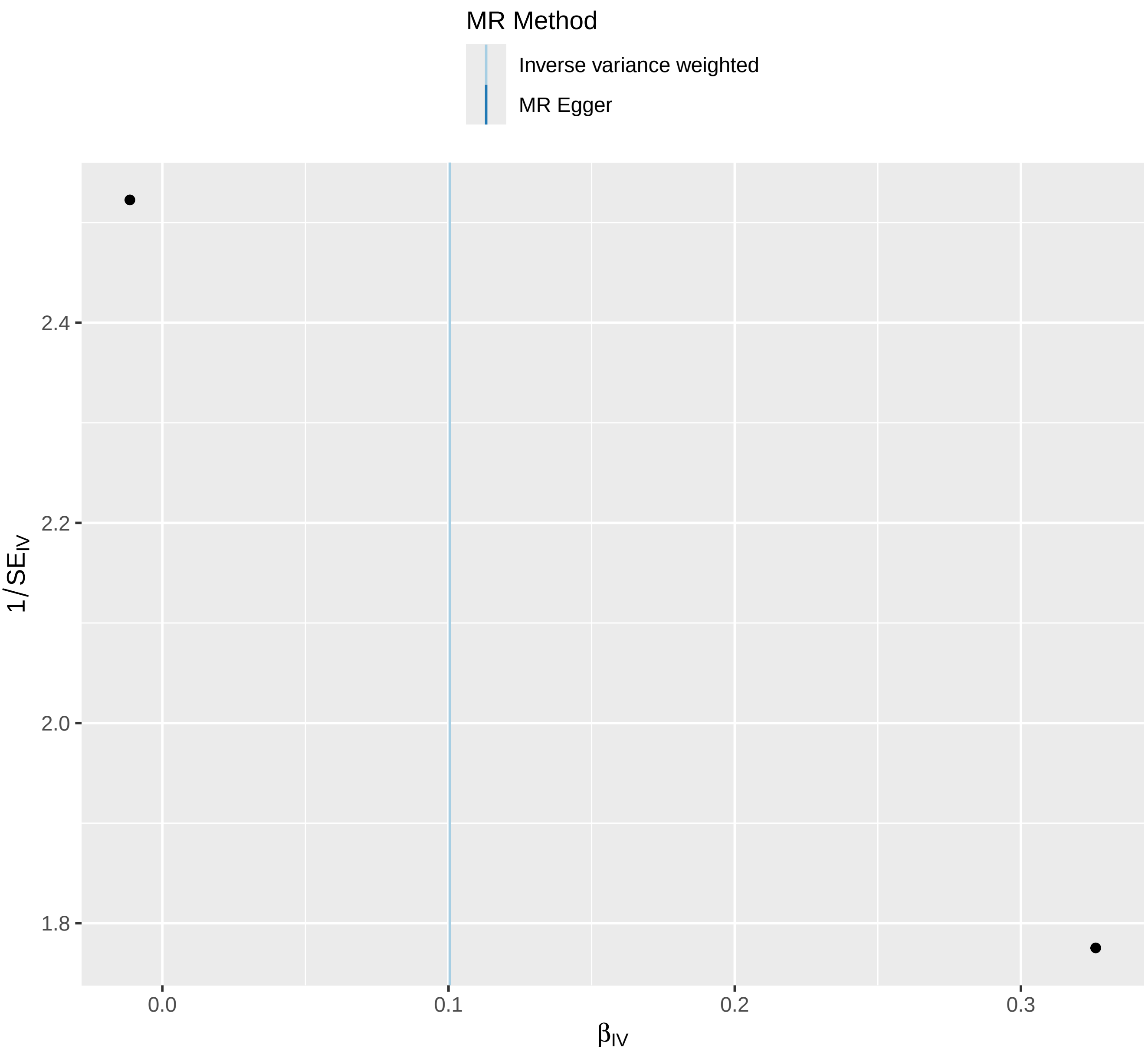
MR Method
Inverse variance weighted
MR Egger
2.4
2.2
2.0
1.8
1/SE_IV
0.0
0.1
0.2
0.3
β_IV

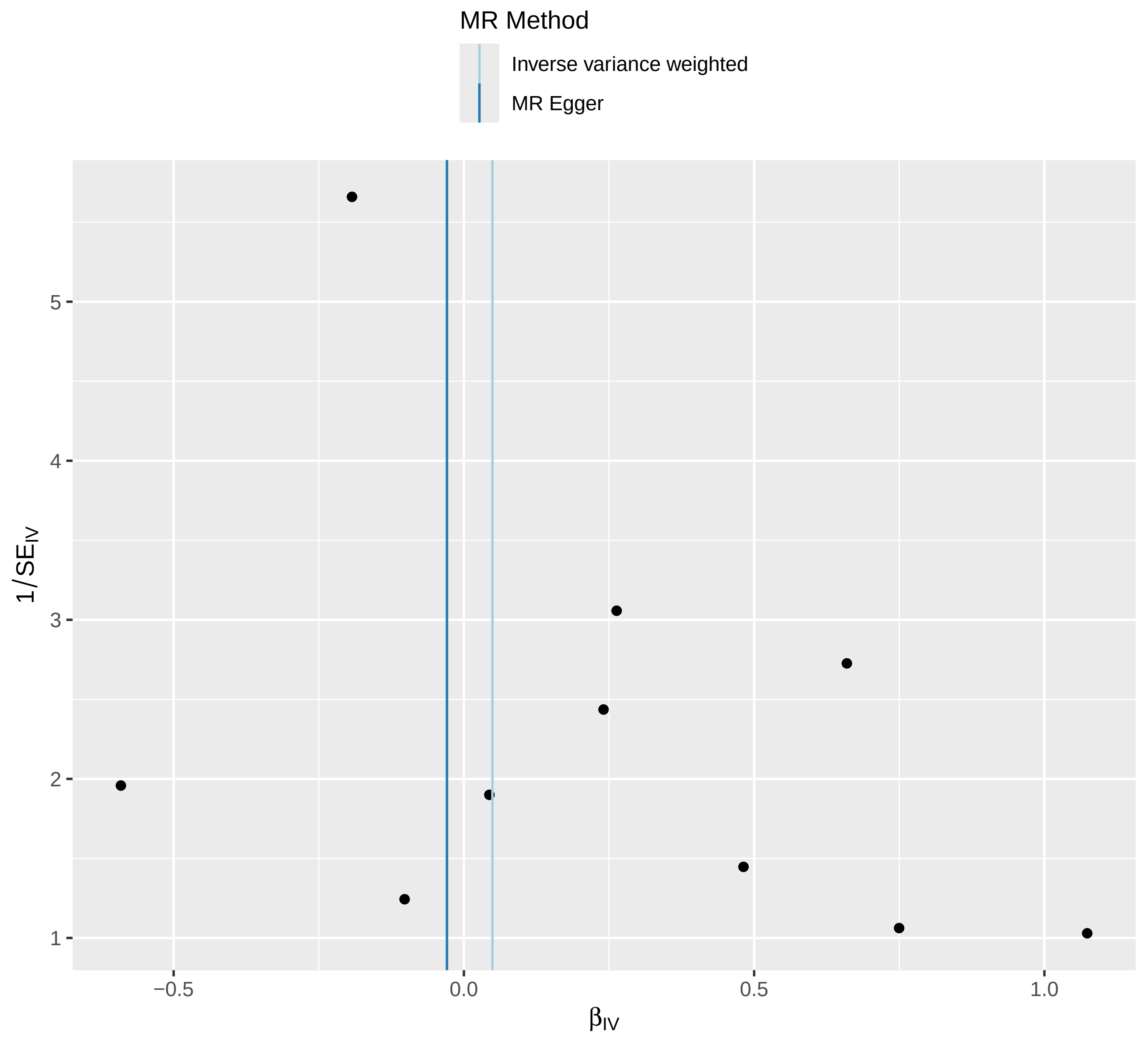
MR Method
Inverse variance weighted
MR Egger
1/SE$_{IV}$
β$_{IV}$
5
4
3
2
1
−0.5
0.0
0.5
1.0

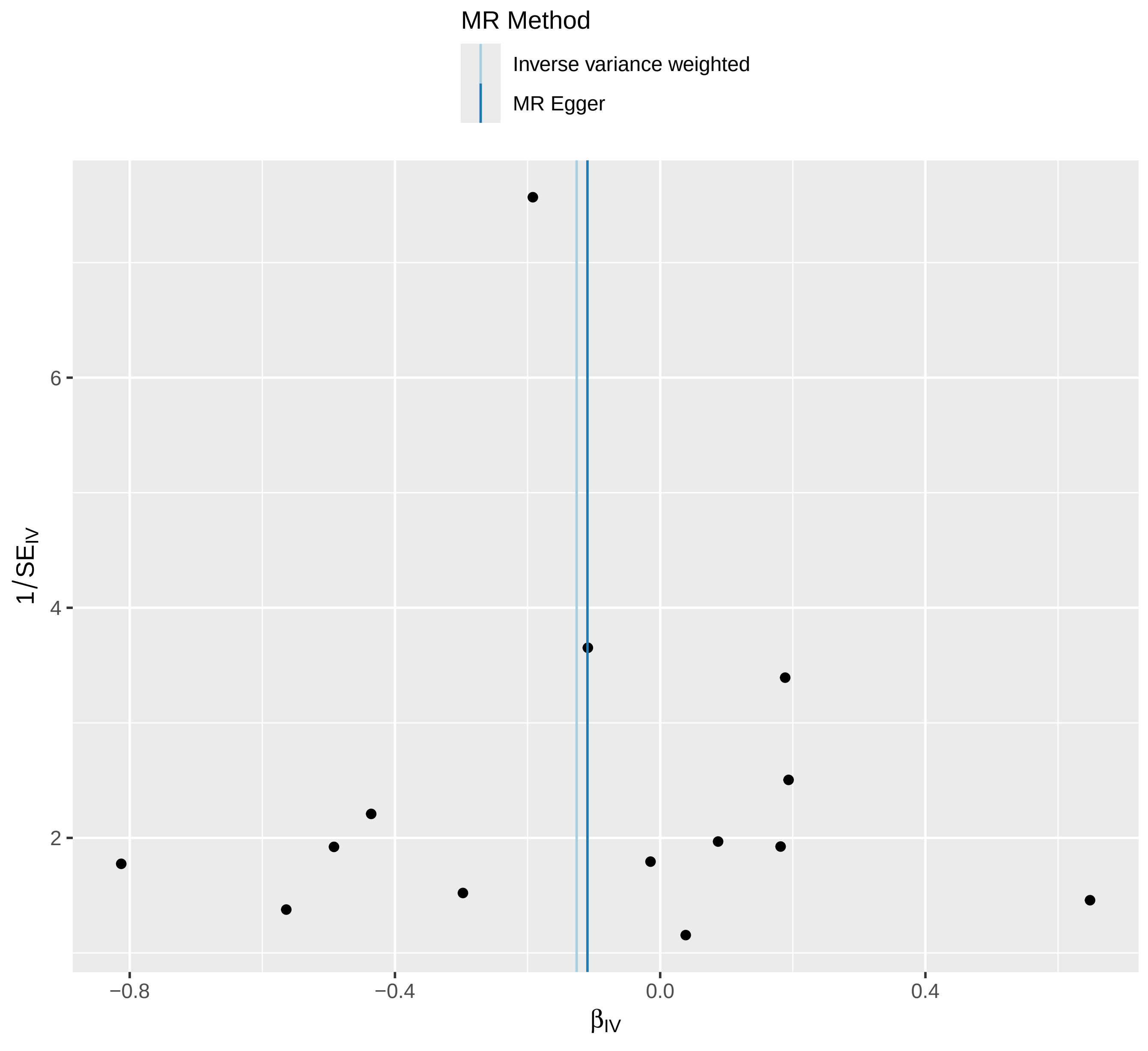
MR Method
Inverse variance weighted
MR Egger
6
4
2
1/SE$_{IV}$
−0.8
−0.4
0.0
0.4
β_{IV}

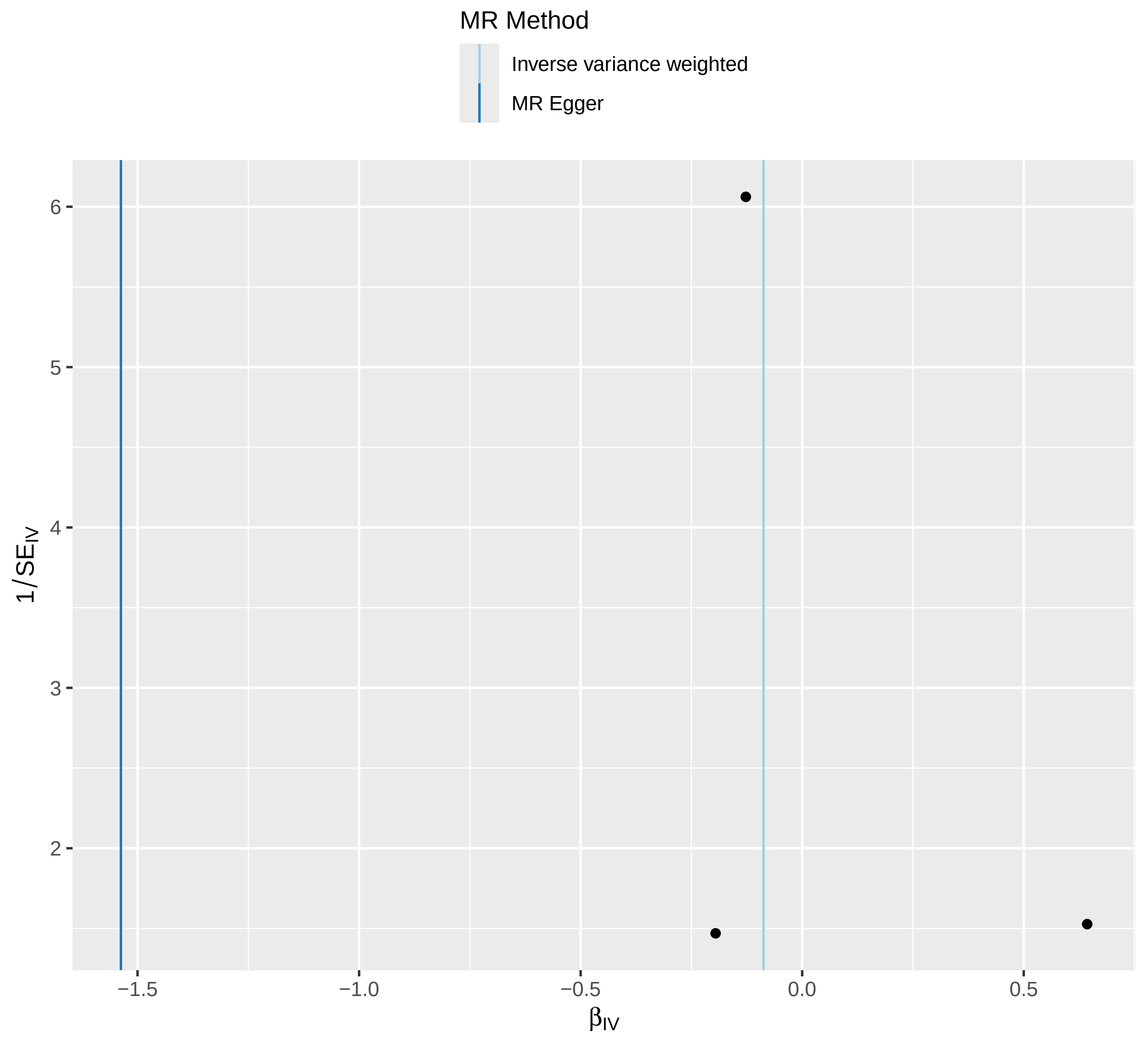
MR Method
Inverse variance weighted
MR Egger
1/SE_IV
6
5
4
3
2
β_IV
−1.5
−1.0
−0.5
0.0
0.5

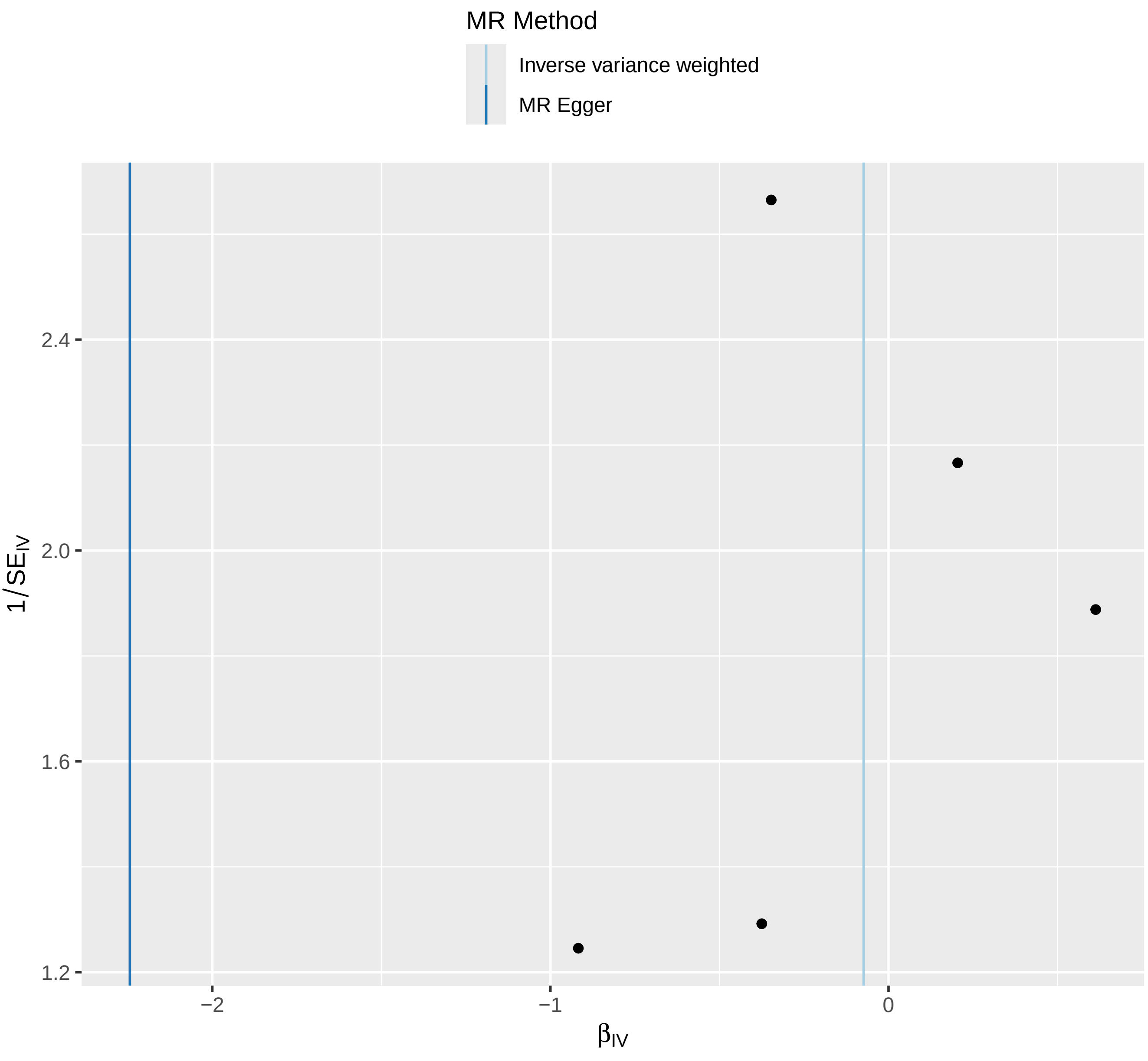
MR Method
Inverse variance weighted
MR Egger
2.4
2.0
1.6
1.2
1/SE_IV
−2
−1
0
β_IV

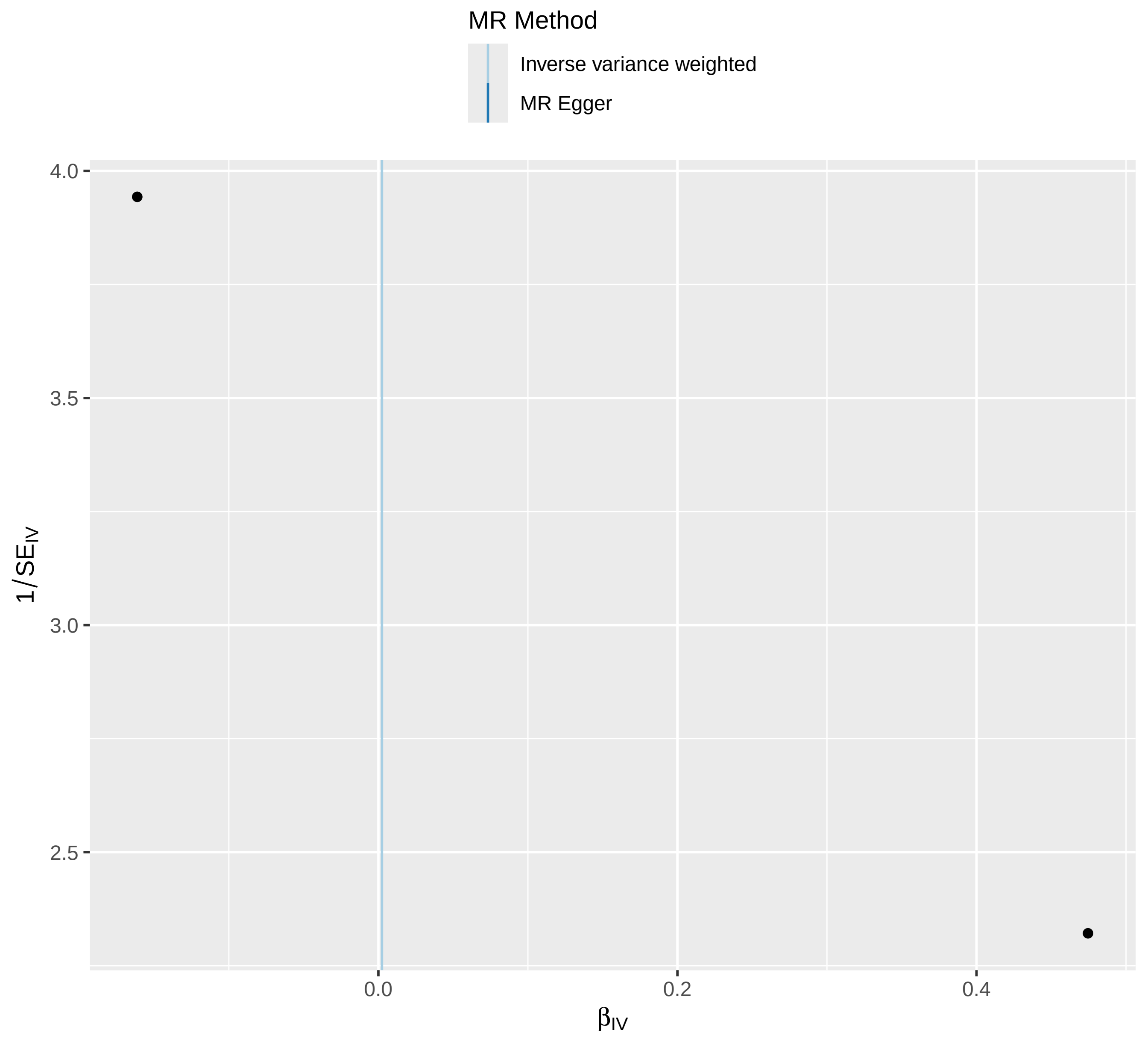

MR Method
Inverse variance weighted
MR Egger
4.0
3.5
3.0
2.5
1/SE$_{IV}$
0.0
0.2
0.4
β$_{IV}$

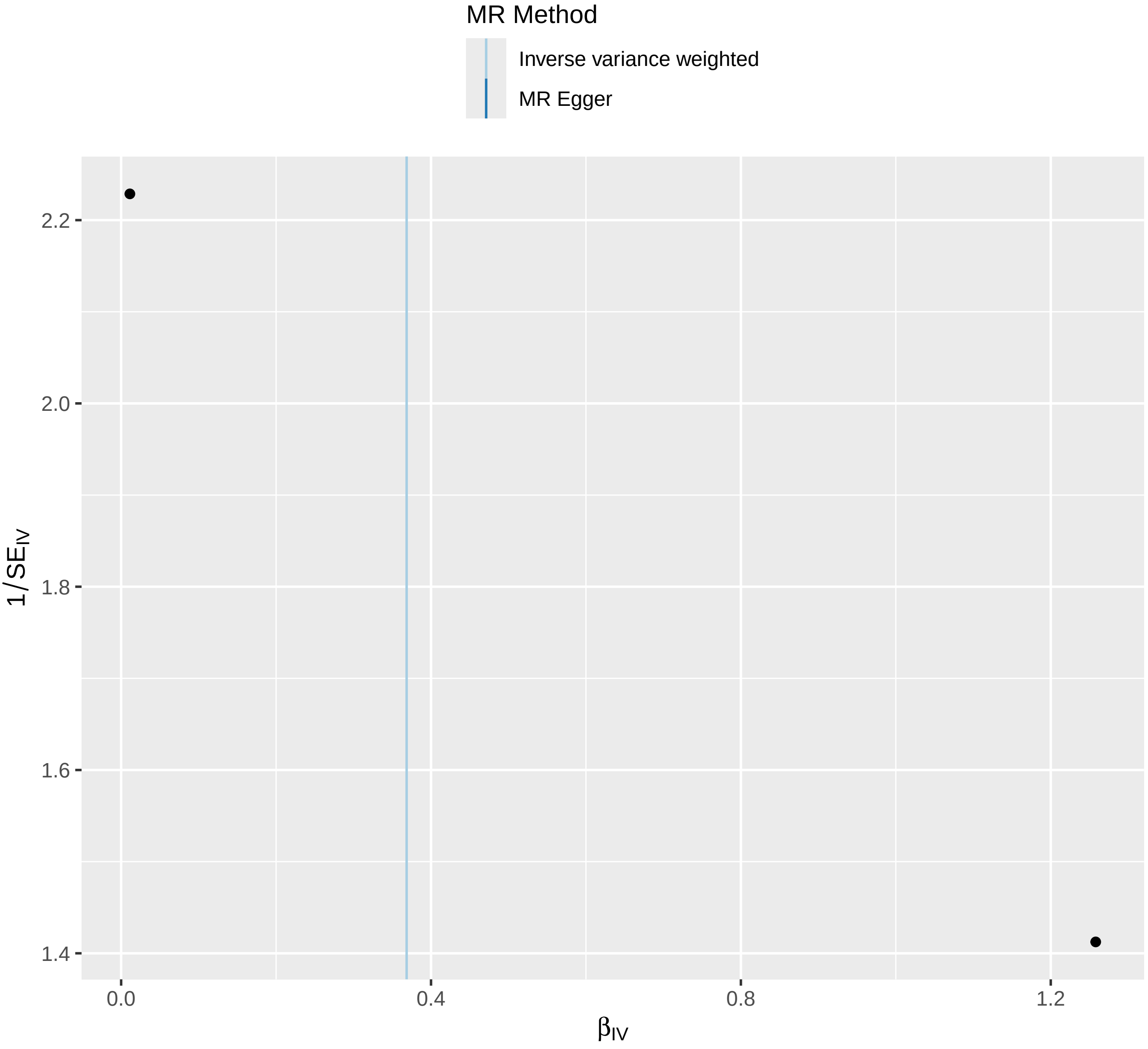
MR Method
Inverse variance weighted
MR Egger
2.2
2.0
1.8
1.6
1.4
$1/SE_{IV}$
0.0
0.4
0.8
1.2
β_{IV}

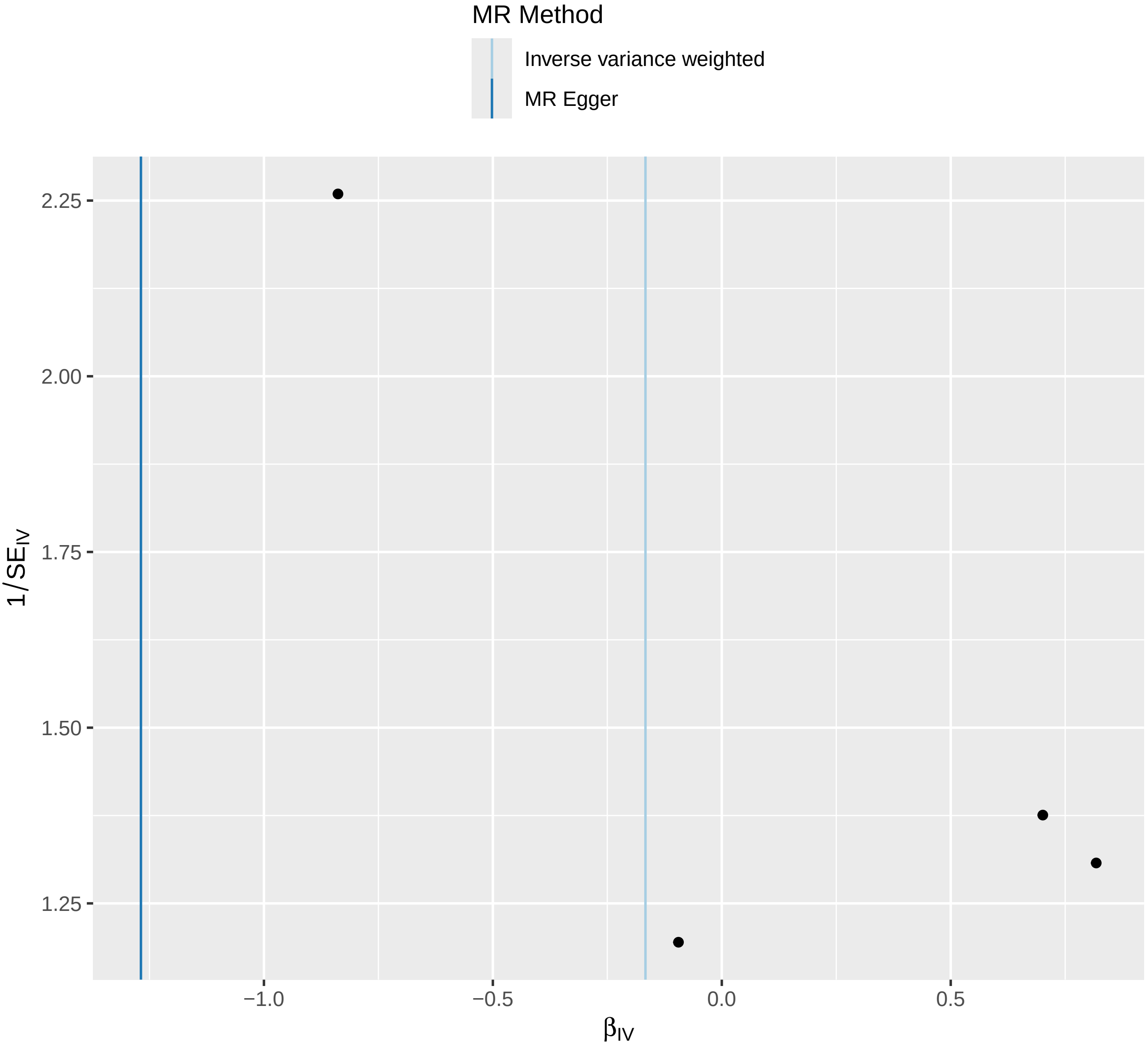

MR Method
Inverse variance weighted
MR Egger
2.25
2.00
1.75
1.50
1.25
$1/SE_{IV}$
−1.0
−0.5
0.0
0.5
β_{IV}

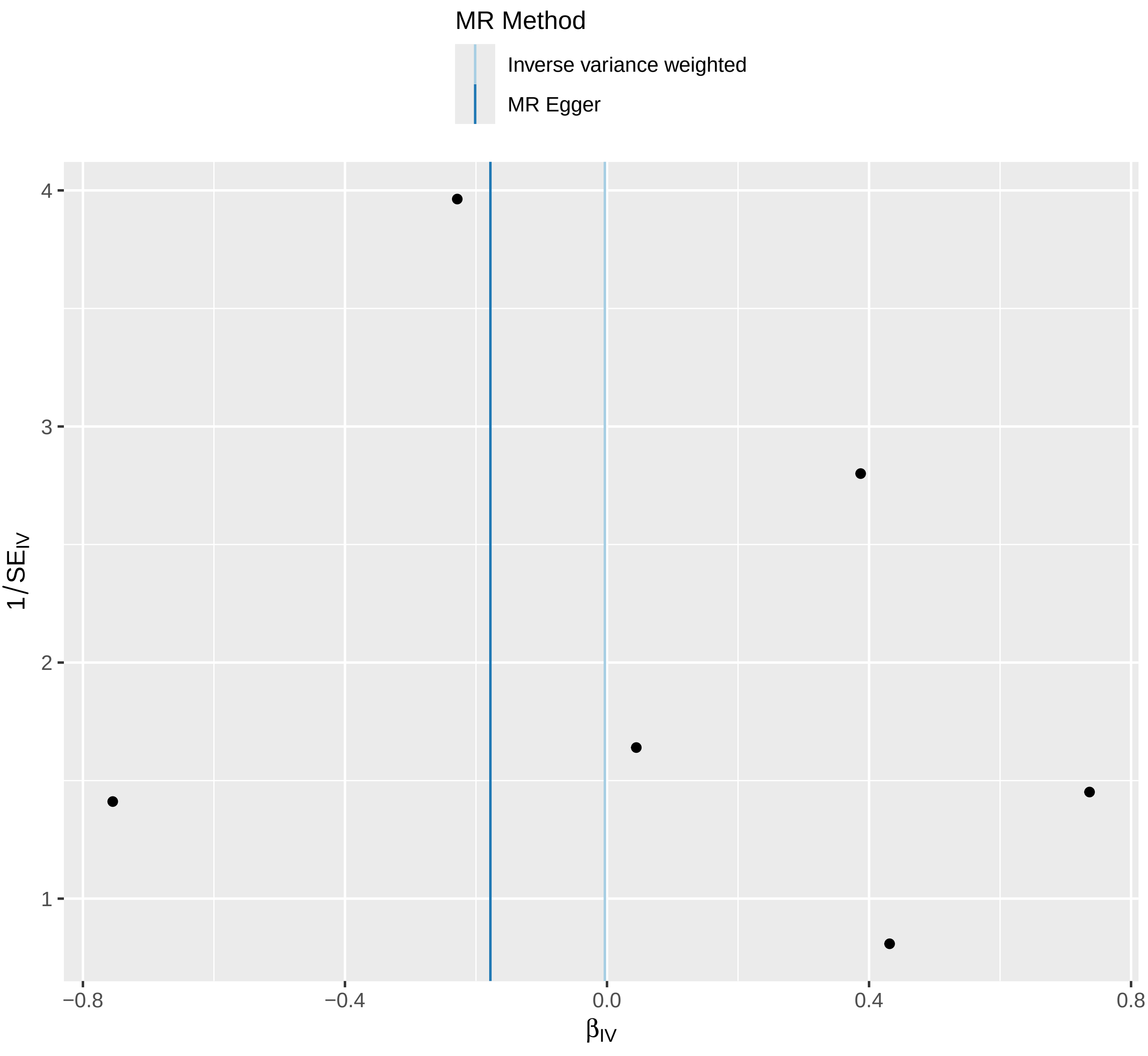
MR Method
Inverse variance weighted
MR Egger
4
3
2
1
1/SE_IV
−0.8
−0.4
0.0
0.4
0.8
β_IV

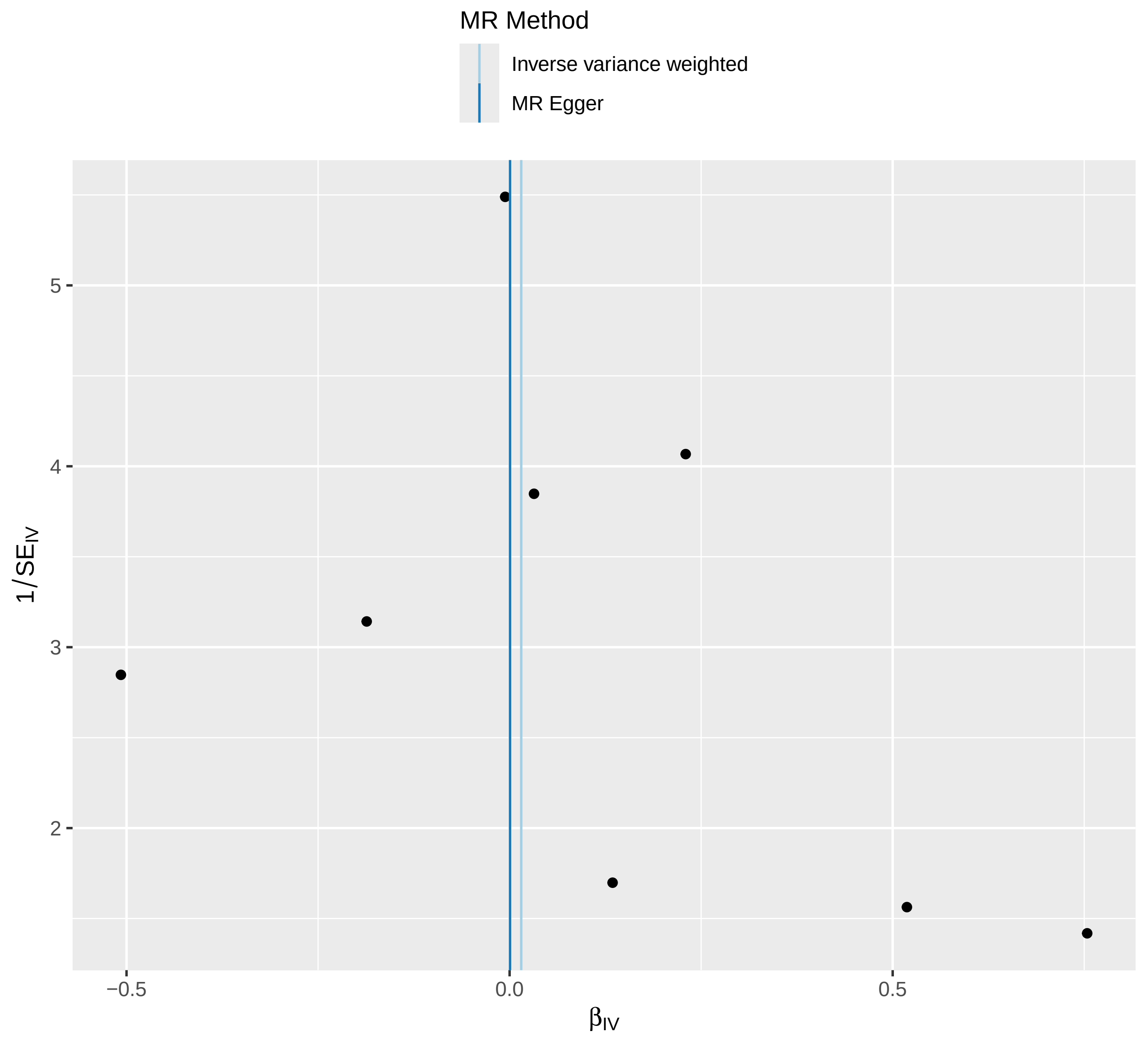

MR Method
Inverse variance weighted
MR Egger
5
4
3
2
1/SE_IV
−0.5
0.0
0.5
β_IV

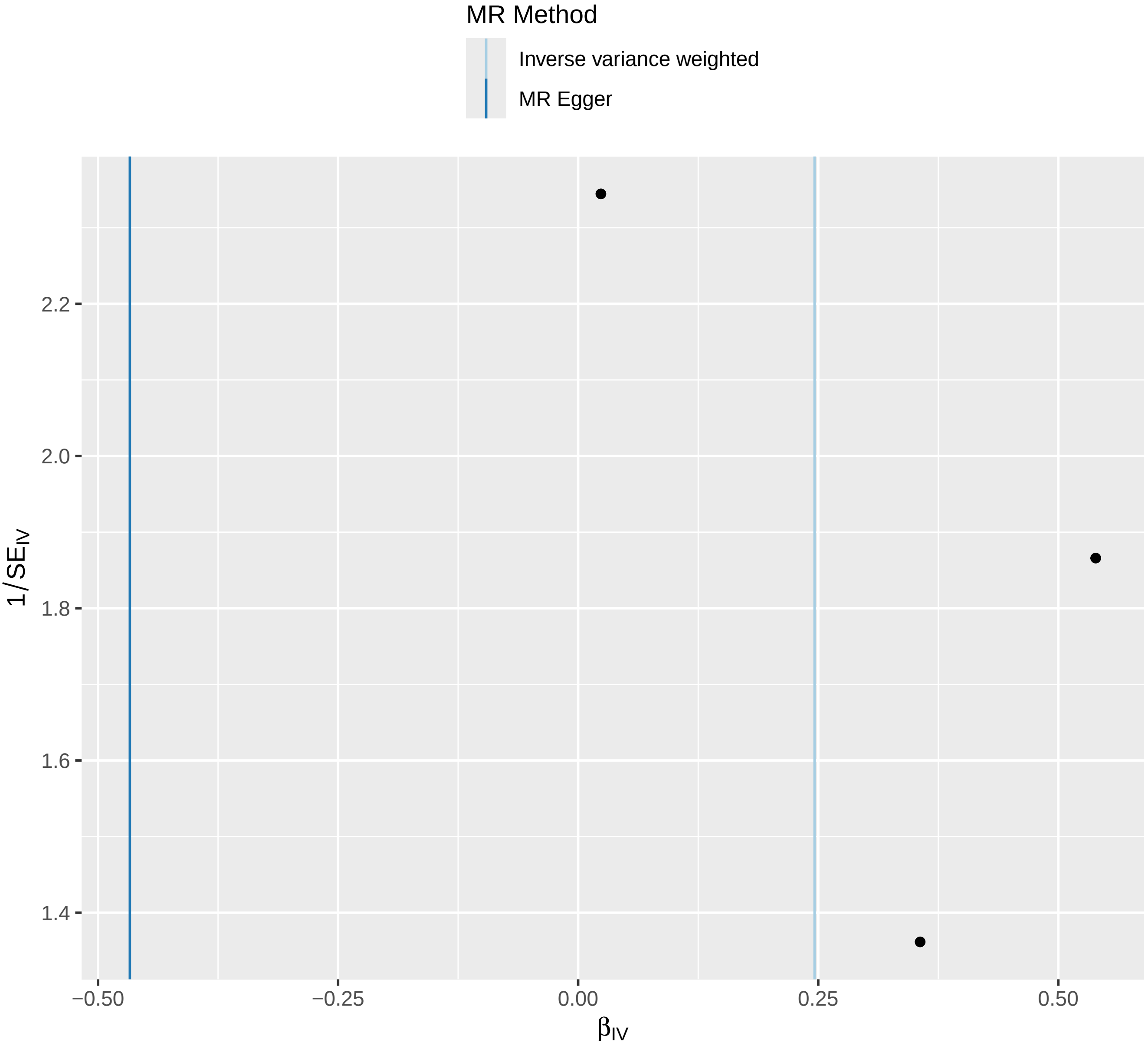
MR Method
Inverse variance weighted
MR Egger
2.2
2.0
1.8
1.6
1.4
1/SE_IV
−0.50
−0.25
0.00
0.25
0.50
β_IV

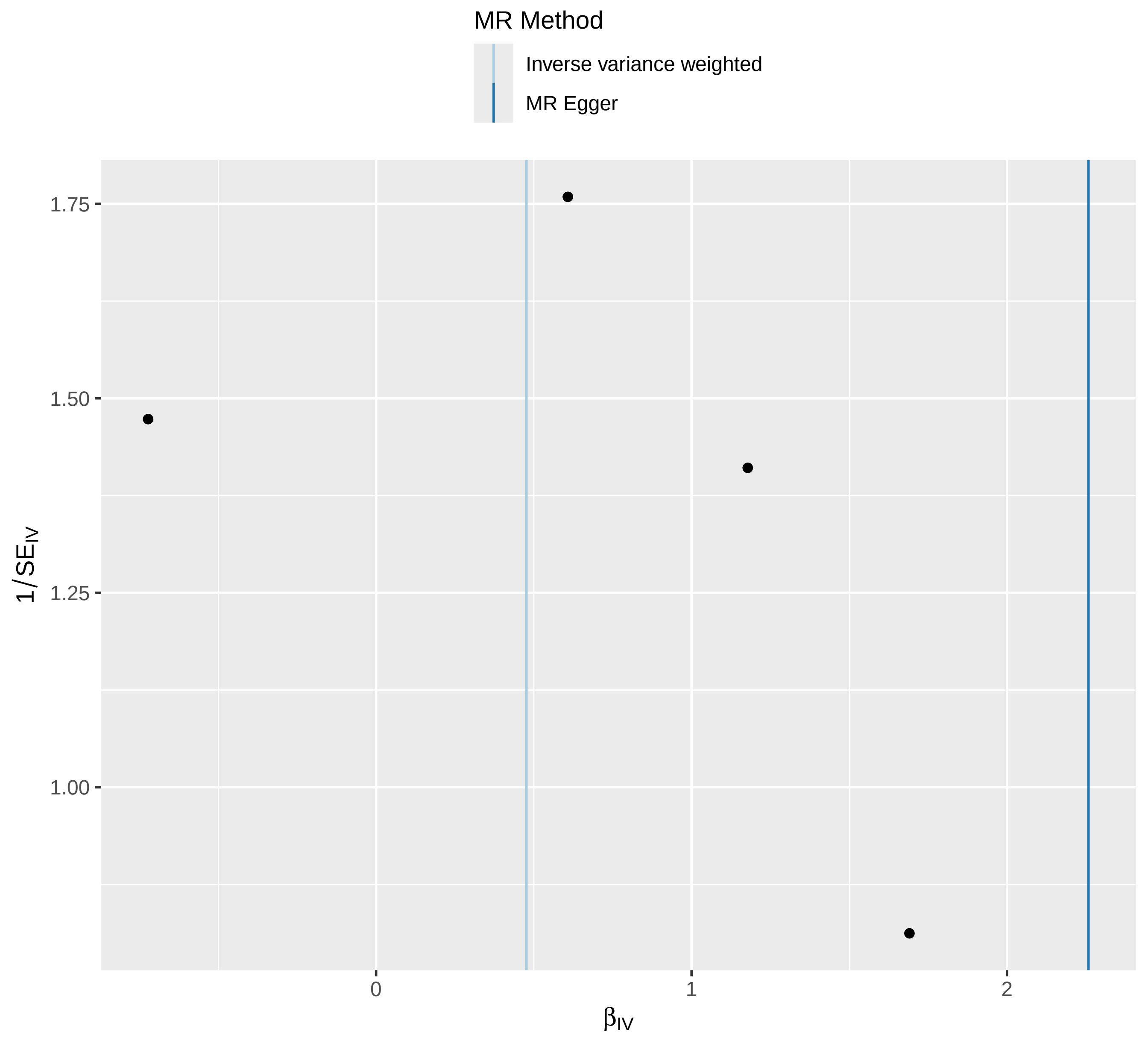
MR Method
Inverse variance weighted
MR Egger
1.75
1.50
1.25
1.00
1/SE_IV
0
1
2
β_IV

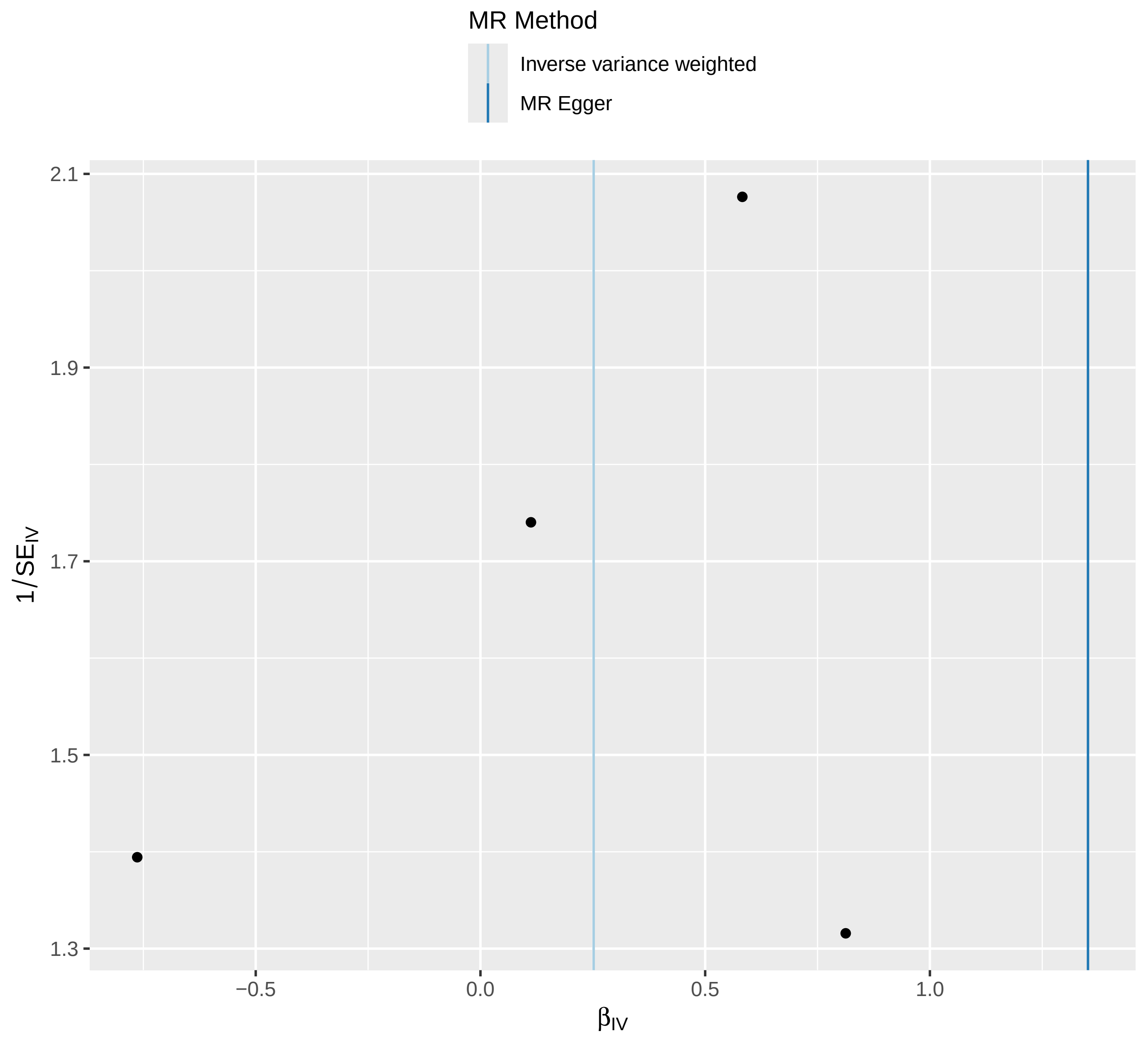
MR Method
Inverse variance weighted
MR Egger
2.1
1.9
1.7
1.5
1.3
1/SE_IV
−0.5
0.0
0.5
1.0
β_IV

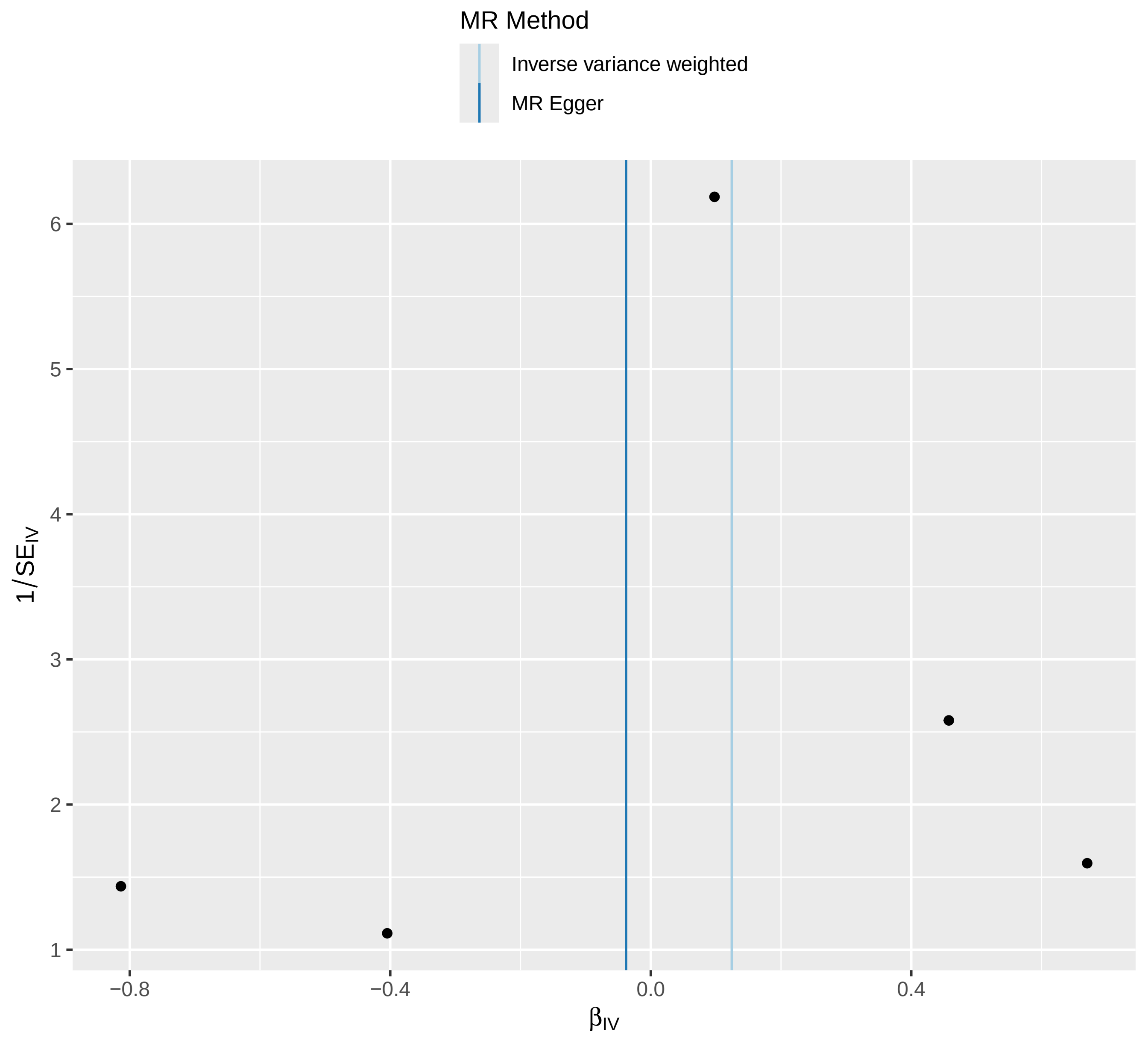
MR Method
Inverse variance weighted
MR Egger
1/SE_IV
β_IV
−0.8
−0.4
0.0
0.4
1
2
3
4
5
6

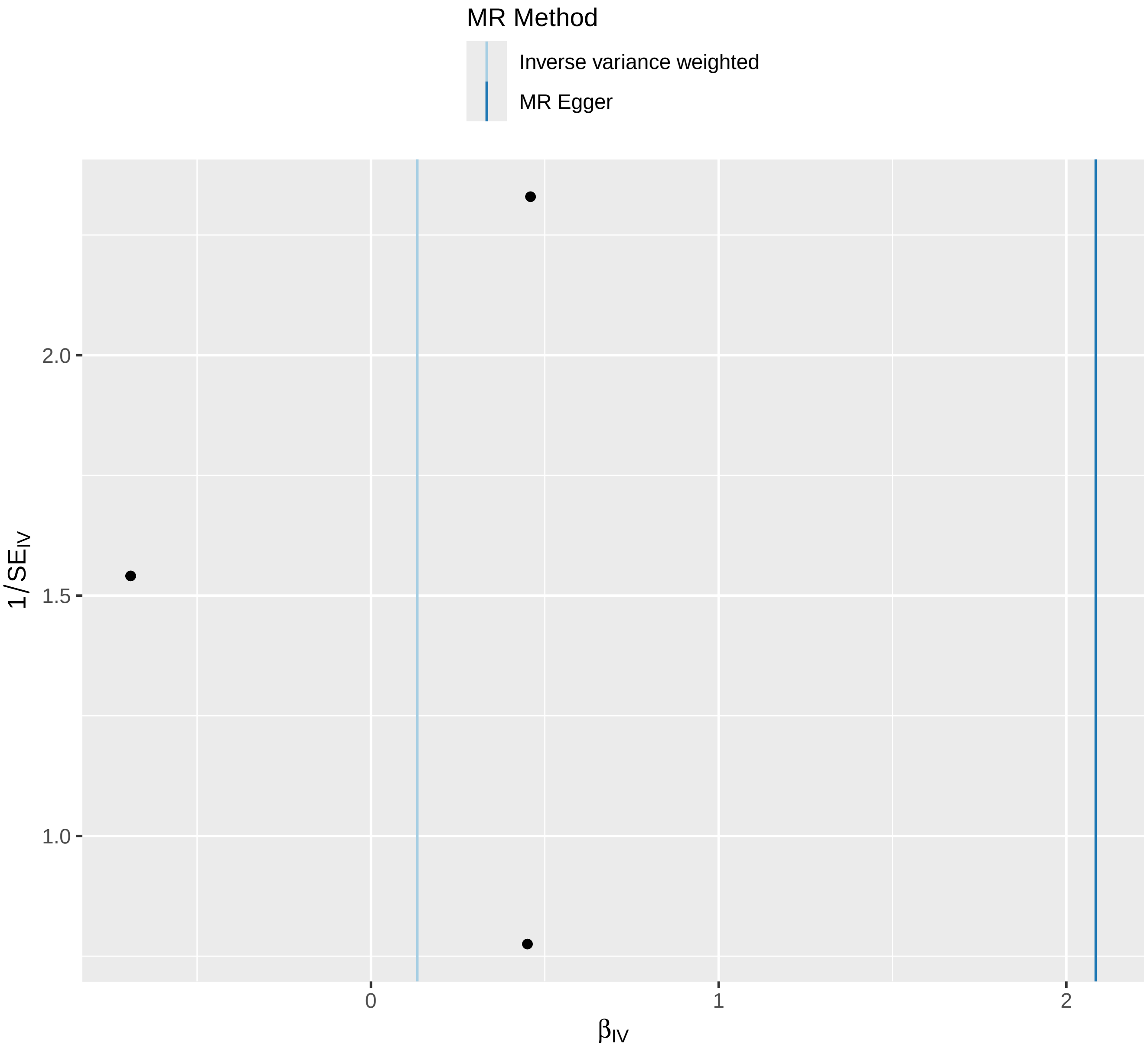

MR Method
Inverse variance weighted
MR Egger
2.0
1.5
1.0
$1/SE_{IV}$
0
1
2
β_{IV}

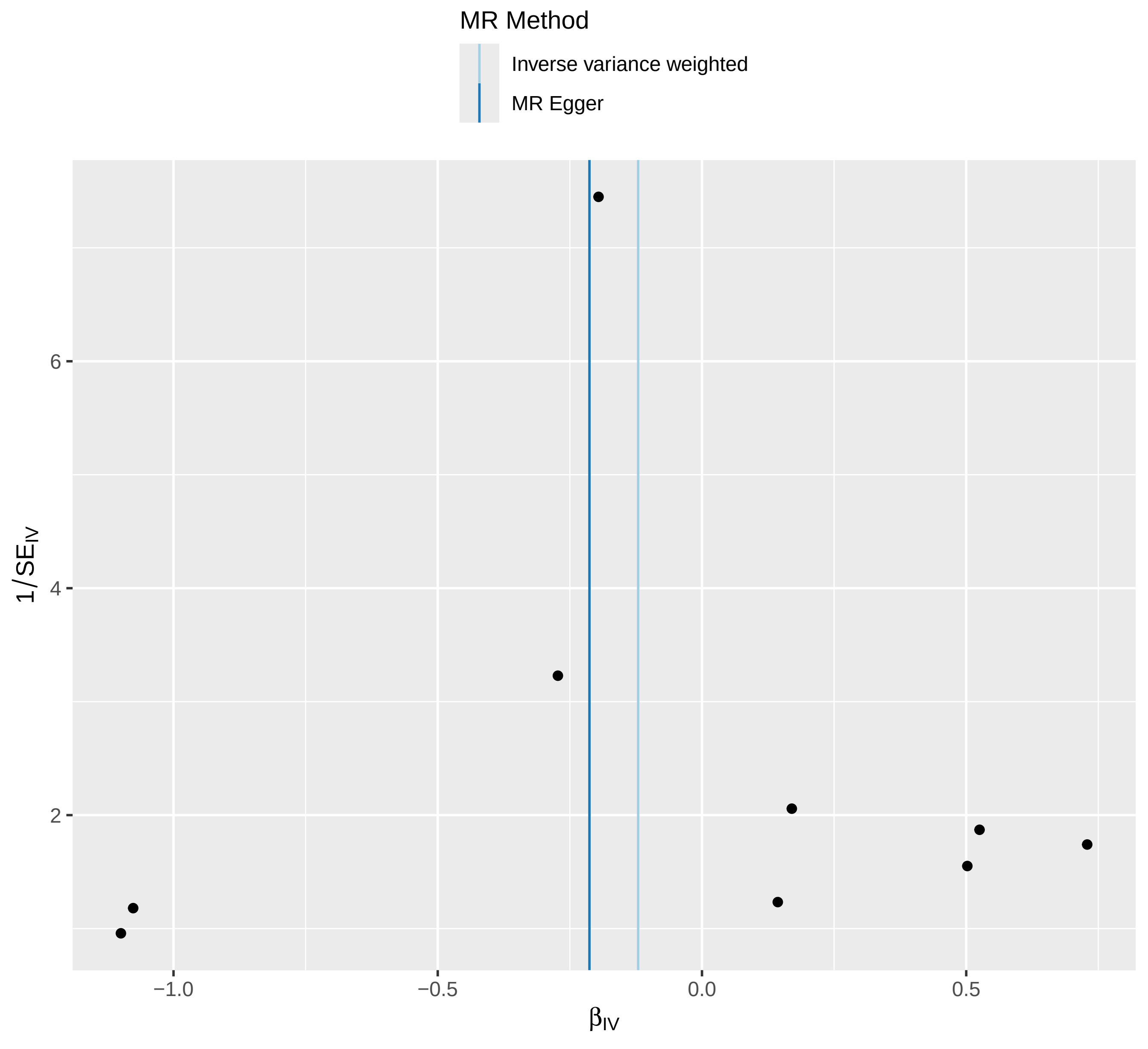
MR Method
Inverse variance weighted
MR Egger
6
4
2
1/SE_IV
−1.0
−0.5
0.0
0.5
β_IV

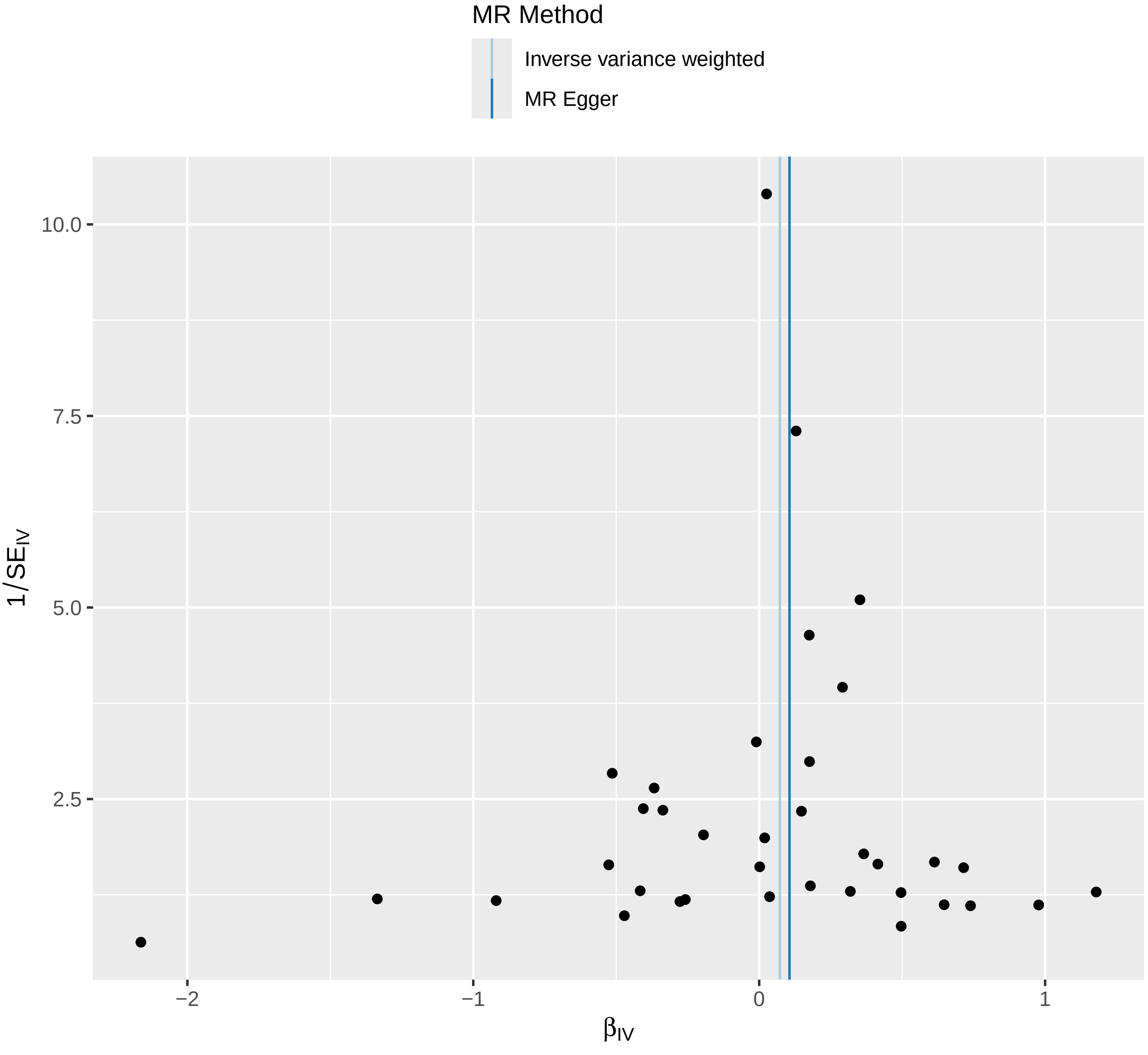

MR Method
Inverse variance weighted
MR Egger
10.0
7.5
5.0
2.5
1/SE_IV
−2
−1
0
1
β_IV

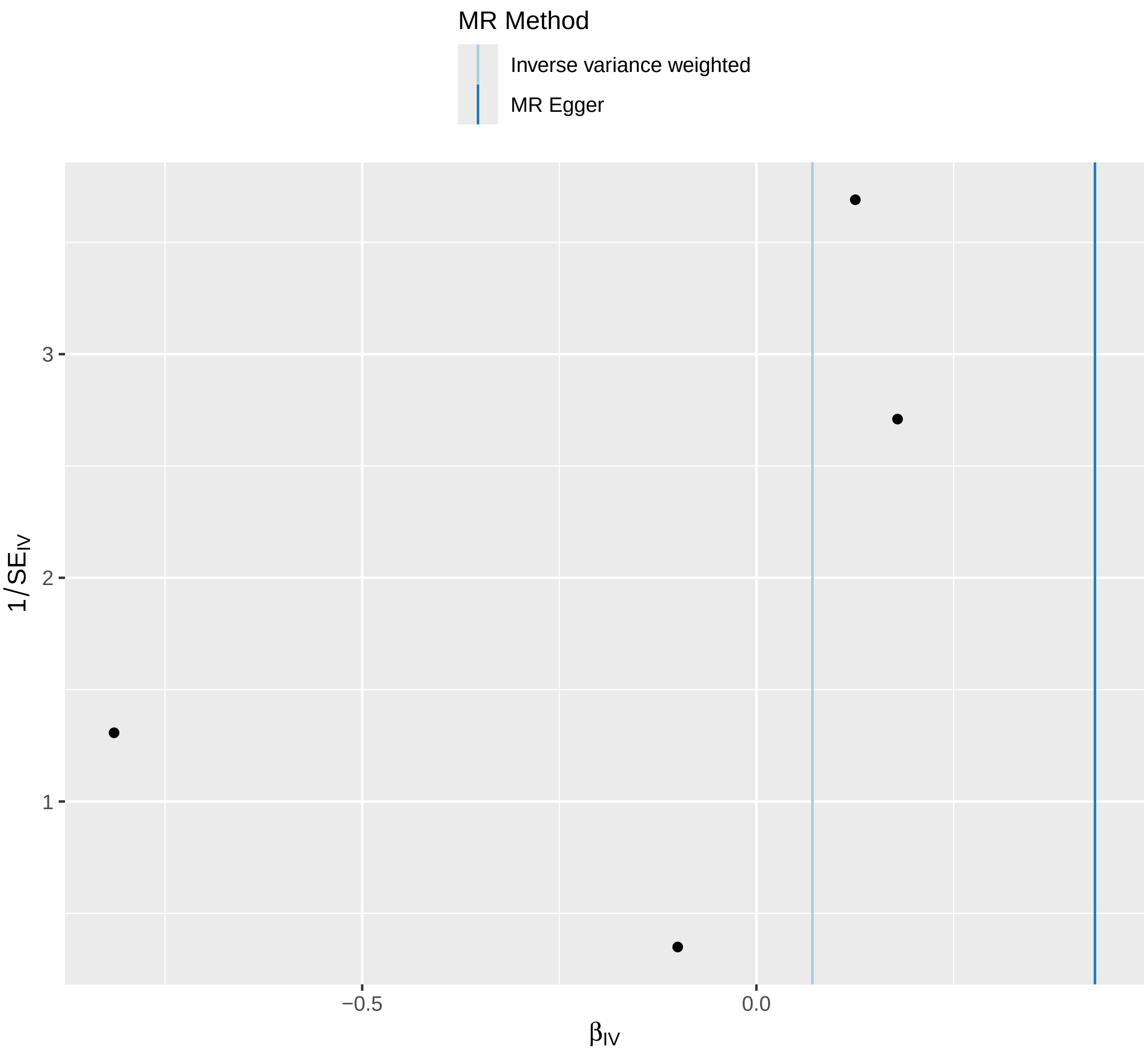

MR Method
Inverse variance weighted
MR Egger
3
2
1
1/SE_IV
−0.5
0.0
β_IV

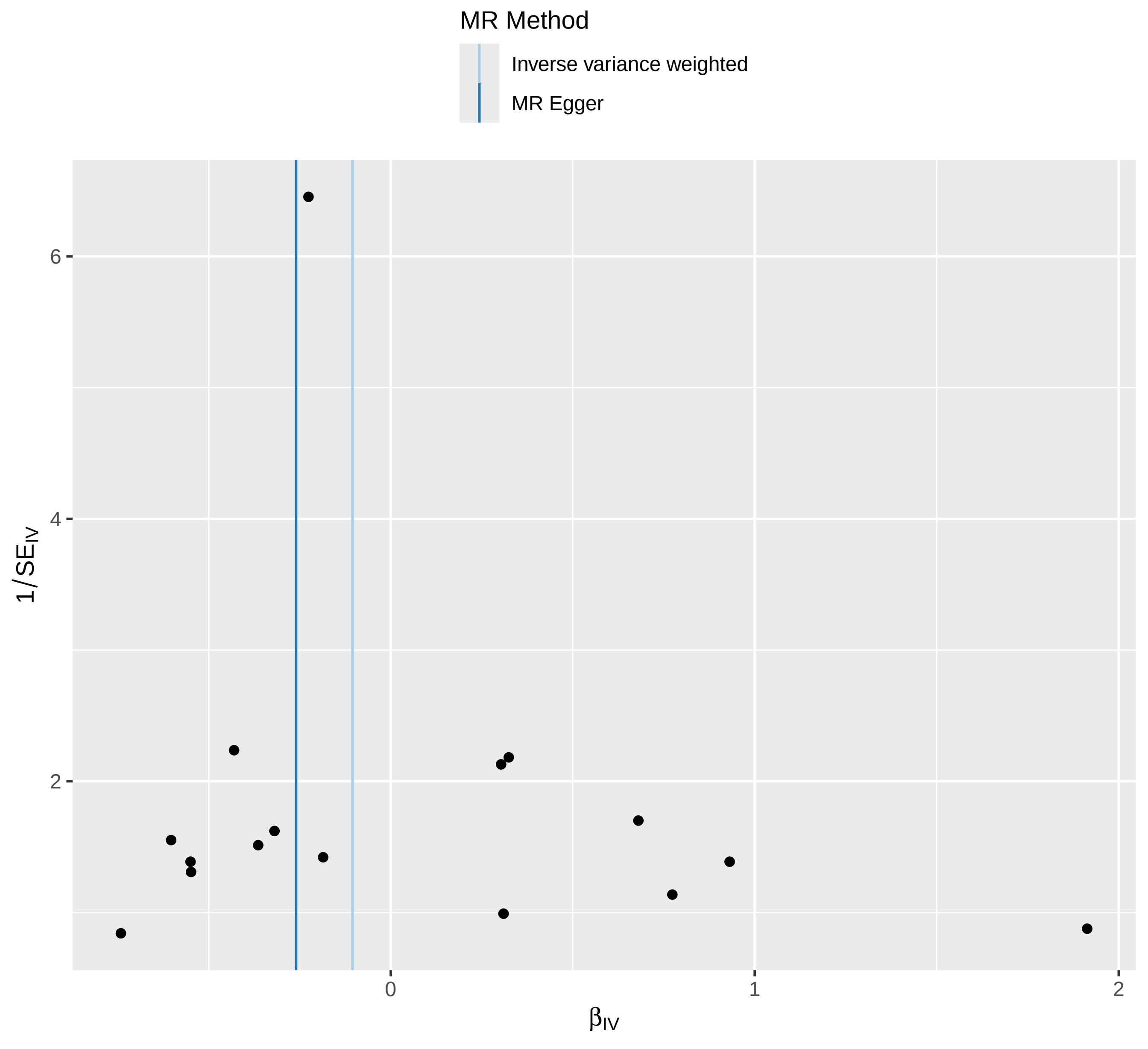

MR Method
Inverse variance weighted
MR Egger
6
4
2
1/SE$_{IV}$
0
1
2
β$_{IV}$

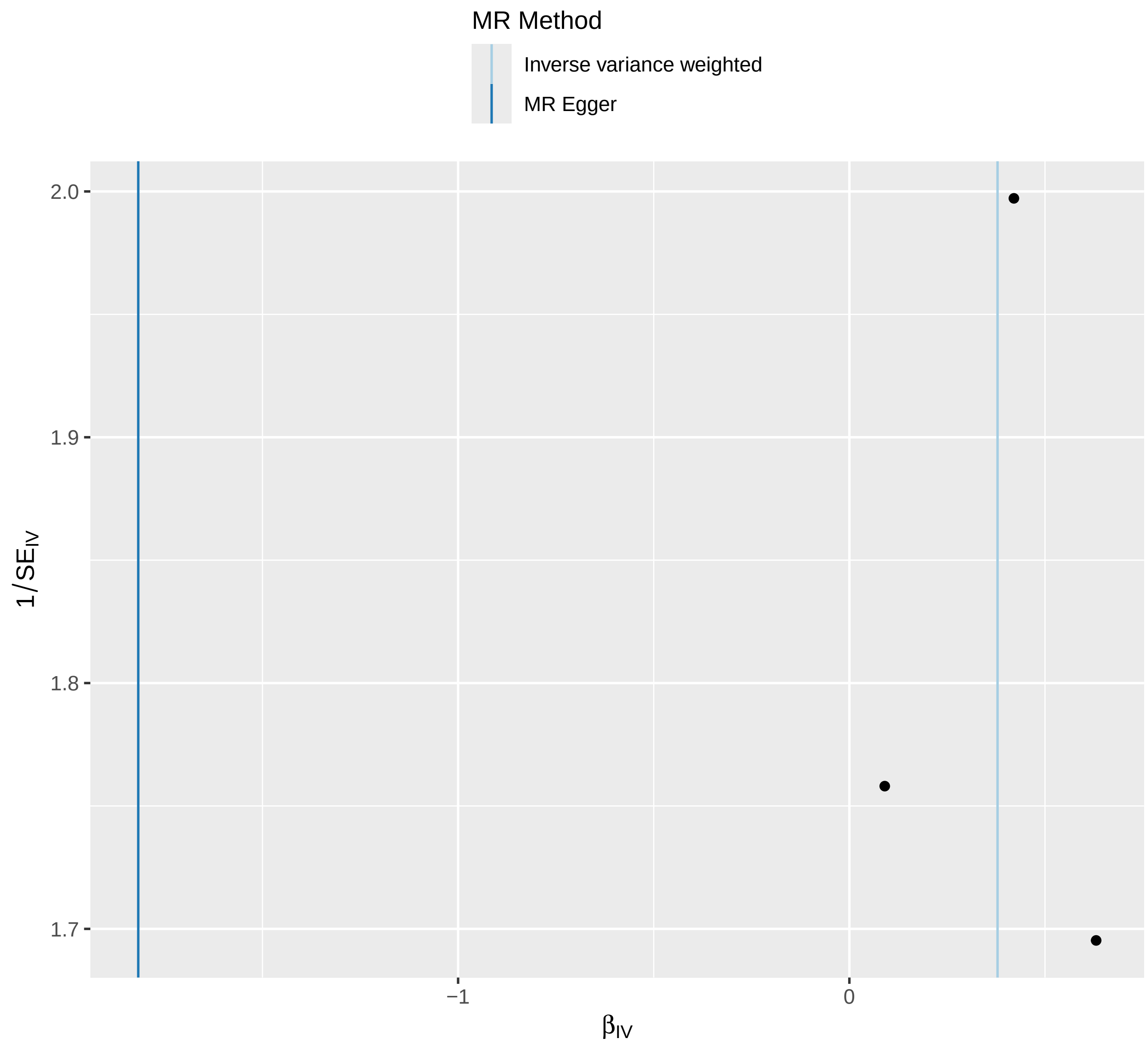
MR Method
Inverse variance weighted
MR Egger
2.0
1.9
1.8
1.7
1/SE_IV
−1
0
β_IV

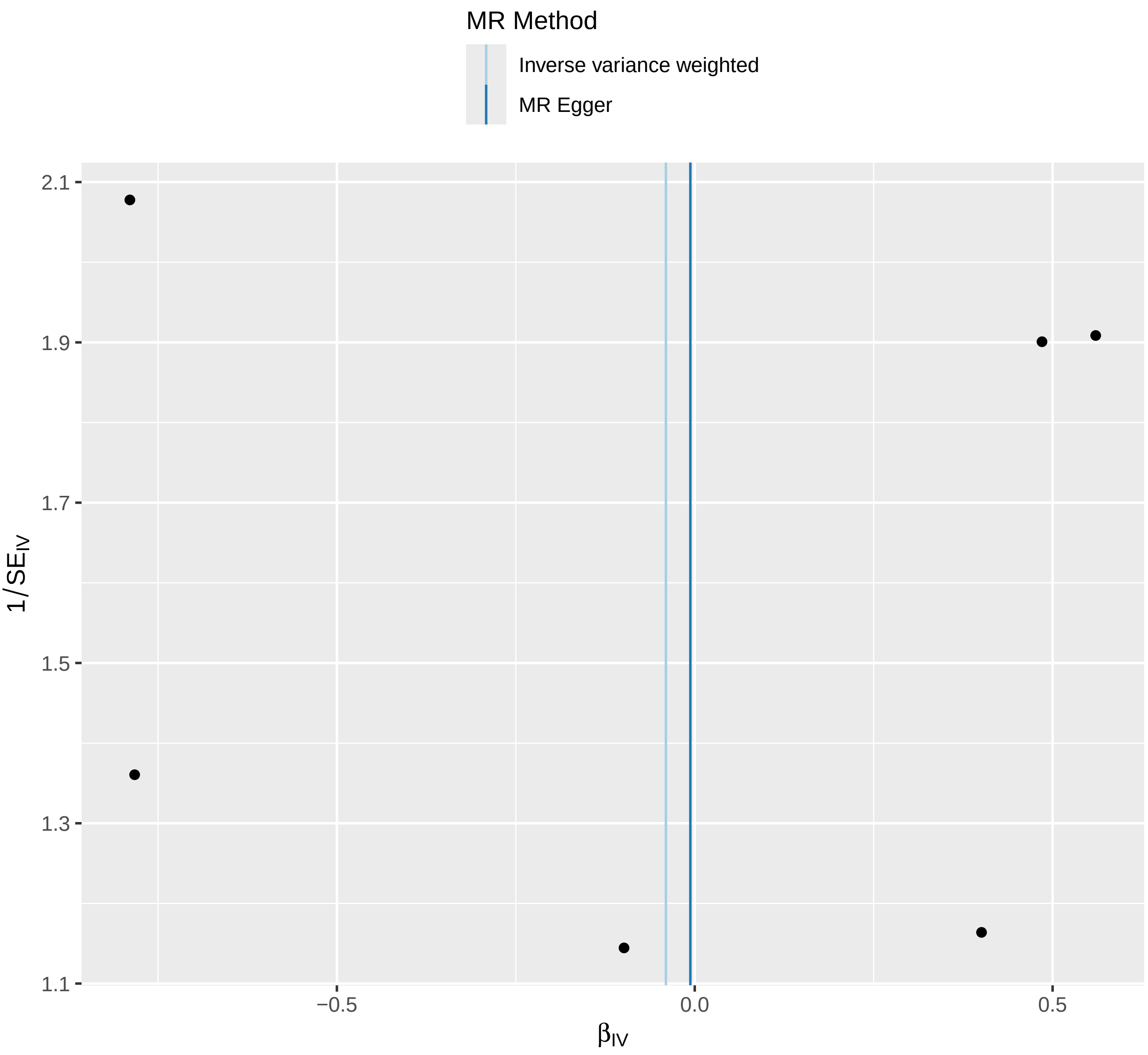

MR Method
Inverse variance weighted
MR Egger
2.1
1.9
1.7
1.5
1.3
1.1
1/SE_IV
−0.5
0.0
0.5
β_IV

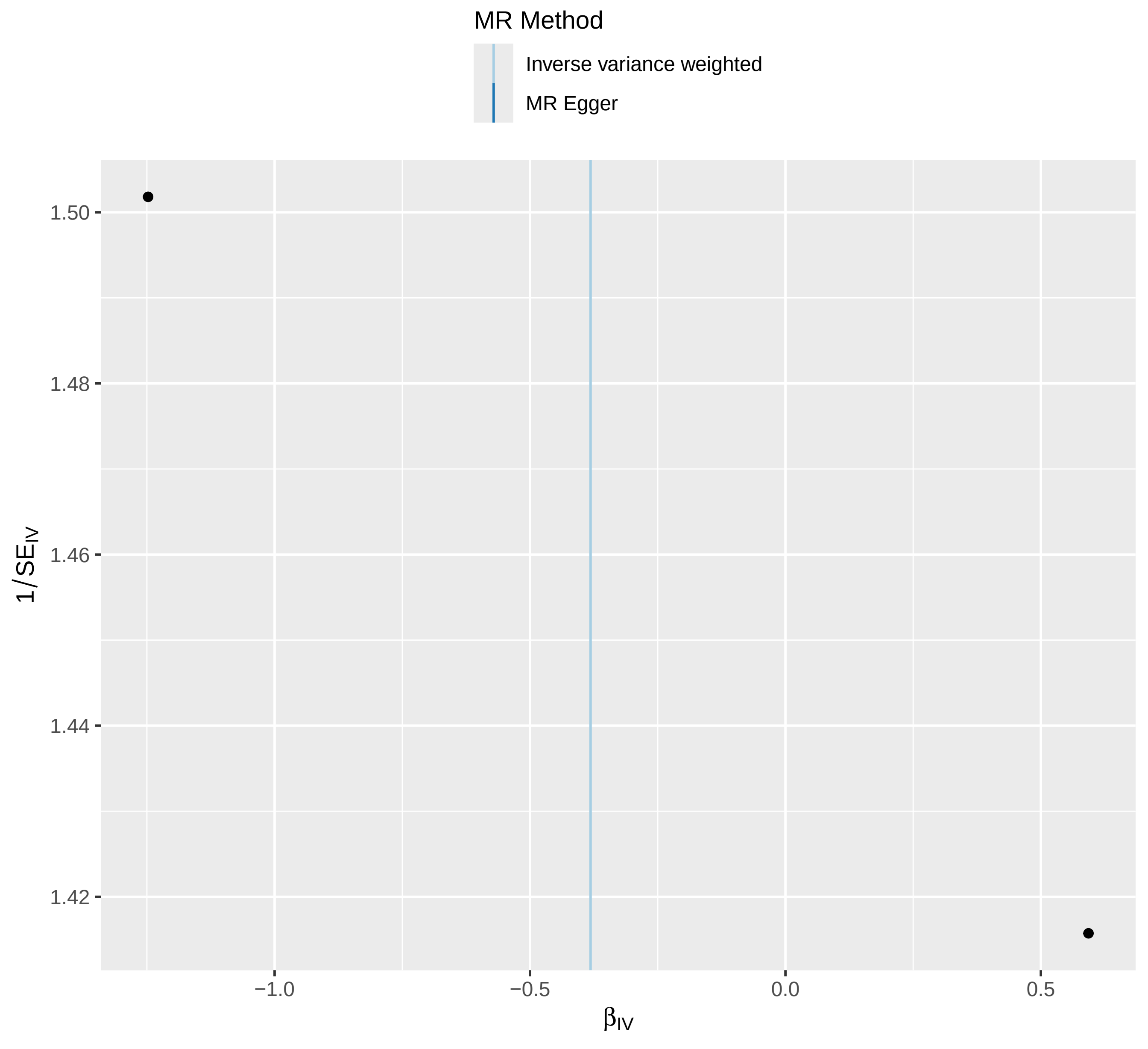
MR Method
Inverse variance weighted
MR Egger
1.50
1.48
1.46
1.44
1.42
$1/SE_{IV}$
−1.0
−0.5
0.0
0.5
β_{IV}

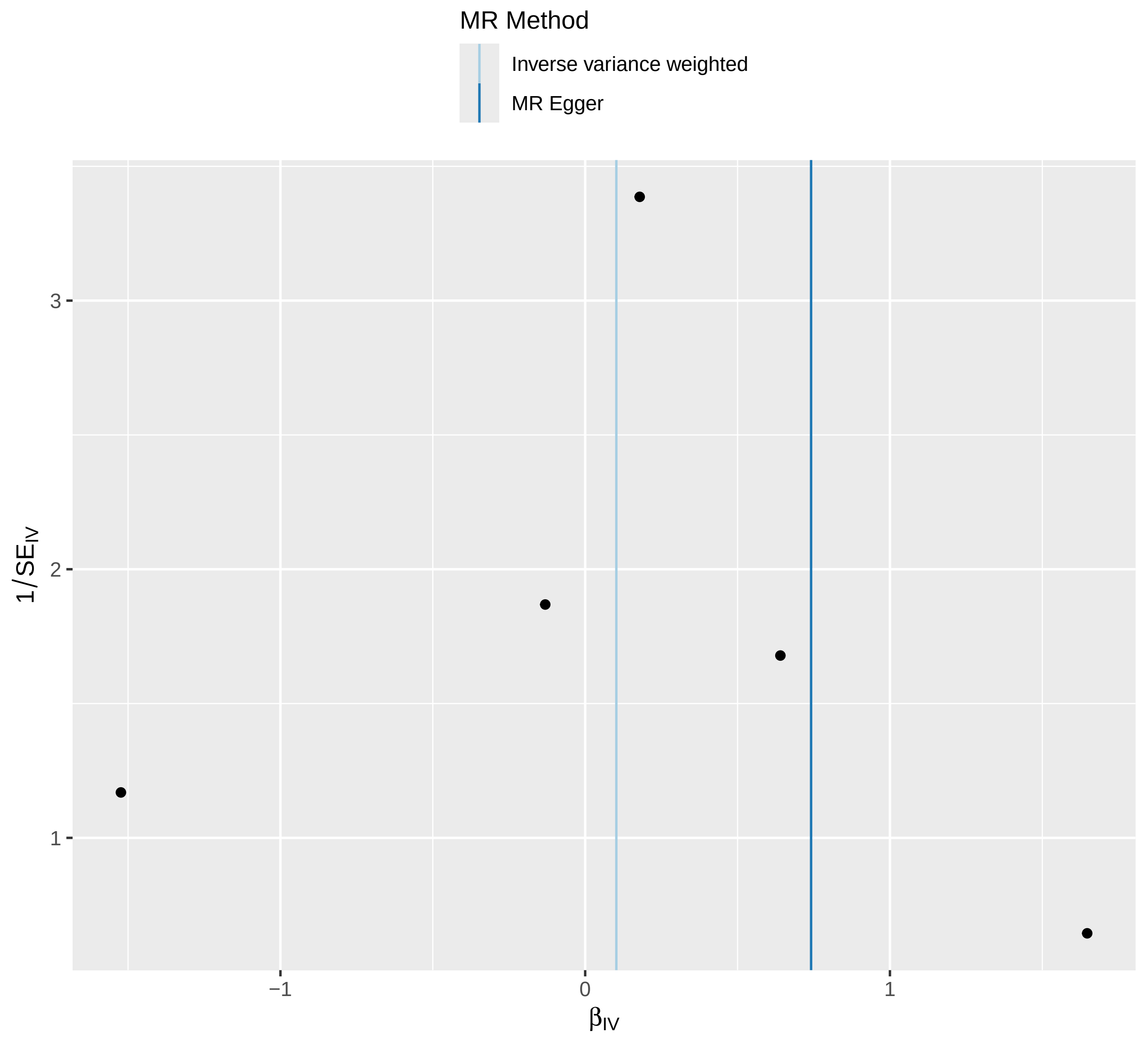

MR Method
Inverse variance weighted
MR Egger
3
2
1
1/SE_IV
−1
0
1
β_IV

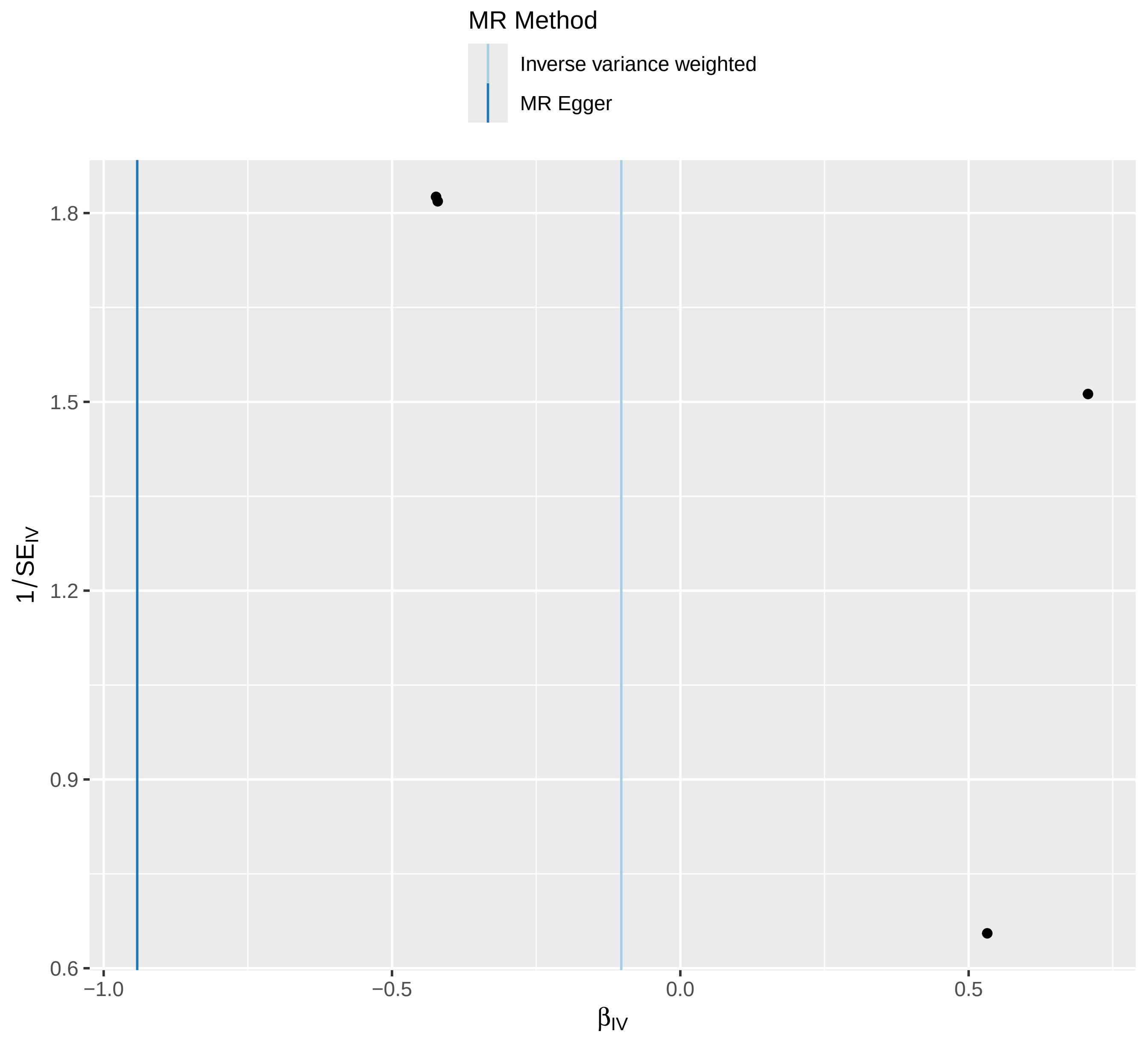
MR Method
Inverse variance weighted
MR Egger
1.8
1.5
1.2
0.9
0.6
1/SE_IV
−1.0
−0.5
0.0
0.5
β_IV

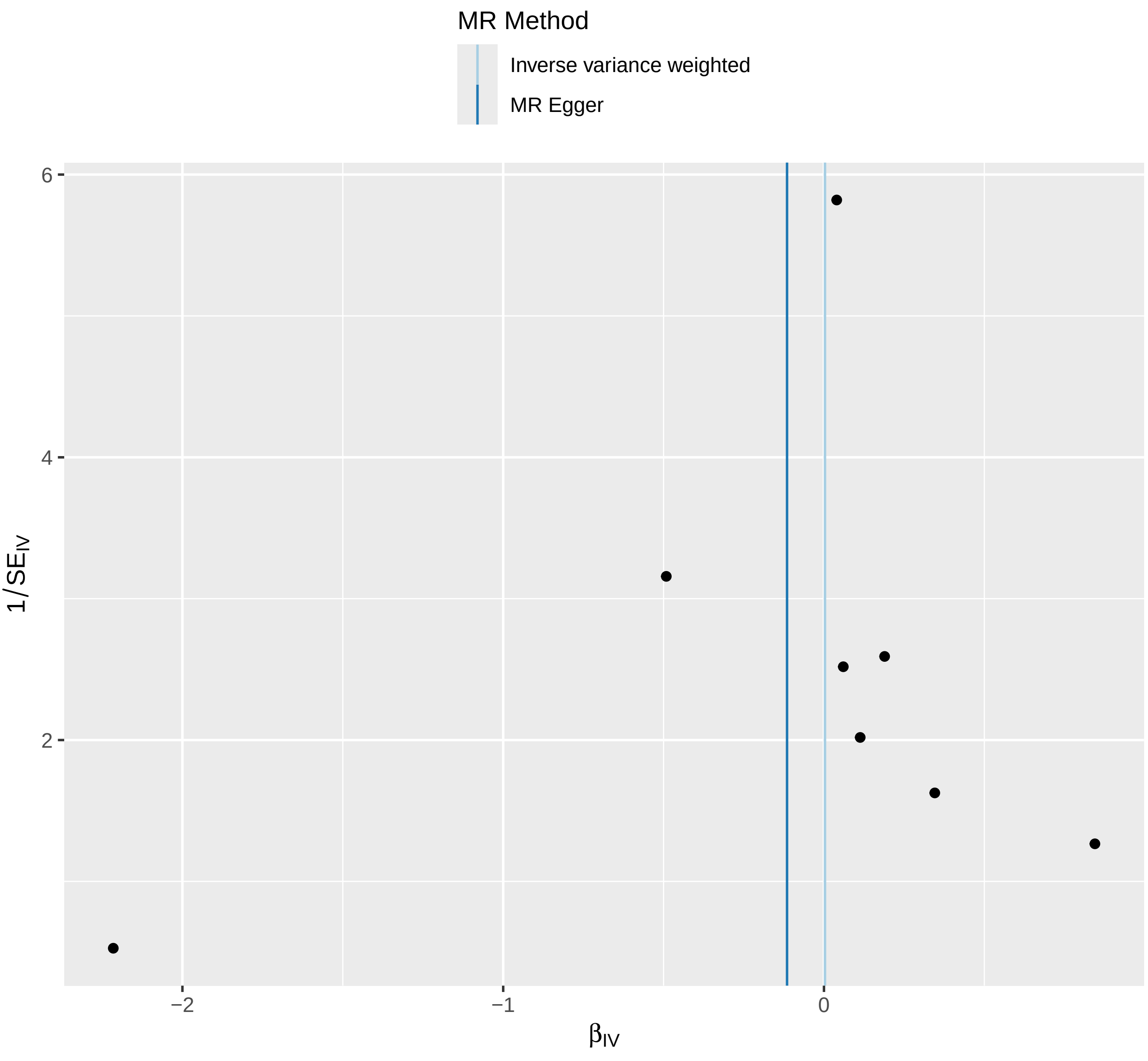
MR Method
Inverse variance weighted
MR Egger
6
4
2
1/SE_IV
−2
−1
0
β_IV

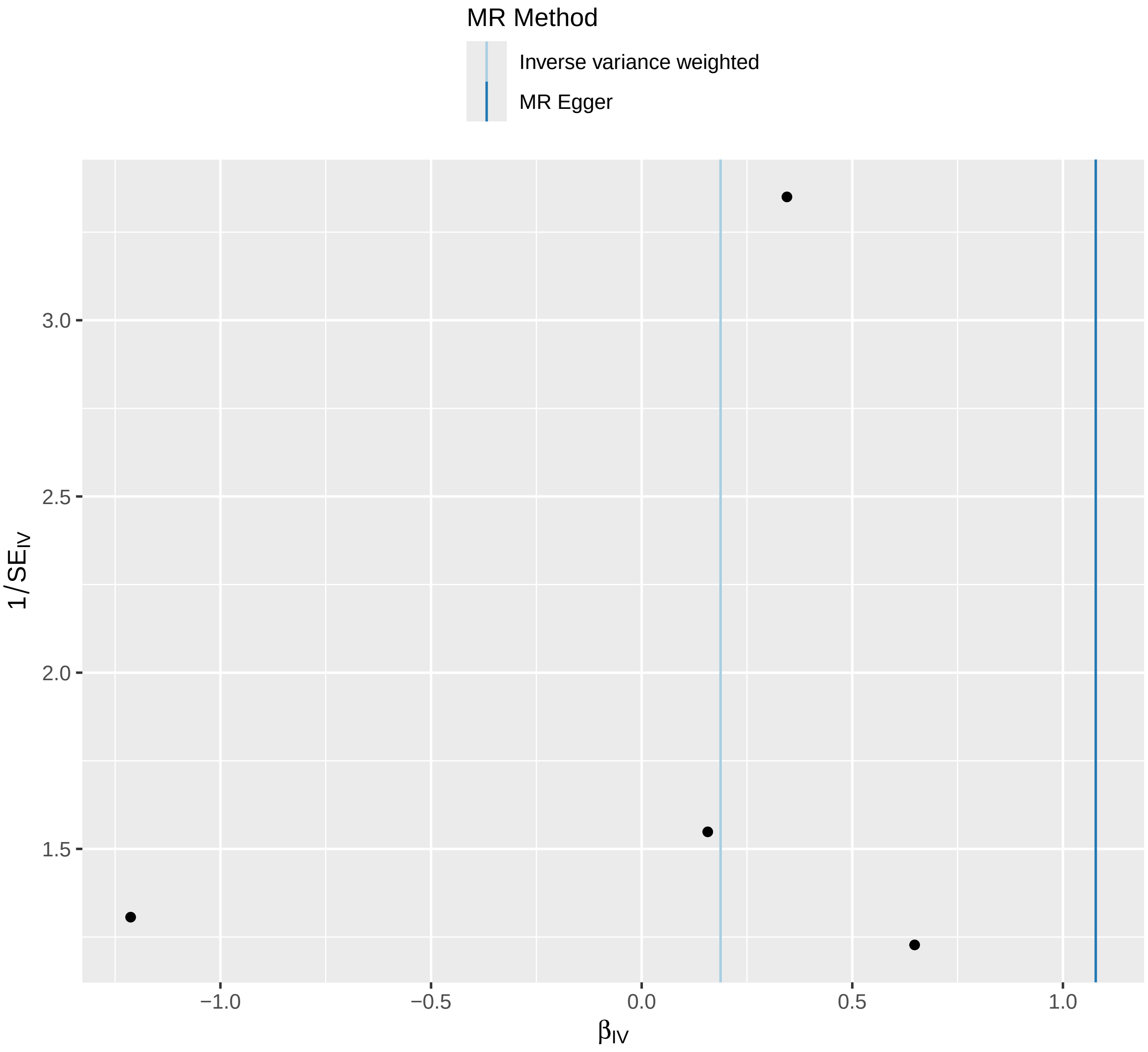
MR Method
Inverse variance weighted
MR Egger
3.0
2.5
2.0
1.5
1/SE$_{IV}$
−1.0
−0.5
0.0
0.5
1.0
β$_{IV}$

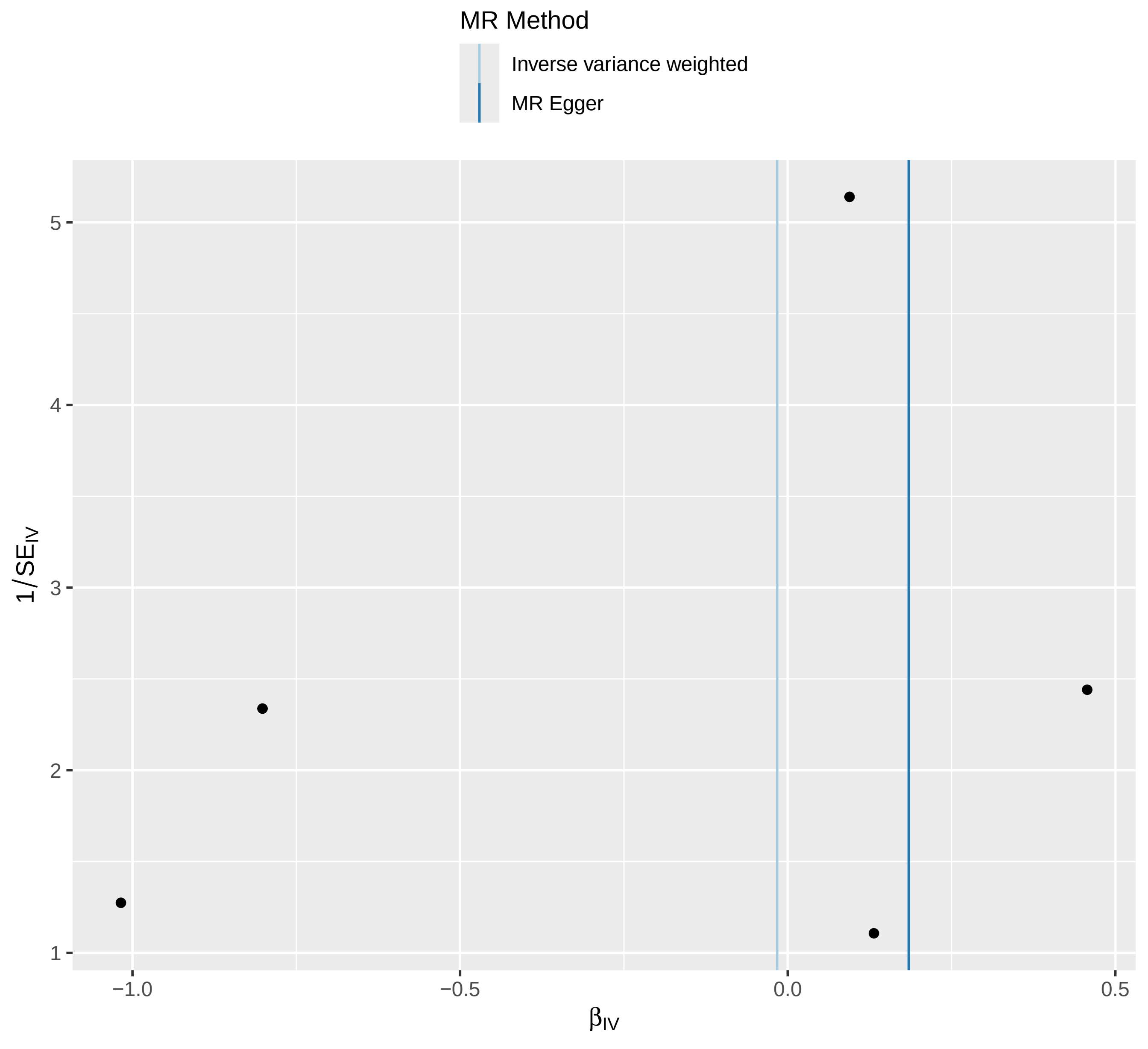
MR Method
Inverse variance weighted
MR Egger
1/SE_IV
β_IV
−1.0
−0.5
0.0
0.5
1
2
3
4
5

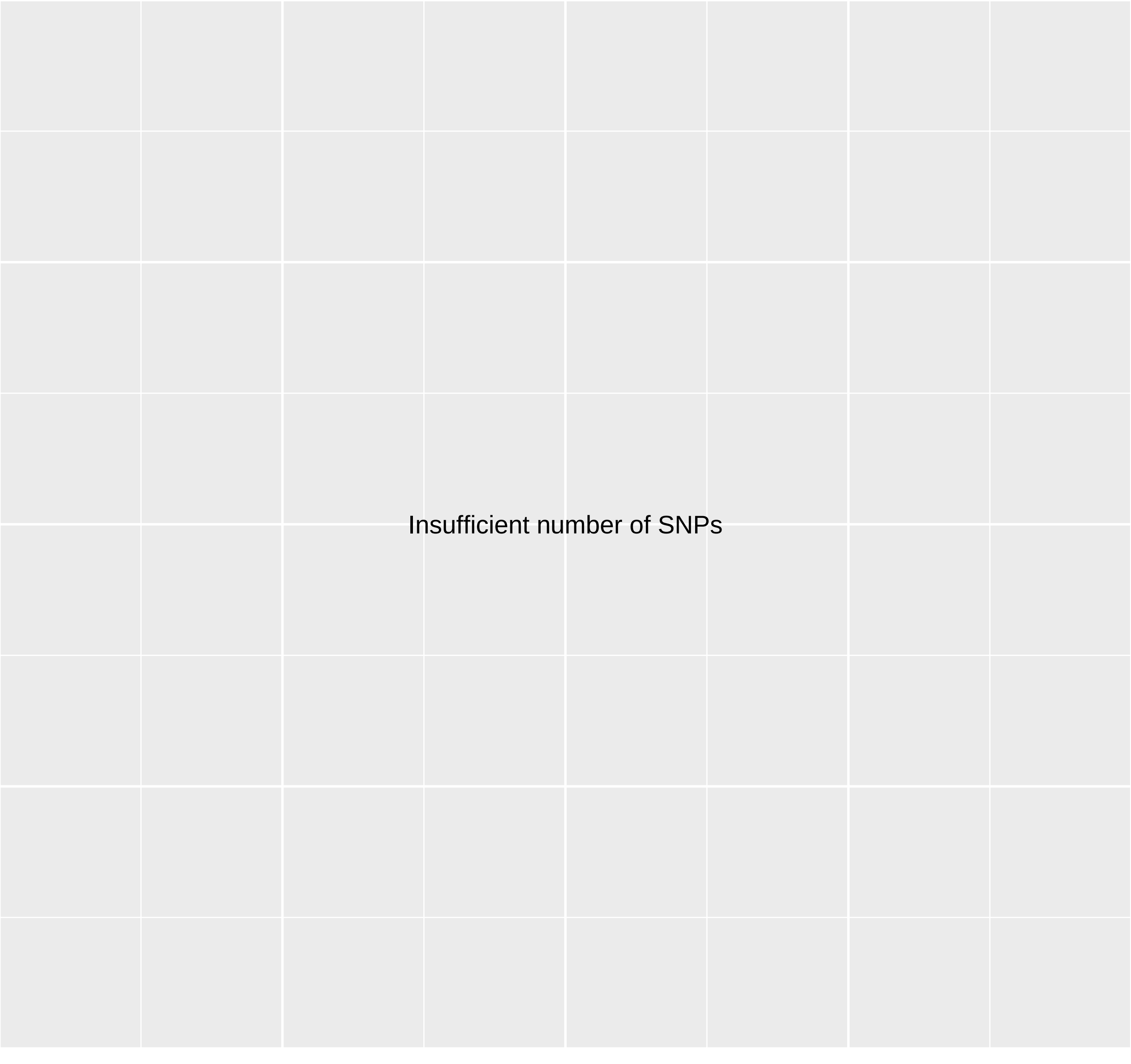
Insufficient number of SNPs

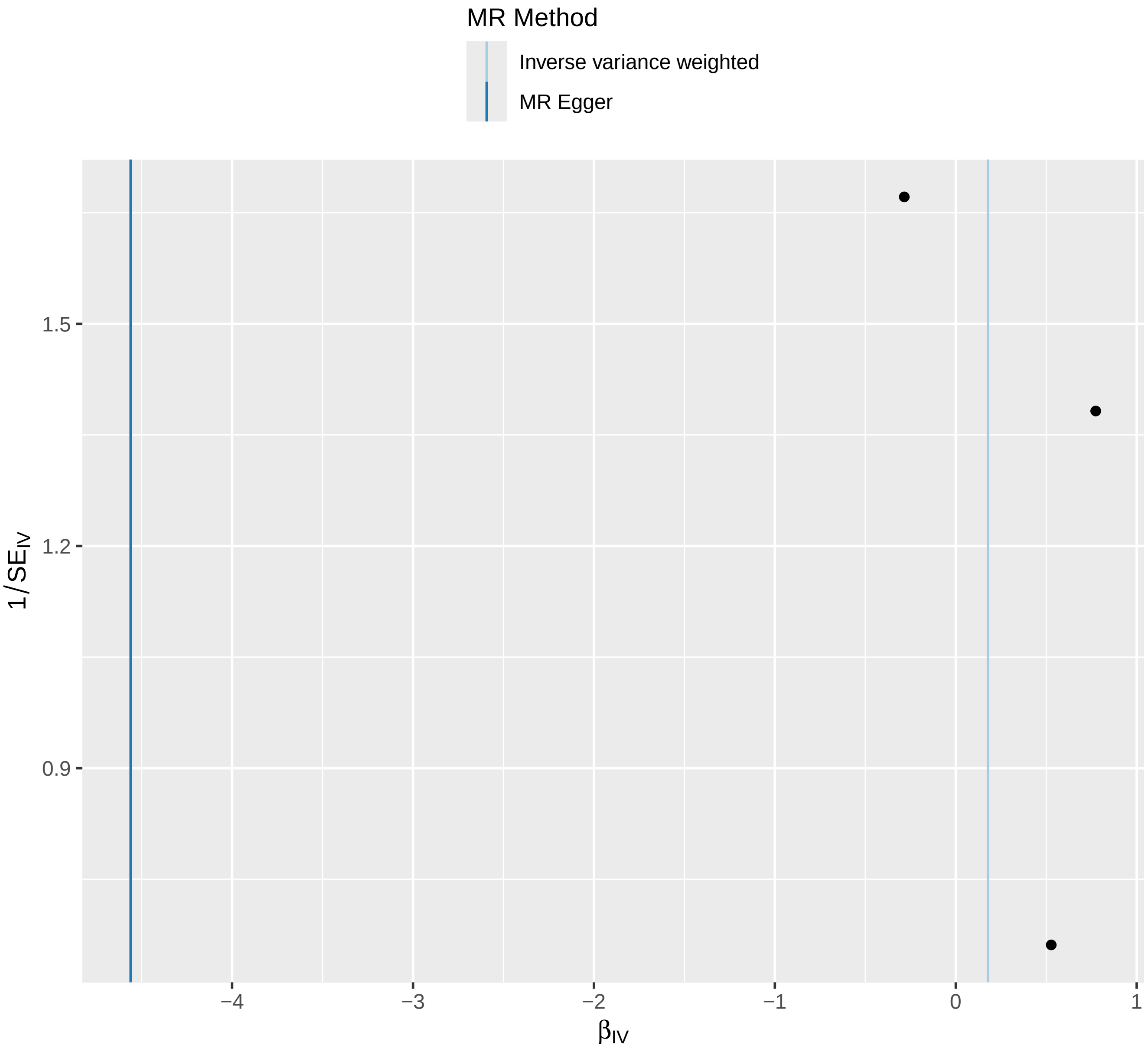

MR Method
Inverse variance weighted
MR Egger
1.5
1.2
0.9
1/SE$_{IV}$
−4
−3
−2
−1
0
1
β$_{IV}$

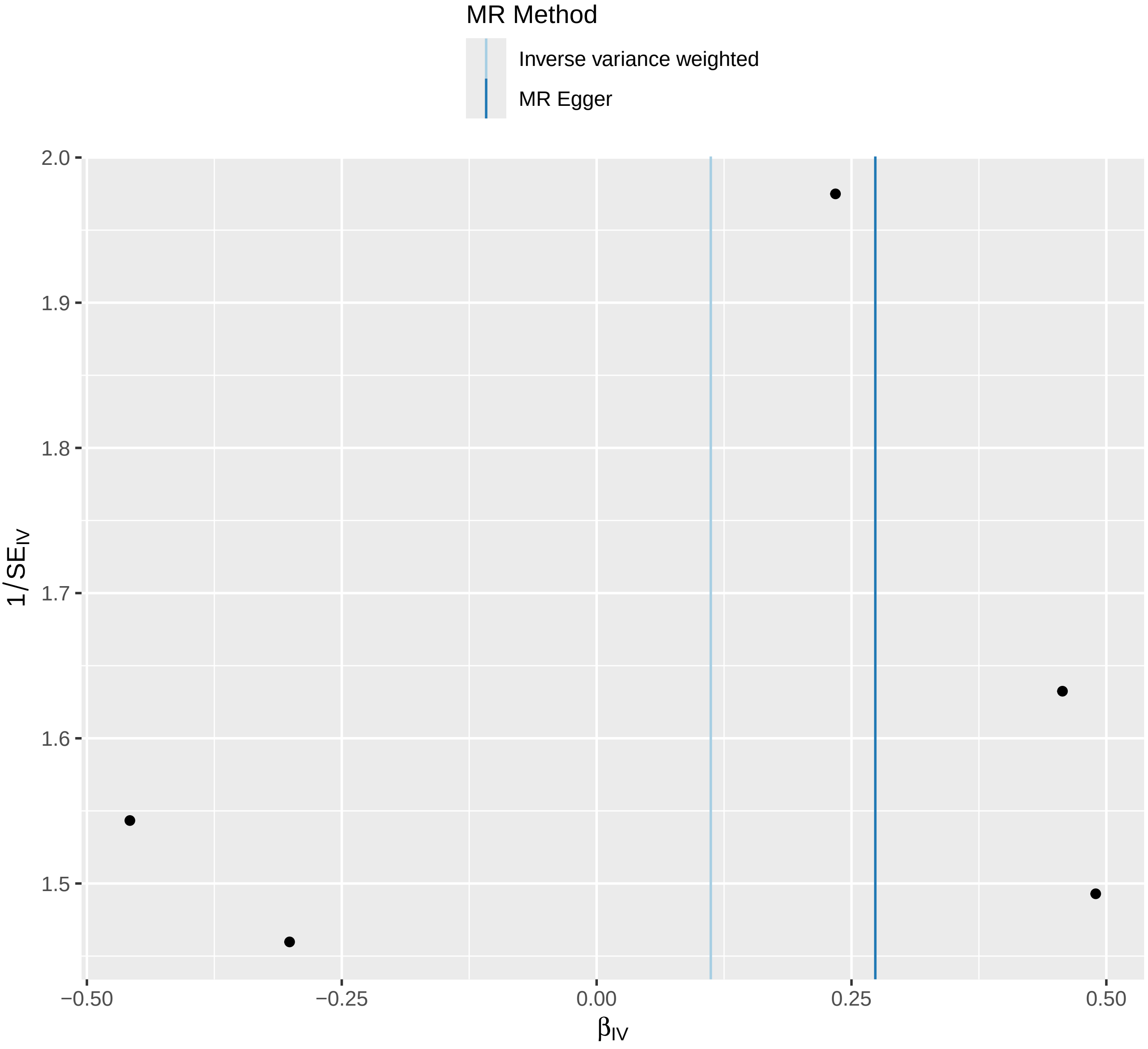
MR Method
Inverse variance weighted
MR Egger
2.0
1.9
1.8
1.7
1.6
1.5
1/SE_IV
−0.50
−0.25
0.00
0.25
0.50
β_IV

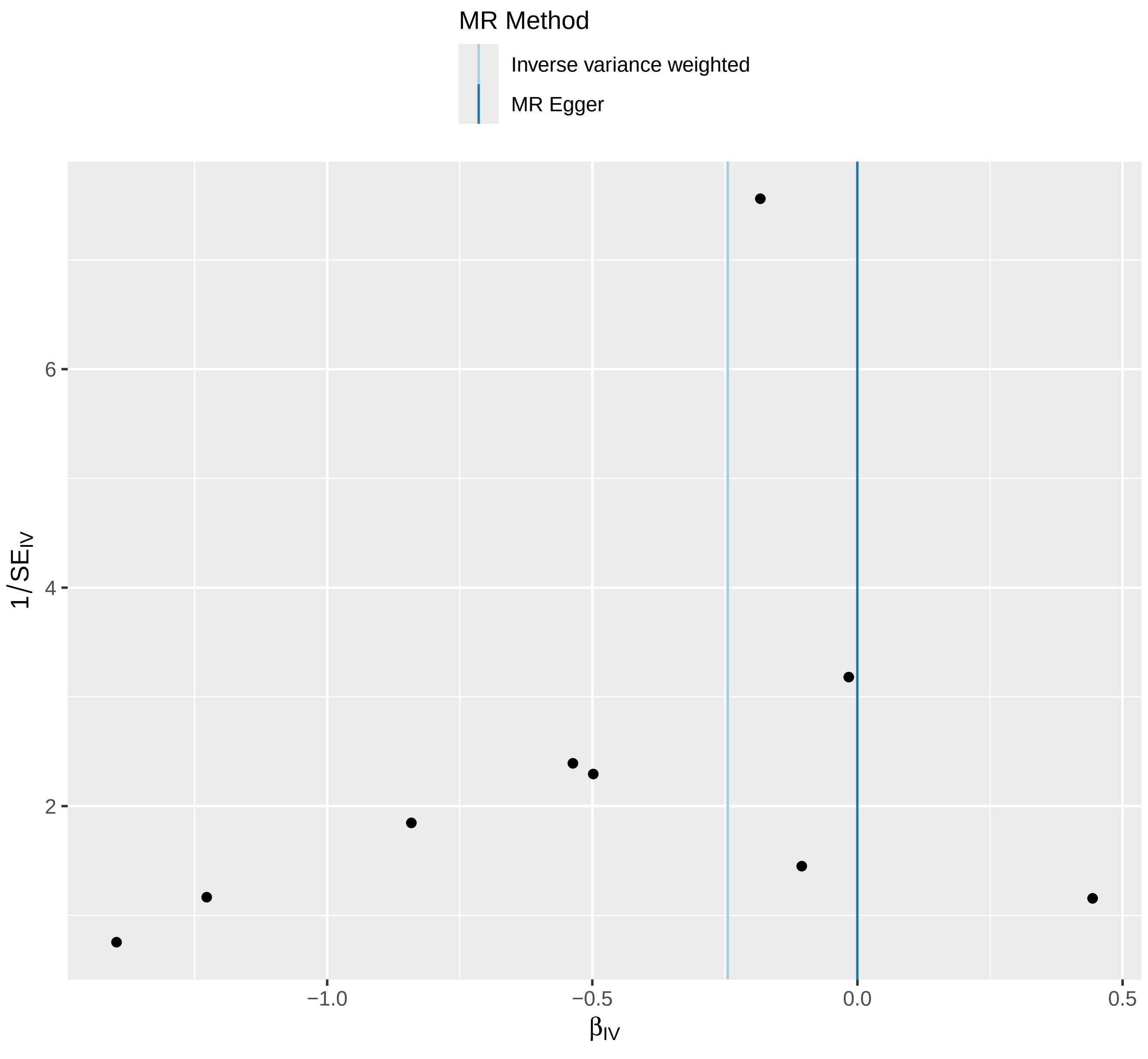

MR Method
Inverse variance weighted
MR Egger
6
4
2
1/SE_IV
−1.0
−0.5
0.0
0.5
β_IV

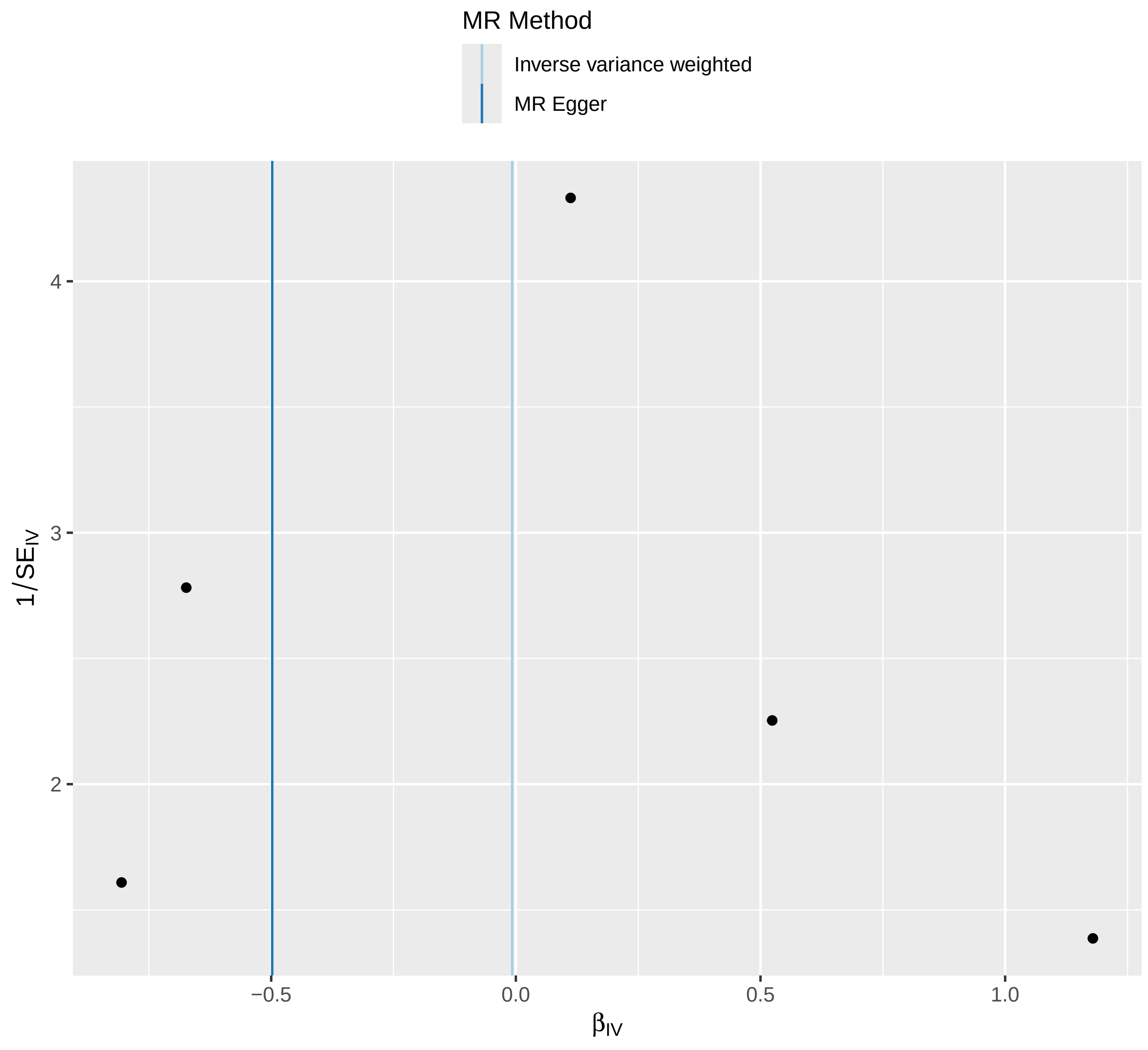

MR Method
Inverse variance weighted
MR Egger
4
3
2
1/SE$_{IV}$
−0.5
0.0
0.5
1.0
β$_{IV}$

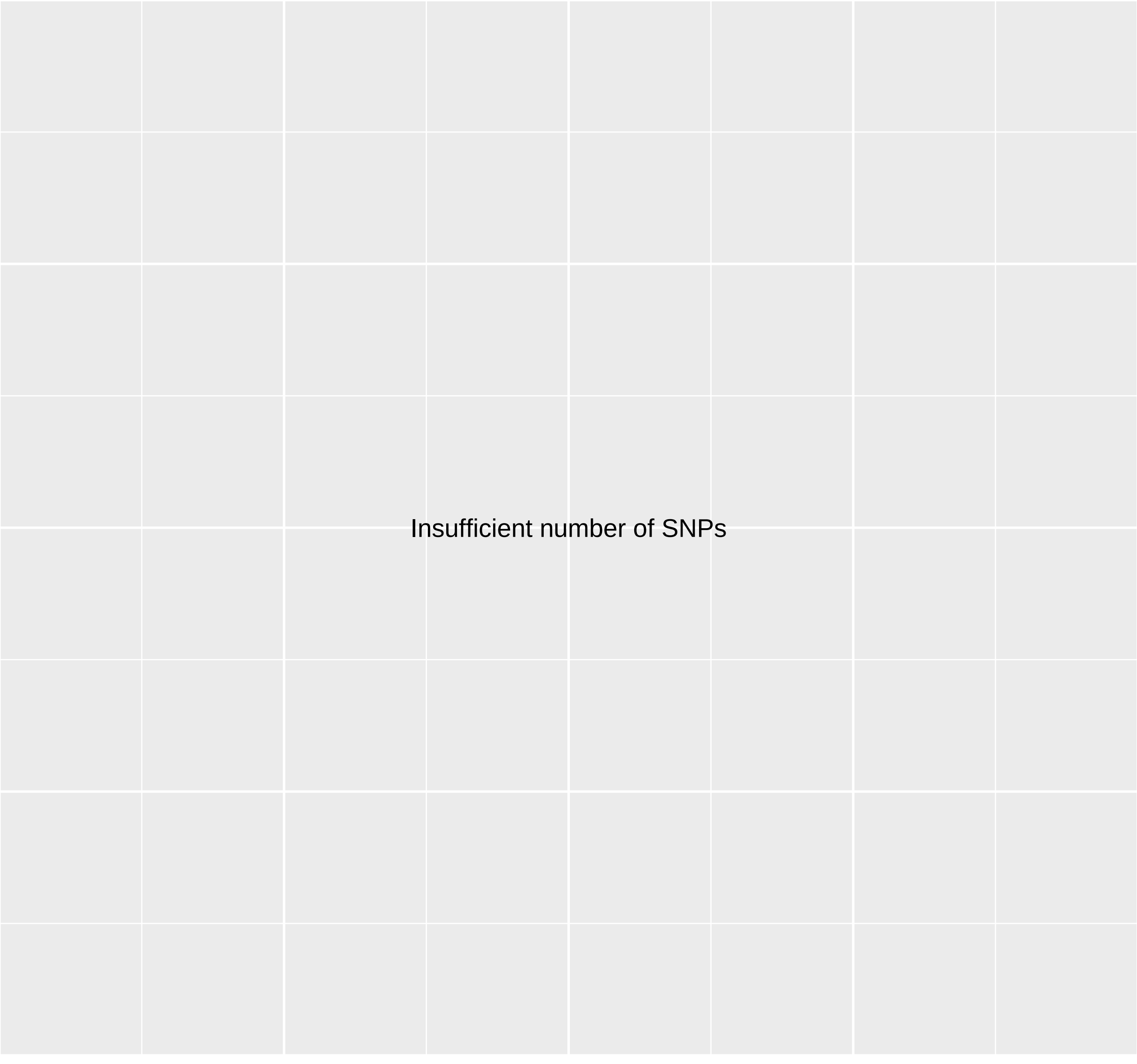
Insufficient number of SNPs

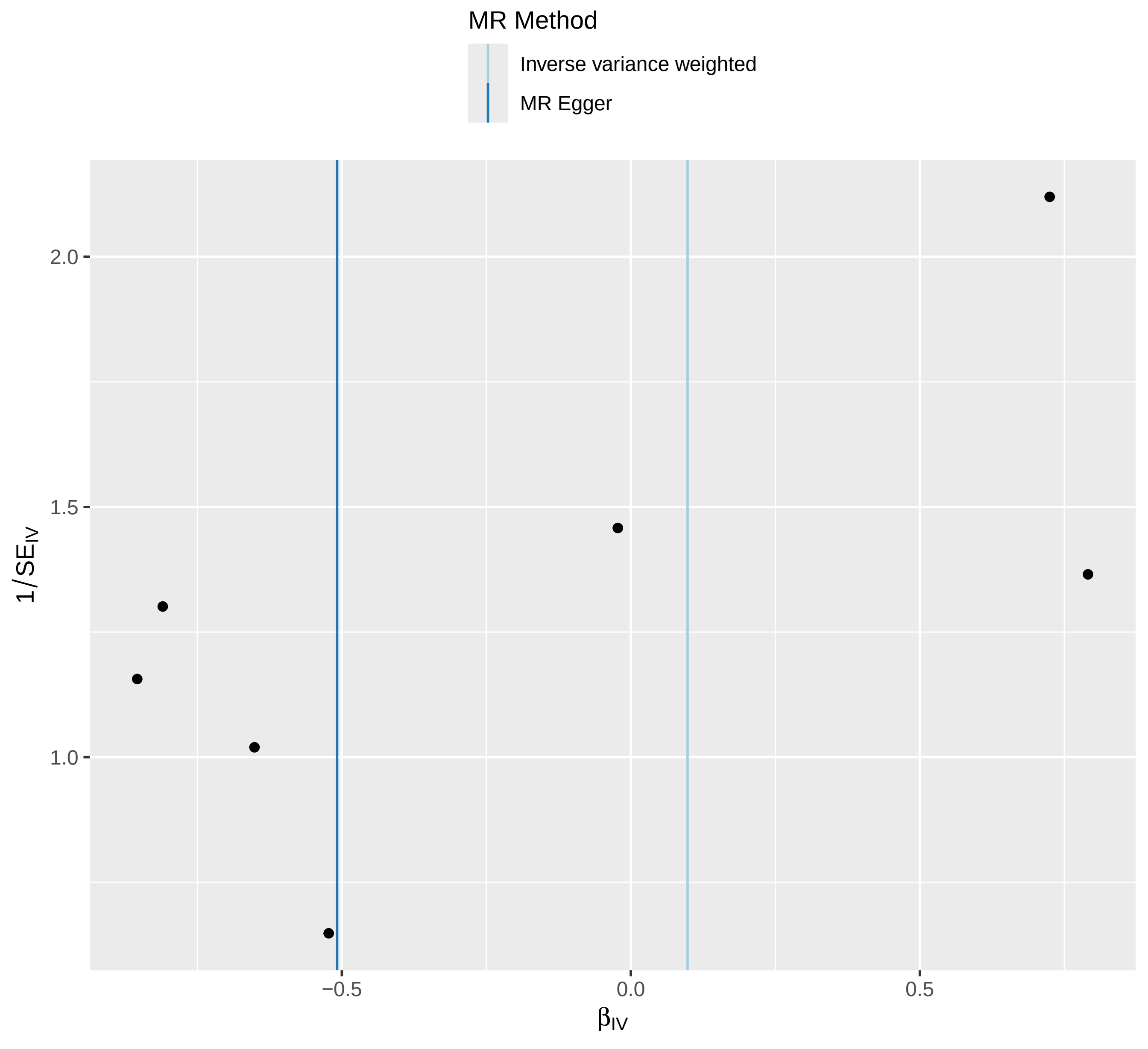
MR Method
Inverse variance weighted
MR Egger
1/SE_IV
2.0
1.5
1.0
−0.5
0.0
0.5
β_IV

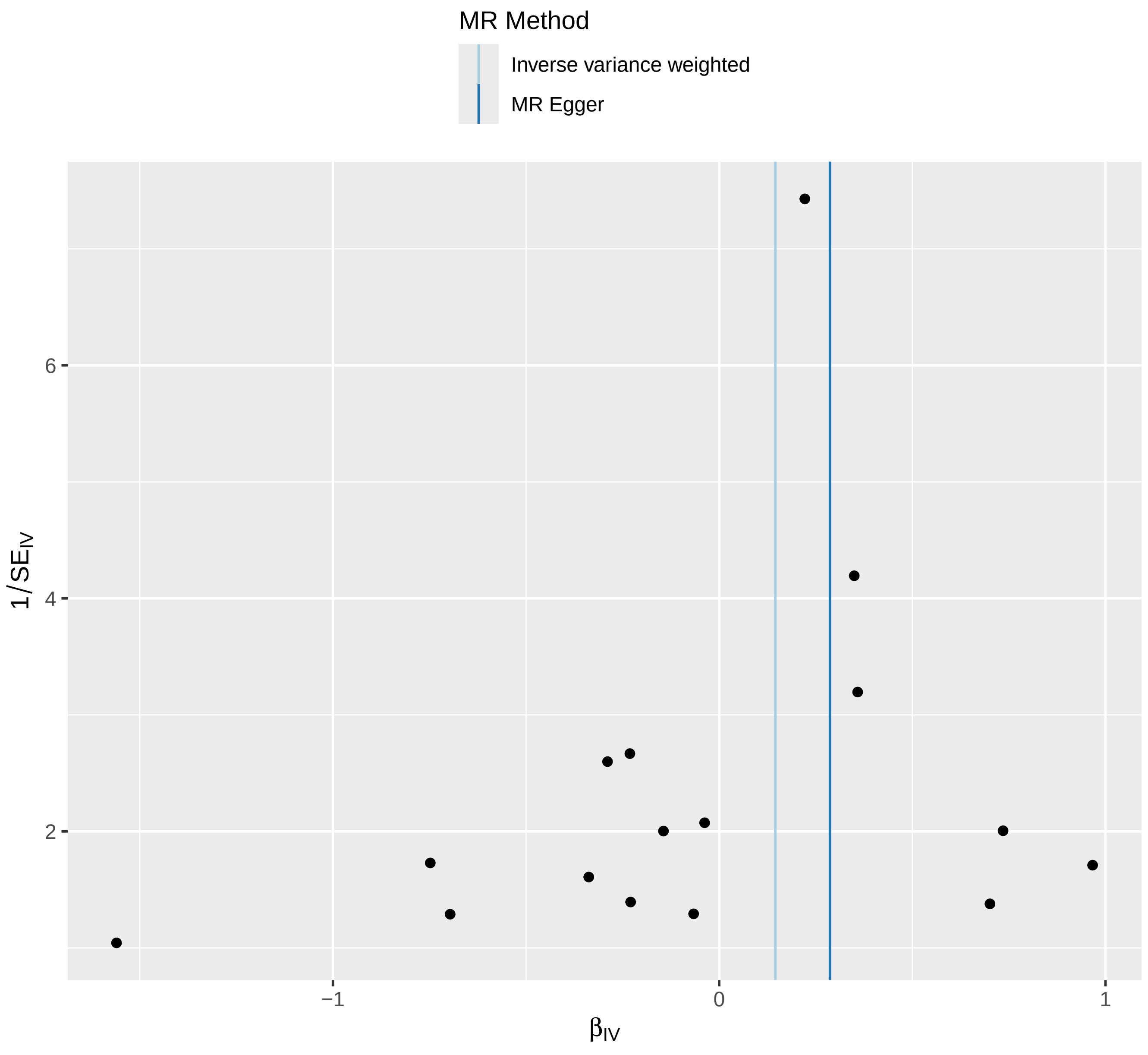
MR Method
Inverse variance weighted
MR Egger
1/SE_IV
6
4
2
−1
0
1
β_IV

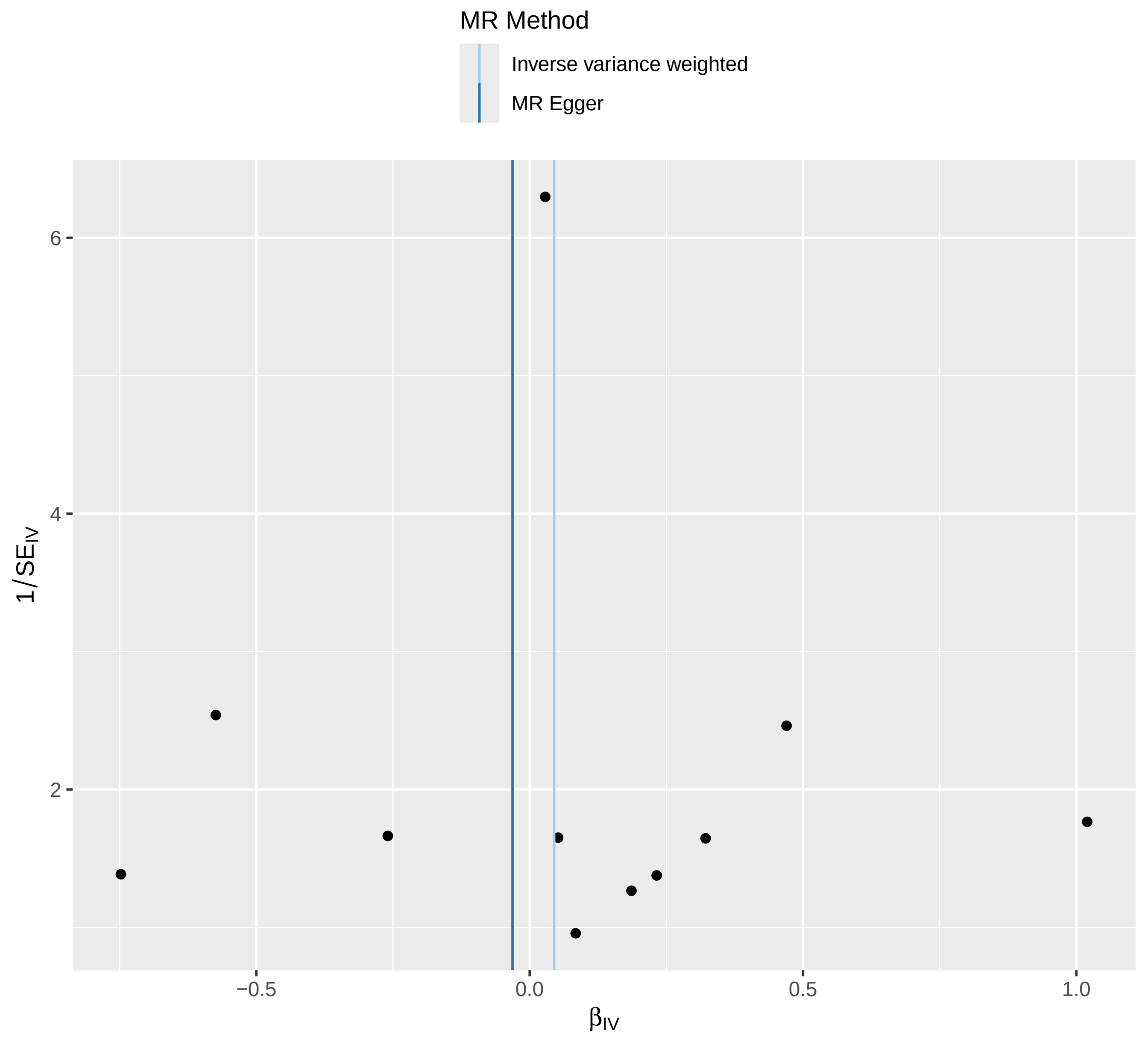

MR Method
Inverse variance weighted
MR Egger
6
4
2
1/SE_IV
−0.5
0.0
0.5
1.0
β_IV

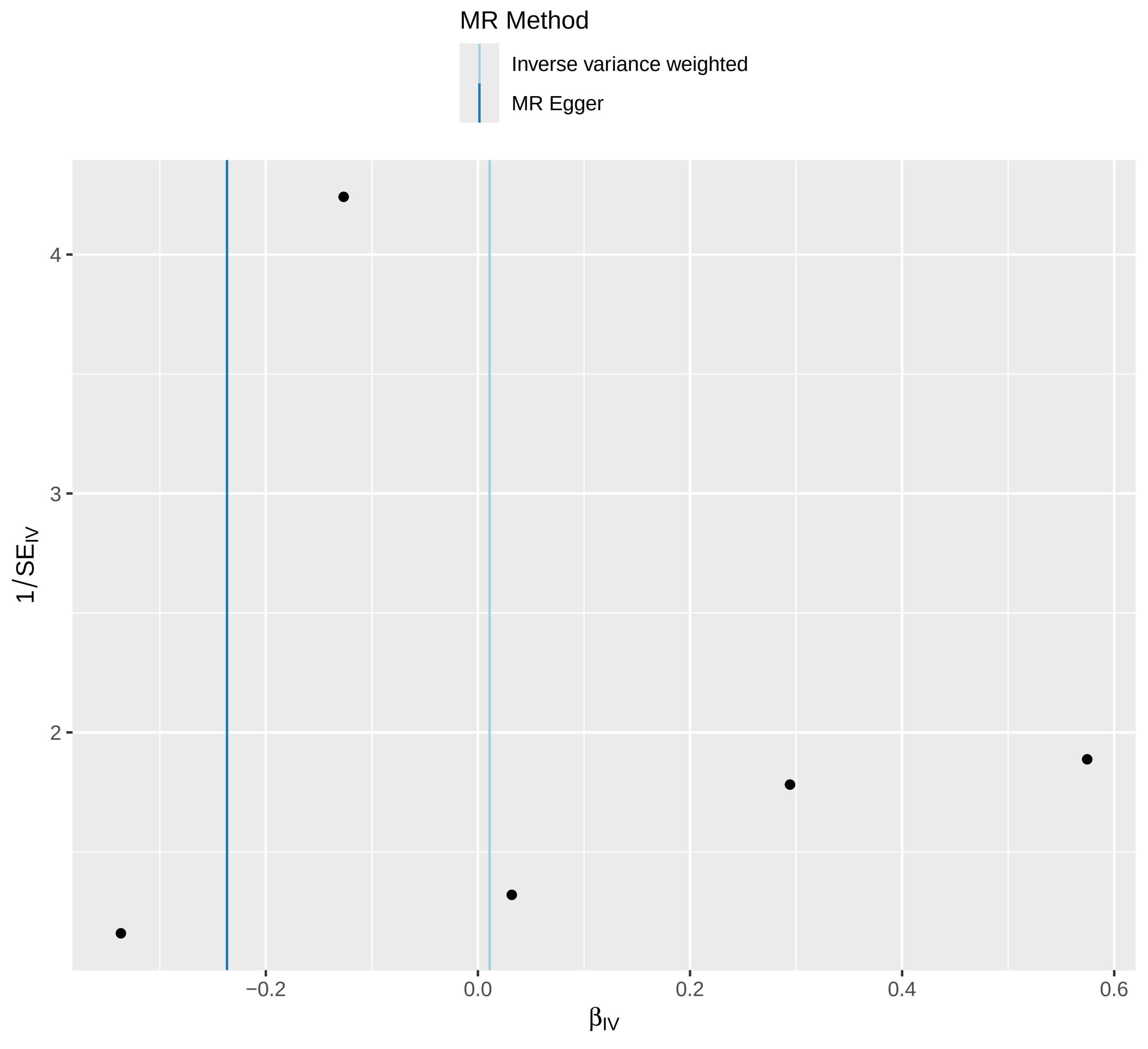
MR Method
Inverse variance weighted
MR Egger
4
3
2
1/SE_IV
−0.2
0.0
0.2
0.4
0.6
β_IV

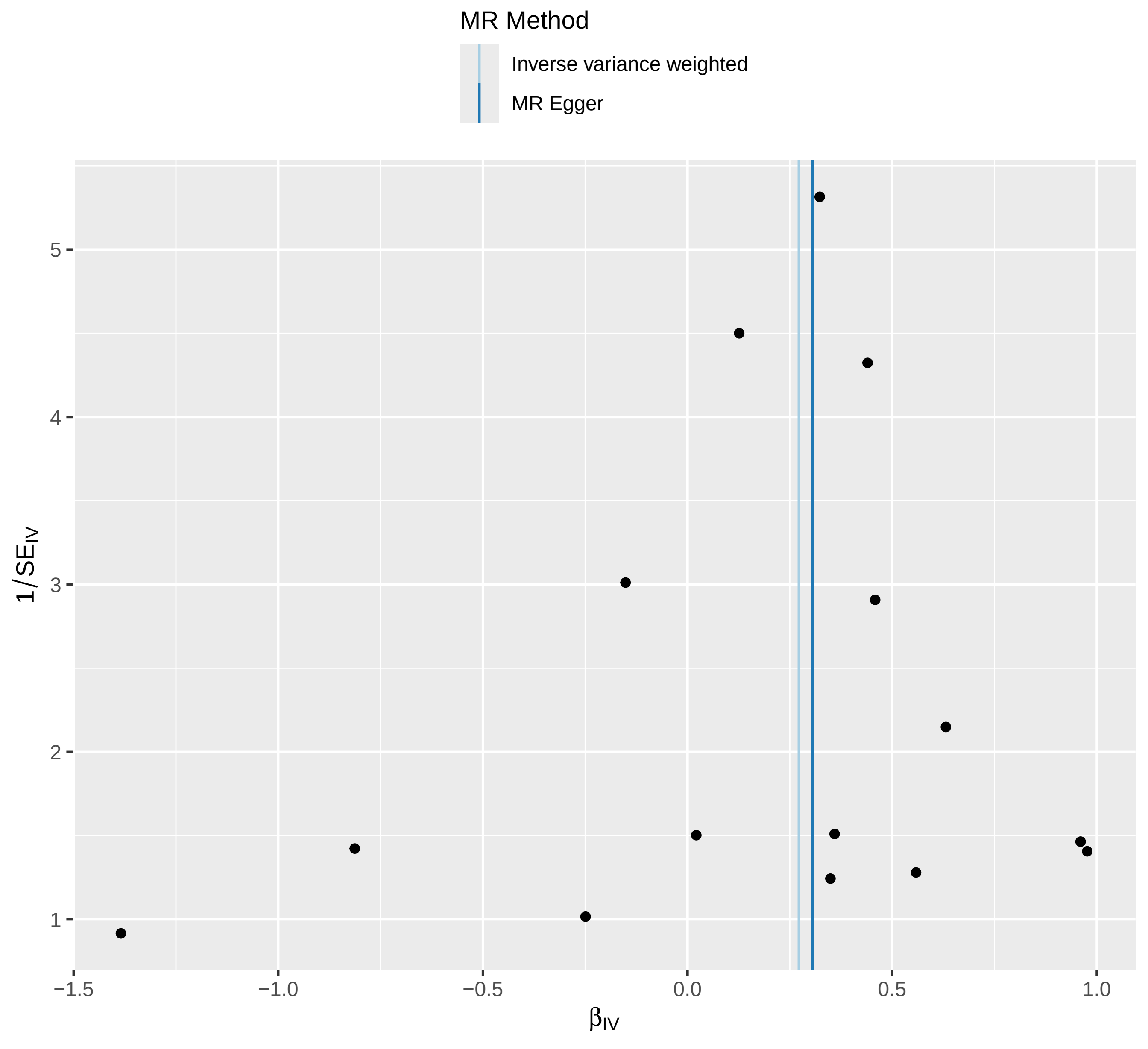
MR Method
Inverse variance weighted
MR Egger
1/SE_IV
5
4
3
2
1
−1.5
−1.0
−0.5
0.0
0.5
1.0
β_IV

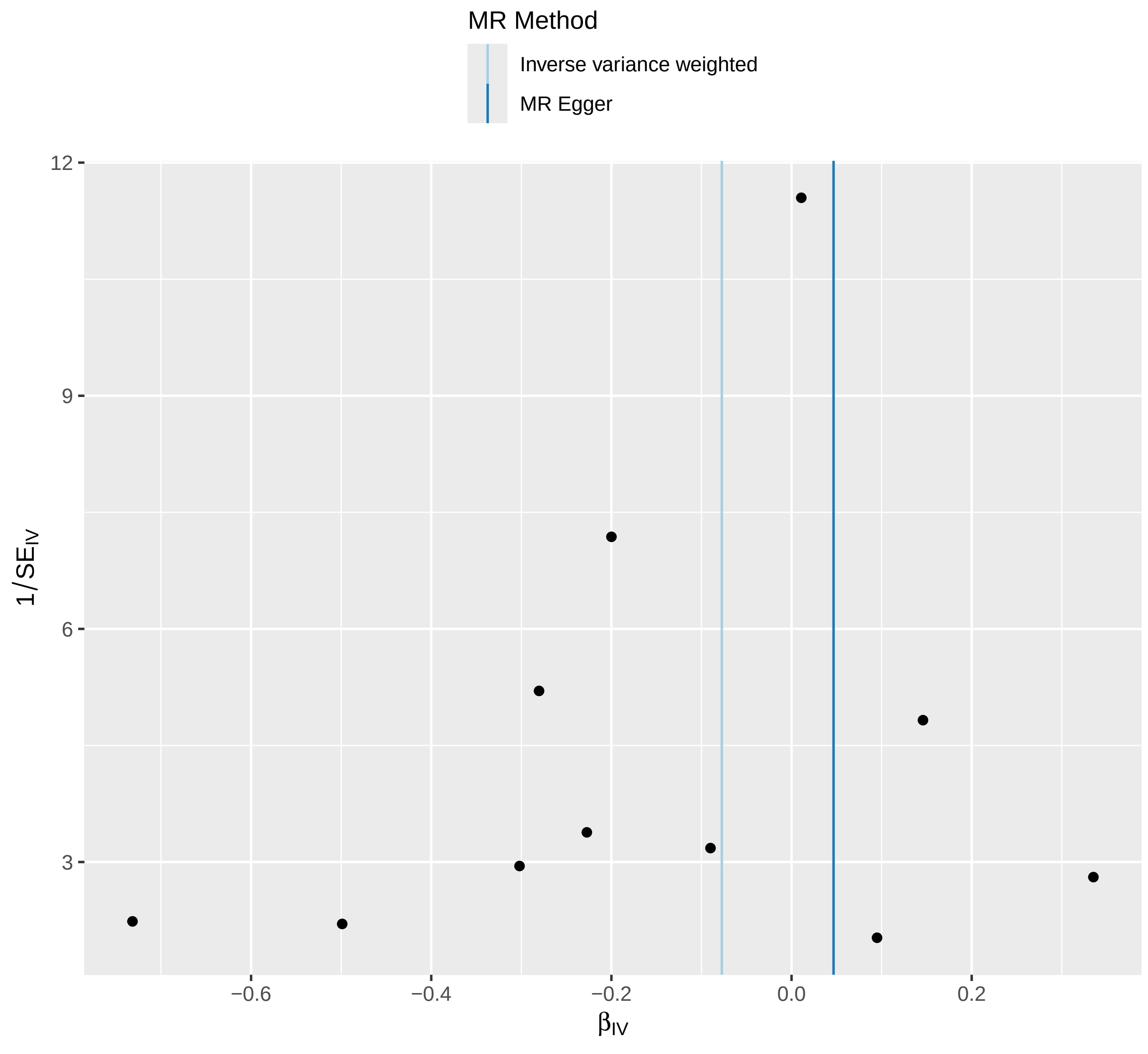

MR Method
Inverse variance weighted
MR Egger
12
9
6
3
1/SE_IV
−0.6
−0.4
−0.2
0.0
0.2
β_IV

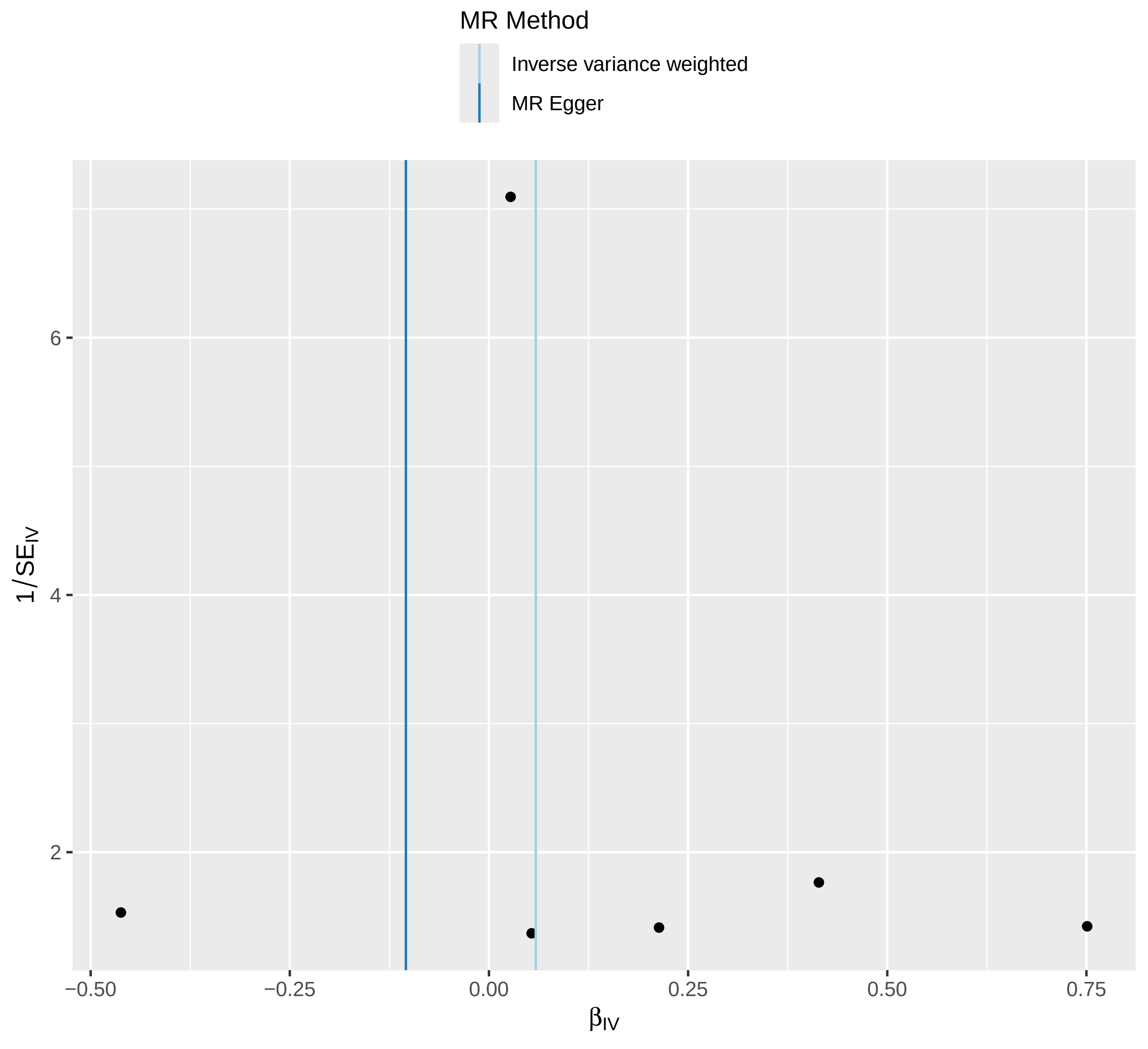
MR Method
Inverse variance weighted
MR Egger
6
4
2
$1/SE_{IV}$
−0.50
−0.25
0.00
0.25
0.50
0.75
β_{IV}

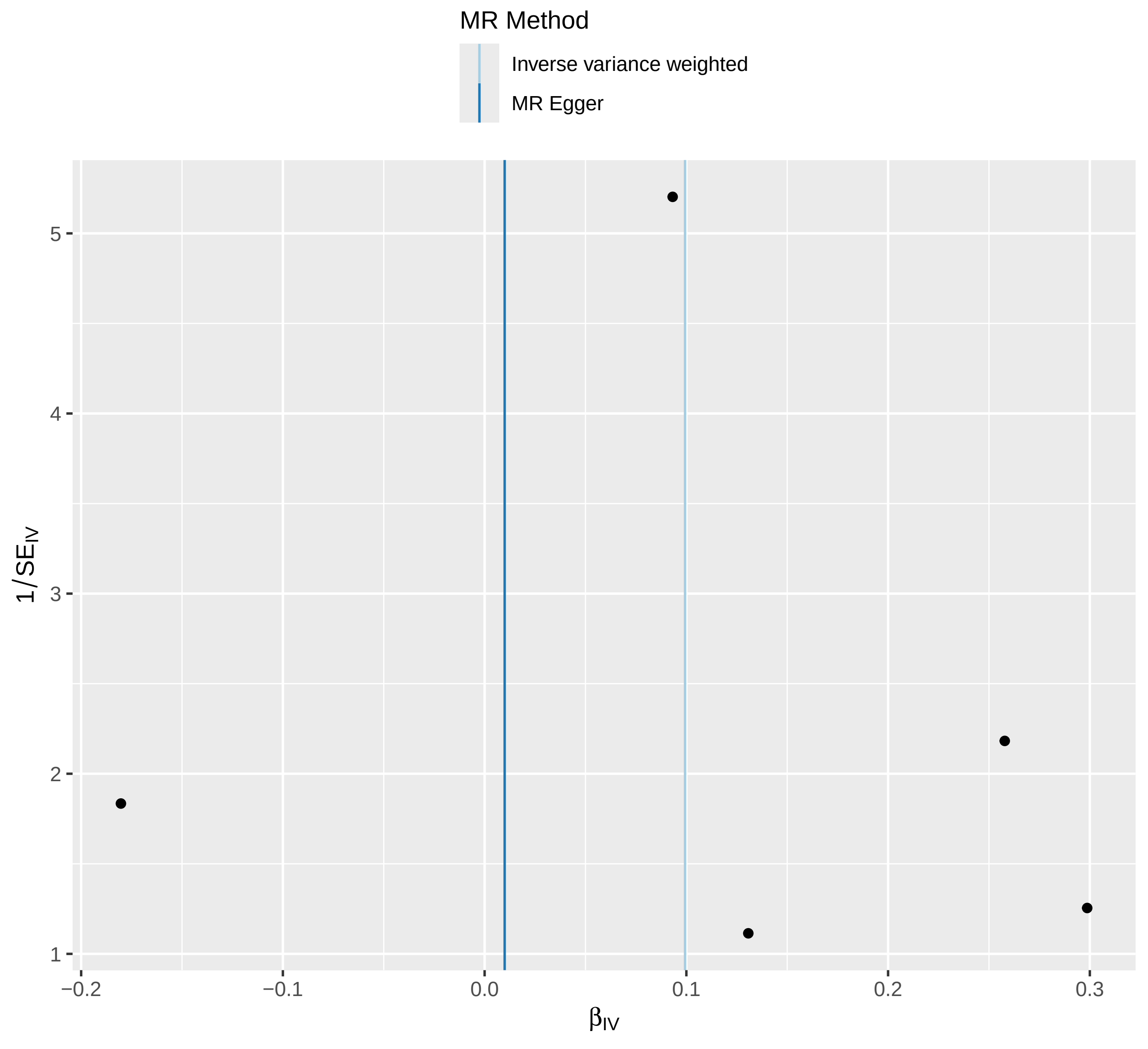

MR Method
Inverse variance weighted
MR Egger
1/SE_IV
5
4
3
2
1
−0.2
−0.1
0.0
0.1
0.2
0.3
β_IV

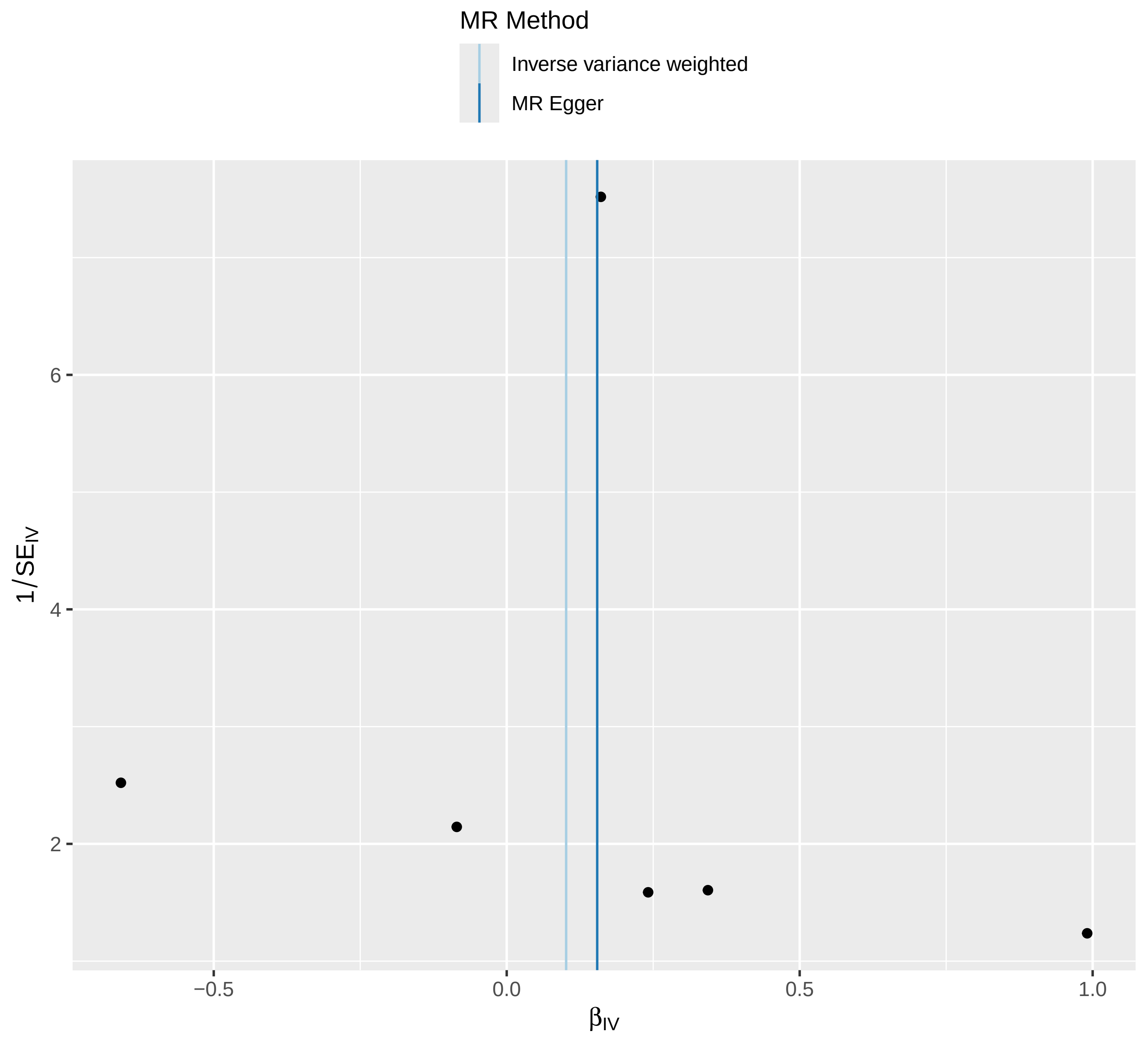
MR Method
Inverse variance weighted
MR Egger
6
4
2
1/SE_IV
−0.5
0.0
0.5
1.0
β_IV

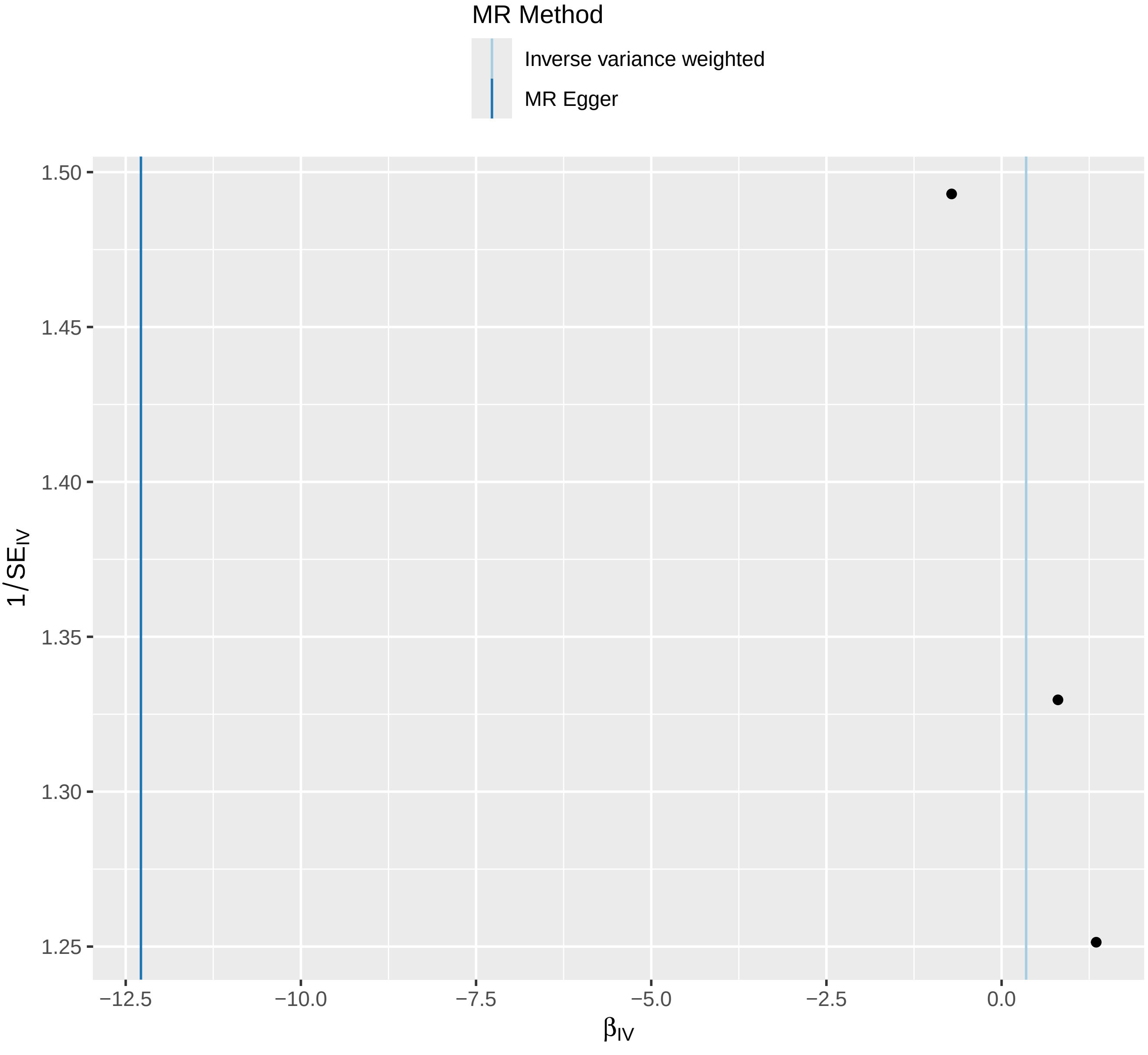
MR Method
Inverse variance weighted
MR Egger
1.50
1.45
1.40
1.35
1.30
1.25
1/SE_IV
−12.5
−10.0
−7.5
−5.0
−2.5
0.0
β_IV

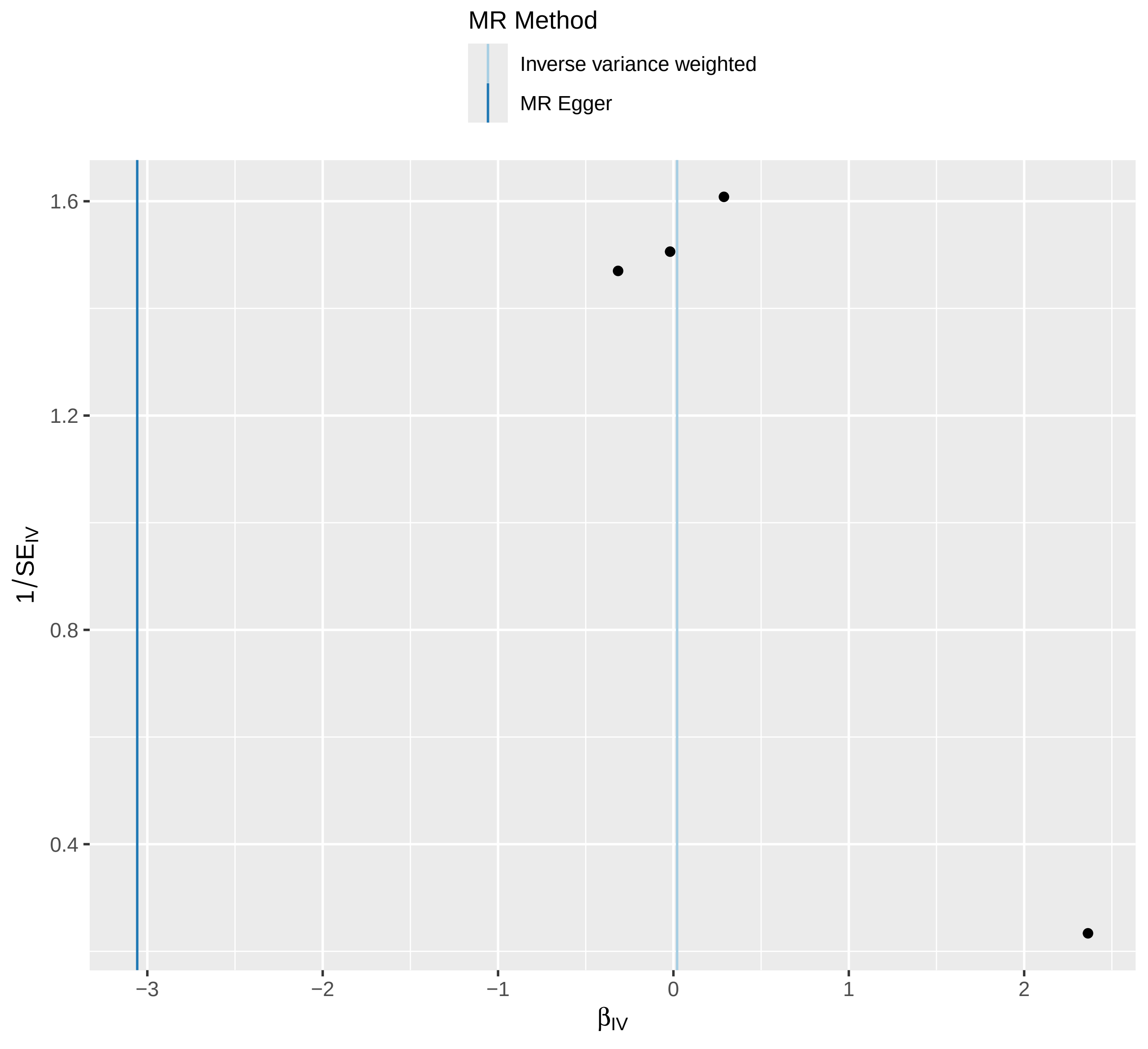
MR Method
Inverse variance weighted
MR Egger
1.6
1.2
0.8
0.4
1/SE$_{IV}$
−3
−2
−1
0
1
2
β$_{IV}$

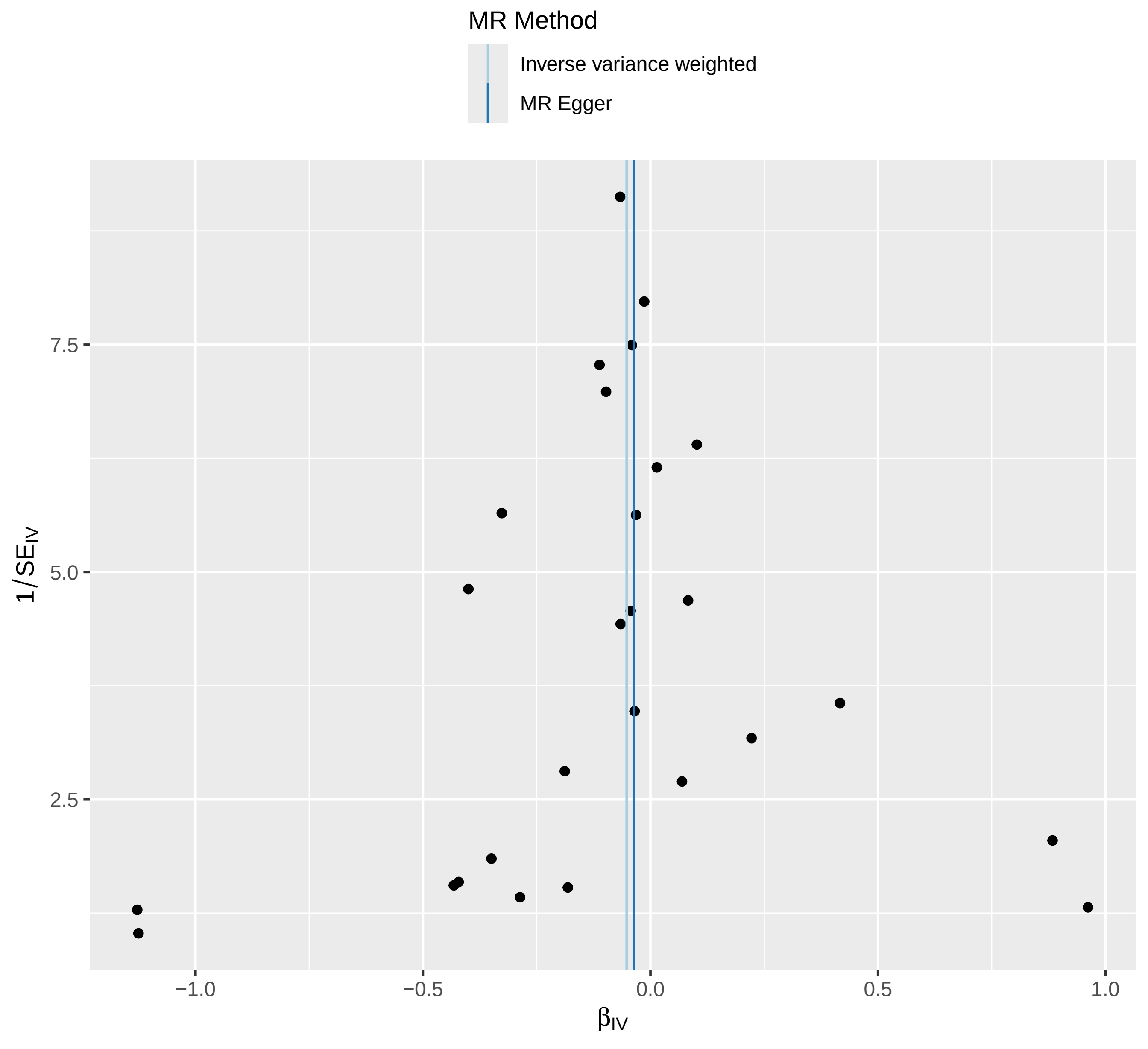
MR Method
Inverse variance weighted
MR Egger
7.5
5.0
2.5
1/SE_IV
−1.0
−0.5
0.0
0.5
1.0
β_IV

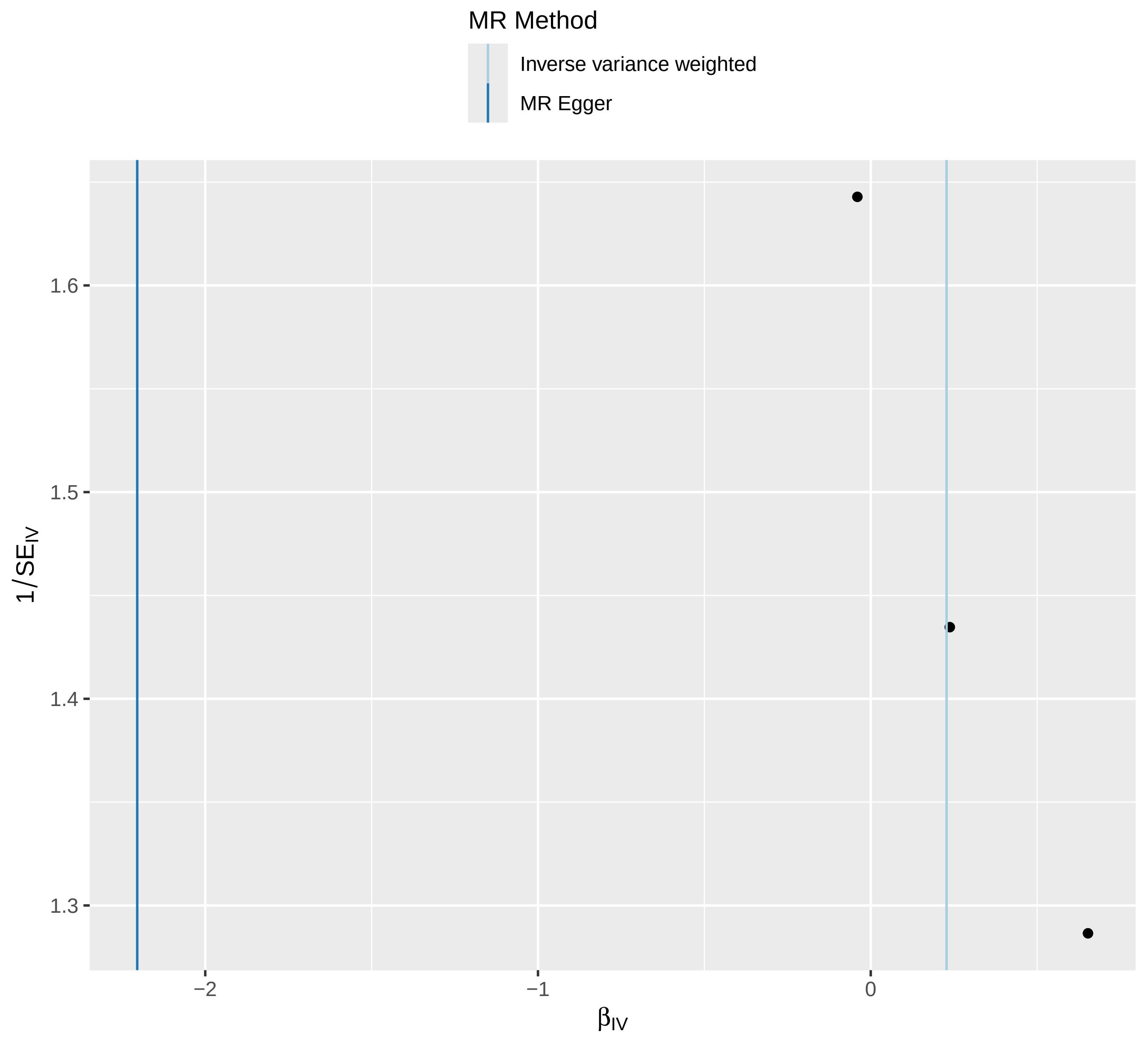

MR Method
Inverse variance weighted
MR Egger
1.6
1.5
1.4
1.3
1/SE_IV
−2
−1
0
β_IV

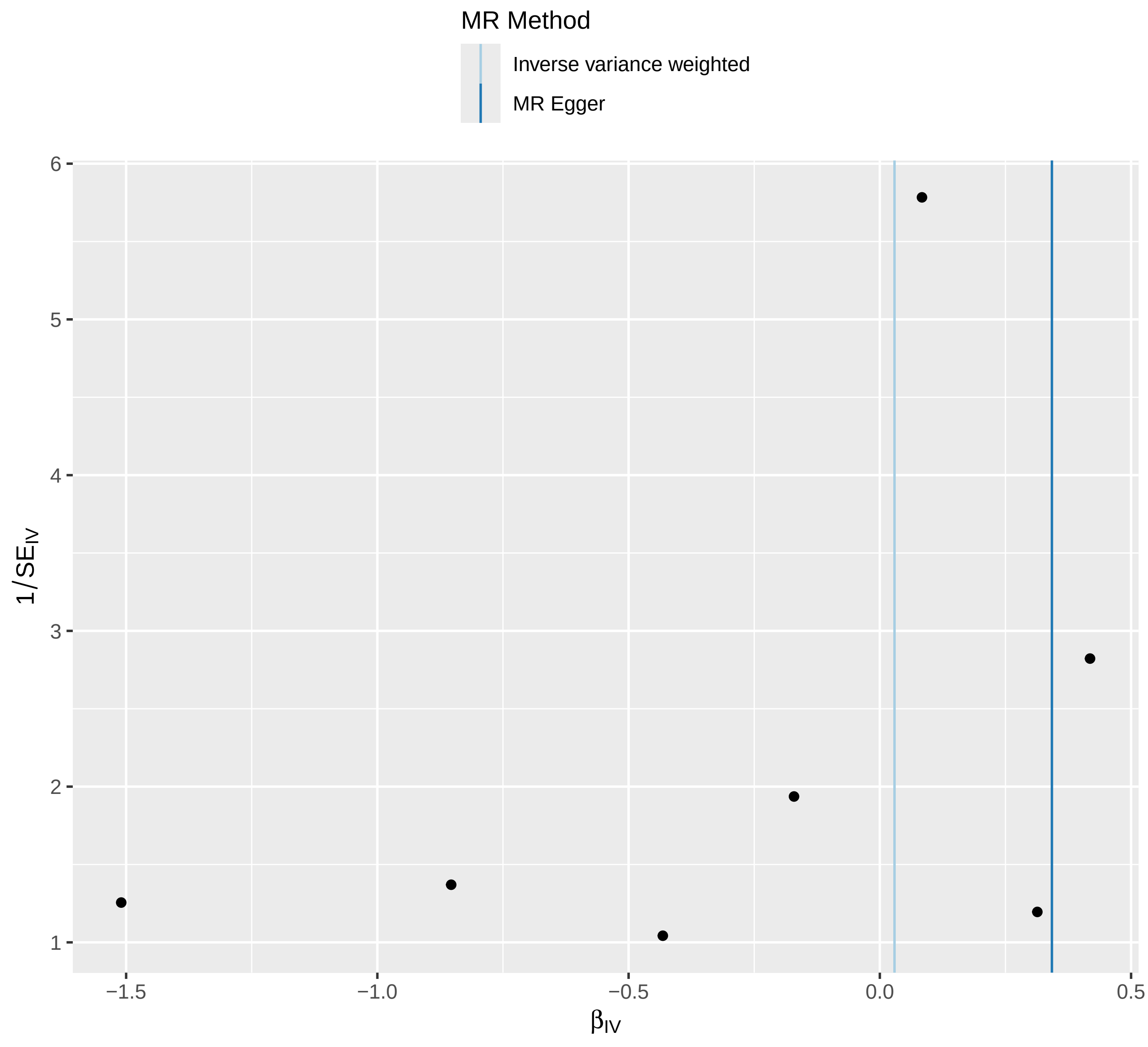
MR Method
Inverse variance weighted
MR Egger
6
5
4
3
2
1
$1/SE_{IV}$
−1.5
−1.0
−0.5
0.0
0.5
β_{IV}

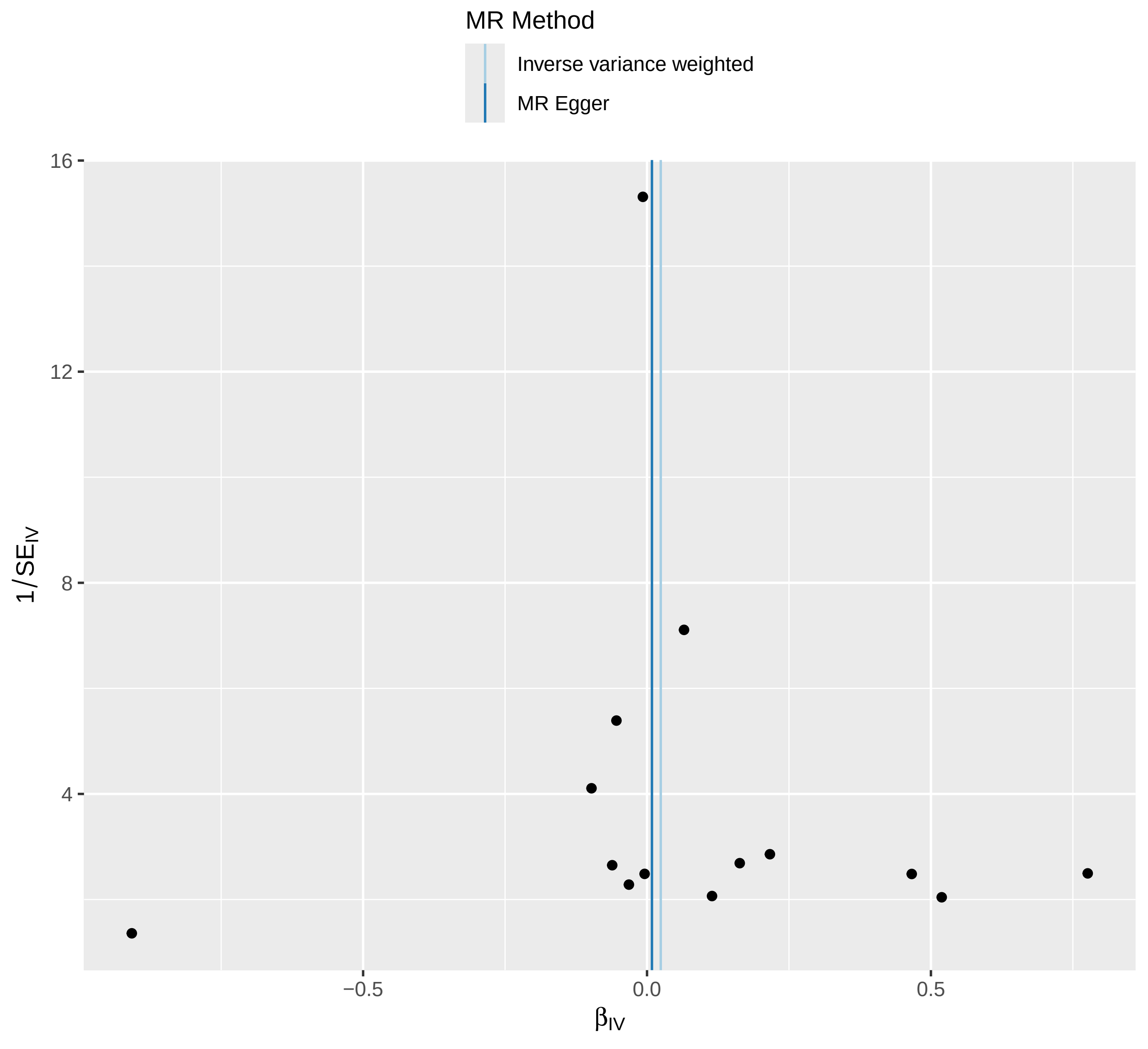

MR Method
Inverse variance weighted
MR Egger
16
12
8
4
1/SE_IV
−0.5
0.0
0.5
β_IV

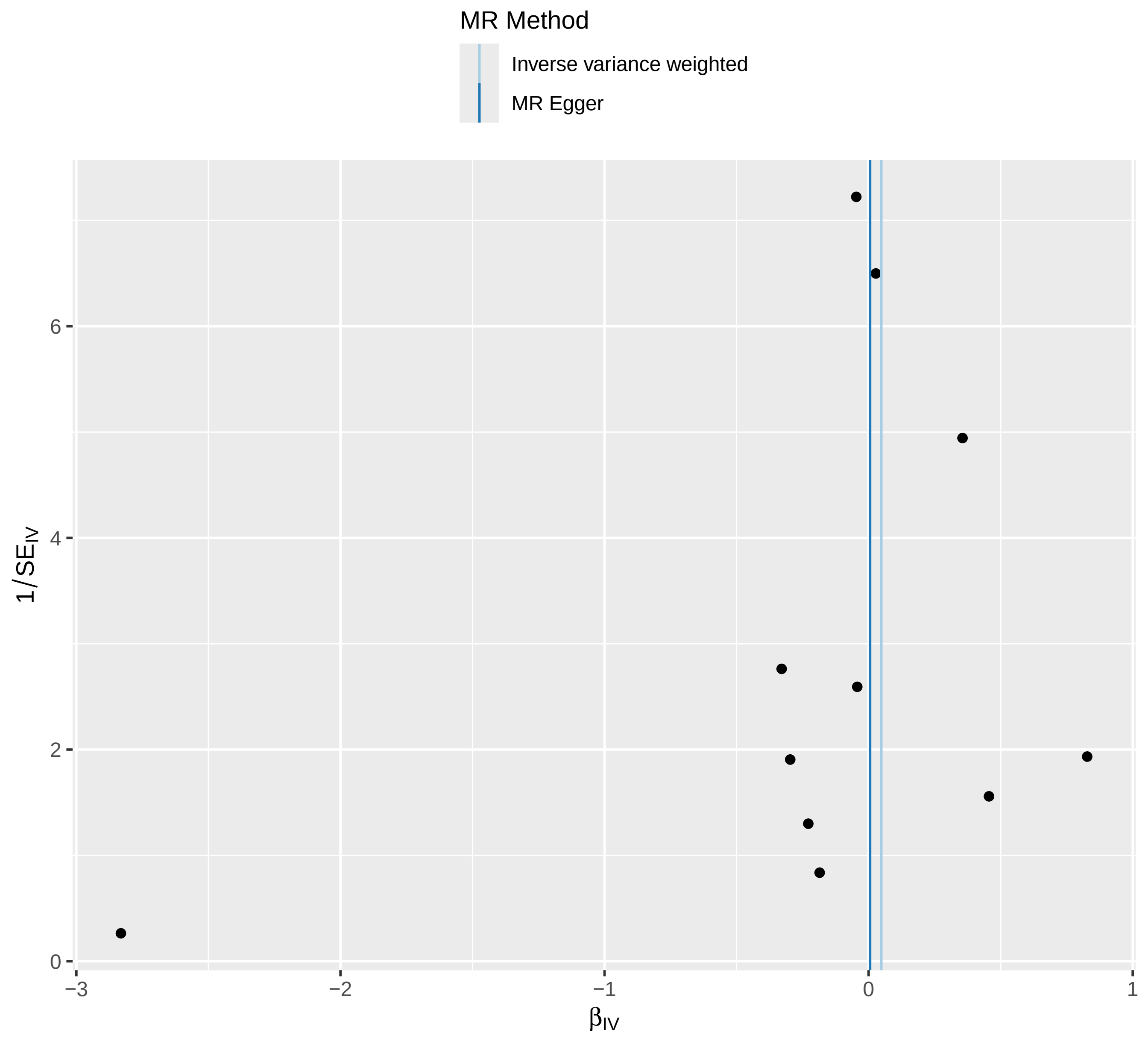
MR Method
Inverse variance weighted
MR Egger
1/SE$_{IV}$
β_{IV}
0
2
4
6
−3
−2
−1
0
1

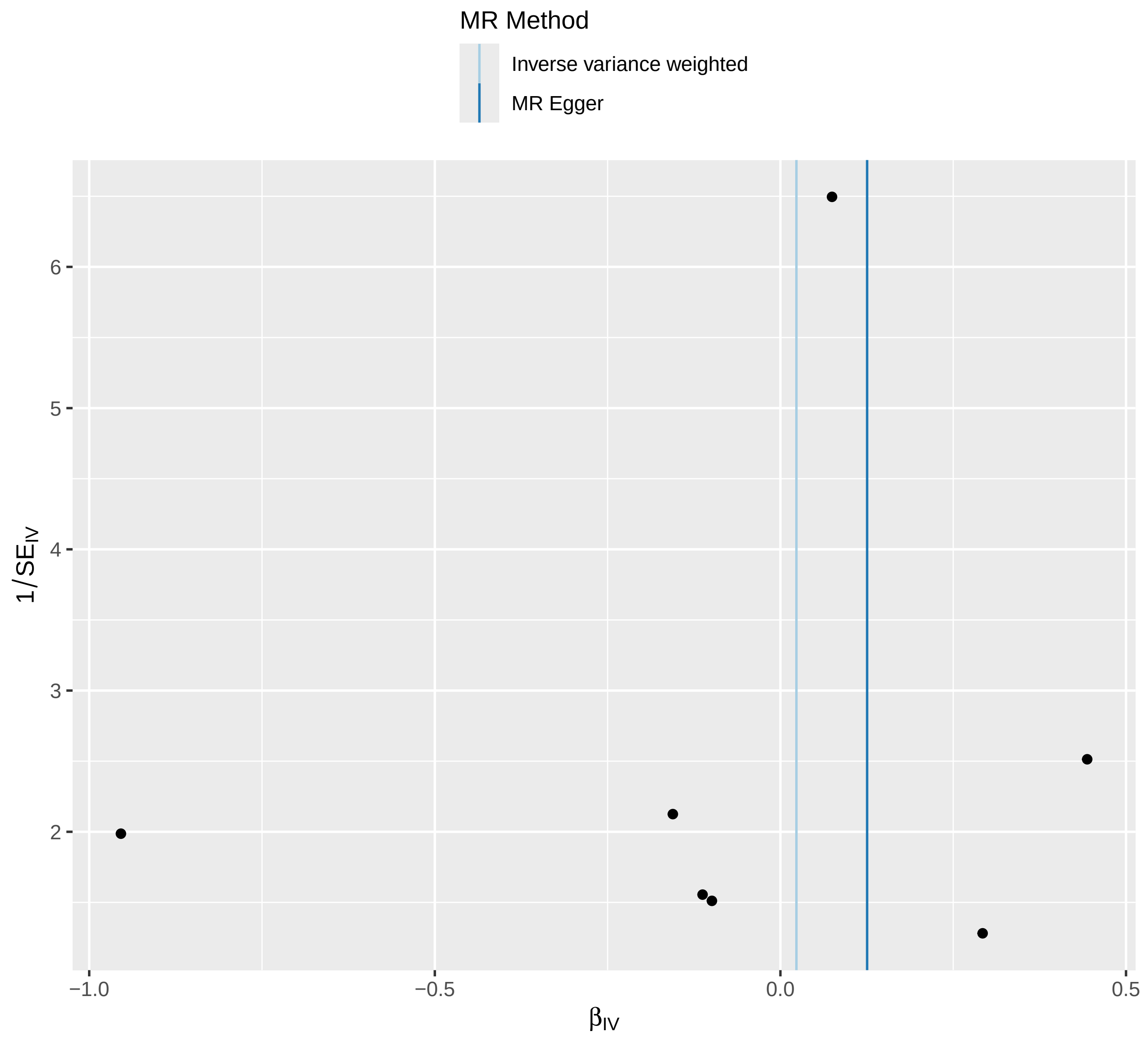

MR Method
Inverse variance weighted
MR Egger
1/SE$_{IV}$
6
5
4
3
2
−1.0
−0.5
0.0
0.5
β$_{IV}$

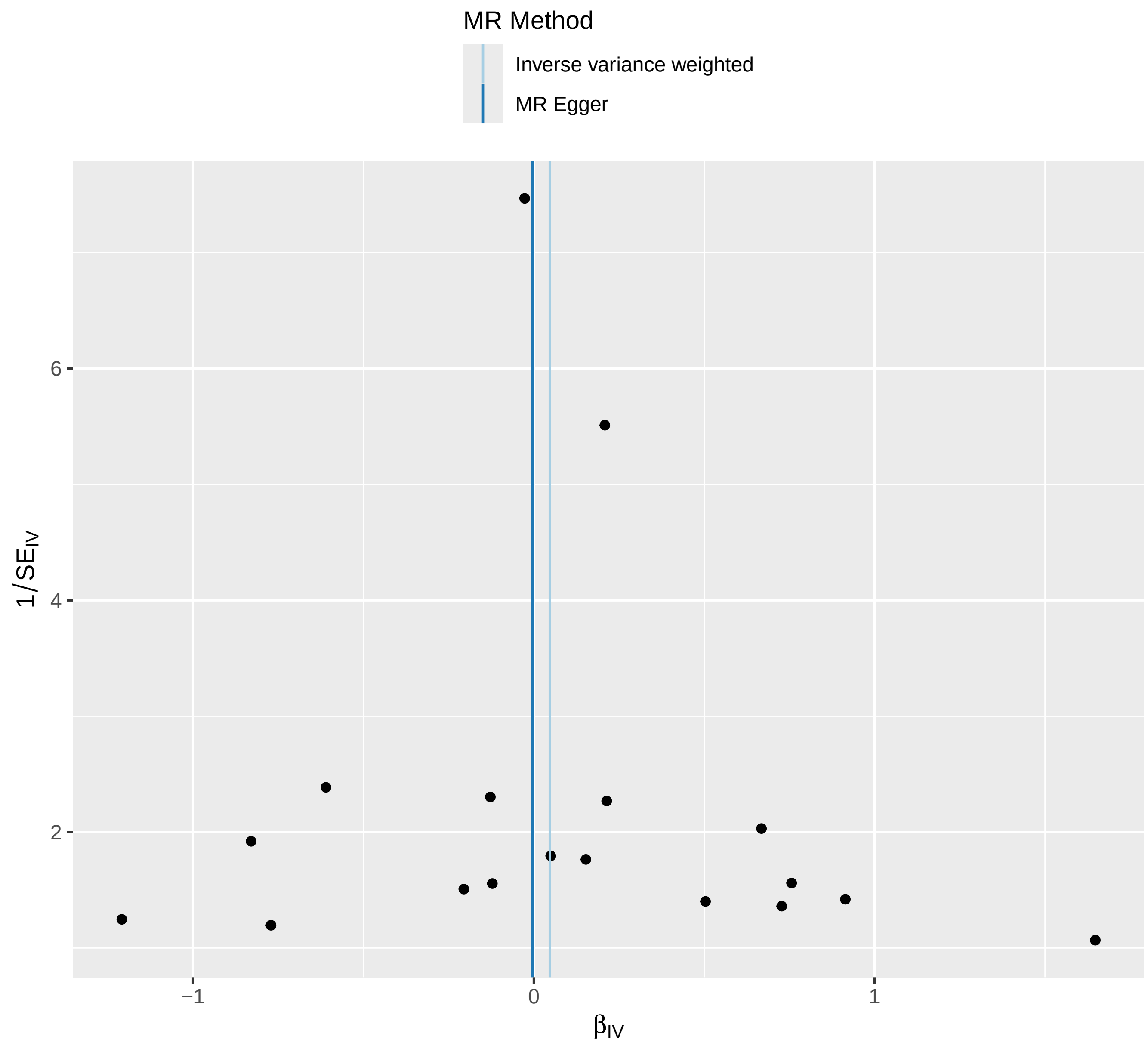
MR Method
Inverse variance weighted
MR Egger
6
4
2
1/SE$_{IV}$
−1
0
1
β$_{IV}$

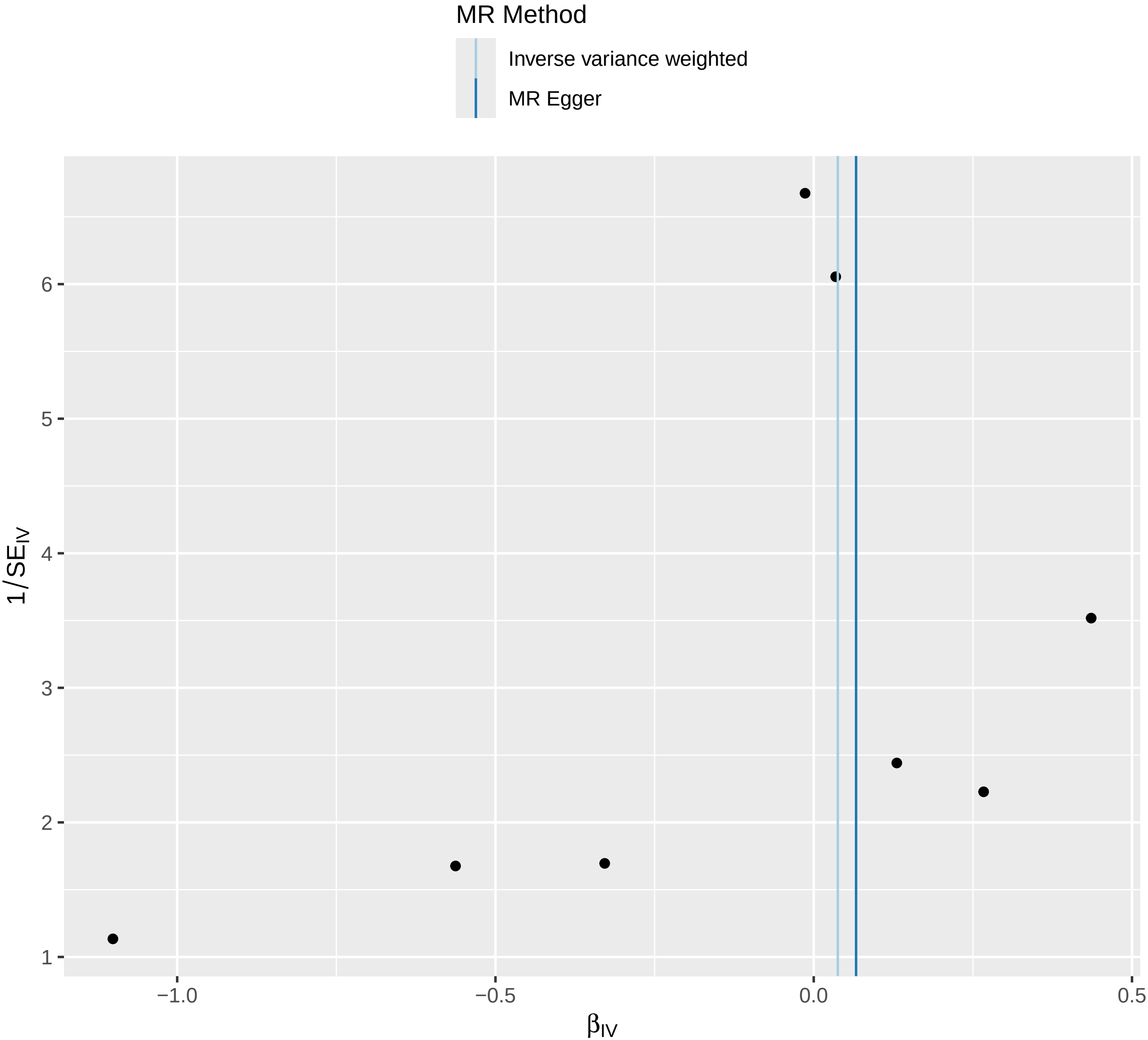

MR Method
Inverse variance weighted
MR Egger
1/SE_IV
β_IV
−1.0
−0.5
0.0
0.5
1
2
3
4
5
6

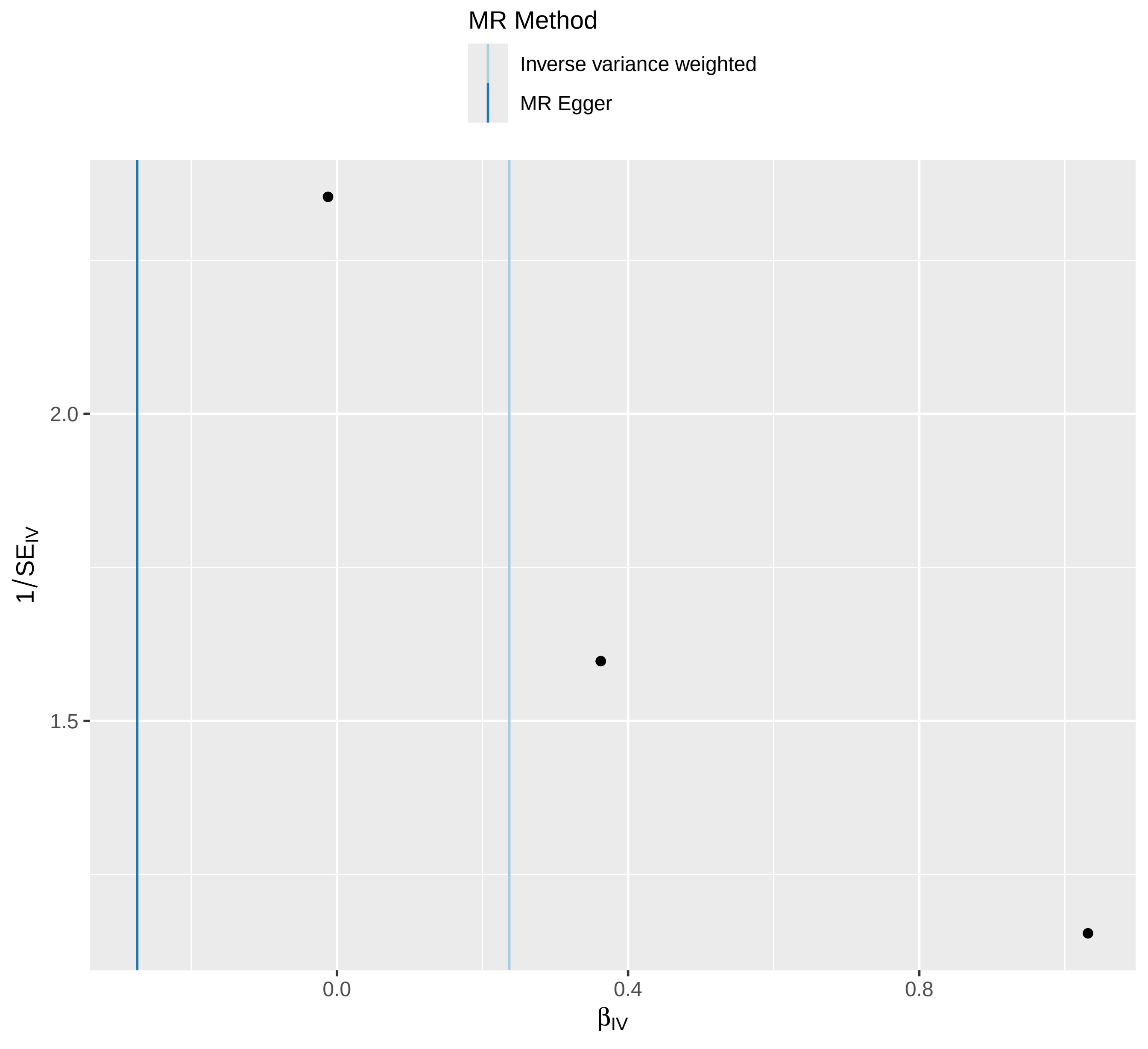

MR Method
Inverse variance weighted
MR Egger
2.0
1.5
1/SE_IV
0.0
0.4
0.8
β_IV

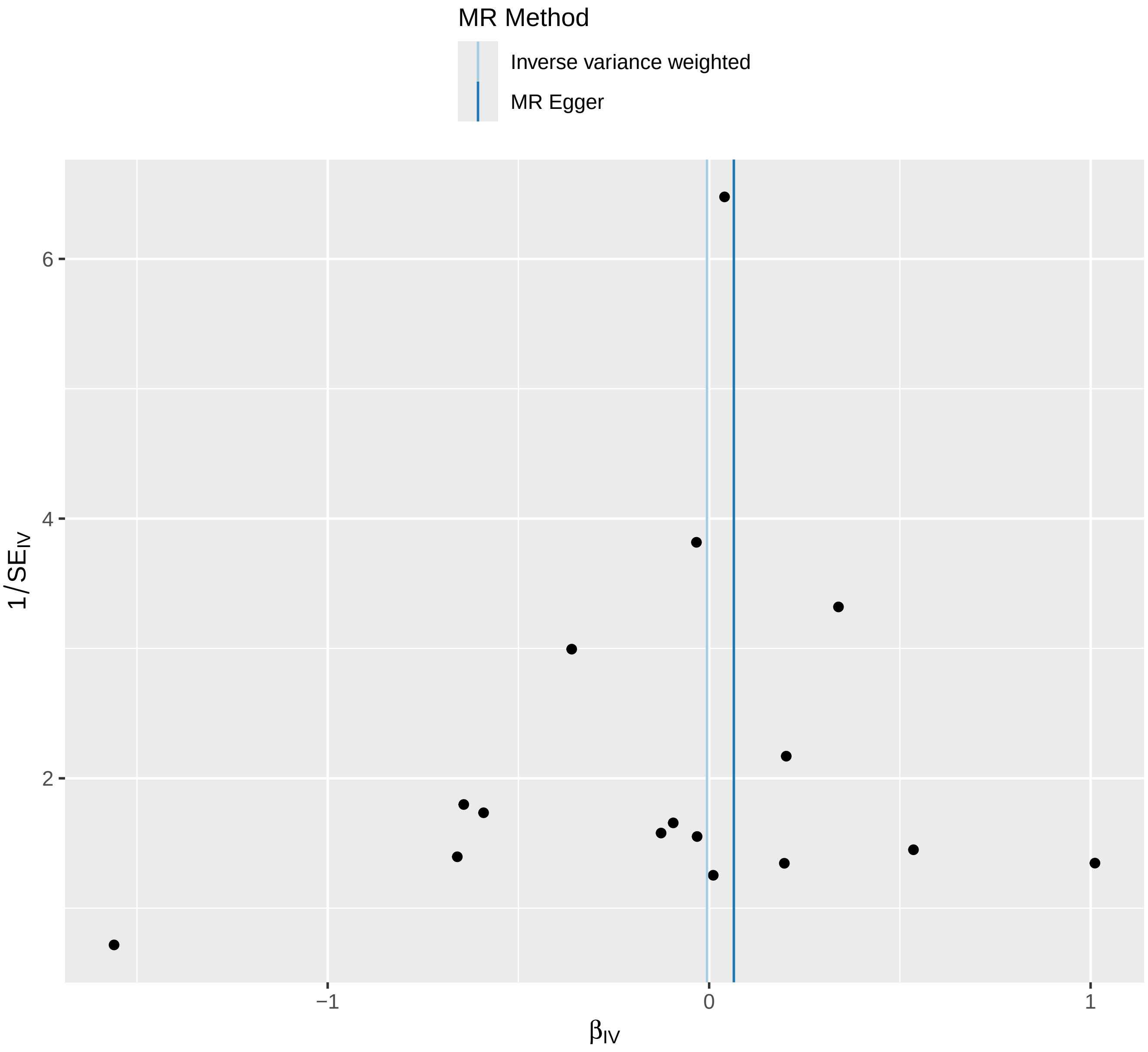

MR Method
Inverse variance weighted
MR Egger
6
4
2
1/SE_IV
−1
0
1
β_IV

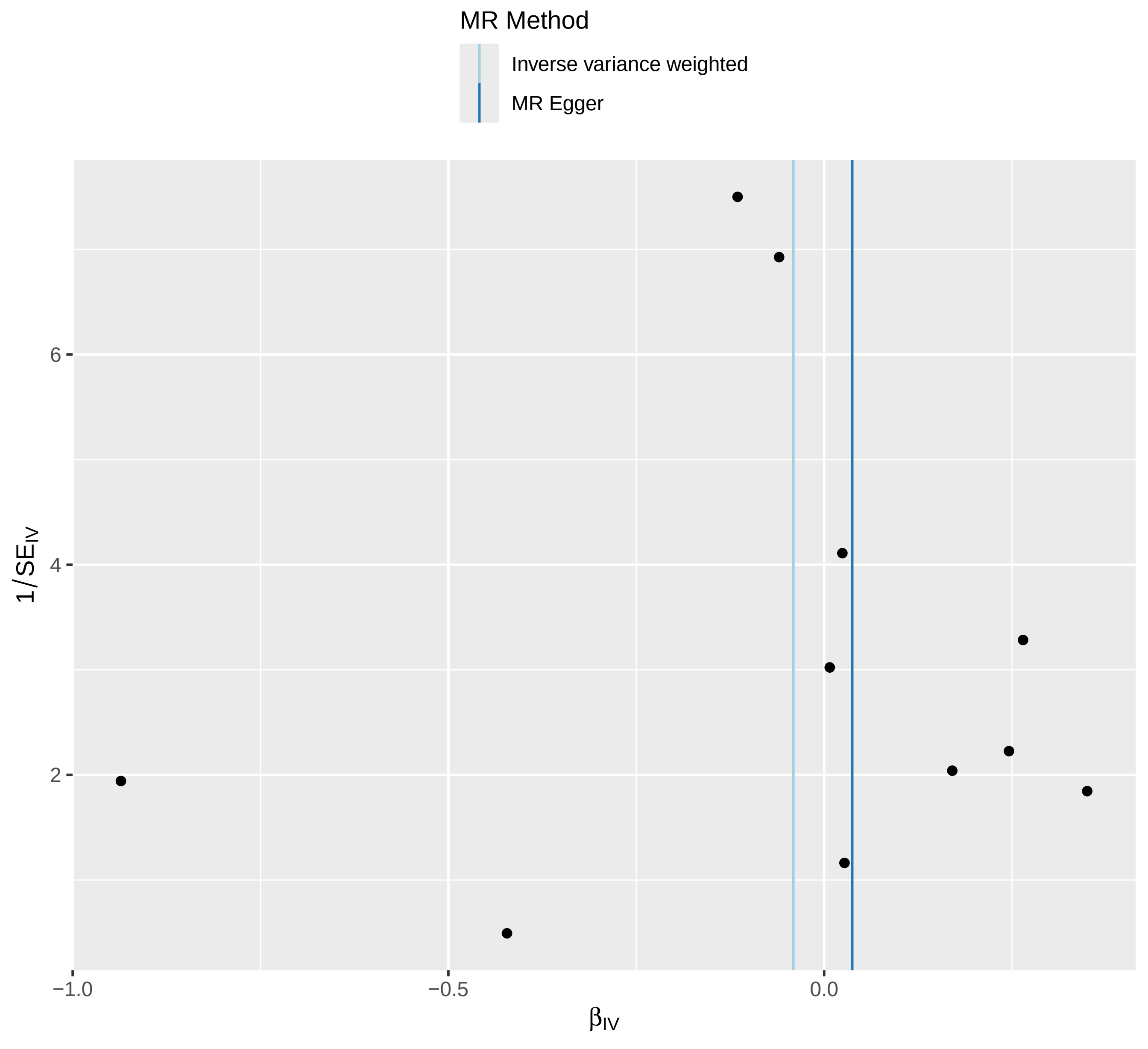
MR Method
Inverse variance weighted
MR Egger
6
4
2
1/SE$_{IV}$
−1.0
−0.5
0.0
β$_{IV}$

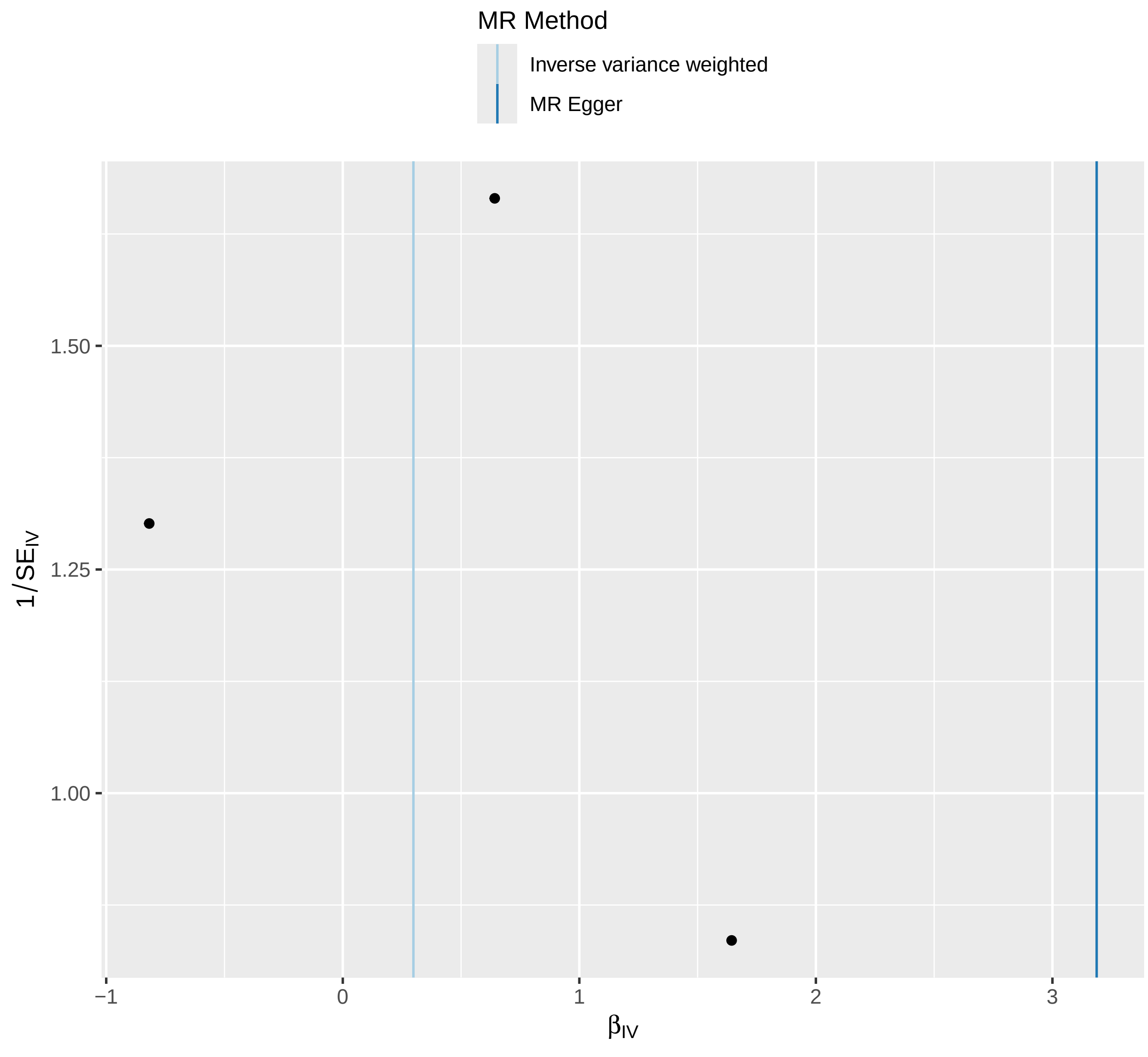

MR Method
Inverse variance weighted
MR Egger
1.50
1.25
1.00
1/SE_IV
−1
0
1
2
3
β_IV

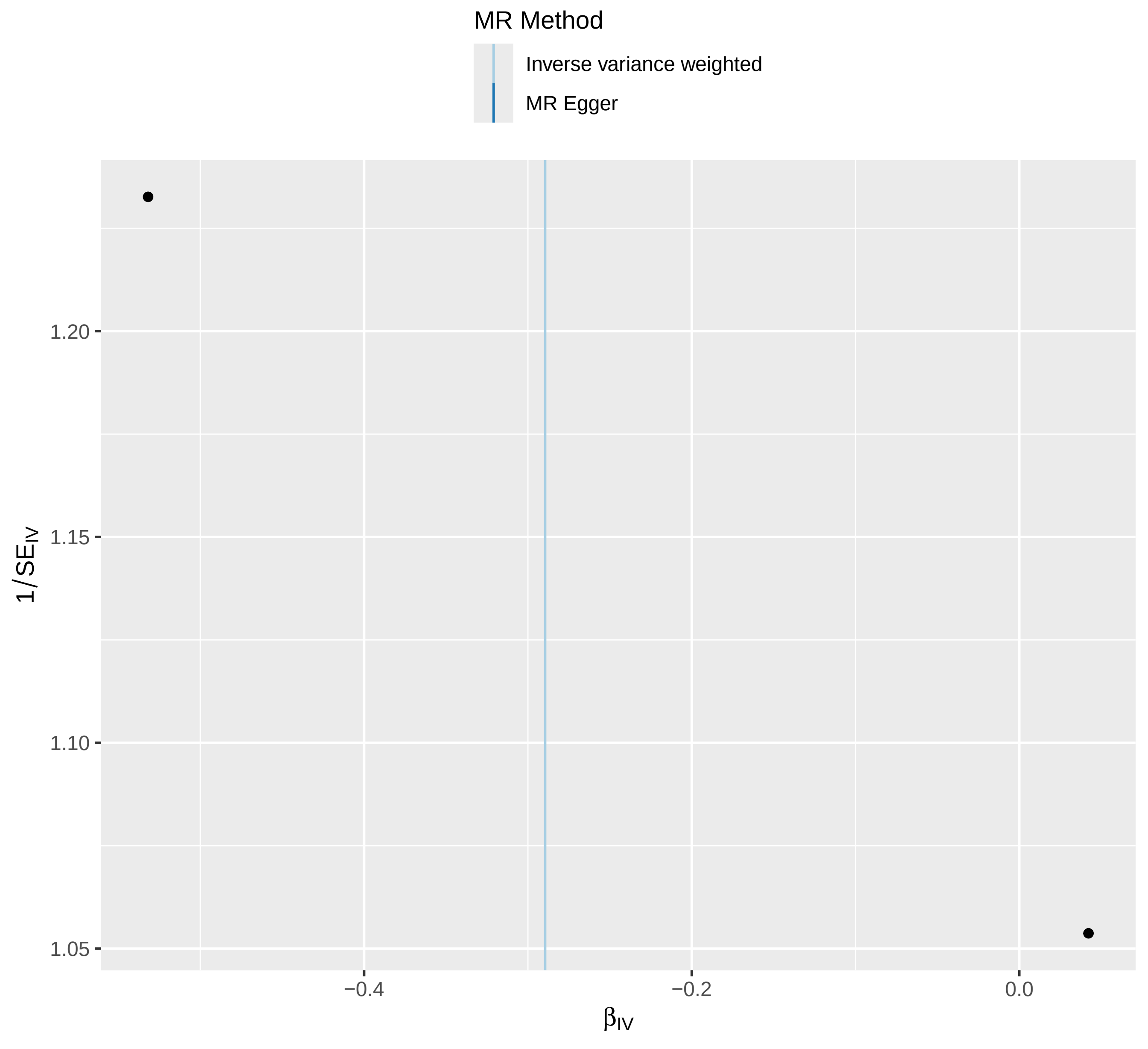

MR Method
Inverse variance weighted
MR Egger
1.20
1.15
1.10
1.05
1/SE$_{IV}$
−0.4
−0.2
0.0
β$_{IV}$

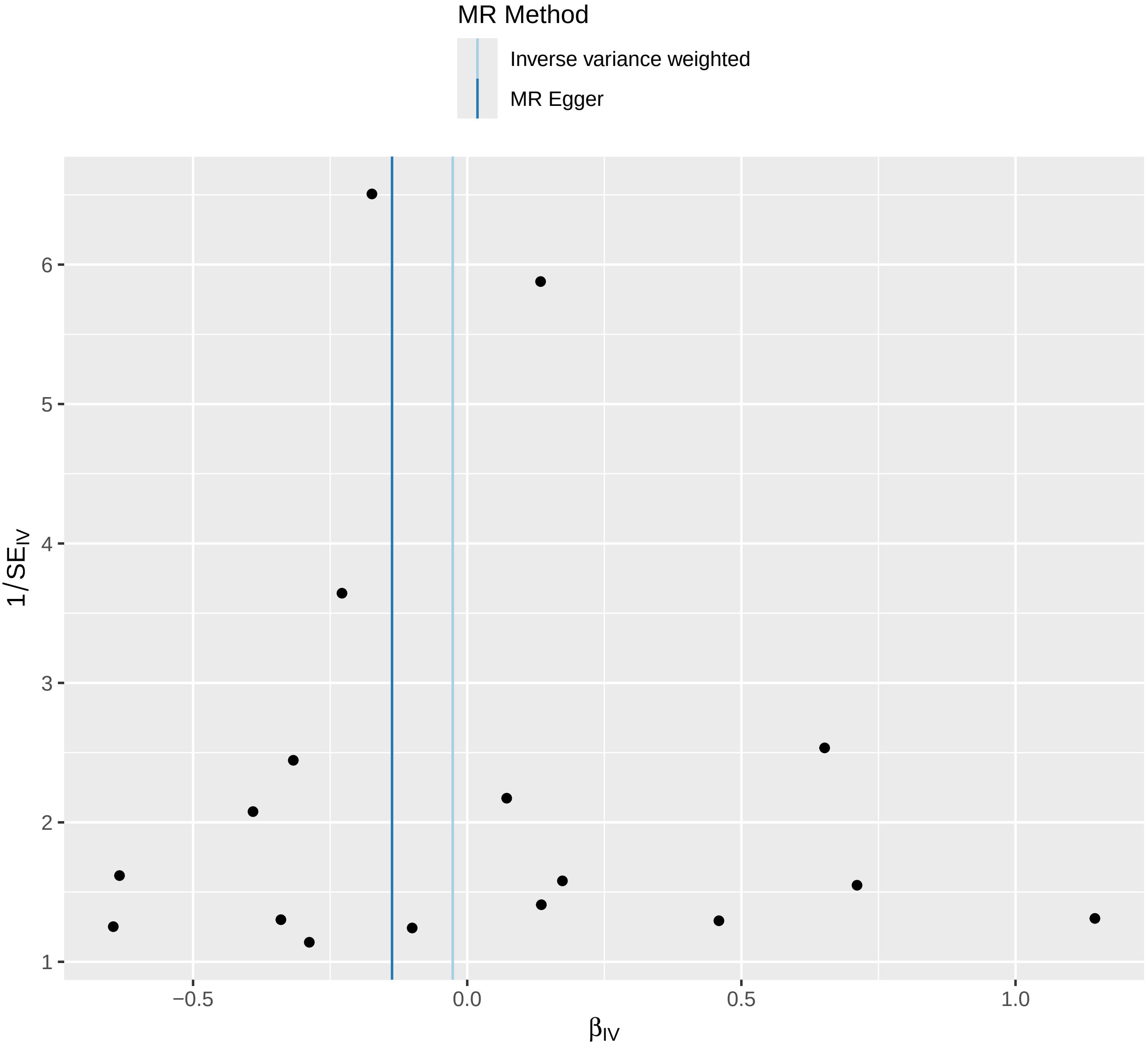

MR Method
Inverse variance weighted
MR Egger
1/SE$_{IV}$
1
2
3
4
5
6
−0.5
0.0
0.5
1.0
β_{IV}

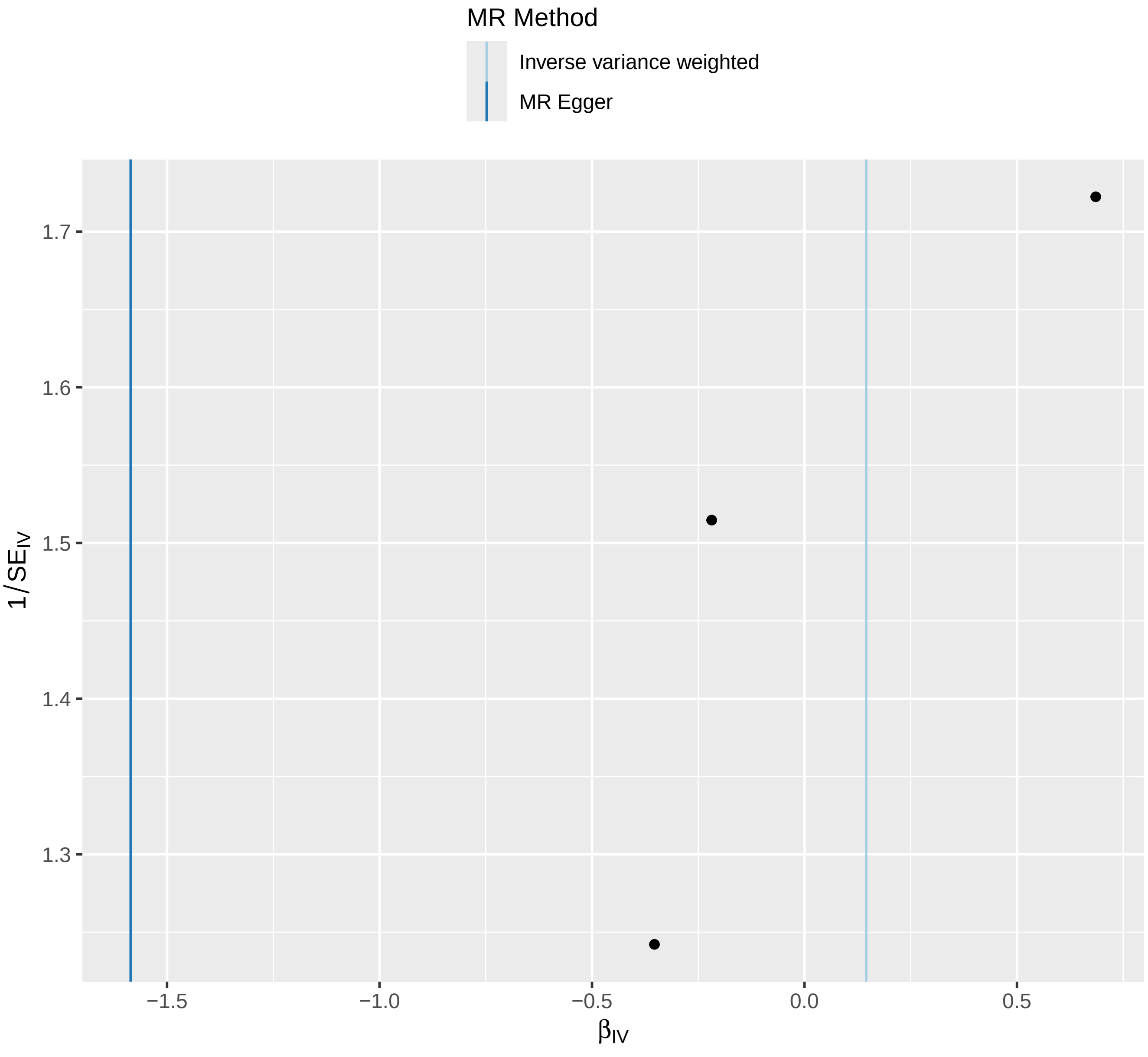

MR Method
Inverse variance weighted
MR Egger
1.7
1.6
1.5
1.4
1.3
$1/SE_{IV}$
−1.5
−1.0
−0.5
0.0
0.5
β_{IV}

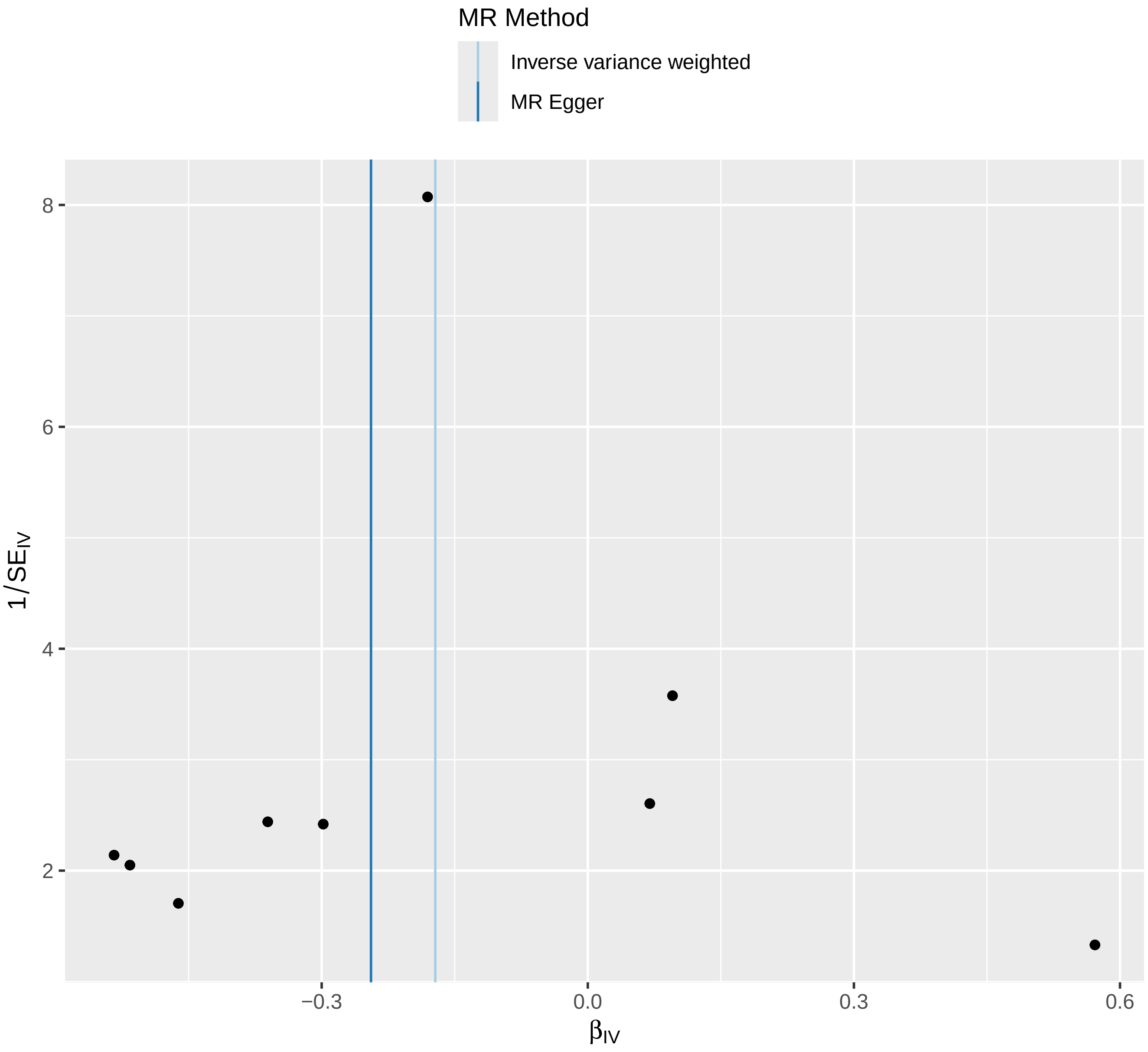

MR Method
Inverse variance weighted
MR Egger
8
6
4
2
$1/SE_{IV}$
−0.3
0.0
0.3
0.6
β_{IV}

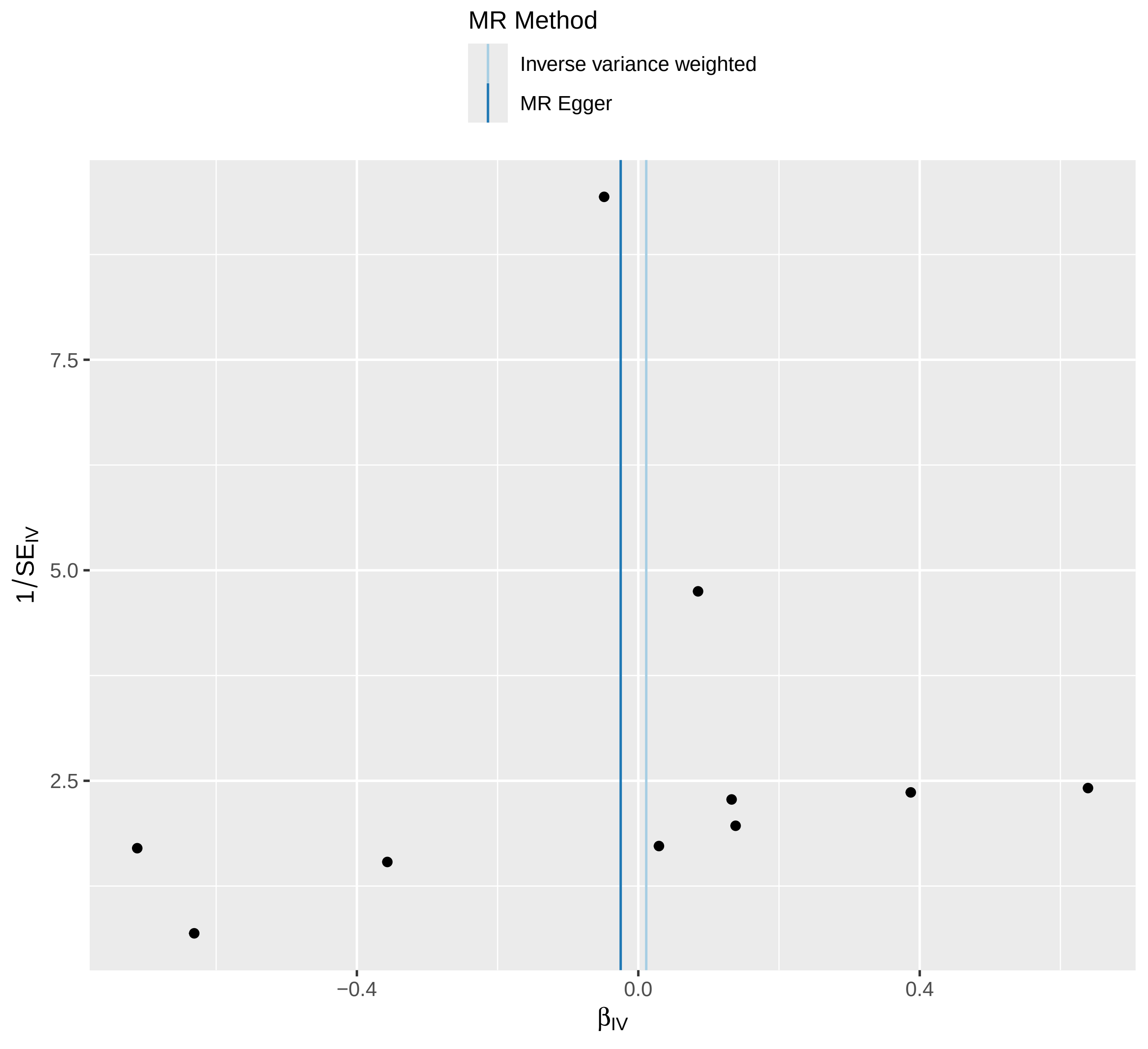
MR Method
Inverse variance weighted
MR Egger
7.5
5.0
2.5
1/SE_IV
−0.4
0.0
0.4
β_IV

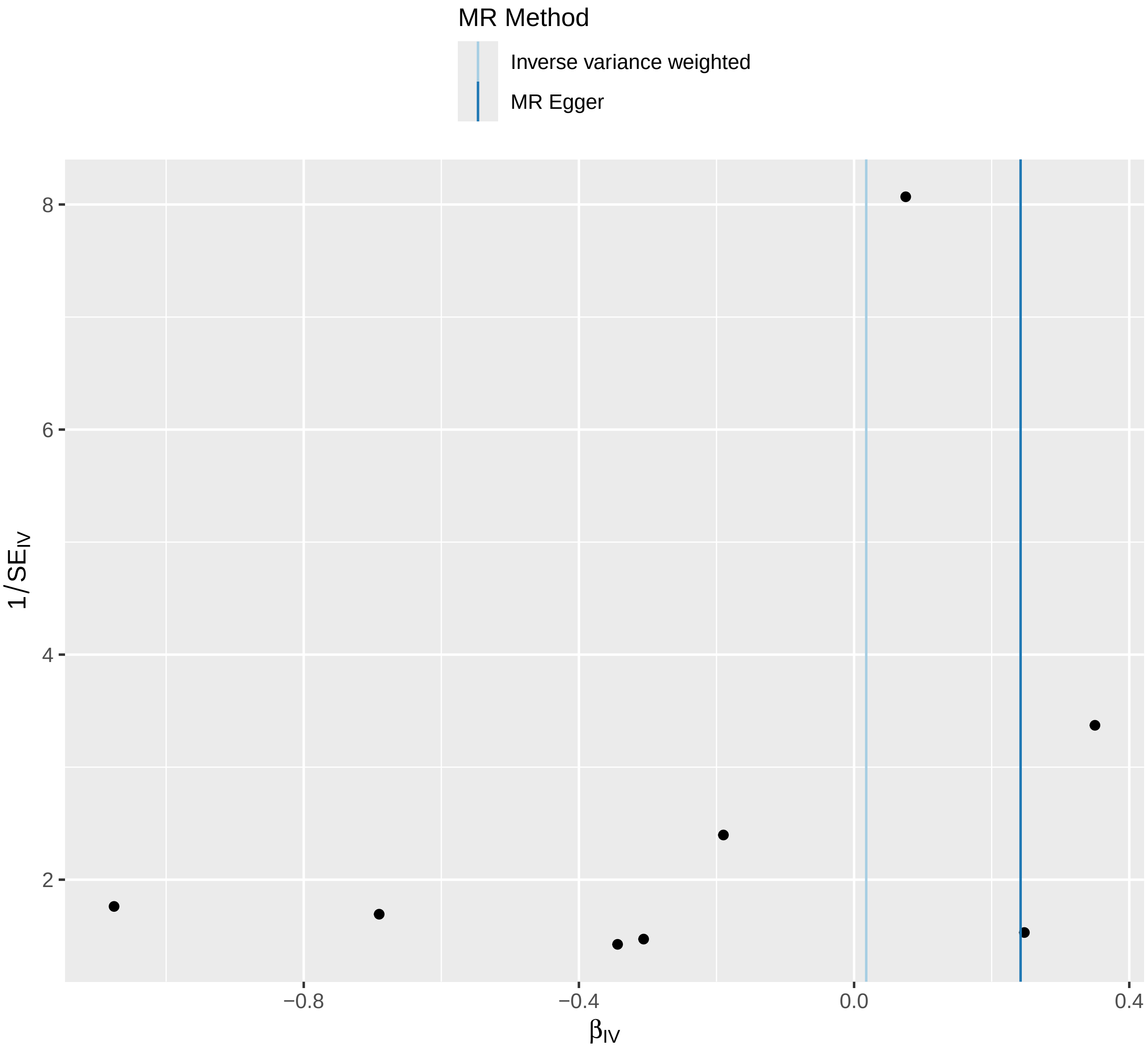
MR Method
Inverse variance weighted
MR Egger
8
6
4
2
1/SE_IV
−0.8
−0.4
0.0
0.4
β_IV

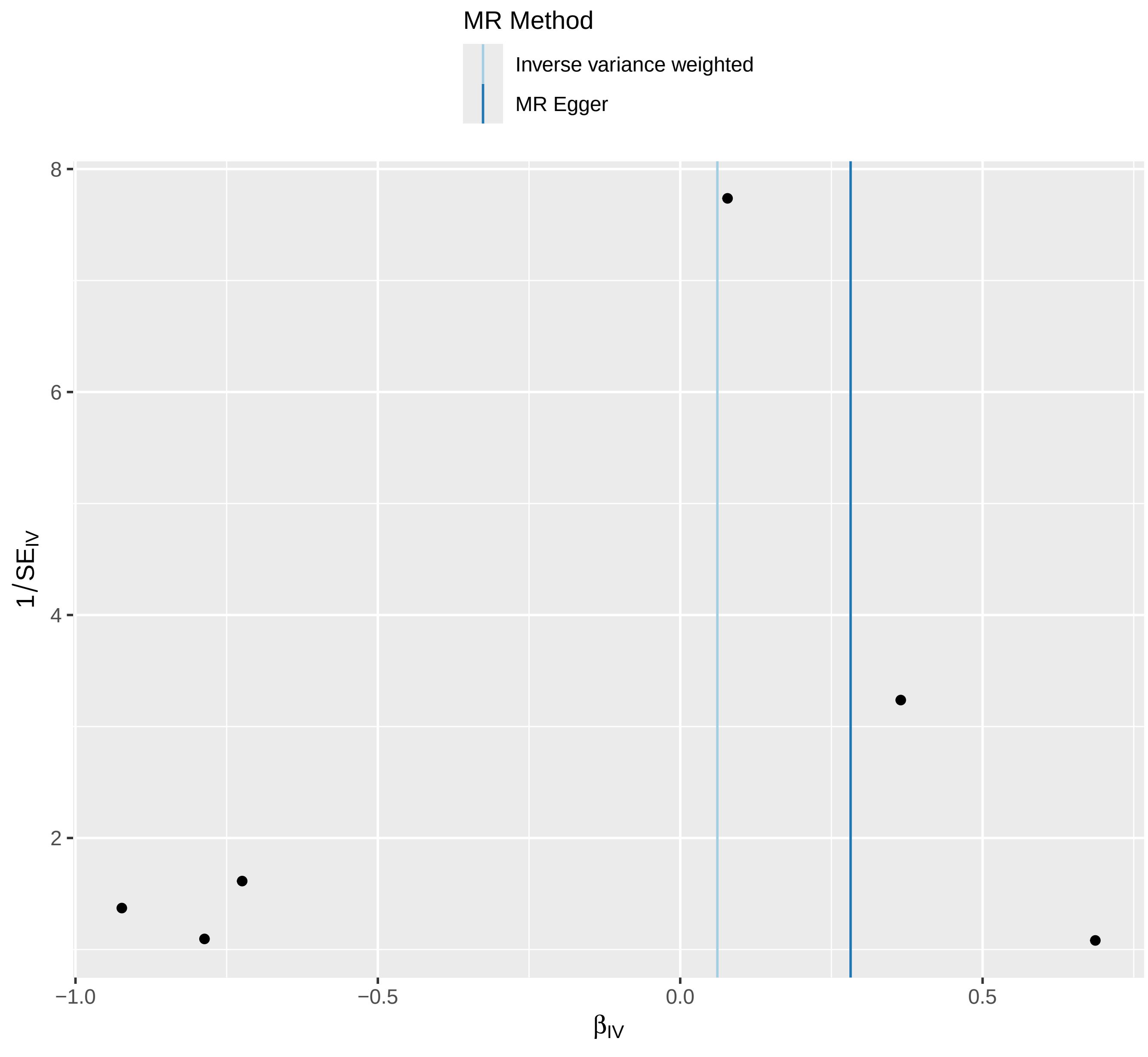

MR Method
Inverse variance weighted
MR Egger
8
6
4
2
1/SE$_{IV}$
−1.0
−0.5
0.0
0.5
β$_{IV}$

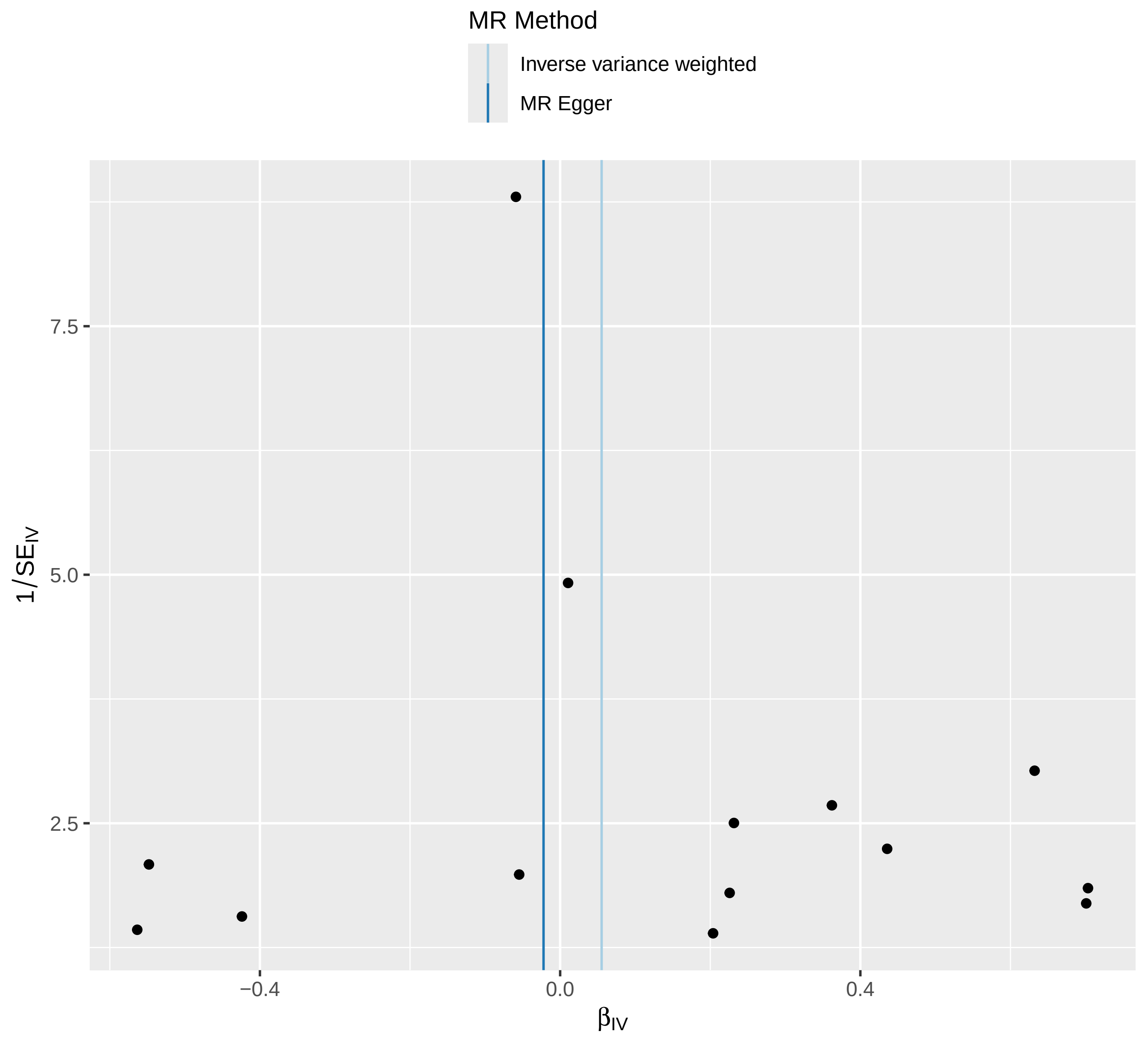
MR Method
Inverse variance weighted
MR Egger
7.5
5.0
2.5
1/SE$_{IV}$
−0.4
0.0
0.4
β$_{IV}$

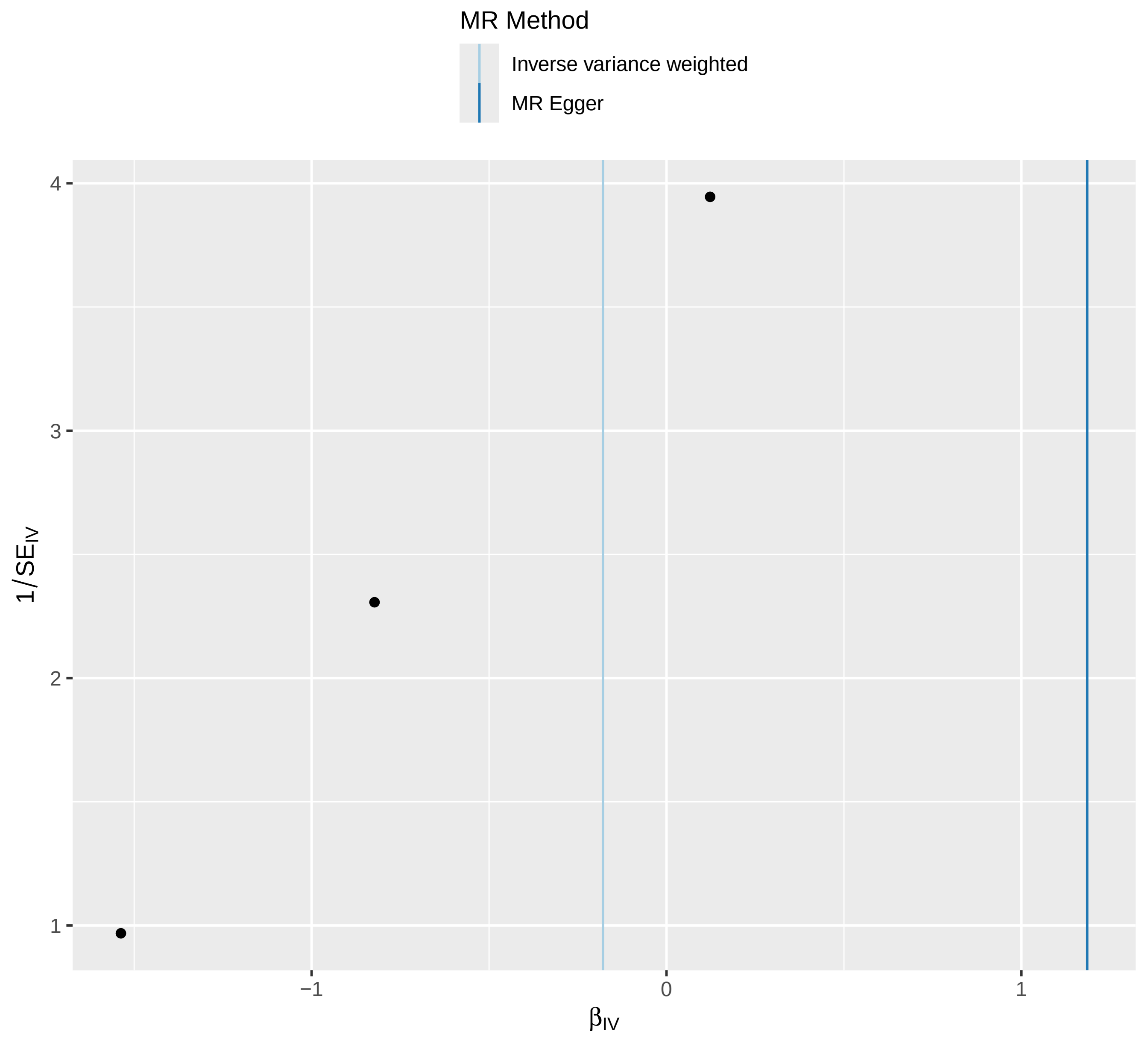
MR Method
Inverse variance weighted
MR Egger
4
3
2
1
1/SE_IV
−1
0
1
β_IV

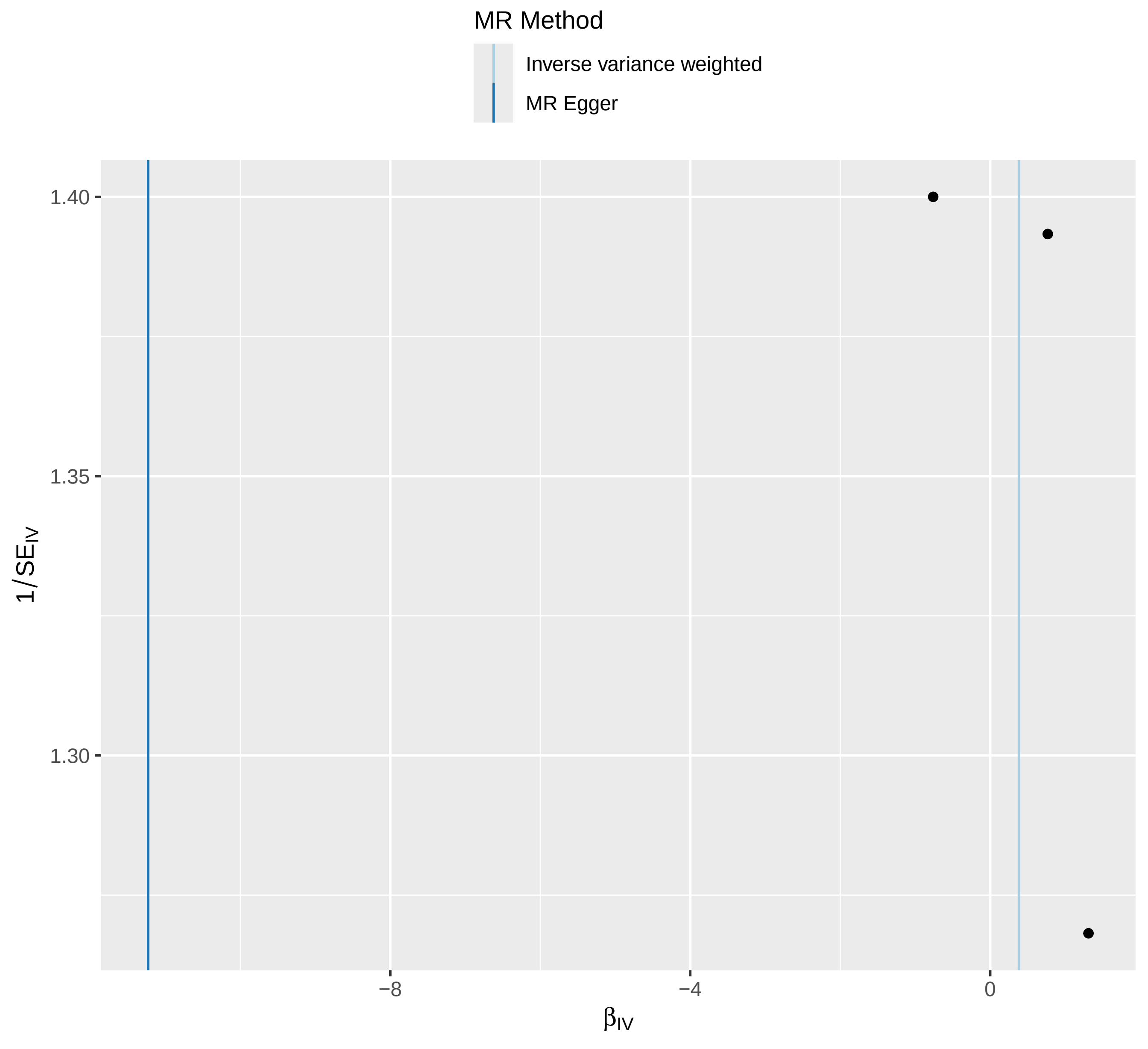
MR Method
Inverse variance weighted
MR Egger
1.40
1.35
1.30
1/SE_IV
−8
−4
0
β_IV

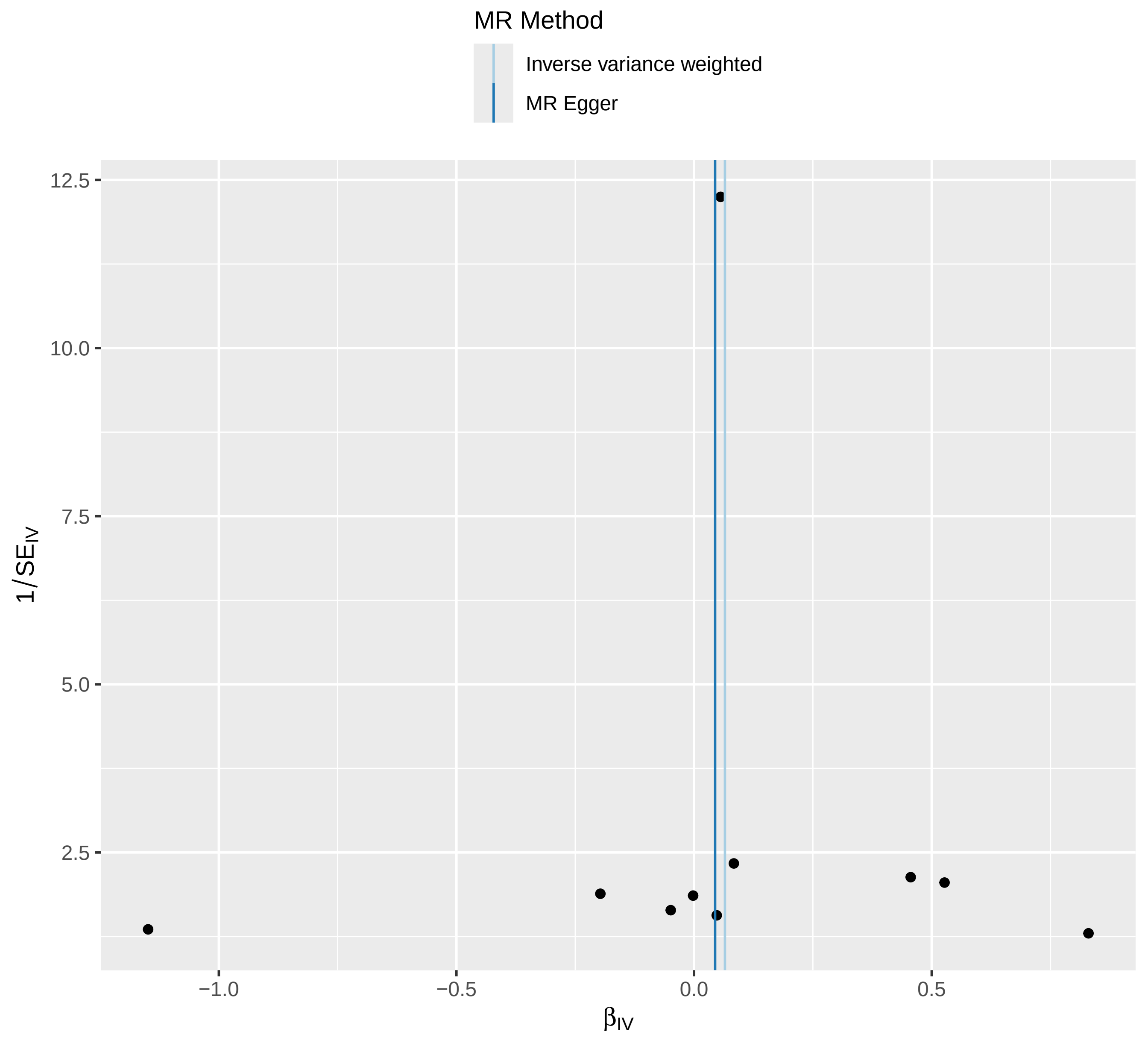

MR Method
Inverse variance weighted
MR Egger
12.5
10.0
7.5
5.0
2.5
−1.0
−0.5
0.0
0.5
$1/SE_{IV}$
β_{IV}

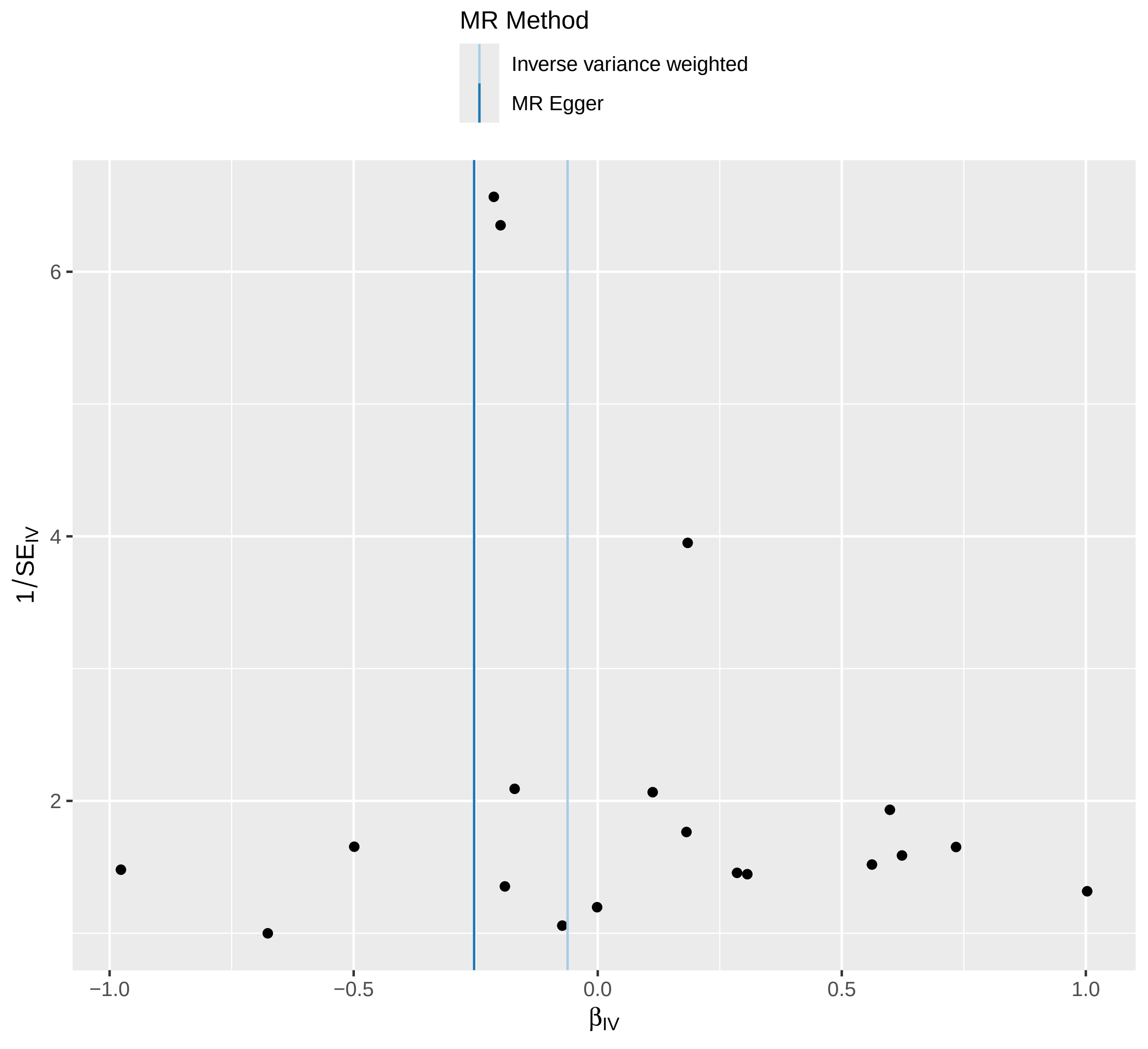

MR Method
Inverse variance weighted
MR Egger
6
4
2
1/SE$_{IV}$
−1.0
−0.5
0.0
0.5
1.0
β$_{IV}$

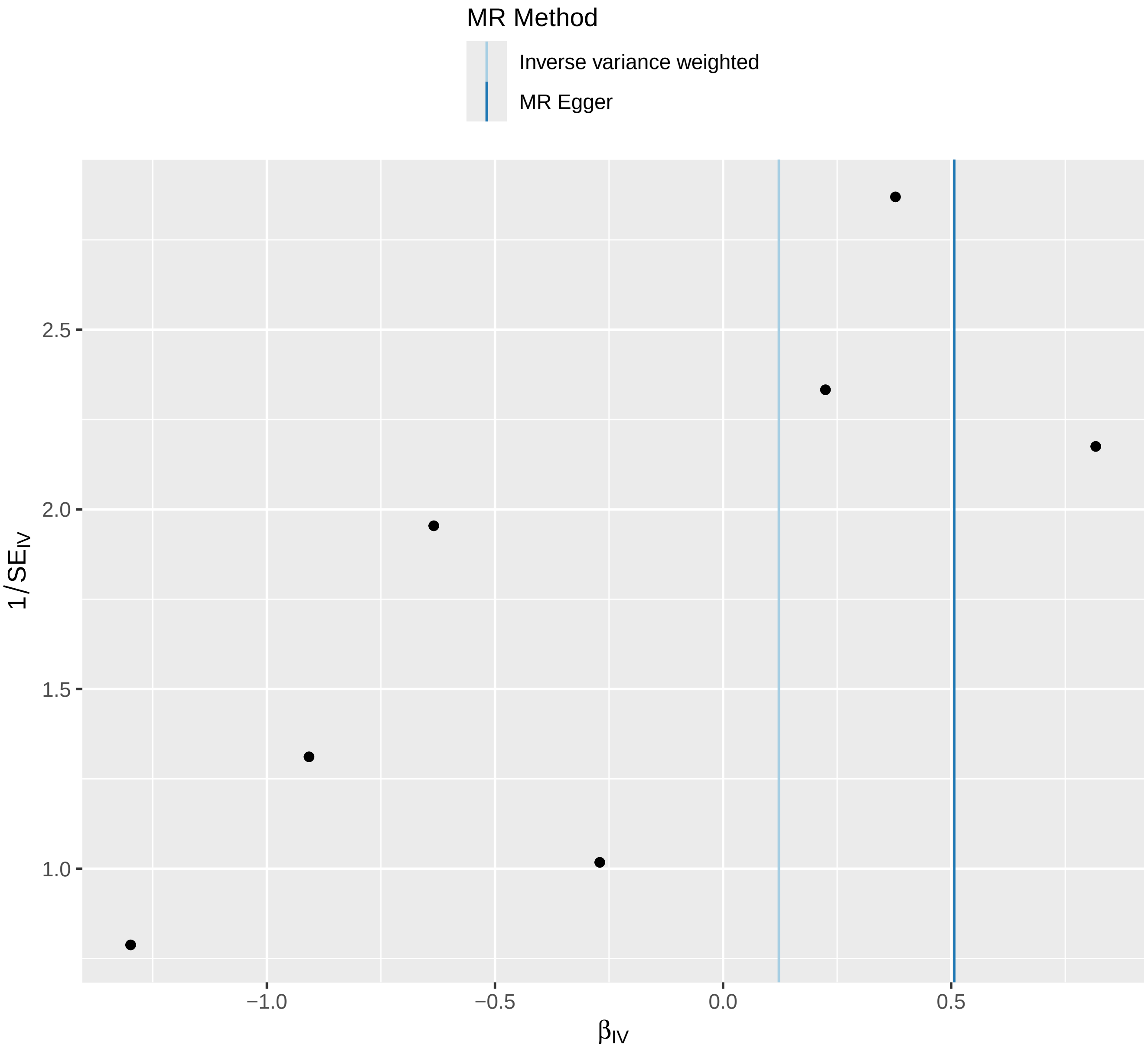
MR Method
Inverse variance weighted
MR Egger
2.5
2.0
1.5
1.0
1/SE$_{IV}$
−1.0
−0.5
0.0
0.5
β$_{IV}$

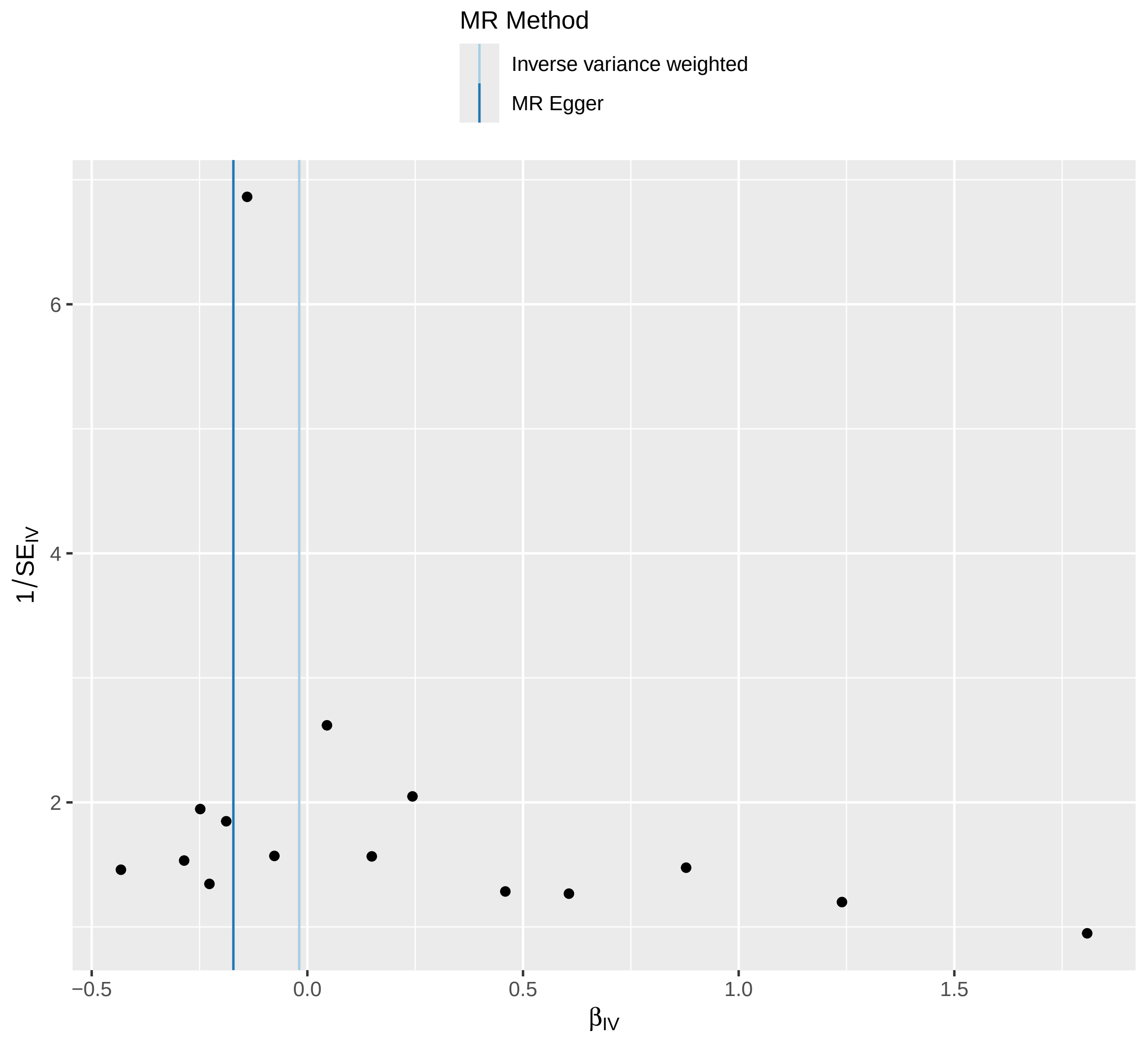

MR Method
Inverse variance weighted
MR Egger
6
4
2
1/SE$_{IV}$
−0.5
0.0
0.5
1.0
1.5
β$_{IV}$

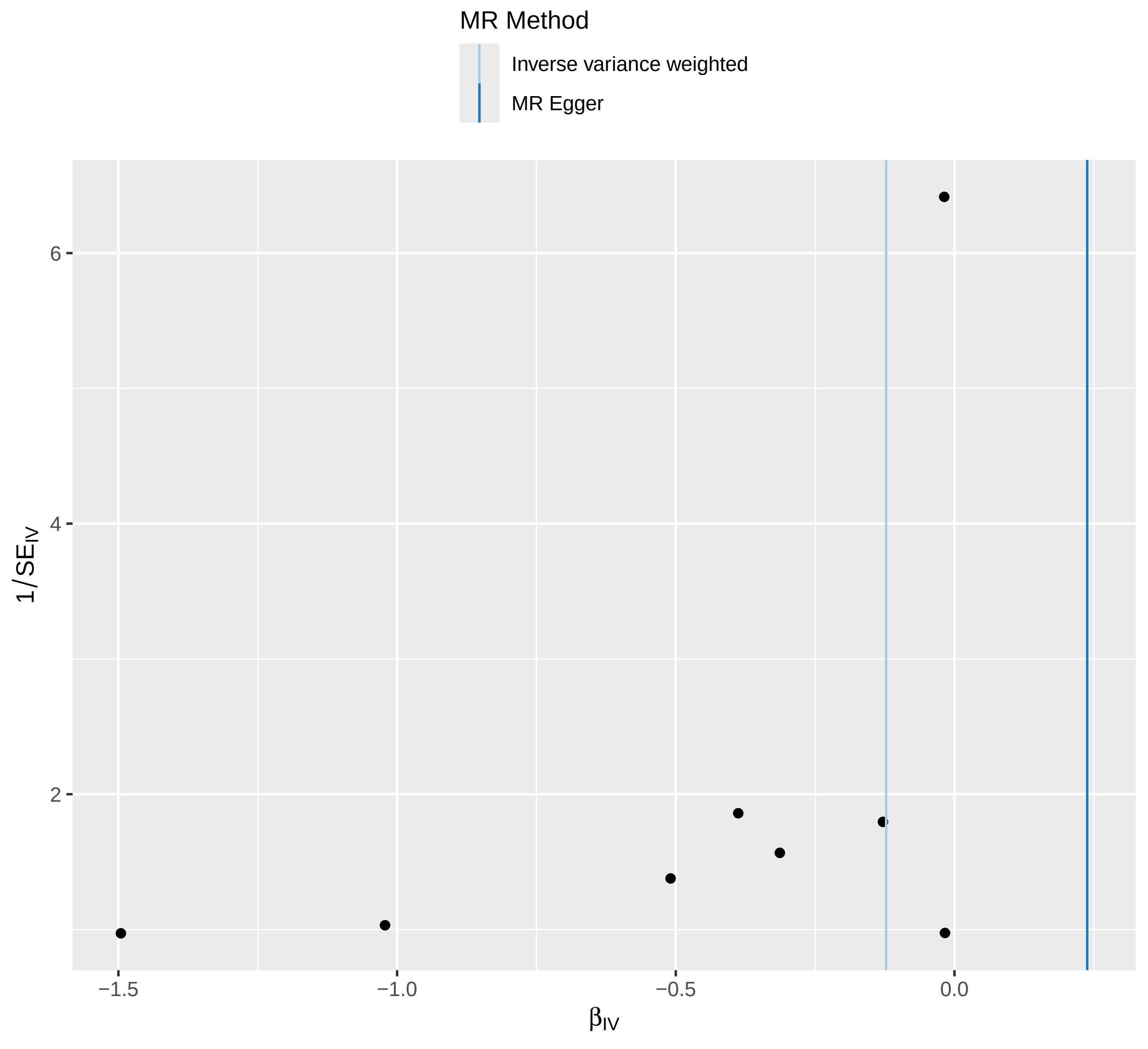
MR Method
Inverse variance weighted
MR Egger
6
4
2
1/SE$_{IV}$
−1.5
−1.0
−0.5
0.0
β$_{IV}$

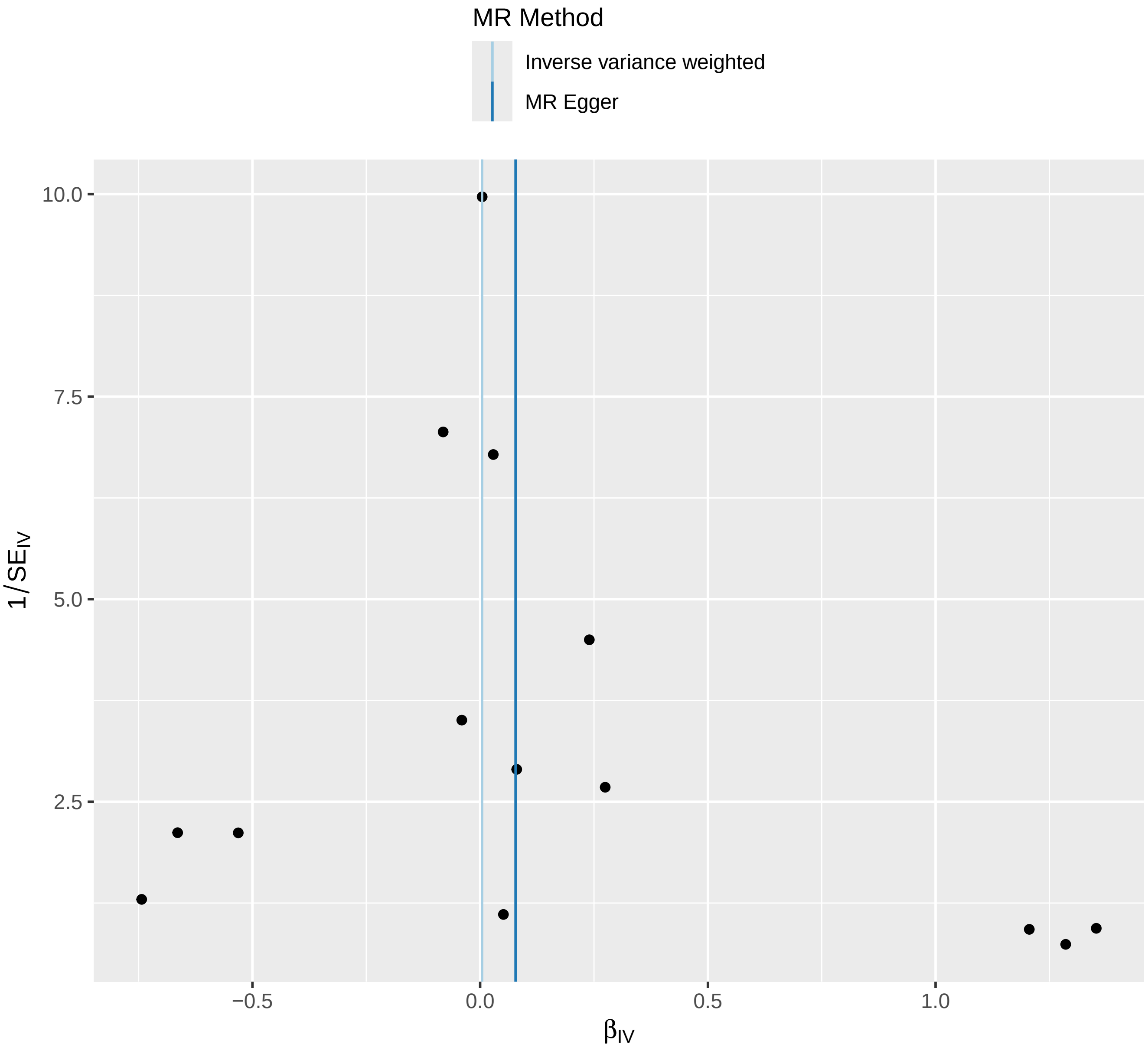

MR Method
Inverse variance weighted
MR Egger
10.0
7.5
5.0
2.5
−0.5
0.0
0.5
1.0
$1/SE_{IV}$
β_{IV}

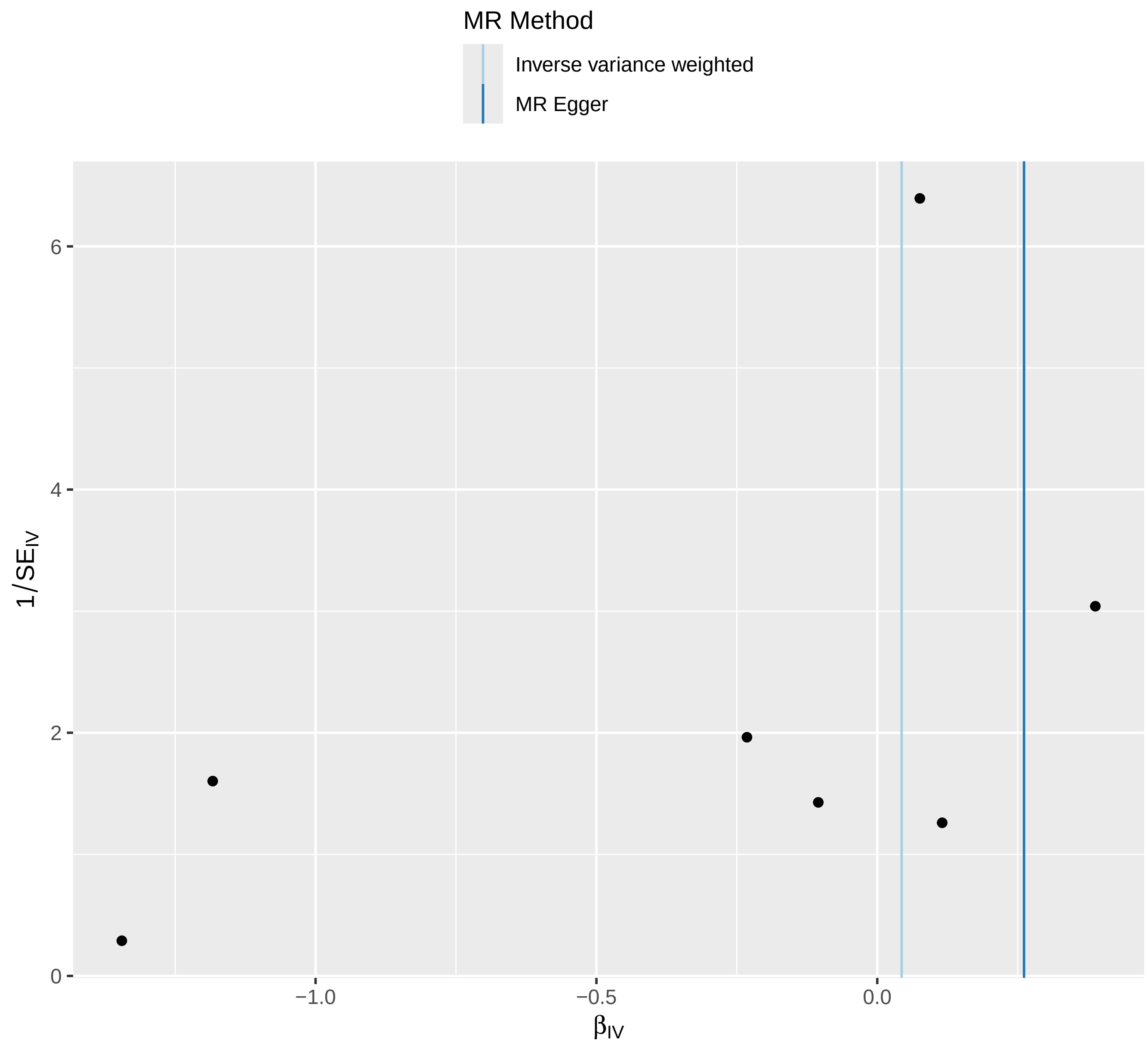

MR Method
Inverse variance weighted
MR Egger
6
4
2
0
−1.0
−0.5
0.0
$1/SE_{IV}$
β_{IV}

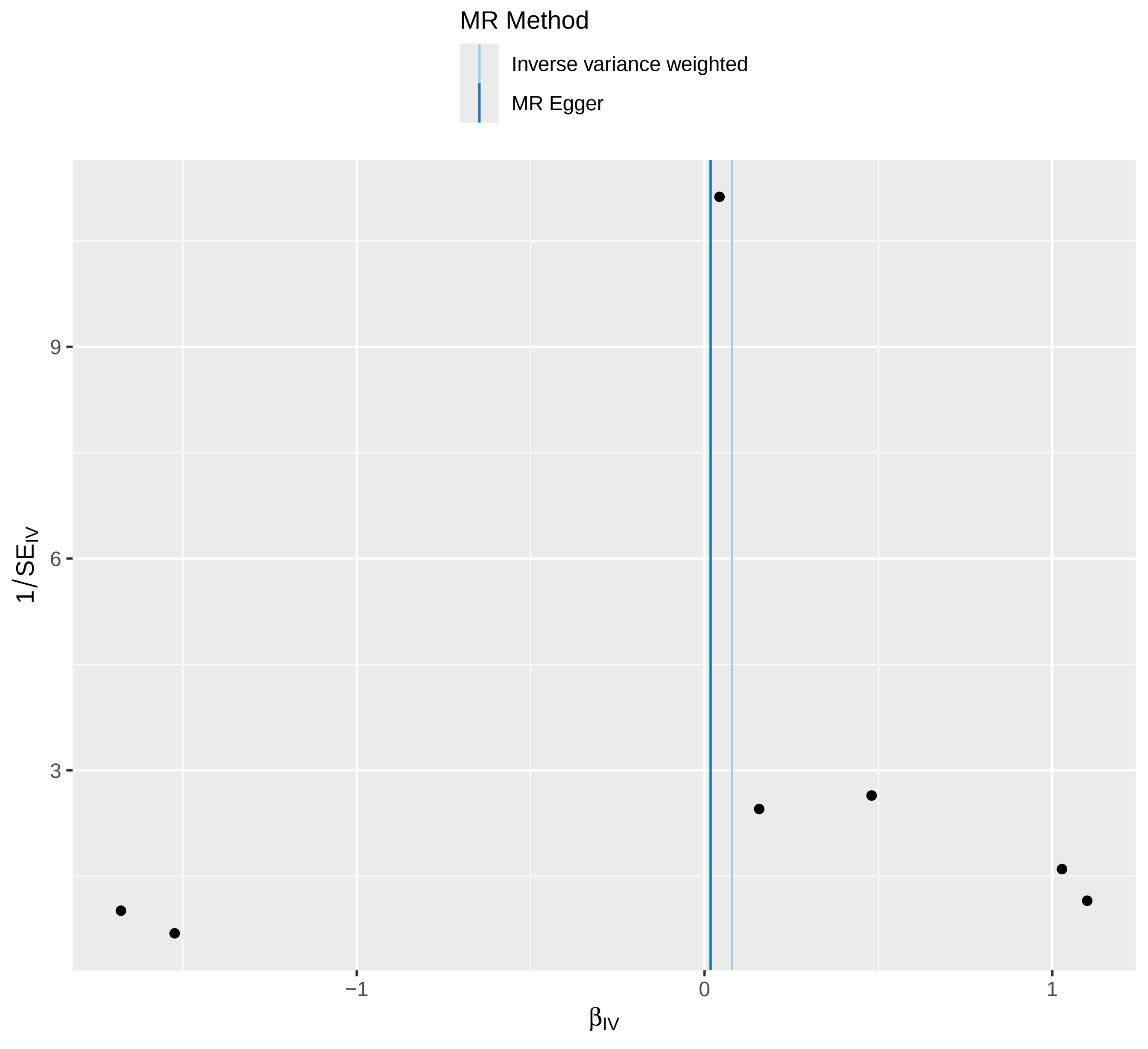
MR Method
Inverse variance weighted
MR Egger
1/SE_IV
9
6
3
β_IV
−1
0
1

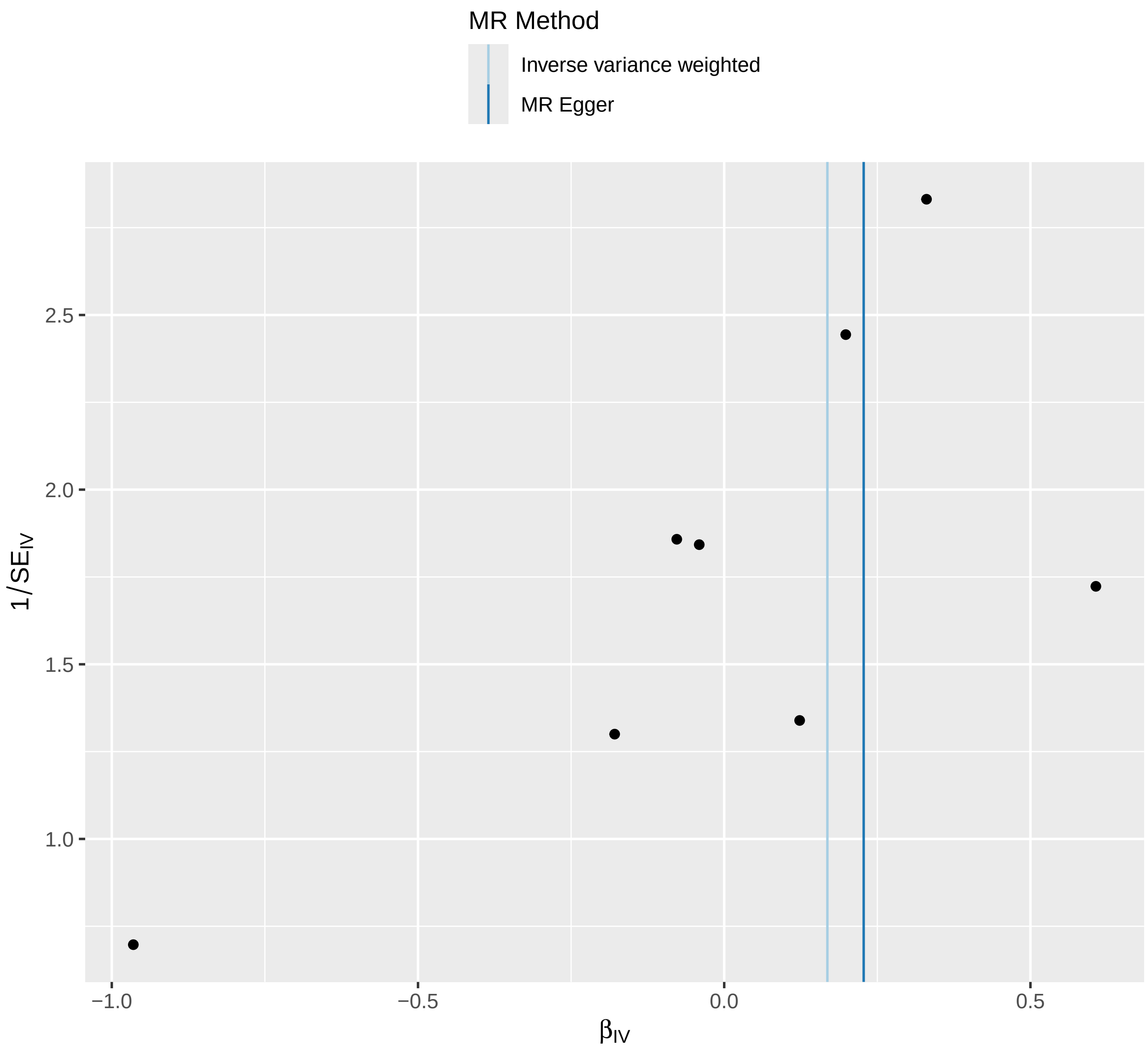
MR Method
Inverse variance weighted
MR Egger
2.5
2.0
1.5
1.0
1/SE_IV
−1.0
−0.5
0.0
0.5
β_IV

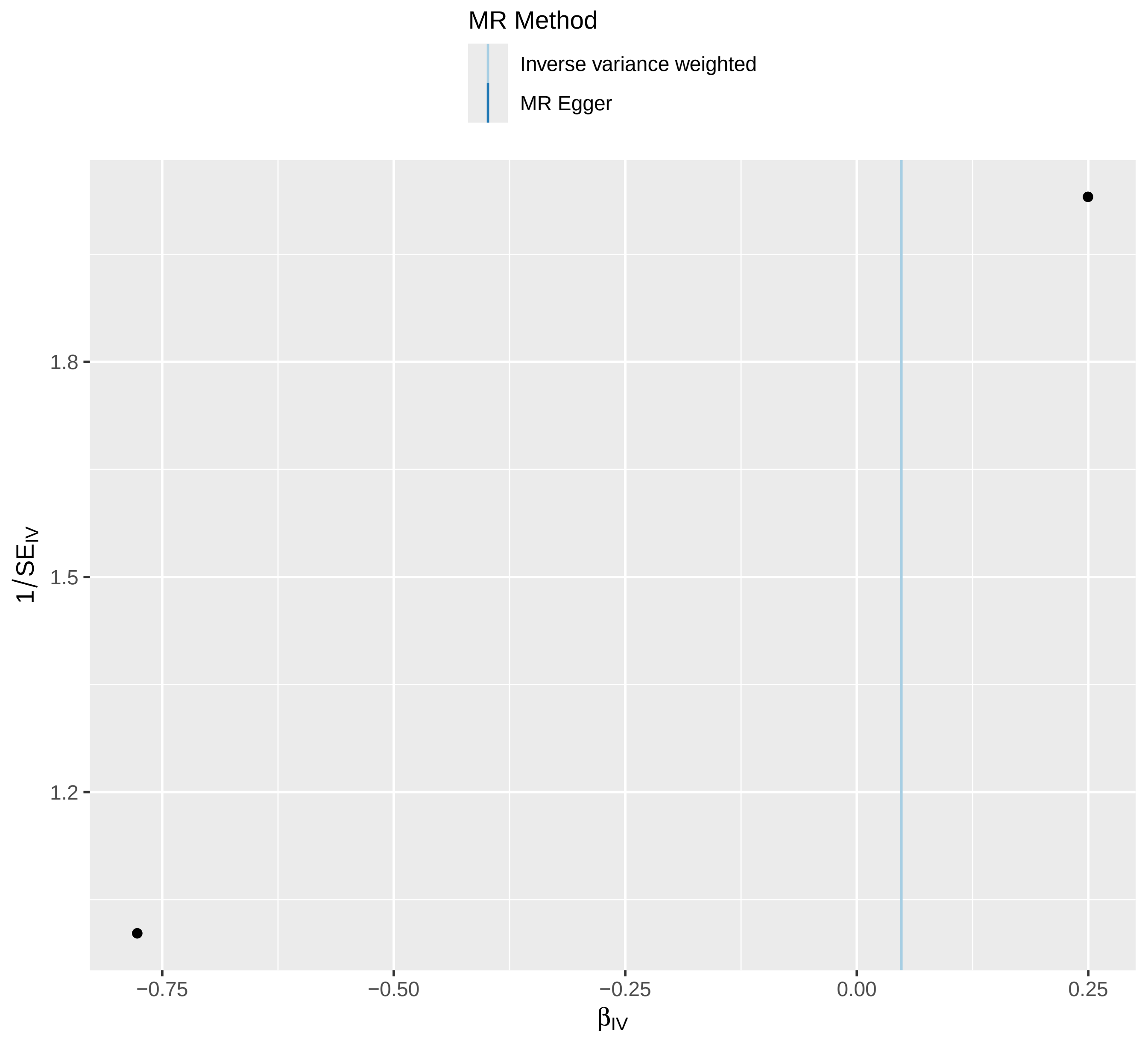
MR Method
Inverse variance weighted
MR Egger
1.8
1.5
1.2
1/SE_IV
−0.75
−0.50
−0.25
0.00
0.25
β_IV

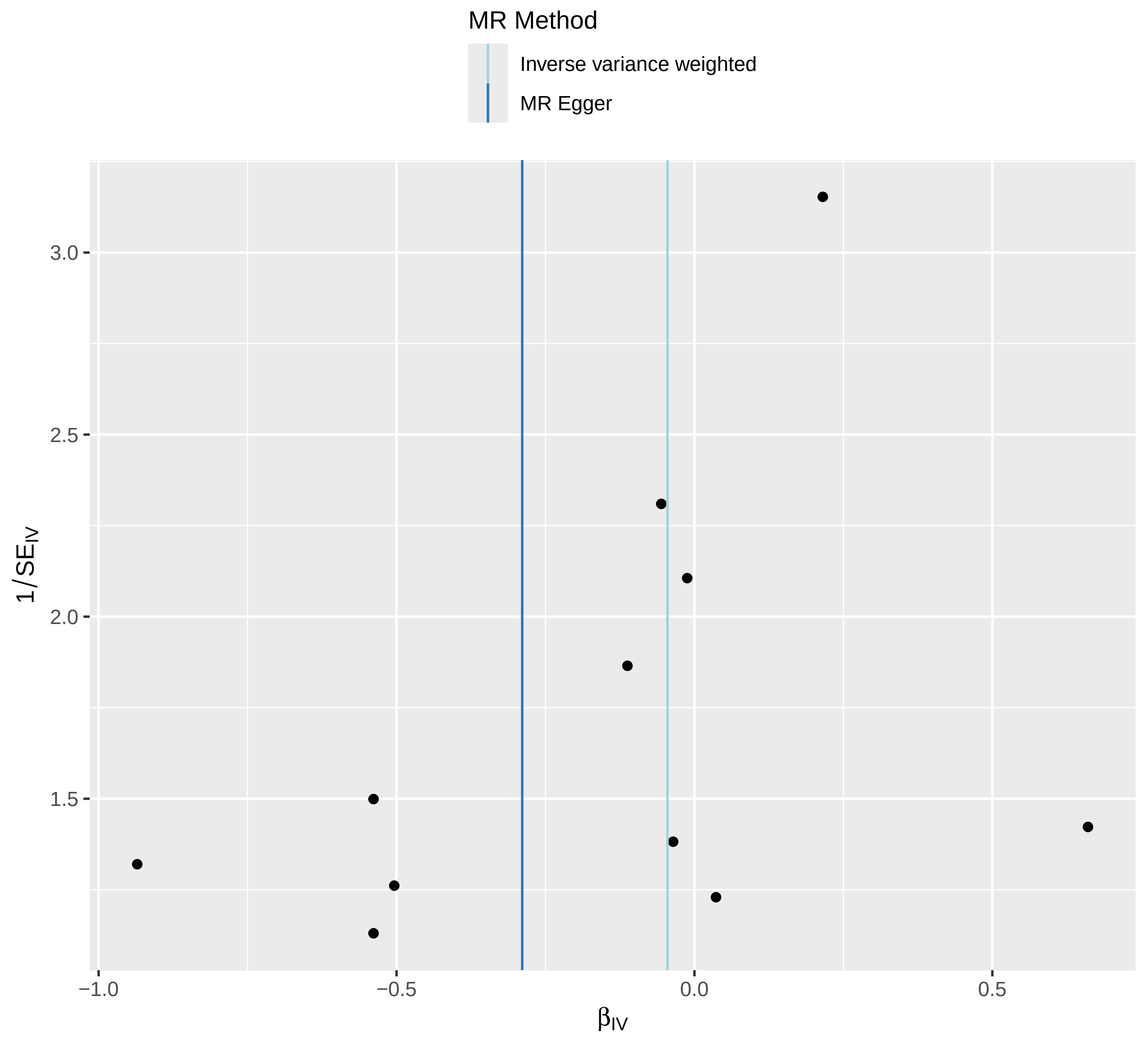
MR Method
Inverse variance weighted
MR Egger
3.0
2.5
2.0
1.5
1/SE_IV
−1.0
−0.5
0.0
0.5
β_IV

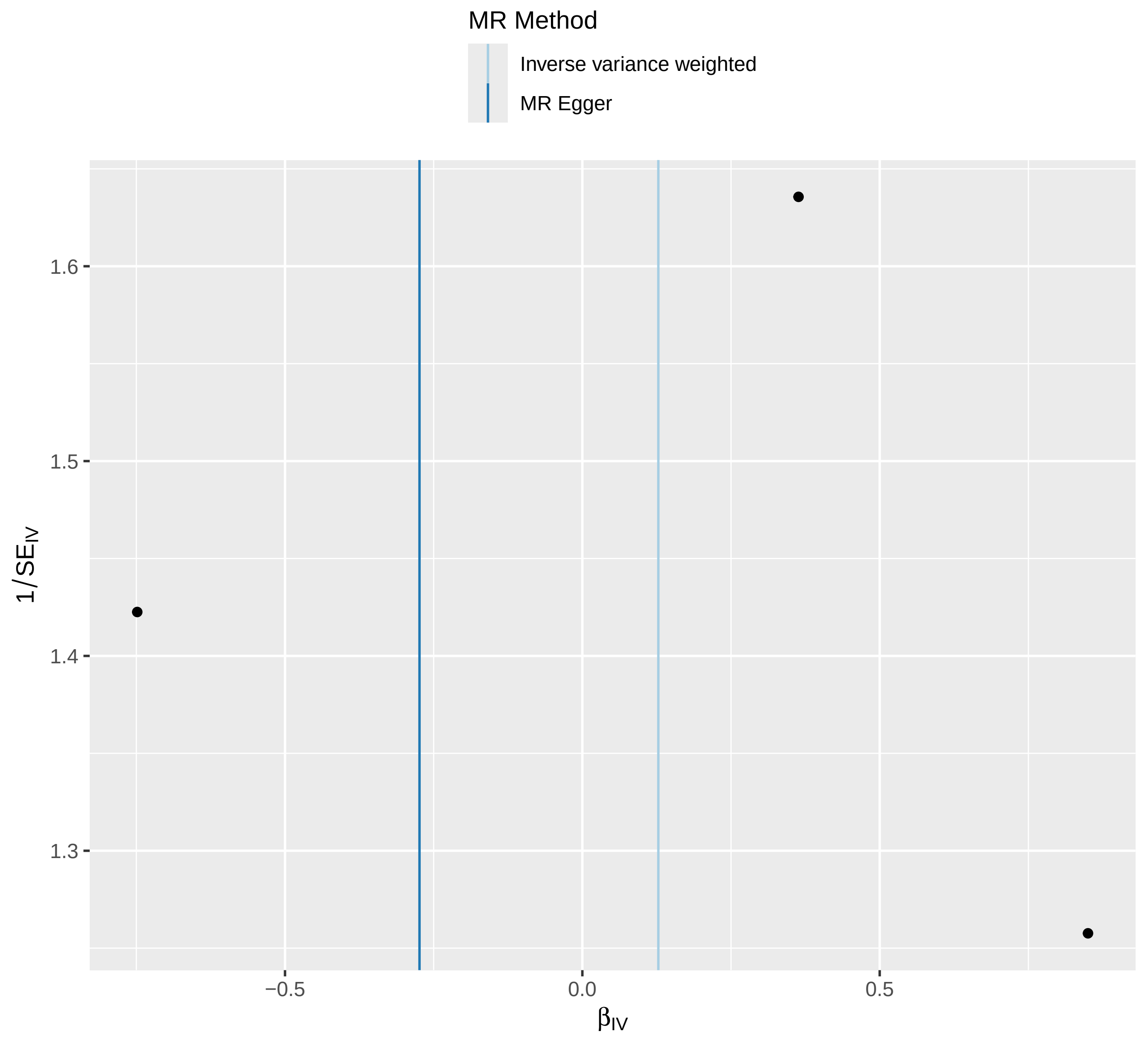
MR Method
Inverse variance weighted
MR Egger
1.6
1.5
1.4
1.3
−0.5
0.0
0.5
$1/SE_{IV}$
β_{IV}

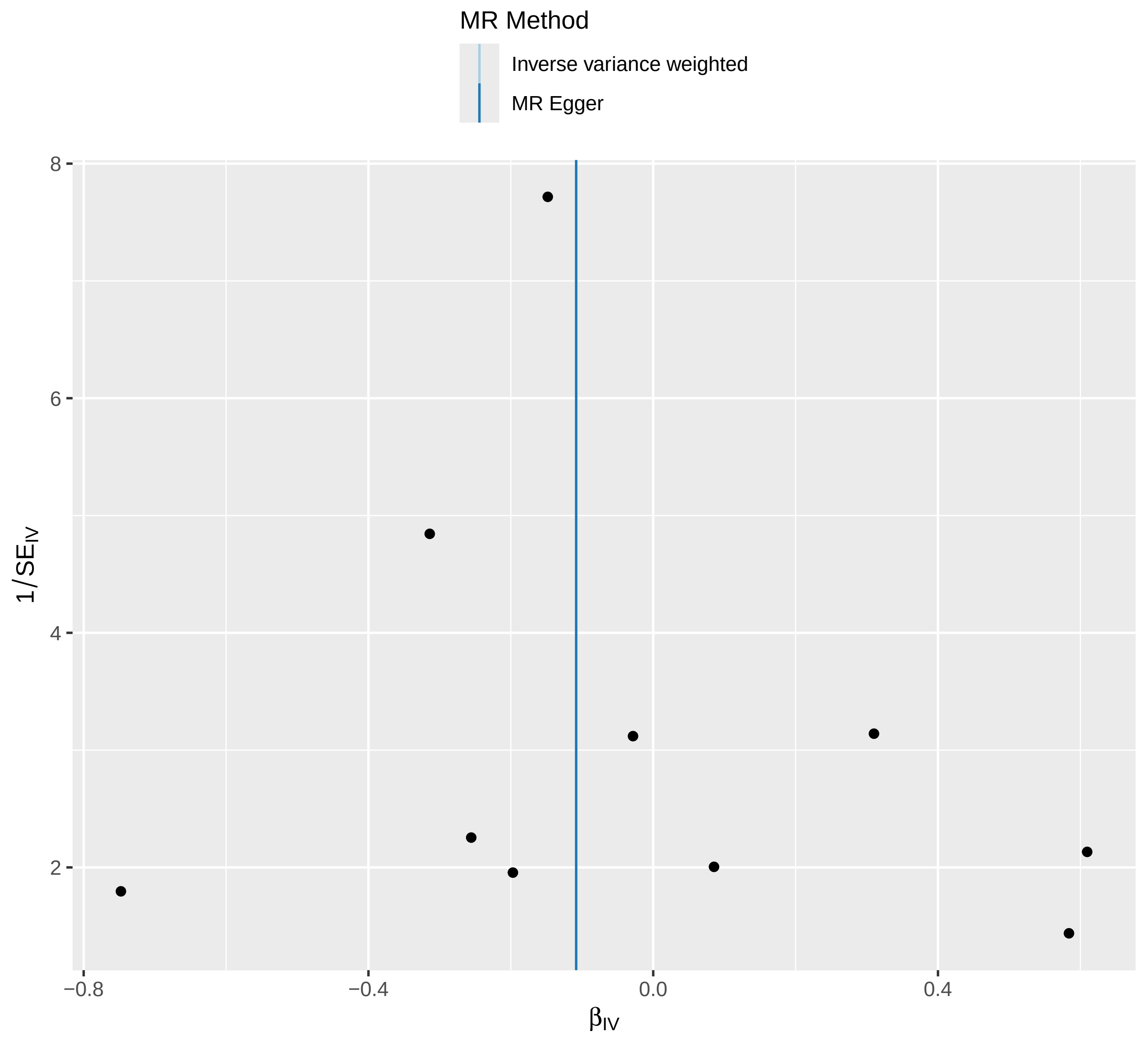
MR Method
Inverse variance weighted
MR Egger
8
6
4
2
1/SE_IV
−0.8
−0.4
0.0
0.4
β_IV

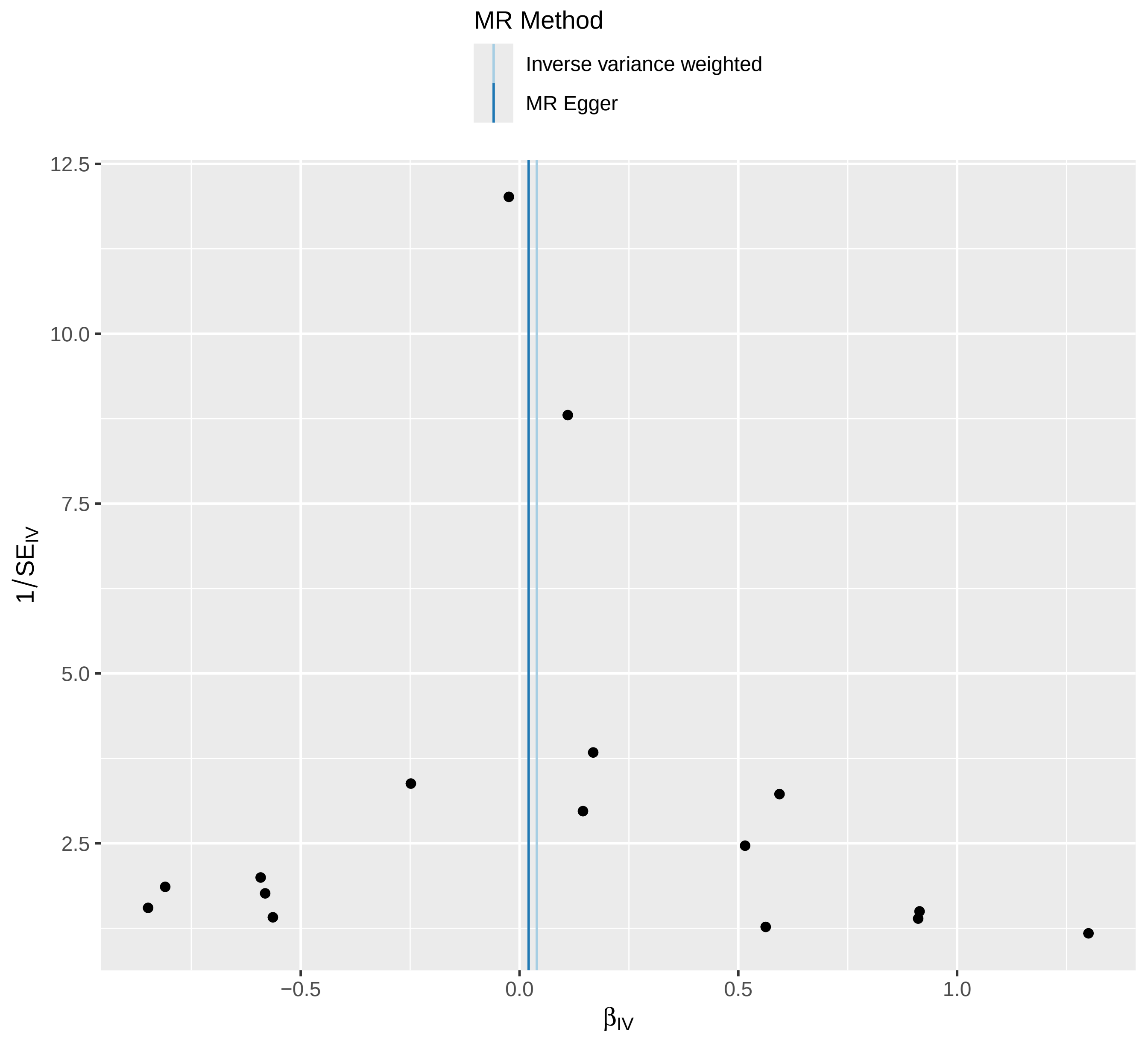
MR Method
Inverse variance weighted
MR Egger
12.5
10.0
7.5
5.0
2.5
1/SE$_{IV}$
−0.5
0.0
0.5
1.0
β$_{IV}$

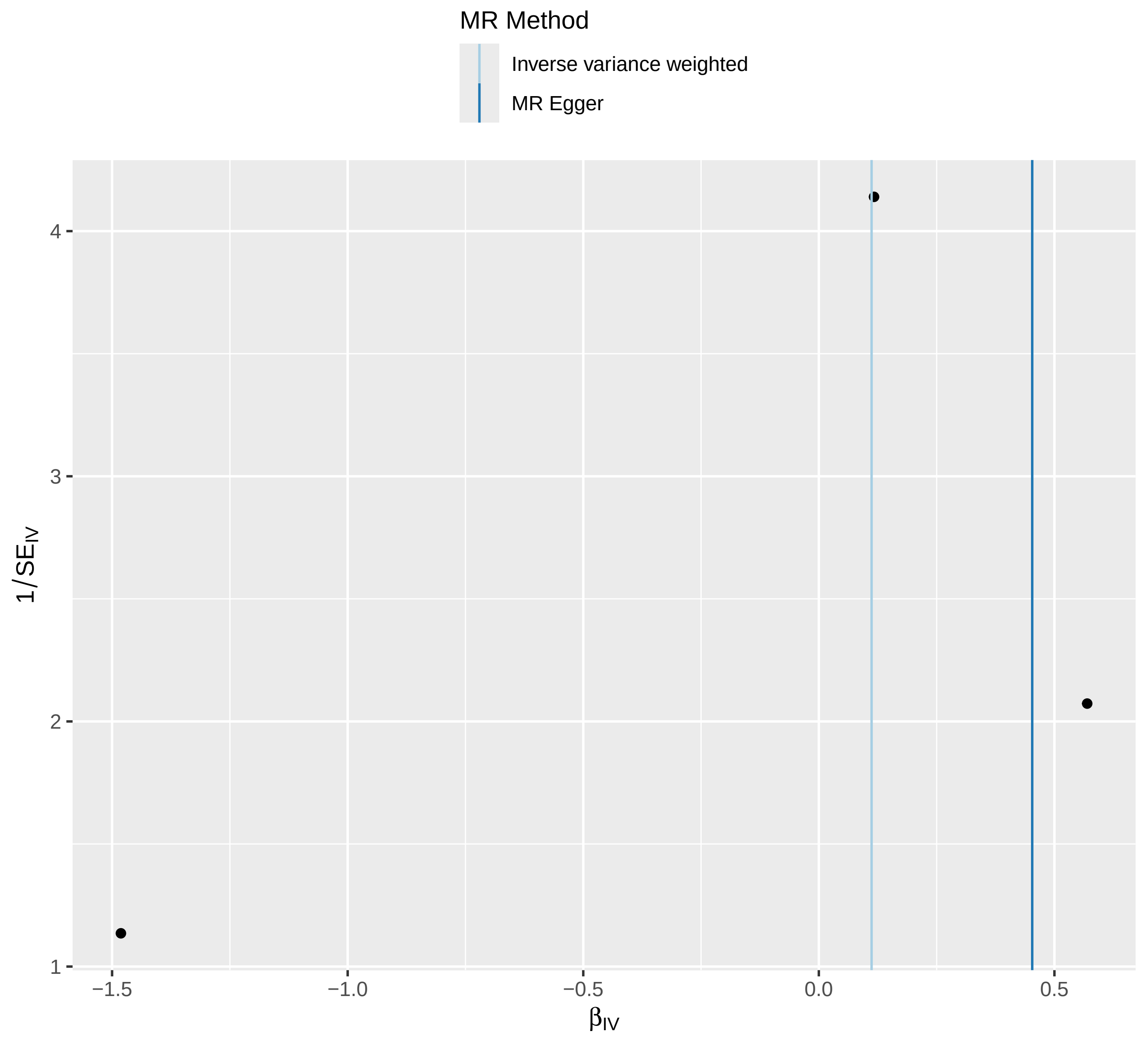
MR Method
Inverse variance weighted
MR Egger
1/SE_IV
4
3
2
1
−1.5
−1.0
−0.5
0.0
0.5
β_IV

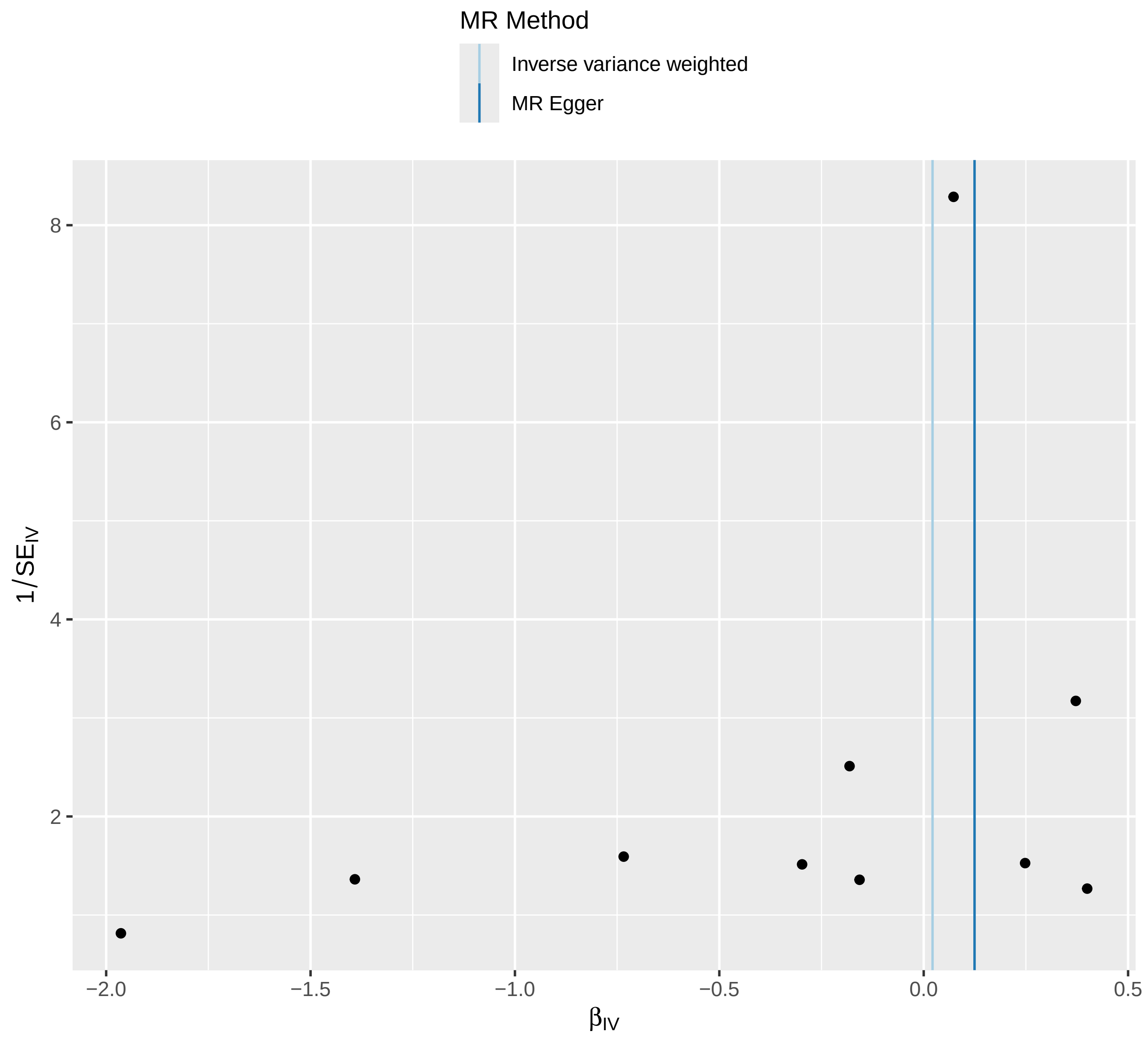

MR Method
Inverse variance weighted
MR Egger
8
6
4
2
1/SE$_{IV}$
−2.0
−1.5
−1.0
−0.5
0.0
0.5
β$_{IV}$

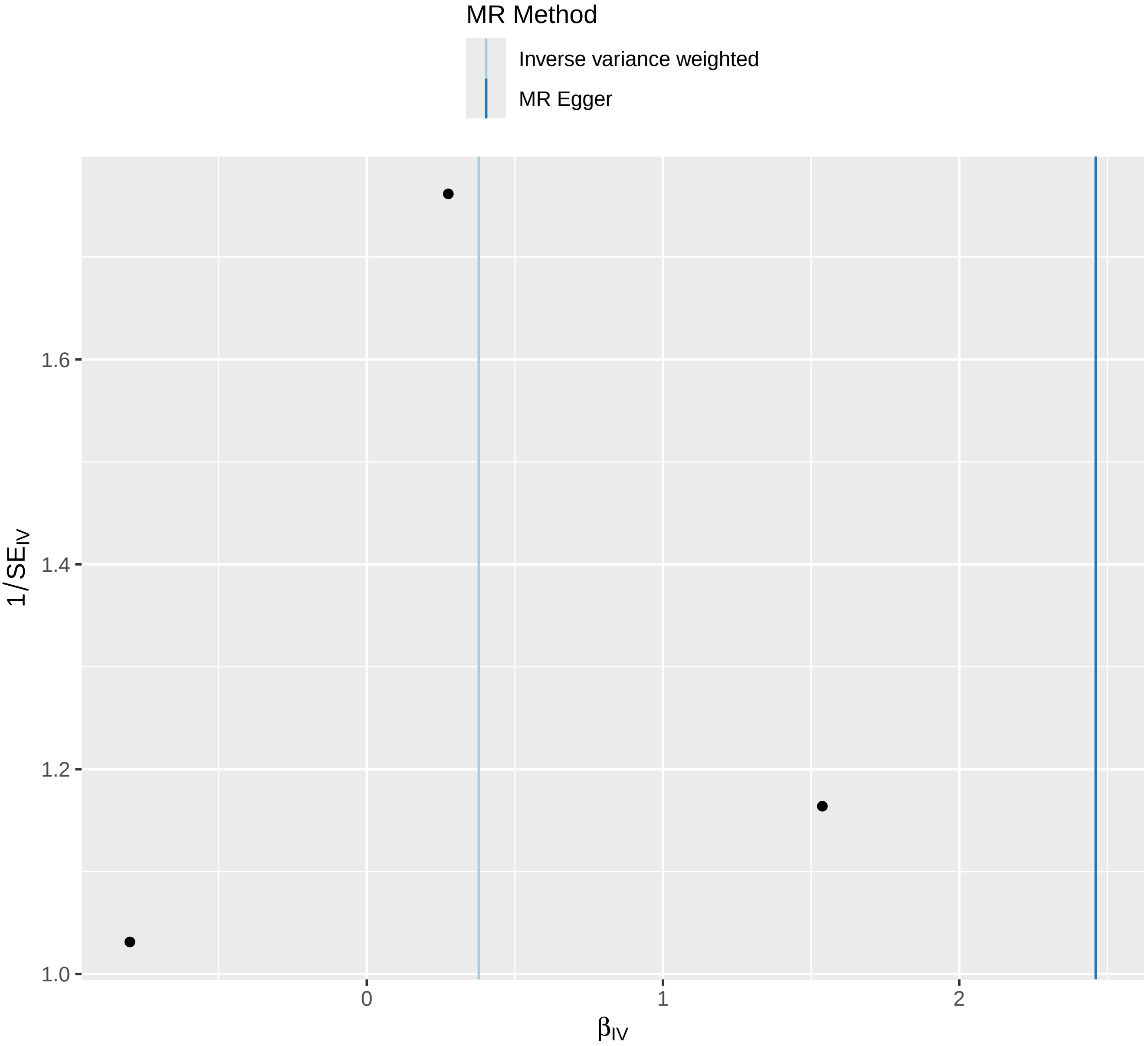

MR Method
Inverse variance weighted
MR Egger
1.0
1.2
1.4
1.6
0
1
2
1/SE_IV
β_IV

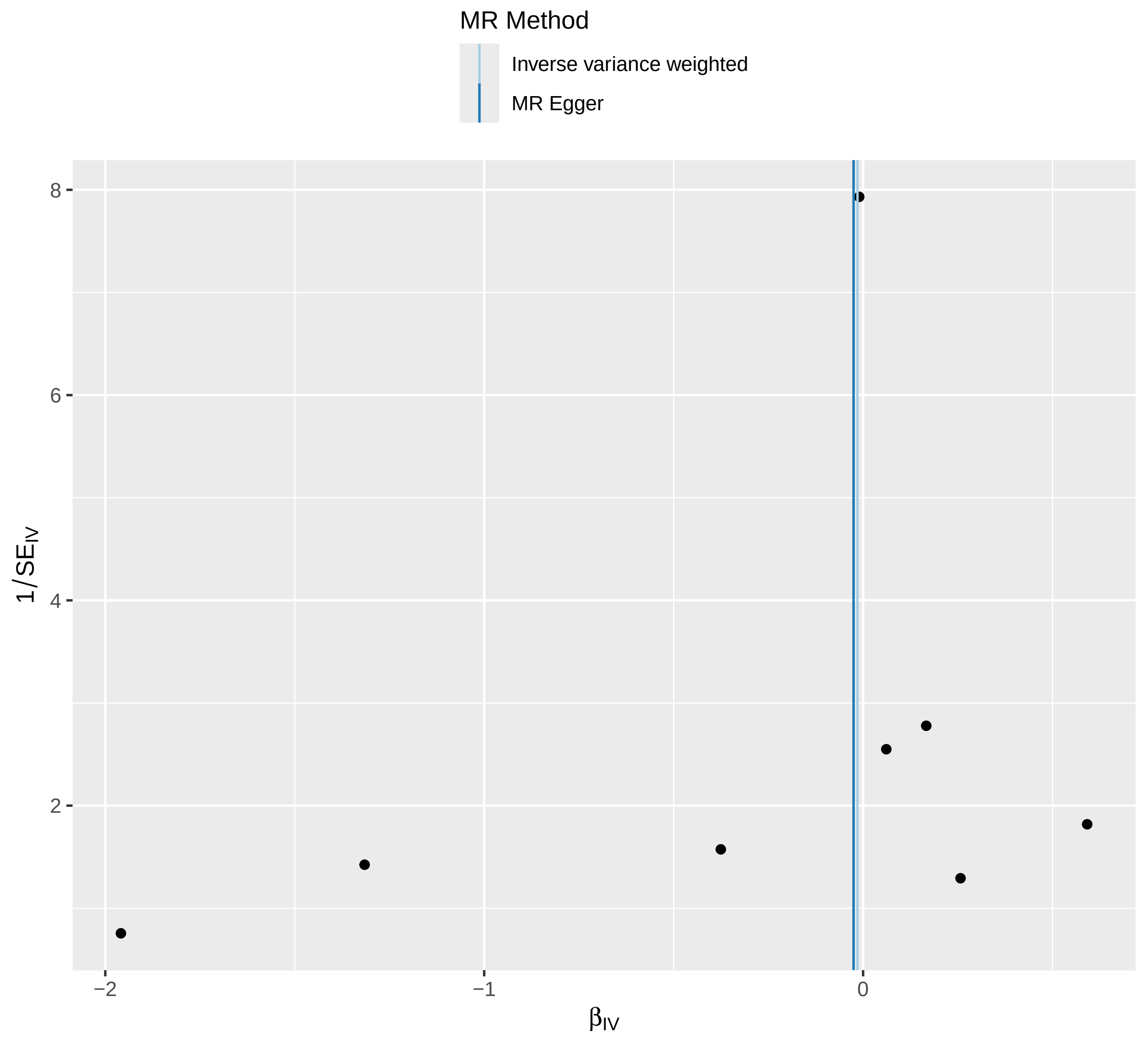

MR Method
Inverse variance weighted
MR Egger
8
6
4
2
1/SE_IV
−2
−1
0
β_IV

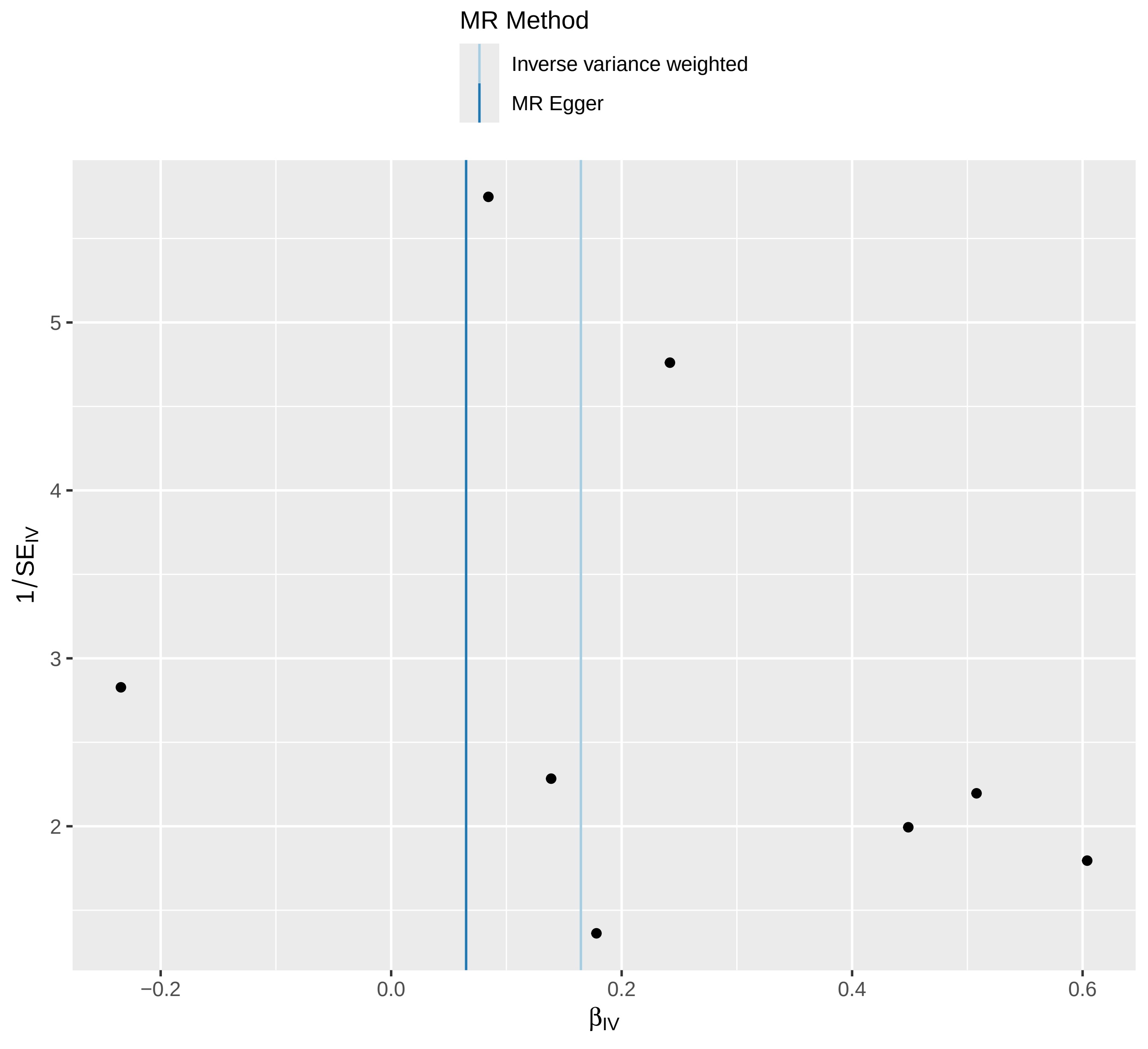
MR Method
Inverse variance weighted
MR Egger
5
4
3
2
1/SE$_{IV}$
−0.2
0.0
0.2
0.4
0.6
β$_{IV}$

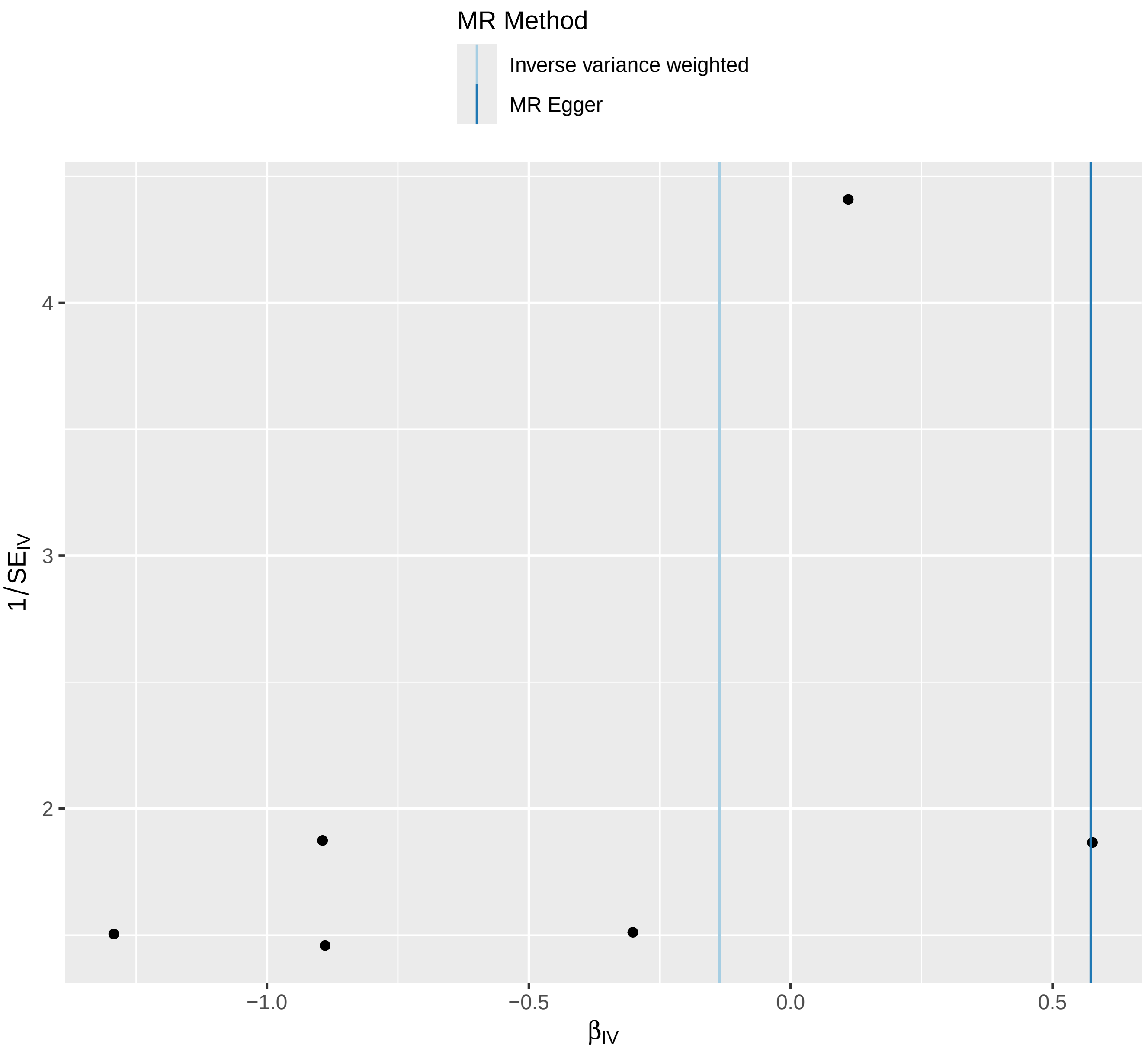

MR Method
Inverse variance weighted
MR Egger
4
3
2
1/SE_IV
−1.0
−0.5
0.0
0.5
β_IV

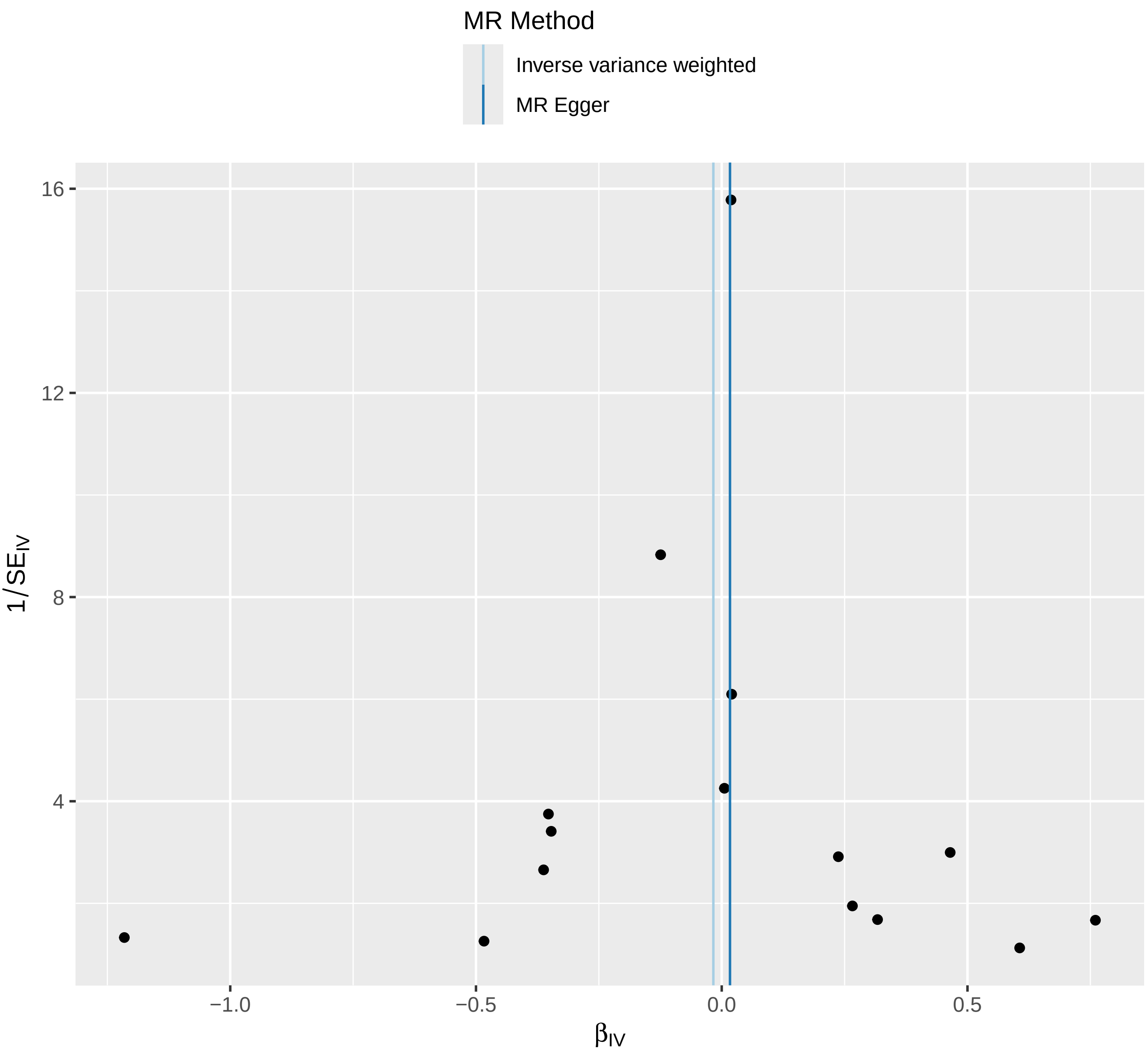
MR Method
Inverse variance weighted
MR Egger
16
12
8
4
1/SE_IV
−1.0
−0.5
0.0
0.5
β_IV

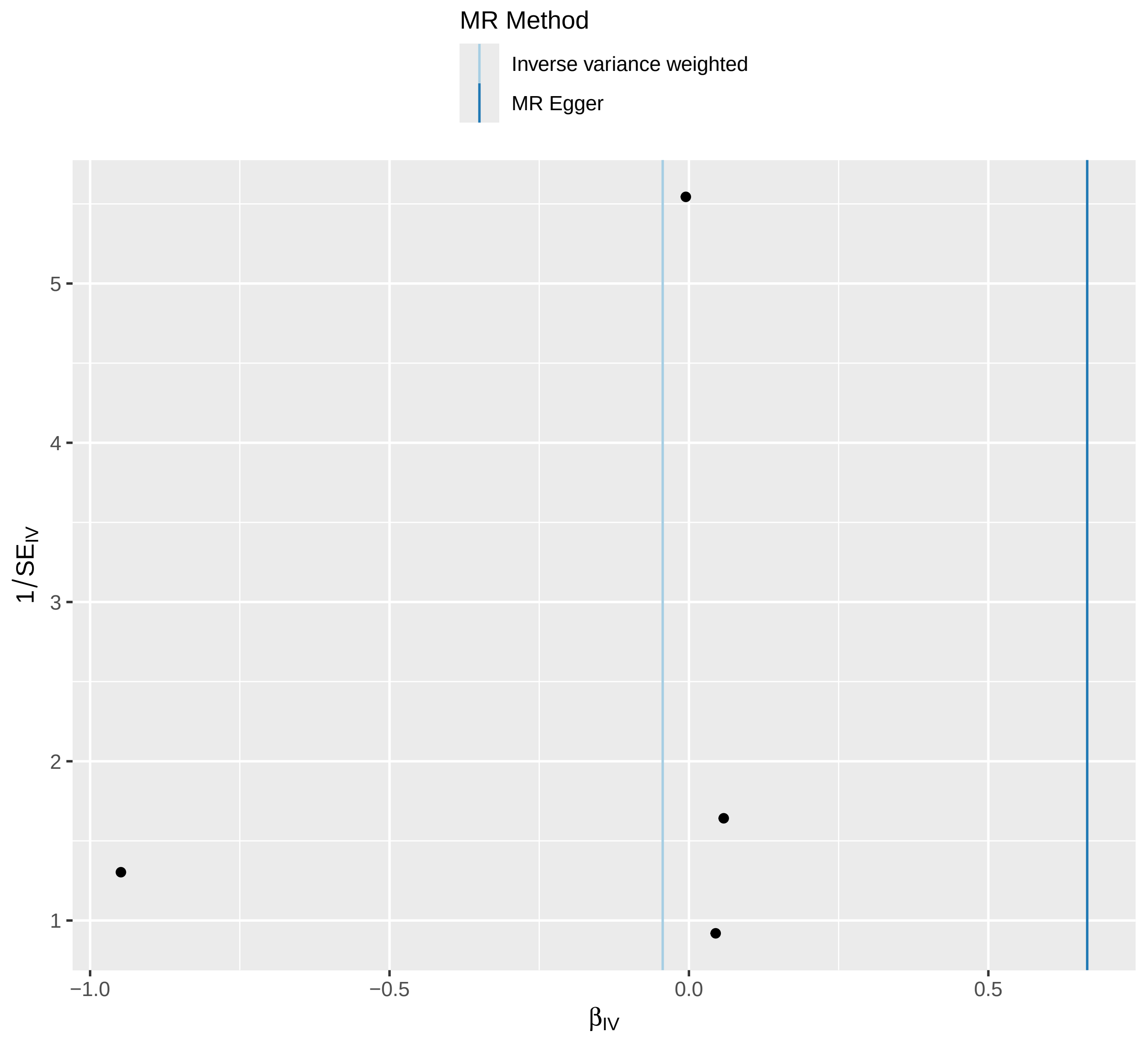

MR Method
Inverse variance weighted
MR Egger
1/SE_IV
β_IV
5
4
3
2
1
−1.0
−0.5
0.0
0.5

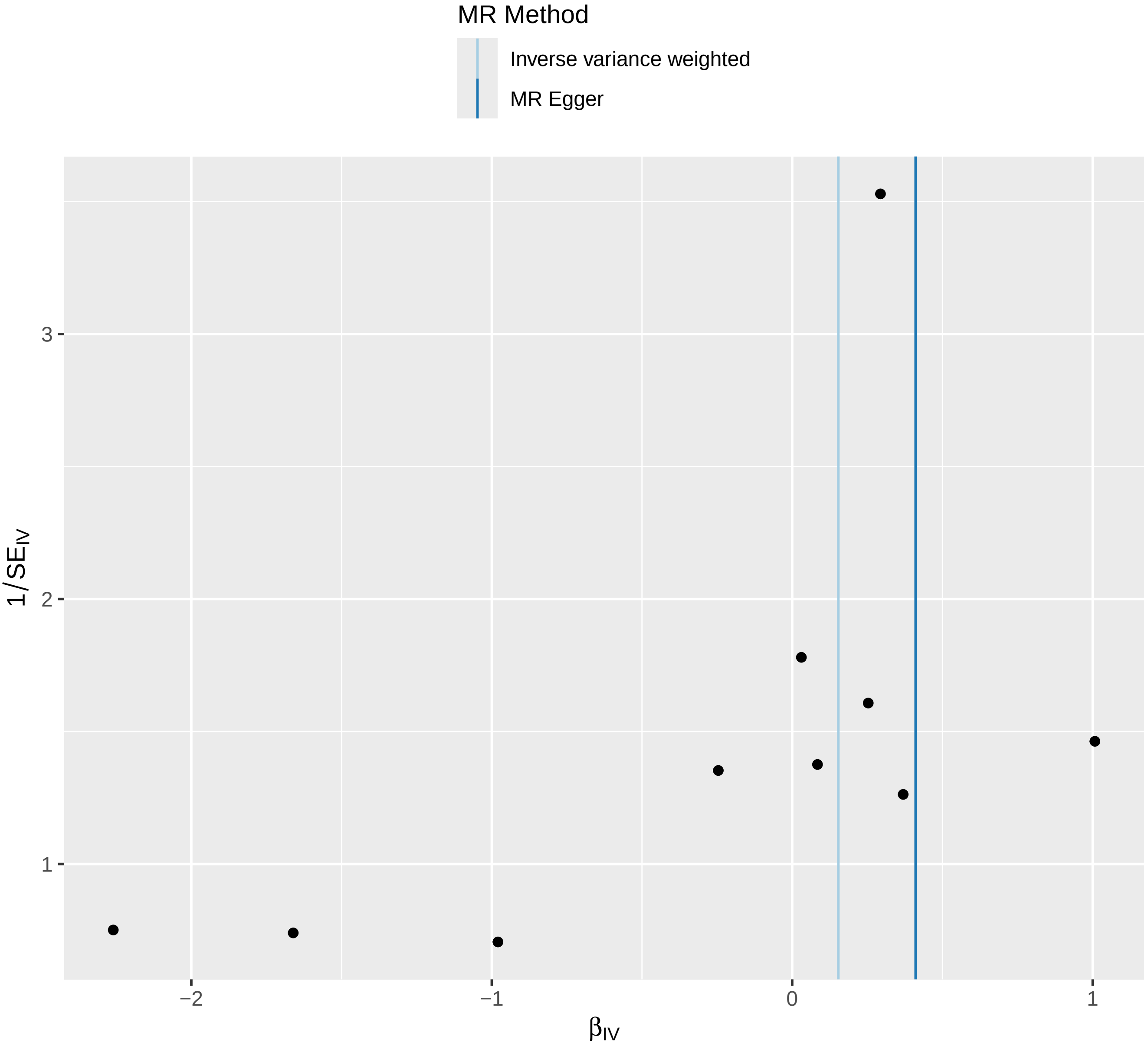

MR Method
Inverse variance weighted
MR Egger
3
2
1
1/SE_IV
−2
−1
0
1
β_IV

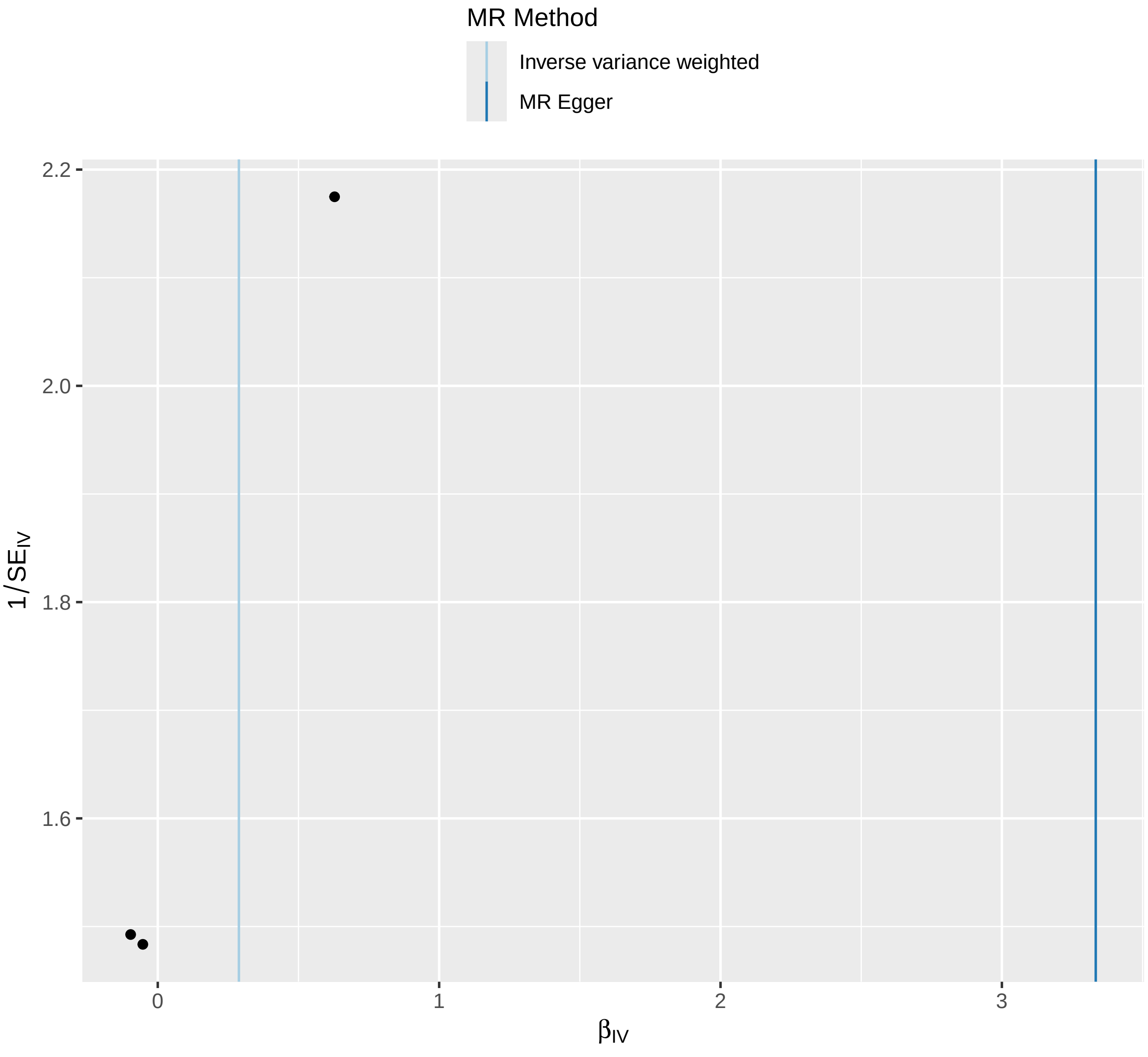
MR Method
Inverse variance weighted
MR Egger
2.2
2.0
1.8
1.6
1/SE_IV
0
1
2
3
β_IV

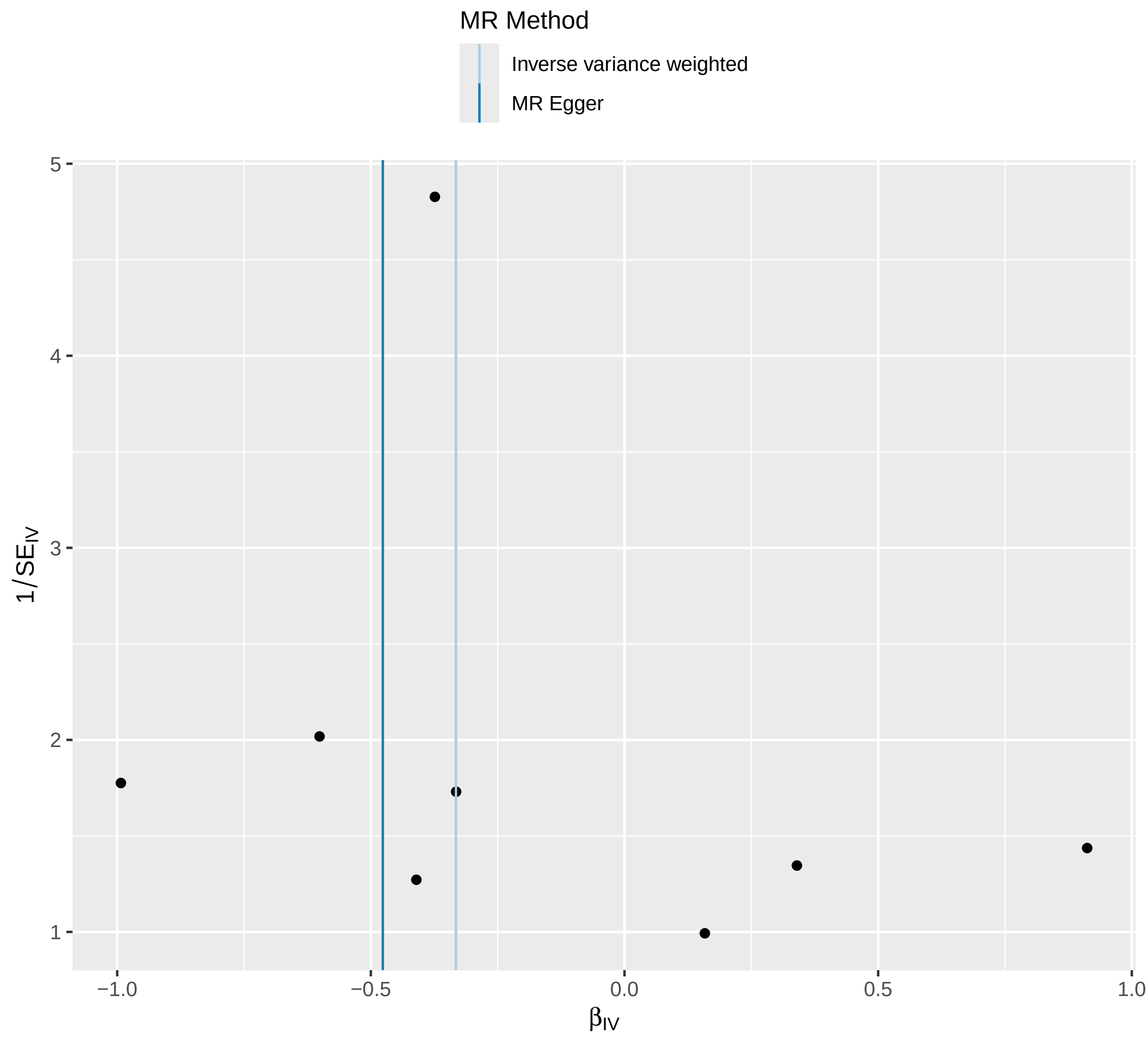

MR Method
Inverse variance weighted
MR Egger
5
4
3
2
1
1/SE_IV
−1.0
−0.5
0.0
0.5
1.0
β_IV

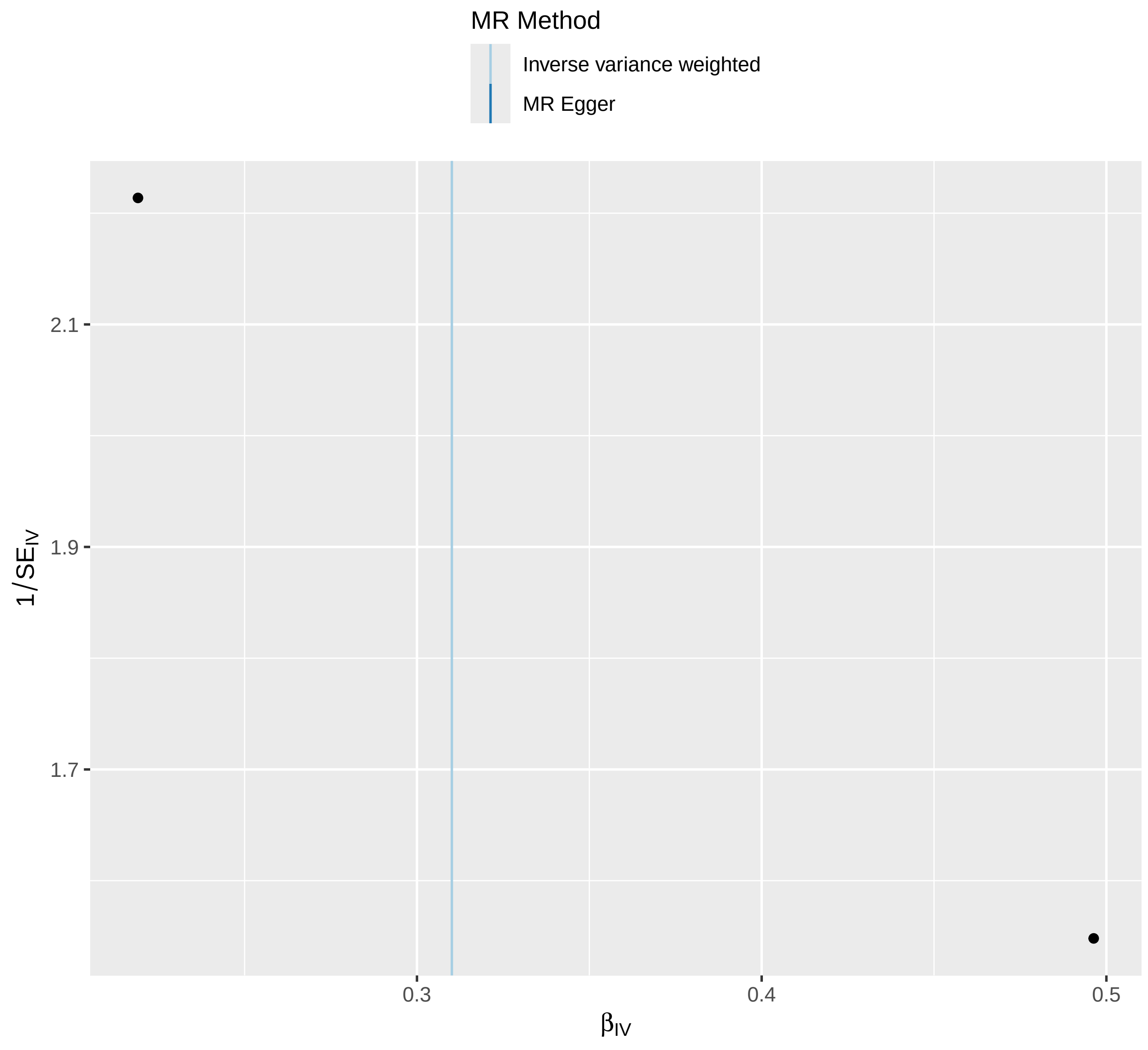

MR Method
Inverse variance weighted
MR Egger
2.1
1.9
1.7
1/SE_IV
0.3
0.4
0.5
β_IV

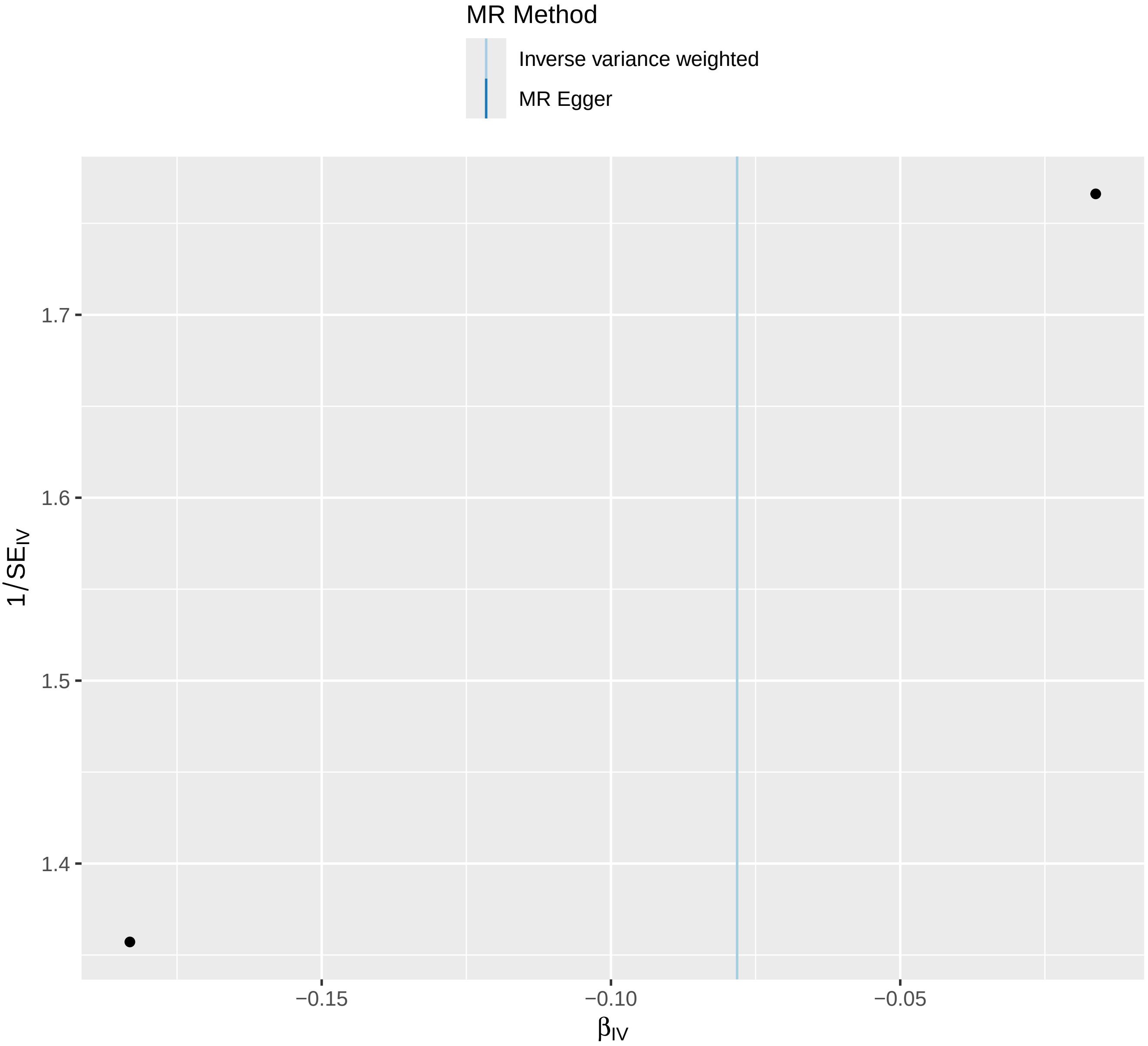

MR Method
Inverse variance weighted
MR Egger
1.7
1.6
1.5
1.4
$1/SE_{IV}$
−0.15
−0.10
−0.05
β_{IV}

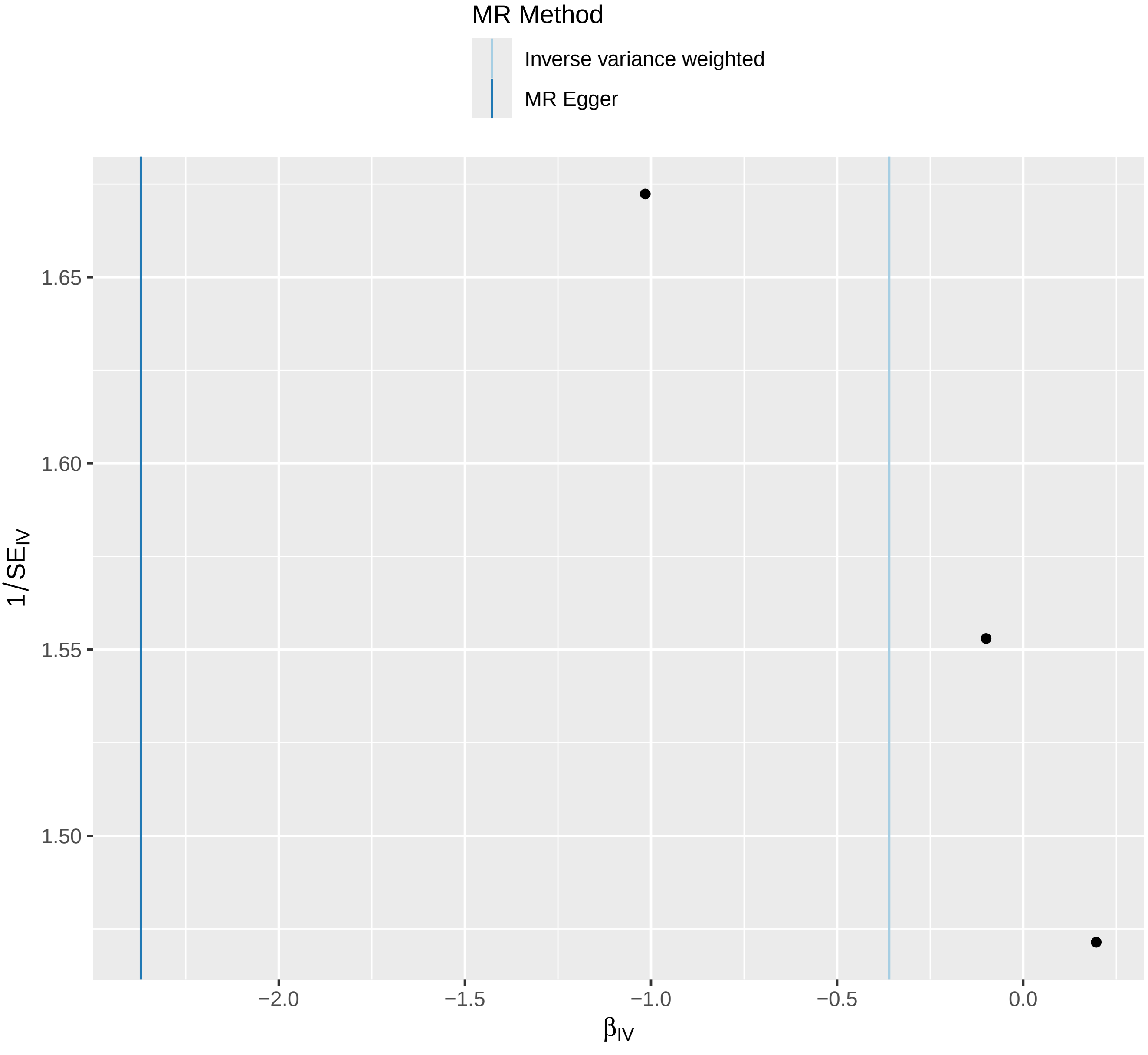

MR Method
Inverse variance weighted
MR Egger
1.65
1.60
1.55
1.50
1/SE$_{IV}$
−2.0
−1.5
−1.0
−0.5
0.0
β$_{IV}$

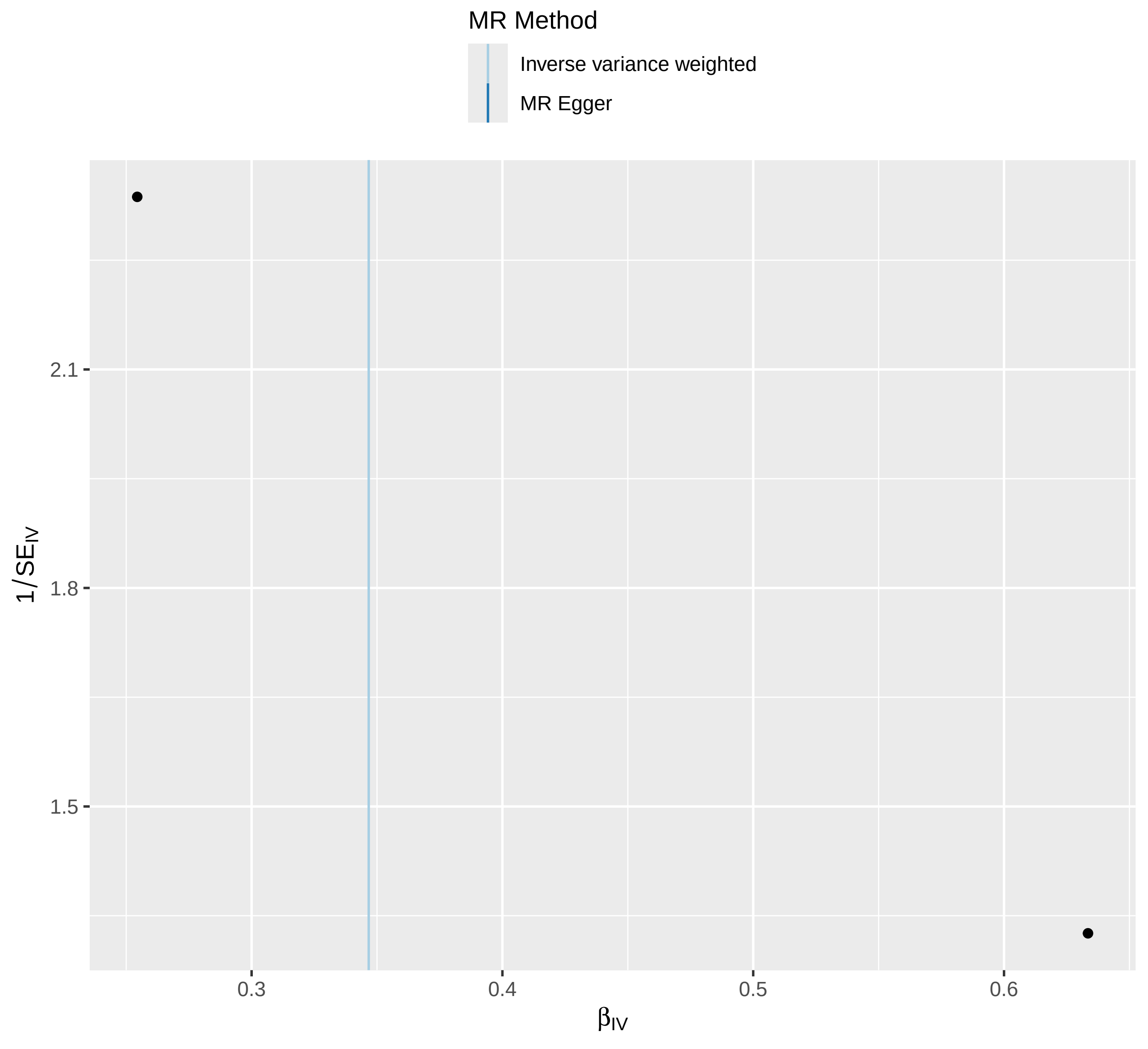
MR Method
Inverse variance weighted
MR Egger
2.1
1.8
1.5
1/SE_IV
0.3
0.4
0.5
0.6
β_IV

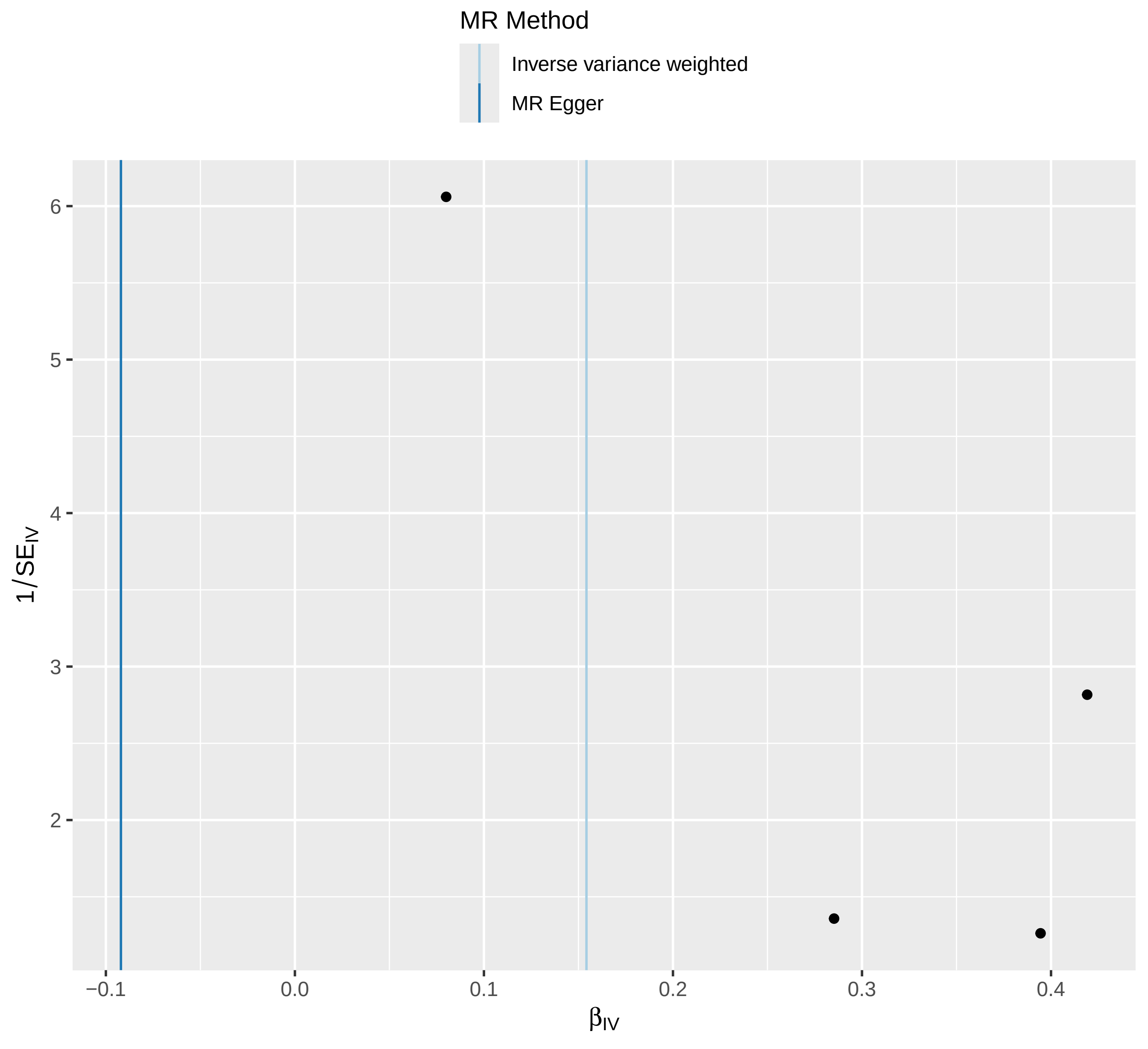
MR Method
Inverse variance weighted
MR Egger
1/SE_IV
β_IV
6
5
4
3
2
−0.1
0.0
0.1
0.2
0.3
0.4

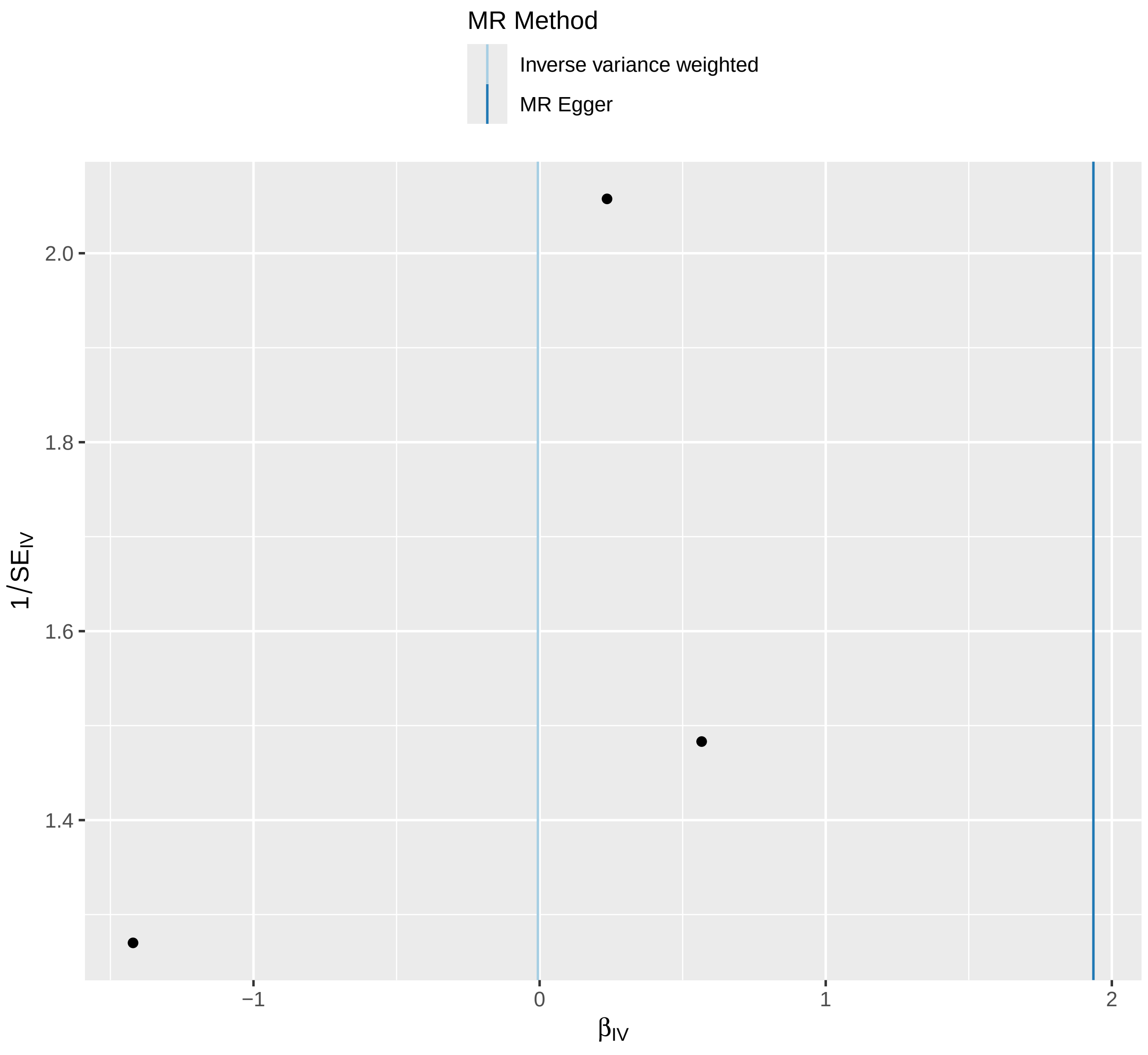

MR Method
Inverse variance weighted
MR Egger
2.0
1.8
1.6
1.4
−1
0
1
2
β_IV
1/SE_IV

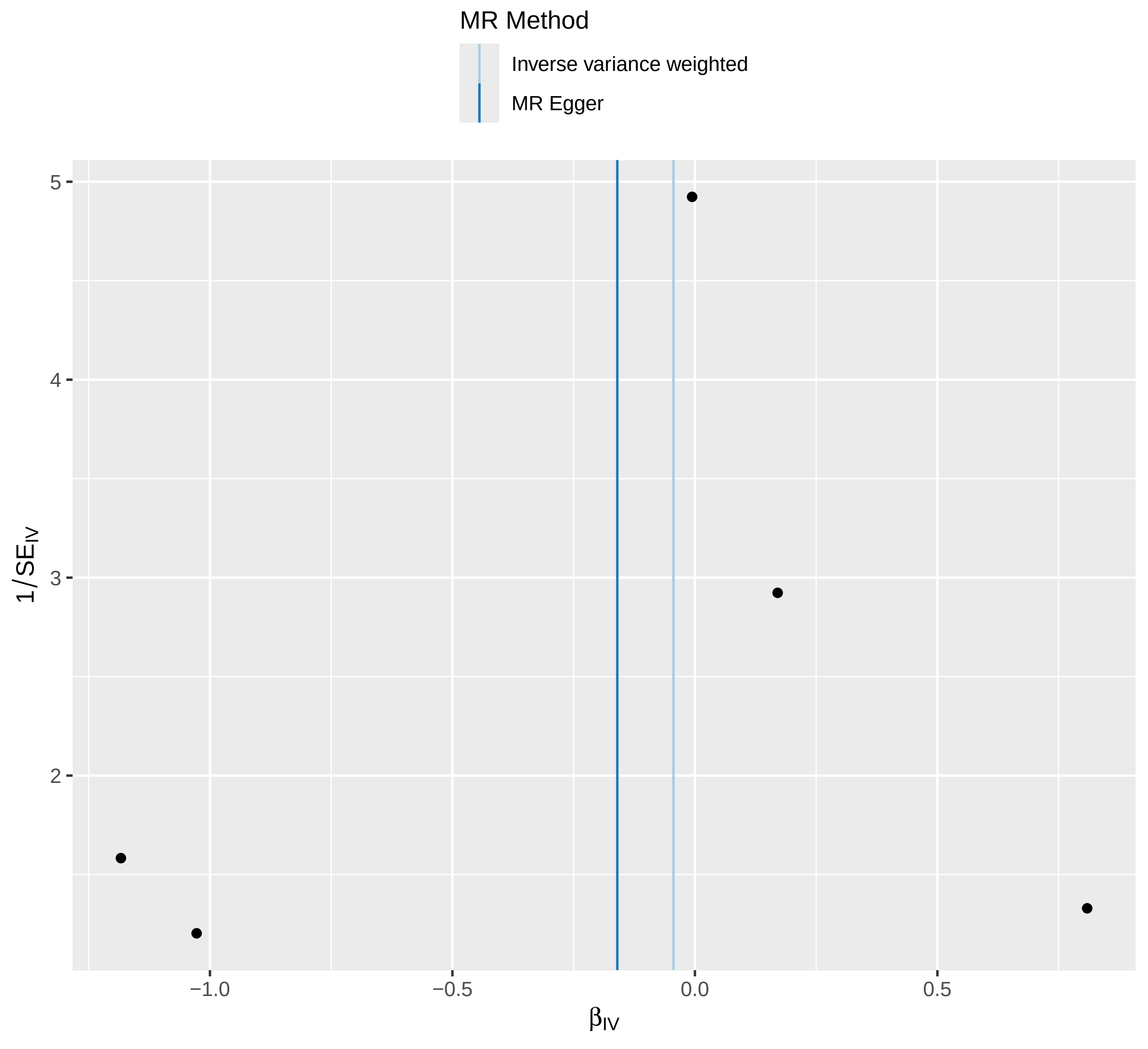
MR Method
Inverse variance weighted
MR Egger
5
4
3
2
1/SE_IV
−1.0
−0.5
0.0
0.5
β_IV

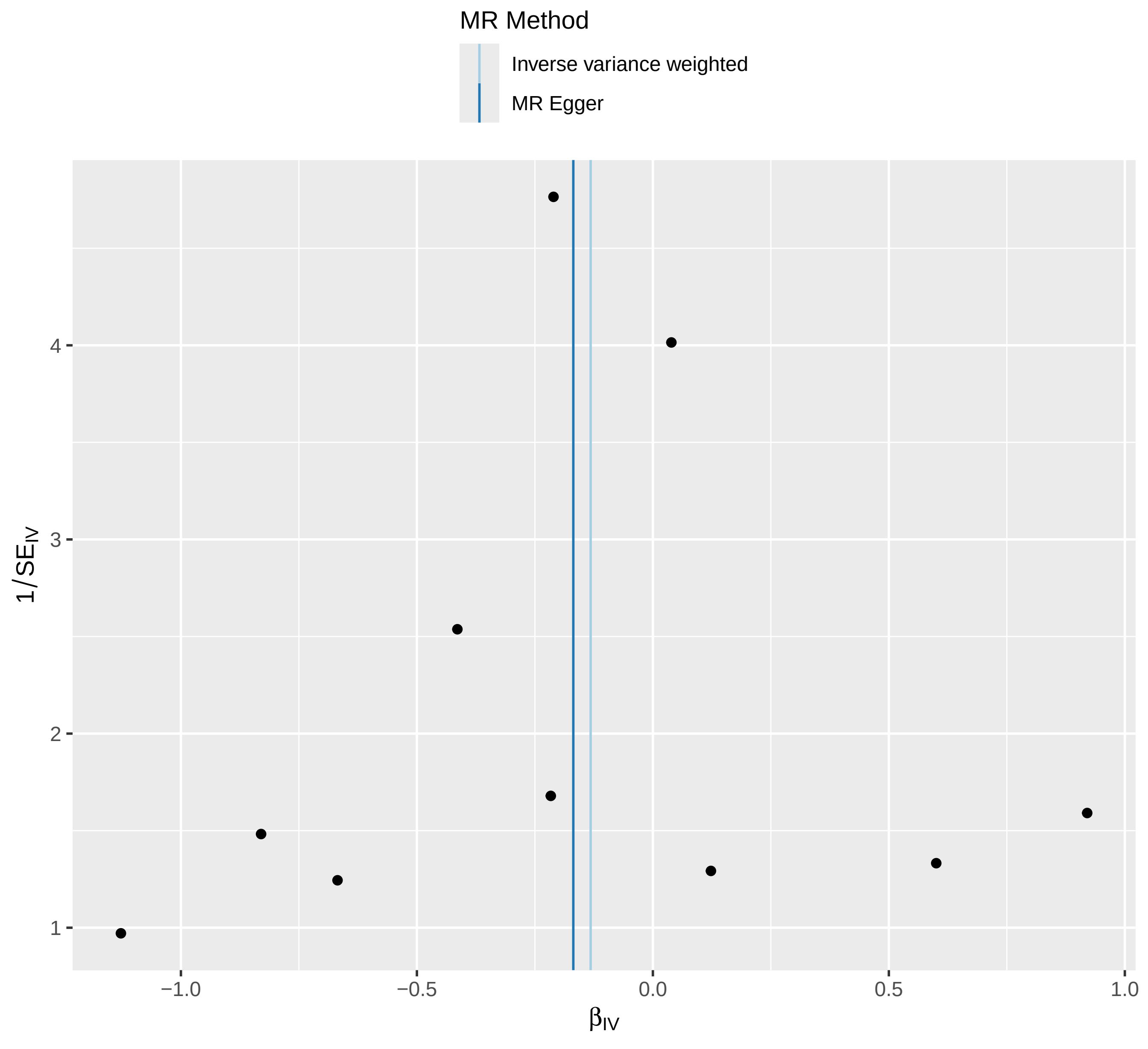
MR Method
Inverse variance weighted
MR Egger
1/SE_IV
4
3
2
1
−1.0
−0.5
0.0
0.5
1.0
β_IV

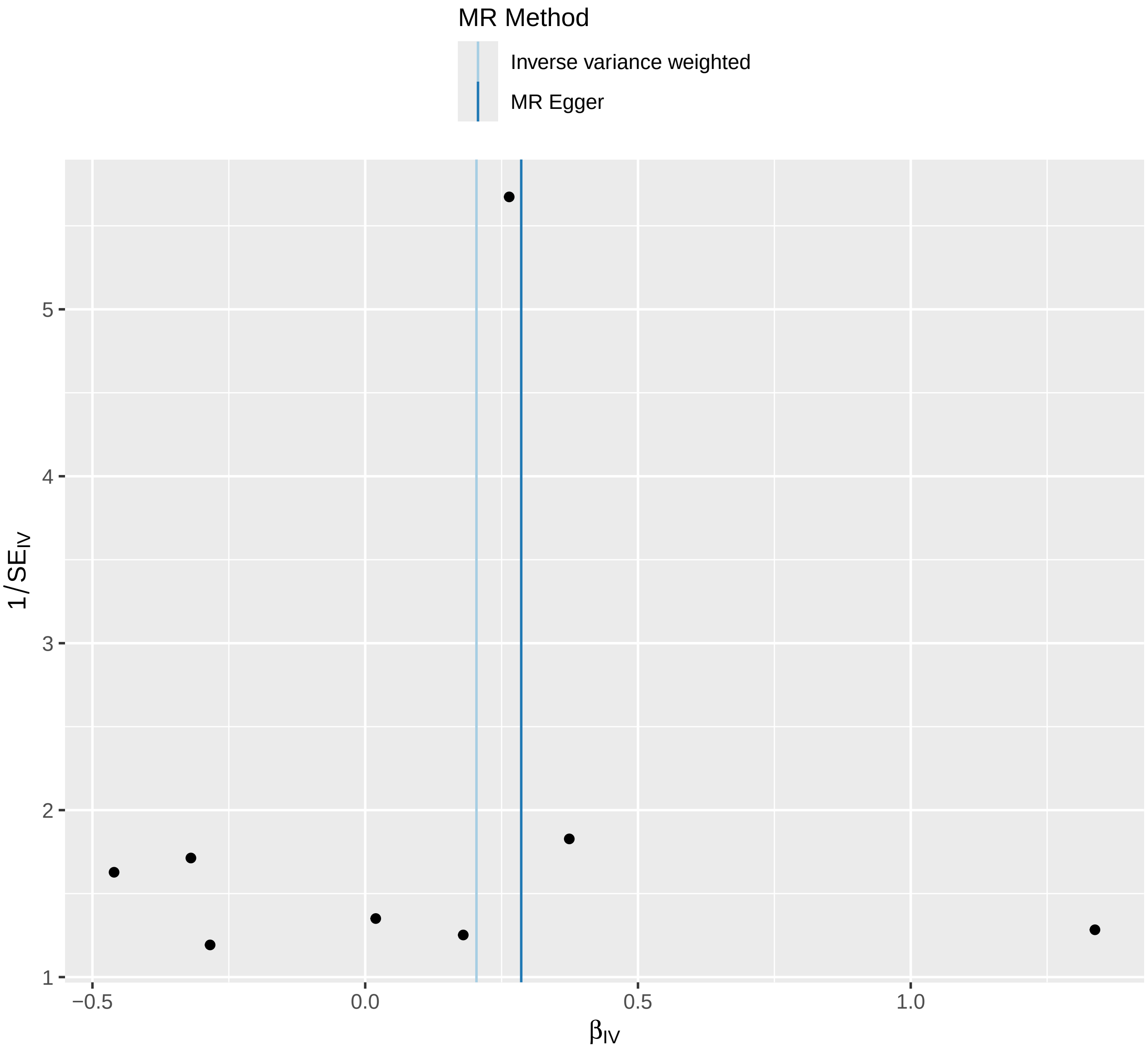
MR Method
Inverse variance weighted
MR Egger
5
4
3
2
1
1/SE_IV
−0.5
0.0
0.5
1.0
β_IV

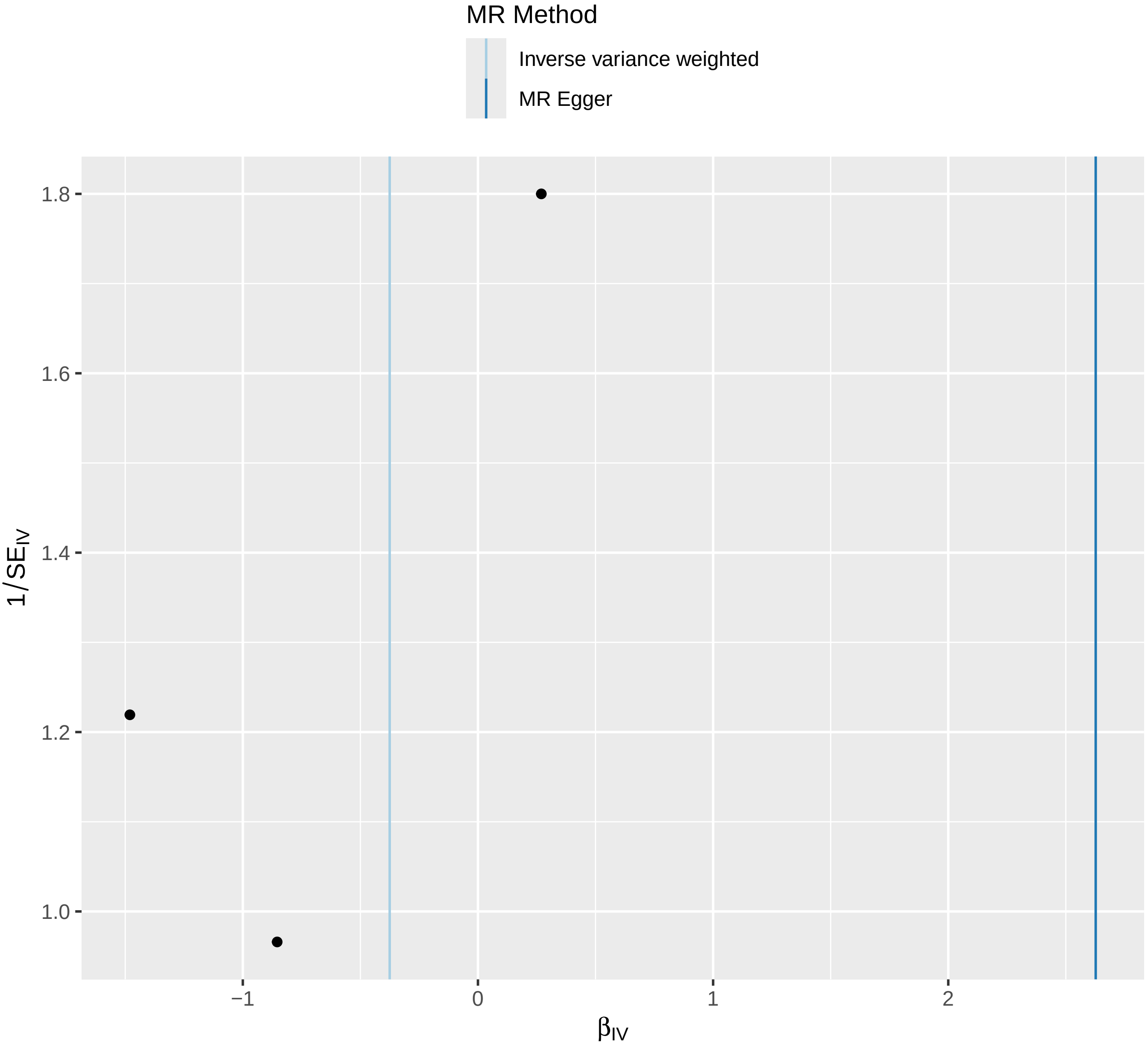
MR Method
Inverse variance weighted
MR Egger
1.8
1.6
1.4
1.2
1.0
$1/SE_{IV}$
−1
0
1
2
β_{IV}

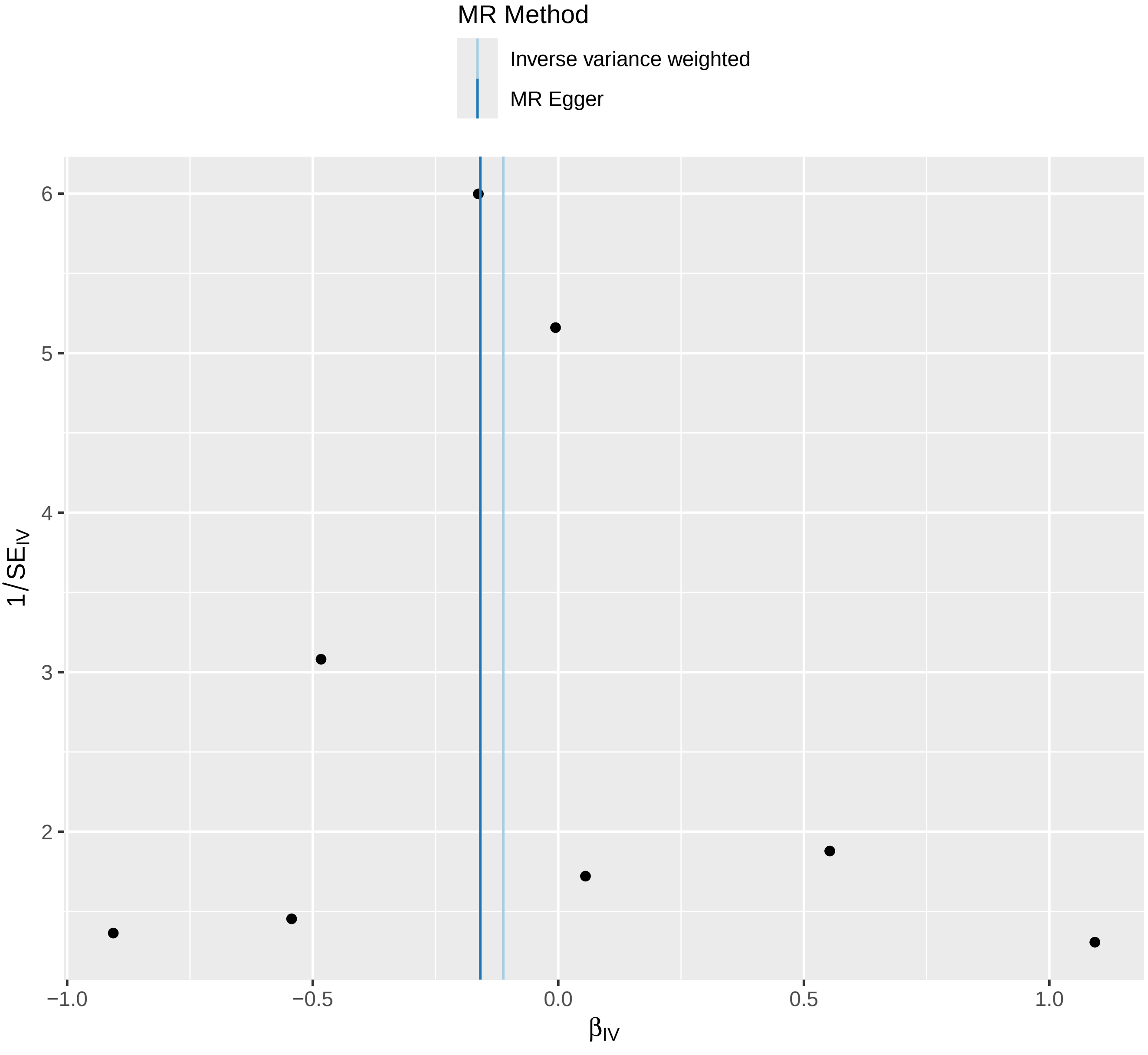

MR Method
Inverse variance weighted
MR Egger
6
5
4
3
2
$1/SE_{IV}$
−1.0
−0.5
0.0
0.5
1.0
β_{IV}

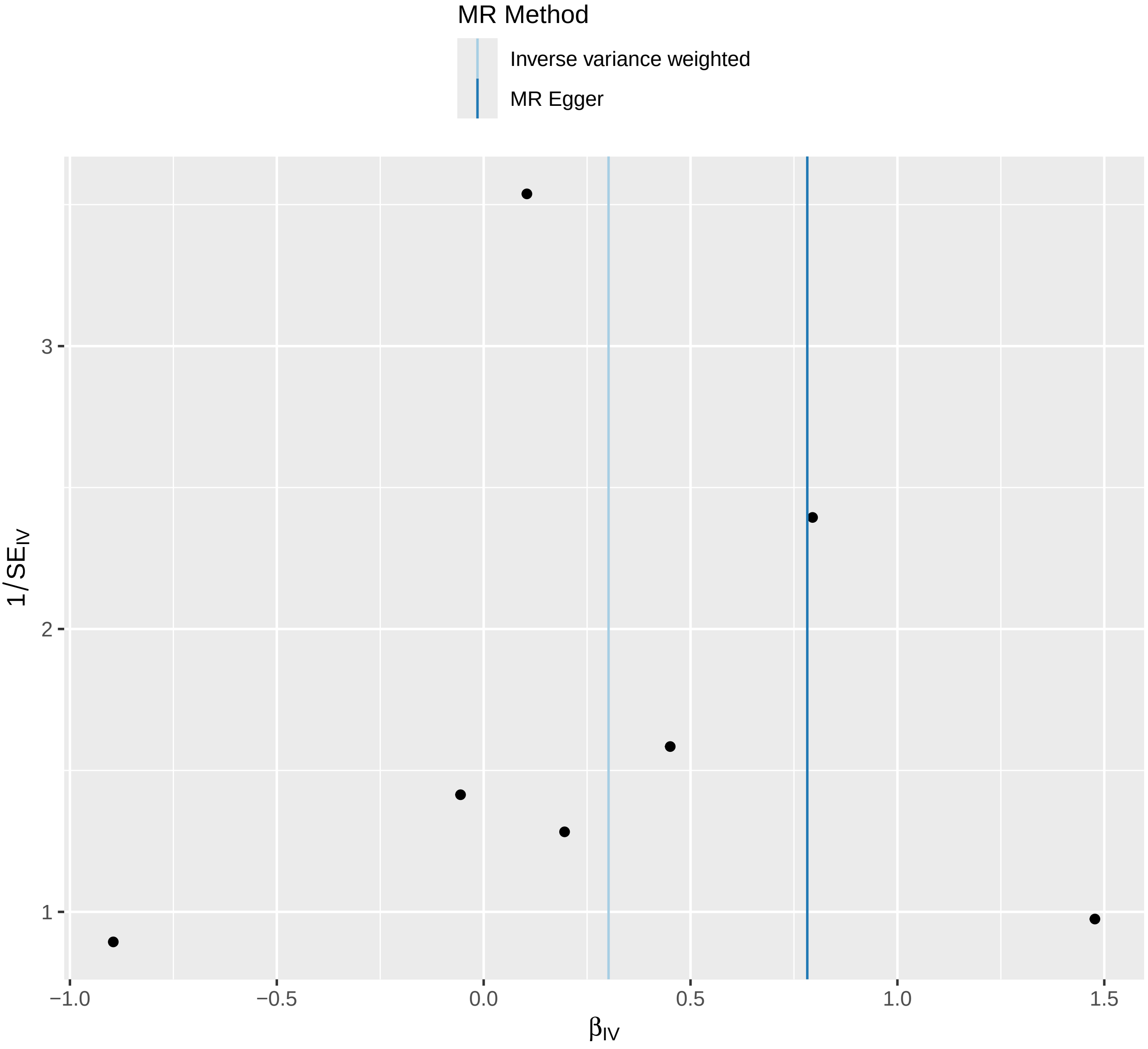
MR Method
Inverse variance weighted
MR Egger
1/SE_{IV}
3
2
1
−1.0
−0.5
0.0
0.5
1.0
1.5
β_{IV}

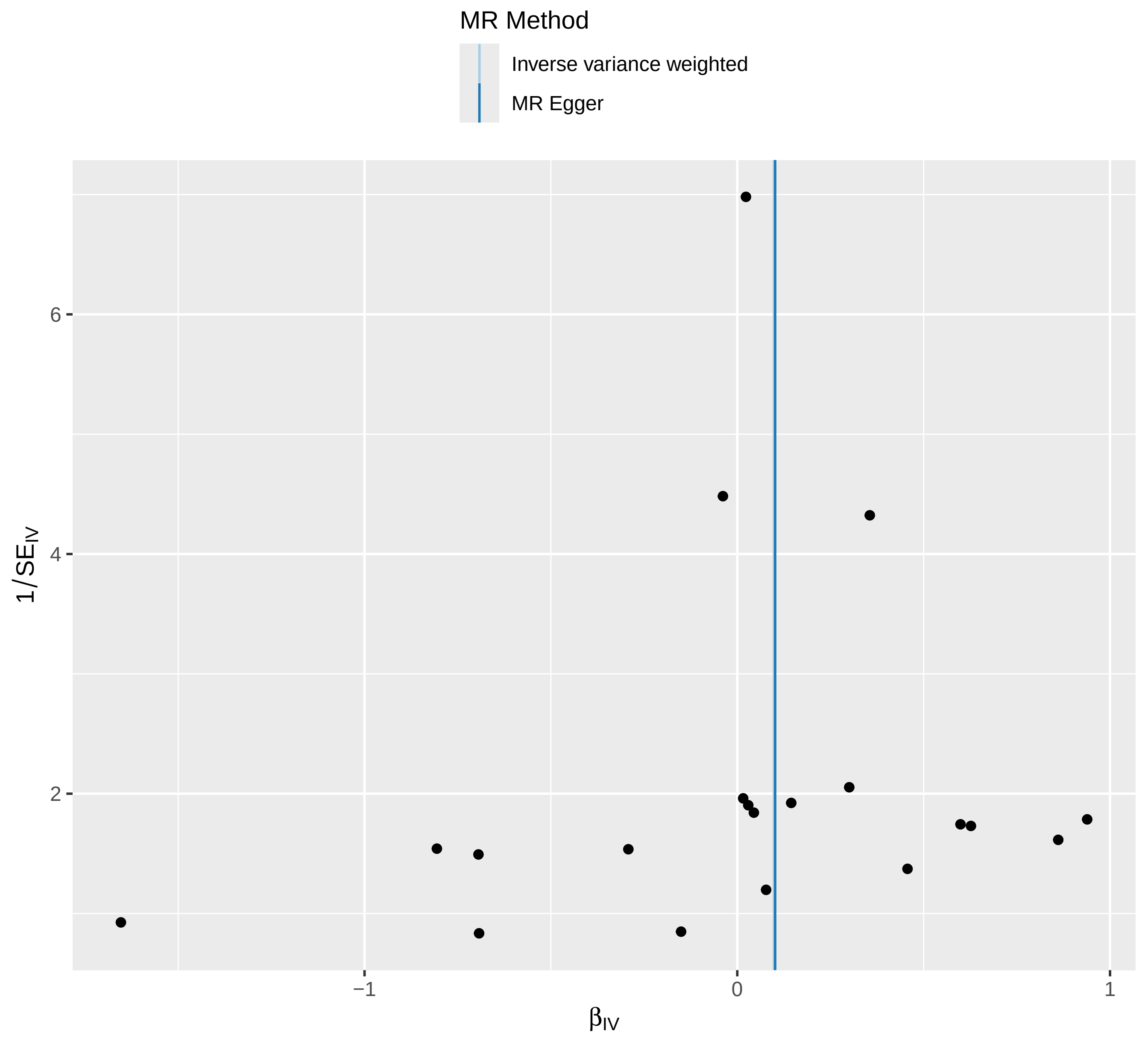
MR Method
Inverse variance weighted
MR Egger
6
4
2
1/SE$_{IV}$
−1
0
1
β$_{IV}$

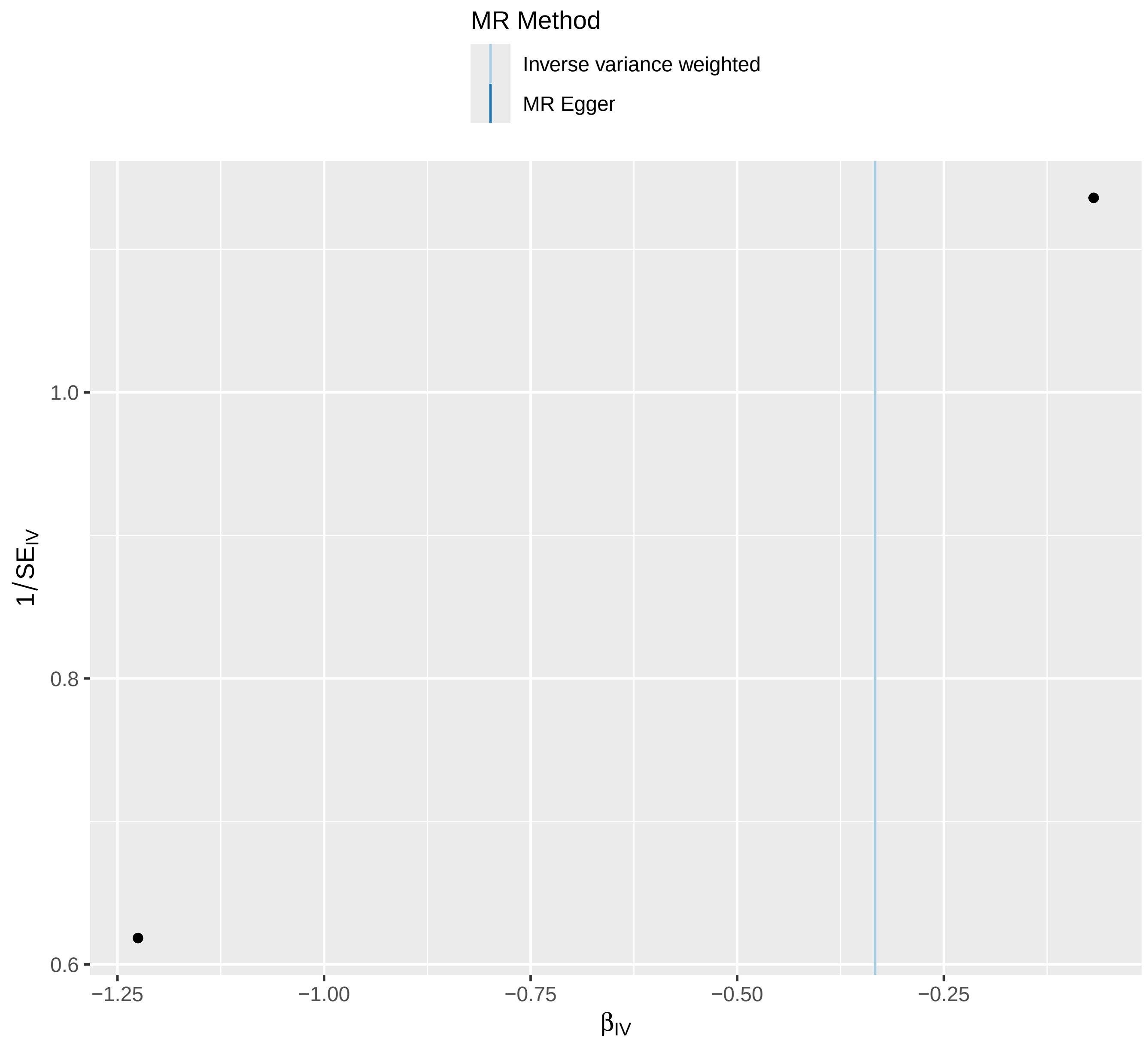

MR Method
Inverse variance weighted
MR Egger
1.0
0.8
0.6
1/SE$_{IV}$
−1.25
−1.00
−0.75
−0.50
−0.25
β$_{IV}$

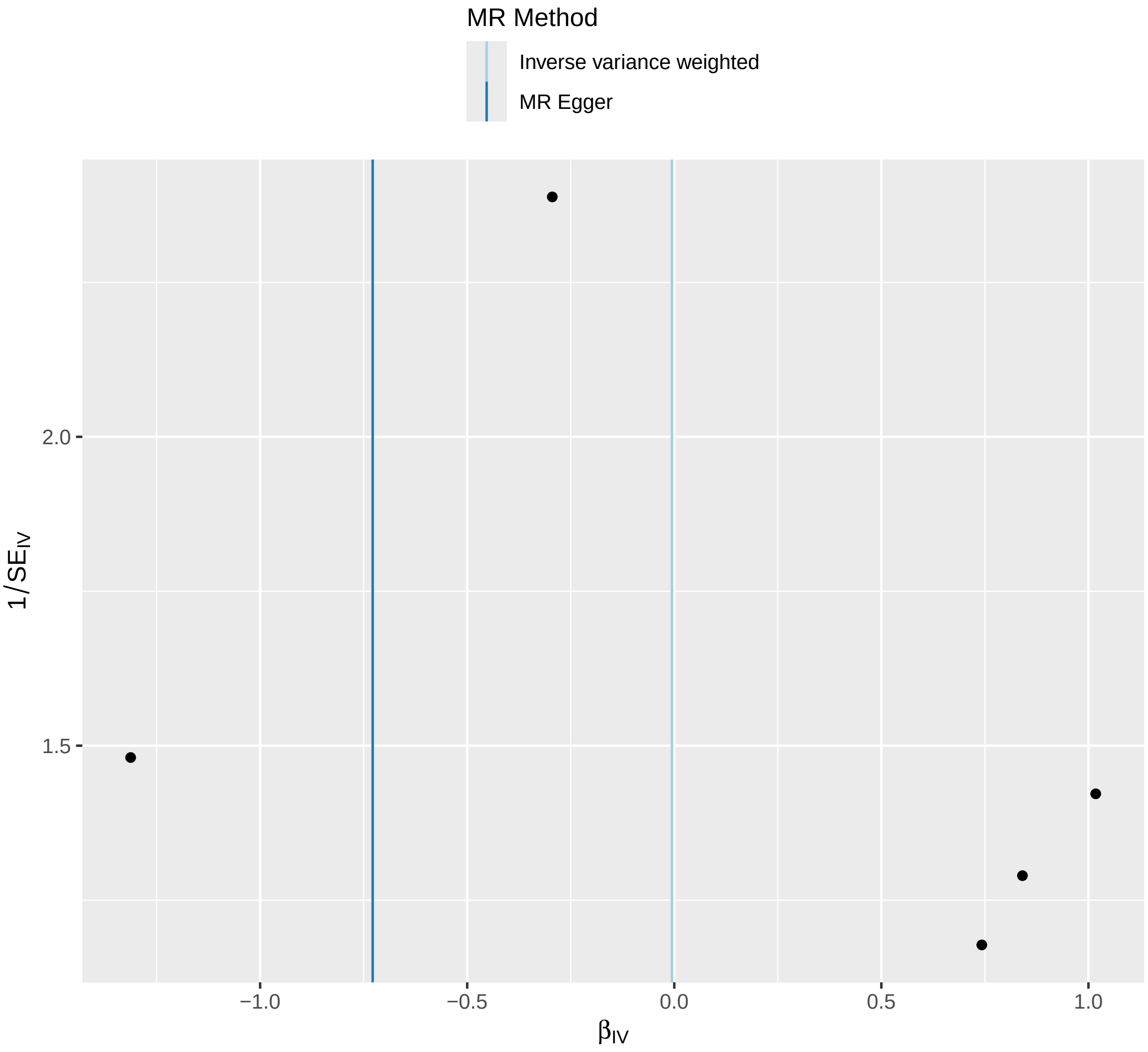
MR Method
Inverse variance weighted
MR Egger
2.0
1.5
$1/SE_{IV}$
−1.0
−0.5
0.0
0.5
1.0
β_{IV}

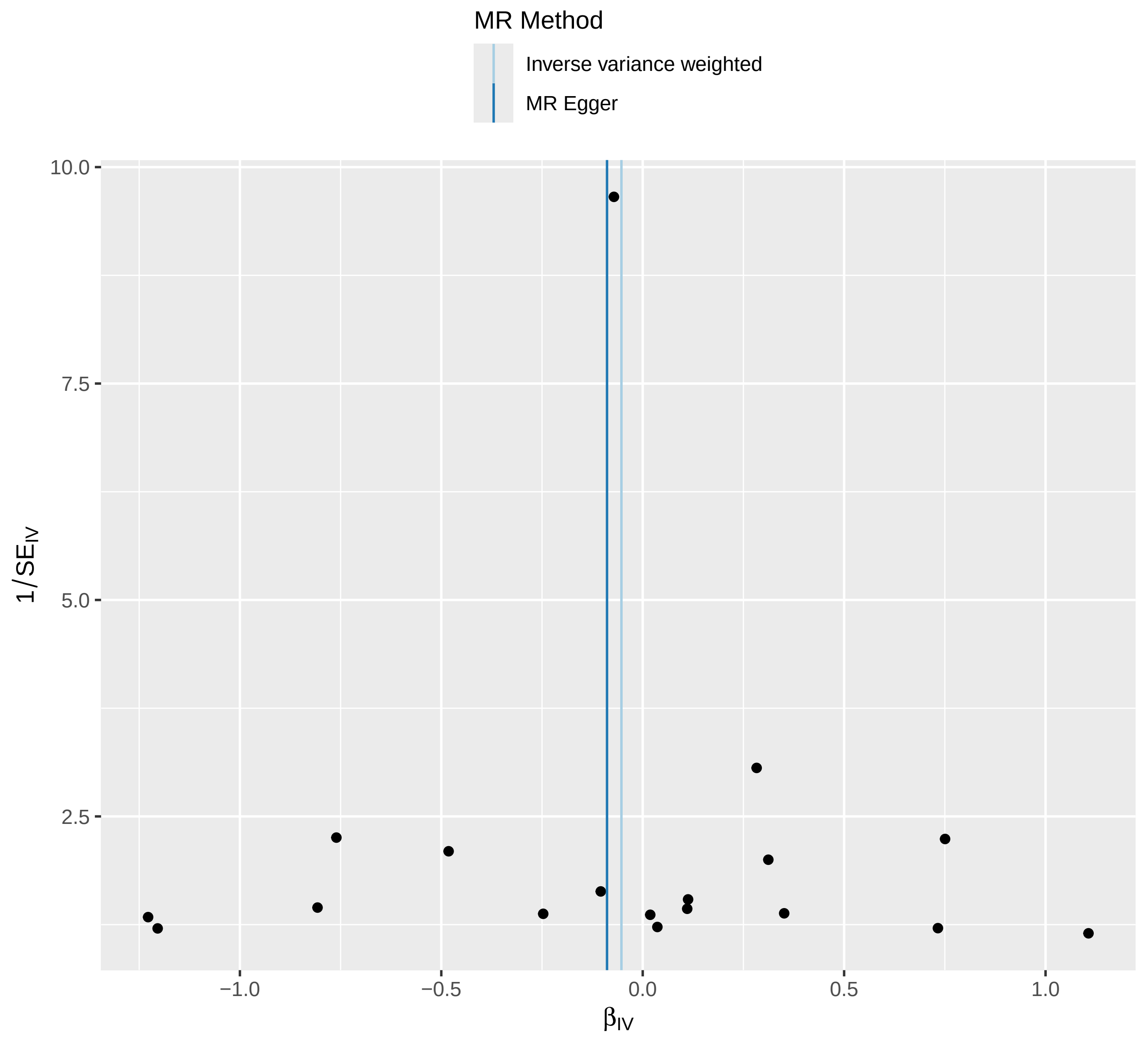

MR Method
Inverse variance weighted
MR Egger
10.0
7.5
5.0
2.5
$1/SE_{IV}$
−1.0
−0.5
0.0
0.5
1.0
β_{IV}

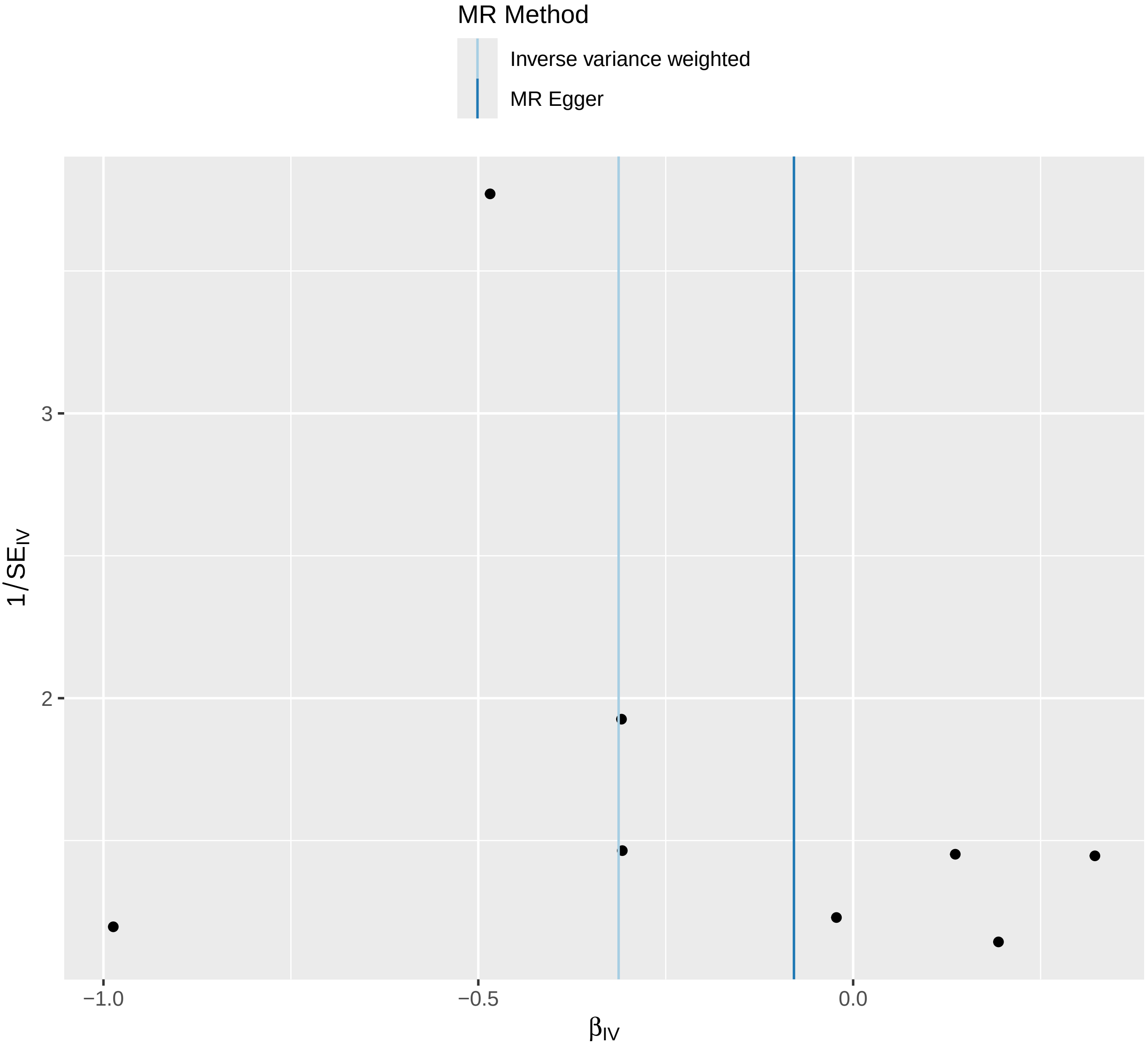
MR Method
Inverse variance weighted
MR Egger
3
2
1/SE_IV
−1.0
−0.5
0.0
β_IV

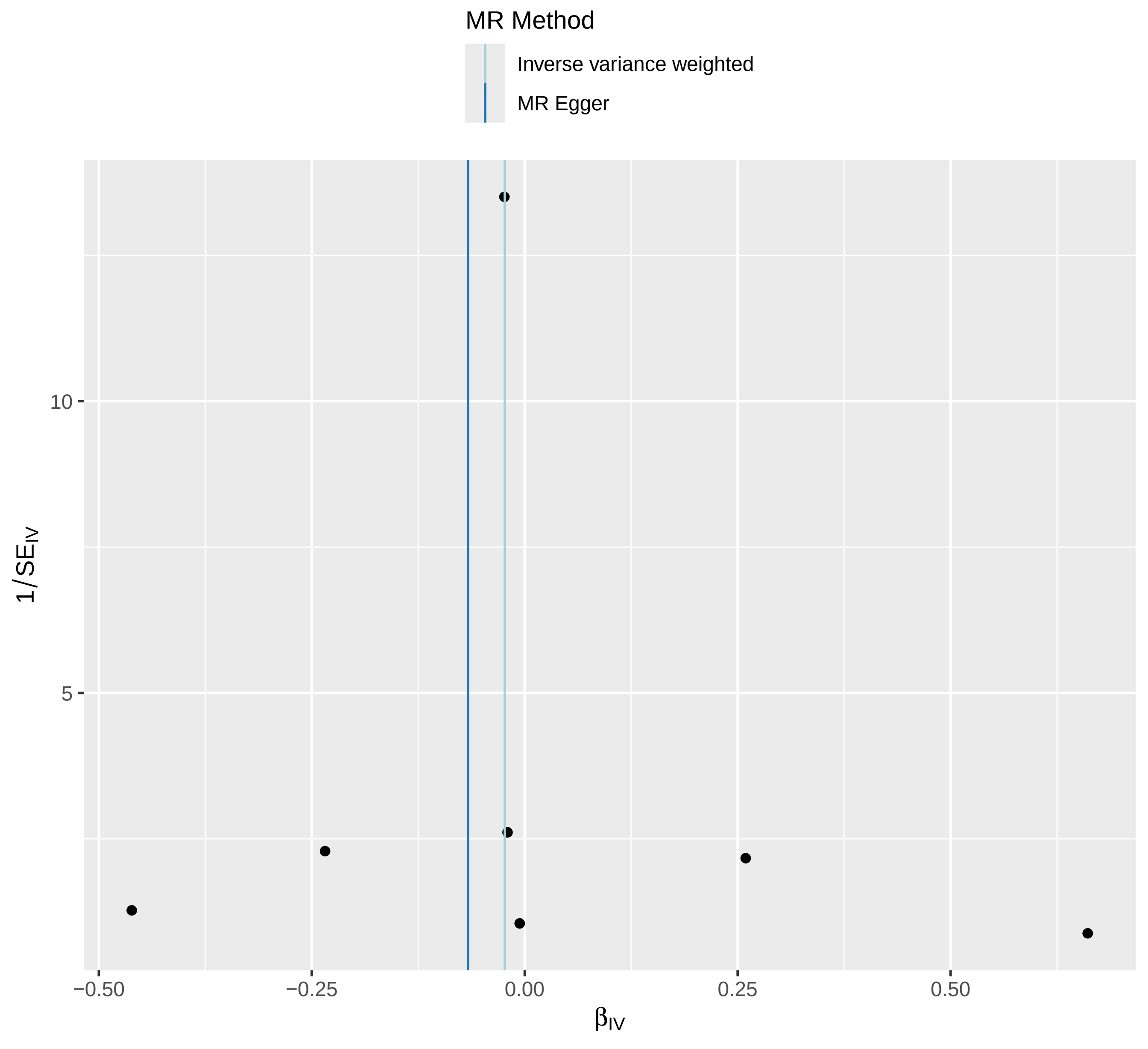
MR Method
Inverse variance weighted
MR Egger
10
5
1/SE_IV
−0.50
−0.25
0.00
0.25
0.50
β_IV

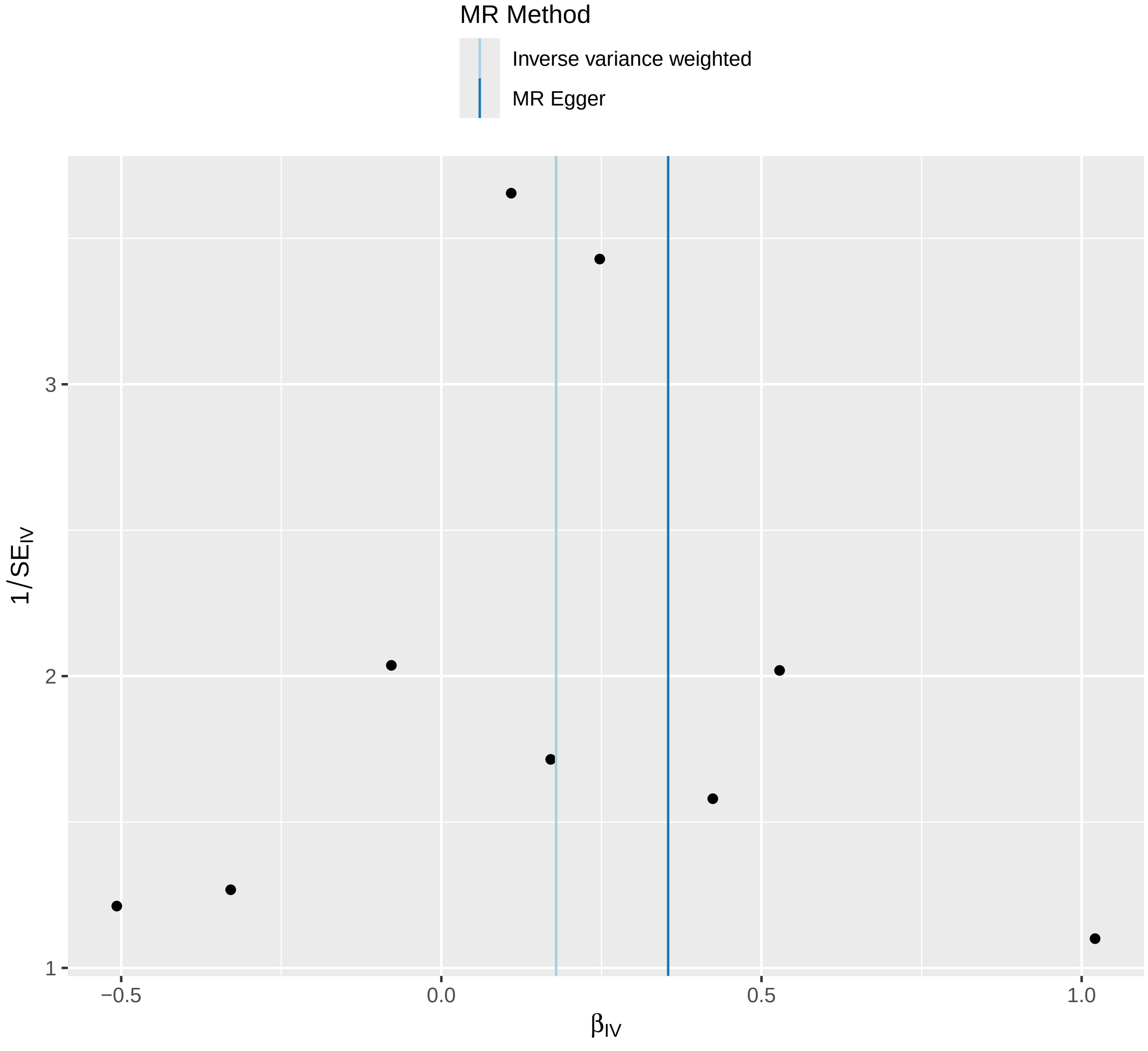

MR Method
Inverse variance weighted
MR Egger
3
2
1
1/SE$_{IV}$
−0.5
0.0
0.5
1.0
β$_{IV}$

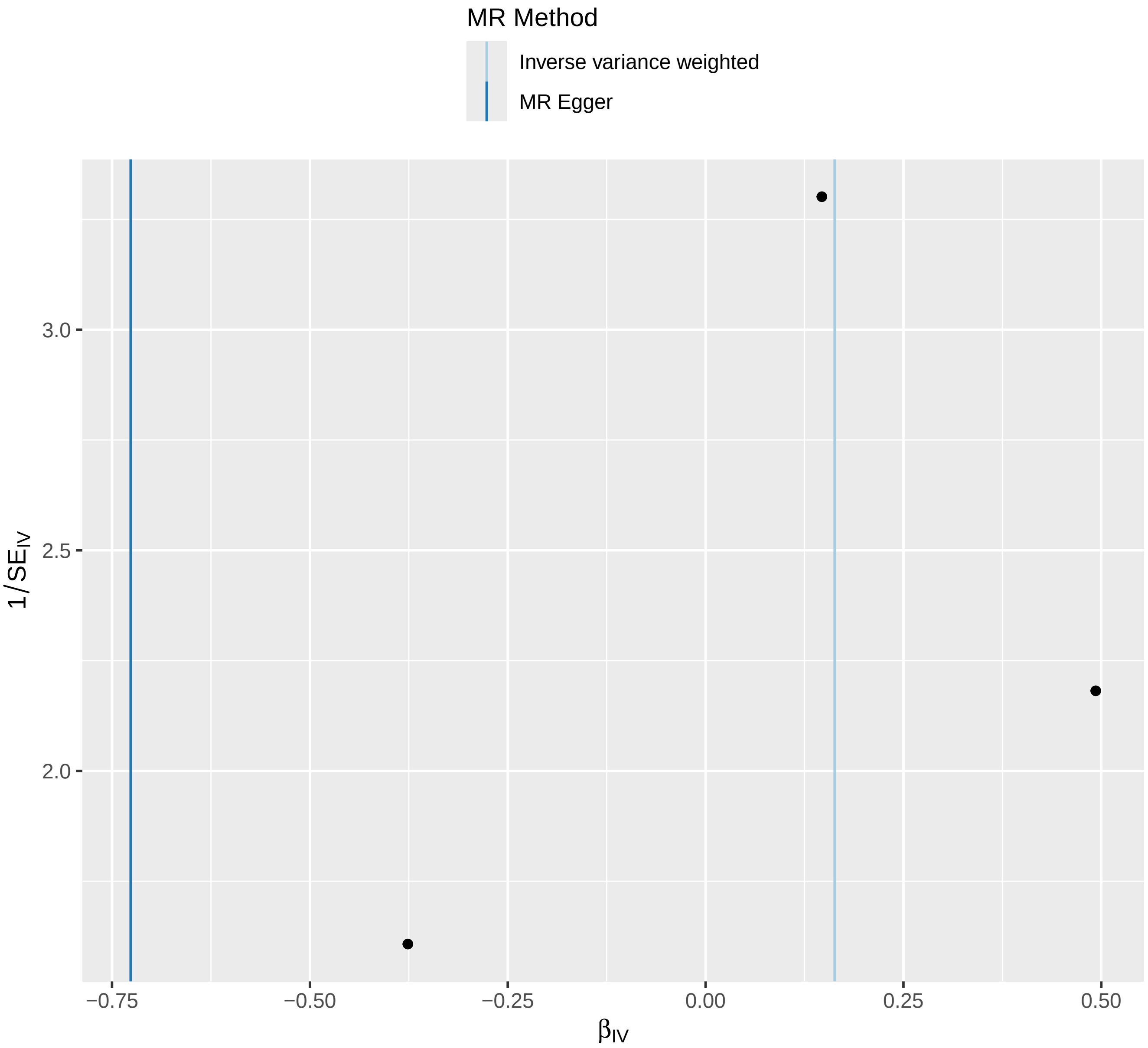
MR Method
Inverse variance weighted
MR Egger
3.0
2.5
2.0
1/SE_IV
−0.75
−0.50
−0.25
0.00
0.25
0.50
β_IV

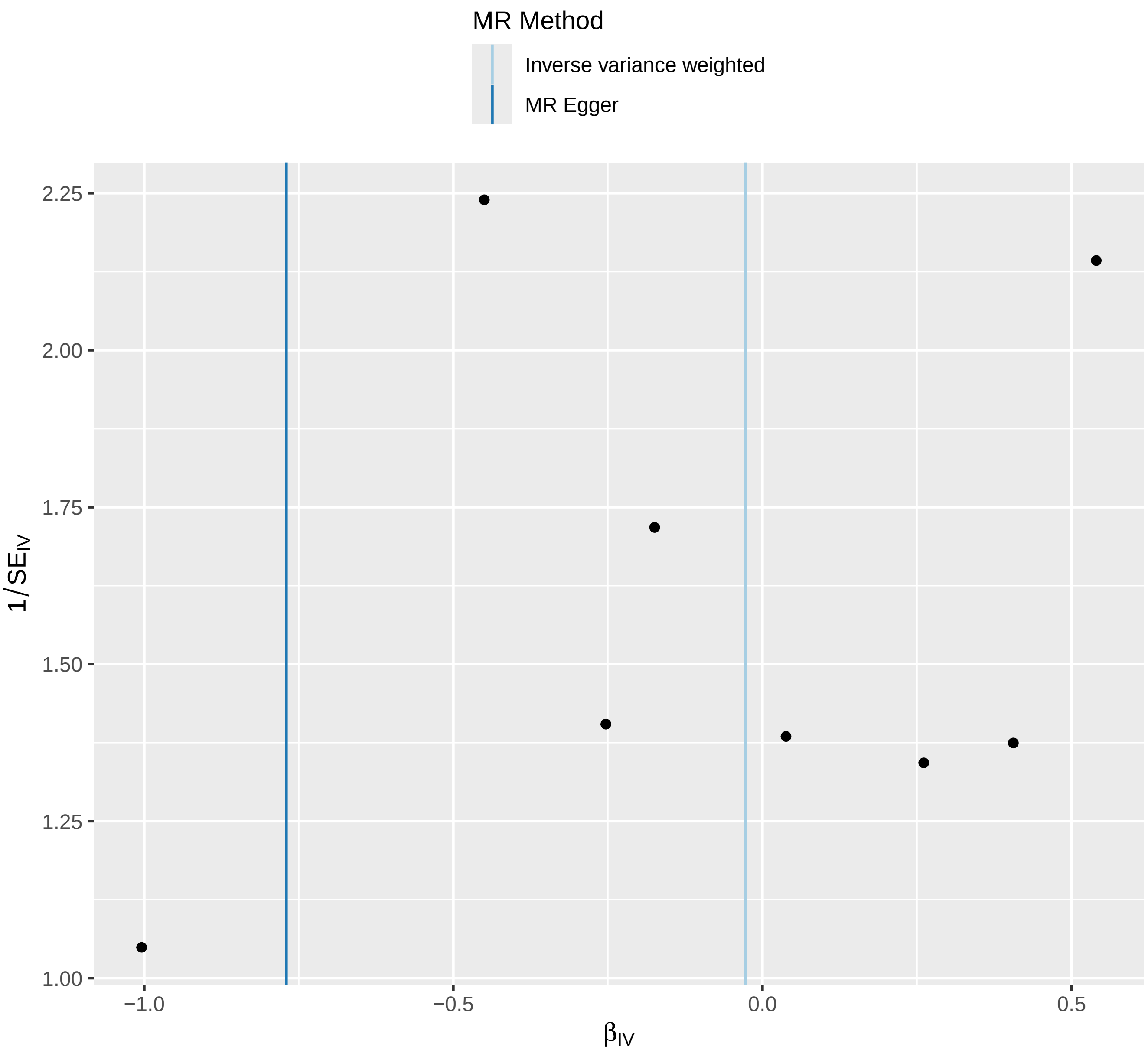
MR Method
Inverse variance weighted
MR Egger
2.25
2.00
1.75
1.50
1.25
1.00
1/SE_IV
−1.0
−0.5
0.0
0.5
β_IV

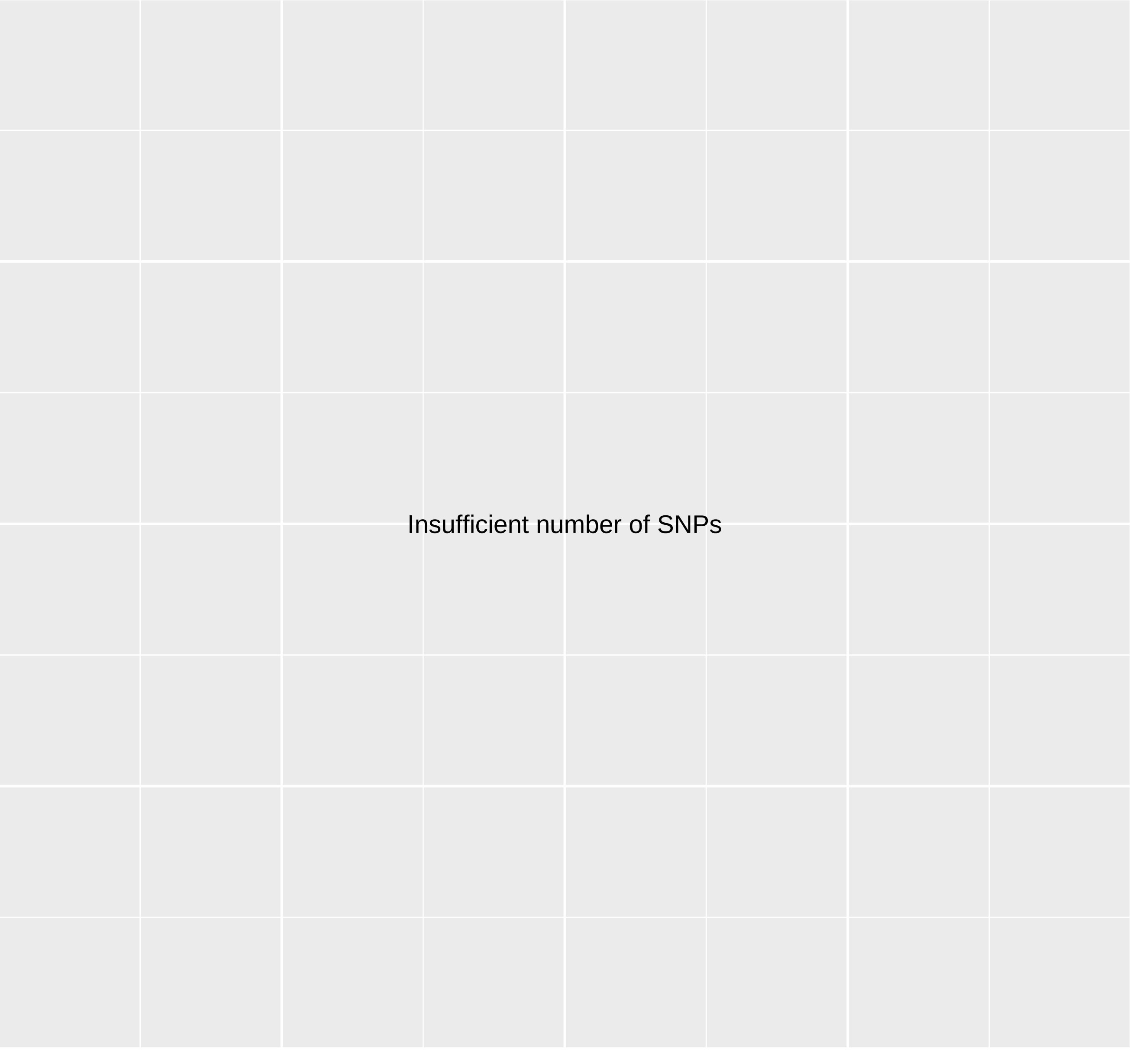
Insufficient number of SNPs

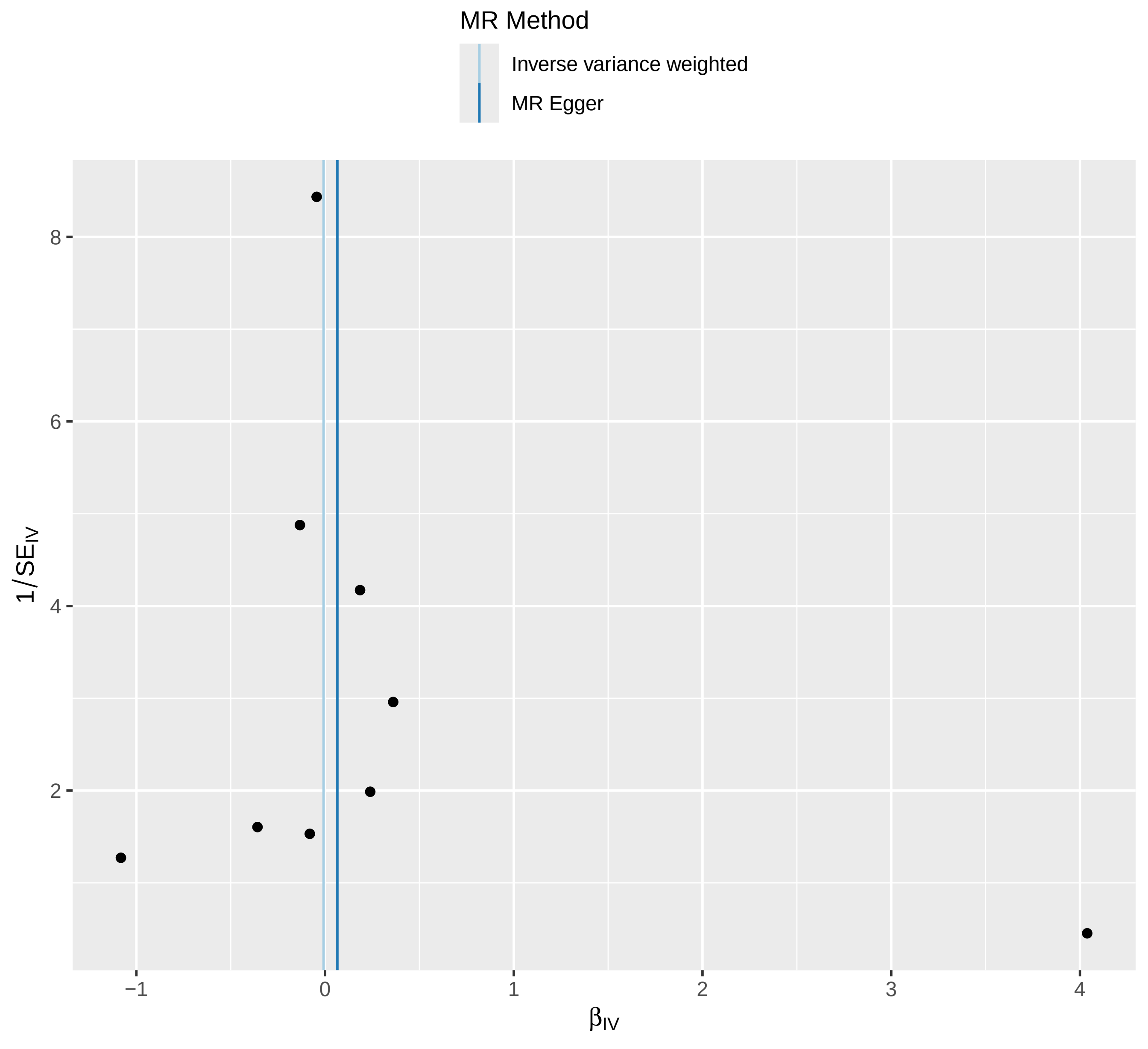
MR Method
Inverse variance weighted
MR Egger
8
6
4
2
1/SE_IV
−1
0
1
2
3
4
β_IV

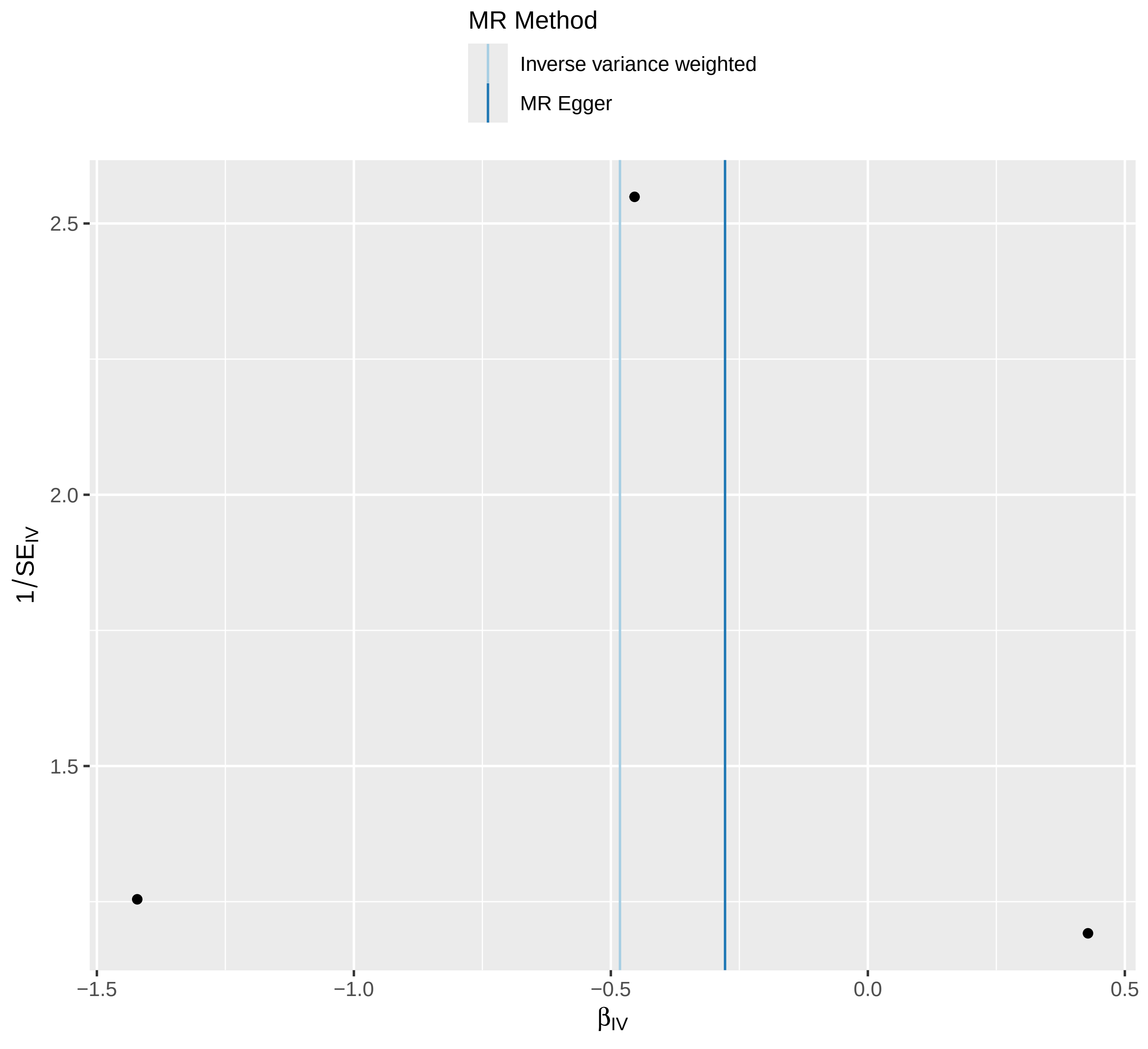

MR Method
Inverse variance weighted
MR Egger
2.5
2.0
1.5
1/SE_IV
−1.5
−1.0
−0.5
0.0
0.5
β_IV

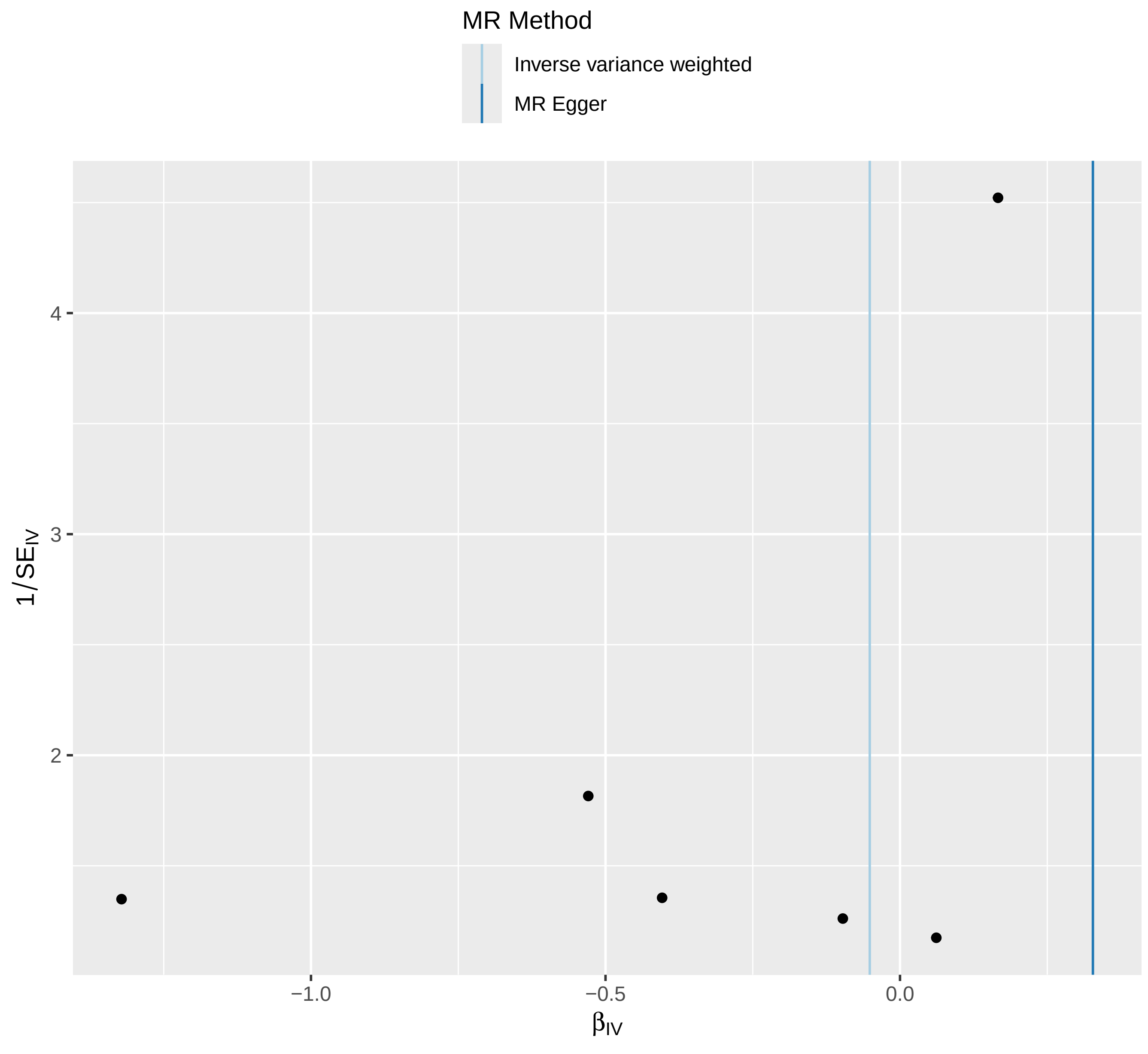
MR Method
Inverse variance weighted
MR Egger
4
3
2
1/SE$_{IV}$
−1.0
−0.5
0.0
β$_{IV}$

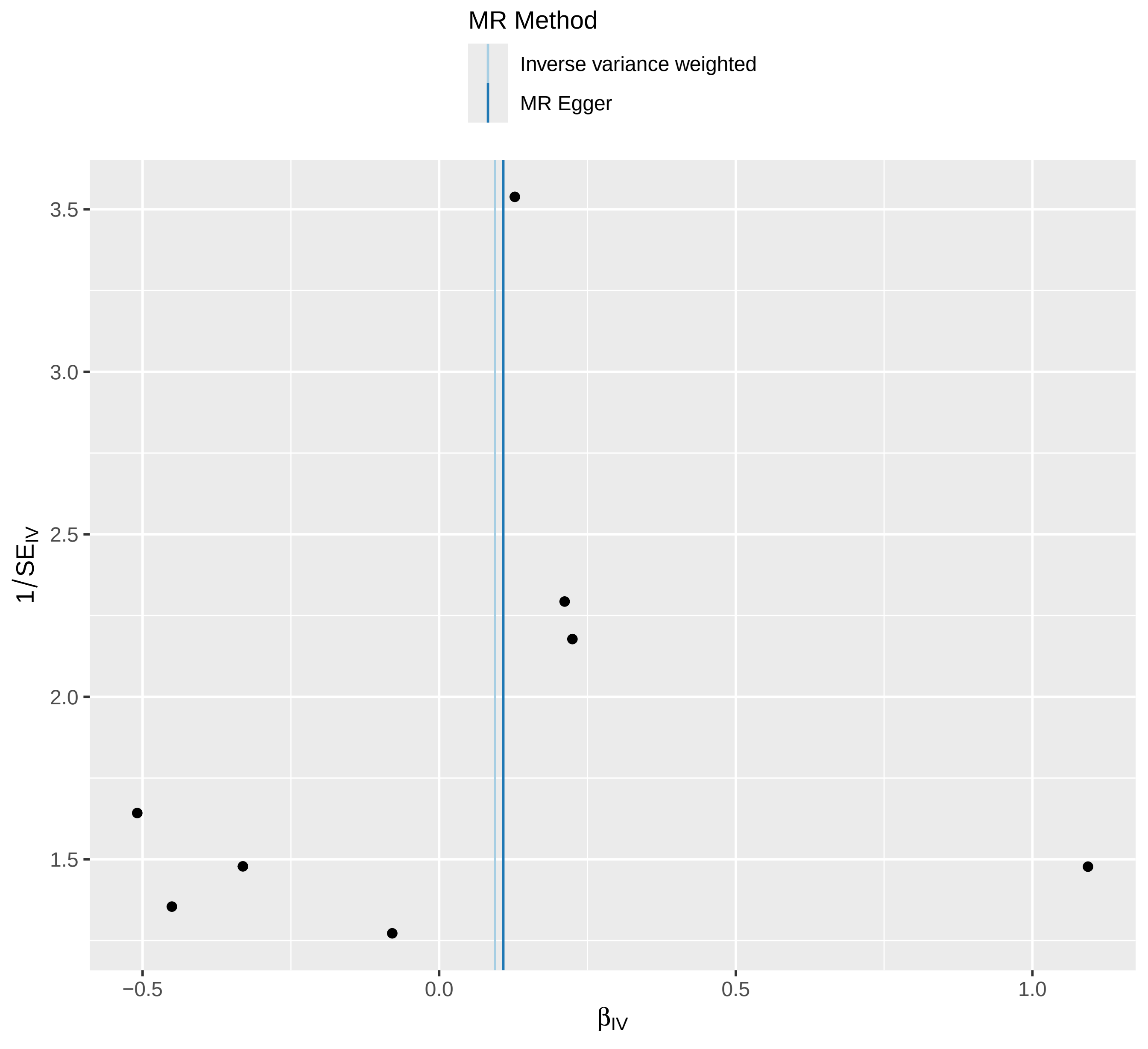

MR Method
Inverse variance weighted
MR Egger
3.5
3.0
2.5
2.0
1.5
1/SE$_{IV}$
−0.5
0.0
0.5
1.0
β$_{IV}$

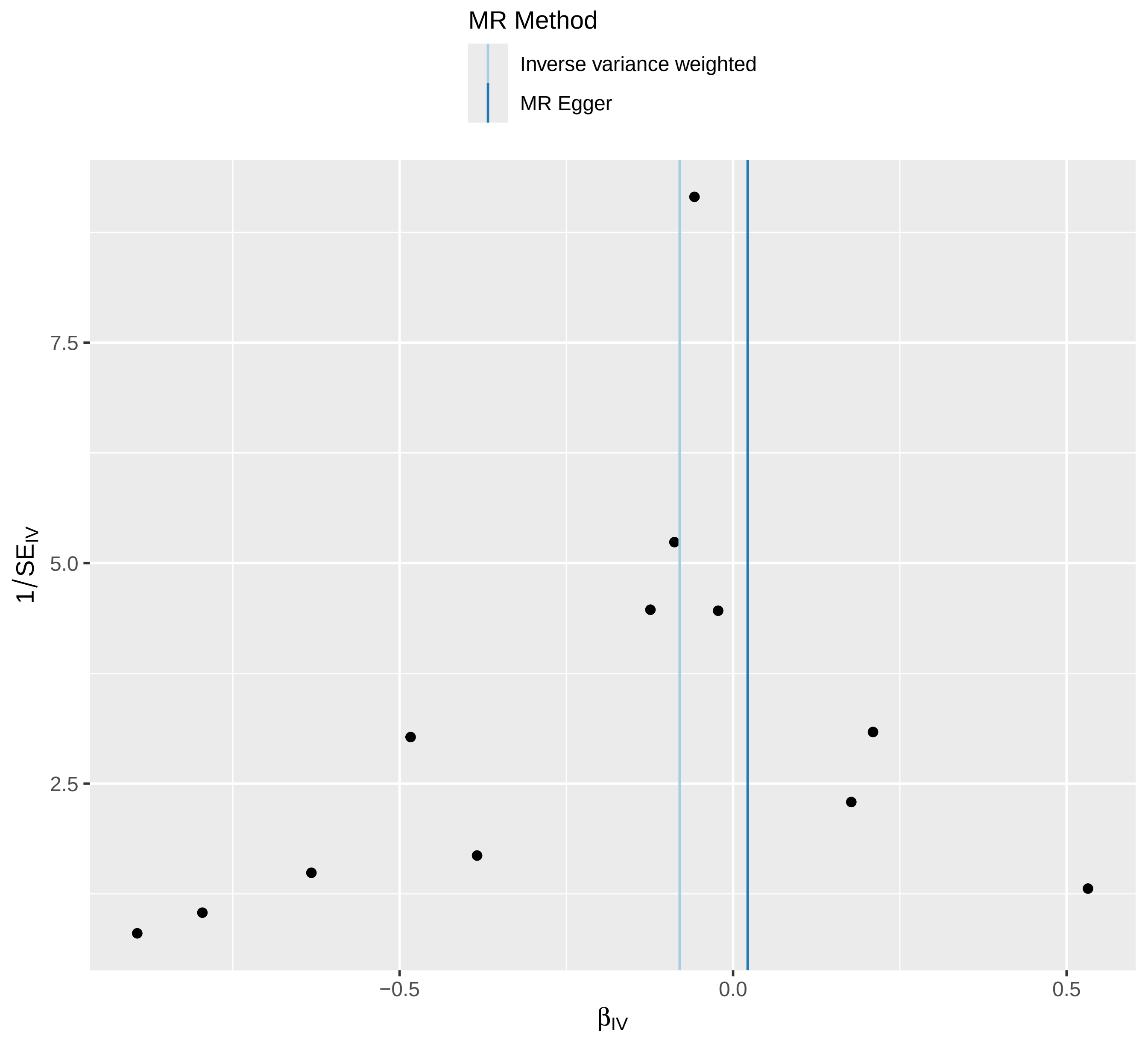

MR Method
Inverse variance weighted
MR Egger
7.5
5.0
2.5
1/SE_IV
−0.5
0.0
0.5
β_IV

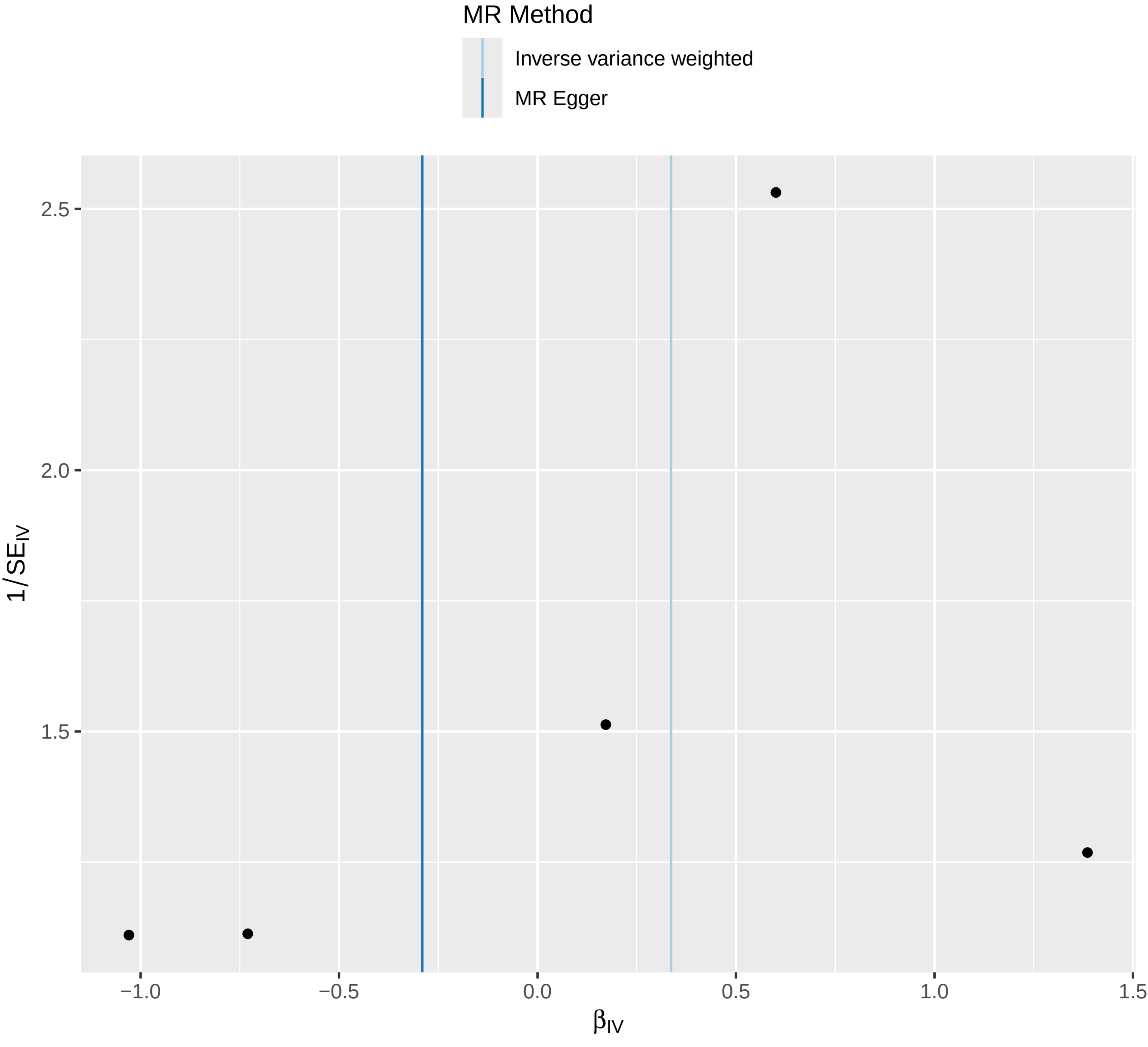

MR Method
Inverse variance weighted
MR Egger
2.5
2.0
1.5
1/SE$_{IV}$
−1.0
−0.5
0.0
0.5
1.0
1.5
β$_{IV}$

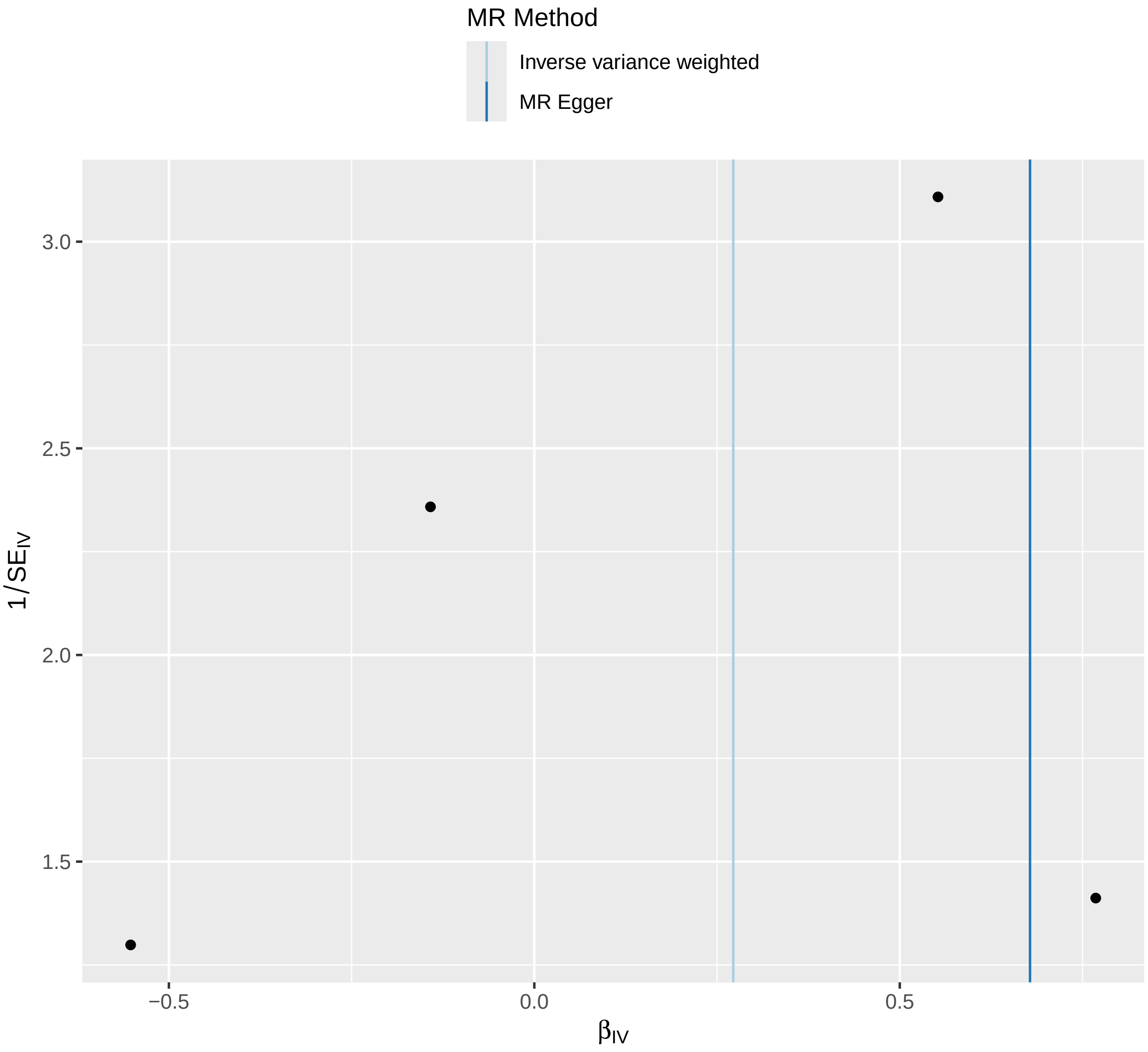

MR Method
Inverse variance weighted
MR Egger
3.0
2.5
2.0
1.5
1/SE_IV
−0.5
0.0
0.5
β_IV

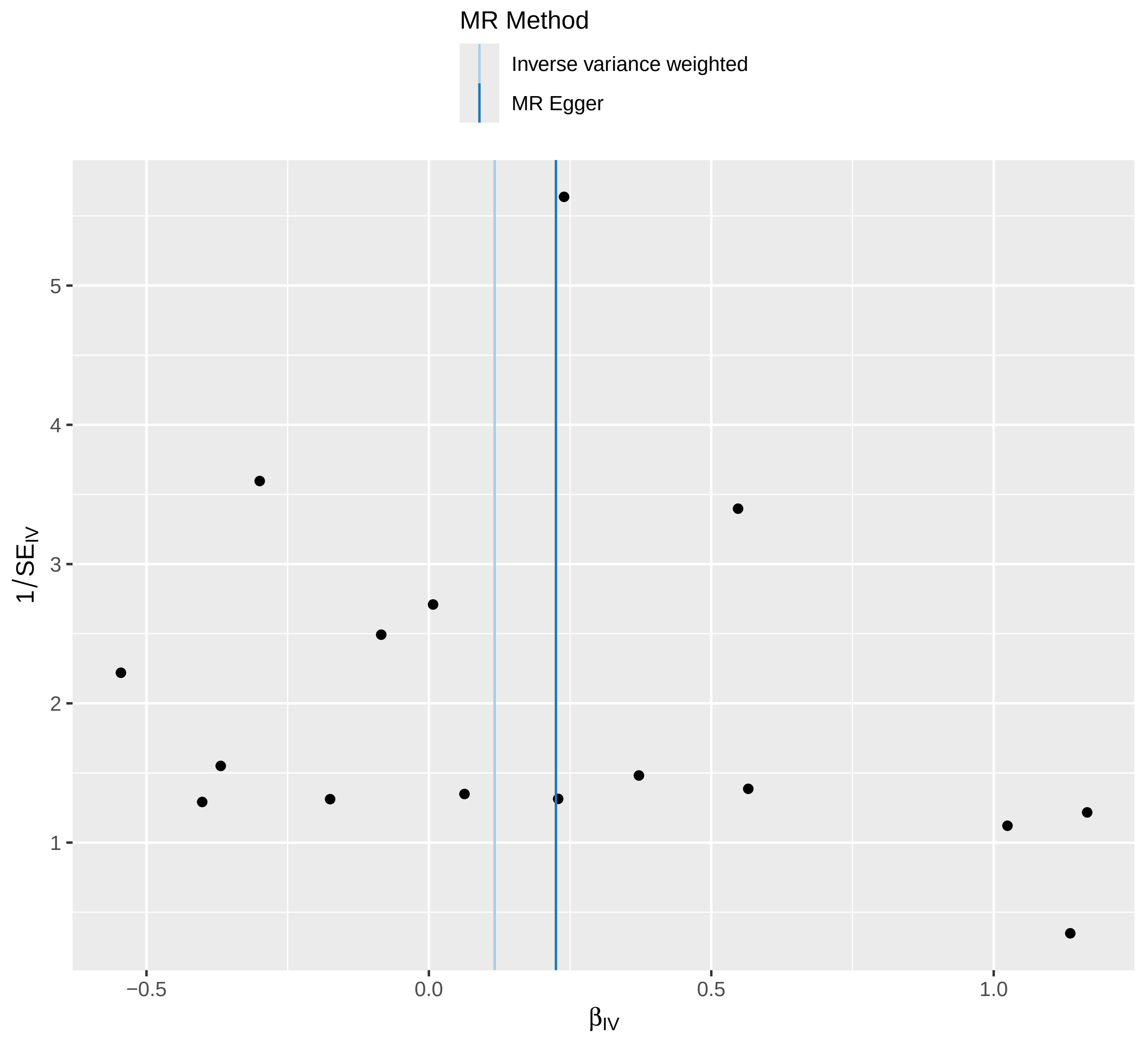
MR Method
Inverse variance weighted
MR Egger
5
4
3
2
1
1/SE_IV
−0.5
0.0
0.5
1.0
β_IV

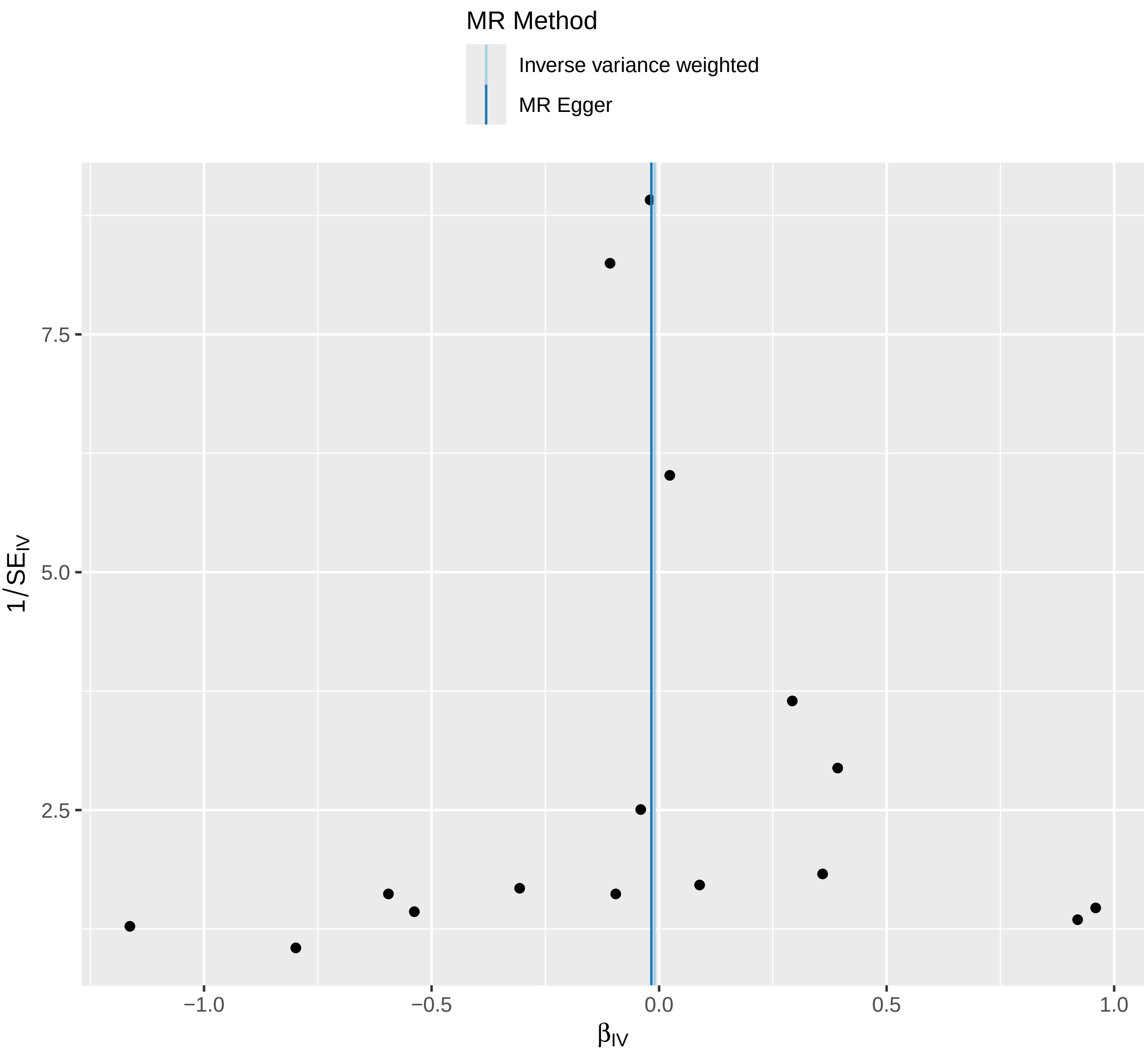
MR Method
Inverse variance weighted
MR Egger
7.5
5.0
2.5
1/SE_IV
−1.0
−0.5
0.0
0.5
1.0
β_IV

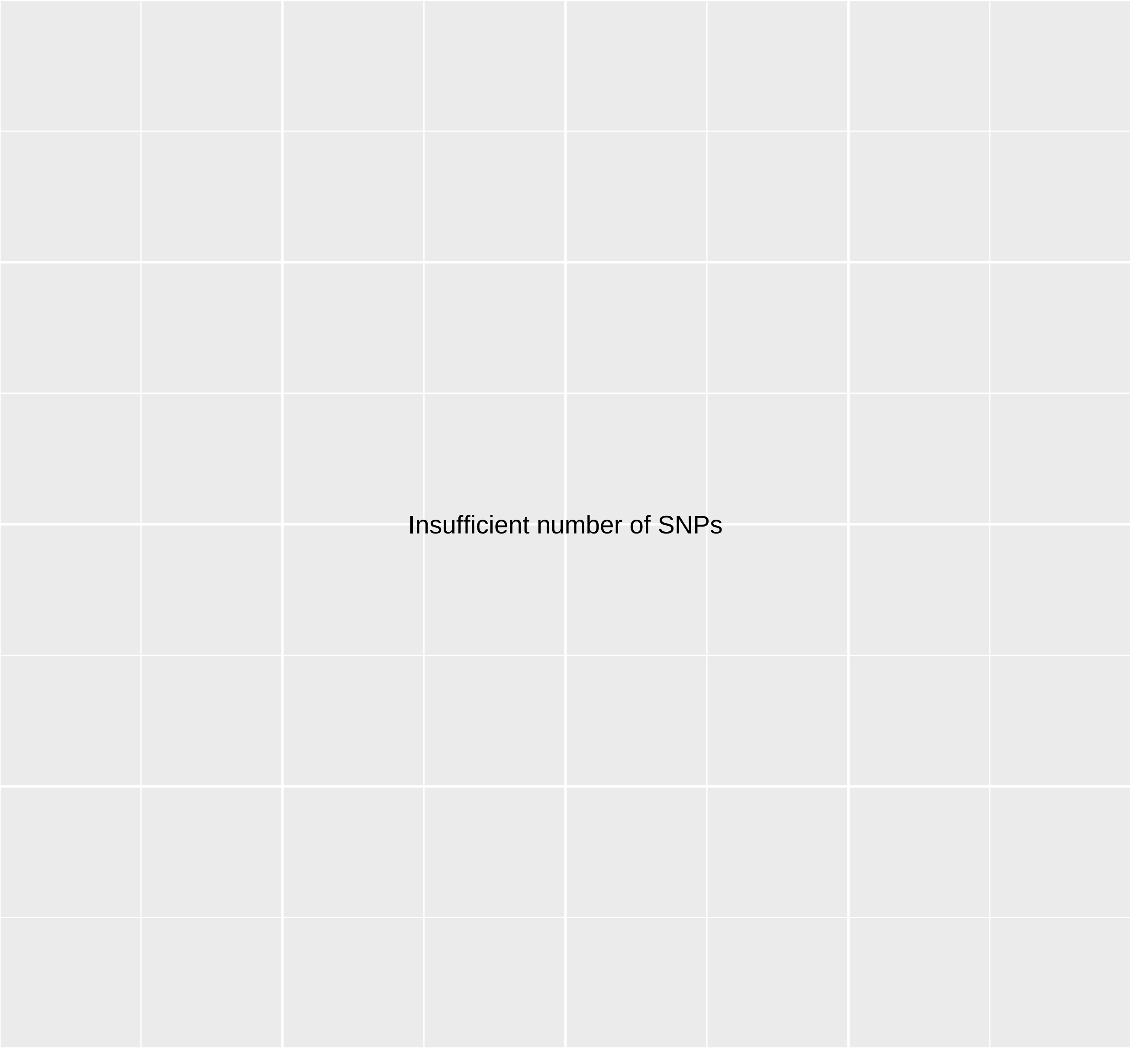
Insufficient number of SNPs

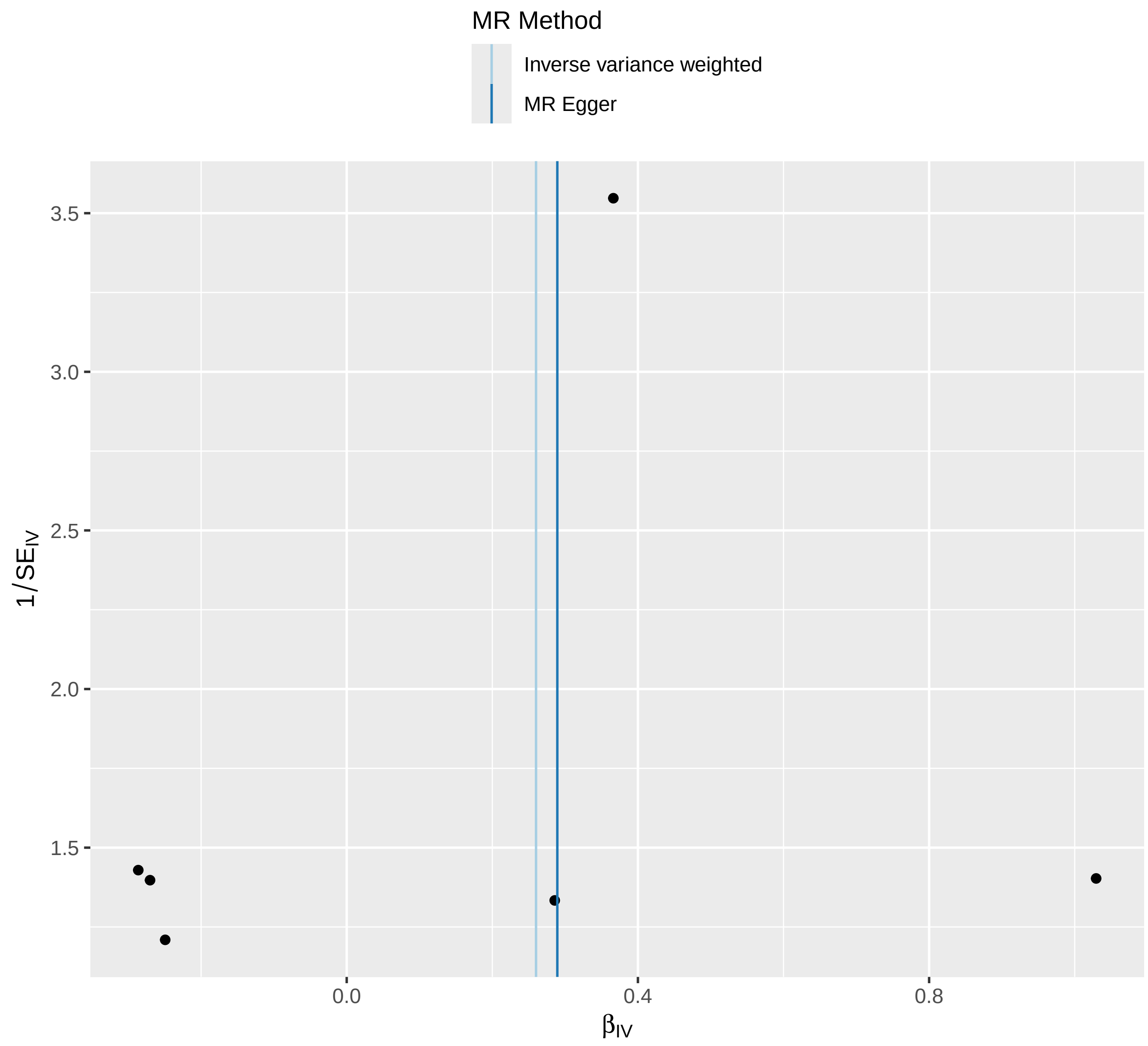
MR Method
Inverse variance weighted
MR Egger
3.5
3.0
2.5
2.0
1.5
1/SE_IV
0.0
0.4
0.8
β_IV

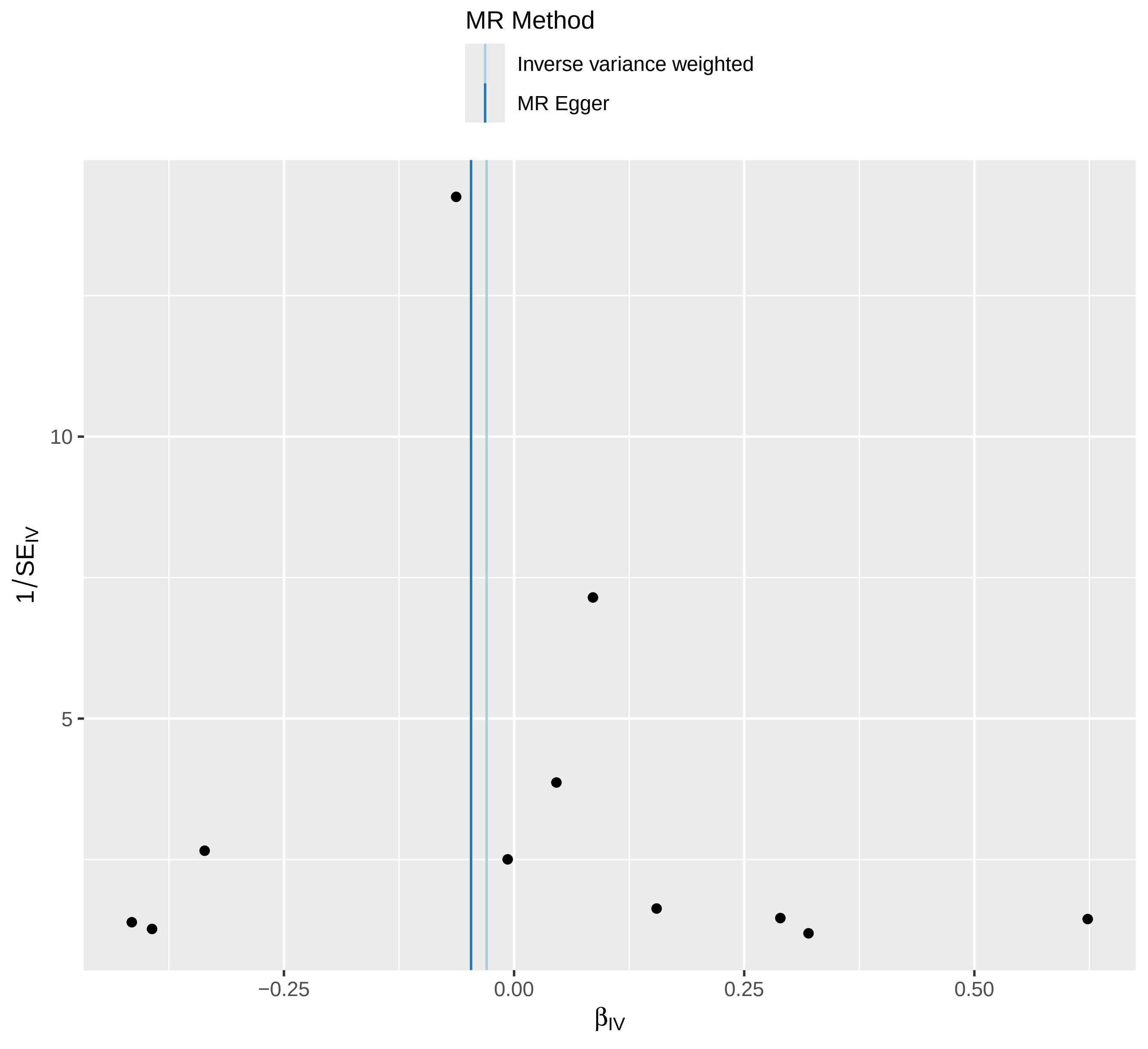
MR Method
Inverse variance weighted
MR Egger
10
5
$1/SE_{IV}$
−0.25
0.00
0.25
0.50
β_{IV}

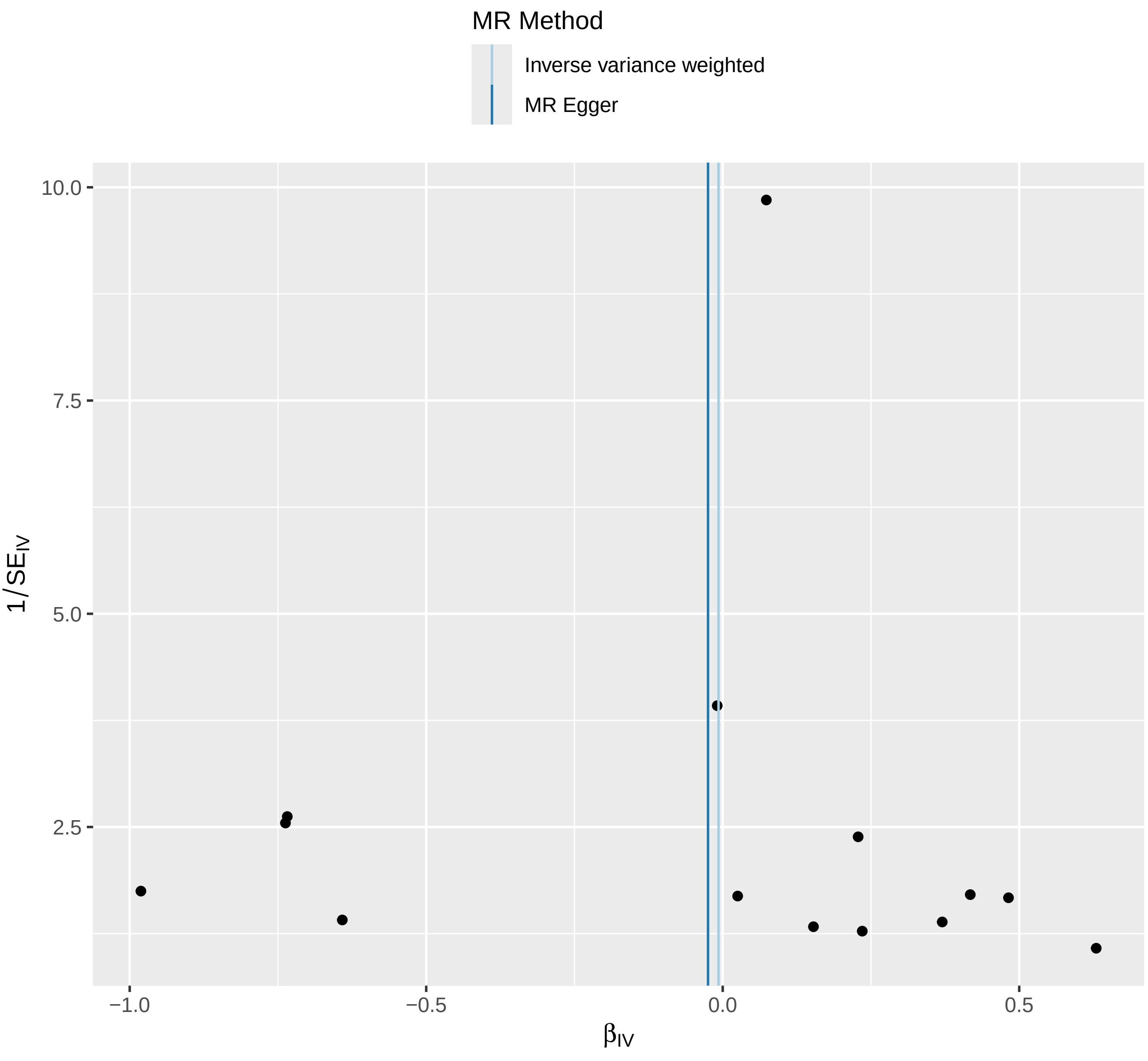
MR Method
Inverse variance weighted
MR Egger
10.0
7.5
5.0
2.5
1/SE_{IV}
−1.0
−0.5
0.0
0.5
β_{IV}

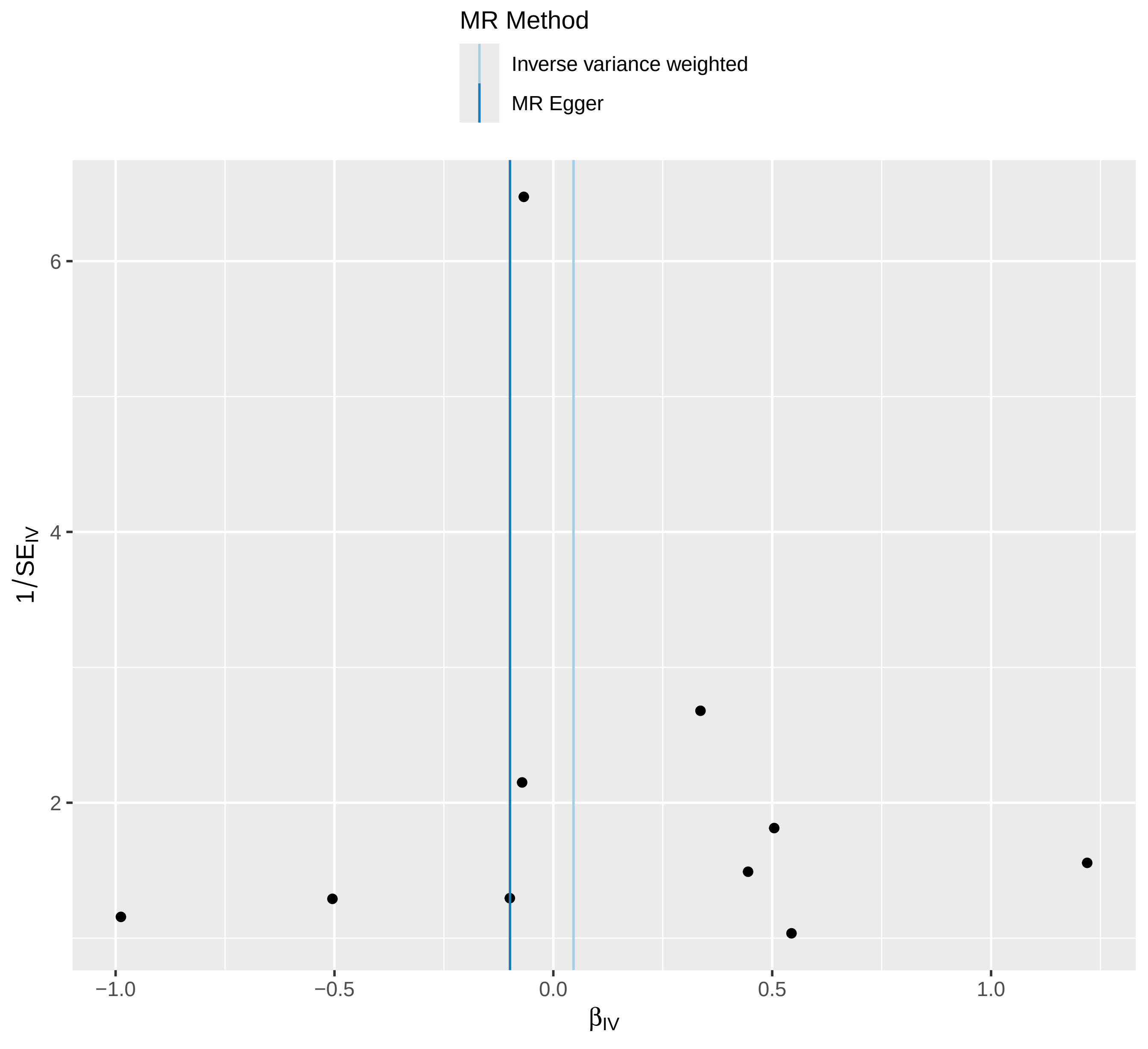
MR Method
Inverse variance weighted
MR Egger
1/SE$_{IV}$
6
4
2
−1.0
−0.5
0.0
0.5
1.0
β$_{IV}$

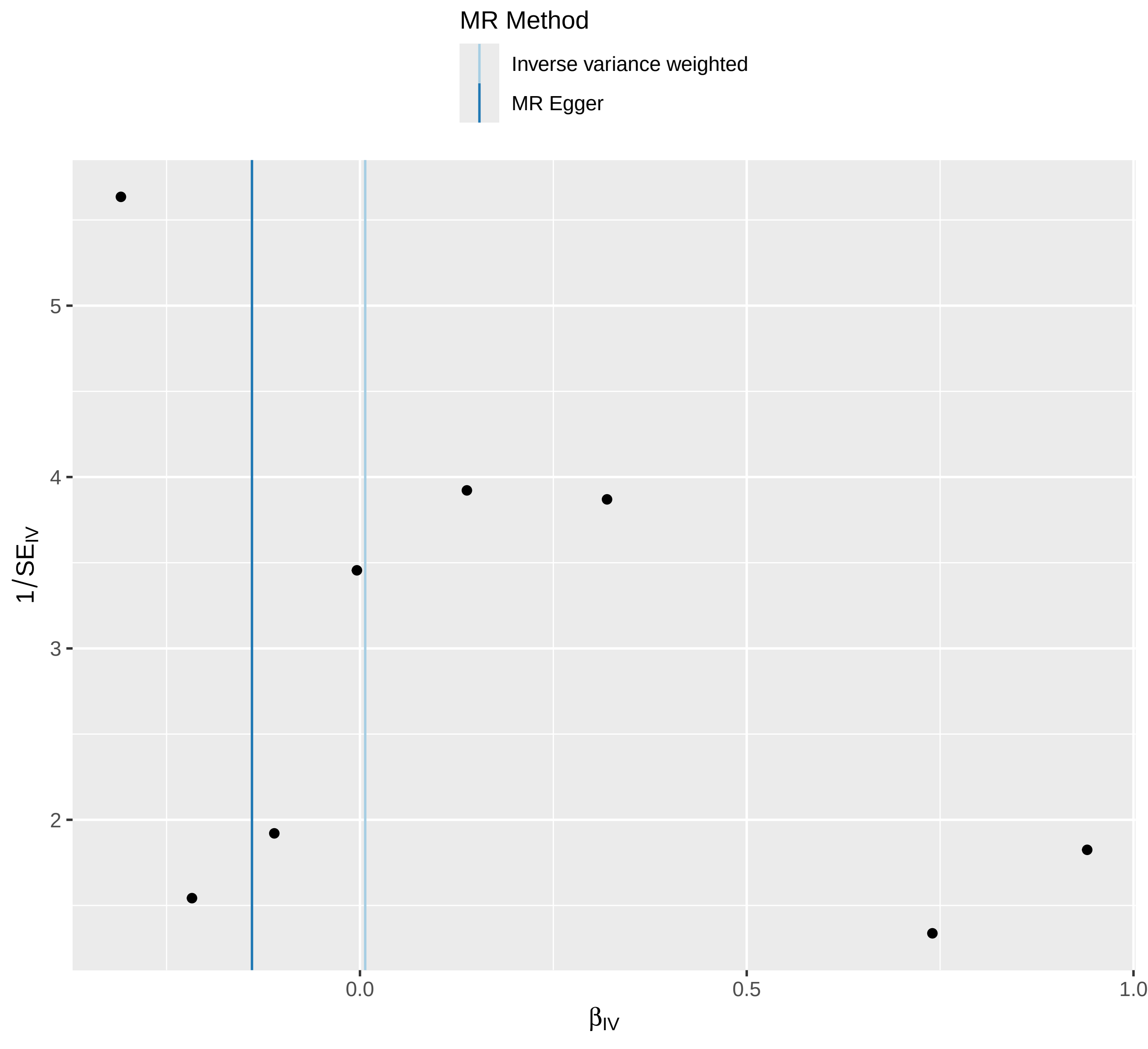
MR Method
Inverse variance weighted
MR Egger
1/SE_IV
5
4
3
2
0.0
0.5
1.0
β_IV

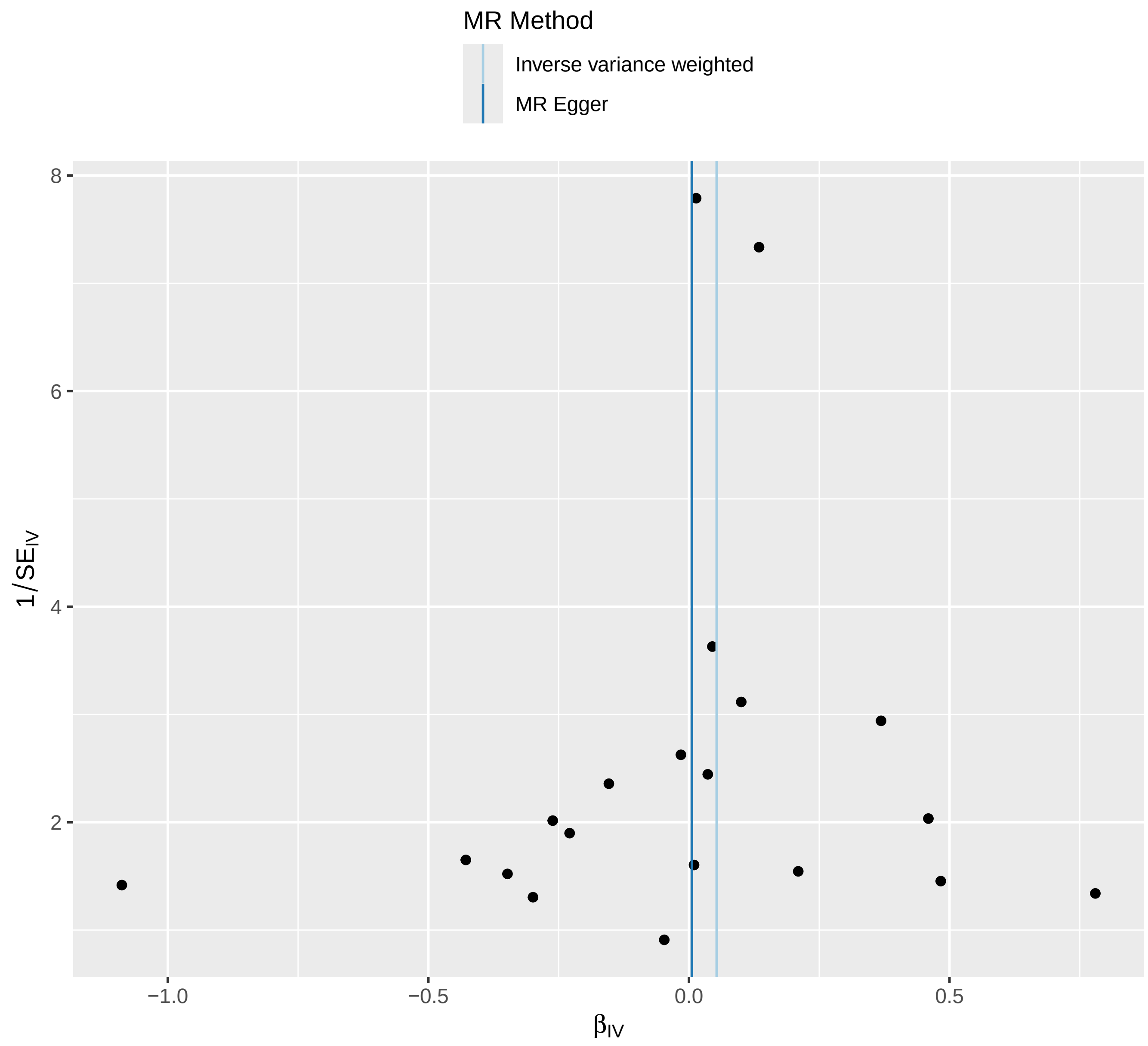
MR Method
Inverse variance weighted
MR Egger
8
6
4
2
1/SE_IV
−1.0
−0.5
0.0
0.5
β_IV

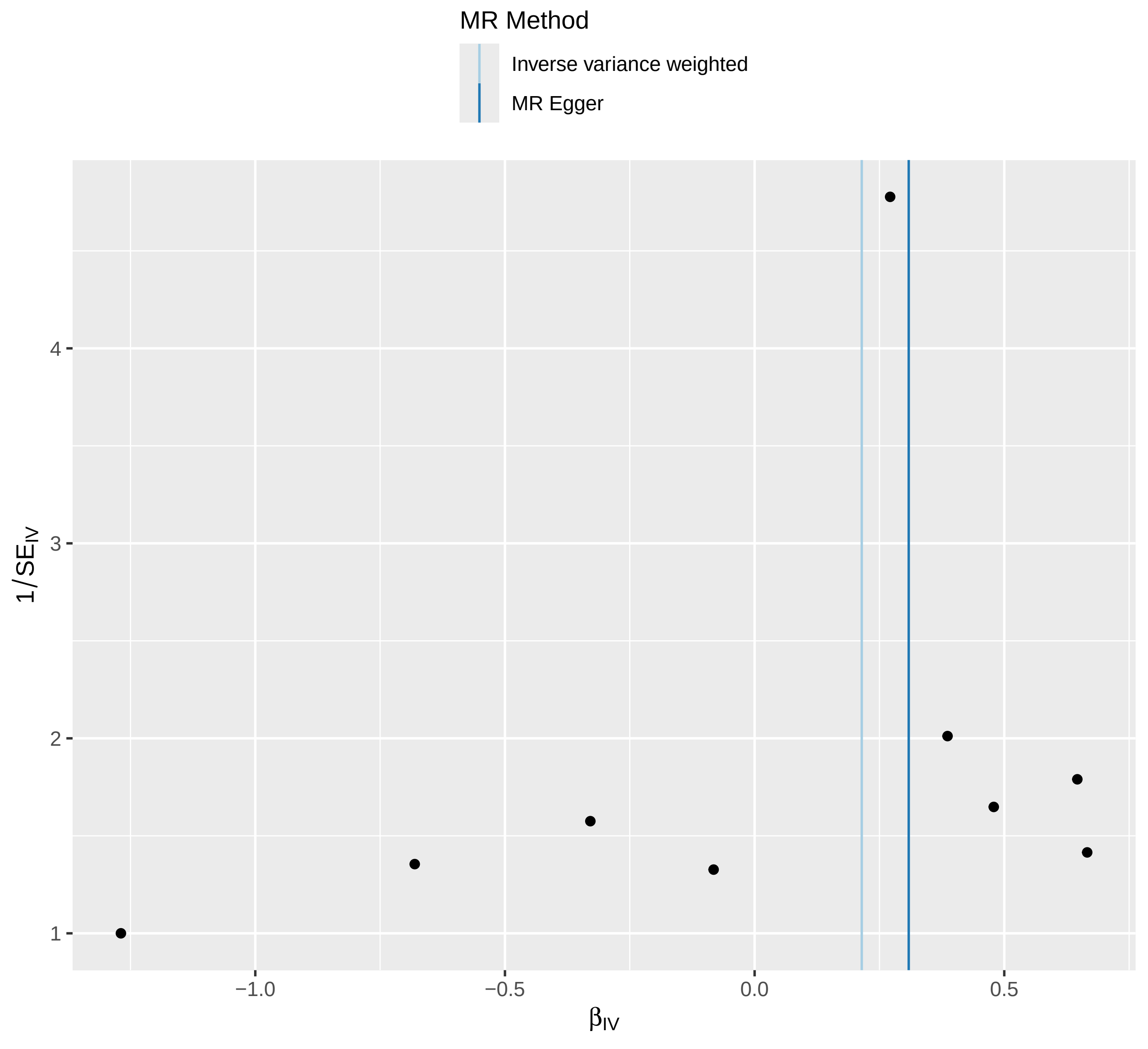
MR Method
Inverse variance weighted
MR Egger
1/SE_IV
β_IV
4
3
2
1
−1.0
−0.5
0.0
0.5

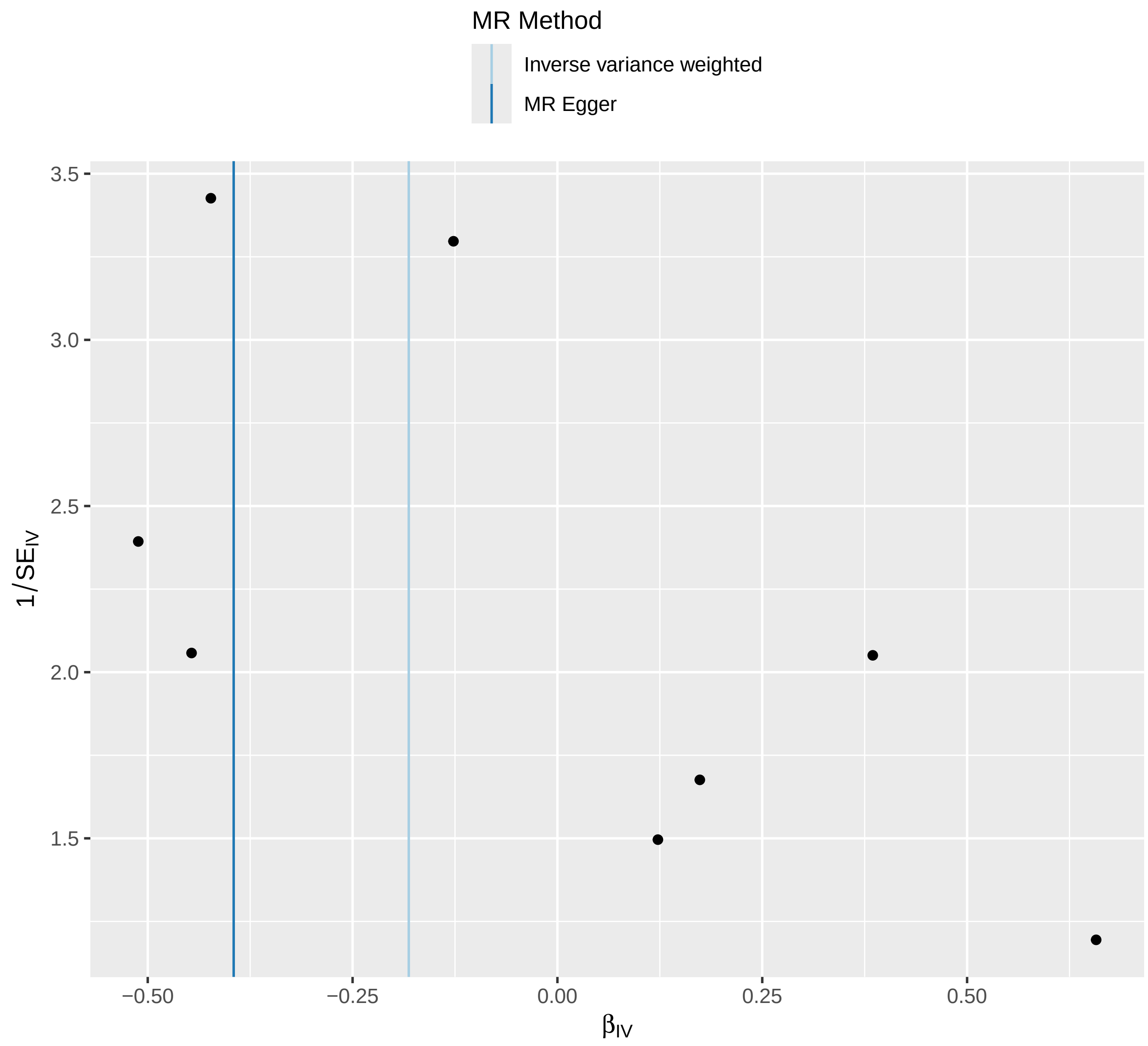

MR Method
Inverse variance weighted
MR Egger
3.5
3.0
2.5
2.0
1.5
1/SE$_{IV}$
−0.50
−0.25
0.00
0.25
0.50
β$_{IV}$

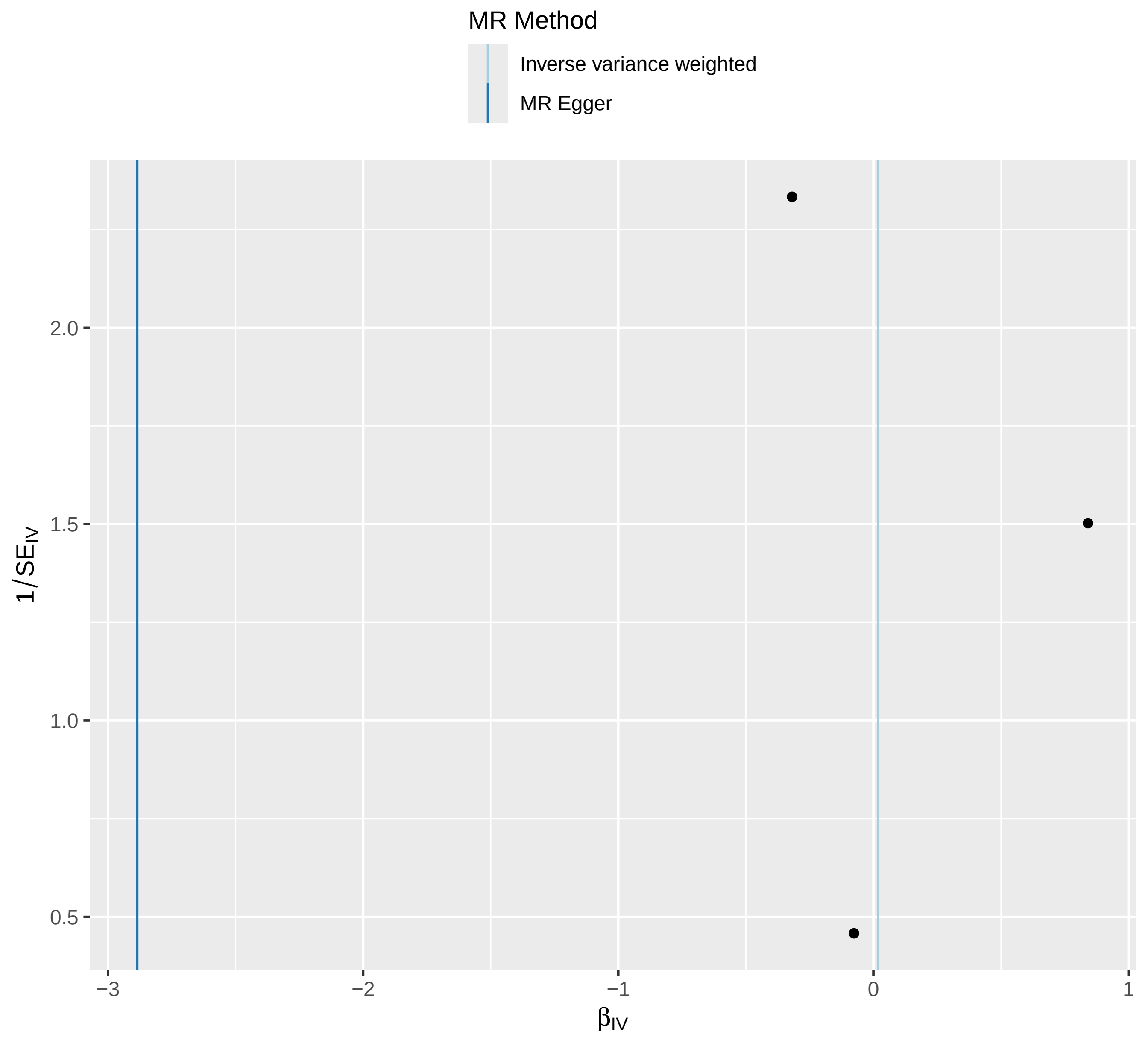
MR Method
Inverse variance weighted
MR Egger
2.0
1.5
1.0
0.5
1/SE$_{IV}$
−3
−2
−1
0
1
β$_{IV}$

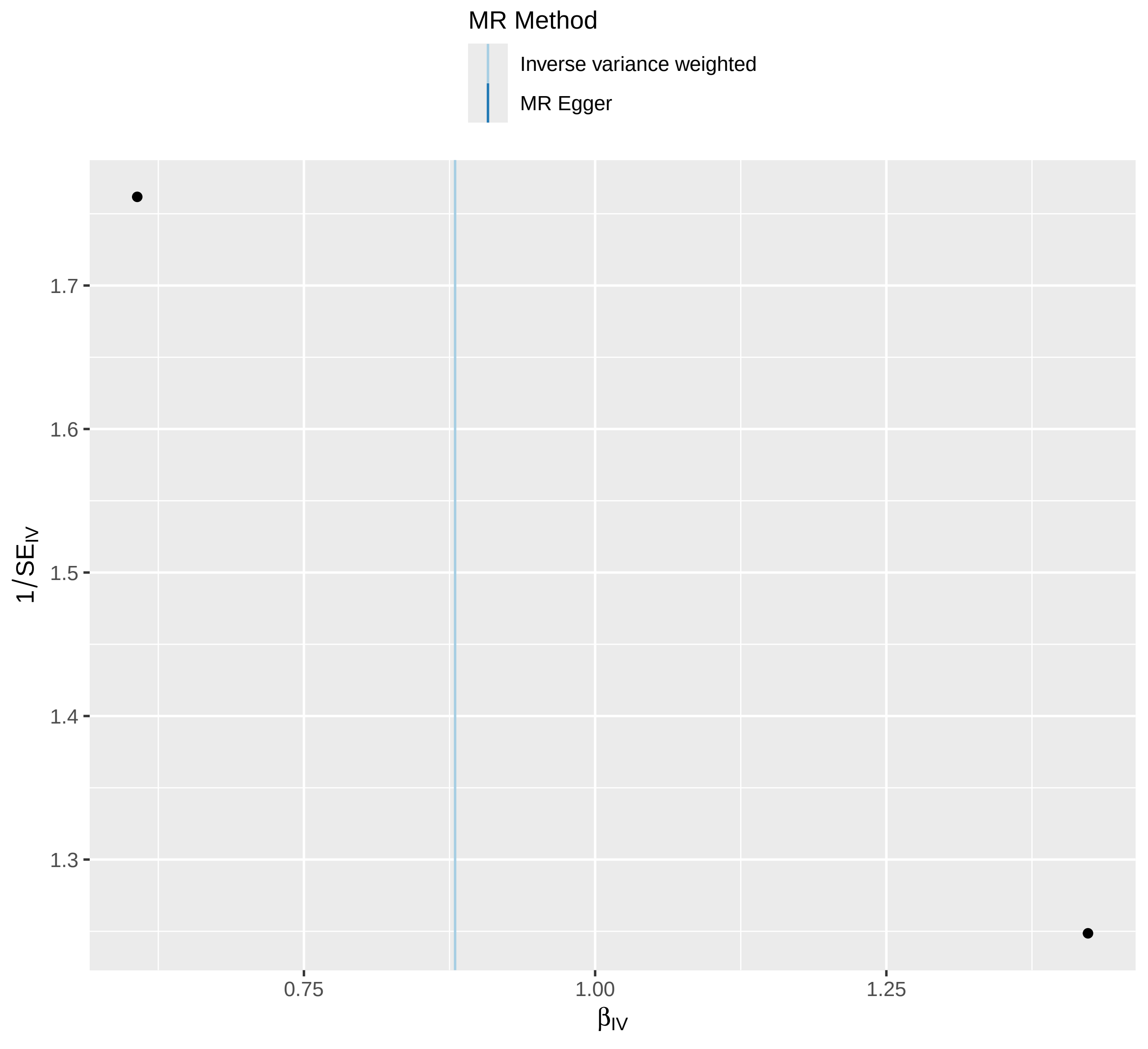
MR Method
Inverse variance weighted
MR Egger
1.7
1.6
1.5
1.4
1.3
$1/SE_{IV}$
0.75
1.00
1.25
β_{IV}

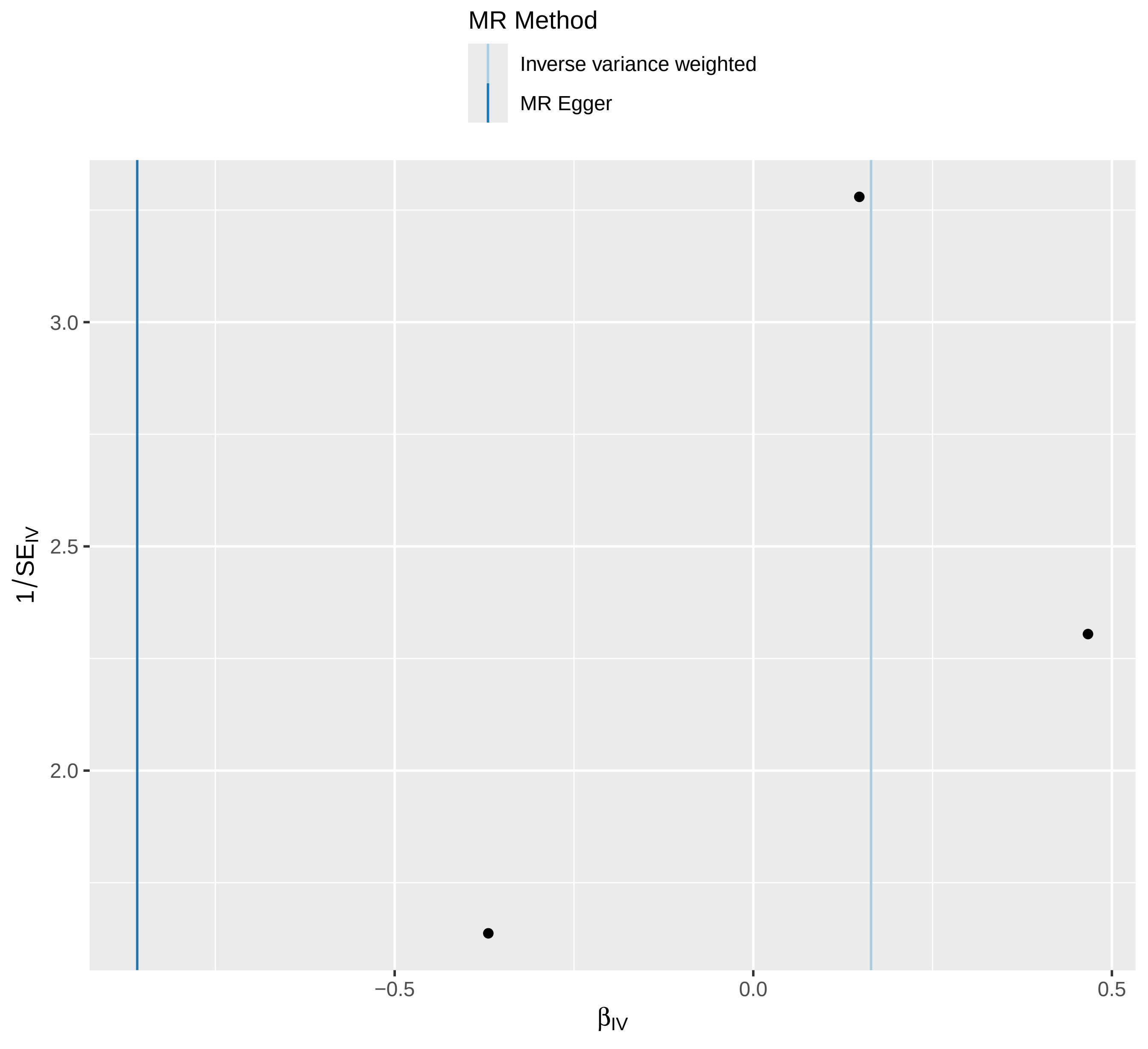
MR Method
Inverse variance weighted
MR Egger
3.0
2.5
2.0
1/SE$_{IV}$
−0.5
0.0
0.5
β$_{IV}$

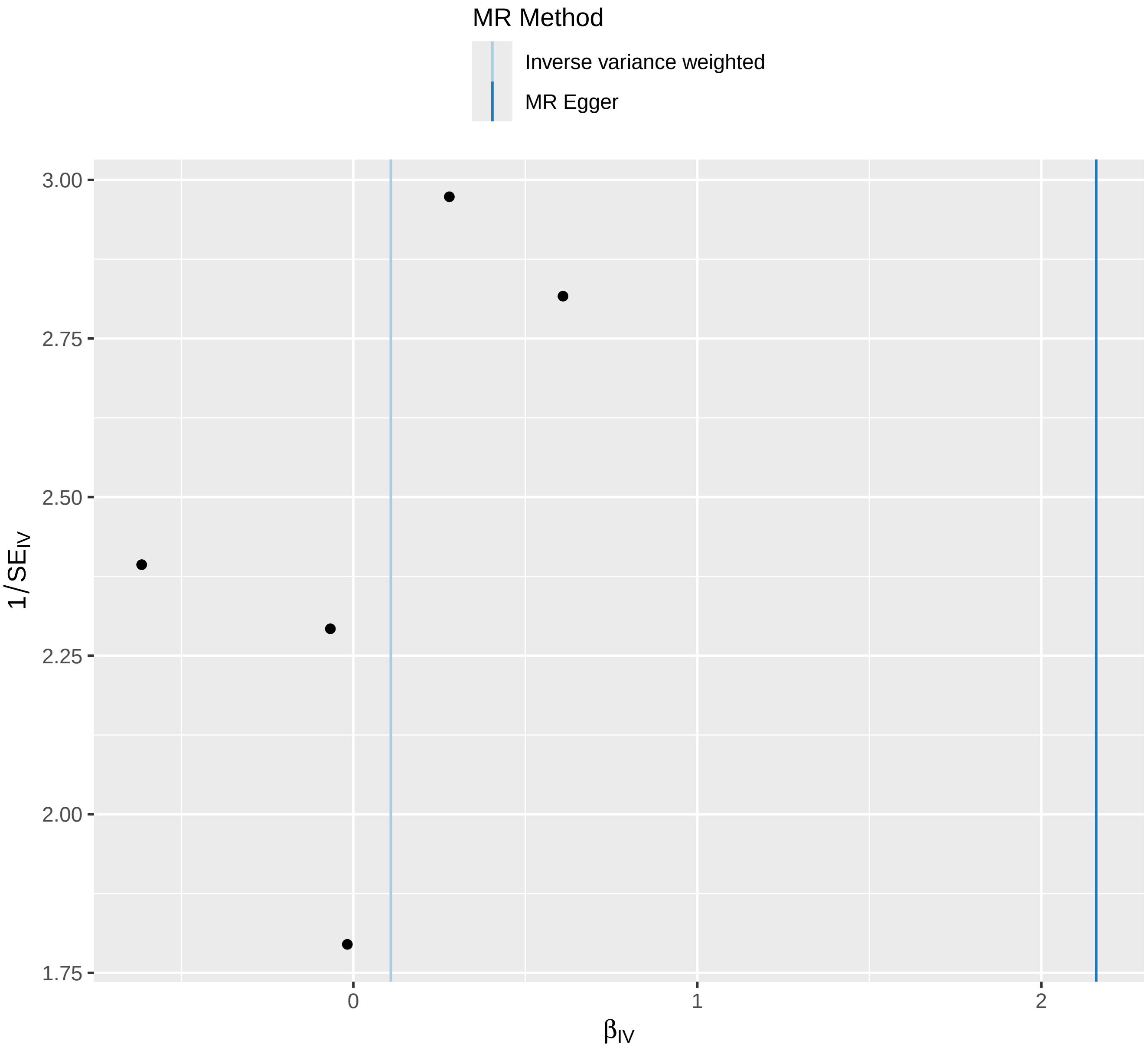
MR Method
Inverse variance weighted
MR Egger
3.00
2.75
2.50
2.25
2.00
1.75
$1/SE_{IV}$
0
1
2
β_{IV}

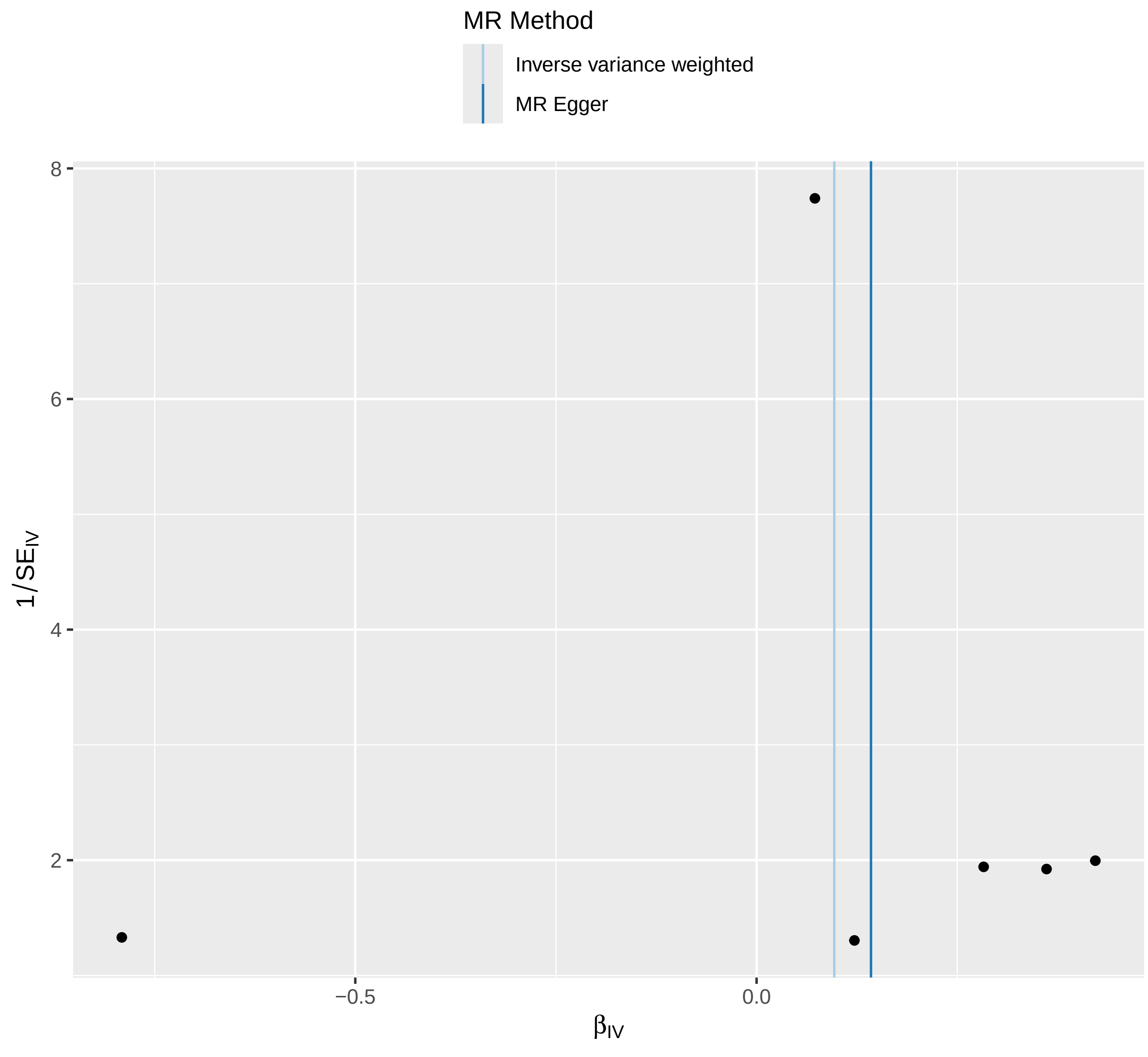
MR Method
Inverse variance weighted
MR Egger
8
6
4
2
1/SE_IV
−0.5
0.0
β_IV

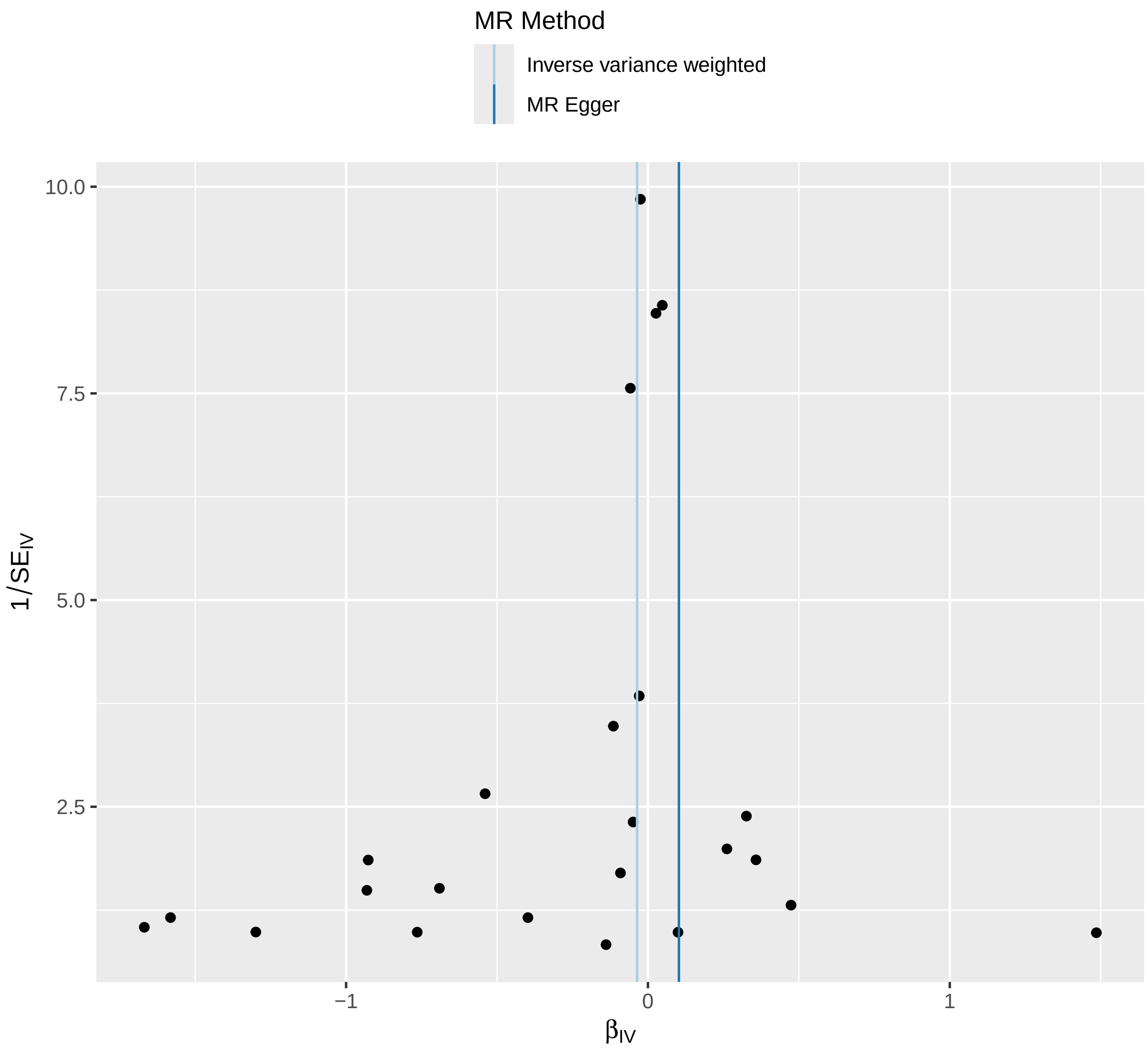

MR Method
Inverse variance weighted
MR Egger
10.0
7.5
5.0
2.5
$1/SE_{IV}$
−1
0
1
β_{IV}

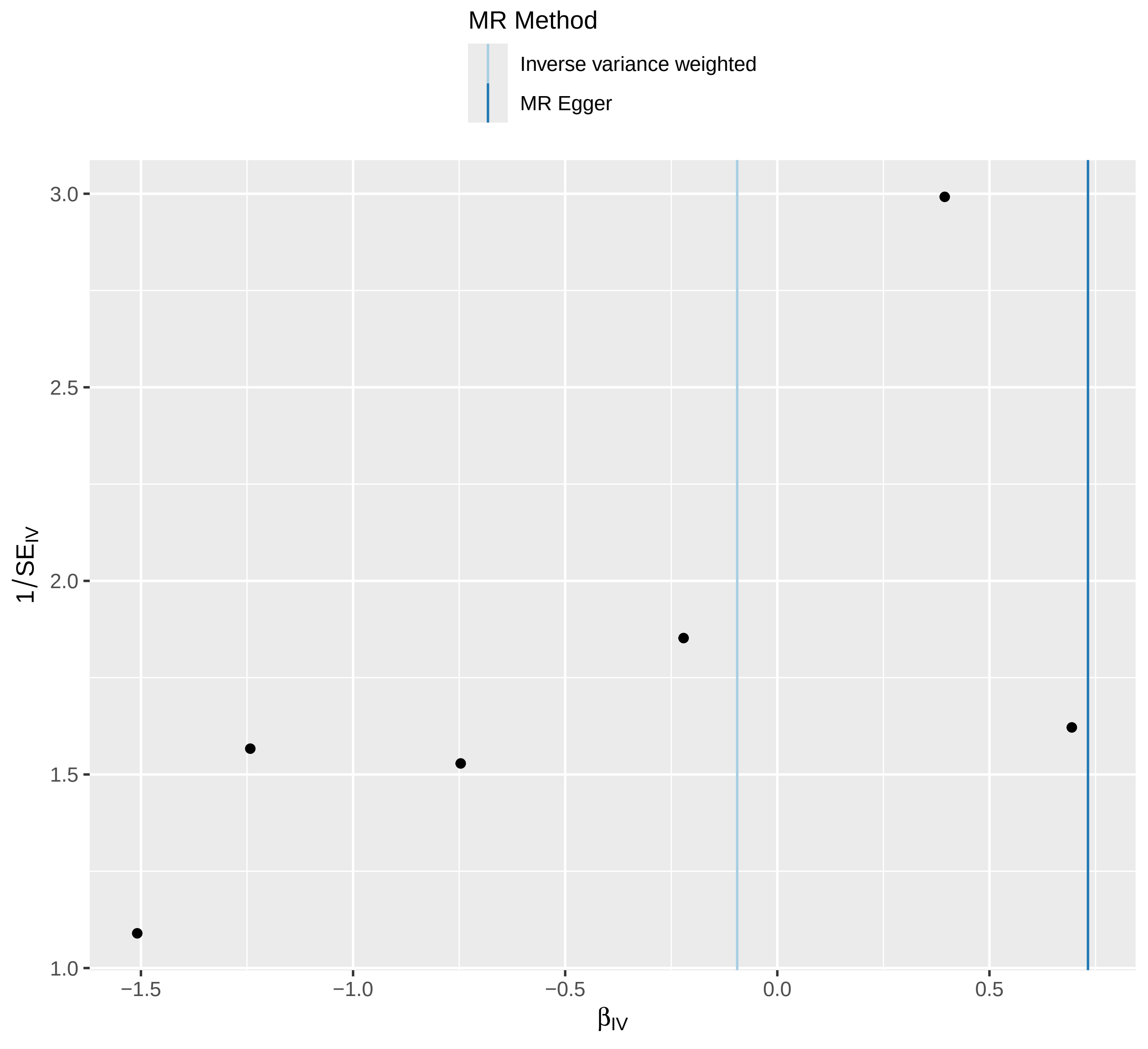

MR Method
Inverse variance weighted
MR Egger
3.0
2.5
2.0
1.5
1.0
1/SE_IV
−1.5
−1.0
−0.5
0.0
0.5
β_IV

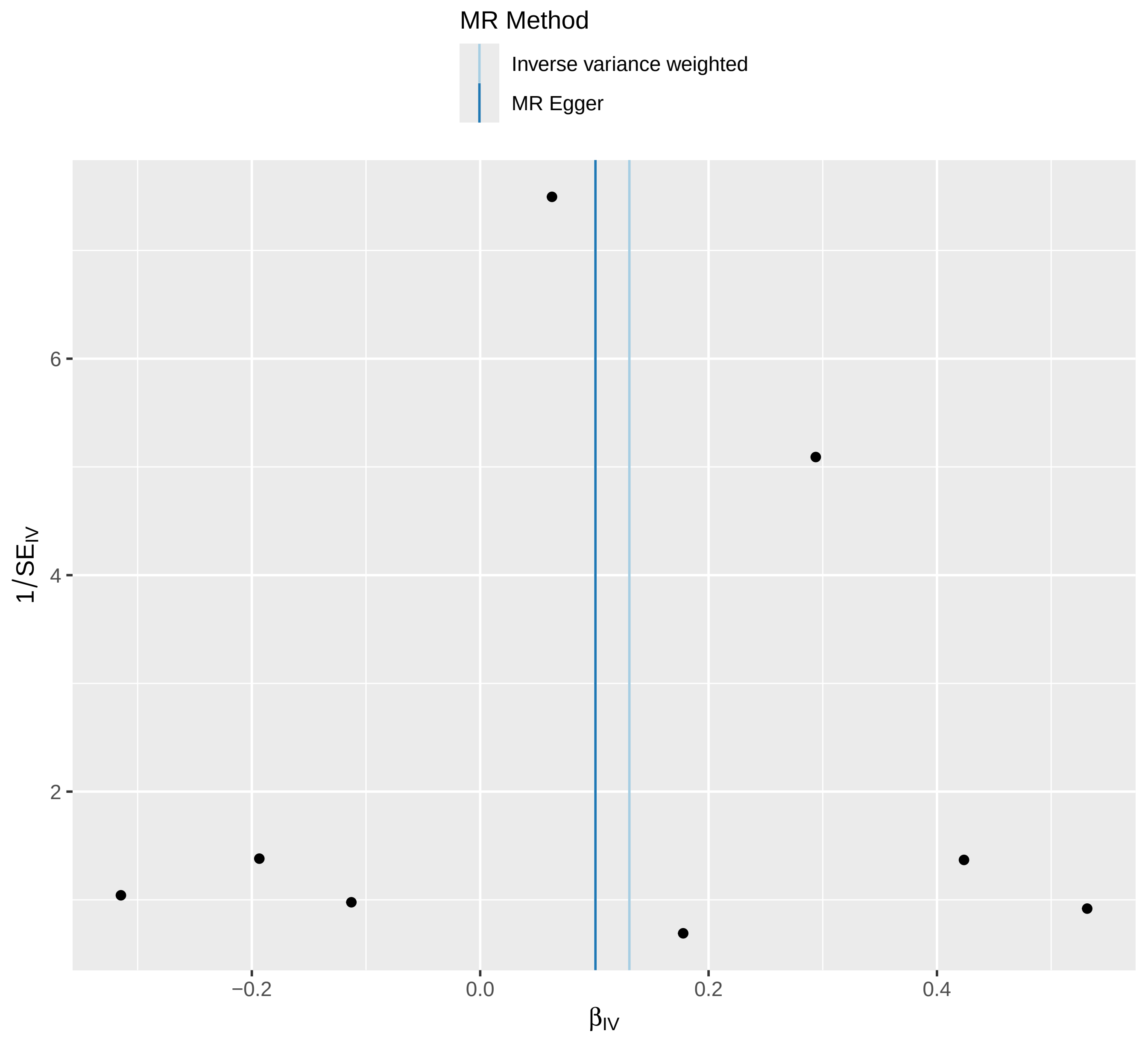

MR Method
Inverse variance weighted
MR Egger
1/SE_IV
6
4
2
−0.2
0.0
0.2
0.4
β_IV

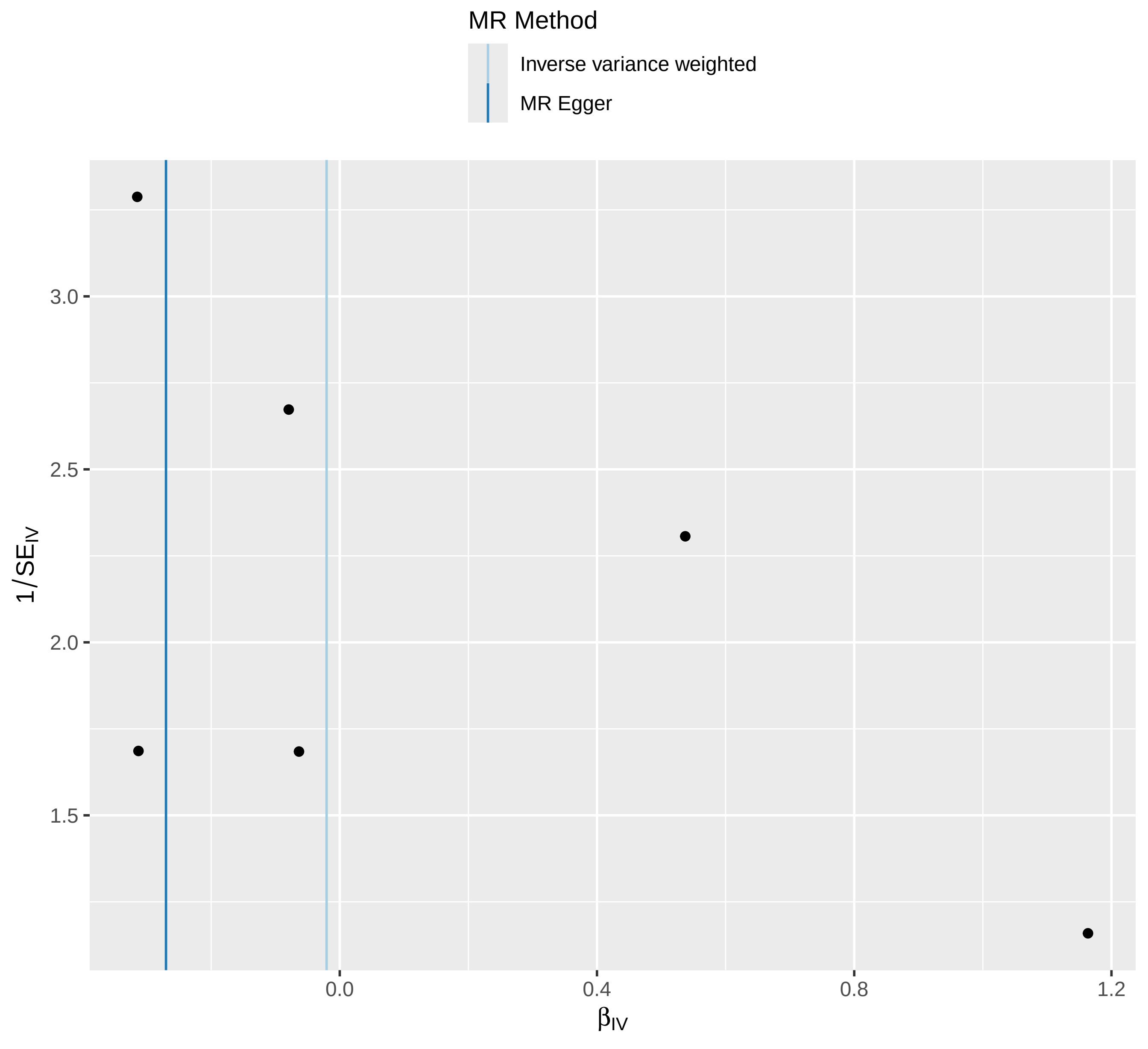

MR Method
Inverse variance weighted
MR Egger
3.0
2.5
2.0
1.5
1/SE$_{IV}$
0.0
0.4
0.8
1.2
β$_{IV}$

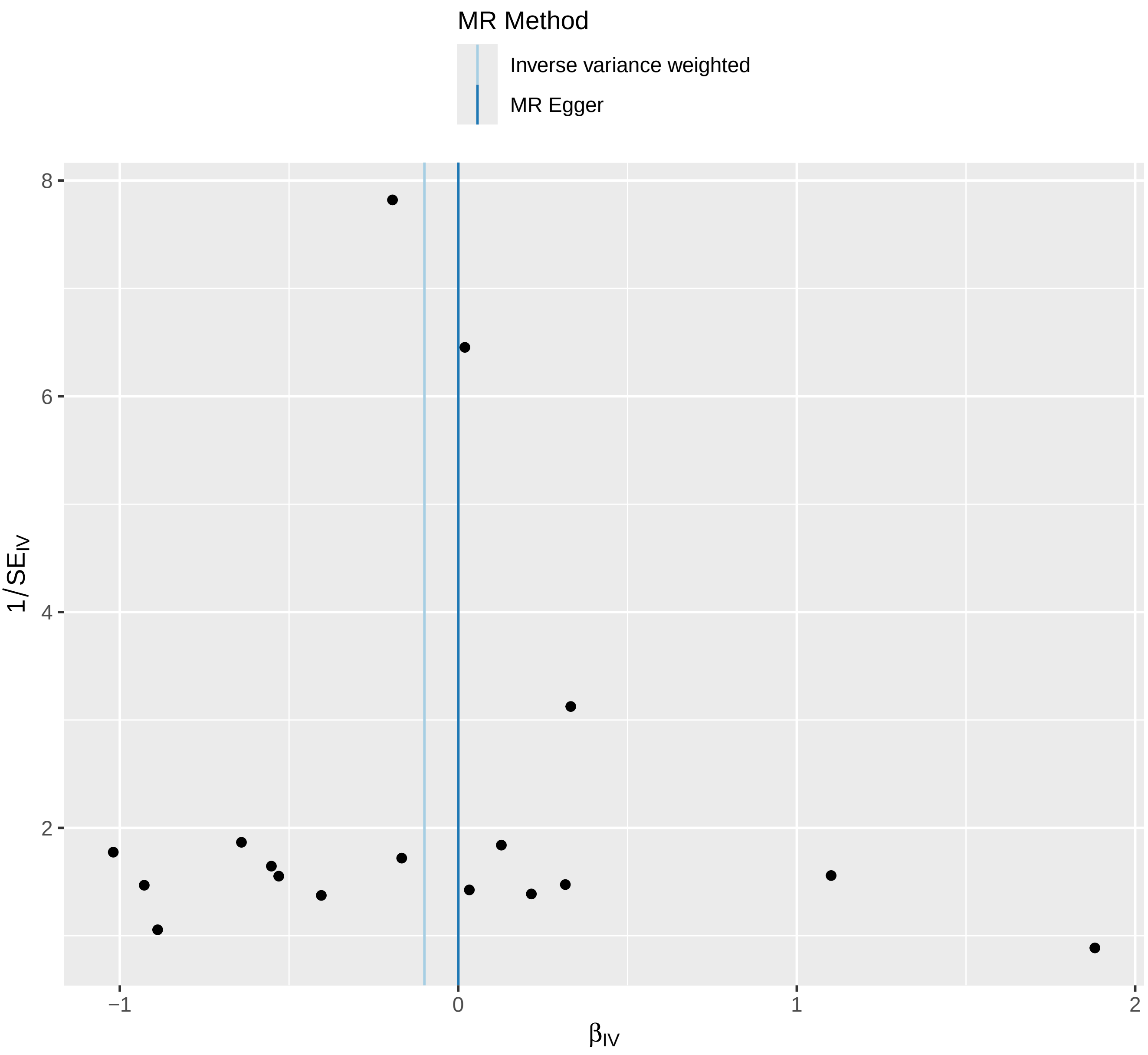
MR Method
Inverse variance weighted
MR Egger
8
6
4
2
1/SE_IV
−1
0
1
2
β_IV

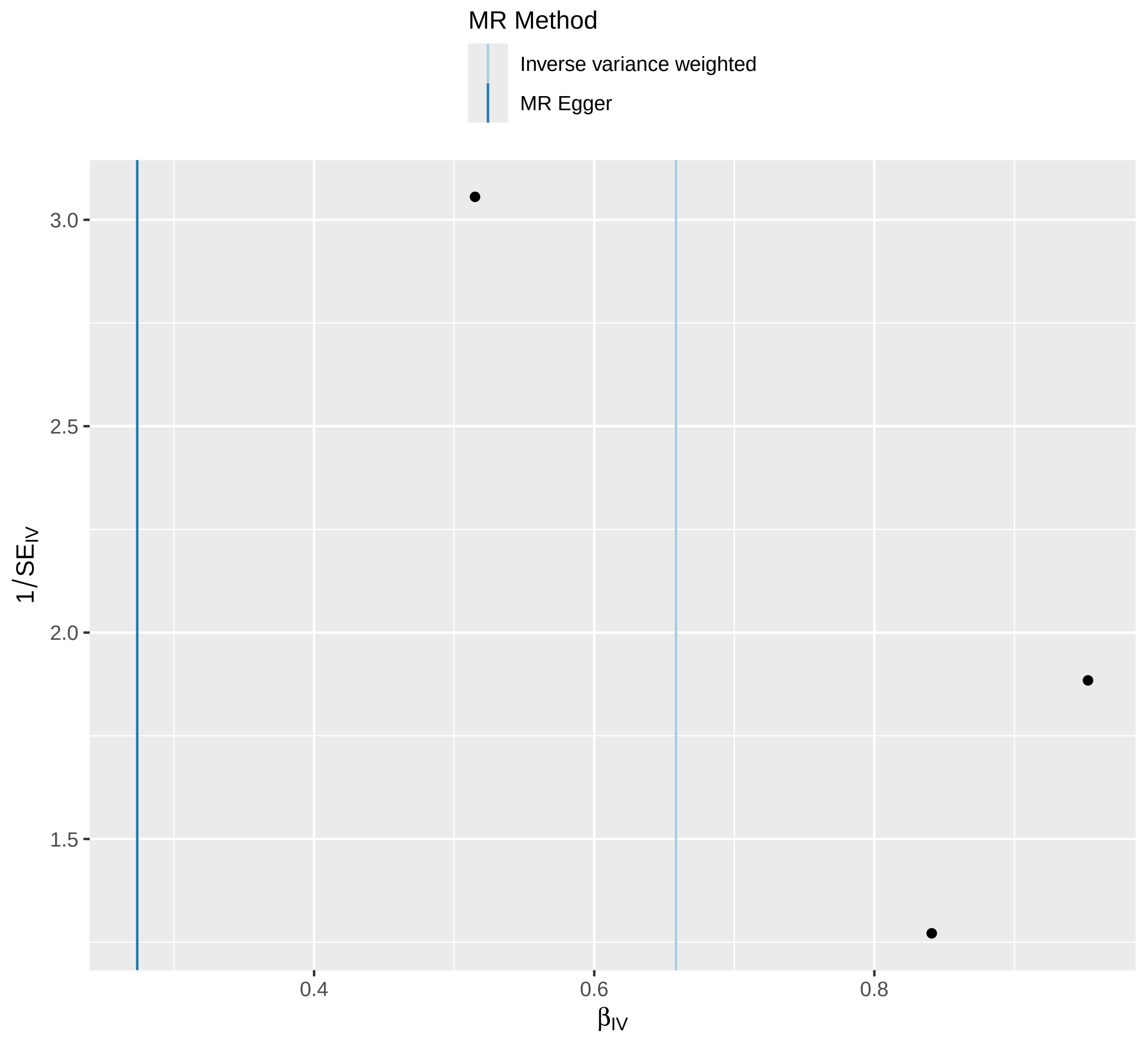
MR Method
Inverse variance weighted
MR Egger
3.0
2.5
2.0
1.5
0.4
0.6
0.8
$1/SE_{IV}$
β_{IV}

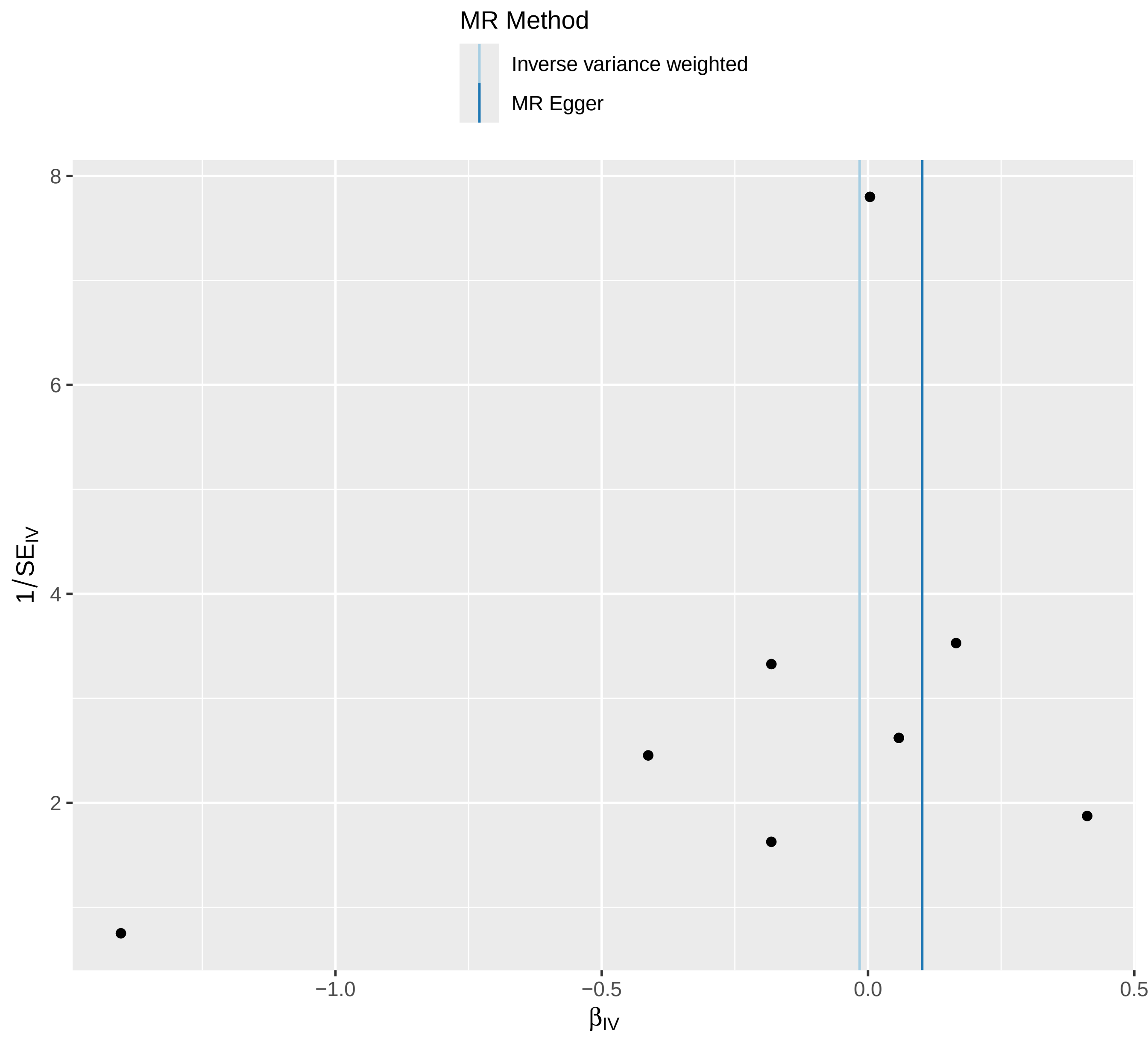

MR Method
Inverse variance weighted
MR Egger
8
6
4
2
1/SE_IV
−1.0
−0.5
0.0
0.5
β_IV

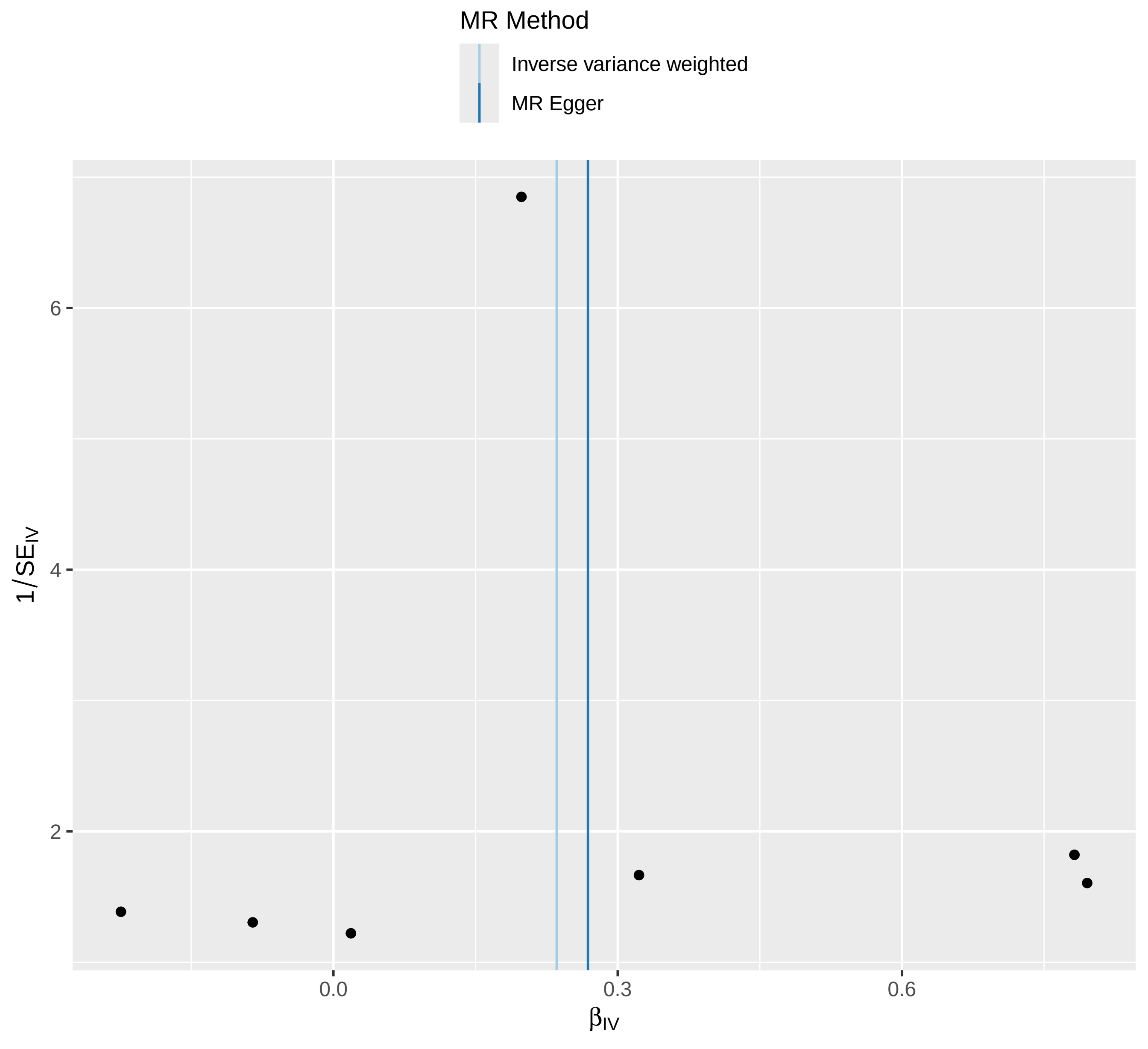

MR Method
Inverse variance weighted
MR Egger
6
4
2
0.0
0.3
0.6
$1/SE_{IV}$
β_{IV}

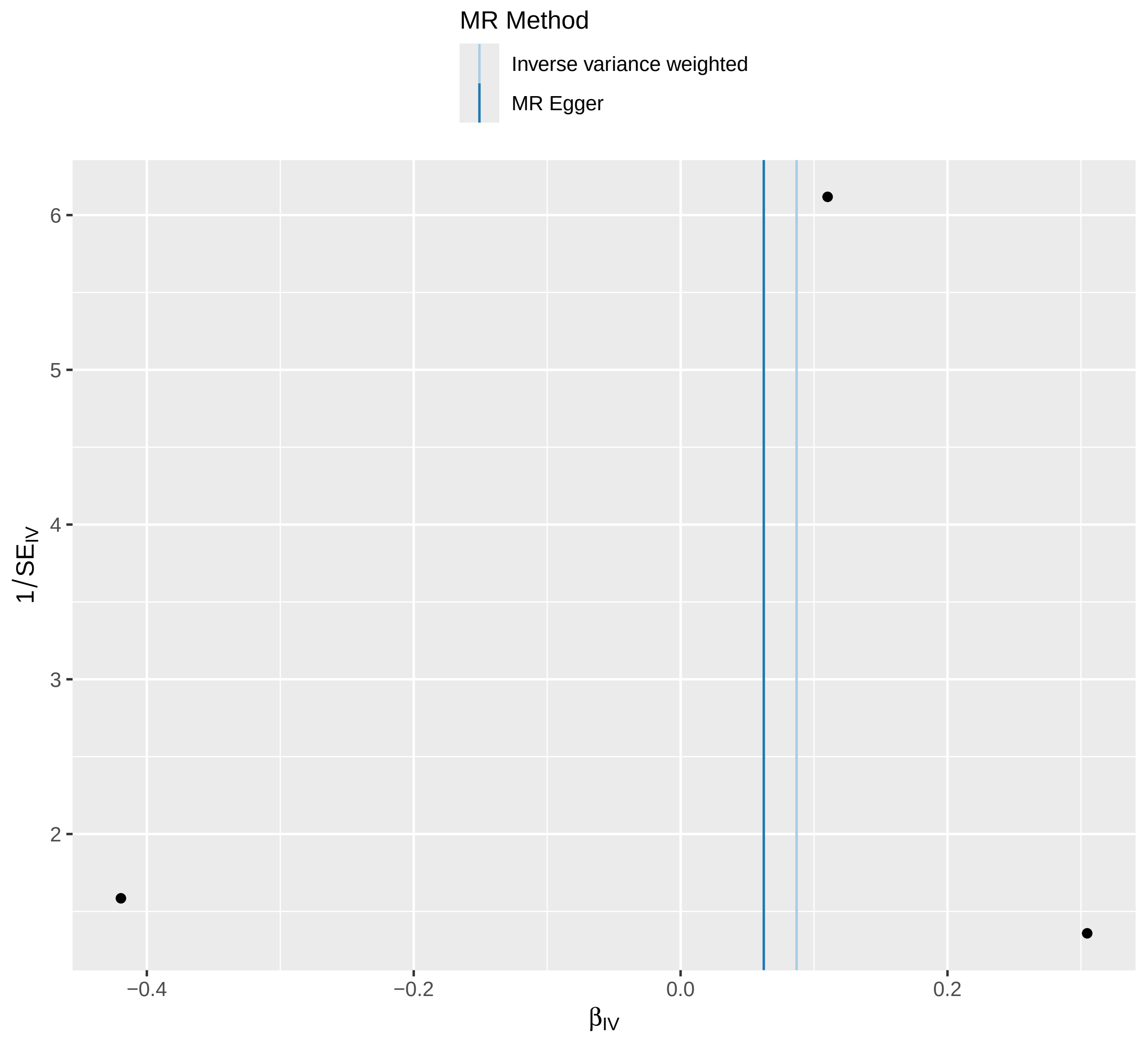

MR Method
Inverse variance weighted
MR Egger
6
5
4
3
2
1/SE_IV
−0.4
−0.2
0.0
0.2
β_IV

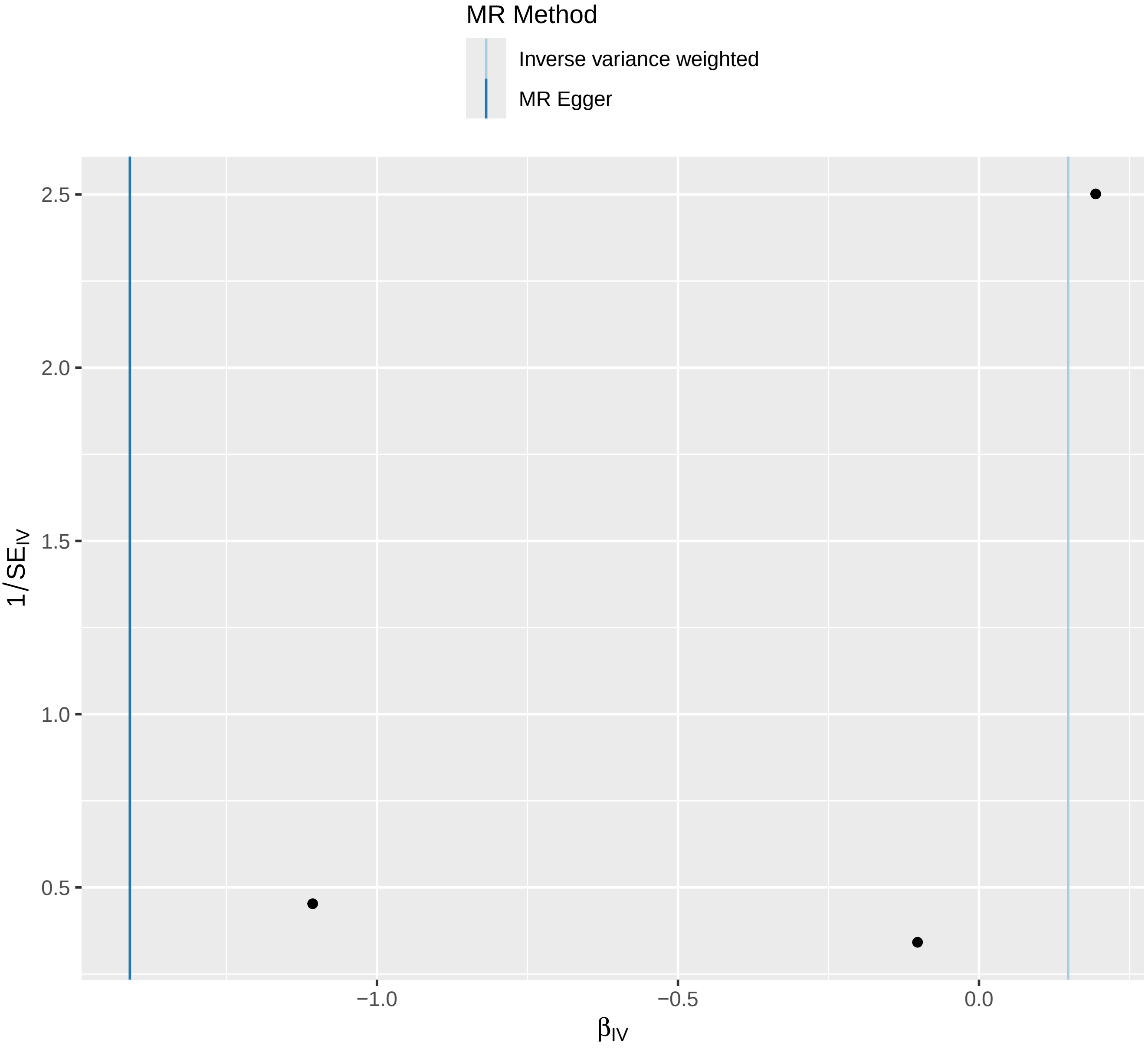

MR Method
Inverse variance weighted
MR Egger
1/SE$_{IV}$
2.5
2.0
1.5
1.0
0.5
−1.0
−0.5
0.0
β$_{IV}$

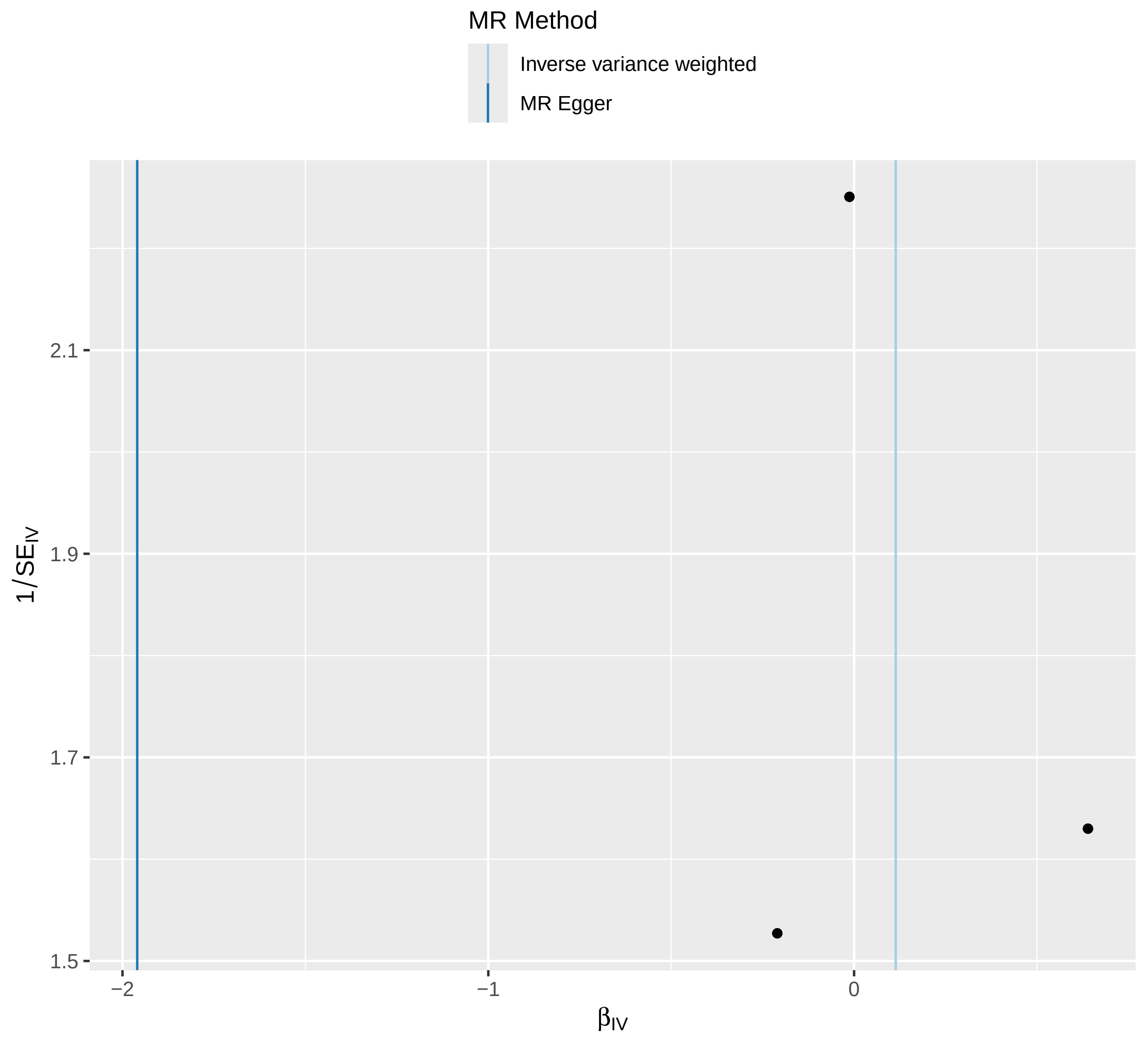

MR Method
Inverse variance weighted
MR Egger
2.1
1.9
1.7
1.5
1/SE_IV
−2
−1
0
β_IV

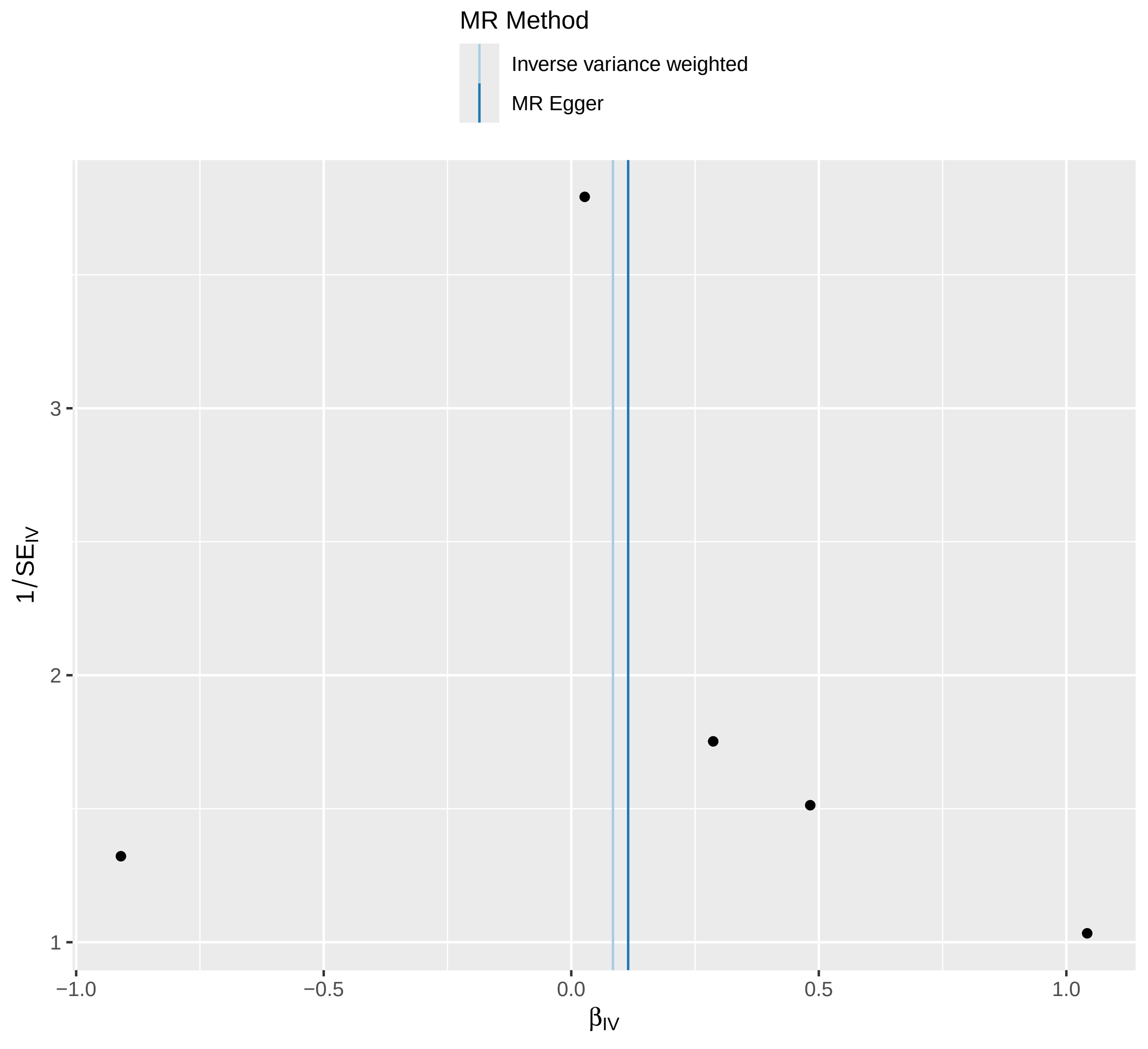
MR Method
Inverse variance weighted
MR Egger
$1/SE_{IV}$
3
2
1
−1.0
−0.5
0.0
0.5
1.0
β_{IV}

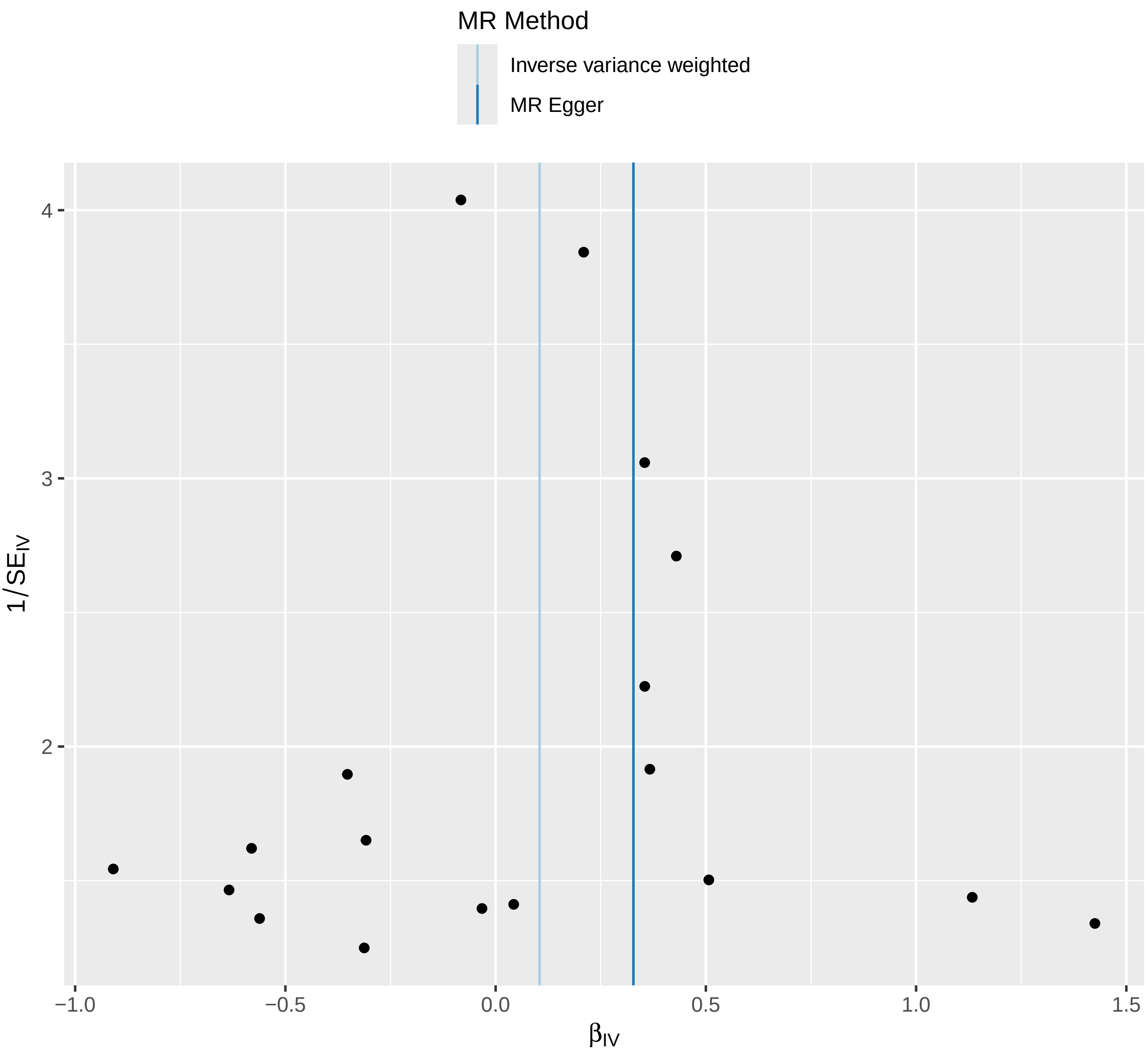

MR Method
Inverse variance weighted
MR Egger
4
3
2
1/SE_IV
−1.0
−0.5
0.0
0.5
1.0
1.5
β_IV

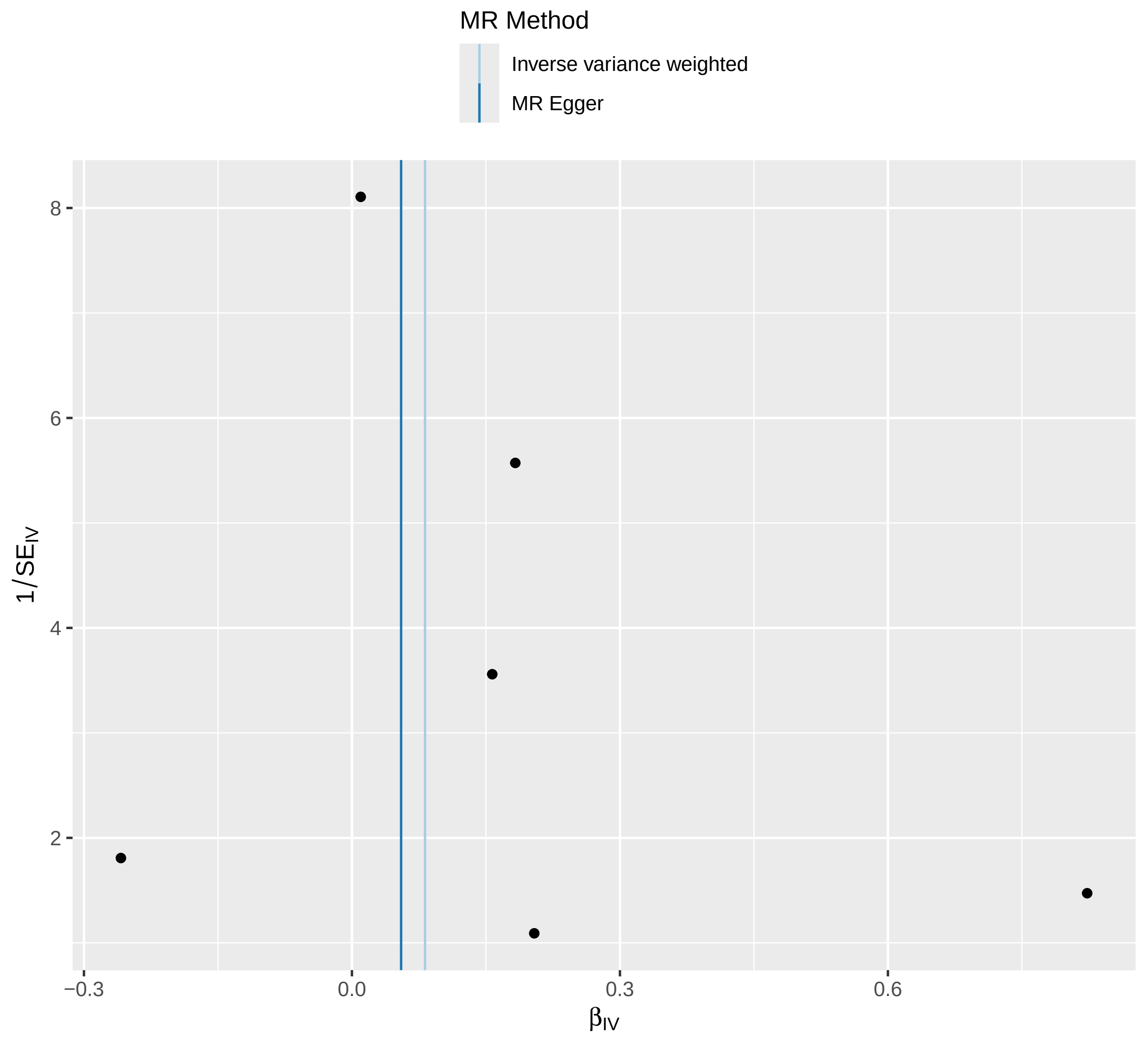

MR Method
Inverse variance weighted
MR Egger
8
6
4
2
1/SE_IV
−0.3
0.0
0.3
0.6
β_IV

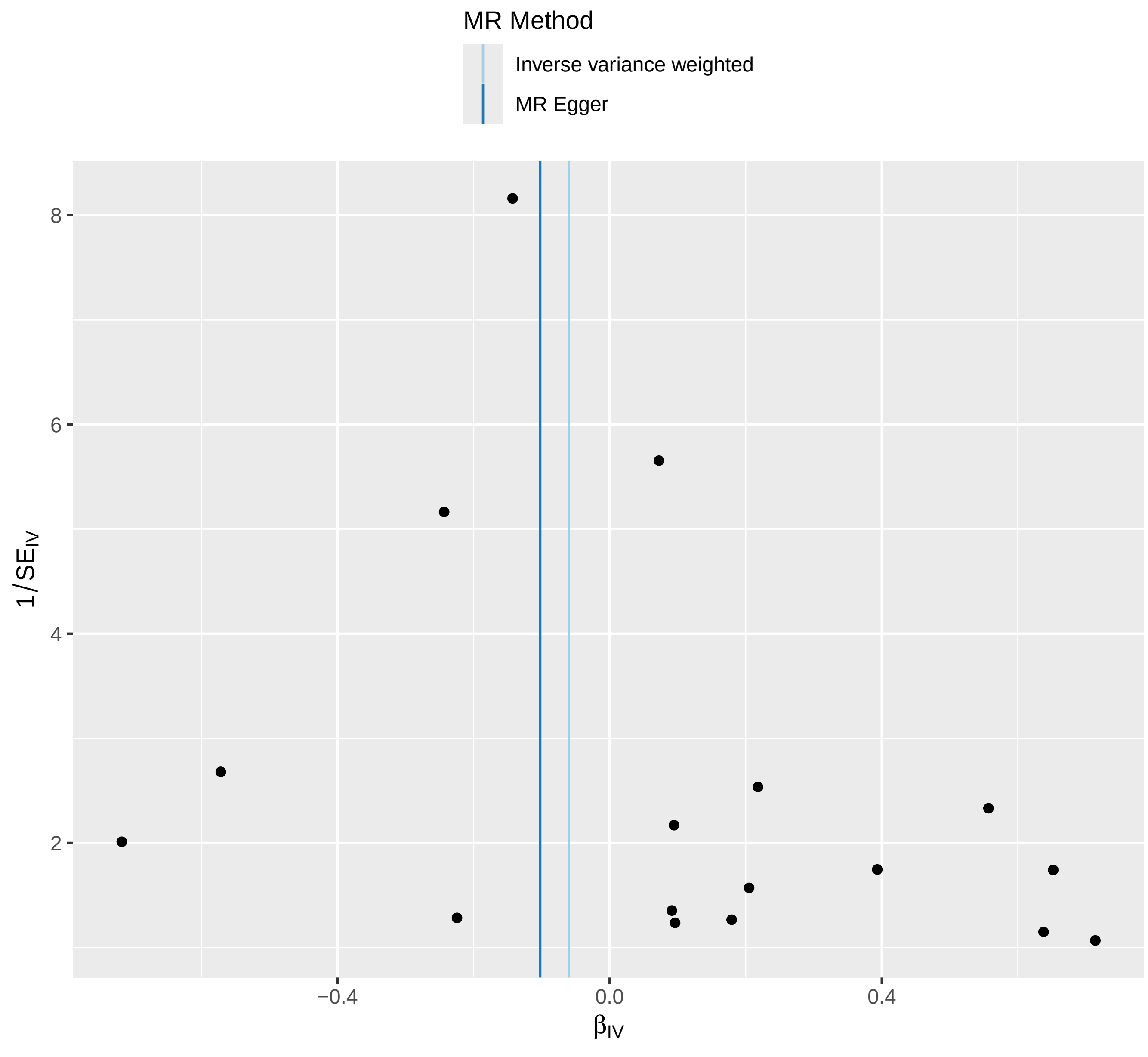
MR Method
Inverse variance weighted
MR Egger
8
6
4
2
1/SE_IV
−0.4
0.0
0.4
β_IV

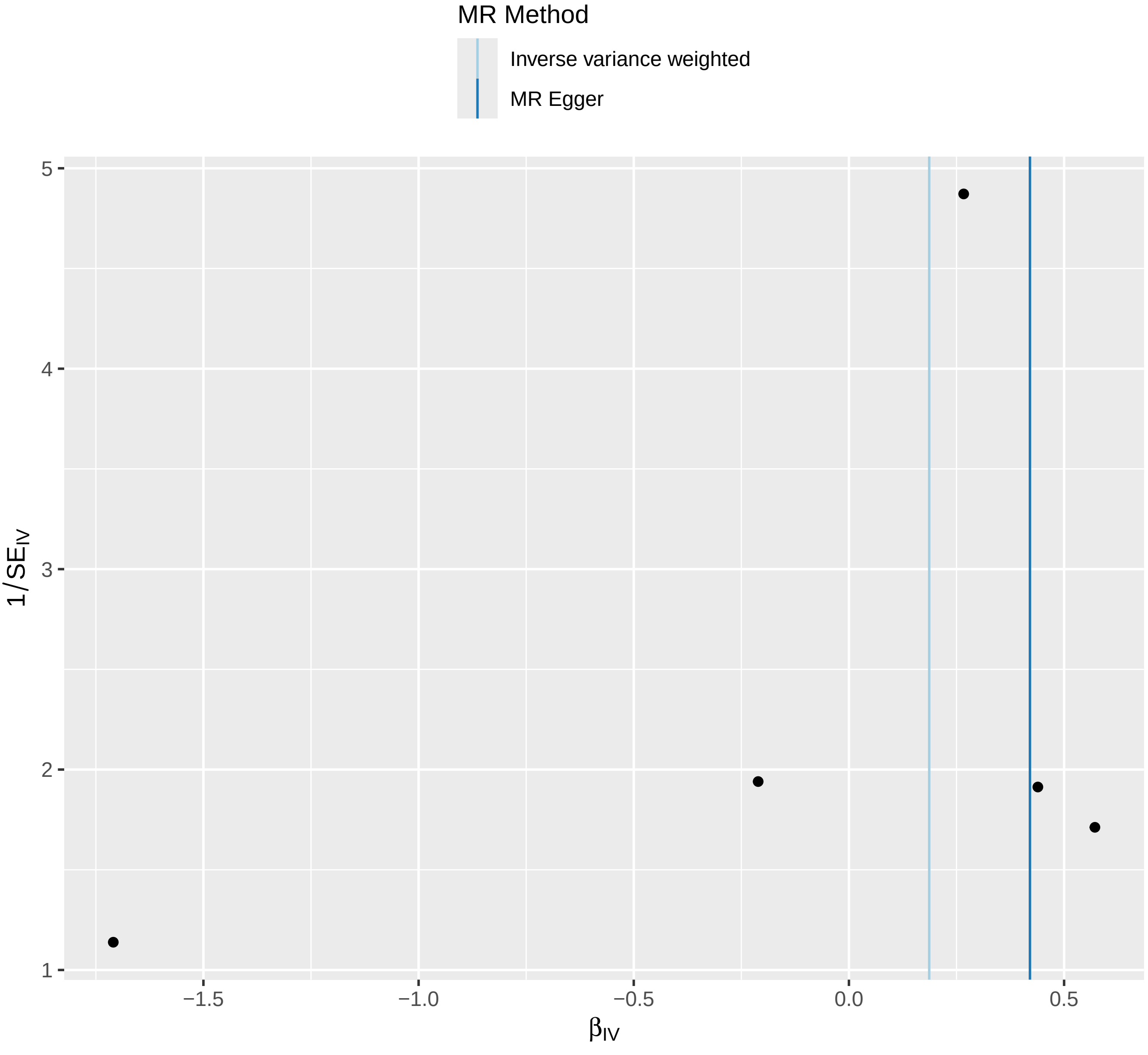

MR Method
Inverse variance weighted
MR Egger
1/SE_IV
β_IV
5
4
3
2
1
−1.5
−1.0
−0.5
0.0
0.5

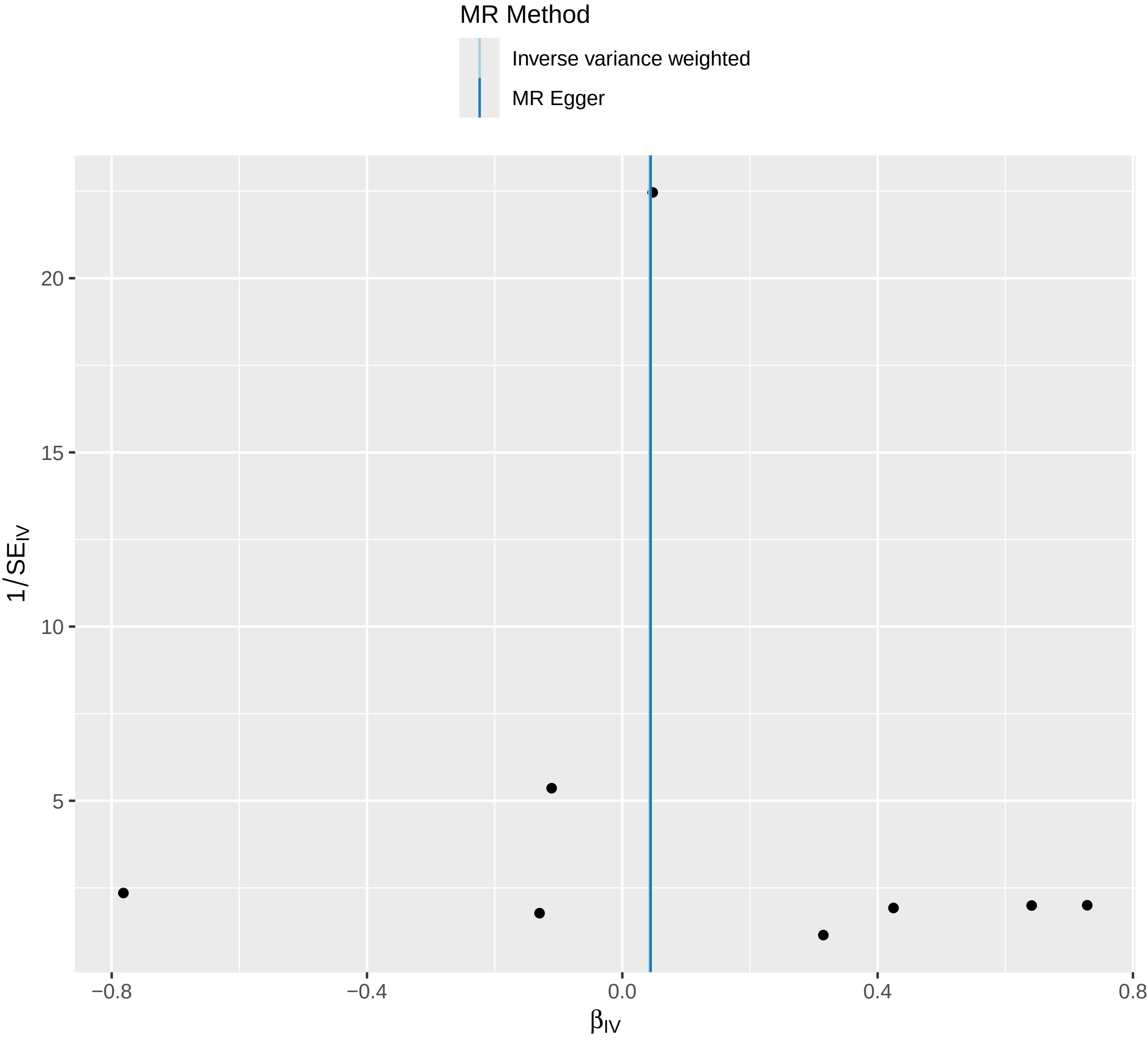
MR Method
Inverse variance weighted
MR Egger
20
15
10
5
1/SE_IV
−0.8
−0.4
0.0
0.4
0.8
β_IV

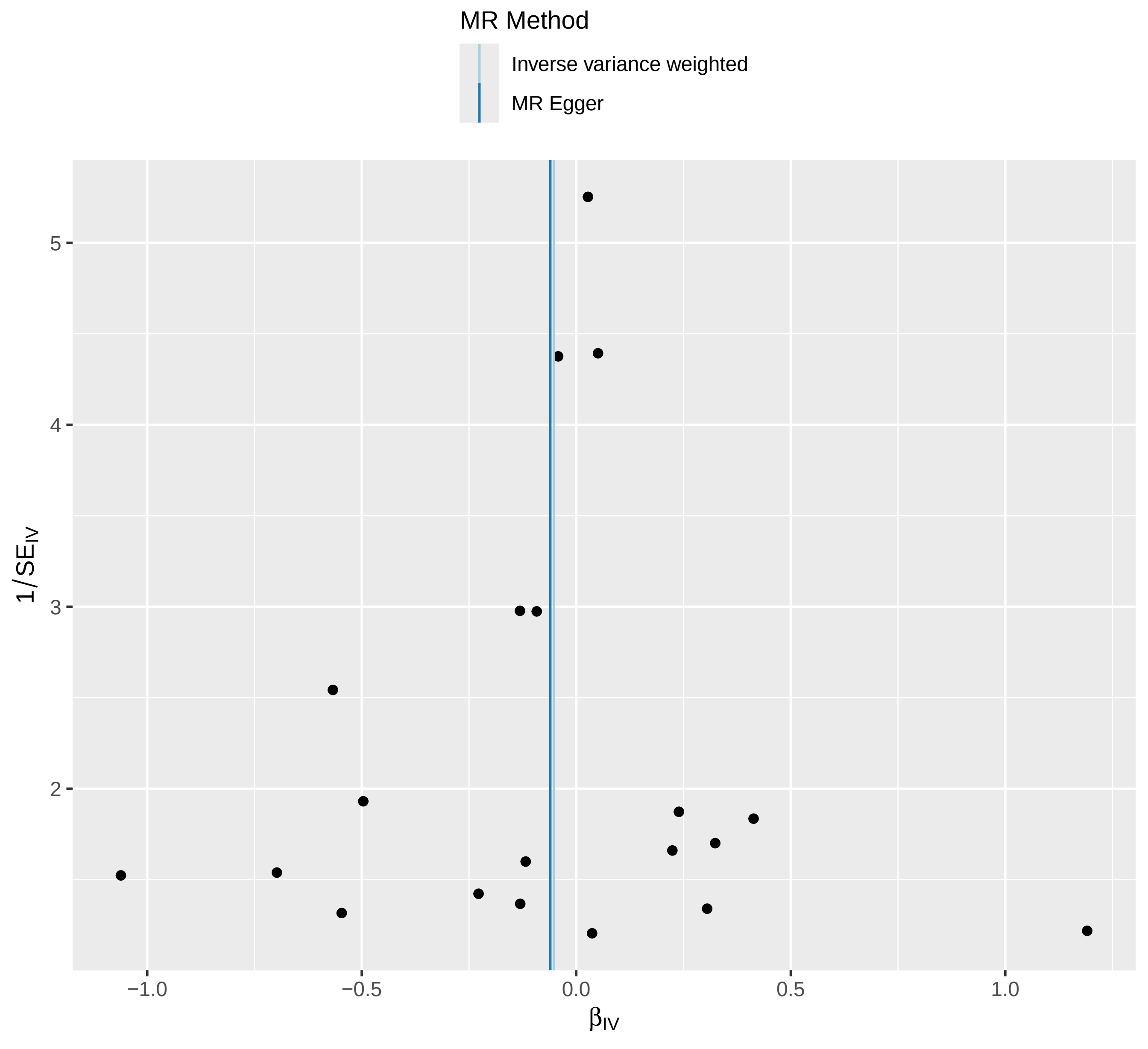

MR Method
Inverse variance weighted
MR Egger
5
4
3
2
1/SE$_{IV}$
−1.0
−0.5
0.0
0.5
1.0
β$_{IV}$

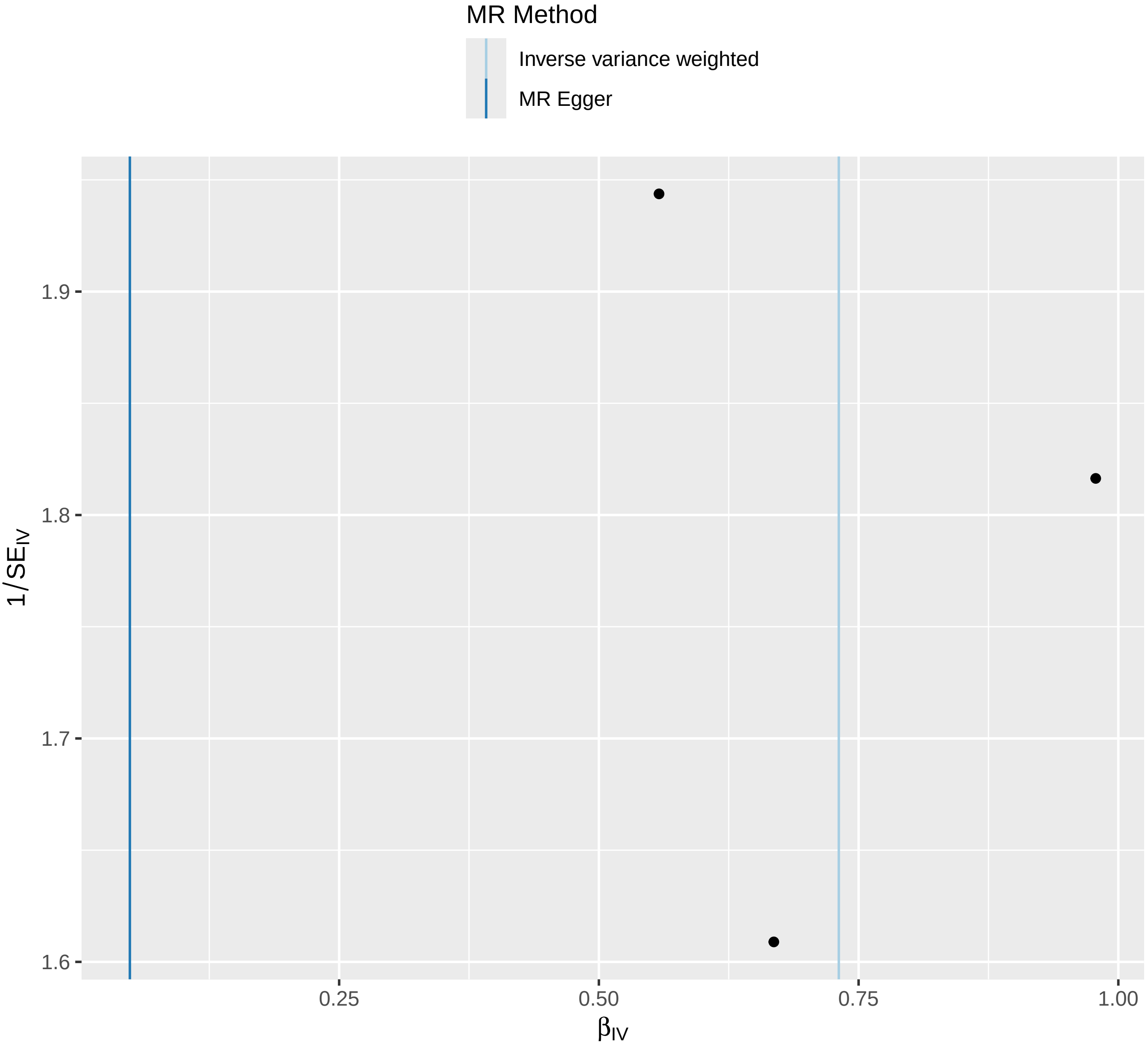
MR Method
Inverse variance weighted
MR Egger
1.9
1.8
1.7
1.6
$1/SE_{IV}$
0.25
0.50
0.75
1.00
β_{IV}

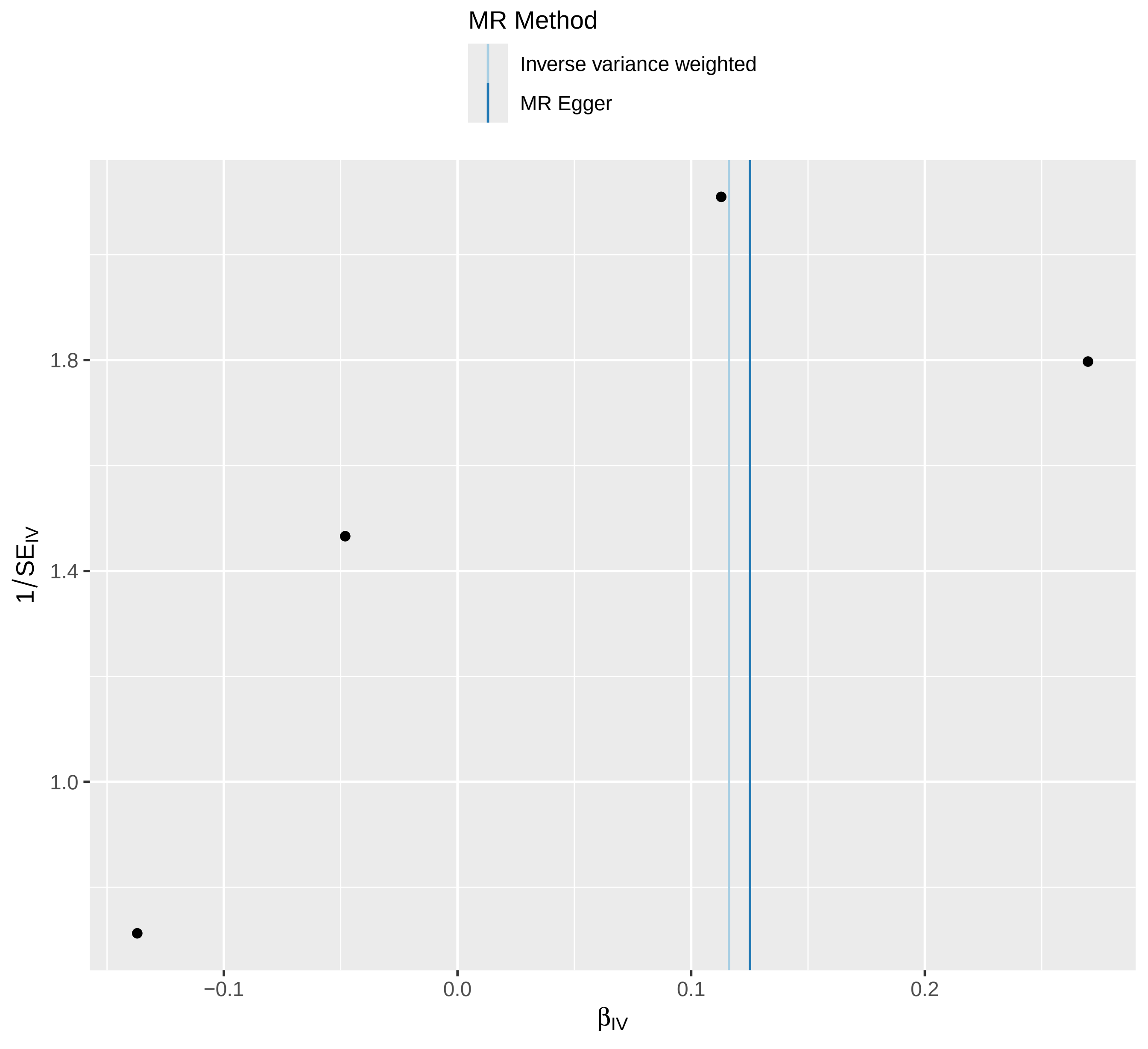

MR Method
Inverse variance weighted
MR Egger
1.8
1.4
1.0
1/SE_IV
−0.1
0.0
0.1
0.2
β_IV

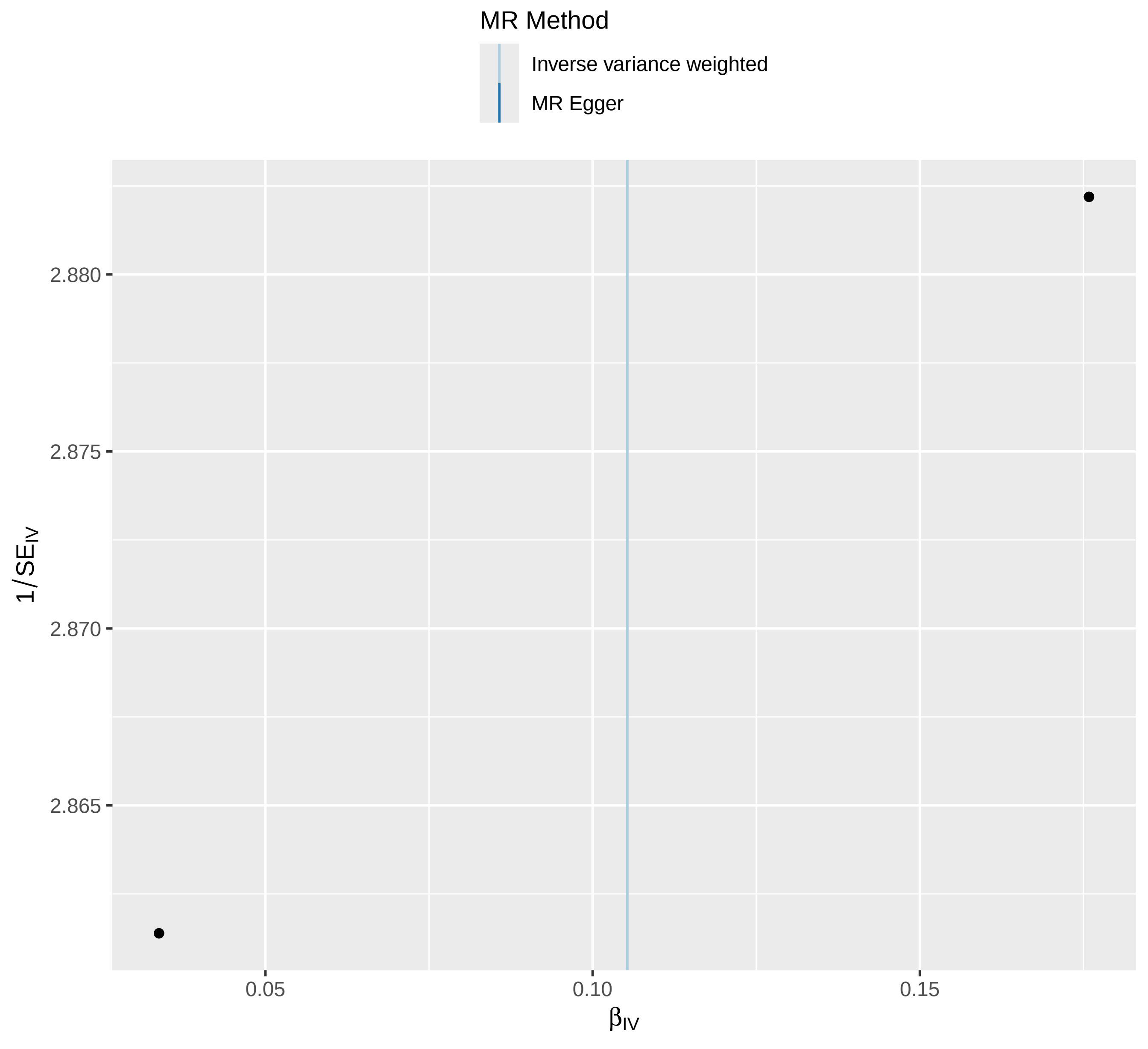
MR Method
Inverse variance weighted
MR Egger
2.880
2.875
2.870
2.865
1/SE$_{IV}$
0.05
0.10
0.15
β$_{IV}$

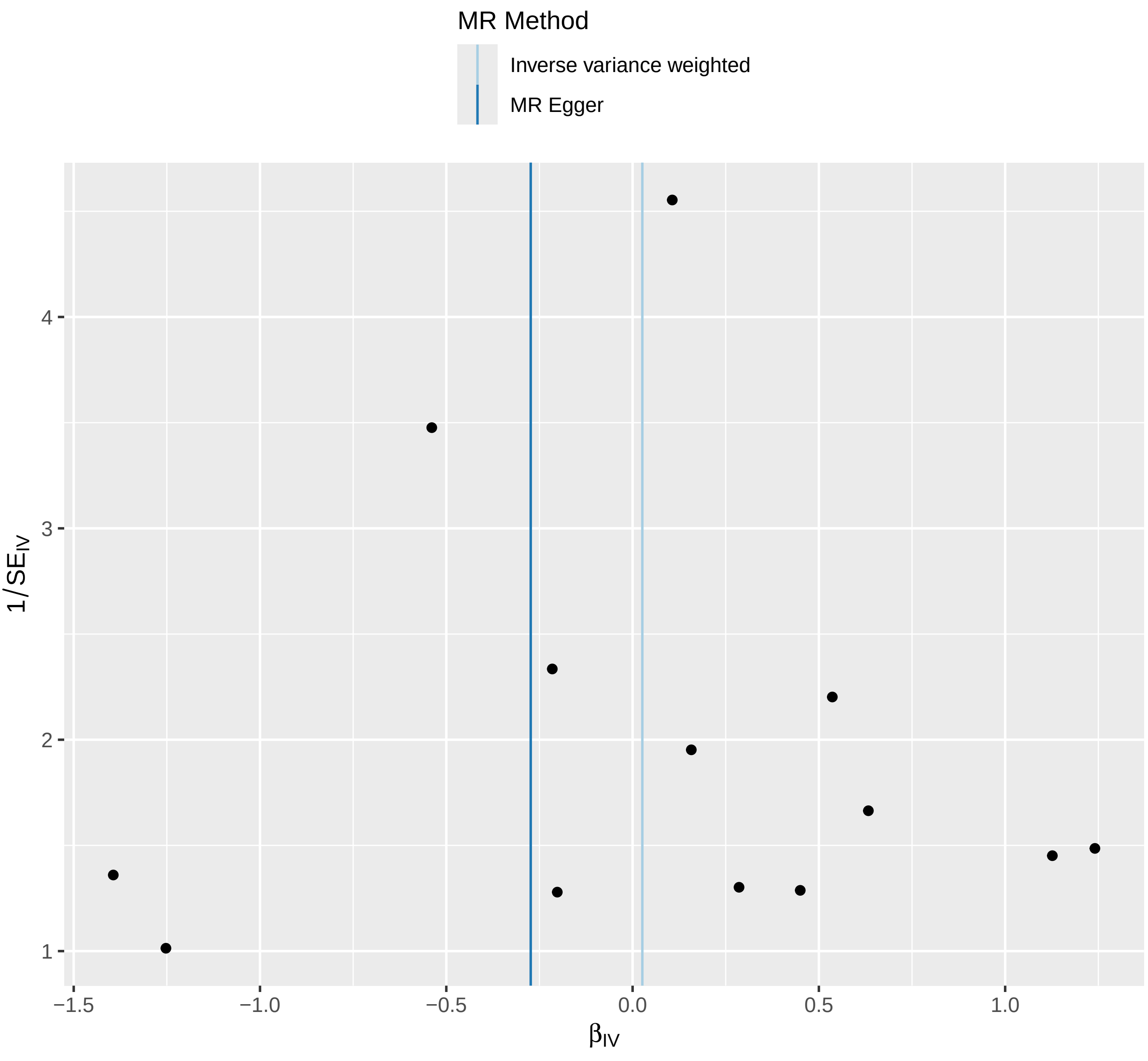

MR Method
Inverse variance weighted
MR Egger
1/SE_IV
β_IV
4
3
2
1
−1.5
−1.0
−0.5
0.0
0.5
1.0

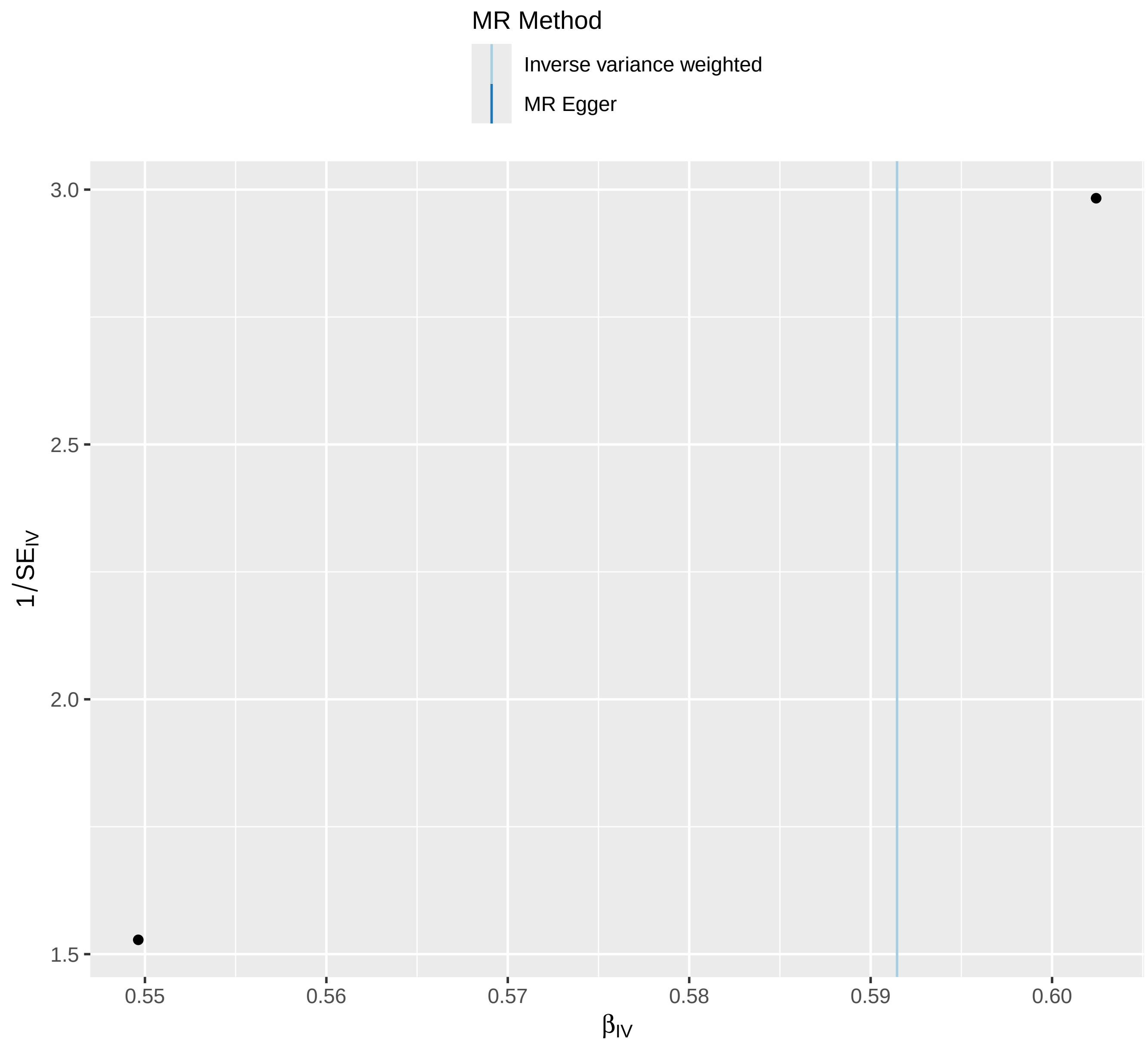
MR Method
Inverse variance weighted
MR Egger
3.0
2.5
2.0
1.5
$1/SE_{IV}$
0.55
0.56
0.57
0.58
0.59
0.60
β_{IV}

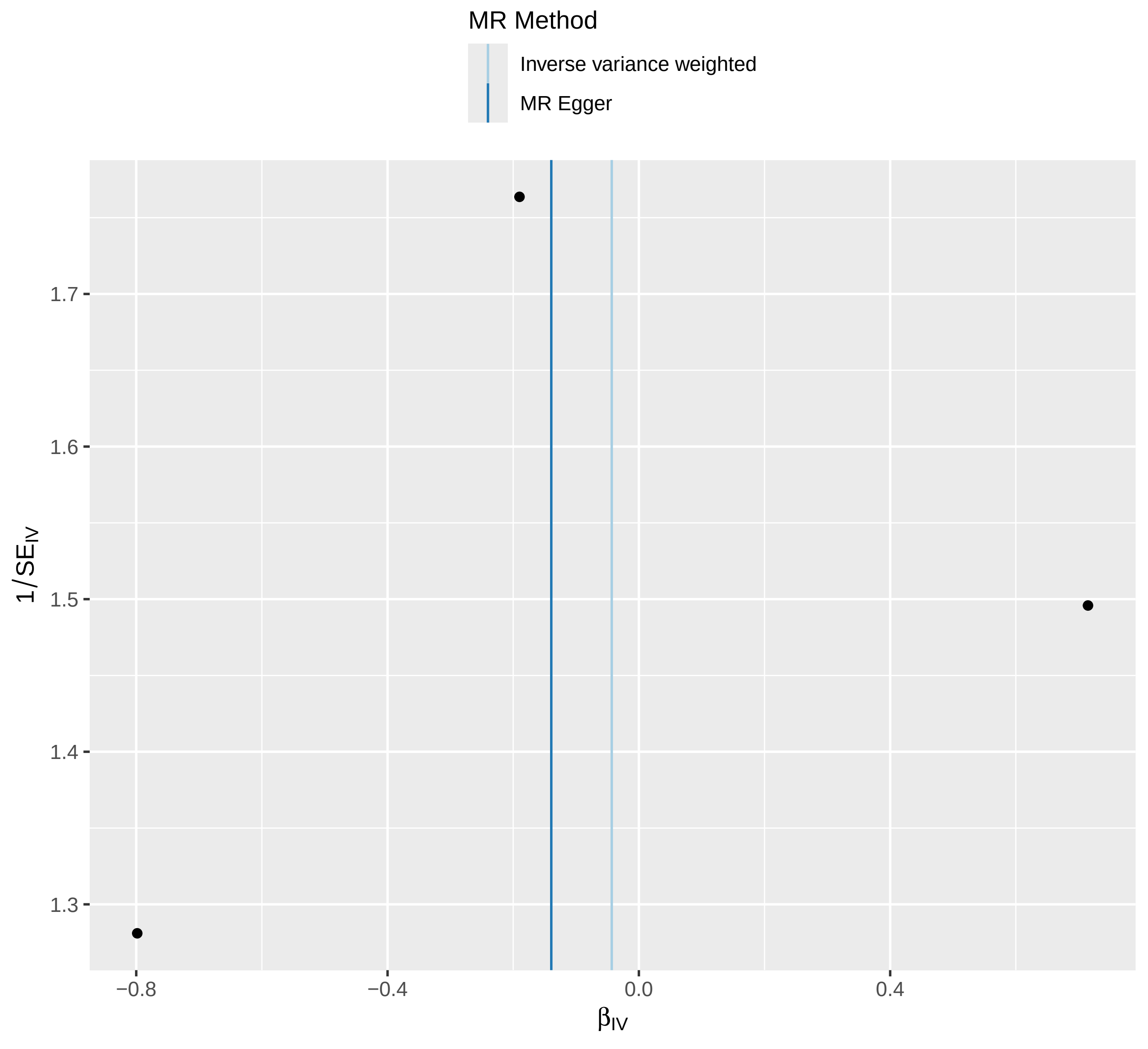

MR Method
Inverse variance weighted
MR Egger
1.7
1.6
1.5
1.4
1.3
1/SE_IV
−0.8
−0.4
0.0
0.4
β_IV

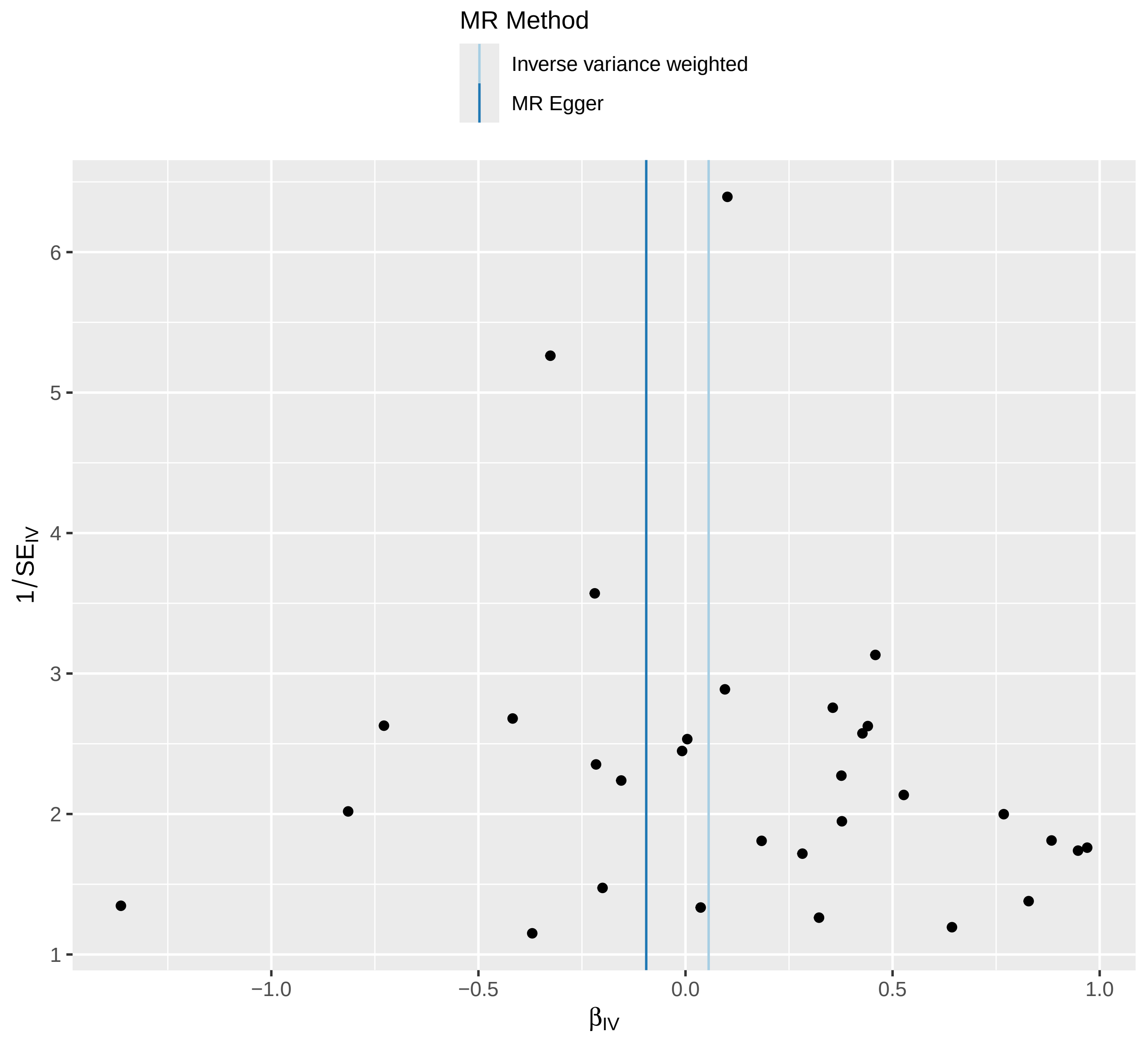

MR Method
Inverse variance weighted
MR Egger
1/SE_IV
β_IV
6
5
4
3
2
1
−1.0
−0.5
0.0
0.5
1.0

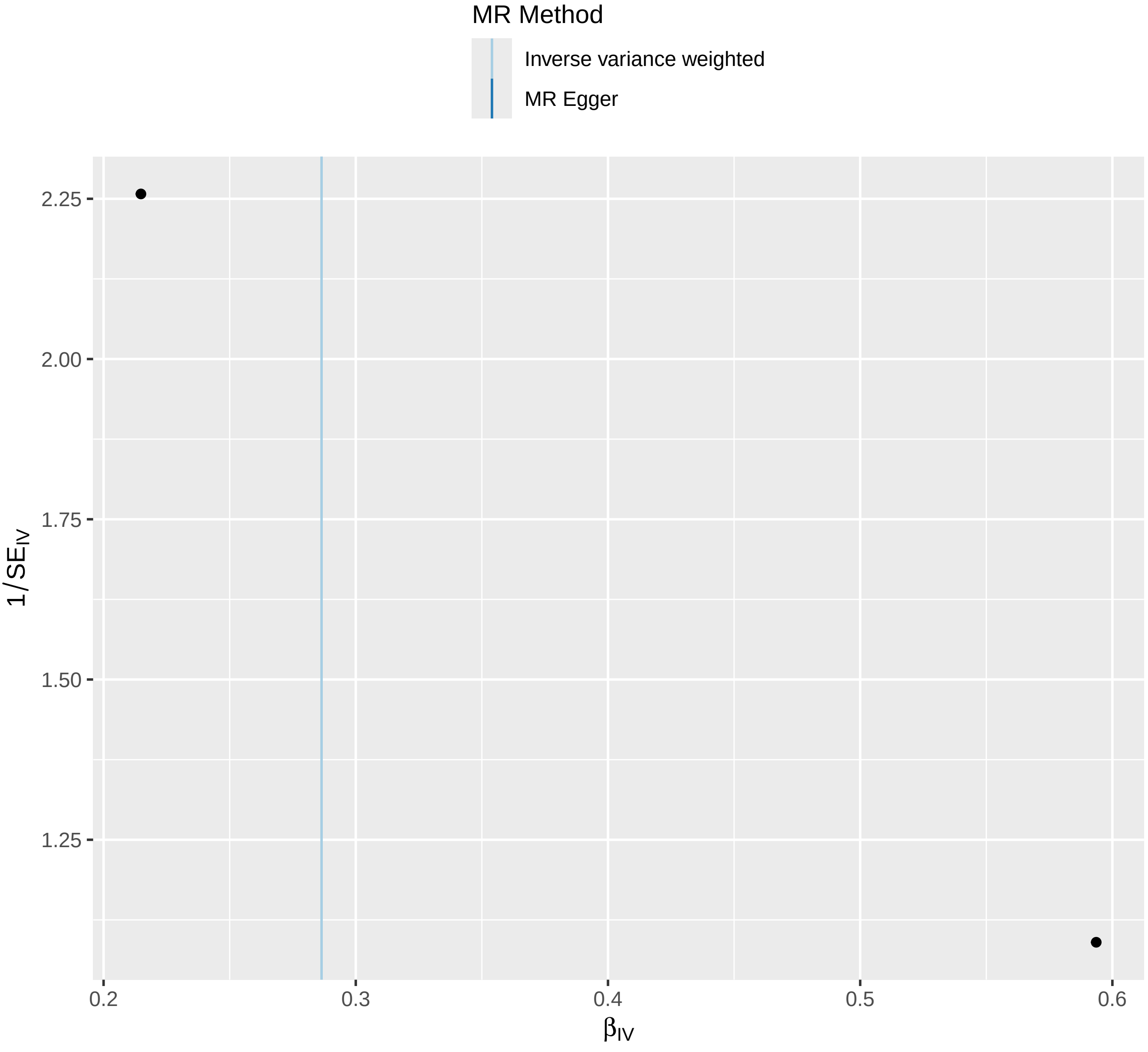

MR Method
Inverse variance weighted
MR Egger
2.25
2.00
1.75
1.50
1.25
1/SE_IV
0.2
0.3
0.4
0.5
0.6
β_IV

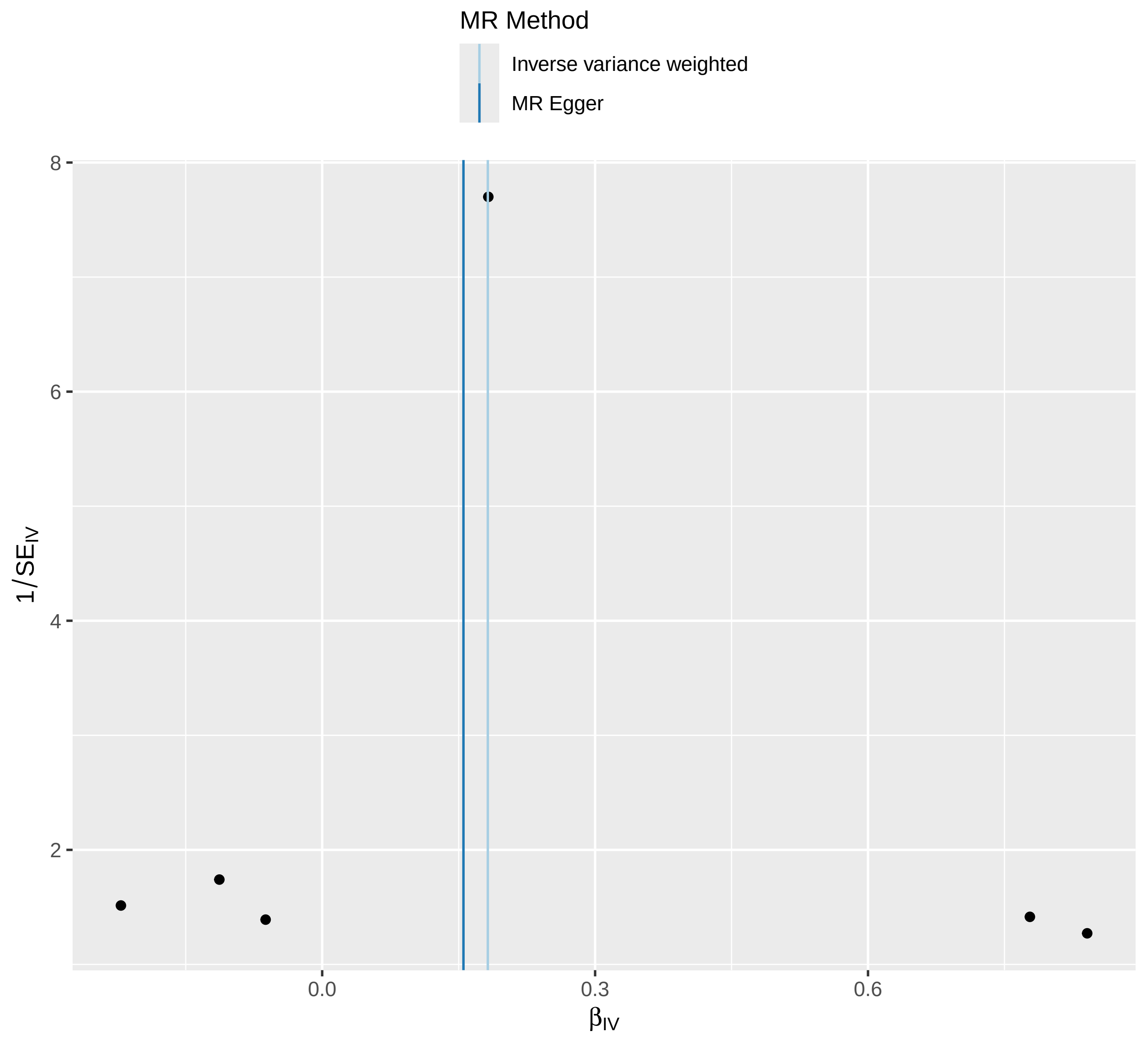

MR Method
Inverse variance weighted
MR Egger
8
6
4
2
1/SE_IV
0.0
0.3
0.6
β_IV

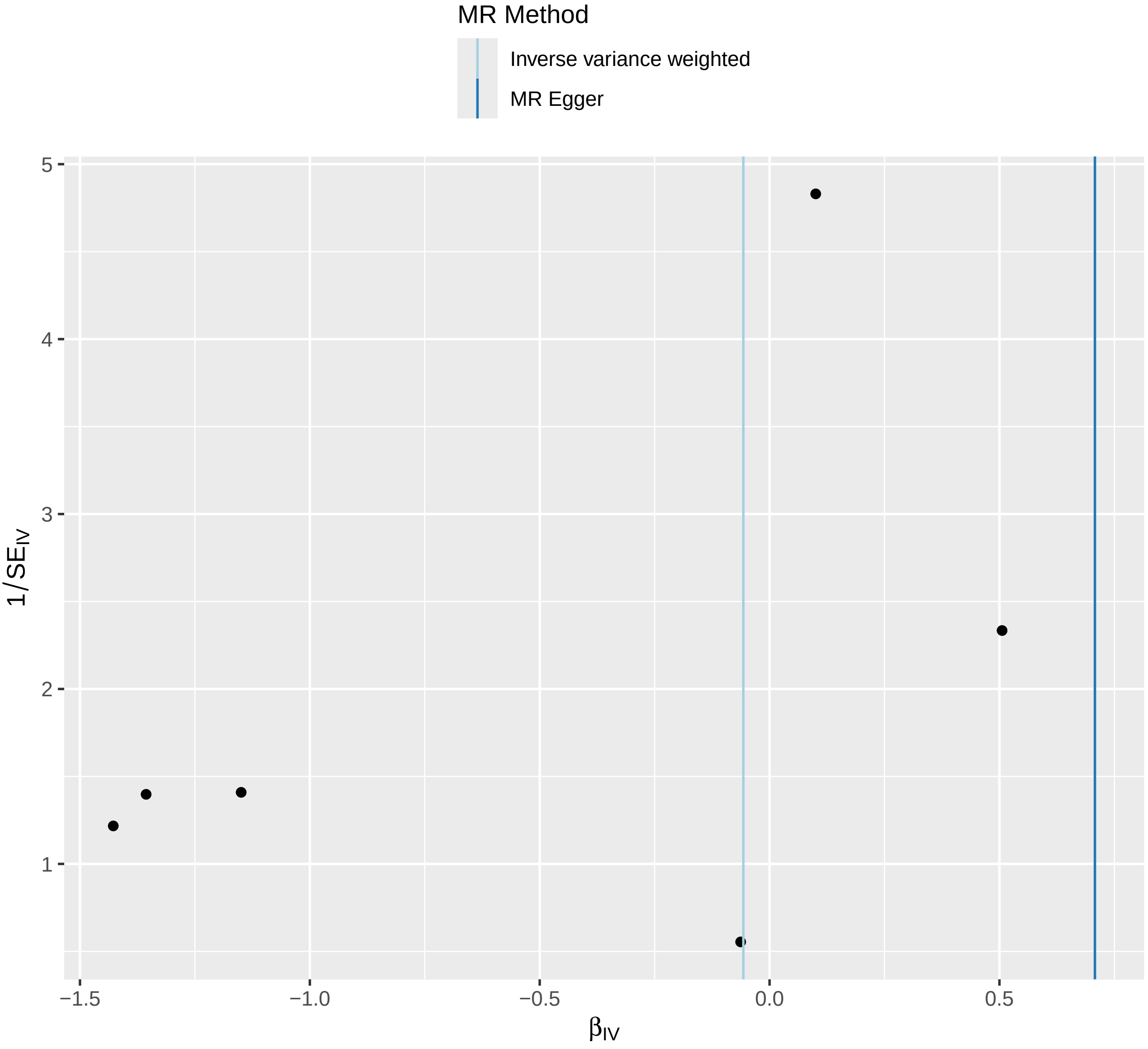
MR Method
Inverse variance weighted
MR Egger
1/SE$_{IV}$
β$_{IV}$
5
4
3
2
1
−1.5
−1.0
−0.5
0.0
0.5

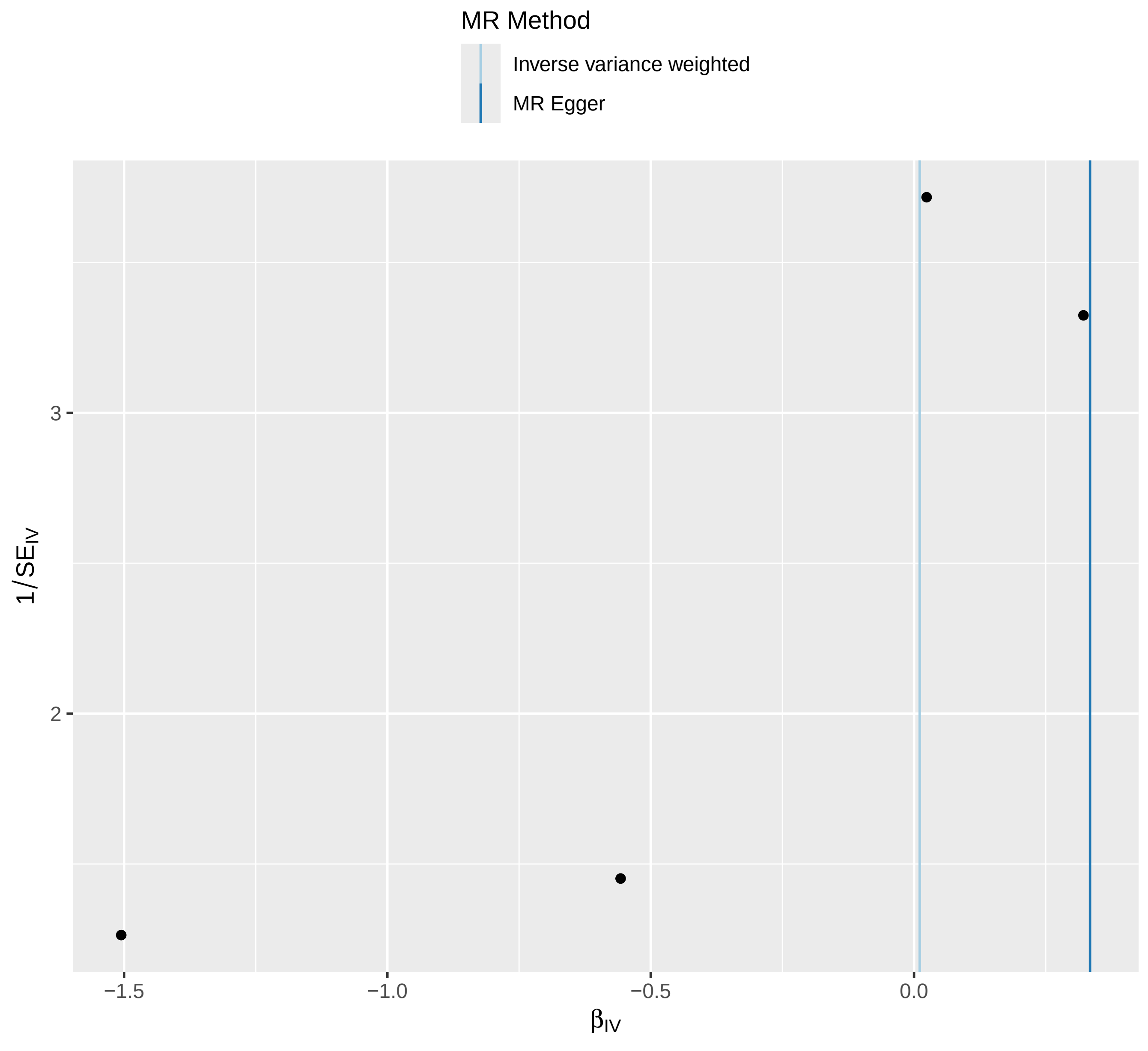
MR Method
Inverse variance weighted
MR Egger
3
2
1/SE_IV
−1.5
−1.0
−0.5
0.0
β_IV

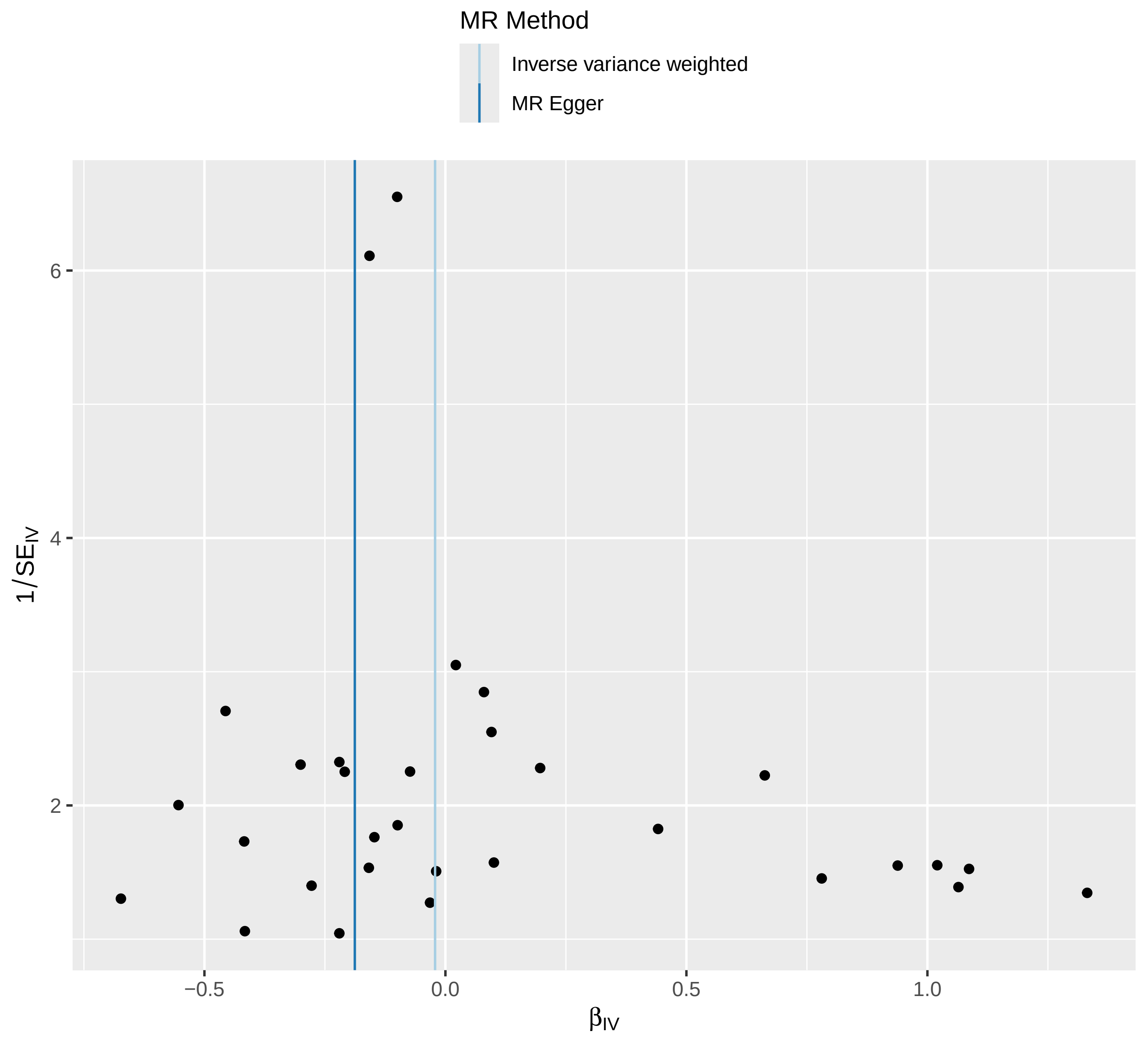
MR Method
Inverse variance weighted
MR Egger
6
4
2
1/SE$_{IV}$
−0.5
0.0
0.5
1.0
β$_{IV}$

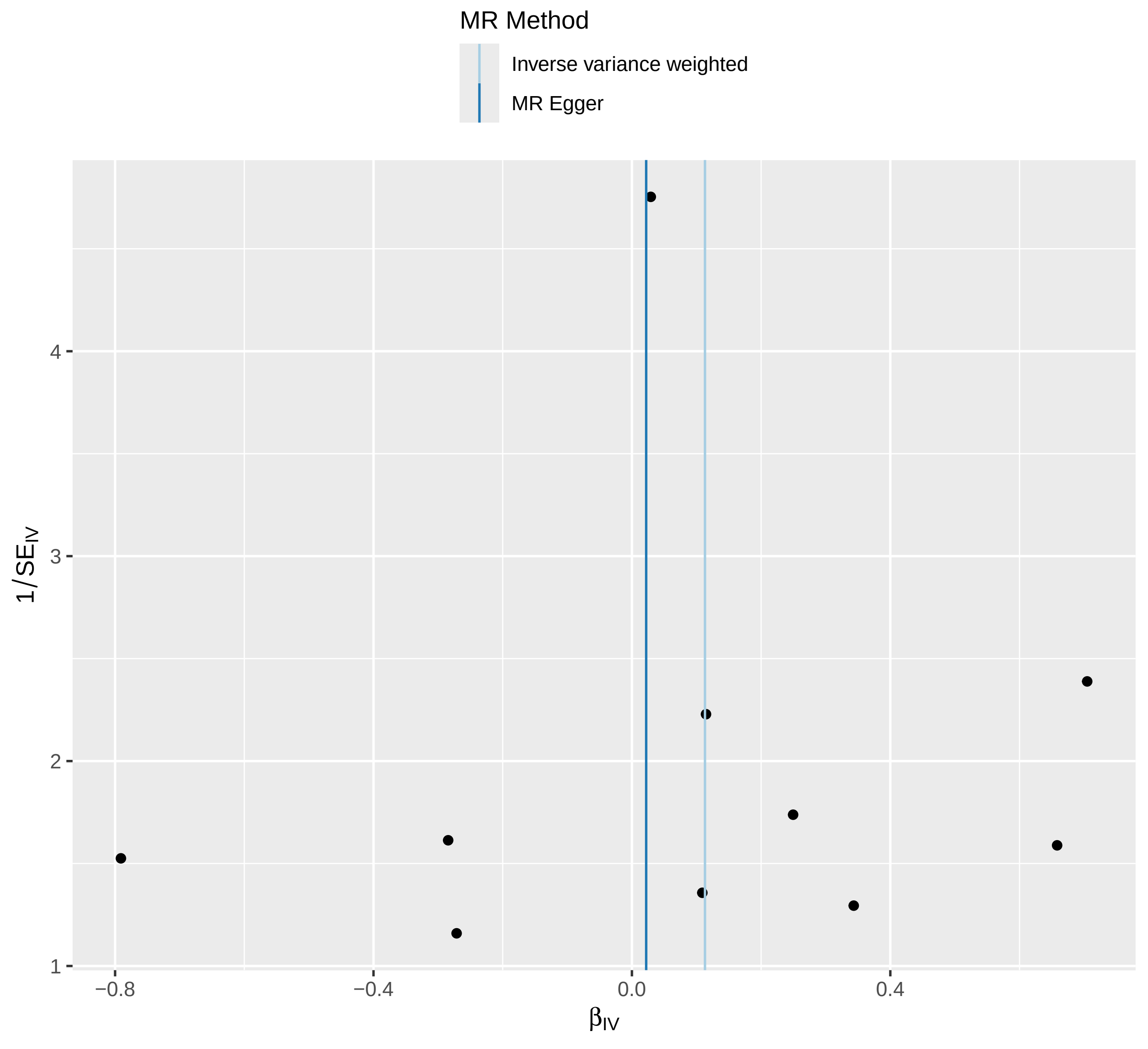

MR Method
Inverse variance weighted
MR Egger
1/SE_IV
4
3
2
1
−0.8
−0.4
0.0
0.4
β_IV

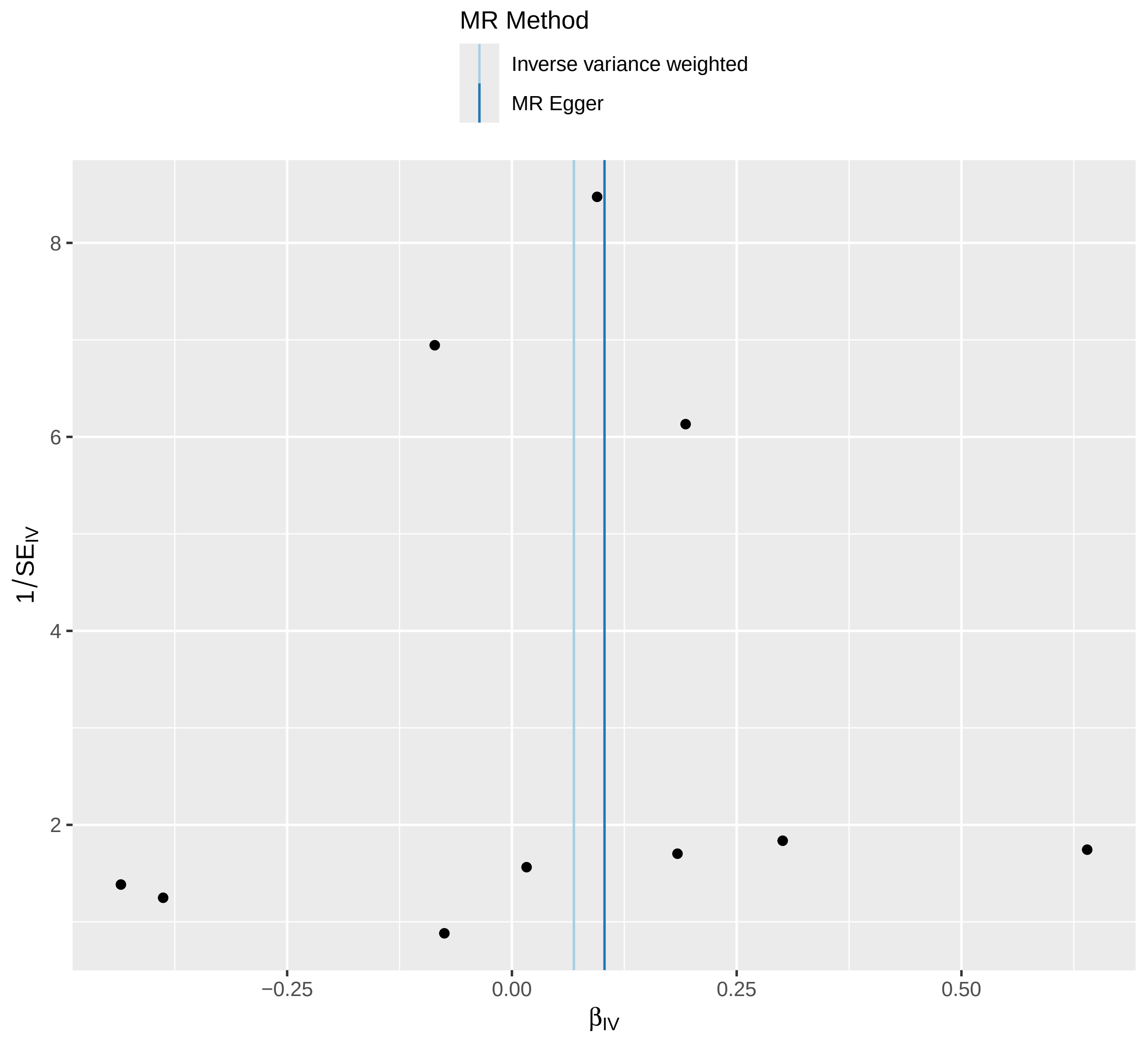
MR Method
Inverse variance weighted
MR Egger
8
6
4
2
1/SE$_{IV}$
−0.25
0.00
0.25
0.50
β$_{IV}$

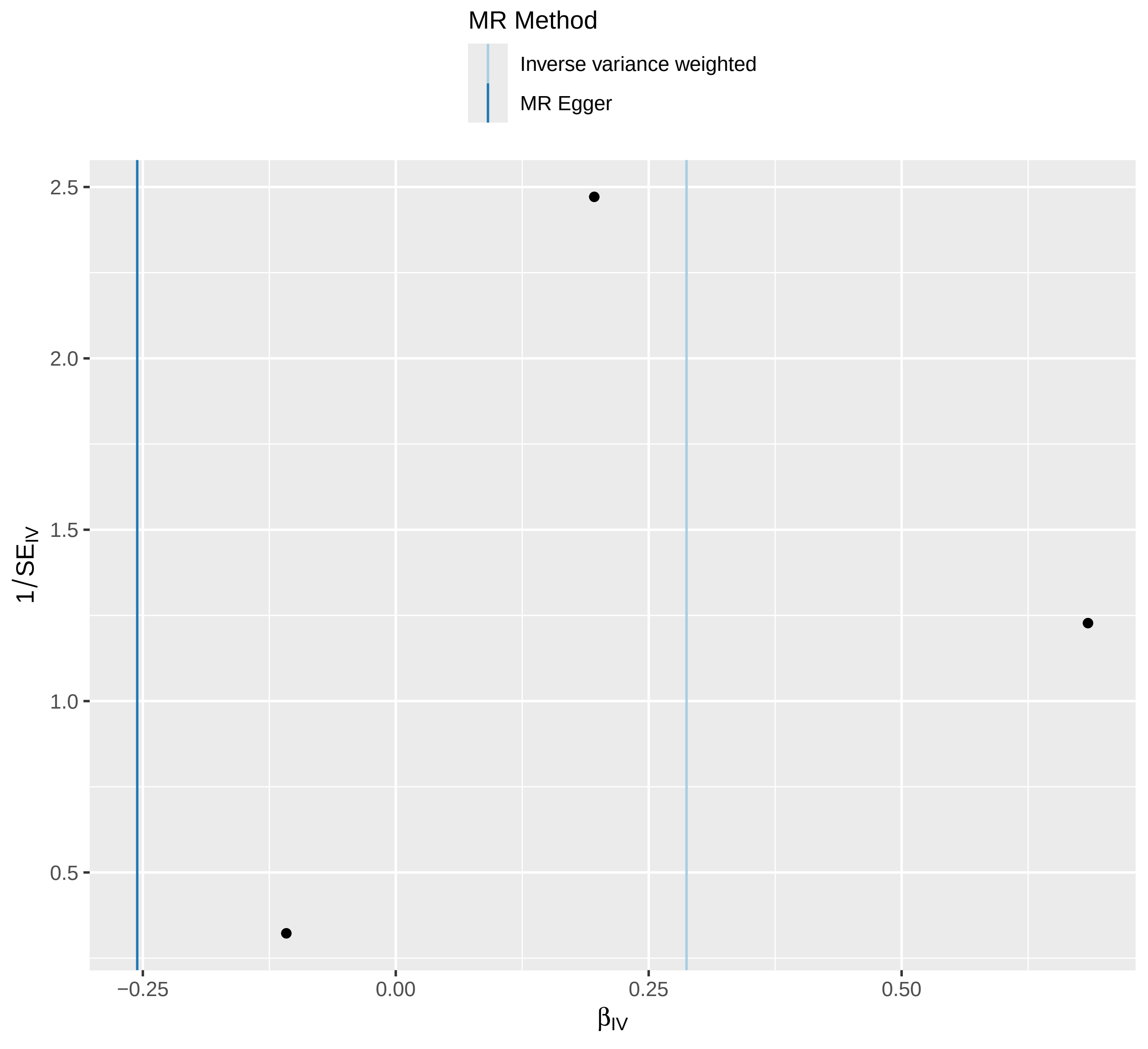

MR Method
Inverse variance weighted
MR Egger
2.5
2.0
1.5
1.0
0.5
$1/SE_{IV}$
−0.25
0.00
0.25
0.50
β_{IV}

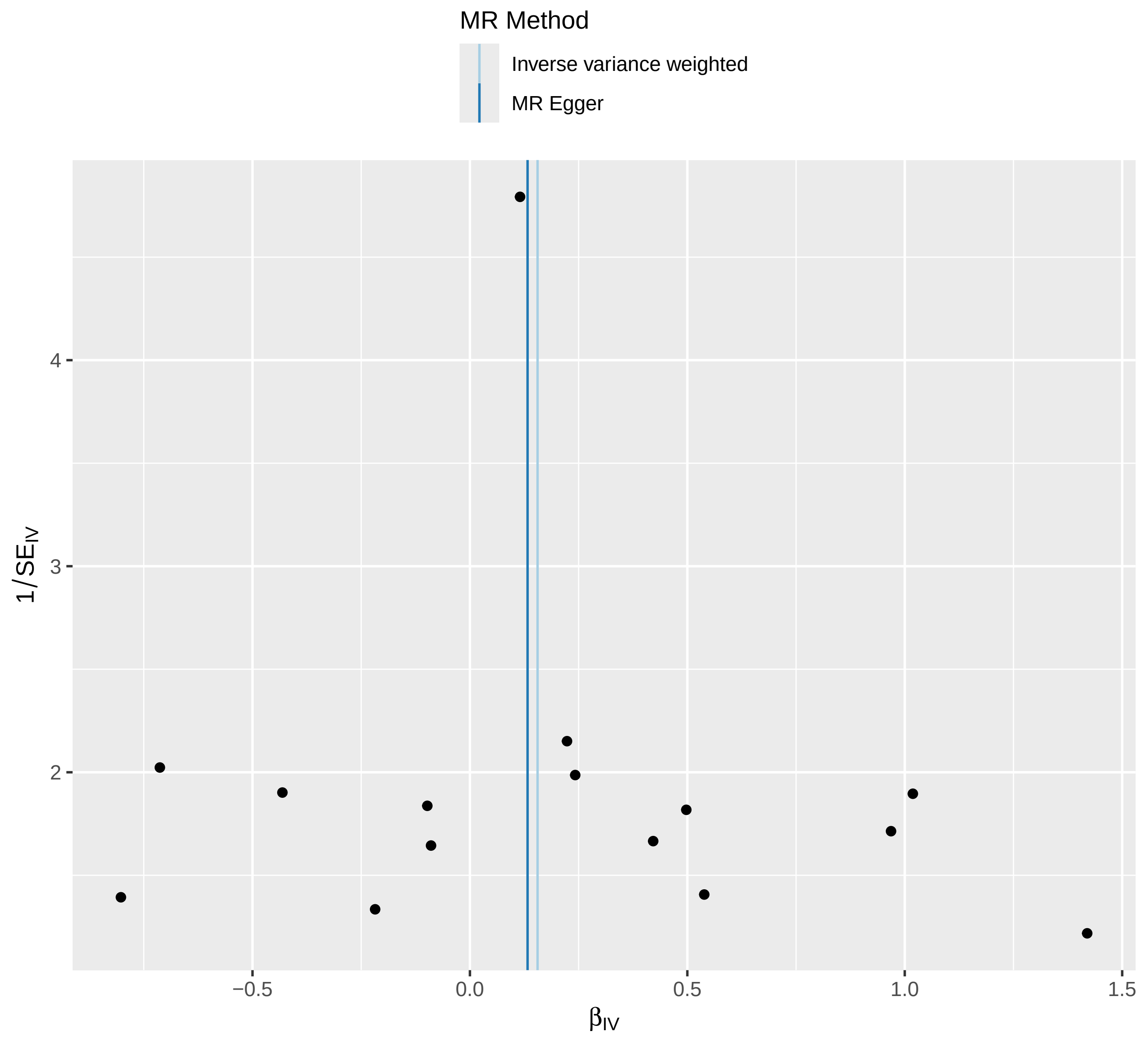
MR Method
Inverse variance weighted
MR Egger
4
3
2
1/SE_IV
−0.5
0.0
0.5
1.0
1.5
β_IV

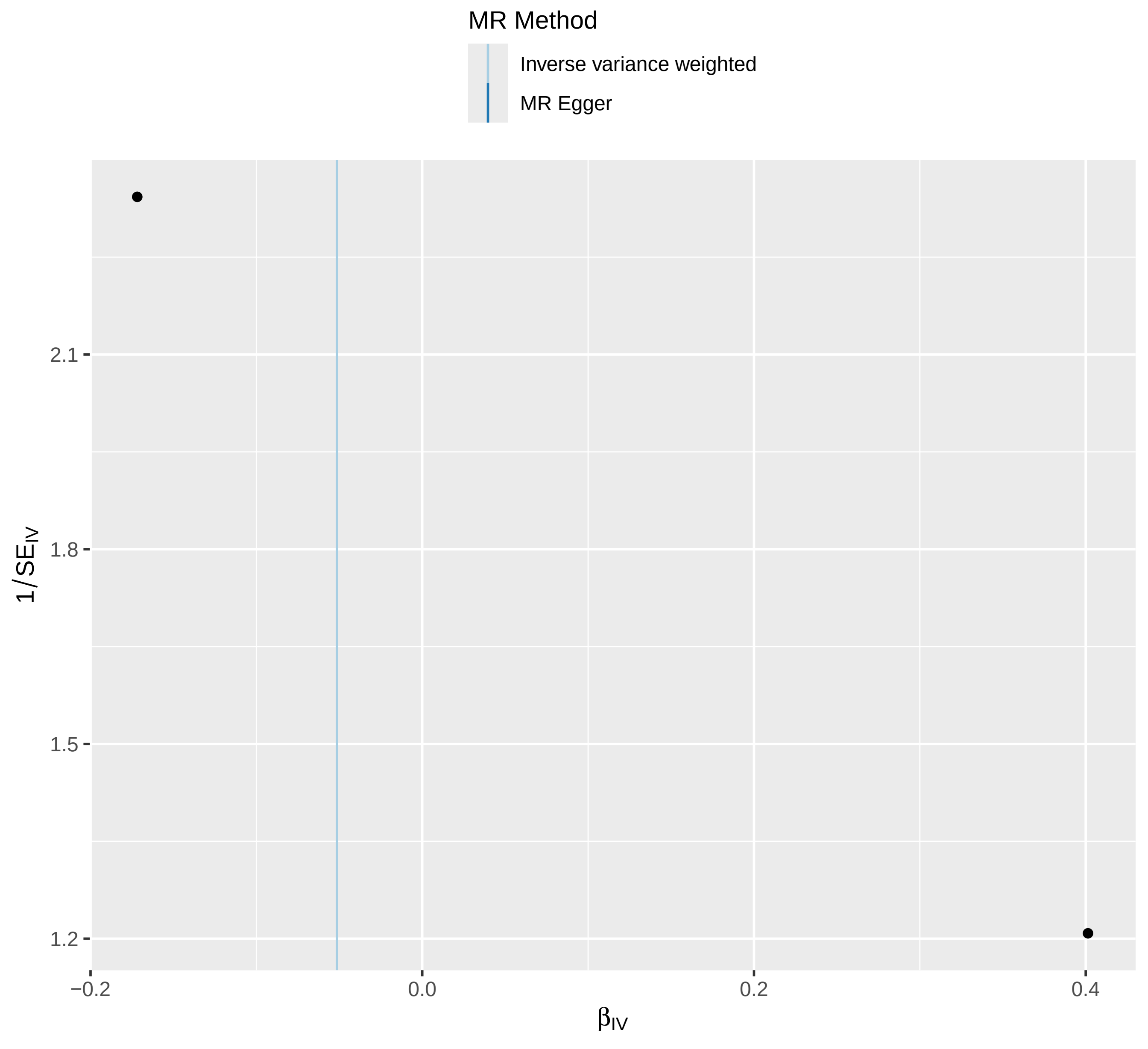

MR Method
Inverse variance weighted
MR Egger
2.1
1.8
1.5
1.2
1/SE_IV
−0.2
0.0
0.2
0.4
β_IV

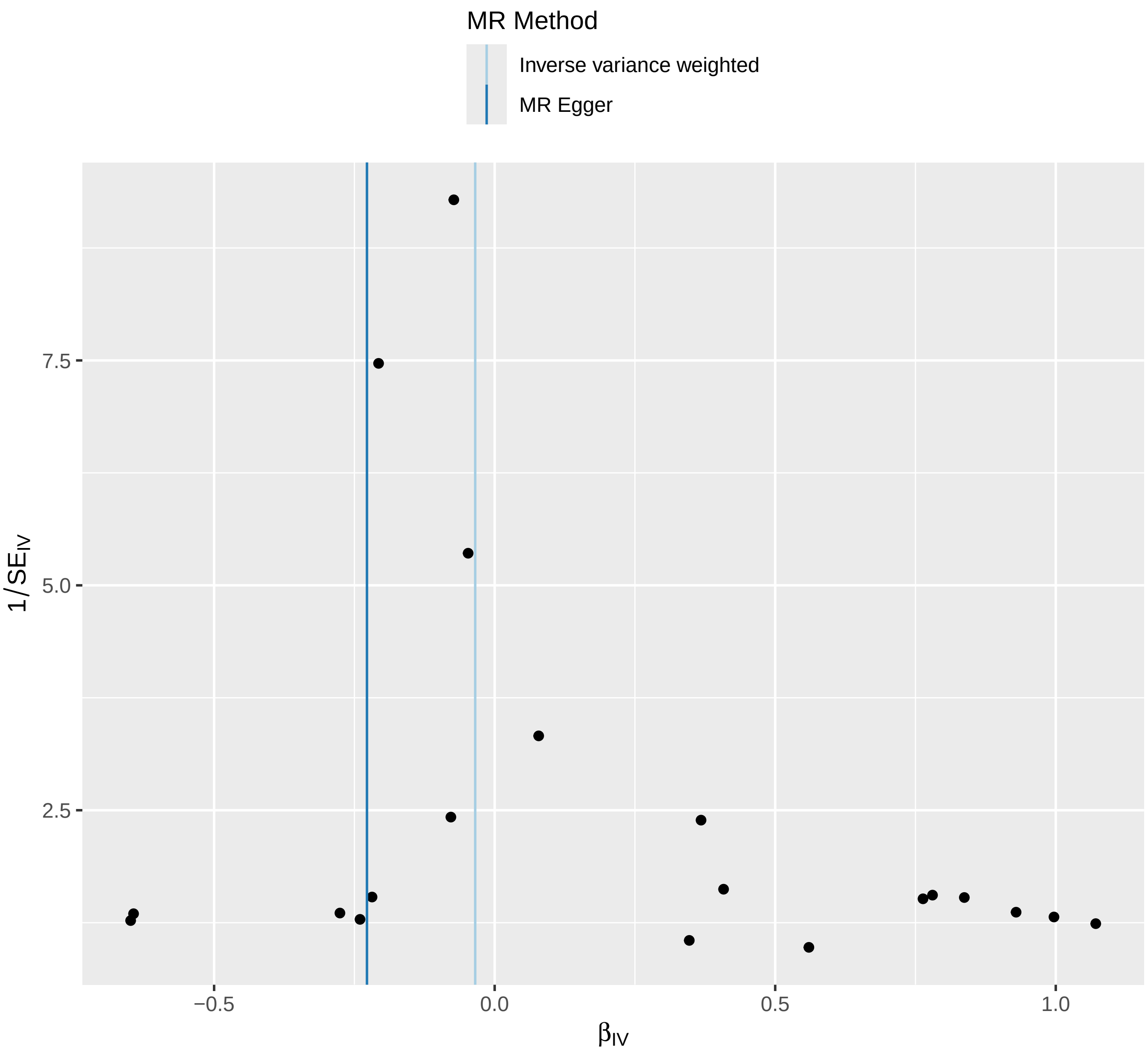

MR Method
Inverse variance weighted
MR Egger
7.5
5.0
2.5
1/SE_IV
−0.5
0.0
0.5
1.0
β_IV

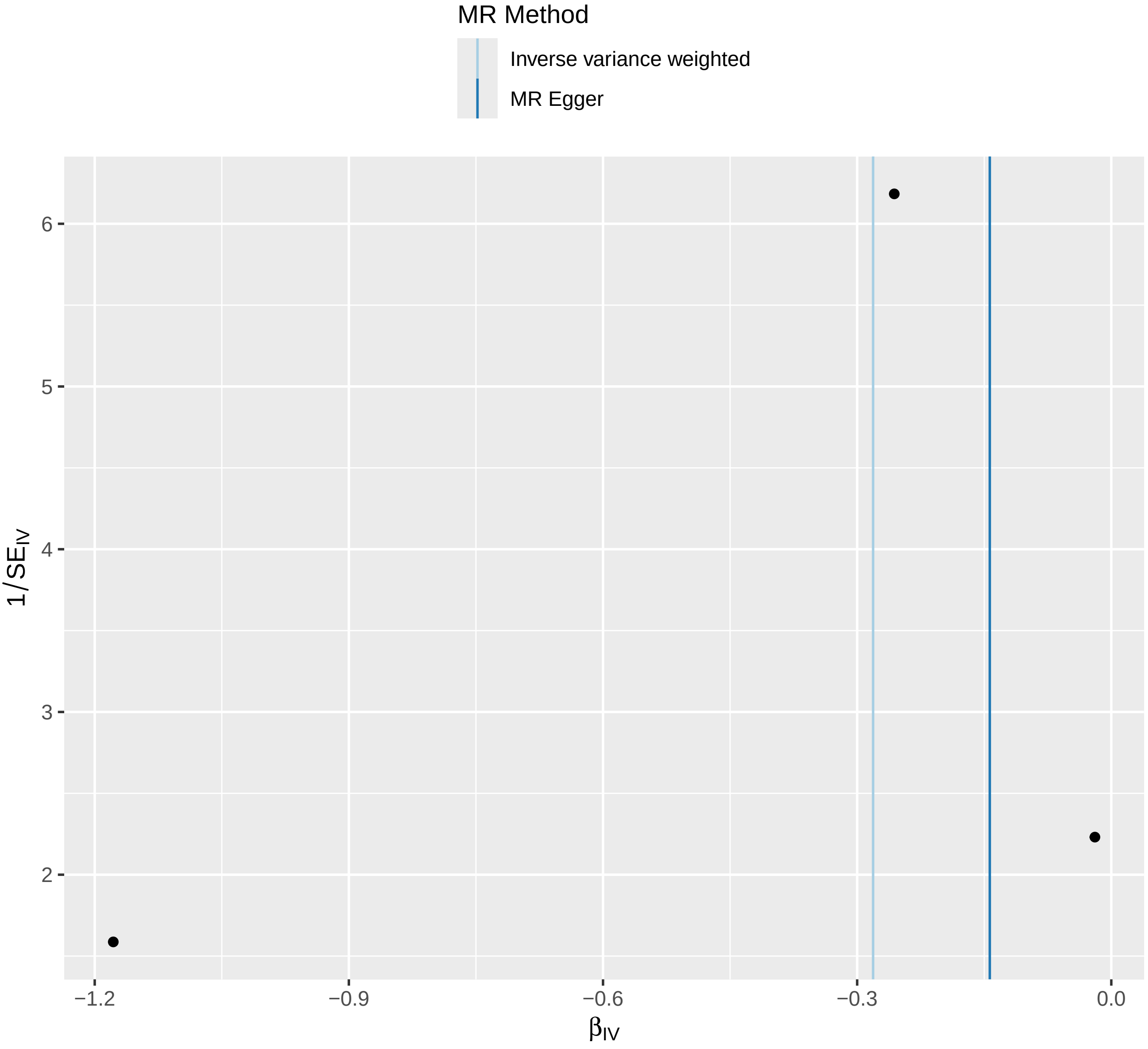
MR Method
Inverse variance weighted
MR Egger
1/SE_IV
6
5
4
3
2
−1.2
−0.9
−0.6
−0.3
0.0
β_IV

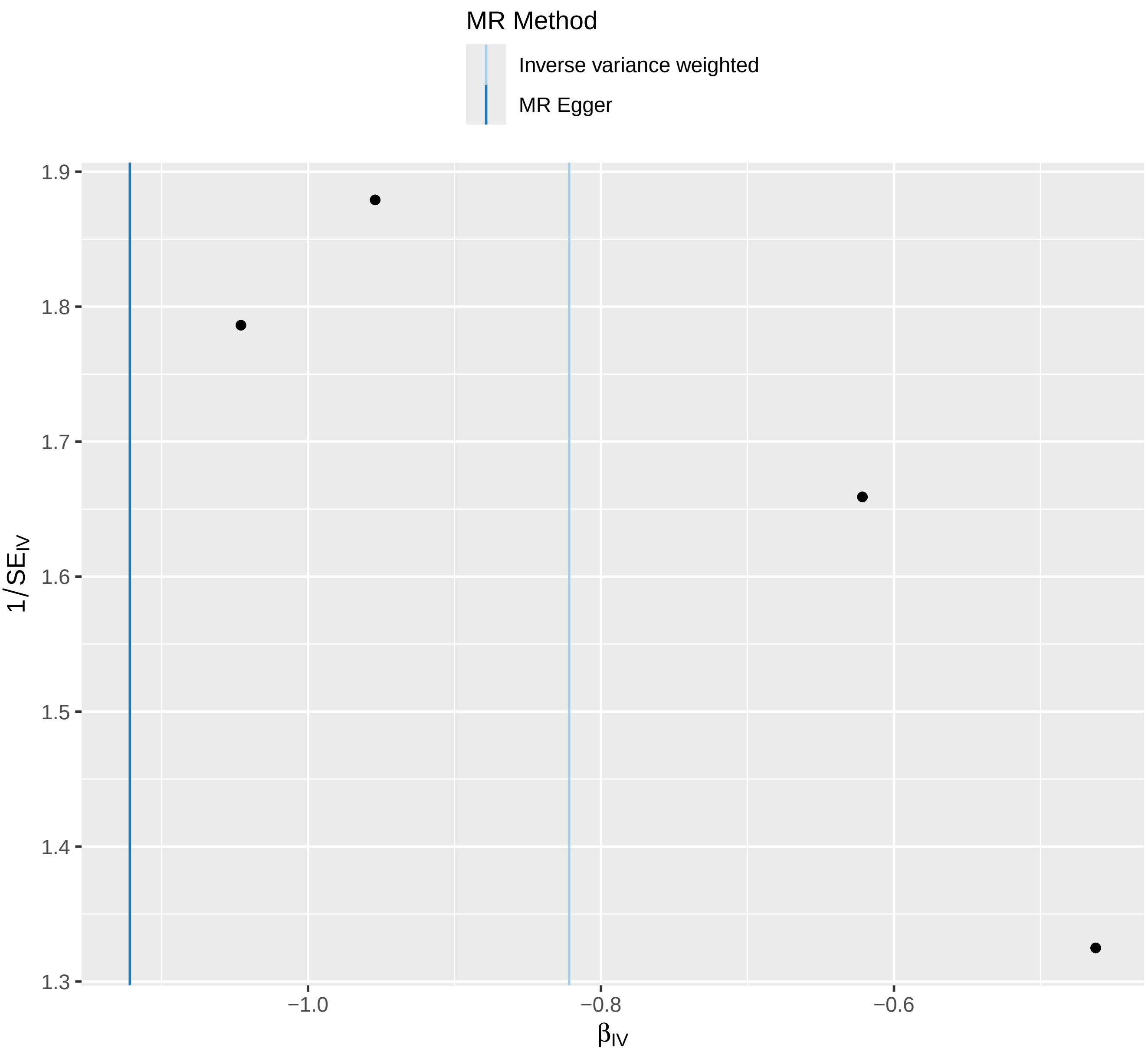

MR Method
Inverse variance weighted
MR Egger
1.9
1.8
1.7
1.6
1.5
1.4
1.3
1/SE_IV
−1.0
−0.8
−0.6
β_IV

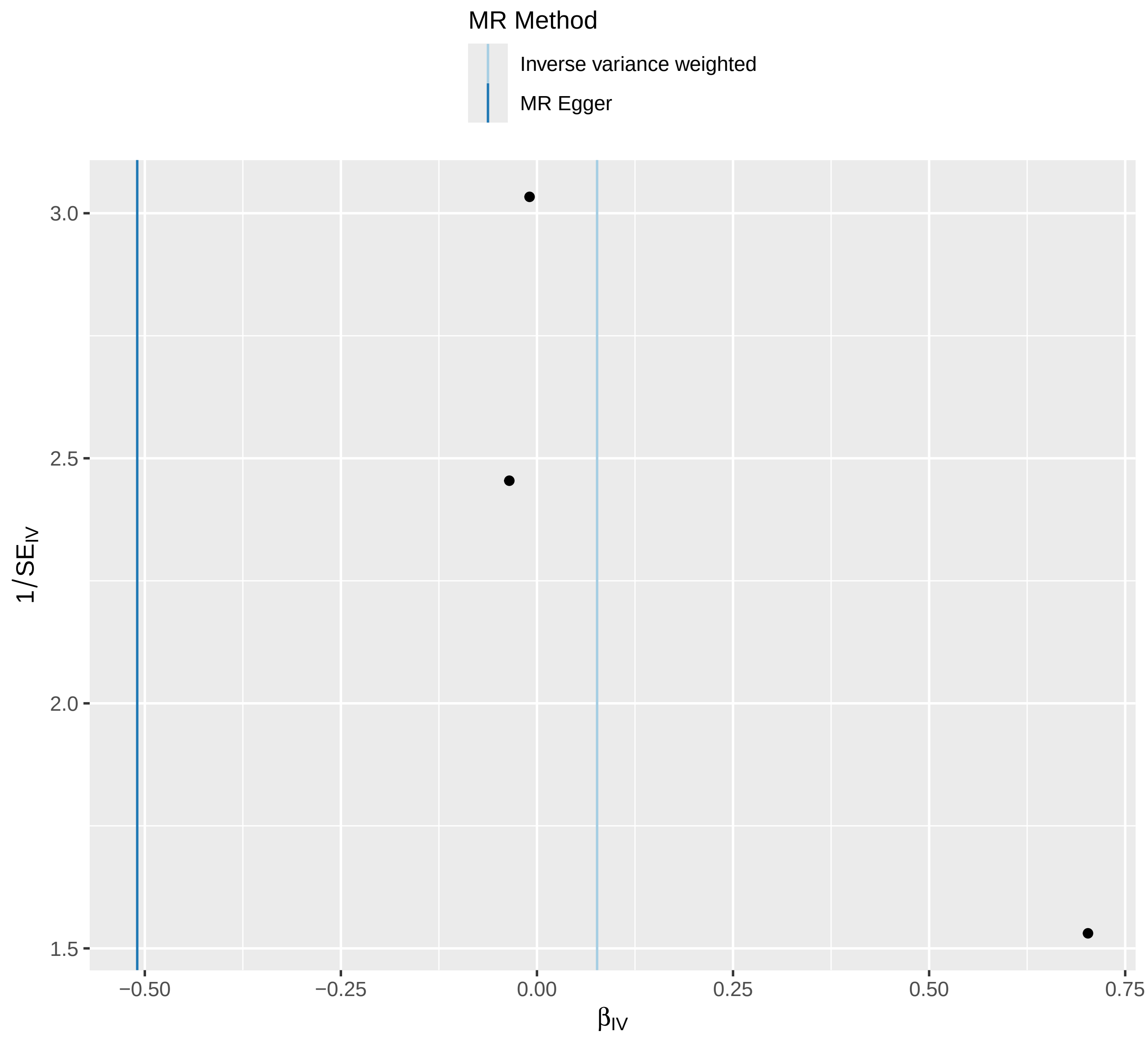
MR Method
Inverse variance weighted
MR Egger
3.0
2.5
2.0
1.5
1/SE_IV
−0.50
−0.25
0.00
0.25
0.50
0.75
β_IV

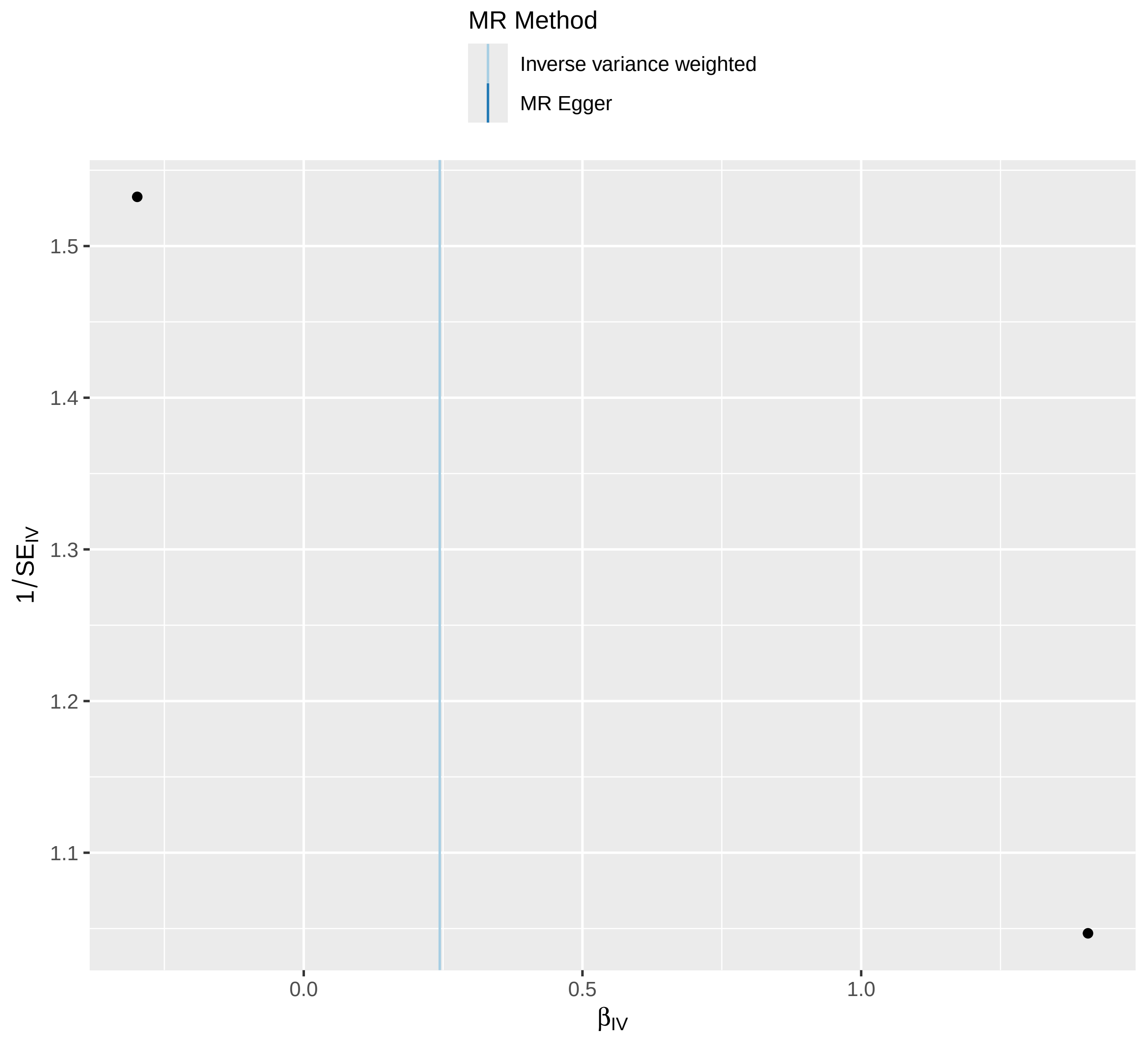
MR Method
Inverse variance weighted
MR Egger
1.5
1.4
1.3
1.2
1.1
$1/SE_{IV}$
0.0
0.5
1.0
β_{IV}

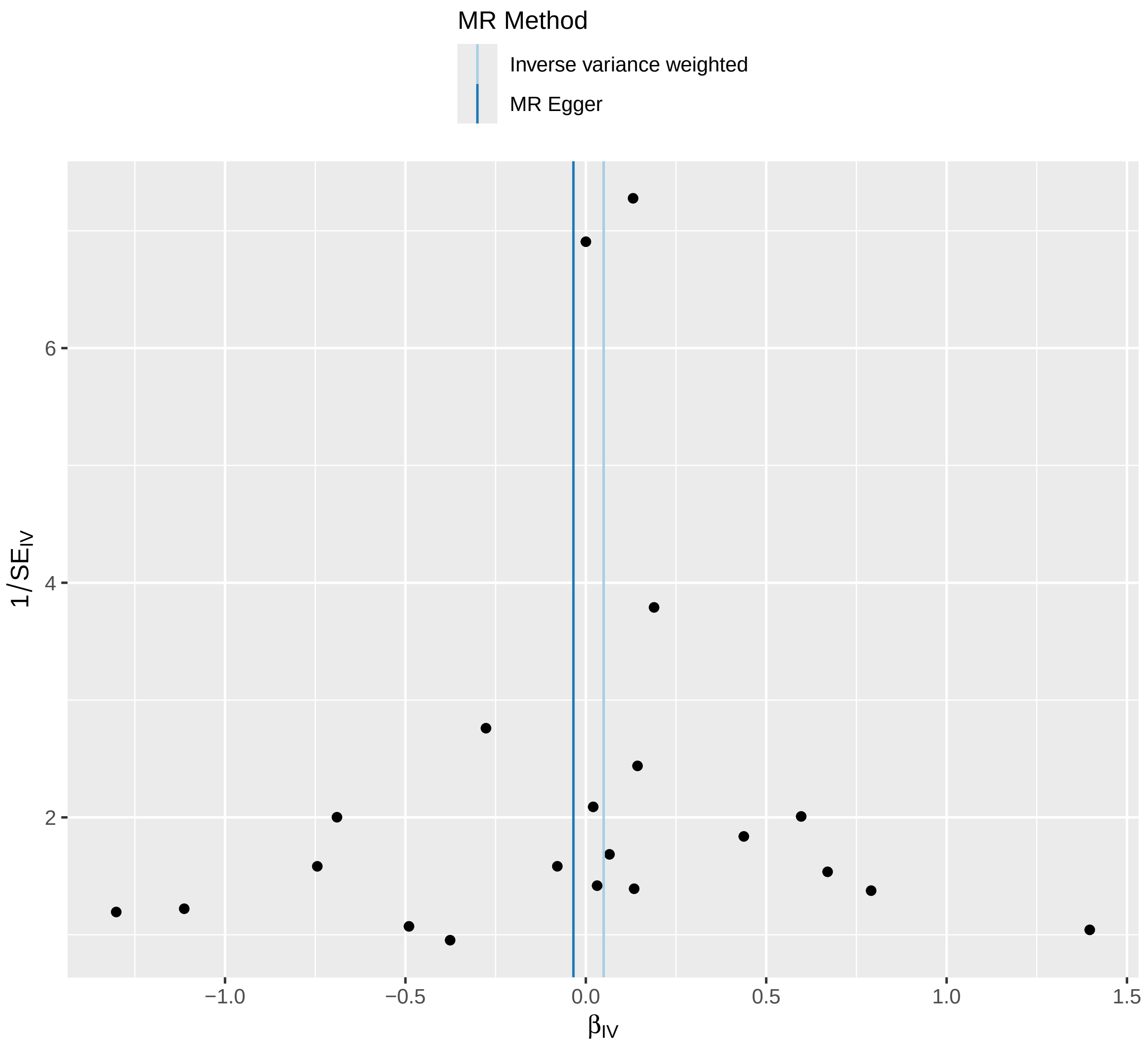
MR Method
Inverse variance weighted
MR Egger
1/SE$_{IV}$
2
4
6
−1.0
−0.5
0.0
0.5
1.0
1.5
β$_{IV}$

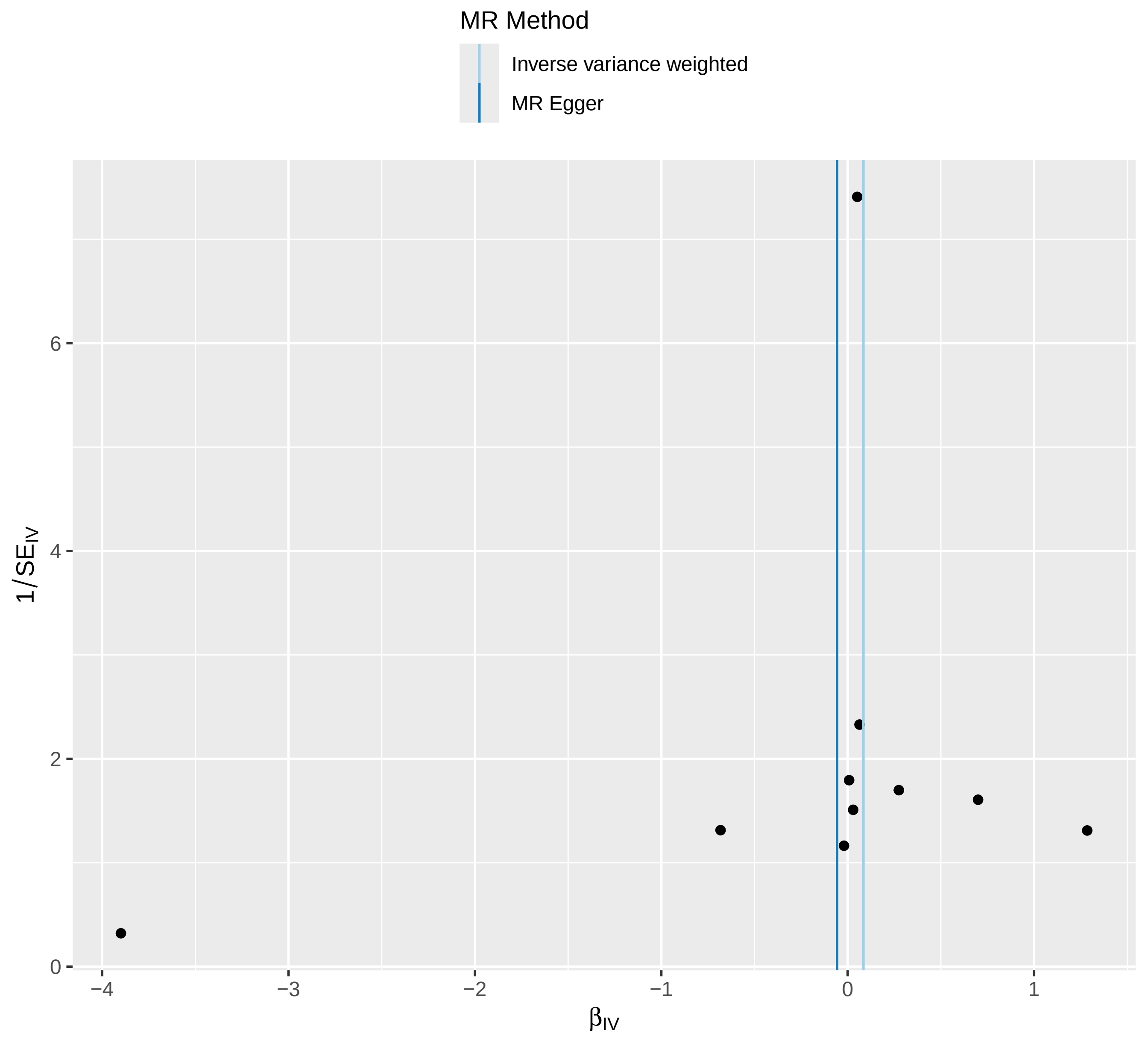

MR Method
Inverse variance weighted
MR Egger
1/SE_IV
6
4
2
0
−4
−3
−2
−1
0
1
β_IV

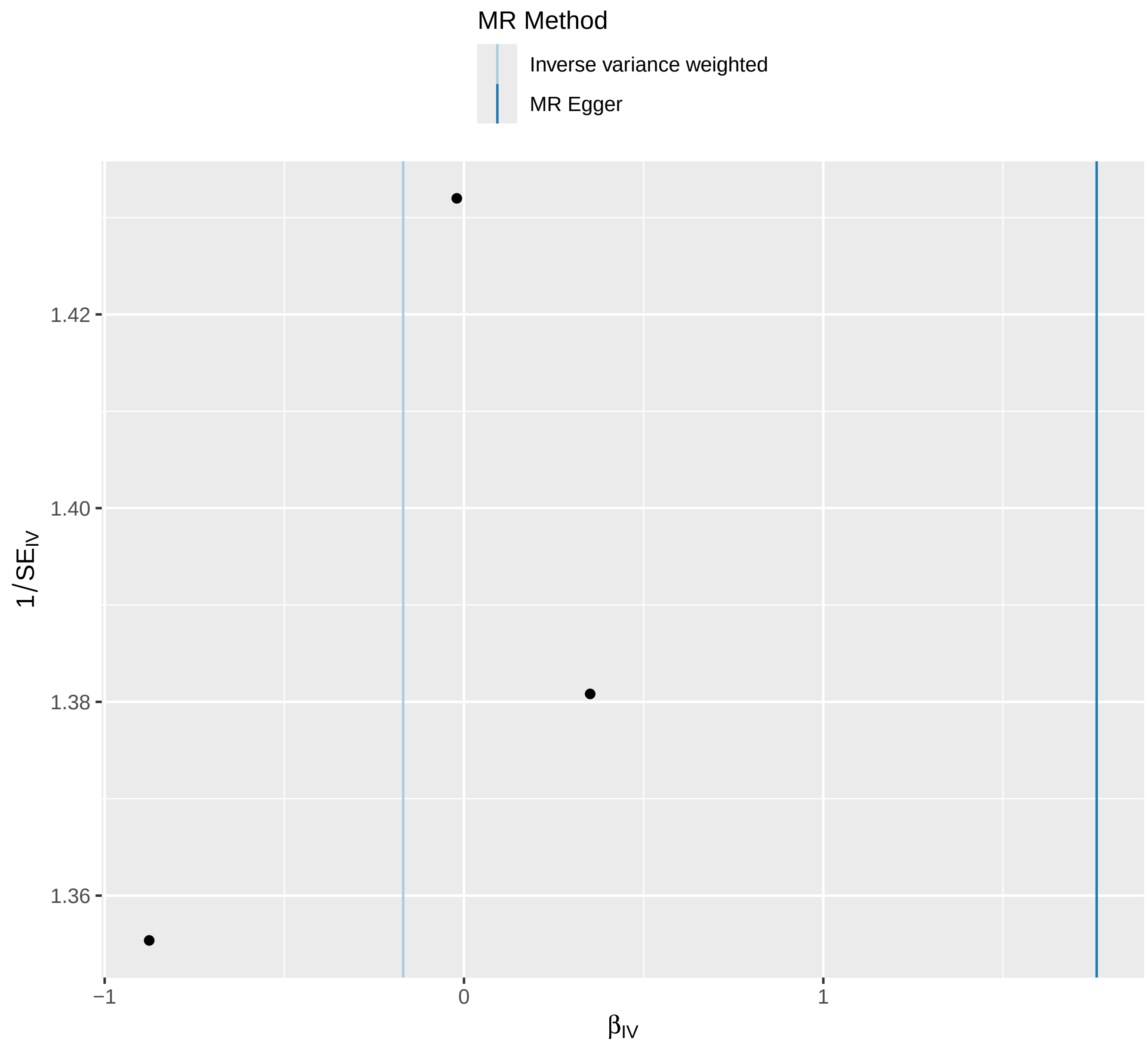
MR Method
Inverse variance weighted
MR Egger
1.42
1.40
1.38
1.36
$1/SE_{IV}$
−1
0
1
β_{IV}

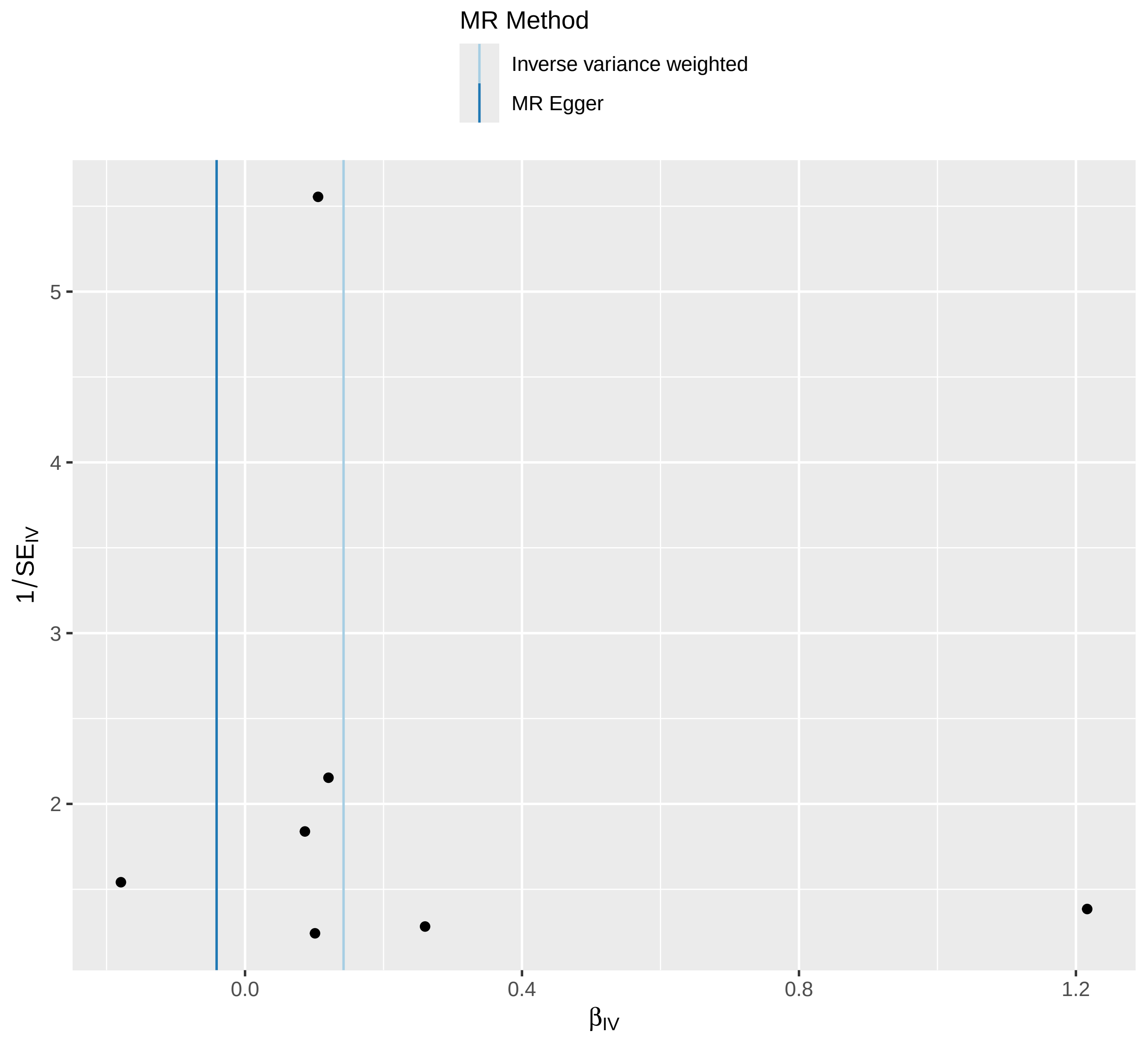
MR Method
Inverse variance weighted
MR Egger
1/SE_IV
5
4
3
2
0.0
0.4
0.8
1.2
β_IV

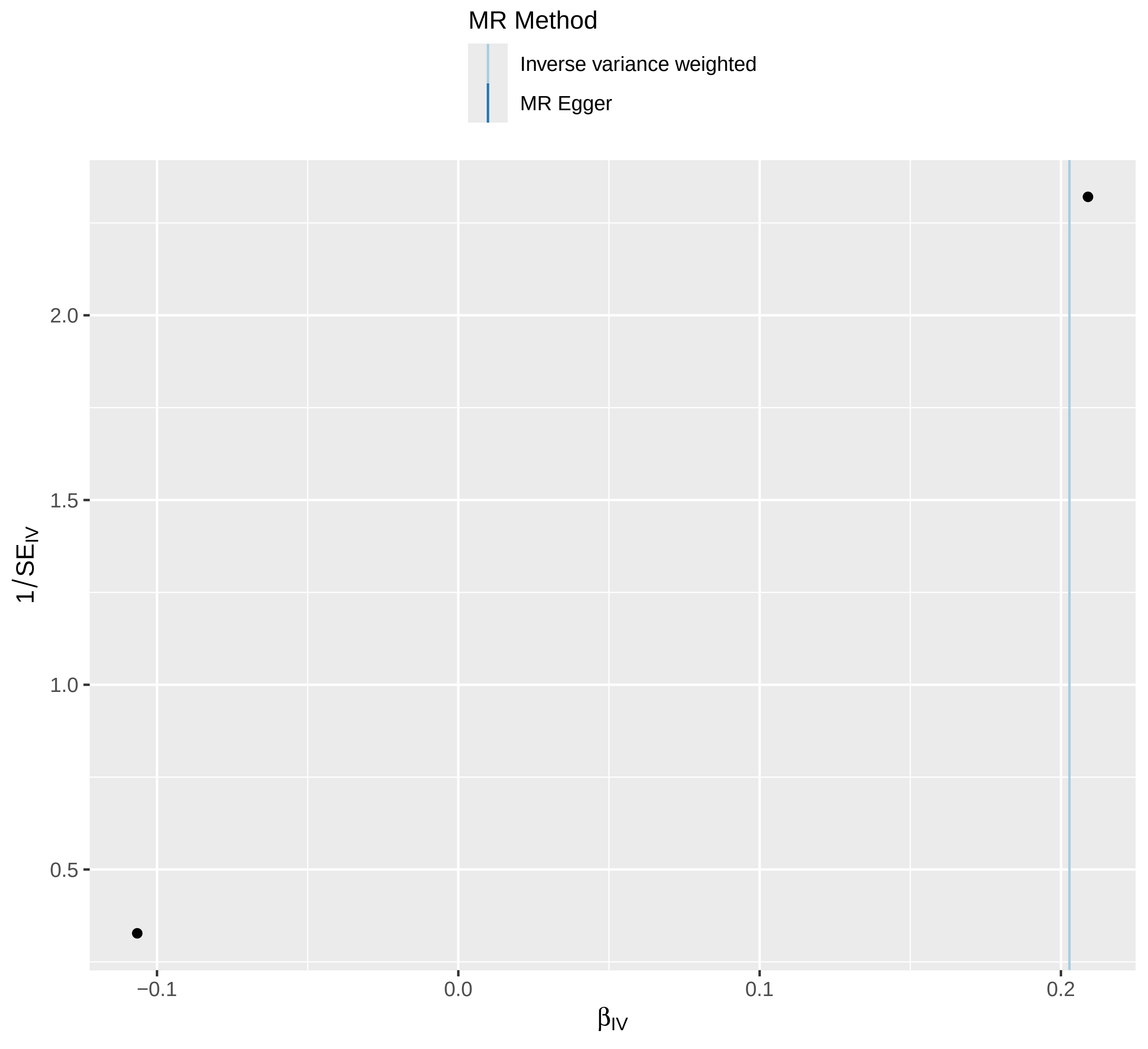
MR Method
Inverse variance weighted
MR Egger
2.0
1.5
1.0
0.5
1/SE_{IV}
−0.1
0.0
0.1
0.2
β_{IV}

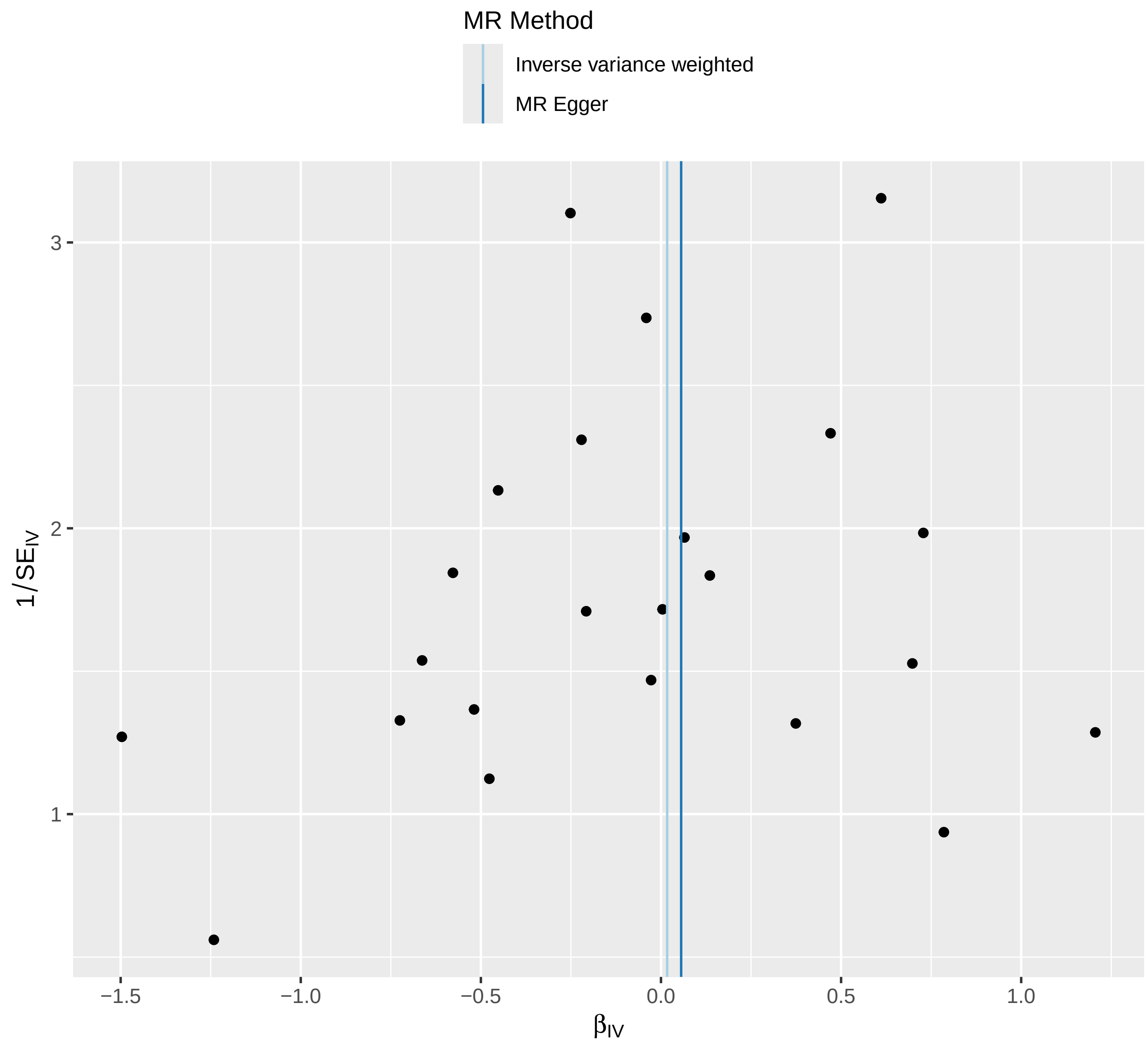

MR Method
Inverse variance weighted
MR Egger
3
2
1
1/SE_IV
−1.5
−1.0
−0.5
0.0
0.5
1.0
β_IV

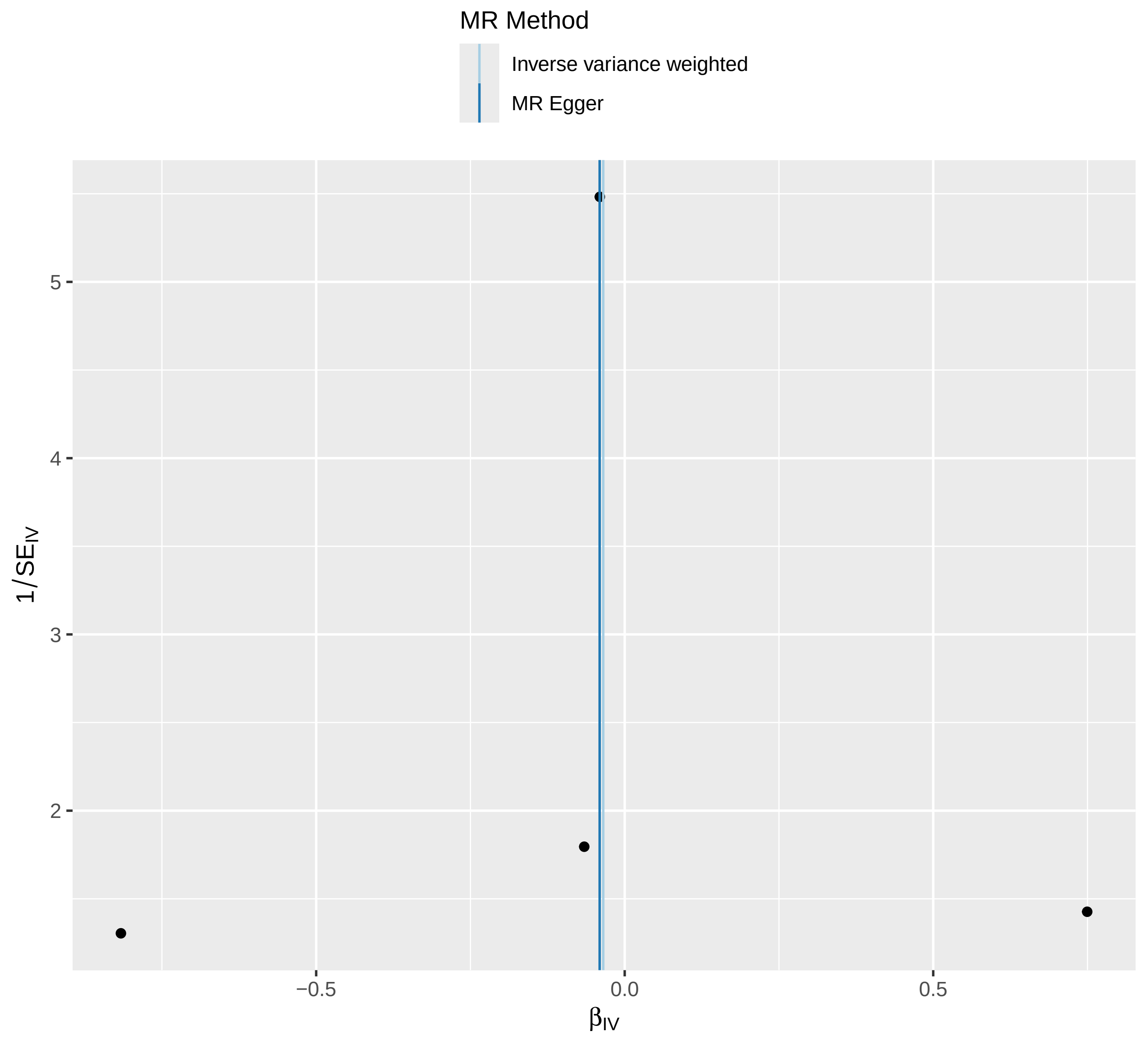
MR Method
Inverse variance weighted
MR Egger
5
4
3
2
1/SE_IV
−0.5
0.0
0.5
β_IV

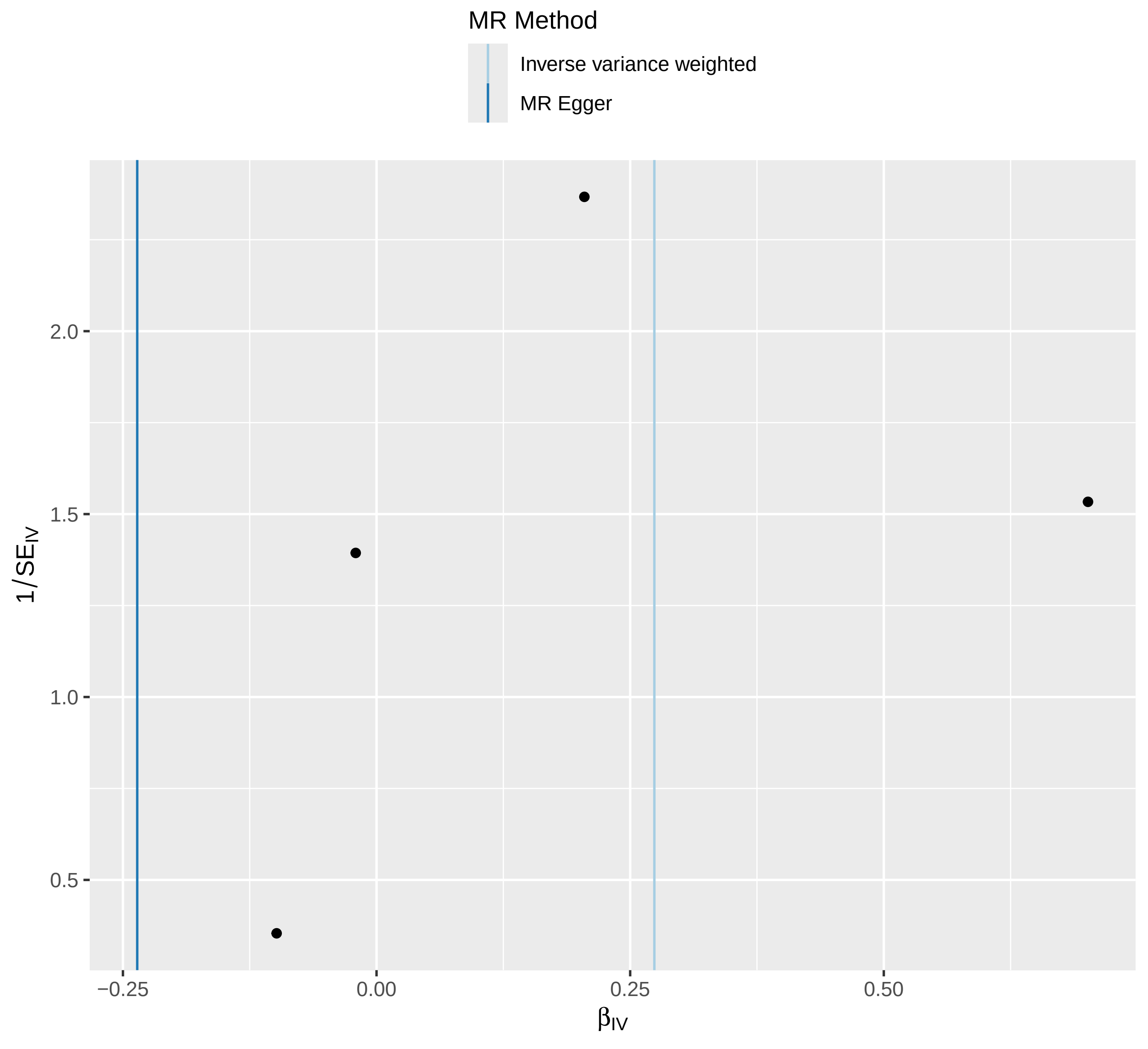

MR Method
Inverse variance weighted
MR Egger
2.0
1.5
1.0
0.5
1/SE_IV
−0.25
0.00
0.25
0.50
β_IV

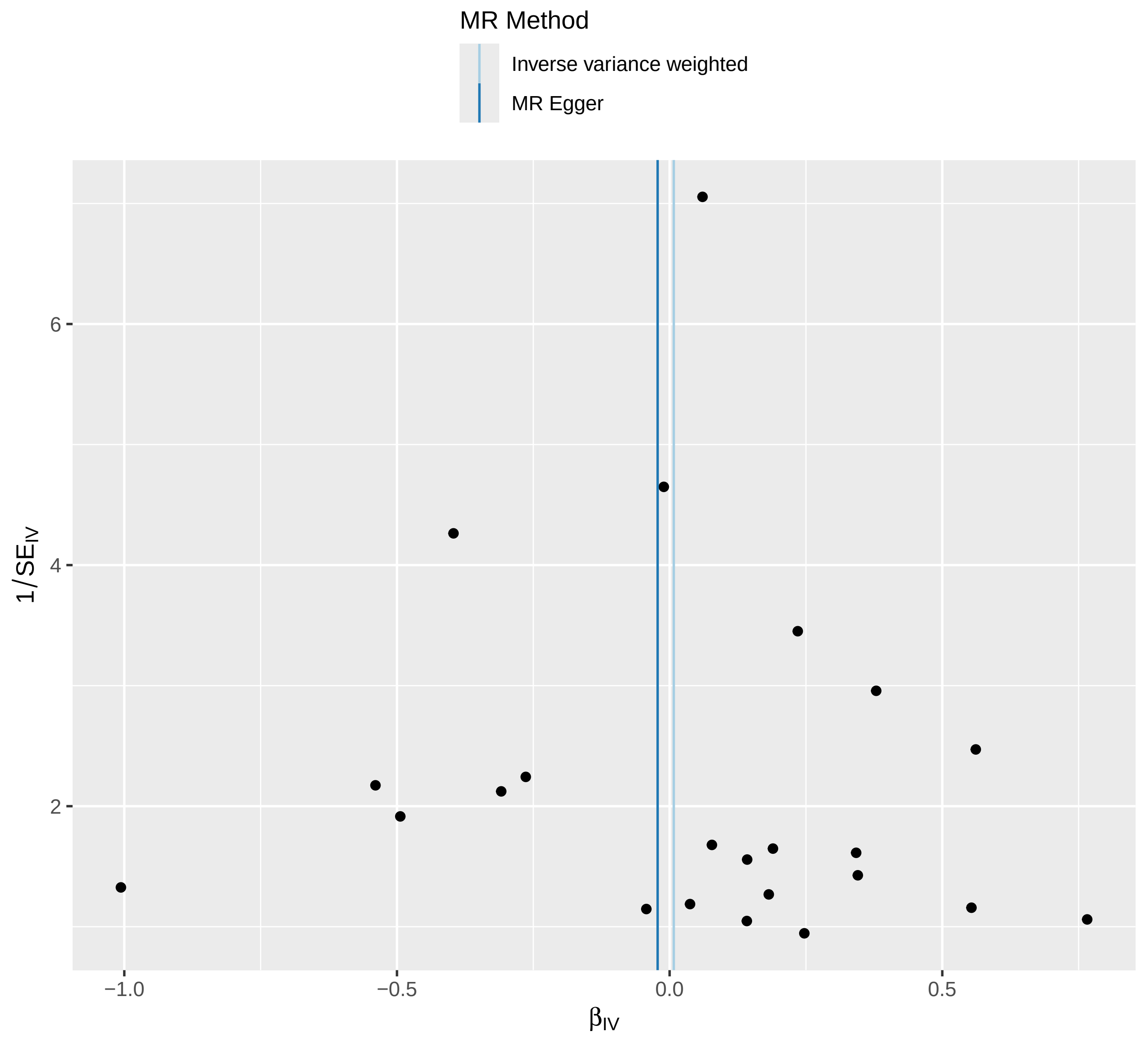

MR Method
Inverse variance weighted
MR Egger
6
4
2
1/SE_IV
−1.0
−0.5
0.0
0.5
β_IV

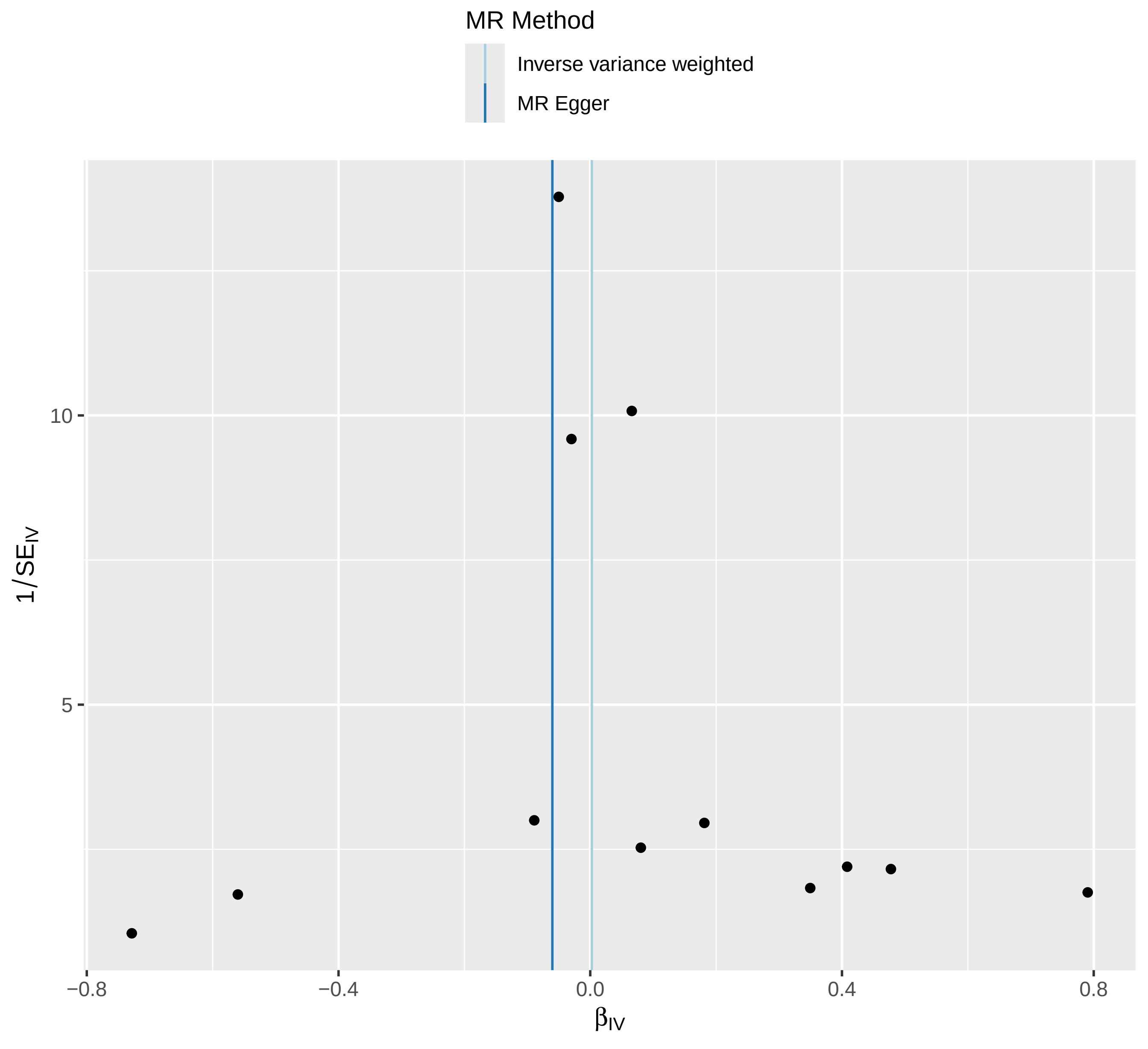
MR Method
Inverse variance weighted
MR Egger
1/SE_IV
10
5
−0.8
−0.4
0.0
0.4
0.8
β_IV

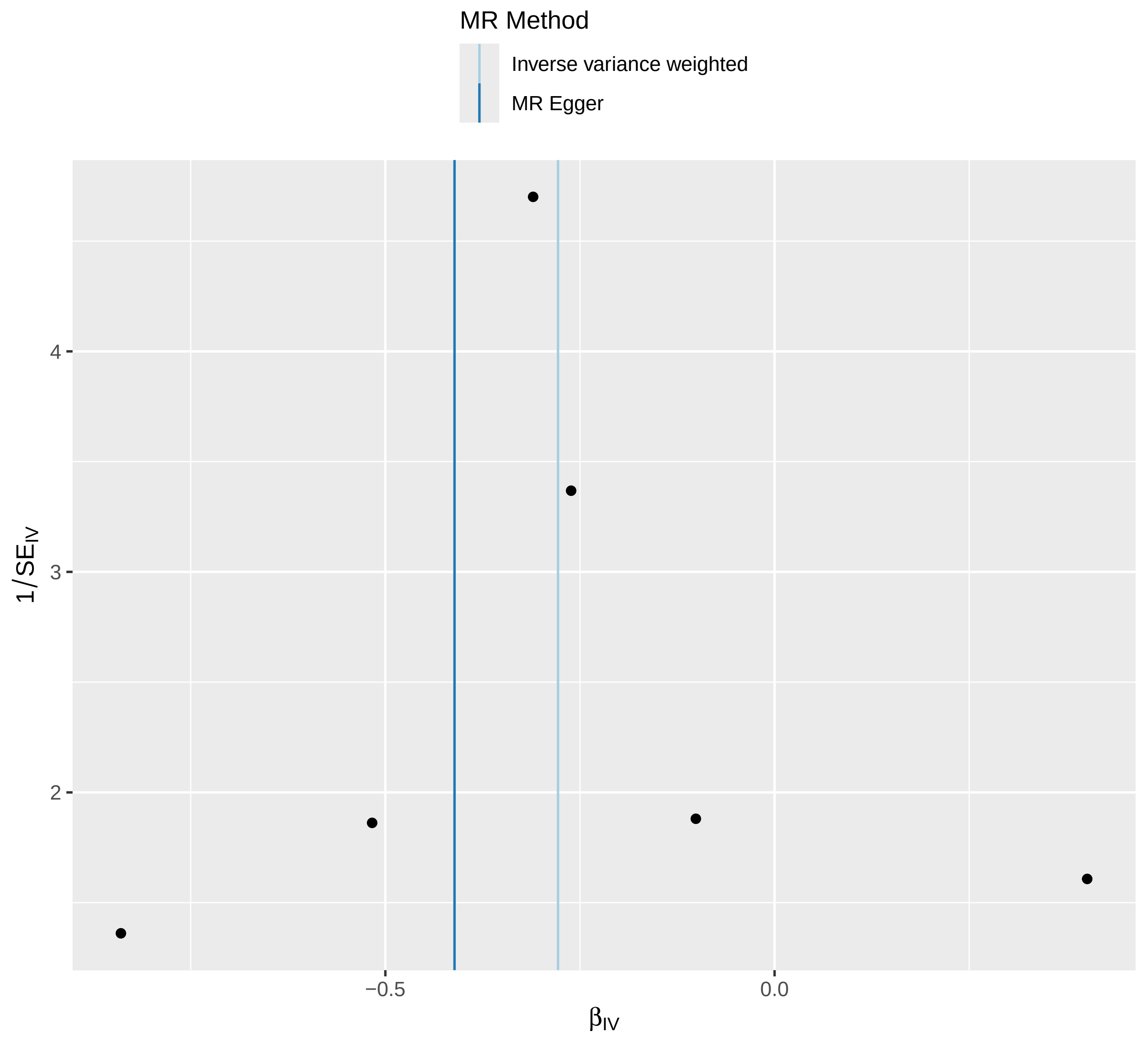

MR Method
Inverse variance weighted
MR Egger
4
3
2
$1/SE_{IV}$
−0.5
0.0
β_{IV}

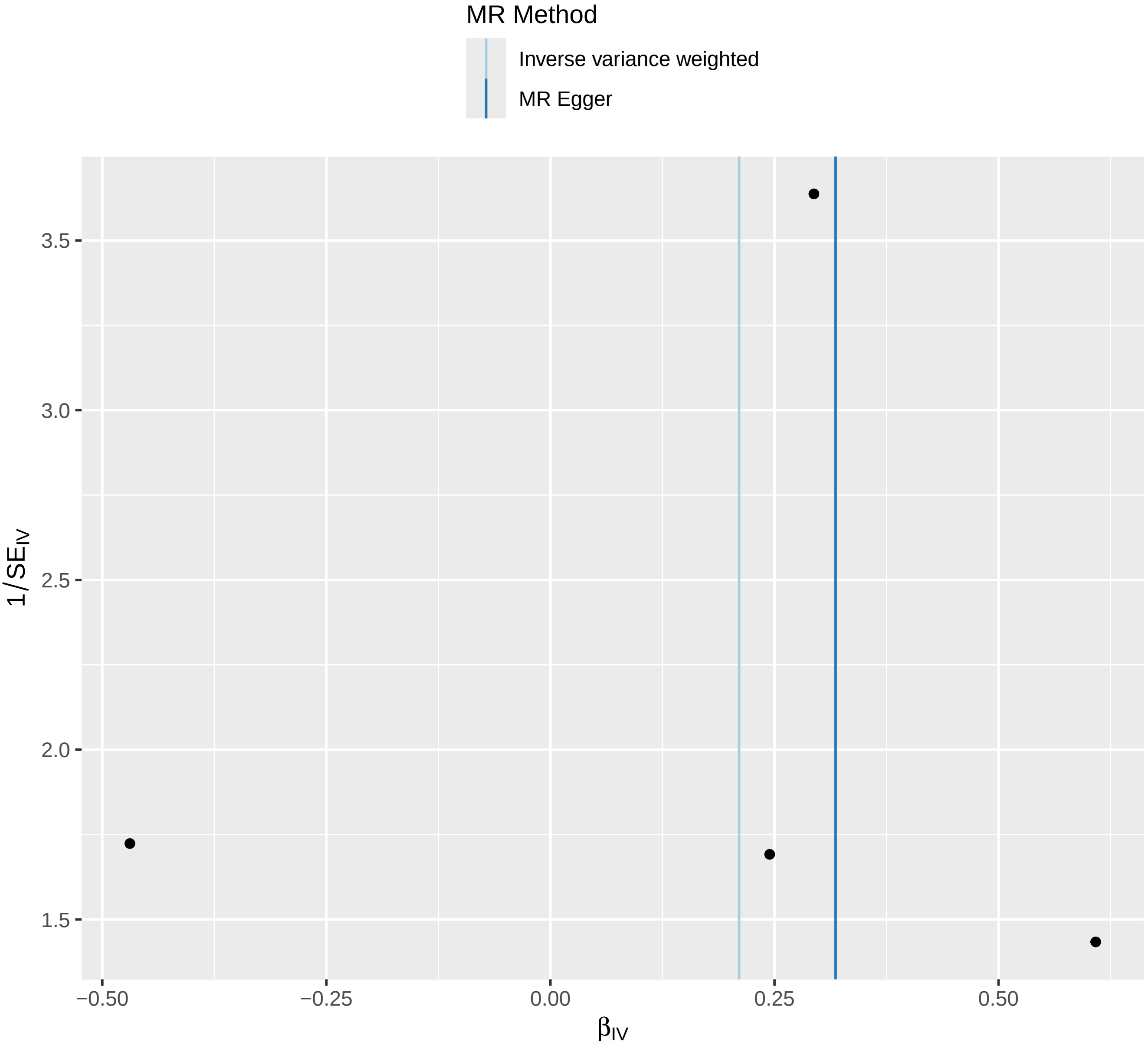

MR Method
Inverse variance weighted
MR Egger
3.5
3.0
2.5
2.0
1.5
1/SE_IV
−0.50
−0.25
0.00
0.25
0.50
β_IV

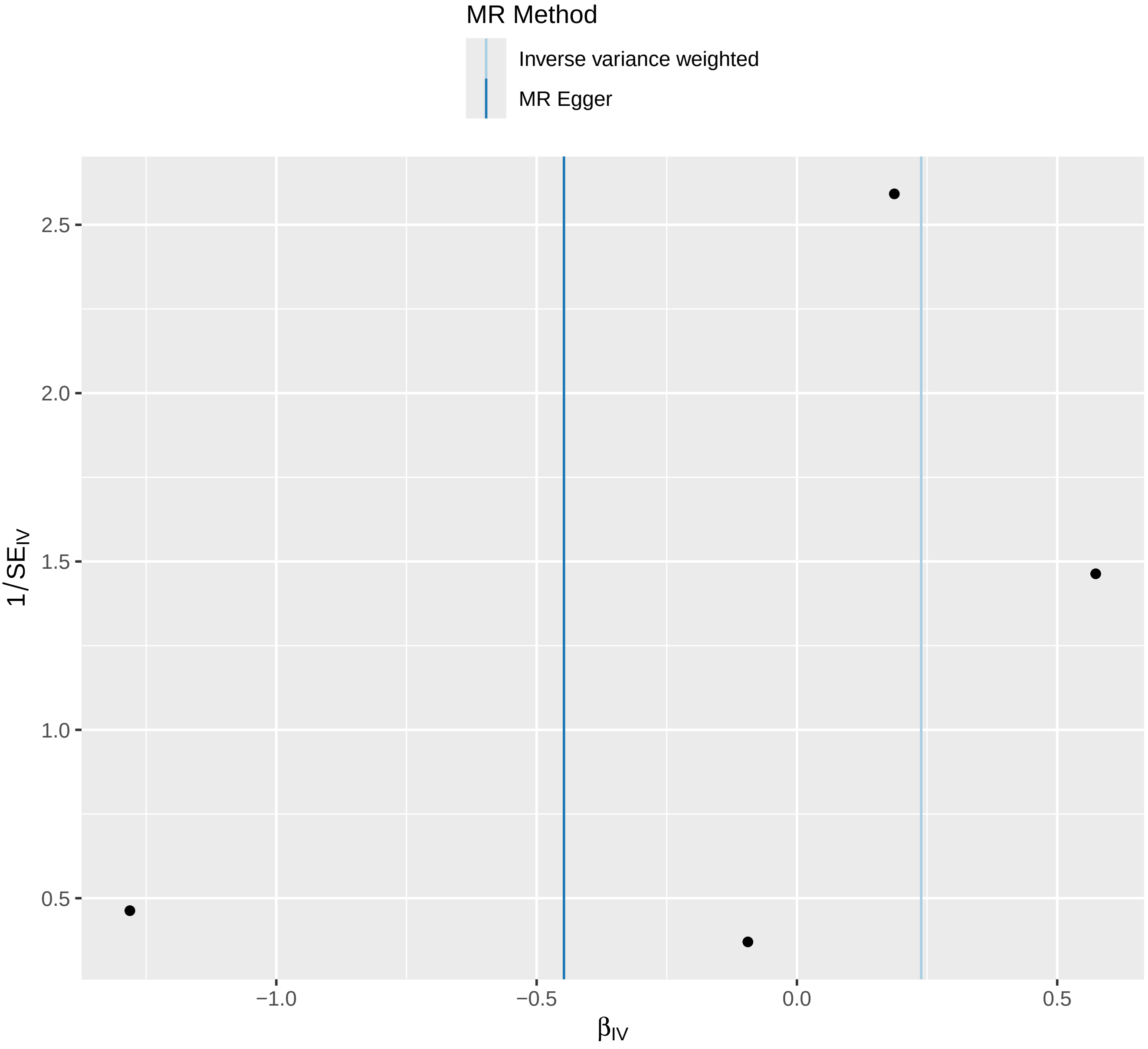

MR Method
Inverse variance weighted
MR Egger
2.5
2.0
1.5
1.0
0.5
1/SE_IV
−1.0
−0.5
0.0
0.5
β_IV

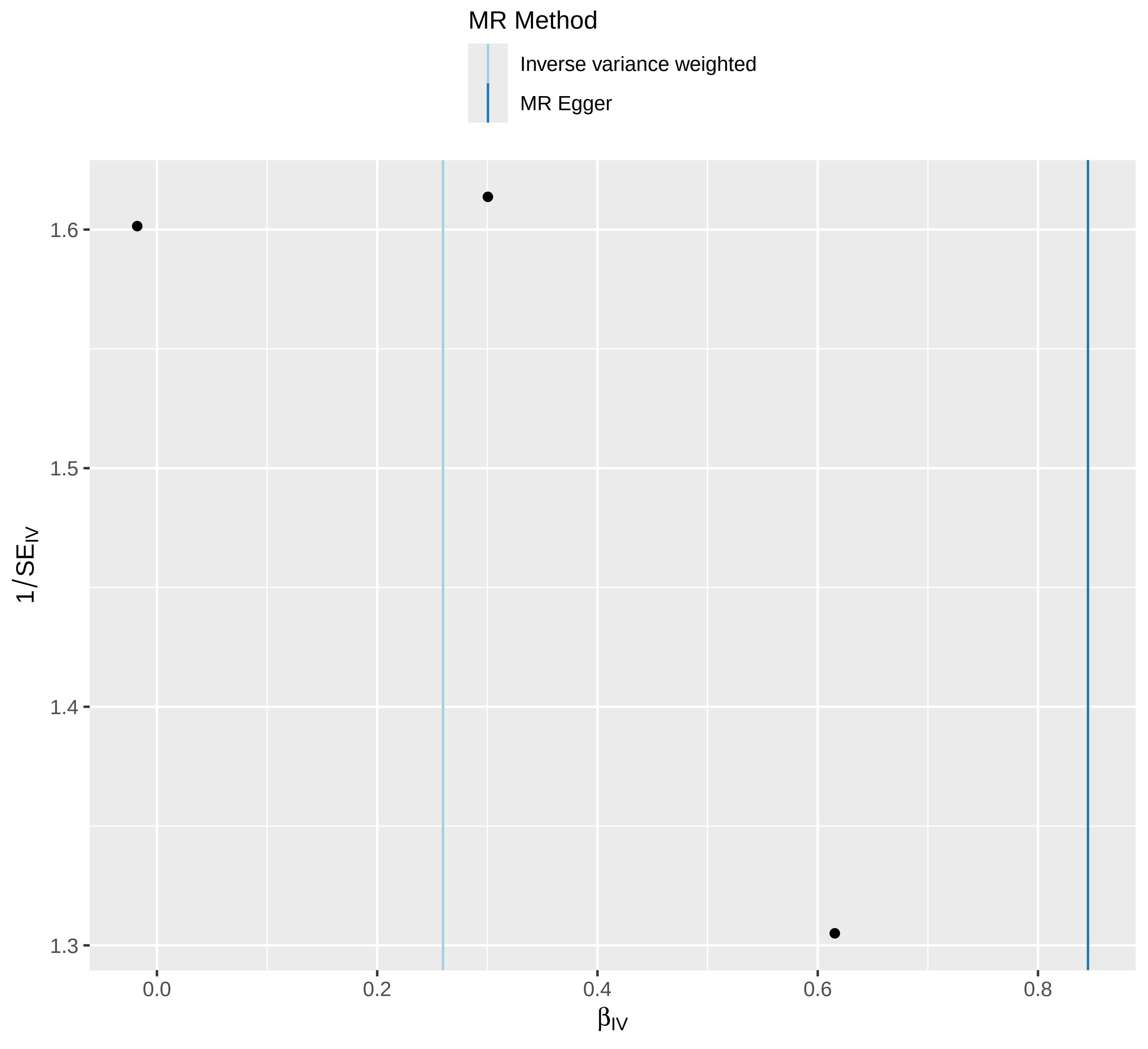
MR Method
Inverse variance weighted
MR Egger
1.6
1.5
1.4
1.3
$1/SE_{IV}$
0.0
0.2
0.4
0.6
0.8
β_{IV}

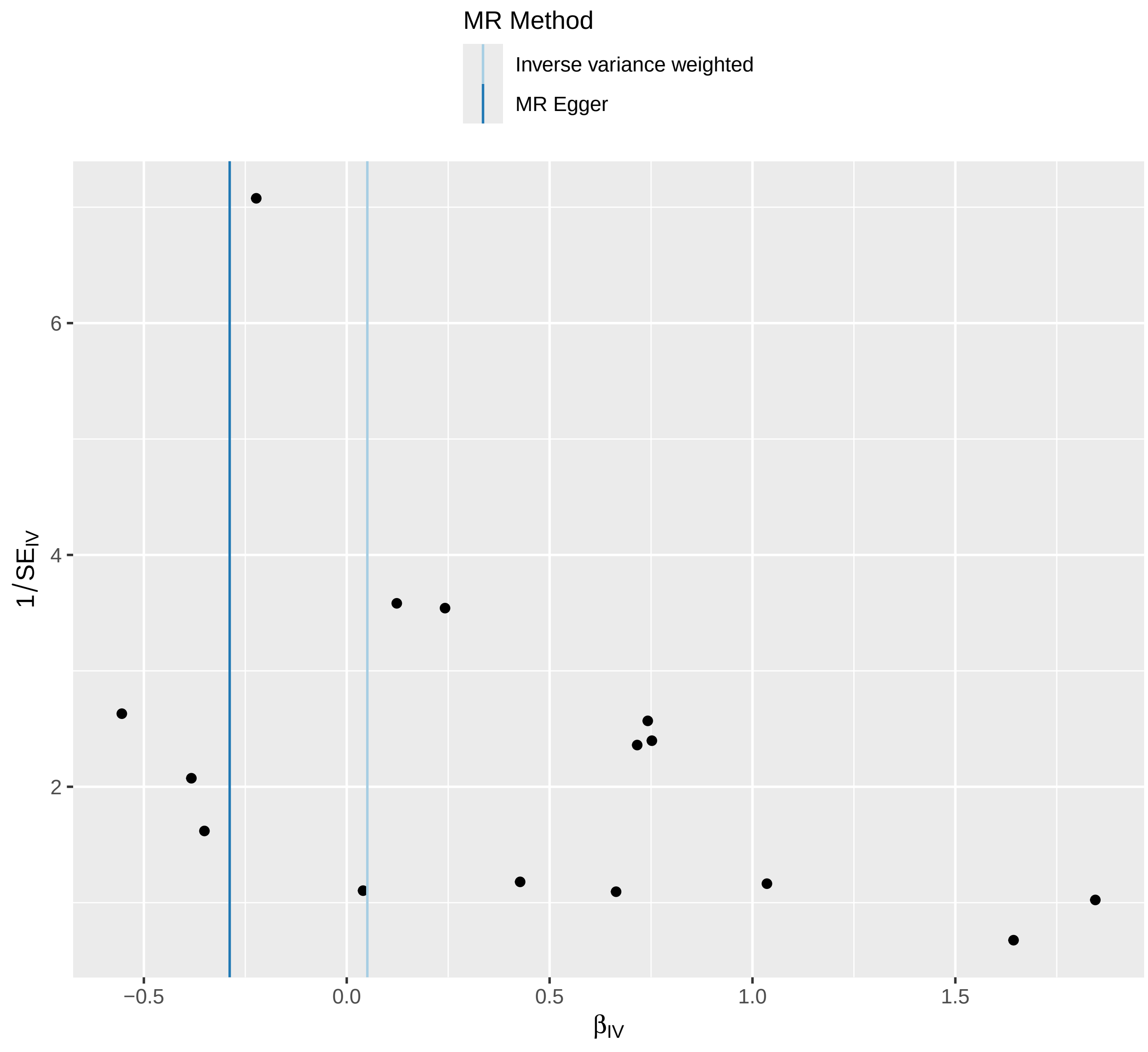
MR Method
Inverse variance weighted
MR Egger
6
4
2
−0.5
0.0
0.5
1.0
1.5
$1/SE_{IV}$
β_{IV}

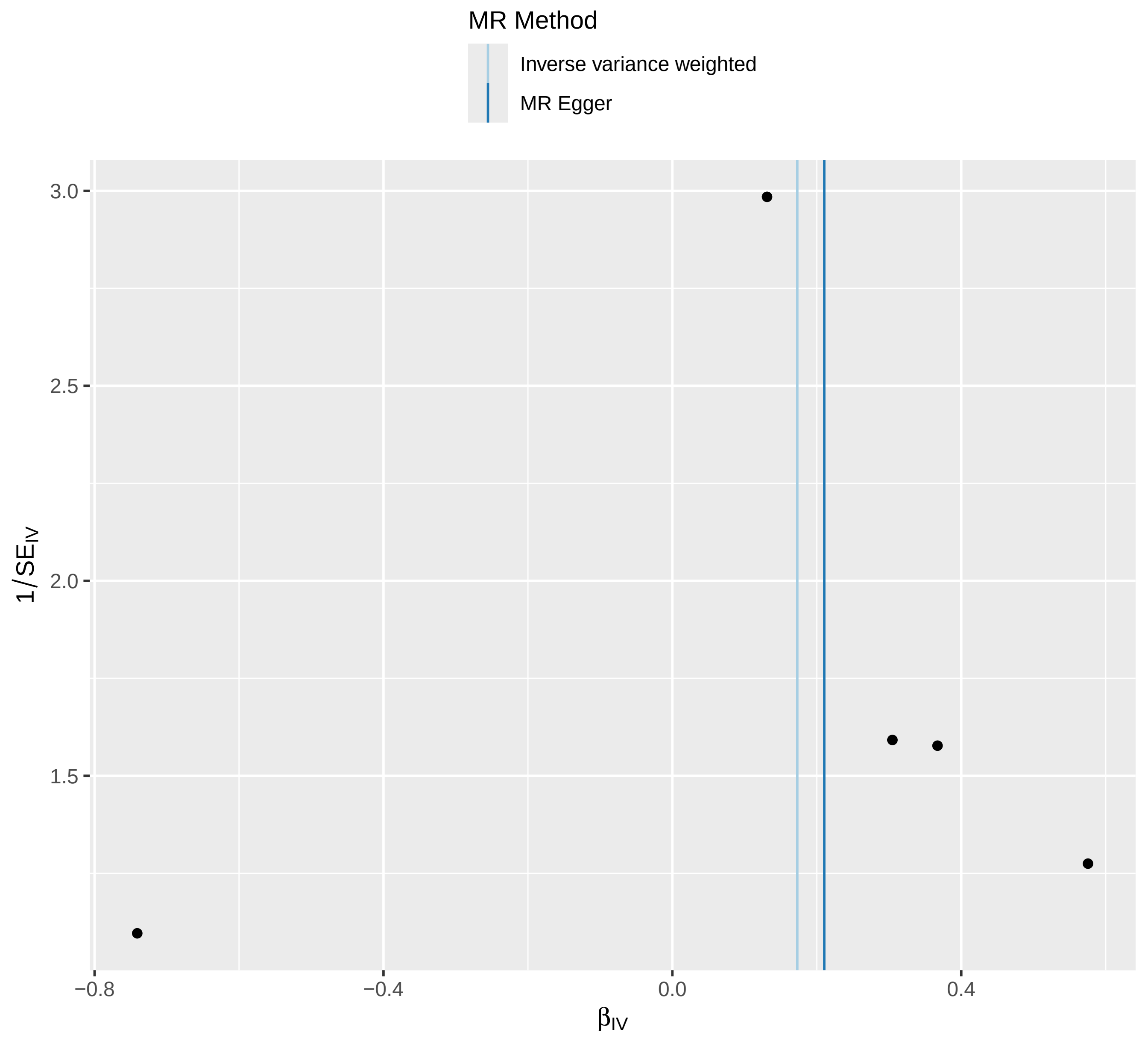

MR Method
Inverse variance weighted
MR Egger
3.0
2.5
2.0
1.5
$1/SE_{IV}$
−0.8
−0.4
0.0
0.4
β_{IV}

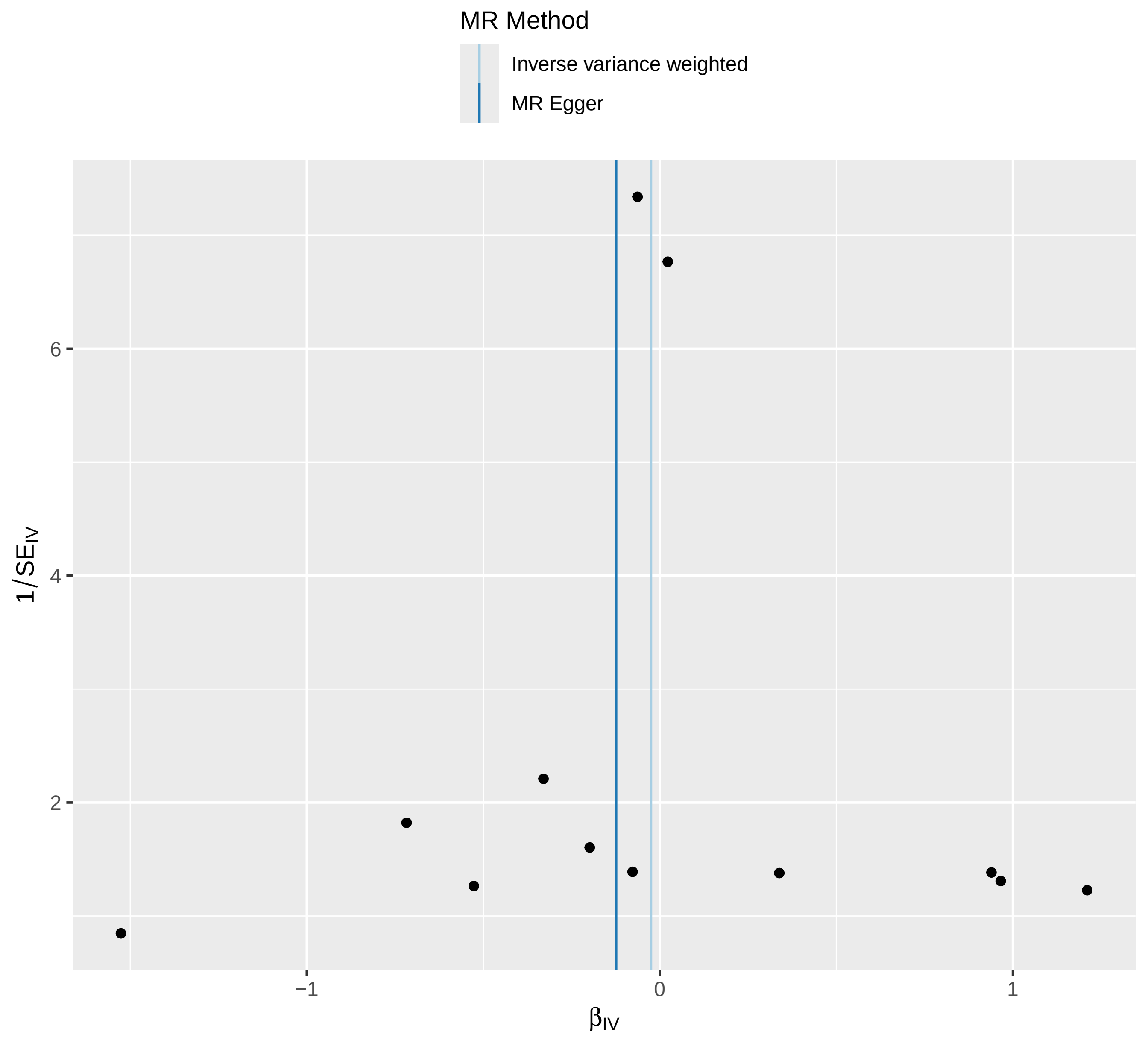
MR Method
Inverse variance weighted
MR Egger
6
4
2
1/SE$_{IV}$
−1
0
1
β$_{IV}$

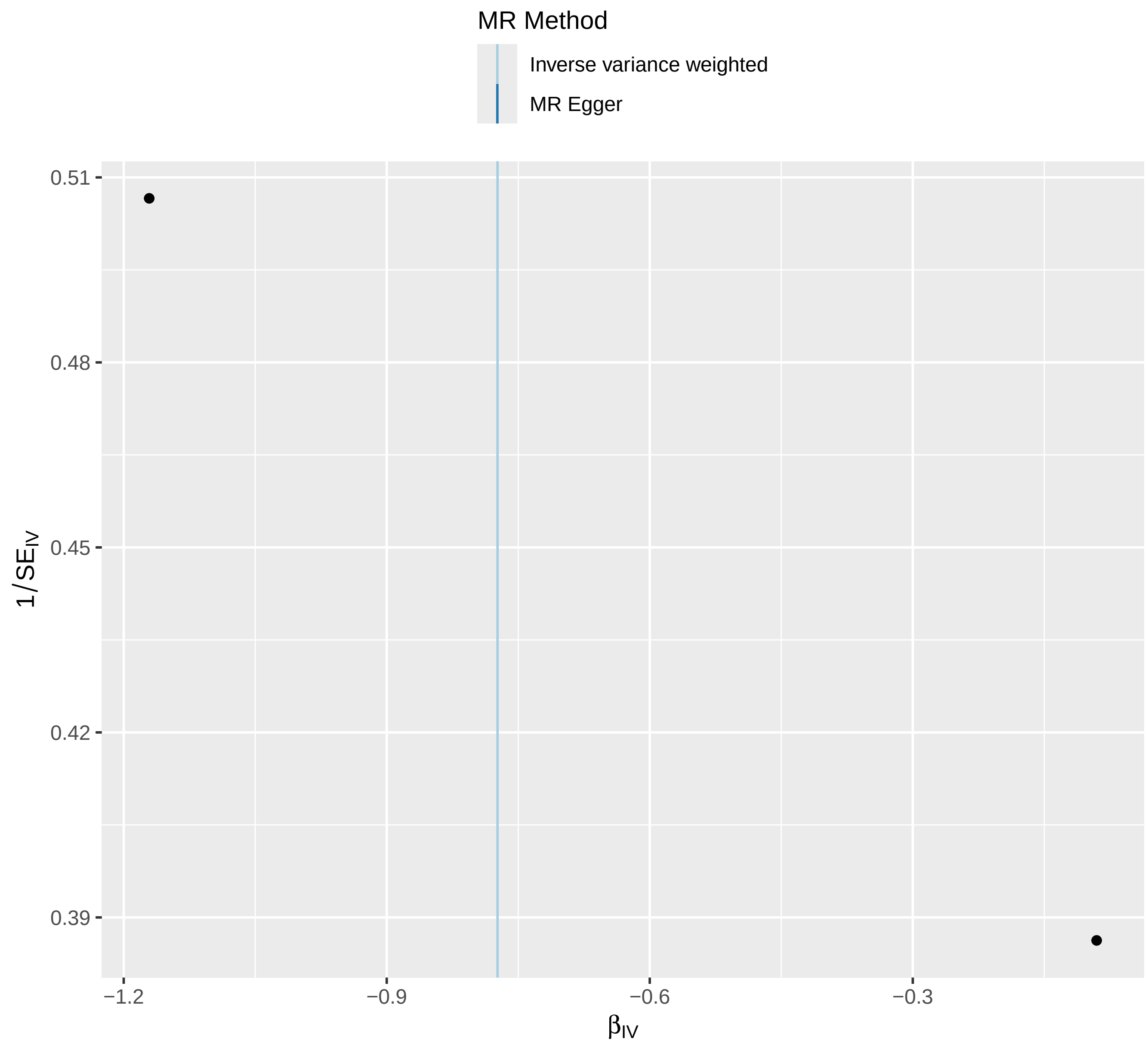

MR Method
Inverse variance weighted
MR Egger
0.51
0.48
0.45
0.42
0.39
$1/SE_{IV}$
−1.2
−0.9
−0.6
−0.3
β_{IV}

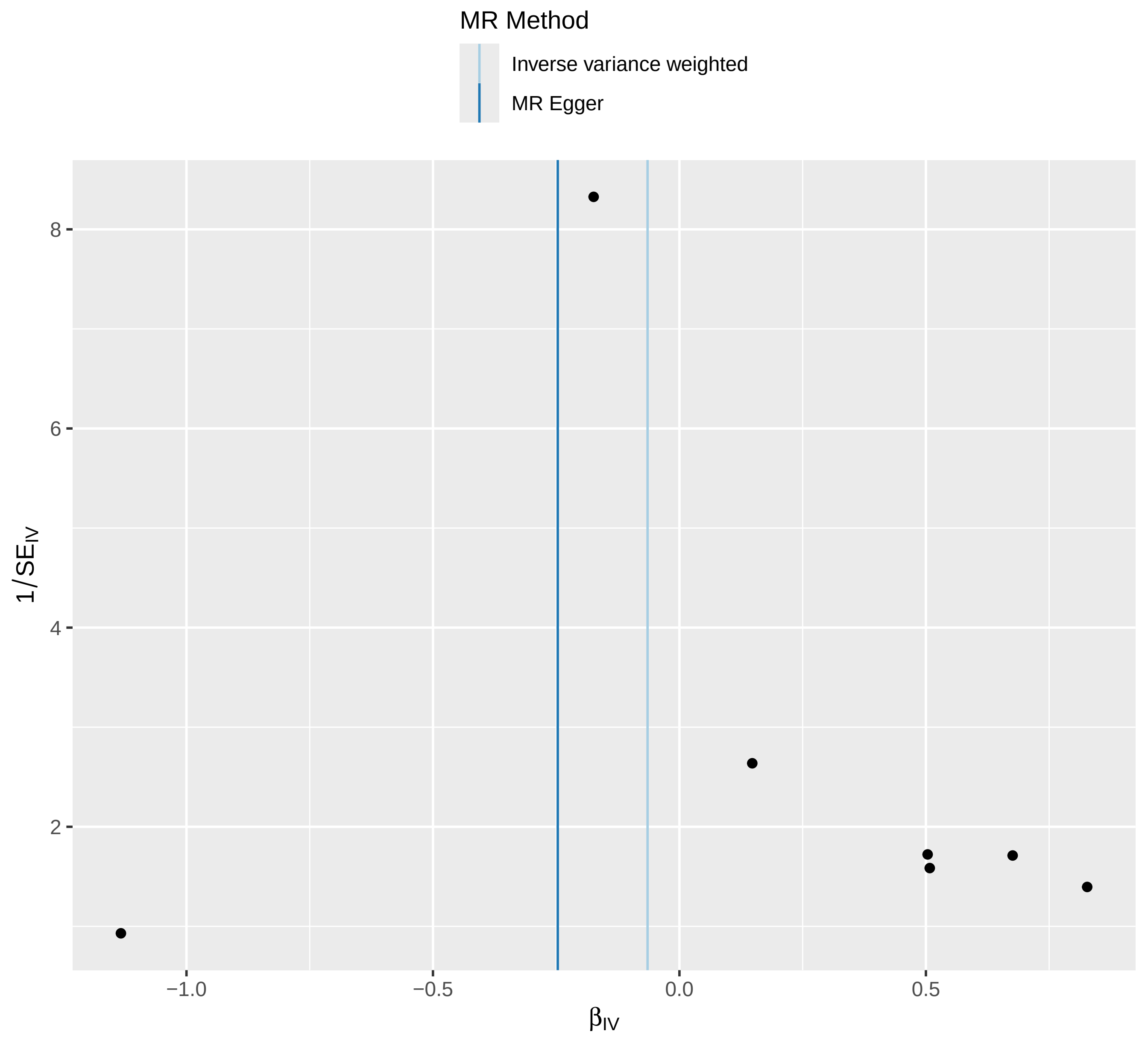
MR Method
Inverse variance weighted
MR Egger
8
6
4
2
1/SE_IV
−1.0
−0.5
0.0
0.5
β_IV

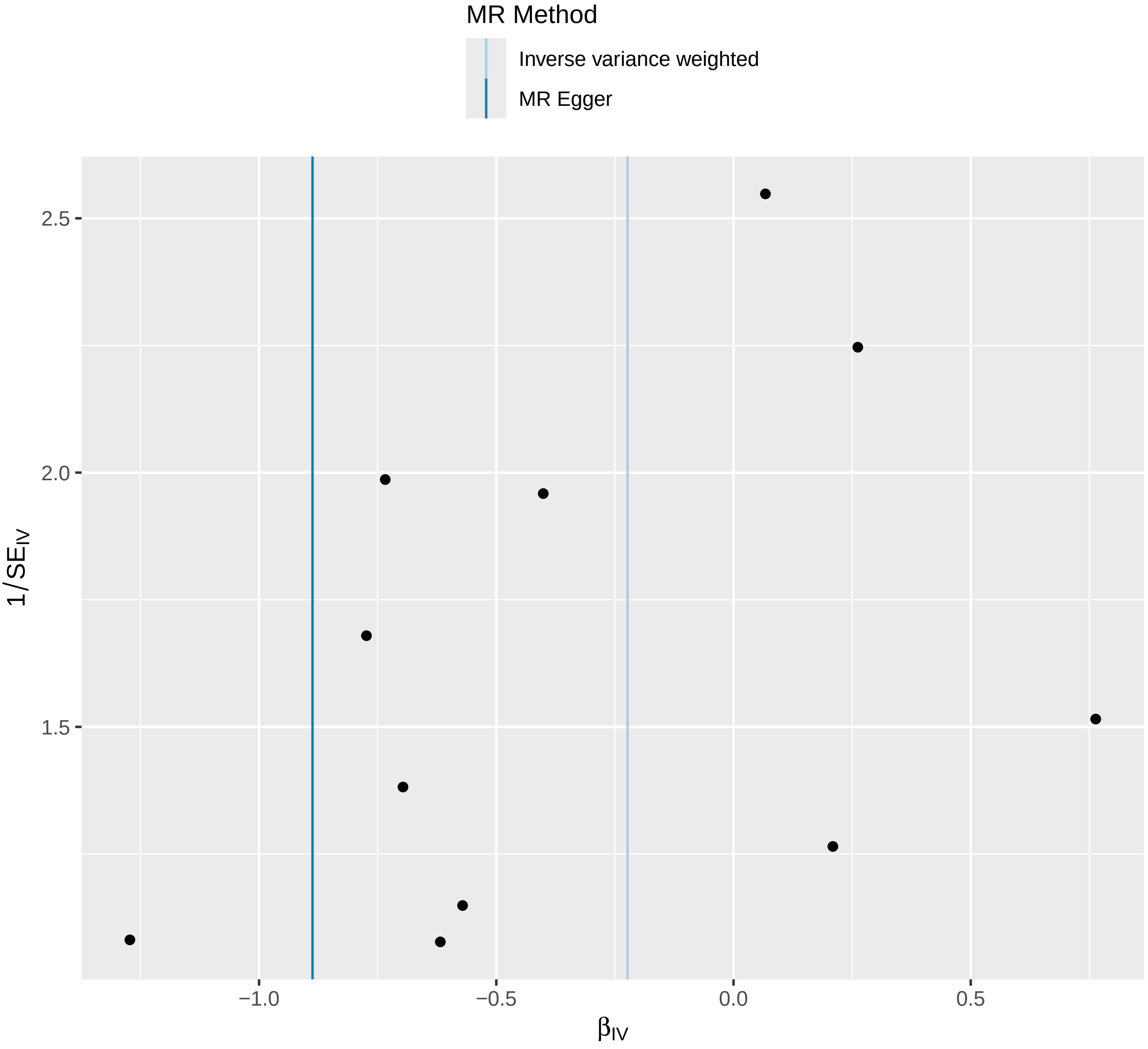
MR Method
Inverse variance weighted
MR Egger
2.5
2.0
1.5
$1/SE_{IV}$
−1.0
−0.5
0.0
0.5
β_{IV}

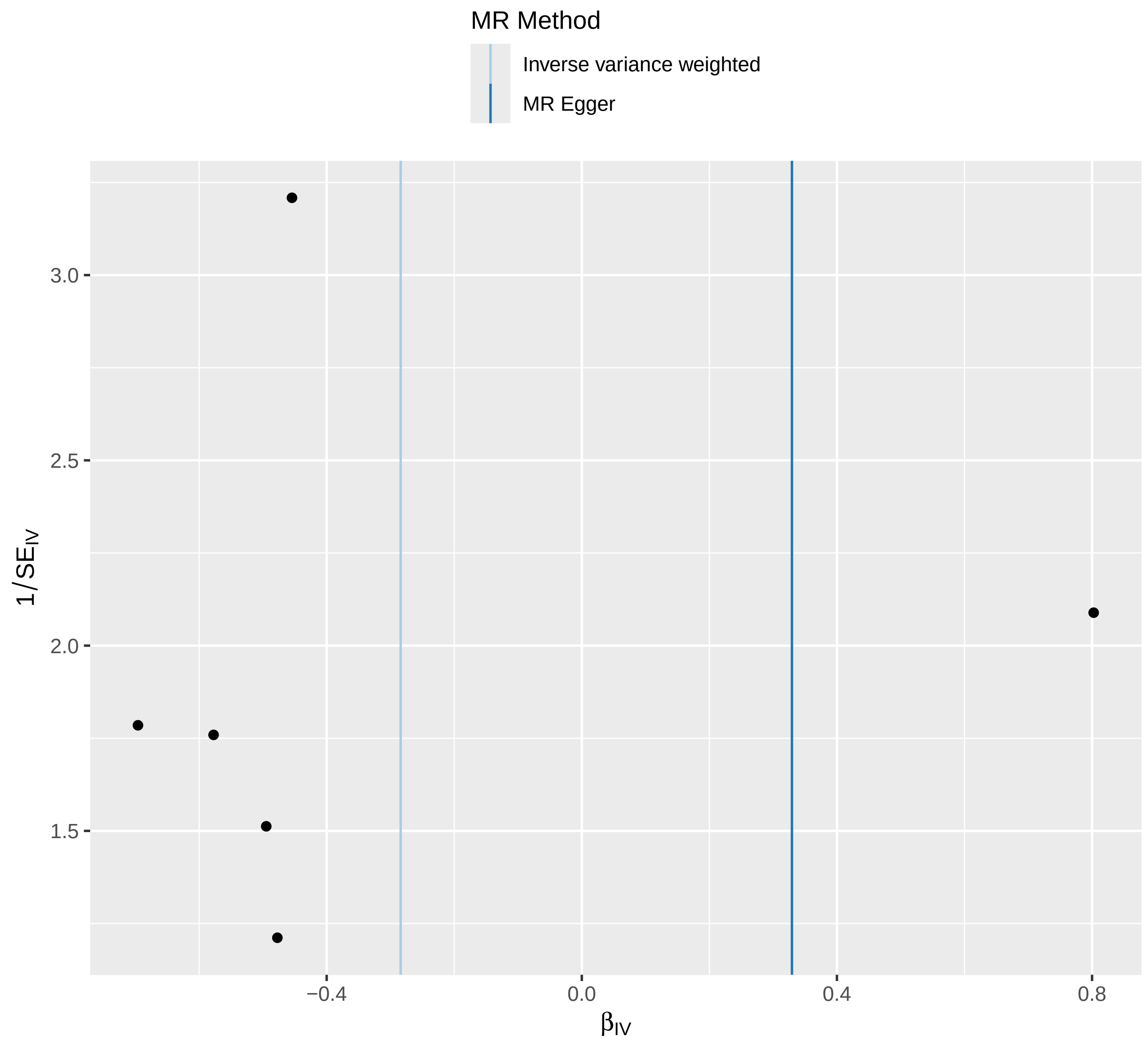

MR Method
Inverse variance weighted
MR Egger
1/SE_IV
3.0
2.5
2.0
1.5
−0.4
0.0
0.4
0.8
β_IV

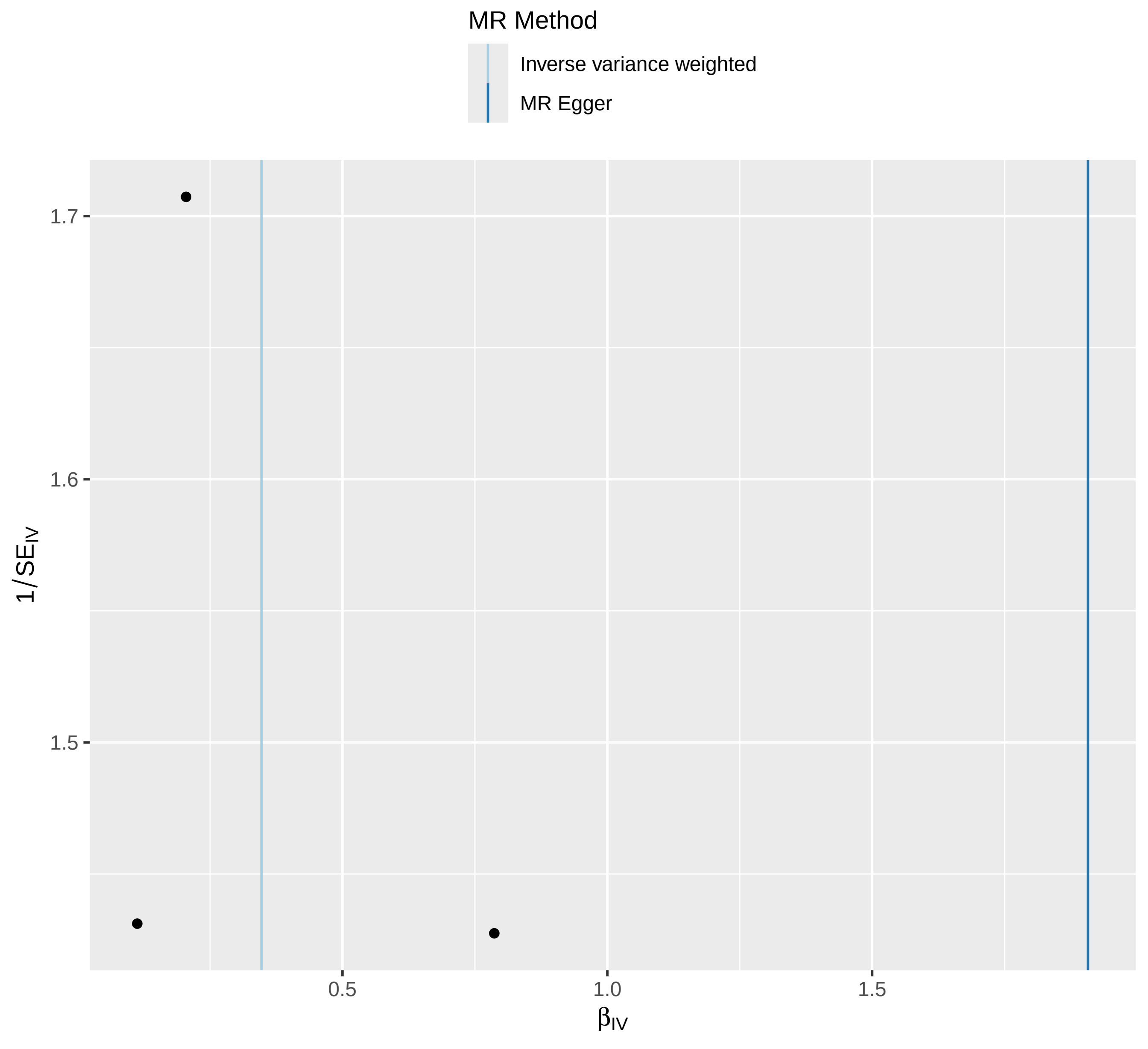

MR Method
Inverse variance weighted
MR Egger
1.7
1.6
1.5
1/SE$_{IV}$
0.5
1.0
1.5
β$_{IV}$

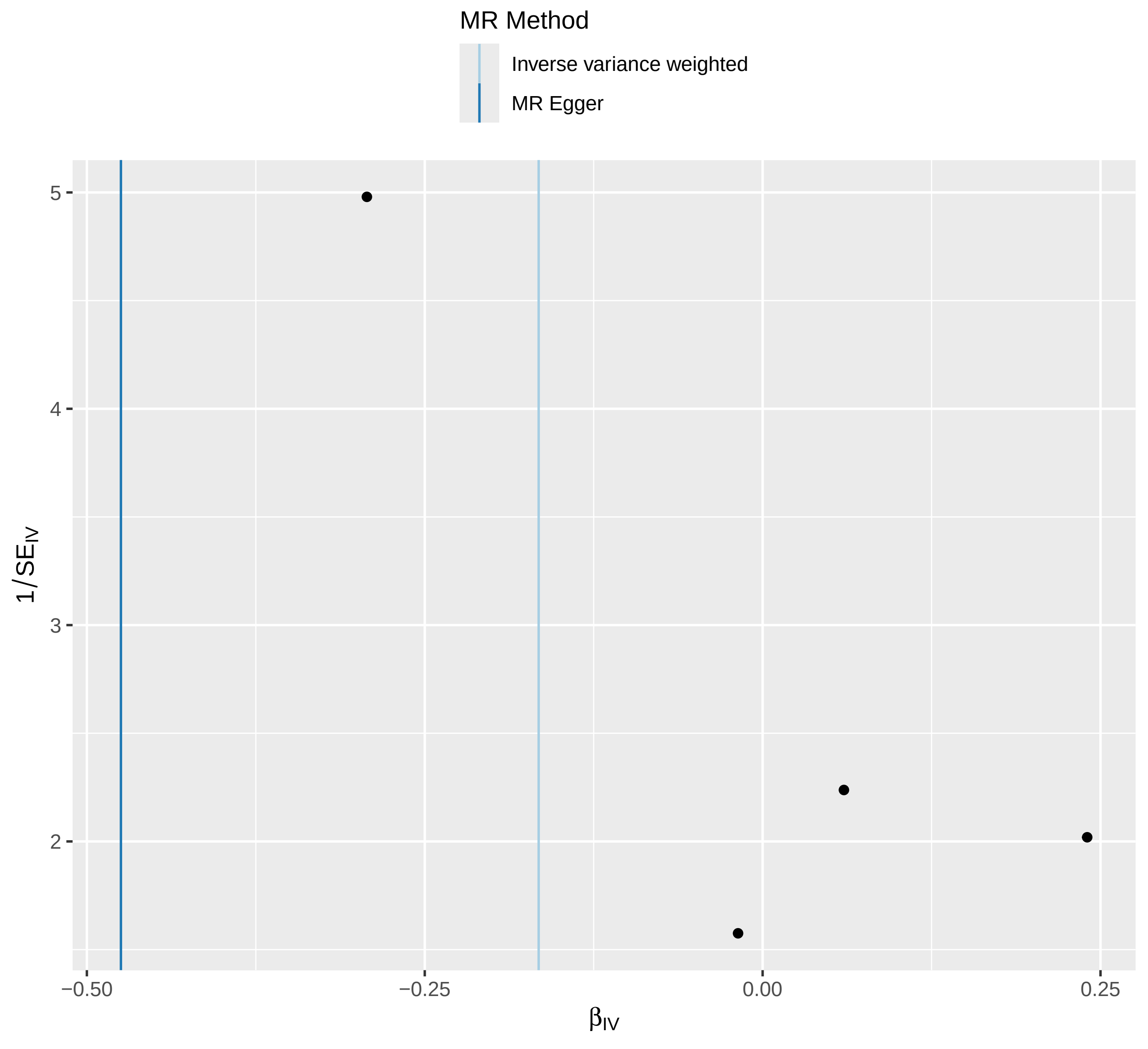
MR Method
Inverse variance weighted
MR Egger
5
4
3
2
1/SE_IV
−0.50
−0.25
0.00
0.25
β_IV

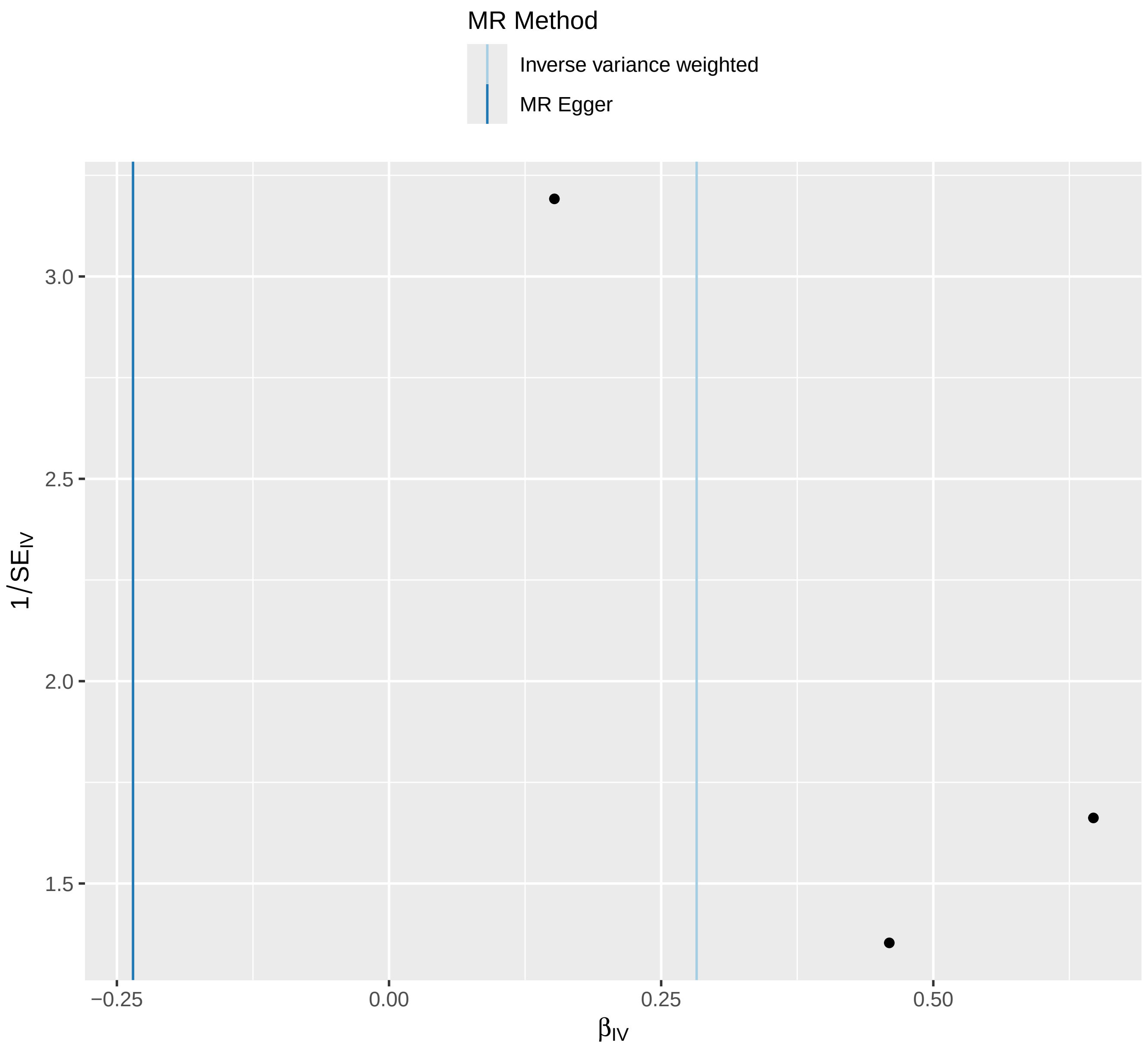
MR Method
Inverse variance weighted
MR Egger
1/SE$_{IV}$
3.0
2.5
2.0
1.5
−0.25
0.00
0.25
0.50
β$_{IV}$

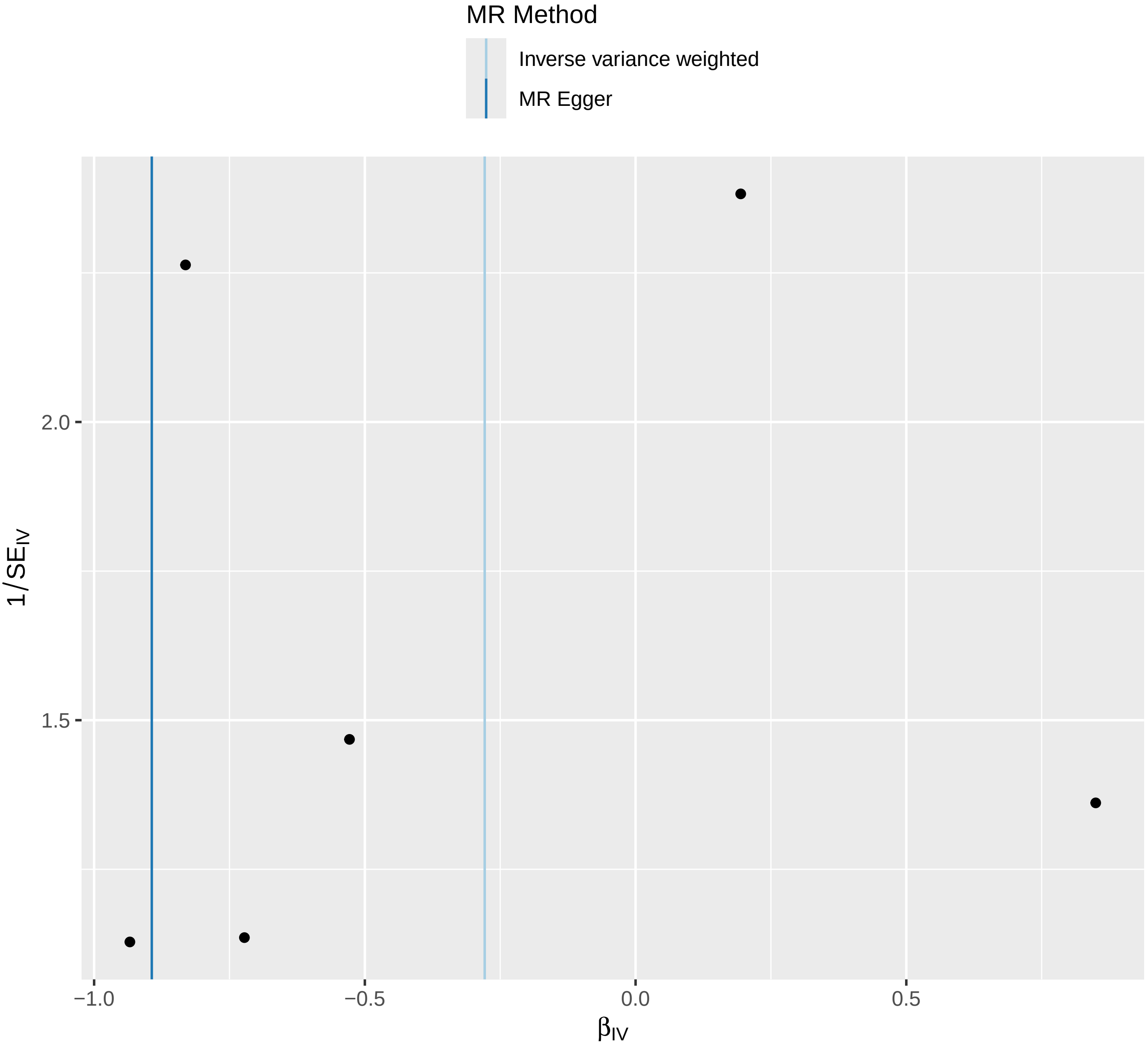
MR Method
Inverse variance weighted
MR Egger
2.0
1.5
1/SE$_{IV}$
−1.0
−0.5
0.0
0.5
β$_{IV}$

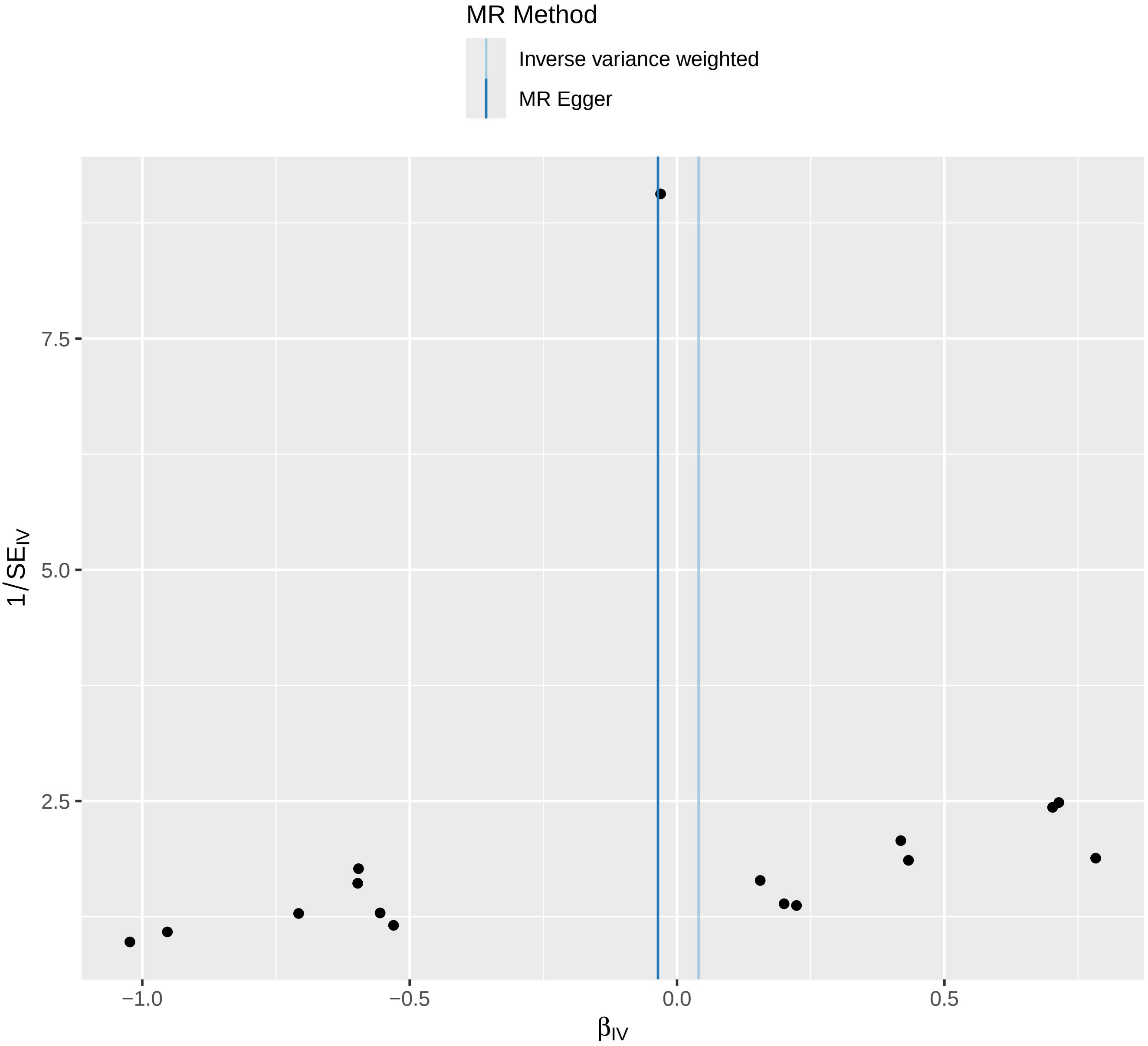

MR Method
Inverse variance weighted
MR Egger
7.5
5.0
2.5
1/SE_IV
−1.0
−0.5
0.0
0.5
β_IV

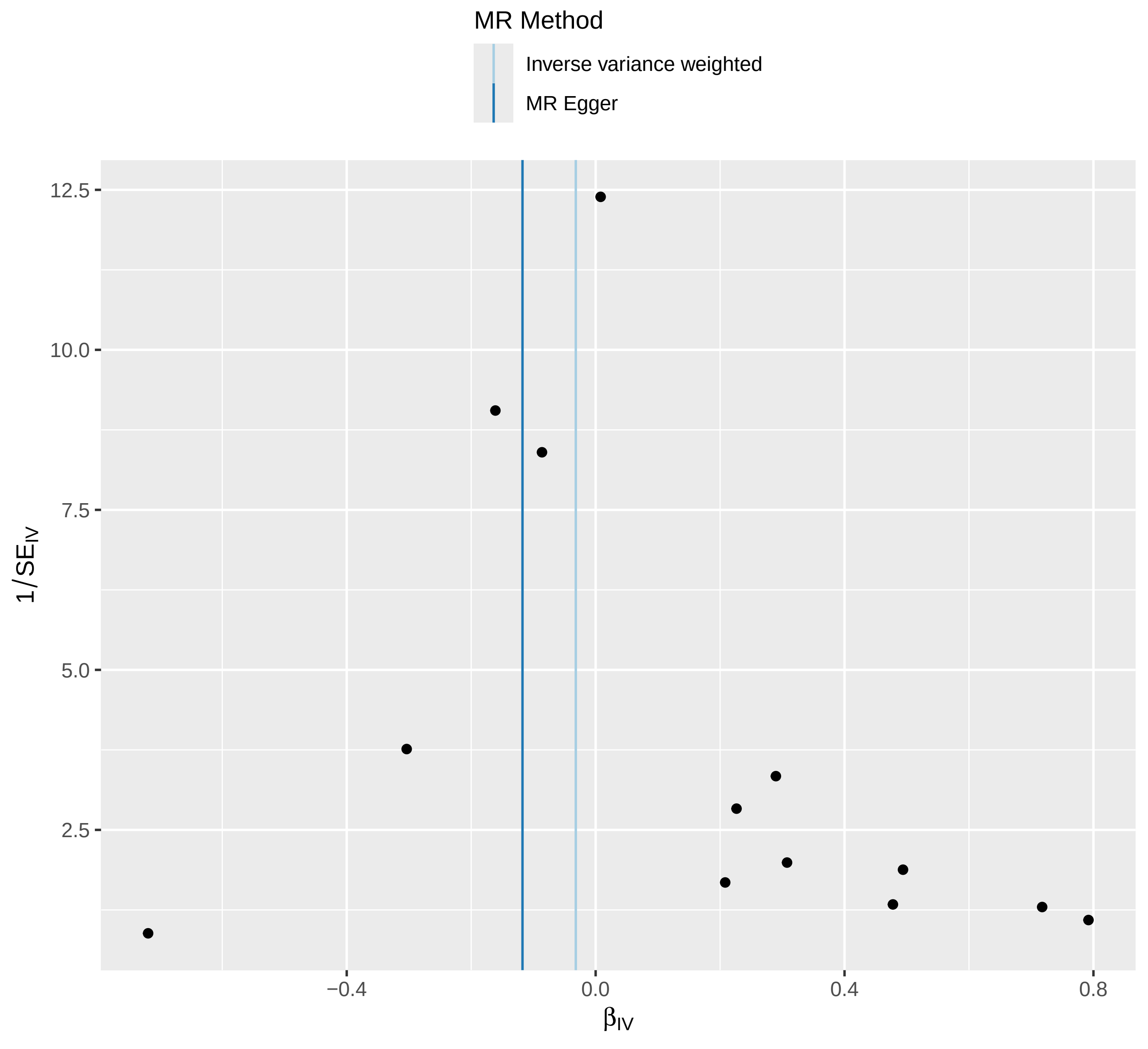
MR Method
Inverse variance weighted
MR Egger
12.5
10.0
7.5
5.0
2.5
1/SE_IV
−0.4
0.0
0.4
0.8
β_IV

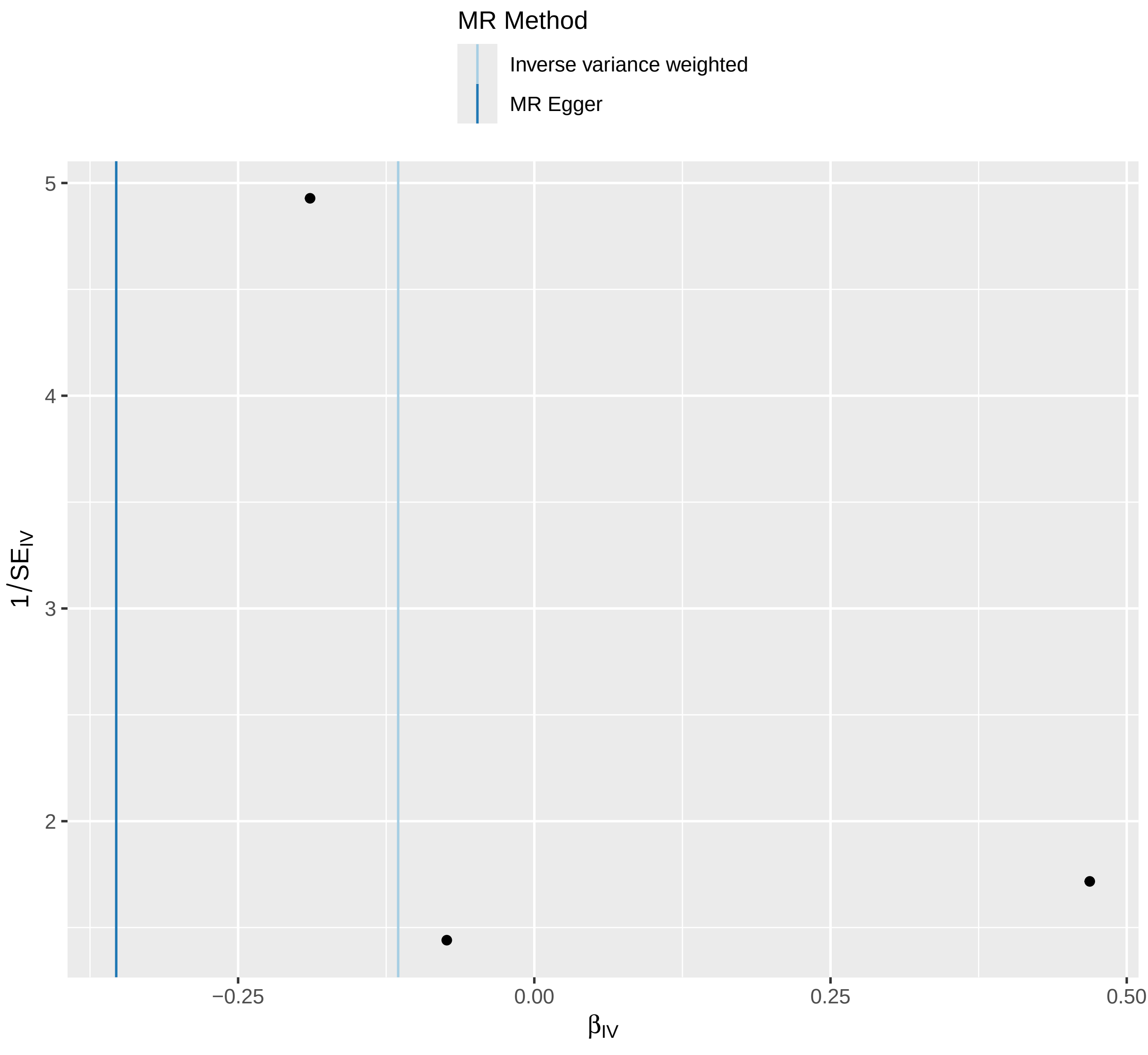
MR Method
Inverse variance weighted
MR Egger
1/SE_IV
5
4
3
2
−0.25
0.00
0.25
0.50
β_IV

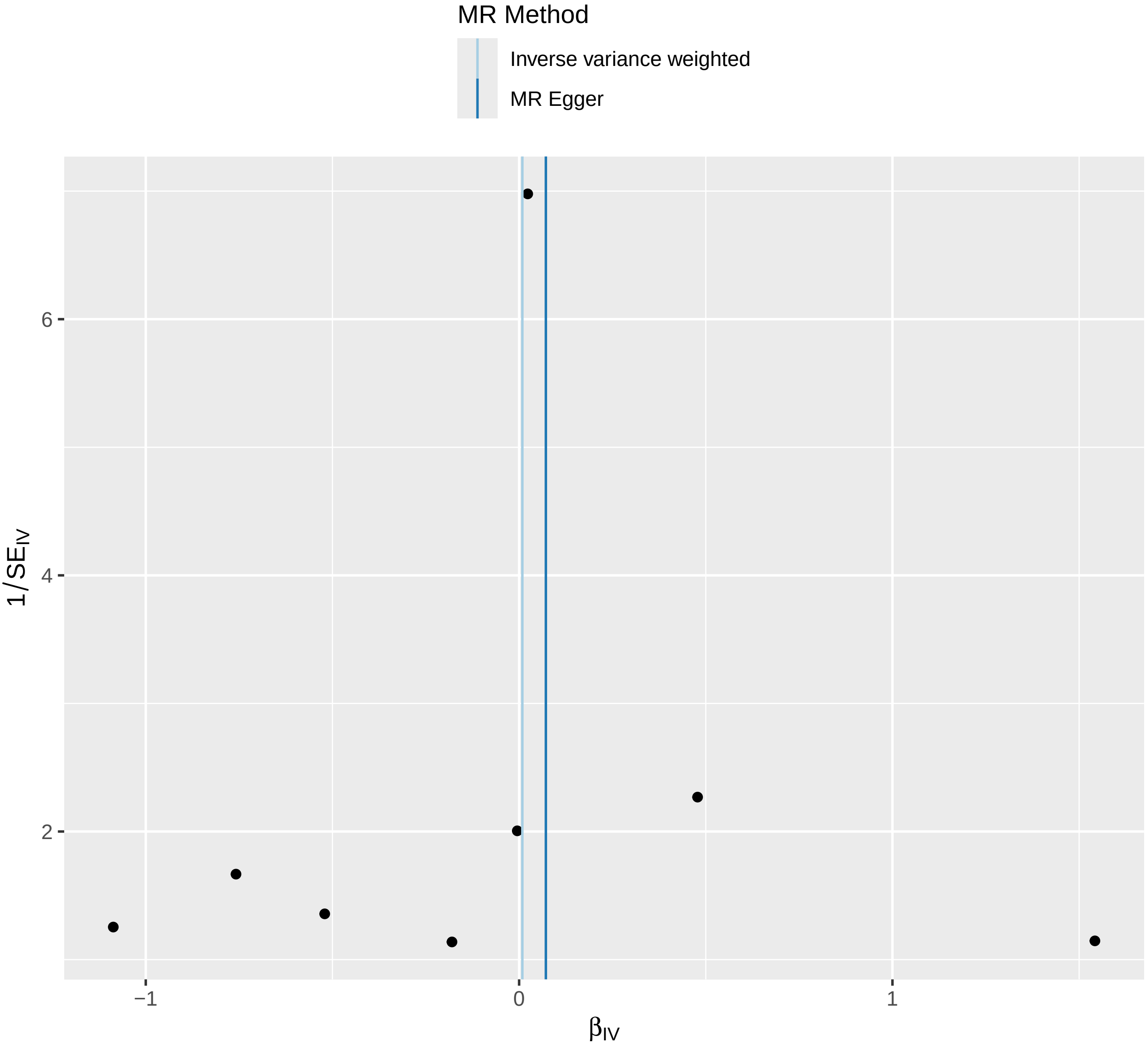

MR Method
Inverse variance weighted
MR Egger
6
4
2
1/SE_IV
−1
0
1
β_IV

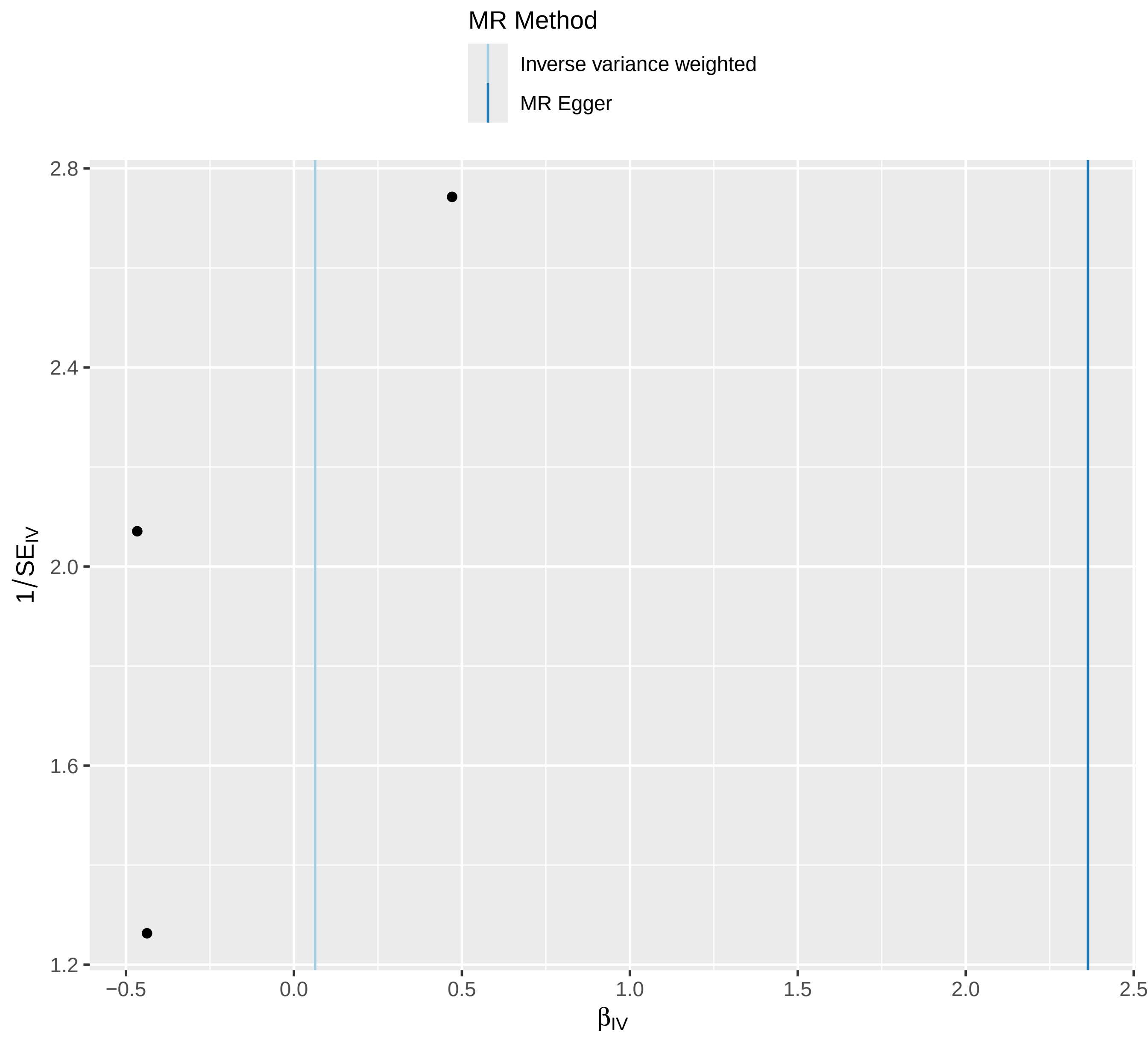
MR Method
Inverse variance weighted
MR Egger
2.8
2.4
2.0
1.6
1.2
1/SE_IV
−0.5
0.0
0.5
1.0
1.5
2.0
2.5
β_IV

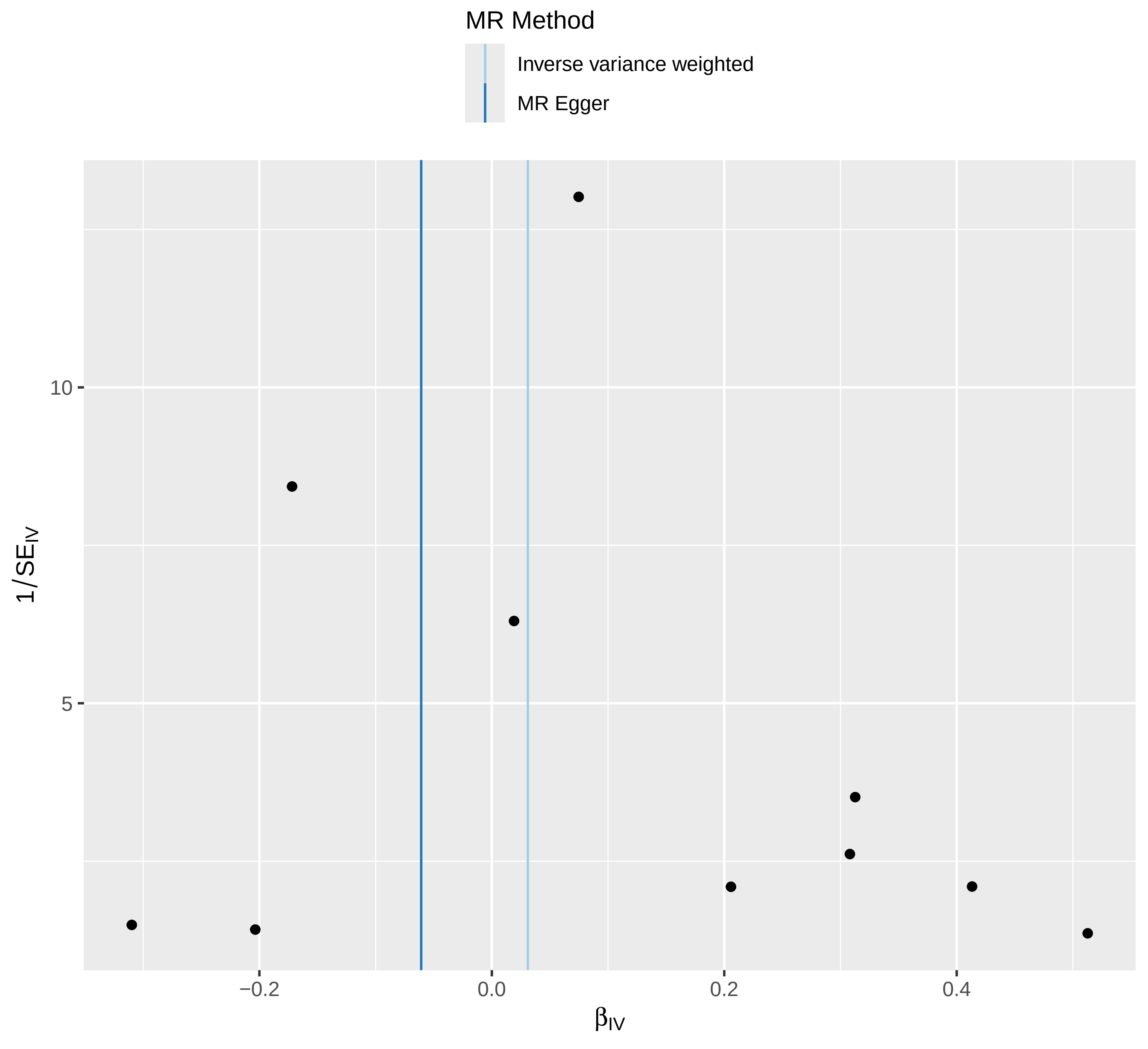
MR Method
Inverse variance weighted
MR Egger
10
5
1/SE_IV
−0.2
0.0
0.2
0.4
β_IV

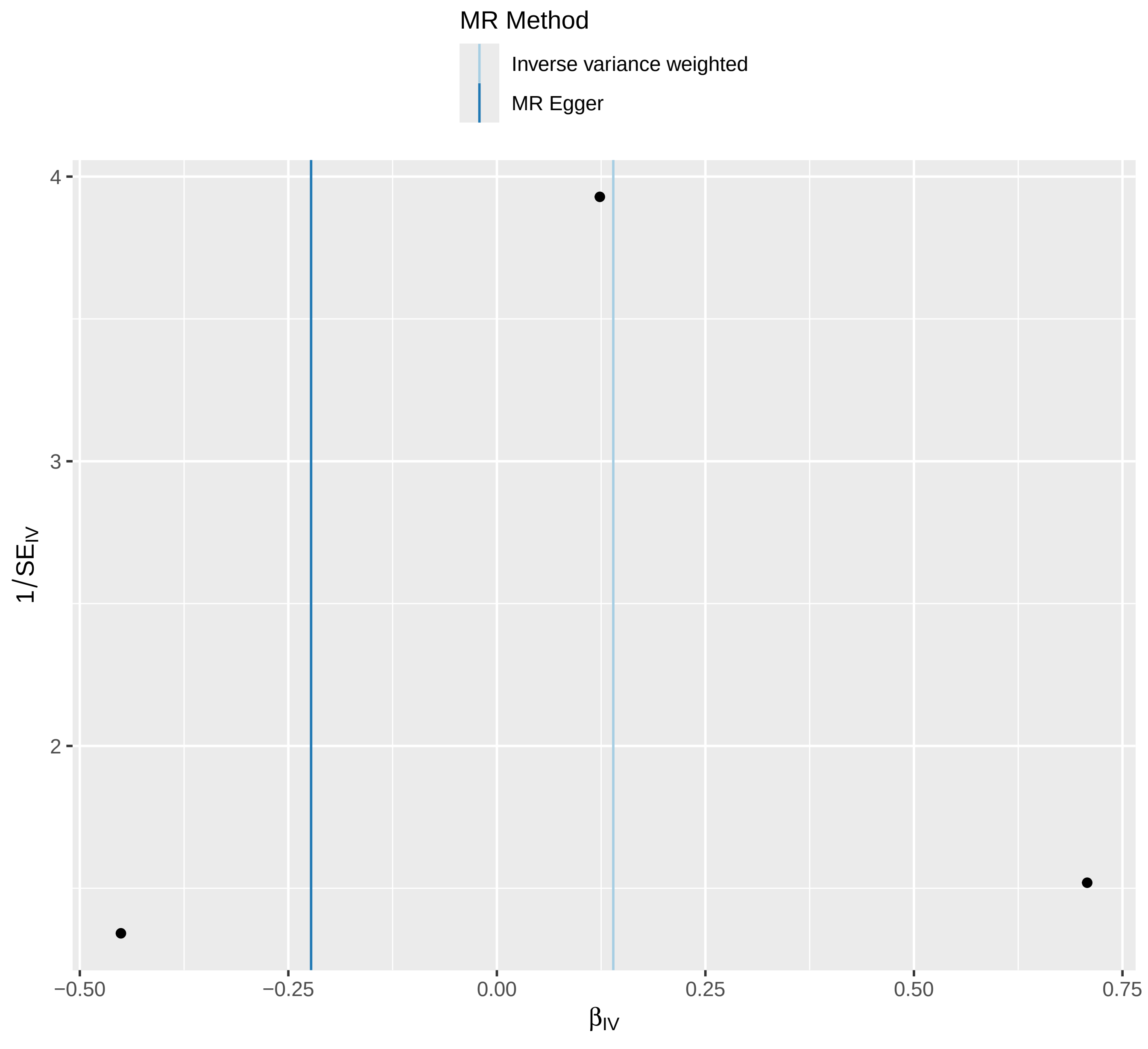

MR Method
Inverse variance weighted
MR Egger
4
3
2
1/SE_IV
−0.50
−0.25
0.00
0.25
0.50
0.75
β_IV

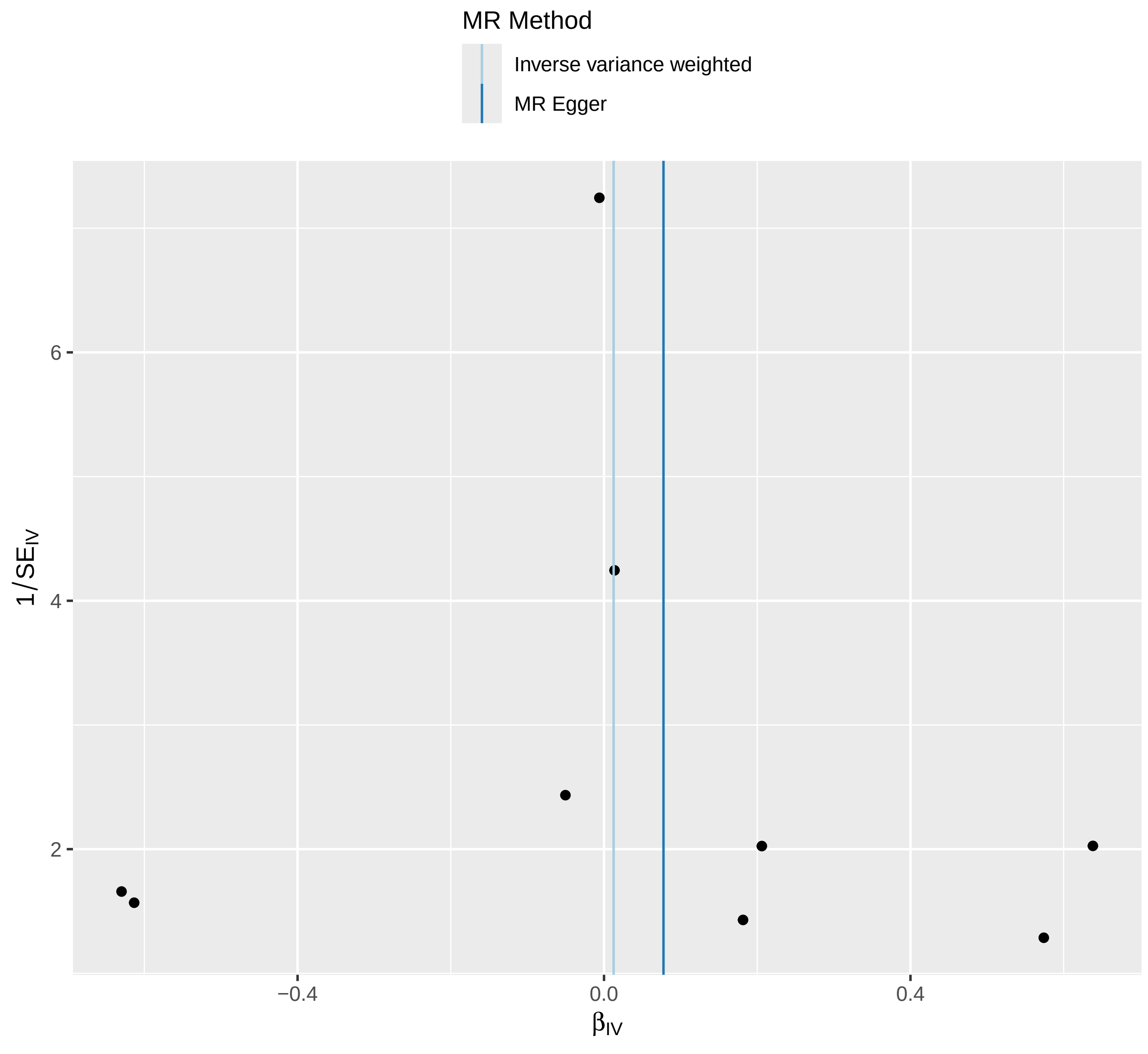

MR Method
Inverse variance weighted
MR Egger
1/SE_IV
6
4
2
−0.4
0.0
0.4
β_IV

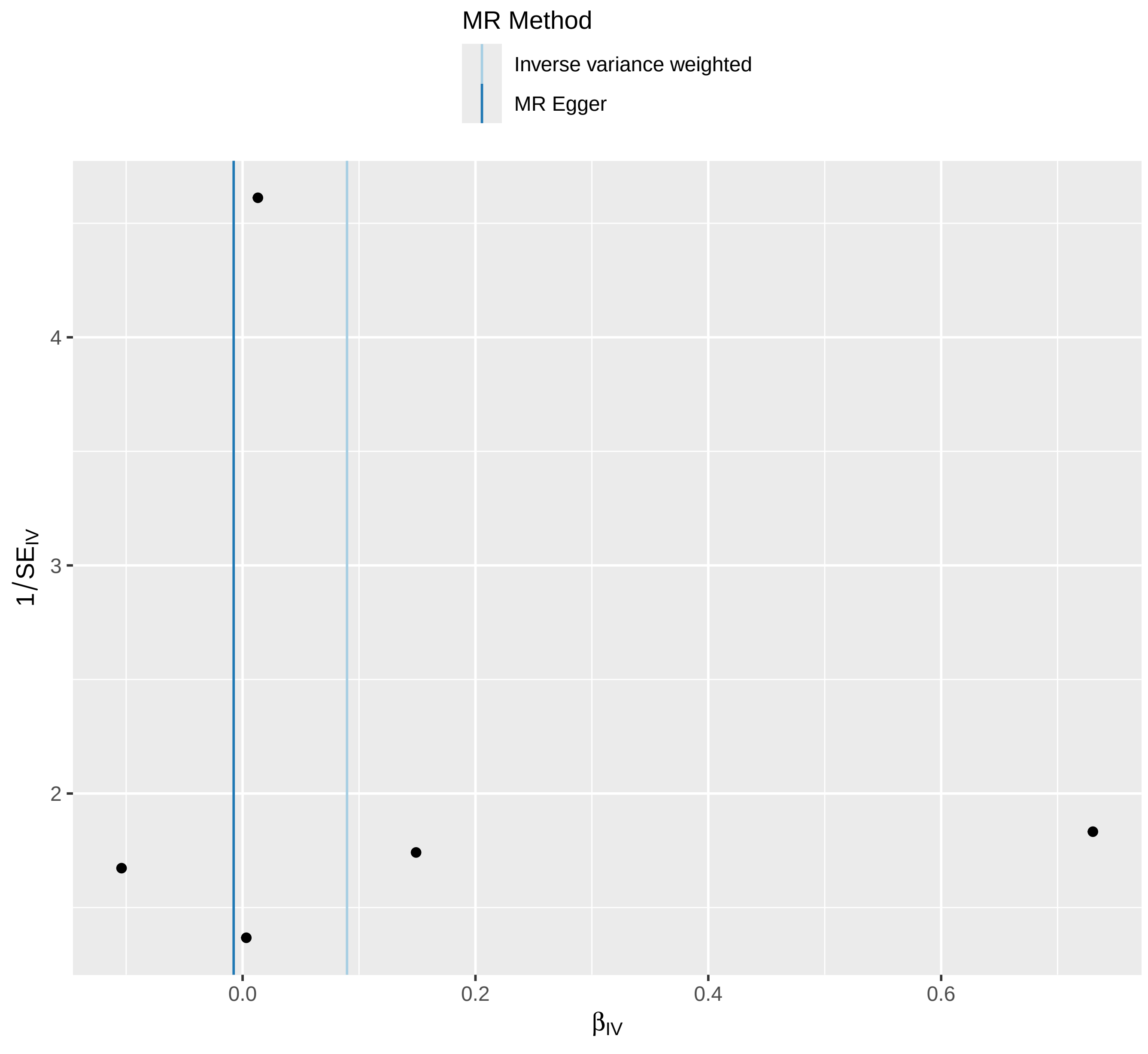
MR Method
Inverse variance weighted
MR Egger
4
3
2
$1/SE_{IV}$
0.0
0.2
0.4
0.6
β_{IV}

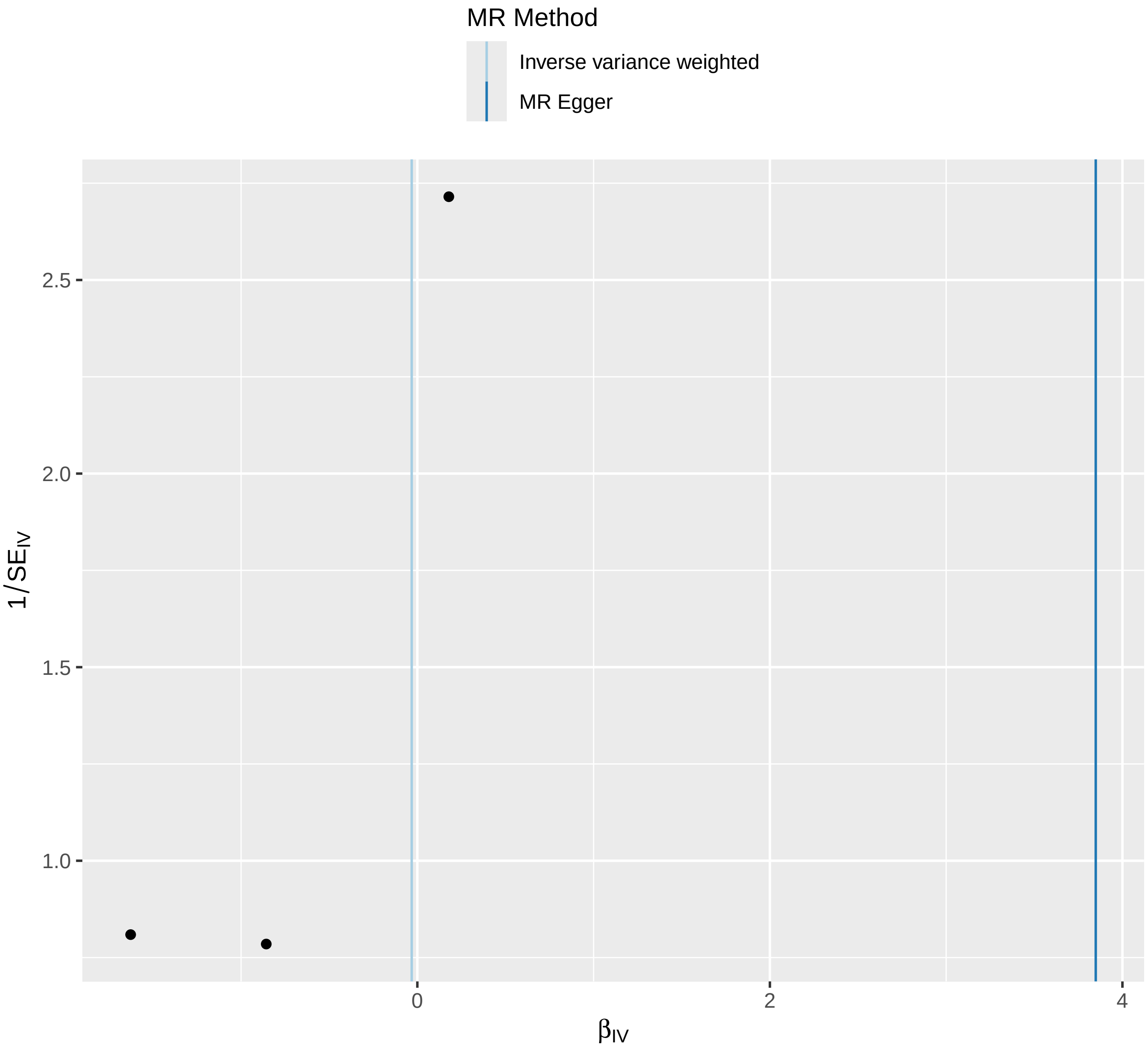
MR Method
Inverse variance weighted
MR Egger
2.5
2.0
1.5
1.0
1/SE_IV
0
2
4
β_IV

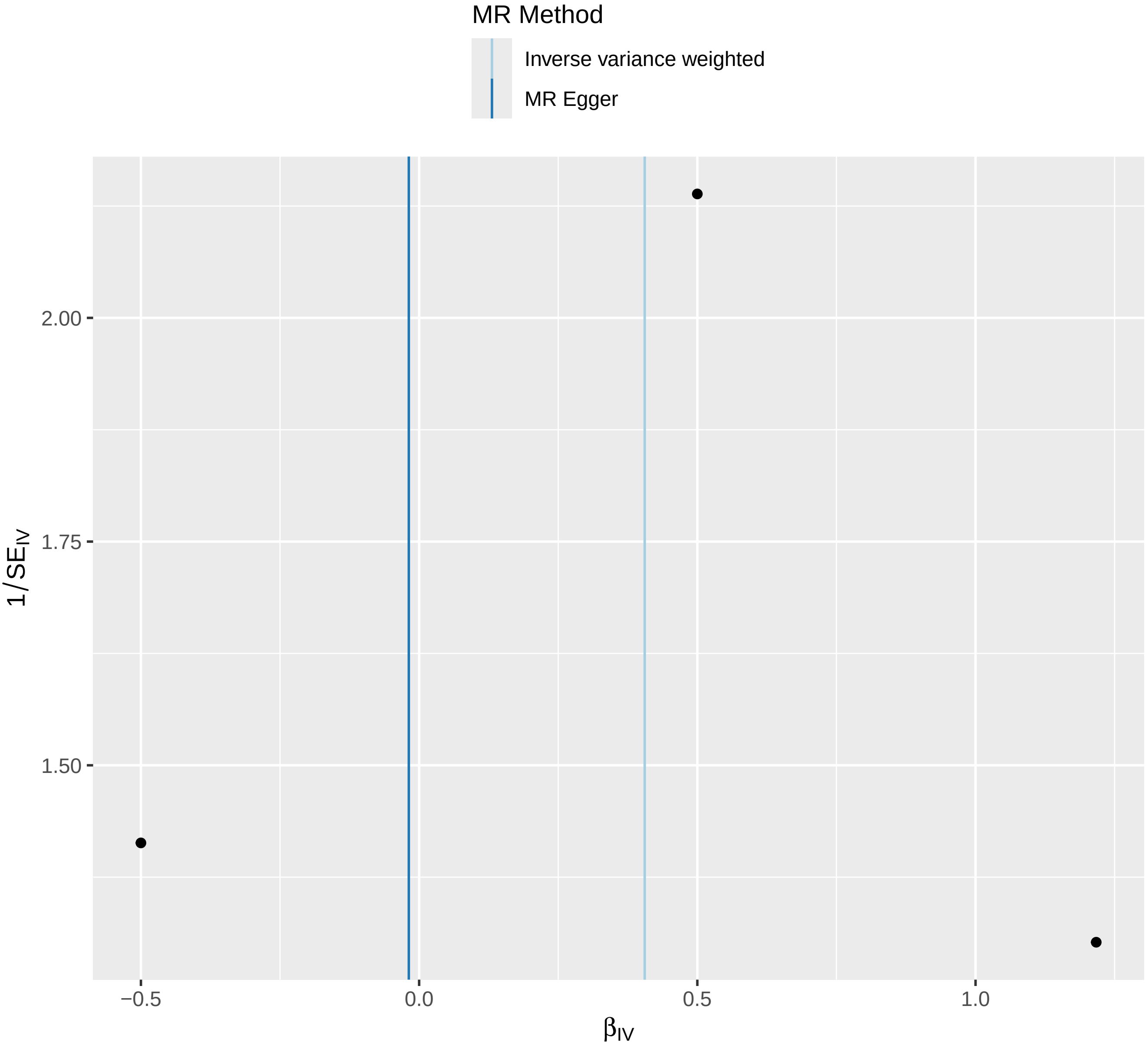

MR Method
Inverse variance weighted
MR Egger
2.00
1.75
1.50
1/SE_IV
−0.5
0.0
0.5
1.0
β_IV

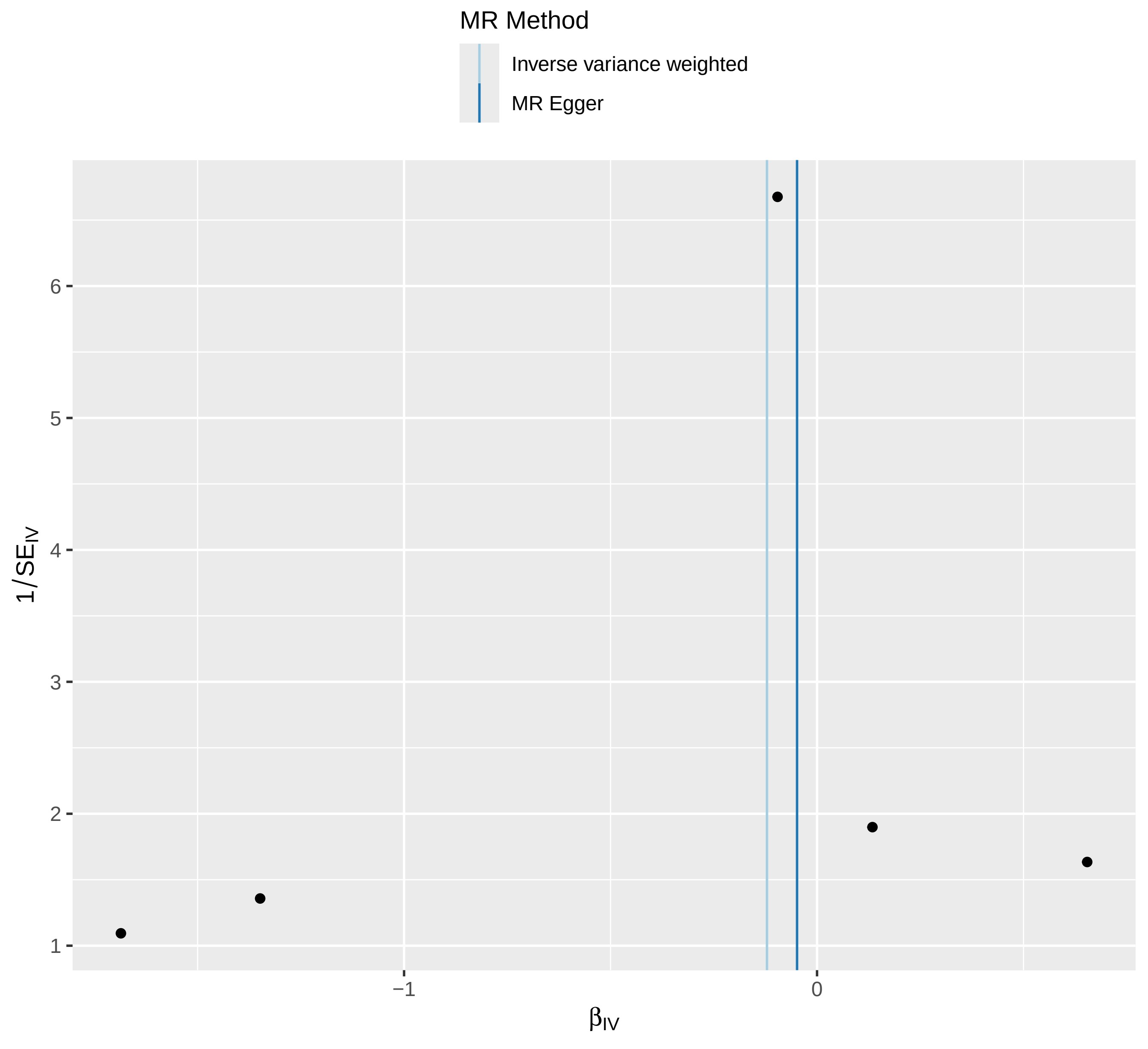
MR Method
Inverse variance weighted
MR Egger
$1/SE_{IV}$
6
5
4
3
2
1
−1
0
β_{IV}

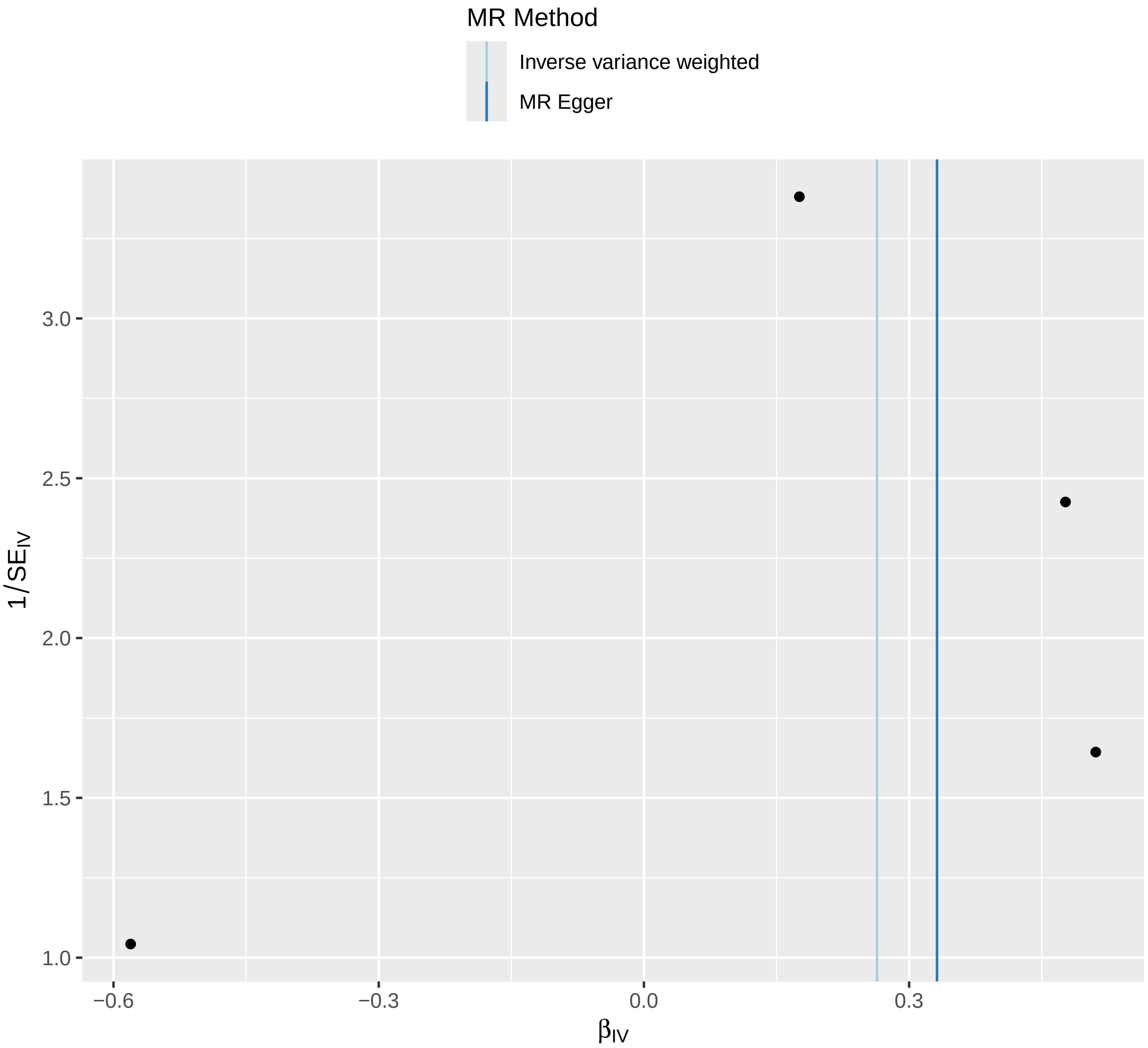
MR Method
Inverse variance weighted
MR Egger
1/SE_IV
3.0
2.5
2.0
1.5
1.0
−0.6
−0.3
0.0
0.3
β_IV

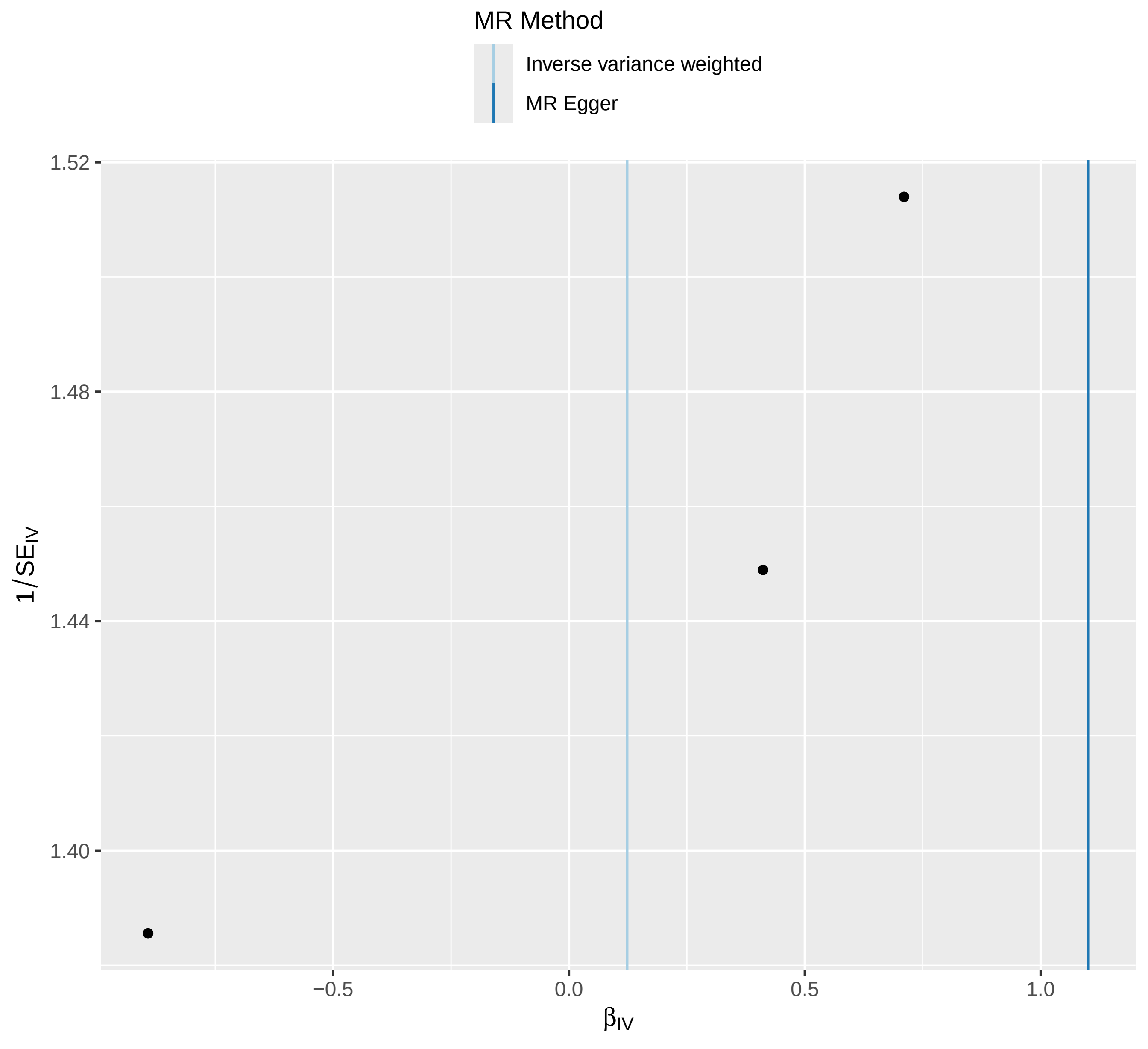
MR Method
Inverse variance weighted
MR Egger
1.52
1.48
1.44
1.40
$1/SE_{IV}$
−0.5
0.0
0.5
1.0
β_{IV}

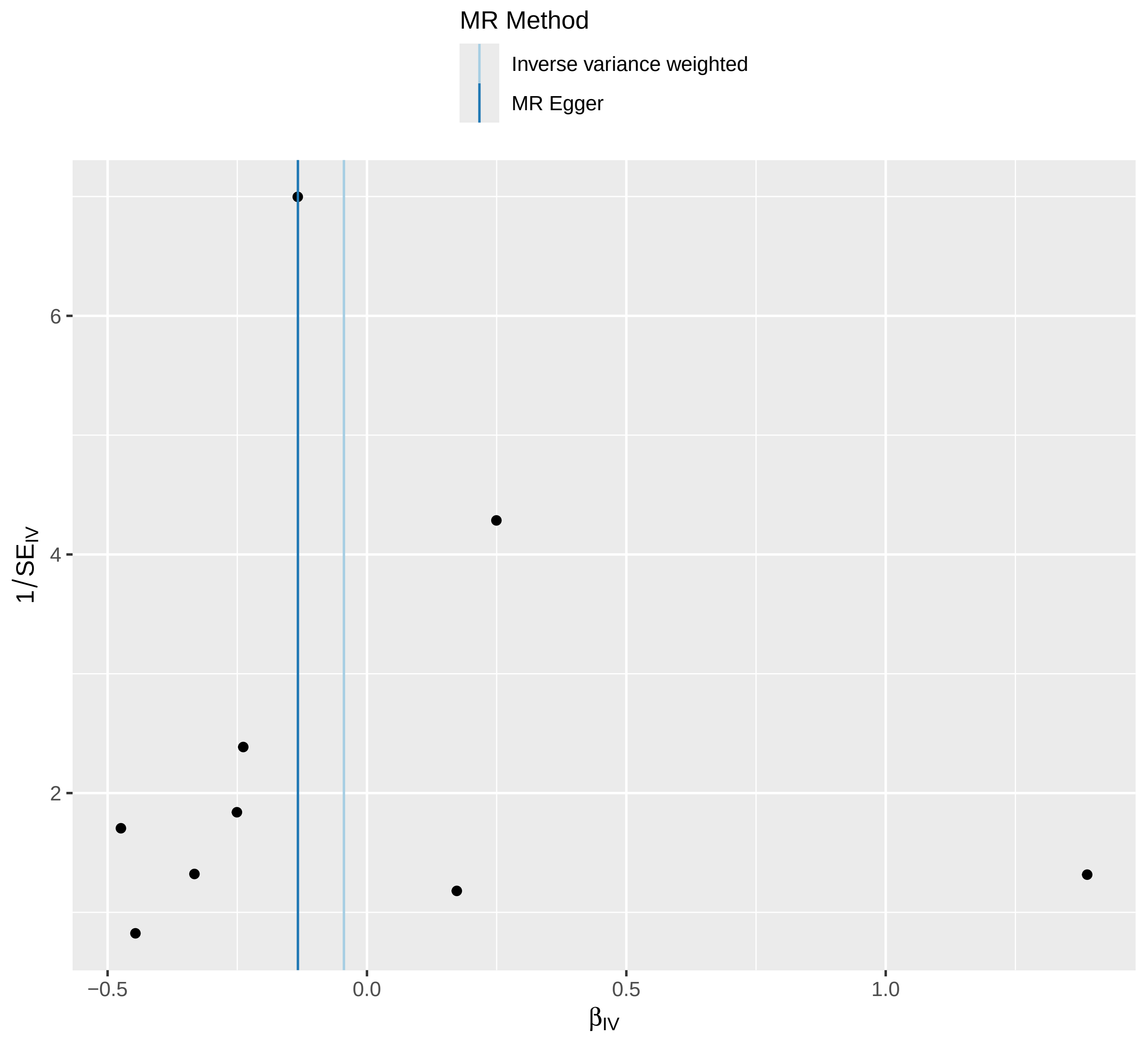

MR Method
Inverse variance weighted
MR Egger
6
4
2
1/SE_IV
−0.5
0.0
0.5
1.0
β_IV

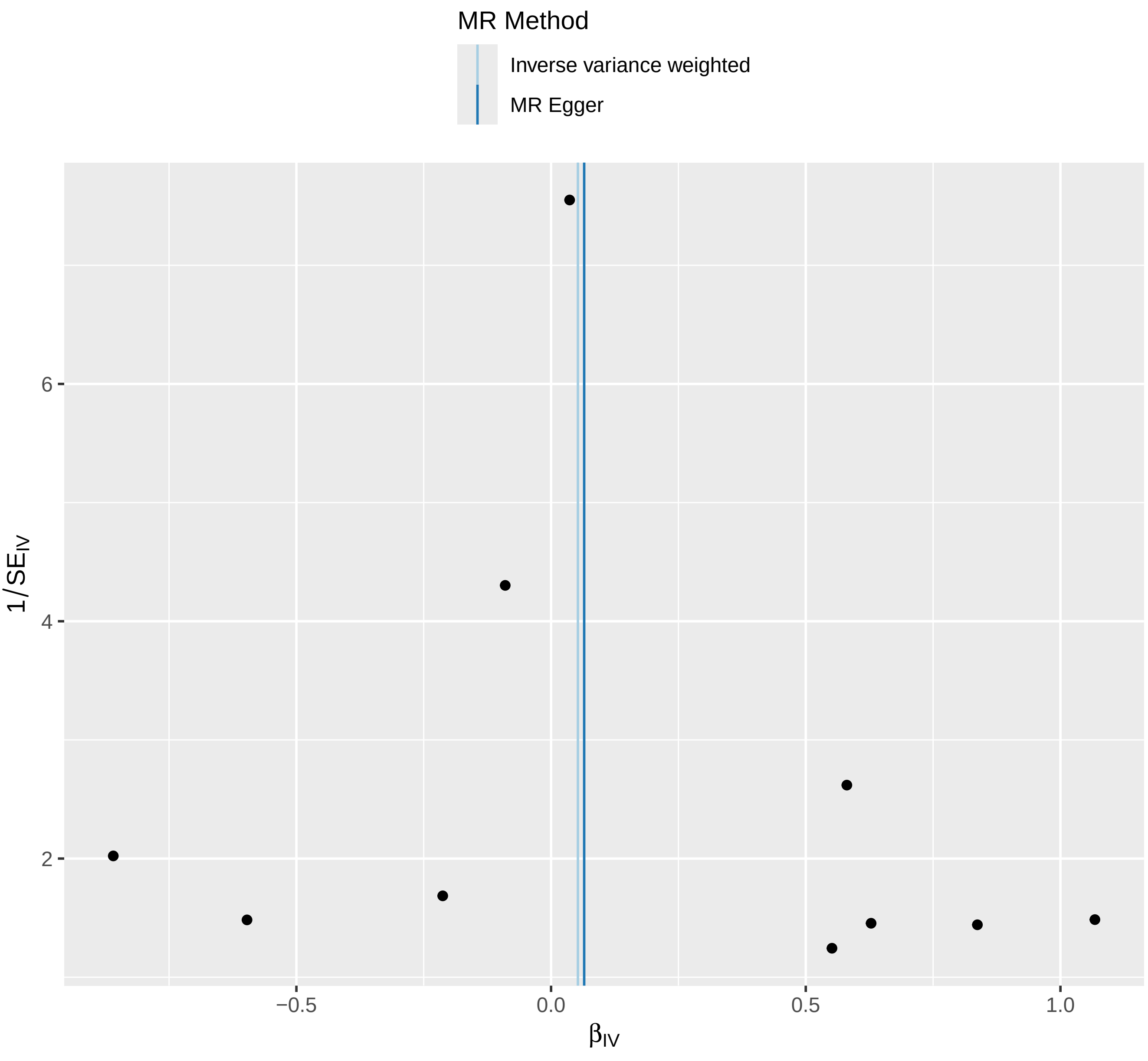

MR Method
Inverse variance weighted
MR Egger
6
4
2
1/SE_IV
−0.5
0.0
0.5
1.0
β_IV

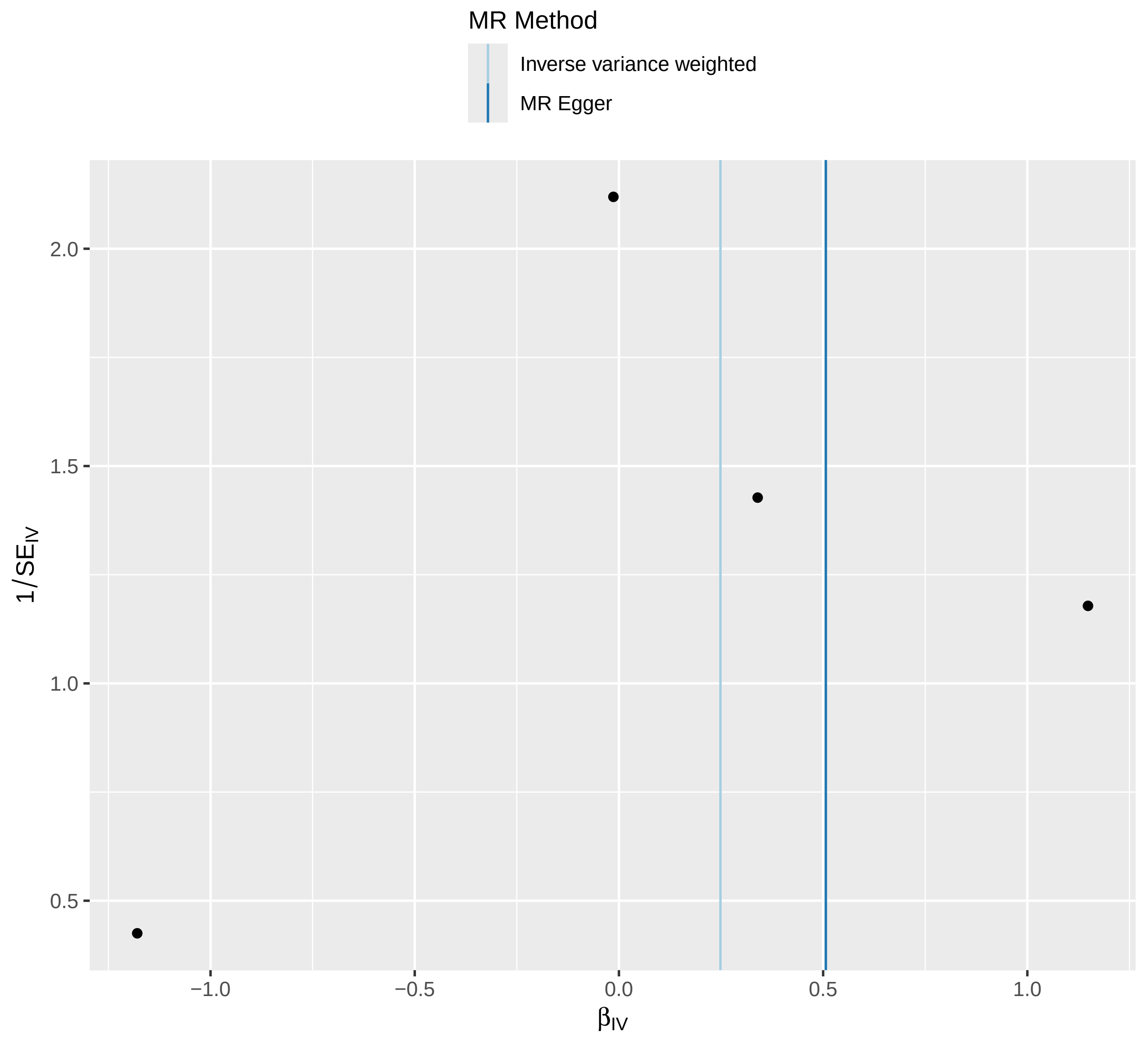
MR Method
Inverse variance weighted
MR Egger
2.0
1.5
1.0
0.5
1/SE_IV
−1.0
−0.5
0.0
0.5
1.0
β_IV

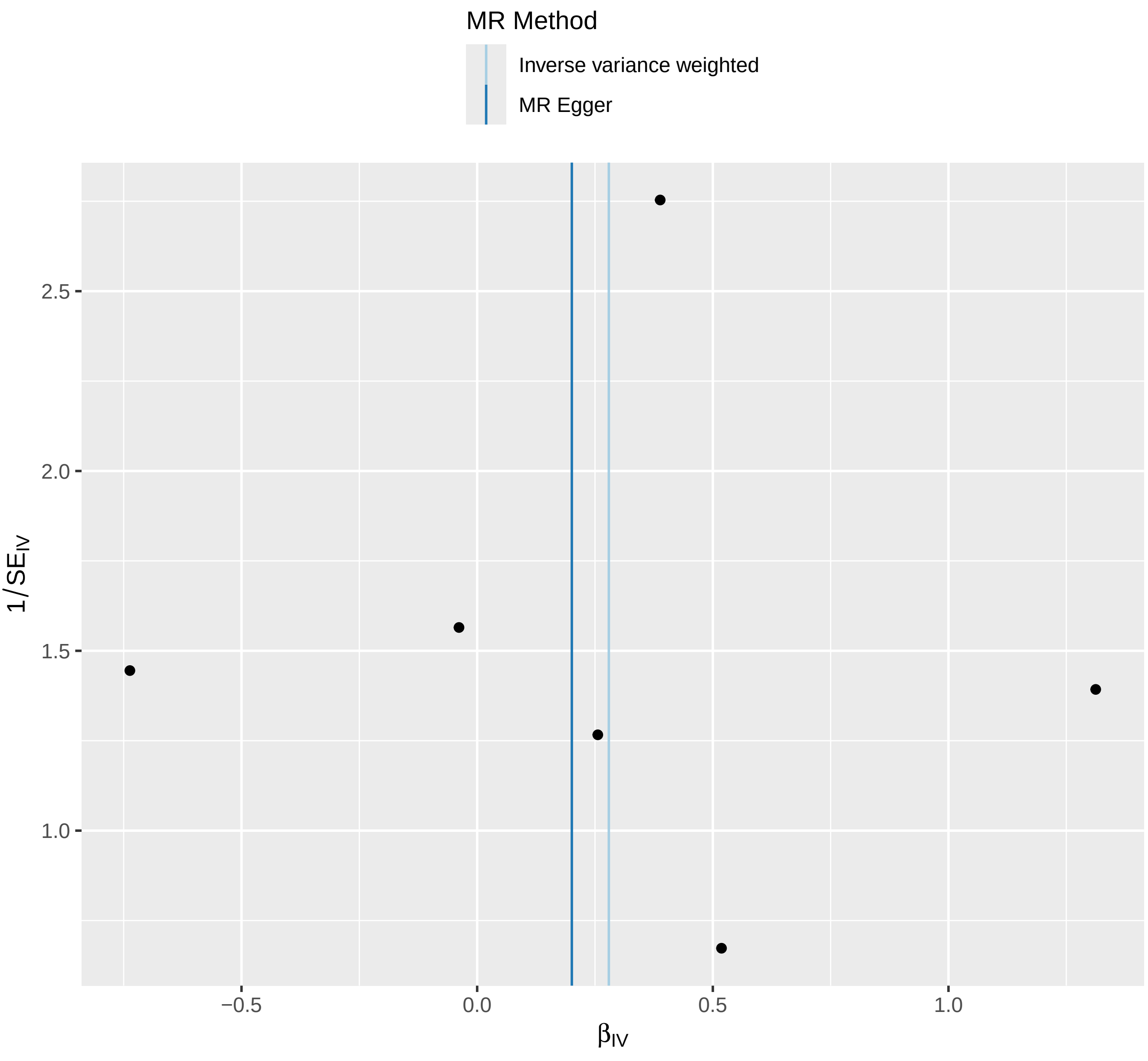

MR Method
Inverse variance weighted
MR Egger
2.5
2.0
1.5
1.0
1/SE_IV
−0.5
0.0
0.5
1.0
β_IV

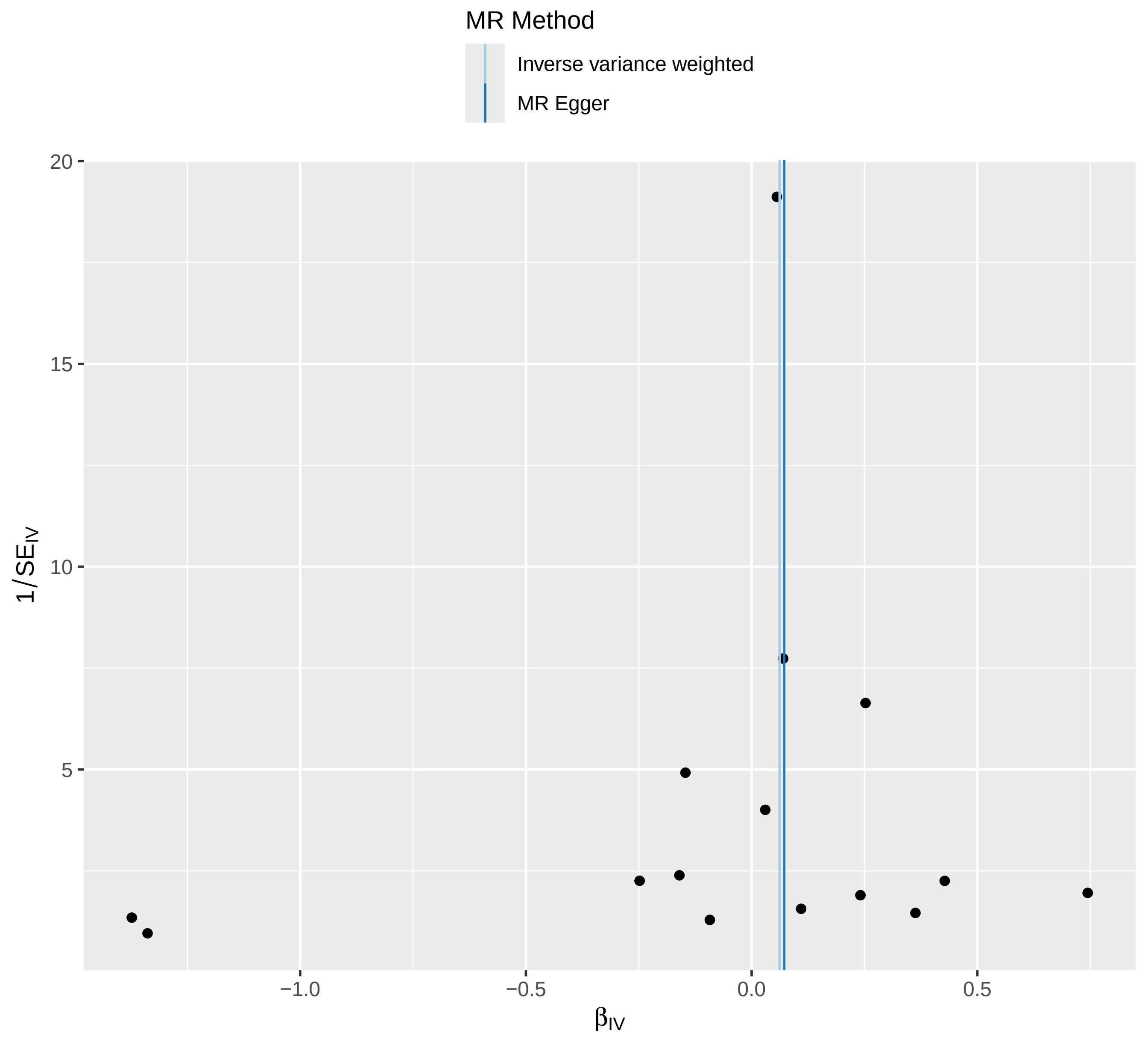
MR Method
Inverse variance weighted
MR Egger
20
15
10
5
1/SE_IV
−1.0
−0.5
0.0
0.5
β_IV

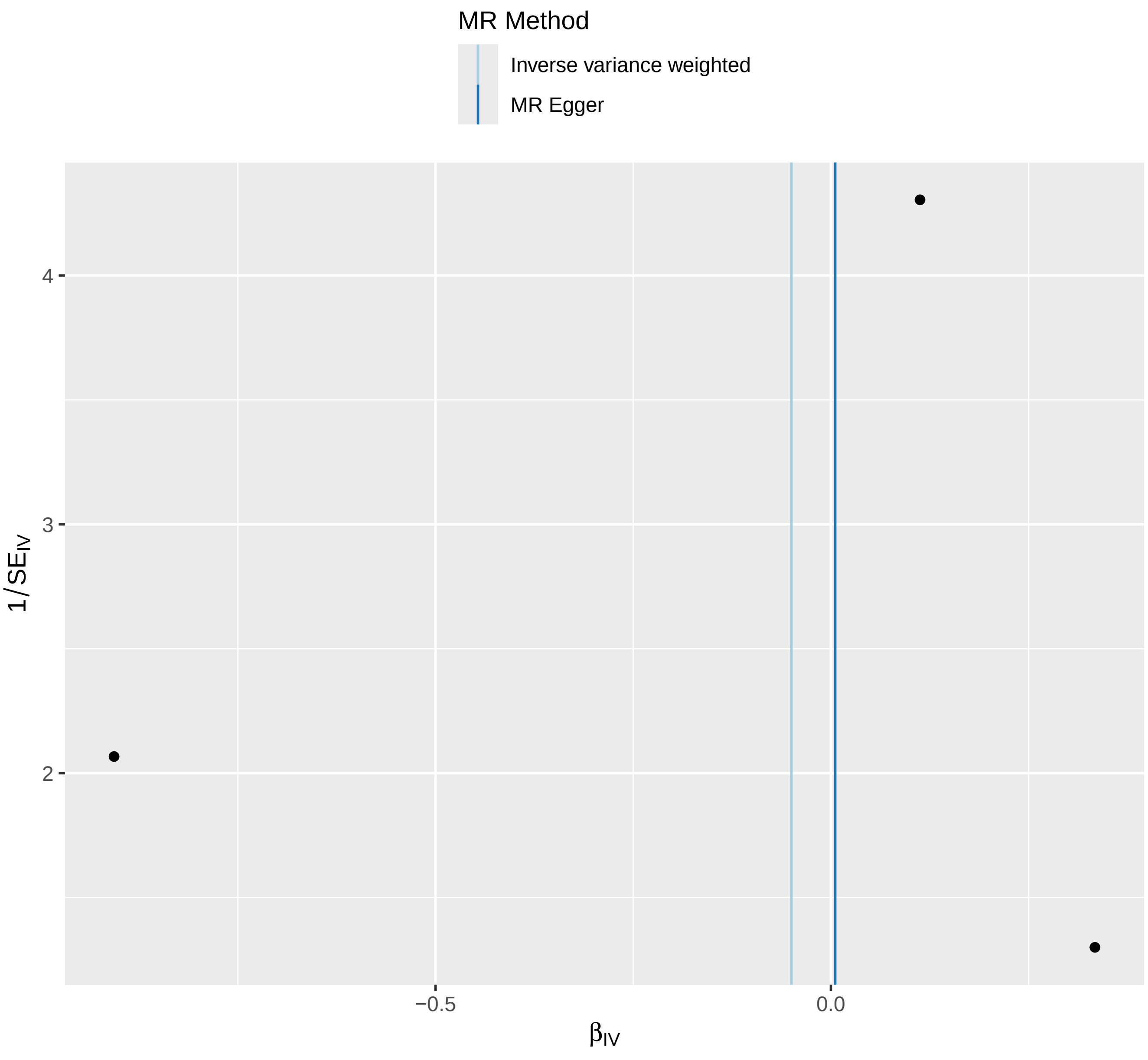

MR Method
Inverse variance weighted
MR Egger
4
3
2
1/SE_IV
−0.5
0.0
β_IV

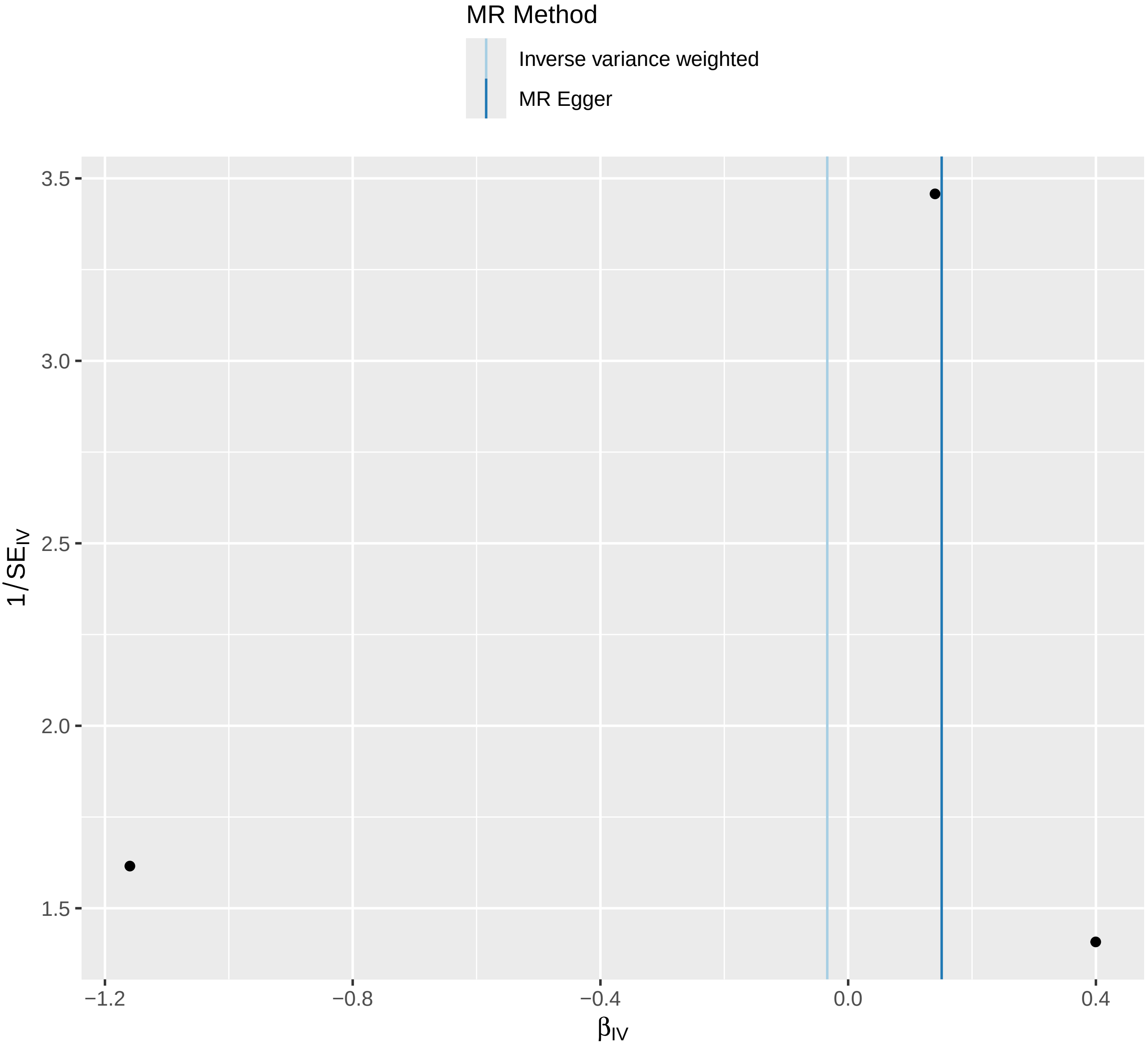

MR Method
Inverse variance weighted
MR Egger
3.5
3.0
2.5
2.0
1.5
1/SE$_{IV}$
−1.2
−0.8
−0.4
0.0
0.4
β$_{IV}$

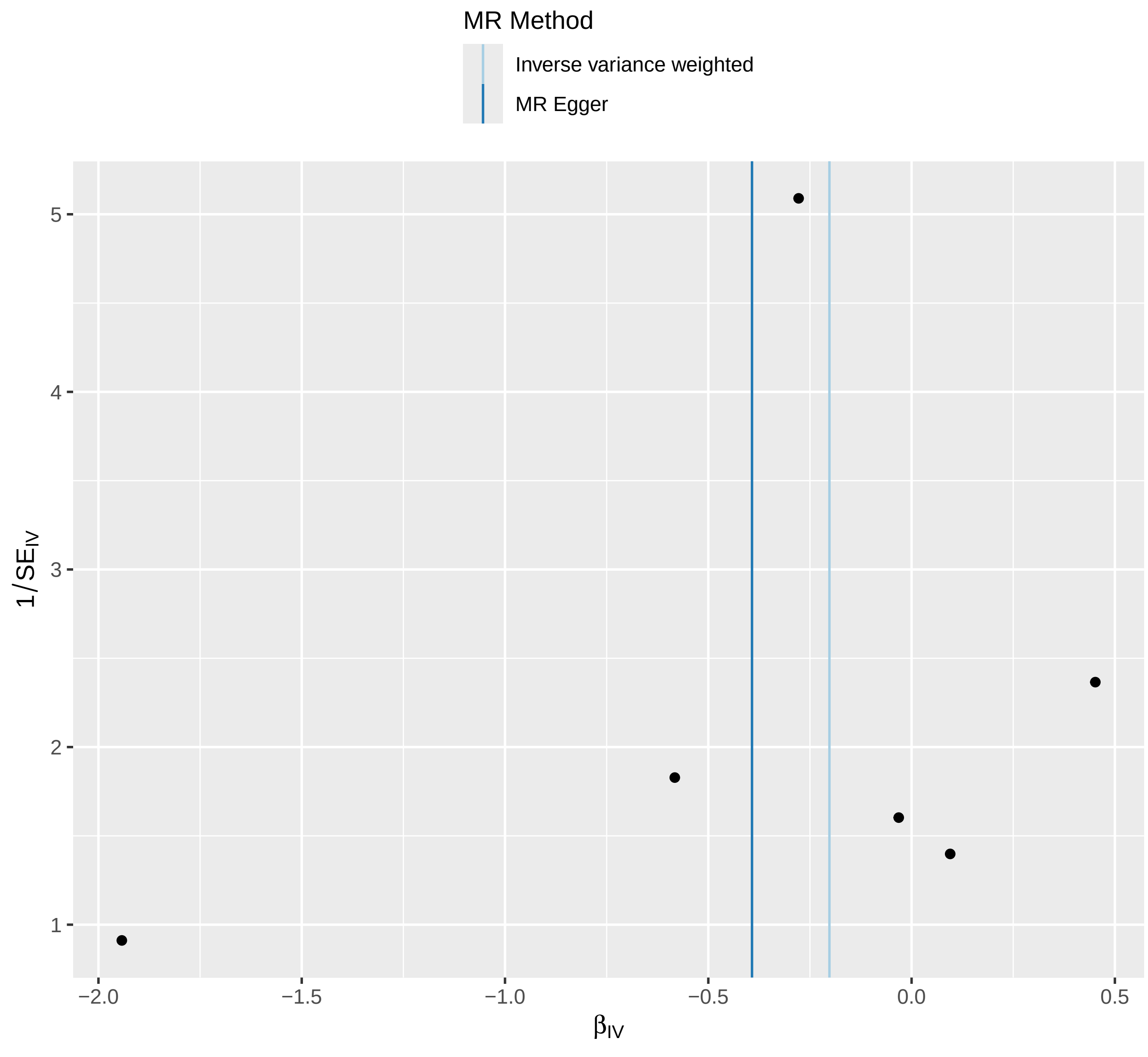

MR Method
Inverse variance weighted
MR Egger
5
4
3
2
1
1/SE_IV
−2.0
−1.5
−1.0
−0.5
0.0
0.5
β_IV

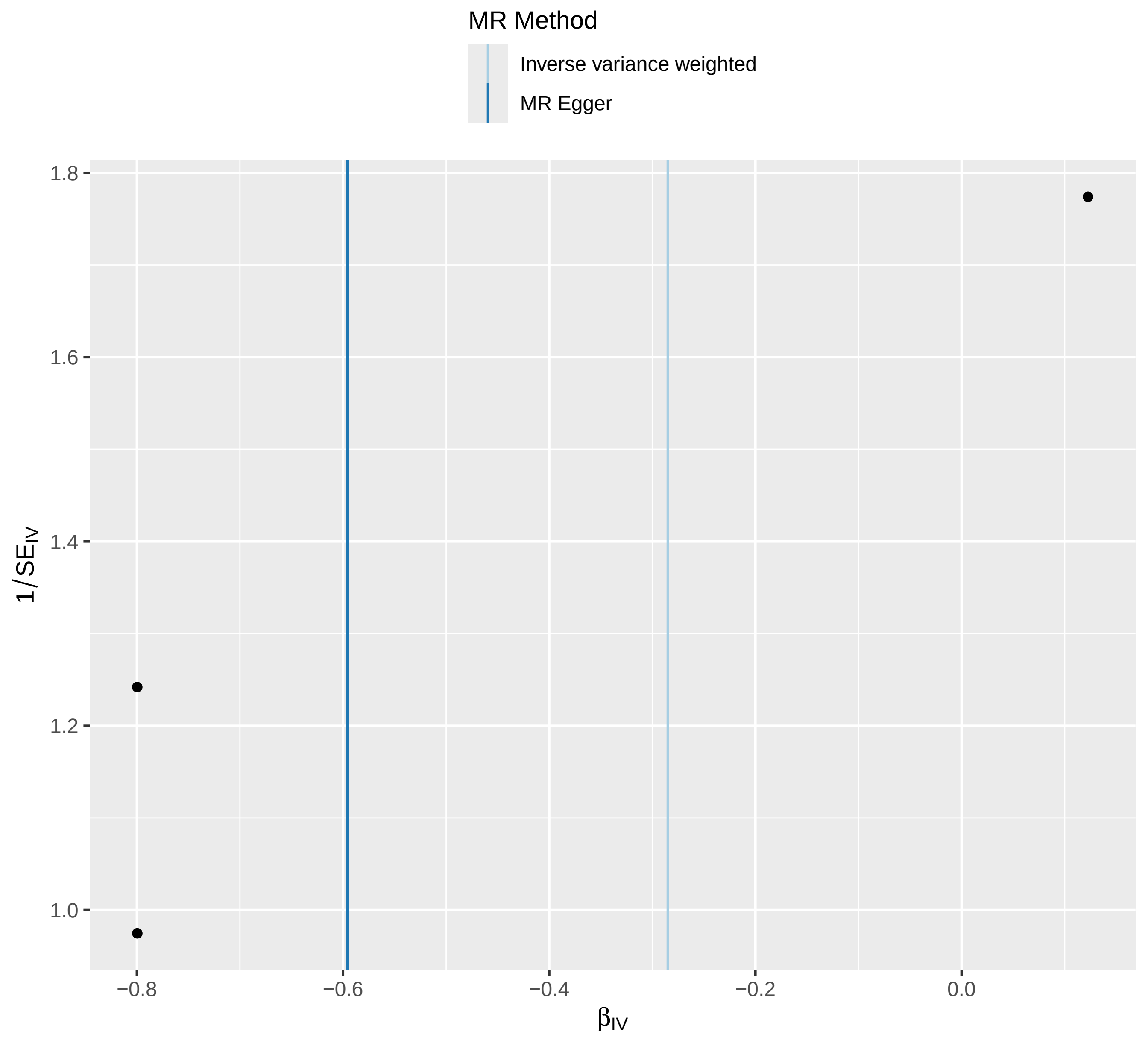

MR Method
Inverse variance weighted
MR Egger
1.8
1.6
1.4
1.2
1.0
$1/SE_{IV}$
−0.8
−0.6
−0.4
−0.2
0.0
β_{IV}

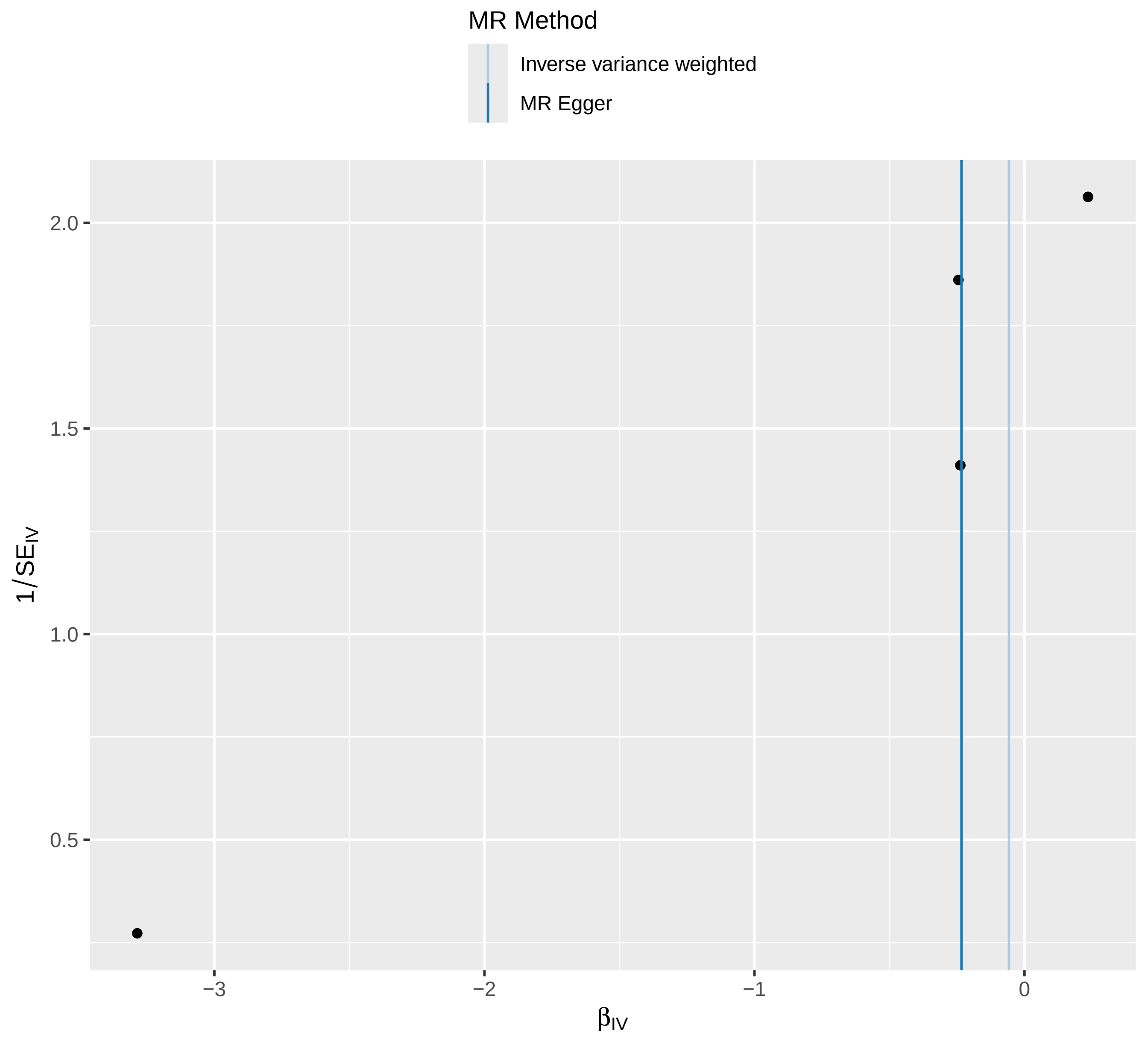

MR Method
Inverse variance weighted
MR Egger
2.0
1.5
1.0
0.5
1/SE$_{IV}$
−3
−2
−1
0
β$_{IV}$

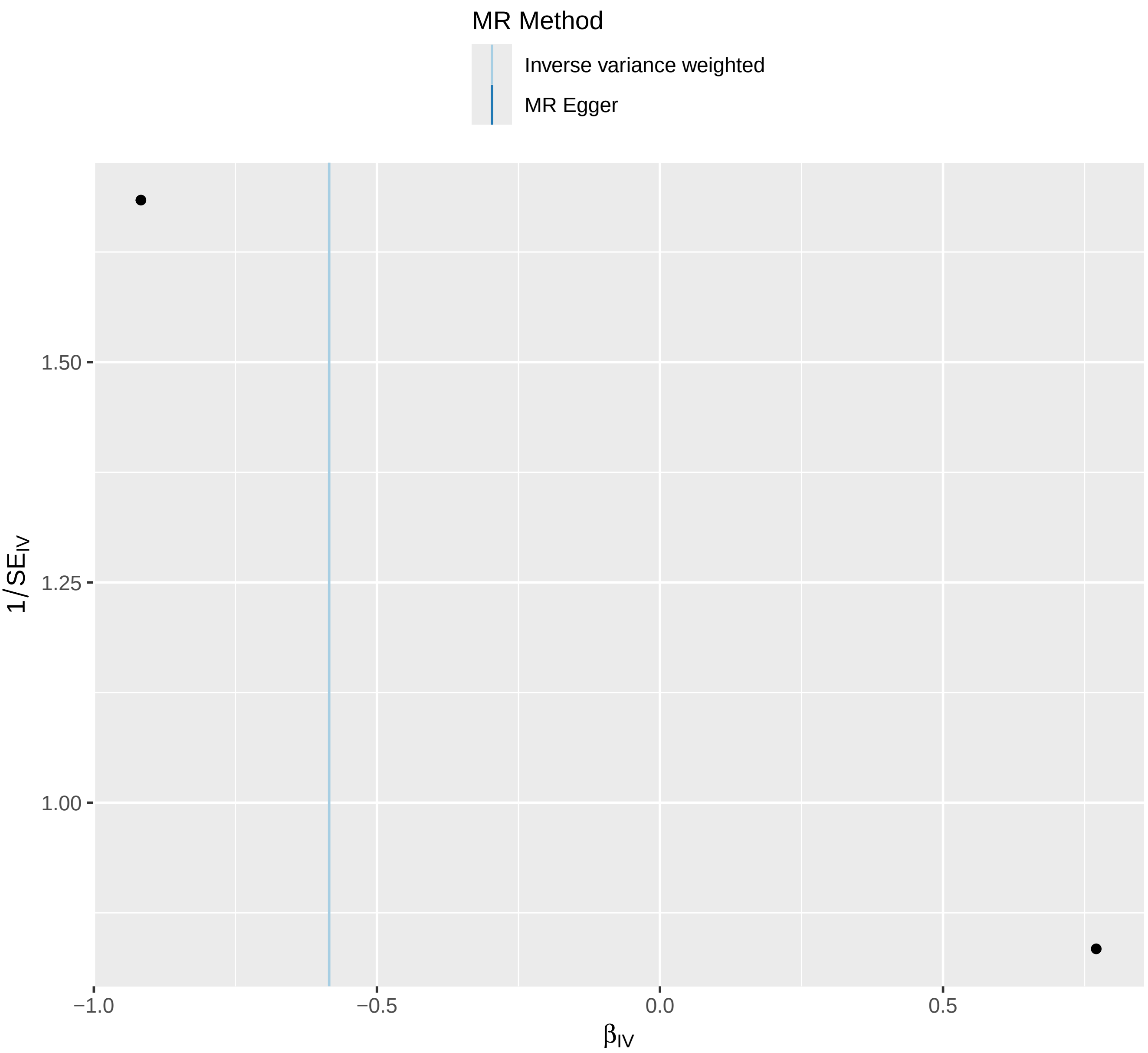
MR Method
Inverse variance weighted
MR Egger
1.50
1.25
1.00
−1.0
−0.5
0.0
0.5
$1/SE_{IV}$
β_{IV}

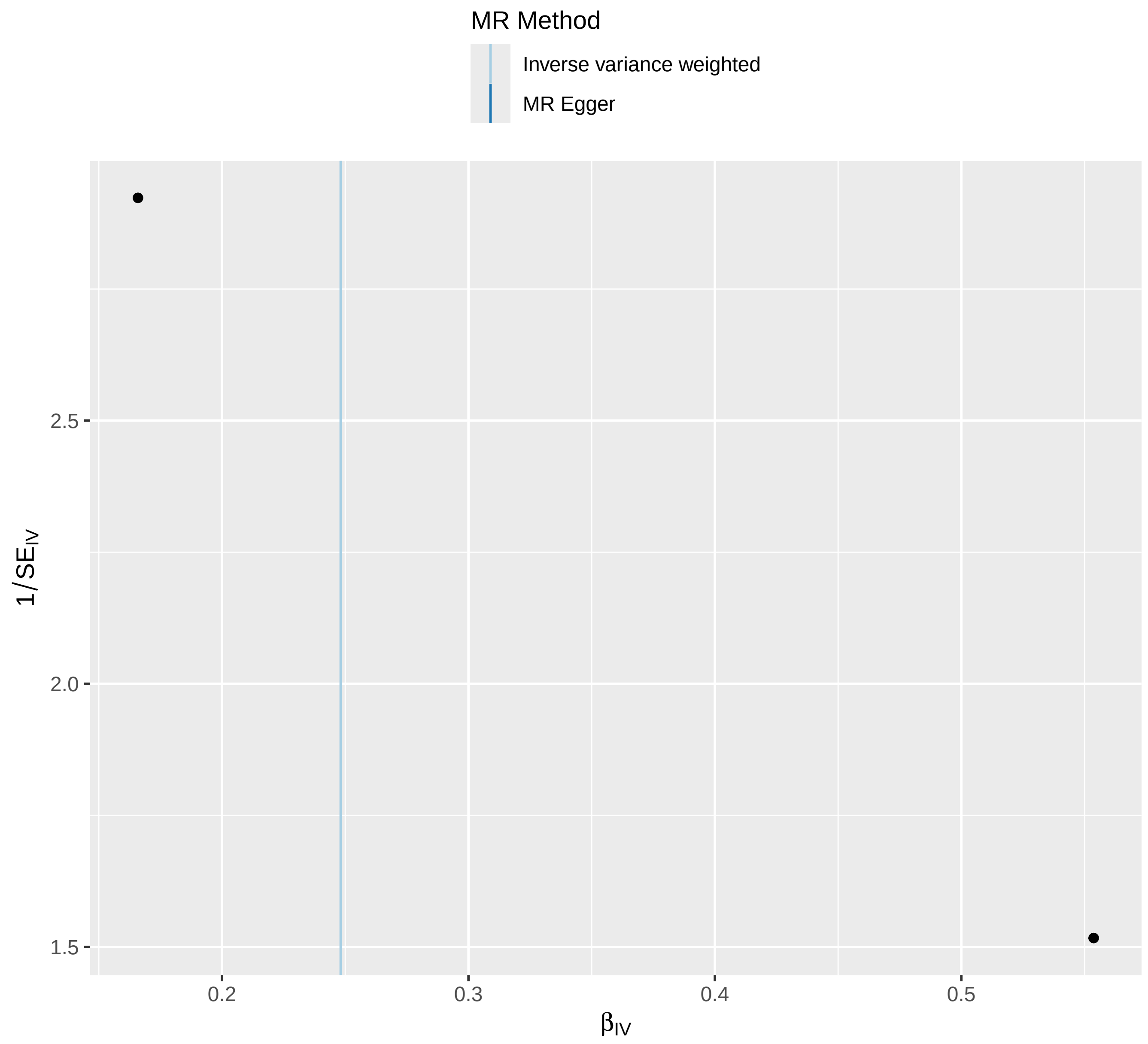

MR Method
Inverse variance weighted
MR Egger
2.5
2.0
1.5
0.2
0.3
0.4
0.5
1/SE_IV
β_IV

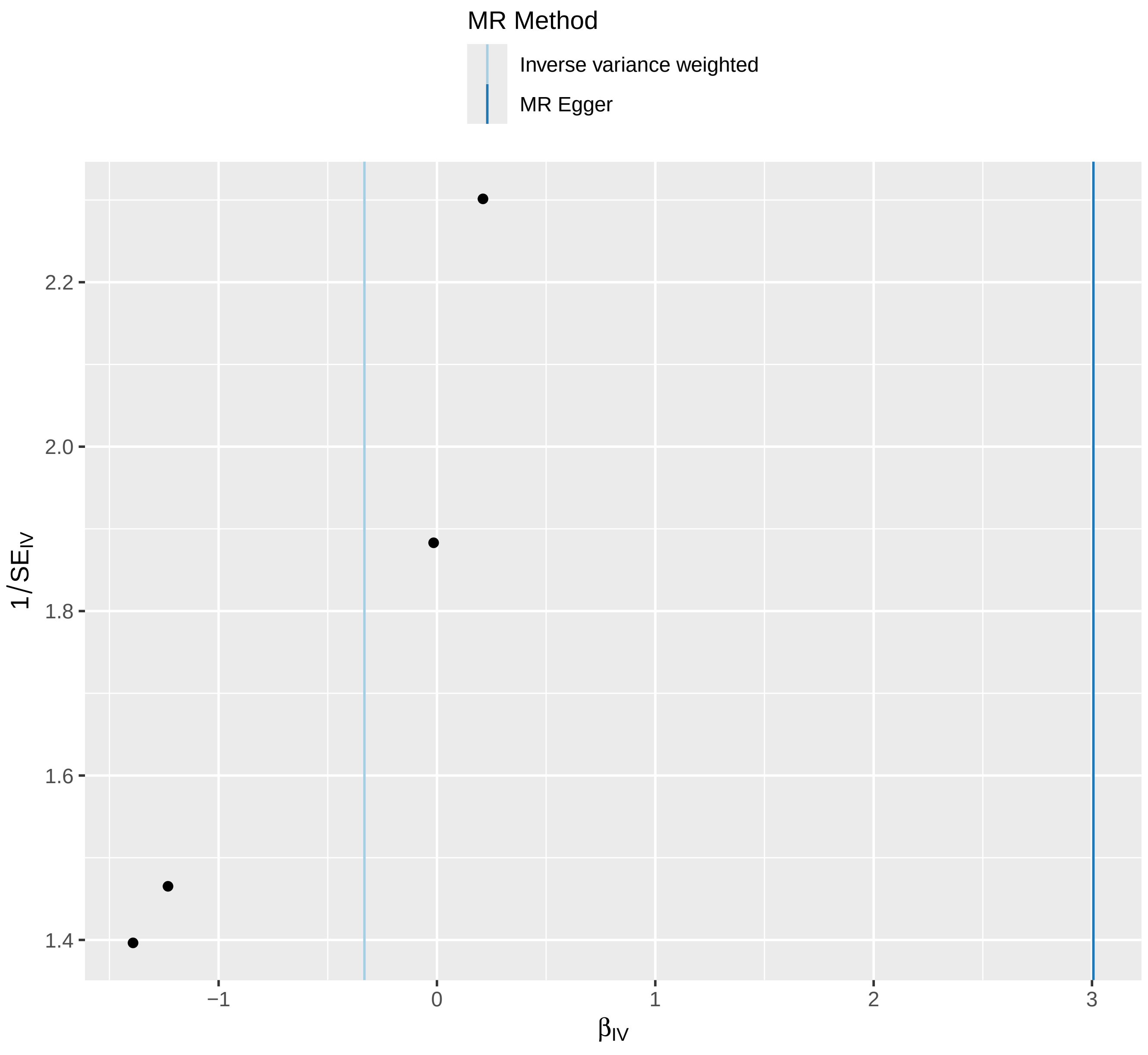

MR Method
Inverse variance weighted
MR Egger
2.2
2.0
1.8
1.6
1.4
1/SE_IV
−1
0
1
2
3
β_IV

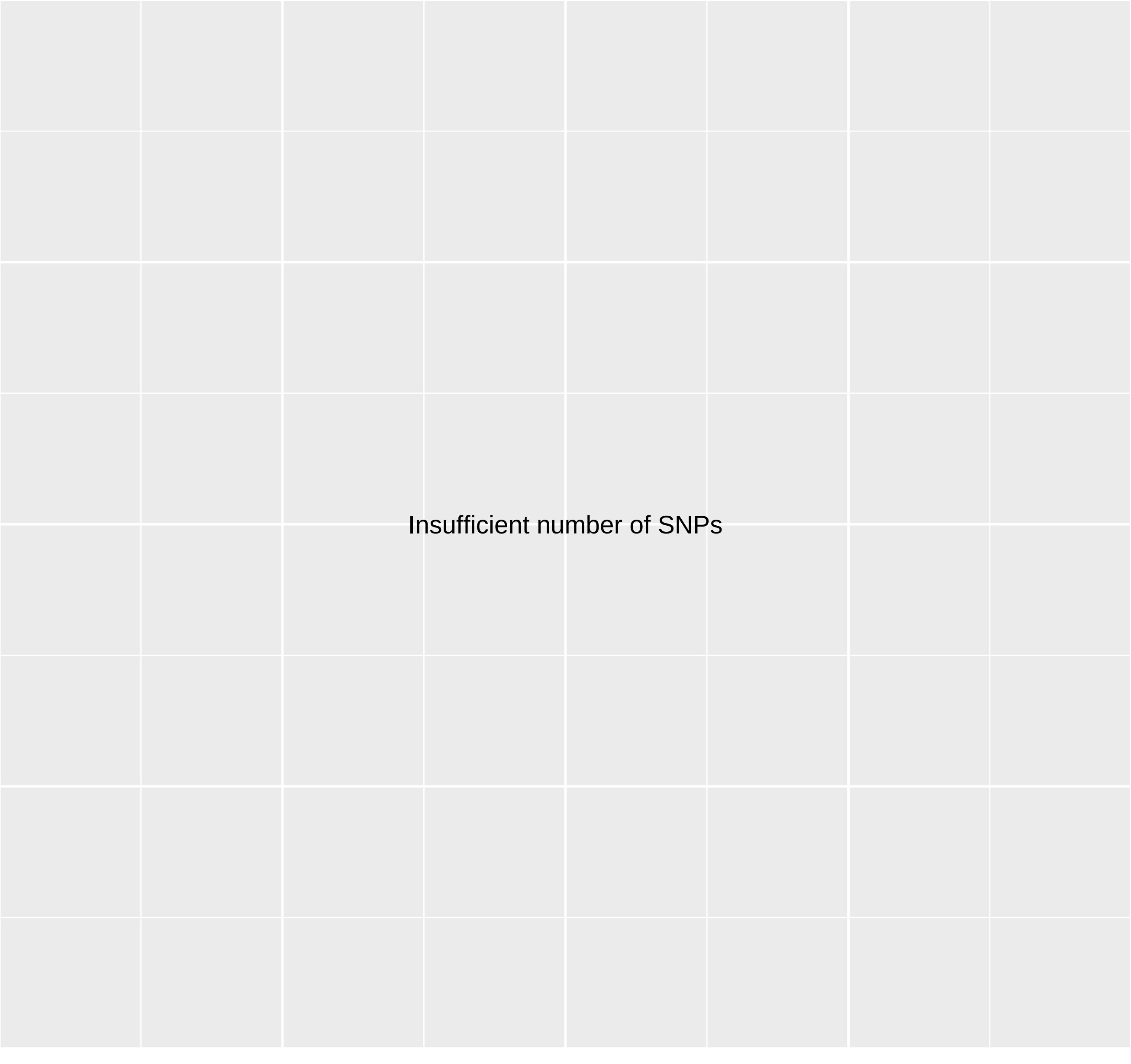
Insufficient number of SNPs

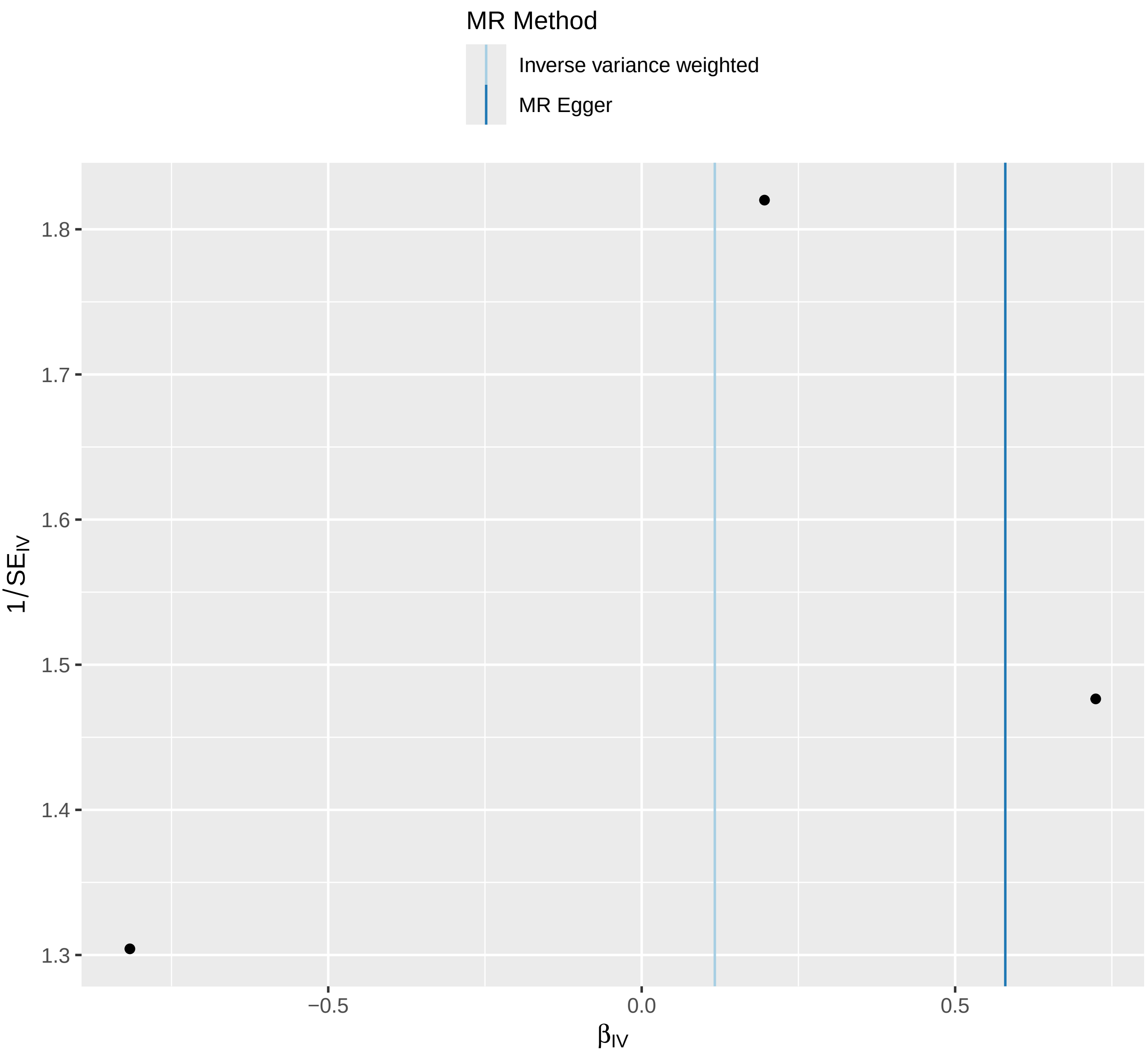
MR Method
Inverse variance weighted
MR Egger
1.8
1.7
1.6
1.5
1.4
1.3
$1/SE_{IV}$
−0.5
0.0
0.5
β_{IV}

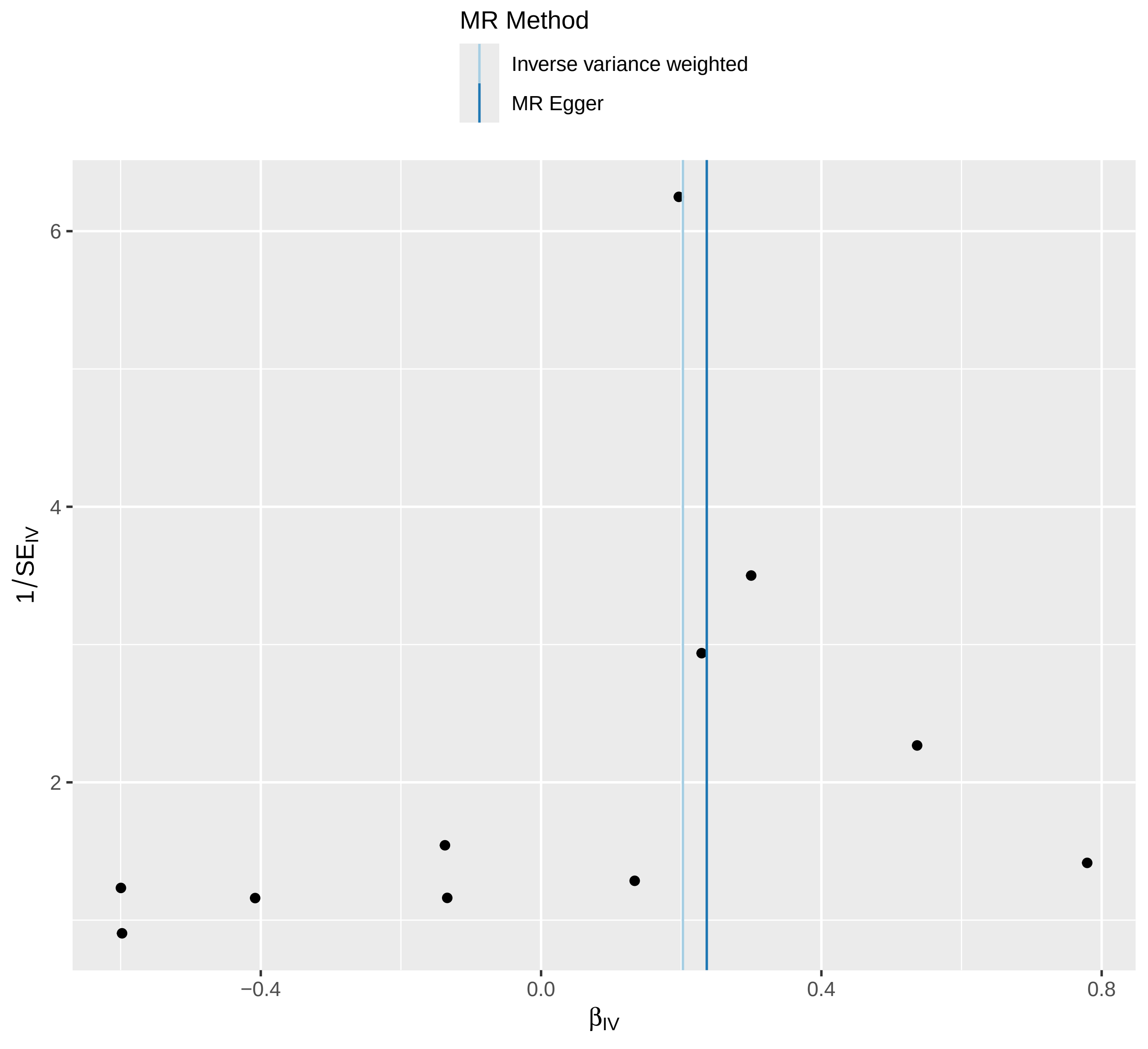
MR Method
Inverse variance weighted
MR Egger
1/SE_IV
6
4
2
−0.4
0.0
0.4
0.8
β_IV

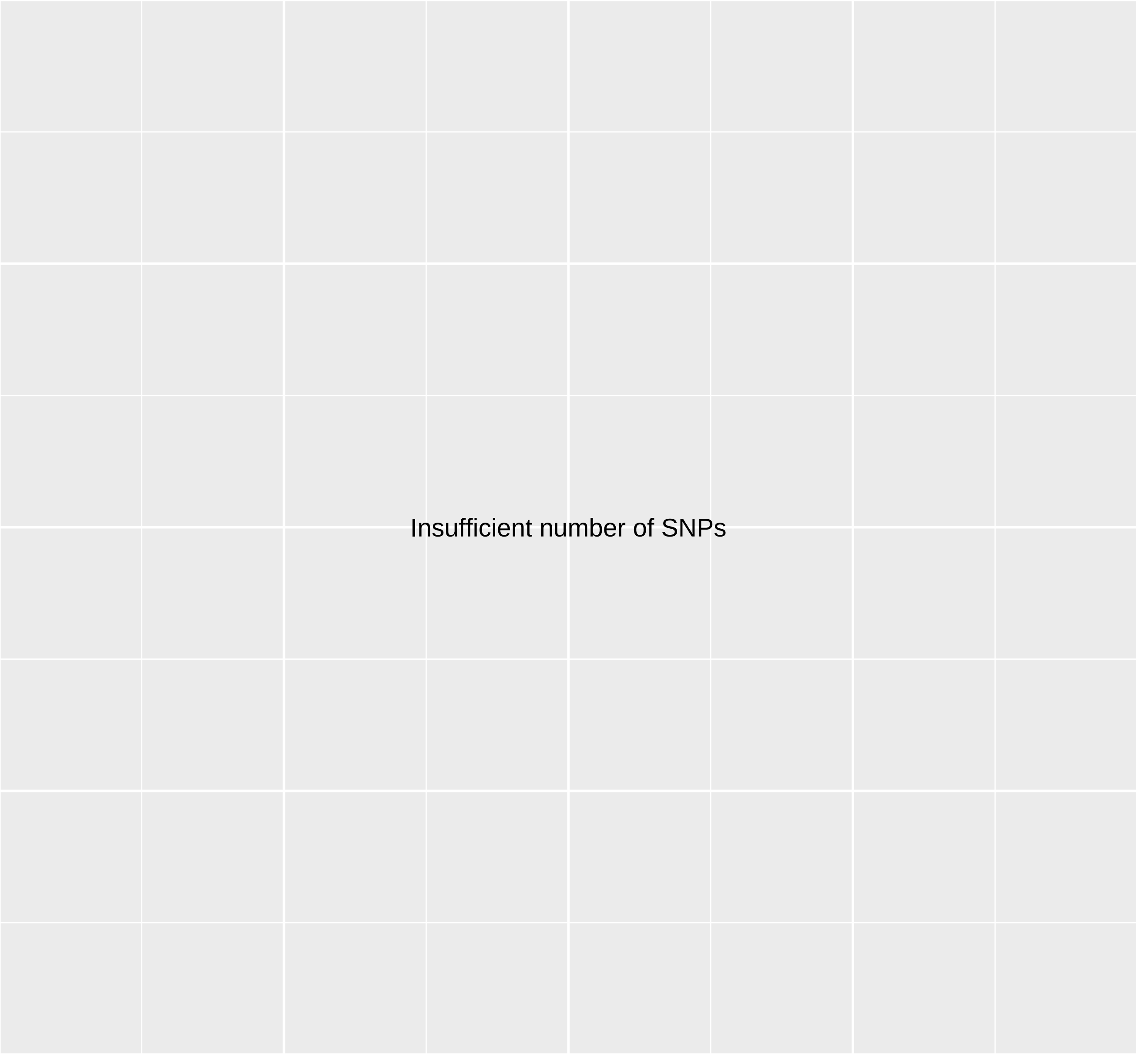
Insufficient number of SNPs

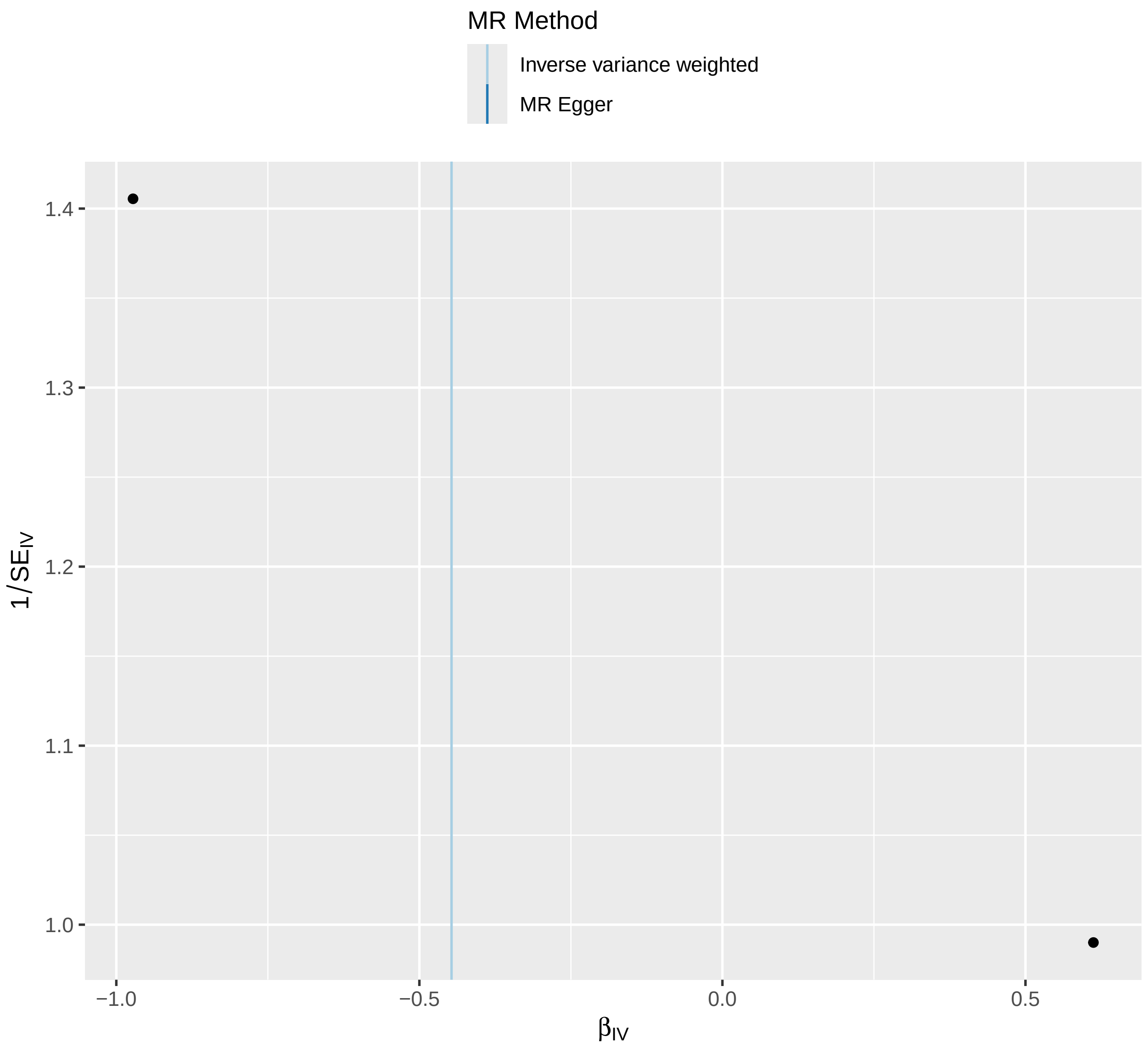
MR Method
Inverse variance weighted
MR Egger
1.4
1.3
1.2
1.1
1.0
1/SE_IV
−1.0
−0.5
0.0
0.5
β_IV

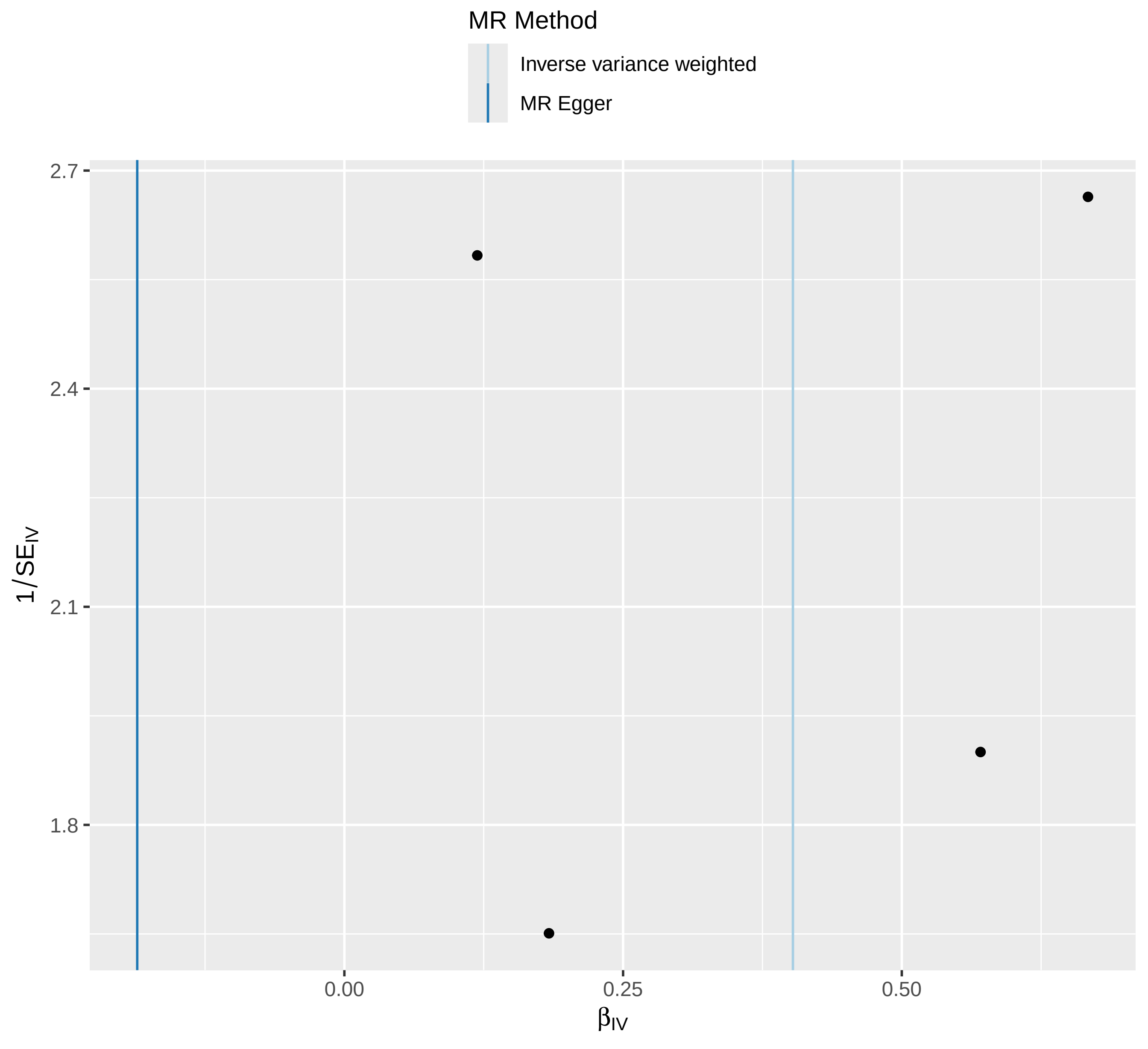

MR Method
Inverse variance weighted
MR Egger
2.7
2.4
2.1
1.8
1/SE_IV
0.00
0.25
0.50
β_IV

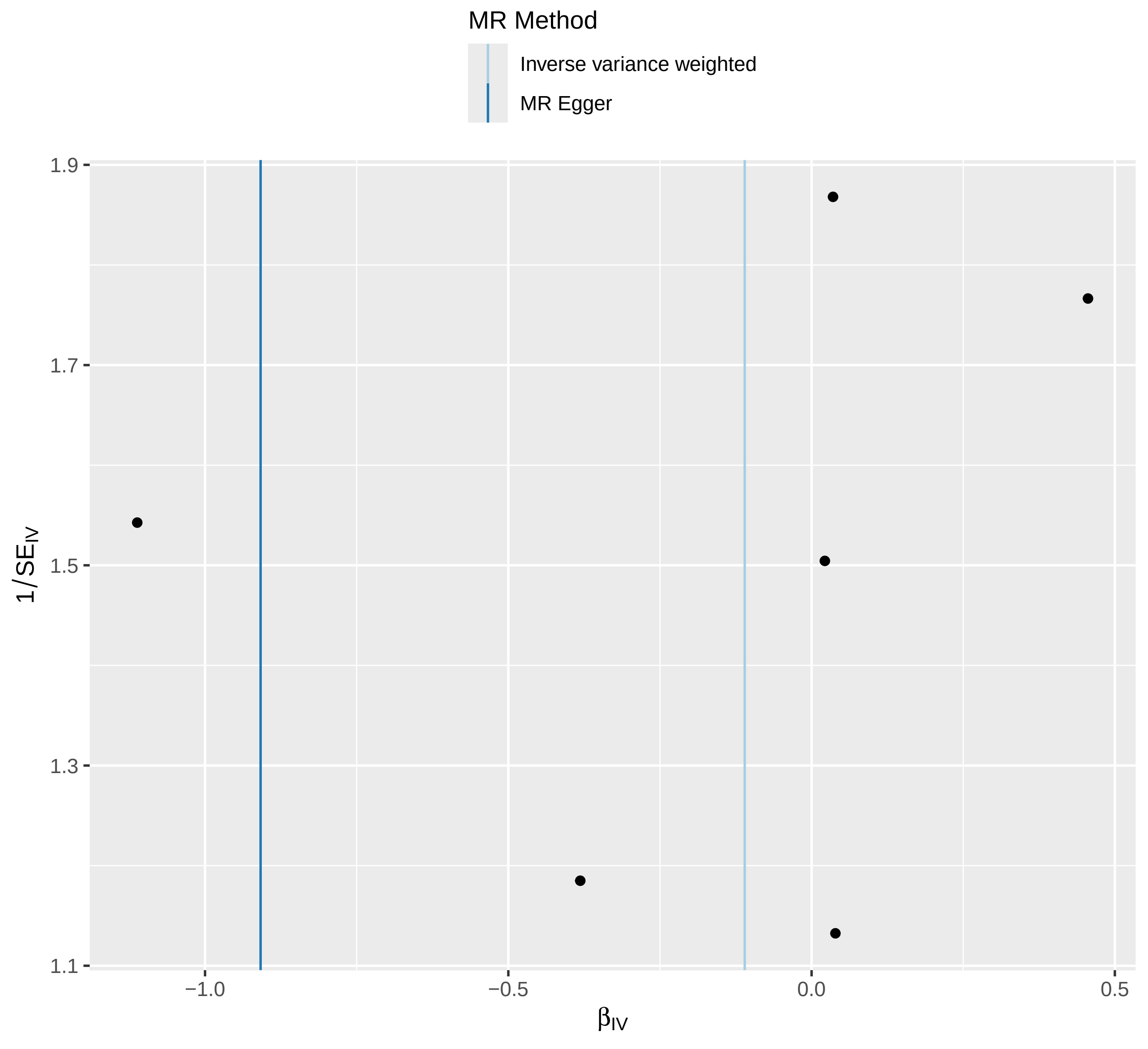

MR Method
Inverse variance weighted
MR Egger
1.9
1.7
1.5
1.3
1.1
$1/SE_{IV}$
−1.0
−0.5
0.0
0.5
β_{IV}

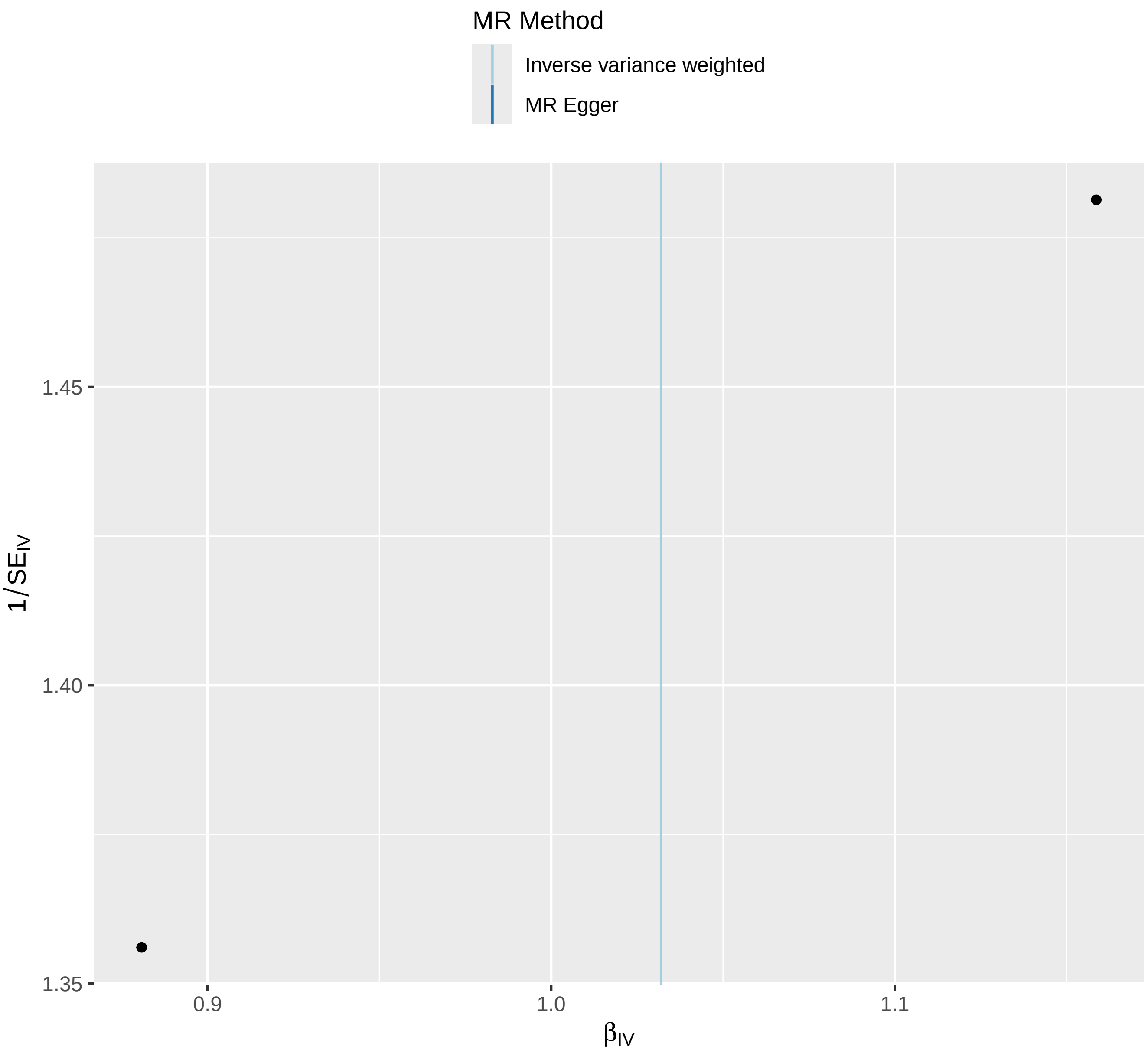

MR Method
Inverse variance weighted
MR Egger
1.45
1.40
1.35
0.9
1.0
1.1
$1/SE_{IV}$
β_{IV}

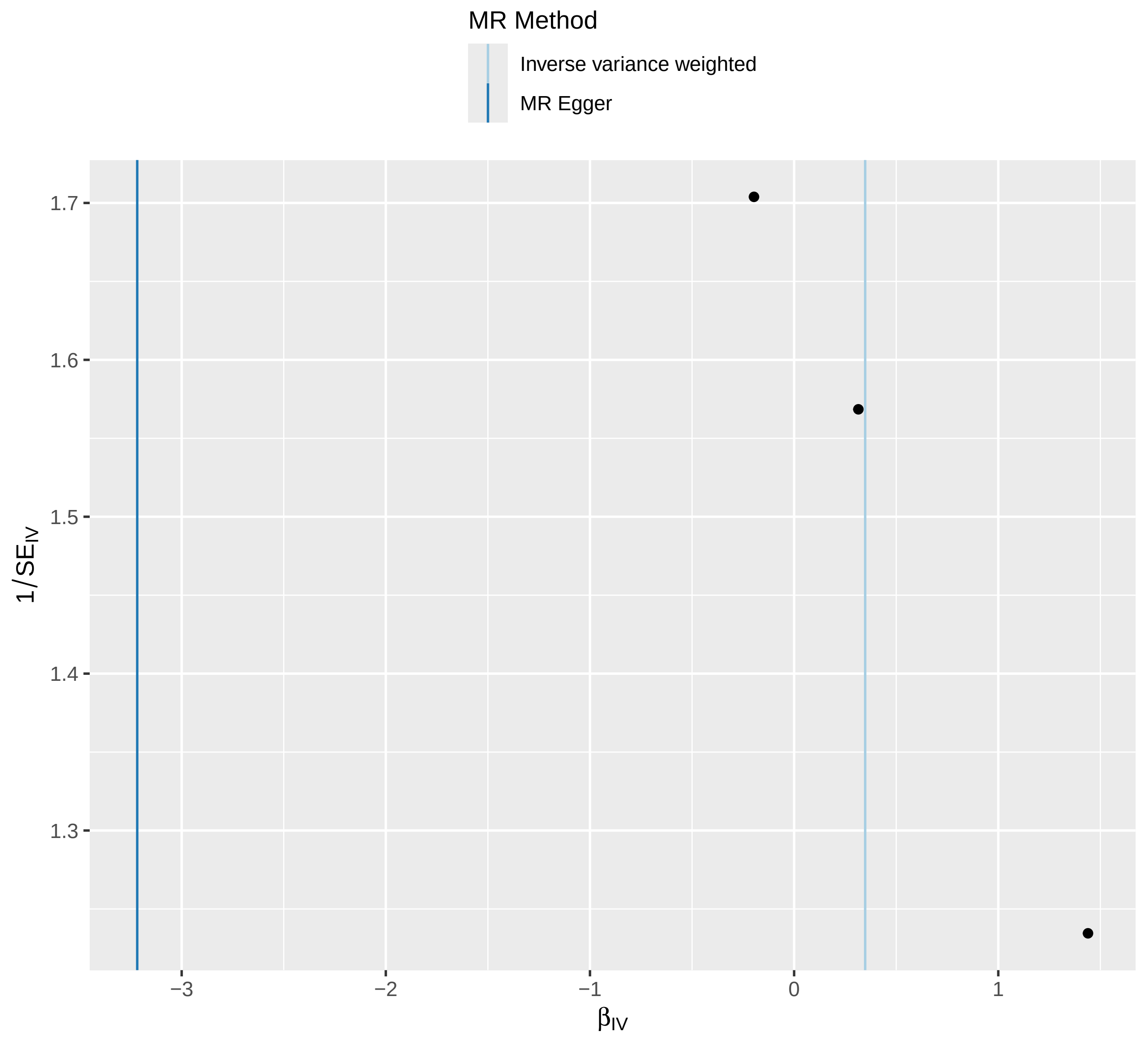
MR Method
Inverse variance weighted
MR Egger
1.7
1.6
1.5
1.4
1.3
$1/SE_{IV}$
−3
−2
−1
0
1
β_{IV}

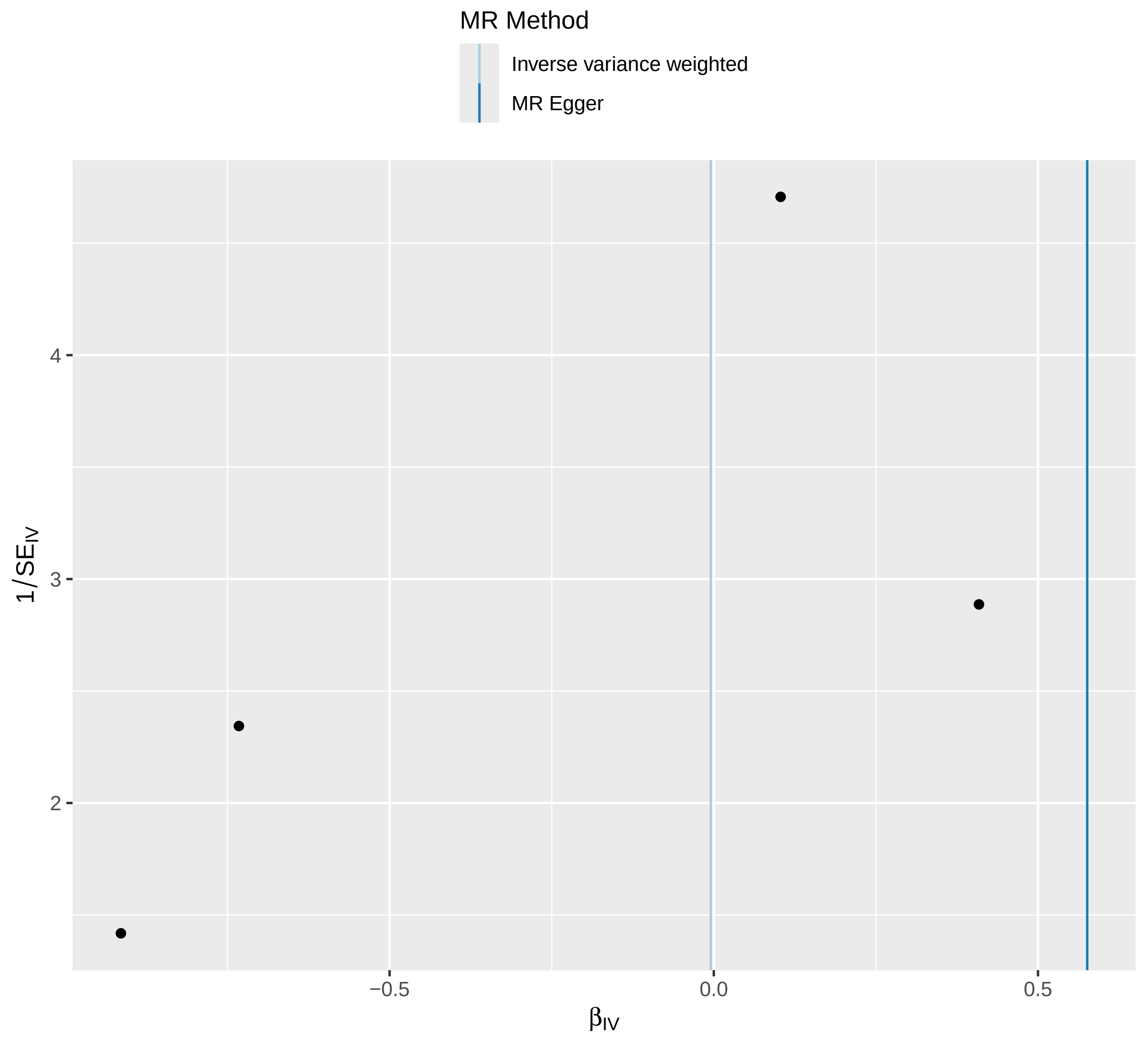

MR Method
Inverse variance weighted
MR Egger
4
3
2
1/SE_IV
−0.5
0.0
0.5
β_IV

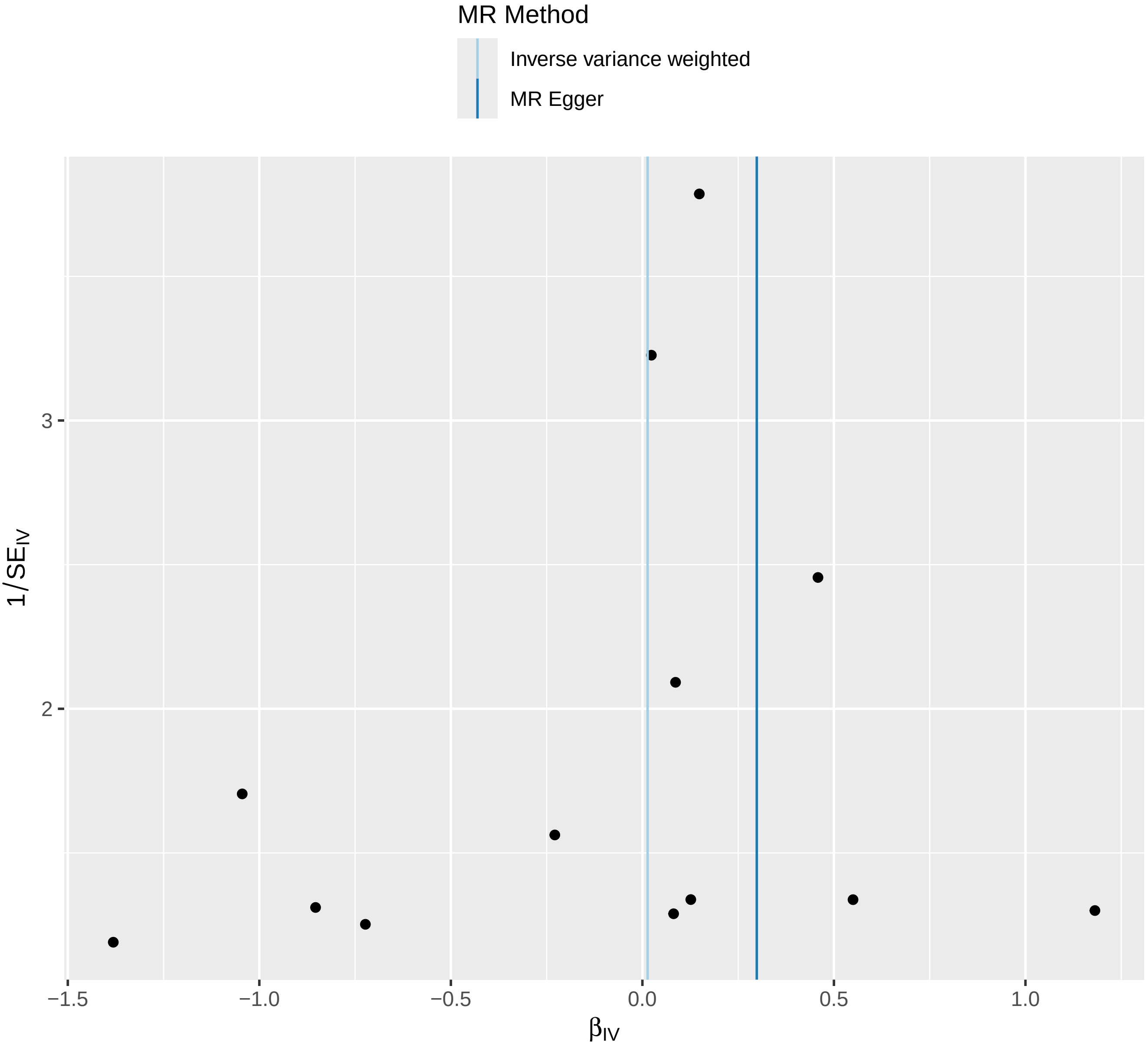

MR Method
Inverse variance weighted
MR Egger
1/SE_{IV}
3
2
−1.5
−1.0
−0.5
0.0
0.5
1.0
β_{IV}

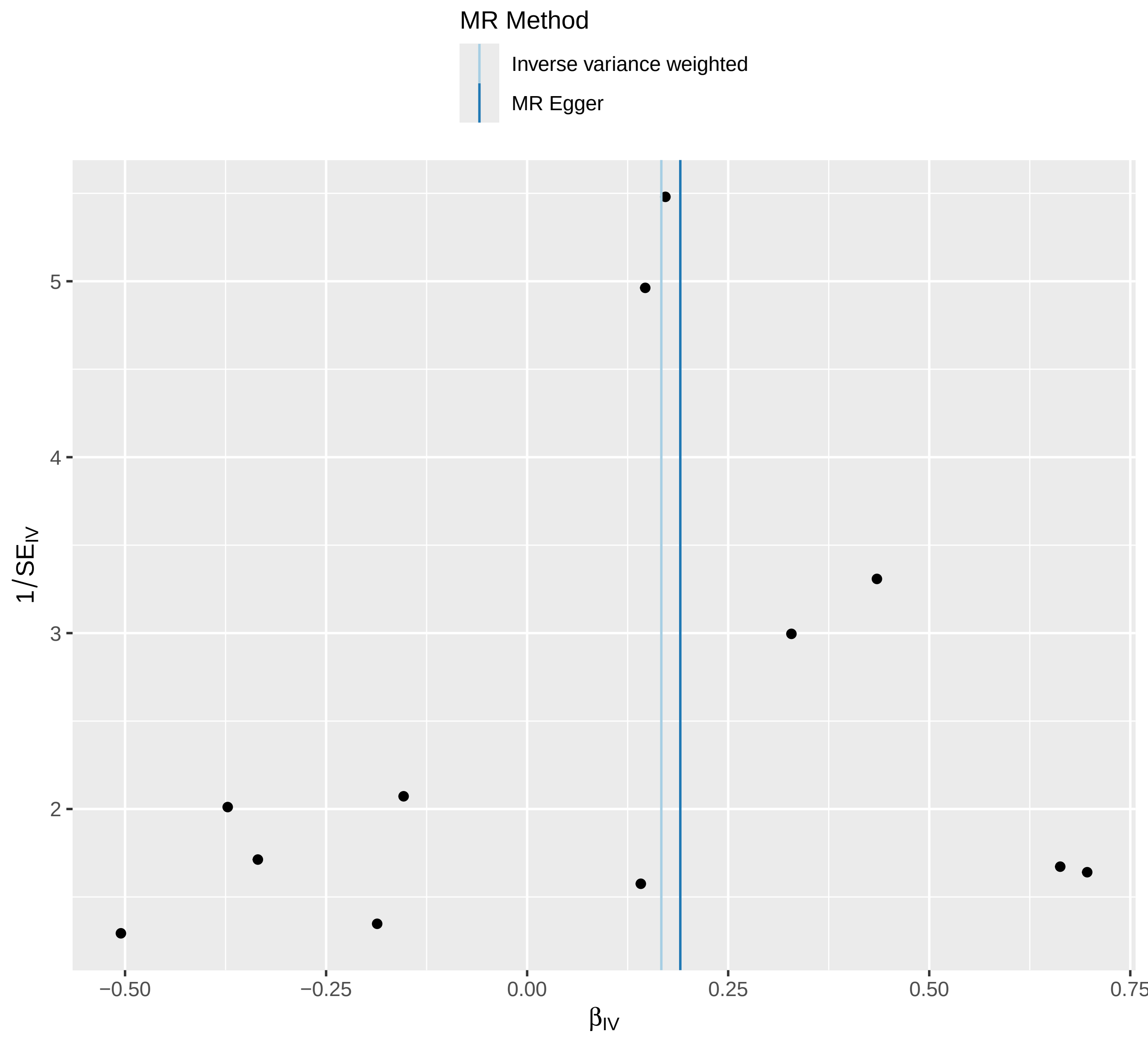

MR Method
Inverse variance weighted
MR Egger
5
4
3
2
1/SE$_{IV}$
−0.50
−0.25
0.00
0.25
0.50
0.75
β$_{IV}$

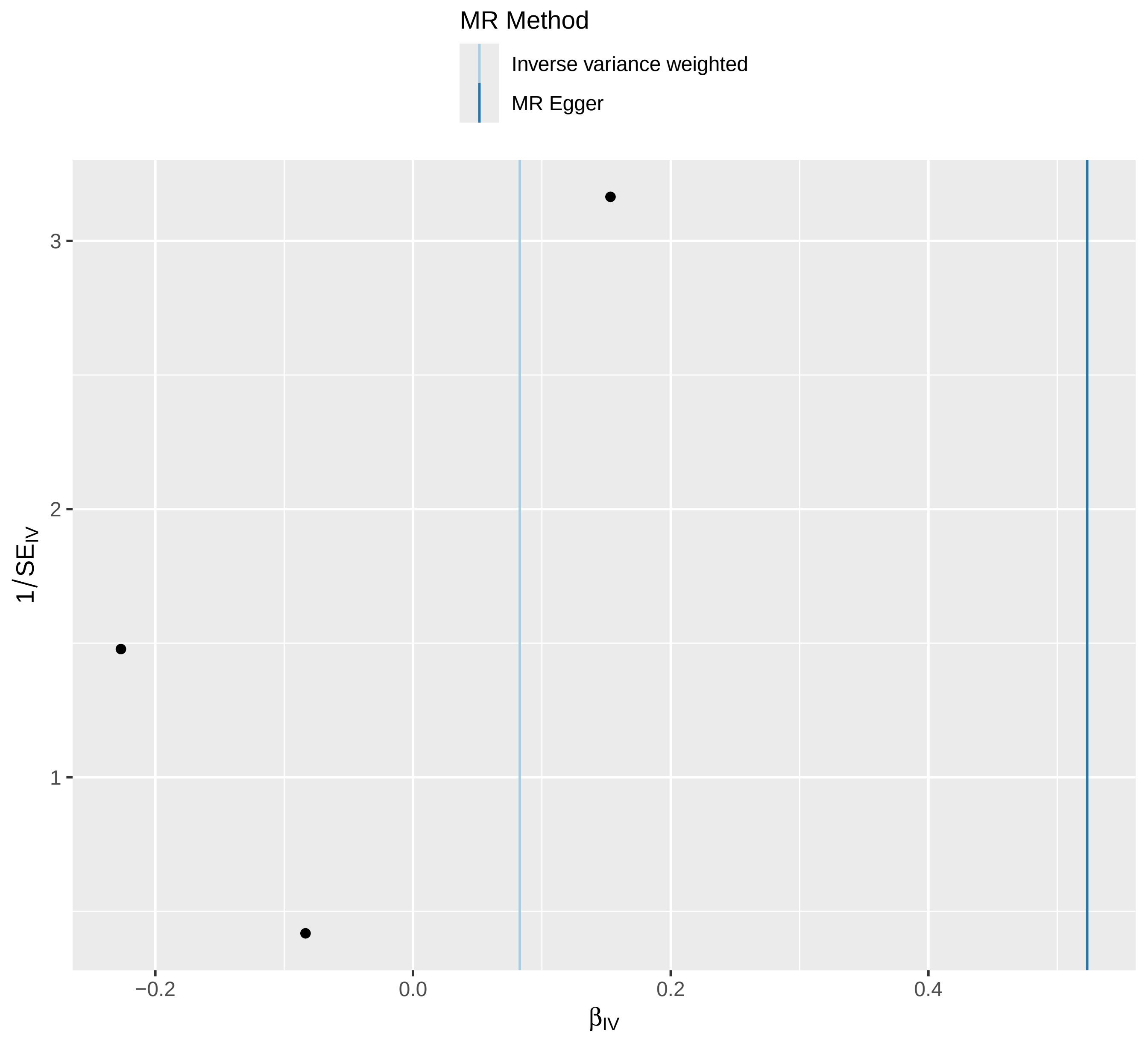

MR Method
Inverse variance weighted
MR Egger
3
2
1
1/SE_IV
−0.2
0.0
0.2
0.4
β_IV

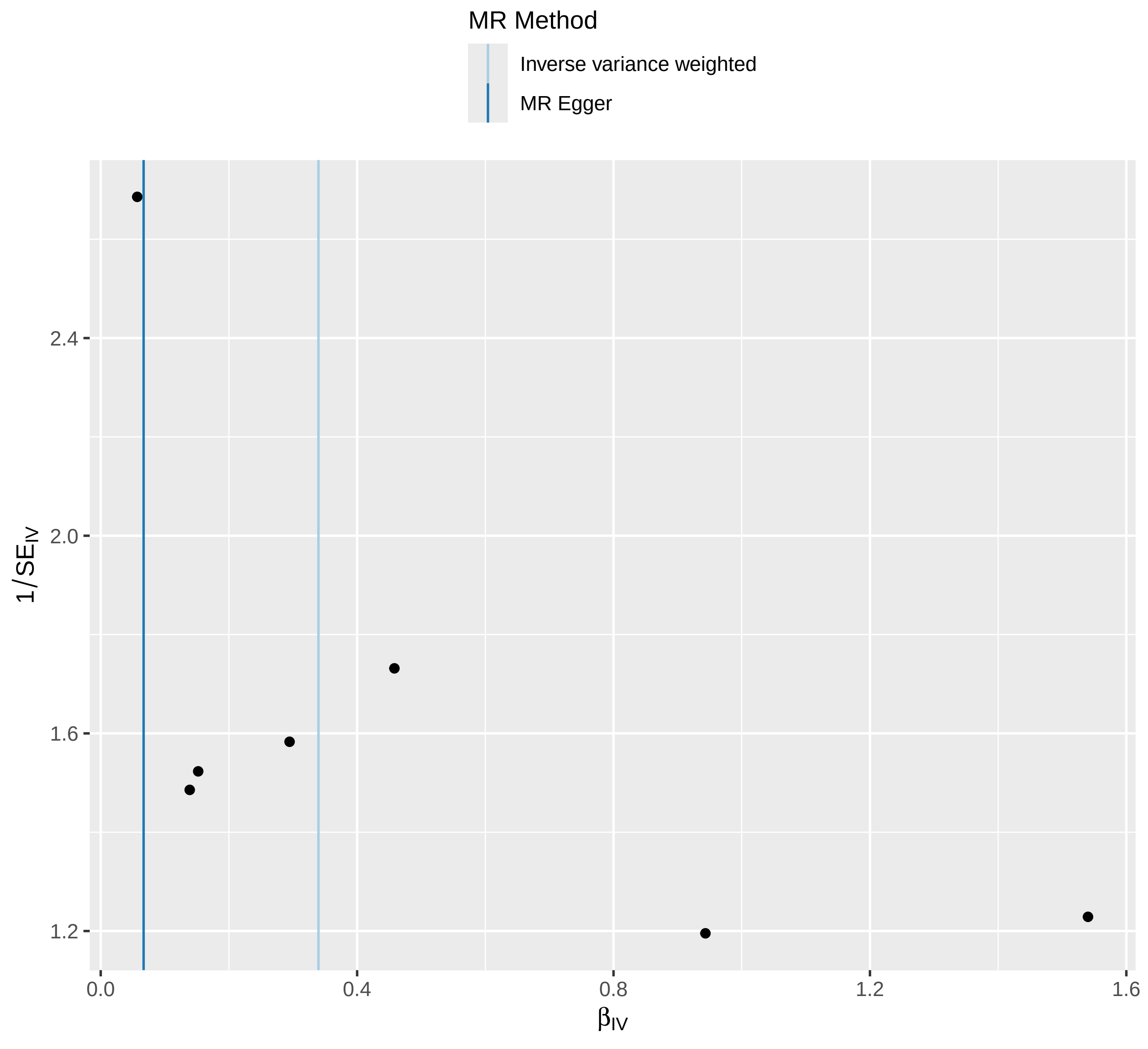

MR Method
Inverse variance weighted
MR Egger
2.4
2.0
1.6
1.2
1/SE_IV
0.0
0.4
0.8
1.2
1.6
β_IV

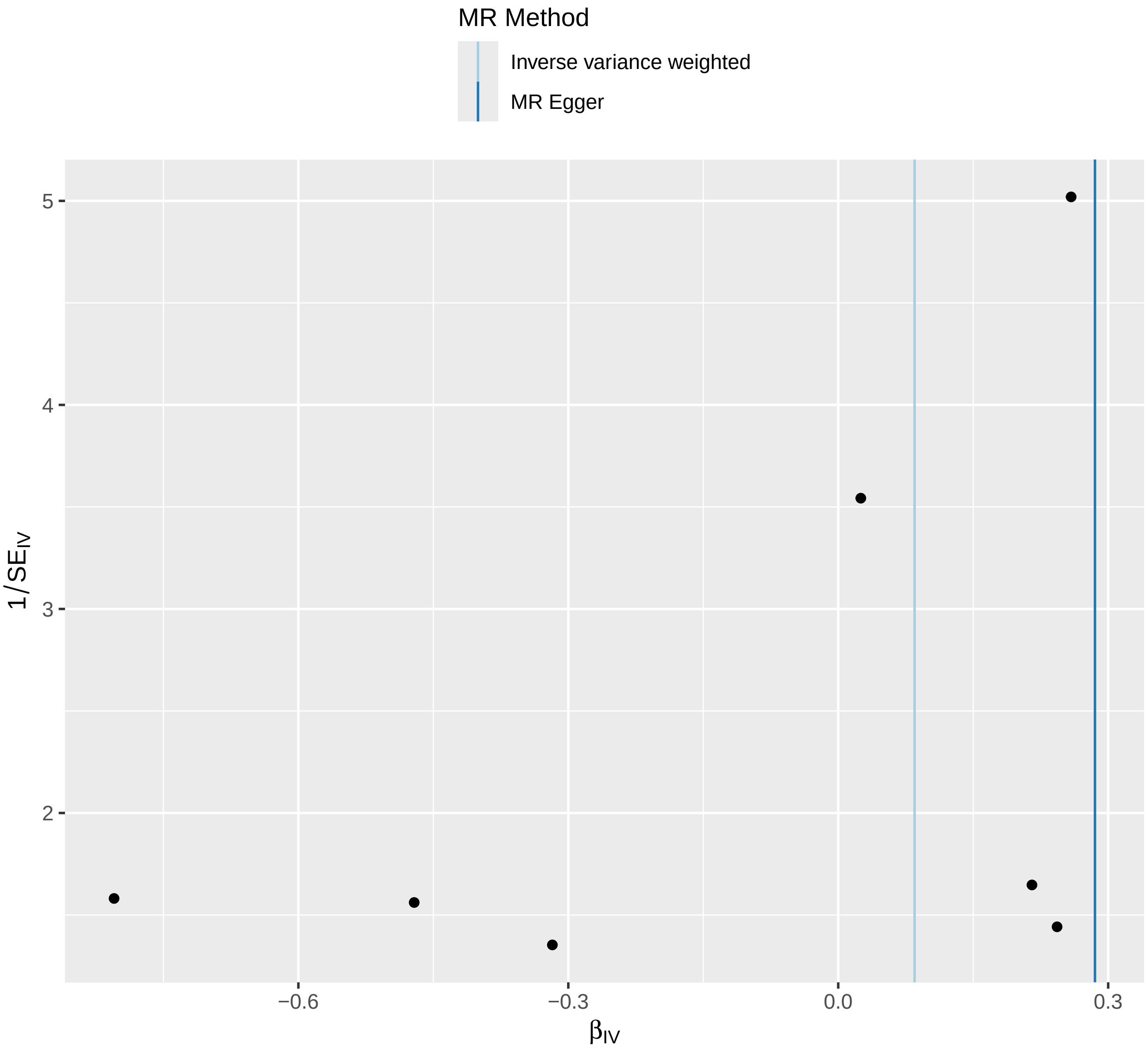
MR Method
Inverse variance weighted
MR Egger
5
4
3
2
1/SE_IV
−0.6
−0.3
0.0
0.3
β_IV

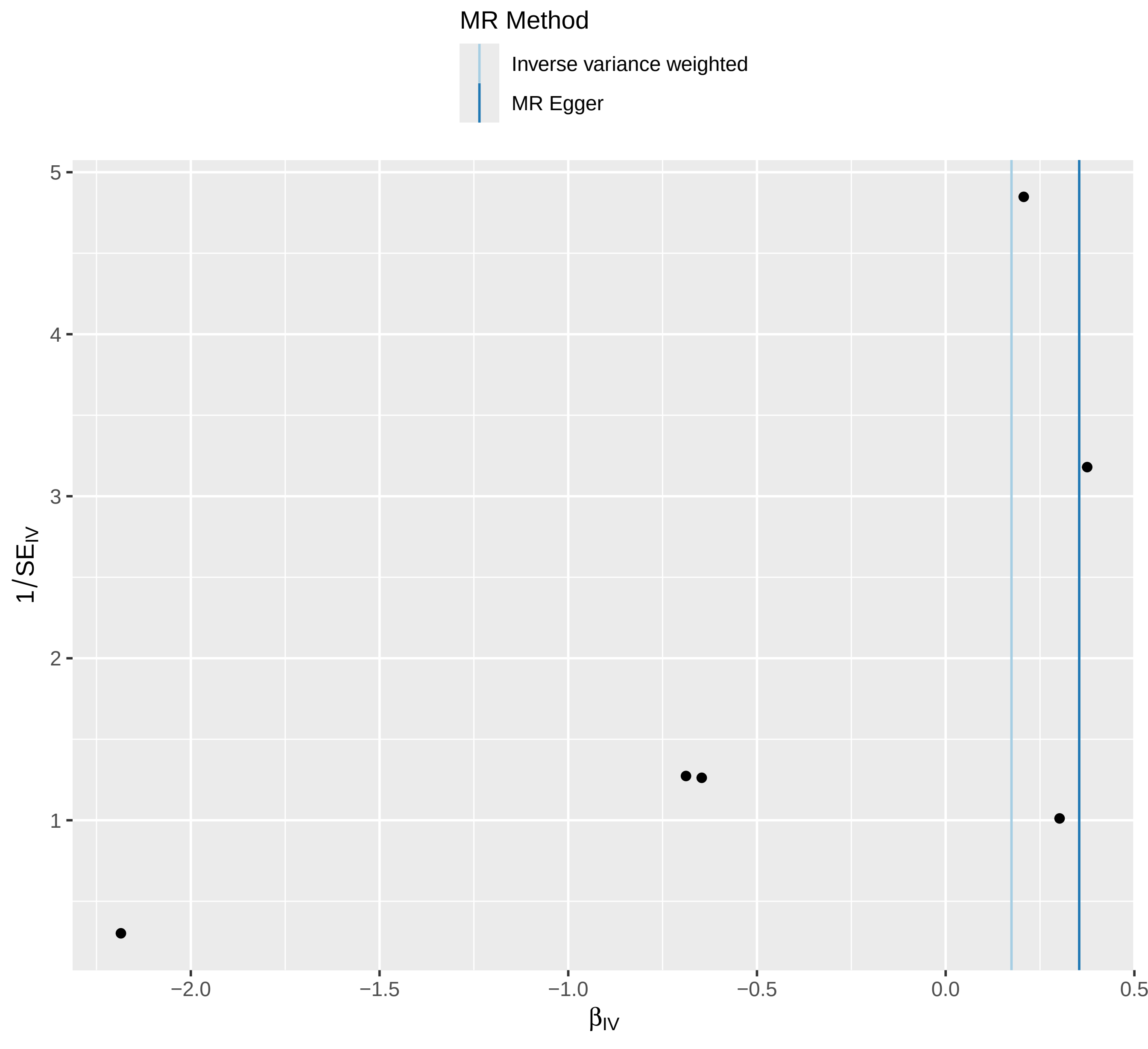
MR Method
Inverse variance weighted
MR Egger
5
4
3
2
1
$1/SE_{IV}$
−2.0
−1.5
−1.0
−0.5
0.0
0.5
β_{IV}

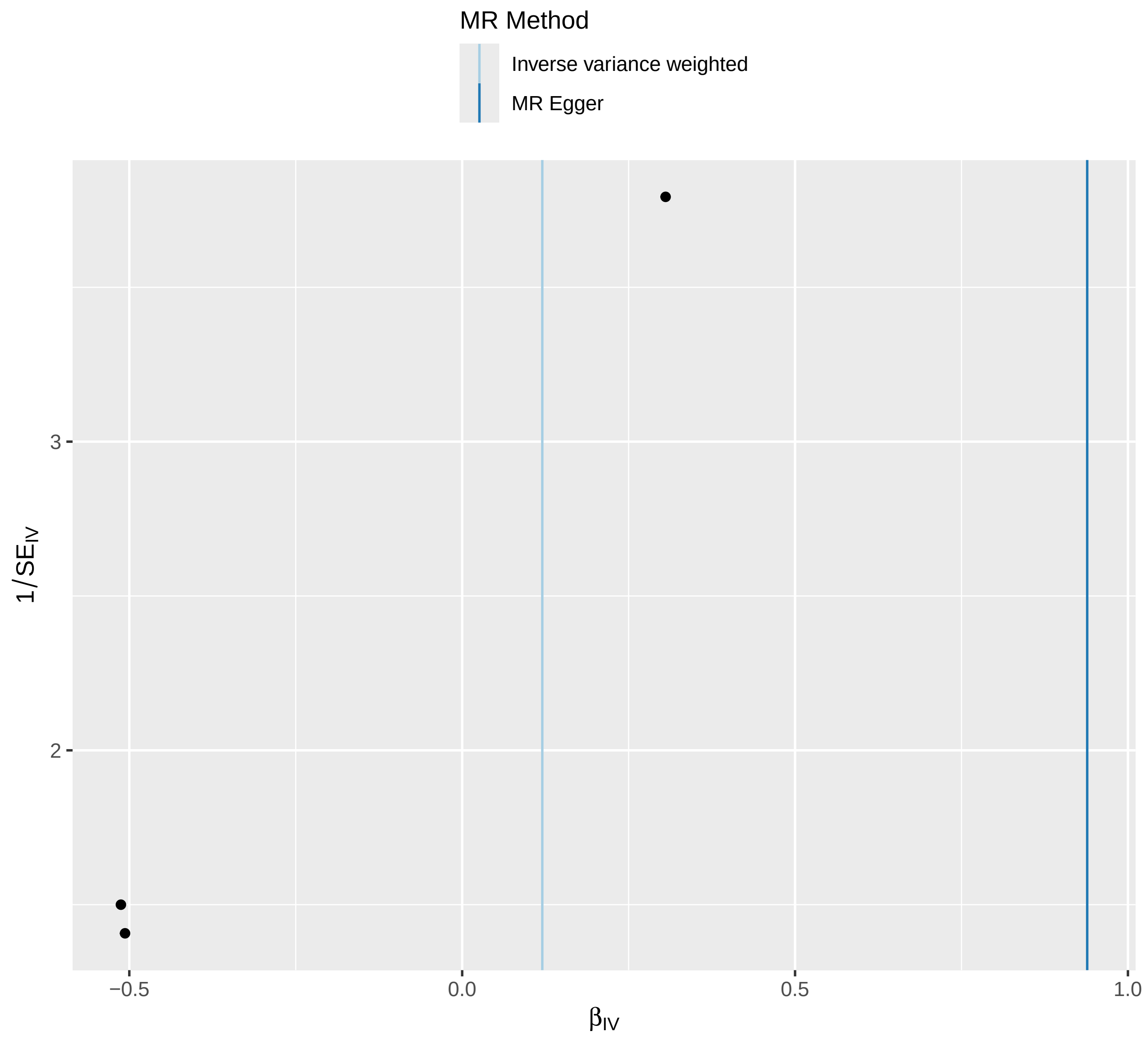

MR Method
Inverse variance weighted
MR Egger
3
2
1/SE_IV
−0.5
0.0
0.5
1.0
β_IV

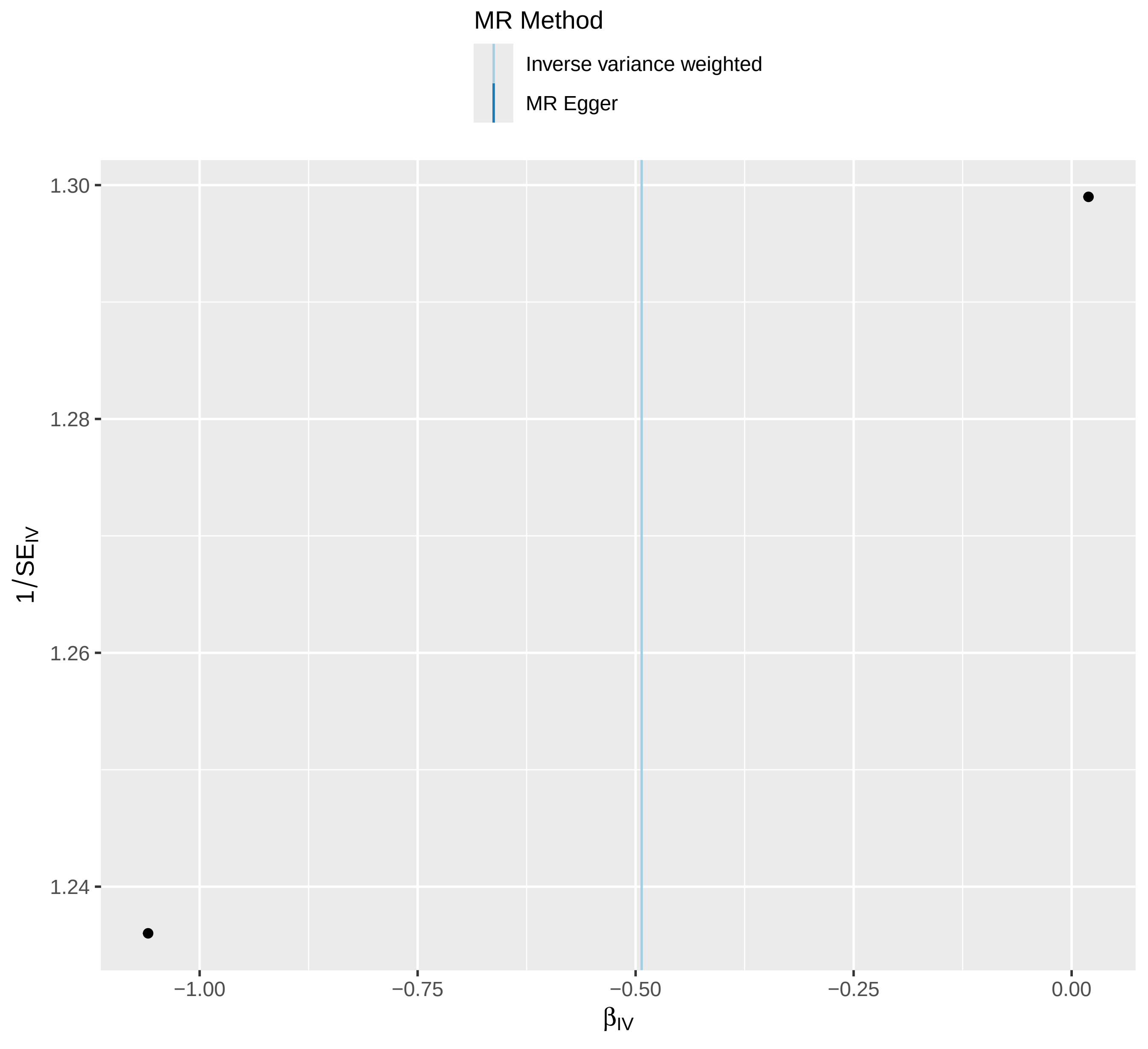

MR Method
Inverse variance weighted
MR Egger
1.30
1.28
1.26
1.24
$1/SE_{IV}$
−1.00
−0.75
−0.50
−0.25
0.00
β_{IV}

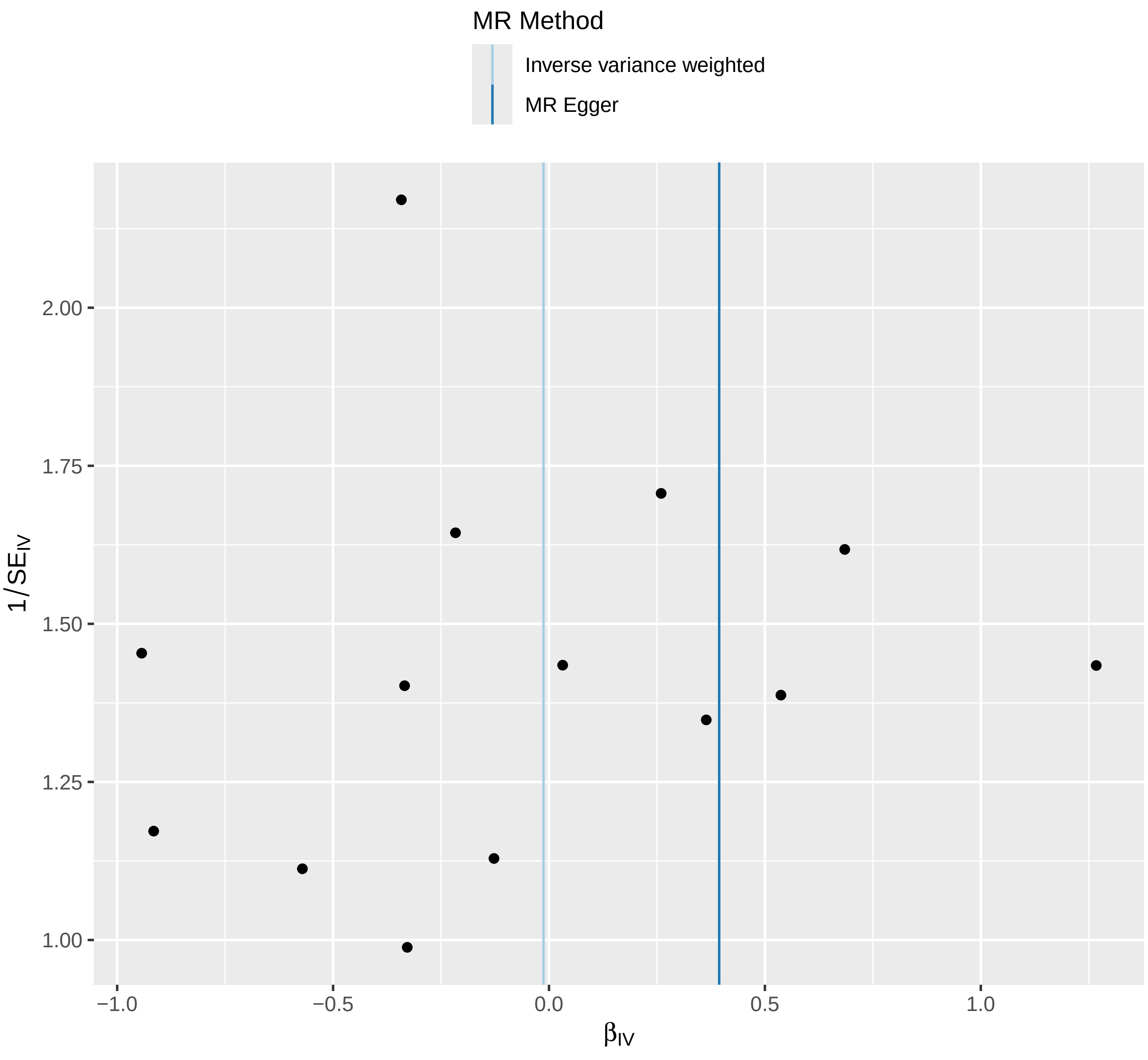

MR Method
Inverse variance weighted
MR Egger
2.00
1.75
1.50
1.25
1.00
$1/SE_{IV}$
−1.0
−0.5
0.0
0.5
1.0
β_{IV}

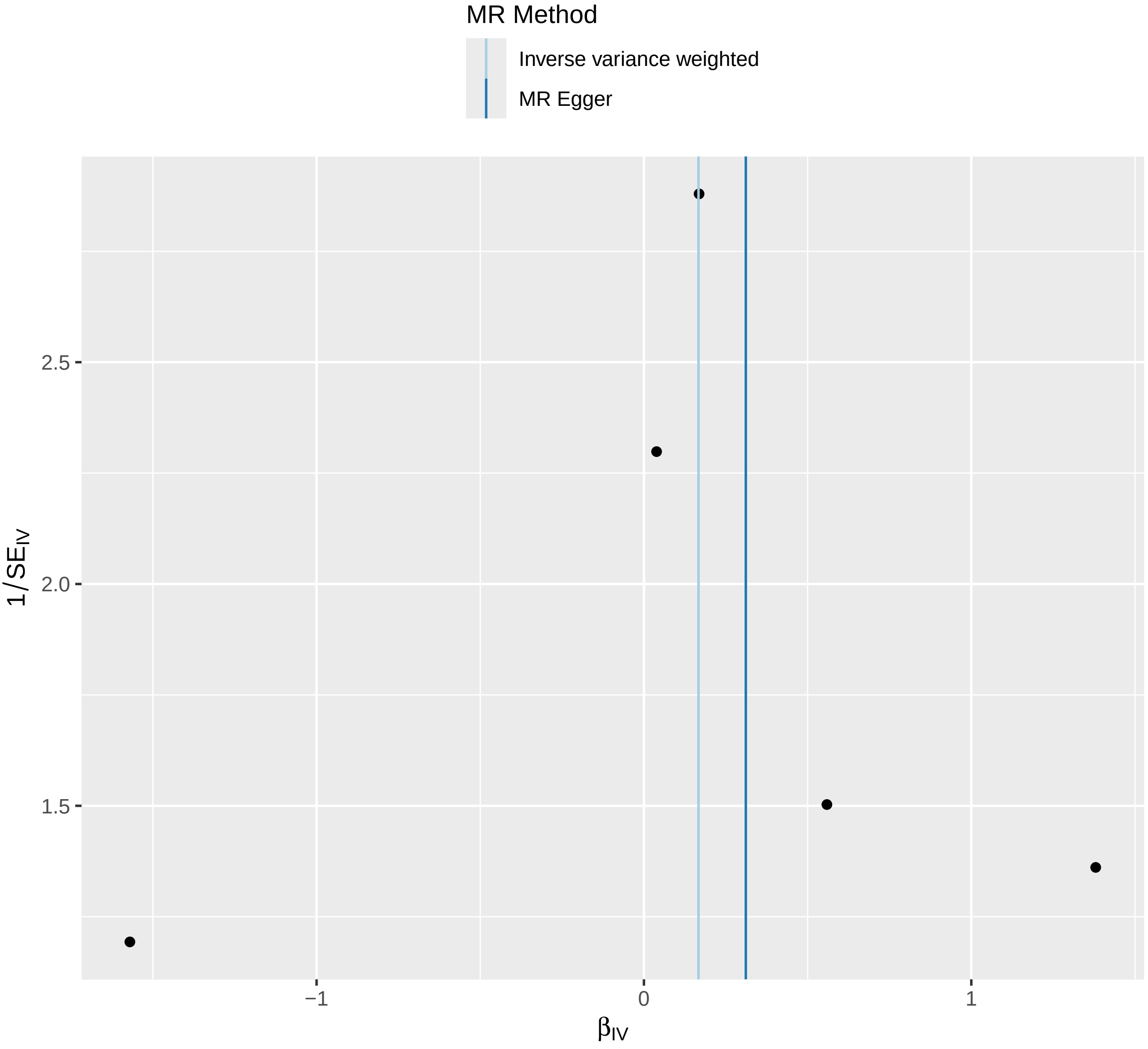
MR Method
Inverse variance weighted
MR Egger
2.5
2.0
1.5
1/SE_IV
−1
0
1
β_IV

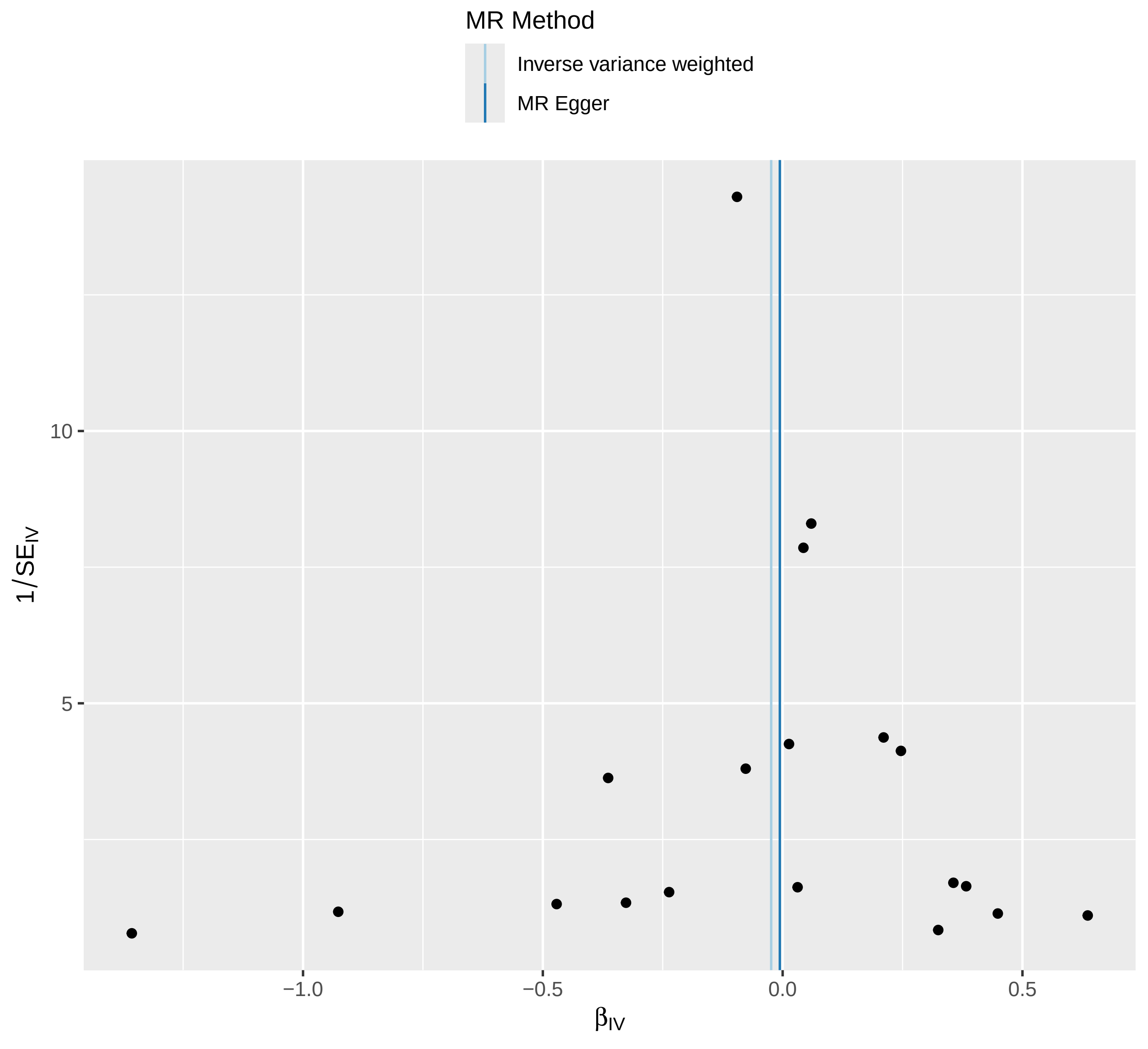
MR Method
Inverse variance weighted
MR Egger
10
5
1/SE_IV
−1.0
−0.5
0.0
0.5
β_IV

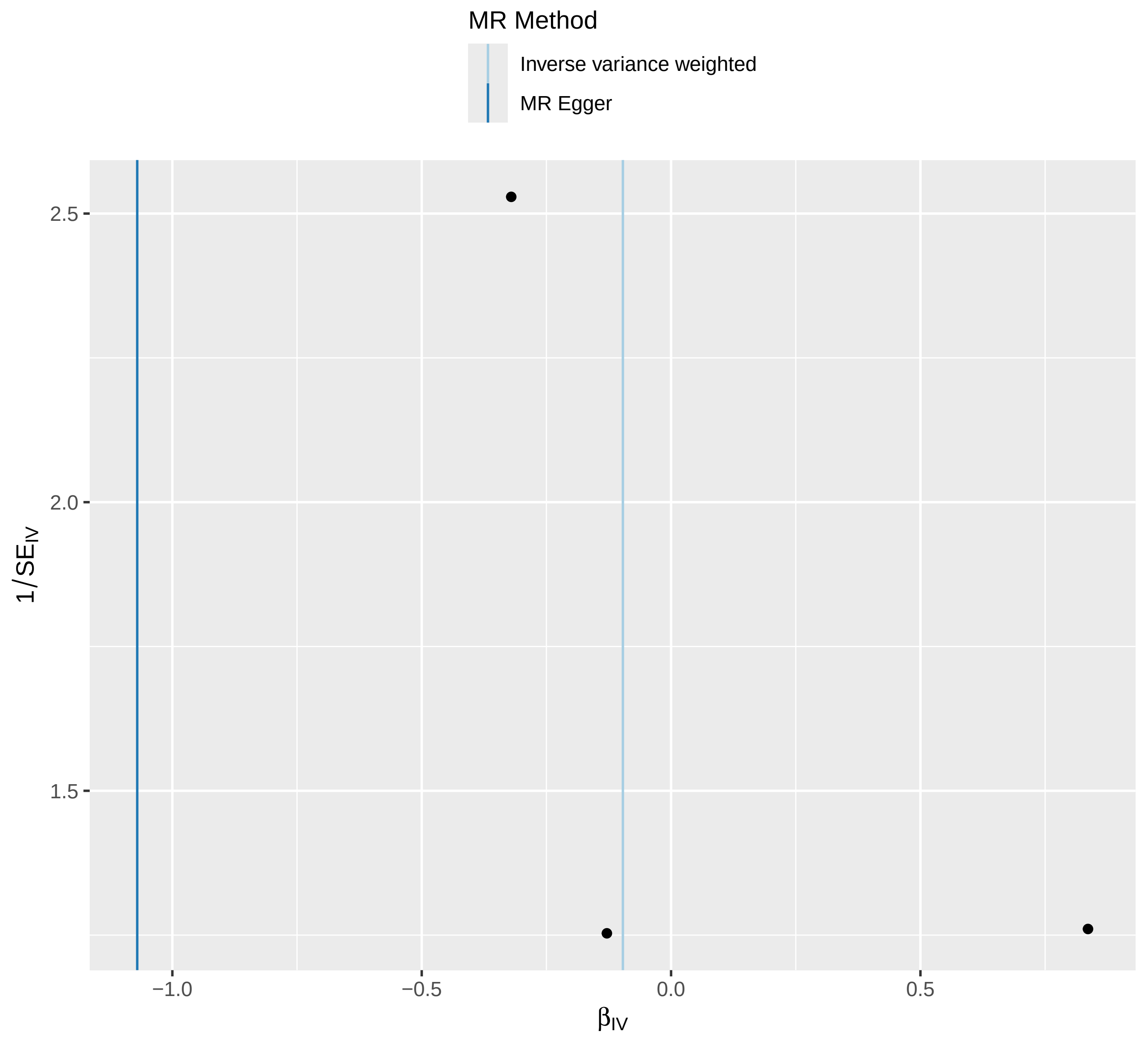

MR Method
Inverse variance weighted
MR Egger
2.5
2.0
1.5
$1/SE_{IV}$
−1.0
−0.5
0.0
0.5
β_{IV}

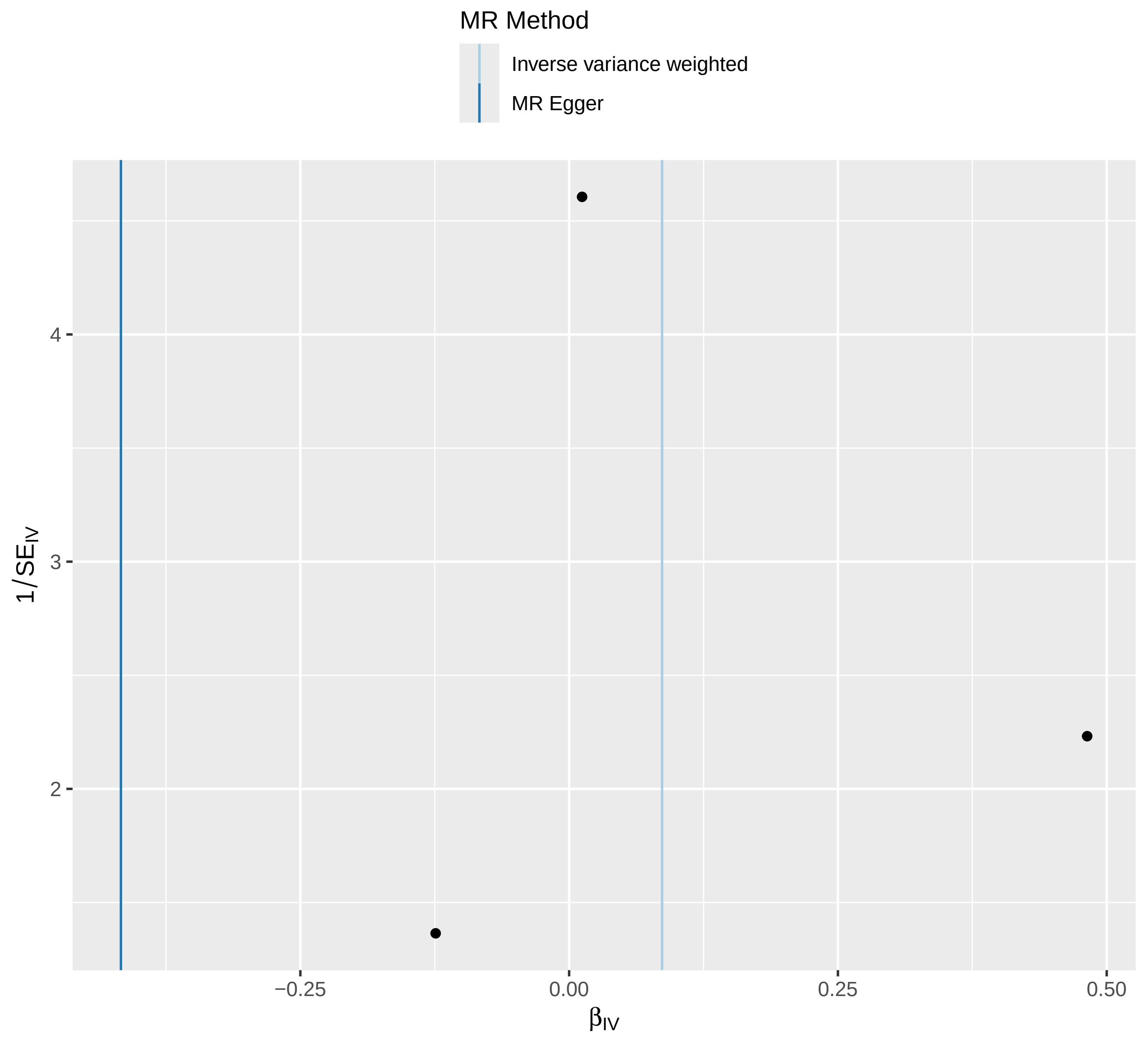
MR Method
Inverse variance weighted
MR Egger
4
3
2
1/SE_IV
−0.25
0.00
0.25
0.50
β_IV

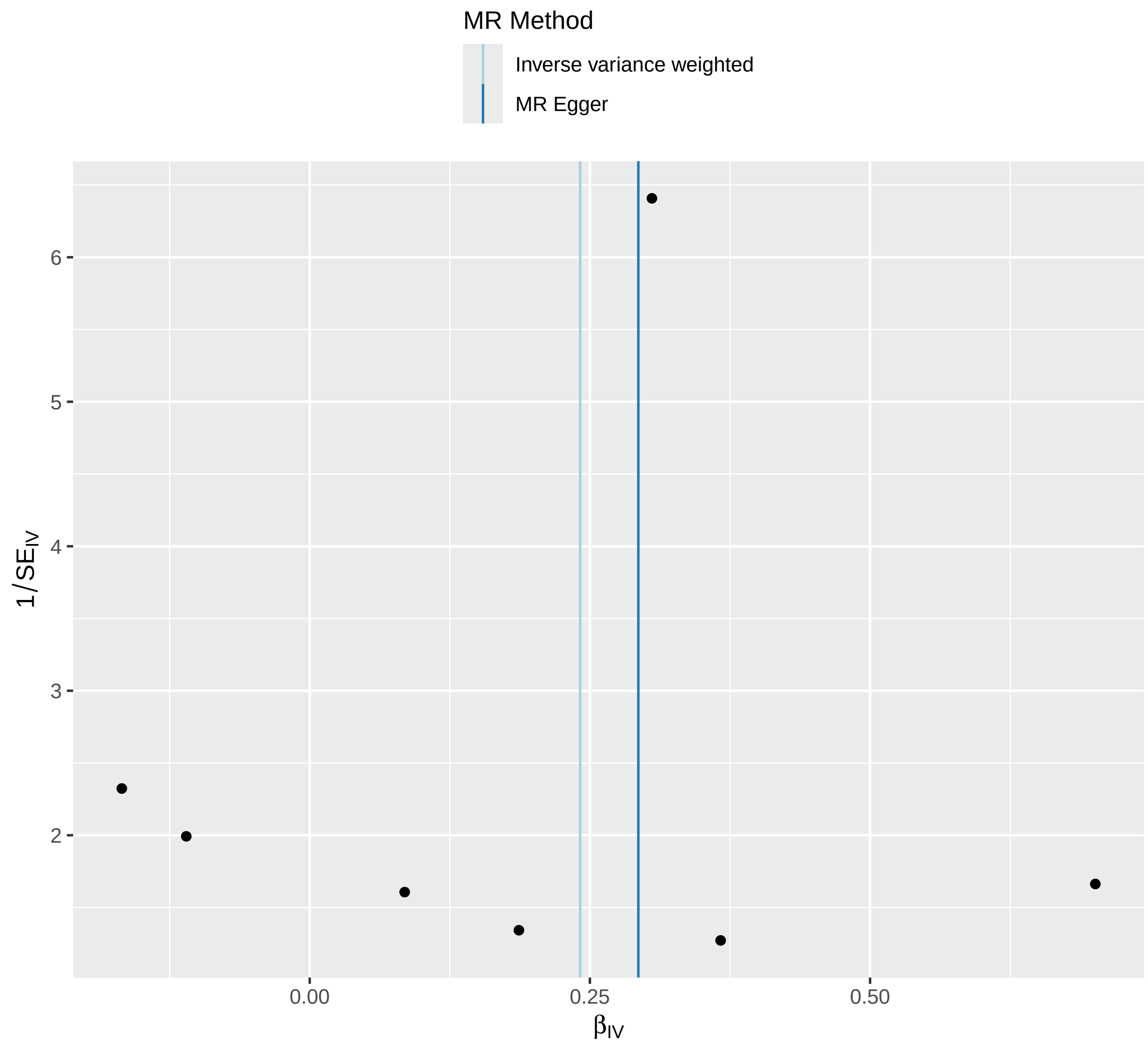

MR Method
Inverse variance weighted
MR Egger
6
5
4
3
2
1/SE_IV
0.00
0.25
0.50
β_IV

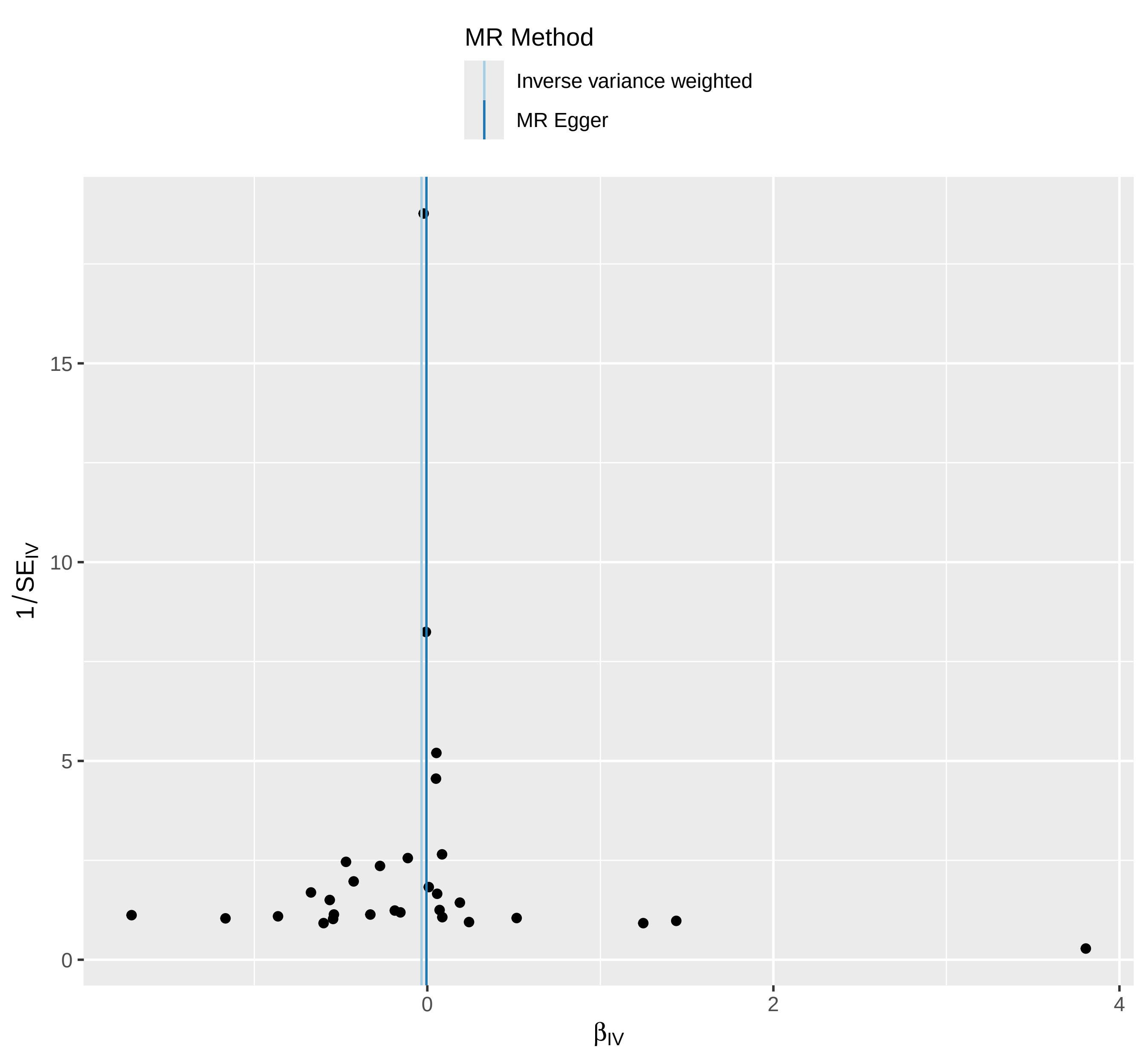
MR Method
Inverse variance weighted
MR Egger
1/SE_IV
β_IV
0
5
10
15
0
2
4

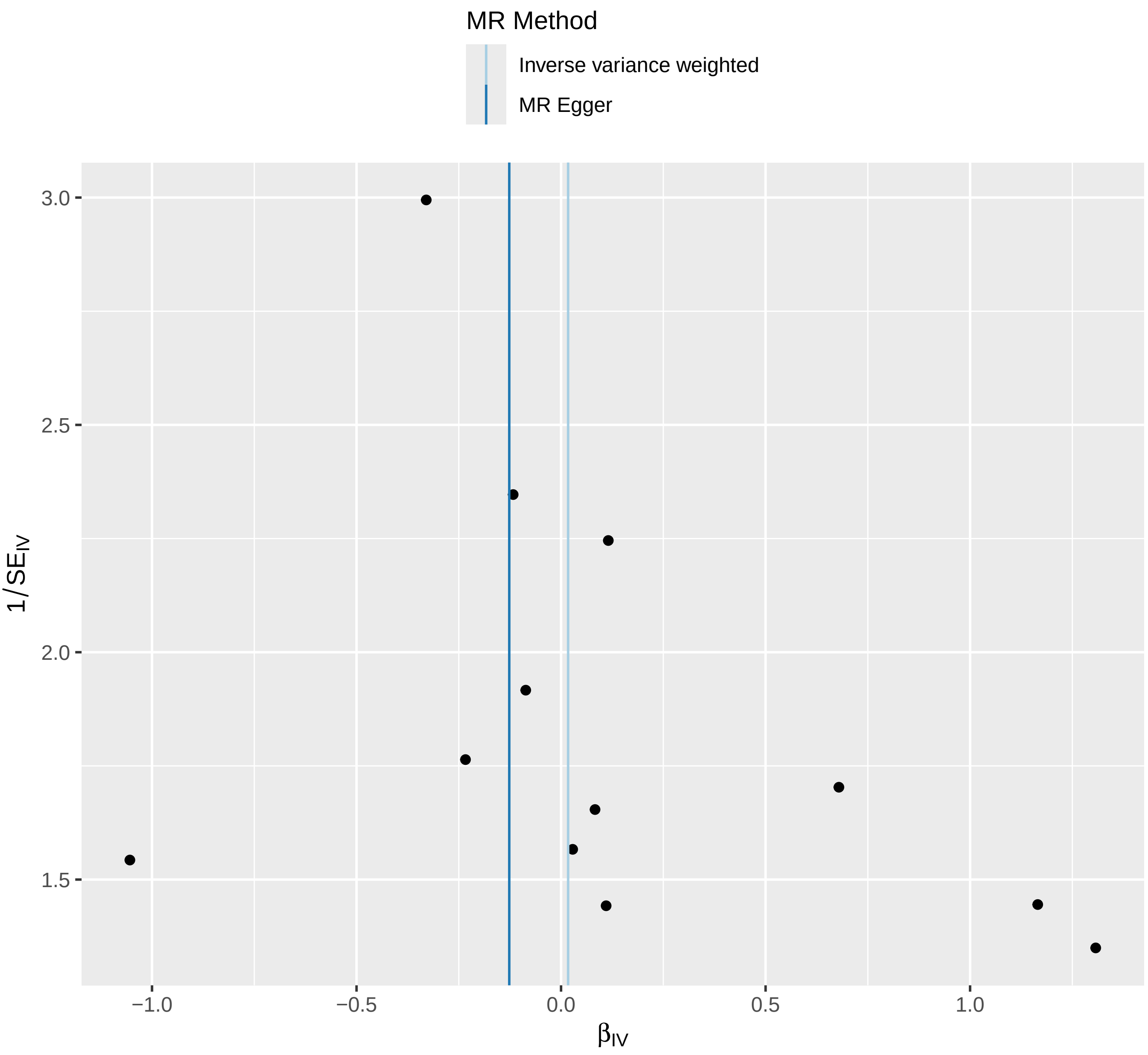
MR Method
Inverse variance weighted
MR Egger
3.0
2.5
2.0
1.5
$1/SE_{IV}$
−1.0
−0.5
0.0
0.5
1.0
β_{IV}

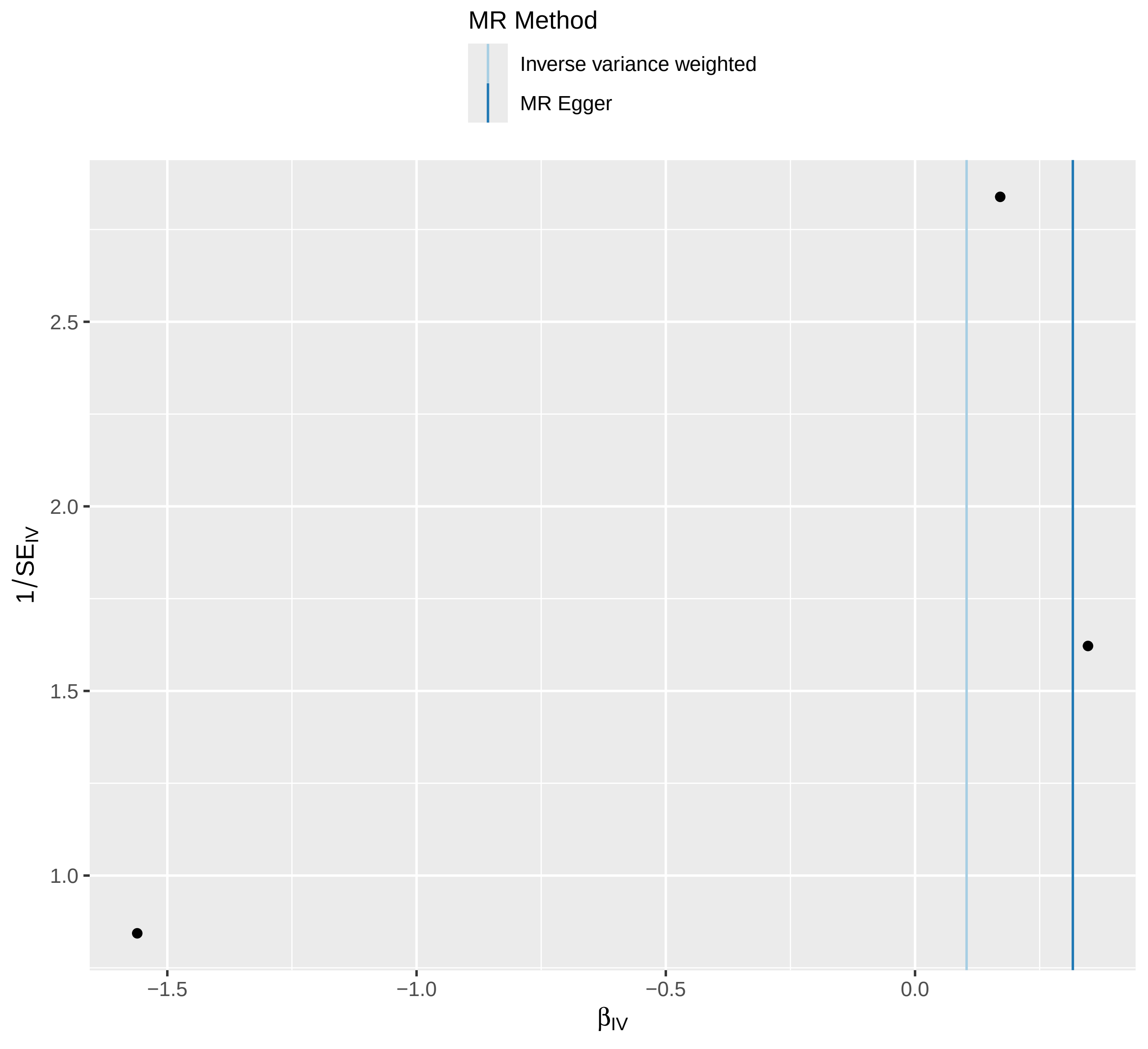
MR Method
Inverse variance weighted
MR Egger
1/SE_IV
β_IV
2.5
2.0
1.5
1.0
−1.5
−1.0
−0.5
0.0

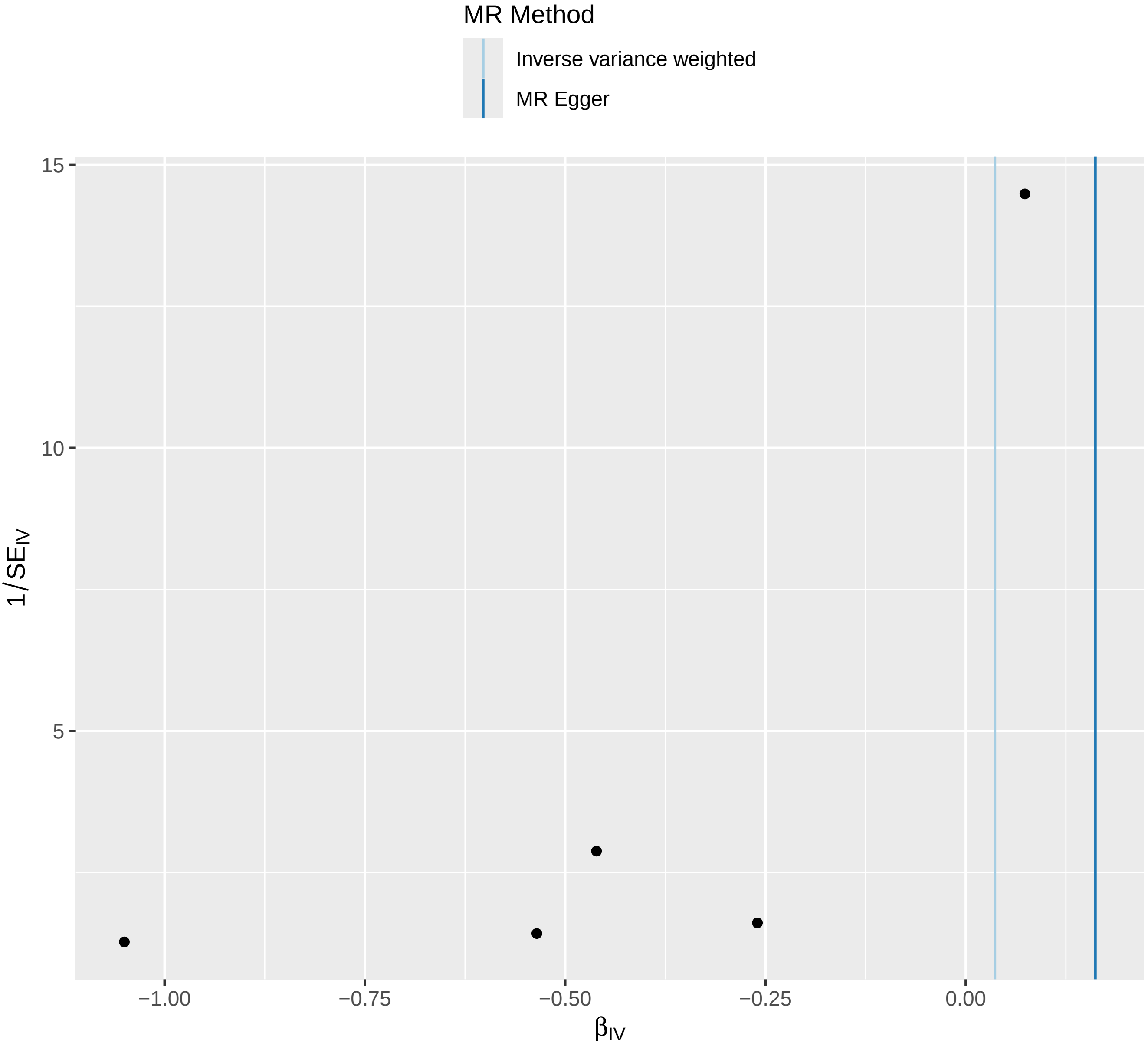

MR Method
Inverse variance weighted
MR Egger
15
10
5
1/SE_IV
−1.00
−0.75
−0.50
−0.25
0.00
β_IV

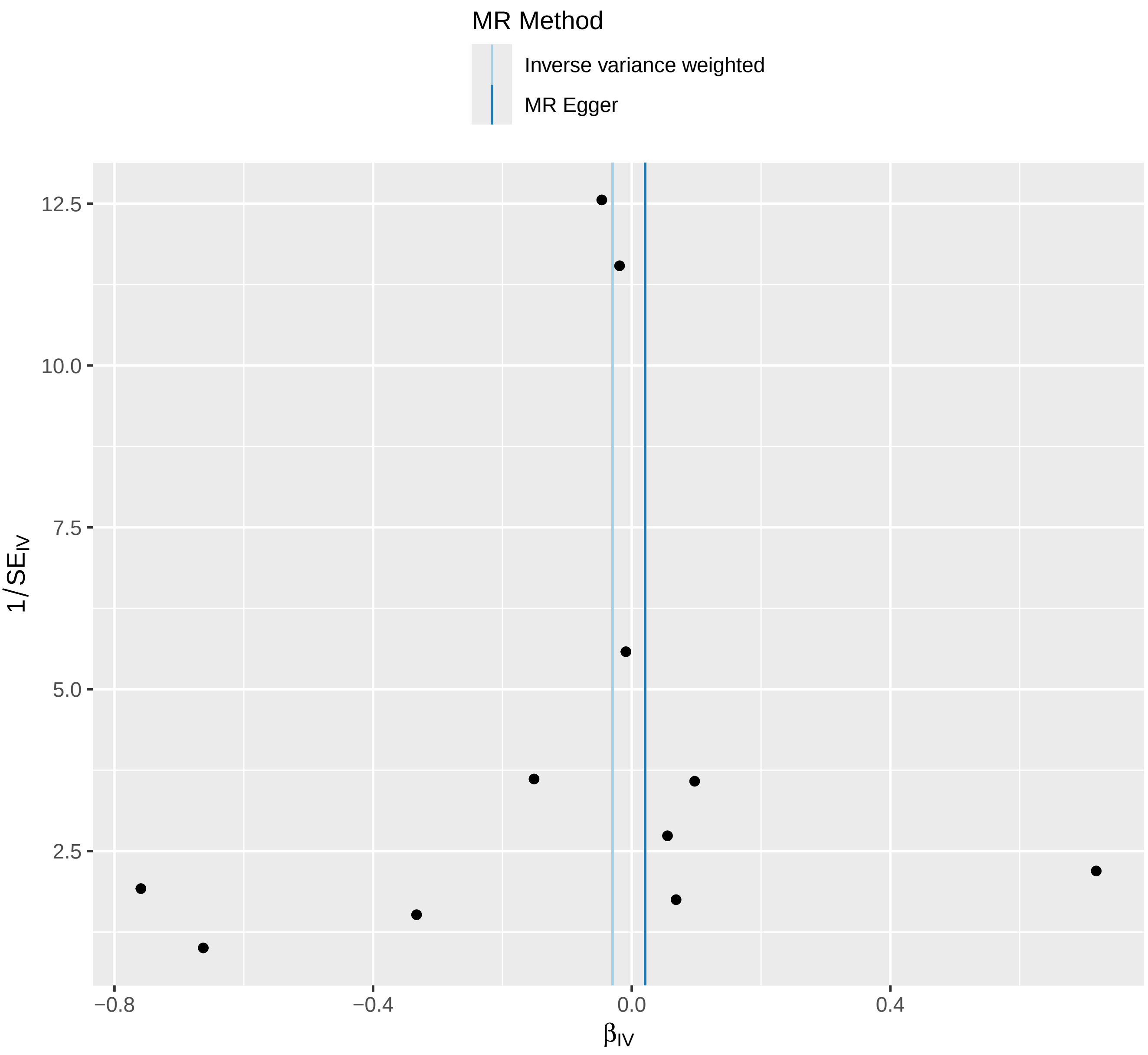

MR Method
Inverse variance weighted
MR Egger
12.5
10.0
7.5
5.0
2.5
1/SE_IV
−0.8
−0.4
0.0
0.4
β_IV

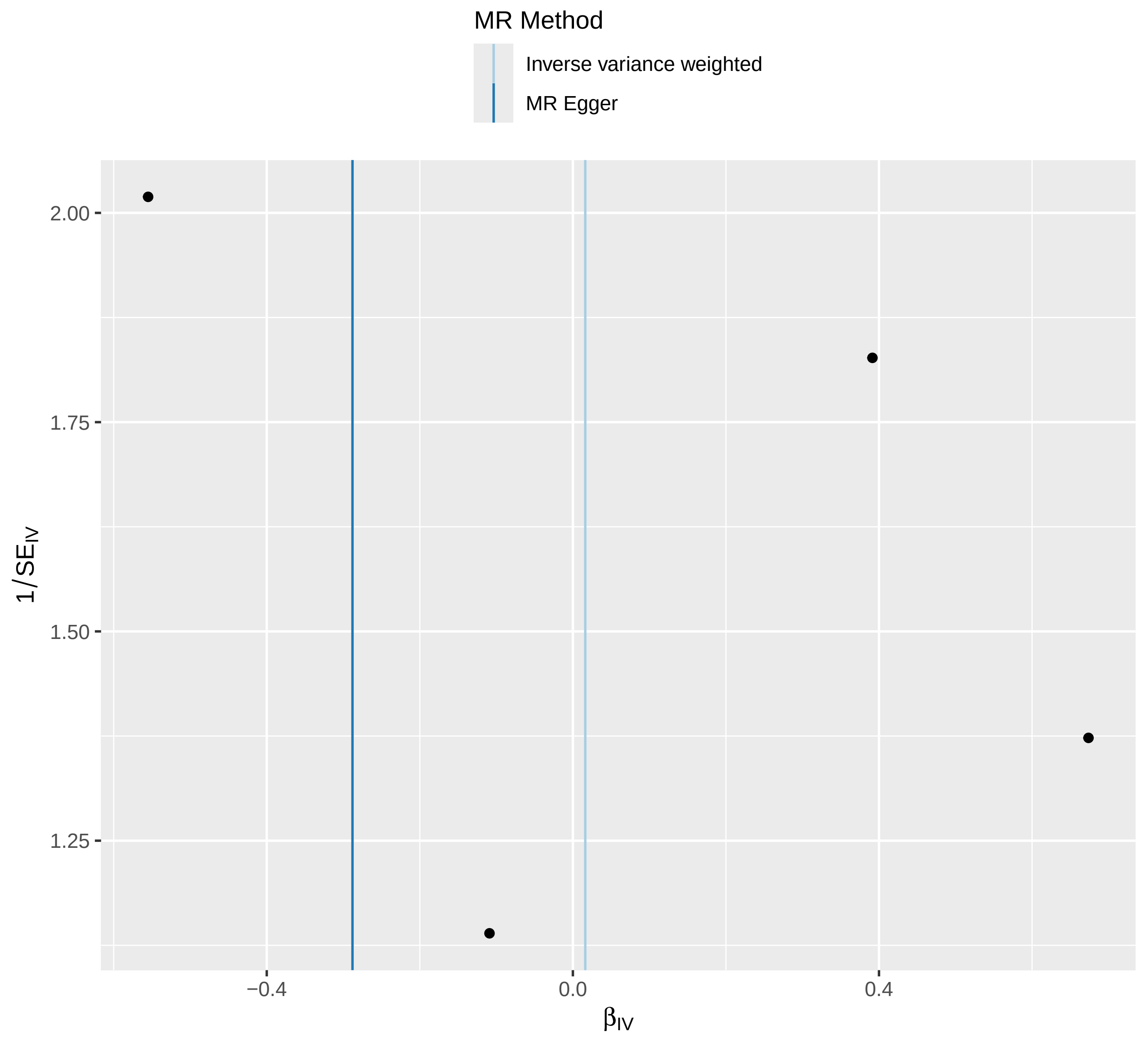
MR Method
Inverse variance weighted
MR Egger
2.00
1.75
1.50
1.25
1/SE_IV
−0.4
0.0
0.4
β_IV

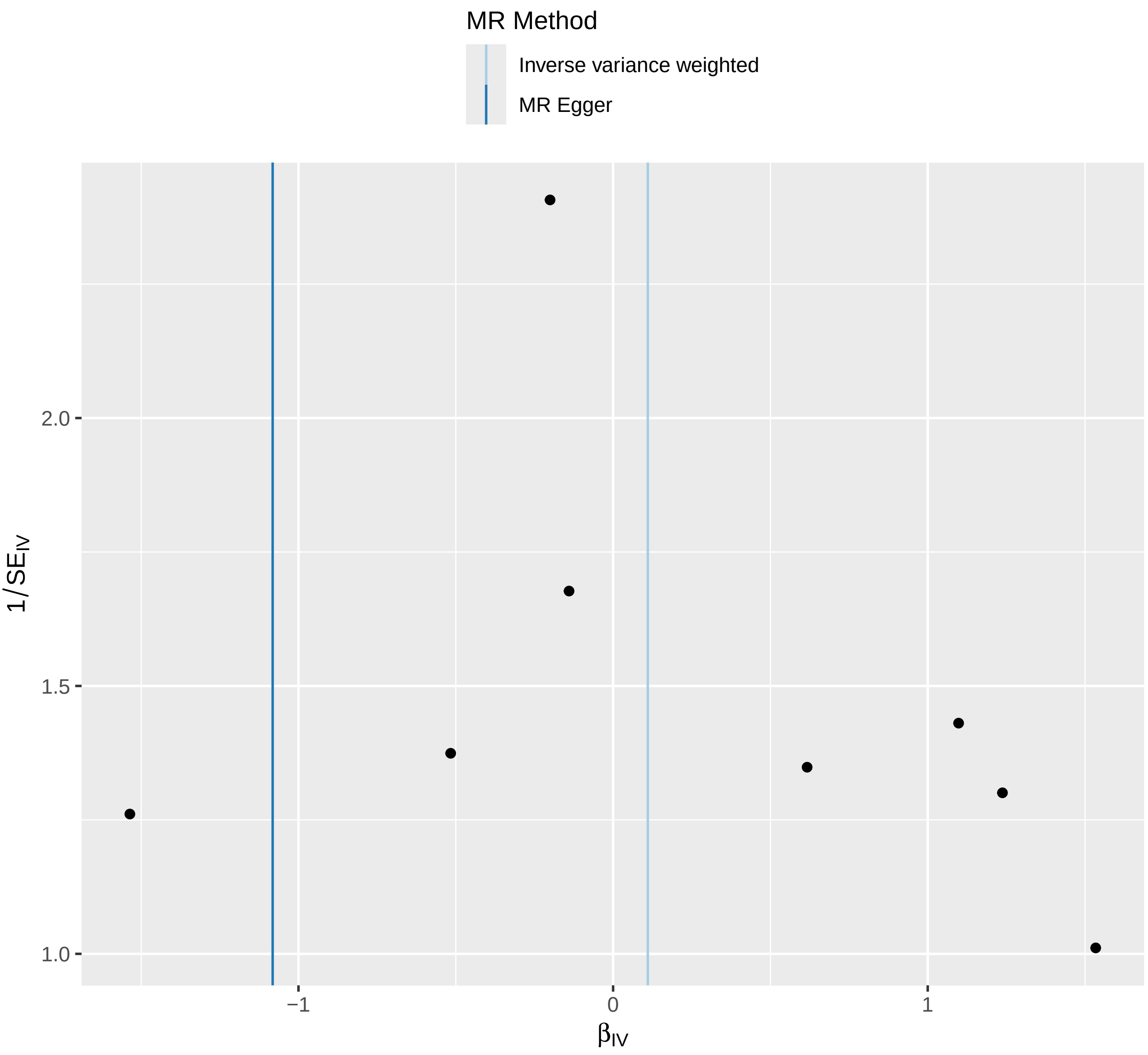
MR Method
Inverse variance weighted
MR Egger
2.0
1.5
1.0
1/SE_IV
−1
0
1
β_IV

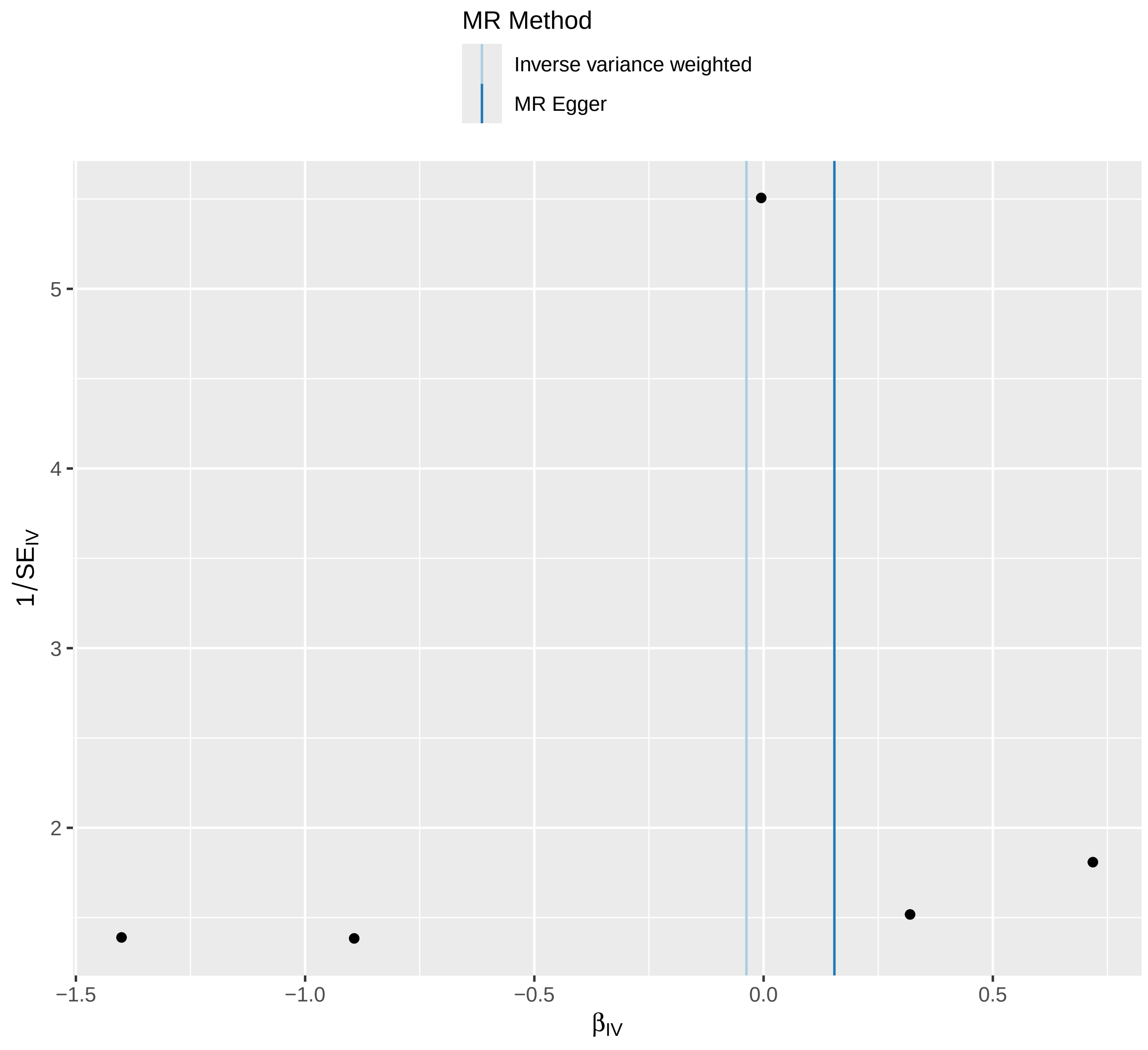
MR Method
Inverse variance weighted
MR Egger
1/SE_IV
5
4
3
2
−1.5
−1.0
−0.5
0.0
0.5
β_IV

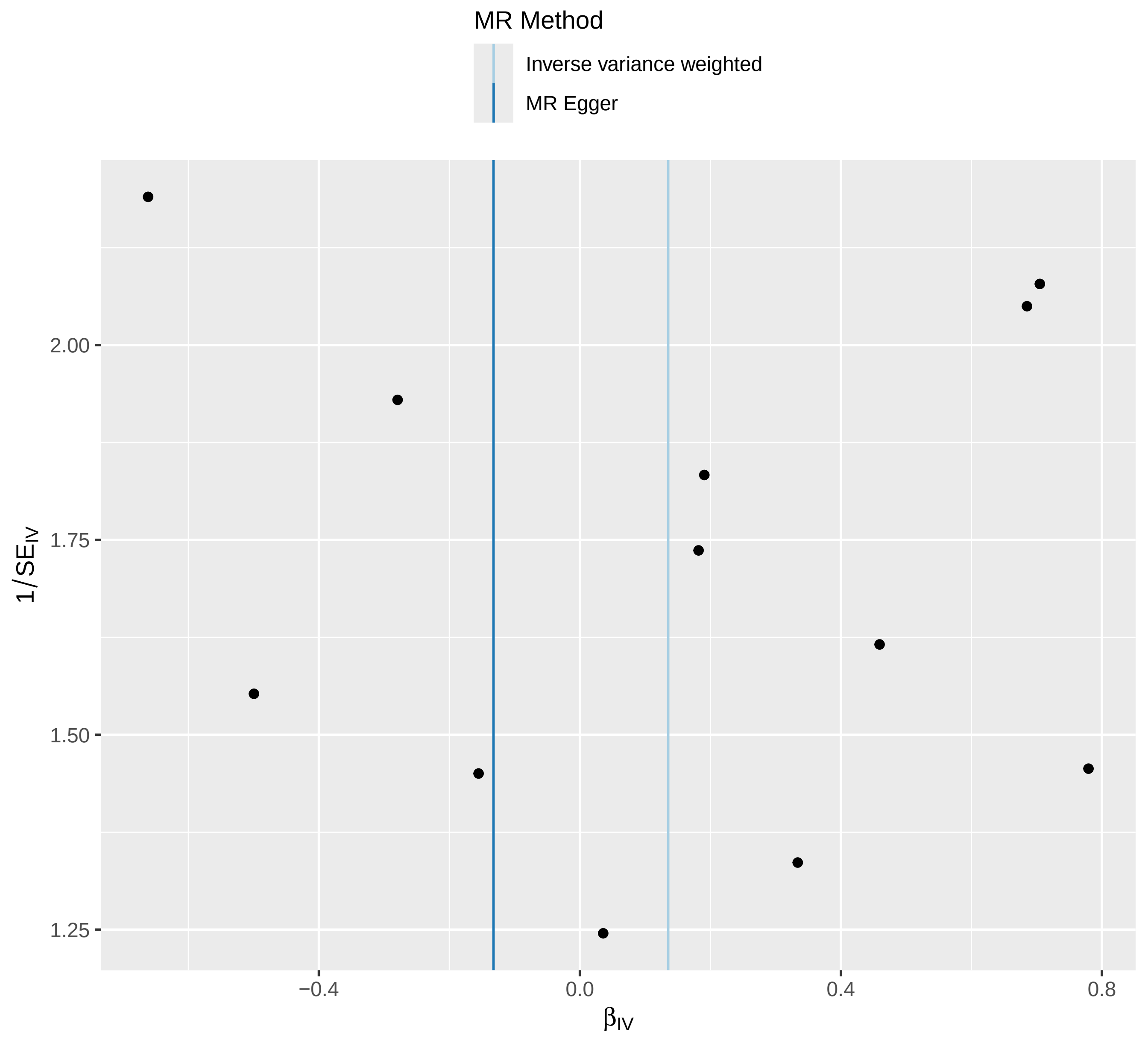
MR Method
Inverse variance weighted
MR Egger
2.00
1.75
1.50
1.25
1/SE_IV
−0.4
0.0
0.4
0.8
β_IV

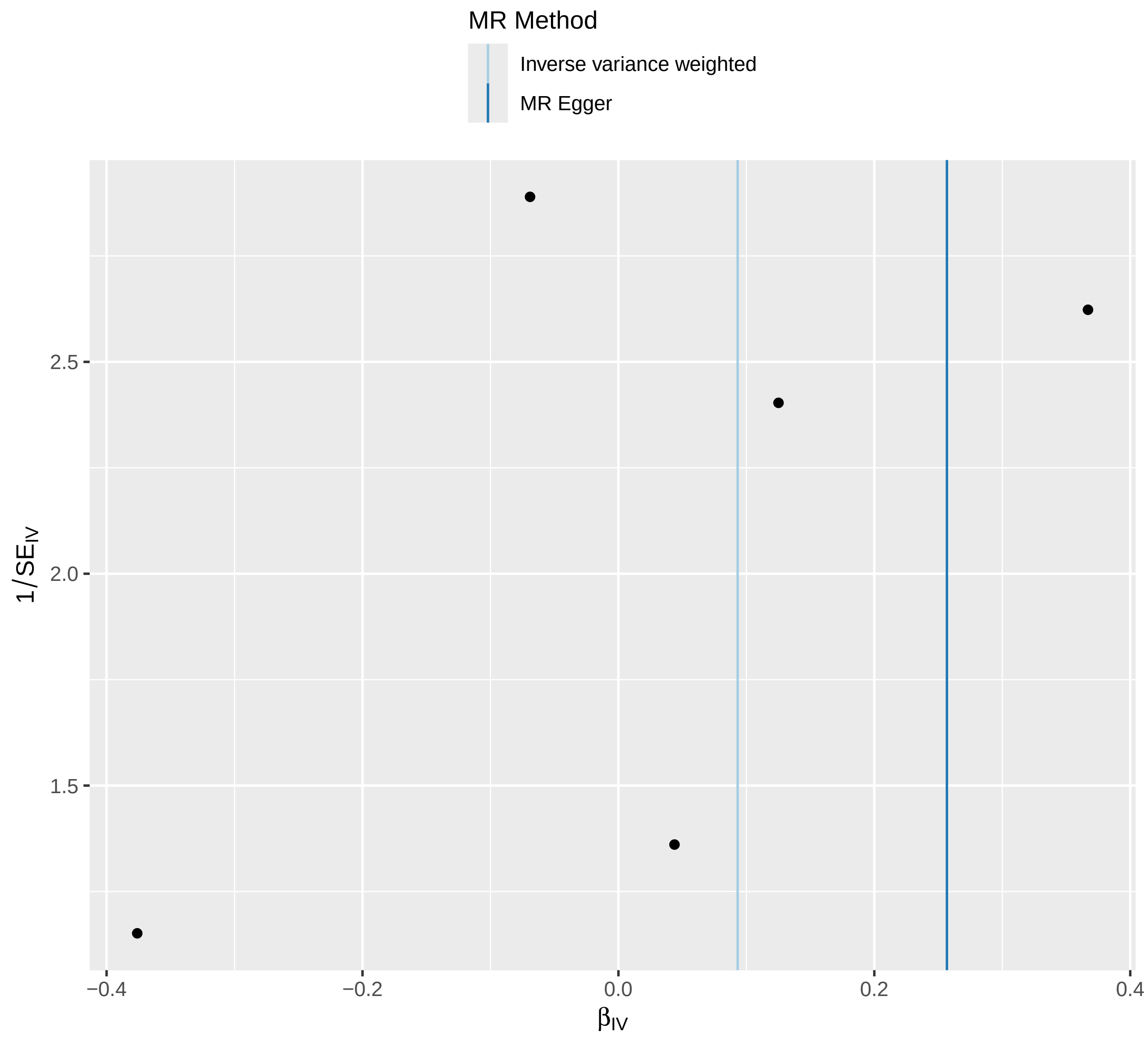
MR Method
Inverse variance weighted
MR Egger
2.5
2.0
1.5
1/SE_IV
−0.4
−0.2
0.0
0.2
0.4
β_IV

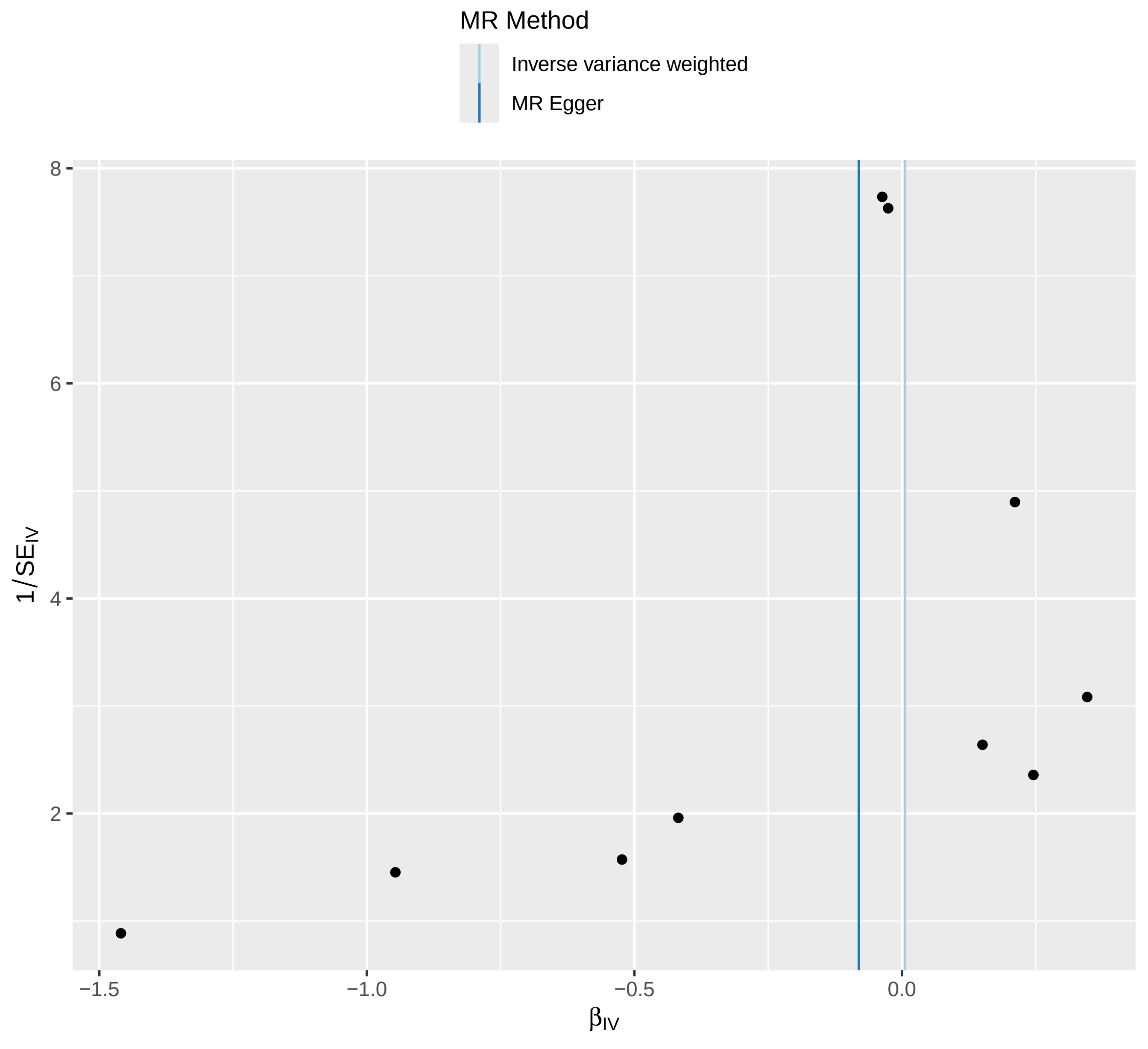

MR Method
Inverse variance weighted
MR Egger
8
6
4
2
1/SE_IV
−1.5
−1.0
−0.5
0.0
β_IV

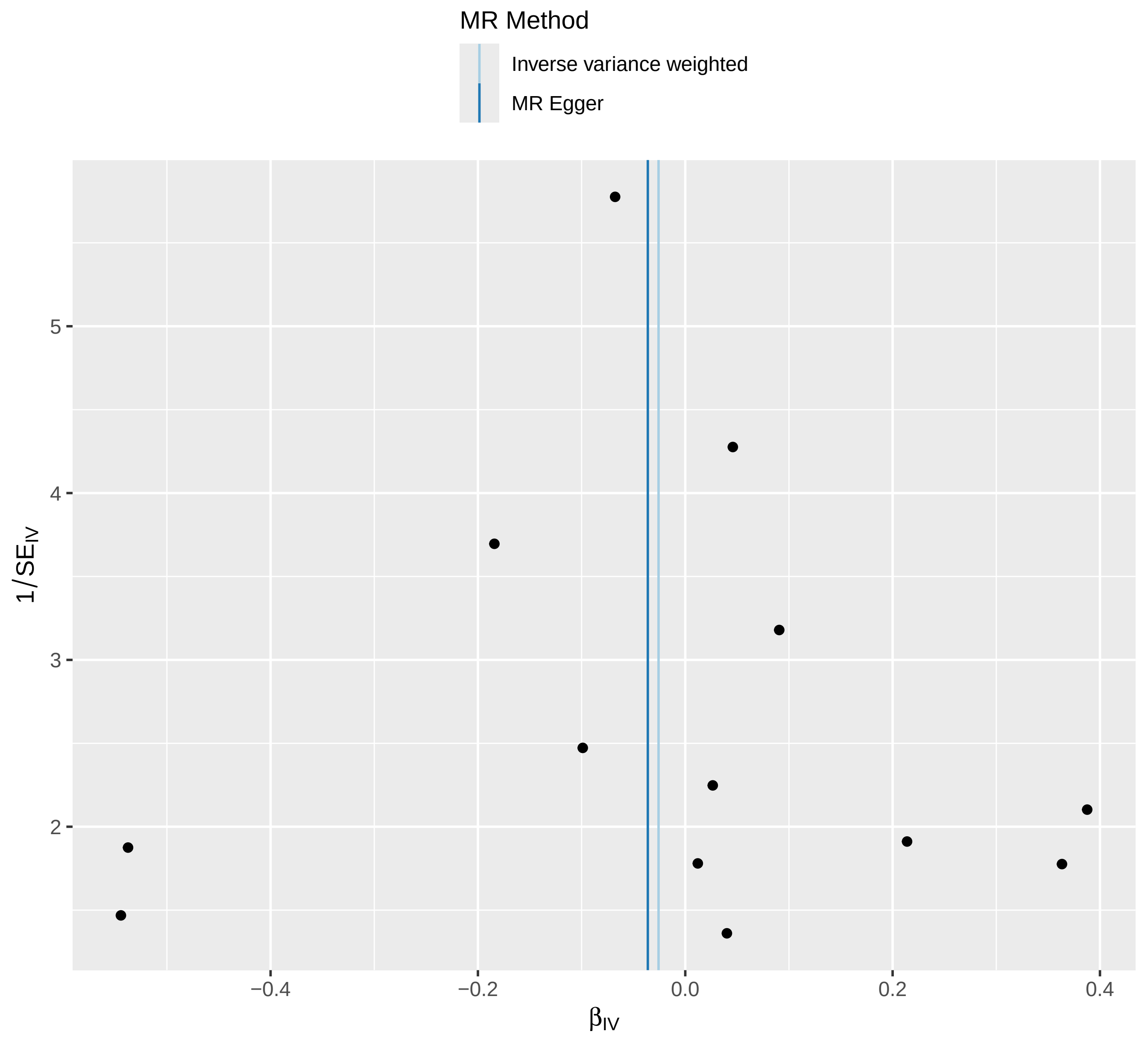
MR Method
Inverse variance weighted
MR Egger
5
4
3
2
1/SE_IV
−0.4
−0.2
0.0
0.2
0.4
β_IV

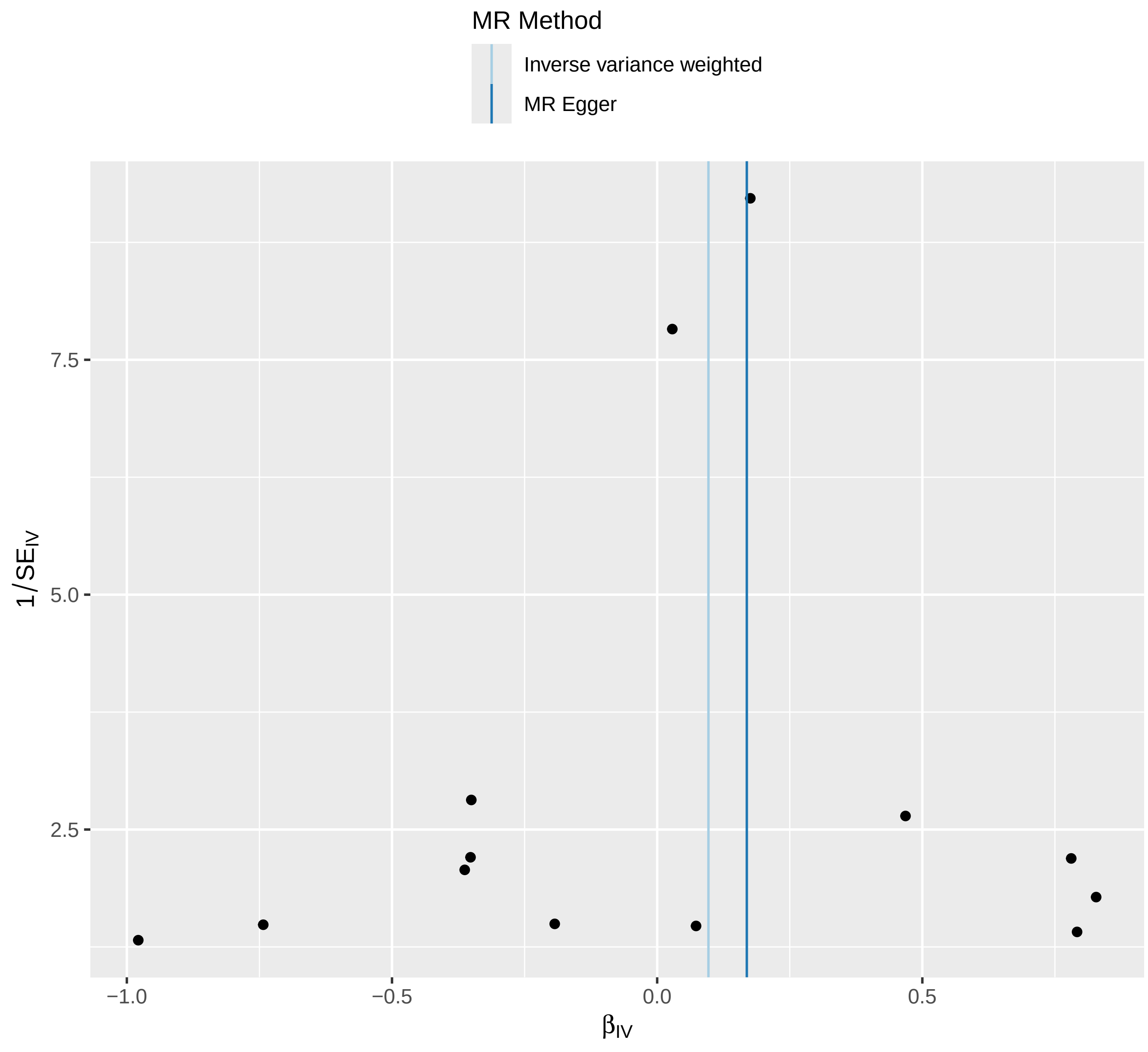

MR Method
Inverse variance weighted
MR Egger
7.5
5.0
2.5
−1.0
−0.5
0.0
0.5
$1/SE_{IV}$
β_{IV}

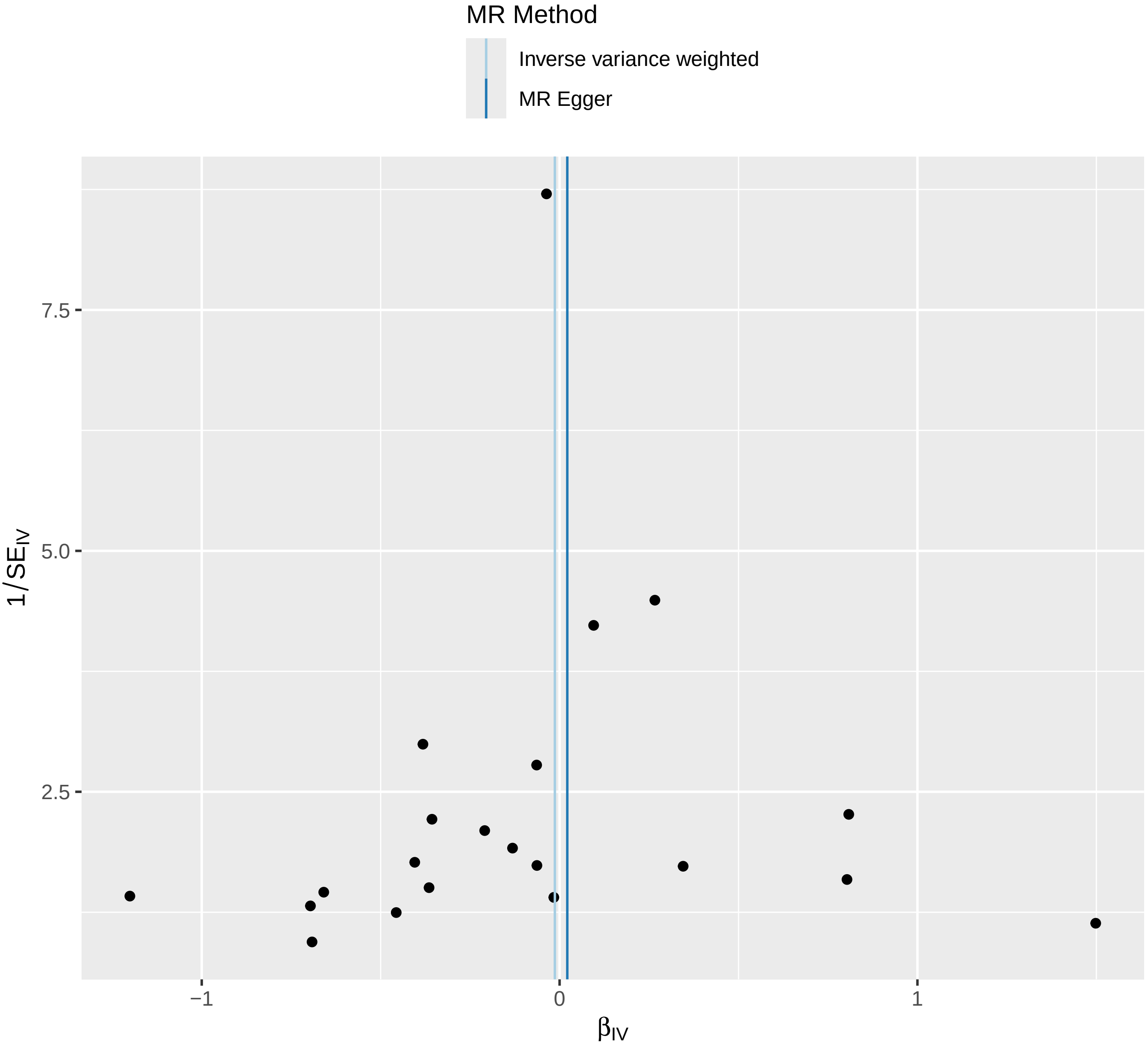

MR Method
Inverse variance weighted
MR Egger
7.5
5.0
2.5
1/SE$_{IV}$
−1
0
1
β$_{IV}$

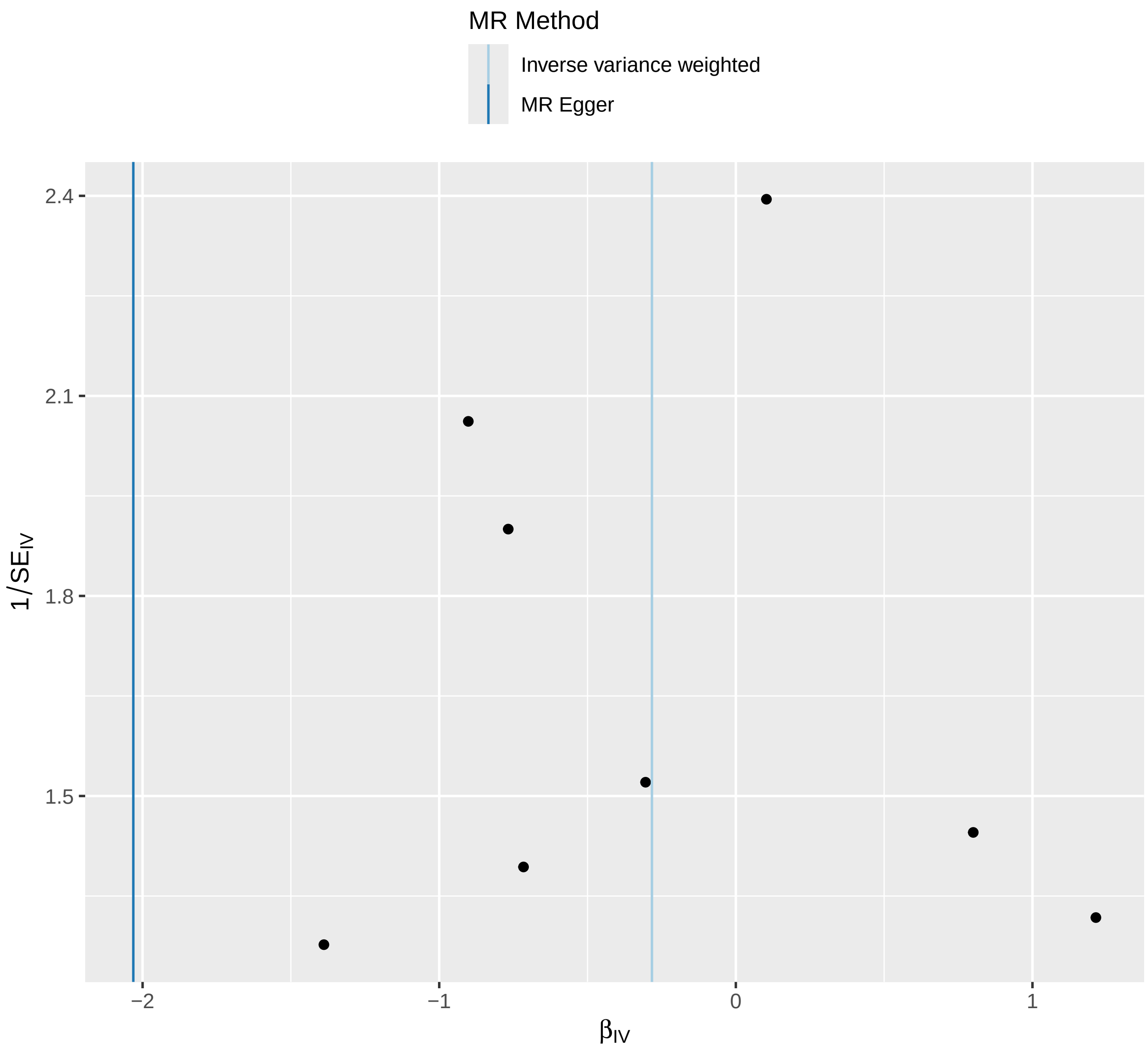
MR Method
Inverse variance weighted
MR Egger
2.4
2.1
1.8
1.5
−2
−1
0
1
1/SE$_{IV}$
β$_{IV}$

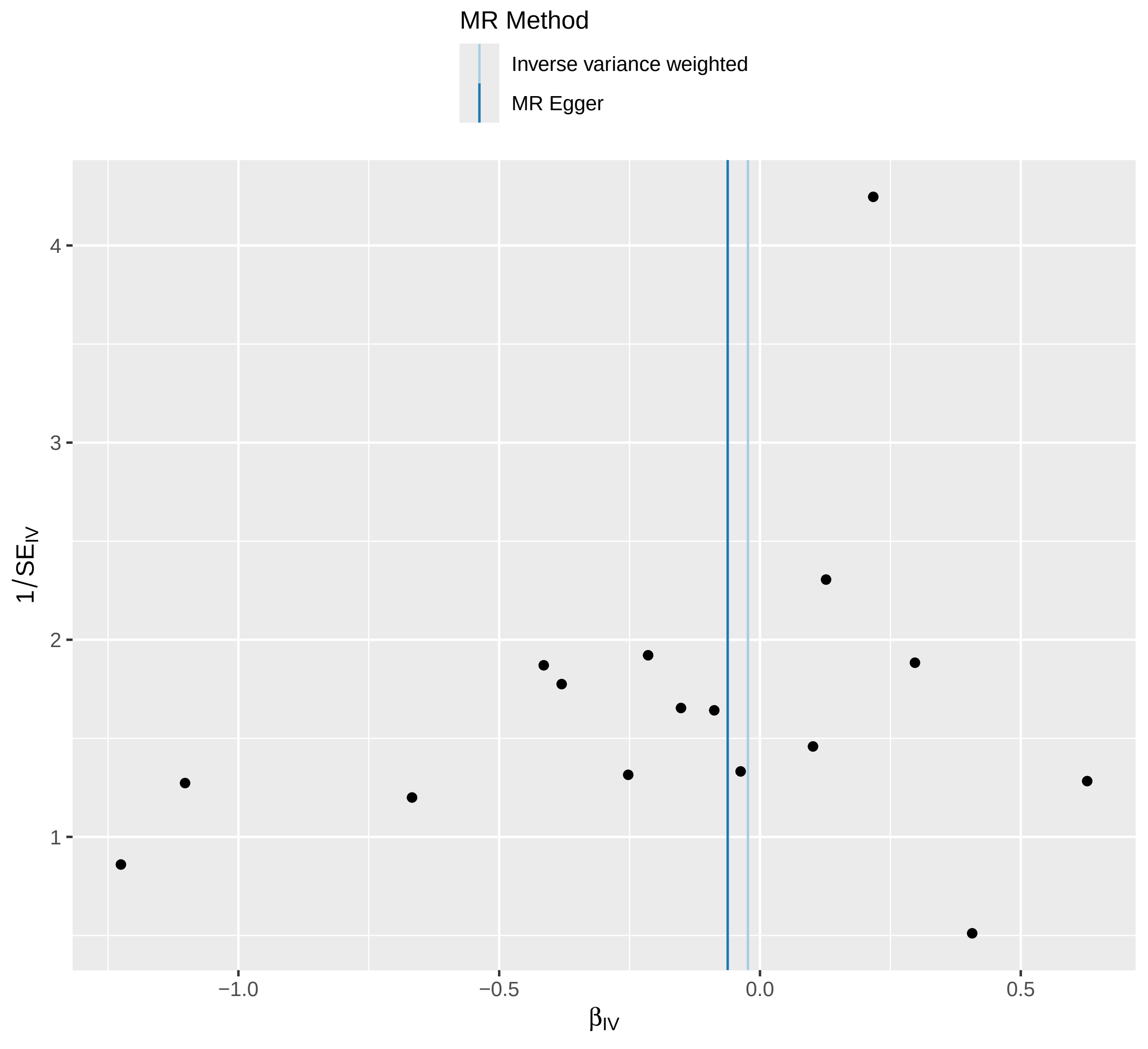
MR Method
Inverse variance weighted
MR Egger
1/SE$_{IV}$
4
3
2
1
−1.0
−0.5
0.0
0.5
β$_{IV}$

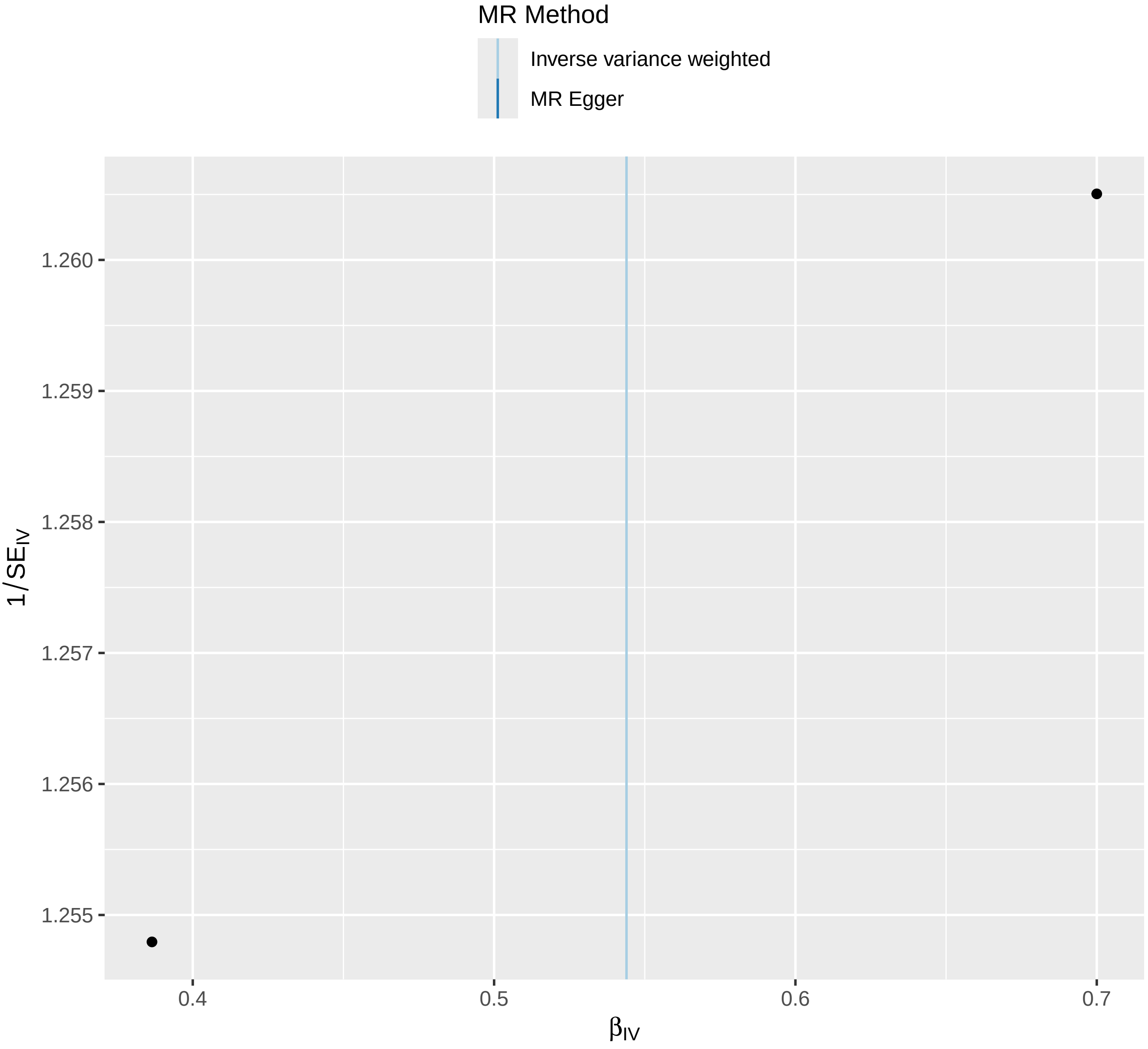
MR Method
Inverse variance weighted
MR Egger
1.260
1.259
1.258
1.257
1.256
1.255
$1/SE_{IV}$
0.4
0.5
0.6
0.7
β_{IV}

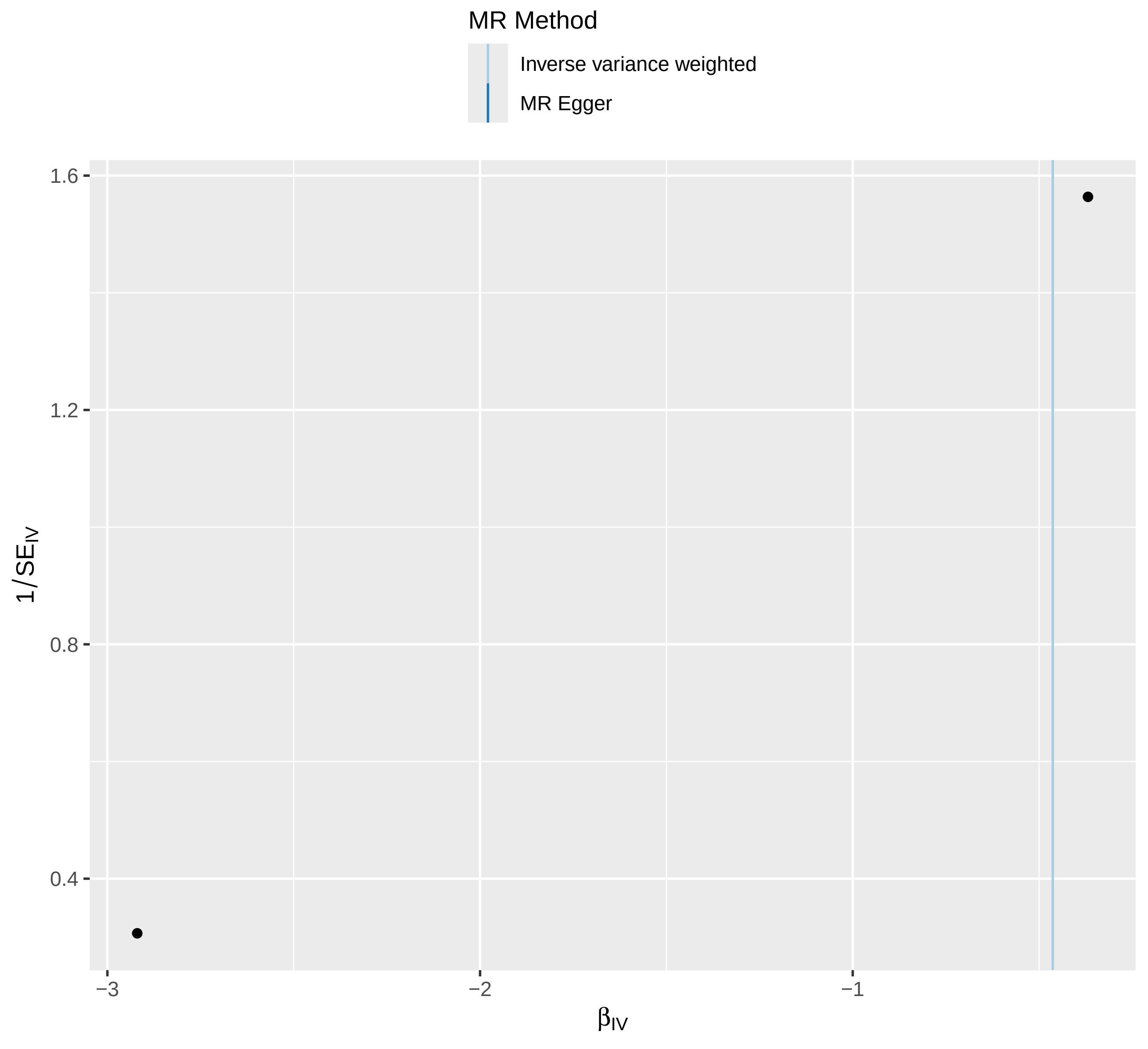
MR Method
Inverse variance weighted
MR Egger
1.6
1.2
0.8
0.4
1/SE_IV
−3
−2
−1
β_IV

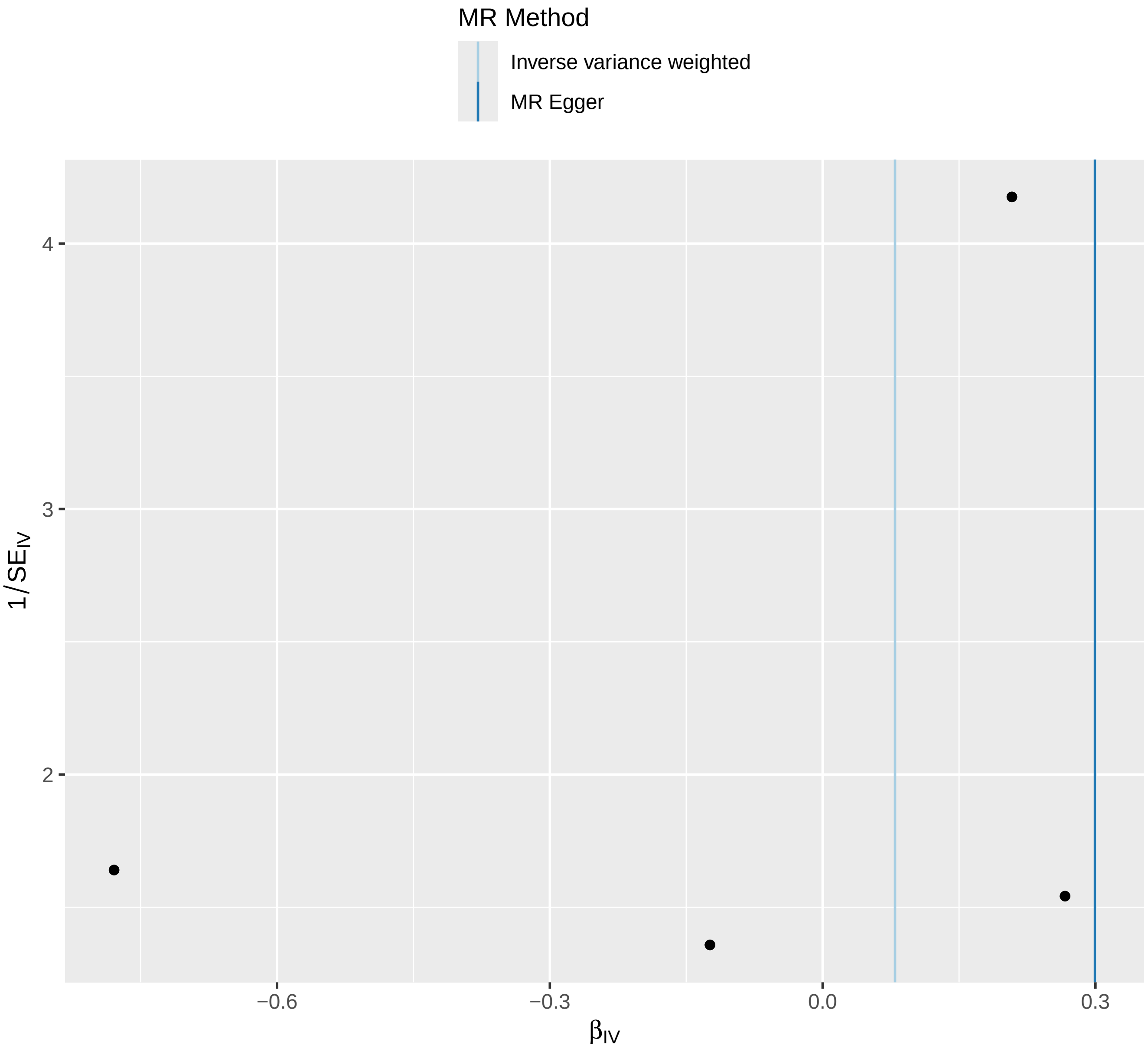

MR Method
Inverse variance weighted
MR Egger
4
3
2
1/SE$_{IV}$
−0.6
−0.3
0.0
0.3
β$_{IV}$

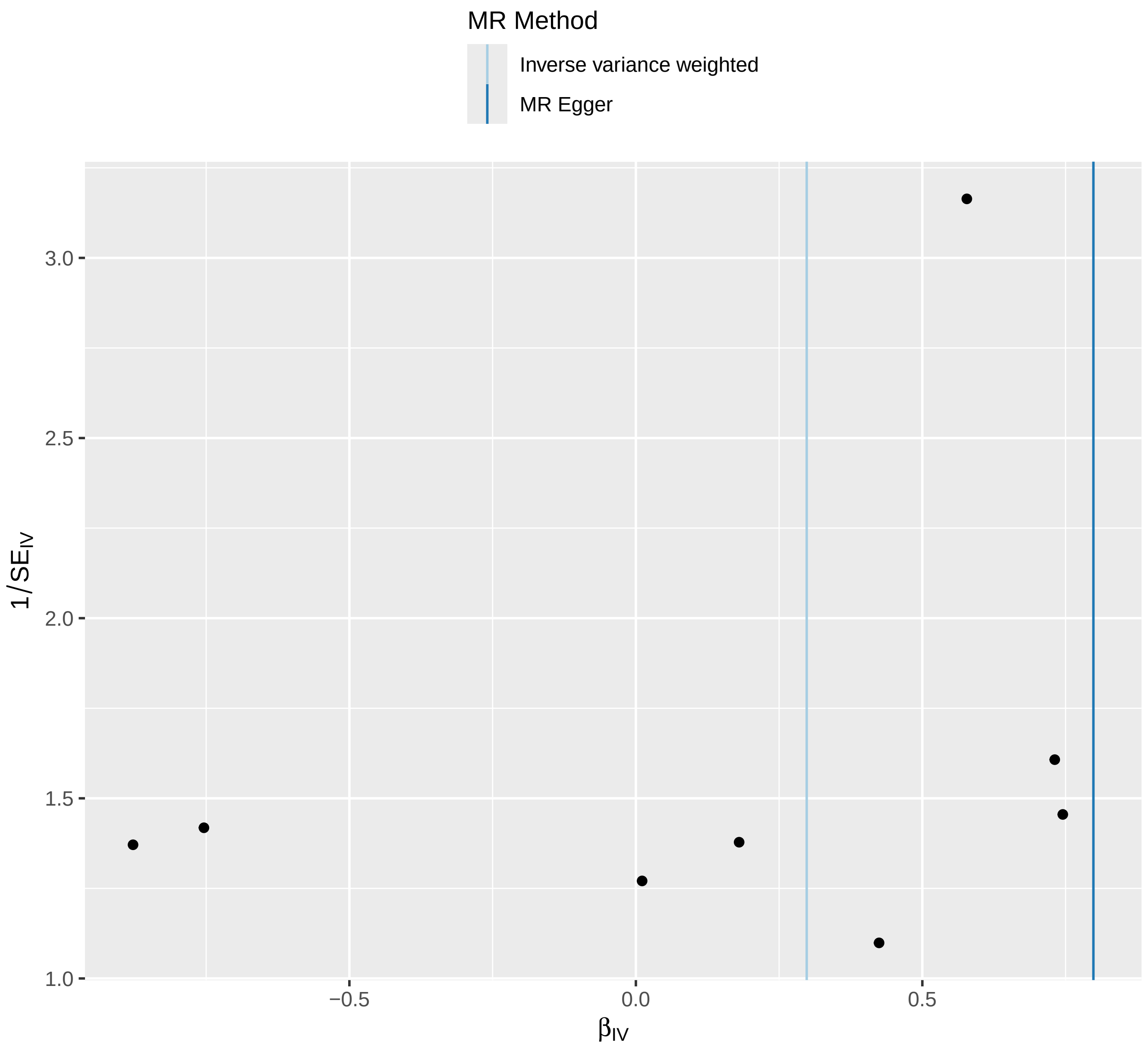

MR Method
Inverse variance weighted
MR Egger
3.0
2.5
2.0
1.5
1.0
1/SE_IV
−0.5
0.0
0.5
β_IV

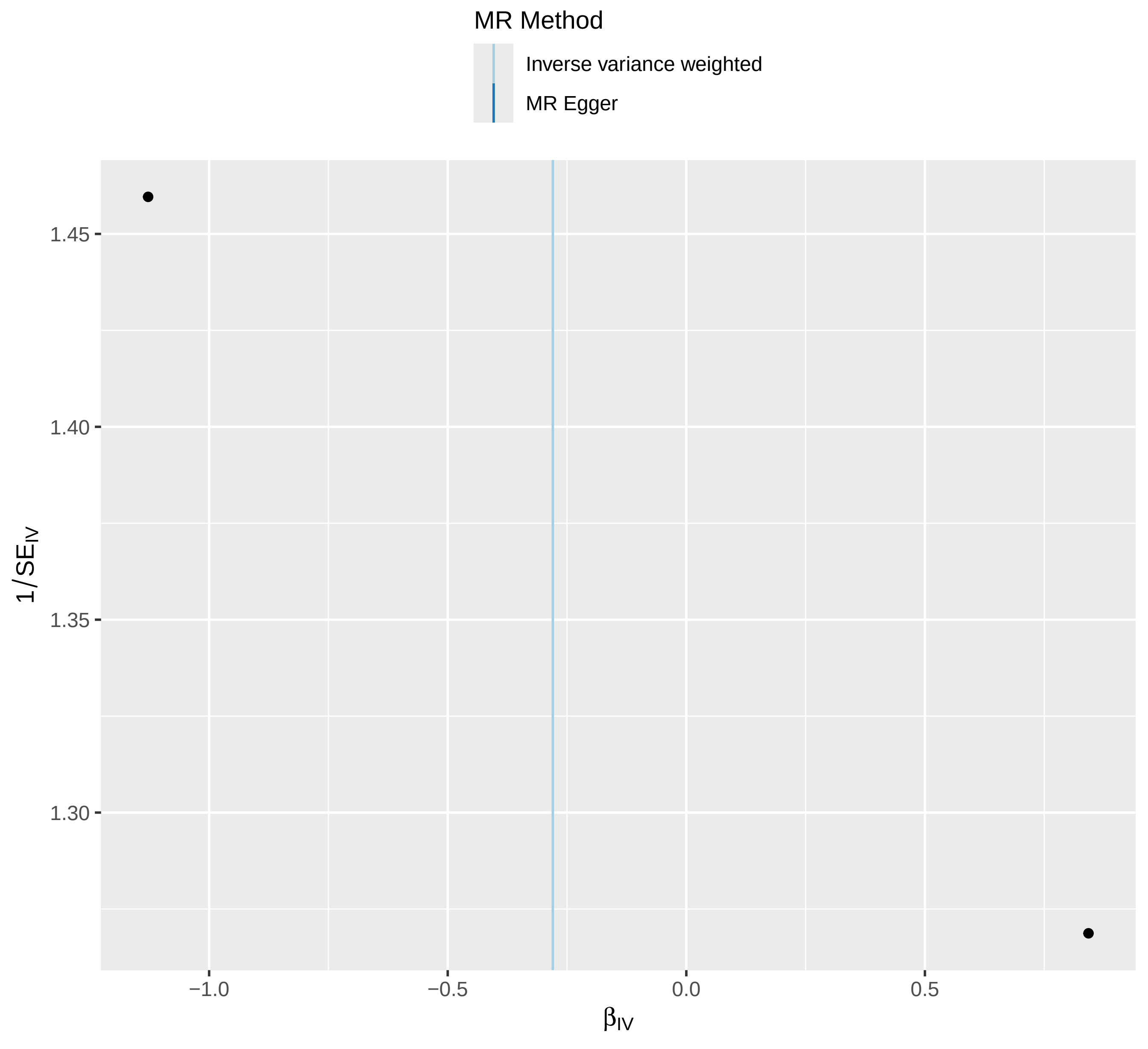
MR Method
Inverse variance weighted
MR Egger
1.45
1.40
1.35
1.30
1/SE_IV
−1.0
−0.5
0.0
0.5
β_IV

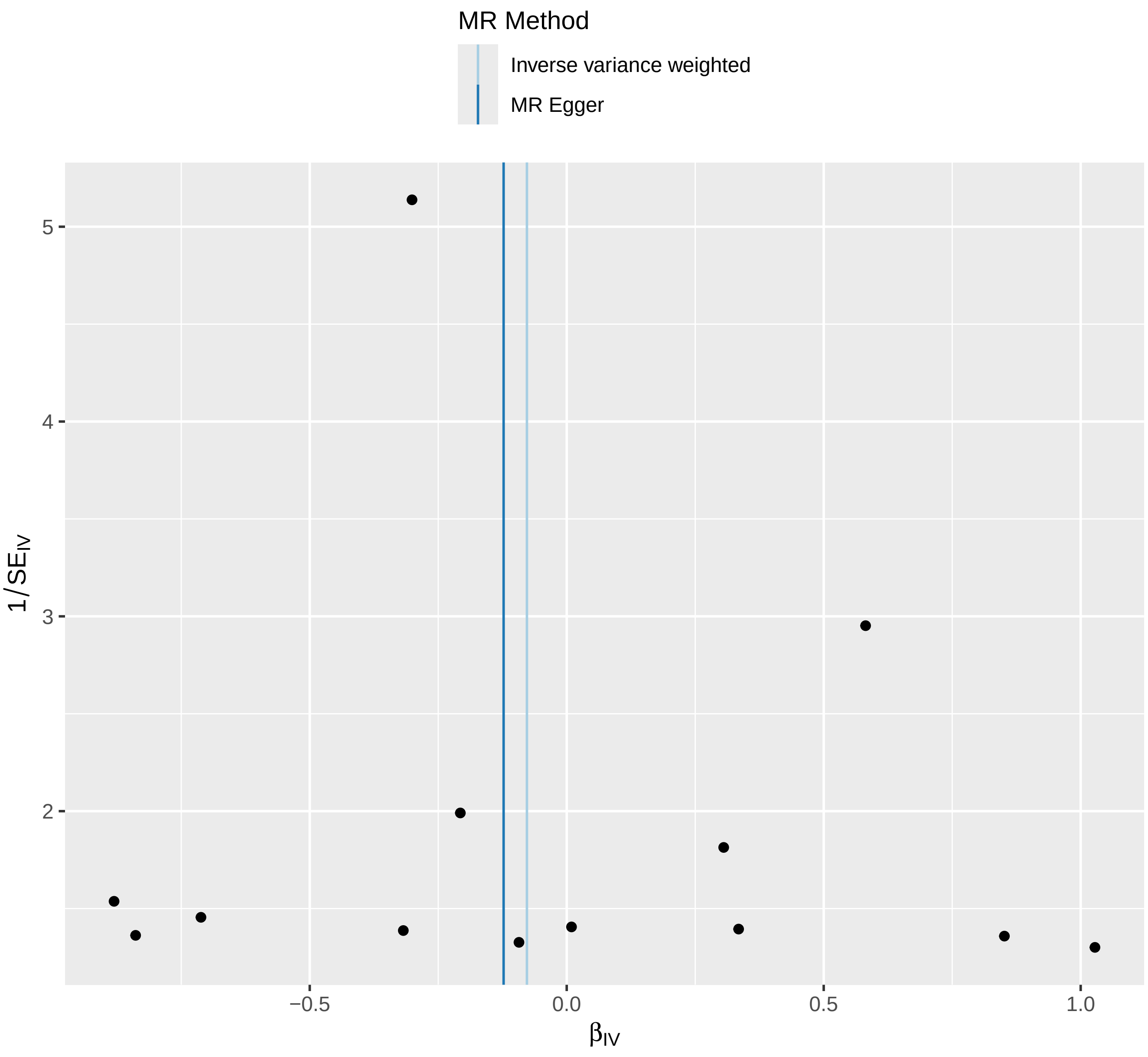

MR Method
Inverse variance weighted
MR Egger
5
4
3
2
1/SE_IV
−0.5
0.0
0.5
1.0
β_IV

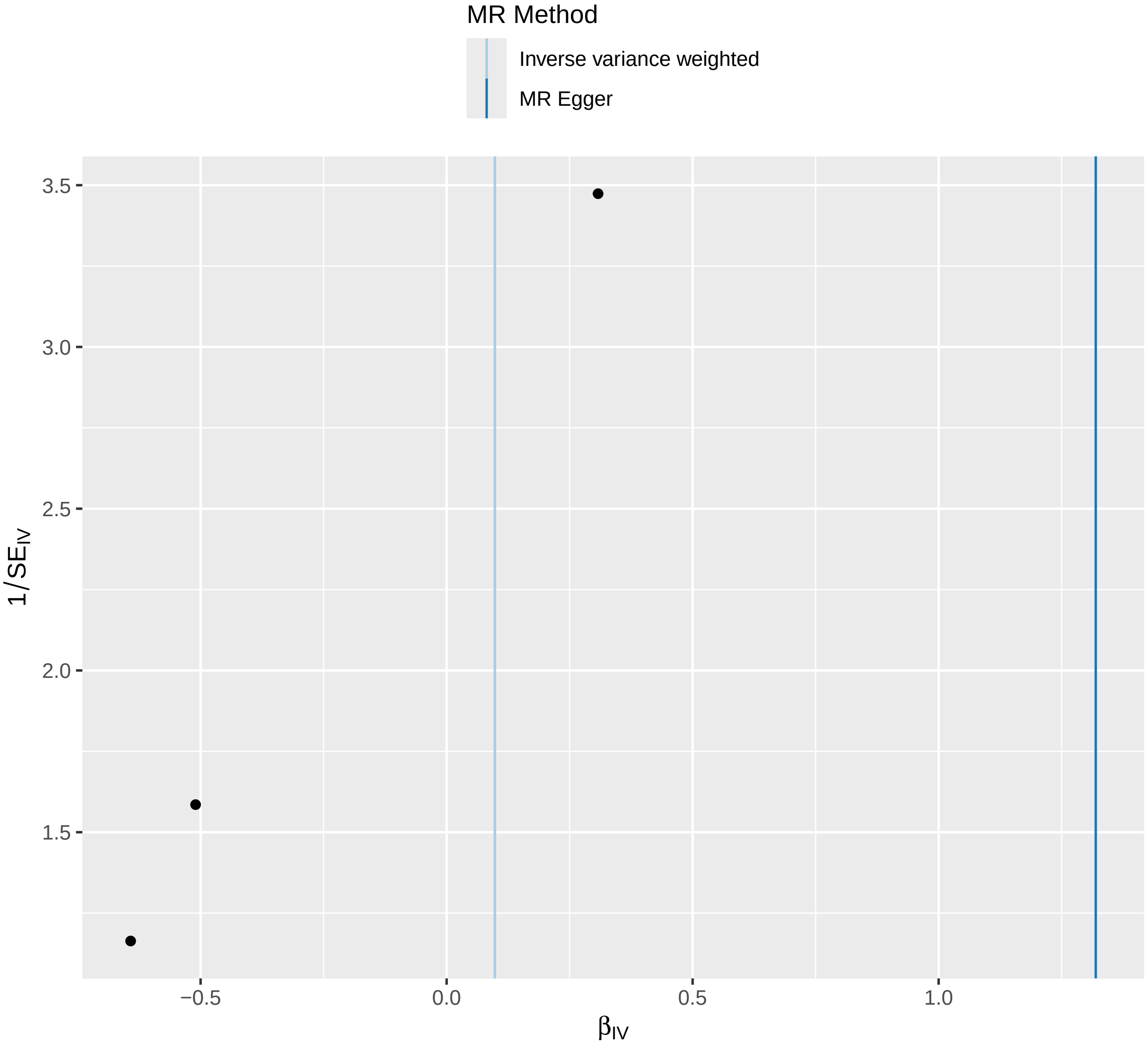
MR Method
Inverse variance weighted
MR Egger
3.5
3.0
2.5
2.0
1.5
1/SE$_{IV}$
−0.5
0.0
0.5
1.0
β$_{IV}$

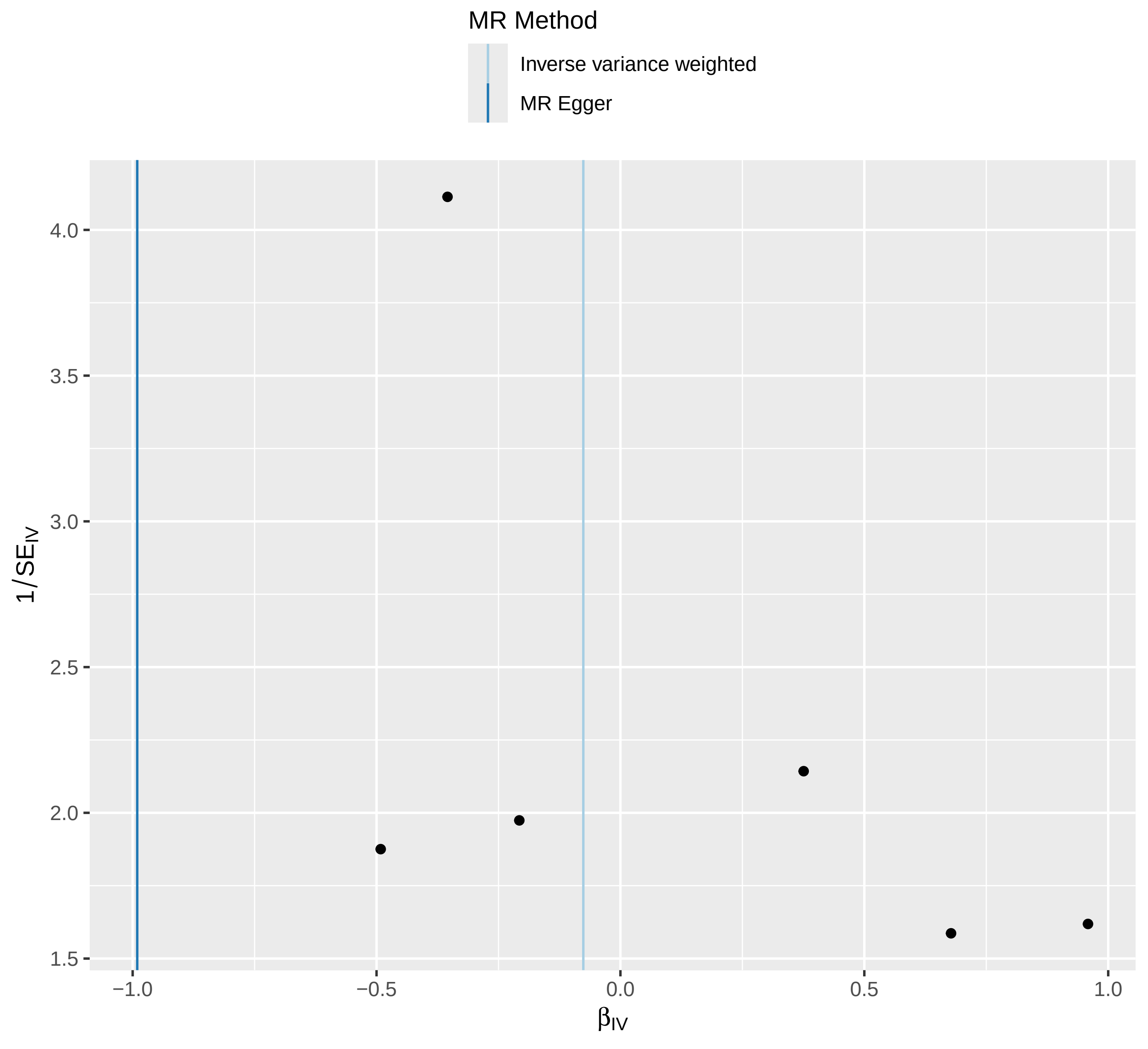

MR Method
Inverse variance weighted
MR Egger
4.0
3.5
3.0
2.5
2.0
1.5
1/SE$_{IV}$
−1.0
−0.5
0.0
0.5
1.0
β_{IV}

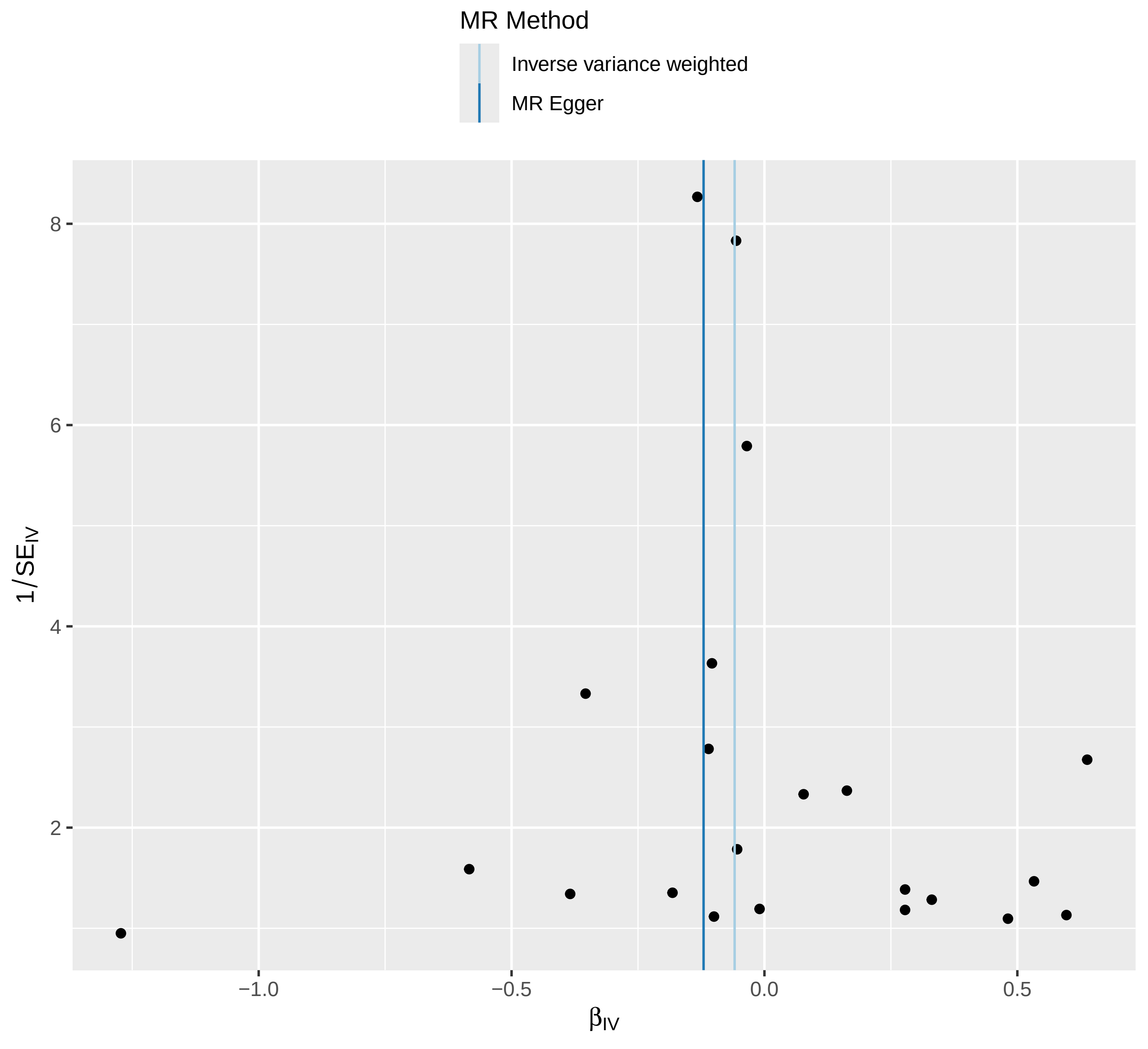
MR Method
Inverse variance weighted
MR Egger
8
6
4
2
1/SE$_{IV}$
−1.0
−0.5
0.0
0.5
β$_{IV}$

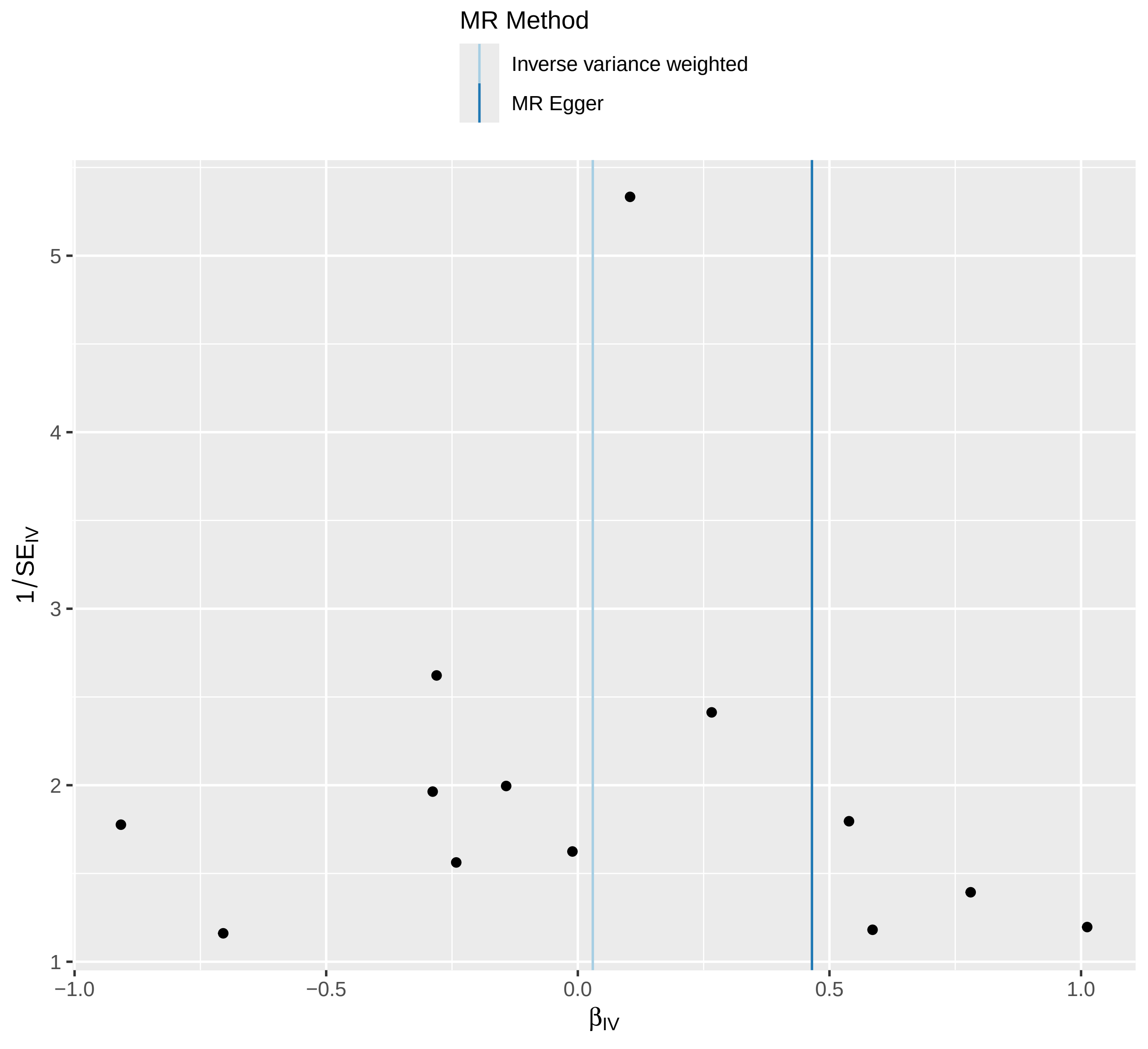
MR Method
Inverse variance weighted
MR Egger
1/SE_IV
5
4
3
2
1
−1.0
−0.5
0.0
0.5
1.0
β_IV

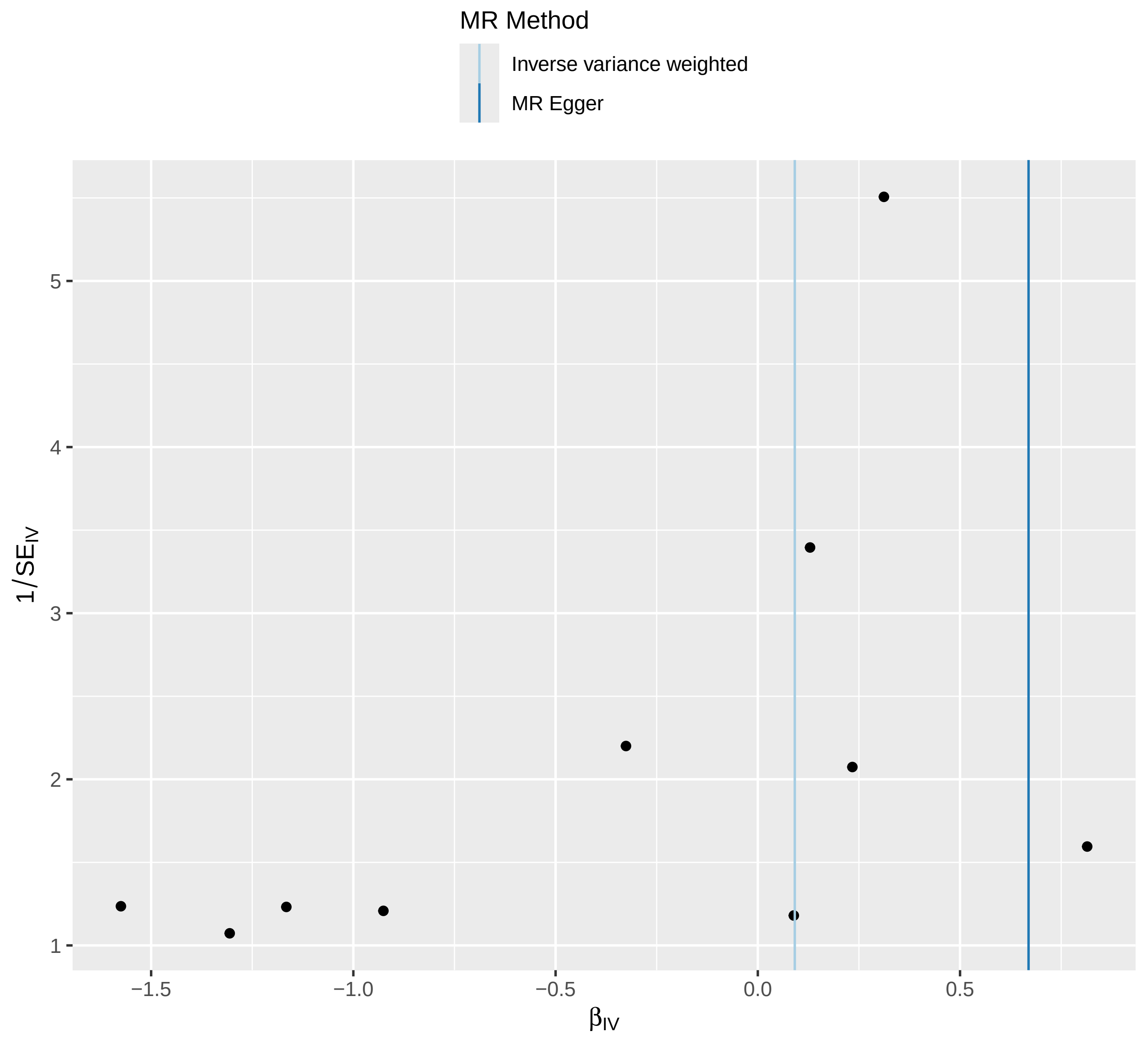
MR Method
Inverse variance weighted
MR Egger
1/SE_IV
5
4
3
2
1
−1.5
−1.0
−0.5
0.0
0.5
β_IV

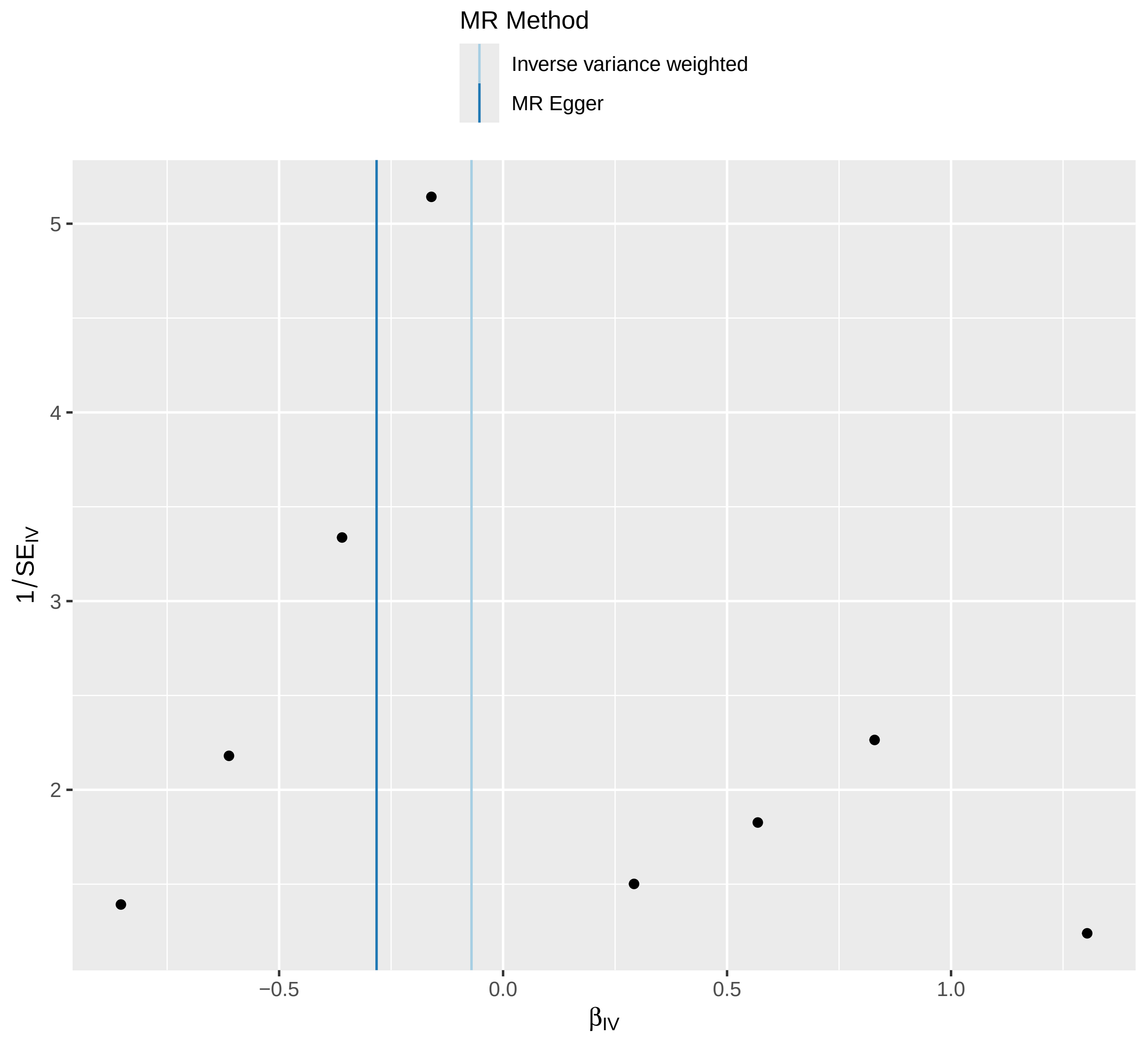

MR Method
Inverse variance weighted
MR Egger
5
4
3
2
1/SE_IV
−0.5
0.0
0.5
1.0
β_IV

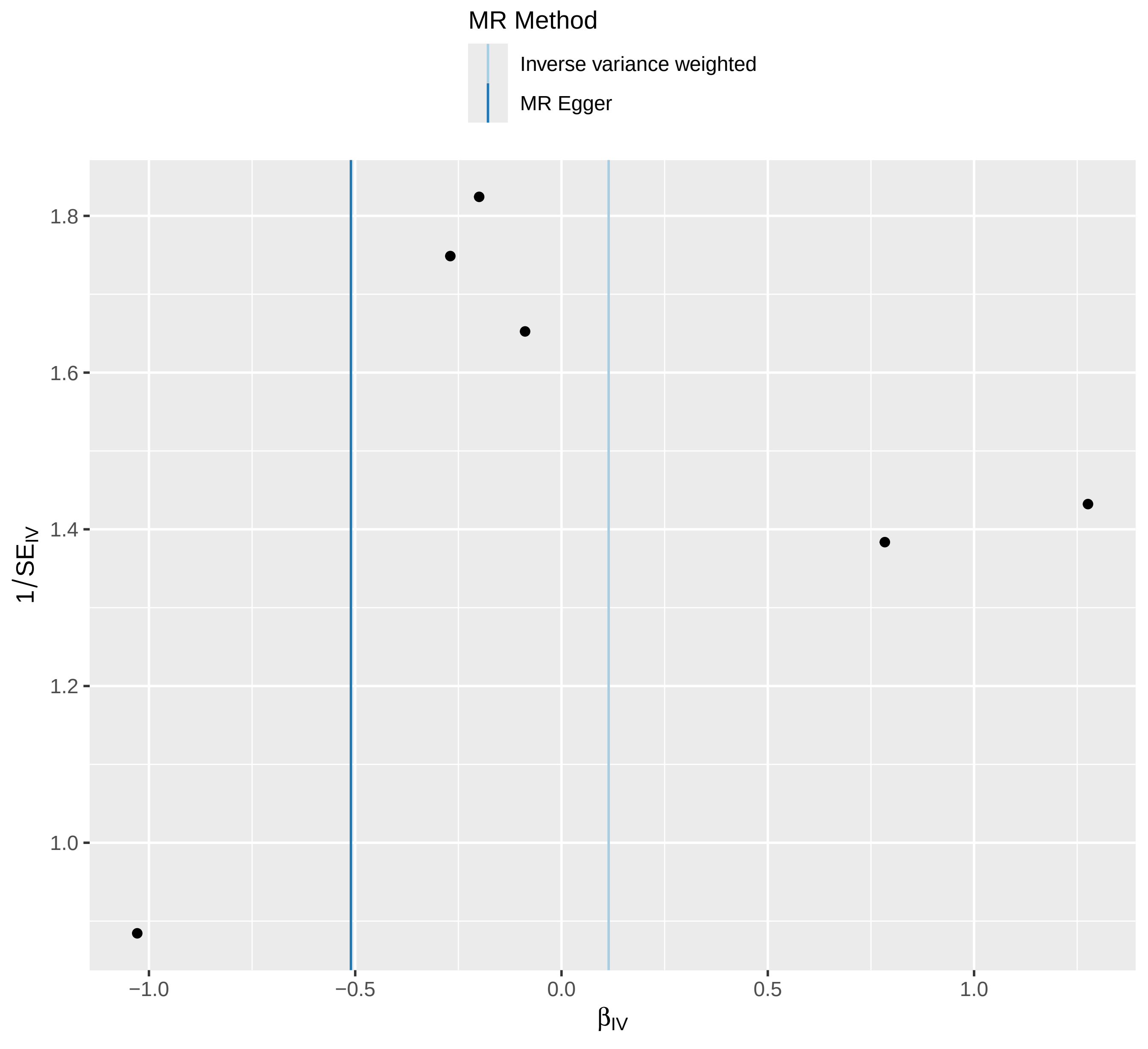

MR Method
Inverse variance weighted
MR Egger
1.8
1.6
1.4
1.2
1.0
$1/SE_{IV}$
−1.0
−0.5
0.0
0.5
1.0
β_{IV}

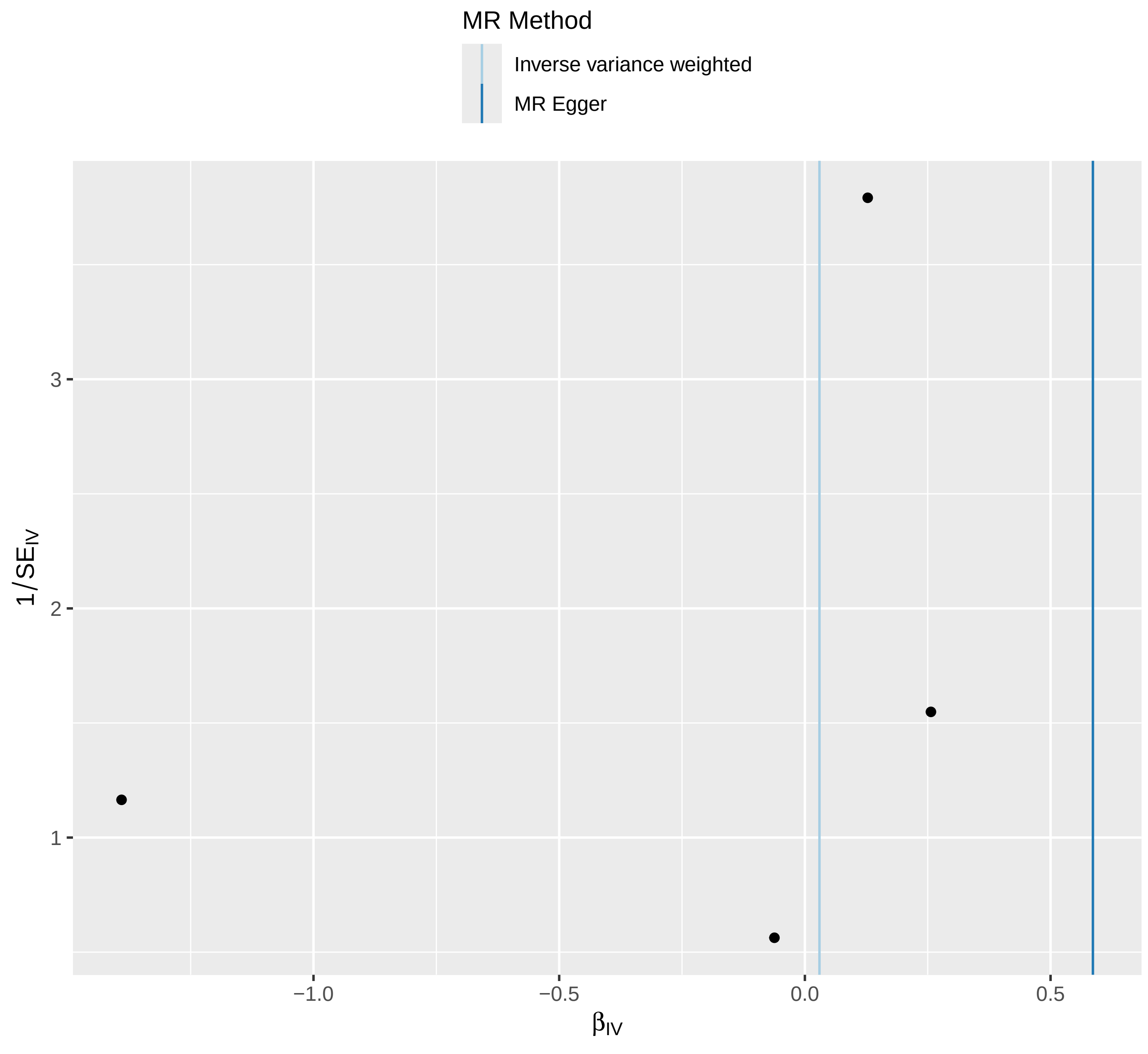

MR Method
Inverse variance weighted
MR Egger
1/SE$_{IV}$
3
2
1
−1.0
−0.5
0.0
0.5
β$_{IV}$

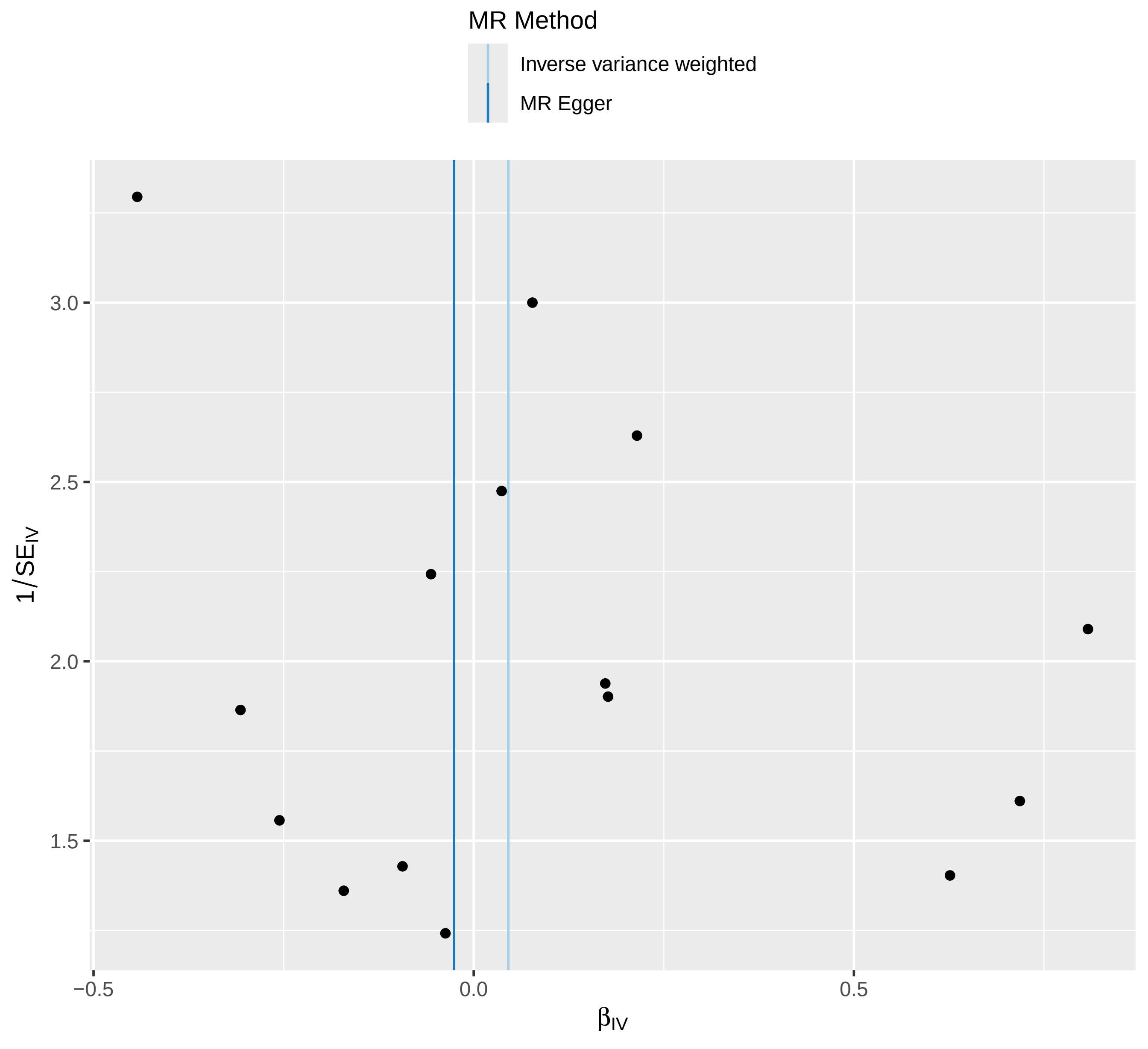

MR Method
Inverse variance weighted
MR Egger
1/SE_IV
3.0
2.5
2.0
1.5
−0.5
0.0
0.5
β_IV

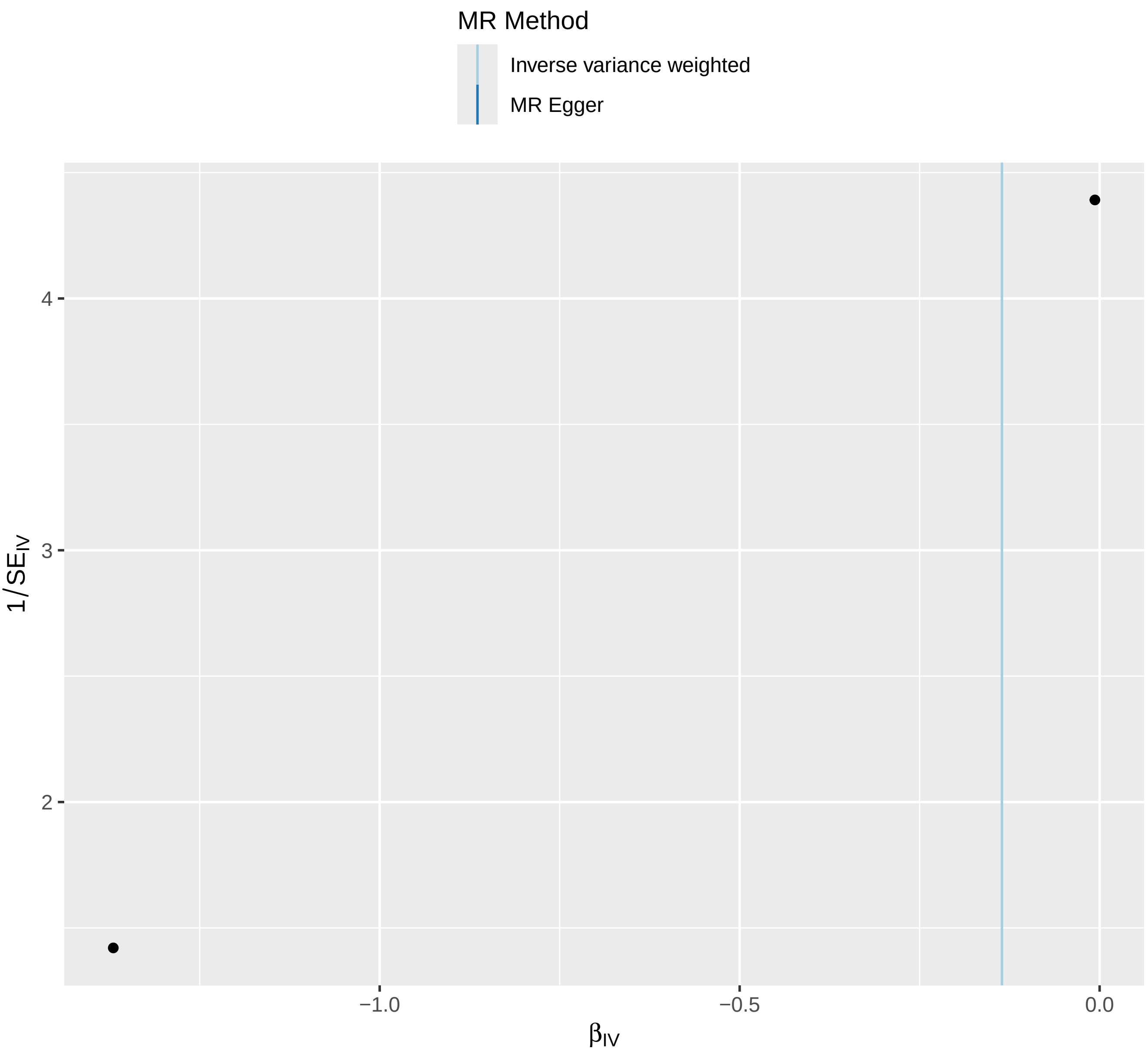

MR Method
Inverse variance weighted
MR Egger
4
3
2
1/SE_IV
−1.0
−0.5
0.0
β_IV

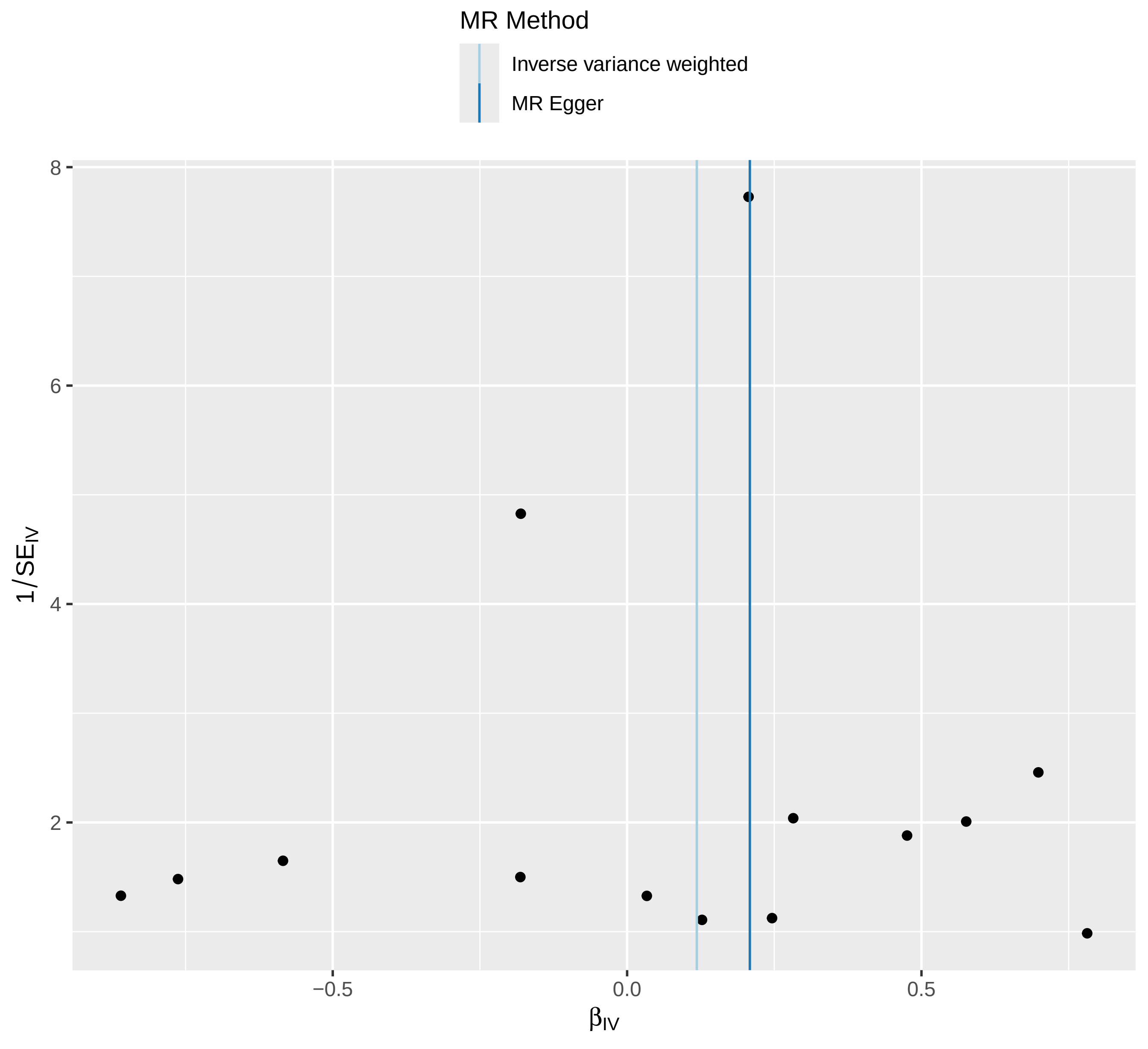
MR Method
Inverse variance weighted
MR Egger
8
6
4
2
1/SE_IV
−0.5
0.0
0.5
β_IV

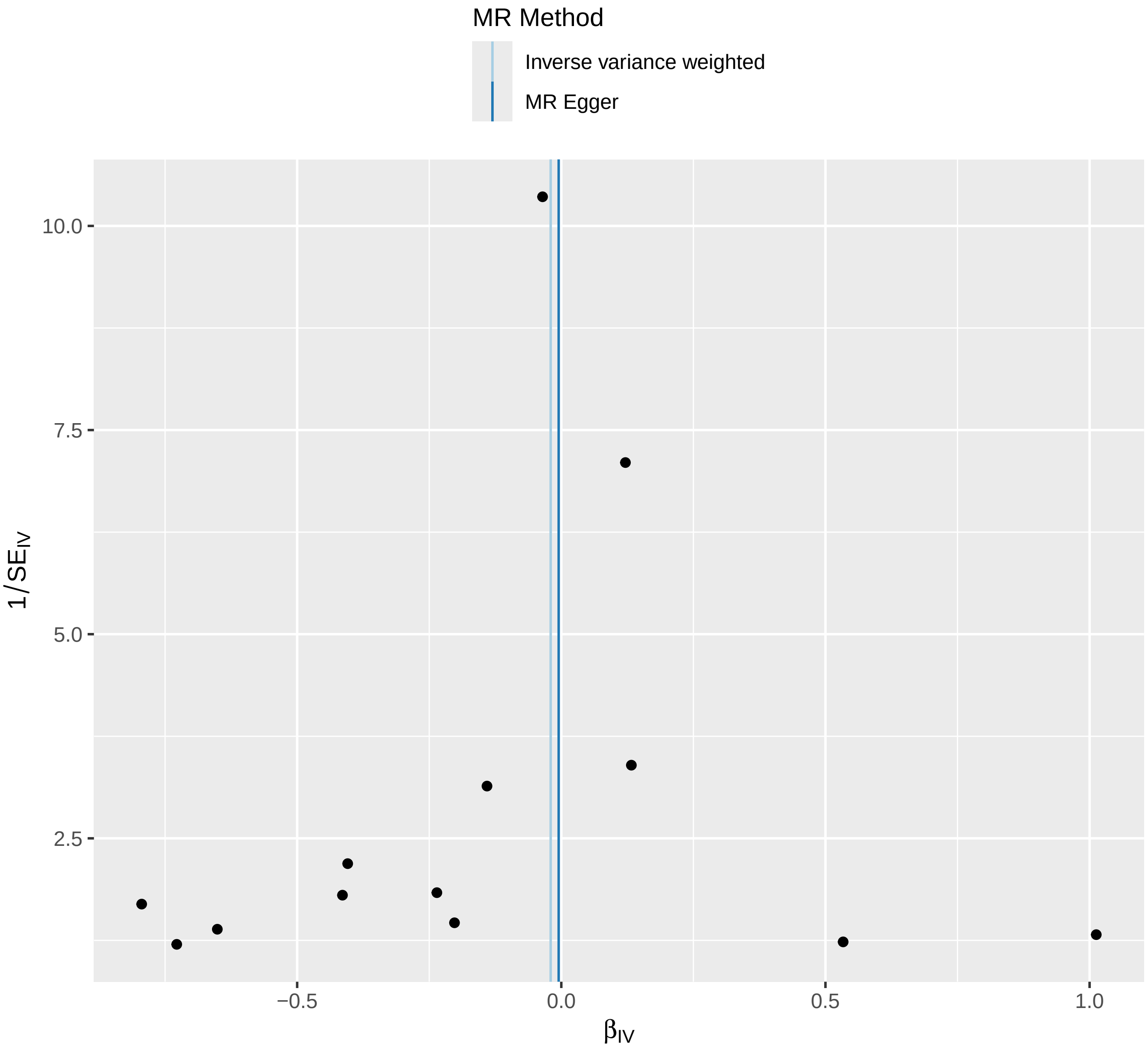
MR Method
Inverse variance weighted
MR Egger
10.0
7.5
5.0
2.5
$1/SE_{IV}$
−0.5
0.0
0.5
1.0
β_{IV}

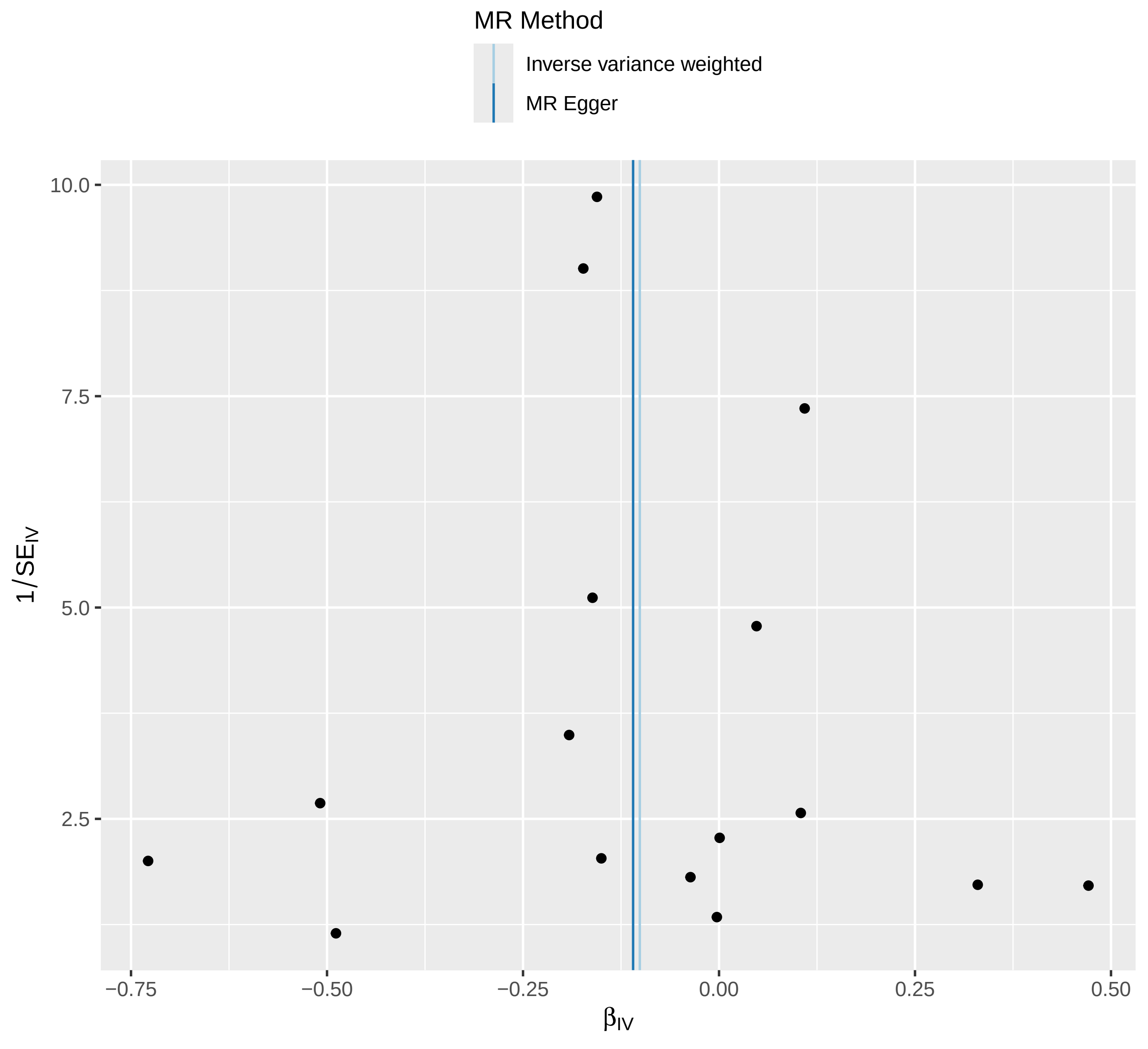

MR Method
Inverse variance weighted
MR Egger
10.0
7.5
5.0
2.5
1/SE_IV
−0.75
−0.50
−0.25
0.00
0.25
0.50
β_IV

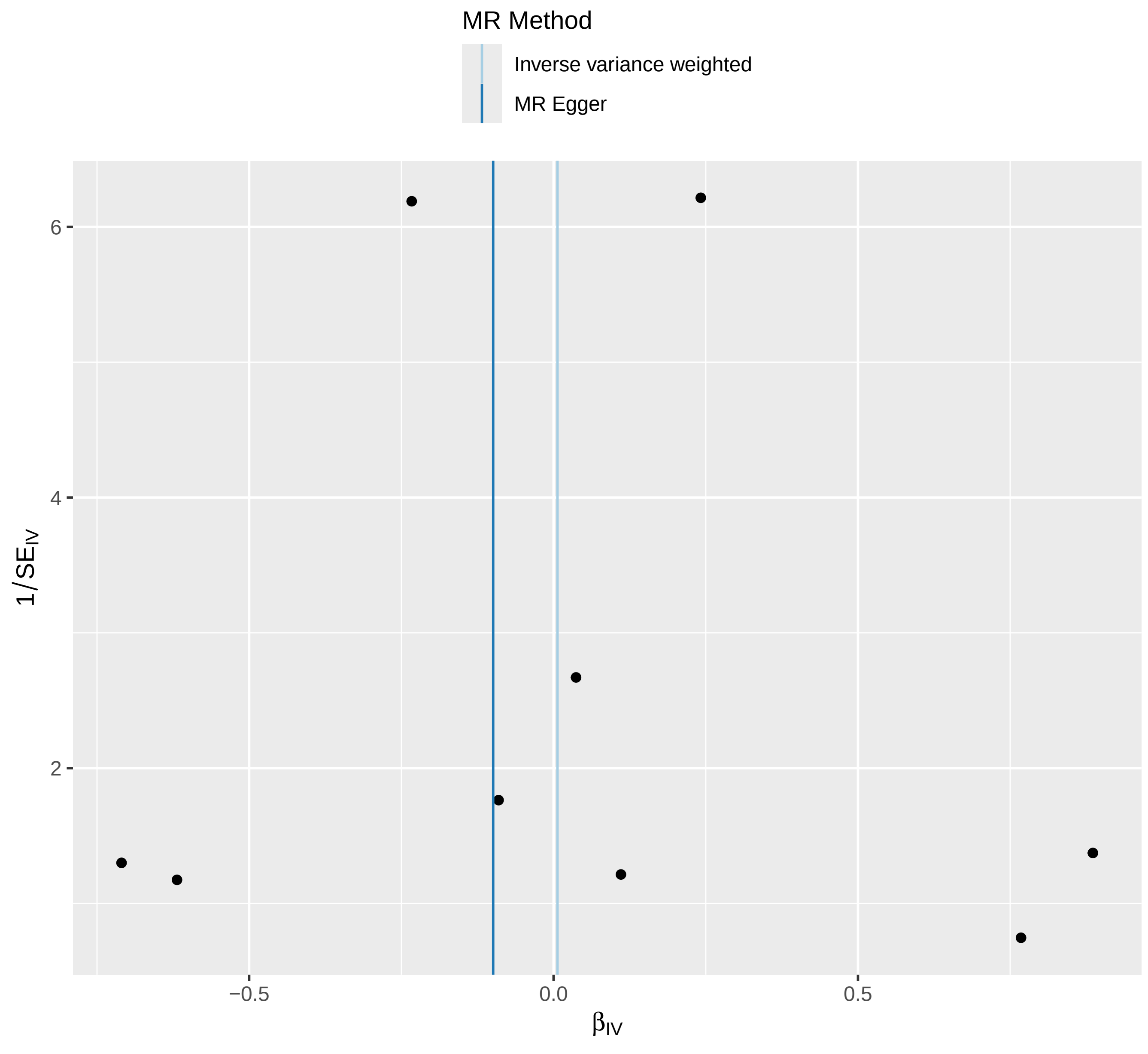
MR Method
Inverse variance weighted
MR Egger
6
4
2
1/SE_IV
−0.5
0.0
0.5
β_IV

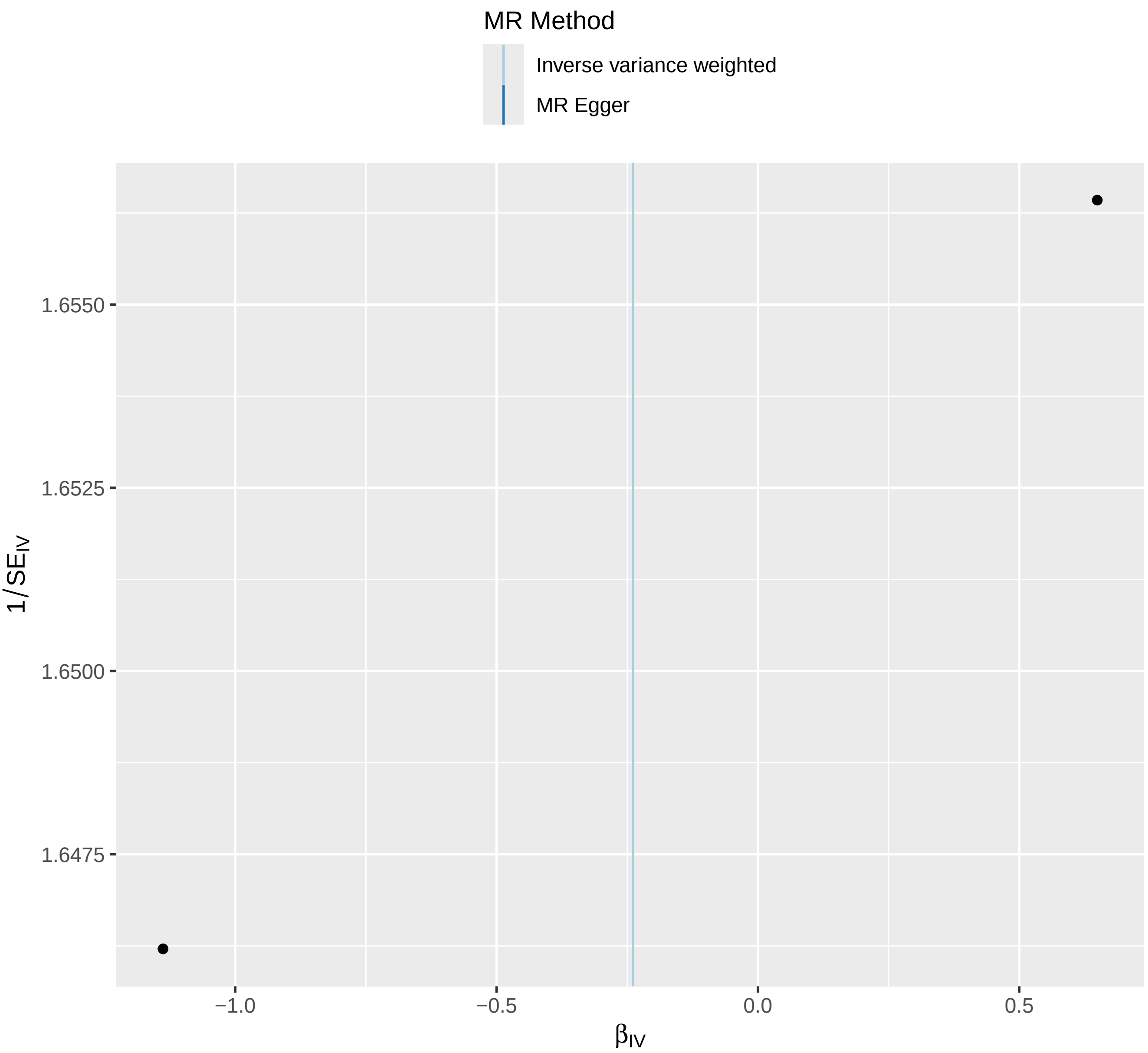
MR Method
Inverse variance weighted
MR Egger
1.6550
1.6525
1.6500
1.6475
1/SE_IV
−1.0
−0.5
0.0
0.5
β_IV

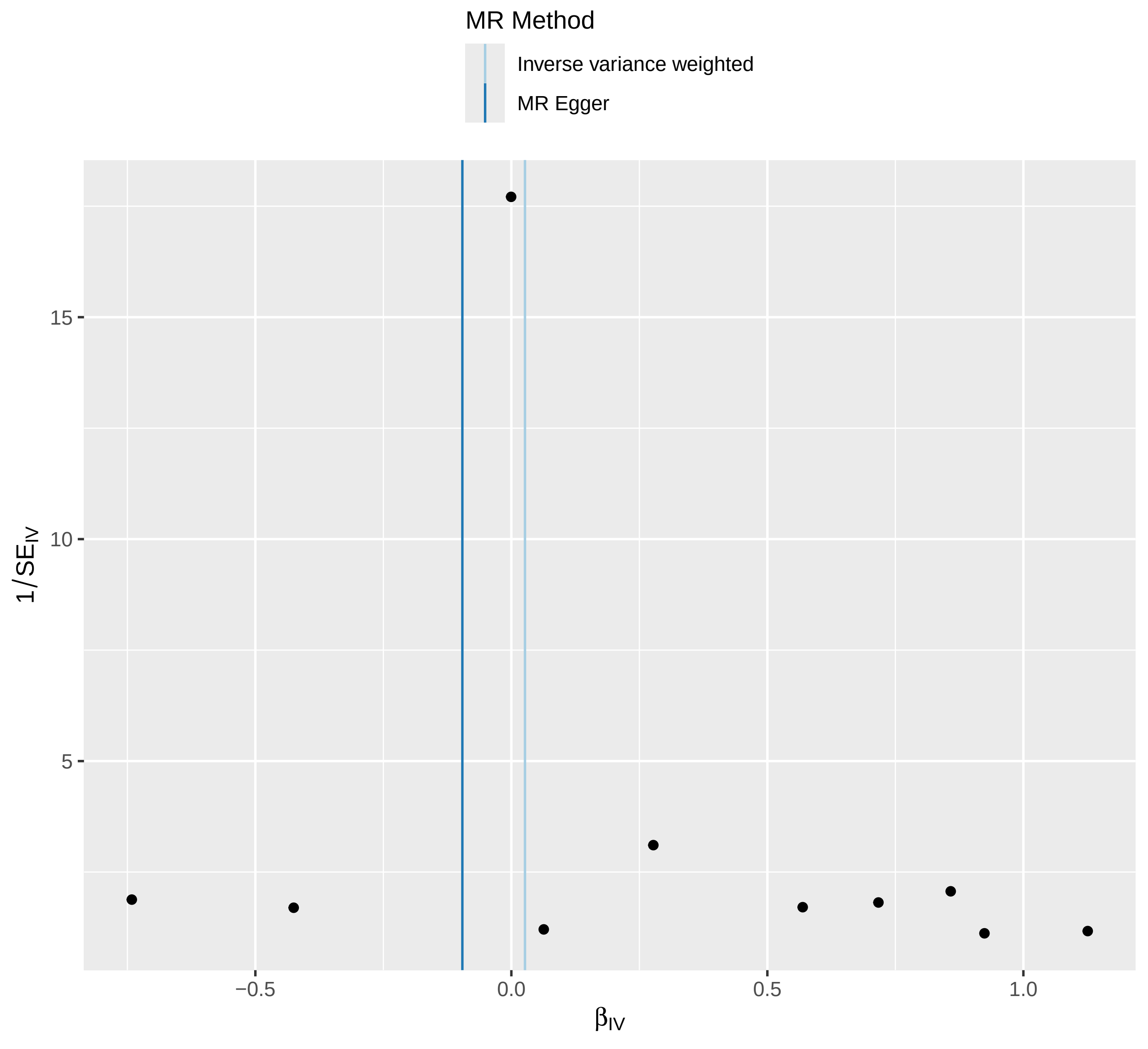

MR Method
Inverse variance weighted
MR Egger
15
10
5
−0.5
0.0
0.5
1.0
1/SE_IV
β_IV

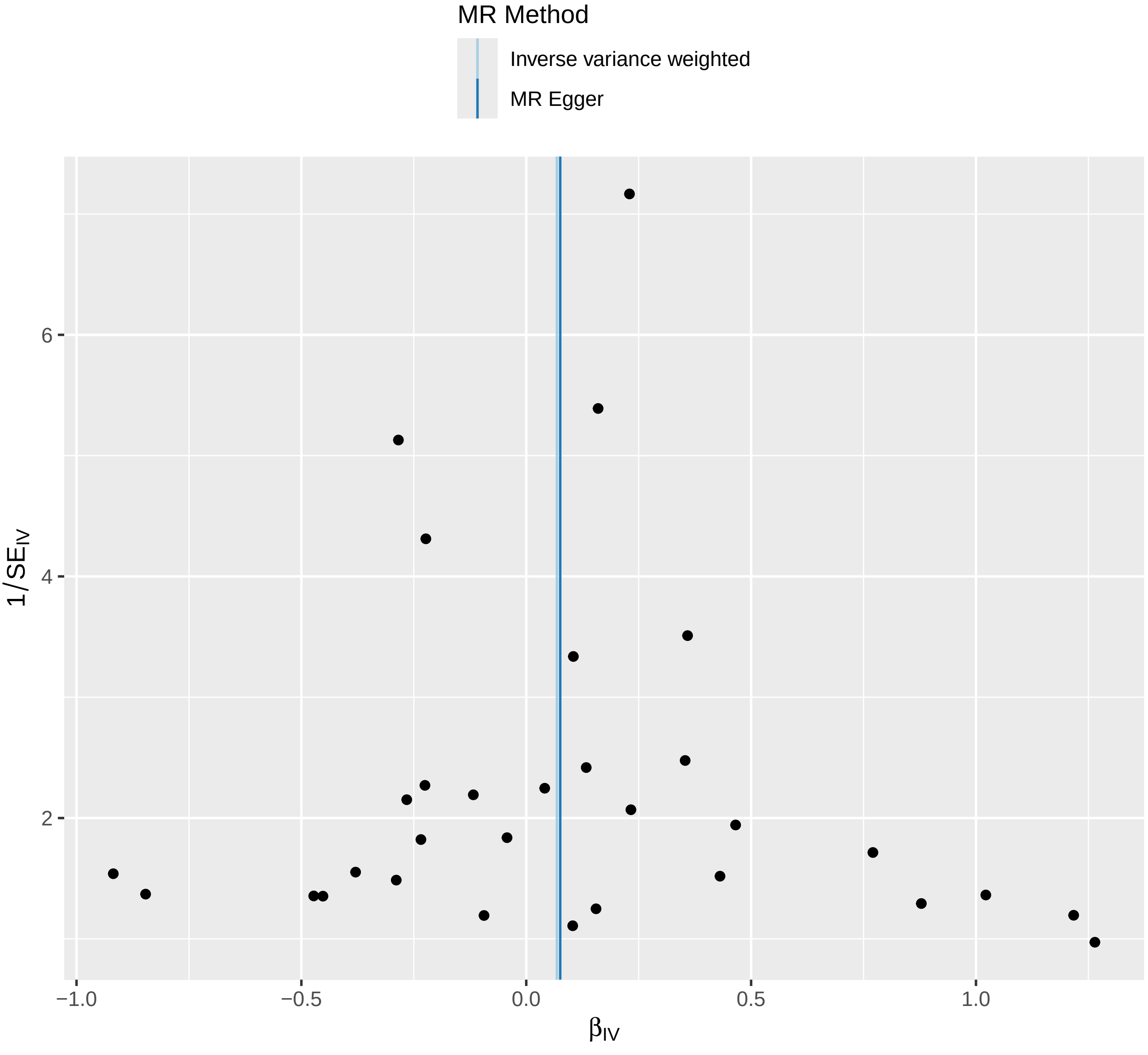
MR Method
Inverse variance weighted
MR Egger
6
4
2
1/SE$_{IV}$
−1.0
−0.5
0.0
0.5
1.0
β$_{IV}$

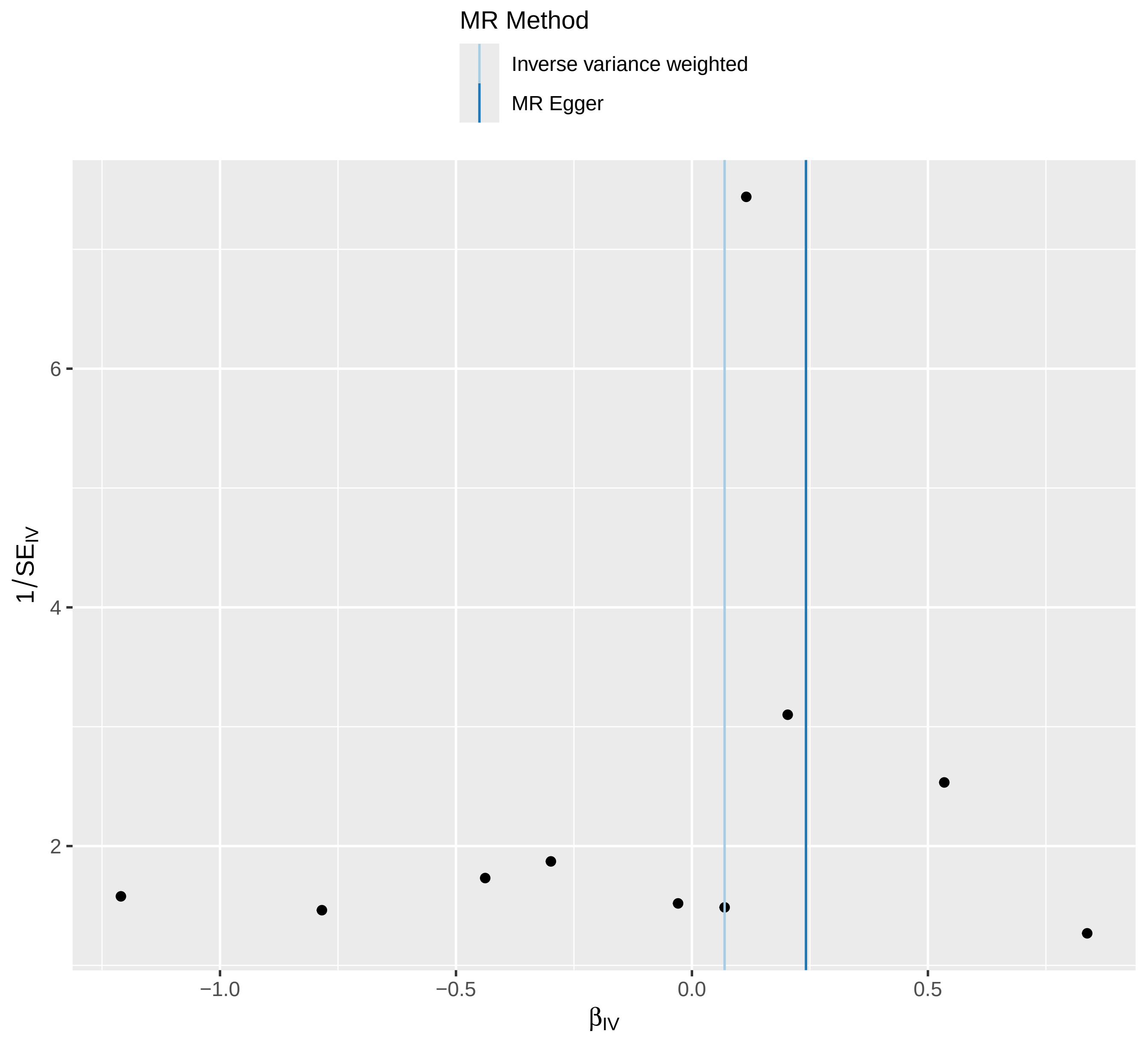

MR Method
Inverse variance weighted
MR Egger
6
4
2
1/SE$_{IV}$
−1.0
−0.5
0.0
0.5
β$_{IV}$

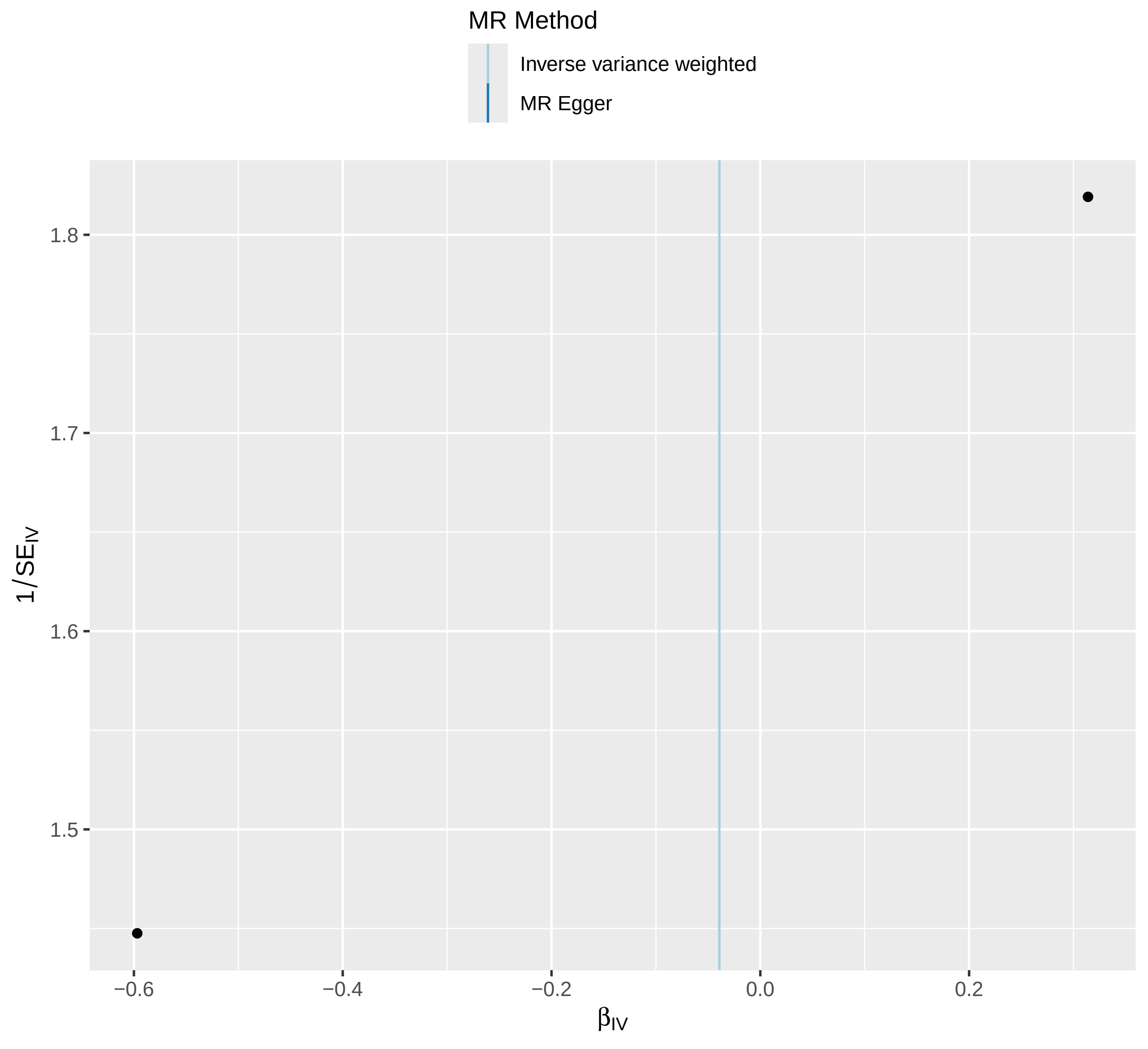

MR Method
Inverse variance weighted
MR Egger
1.8
1.7
1.6
1.5
1/SE_IV
−0.6
−0.4
−0.2
0.0
0.2
β_IV

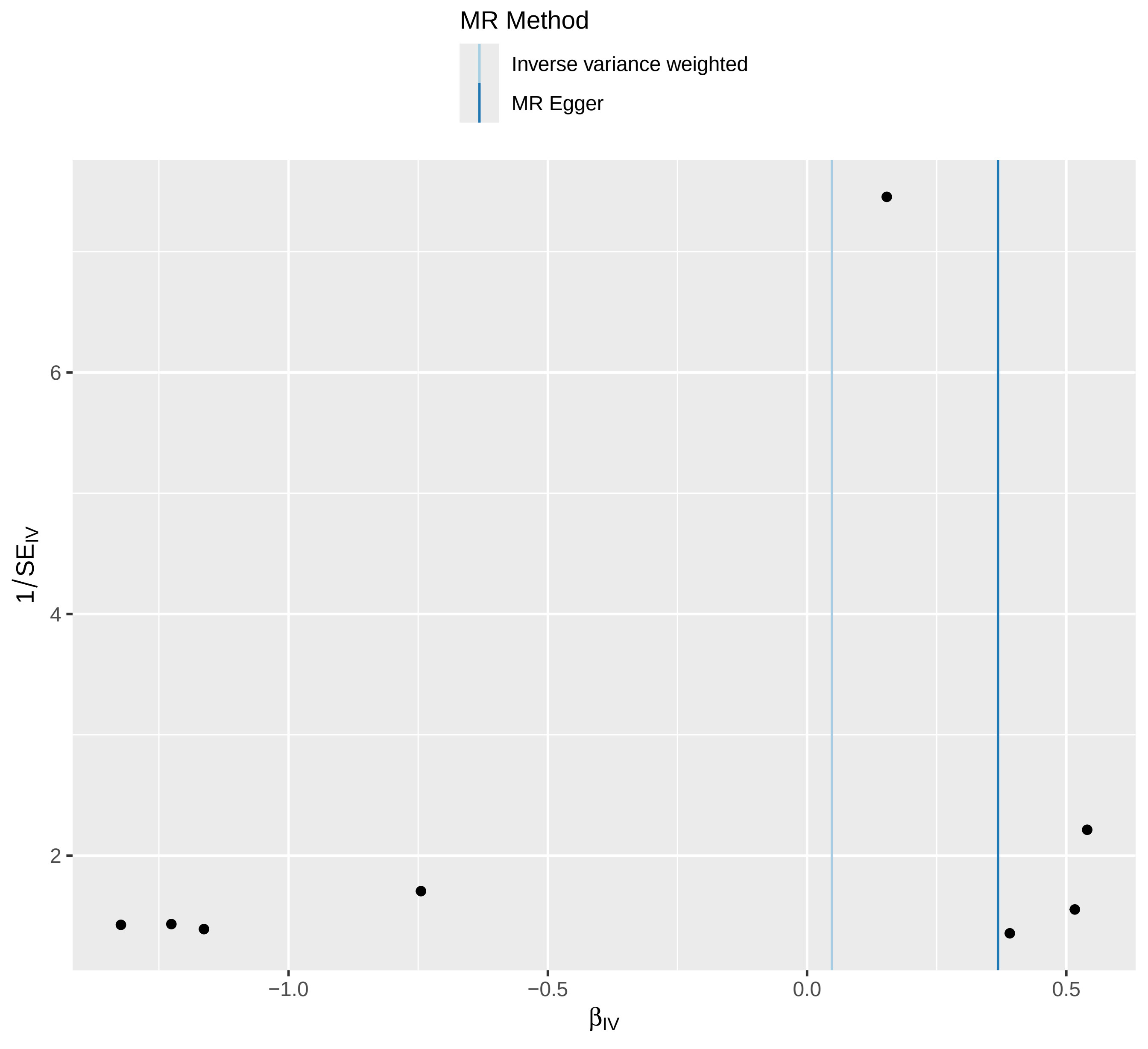

MR Method
Inverse variance weighted
MR Egger
1/SE_IV
6
4
2
−1.0
−0.5
0.0
0.5
β_IV

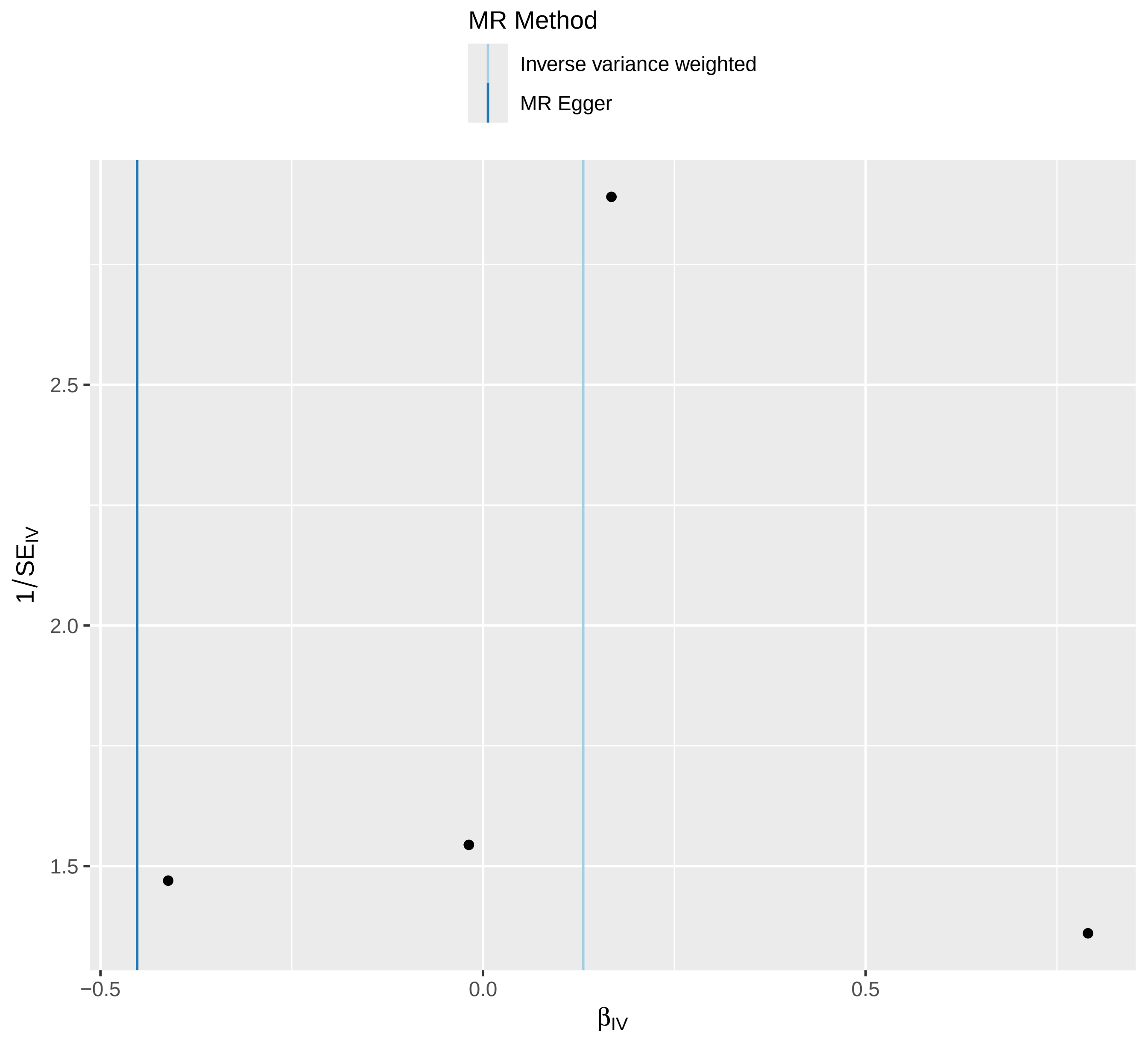
MR Method
Inverse variance weighted
MR Egger
2.5
2.0
1.5
1/SE_IV
−0.5
0.0
0.5
β_IV

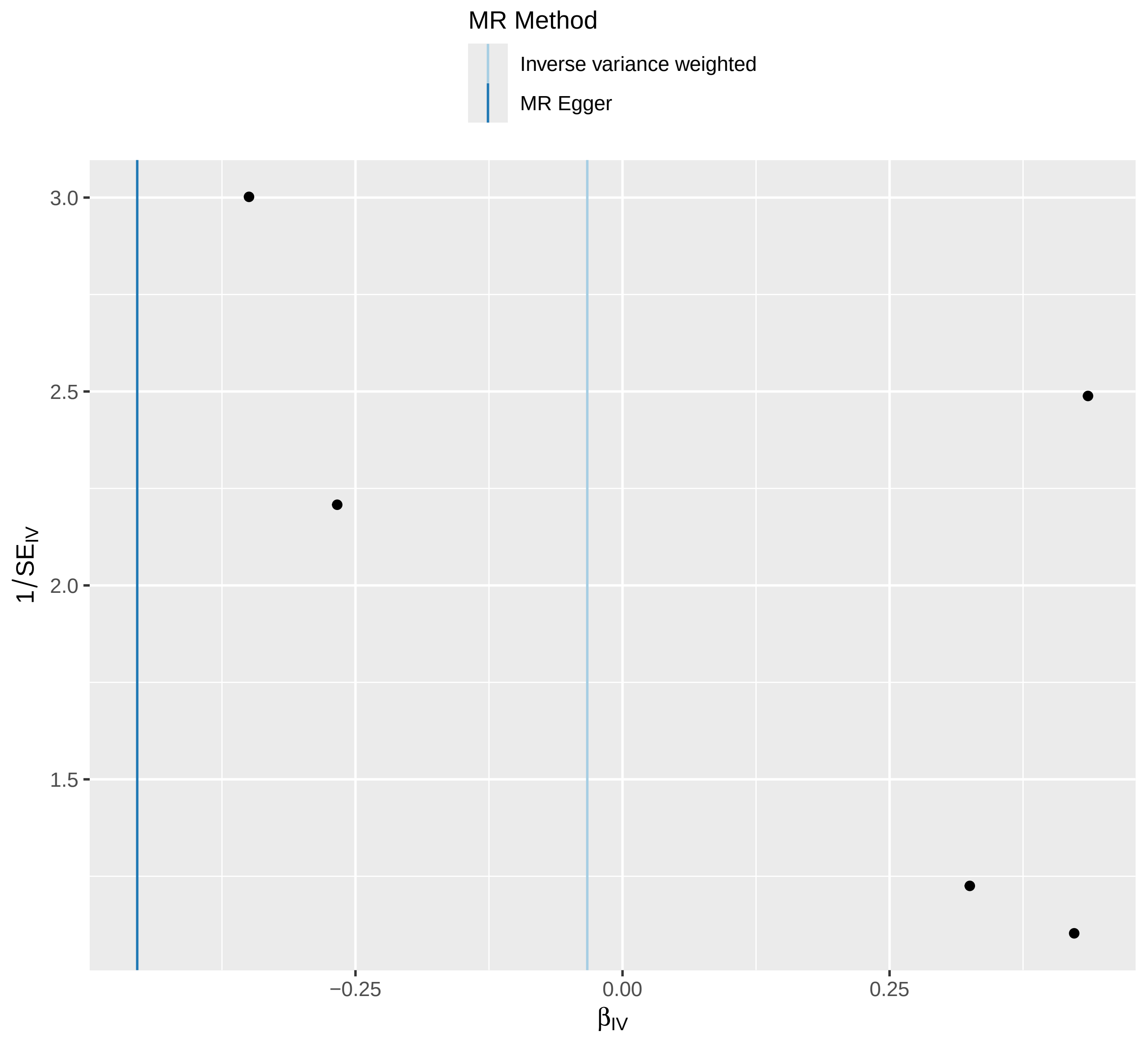

MR Method
Inverse variance weighted
MR Egger
3.0
2.5
2.0
1.5
1/SE$_{IV}$
−0.25
0.00
0.25
β$_{IV}$

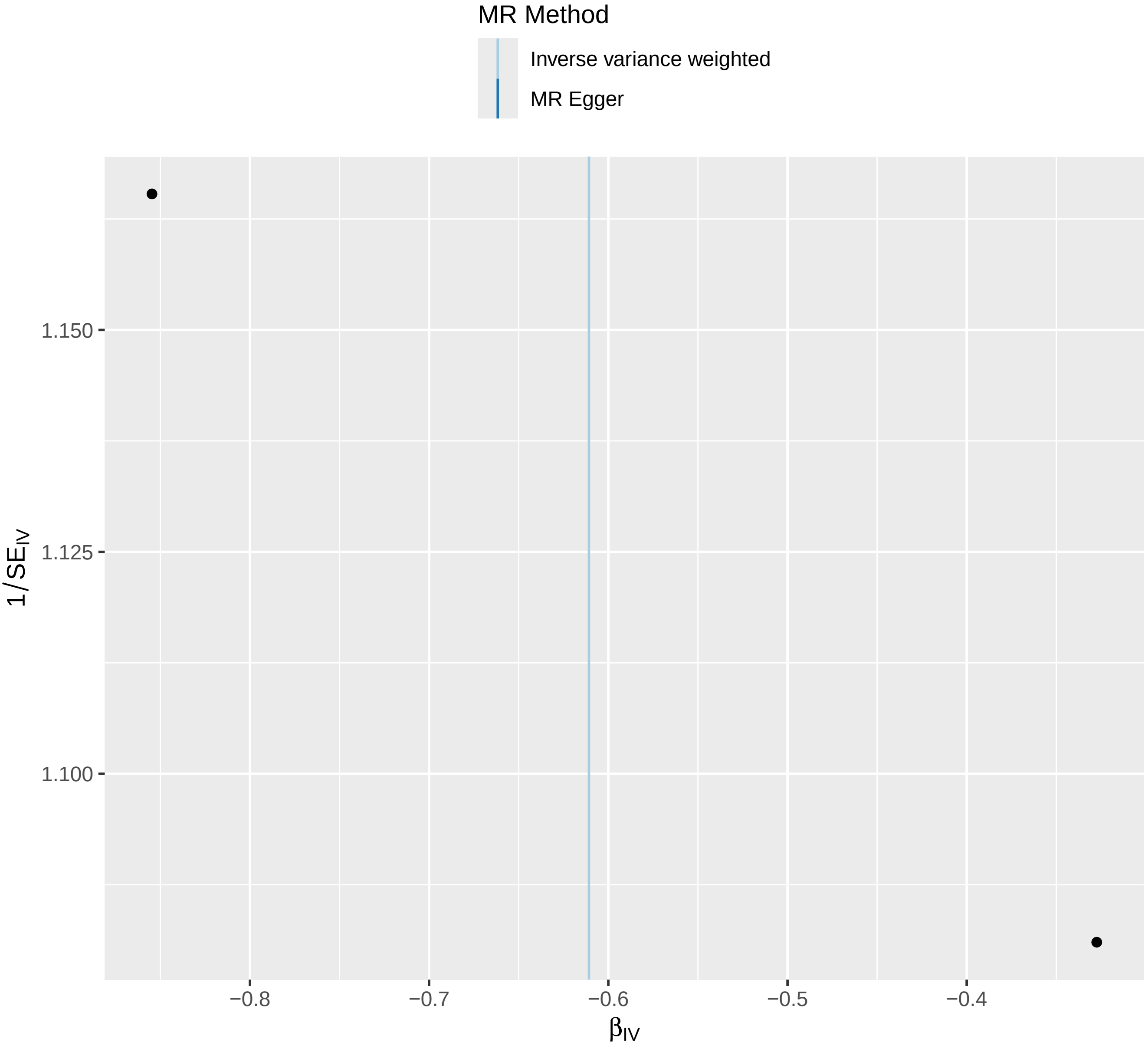
MR Method
Inverse variance weighted
MR Egger
1.150
1.125
1.100
1/SE_IV
−0.8
−0.7
−0.6
−0.5
−0.4
β_IV

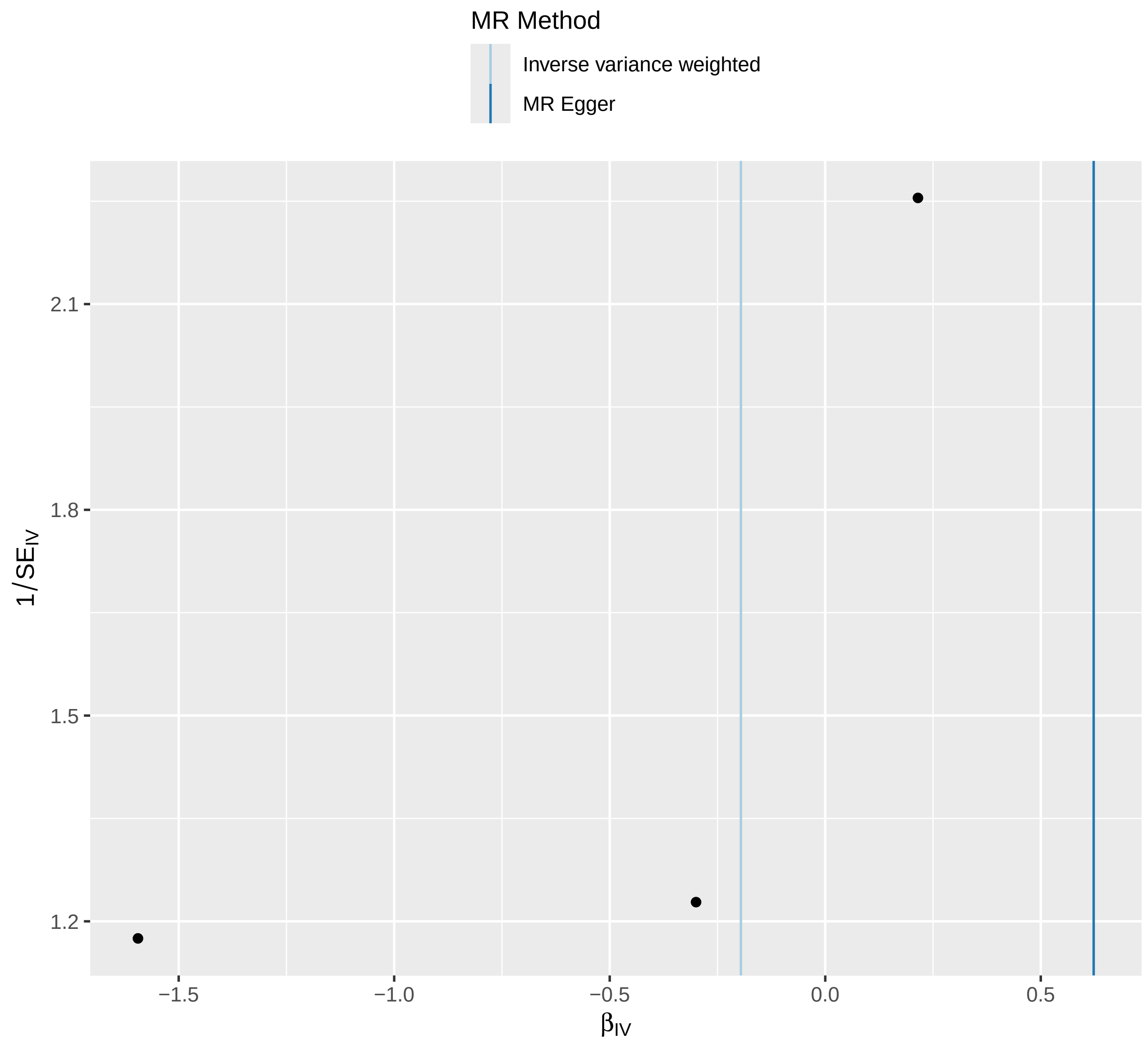

MR Method
Inverse variance weighted
MR Egger
2.1
1.8
1.5
1.2
$1/SE_{IV}$
−1.5
−1.0
−0.5
0.0
0.5
β_{IV}

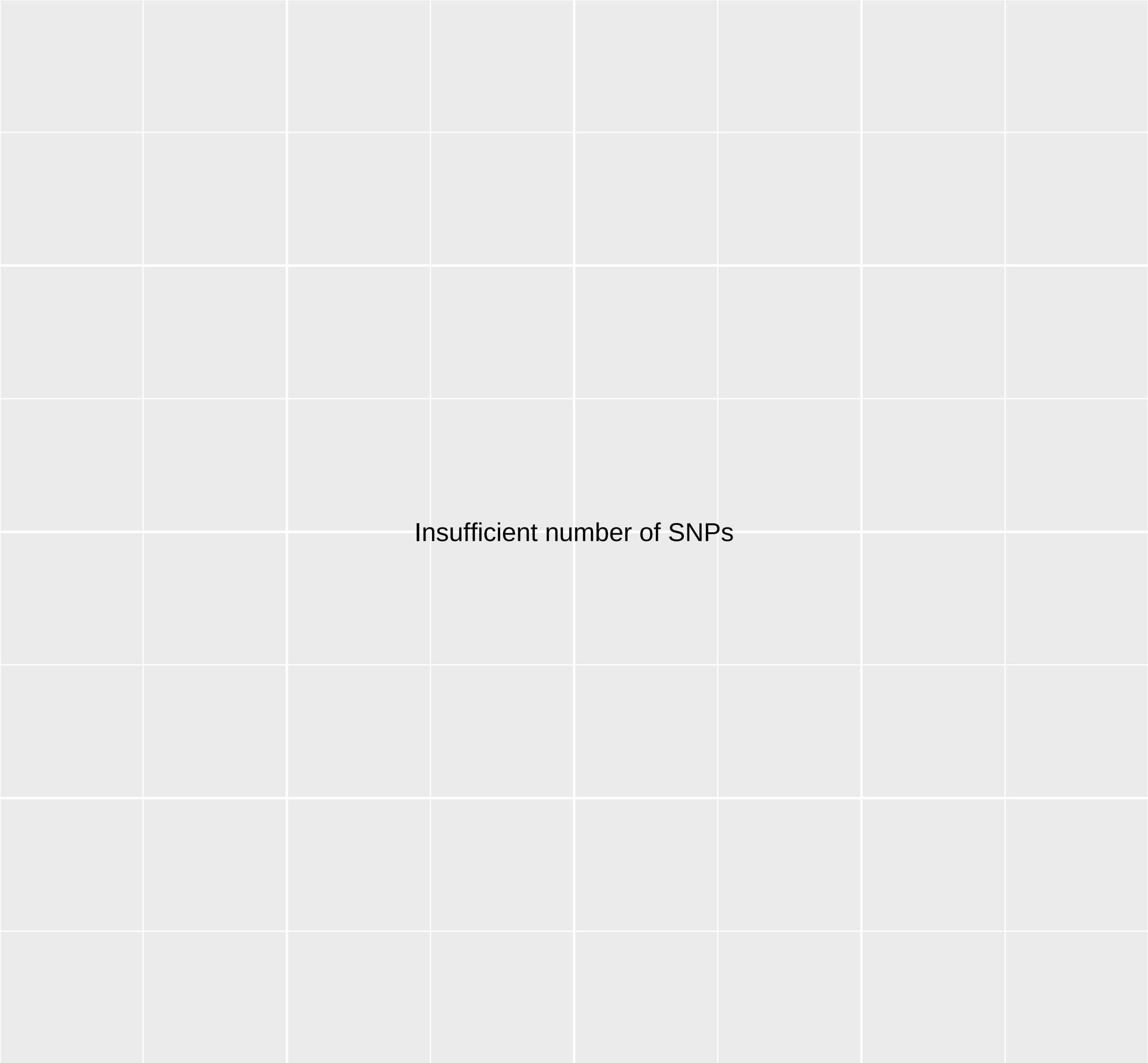
Insufficient number of SNPs

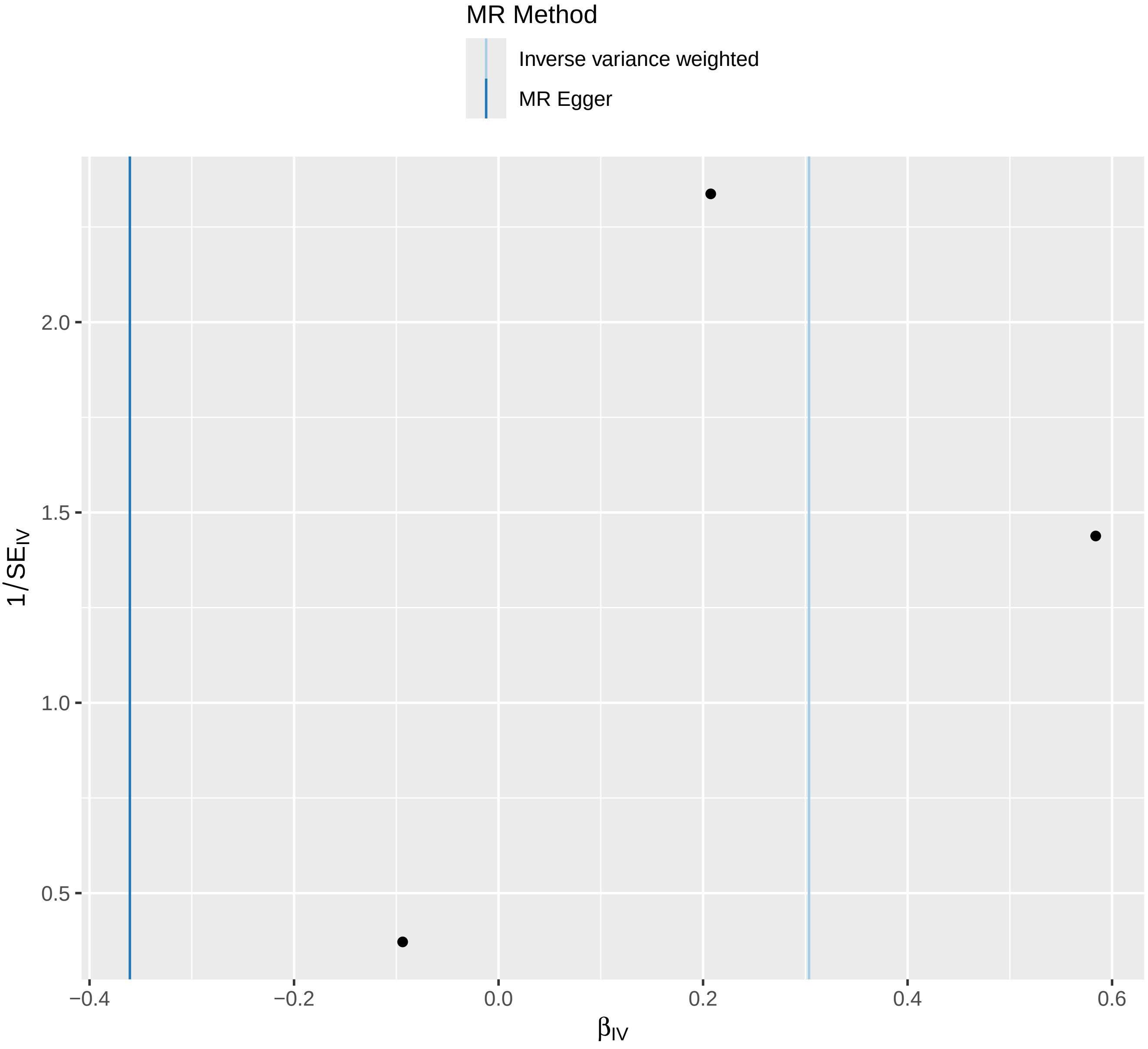
MR Method
Inverse variance weighted
MR Egger
2.0
1.5
1.0
0.5
1/SE_IV
−0.4
−0.2
0.0
0.2
0.4
0.6
β_IV

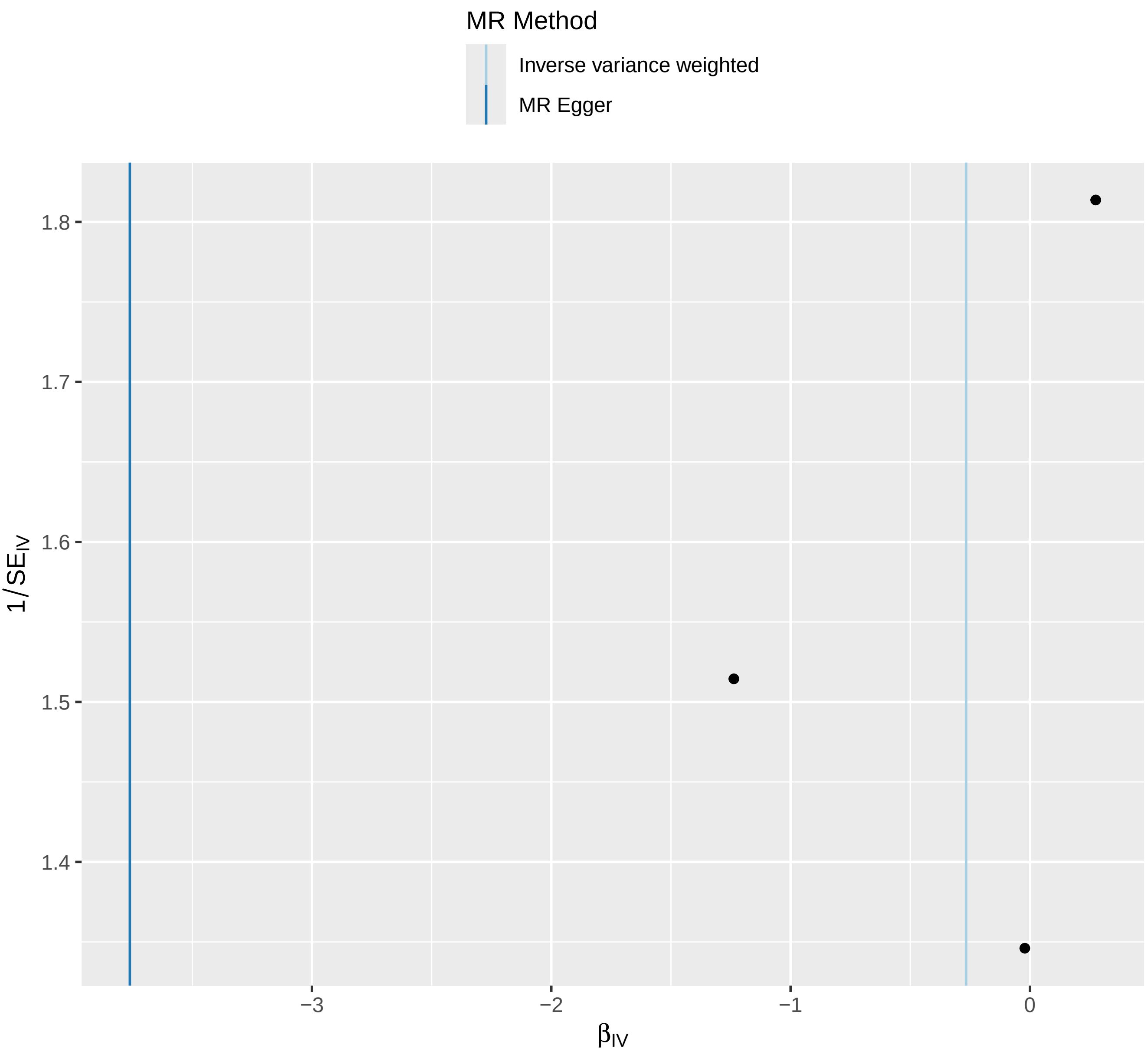
MR Method
Inverse variance weighted
MR Egger
1.8
1.7
1.6
1.5
1.4
1/SE_IV
−3
−2
−1
0
β_IV

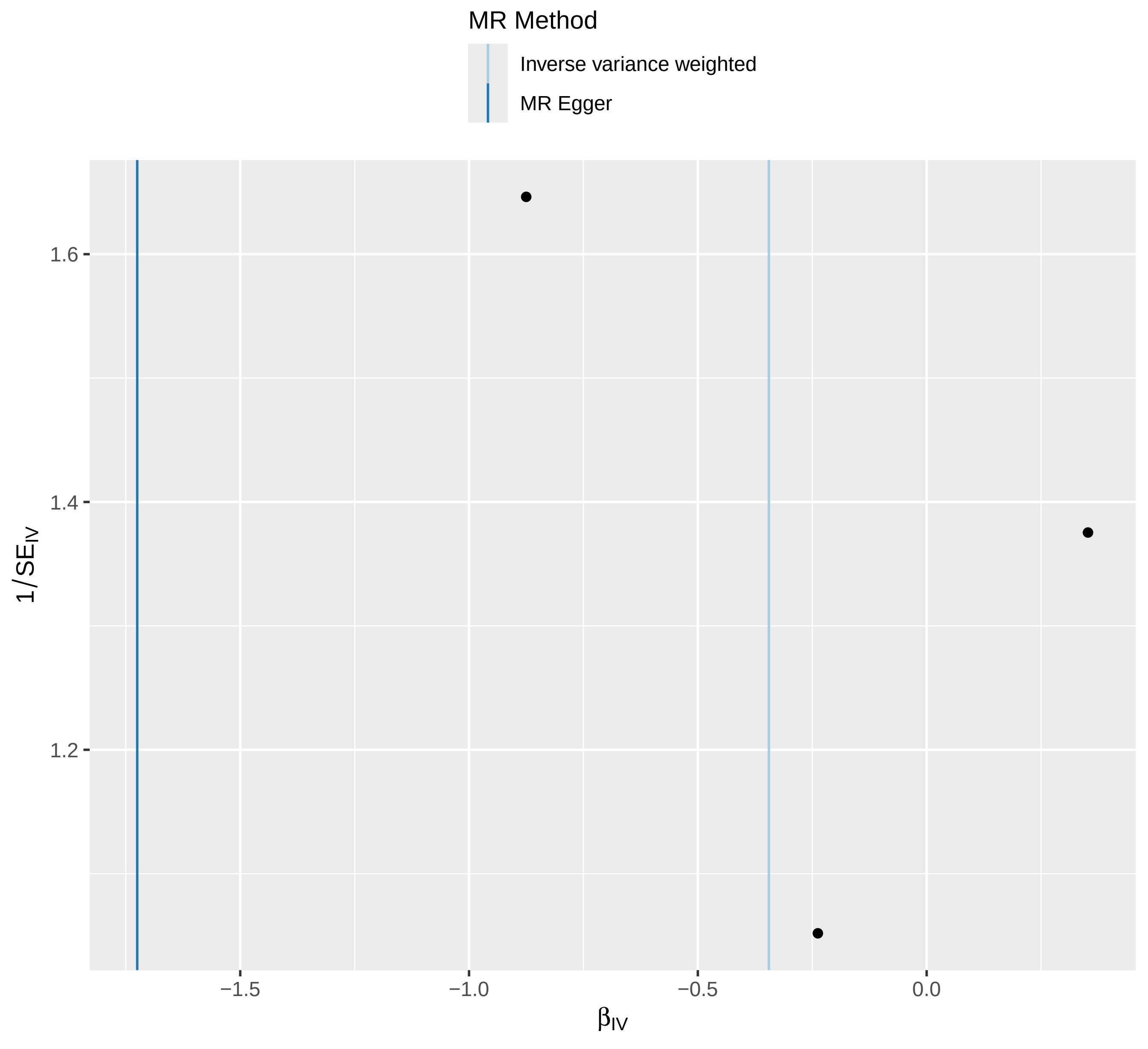

MR Method
Inverse variance weighted
MR Egger
1.6
1.4
1.2
−1.5
−1.0
−0.5
0.0
$1/SE_{IV}$
β_{IV}

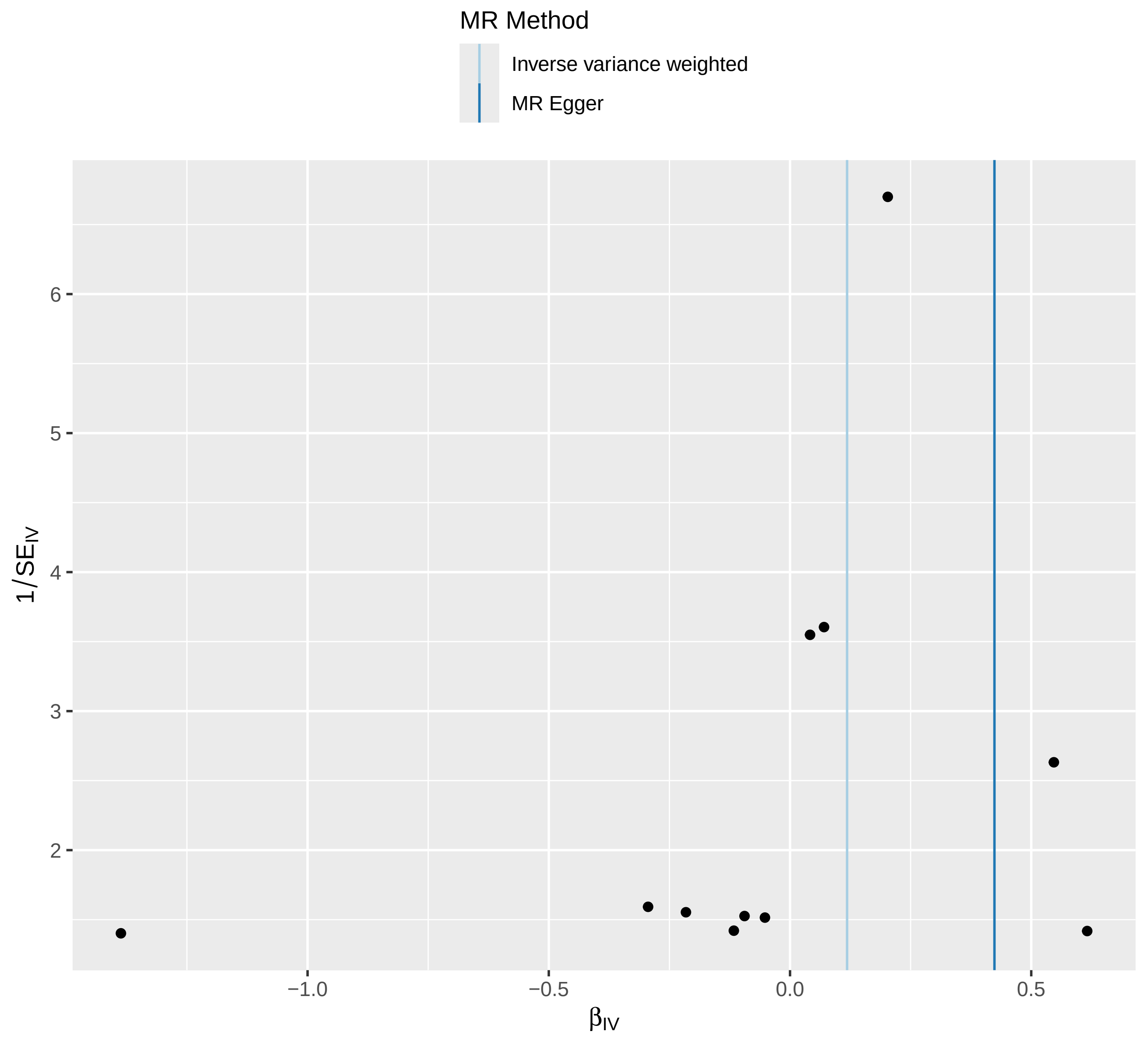

MR Method
Inverse variance weighted
MR Egger
6
5
4
3
2
1/SE$_{IV}$
−1.0
−0.5
0.0
0.5
β$_{IV}$

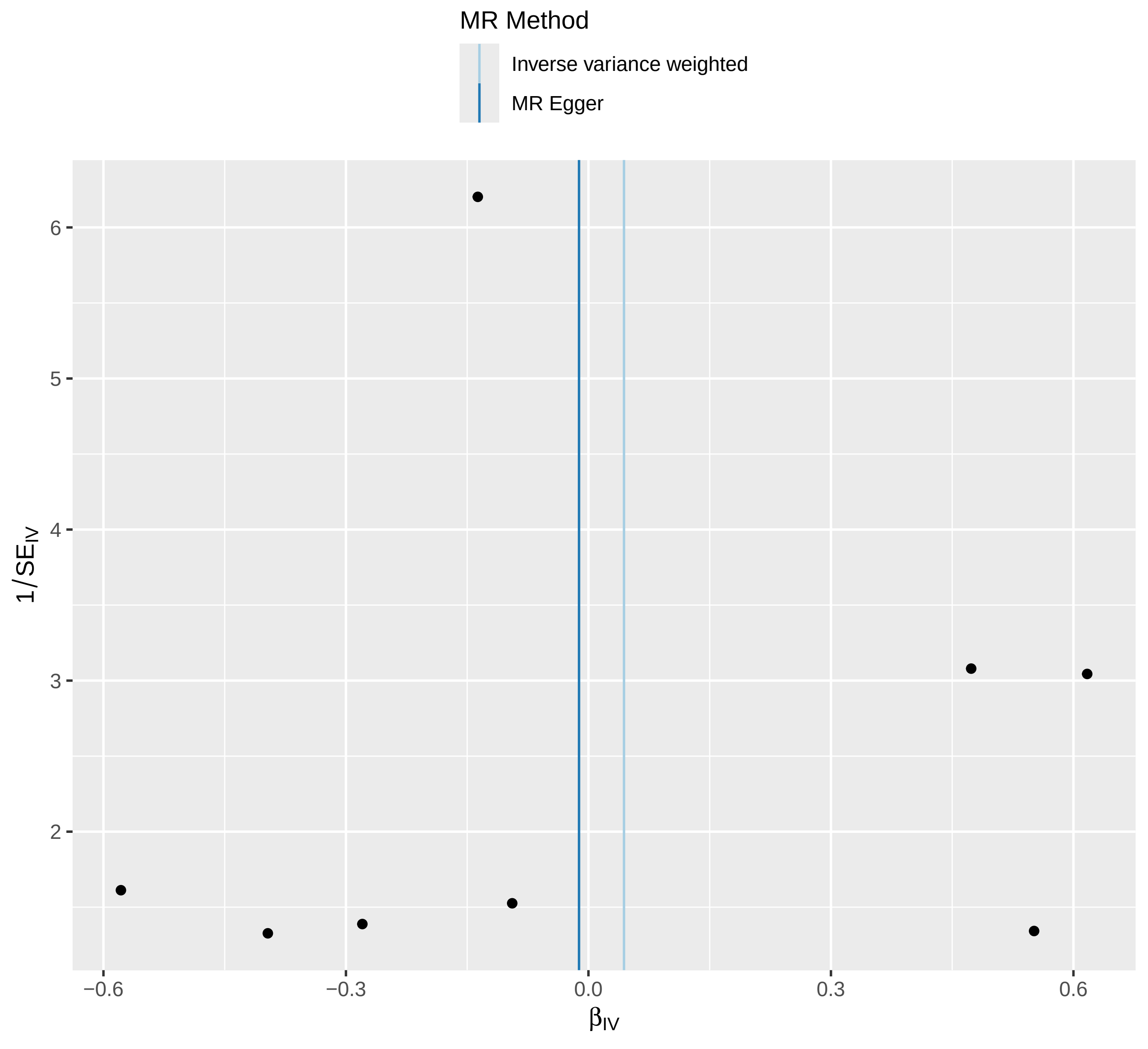
MR Method
Inverse variance weighted
MR Egger
6
5
4
3
2
1/SE_IV
−0.6
−0.3
0.0
0.3
0.6
β_IV

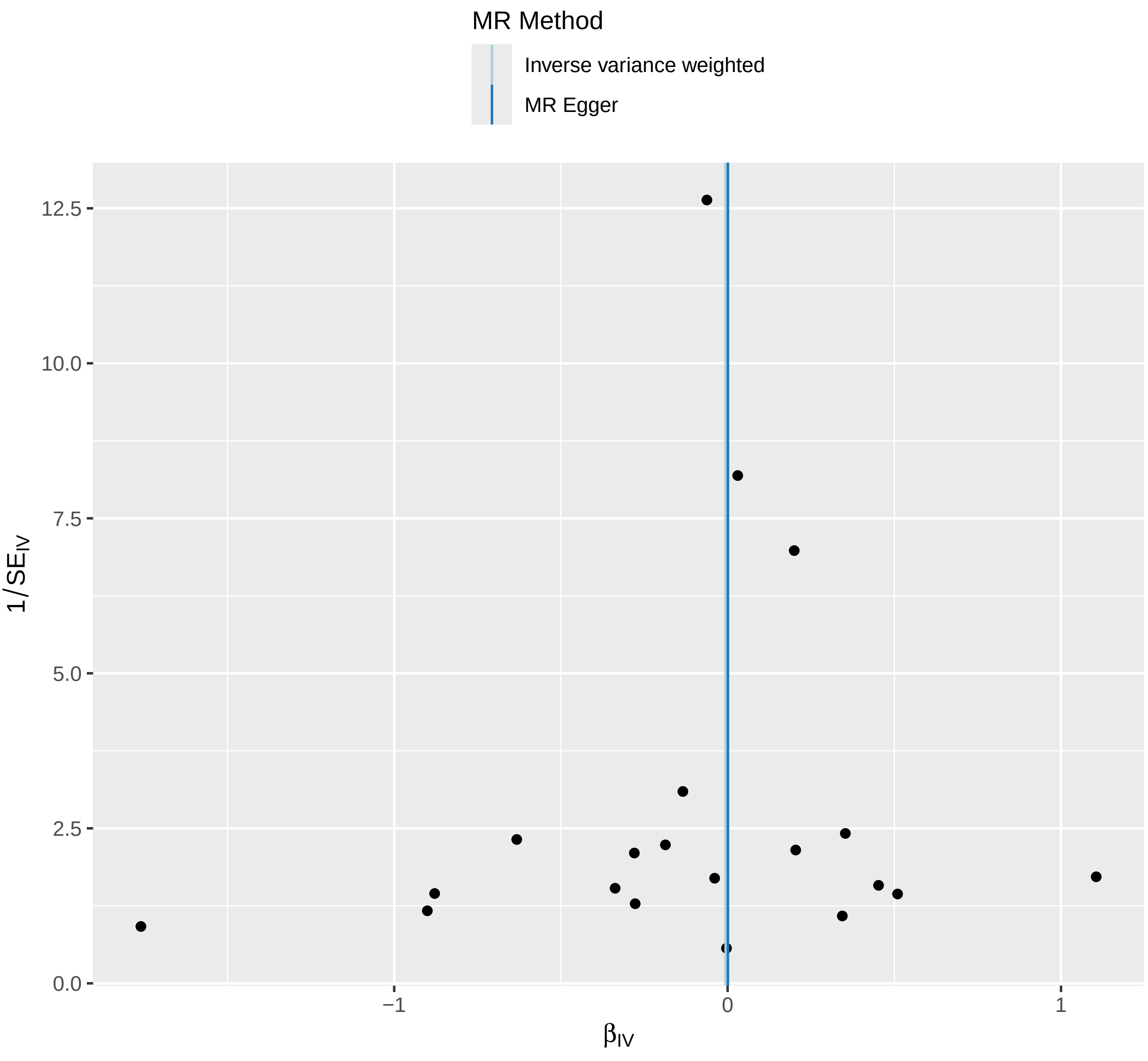

MR Method
Inverse variance weighted
MR Egger
12.5
10.0
7.5
5.0
2.5
0.0
1/SE_IV
−1
0
1
β_IV

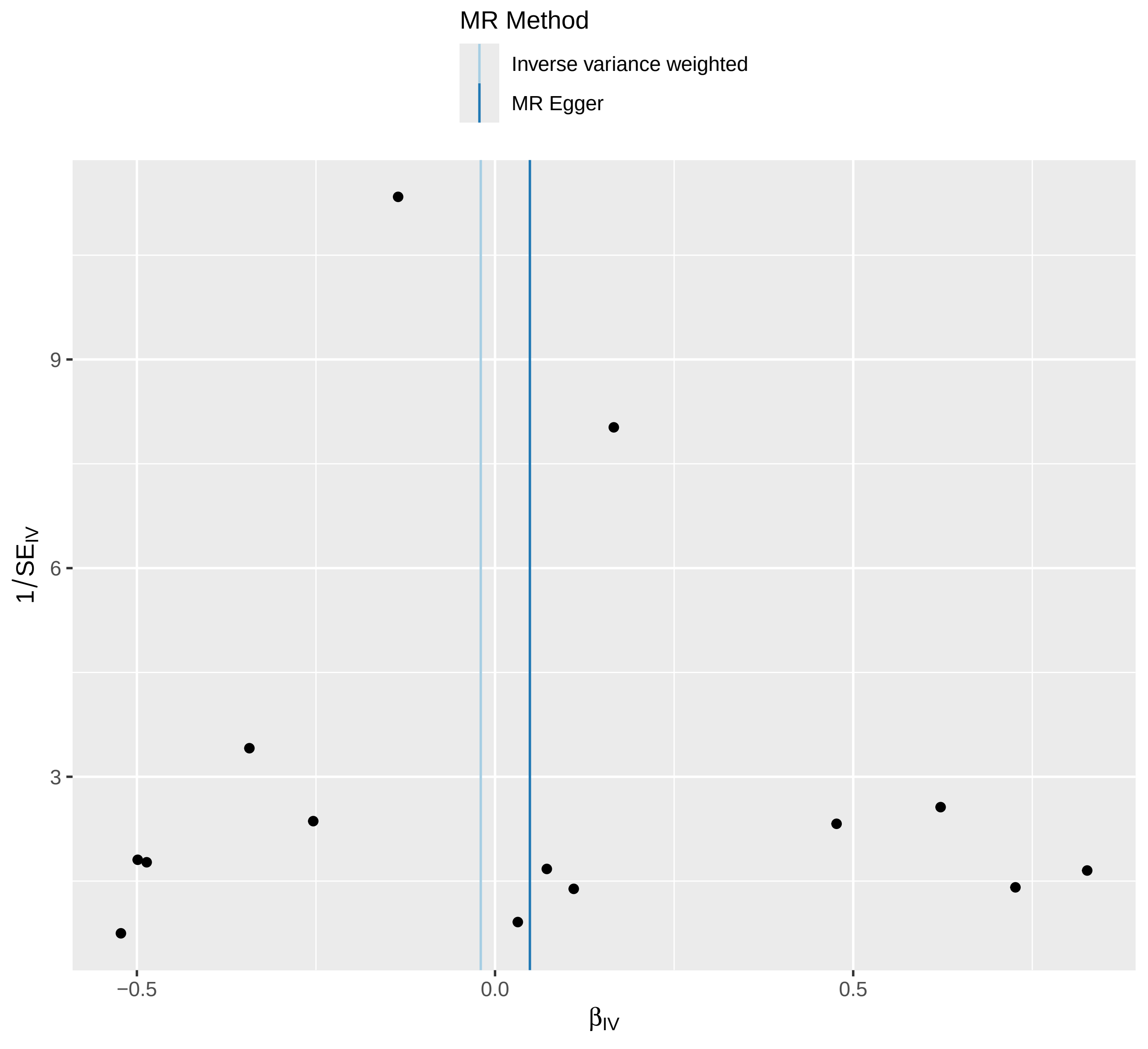

MR Method
Inverse variance weighted
MR Egger
9
6
3
1/SE_IV
−0.5
0.0
0.5
β_IV

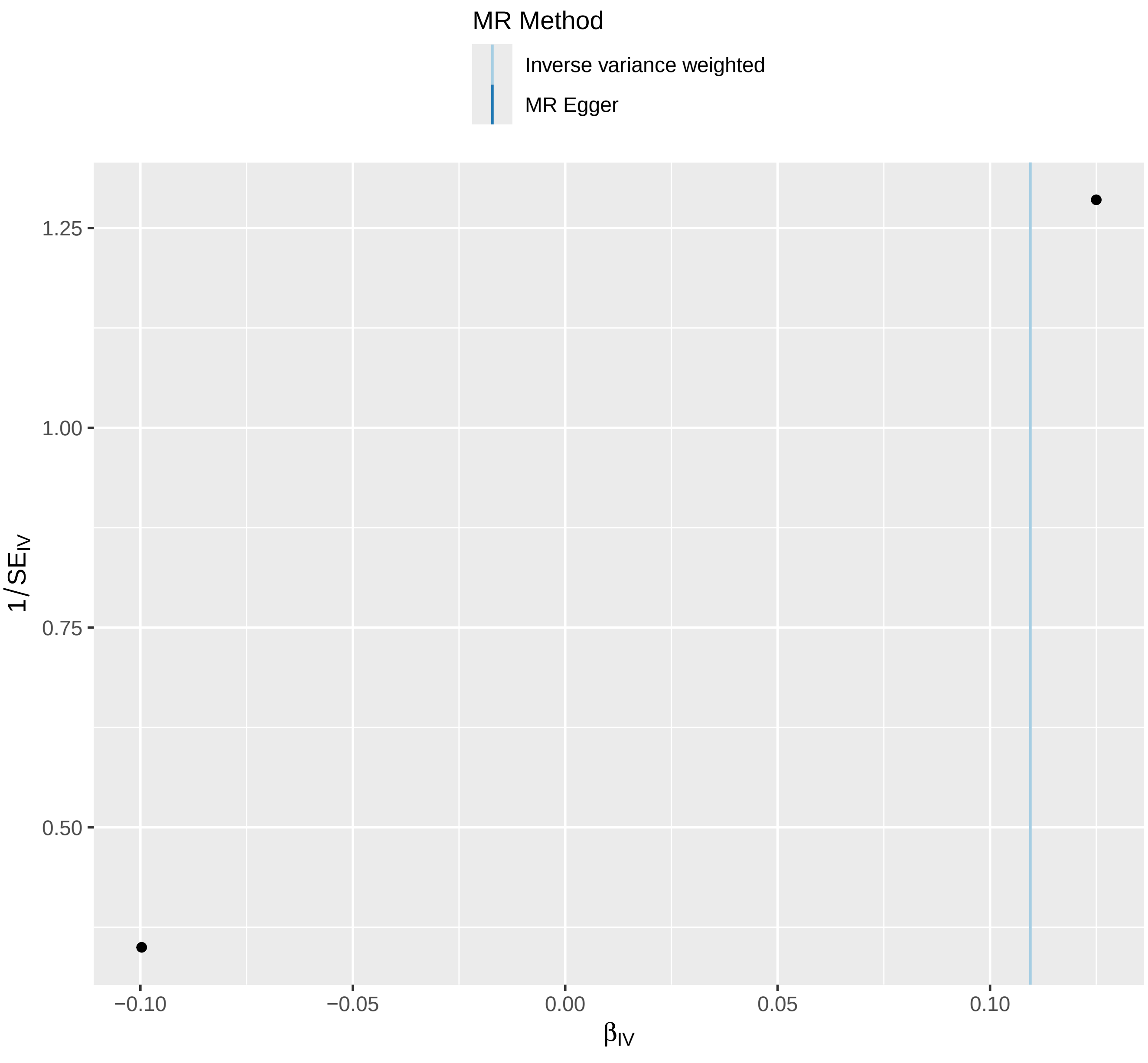
MR Method
Inverse variance weighted
MR Egger
1.25
1.00
0.75
0.50
1/SE_IV
−0.10
−0.05
0.00
0.05
0.10
β_IV

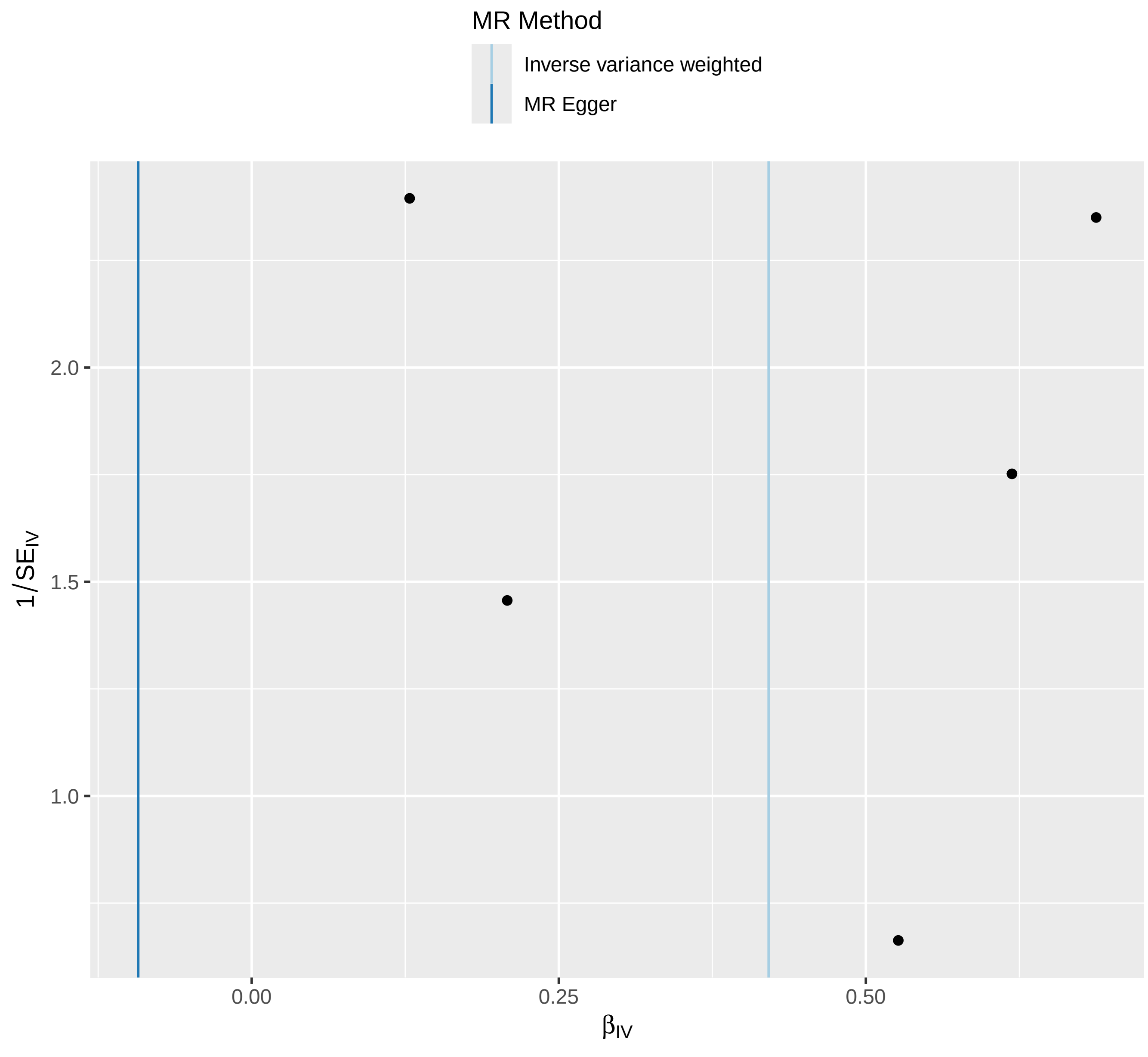
MR Method
Inverse variance weighted
MR Egger
1/SE_IV
2.0
1.5
1.0
0.00
0.25
0.50
β_IV

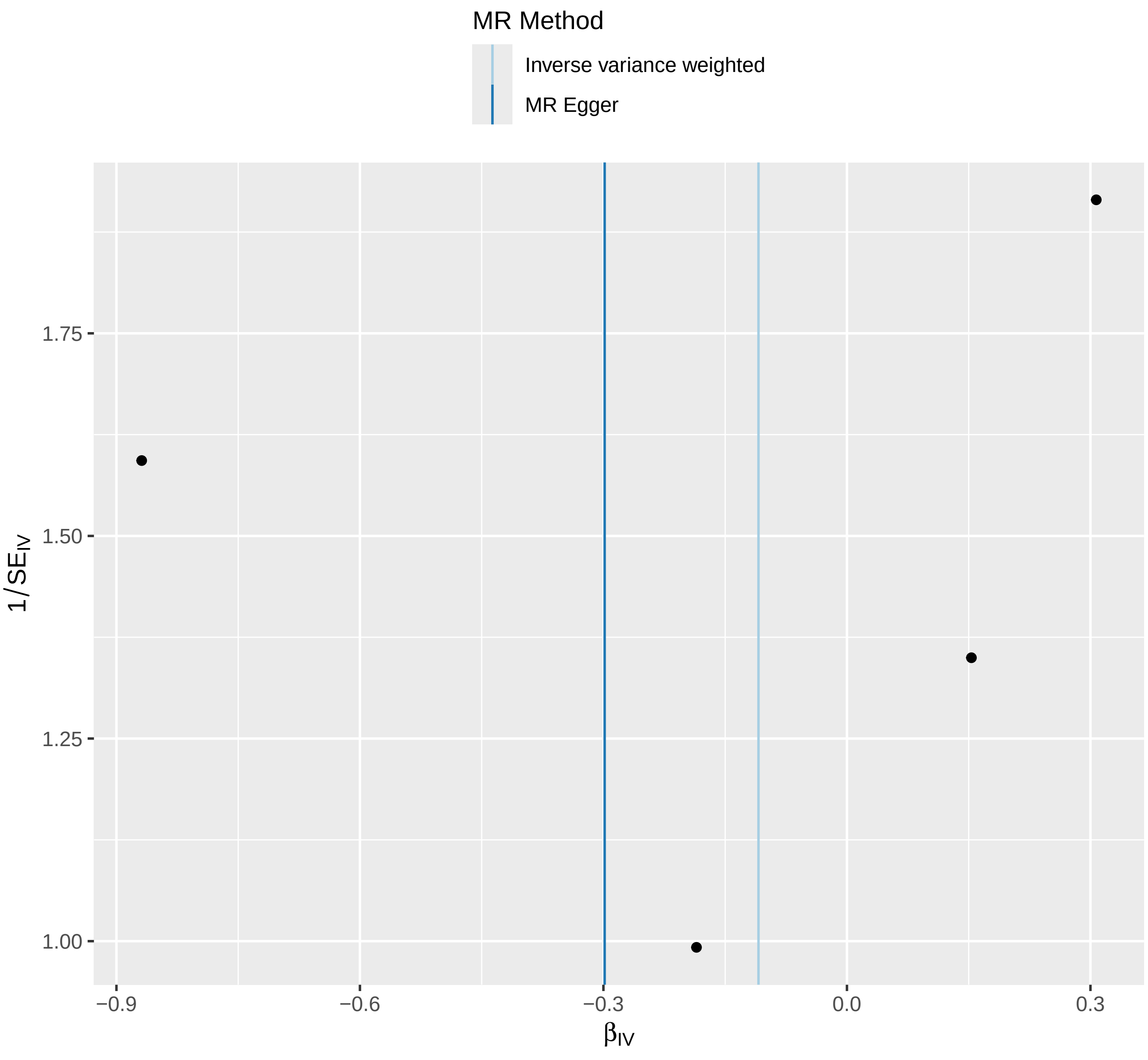
MR Method
Inverse variance weighted
MR Egger
1.75
1.50
1.25
1.00
1/SE$_{IV}$
−0.9
−0.6
−0.3
0.0
0.3
β$_{IV}$

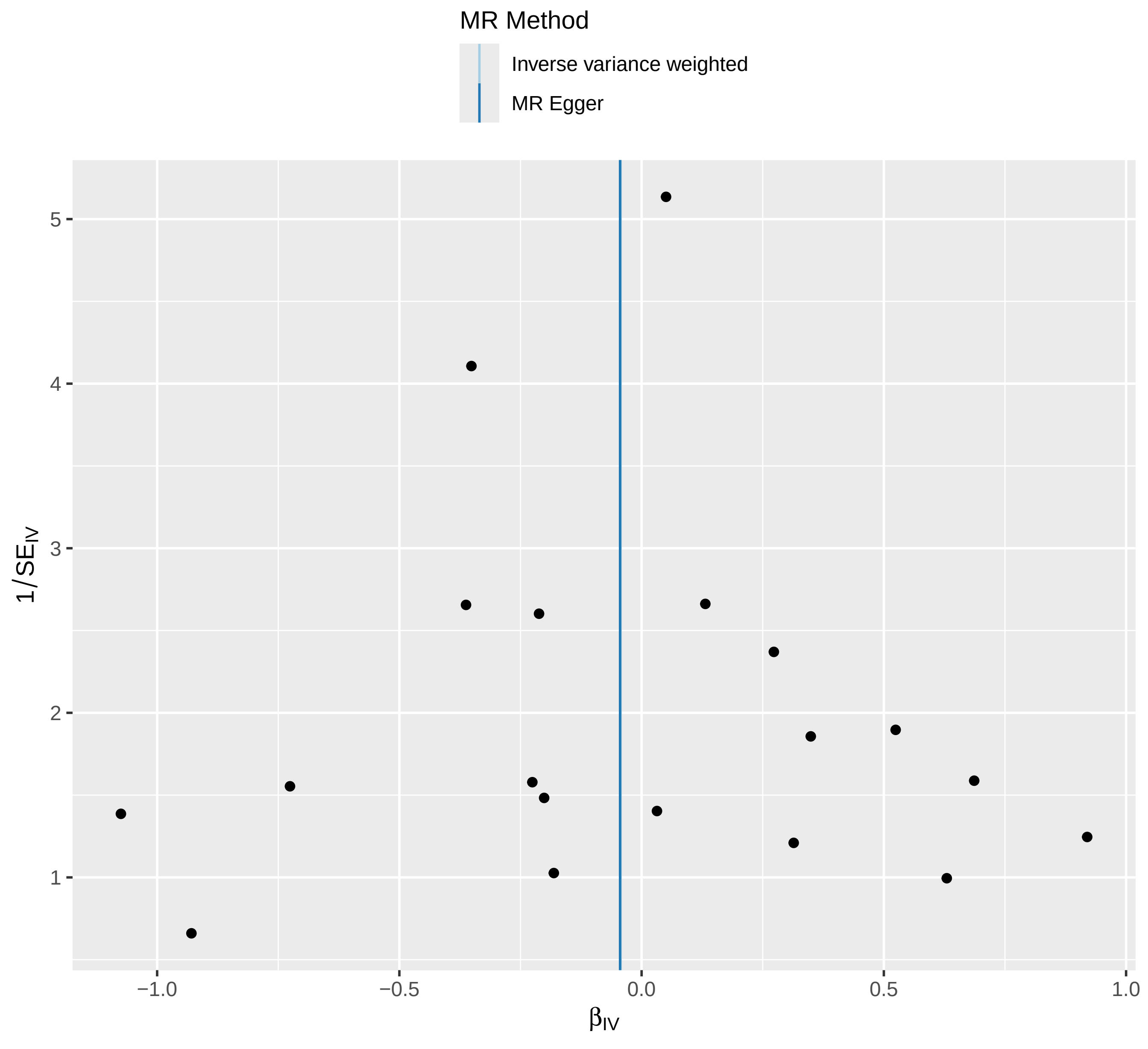

MR Method
Inverse variance weighted
MR Egger
5
4
3
2
1
1/SE_IV
−1.0
−0.5
0.0
0.5
1.0
β_IV

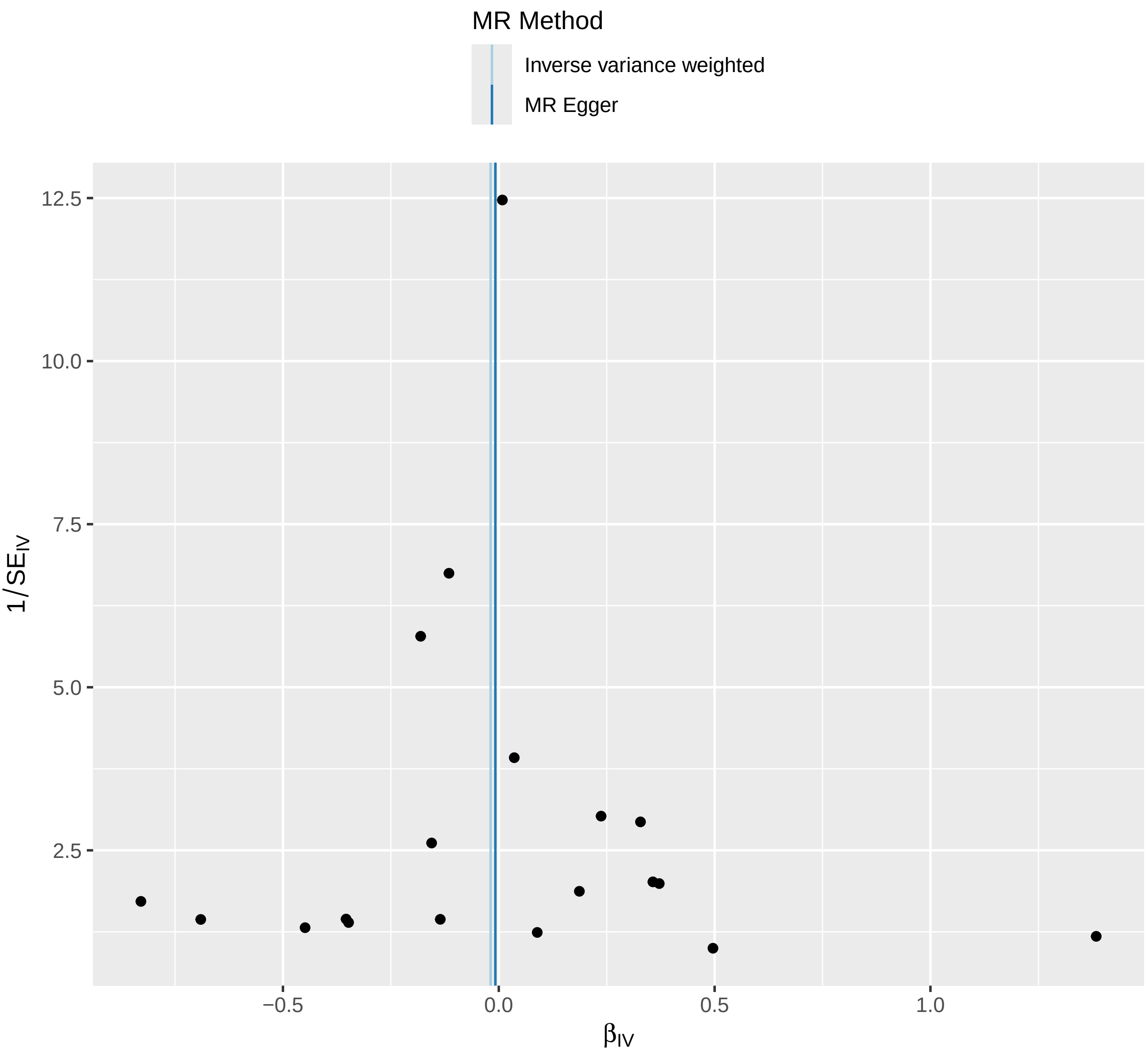

MR Method
Inverse variance weighted
MR Egger
12.5
10.0
7.5
5.0
2.5
$1/SE_{IV}$
−0.5
0.0
0.5
1.0
β_{IV}

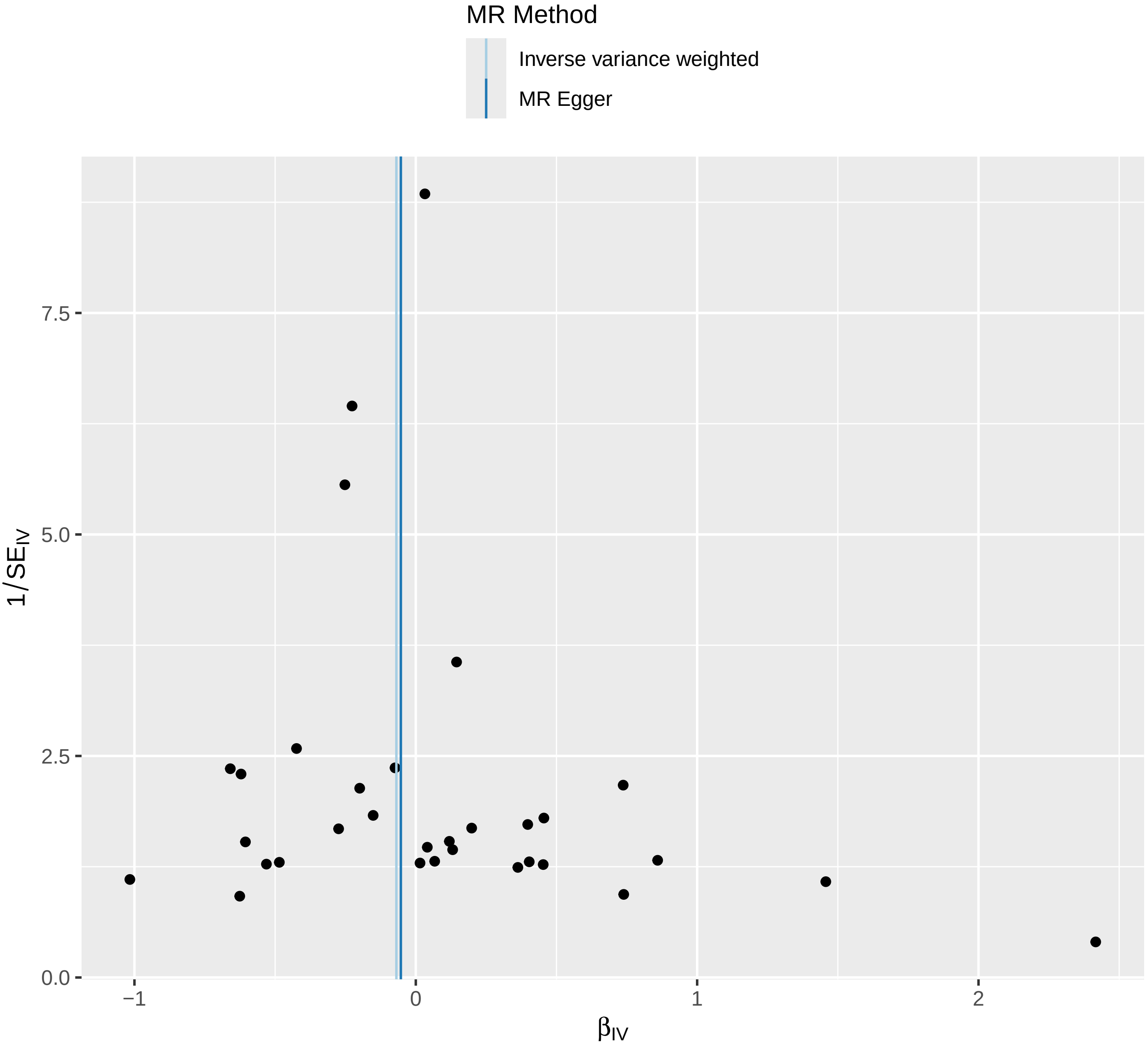

MR Method
Inverse variance weighted
MR Egger
7.5
5.0
2.5
0.0
1/SE_{IV}
−1
0
1
2
β_{IV}

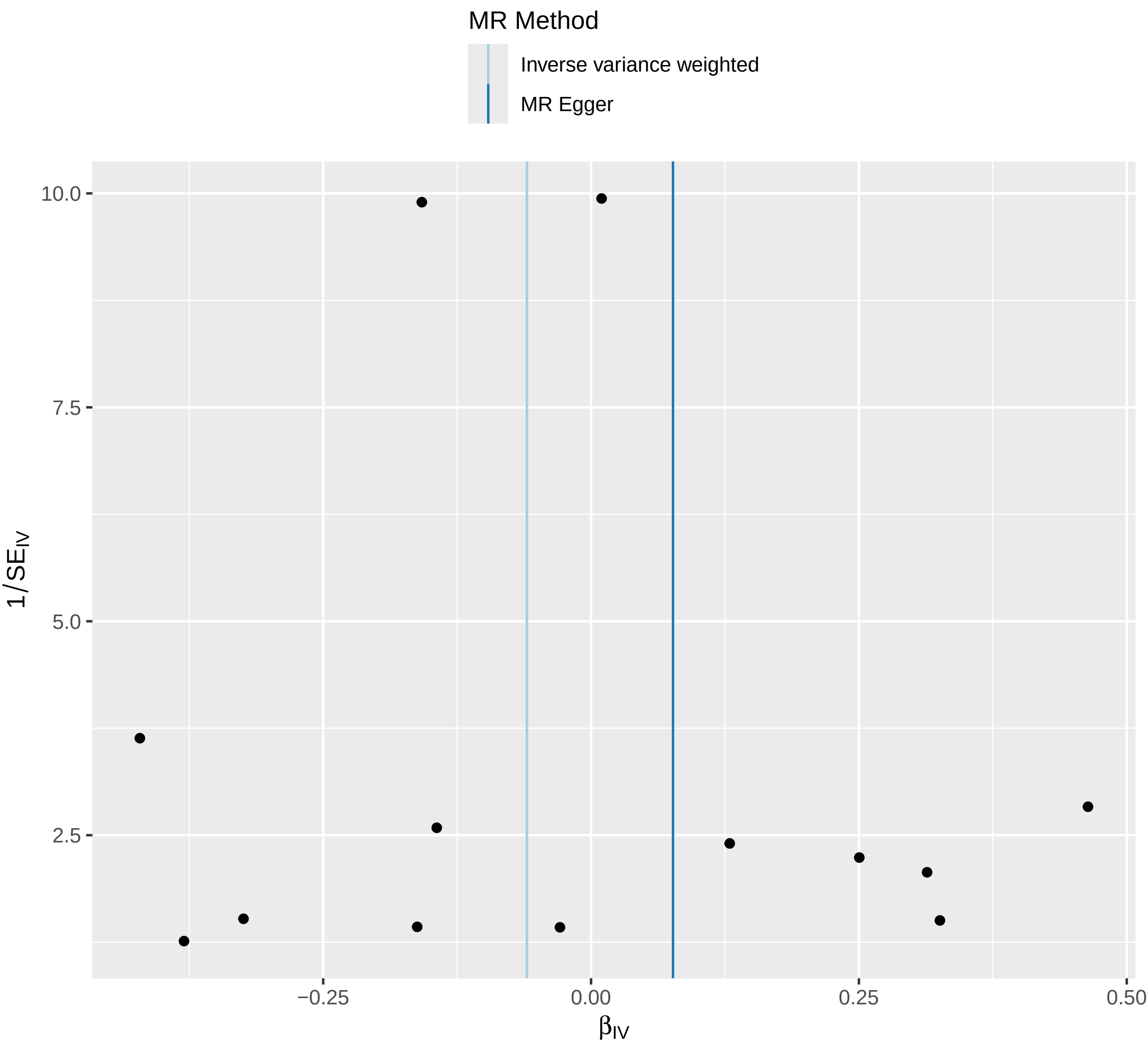
MR Method
Inverse variance weighted
MR Egger
10.0
7.5
5.0
2.5
1/SE$_{IV}$
−0.25
0.00
0.25
0.50
β$_{IV}$

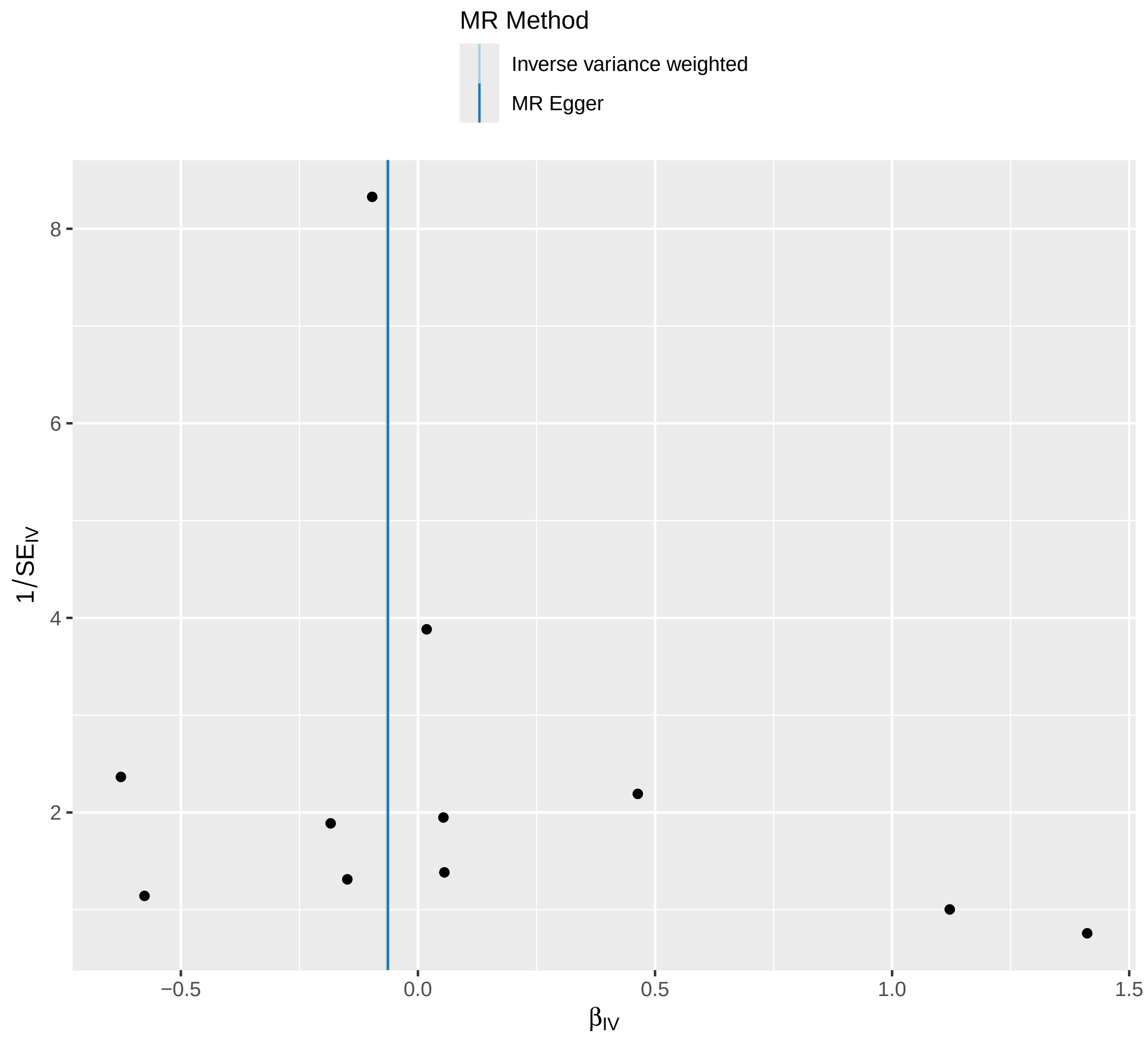
MR Method
Inverse variance weighted
MR Egger
8
6
4
2
1/SE$_{IV}$
−0.5
0.0
0.5
1.0
1.5
β$_{IV}$

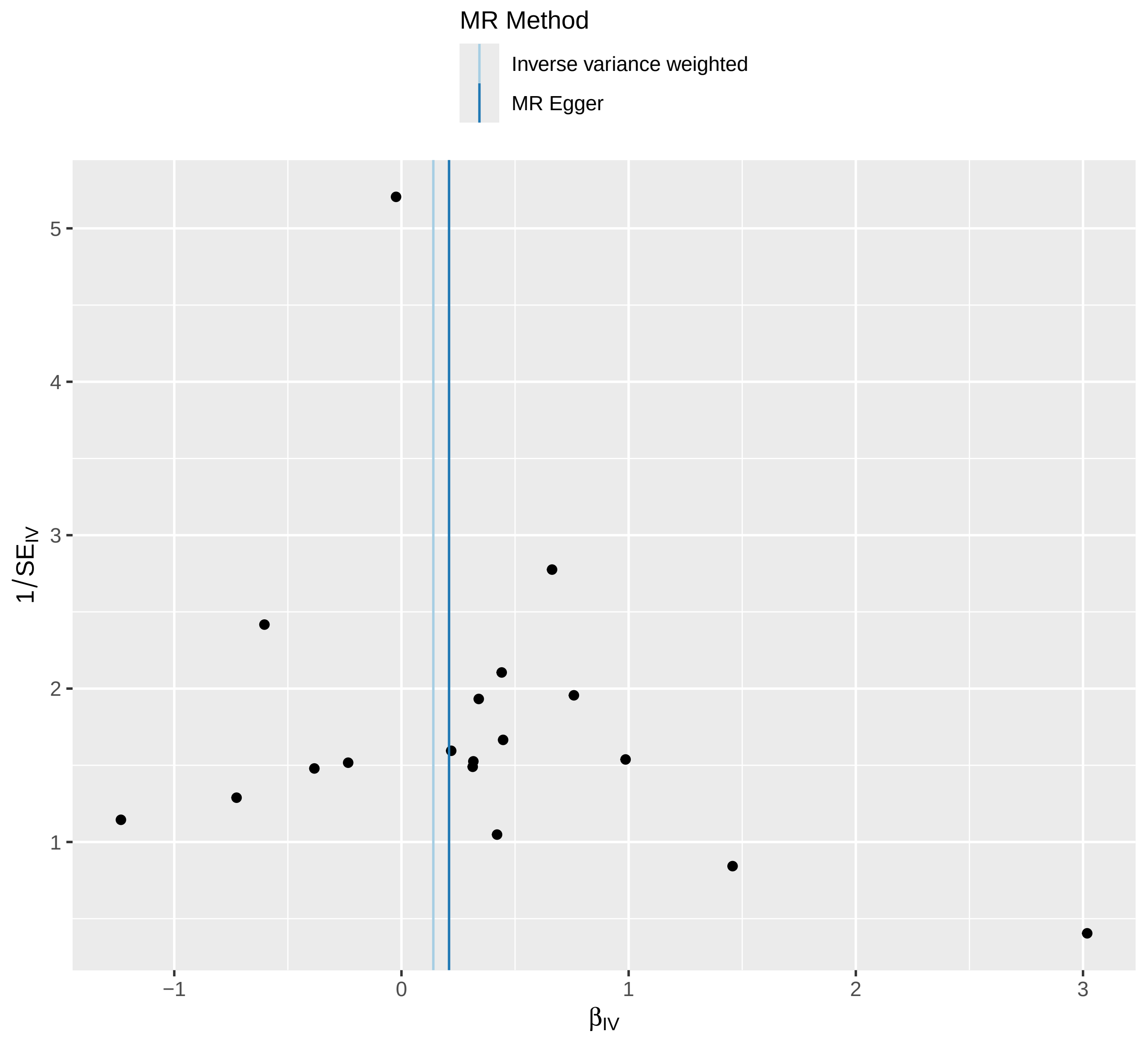
MR Method
Inverse variance weighted
MR Egger
5
4
3
2
1
1/SE$_{IV}$
−1
0
1
2
3
β$_{IV}$

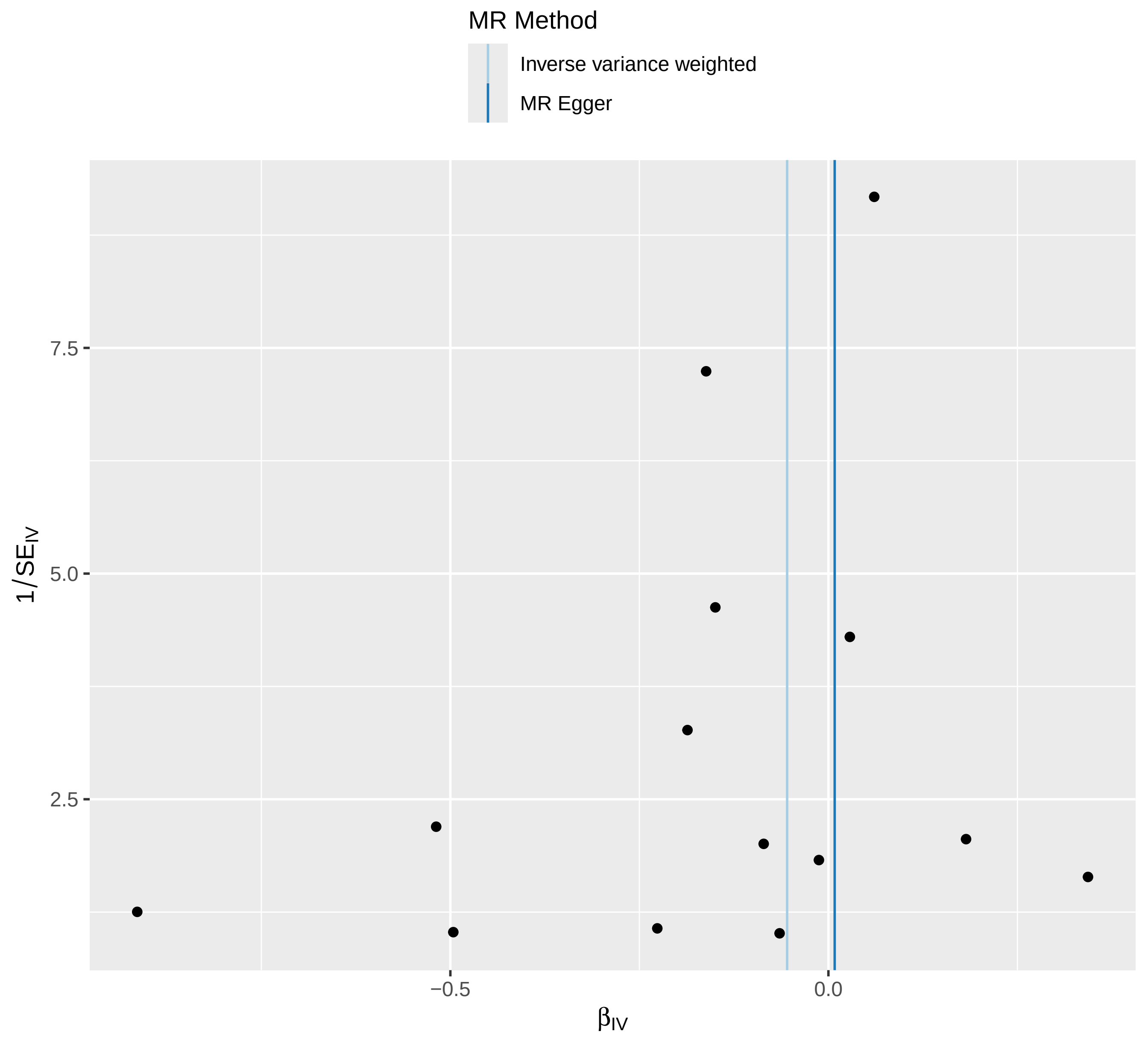
MR Method
Inverse variance weighted
MR Egger
7.5
5.0
2.5
1/SE$_{IV}$
−0.5
0.0
β$_{IV}$

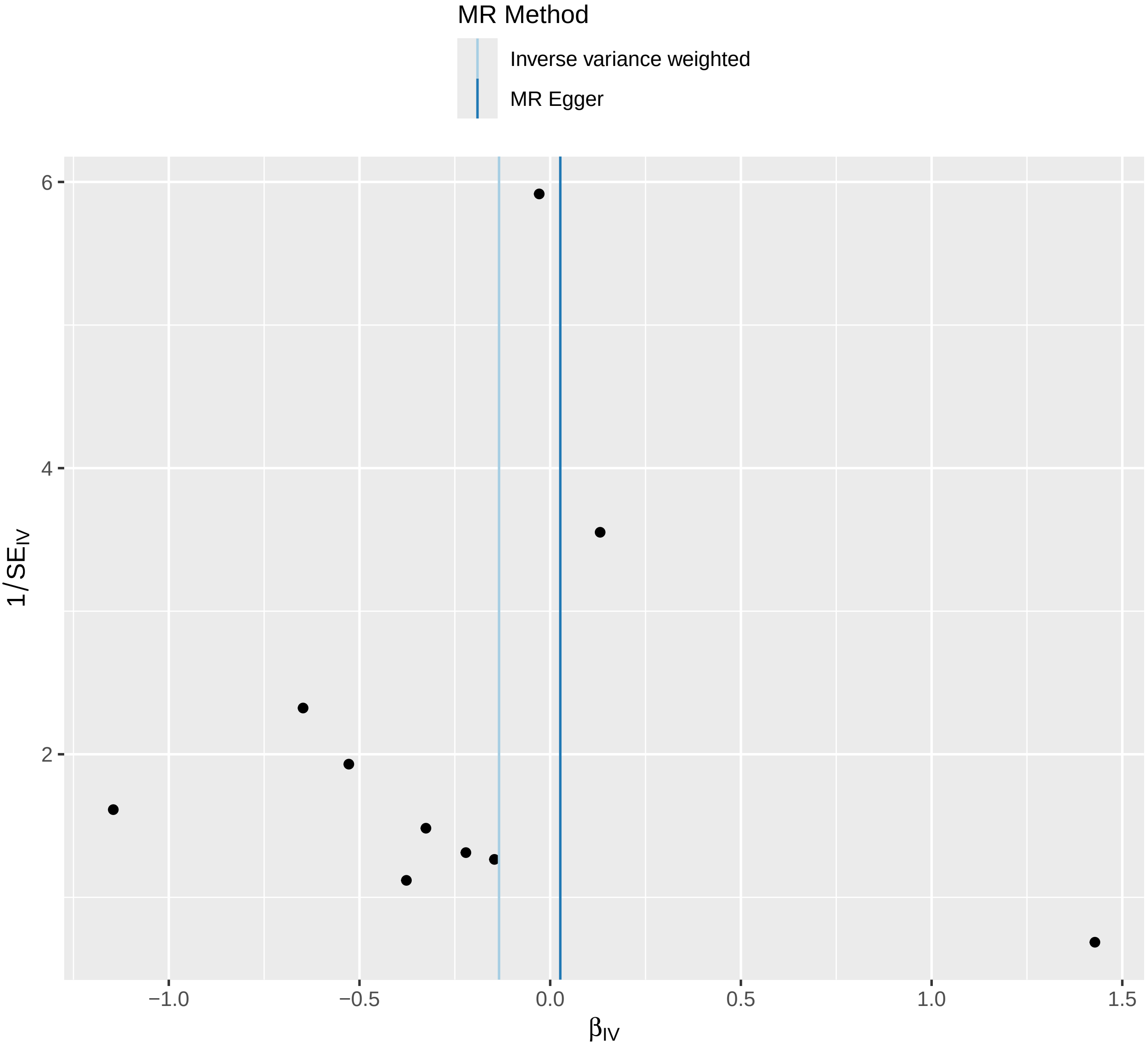
MR Method
Inverse variance weighted
MR Egger
1/SE_IV
β_IV
6
4
2
−1.0
−0.5
0.0
0.5
1.0
1.5

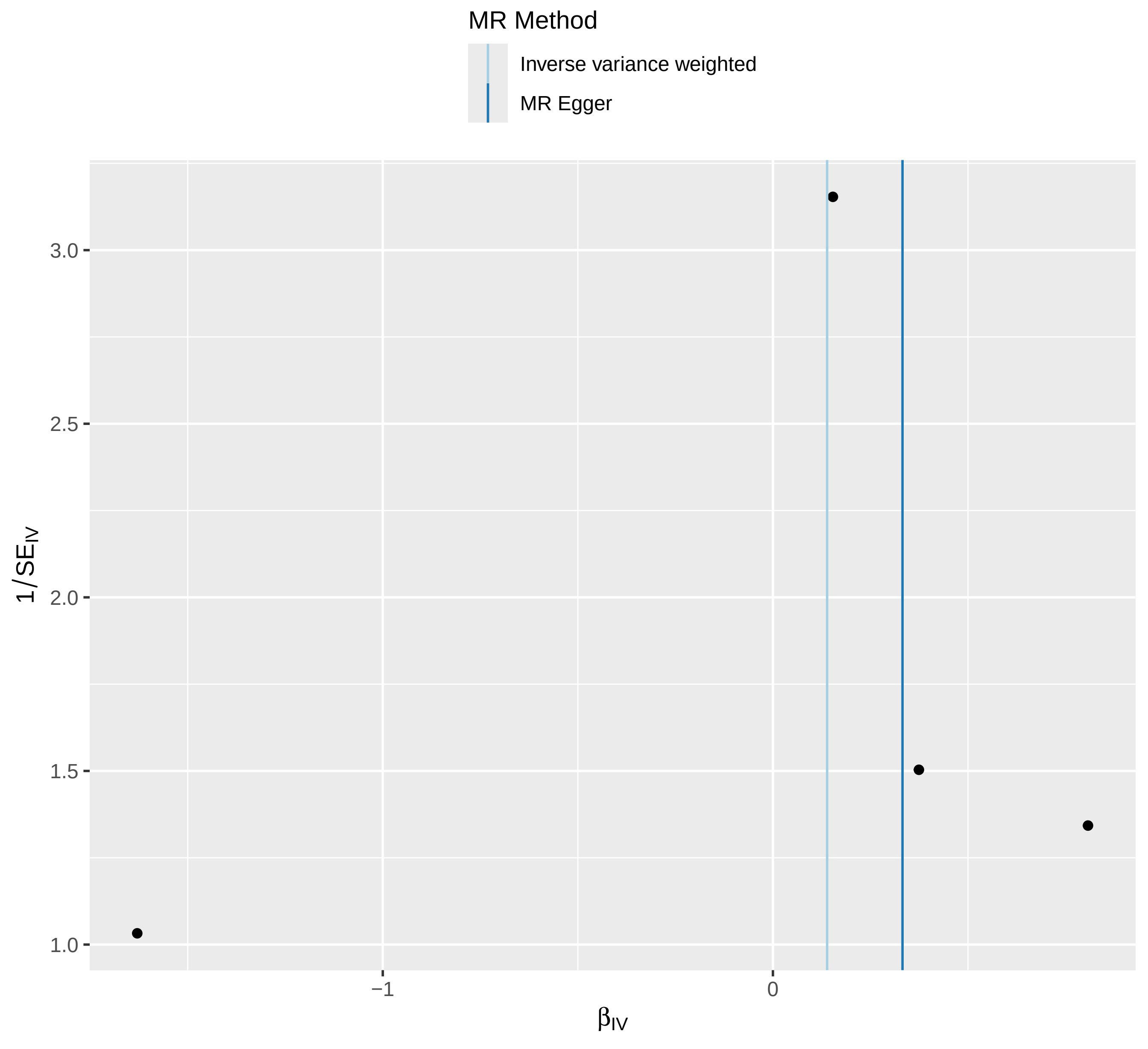

MR Method
Inverse variance weighted
MR Egger
3.0
2.5
2.0
1.5
1.0
1/SE_IV
−1
0
β_IV

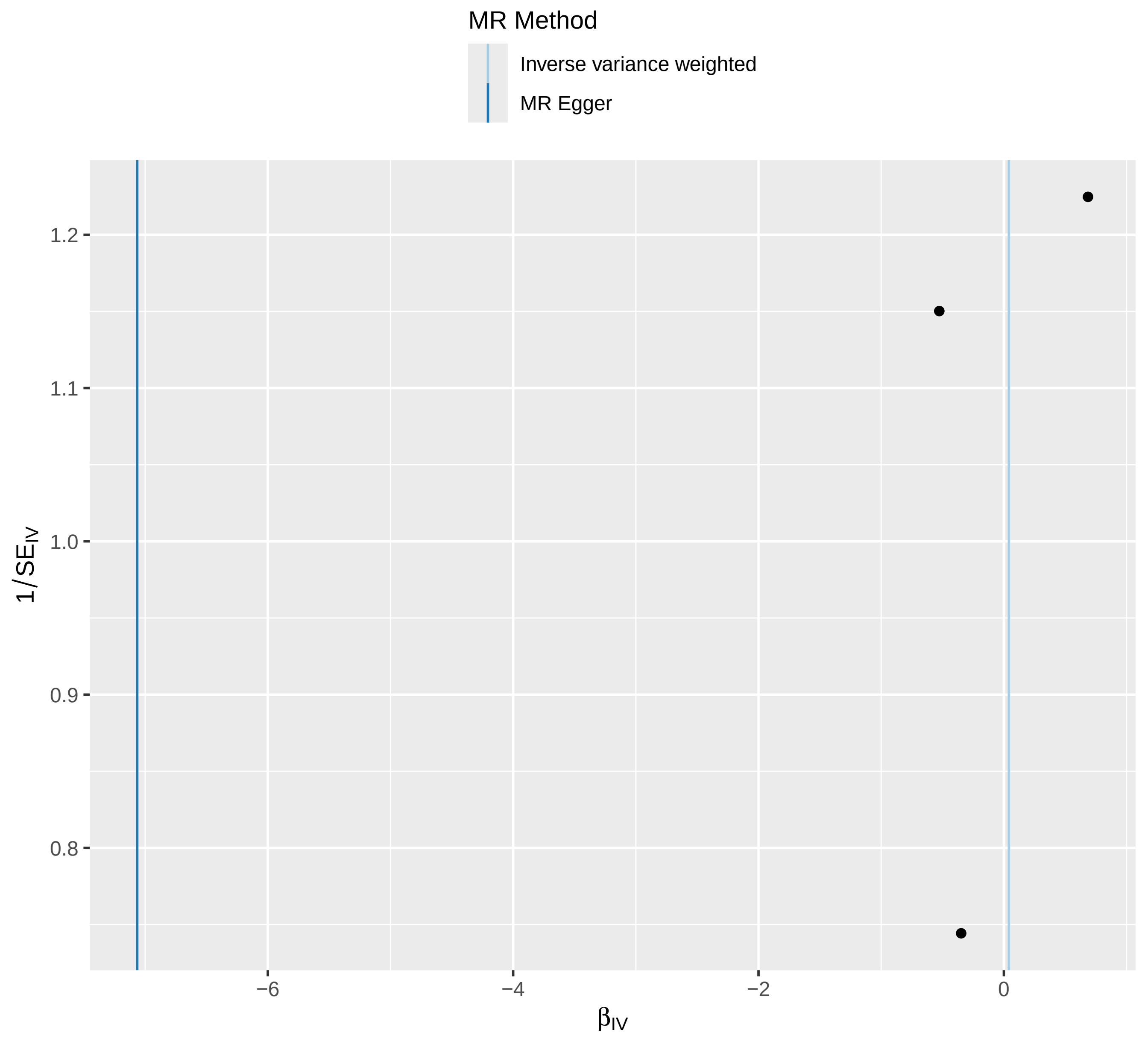

MR Method
Inverse variance weighted
MR Egger
1.2
1.1
1.0
0.9
0.8
1/SE_IV
−6
−4
−2
0
β_IV

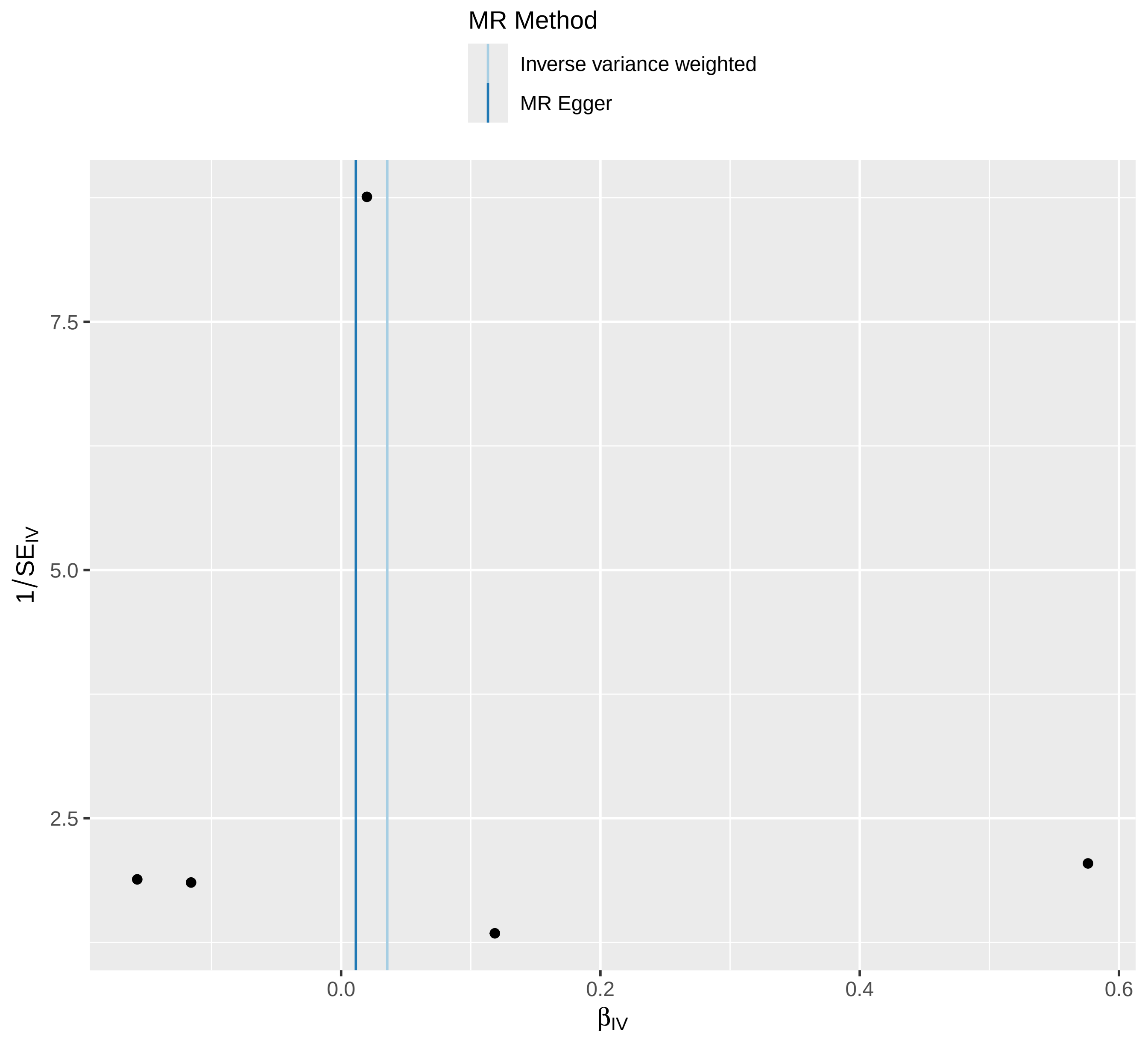

MR Method
Inverse variance weighted
MR Egger
7.5
5.0
2.5
0.0
0.2
0.4
0.6
1/SE_IV
β_IV

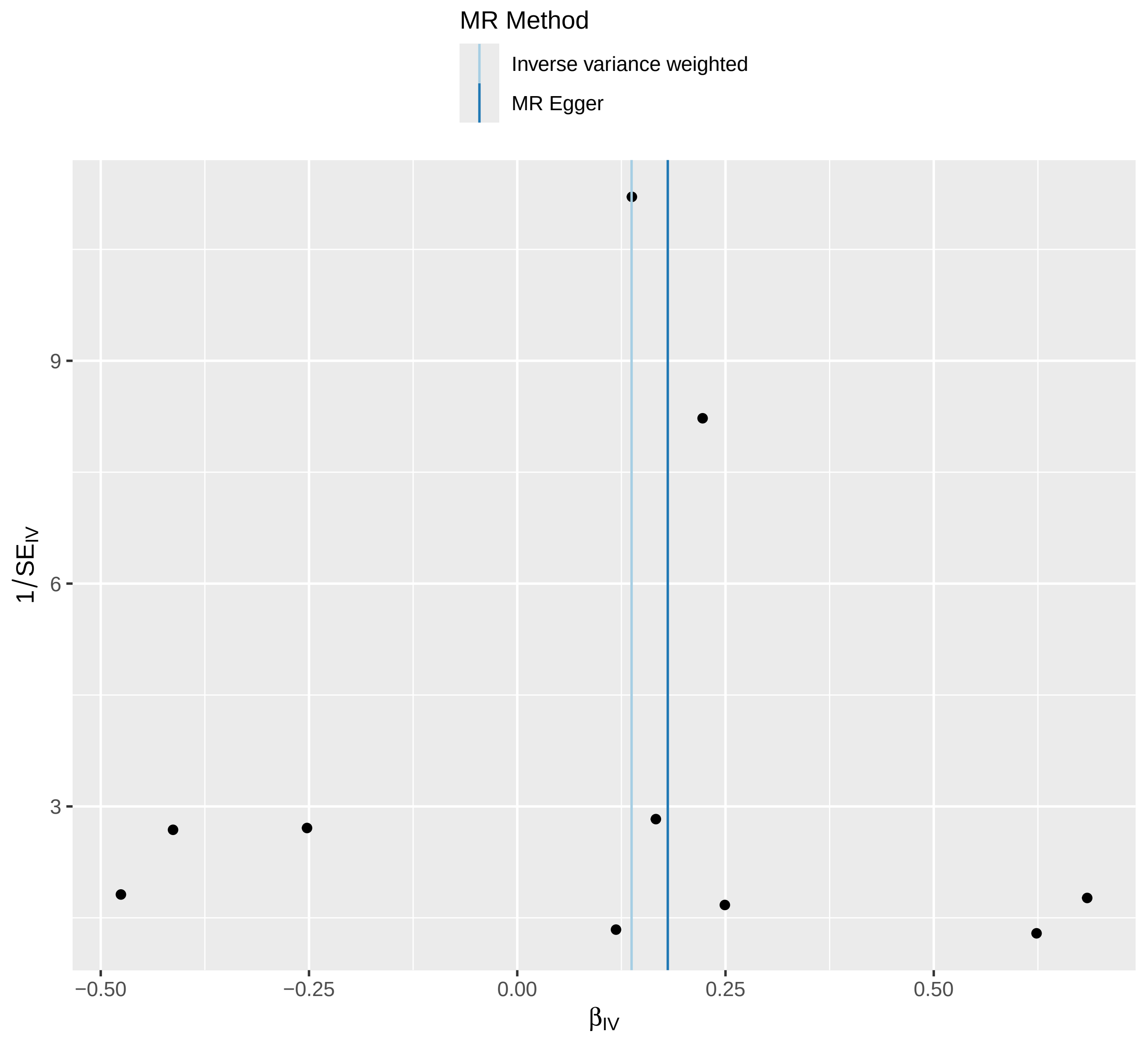
MR Method
Inverse variance weighted
MR Egger
9
6
3
1/SE_IV
−0.50
−0.25
0.00
0.25
0.50
β_IV

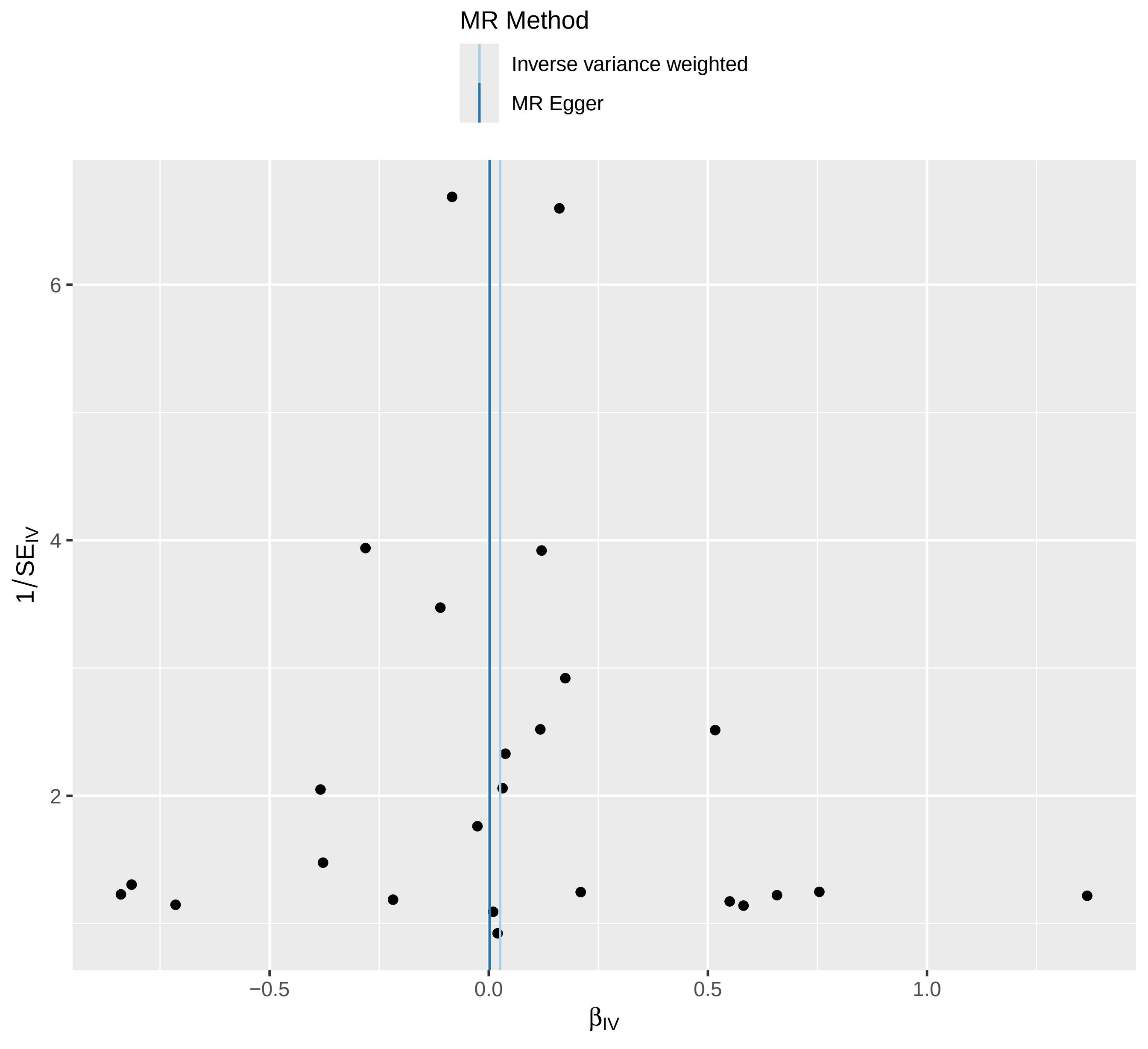
MR Method
Inverse variance weighted
MR Egger
6
4
2
1/SE_IV
−0.5
0.0
0.5
1.0
β_IV

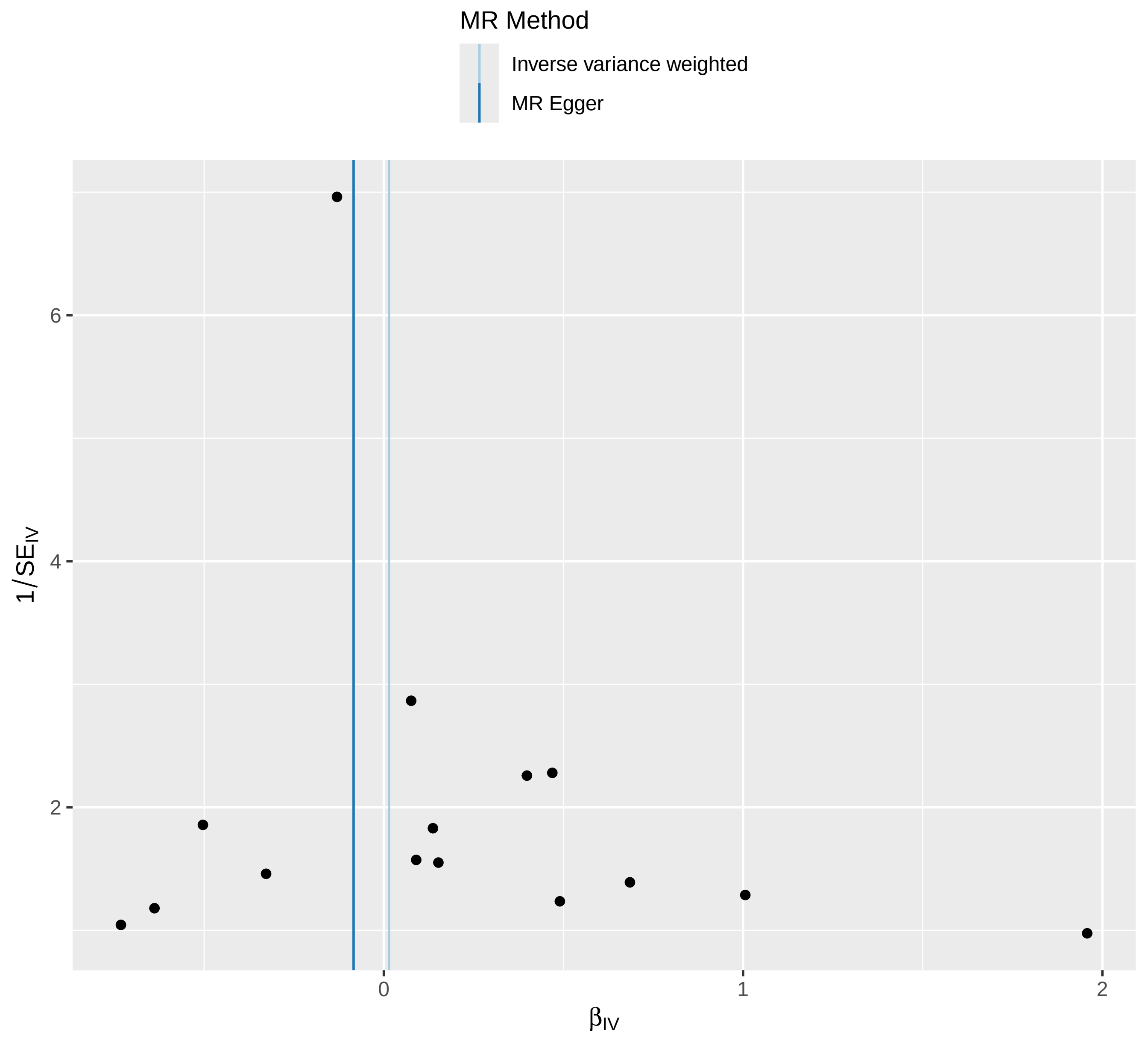

MR Method
Inverse variance weighted
MR Egger
6
4
2
0
1
2
1/SE_IV
β_IV

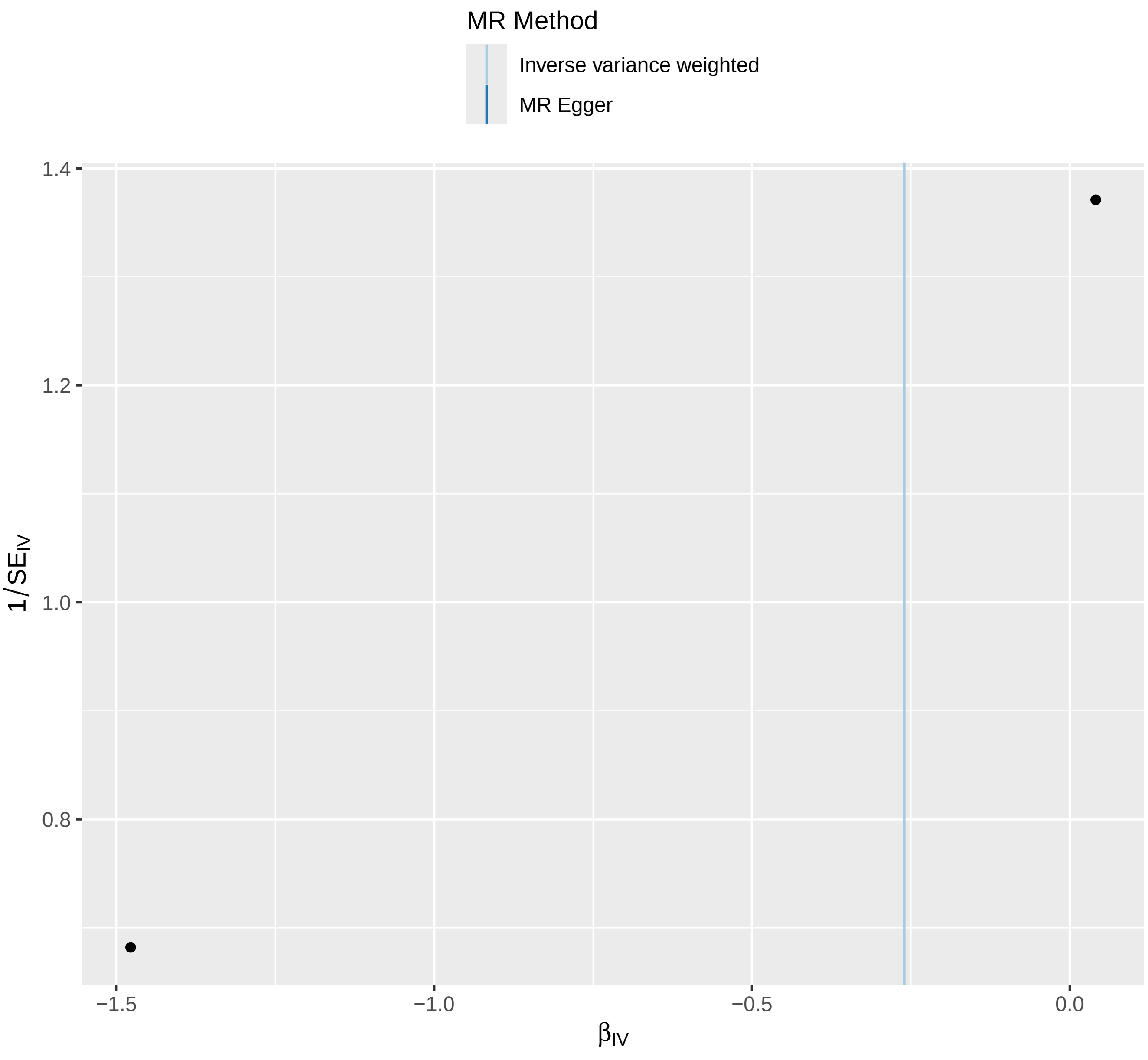
MR Method
Inverse variance weighted
MR Egger
1.4
1.2
1.0
0.8
1/SE$_{IV}$
−1.5
−1.0
−0.5
0.0
β$_{IV}$

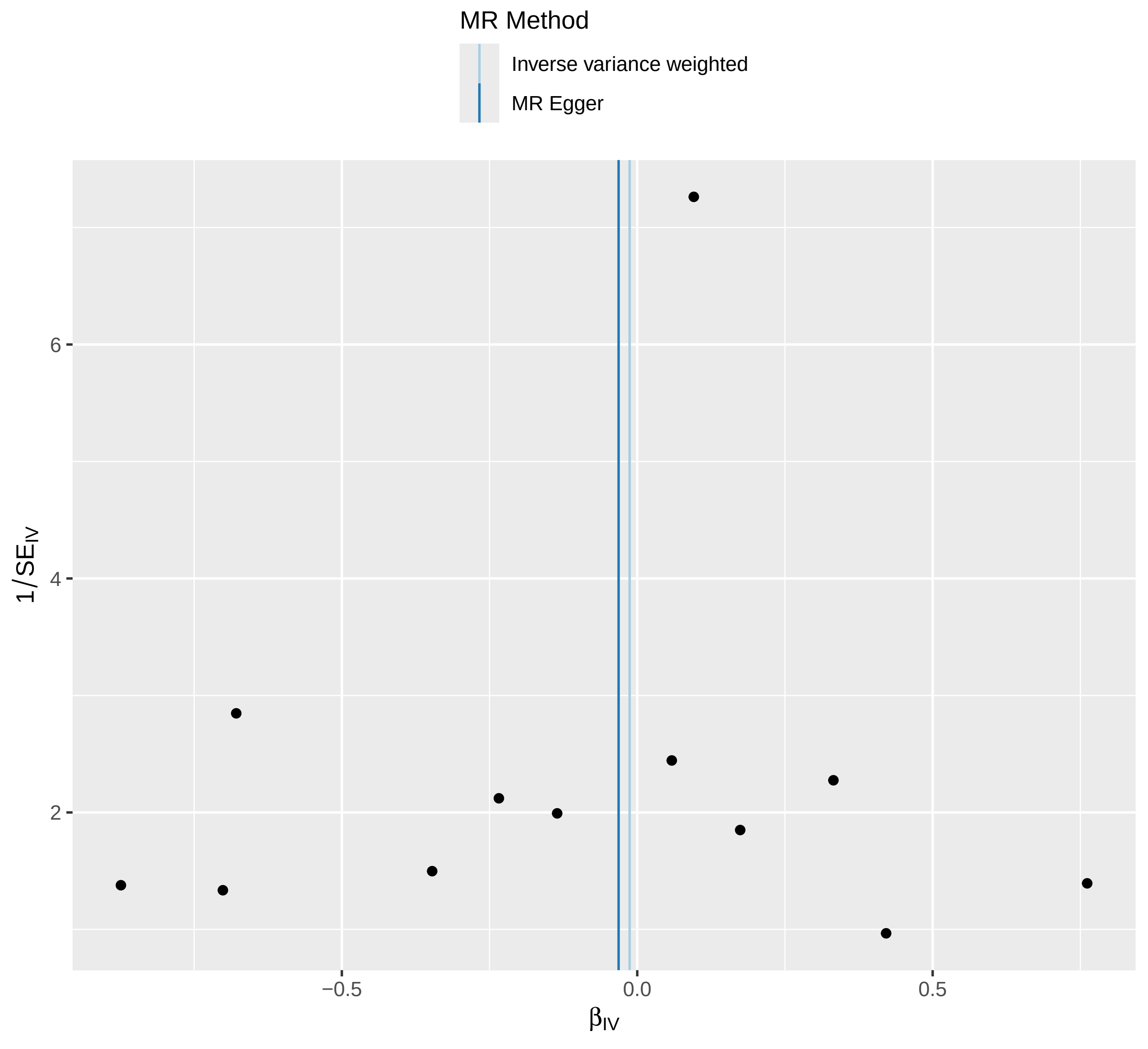

MR Method
Inverse variance weighted
MR Egger
6
4
2
1/SE_IV
−0.5
0.0
0.5
β_IV

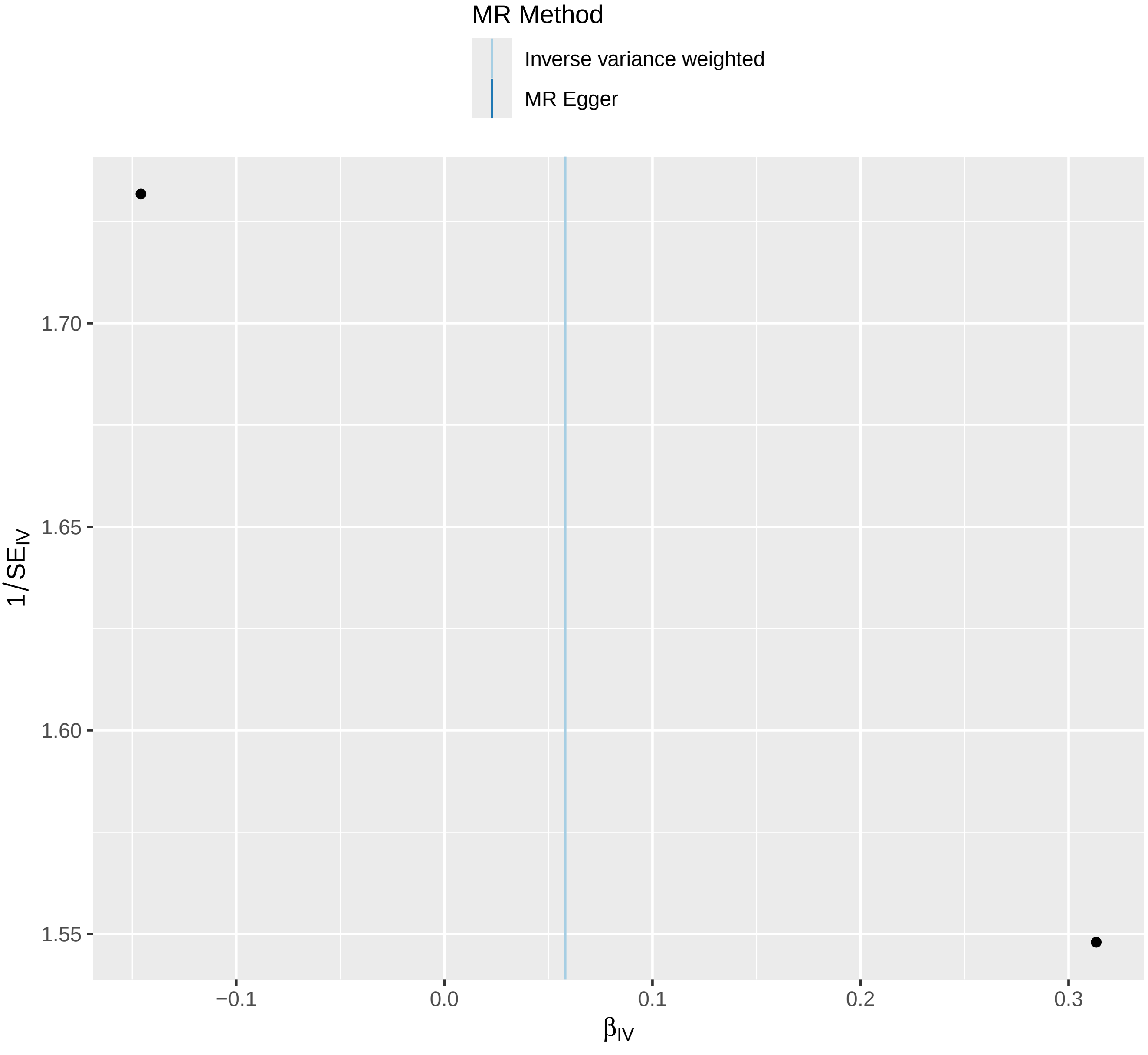
MR Method
Inverse variance weighted
MR Egger
1.70
1.65
1.60
1.55
1/SE_IV
−0.1
0.0
0.1
0.2
0.3
β_IV

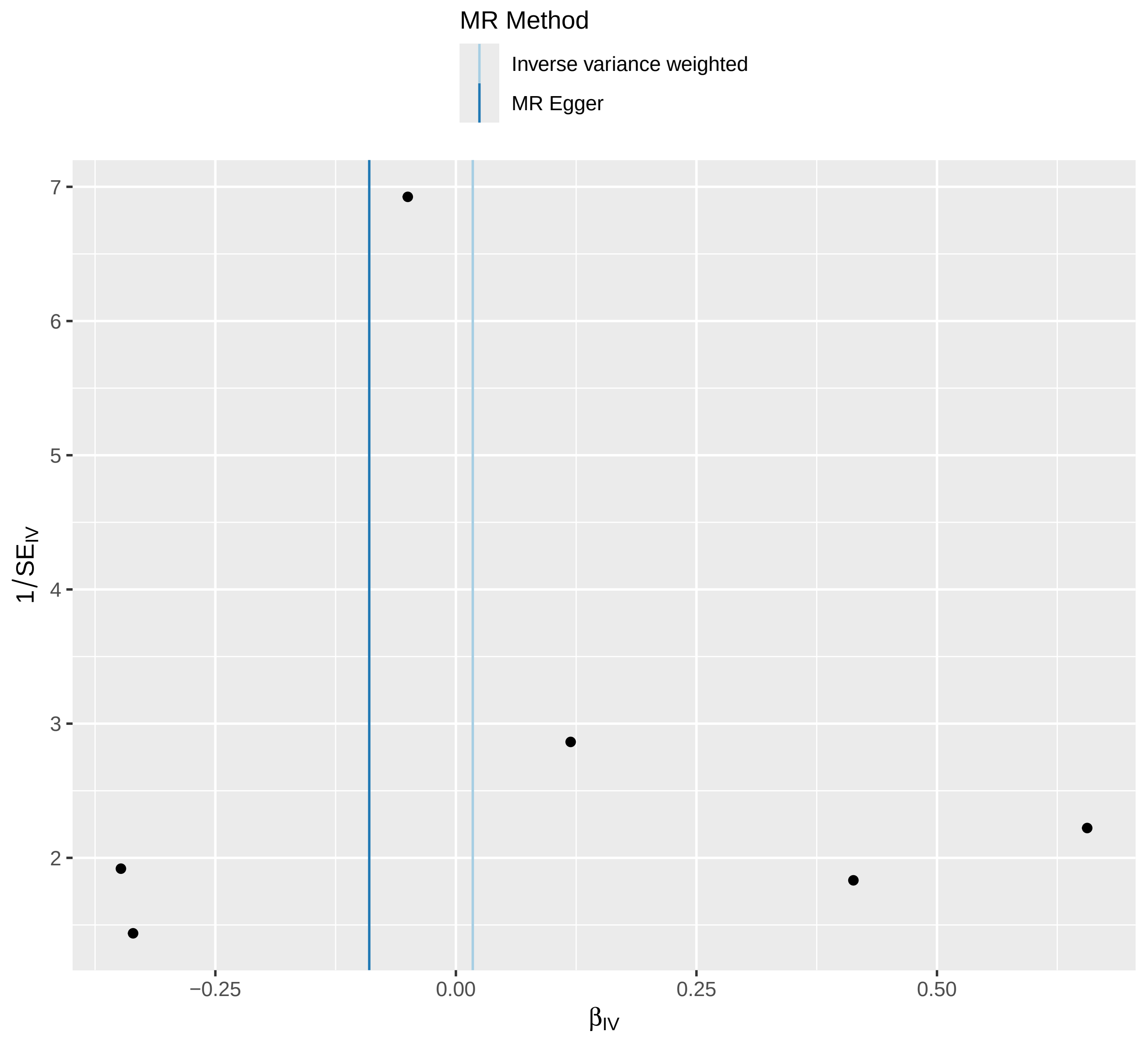

MR Method
Inverse variance weighted
MR Egger
7
6
5
4
3
2
1/SE_IV
−0.25
0.00
0.25
0.50
β_IV

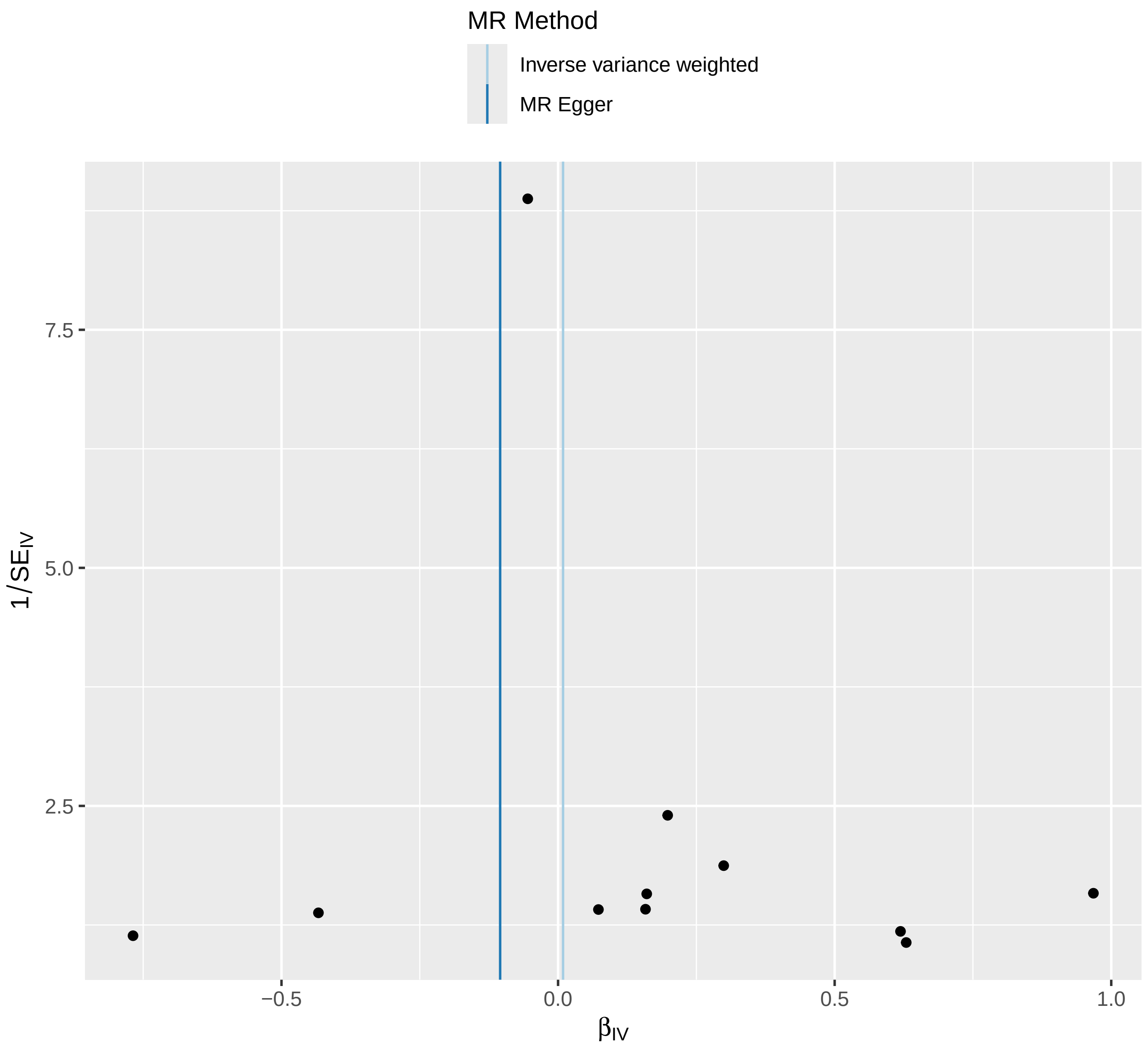
MR Method
Inverse variance weighted
MR Egger
7.5
5.0
2.5
−0.5
0.0
0.5
1.0
$1/SE_{IV}$
β_{IV}

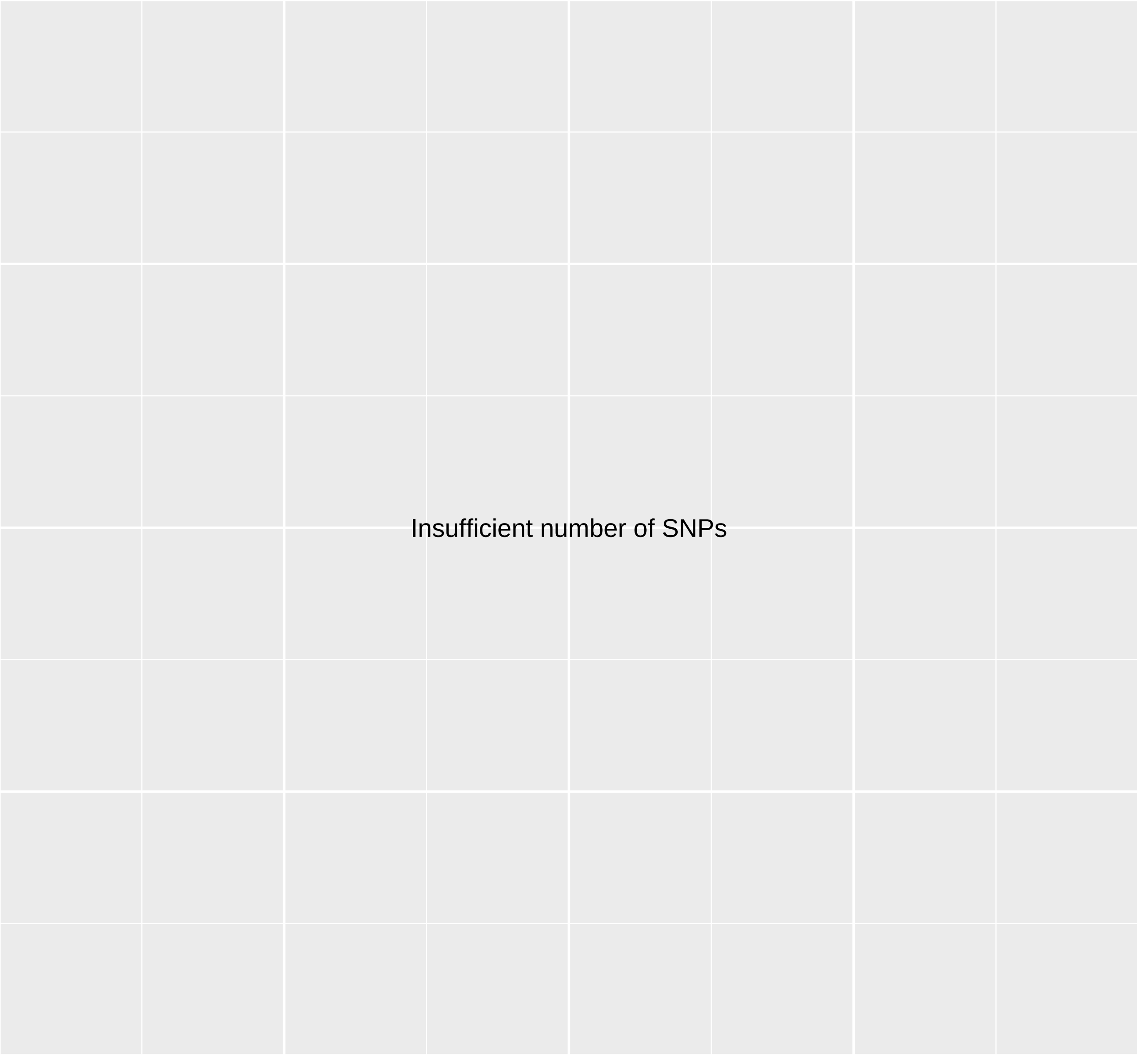
Insufficient number of SNPs

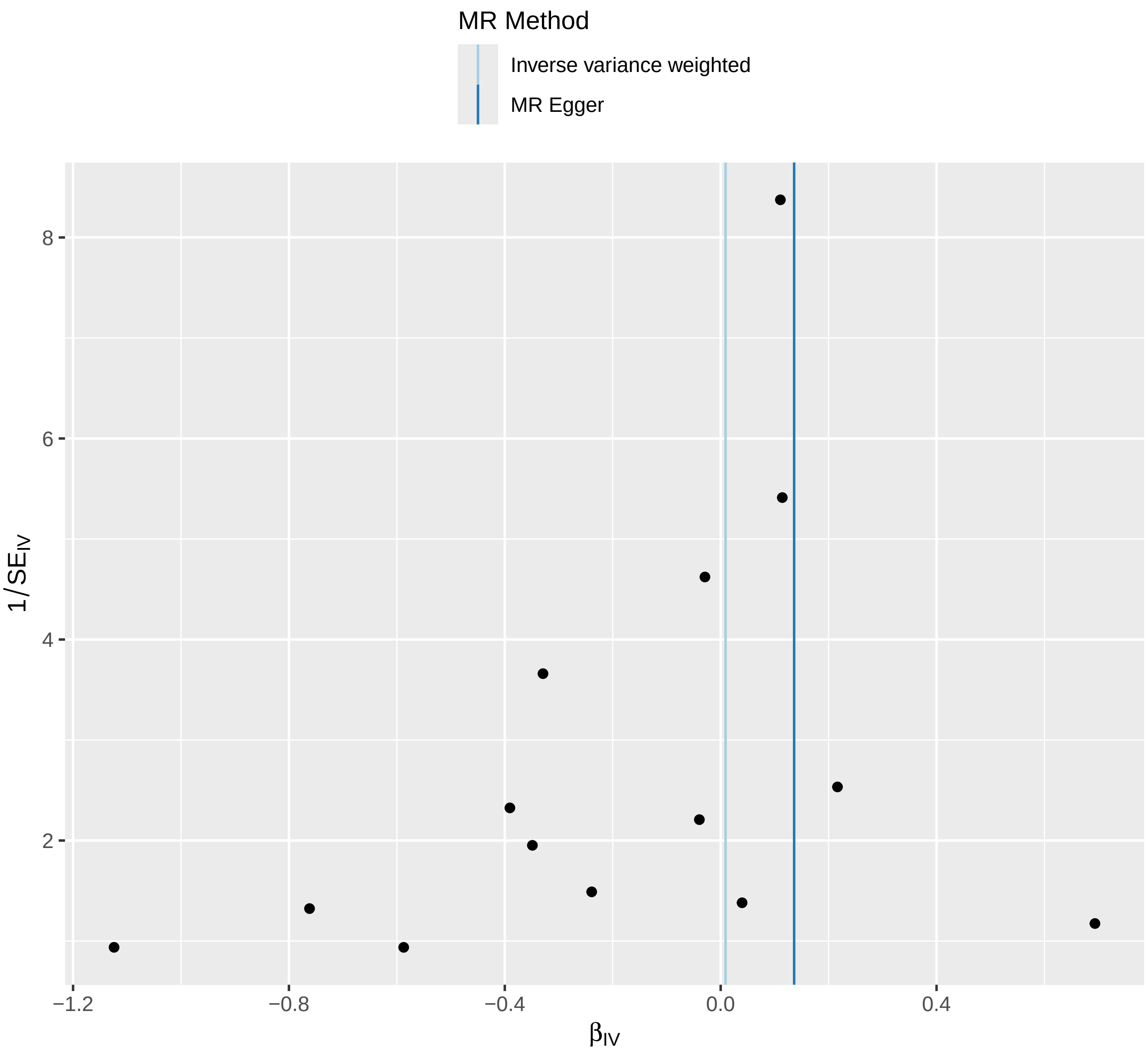
MR Method
Inverse variance weighted
MR Egger
8
6
4
2
$1/SE_{IV}$
−1.2
−0.8
−0.4
0.0
0.4
β_{IV}

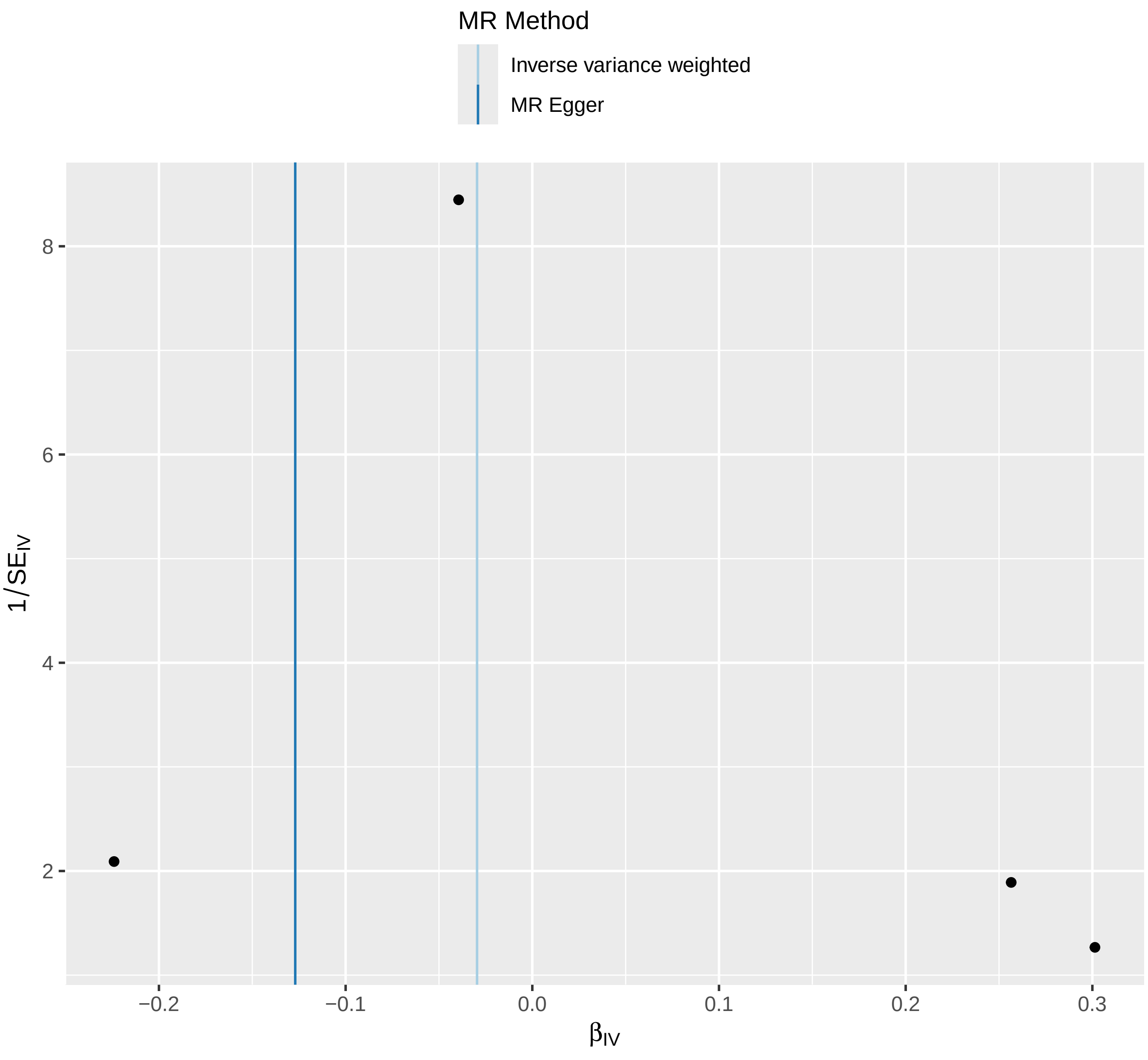
MR Method
Inverse variance weighted
MR Egger
8
6
4
2
1/SE_IV
−0.2
−0.1
0.0
0.1
0.2
0.3
β_IV

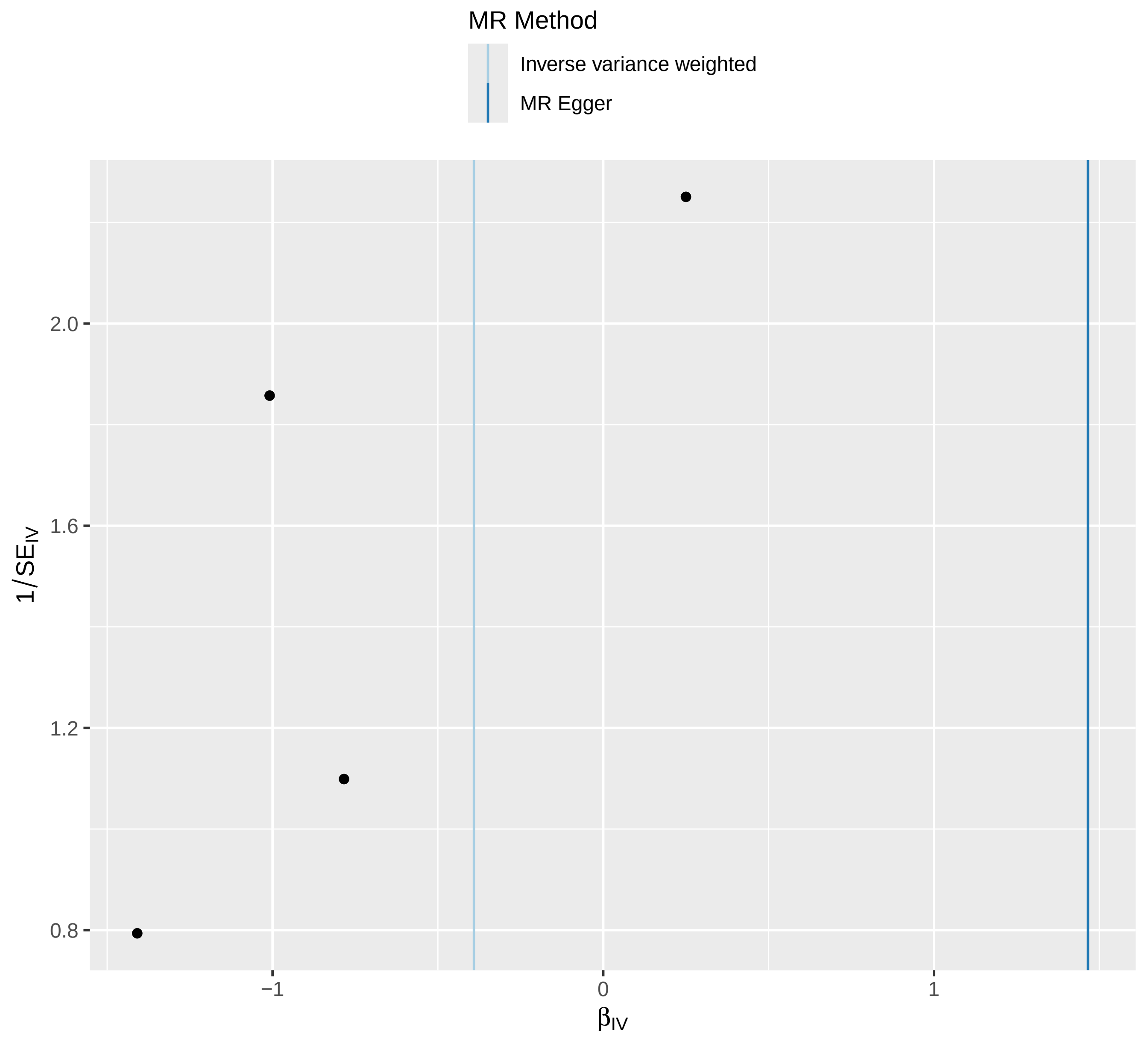

MR Method
Inverse variance weighted
MR Egger
2.0
1.6
1.2
0.8
1/SE$_{IV}$
−1
0
1
β$_{IV}$

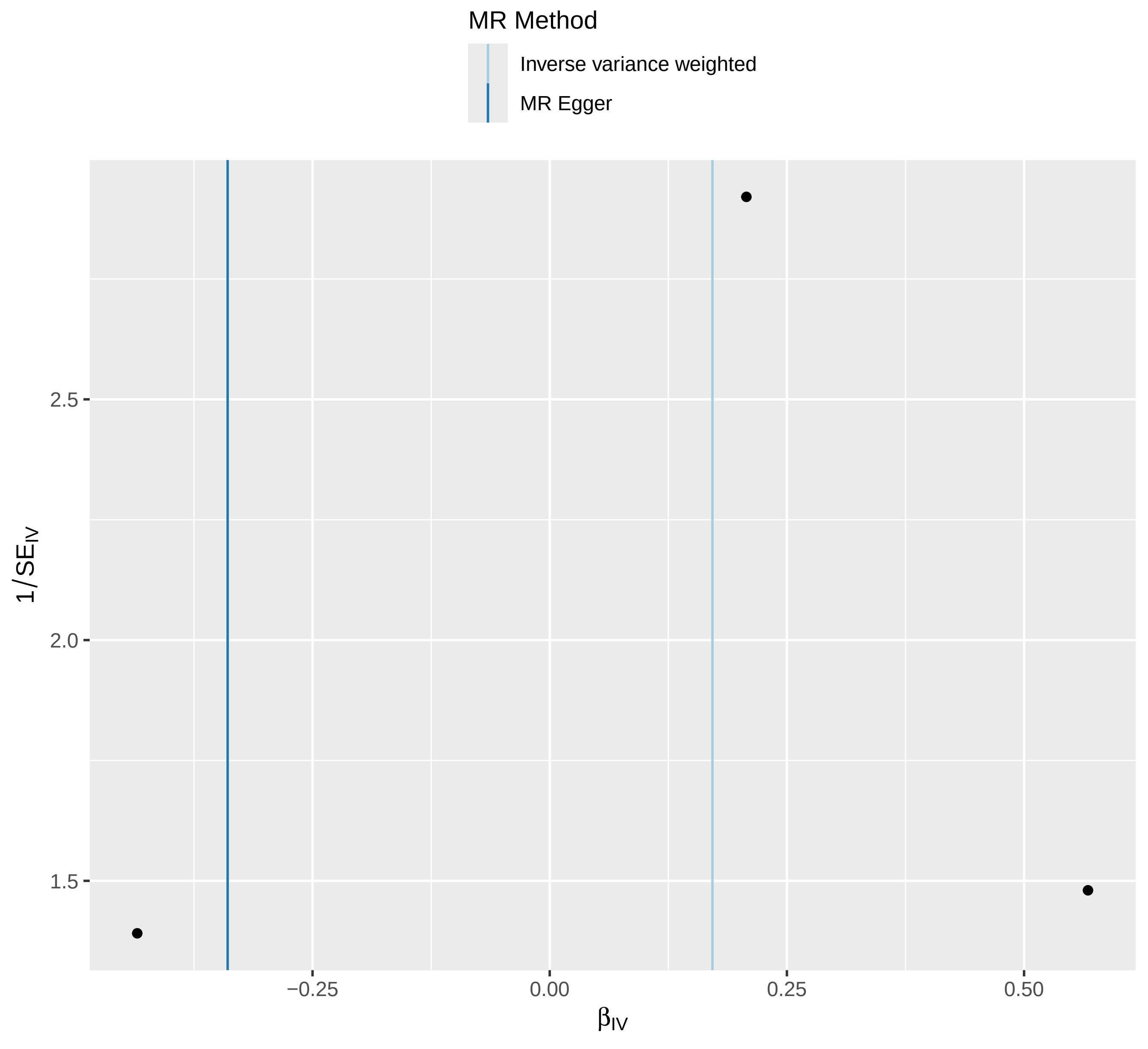
MR Method
Inverse variance weighted
MR Egger
1/SE_IV
2.5
2.0
1.5
−0.25
0.00
0.25
0.50
β_IV

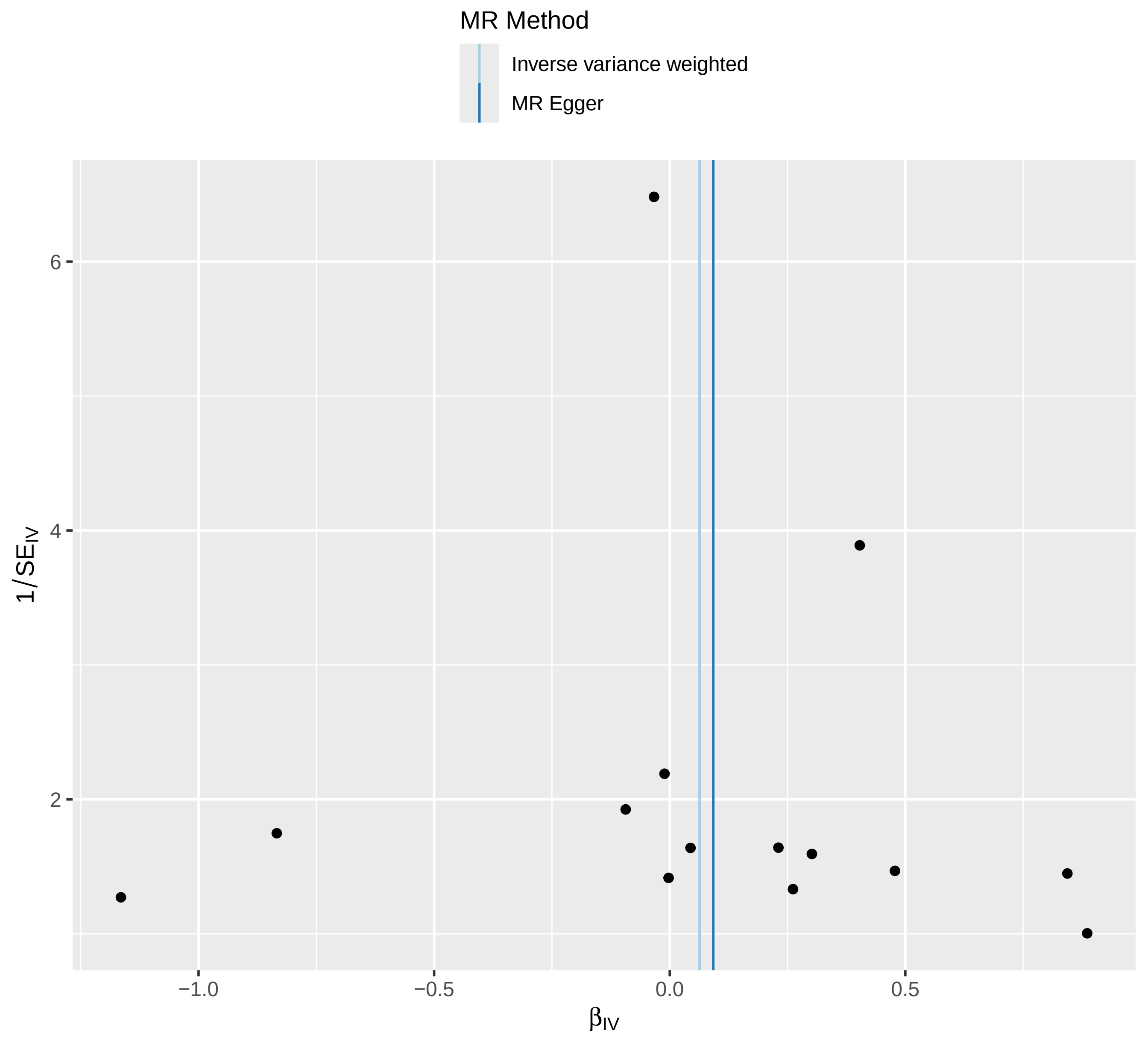
MR Method
Inverse variance weighted
MR Egger
6
4
2
1/SE$_{IV}$
−1.0
−0.5
0.0
0.5
β$_{IV}$

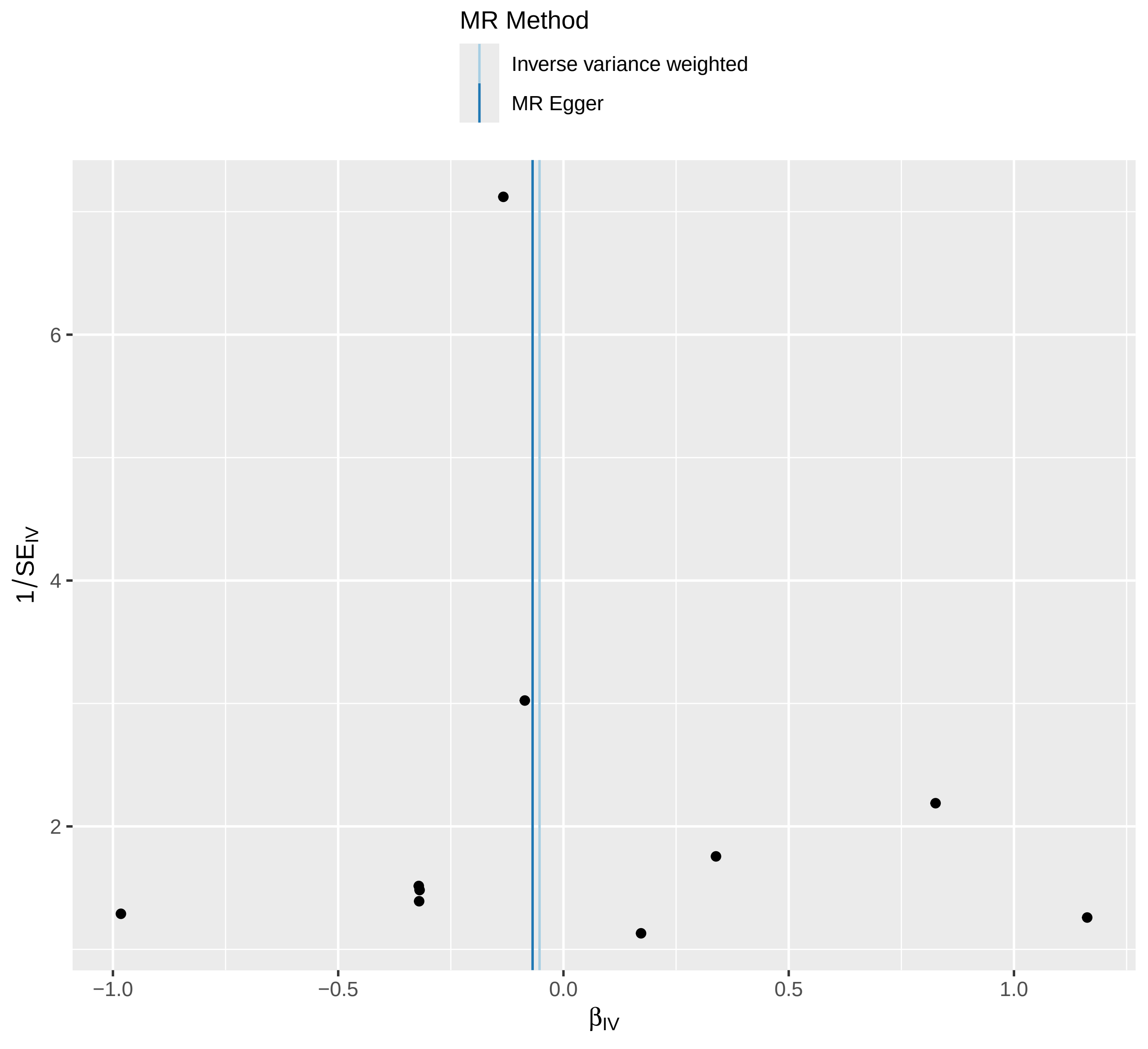

MR Method
Inverse variance weighted
MR Egger
6
4
2
1/SE_IV
−1.0
−0.5
0.0
0.5
1.0
β_IV

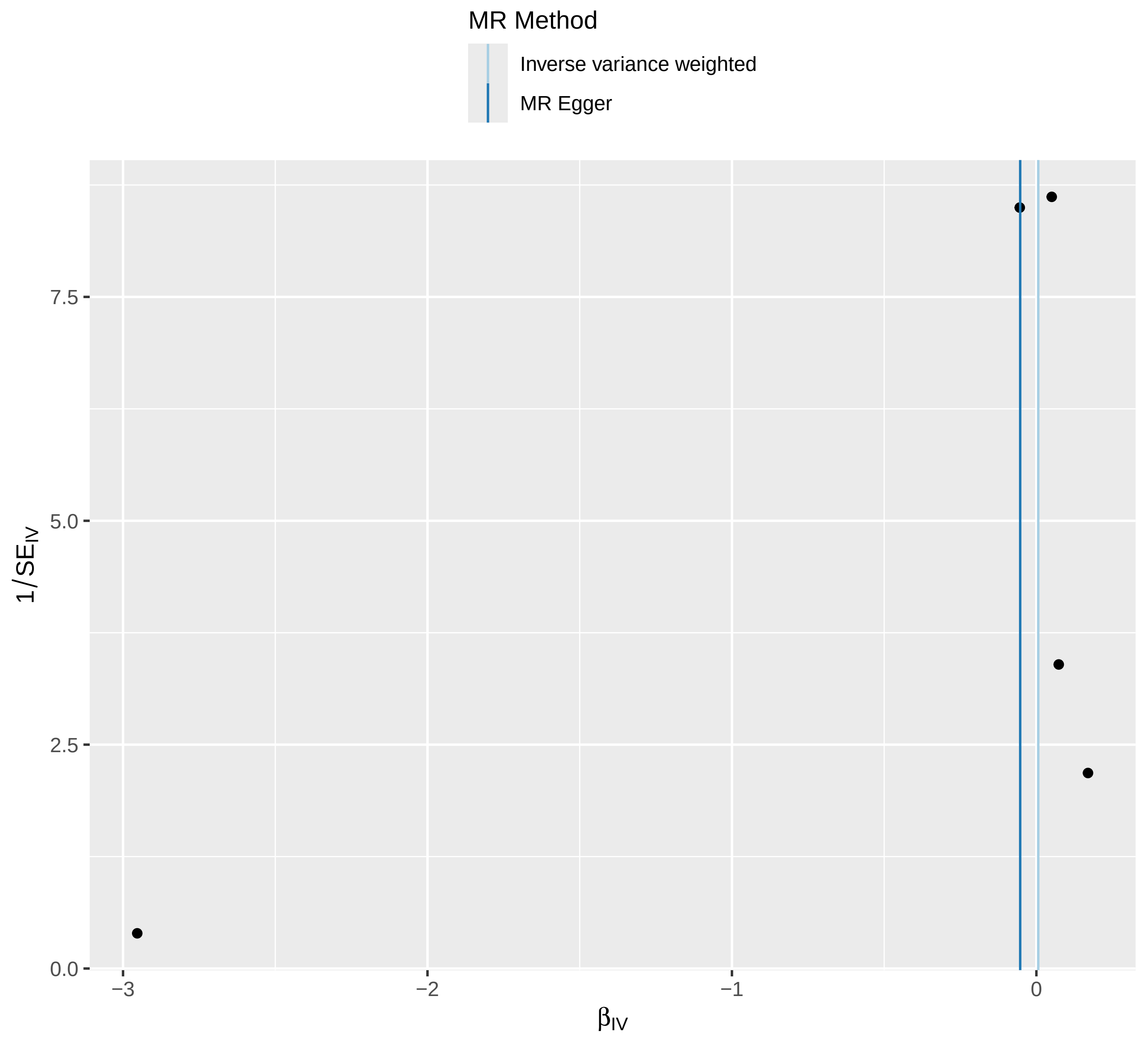
MR Method
Inverse variance weighted
MR Egger
7.5
5.0
2.5
0.0
1/SE_IV
−3
−2
−1
0
β_IV

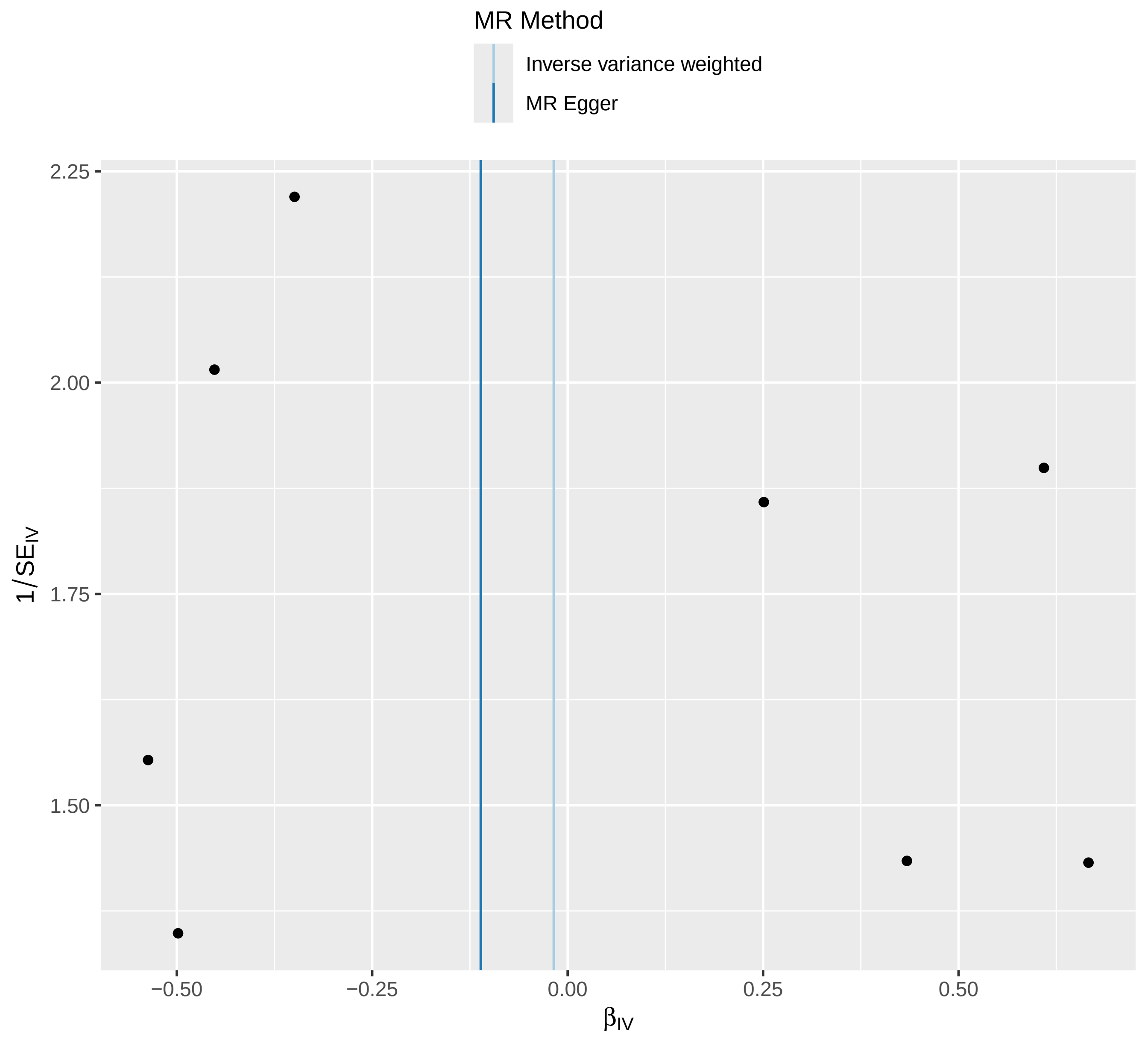

MR Method
Inverse variance weighted
MR Egger
2.25
2.00
1.75
1.50
1/SE_IV
−0.50
−0.25
0.00
0.25
0.50
β_IV

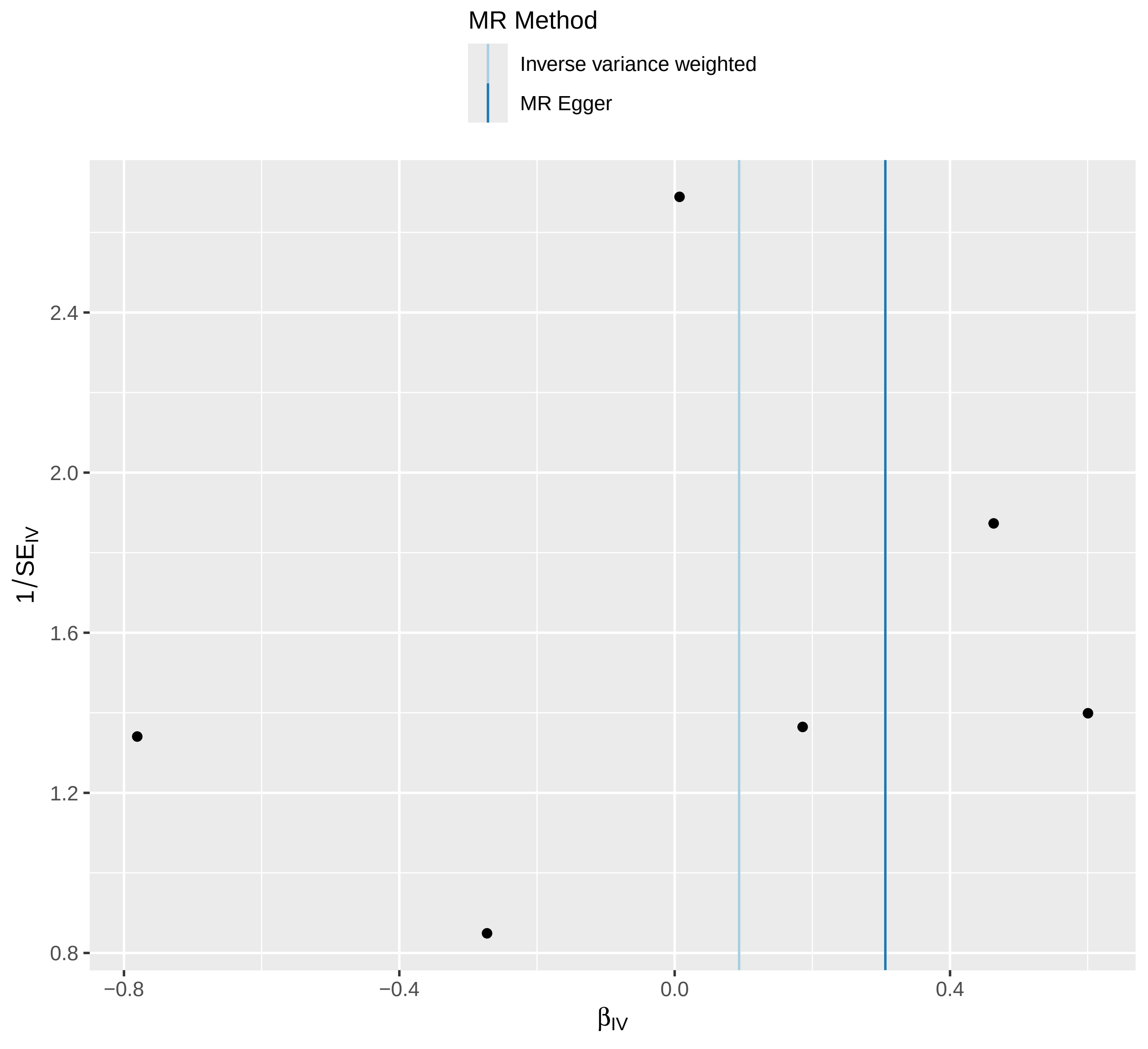

MR Method
Inverse variance weighted
MR Egger
2.4
2.0
1.6
1.2
0.8
1/SE_IV
−0.8
−0.4
0.0
0.4
β_IV

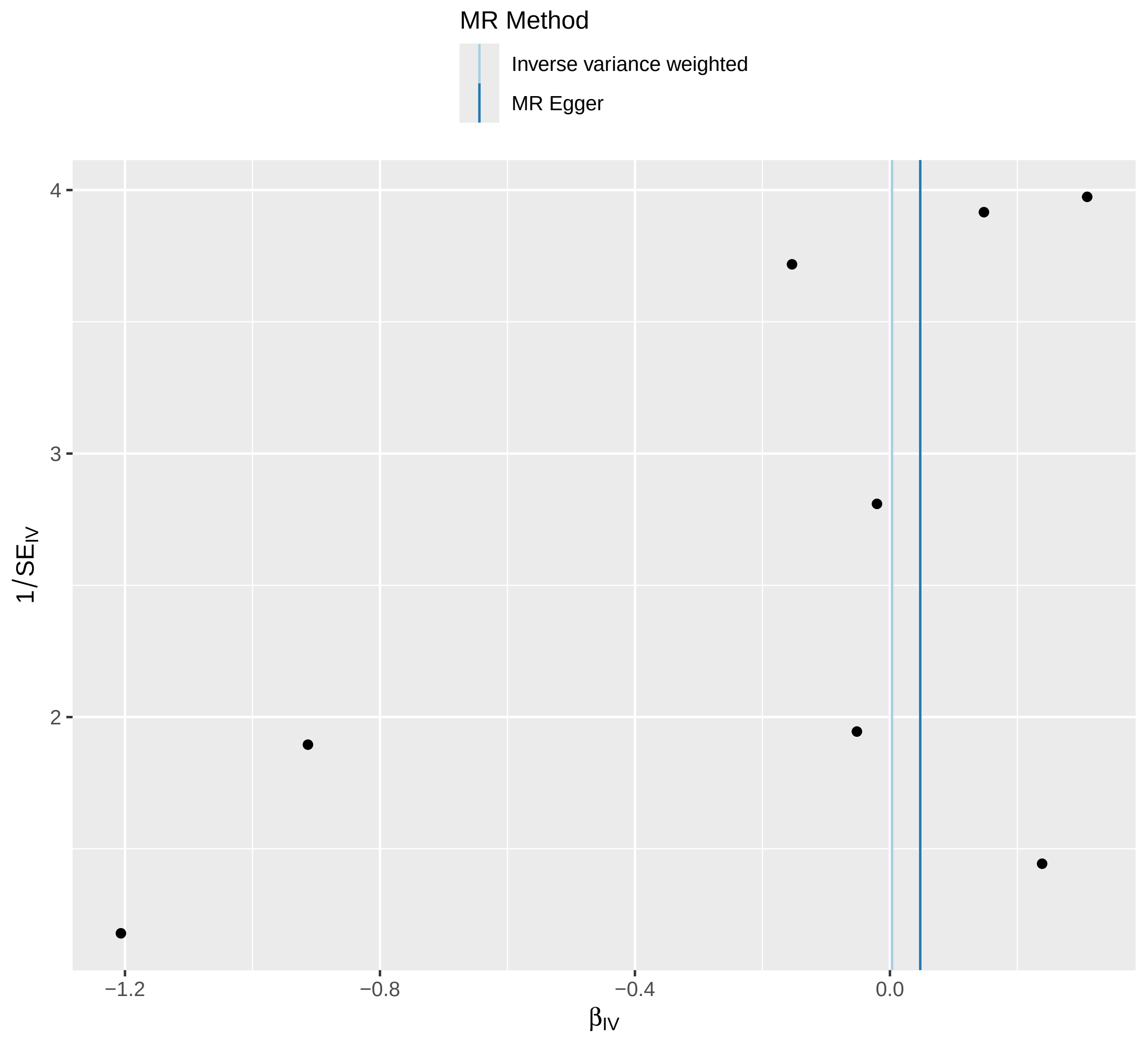
MR Method
Inverse variance weighted
MR Egger
4
3
2
$1/SE_{IV}$
−1.2
−0.8
−0.4
0.0
β_{IV}

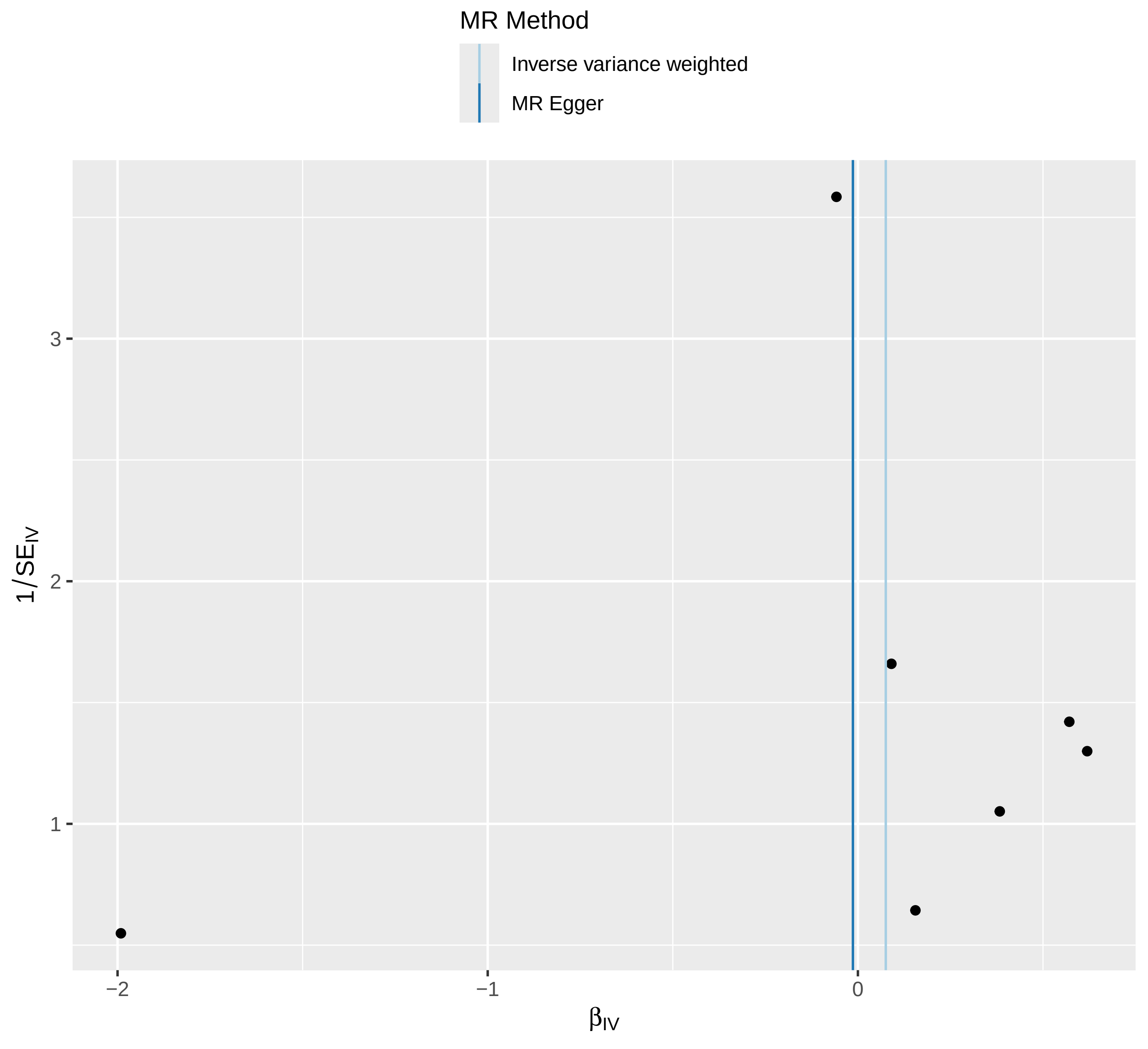
MR Method
Inverse variance weighted
MR Egger
3
2
1
1/SE$_{IV}$
−2
−1
0
β$_{IV}$

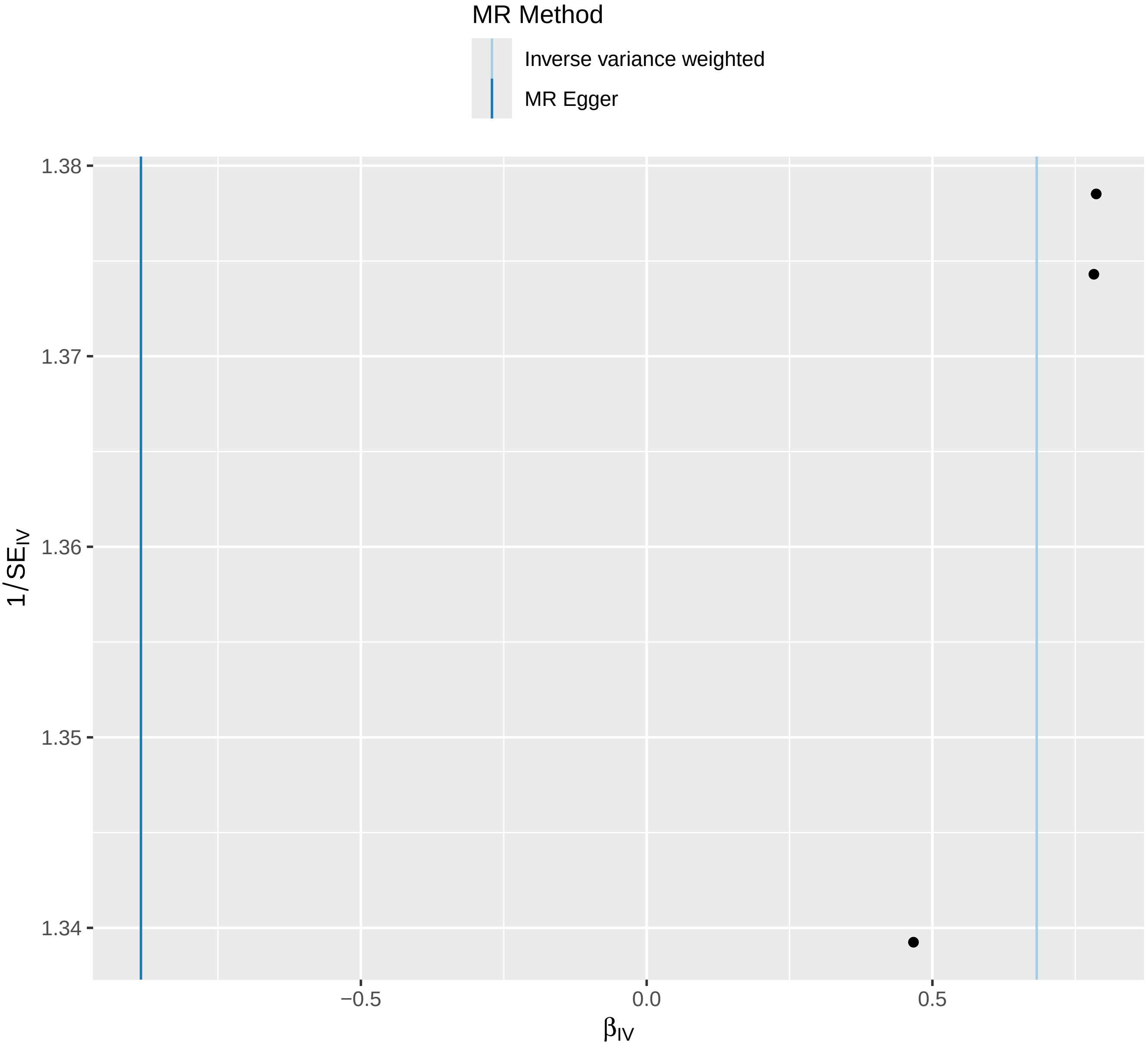
MR Method
Inverse variance weighted
MR Egger
1.38
1.37
1.36
1.35
1.34
$1/SE_{IV}$
−0.5
0.0
0.5
β_{IV}

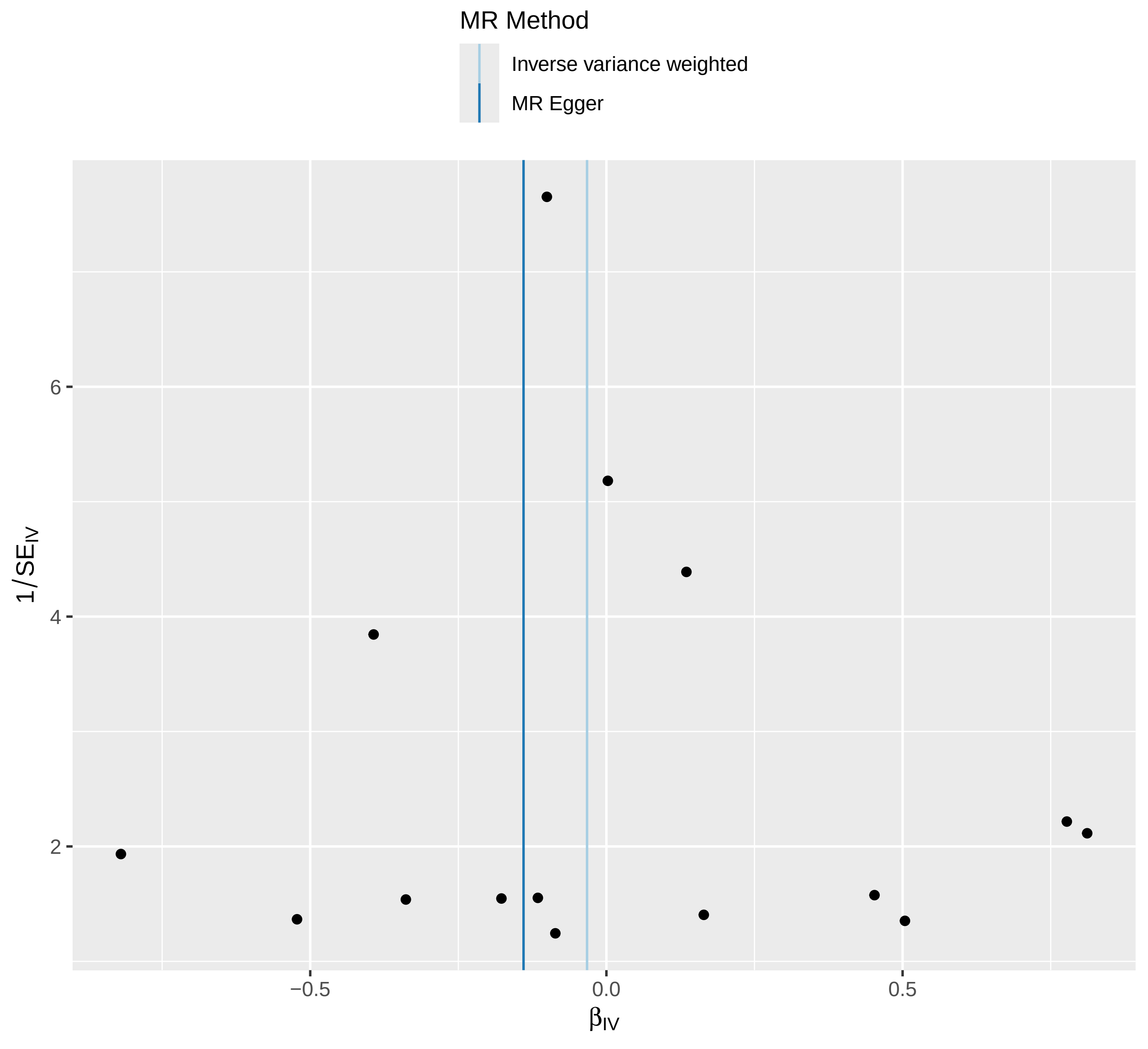
MR Method
Inverse variance weighted
MR Egger
1/SE_IV
6
4
2
−0.5
0.0
0.5
β_IV

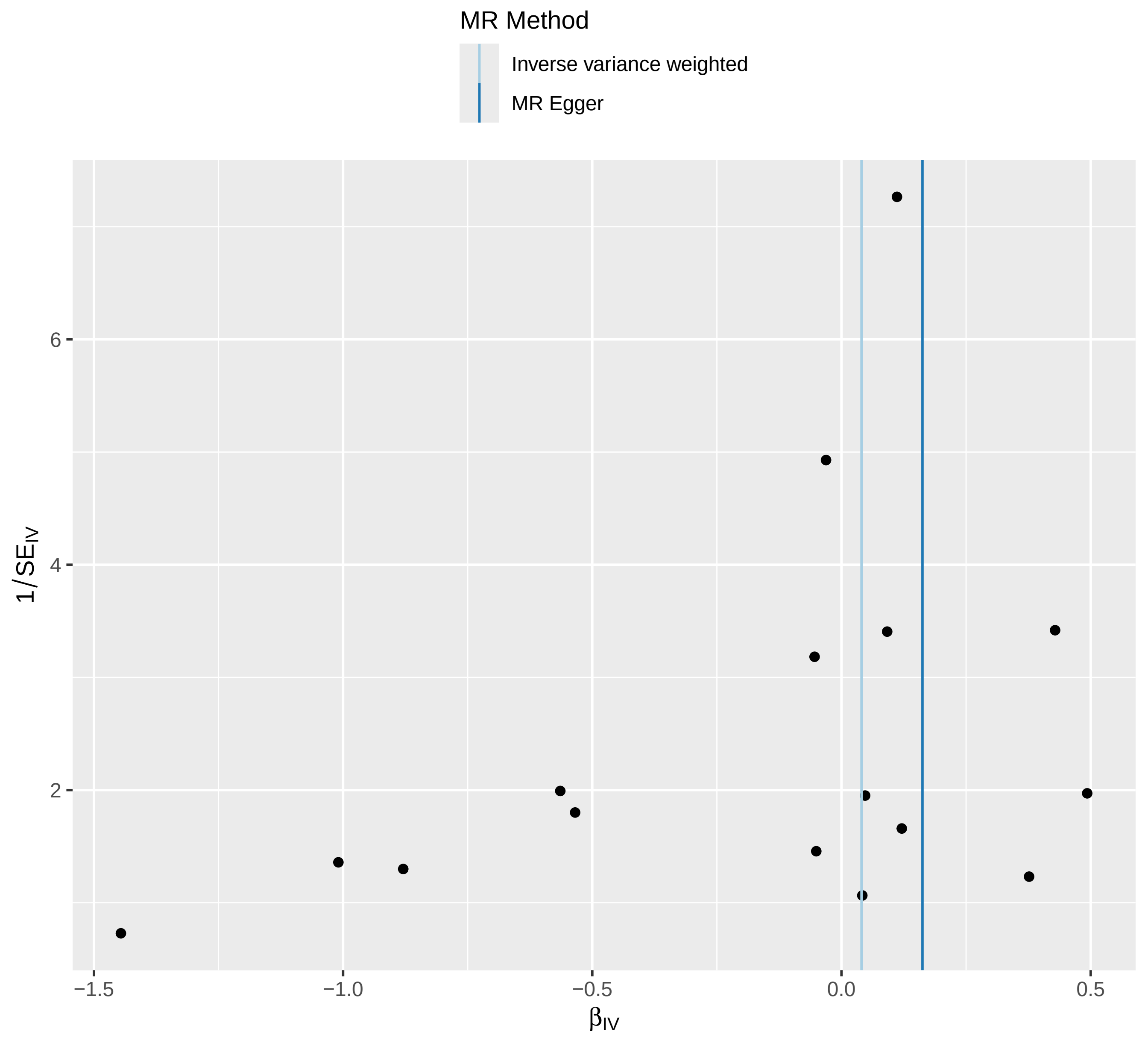
MR Method
Inverse variance weighted
MR Egger
1/SE$_{IV}$
6
4
2
−1.5
−1.0
−0.5
0.0
0.5
β$_{IV}$

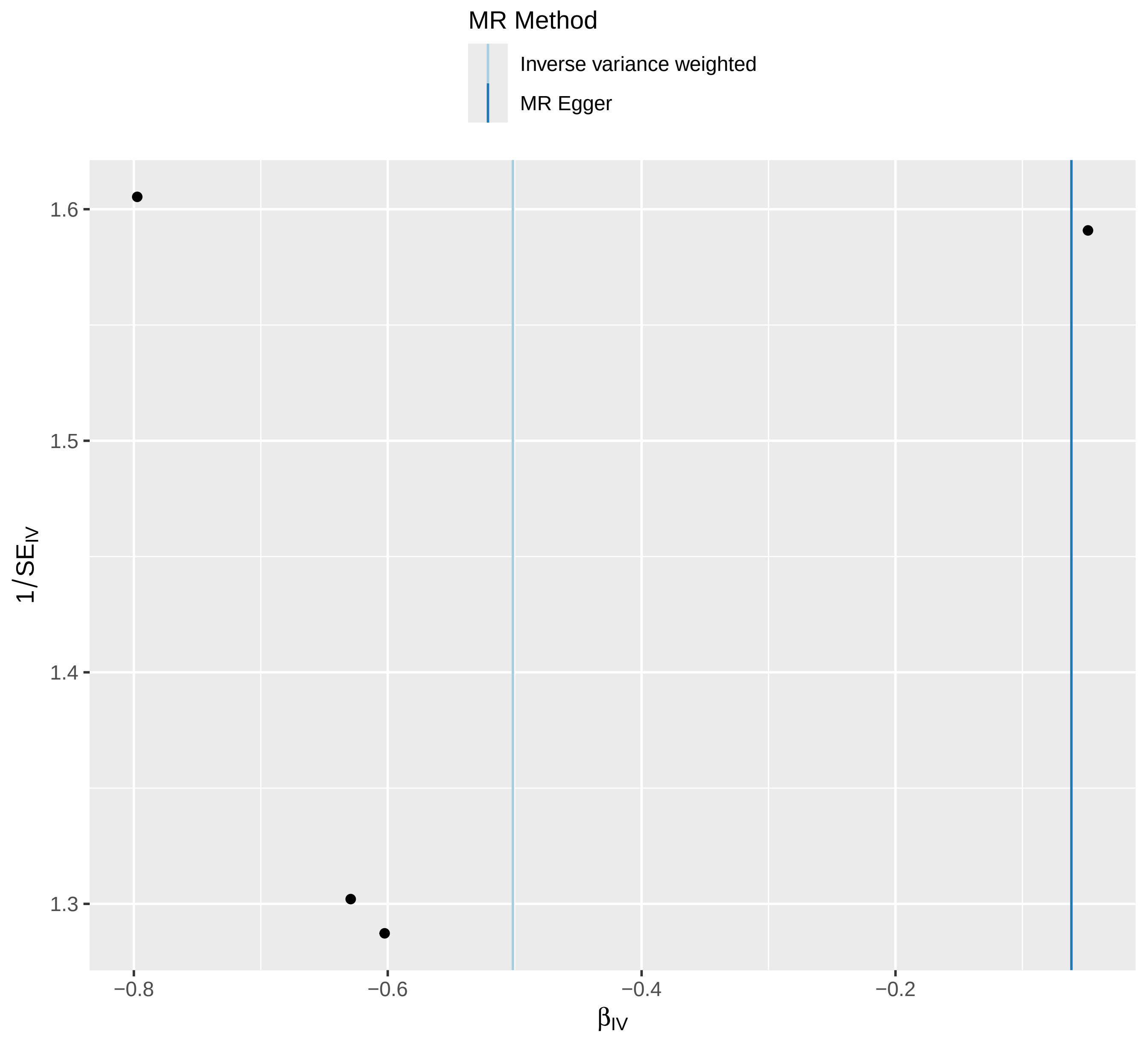
MR Method
Inverse variance weighted
MR Egger
1.6
1.5
1.4
1.3
$1/SE_{IV}$
−0.8
−0.6
−0.4
−0.2
β_{IV}

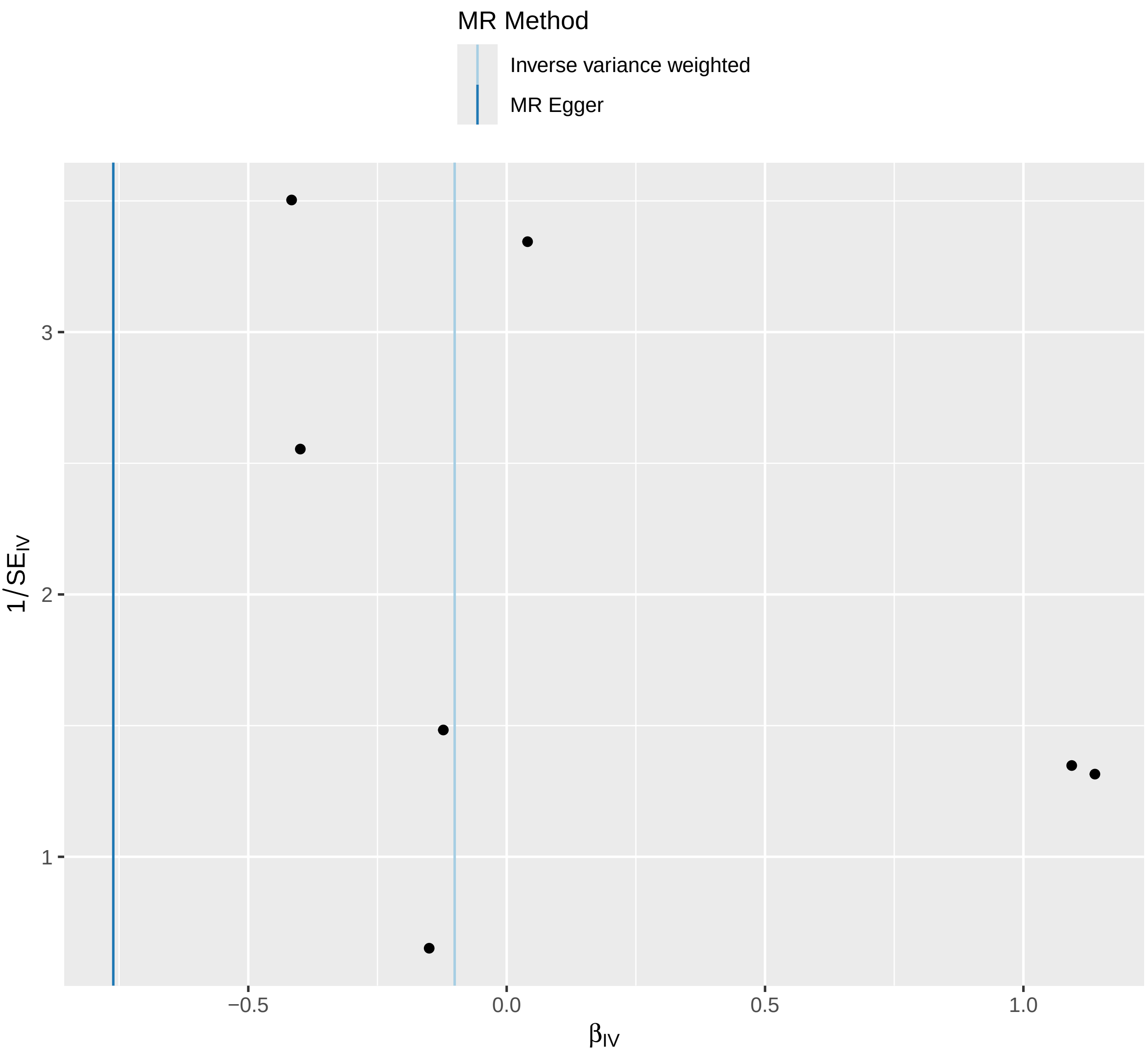
MR Method
Inverse variance weighted
MR Egger
1/SE_IV
3
2
1
−0.5
0.0
0.5
1.0
β_IV

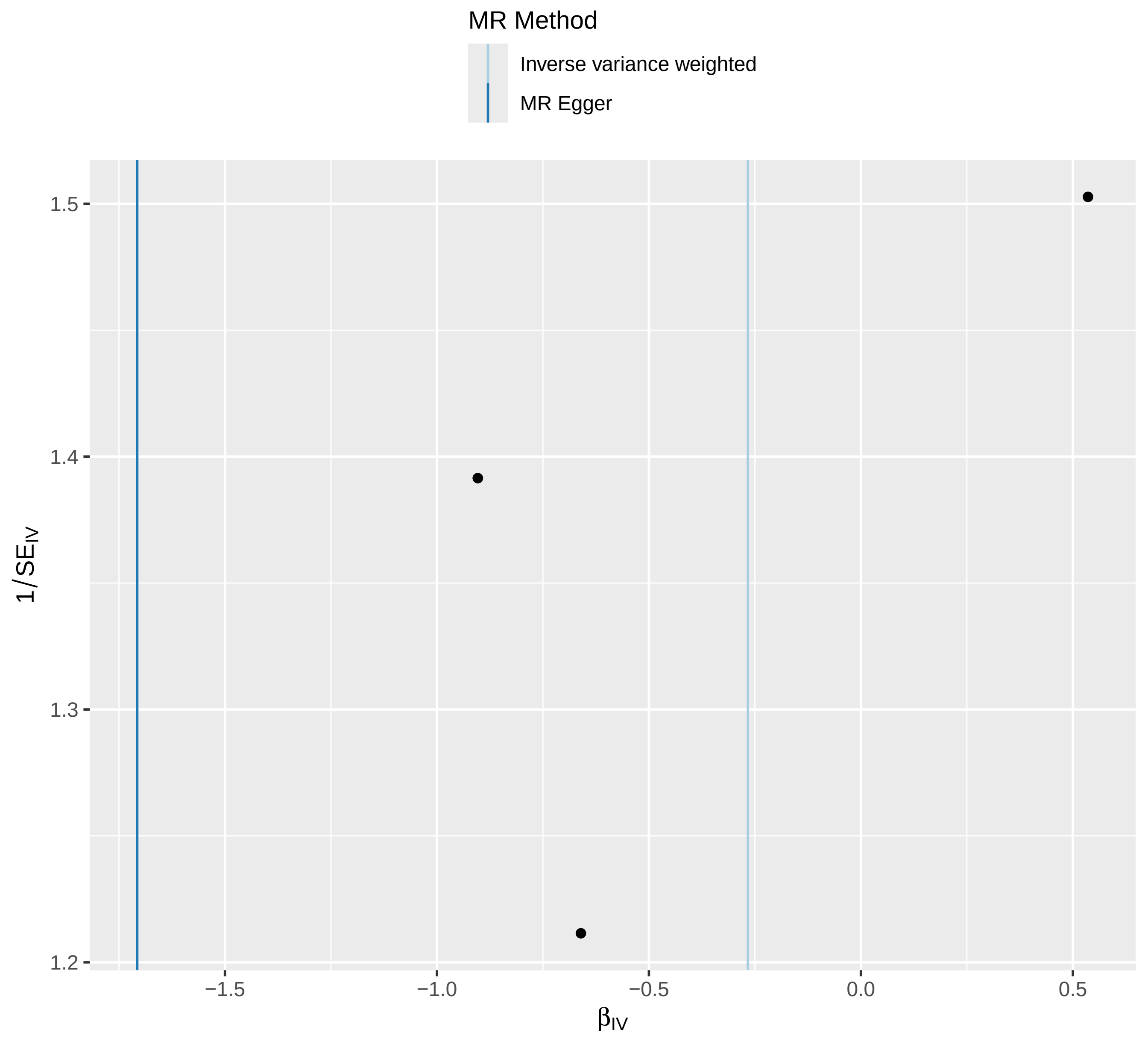

MR Method
Inverse variance weighted
MR Egger
1.5
1.4
1.3
1.2
−1.5
−1.0
−0.5
0.0
0.5
$1/SE_{IV}$
β_{IV}

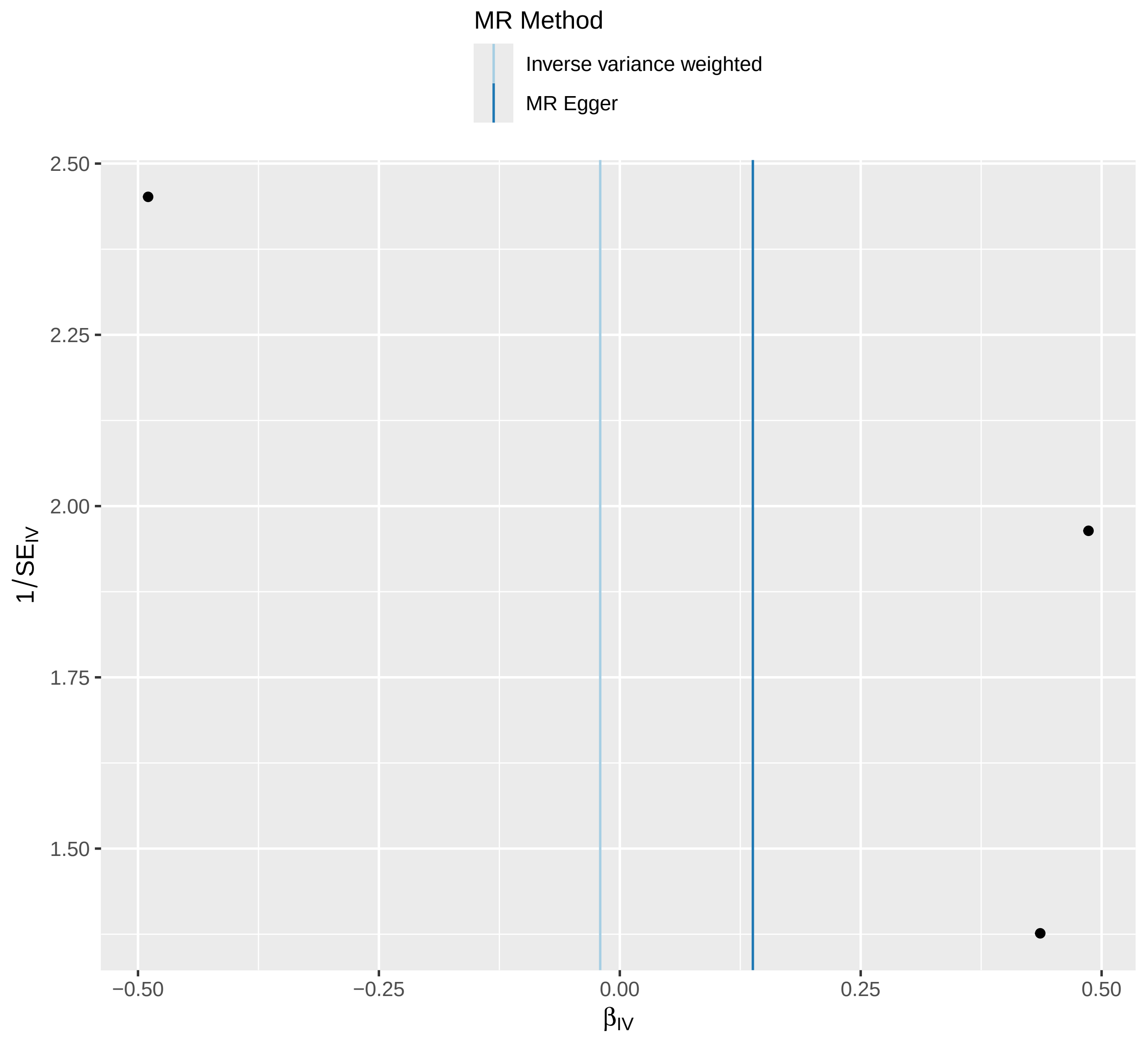
MR Method
Inverse variance weighted
MR Egger
2.50
2.25
2.00
1.75
1.50
1/SE$_{IV}$
−0.50
−0.25
0.00
0.25
0.50
β$_{IV}$

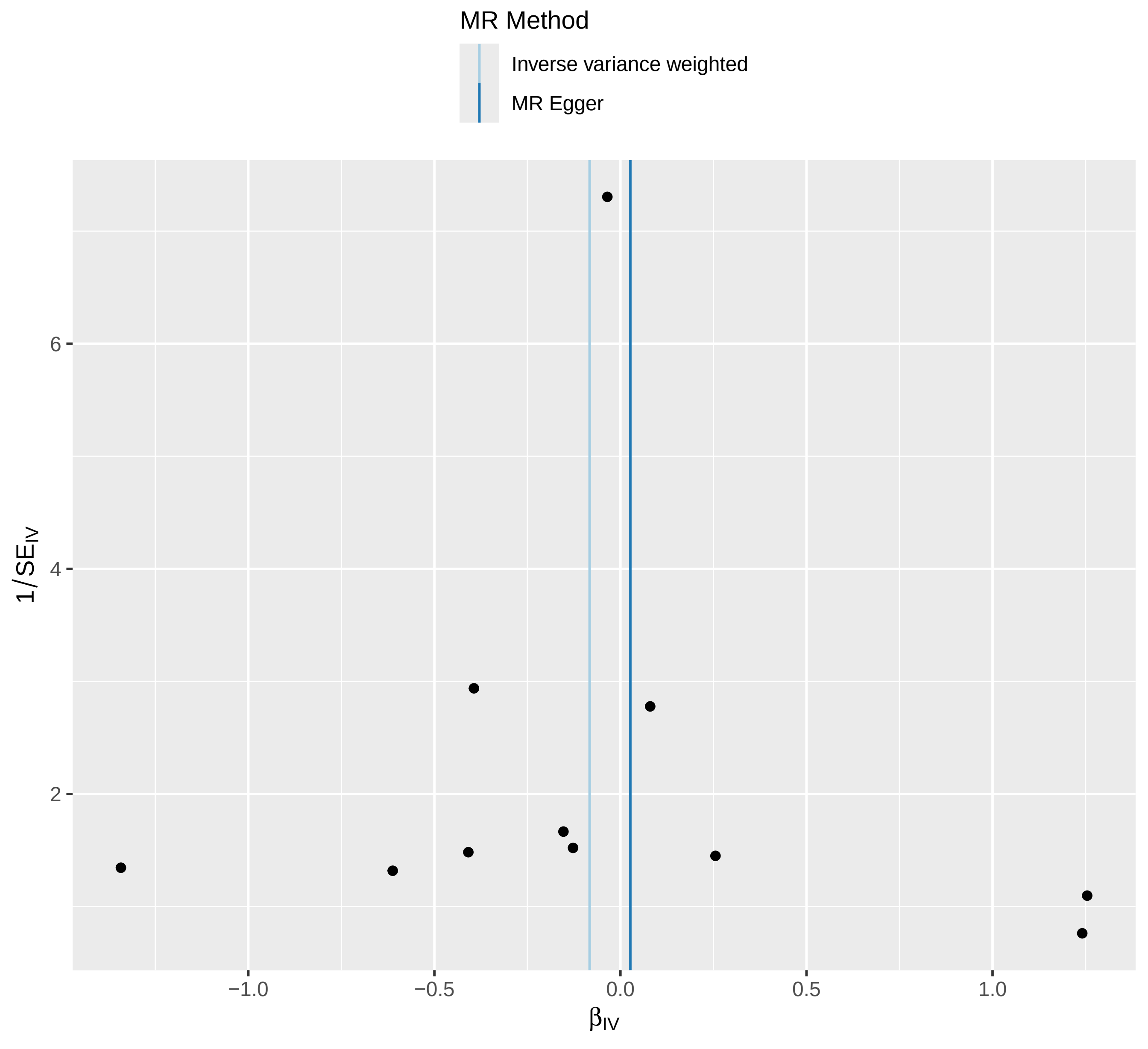
MR Method
Inverse variance weighted
MR Egger
6
4
2
$1/SE_{IV}$
−1.0
−0.5
0.0
0.5
1.0
β_{IV}

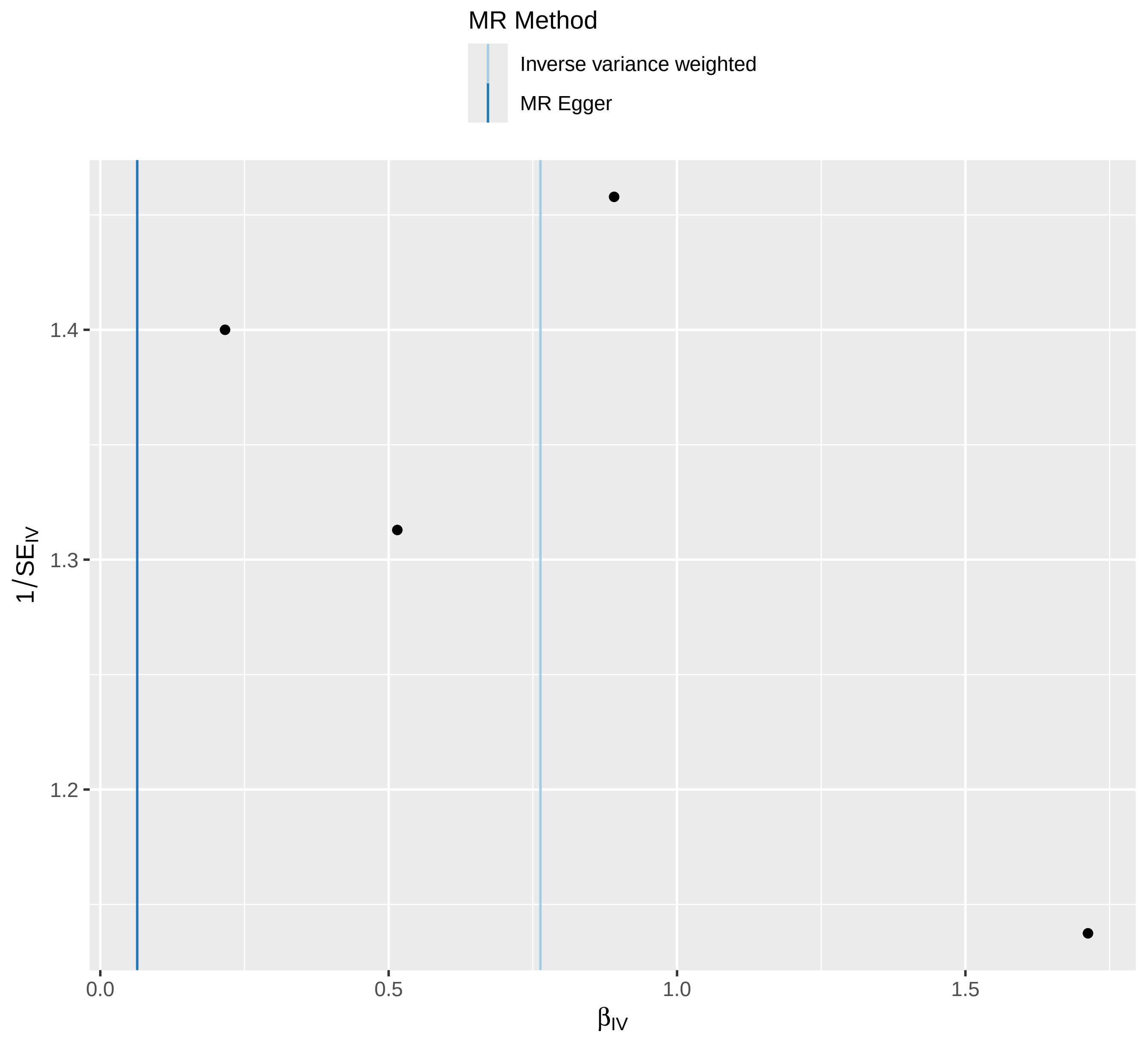
MR Method
Inverse variance weighted
MR Egger
1.4
1.3
1.2
0.0
0.5
1.0
1.5
$1/SE_{IV}$
β_{IV}

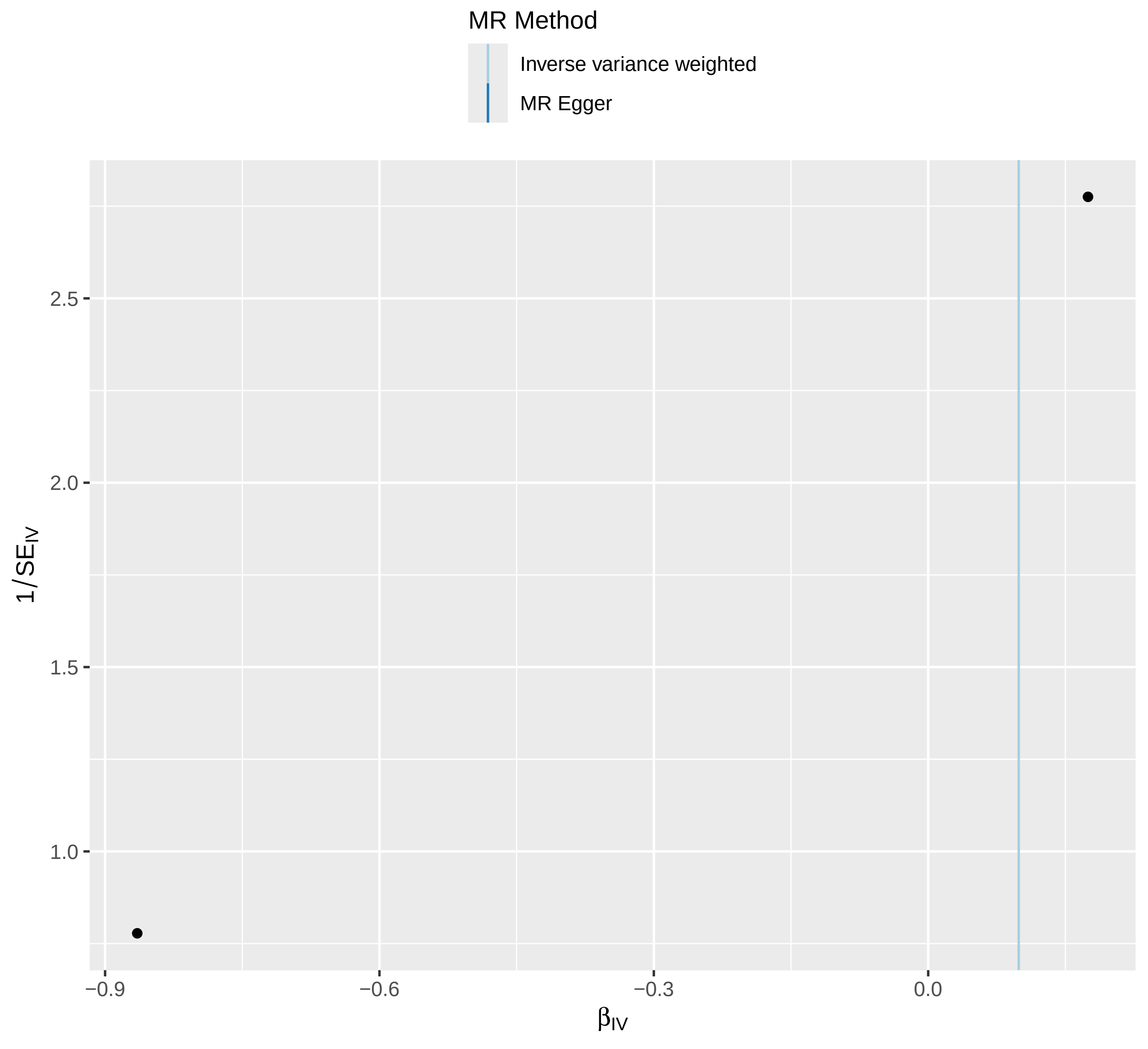

MR Method
Inverse variance weighted
MR Egger
2.5
2.0
1.5
1.0
1/SE$_{IV}$
−0.9
−0.6
−0.3
0.0
β$_{IV}$

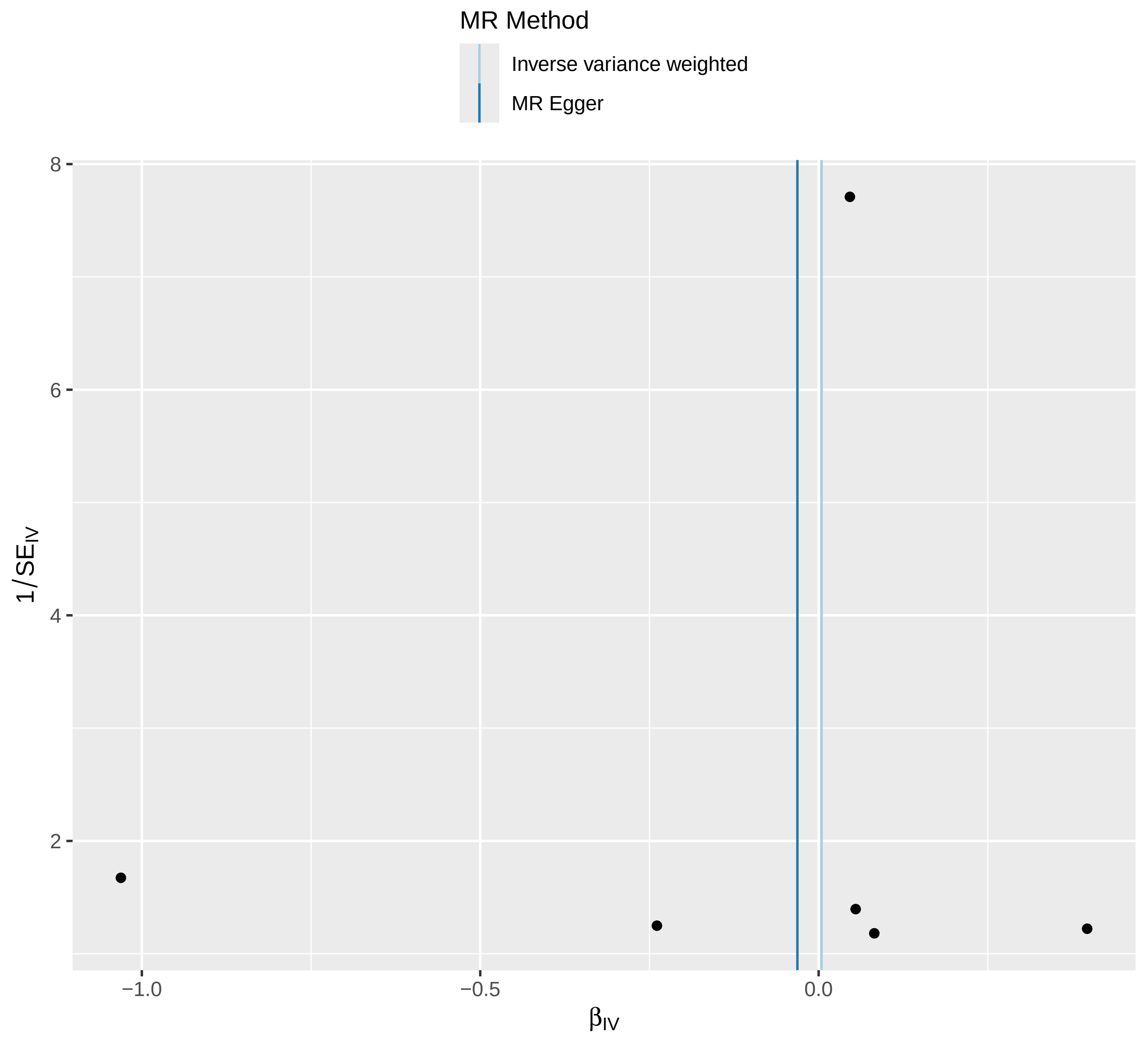
MR Method
Inverse variance weighted
MR Egger
8
6
4
2
1/SE$_{IV}$
−1.0
−0.5
0.0
β$_{IV}$

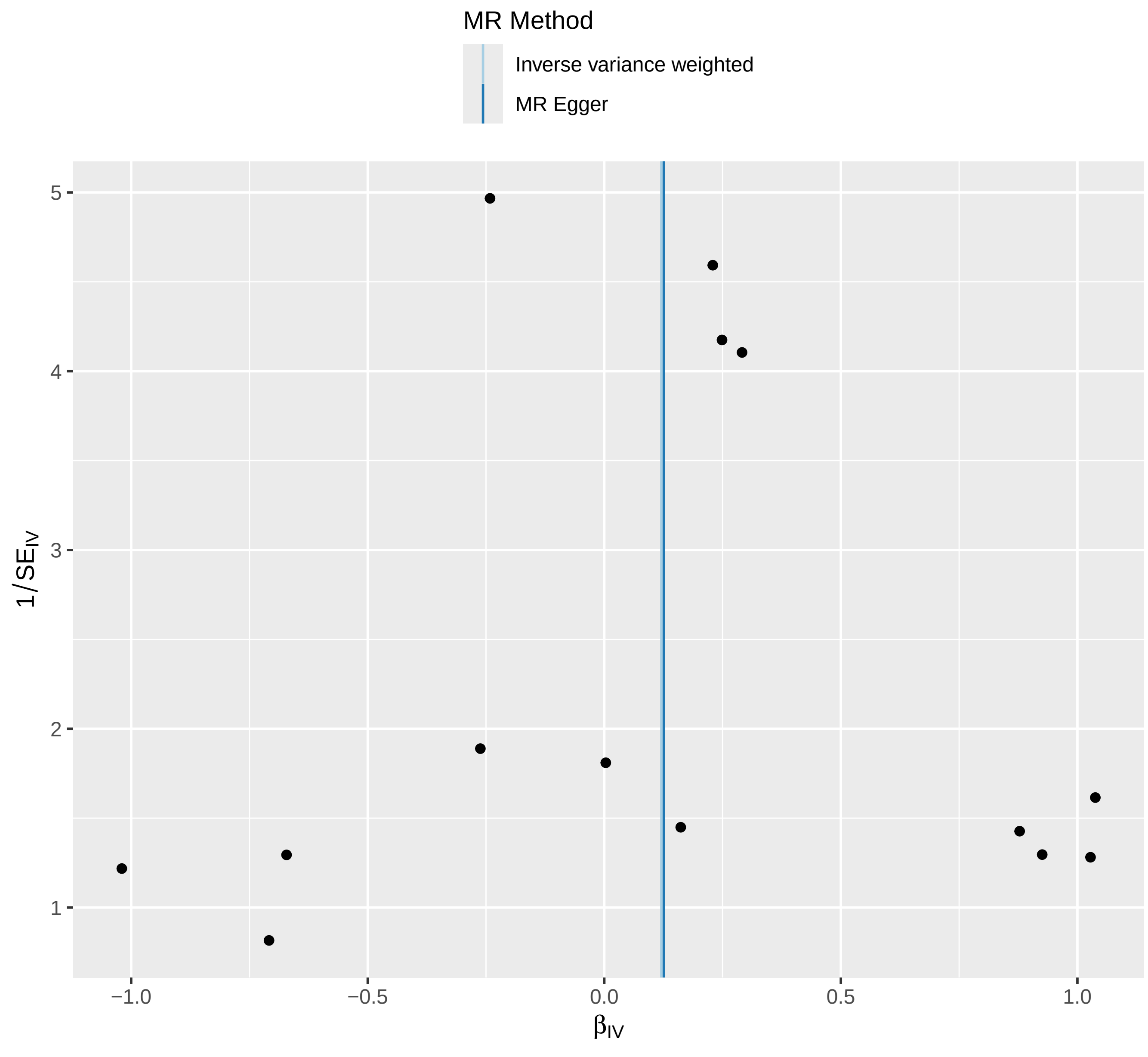
MR Method
Inverse variance weighted
MR Egger
5
4
3
2
1
1/SE$_{IV}$
−1.0
−0.5
0.0
0.5
1.0
β$_{IV}$

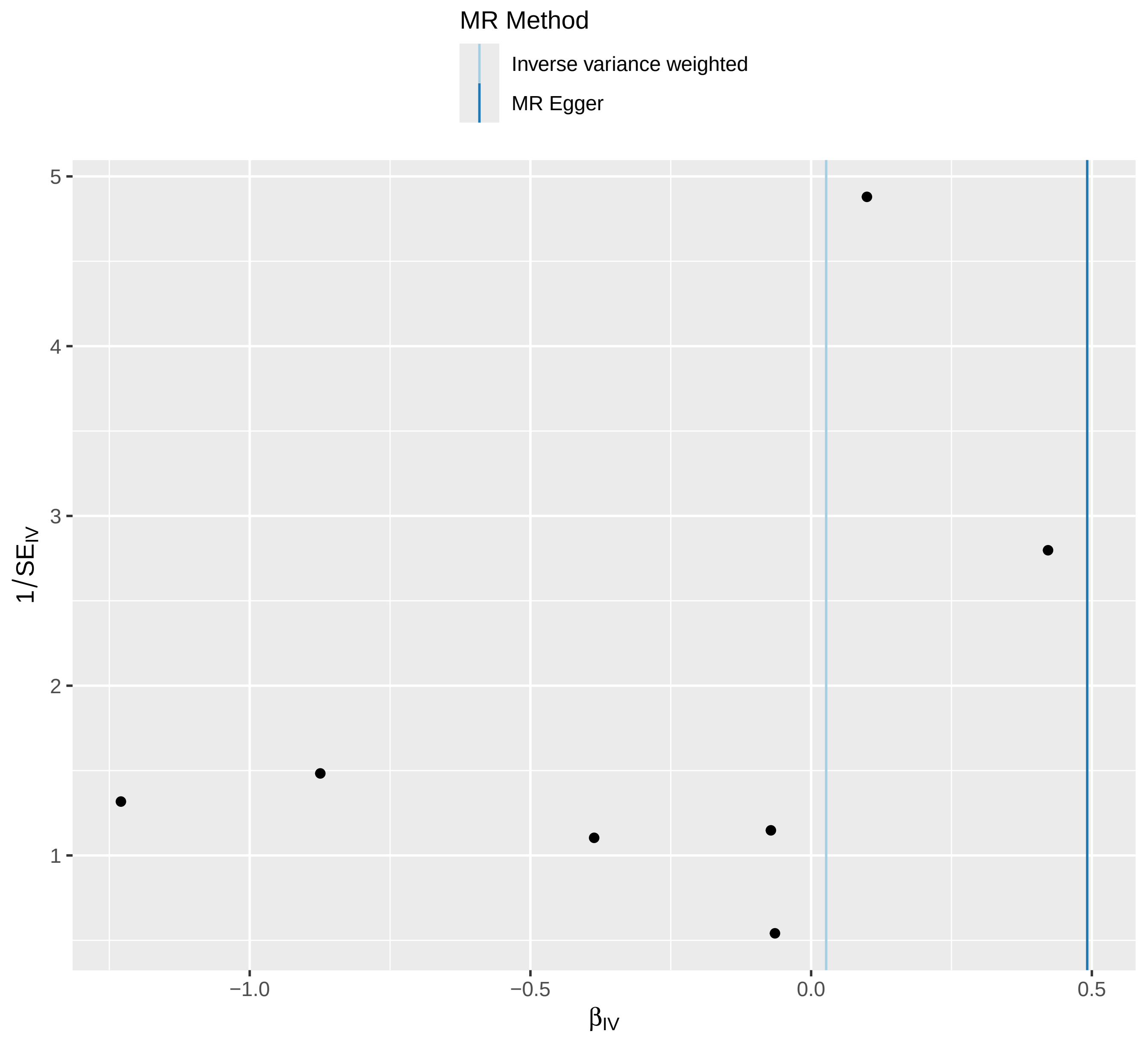

MR Method
Inverse variance weighted
MR Egger
5
4
3
2
1
1/SE_IV
−1.0
−0.5
0.0
0.5
β_IV

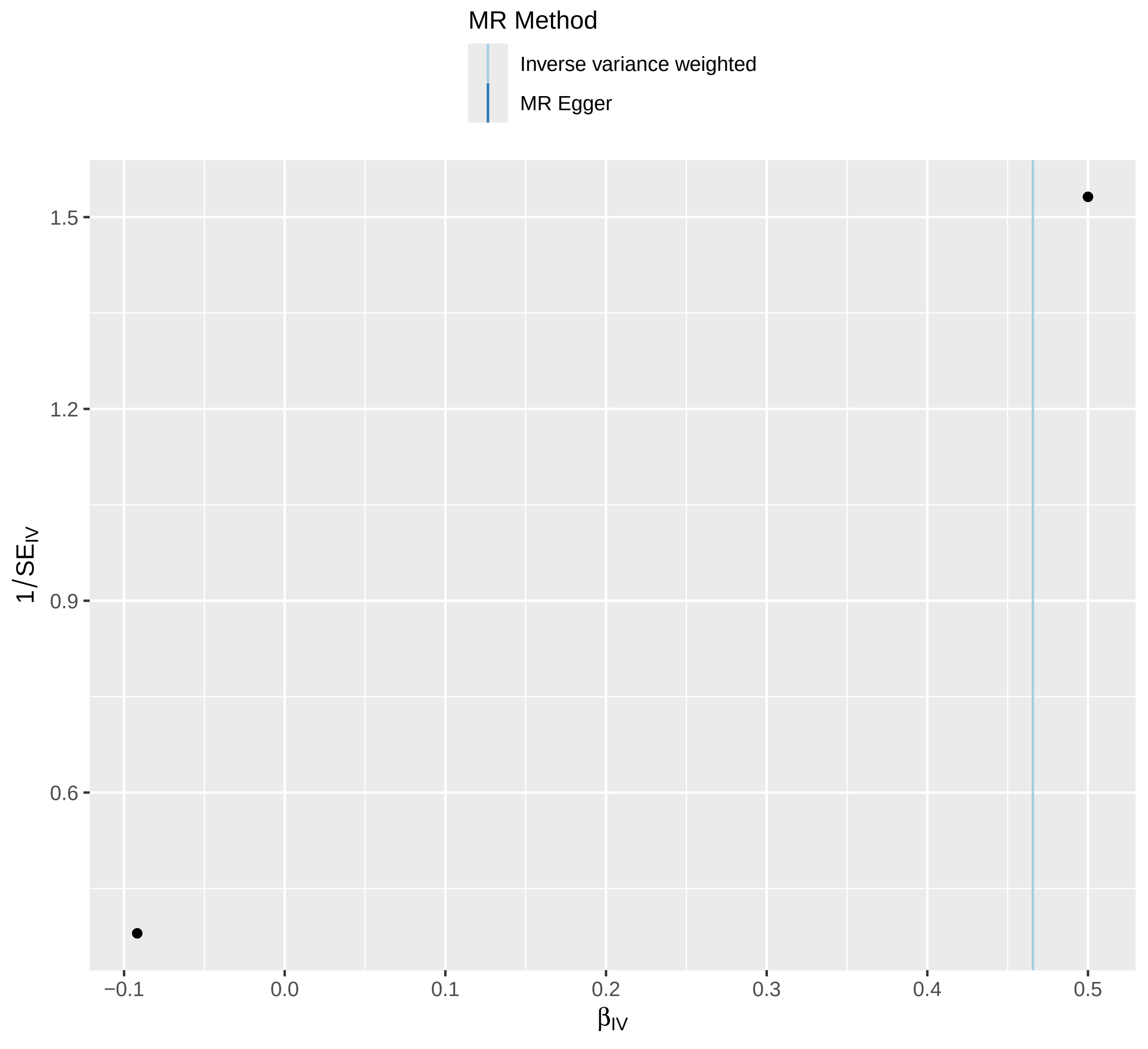

MR Method
Inverse variance weighted
MR Egger
1.5
1.2
0.9
0.6
1/SE_IV
−0.1
0.0
0.1
0.2
0.3
0.4
0.5
β_IV

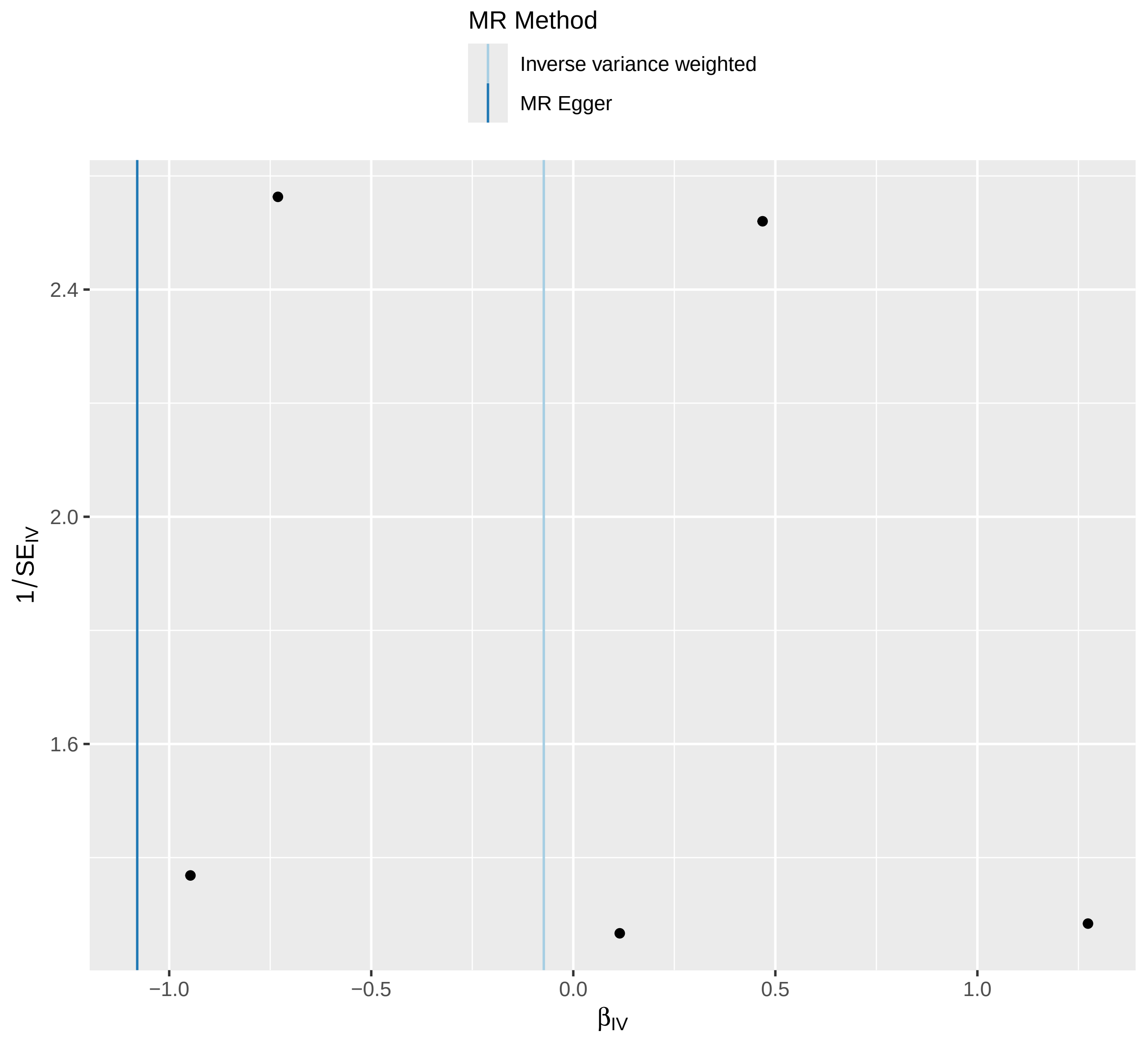
MR Method
Inverse variance weighted
MR Egger
2.4
2.0
1.6
$1/SE_{IV}$
−1.0
−0.5
0.0
0.5
1.0
β_{IV}

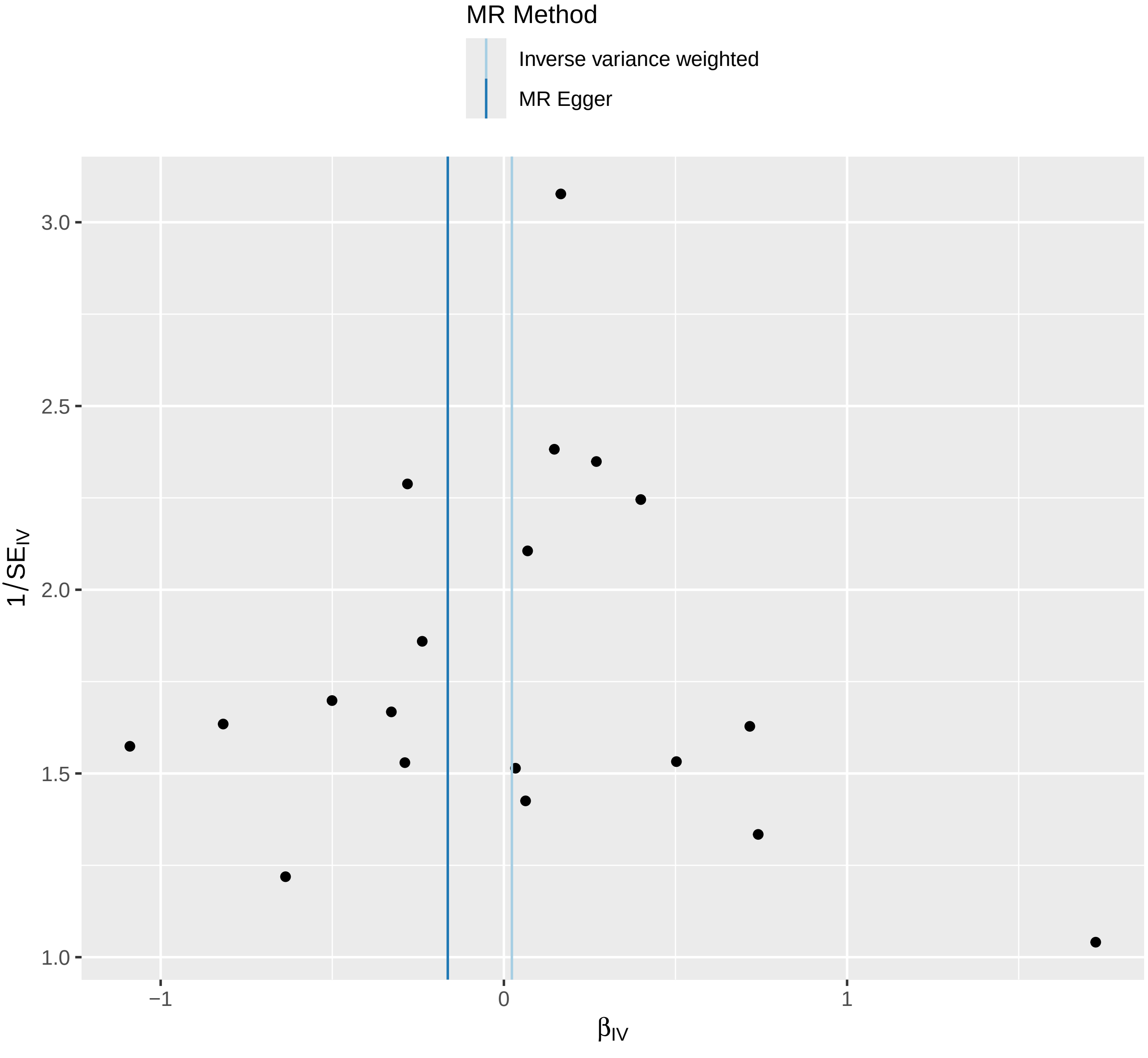
MR Method
Inverse variance weighted
MR Egger
3.0
2.5
2.0
1.5
1.0
1/SE_IV
−1
0
1
β_IV

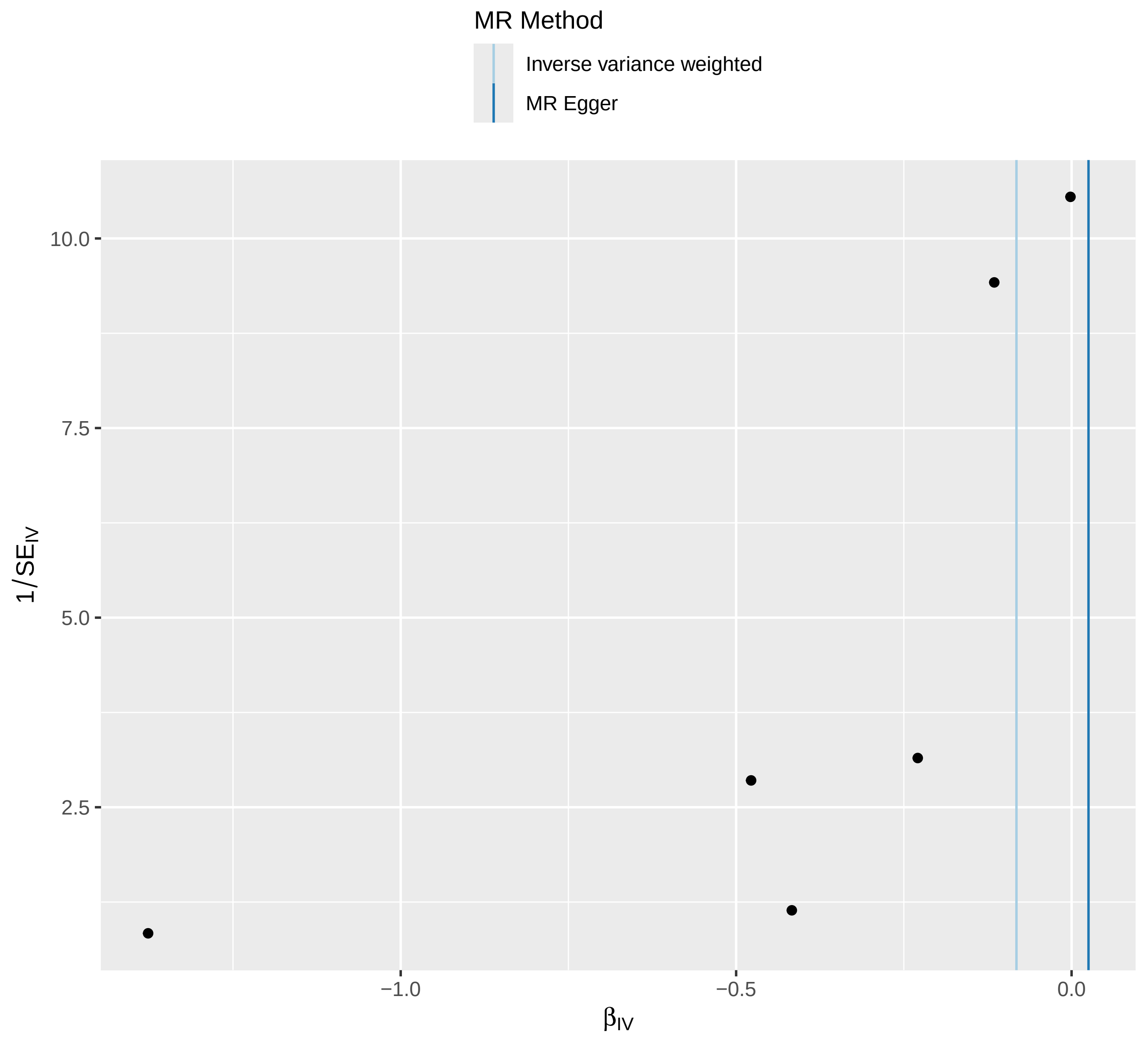

MR Method
Inverse variance weighted
MR Egger
10.0
7.5
5.0
2.5
1/SE_IV
−1.0
−0.5
0.0
β_IV

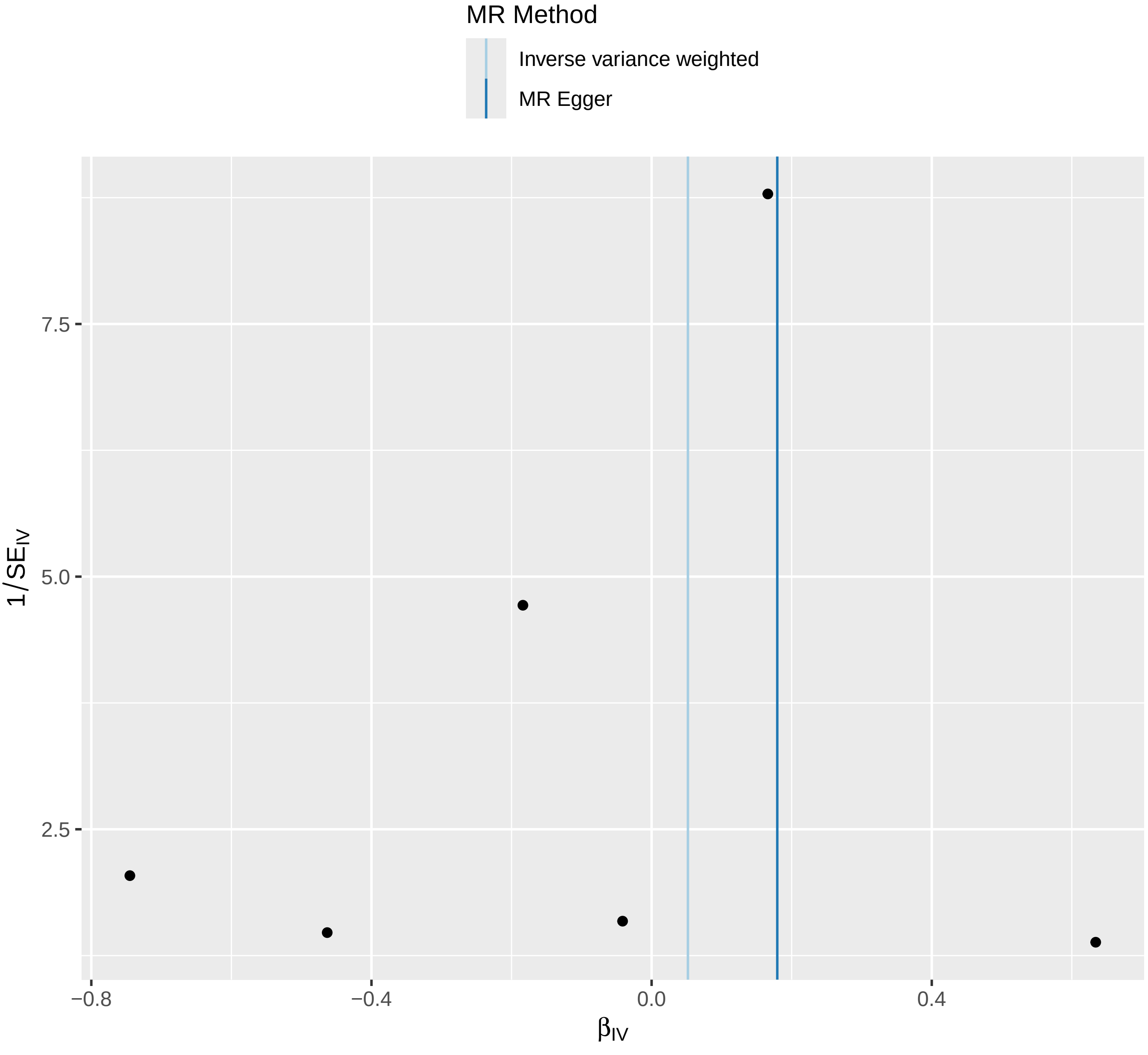
MR Method
Inverse variance weighted
MR Egger
7.5
5.0
2.5
1/SE_IV
−0.8
−0.4
0.0
0.4
β_IV

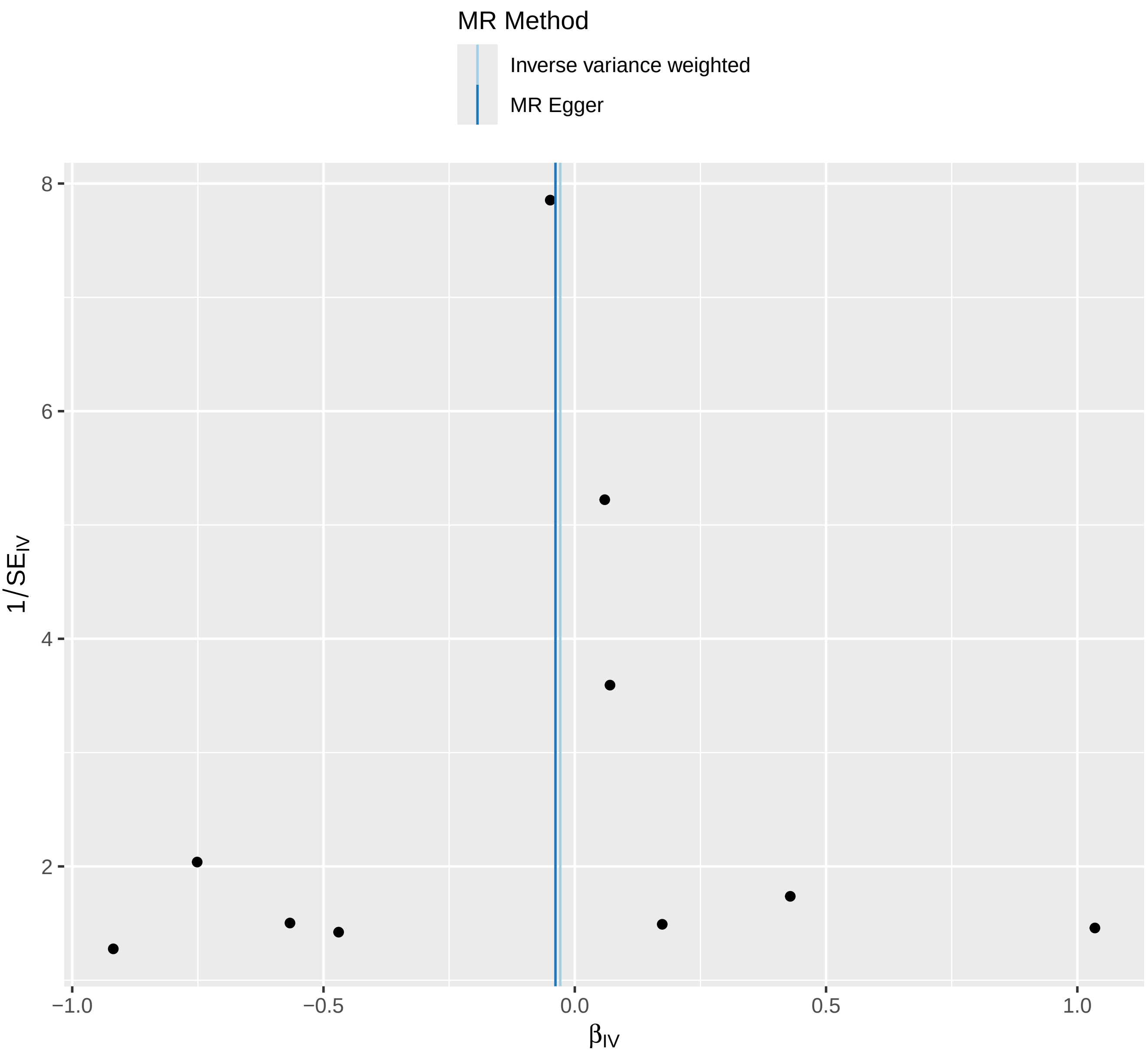
MR Method
Inverse variance weighted
MR Egger
1/SE_IV
β_IV
8
6
4
2
−1.0
−0.5
0.0
0.5
1.0

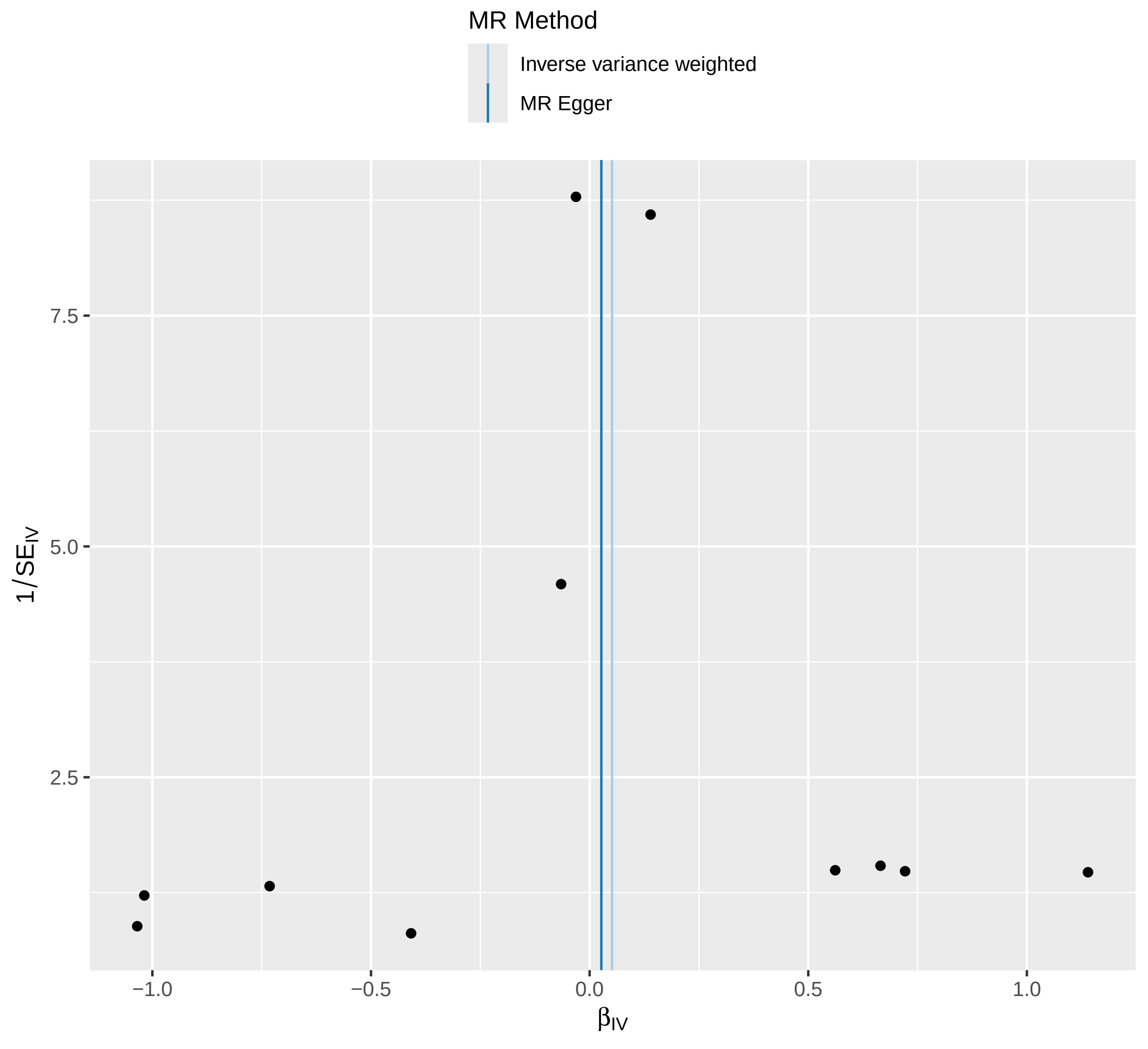

MR Method
Inverse variance weighted
MR Egger
7.5
5.0
2.5
1/SE_IV
−1.0
−0.5
0.0
0.5
1.0
β_IV

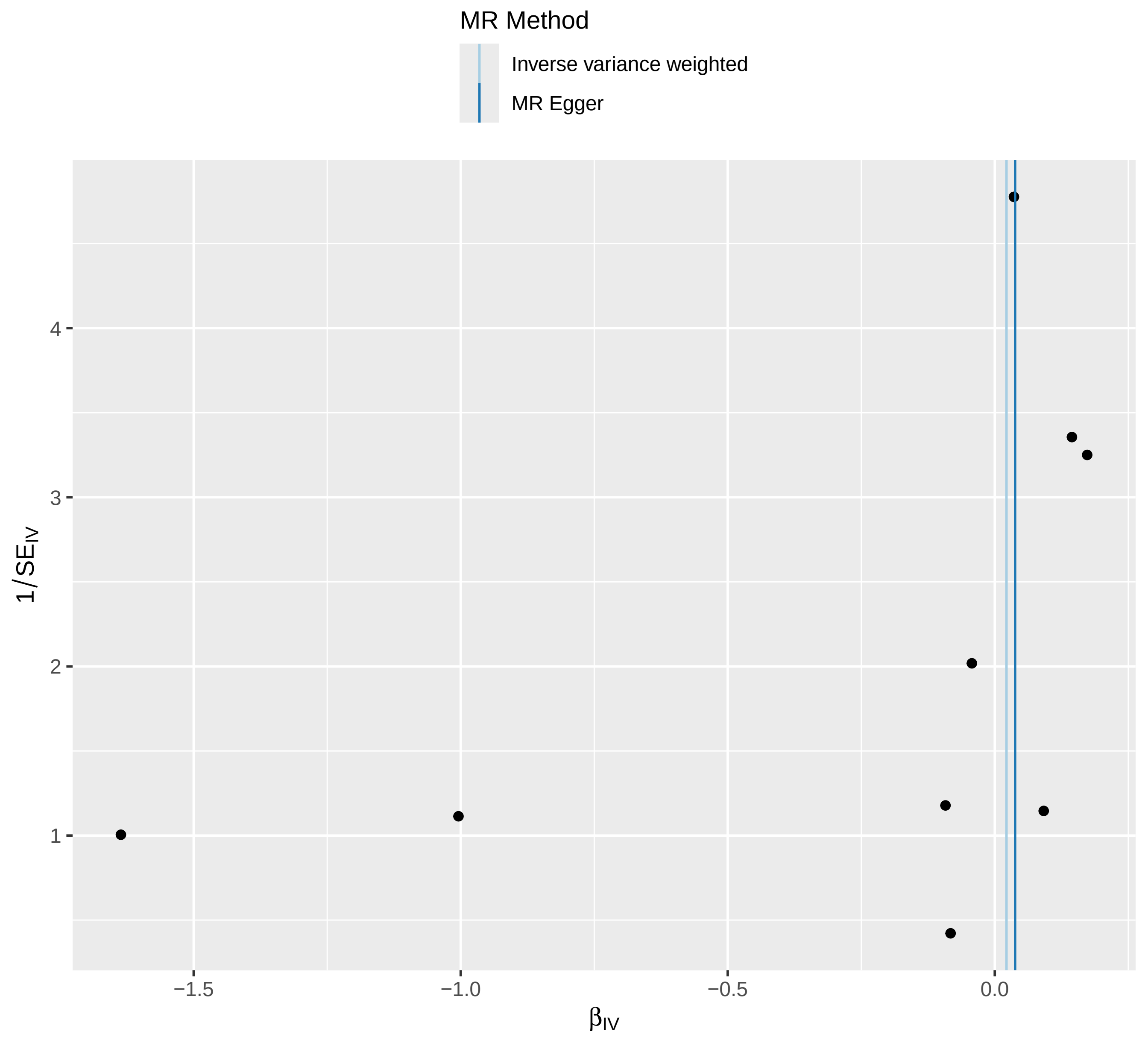
MR Method
Inverse variance weighted
MR Egger
1/SE_IV
4
3
2
1
−1.5
−1.0
−0.5
0.0
β_IV

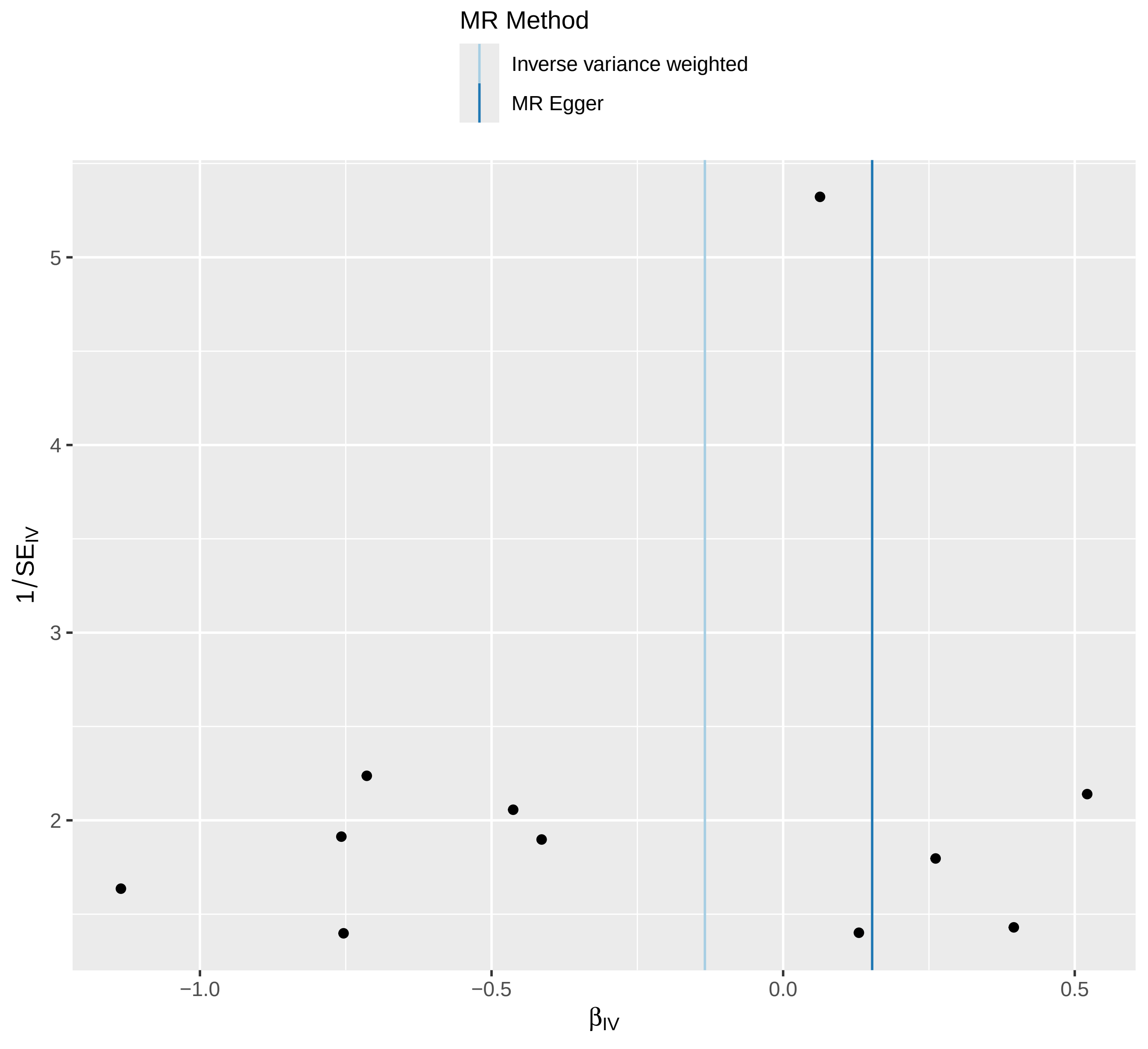

MR Method
Inverse variance weighted
MR Egger
5
4
3
2
1/SE$_{IV}$
−1.0
−0.5
0.0
0.5
β$_{IV}$

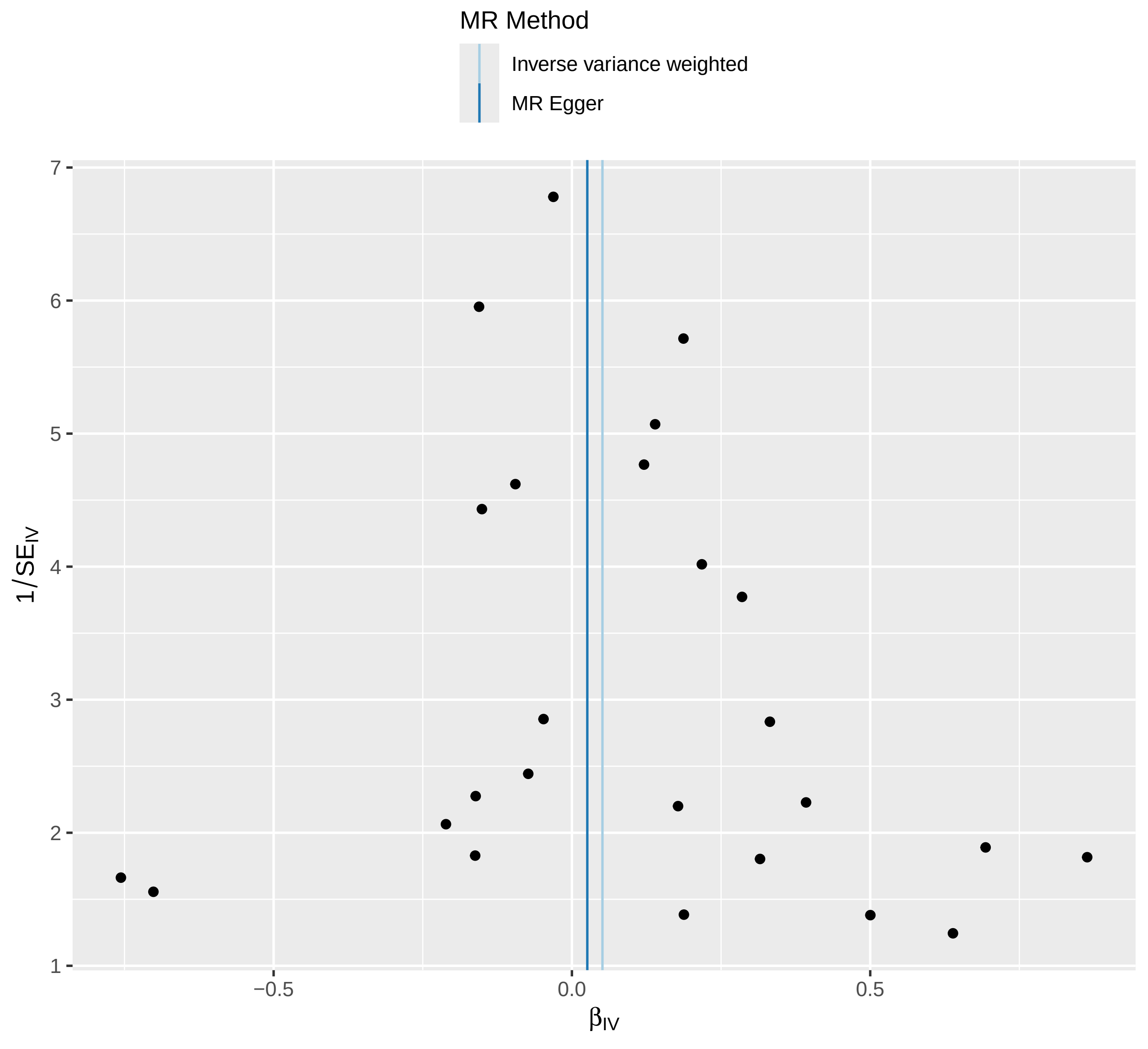
MR Method
Inverse variance weighted
MR Egger
1/SE_IV
7
6
5
4
3
2
1
−0.5
0.0
0.5
β_IV

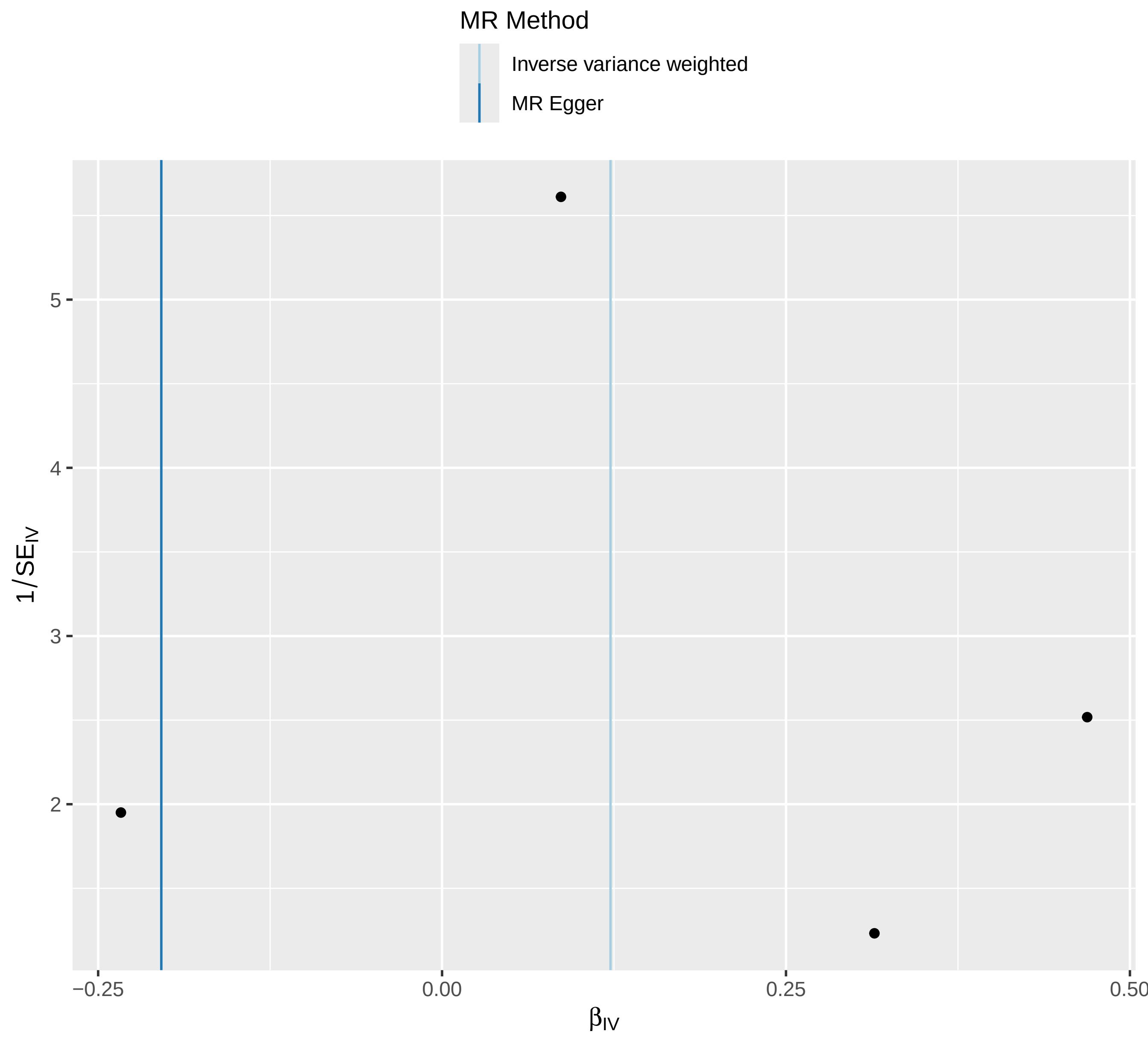
MR Method
Inverse variance weighted
MR Egger
1/SE$_{IV}$
5
4
3
2
−0.25
0.00
0.25
0.50
β_{IV}

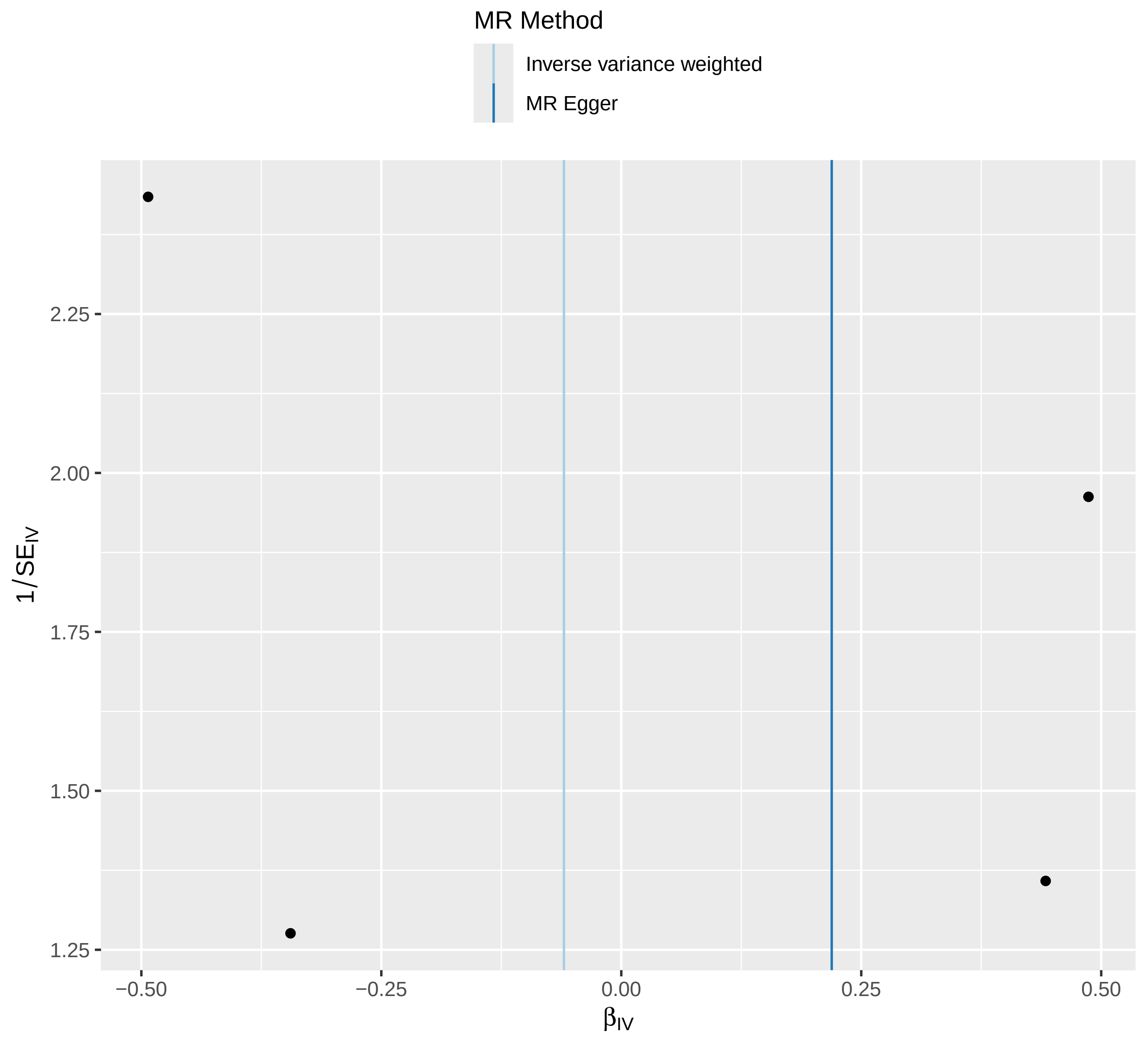
MR Method
Inverse variance weighted
MR Egger
2.25
2.00
1.75
1.50
1.25
$1/SE_{IV}$
−0.50
−0.25
0.00
0.25
0.50
β_{IV}

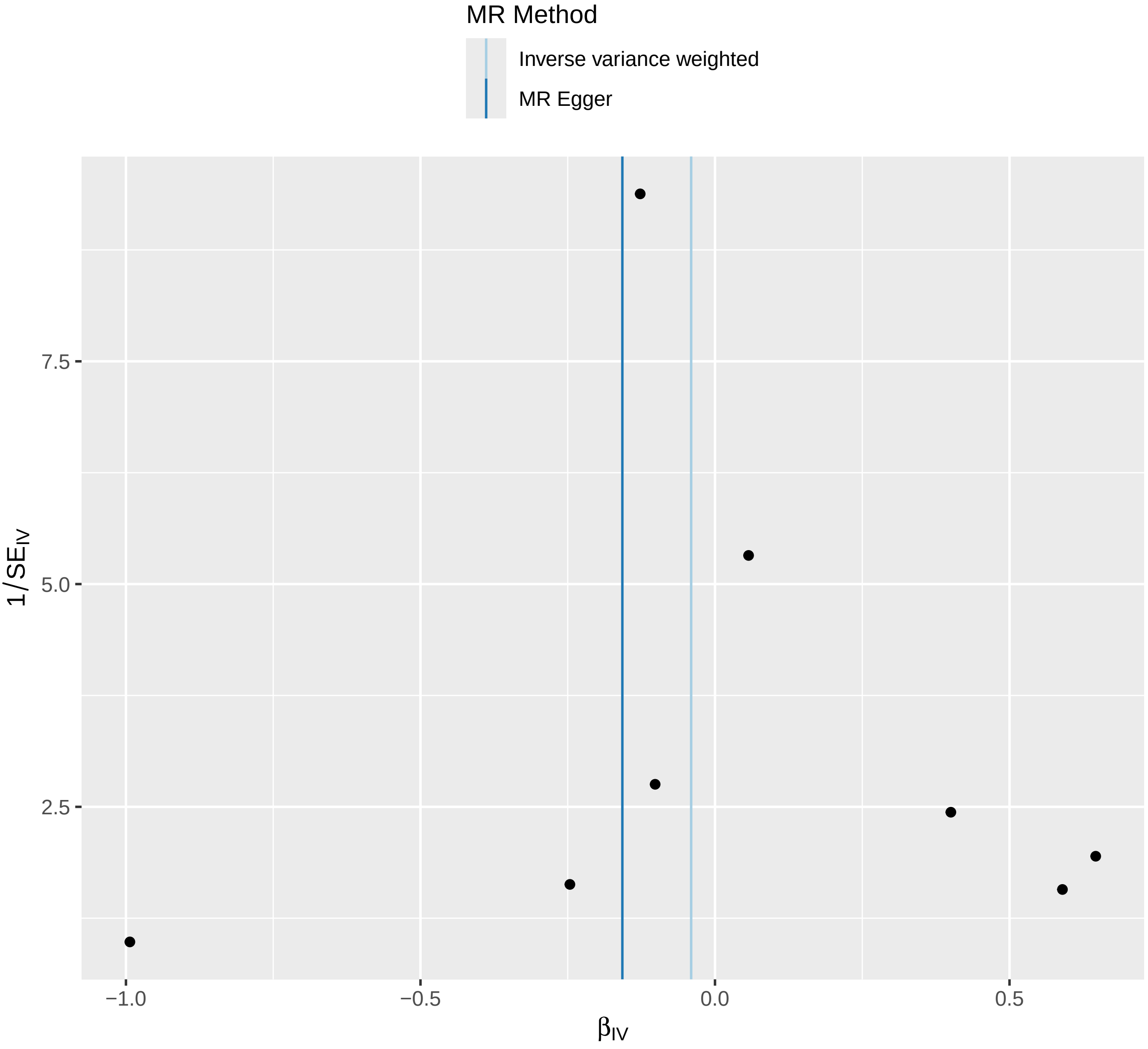

MR Method
Inverse variance weighted
MR Egger
7.5
5.0
2.5
1/SE$_{IV}$
−1.0
−0.5
0.0
0.5
β$_{IV}$

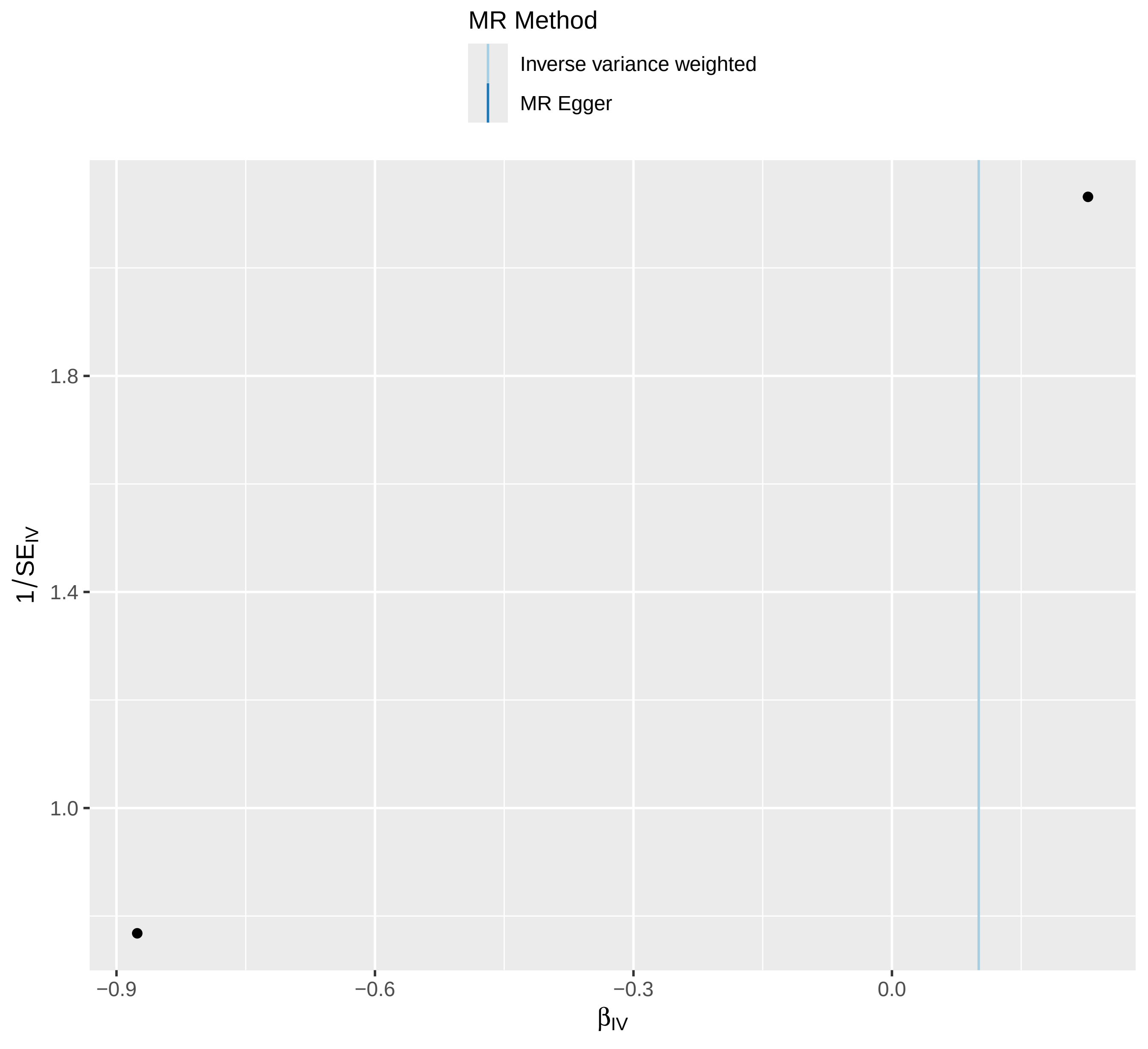
MR Method
Inverse variance weighted
MR Egger
1.8
1.4
1.0
1/SE$_{IV}$
−0.9
−0.6
−0.3
0.0
β$_{IV}$

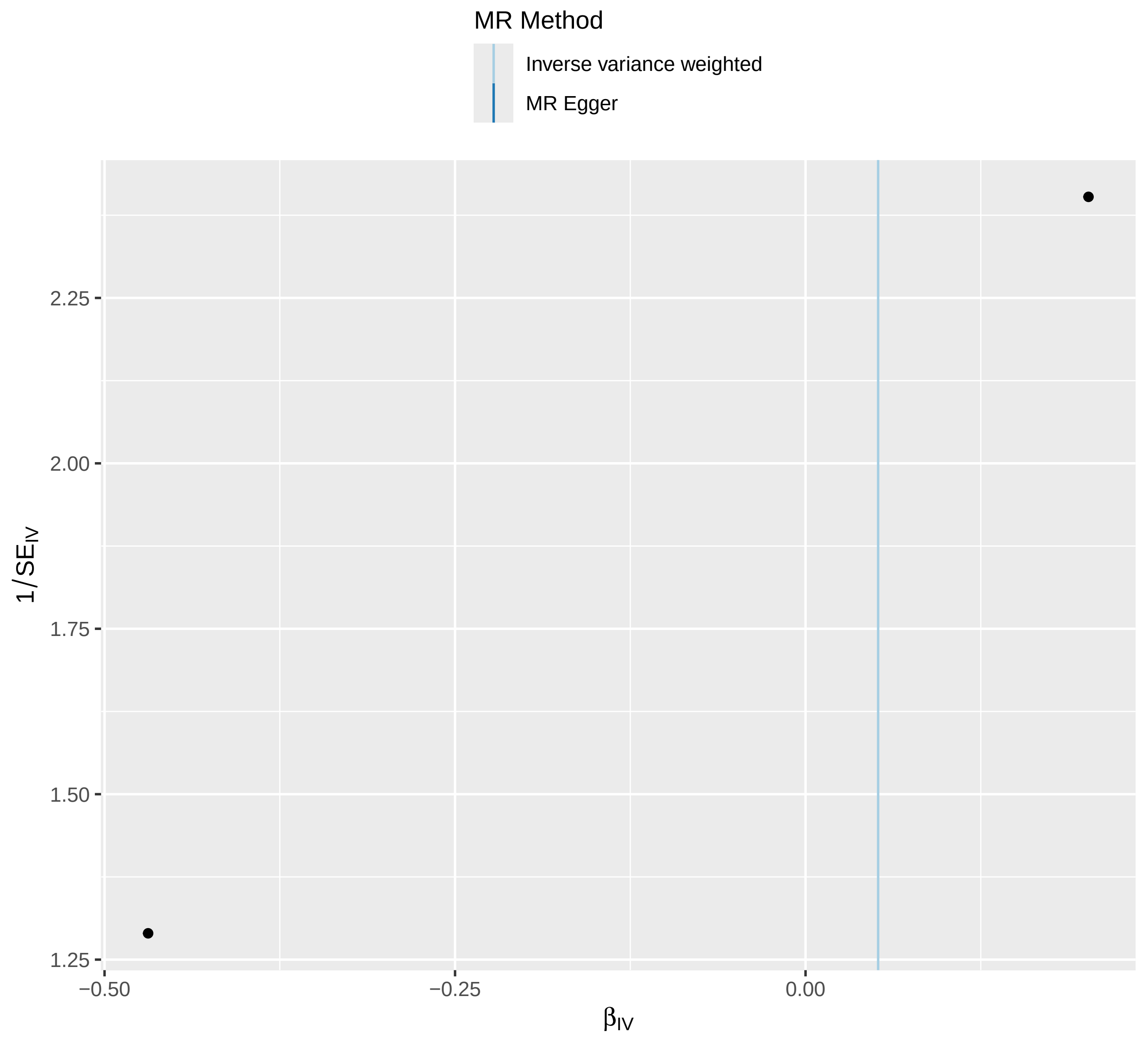

MR Method
Inverse variance weighted
MR Egger
2.25
2.00
1.75
1.50
1.25
1/SE_IV
−0.50
−0.25
0.00
β_IV

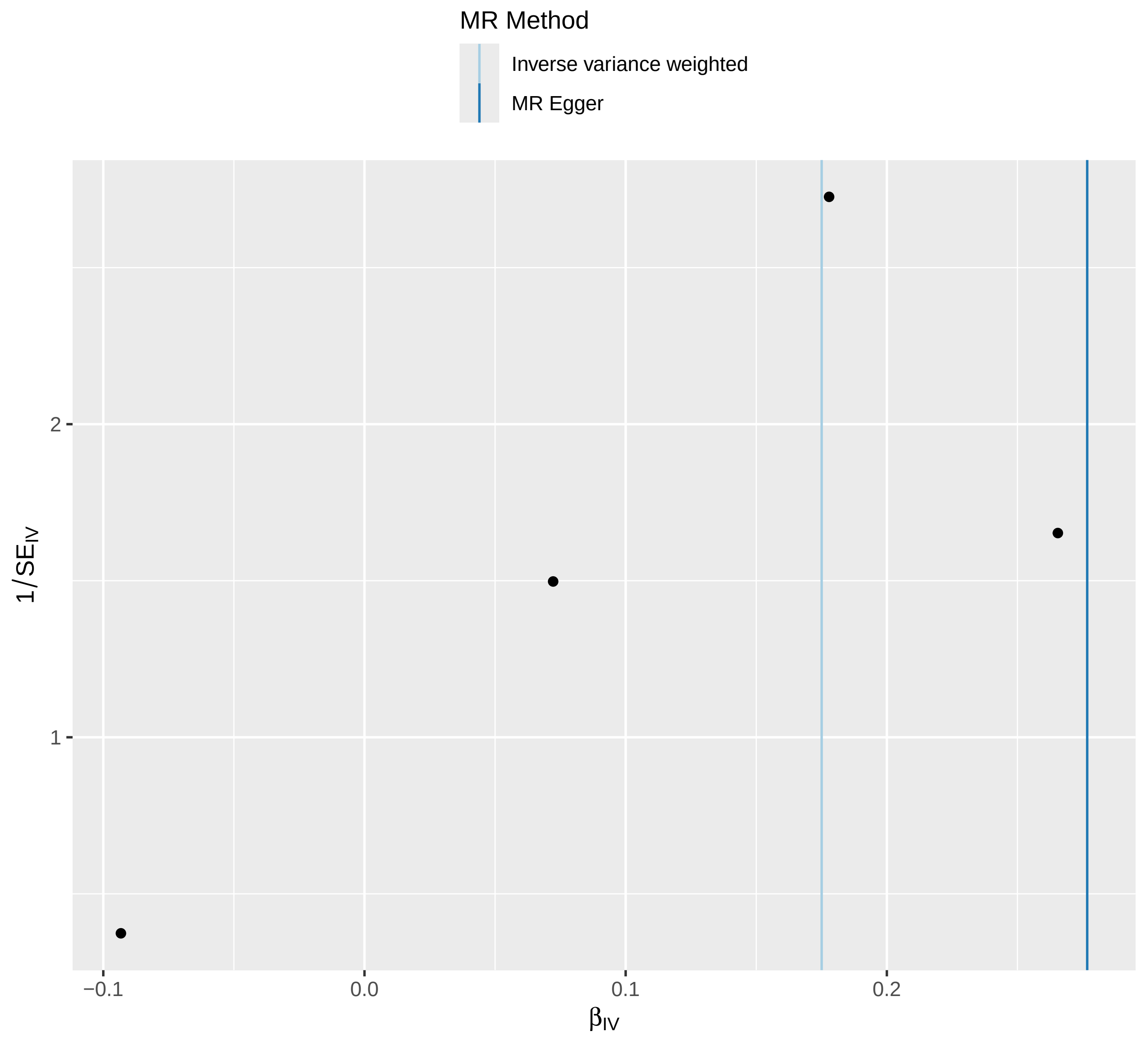
MR Method
Inverse variance weighted
MR Egger
2
1
1/SE_{IV}
−0.1
0.0
0.1
0.2
β_{IV}

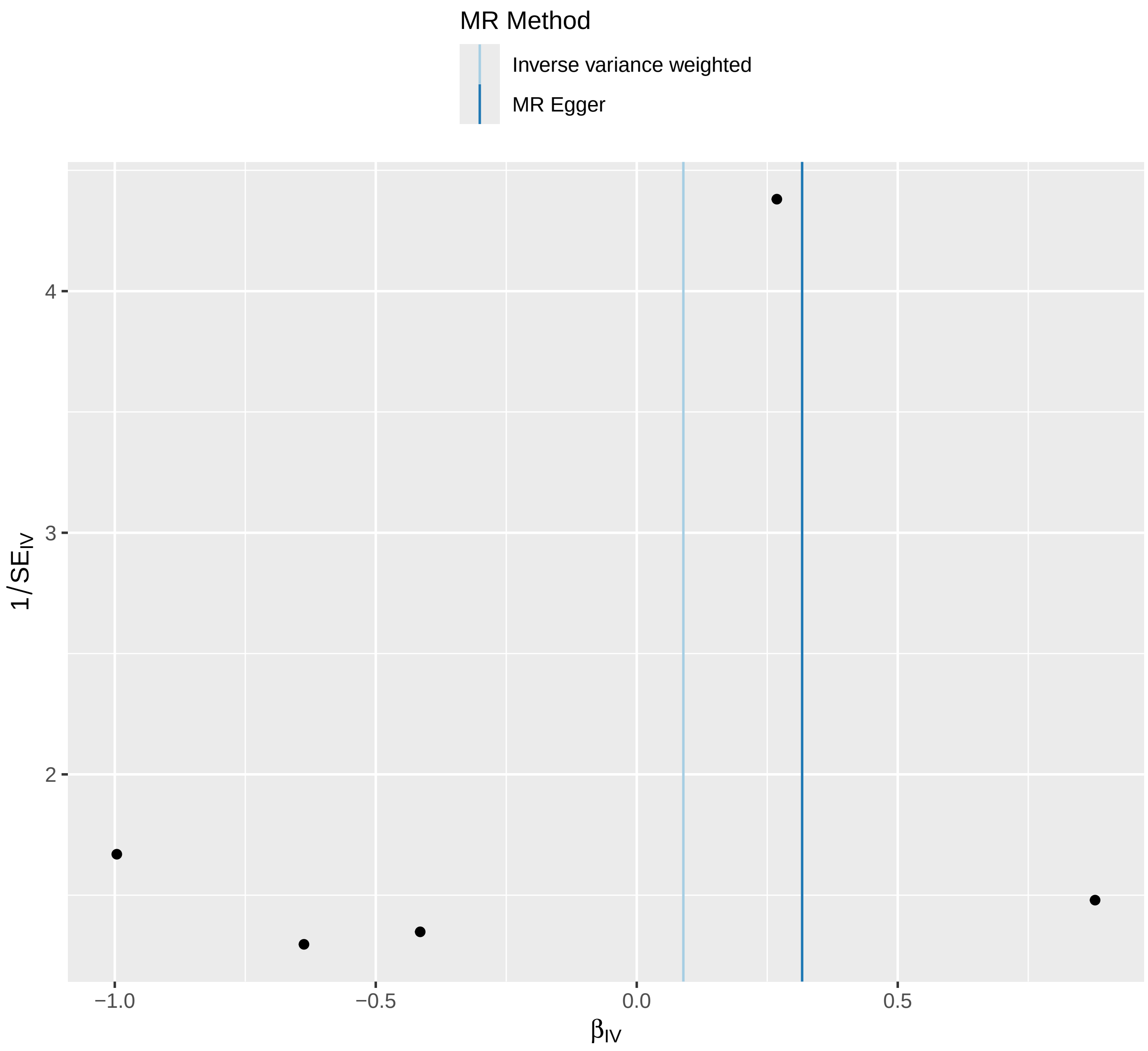

MR Method
Inverse variance weighted
MR Egger
4
3
2
1/SE_IV
−1.0
−0.5
0.0
0.5
β_IV

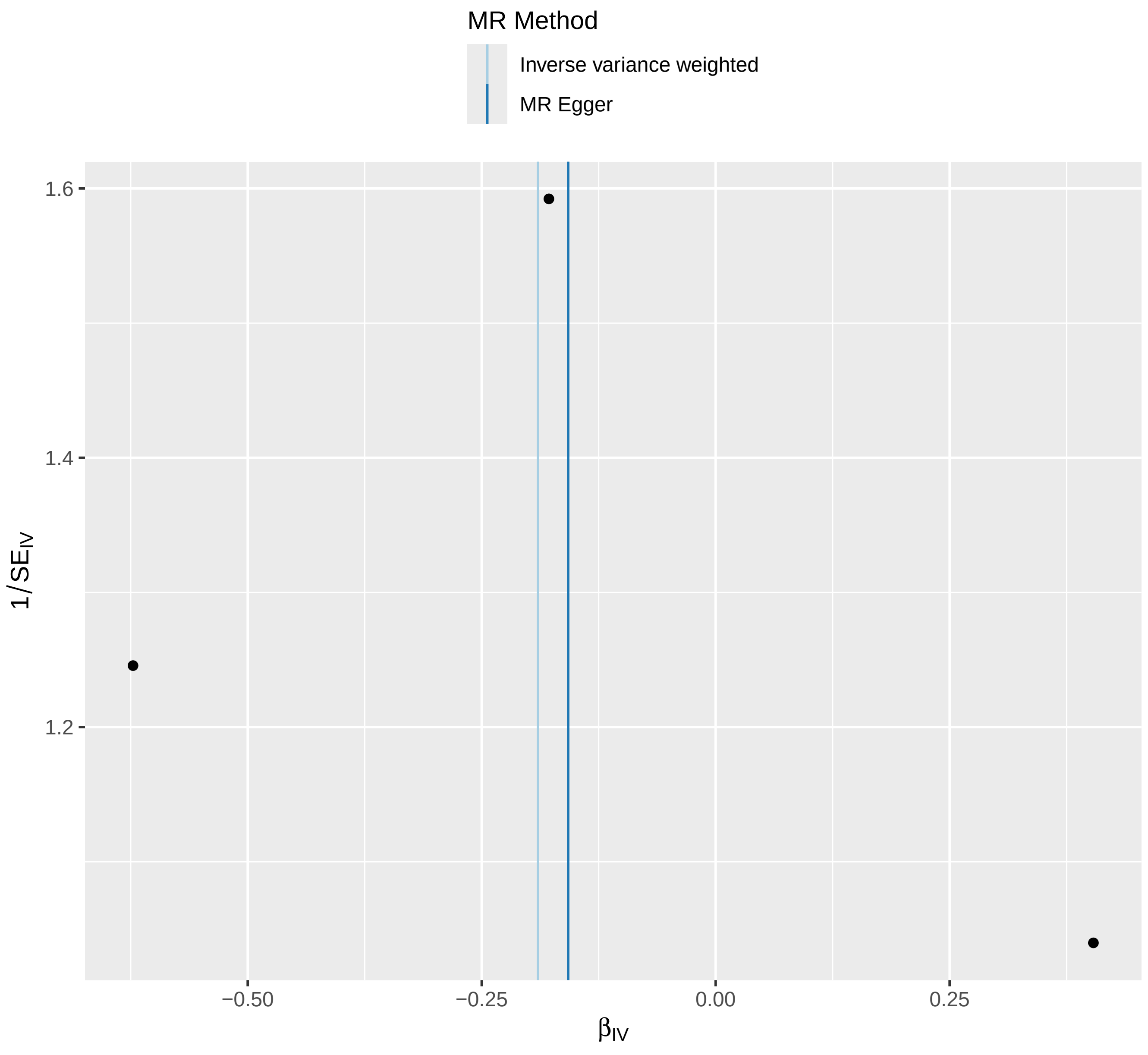

MR Method
Inverse variance weighted
MR Egger
1.6
1.4
1.2
1/SE$_{IV}$
−0.50
−0.25
0.00
0.25
β$_{IV}$

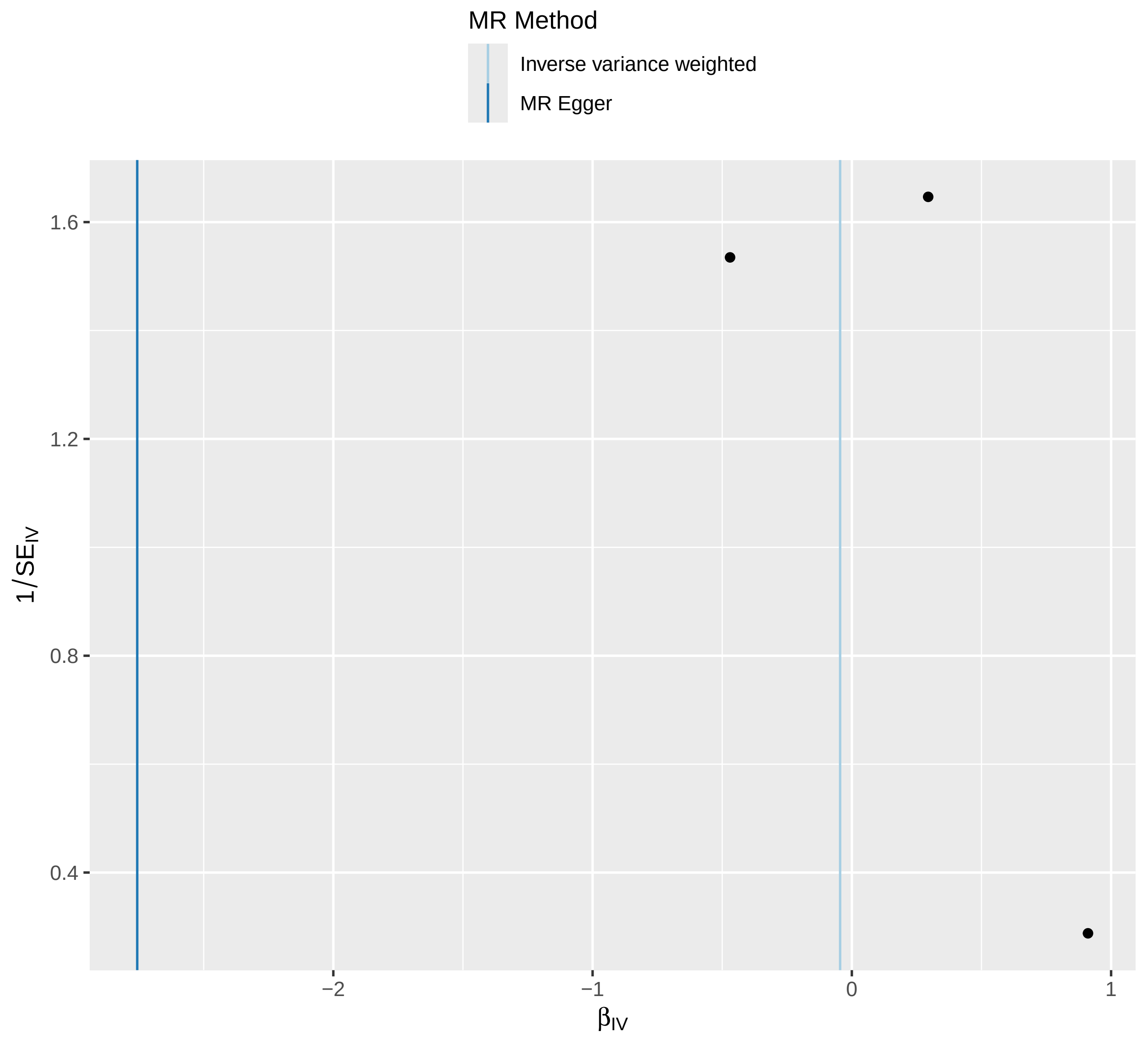

MR Method
Inverse variance weighted
MR Egger
1.6
1.2
0.8
0.4
1/SE_IV
−2
−1
0
1
β_IV

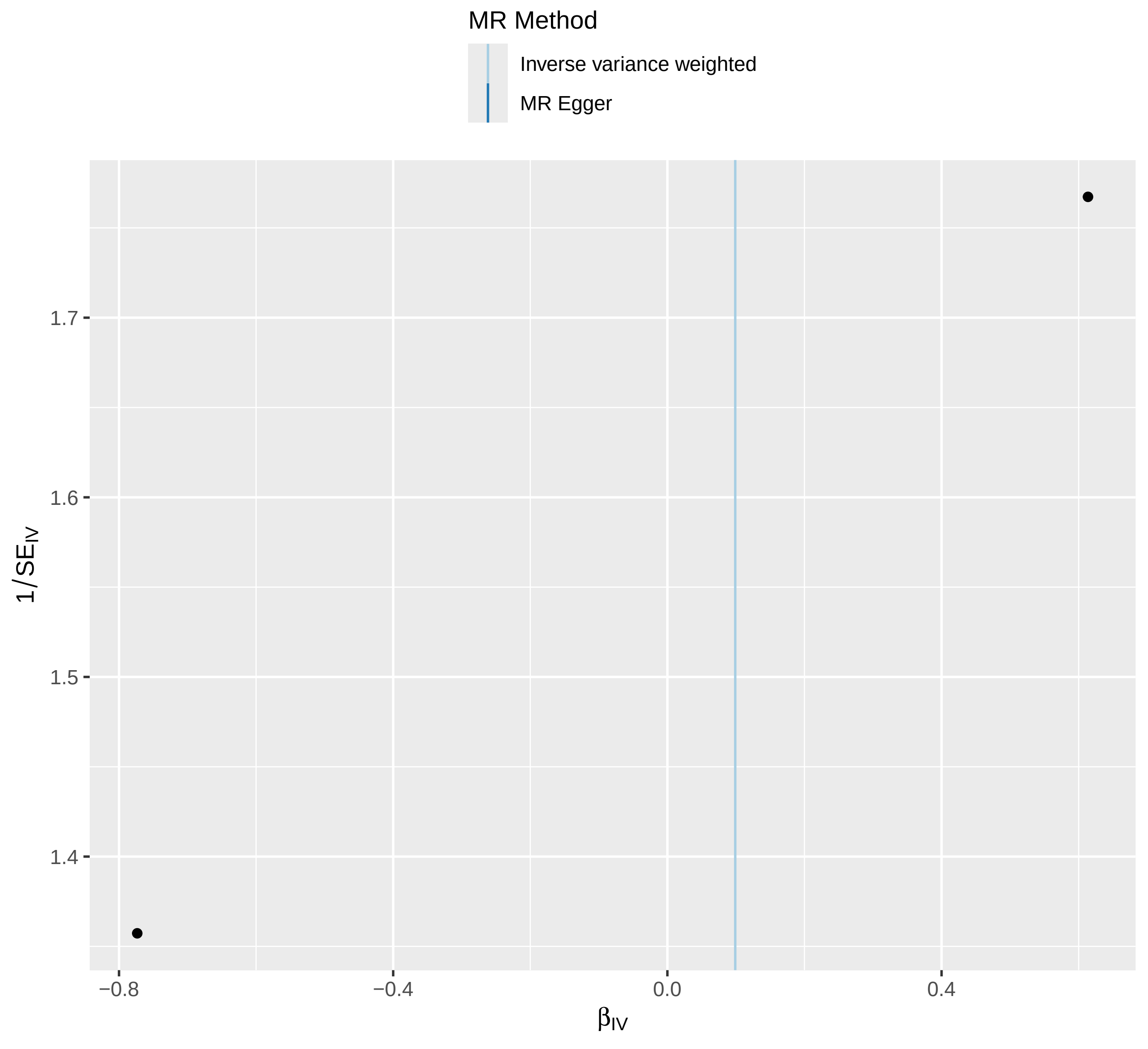

MR Method
Inverse variance weighted
MR Egger
1.7
1.6
1.5
1.4
−0.8
−0.4
0.0
0.4
β_{IV}
$1/SE_{IV}$

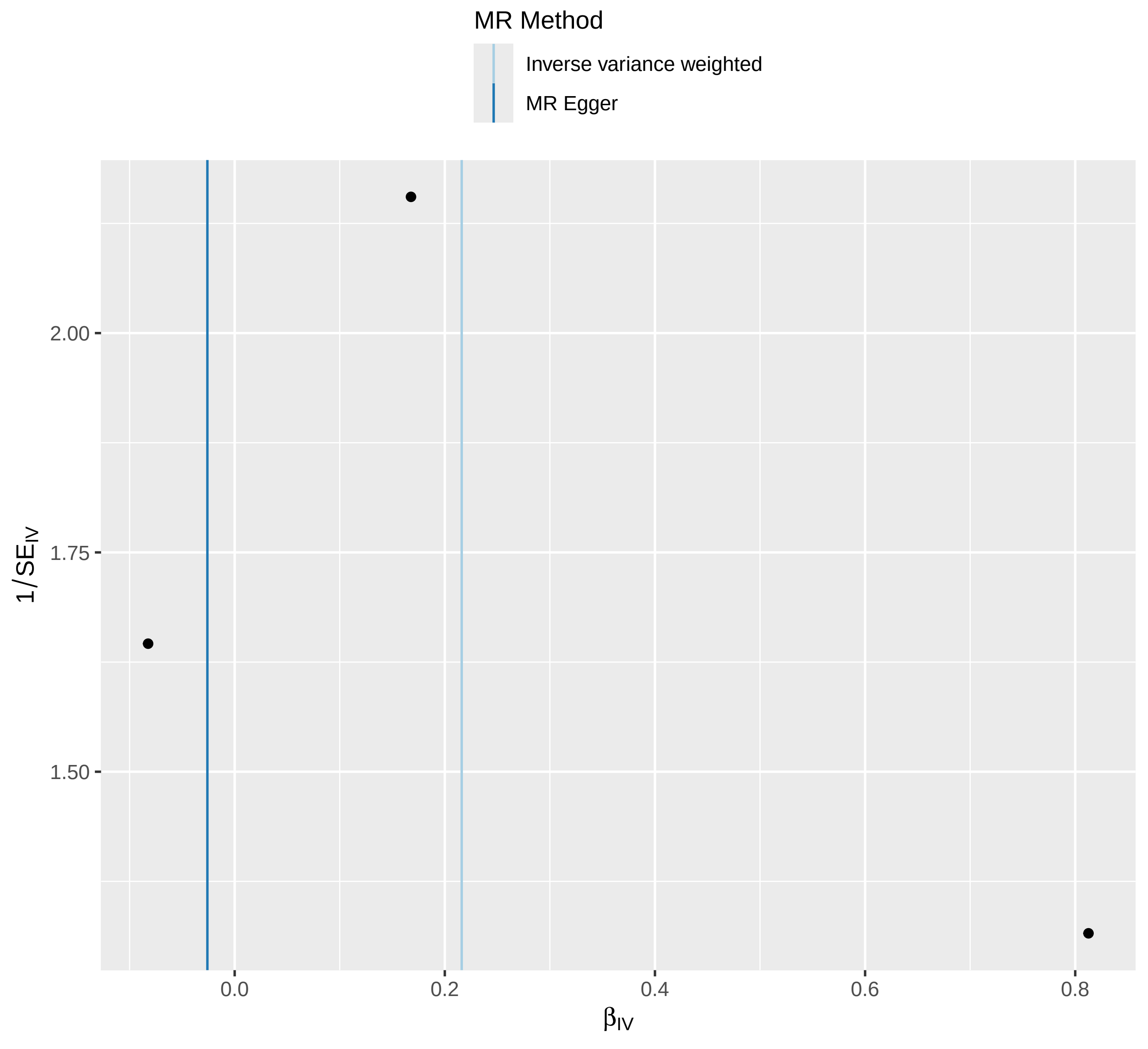

MR Method
Inverse variance weighted
MR Egger
2.00
1.75
1.50
1/SE_IV
0.0
0.2
0.4
0.6
0.8
β_IV

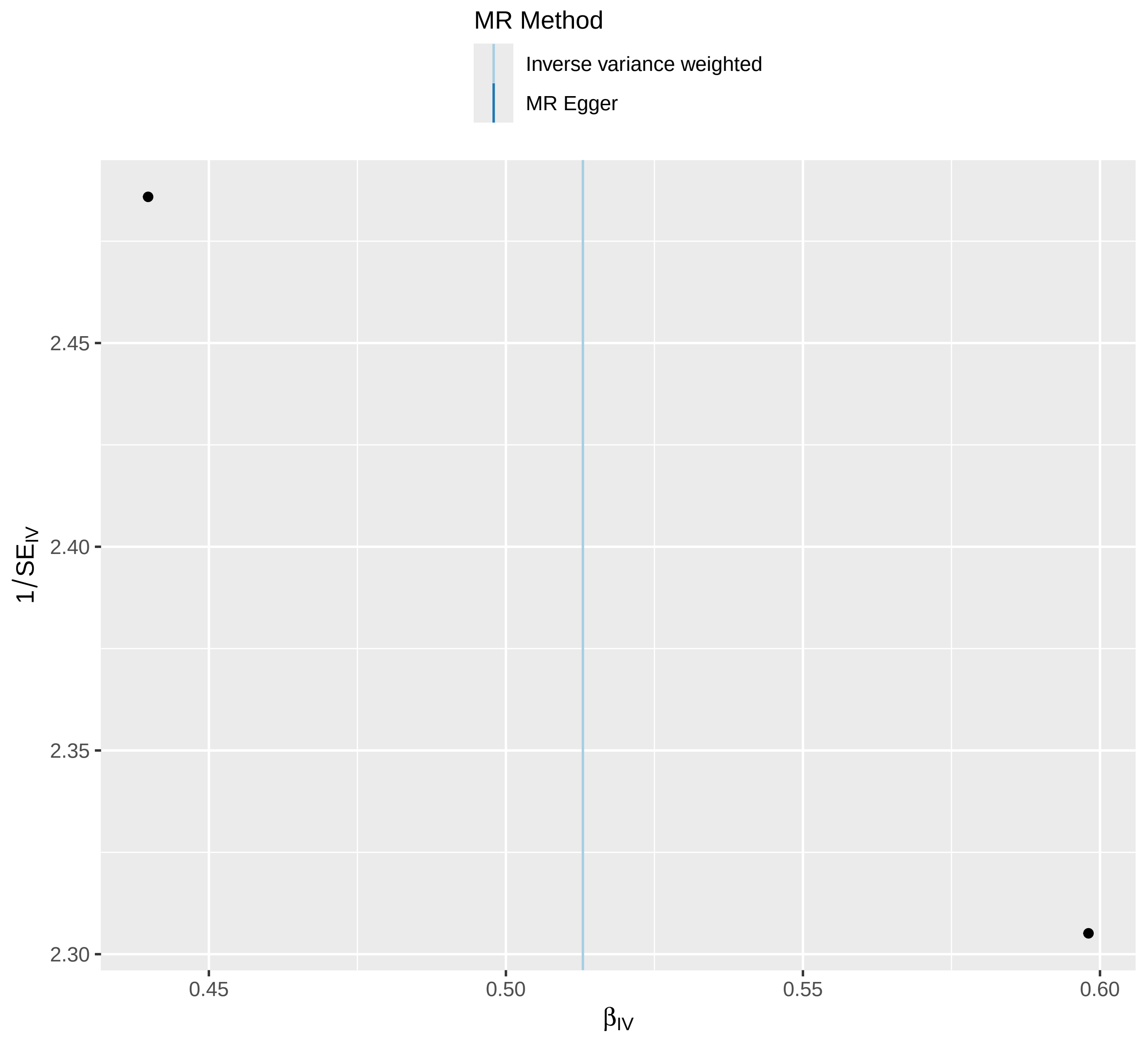
MR Method
Inverse variance weighted
MR Egger
1/SE_IV
2.45
2.40
2.35
2.30
0.45
0.50
0.55
0.60
β_IV

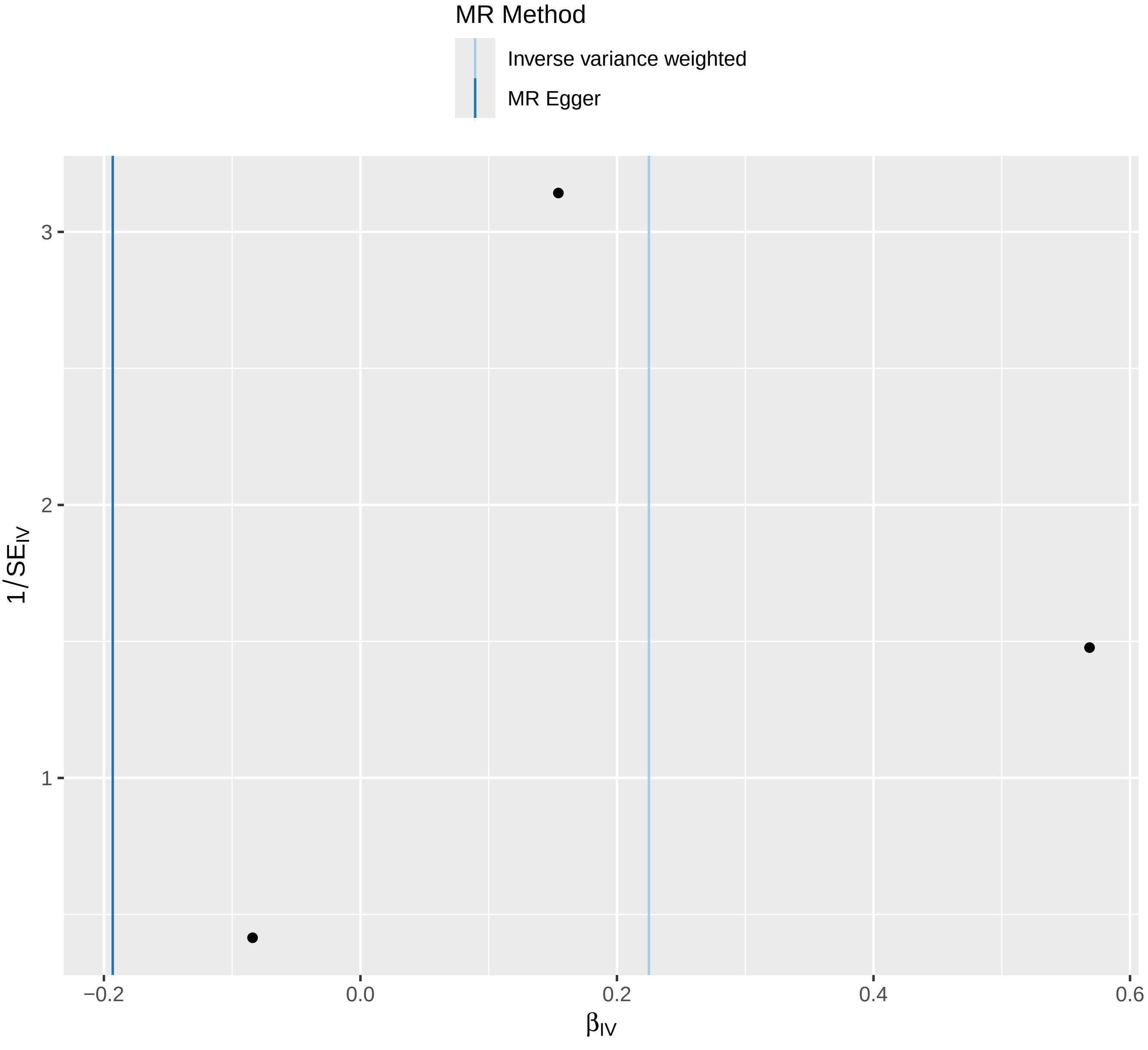

MR Method
Inverse variance weighted
MR Egger
3
2
1
1/SE$_{IV}$
−0.2
0.0
0.2
0.4
0.6
β$_{IV}$

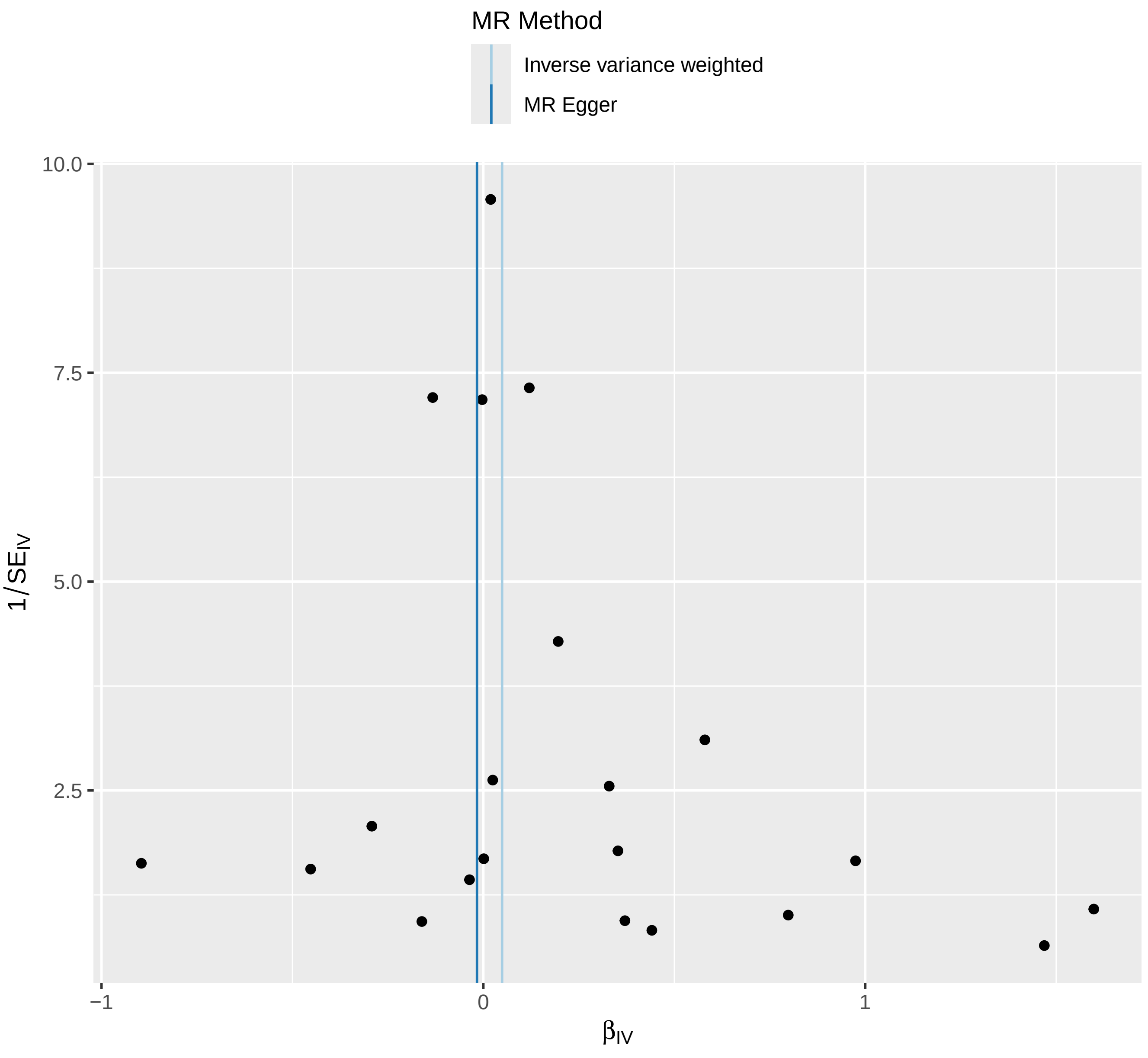
MR Method
Inverse variance weighted
MR Egger
10.0
7.5
5.0
2.5
1/SE$_{IV}$
−1
0
1
β$_{IV}$

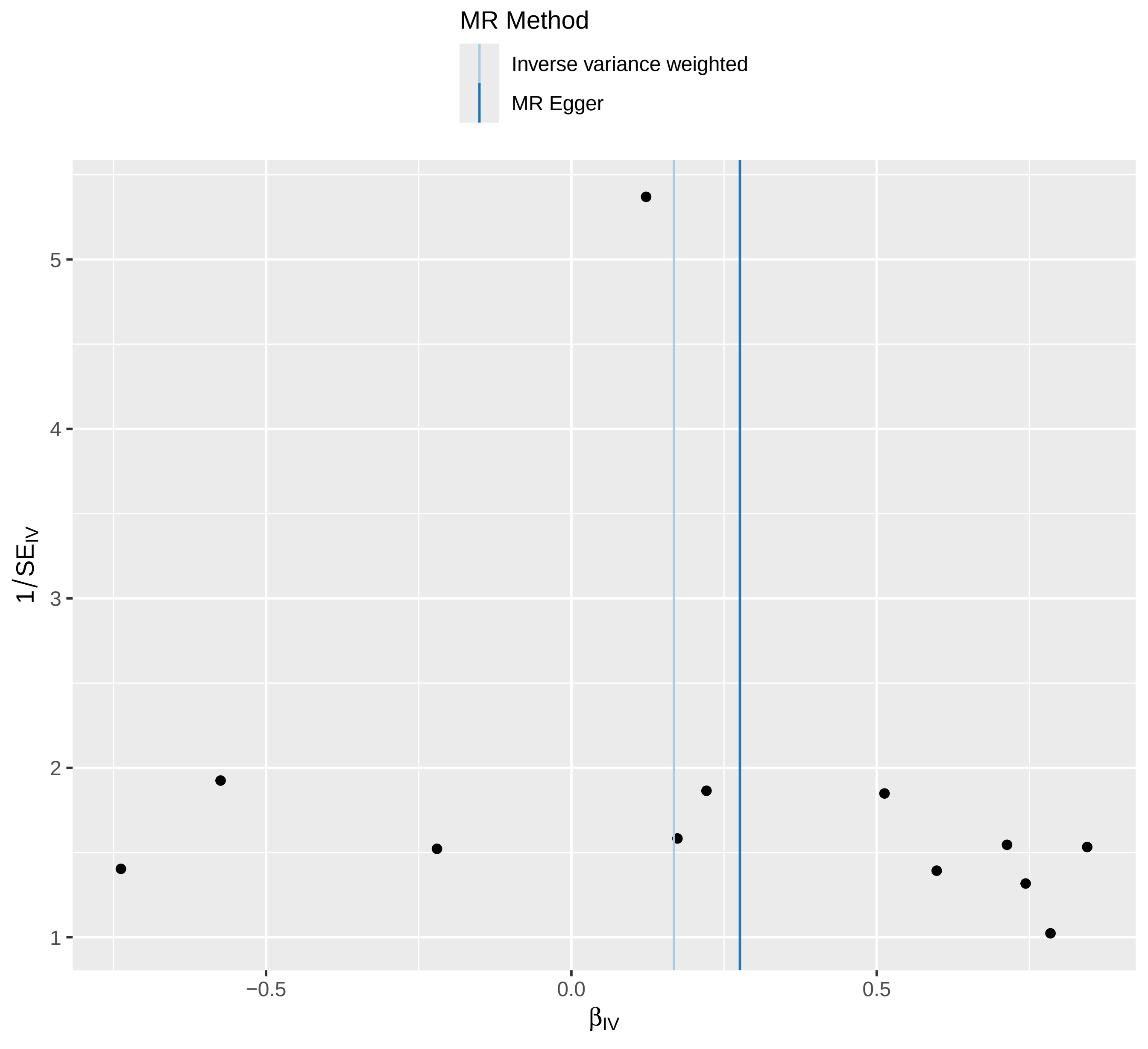

MR Method
Inverse variance weighted
MR Egger
1/SE_{IV}
5
4
3
2
1
−0.5
0.0
0.5
β_{IV}

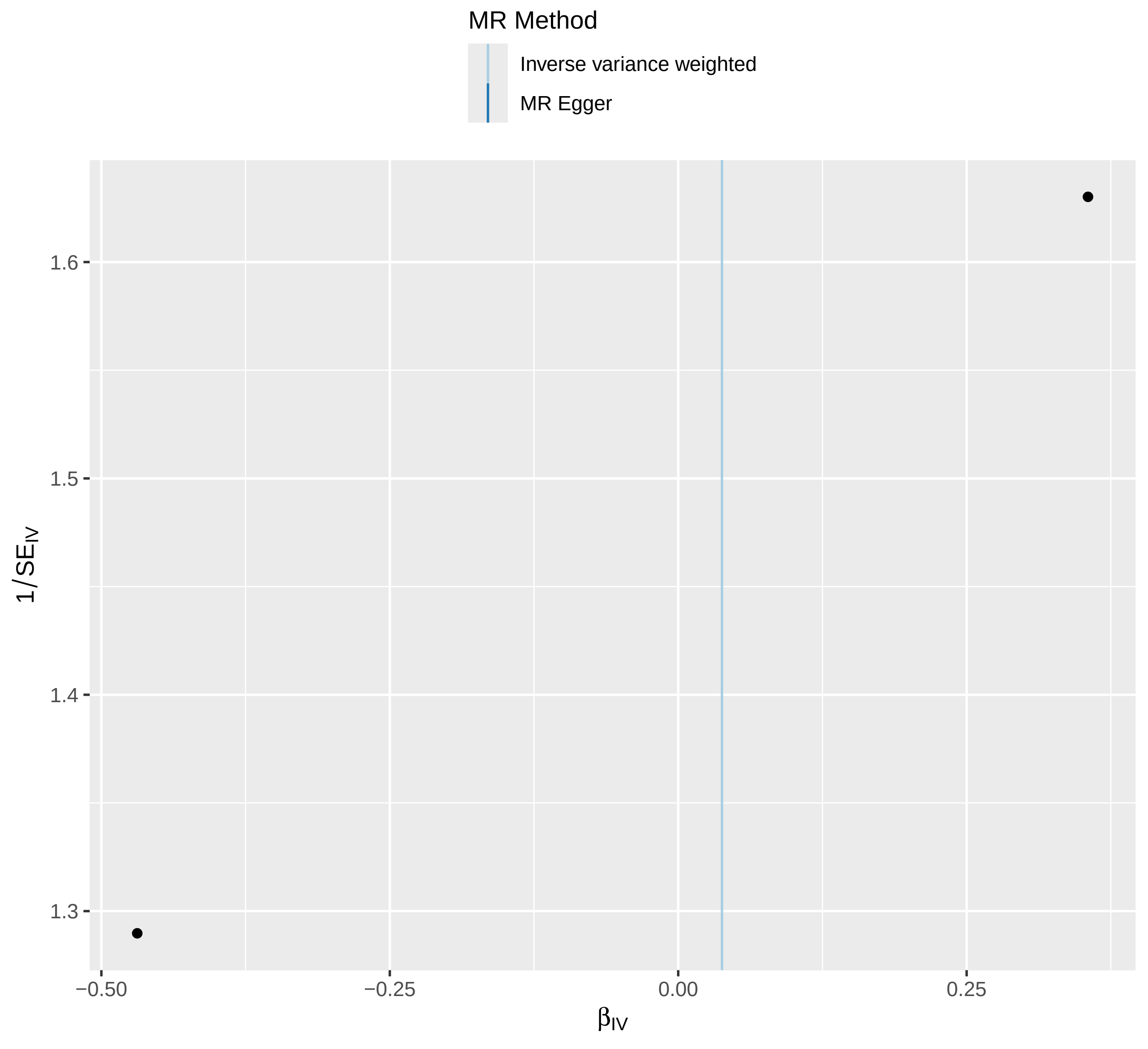

MR Method
Inverse variance weighted
MR Egger
1.6
1.5
1.4
1.3
1/SE$_{IV}$
−0.50
−0.25
0.00
0.25
β$_{IV}$

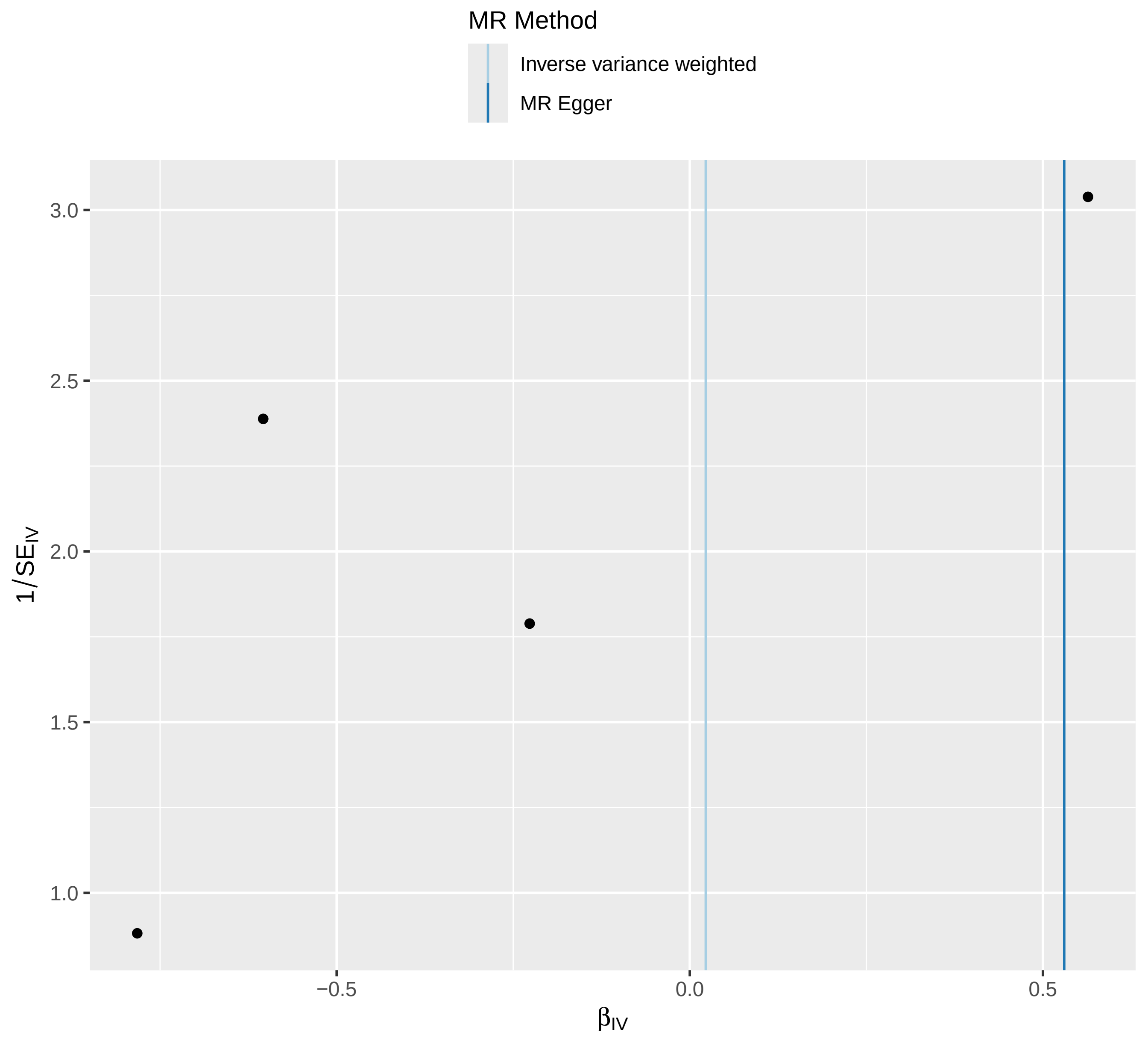

MR Method
Inverse variance weighted
MR Egger
3.0
2.5
2.0
1.5
1.0
1/SE$_{IV}$
−0.5
0.0
0.5
β$_{IV}$

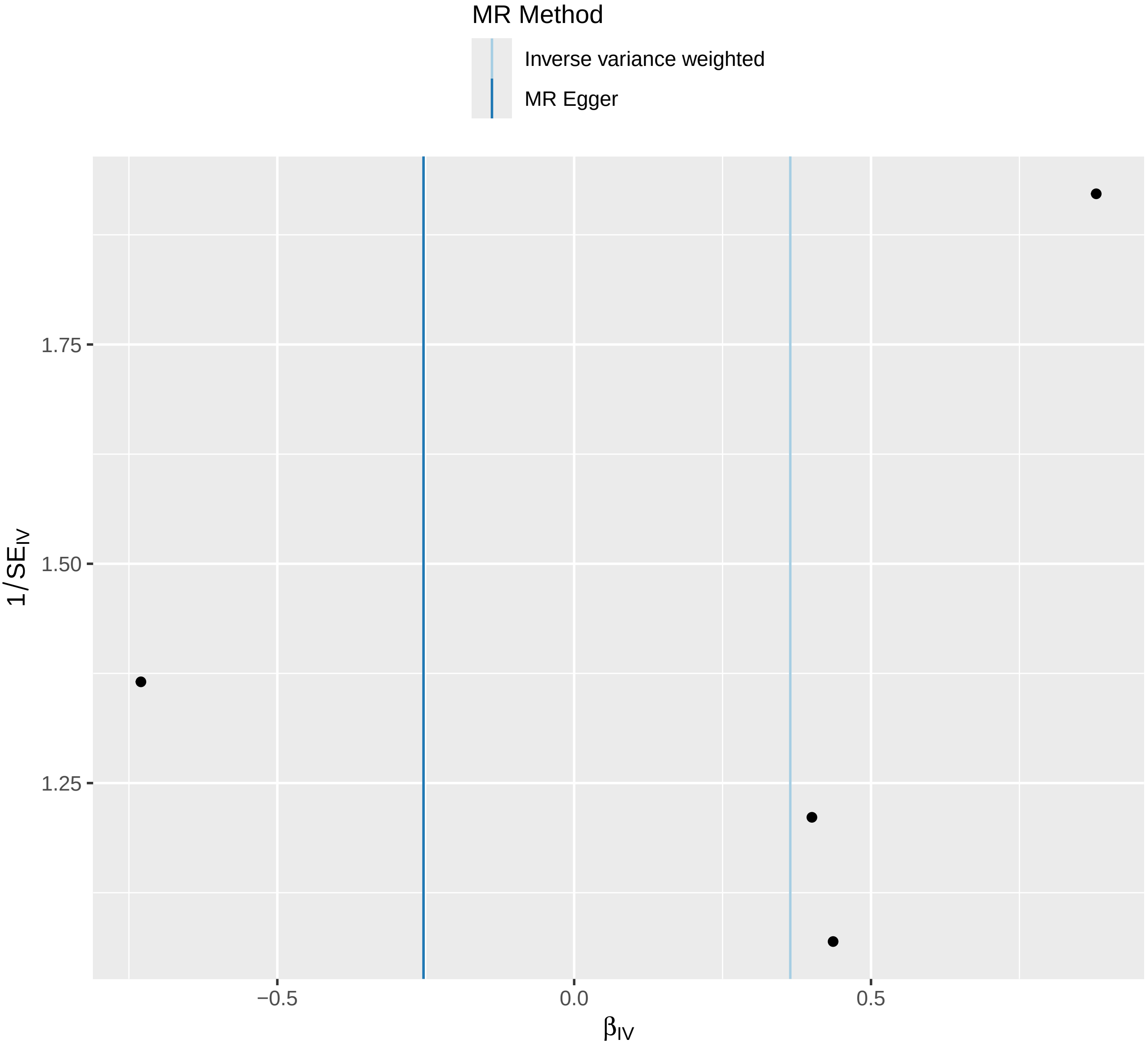

MR Method
Inverse variance weighted
MR Egger
1.75
1.50
1.25
1/SE_IV
−0.5
0.0
0.5
β_IV

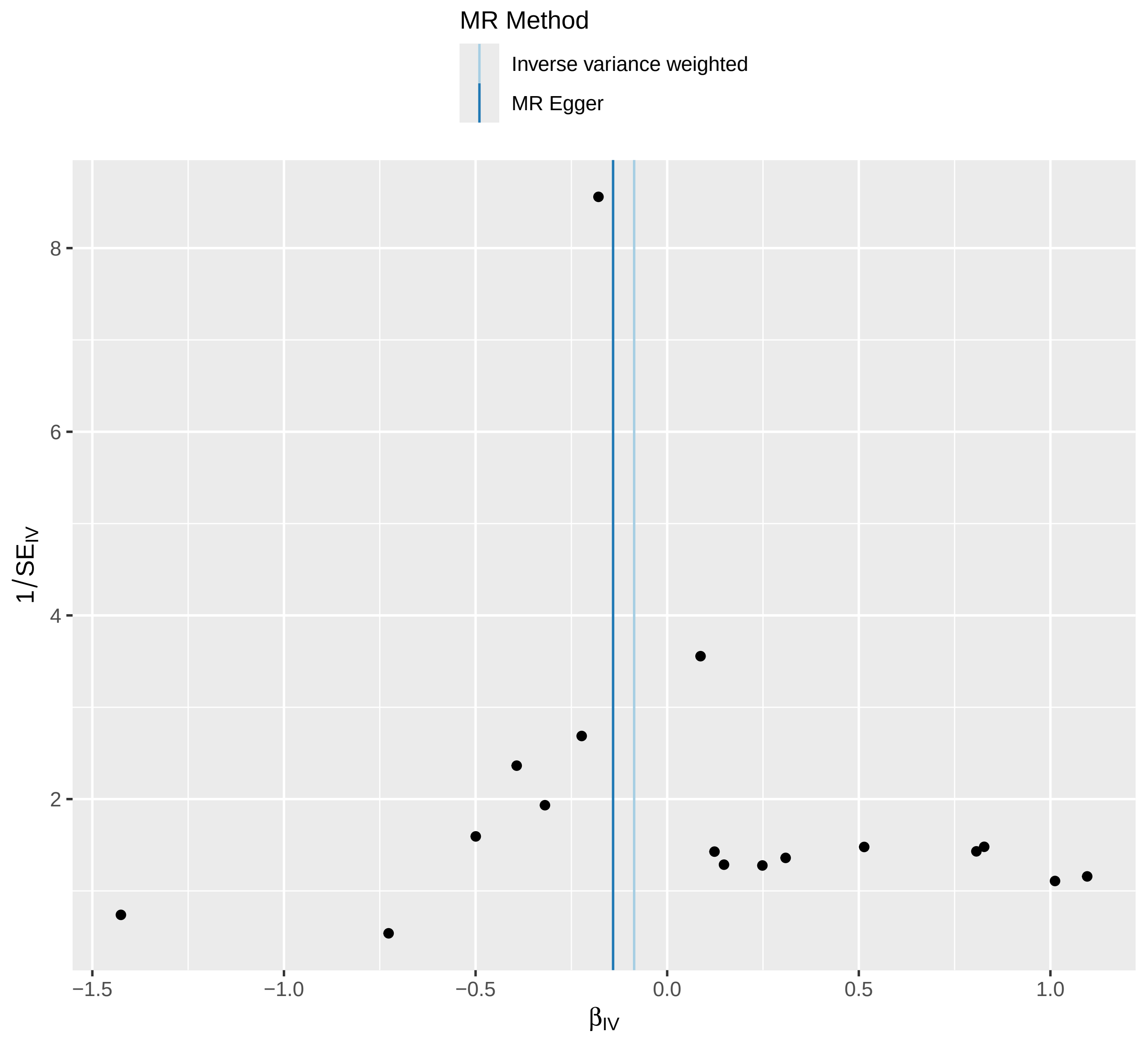

MR Method
Inverse variance weighted
MR Egger
8
6
4
2
$1/SE_{IV}$
−1.5
−1.0
−0.5
0.0
0.5
1.0
β_{IV}

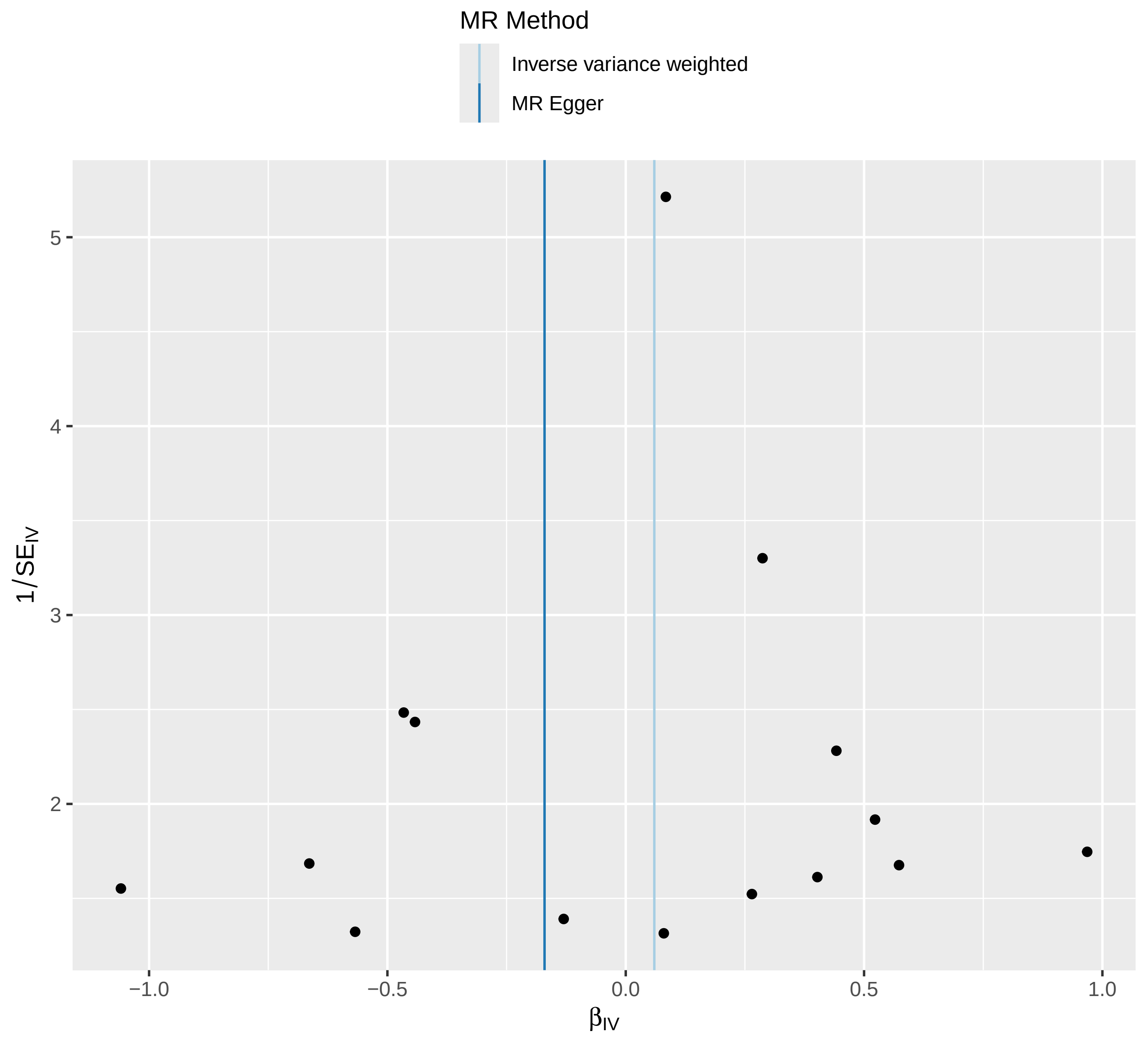
MR Method
Inverse variance weighted
MR Egger
1/SE_IV
5
4
3
2
−1.0
−0.5
0.0
0.5
1.0
β_IV

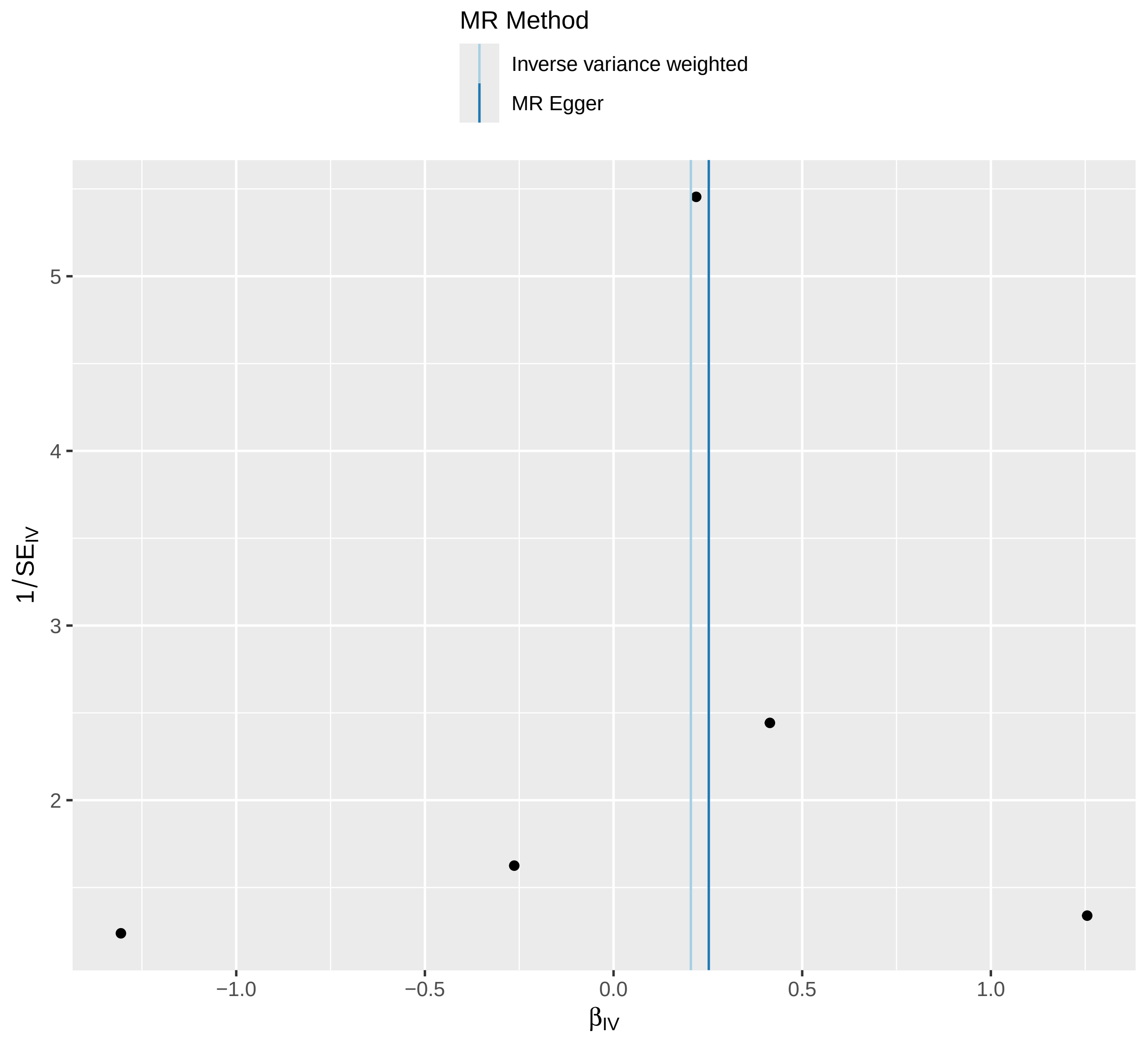

MR Method
Inverse variance weighted
MR Egger
5
4
3
2
1/SE_IV
−1.0
−0.5
0.0
0.5
1.0
β_IV

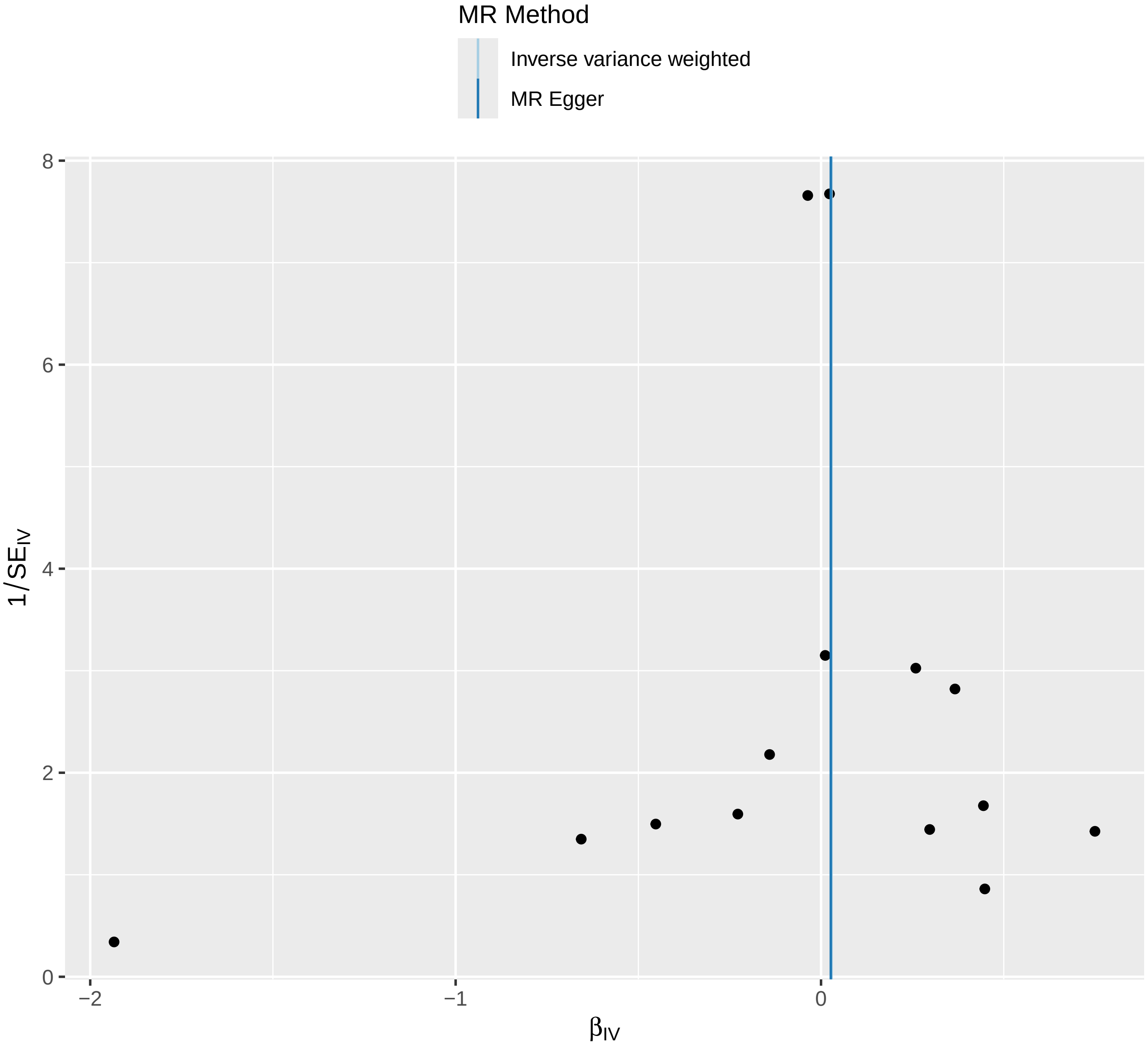
MR Method
Inverse variance weighted
MR Egger
8
6
4
2
0
−2
−1
0
1/SE_IV
β_IV

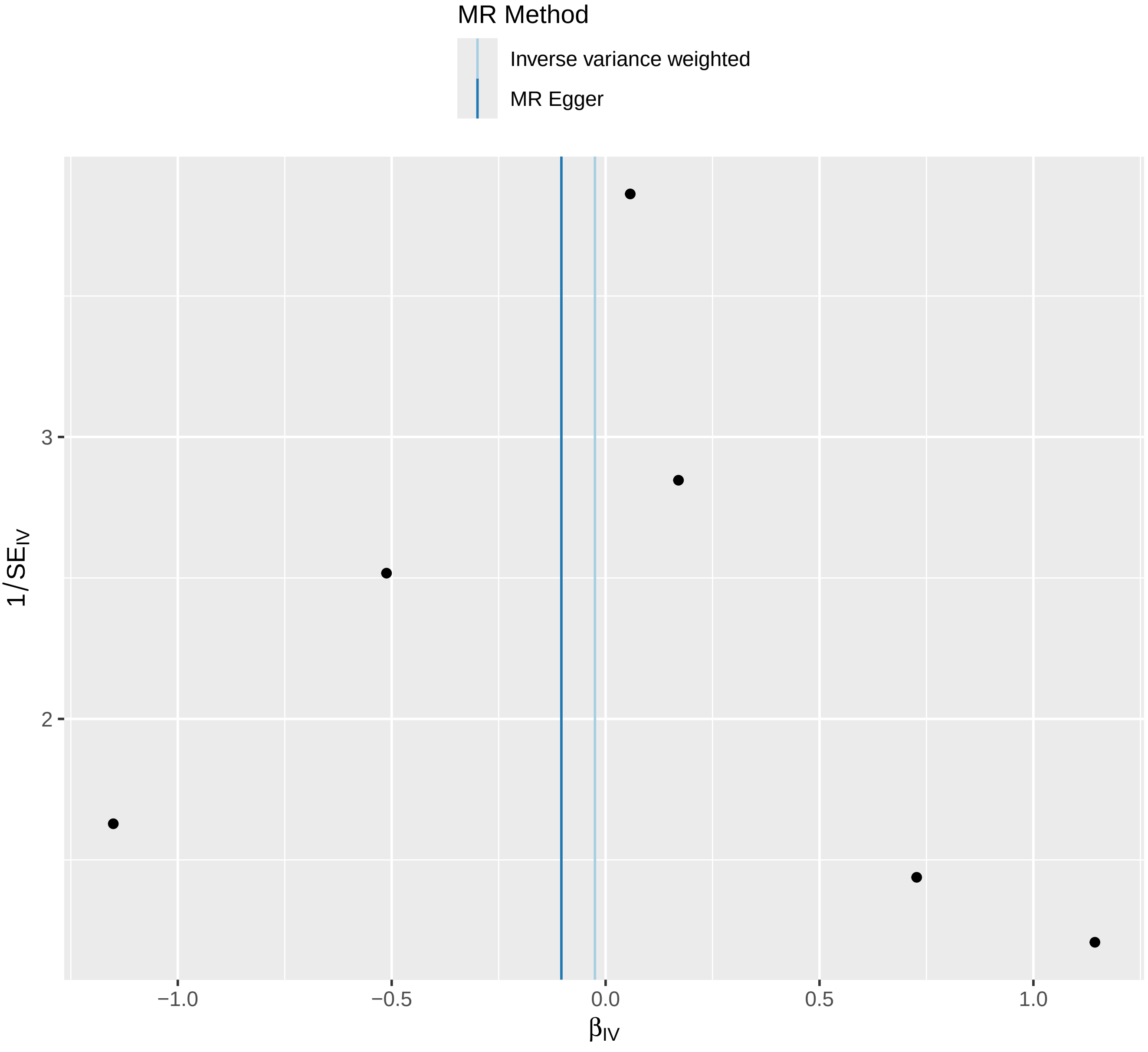
MR Method
Inverse variance weighted
MR Egger
3
2
1/SE_IV
−1.0
−0.5
0.0
0.5
1.0
β_IV

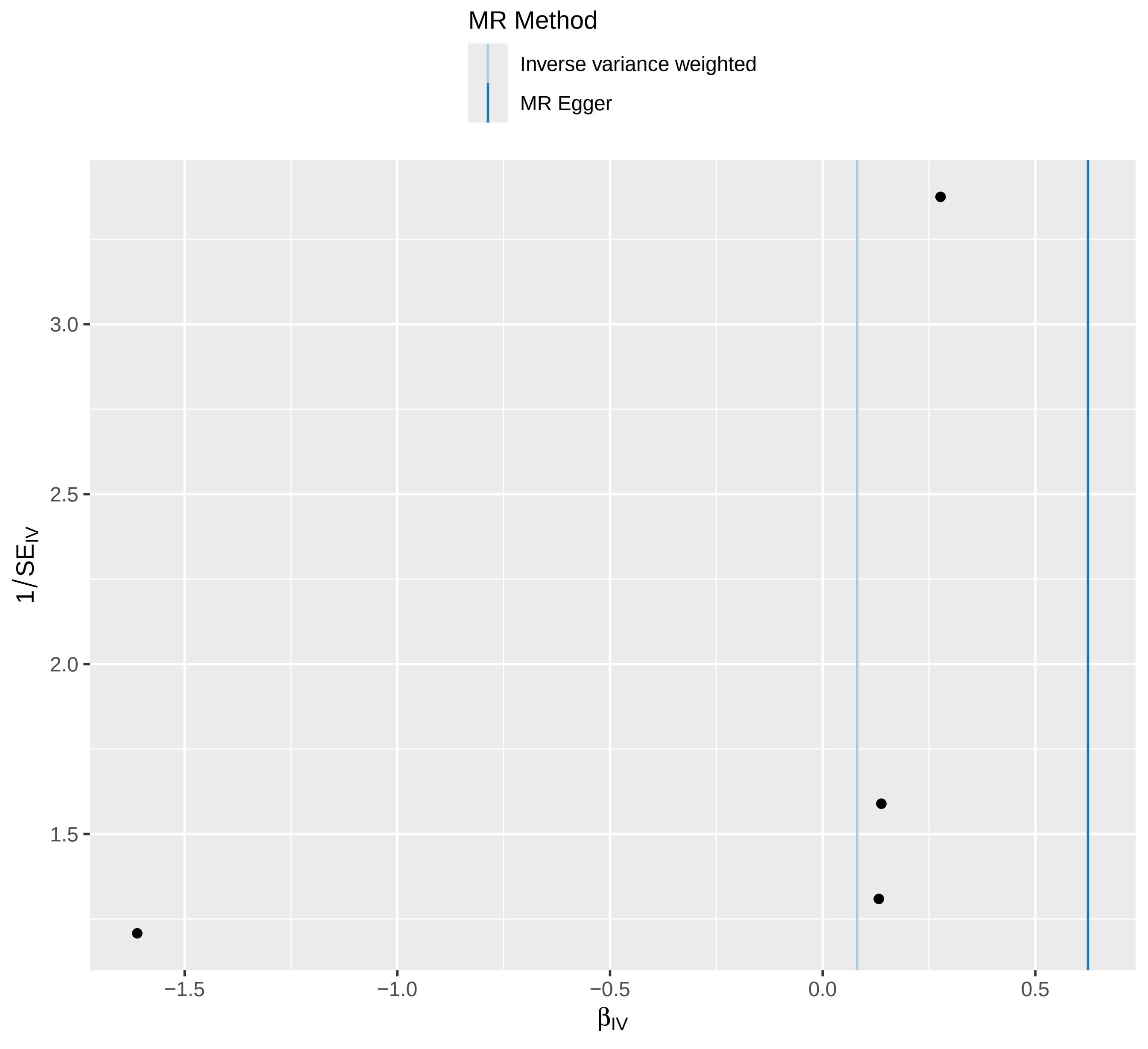

MR Method
Inverse variance weighted
MR Egger
3.0
2.5
2.0
1.5
1/SE$_{IV}$
−1.5
−1.0
−0.5
0.0
0.5
β$_{IV}$

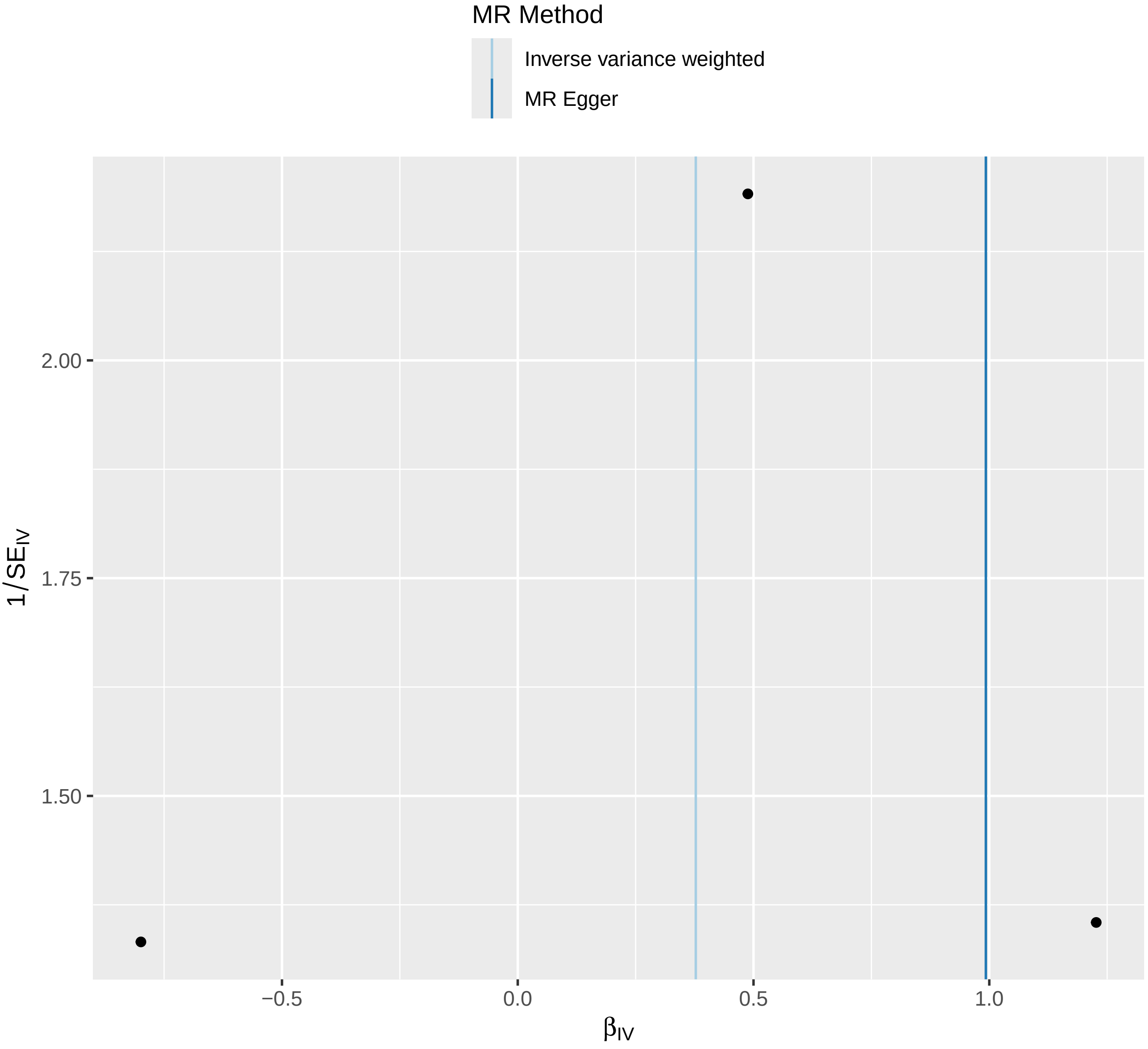

MR Method
Inverse variance weighted
MR Egger
2.00
1.75
1.50
1/SE_IV
−0.5
0.0
0.5
1.0
β_IV

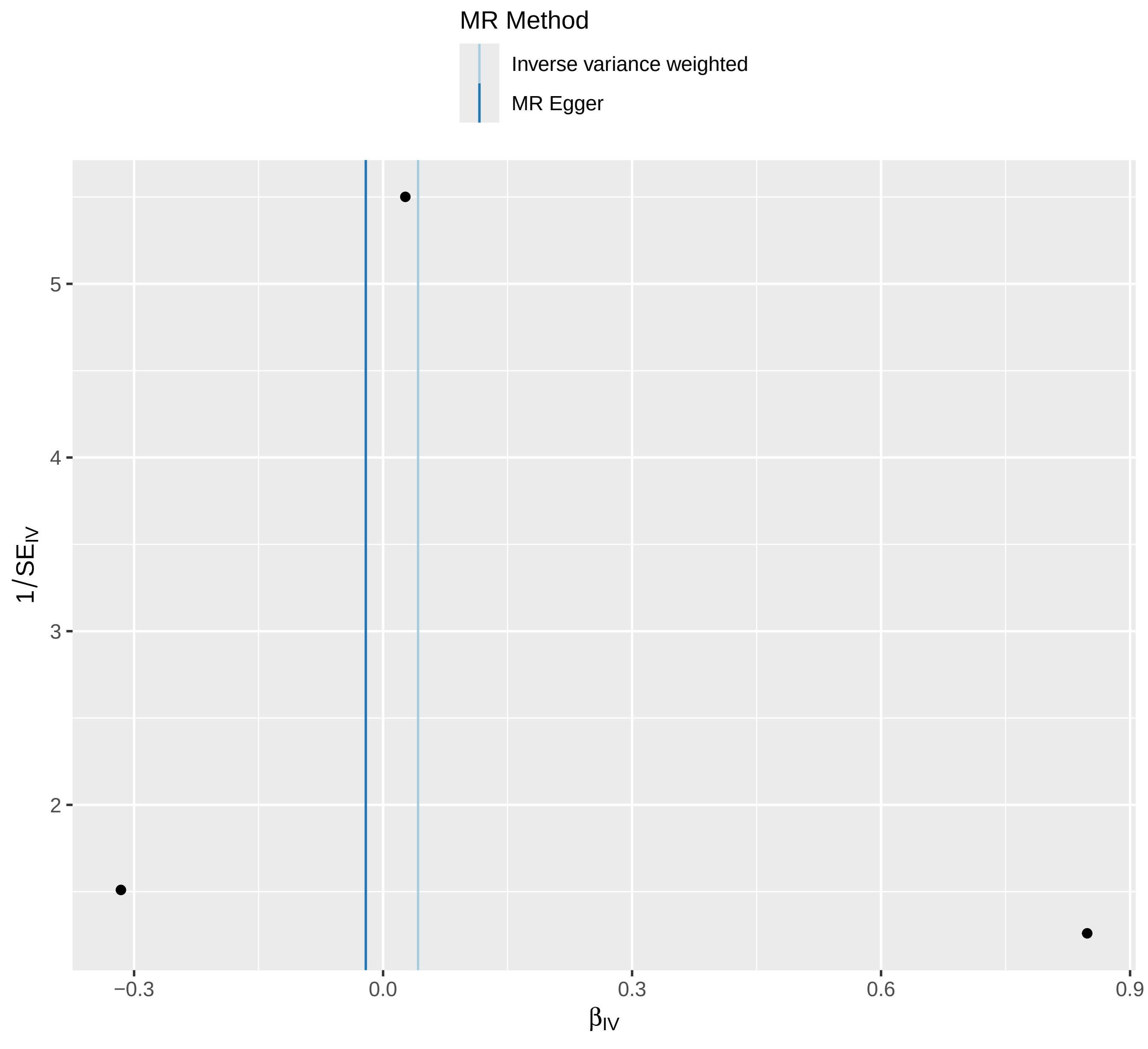
MR Method
Inverse variance weighted
MR Egger
1/SE_IV
5
4
3
2
−0.3
0.0
0.3
0.6
0.9
β_IV

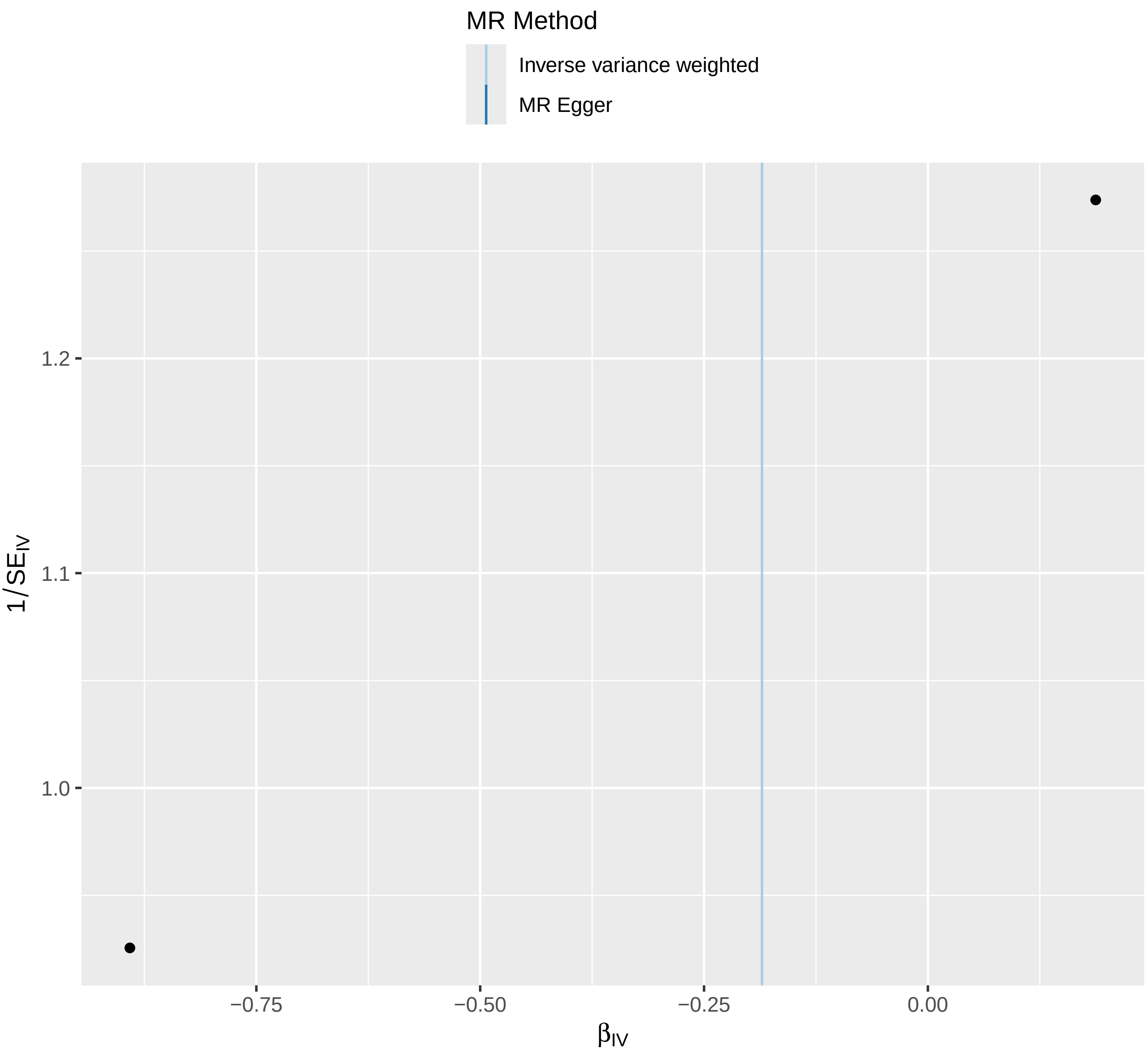

MR Method
Inverse variance weighted
MR Egger
1.2
1.1
1.0
−0.75
−0.50
−0.25
0.00
$1/SE_{IV}$
β_{IV}

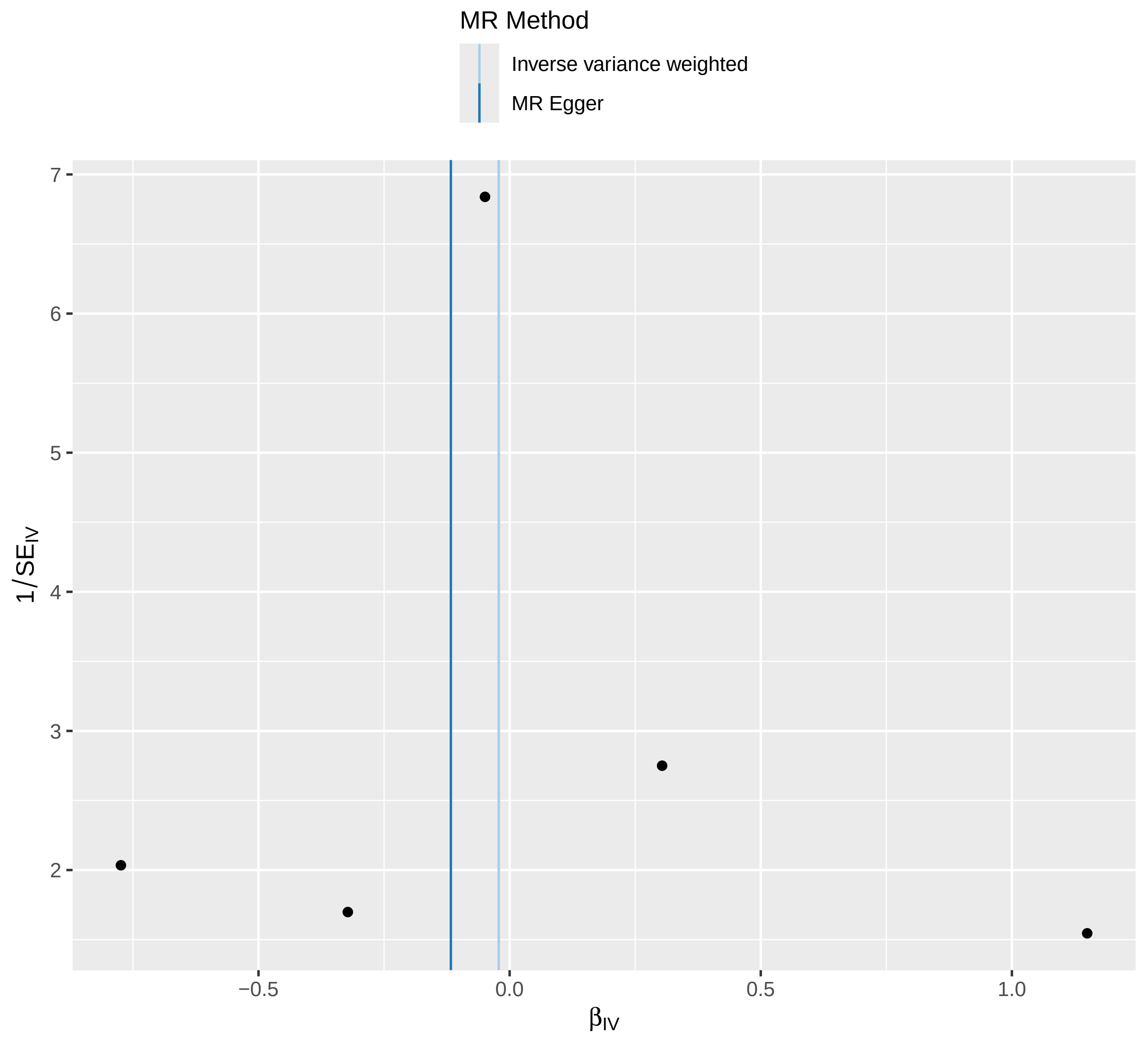
MR Method
Inverse variance weighted
MR Egger
7
6
5
4
3
2
1/SE_IV
−0.5
0.0
0.5
1.0
β_IV

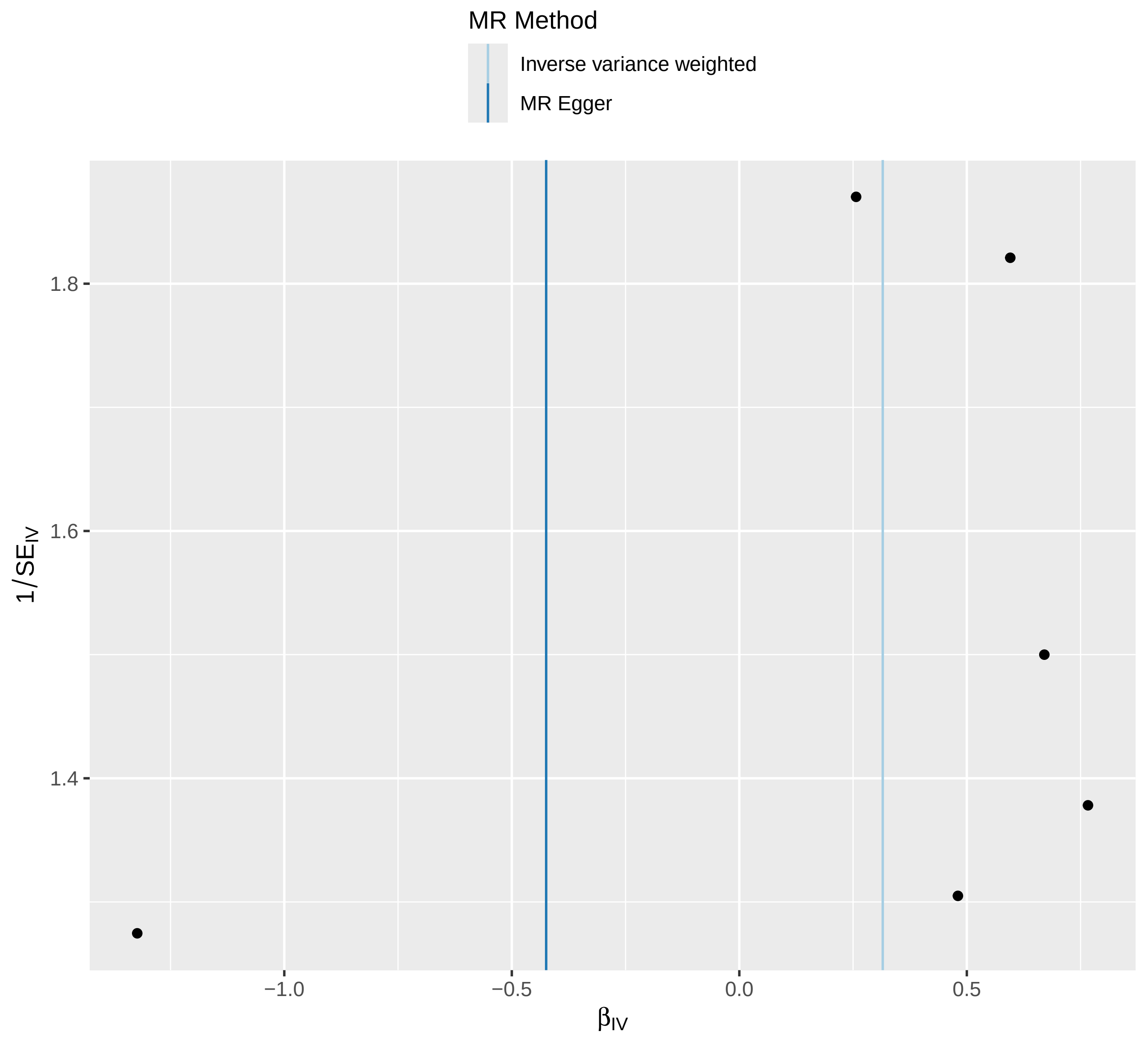
MR Method
Inverse variance weighted
MR Egger
1.8
1.6
1.4
1/SE_IV
−1.0
−0.5
0.0
0.5
β_IV

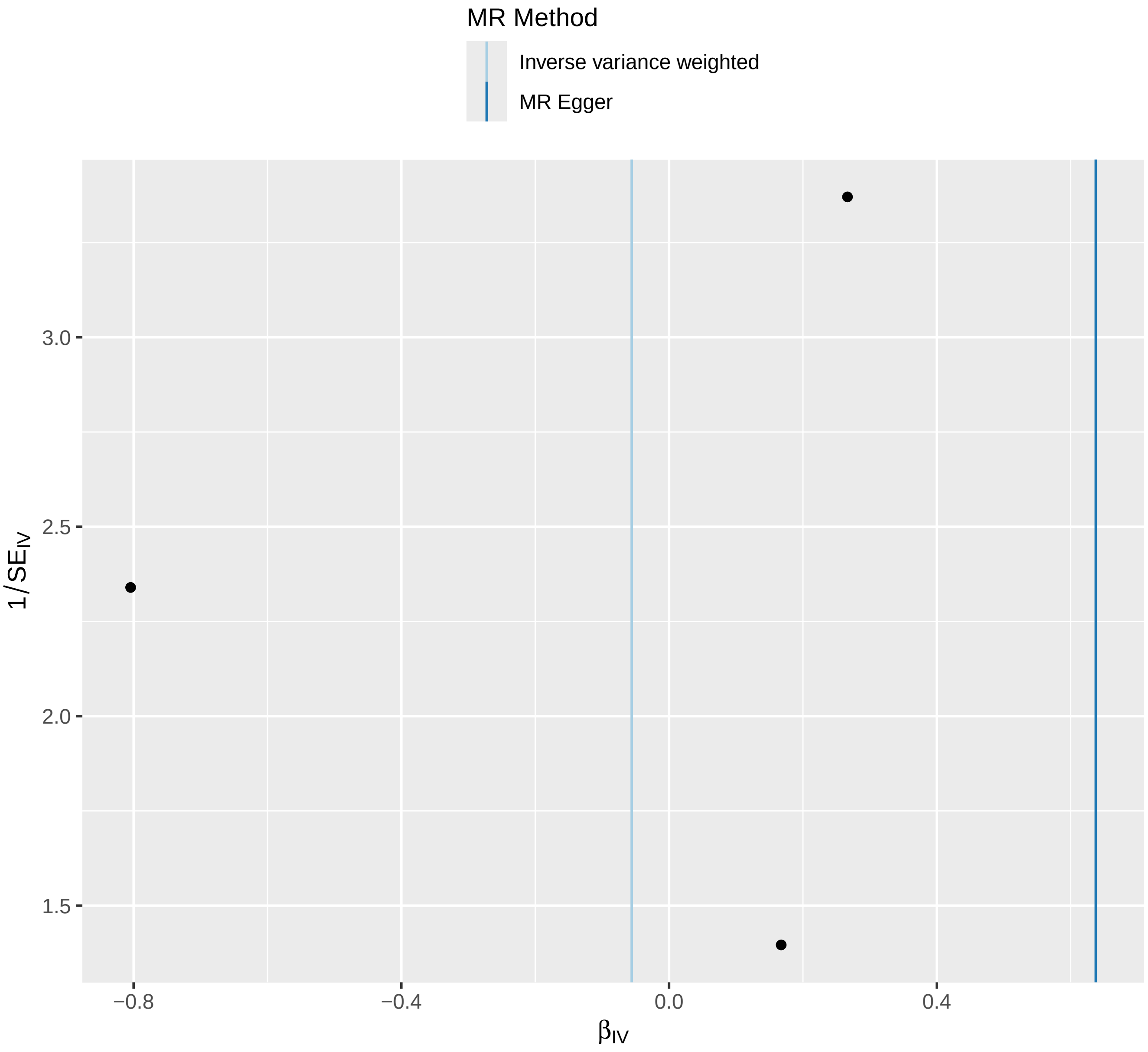
MR Method
Inverse variance weighted
MR Egger
3.0
2.5
2.0
1.5
$1/SE_{IV}$
−0.8
−0.4
0.0
0.4
β_{IV}

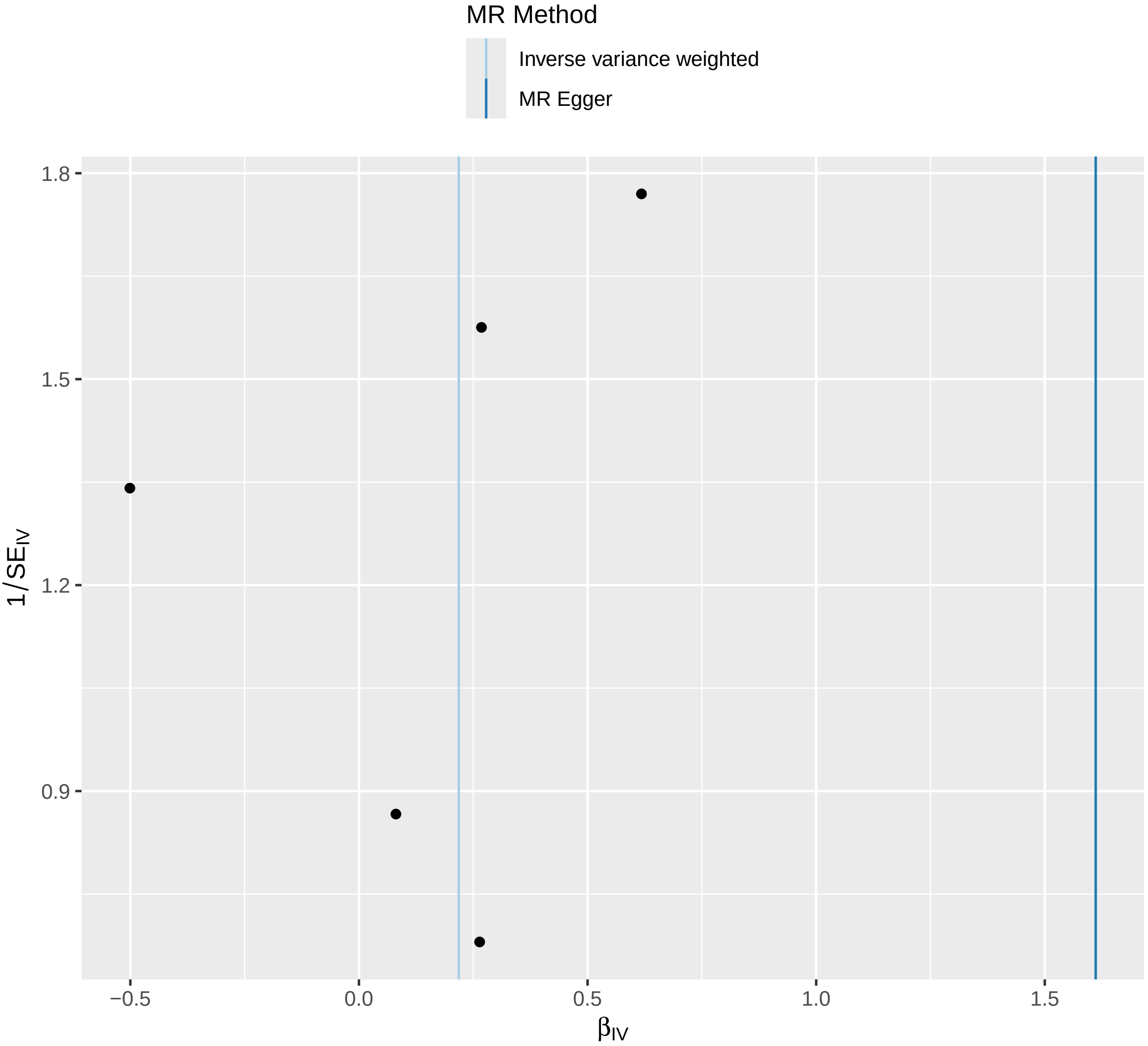

MR Method
Inverse variance weighted
MR Egger
1.8
1.5
1.2
0.9
1/SE_IV
−0.5
0.0
0.5
1.0
1.5
β_IV

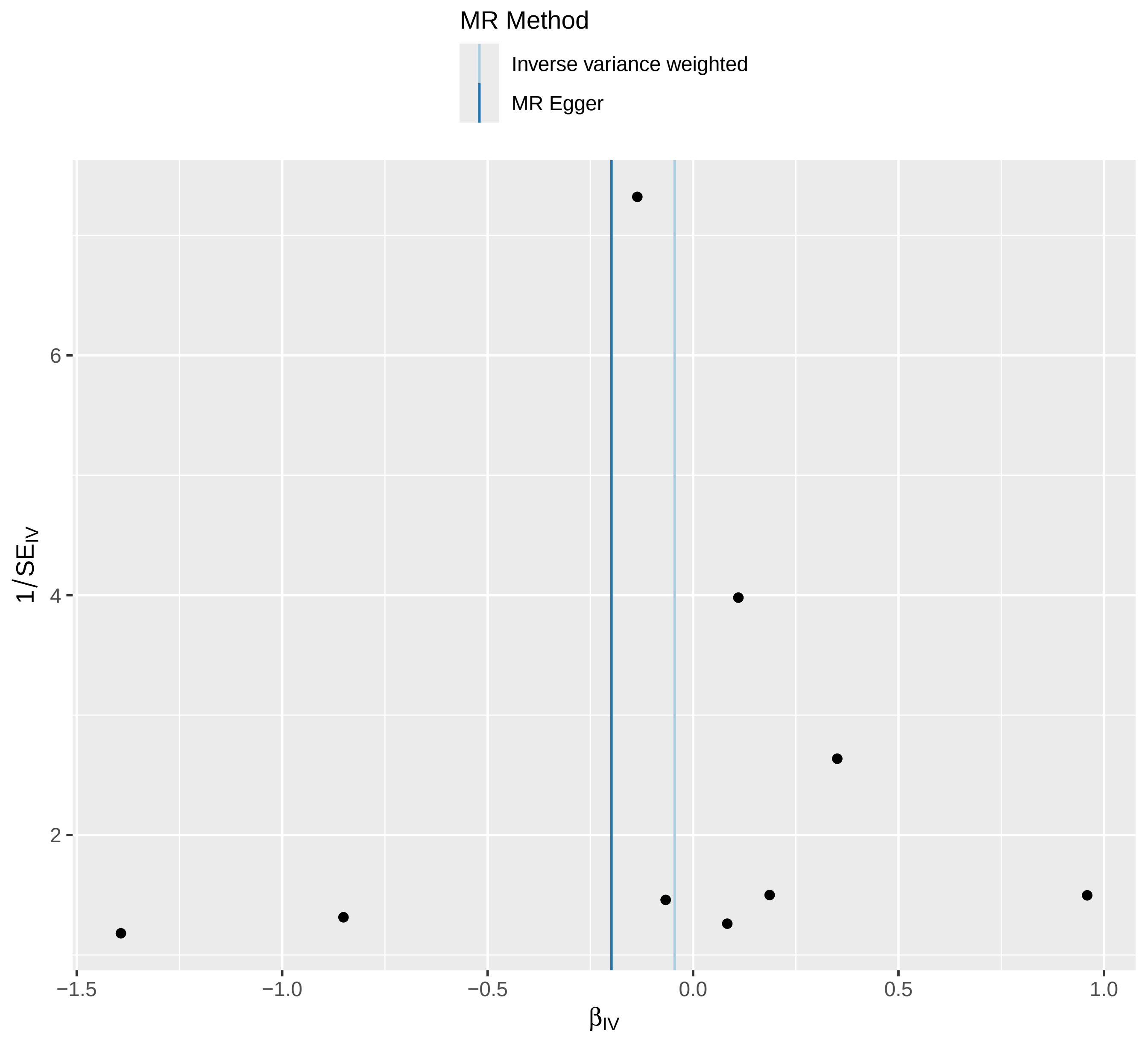
MR Method
Inverse variance weighted
MR Egger
1/SE_IV
6
4
2
−1.5
−1.0
−0.5
0.0
0.5
1.0
β_IV

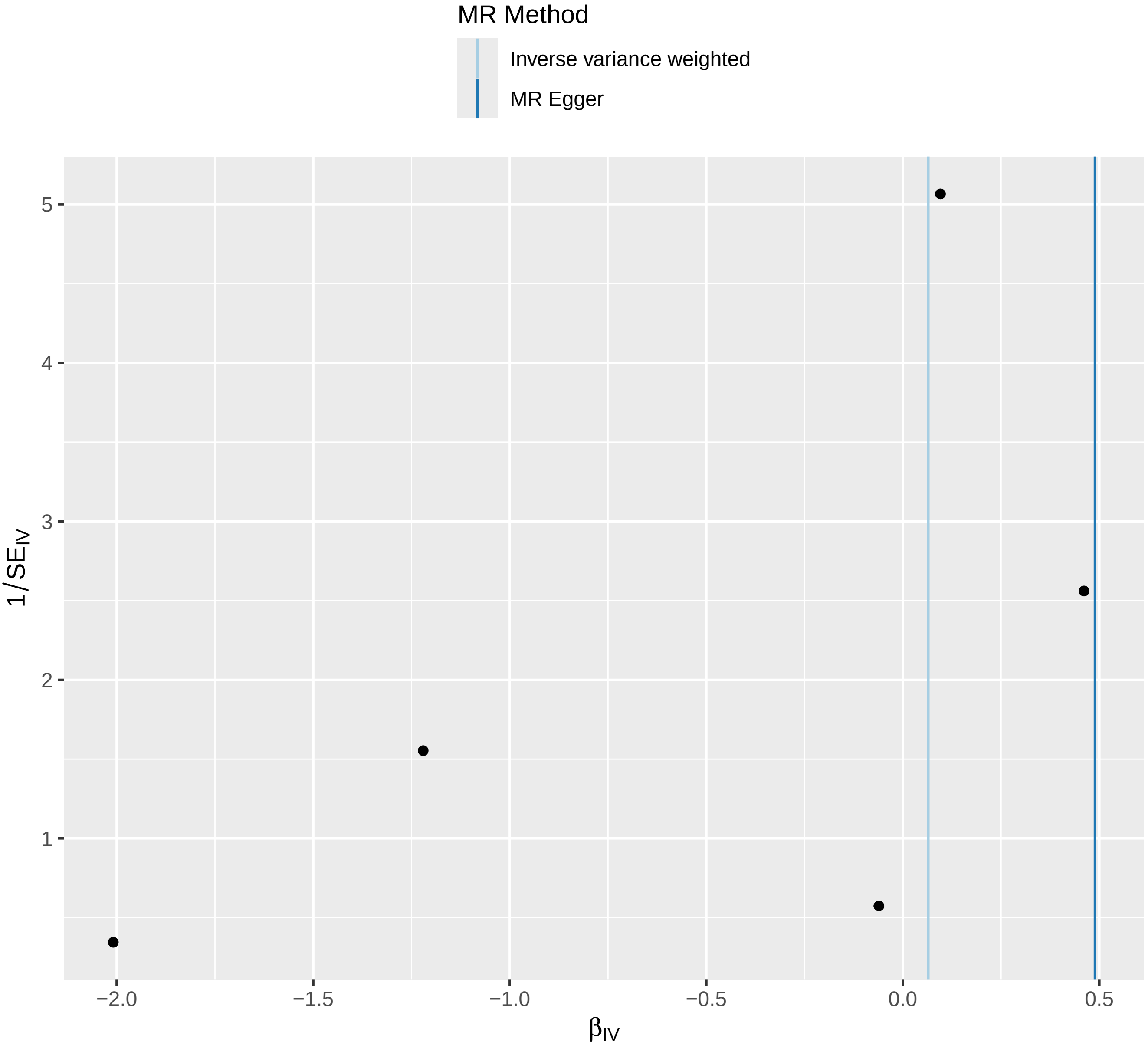
MR Method
Inverse variance weighted
MR Egger
1/SE_IV
β_IV
5
4
3
2
1
−2.0
−1.5
−1.0
−0.5
0.0
0.5

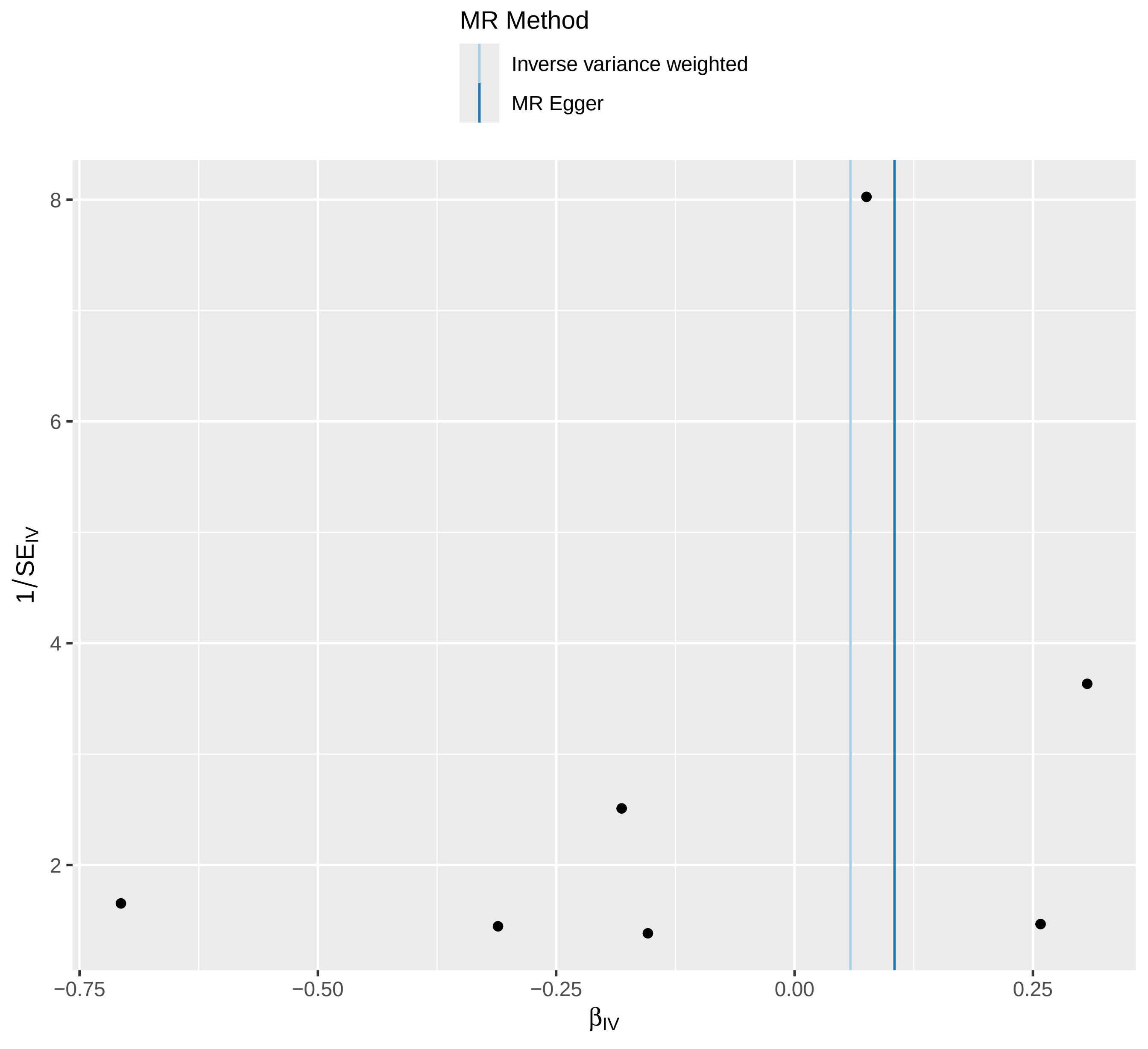

MR Method
Inverse variance weighted
MR Egger
8
6
4
2
$1/SE_{IV}$
−0.75
−0.50
−0.25
0.00
0.25
β_{IV}

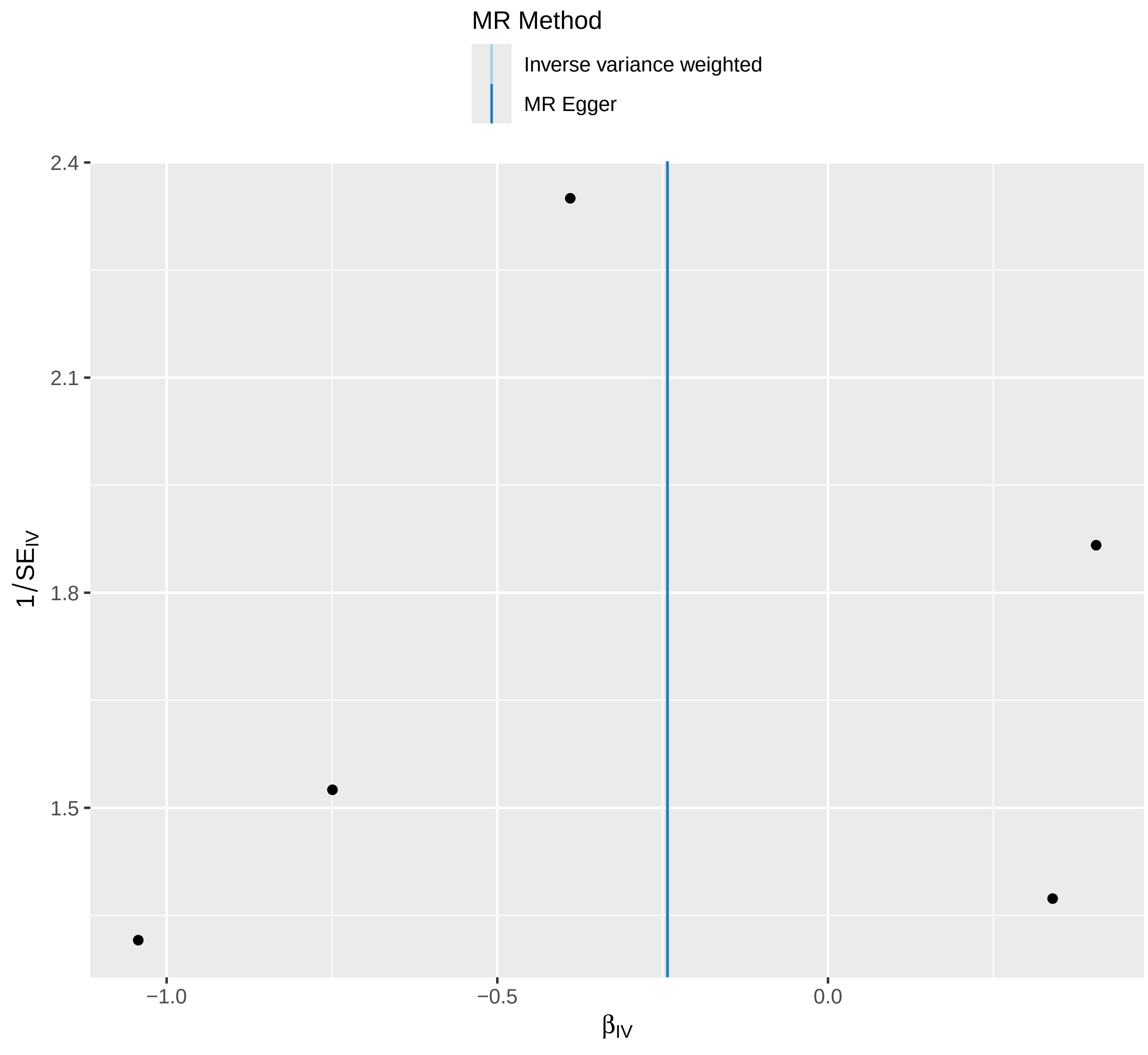
MR Method
Inverse variance weighted
MR Egger
2.4
2.1
1.8
1.5
1/SE_IV
−1.0
−0.5
0.0
β_IV

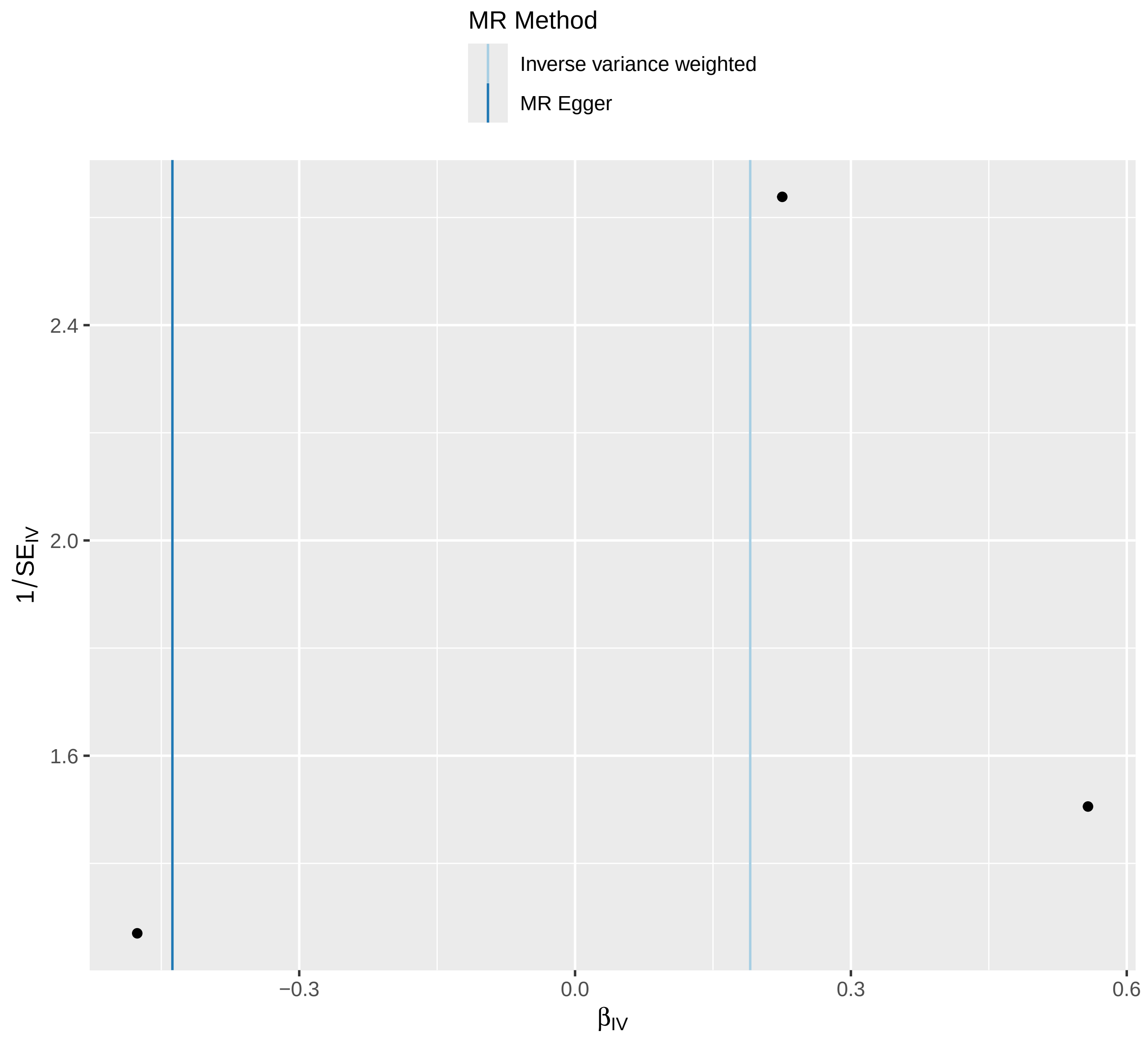
MR Method
Inverse variance weighted
MR Egger
2.4
2.0
1.6
1/SE_IV
−0.3
0.0
0.3
0.6
β_IV

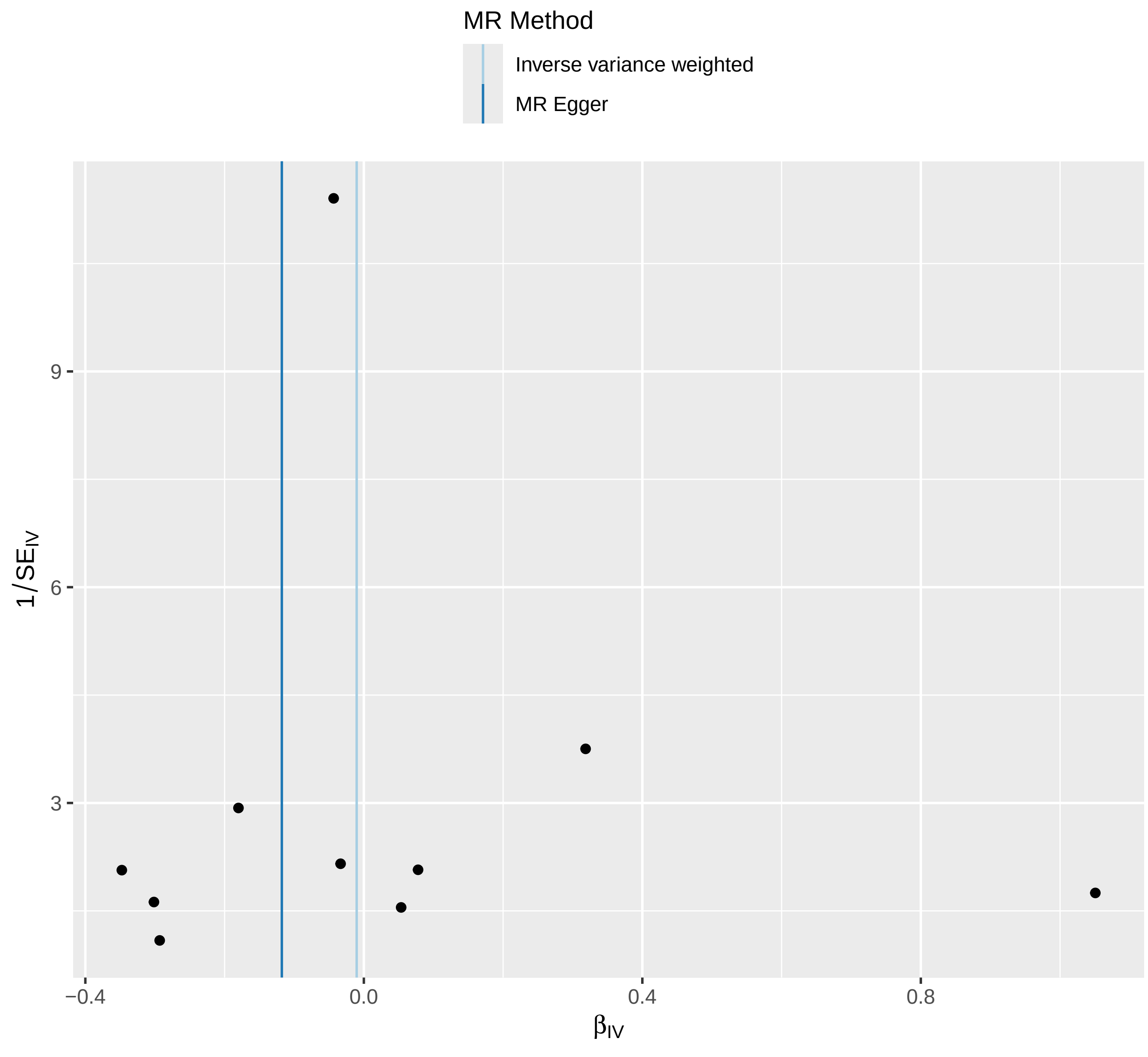
MR Method
Inverse variance weighted
MR Egger
9
6
3
1/SE_IV
−0.4
0.0
0.4
0.8
β_IV

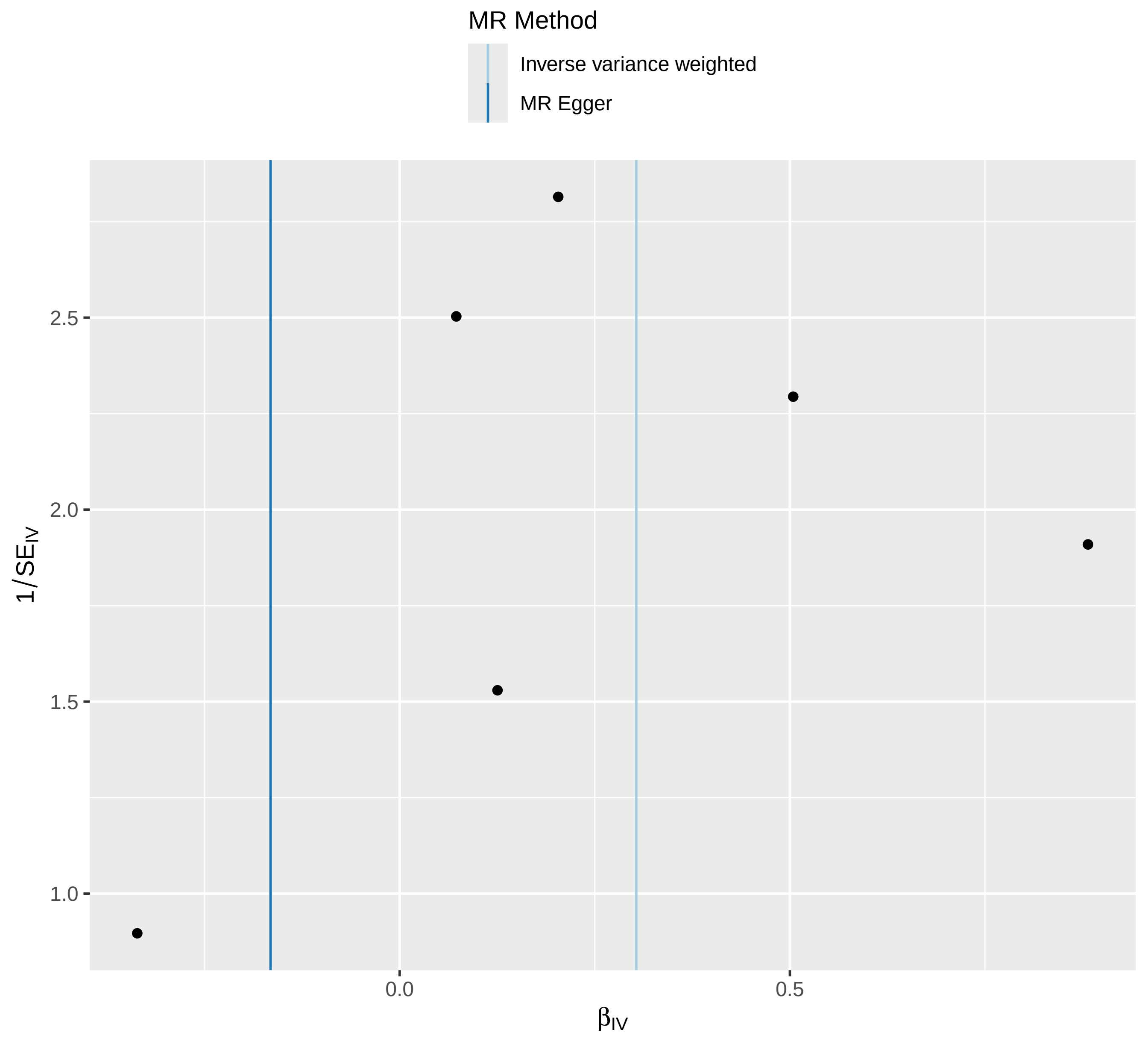

MR Method
Inverse variance weighted
MR Egger
2.5
2.0
1.5
1.0
1/SE_IV
0.0
0.5
β_IV

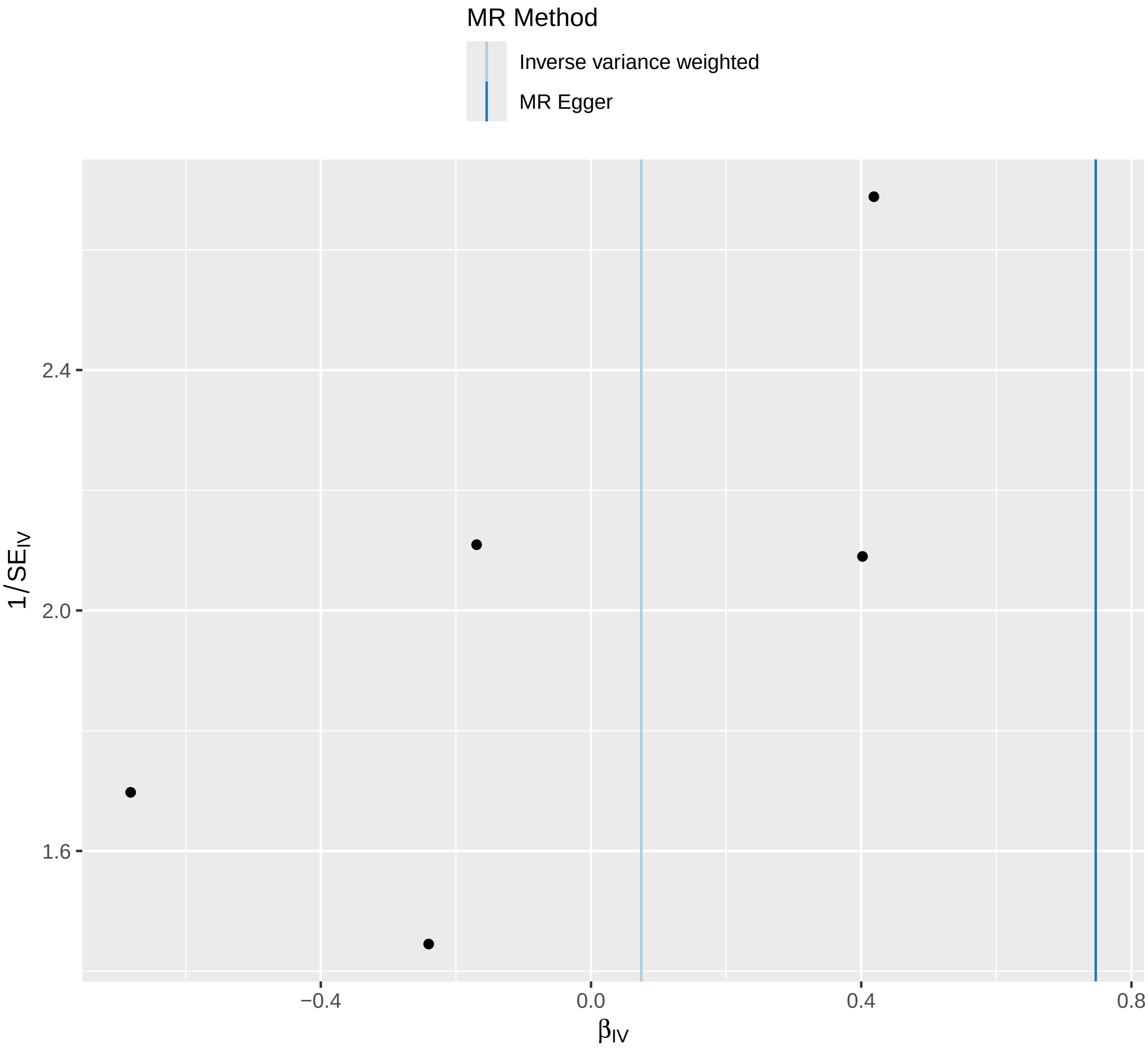

MR Method
Inverse variance weighted
MR Egger
2.4
2.0
1.6
1/SE_IV
−0.4
0.0
0.4
0.8
β_IV

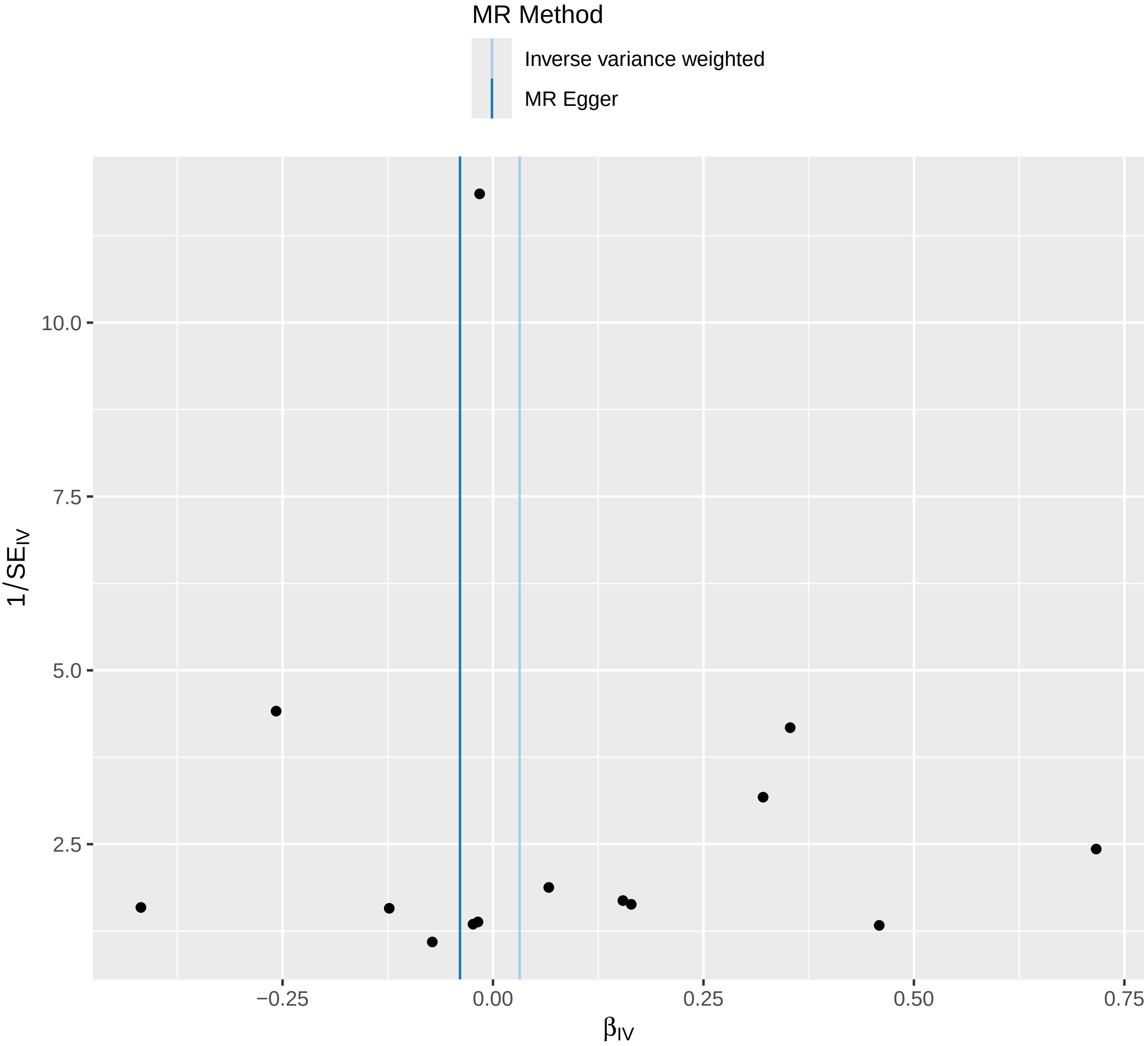
MR Method
Inverse variance weighted
MR Egger
10.0
7.5
5.0
2.5
1/SE$_{IV}$
−0.25
0.00
0.25
0.50
0.75
β$_{IV}$

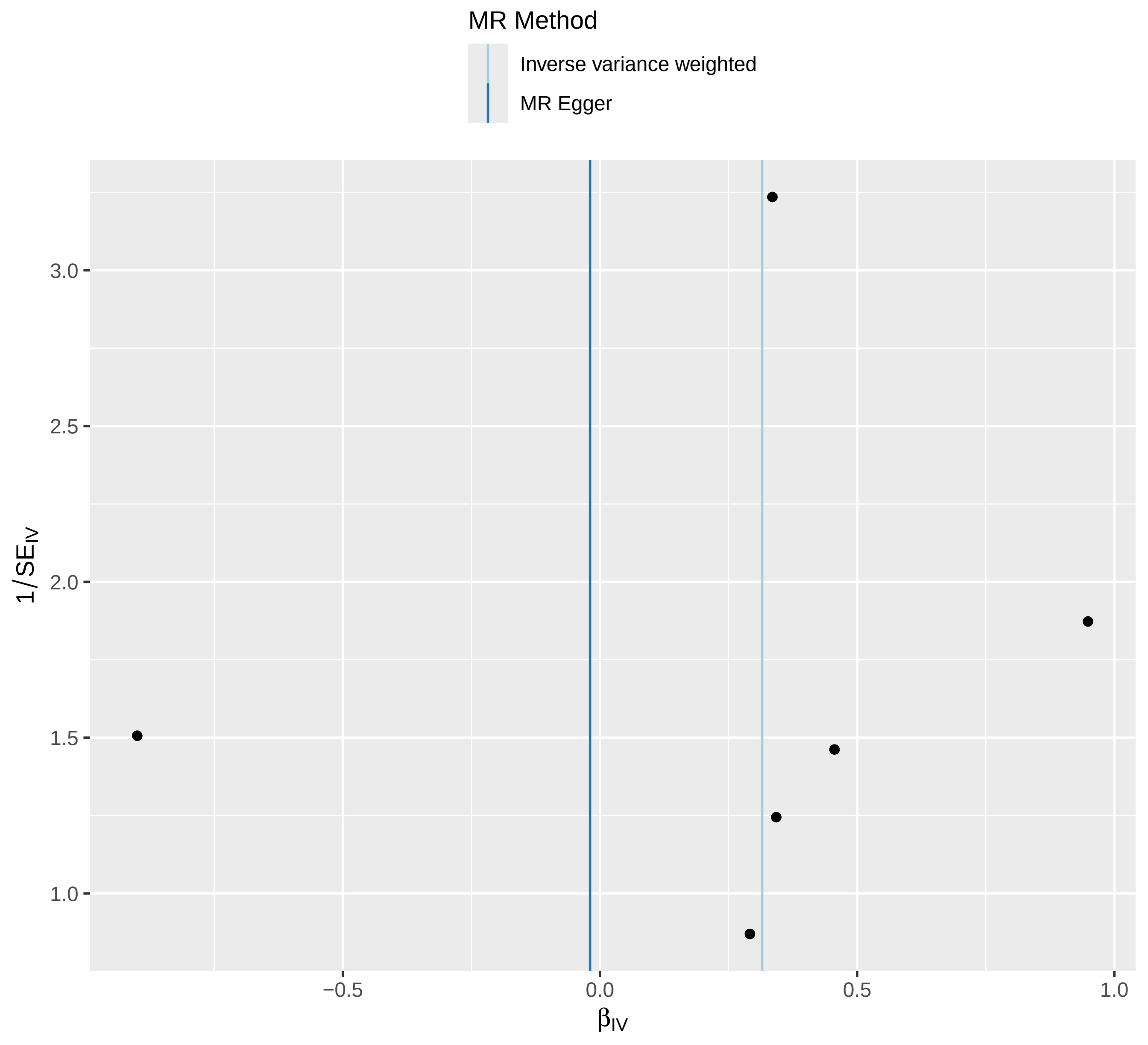

MR Method
Inverse variance weighted
MR Egger
3.0
2.5
2.0
1.5
1.0
1/SE$_{IV}$
−0.5
0.0
0.5
1.0
β$_{IV}$

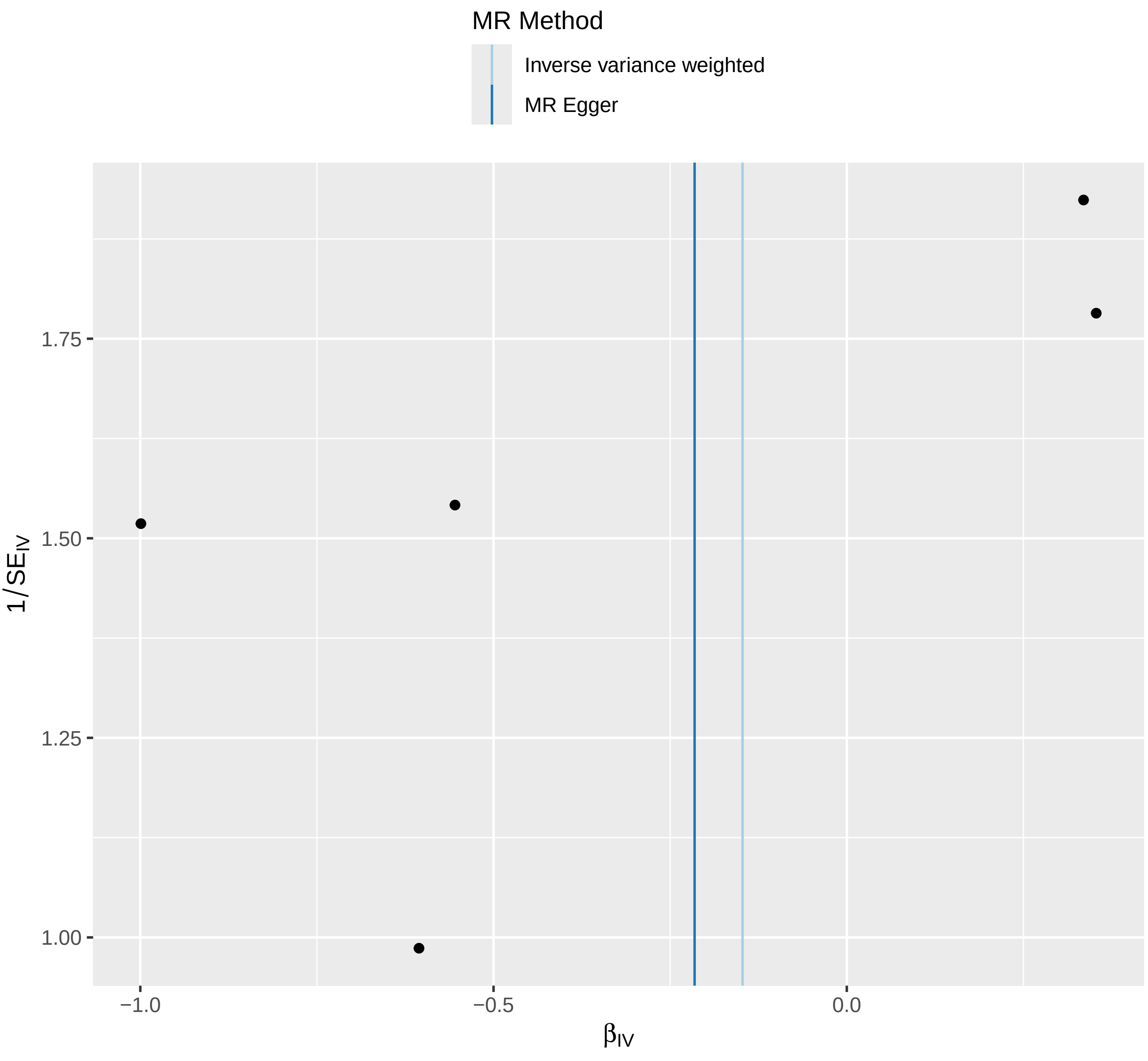
MR Method
Inverse variance weighted
MR Egger
1.75
1.50
1.25
1.00
1/SE$_{IV}$
−1.0
−0.5
0.0
β$_{IV}$

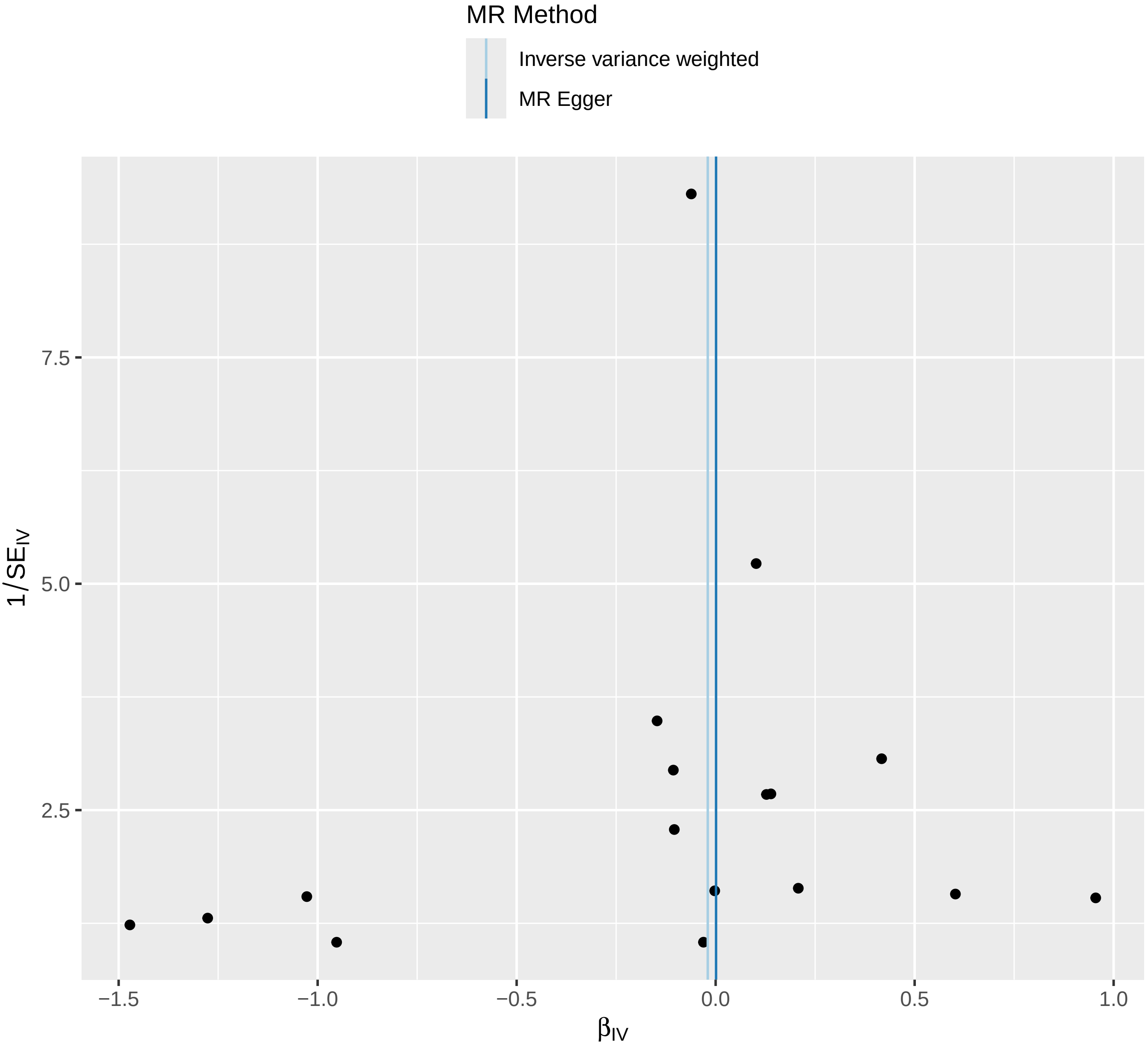

MR Method
Inverse variance weighted
MR Egger
7.5
5.0
2.5
1/SE_IV
−1.5
−1.0
−0.5
0.0
0.5
1.0
β_IV

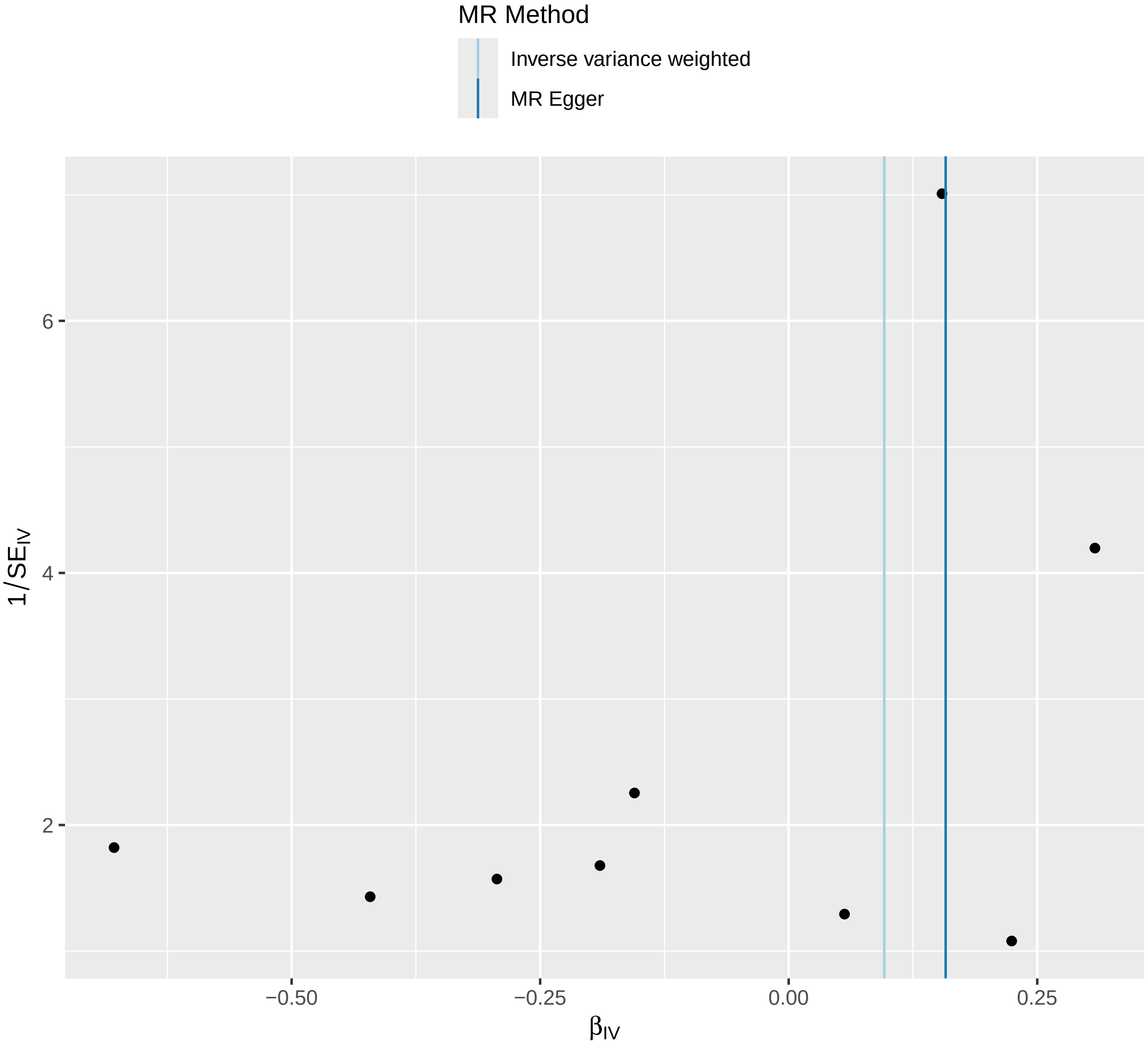

MR Method
Inverse variance weighted
MR Egger
6
4
2
1/SE$_{IV}$
−0.50
−0.25
0.00
0.25
β$_{IV}$

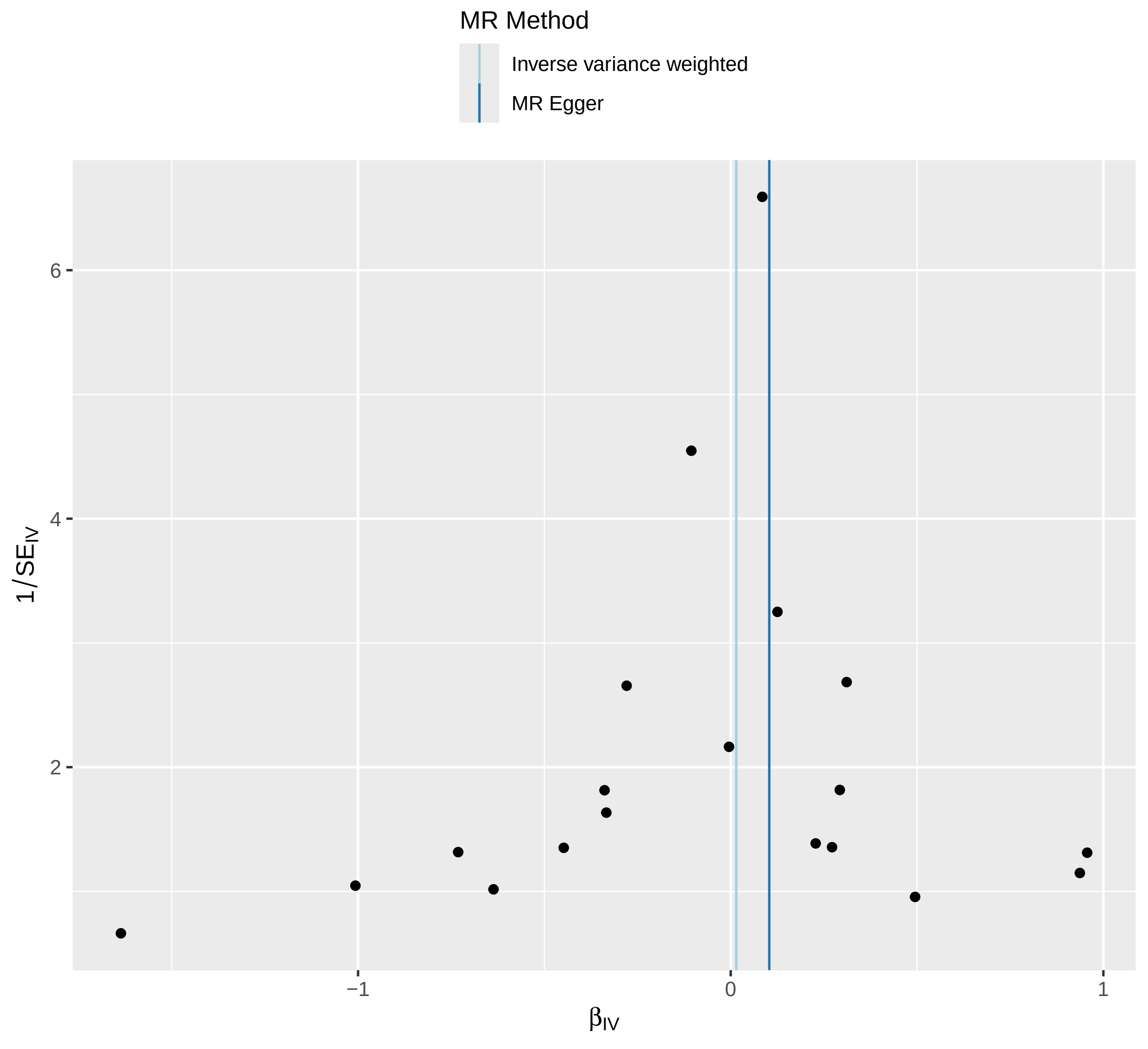
MR Method
Inverse variance weighted
MR Egger
6
4
2
1/SE_IV
−1
0
1
β_IV

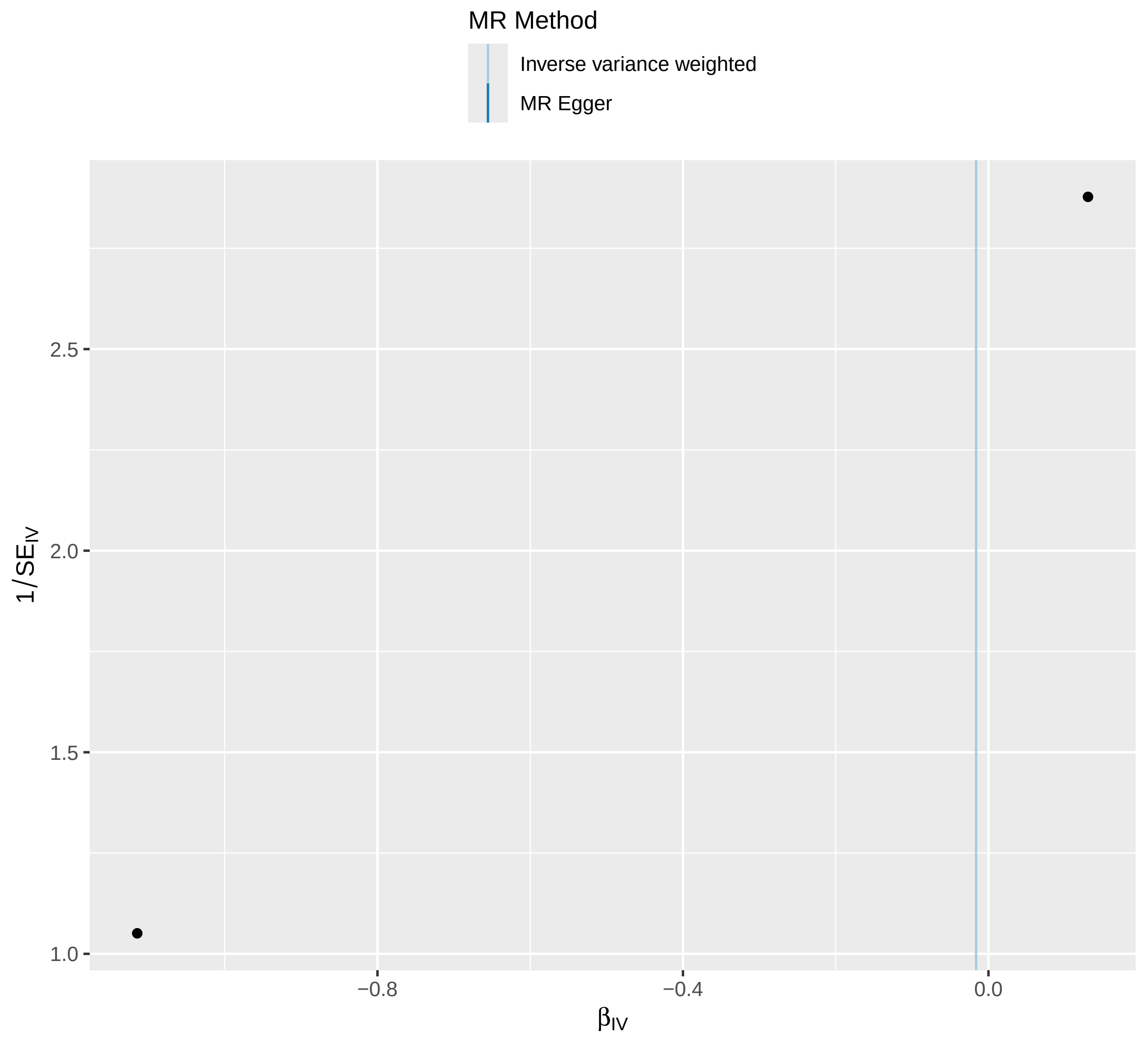

MR Method
Inverse variance weighted
MR Egger
2.5
2.0
1.5
1.0
−0.8
−0.4
0.0
1/SE_IV
β_IV

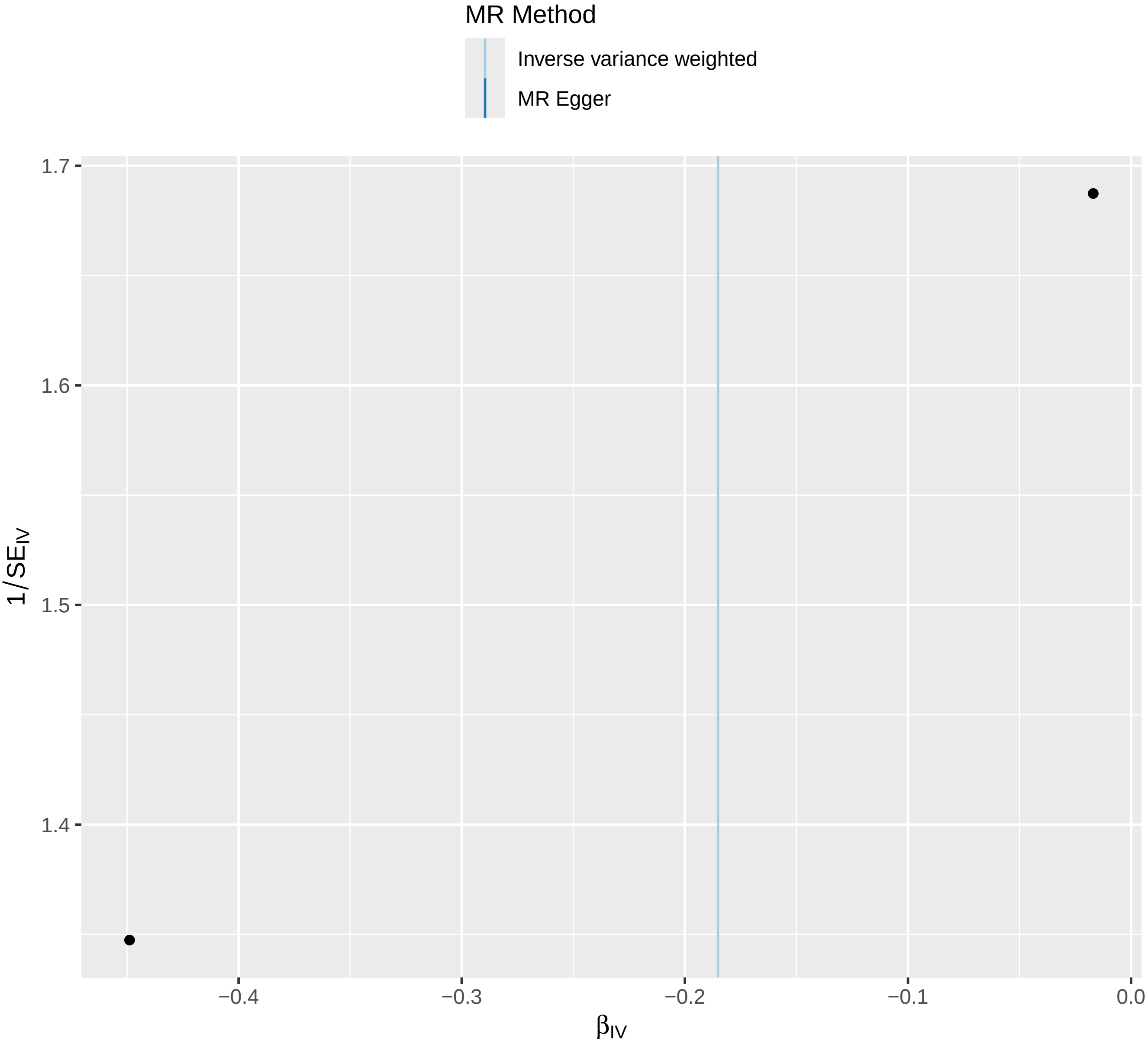
MR Method
Inverse variance weighted
MR Egger
1.7
1.6
1.5
1.4
1/SE$_{IV}$
−0.4
−0.3
−0.2
−0.1
0.0
β$_{IV}$

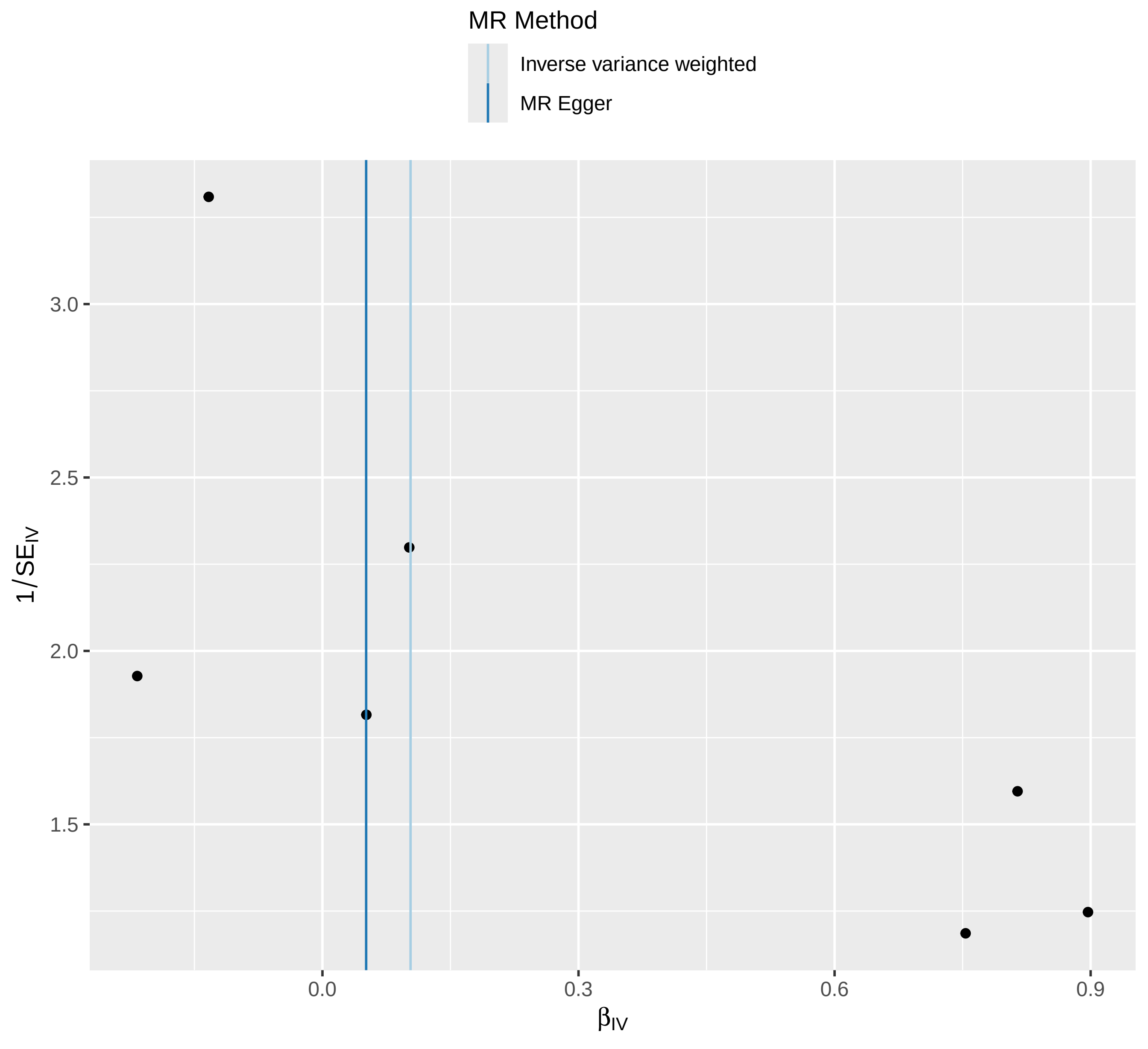
MR Method
Inverse variance weighted
MR Egger
3.0
2.5
2.0
1.5
1/SE_IV
0.0
0.3
0.6
0.9
β_IV

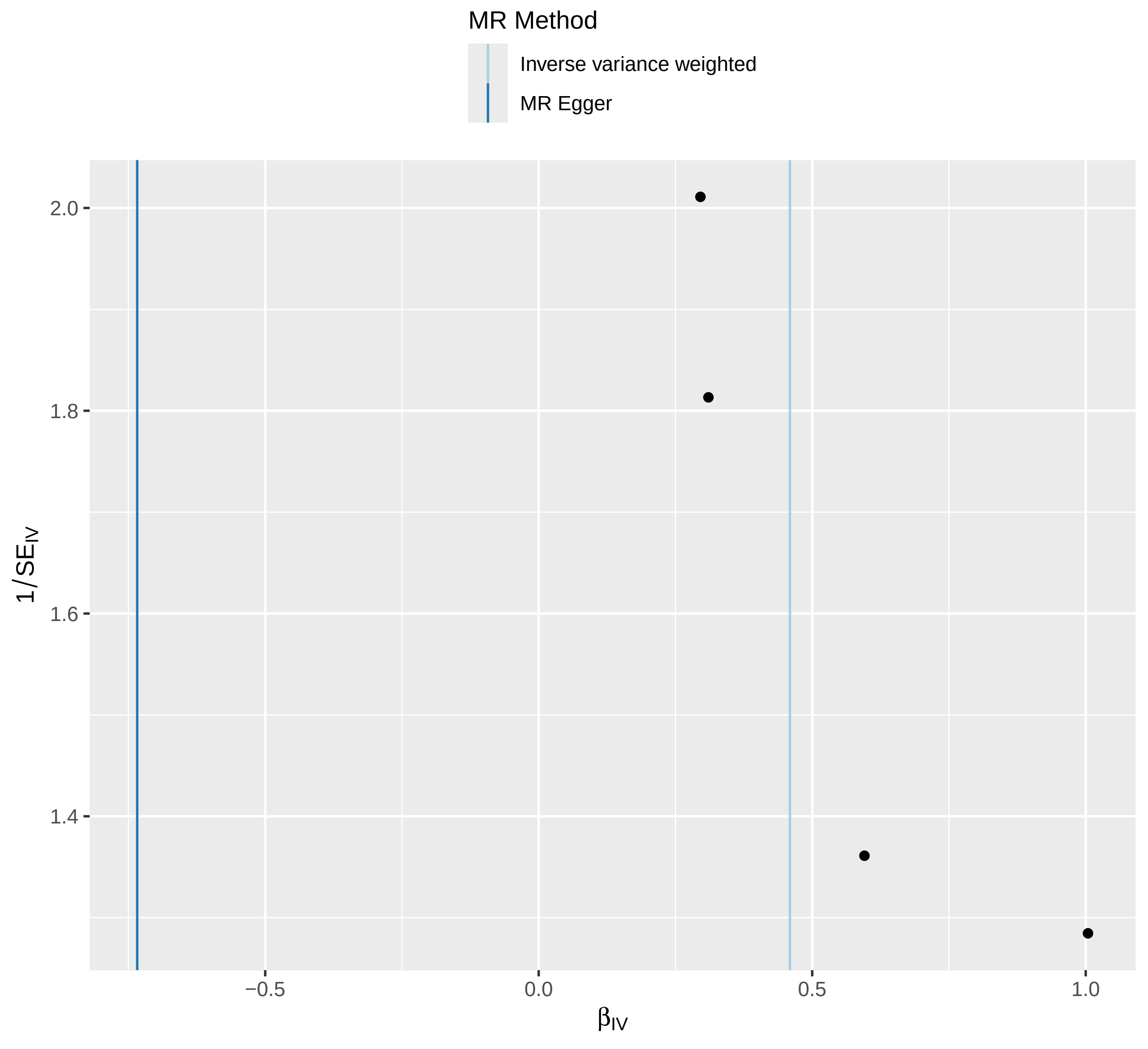
MR Method
Inverse variance weighted
MR Egger
2.0
1.8
1.6
1.4
1/SE$_{IV}$
−0.5
0.0
0.5
1.0
β_{IV}

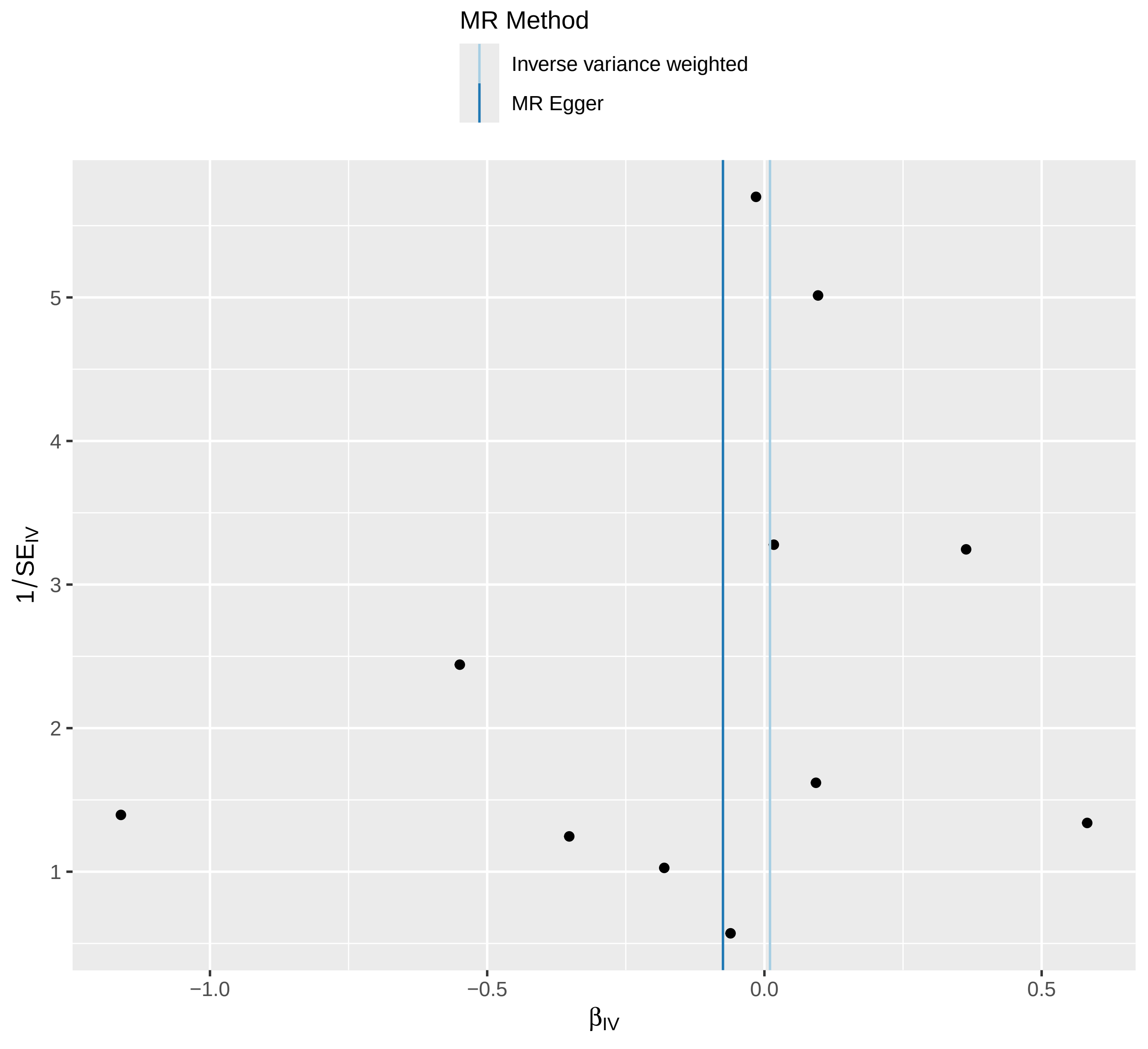
MR Method
Inverse variance weighted
MR Egger
1/SE$_{IV}$
5
4
3
2
1
−1.0
−0.5
0.0
0.5
β$_{IV}$

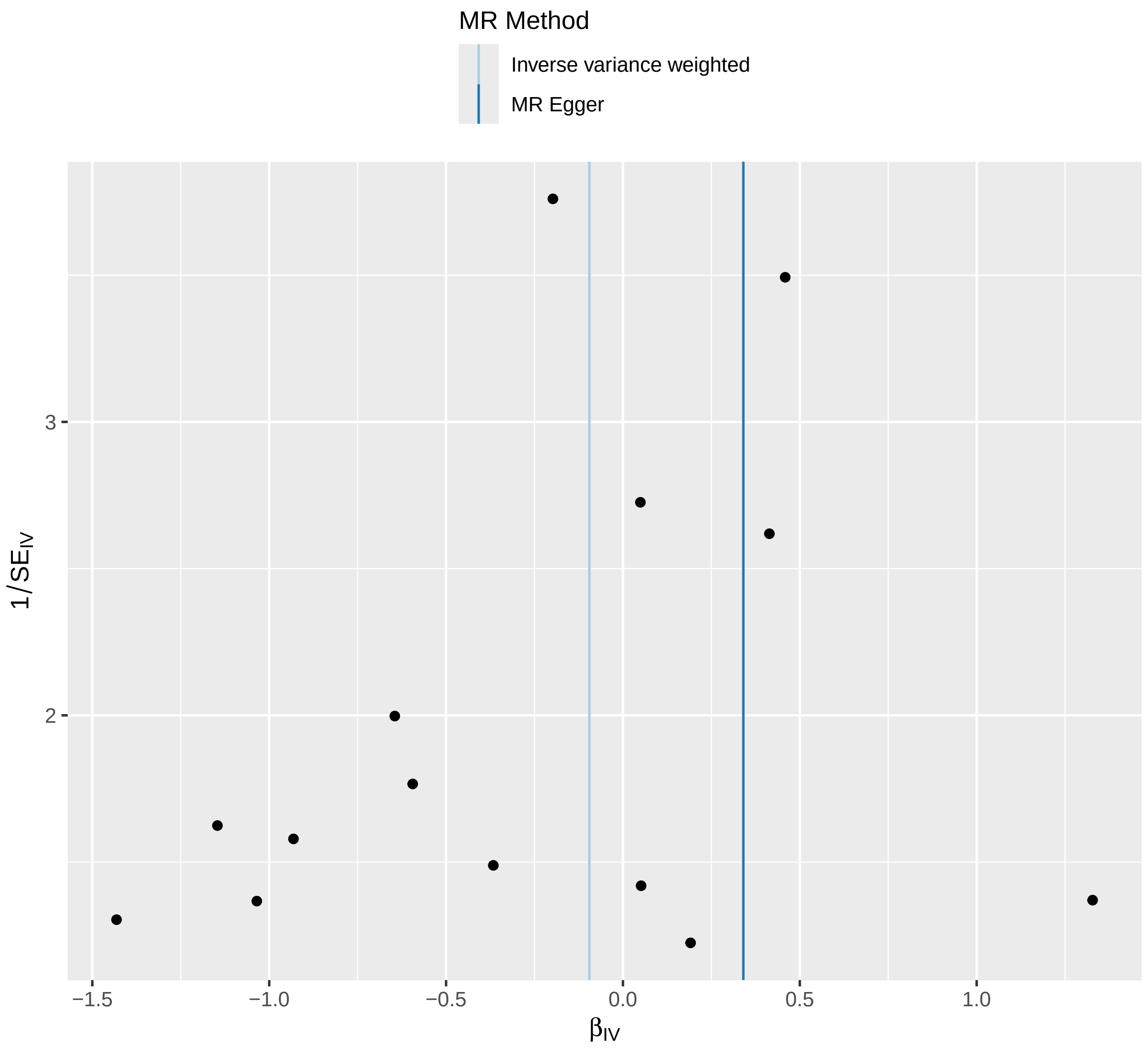
MR Method
Inverse variance weighted
MR Egger
1/SE_IV
β_IV
3
2
−1.5
−1.0
−0.5
0.0
0.5
1.0

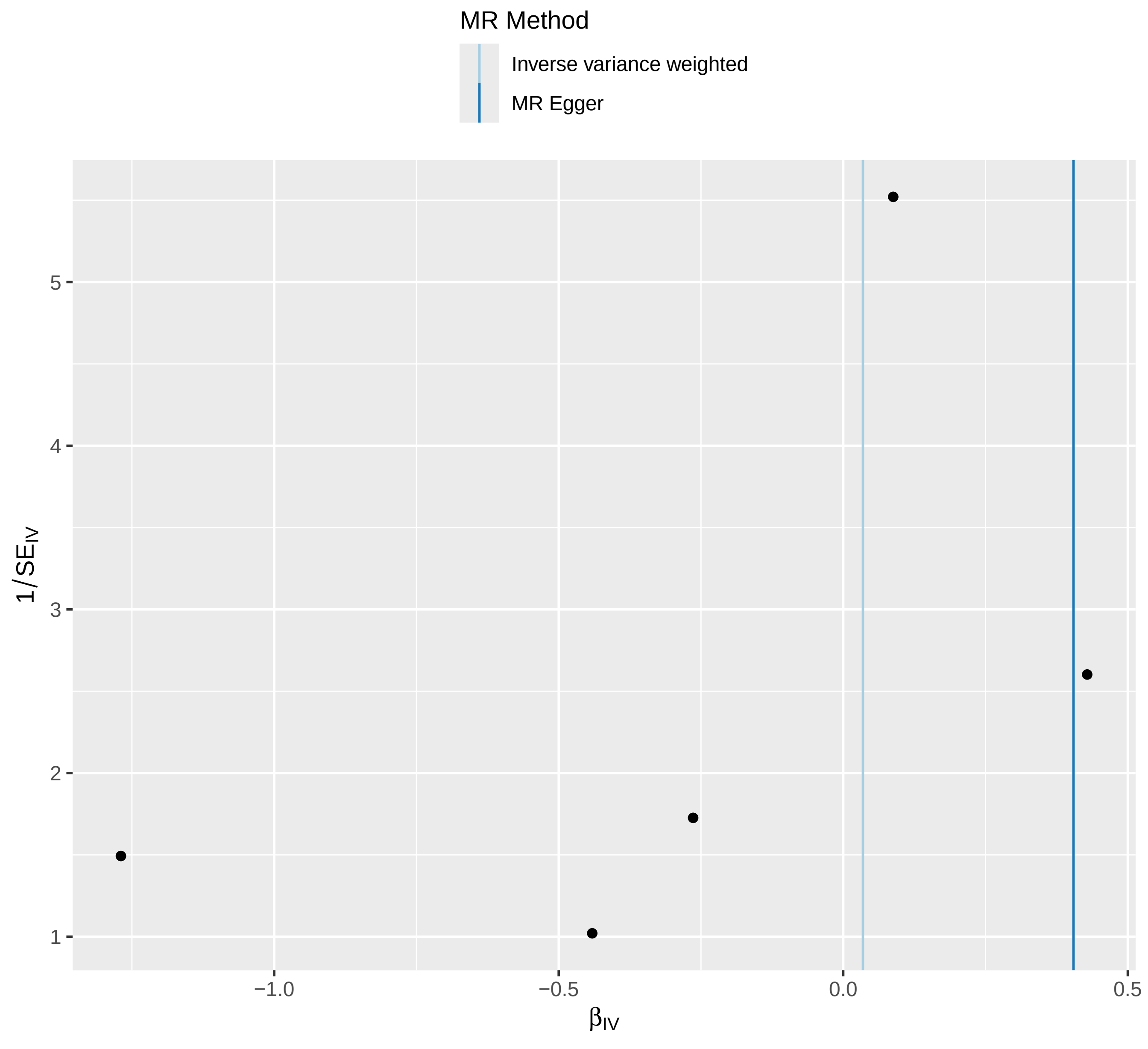

MR Method
Inverse variance weighted
MR Egger
5
4
3
2
1
1/SE_IV
−1.0
−0.5
0.0
0.5
β_IV

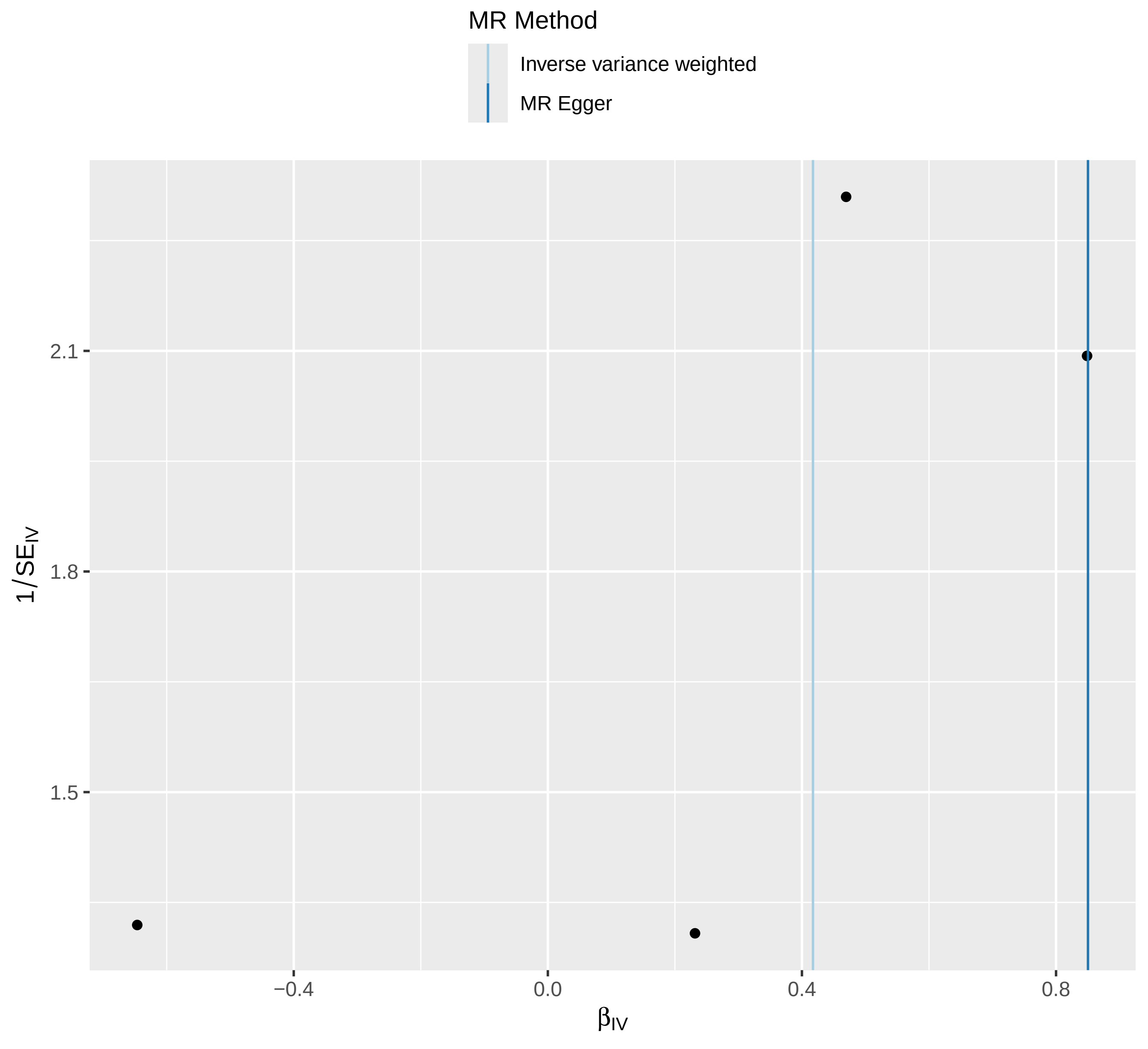
MR Method
Inverse variance weighted
MR Egger
2.1
1.8
1.5
1/SE$_{IV}$
−0.4
0.0
0.4
0.8
β$_{IV}$

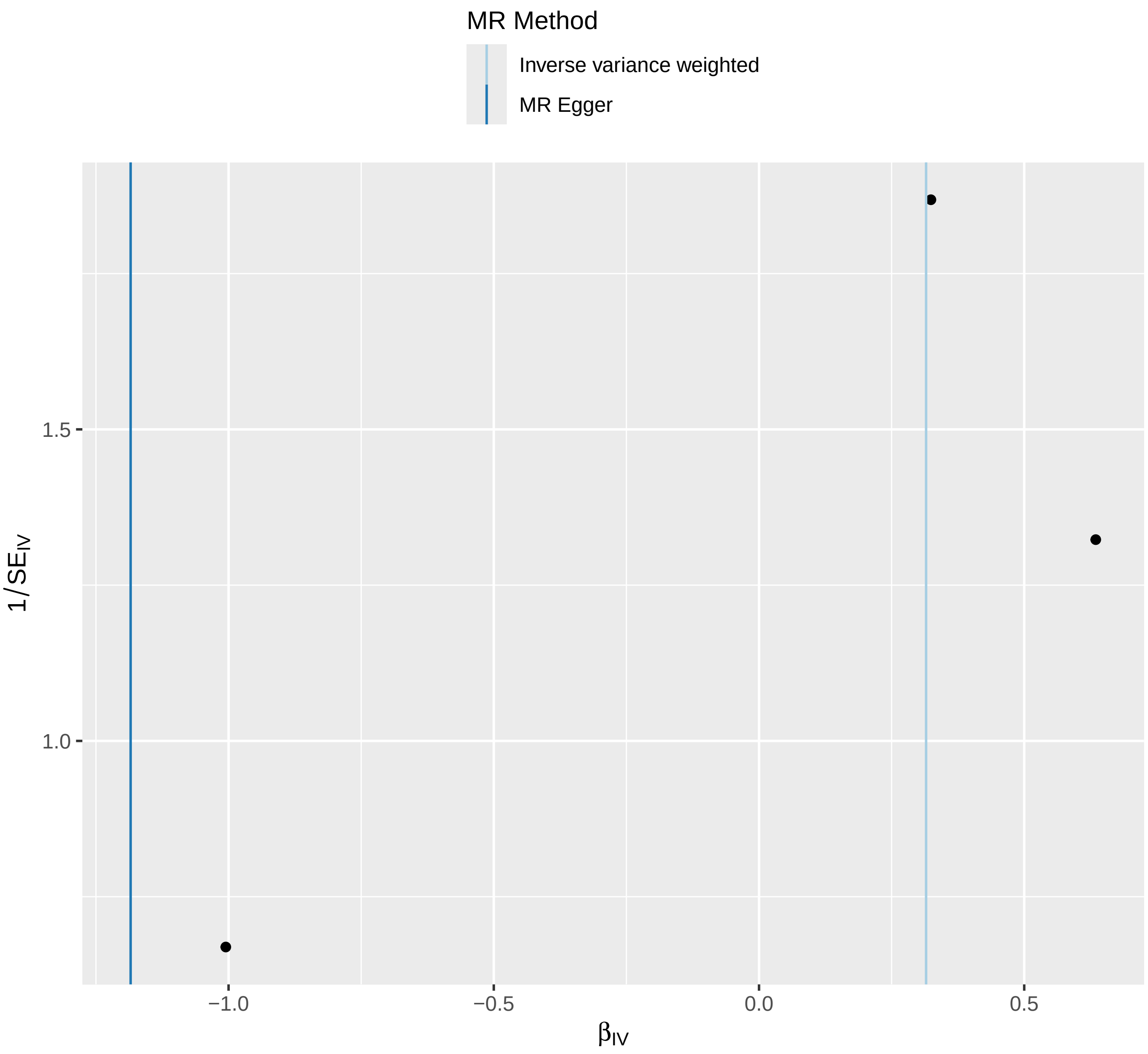
MR Method
Inverse variance weighted
MR Egger
1.5
1.0
1/SE_IV
−1.0
−0.5
0.0
0.5
β_IV

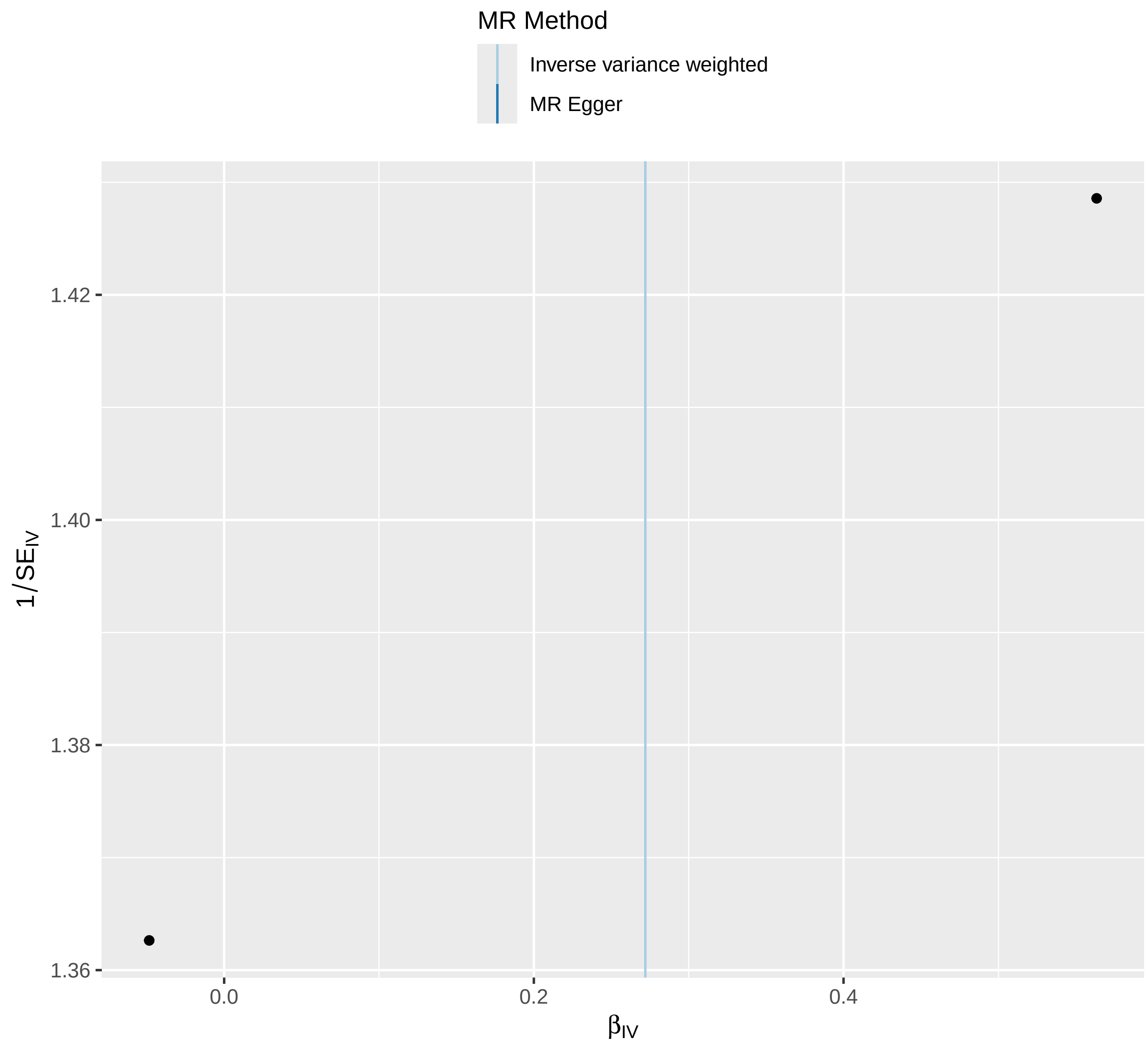

MR Method
Inverse variance weighted
MR Egger
1.42
1.40
1.38
1.36
1/SE_IV
0.0
0.2
0.4
β_IV

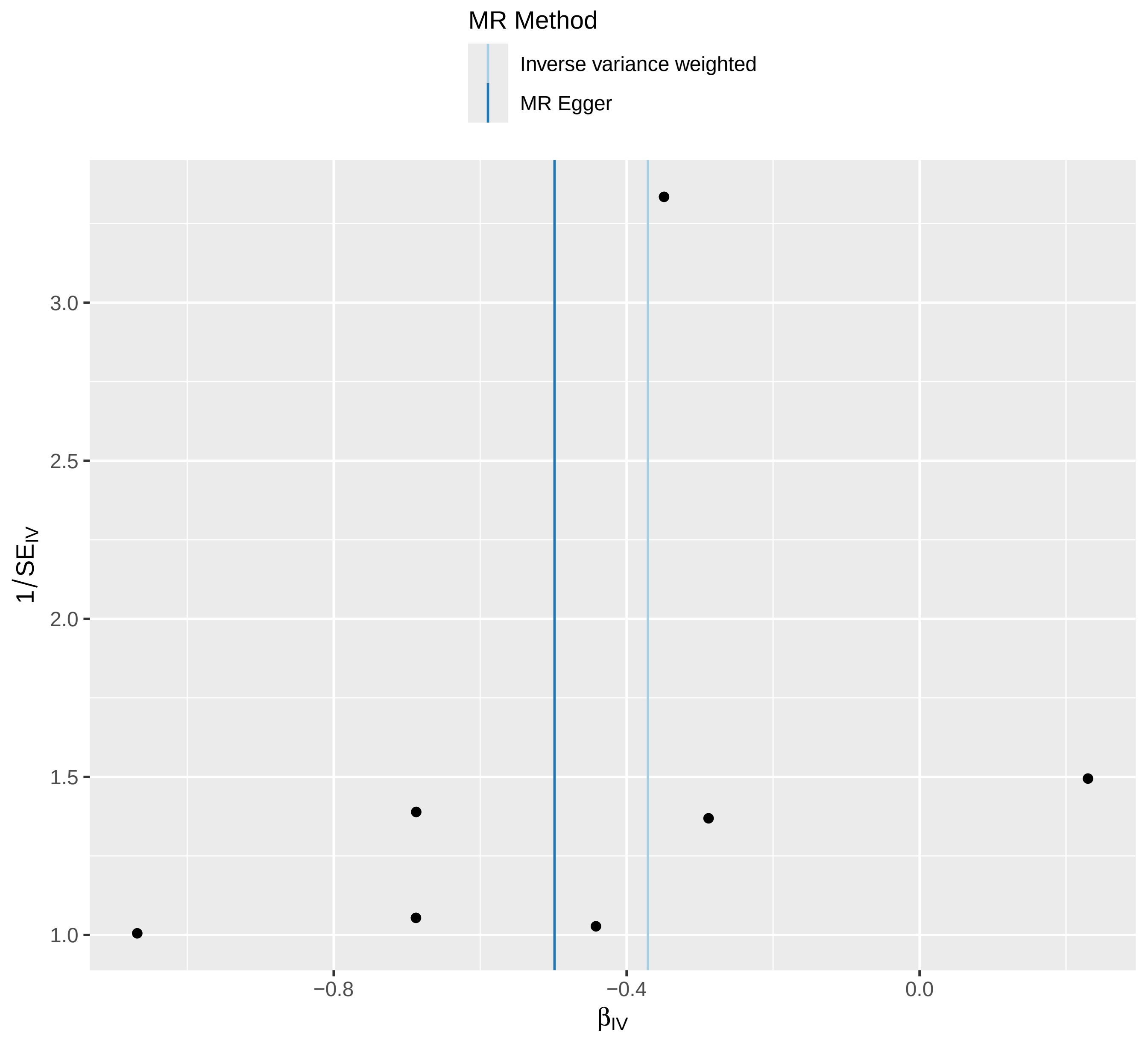

MR Method
Inverse variance weighted
MR Egger
3.0
2.5
2.0
1.5
1.0
1/SE_IV
−0.8
−0.4
0.0
β_IV

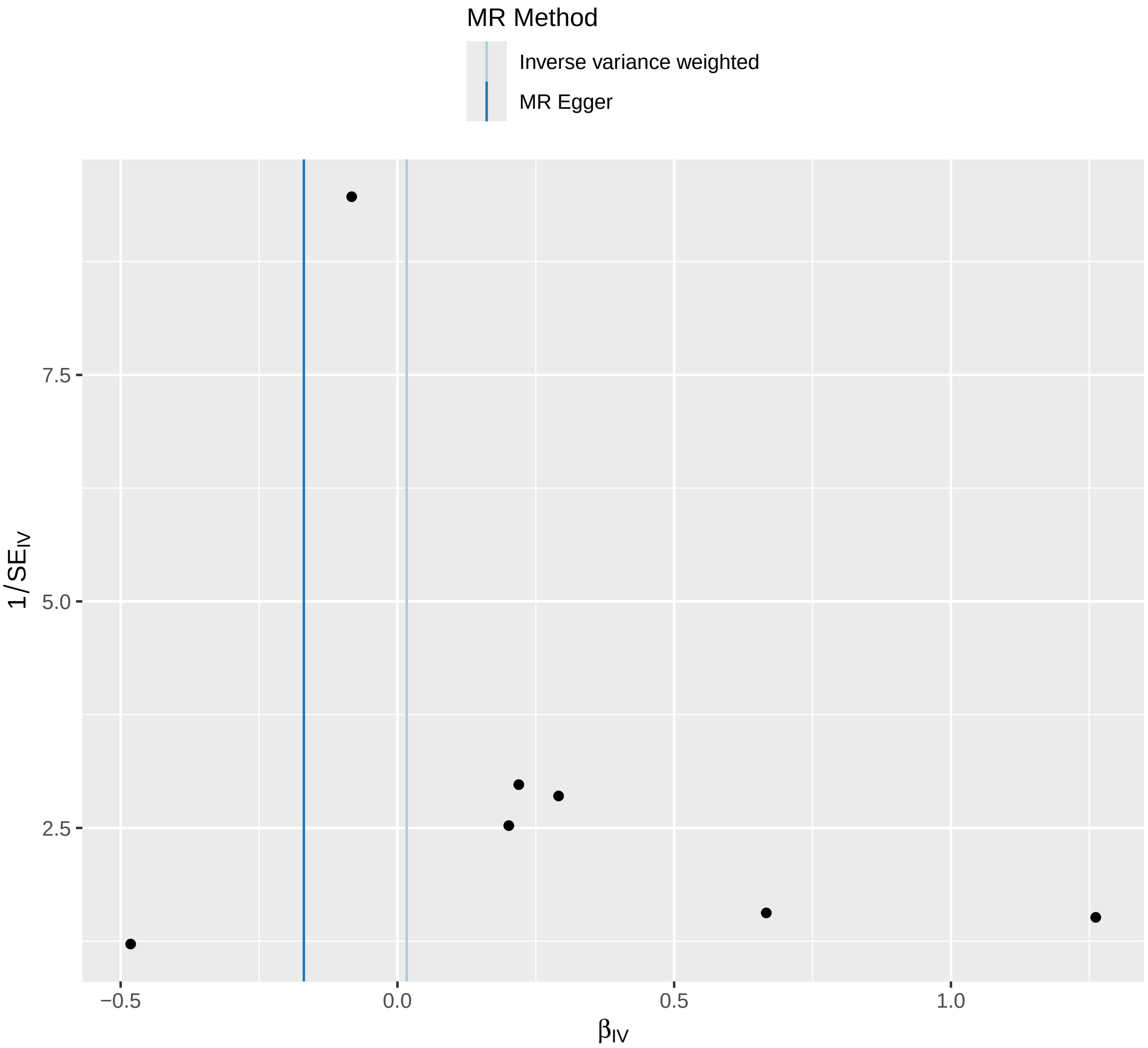
MR Method
Inverse variance weighted
MR Egger
7.5
5.0
2.5
1/SE$_{IV}$
−0.5
0.0
0.5
1.0
β$_{IV}$

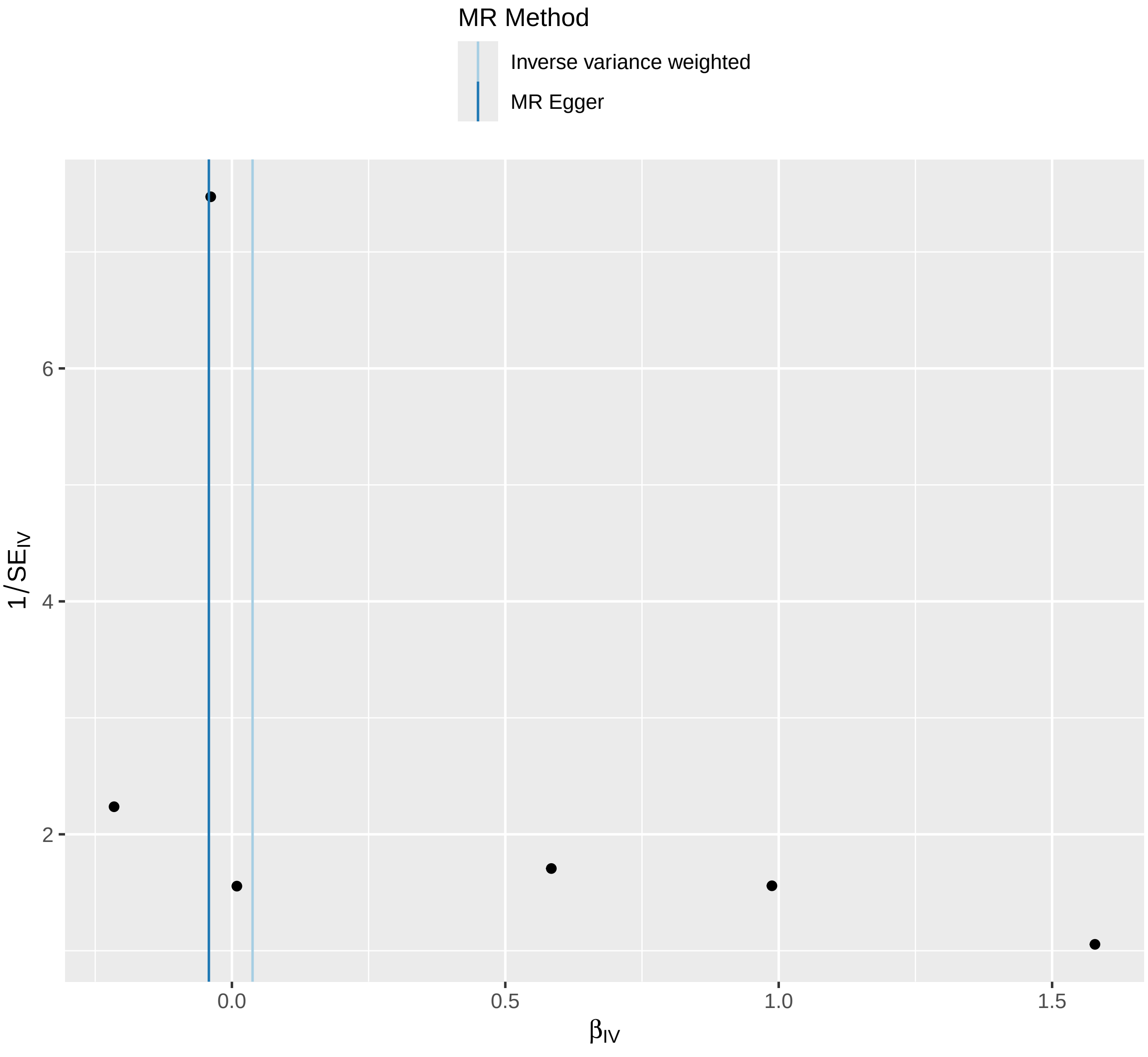
MR Method
Inverse variance weighted
MR Egger
6
4
2
$1/SE_{IV}$
0.0
0.5
1.0
1.5
β_{IV}

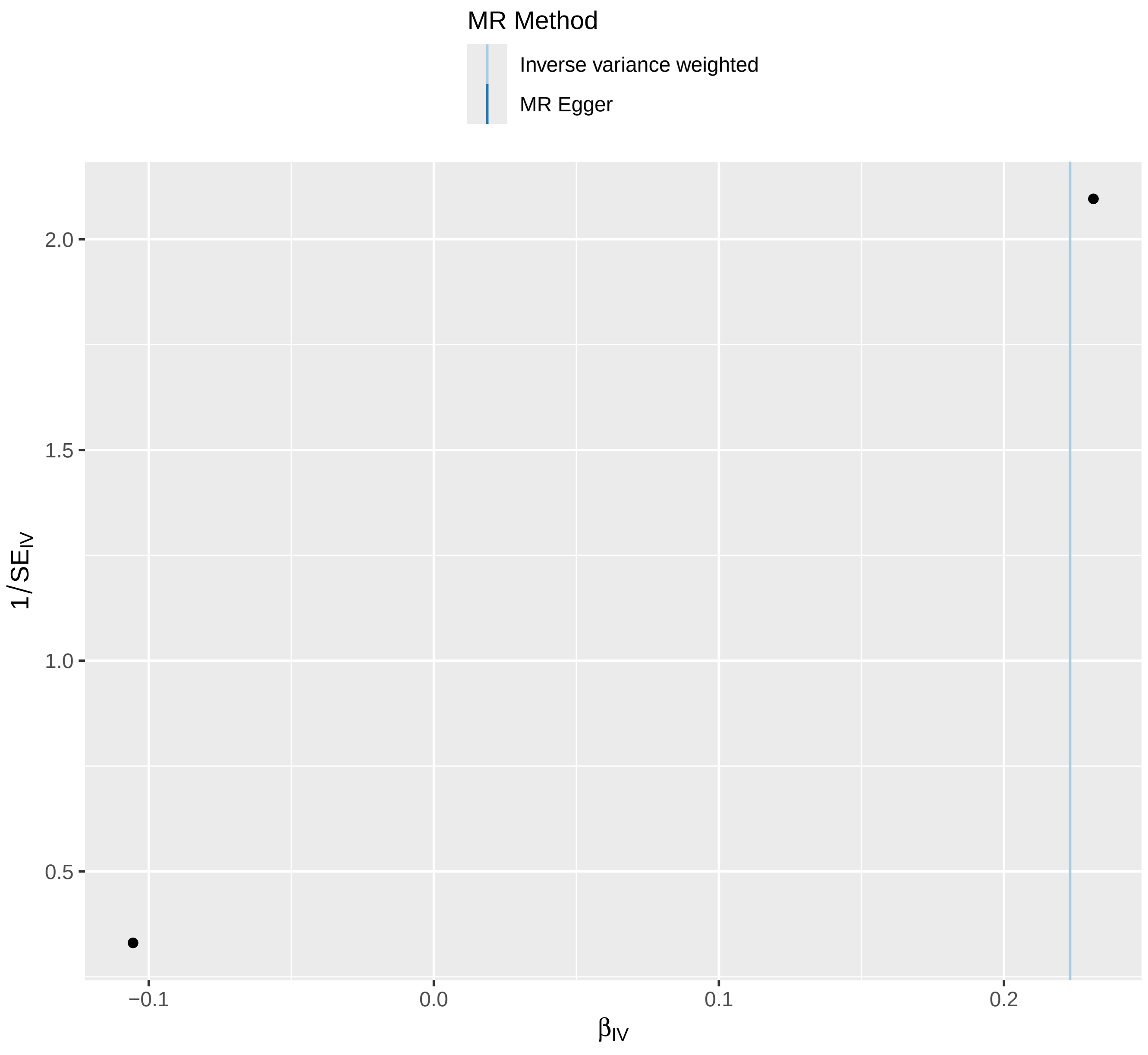
MR Method
Inverse variance weighted
MR Egger
2.0
1.5
1.0
0.5
1/SE$_{IV}$
−0.1
0.0
0.1
0.2
β$_{IV}$

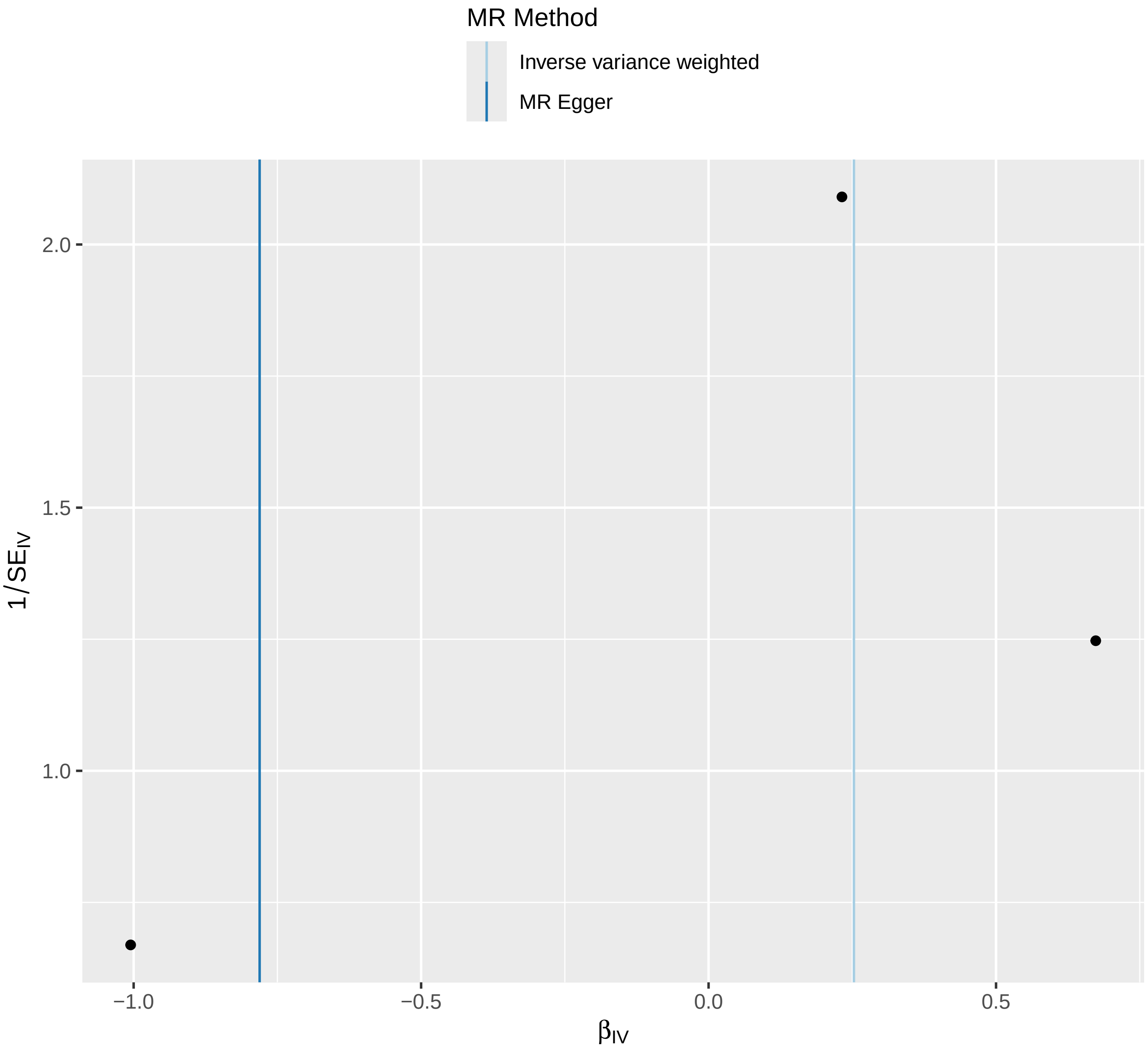
MR Method
Inverse variance weighted
MR Egger
2.0
1.5
1.0
1/SE$_{IV}$
−1.0
−0.5
0.0
0.5
β$_{IV}$

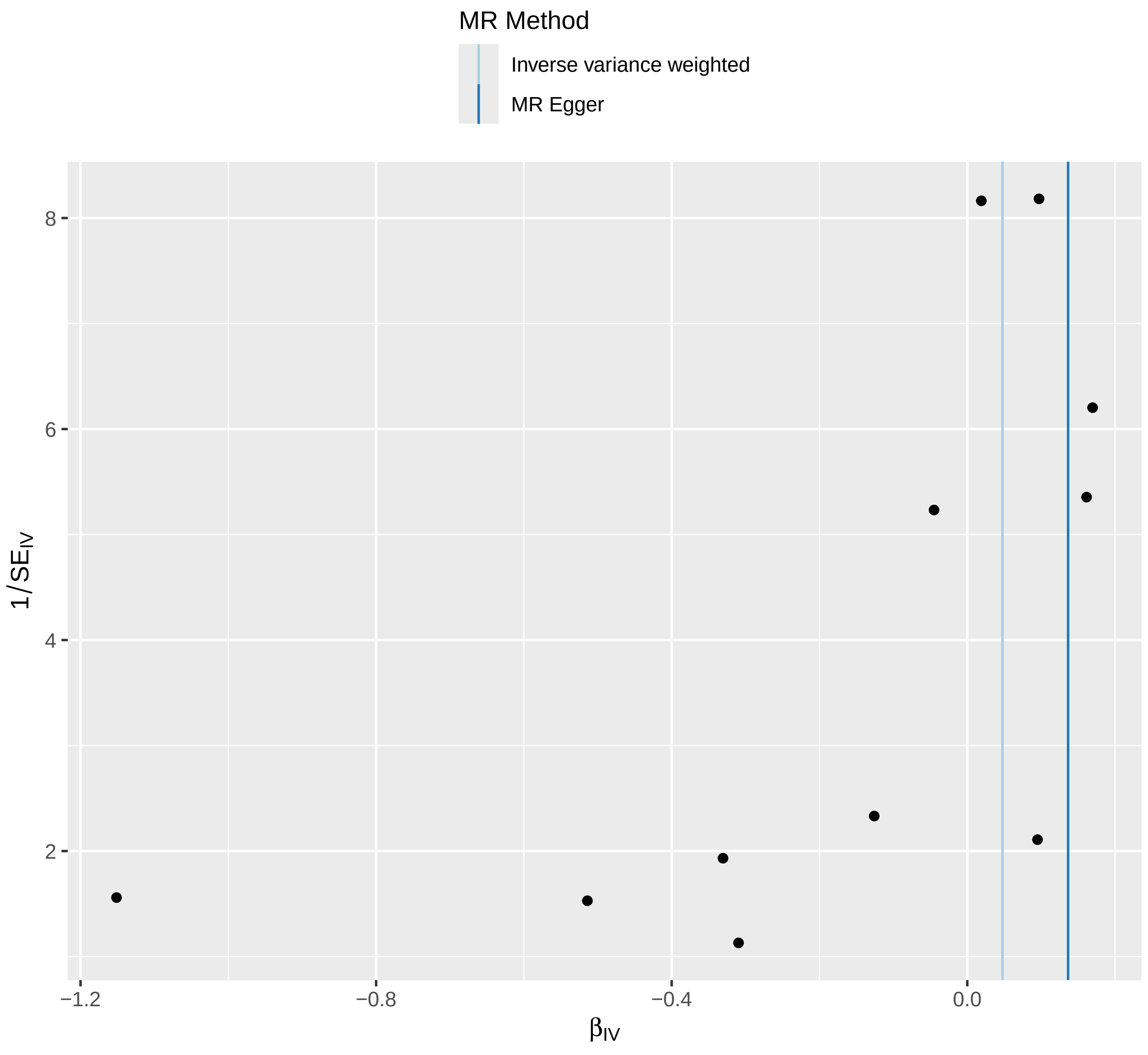

MR Method
Inverse variance weighted
MR Egger
8
6
4
2
$1/SE_{IV}$
−1.2
−0.8
−0.4
0.0
β_{IV}

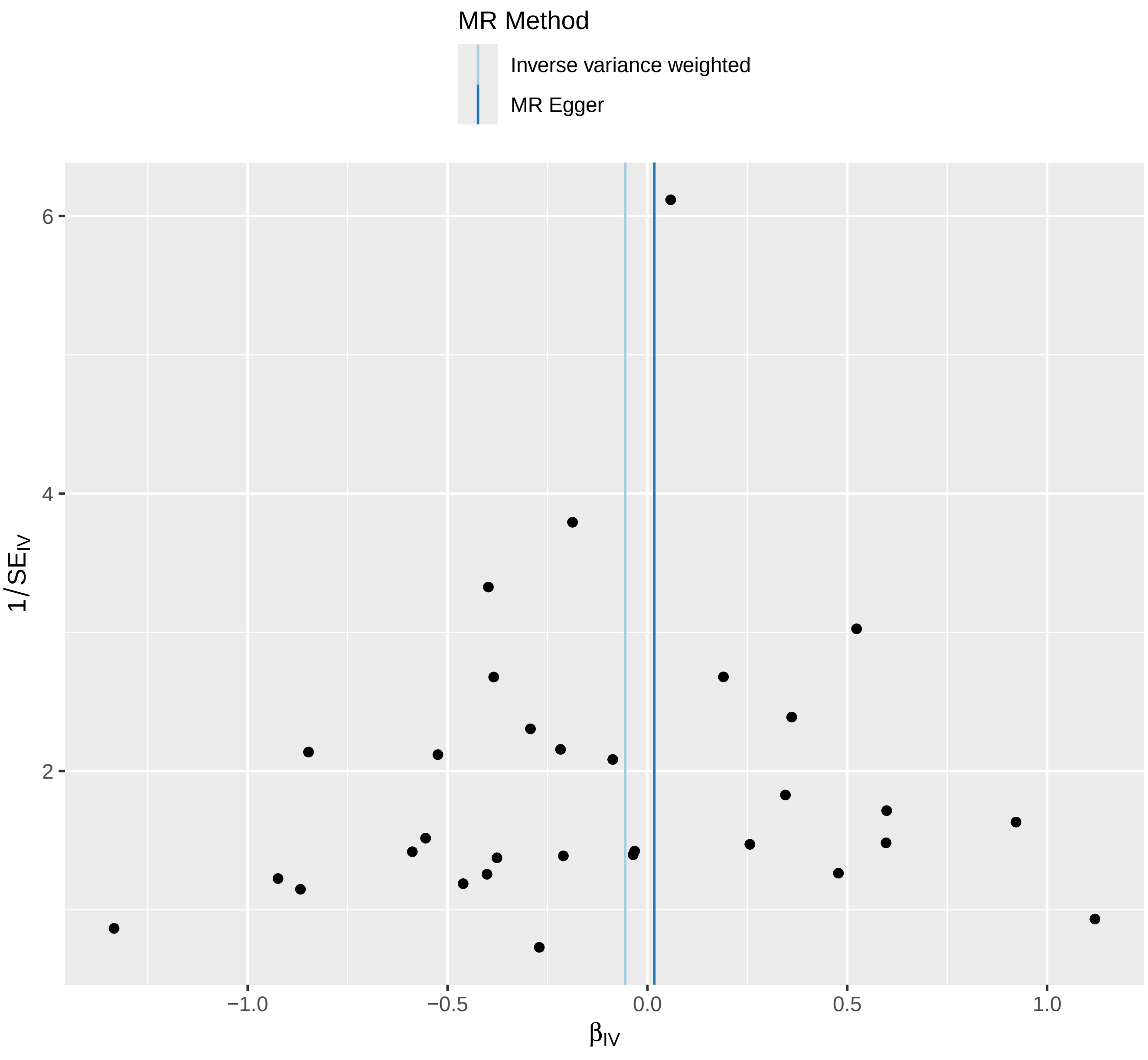

MR Method
Inverse variance weighted
MR Egger
6
4
2
1/SE_IV
−1.0
−0.5
0.0
0.5
1.0
β_IV

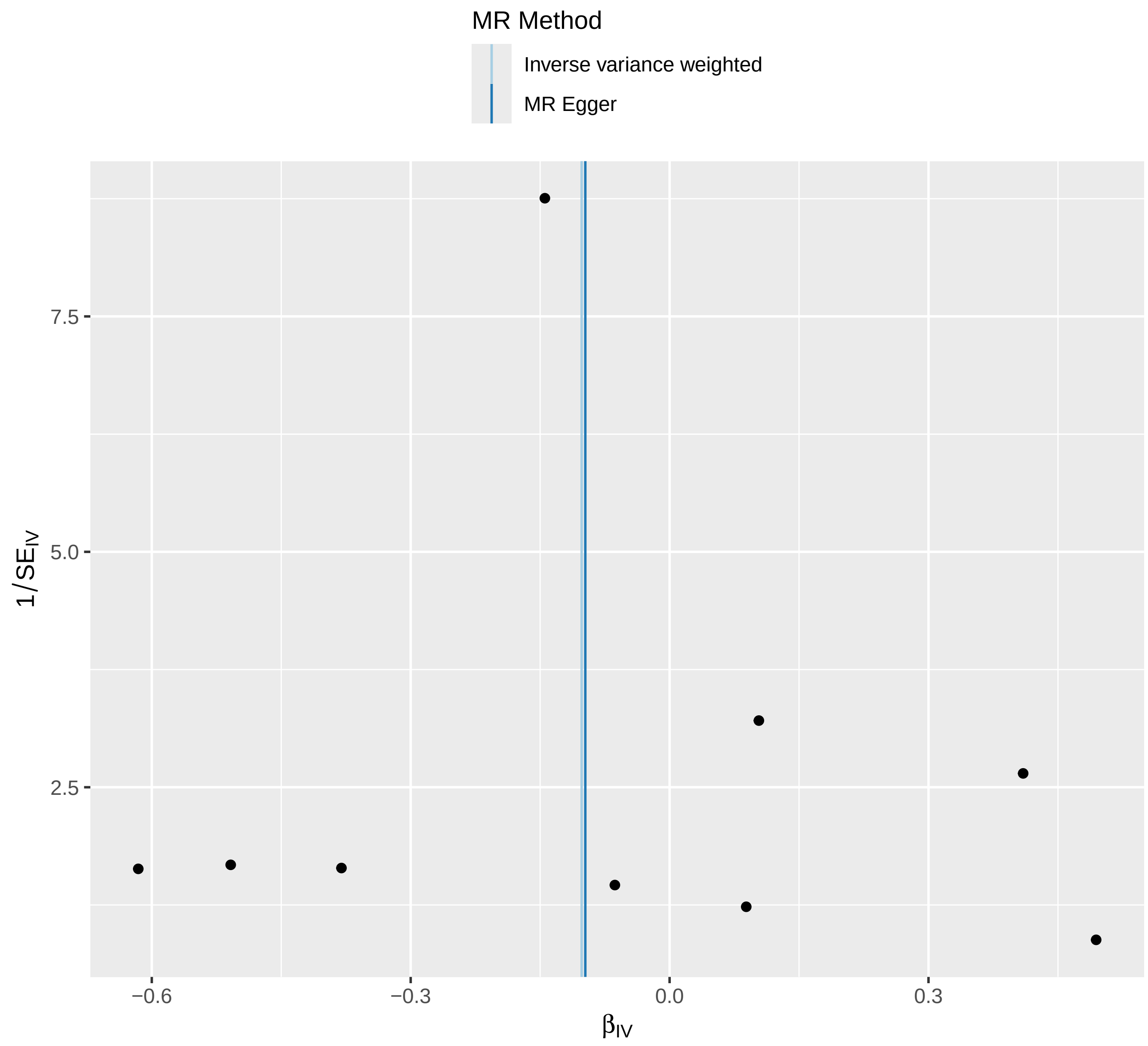

MR Method
Inverse variance weighted
MR Egger
7.5
5.0
2.5
1/SE$_{IV}$
−0.6
−0.3
0.0
0.3
β$_{IV}$

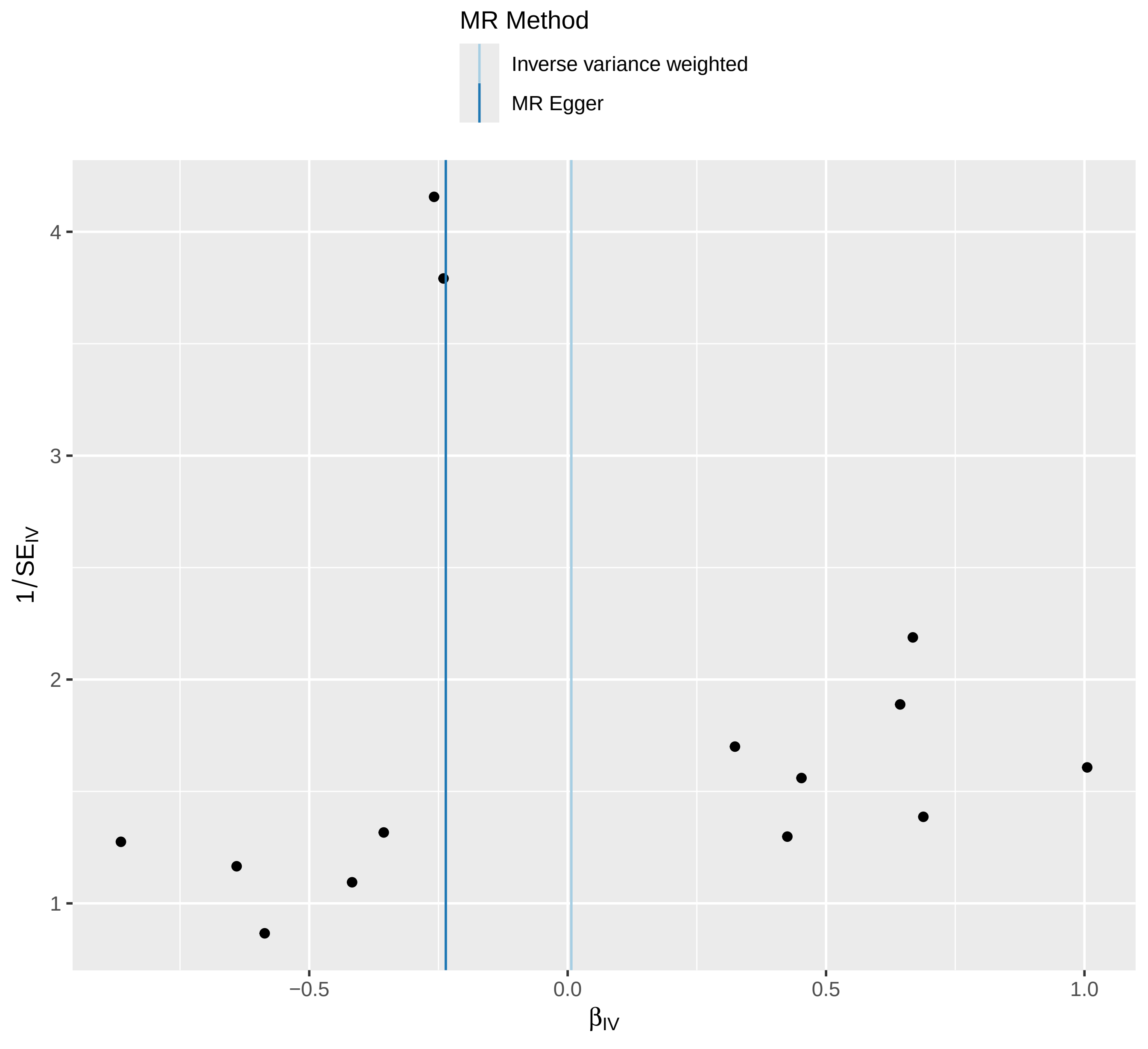

MR Method
Inverse variance weighted
MR Egger
4
3
2
1
1/SE_IV
−0.5
0.0
0.5
1.0
β_IV

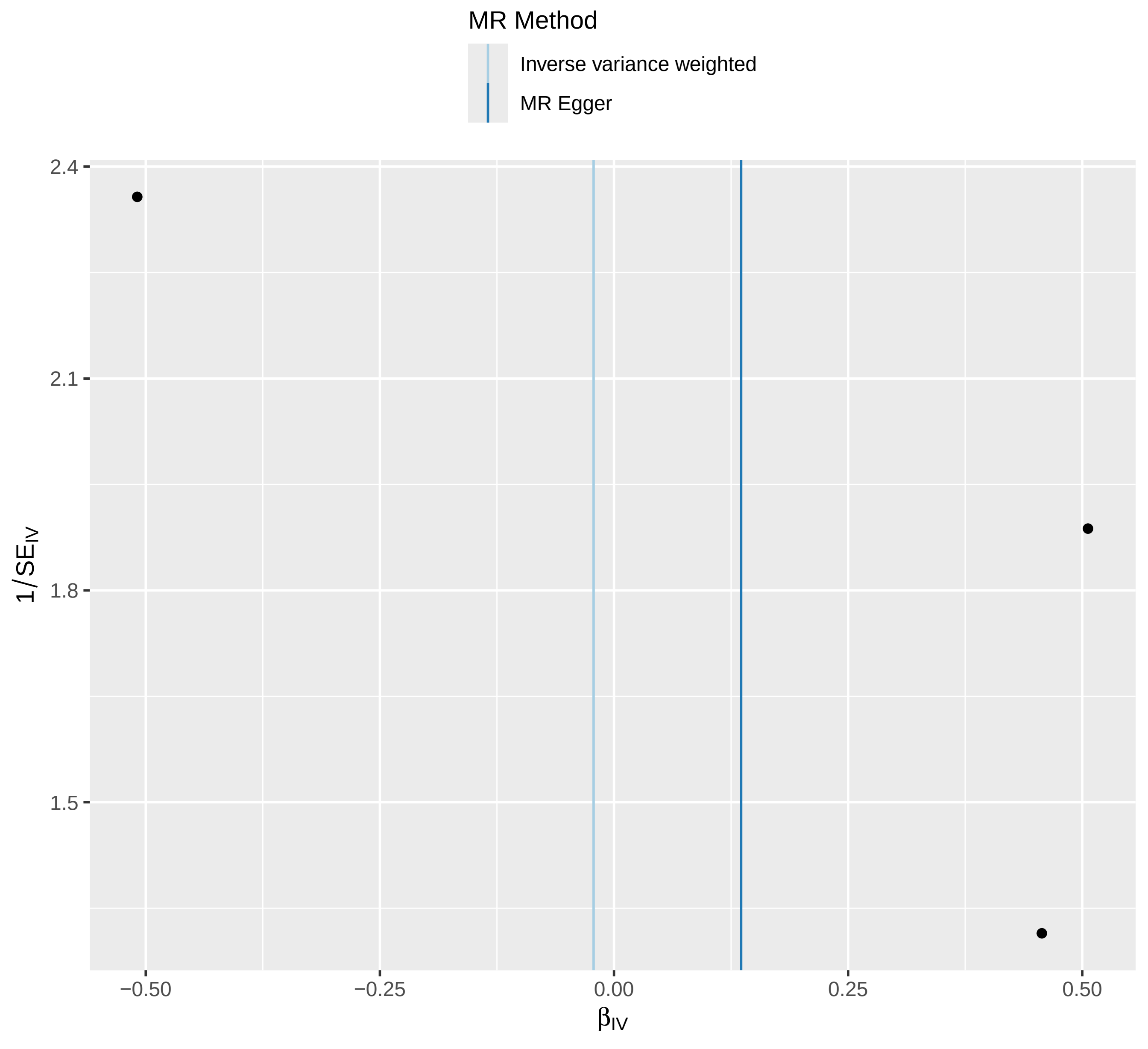
MR Method
Inverse variance weighted
MR Egger
2.4
2.1
1.8
1.5
1/SE_IV
−0.50
−0.25
0.00
0.25
0.50
β_IV

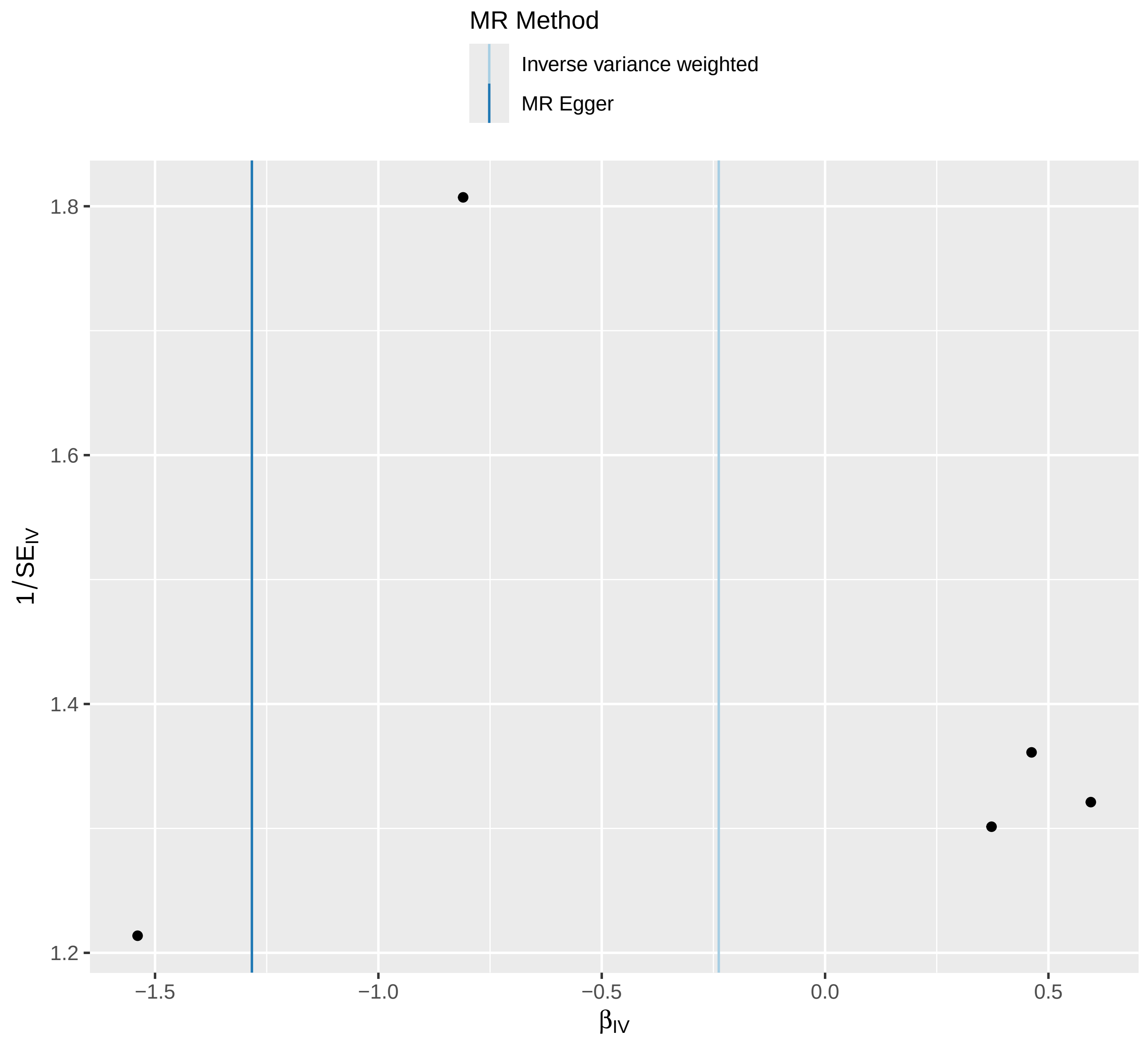
MR Method
Inverse variance weighted
MR Egger
1.8
1.6
1.4
1.2
1/SE_IV
−1.5
−1.0
−0.5
0.0
0.5
β_IV

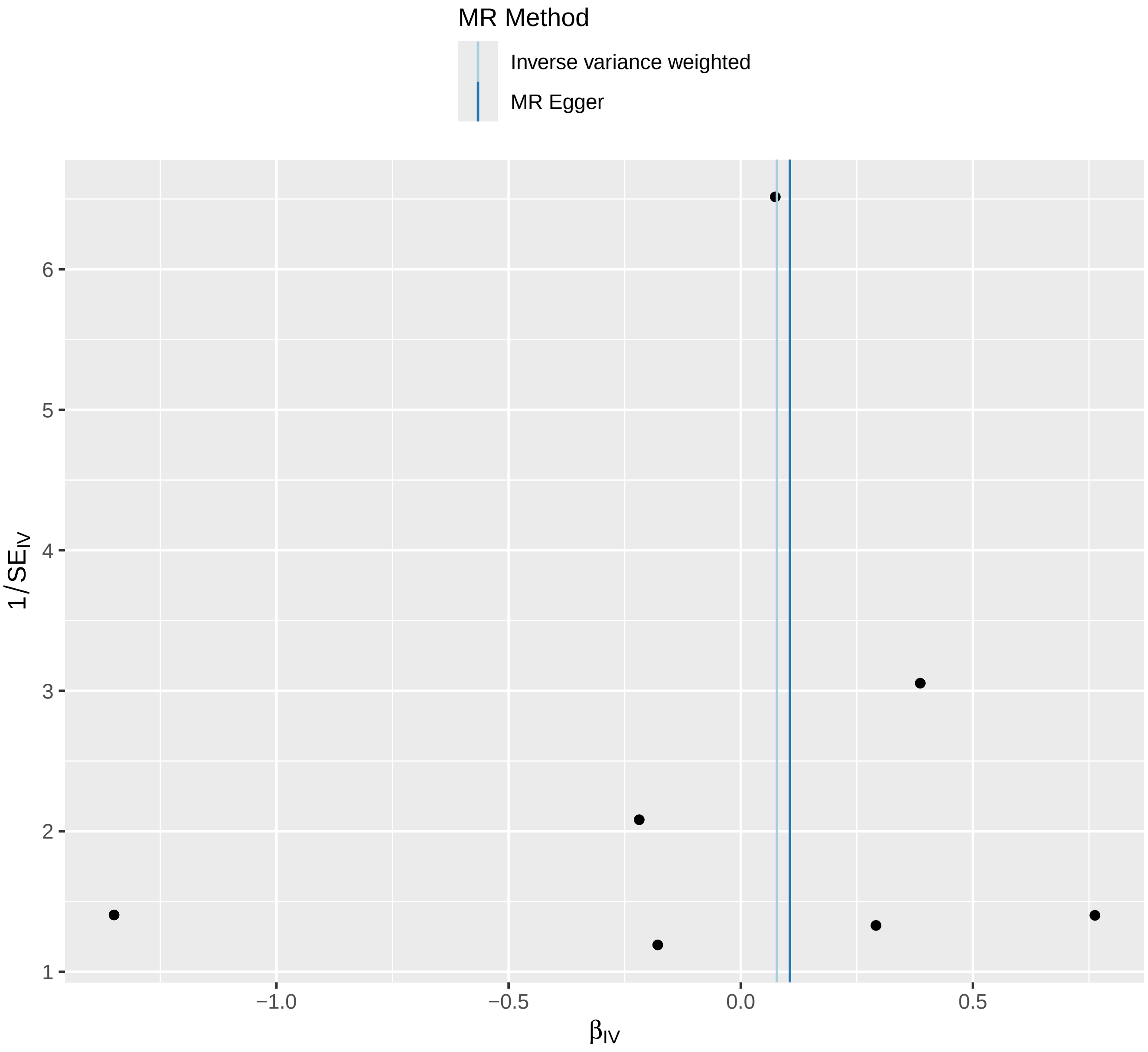

MR Method
Inverse variance weighted
MR Egger
6
5
4
3
2
1
1/SE$_{IV}$
−1.0
−0.5
0.0
0.5
β$_{IV}$

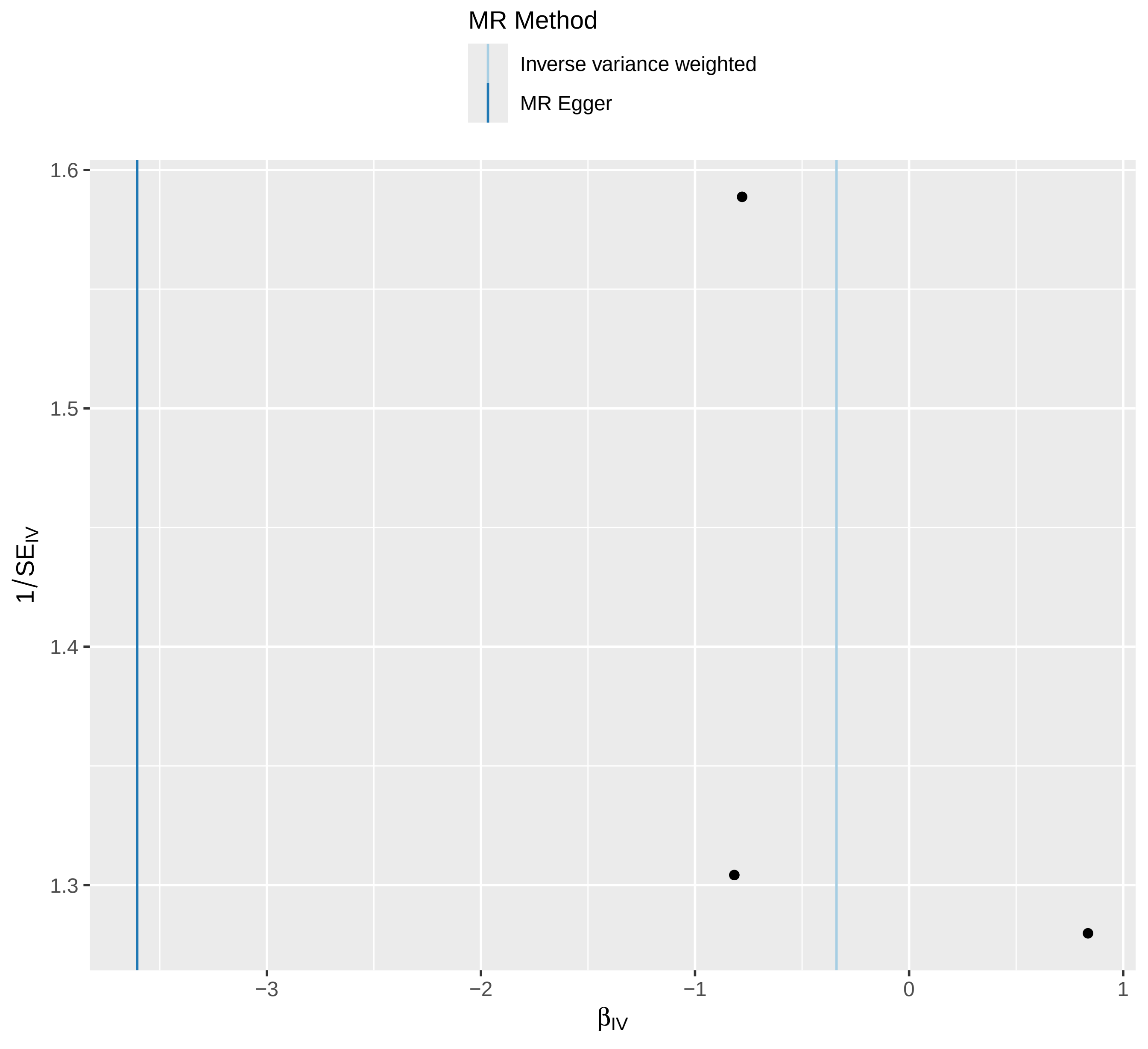

MR Method
Inverse variance weighted
MR Egger
1.6
1.5
1.4
1.3
1/SE_IV
−3
−2
−1
0
1
β_IV

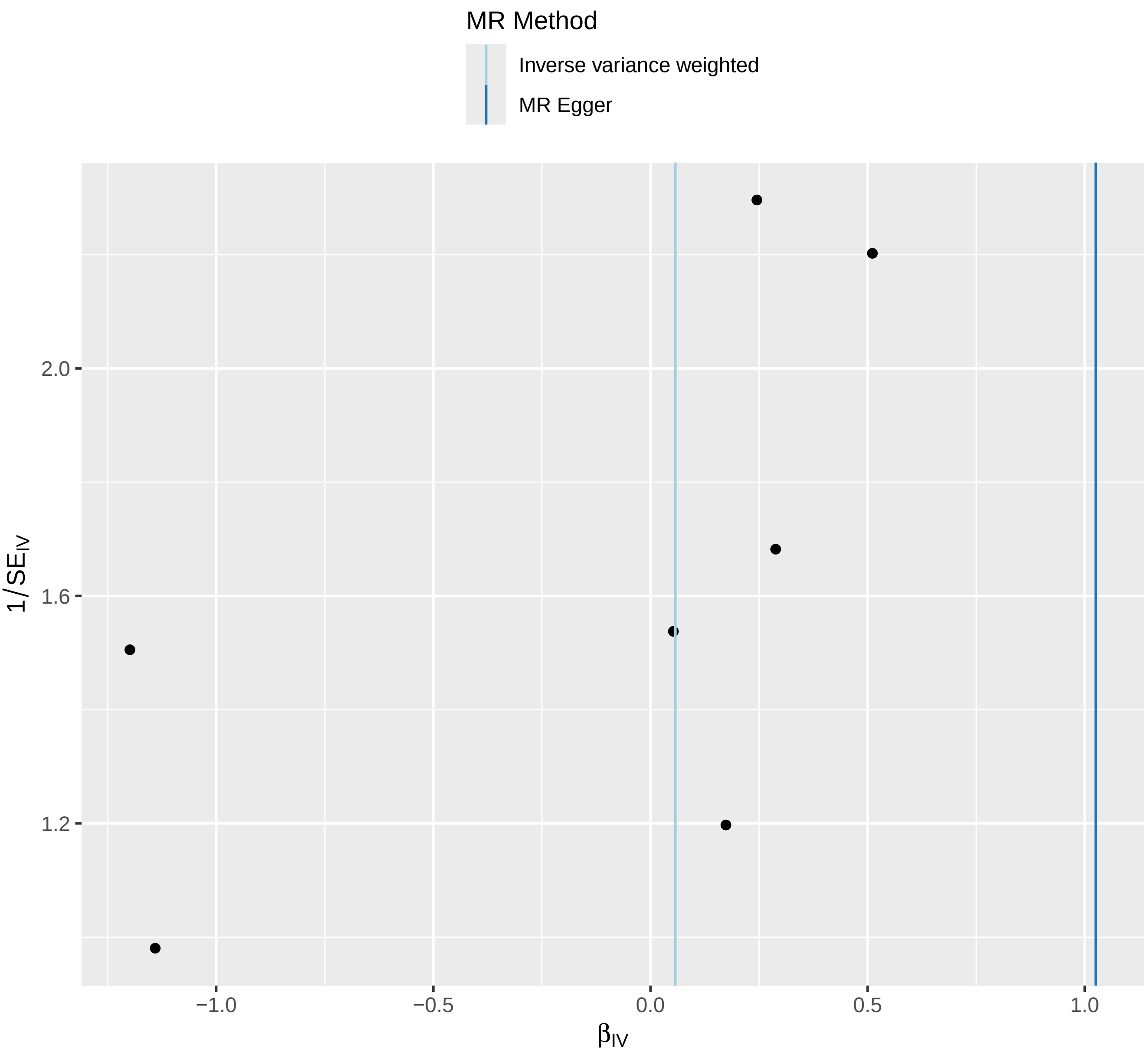
MR Method
Inverse variance weighted
MR Egger
2.0
1.6
1.2
1/SE_IV
−1.0
−0.5
0.0
0.5
1.0
β_IV

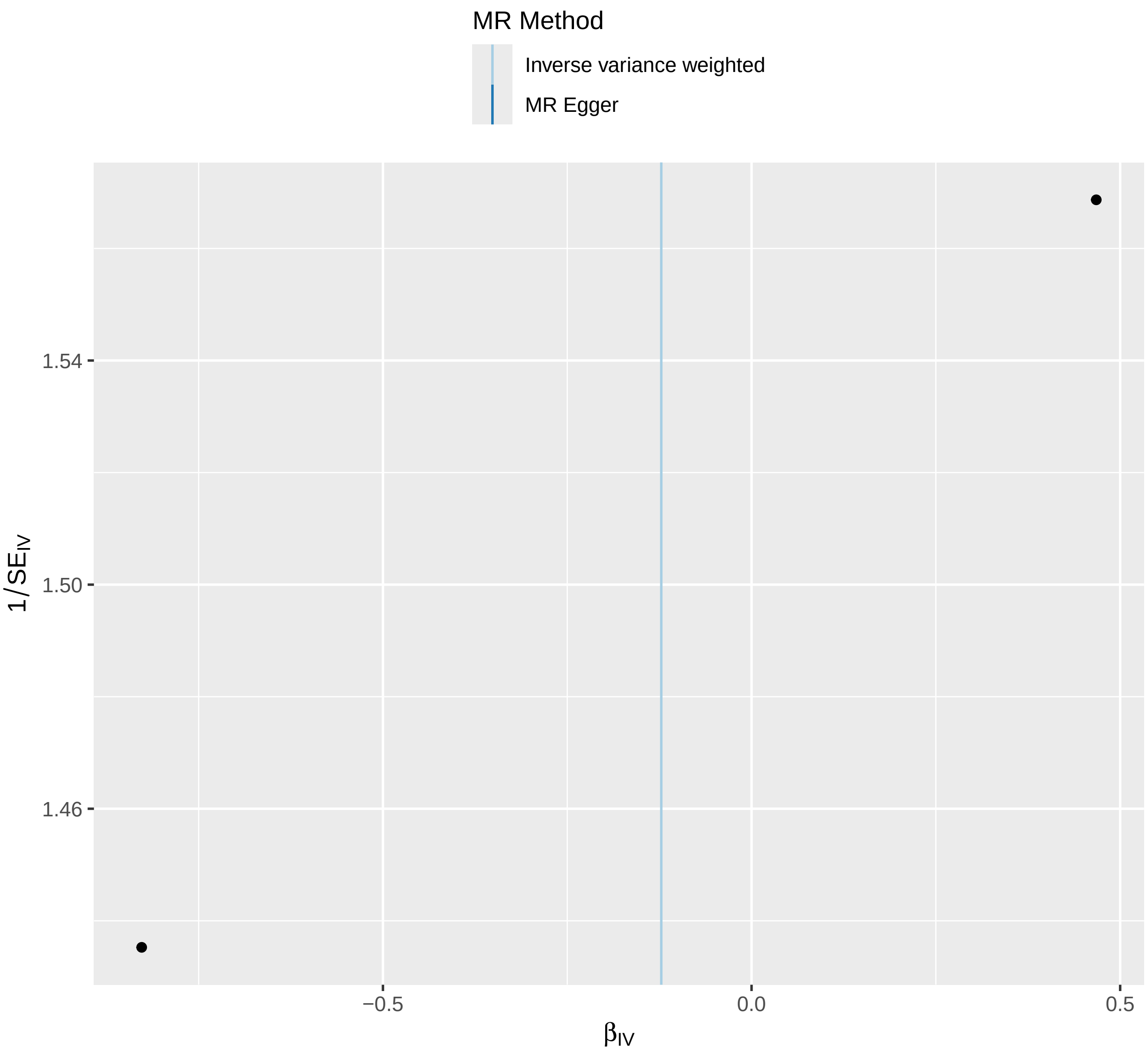
MR Method
Inverse variance weighted
MR Egger
1.54
1.50
1.46
1/SE_IV
−0.5
0.0
0.5
β_IV

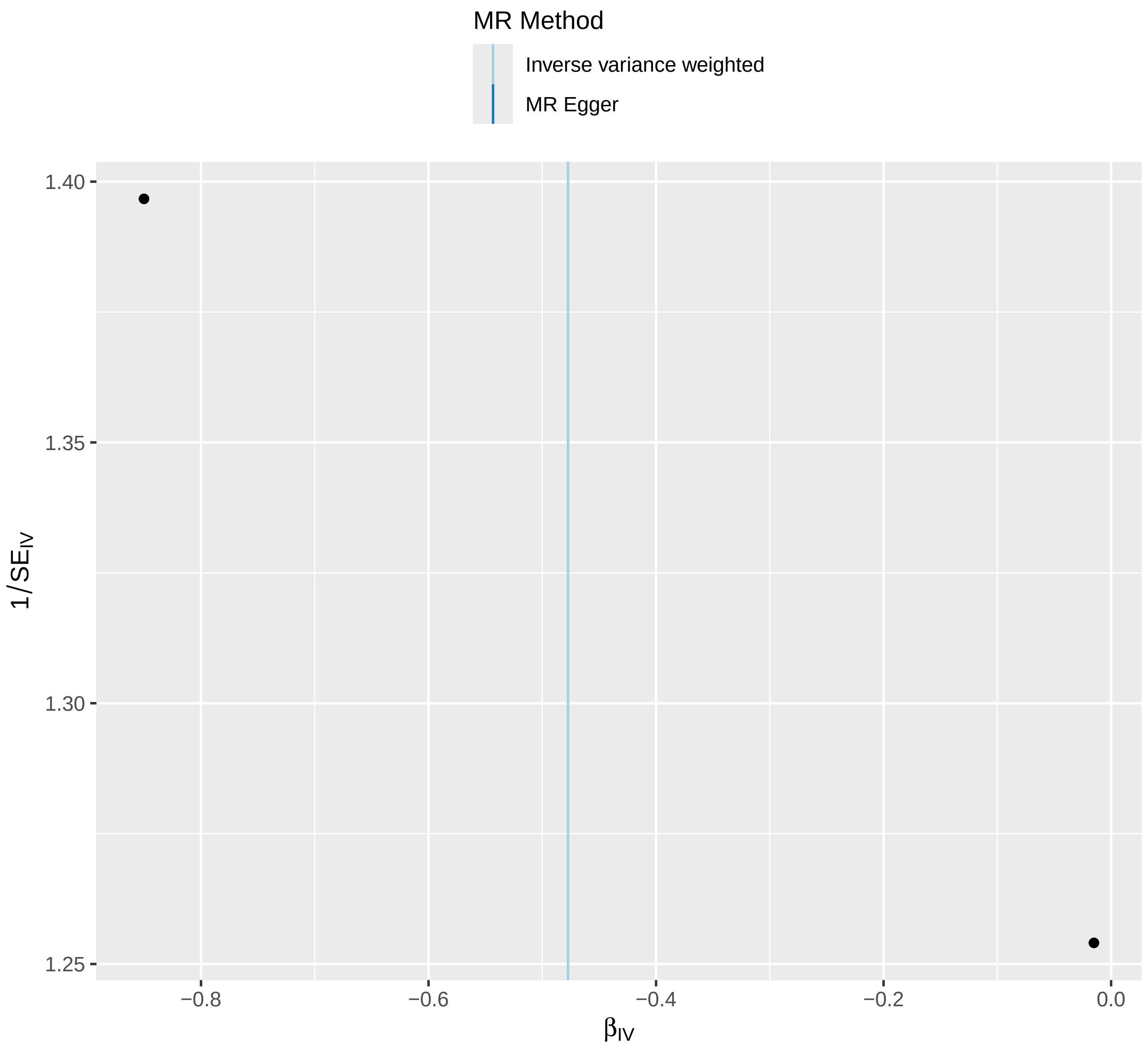

MR Method
Inverse variance weighted
MR Egger
1.40
1.35
1.30
1.25
1/SE_IV
−0.8
−0.6
−0.4
−0.2
0.0
β_IV

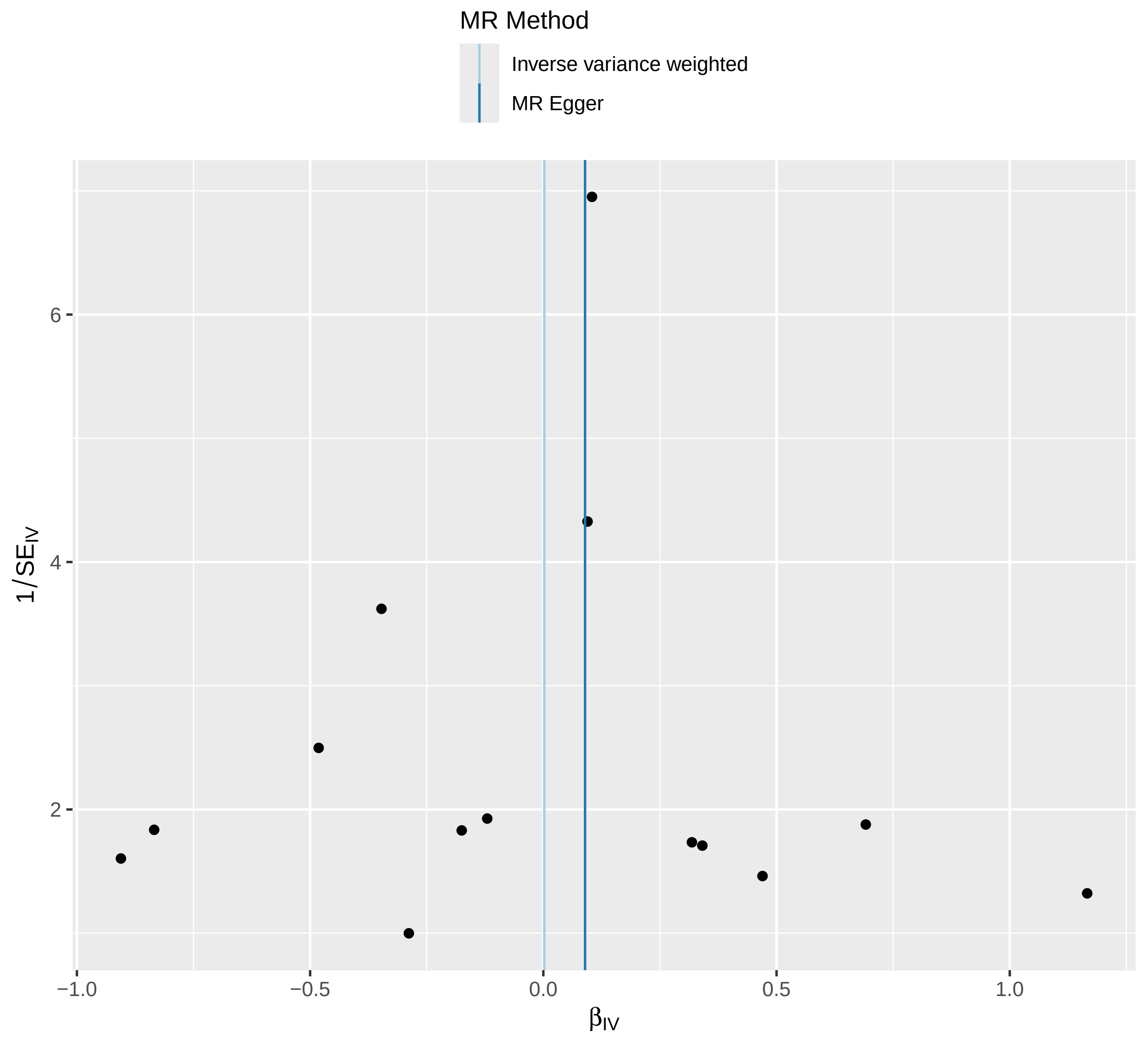
MR Method
Inverse variance weighted
MR Egger
1/SE_IV
6
4
2
−1.0
−0.5
0.0
0.5
1.0
β_IV

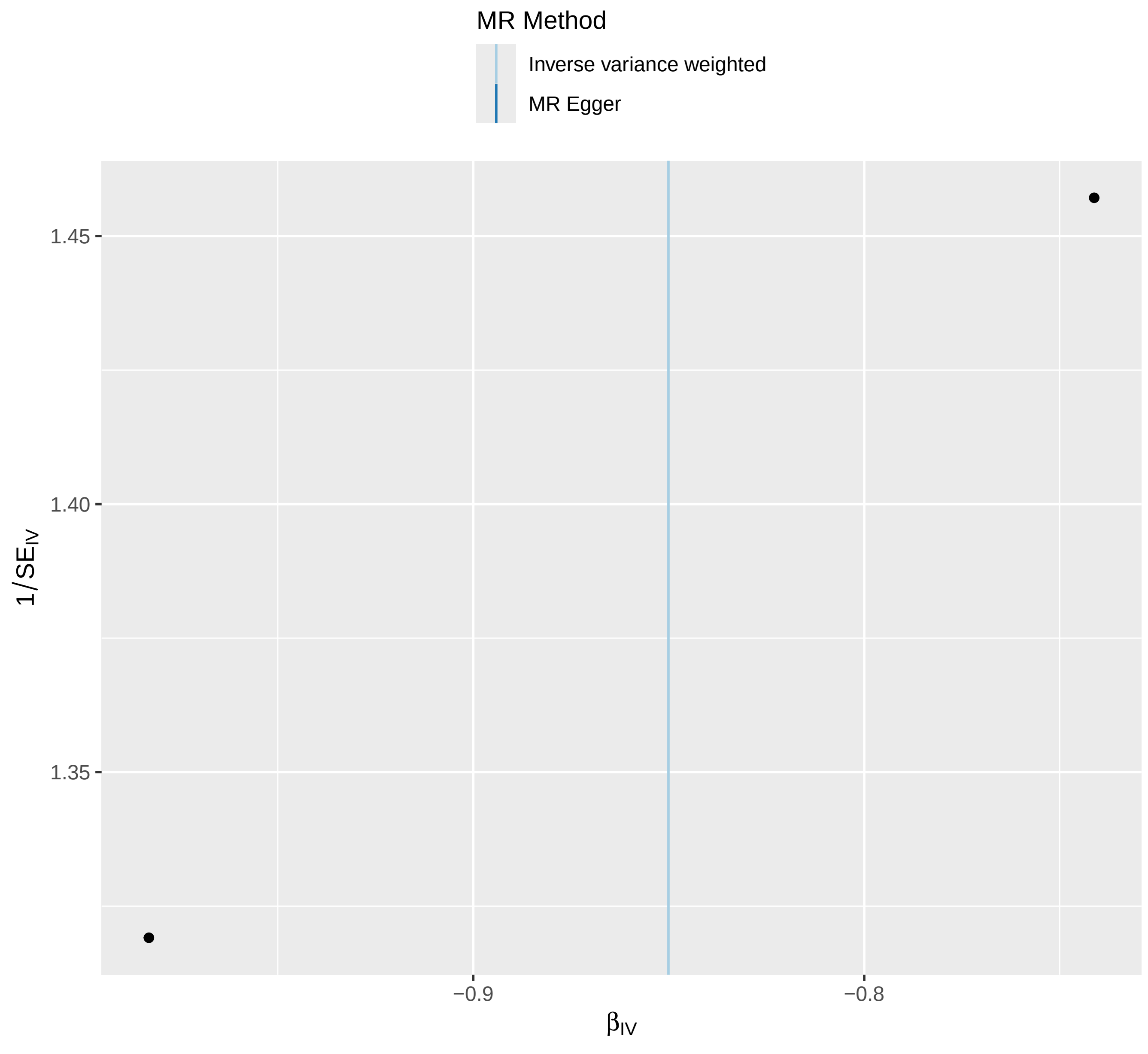

MR Method
Inverse variance weighted
MR Egger
1.45
1.40
1.35
1/SE_IV
−0.9
−0.8
β_IV

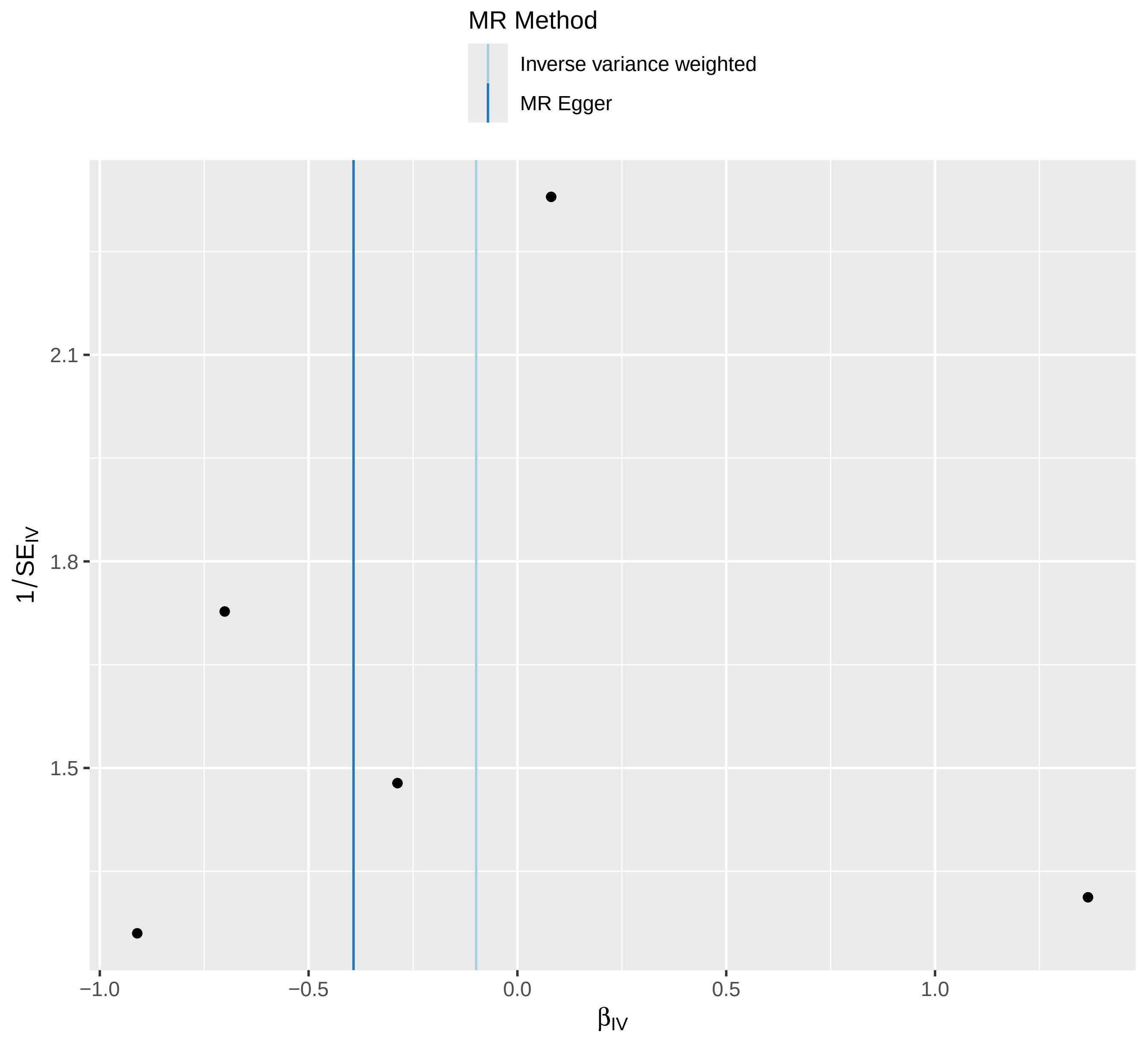

MR Method
Inverse variance weighted
MR Egger
2.1
1.8
1.5
1/SE_IV
−1.0
−0.5
0.0
0.5
1.0
β_IV

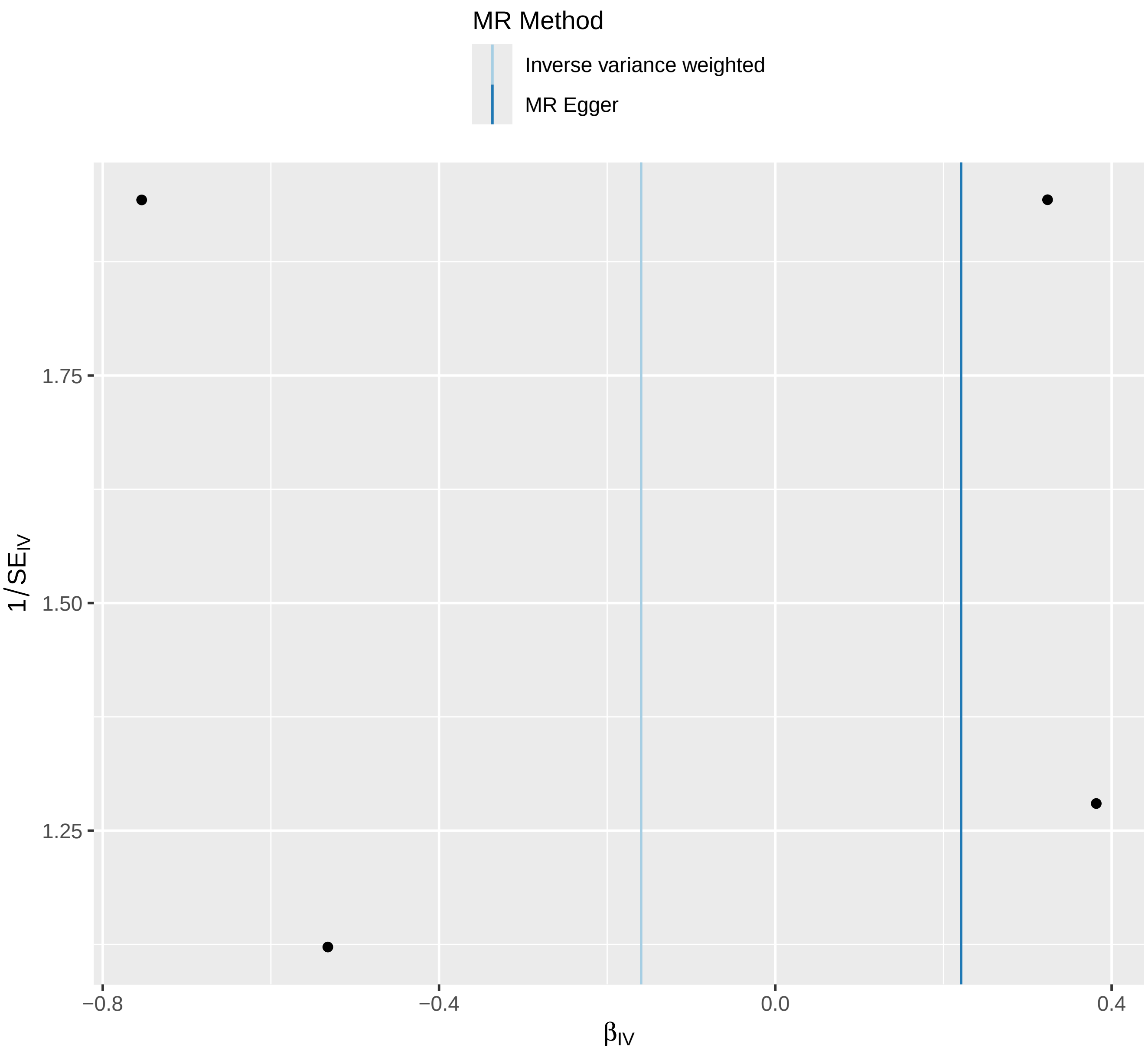

MR Method
Inverse variance weighted
MR Egger
1.75
1.50
1.25
$1/SE_{IV}$
−0.8
−0.4
0.0
0.4
β_{IV}

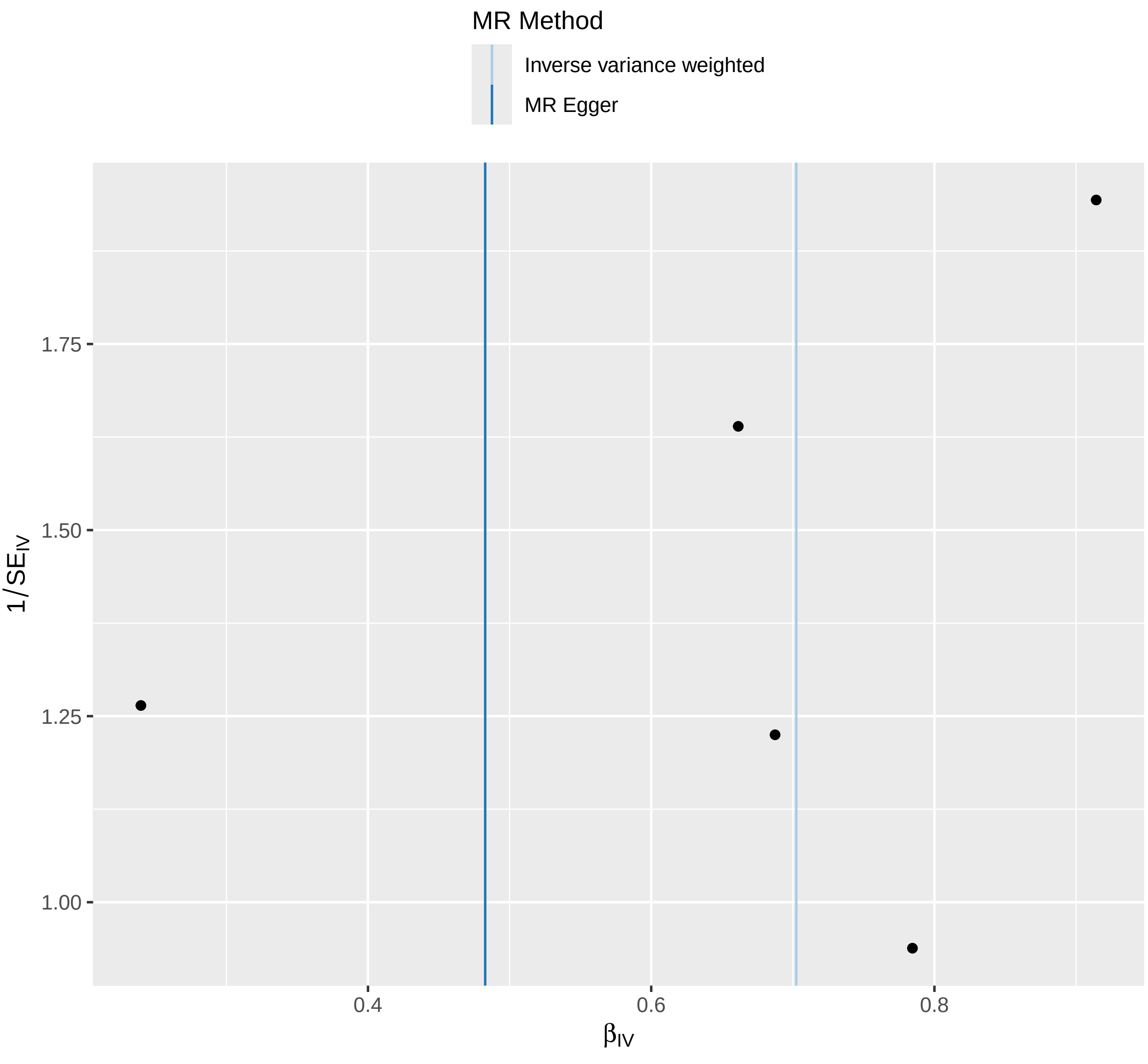

MR Method
Inverse variance weighted
MR Egger
1.75
1.50
1.25
1.00
1/SE_IV
0.4
0.6
0.8
β_IV

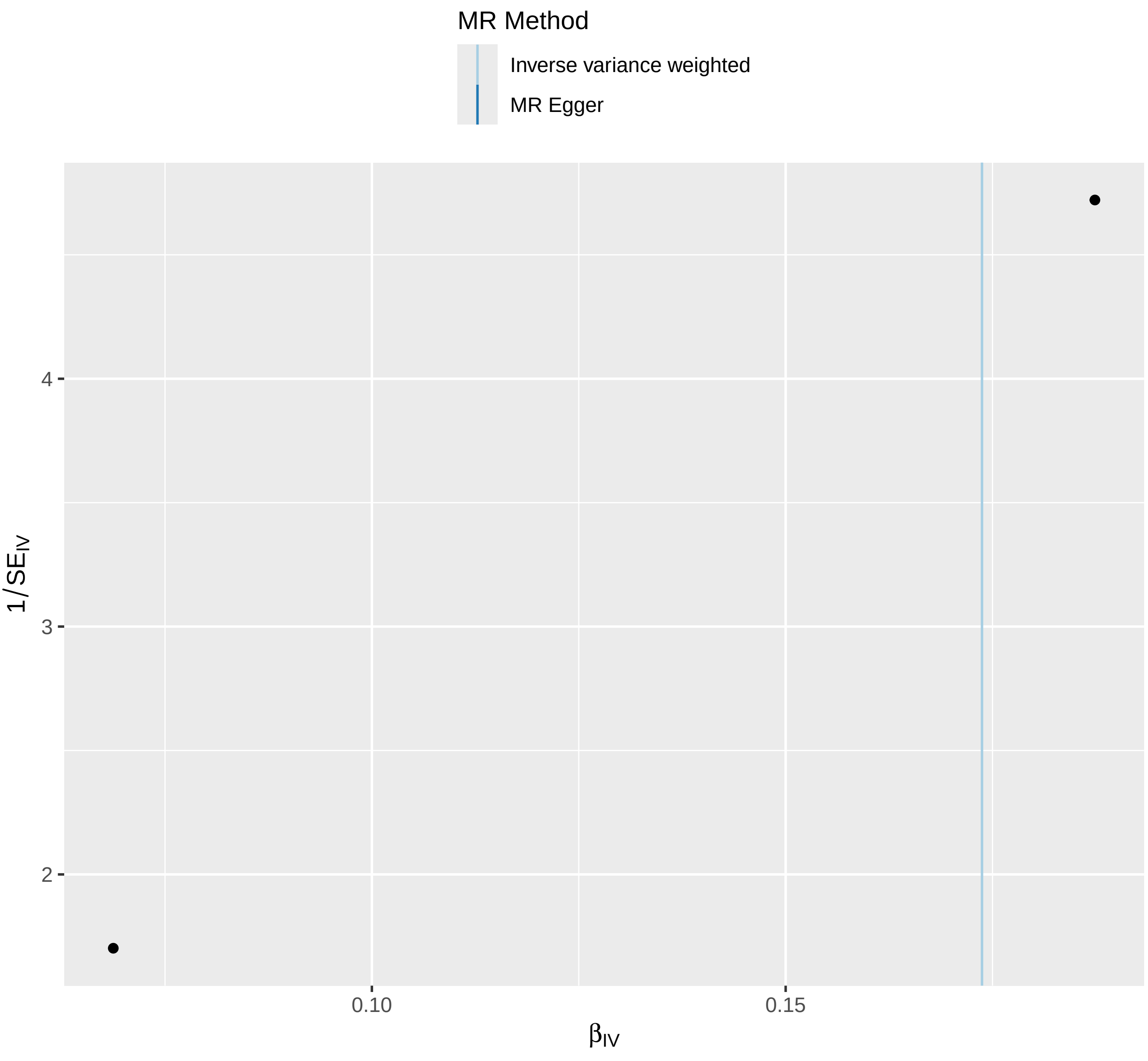
MR Method
Inverse variance weighted
MR Egger
4
3
2
1/SE_IV
0.10
0.15
β_IV

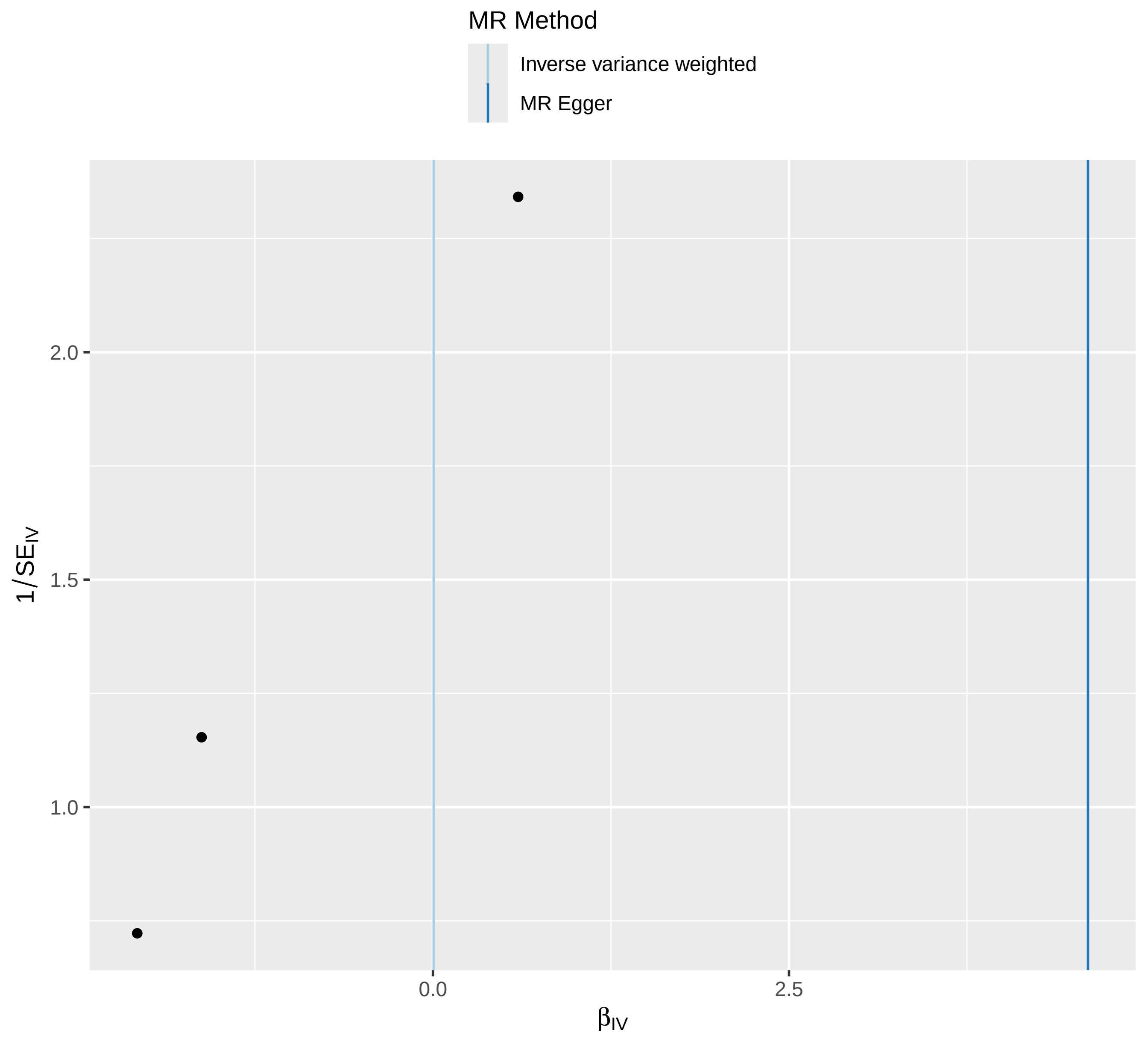
MR Method
Inverse variance weighted
MR Egger
2.0
1.5
1.0
1/SE_IV
0.0
2.5
β_IV

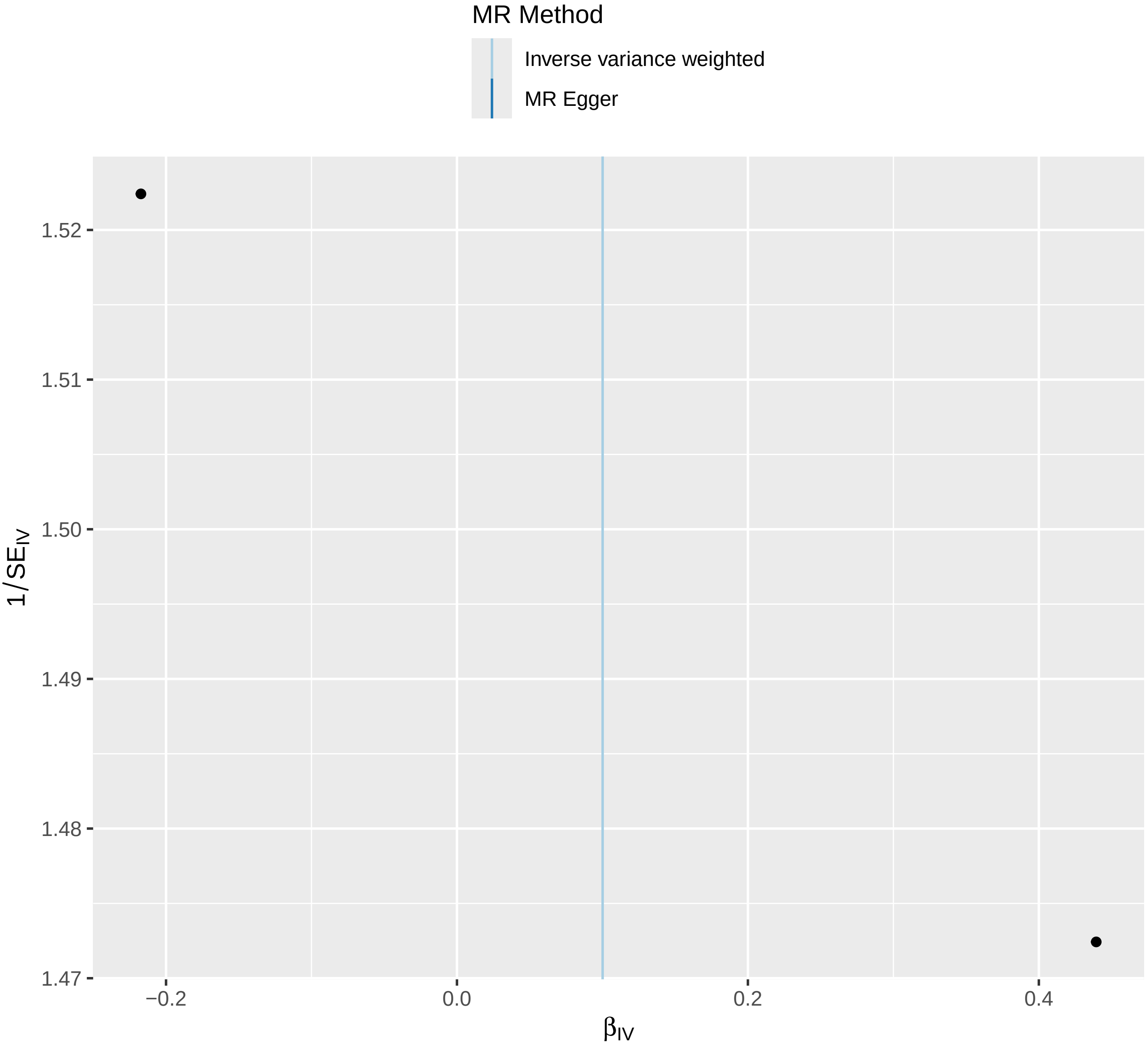

MR Method
Inverse variance weighted
MR Egger
1.52
1.51
1.50
1.49
1.48
1.47
$1/SE_{IV}$
−0.2
0.0
0.2
0.4
β_{IV}

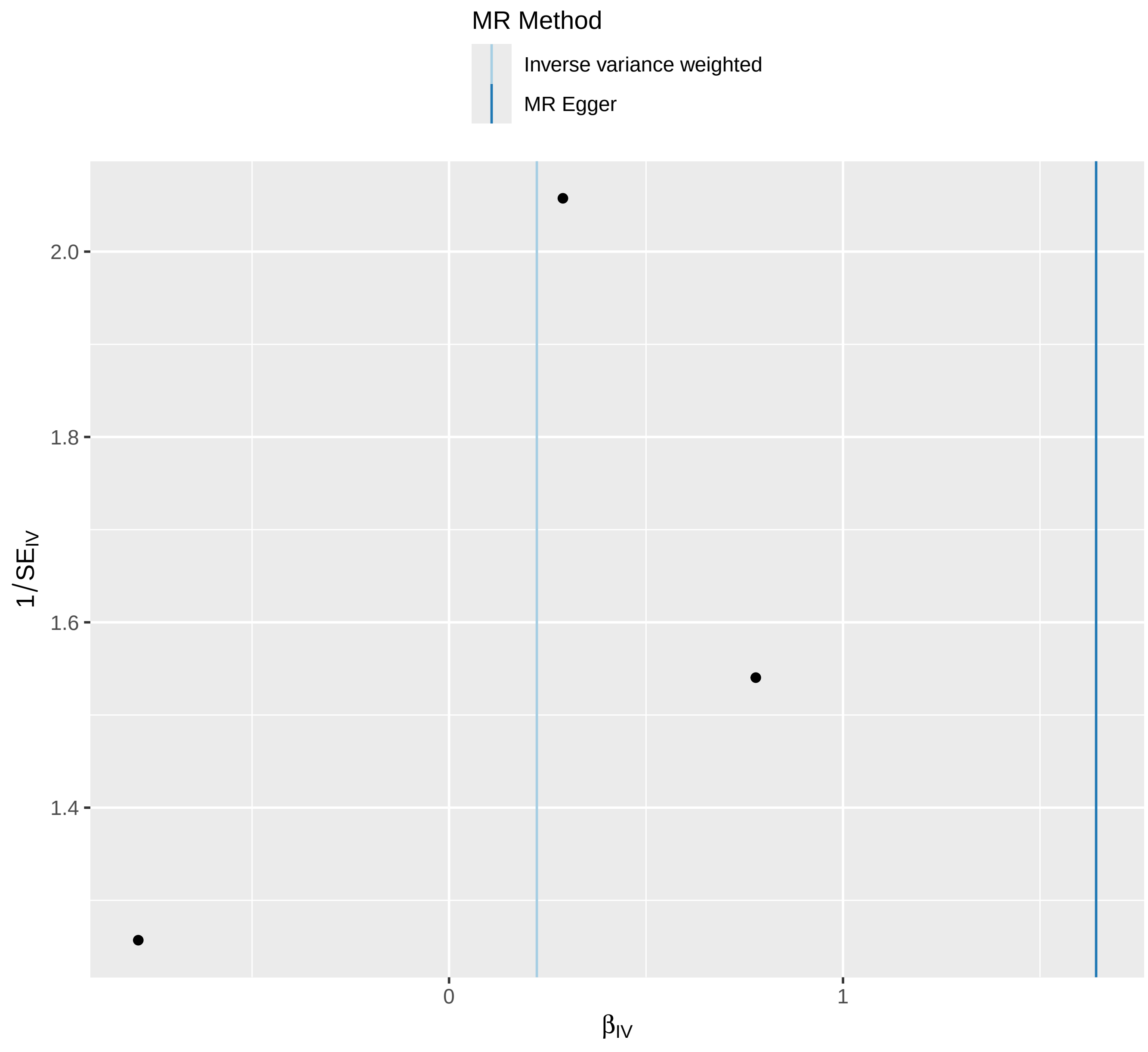
MR Method
Inverse variance weighted
MR Egger
2.0
1.8
1.6
1.4
$1/SE_{IV}$
0
1
β_{IV}

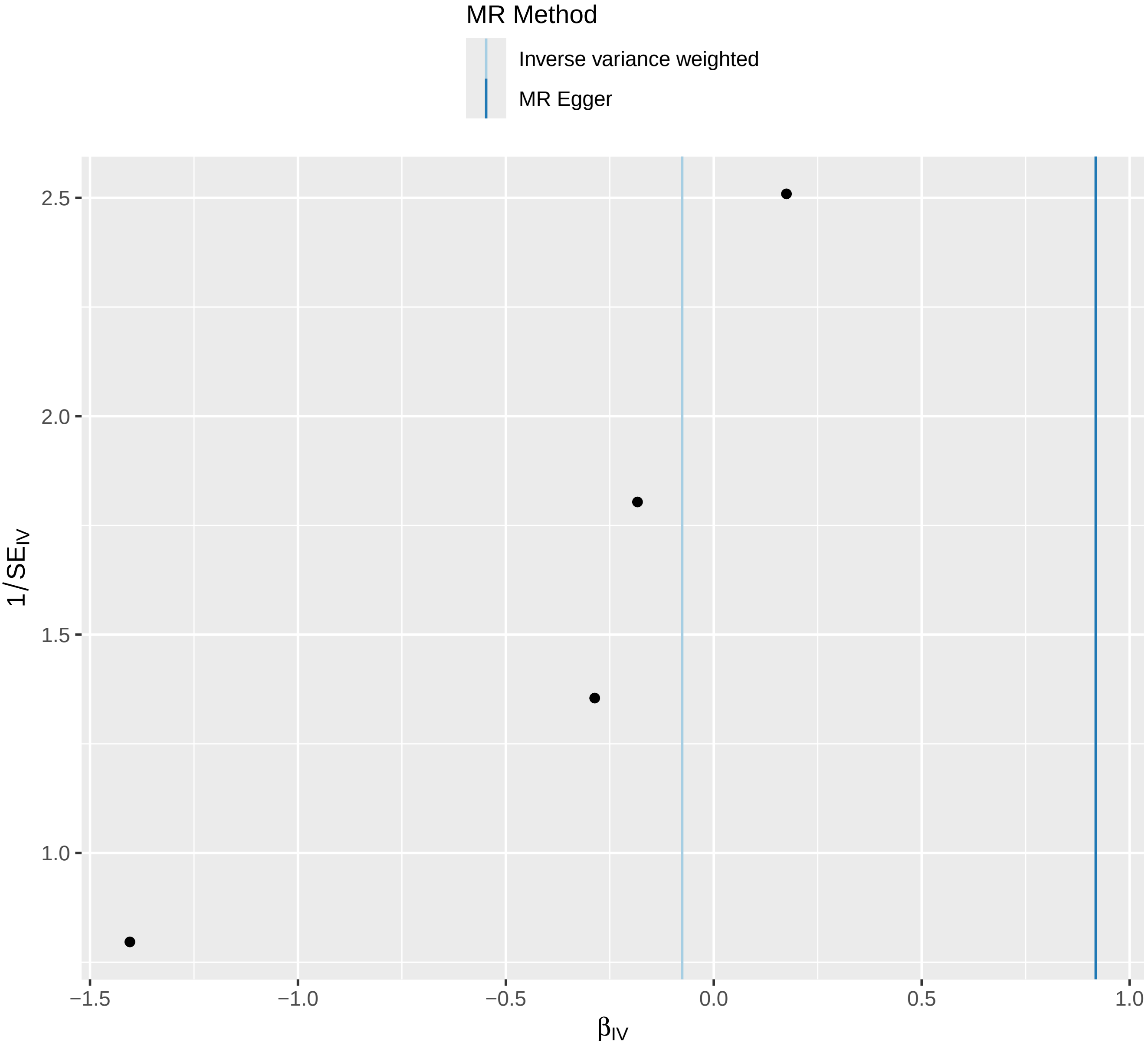

MR Method
Inverse variance weighted
MR Egger
2.5
2.0
1.5
1.0
1/SE_IV
−1.5
−1.0
−0.5
0.0
0.5
1.0
β_IV

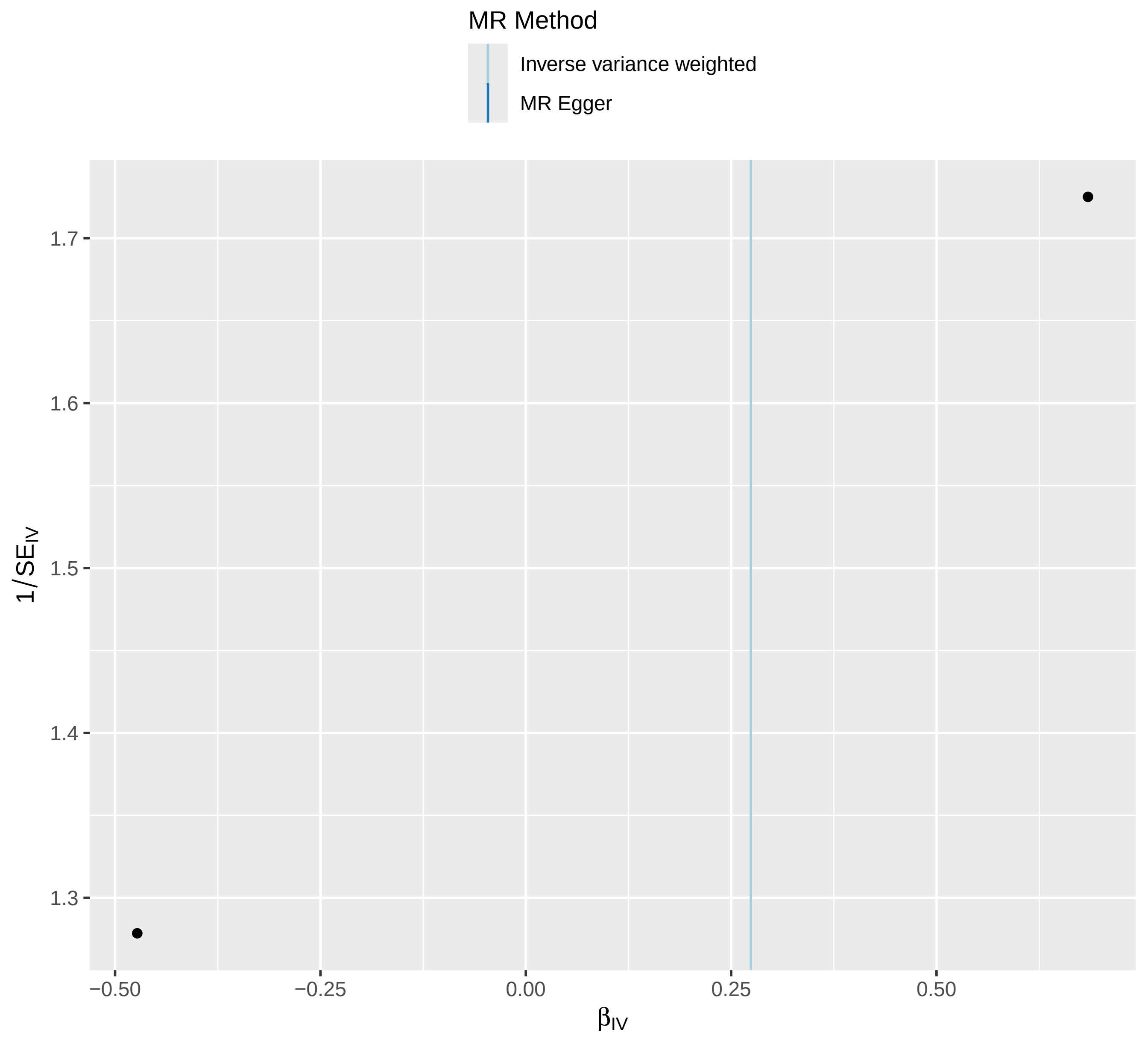

MR Method
Inverse variance weighted
MR Egger
1.7
1.6
1.5
1.4
1.3
1/SE$_{IV}$
−0.50
−0.25
0.00
0.25
0.50
β$_{IV}$

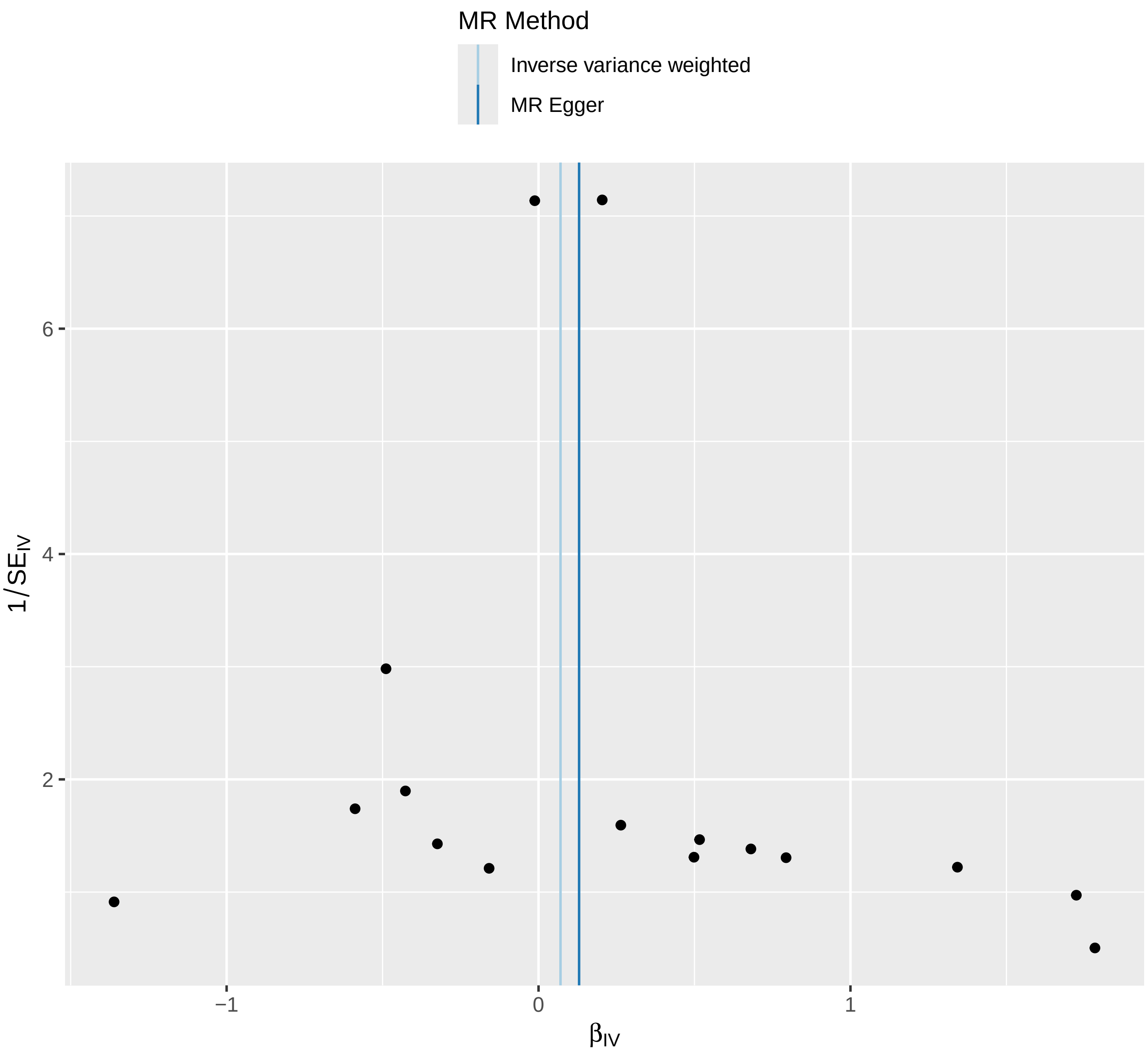

MR Method
Inverse variance weighted
MR Egger
6
4
2
1/SE_IV
−1
0
1
β_IV

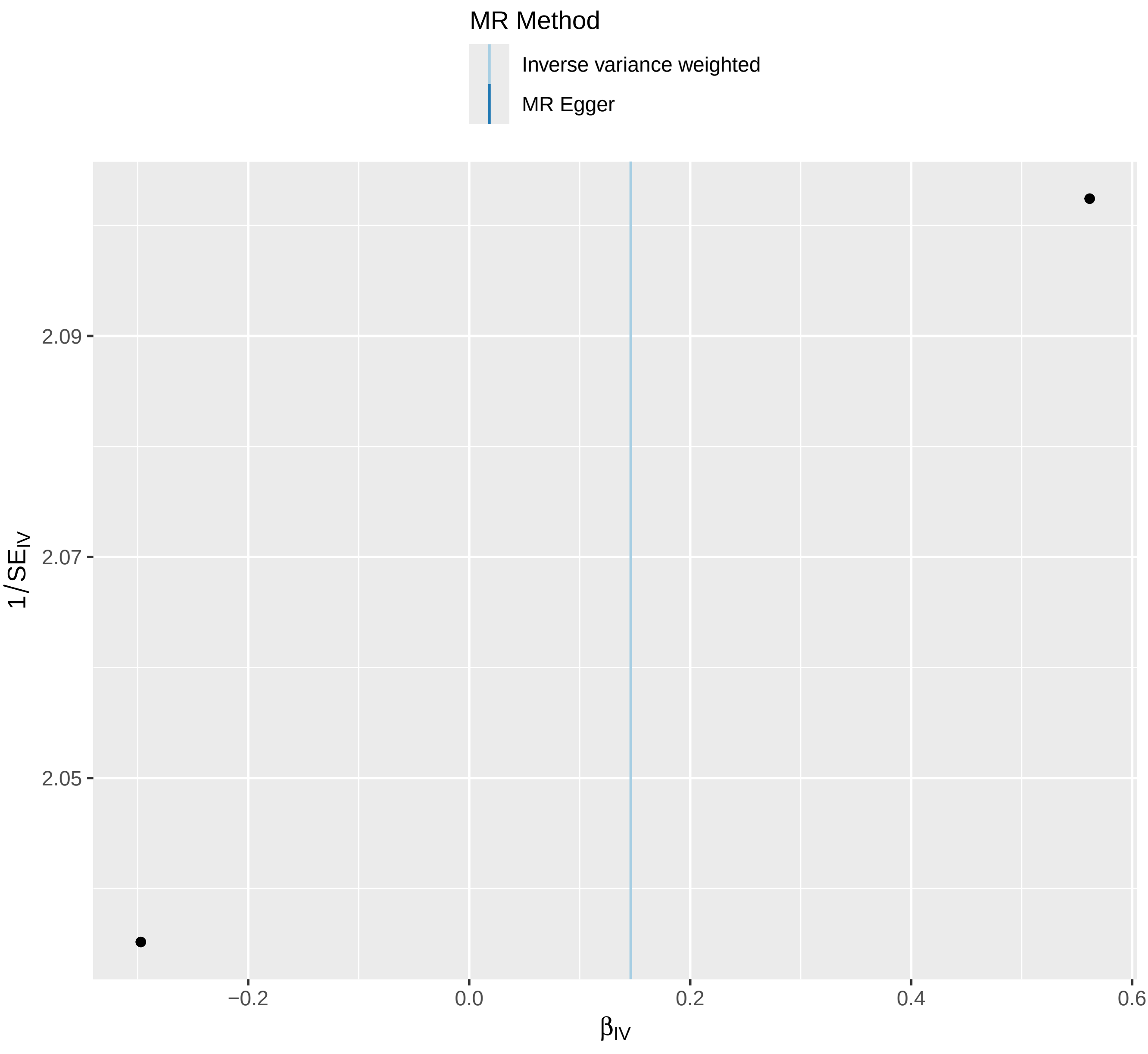

MR Method
Inverse variance weighted
MR Egger
2.09
2.07
2.05
1/SE_IV
−0.2
0.0
0.2
0.4
0.6
β_IV

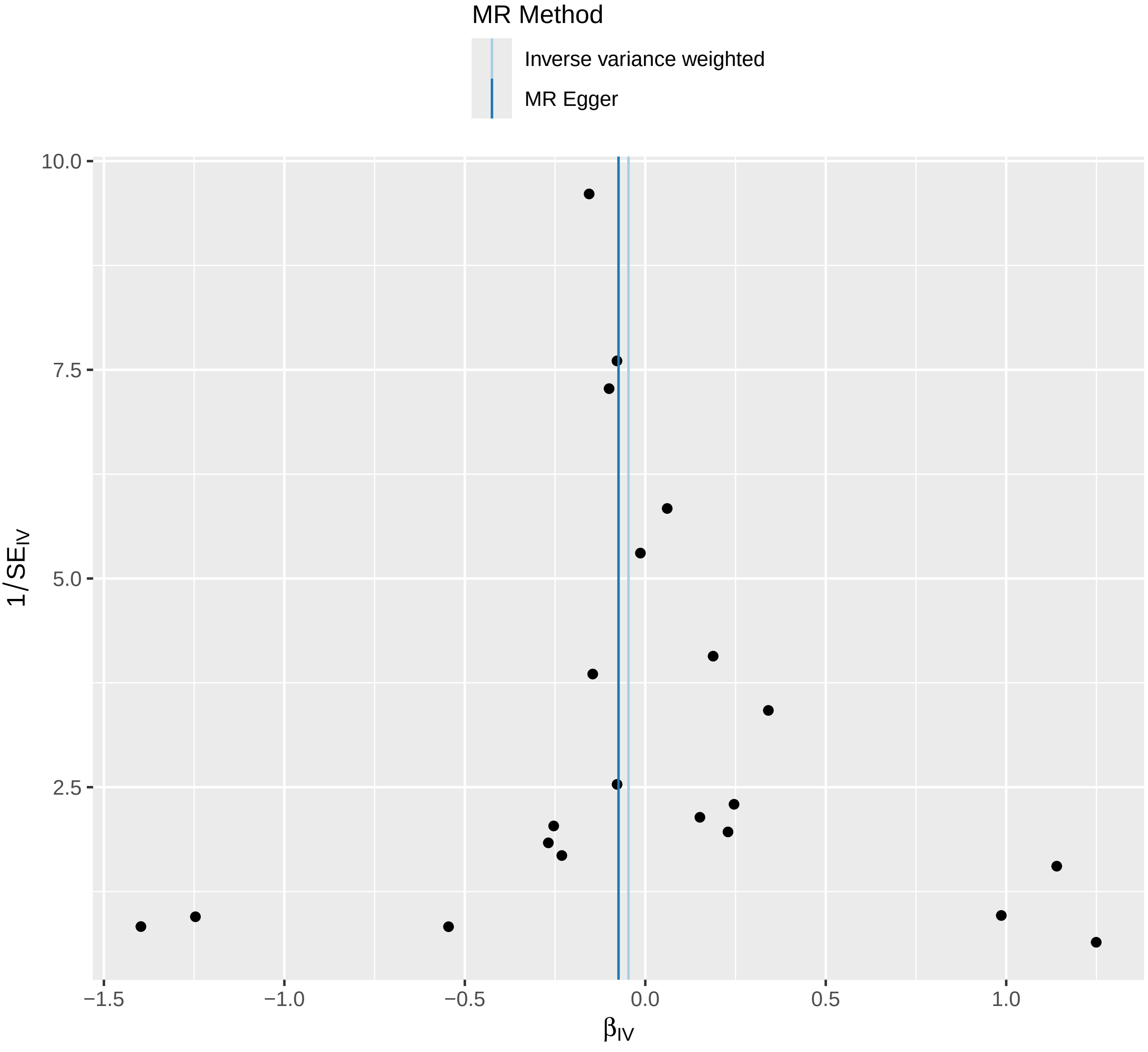

MR Method
Inverse variance weighted
MR Egger
10.0
7.5
5.0
2.5
1/SE_IV
−1.5
−1.0
−0.5
0.0
0.5
1.0
β_IV

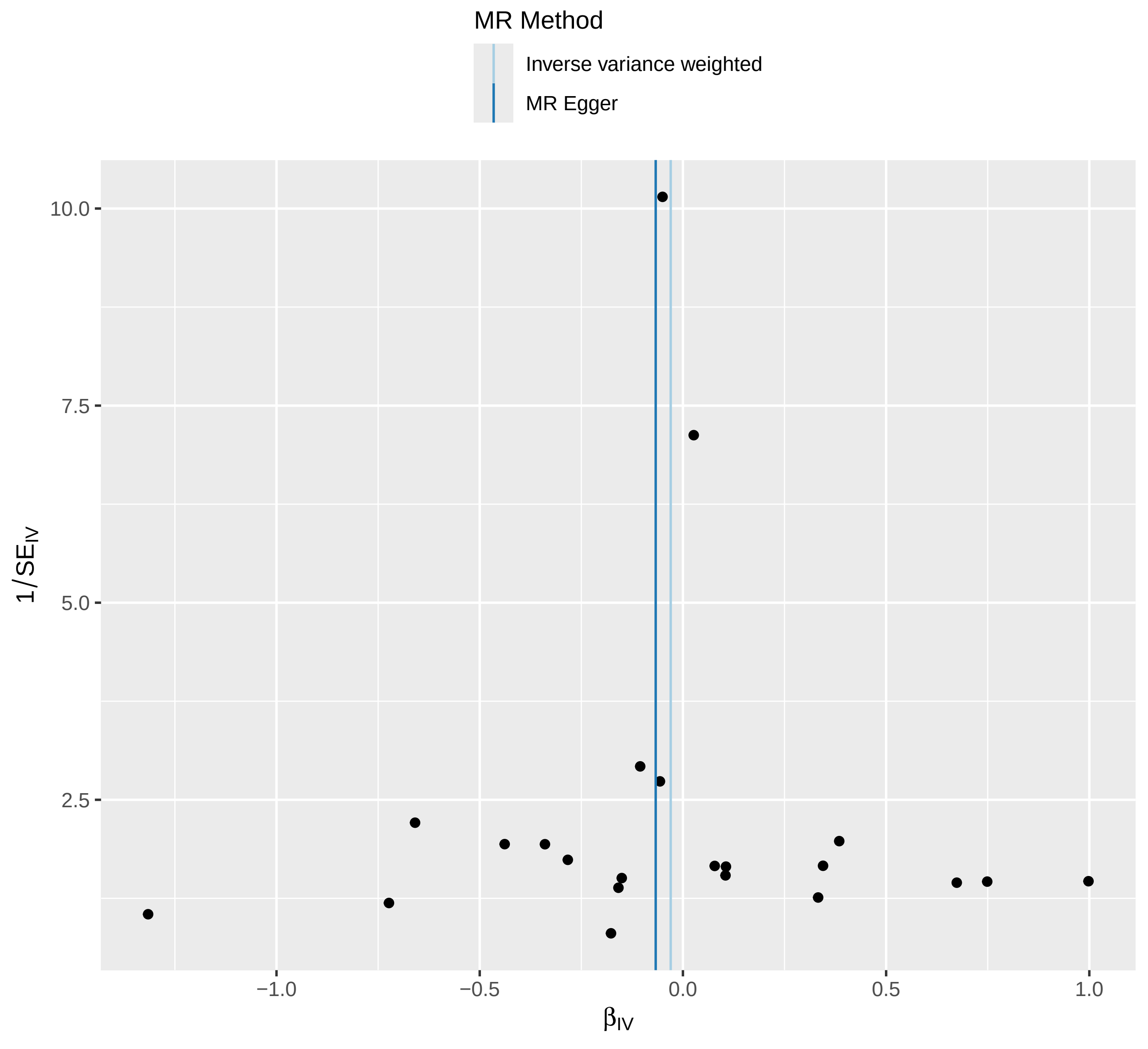

MR Method
Inverse variance weighted
MR Egger
10.0
7.5
5.0
2.5
$1/SE_{IV}$
−1.0
−0.5
0.0
0.5
1.0
β_{IV}

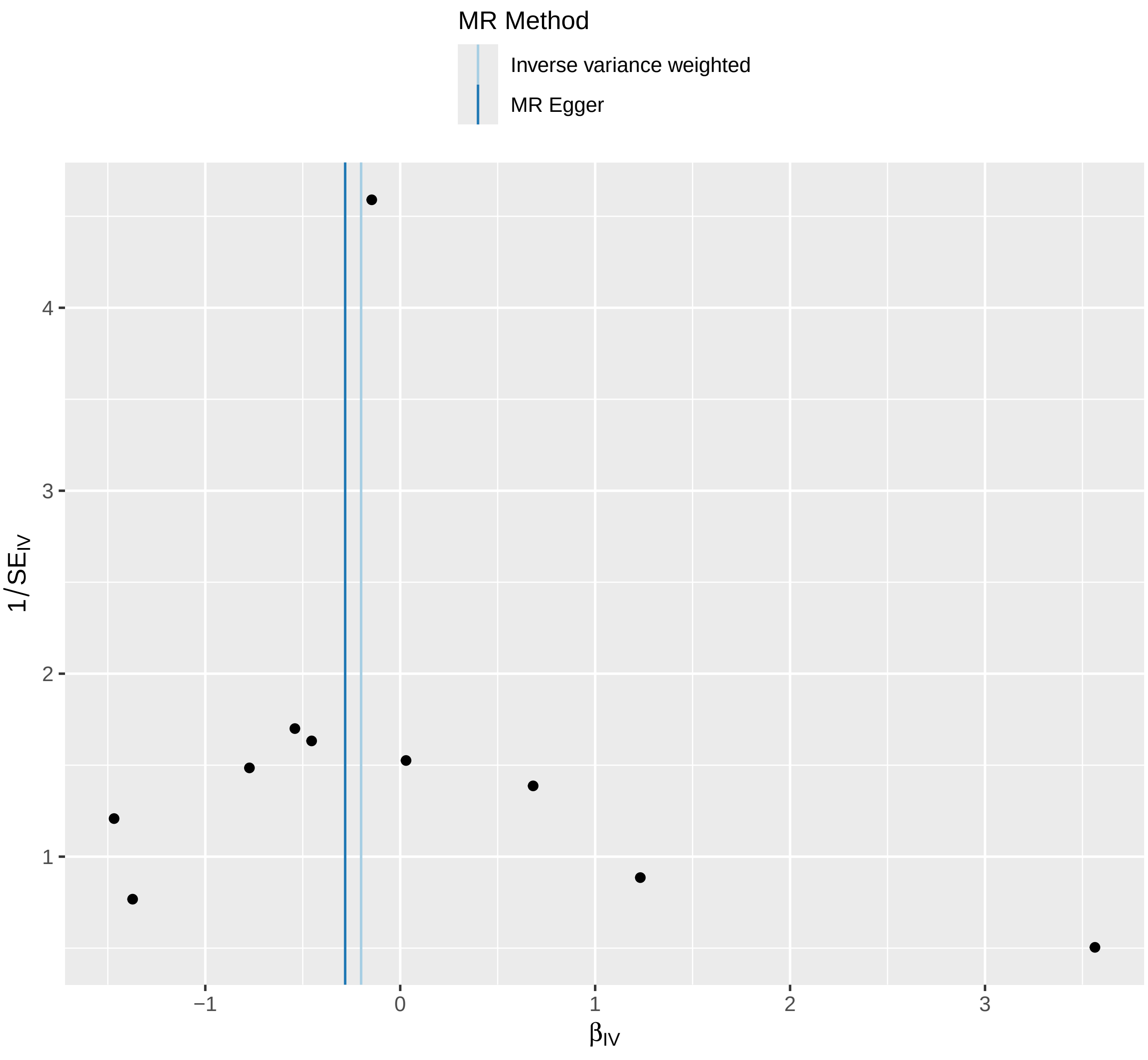
MR Method
Inverse variance weighted
MR Egger
1/SE_IV
β_IV
4
3
2
1
−1
0
1
2
3

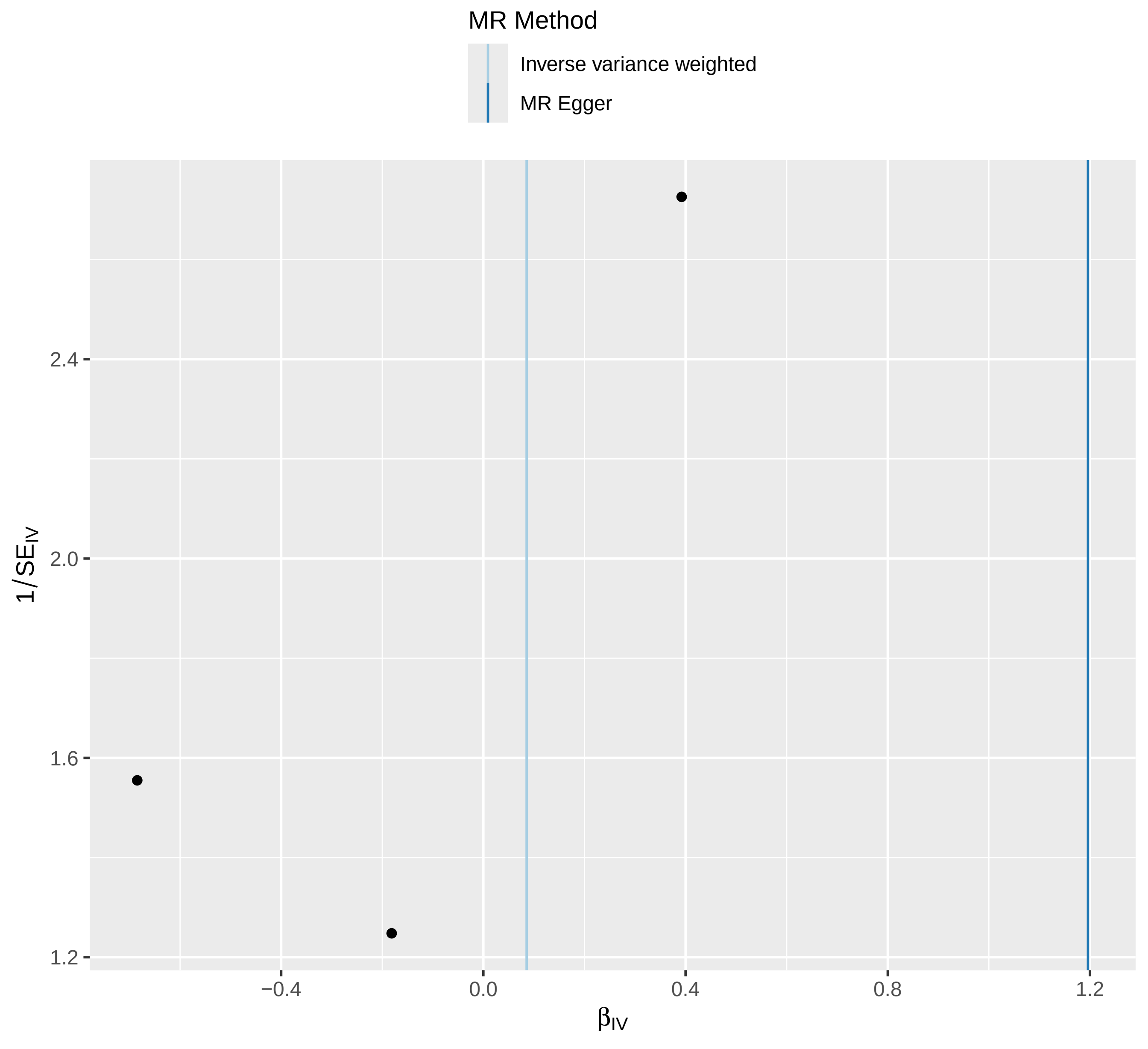

MR Method
Inverse variance weighted
MR Egger
1/SE_IV
2.4
2.0
1.6
1.2
−0.4
0.0
0.4
0.8
1.2
β_IV

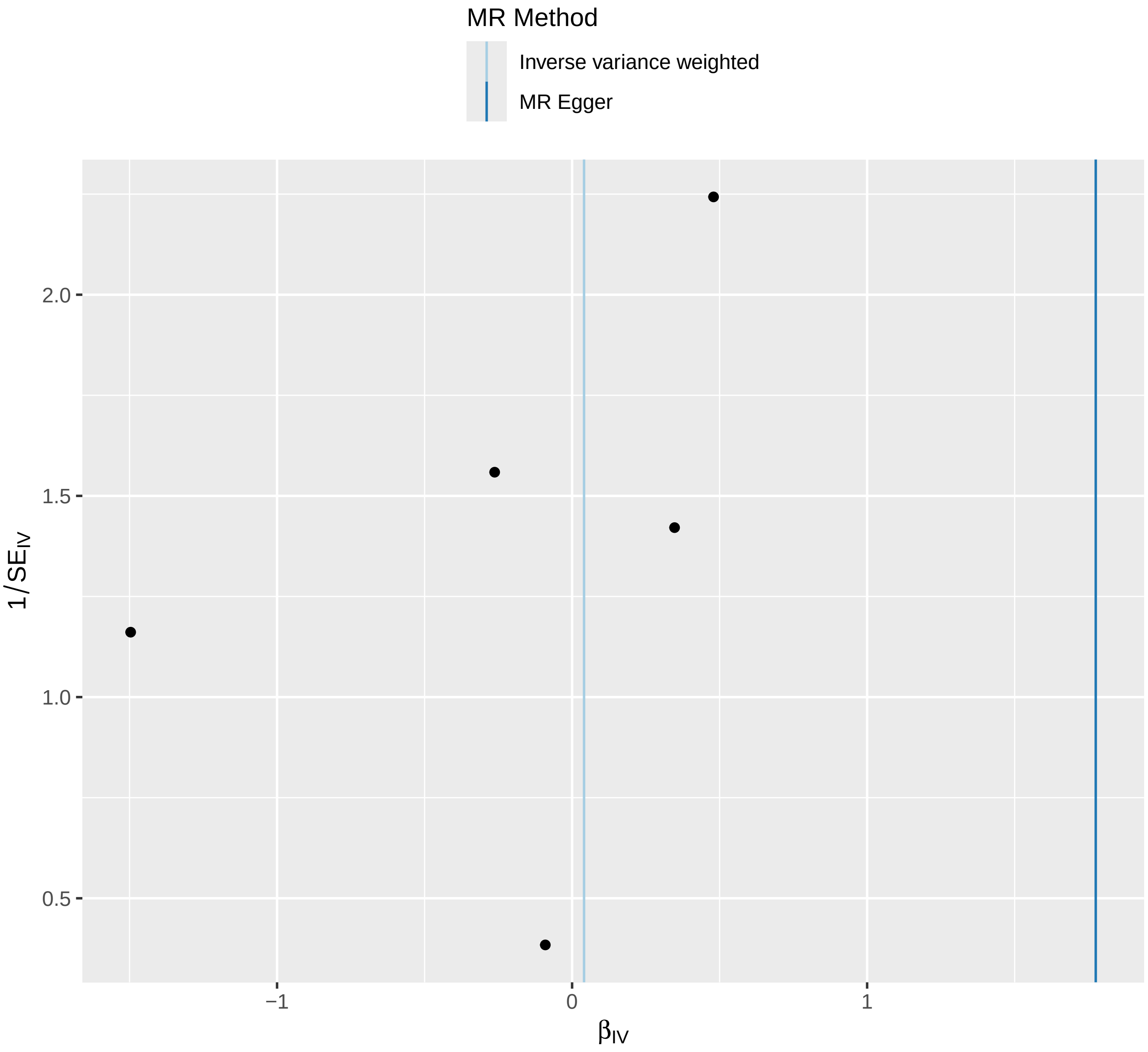
MR Method
Inverse variance weighted
MR Egger
2.0
1.5
1.0
0.5
−1
0
1
$1/SE_{IV}$
β_{IV}

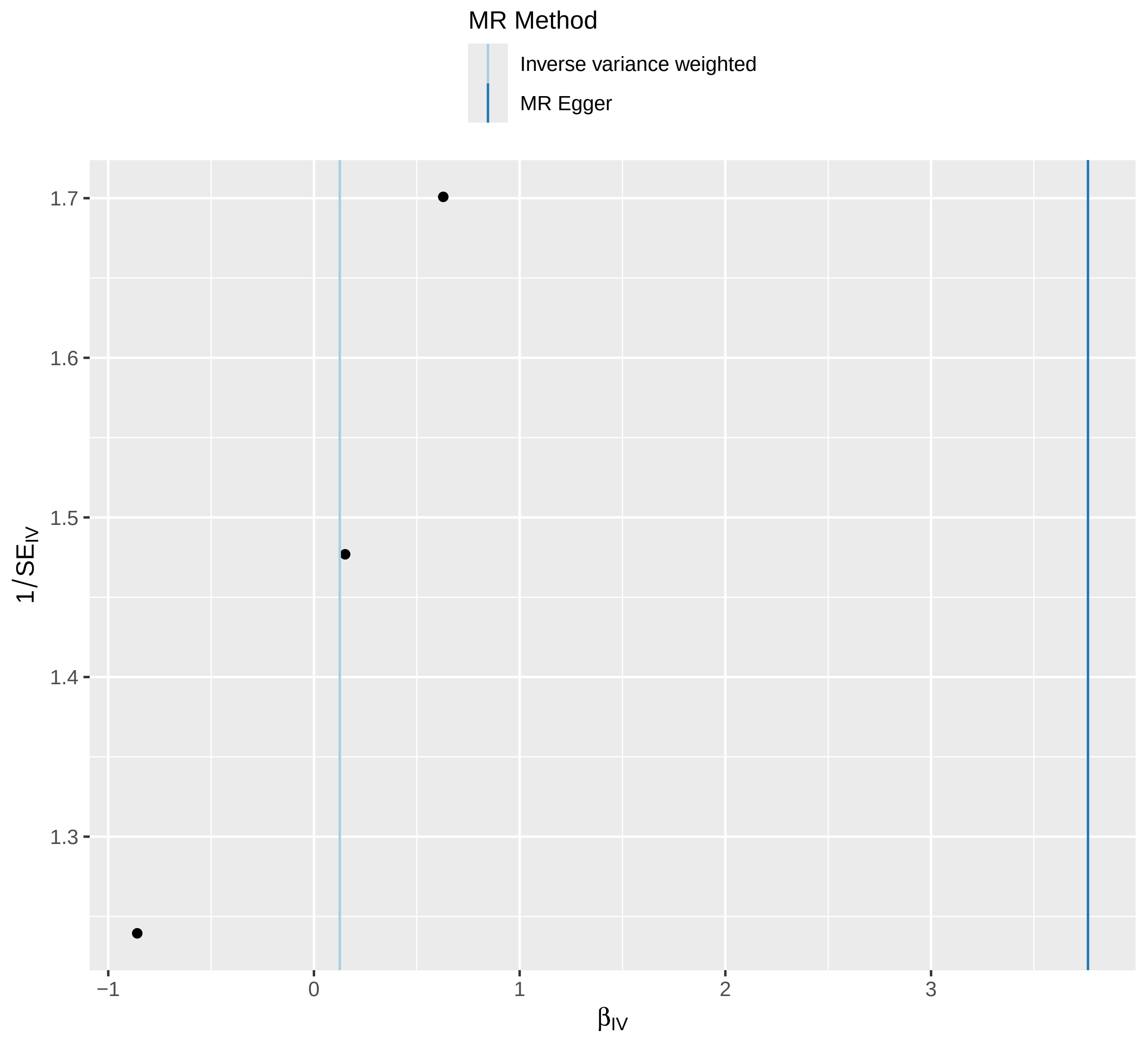
MR Method
Inverse variance weighted
MR Egger
1.7
1.6
1.5
1.4
1.3
$1/SE_{IV}$
−1
0
1
2
3
β_{IV}

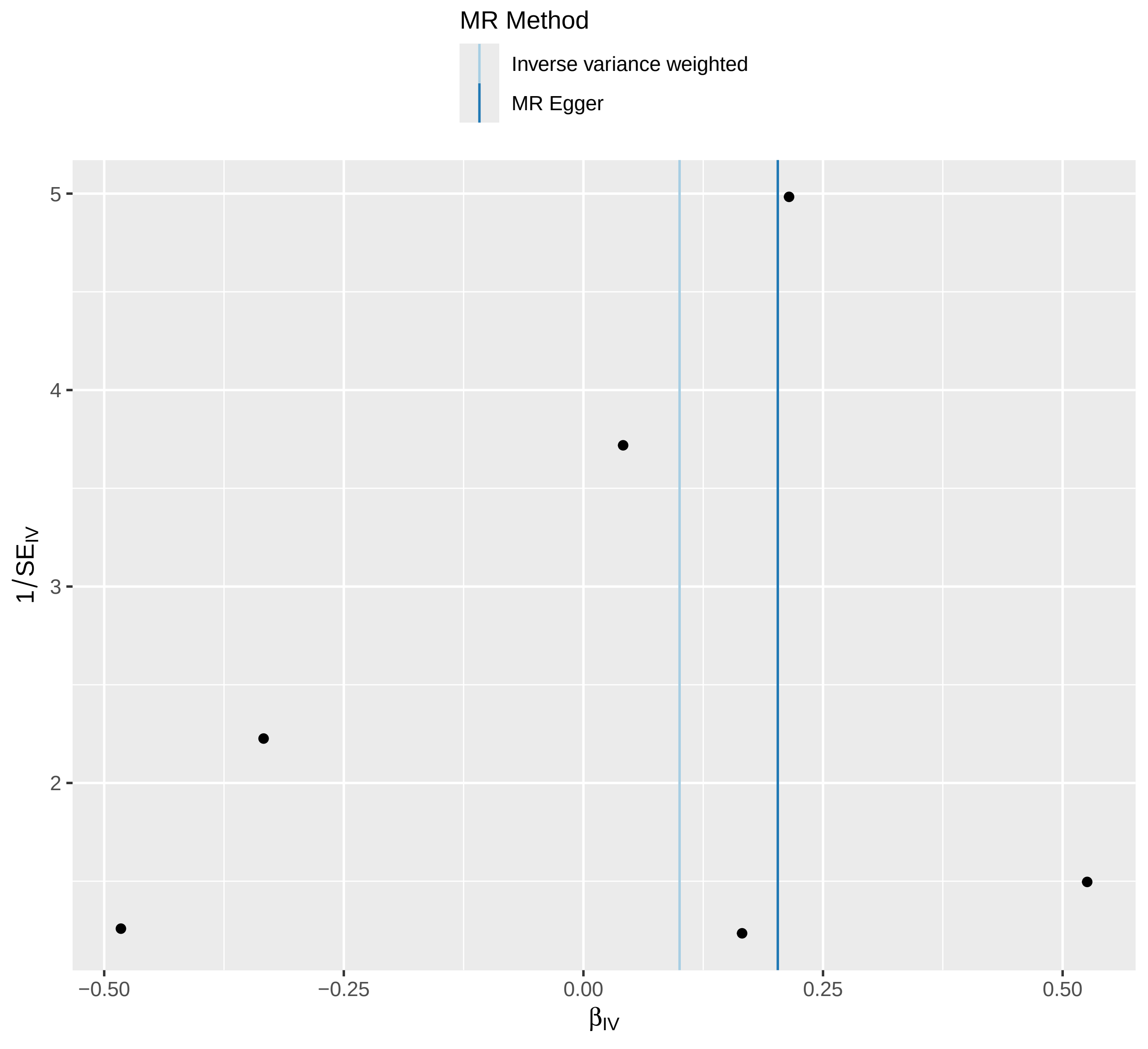
MR Method
Inverse variance weighted
MR Egger
5
4
3
2
1/SE$_{IV}$
−0.50
−0.25
0.00
0.25
0.50
β$_{IV}$

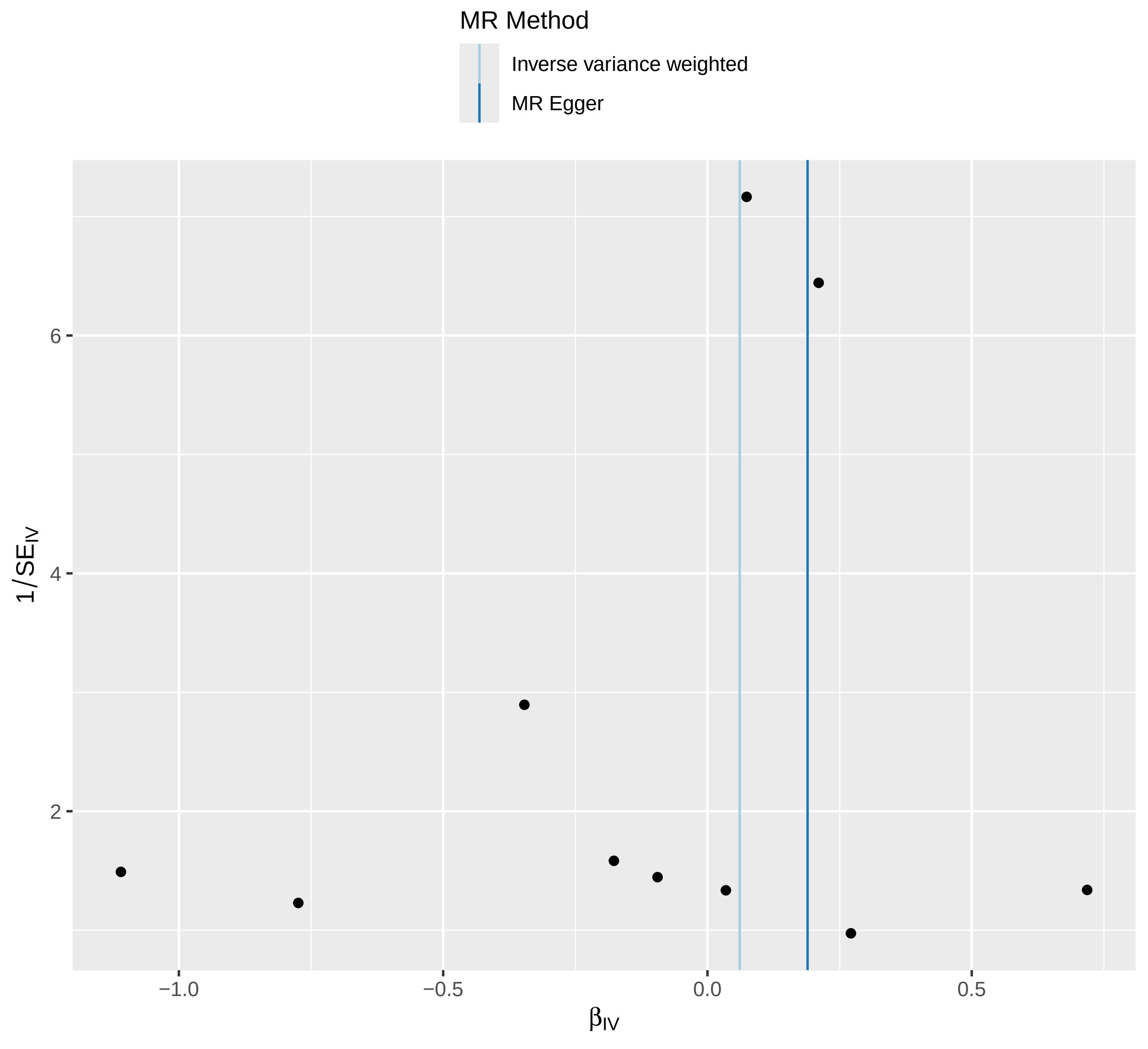

MR Method
Inverse variance weighted
MR Egger
6
4
2
1/SE_IV
−1.0
−0.5
0.0
0.5
β_IV

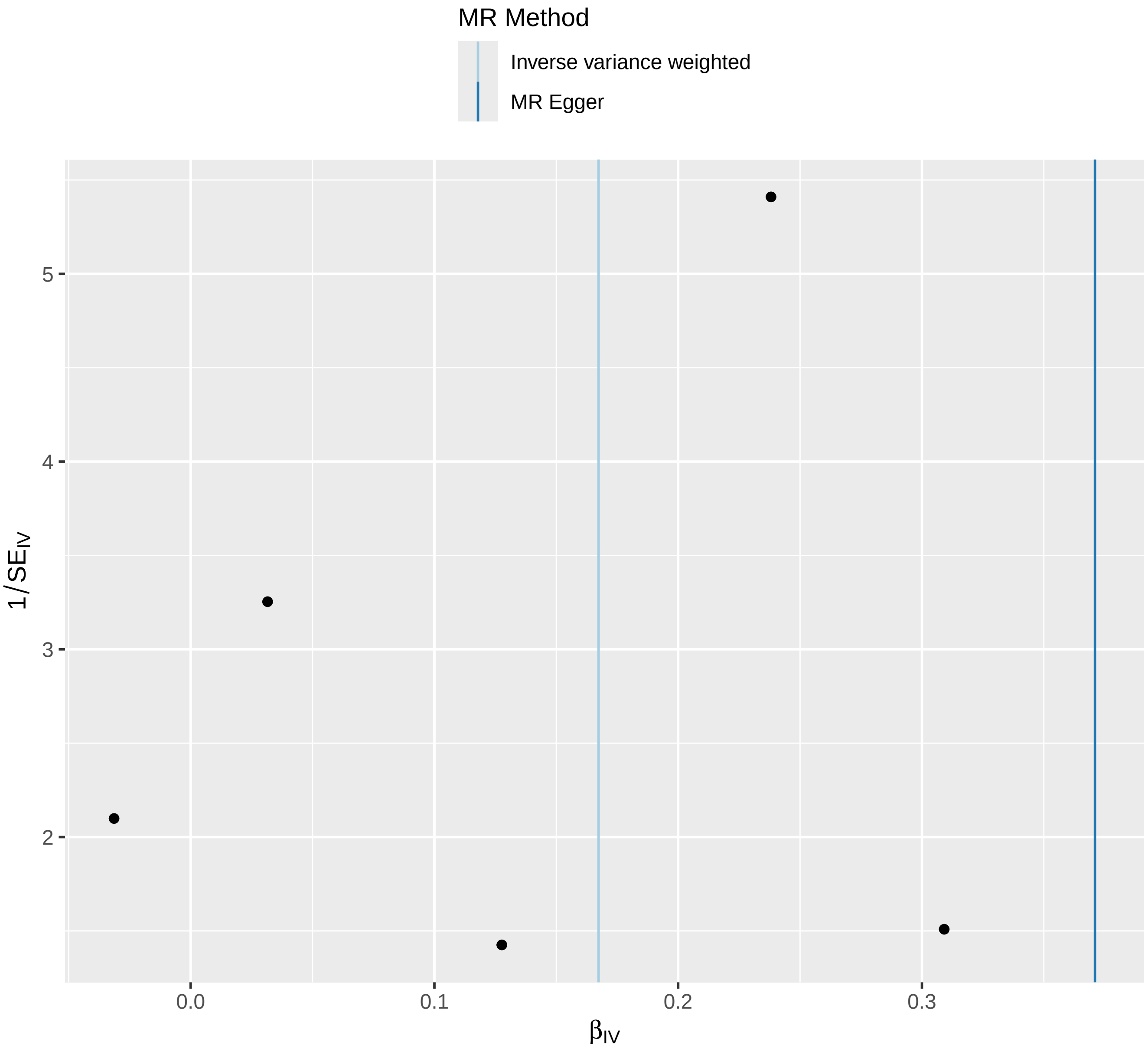
MR Method
Inverse variance weighted
MR Egger
5
4
3
2
1/SE$_{IV}$
0.0
0.1
0.2
0.3
β$_{IV}$

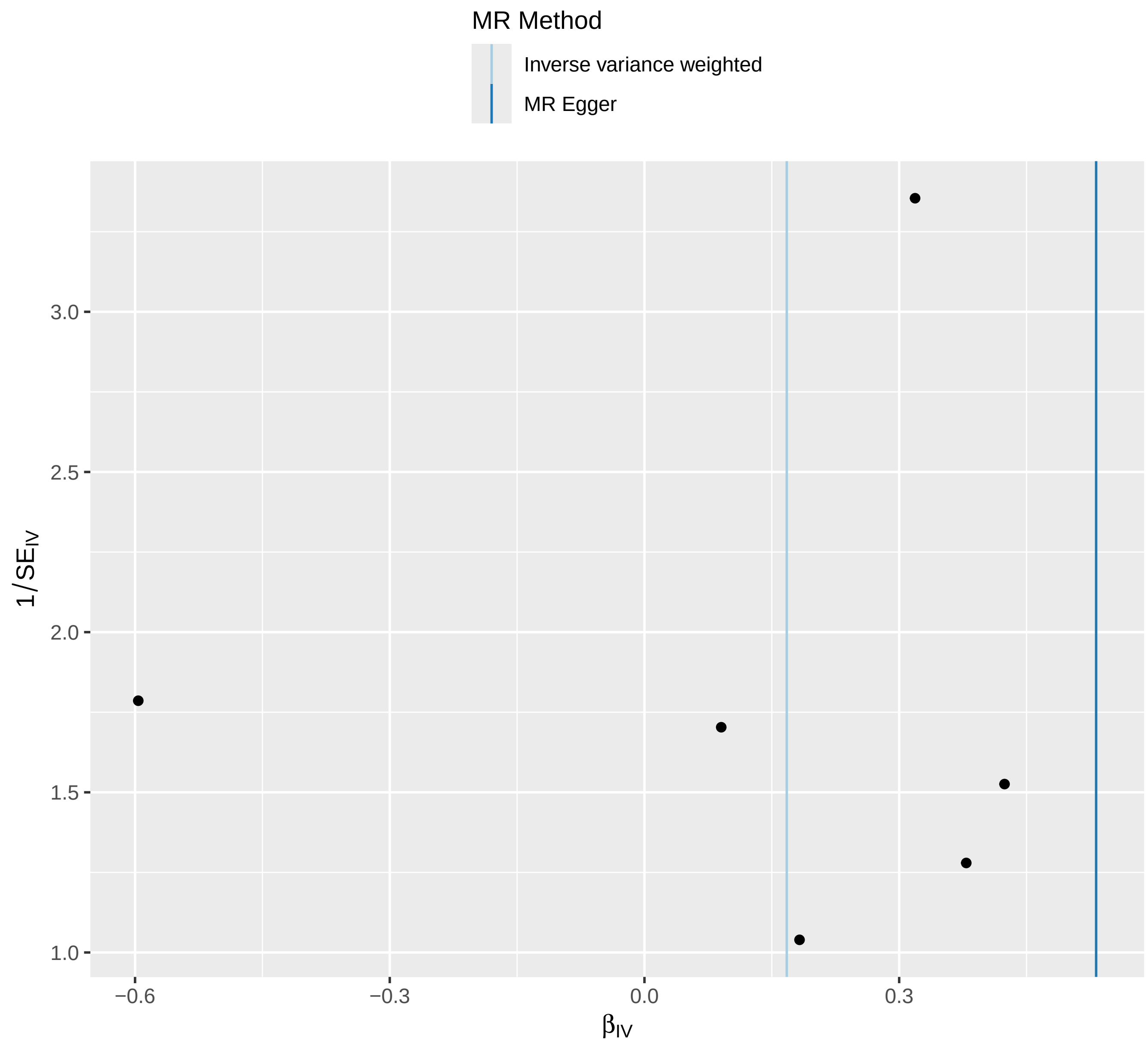
MR Method
Inverse variance weighted
MR Egger
1/SE_{IV}
3.0
2.5
2.0
1.5
1.0
−0.6
−0.3
0.0
0.3
β_{IV}

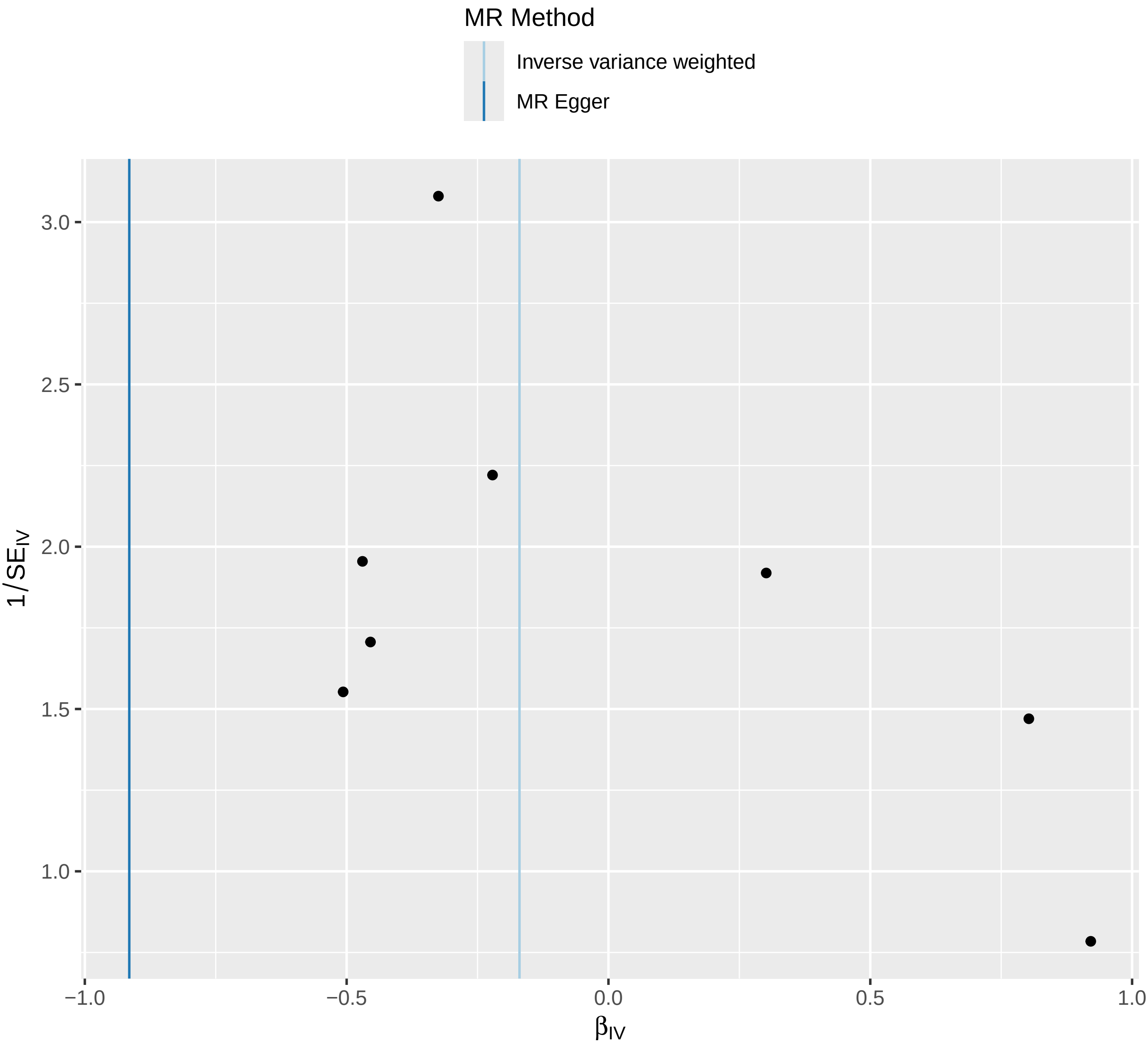

MR Method
Inverse variance weighted
MR Egger
3.0
2.5
2.0
1.5
1.0
1/SE$_{IV}$
−1.0
−0.5
0.0
0.5
1.0
β$_{IV}$

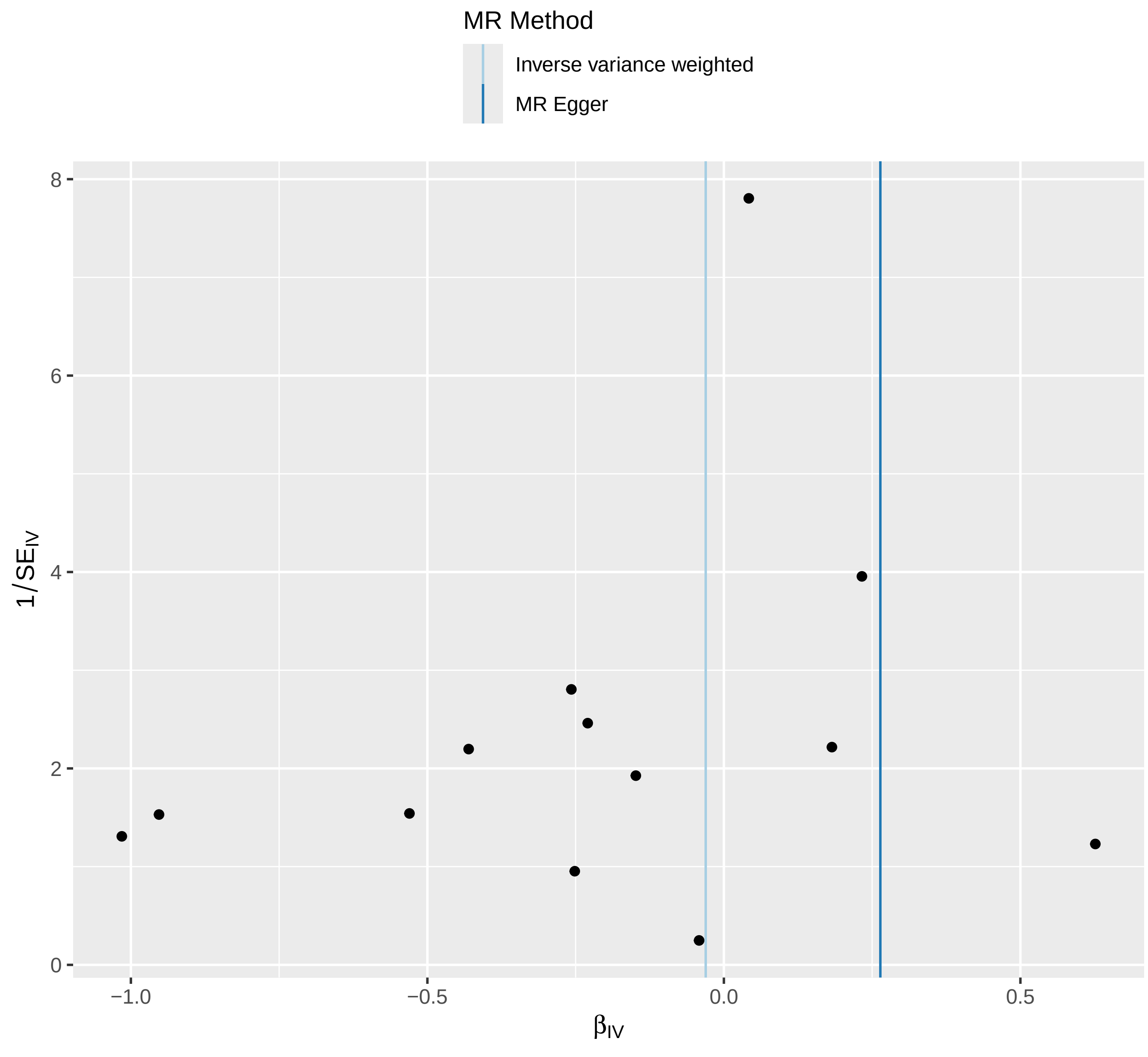
MR Method
Inverse variance weighted
MR Egger
8
6
4
2
0
1/SE_IV
−1.0
−0.5
0.0
0.5
β_IV

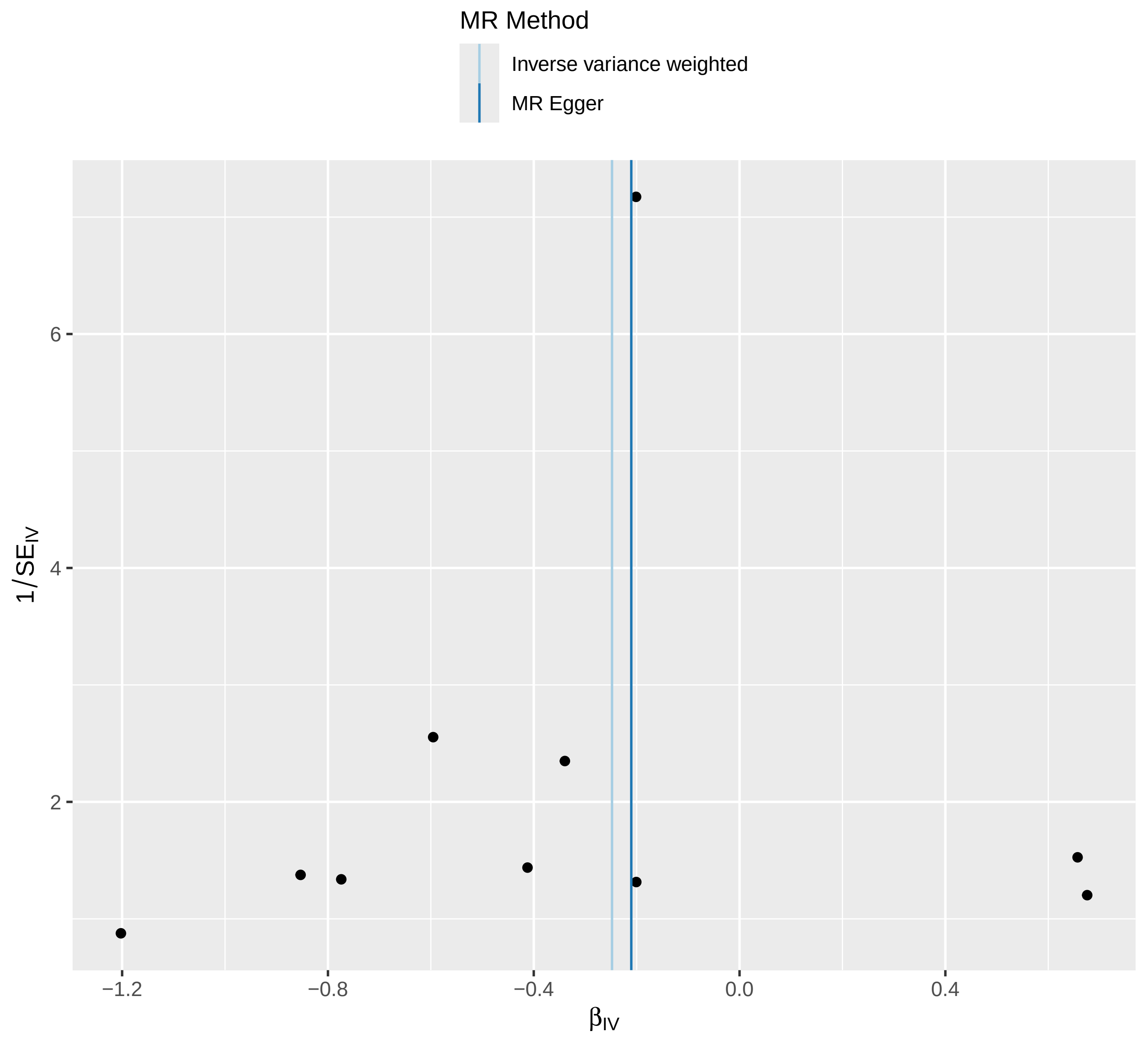
MR Method
Inverse variance weighted
MR Egger
6
4
2
1/SE_IV
−1.2
−0.8
−0.4
0.0
0.4
β_IV

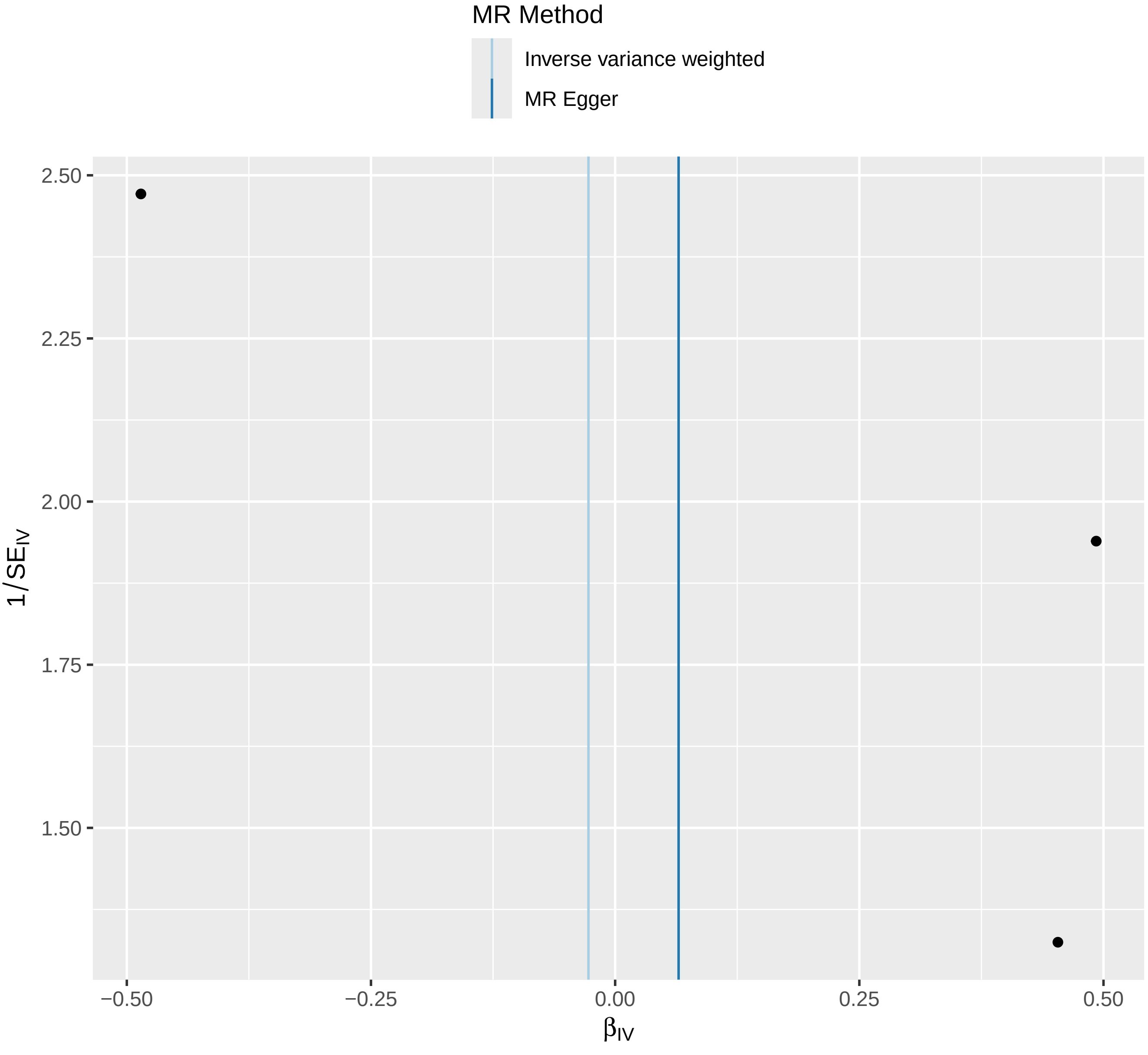
MR Method
Inverse variance weighted
MR Egger
2.50
2.25
2.00
1.75
1.50
1/SE_IV
−0.50
−0.25
0.00
0.25
0.50
β_IV

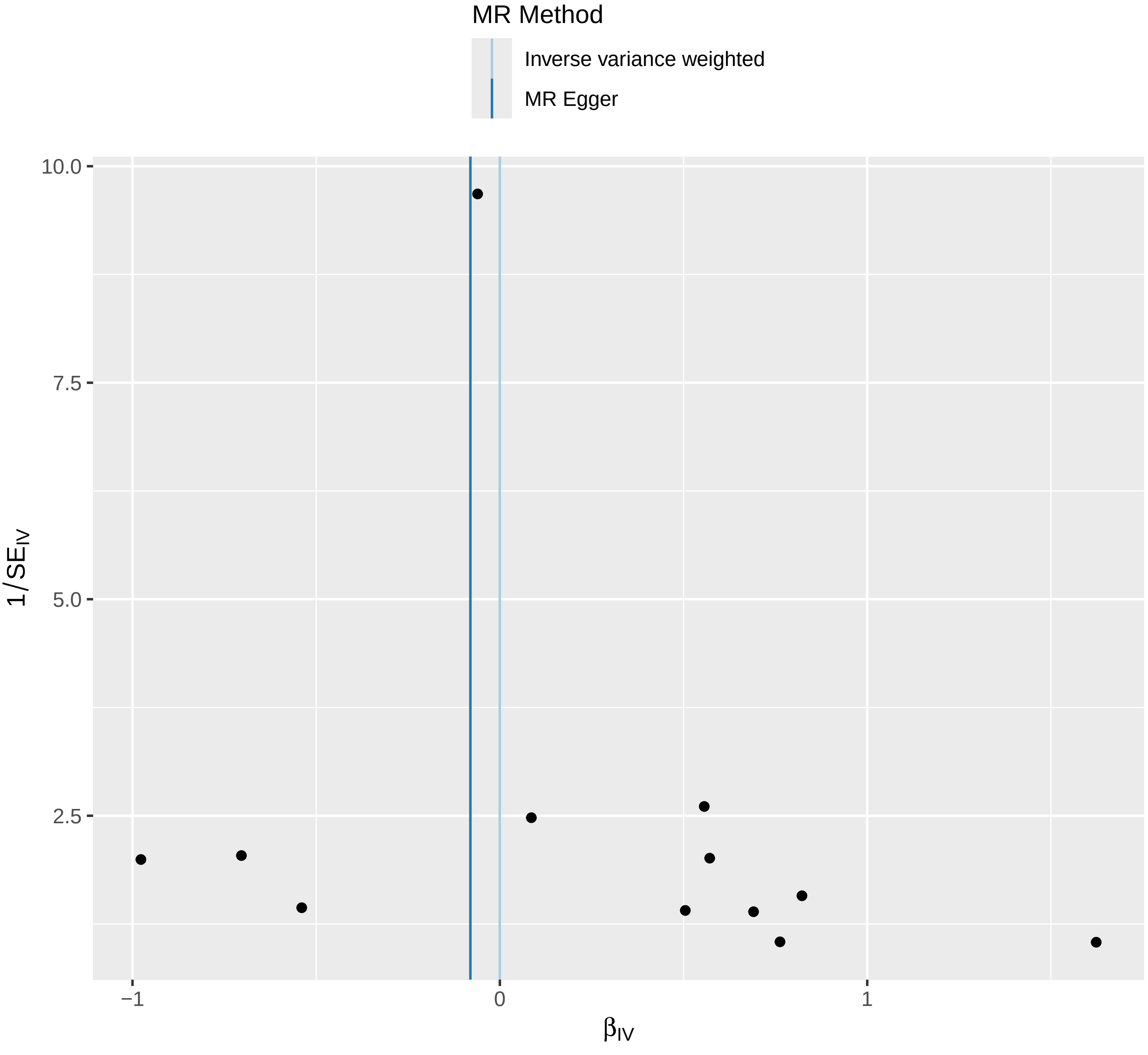
MR Method
Inverse variance weighted
MR Egger
10.0
7.5
5.0
2.5
$1/SE_{IV}$
−1
0
1
β_{IV}

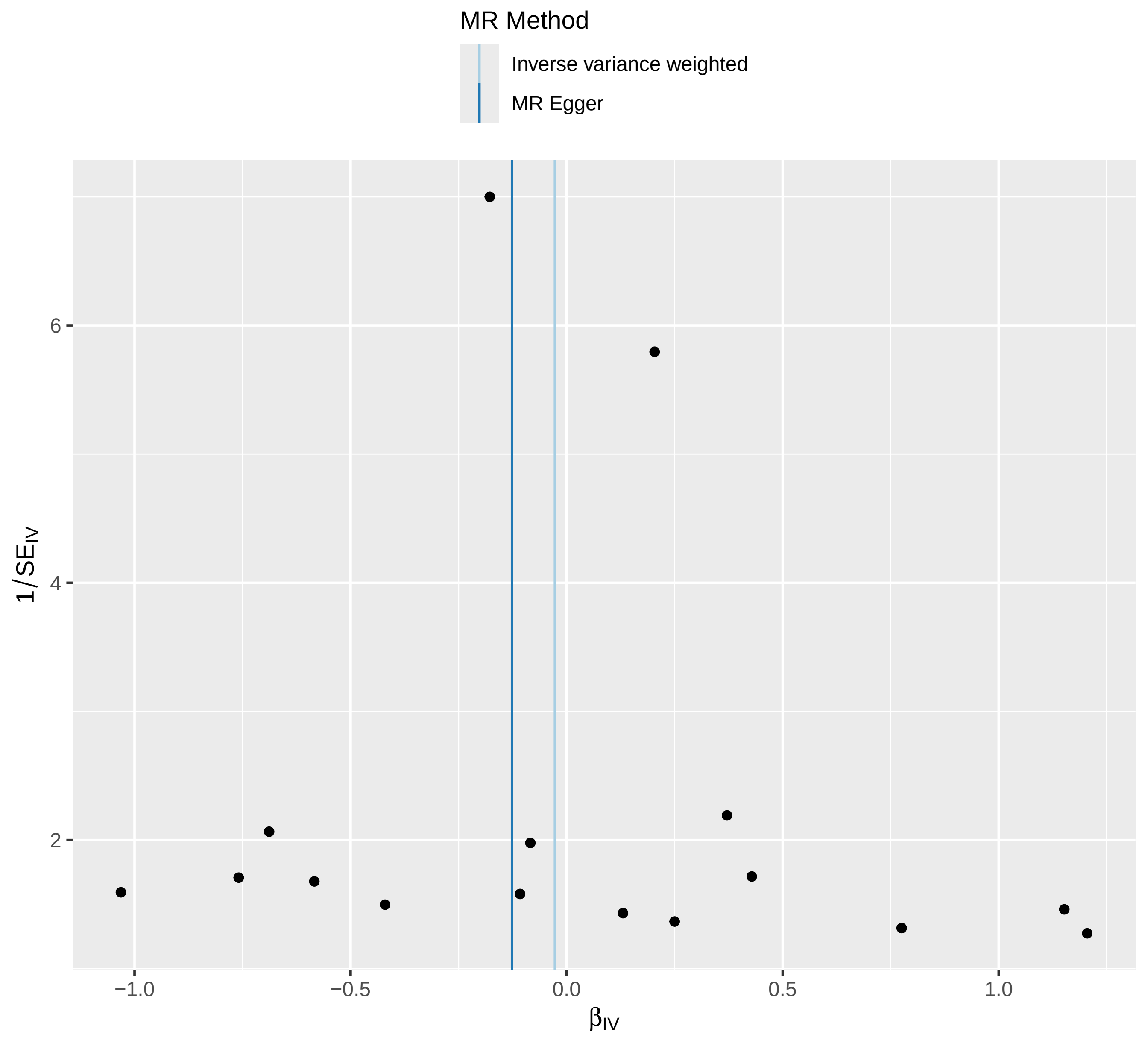
MR Method
Inverse variance weighted
MR Egger
1/SE_IV
6
4
2
−1.0
−0.5
0.0
0.5
1.0
β_IV

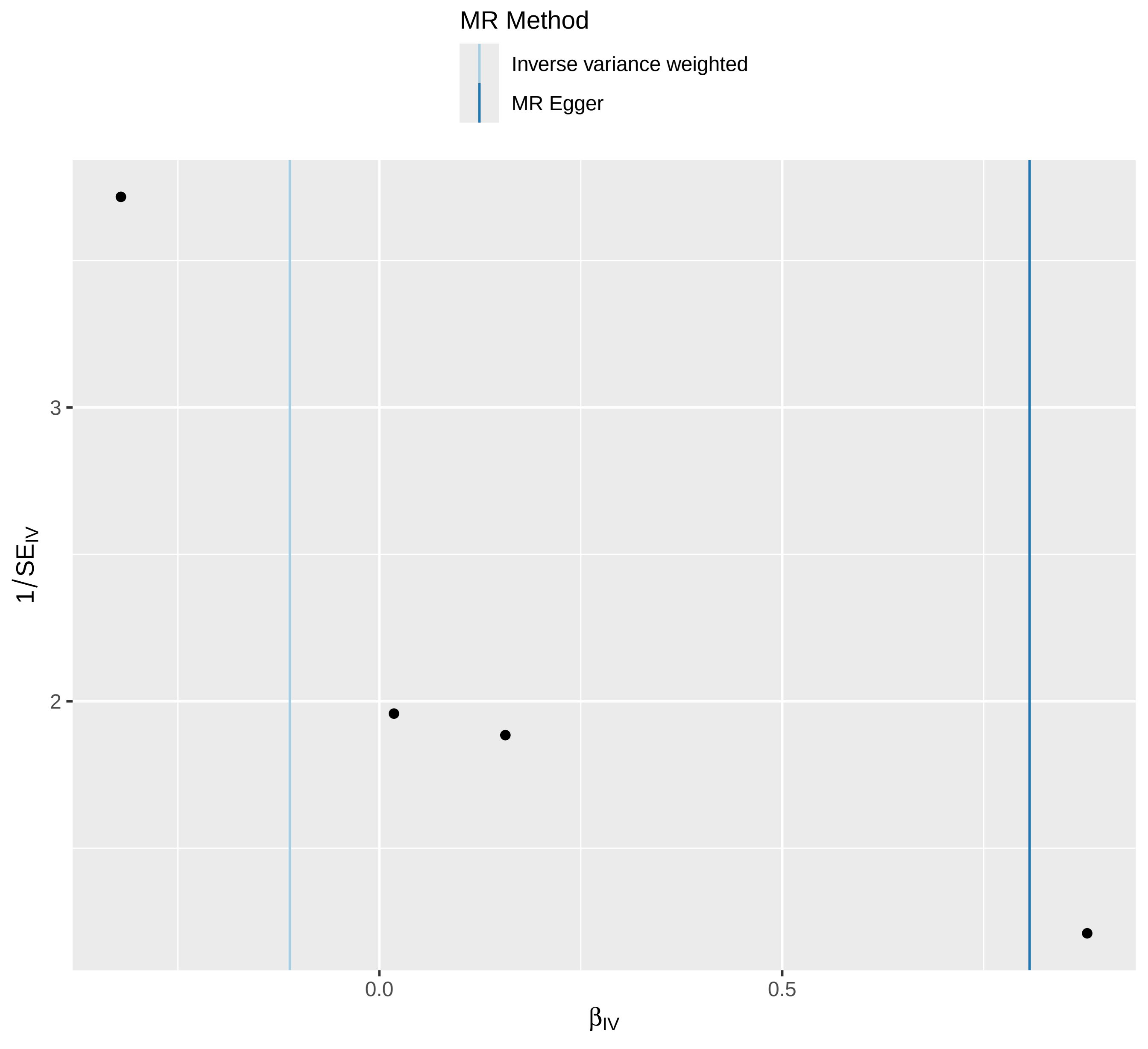
MR Method
Inverse variance weighted
MR Egger
3
2
0.0
0.5
1/SE_IV
β_IV

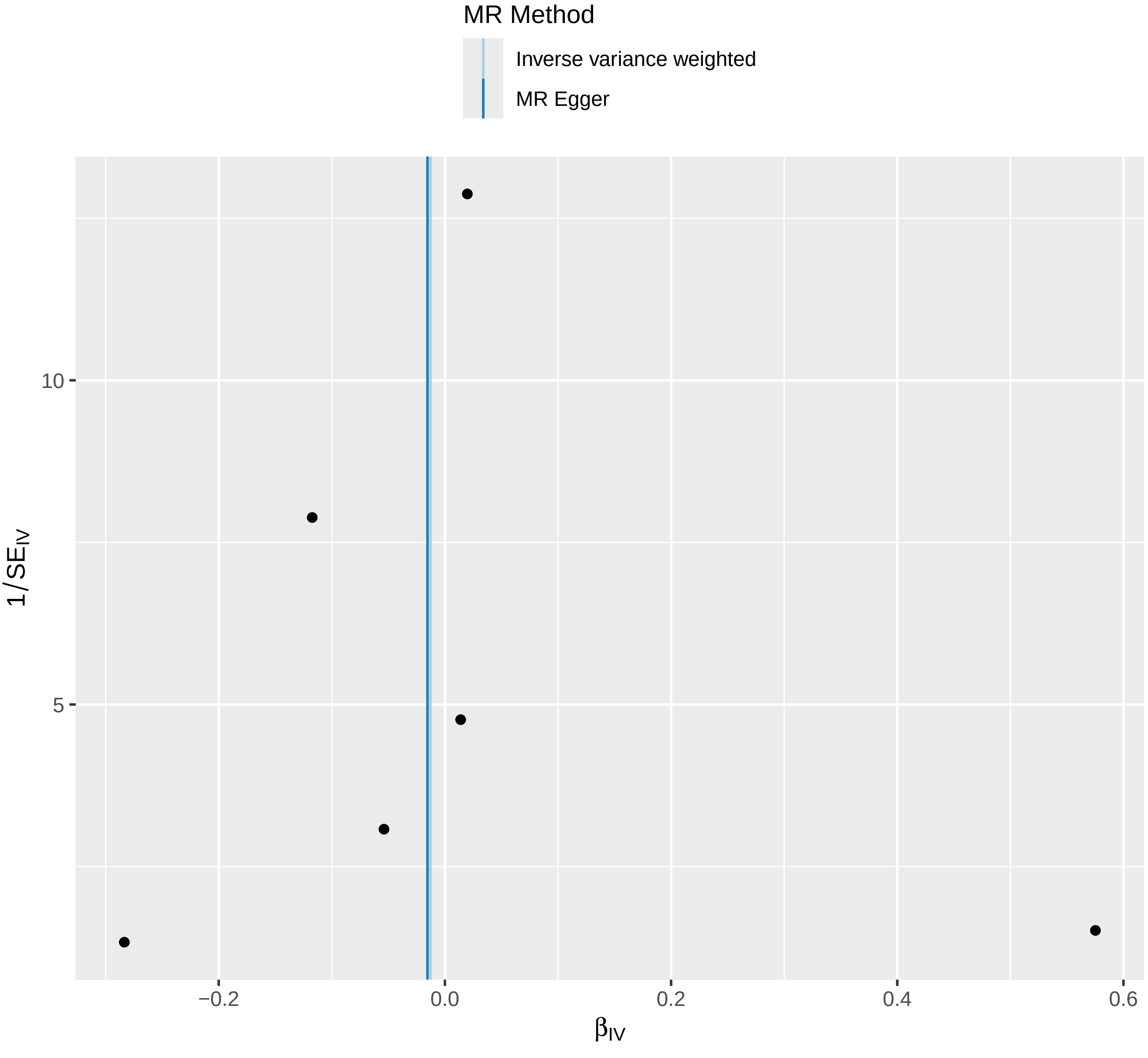

MR Method
Inverse variance weighted
MR Egger
10
5
1/SE_IV
−0.2
0.0
0.2
0.4
0.6
β_IV

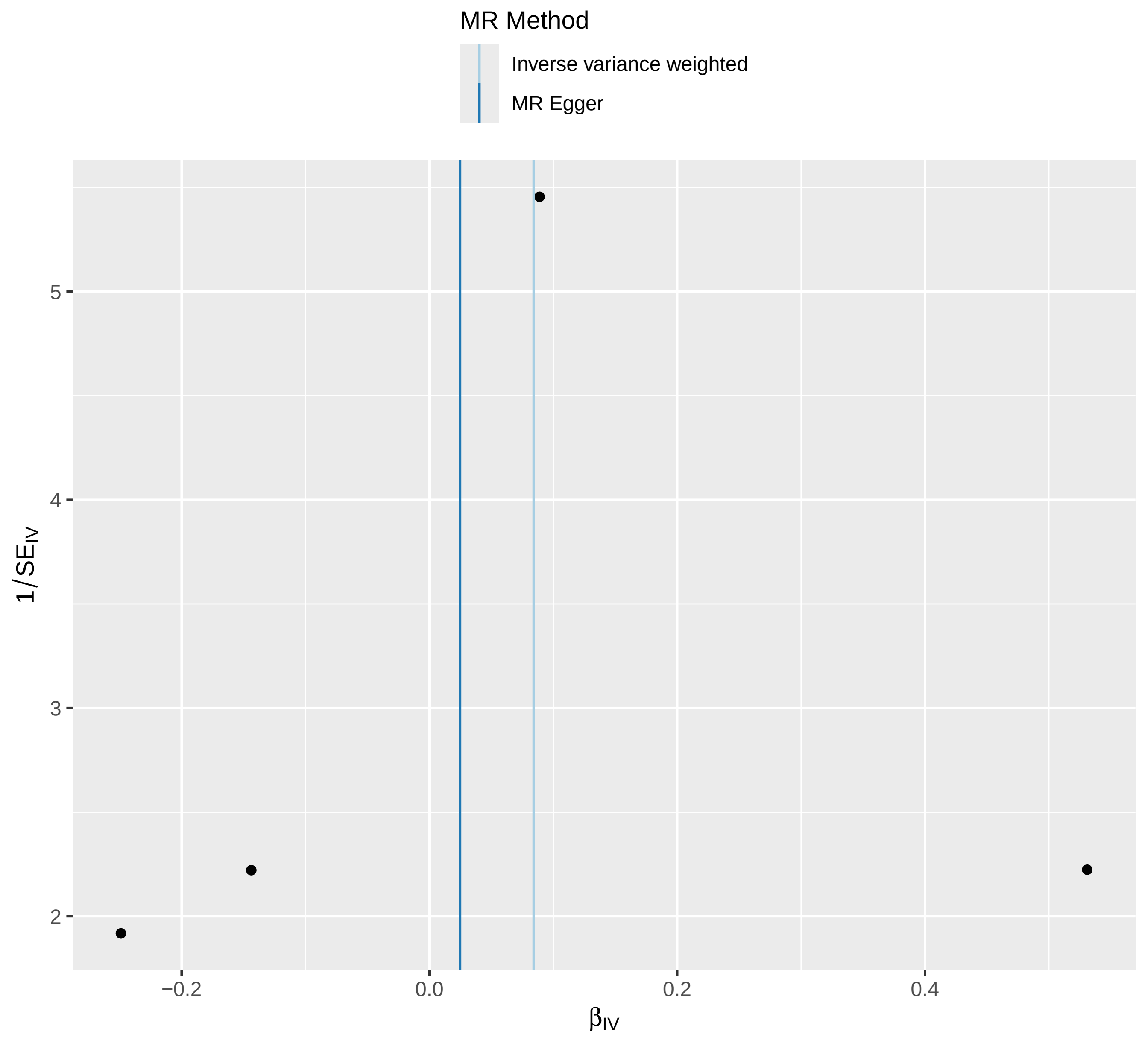

MR Method
Inverse variance weighted
MR Egger
5
4
3
2
1/SE$_{IV}$
−0.2
0.0
0.2
0.4
β$_{IV}$

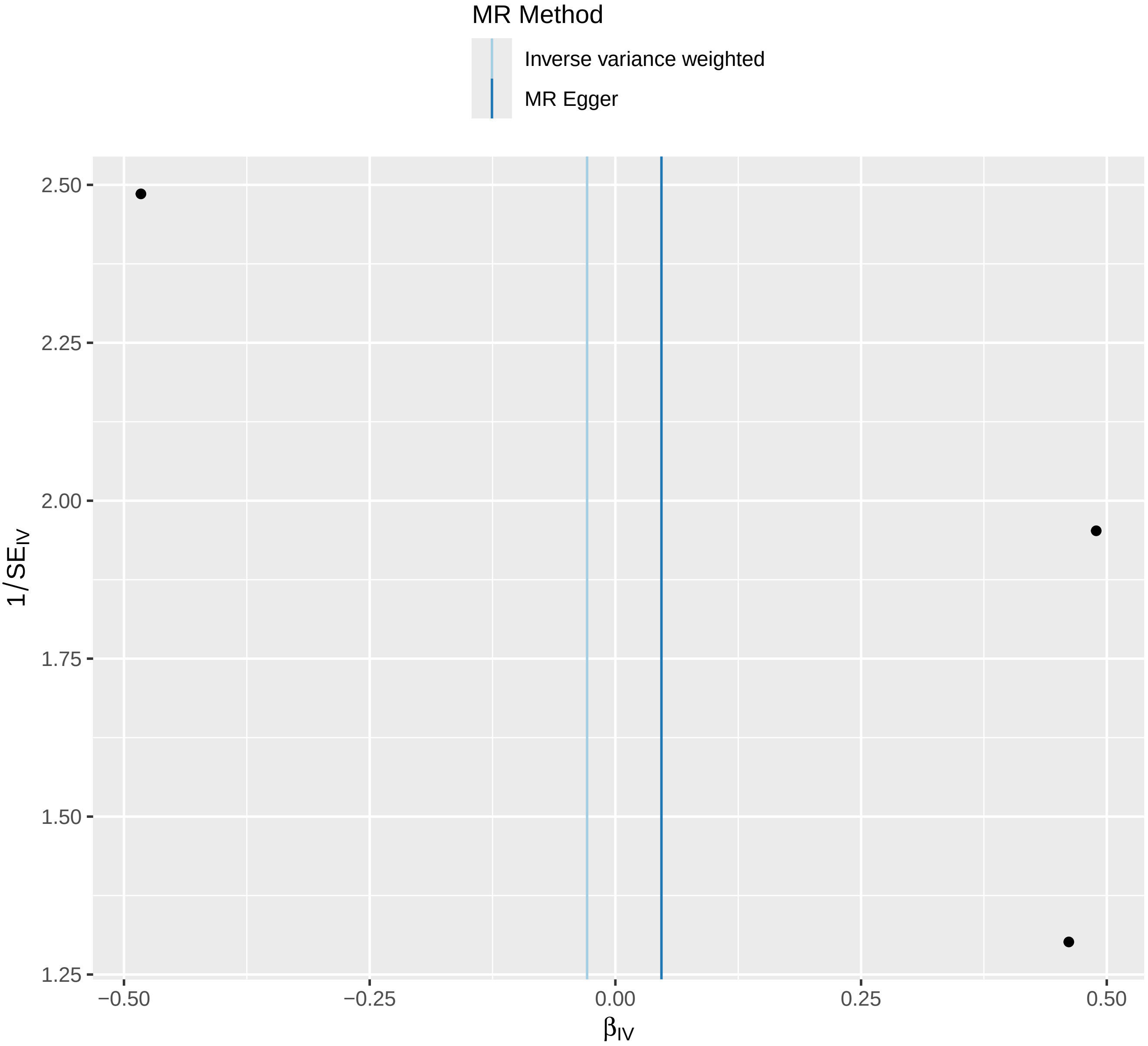
MR Method
Inverse variance weighted
MR Egger
2.50
2.25
2.00
1.75
1.50
1.25
−0.50
−0.25
0.00
0.25
0.50
1/SE_IV
β_IV

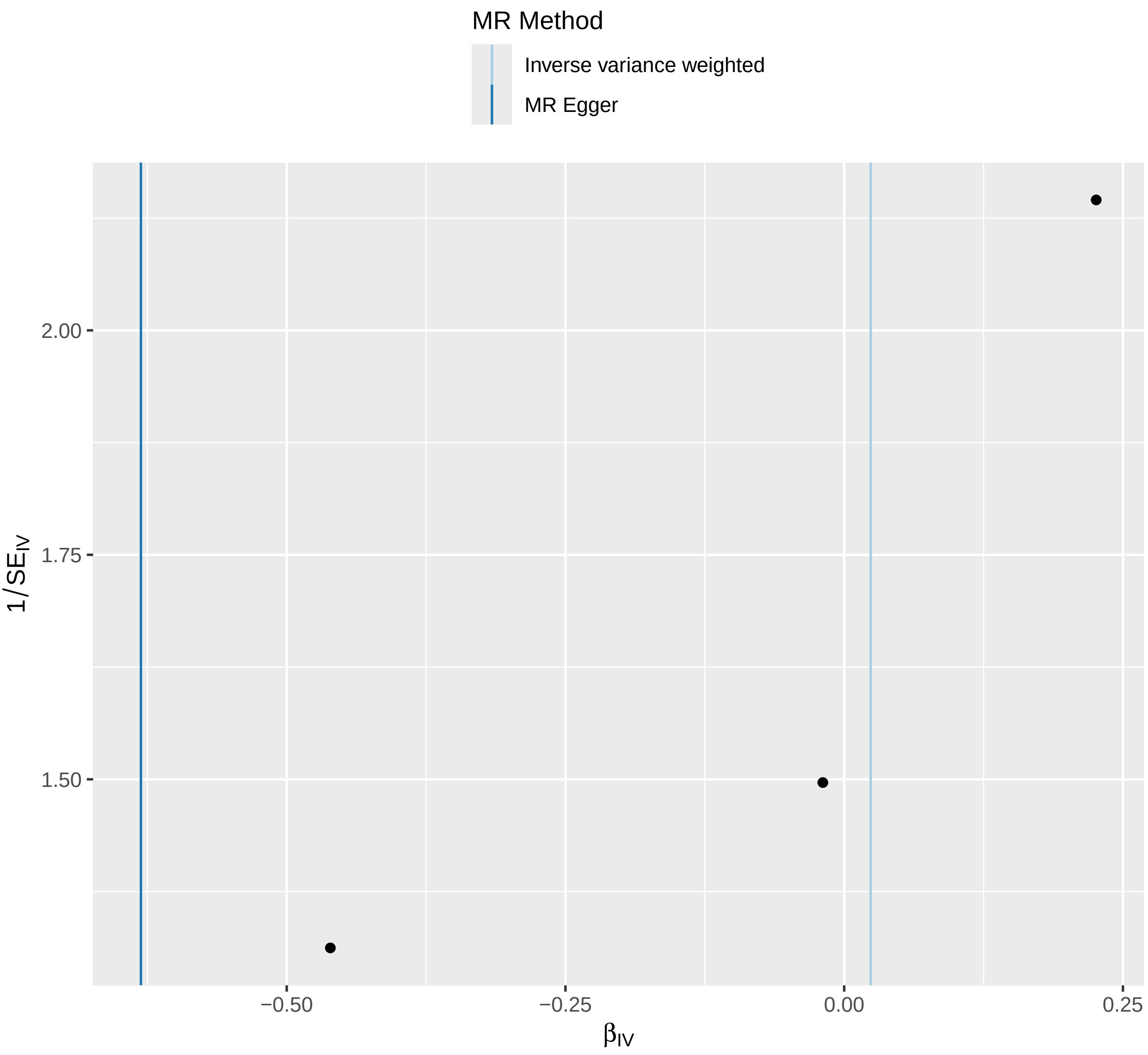

MR Method
Inverse variance weighted
MR Egger
2.00
1.75
1.50
$1/SE_{IV}$
−0.50
−0.25
0.00
0.25
β_{IV}

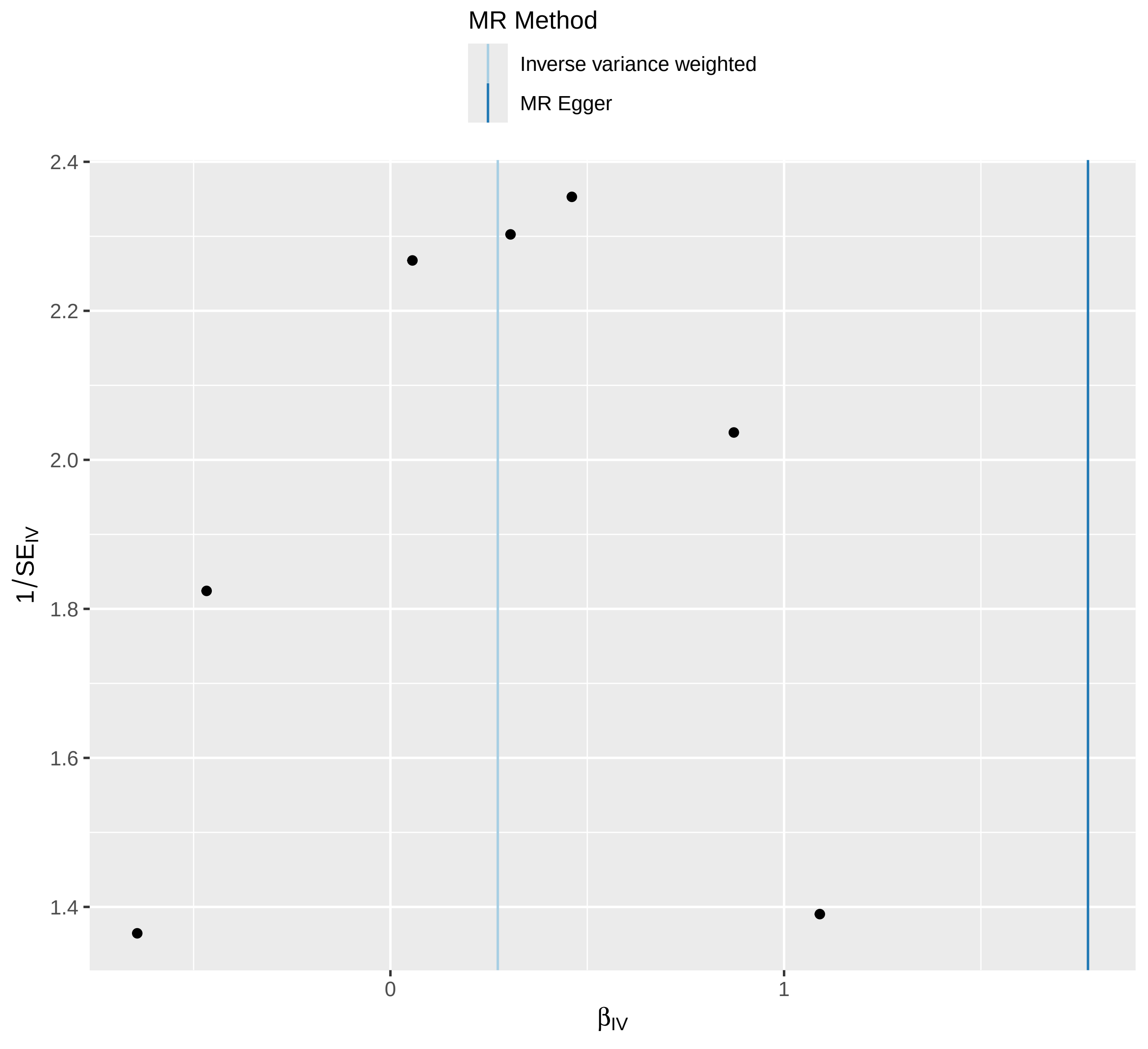
MR Method
Inverse variance weighted
MR Egger
2.4
2.2
2.0
1.8
1.6
1.4
1/SE_IV
0
1
β_IV

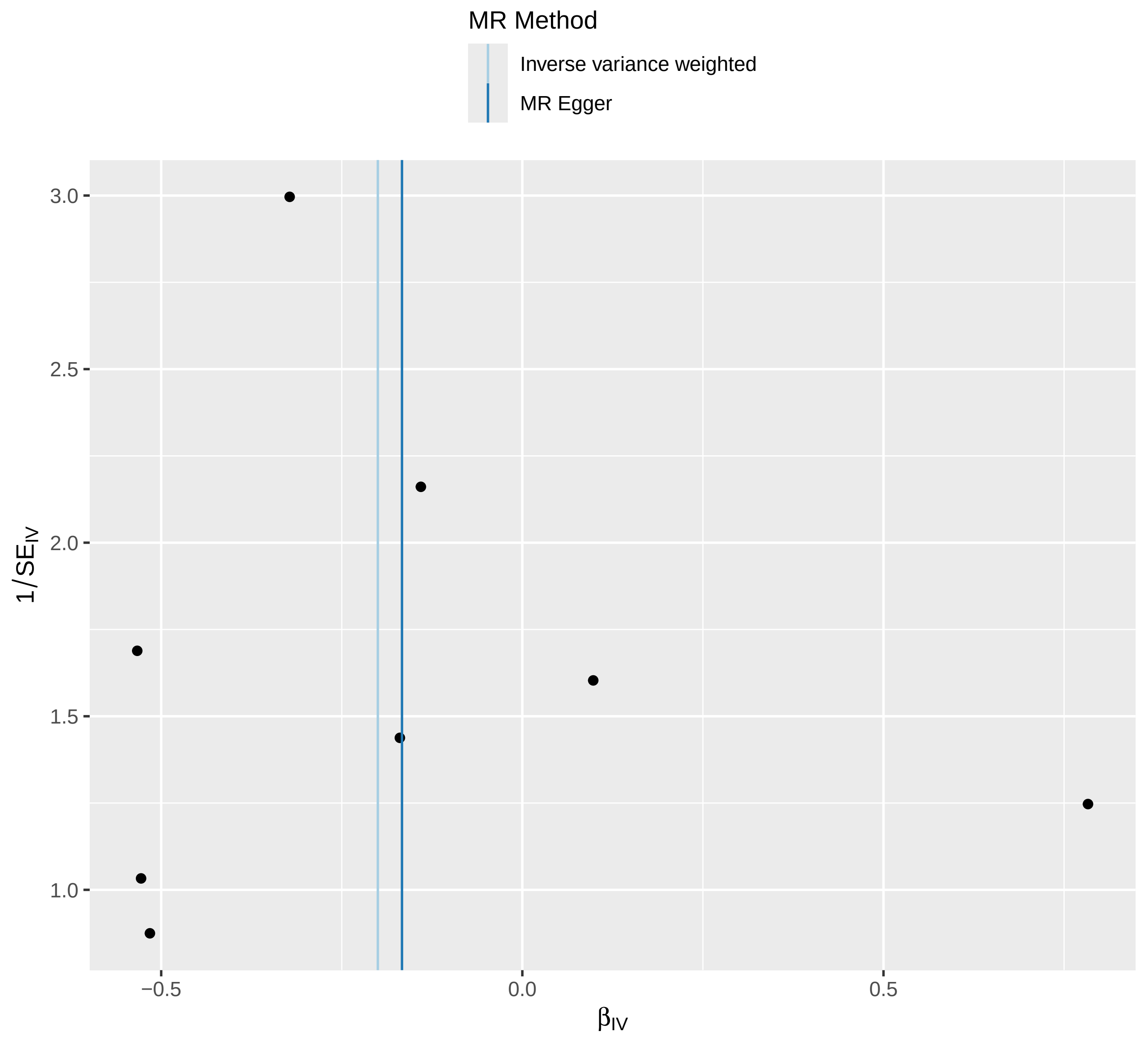
MR Method
Inverse variance weighted
MR Egger
3.0
2.5
2.0
1.5
1.0
1/SE_IV
−0.5
0.0
0.5
β_IV

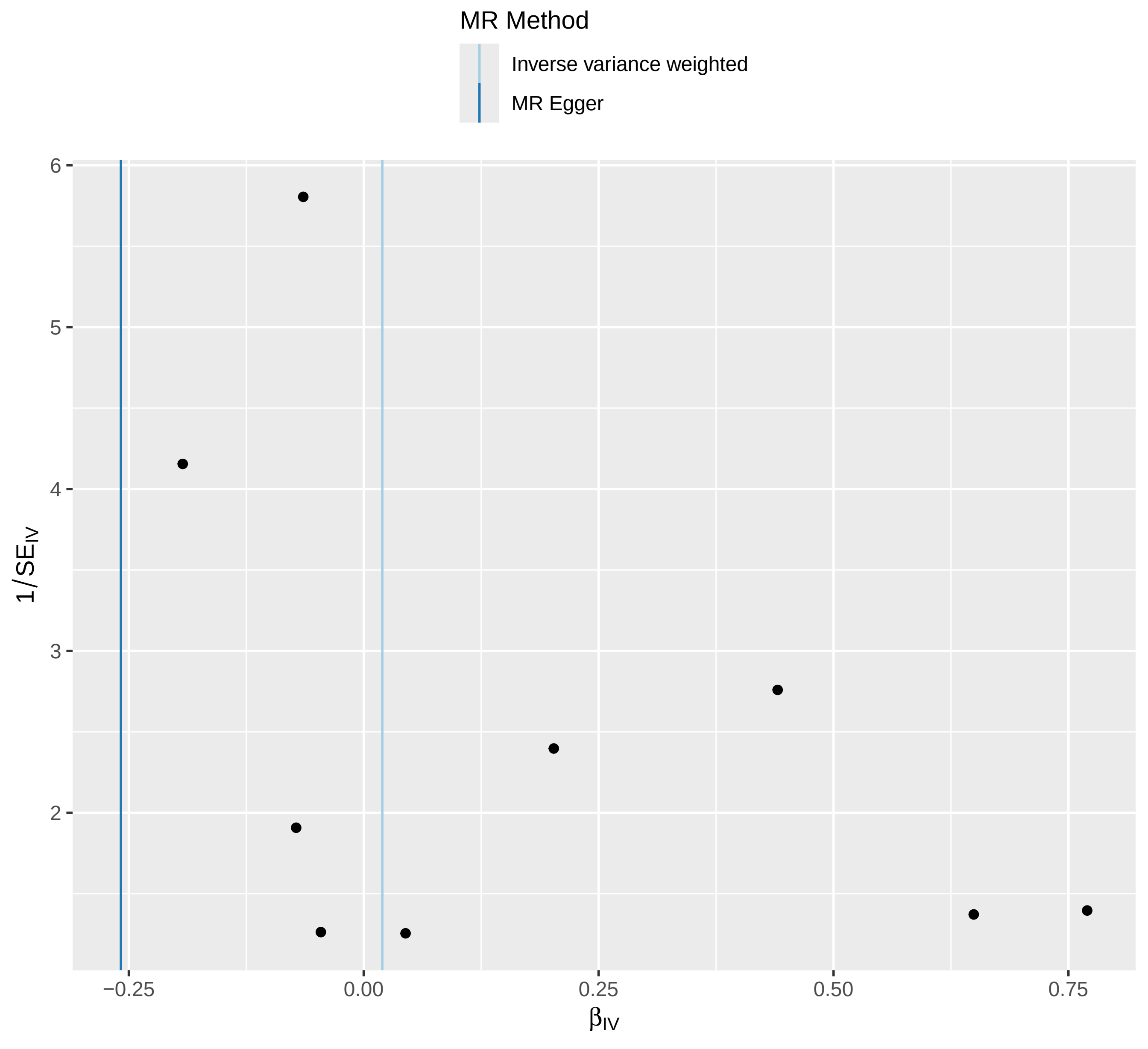

MR Method
Inverse variance weighted
MR Egger
6
5
4
3
2
$1/SE_{IV}$
−0.25
0.00
0.25
0.50
0.75
β_{IV}

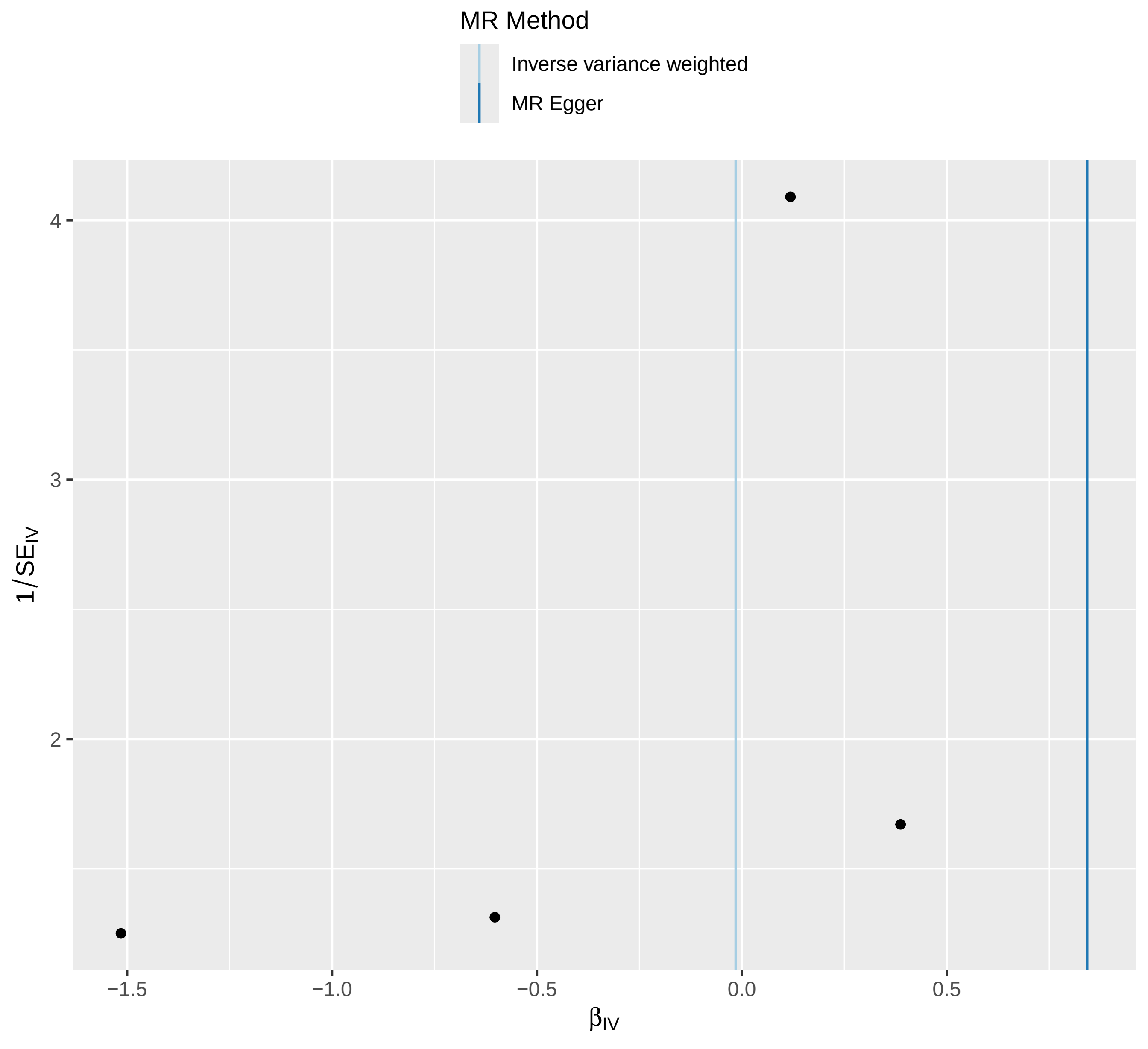
MR Method
Inverse variance weighted
MR Egger
4
3
2
1/SE_IV
−1.5
−1.0
−0.5
0.0
0.5
β_IV

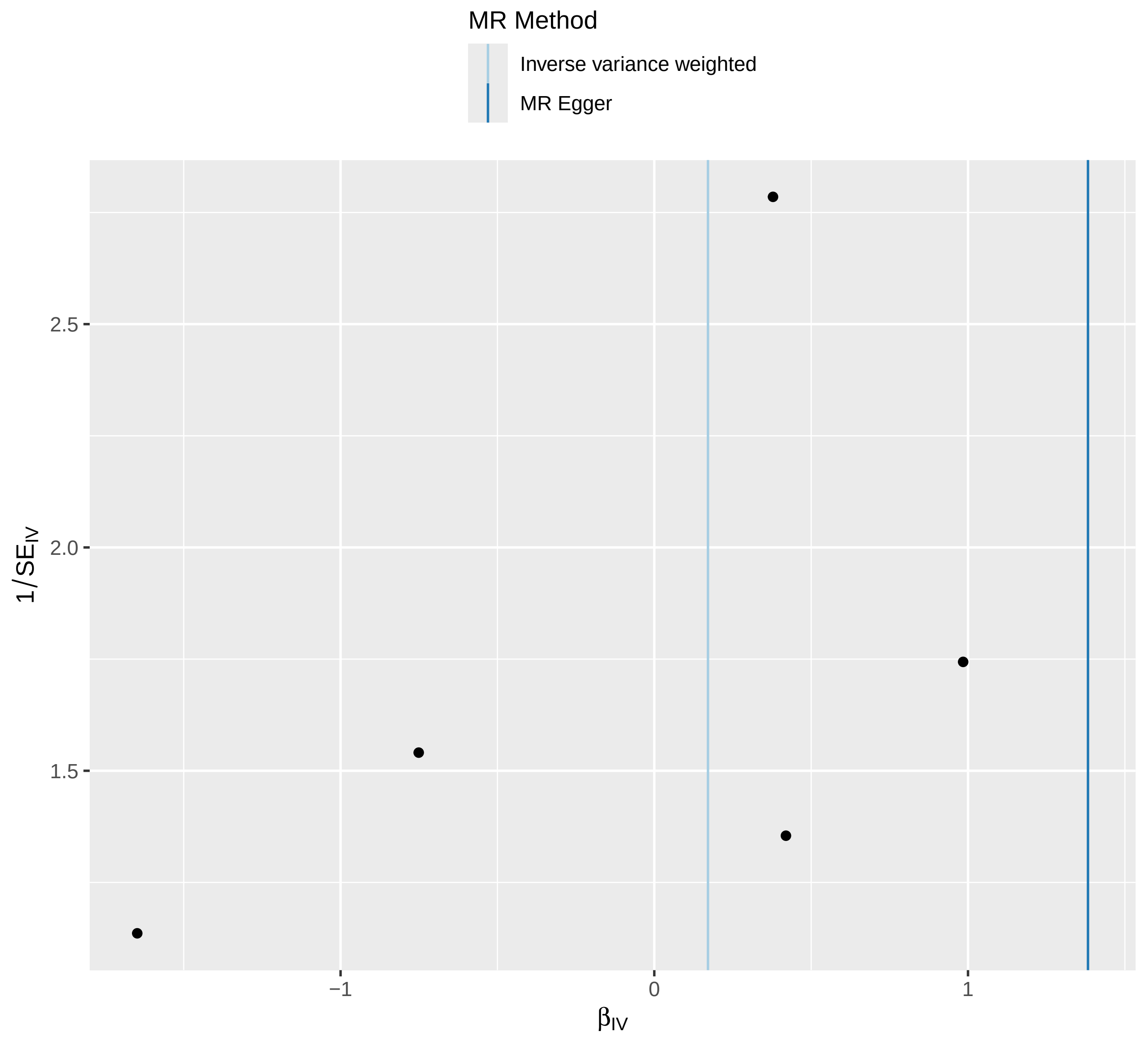
MR Method
Inverse variance weighted
MR Egger
2.5
2.0
1.5
1/SE_IV
−1
0
1
β_IV

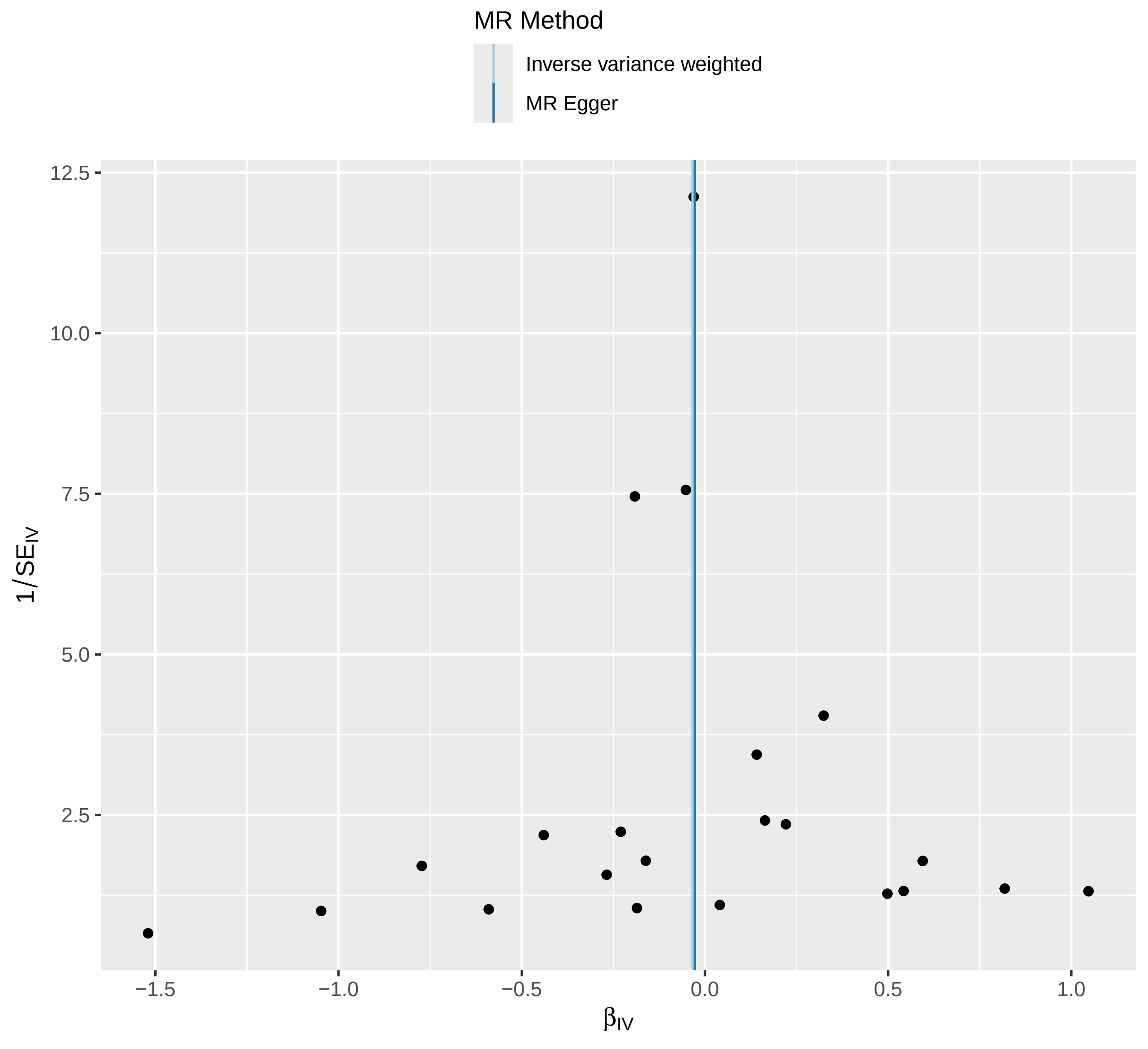

MR Method
Inverse variance weighted
MR Egger
12.5
10.0
7.5
5.0
2.5
1/SE_IV
−1.5
−1.0
−0.5
0.0
0.5
1.0
β_IV

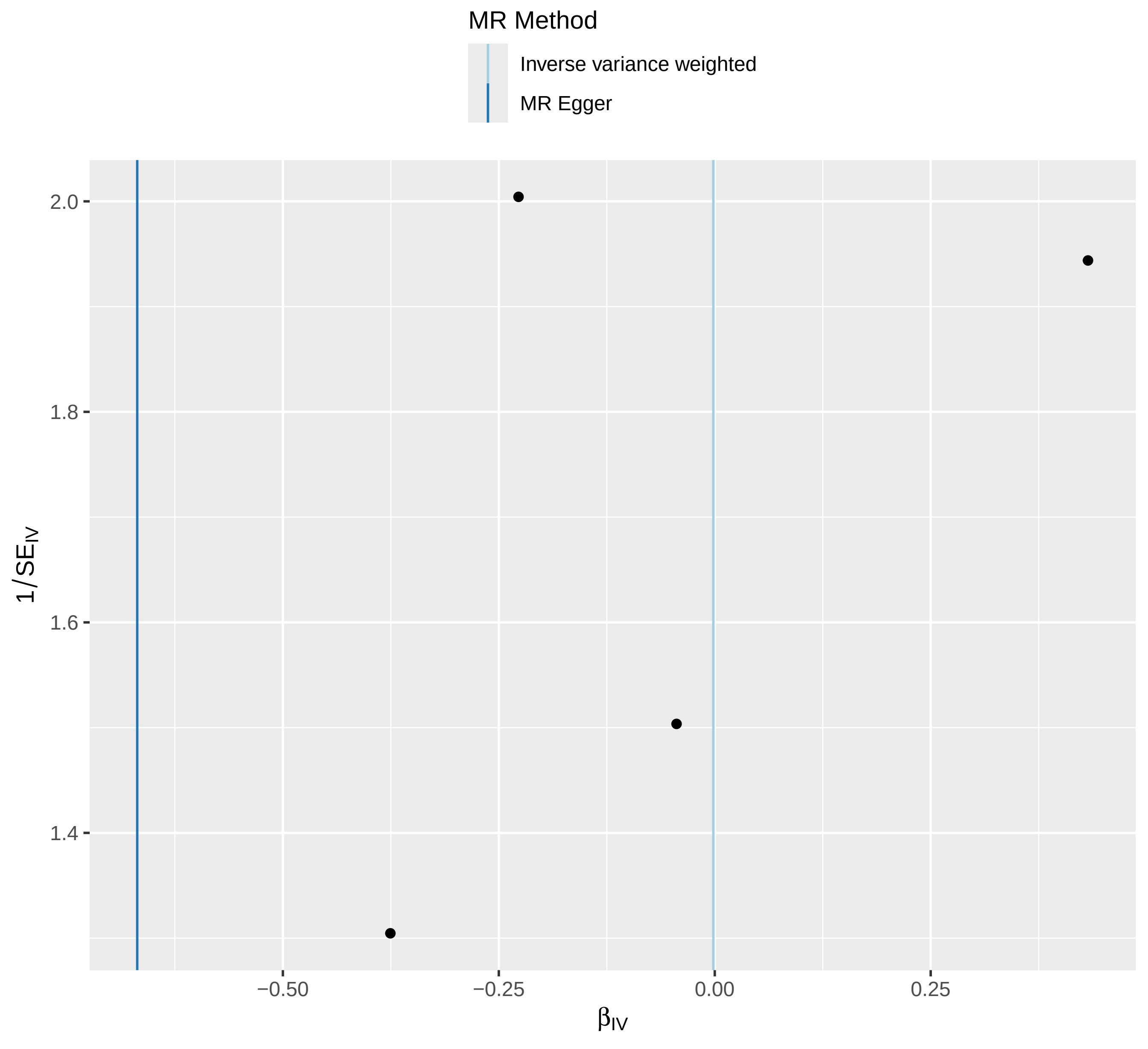
MR Method
Inverse variance weighted
MR Egger
2.0
1.8
1.6
1.4
−0.50
−0.25
0.00
0.25
β_{IV}
$1/SE_{IV}$

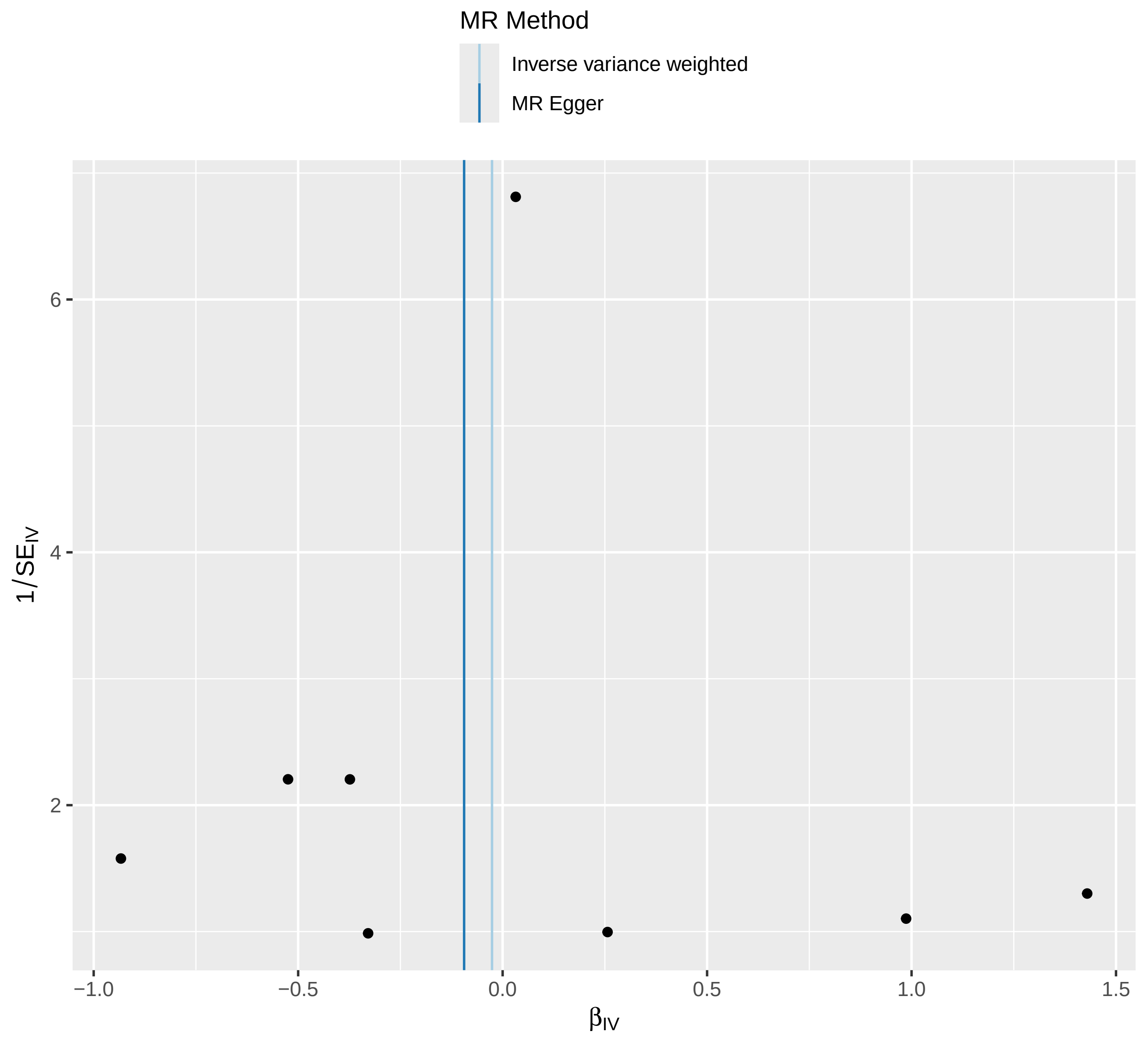

MR Method
Inverse variance weighted
MR Egger
6
4
2
$1/SE_{IV}$
−1.0
−0.5
0.0
0.5
1.0
1.5
β_{IV}

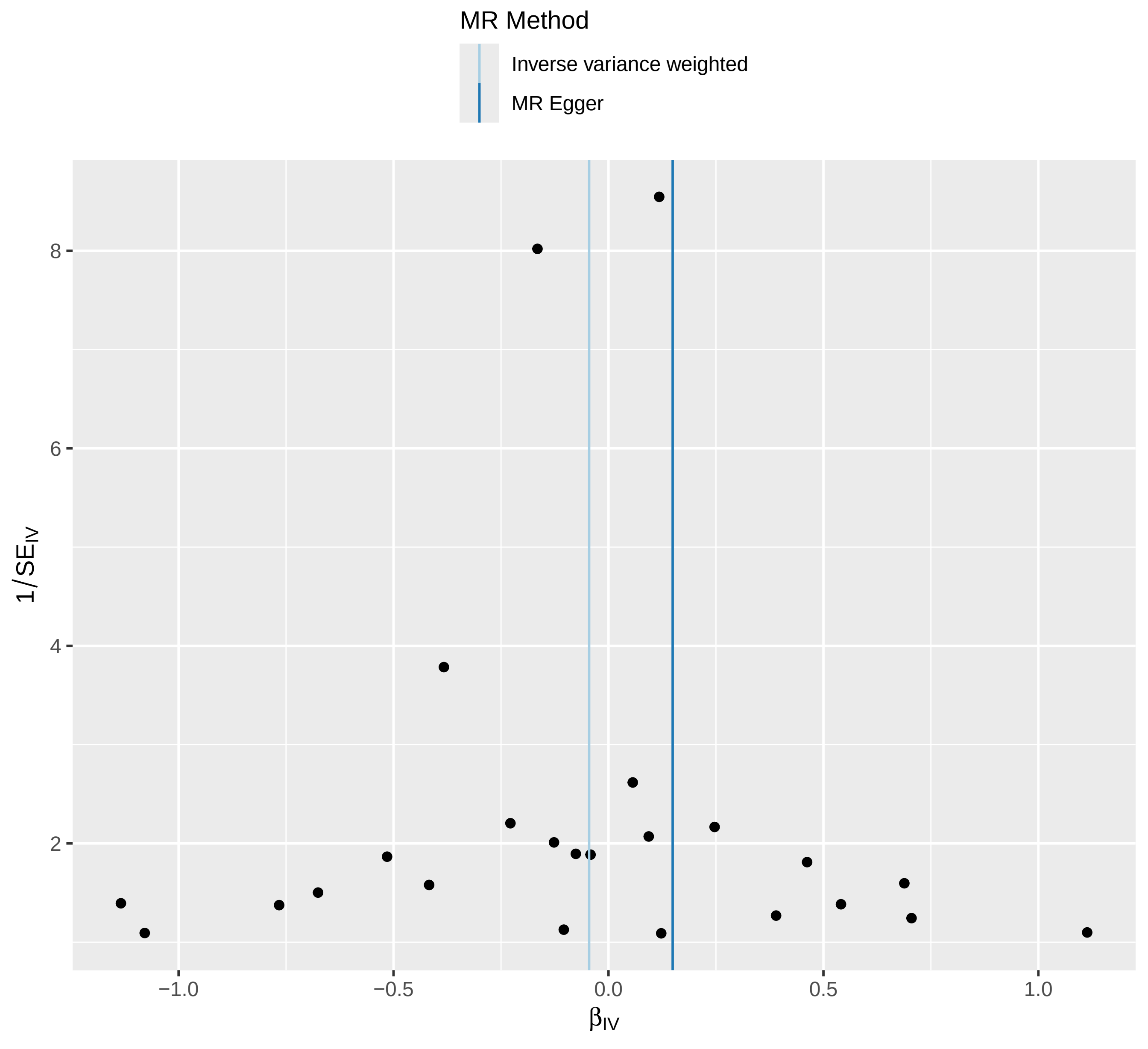
MR Method
Inverse variance weighted
MR Egger
8
6
4
2
$1/SE_{IV}$
−1.0
−0.5
0.0
0.5
1.0
β_{IV}

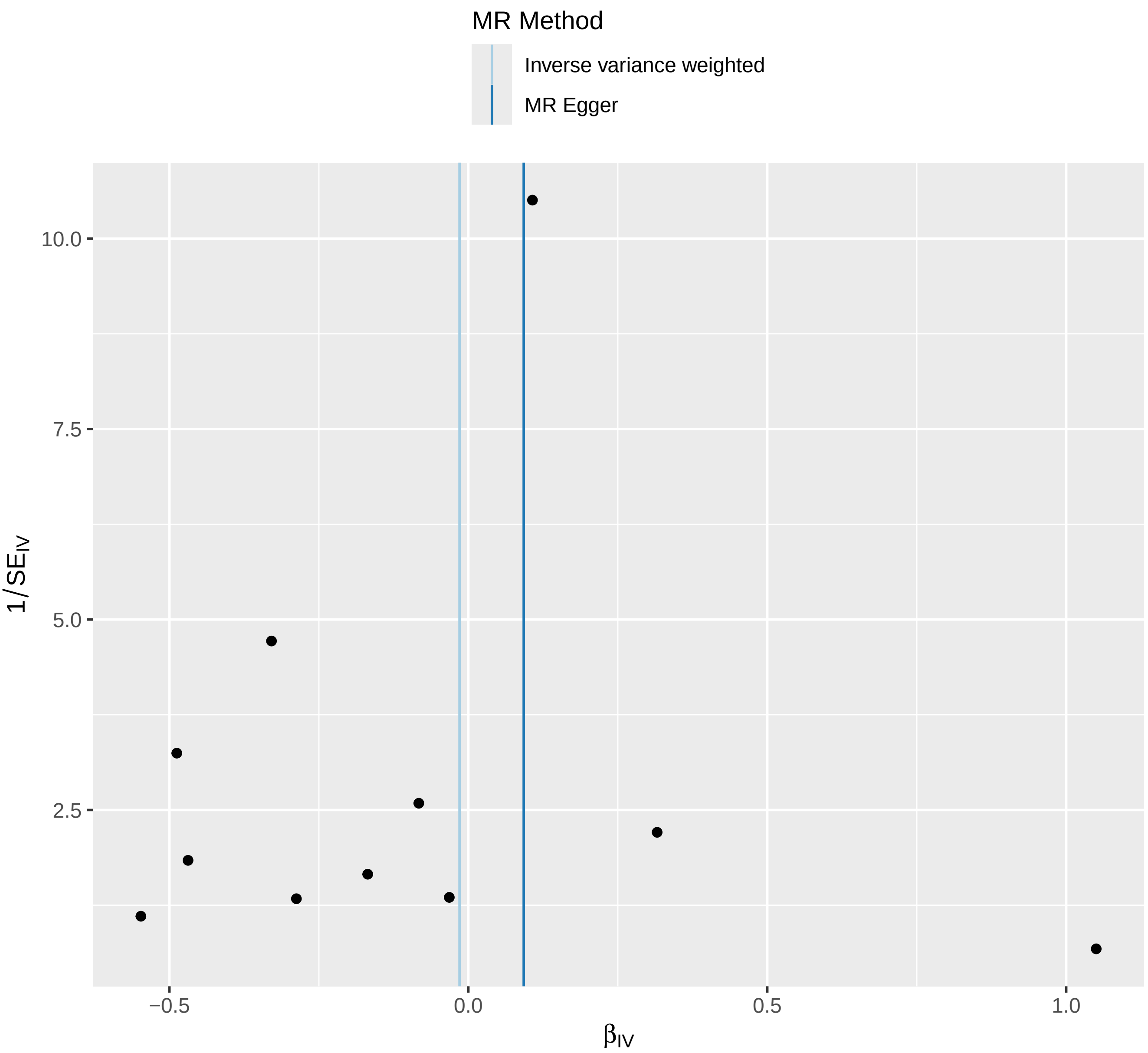
MR Method
Inverse variance weighted
MR Egger
10.0
7.5
5.0
2.5
$1/SE_{IV}$
−0.5
0.0
0.5
1.0
β_{IV}

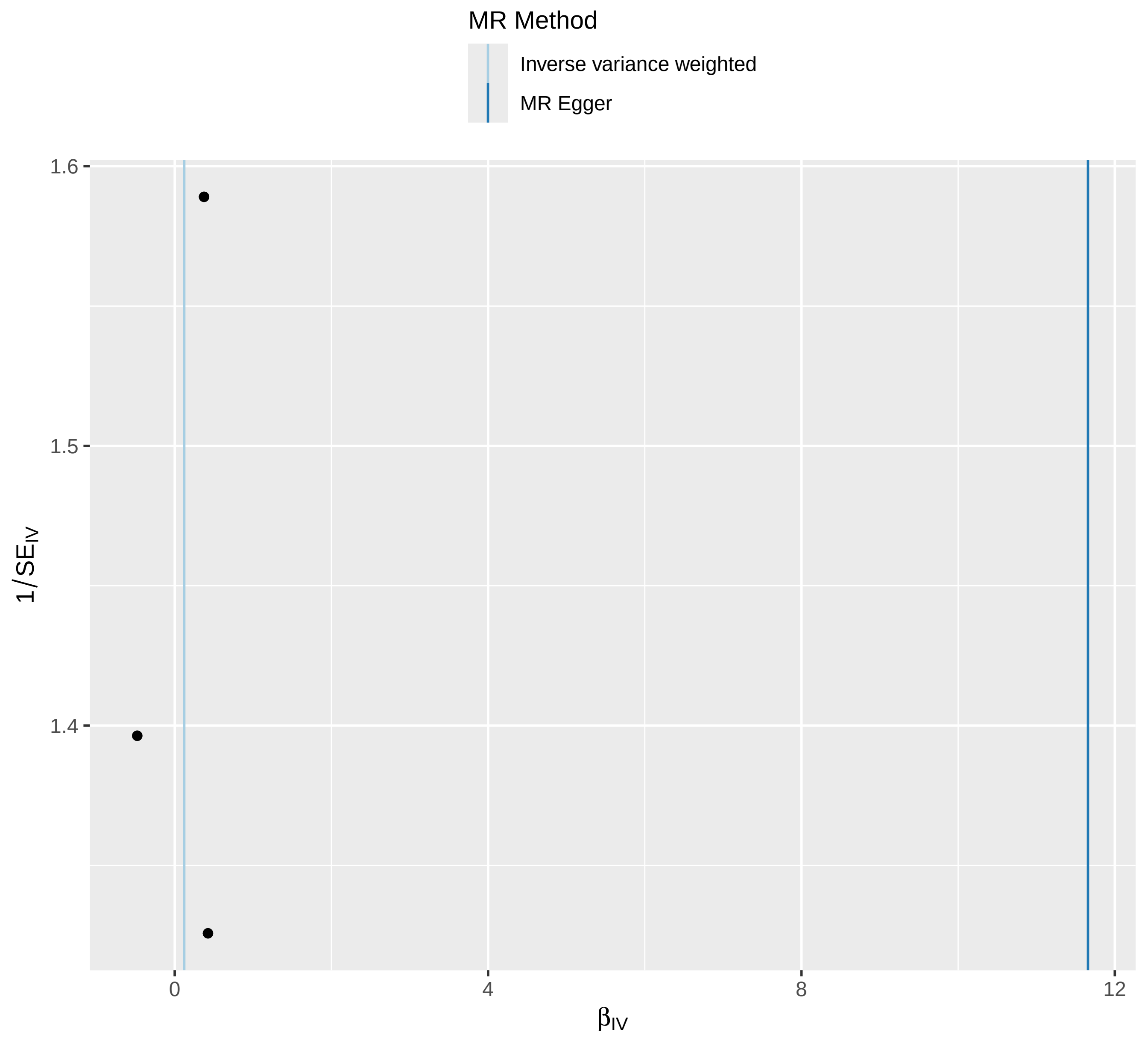
MR Method
Inverse variance weighted
MR Egger
1.6
1.5
1.4
1/SE$_{IV}$
0
4
8
12
β$_{IV}$

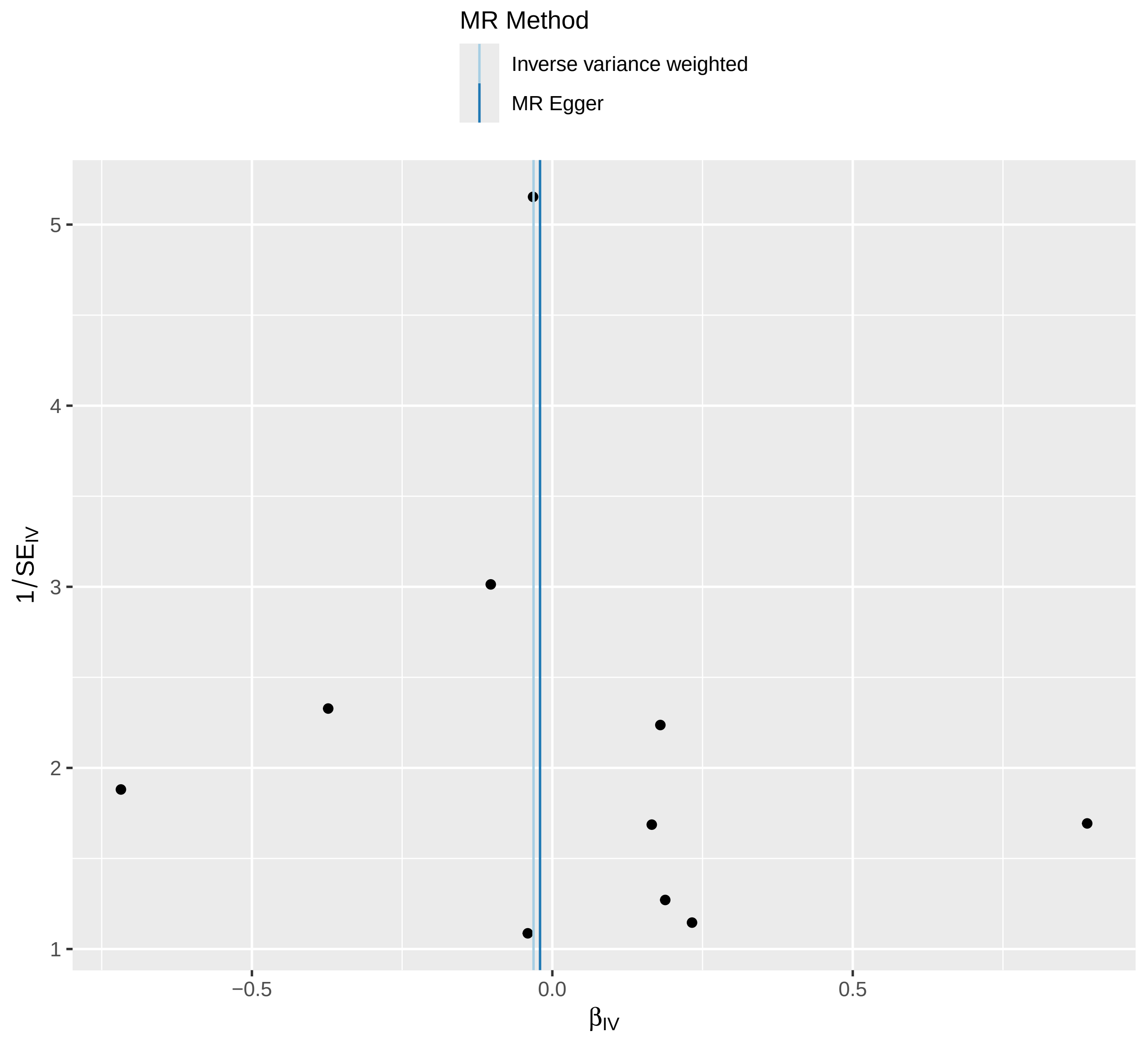
MR Method
Inverse variance weighted
MR Egger
1/SE$_{IV}$
5
4
3
2
1
−0.5
0.0
0.5
β$_{IV}$

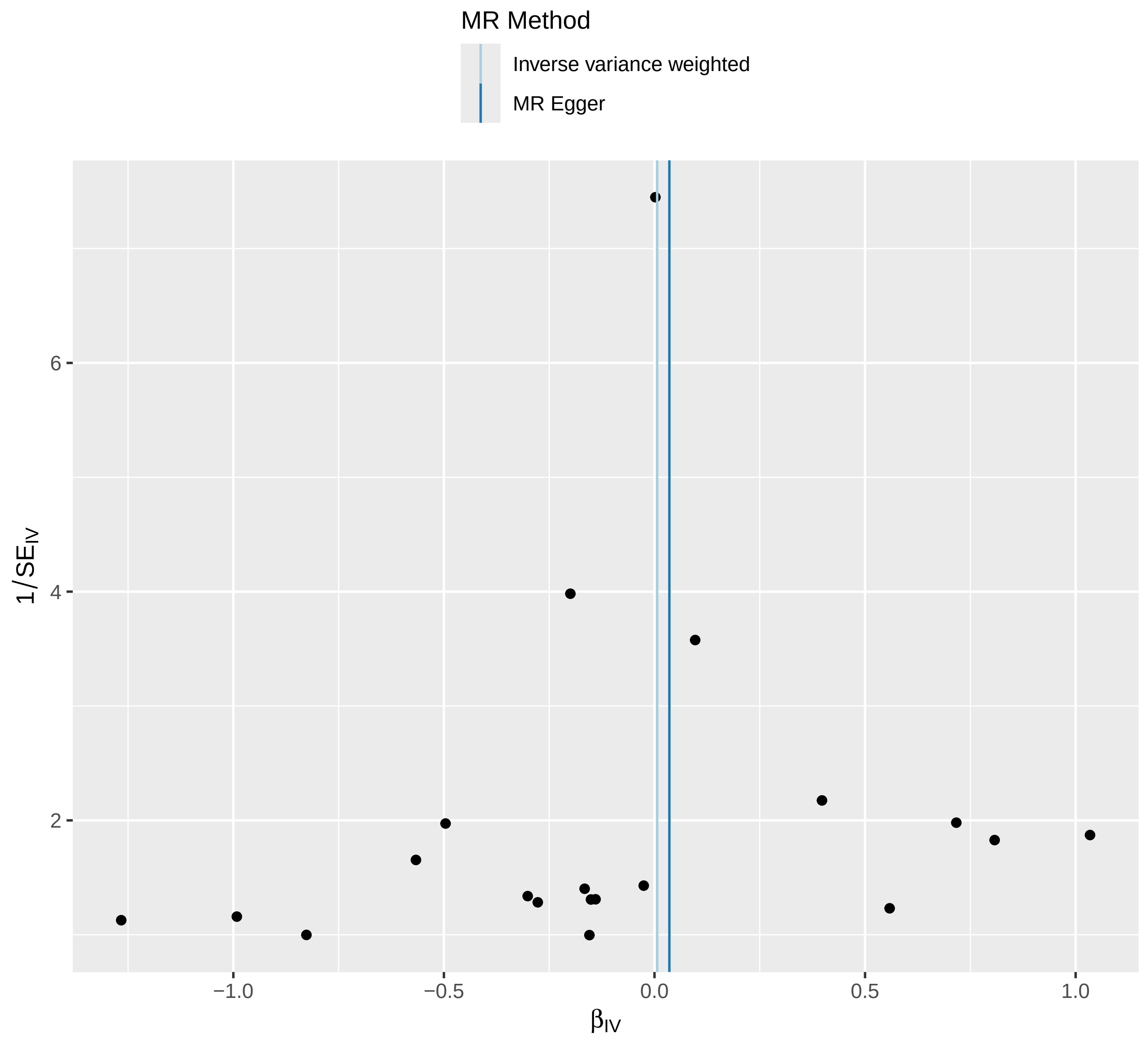
MR Method
Inverse variance weighted
MR Egger
6
4
2
1/SE$_{IV}$
−1.0
−0.5
0.0
0.5
1.0
β$_{IV}$

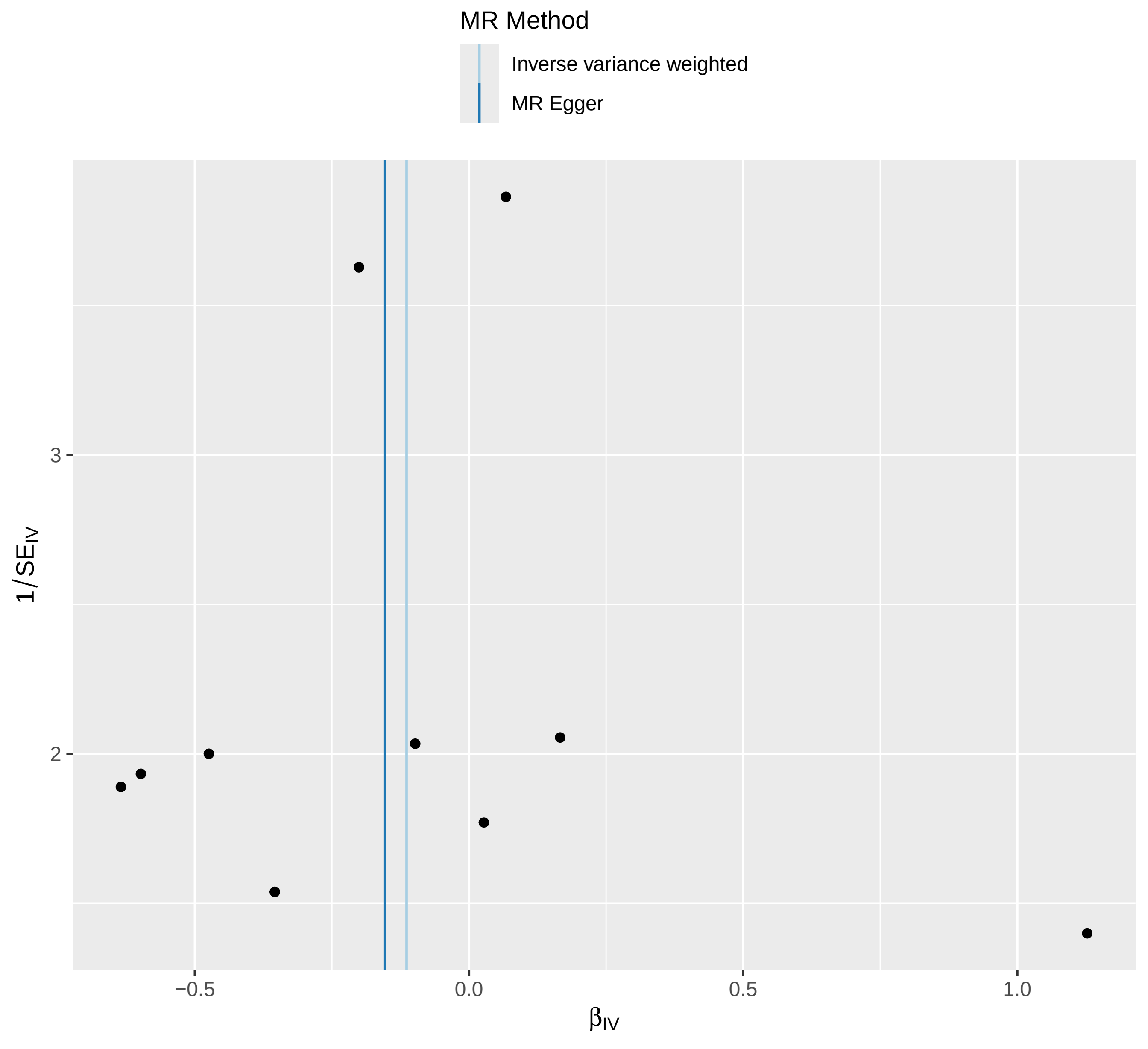
MR Method
Inverse variance weighted
MR Egger
1/SE_IV
3
2
−0.5
0.0
0.5
1.0
β_IV

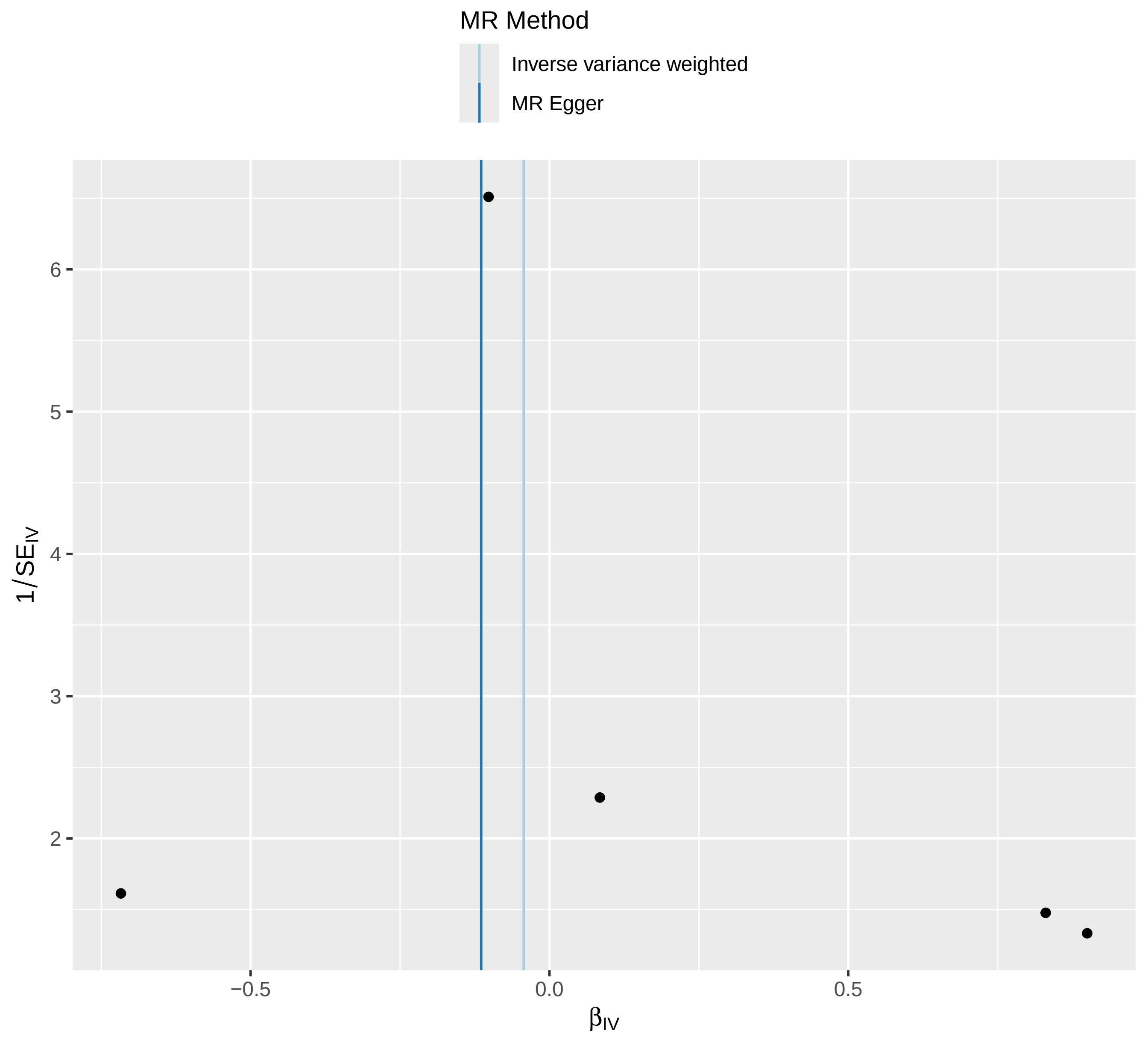

MR Method
Inverse variance weighted
MR Egger
6
5
4
3
2
$1/SE_{IV}$
−0.5
0.0
0.5
β_{IV}

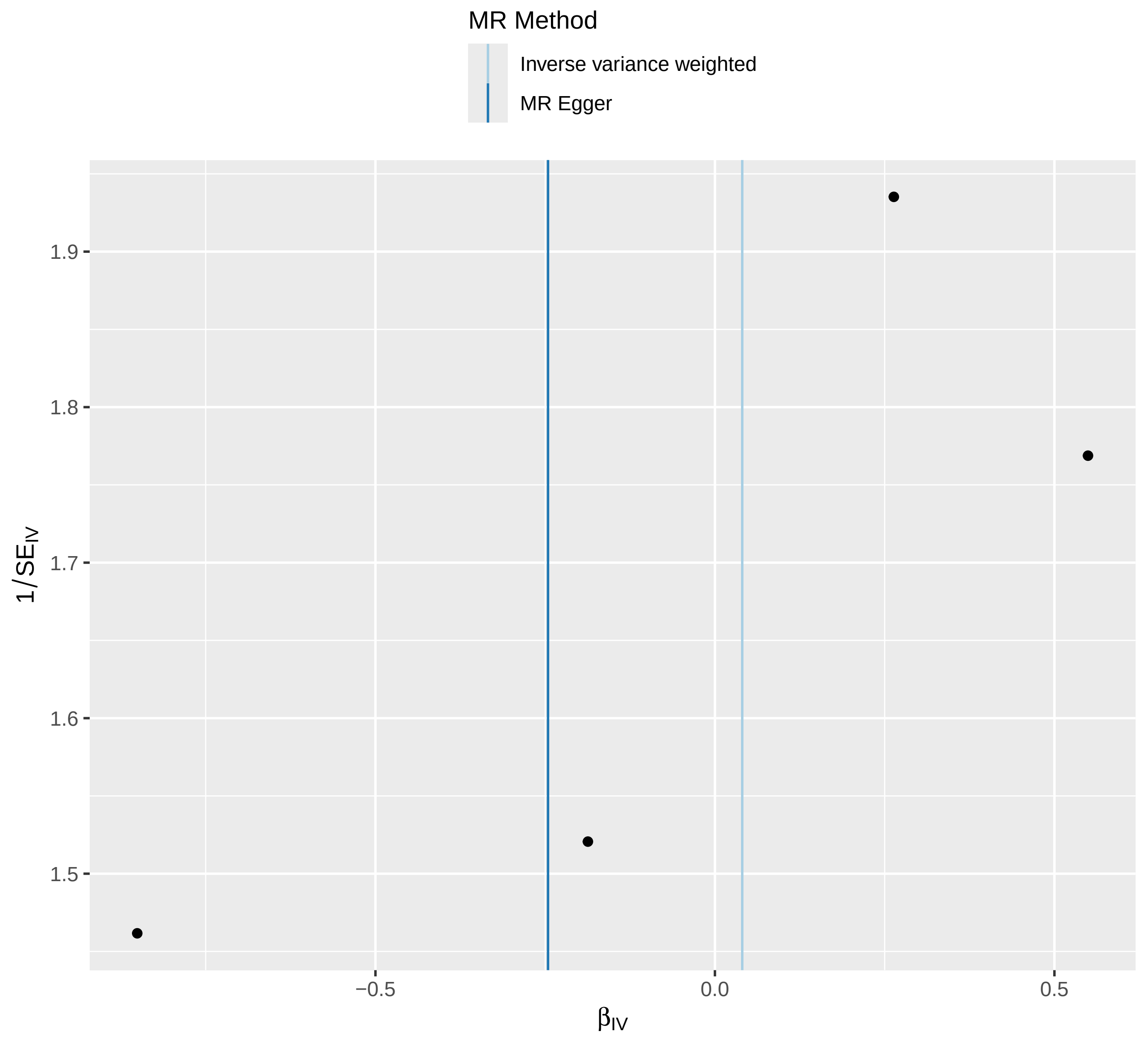
MR Method
Inverse variance weighted
MR Egger
1.9
1.8
1.7
1.6
1.5
1/SE_IV
−0.5
0.0
0.5
β_IV

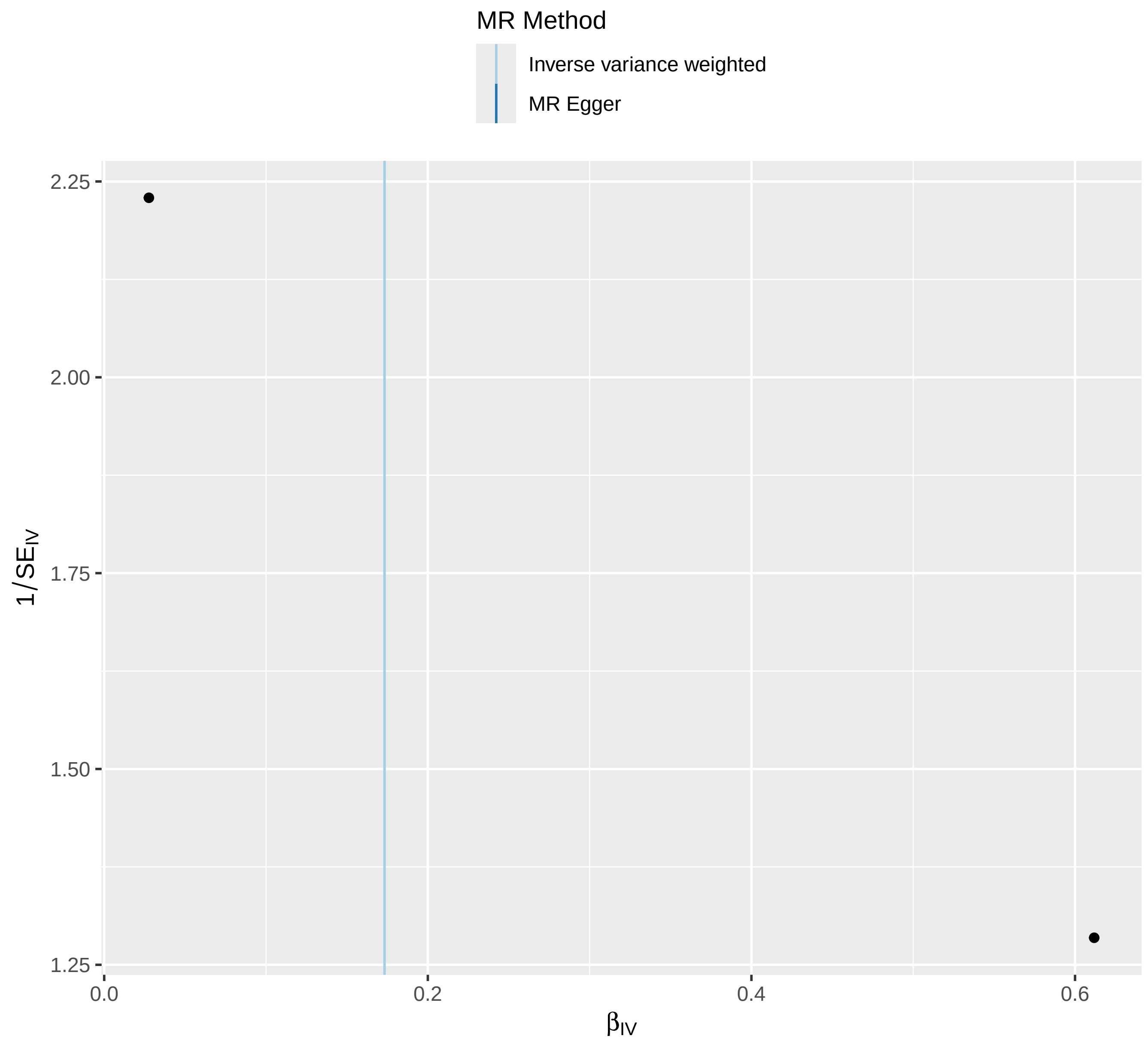
MR Method
Inverse variance weighted
MR Egger
2.25
2.00
1.75
1.50
1.25
0.0
0.2
0.4
0.6
1/SE_IV
β_IV

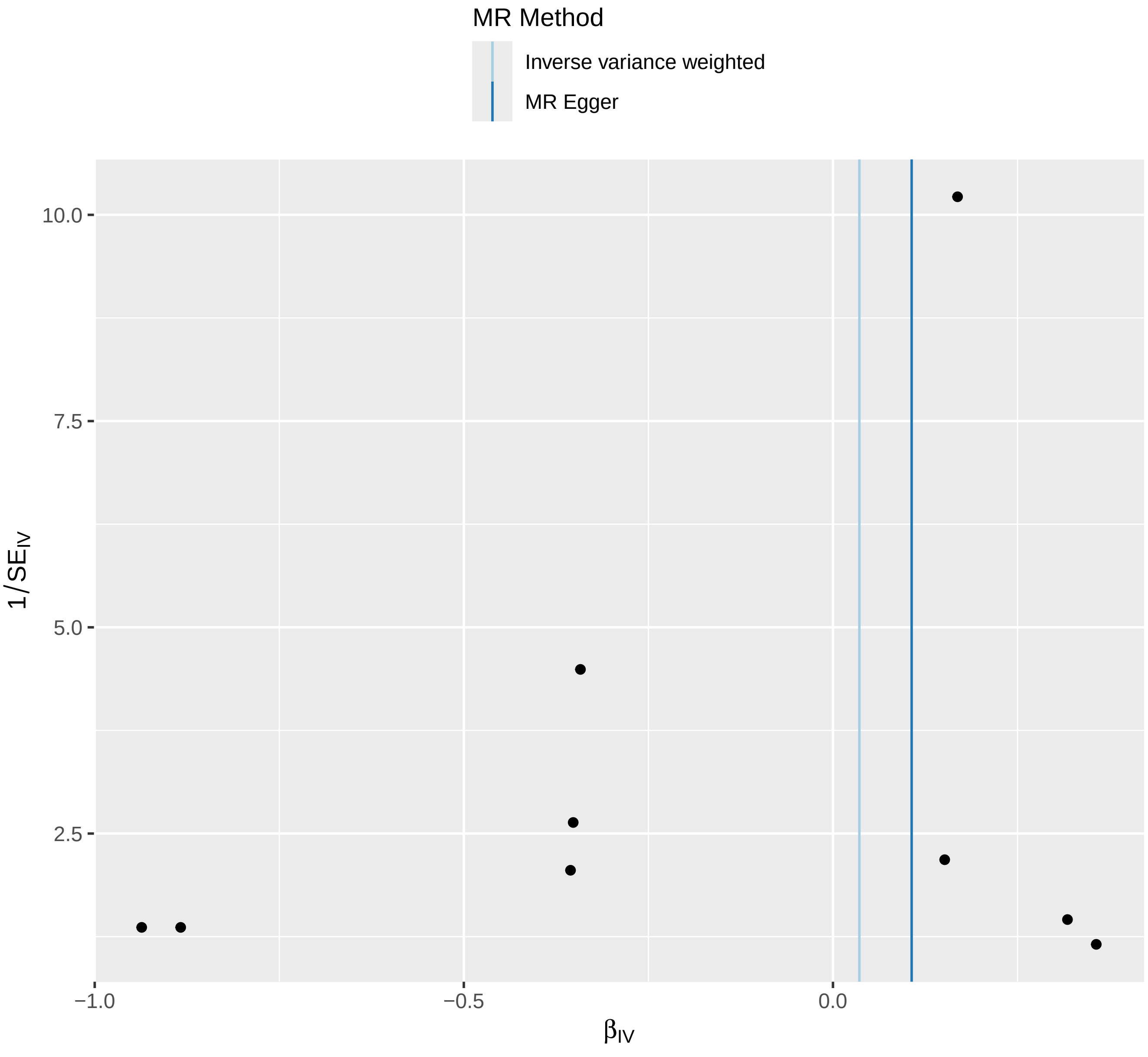

MR Method
Inverse variance weighted
MR Egger
10.0
7.5
5.0
2.5
1/SE_IV
−1.0
−0.5
0.0
β_IV

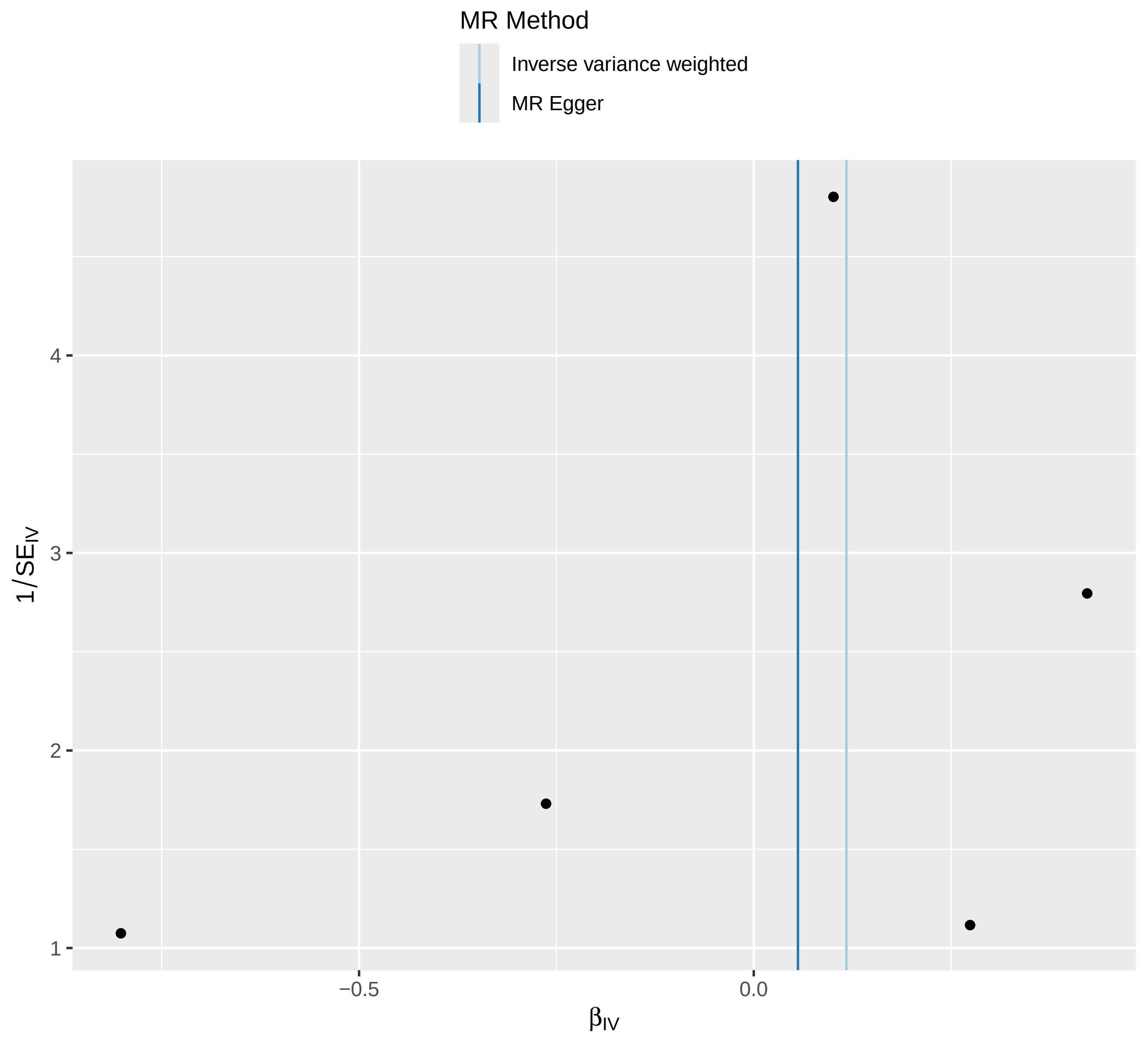

MR Method
Inverse variance weighted
MR Egger
4
3
2
1
1/SE_IV
−0.5
0.0
β_IV

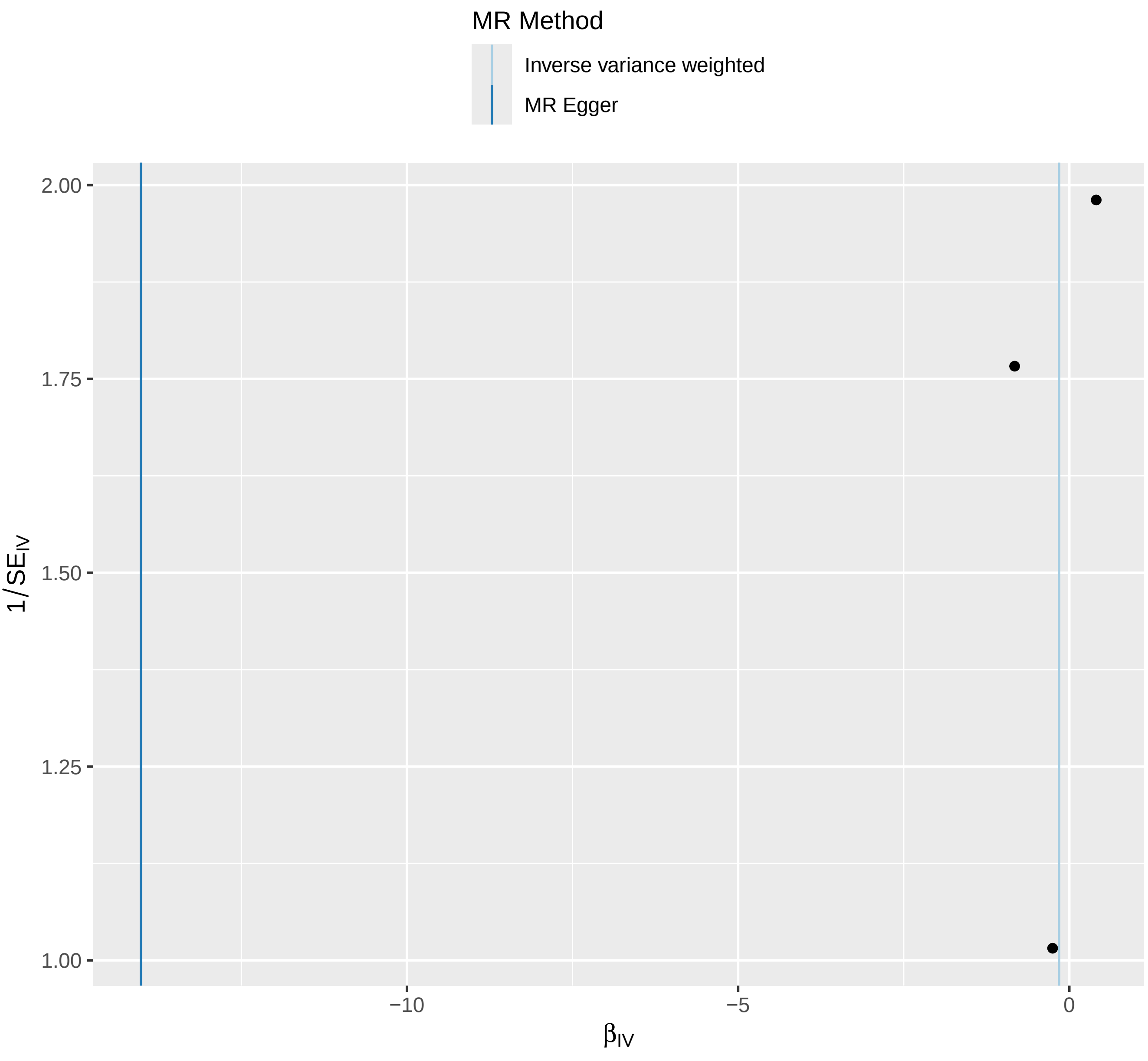

MR Method
Inverse variance weighted
MR Egger
2.00
1.75
1.50
1.25
1.00
1/SE_IV
−10
−5
0
β_IV

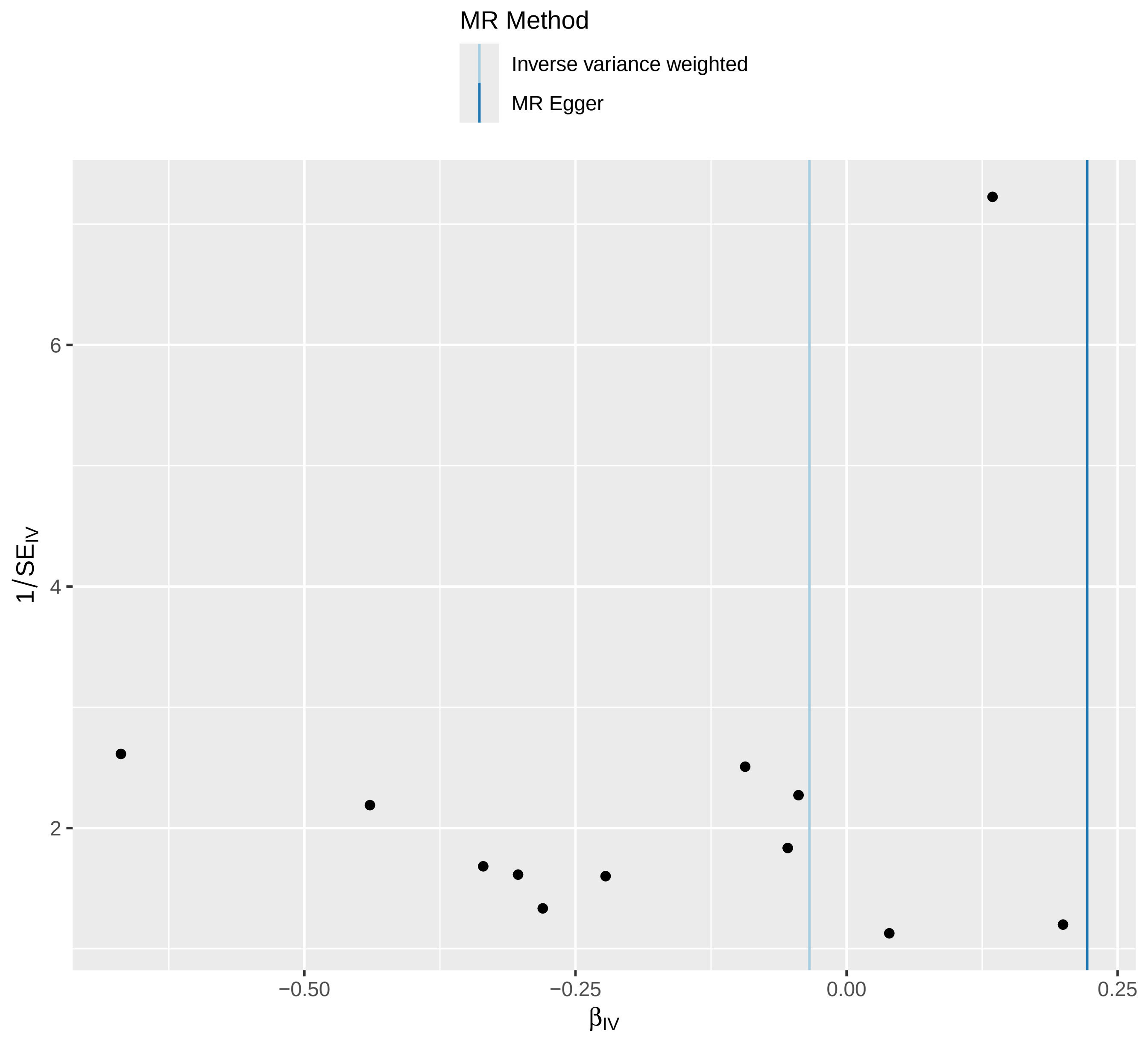

MR Method
Inverse variance weighted
MR Egger
6
4
2
1/SE_IV
−0.50
−0.25
0.00
0.25
β_IV

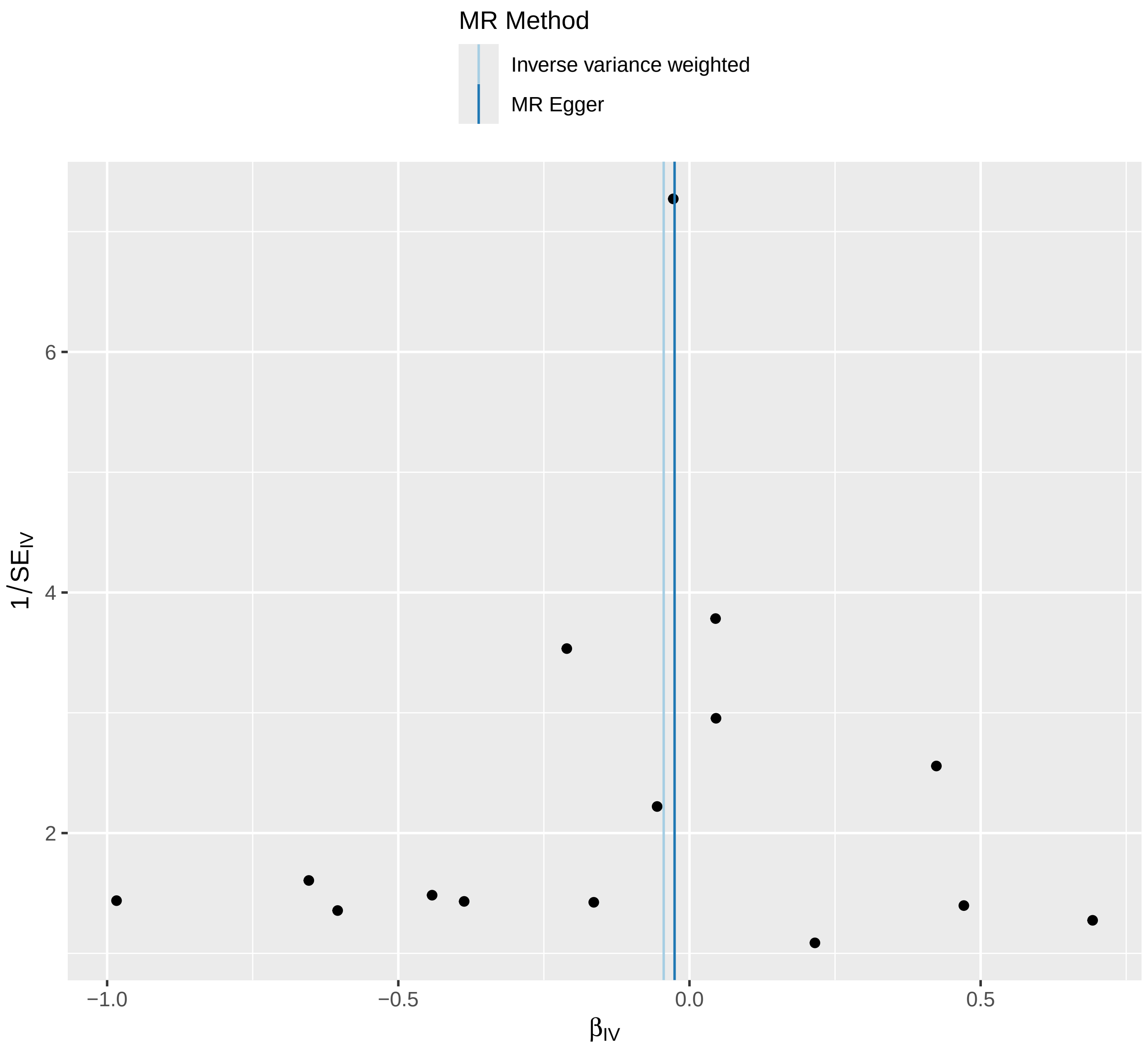
MR Method
Inverse variance weighted
MR Egger
6
4
2
1/SE_IV
−1.0
−0.5
0.0
0.5
β_IV

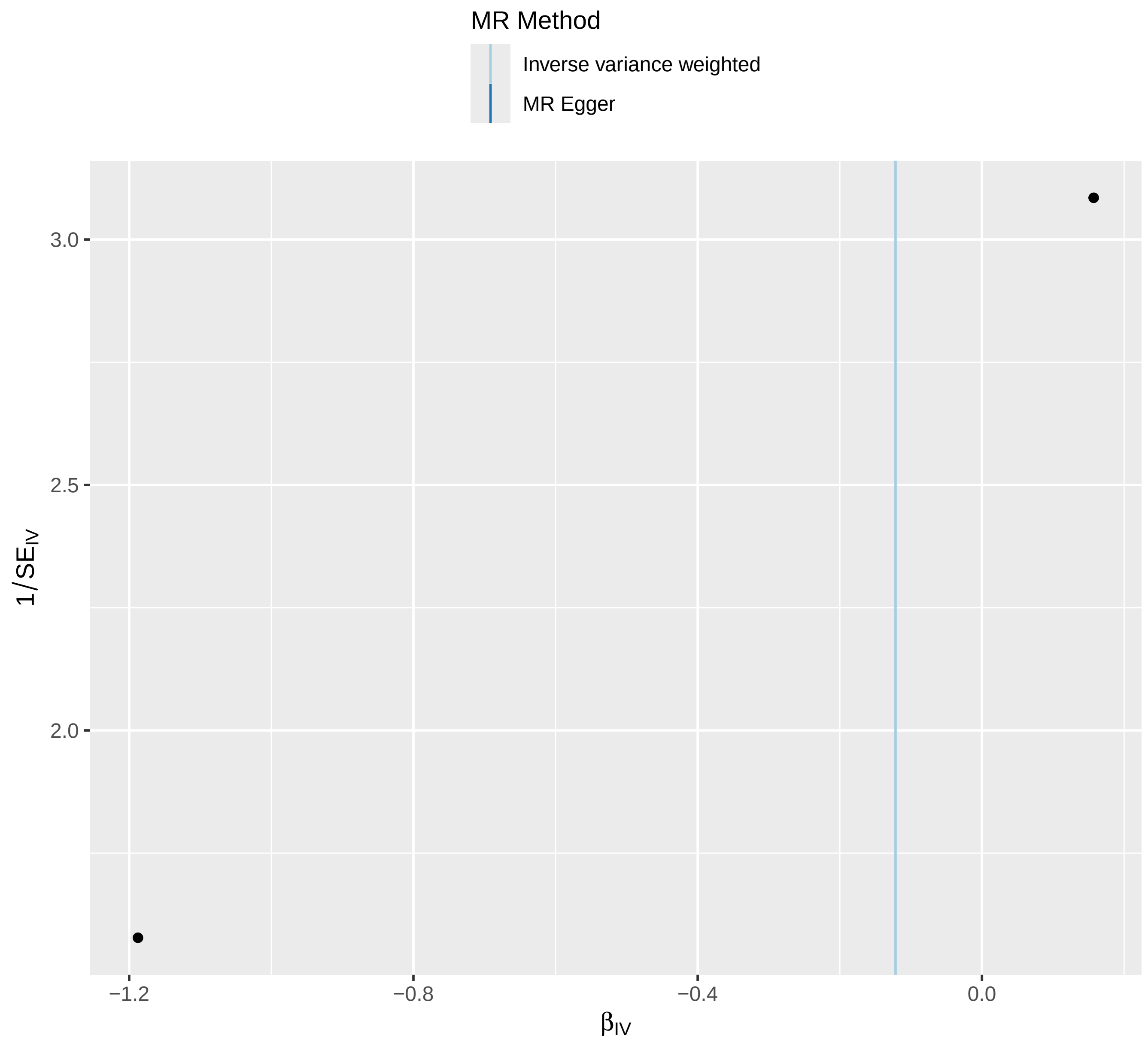
MR Method
Inverse variance weighted
MR Egger
3.0
2.5
2.0
1/SE_IV
−1.2
−0.8
−0.4
0.0
β_IV

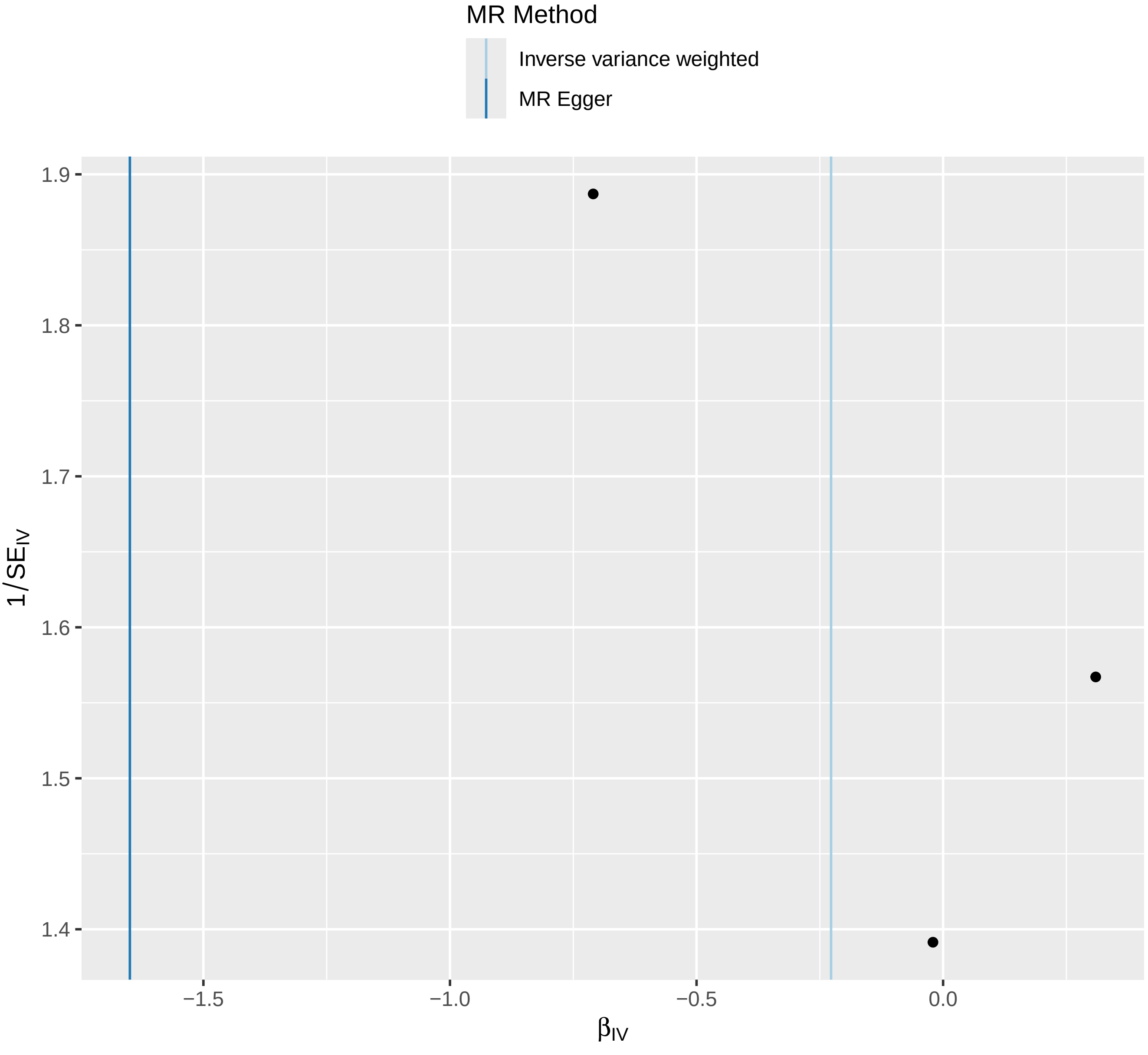
MR Method
Inverse variance weighted
MR Egger
1/SE_IV
1.9
1.8
1.7
1.6
1.5
1.4
−1.5
−1.0
−0.5
0.0
β_IV

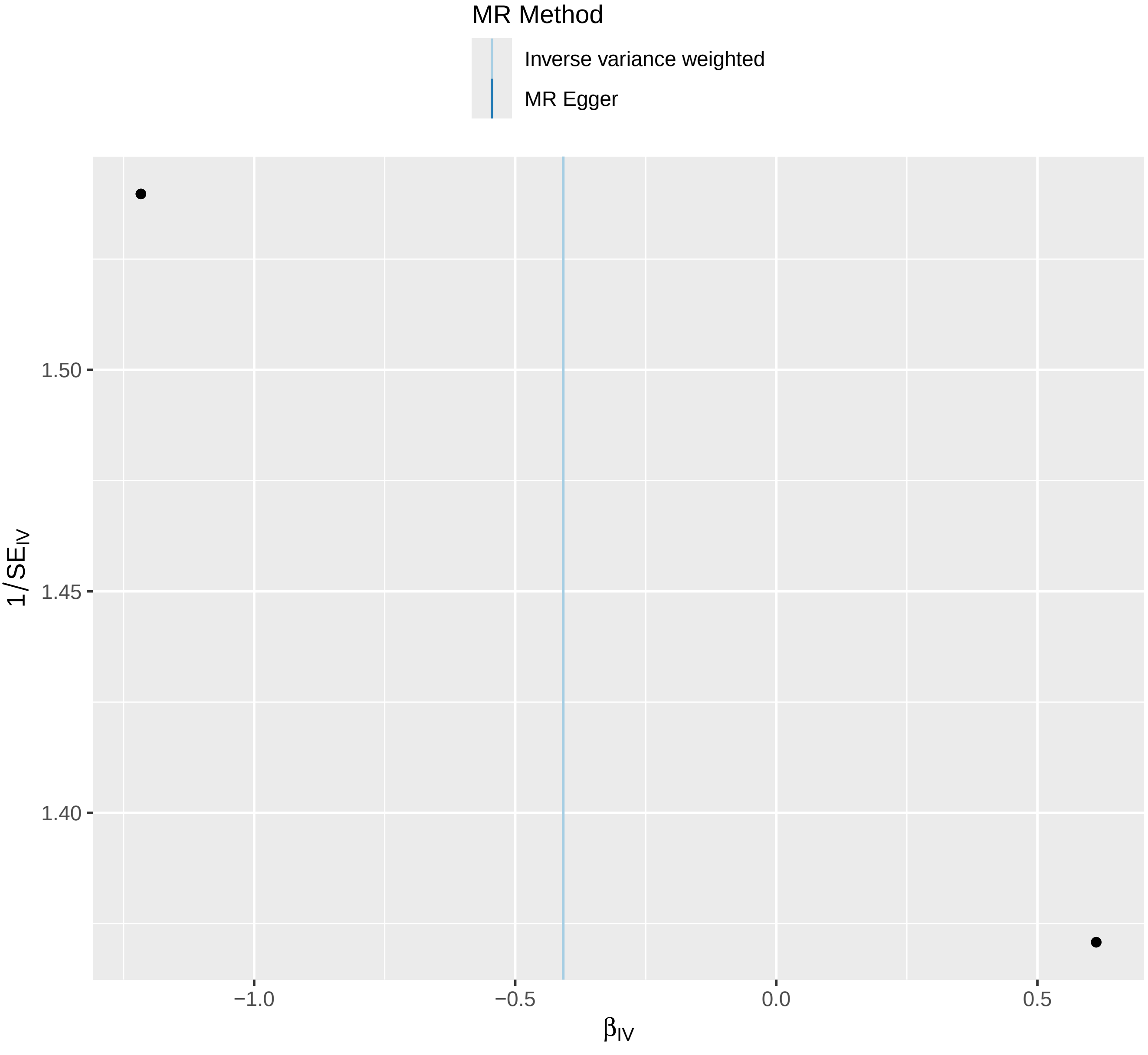
MR Method
Inverse variance weighted
MR Egger
1.50
1.45
1.40
$1/SE_{IV}$
−1.0
−0.5
0.0
0.5
β_{IV}

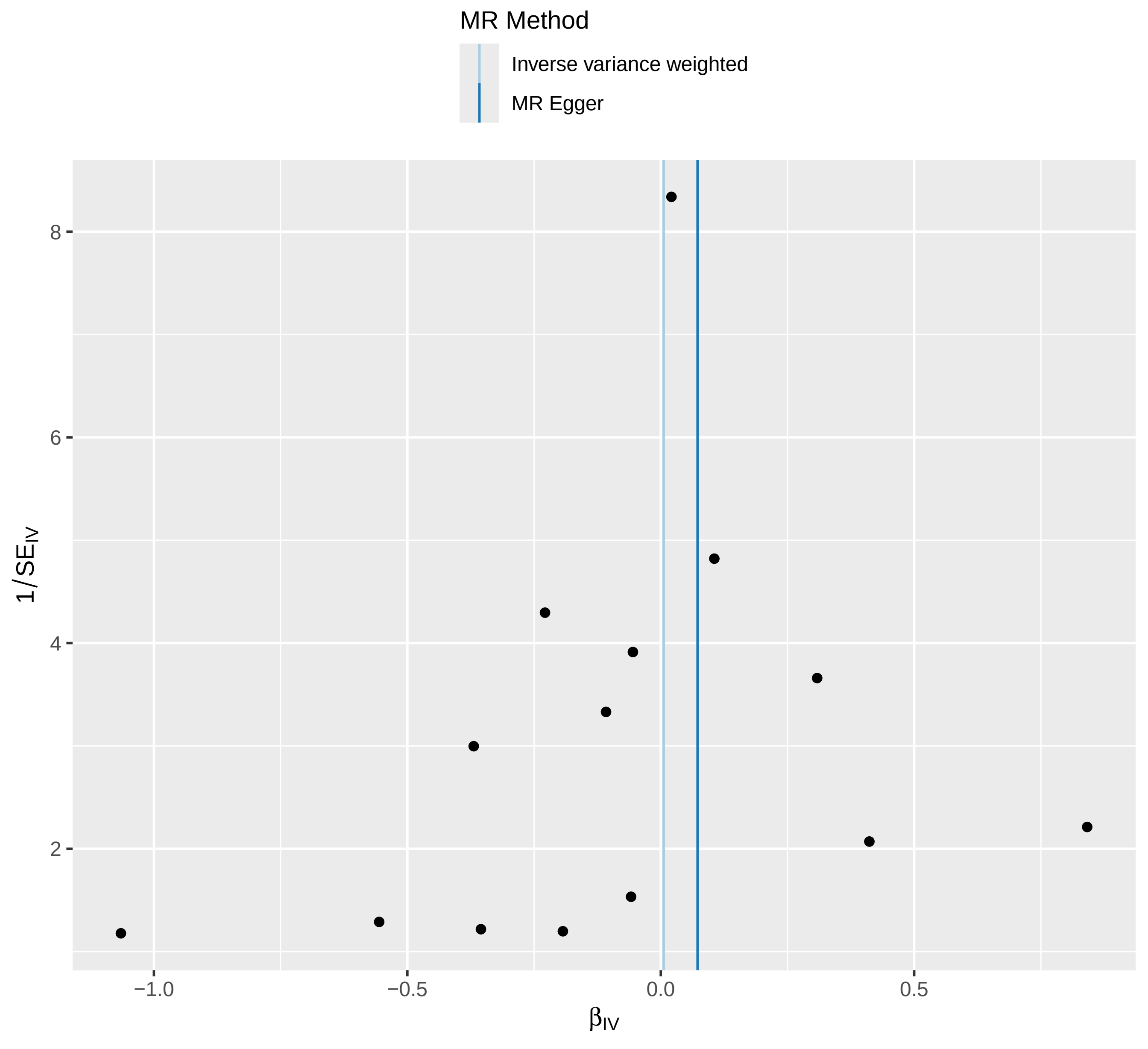
MR Method
Inverse variance weighted
MR Egger
8
6
4
2
1/SE_IV
−1.0
−0.5
0.0
0.5
β_IV

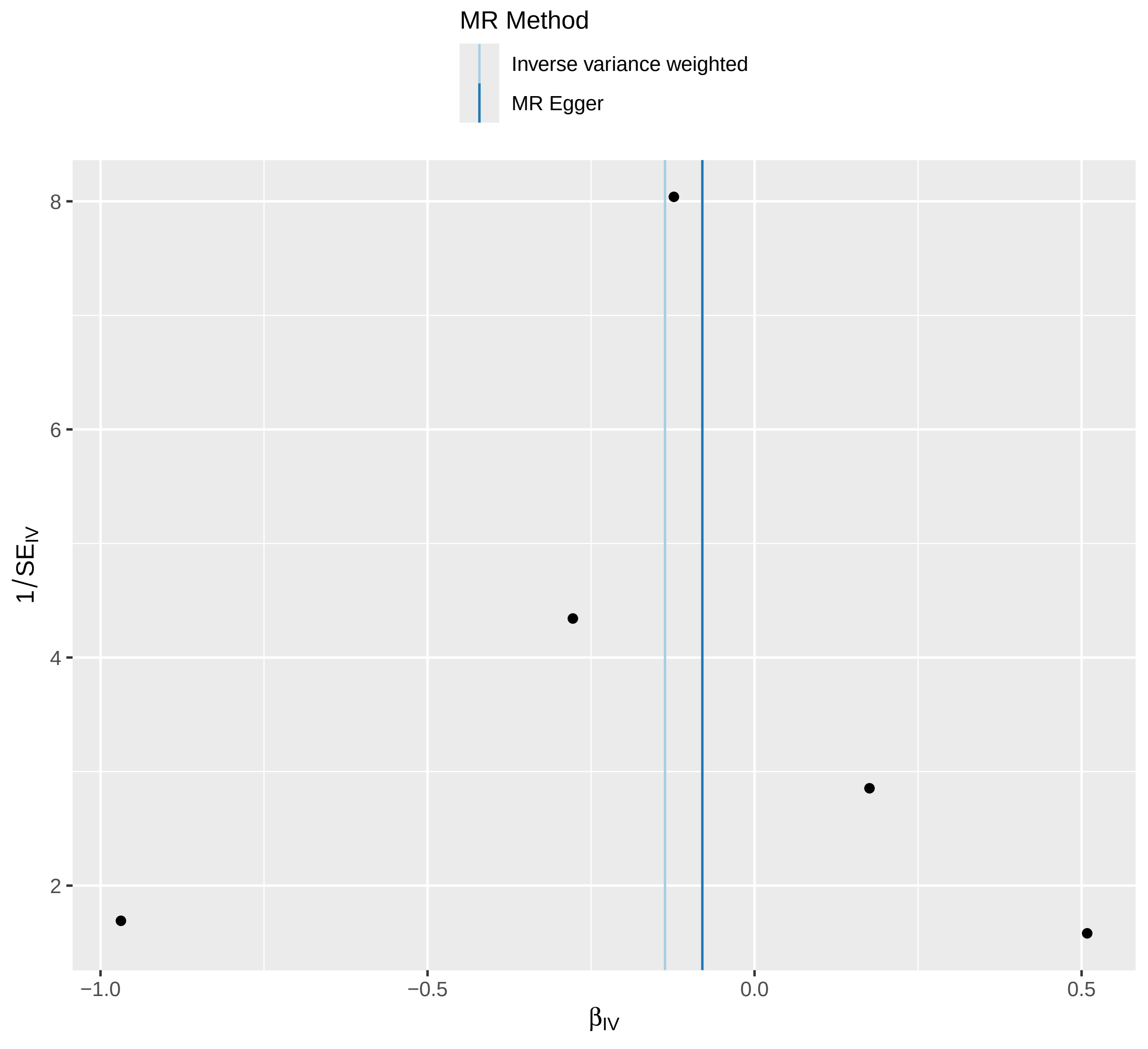
MR Method
Inverse variance weighted
MR Egger
8
6
4
2
1/SE$_{IV}$
−1.0
−0.5
0.0
0.5
β$_{IV}$

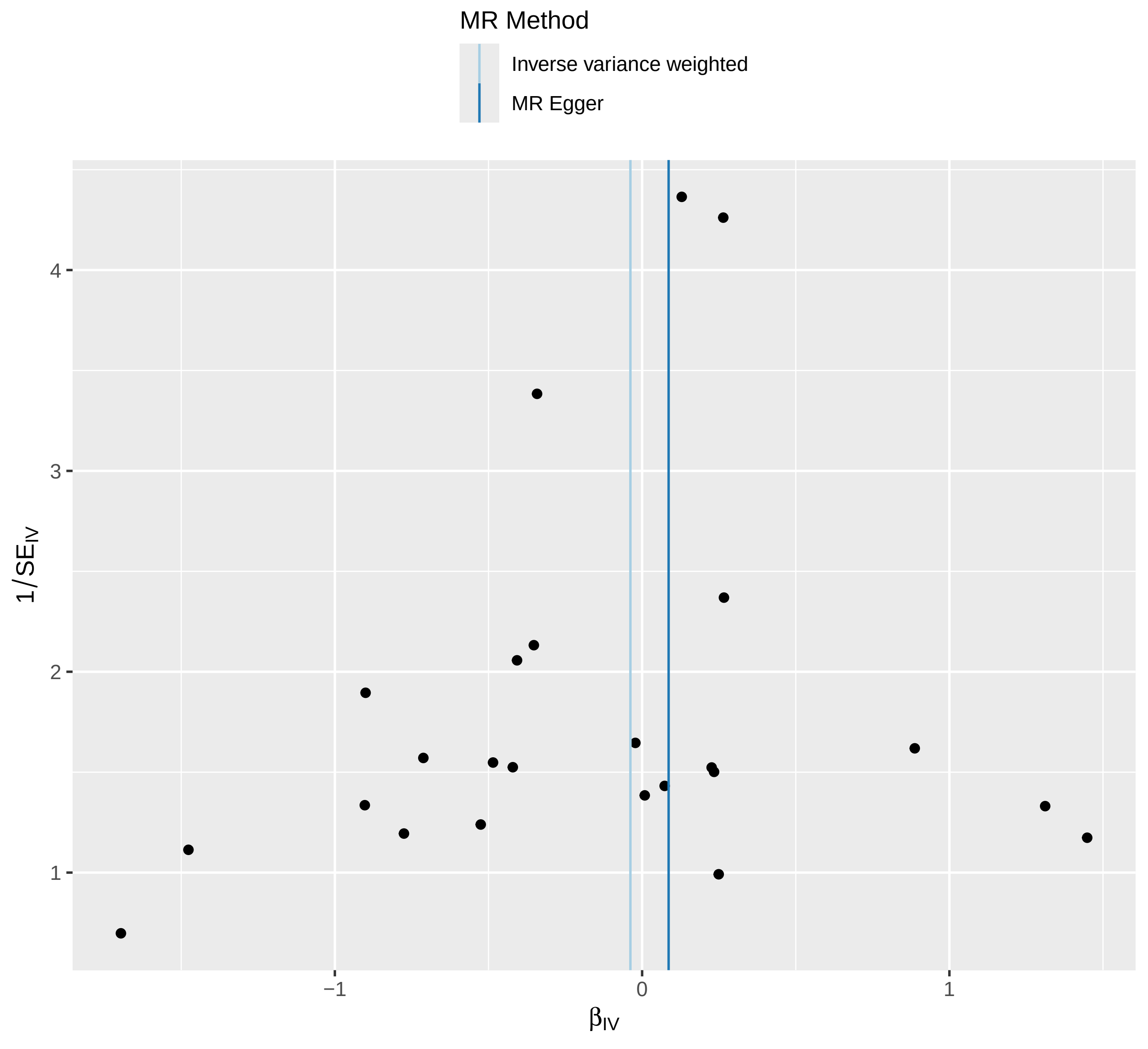
MR Method
Inverse variance weighted
MR Egger
4
3
2
1
1/SE$_{IV}$
−1
0
1
β$_{IV}$

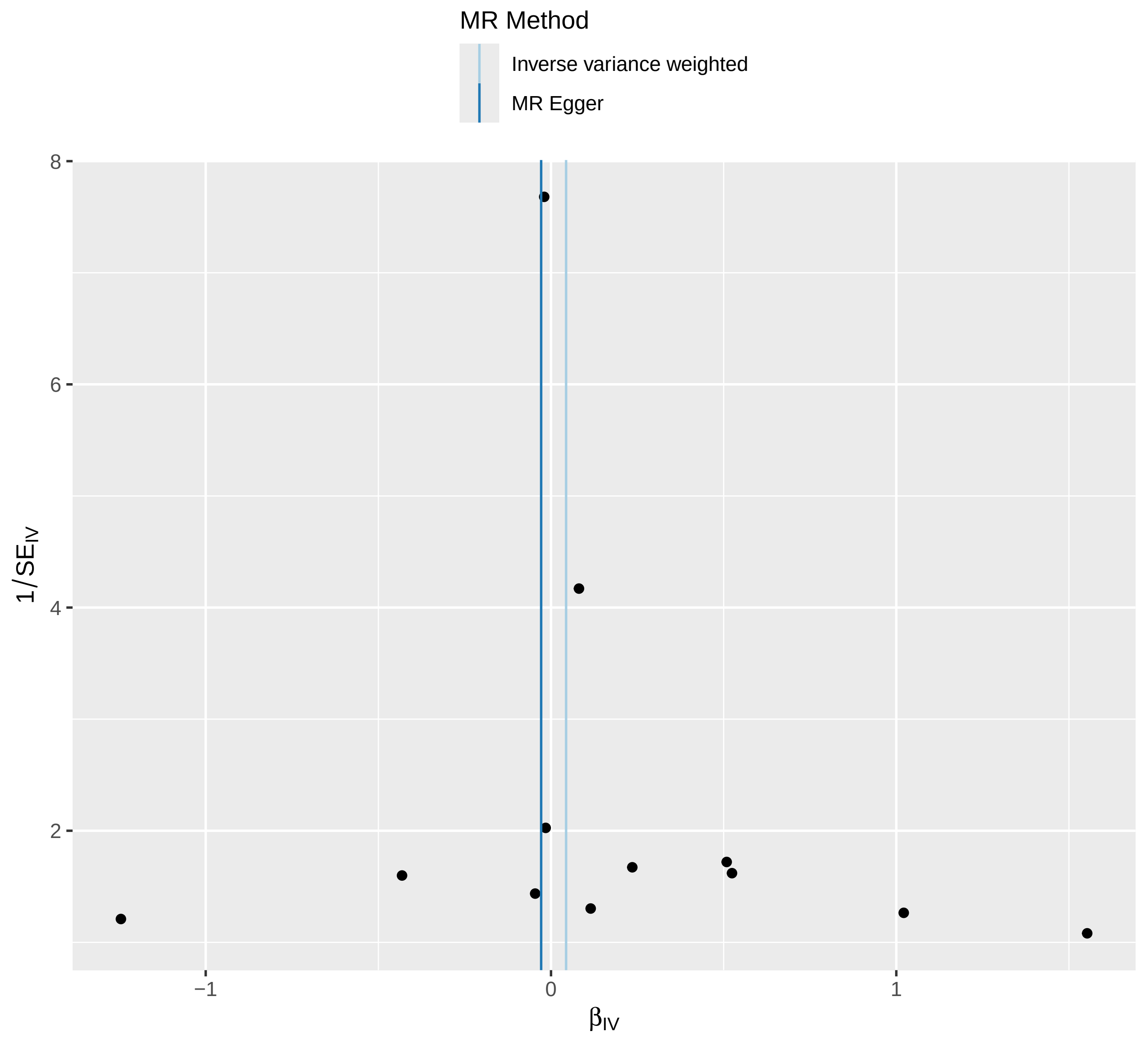

MR Method
Inverse variance weighted
MR Egger
8
6
4
2
1/SE_IV
−1
0
1
β_IV

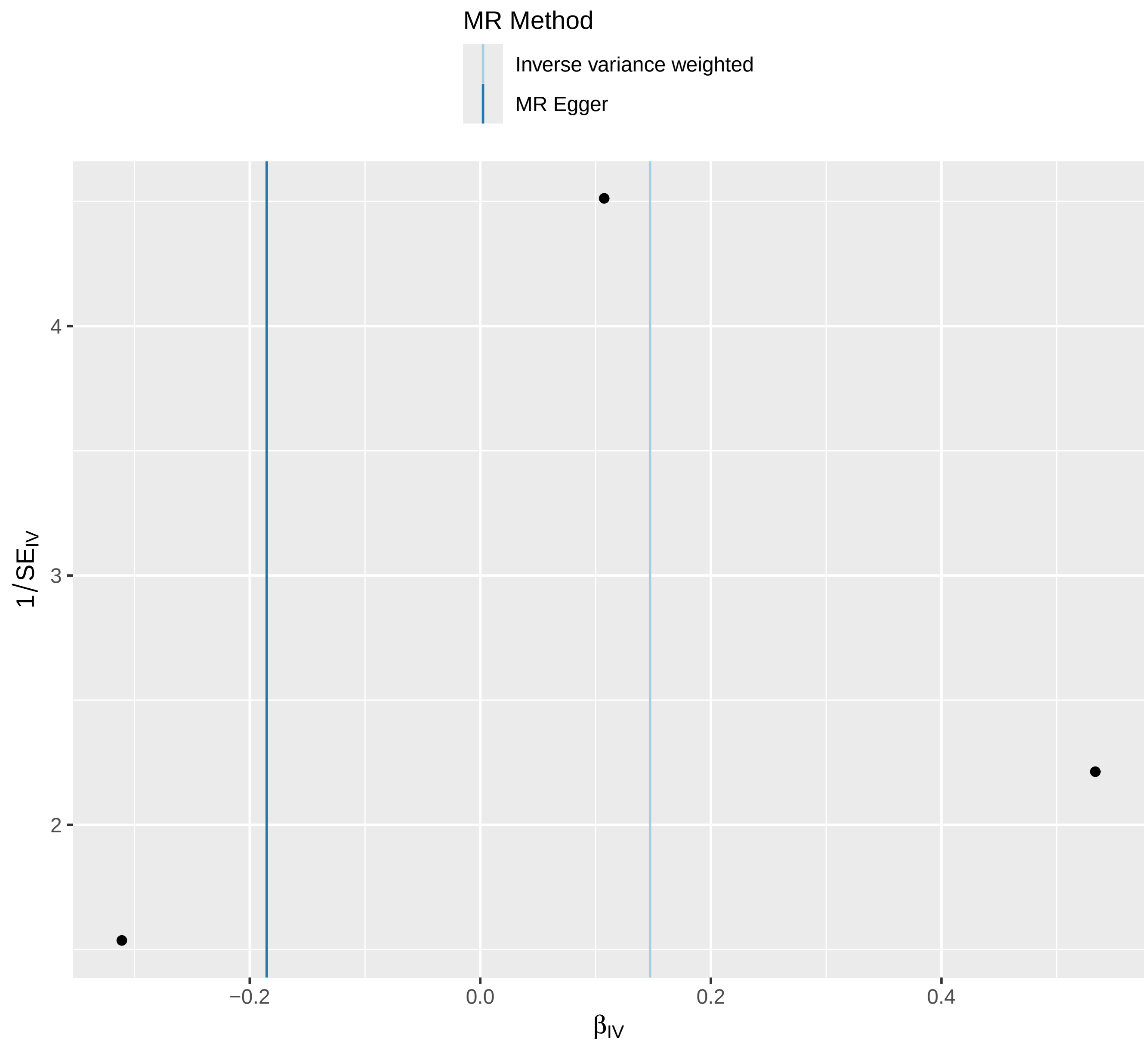
MR Method
Inverse variance weighted
MR Egger
4
3
2
1/SE_IV
−0.2
0.0
0.2
0.4
β_IV

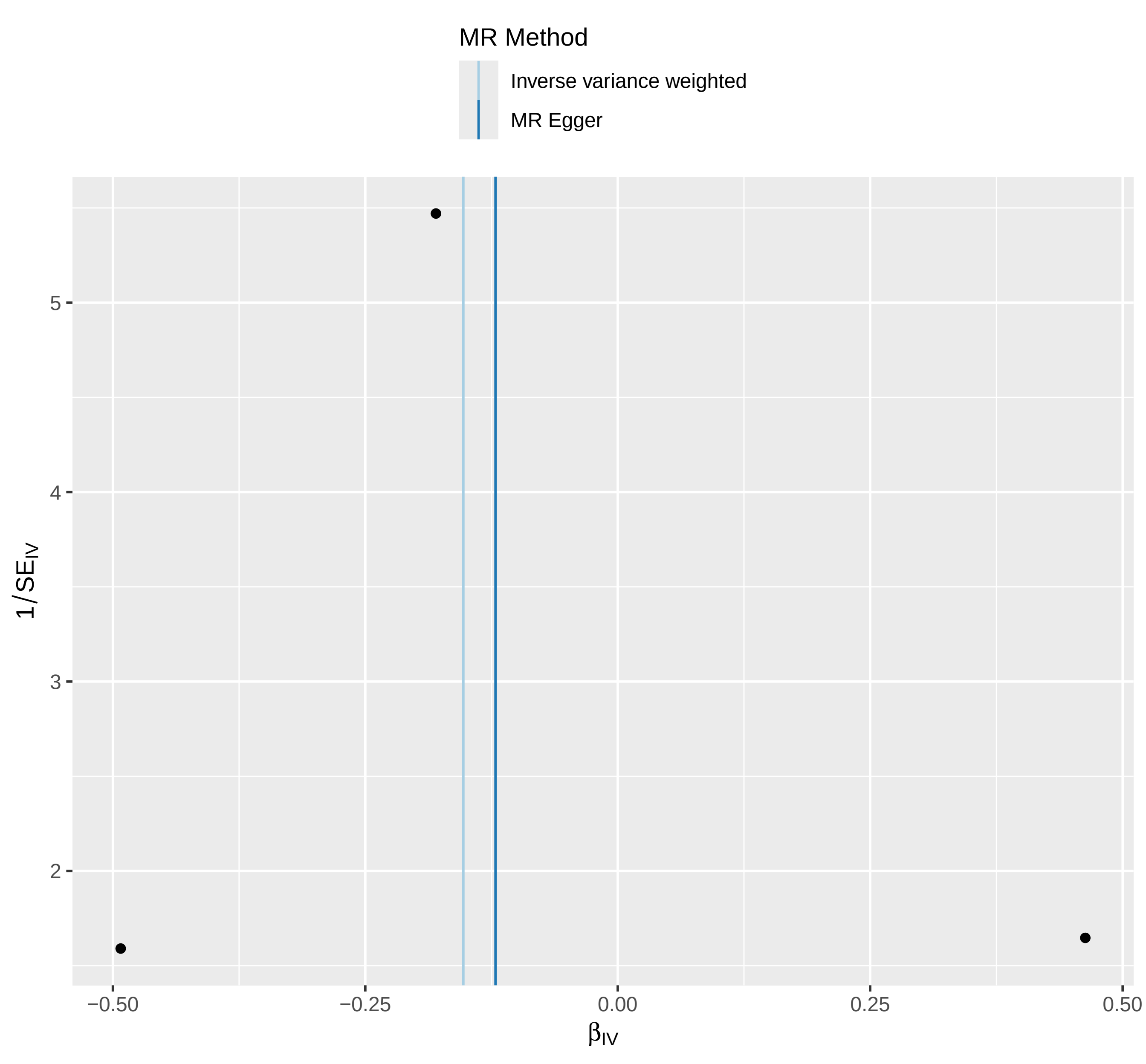

MR Method
Inverse variance weighted
MR Egger
5
4
3
2
1/SE_IV
−0.50
−0.25
0.00
0.25
0.50
β_IV

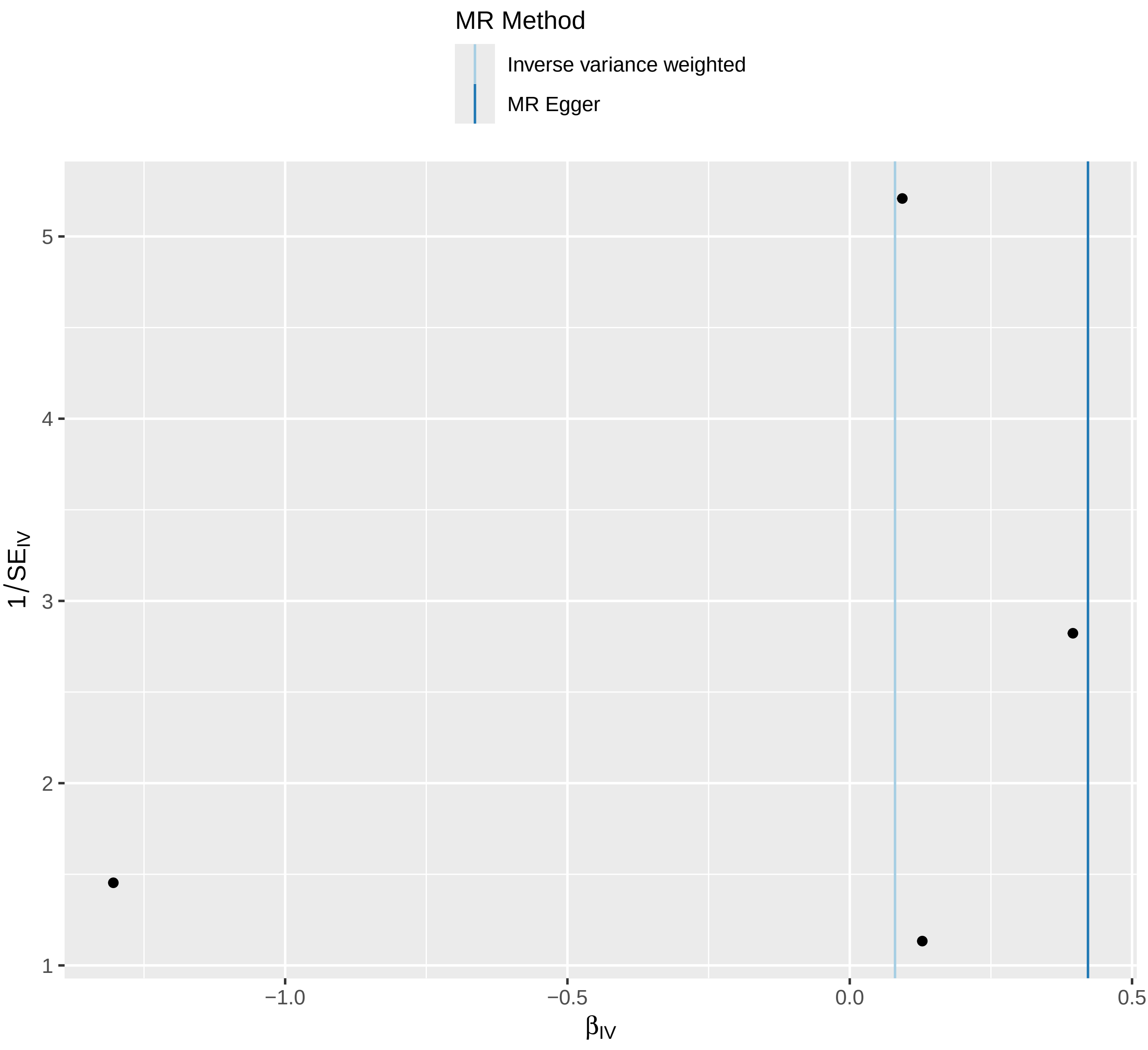

MR Method
Inverse variance weighted
MR Egger
1/SE$_{IV}$
5
4
3
2
1
−1.0
−0.5
0.0
0.5
β$_{IV}$

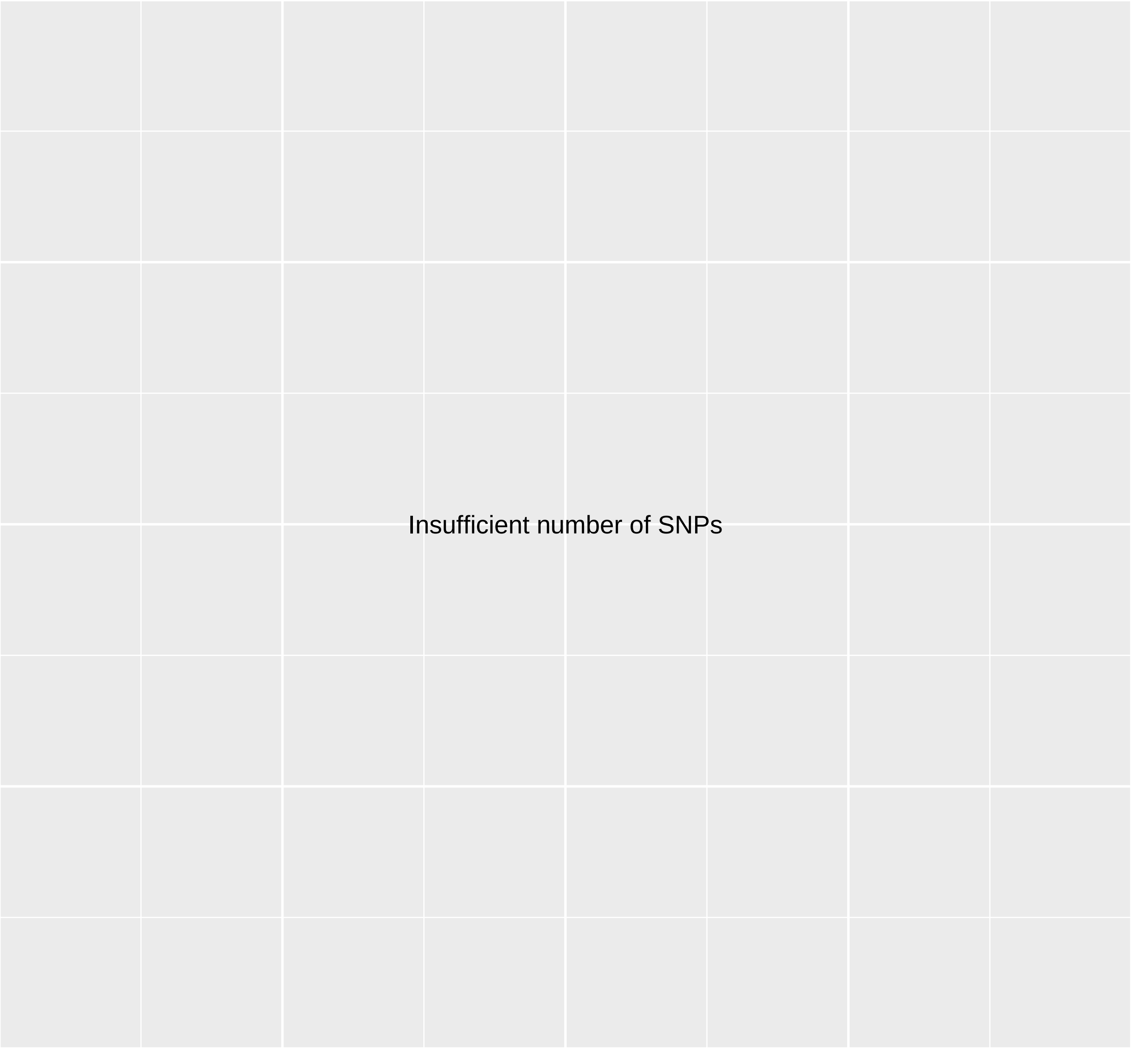
Insufficient number of SNPs

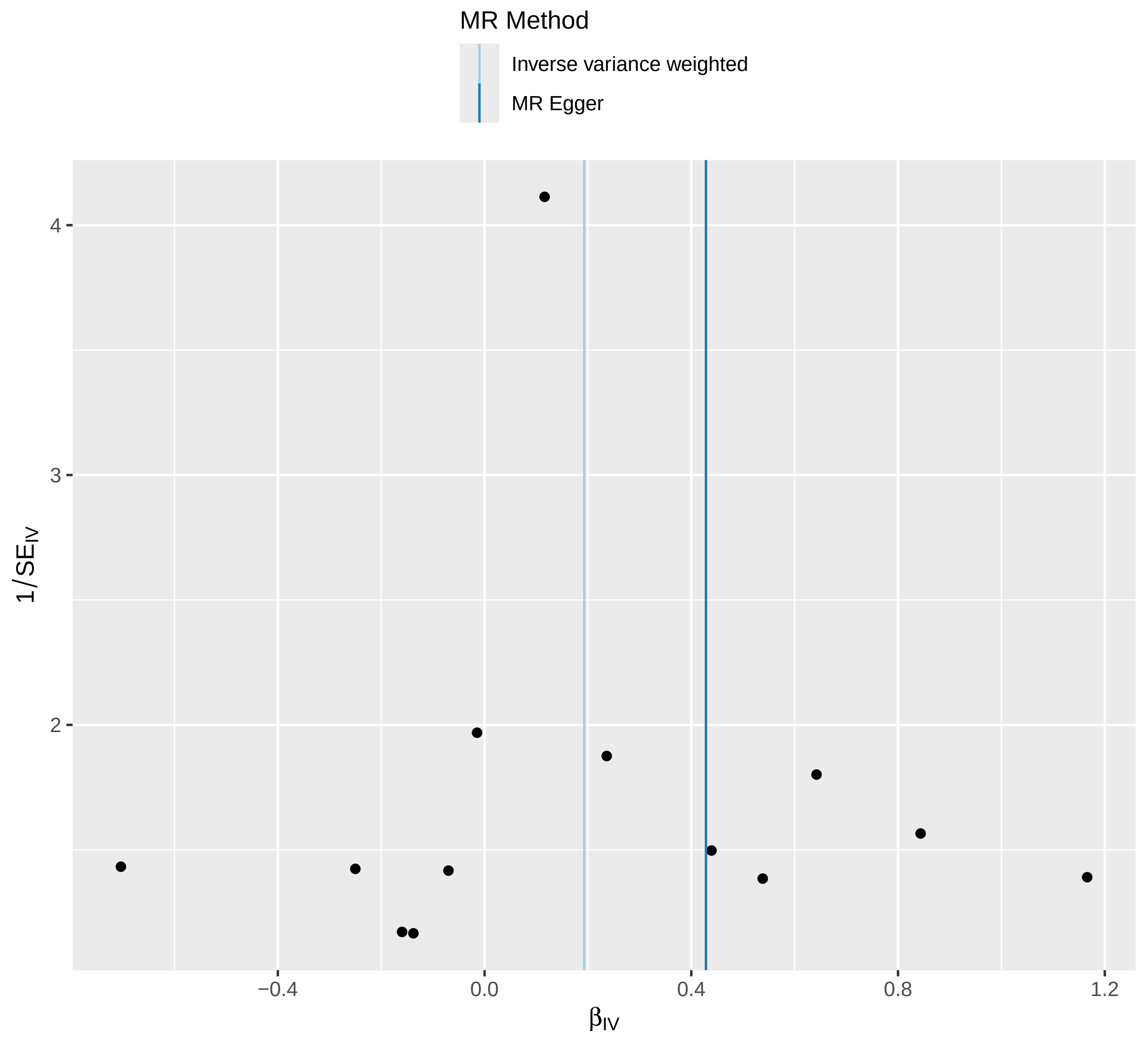

MR Method
Inverse variance weighted
MR Egger
4
3
2
1/SE_IV
−0.4
0.0
0.4
0.8
1.2
β_IV

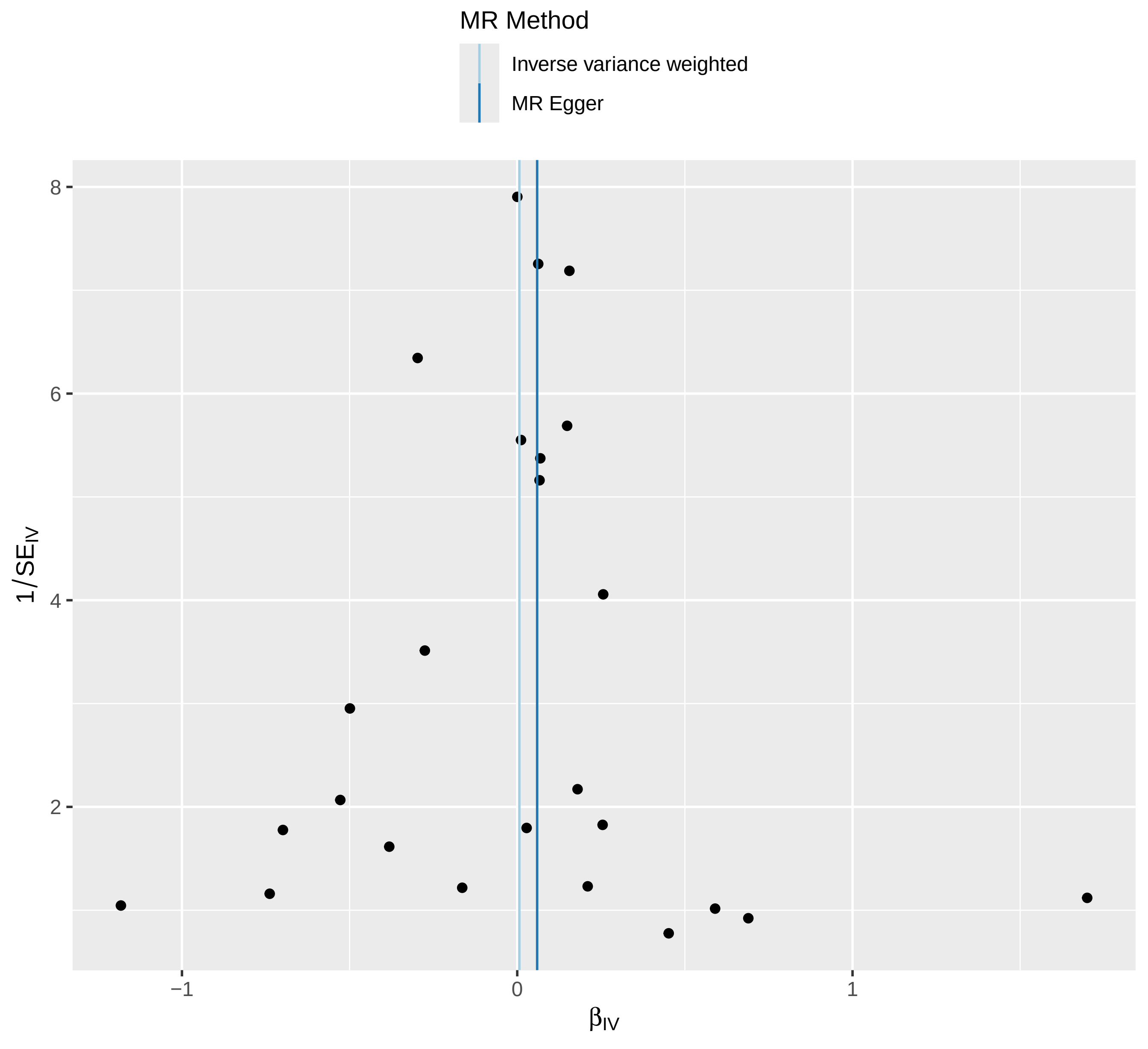

MR Method
Inverse variance weighted
MR Egger
8
6
4
2
1/SE$_{IV}$
−1
0
1
β$_{IV}$

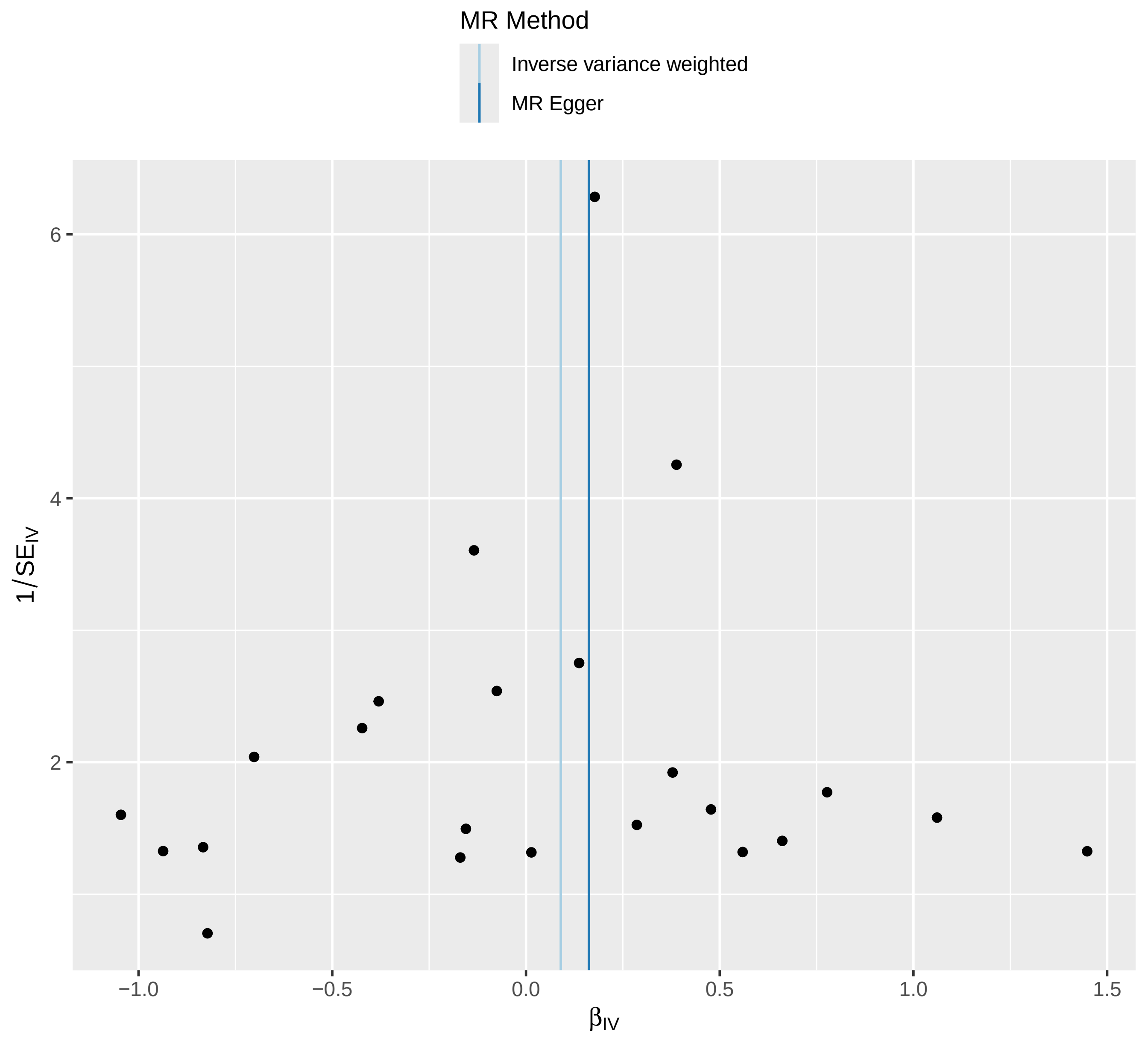

MR Method
Inverse variance weighted
MR Egger
6
4
2
1/SE$_{IV}$
−1.0
−0.5
0.0
0.5
1.0
1.5
β$_{IV}$

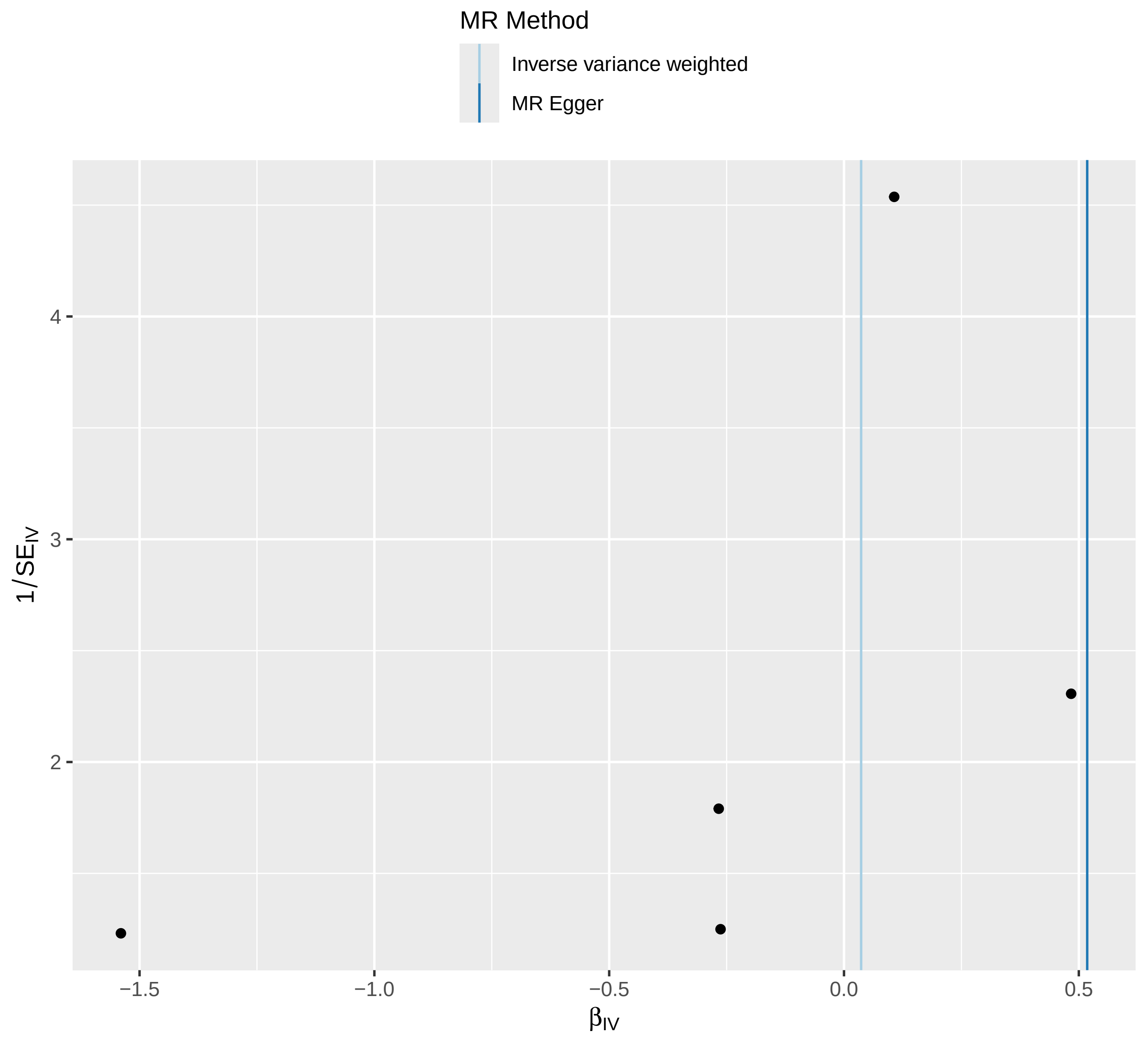

MR Method
Inverse variance weighted
MR Egger
4
3
2
1/SE_IV
−1.5
−1.0
−0.5
0.0
0.5
β_IV

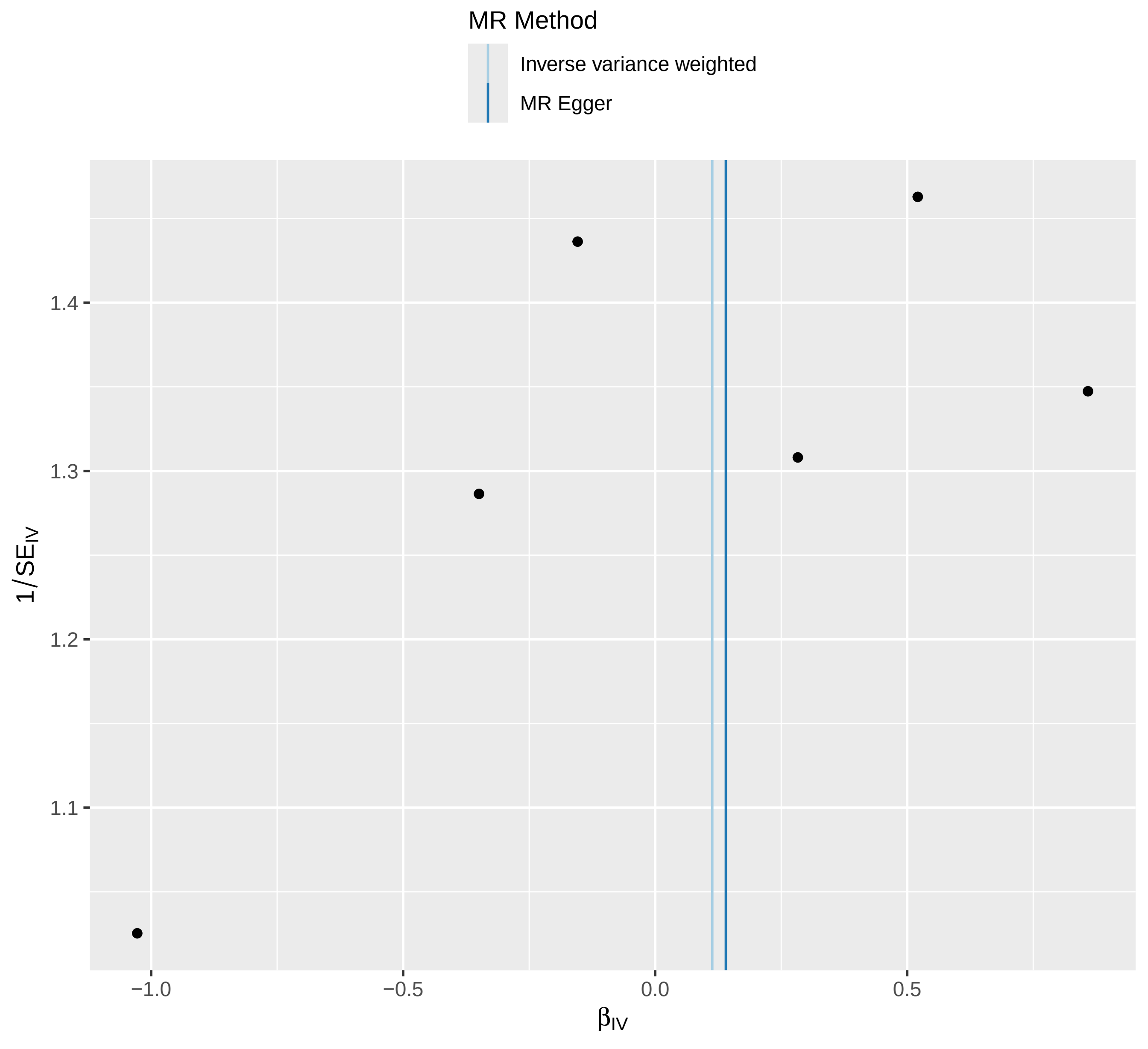

MR Method
Inverse variance weighted
MR Egger
1.4
1.3
1.2
1.1
$1/SE_{IV}$
−1.0
−0.5
0.0
0.5
β_{IV}

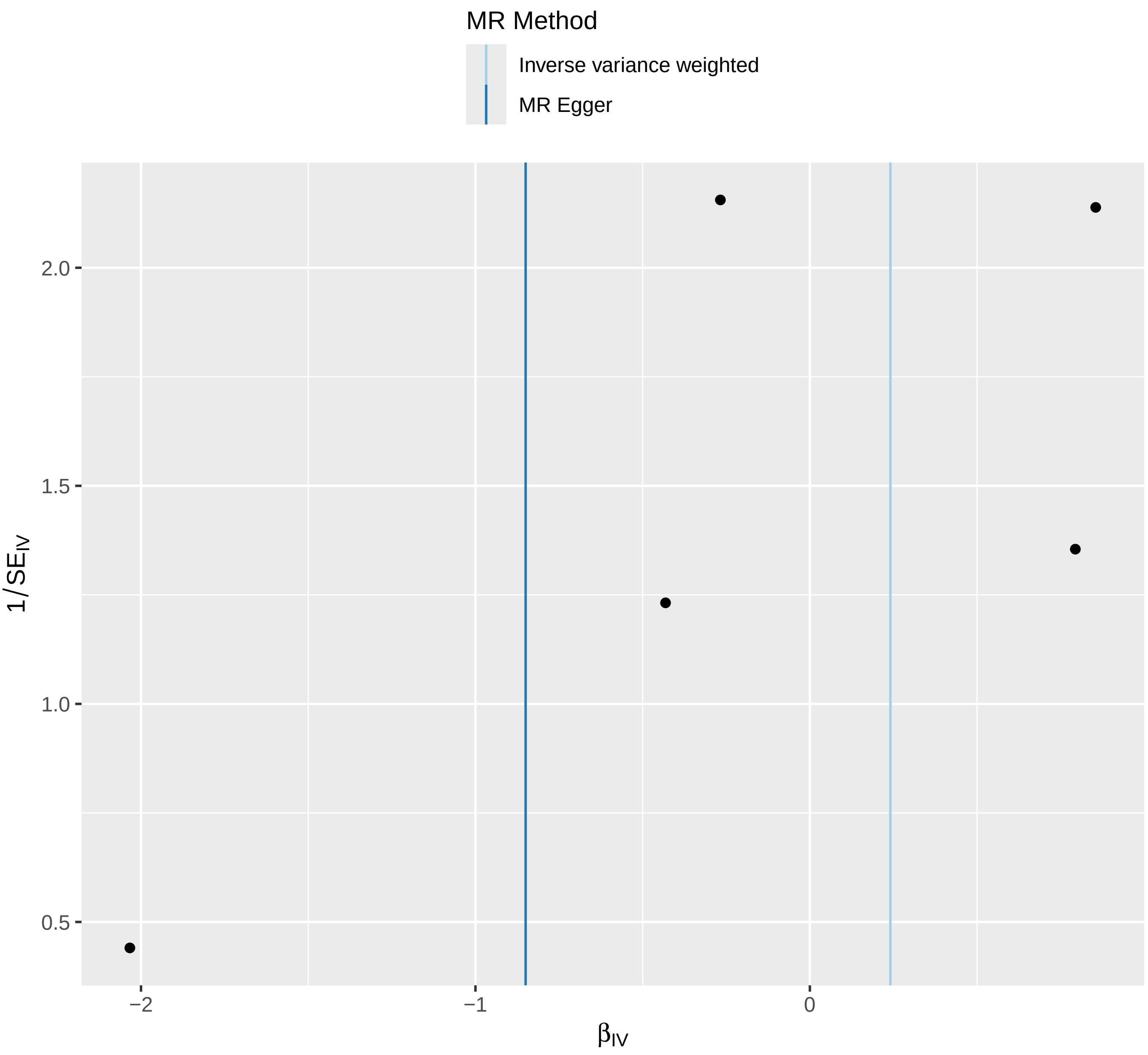

MR Method
Inverse variance weighted
MR Egger
2.0
1.5
1.0
0.5
1/SE_IV
−2
−1
0
β_IV

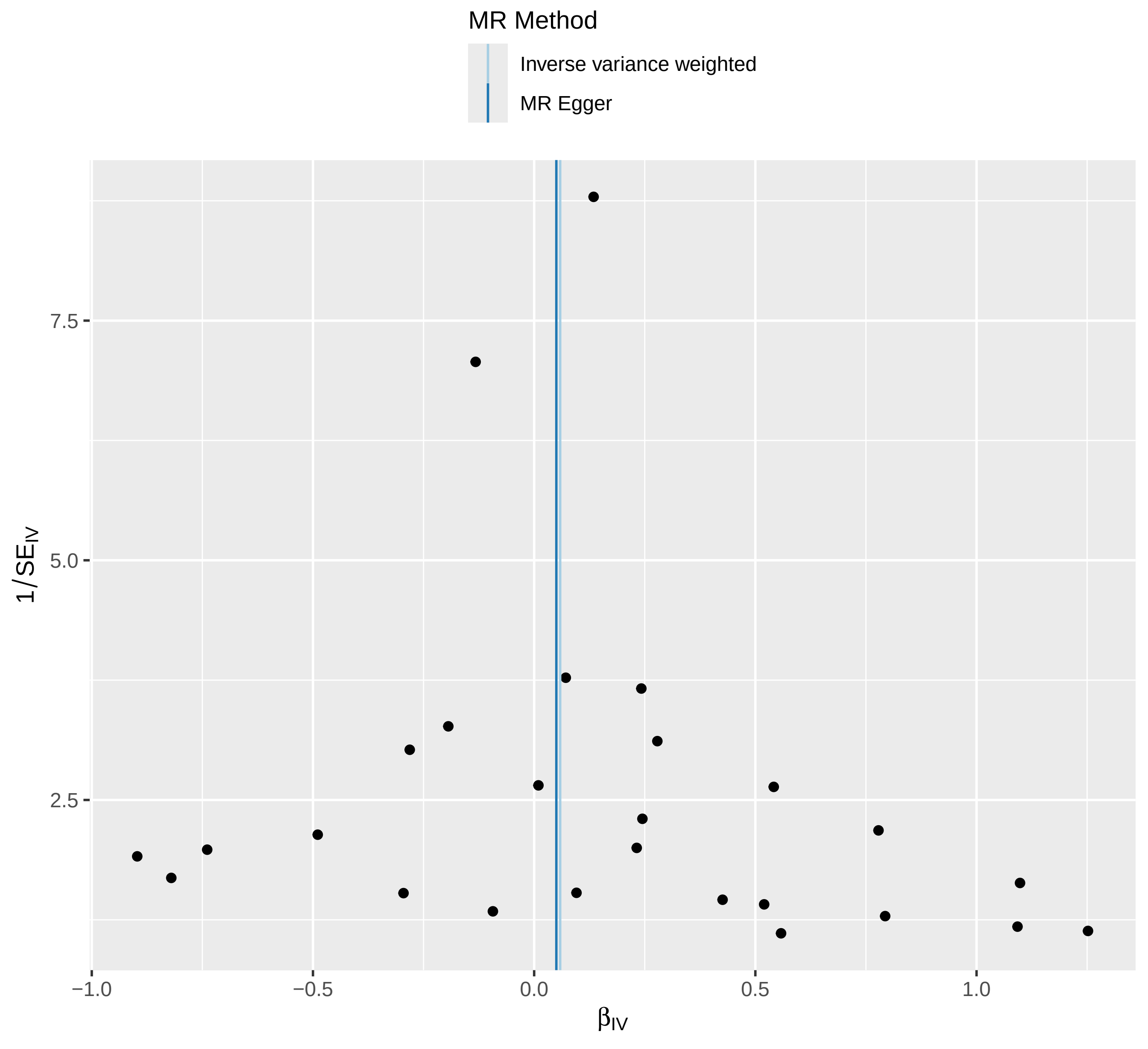
MR Method
Inverse variance weighted
MR Egger
7.5
5.0
2.5
1/SE$_{IV}$
−1.0
−0.5
0.0
0.5
1.0
β$_{IV}$

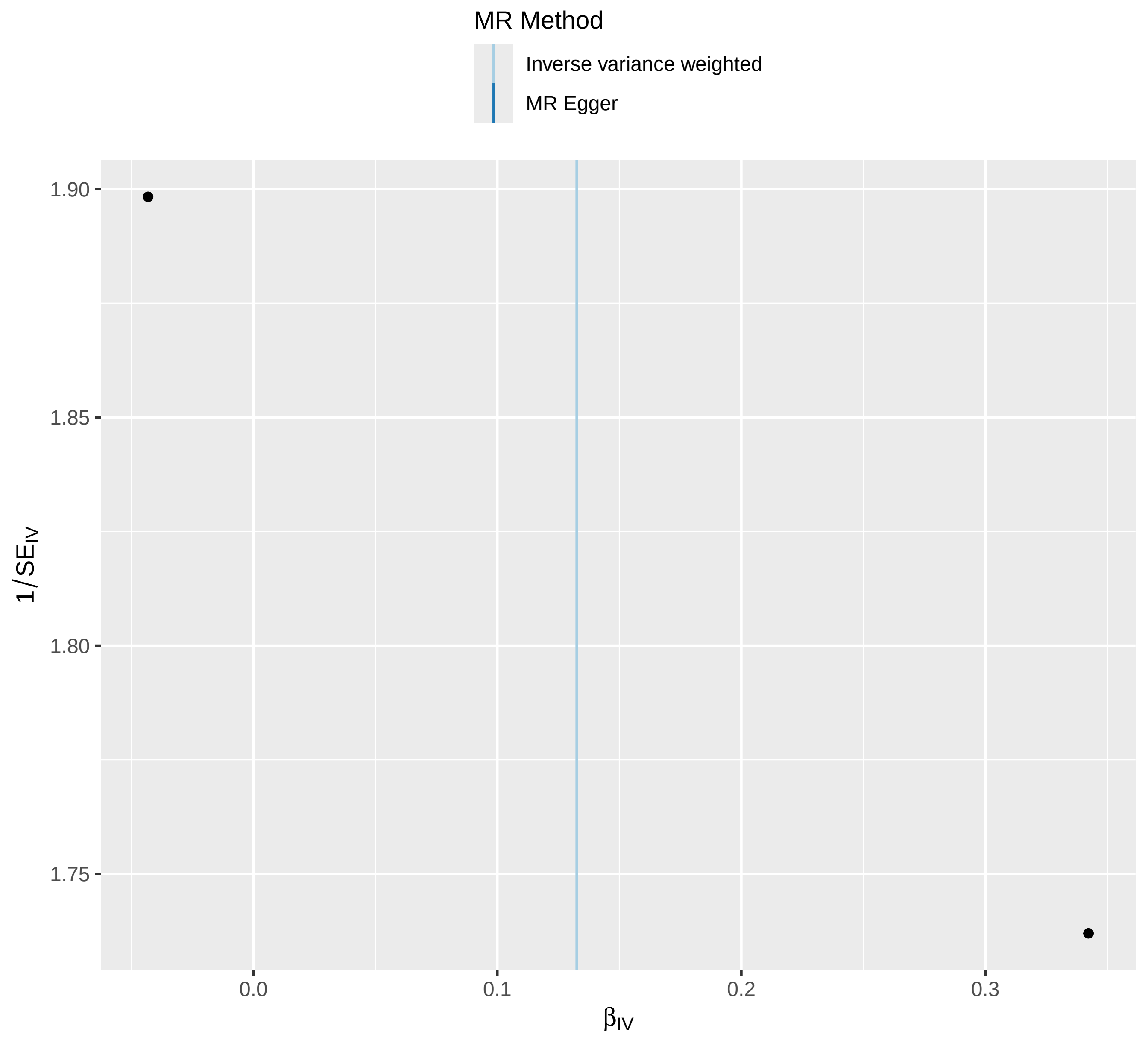

MR Method
Inverse variance weighted
MR Egger
1.90
1.85
1.80
1.75
1/SE_IV
0.0
0.1
0.2
0.3
β_IV

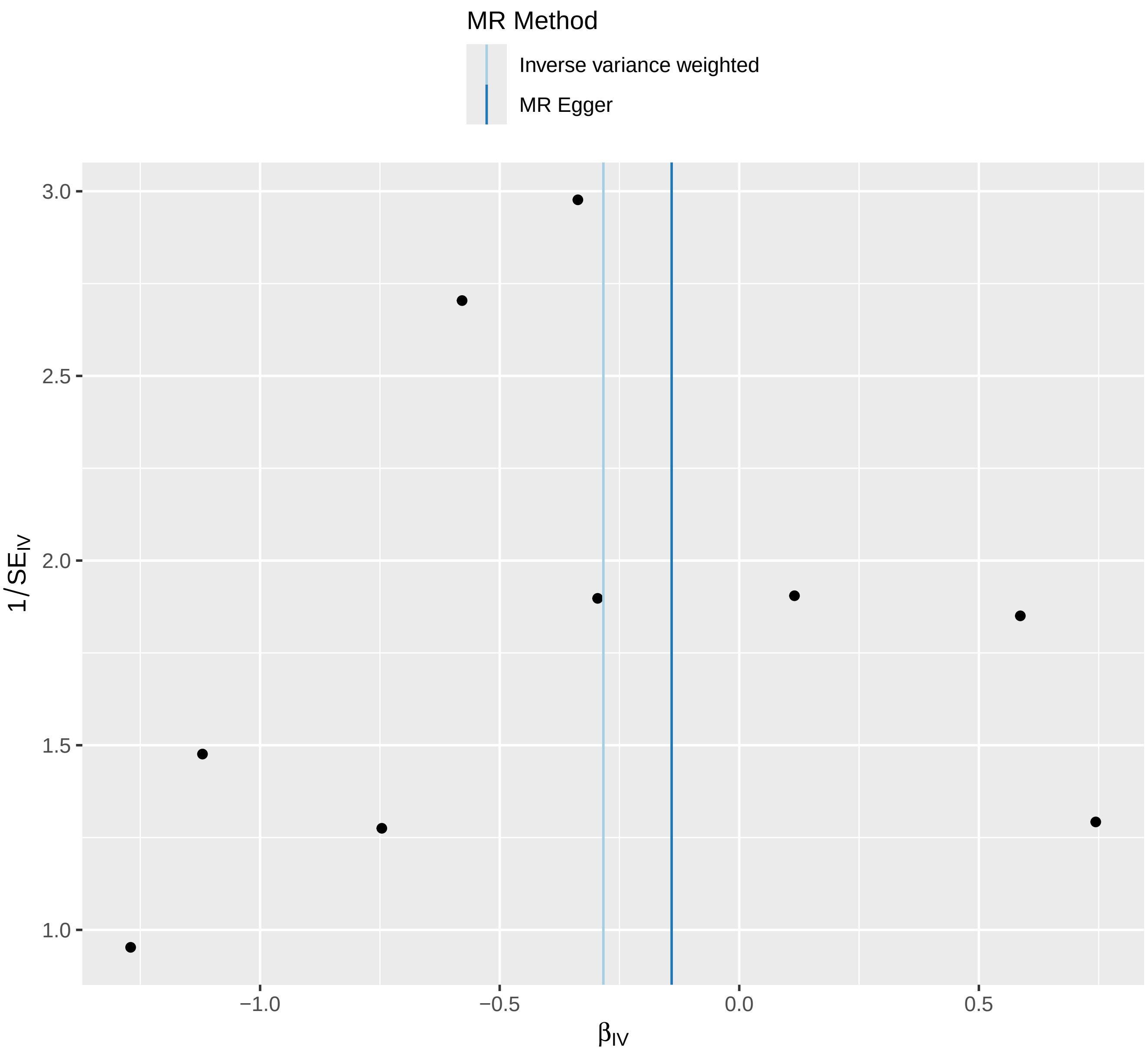
MR Method
Inverse variance weighted
MR Egger
3.0
2.5
2.0
1.5
1.0
−1.0
−0.5
0.0
0.5
1/SE_IV
β_IV

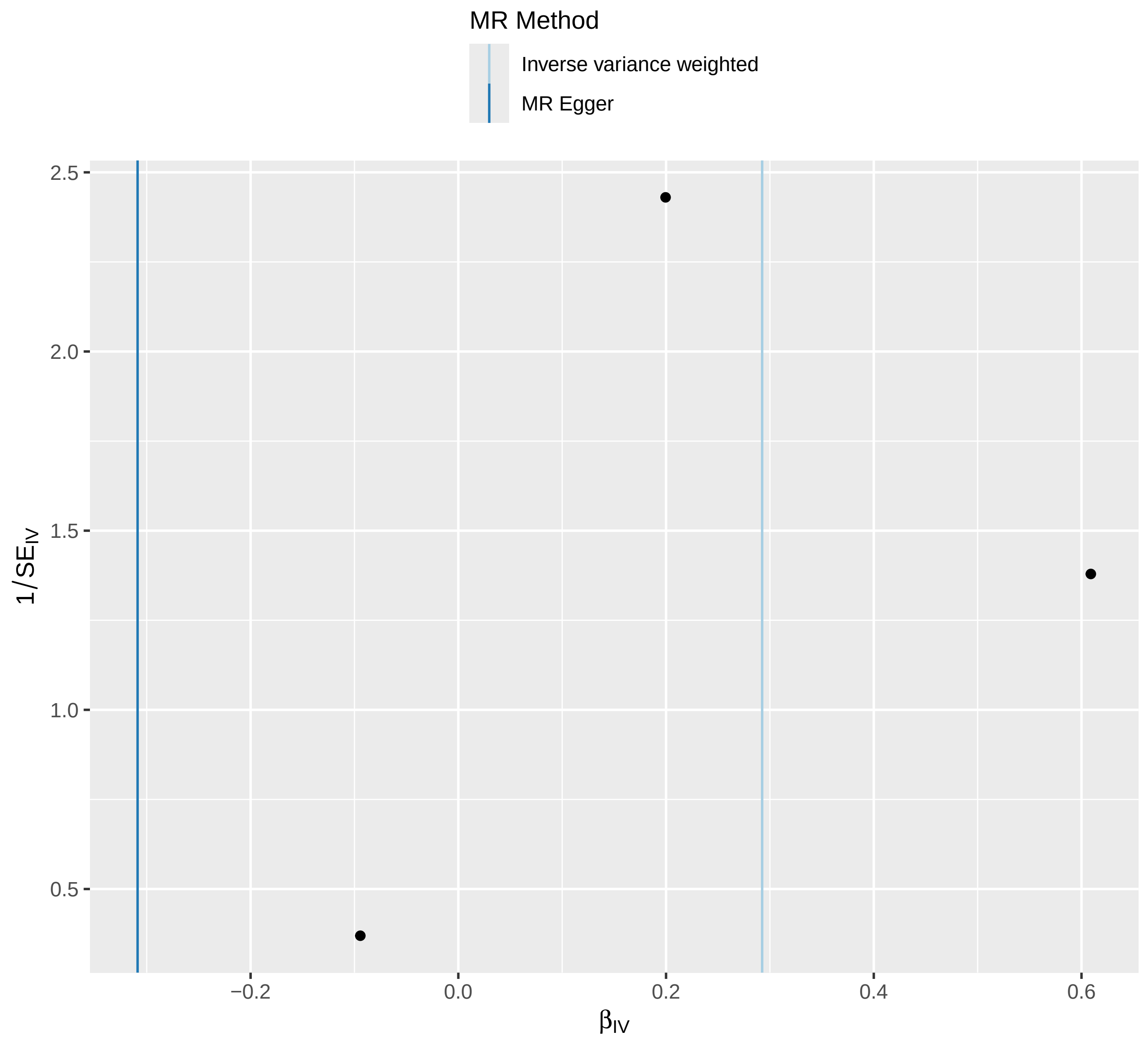

MR Method
Inverse variance weighted
MR Egger
2.5
2.0
1.5
1.0
0.5
$1/SE_{IV}$
−0.2
0.0
0.2
0.4
0.6
β_{IV}

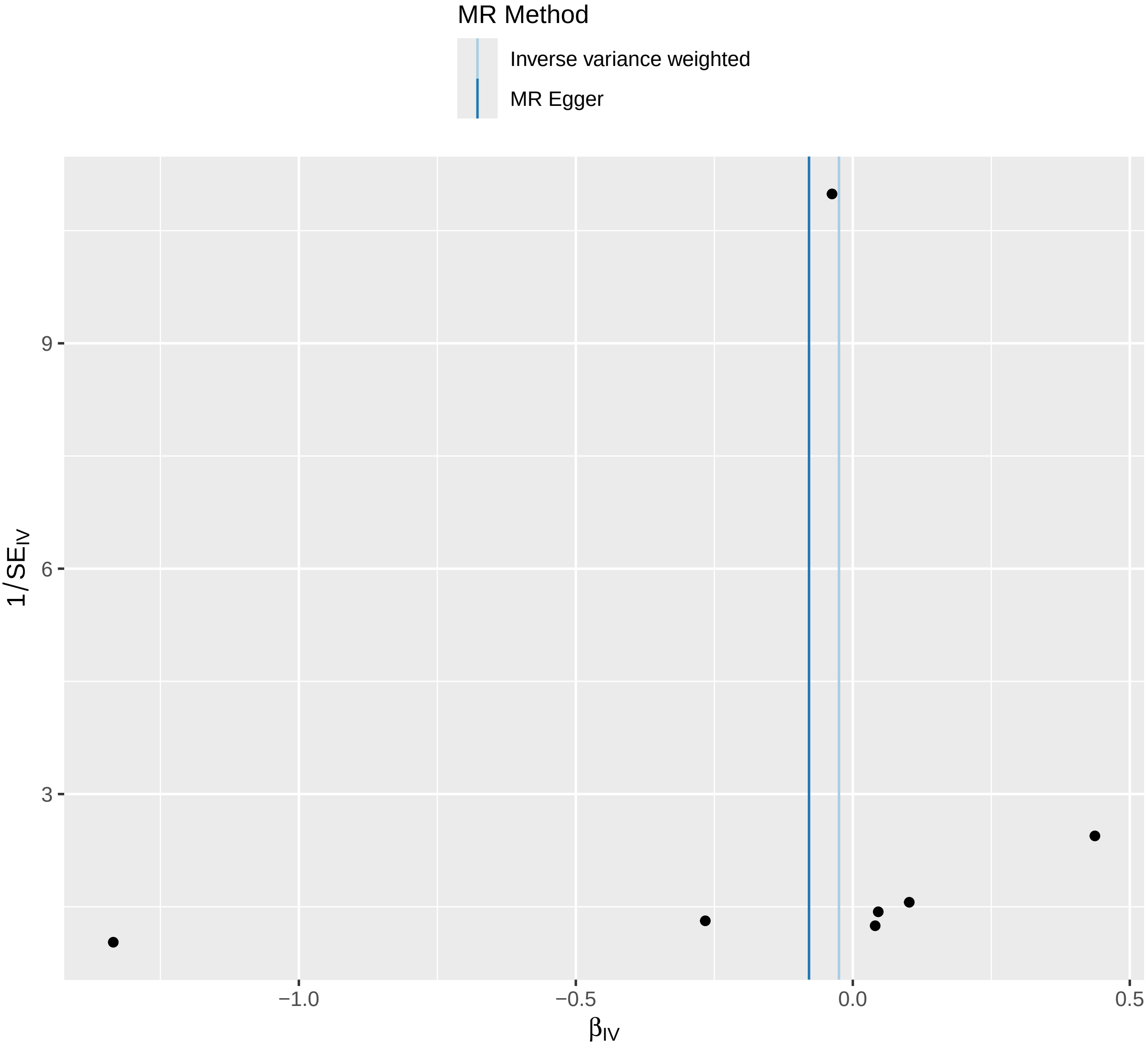
MR Method
Inverse variance weighted
MR Egger
1/SE$_{IV}$
9
6
3
−1.0
−0.5
0.0
0.5
β$_{IV}$

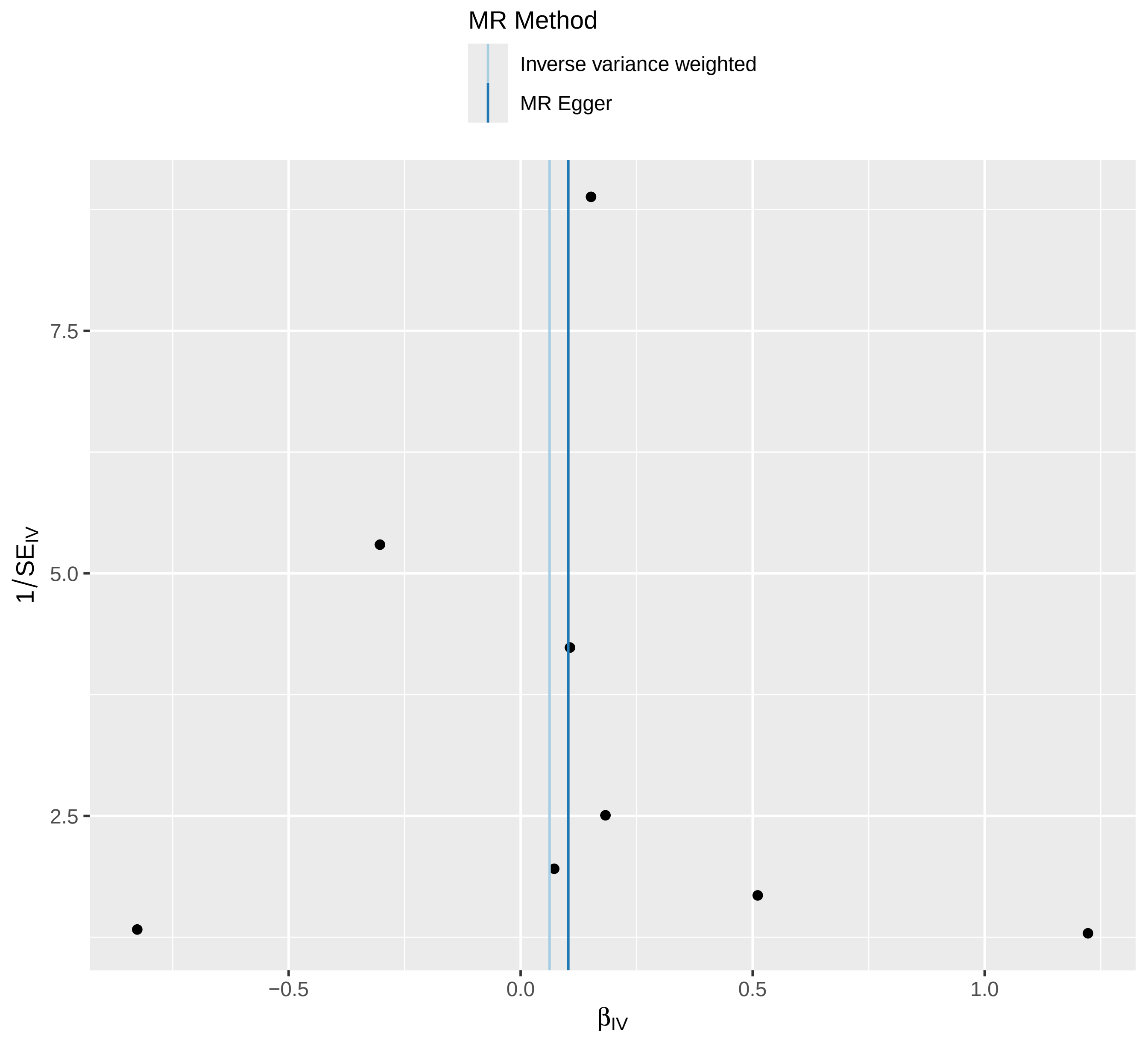

MR Method
Inverse variance weighted
MR Egger
1/SE_IV
7.5
5.0
2.5
−0.5
0.0
0.5
1.0
β_IV

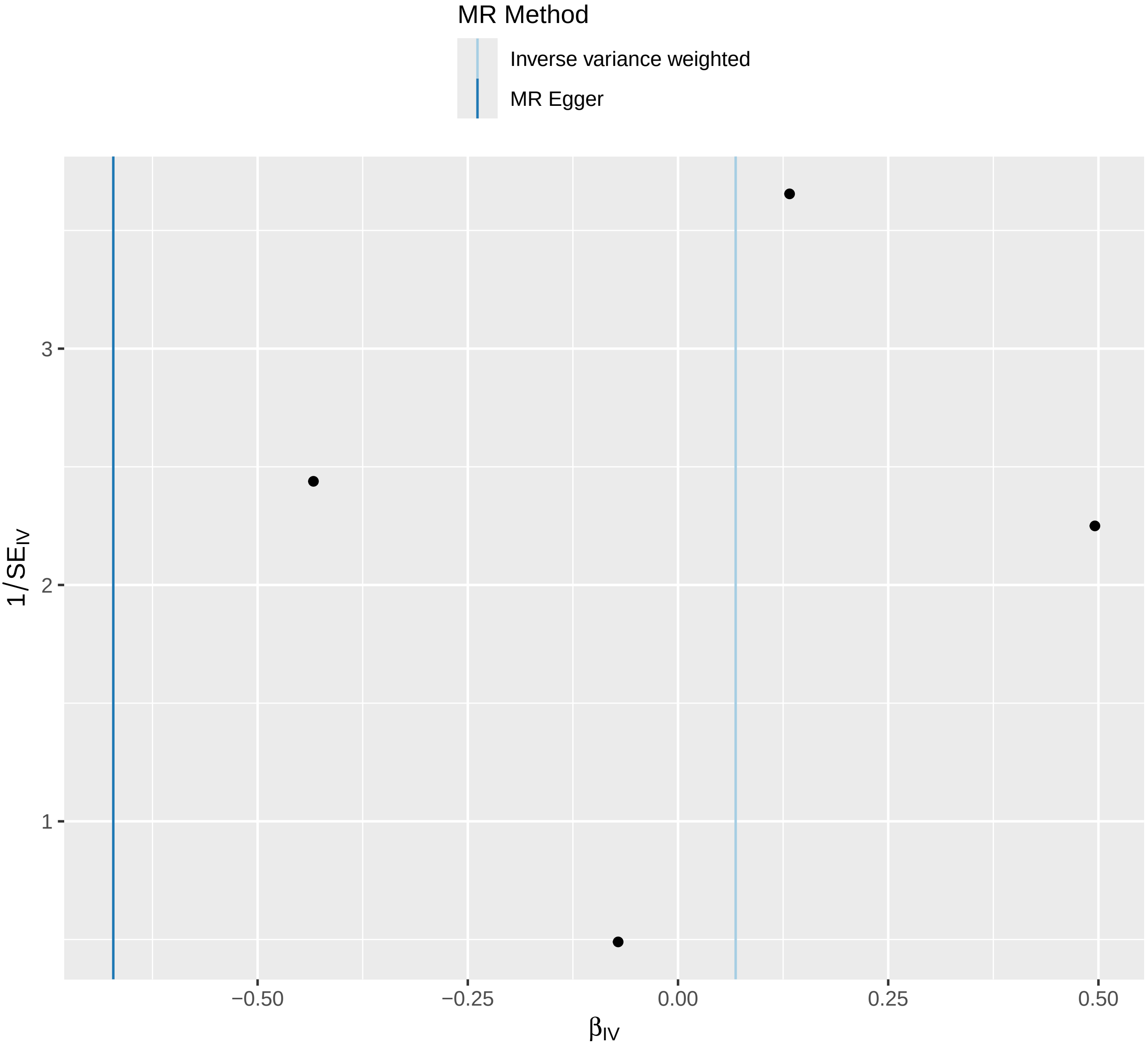
MR Method
Inverse variance weighted
MR Egger
1/SE$_{IV}$
3
2
1
−0.50
−0.25
0.00
0.25
0.50
β$_{IV}$

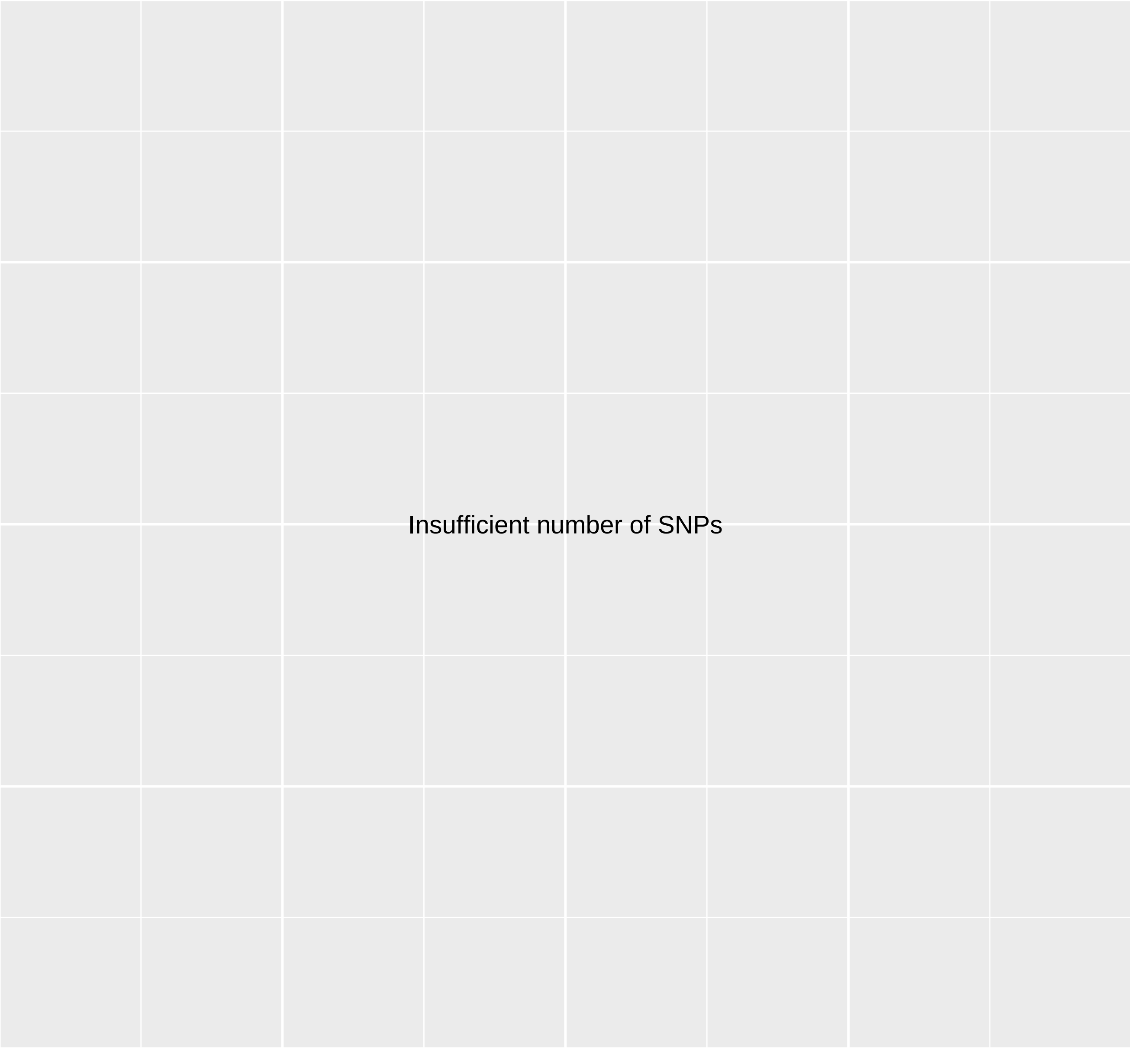
Insufficient number of SNPs

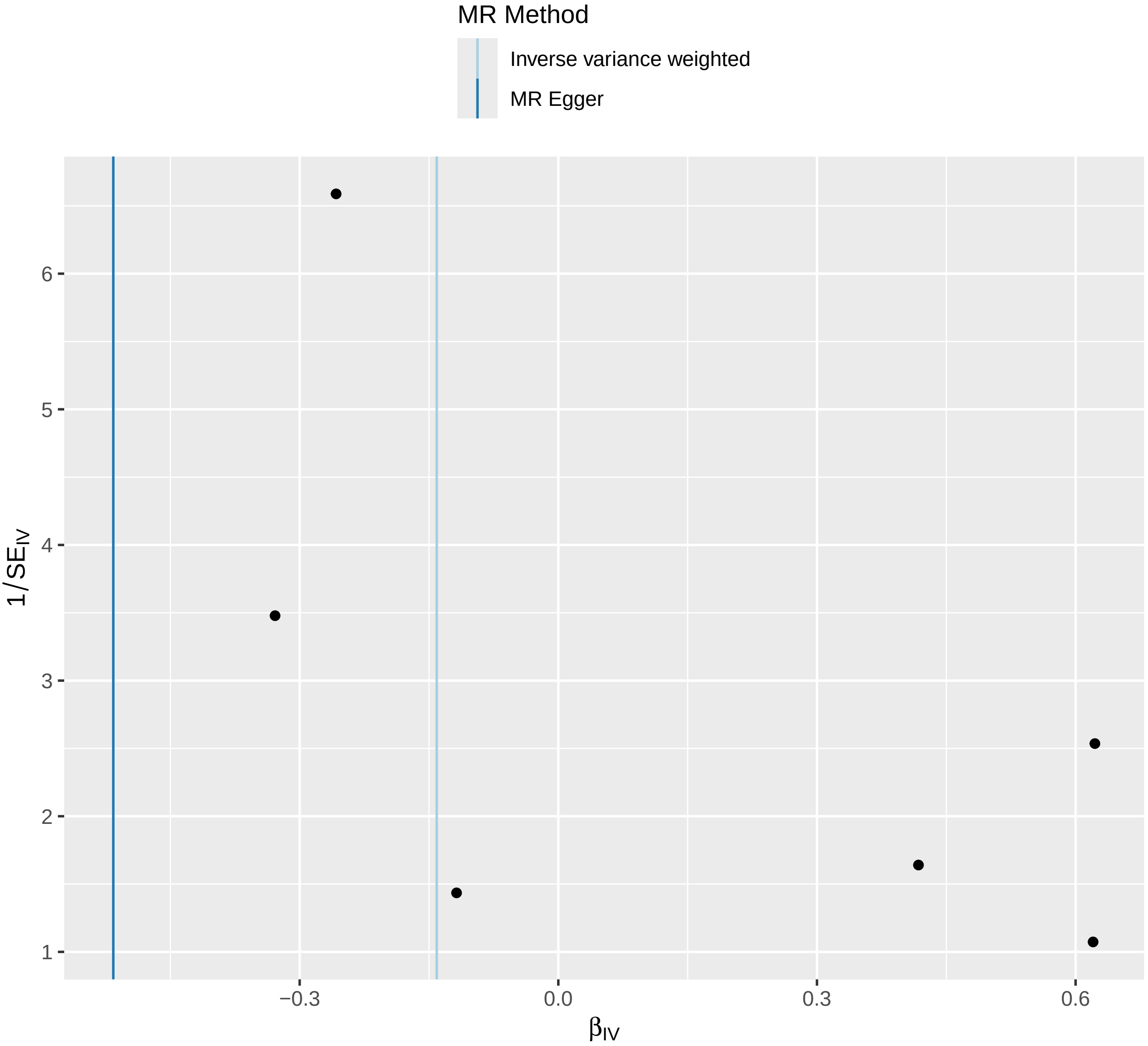
MR Method
Inverse variance weighted
MR Egger
6
5
4
3
2
1
1/SE$_{IV}$
−0.3
0.0
0.3
0.6
β$_{IV}$

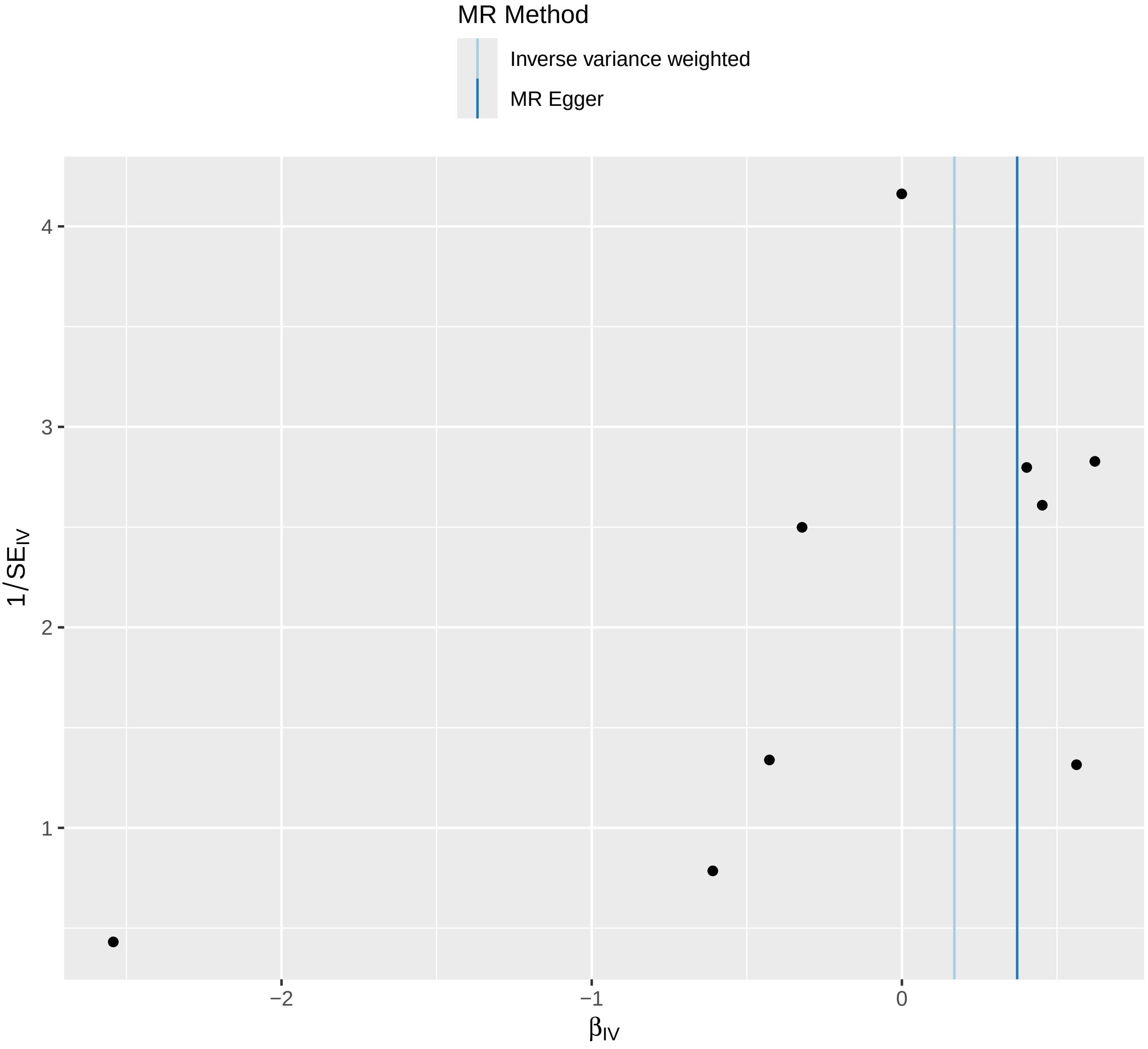
MR Method
Inverse variance weighted
MR Egger
1/SE_{IV}
4
3
2
1
−2
−1
0
β_{IV}

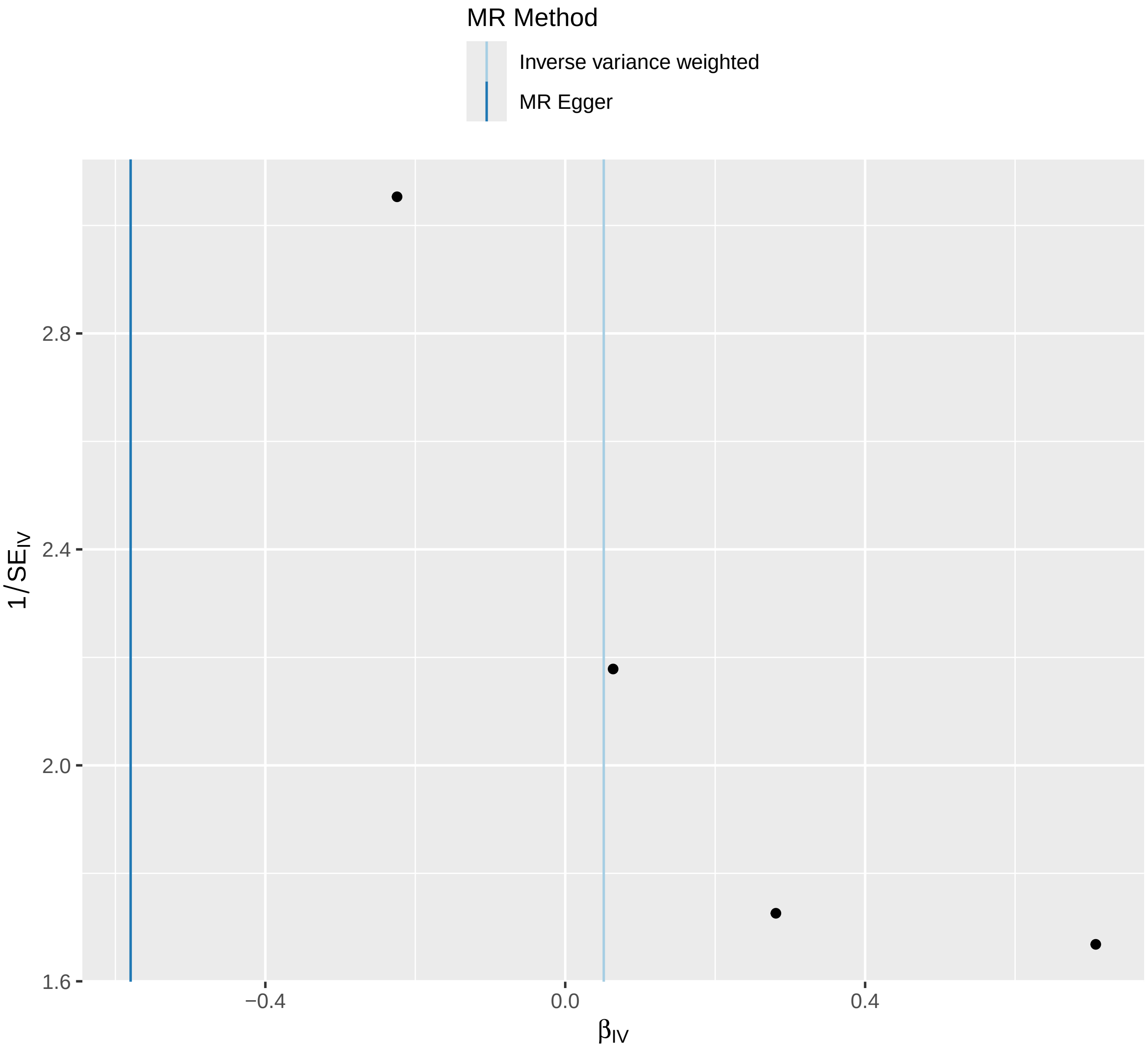
MR Method
Inverse variance weighted
MR Egger
2.8
2.4
2.0
1.6
1/SE_IV
−0.4
0.0
0.4
β_IV

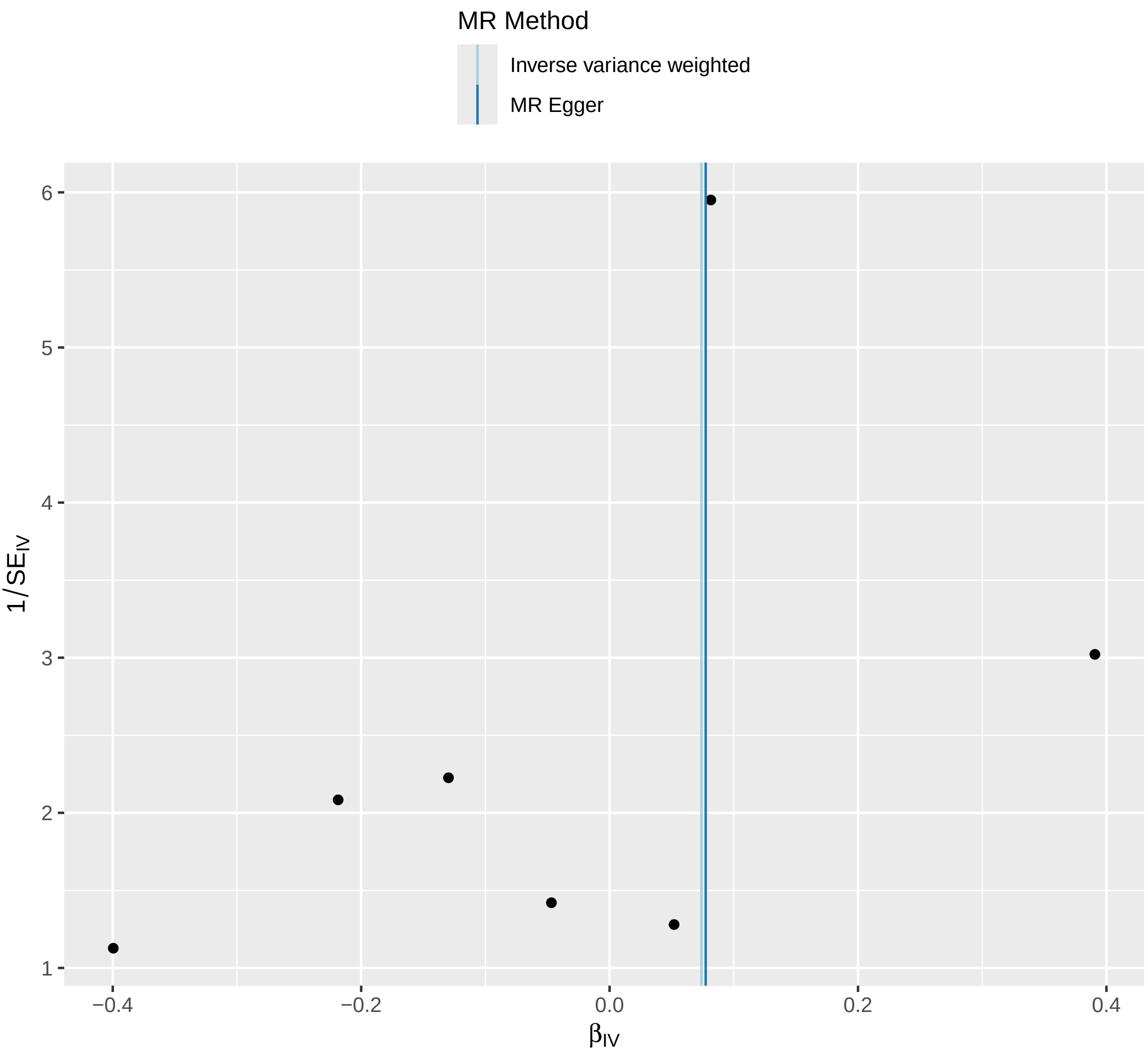

MR Method
Inverse variance weighted
MR Egger
6
5
4
3
2
1
$1/SE_{IV}$
−0.4
−0.2
0.0
0.2
0.4
β_{IV}

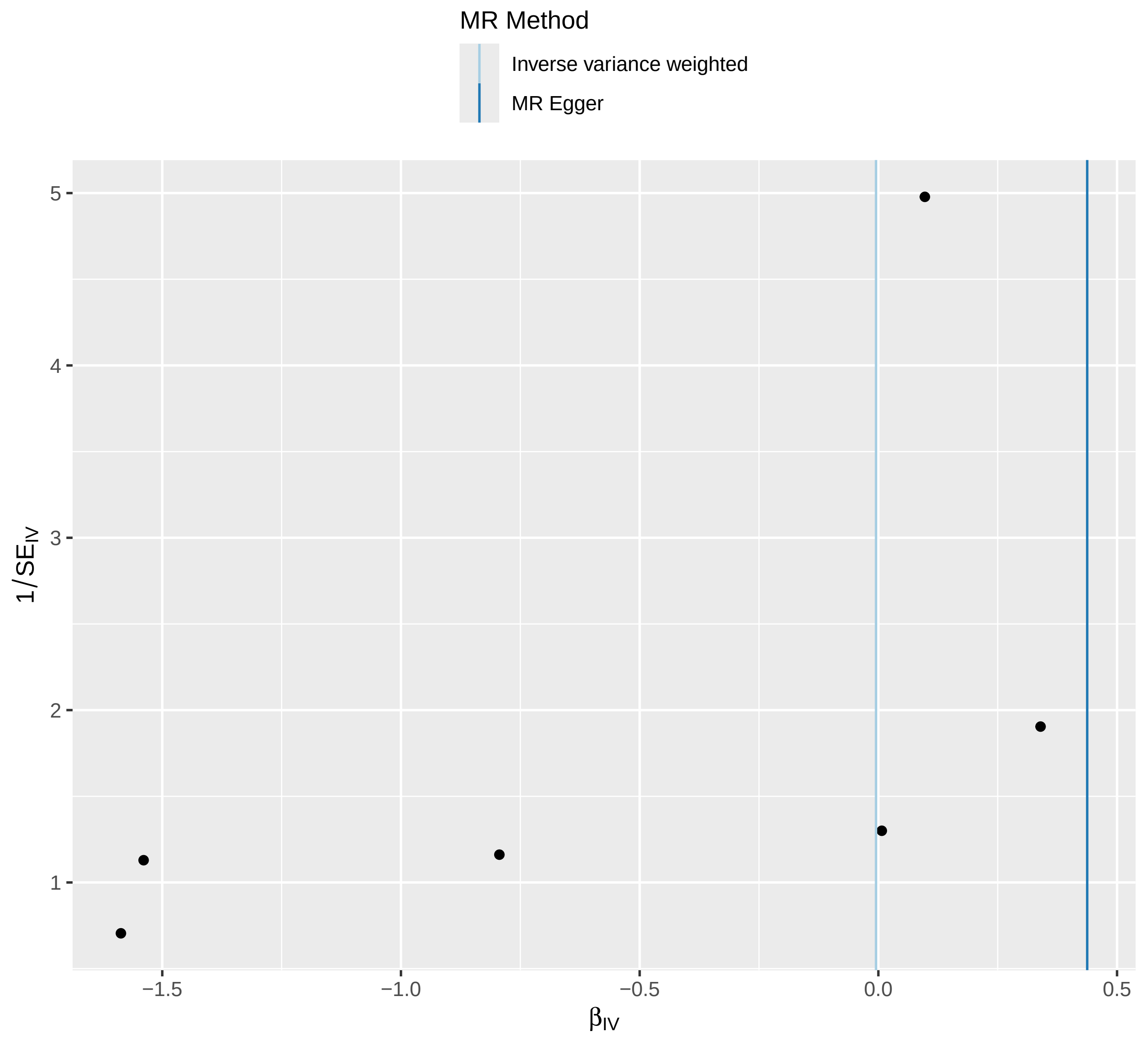

MR Method
Inverse variance weighted
MR Egger
5
4
3
2
1
1/SE_IV
−1.5
−1.0
−0.5
0.0
0.5
β_IV

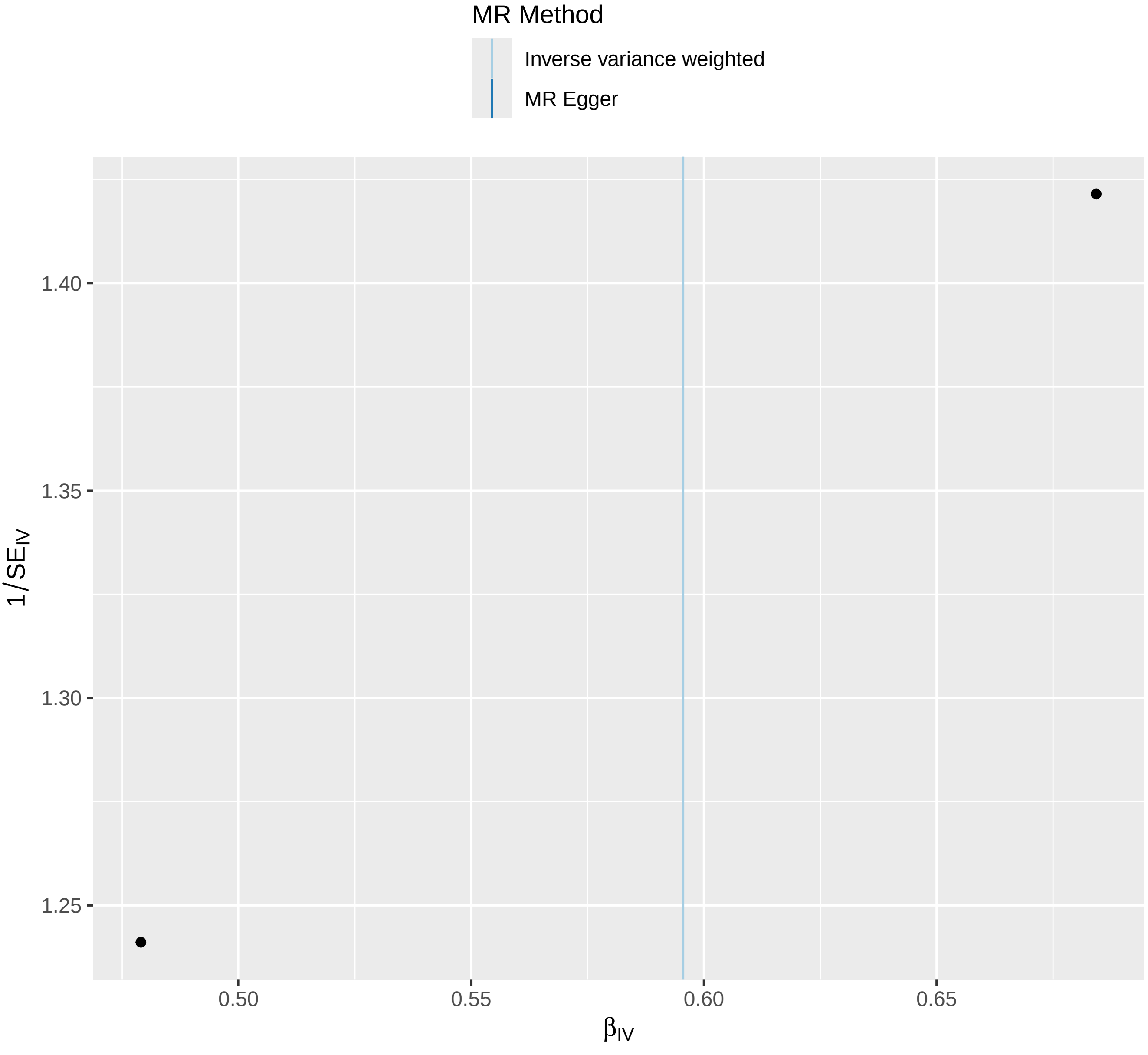
MR Method
Inverse variance weighted
MR Egger
1.40
1.35
1.30
1.25
1/SE_IV
0.50
0.55
0.60
0.65
β_IV

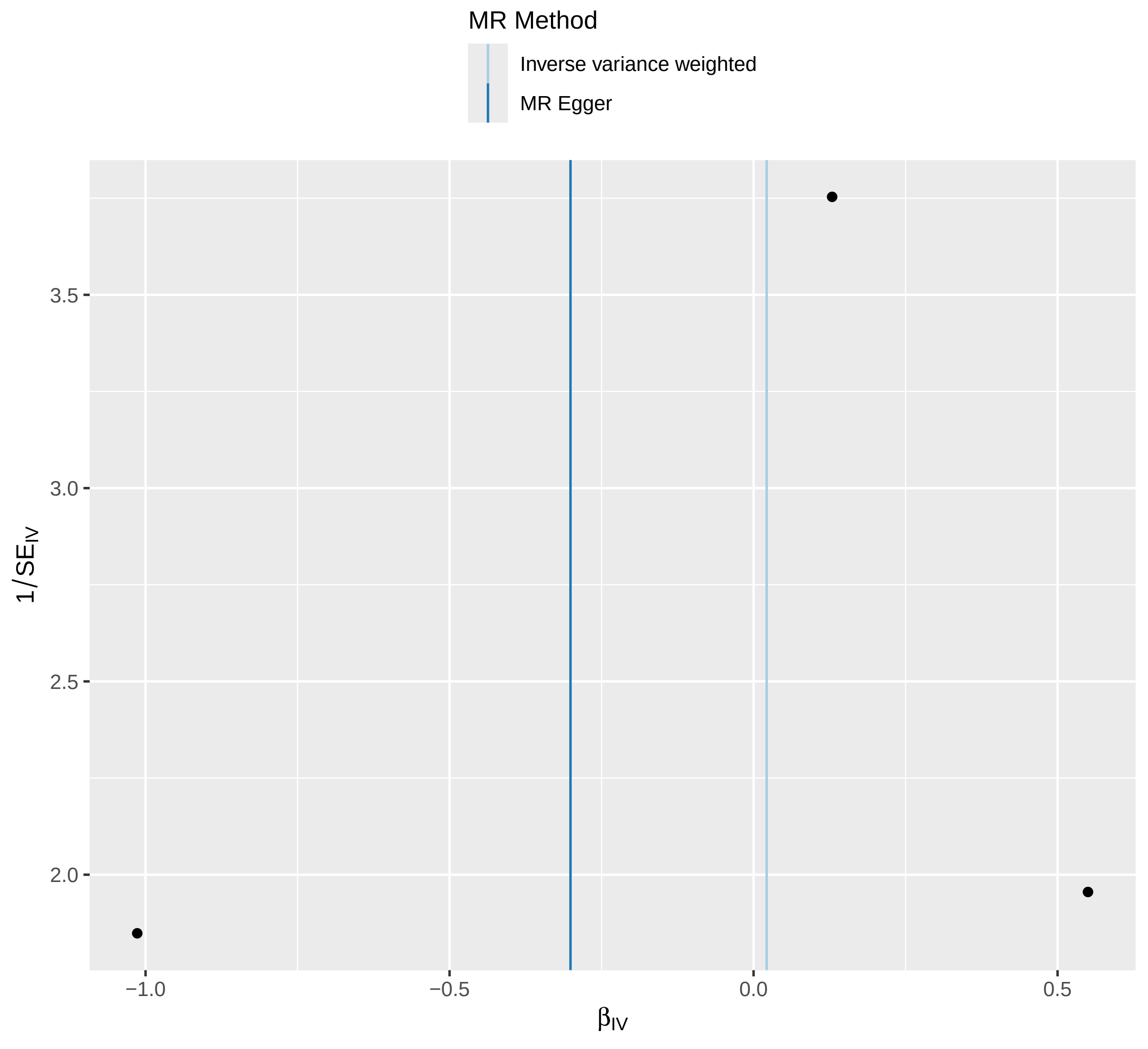

MR Method
Inverse variance weighted
MR Egger
3.5
3.0
2.5
2.0
1/SE_IV
−1.0
−0.5
0.0
0.5
β_IV

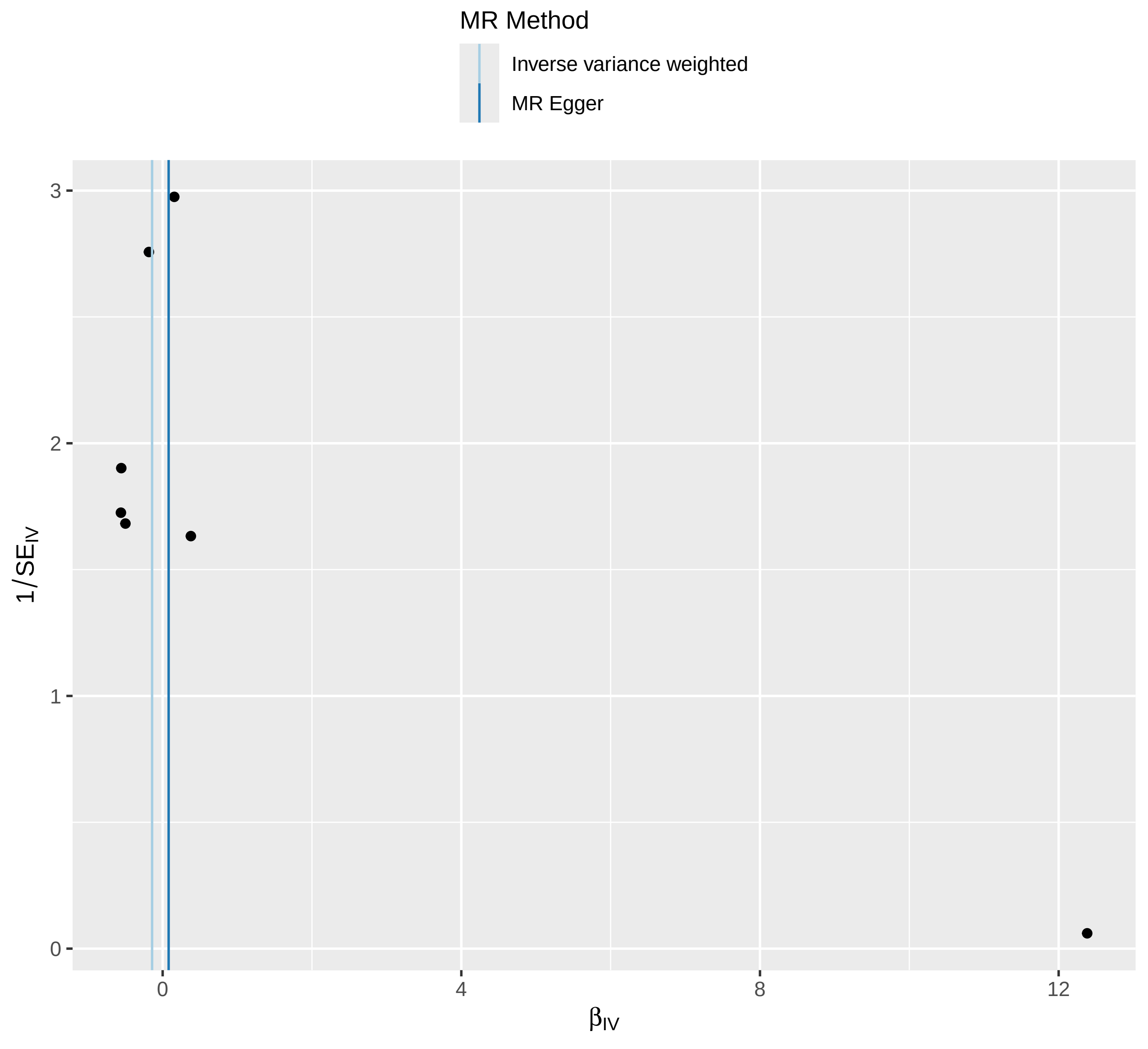

MR Method
Inverse variance weighted
MR Egger
3
2
1
0
1/SE$_{IV}$
0
4
8
12
β$_{IV}$

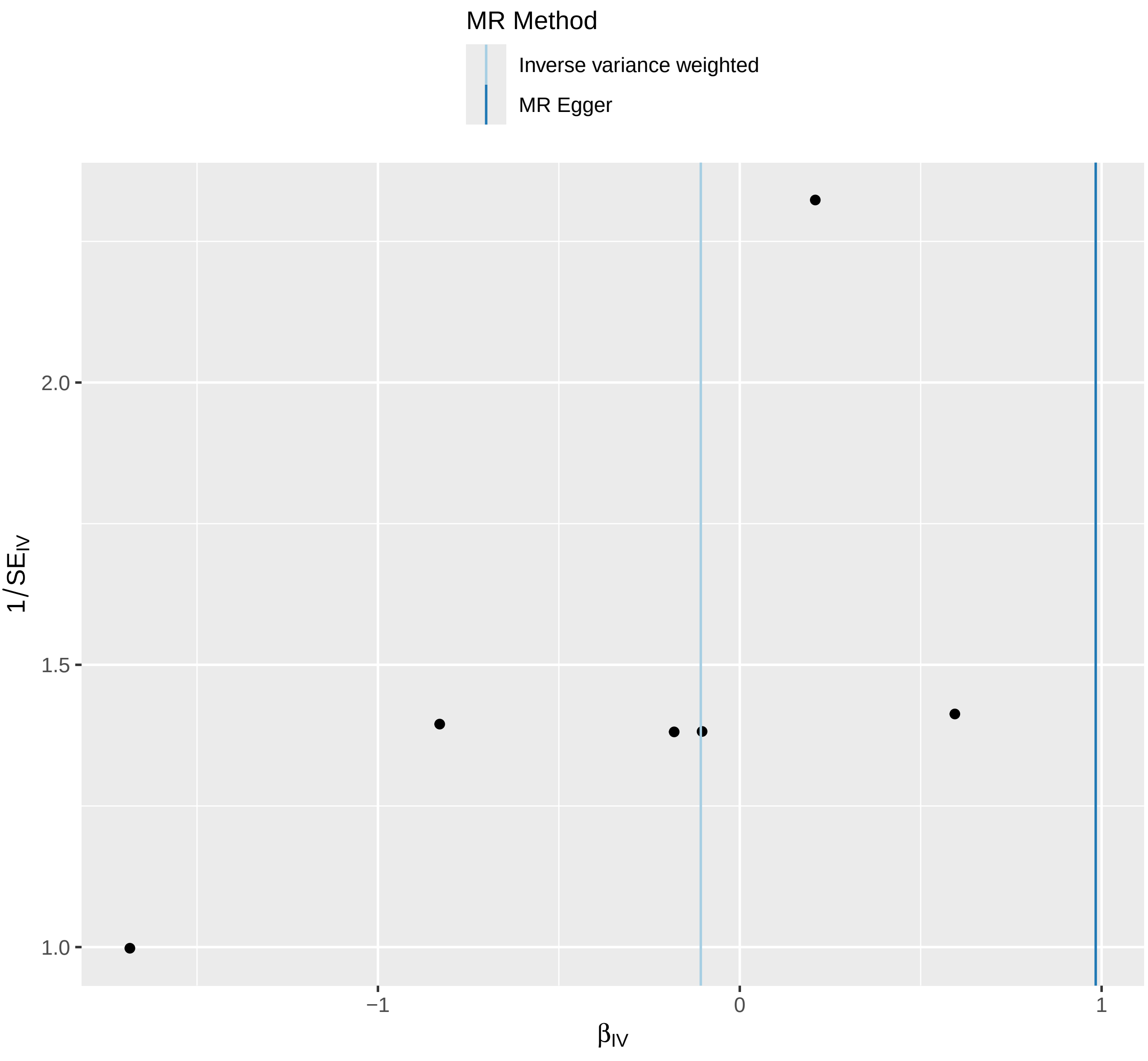
MR Method
Inverse variance weighted
MR Egger
2.0
1.5
1.0
1/SE$_{IV}$
−1
0
1
β$_{IV}$

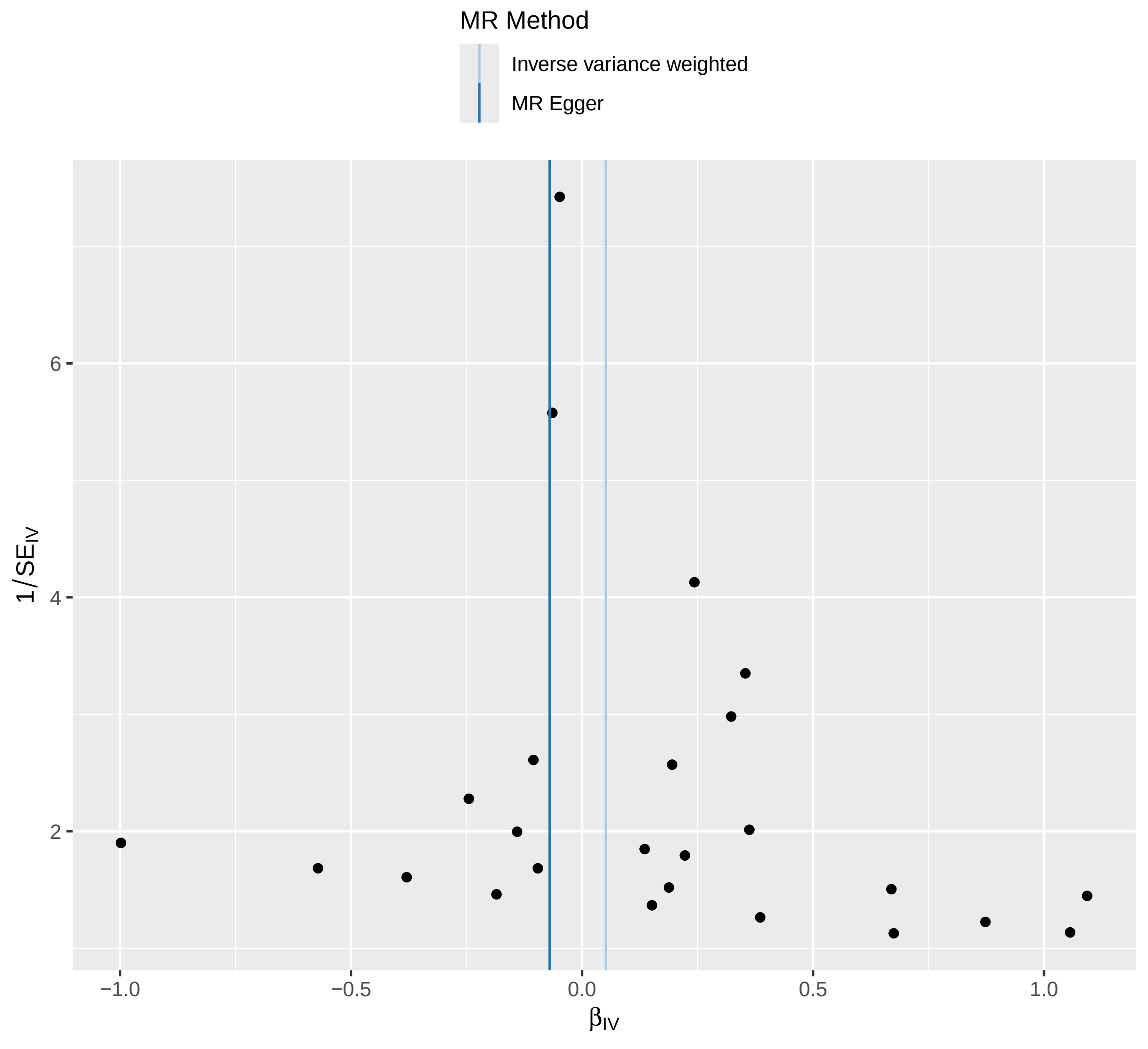

MR Method
Inverse variance weighted
MR Egger
6
4
2
1/SE_IV
−1.0
−0.5
0.0
0.5
1.0
β_IV

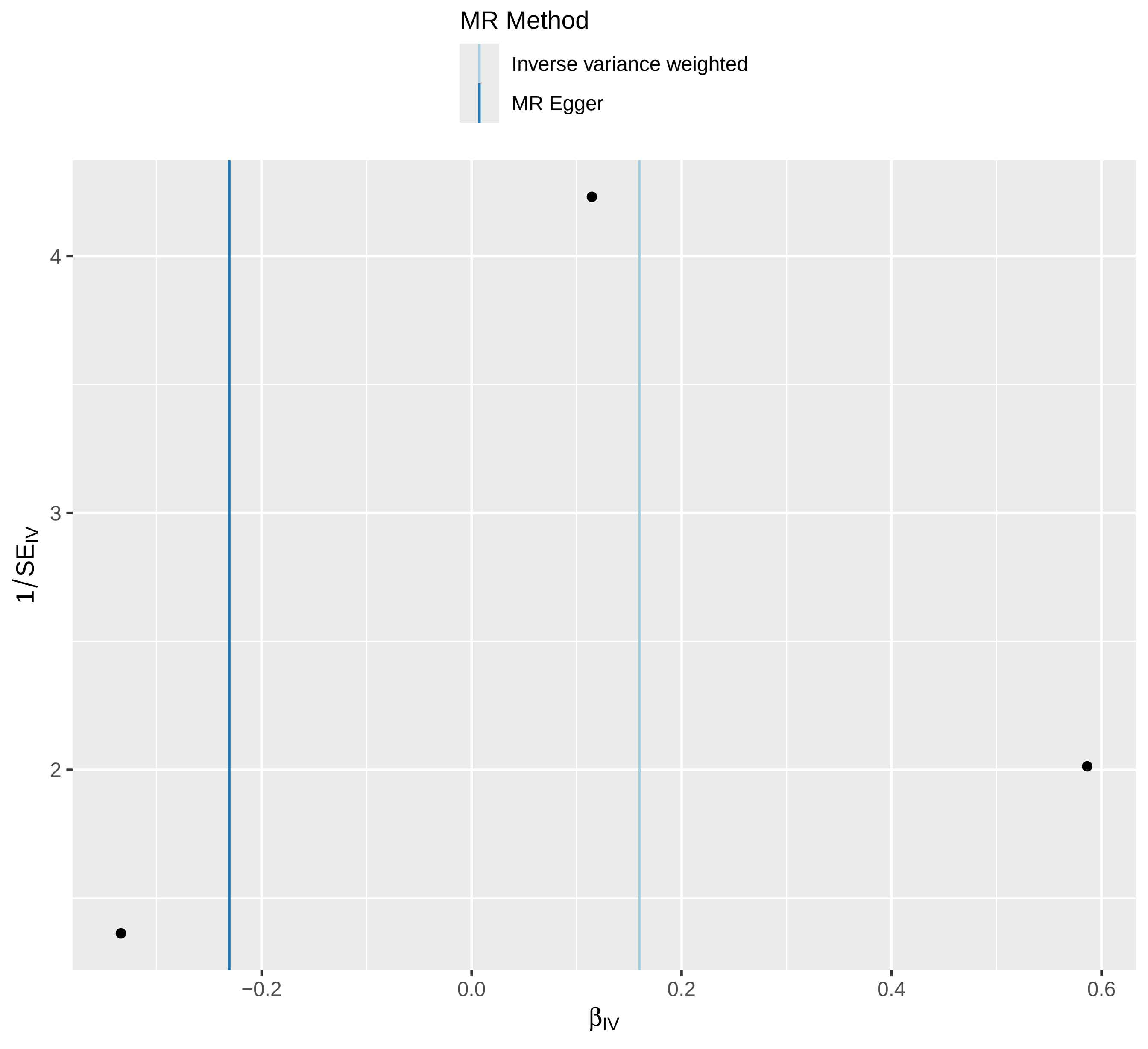
MR Method
Inverse variance weighted
MR Egger
4
3
2
1/SE_IV
−0.2
0.0
0.2
0.4
0.6
β_IV

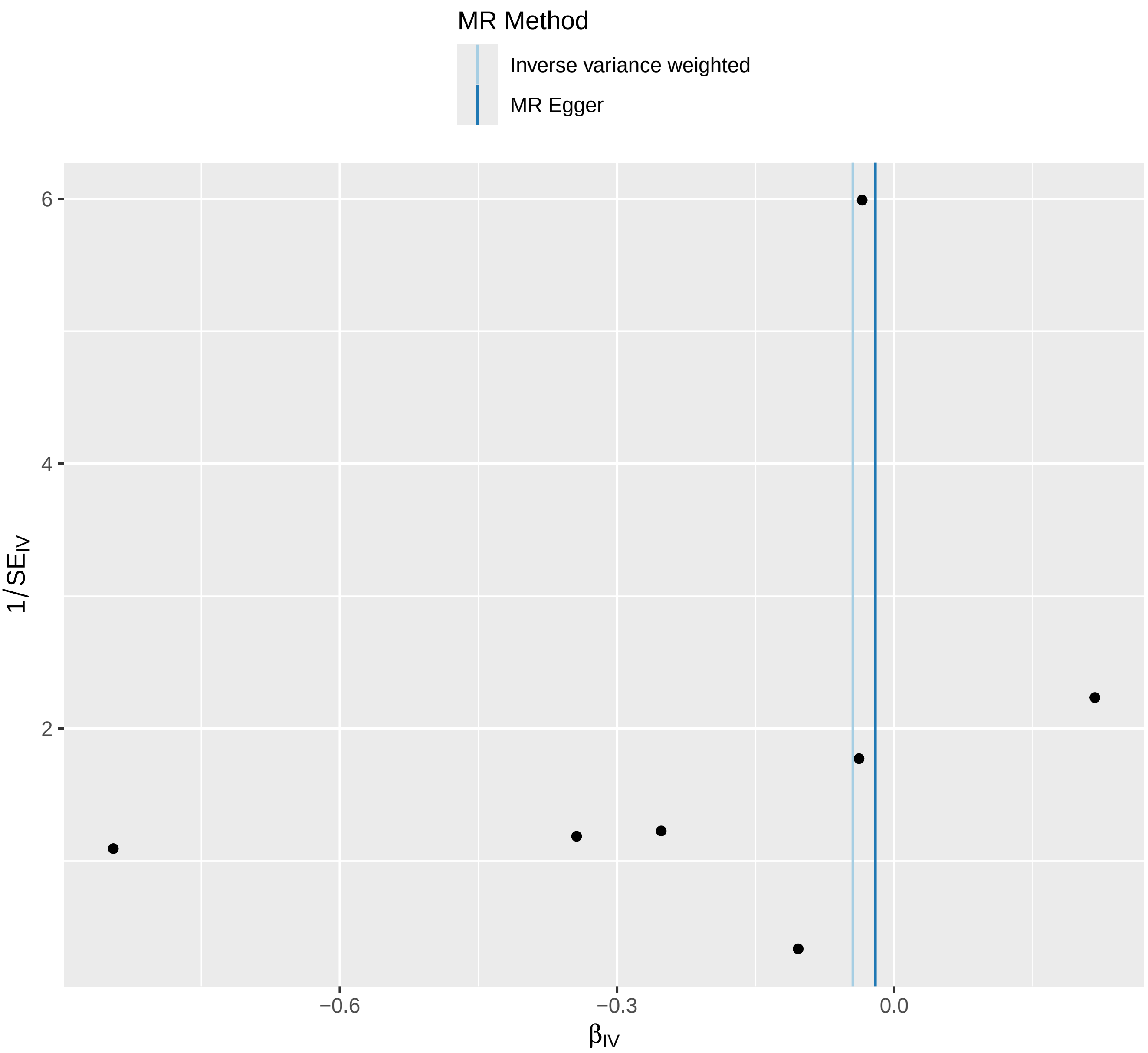
MR Method
Inverse variance weighted
MR Egger
6
4
2
1/SE_IV
−0.6
−0.3
0.0
β_IV

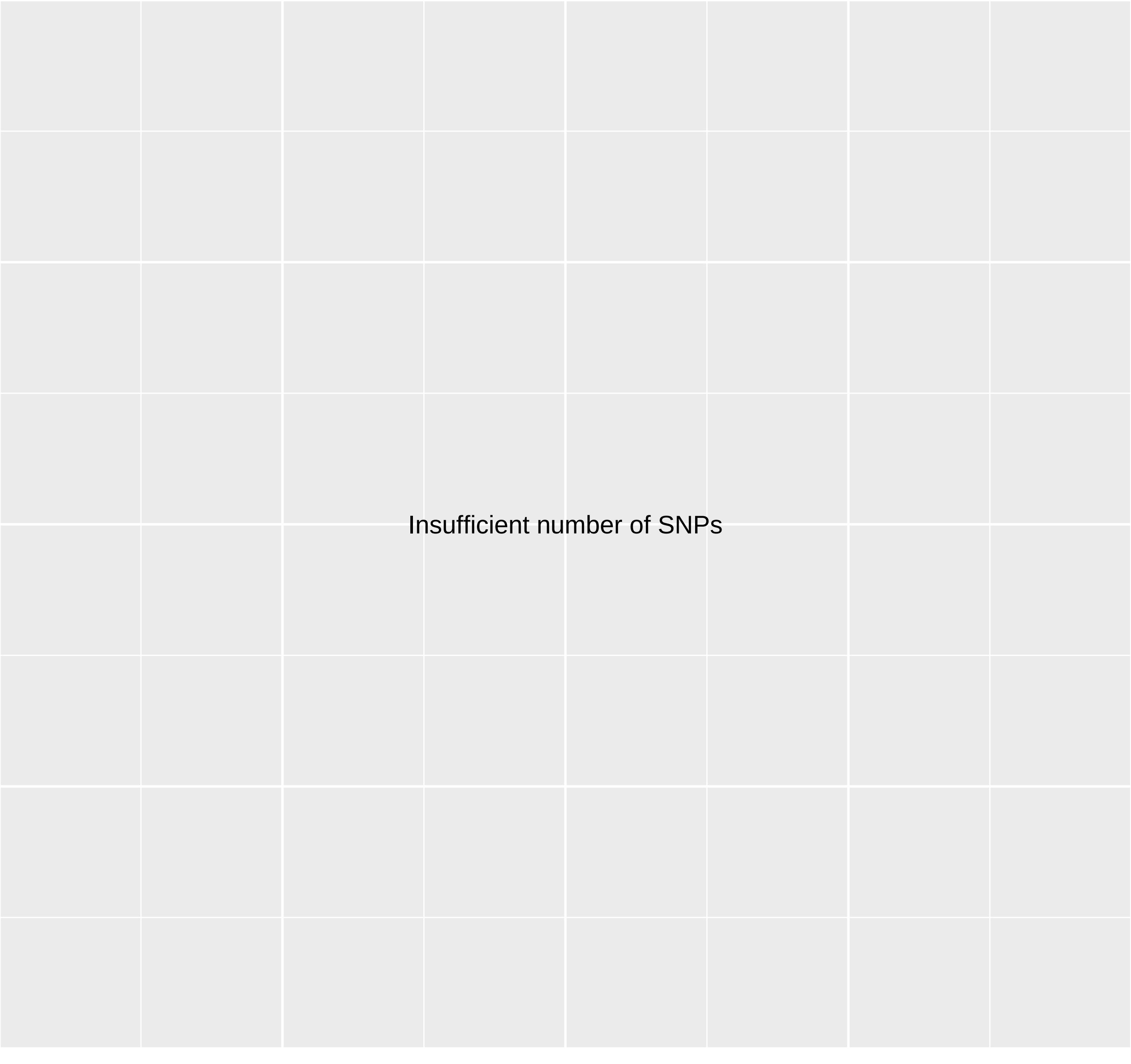
Insufficient number of SNPs

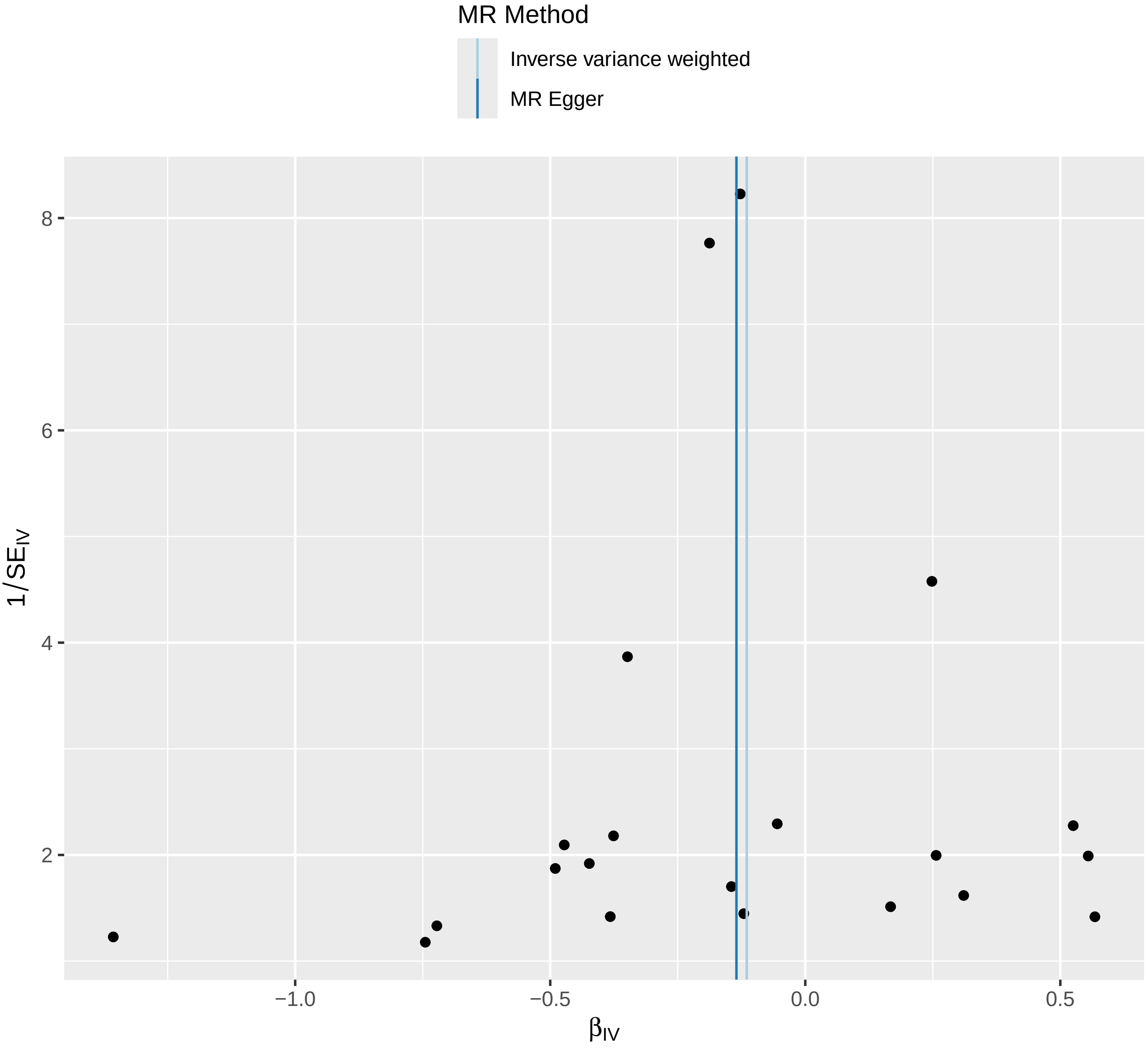

MR Method
Inverse variance weighted
MR Egger
8
6
4
2
1/SE_IV
−1.0
−0.5
0.0
0.5
β_IV

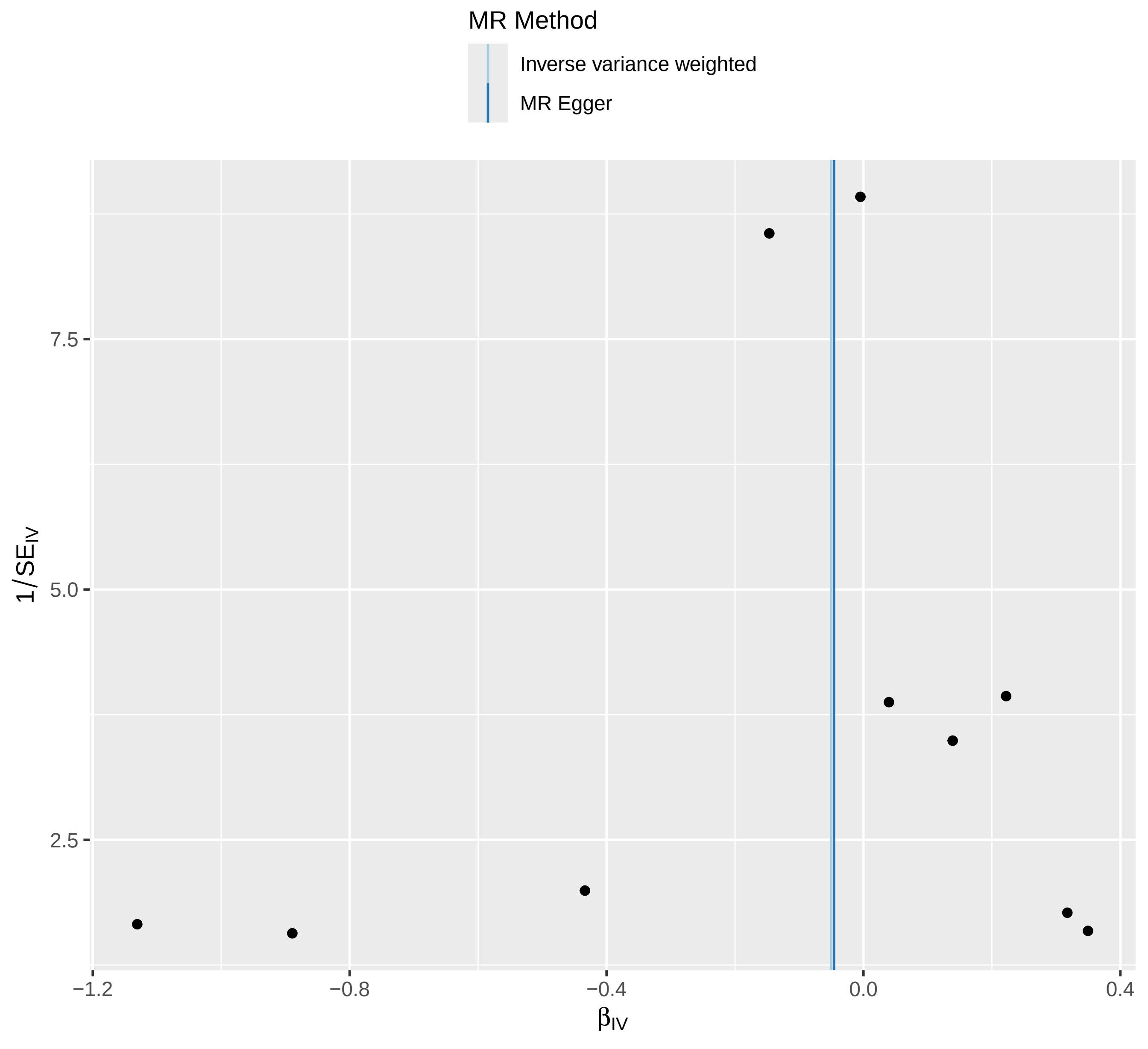
MR Method
Inverse variance weighted
MR Egger
7.5
5.0
2.5
1/SE$_{IV}$
−1.2
−0.8
−0.4
0.0
0.4
β$_{IV}$

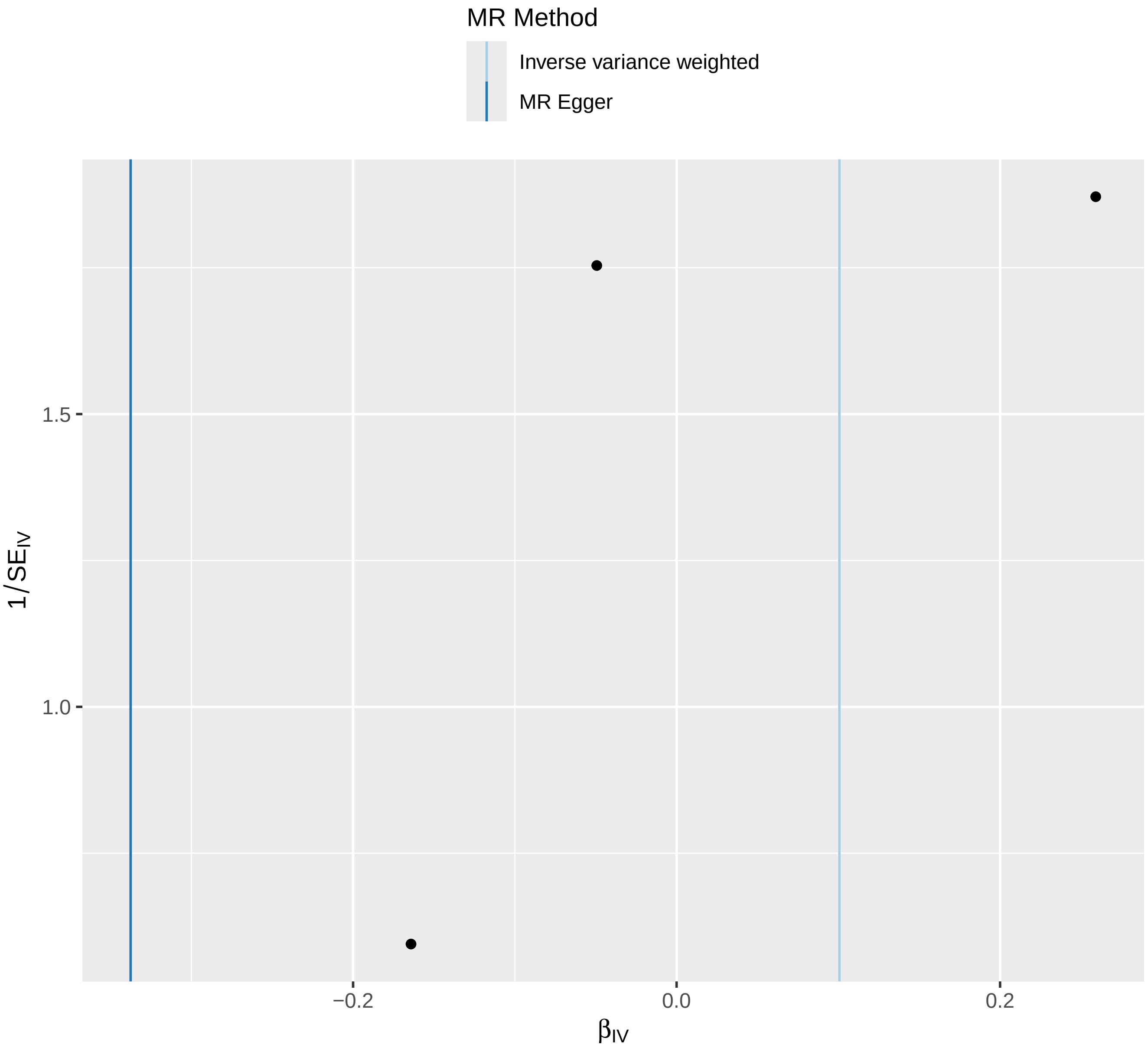
MR Method
Inverse variance weighted
MR Egger
1.5
1.0
−0.2
0.0
0.2
$1/SE_{IV}$
β_{IV}

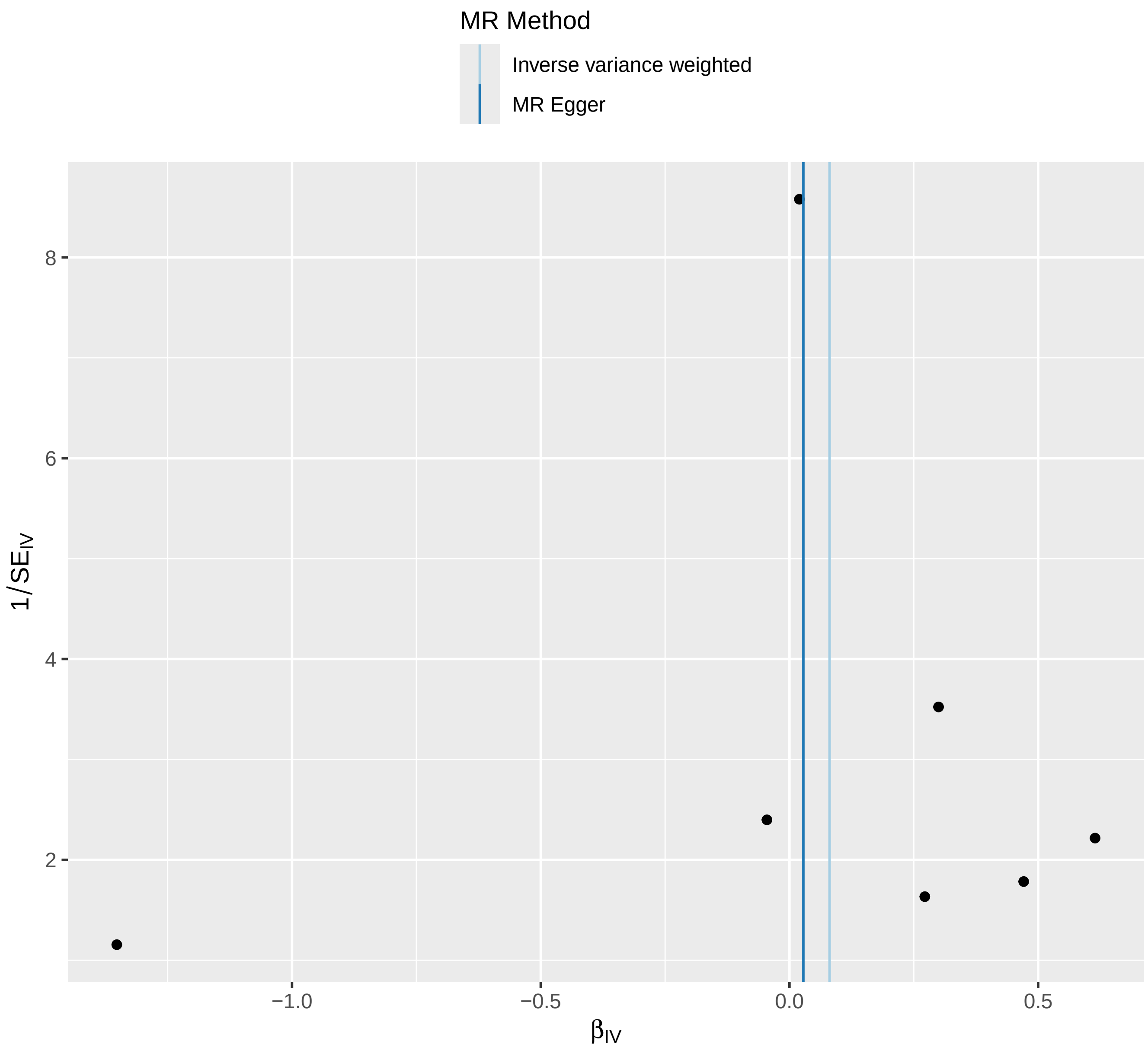

MR Method
Inverse variance weighted
MR Egger
8
6
4
2
1/SE_IV
−1.0
−0.5
0.0
0.5
β_IV

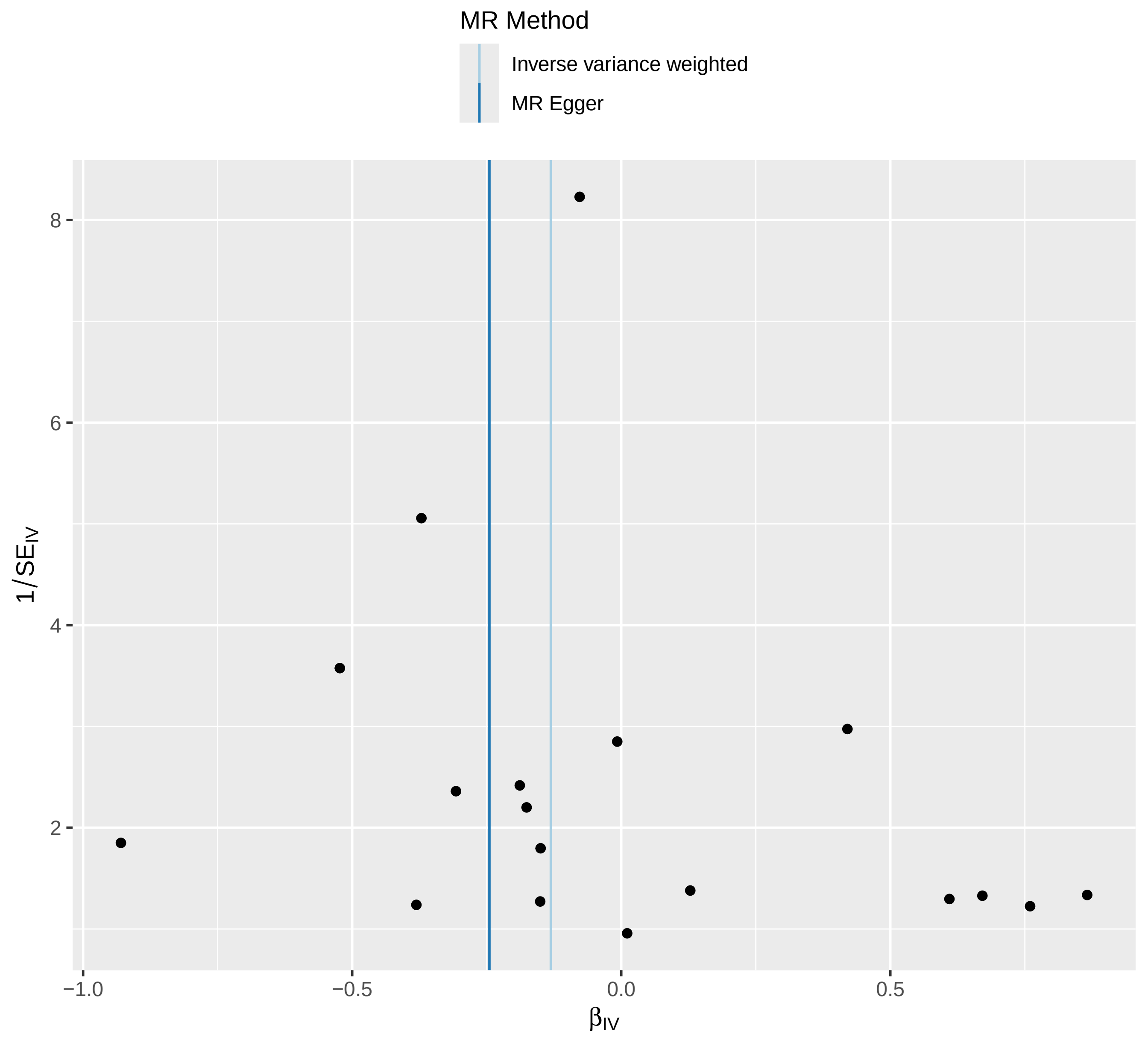

MR Method
Inverse variance weighted
MR Egger
8
6
4
2
−1.0
−0.5
0.0
0.5
$1/SE_{IV}$
β_{IV}

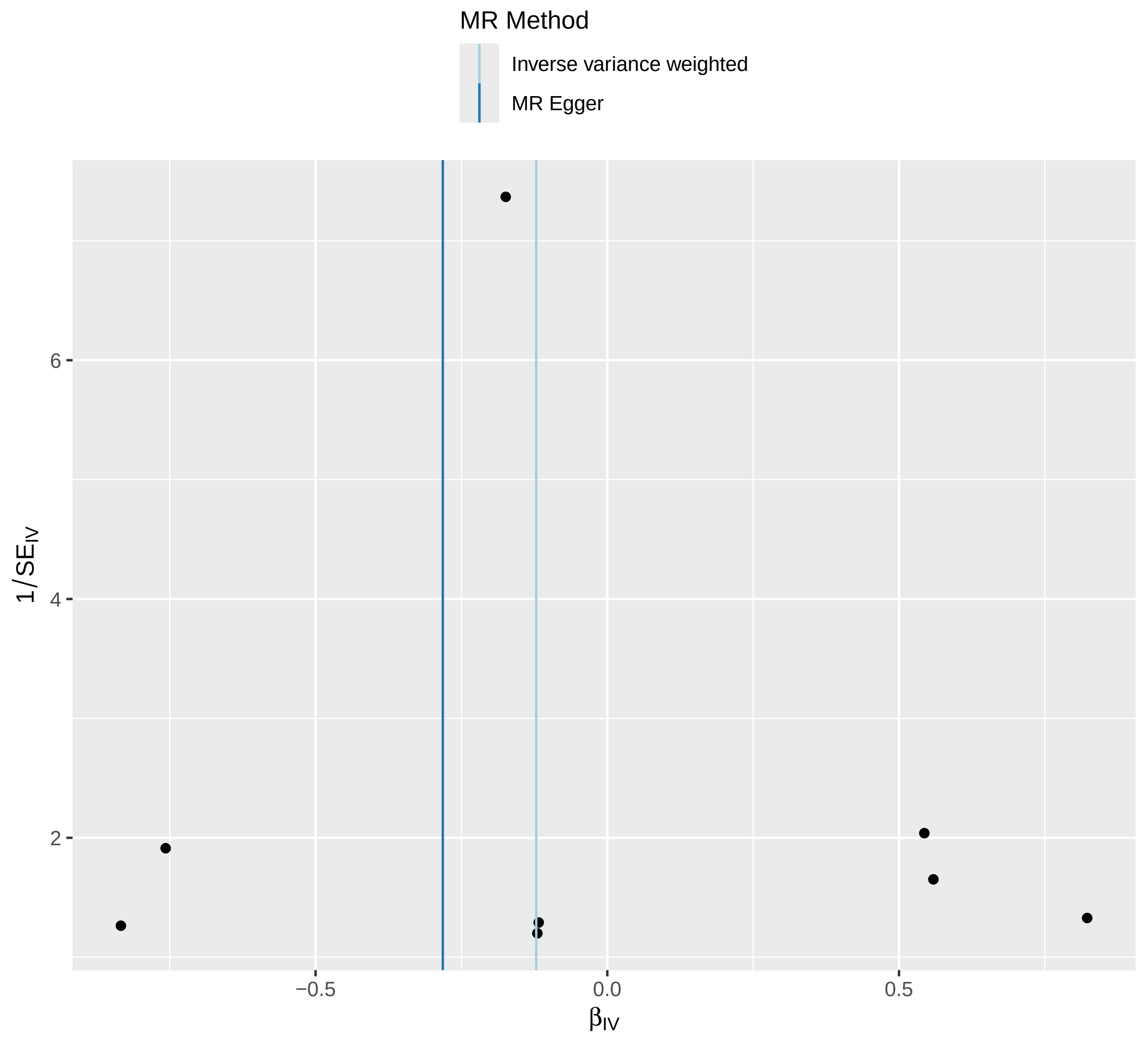
MR Method
Inverse variance weighted
MR Egger
1/SE_IV
6
4
2
−0.5
0.0
0.5
β_IV

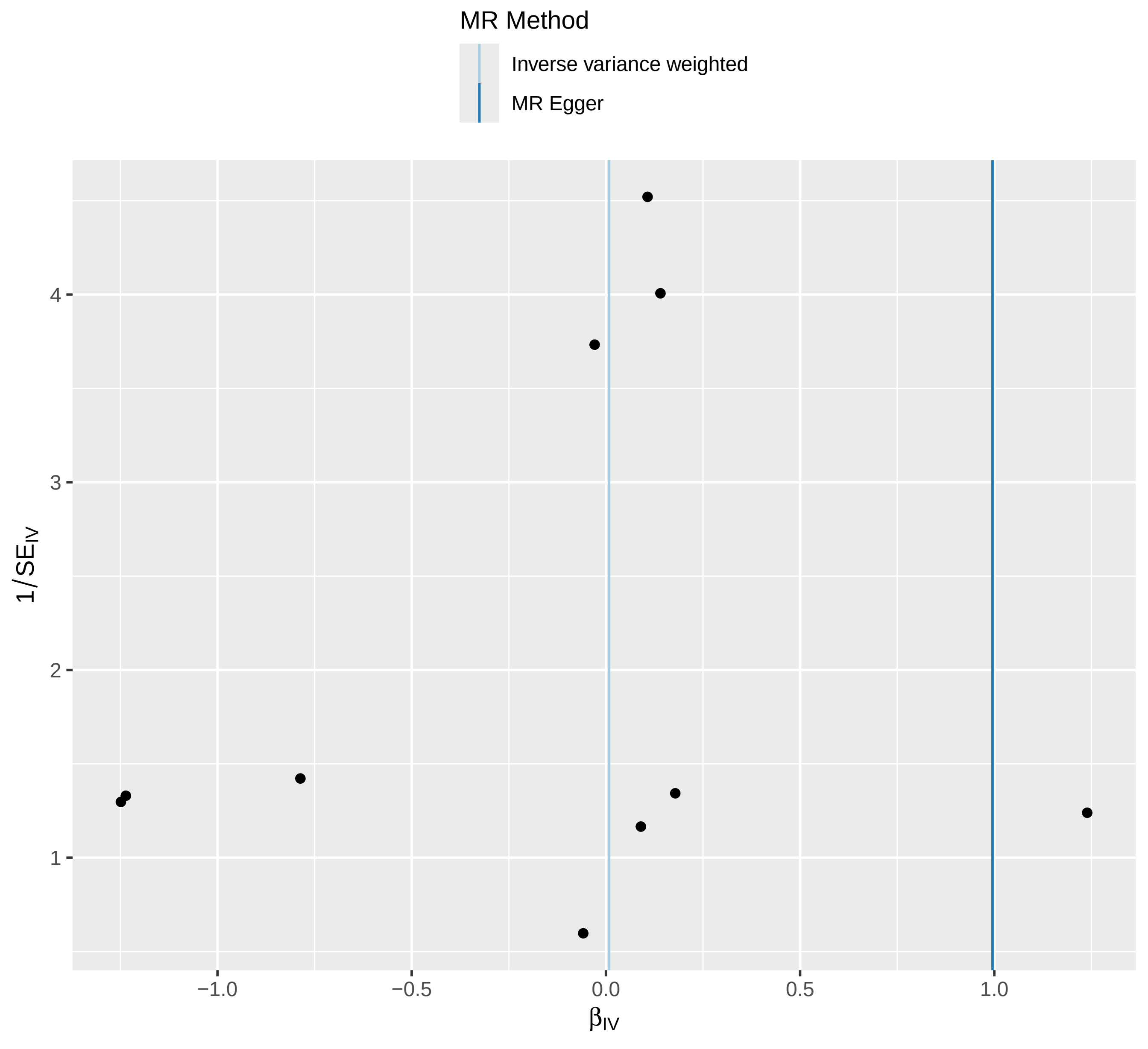

MR Method
Inverse variance weighted
MR Egger
4
3
2
1
$1/SE_{IV}$
−1.0
−0.5
0.0
0.5
1.0
β_{IV}

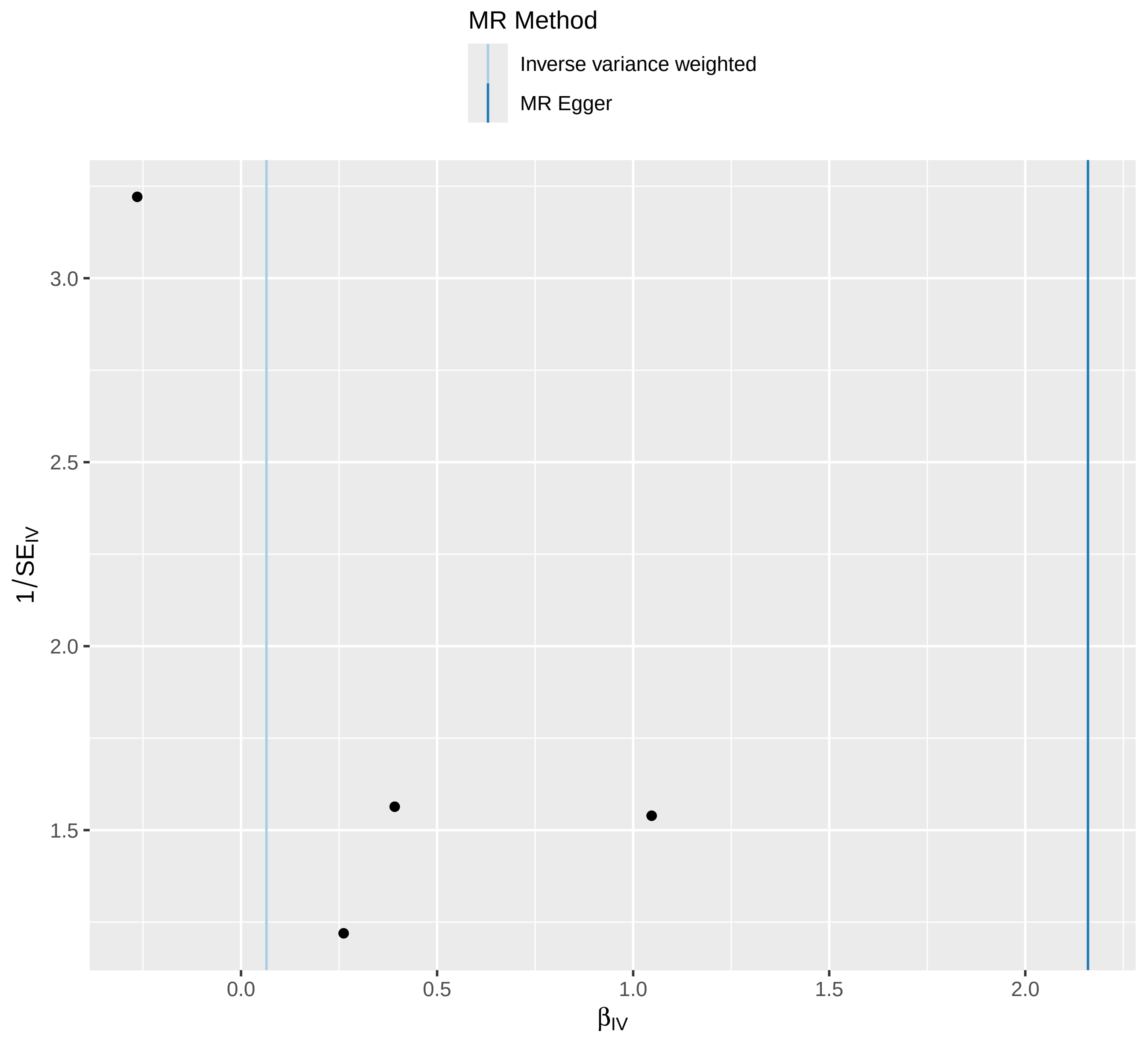

MR Method
Inverse variance weighted
MR Egger
3.0
2.5
2.0
1.5
1/SE_IV
0.0
0.5
1.0
1.5
2.0
β_IV

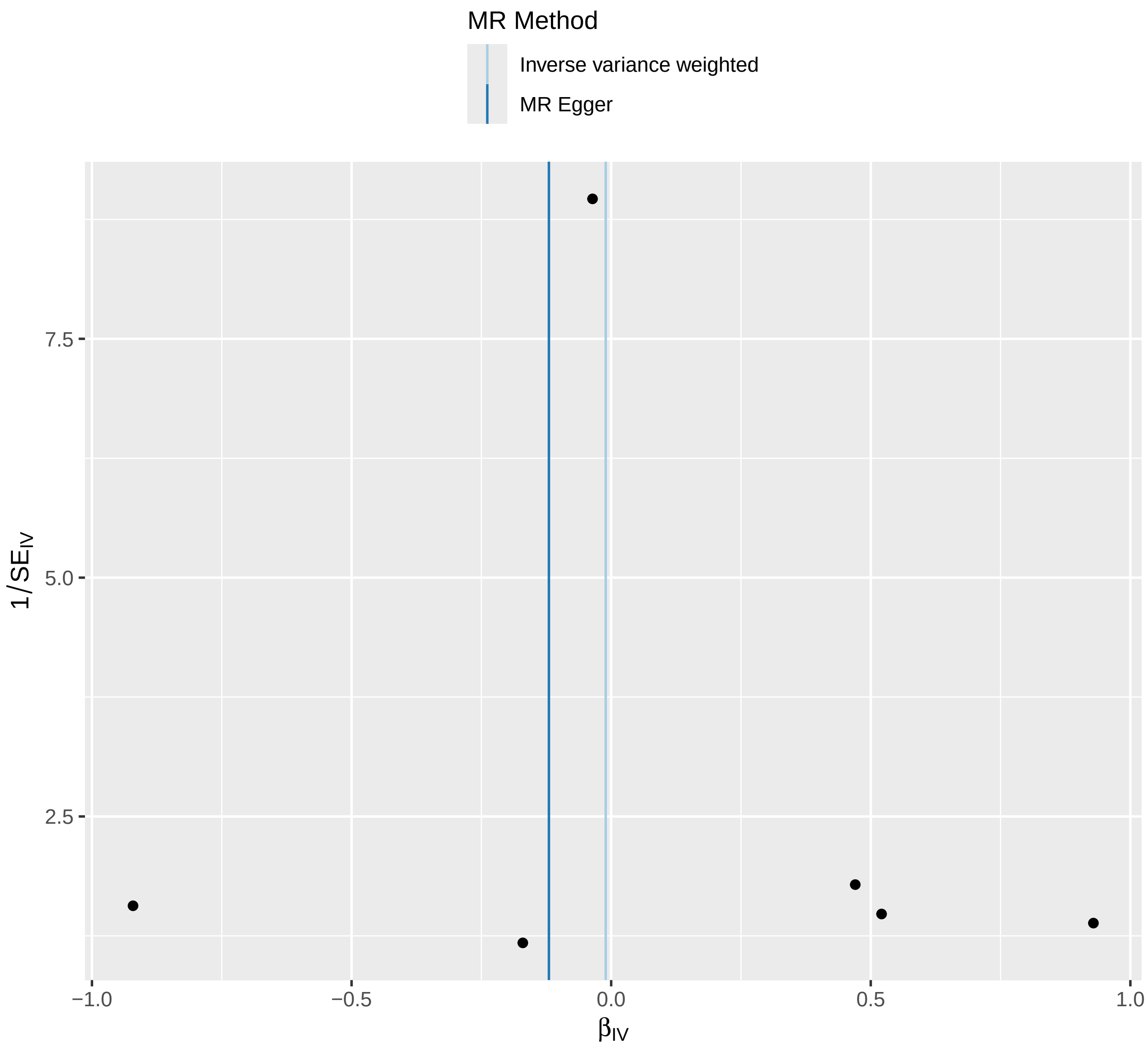
MR Method
Inverse variance weighted
MR Egger
7.5
5.0
2.5
1/SE_IV
−1.0
−0.5
0.0
0.5
1.0
β_IV

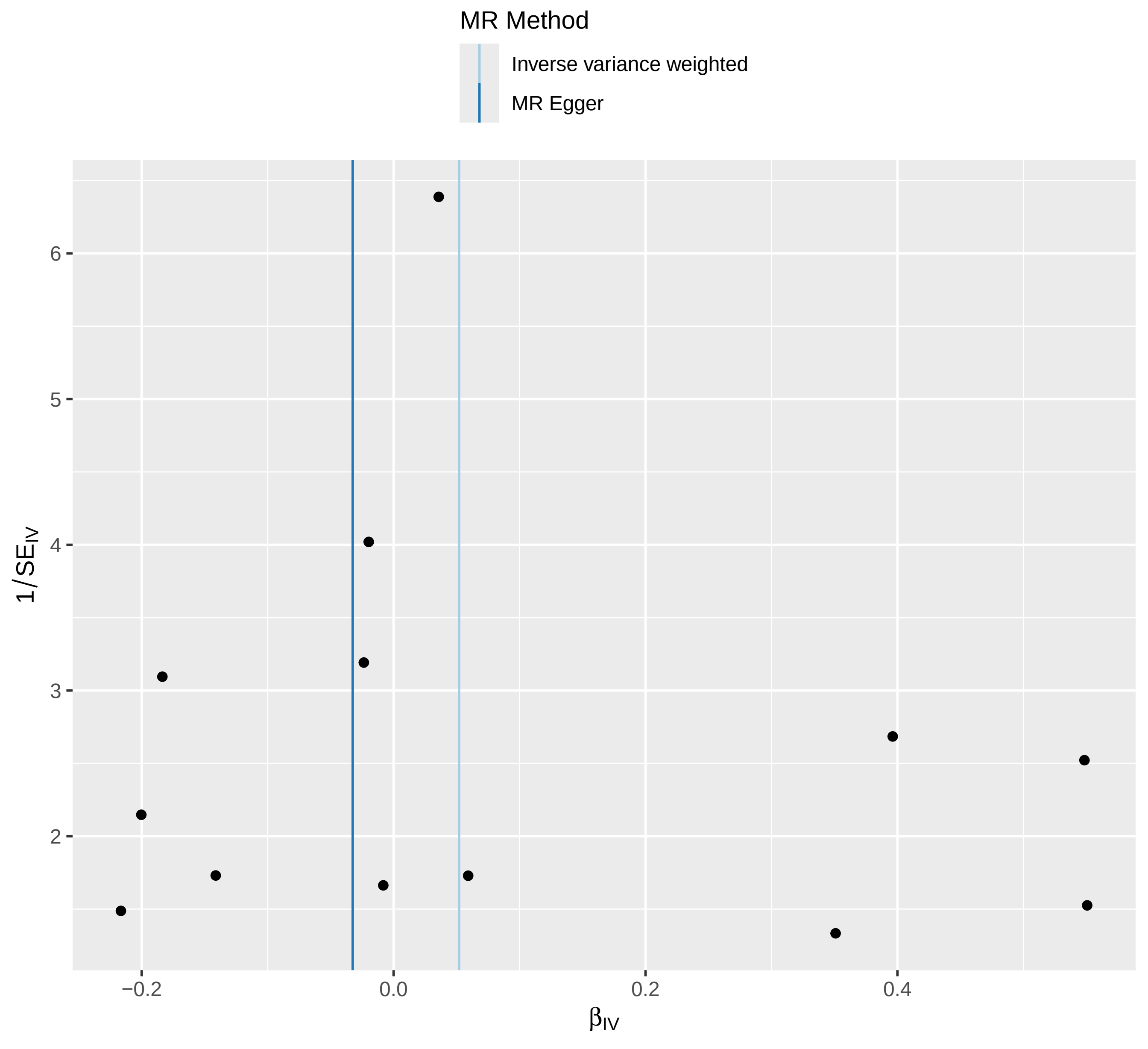

MR Method
Inverse variance weighted
MR Egger
6
5
4
3
2
1/SE$_{IV}$
−0.2
0.0
0.2
0.4
β$_{IV}$

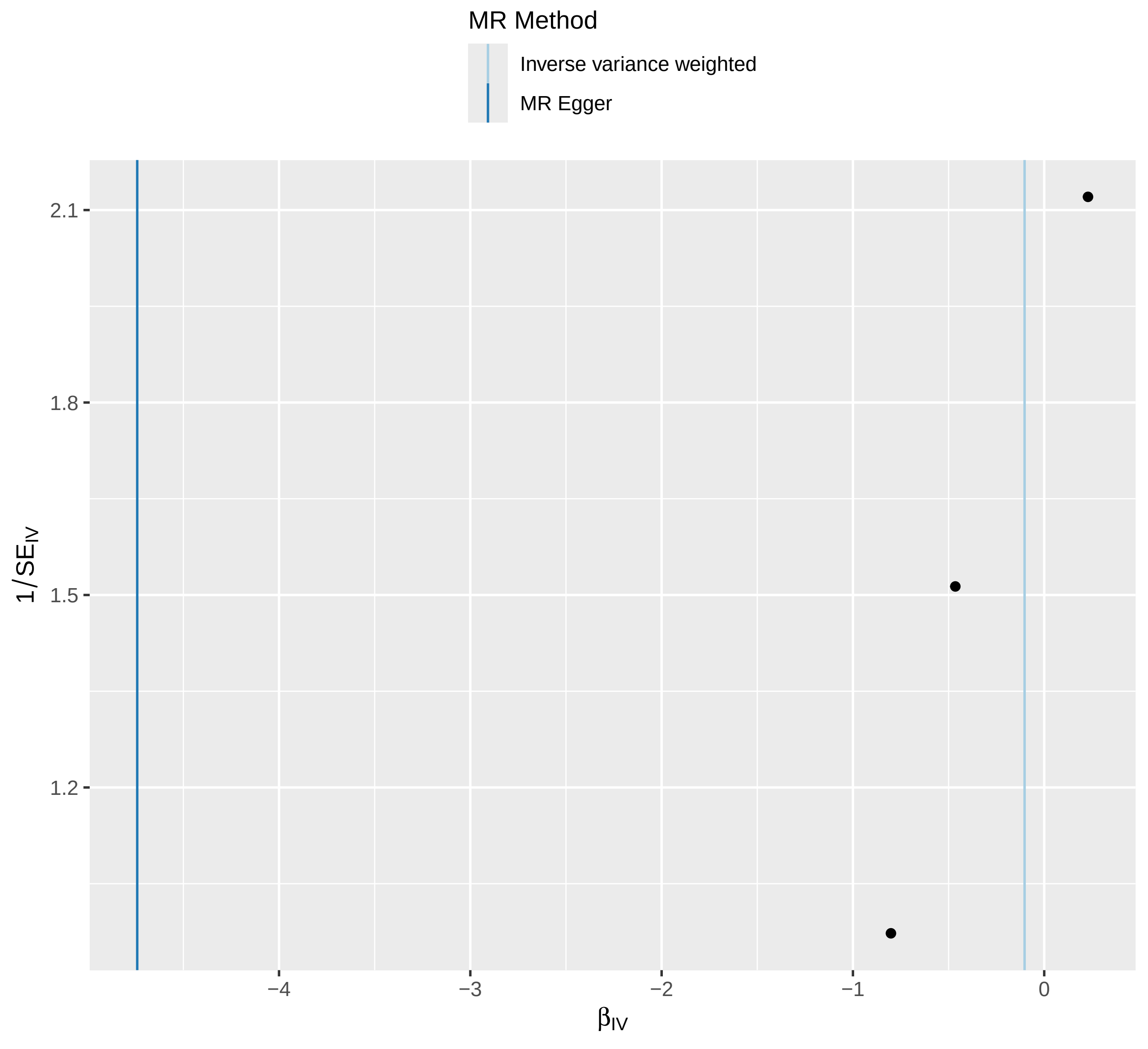
MR Method
Inverse variance weighted
MR Egger
2.1
1.8
1.5
1.2
1/SE$_{IV}$
−4
−3
−2
−1
0
β$_{IV}$

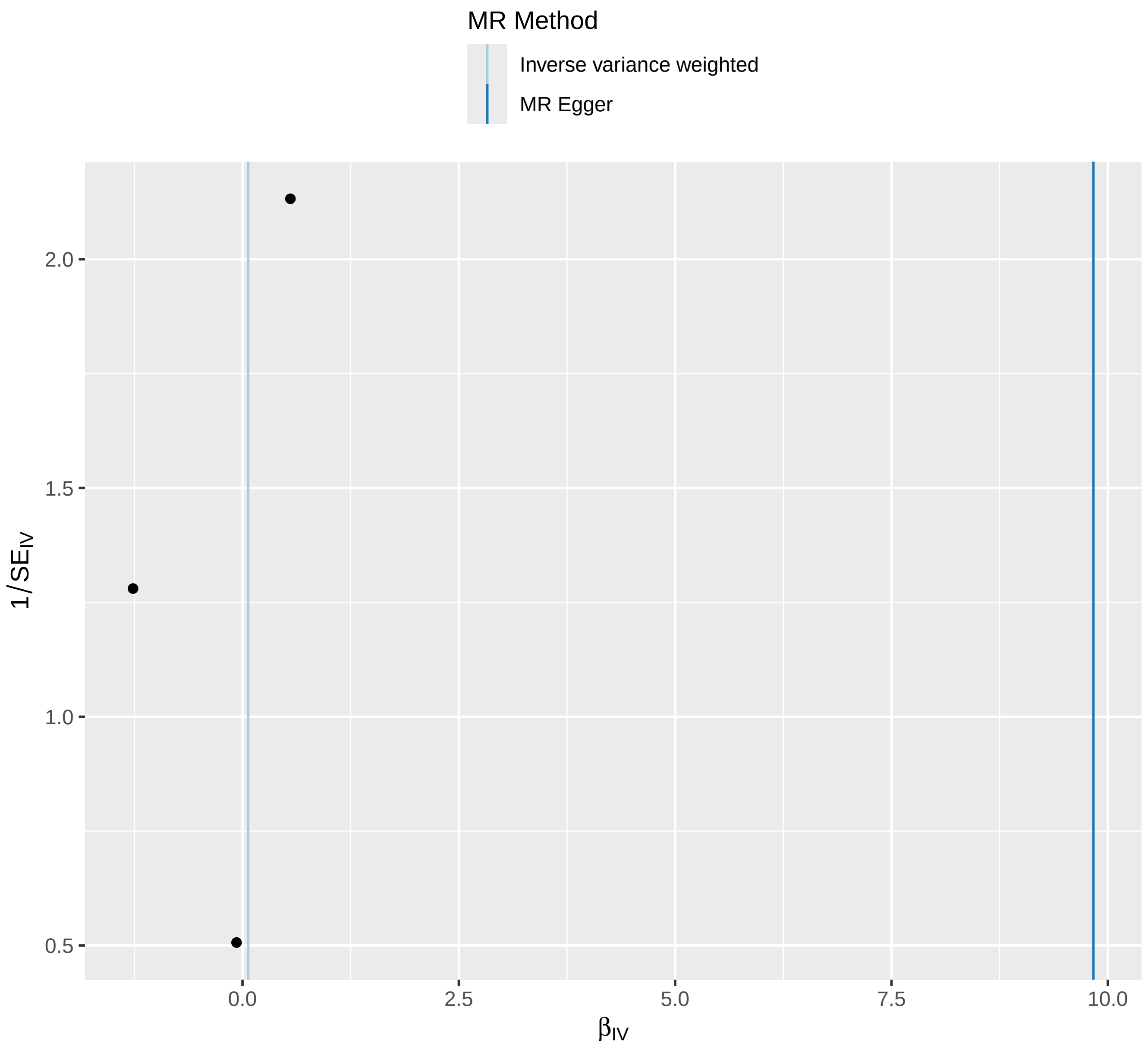
MR Method
Inverse variance weighted
MR Egger
2.0
1.5
1.0
0.5
1/SE_IV
0.0
2.5
5.0
7.5
10.0
β_IV

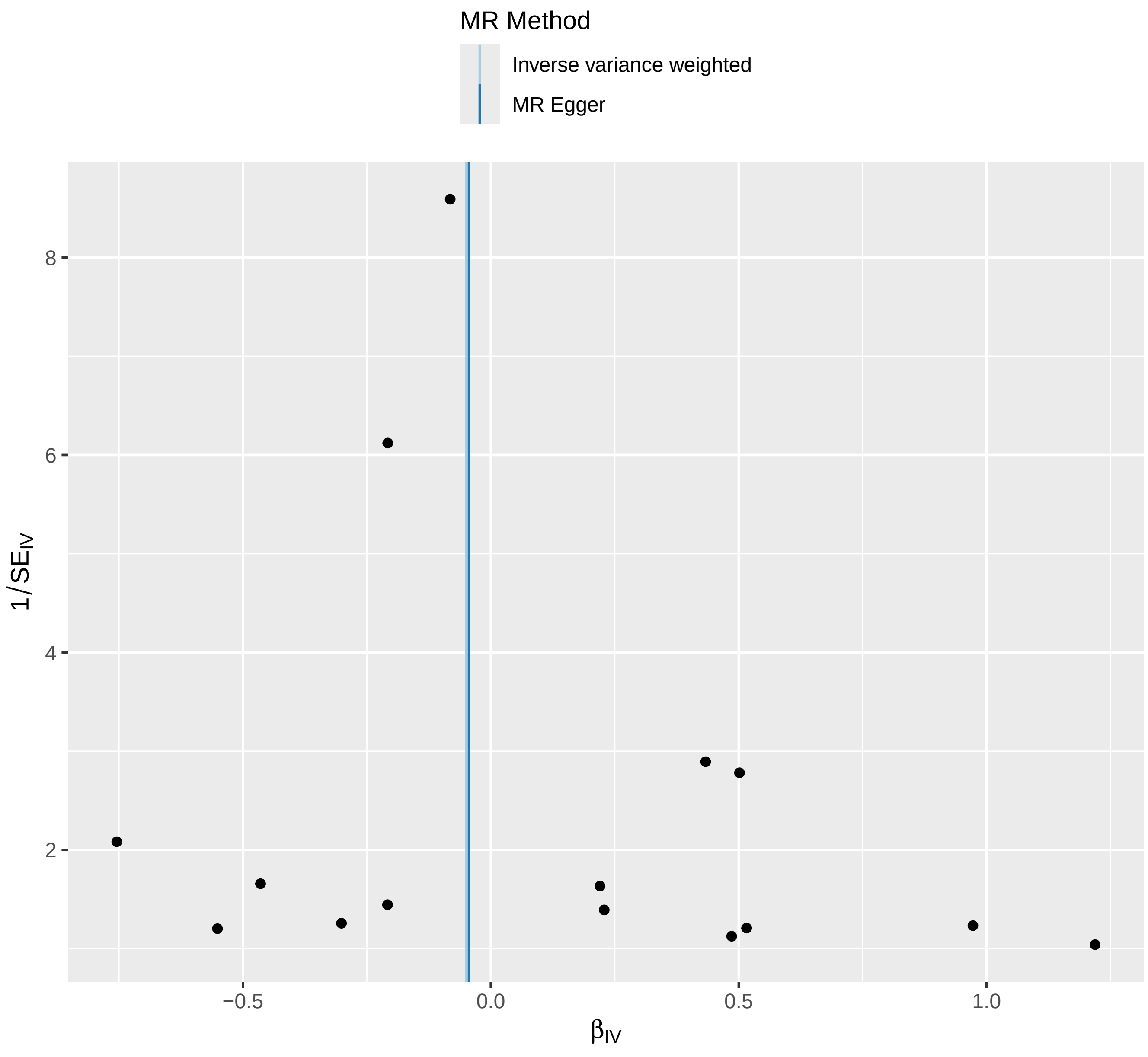
MR Method
Inverse variance weighted
MR Egger
1/SE_IV
8
6
4
2
−0.5
0.0
0.5
1.0
β_IV

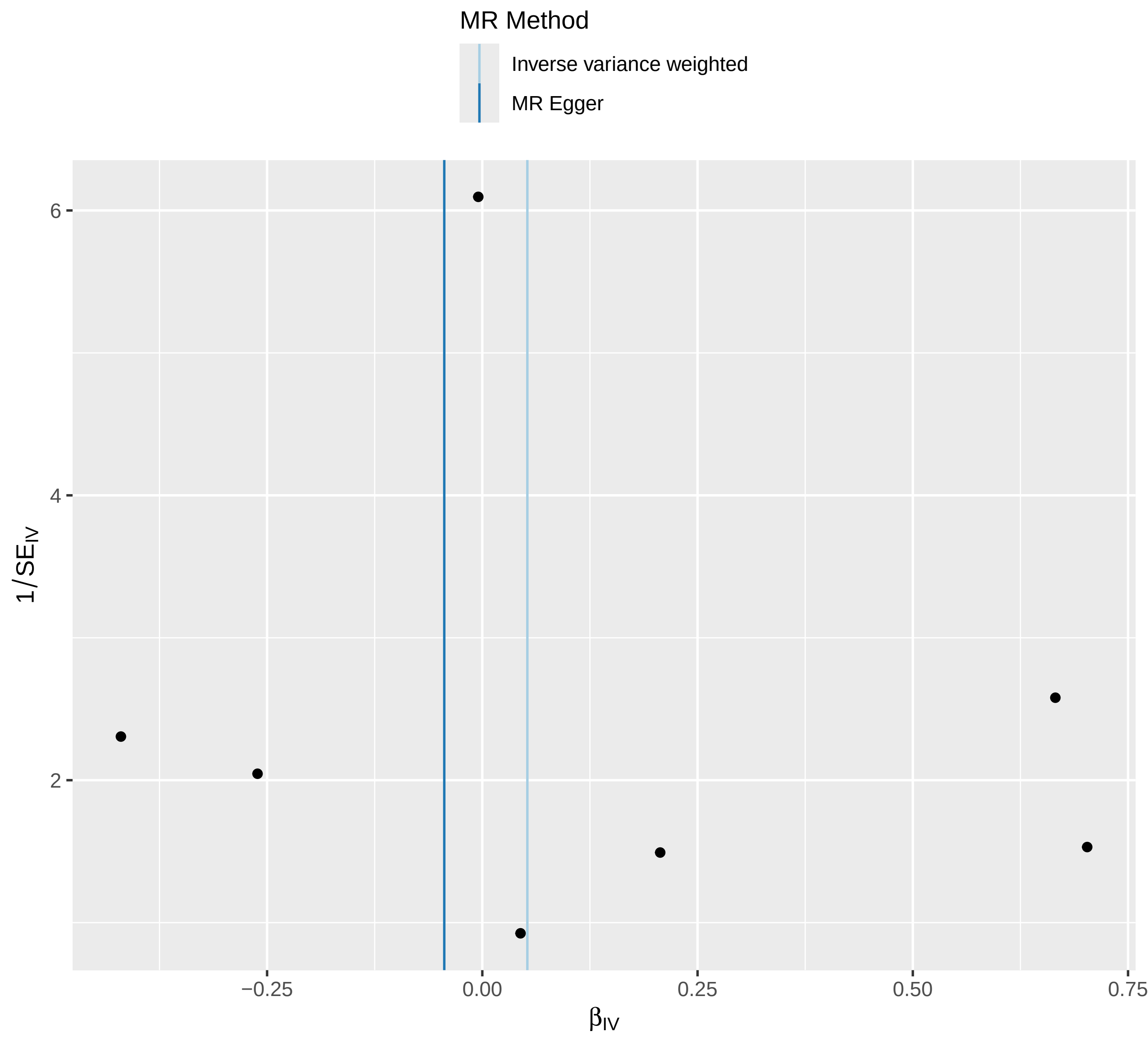

MR Method
Inverse variance weighted
MR Egger
6
4
2
1/SE_IV
−0.25
0.00
0.25
0.50
0.75
β_IV

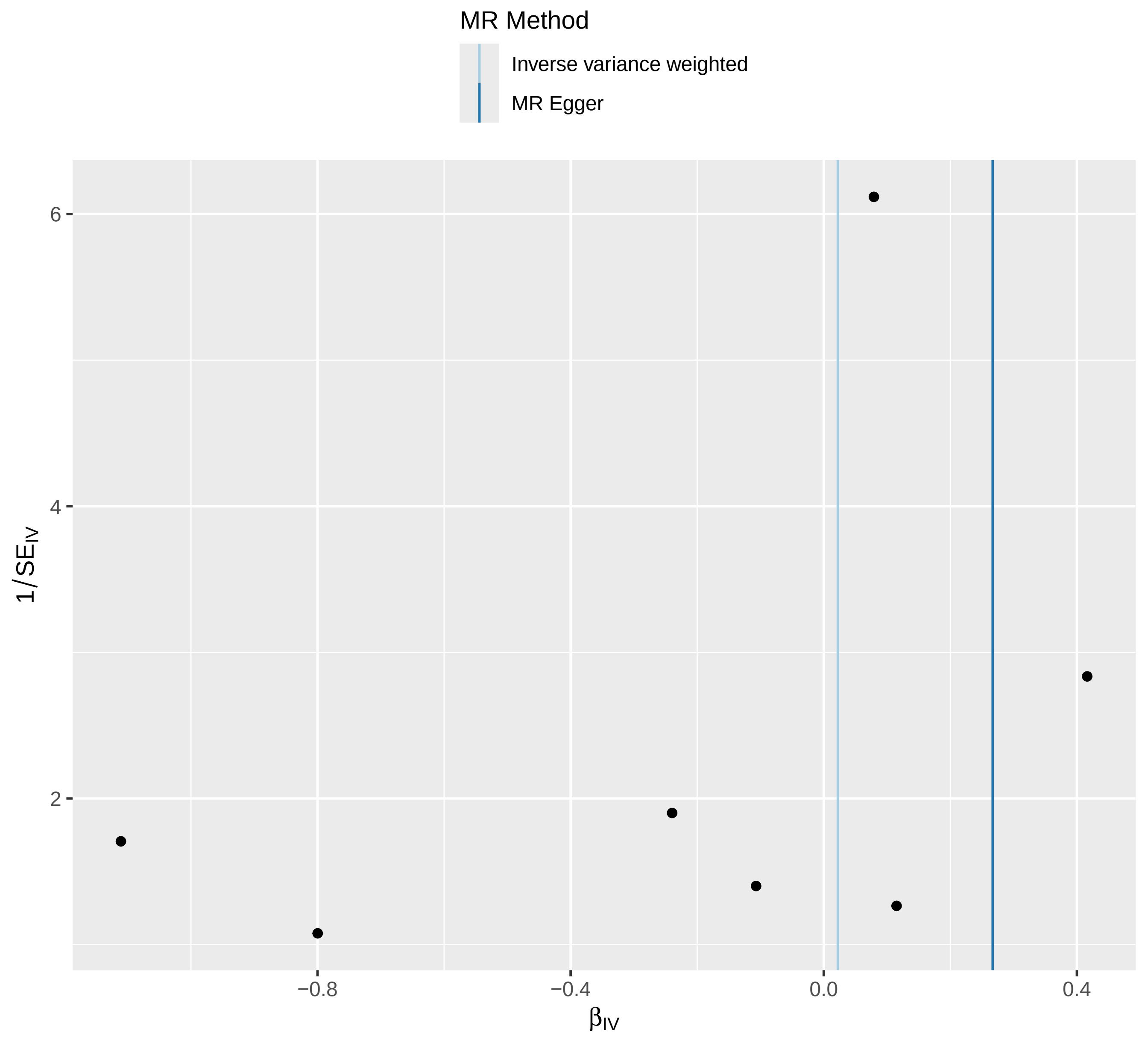

MR Method
Inverse variance weighted
MR Egger
6
4
2
1/SE$_{IV}$
−0.8
−0.4
0.0
0.4
β$_{IV}$

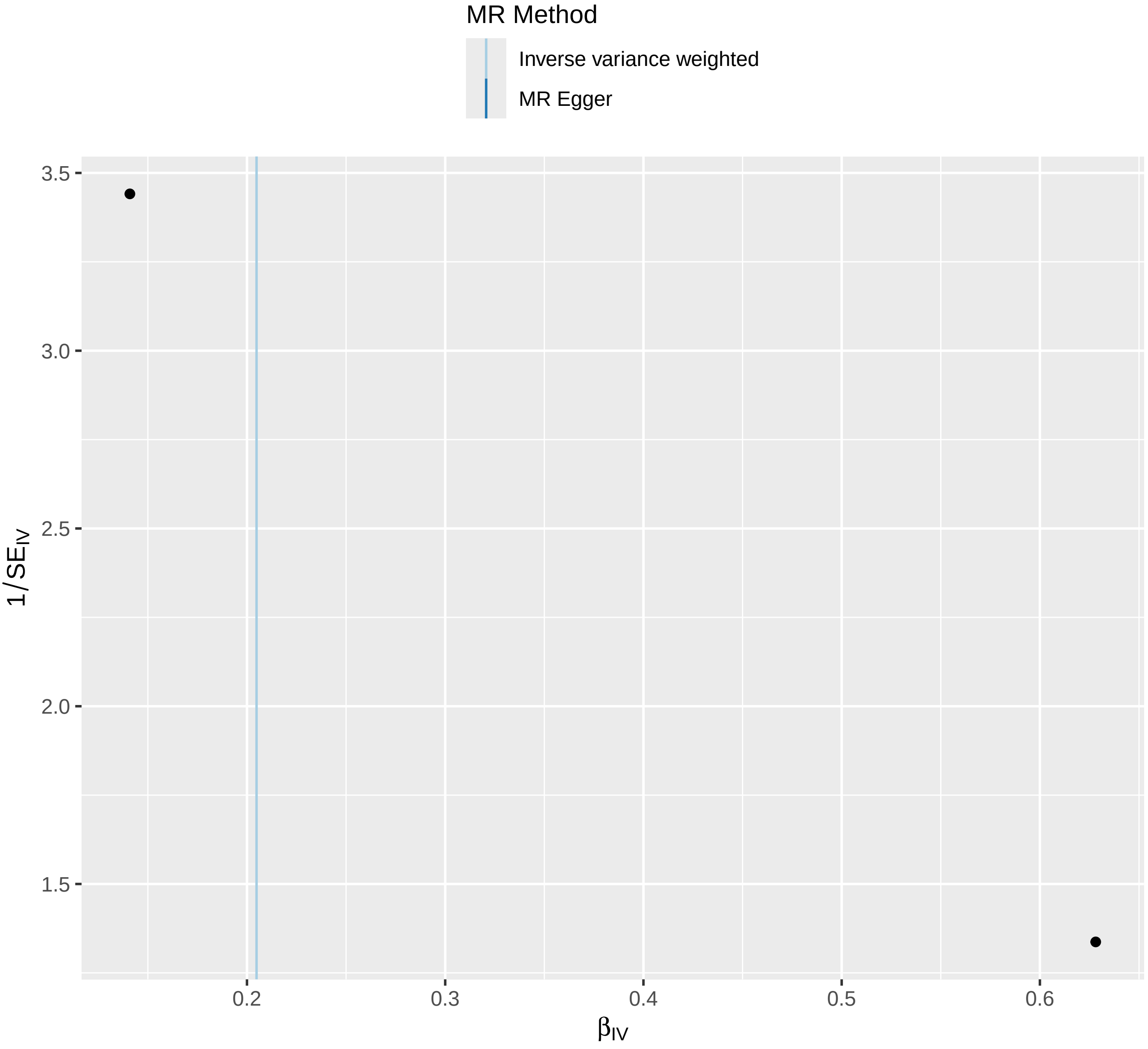

MR Method
Inverse variance weighted
MR Egger
3.5
3.0
2.5
2.0
1.5
1/SE_IV
0.2
0.3
0.4
0.5
0.6
β_IV

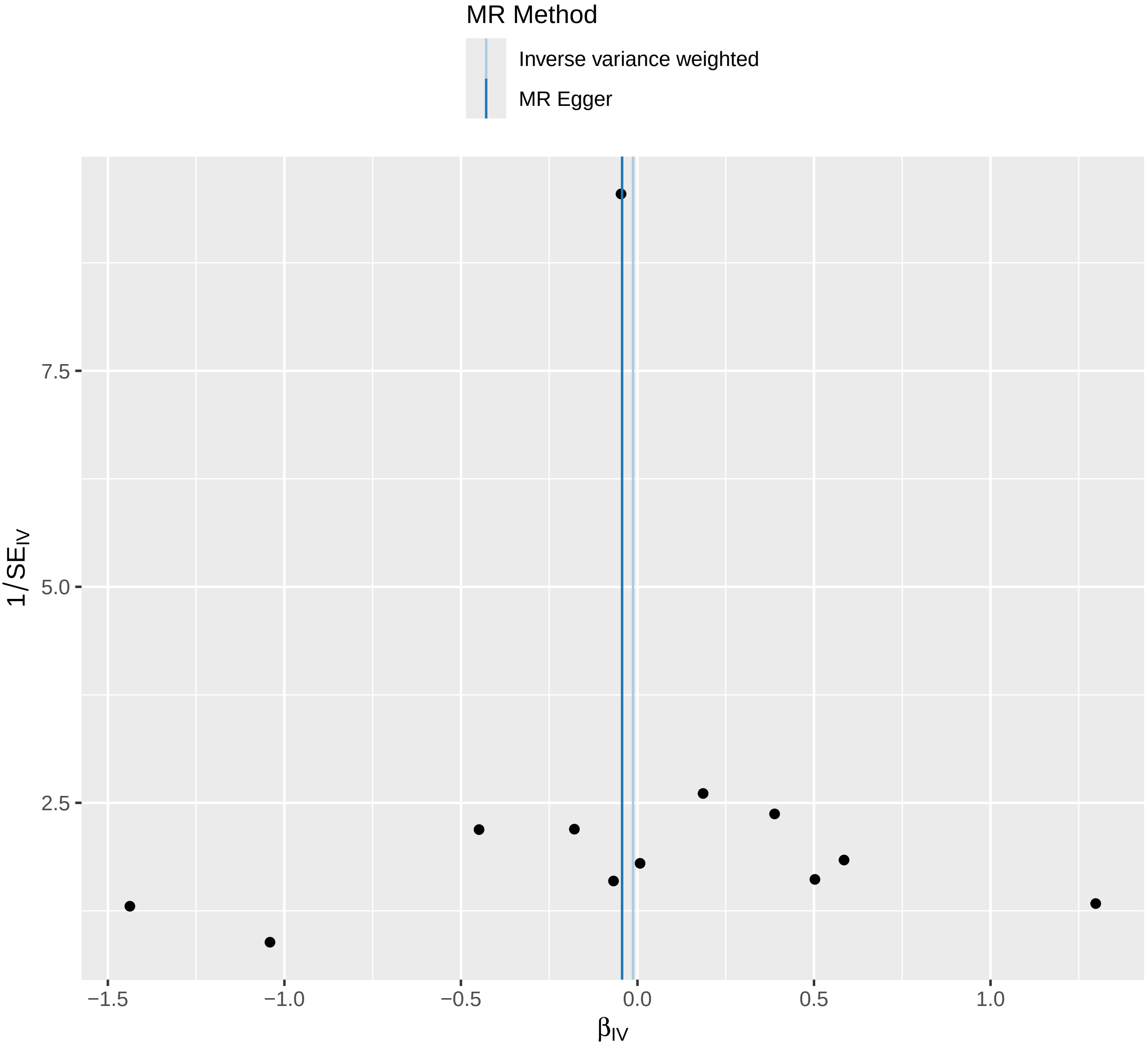

MR Method
Inverse variance weighted
MR Egger
7.5
5.0
2.5
1/SE_IV
−1.5
−1.0
−0.5
0.0
0.5
1.0
β_IV

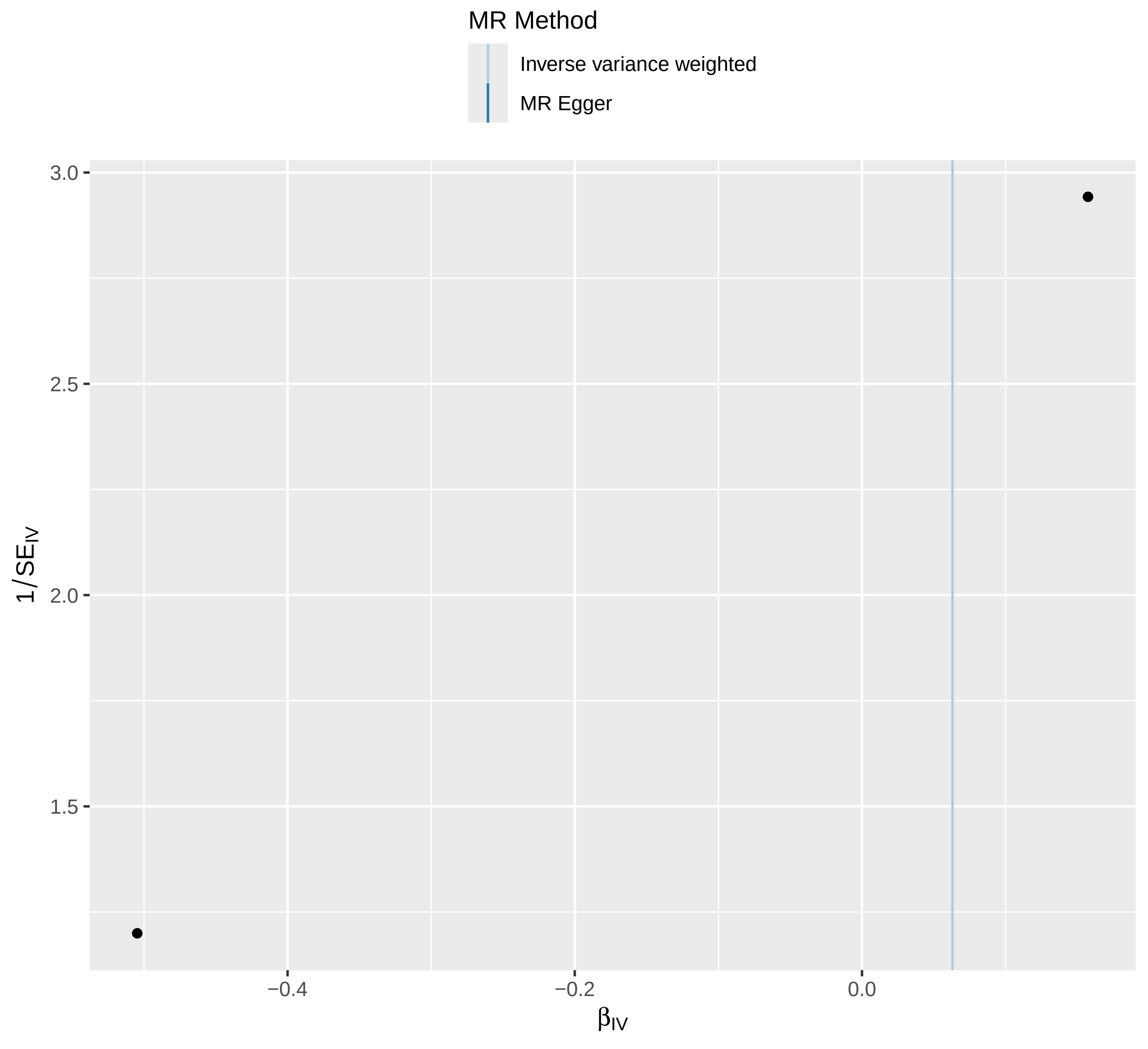
MR Method
Inverse variance weighted
MR Egger
3.0
2.5
2.0
1.5
1/SE_IV
−0.4
−0.2
0.0
β_IV

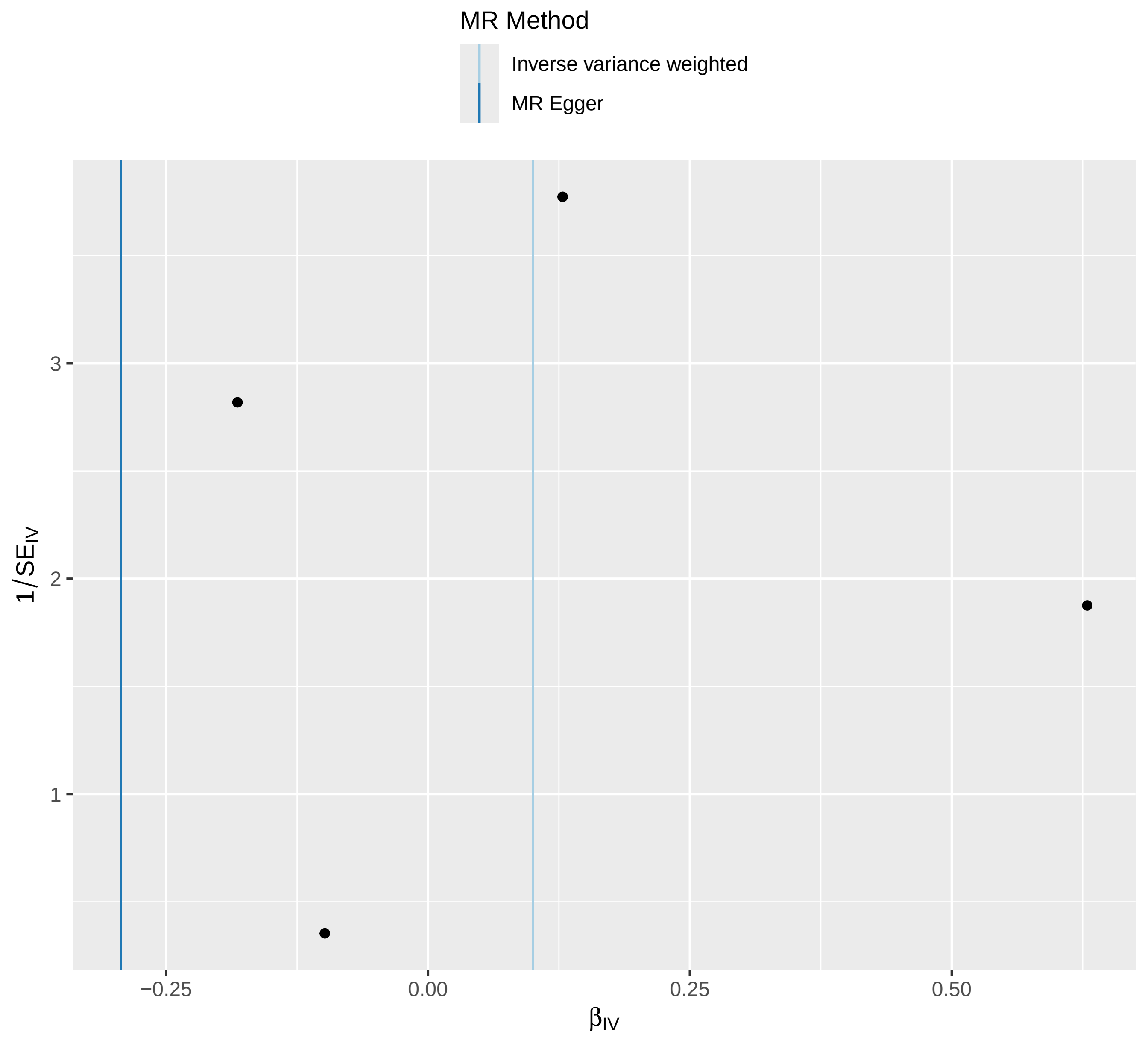

MR Method
Inverse variance weighted
MR Egger
3
2
1
1/SE_IV
−0.25
0.00
0.25
0.50
β_IV

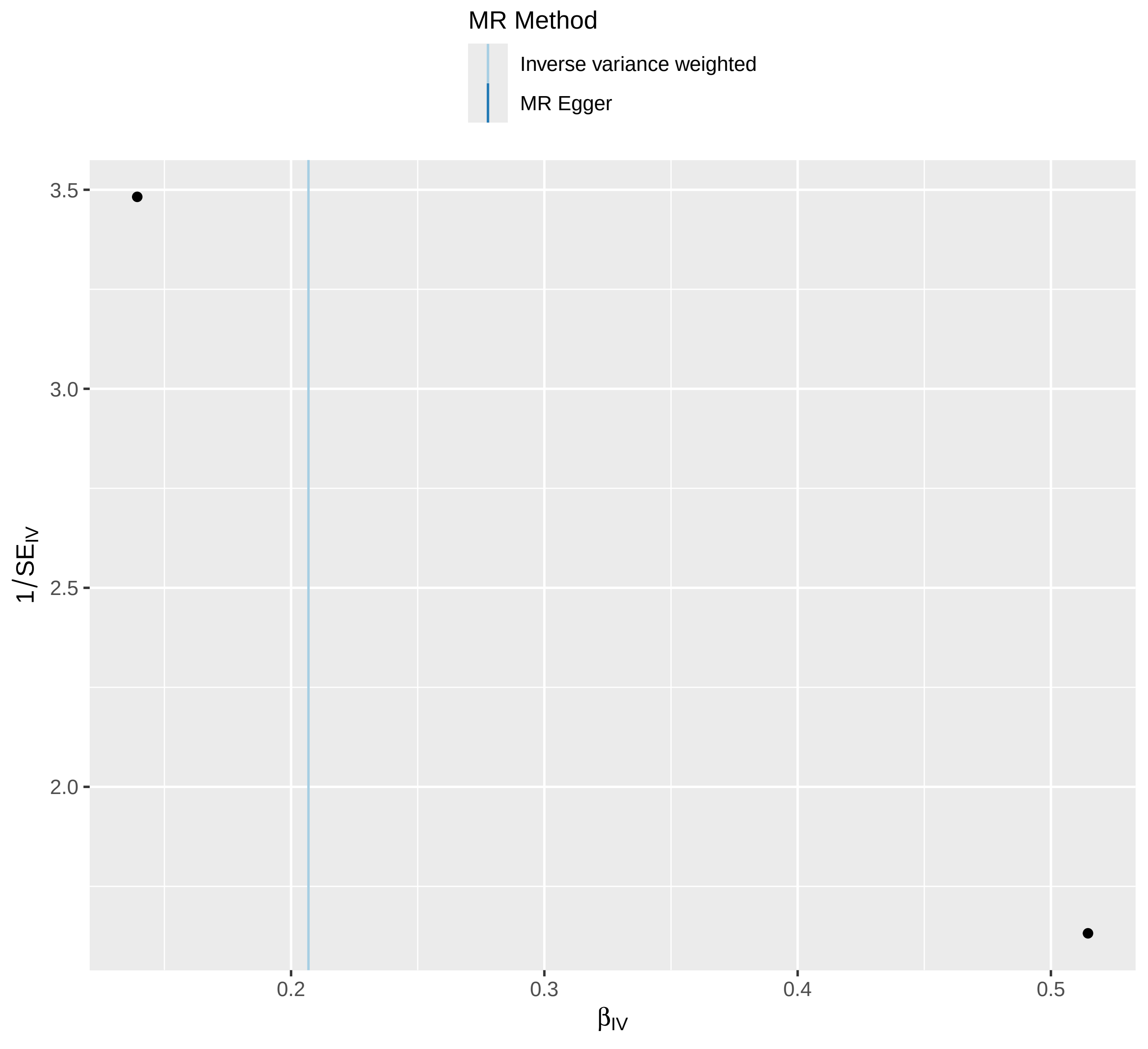

MR Method
Inverse variance weighted
MR Egger
3.5
3.0
2.5
2.0
1/SE$_{IV}$
0.2
0.3
0.4
0.5
β$_{IV}$

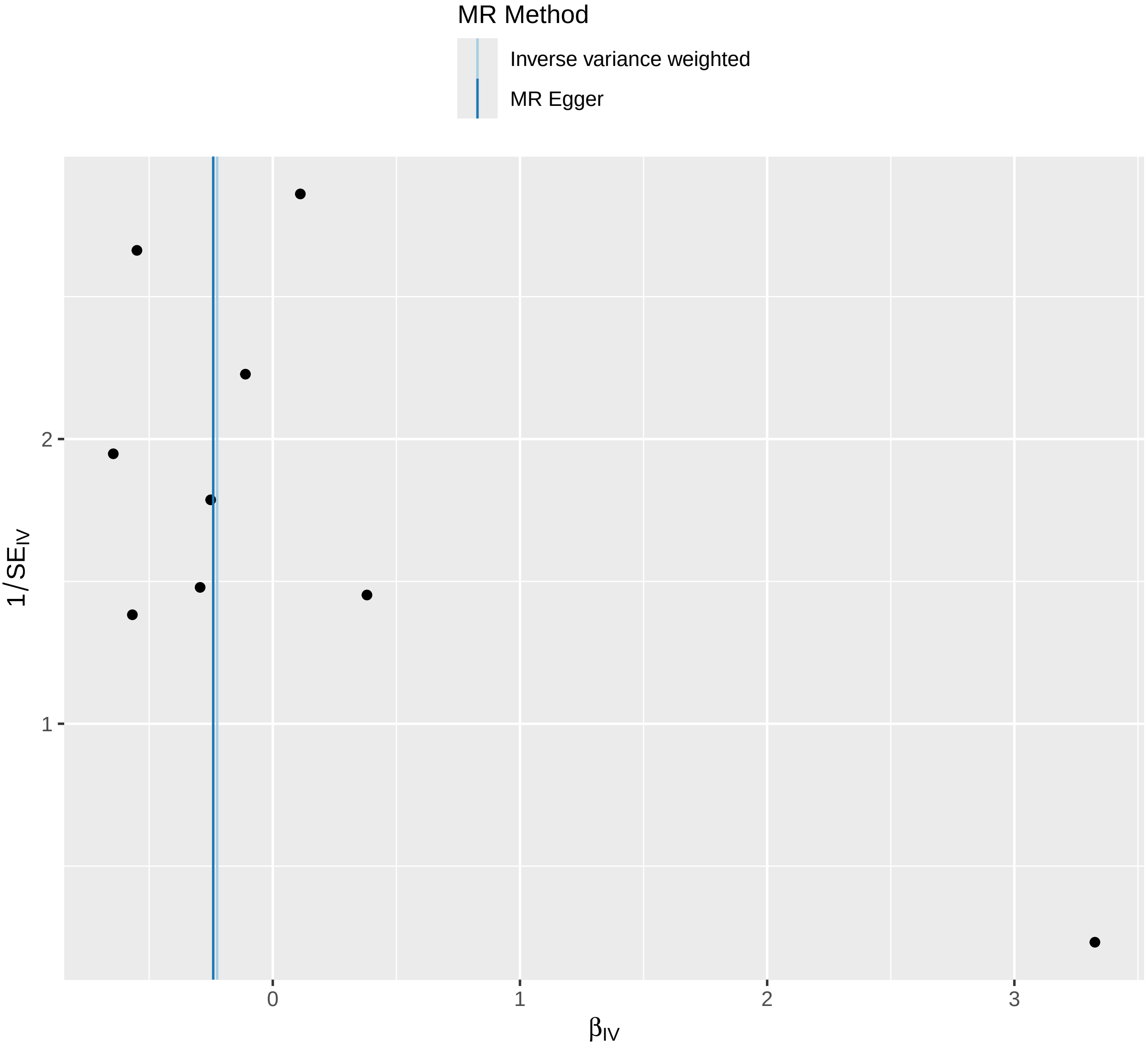
MR Method
Inverse variance weighted
MR Egger
2
1
1/SE_IV
0
1
2
3
β_IV

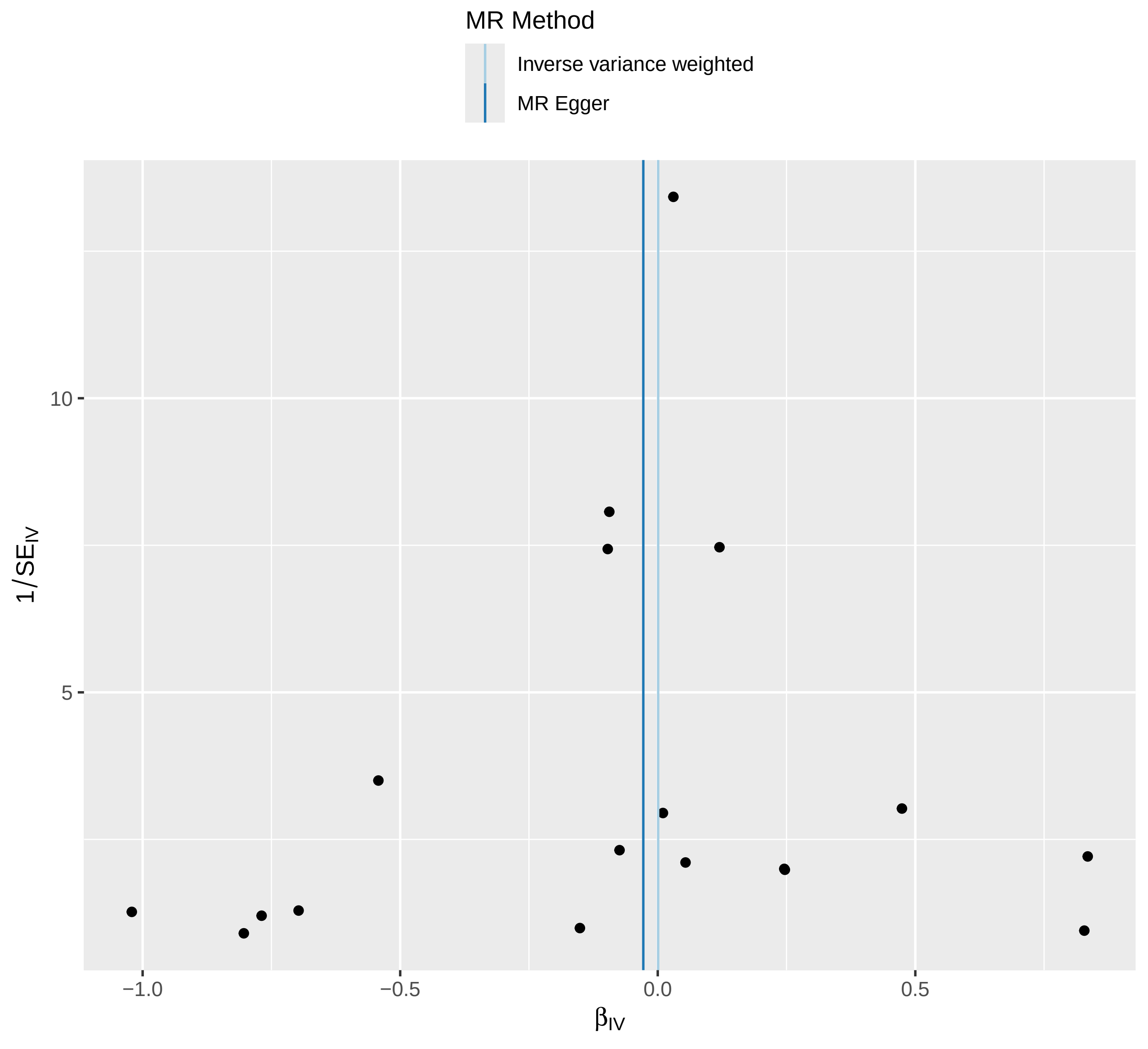
MR Method
Inverse variance weighted
MR Egger
10
5
$1/SE_{IV}$
−1.0
−0.5
0.0
0.5
β_{IV}

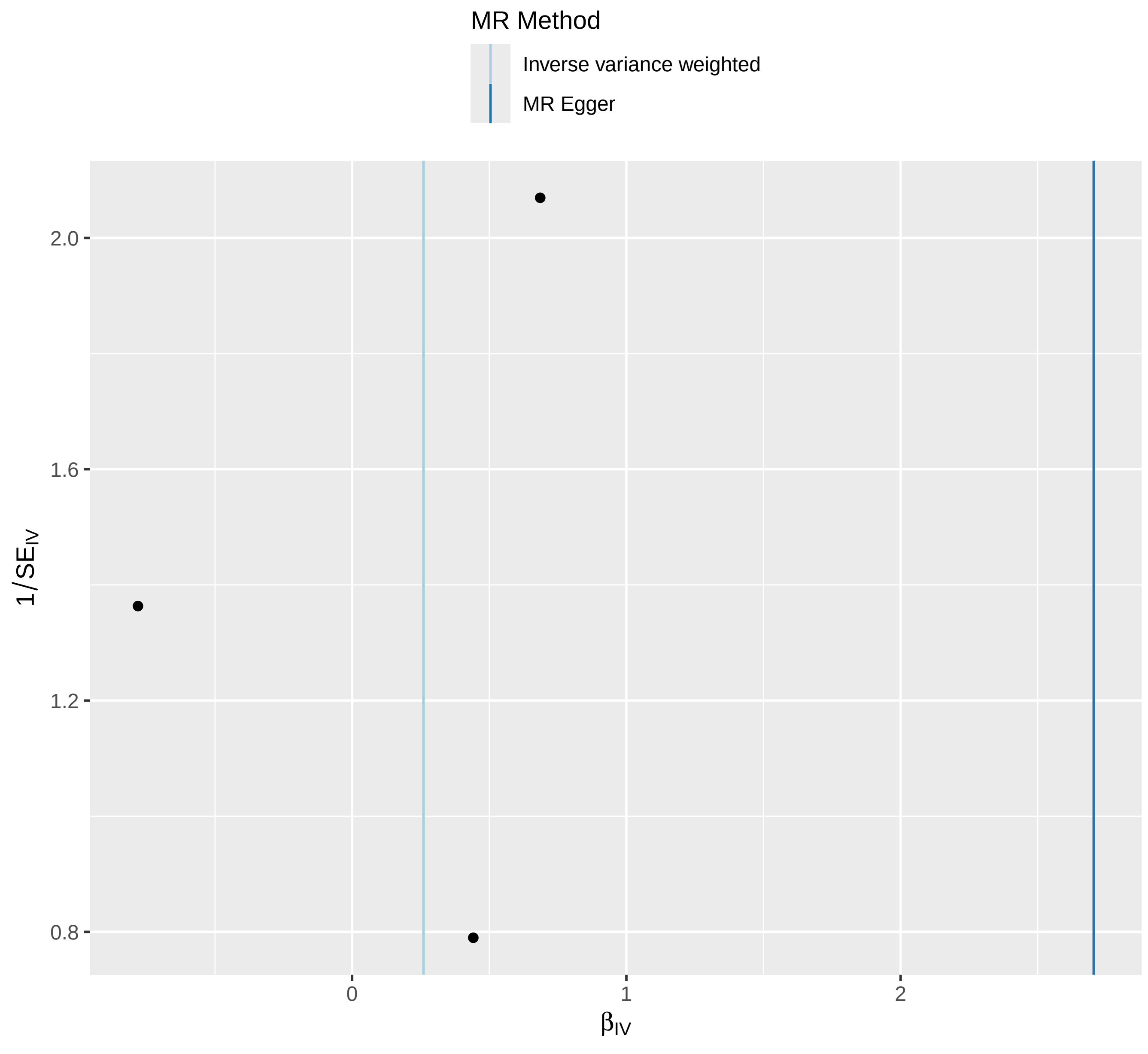
MR Method
Inverse variance weighted
MR Egger
2.0
1.6
1.2
0.8
0
1
2
$1/SE_{IV}$
$β_{IV}$

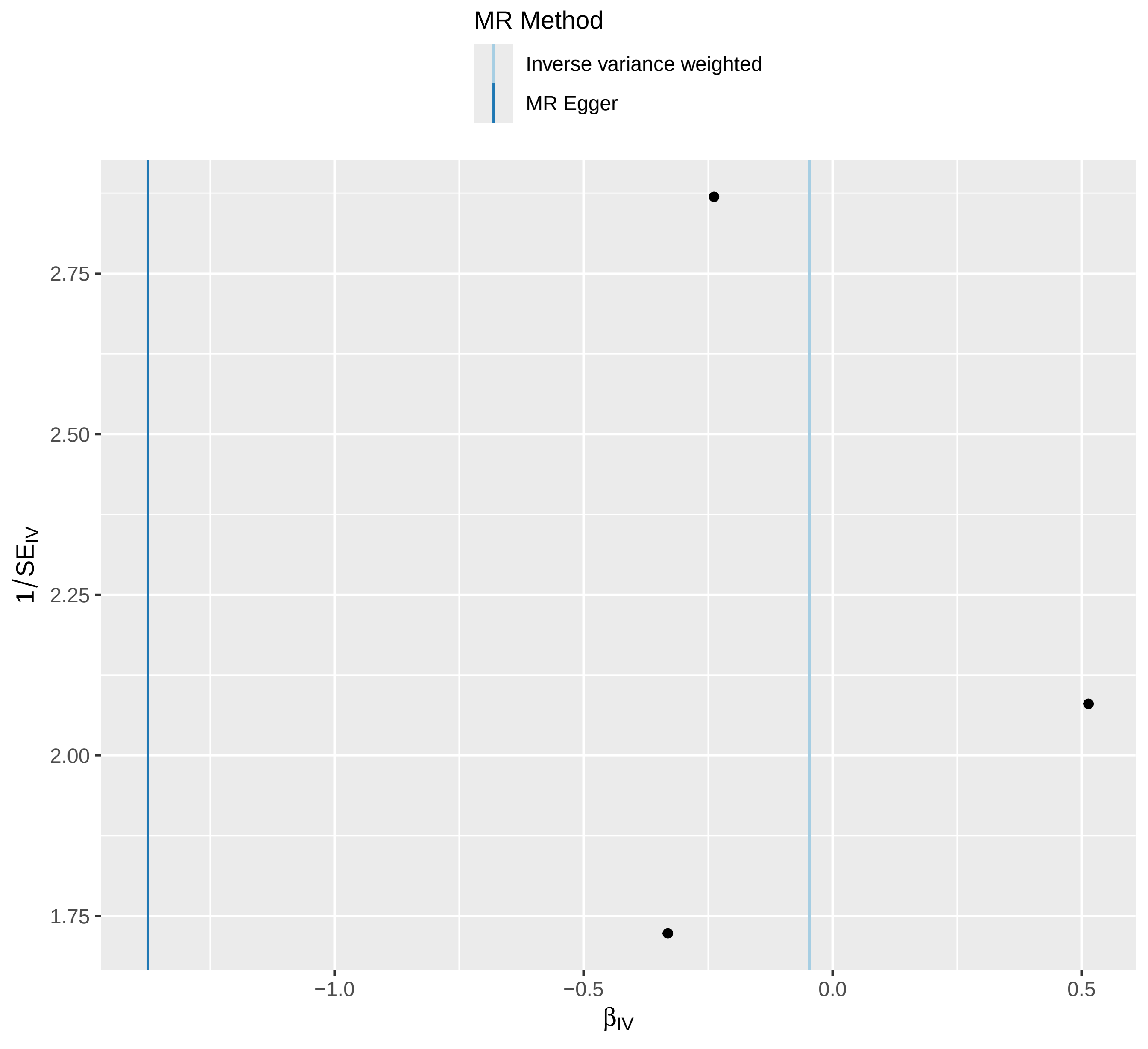
MR Method
Inverse variance weighted
MR Egger
2.75
2.50
2.25
2.00
1.75
$1/SE_{IV}$
−1.0
−0.5
0.0
0.5
β_{IV}

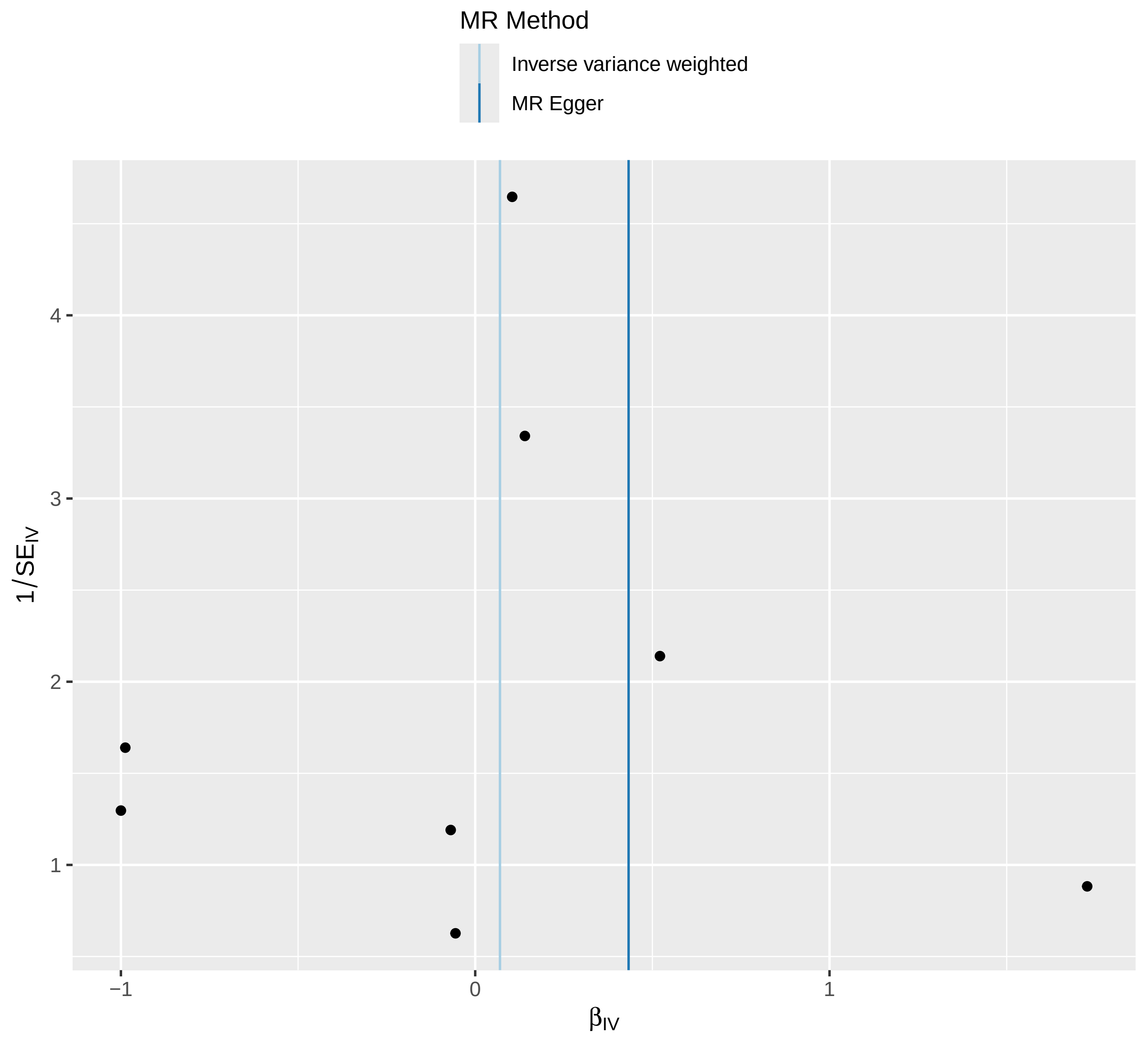
MR Method
Inverse variance weighted
MR Egger
$1/SE_{IV}$
β_{IV}
4
3
2
1
−1
0
1

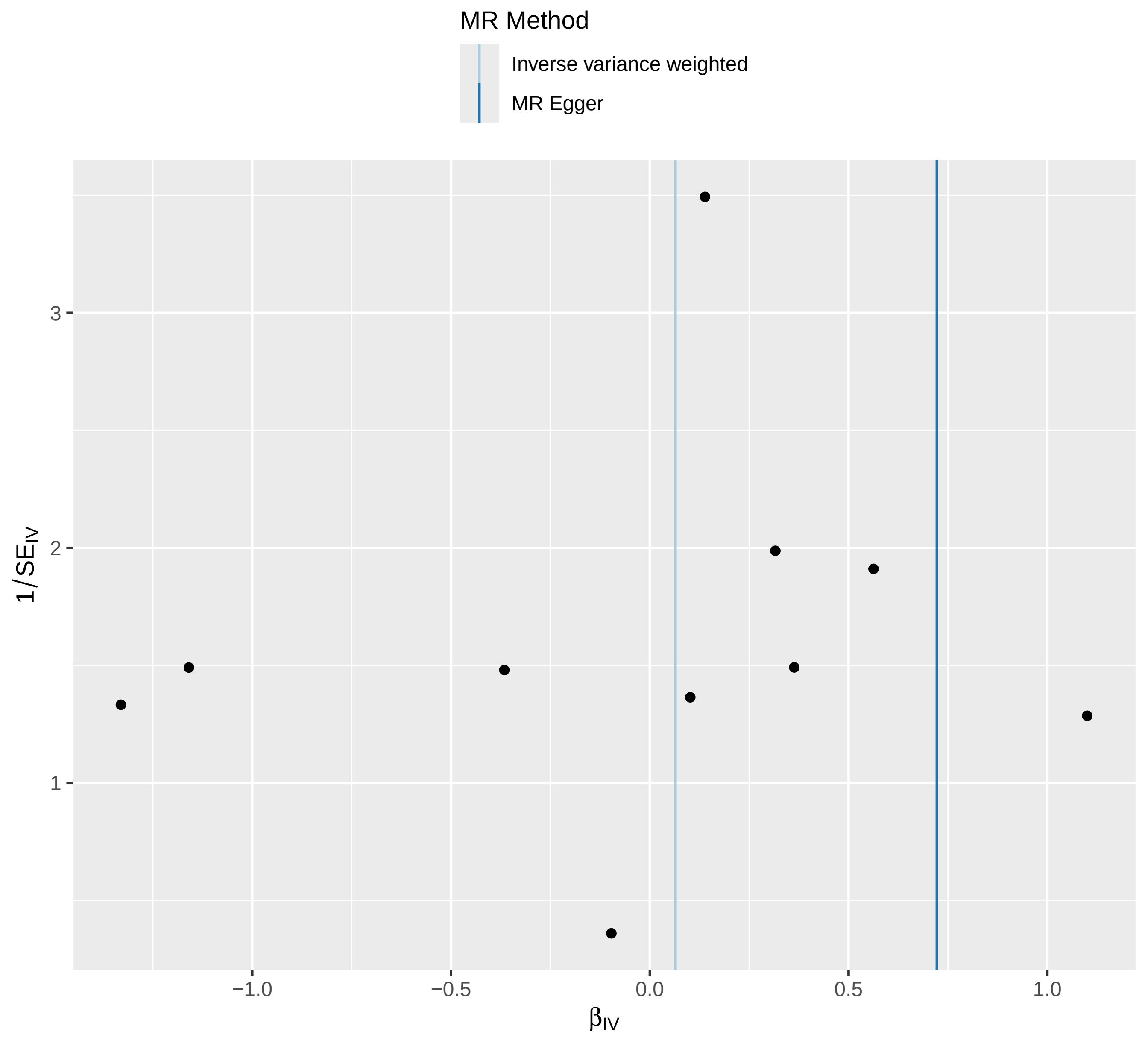
MR Method
Inverse variance weighted
MR Egger
1/SE_{IV}
3
2
1
−1.0
−0.5
0.0
0.5
1.0
β_{IV}

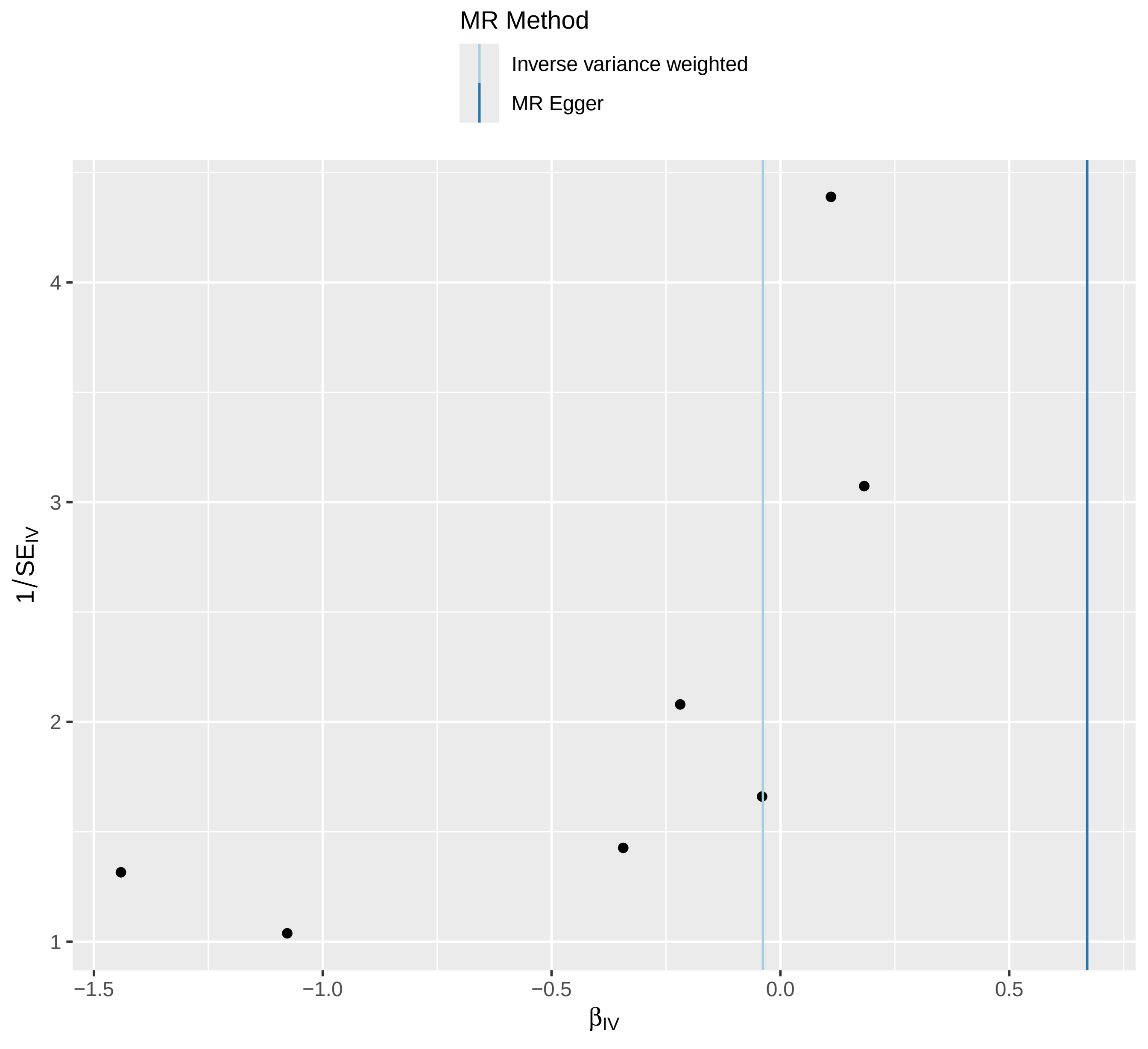

MR Method
Inverse variance weighted
MR Egger
4
3
2
1
1/SE_IV
−1.5
−1.0
−0.5
0.0
0.5
β_IV

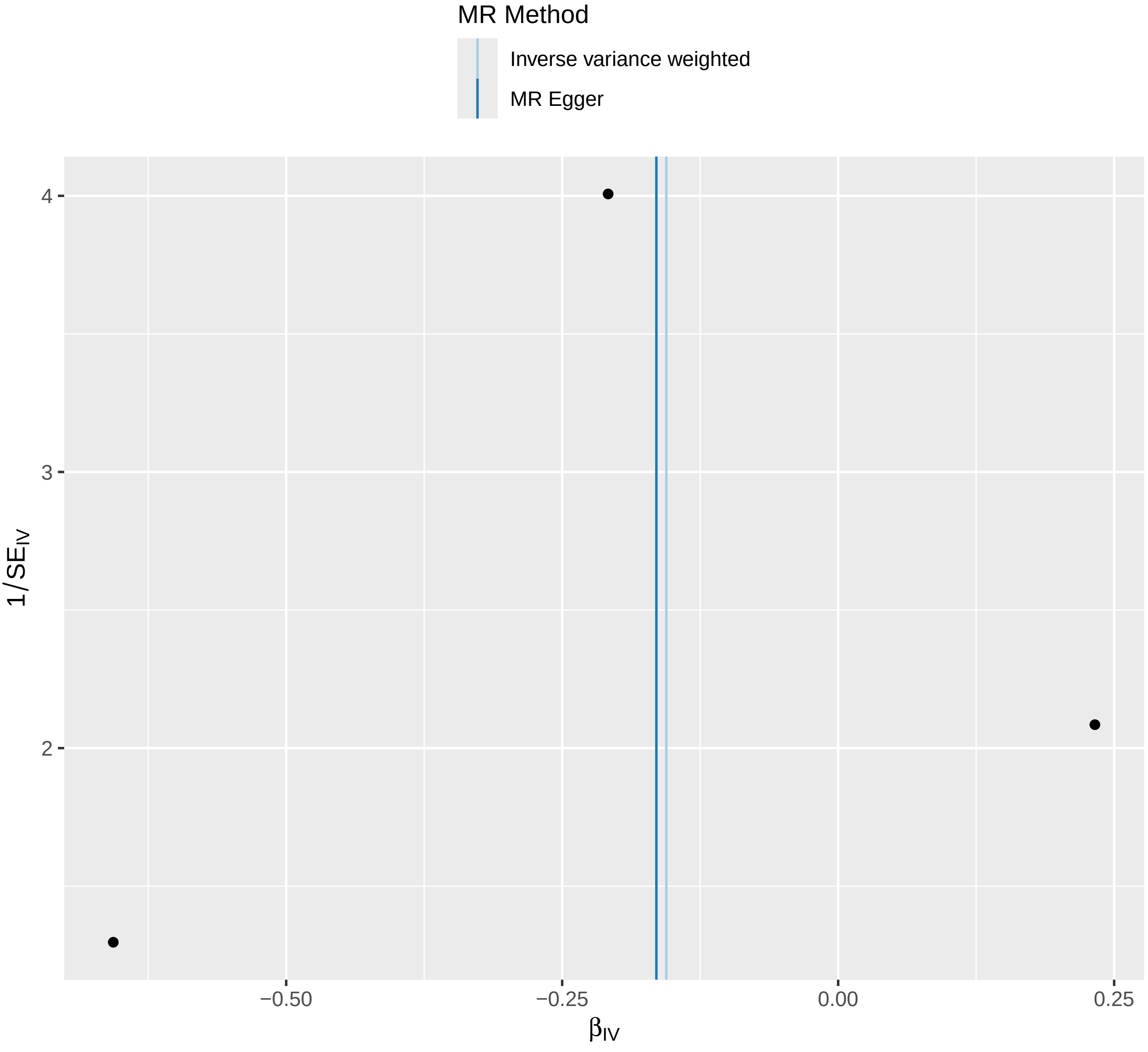

MR Method
Inverse variance weighted
MR Egger
4
3
2
1/SE_IV
−0.50
−0.25
0.00
0.25
β_IV

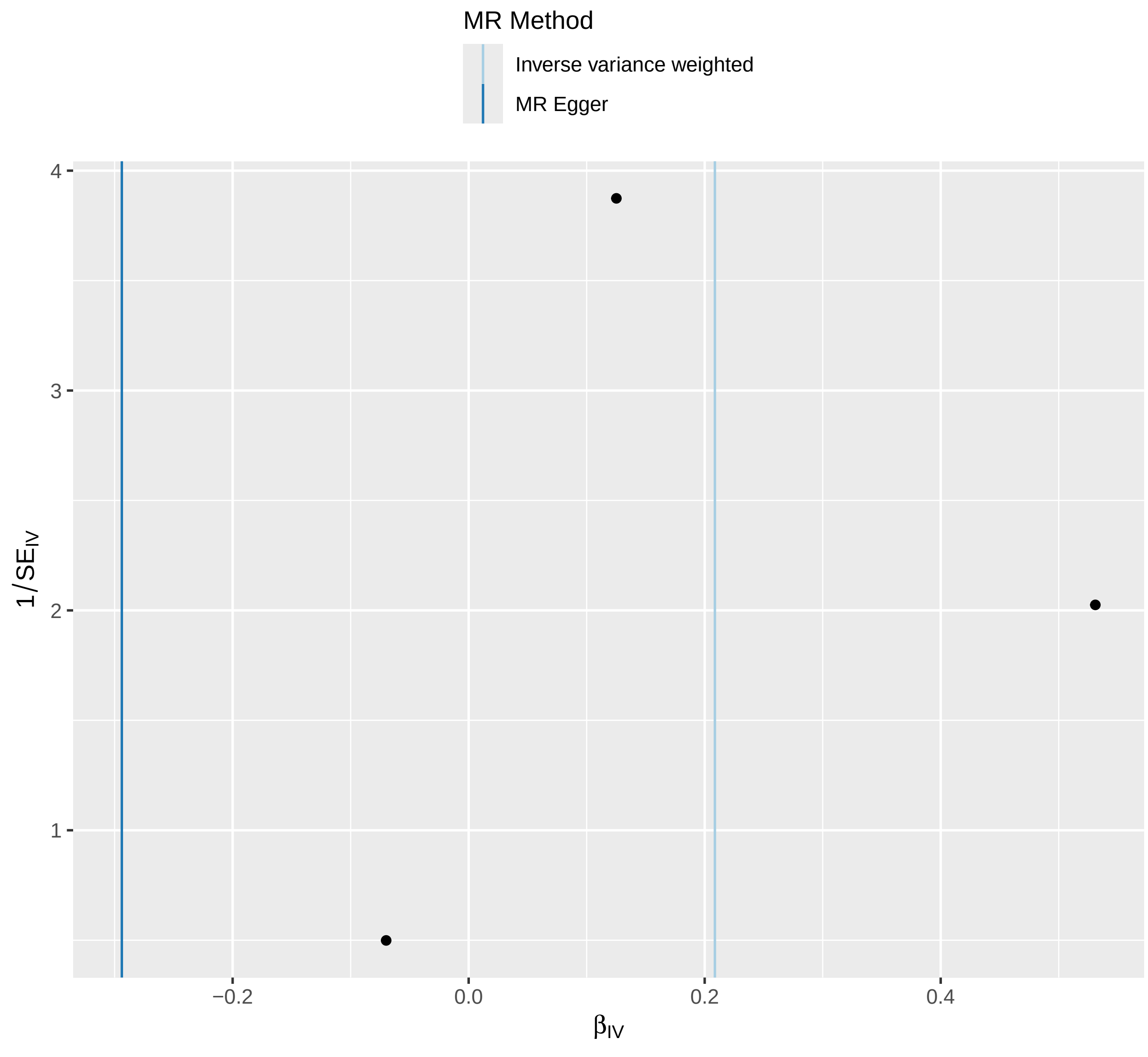

MR Method
Inverse variance weighted
MR Egger
4
3
2
1
$1/SE_{IV}$
−0.2
0.0
0.2
0.4
β_{IV}

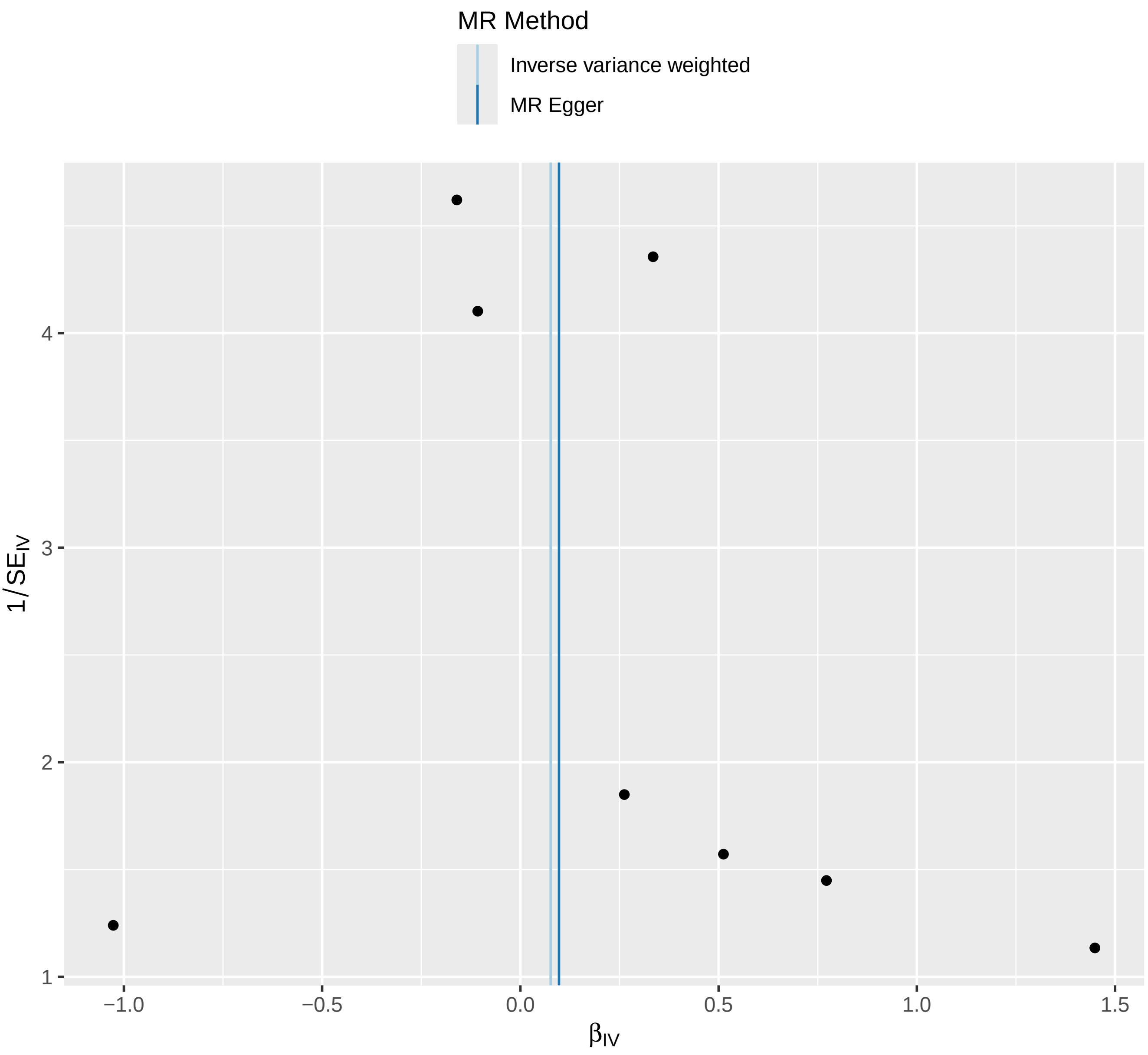

MR Method
Inverse variance weighted
MR Egger
1/SE_IV
β_IV
4
3
2
1
−1.0
−0.5
0.0
0.5
1.0
1.5

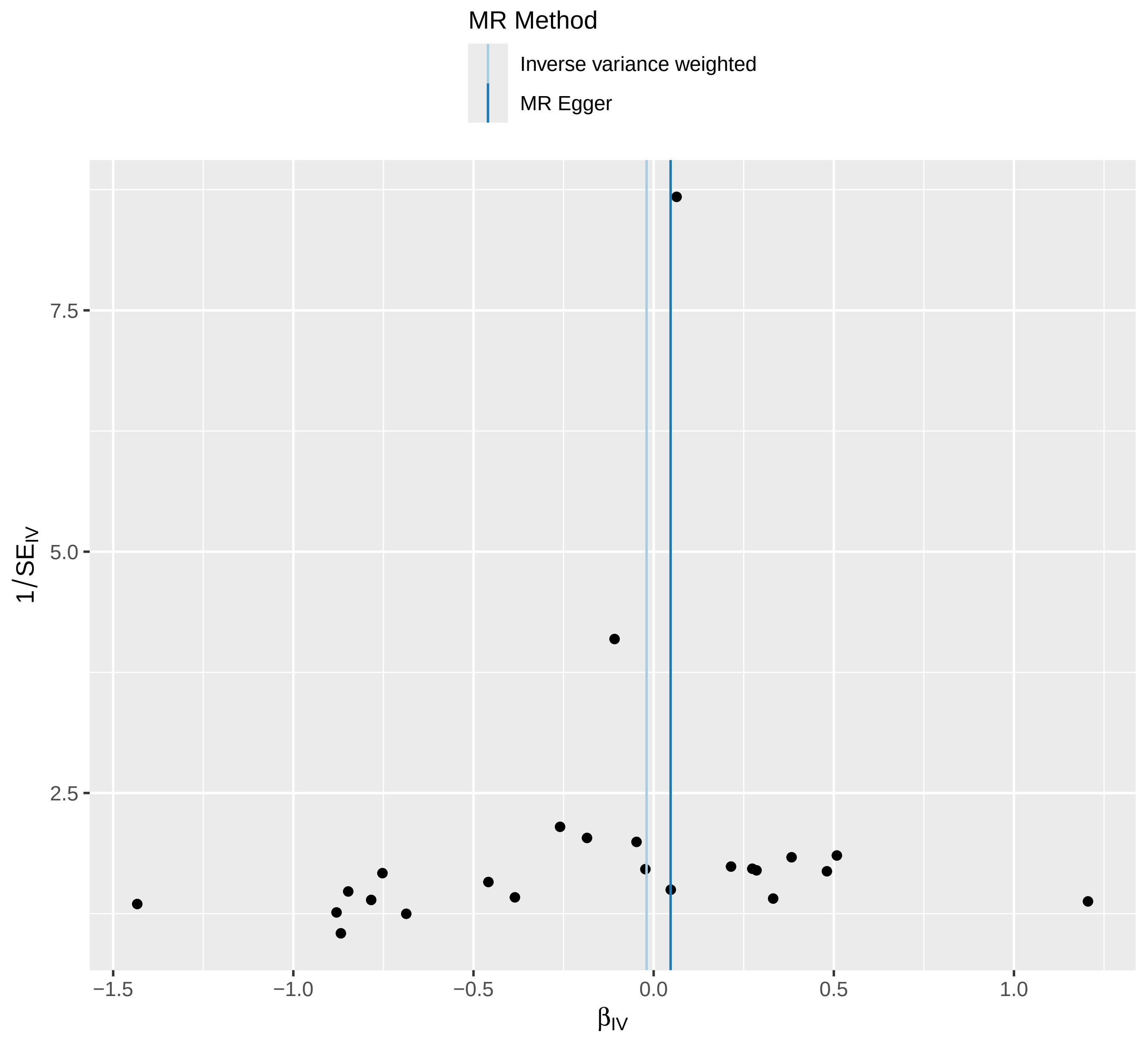
MR Method
Inverse variance weighted
MR Egger
1/SE_IV
7.5
5.0
2.5
−1.5
−1.0
−0.5
0.0
0.5
1.0
β_IV

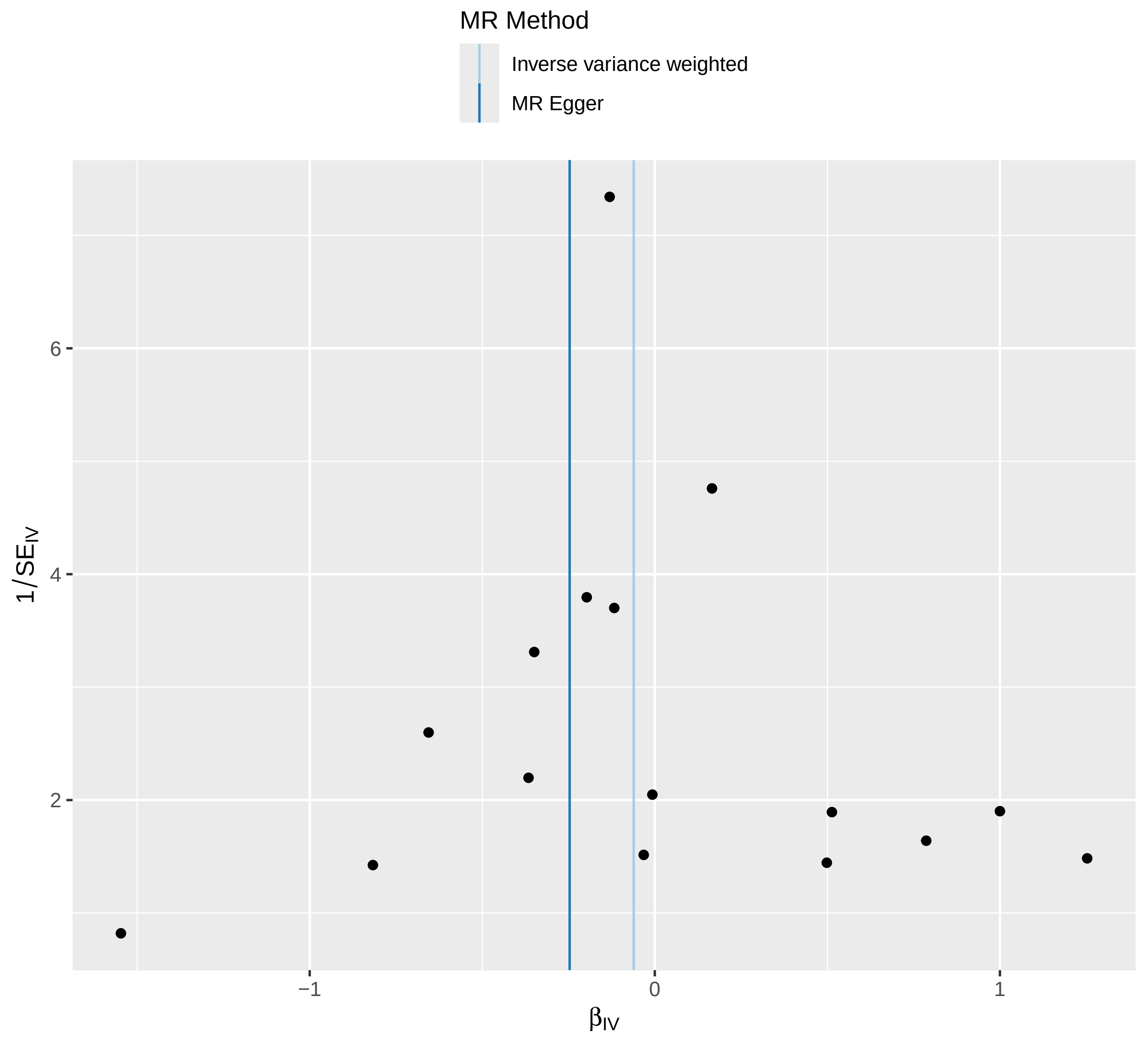
MR Method
Inverse variance weighted
MR Egger
1/SE_IV
6
4
2
−1
0
1
β_IV

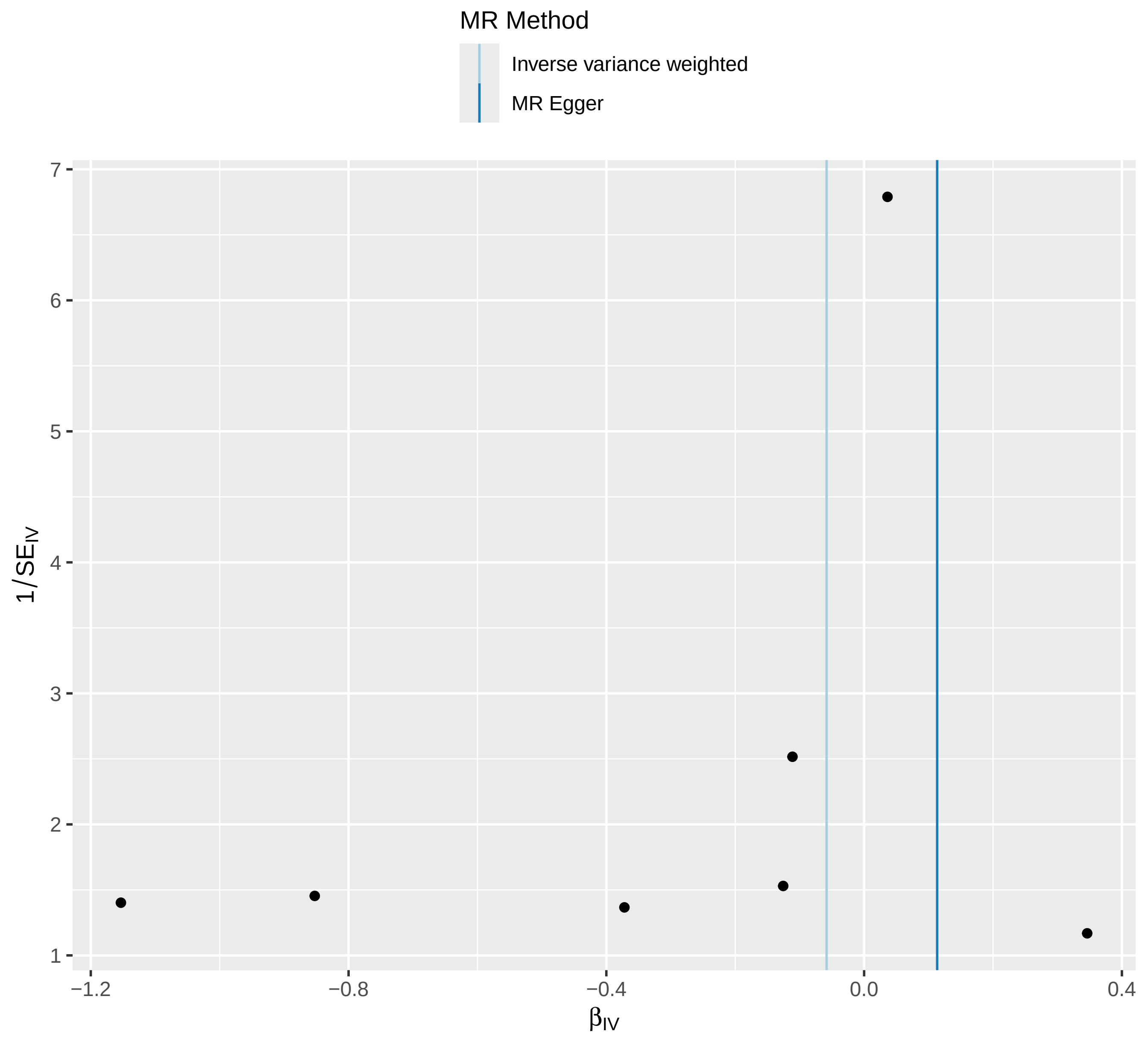
MR Method
Inverse variance weighted
MR Egger
1/SE_IV
7
6
5
4
3
2
1
−1.2
−0.8
−0.4
0.0
0.4
β_IV

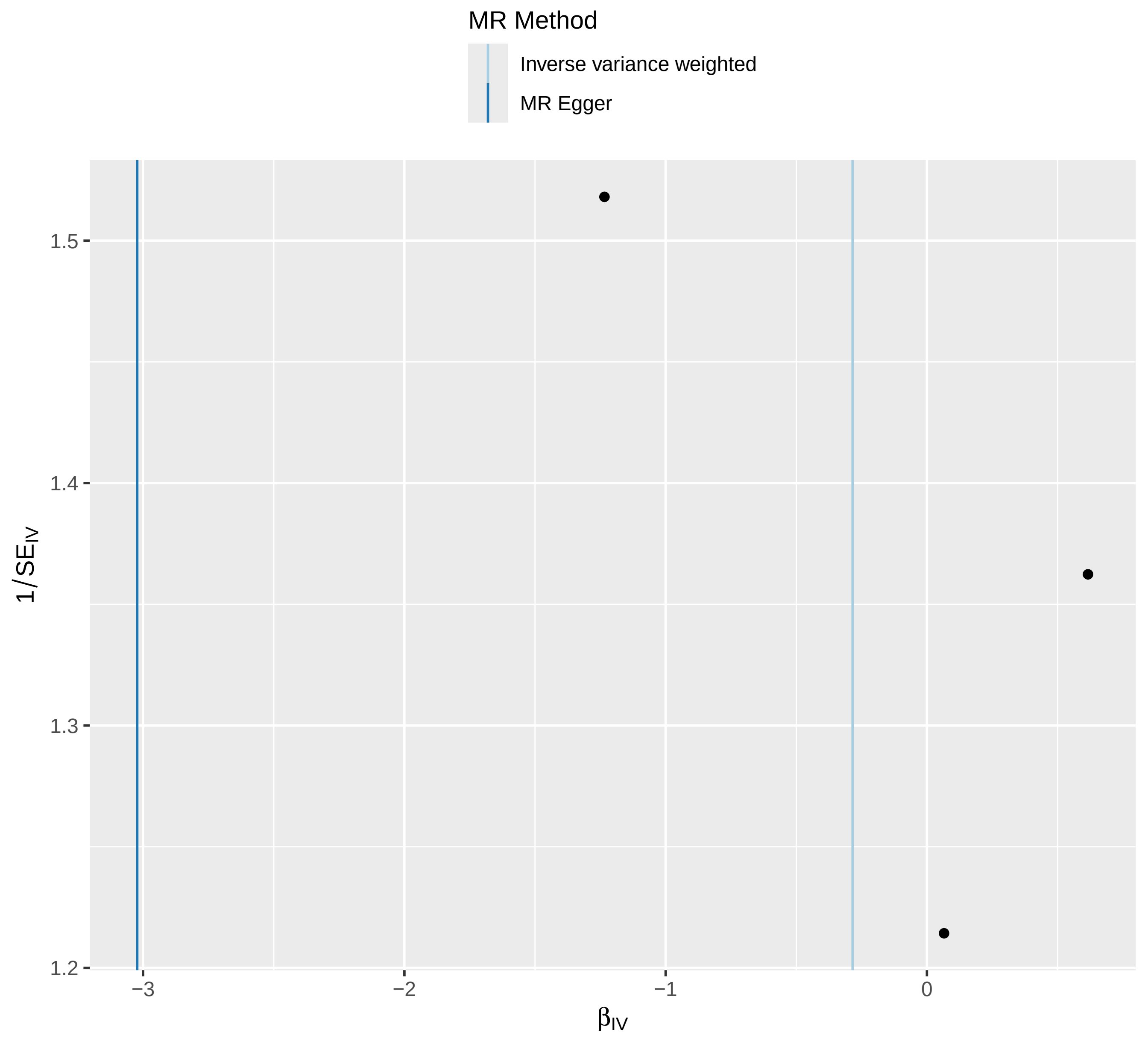
MR Method
Inverse variance weighted
MR Egger
1.5
1.4
1.3
1.2
1/SE_IV
−3
−2
−1
0
β_IV

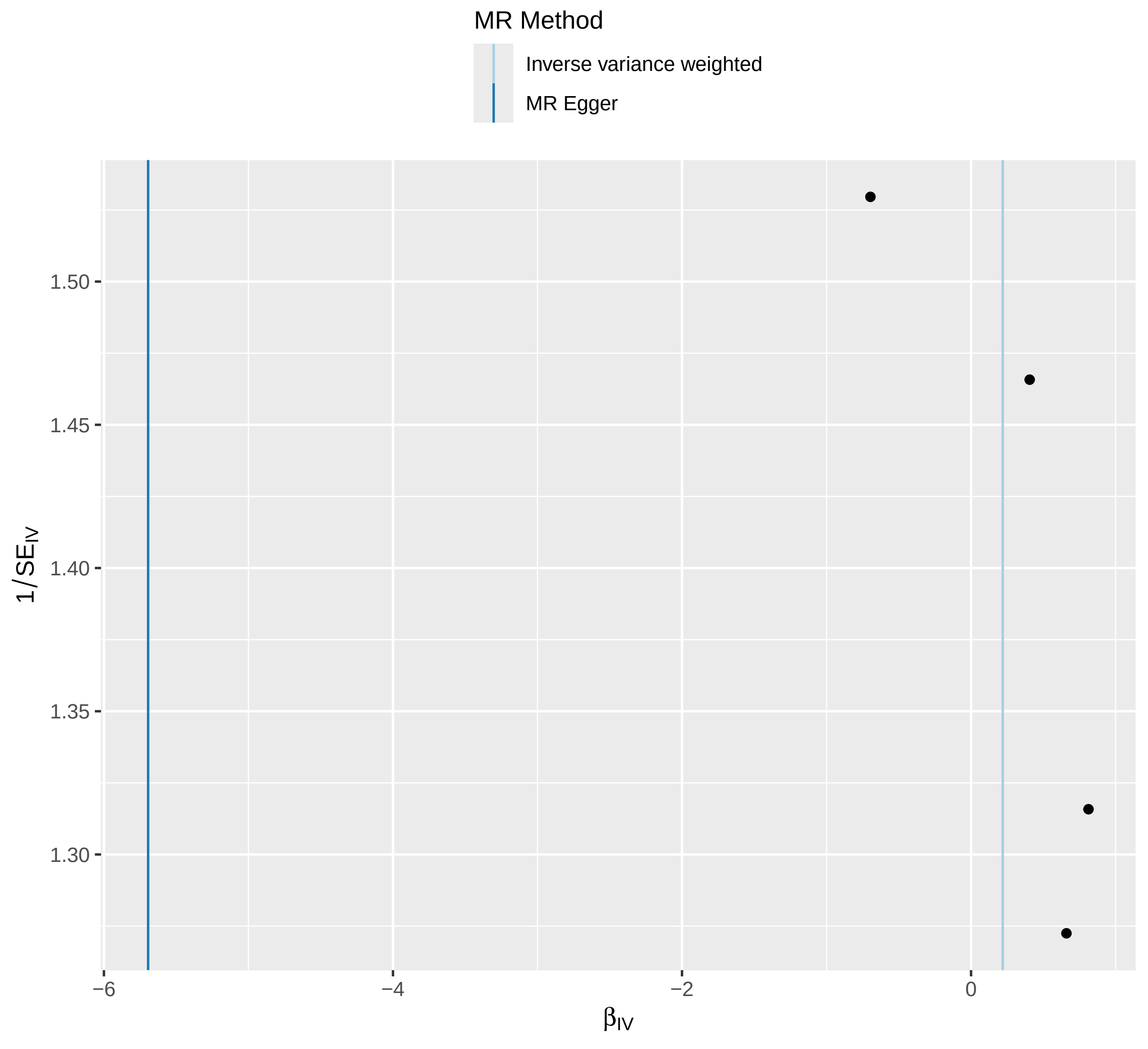
MR Method
Inverse variance weighted
MR Egger
1.50
1.45
1.40
1.35
1.30
1/SE_IV
−6
−4
−2
0
β_IV

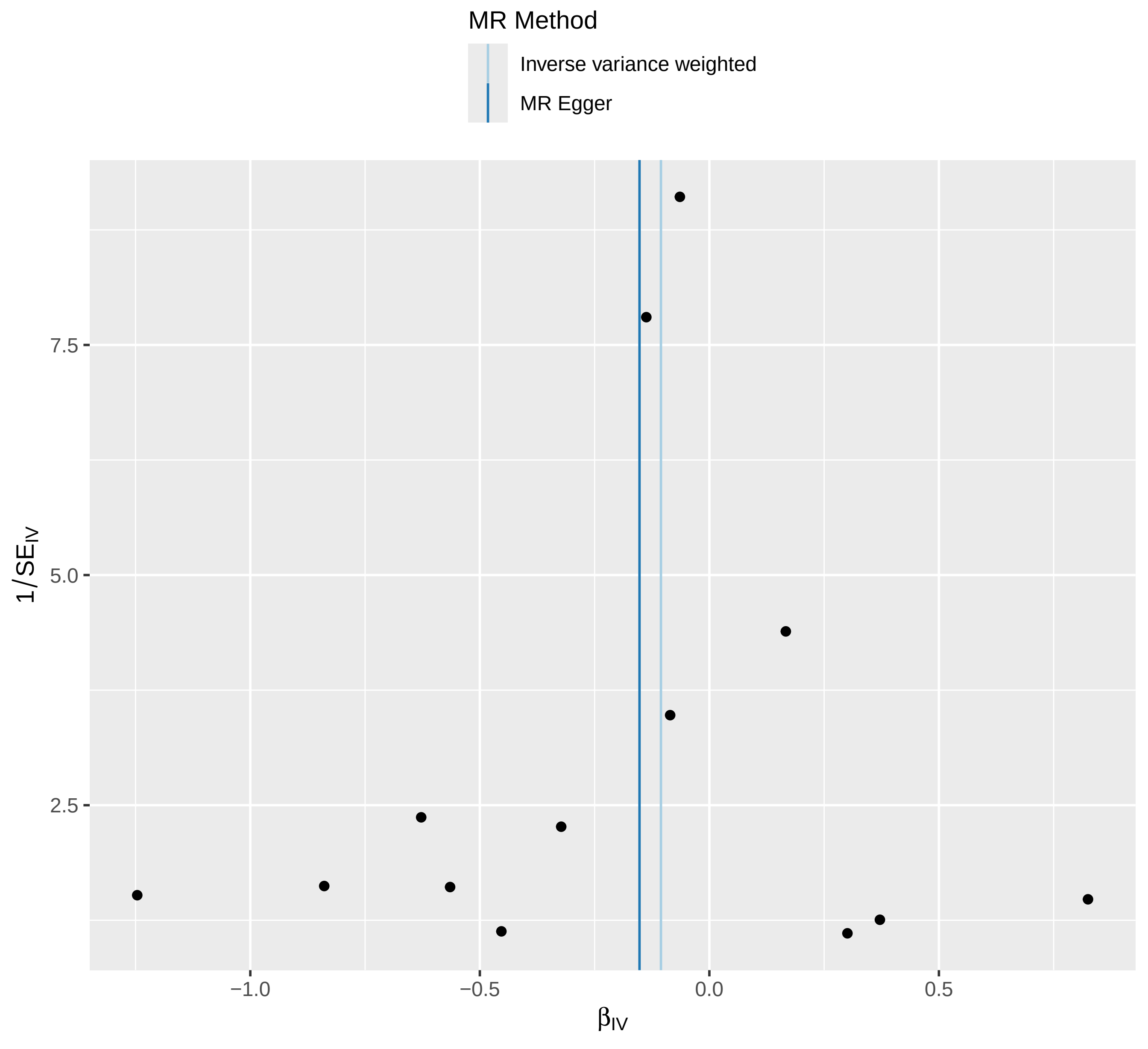

MR Method
Inverse variance weighted
MR Egger
7.5
5.0
2.5
−1.0
−0.5
0.0
0.5
$1/SE_{IV}$
β_{IV}

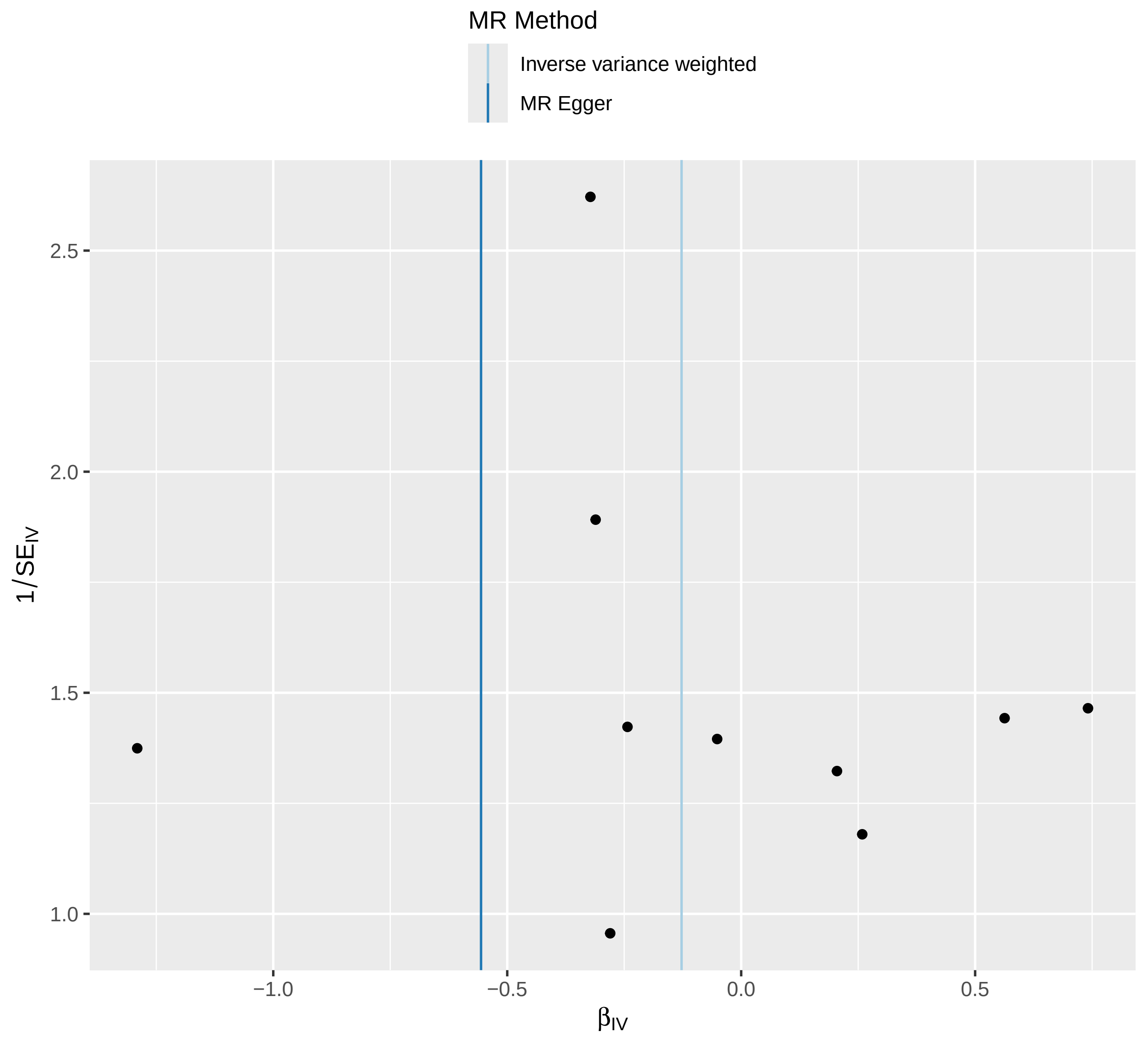
MR Method
Inverse variance weighted
MR Egger
1/SE_{IV}
2.5
2.0
1.5
1.0
−1.0
−0.5
0.0
0.5
β_{IV}

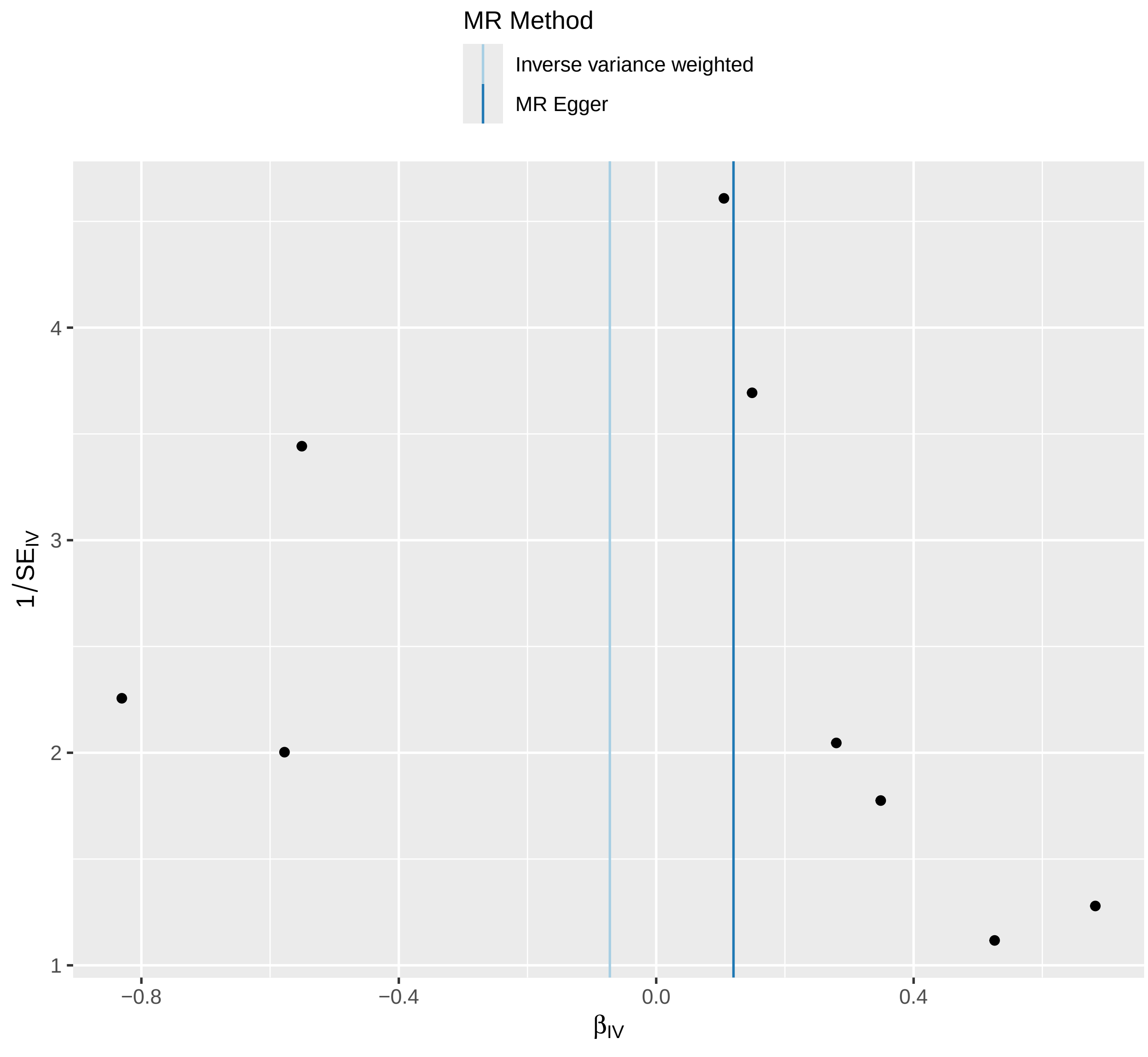

MR Method
Inverse variance weighted
MR Egger
4
3
2
1
1/SE_IV
−0.8
−0.4
0.0
0.4
β_IV

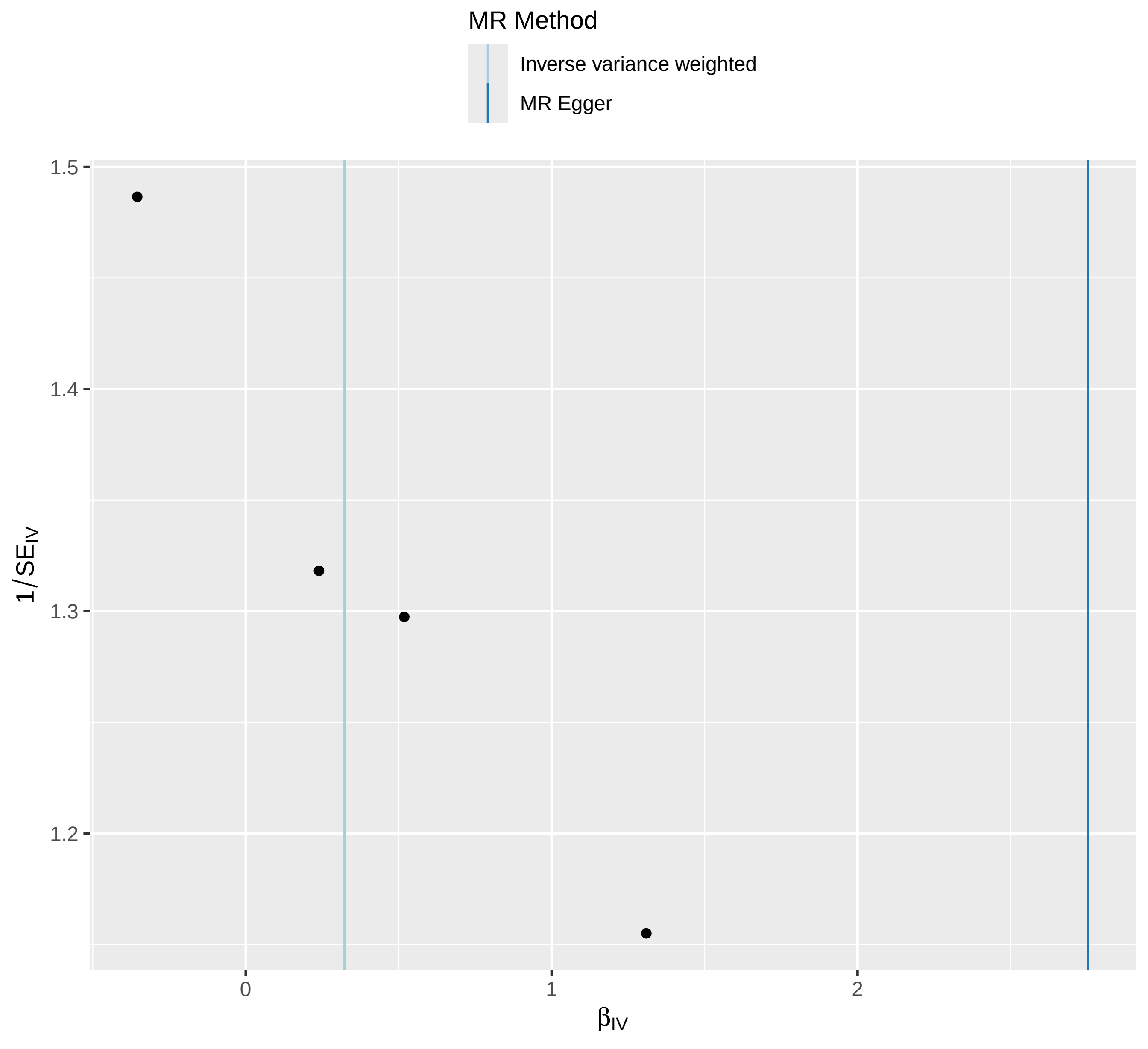
MR Method
Inverse variance weighted
MR Egger
1.5
1.4
1.3
1.2
1/SE_IV
0
1
2
β_IV

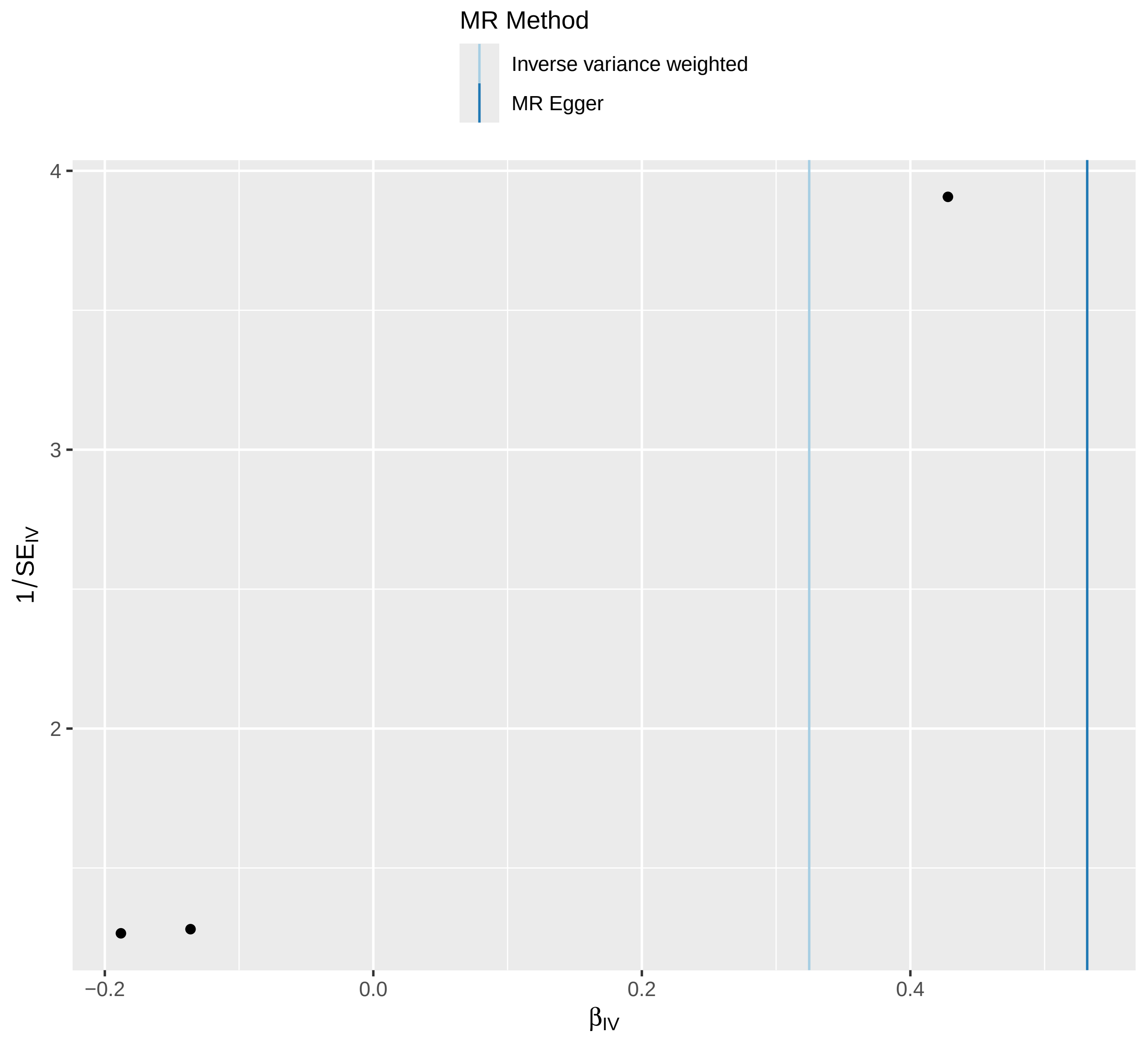

MR Method
Inverse variance weighted
MR Egger
4
3
2
1/SE_IV
−0.2
0.0
0.2
0.4
β_IV

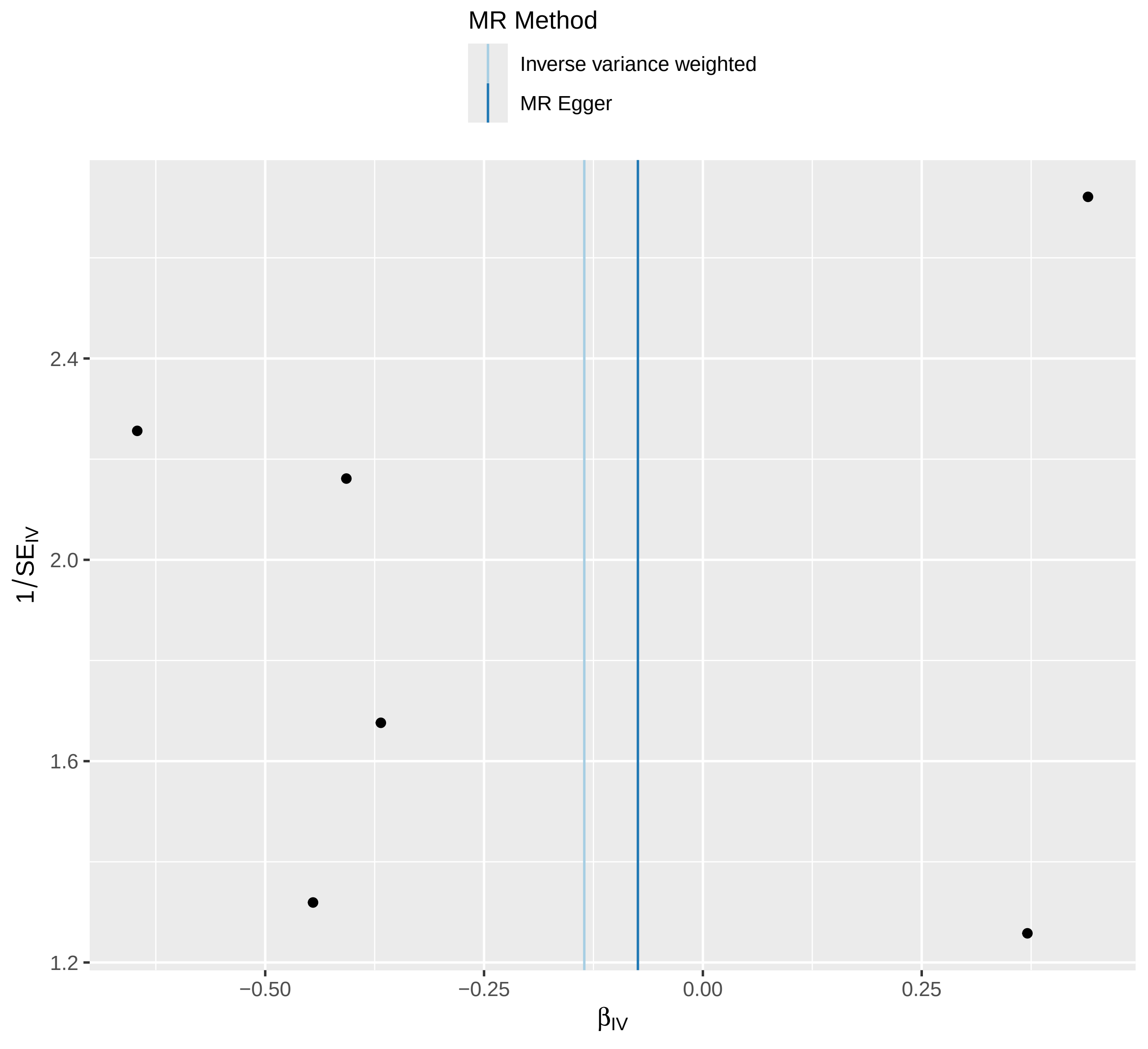

MR Method
Inverse variance weighted
MR Egger
2.4
2.0
1.6
1.2
1/SE_IV
−0.50
−0.25
0.00
0.25
β_IV

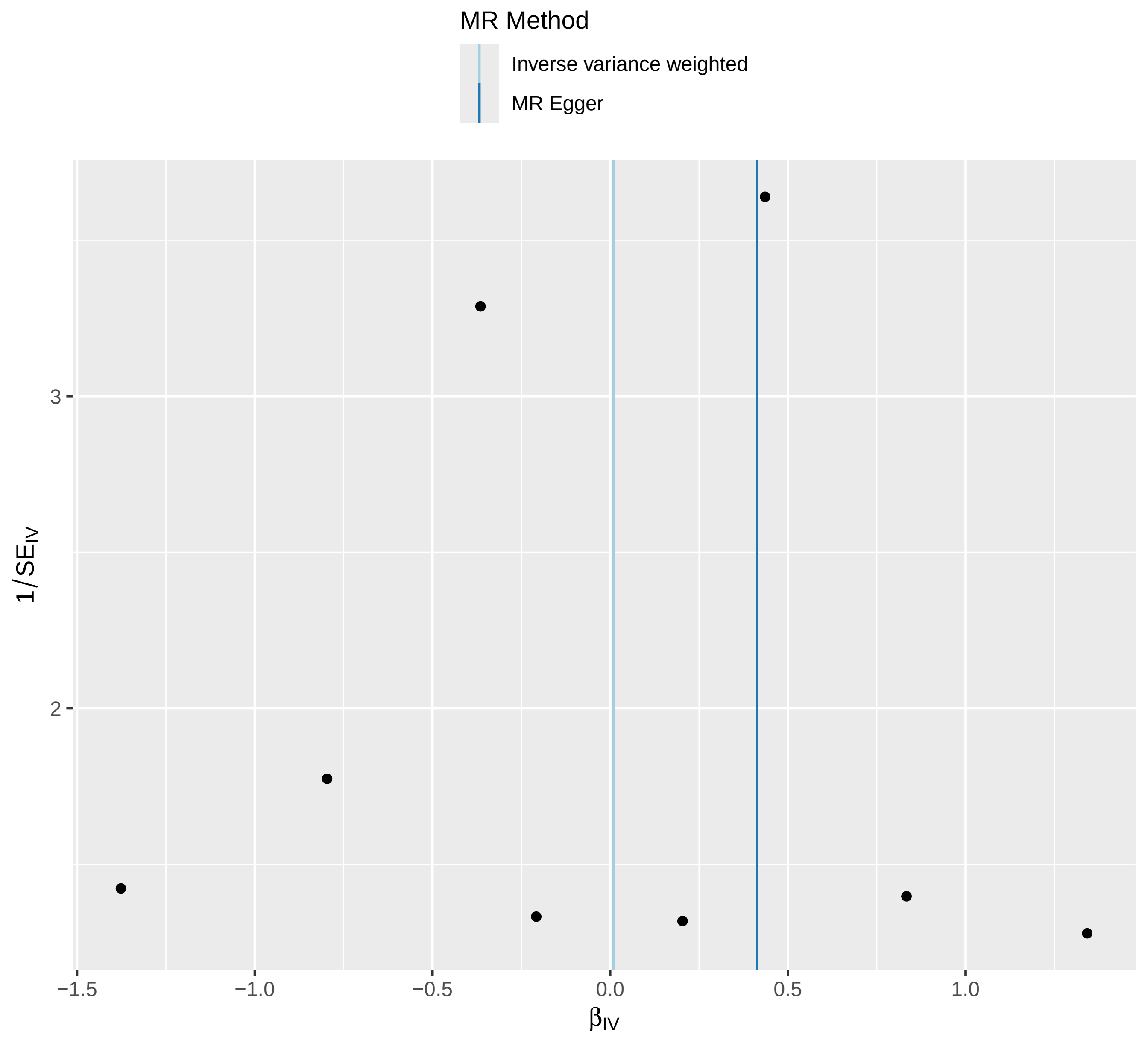
MR Method
Inverse variance weighted
MR Egger
3
2
1/SE_IV
−1.5
−1.0
−0.5
0.0
0.5
1.0
β_IV

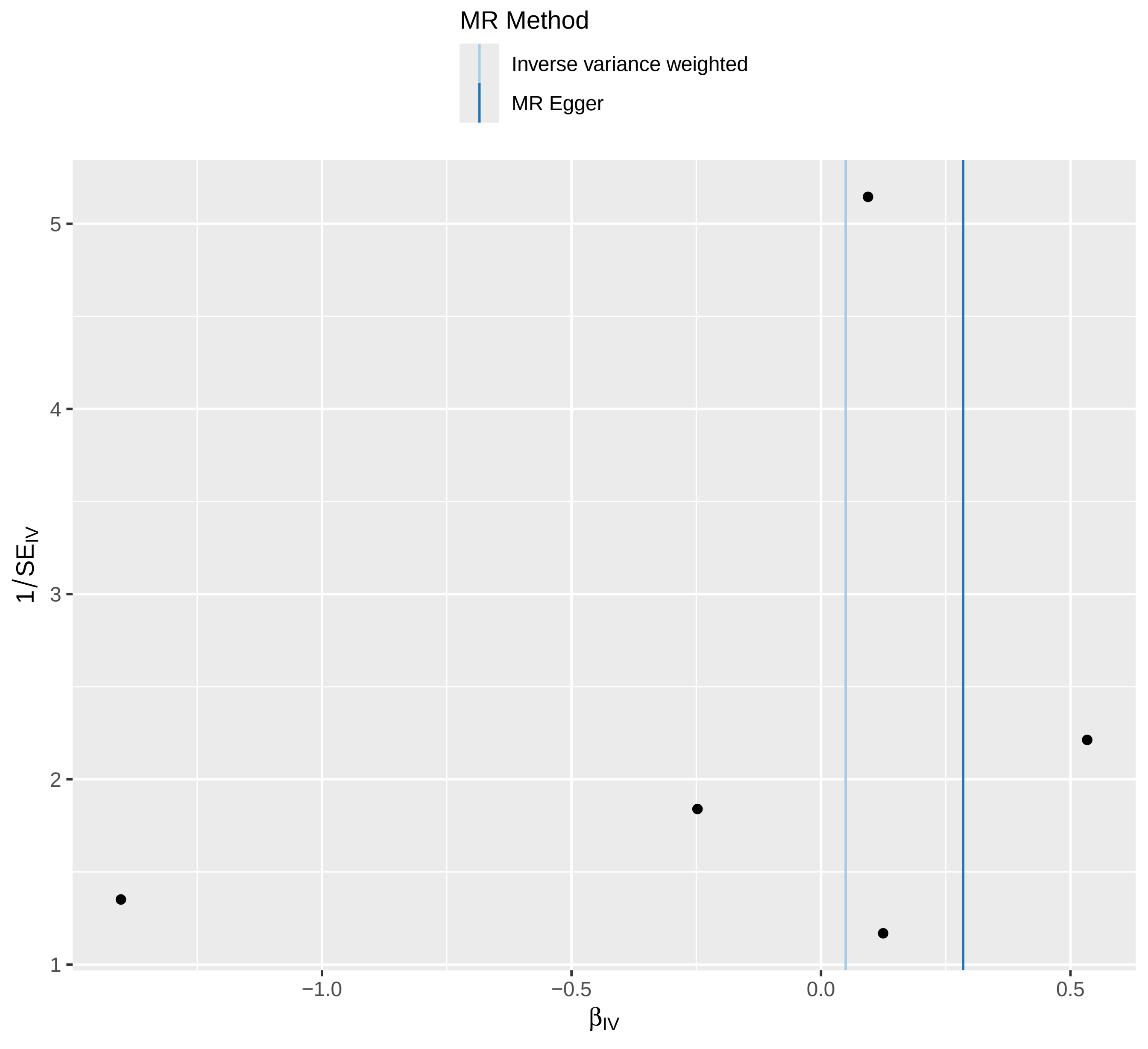
MR Method
Inverse variance weighted
MR Egger
5
4
3
2
1
1/SE_IV
−1.0
−0.5
0.0
0.5
β_IV

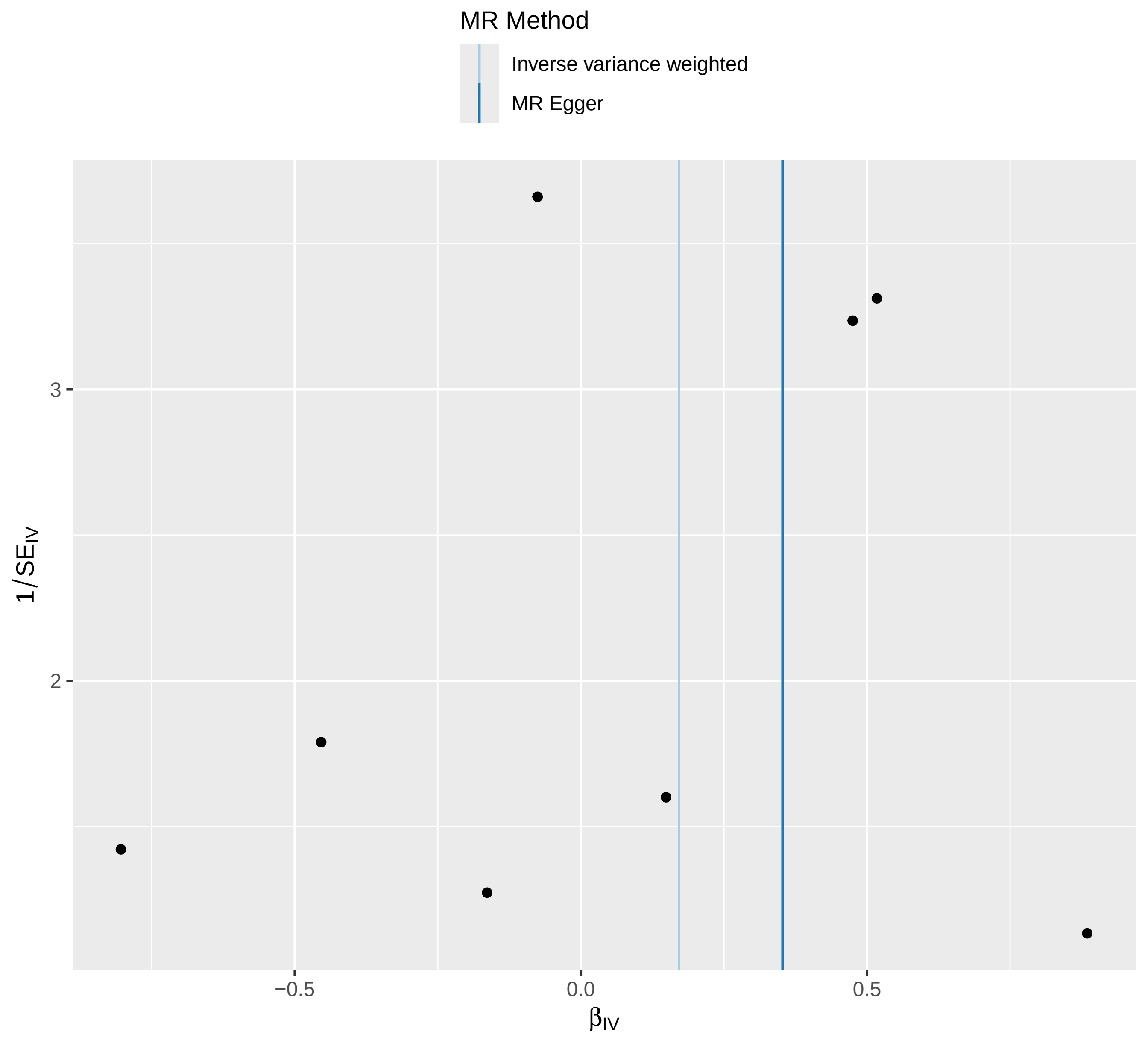
MR Method
Inverse variance weighted
MR Egger
3
2
1/SE$_{IV}$
−0.5
0.0
0.5
β$_{IV}$

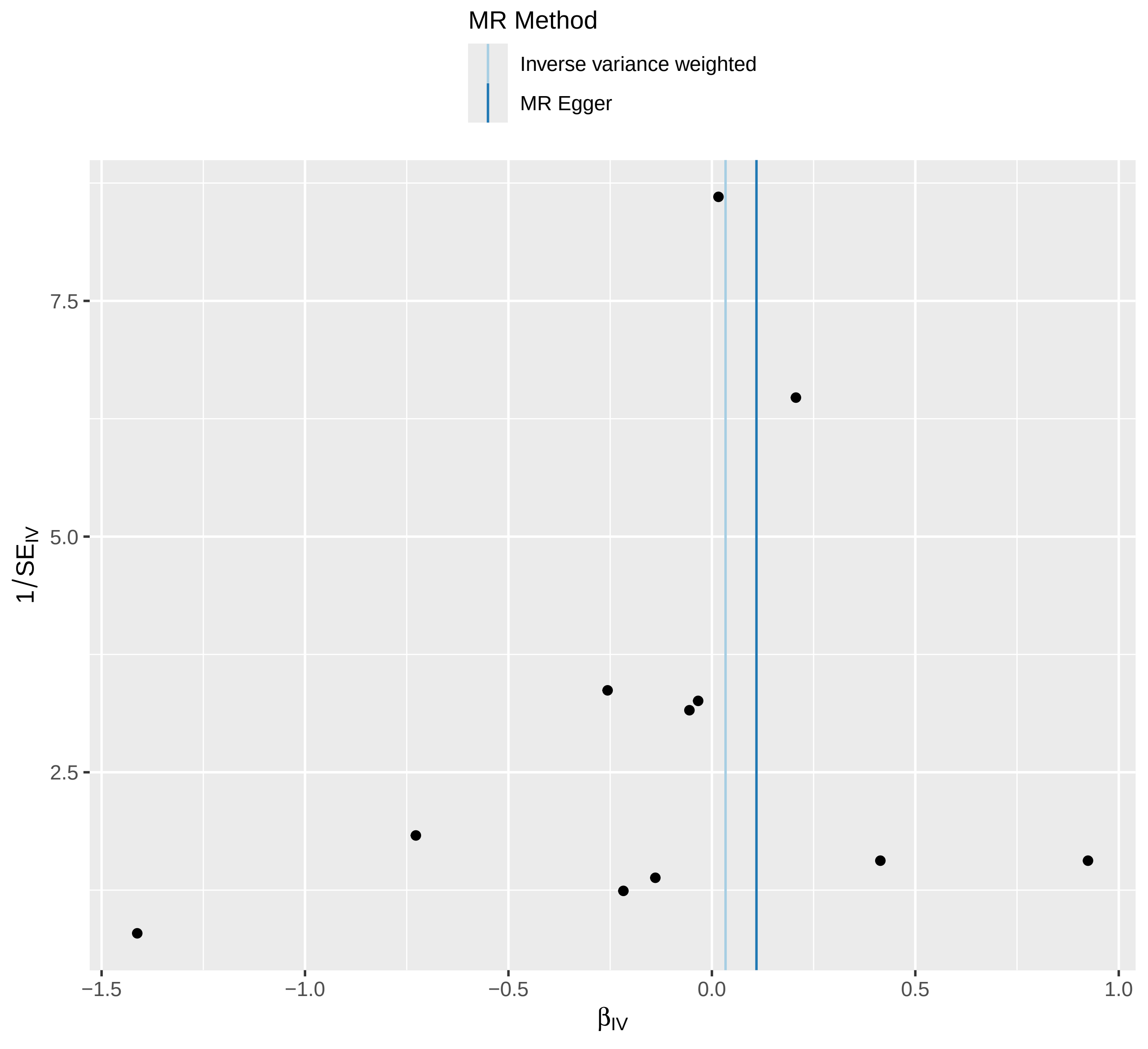

MR Method
Inverse variance weighted
MR Egger
7.5
5.0
2.5
1/SE$_{IV}$
−1.5
−1.0
−0.5
0.0
0.5
1.0
β$_{IV}$

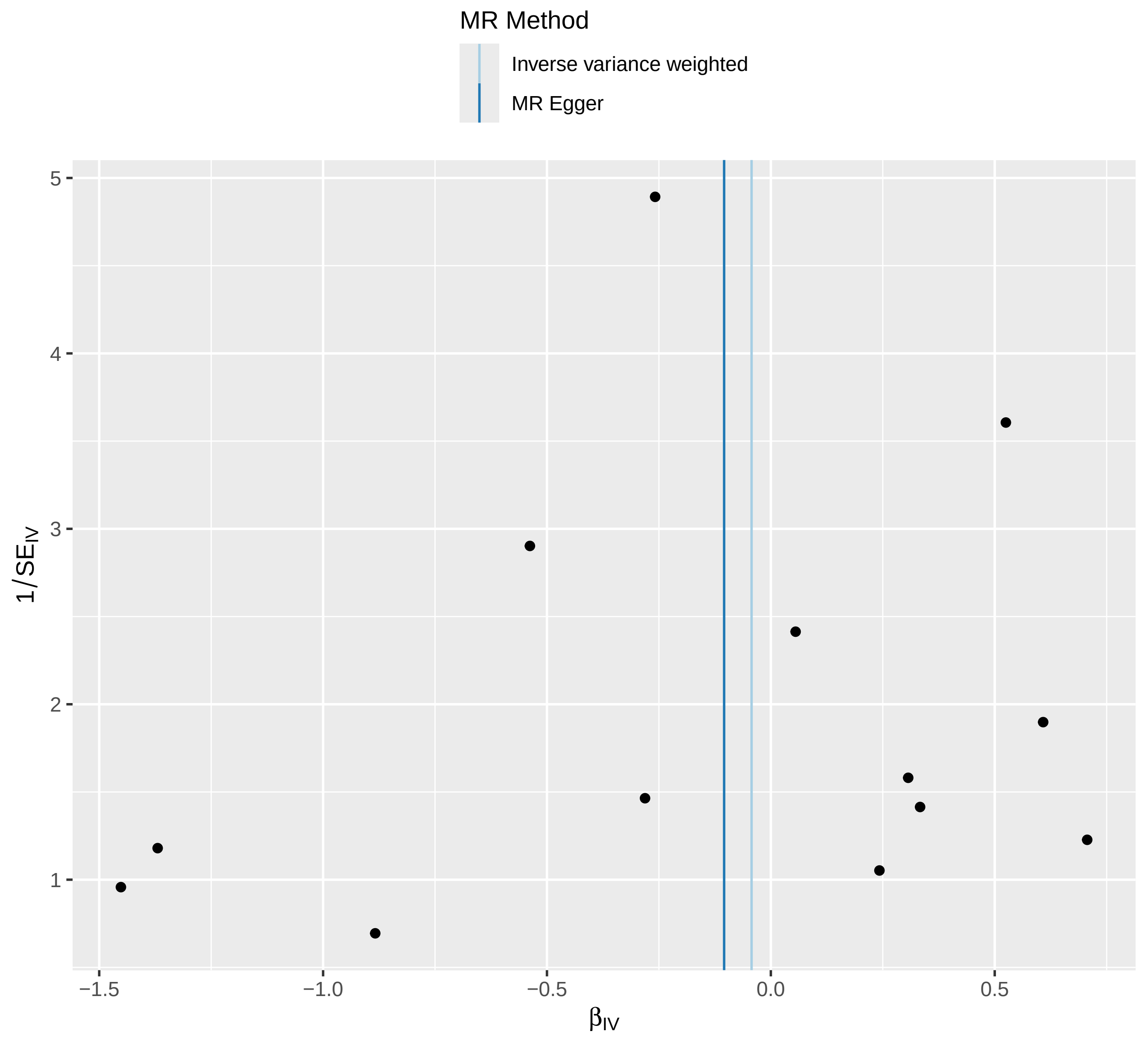
MR Method
Inverse variance weighted
MR Egger
1/SE$_{IV}$
β$_{IV}$
5
4
3
2
1
−1.5
−1.0
−0.5
0.0
0.5

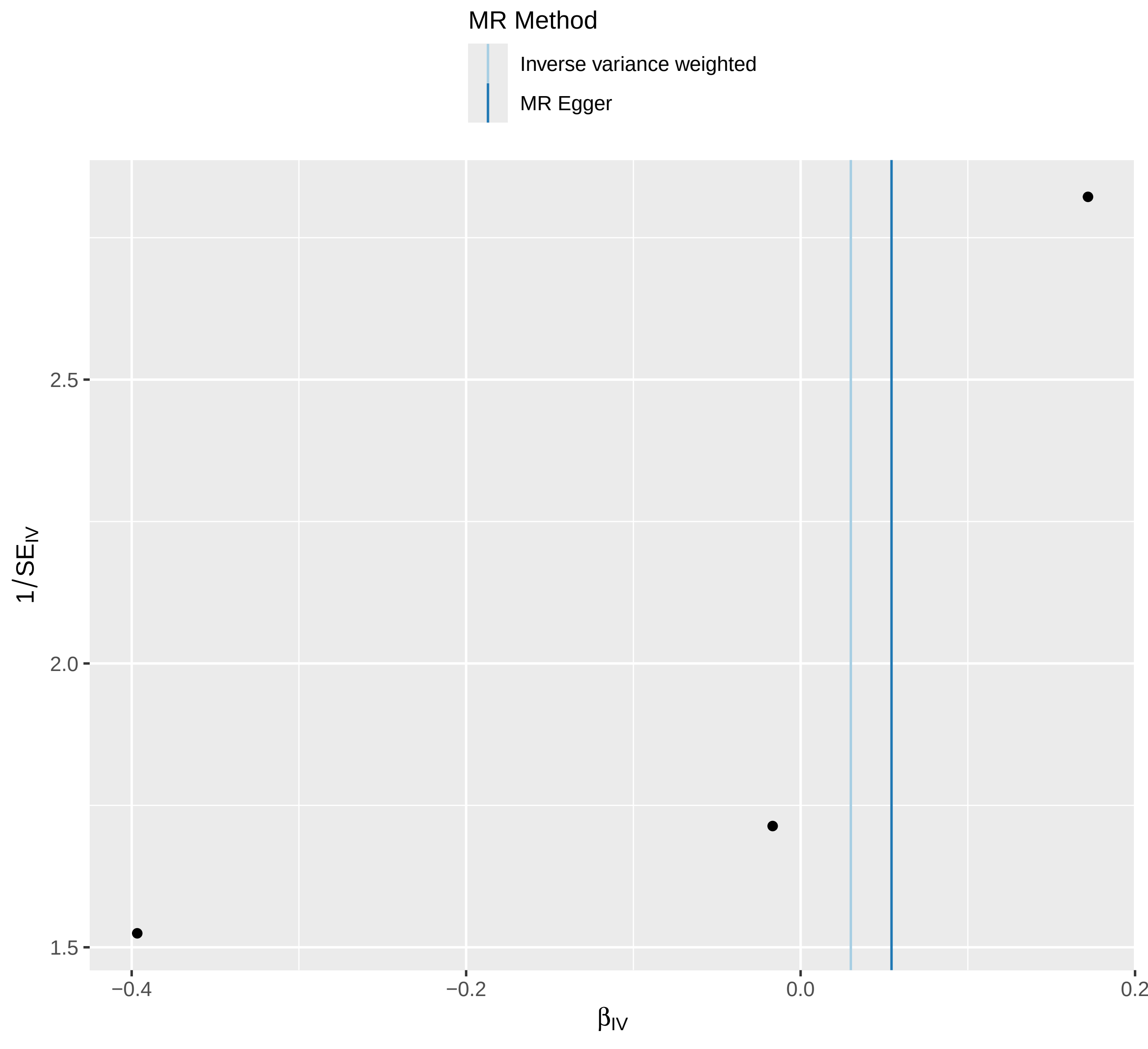
MR Method
Inverse variance weighted
MR Egger
1/SE_IV
2.5
2.0
1.5
−0.4
−0.2
0.0
0.2
β_IV

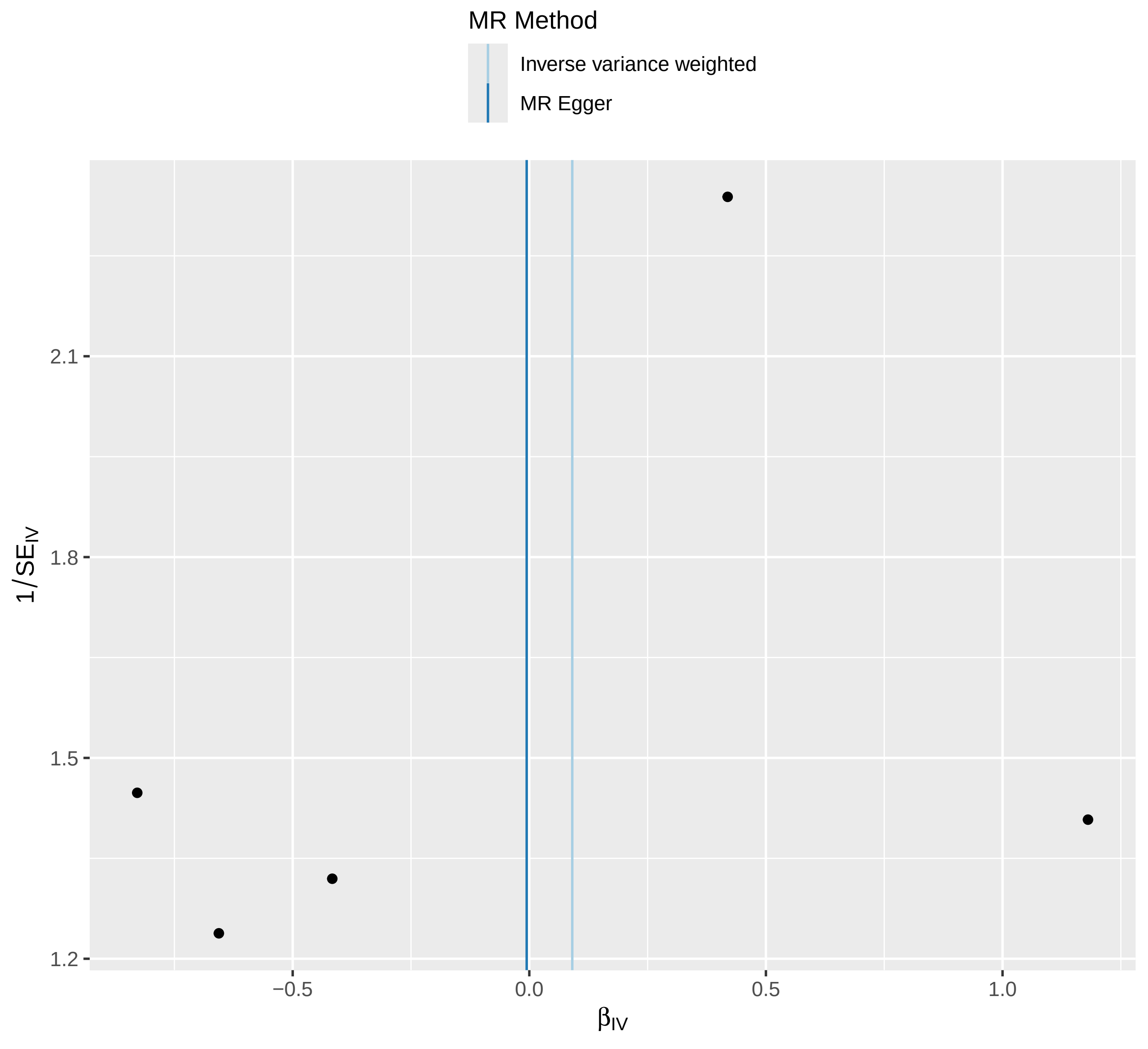
MR Method
Inverse variance weighted
MR Egger
2.1
1.8
1.5
1.2
1/SE_IV
−0.5
0.0
0.5
1.0
β_IV

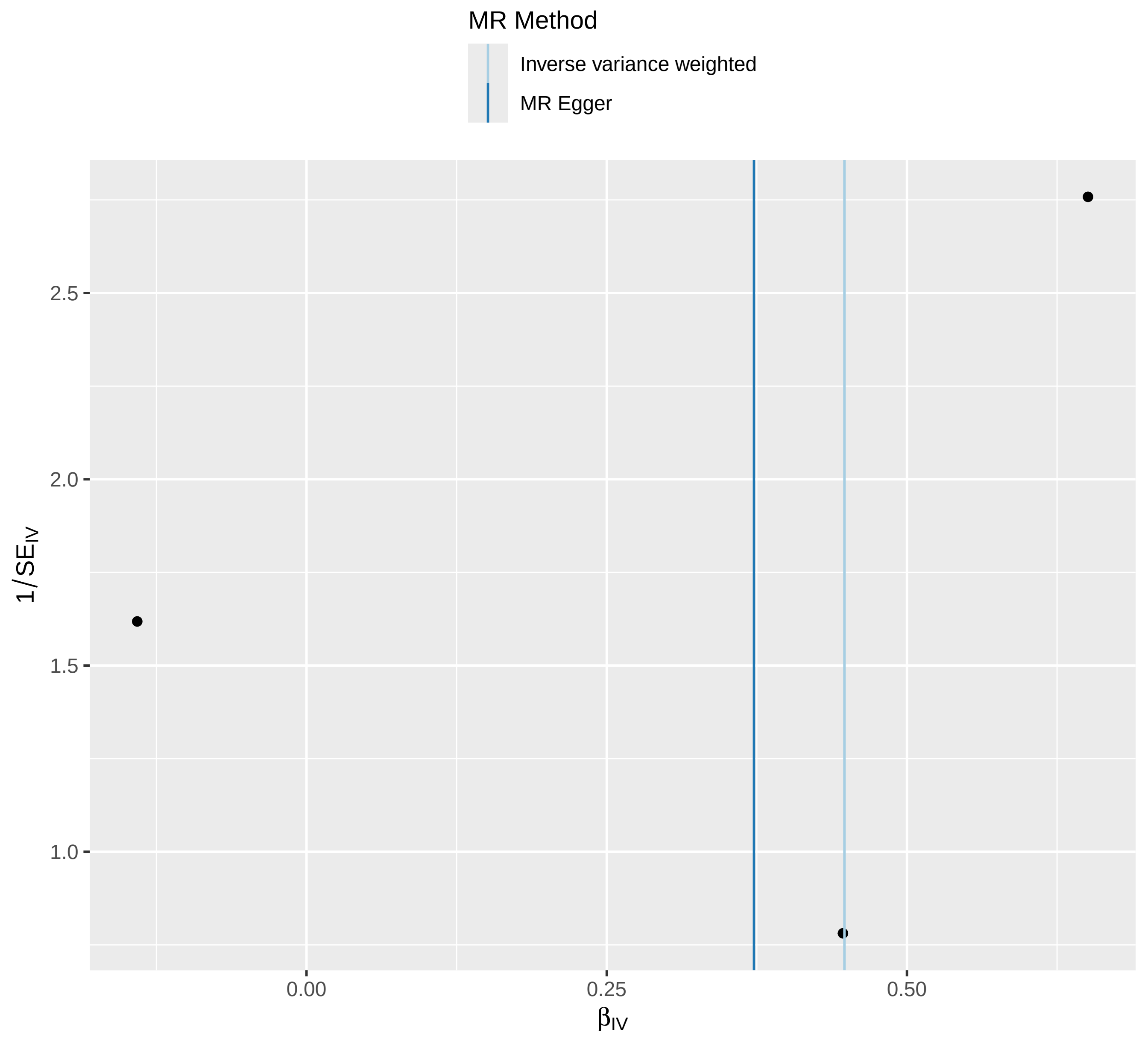
MR Method
Inverse variance weighted
MR Egger
2.5
2.0
1.5
1.0
1/SE_IV
0.00
0.25
0.50
β_IV

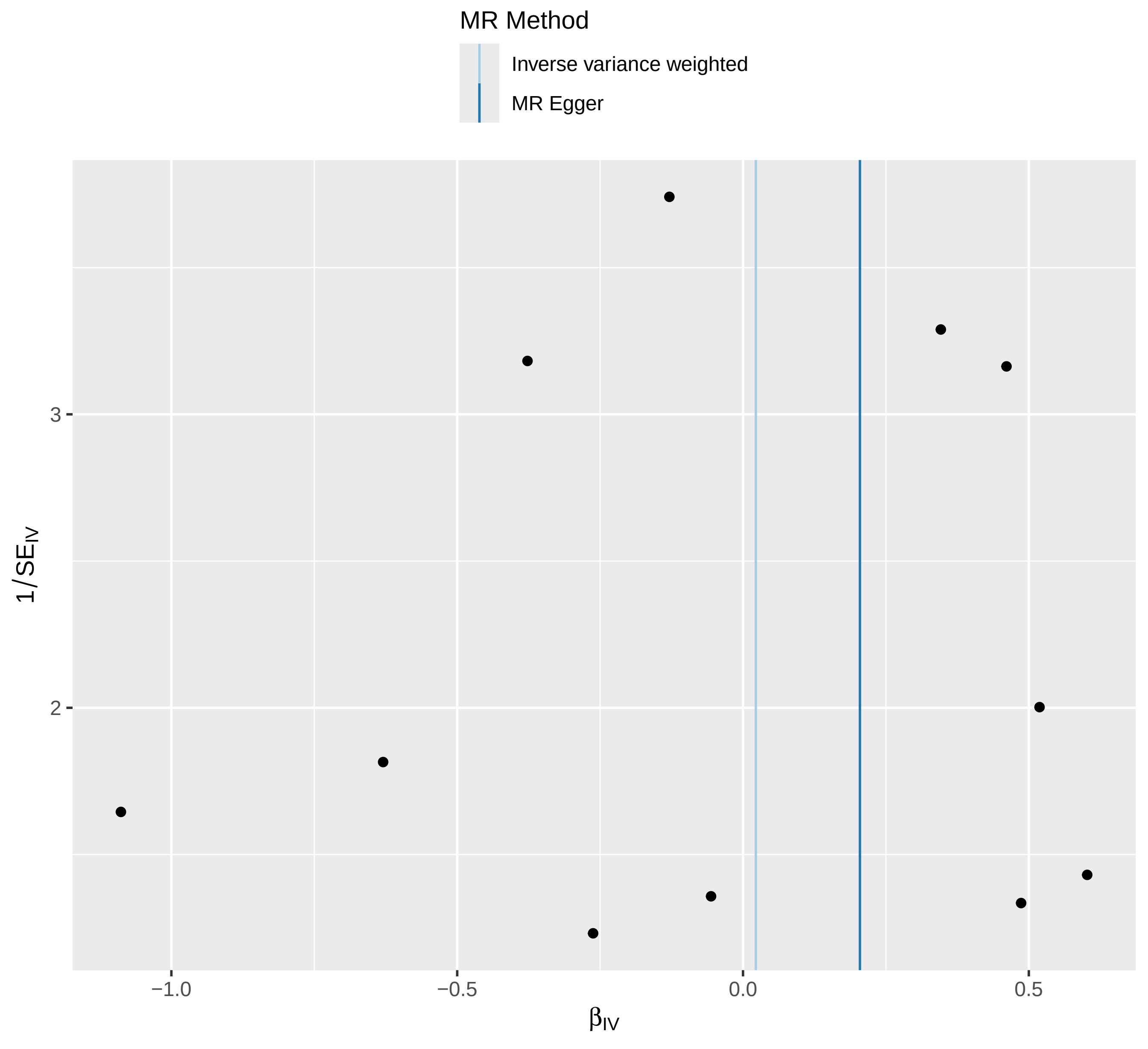
MR Method
Inverse variance weighted
MR Egger
3
2
1/SE$_{IV}$
−1.0
−0.5
0.0
0.5
β$_{IV}$

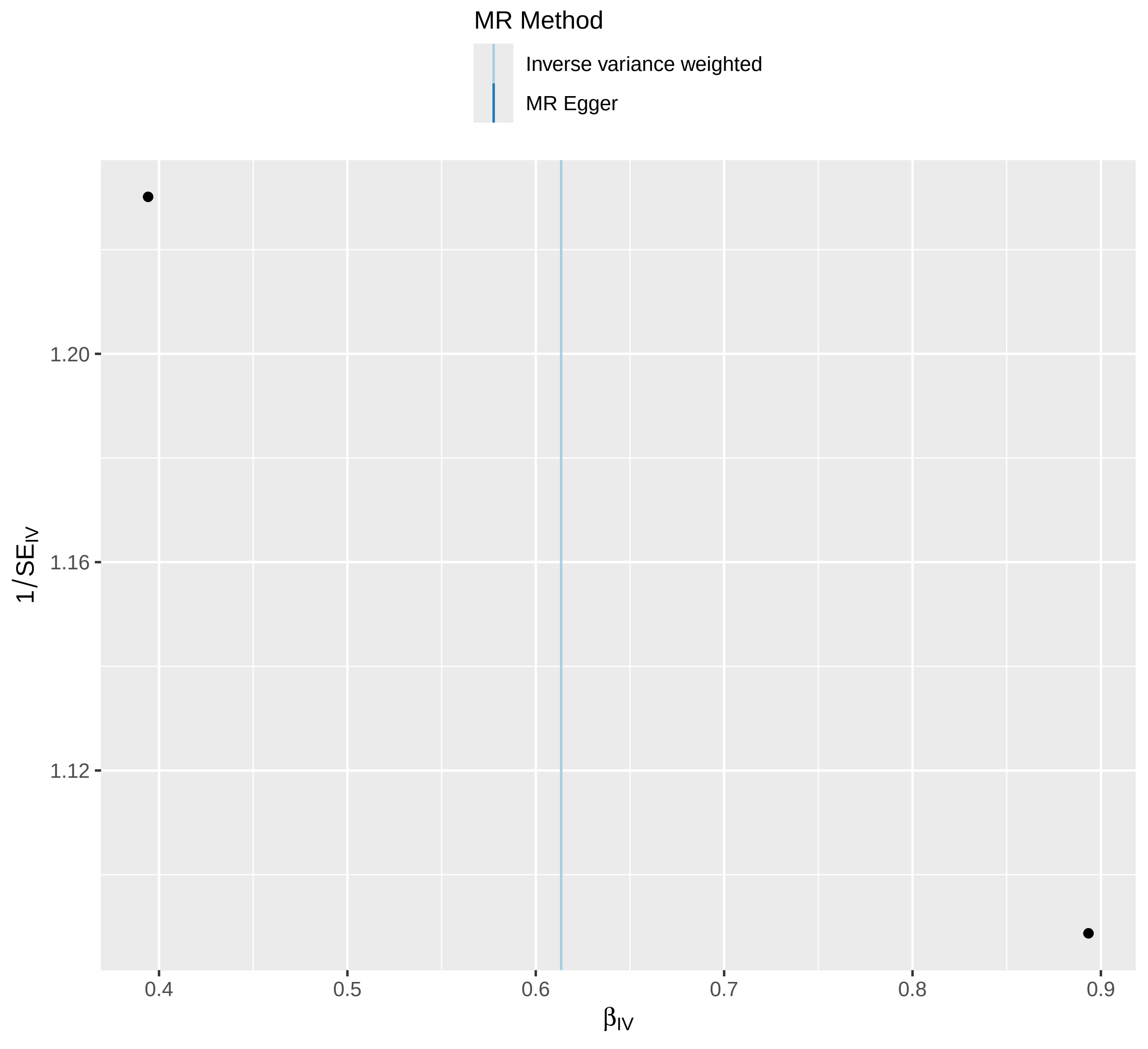

MR Method
Inverse variance weighted
MR Egger
1.20
1.16
1.12
1/SE_IV
0.4
0.5
0.6
0.7
0.8
0.9
β_IV

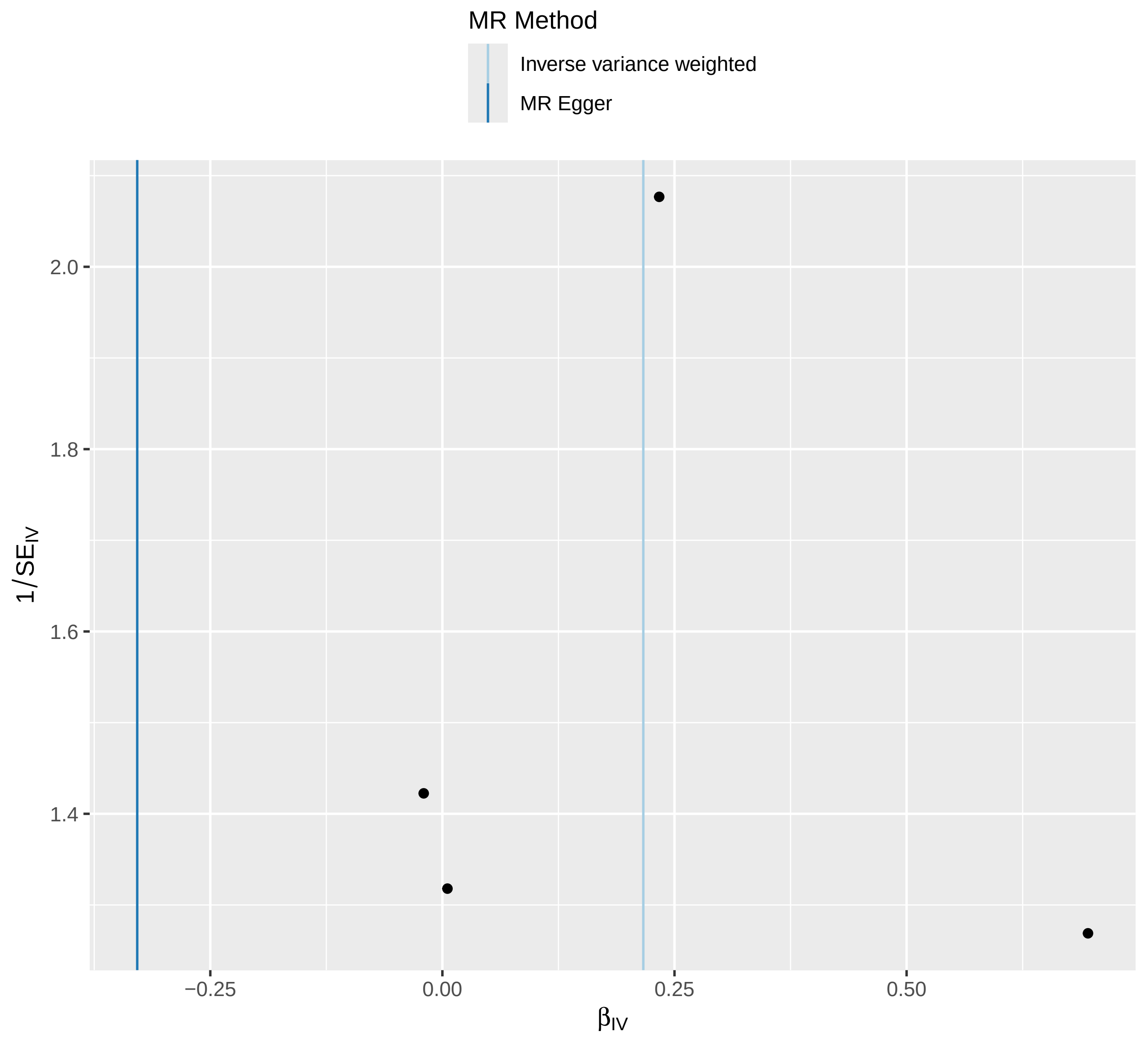

MR Method
Inverse variance weighted
MR Egger
1/SE$_{IV}$
2.0
1.8
1.6
1.4
β$_{IV}$
−0.25
0.00
0.25
0.50

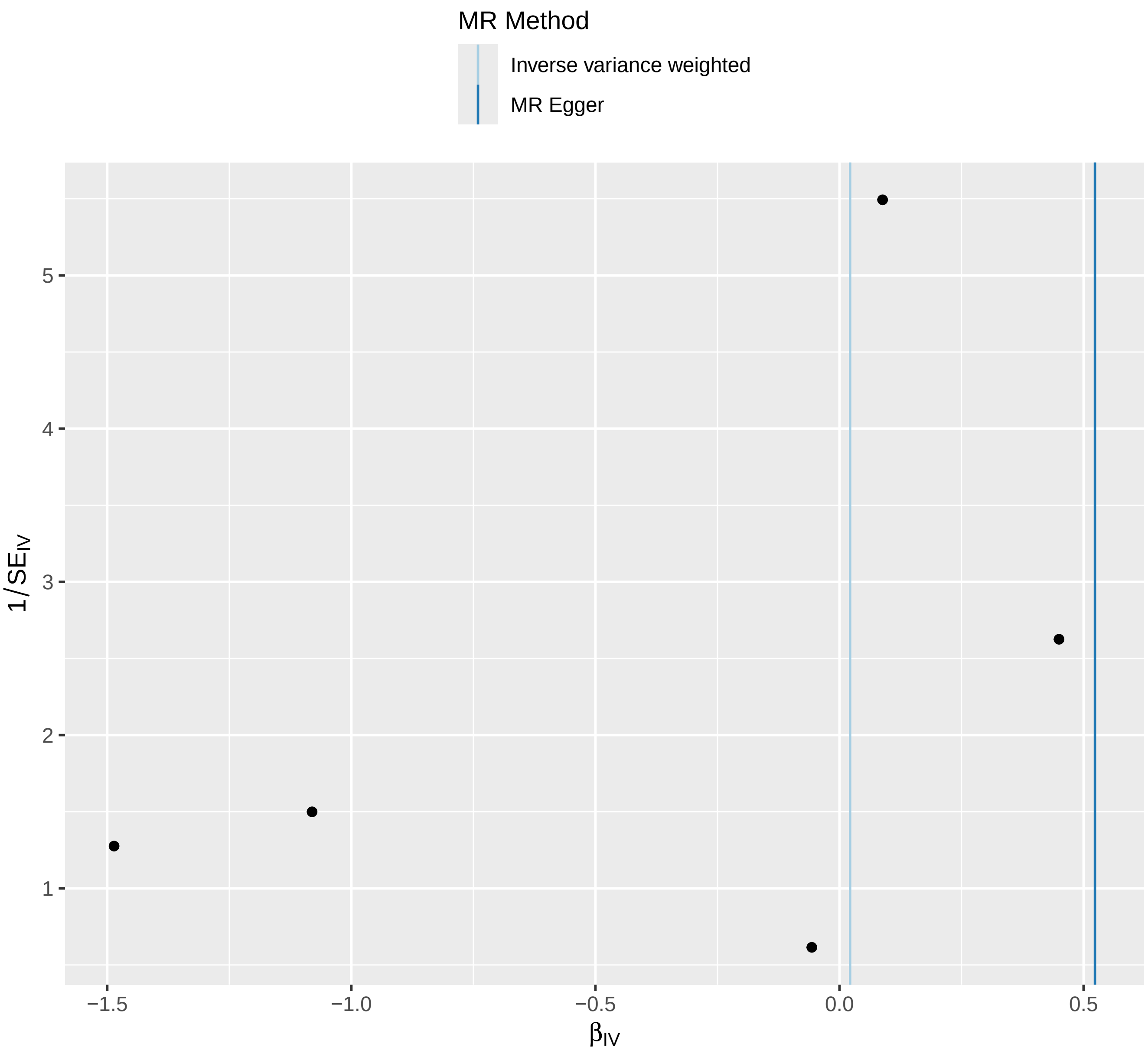
MR Method
Inverse variance weighted
MR Egger
1/SE$_{IV}$
5
4
3
2
1
−1.5
−1.0
−0.5
0.0
0.5
β$_{IV}$

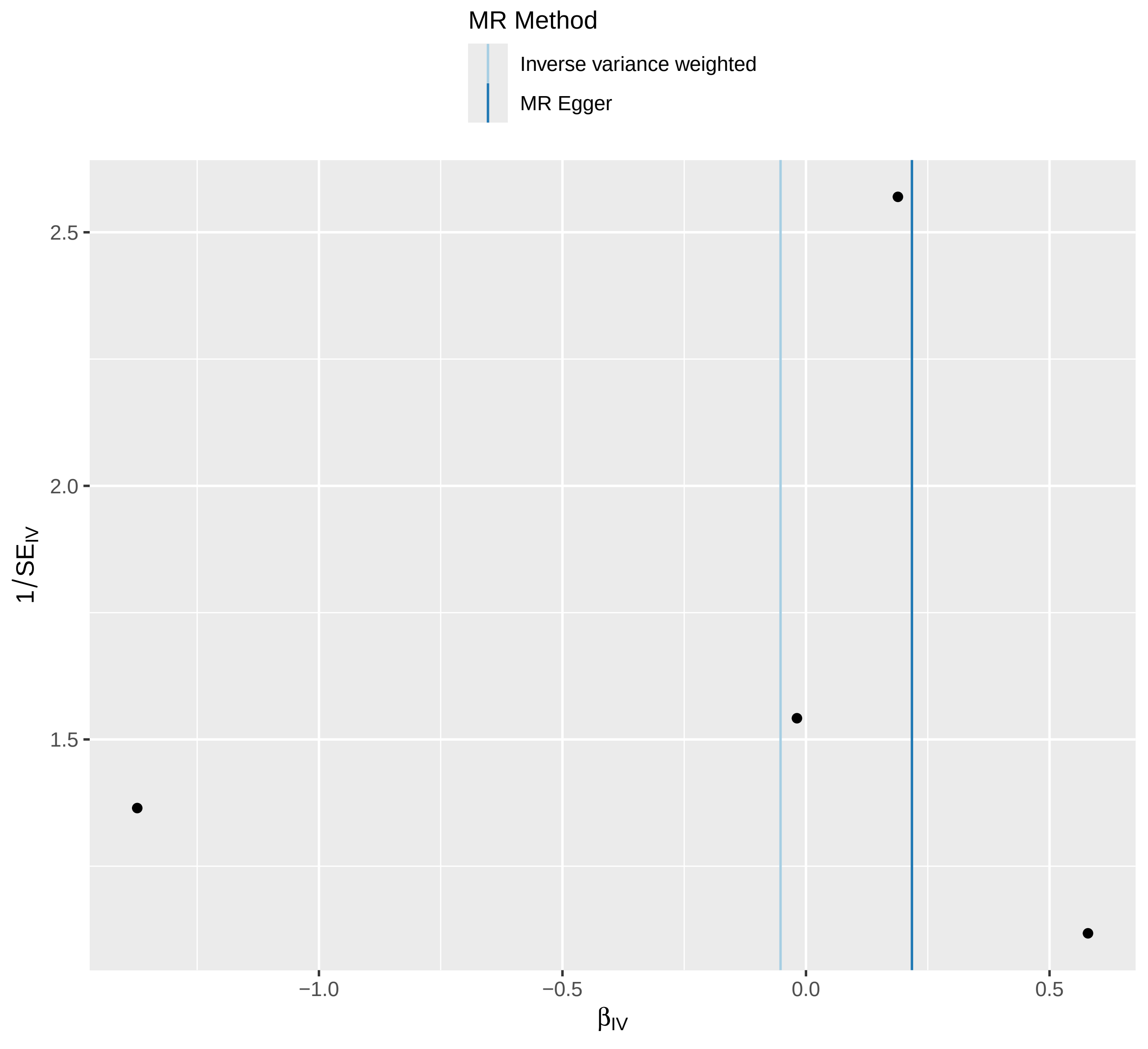

MR Method
Inverse variance weighted
MR Egger
2.5
2.0
1.5
1/SE$_{IV}$
−1.0
−0.5
0.0
0.5
β$_{IV}$

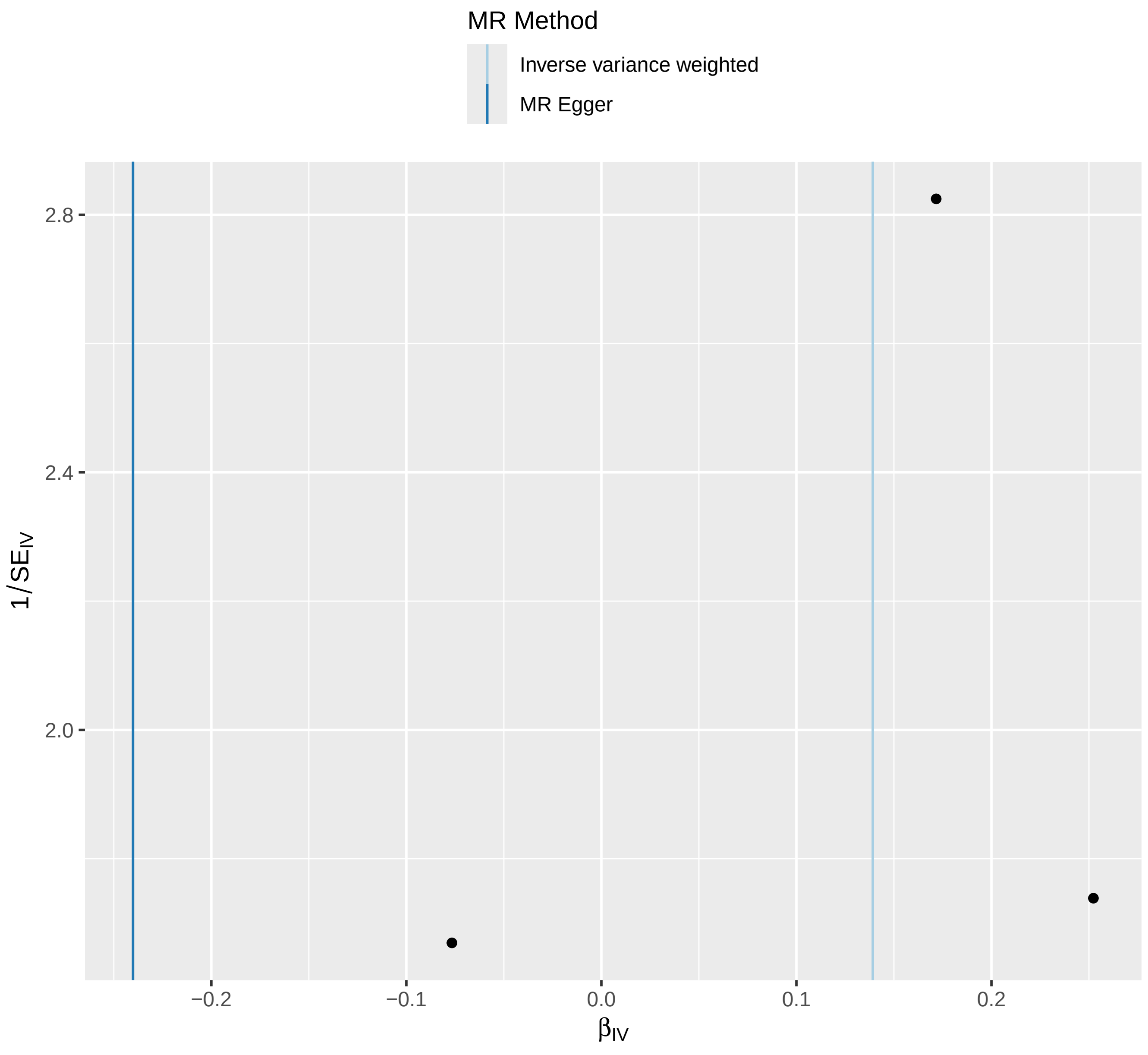

MR Method
Inverse variance weighted
MR Egger
2.8
2.4
2.0
1/SE_IV
−0.2
−0.1
0.0
0.1
0.2
β_IV

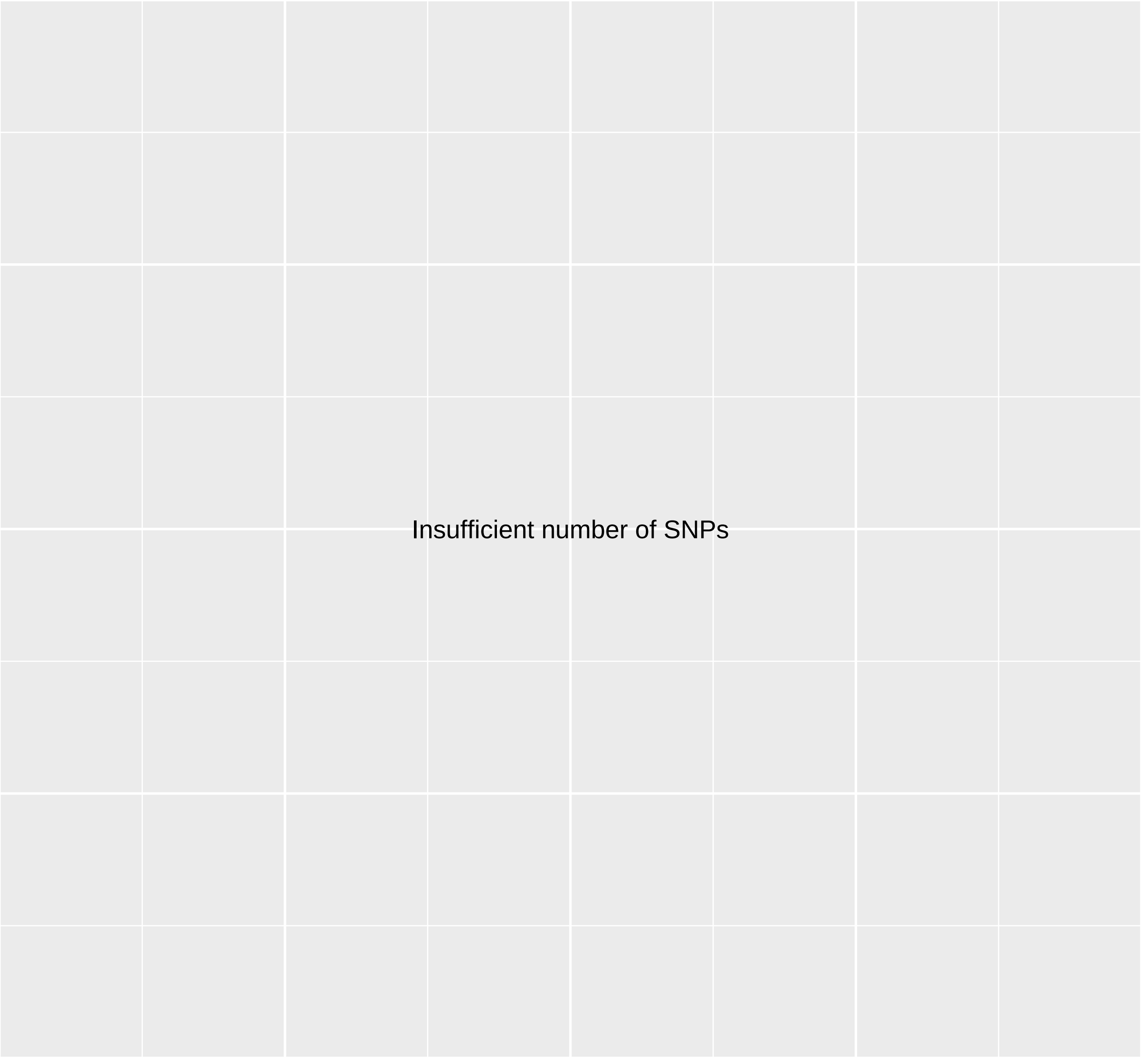
Insufficient number of SNPs

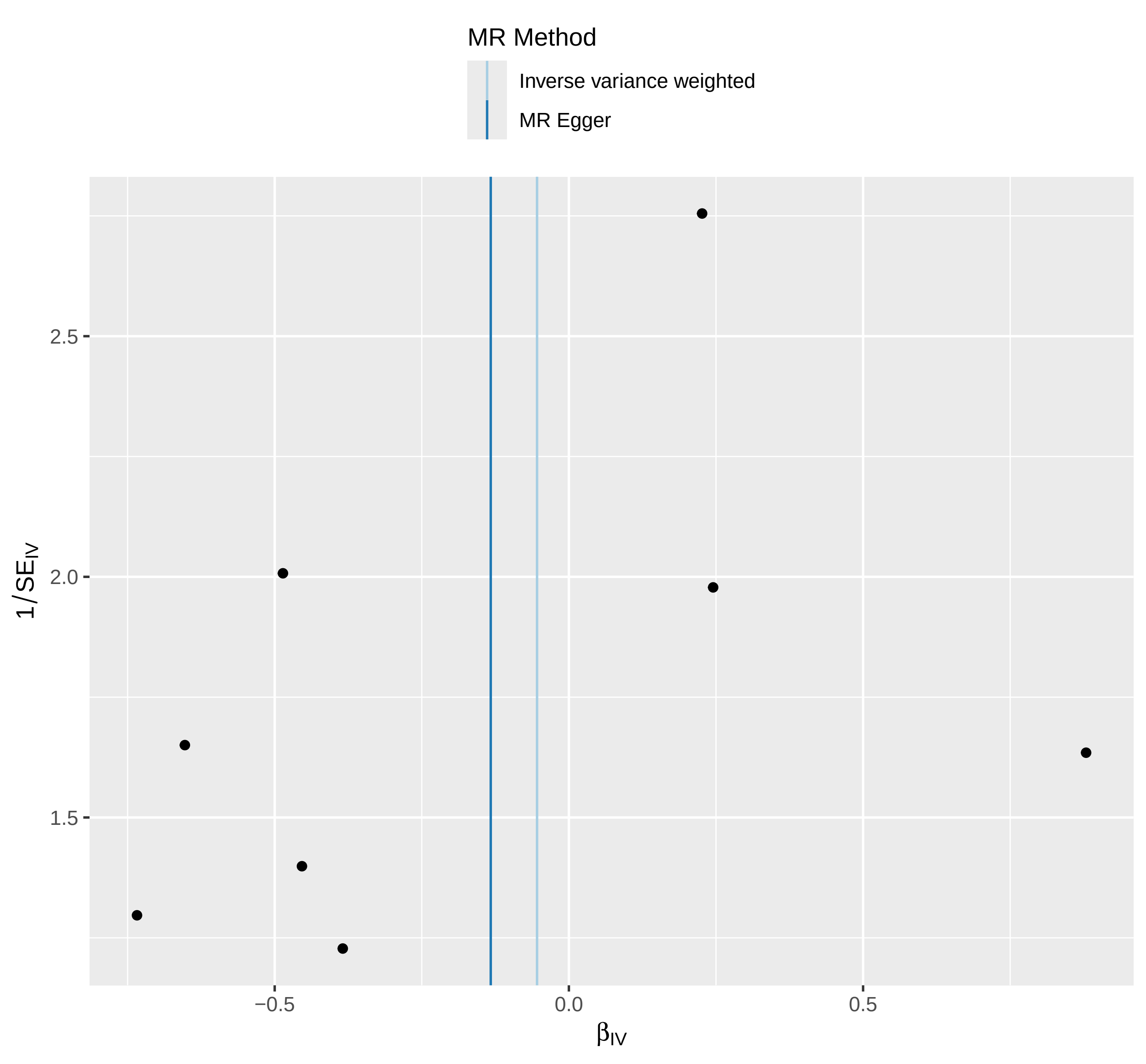

MR Method
Inverse variance weighted
MR Egger
2.5
2.0
1.5
1/SE_IV
−0.5
0.0
0.5
β_IV

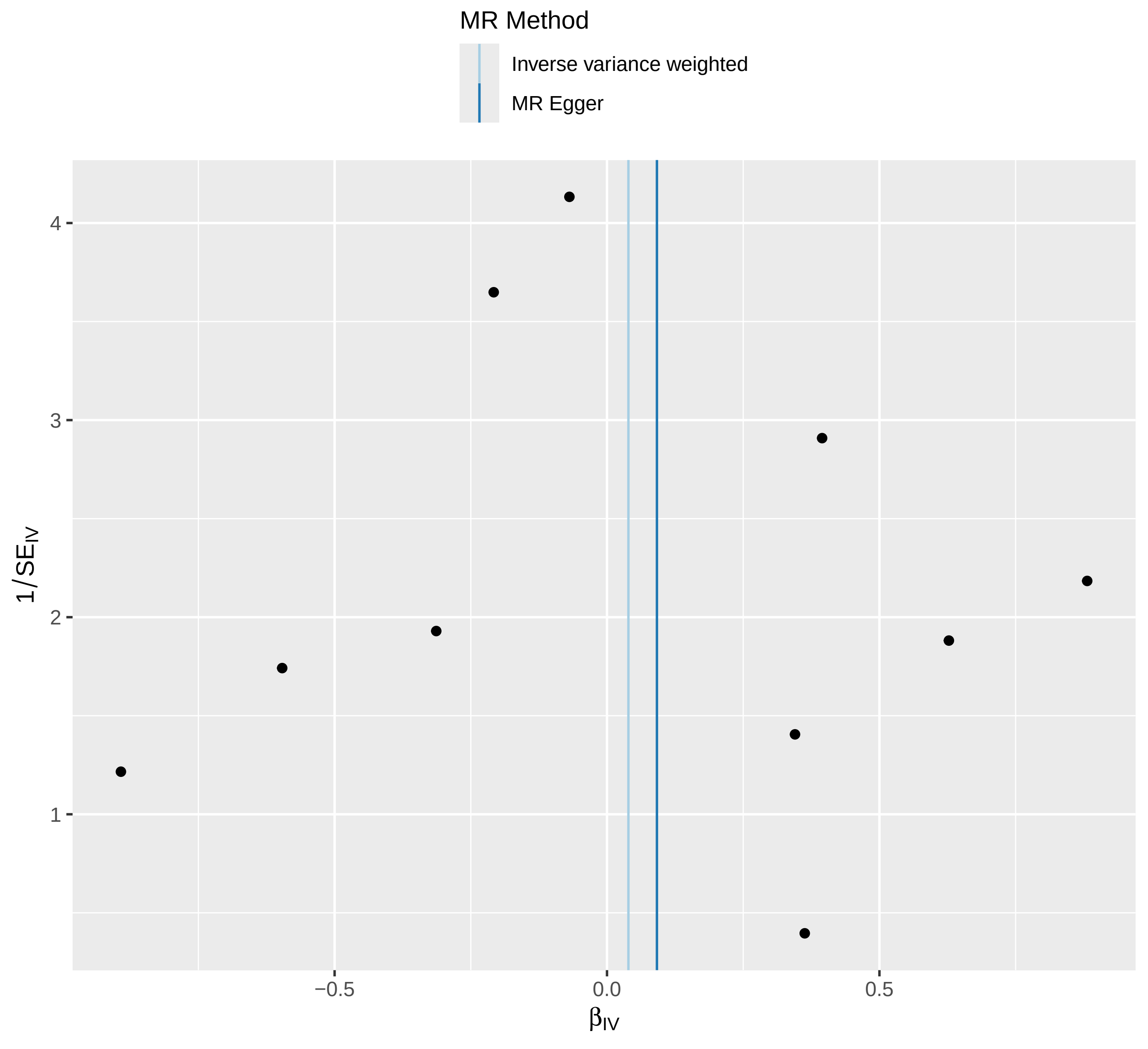

MR Method
Inverse variance weighted
MR Egger
4
3
2
1
1/SE$_{IV}$
−0.5
0.0
0.5
β$_{IV}$

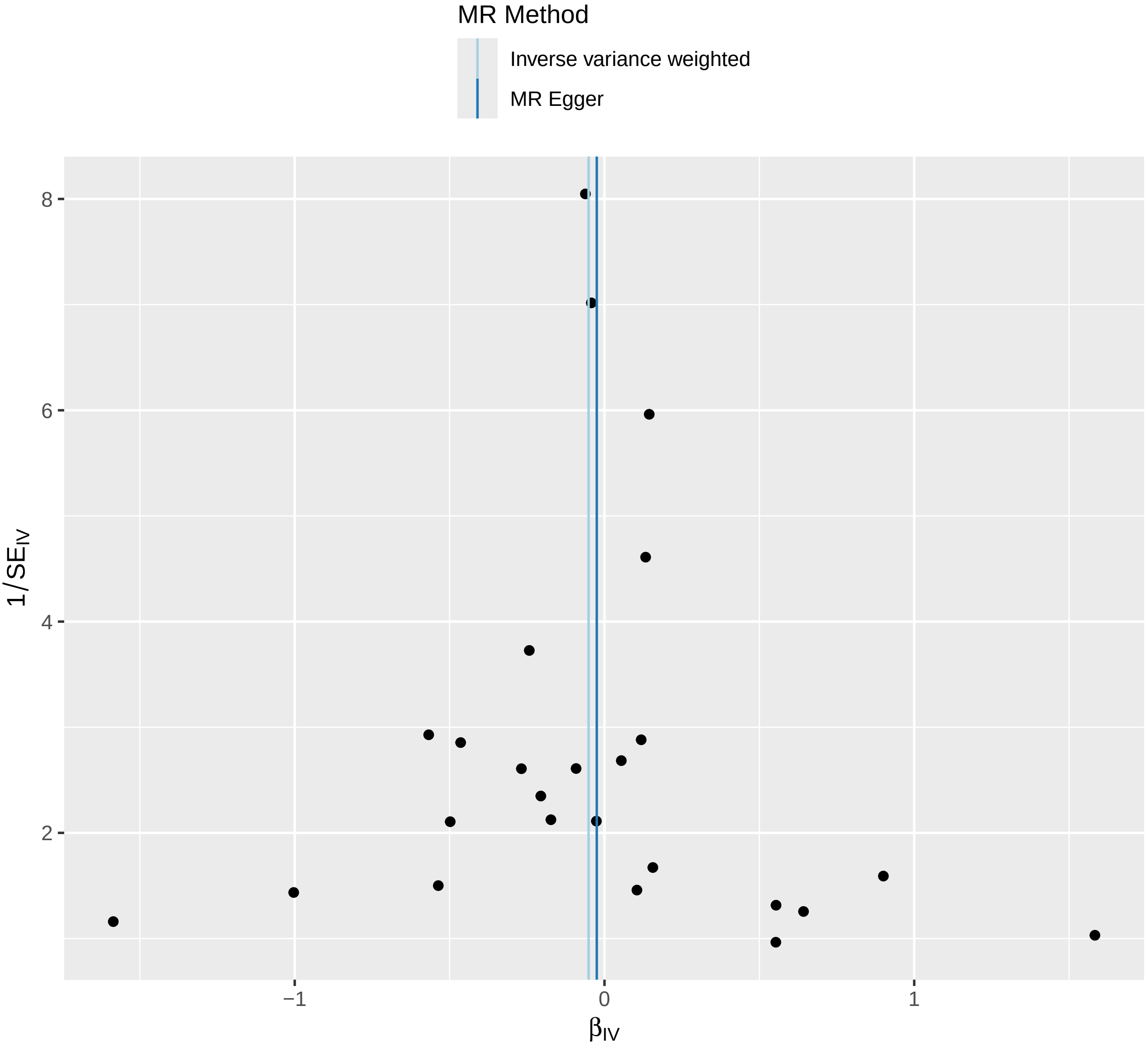

MR Method
Inverse variance weighted
MR Egger
8
6
4
2
1/SE_IV
−1
0
1
β_IV

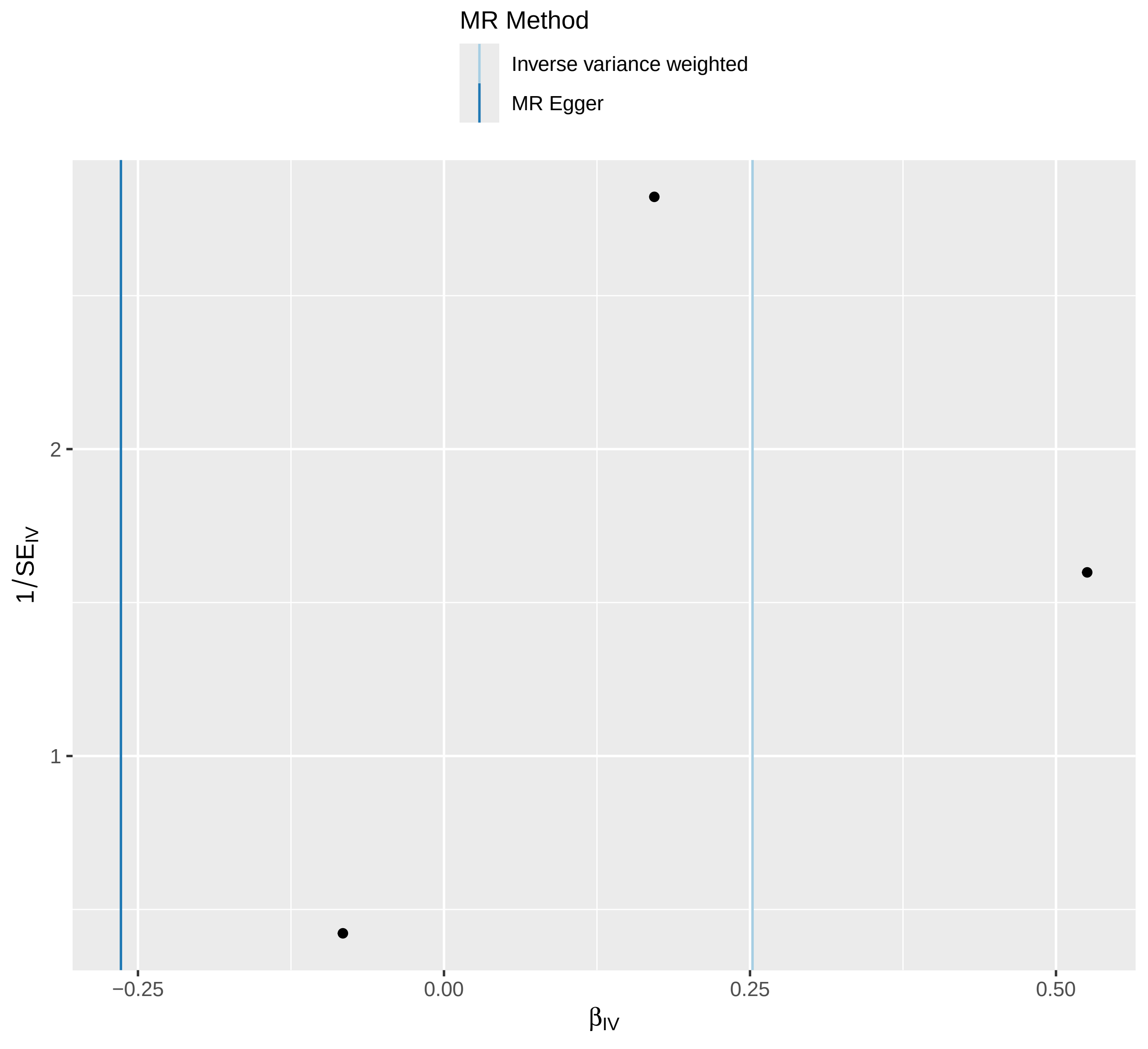

MR Method
Inverse variance weighted
MR Egger
2
1
1/SE_IV
−0.25
0.00
0.25
0.50
β_IV

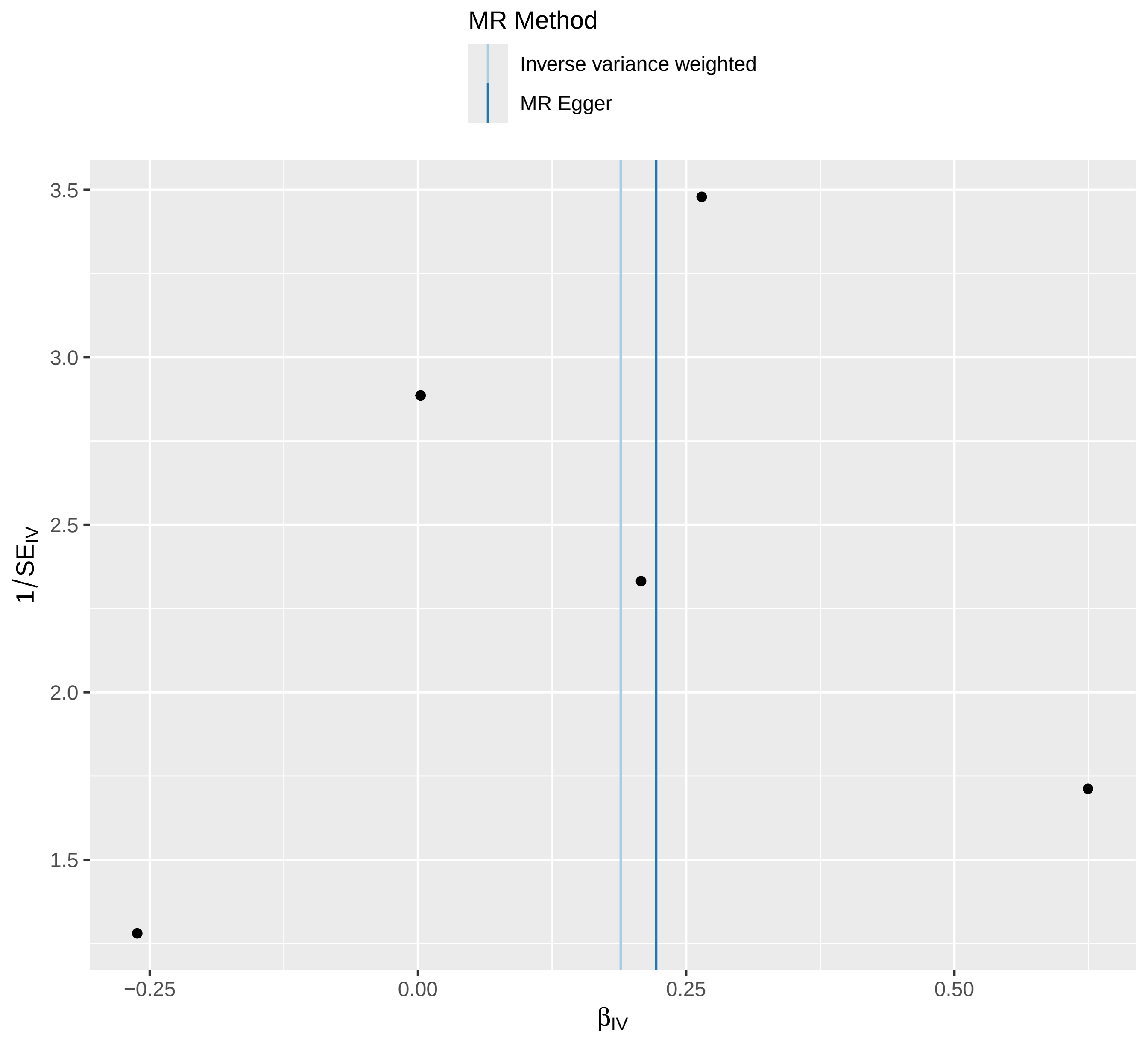

MR Method
Inverse variance weighted
MR Egger
3.5
3.0
2.5
2.0
1.5
1/SE_IV
−0.25
0.00
0.25
0.50
β_IV

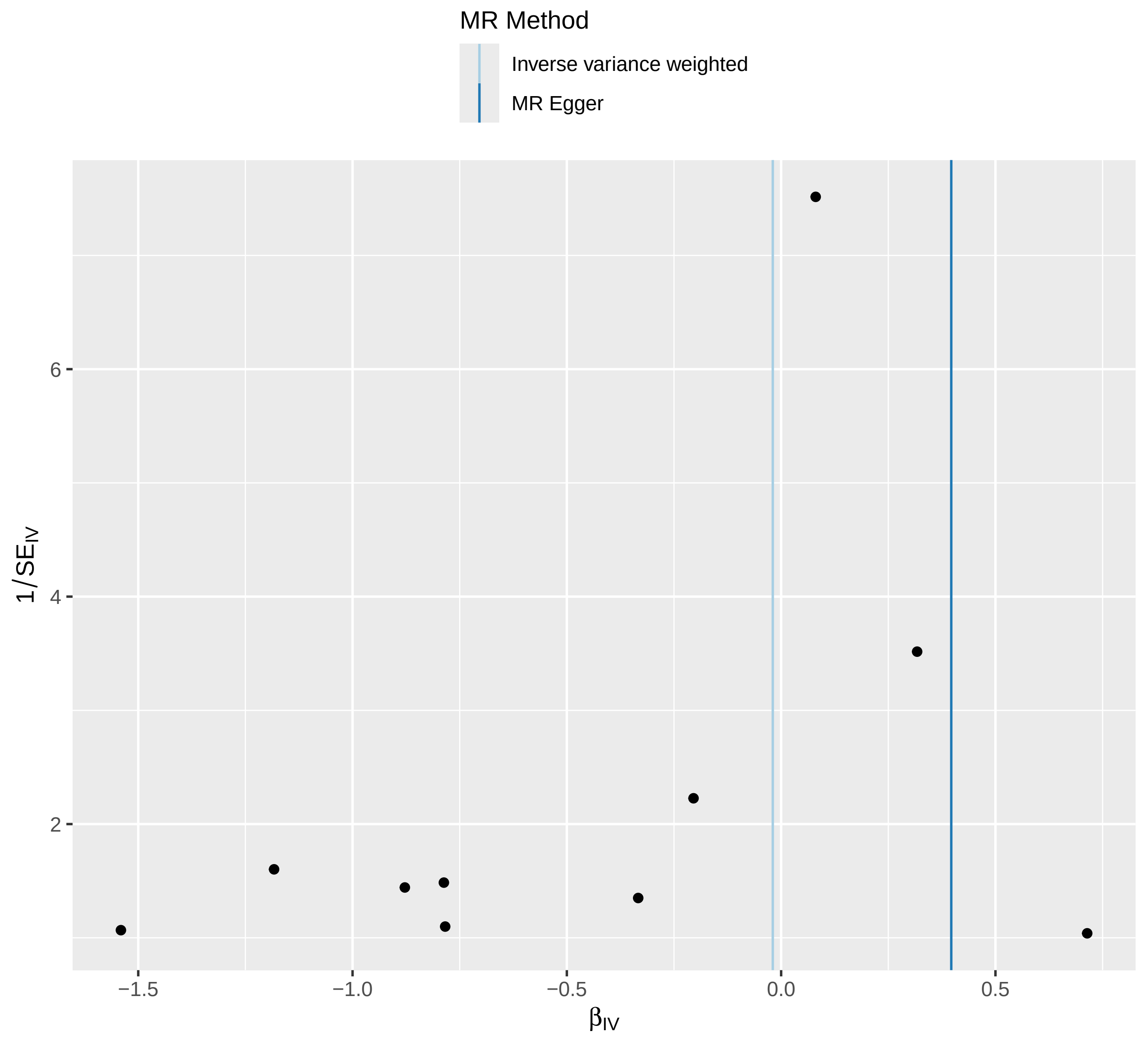
MR Method
Inverse variance weighted
MR Egger
1/SE$_{IV}$
6
4
2
−1.5
−1.0
−0.5
0.0
0.5
β$_{IV}$

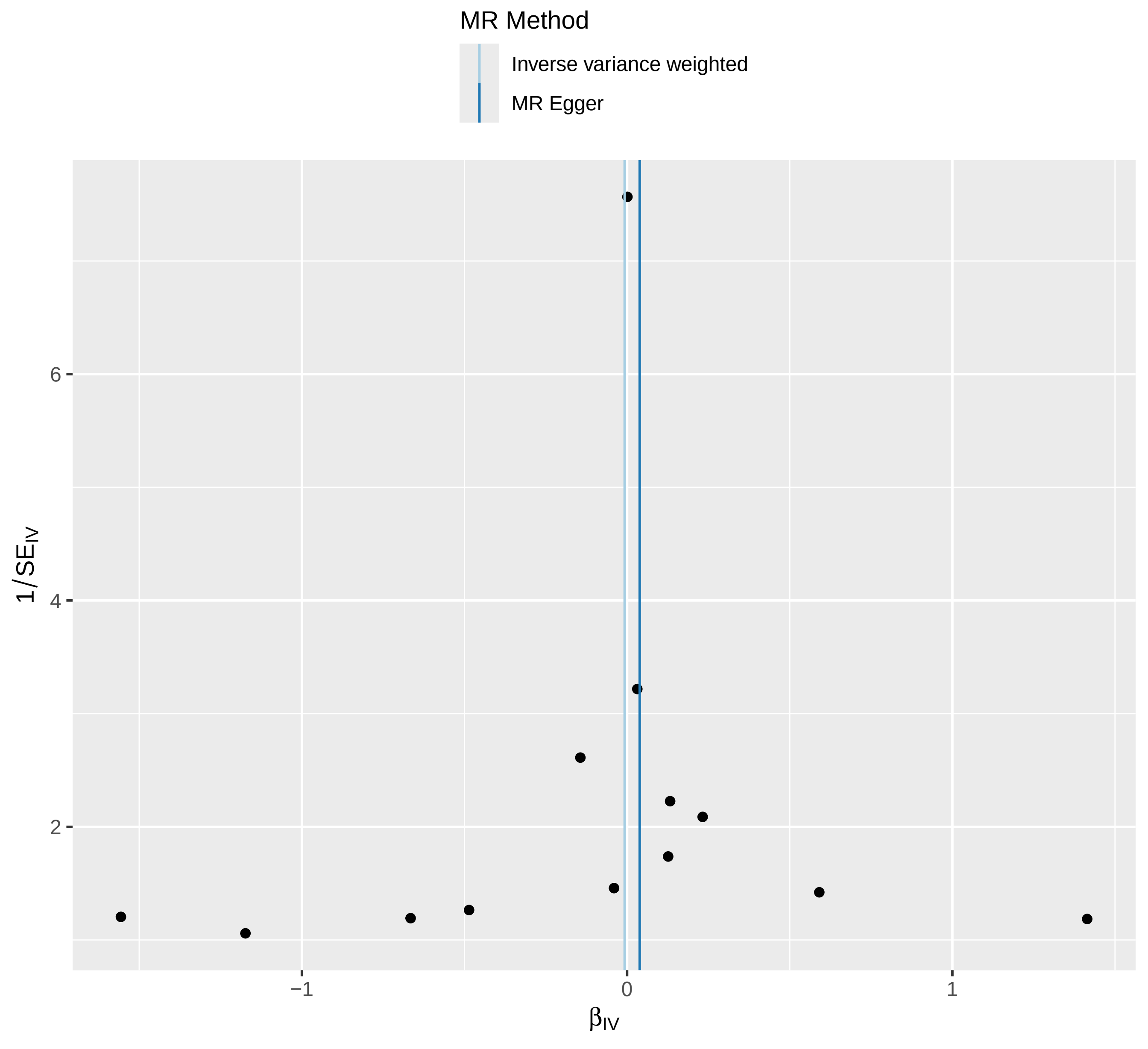

MR Method
Inverse variance weighted
MR Egger
6
4
2
1/SE_IV
−1
0
1
β_IV

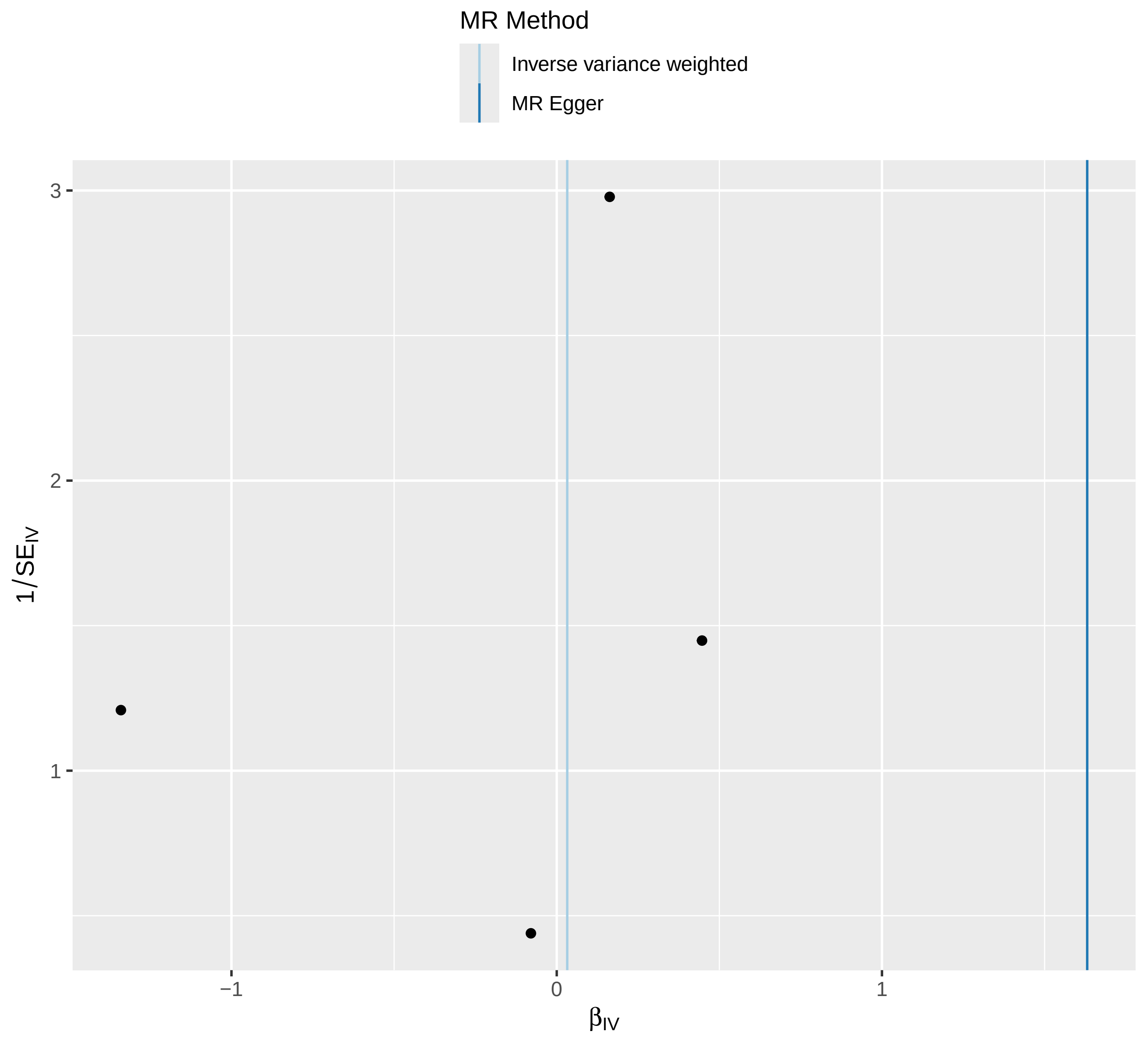
MR Method
Inverse variance weighted
MR Egger
1/SE_IV
3
2
1
−1
0
1
β_IV

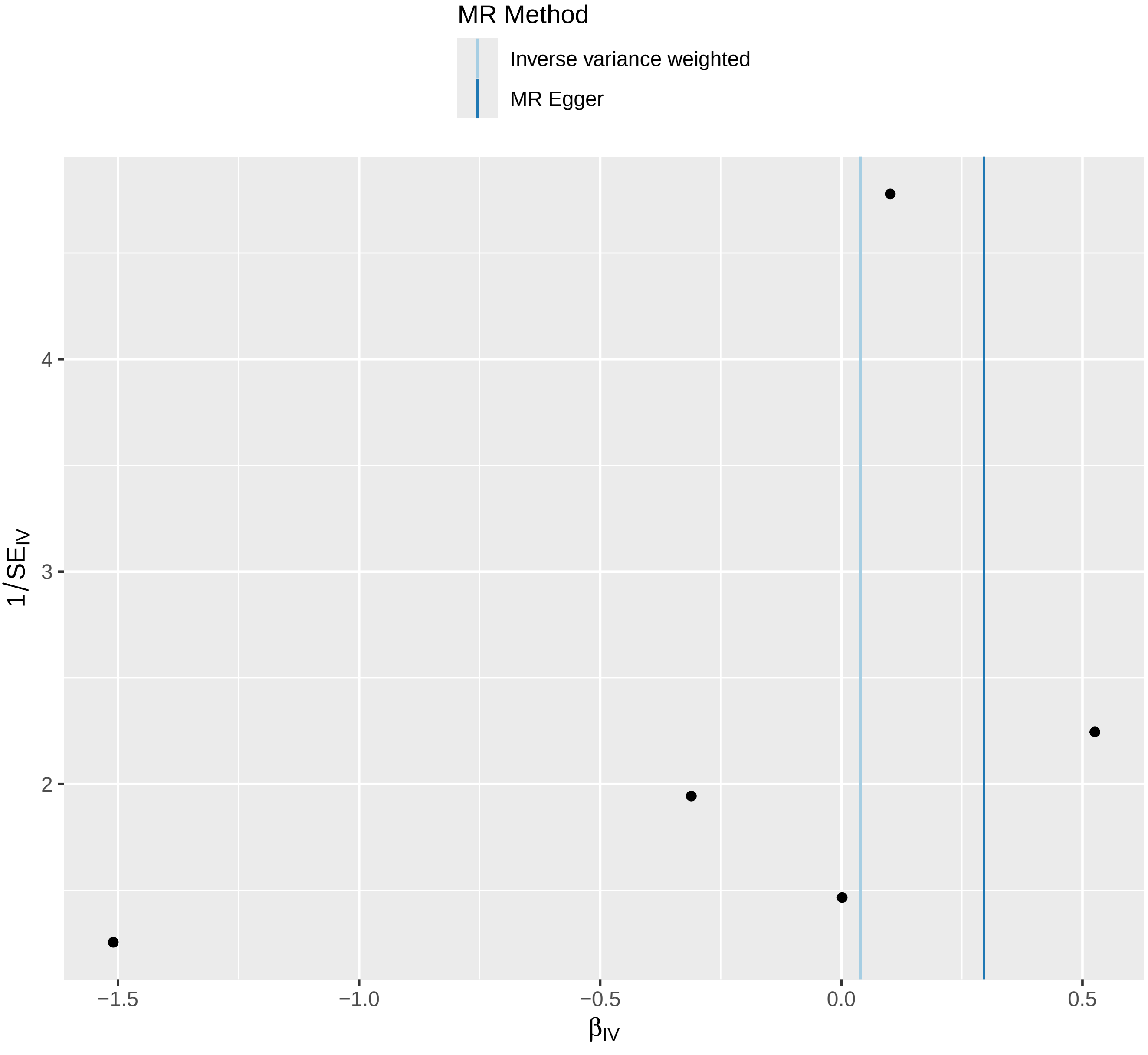
MR Method
Inverse variance weighted
MR Egger
4
3
2
1/SE_IV
−1.5
−1.0
−0.5
0.0
0.5
β_IV

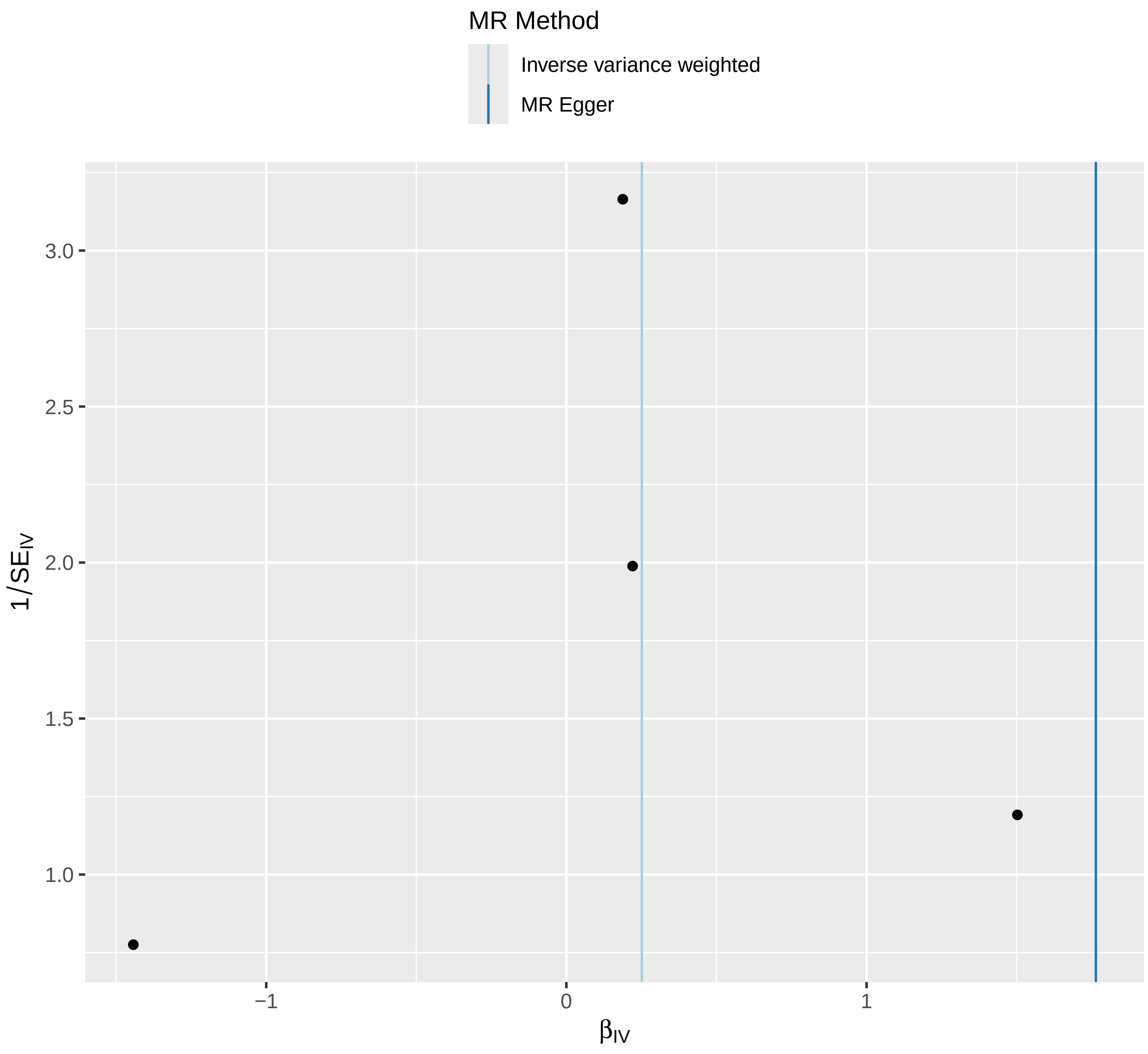
MR Method
Inverse variance weighted
MR Egger
3.0
2.5
2.0
1.5
1.0
$1/SE_{IV}$
−1
0
1
β_{IV}

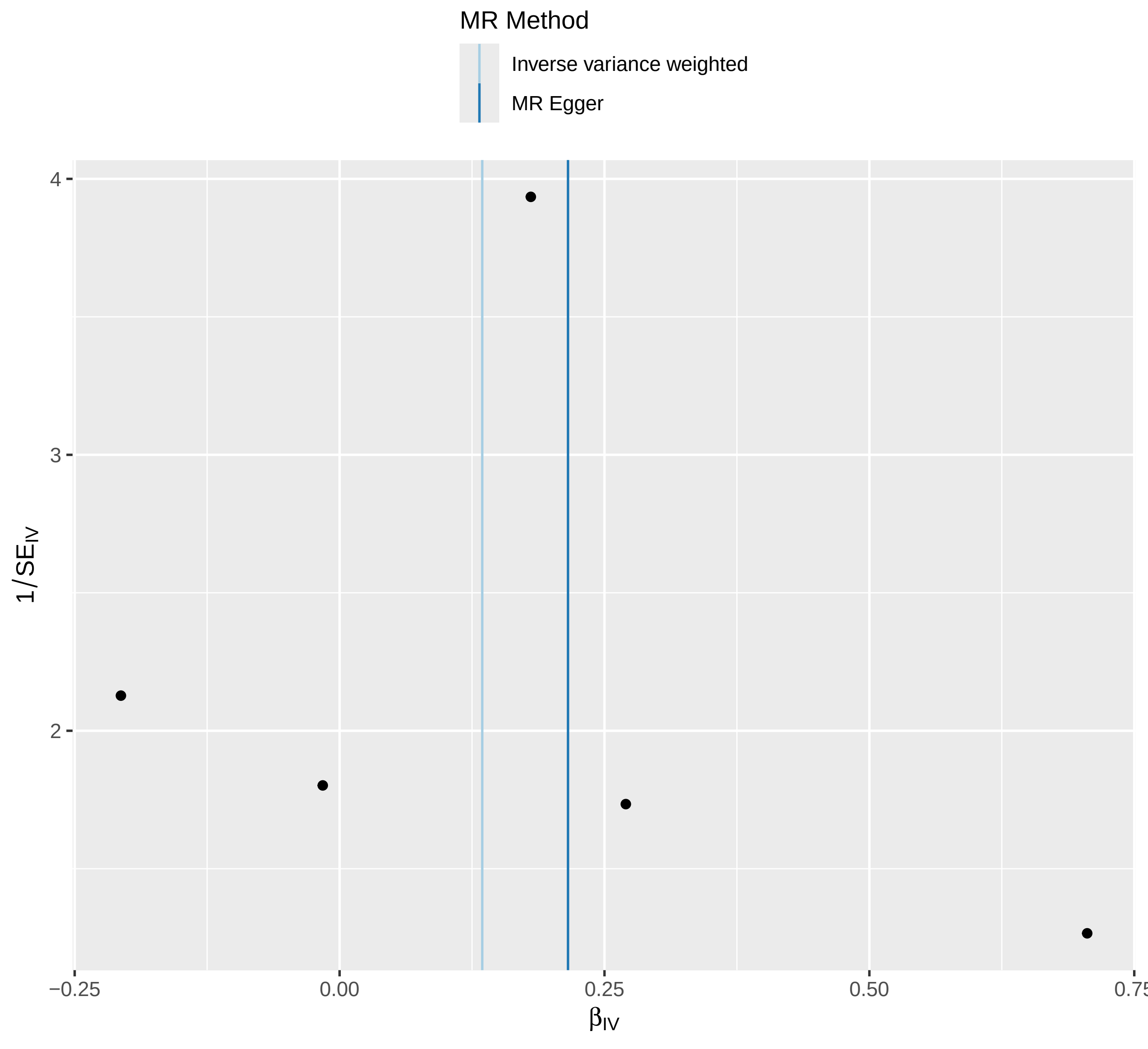
MR Method
Inverse variance weighted
MR Egger
4
3
2
1/SE_IV
−0.25
0.00
0.25
0.50
0.75
β_IV

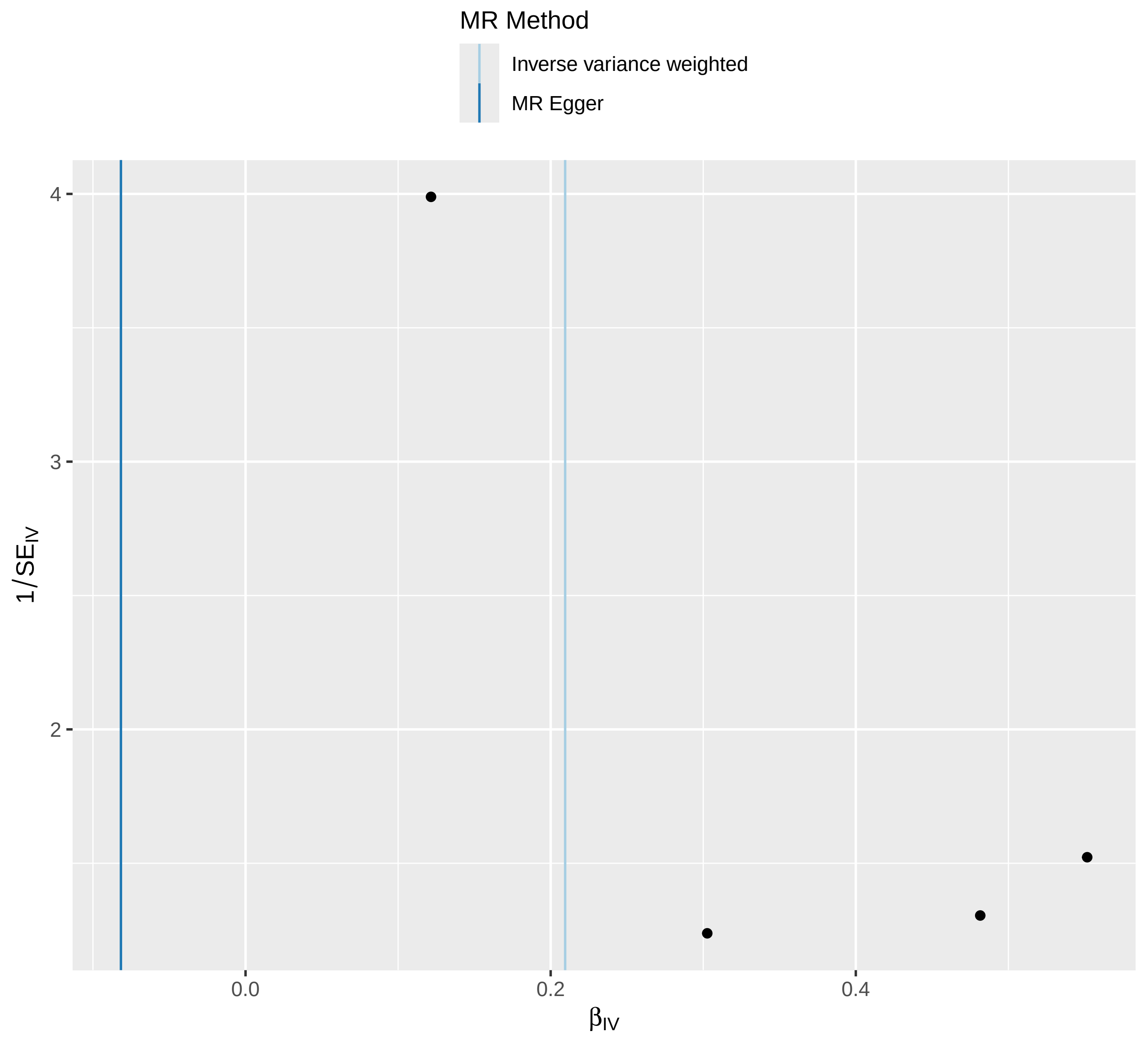

MR Method
Inverse variance weighted
MR Egger
4
3
2
1/SE_IV
0.0
0.2
0.4
β_IV

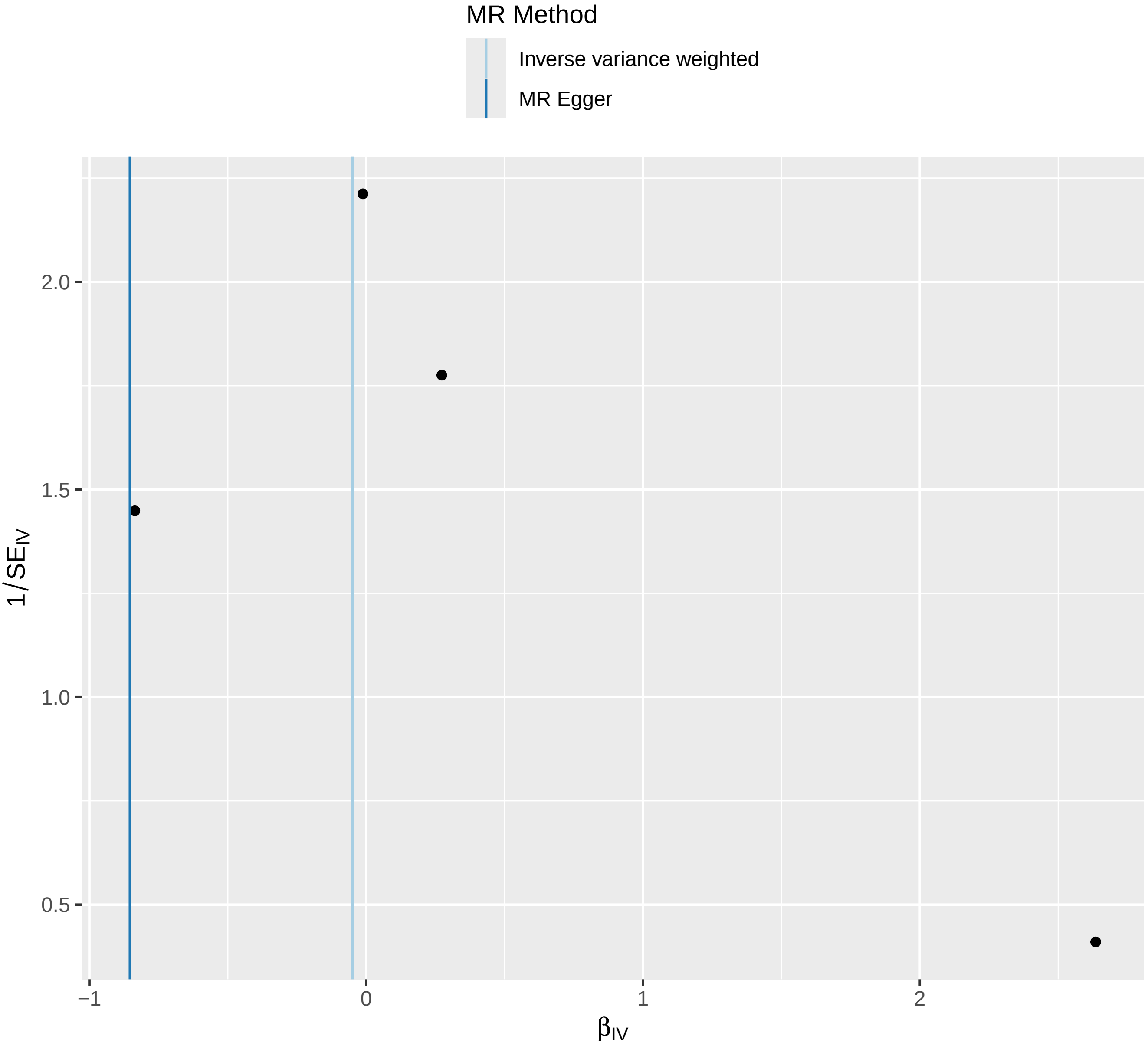
MR Method
Inverse variance weighted
MR Egger
2.0
1.5
1.0
0.5
$1/SE_{IV}$
−1
0
1
2
β_{IV}

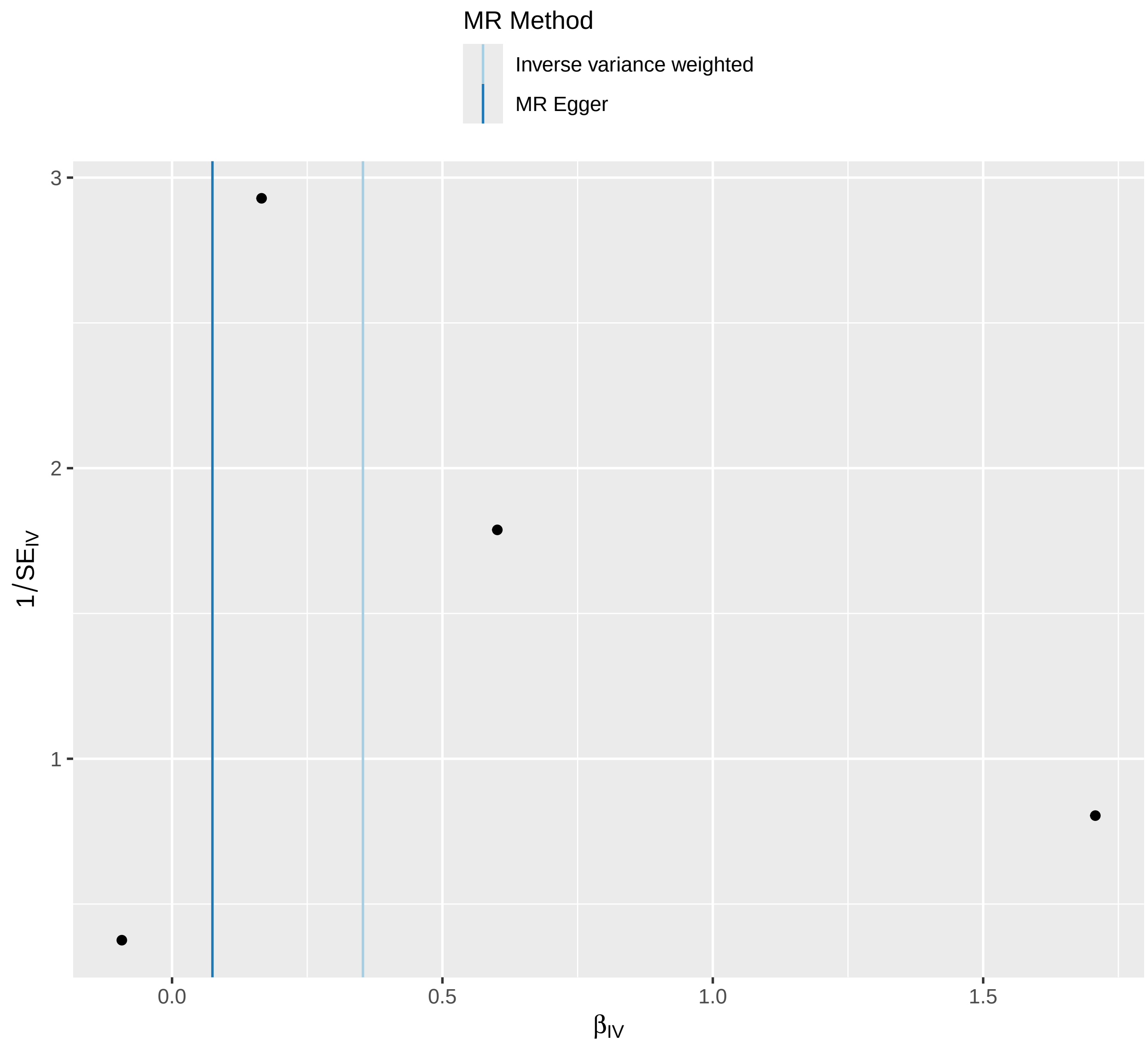

MR Method
Inverse variance weighted
MR Egger
3
2
1
1/SE_{IV}
0.0
0.5
1.0
1.5
β_{IV}

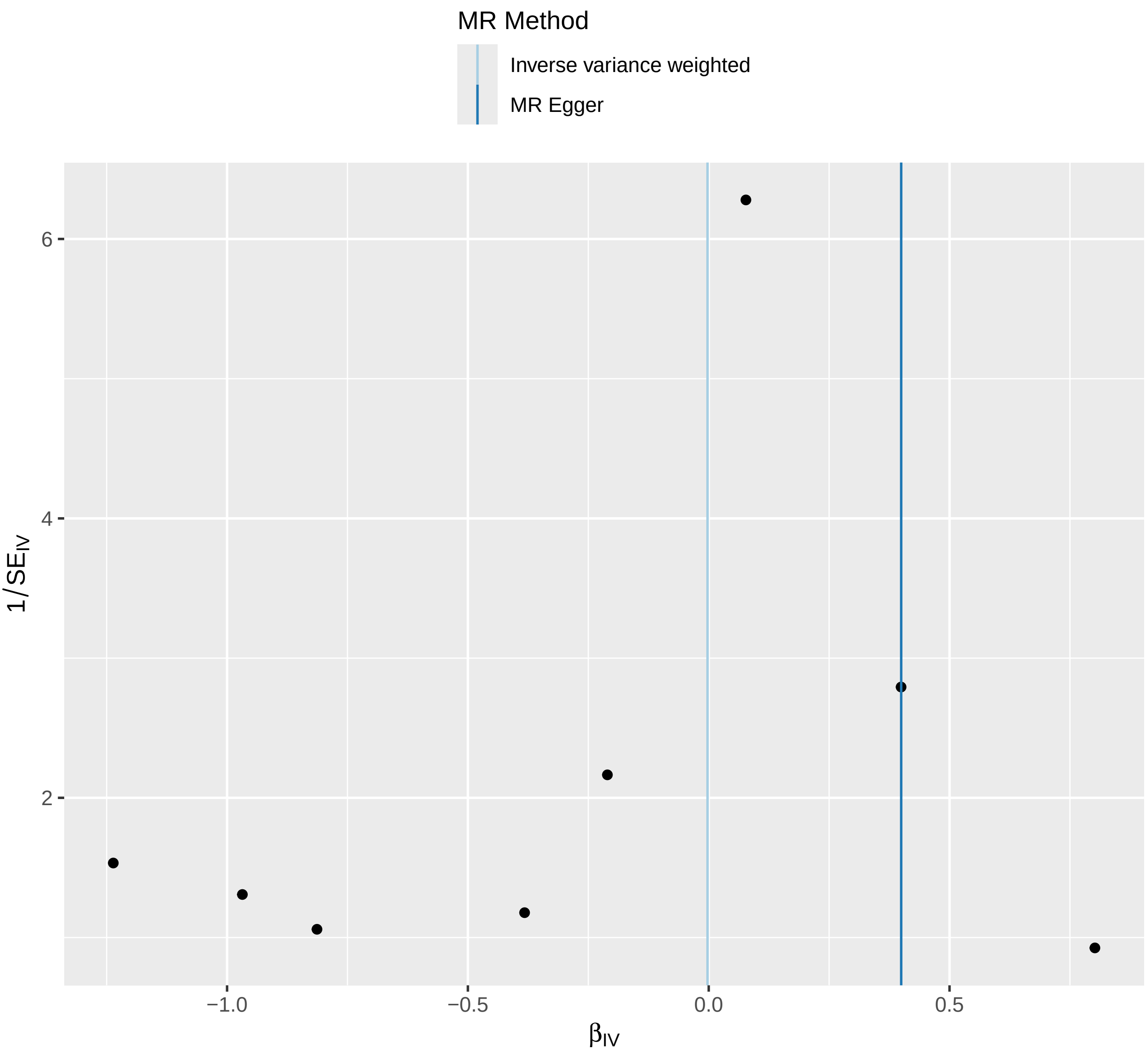
MR Method
Inverse variance weighted
MR Egger
6
4
2
1/SE$_{IV}$
−1.0
−0.5
0.0
0.5
β_{IV}

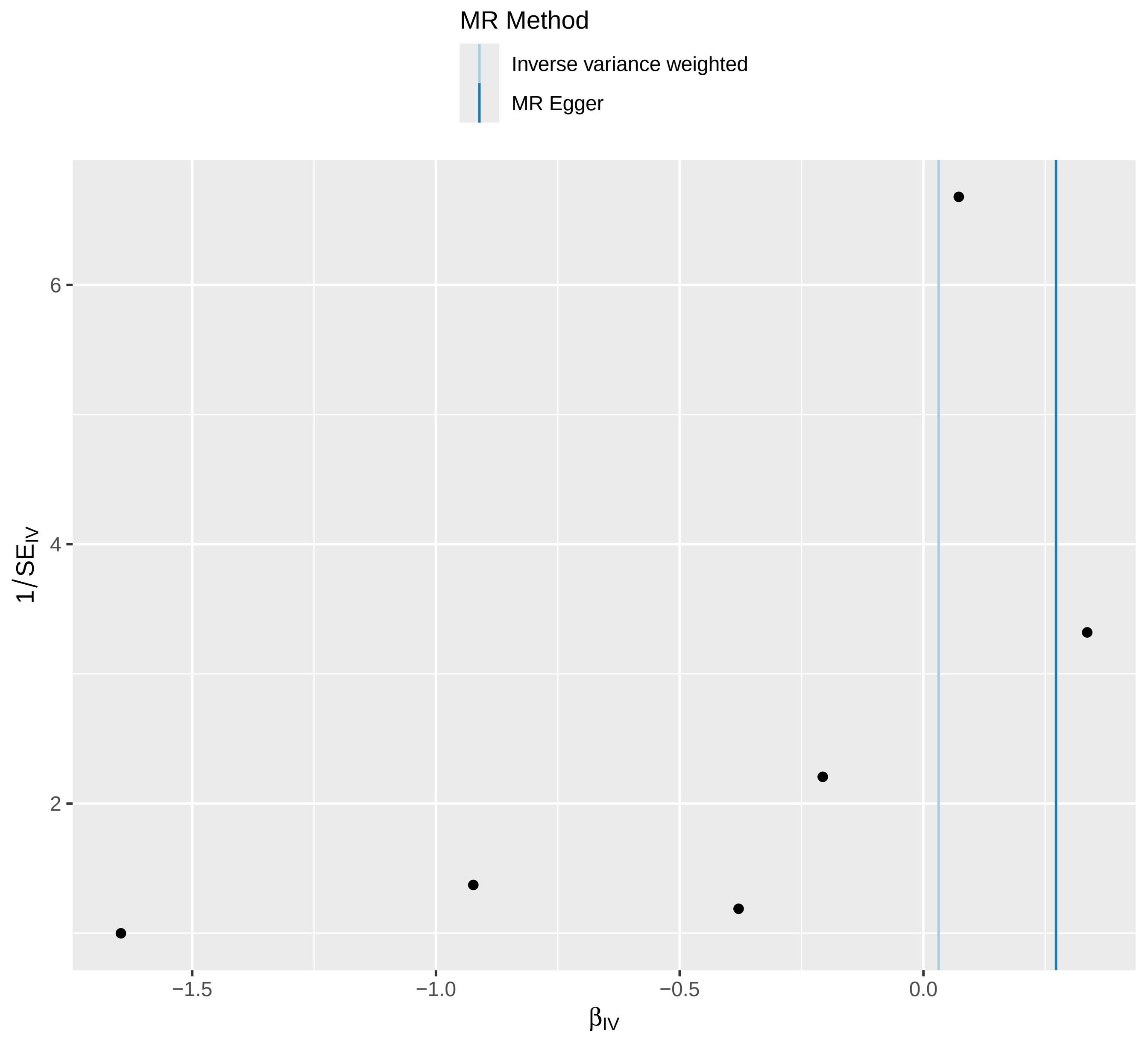

MR Method
Inverse variance weighted
MR Egger
6
4
2
1/SE$_{IV}$
−1.5
−1.0
−0.5
0.0
β$_{IV}$

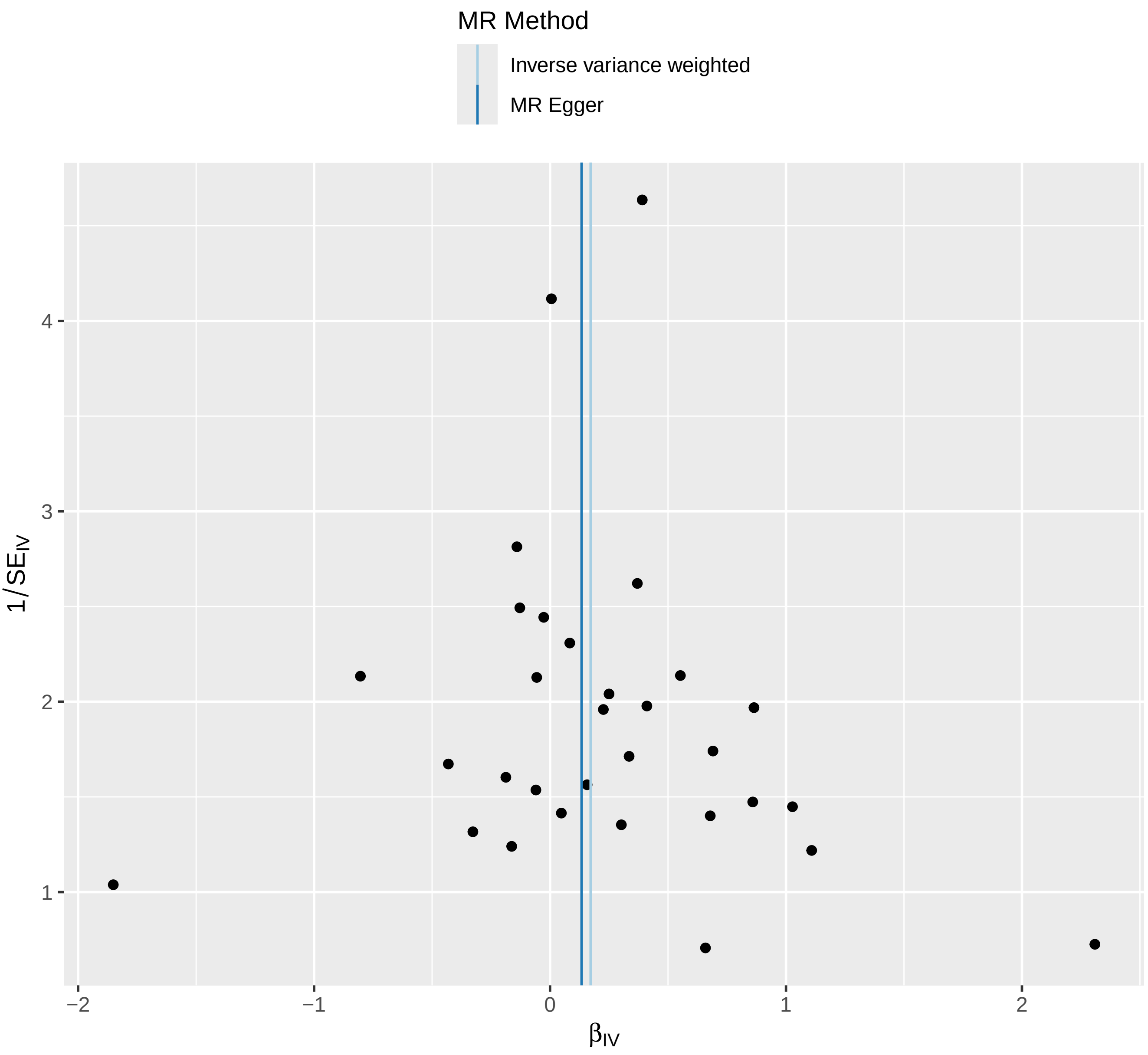
MR Method
Inverse variance weighted
MR Egger
1/SE$_{IV}$
4
3
2
1
−2
−1
0
1
2
β$_{IV}$

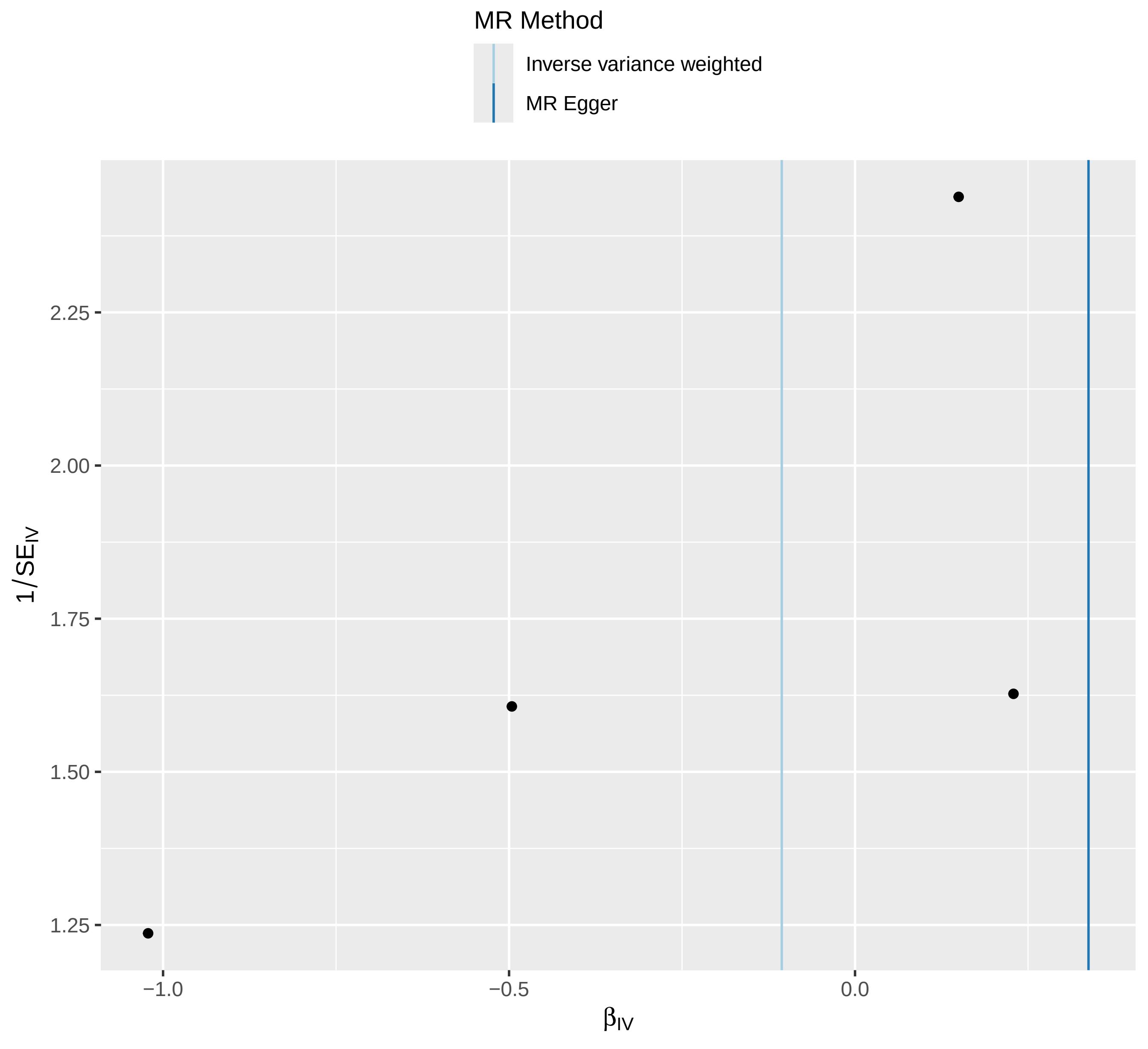

MR Method
Inverse variance weighted
MR Egger
2.25
2.00
1.75
1.50
1.25
1/SE_IV
−1.0
−0.5
0.0
β_IV

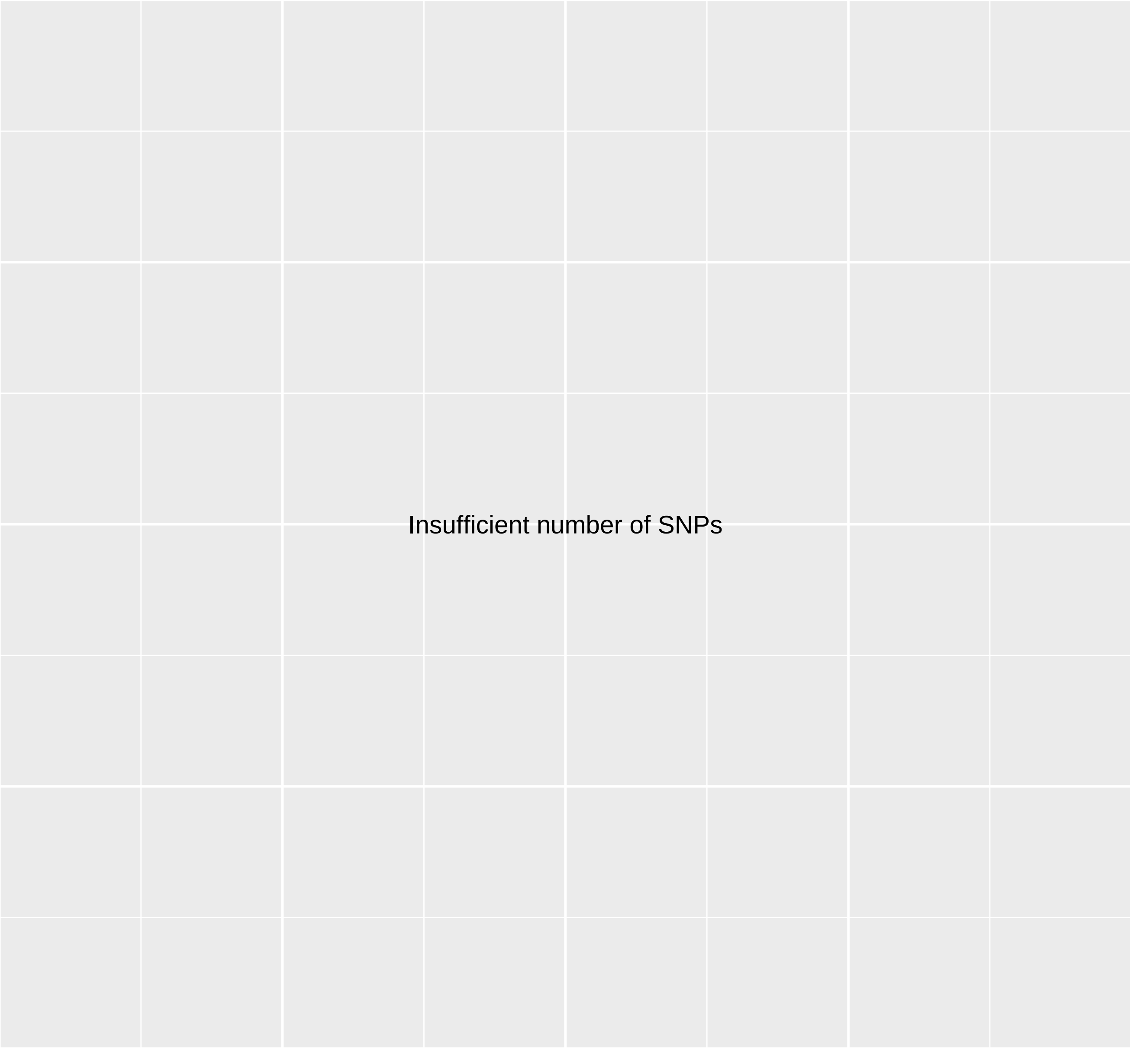
Insufficient number of SNPs

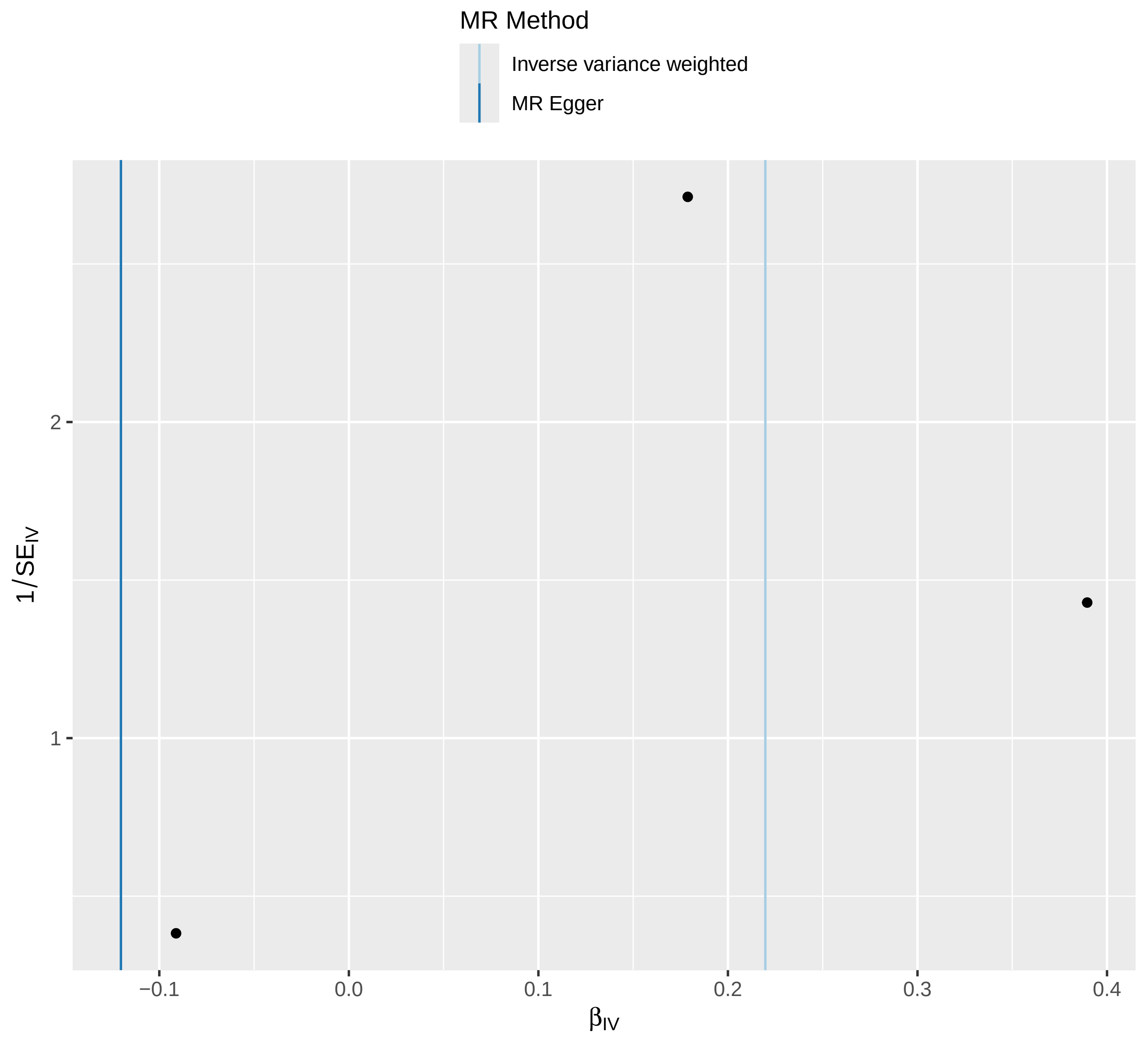

MR Method
Inverse variance weighted
MR Egger
2
1
1/SE_IV
−0.1
0.0
0.1
0.2
0.3
0.4
β_IV

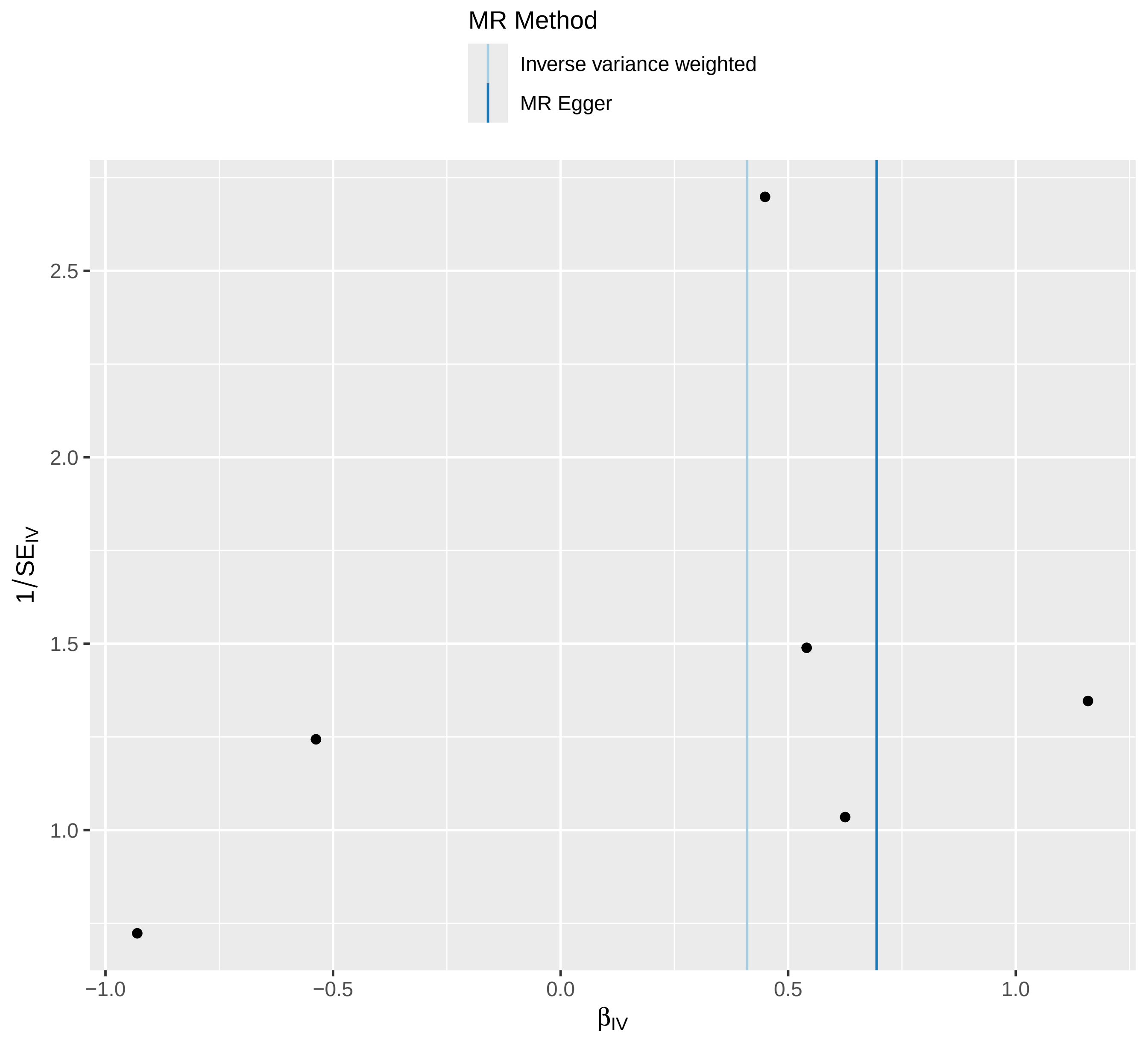

MR Method
Inverse variance weighted
MR Egger
2.5
2.0
1.5
1.0
1/SE_IV
−1.0
−0.5
0.0
0.5
1.0
β_IV

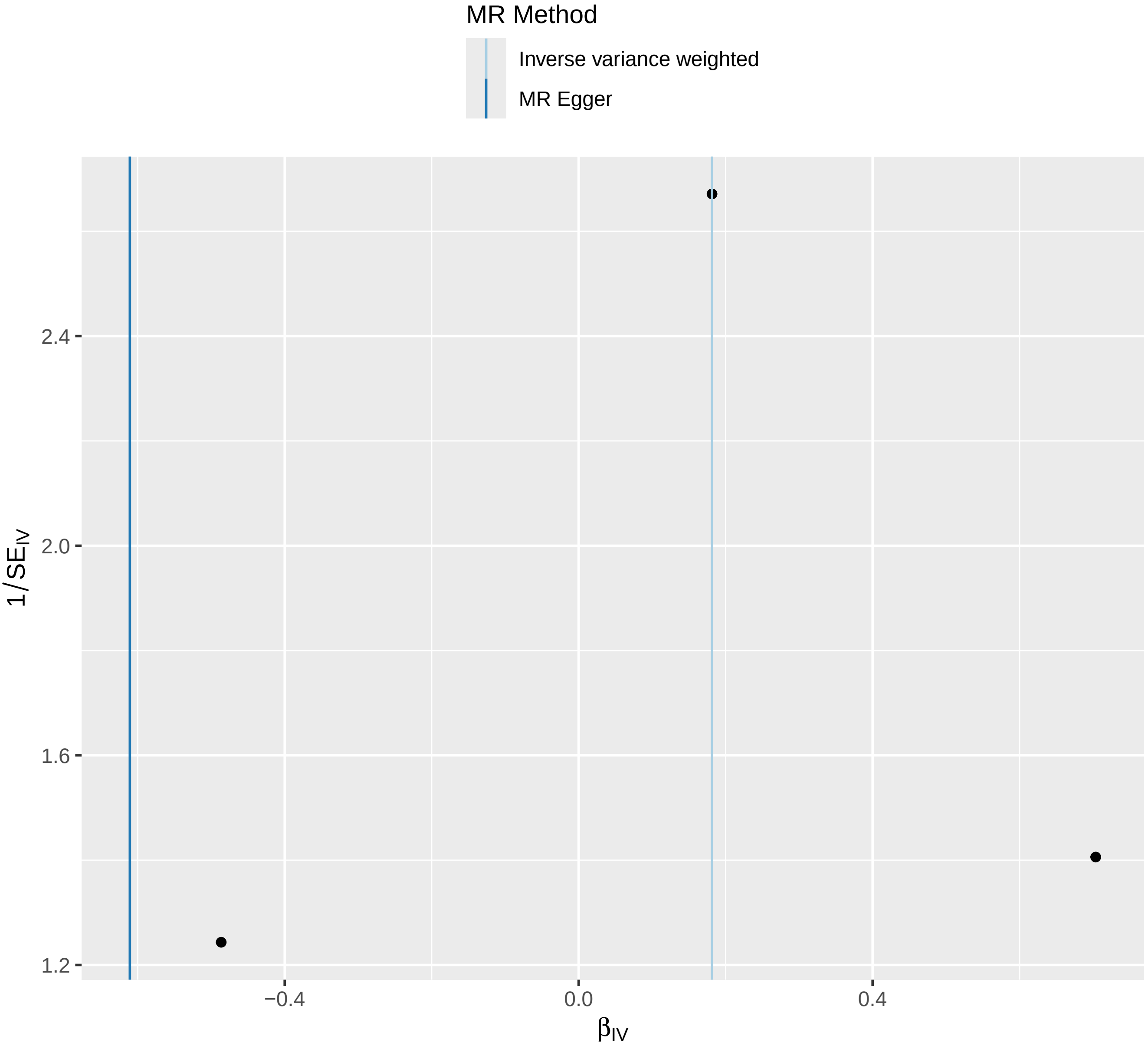
MR Method
Inverse variance weighted
MR Egger
2.4
2.0
1.6
1.2
−0.4
0.0
0.4
1/SE_IV
β_IV

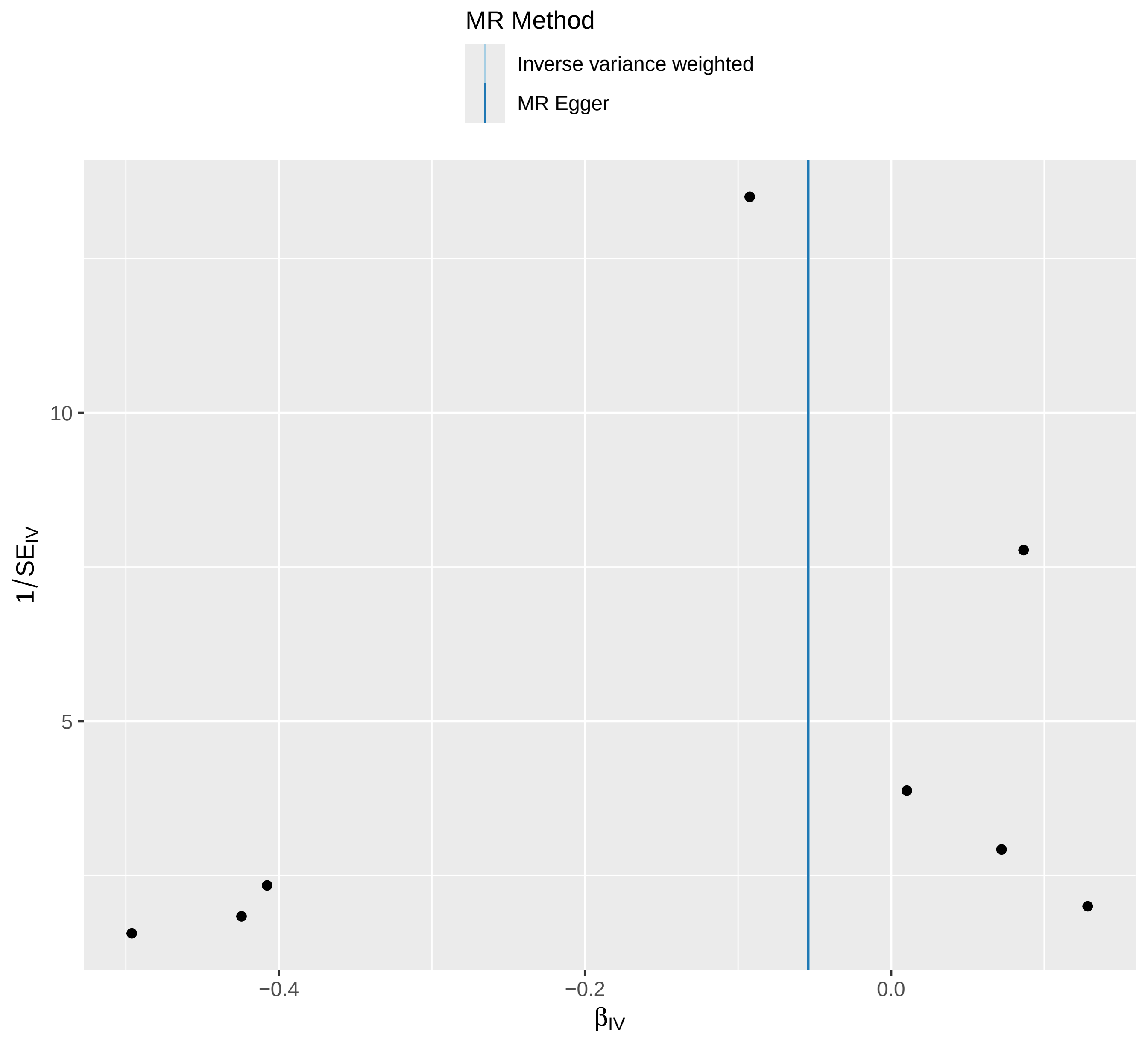
MR Method
Inverse variance weighted
MR Egger
10
5
1/SE$_{IV}$
−0.4
−0.2
0.0
β$_{IV}$

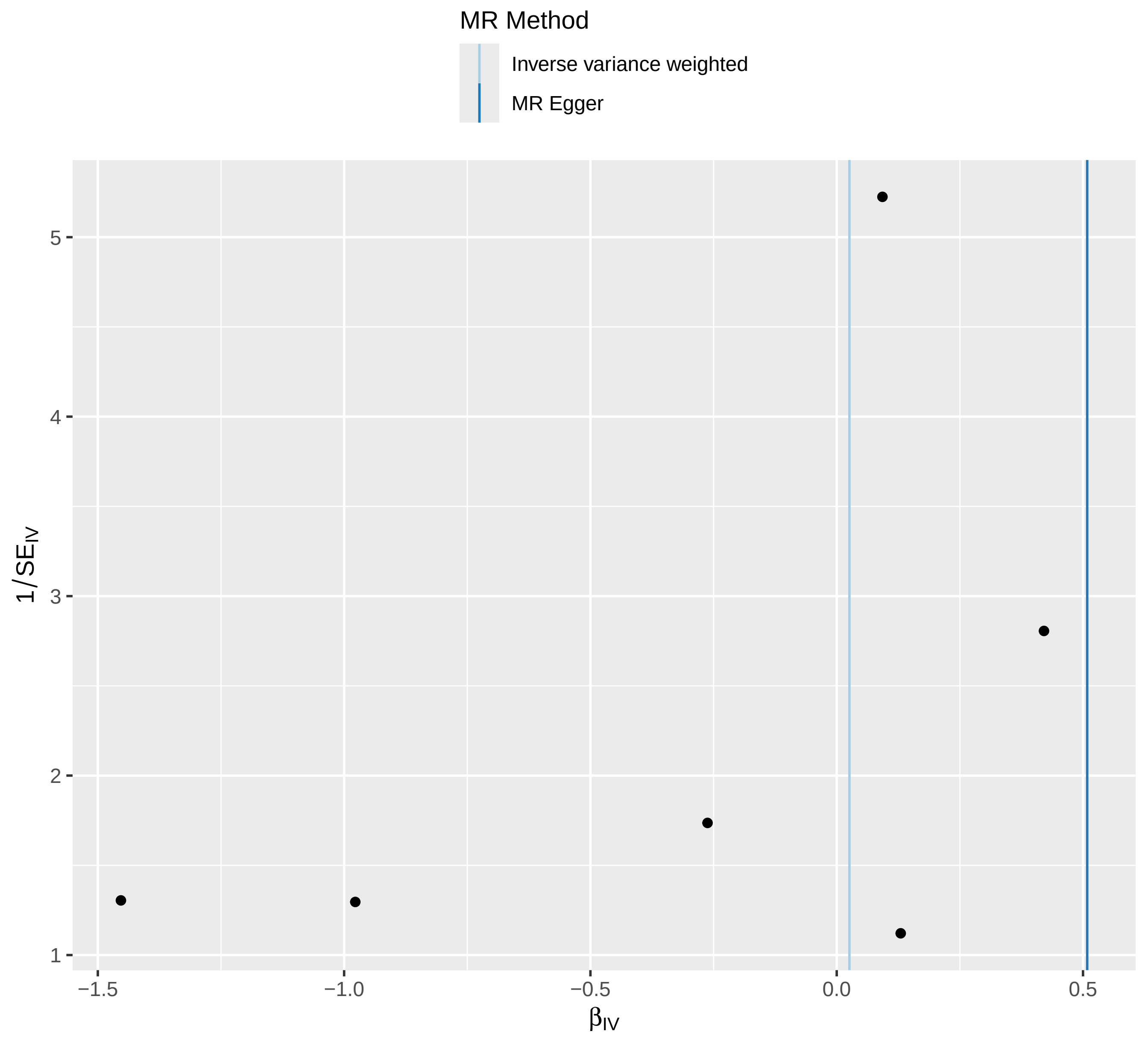
MR Method
Inverse variance weighted
MR Egger
5
4
3
2
1
1/SE_{IV}
−1.5
−1.0
−0.5
0.0
0.5
β_{IV}

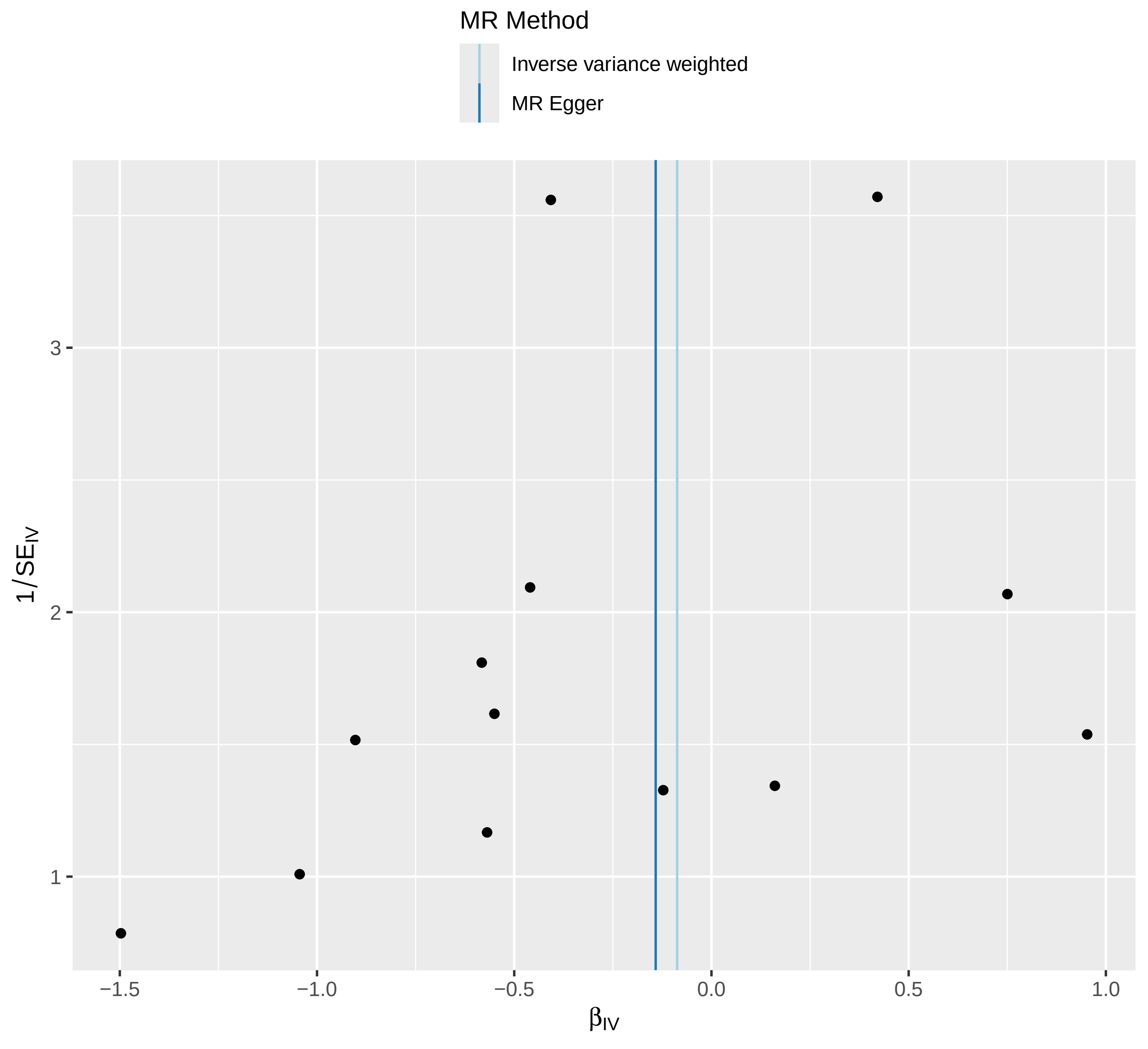

MR Method
Inverse variance weighted
MR Egger
1/SE_IV
3
2
1
−1.5
−1.0
−0.5
0.0
0.5
1.0
β_IV

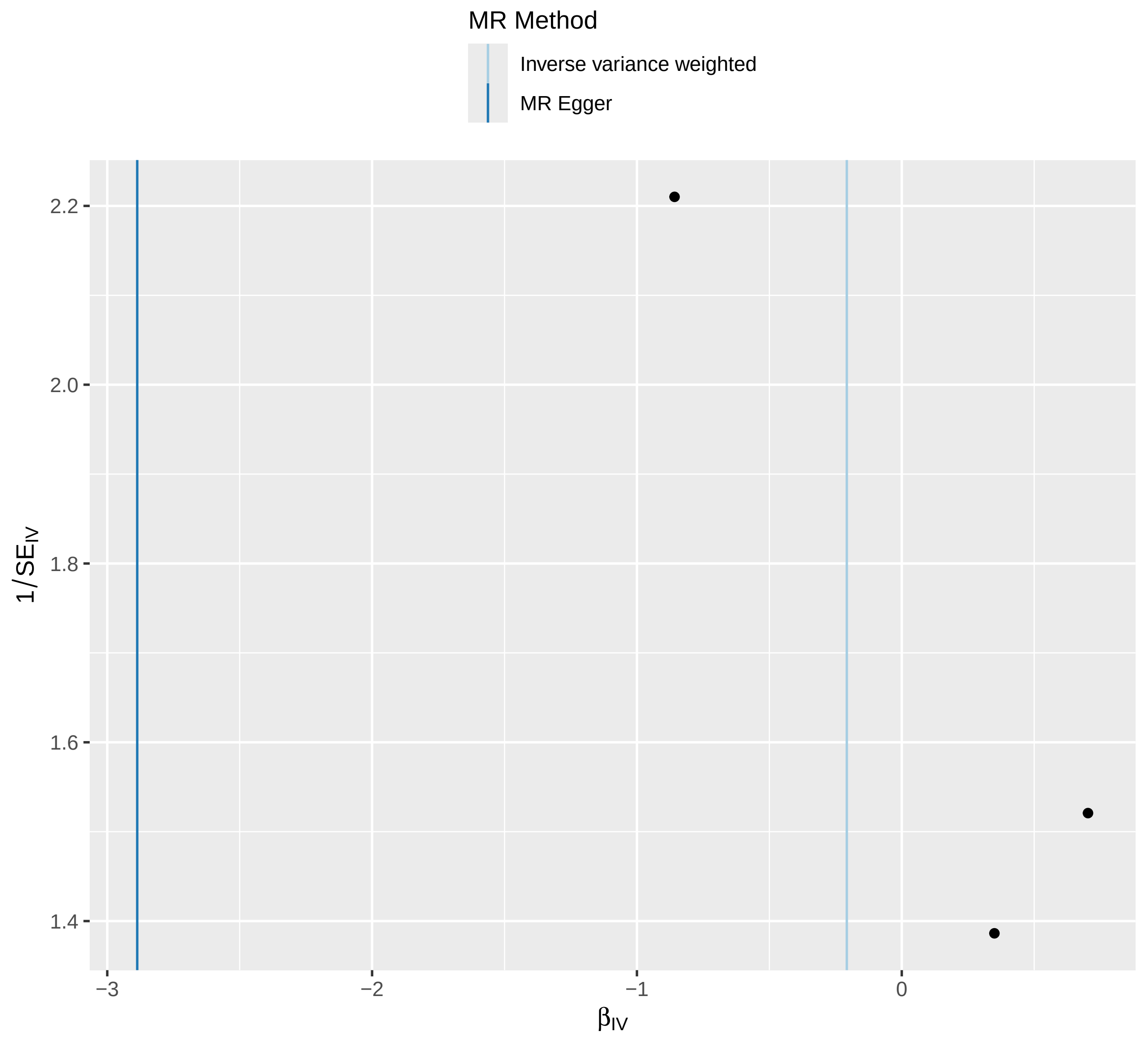

MR Method
Inverse variance weighted
MR Egger
2.2
2.0
1.8
1.6
1.4
1/SE_IV
−3
−2
−1
0
β_IV

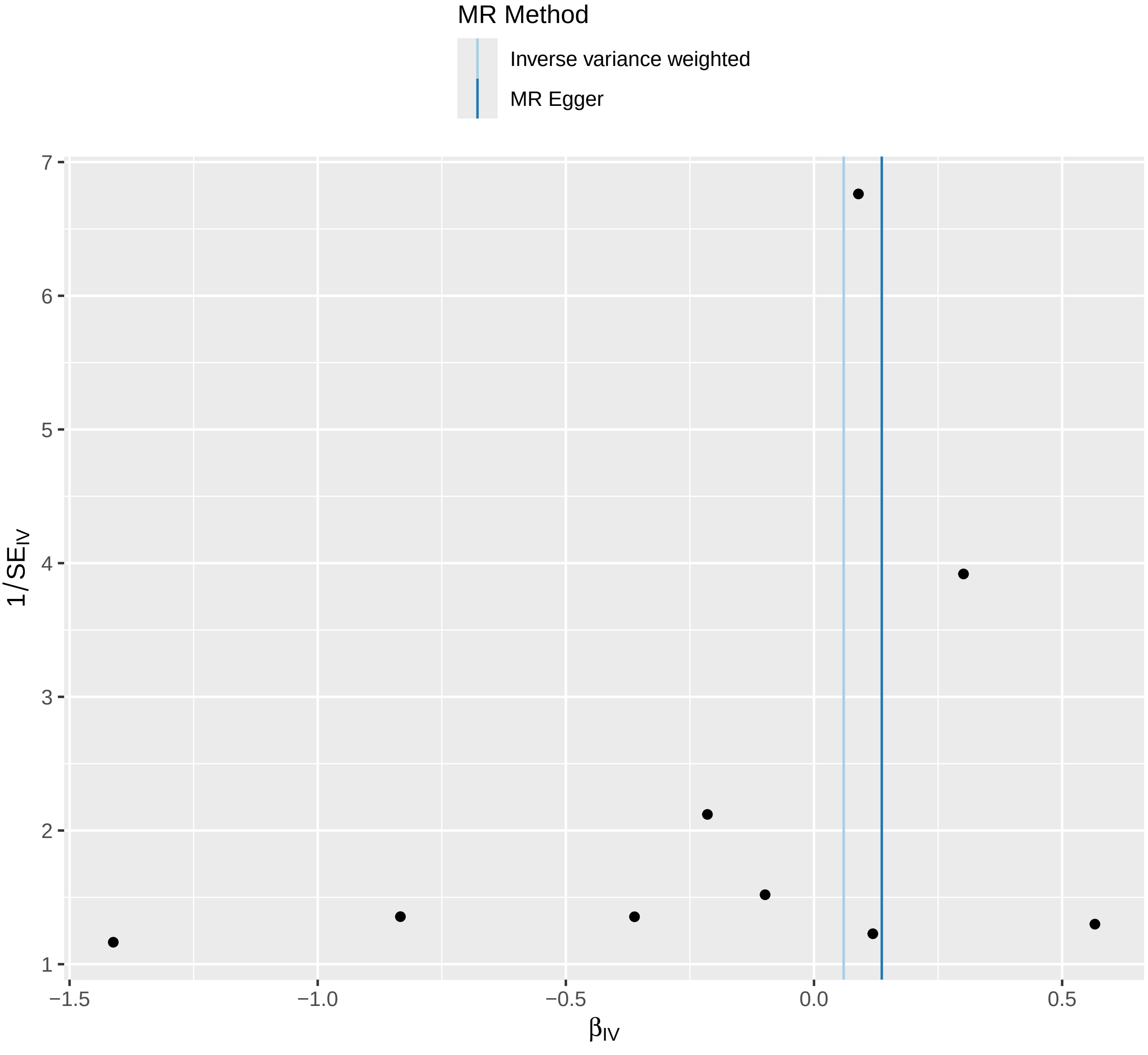
MR Method
Inverse variance weighted
MR Egger
7
6
5
4
3
2
1
$1/SE_{IV}$
−1.5
−1.0
−0.5
0.0
0.5
β_{IV}

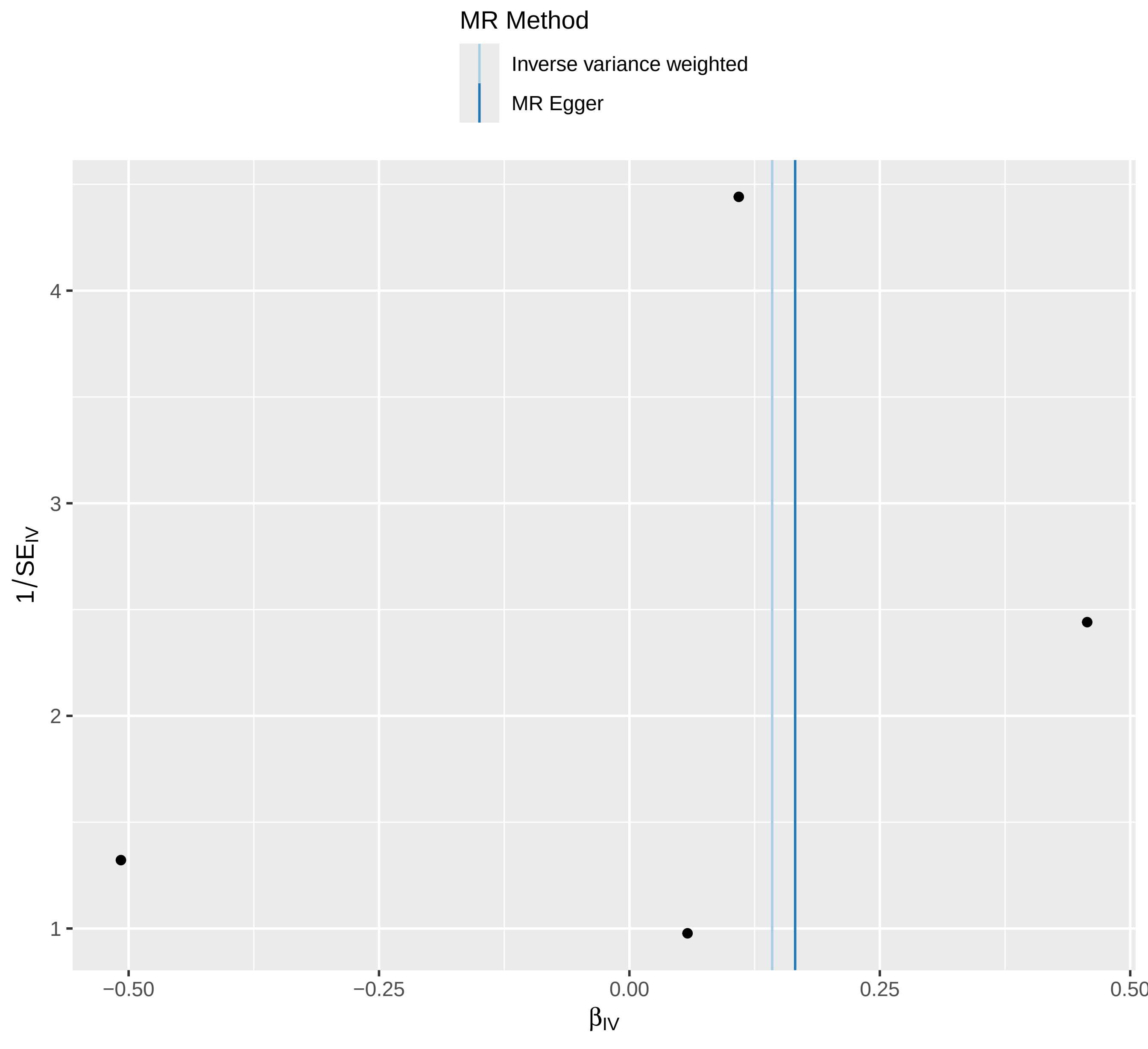

MR Method
Inverse variance weighted
MR Egger
4
3
2
1
1/SE_IV
−0.50
−0.25
0.00
0.25
0.50
β_IV

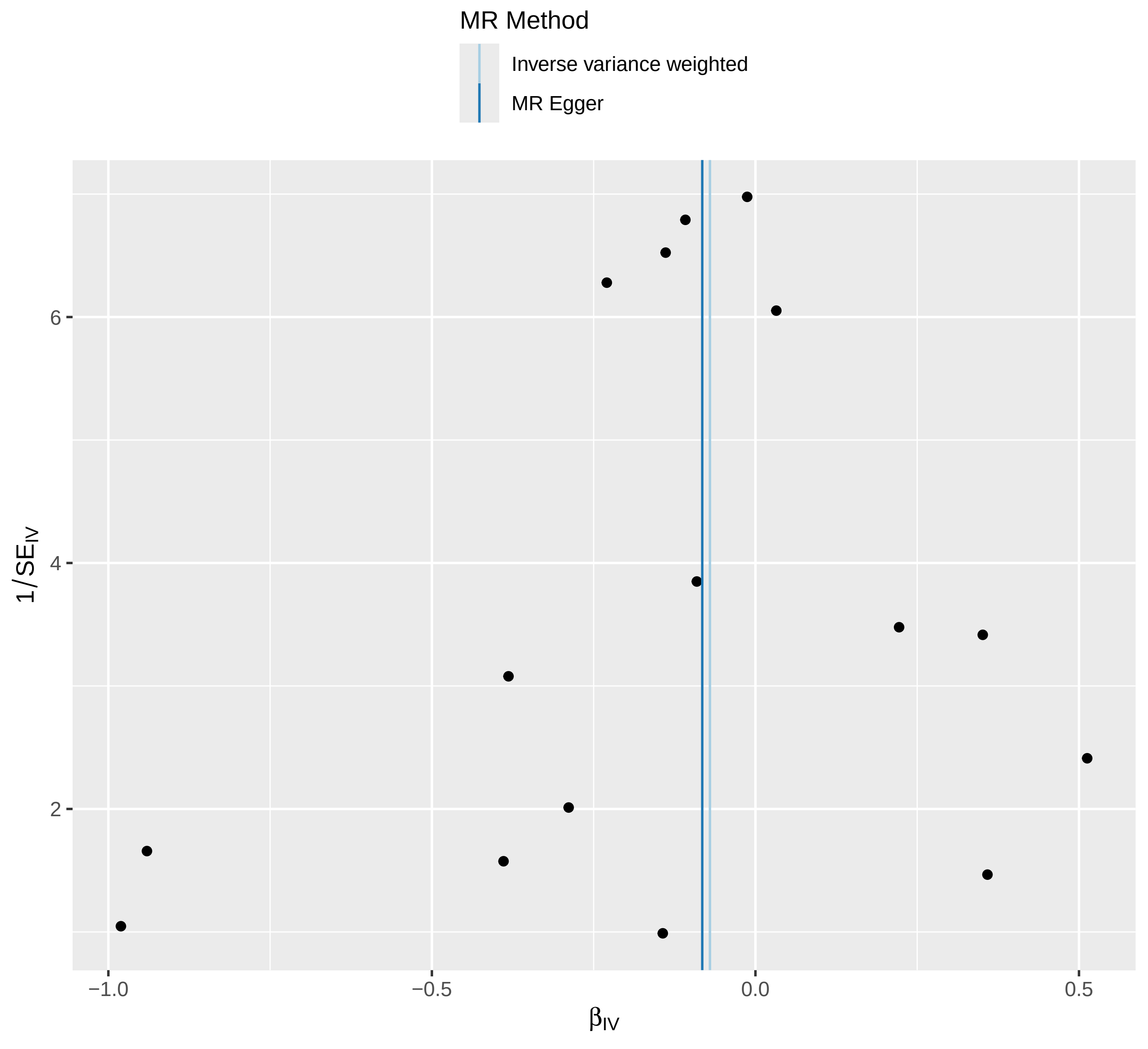

MR Method
Inverse variance weighted
MR Egger
6
4
2
1/SE$_{IV}$
−1.0
−0.5
0.0
0.5
β$_{IV}$

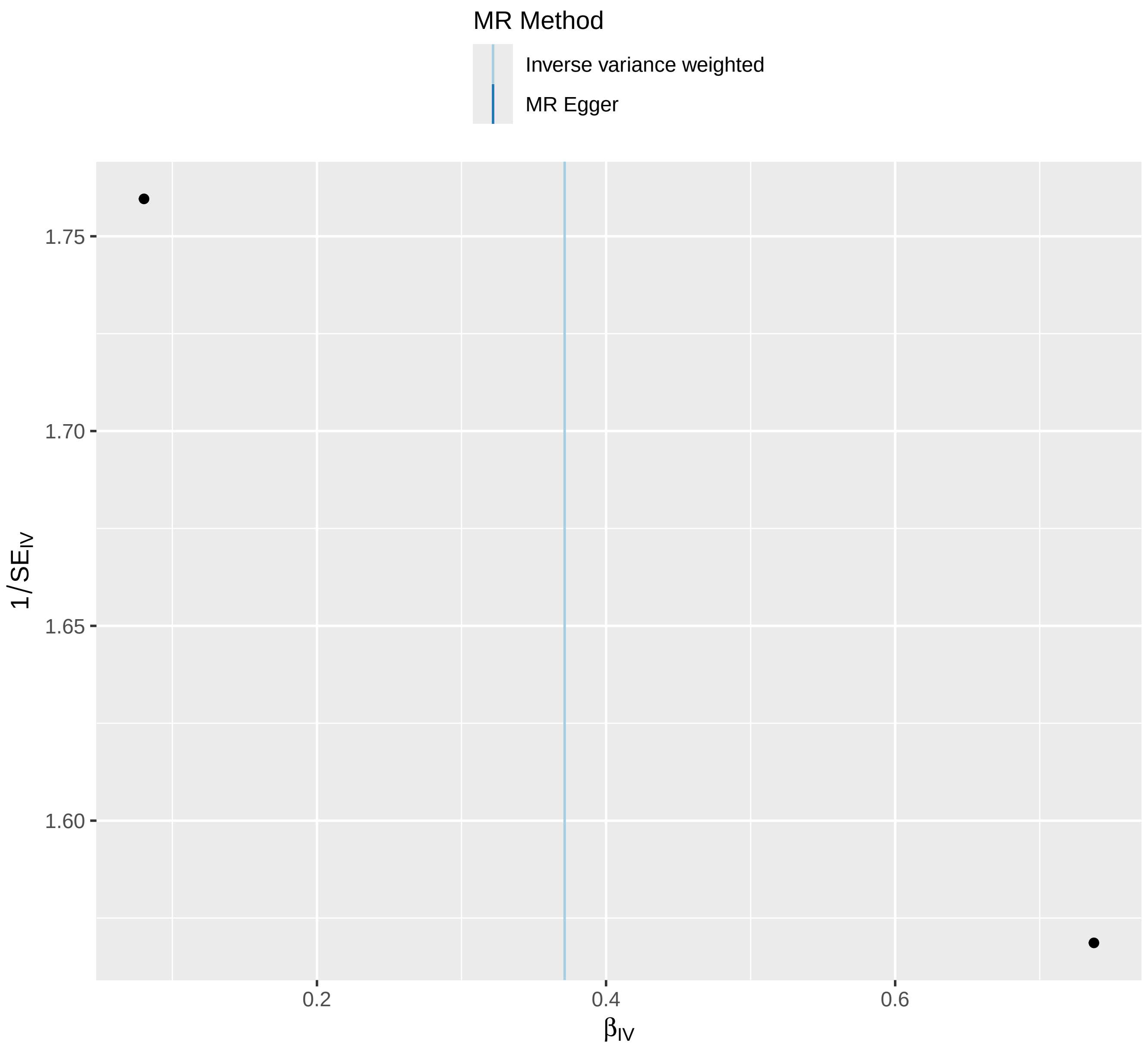
MR Method
Inverse variance weighted
MR Egger
1.75
1.70
1.65
1.60
0.2
0.4
0.6
β_{IV}
$1/SE_{IV}$

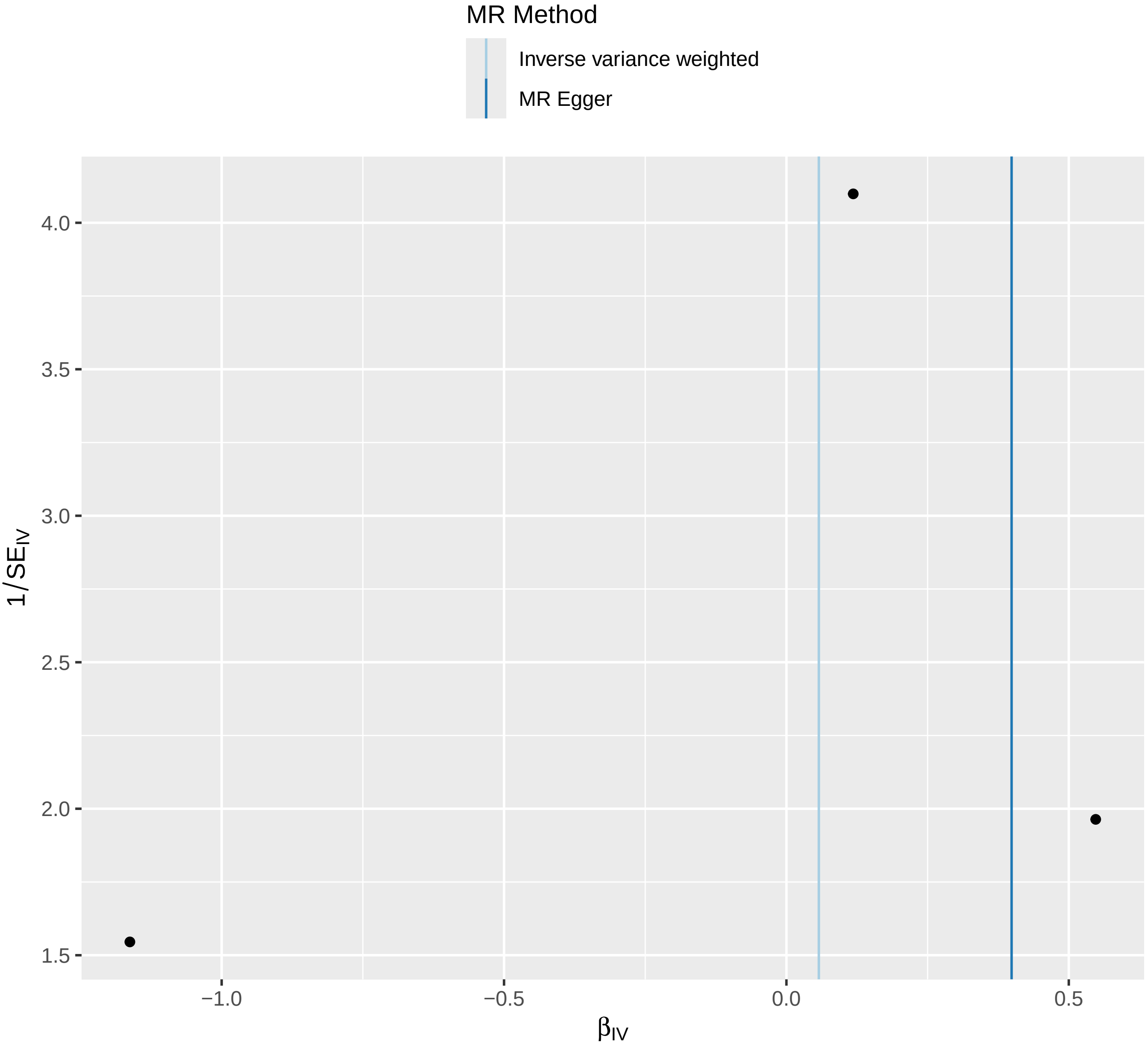

MR Method
Inverse variance weighted
MR Egger
4.0
3.5
3.0
2.5
2.0
1.5
1/SE$_{IV}$
−1.0
−0.5
0.0
0.5
β$_{IV}$

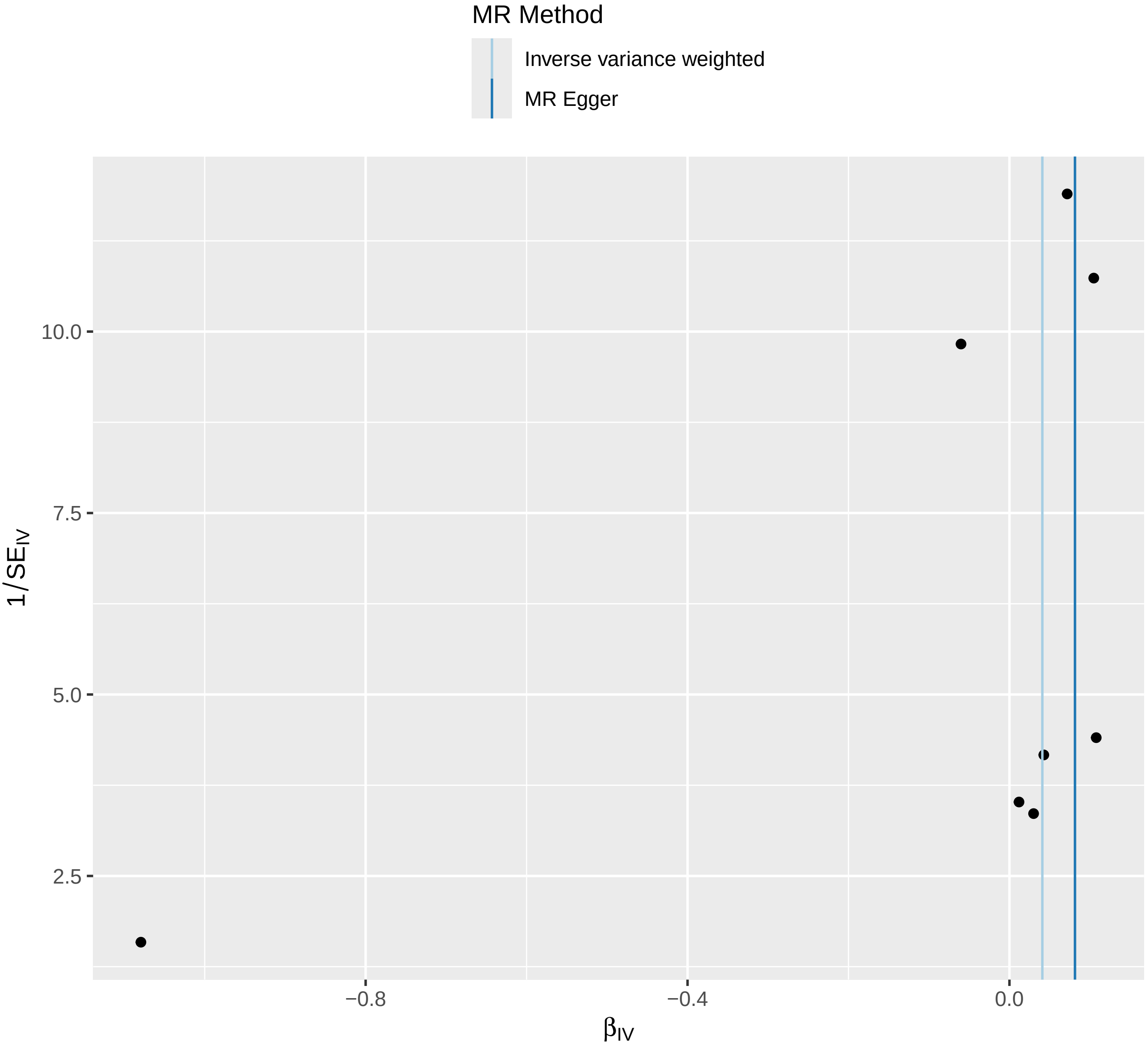

MR Method
Inverse variance weighted
MR Egger
10.0
7.5
5.0
2.5
1/SE_{IV}
−0.8
−0.4
0.0
β_{IV}

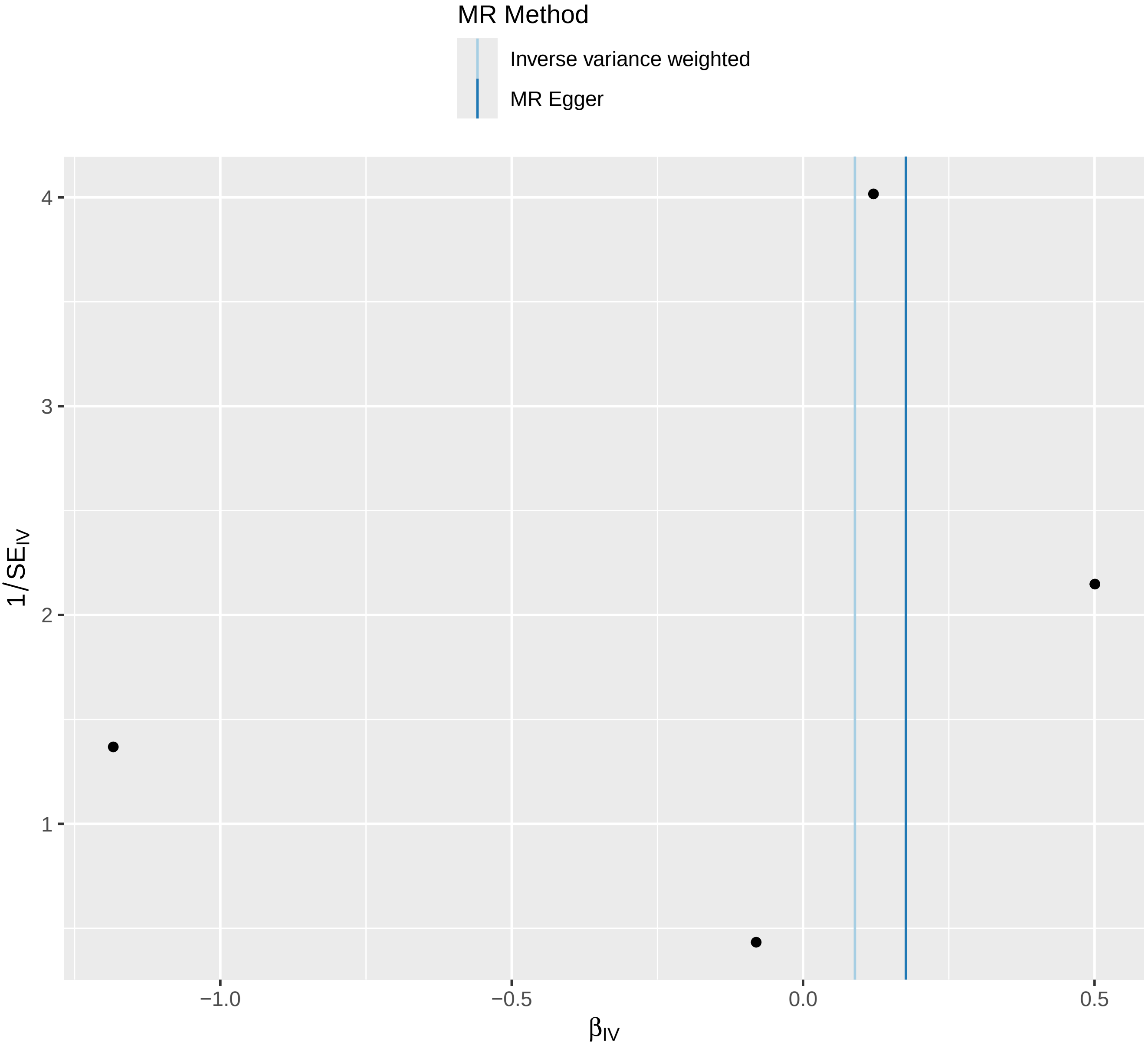

MR Method
Inverse variance weighted
MR Egger
4
3
2
1
1/SE_IV
−1.0
−0.5
0.0
0.5
β_IV

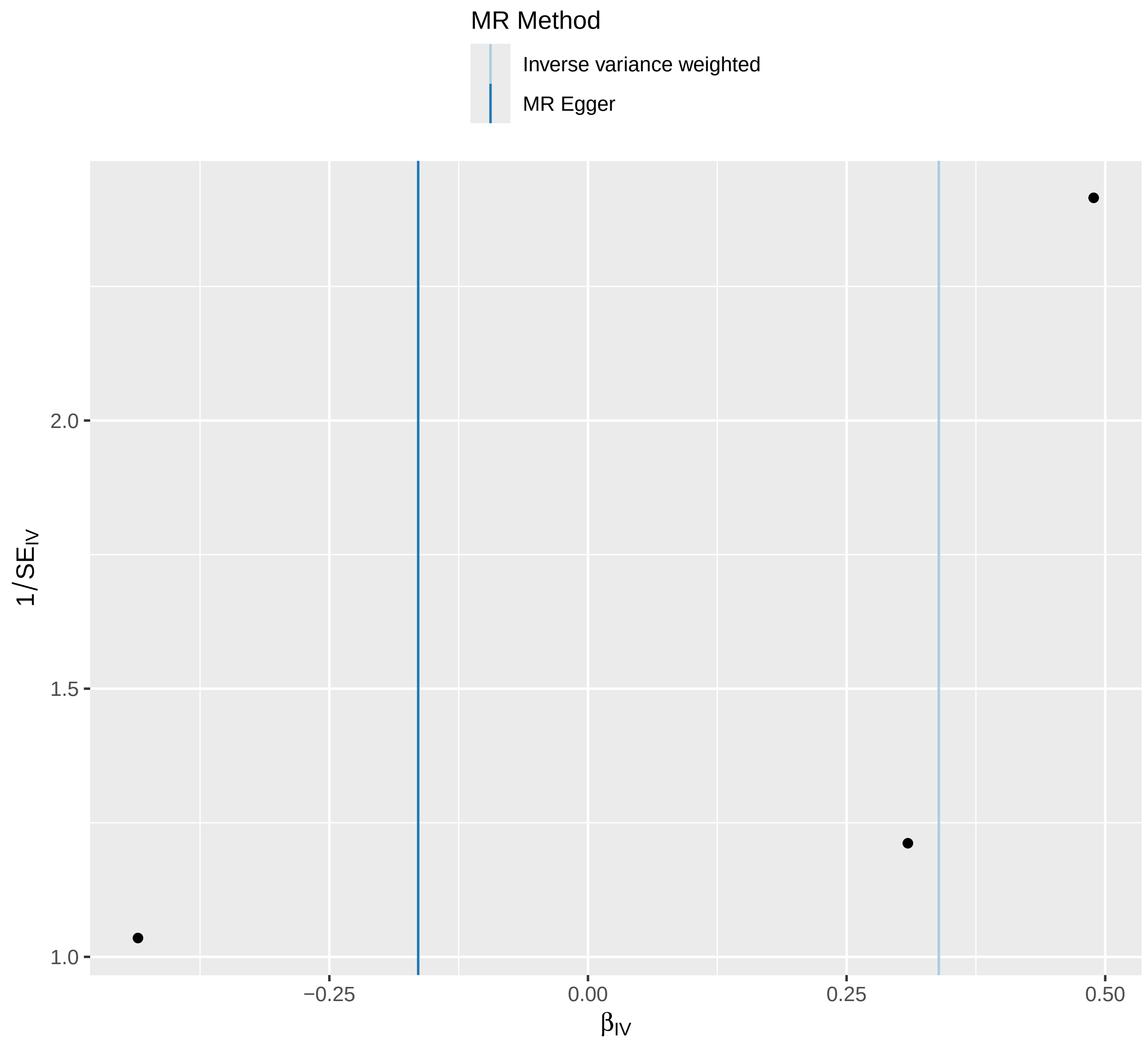
MR Method
Inverse variance weighted
MR Egger
2.0
1.5
1.0
1/SE_IV
−0.25
0.00
0.25
0.50
β_IV

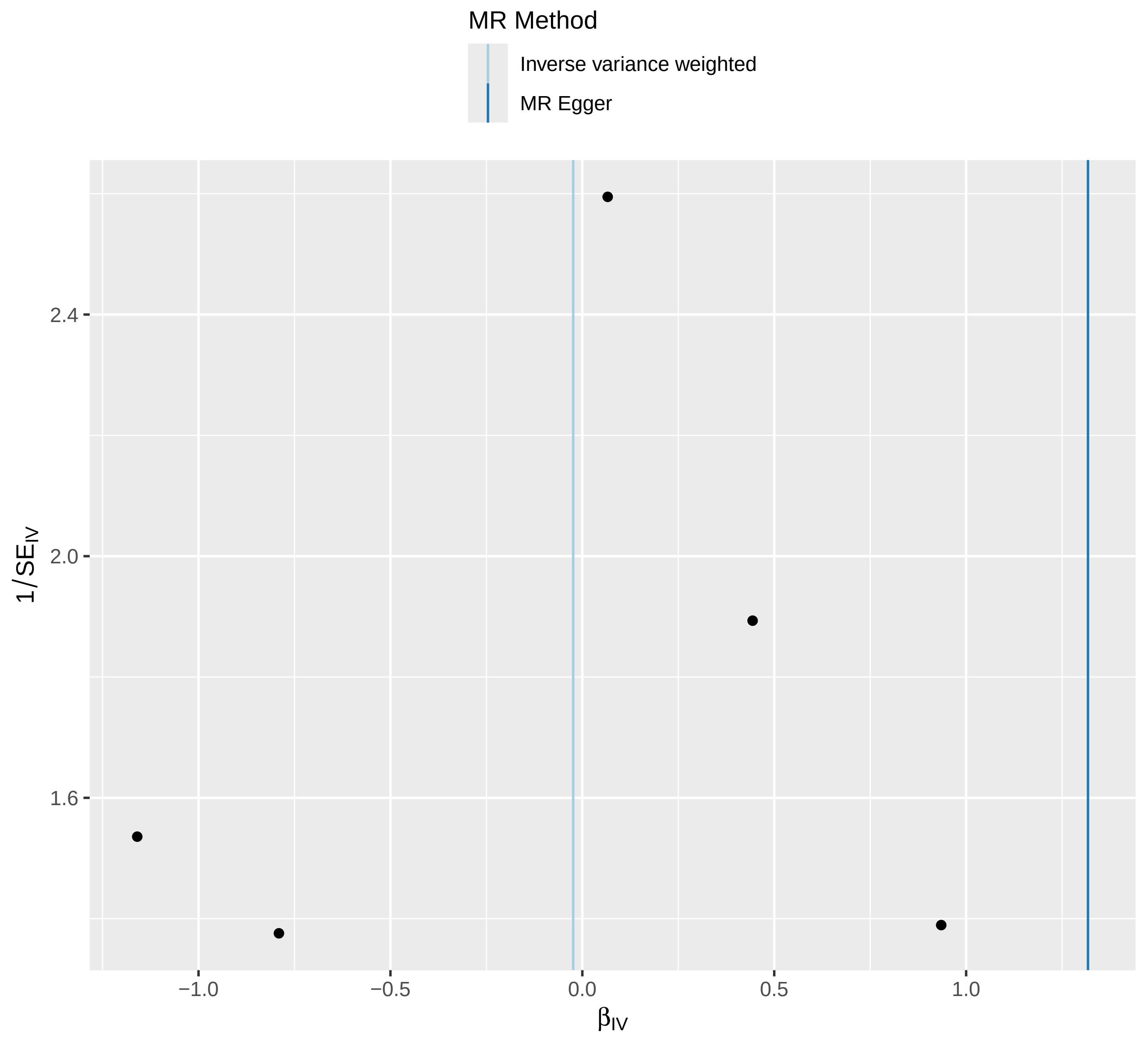
MR Method
Inverse variance weighted
MR Egger
2.4
2.0
1.6
1/SE_IV
−1.0
−0.5
0.0
0.5
1.0
β_IV

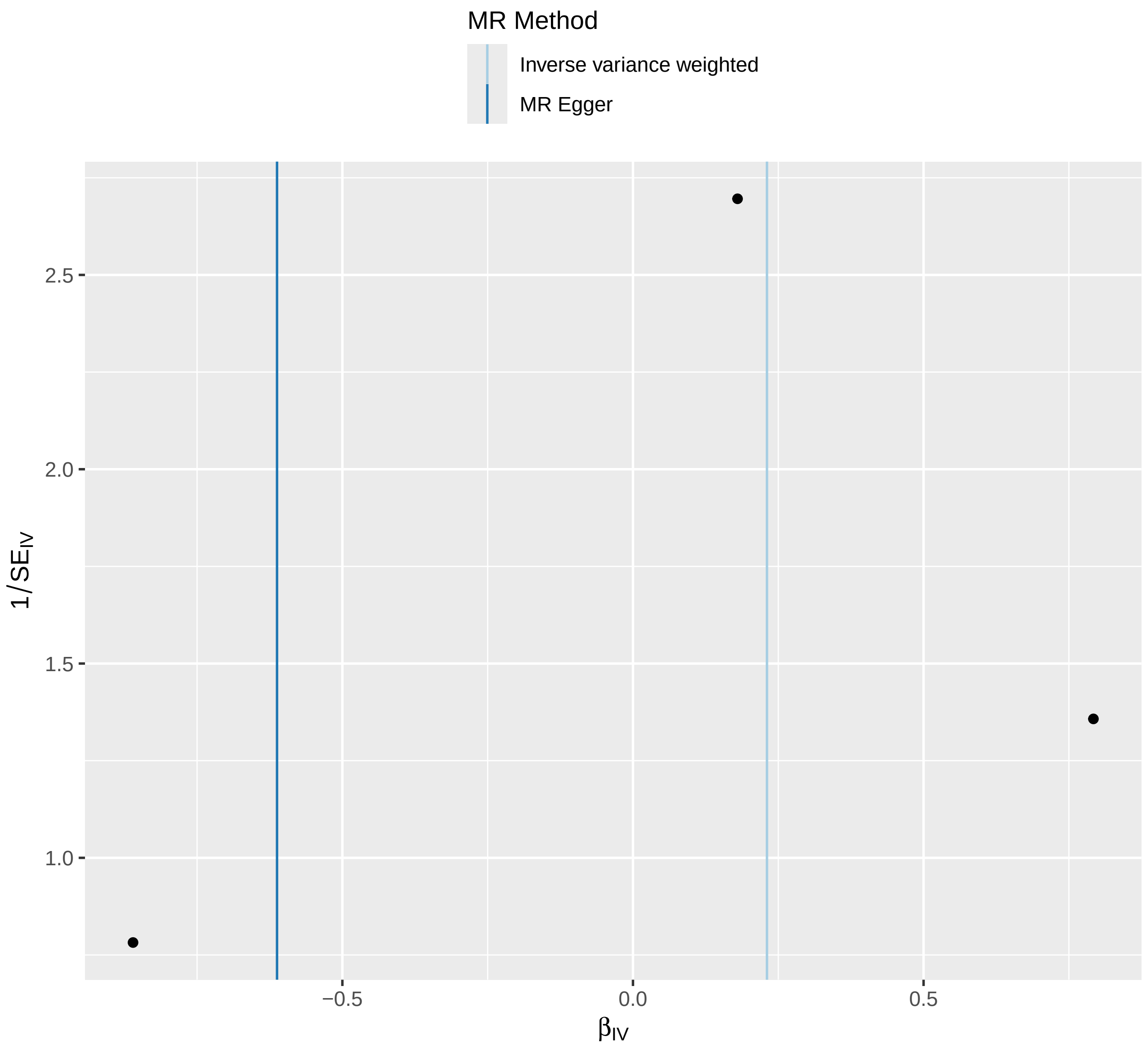
MR Method
Inverse variance weighted
MR Egger
2.5
2.0
1.5
1.0
1/SE$_{IV}$
−0.5
0.0
0.5
β$_{IV}$

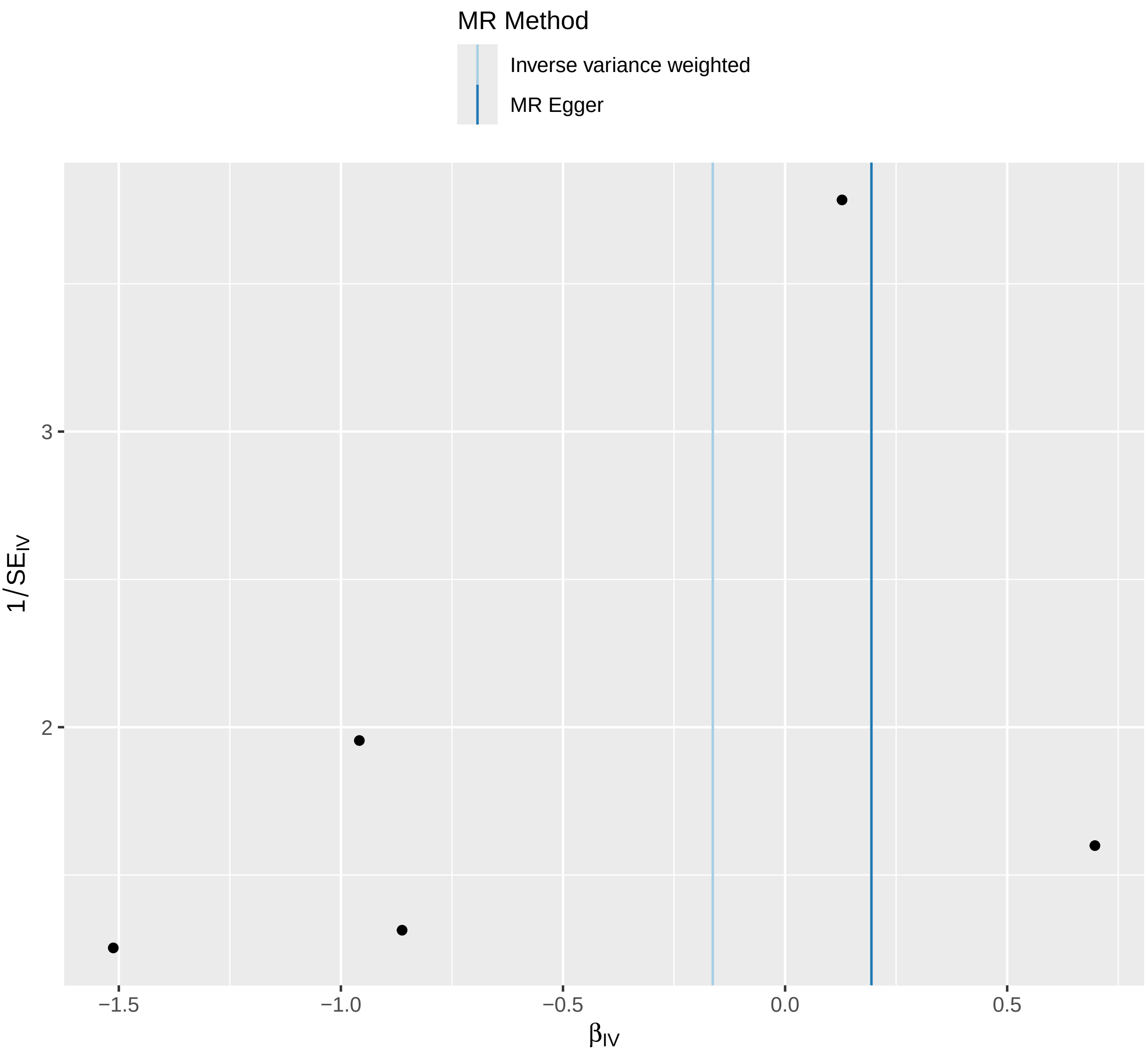

MR Method
Inverse variance weighted
MR Egger
3
2
1/SE_IV
−1.5
−1.0
−0.5
0.0
0.5
β_IV

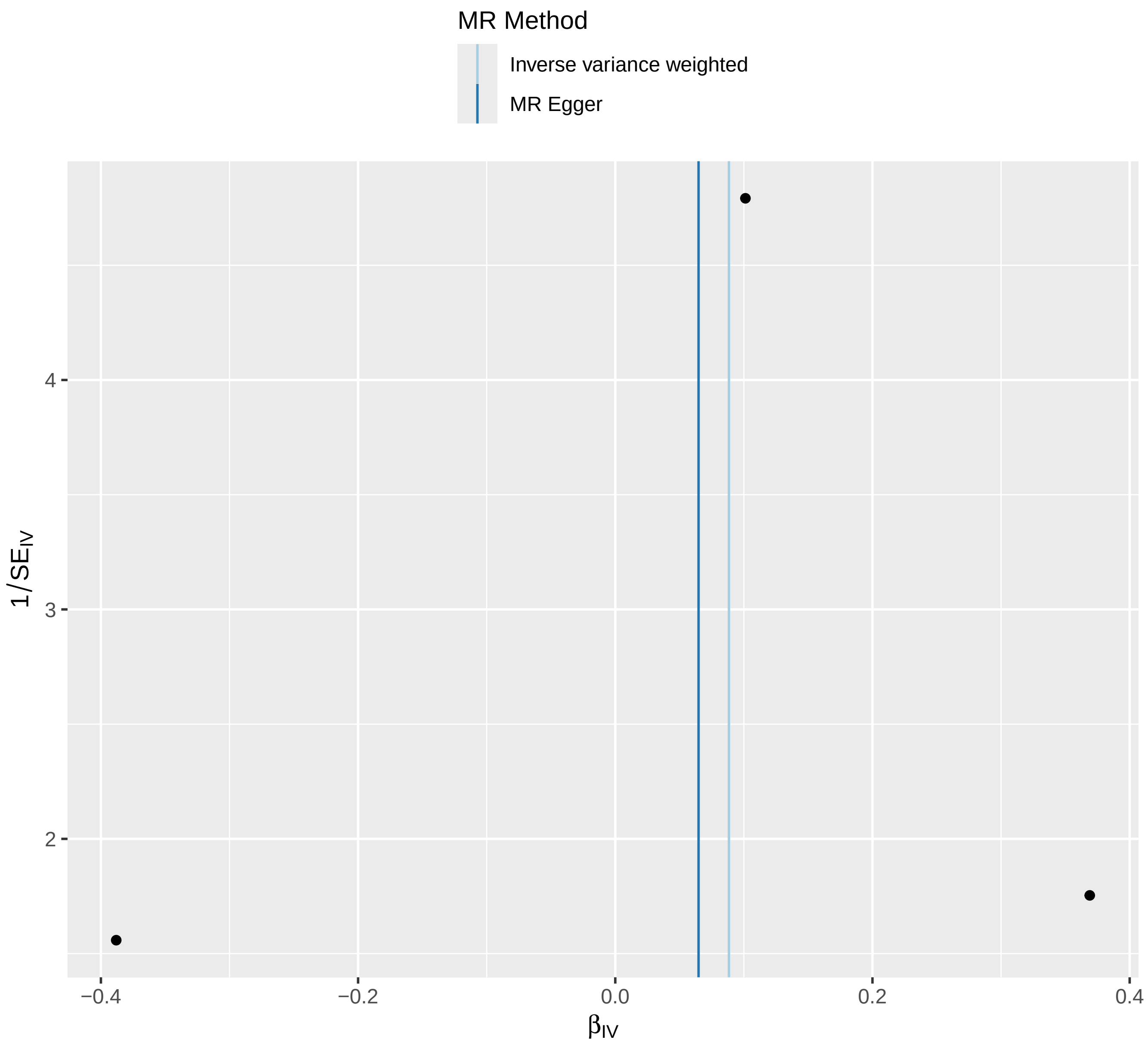

MR Method
Inverse variance weighted
MR Egger
1/SE$_{IV}$
4
3
2
−0.4
−0.2
0.0
0.2
0.4
β$_{IV}$

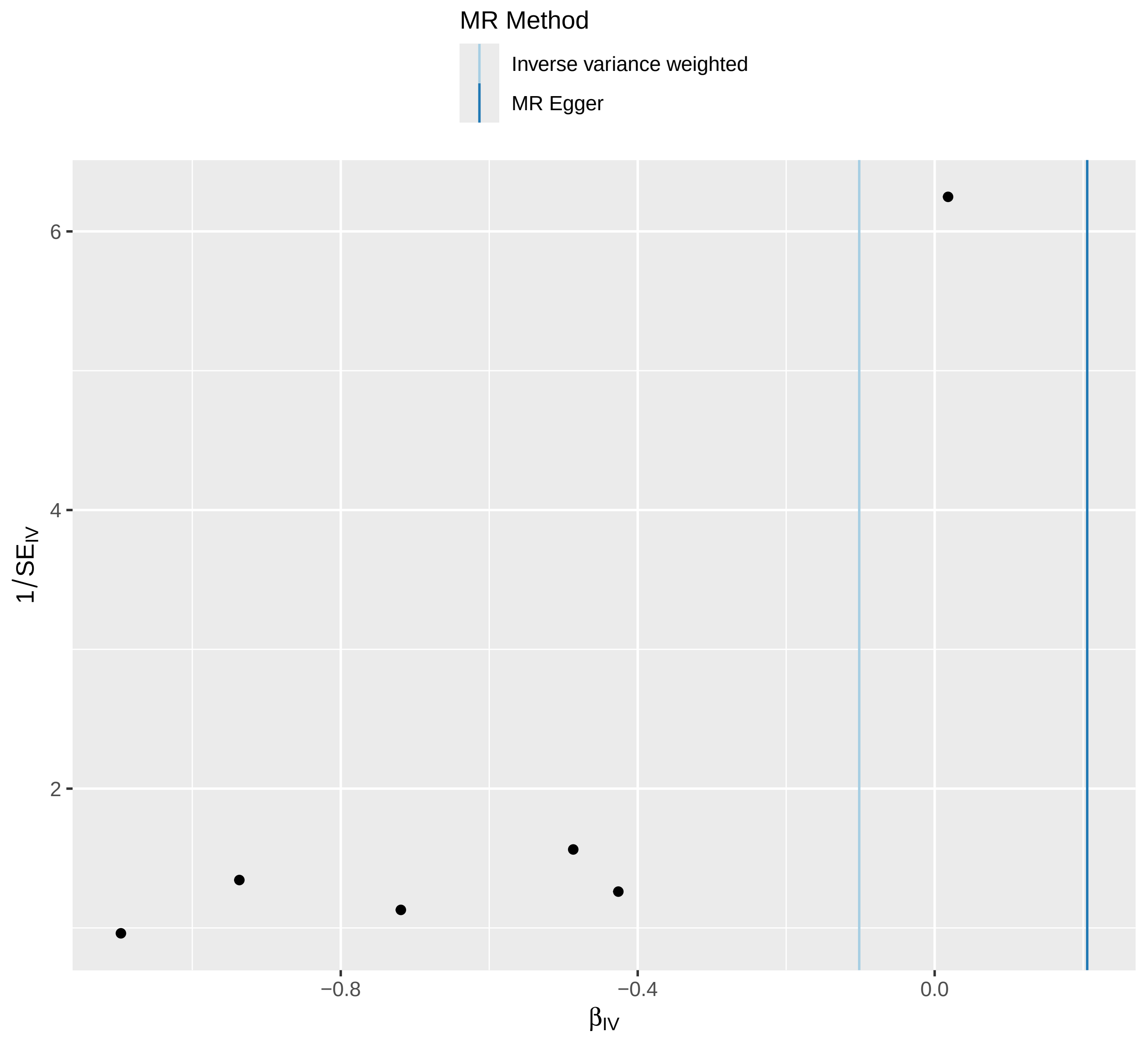
MR Method
Inverse variance weighted
MR Egger
6
4
2
1/SE$_{IV}$
−0.8
−0.4
0.0
β$_{IV}$

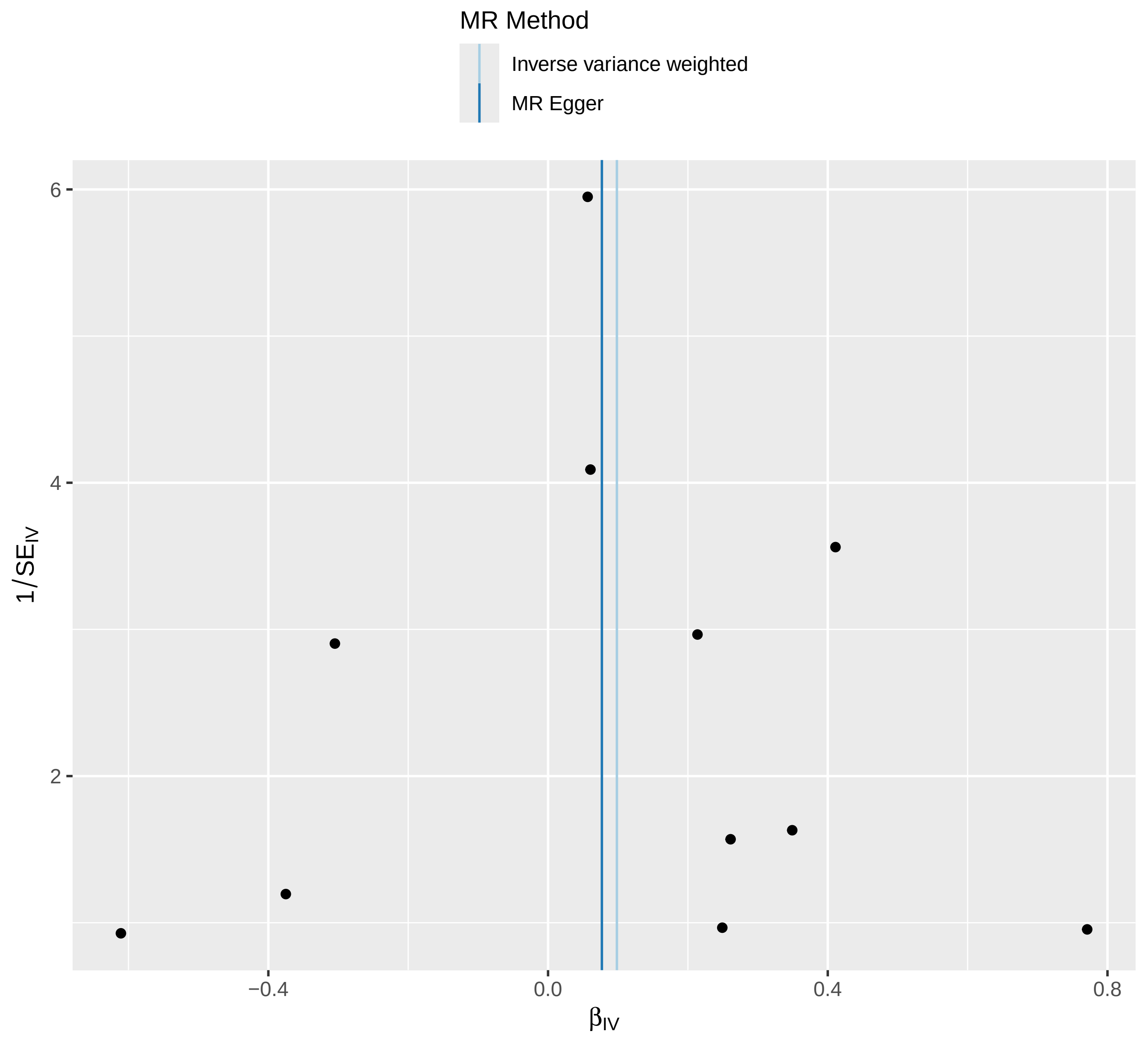
MR Method
Inverse variance weighted
MR Egger
6
4
2
1/SE$_{IV}$
−0.4
0.0
0.4
0.8
β$_{IV}$

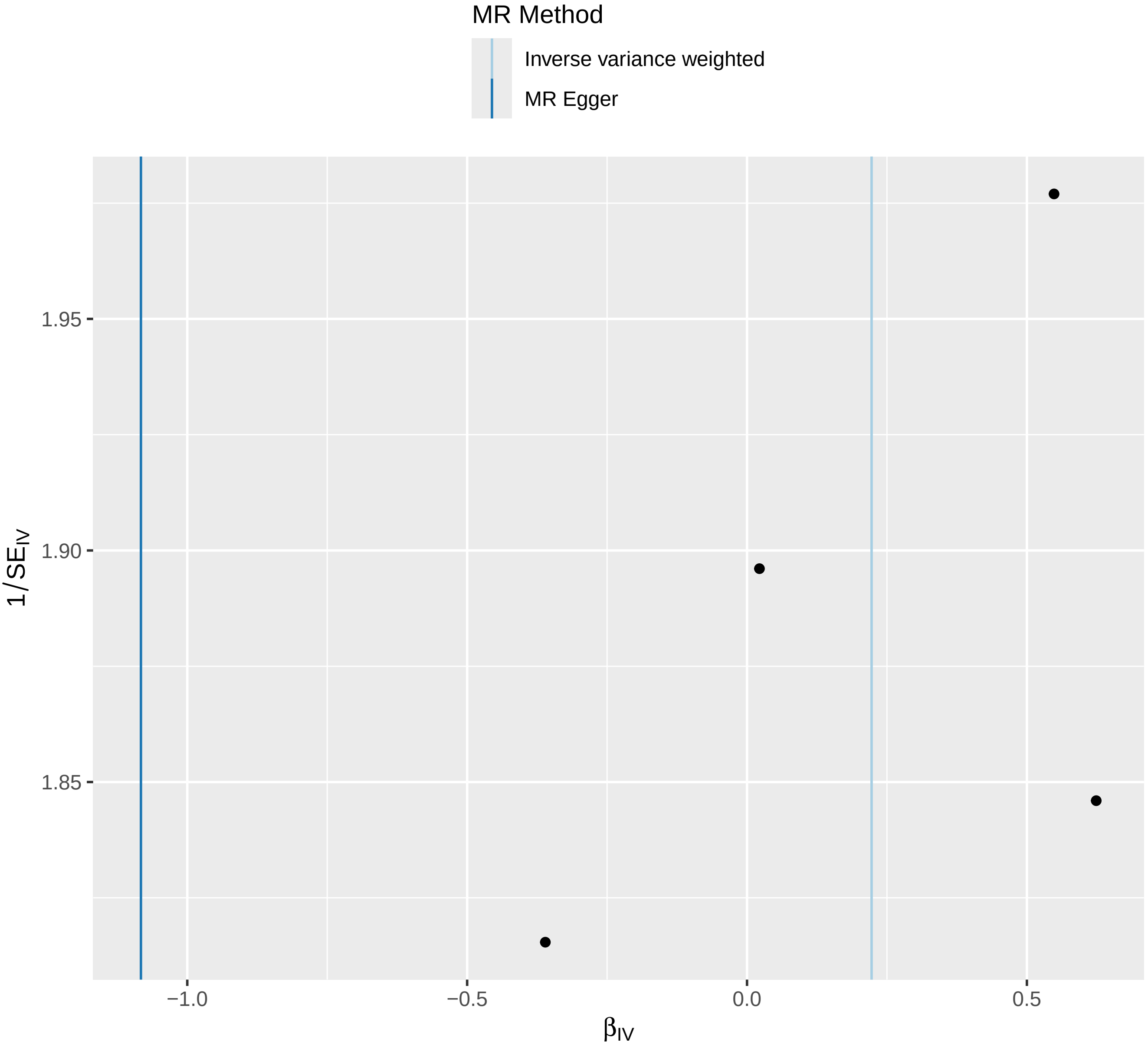

MR Method
Inverse variance weighted
MR Egger
1.95
1.90
1.85
1/SE_IV
−1.0
−0.5
0.0
0.5
β_IV

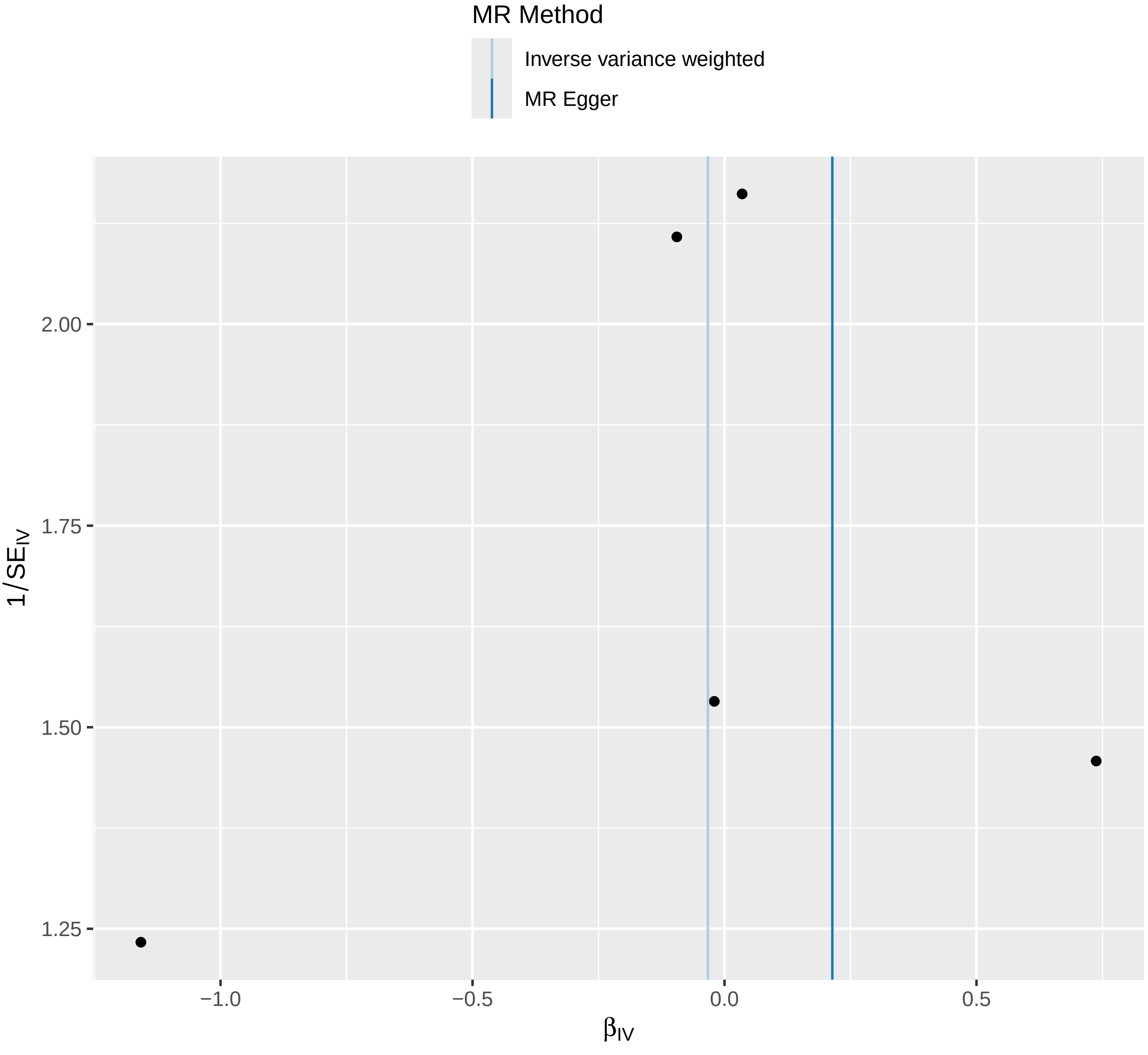
MR Method
Inverse variance weighted
MR Egger
2.00
1.75
1.50
1.25
1/SE$_{IV}$
−1.0
−0.5
0.0
0.5
β$_{IV}$

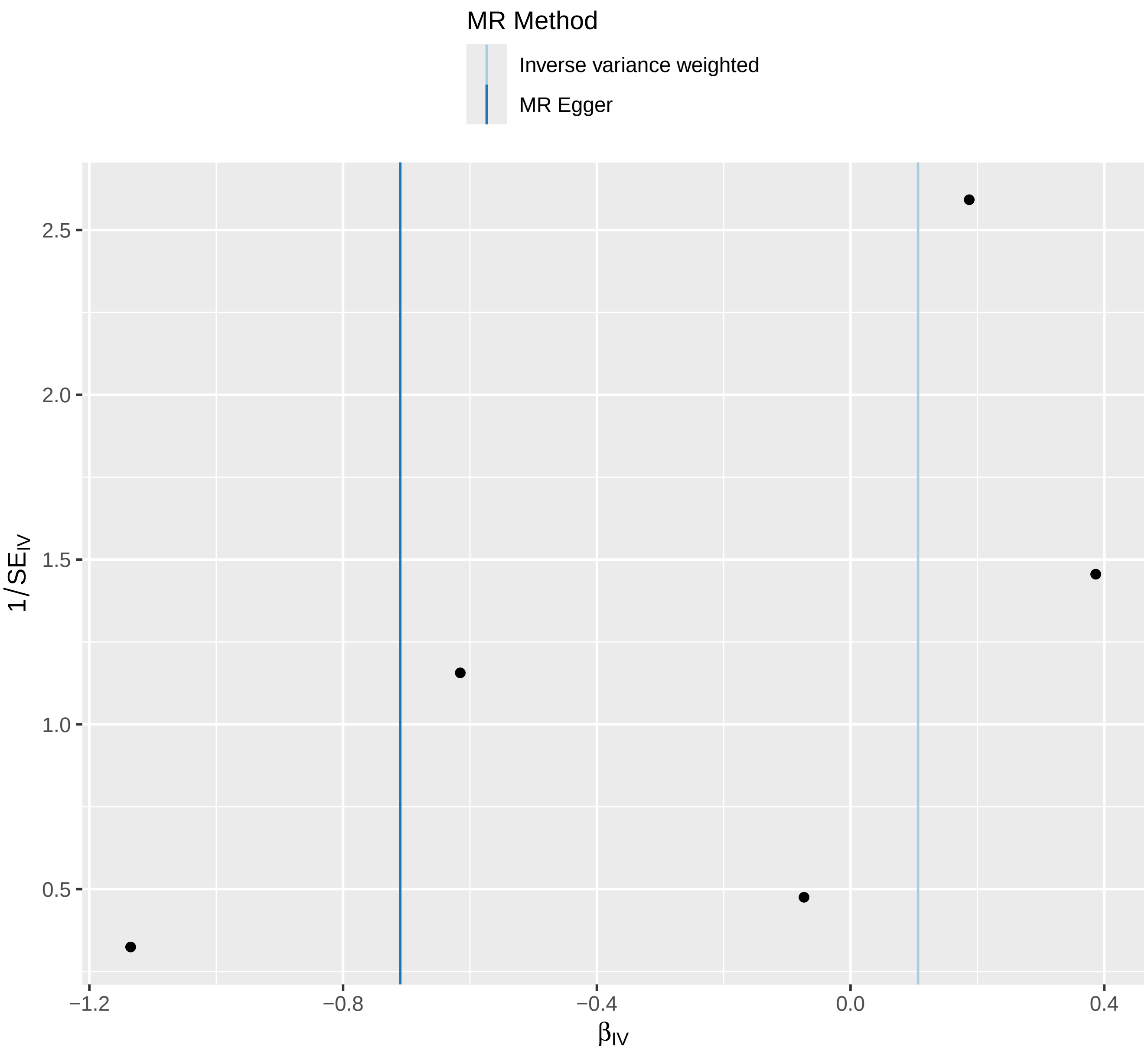
MR Method
Inverse variance weighted
MR Egger
2.5
2.0
1.5
1.0
0.5
1/SE_IV
−1.2
−0.8
−0.4
0.0
0.4
β_IV

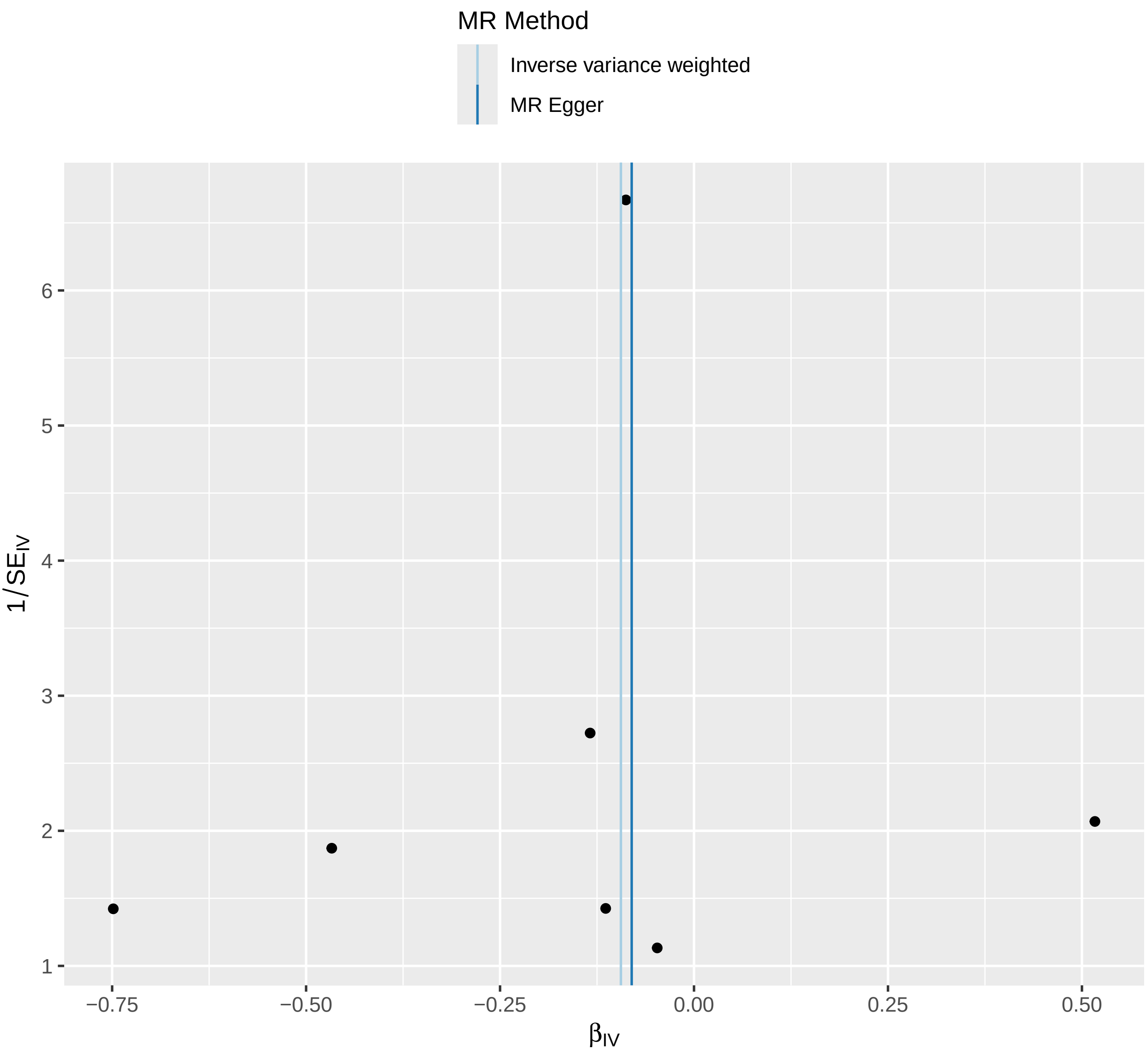
MR Method
Inverse variance weighted
MR Egger
1/SE_IV
6
5
4
3
2
1
−0.75
−0.50
−0.25
0.00
0.25
0.50
β_IV

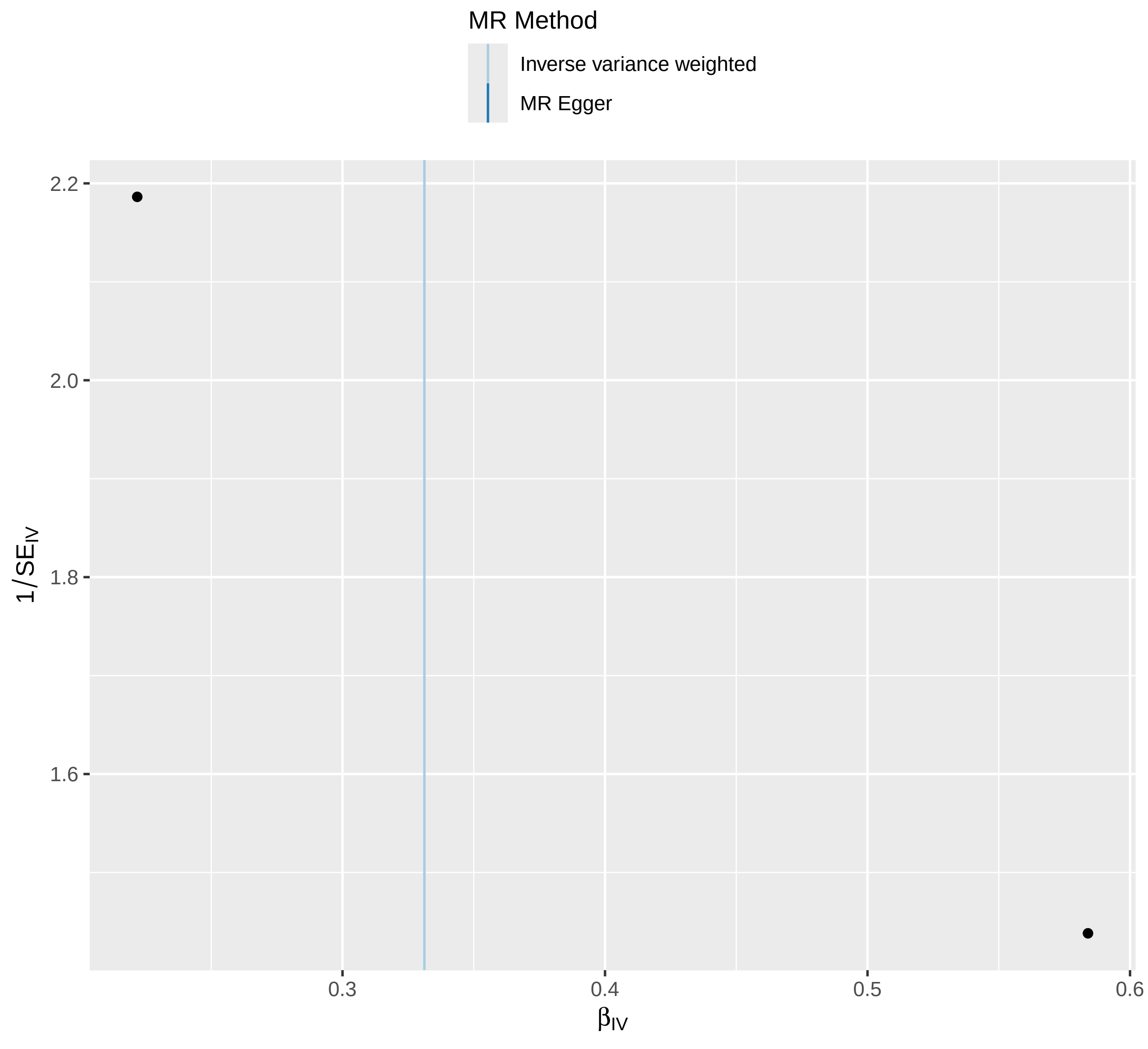
MR Method
Inverse variance weighted
MR Egger
2.2
2.0
1.8
1.6
$1/SE_{IV}$
0.3
0.4
0.5
0.6
β_{IV}

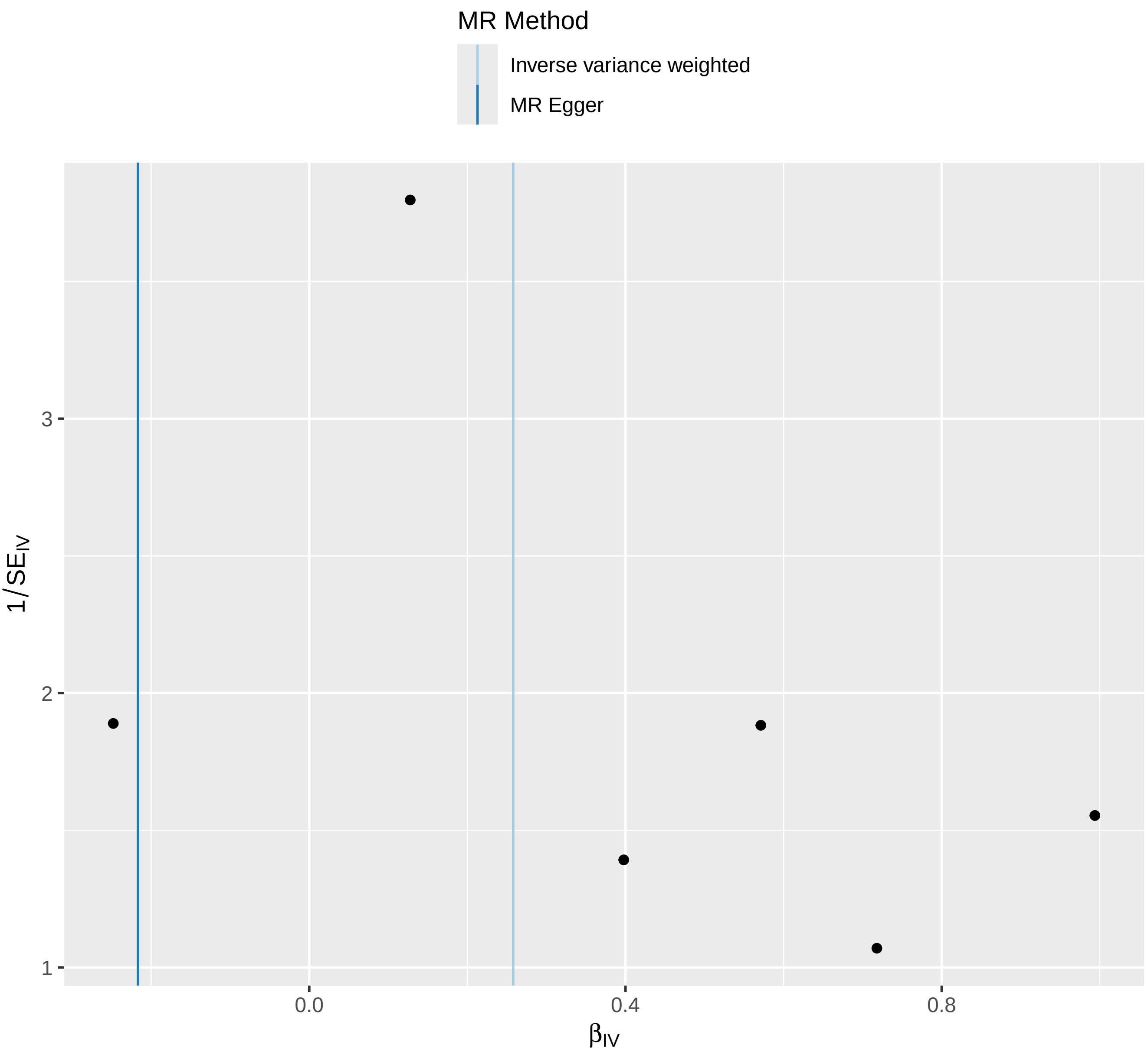

MR Method
Inverse variance weighted
MR Egger
1/SE_IV
3
2
1
0.0
0.4
0.8
β_IV

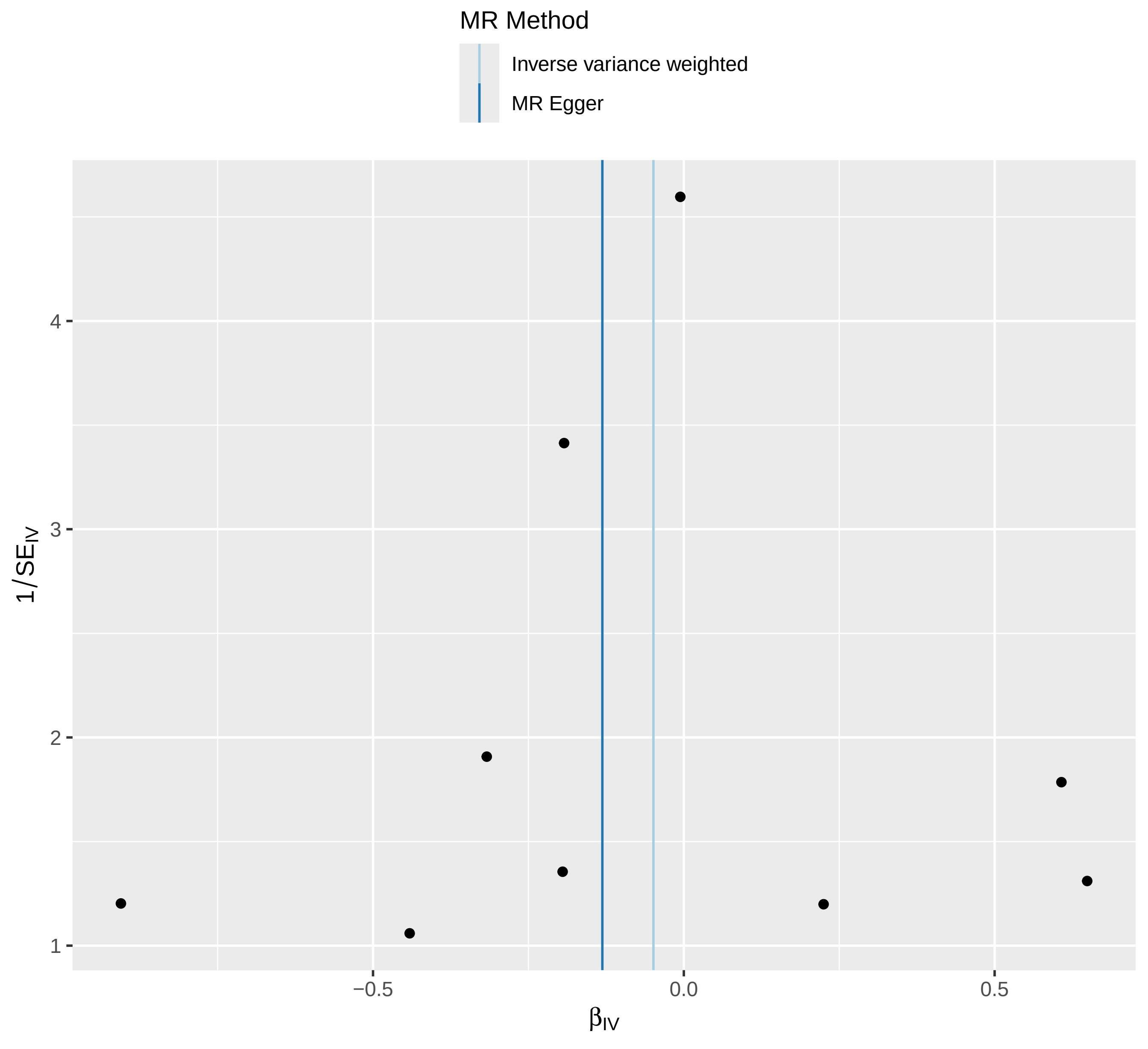
MR Method
Inverse variance weighted
MR Egger
1/SE$_{IV}$
4
3
2
1
−0.5
0.0
0.5
β$_{IV}$

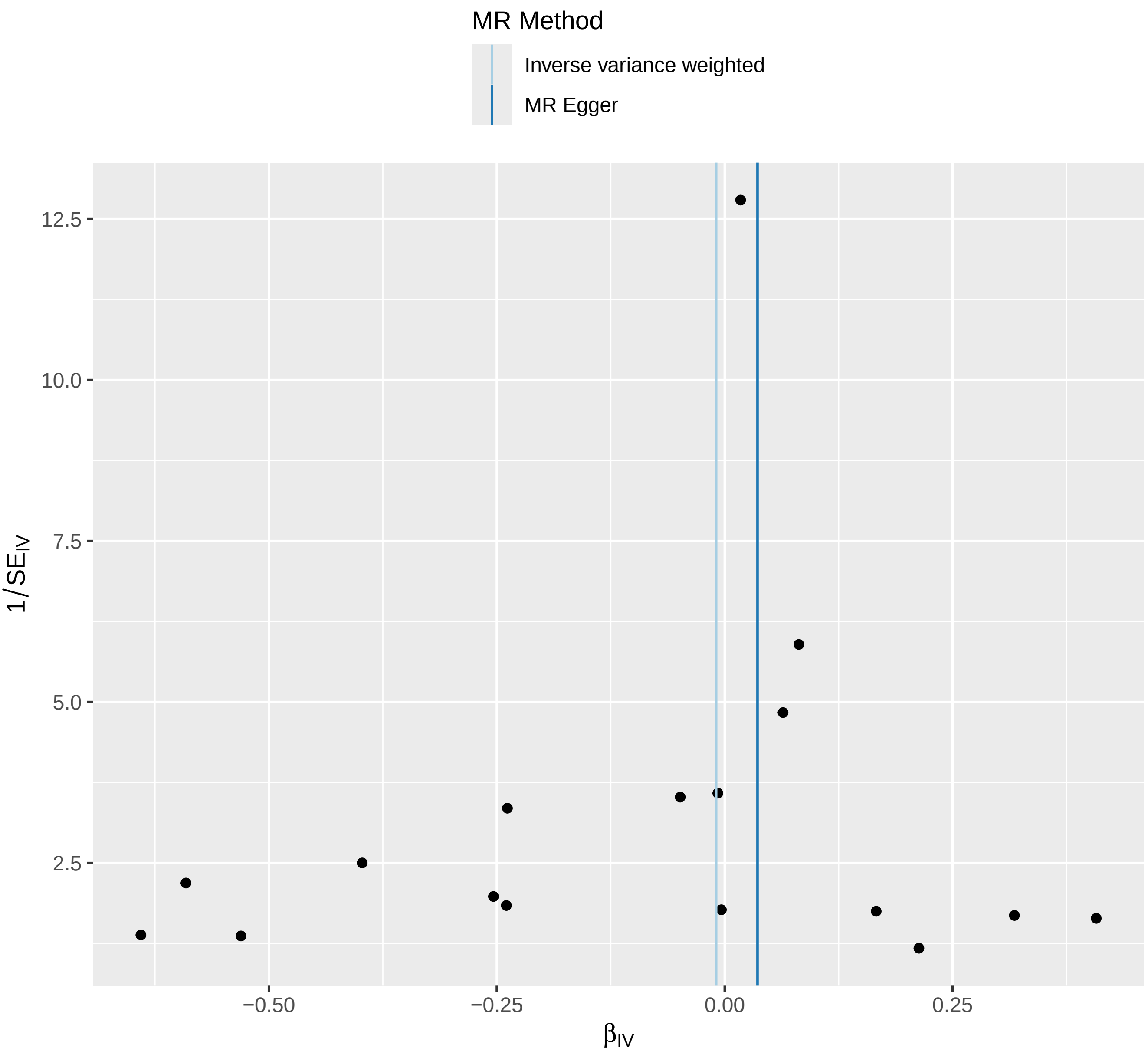

MR Method
Inverse variance weighted
MR Egger
12.5
10.0
7.5
5.0
2.5
1/SE_IV
−0.50
−0.25
0.00
0.25
β_IV

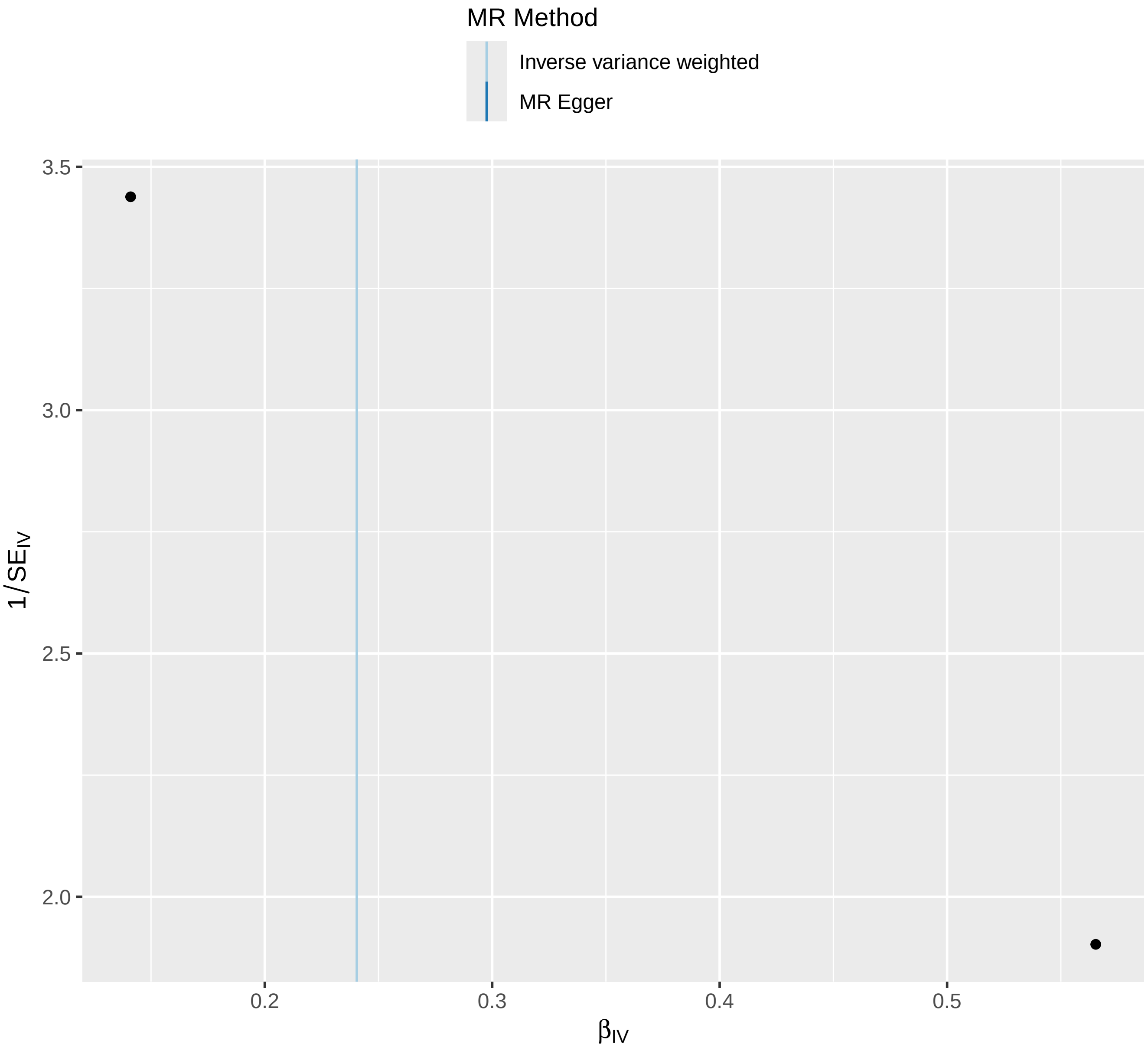
MR Method
Inverse variance weighted
MR Egger
3.5
3.0
2.5
2.0
1/SE_IV
0.2
0.3
0.4
0.5
β_IV

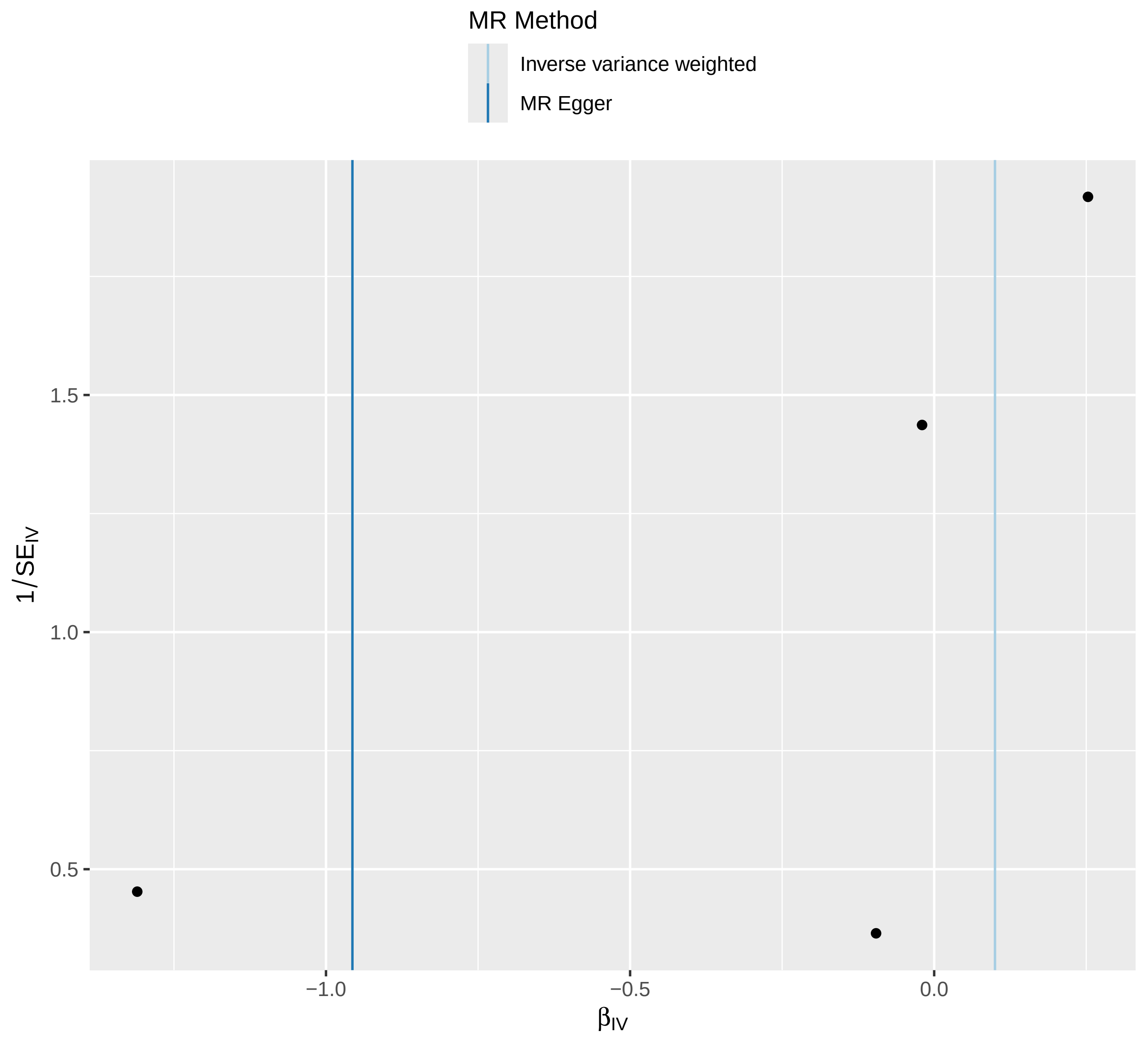

MR Method
Inverse variance weighted
MR Egger
1.5
1.0
0.5
1/SE$_{IV}$
−1.0
−0.5
0.0
β$_{IV}$

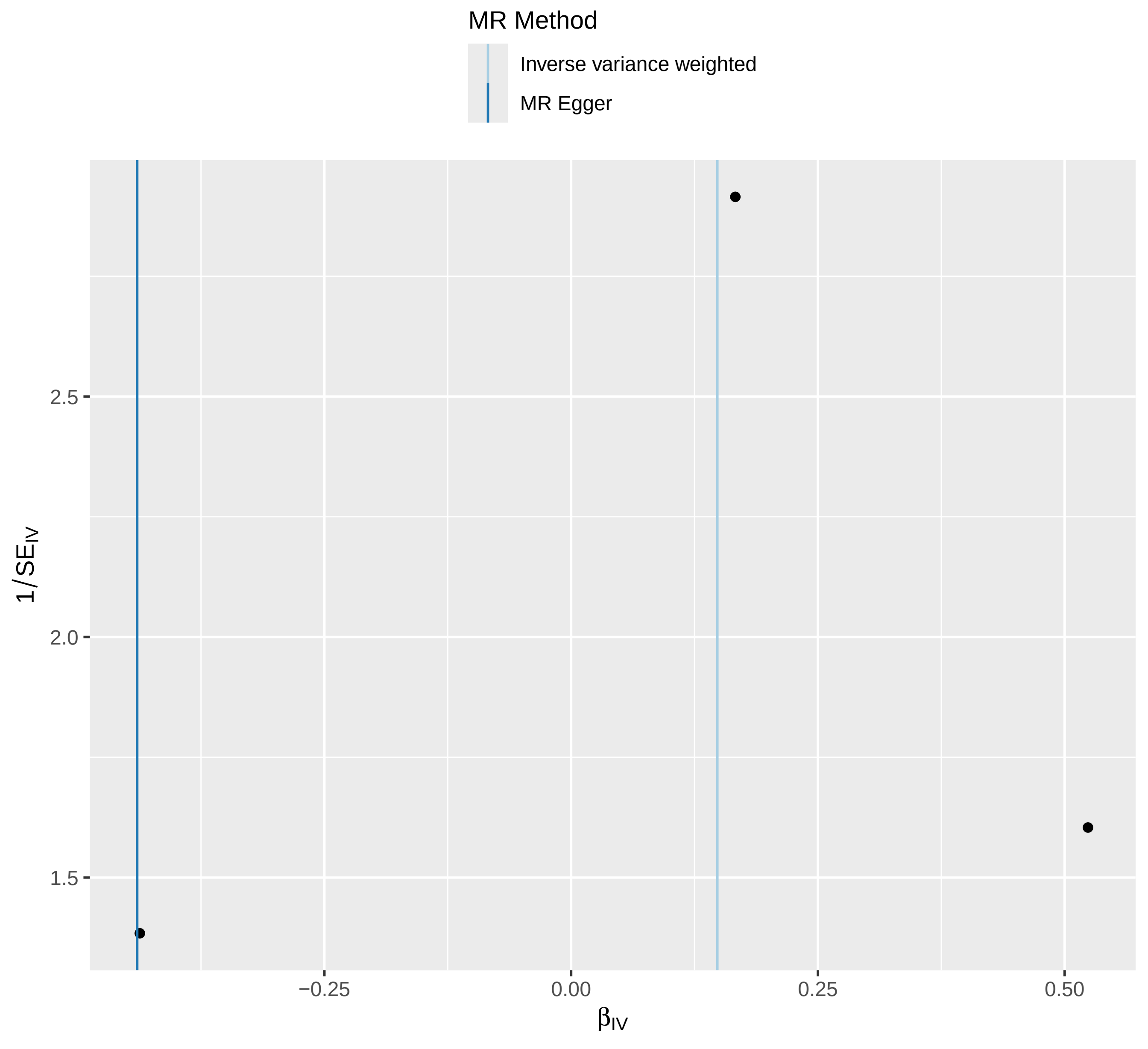

MR Method
Inverse variance weighted
MR Egger
2.5
2.0
1.5
1/SE_IV
−0.25
0.00
0.25
0.50
β_IV

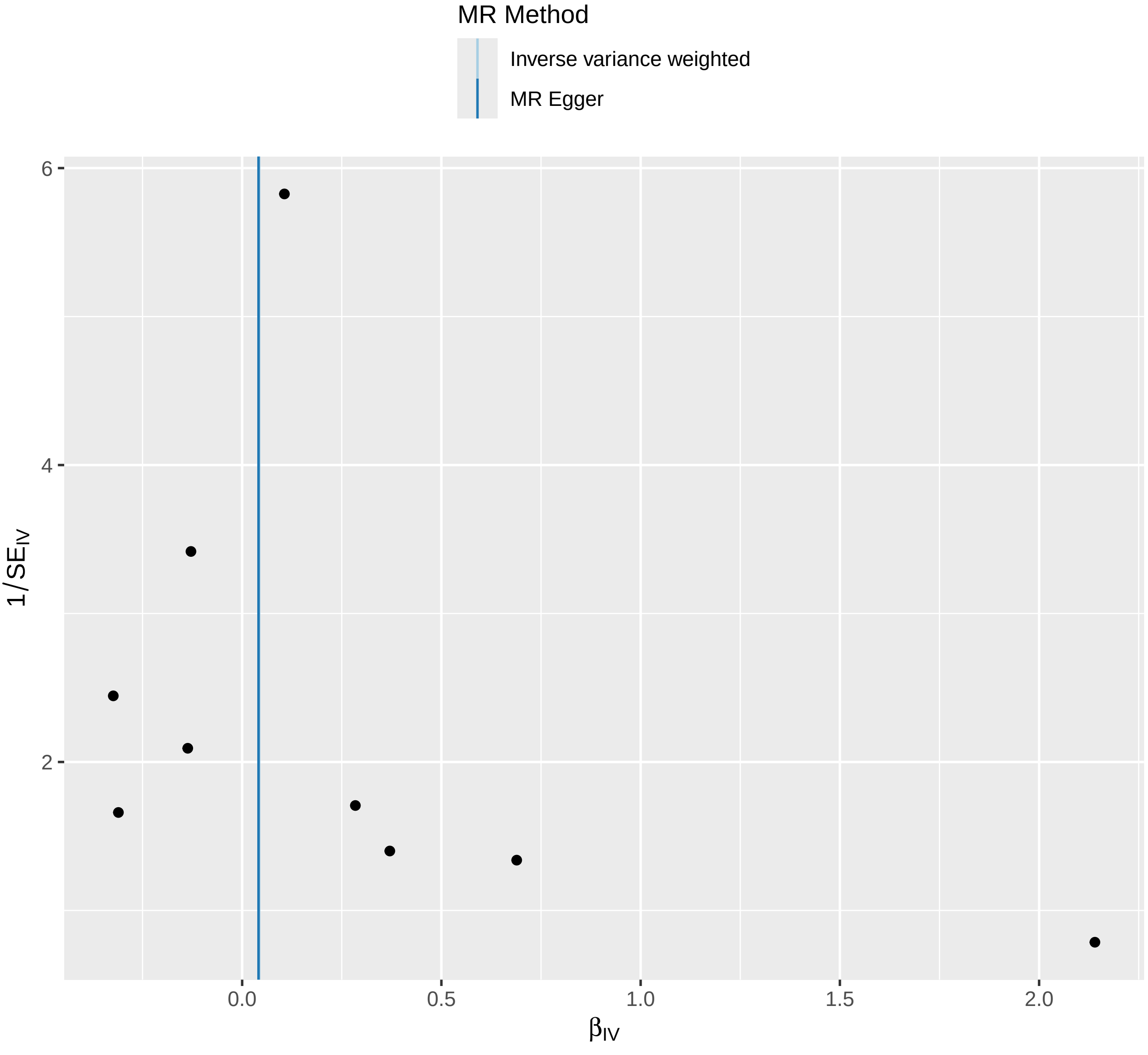

MR Method
Inverse variance weighted
MR Egger
6
4
2
0.0
0.5
1.0
1.5
2.0
$1/SE_{IV}$
β_{IV}

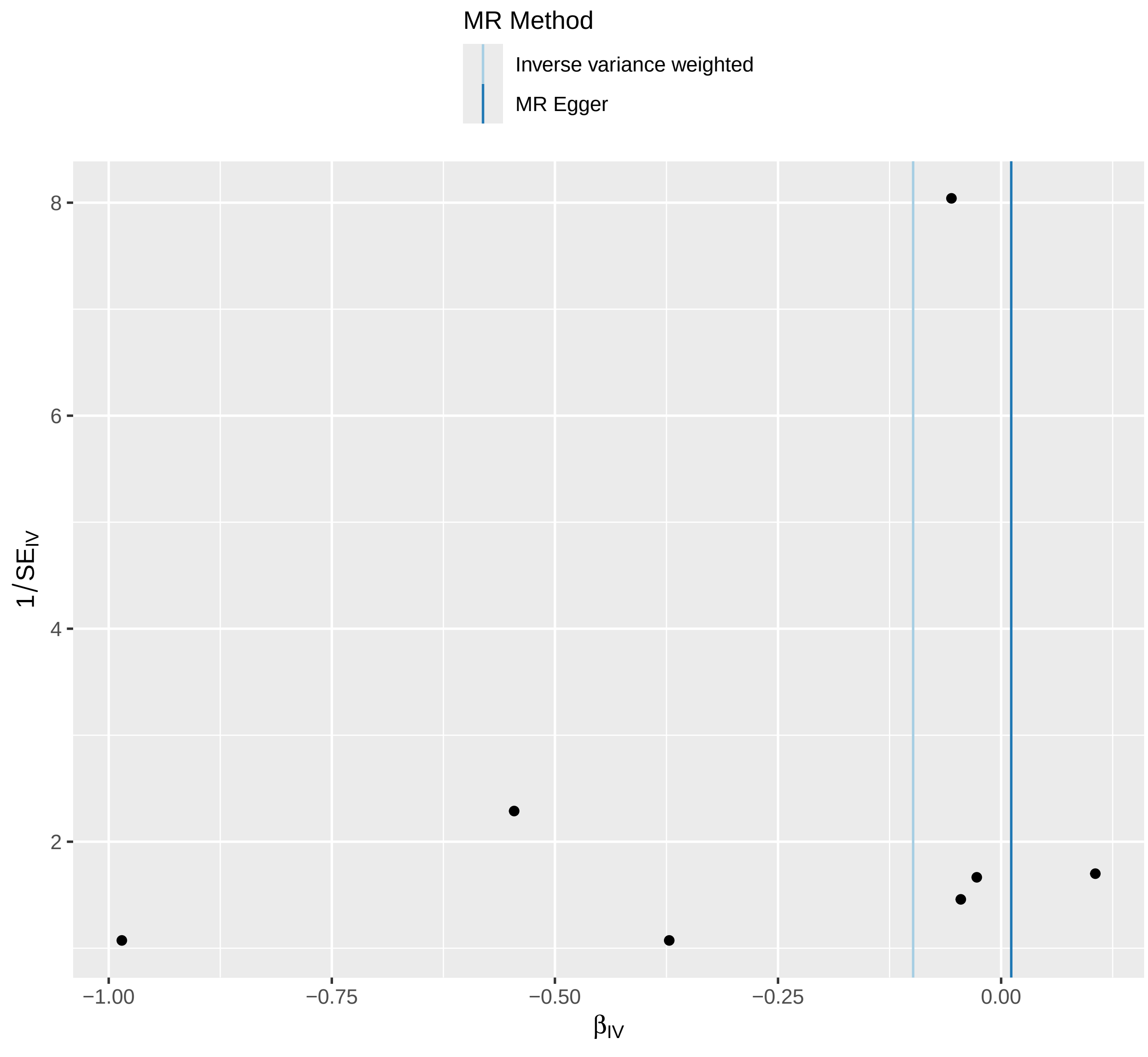
MR Method
Inverse variance weighted
MR Egger
8
6
4
2
1/SE_IV
−1.00
−0.75
−0.50
−0.25
0.00
β_IV

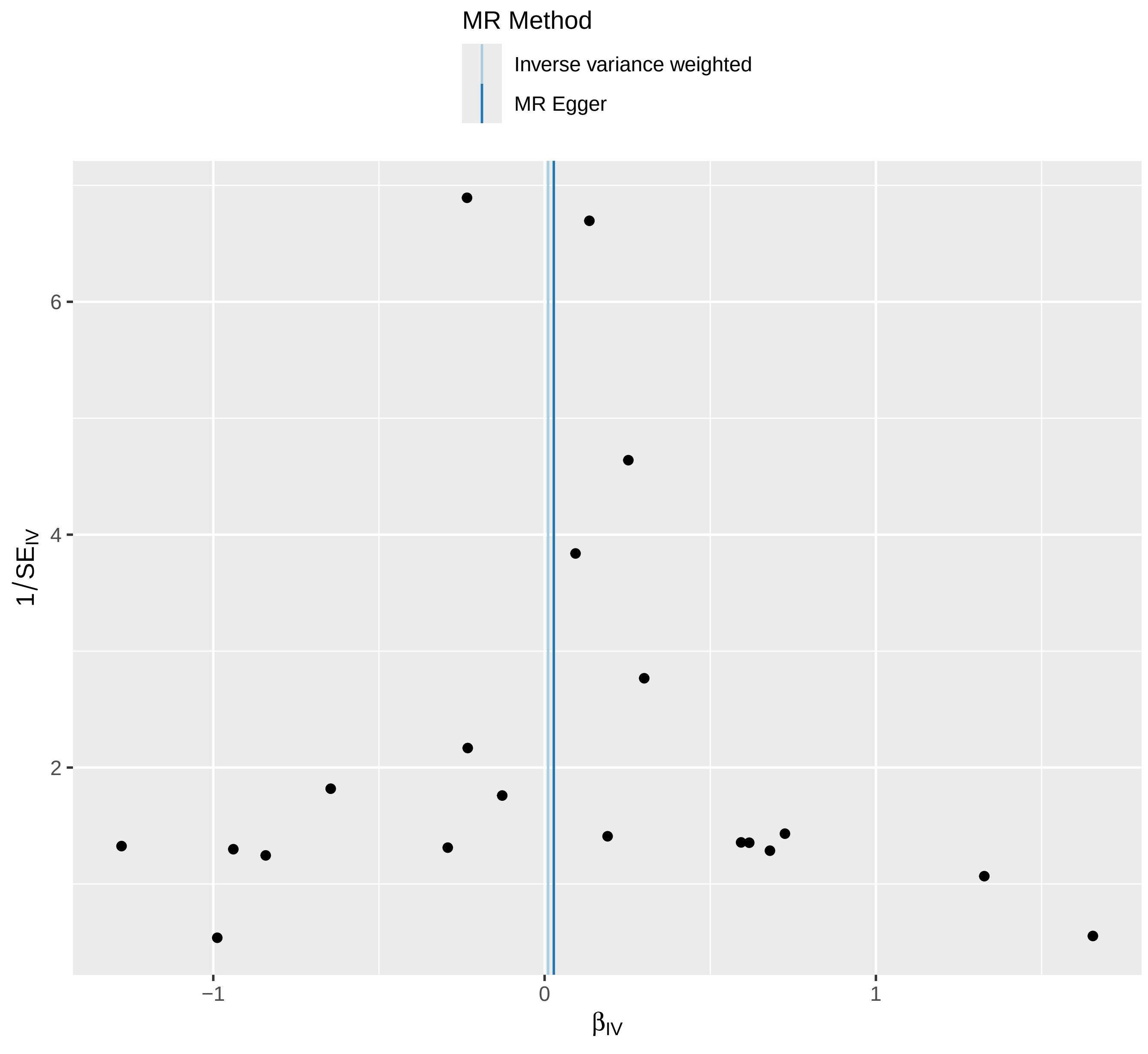
MR Method
Inverse variance weighted
MR Egger
6
4
2
1/SE_IV
−1
0
1
β_IV

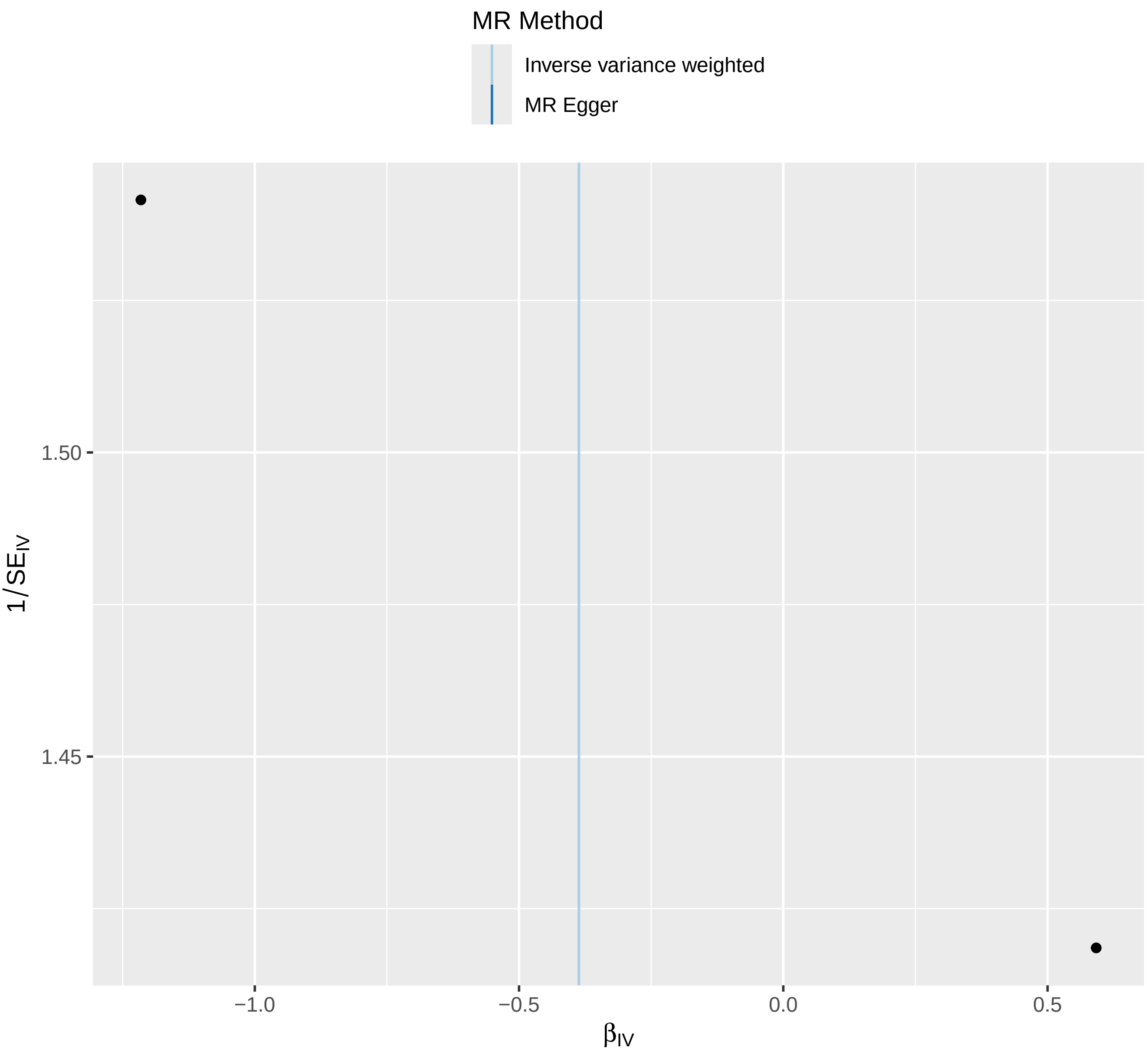
MR Method
Inverse variance weighted
MR Egger
1.50
1.45
$1/SE_{IV}$
−1.0
−0.5
0.0
0.5
β_{IV}

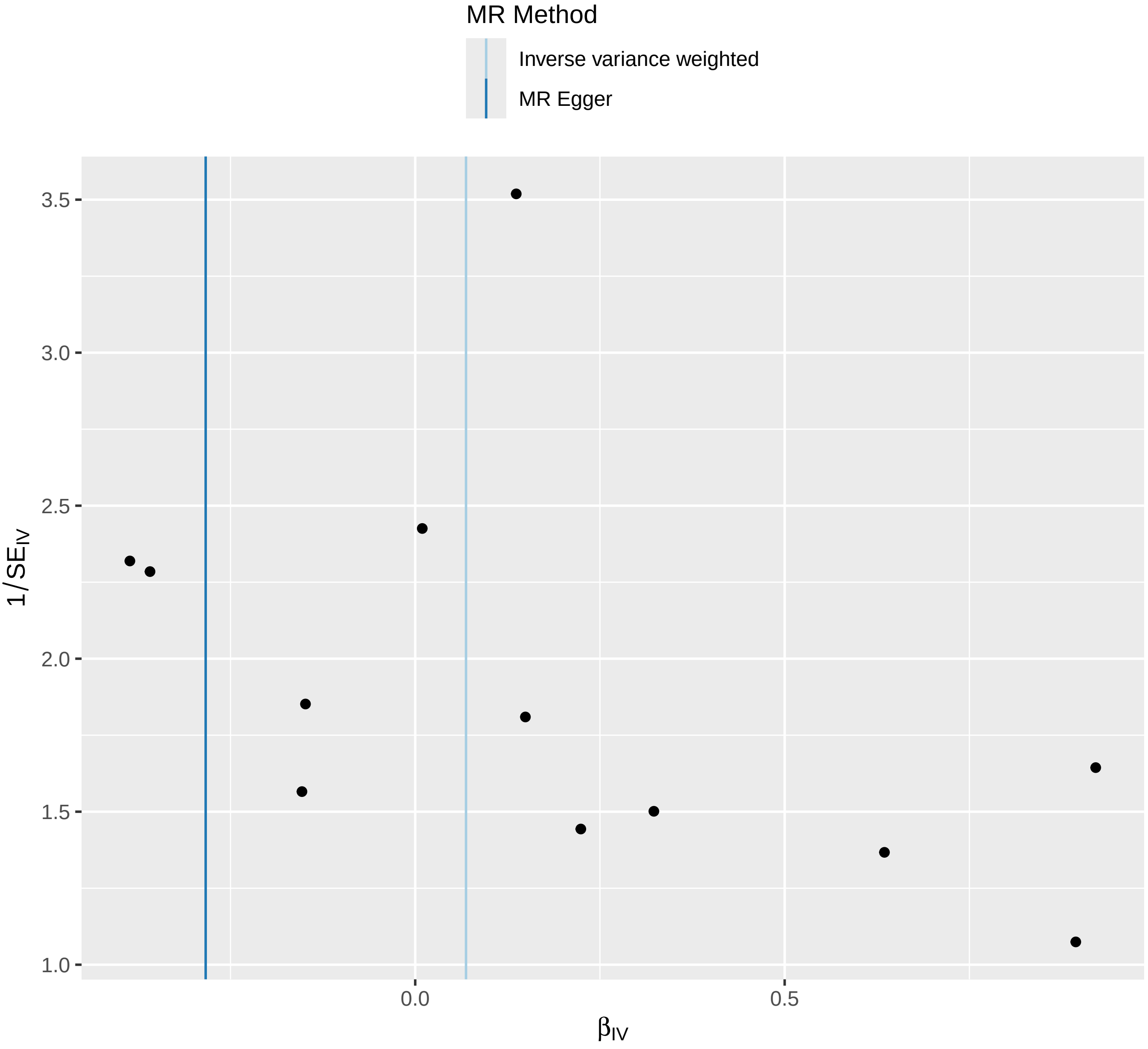

MR Method
Inverse variance weighted
MR Egger
3.5
3.0
2.5
2.0
1.5
1.0
$1/SE_{IV}$
0.0
0.5
β_{IV}

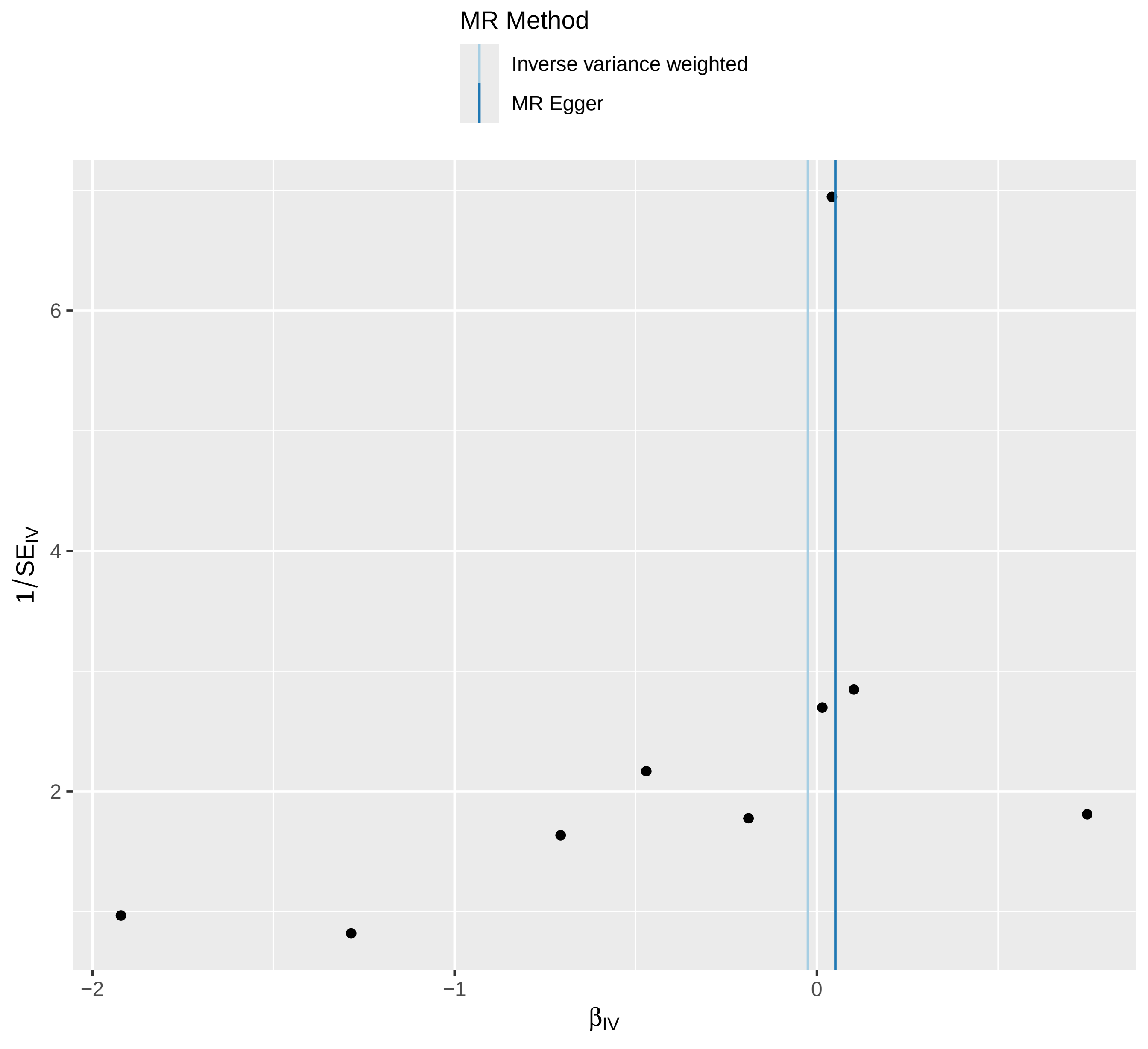
MR Method
Inverse variance weighted
MR Egger
6
4
2
1/SE_IV
−2
−1
0
β_IV

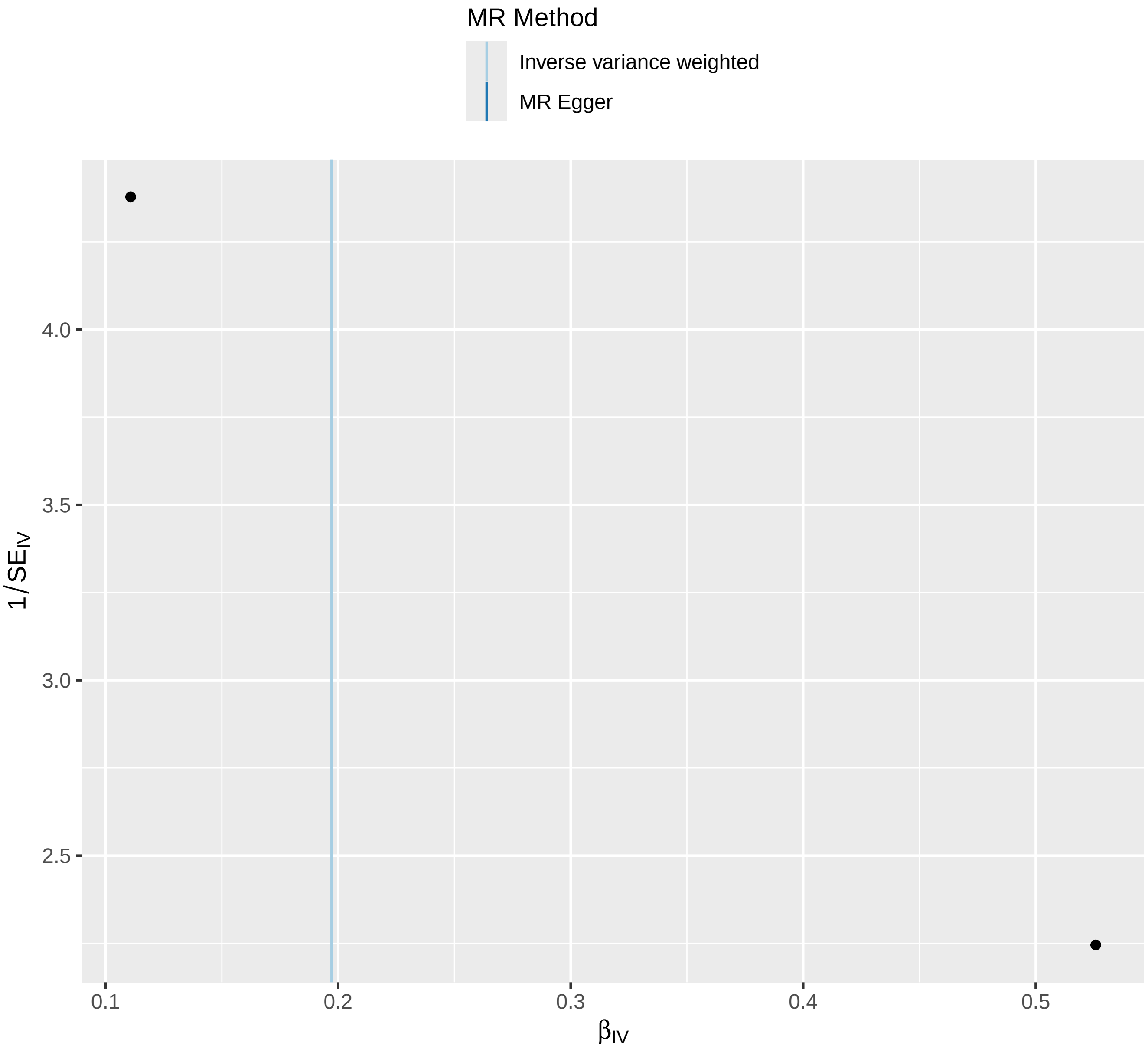

MR Method
Inverse variance weighted
MR Egger
4.0
3.5
3.0
2.5
1/SE_IV
0.1
0.2
0.3
0.4
0.5
β_IV

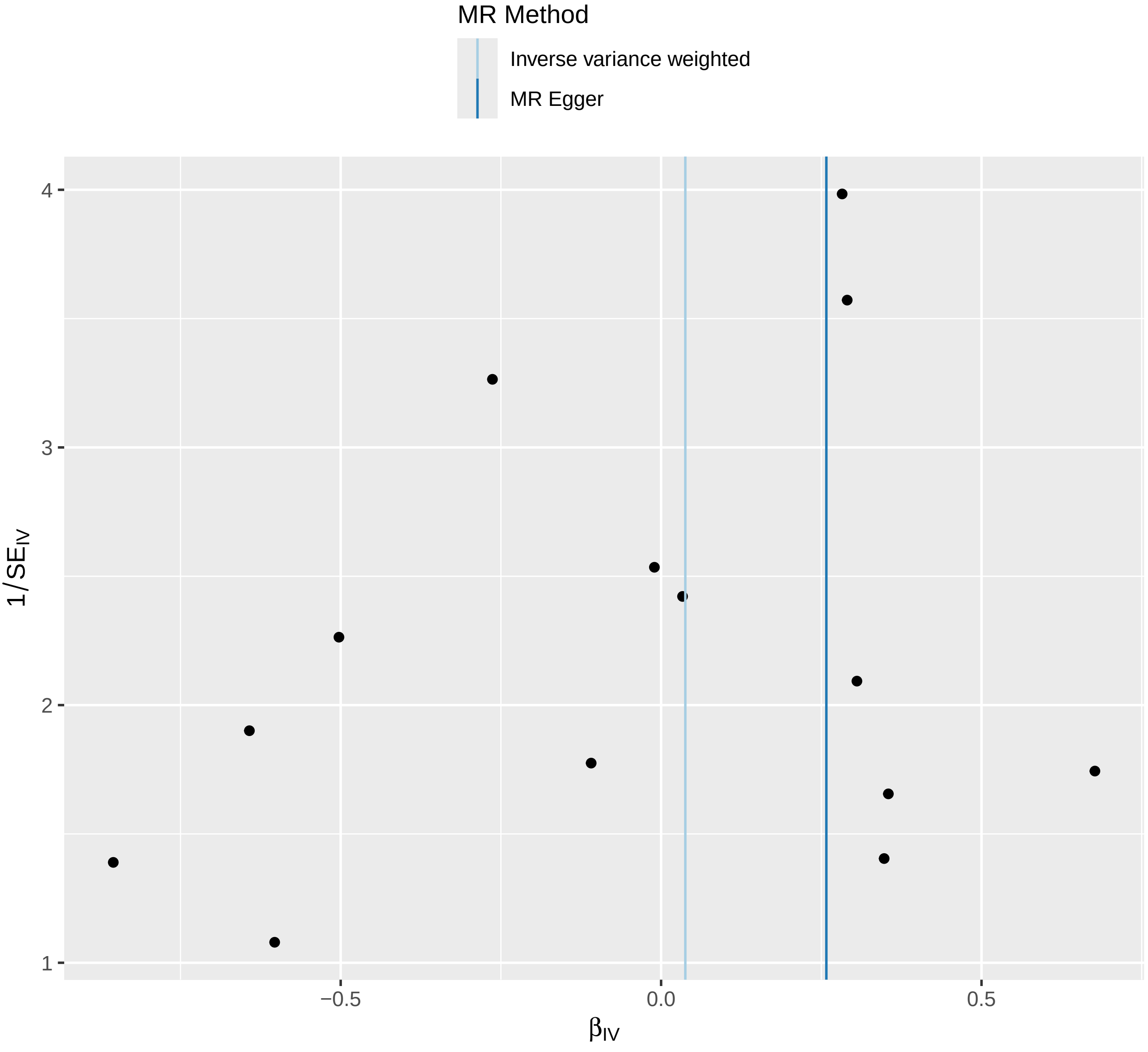
MR Method
Inverse variance weighted
MR Egger
1/SE_IV
4
3
2
1
−0.5
0.0
0.5
β_IV

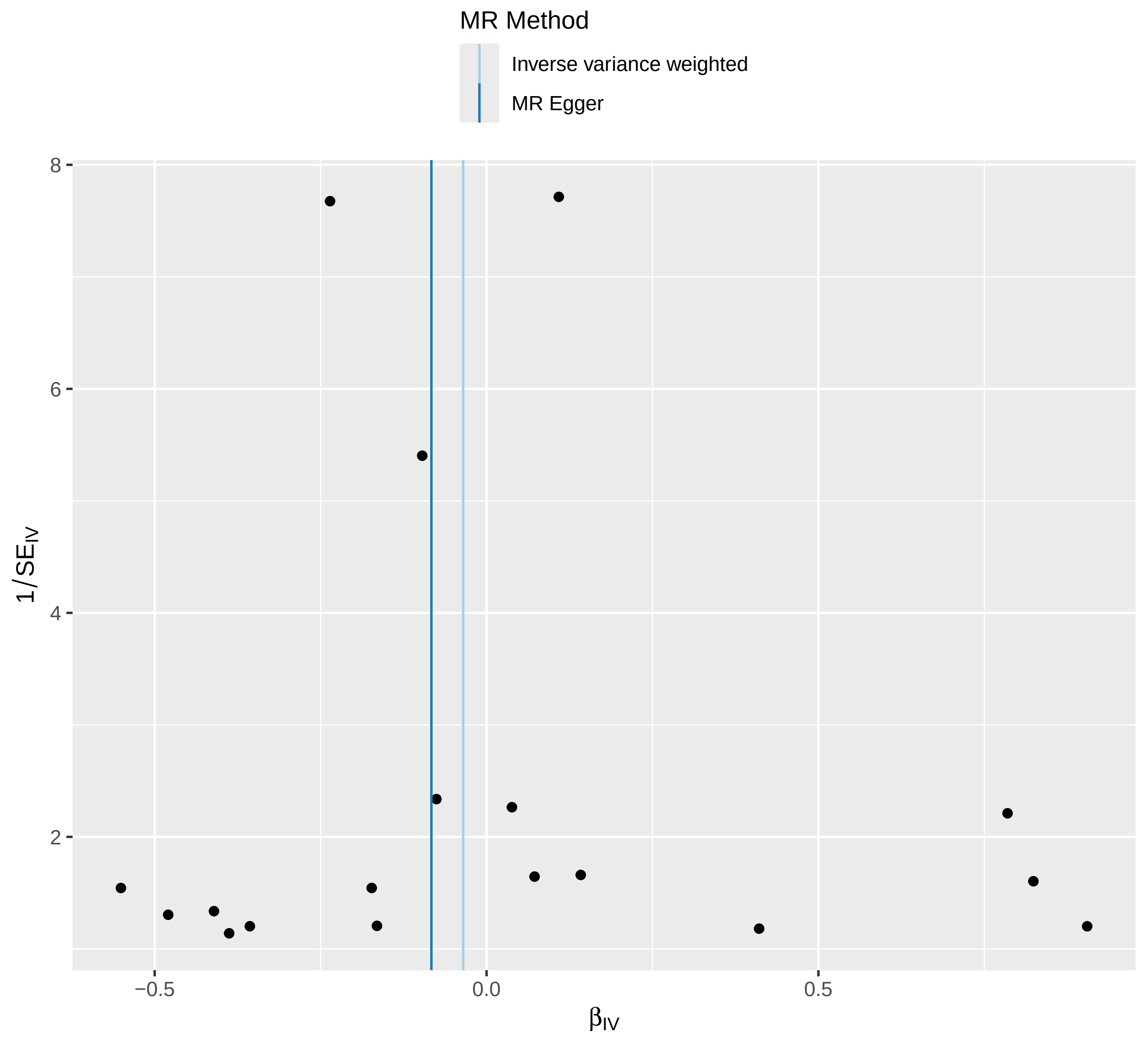
MR Method
Inverse variance weighted
MR Egger
8
6
4
2
1/SE$_{IV}$
−0.5
0.0
0.5
β$_{IV}$

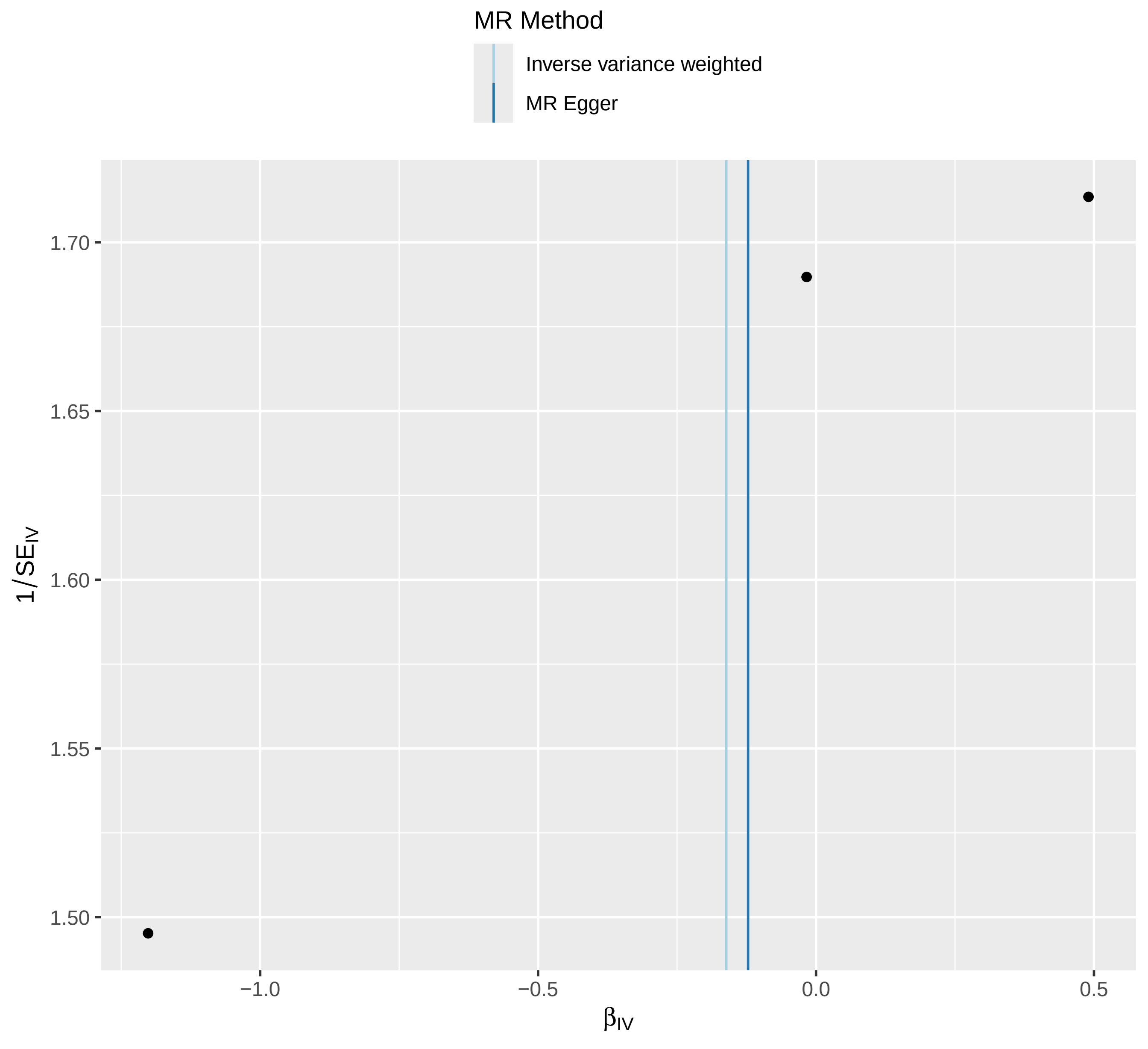

MR Method
Inverse variance weighted
MR Egger
1.70
1.65
1.60
1.55
1.50
$1/SE_{IV}$
−1.0
−0.5
0.0
0.5
β_{IV}

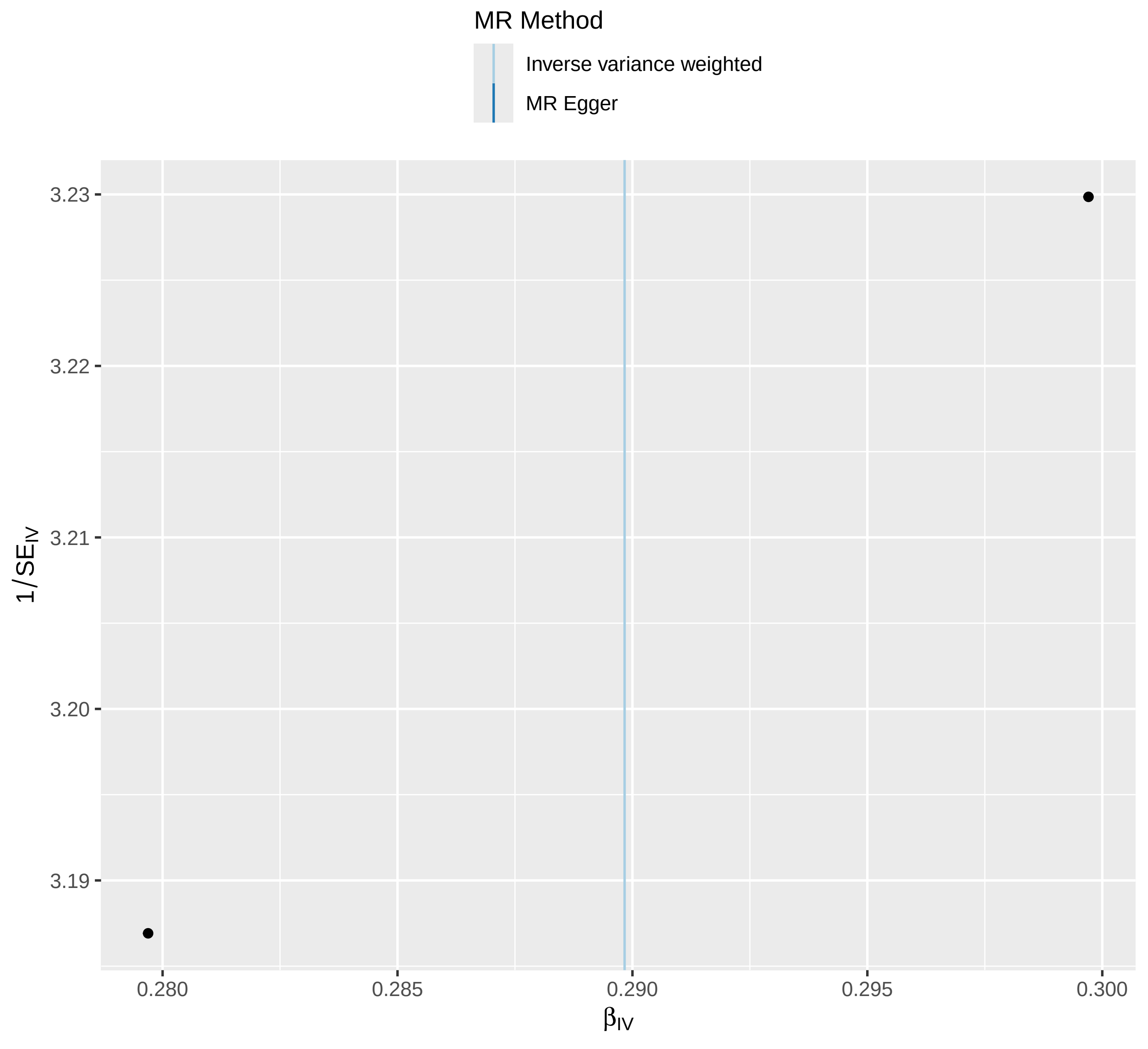
MR Method
Inverse variance weighted
MR Egger
3.23
3.22
3.21
3.20
3.19
$1/SE_{IV}$
0.280
0.285
0.290
0.295
0.300
β_{IV}

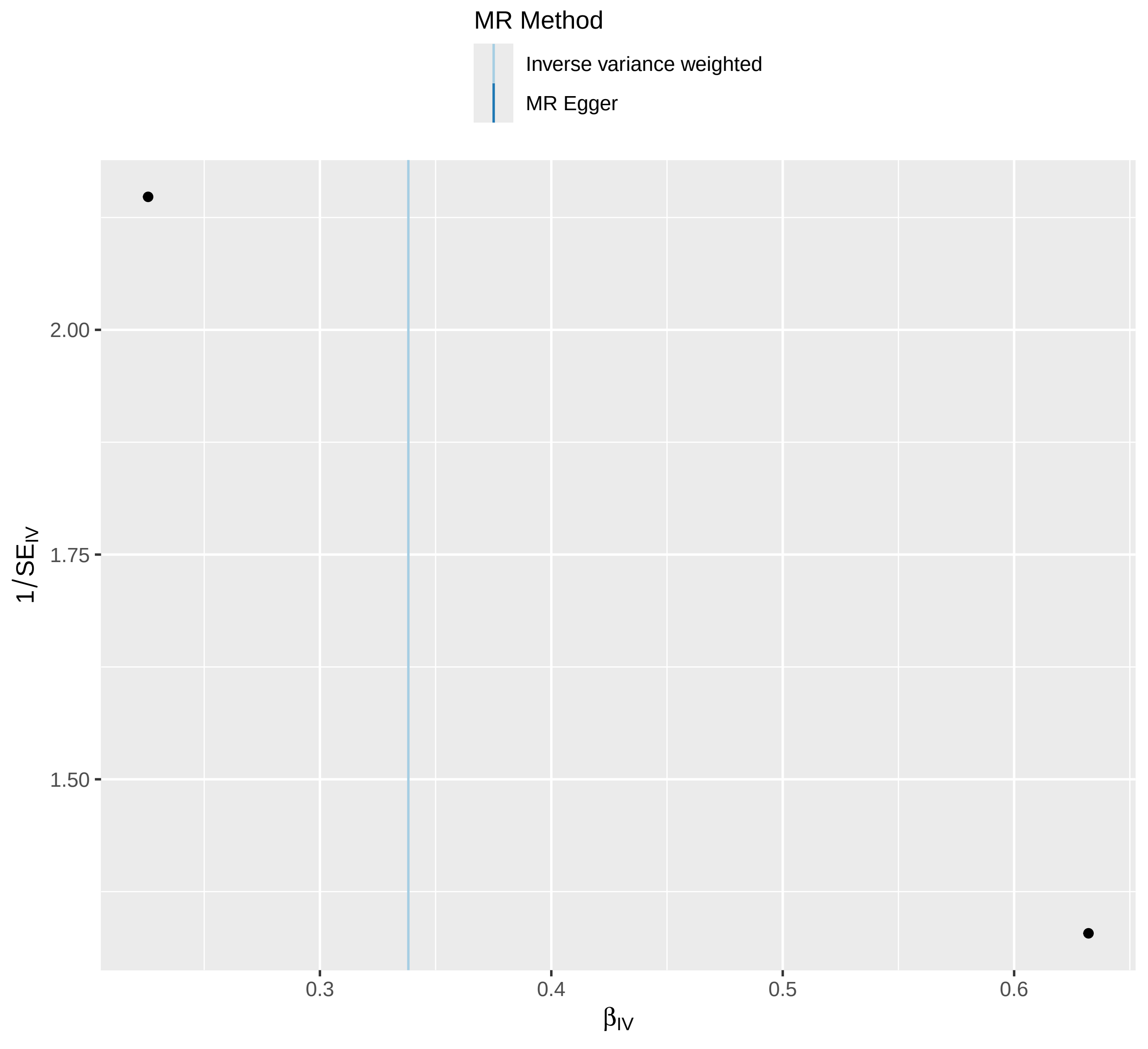

MR Method
Inverse variance weighted
MR Egger
2.00
1.75
1.50
$1/SE_{IV}$
0.3
0.4
0.5
0.6
β_{IV}

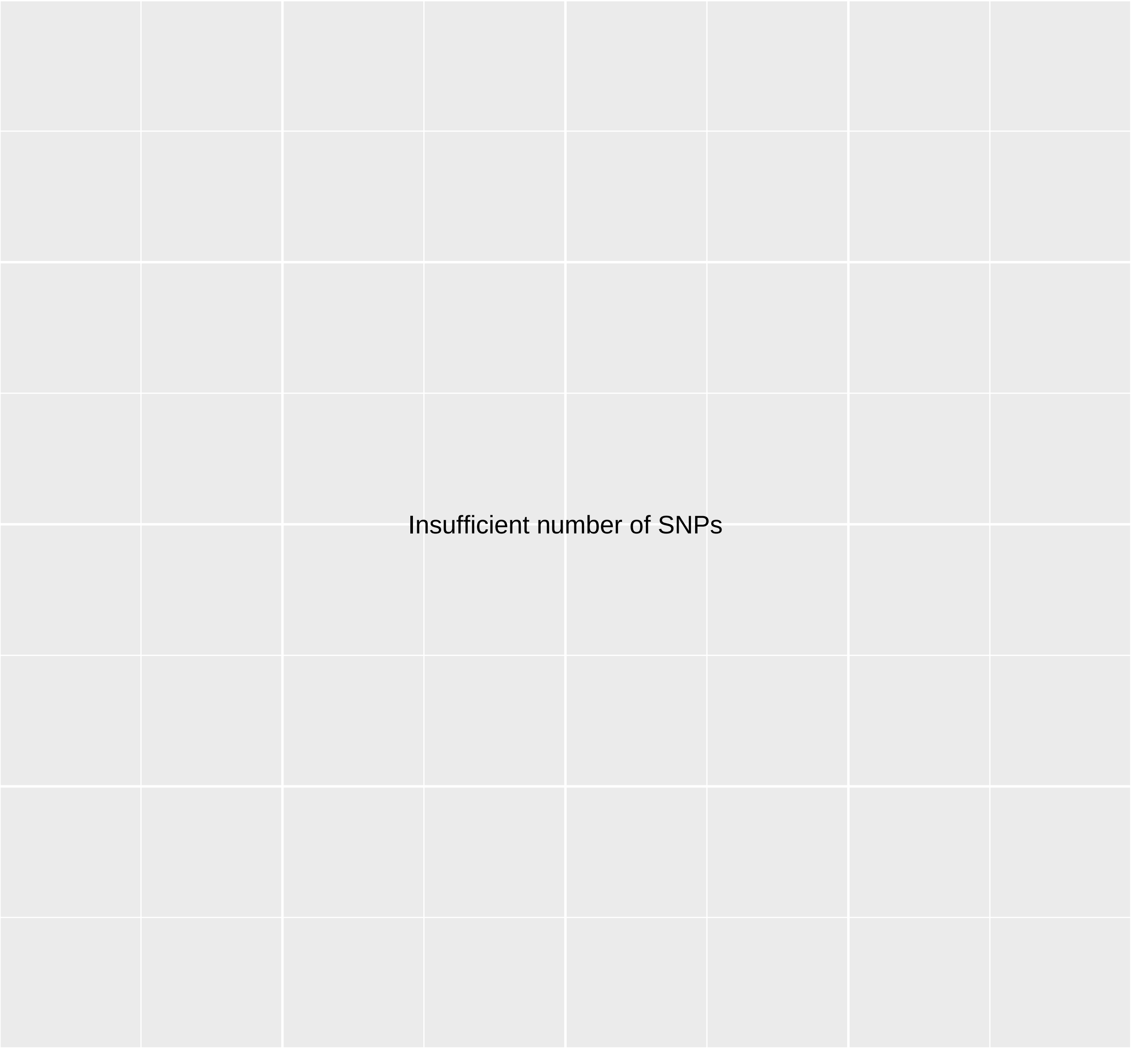
Insufficient number of SNPs

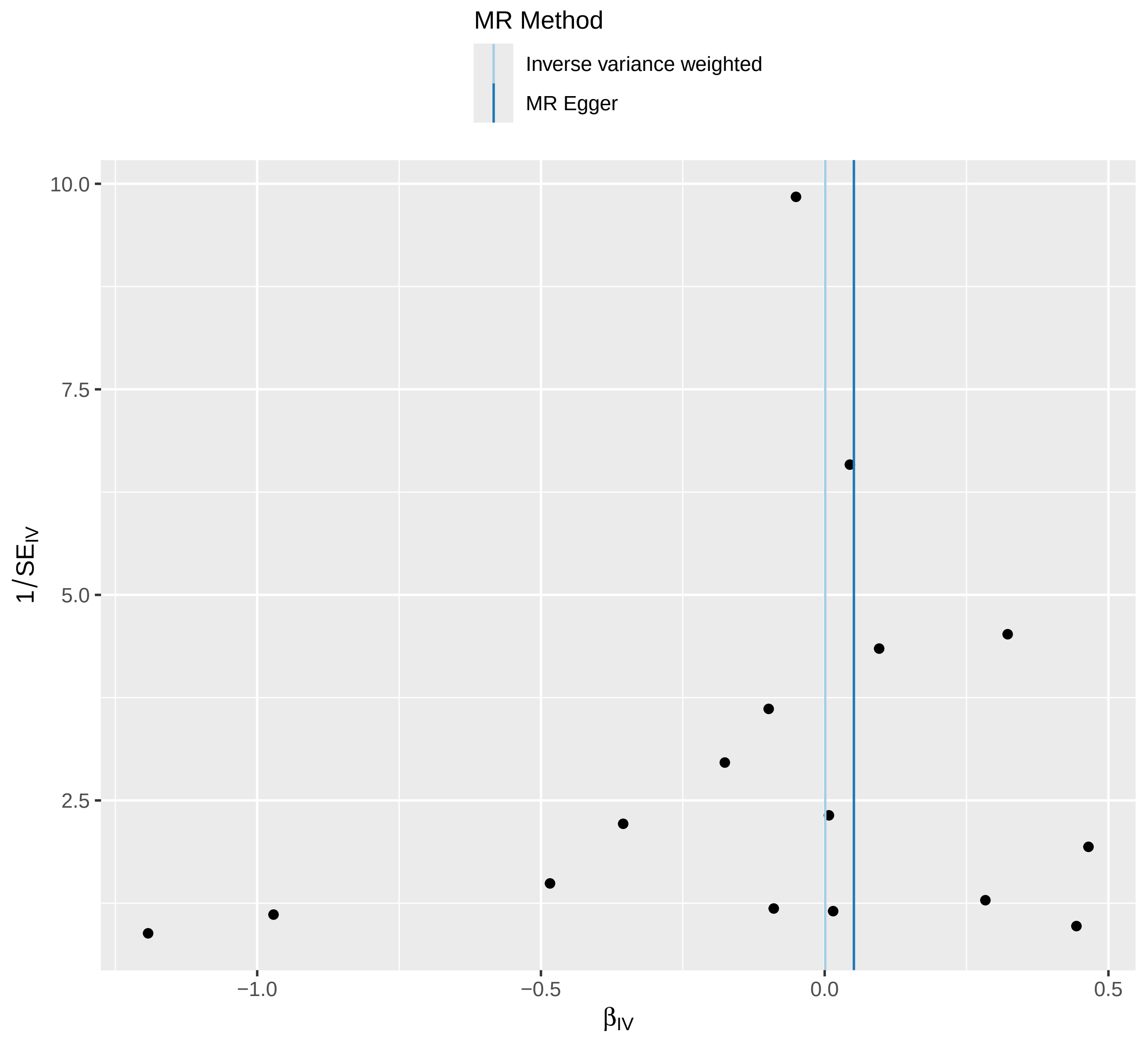
MR Method
Inverse variance weighted
MR Egger
10.0
7.5
5.0
2.5
1/SE_IV
−1.0
−0.5
0.0
0.5
β_IV

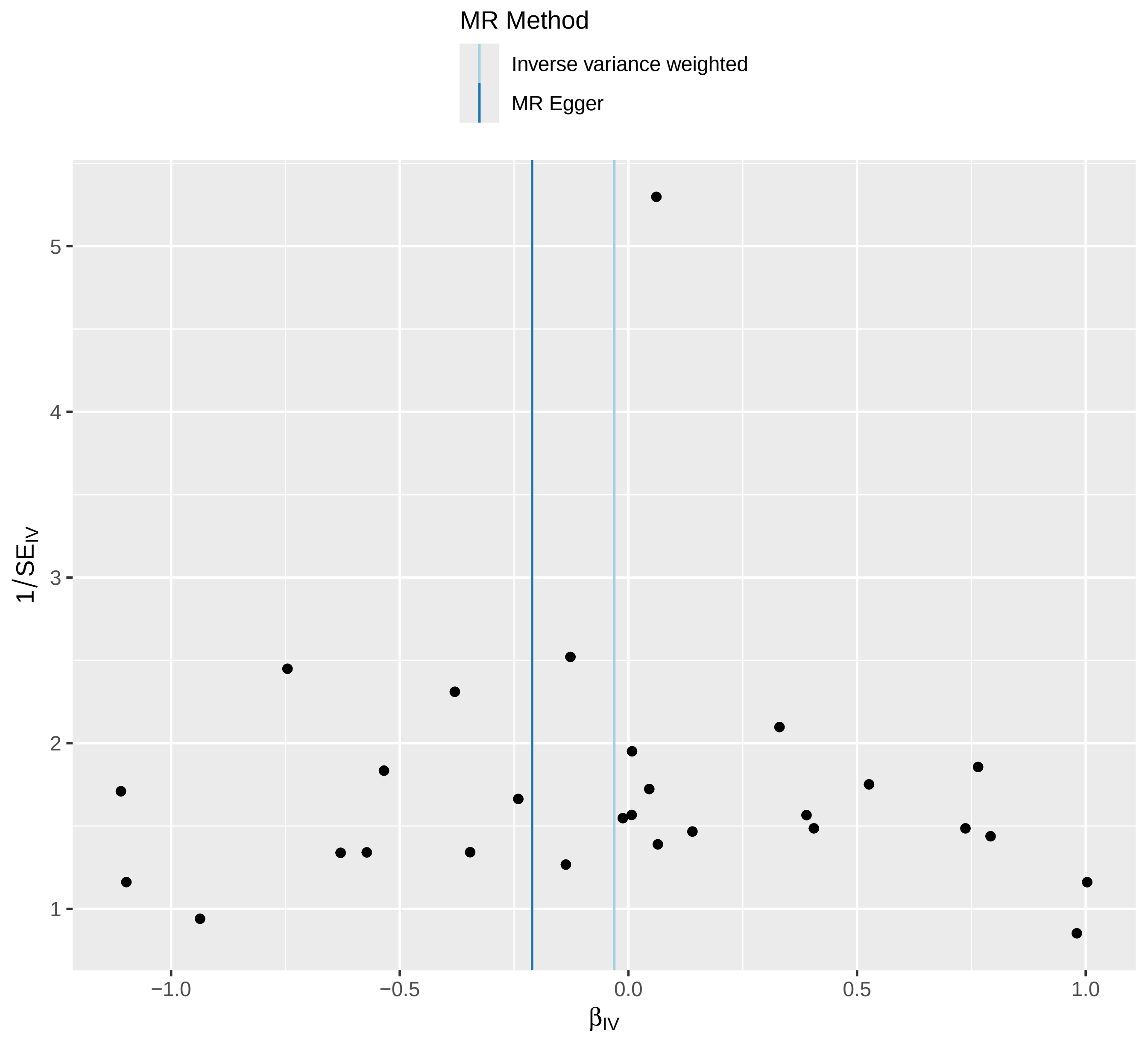
MR Method
Inverse variance weighted
MR Egger
1/SE$_{IV}$
5
4
3
2
1
−1.0
−0.5
0.0
0.5
1.0
β$_{IV}$

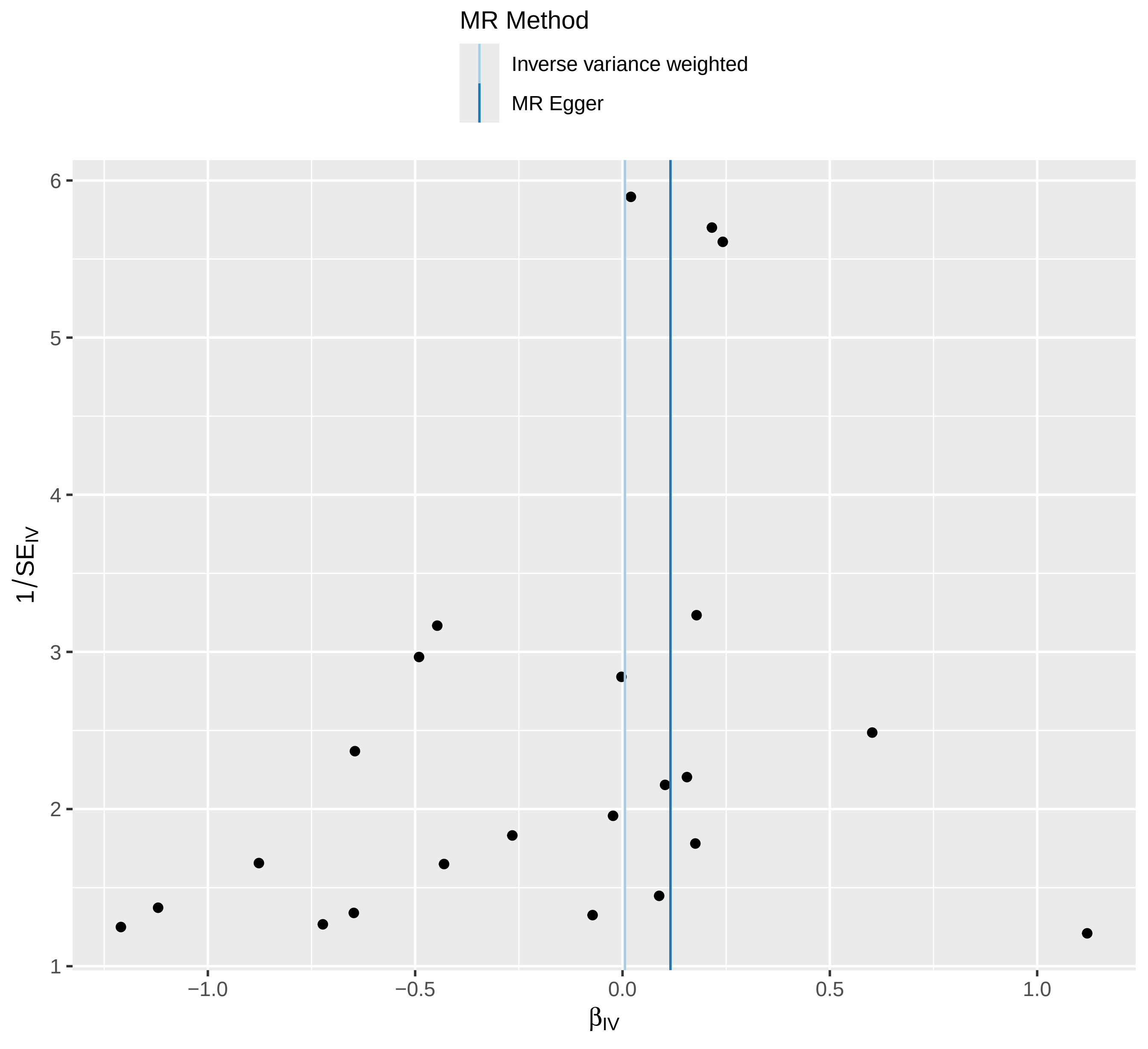
MR Method
Inverse variance weighted
MR Egger
6
5
4
3
2
1
1/SE_IV
−1.0
−0.5
0.0
0.5
1.0
β_IV

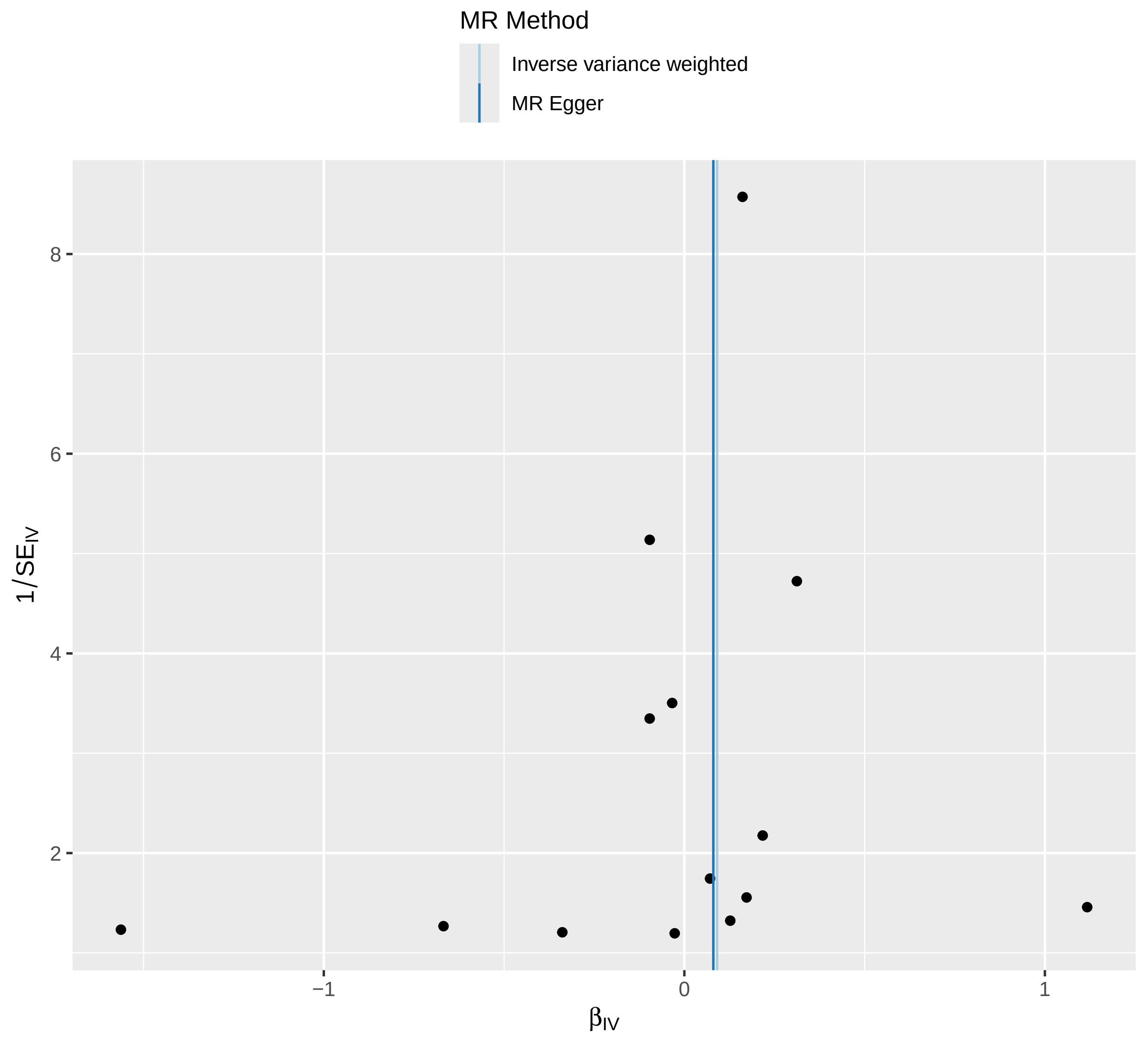
MR Method
Inverse variance weighted
MR Egger
8
6
4
2
1/SE_IV
−1
0
1
β_IV

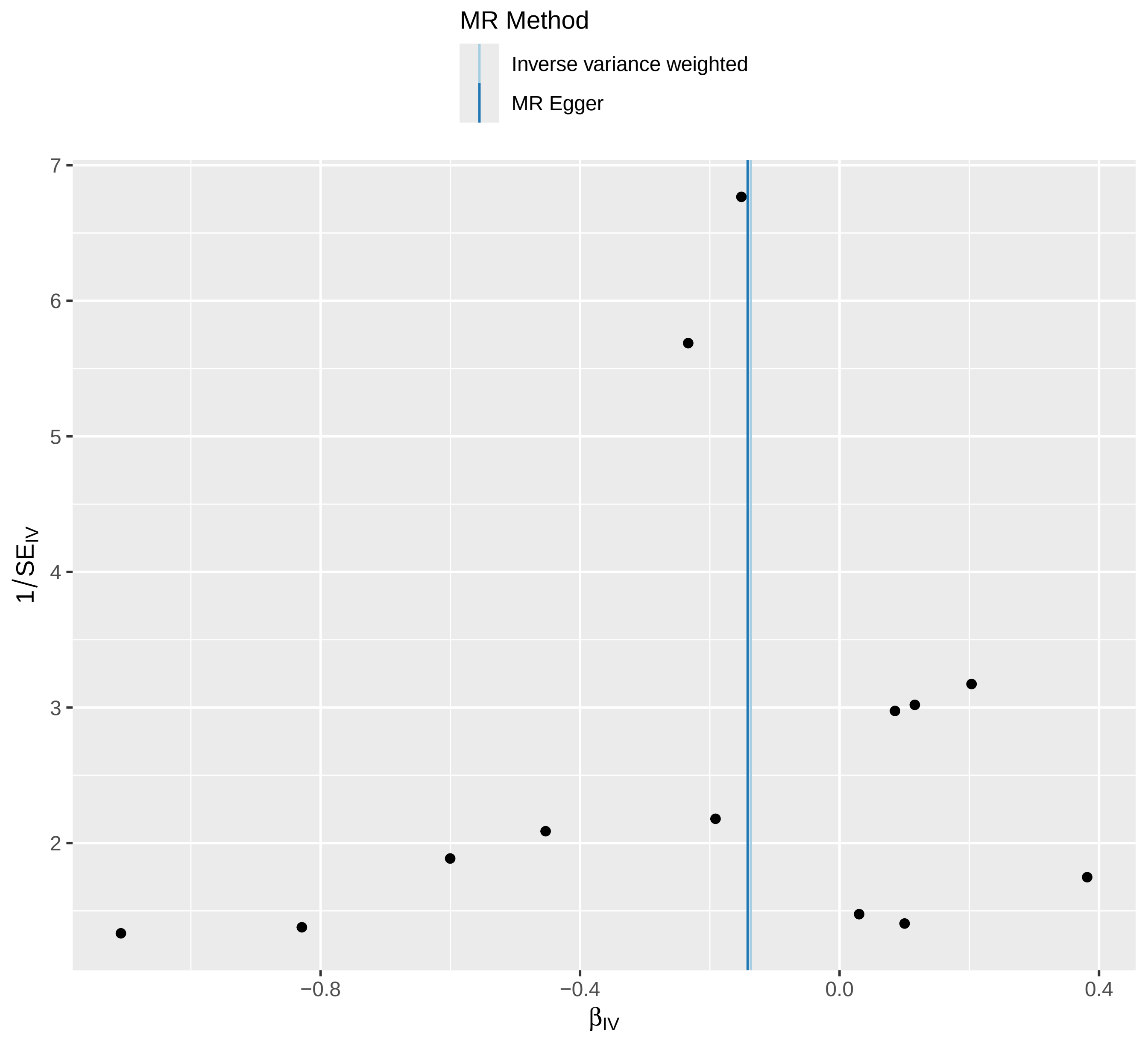
MR Method
Inverse variance weighted
MR Egger
7
6
5
4
3
2
1/SE$_{IV}$
−0.8
−0.4
0.0
0.4
β$_{IV}$

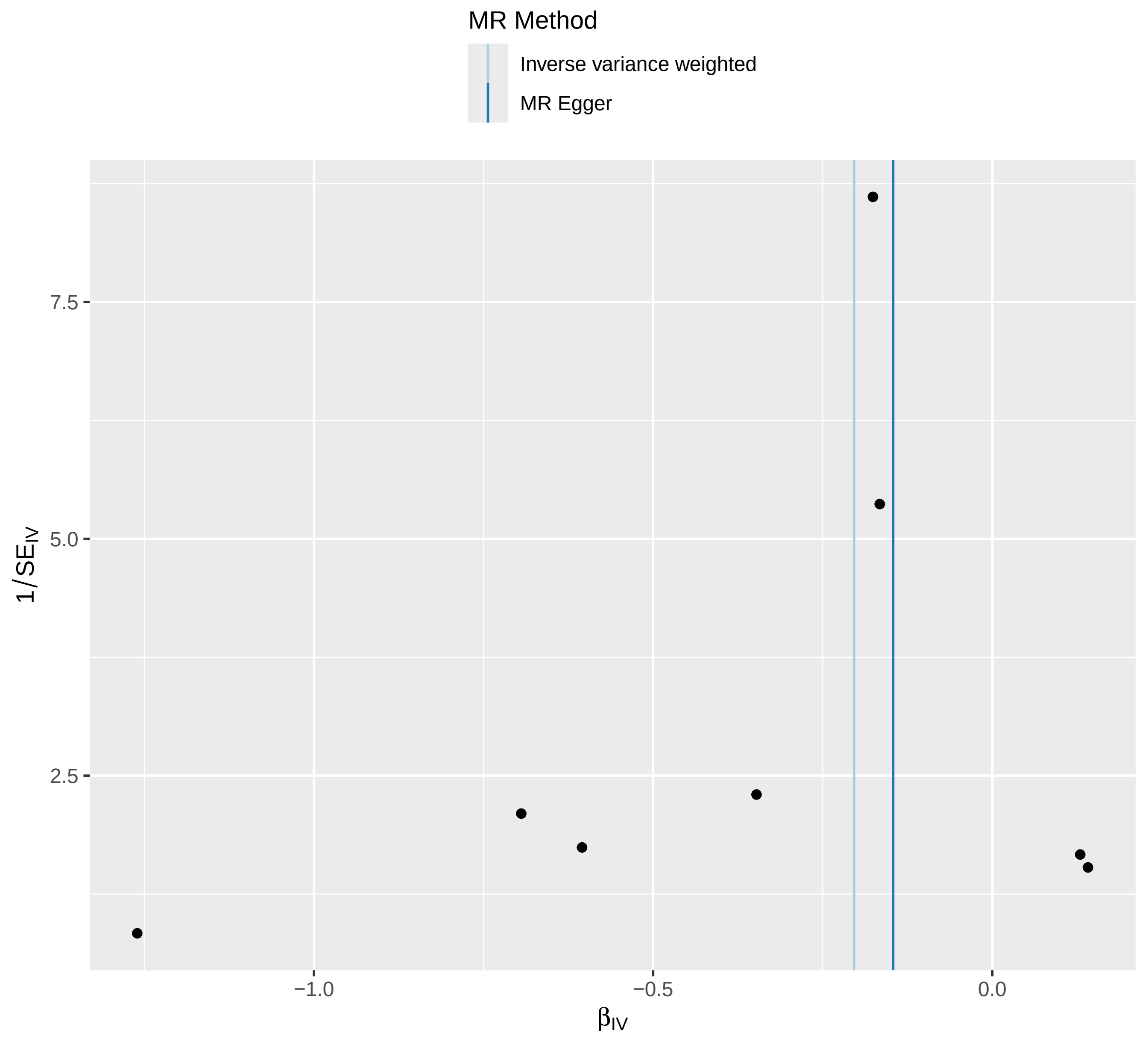
MR Method
Inverse variance weighted
MR Egger
7.5
5.0
2.5
1/SE_IV
−1.0
−0.5
0.0
β_IV

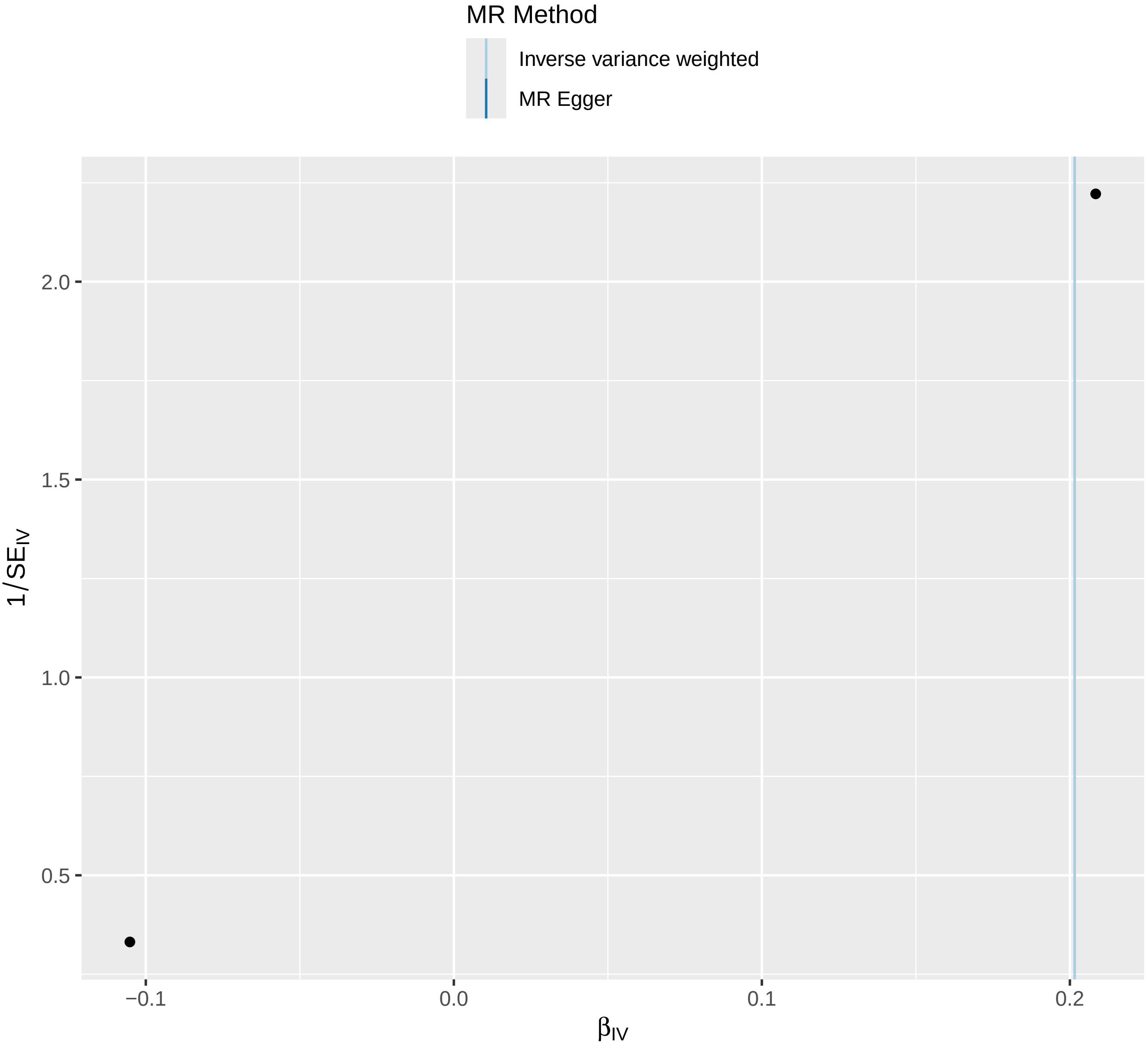
MR Method
Inverse variance weighted
MR Egger
2.0
1.5
1.0
0.5
1/SE_IV
−0.1
0.0
0.1
0.2
β_IV

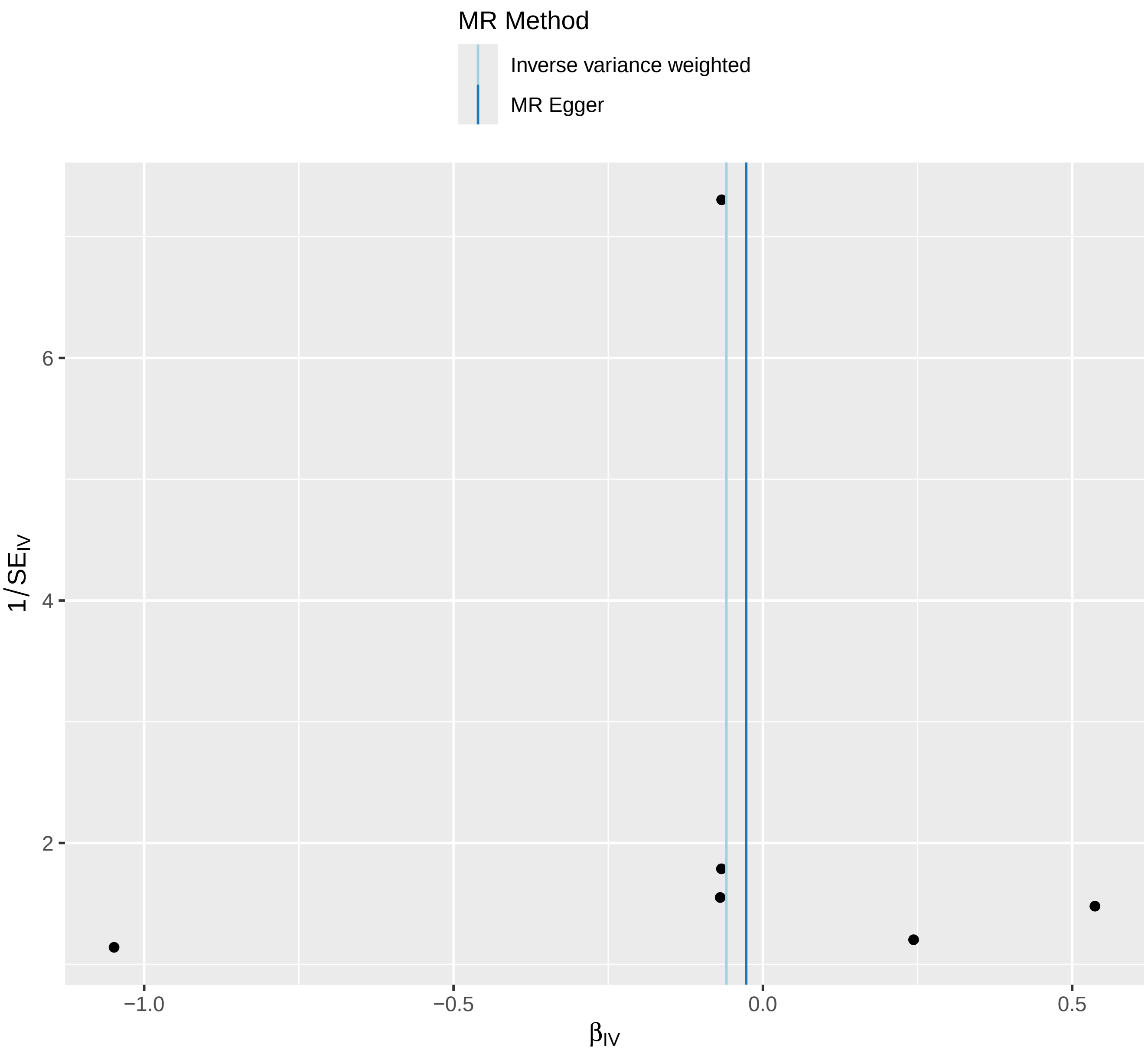
MR Method
Inverse variance weighted
MR Egger
1/SE$_{IV}$
6
4
2
−1.0
−0.5
0.0
0.5
β$_{IV}$

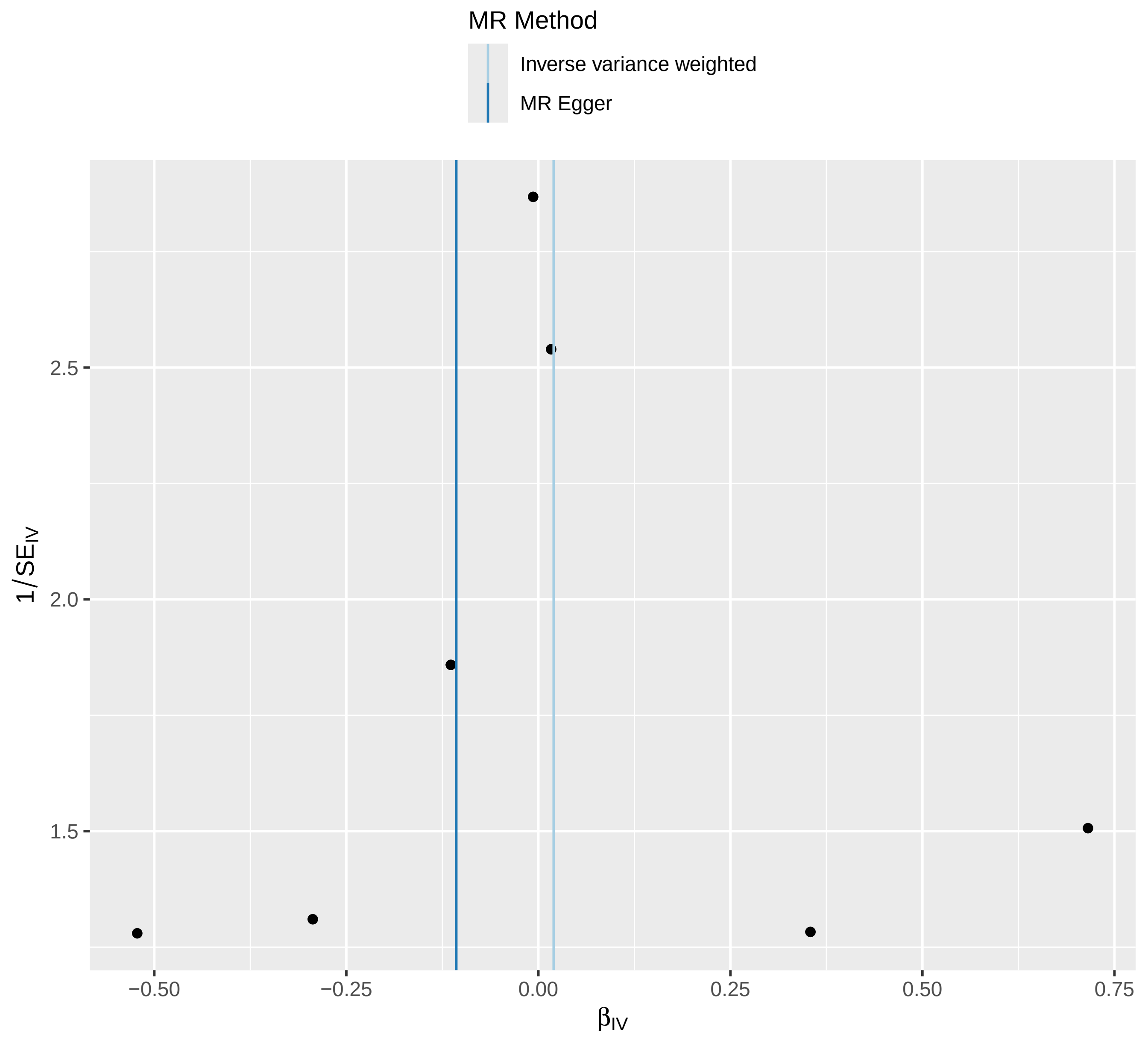

MR Method
Inverse variance weighted
MR Egger
2.5
2.0
1.5
$1/SE_{IV}$
−0.50
−0.25
0.00
0.25
0.50
0.75
β_{IV}

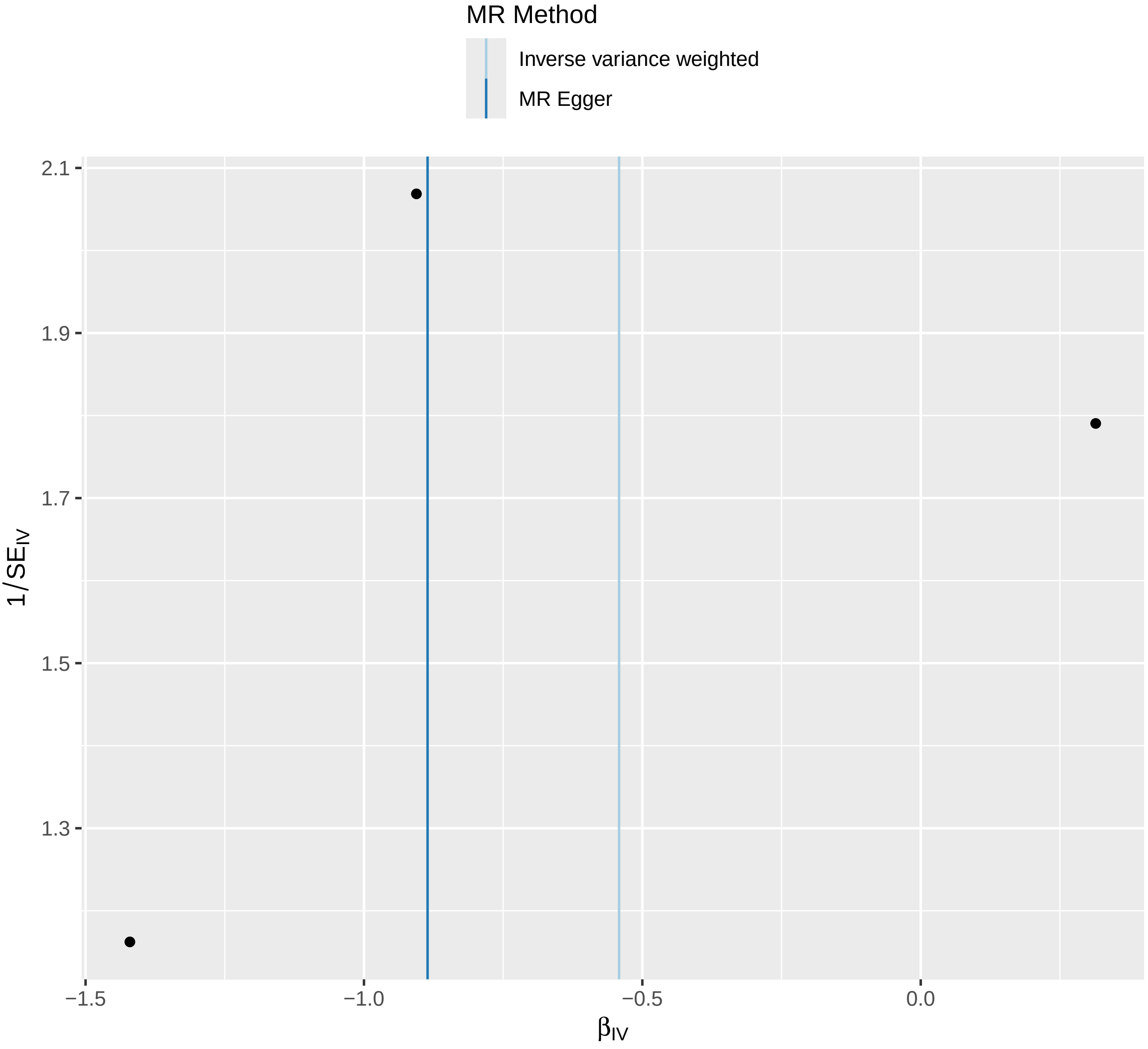
MR Method
Inverse variance weighted
MR Egger
2.1
1.9
1.7
1.5
1.3
1/SE_IV
−1.5
−1.0
−0.5
0.0
β_IV

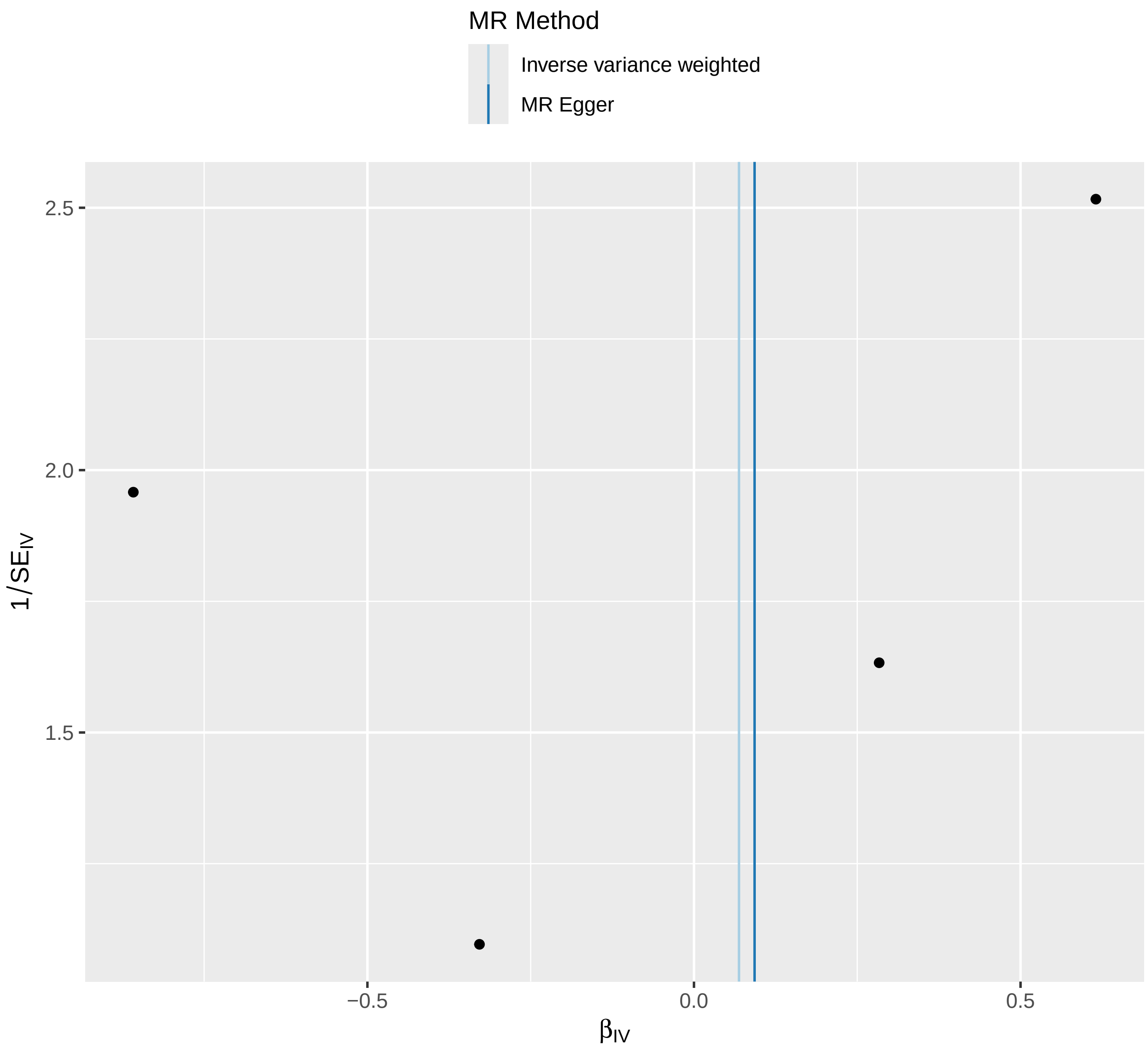

MR Method
Inverse variance weighted
MR Egger
2.5
2.0
1.5
1/SE_IV
−0.5
0.0
0.5
β_IV

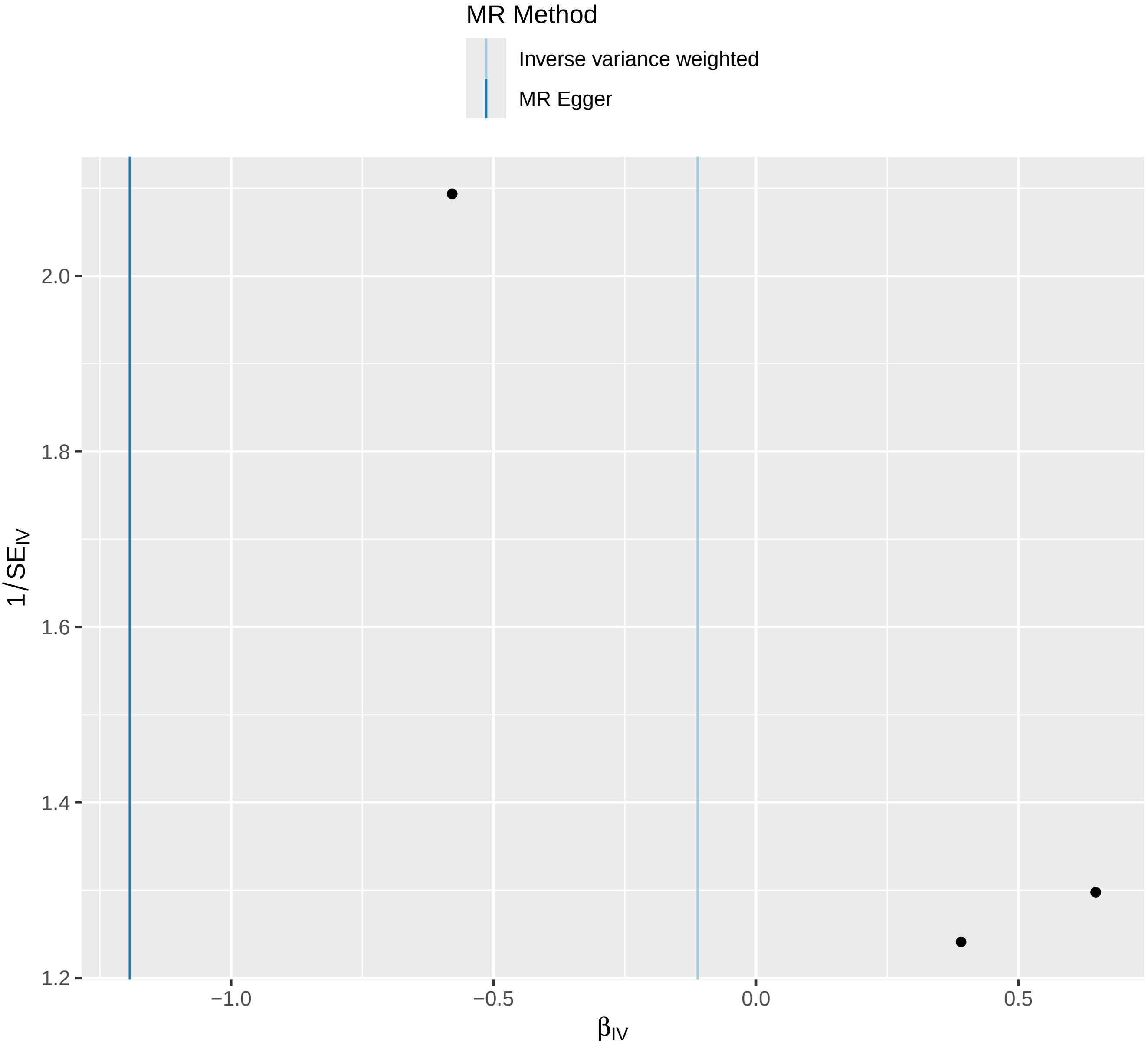

MR Method
Inverse variance weighted
MR Egger
1/SE_IV
2.0
1.8
1.6
1.4
1.2
−1.0
−0.5
0.0
0.5
β_IV

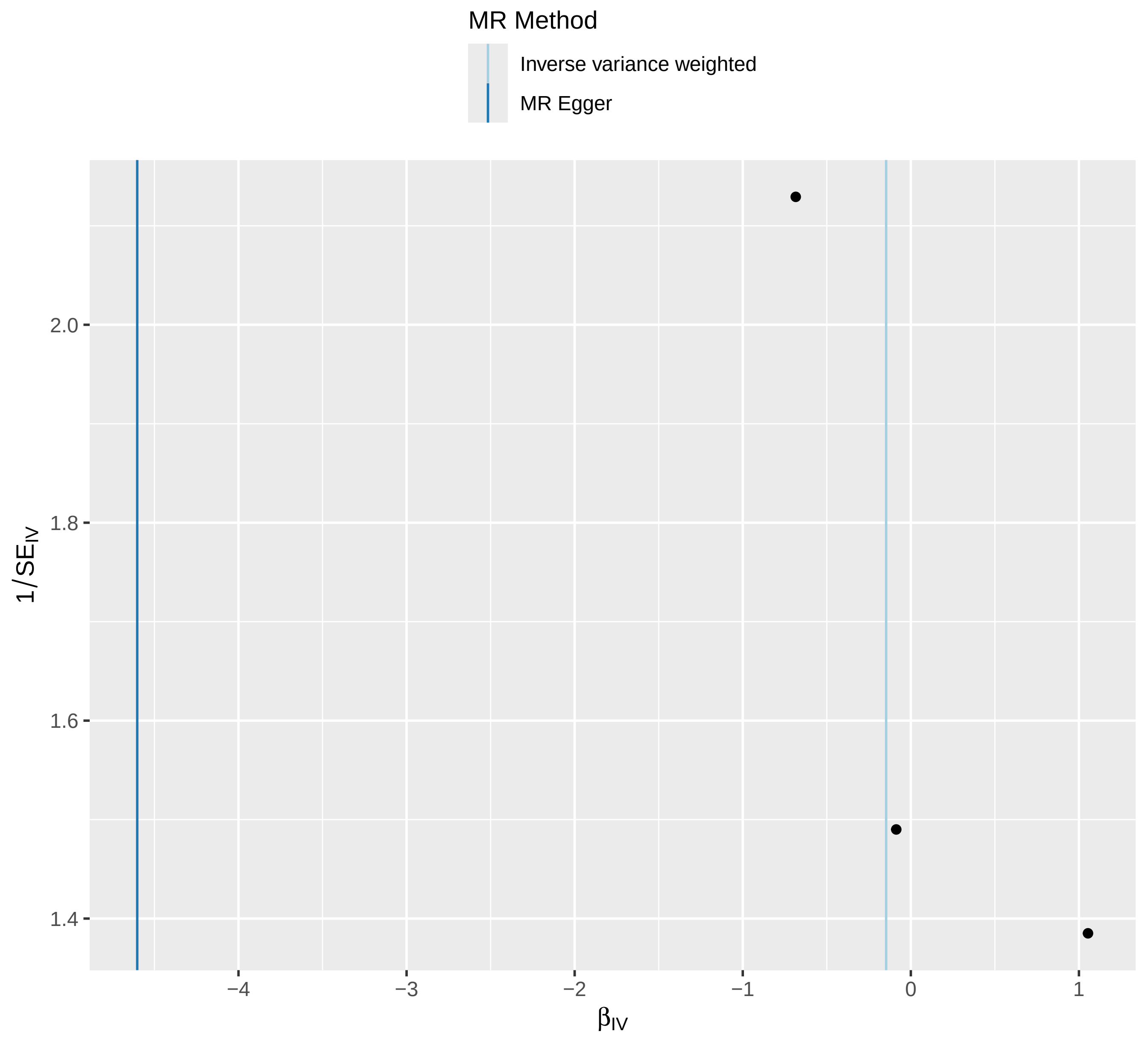
MR Method
Inverse variance weighted
MR Egger
1/SE_IV
2.0
1.8
1.6
1.4
−4
−3
−2
−1
0
1
β_IV

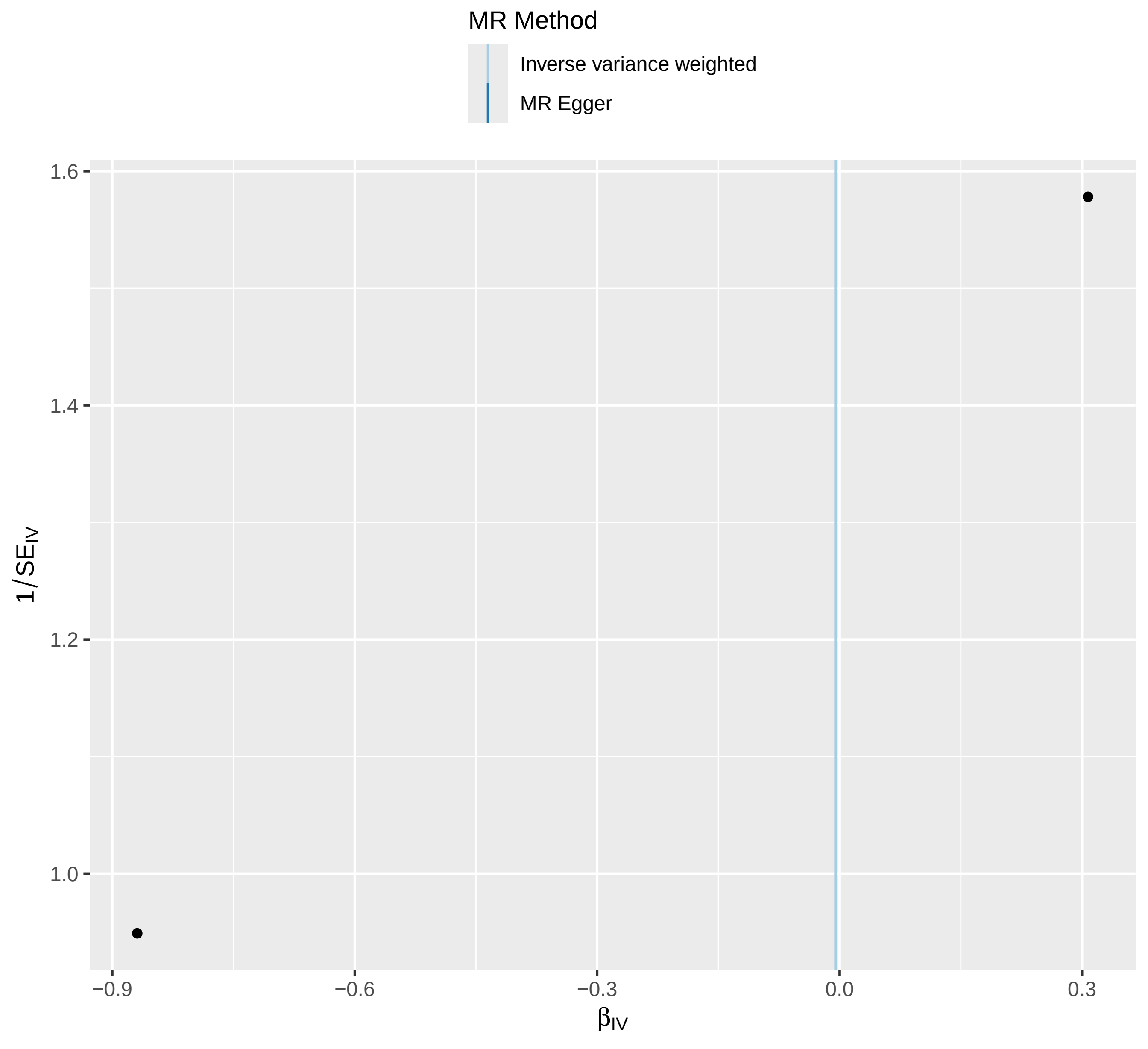
MR Method
Inverse variance weighted
MR Egger
1.6
1.4
1.2
1.0
1/SE_IV
−0.9
−0.6
−0.3
0.0
0.3
β_IV

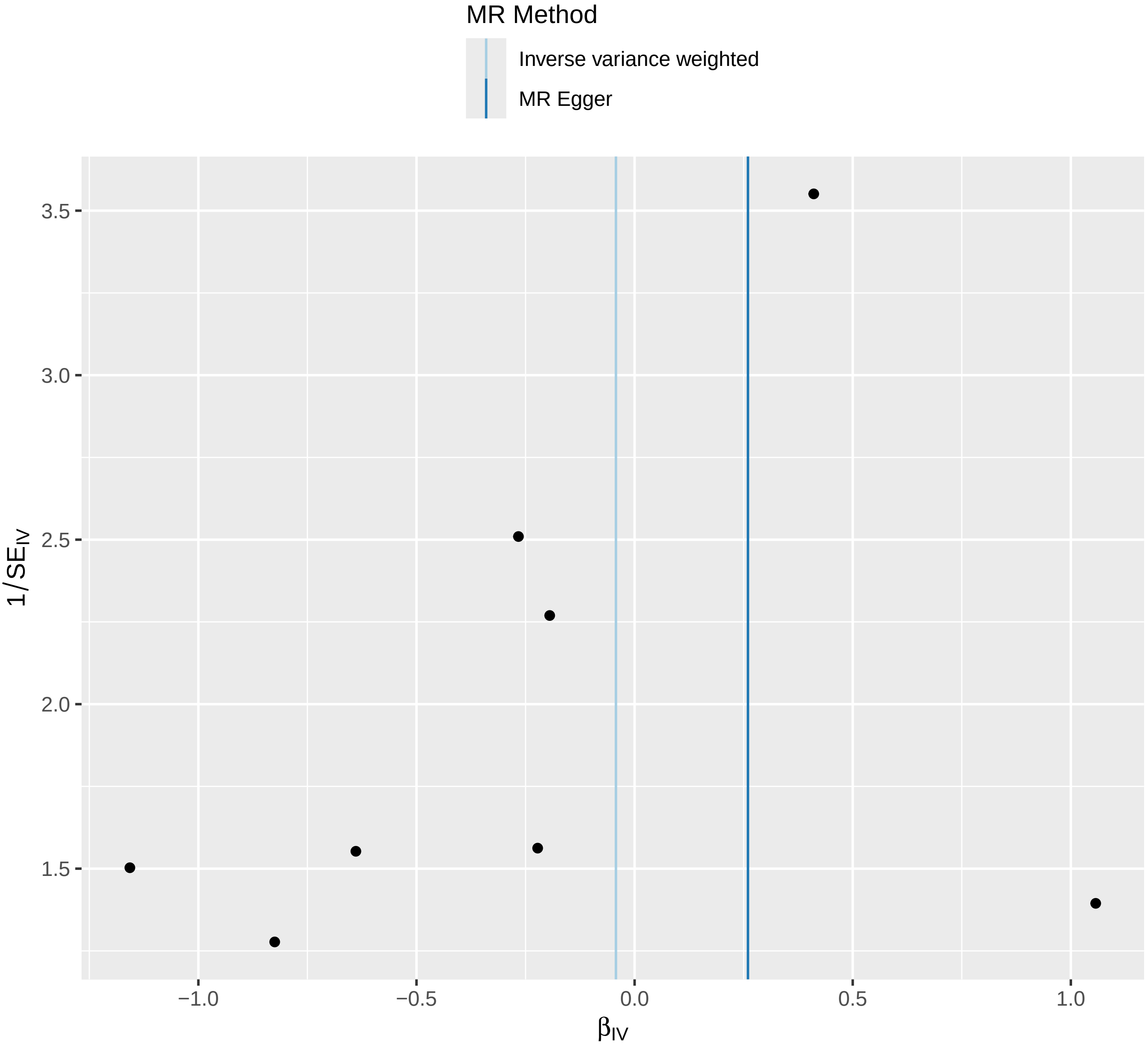
MR Method
Inverse variance weighted
MR Egger
3.5
3.0
2.5
2.0
1.5
1/SE_IV
−1.0
−0.5
0.0
0.5
1.0
β_IV

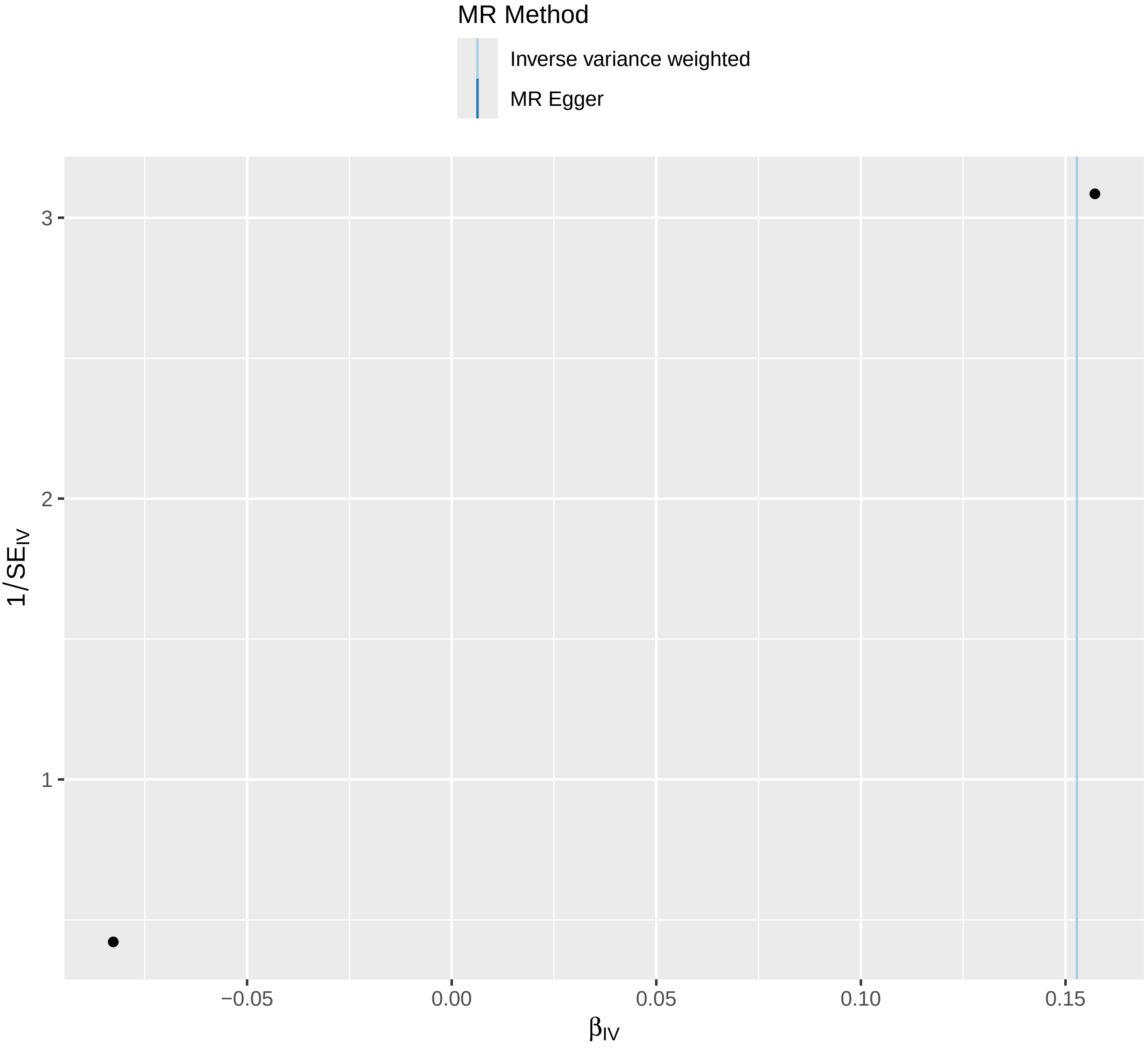

MR Method
Inverse variance weighted
MR Egger
3
2
1
1/SE_IV
−0.05
0.00
0.05
0.10
0.15
β_IV

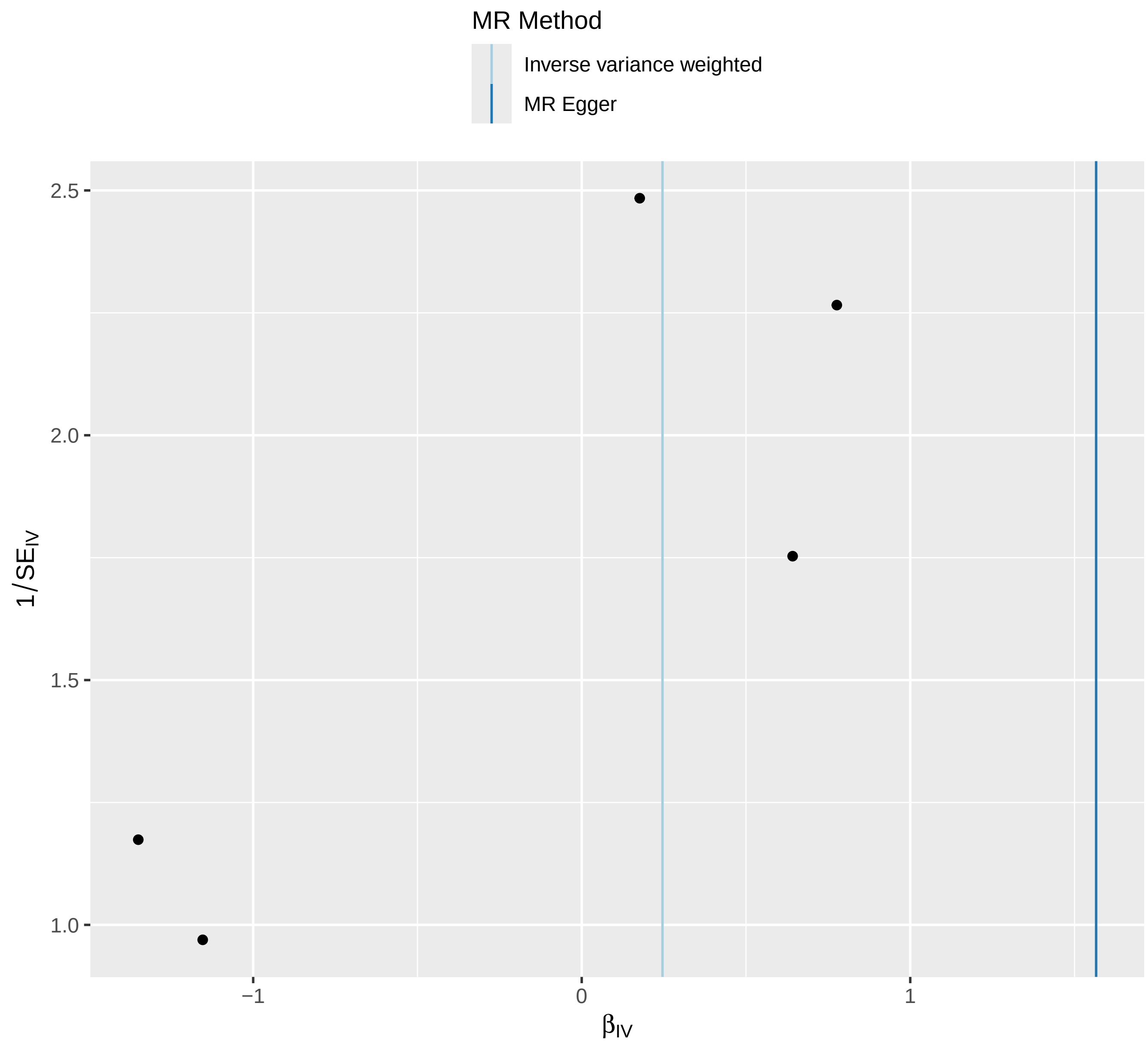
MR Method
Inverse variance weighted
MR Egger
2.5
2.0
1.5
1.0
1/SE_IV
−1
0
1
β_IV

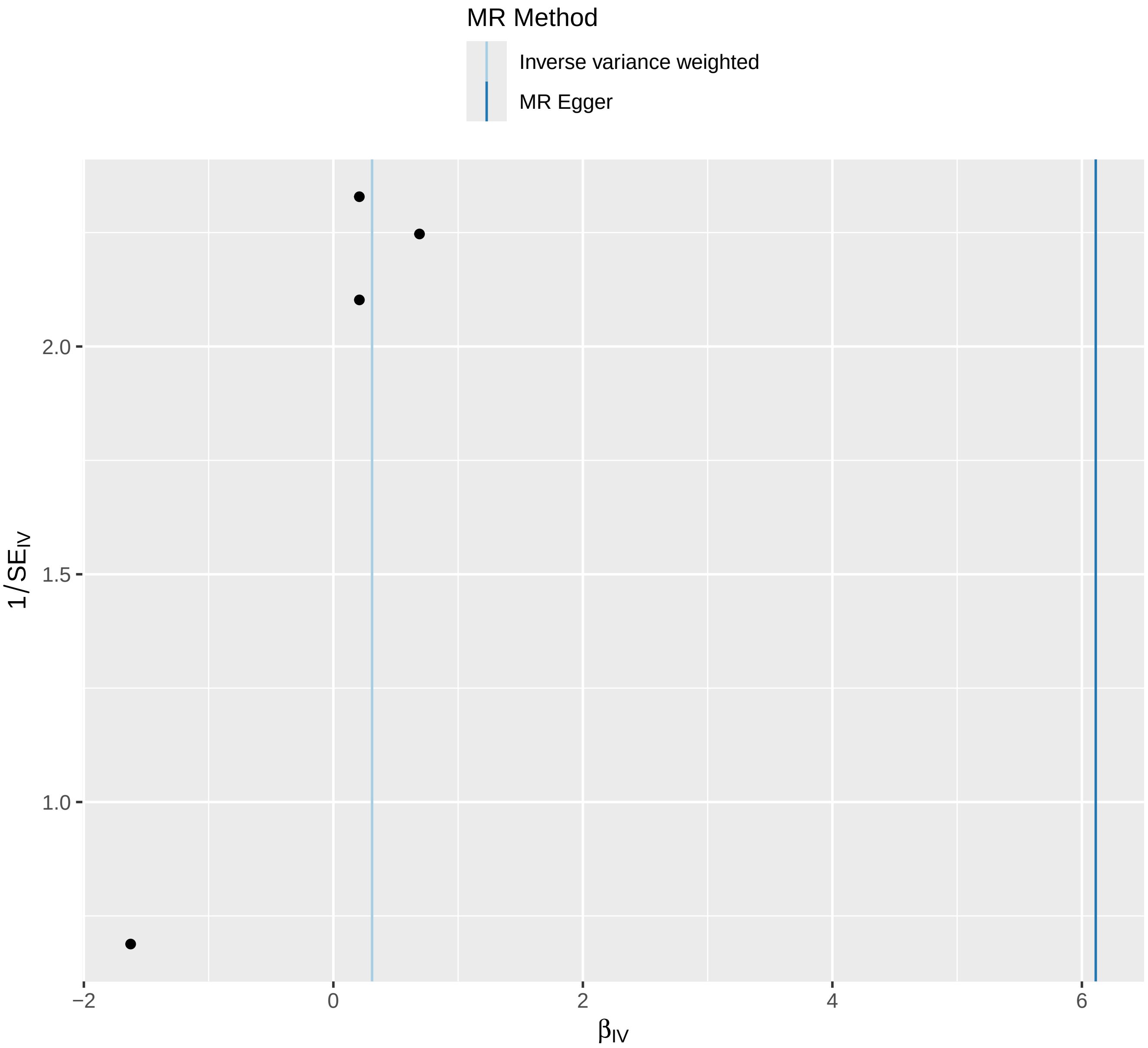
MR Method
Inverse variance weighted
MR Egger
2.0
1.5
1.0
−2
0
2
4
6
β_{IV}
$1/SE_{IV}$

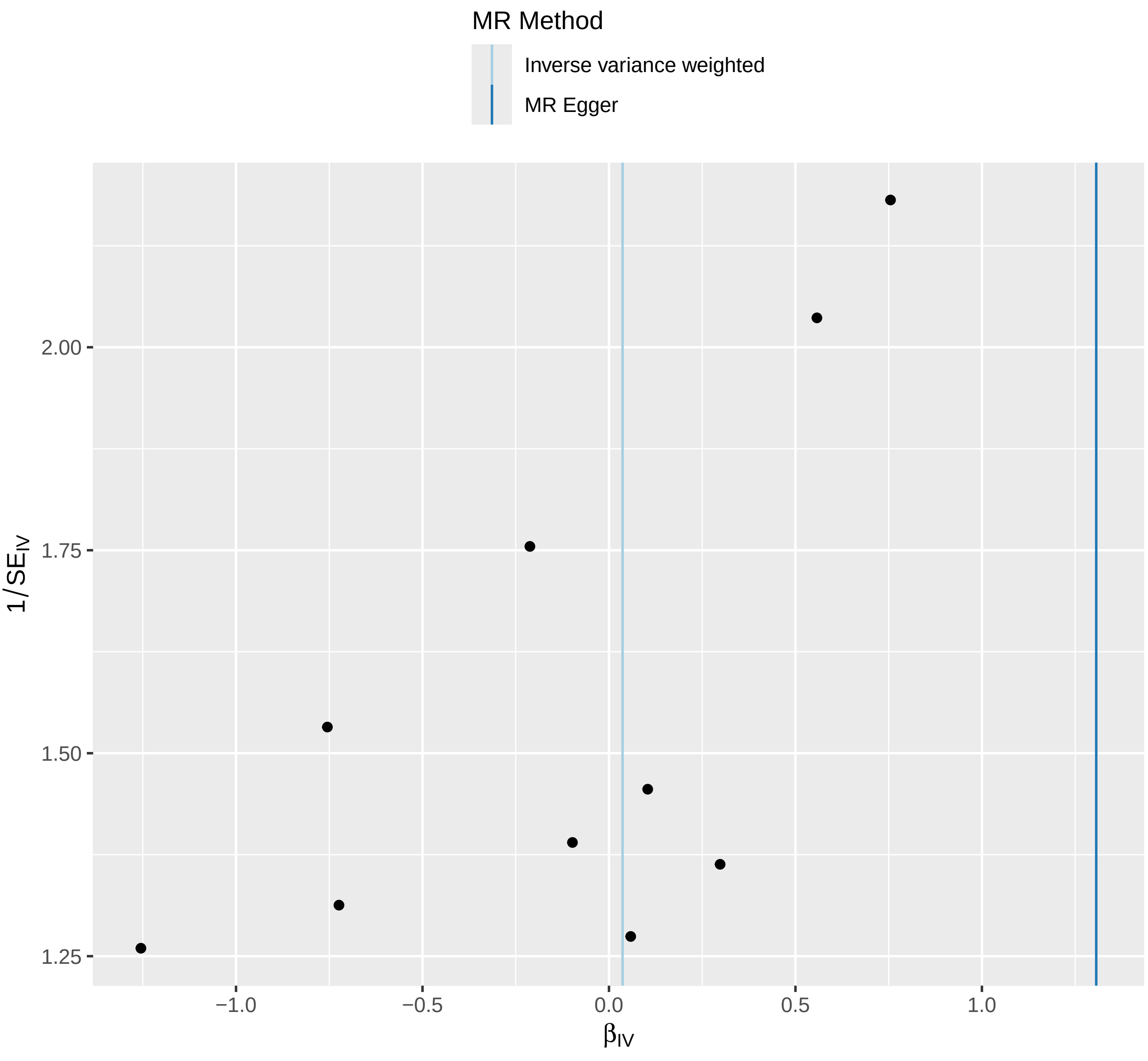

MR Method
Inverse variance weighted
MR Egger
2.00
1.75
1.50
1.25
1/SE$_{IV}$
−1.0
−0.5
0.0
0.5
1.0
β$_{IV}$

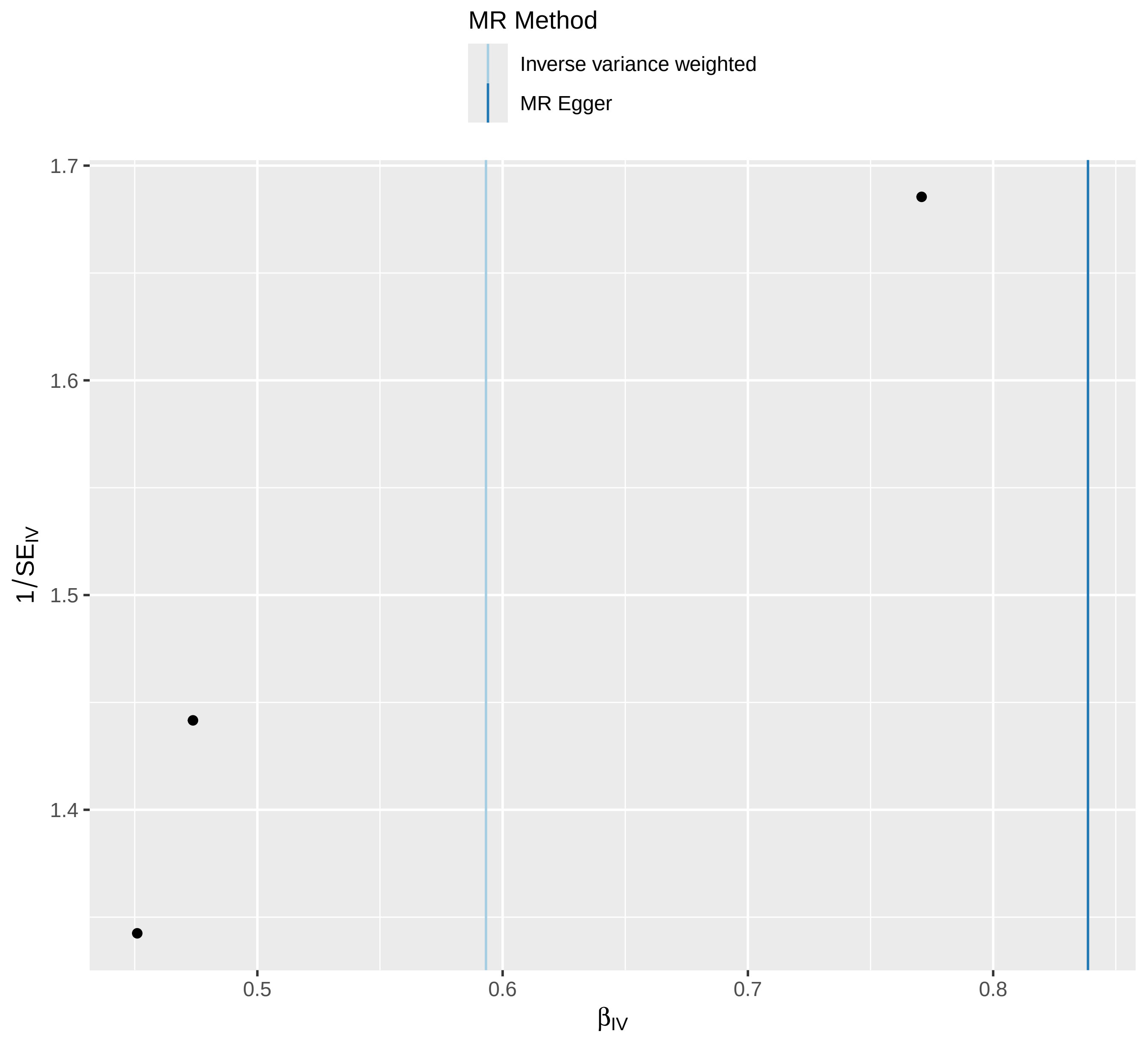
MR Method
Inverse variance weighted
MR Egger
1.7
1.6
1.5
1.4
$1/SE_{IV}$
0.5
0.6
0.7
0.8
β_{IV}

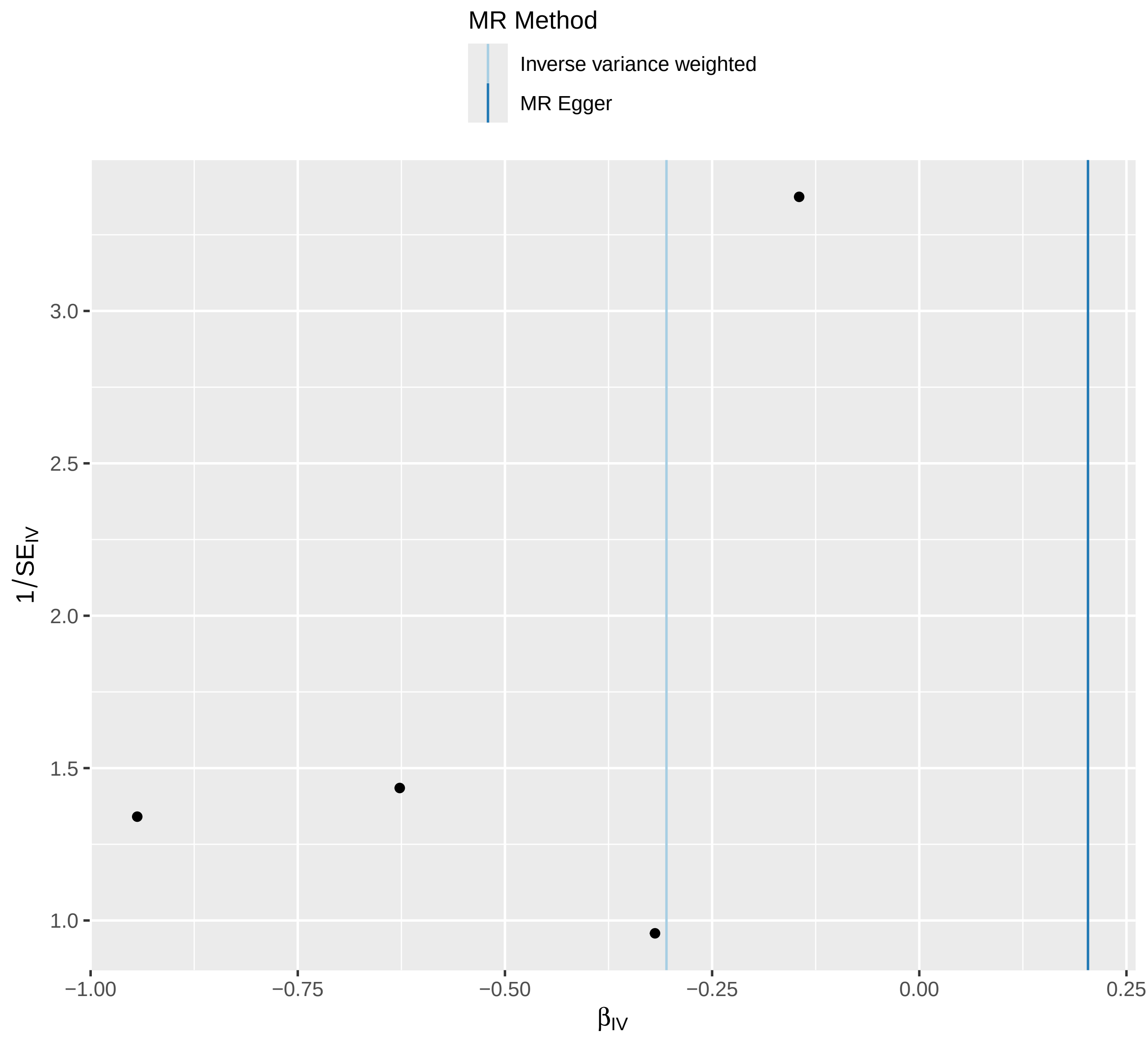
MR Method
Inverse variance weighted
MR Egger
1/SE_IV
3.0
2.5
2.0
1.5
1.0
−1.00
−0.75
−0.50
−0.25
0.00
0.25
β_IV

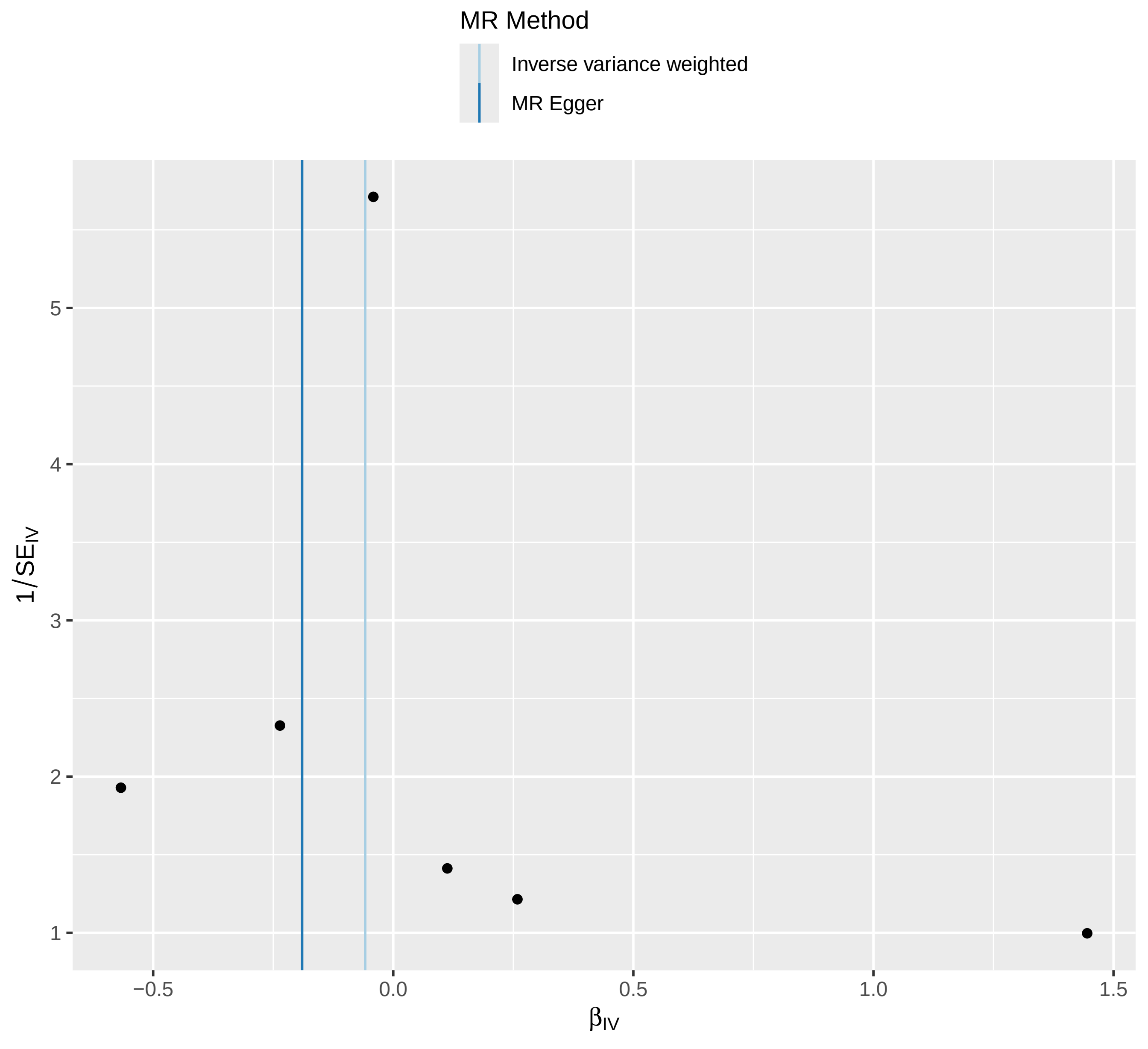
MR Method
Inverse variance weighted
MR Egger
$1/SE_{IV}$
β_{IV}
1
2
3
4
5
−0.5
0.0
0.5
1.0
1.5

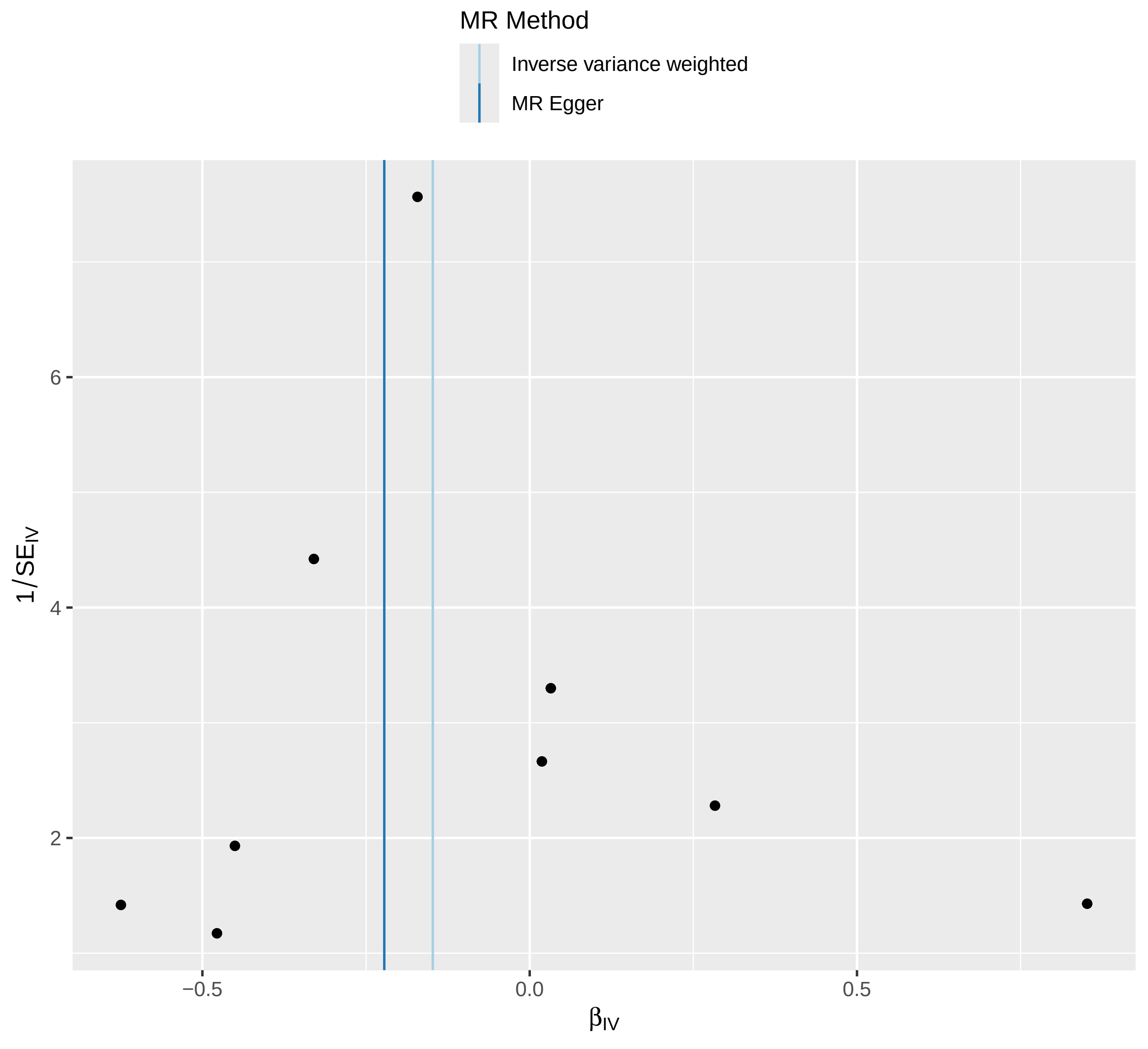

MR Method
Inverse variance weighted
MR Egger
6
4
2
−0.5
0.0
0.5
1/SE_IV
β_IV

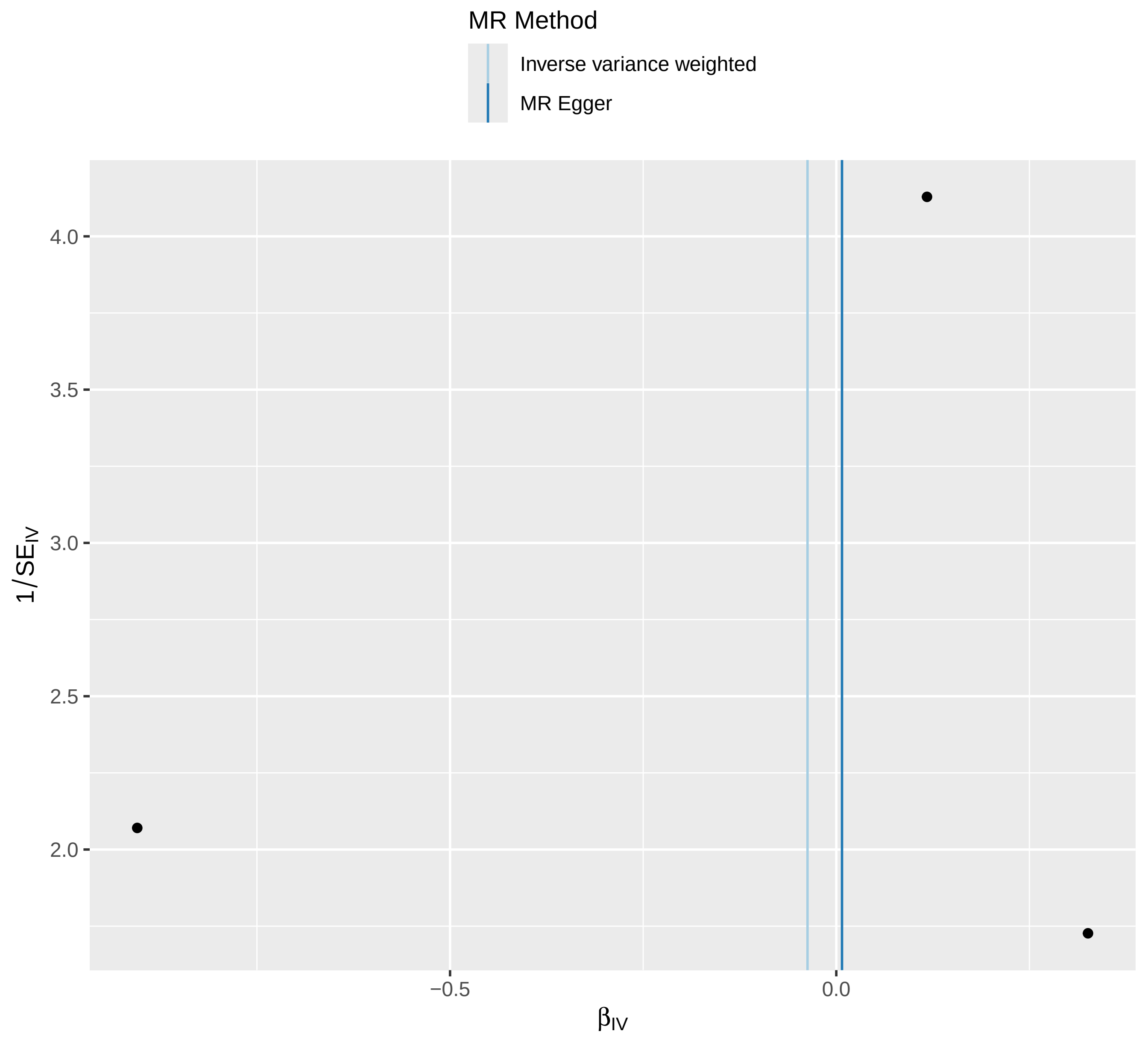
MR Method
Inverse variance weighted
MR Egger
4.0
3.5
3.0
2.5
2.0
1/SE$_{IV}$
−0.5
0.0
β$_{IV}$

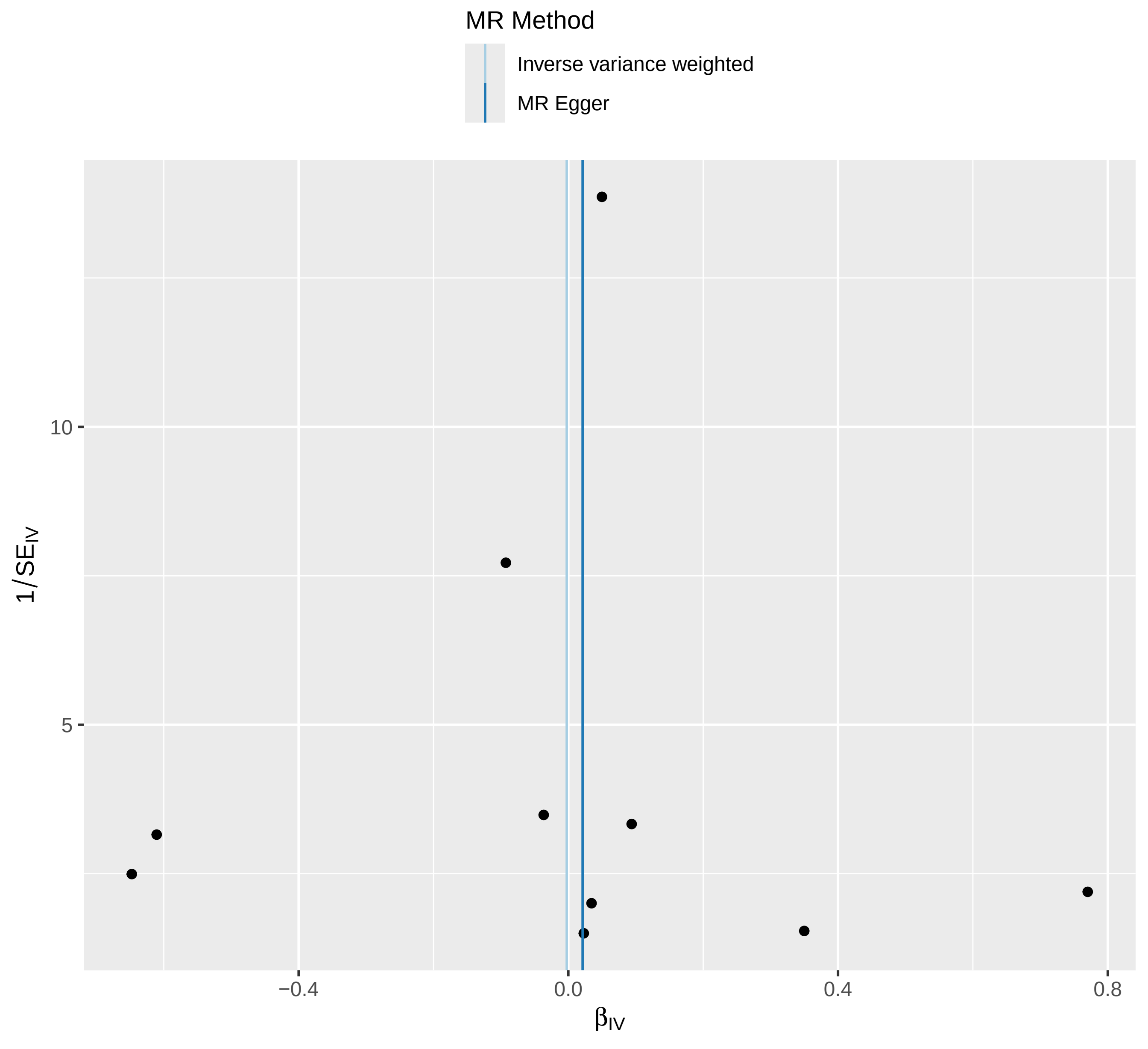

MR Method
Inverse variance weighted
MR Egger
10
5
1/SE_IV
−0.4
0.0
0.4
0.8
β_IV

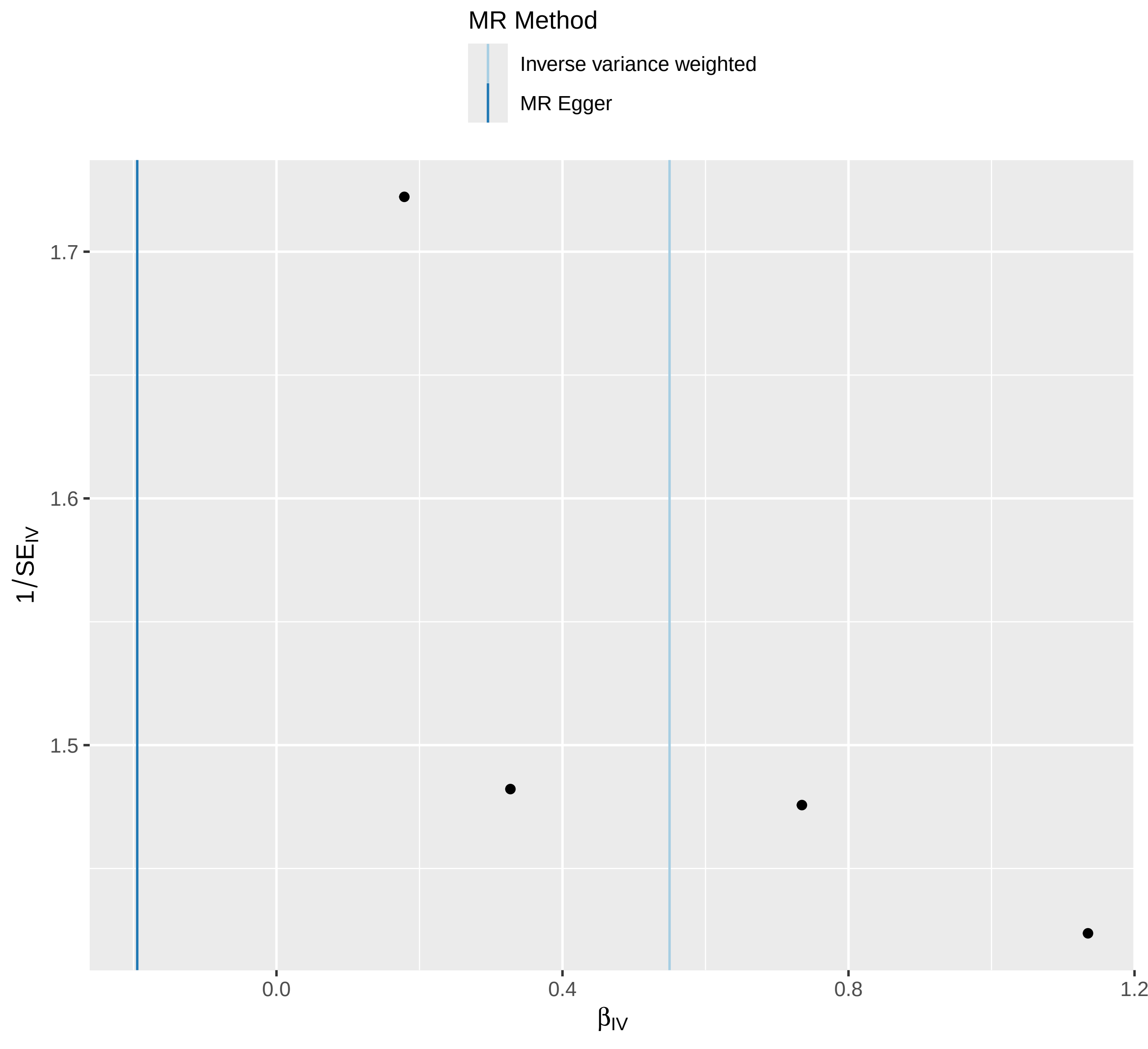
MR Method
Inverse variance weighted
MR Egger
1.7
1.6
1.5
0.0
0.4
0.8
1.2
$1/SE_{IV}$
β_{IV}

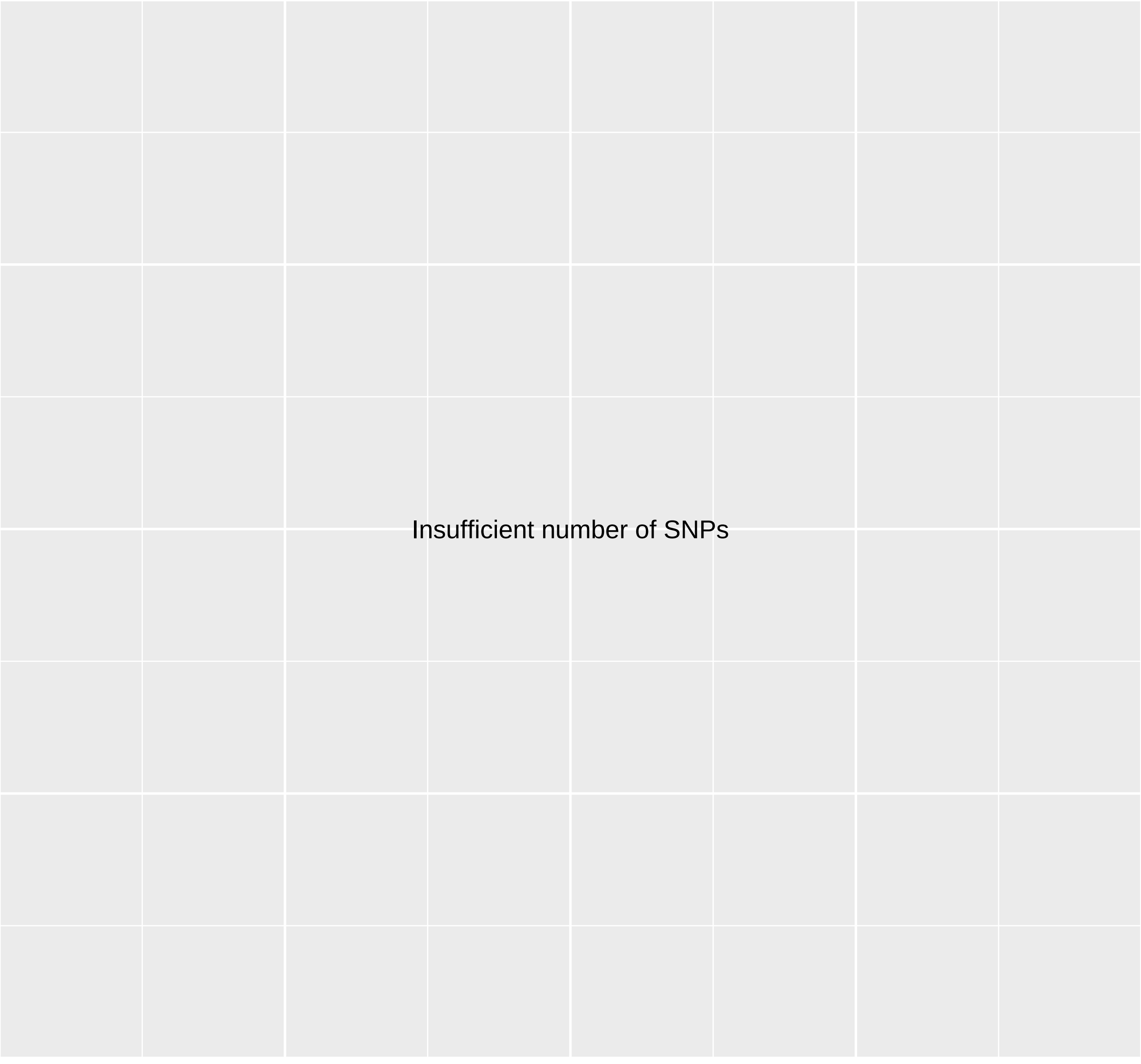
Insufficient number of SNPs

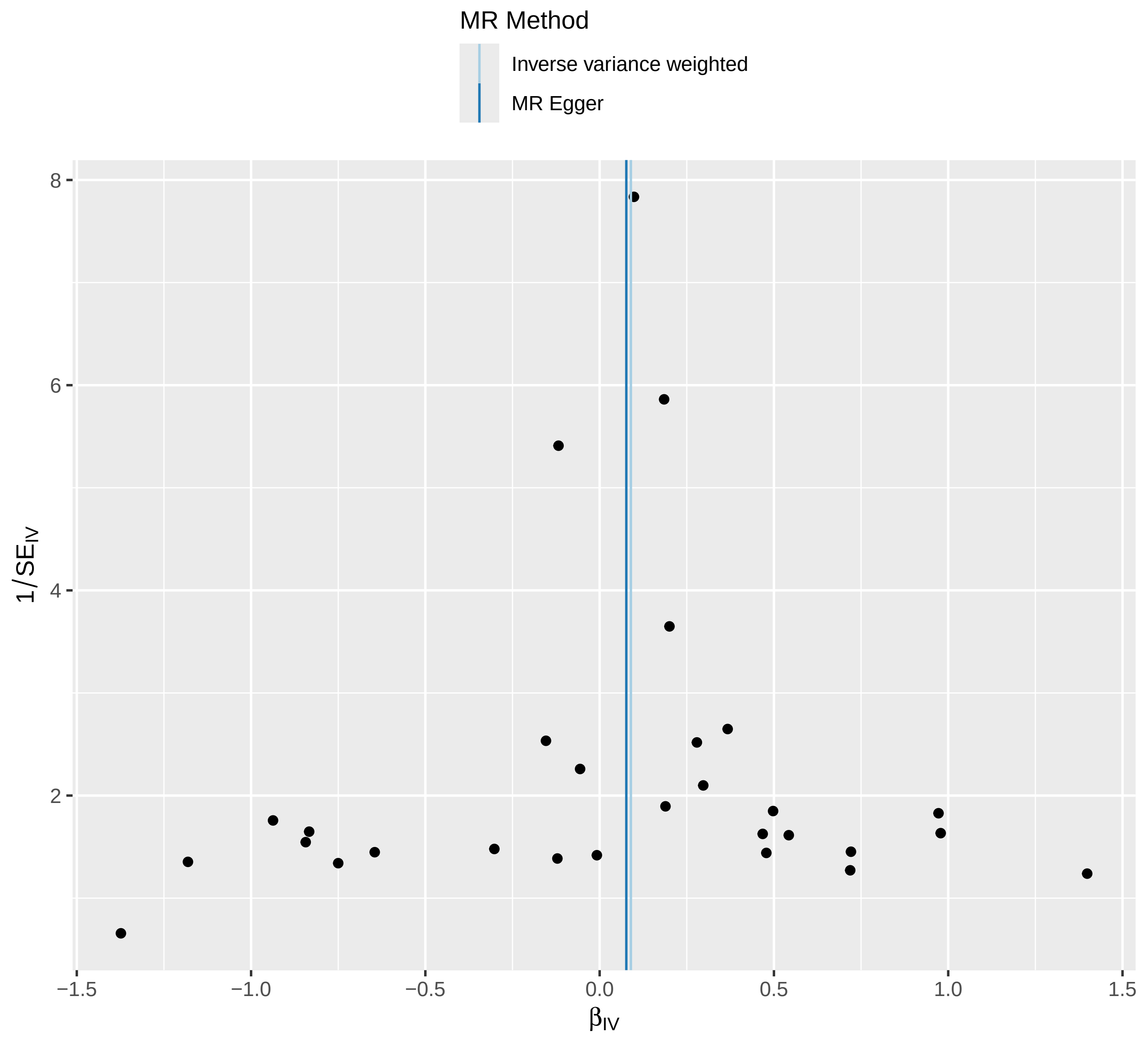

MR Method
Inverse variance weighted
MR Egger
8
6
4
2
1/SE_IV
−1.5
−1.0
−0.5
0.0
0.5
1.0
1.5
β_IV

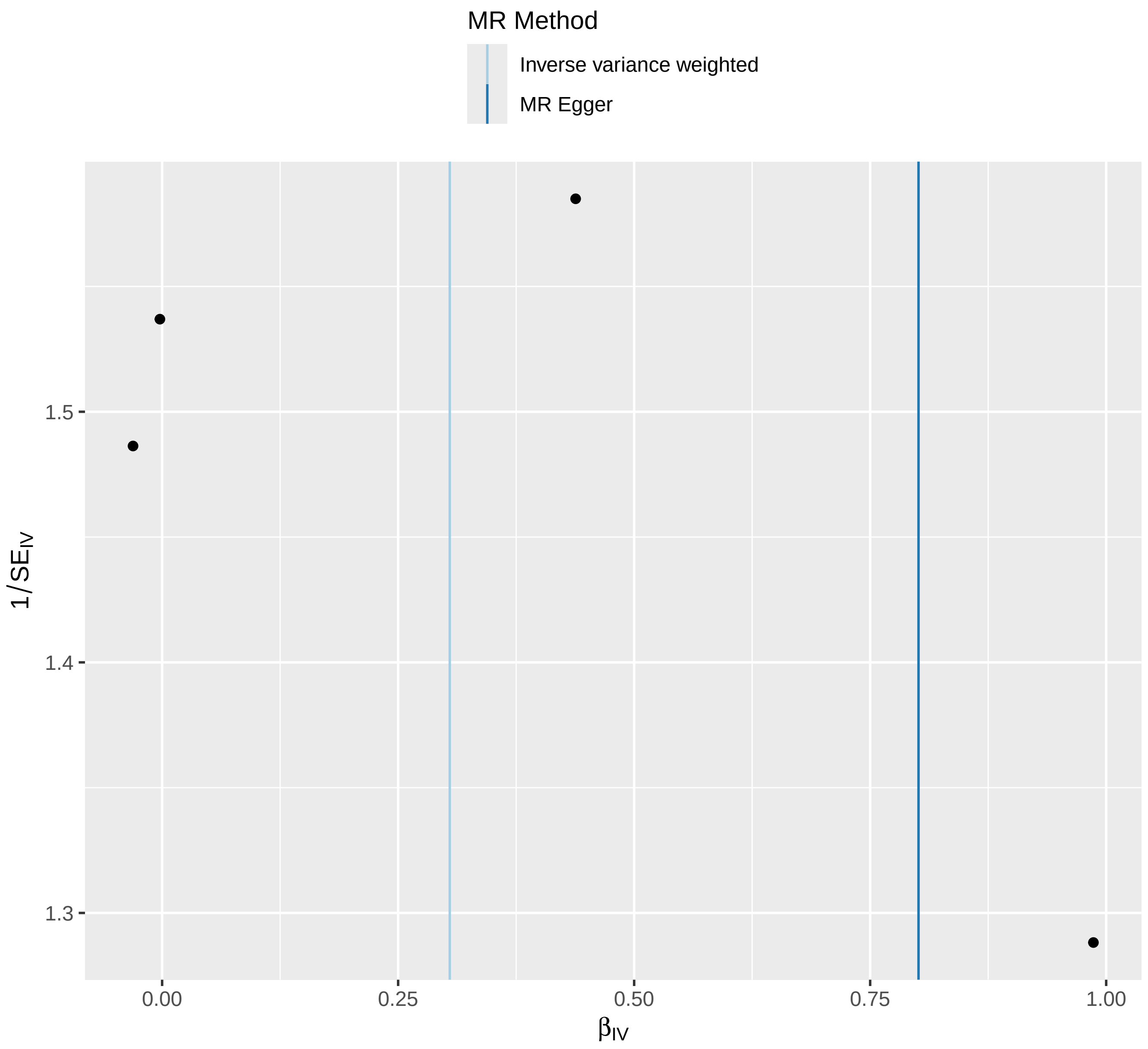
MR Method
Inverse variance weighted
MR Egger
1.5
1.4
1.3
1/SE_IV
0.00
0.25
0.50
0.75
1.00
β_IV

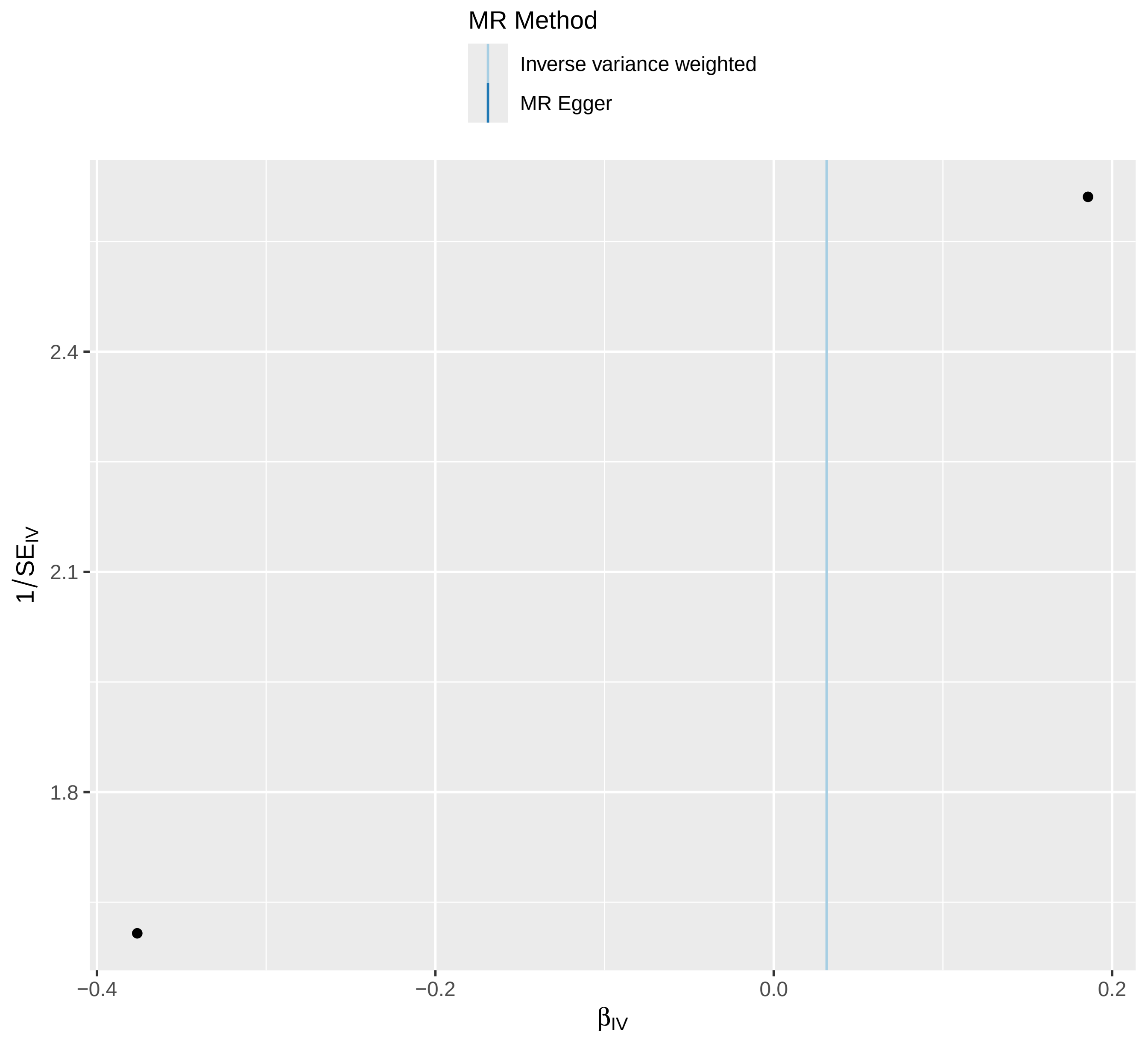
MR Method
Inverse variance weighted
MR Egger
2.4
2.1
1.8
1/SE_IV
−0.4
−0.2
0.0
0.2
β_IV

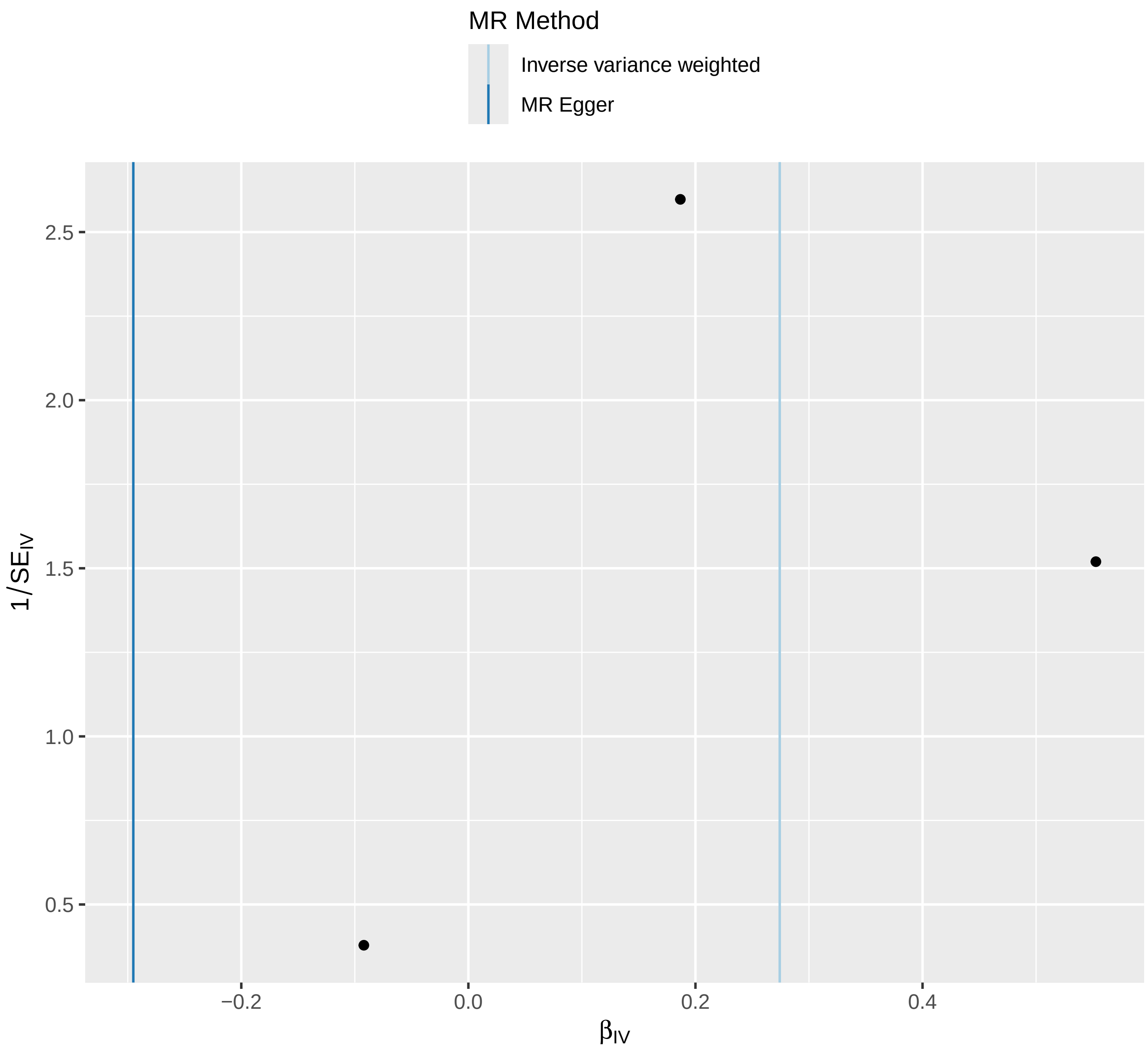

MR Method
Inverse variance weighted
MR Egger
2.5
2.0
1.5
1.0
0.5
1/SE$_{IV}$
−0.2
0.0
0.2
0.4
β$_{IV}$

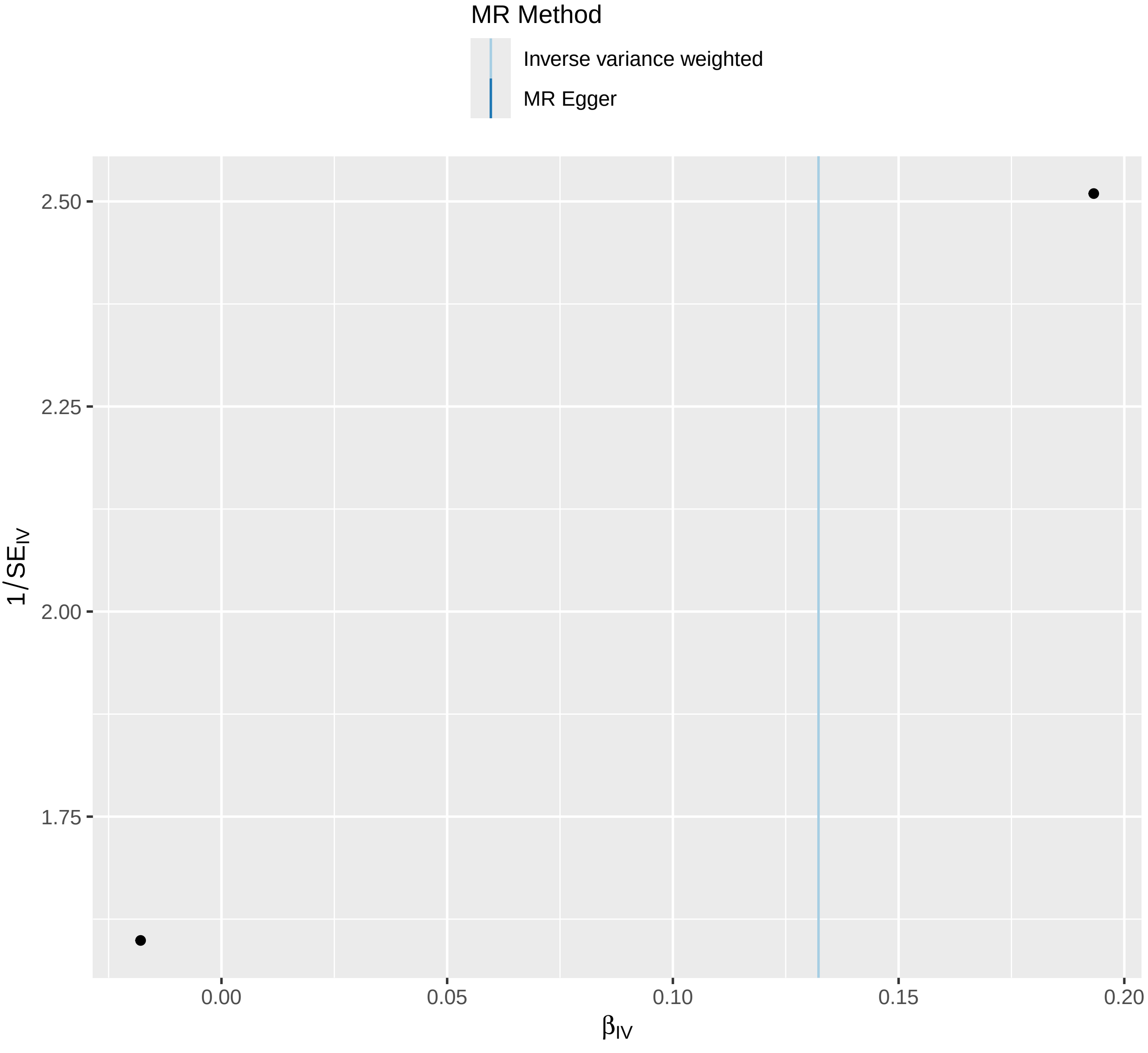
MR Method
Inverse variance weighted
MR Egger
2.50
2.25
2.00
1.75
$1/SE_{IV}$
0.00
0.05
0.10
0.15
0.20
β_{IV}

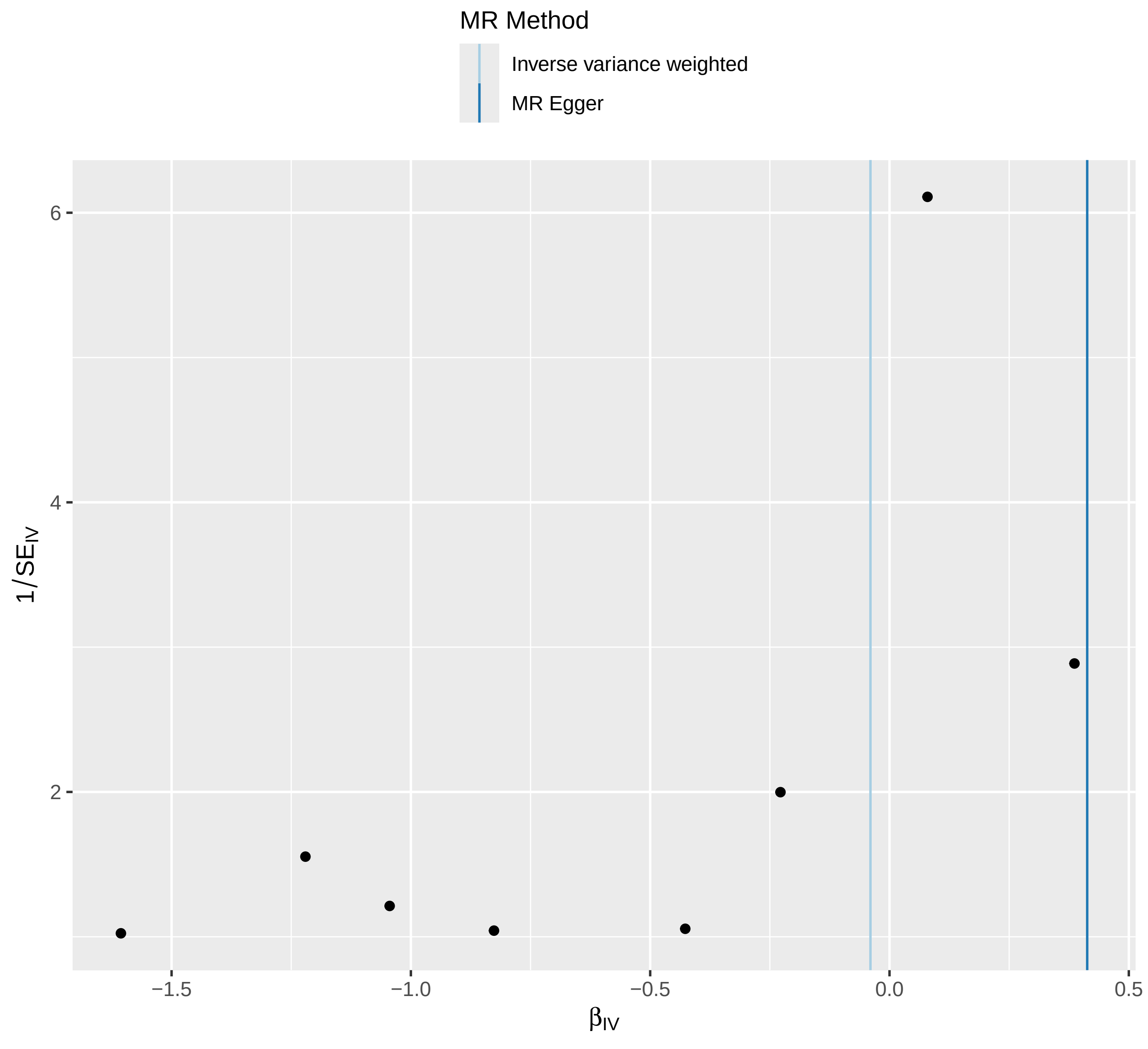
MR Method
Inverse variance weighted
MR Egger
6
4
2
1/SE_IV
−1.5
−1.0
−0.5
0.0
0.5
β_IV

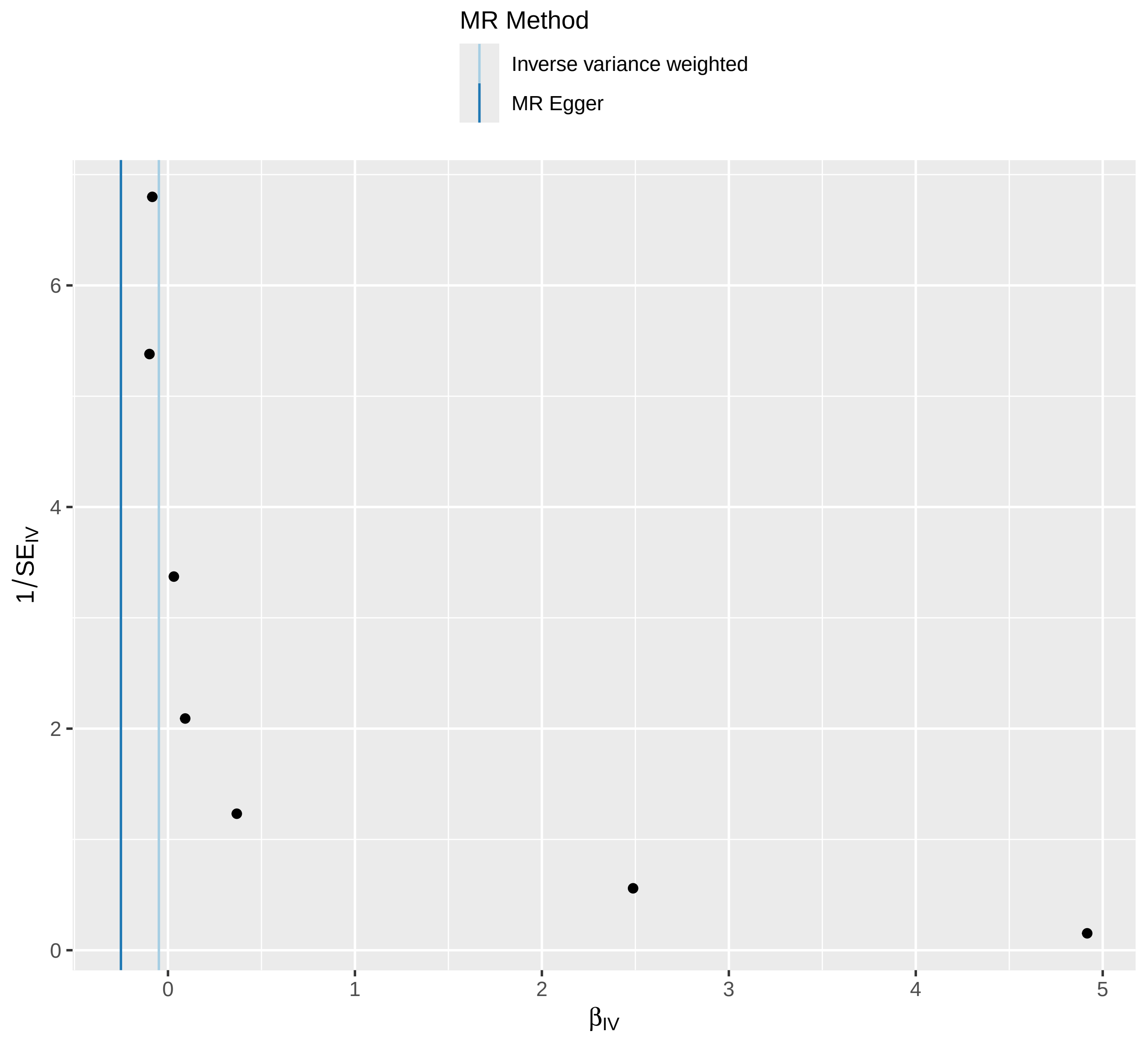

MR Method
Inverse variance weighted
MR Egger
6
4
2
0
1/SE$_{IV}$
0
1
2
3
4
5
β$_{IV}$

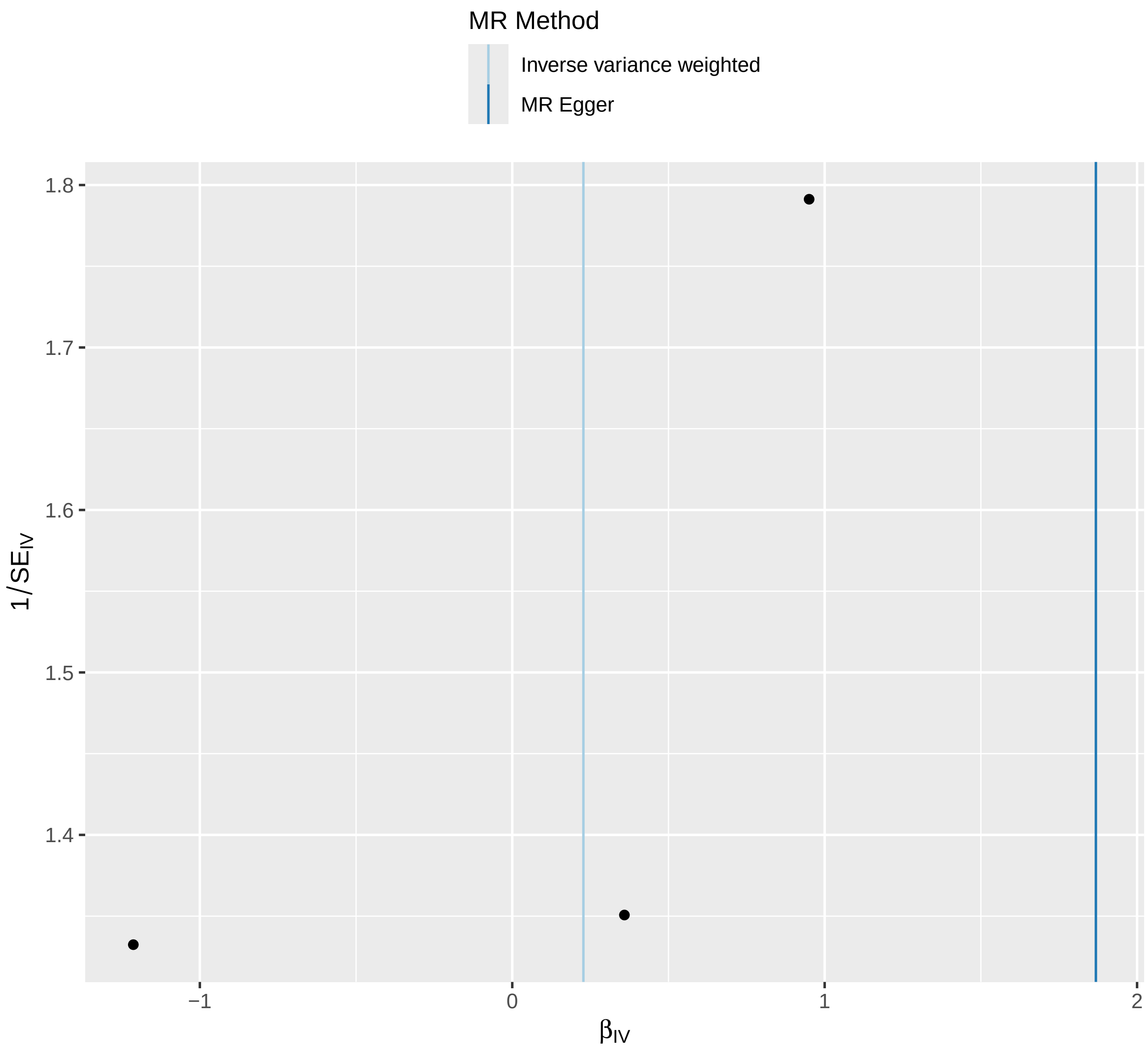
MR Method
Inverse variance weighted
MR Egger
1.8
1.7
1.6
1.5
1.4
$1/SE_{IV}$
−1
0
1
2
β_{IV}

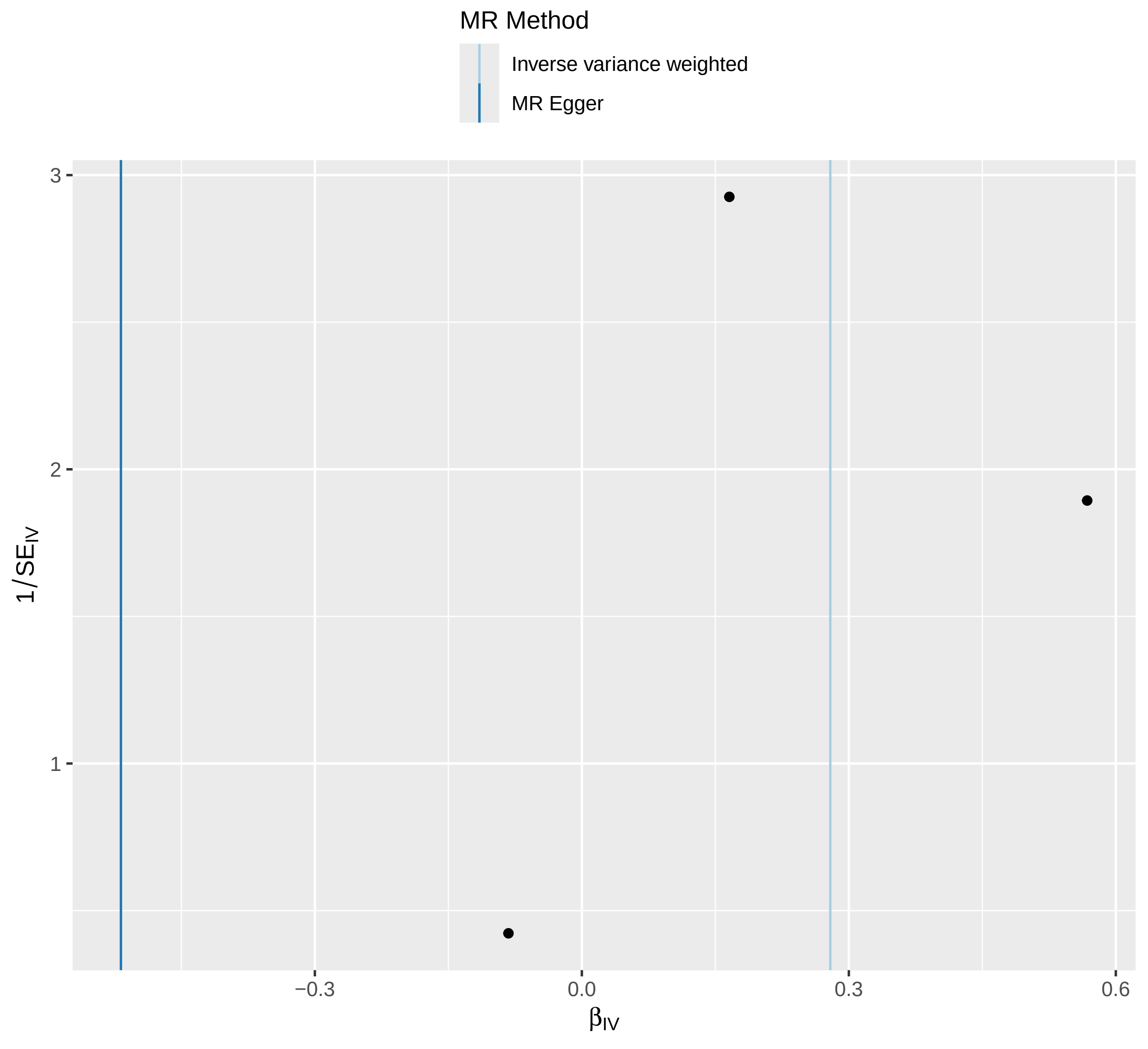
MR Method
Inverse variance weighted
MR Egger
3
2
1
1/SE$_{IV}$
−0.3
0.0
0.3
0.6
β$_{IV}$

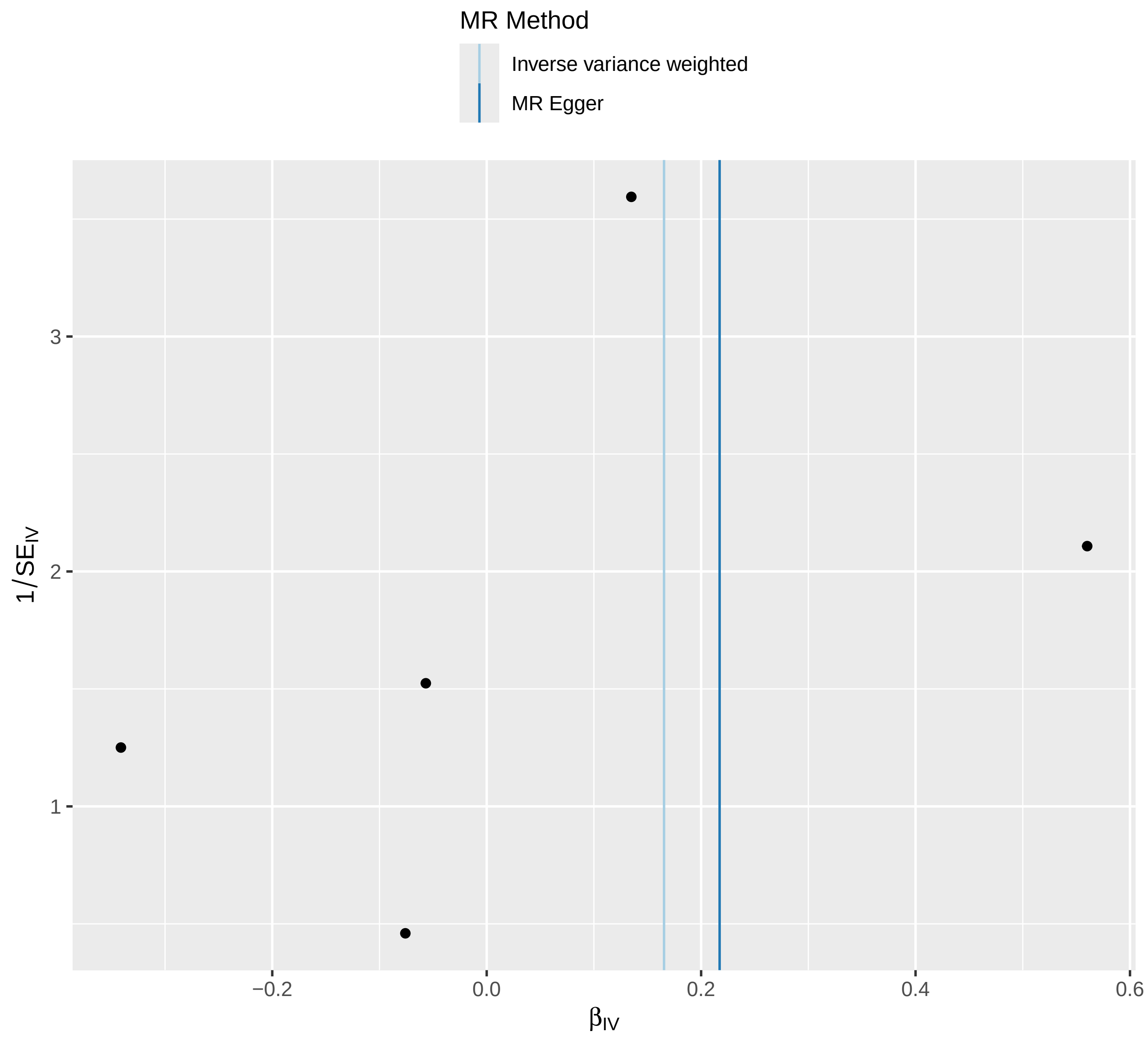
MR Method
Inverse variance weighted
MR Egger
3
2
1
−0.2
0.0
0.2
0.4
0.6
β_{IV}
$1/SE_{IV}$

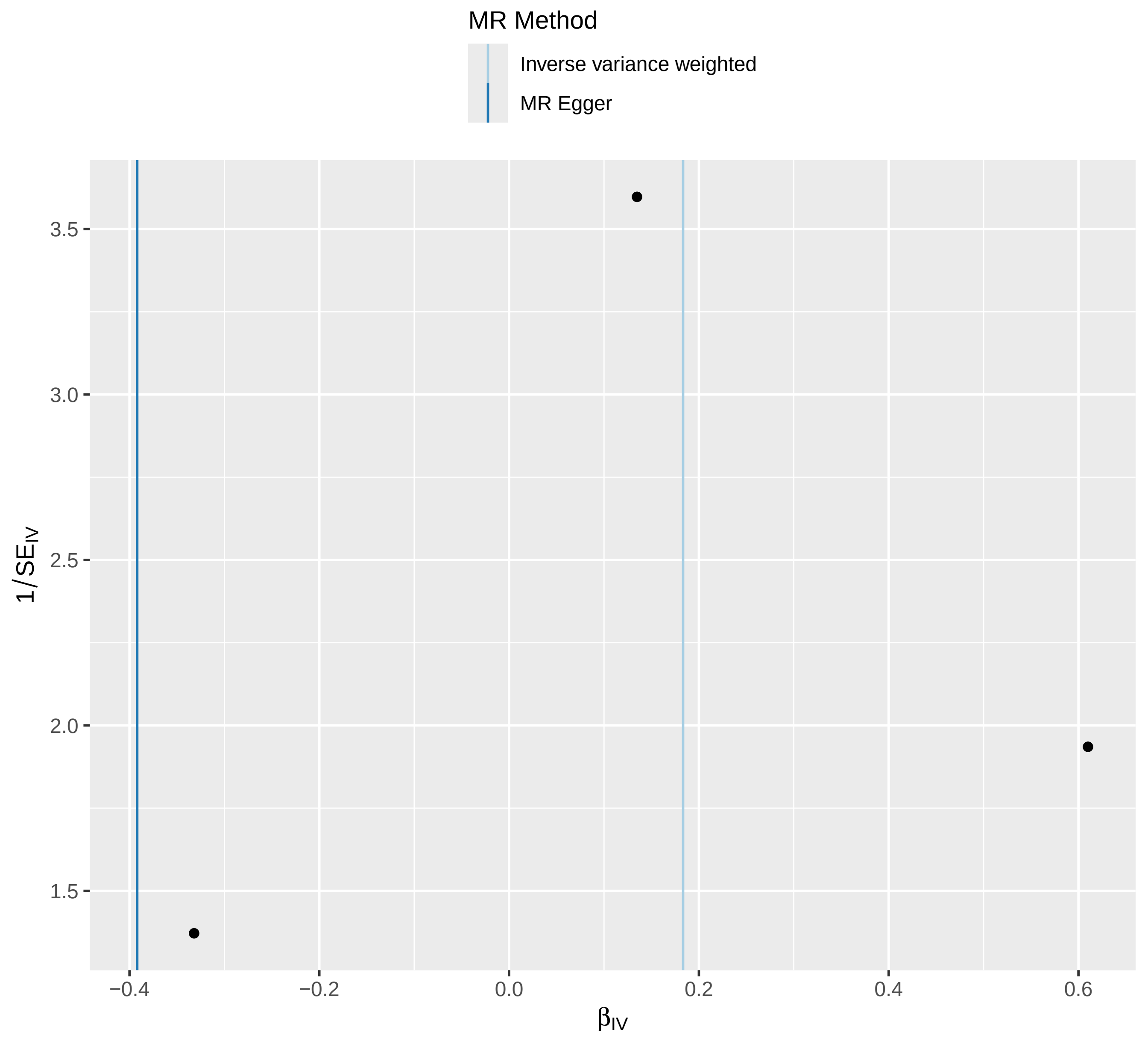

MR Method
Inverse variance weighted
MR Egger
3.5
3.0
2.5
2.0
1.5
1/SE_IV
−0.4
−0.2
0.0
0.2
0.4
0.6
β_IV

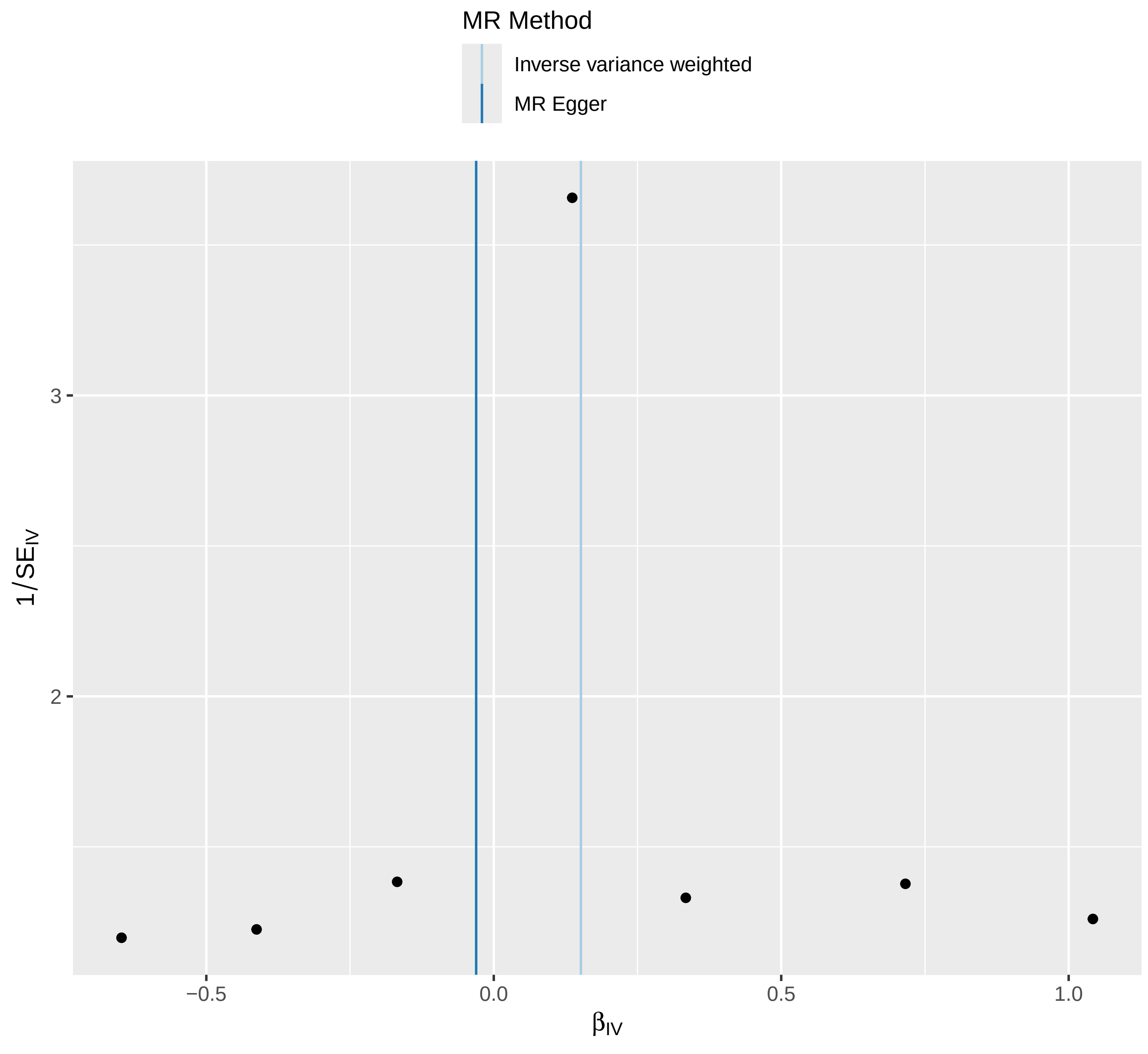

MR Method
Inverse variance weighted
MR Egger
3
2
1/SE_IV
−0.5
0.0
0.5
1.0
β_IV

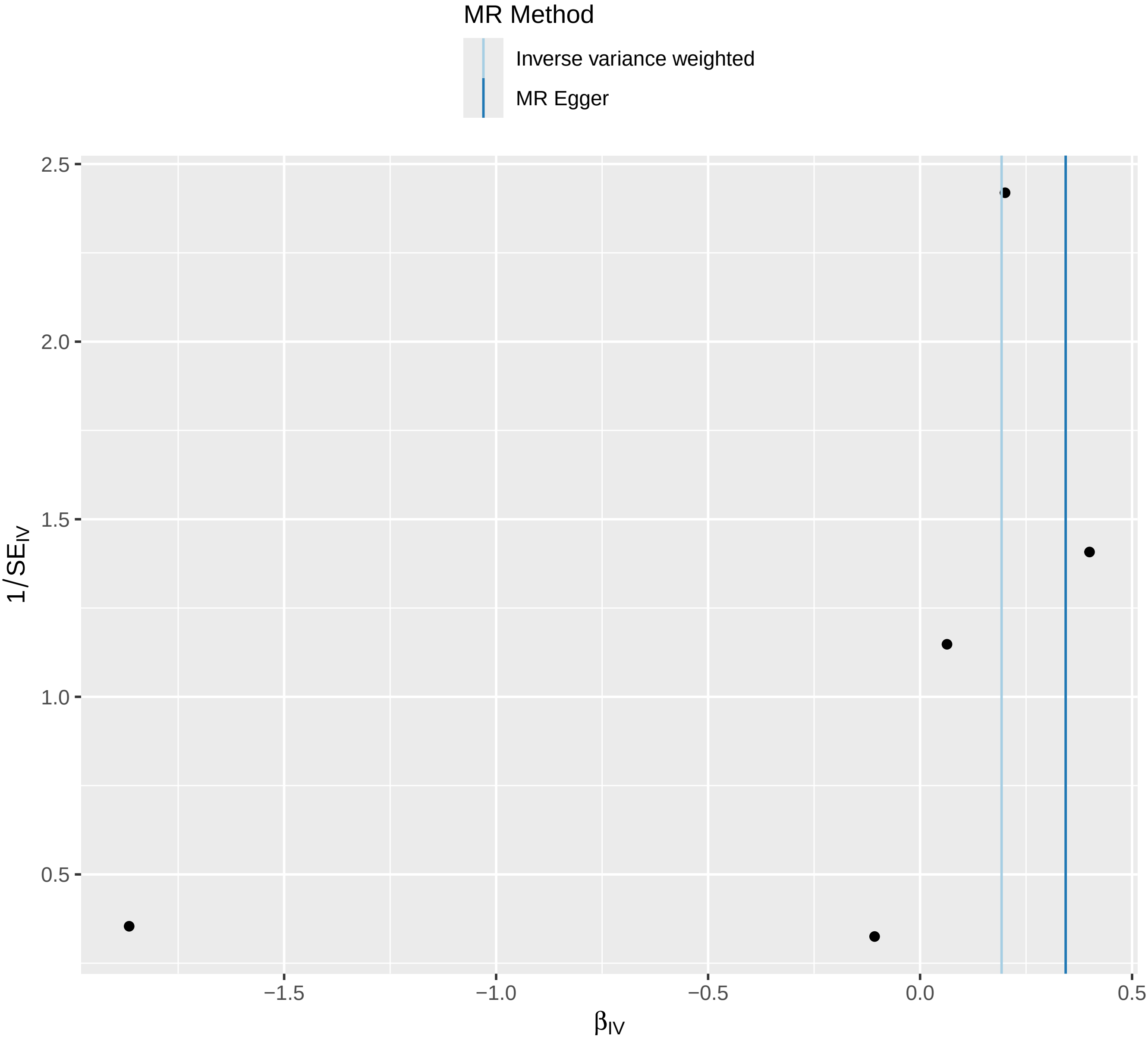
MR Method
Inverse variance weighted
MR Egger
2.5
2.0
1.5
1.0
0.5
$1/SE_{IV}$
−1.5
−1.0
−0.5
0.0
0.5
β_{IV}

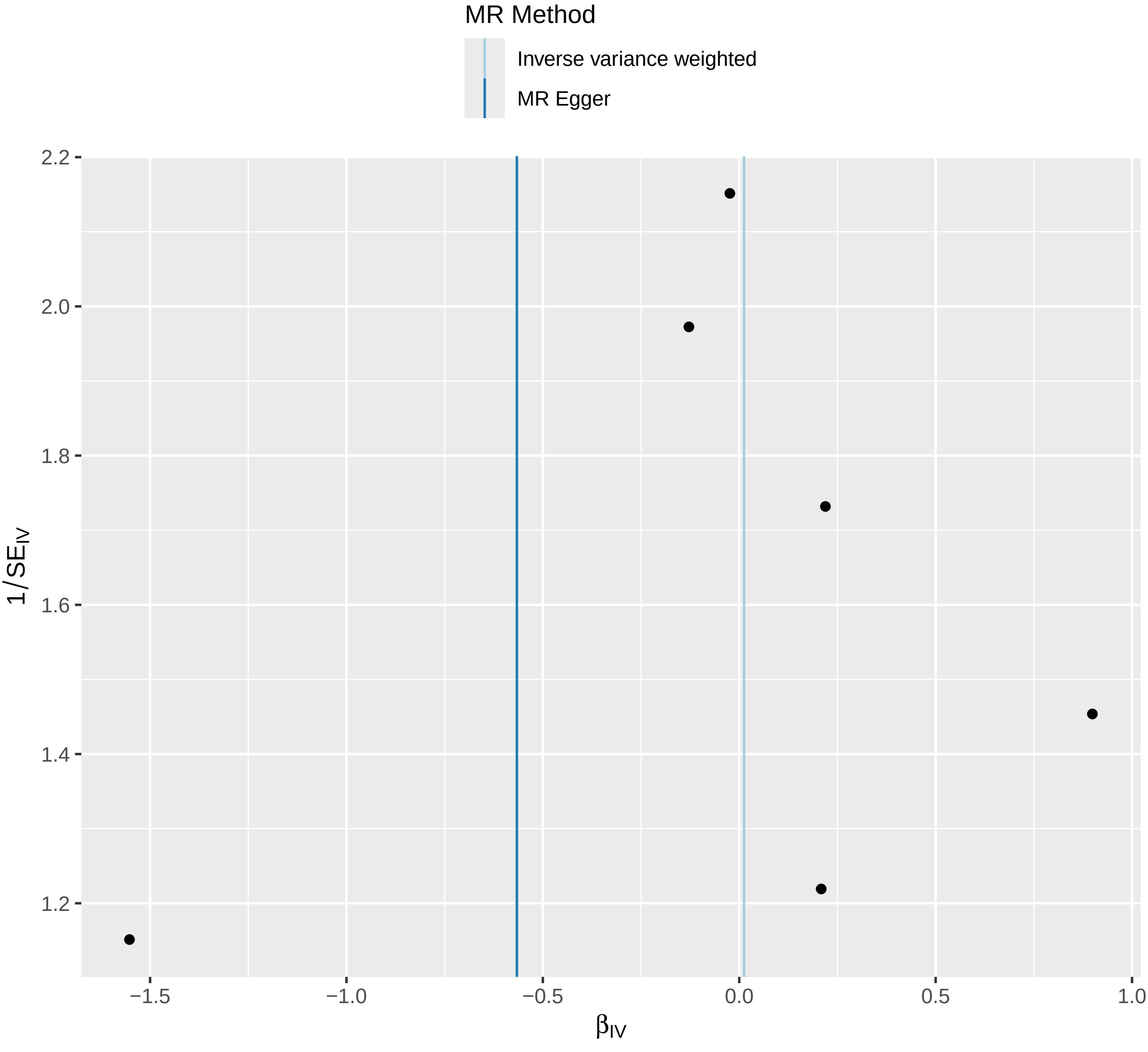
MR Method
Inverse variance weighted
MR Egger
2.2
2.0
1.8
1.6
1.4
1.2
1/SE_IV
−1.5
−1.0
−0.5
0.0
0.5
1.0
β_IV

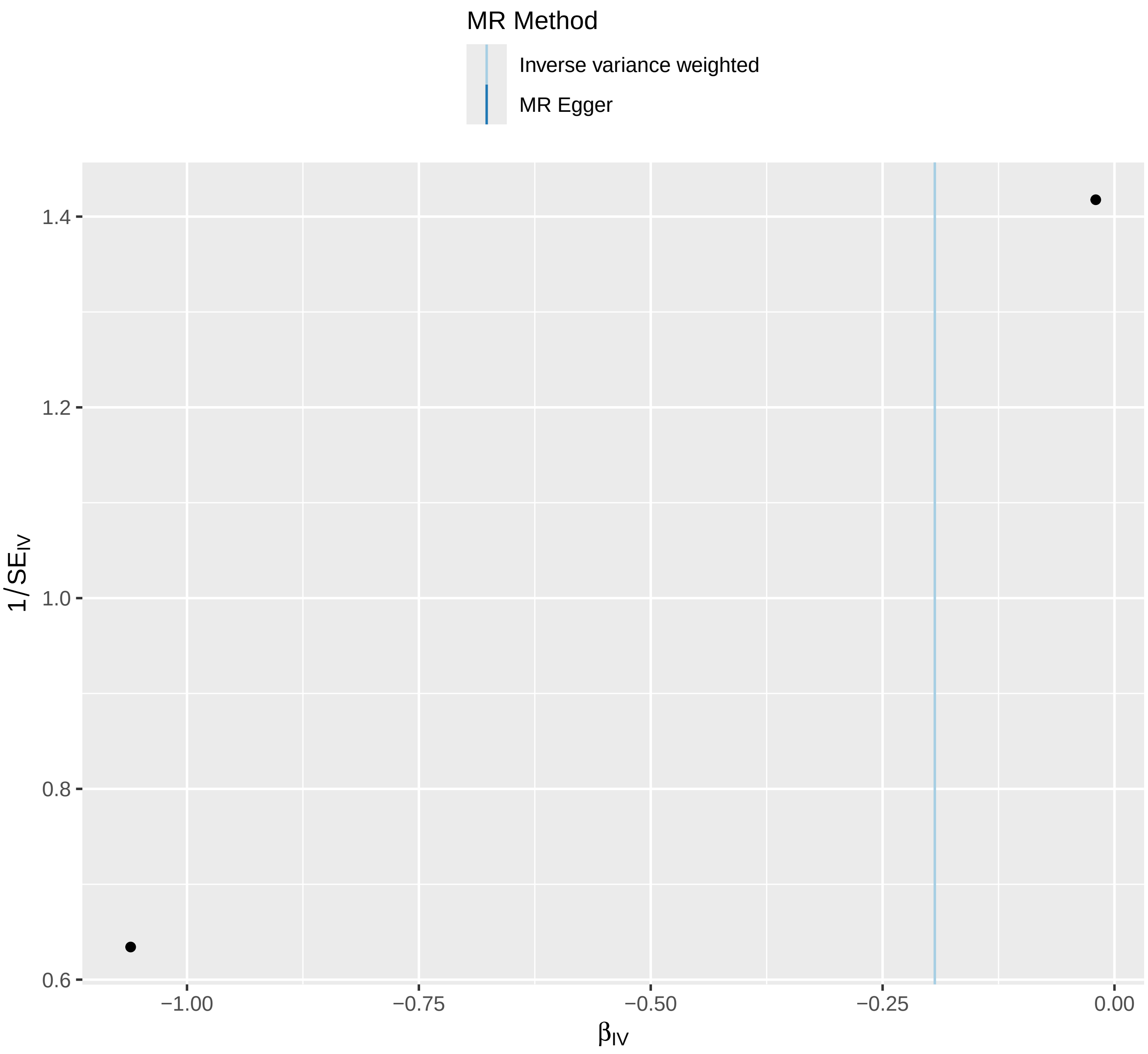

MR Method
Inverse variance weighted
MR Egger
1.4
1.2
1.0
0.8
0.6
1/SE_IV
−1.00
−0.75
−0.50
−0.25
0.00
β_IV

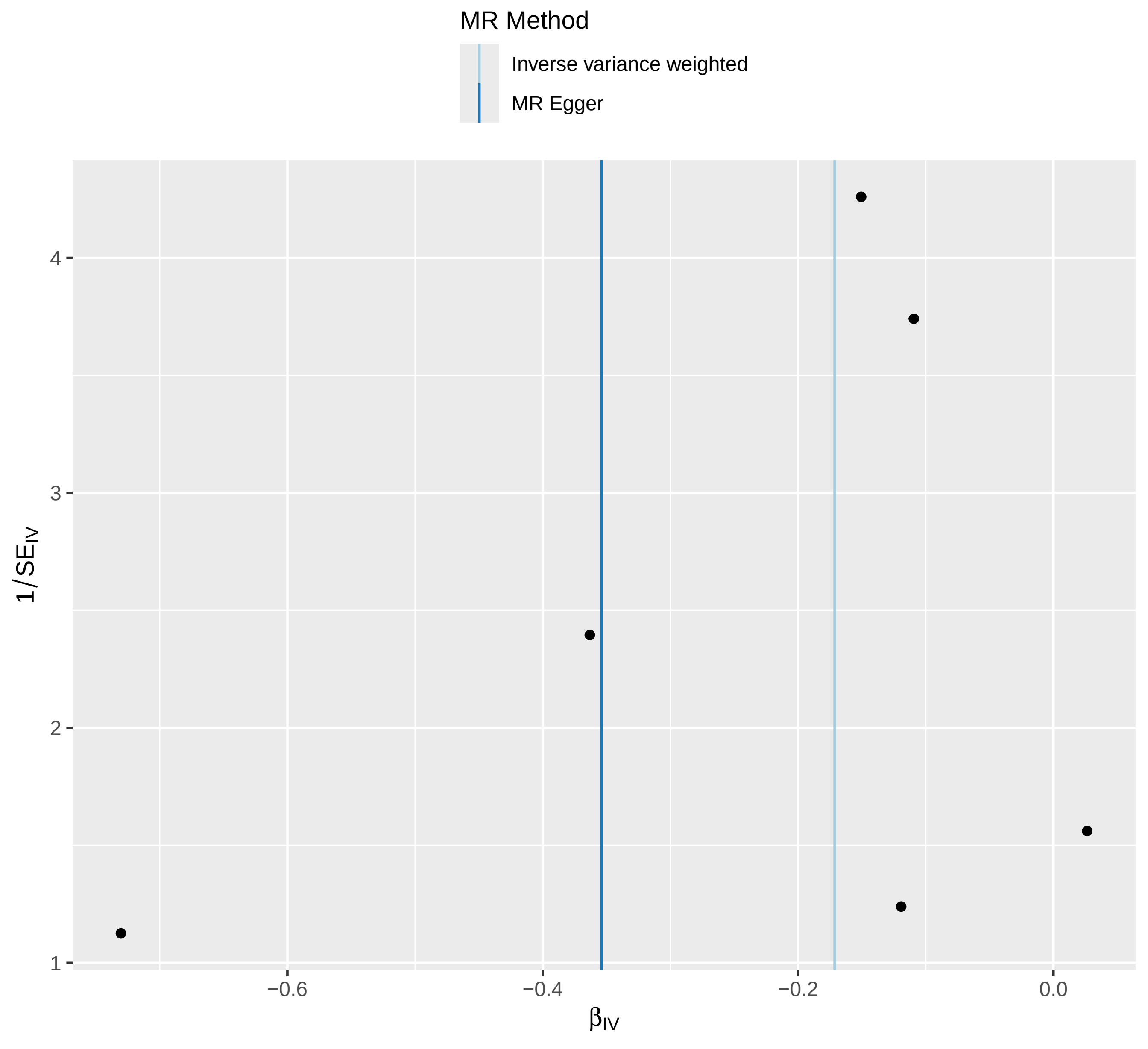
MR Method
Inverse variance weighted
MR Egger
4
3
2
1
1/SE_IV
−0.6
−0.4
−0.2
0.0
β_IV

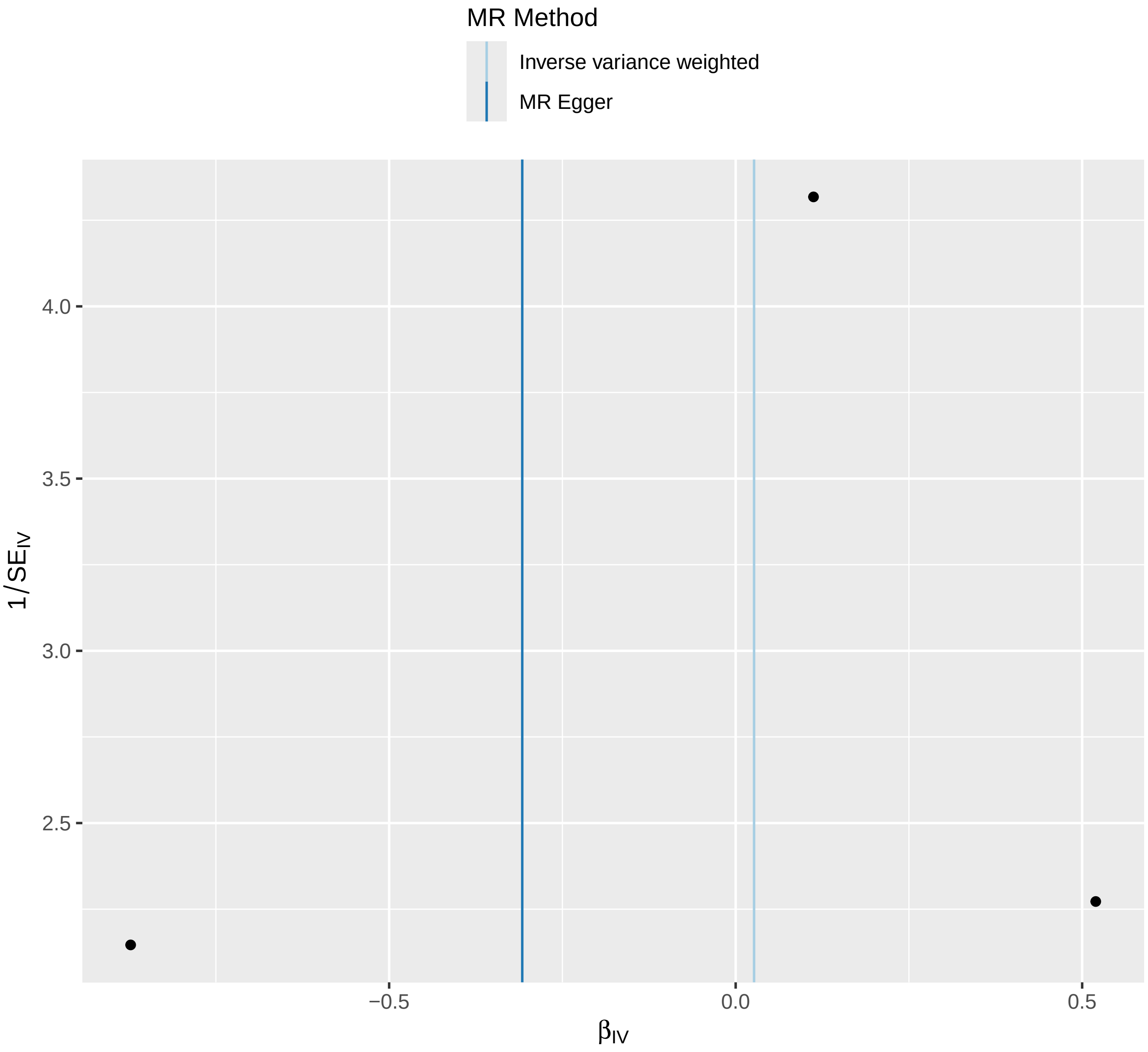

MR Method
Inverse variance weighted
MR Egger
4.0
3.5
3.0
2.5
1/SE_IV
−0.5
0.0
0.5
β_IV

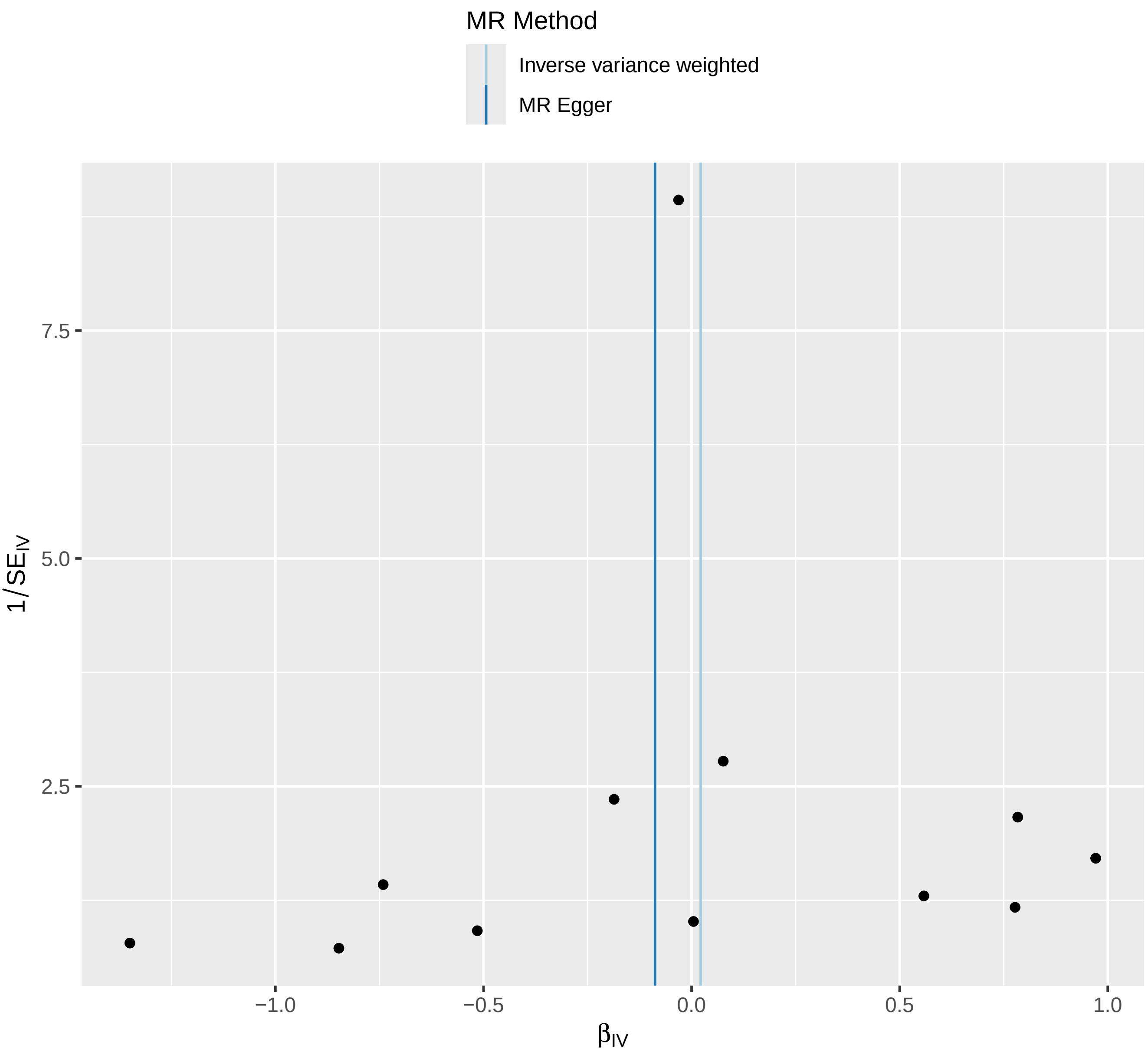
MR Method
Inverse variance weighted
MR Egger
7.5
5.0
2.5
1/SE$_{IV}$
−1.0
−0.5
0.0
0.5
1.0
β$_{IV}$

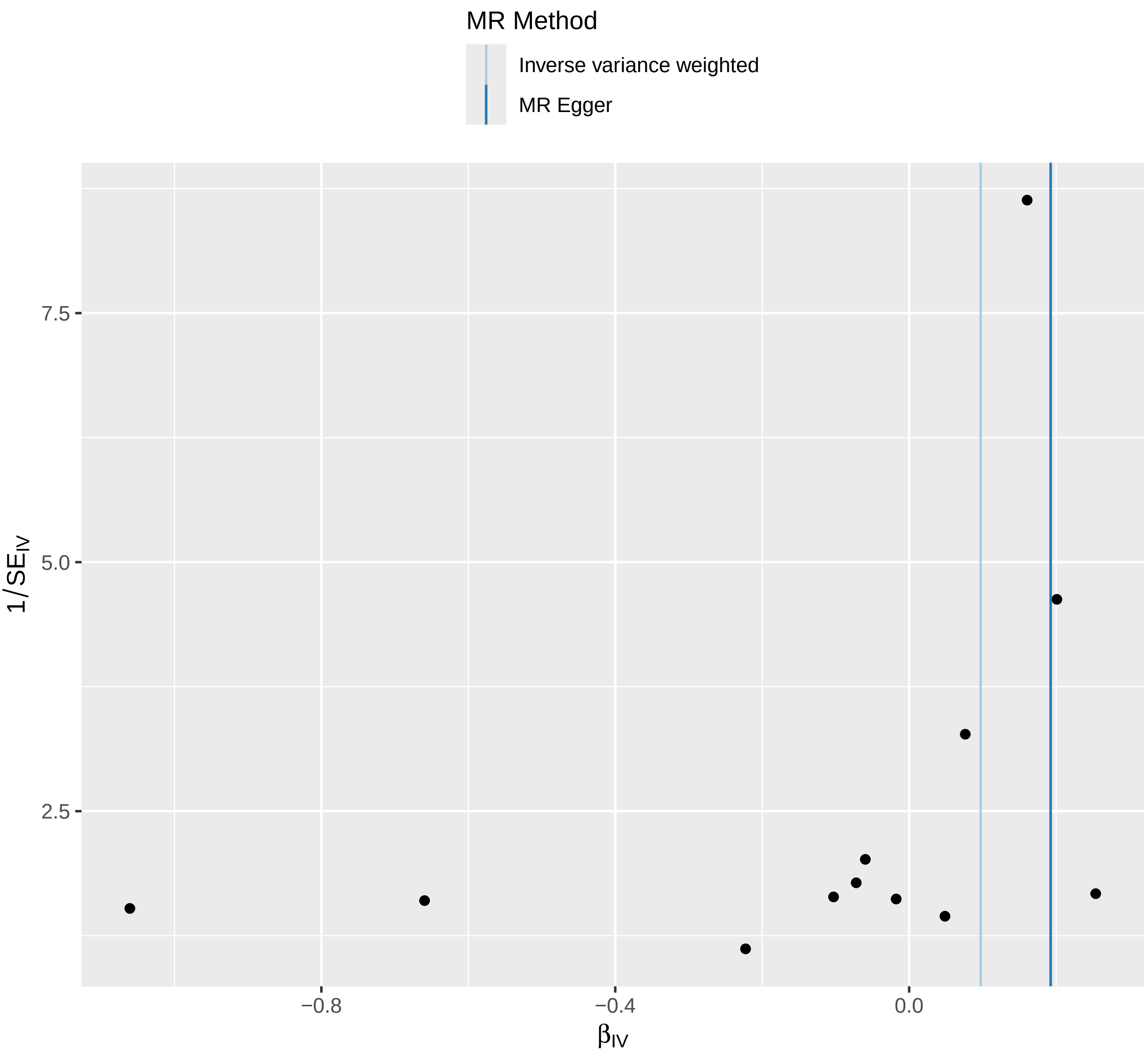

MR Method
Inverse variance weighted
MR Egger
7.5
5.0
2.5
1/SE_IV
−0.8
−0.4
0.0
β_IV

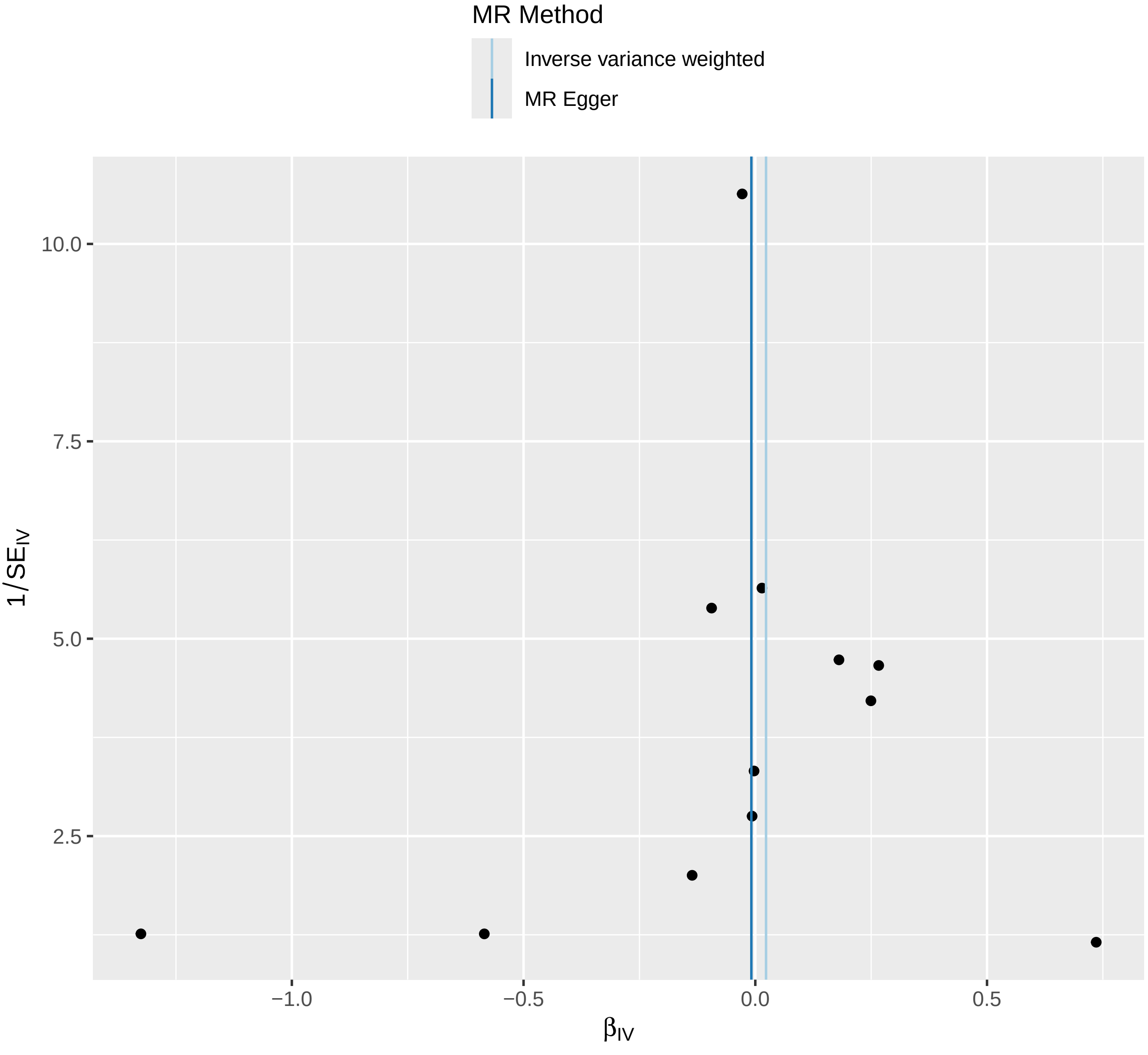

MR Method
Inverse variance weighted
MR Egger
10.0
7.5
5.0
2.5
$1/SE_{IV}$
−1.0
−0.5
0.0
0.5
β_{IV}

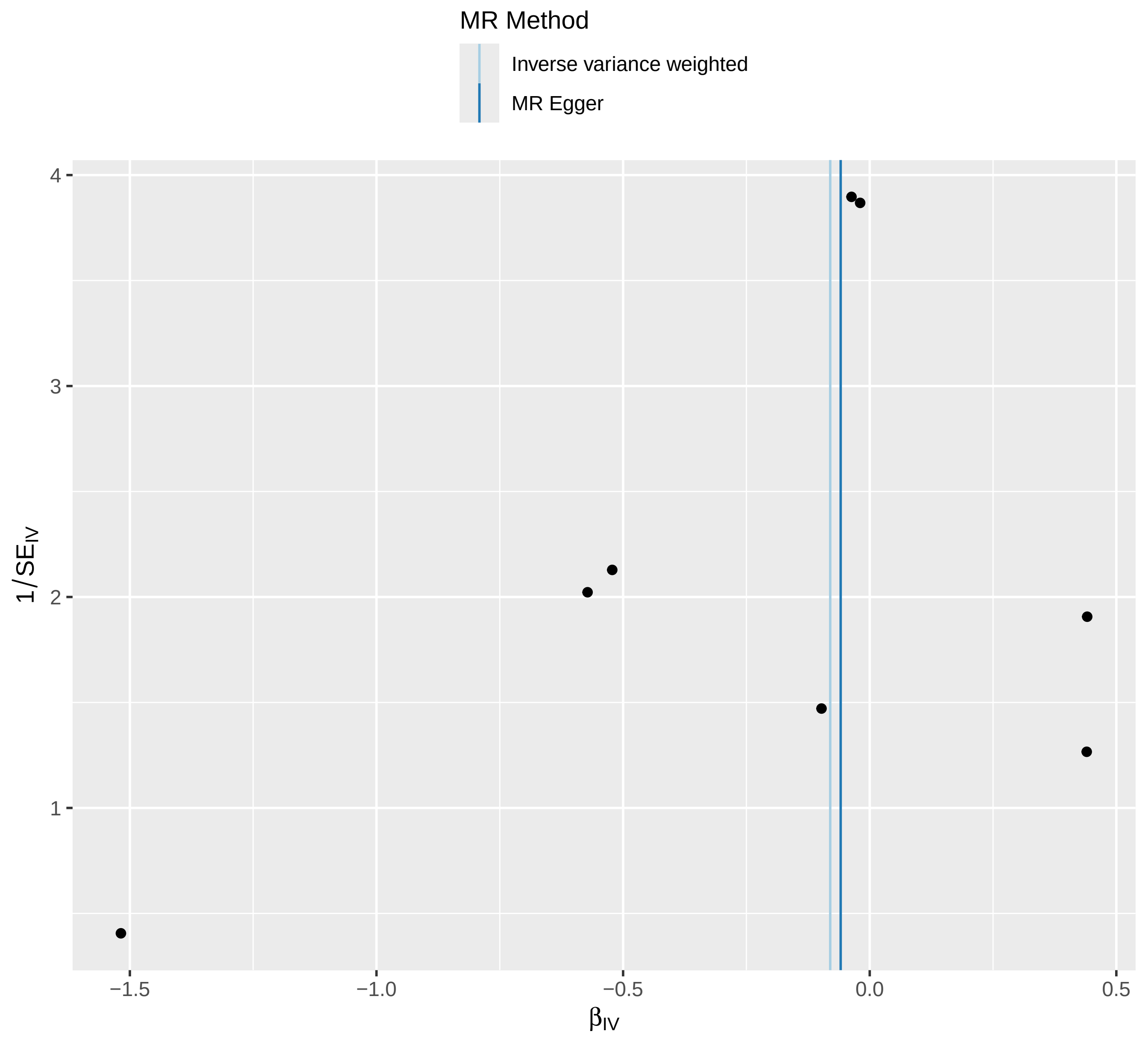
MR Method
Inverse variance weighted
MR Egger
1/SE_IV
β_IV
4
3
2
1
−1.5
−1.0
−0.5
0.0
0.5

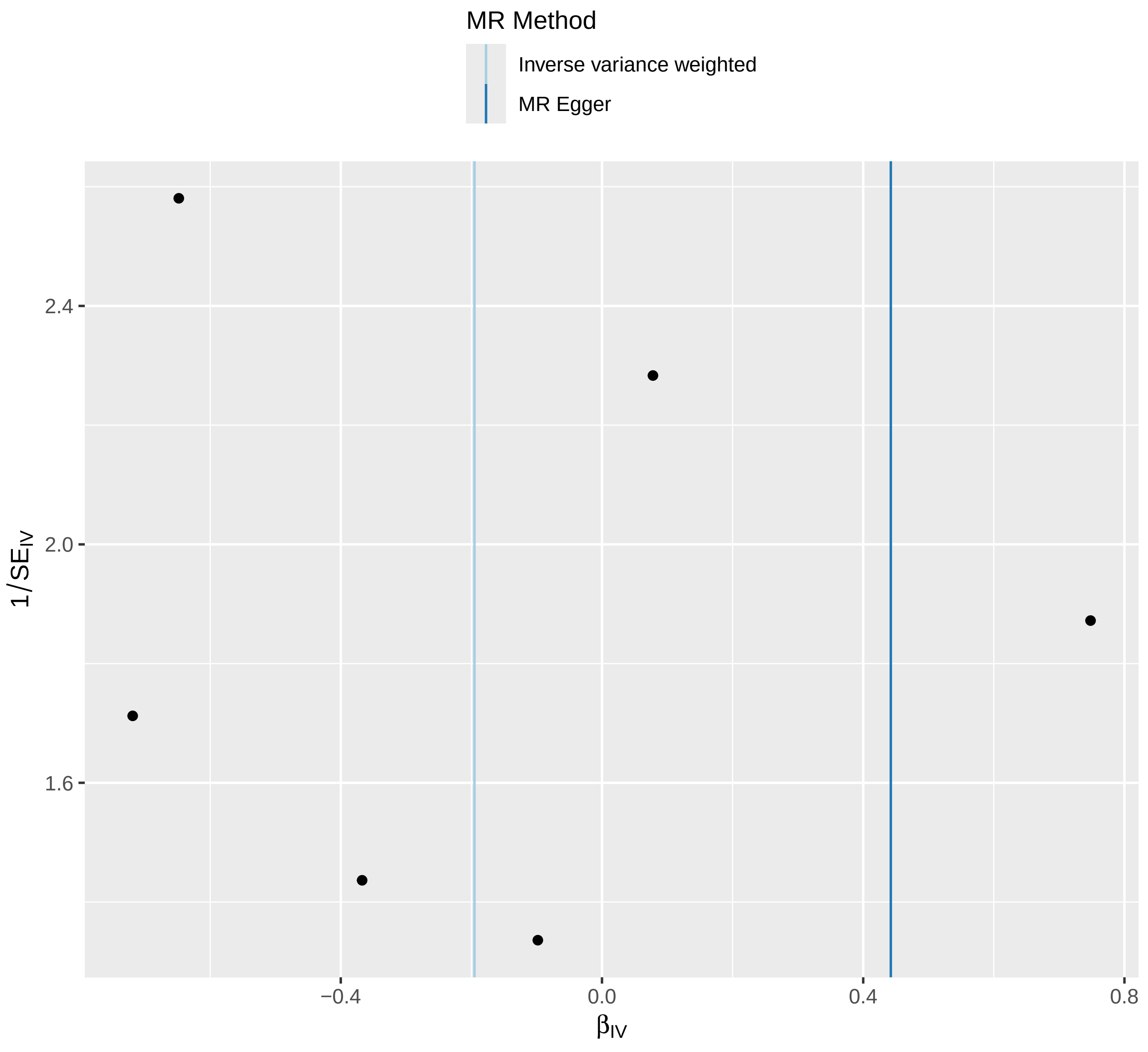
MR Method
Inverse variance weighted
MR Egger
2.4
2.0
1.6
1/SE$_{IV}$
−0.4
0.0
0.4
0.8
β$_{IV}$

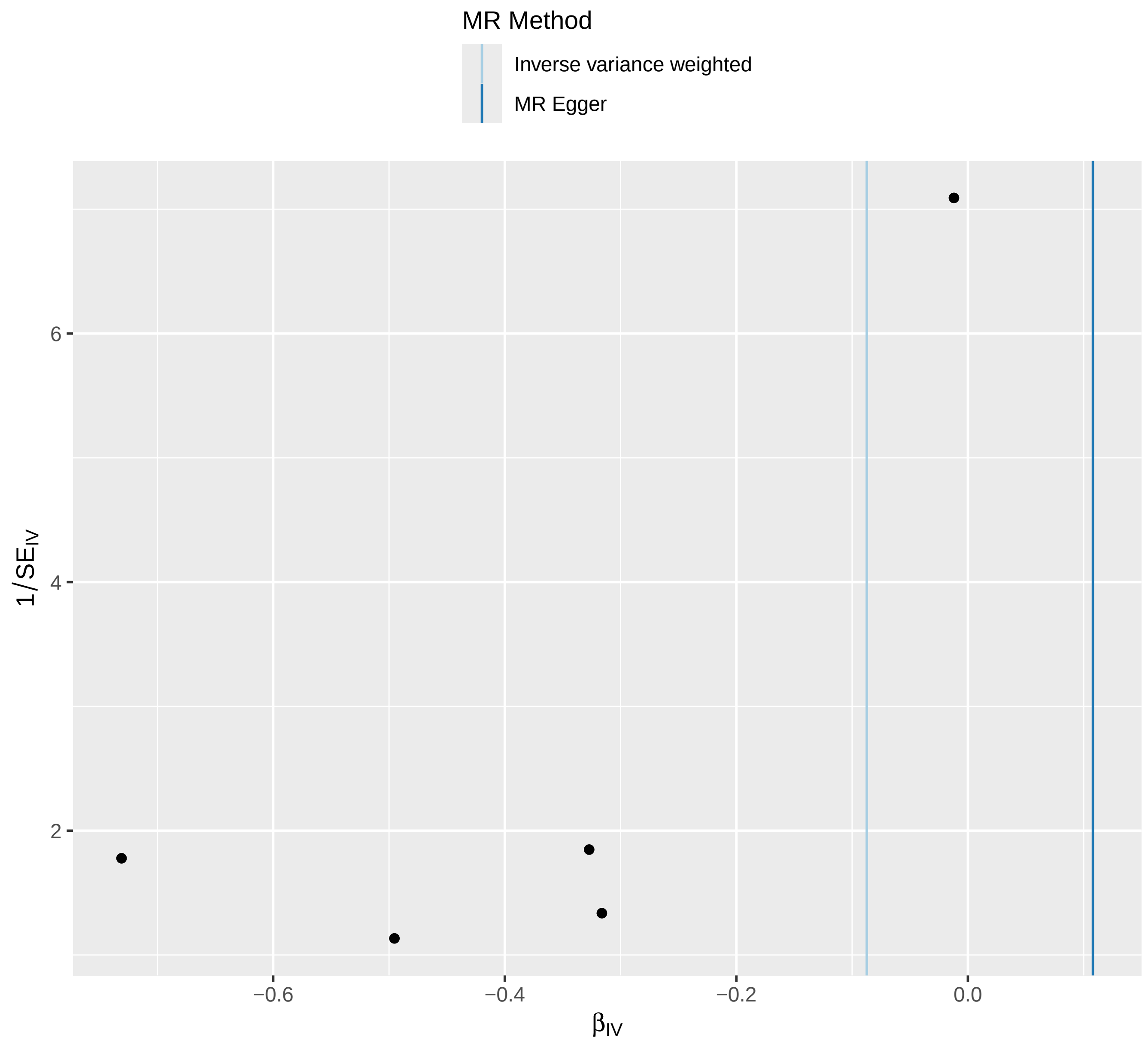

MR Method
Inverse variance weighted
MR Egger
6
4
2
1/SE_IV
−0.6
−0.4
−0.2
0.0
β_IV

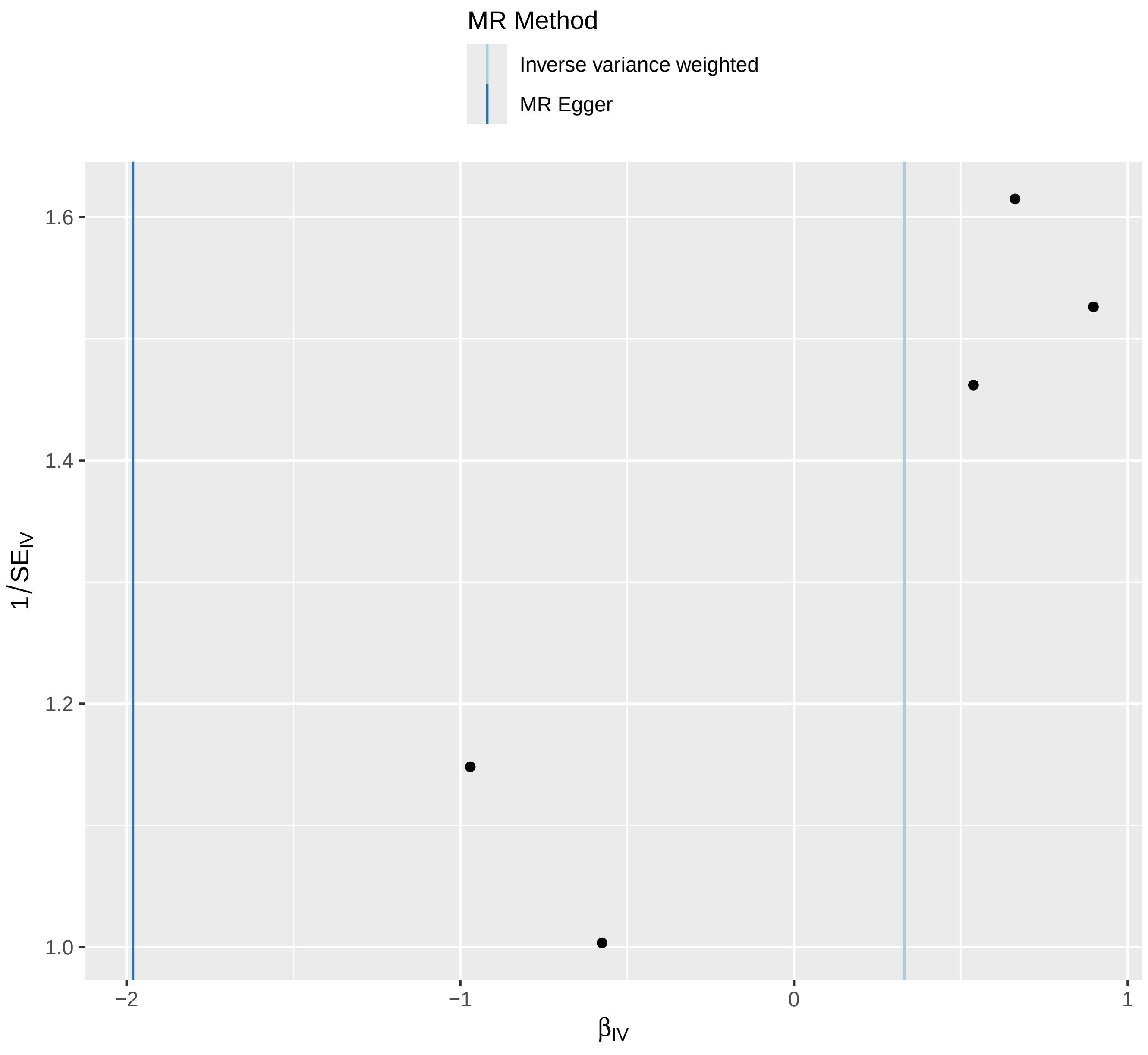

MR Method
Inverse variance weighted
MR Egger
1.6
1.4
1.2
1.0
$1/SE_{IV}$
−2
−1
0
1
β_{IV}

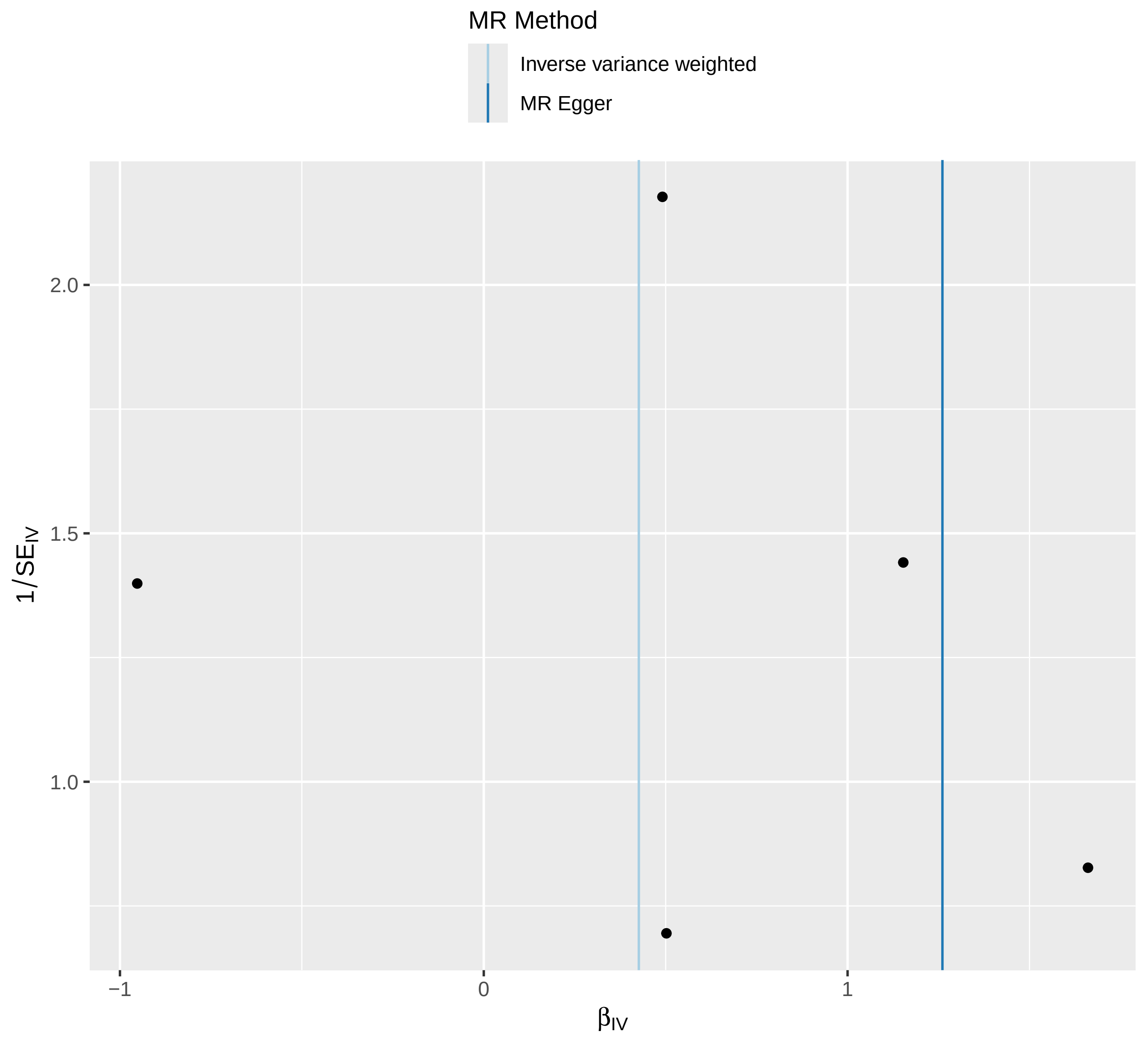
MR Method
Inverse variance weighted
MR Egger
2.0
1.5
1.0
$1/SE_{IV}$
−1
0
1
β_{IV}

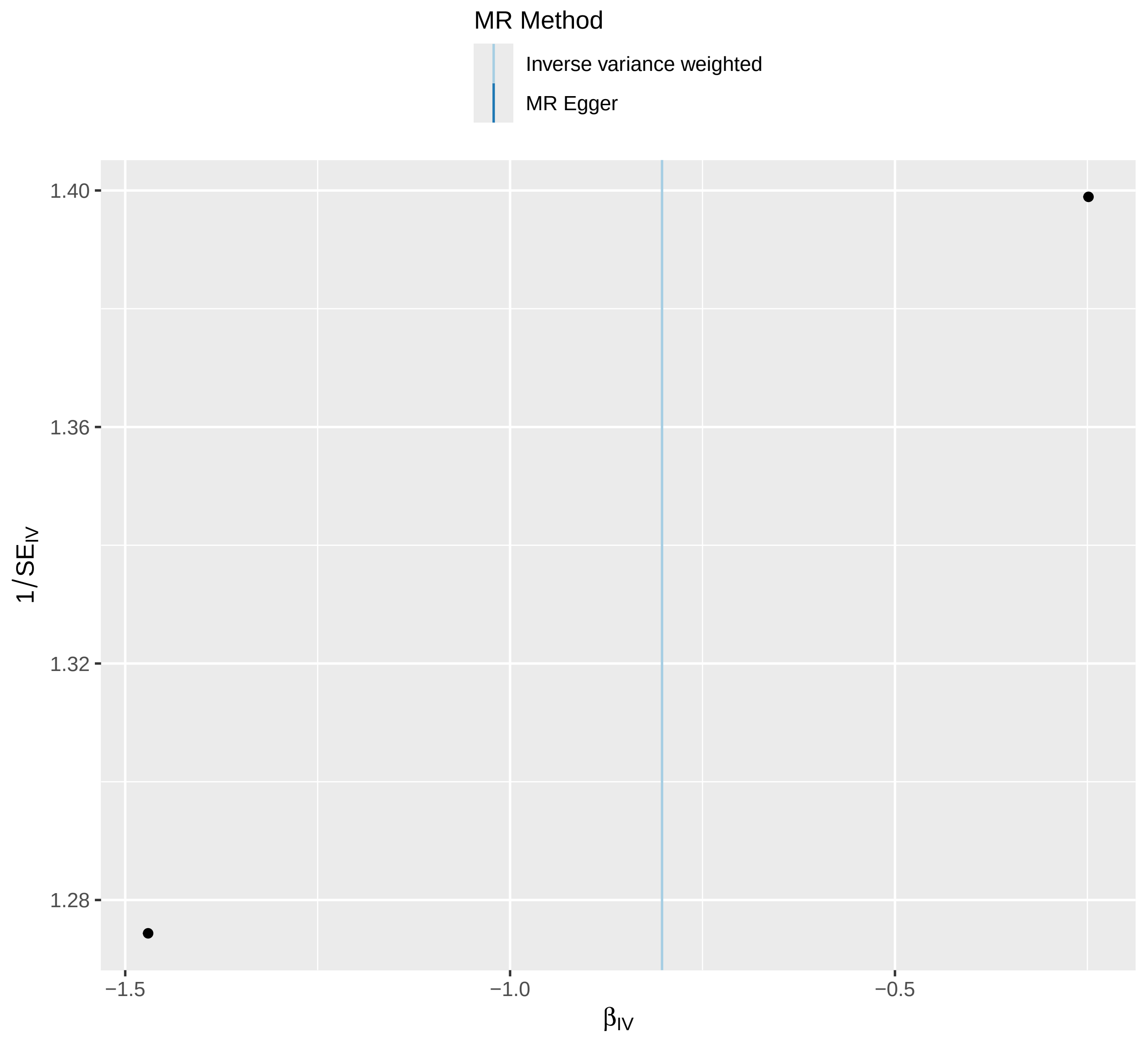

MR Method
Inverse variance weighted
MR Egger
1.40
1.36
1.32
1.28
1/SE_IV
−1.5
−1.0
−0.5
β_IV

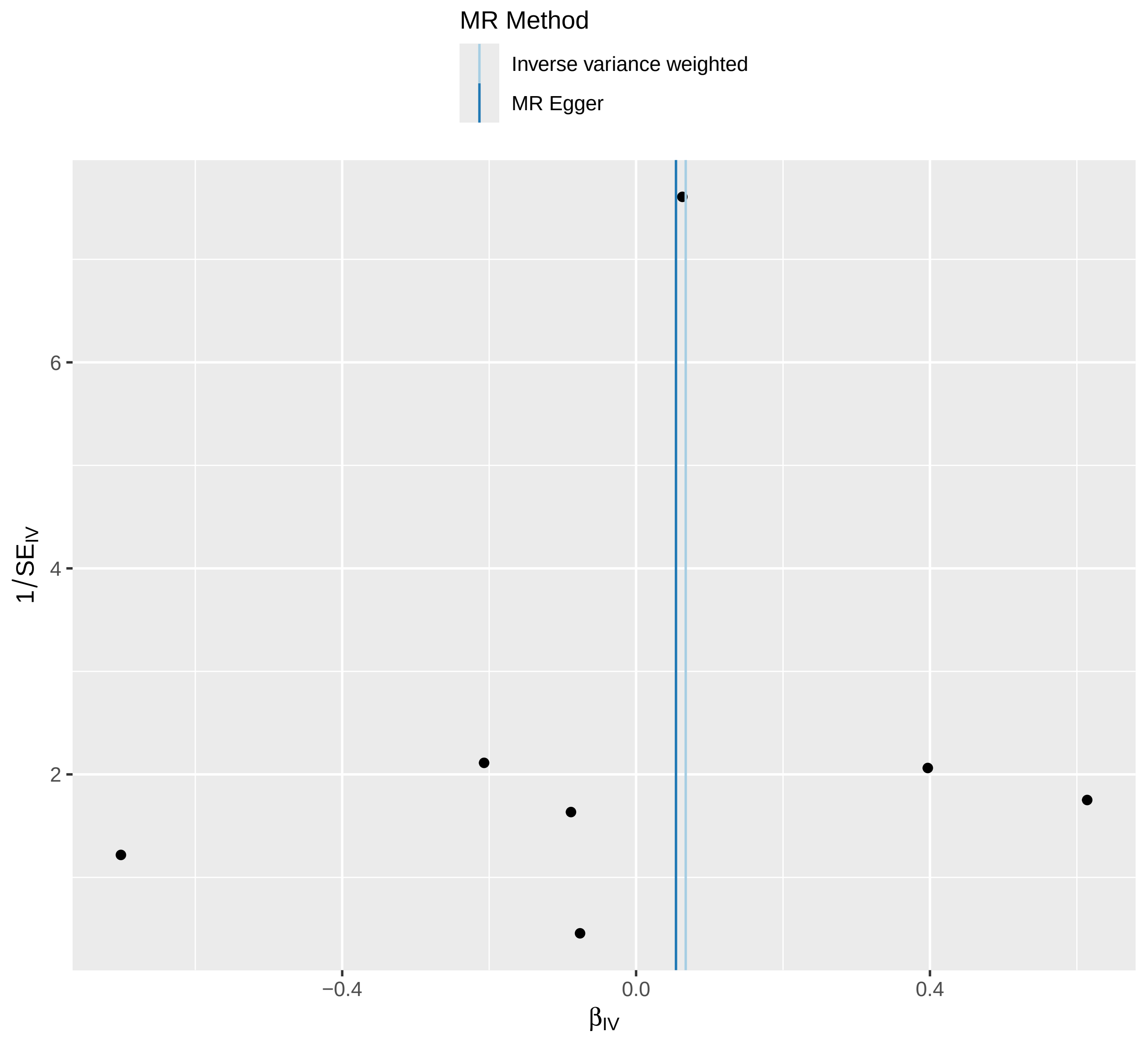
MR Method
Inverse variance weighted
MR Egger
1/SE$_{IV}$
6
4
2
−0.4
0.0
0.4
β$_{IV}$

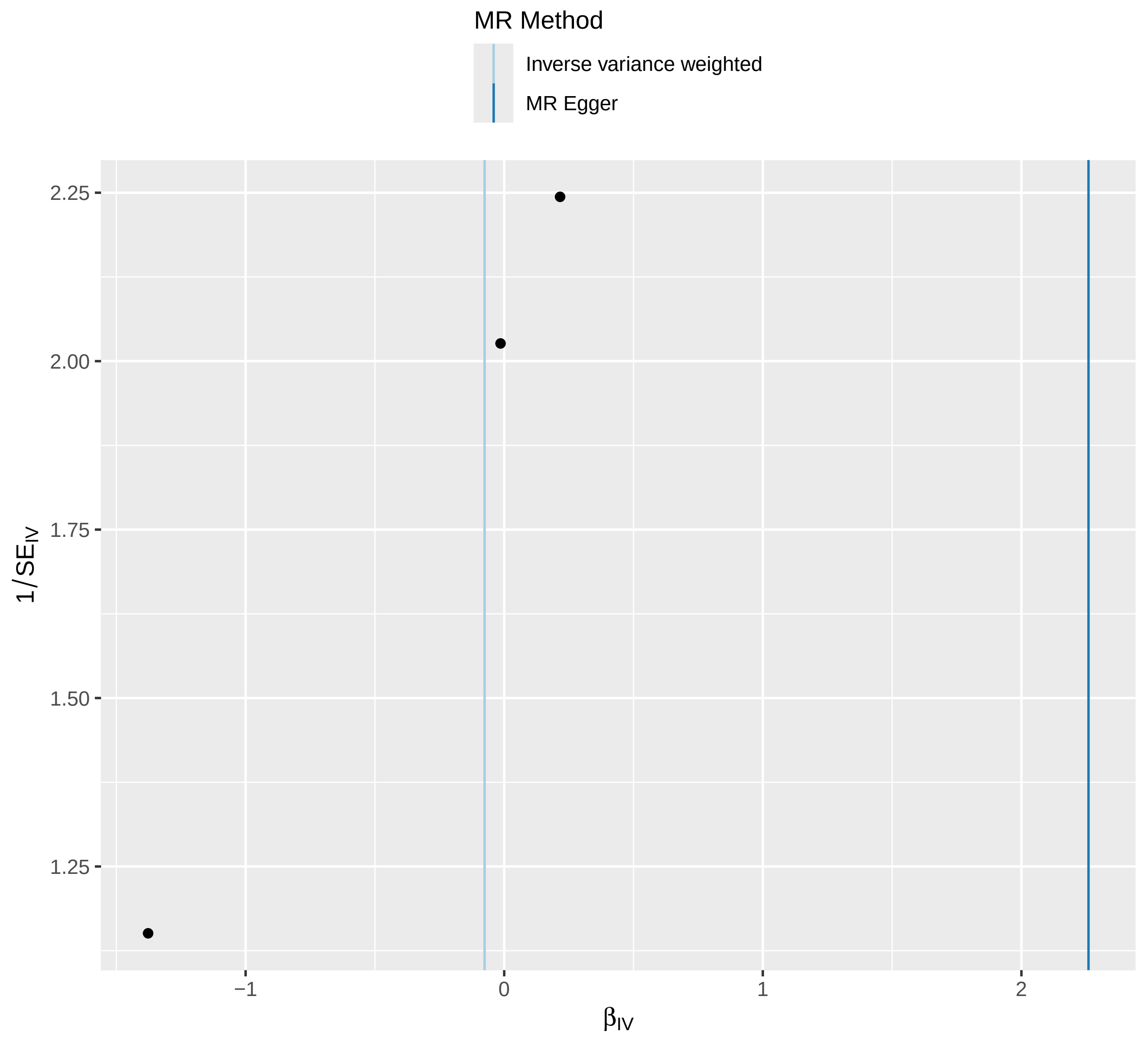

MR Method
Inverse variance weighted
MR Egger
2.25
2.00
1.75
1.50
1.25
1/SE_IV
−1
0
1
2
β_IV

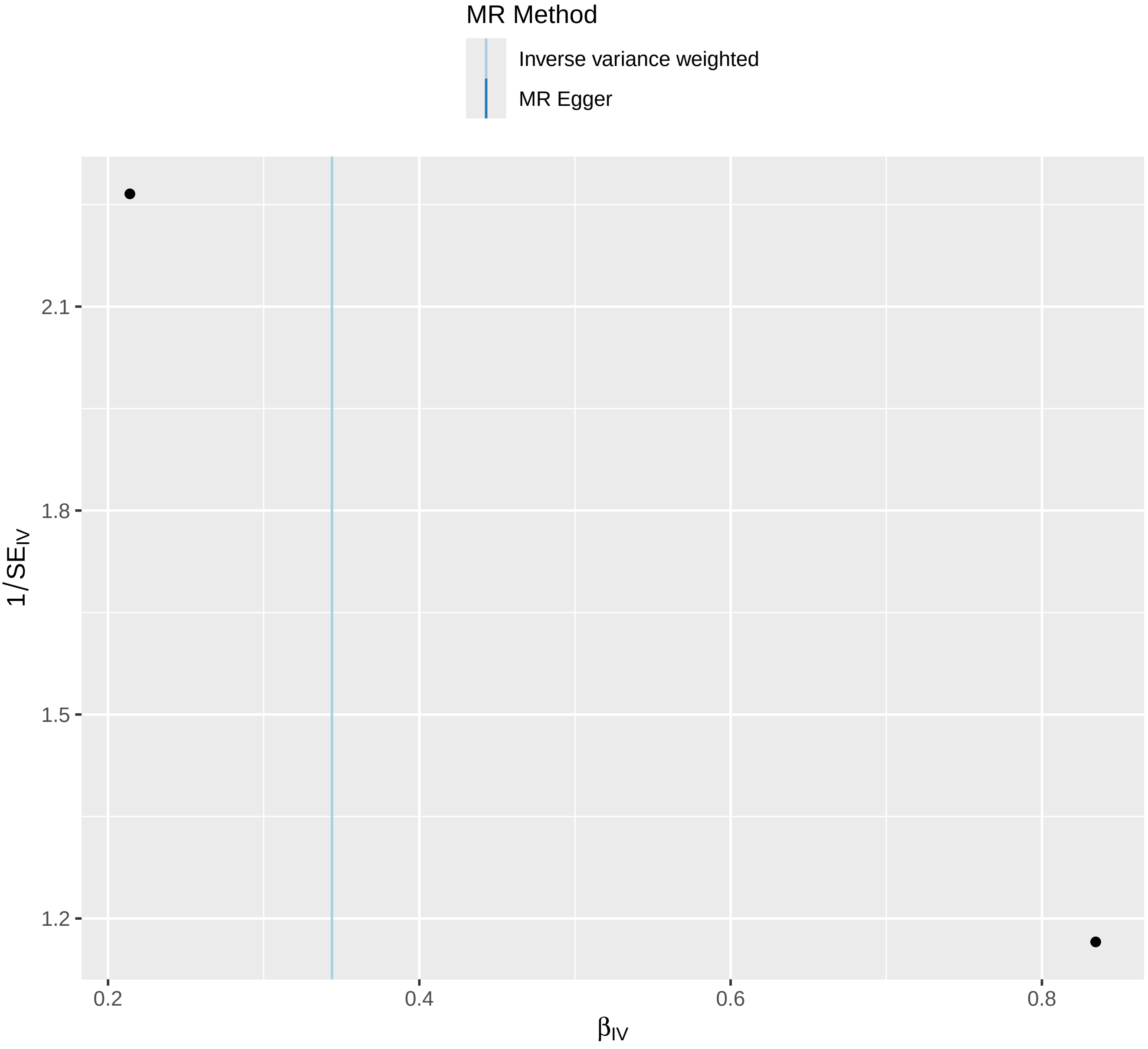

MR Method
Inverse variance weighted
MR Egger
2.1
1.8
1.5
1.2
$1/SE_{IV}$
0.2
0.4
0.6
0.8
β_{IV}

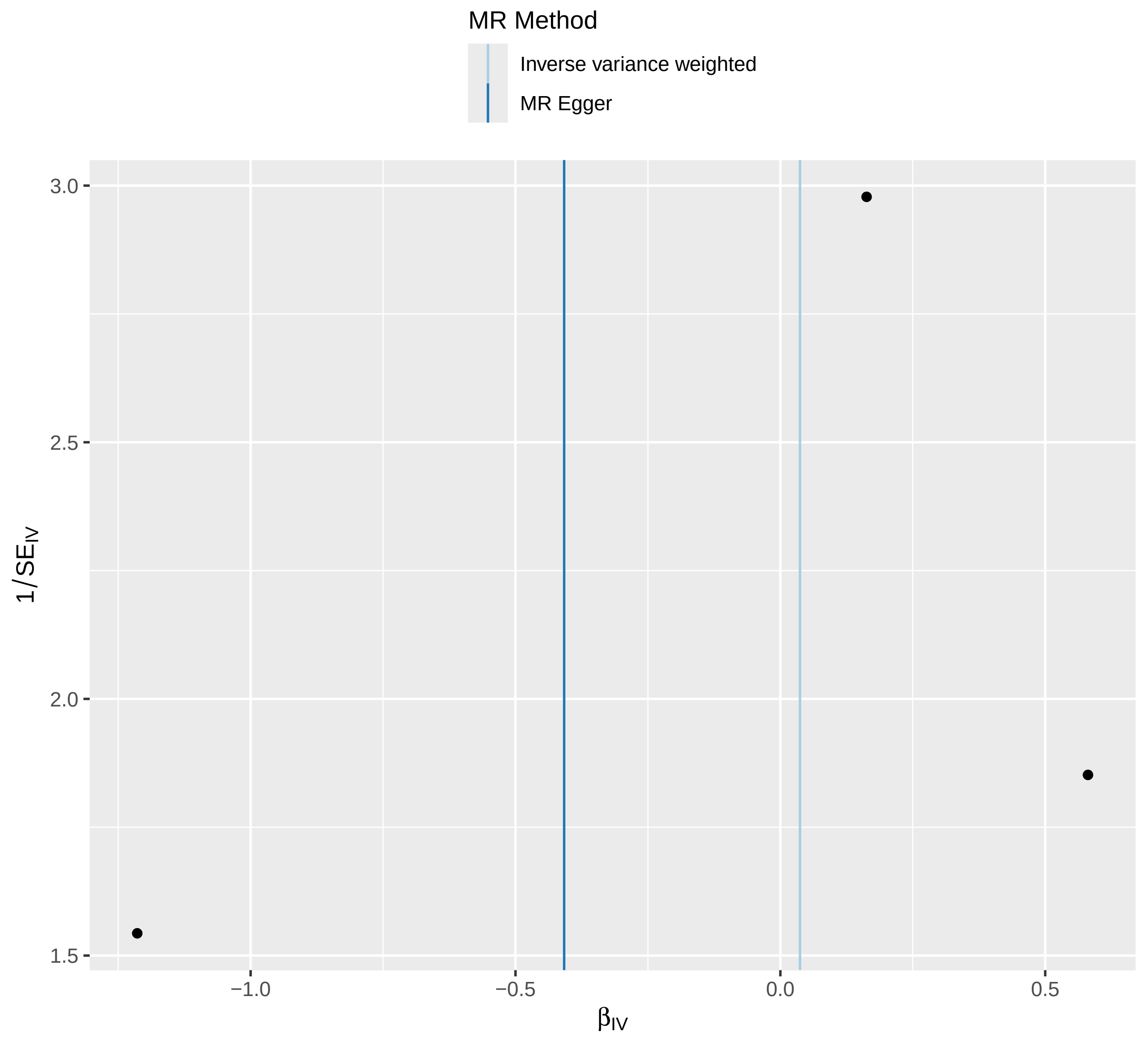
MR Method
Inverse variance weighted
MR Egger
3.0
2.5
2.0
1.5
$1/SE_{IV}$
−1.0
−0.5
0.0
0.5
β_{IV}

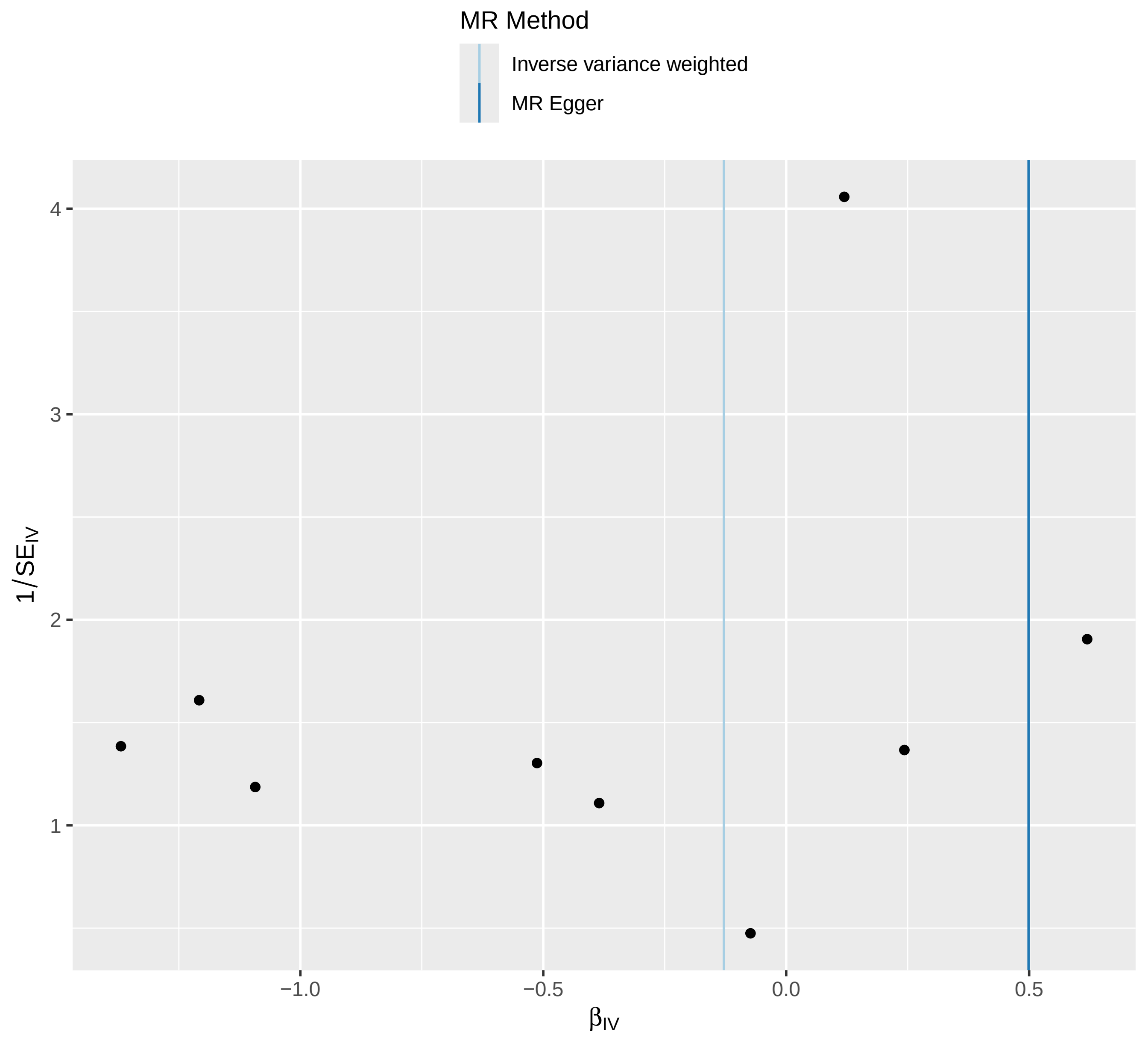
MR Method
Inverse variance weighted
MR Egger
4
3
2
1
$1/SE_{IV}$
−1.0
−0.5
0.0
0.5
β_{IV}

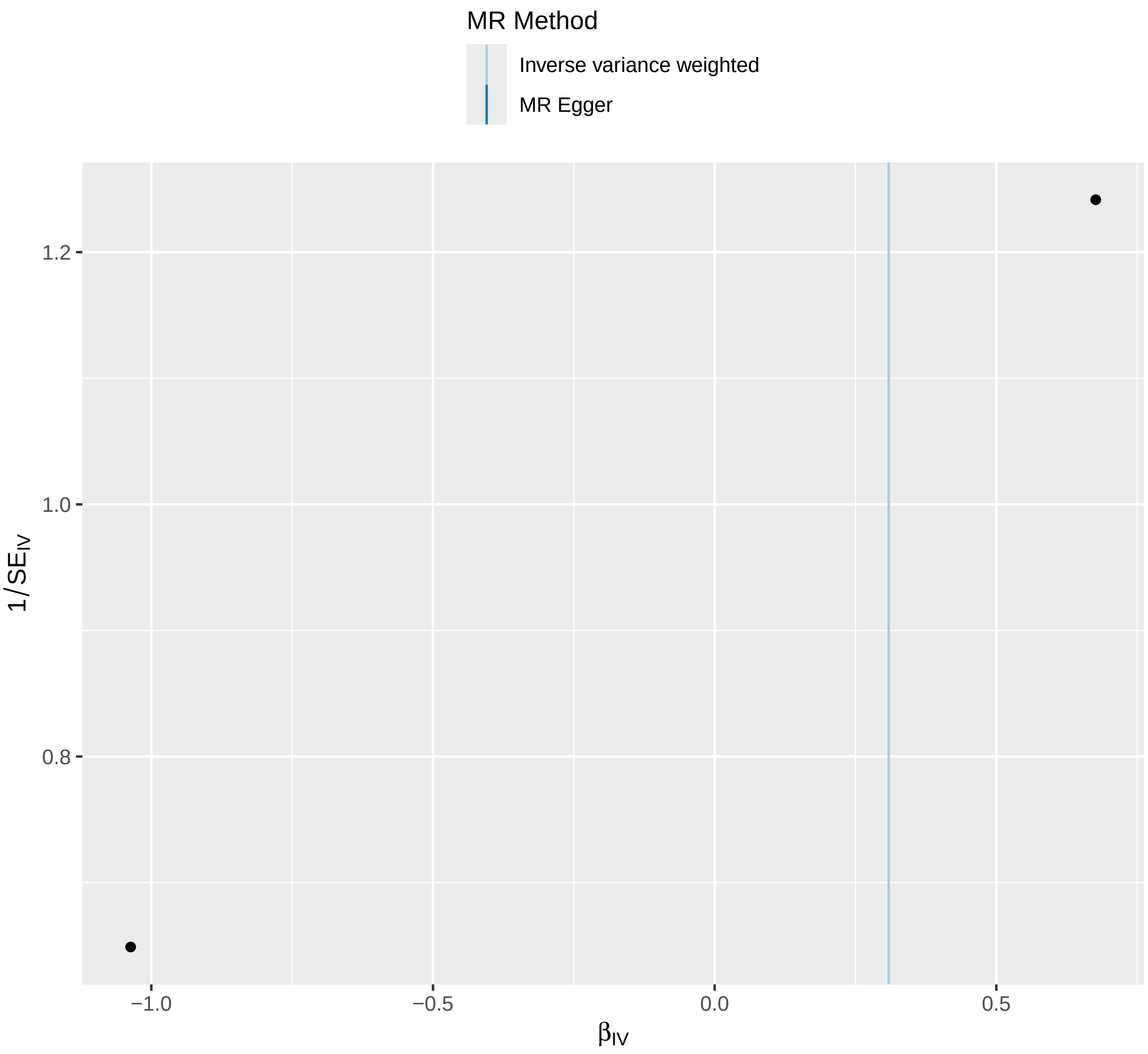

MR Method
Inverse variance weighted
MR Egger
1.2
1.0
0.8
1/SE_{IV}
−1.0
−0.5
0.0
0.5
β_{IV}

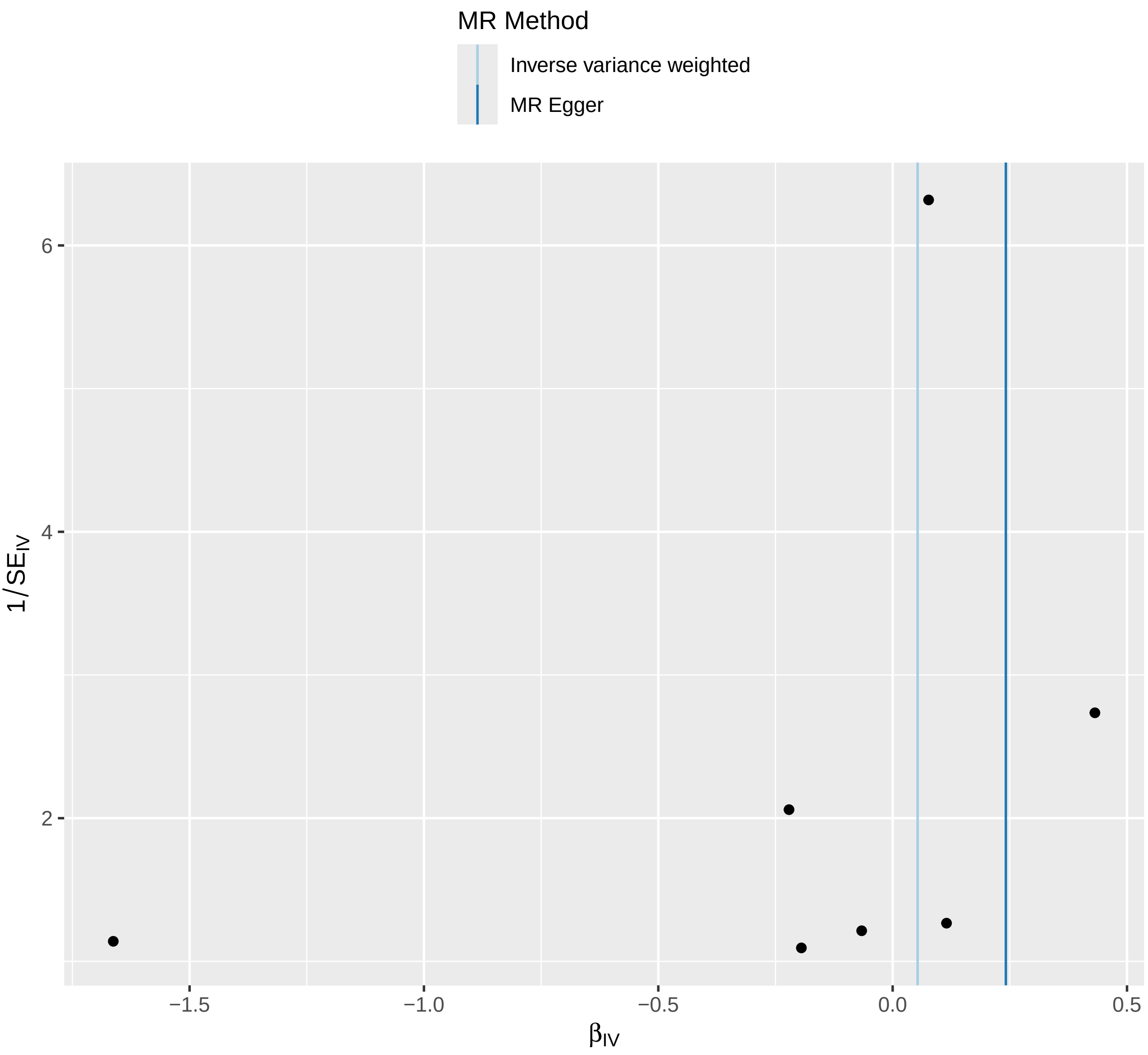
MR Method
Inverse variance weighted
MR Egger
6
4
2
1/SE_IV
−1.5
−1.0
−0.5
0.0
0.5
β_IV

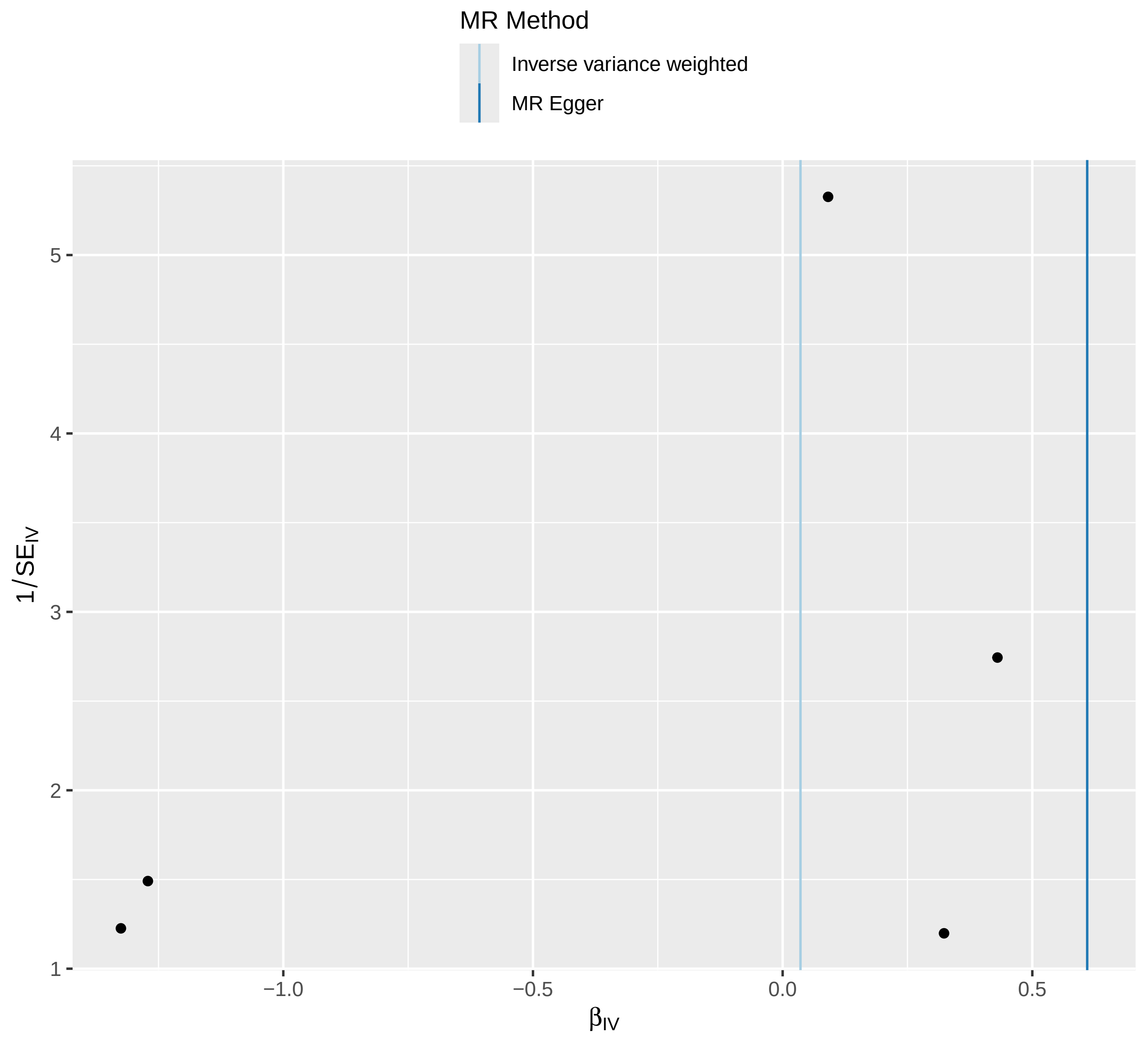
MR Method
Inverse variance weighted
MR Egger
1/SE$_{IV}$
5
4
3
2
1
−1.0
−0.5
0.0
0.5
β_{IV}

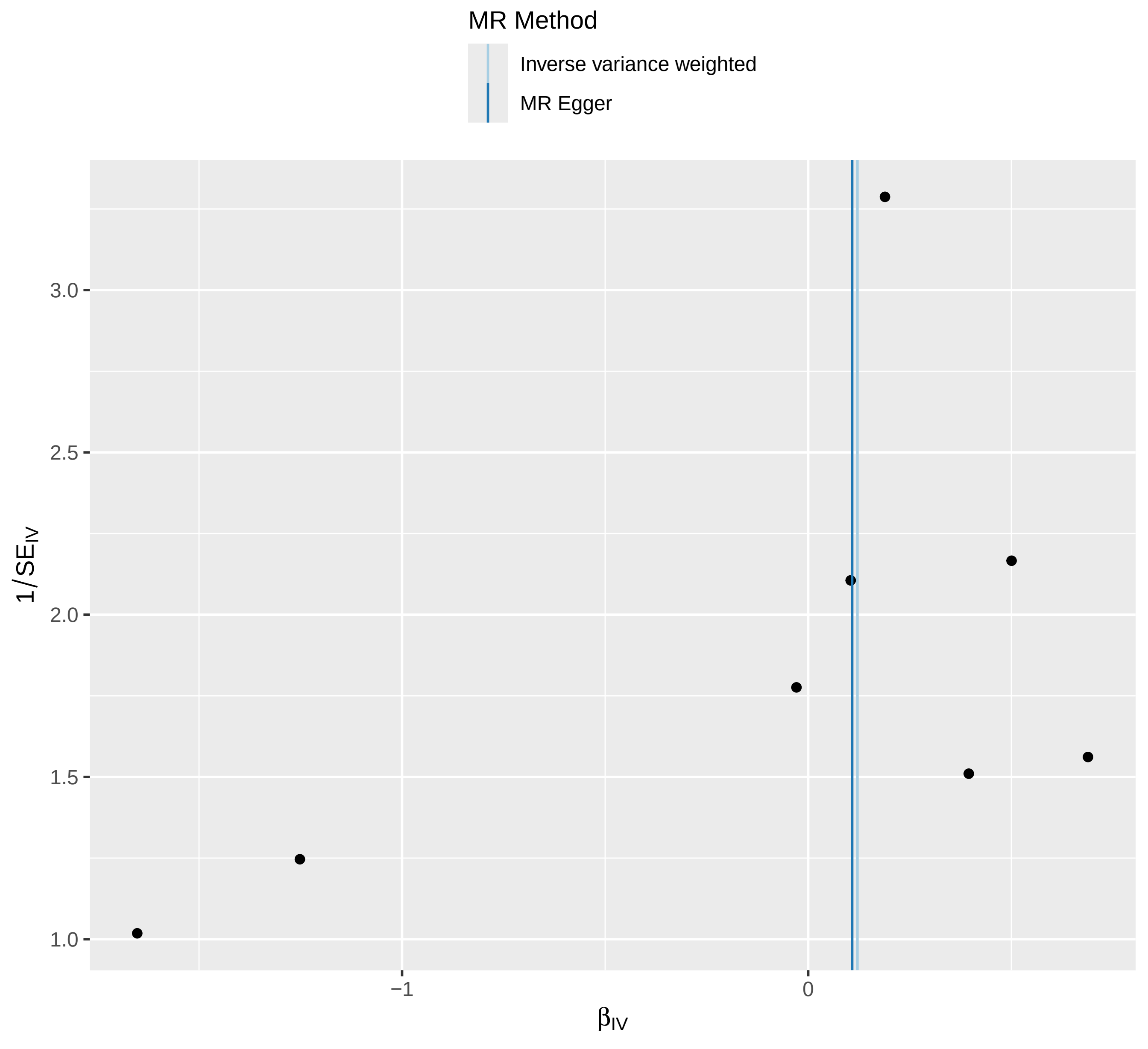

MR Method
Inverse variance weighted
MR Egger
1/SE_IV
3.0
2.5
2.0
1.5
1.0
−1
0
β_IV

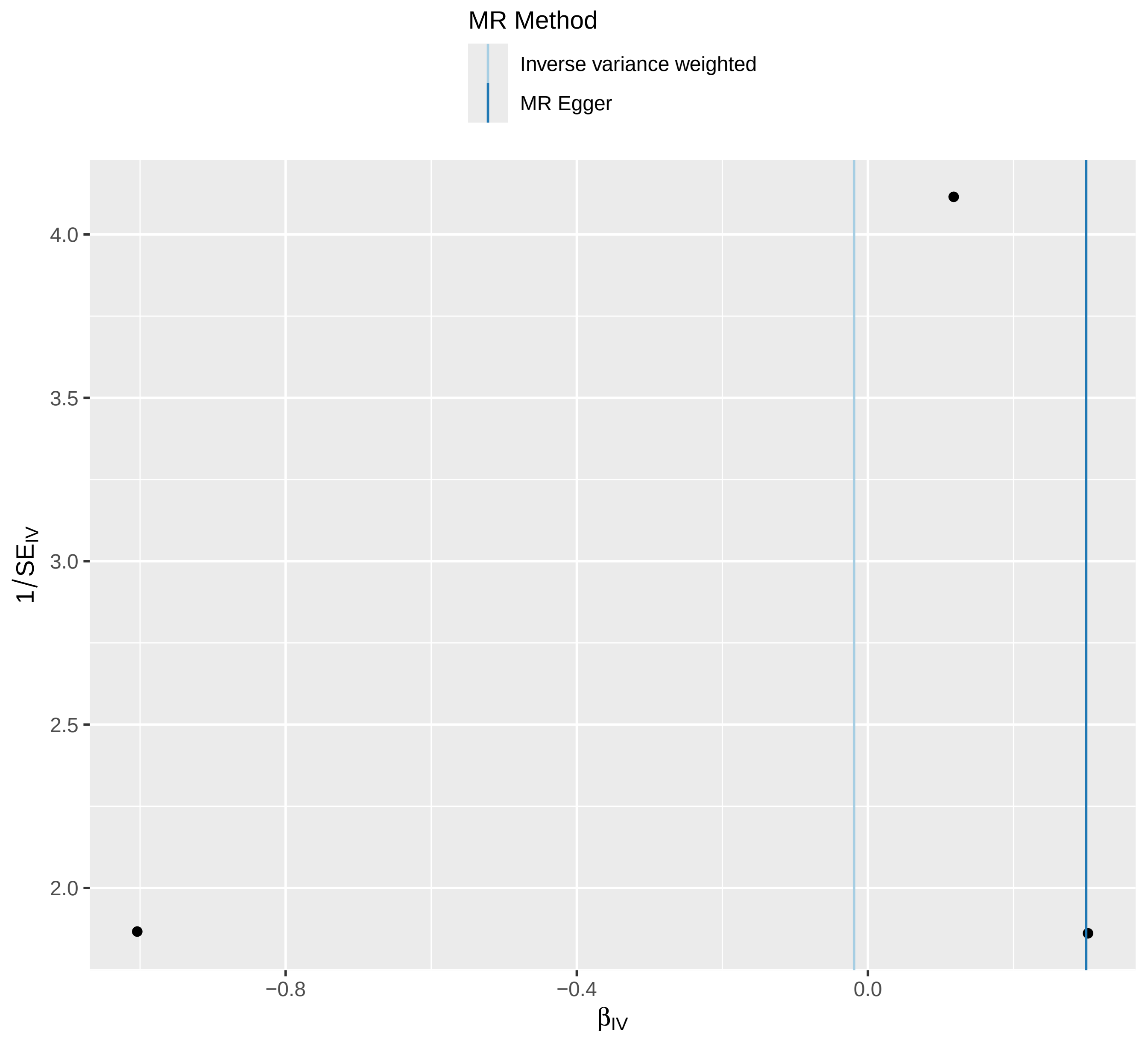
MR Method
Inverse variance weighted
MR Egger
4.0
3.5
3.0
2.5
2.0
1/SE$_{IV}$
−0.8
−0.4
0.0
β$_{IV}$

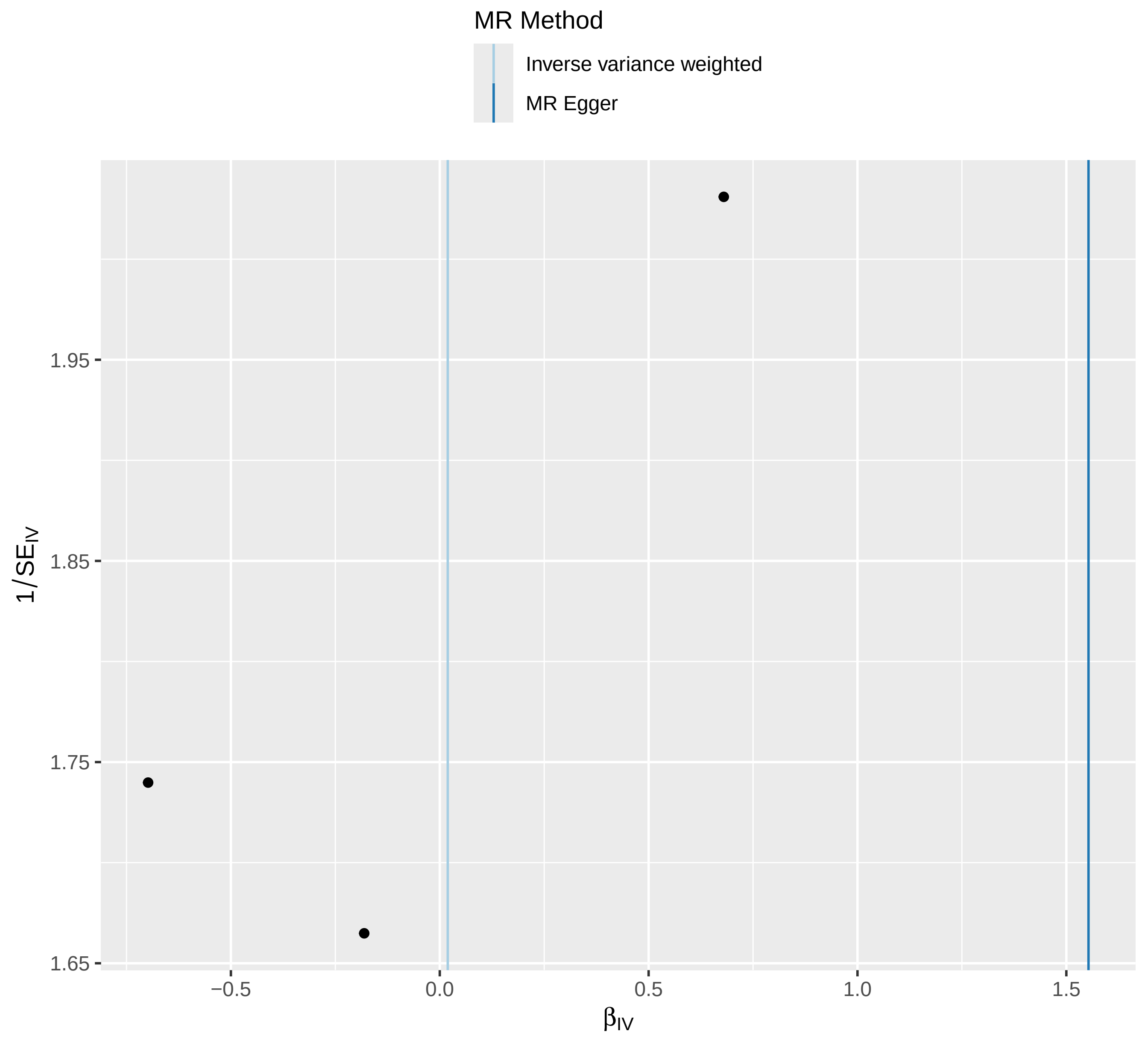
MR Method
Inverse variance weighted
MR Egger
1.95
1.85
1.75
1.65
$1/SE_{IV}$
−0.5
0.0
0.5
1.0
1.5
β_{IV}

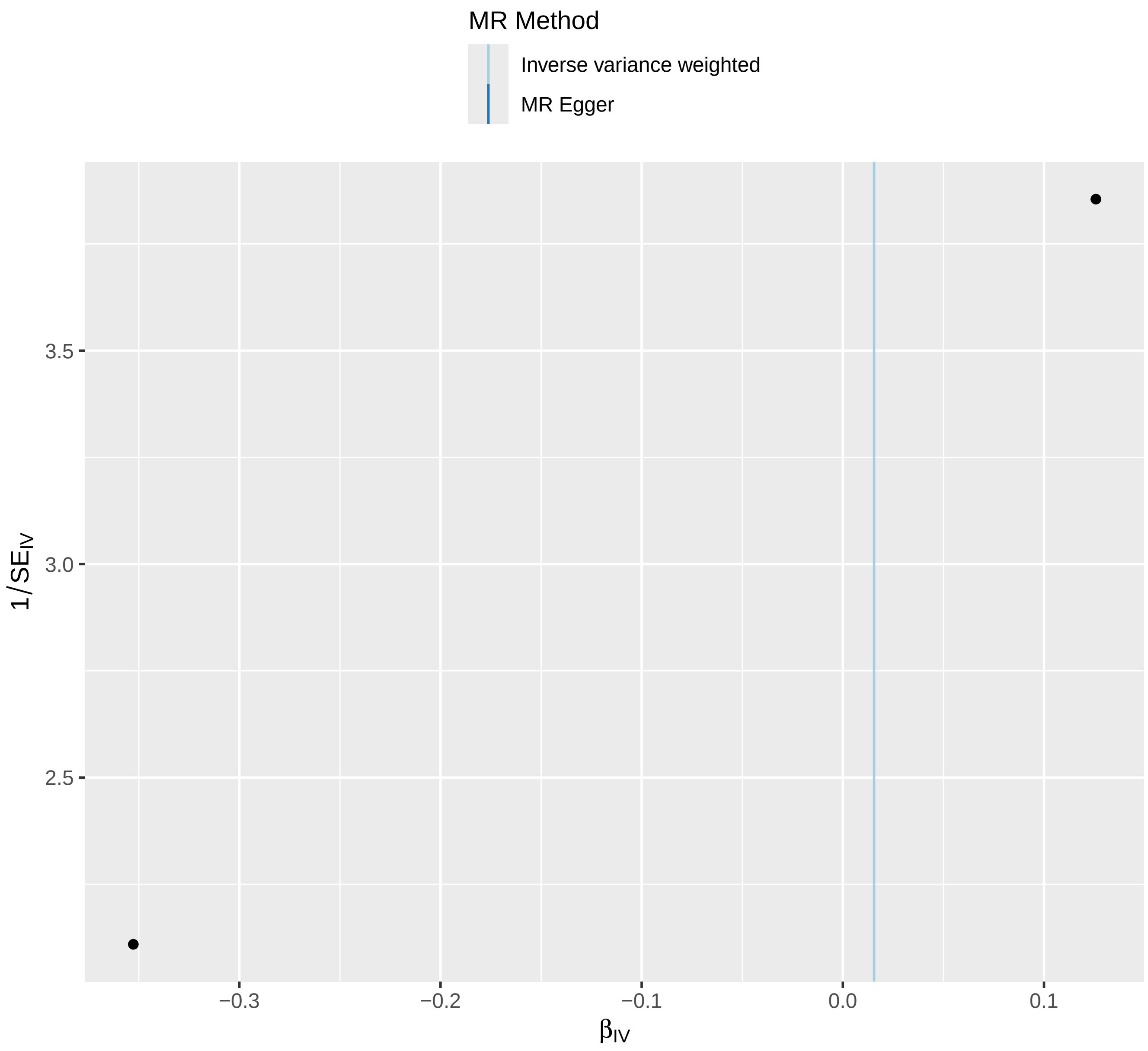
MR Method
Inverse variance weighted
MR Egger
3.5
3.0
2.5
1/SE_IV
−0.3
−0.2
−0.1
0.0
0.1
β_IV

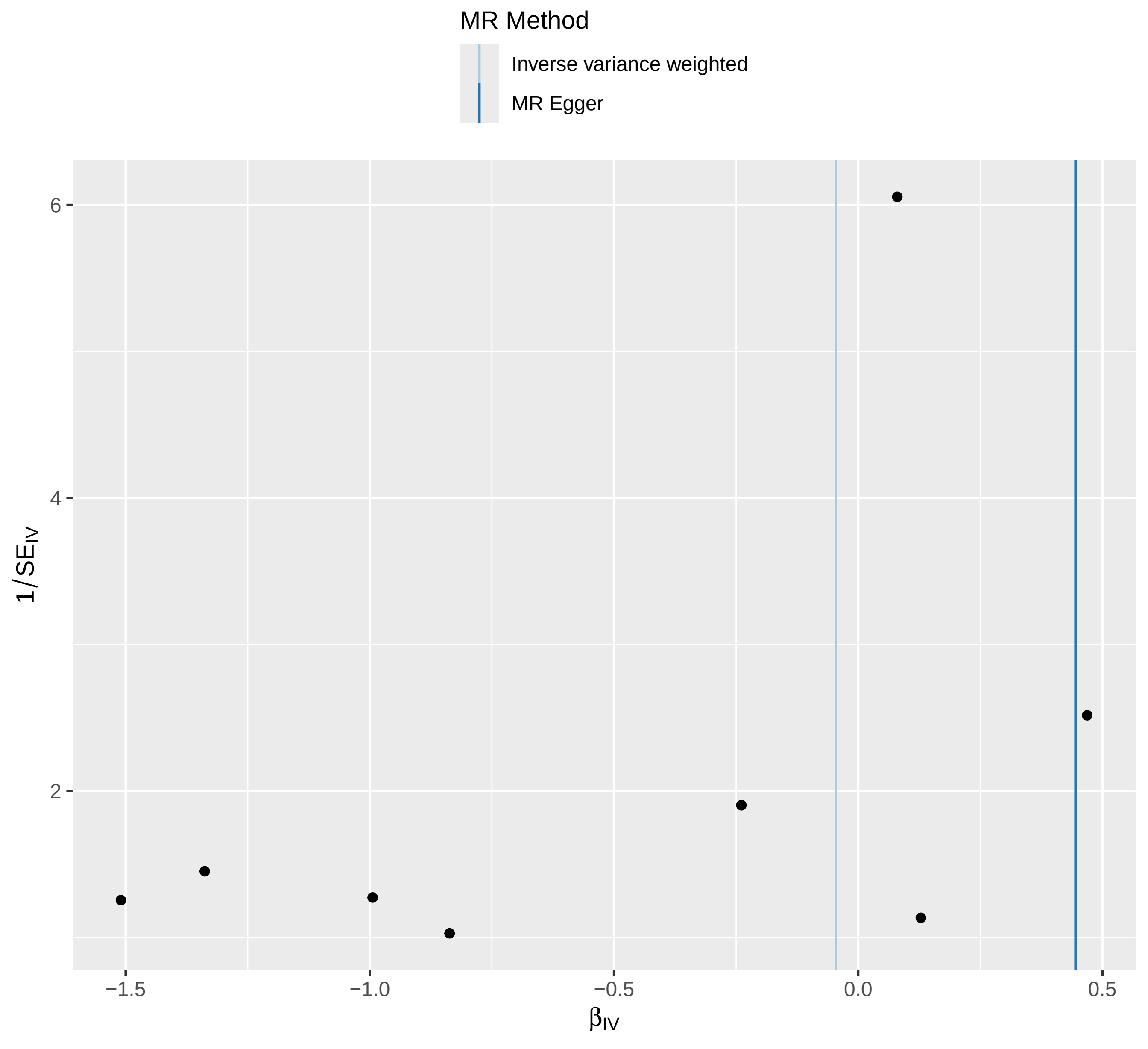

MR Method
Inverse variance weighted
MR Egger
6
4
2
1/SE$_{IV}$
−1.5
−1.0
−0.5
0.0
0.5
β$_{IV}$

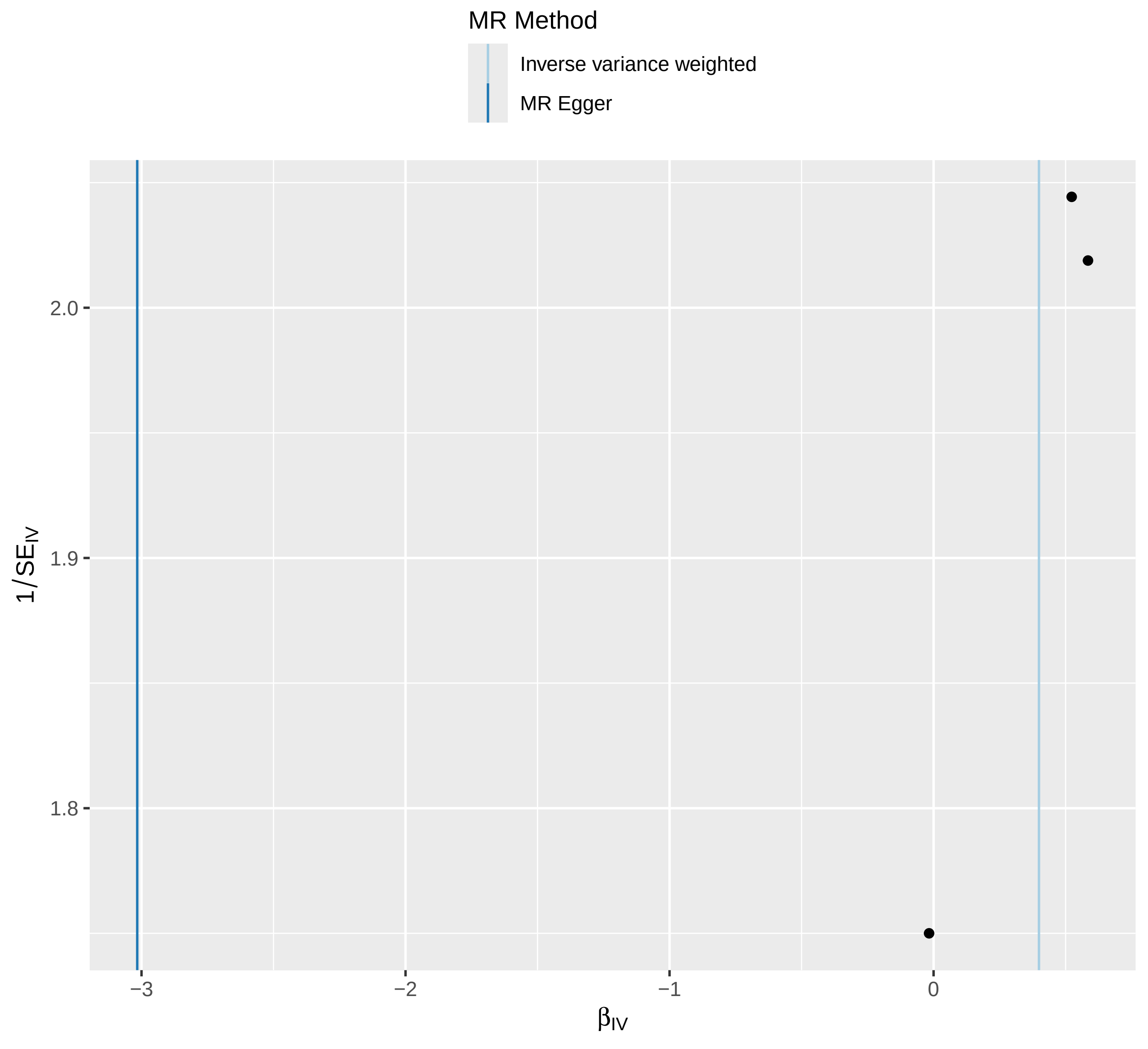
MR Method
Inverse variance weighted
MR Egger
2.0
1.9
1.8
1/SE$_{IV}$
−3
−2
−1
0
β$_{IV}$

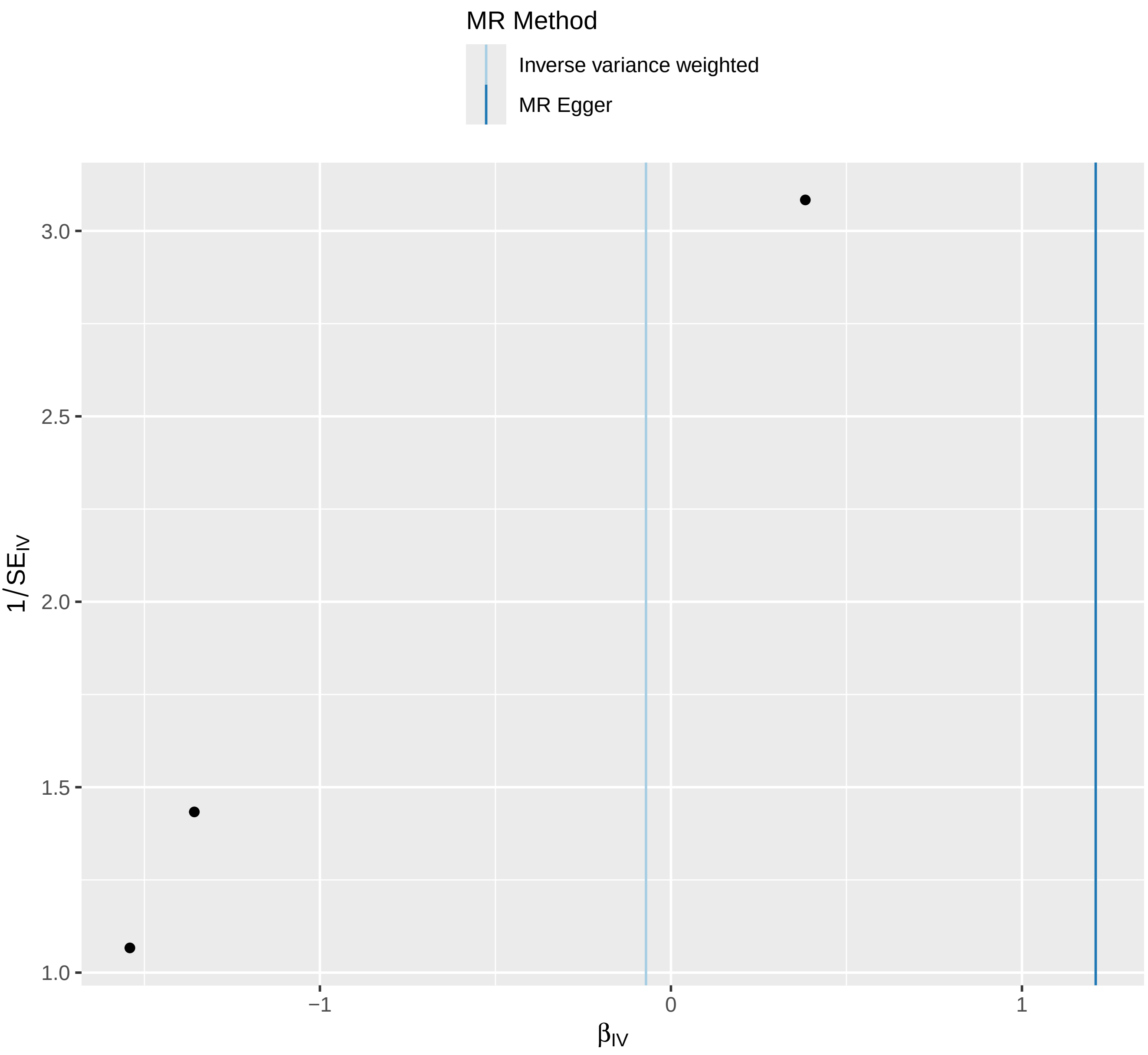

MR Method
Inverse variance weighted
MR Egger
3.0
2.5
2.0
1.5
1.0
$1/SE_{IV}$
−1
0
1
β_{IV}

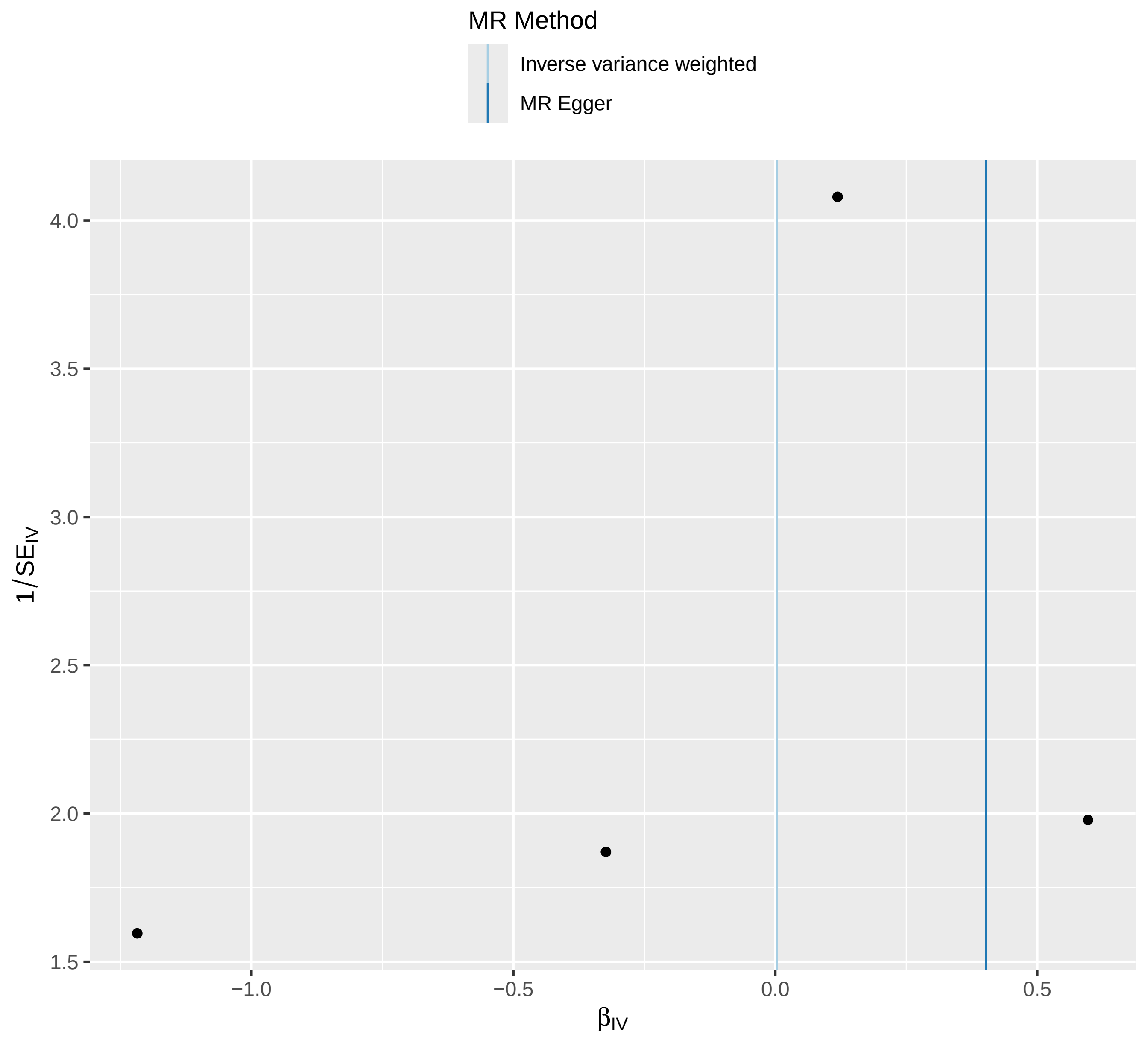
MR Method
Inverse variance weighted
MR Egger
4.0
3.5
3.0
2.5
2.0
1.5
1/SE_IV
−1.0
−0.5
0.0
0.5
β_IV

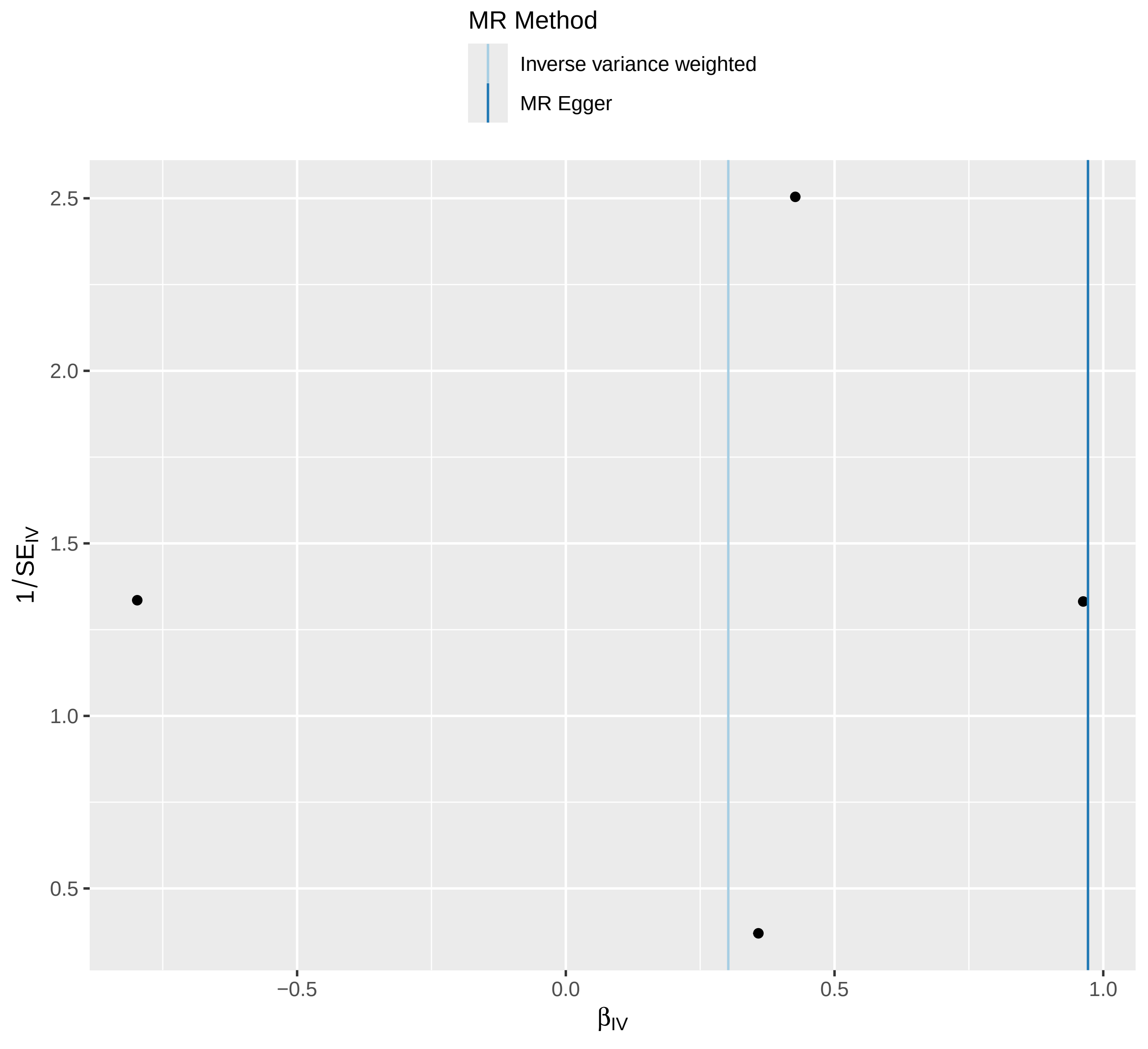
MR Method
Inverse variance weighted
MR Egger
2.5
2.0
1.5
1.0
0.5
$1/SE_{IV}$
−0.5
0.0
0.5
1.0
β_{IV}

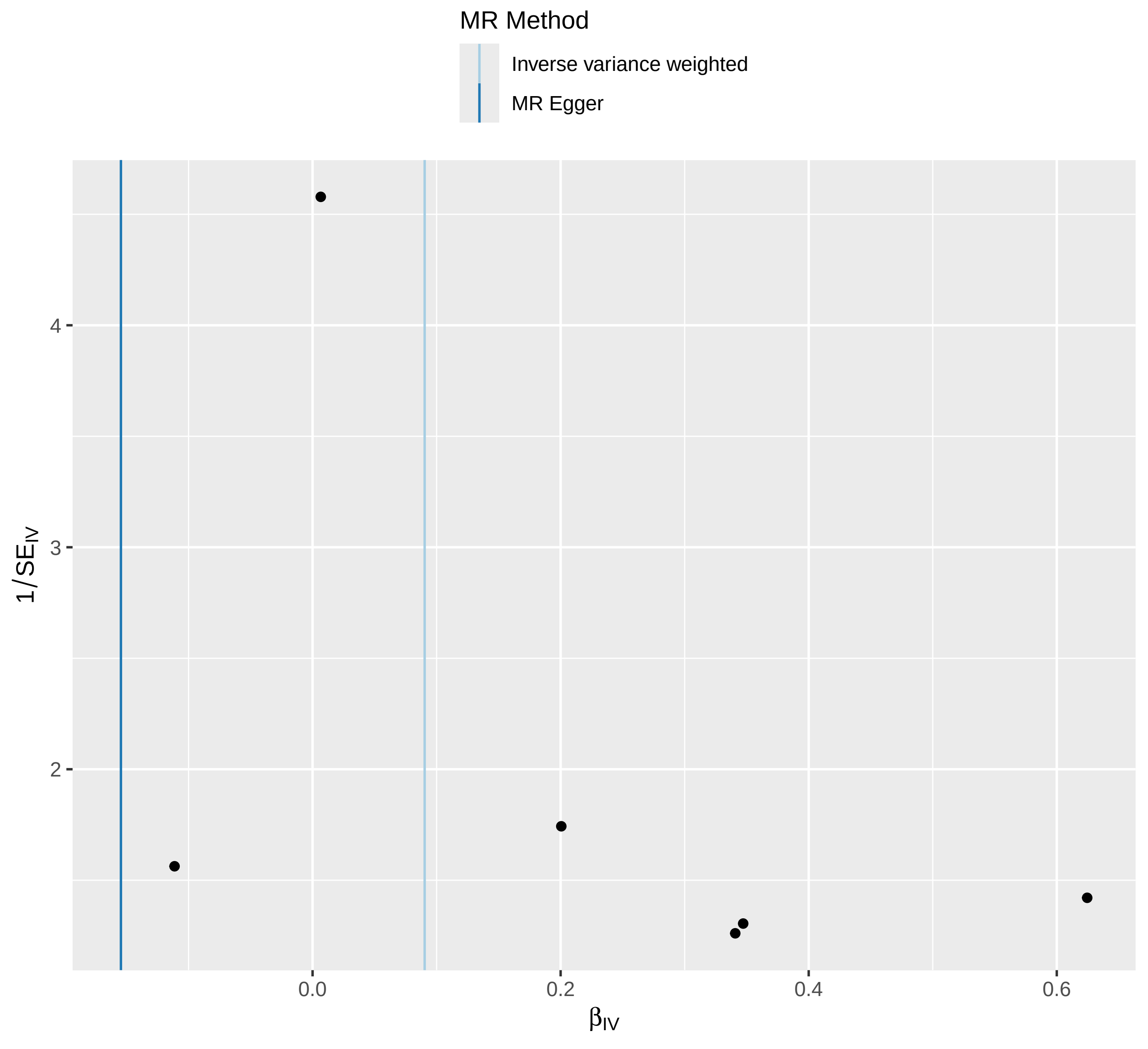

MR Method
Inverse variance weighted
MR Egger
4
3
2
1/SE$_{IV}$
0.0
0.2
0.4
0.6
β$_{IV}$

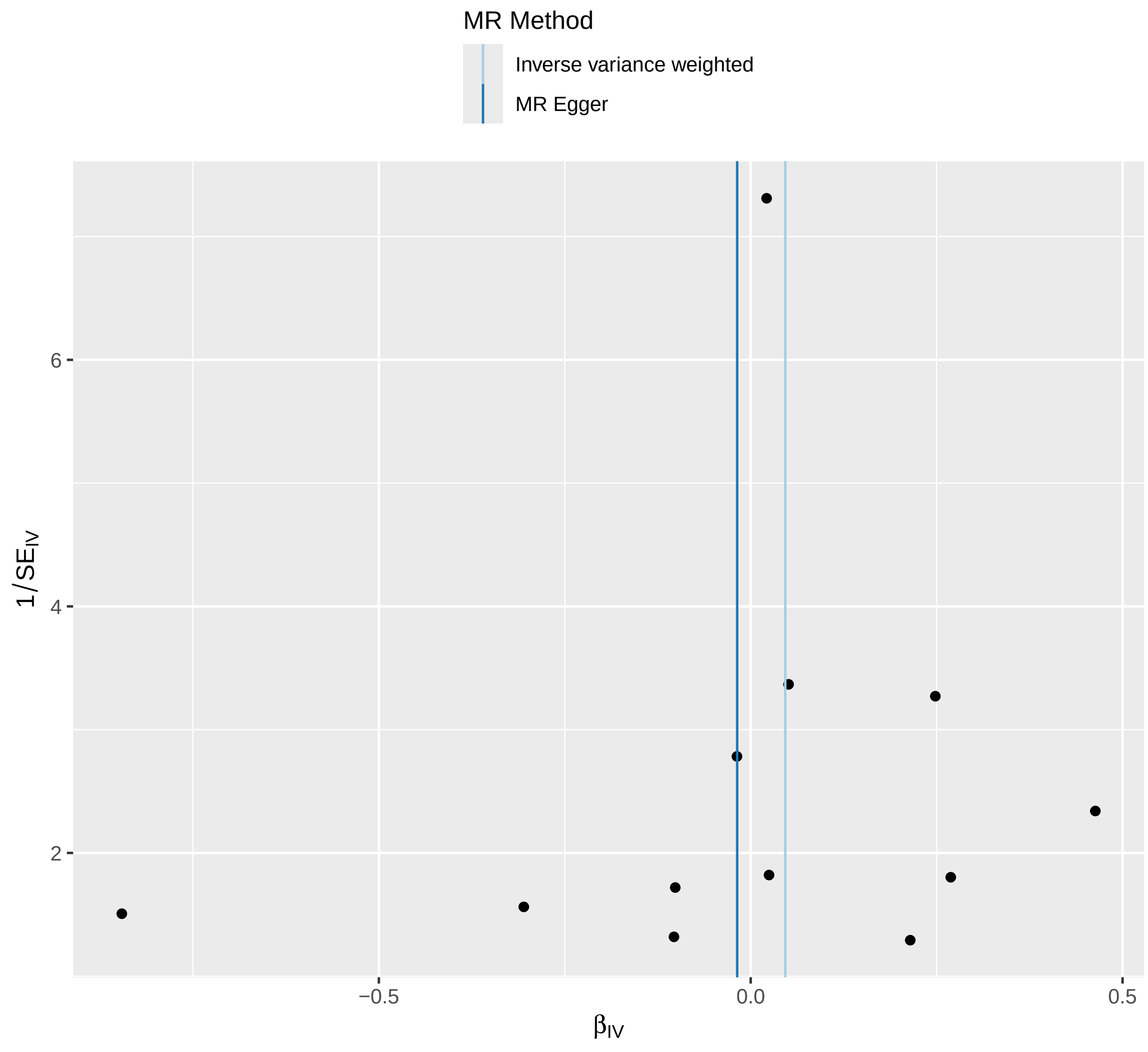
MR Method
Inverse variance weighted
MR Egger
$1/SE_{IV}$
6
4
2
−0.5
0.0
0.5
β_{IV}

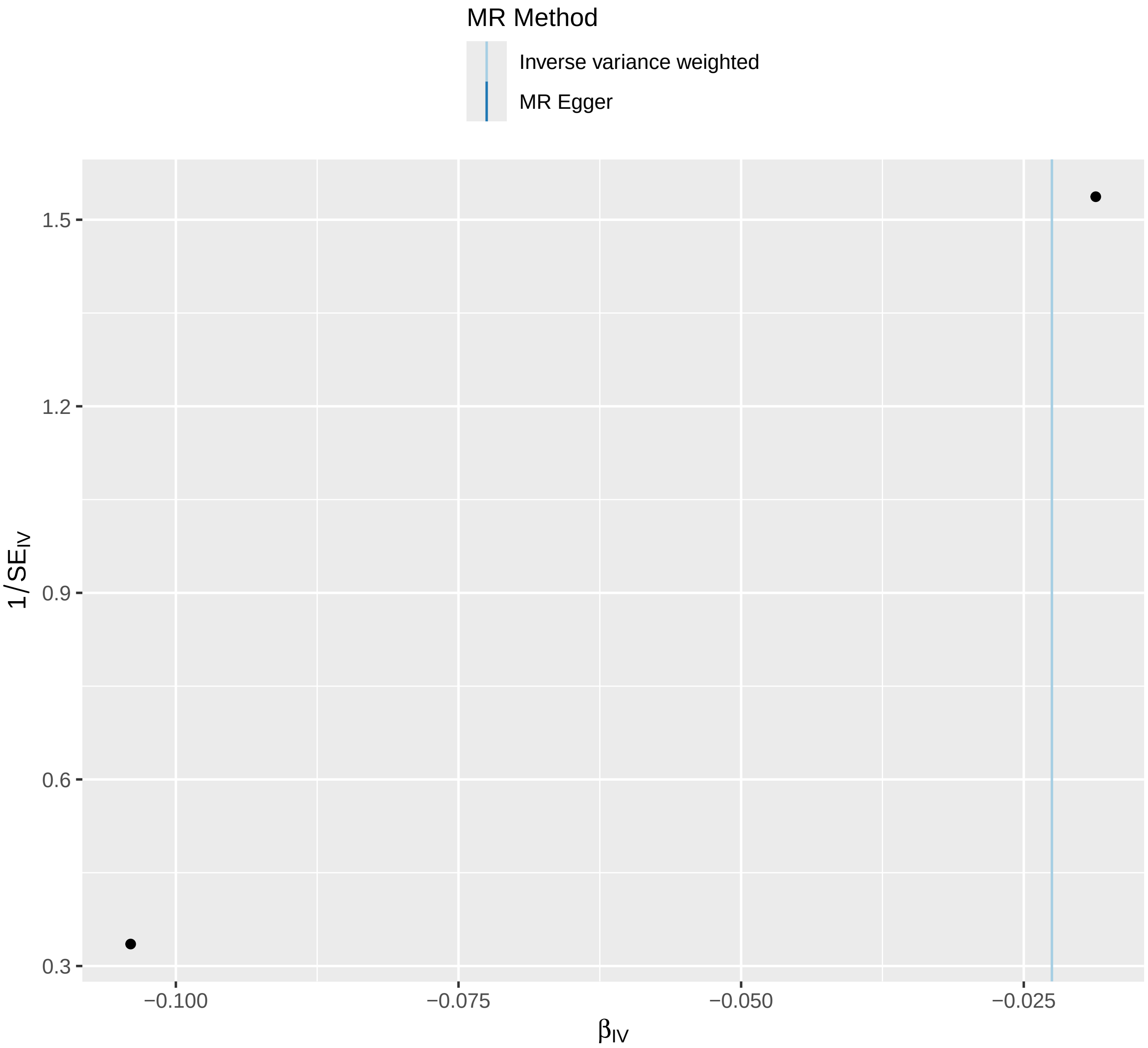
MR Method
Inverse variance weighted
MR Egger
1.5
1.2
0.9
0.6
0.3
−0.100
−0.075
−0.050
−0.025
$1/SE_{IV}$
β_{IV}

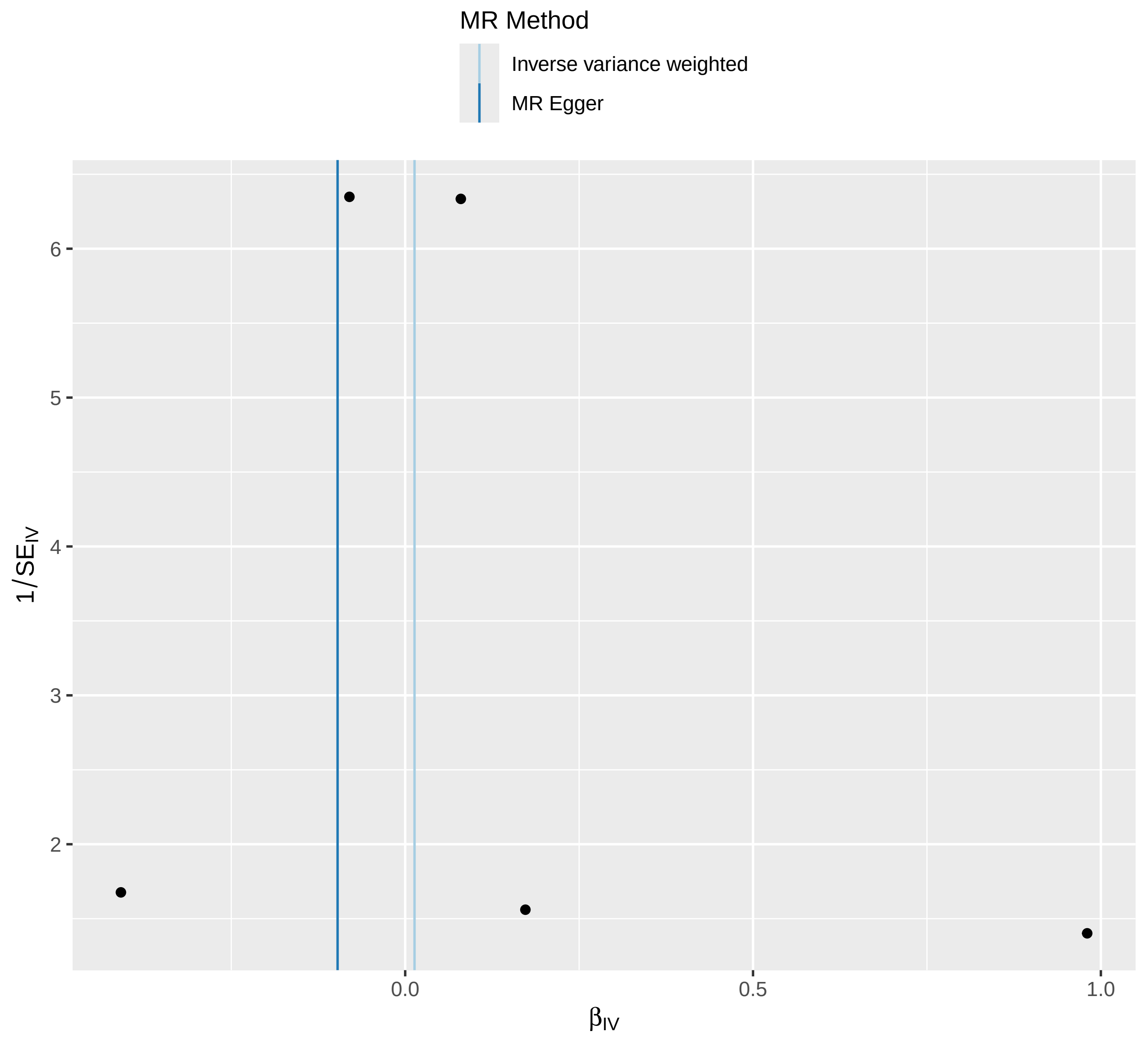

MR Method
Inverse variance weighted
MR Egger
6
5
4
3
2
1/SE_IV
0.0
0.5
1.0
β_IV

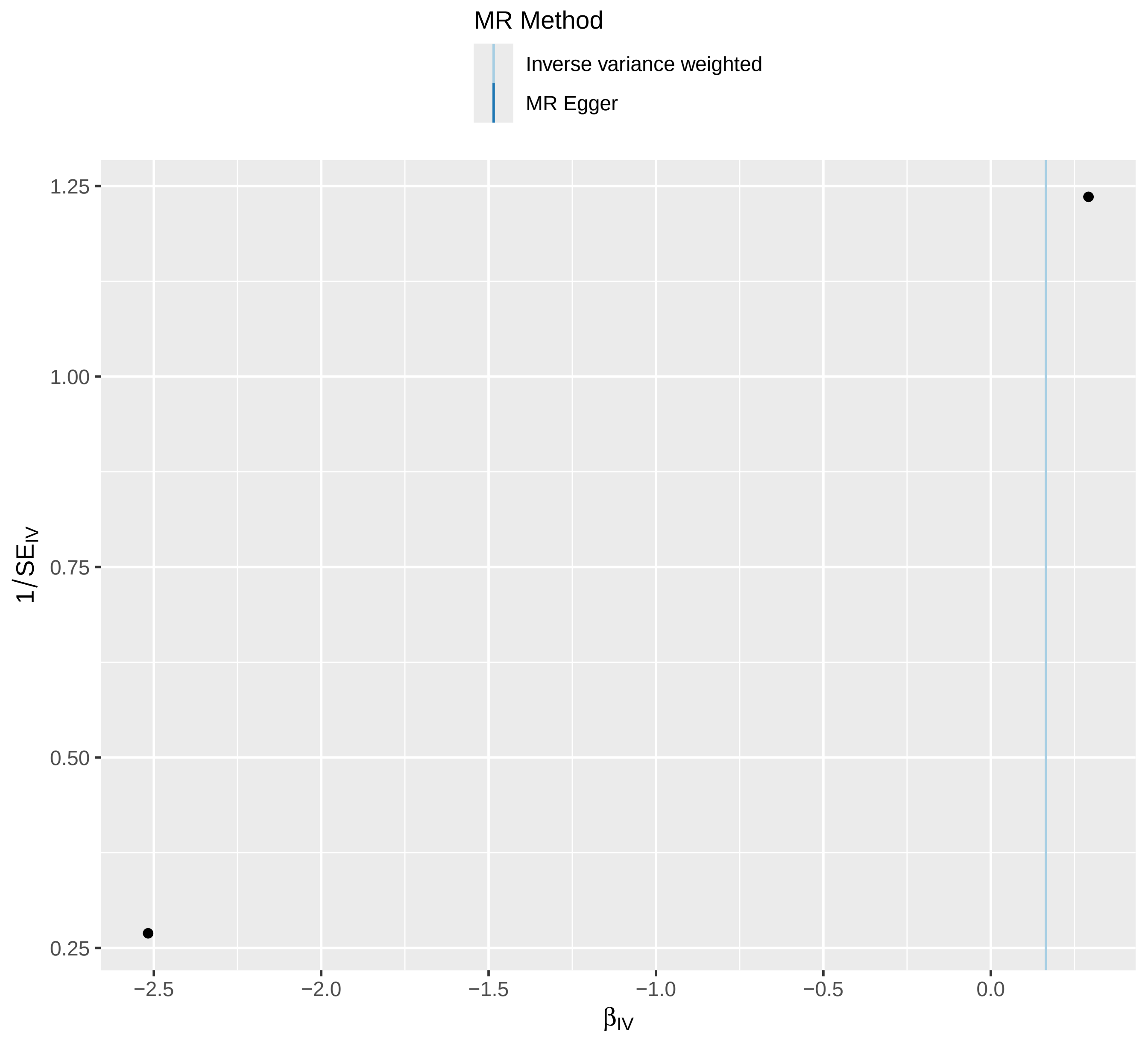

MR Method
Inverse variance weighted
MR Egger
1.25
1.00
0.75
0.50
0.25
1/SE$_{IV}$
−2.5
−2.0
−1.5
−1.0
−0.5
0.0
β$_{IV}$

Insufficient number of SNPs

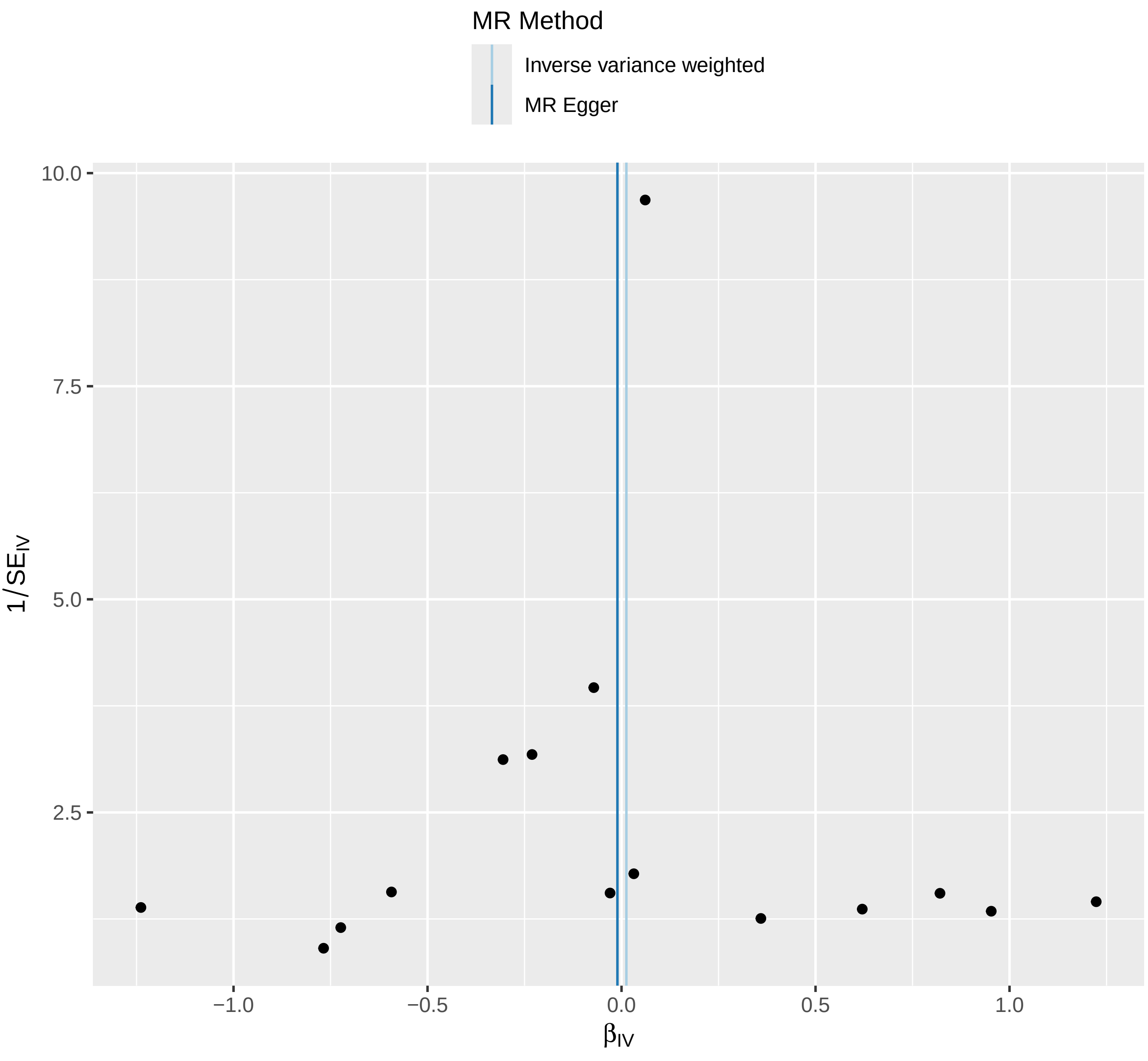

MR Method
Inverse variance weighted
MR Egger
10.0
7.5
5.0
2.5
$1/SE_{IV}$
−1.0
−0.5
0.0
0.5
1.0
β_{IV}

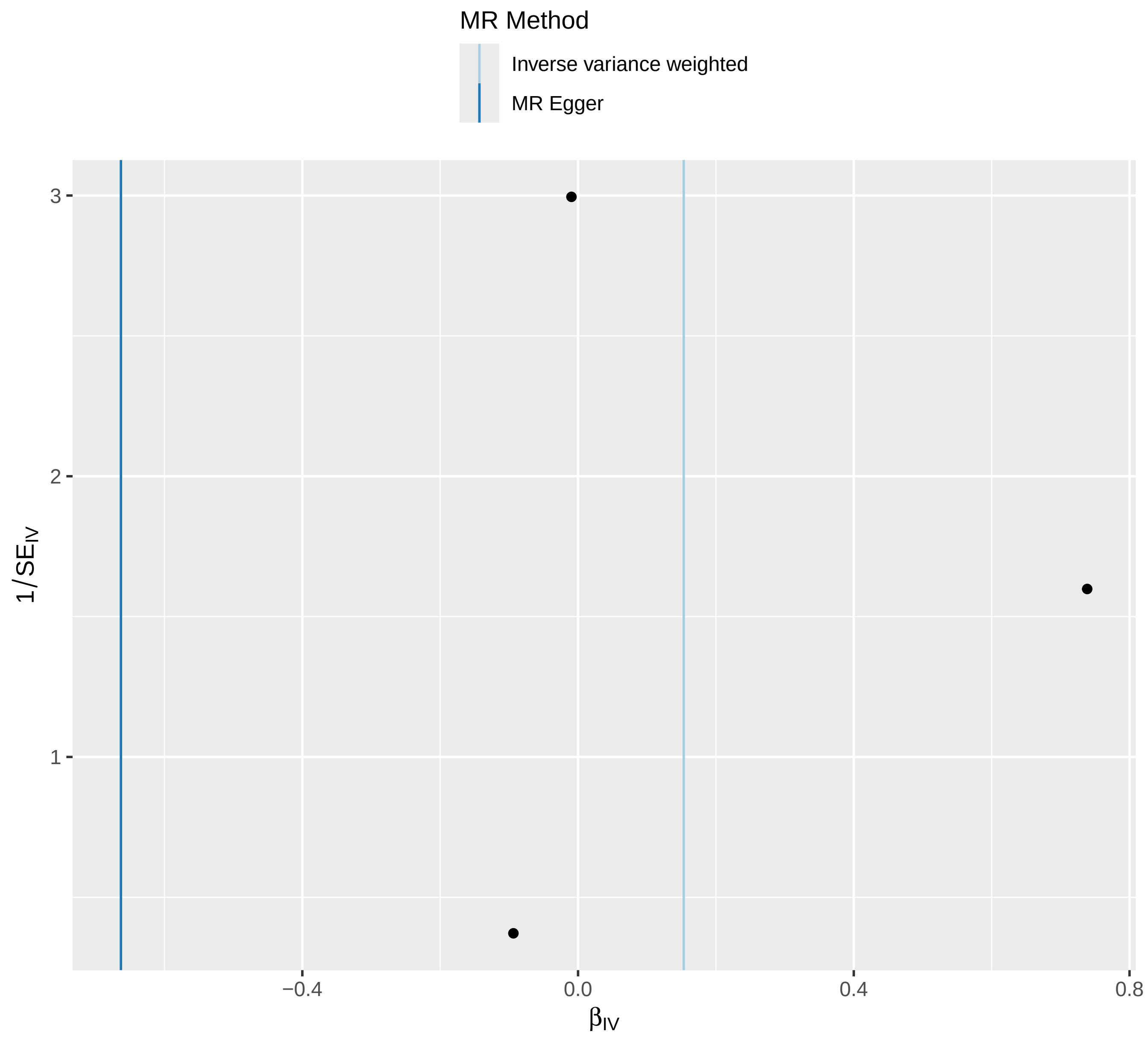

MR Method
Inverse variance weighted
MR Egger
3
2
1
1/SE_IV
−0.4
0.0
0.4
0.8
β_IV

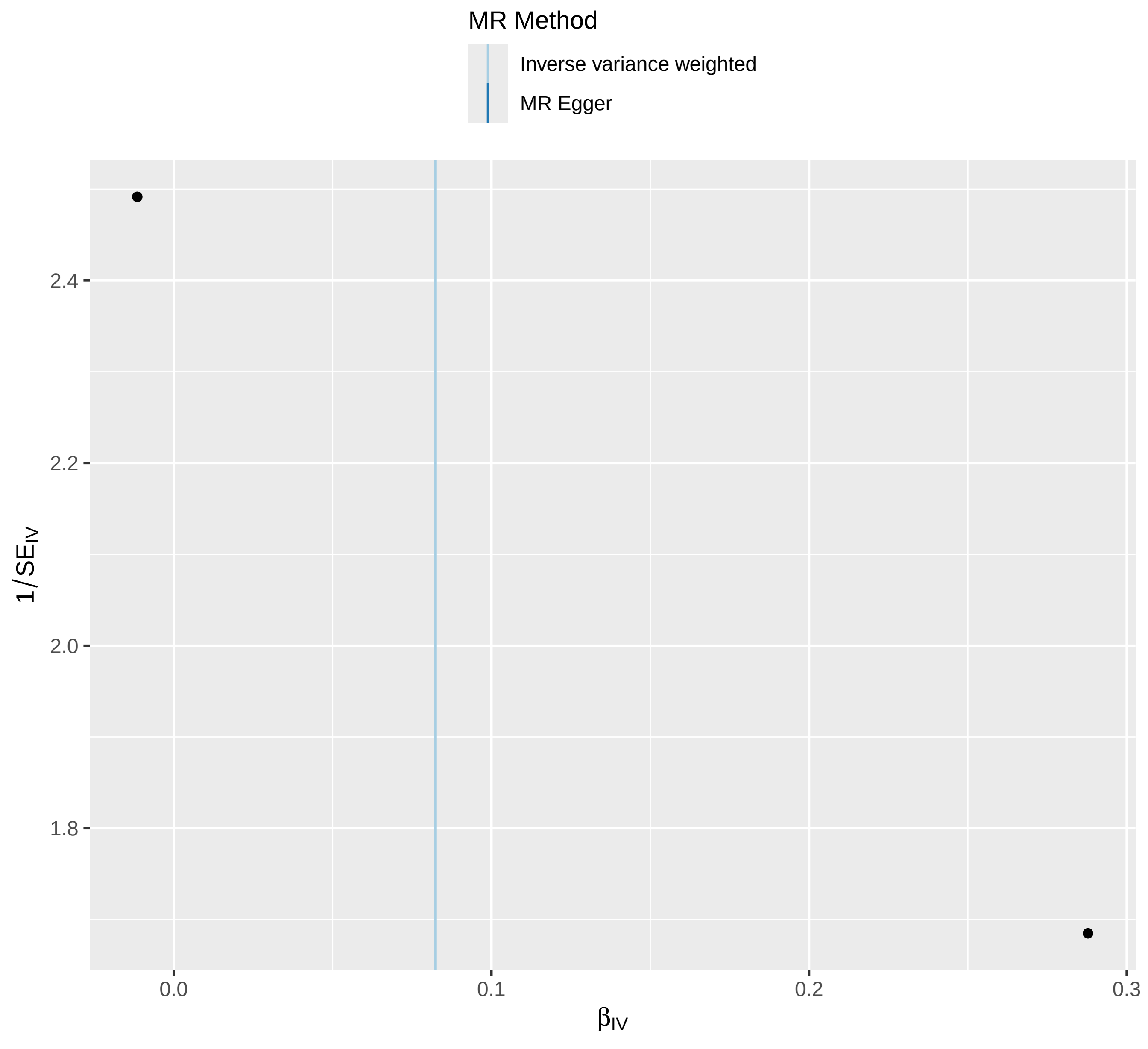

MR Method
Inverse variance weighted
MR Egger
2.4
2.2
2.0
1.8
$1/SE_{IV}$
0.0
0.1
0.2
0.3
β_{IV}

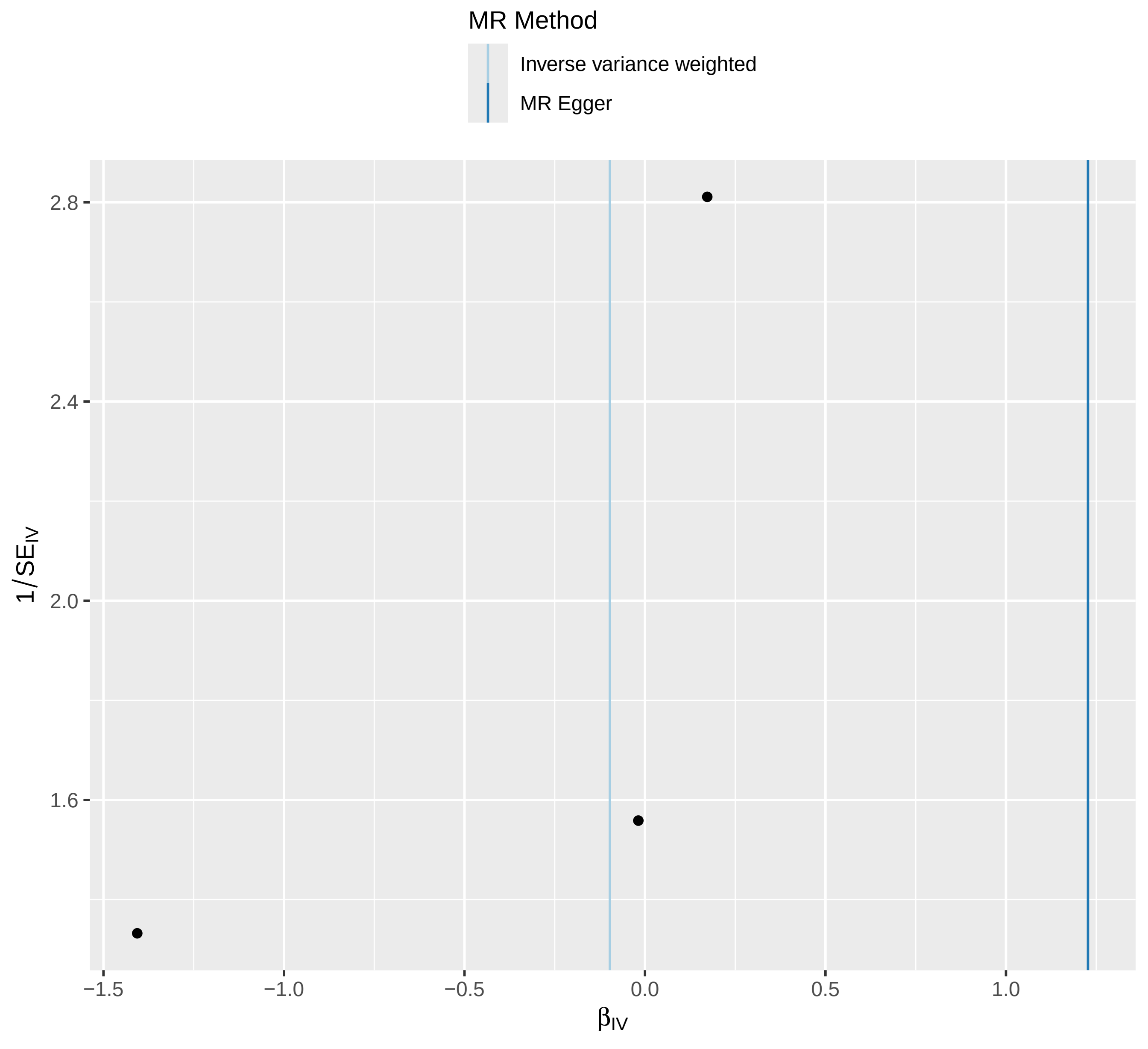

MR Method
Inverse variance weighted
MR Egger
2.8
2.4
2.0
1.6
1/SE_IV
−1.5
−1.0
−0.5
0.0
0.5
1.0
β_IV

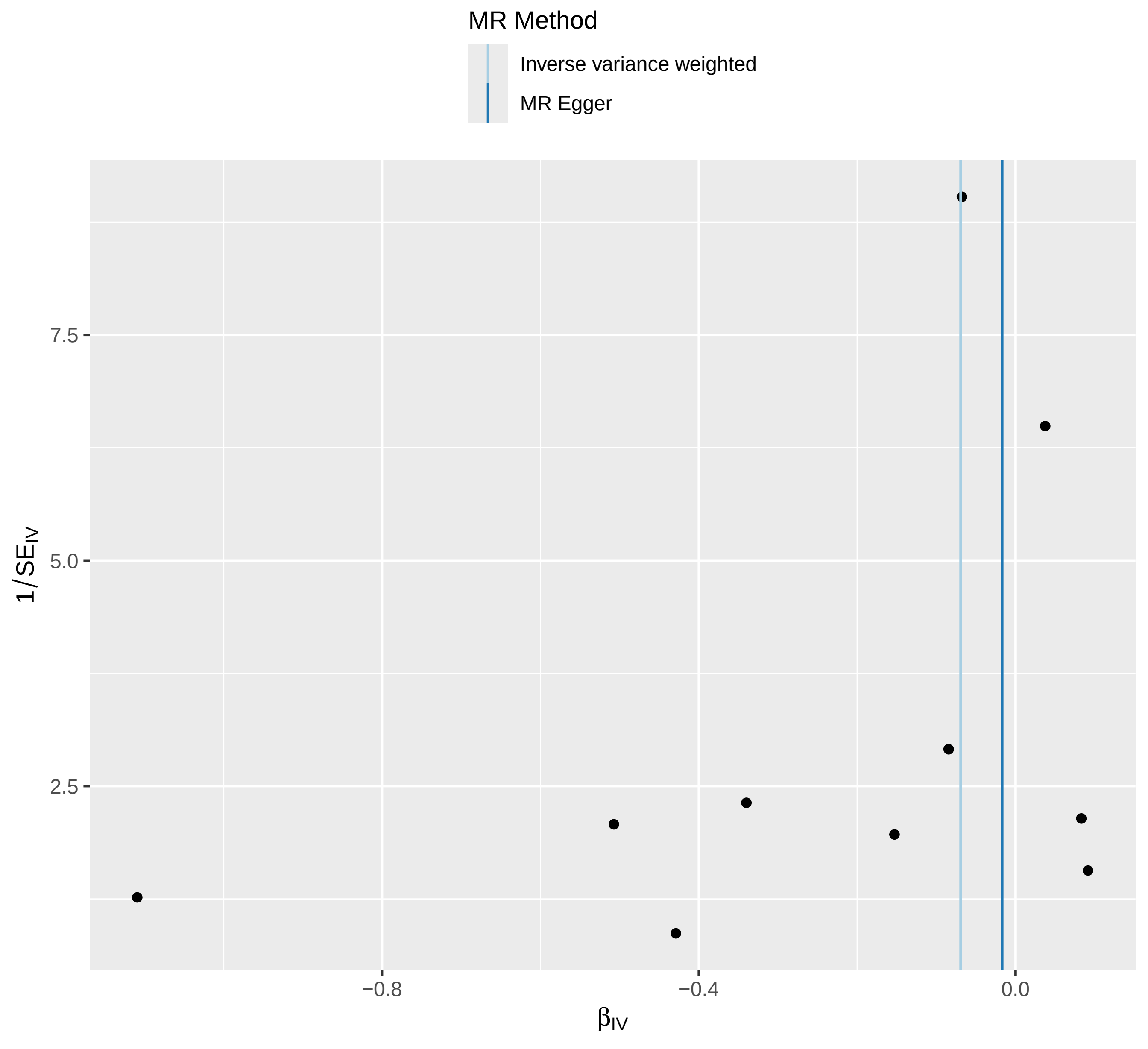

MR Method
Inverse variance weighted
MR Egger
7.5
5.0
2.5
1/SE_IV
−0.8
−0.4
0.0
β_IV

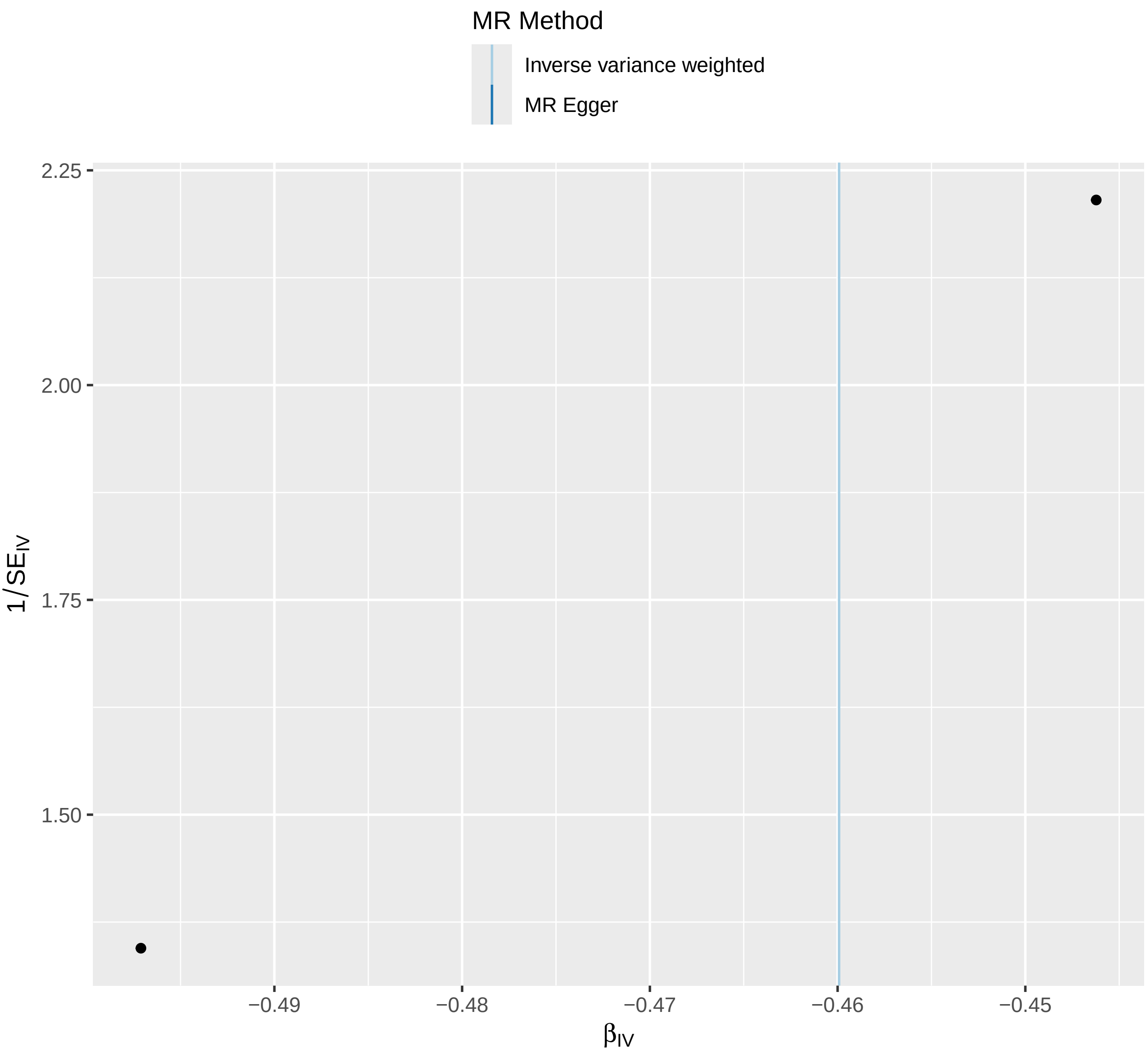
MR Method
Inverse variance weighted
MR Egger
2.25
2.00
1.75
1.50
1/SE_IV
−0.49
−0.48
−0.47
−0.46
−0.45
β_IV

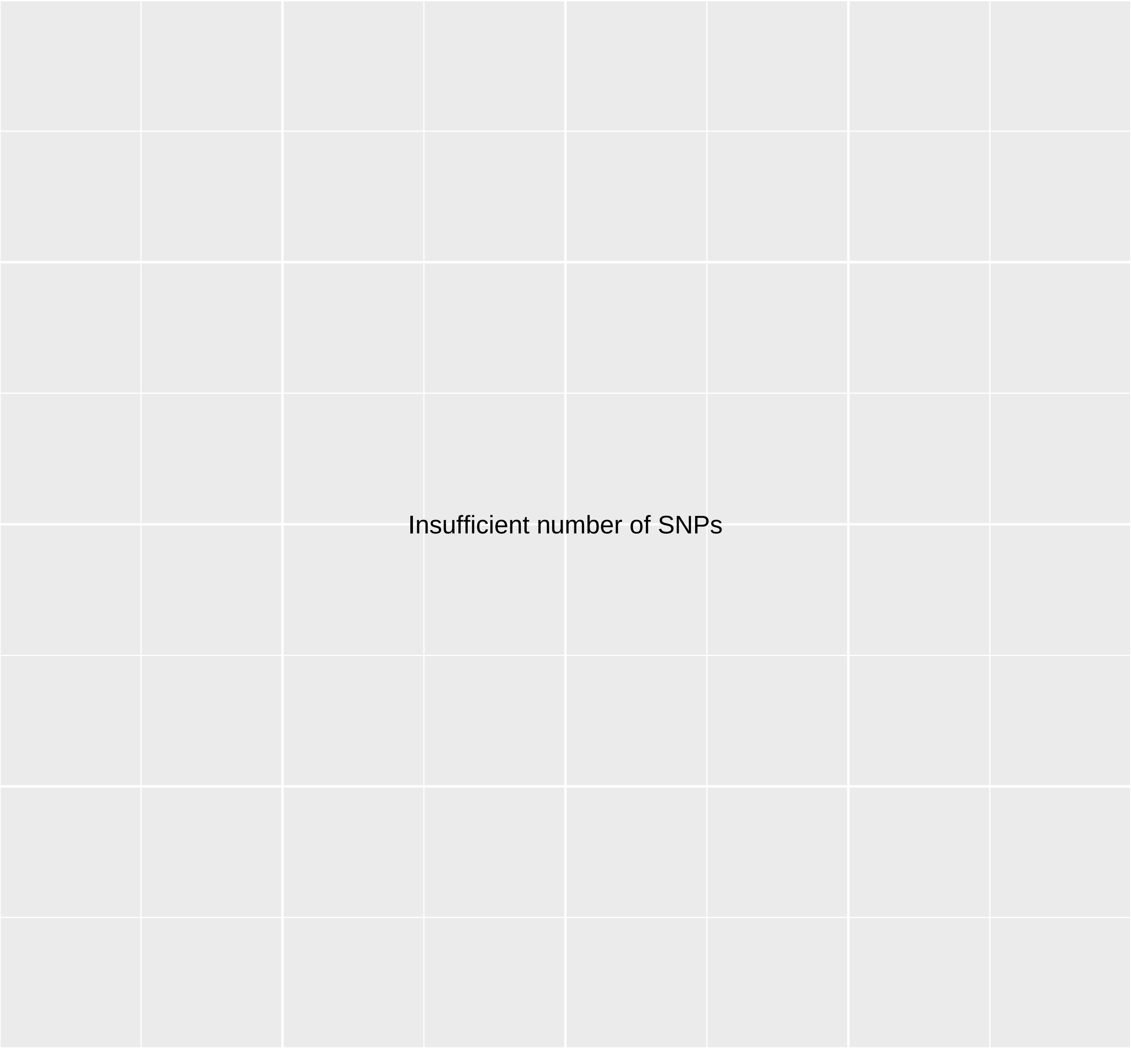
Insufficient number of SNPs

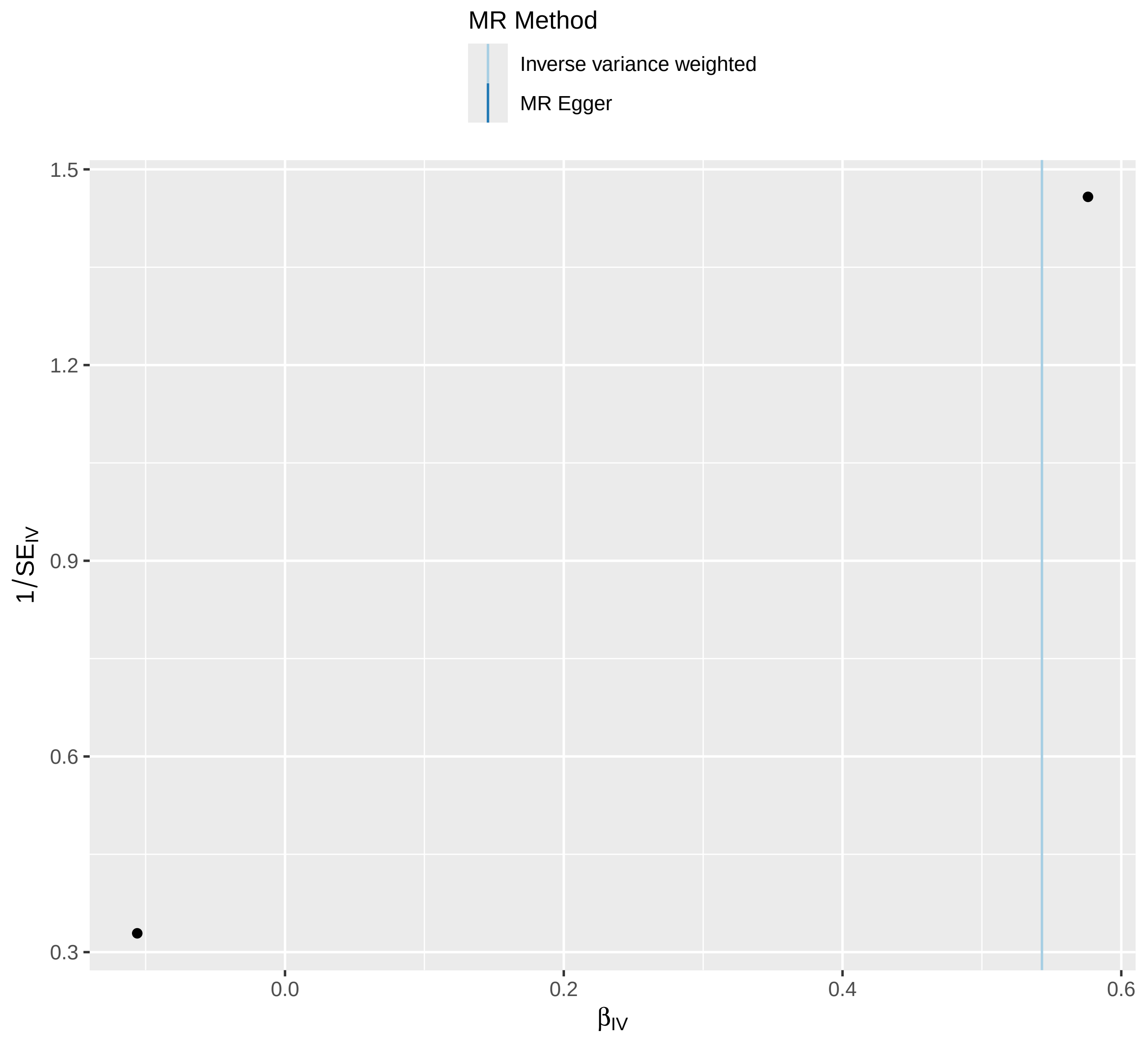

MR Method
Inverse variance weighted
MR Egger
1.5
1.2
0.9
0.6
0.3
1/SE$_{IV}$
0.0
0.2
0.4
0.6
β$_{IV}$

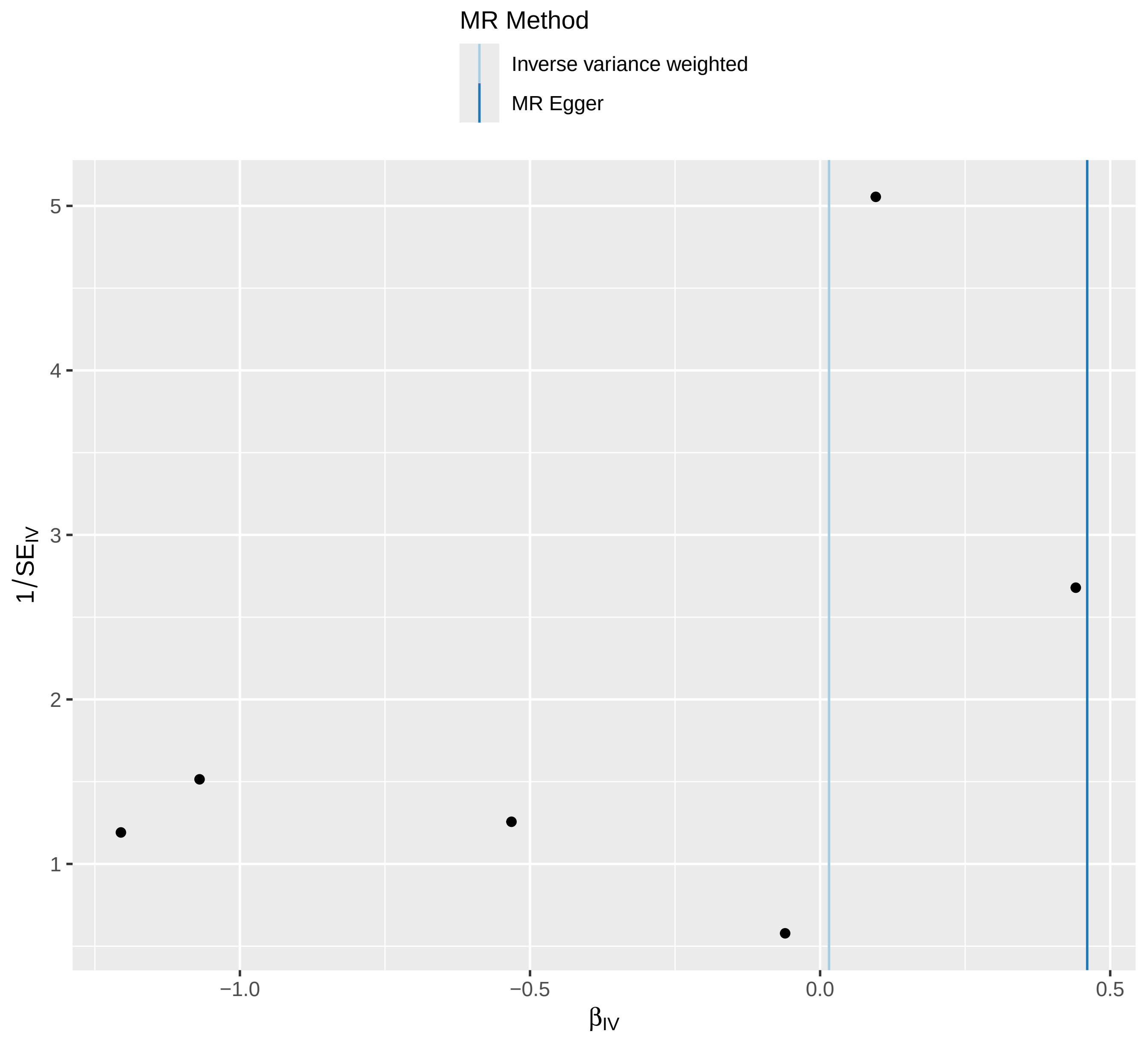
MR Method
Inverse variance weighted
MR Egger
1/SE_IV
β_IV
5
4
3
2
1
−1.0
−0.5
0.0
0.5

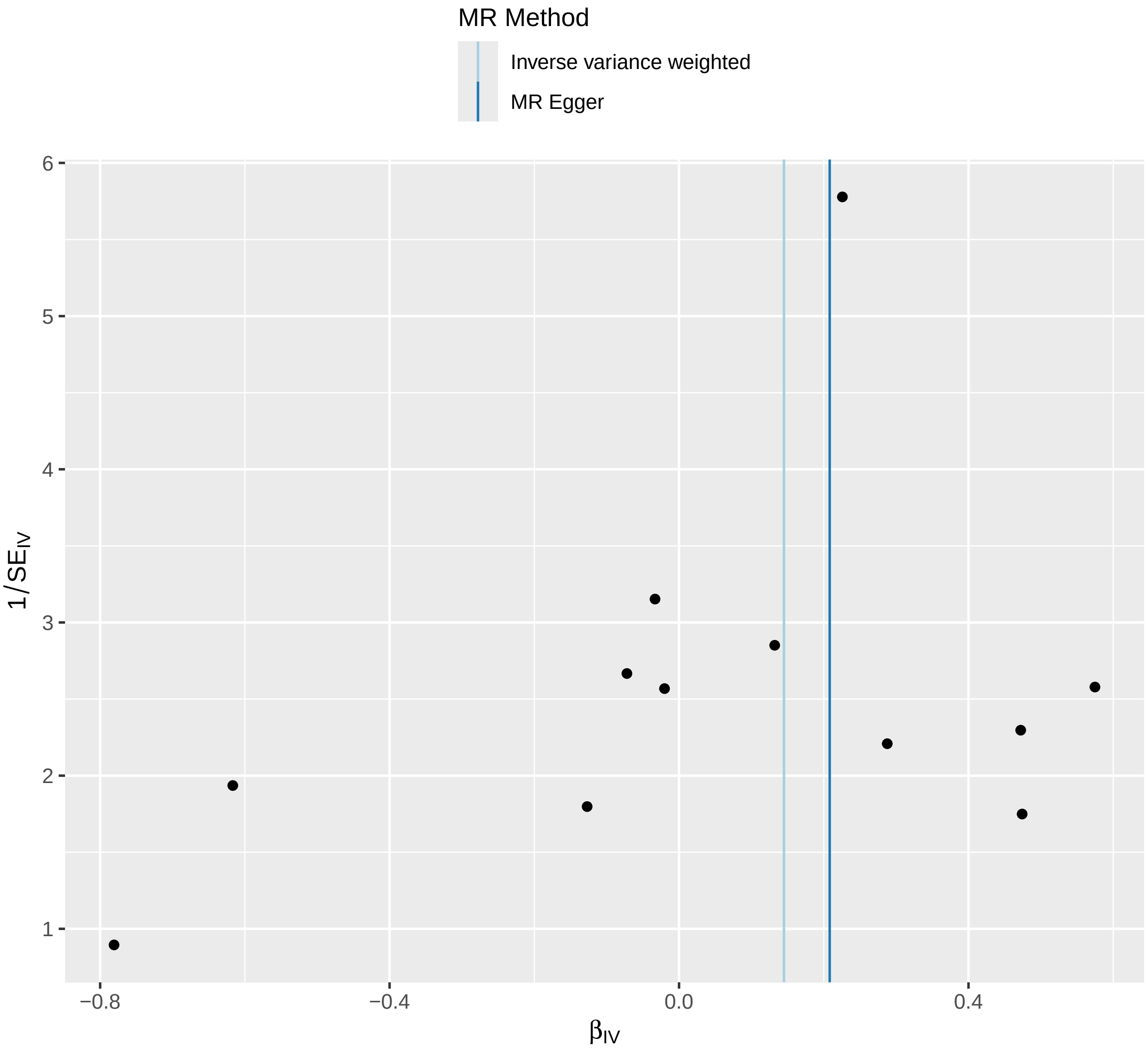
MR Method
Inverse variance weighted
MR Egger
1/SE_IV
β_IV
6
5
4
3
2
1
−0.8
−0.4
0.0
0.4

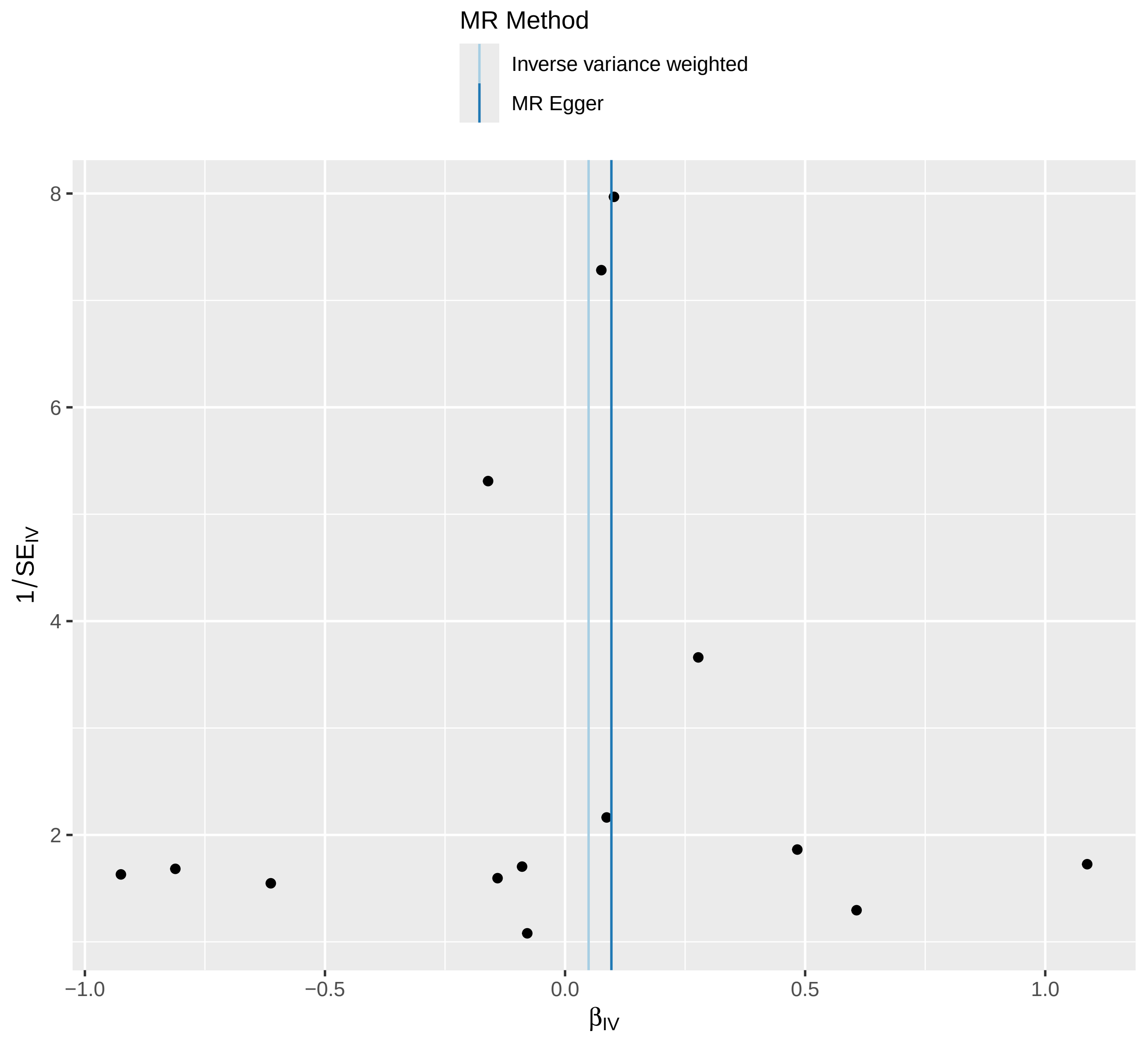

MR Method
Inverse variance weighted
MR Egger
8
6
4
2
1/SE_IV
−1.0
−0.5
0.0
0.5
1.0
β_IV

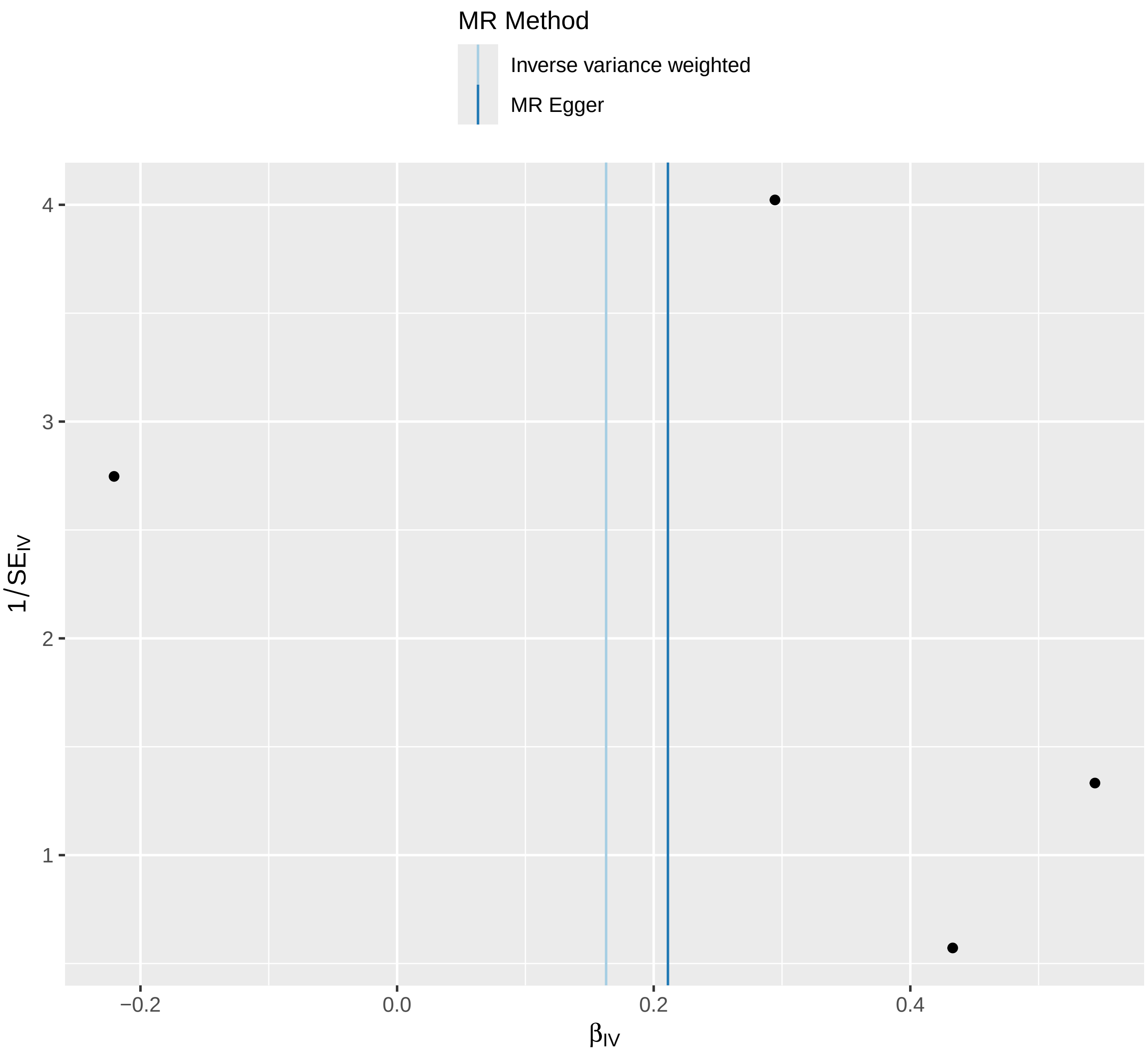

MR Method
Inverse variance weighted
MR Egger
4
3
2
1
1/SE_IV
−0.2
0.0
0.2
0.4
β_IV

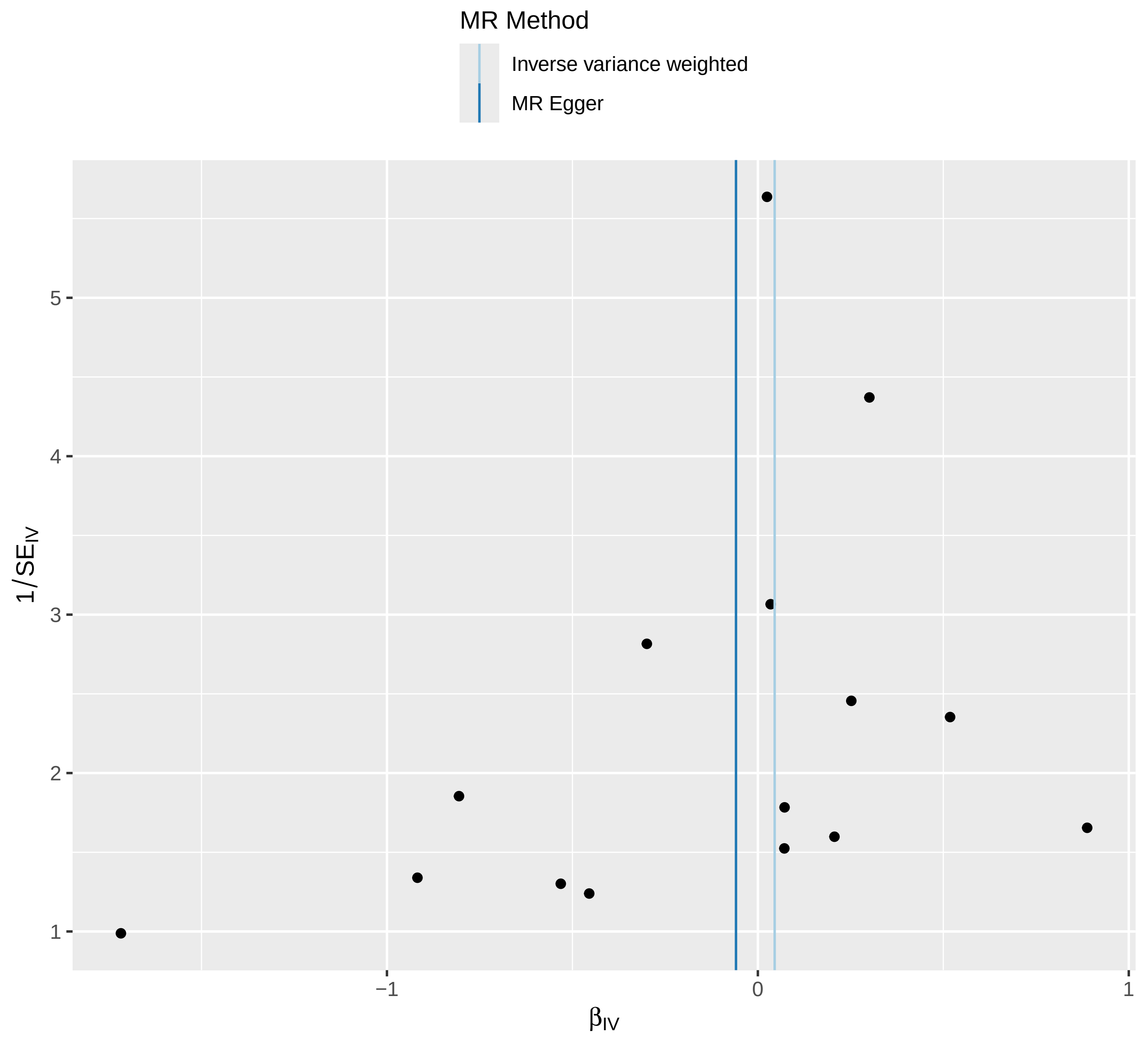
MR Method
Inverse variance weighted
MR Egger
5
4
3
2
1
1/SE_IV
−1
0
1
β_IV

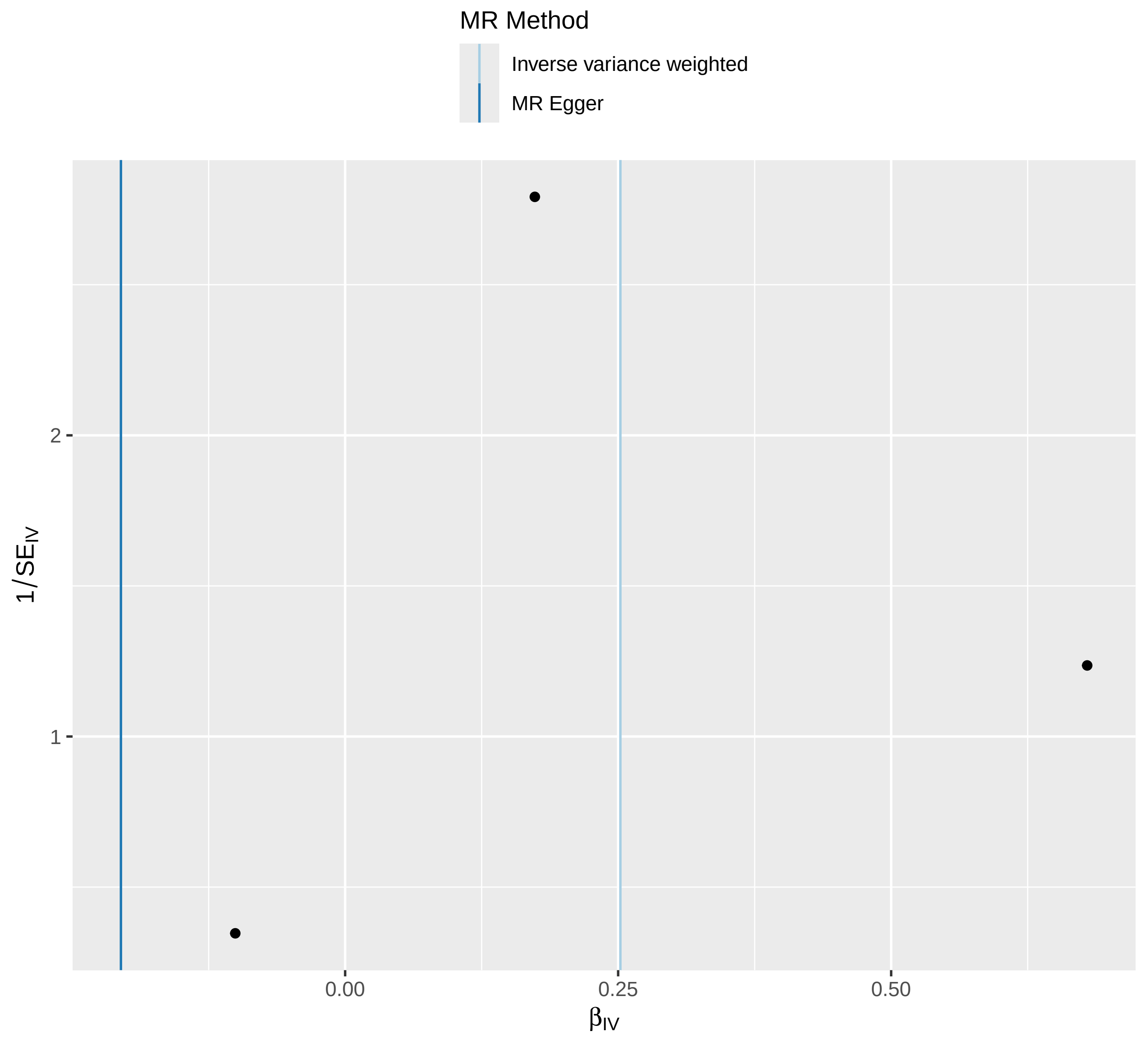
MR Method
Inverse variance weighted
MR Egger
2
1
0.00
0.25
0.50
1/SE$_{IV}$
β$_{IV}$

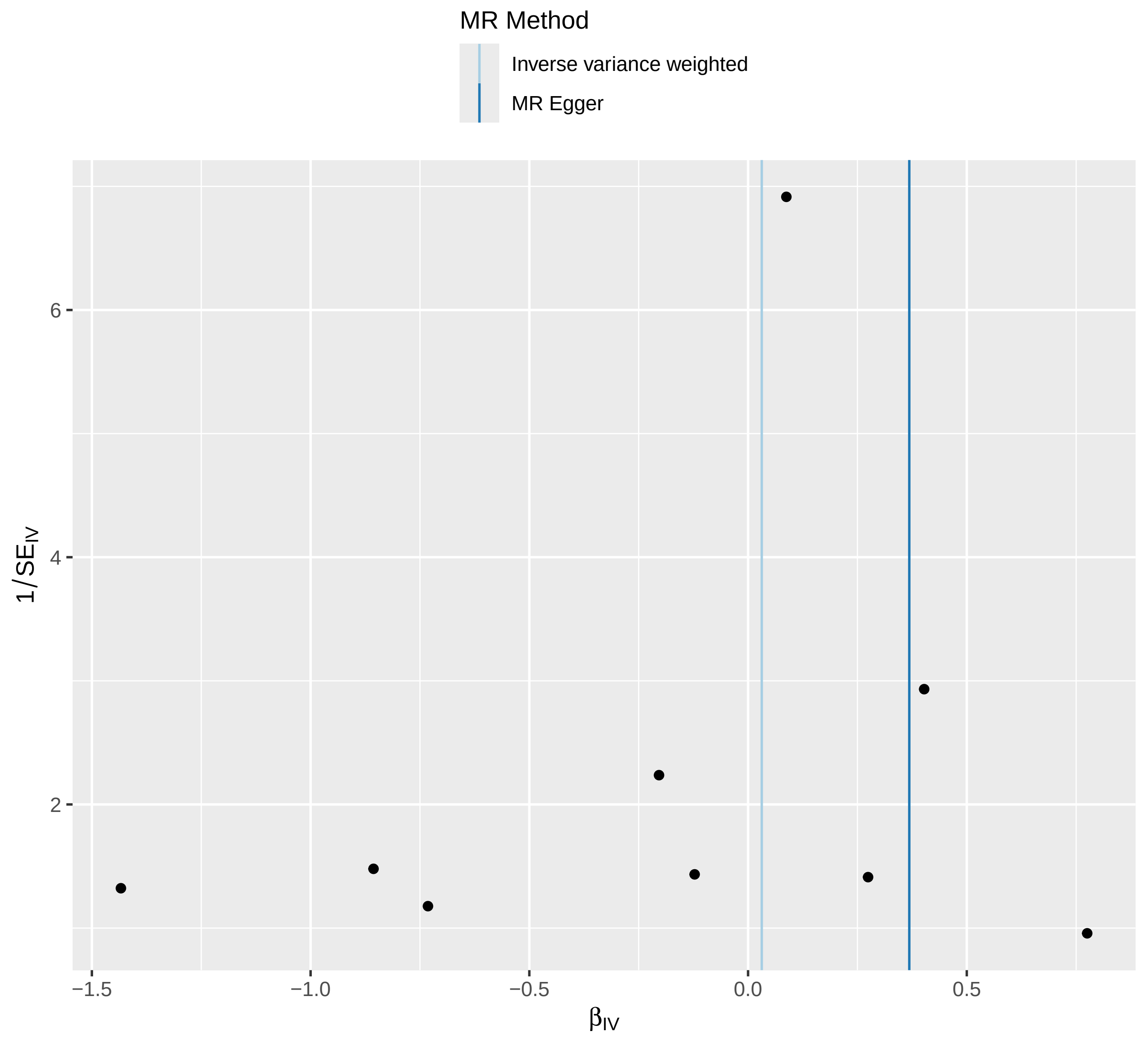
MR Method
Inverse variance weighted
MR Egger
6
4
2
1/SE$_{IV}$
−1.5
−1.0
−0.5
0.0
0.5
β$_{IV}$

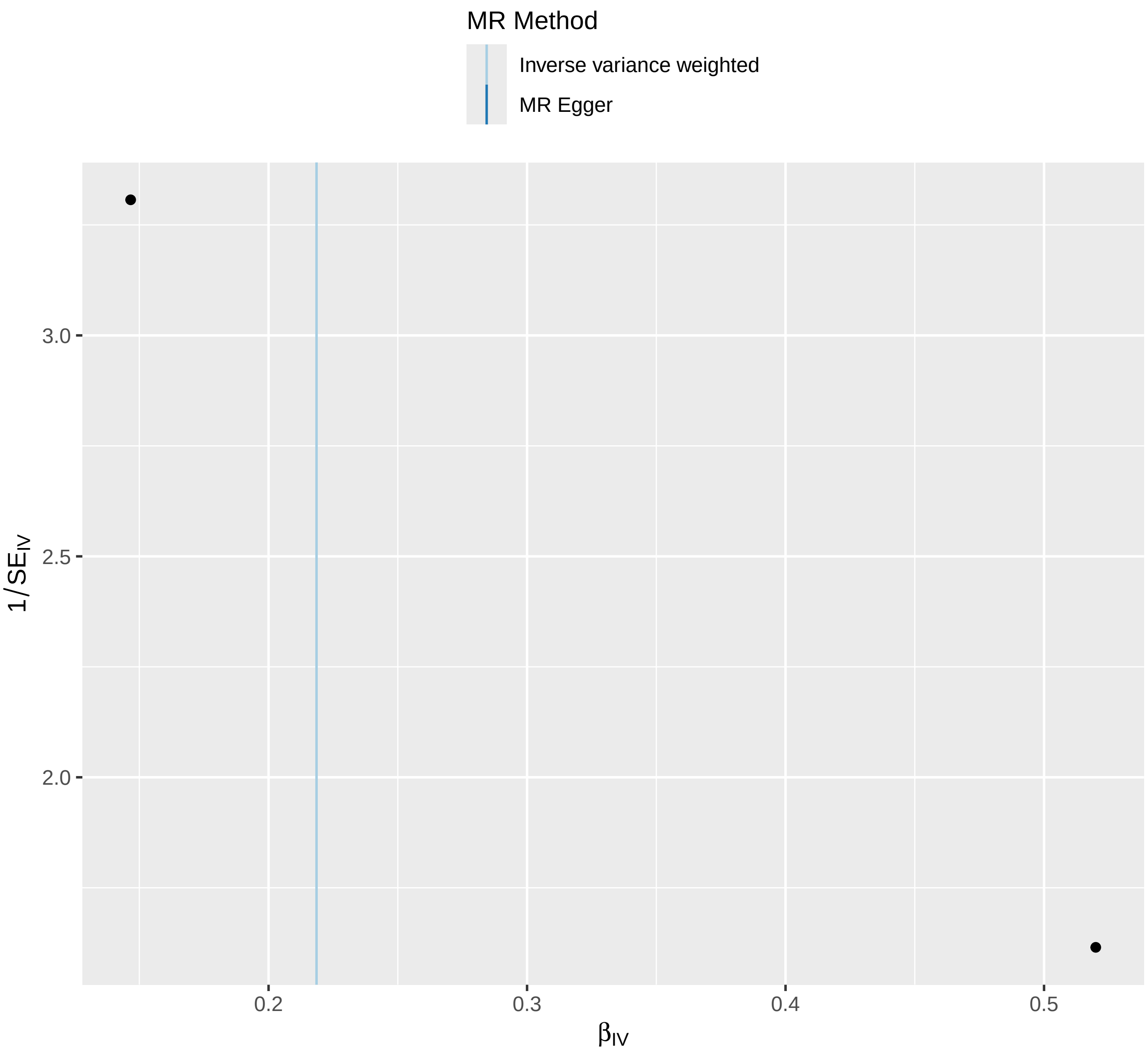
MR Method
Inverse variance weighted
MR Egger
3.0
2.5
2.0
1/SE$_{IV}$
0.2
0.3
0.4
0.5
β$_{IV}$

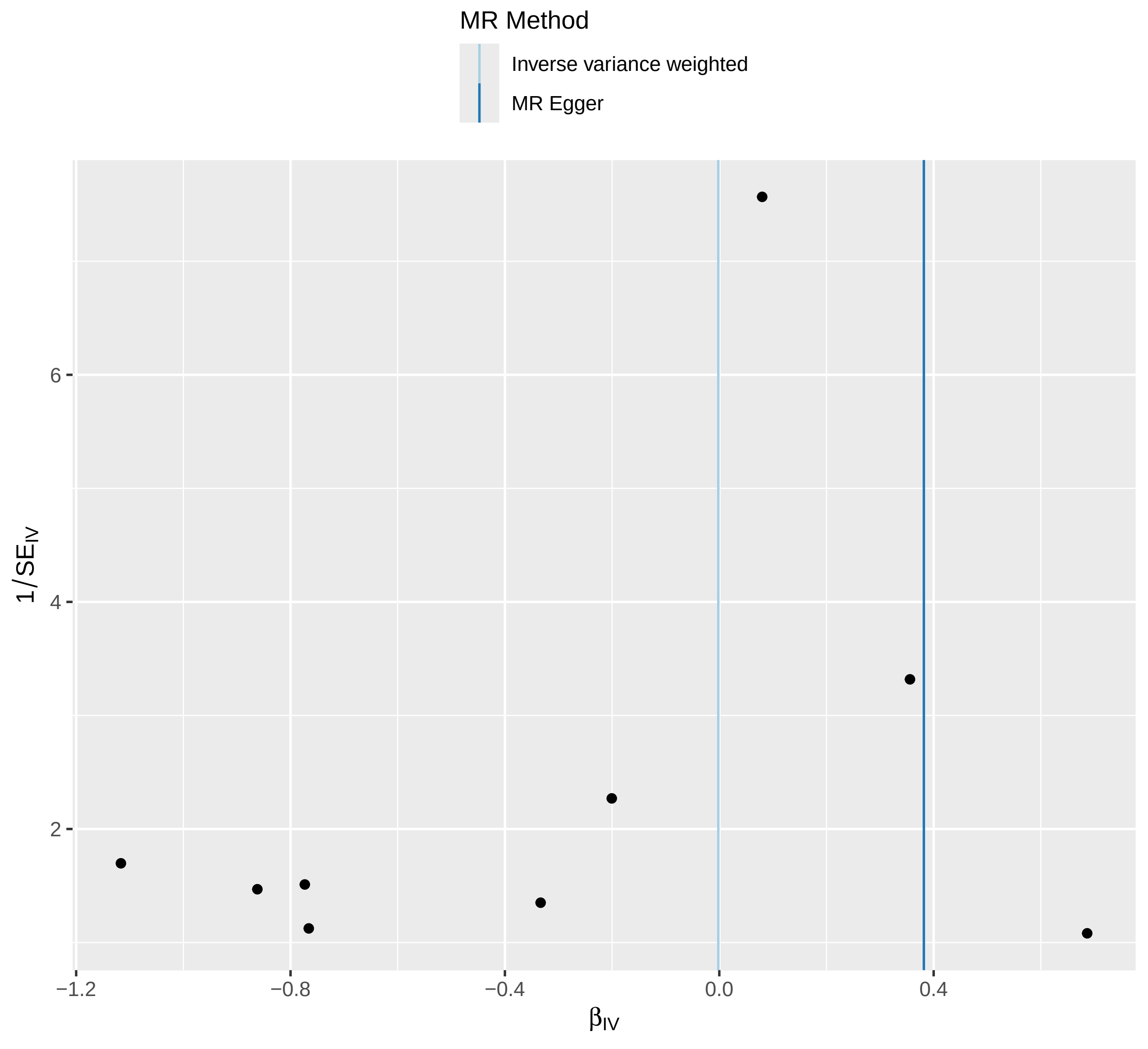
MR Method
Inverse variance weighted
MR Egger
1/SE_{IV}
6
4
2
−1.2
−0.8
−0.4
0.0
0.4
β_{IV}

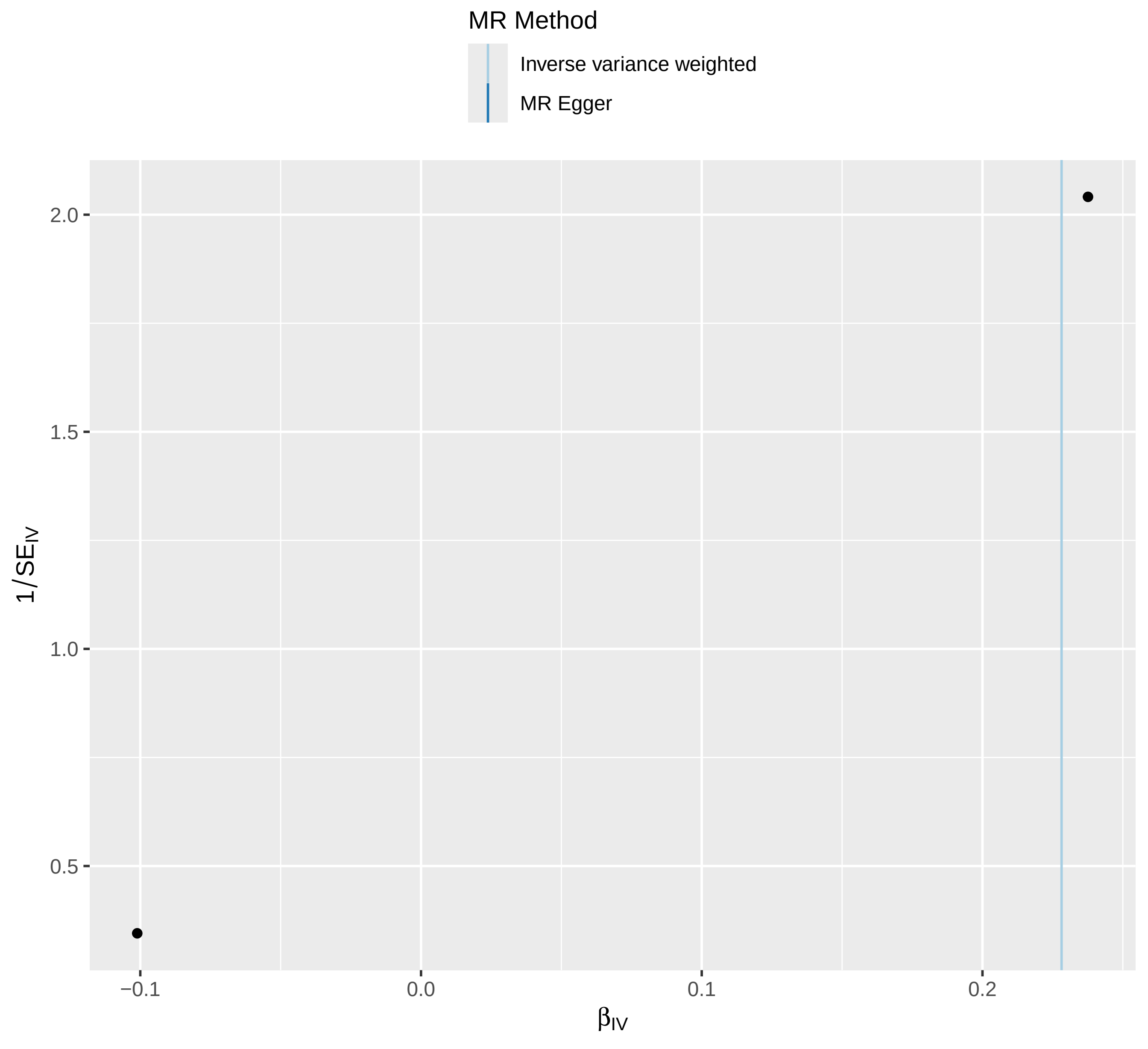

MR Method
Inverse variance weighted
MR Egger
2.0
1.5
1.0
0.5
1/SE_IV
−0.1
0.0
0.1
0.2
β_IV

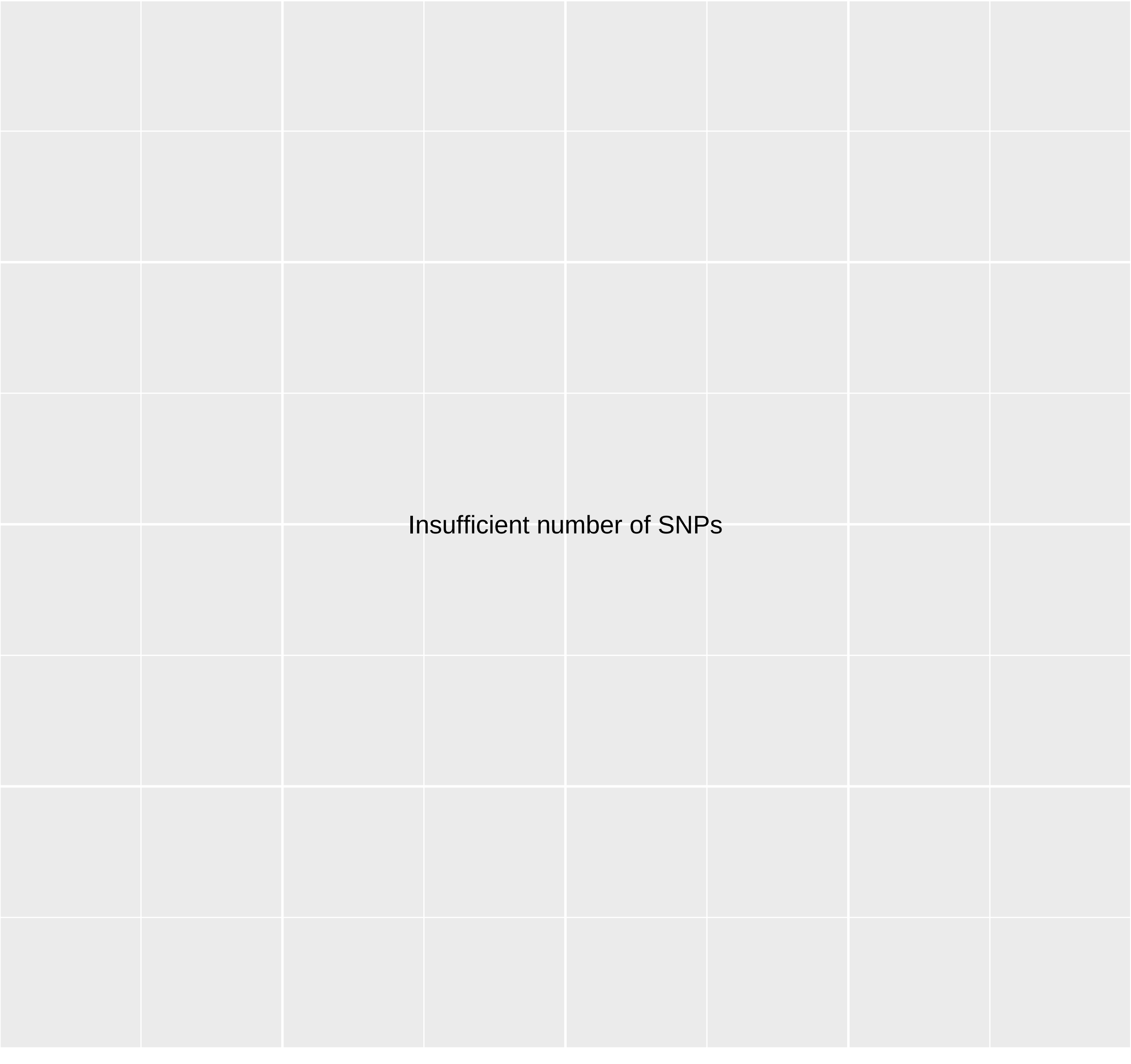
Insufficient number of SNPs

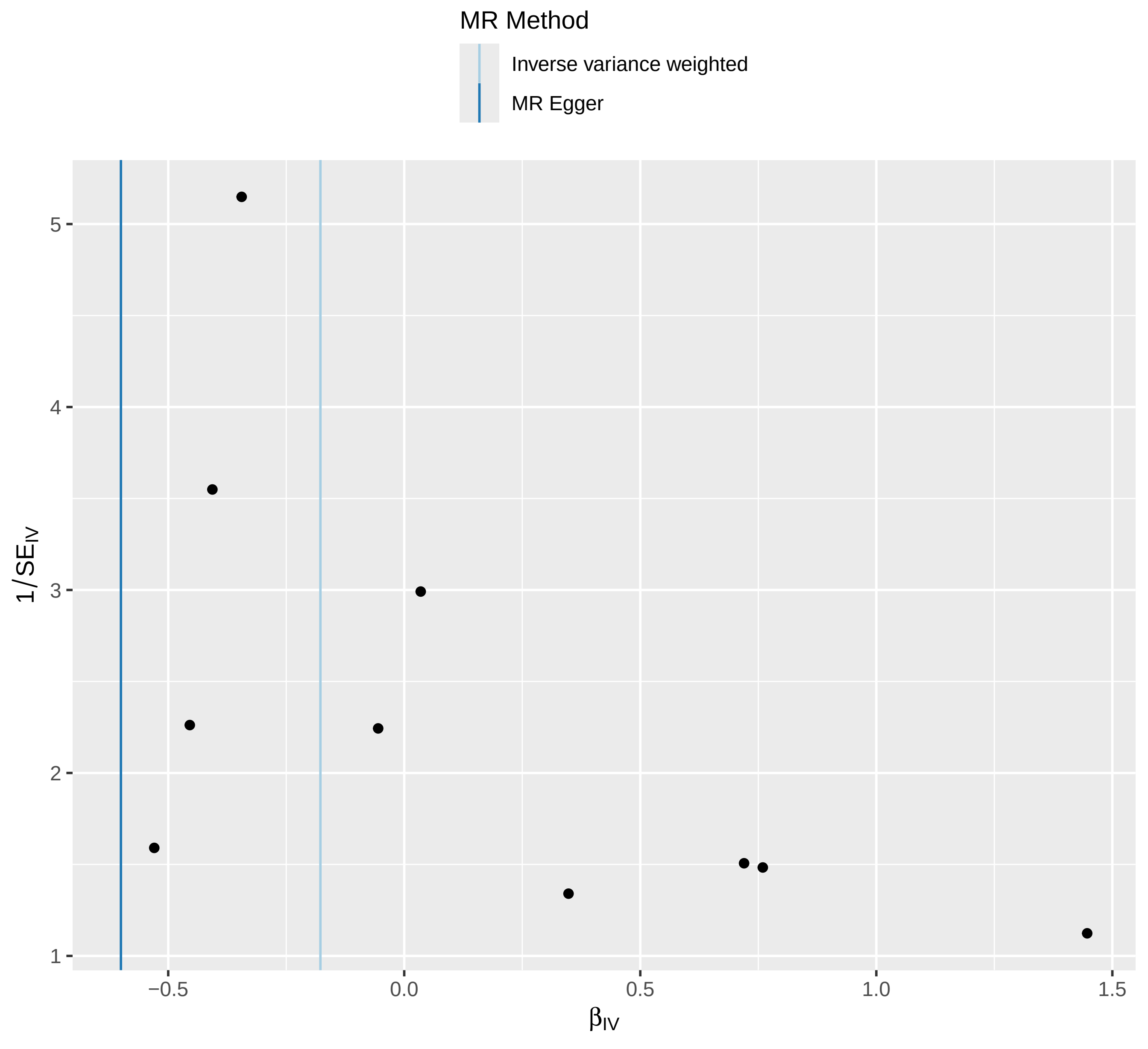

MR Method
Inverse variance weighted
MR Egger
5
4
3
2
1
1/SE_IV
−0.5
0.0
0.5
1.0
1.5
β_IV

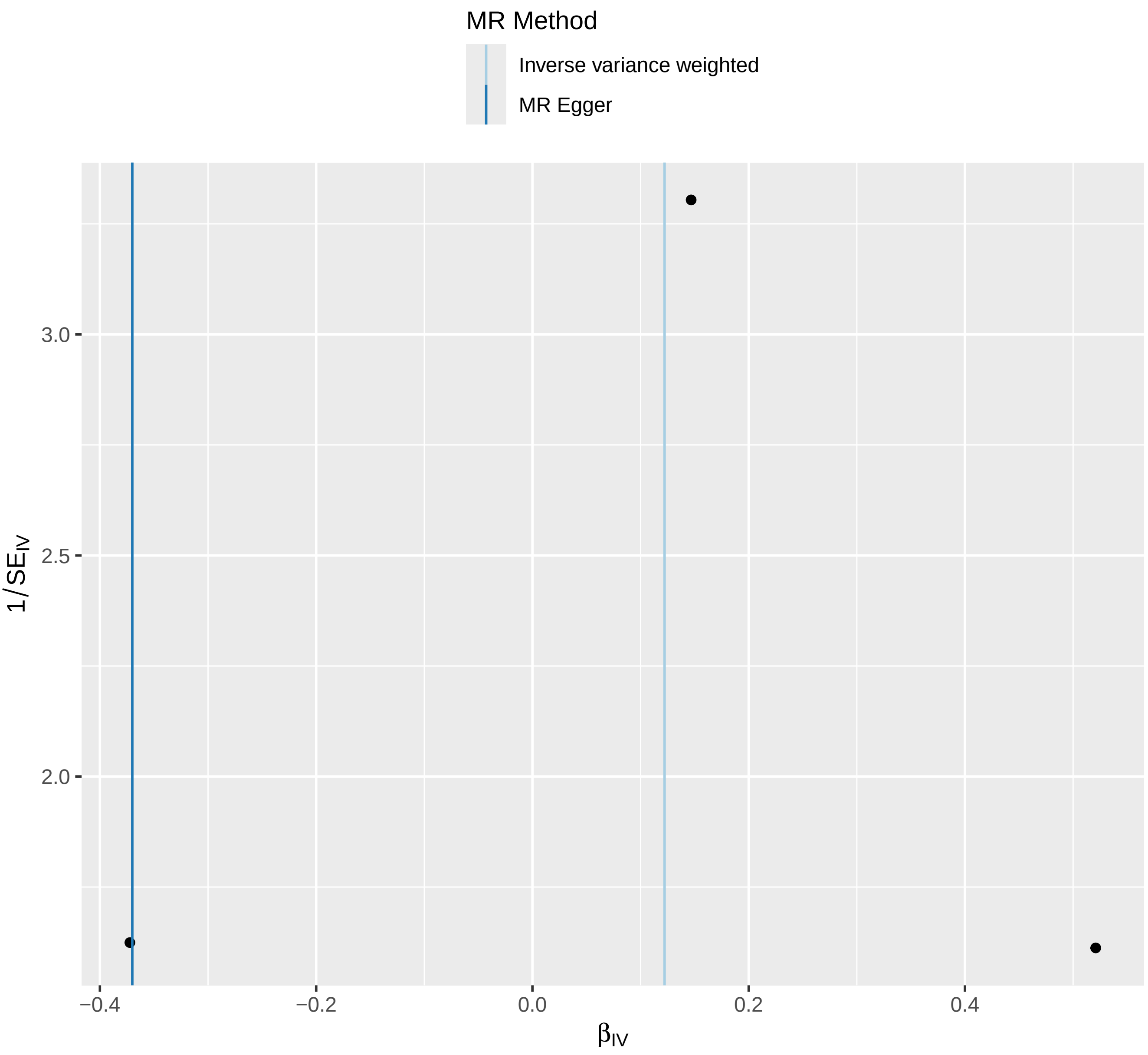

MR Method
Inverse variance weighted
MR Egger
3.0
2.5
2.0
1/SE_IV
−0.4
−0.2
0.0
0.2
0.4
β_IV

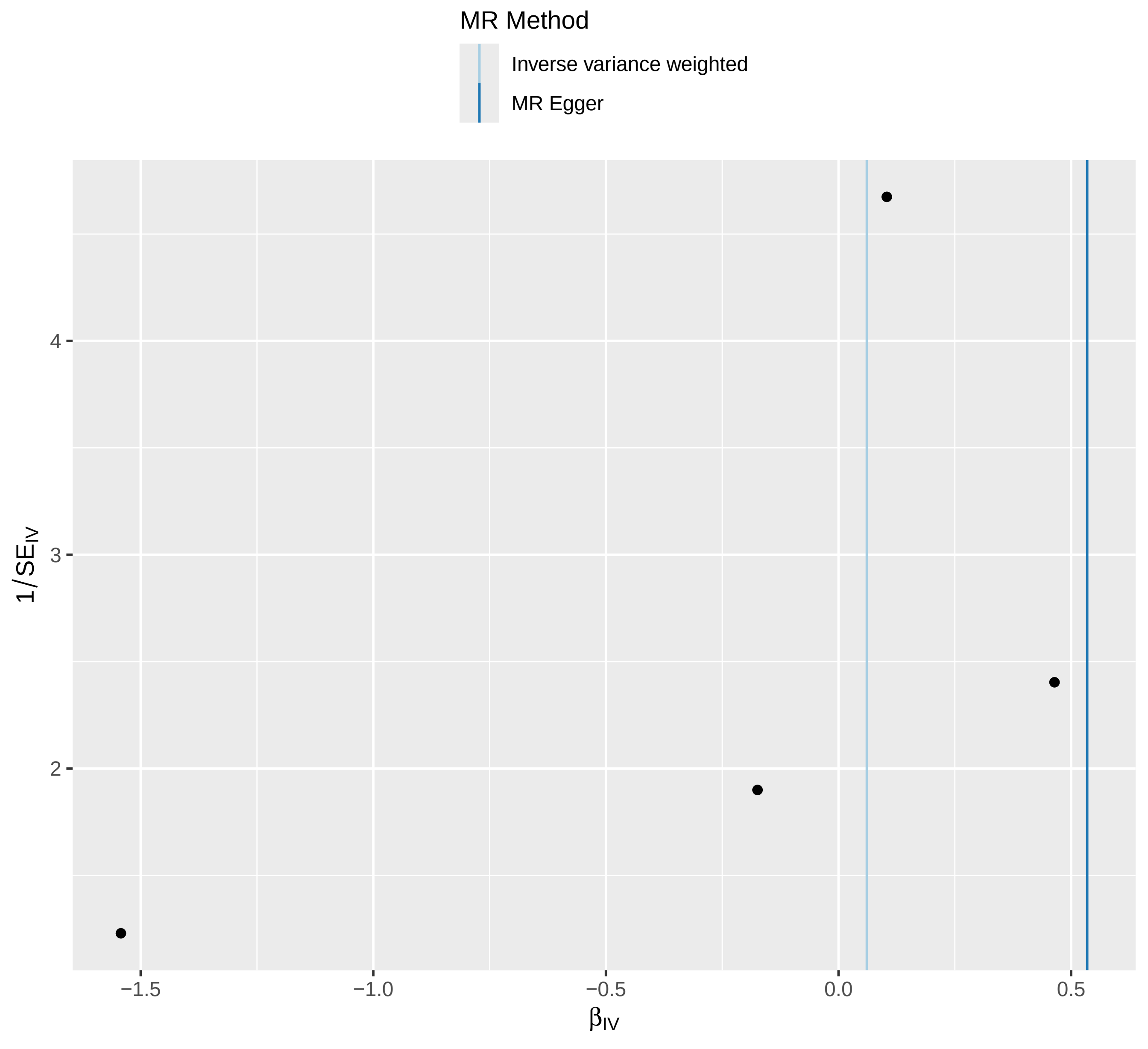
MR Method
Inverse variance weighted
MR Egger
4
3
2
1/SE$_{IV}$
−1.5
−1.0
−0.5
0.0
0.5
β$_{IV}$

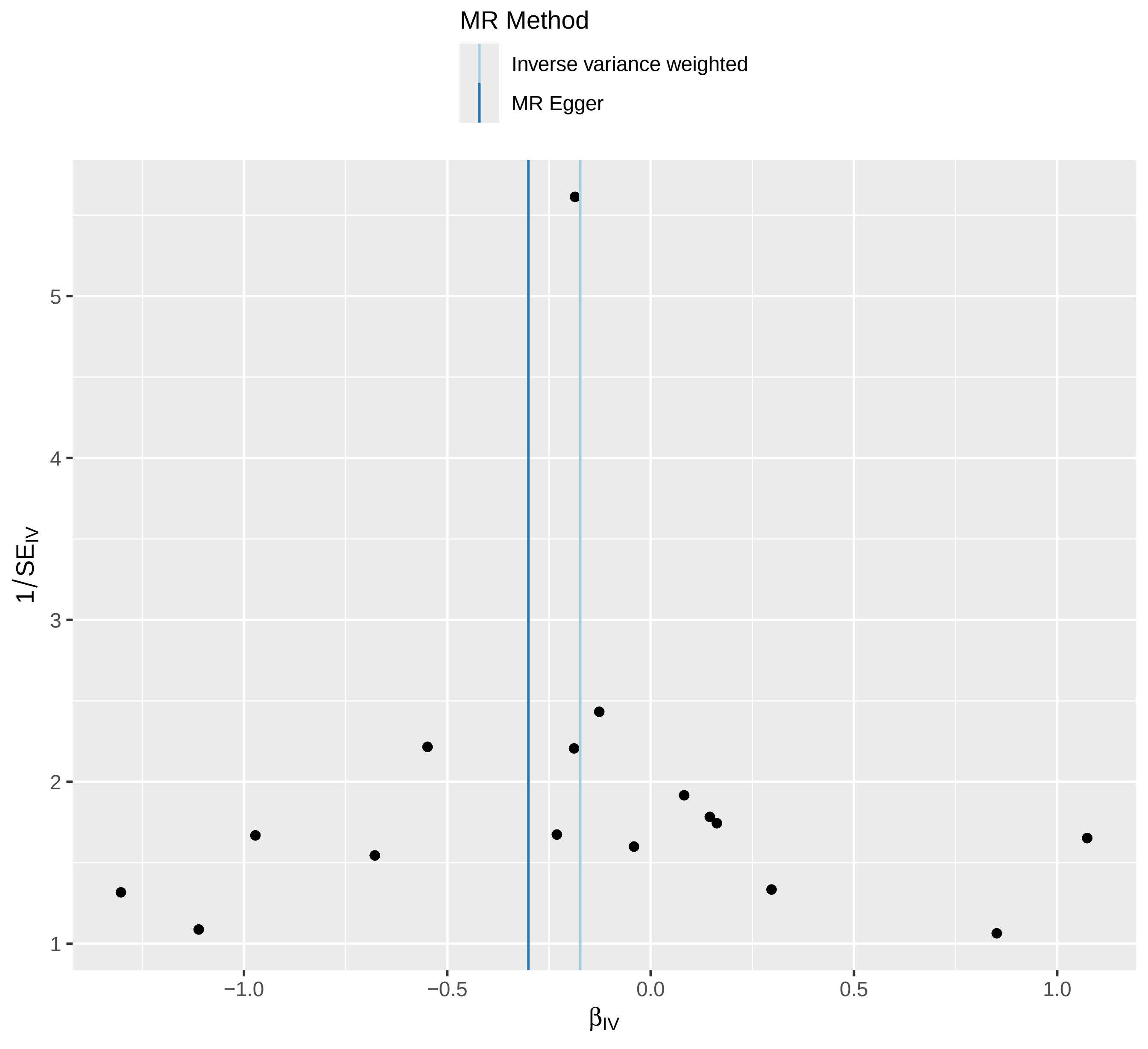

MR Method
Inverse variance weighted
MR Egger
1/SE_IV
5
4
3
2
1
−1.0
−0.5
0.0
0.5
1.0
β_IV

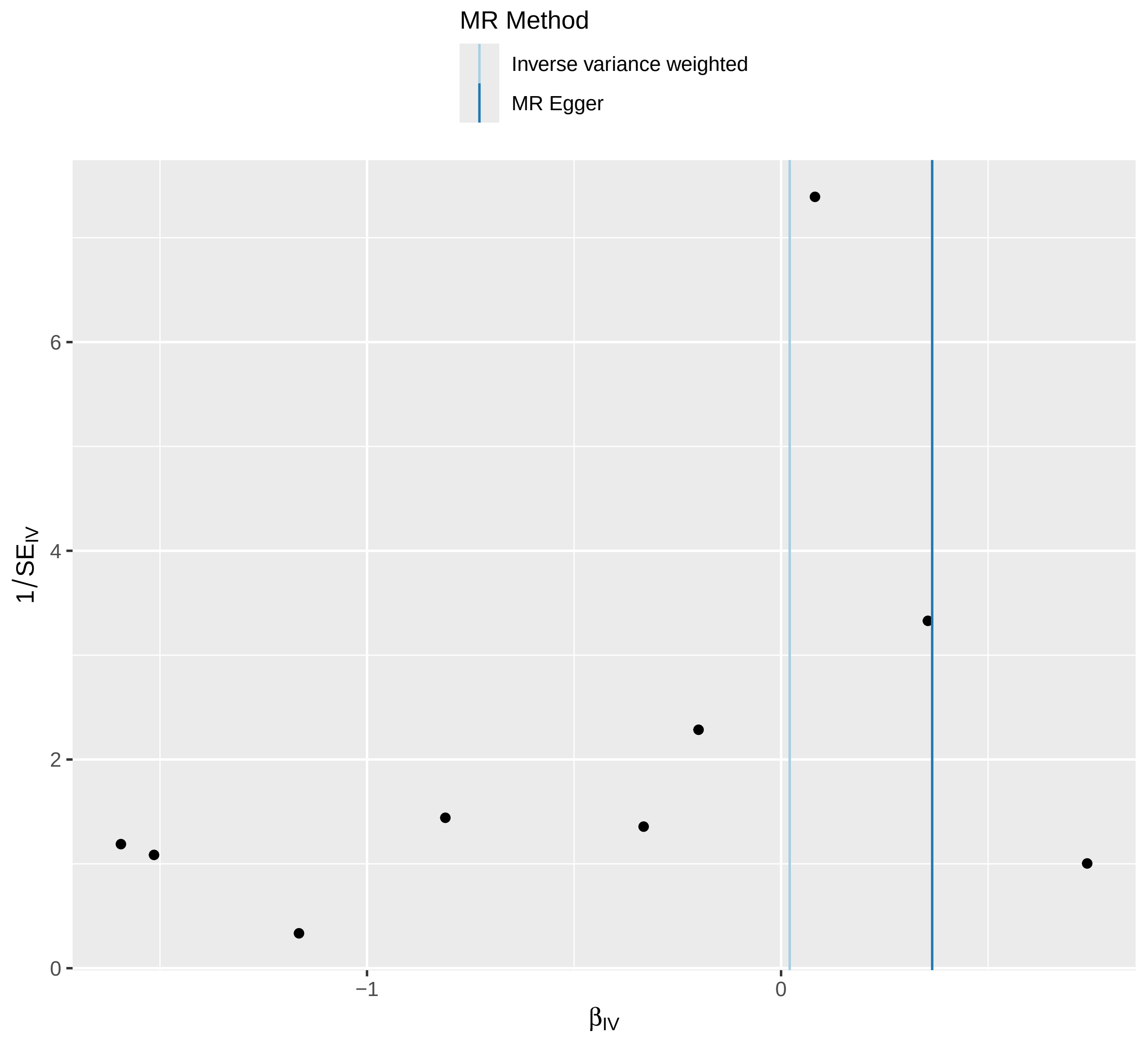

MR Method
Inverse variance weighted
MR Egger
6
4
2
0
1/SE$_{IV}$
−1
0
β$_{IV}$

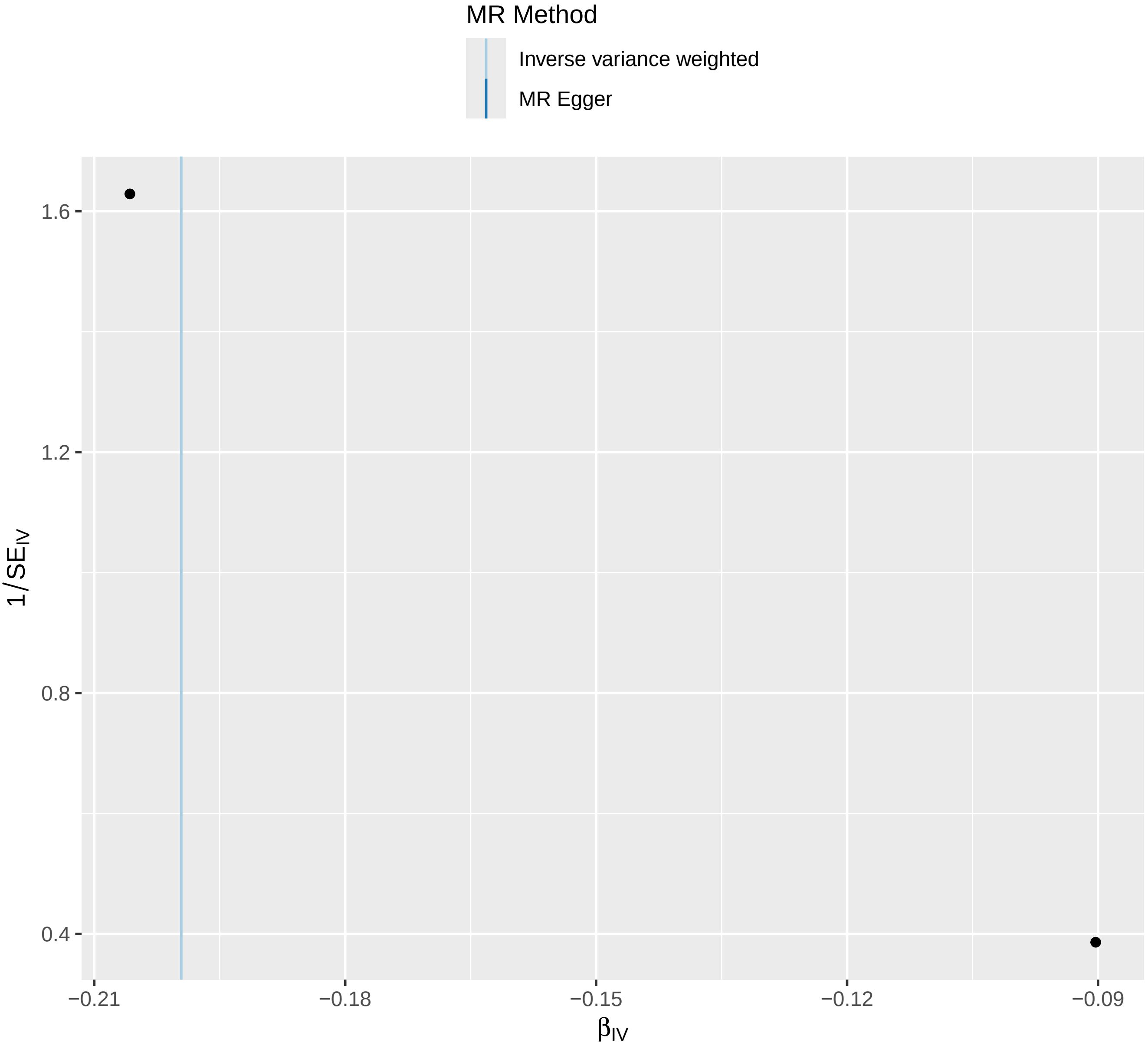
MR Method
Inverse variance weighted
MR Egger
1.6
1.2
0.8
0.4
1/SE$_{IV}$
−0.21
−0.18
−0.15
−0.12
−0.09
β$_{IV}$

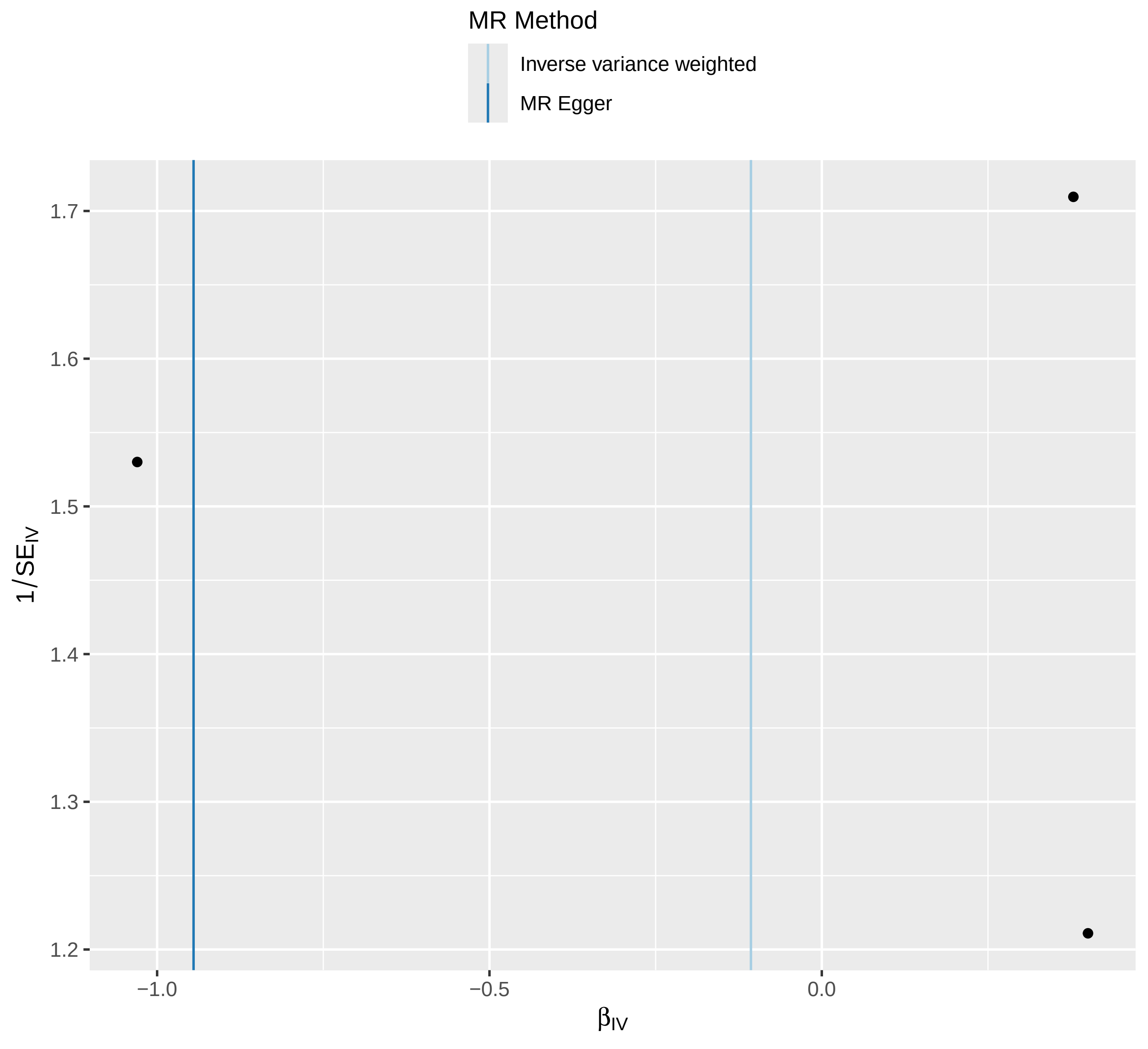
MR Method
Inverse variance weighted
MR Egger
1.7
1.6
1.5
1.4
1.3
1.2
$1/SE_{IV}$
−1.0
−0.5
0.0
β_{IV}

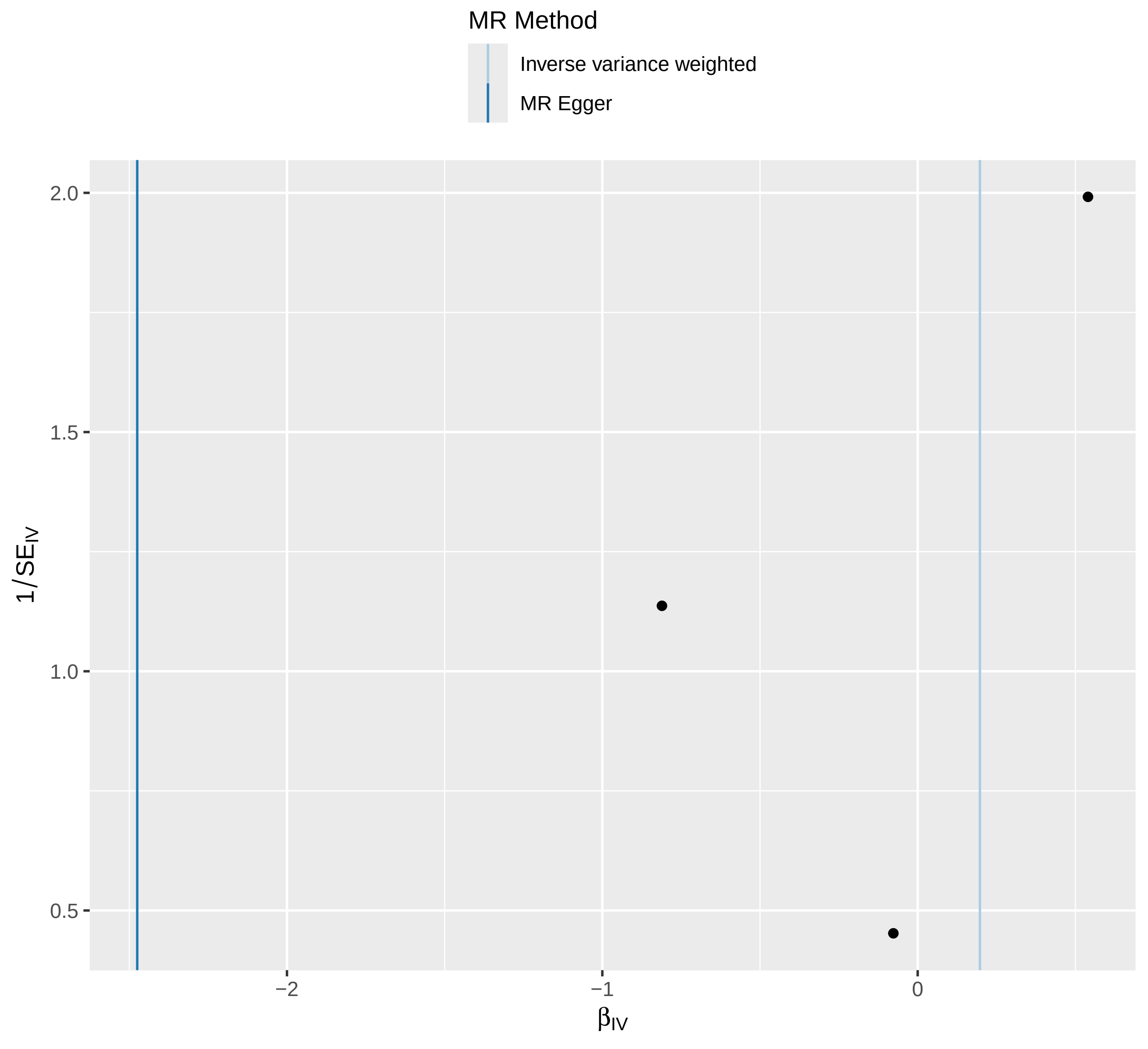

MR Method
Inverse variance weighted
MR Egger
2.0
1.5
1.0
0.5
−2
−1
0
1/SE_IV
β_IV

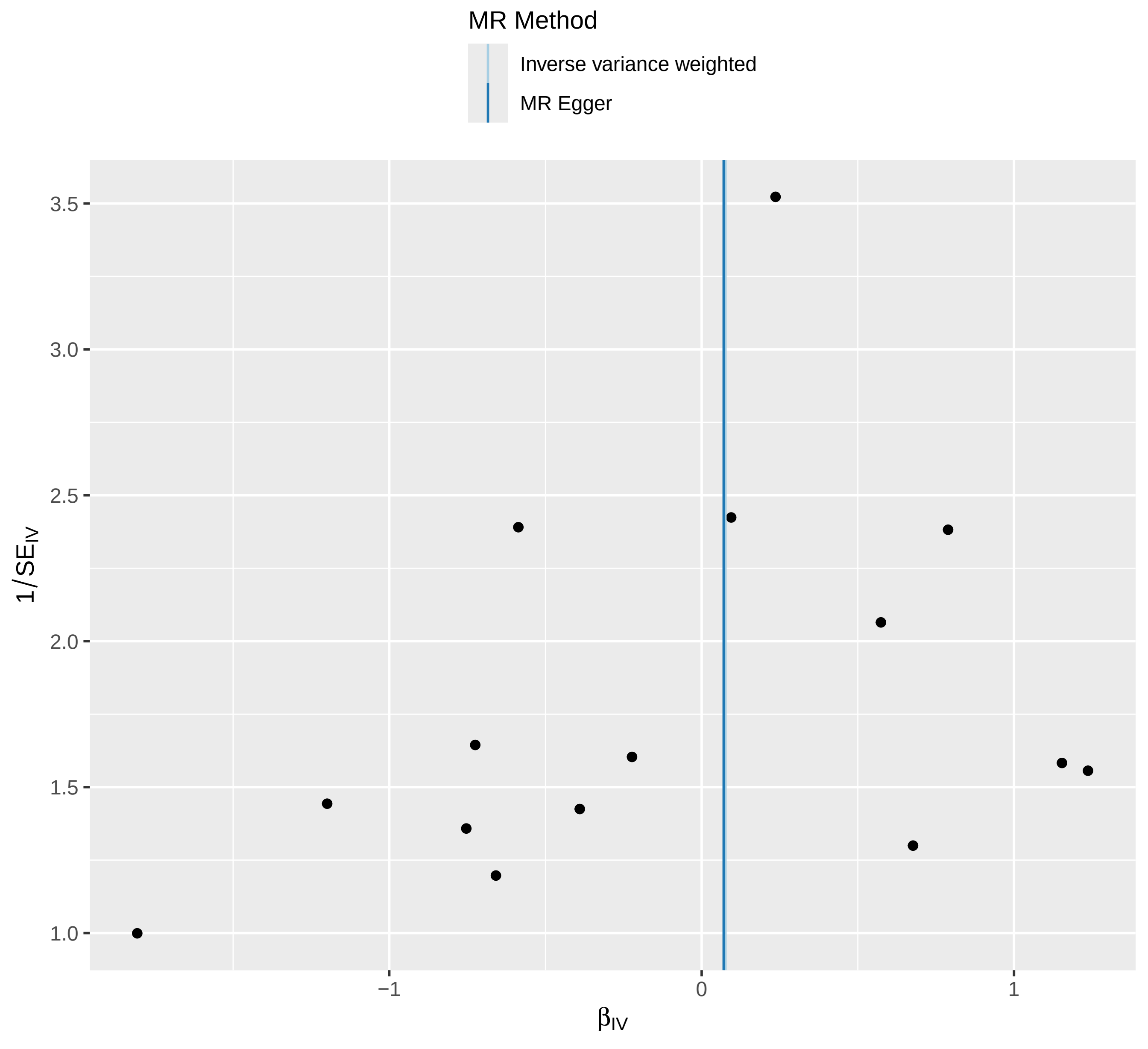

MR Method
Inverse variance weighted
MR Egger
3.5
3.0
2.5
2.0
1.5
1.0
1/SE_IV
−1
0
1
β_IV

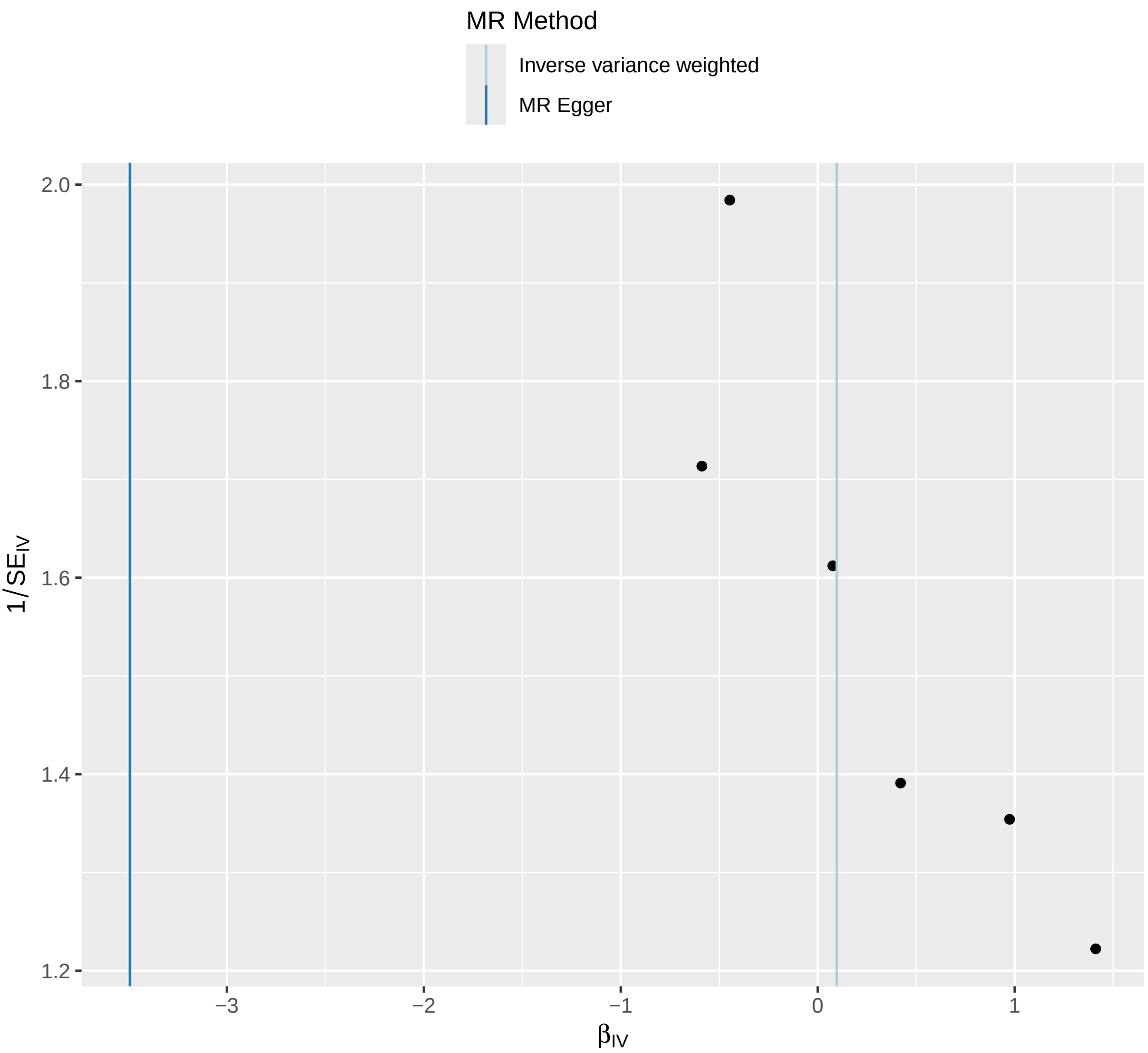

MR Method
Inverse variance weighted
MR Egger
2.0
1.8
1.6
1.4
1.2
1/SE_IV
−3
−2
−1
0
1
β_IV

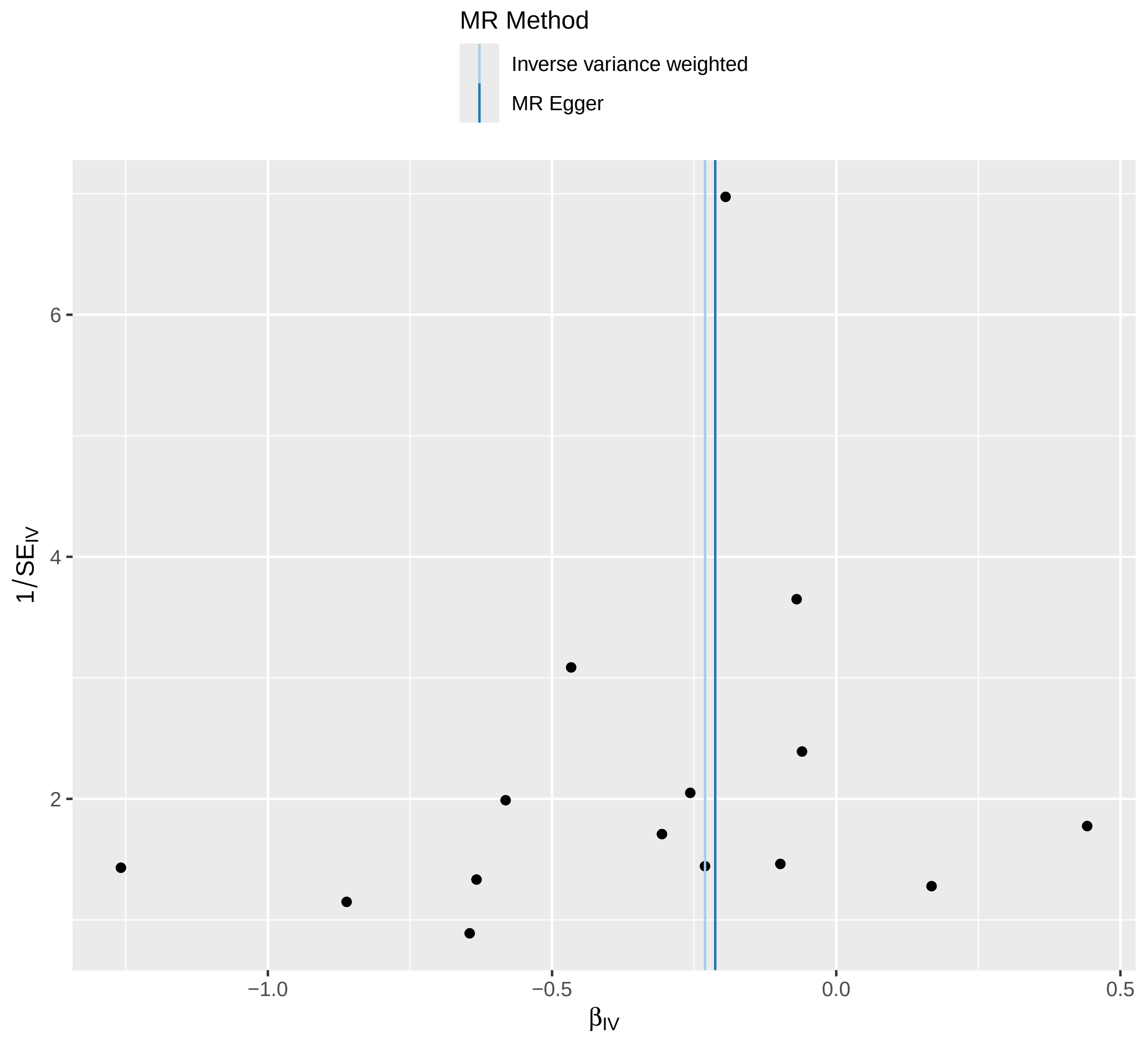
MR Method
Inverse variance weighted
MR Egger
1/SE$_{IV}$
6
4
2
−1.0
−0.5
0.0
0.5
β$_{IV}$

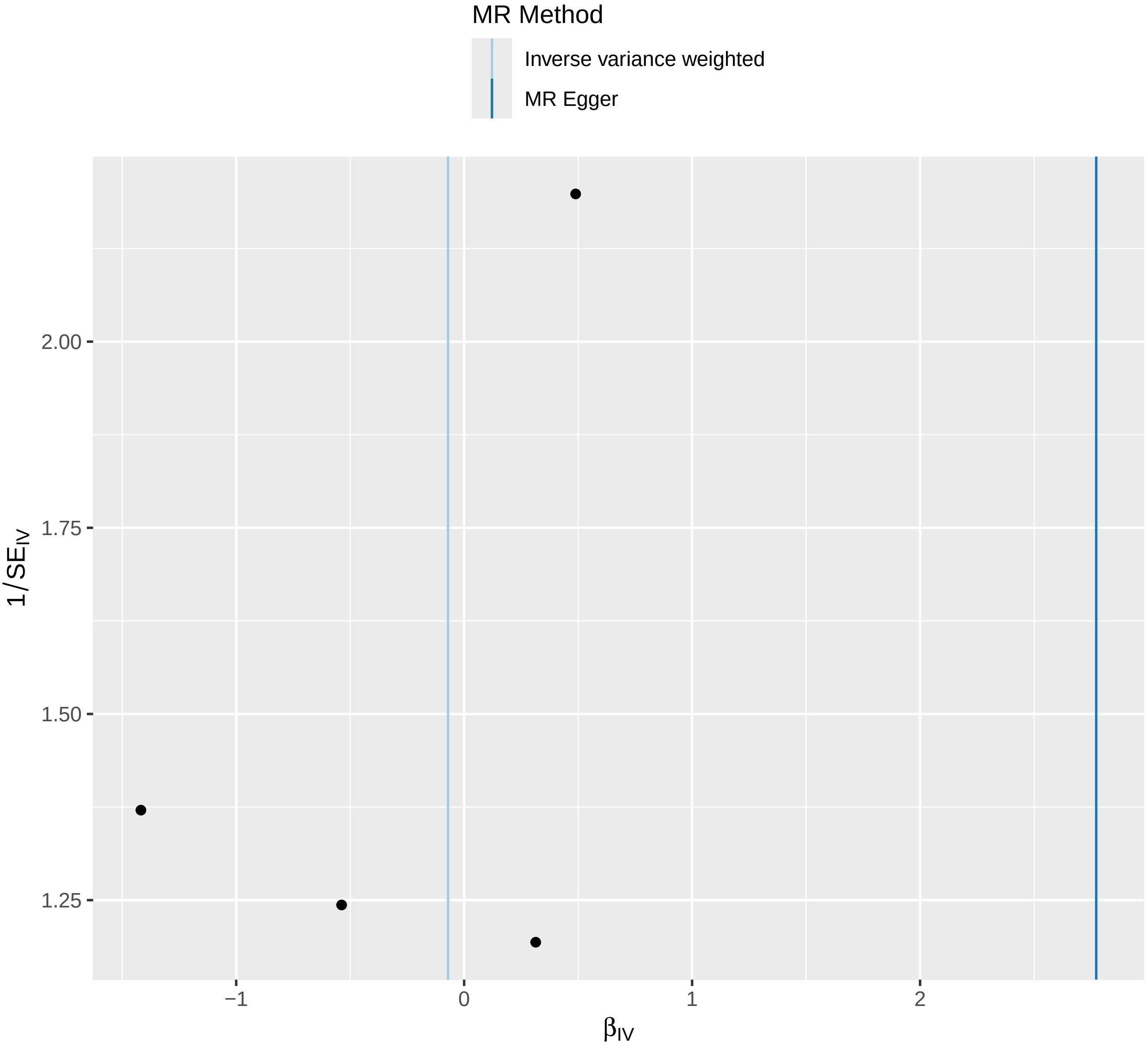
MR Method
Inverse variance weighted
MR Egger
2.00
1.75
1.50
1.25
$1/SE_{IV}$
−1
0
1
2
β_{IV}

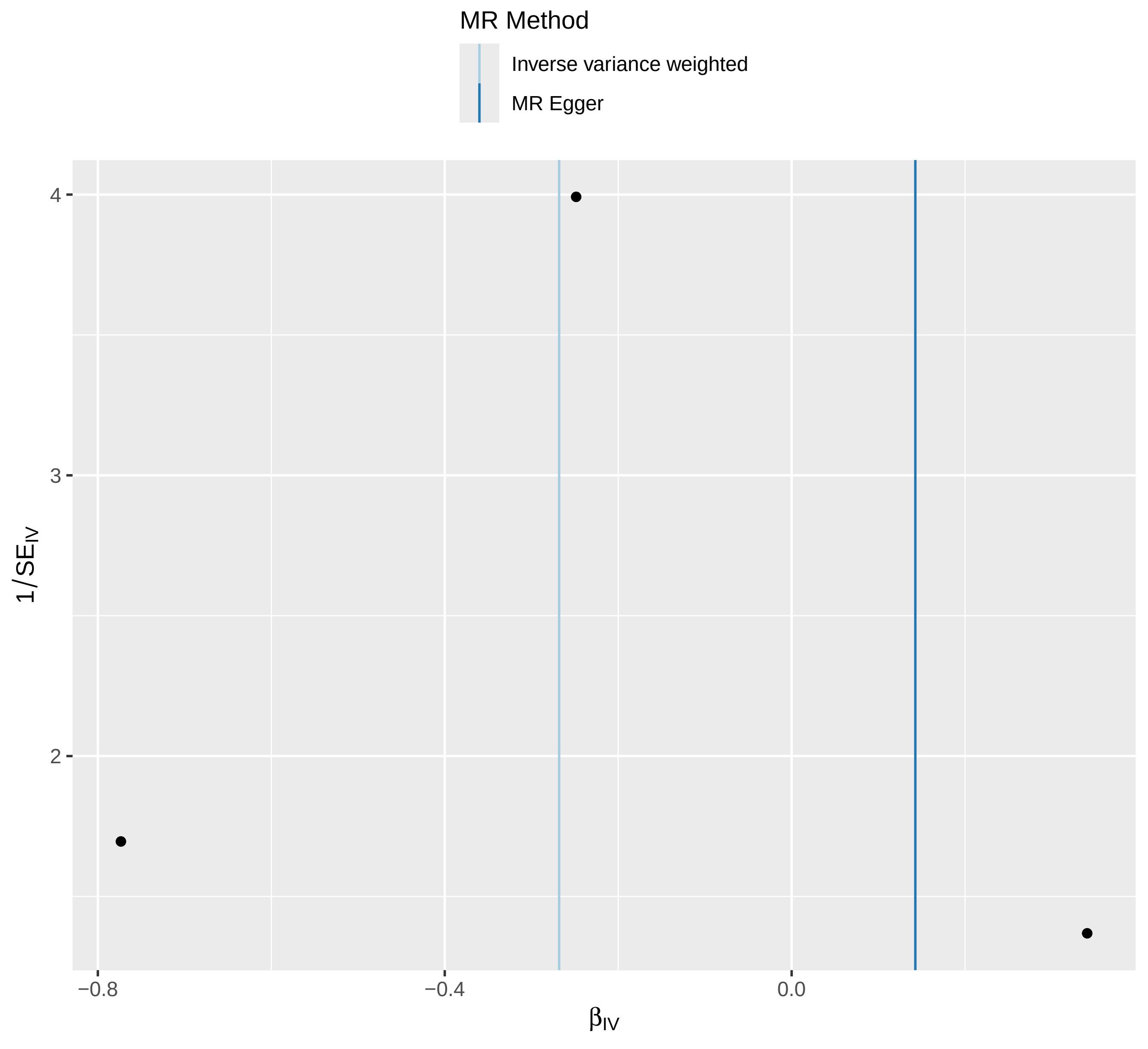

MR Method
Inverse variance weighted
MR Egger
4
3
2
1/SE$_{IV}$
−0.8
−0.4
0.0
β$_{IV}$

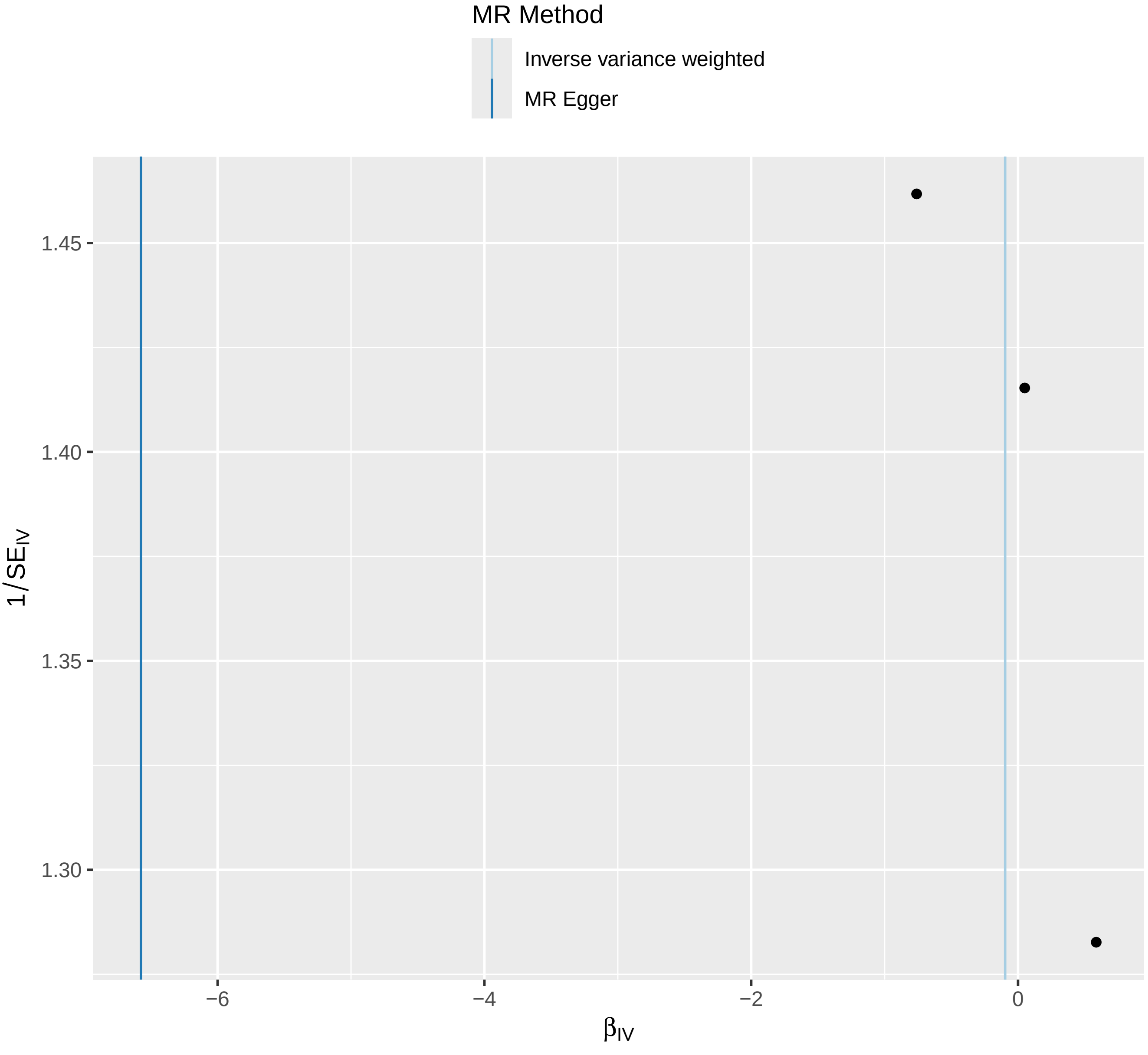

MR Method
Inverse variance weighted
MR Egger
1.45
1.40
1.35
1.30
1/SE_IV
−6
−4
−2
0
β_IV

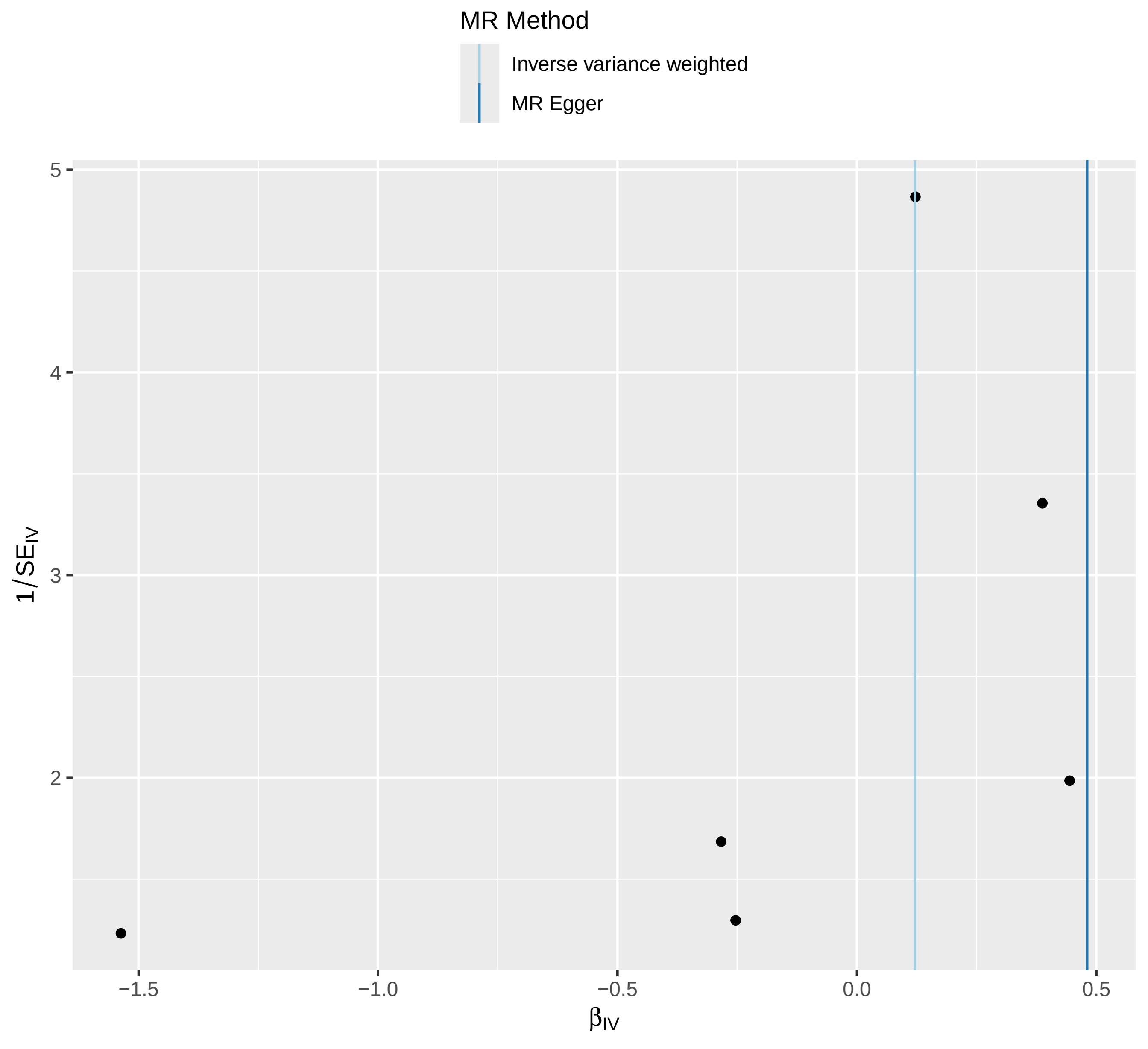

MR Method
Inverse variance weighted
MR Egger
5
4
3
2
1/SE_IV
−1.5
−1.0
−0.5
0.0
0.5
β_IV

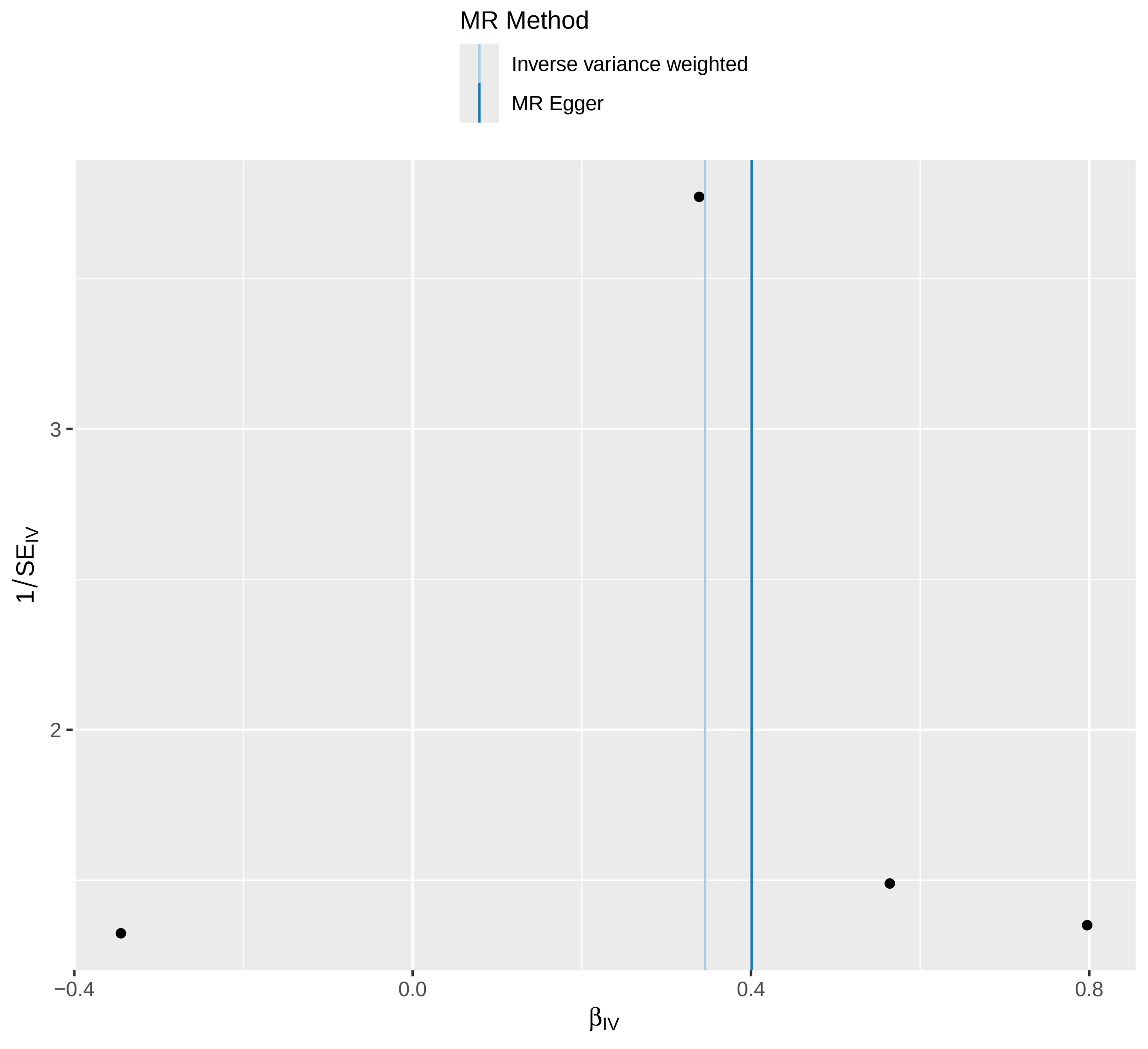

MR Method
Inverse variance weighted
MR Egger
3
2
1/SE_IV
−0.4
0.0
0.4
0.8
β_IV

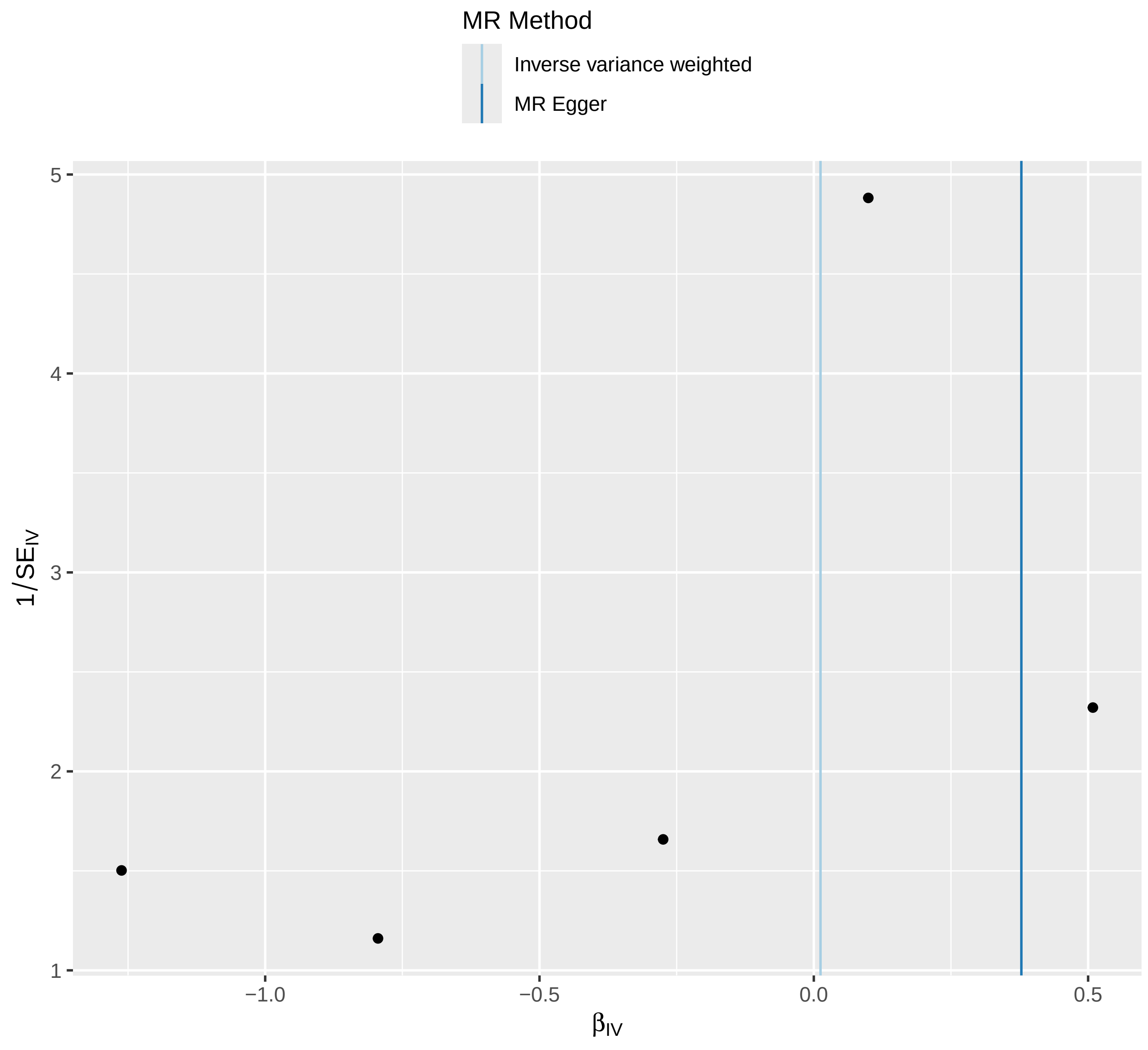

MR Method
Inverse variance weighted
MR Egger
5
4
3
2
1
$1/SE_{IV}$
−1.0
−0.5
0.0
0.5
β_{IV}

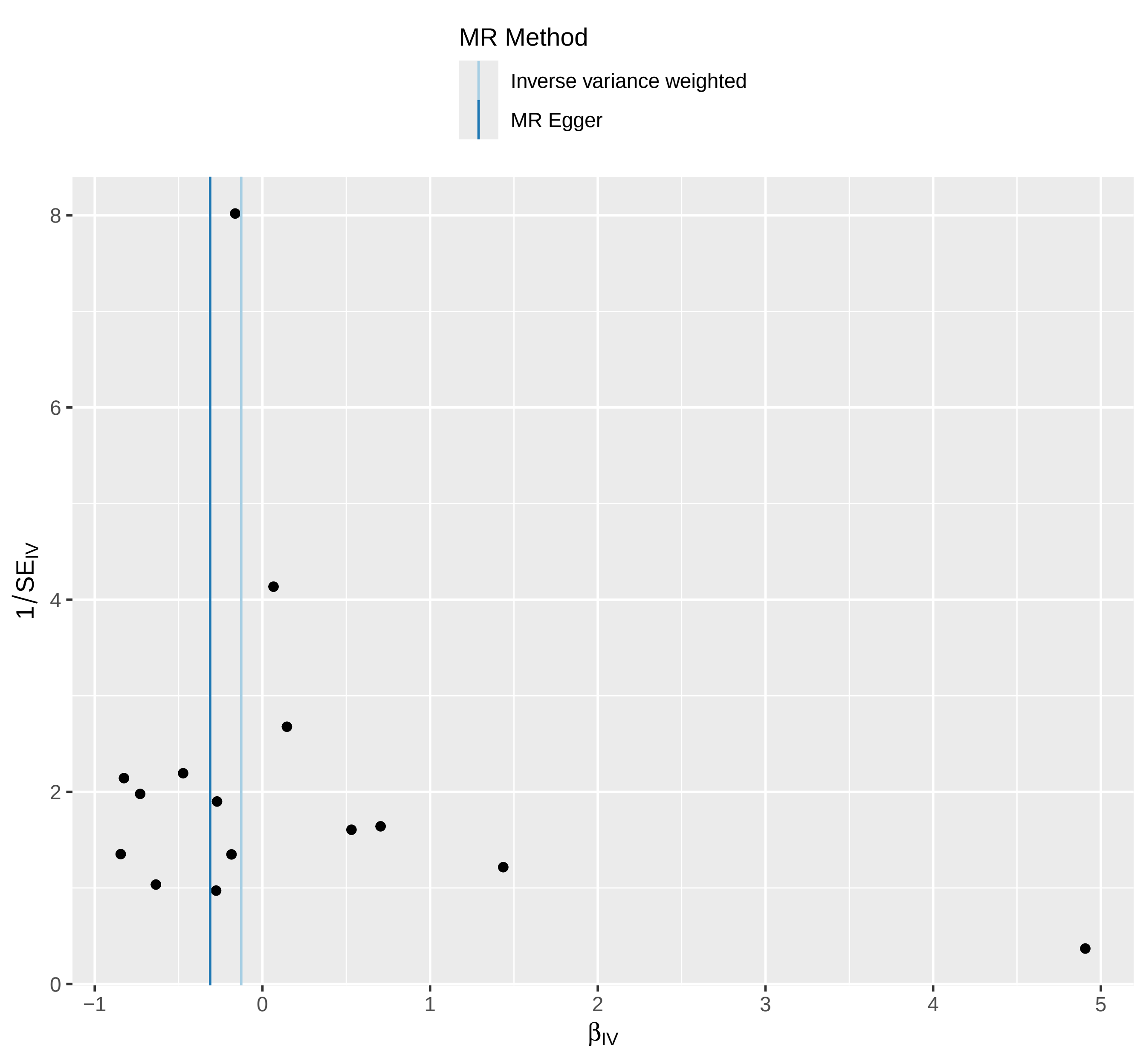
MR Method
Inverse variance weighted
MR Egger
1/SE_IV
8
6
4
2
0
−1
0
1
2
3
4
5
β_IV

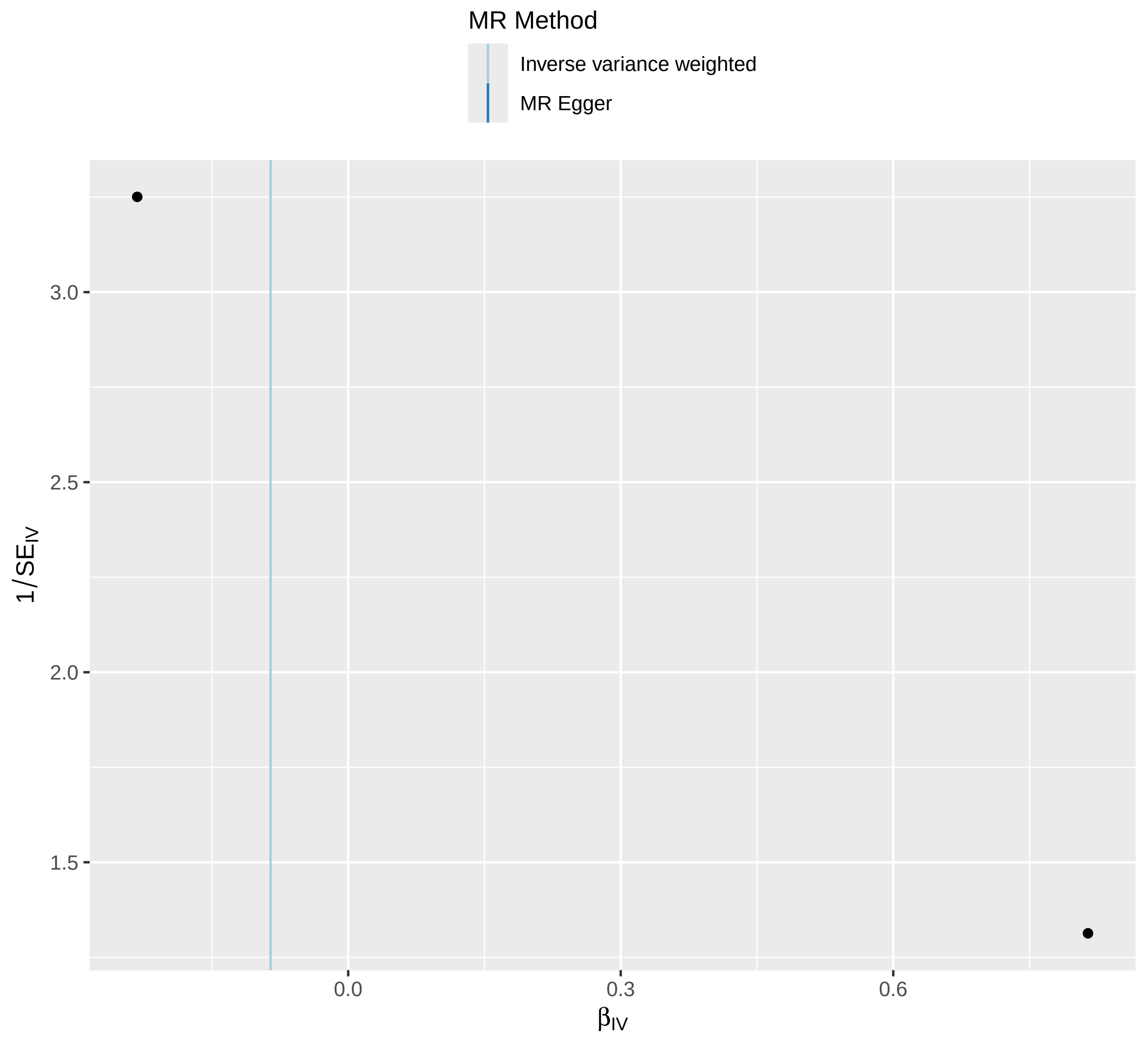

MR Method
Inverse variance weighted
MR Egger
3.0
2.5
2.0
1.5
1/SE_IV
0.0
0.3
0.6
β_IV

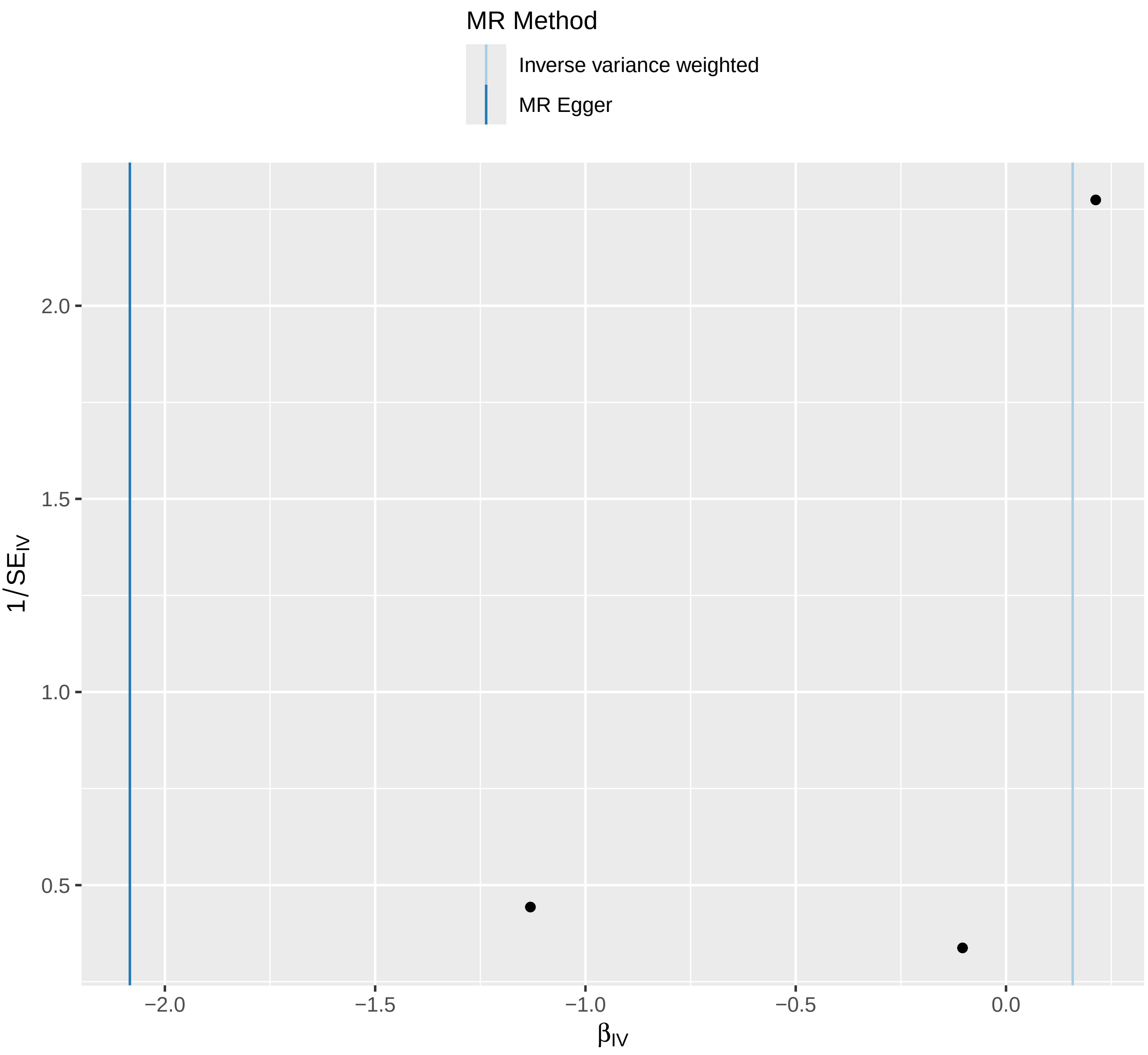
MR Method
Inverse variance weighted
MR Egger
2.0
1.5
1.0
0.5
1/SE_IV
−2.0
−1.5
−1.0
−0.5
0.0
β_IV

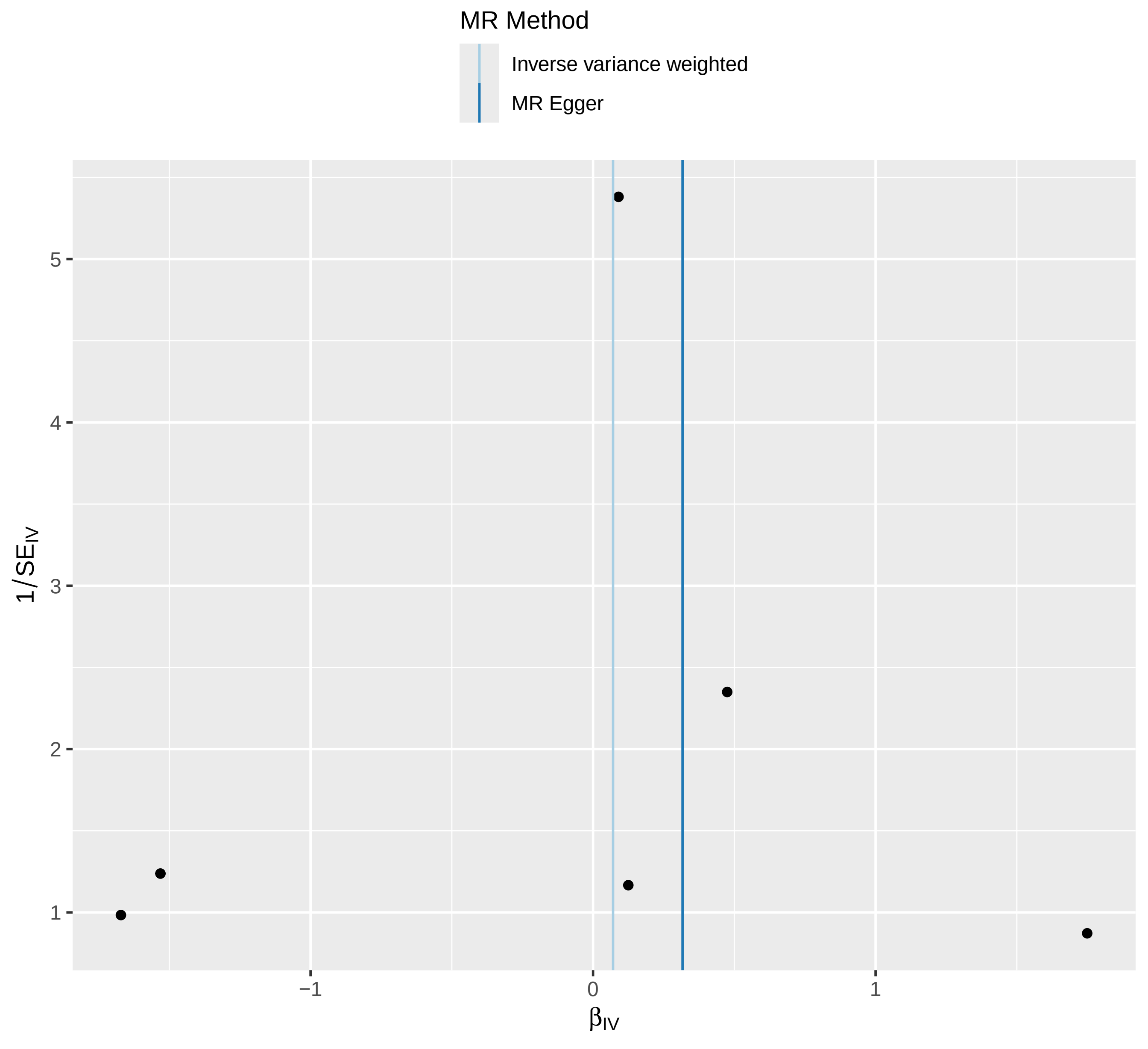

MR Method
Inverse variance weighted
MR Egger
1/SE_IV
5
4
3
2
1
−1
0
1
β_IV

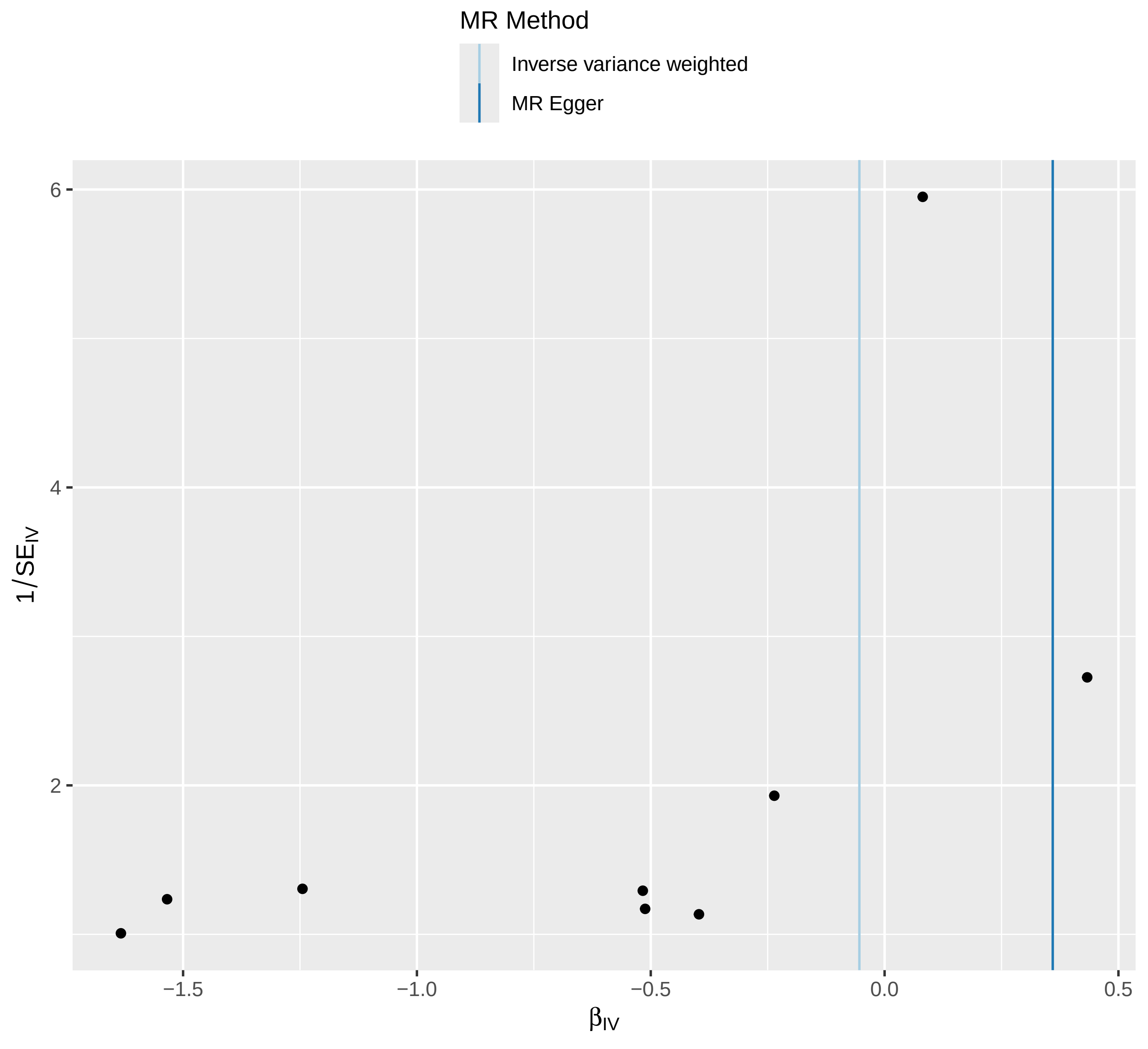
MR Method
Inverse variance weighted
MR Egger
6
4
2
1/SE_IV
−1.5
−1.0
−0.5
0.0
0.5
β_IV

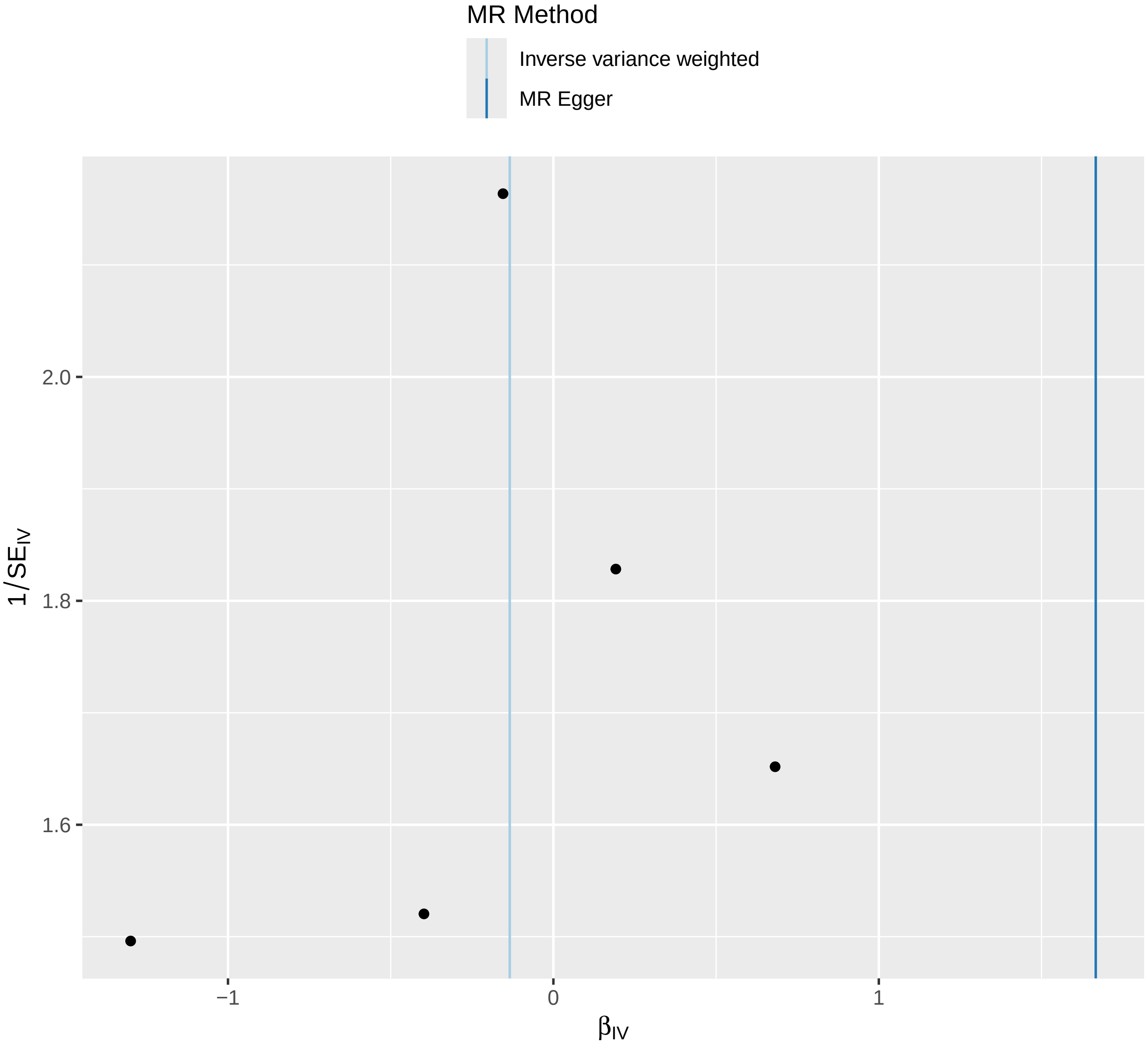
MR Method
Inverse variance weighted
MR Egger
2.0
1.8
1.6
1/SE_IV
−1
0
1
β_IV

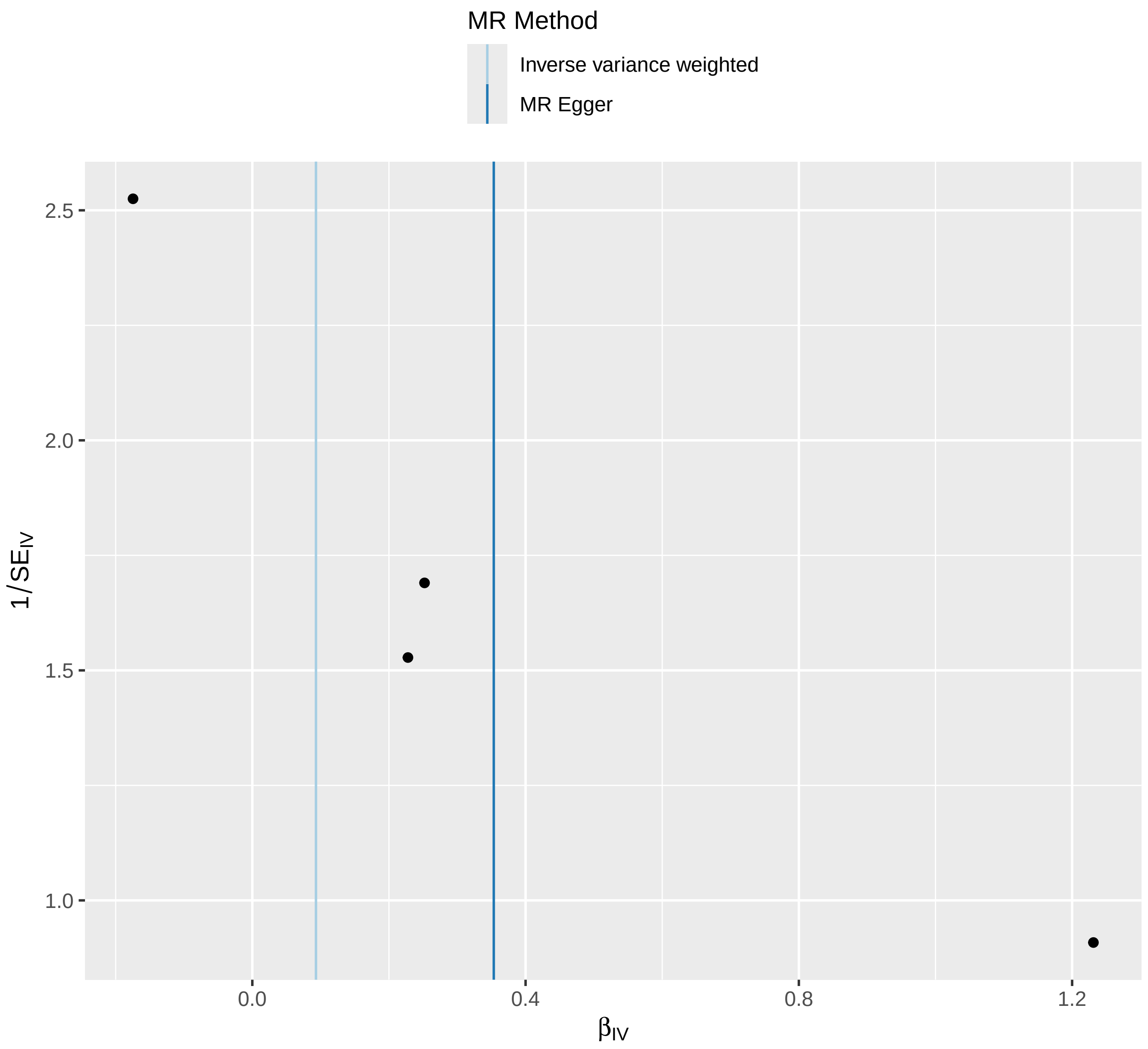
MR Method
Inverse variance weighted
MR Egger
2.5
2.0
1.5
1.0
1/SE_IV
0.0
0.4
0.8
1.2
β_IV

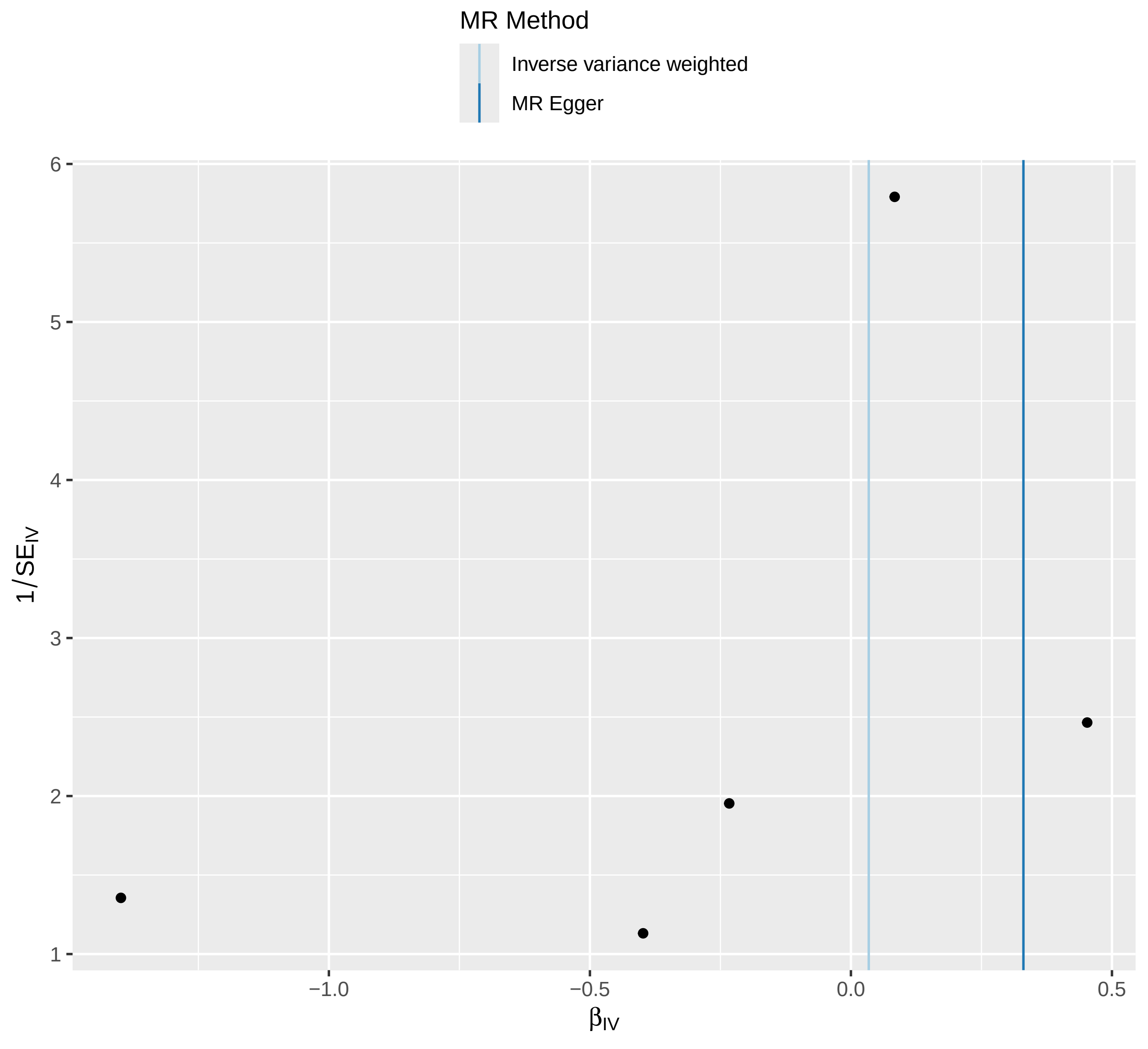
MR Method
Inverse variance weighted
MR Egger
1/SE_IV
6
5
4
3
2
1
−1.0
−0.5
0.0
0.5
β_IV

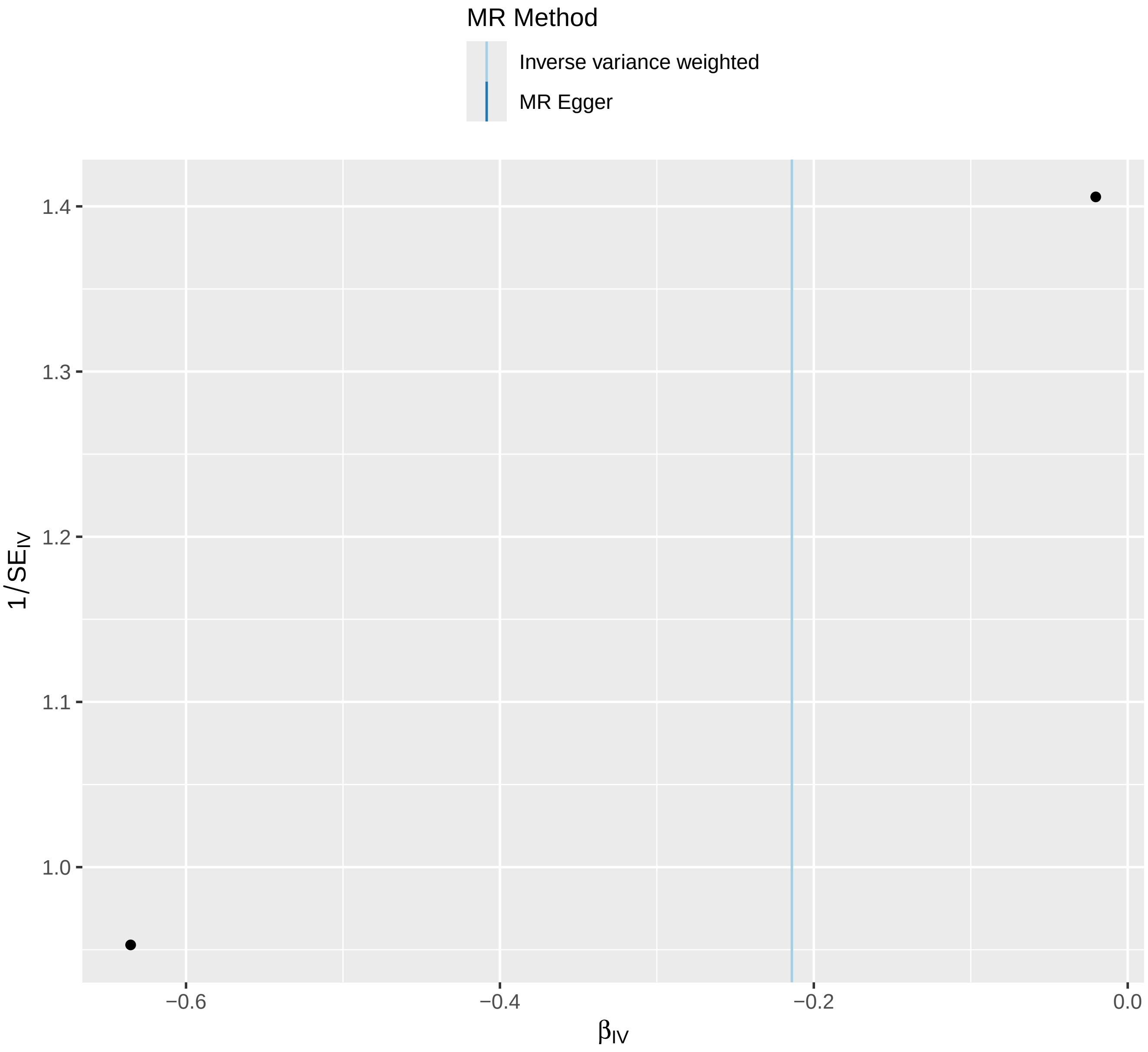

MR Method
Inverse variance weighted
MR Egger
1.4
1.3
1.2
1.1
1.0
$1/SE_{IV}$
−0.6
−0.4
−0.2
0.0
β_{IV}

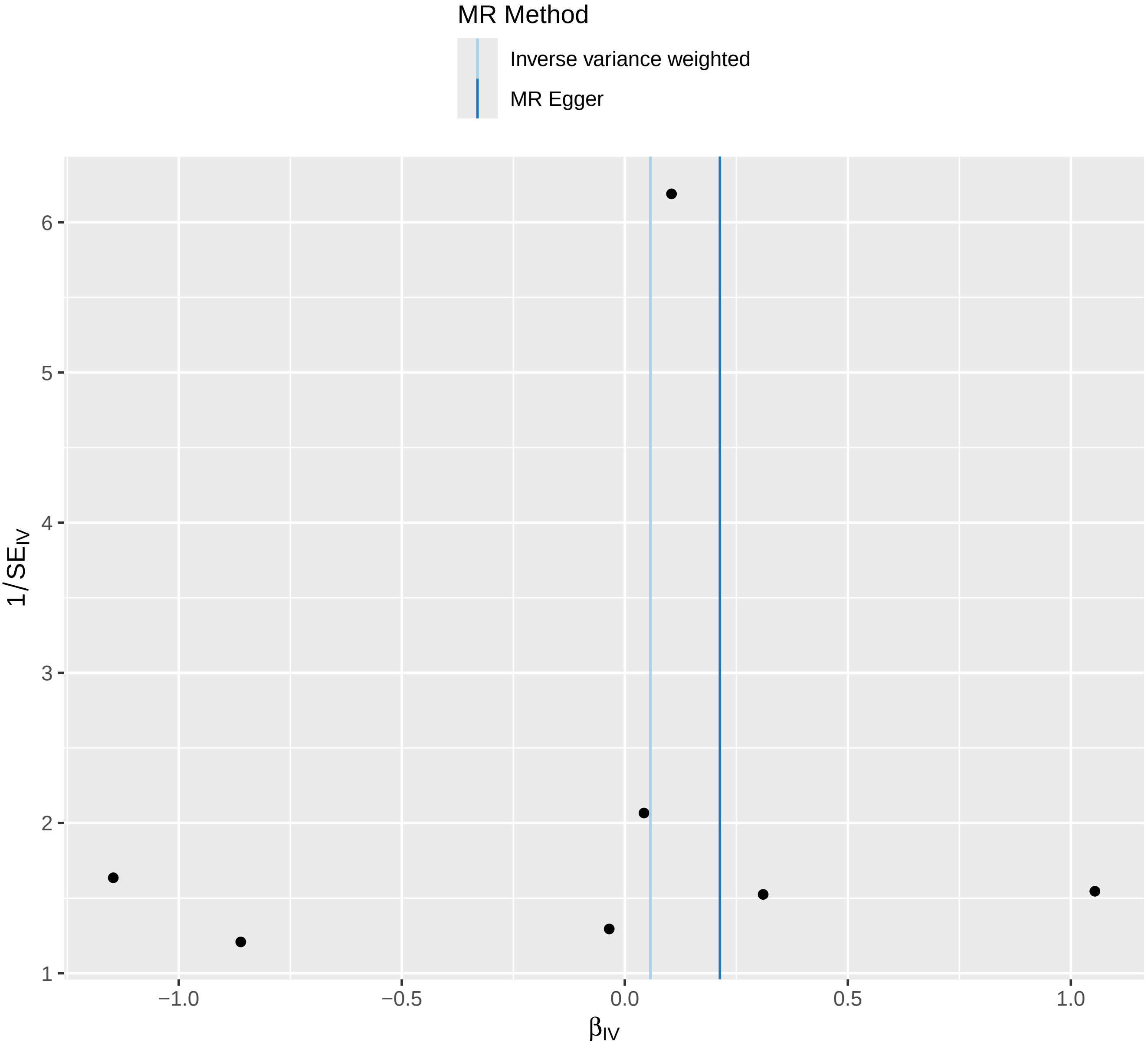
MR Method
Inverse variance weighted
MR Egger
1/SE_IV
β_IV
6
5
4
3
2
1
−1.0
−0.5
0.0
0.5
1.0

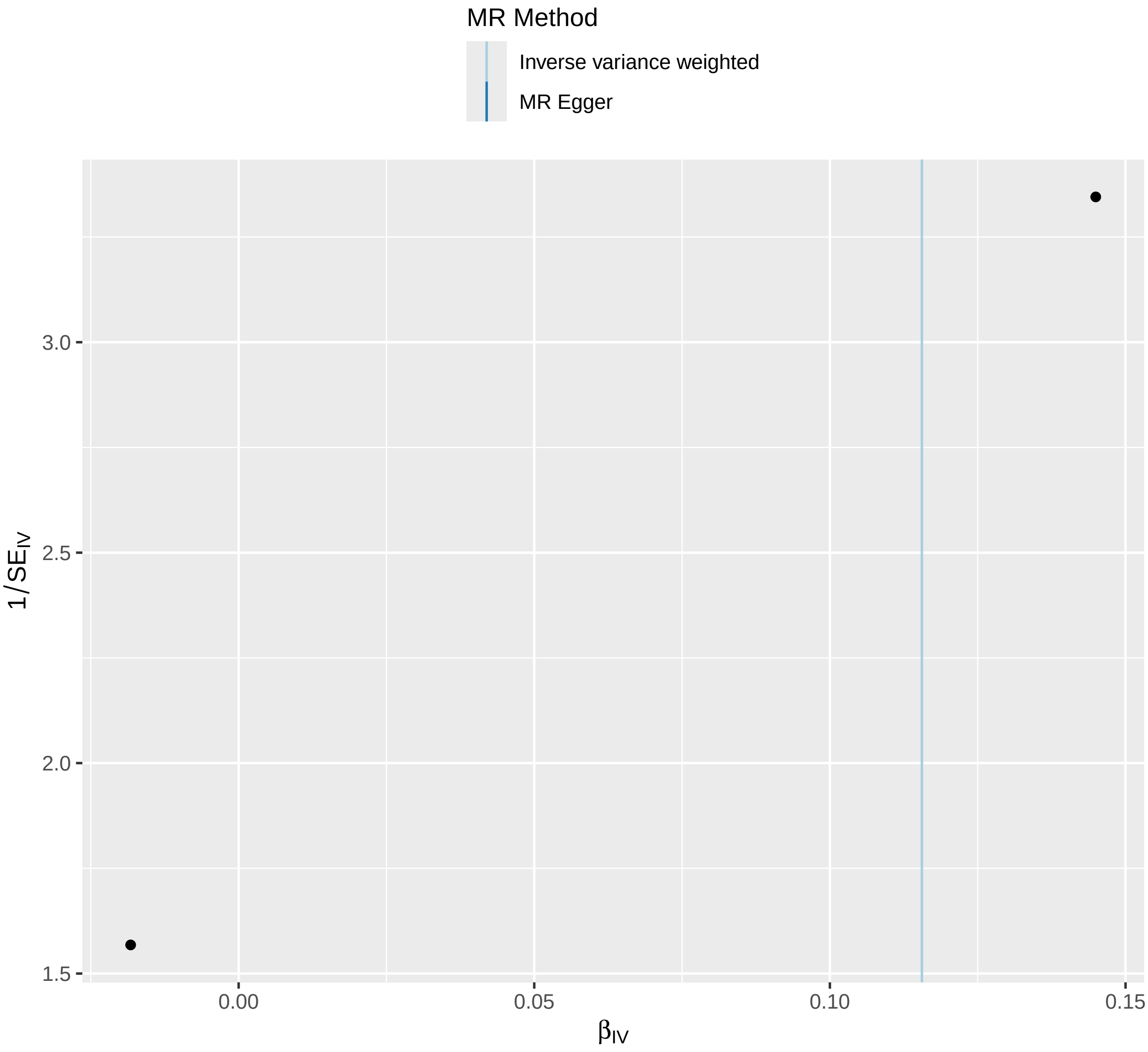
MR Method
Inverse variance weighted
MR Egger
3.0
2.5
2.0
1.5
1/SE_IV
0.00
0.05
0.10
0.15
β_IV

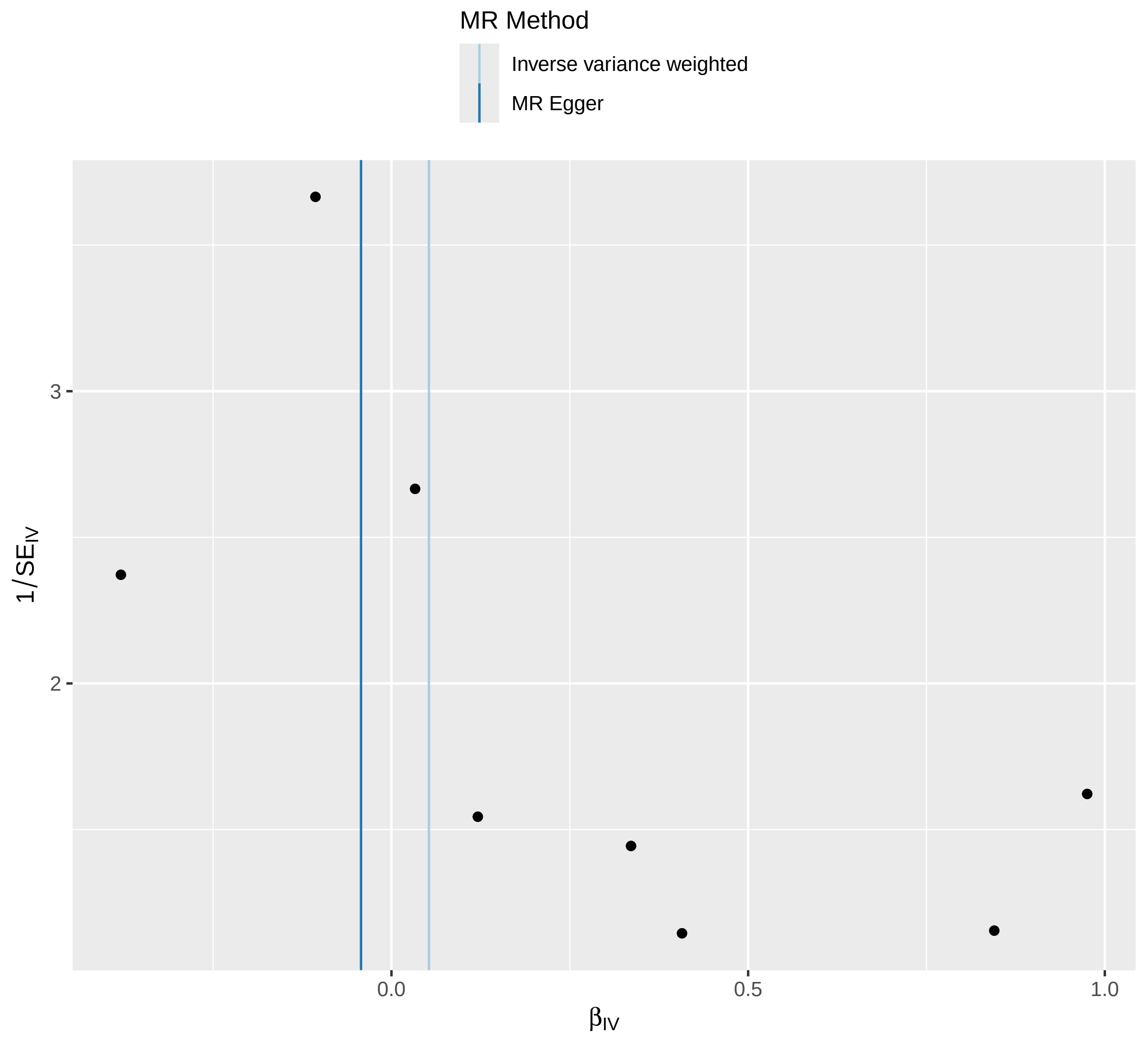

MR Method
Inverse variance weighted
MR Egger
3
2
0.0
0.5
1.0
$1/SE_{IV}$
β_{IV}

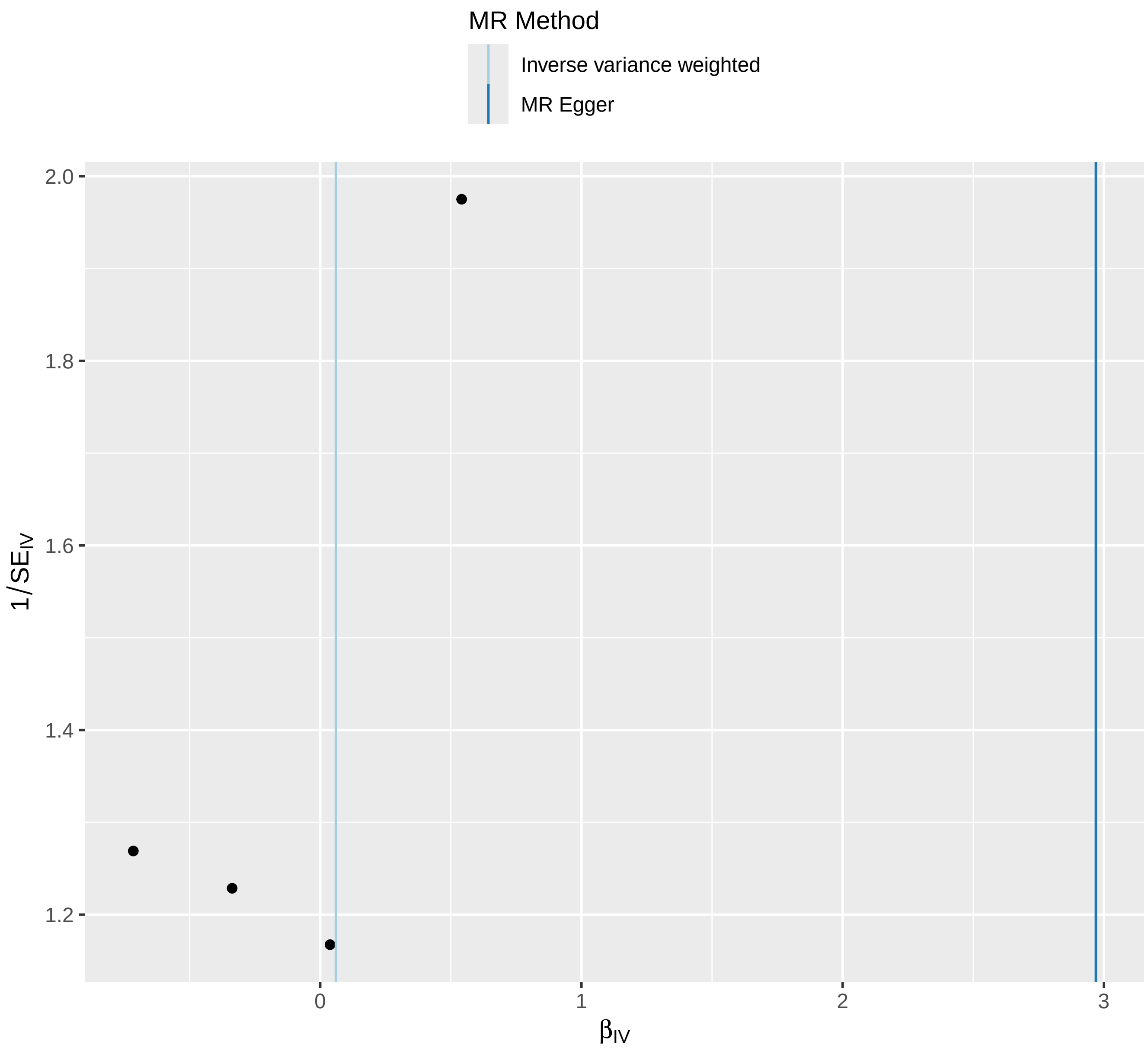

MR Method
Inverse variance weighted
MR Egger
2.0
1.8
1.6
1.4
1.2
$1/SE_{IV}$
0
1
2
3
β_{IV}

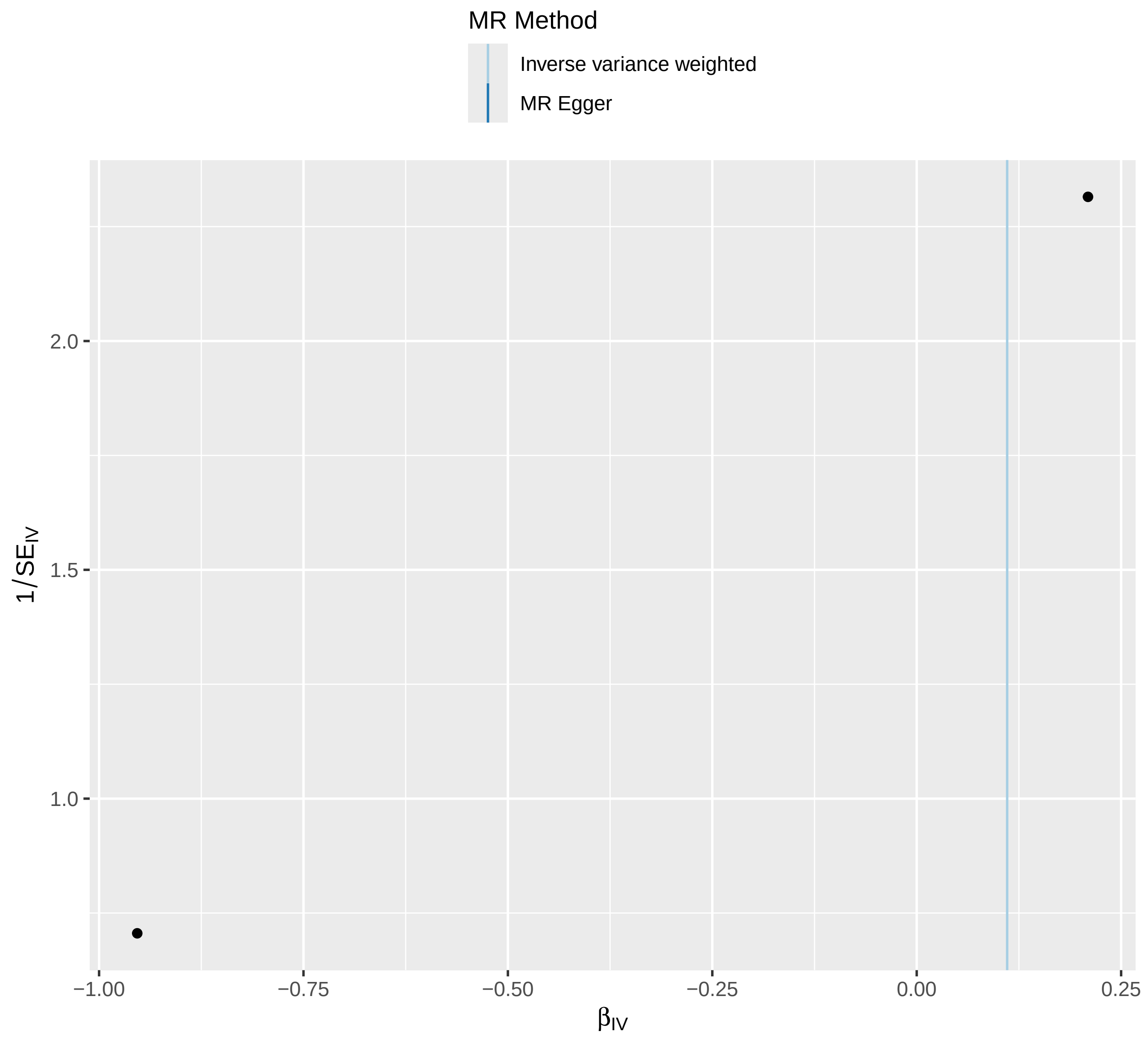

MR Method
Inverse variance weighted
MR Egger
2.0
1.5
1.0
1/SE_IV
−1.00
−0.75
−0.50
−0.25
0.00
0.25
β_IV

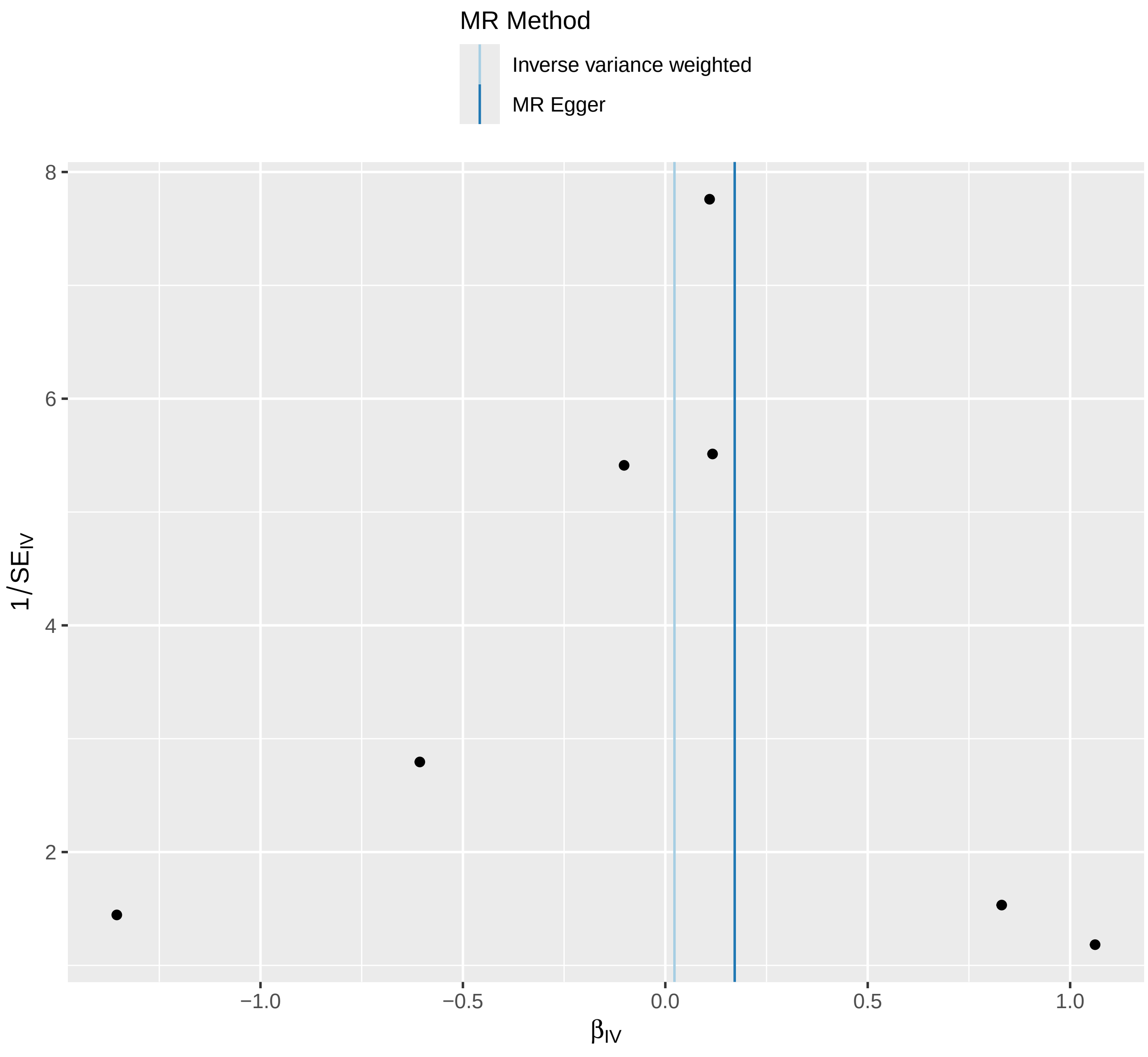

MR Method
Inverse variance weighted
MR Egger
8
6
4
2
1/SE_IV
−1.0
−0.5
0.0
0.5
1.0
β_IV

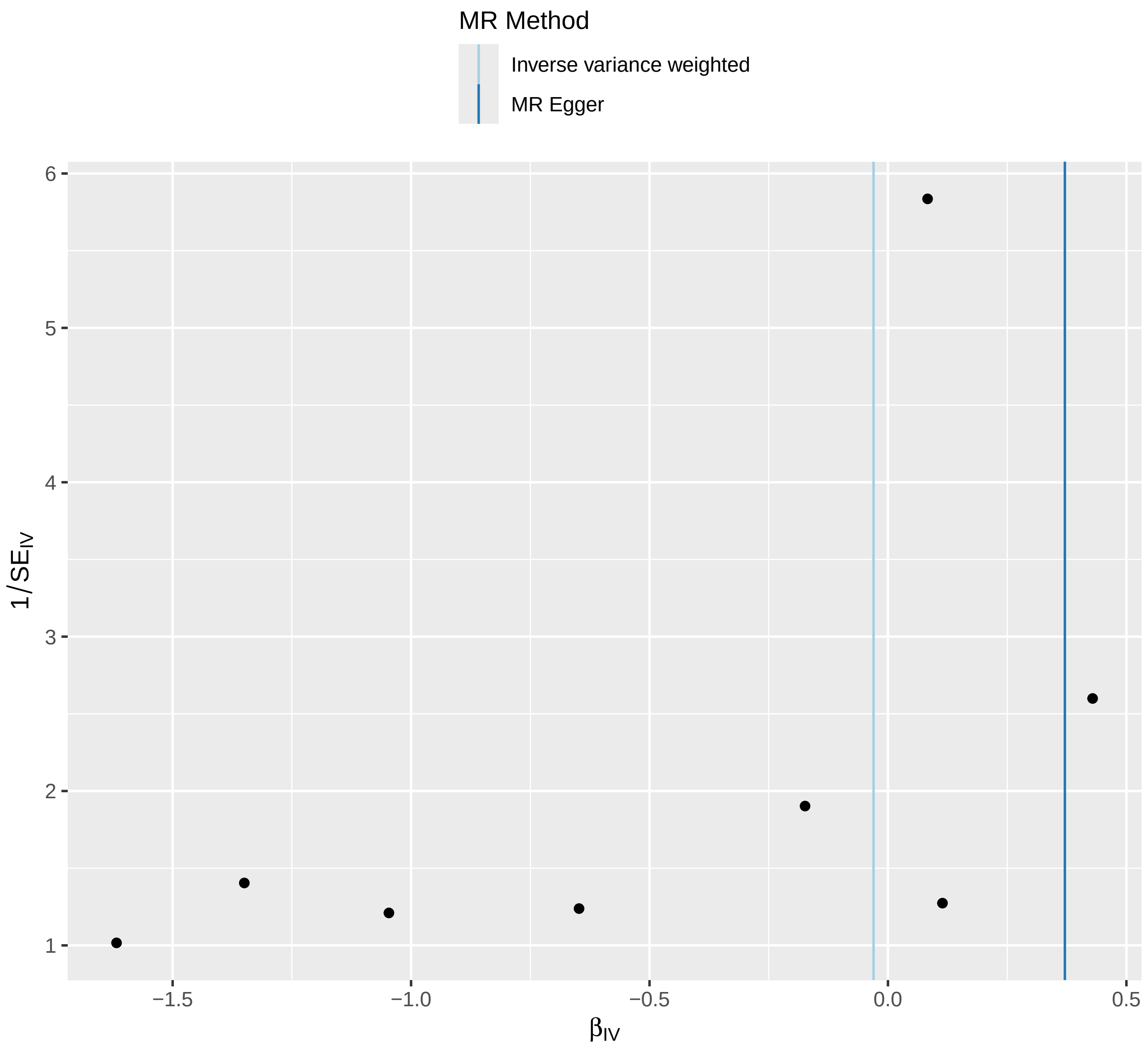

MR Method
Inverse variance weighted
MR Egger
1/SE_IV
6
5
4
3
2
1
β_IV
−1.5
−1.0
−0.5
0.0
0.5

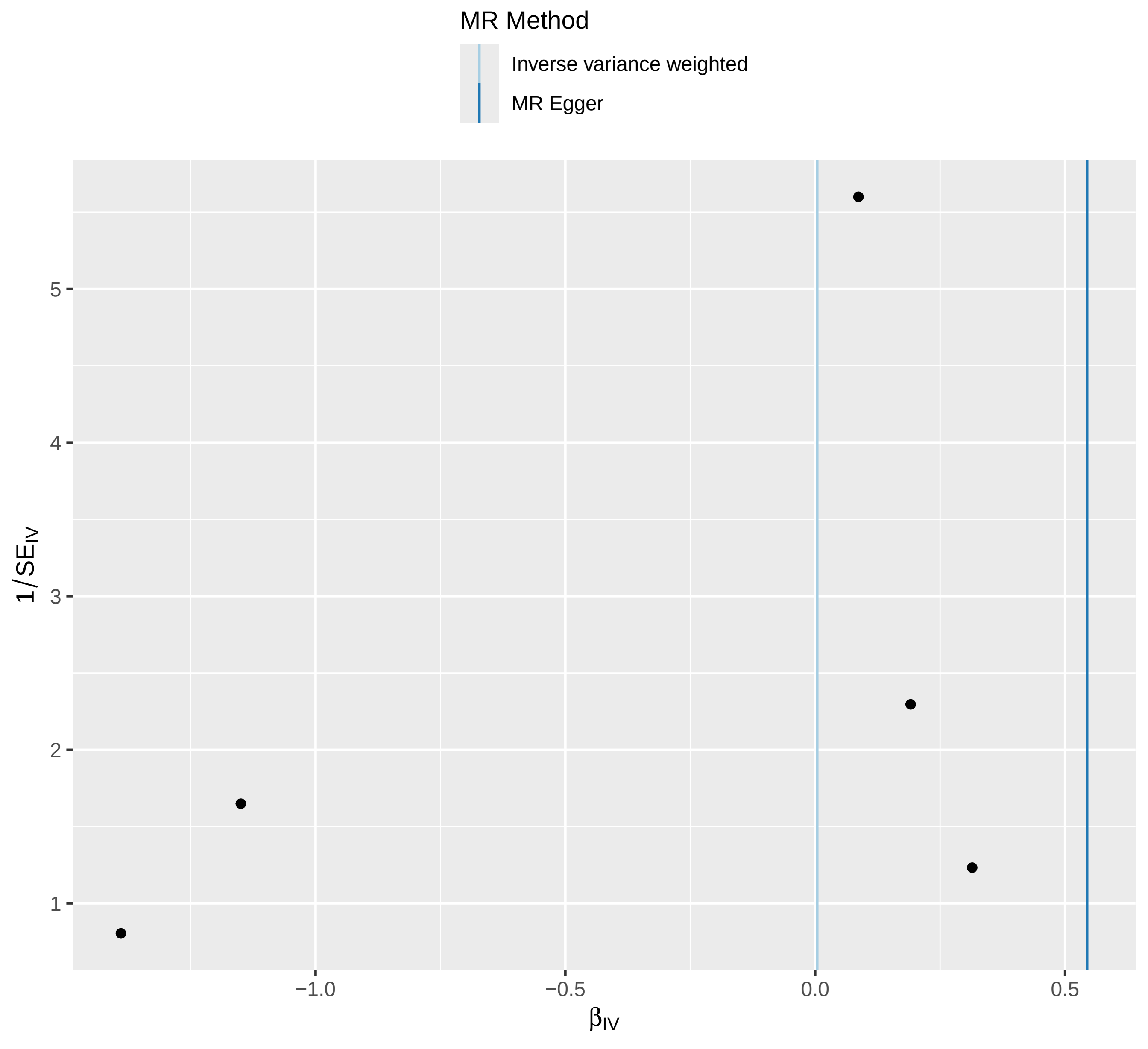
MR Method
Inverse variance weighted
MR Egger
1/SE_IV
5
4
3
2
1
−1.0
−0.5
0.0
0.5
β_IV

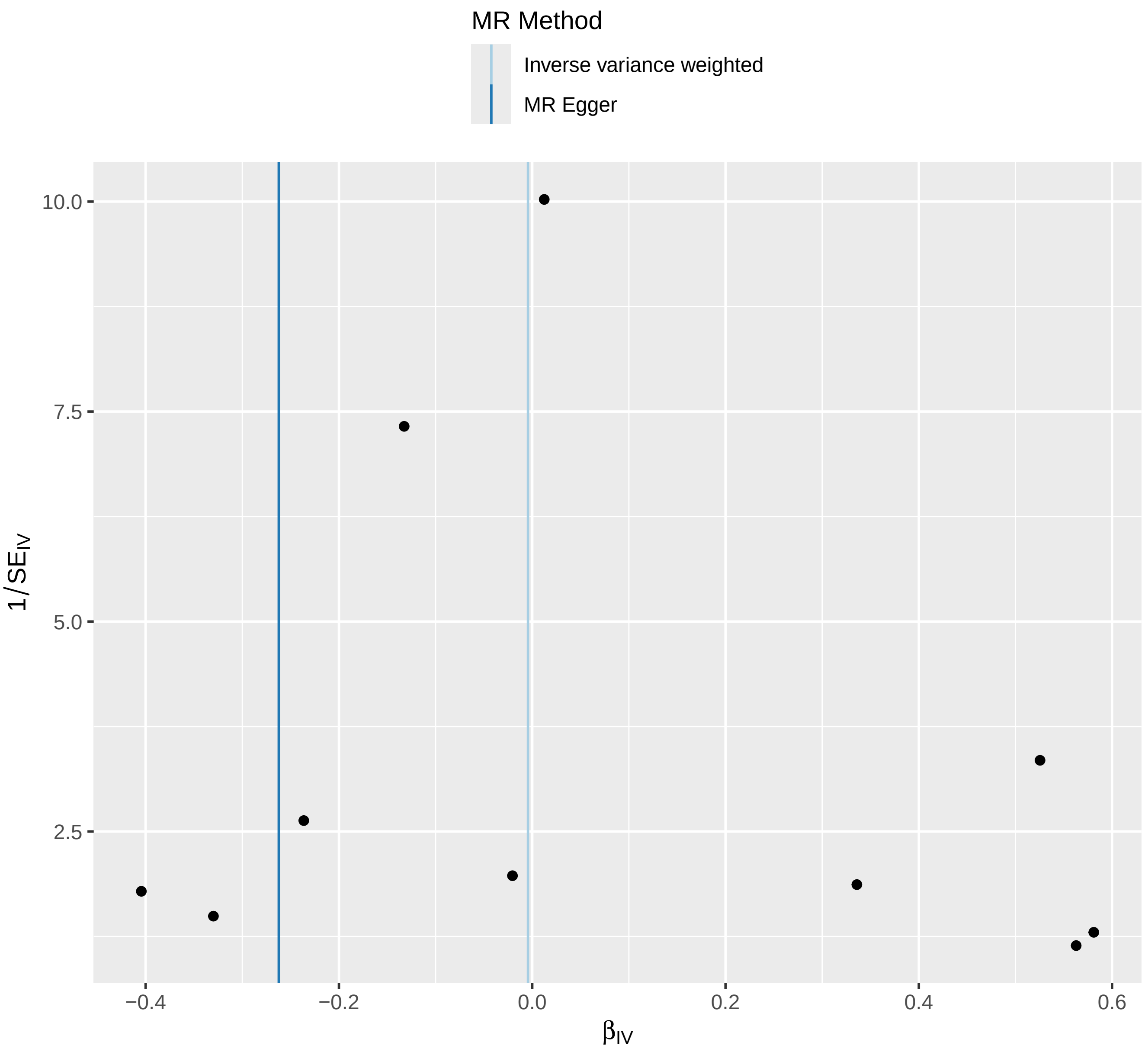

MR Method
Inverse variance weighted
MR Egger
10.0
7.5
5.0
2.5
1/SE_IV
−0.4
−0.2
0.0
0.2
0.4
0.6
β_IV

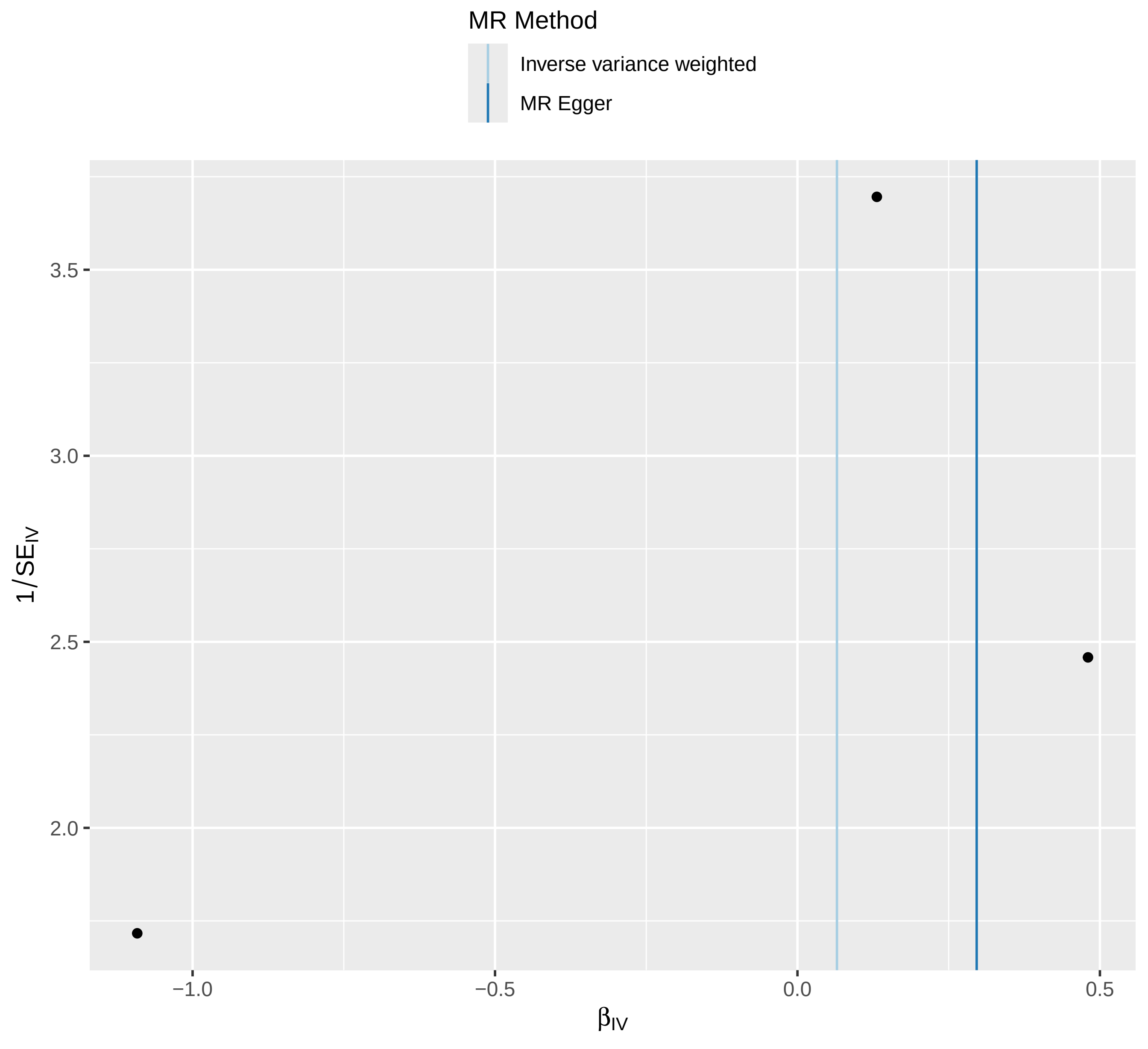
MR Method
Inverse variance weighted
MR Egger
3.5
3.0
2.5
2.0
1/SE_IV
−1.0
−0.5
0.0
0.5
β_IV

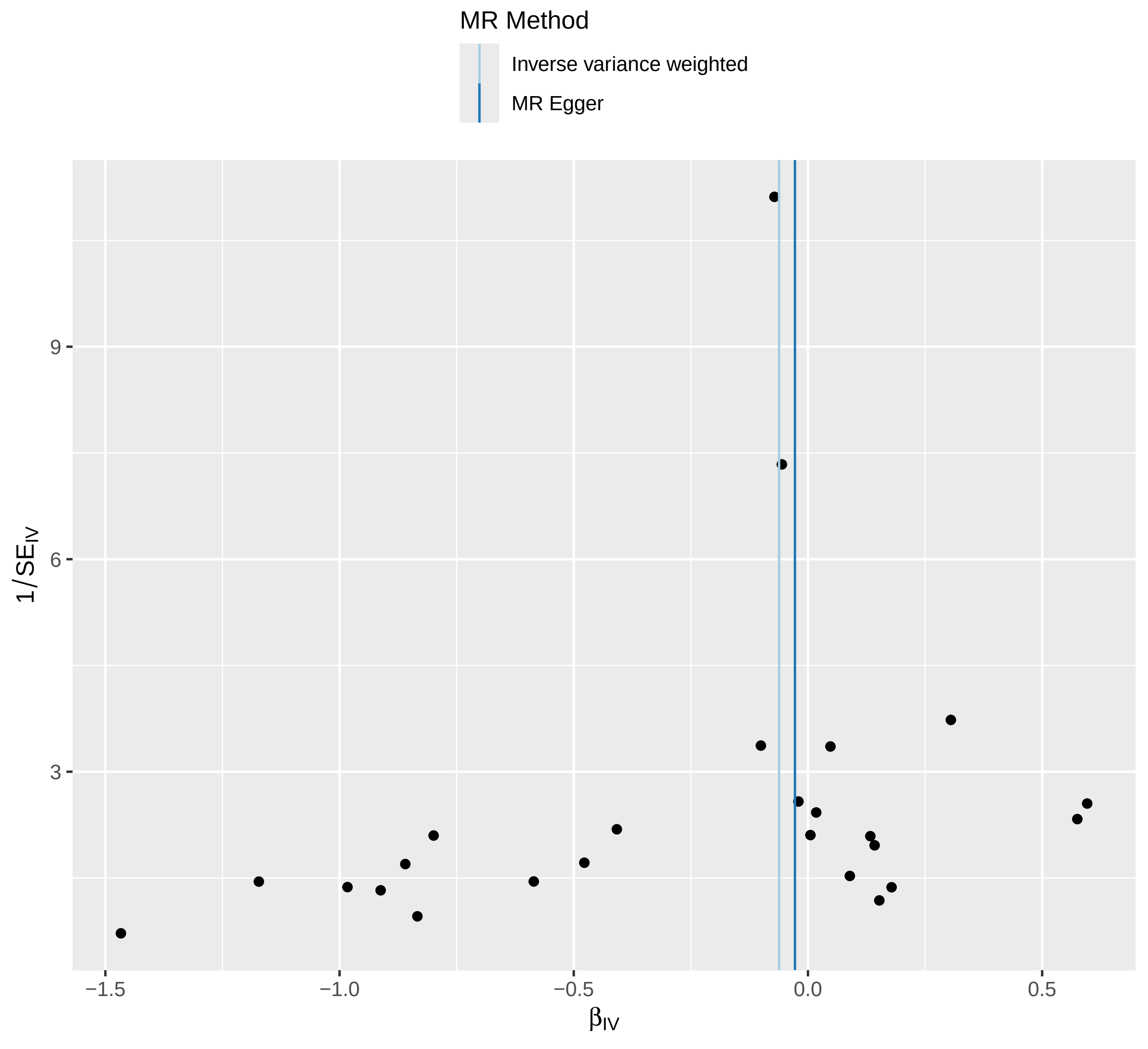
MR Method
Inverse variance weighted
MR Egger
1/SE$_{IV}$
9
6
3
−1.5
−1.0
−0.5
0.0
0.5
β$_{IV}$

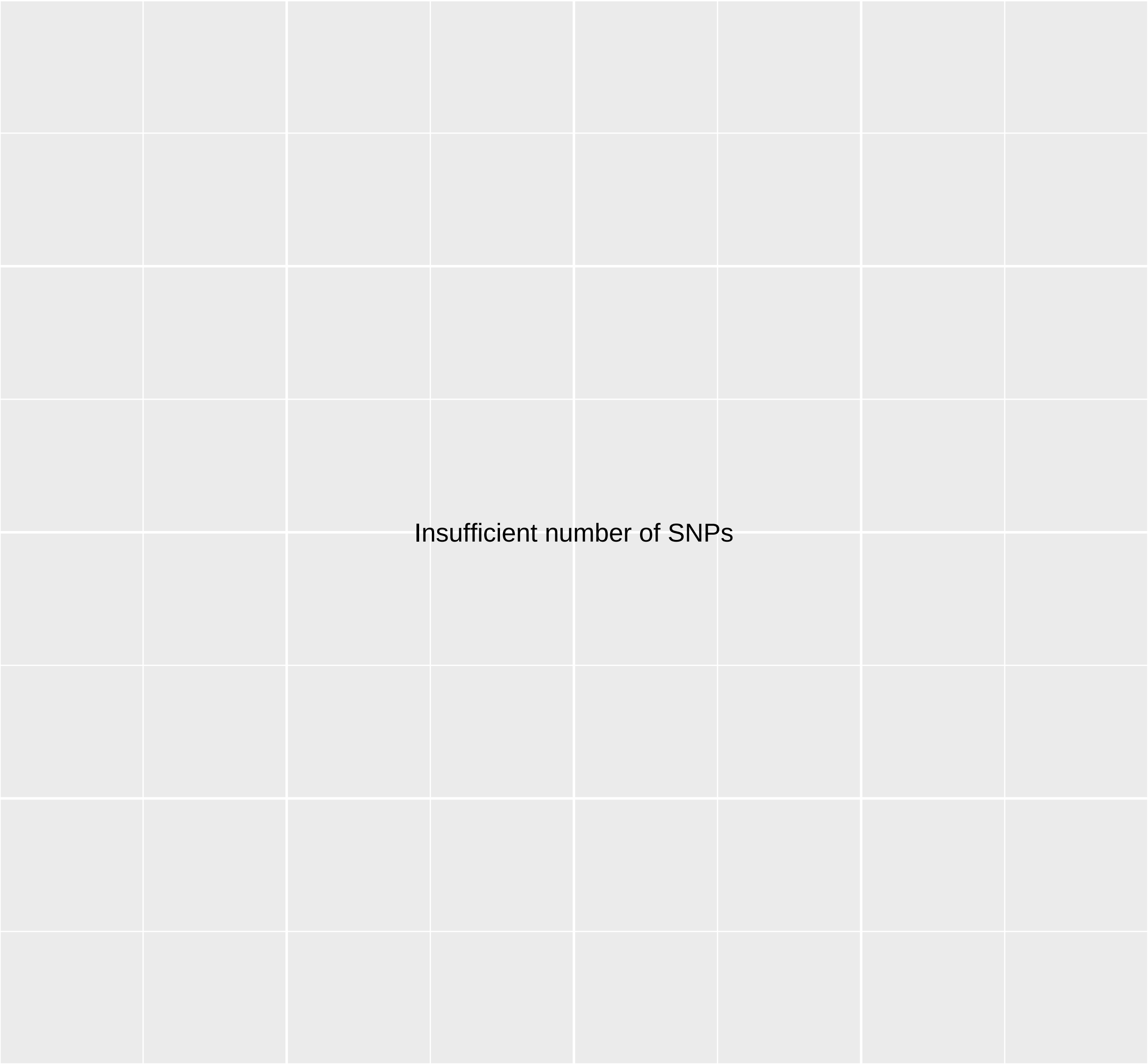
Insufficient number of SNPs

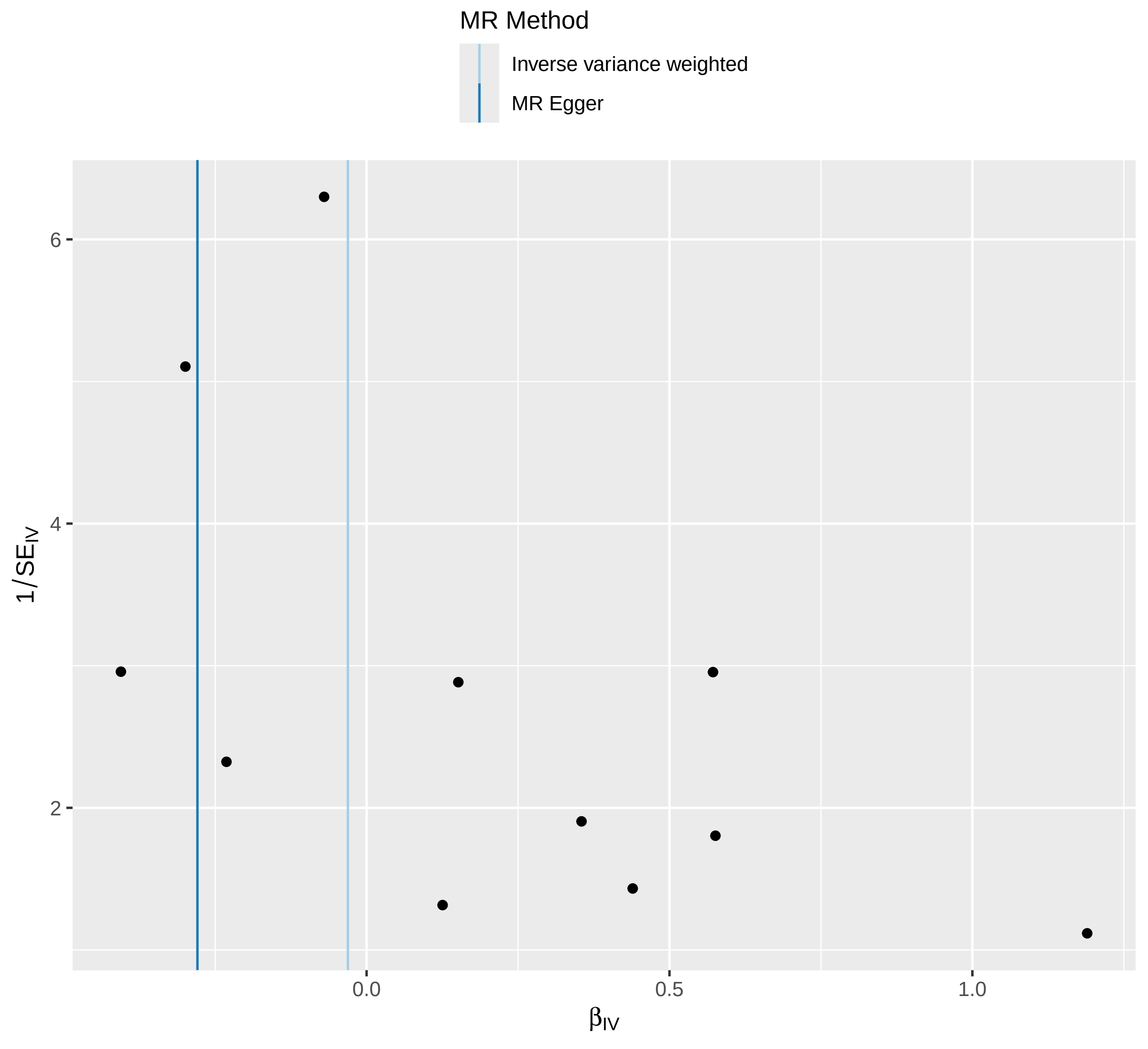
MR Method
Inverse variance weighted
MR Egger
6
4
2
$1/SE_{IV}$
0.0
0.5
1.0
β_{IV}

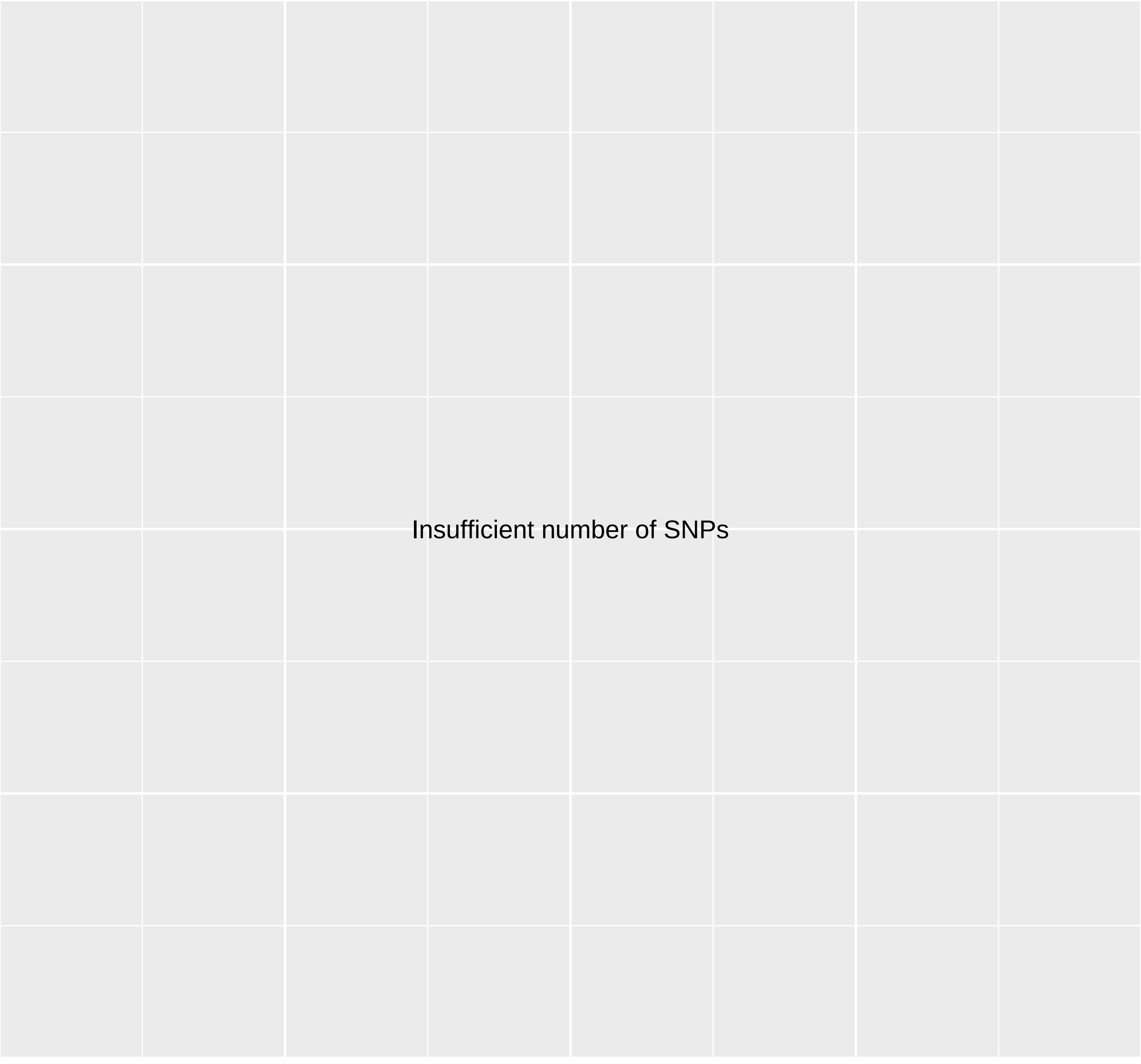
Insufficient number of SNPs

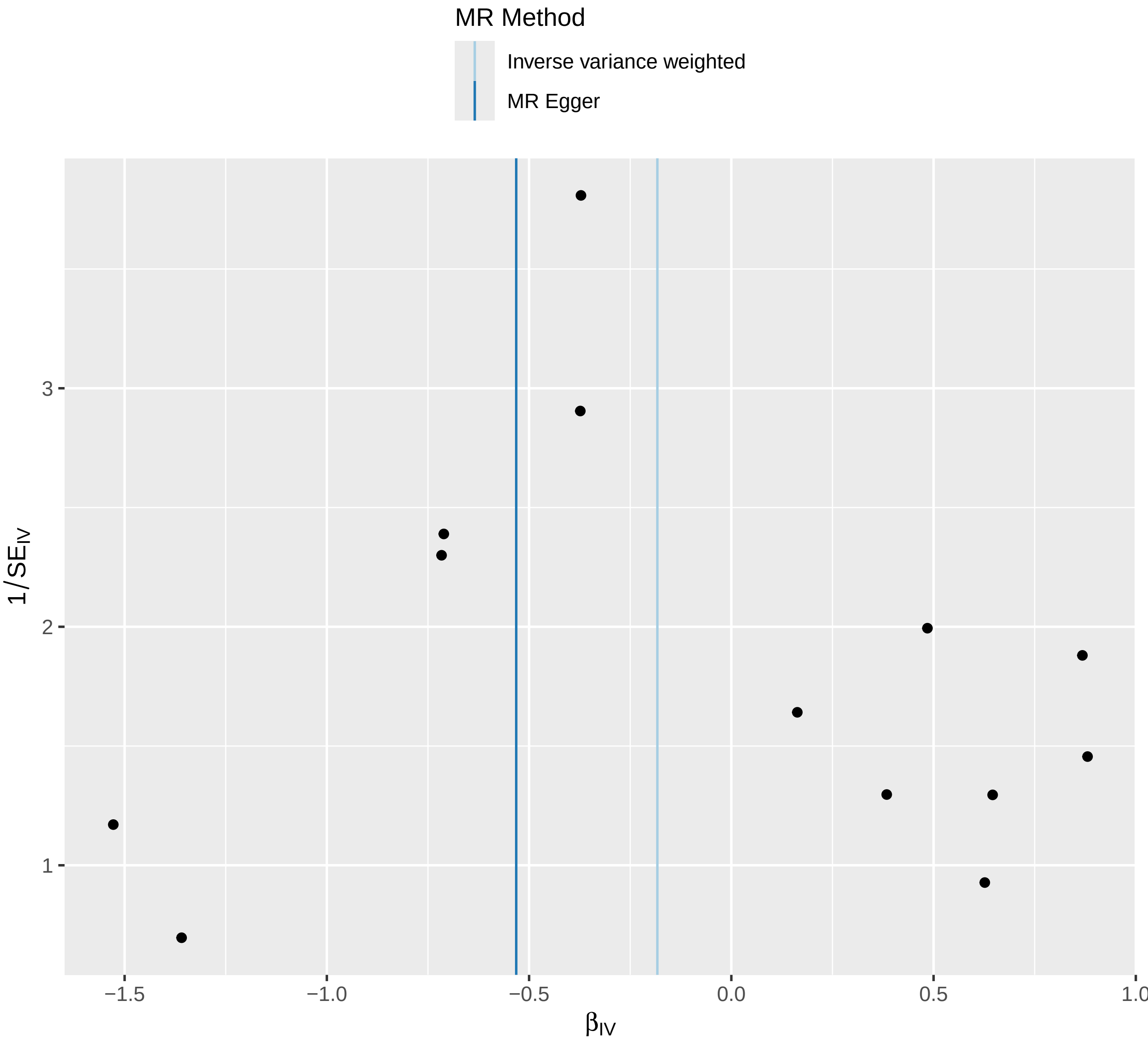
MR Method
Inverse variance weighted
MR Egger
3
2
1
$1/SE_{IV}$
−1.5
−1.0
−0.5
0.0
0.5
1.0
β_{IV}

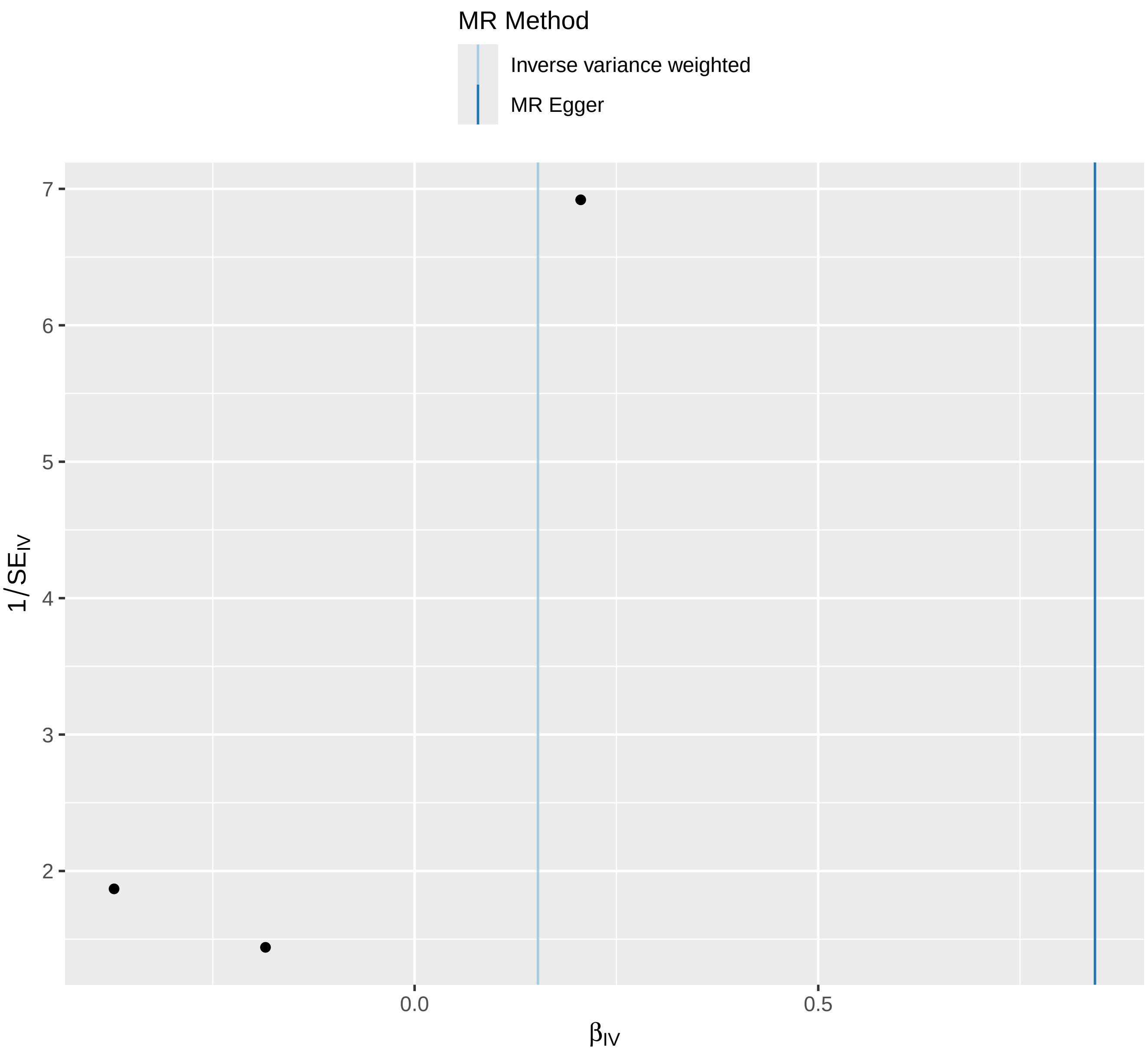

MR Method
Inverse variance weighted
MR Egger
7
6
5
4
3
2
1/SE_IV
0.0
0.5
β_IV

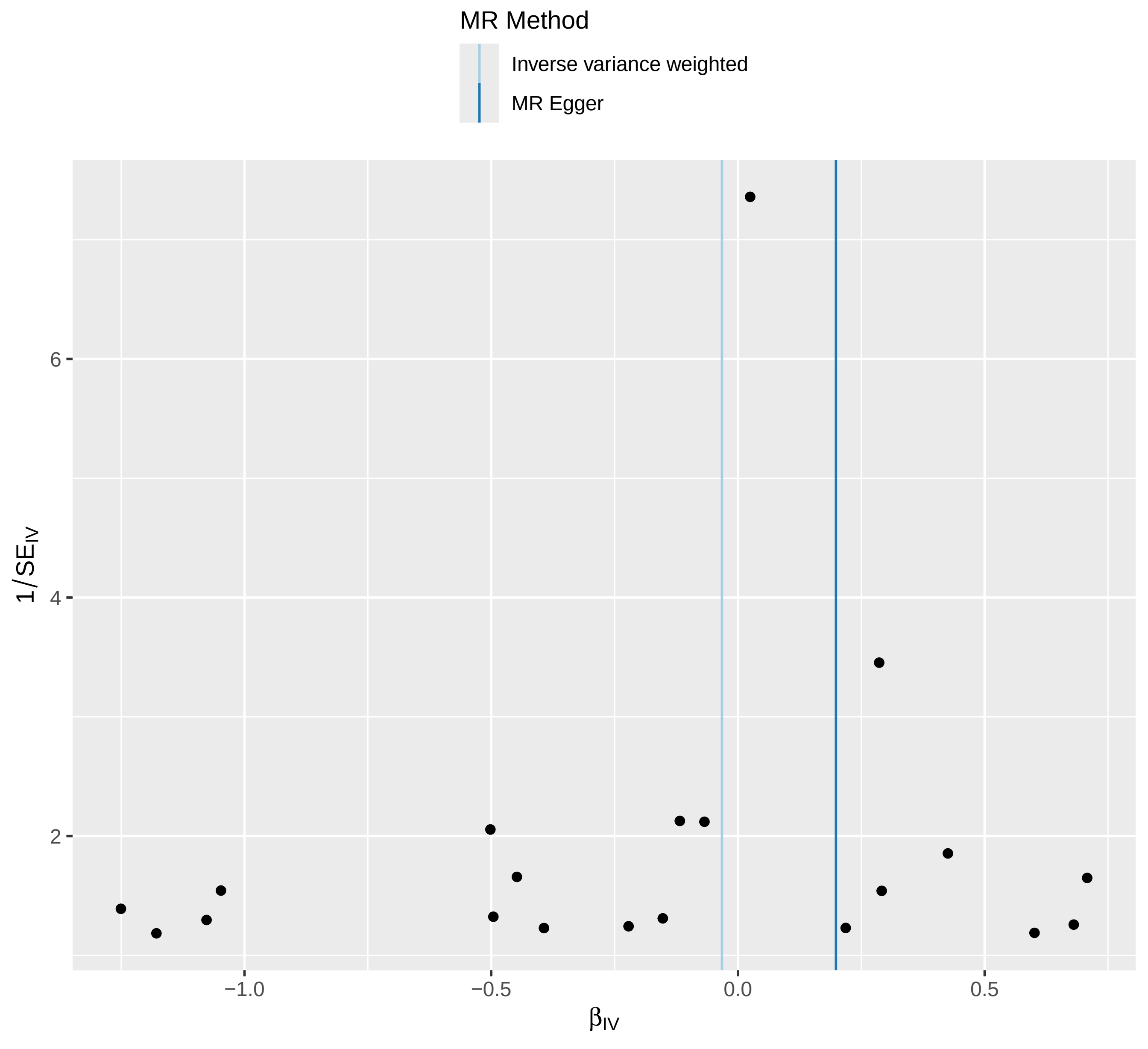
MR Method
Inverse variance weighted
MR Egger
6
4
2
1/SE$_{IV}$
−1.0
−0.5
0.0
0.5
β$_{IV}$

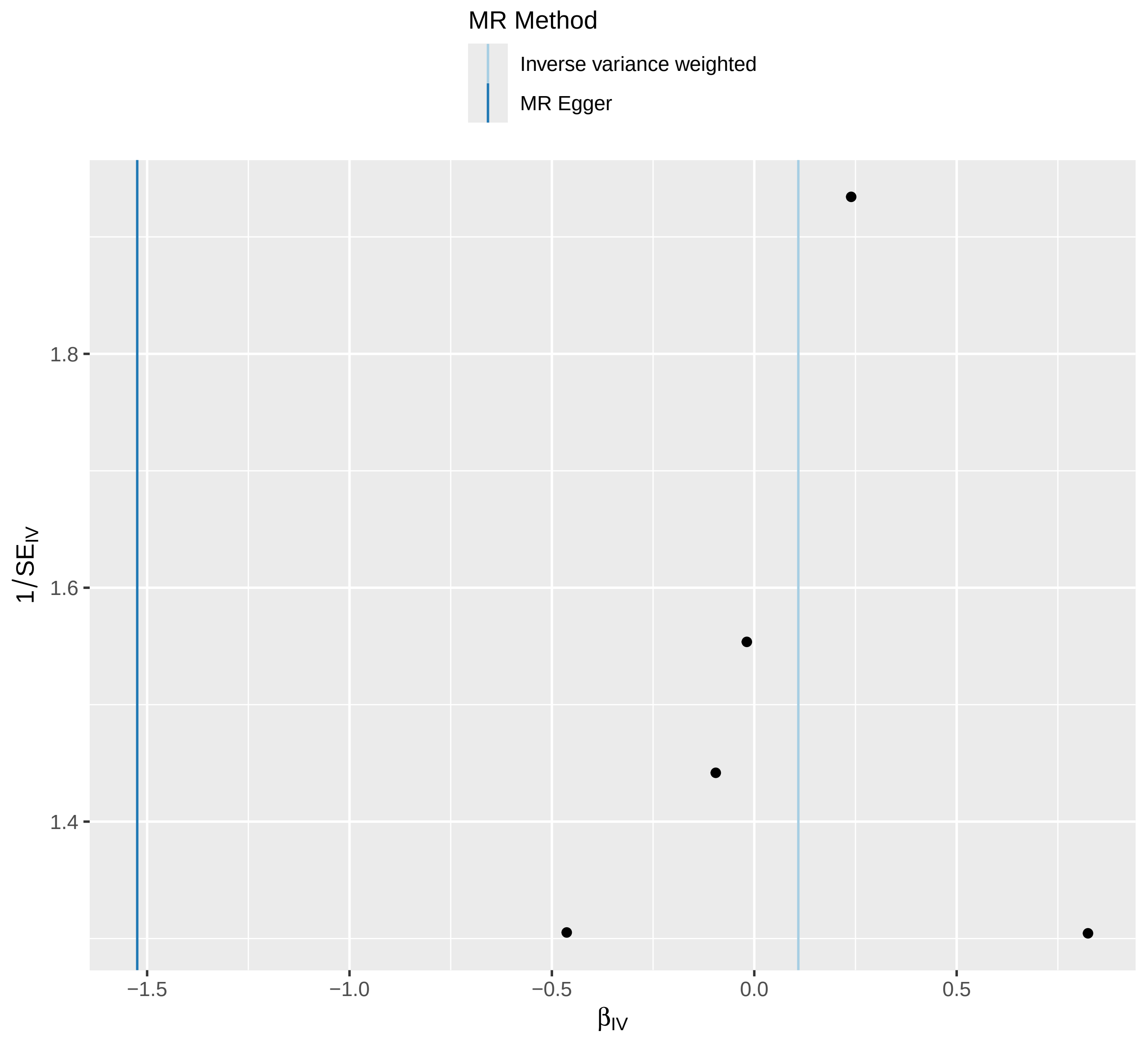

MR Method
Inverse variance weighted
MR Egger
1.8
1.6
1.4
1/SE$_{IV}$
−1.5
−1.0
−0.5
0.0
0.5
β$_{IV}$

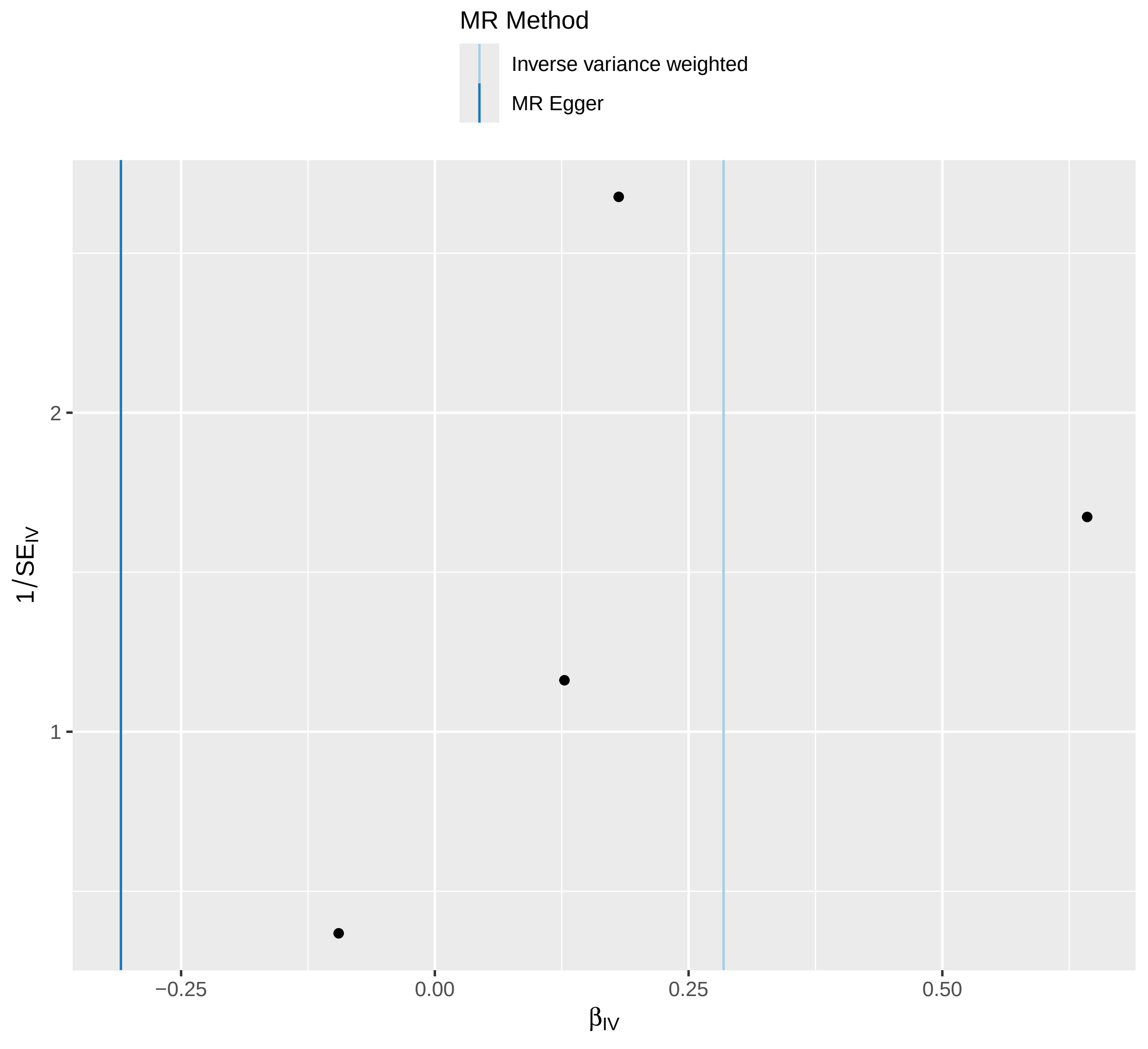

MR Method
Inverse variance weighted
MR Egger
2
1
1/SE_IV
−0.25
0.00
0.25
0.50
β_IV

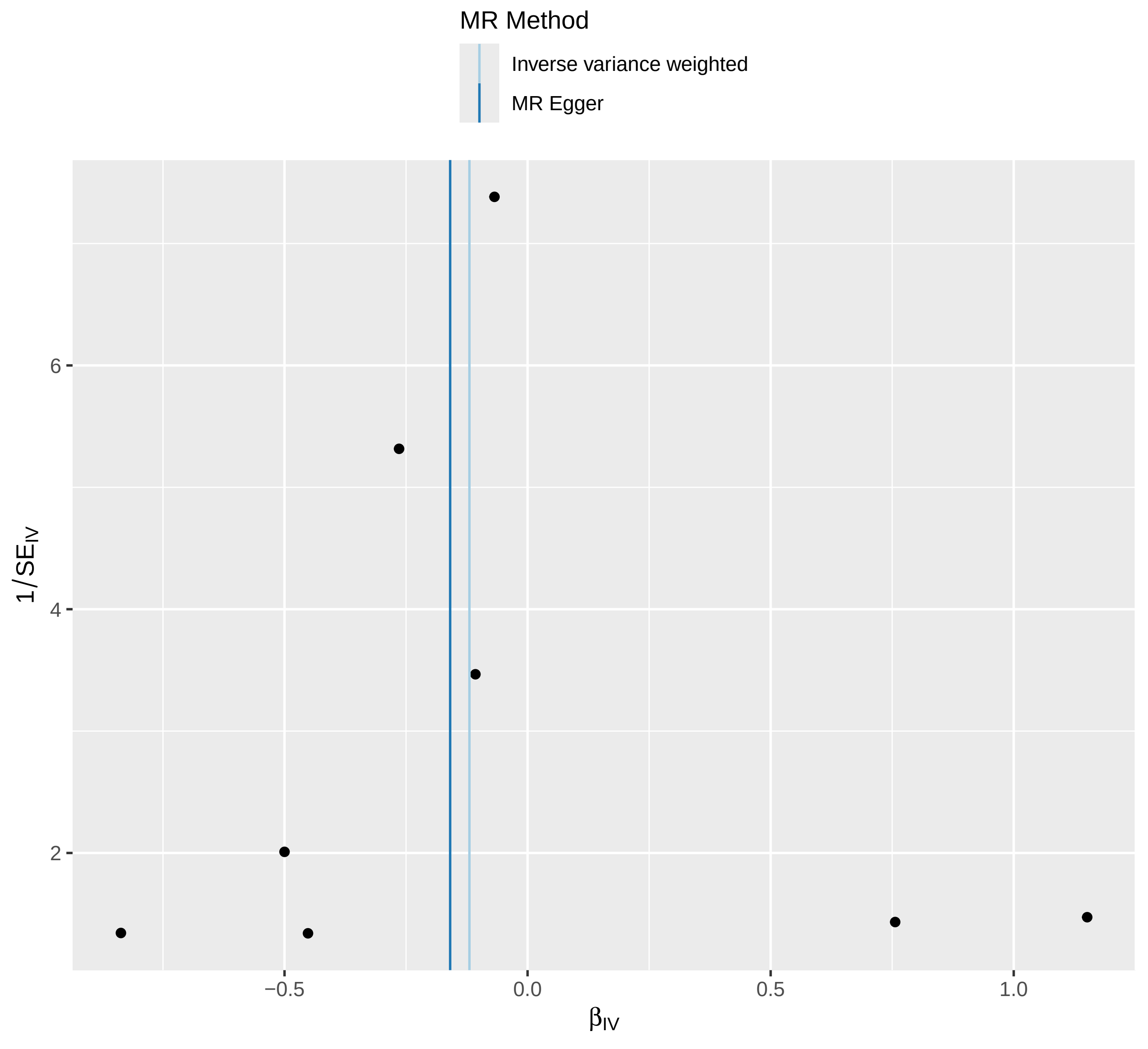

MR Method
Inverse variance weighted
MR Egger
6
4
2
1/SE_IV
−0.5
0.0
0.5
1.0
β_IV

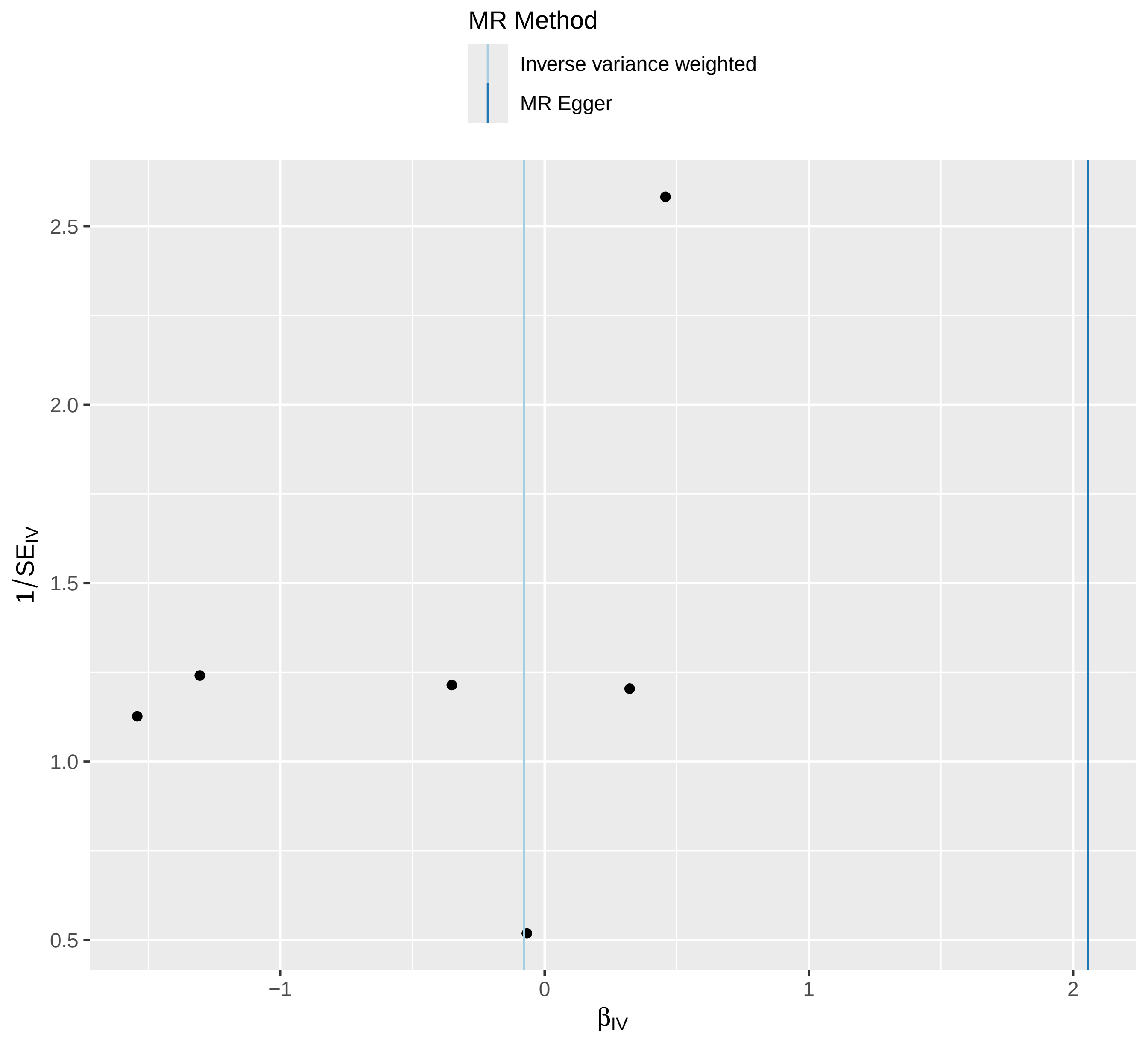
MR Method
Inverse variance weighted
MR Egger
2.5
2.0
1.5
1.0
0.5
1/SE_IV
−1
0
1
2
β_IV

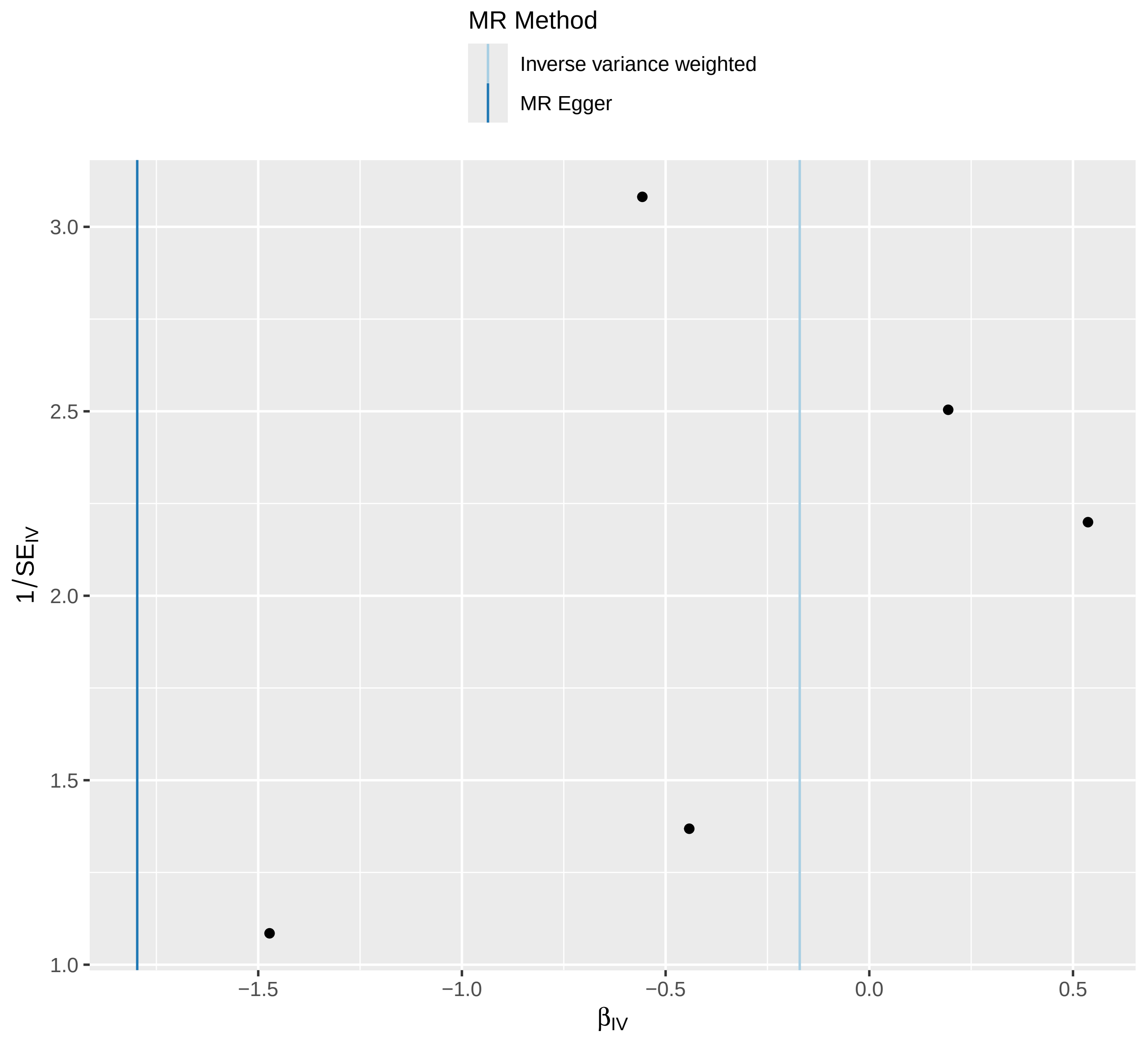
MR Method
Inverse variance weighted
MR Egger
3.0
2.5
2.0
1.5
1.0
1/SE$_{IV}$
−1.5
−1.0
−0.5
0.0
0.5
β$_{IV}$

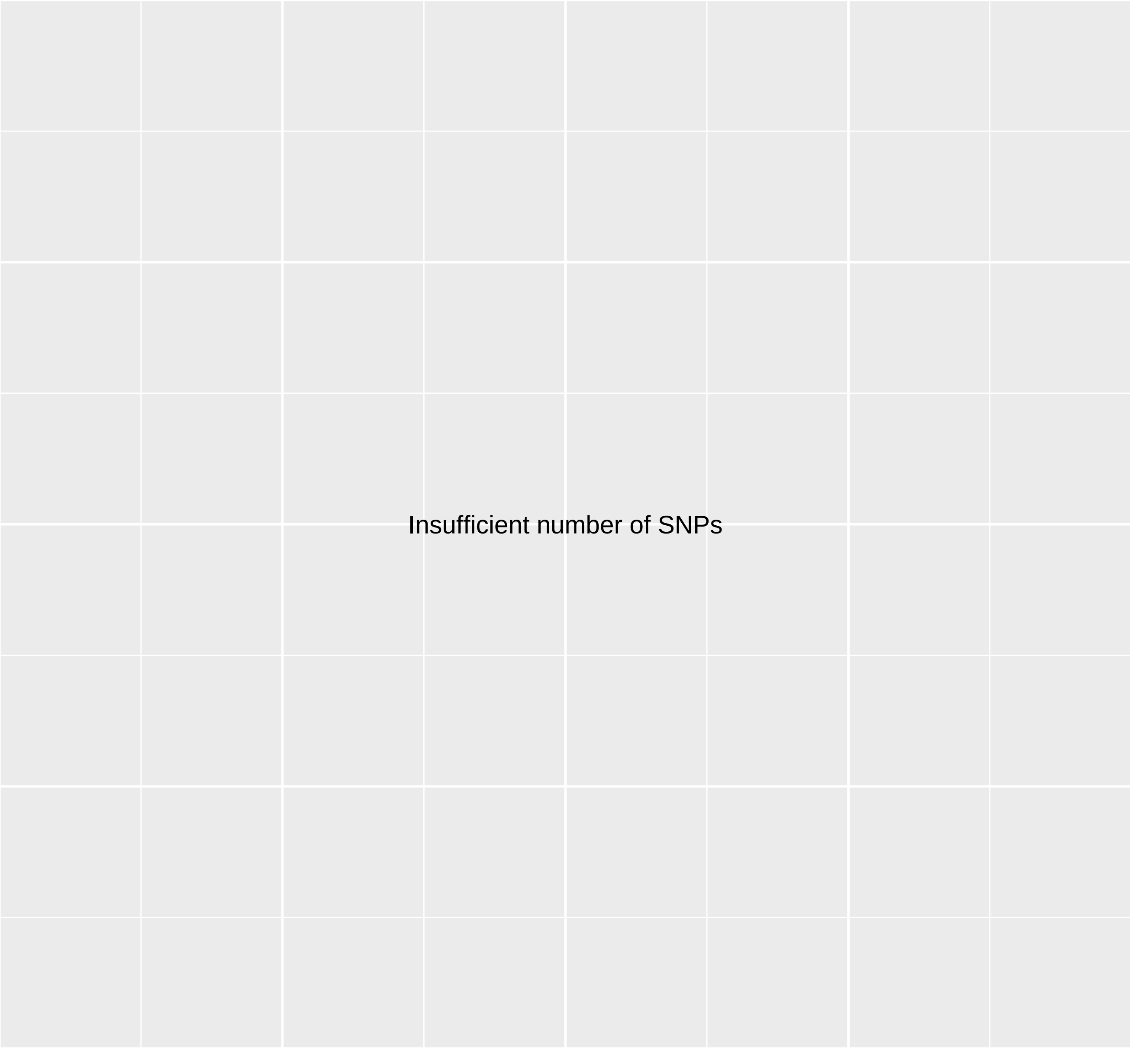
Insufficient number of SNPs

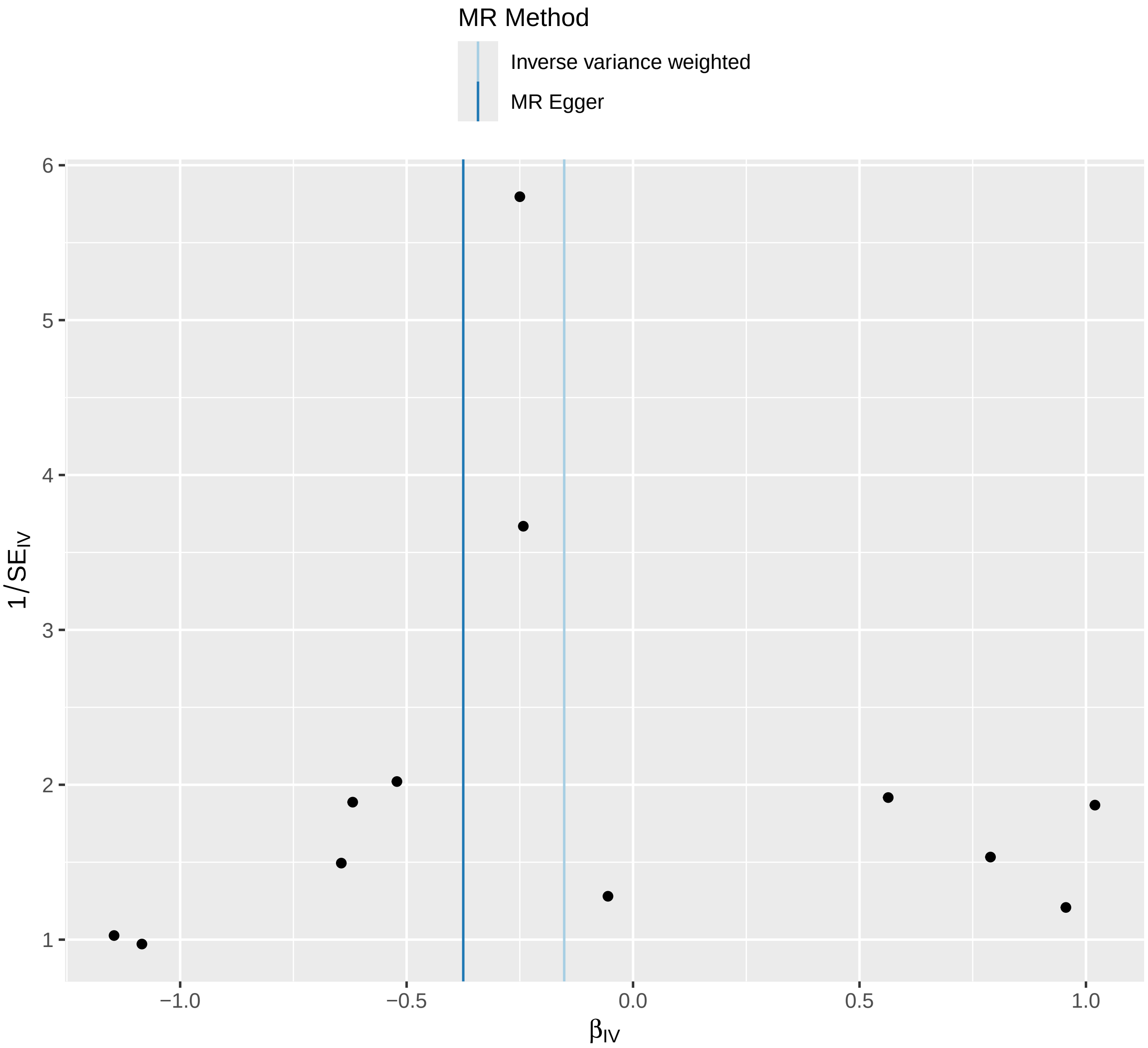
MR Method
Inverse variance weighted
MR Egger
6
5
4
3
2
1
1/SE_IV
−1.0
−0.5
0.0
0.5
1.0
β_IV

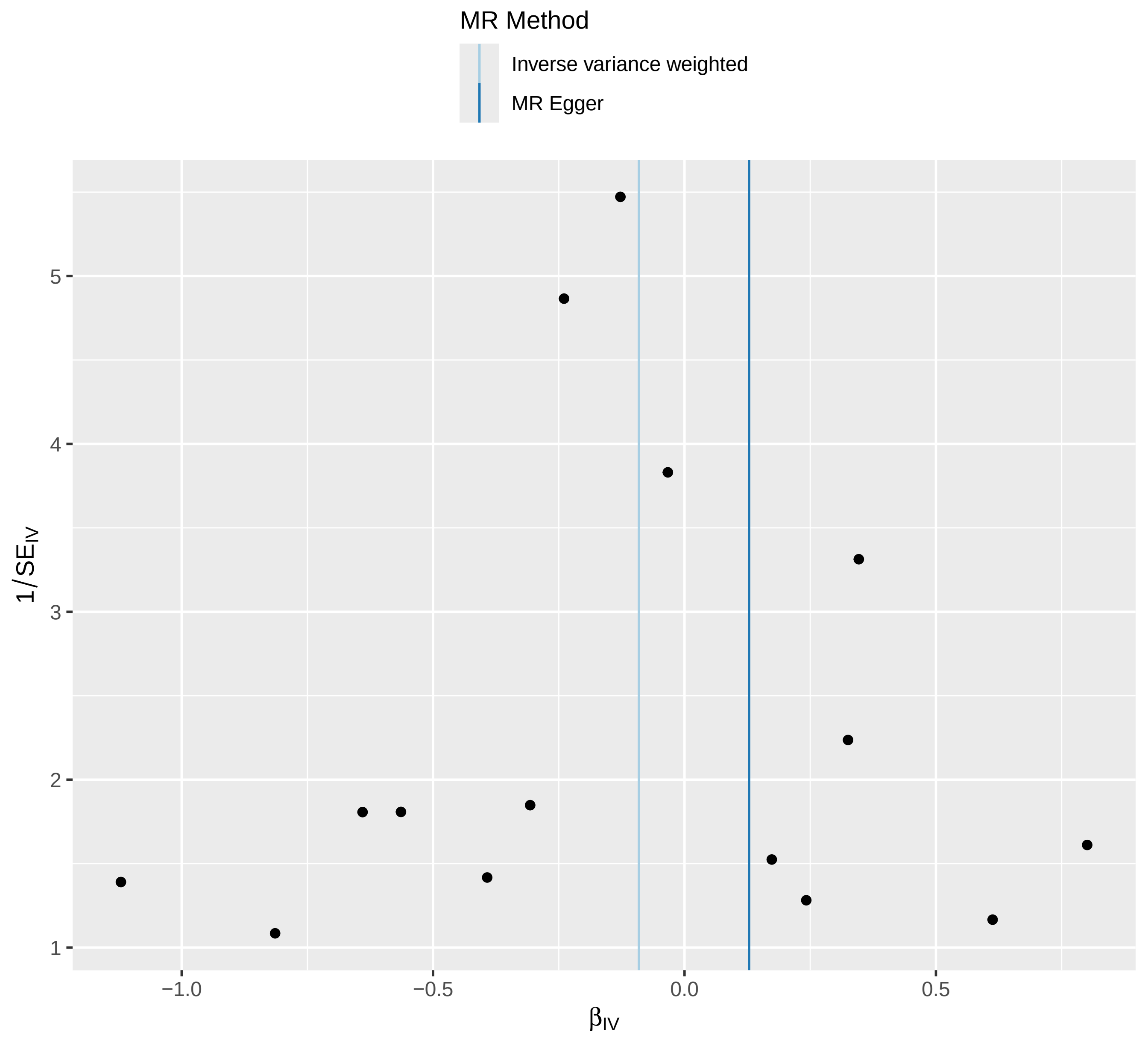

MR Method
Inverse variance weighted
MR Egger
5
4
3
2
1
$1/SE_{IV}$
−1.0
−0.5
0.0
0.5
β_{IV}

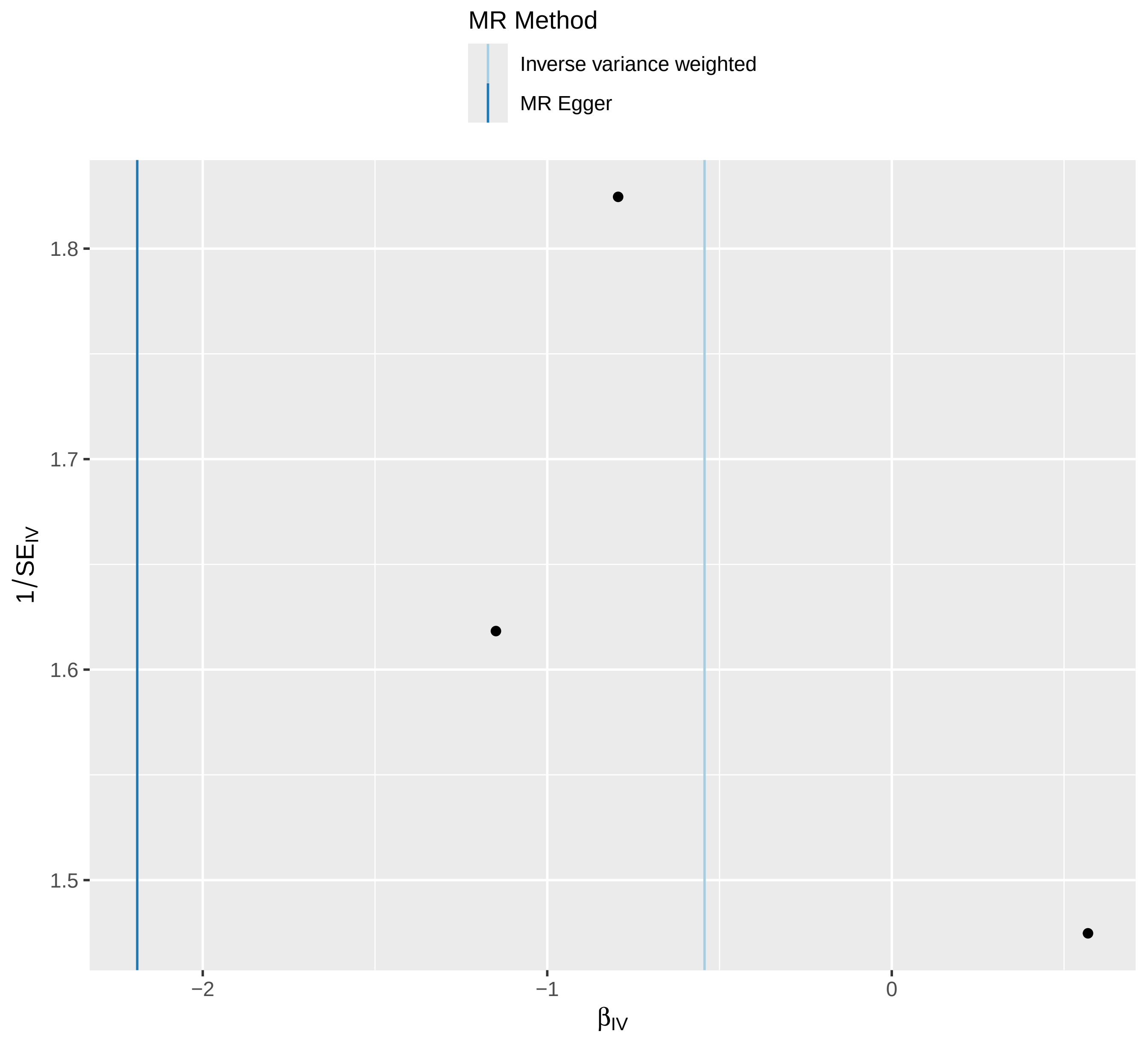

MR Method
Inverse variance weighted
MR Egger
1.8
1.7
1.6
1.5
−2
−1
0
1/SE_IV
β_IV

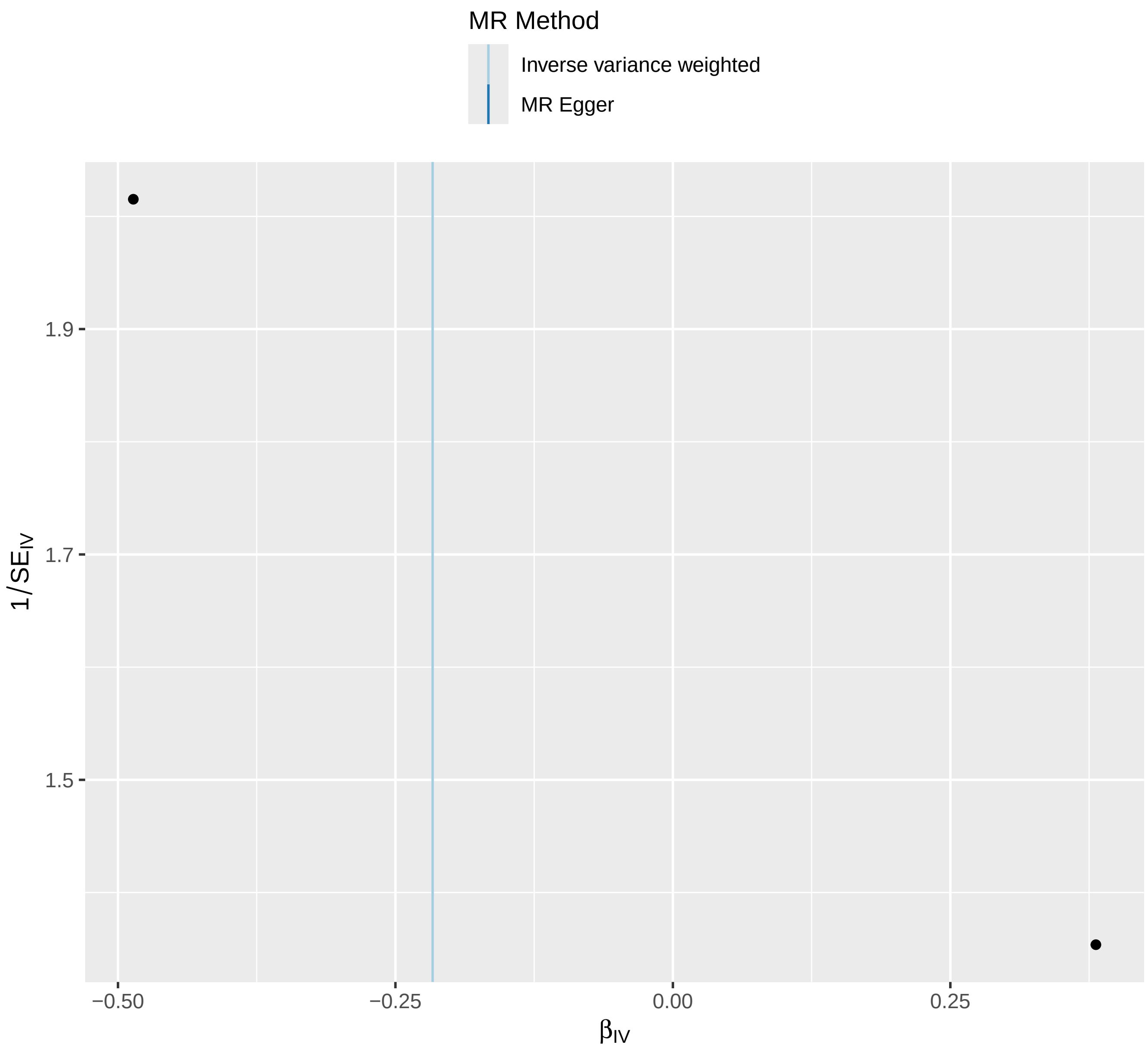

MR Method
Inverse variance weighted
MR Egger
1.9
1.7
1.5
1/SE$_{IV}$
−0.50
−0.25
0.00
0.25
β$_{IV}$

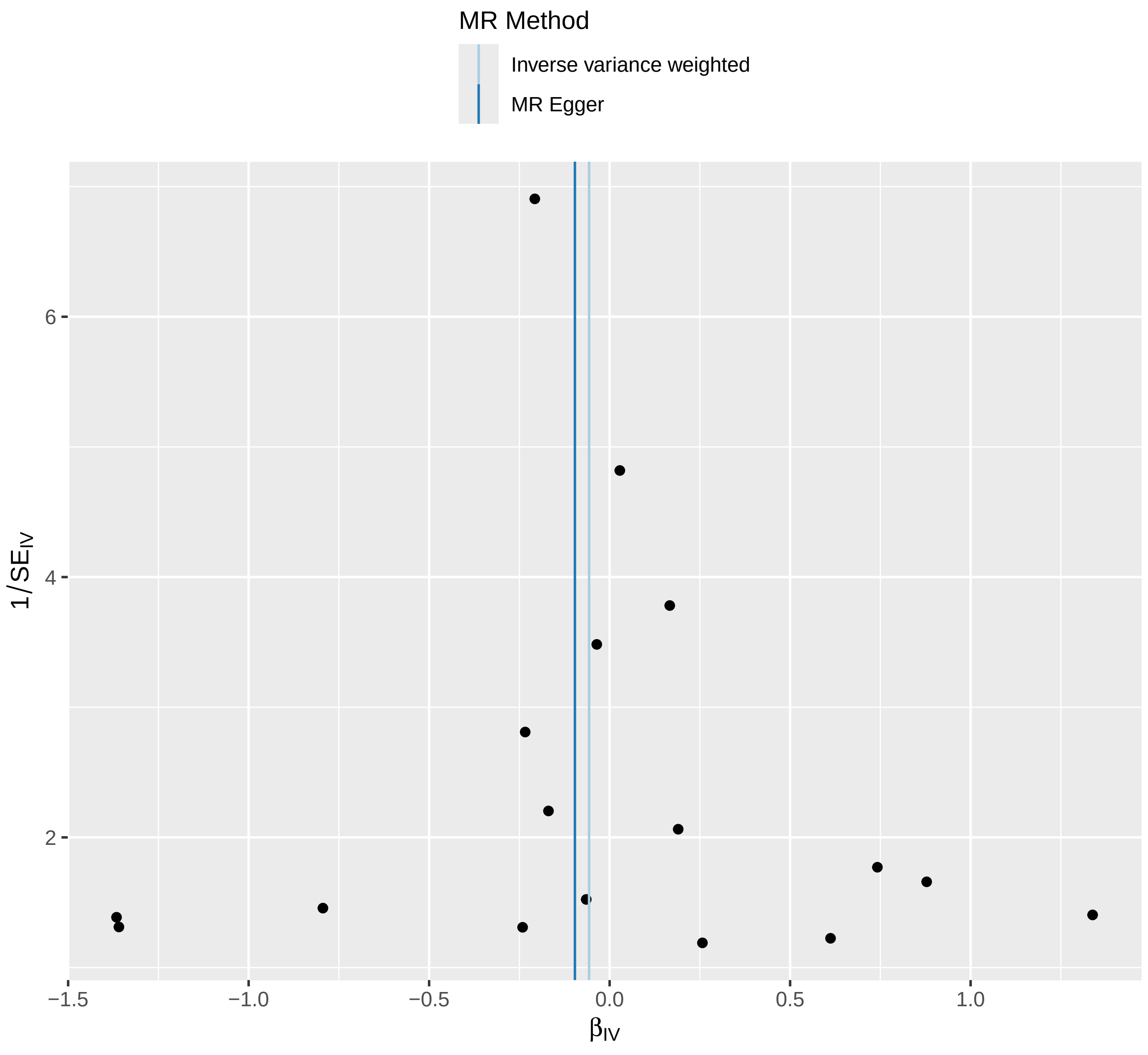

MR Method
Inverse variance weighted
MR Egger
6
4
2
1/SE$_{IV}$
−1.5
−1.0
−0.5
0.0
0.5
1.0
β$_{IV}$

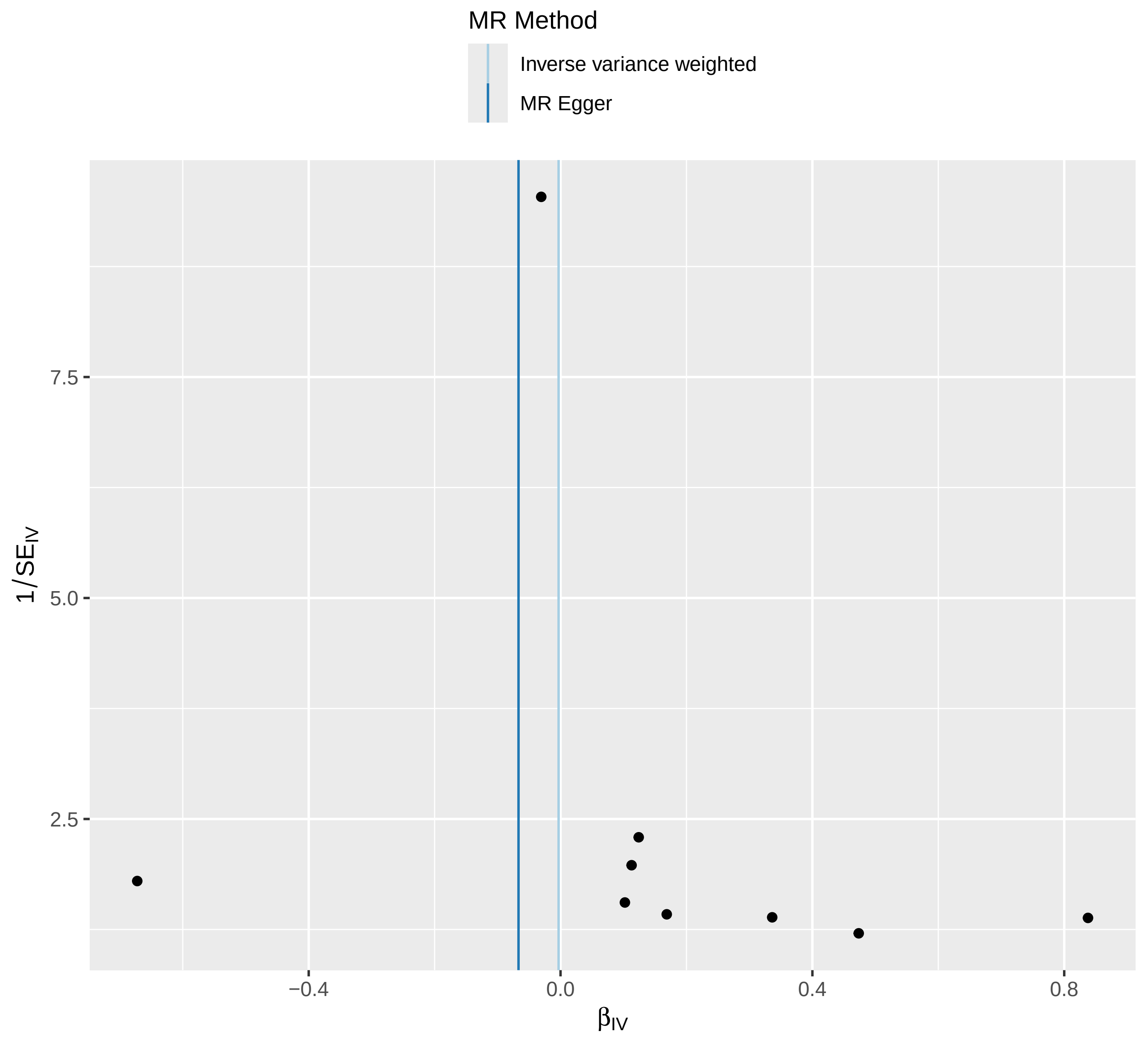
MR Method
Inverse variance weighted
MR Egger
7.5
5.0
2.5
$1/SE_{IV}$
−0.4
0.0
0.4
0.8
β_{IV}

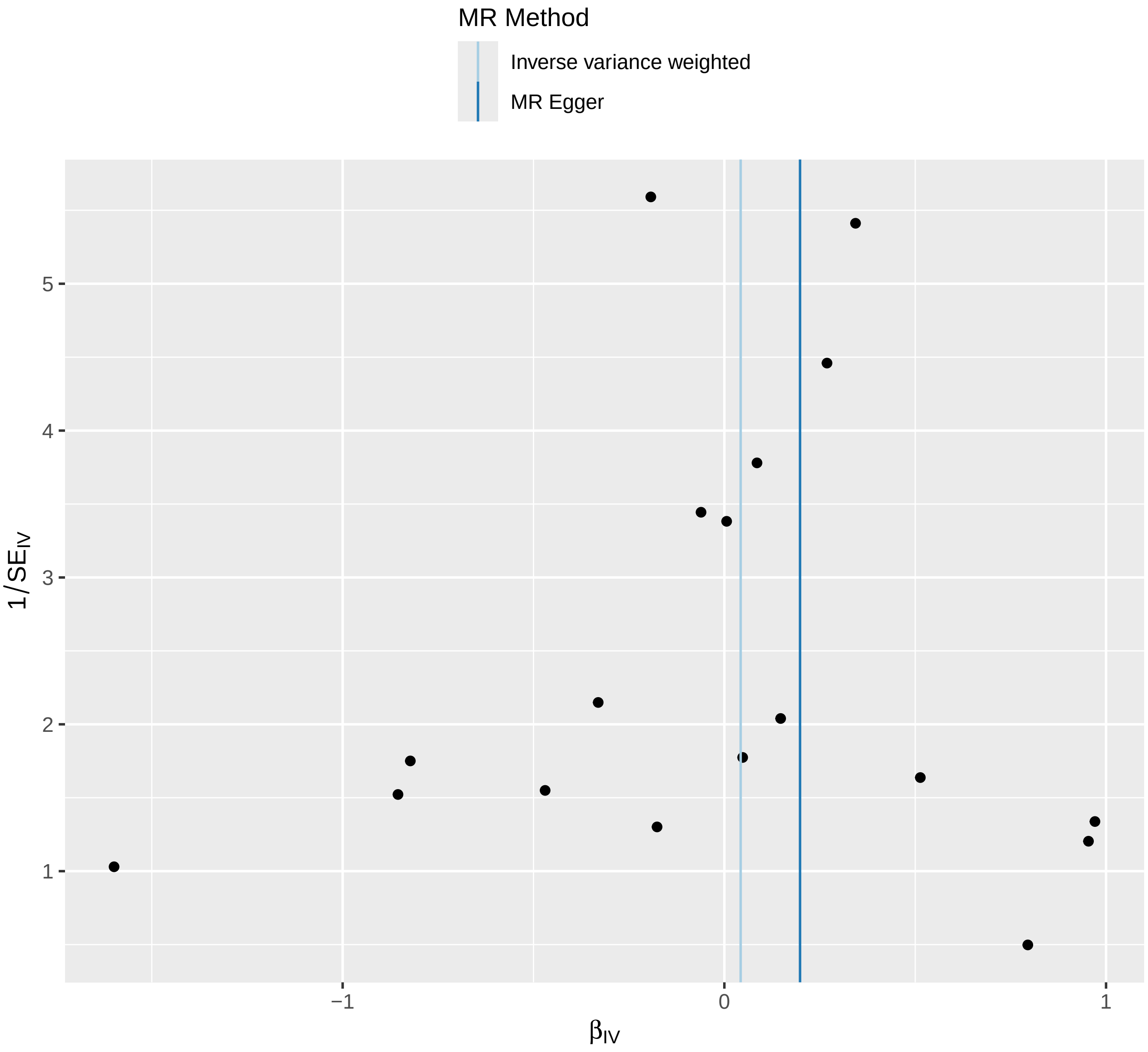

MR Method
Inverse variance weighted
MR Egger
5
4
3
2
1
1/SE_IV
−1
0
1
β_IV

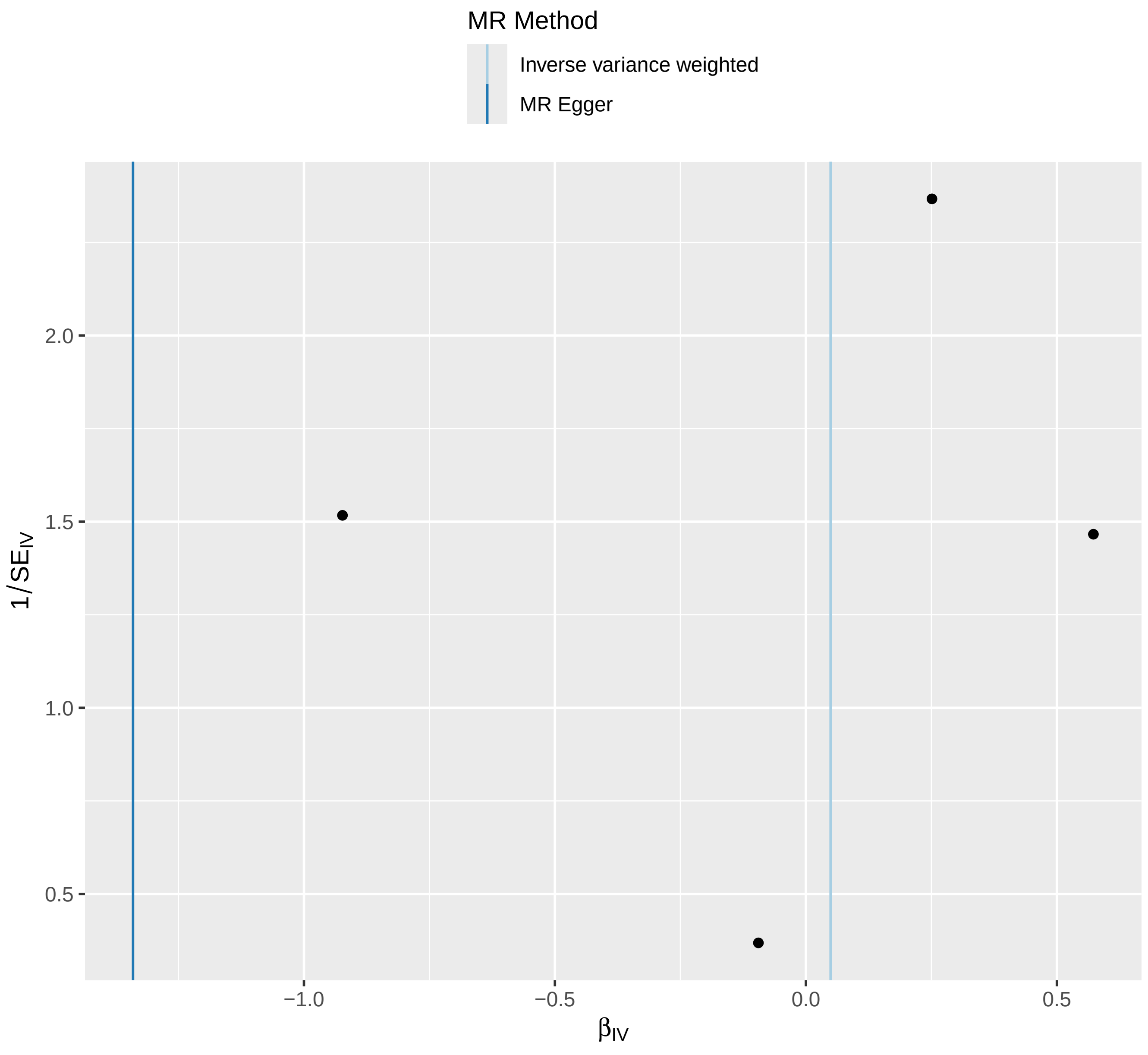
MR Method
Inverse variance weighted
MR Egger
2.0
1.5
1.0
0.5
1/SE$_{IV}$
−1.0
−0.5
0.0
0.5
β_{IV}

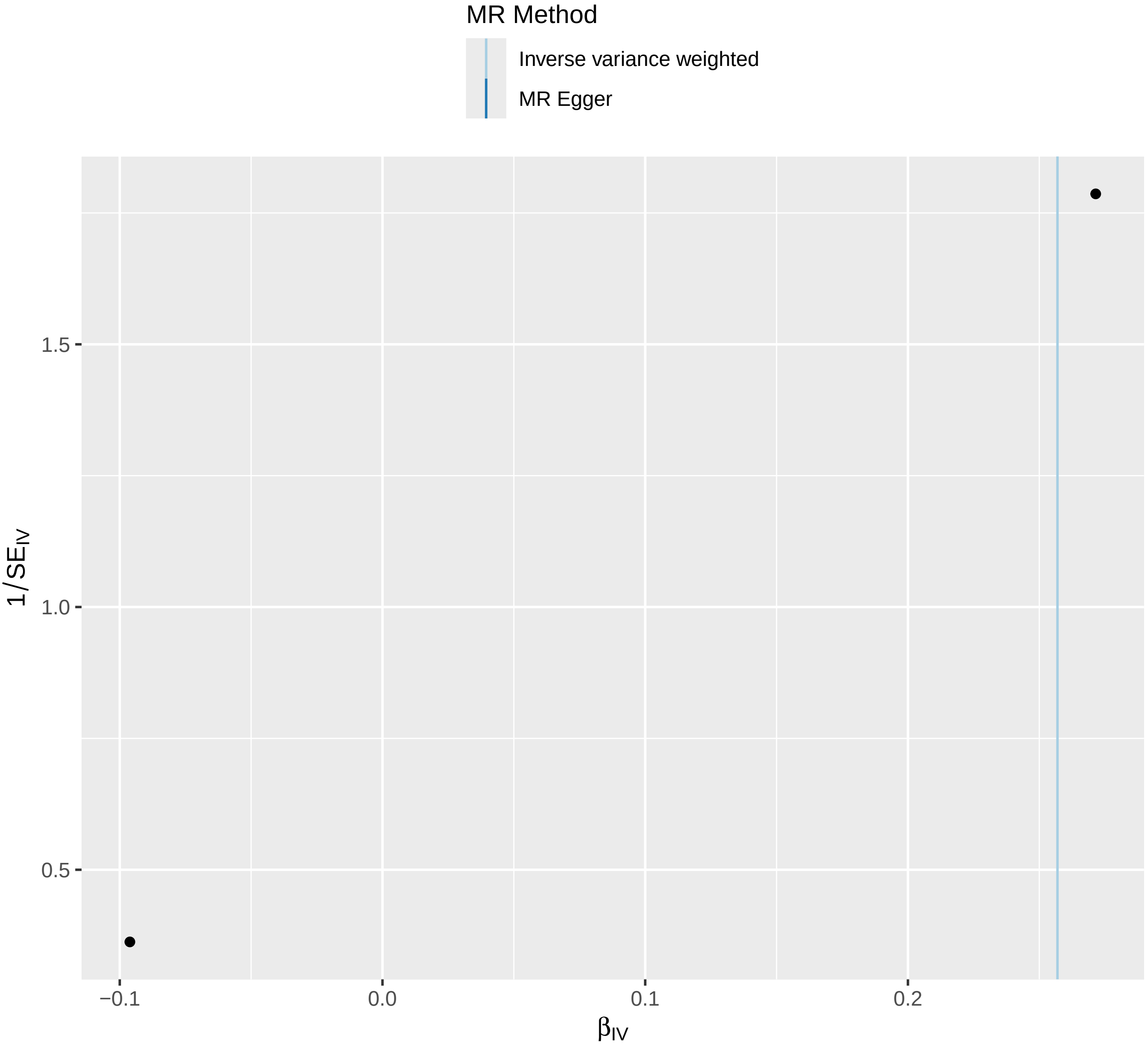
MR Method
Inverse variance weighted
MR Egger
1.5
1.0
0.5
1/SE$_{IV}$
−0.1
0.0
0.1
0.2
β$_{IV}$

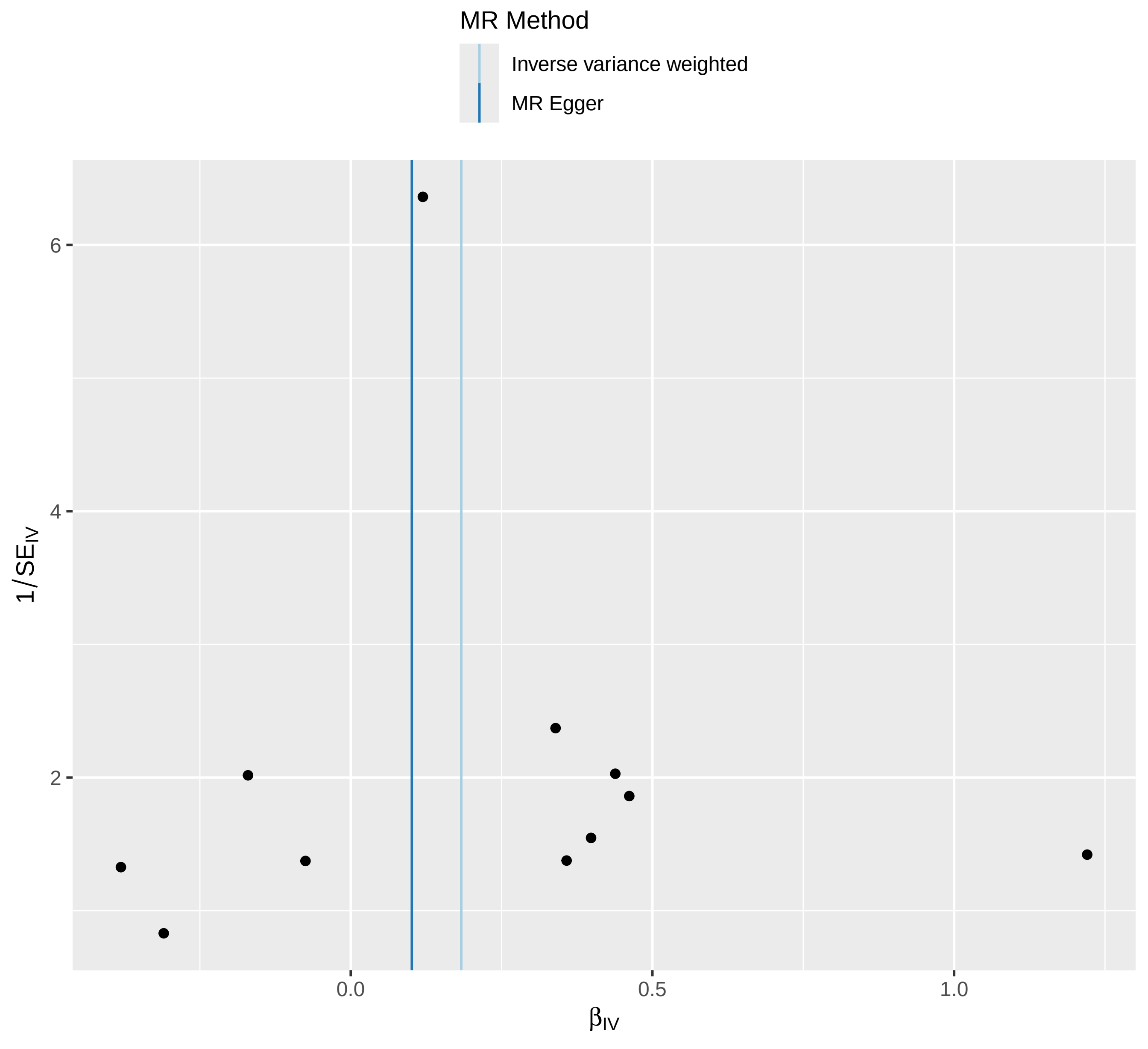

MR Method
Inverse variance weighted
MR Egger
6
4
2
1/SE_IV
0.0
0.5
1.0
β_IV

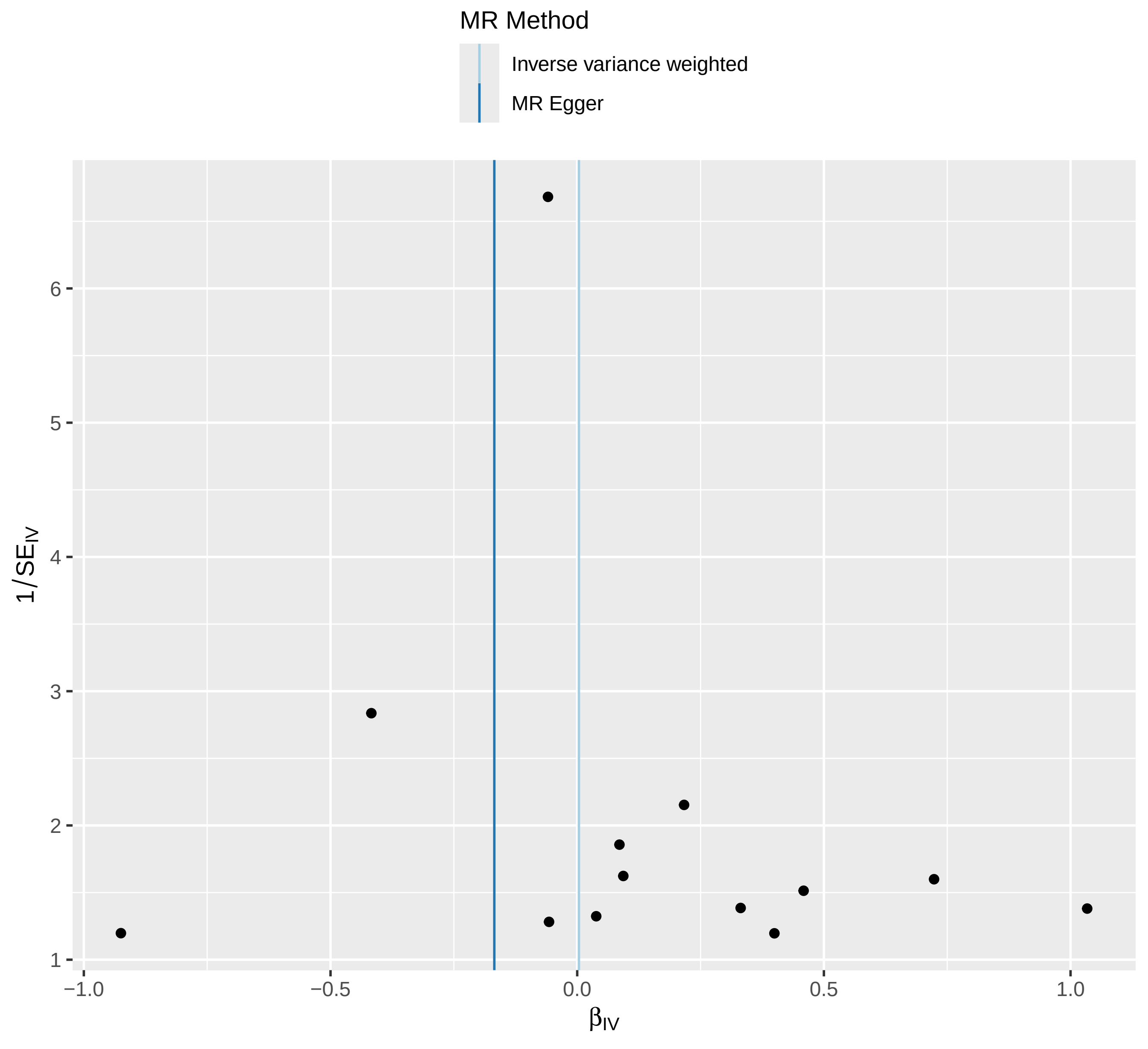
MR Method
Inverse variance weighted
MR Egger
1/SE$_{IV}$
6
5
4
3
2
1
−1.0
−0.5
0.0
0.5
1.0
β$_{IV}$

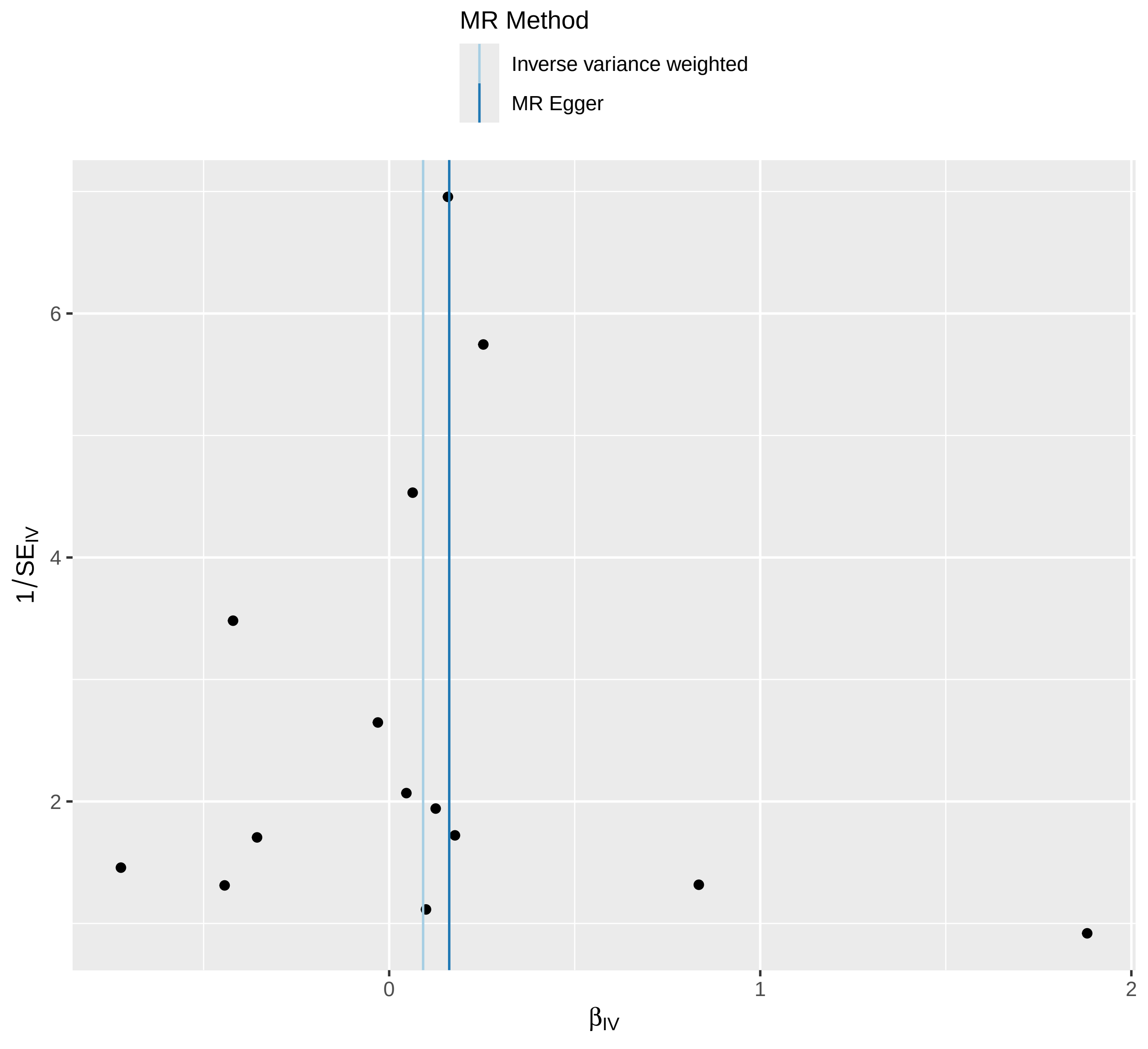

MR Method
Inverse variance weighted
MR Egger
6
4
2
1/SE_IV
0
1
2
β_IV

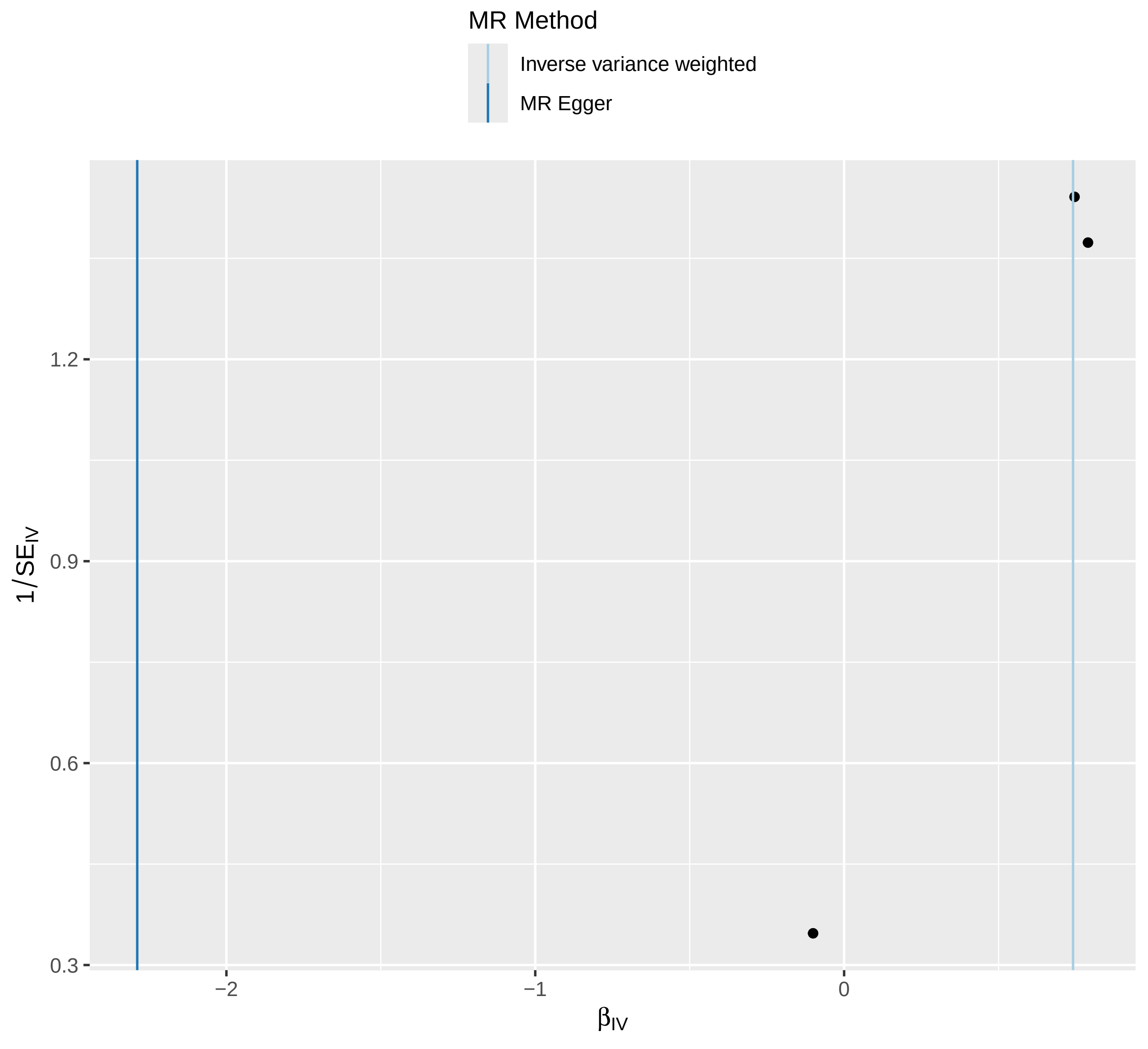
MR Method
Inverse variance weighted
MR Egger
1.2
0.9
0.6
0.3
1/SE_IV
−2
−1
0
β_IV

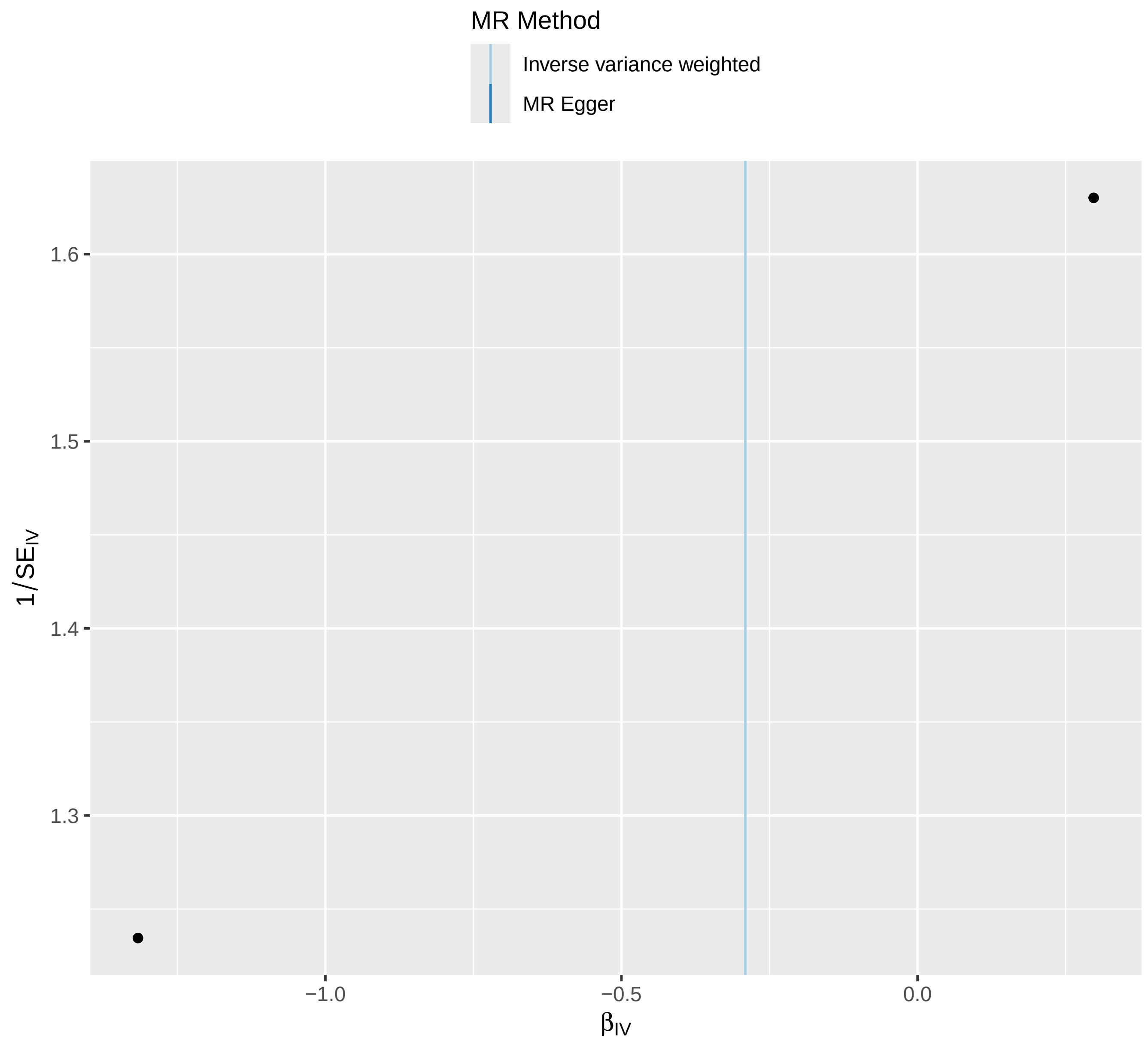

MR Method
Inverse variance weighted
MR Egger
1.6
1.5
1.4
1.3
1/SE_IV
−1.0
−0.5
0.0
β_IV

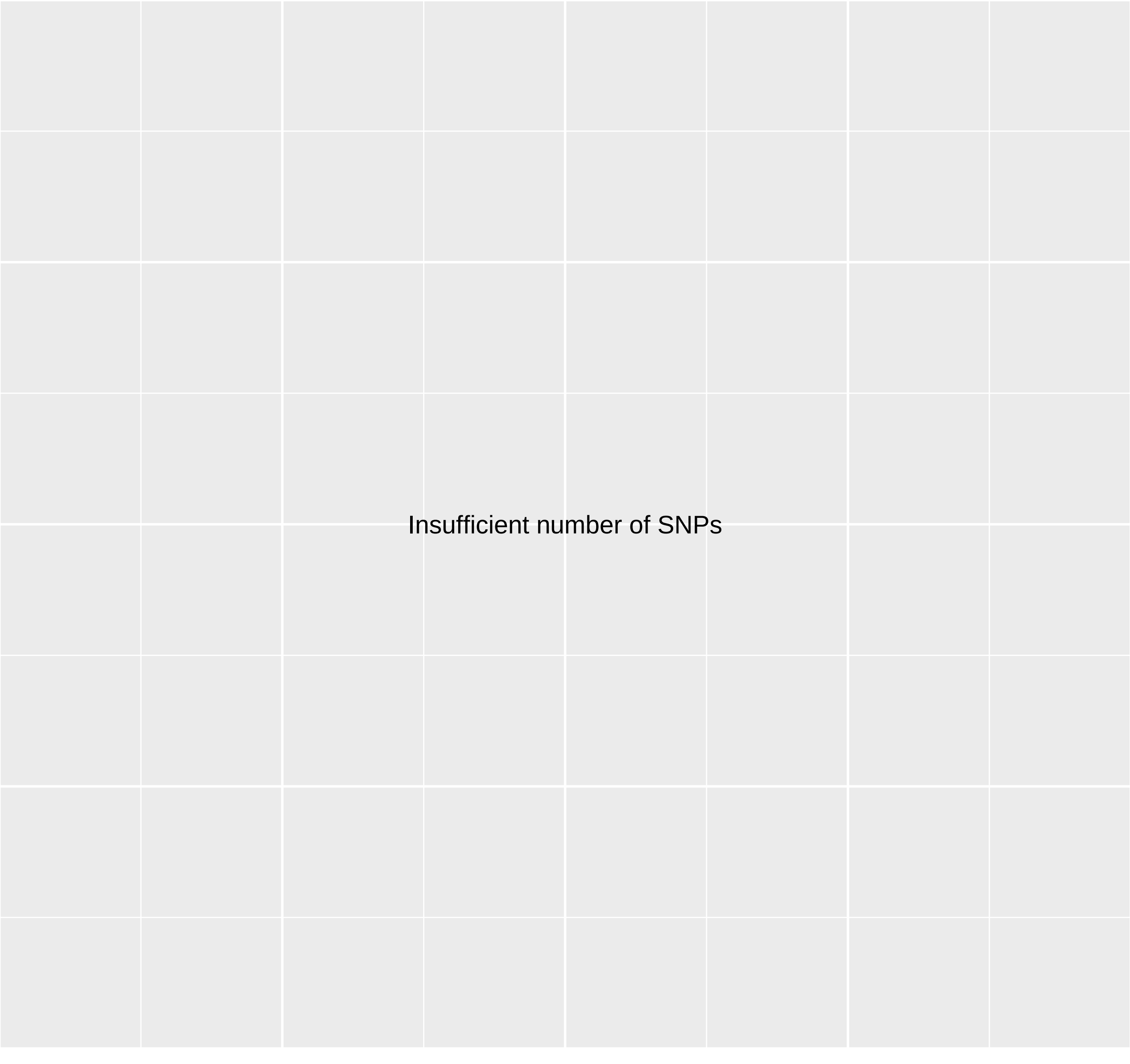
Insufficient number of SNPs

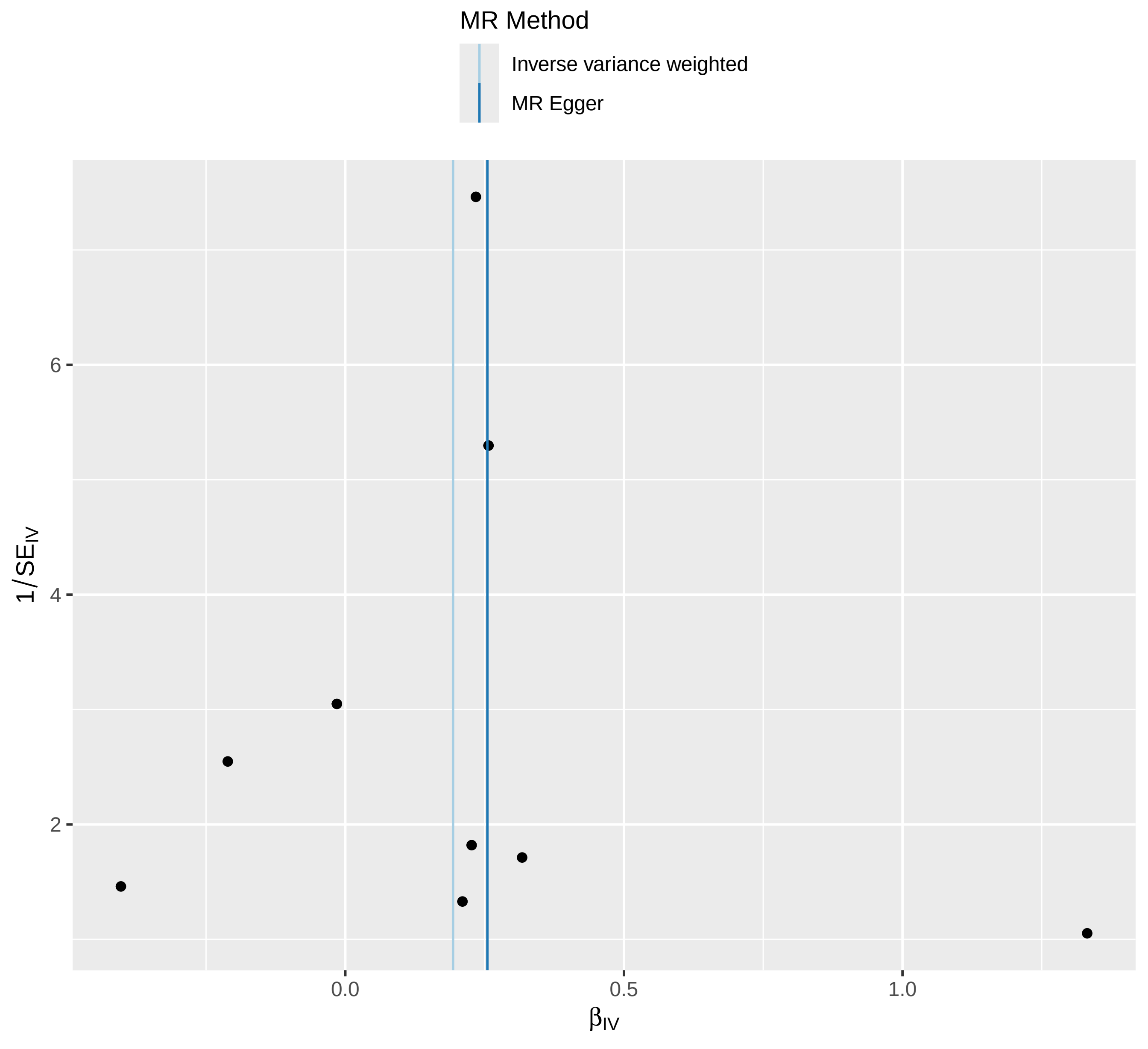

MR Method
Inverse variance weighted
MR Egger
1/SE$_{IV}$
β_{IV}
6
4
2
0.0
0.5
1.0

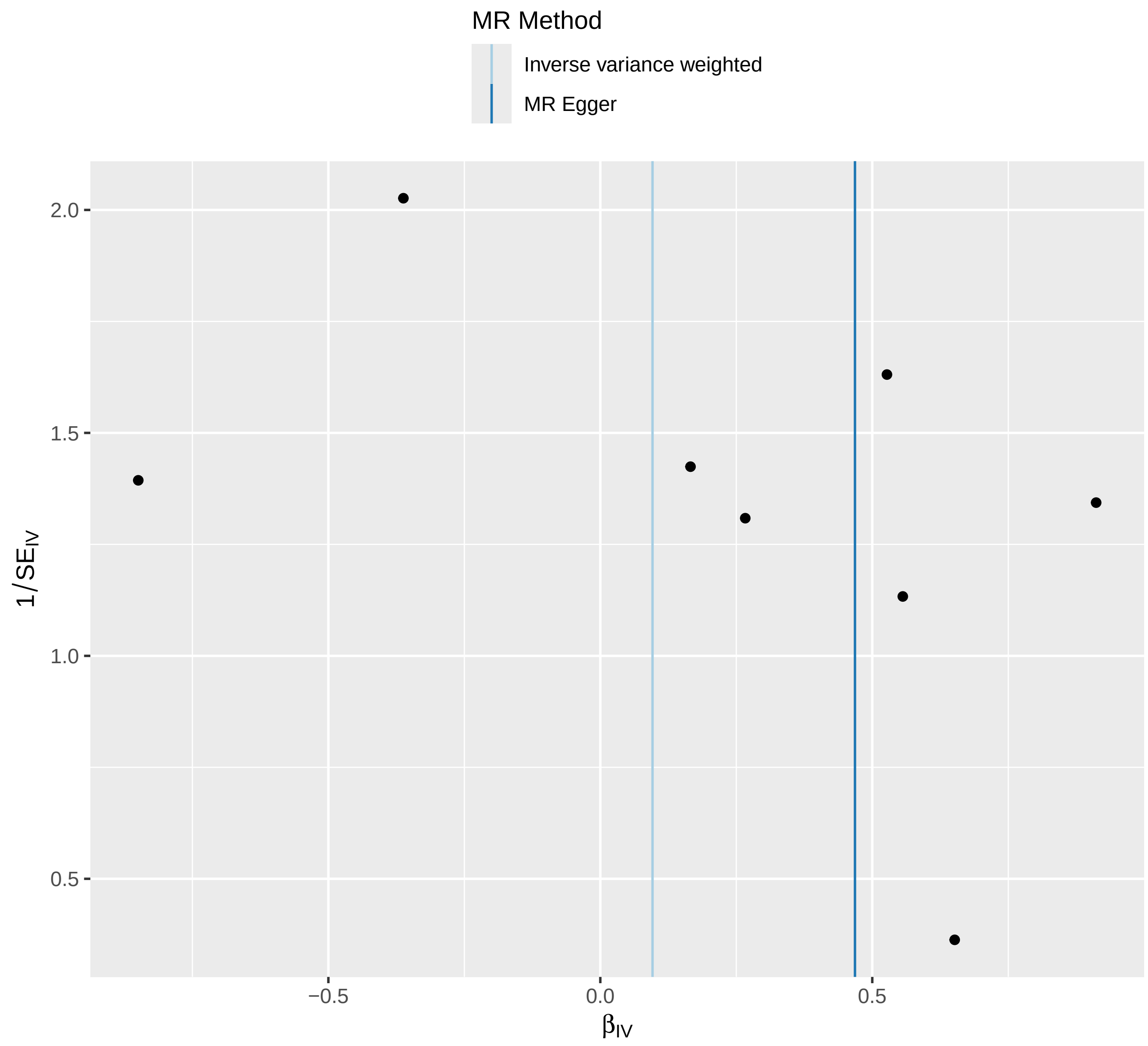
MR Method
Inverse variance weighted
MR Egger
2.0
1.5
1.0
0.5
1/SE_IV
−0.5
0.0
0.5
β_IV

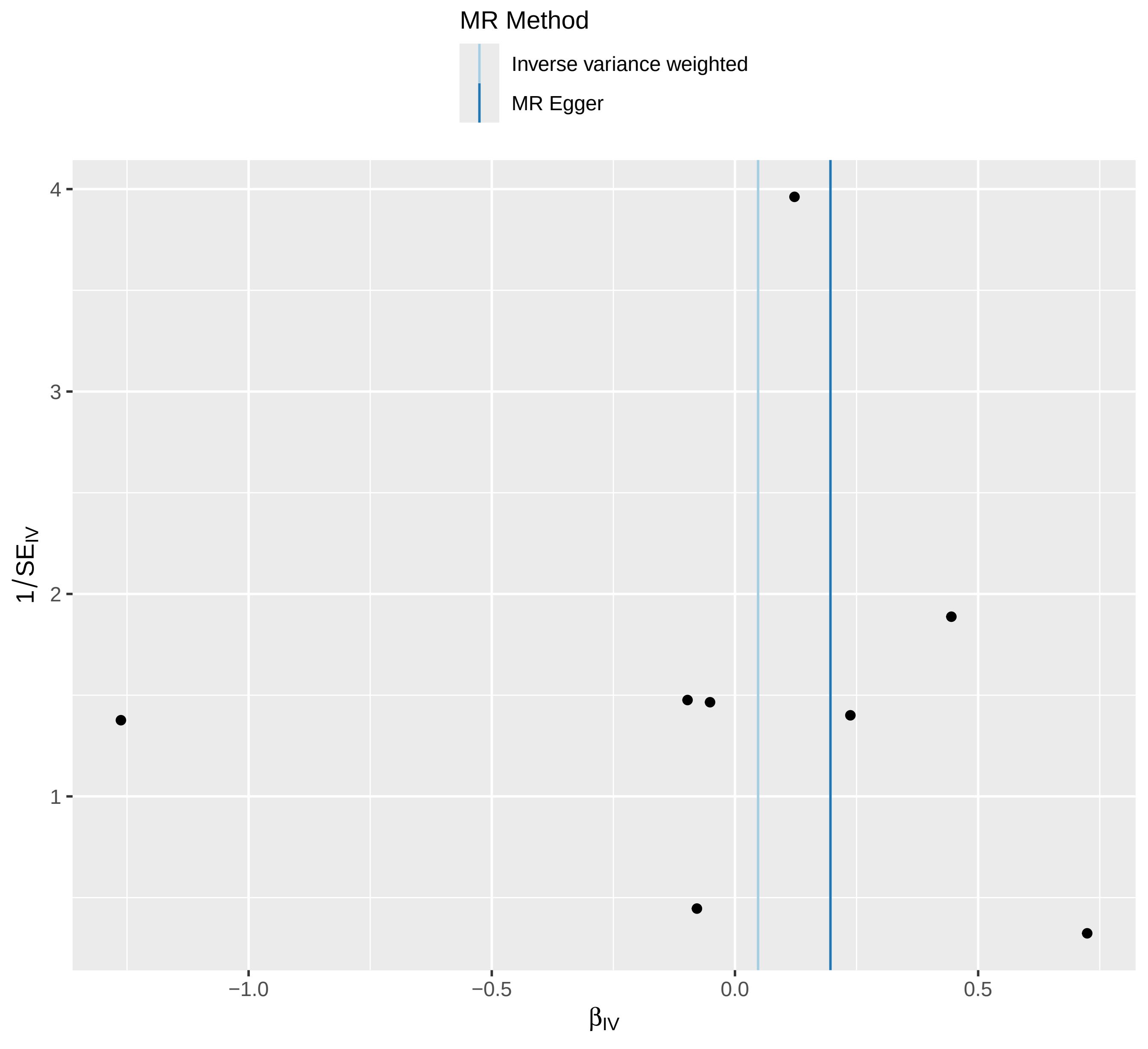
MR Method
Inverse variance weighted
MR Egger
4
3
2
1
1/SE_IV
−1.0
−0.5
0.0
0.5
β_IV

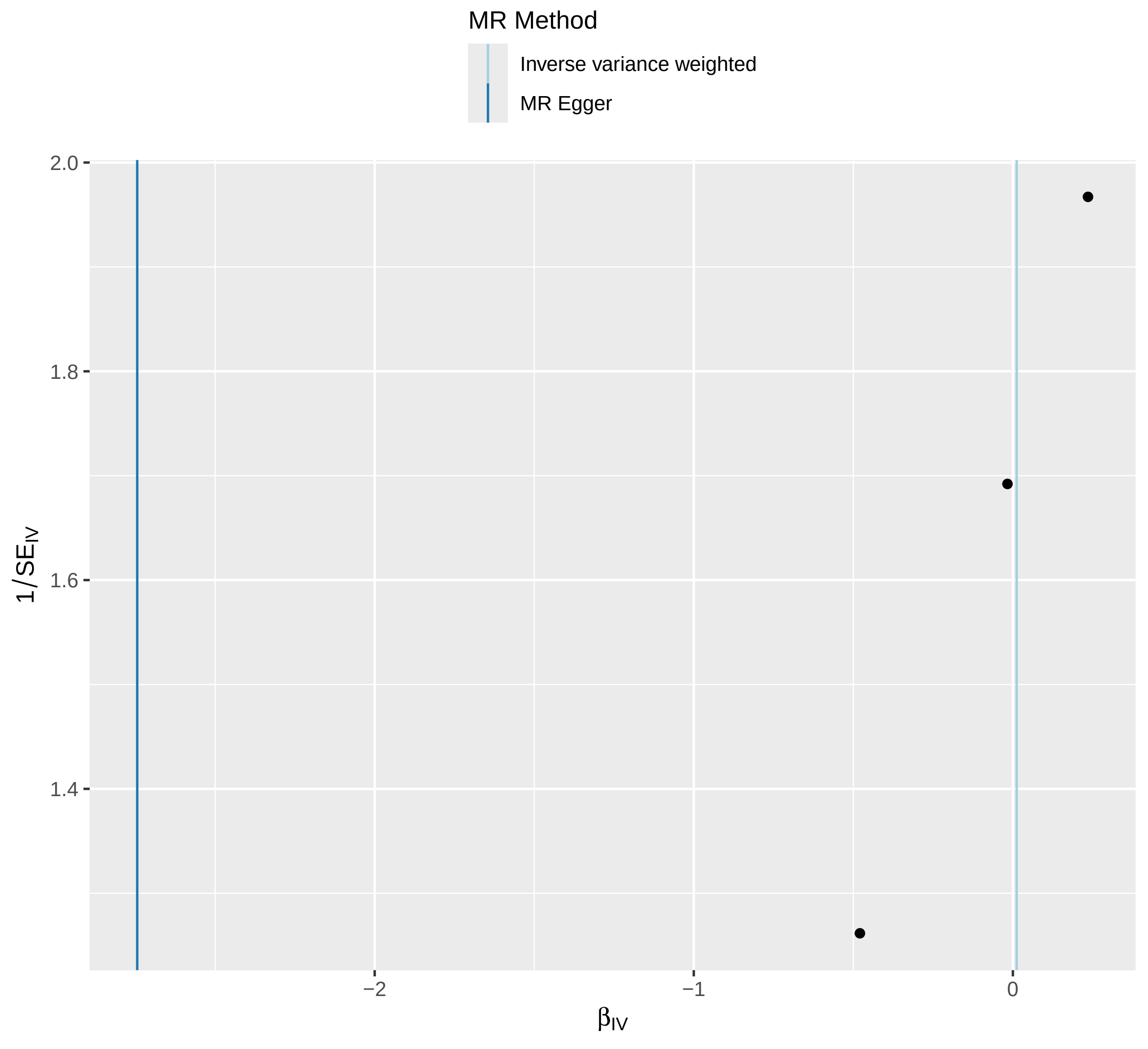
MR Method
Inverse variance weighted
MR Egger
2.0
1.8
1.6
1.4
1/SE_IV
−2
−1
0
β_IV

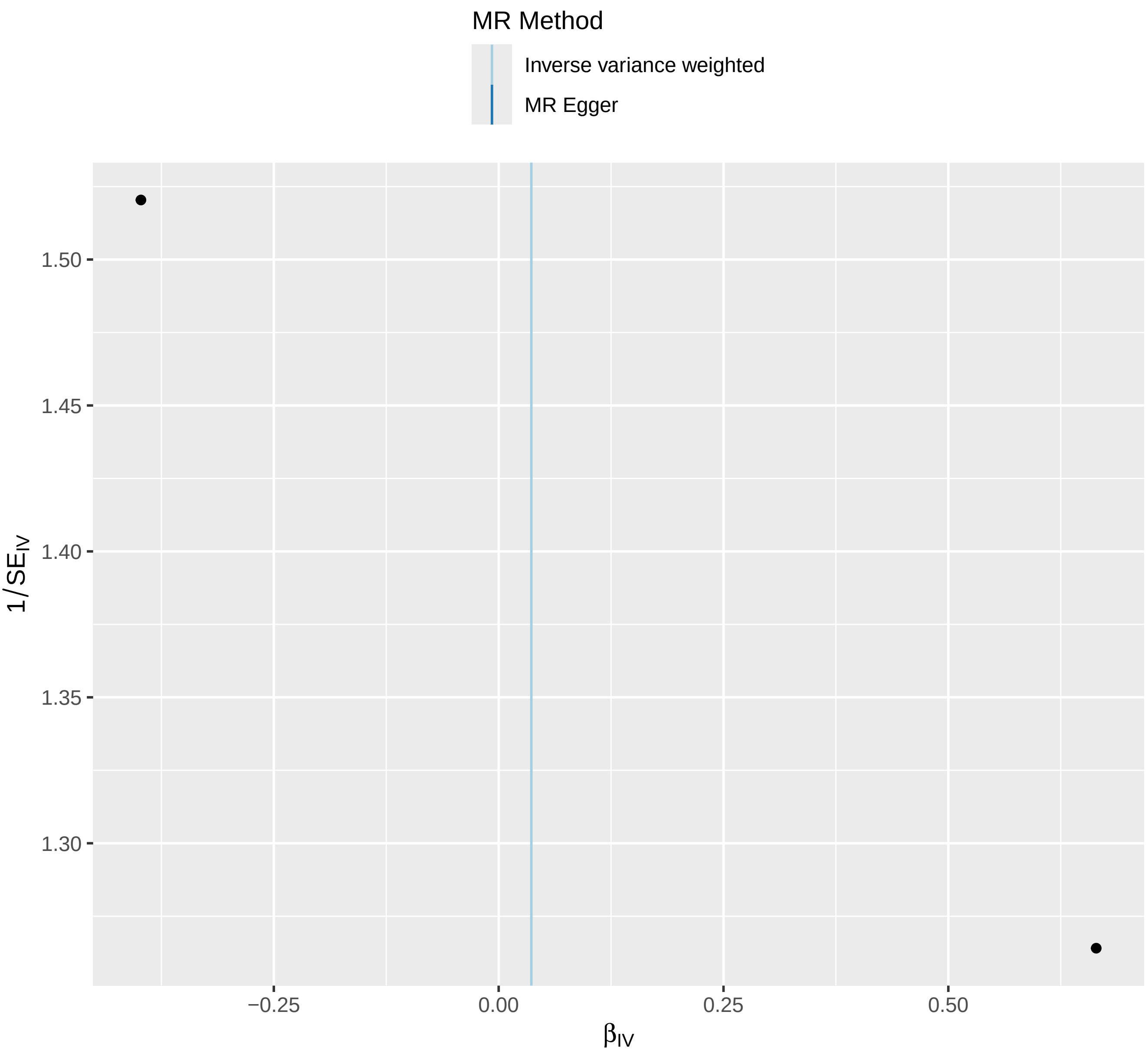
MR Method
Inverse variance weighted
MR Egger
1.50
1.45
1.40
1.35
1.30
1/SE_IV
−0.25
0.00
0.25
0.50
β_IV

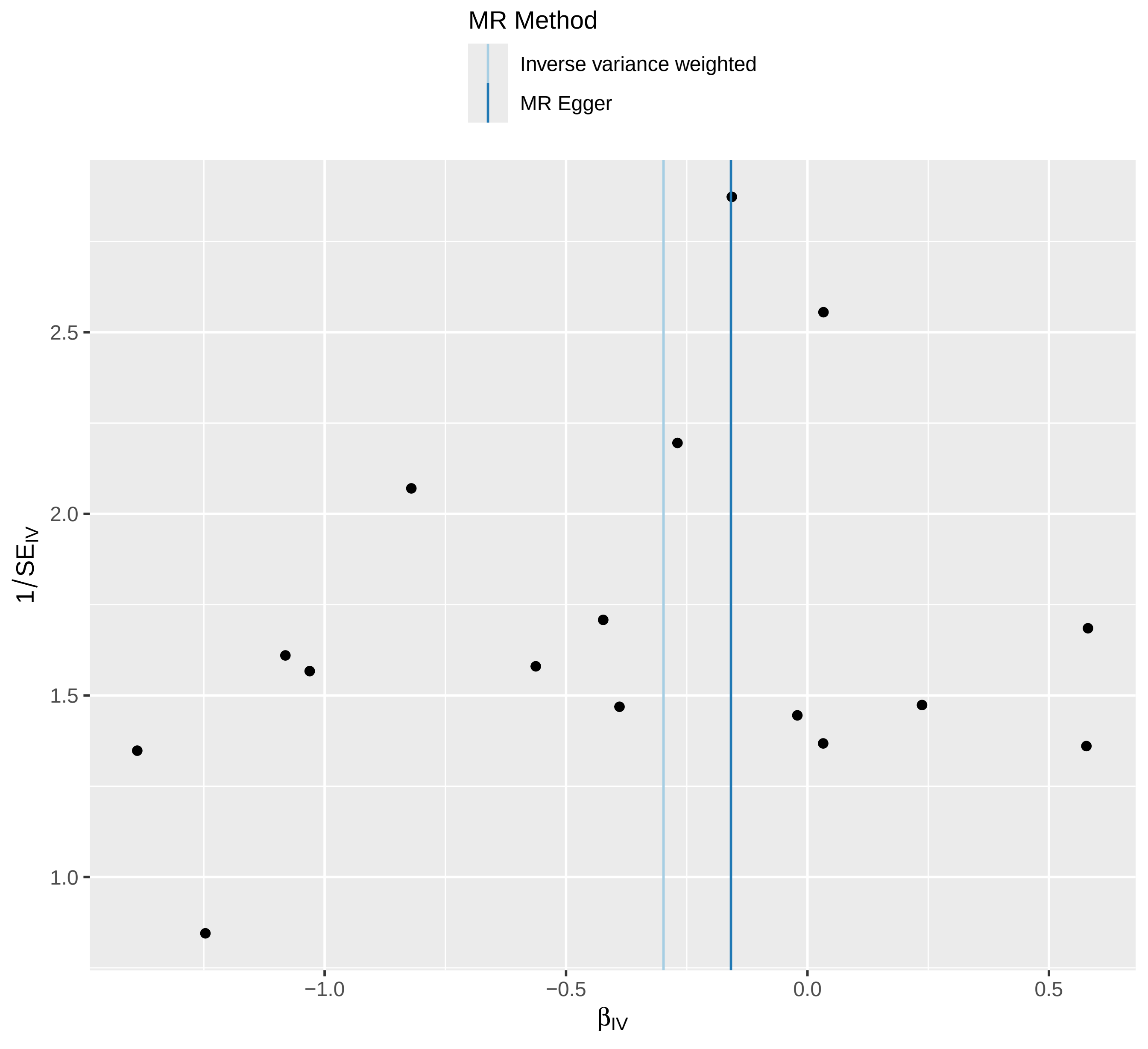
MR Method
Inverse variance weighted
MR Egger
2.5
2.0
1.5
1.0
1/SE$_{IV}$
−1.0
−0.5
0.0
0.5
β$_{IV}$

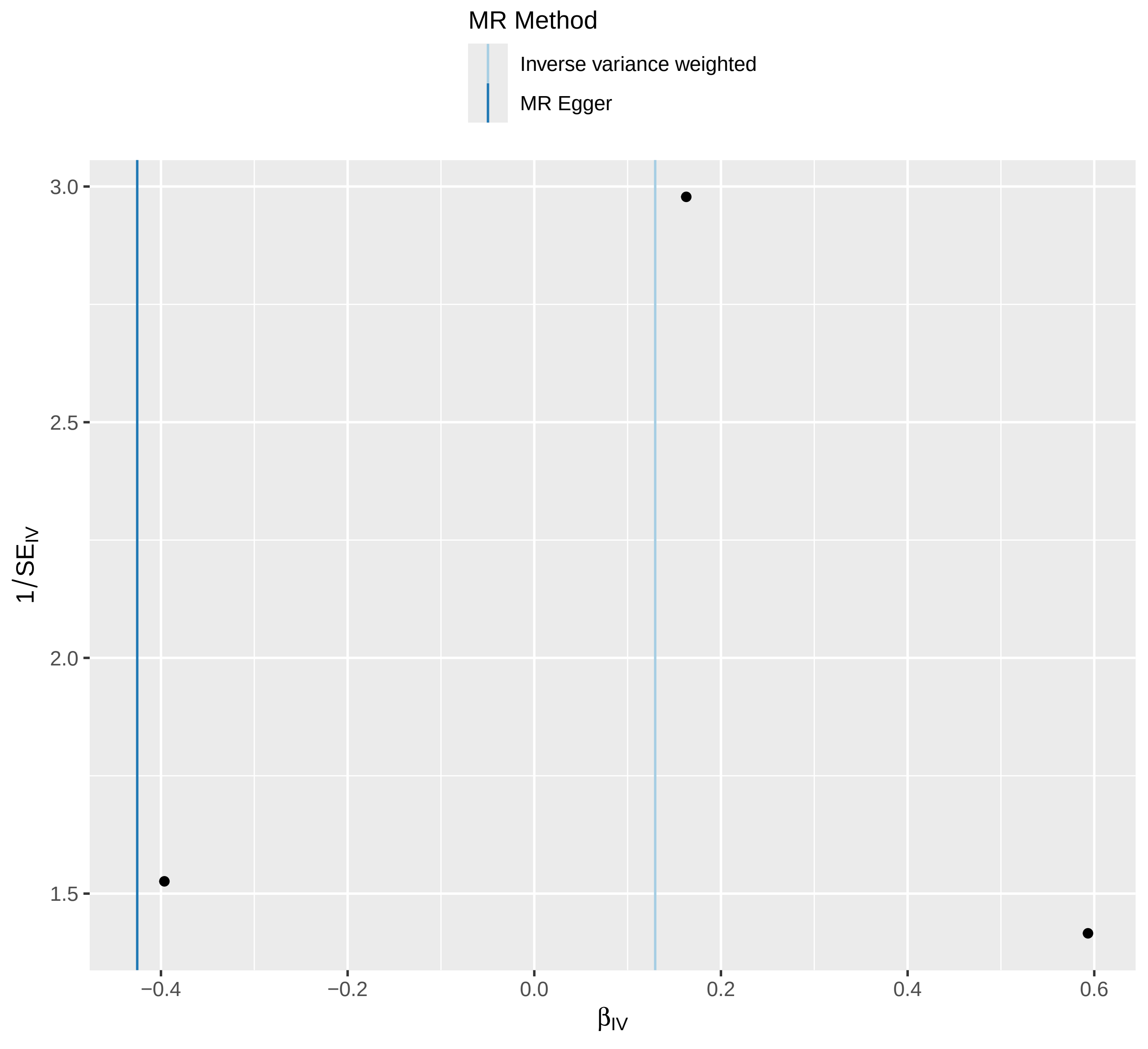

MR Method
Inverse variance weighted
MR Egger
3.0
2.5
2.0
1.5
$1/SE_{IV}$
−0.4
−0.2
0.0
0.2
0.4
0.6
β_{IV}

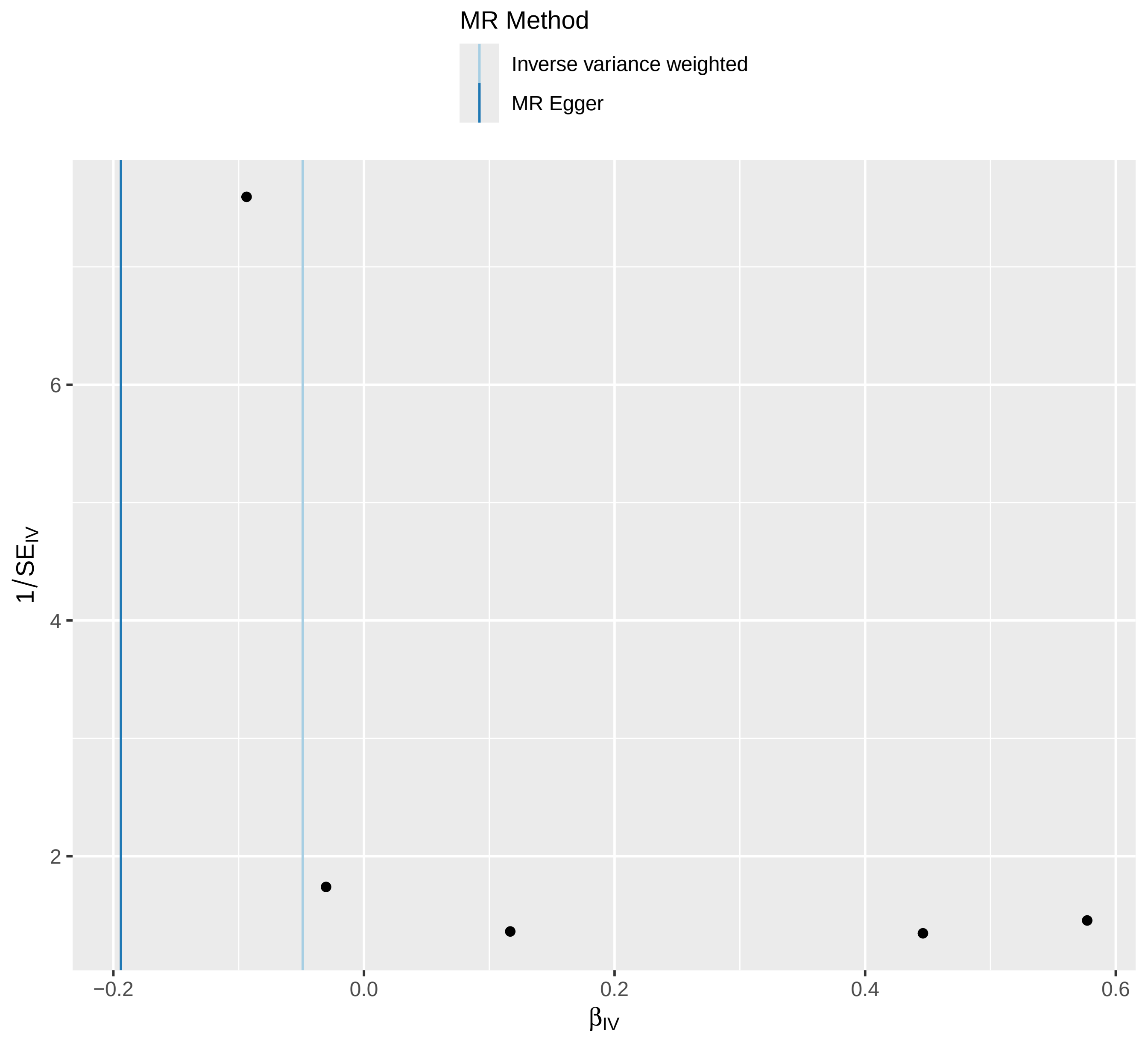

MR Method
Inverse variance weighted
MR Egger
6
4
2
1/SE$_{IV}$
−0.2
0.0
0.2
0.4
0.6
β$_{IV}$

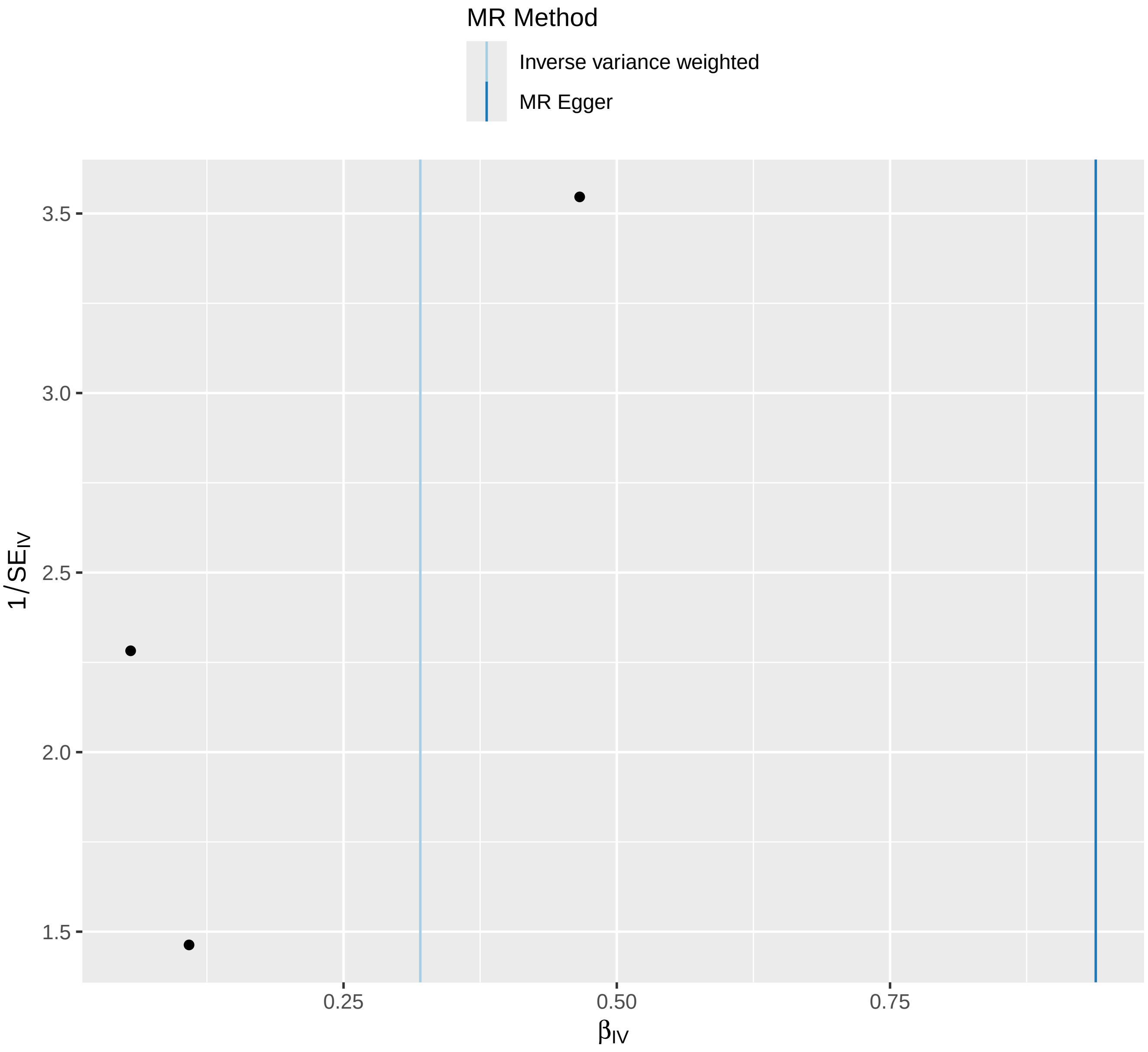
MR Method
Inverse variance weighted
MR Egger
3.5
3.0
2.5
2.0
1.5
$1/SE_{IV}$
0.25
0.50
0.75
β_{IV}

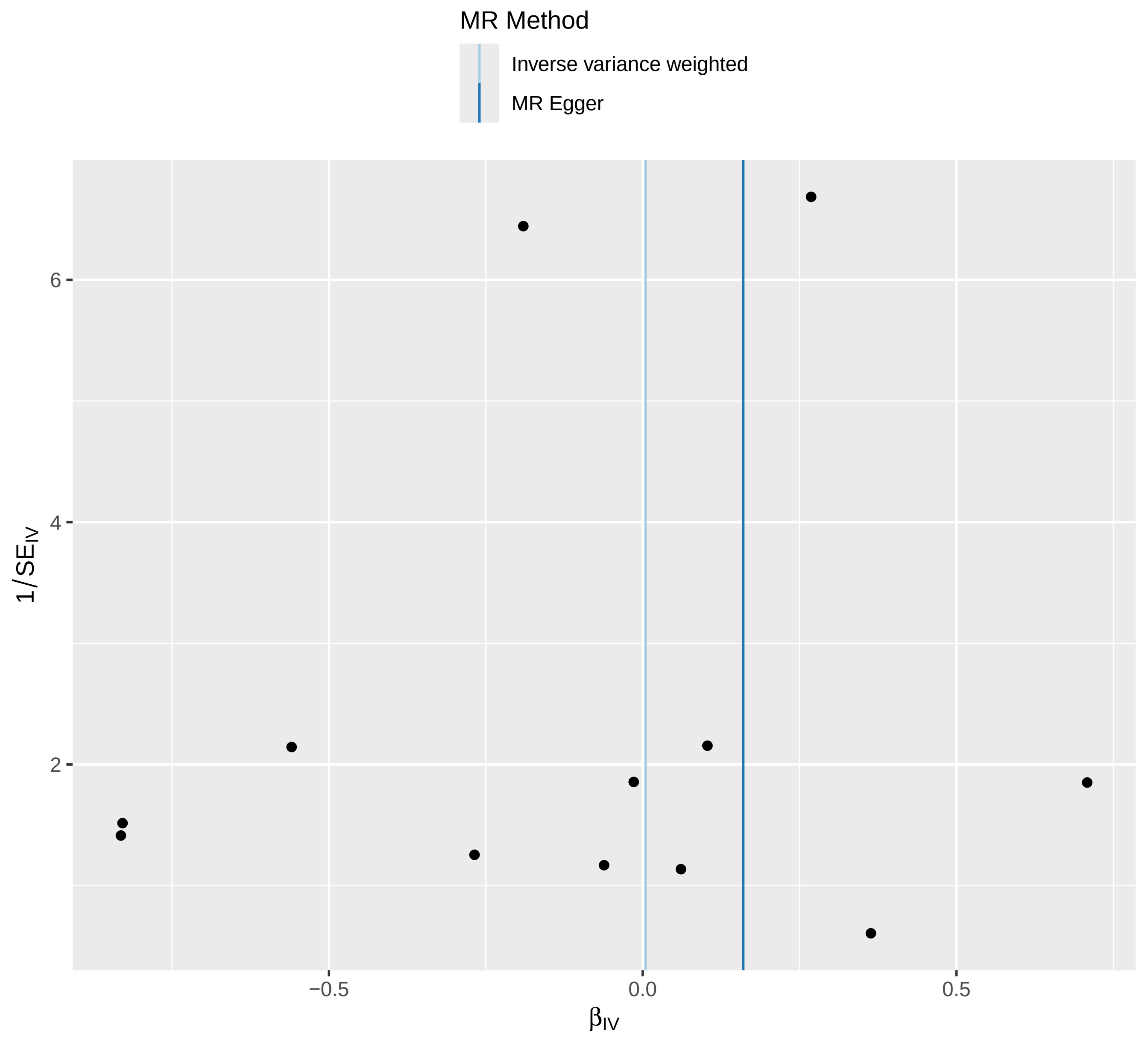
MR Method
Inverse variance weighted
MR Egger
6
4
2
1/SE_{IV}
−0.5
0.0
0.5
β_{IV}

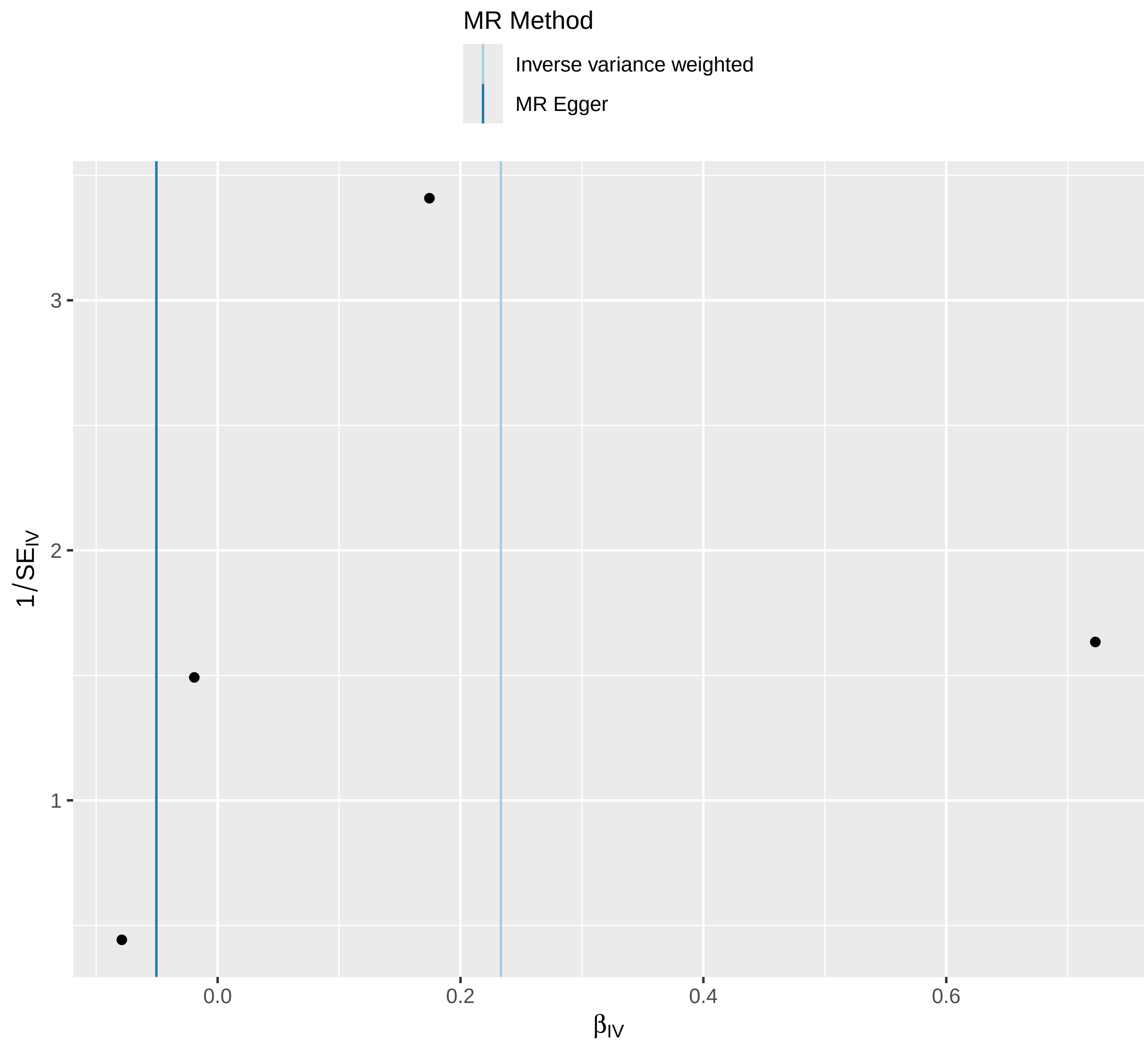
MR Method
Inverse variance weighted
MR Egger
3
2
1
1/SE_IV
0.0
0.2
0.4
0.6
β_IV

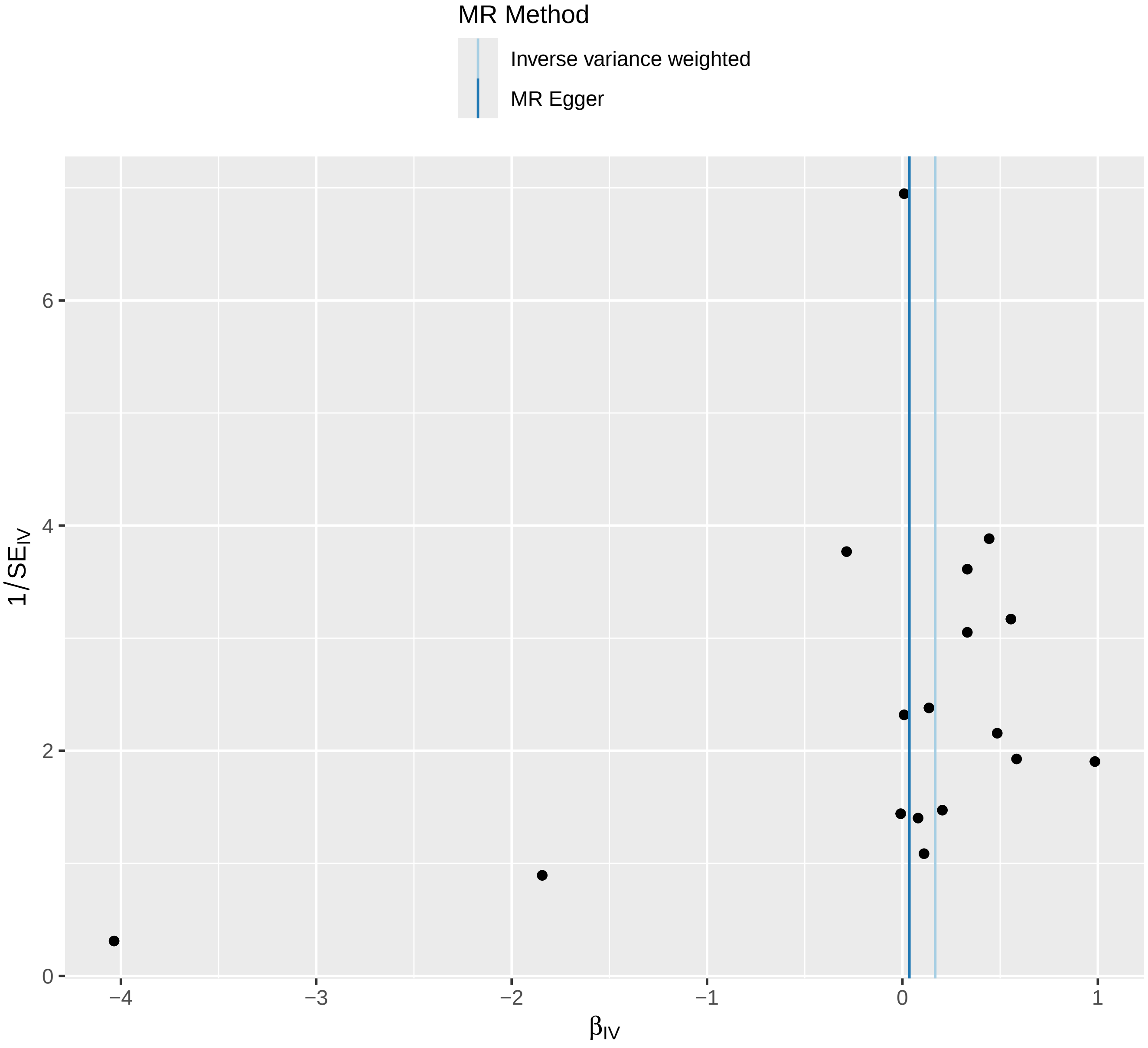
MR Method
Inverse variance weighted
MR Egger
1/SE$_{IV}$
0
2
4
6
−4
−3
−2
−1
0
1
β$_{IV}$

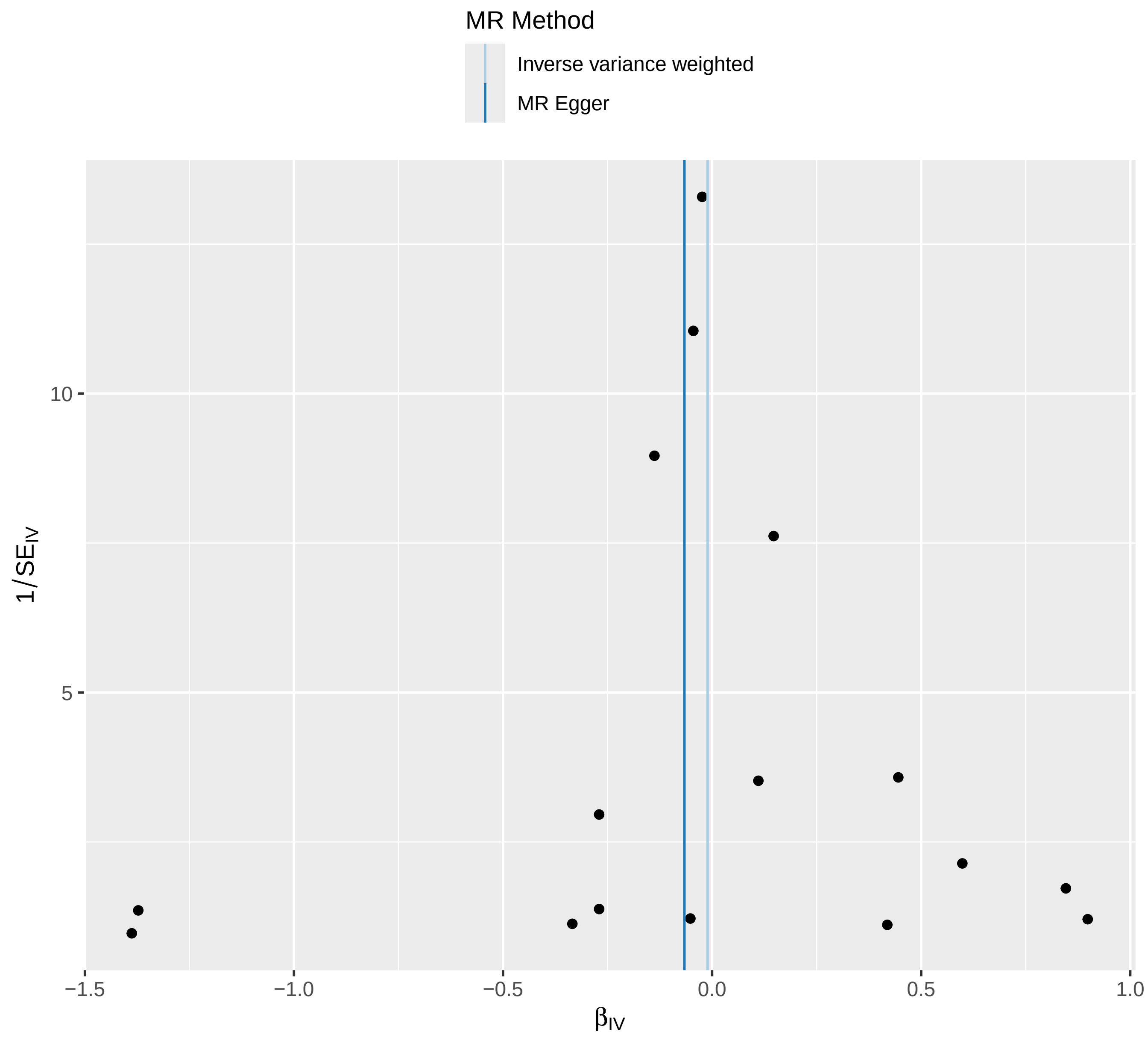
MR Method
Inverse variance weighted
MR Egger
10
5
1/SE_IV
−1.5
−1.0
−0.5
0.0
0.5
1.0
β_IV

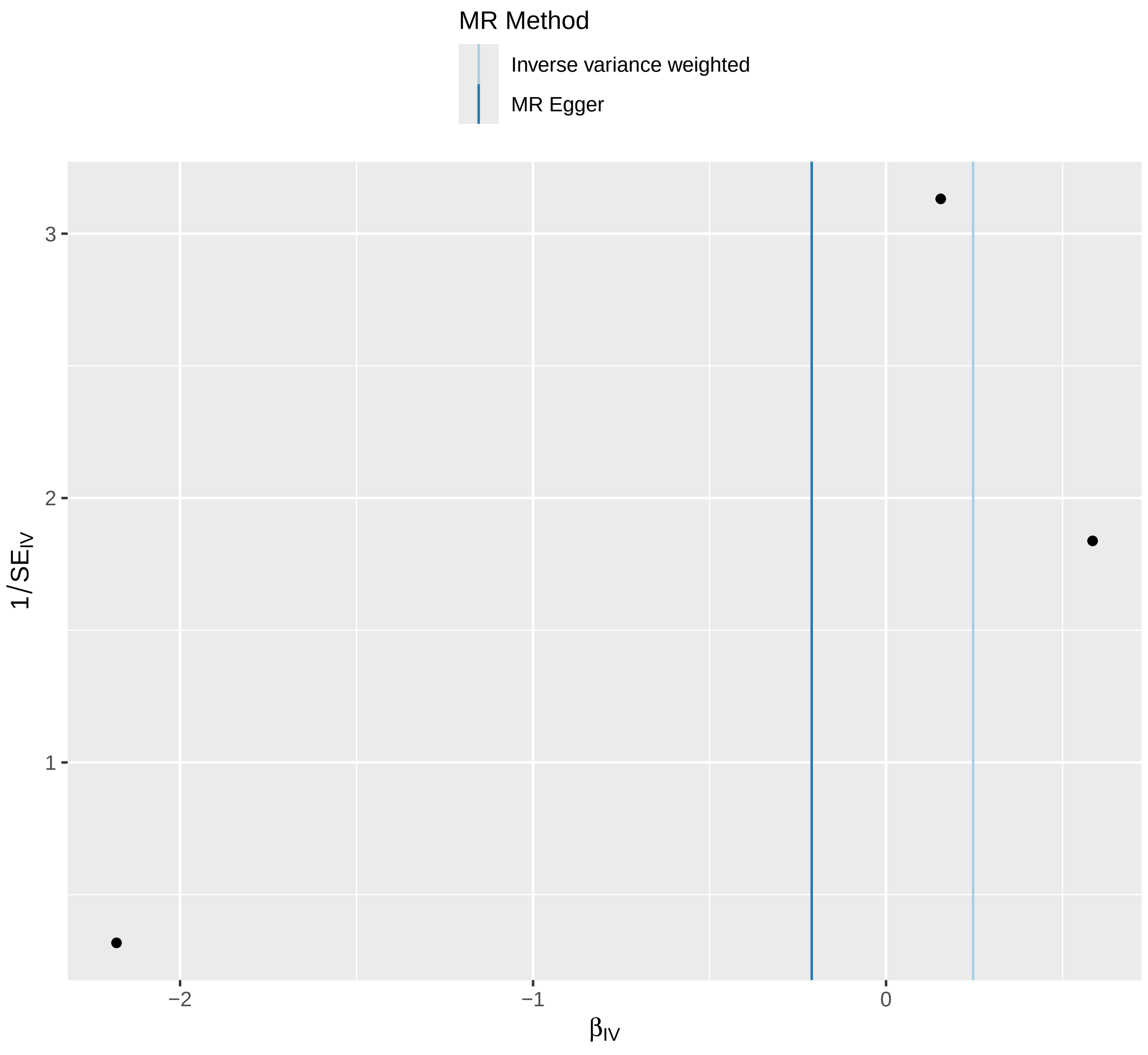
MR Method
Inverse variance weighted
MR Egger
3
2
1
−2
−1
0
1/SE_IV
β_IV

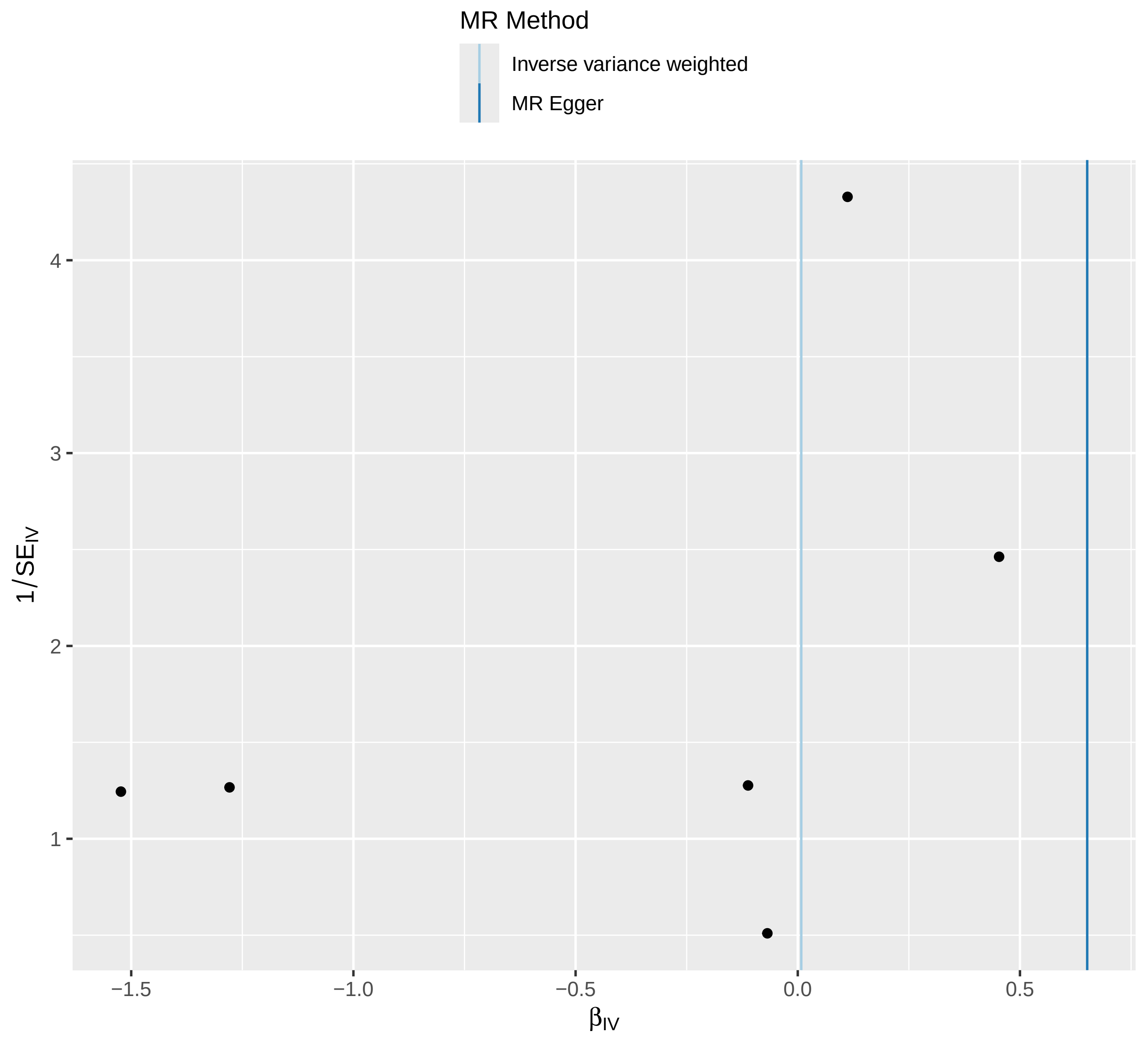

MR Method
Inverse variance weighted
MR Egger
4
3
2
1
1/SE$_{IV}$
−1.5
−1.0
−0.5
0.0
0.5
β$_{IV}$

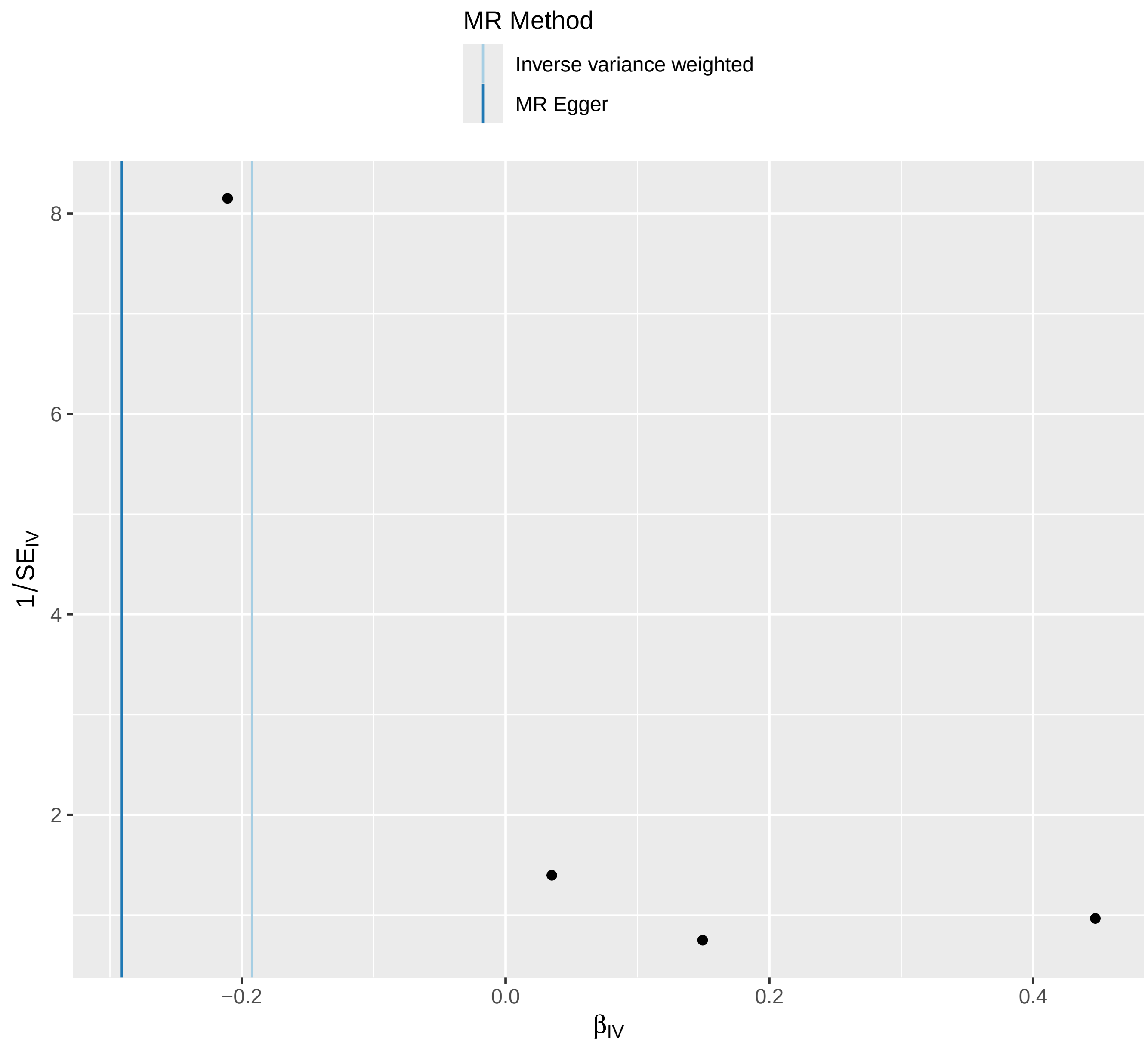

MR Method
Inverse variance weighted
MR Egger
8
6
4
2
1/SE_IV
−0.2
0.0
0.2
0.4
β_IV

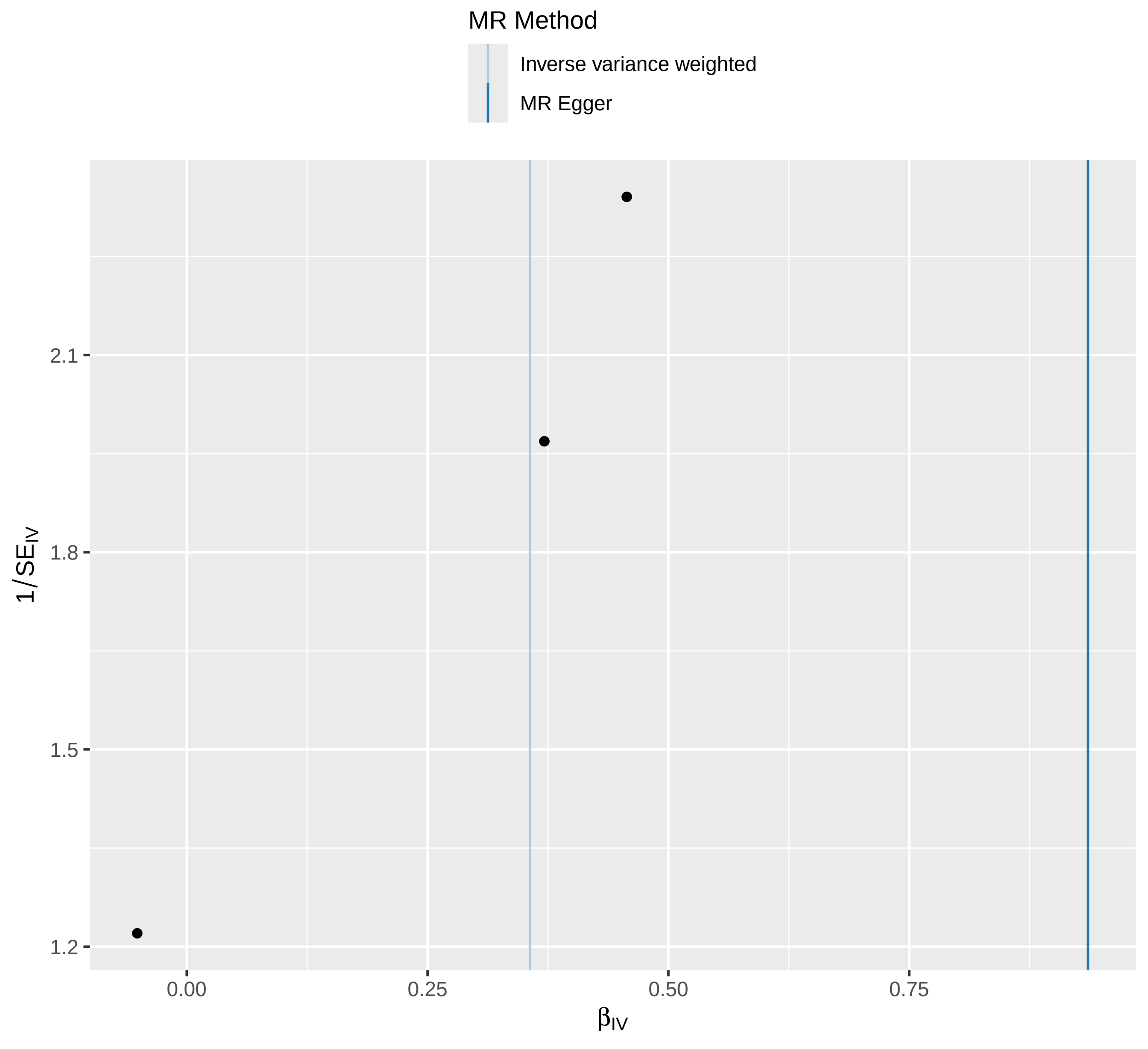
MR Method
Inverse variance weighted
MR Egger
2.1
1.8
1.5
1.2
1/SE_IV
0.00
0.25
0.50
0.75
β_IV

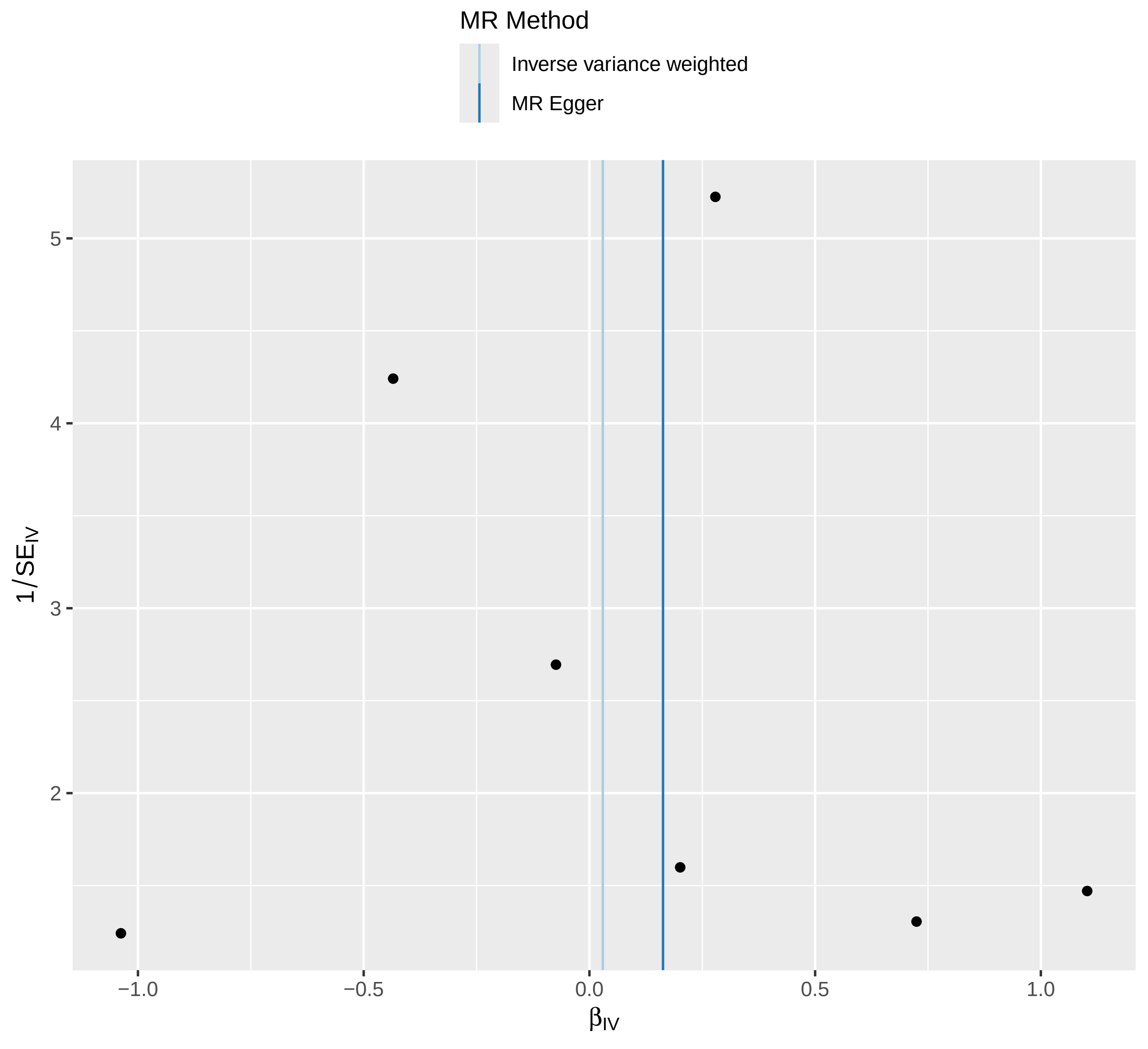
MR Method
Inverse variance weighted
MR Egger
5
4
3
2
1/SE$_{IV}$
−1.0
−0.5
0.0
0.5
1.0
β$_{IV}$

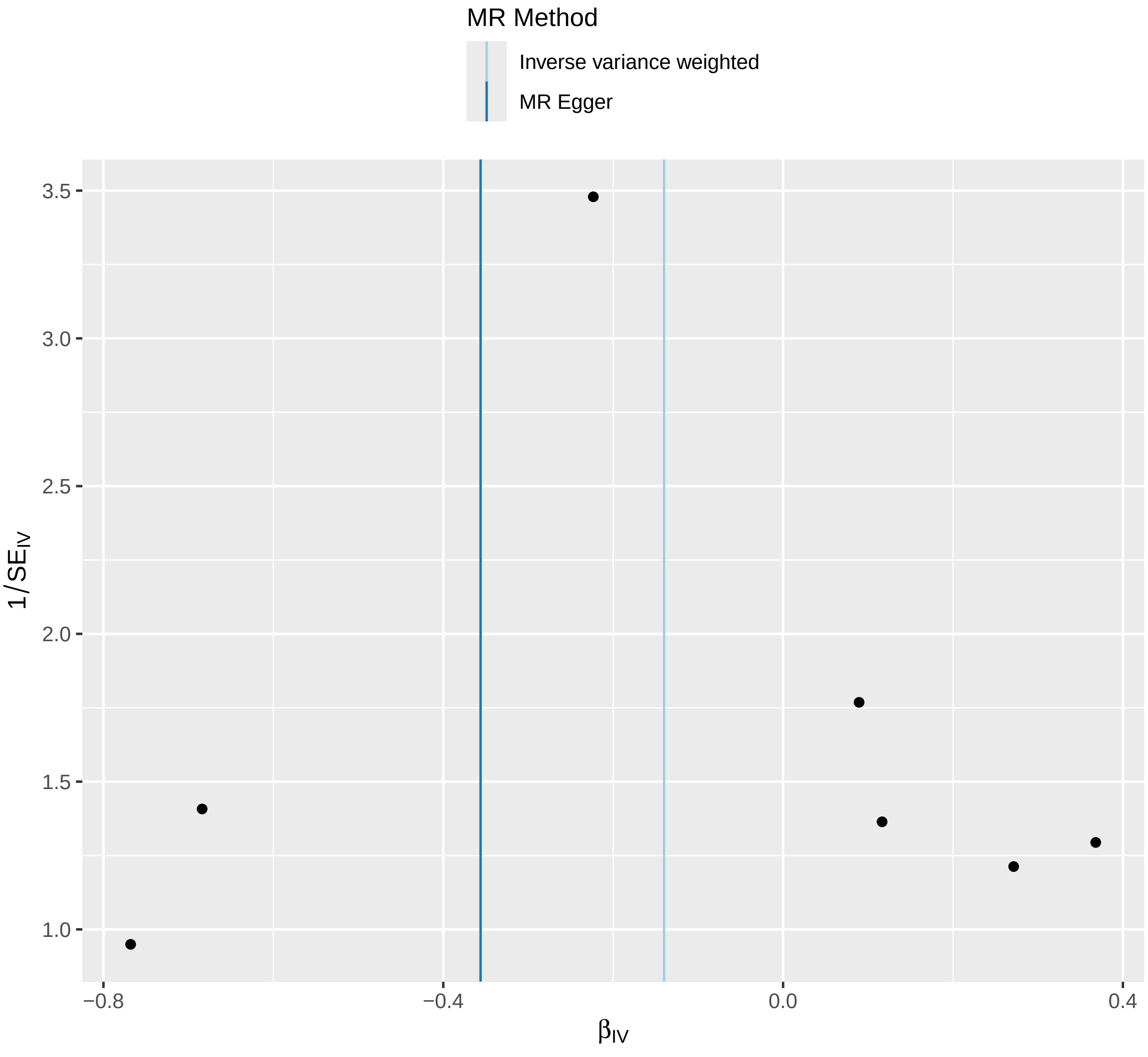
MR Method
Inverse variance weighted
MR Egger
3.5
3.0
2.5
2.0
1.5
1.0
$1/SE_{IV}$
−0.8
−0.4
0.0
0.4
β_{IV}

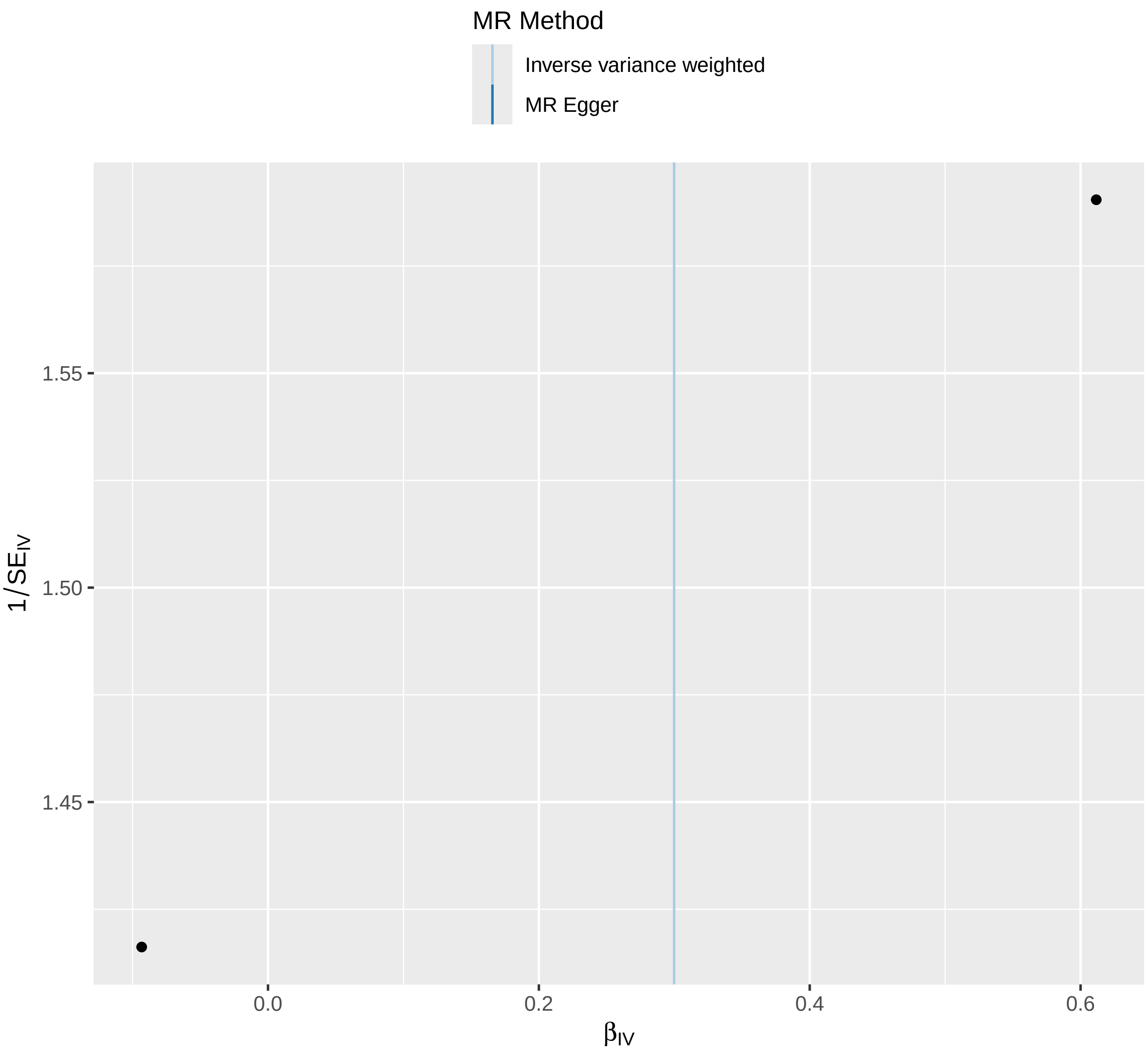
MR Method
Inverse variance weighted
MR Egger
1.55
1.50
1.45
$1/SE_{IV}$
0.0
0.2
0.4
0.6
β_{IV}

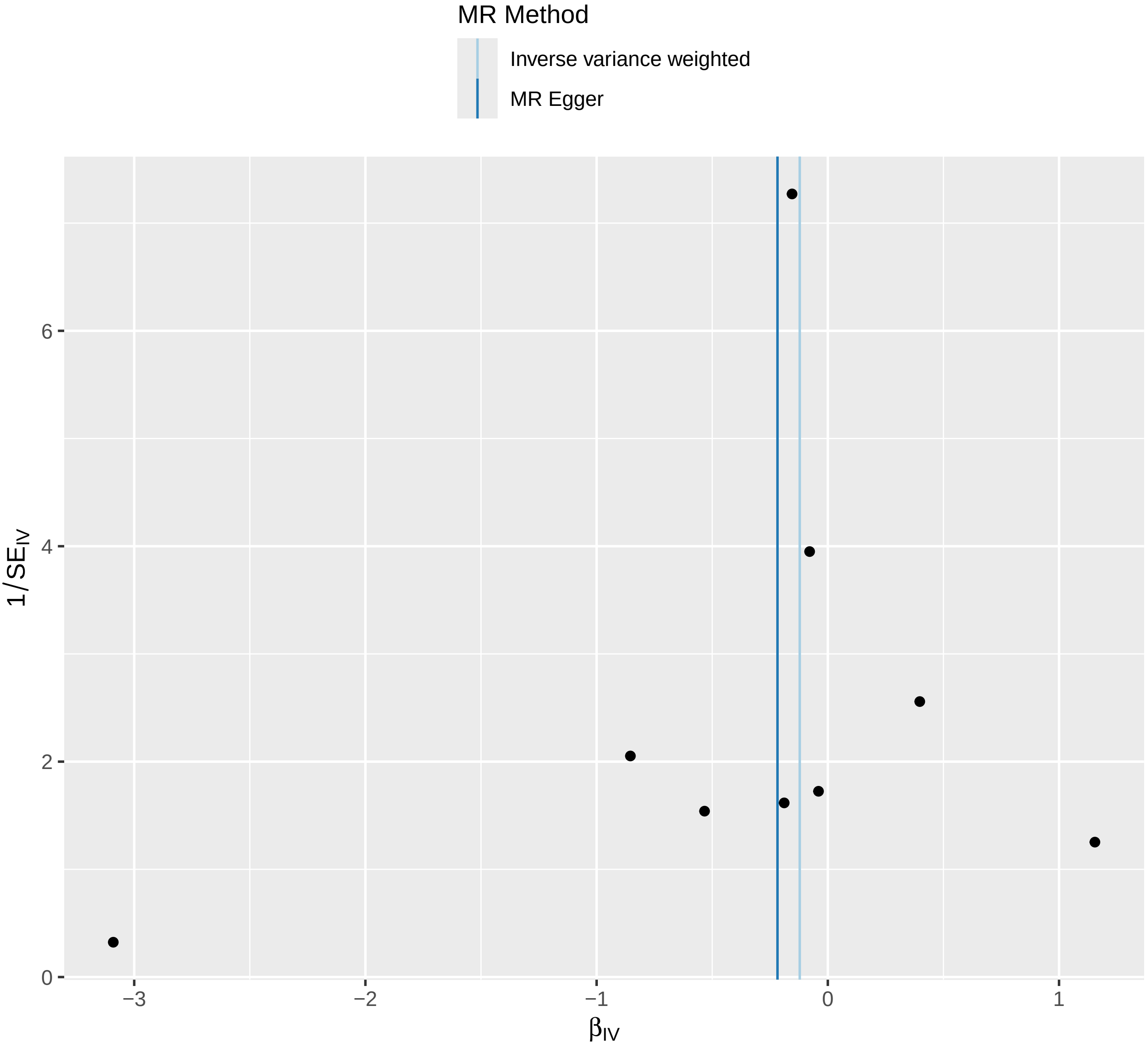
MR Method
Inverse variance weighted
MR Egger
6
4
2
0
1/SE_IV
−3
−2
−1
0
1
β_IV

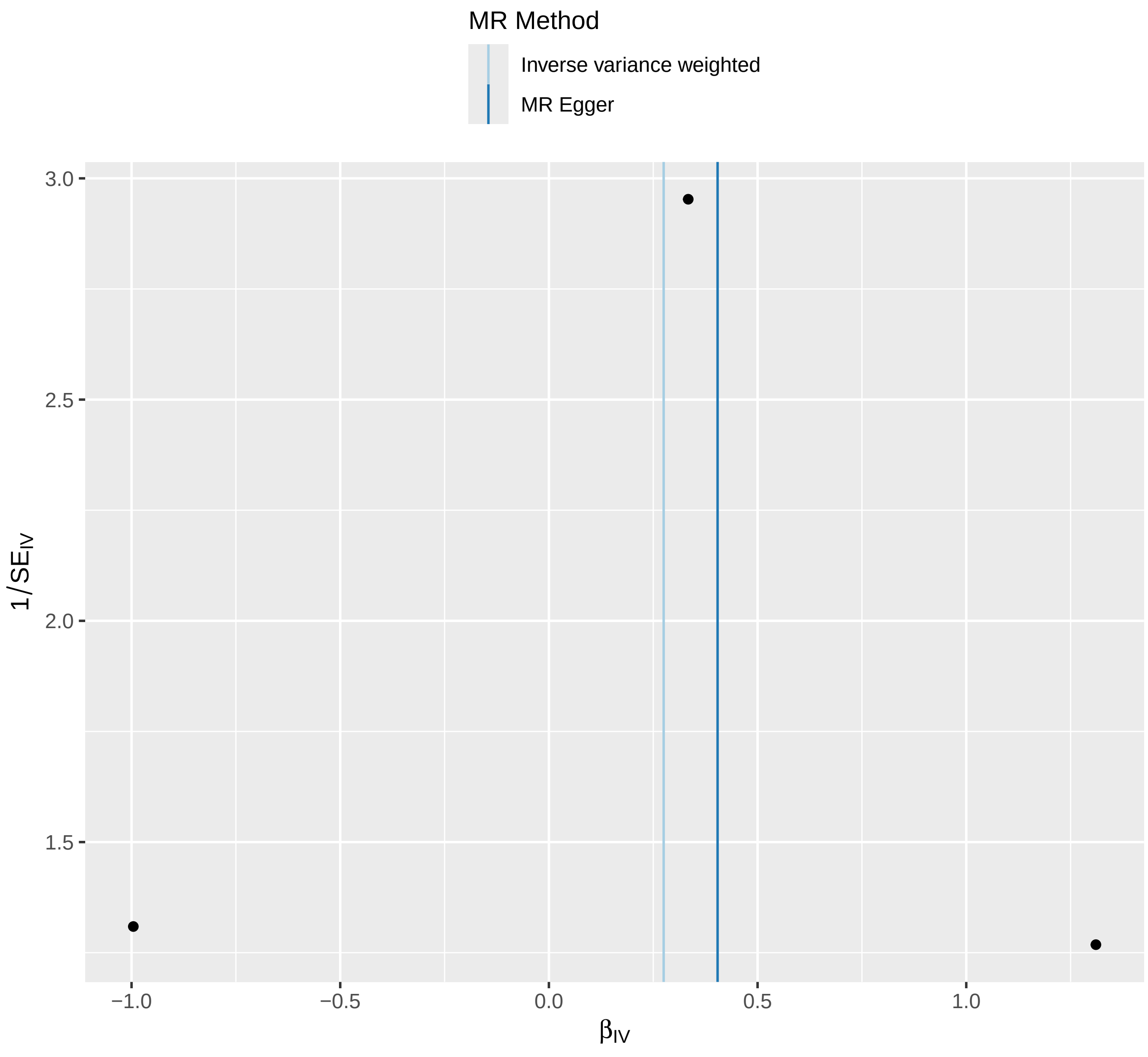
MR Method
Inverse variance weighted
MR Egger
3.0
2.5
2.0
1.5
$1/SE_{IV}$
−1.0
−0.5
0.0
0.5
1.0
β_{IV}

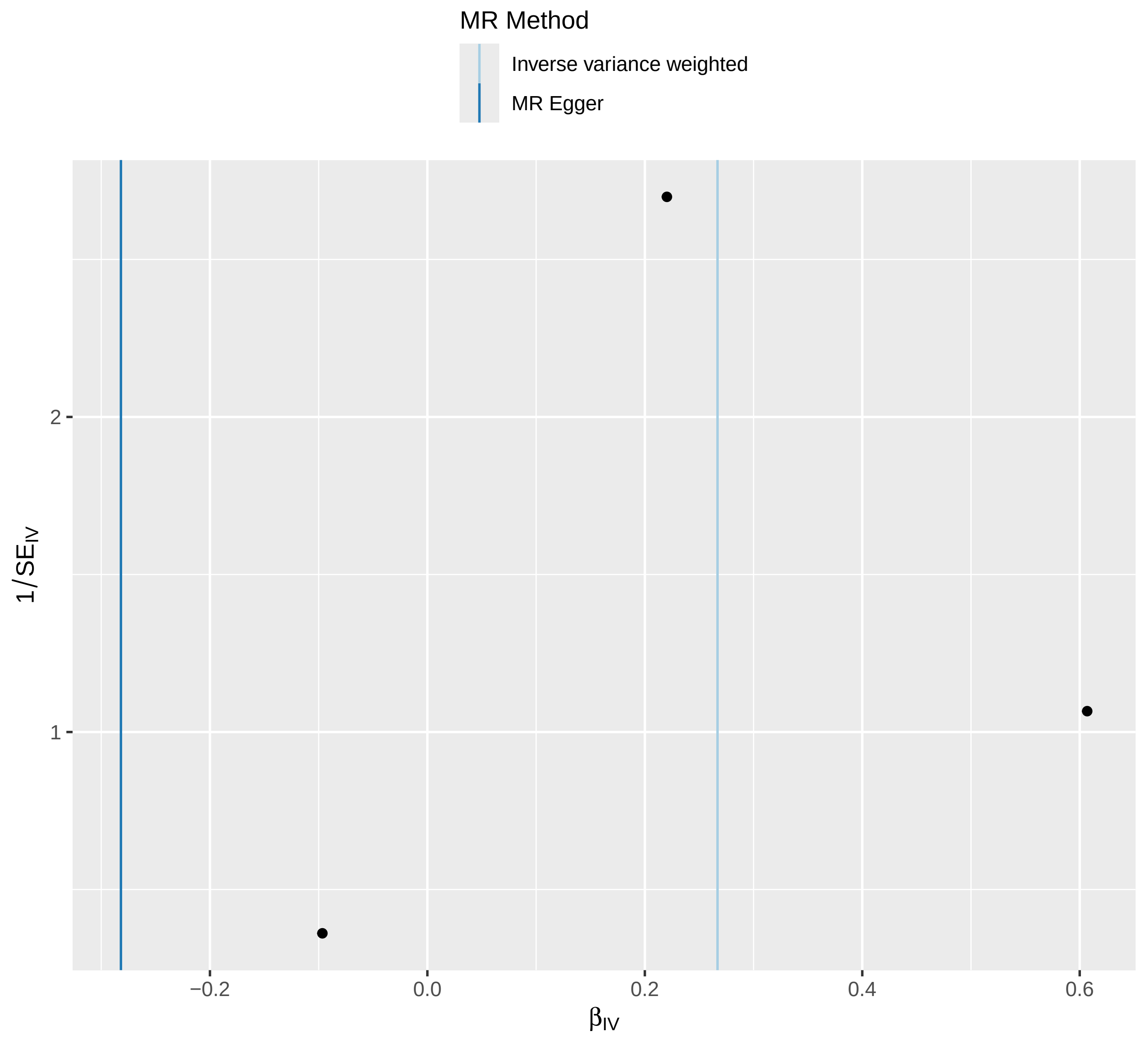
MR Method
Inverse variance weighted
MR Egger
2
1
1/SE_IV
−0.2
0.0
0.2
0.4
0.6
β_IV

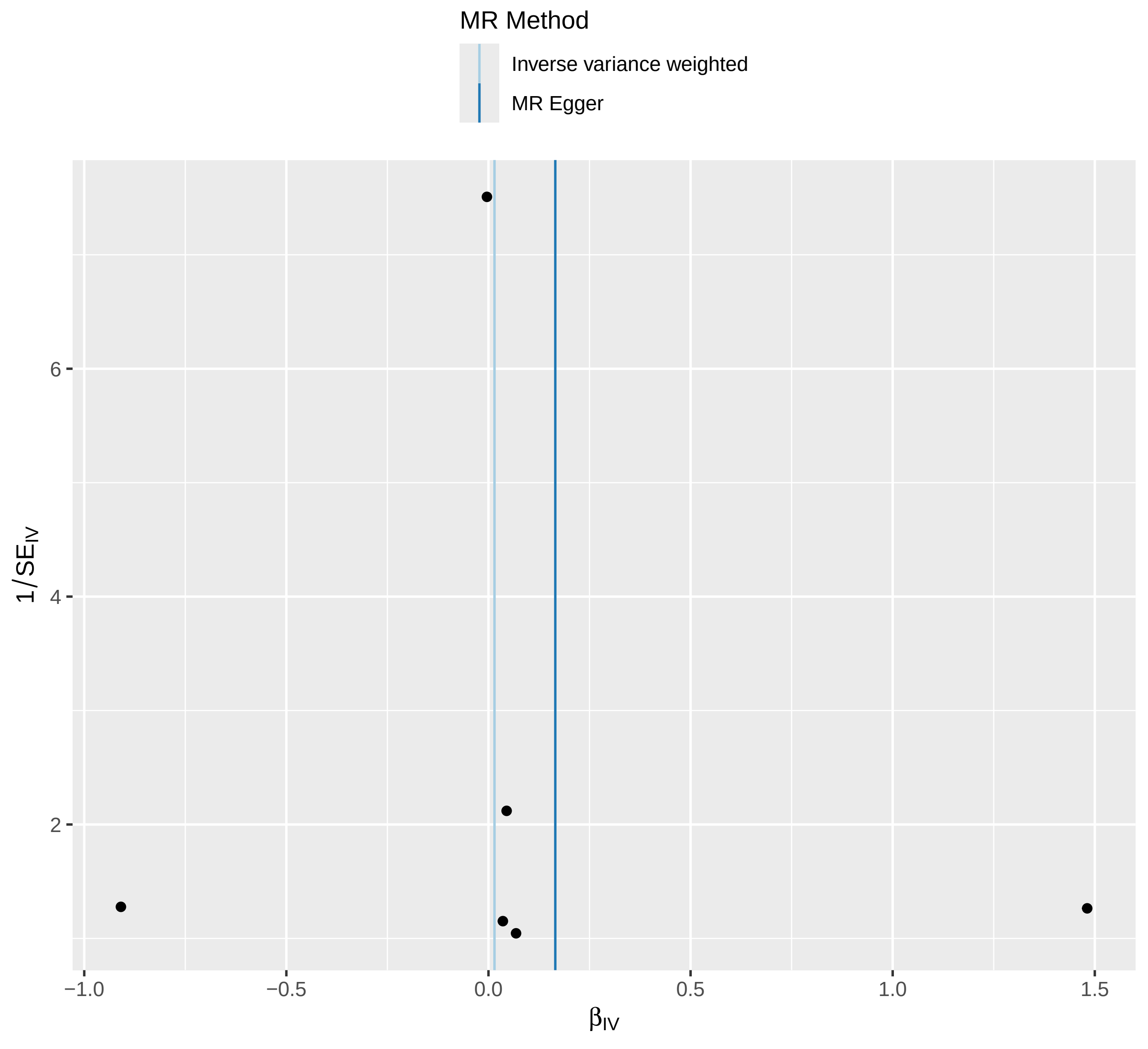
MR Method
Inverse variance weighted
MR Egger
6
4
2
1/SE_IV
−1.0
−0.5
0.0
0.5
1.0
1.5
β_IV

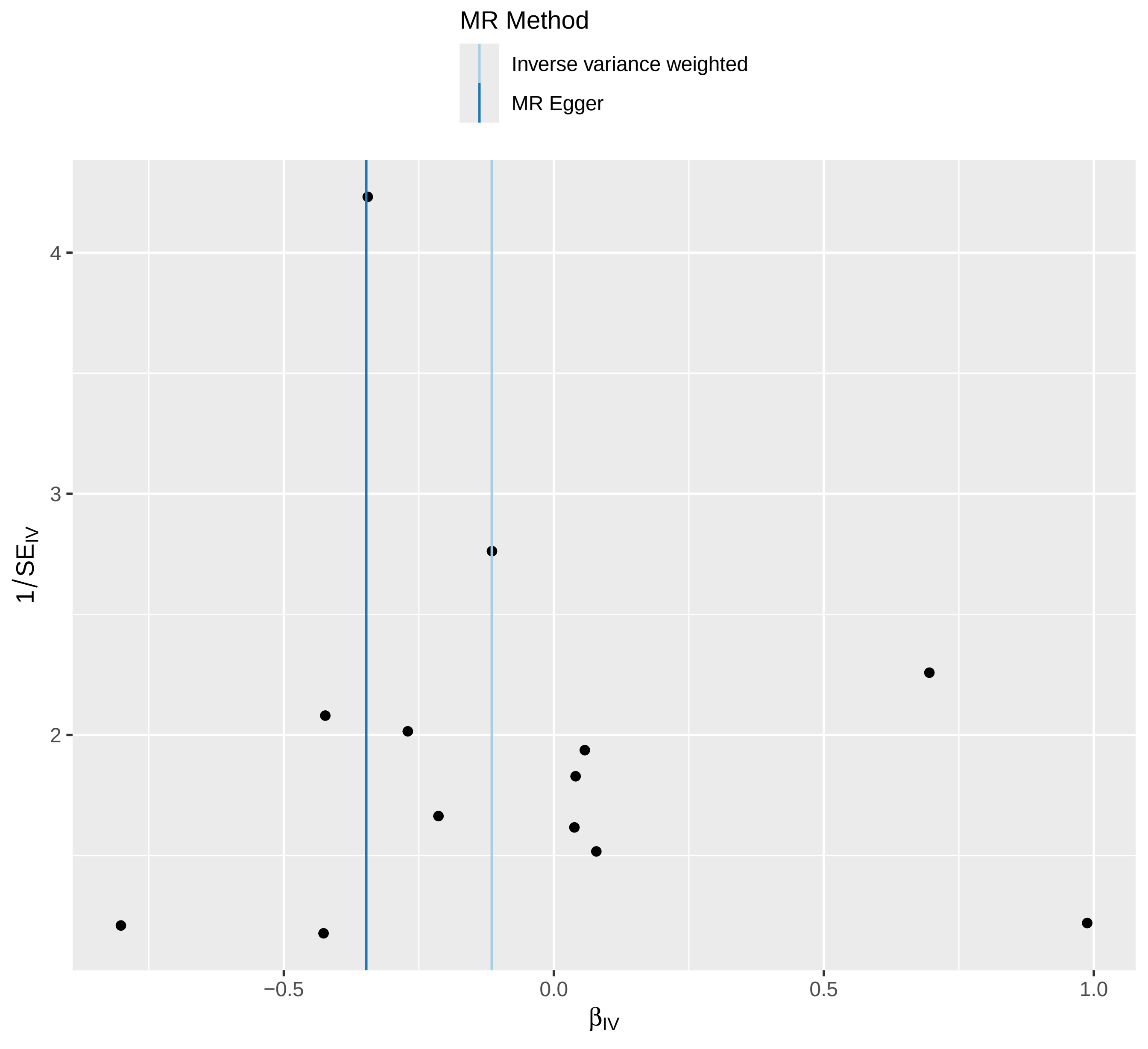

MR Method
Inverse variance weighted
MR Egger
4
3
2
1/SE$_{IV}$
−0.5
0.0
0.5
1.0
β$_{IV}$

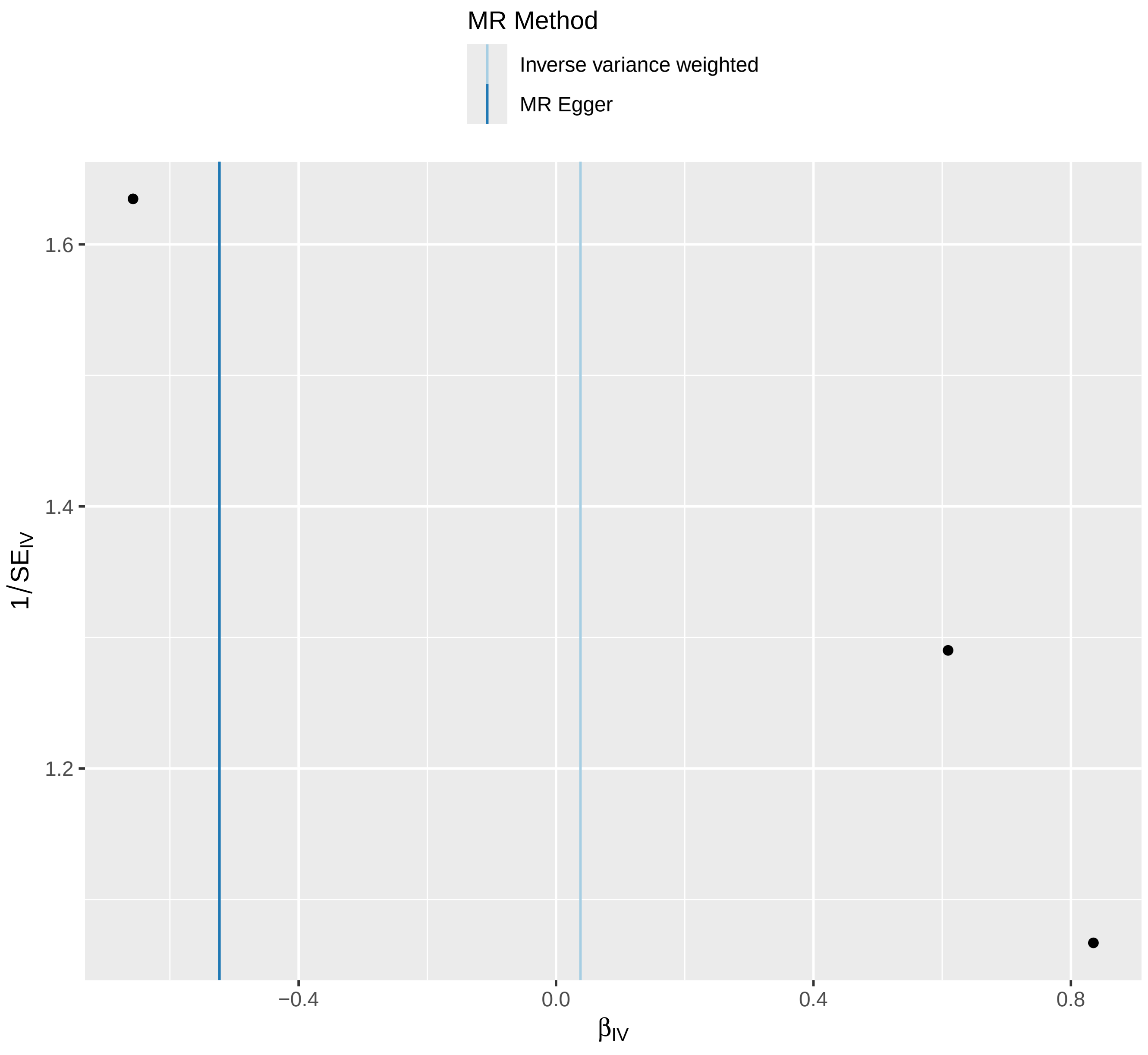
MR Method
Inverse variance weighted
MR Egger
1.6
1.4
1.2
$1/SE_{IV}$
−0.4
0.0
0.4
0.8
β_{IV}

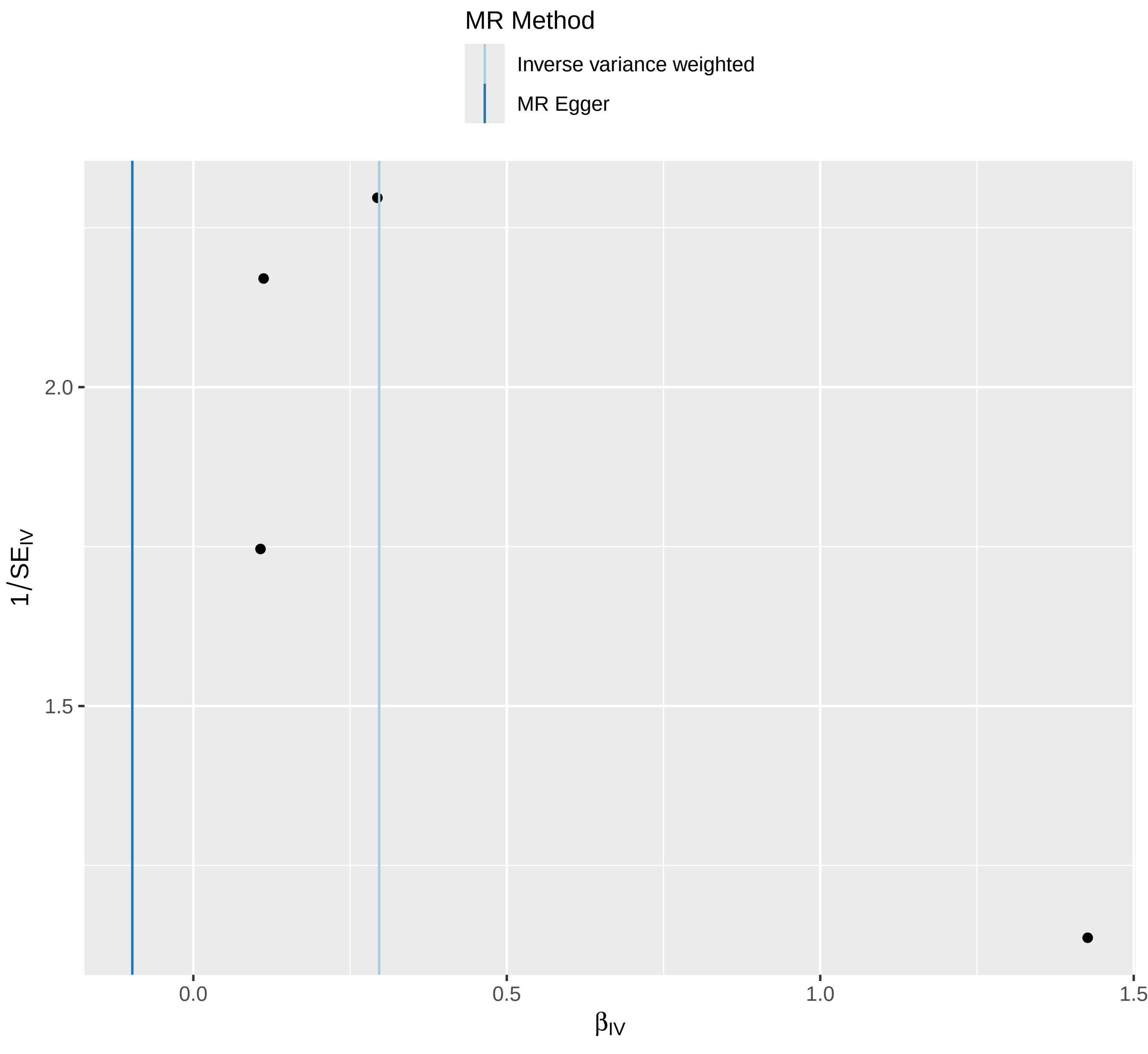
MR Method
Inverse variance weighted
MR Egger
2.0
1.5
1/SE_IV
0.0
0.5
1.0
1.5
β_IV

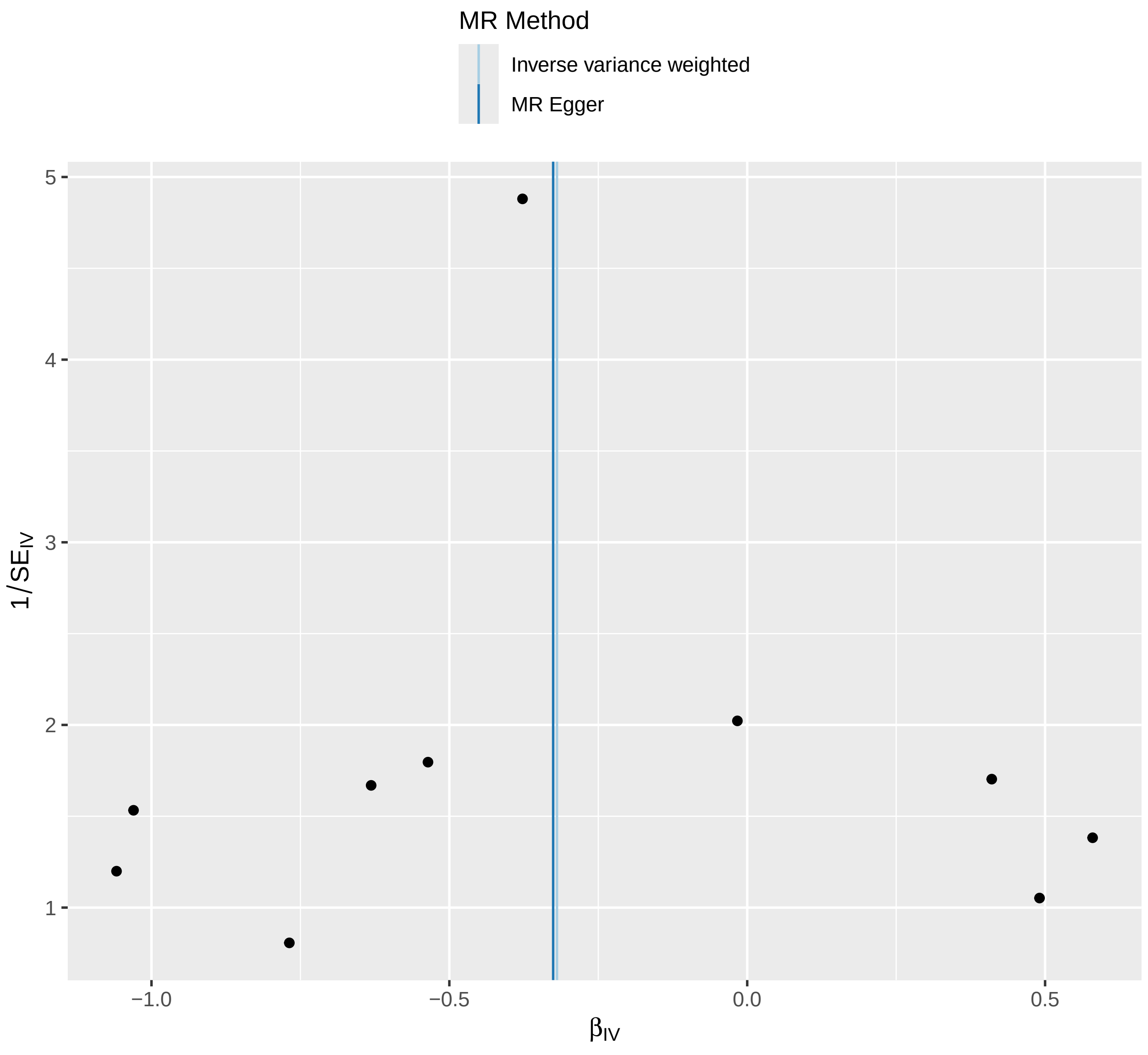

MR Method
Inverse variance weighted
MR Egger
5
4
3
2
1
1/SE_IV
−1.0
−0.5
0.0
0.5
β_IV

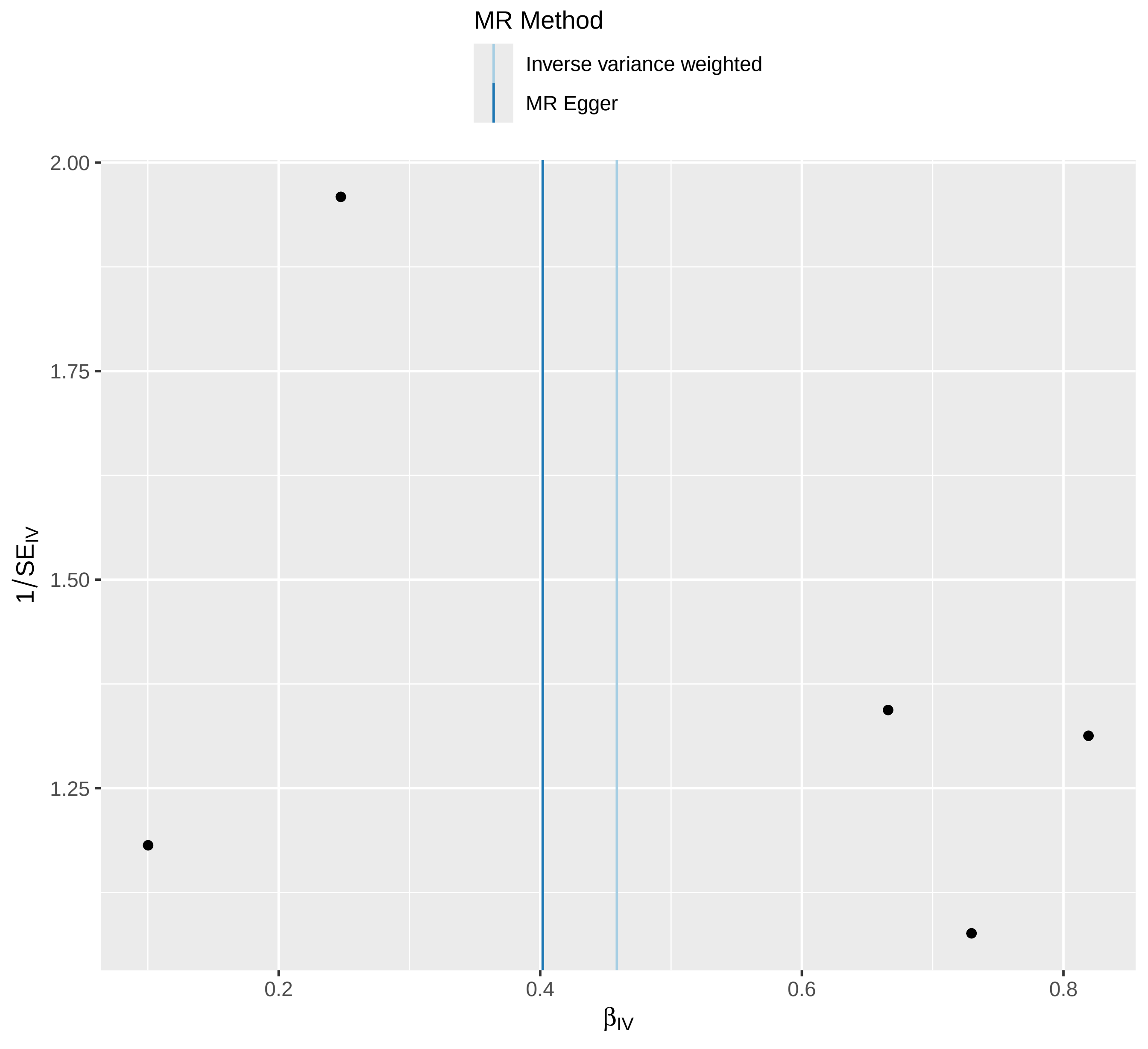
MR Method
Inverse variance weighted
MR Egger
2.00
1.75
1.50
1.25
1/SE_IV
0.2
0.4
0.6
0.8
β_IV

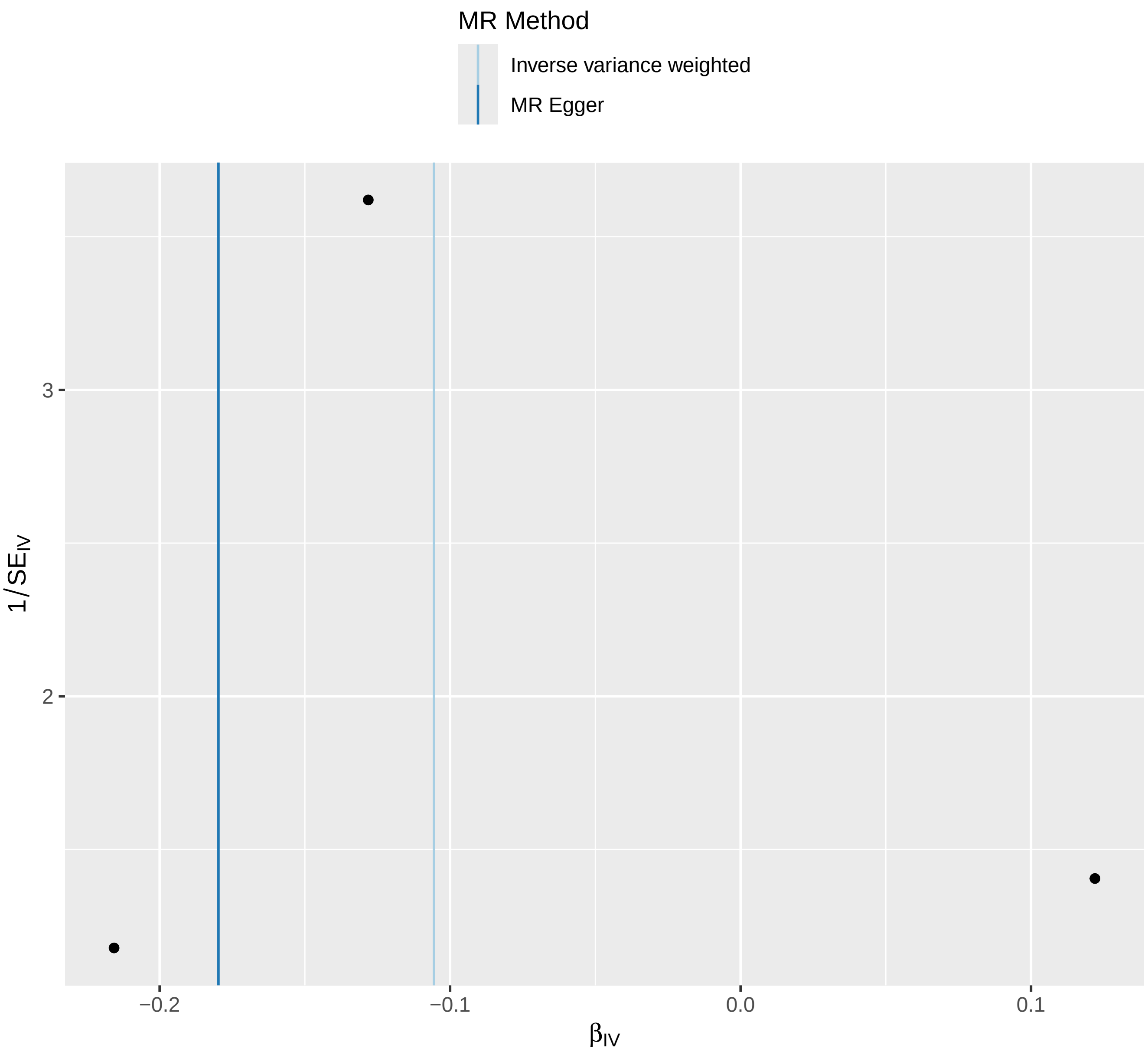

MR Method
Inverse variance weighted
MR Egger
3
2
1/SE$_{IV}$
−0.2
−0.1
0.0
0.1
β$_{IV}$

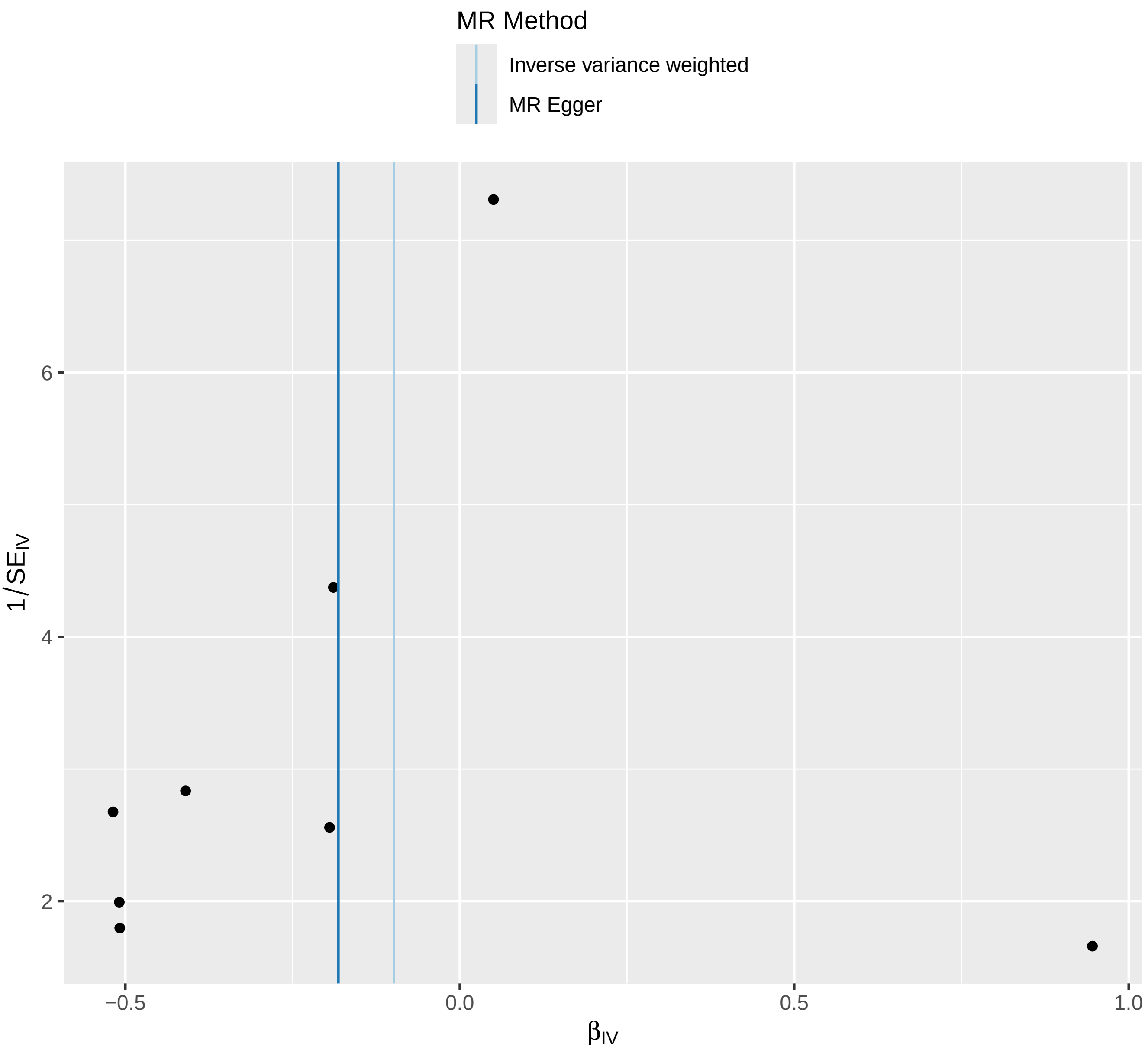

MR Method
Inverse variance weighted
MR Egger
1/SE_IV
6
4
2
−0.5
0.0
0.5
1.0
β_IV

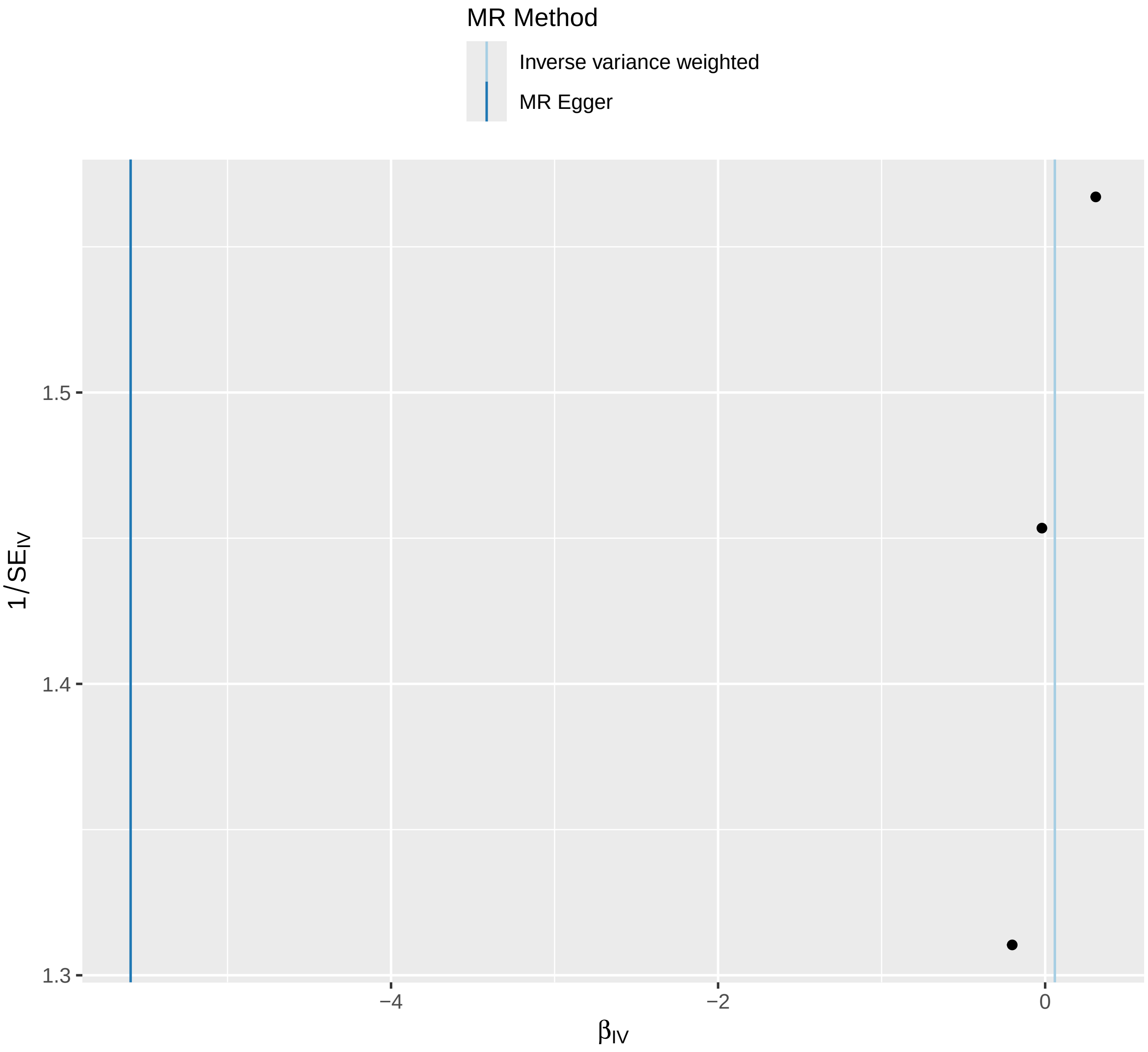
MR Method
Inverse variance weighted
MR Egger
1.5
1.4
1.3
1/SE_IV
−4
−2
0
β_IV

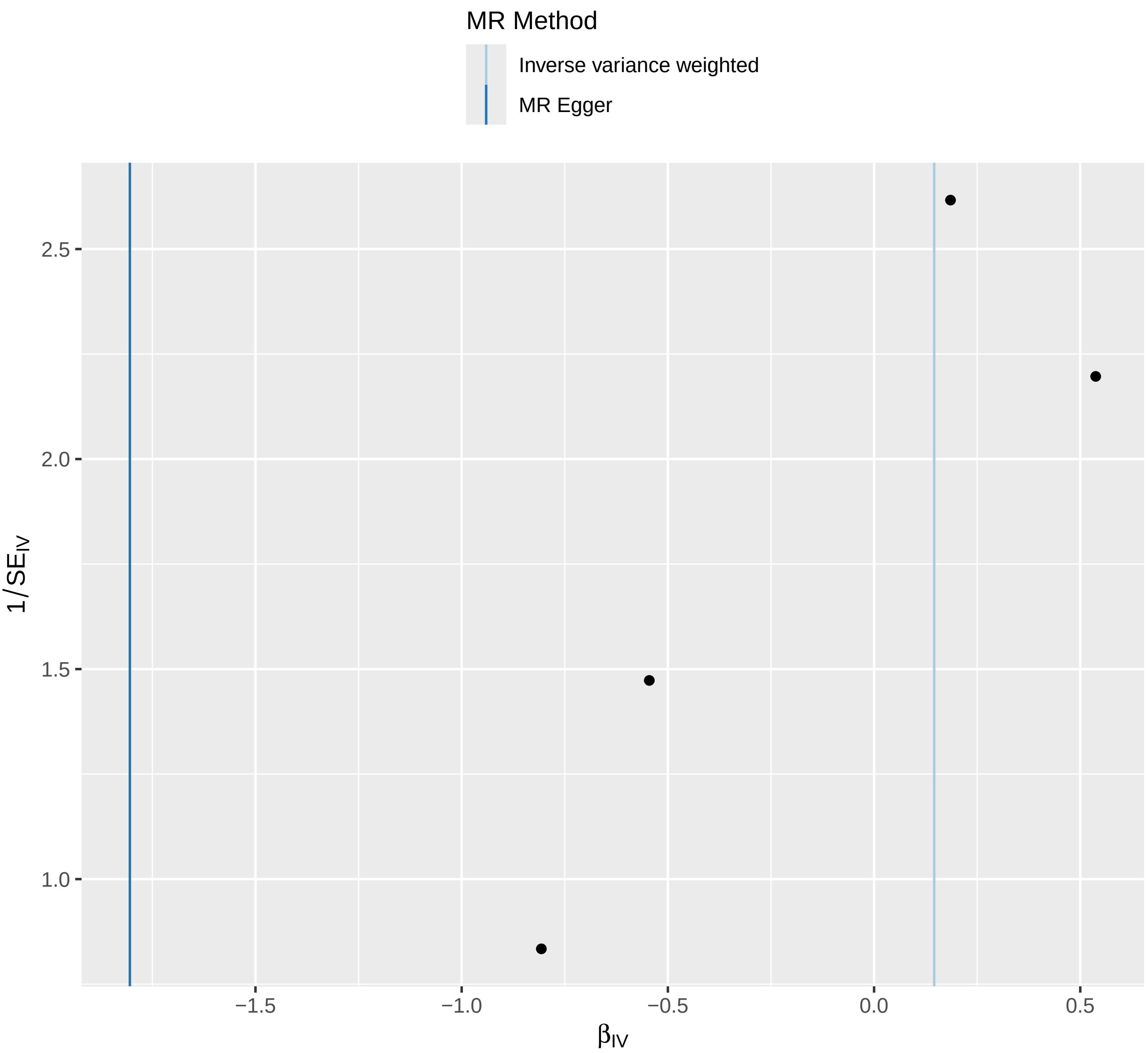
MR Method
Inverse variance weighted
MR Egger
2.5
2.0
1.5
1.0
1/SE$_{IV}$
−1.5
−1.0
−0.5
0.0
0.5
β$_{IV}$

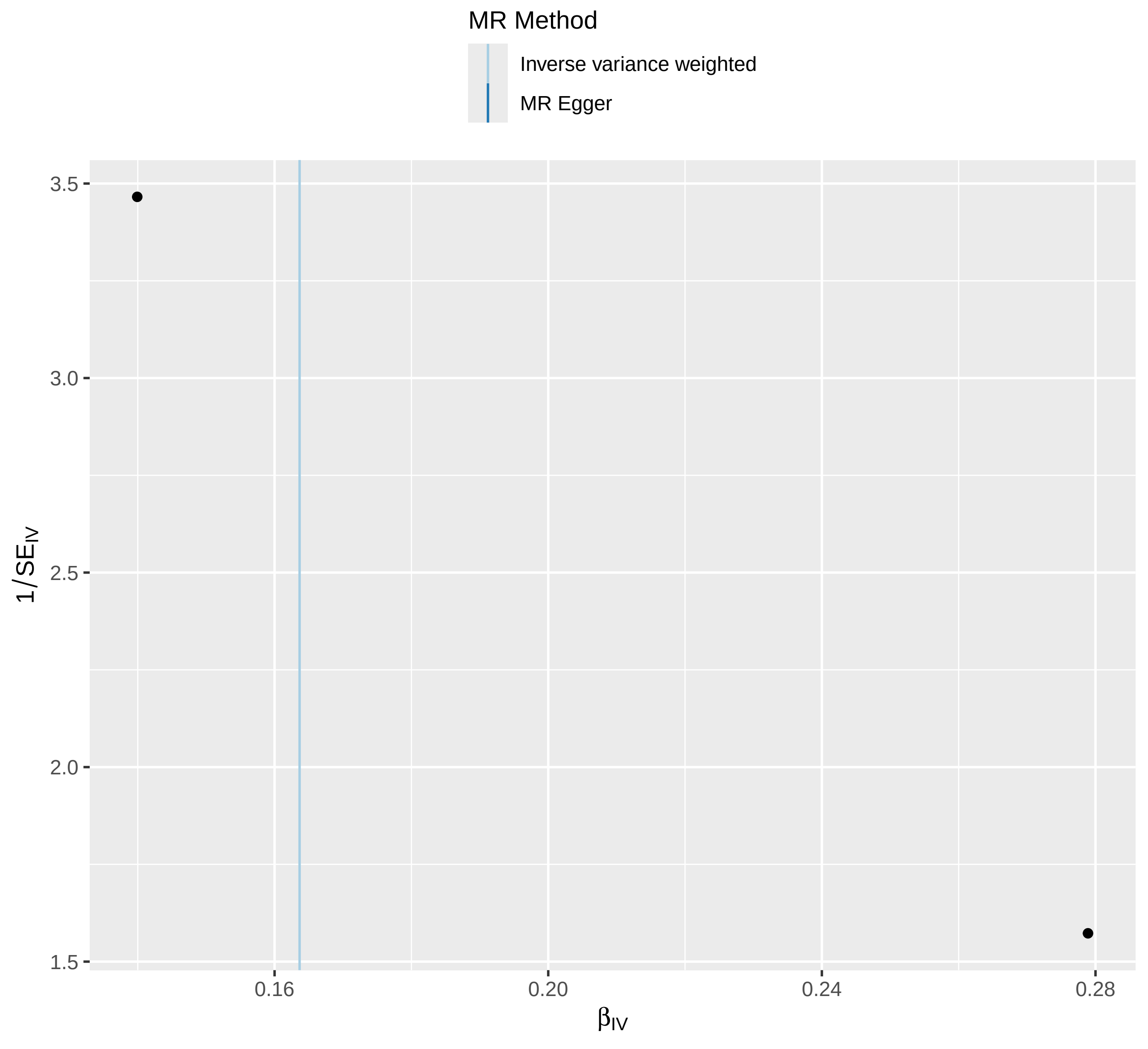
MR Method
Inverse variance weighted
MR Egger
3.5
3.0
2.5
2.0
1.5
$1/SE_{IV}$
0.16
0.20
0.24
0.28
β_{IV}

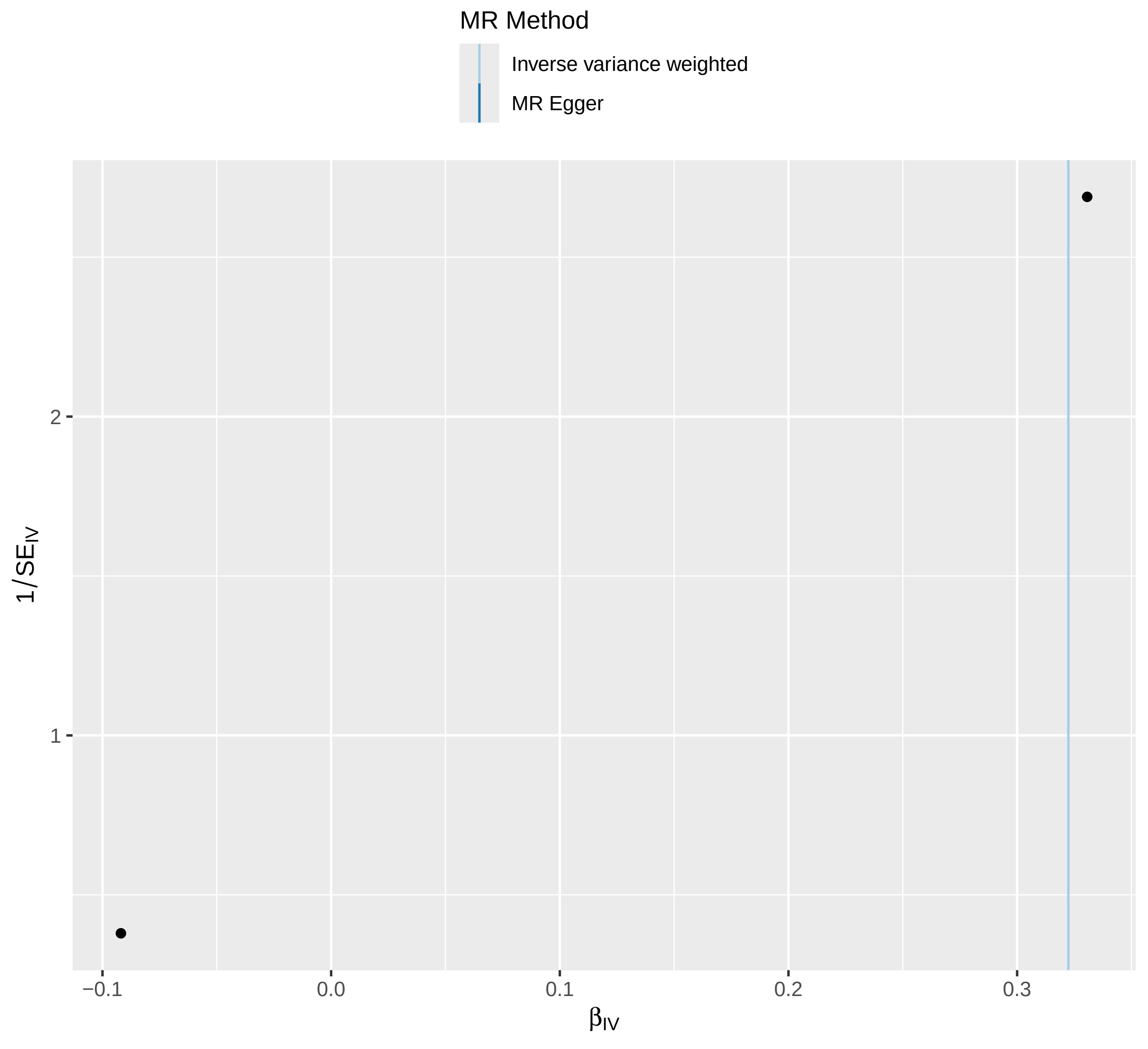

MR Method
Inverse variance weighted
MR Egger
2
1
1/SE_IV
−0.1
0.0
0.1
0.2
0.3
β_IV

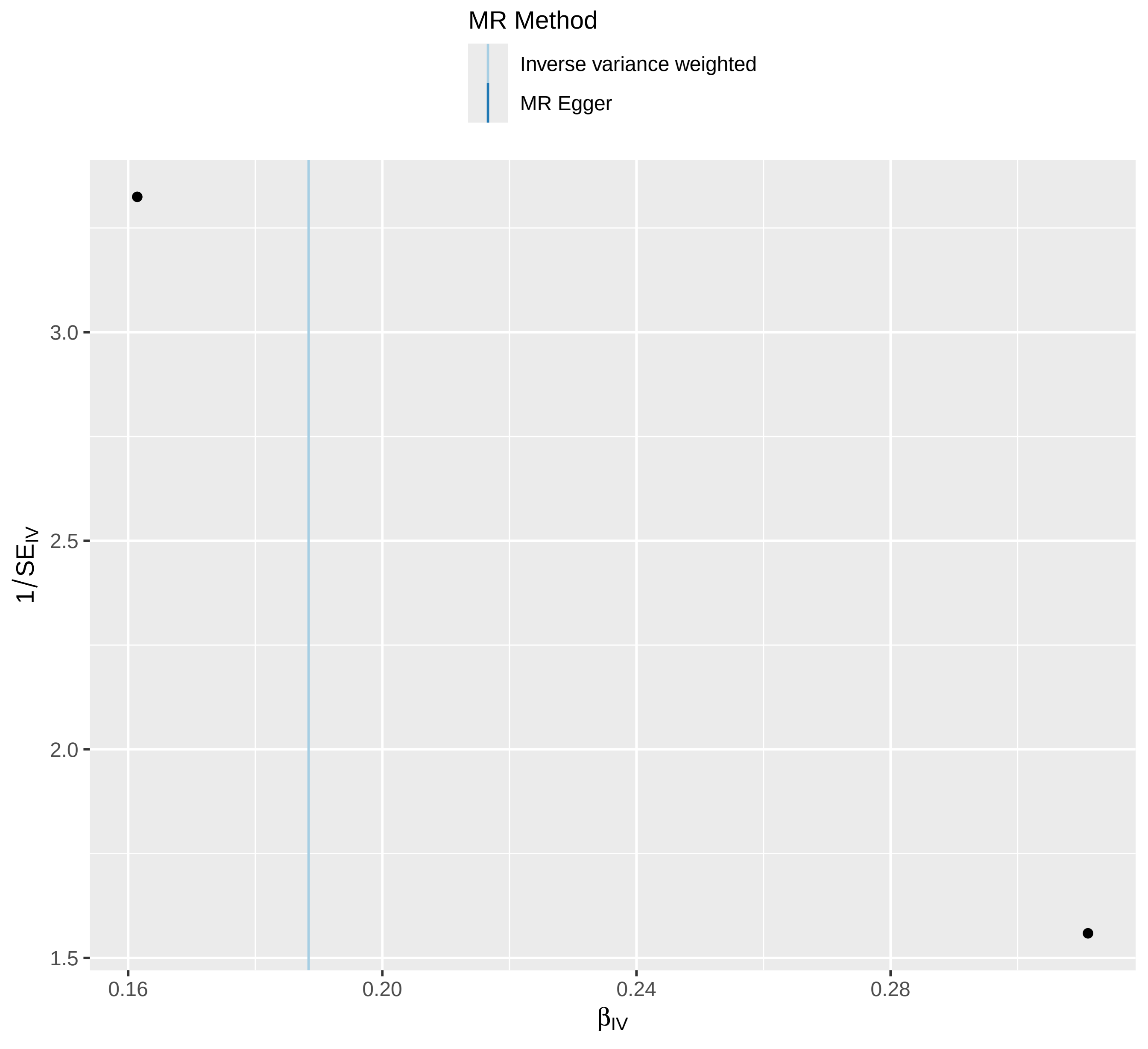
MR Method
Inverse variance weighted
MR Egger
3.0
2.5
2.0
1.5
$1/SE_{IV}$
0.16
0.20
0.24
0.28
β_{IV}

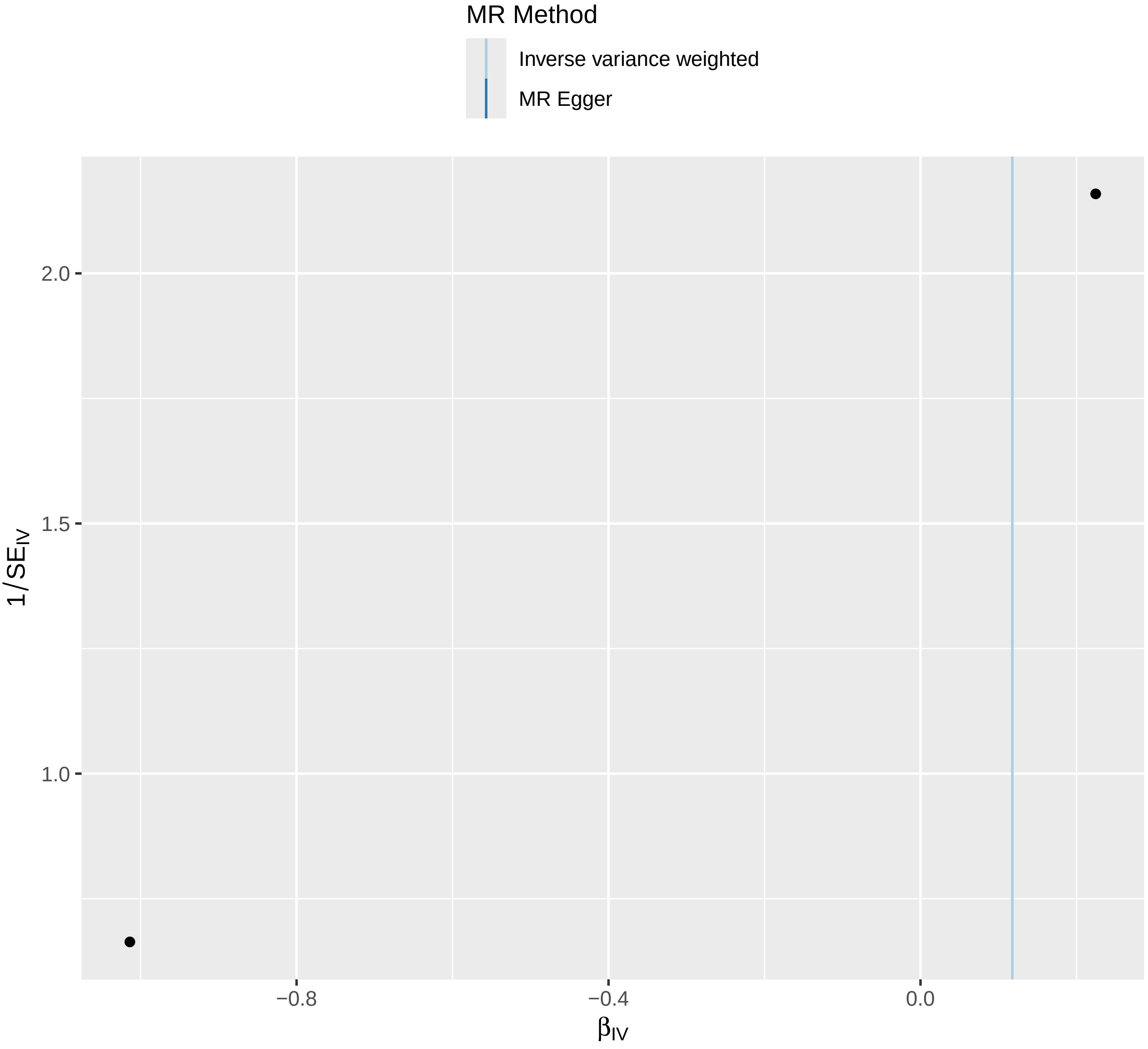

MR Method
Inverse variance weighted
MR Egger
2.0
1.5
1.0
$1/SE_{IV}$
−0.8
−0.4
0.0
β_{IV}

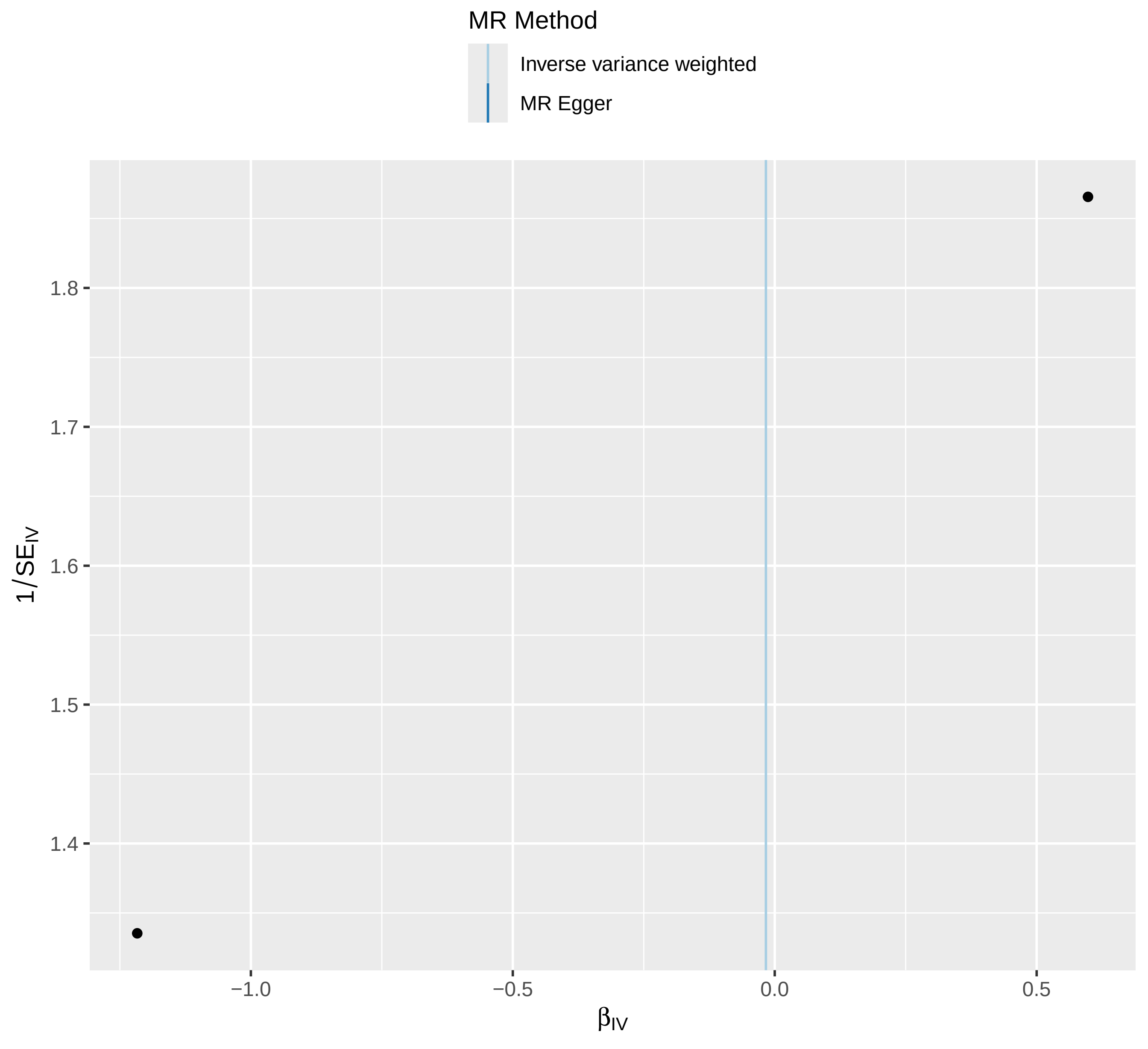
MR Method
Inverse variance weighted
MR Egger
1/SE_IV
1.8
1.7
1.6
1.5
1.4
−1.0
−0.5
0.0
0.5
β_IV

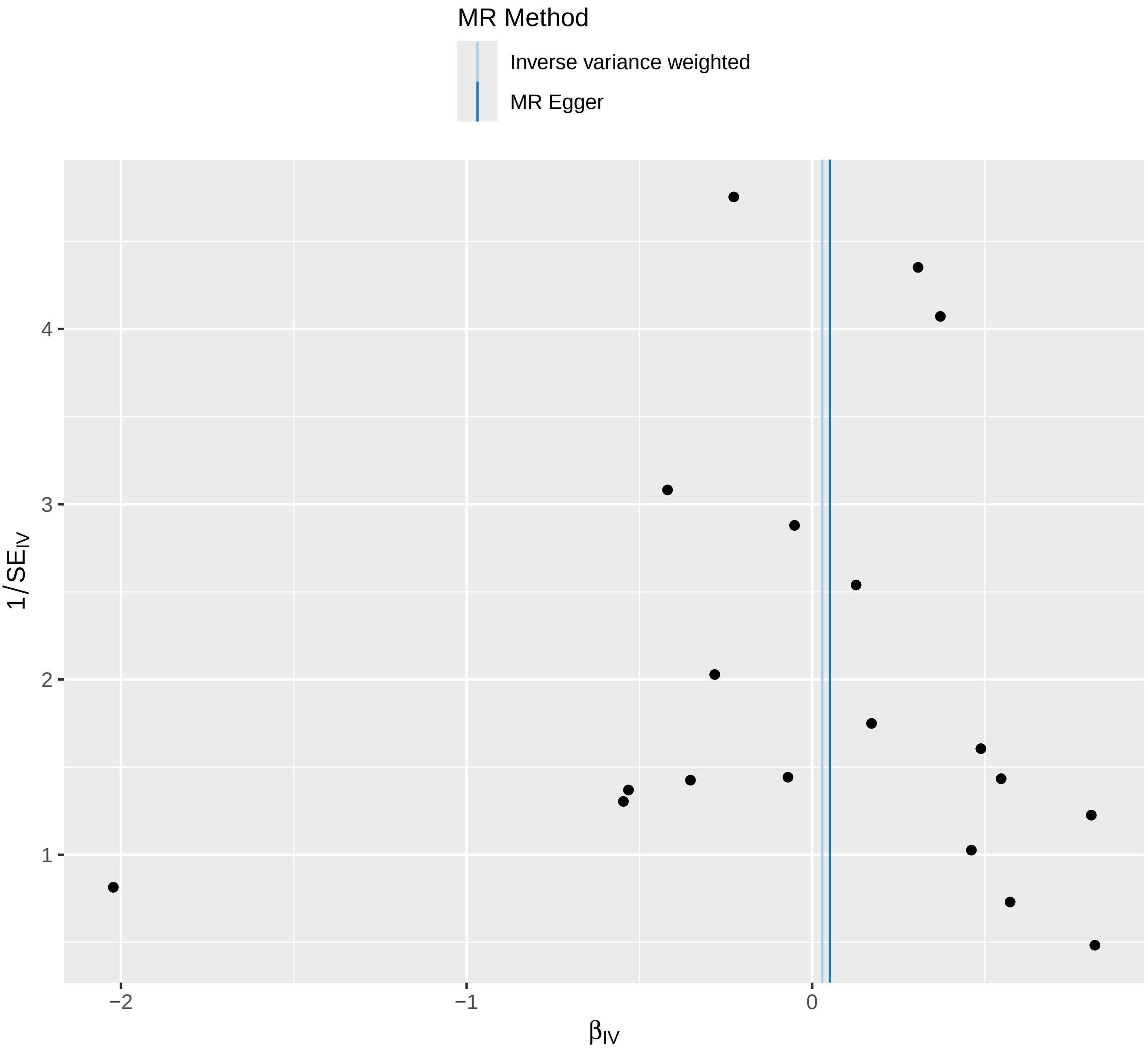

MR Method
Inverse variance weighted
MR Egger
1/SE_IV
4
3
2
1
−2
−1
0
β_IV

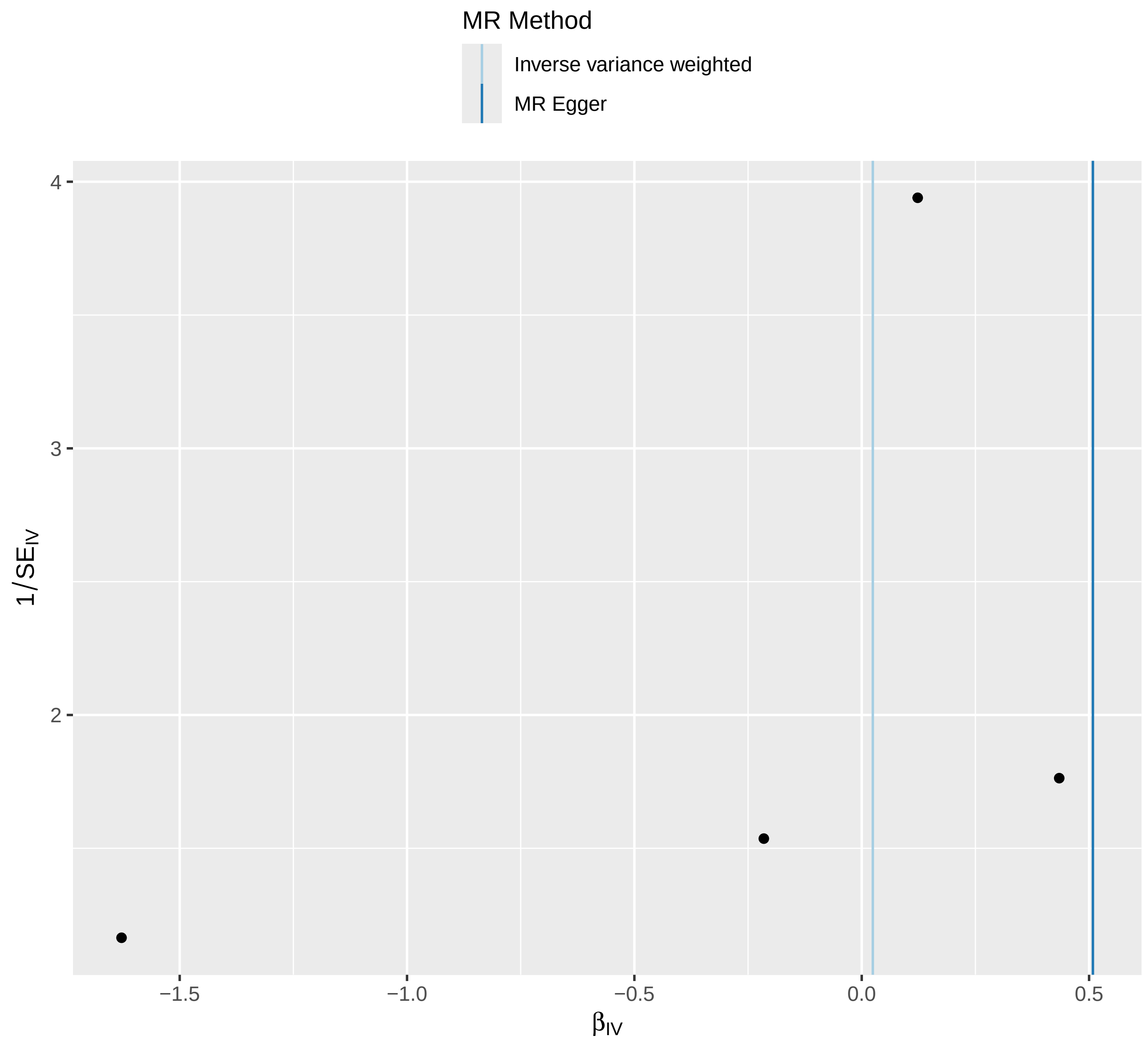

MR Method
Inverse variance weighted
MR Egger
4
3
2
1/SE_IV
−1.5
−1.0
−0.5
0.0
0.5
β_IV

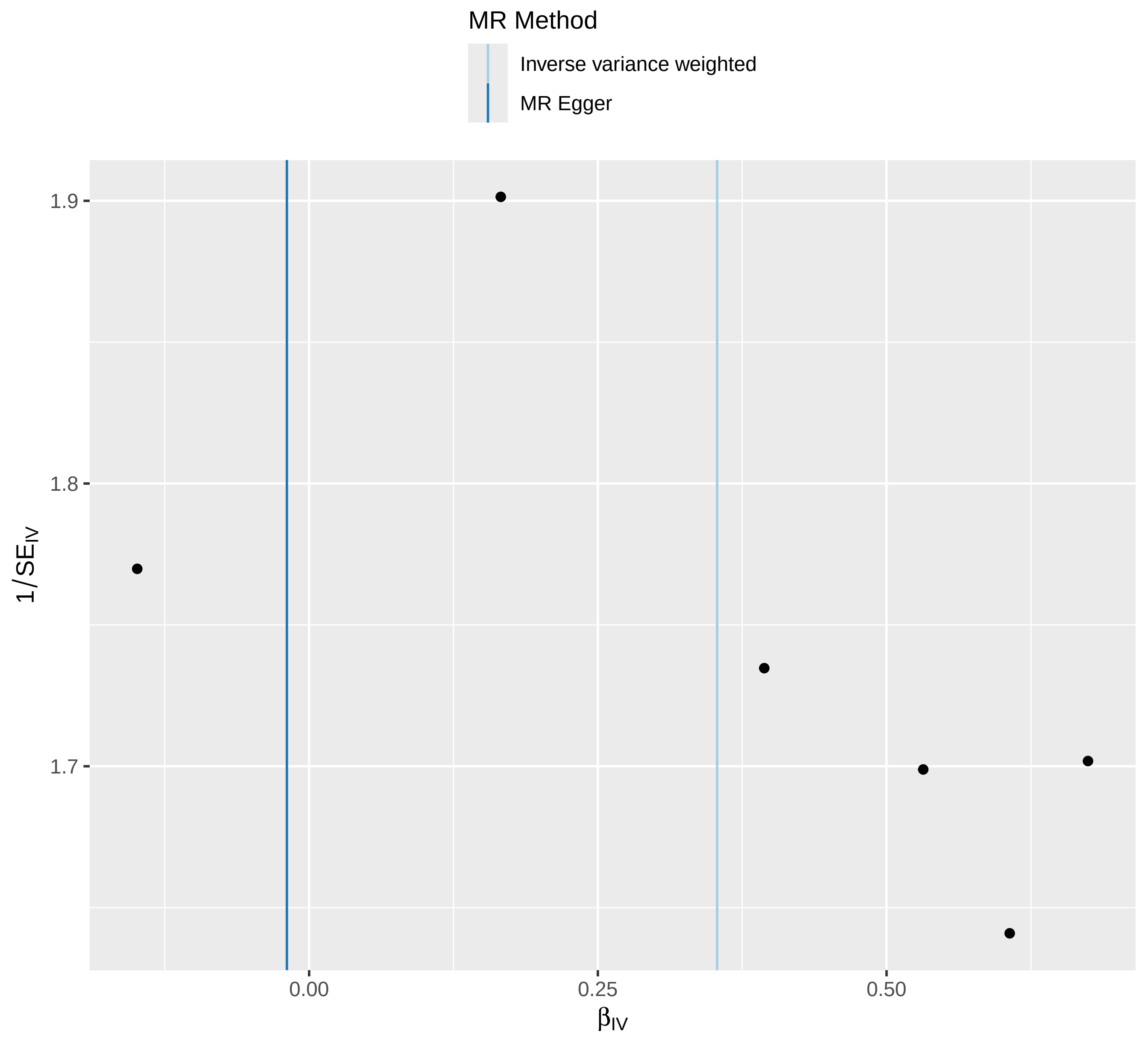

MR Method
Inverse variance weighted
MR Egger
1.9
1.8
1.7
$1/SE_{IV}$
0.00
0.25
0.50
β_{IV}

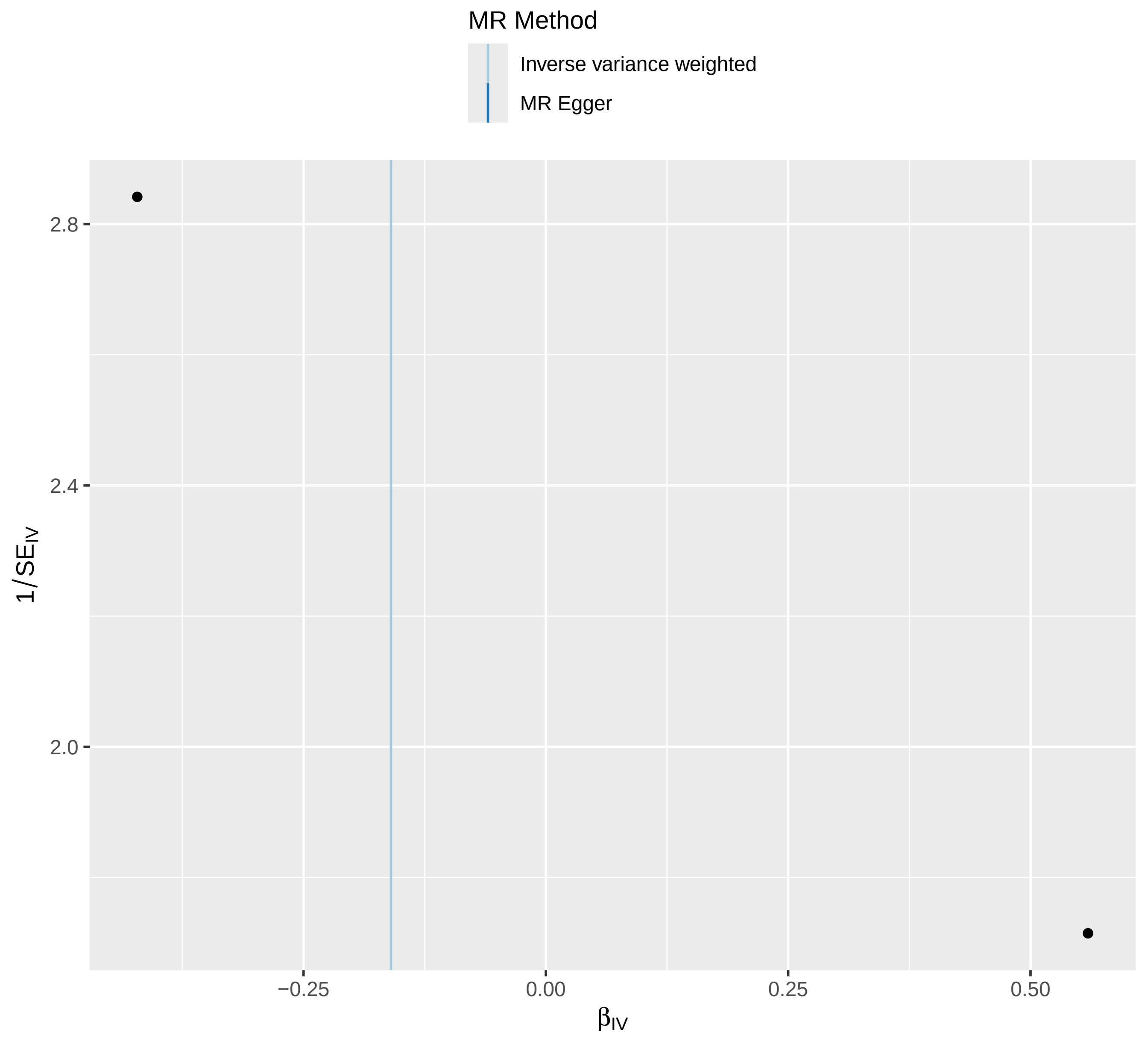

MR Method
Inverse variance weighted
MR Egger
2.8
2.4
2.0
1/SE$_{IV}$
−0.25
0.00
0.25
0.50
β$_{IV}$

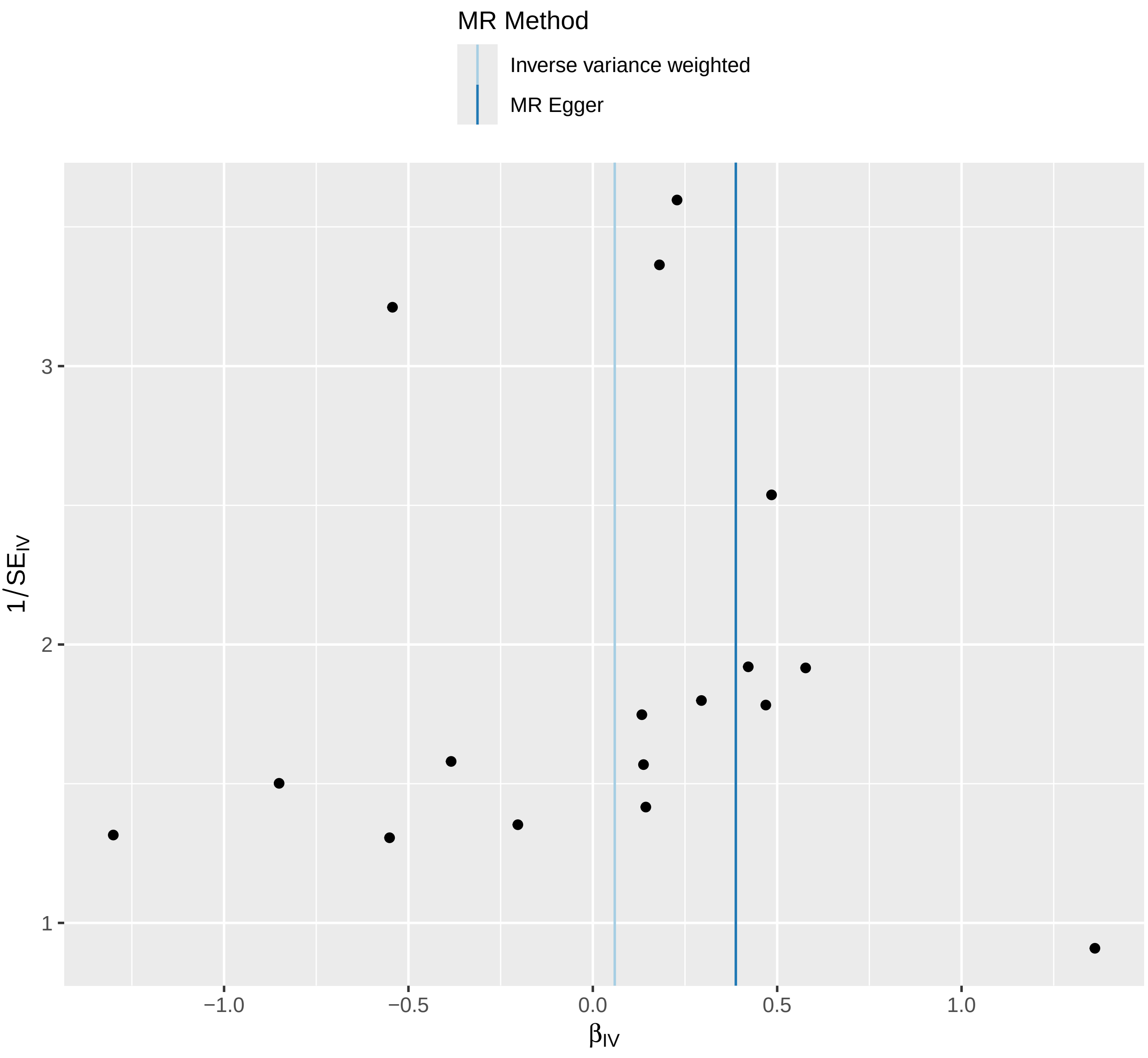
MR Method
Inverse variance weighted
MR Egger
1/SE_IV
3
2
1
−1.0
−0.5
0.0
0.5
1.0
β_IV

Insufficient number of SNPs

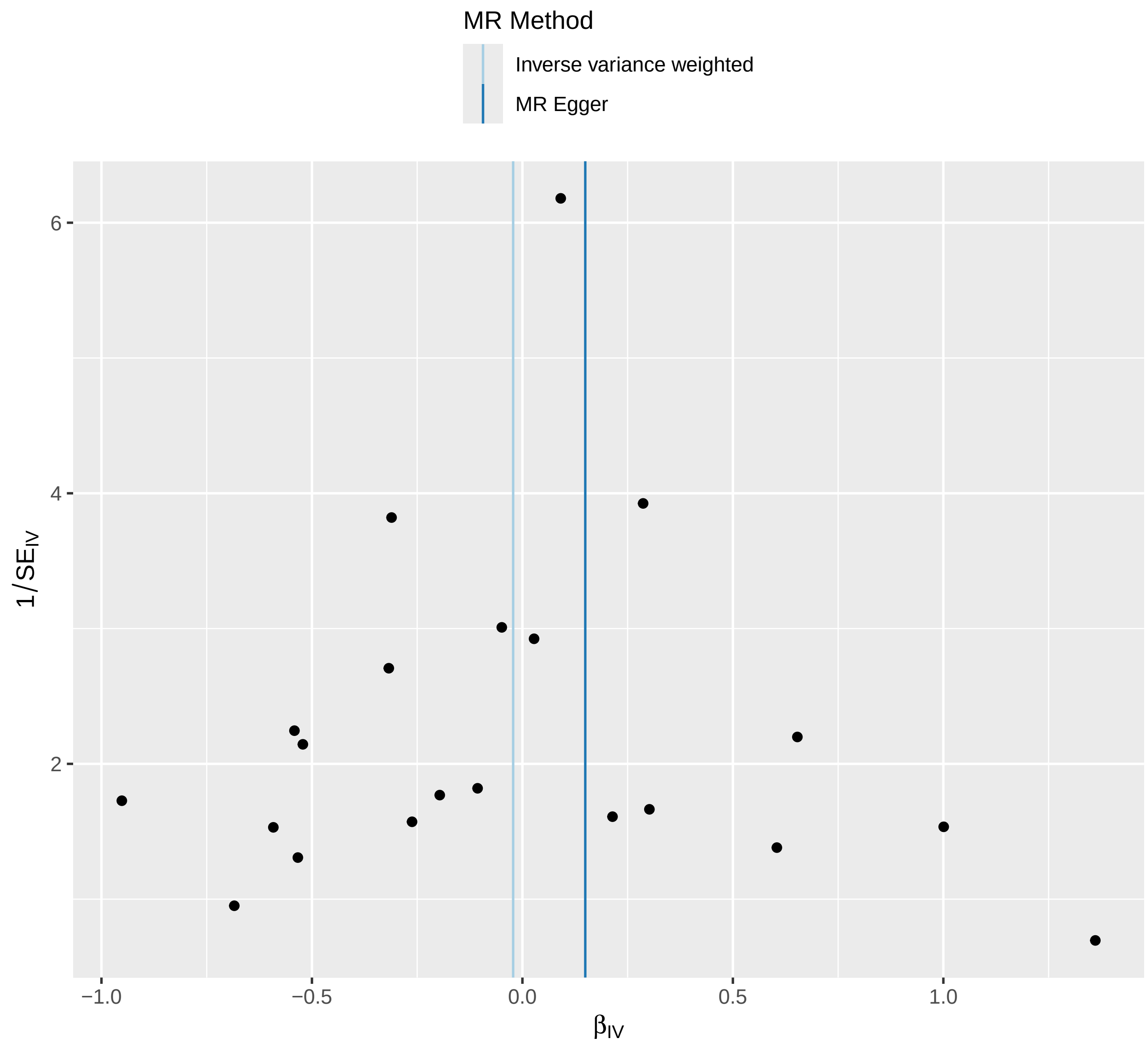
MR Method
Inverse variance weighted
MR Egger
6
4
2
1/SE_IV
−1.0
−0.5
0.0
0.5
1.0
β_IV

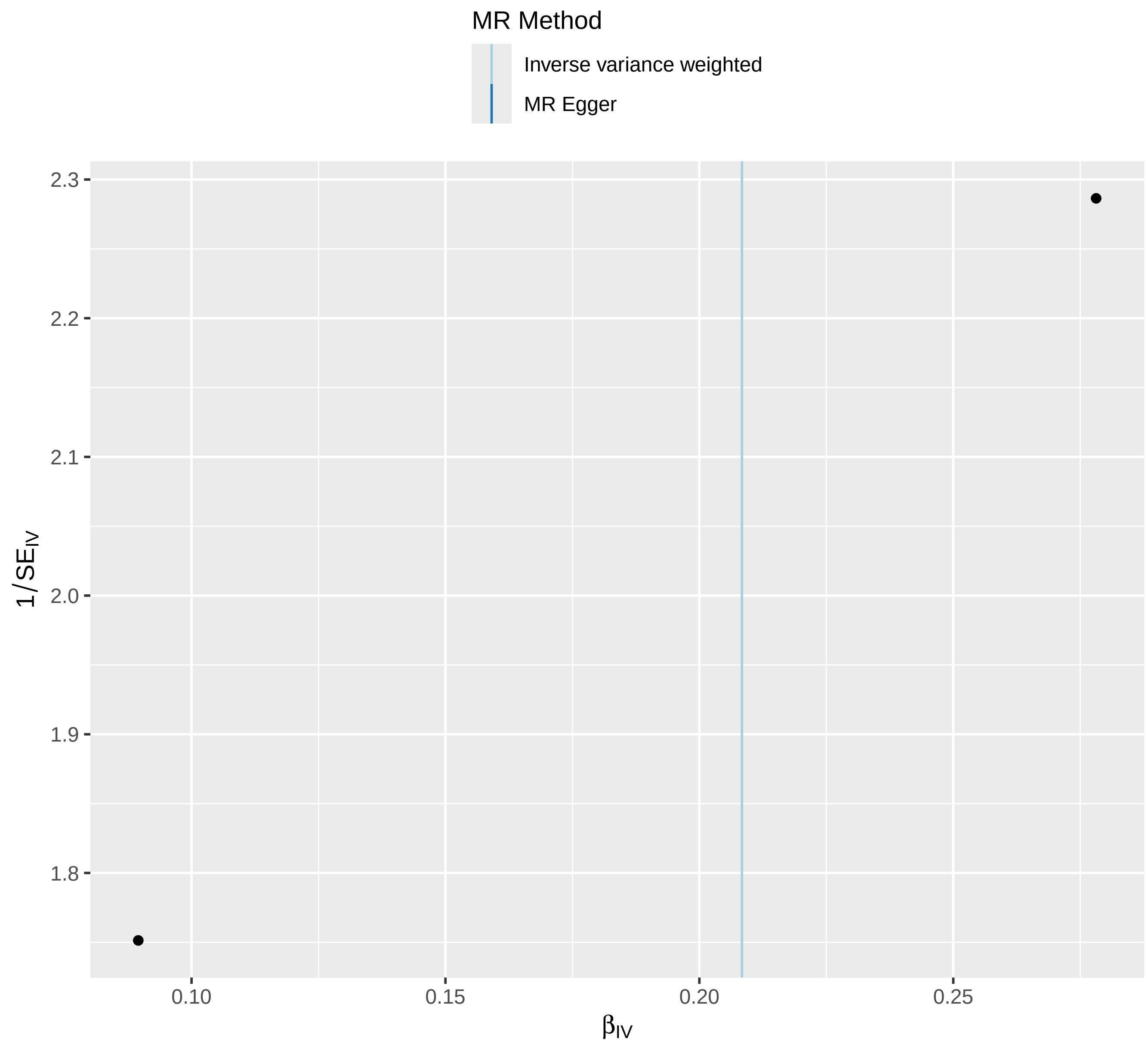
MR Method
Inverse variance weighted
MR Egger
2.3
2.2
2.1
2.0
1.9
1.8
1/SE_IV
0.10
0.15
0.20
0.25
β_IV

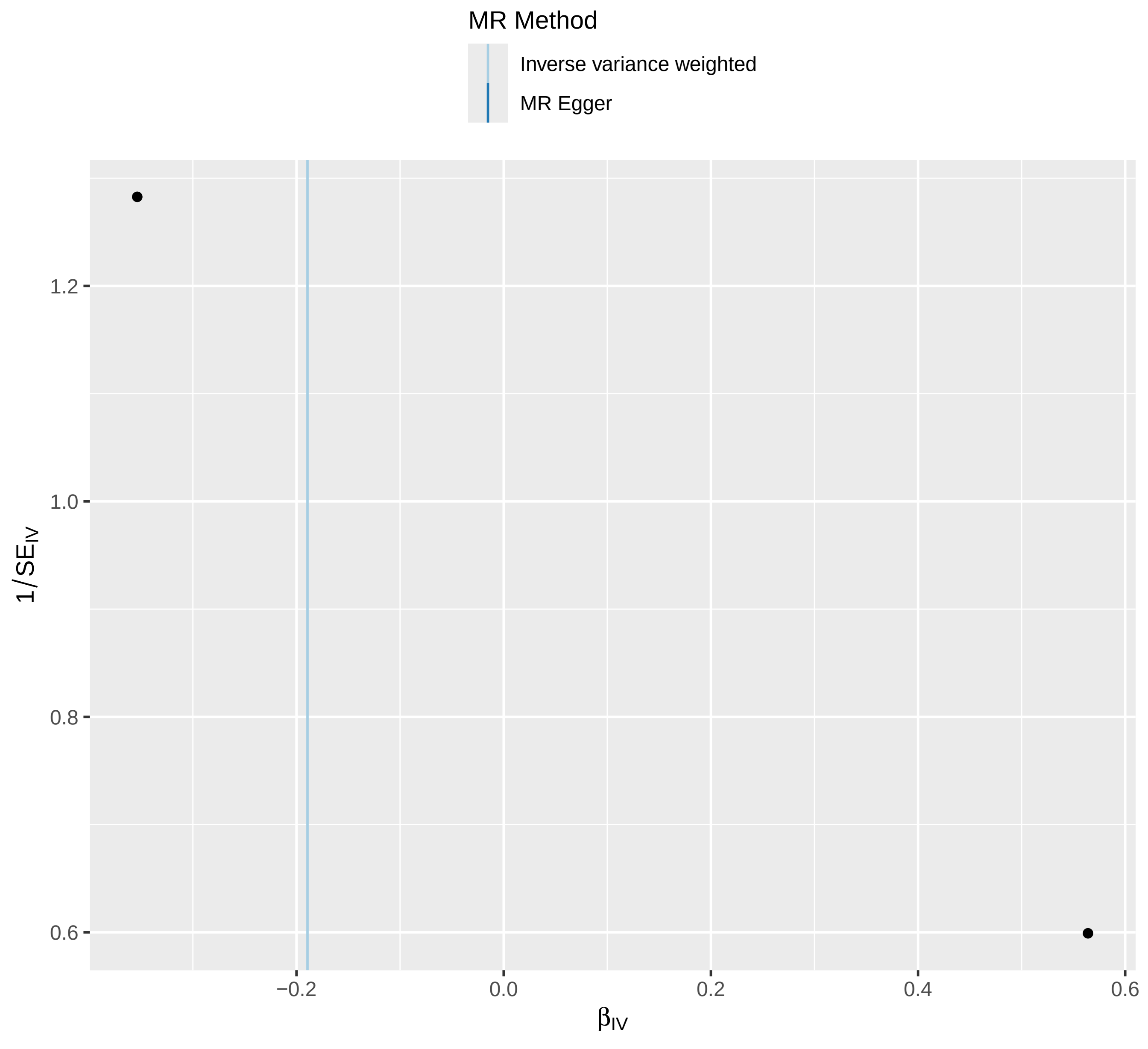

MR Method
Inverse variance weighted
MR Egger
1.2
1.0
0.8
0.6
1/SE_IV
−0.2
0.0
0.2
0.4
0.6
β_IV

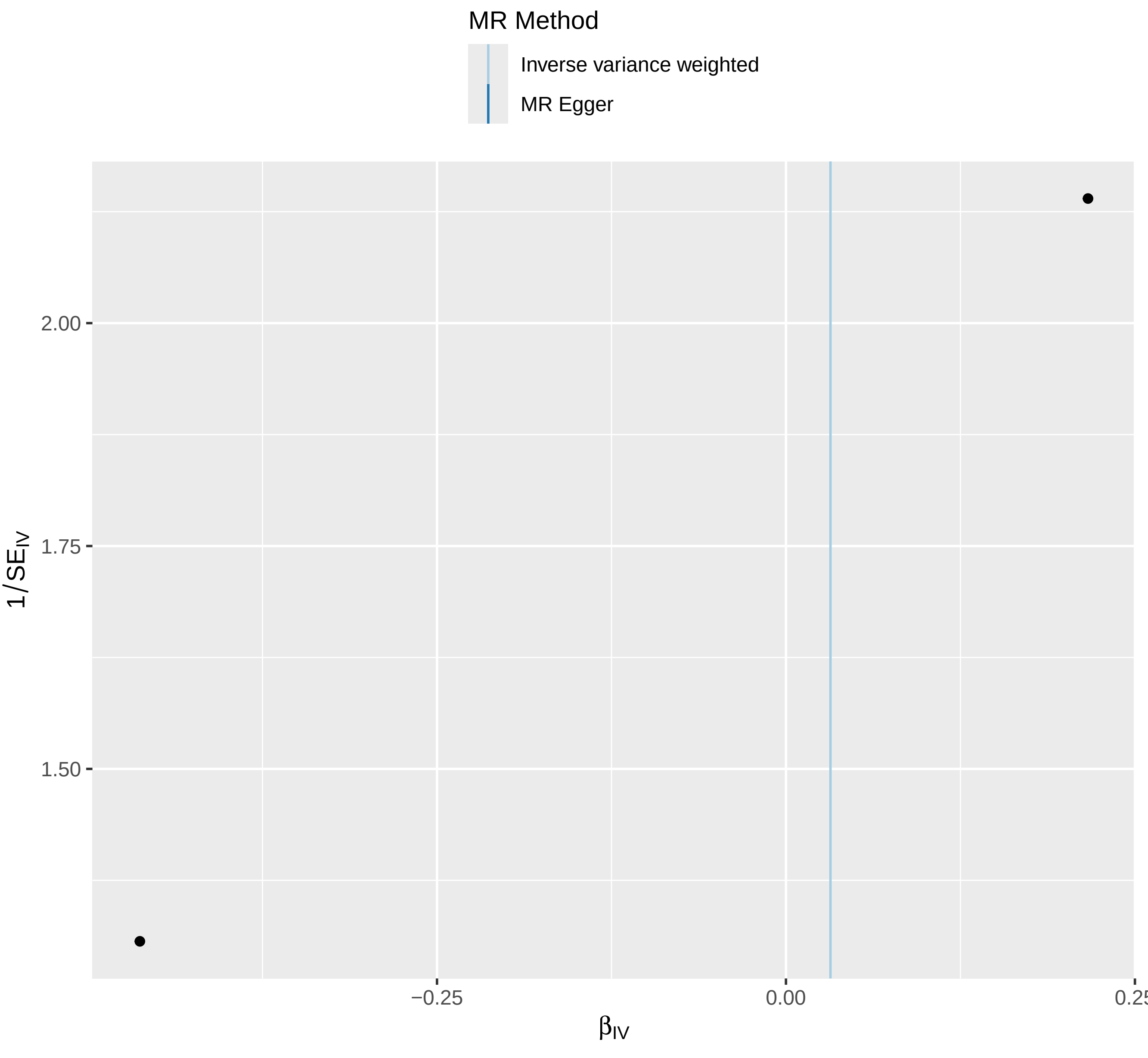
MR Method
Inverse variance weighted
MR Egger
2.00
1.75
1.50
1/SE_IV
−0.25
0.00
0.25
β_IV

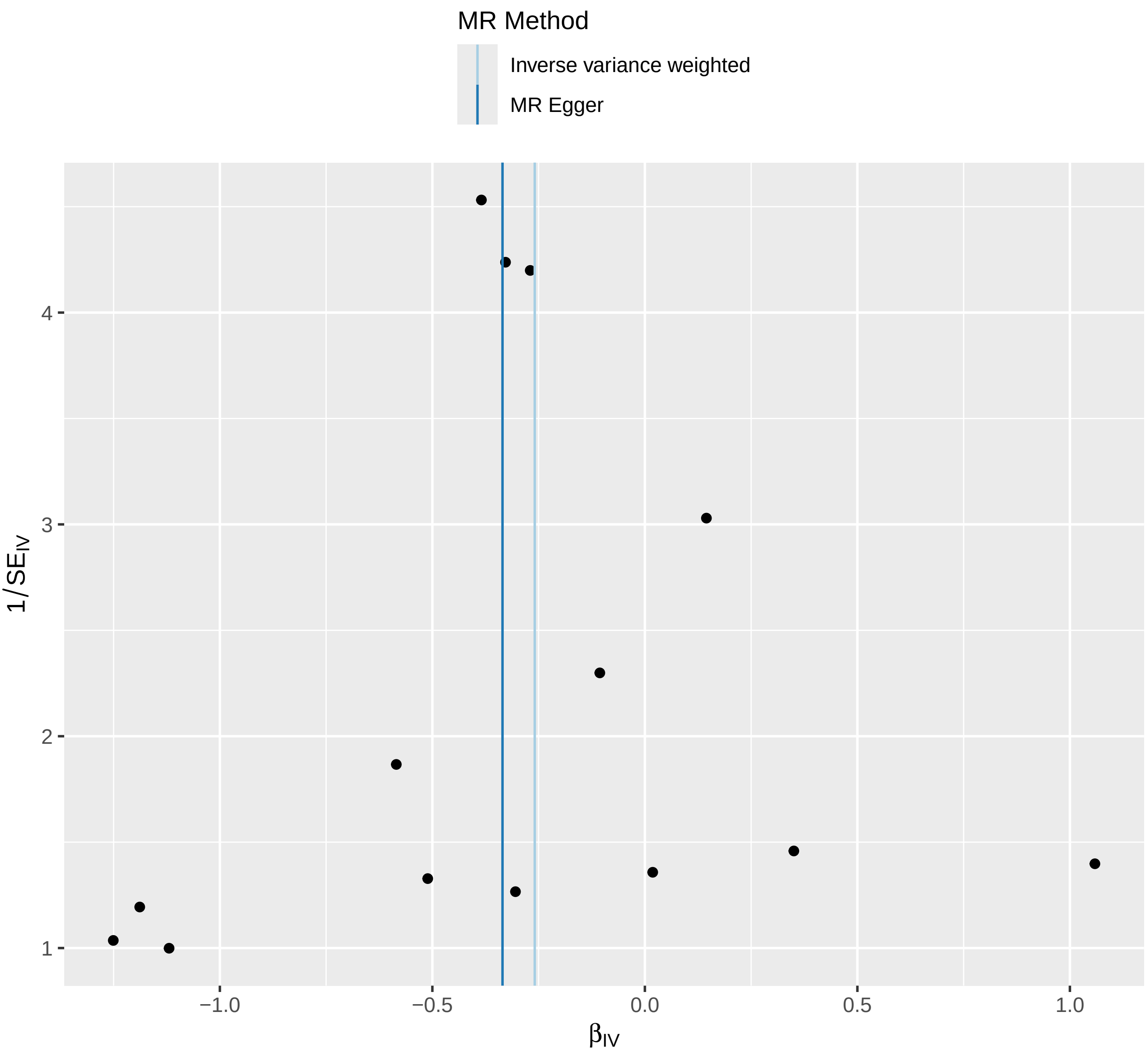

MR Method
Inverse variance weighted
MR Egger
4
3
2
1
1/SE$_{IV}$
−1.0
−0.5
0.0
0.5
1.0
β$_{IV}$

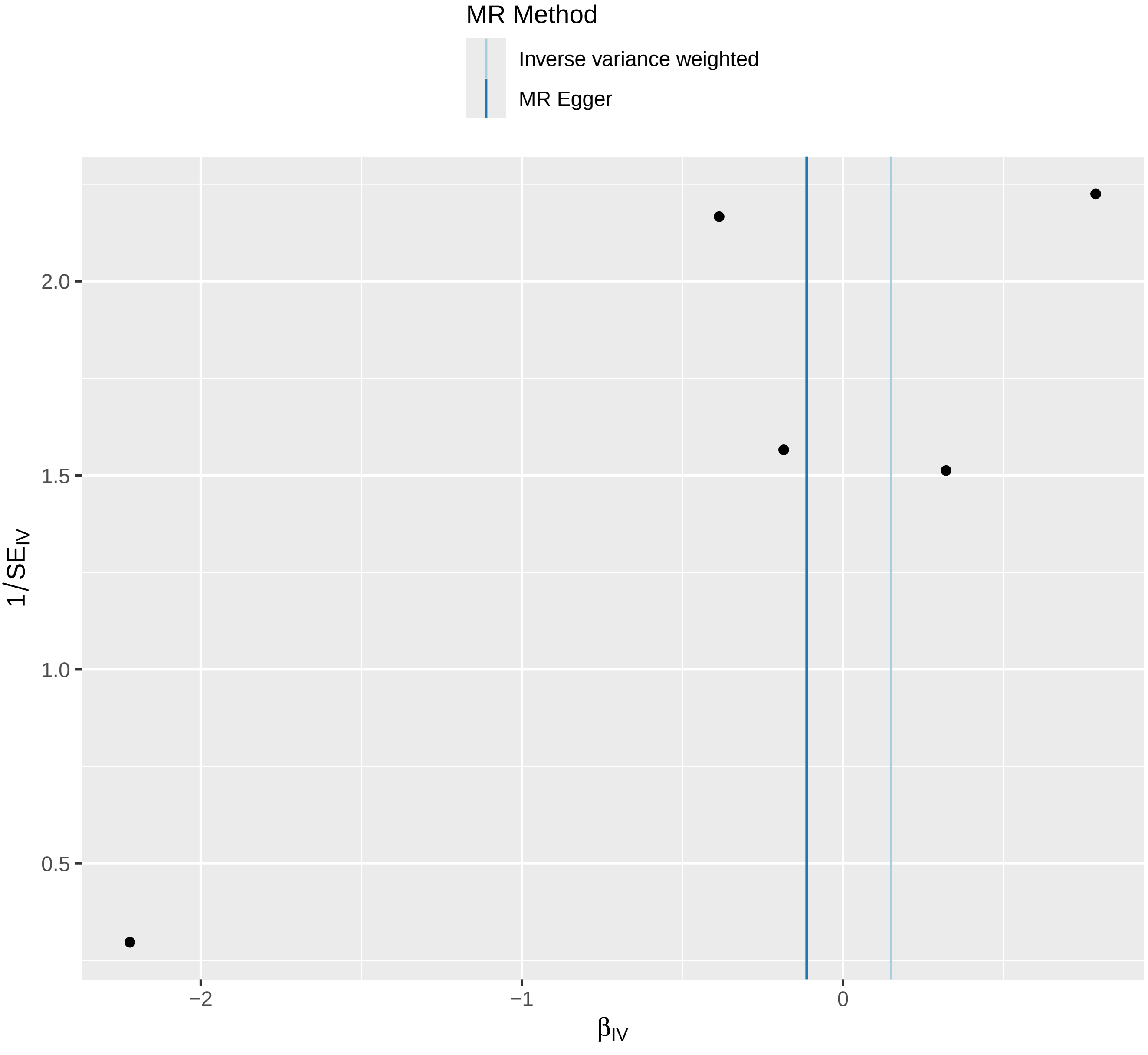

MR Method
Inverse variance weighted
MR Egger
2.0
1.5
1.0
0.5
1/SE_IV
−2
−1
0
β_IV

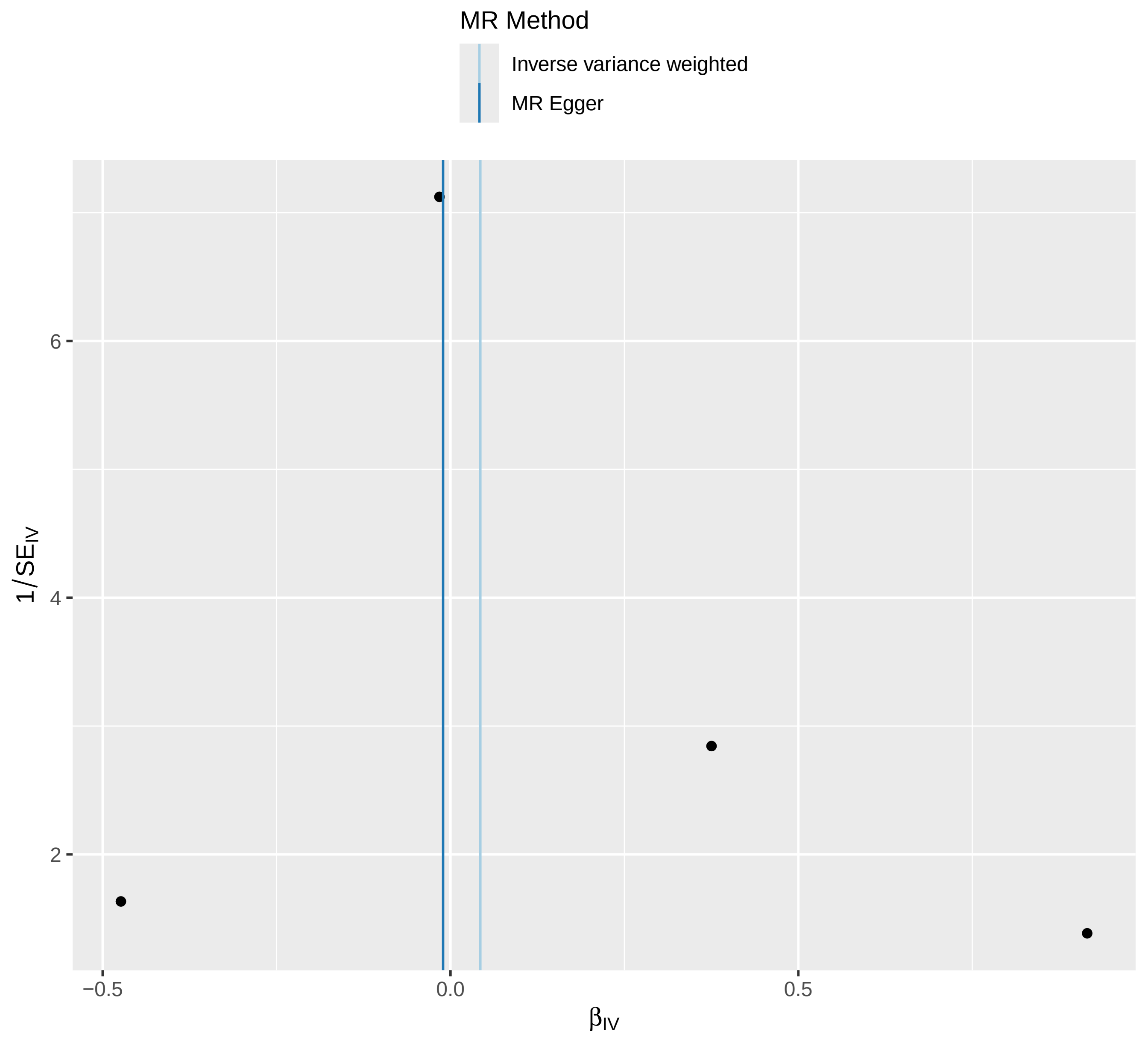
MR Method
Inverse variance weighted
MR Egger
6
4
2
1/SE_IV
−0.5
0.0
0.5
β_IV

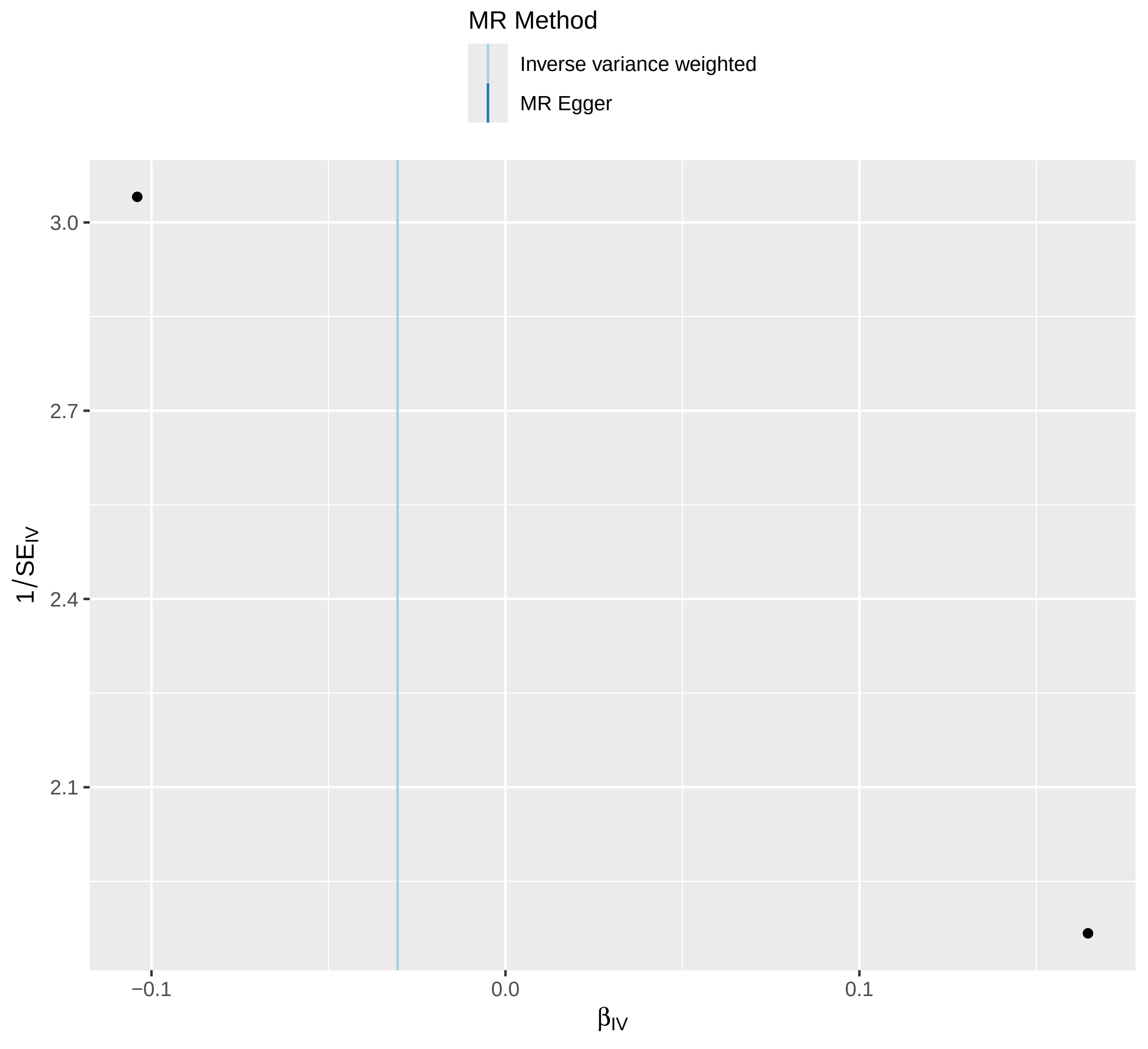

MR Method
Inverse variance weighted
MR Egger
3.0
2.7
2.4
2.1
$1/SE_{IV}$
−0.1
0.0
0.1
β_{IV}

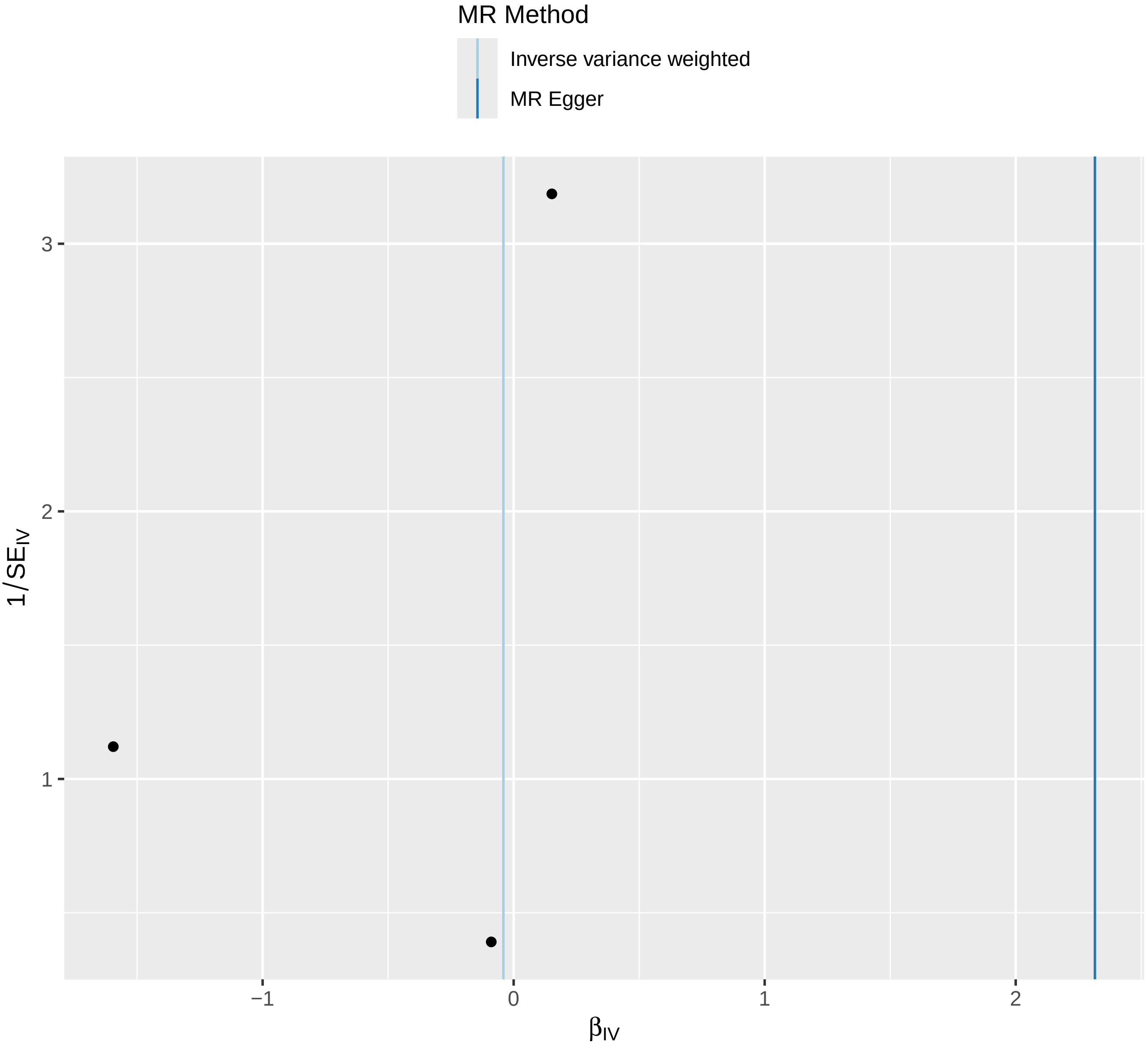
MR Method
Inverse variance weighted
MR Egger
3
2
1
$1/SE_{IV}$
−1
0
1
2
β_{IV}

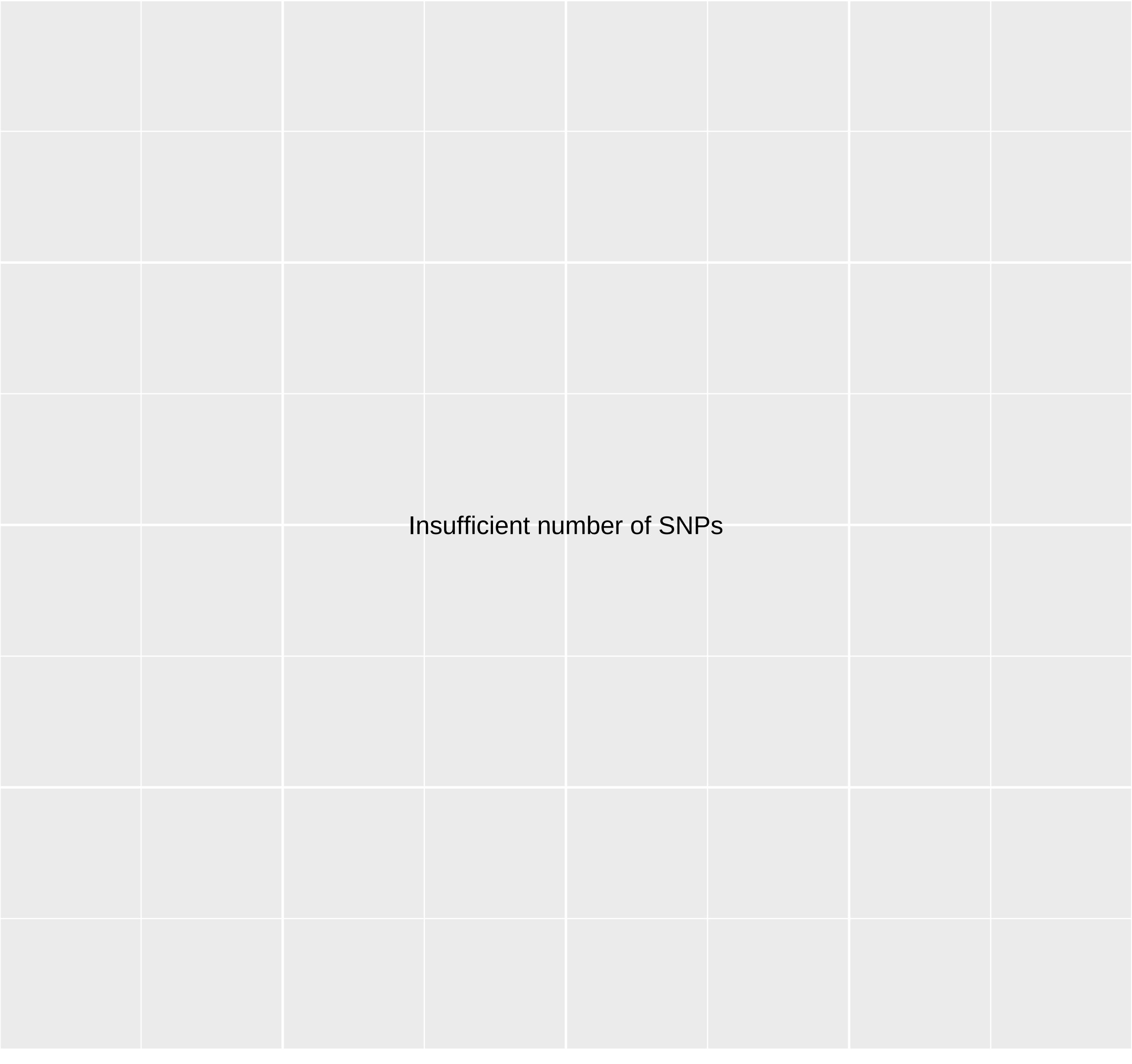
Insufficient number of SNPs

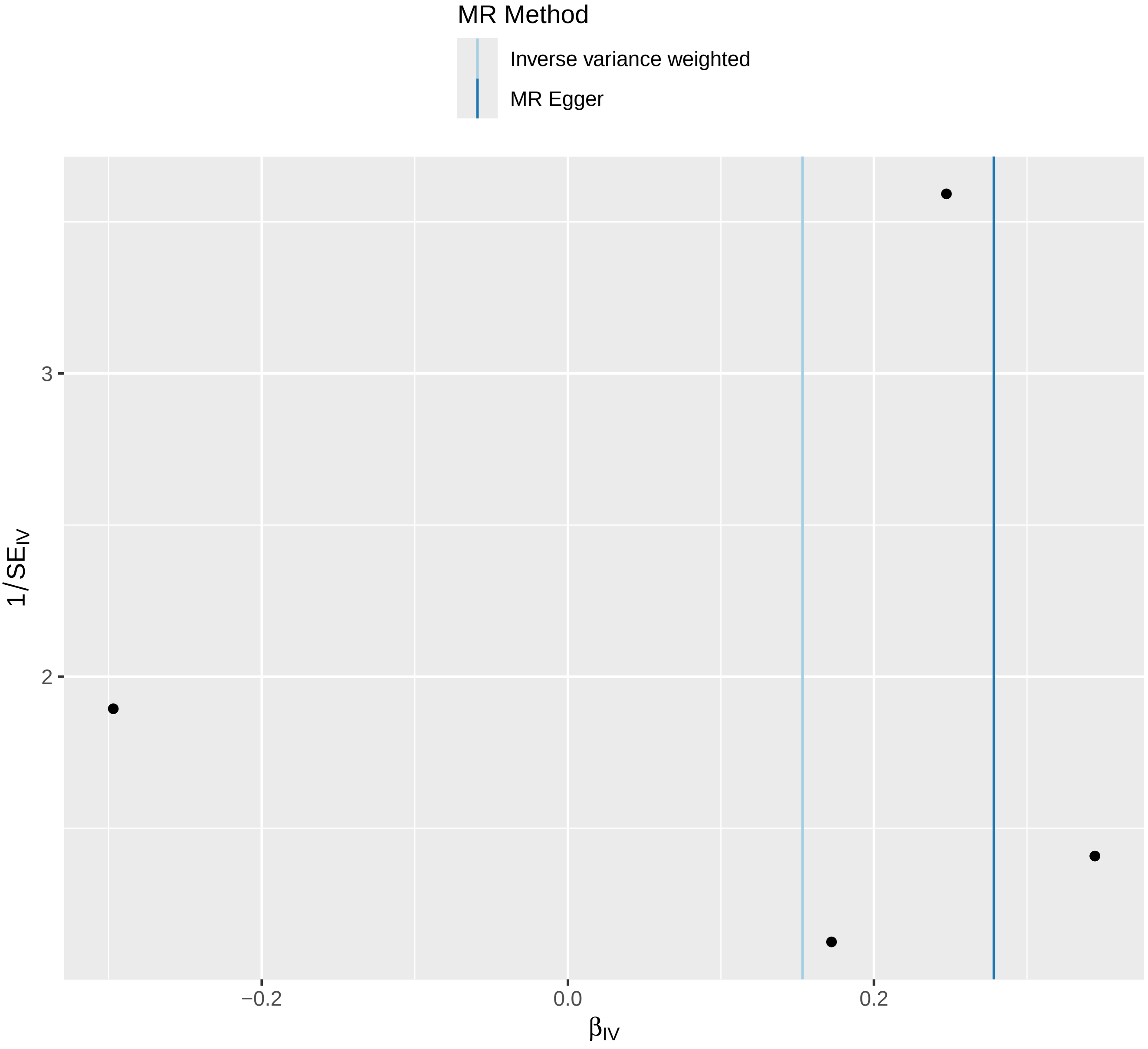
MR Method
Inverse variance weighted
MR Egger
3
2
−0.2
0.0
0.2
$1/SE_{IV}$
β_{IV}

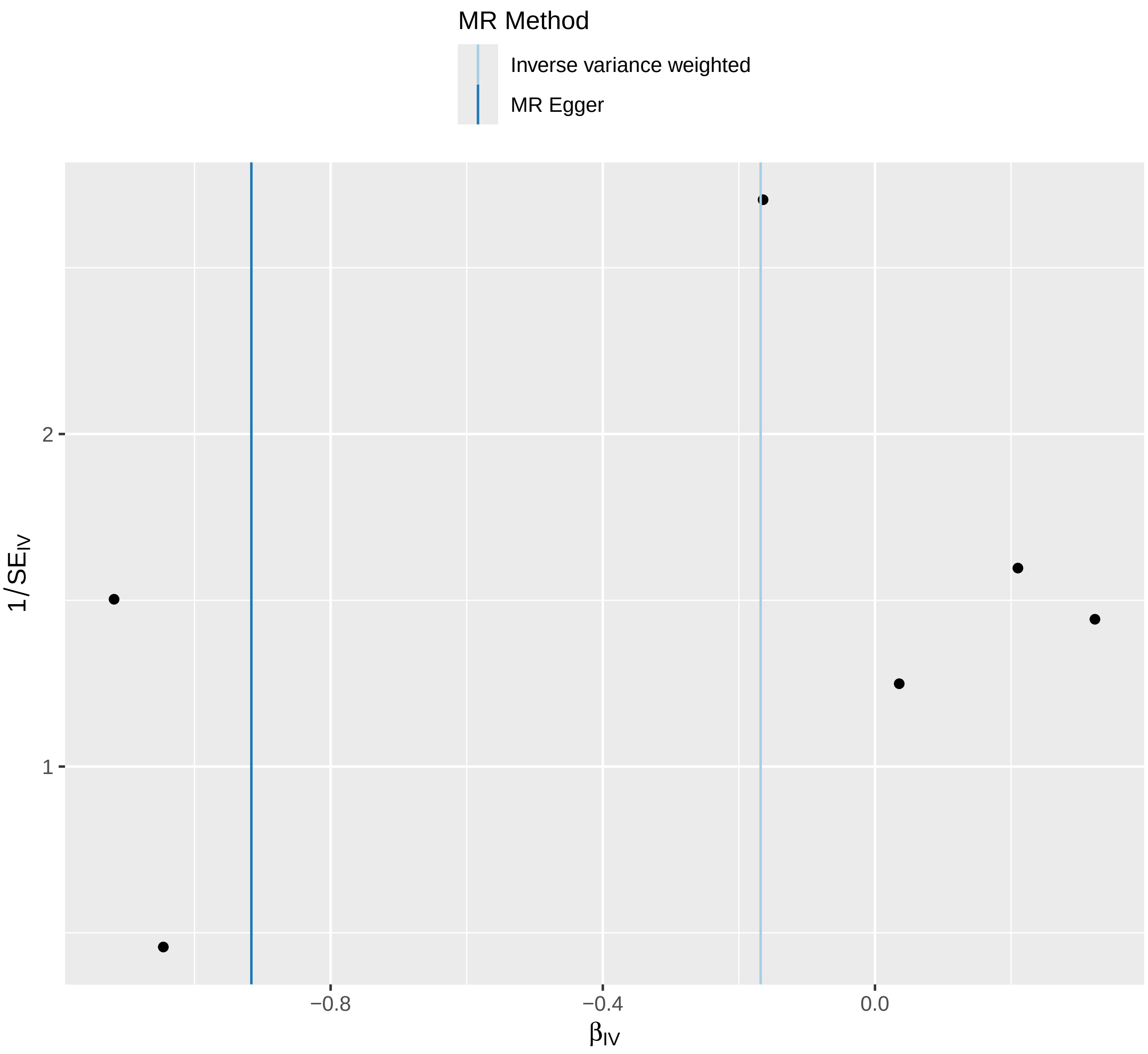

MR Method
Inverse variance weighted
MR Egger
2
1
1/SE_IV
−0.8
−0.4
0.0
β_IV

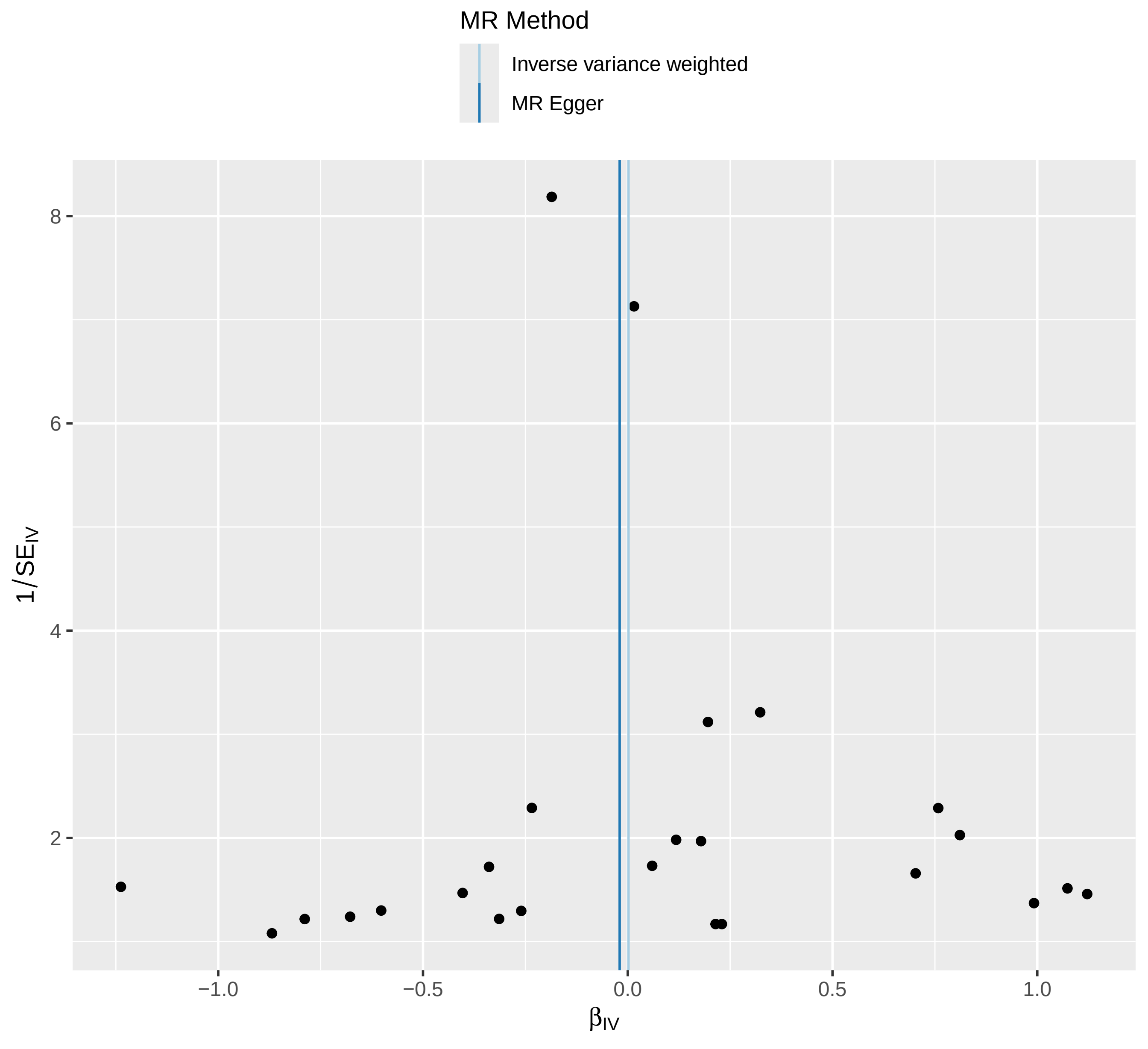

MR Method
Inverse variance weighted
MR Egger
8
6
4
2
1/SE_IV
−1.0
−0.5
0.0
0.5
1.0
β_IV

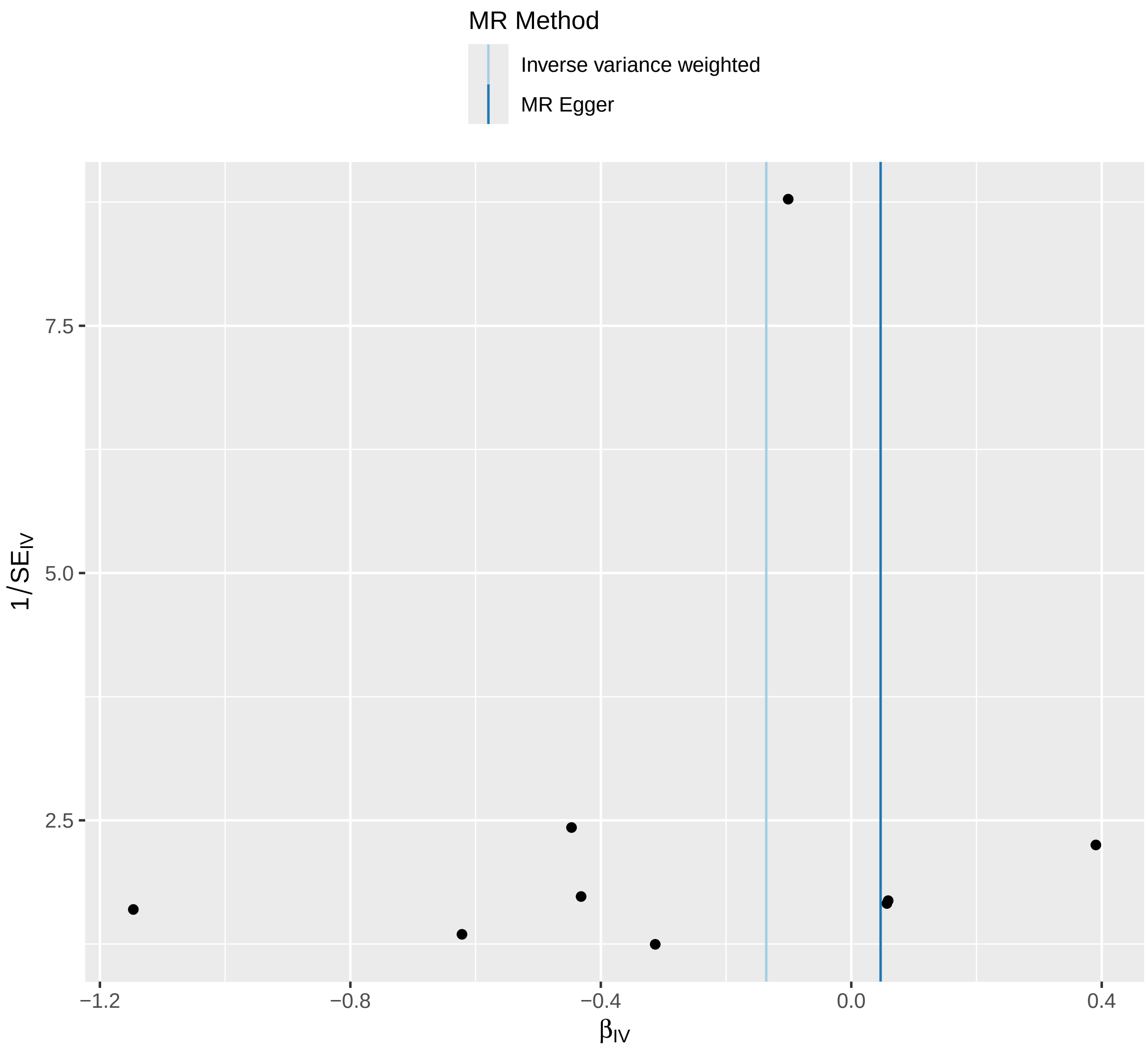
MR Method
Inverse variance weighted
MR Egger
7.5
5.0
2.5
1/SE$_{IV}$
−1.2
−0.8
−0.4
0.0
0.4
β$_{IV}$

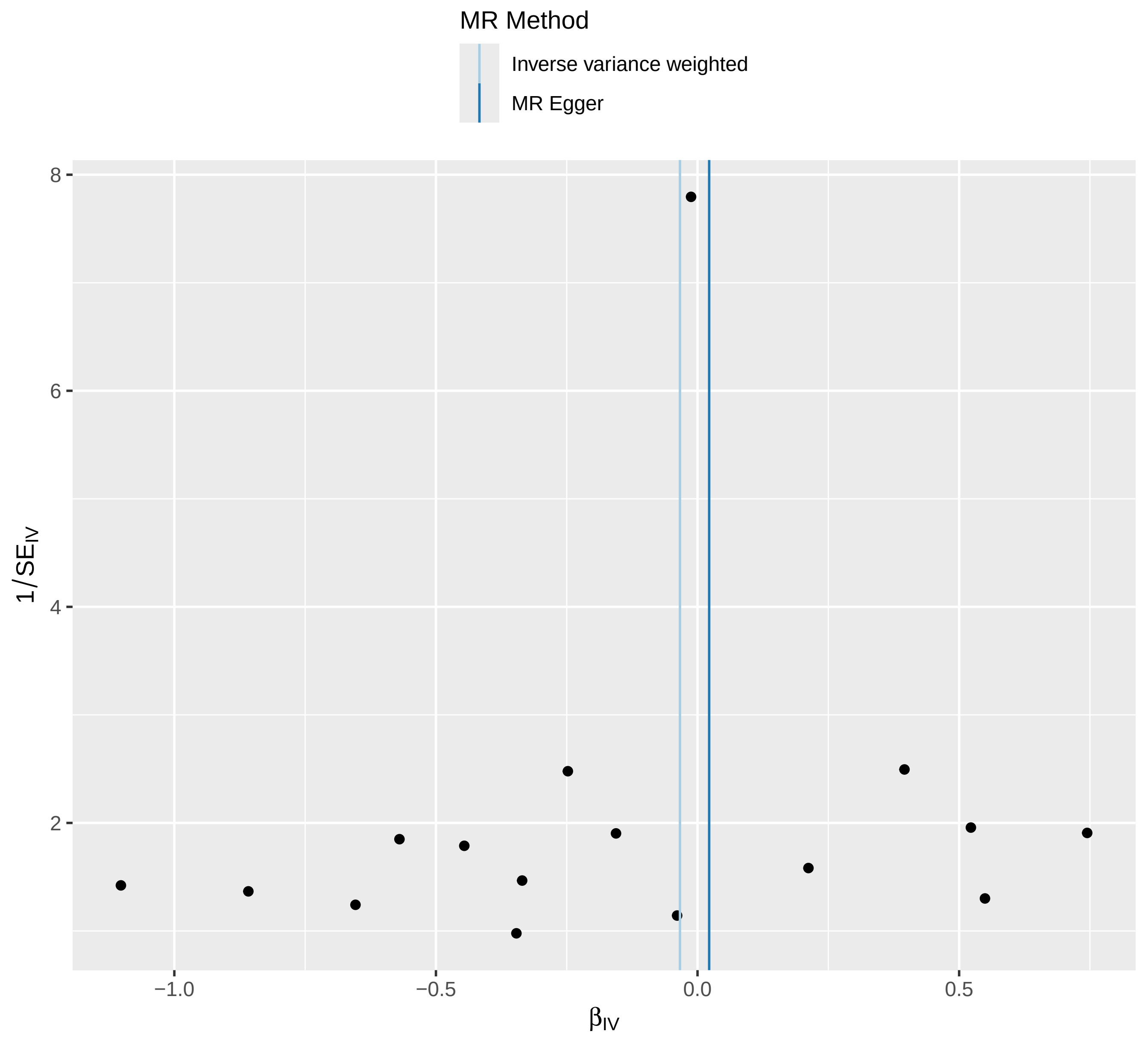
MR Method
Inverse variance weighted
MR Egger
8
6
4
2
1/SE$_{IV}$
−1.0
−0.5
0.0
0.5
β$_{IV}$

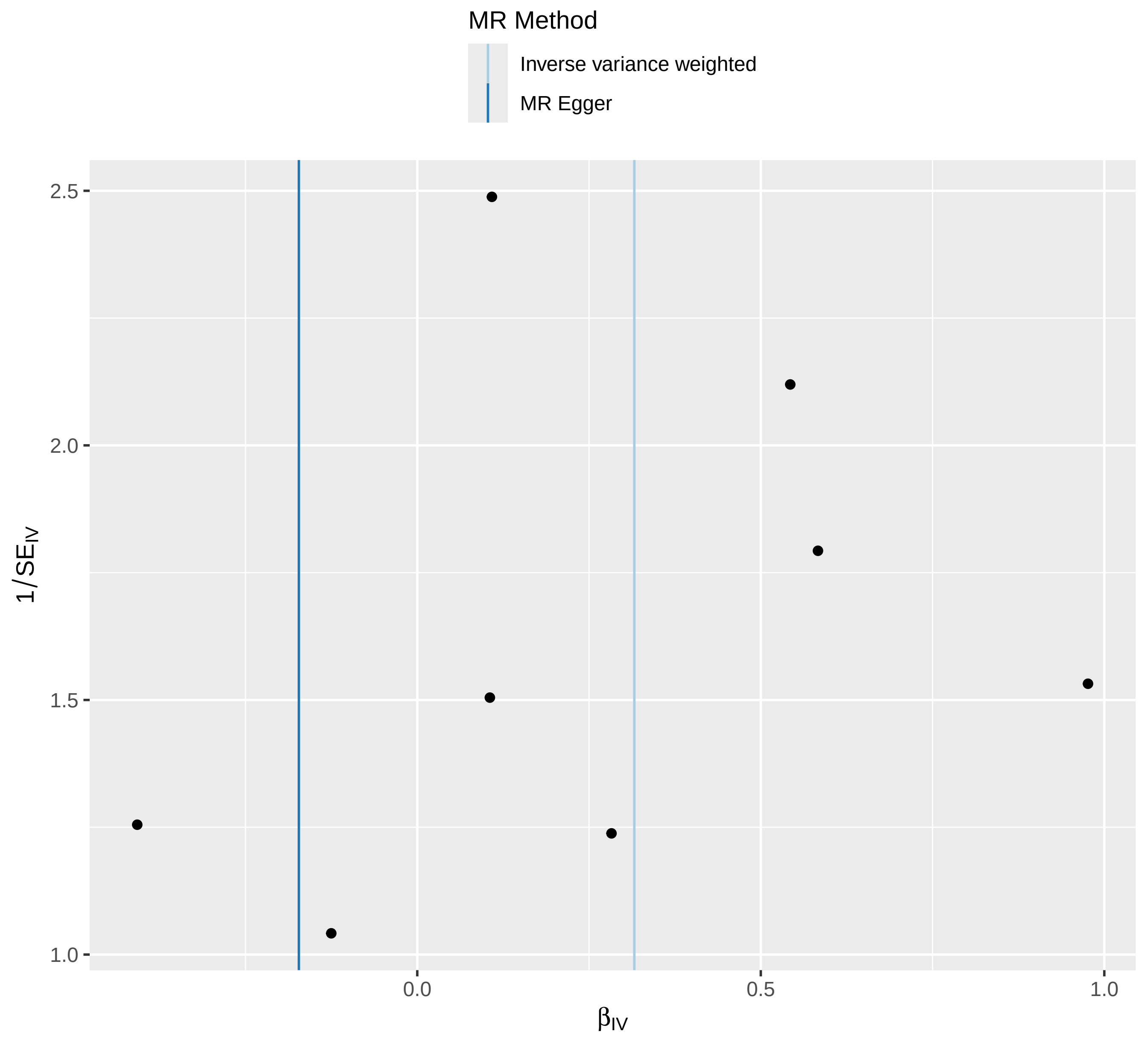

MR Method
Inverse variance weighted
MR Egger
2.5
2.0
1.5
1.0
0.0
0.5
1.0
$1/SE_{IV}$
β_{IV}

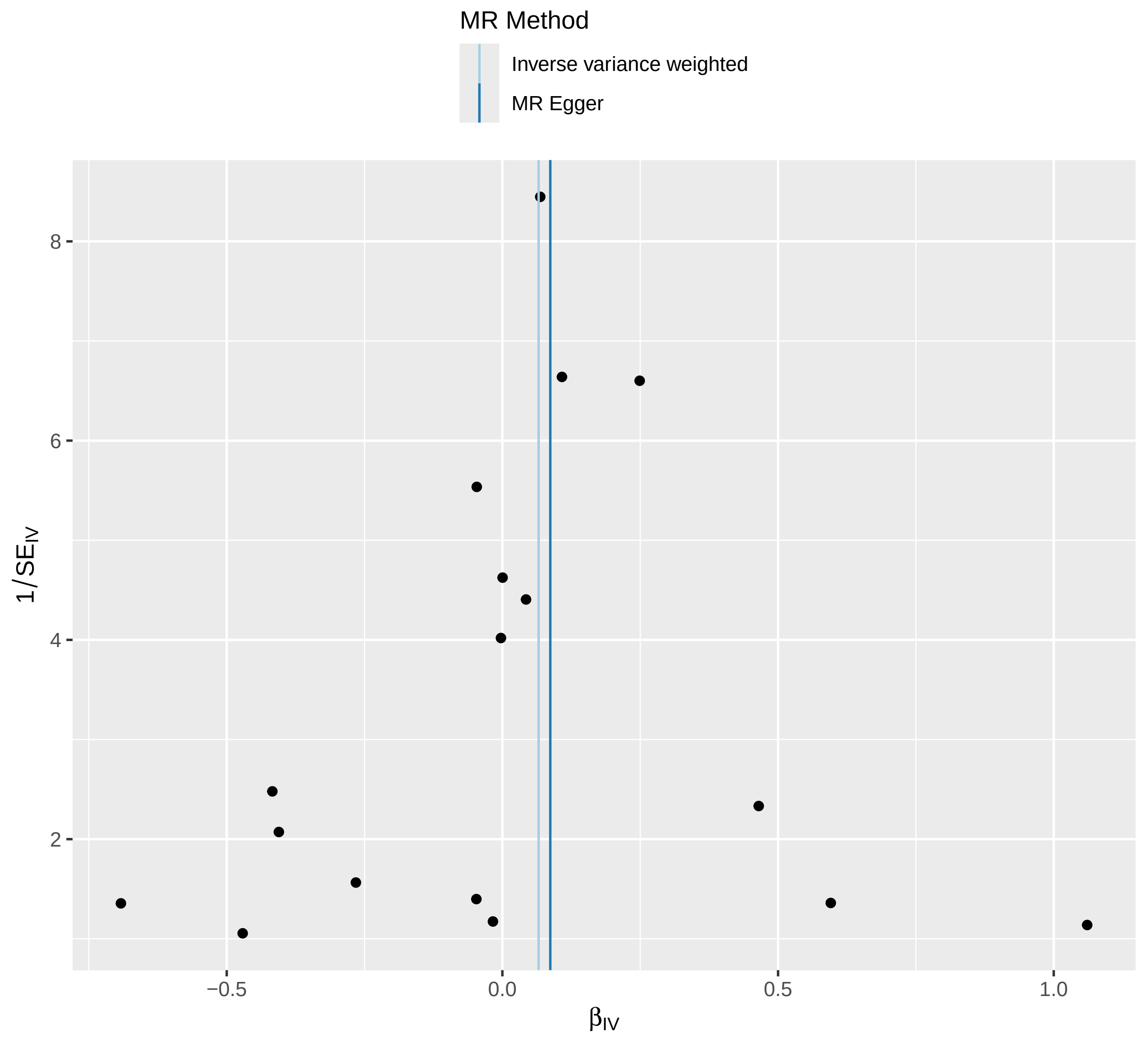
MR Method
Inverse variance weighted
MR Egger
8
6
4
2
1/SE_IV
−0.5
0.0
0.5
1.0
β_IV

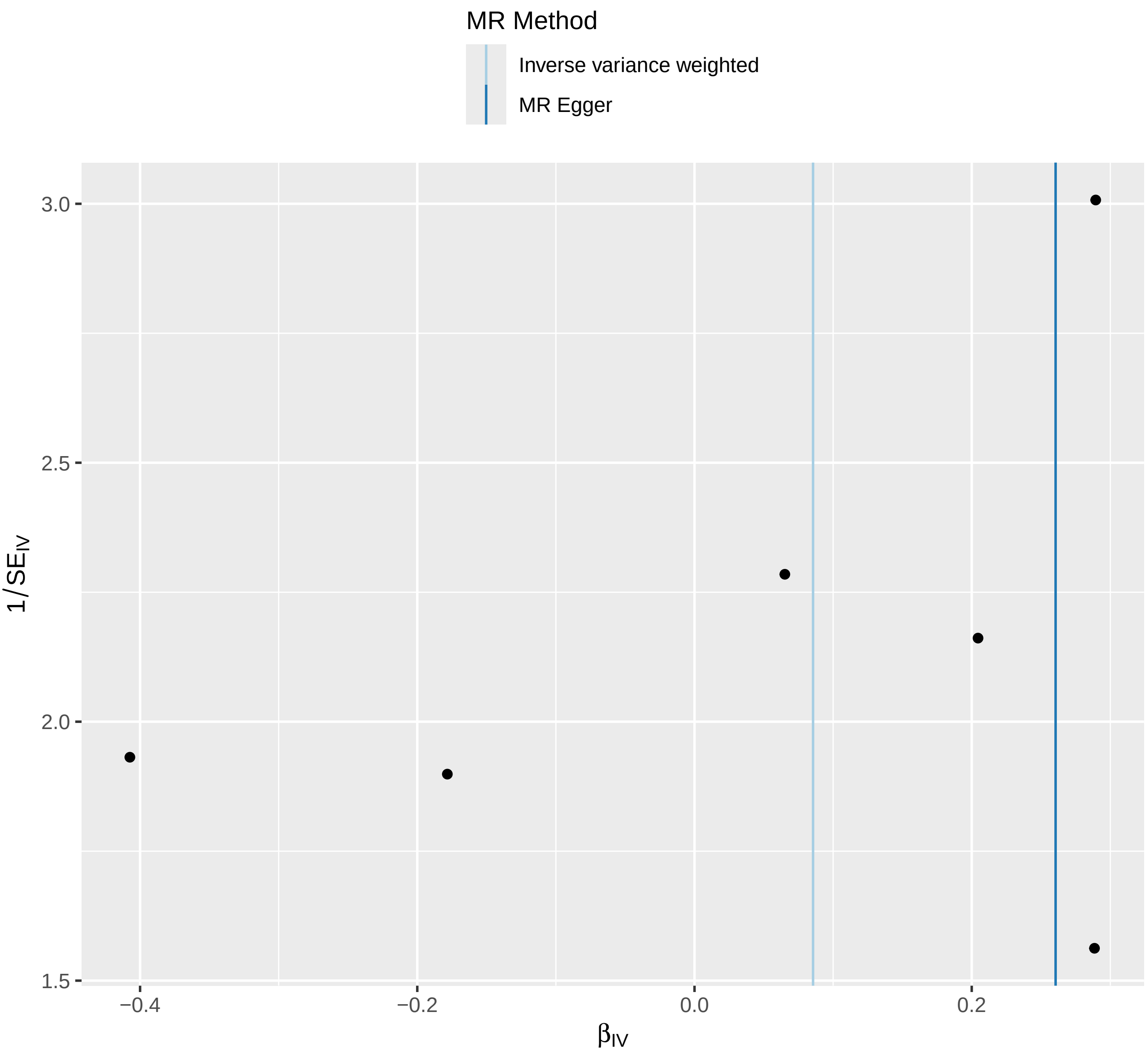

MR Method
Inverse variance weighted
MR Egger
3.0
2.5
2.0
1.5
$1/SE_{IV}$
−0.4
−0.2
0.0
0.2
β_{IV}

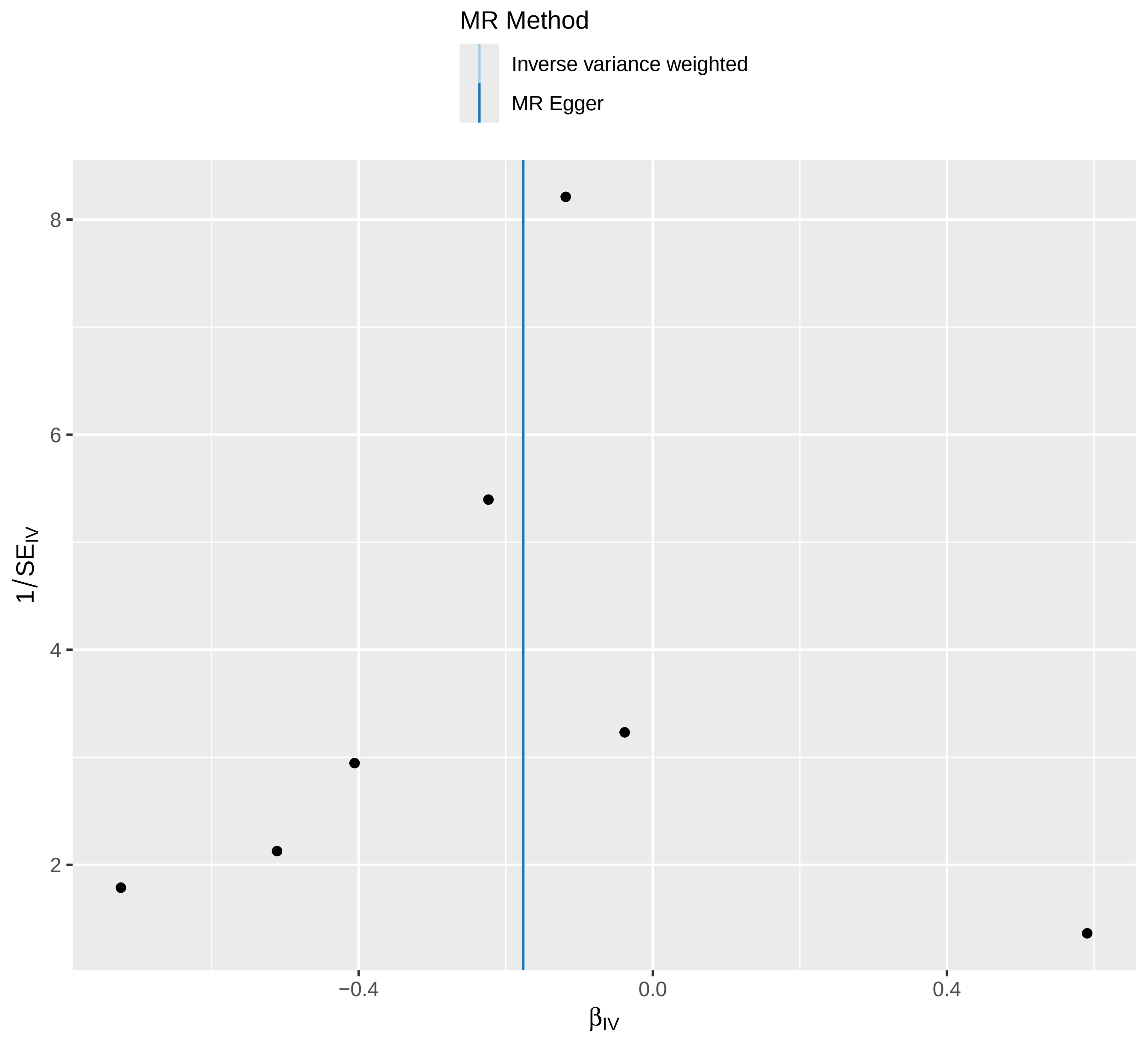
MR Method
Inverse variance weighted
MR Egger
8
6
4
2
$1/SE_{IV}$
−0.4
0.0
0.4
β_{IV}

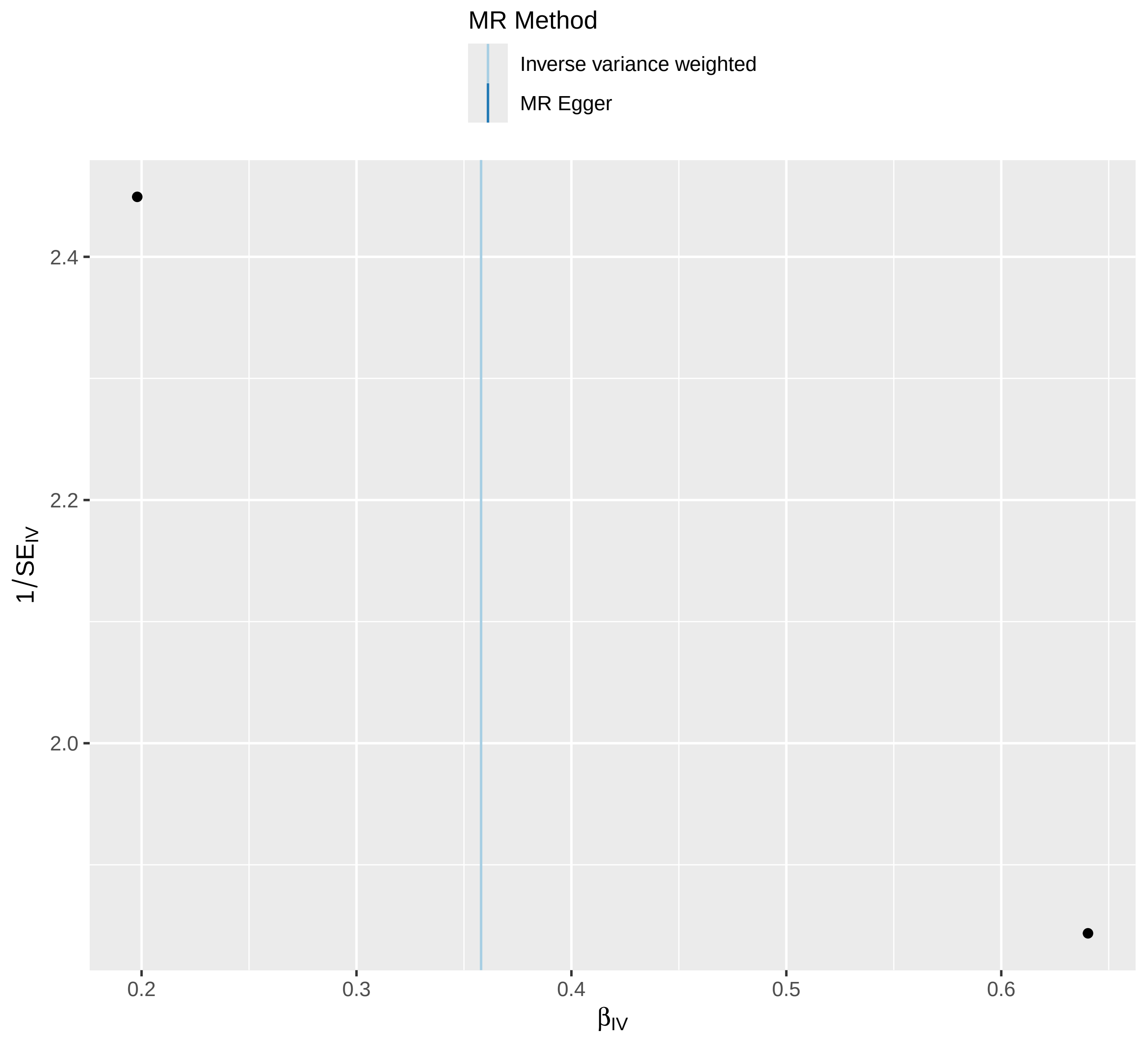
MR Method
Inverse variance weighted
MR Egger
2.4
2.2
2.0
$1/SE_{IV}$
0.2
0.3
0.4
0.5
0.6
β_{IV}

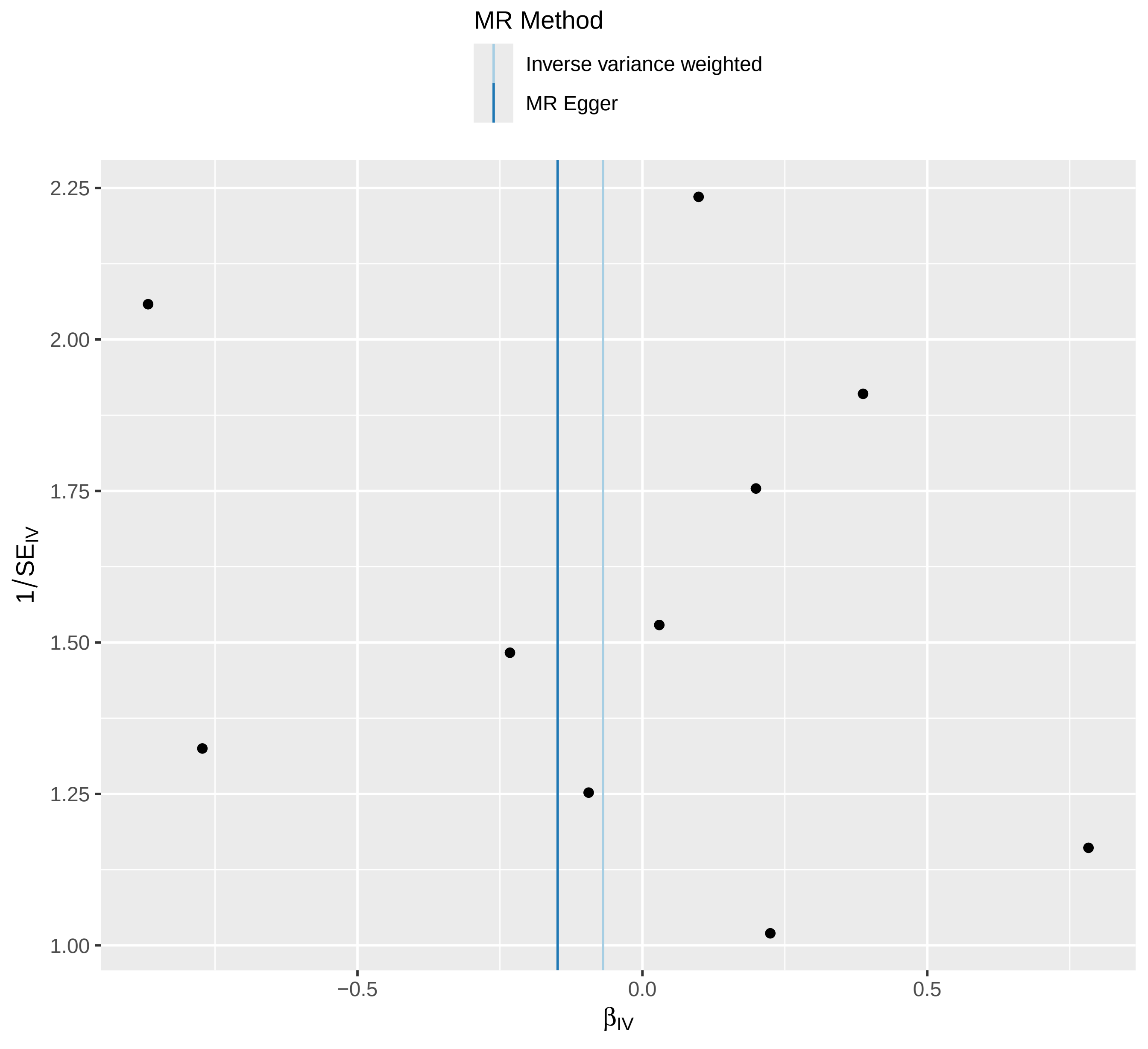

MR Method
Inverse variance weighted
MR Egger
2.25
2.00
1.75
1.50
1.25
1.00
1/SE_IV
−0.5
0.0
0.5
β_IV

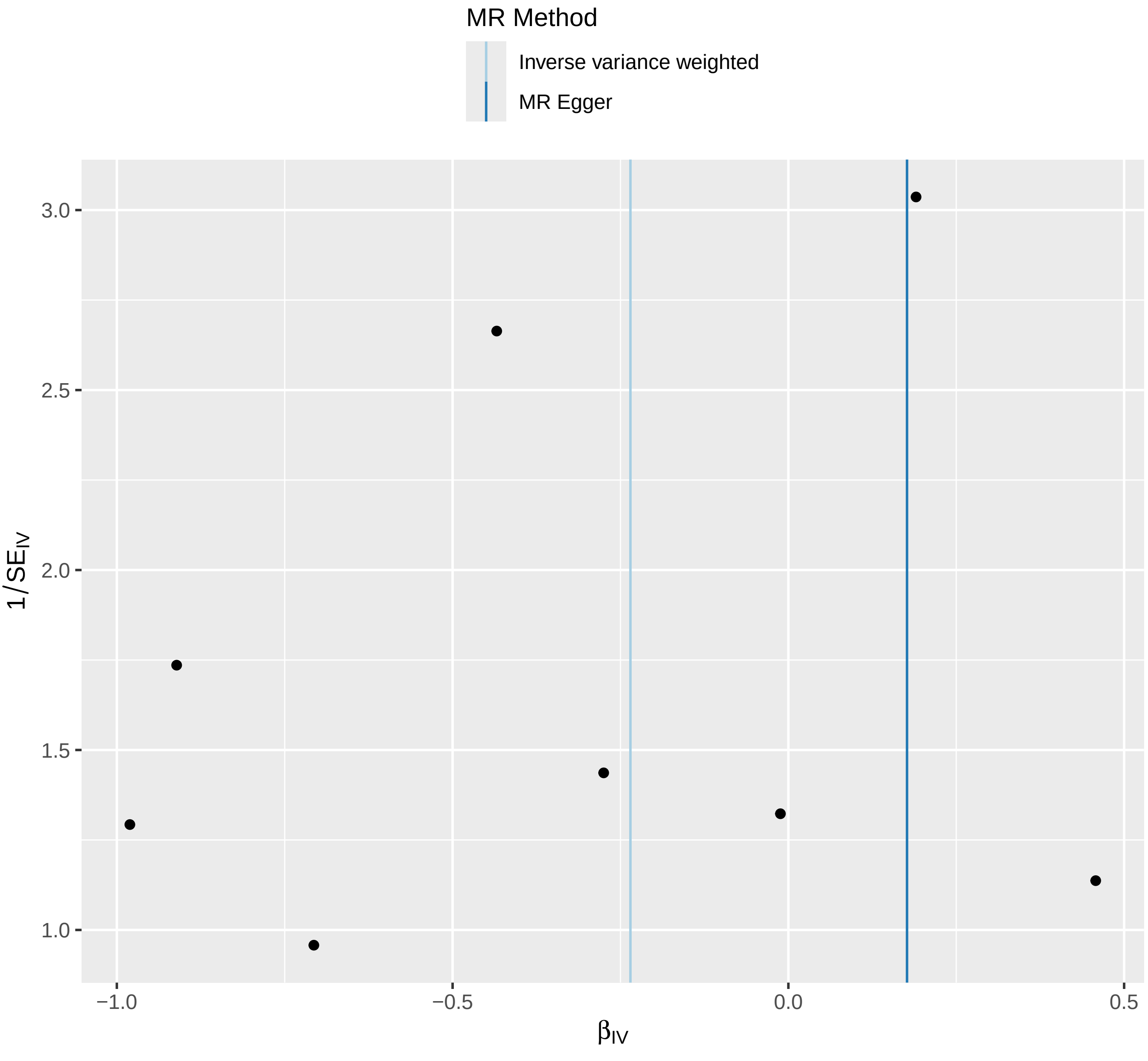
MR Method
Inverse variance weighted
MR Egger
3.0
2.5
2.0
1.5
1.0
1/SE_IV
−1.0
−0.5
0.0
0.5
β_IV

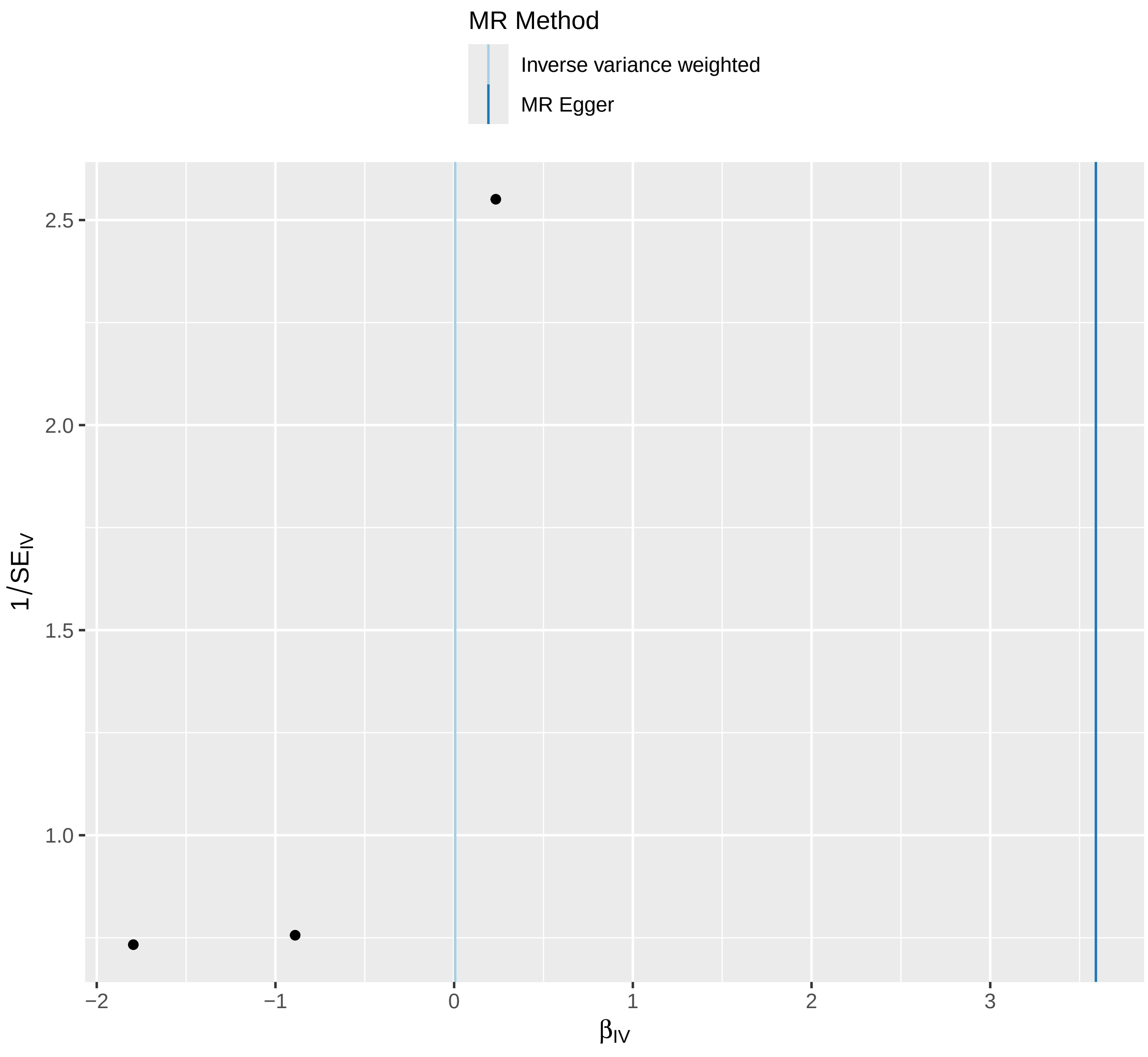
MR Method
Inverse variance weighted
MR Egger
2.5
2.0
1.5
1.0
−2
−1
0
1
2
3
$1/SE_{IV}$
β_{IV}

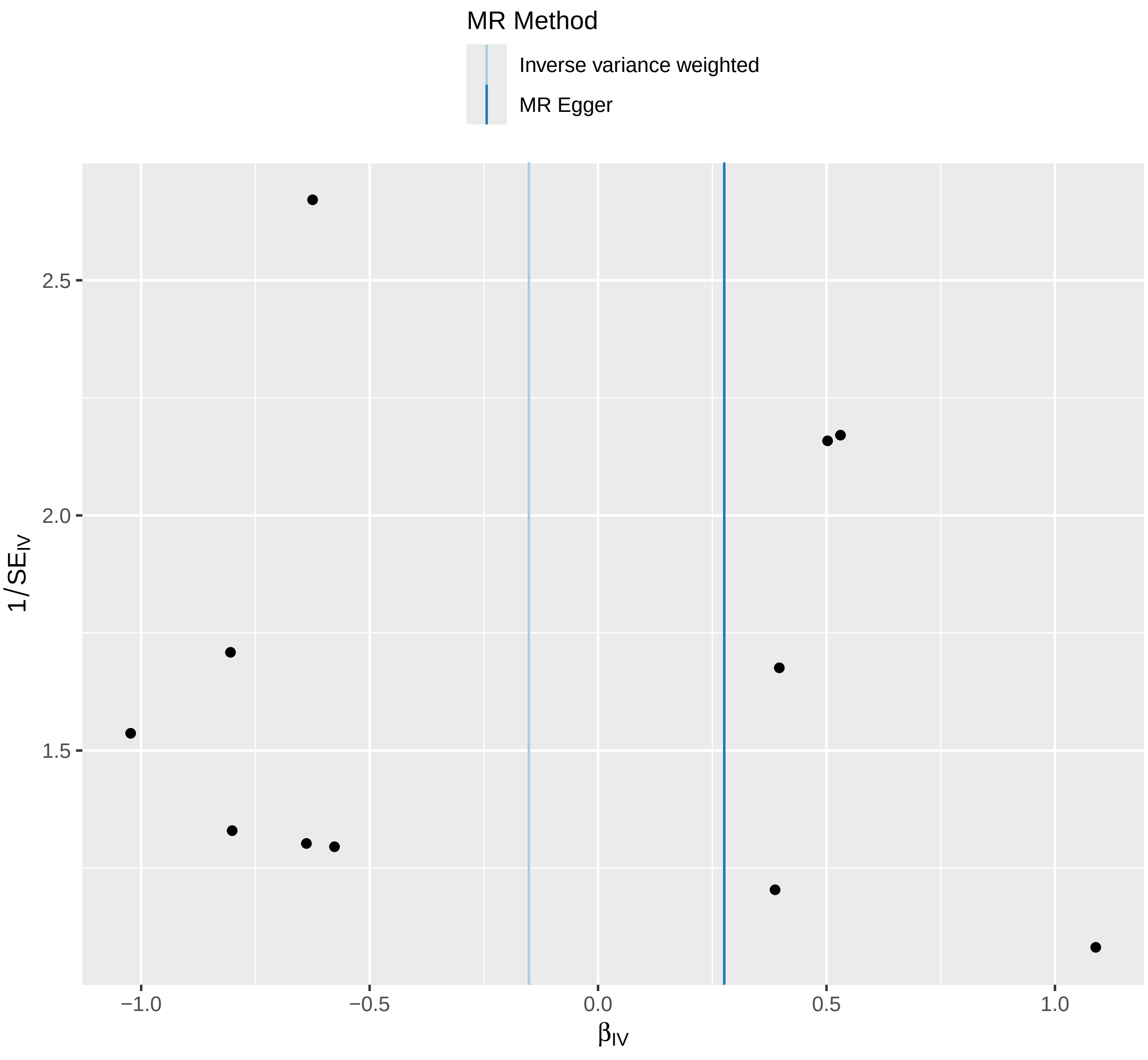
MR Method
Inverse variance weighted
MR Egger
2.5
2.0
1.5
1/SE$_{IV}$
−1.0
−0.5
0.0
0.5
1.0
β_{IV}

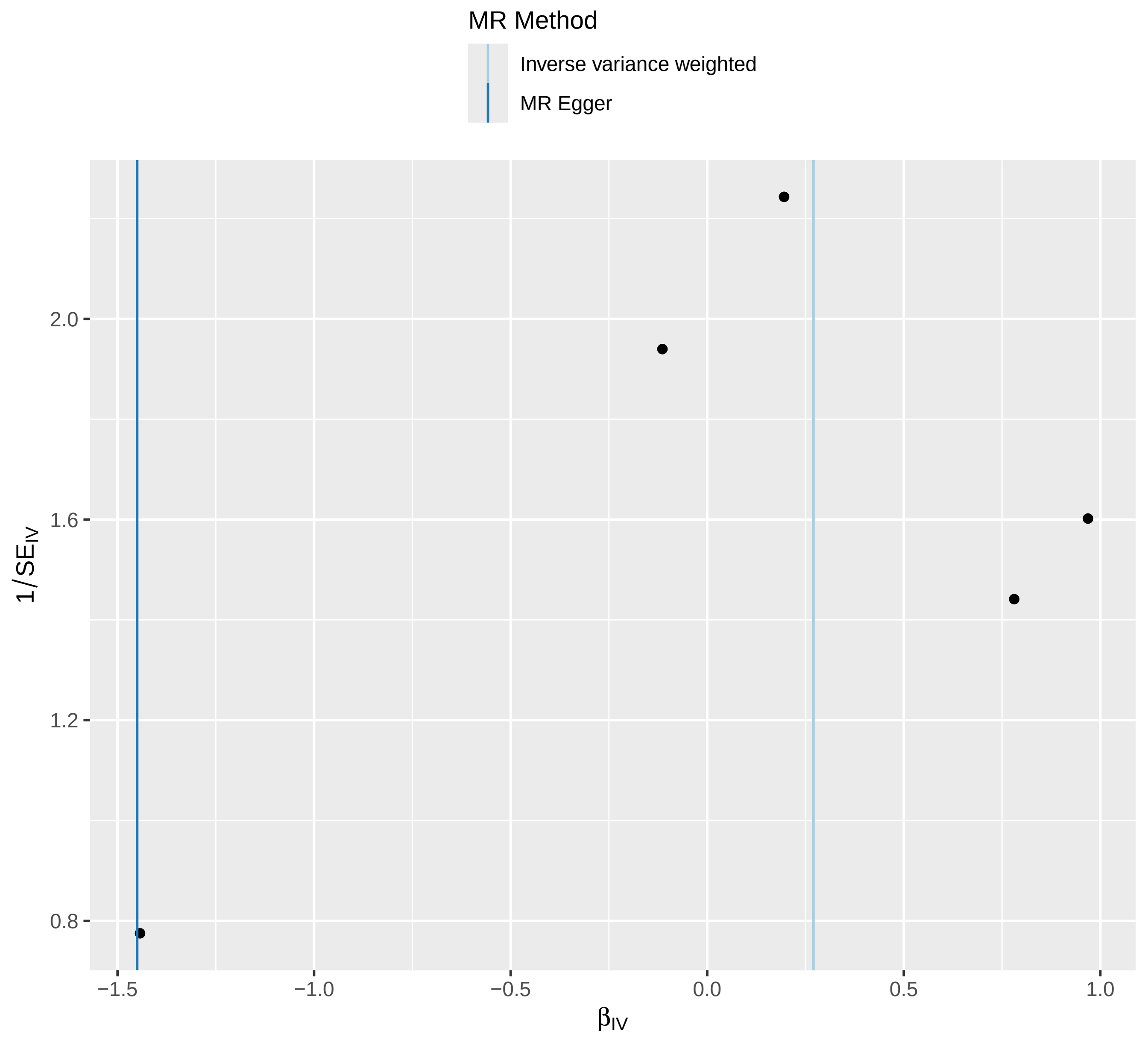

MR Method
Inverse variance weighted
MR Egger
2.0
1.6
1.2
0.8
1/SE$_{IV}$
−1.5
−1.0
−0.5
0.0
0.5
1.0
β$_{IV}$

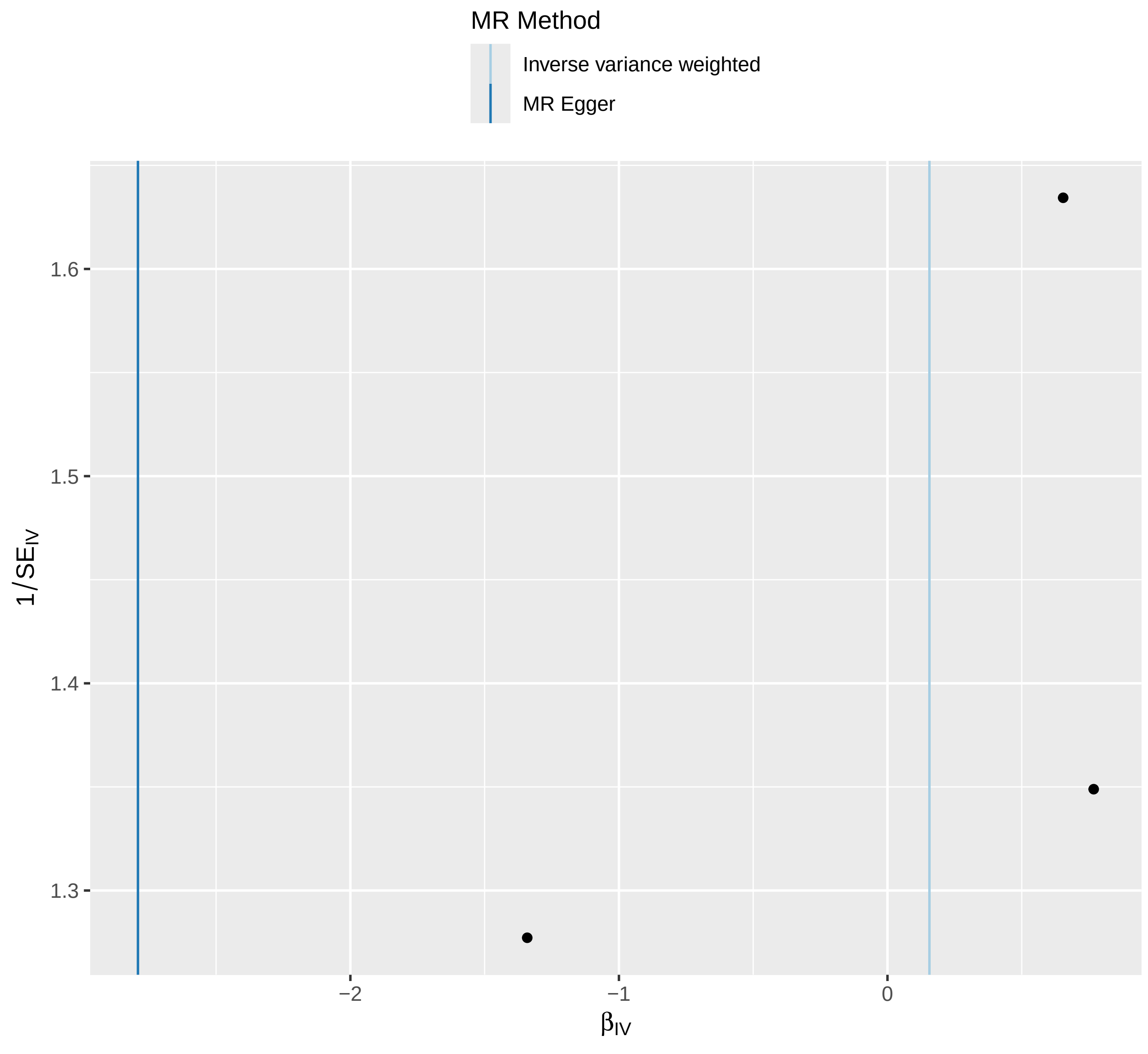
MR Method
Inverse variance weighted
MR Egger
1.6
1.5
1.4
1.3
$1/SE_{IV}$
−2
−1
0
β_{IV}

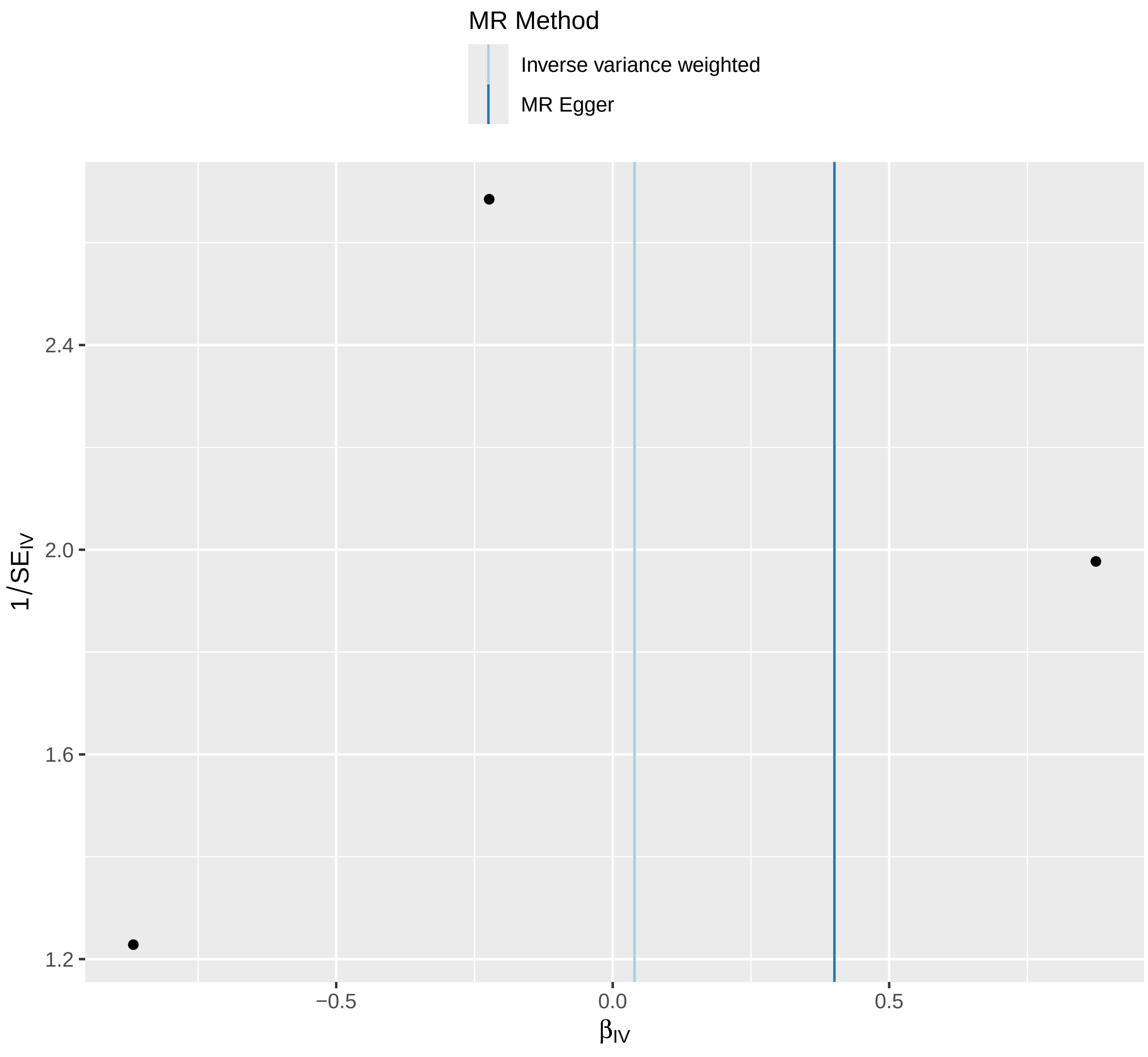

MR Method
Inverse variance weighted
MR Egger
2.4
2.0
1.6
1.2
1/SE_IV
−0.5
0.0
0.5
β_IV

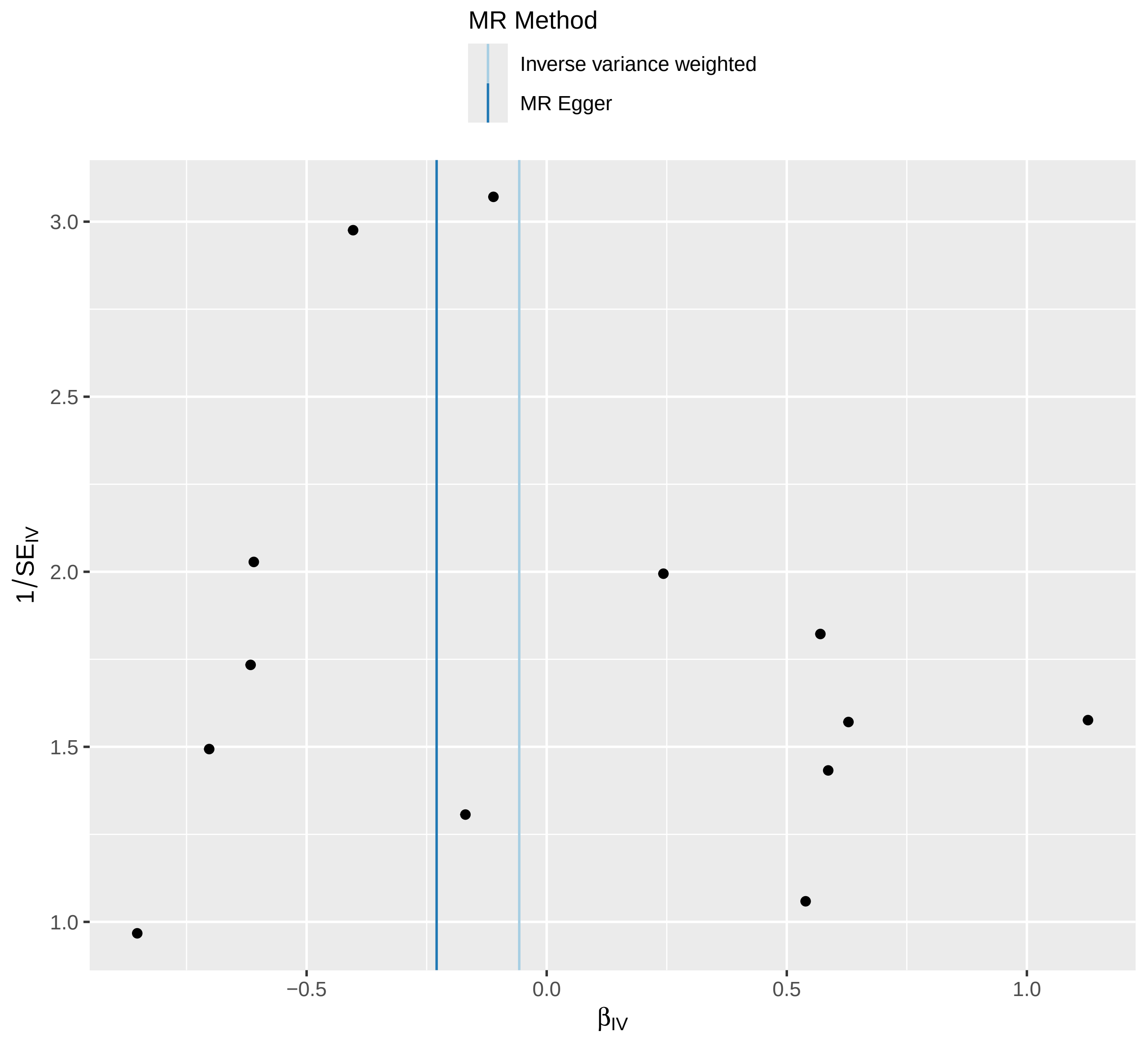
MR Method
Inverse variance weighted
MR Egger
3.0
2.5
2.0
1.5
1.0
1/SE_IV
−0.5
0.0
0.5
1.0
β_IV

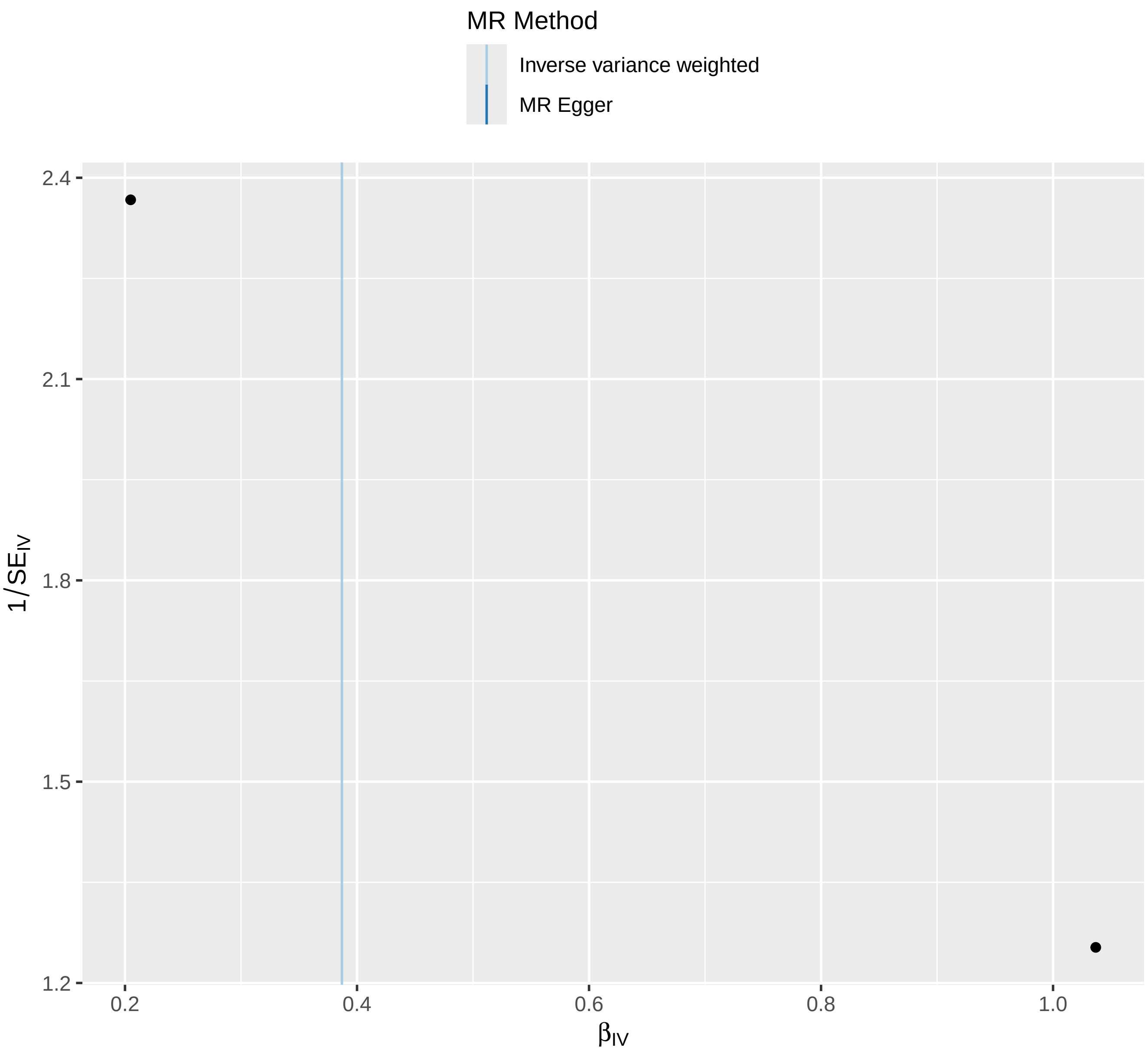
MR Method
Inverse variance weighted
MR Egger
2.4
2.1
1.8
1.5
1.2
1/SE_IV
0.2
0.4
0.6
0.8
1.0
β_IV

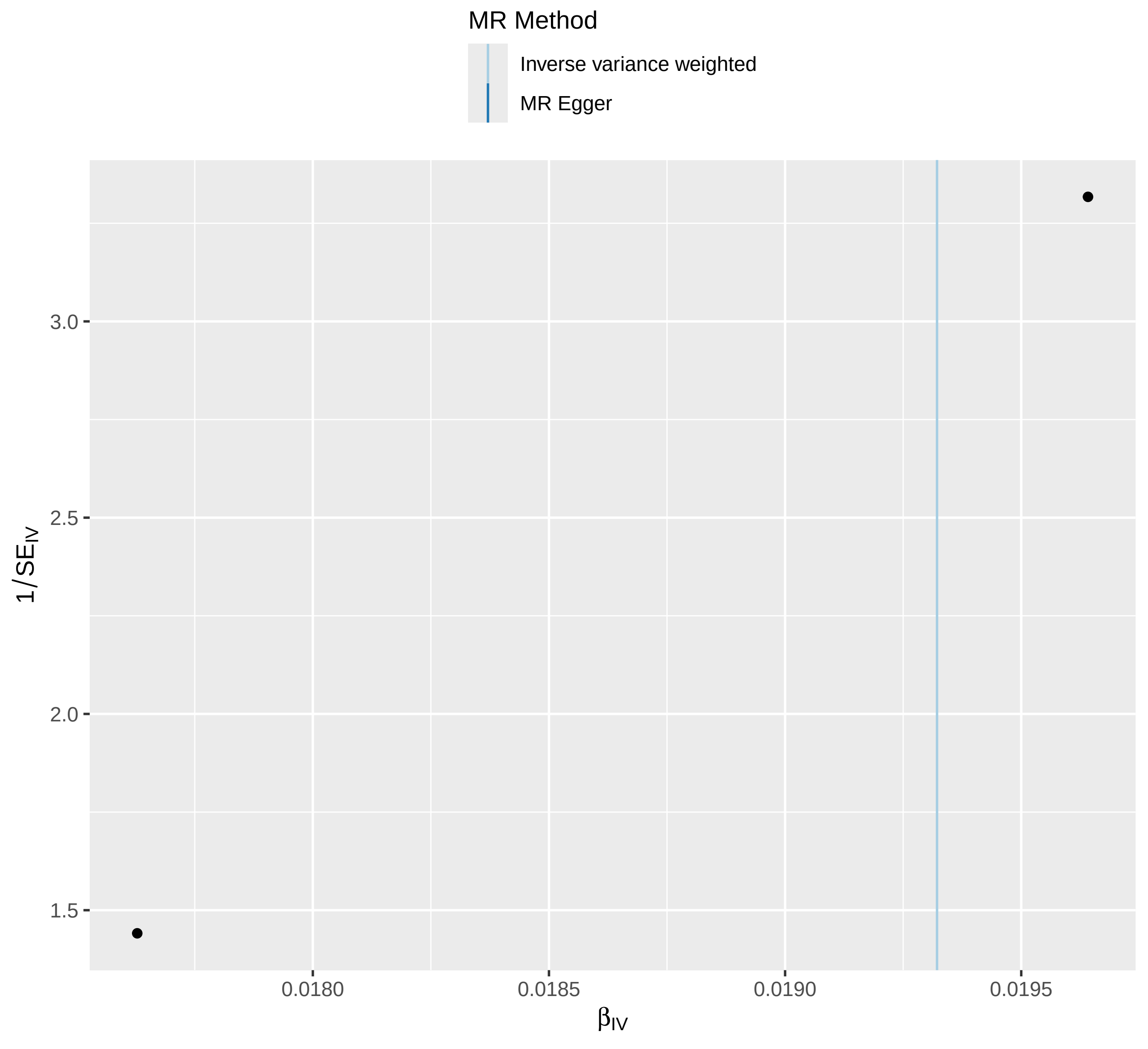
MR Method
Inverse variance weighted
MR Egger
3.0
2.5
2.0
1.5
1/SE$_{IV}$
0.0180
0.0185
0.0190
0.0195
β$_{IV}$

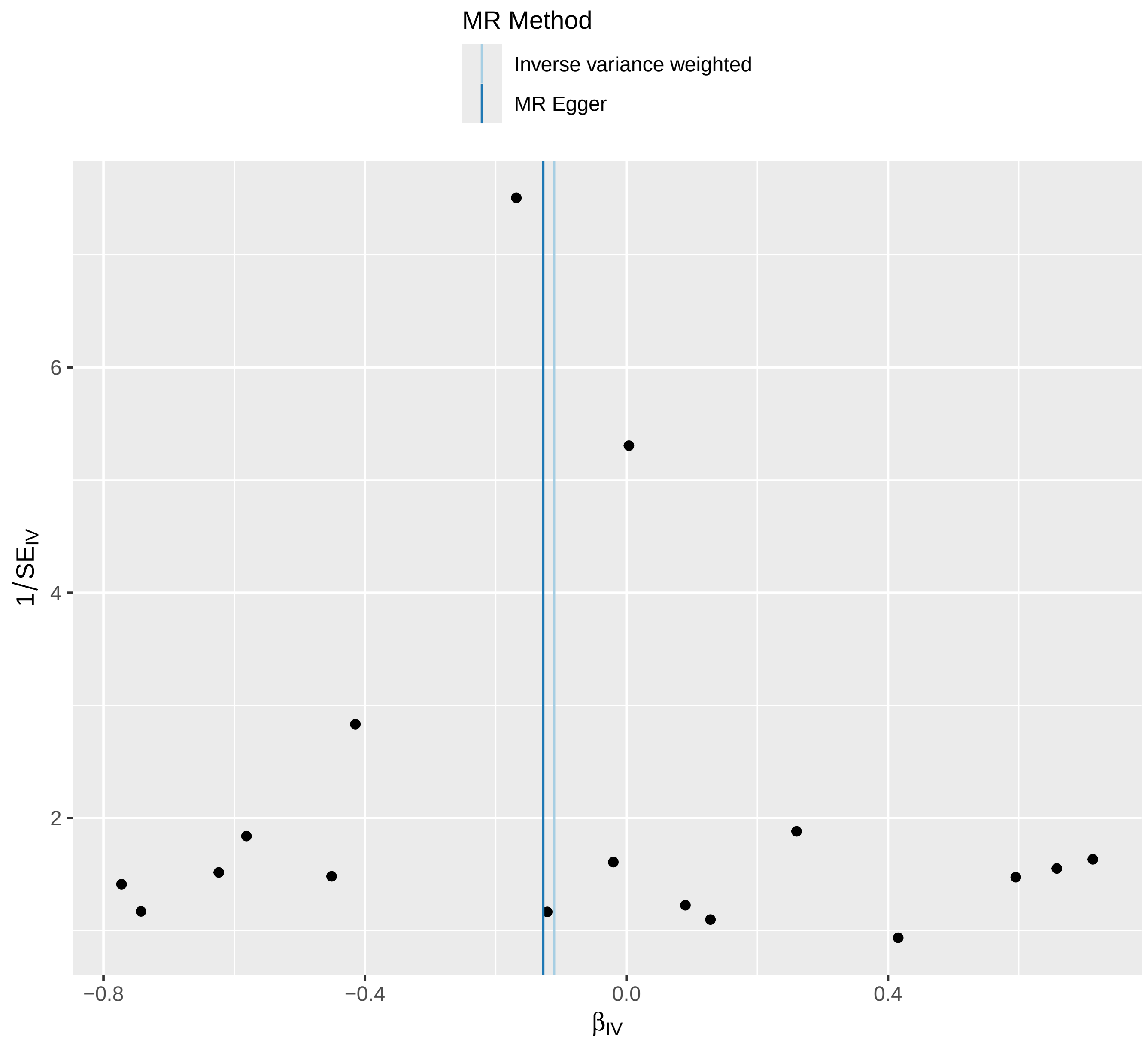
MR Method
Inverse variance weighted
MR Egger
6
4
2
1/SE$_{IV}$
−0.8
−0.4
0.0
0.4
β$_{IV}$

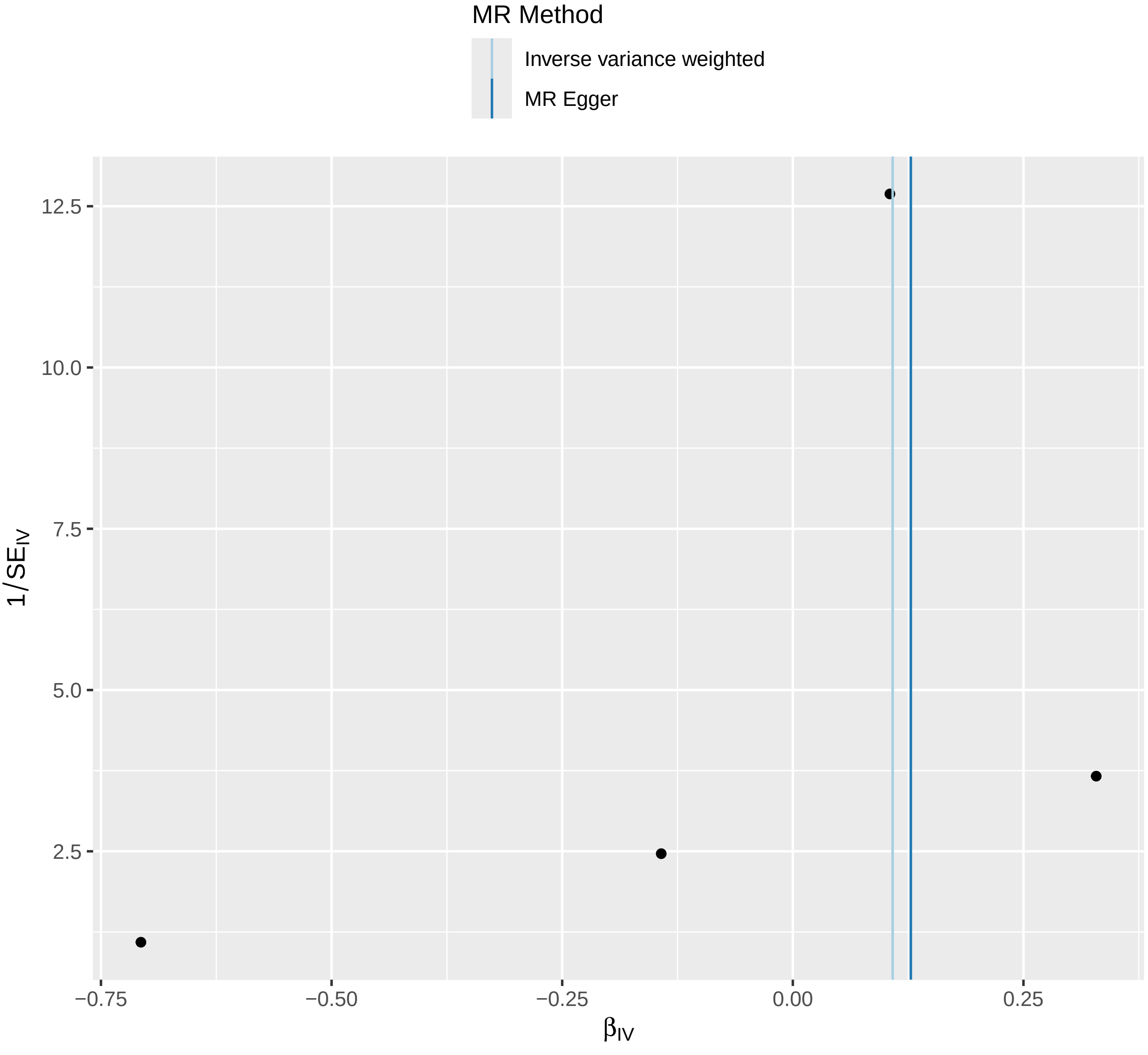
MR Method
Inverse variance weighted
MR Egger
12.5
10.0
7.5
5.0
2.5
1/SE_IV
−0.75
−0.50
−0.25
0.00
0.25
β_IV

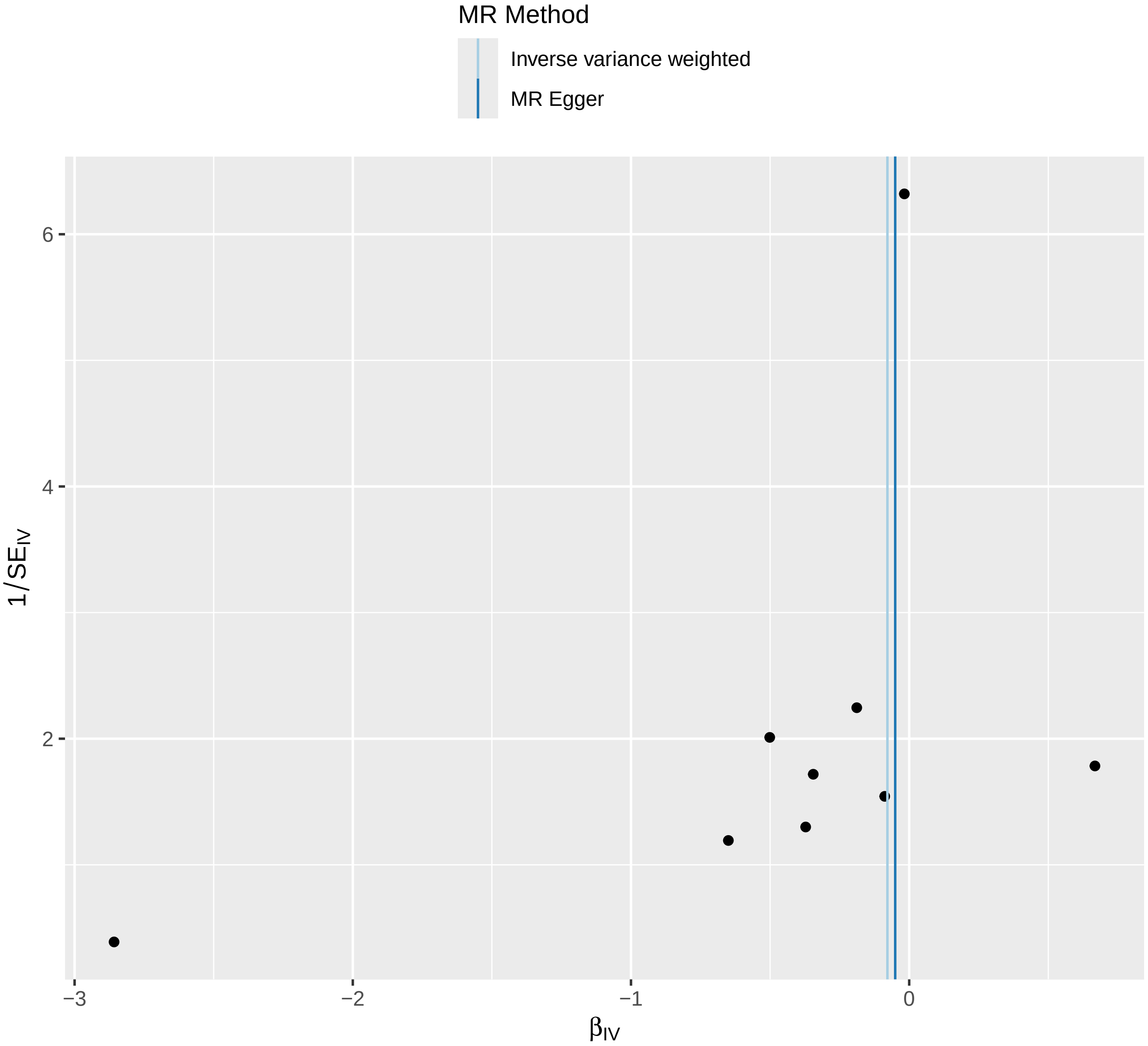
MR Method
Inverse variance weighted
MR Egger
6
4
2
1/SE_IV
−3
−2
−1
0
β_IV

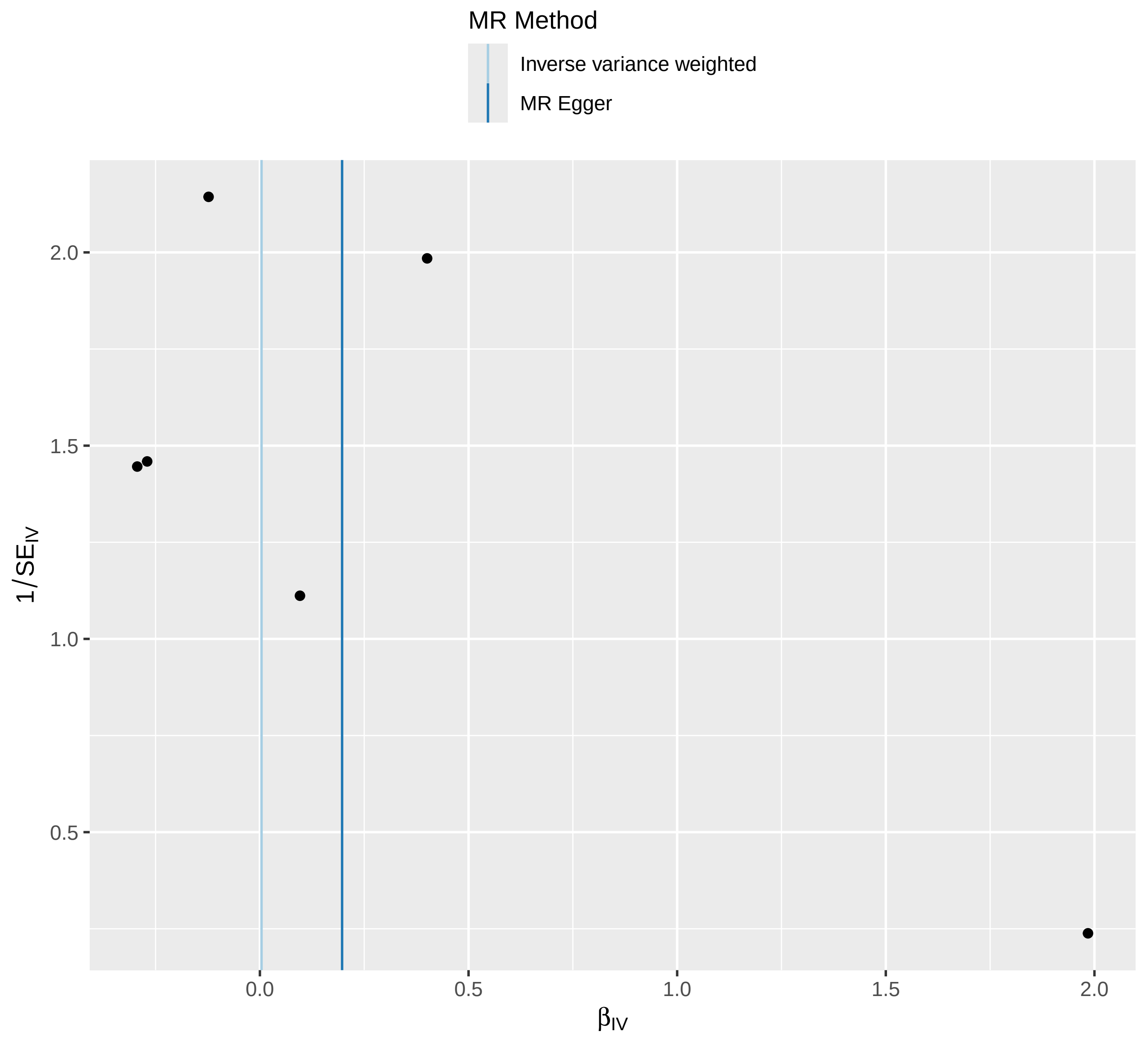
MR Method
Inverse variance weighted
MR Egger
2.0
1.5
1.0
0.5
$1/SE_{IV}$
0.0
0.5
1.0
1.5
2.0
β_{IV}

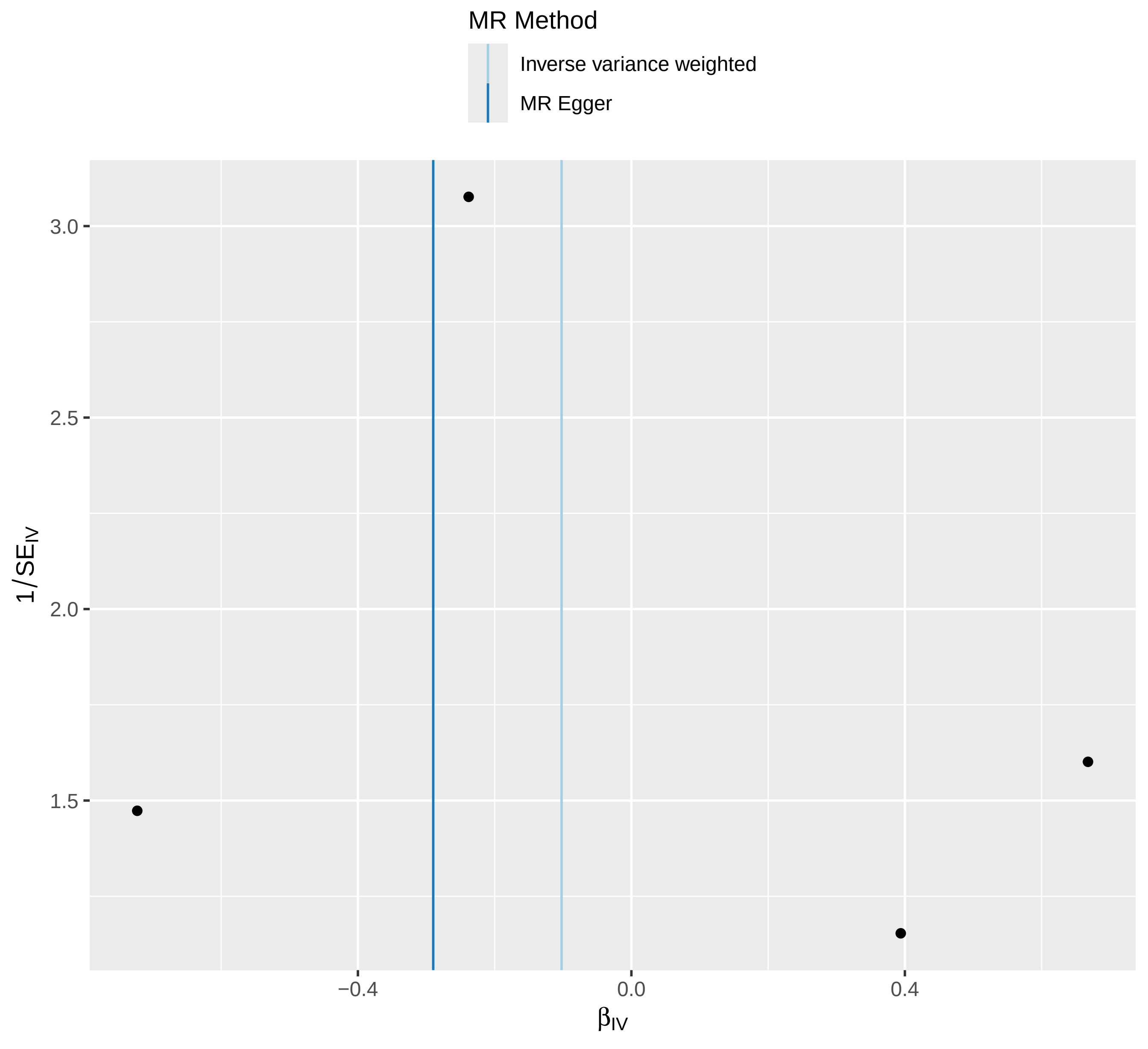
MR Method
Inverse variance weighted
MR Egger
3.0
2.5
2.0
1.5
1/SE_IV
−0.4
0.0
0.4
β_IV

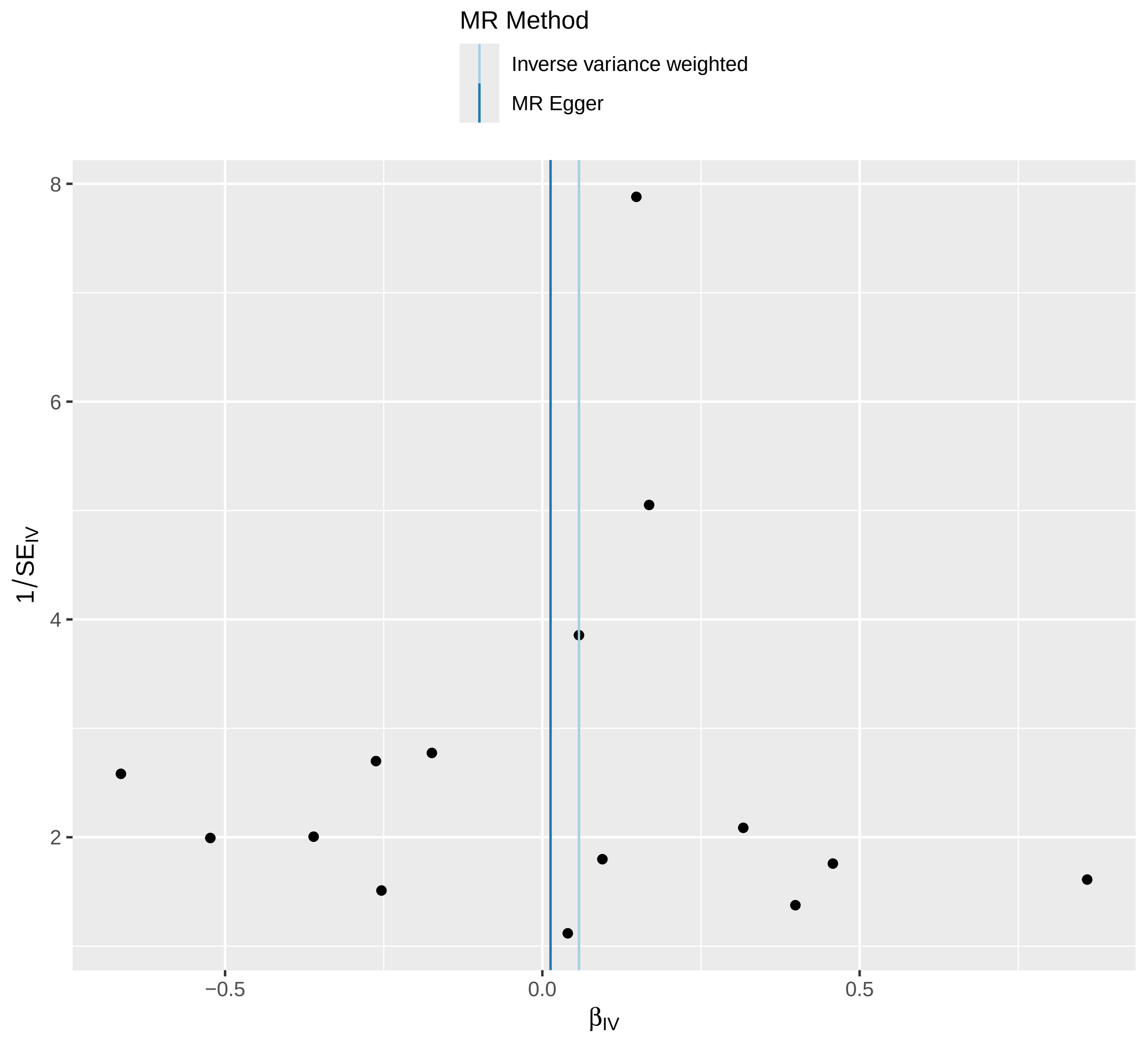

MR Method
Inverse variance weighted
MR Egger
8
6
4
2
1/SE_IV
−0.5
0.0
0.5
β_IV

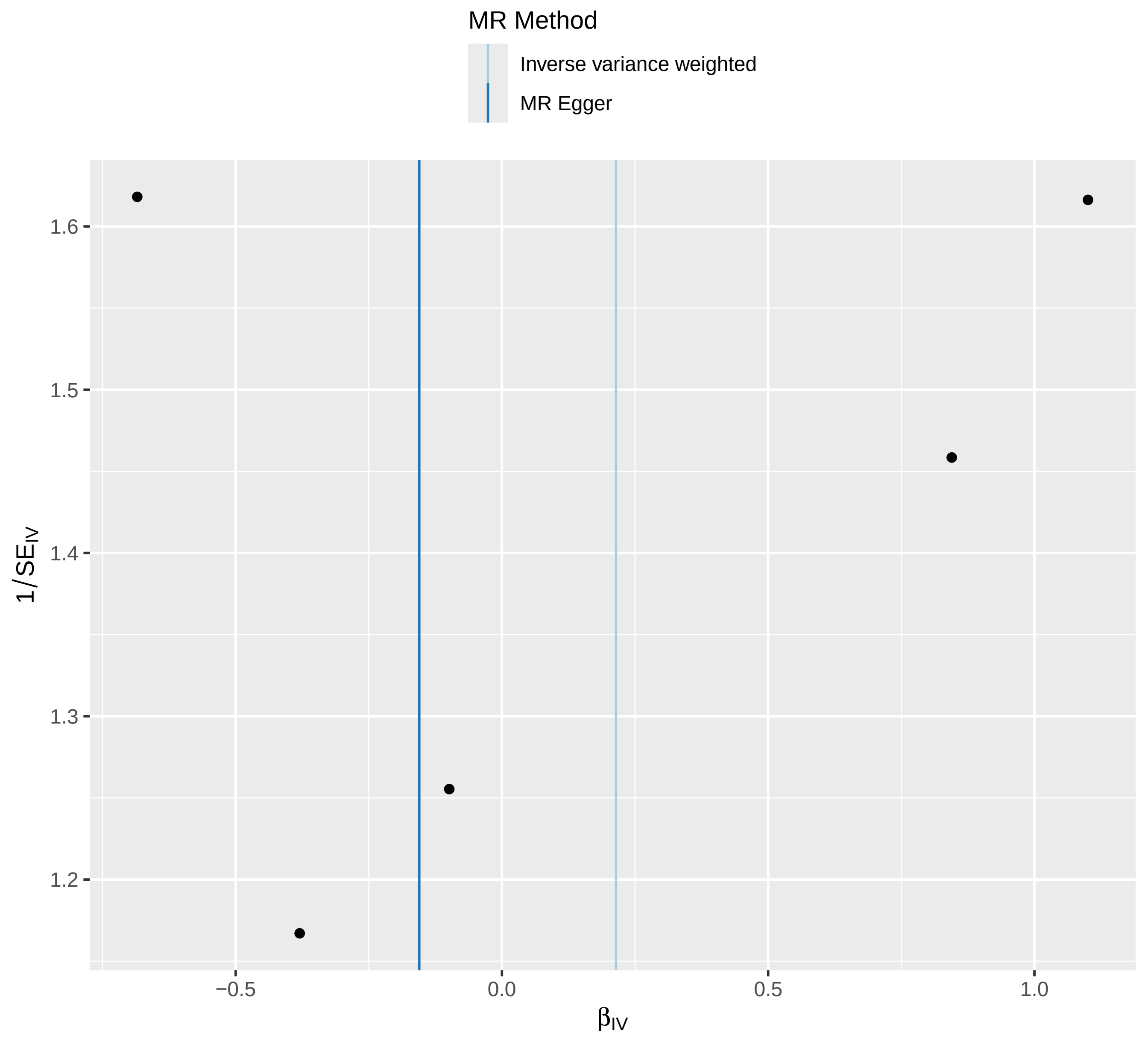
MR Method
Inverse variance weighted
MR Egger
1.6
1.5
1.4
1.3
1.2
1/SE_IV
−0.5
0.0
0.5
1.0
β_IV

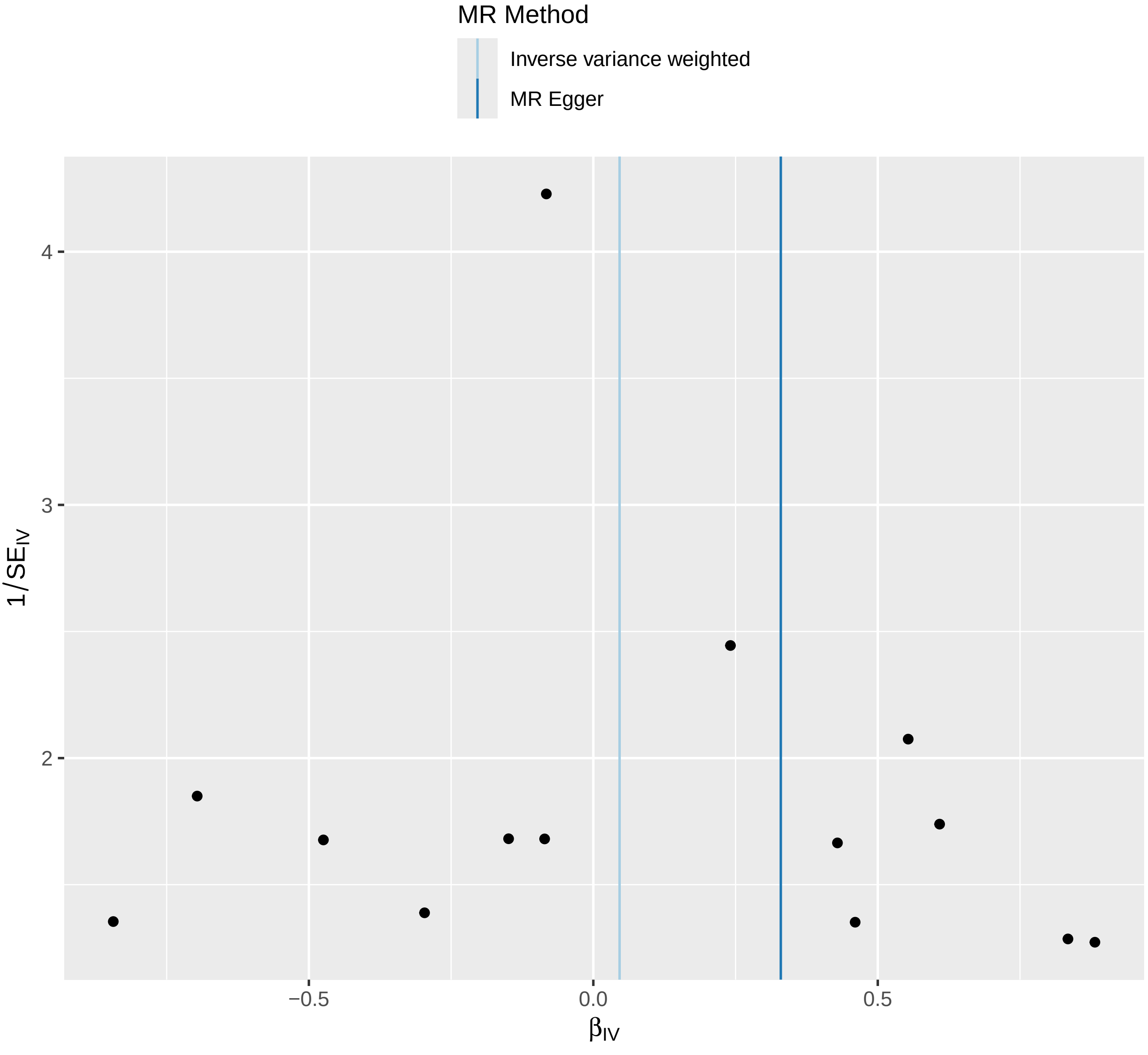
MR Method
Inverse variance weighted
MR Egger
4
3
2
1/SE_IV
−0.5
0.0
0.5
β_IV

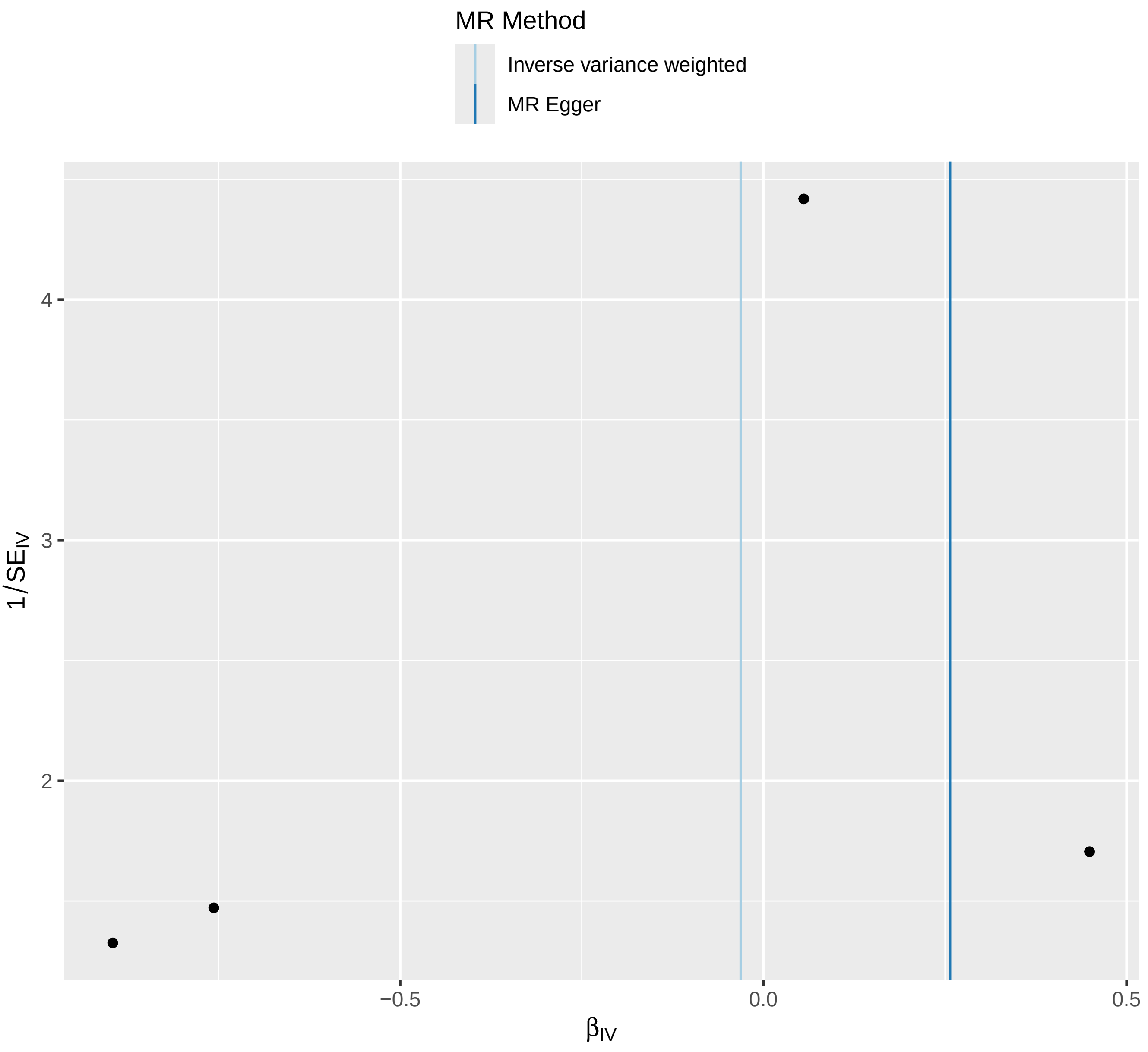

MR Method
Inverse variance weighted
MR Egger
4
3
2
$1/SE_{IV}$
−0.5
0.0
0.5
β_{IV}

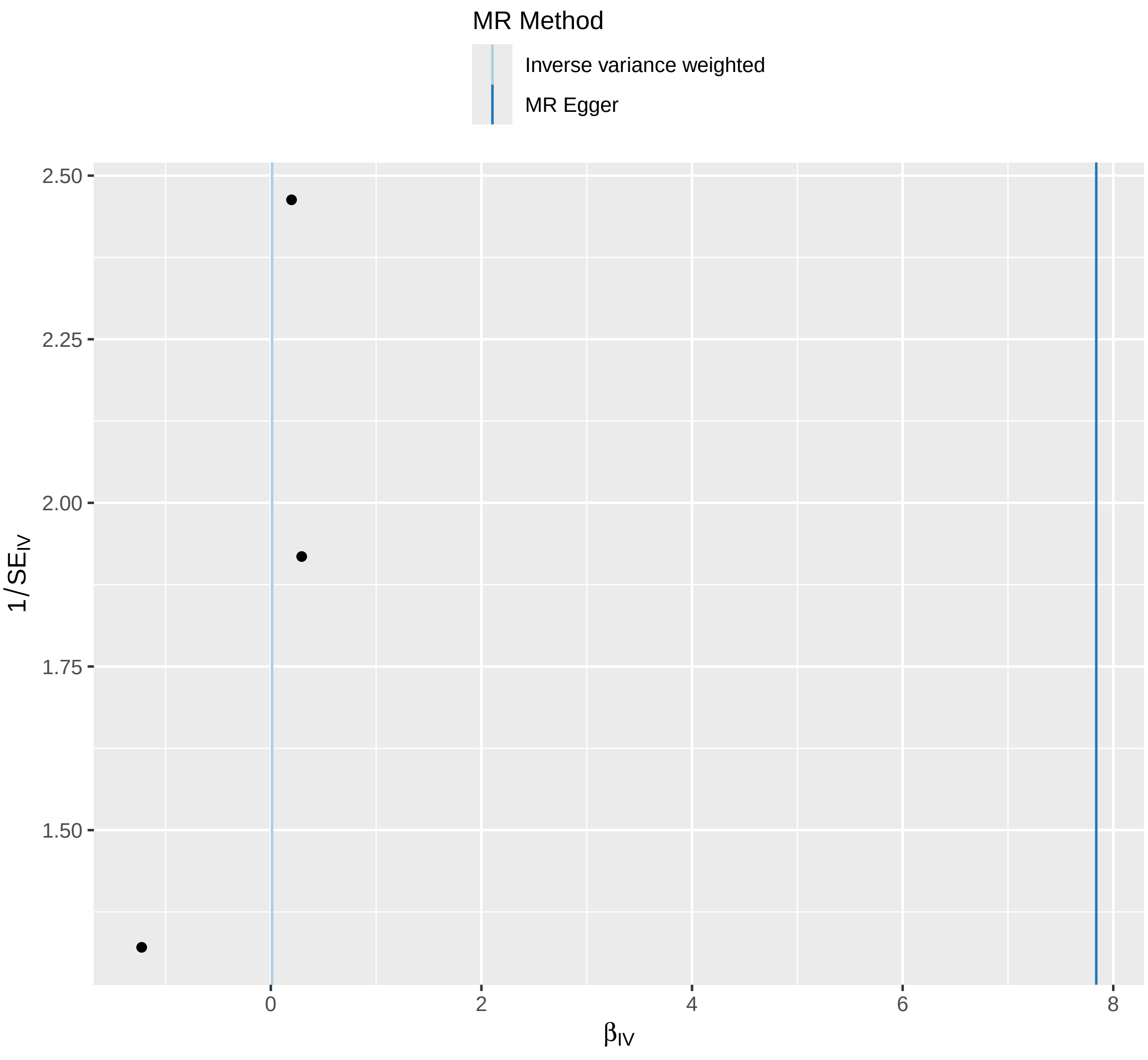
MR Method
Inverse variance weighted
MR Egger
2.50
2.25
2.00
1.75
1.50
1/SE_IV
0
2
4
6
8
β_IV

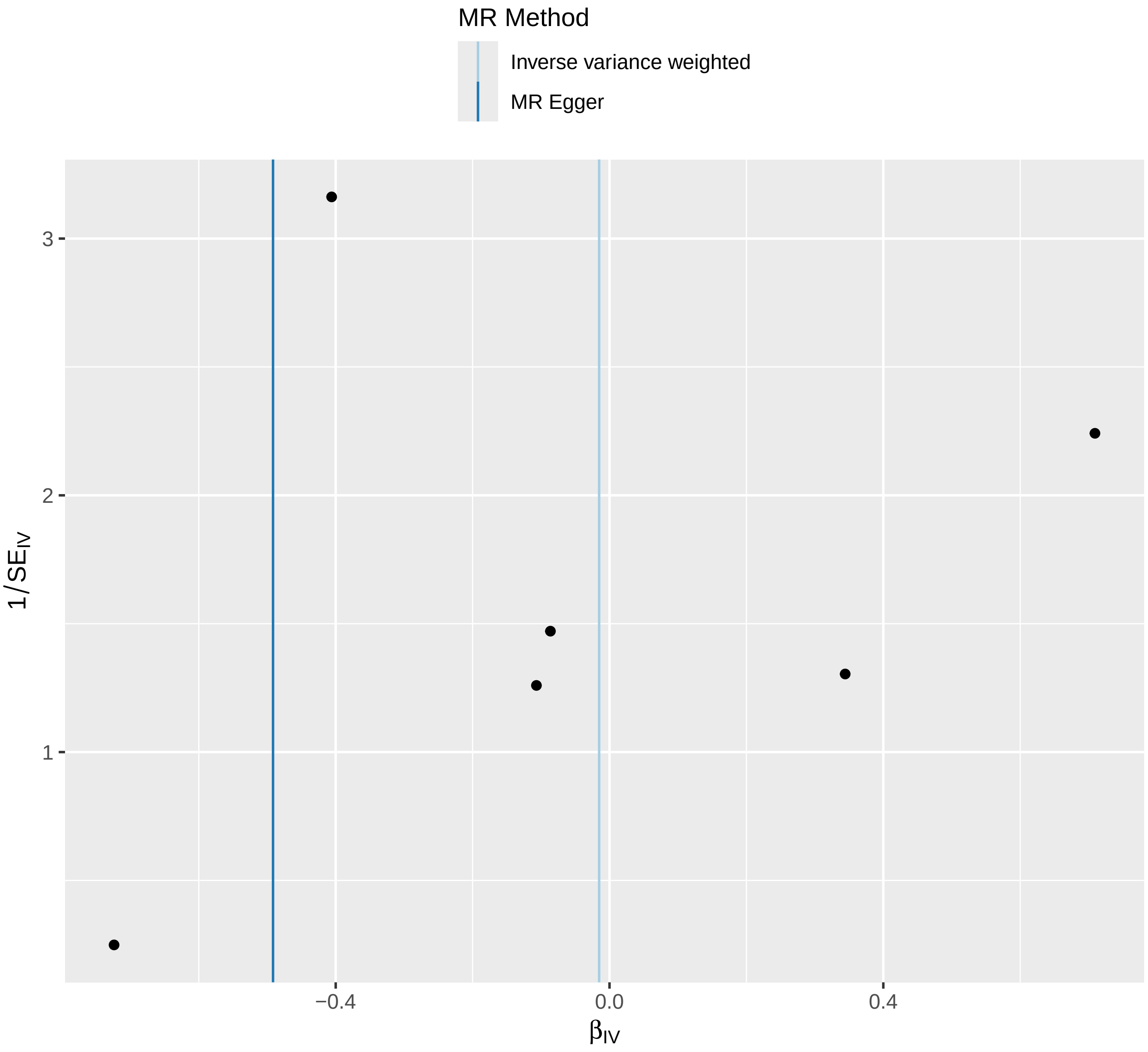
MR Method
Inverse variance weighted
MR Egger
3
2
1
$1/SE_{IV}$
−0.4
0.0
0.4
β_{IV}

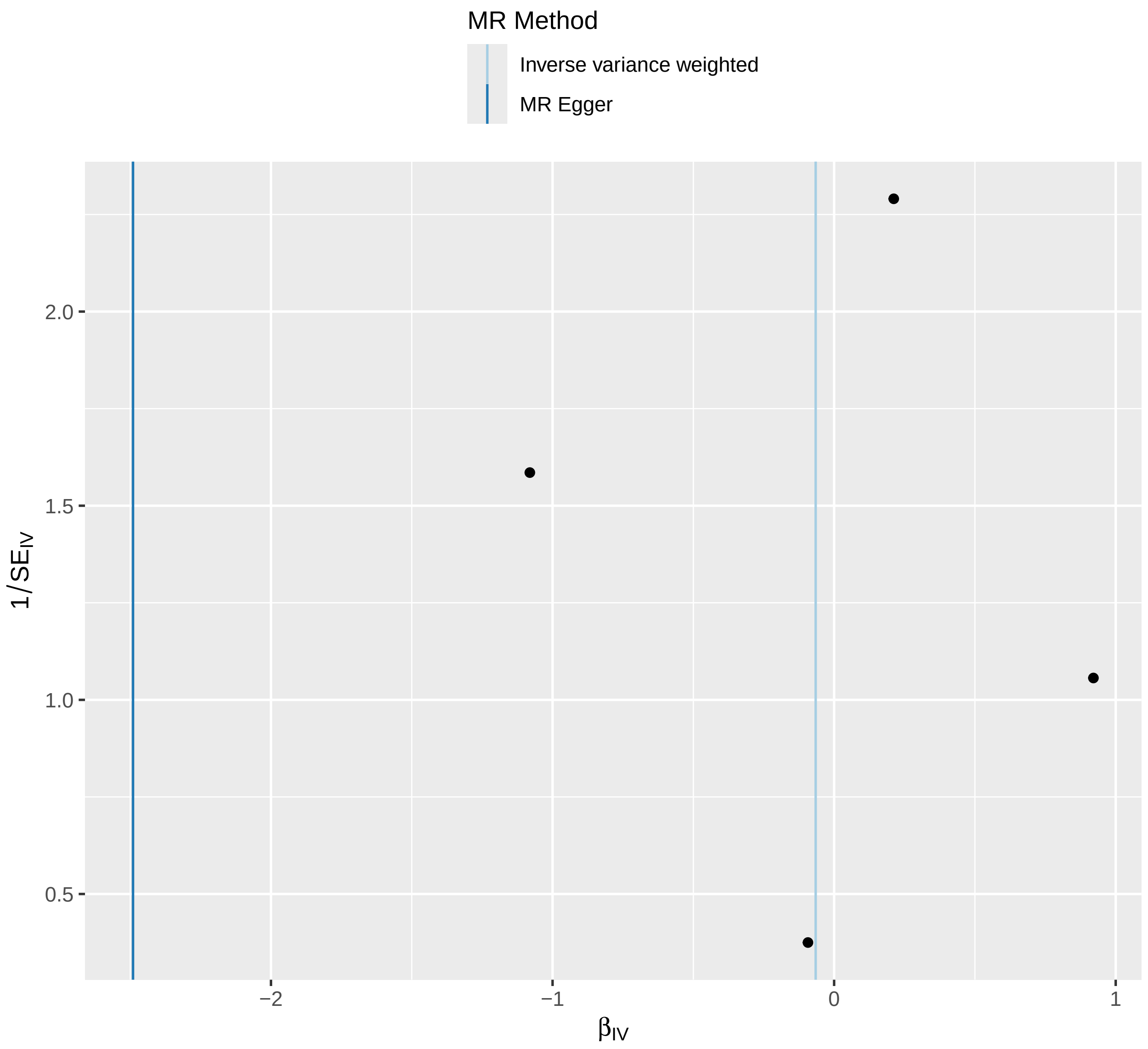
MR Method
Inverse variance weighted
MR Egger
2.0
1.5
1.0
0.5
1/SE$_{IV}$
−2
−1
0
1
β$_{IV}$

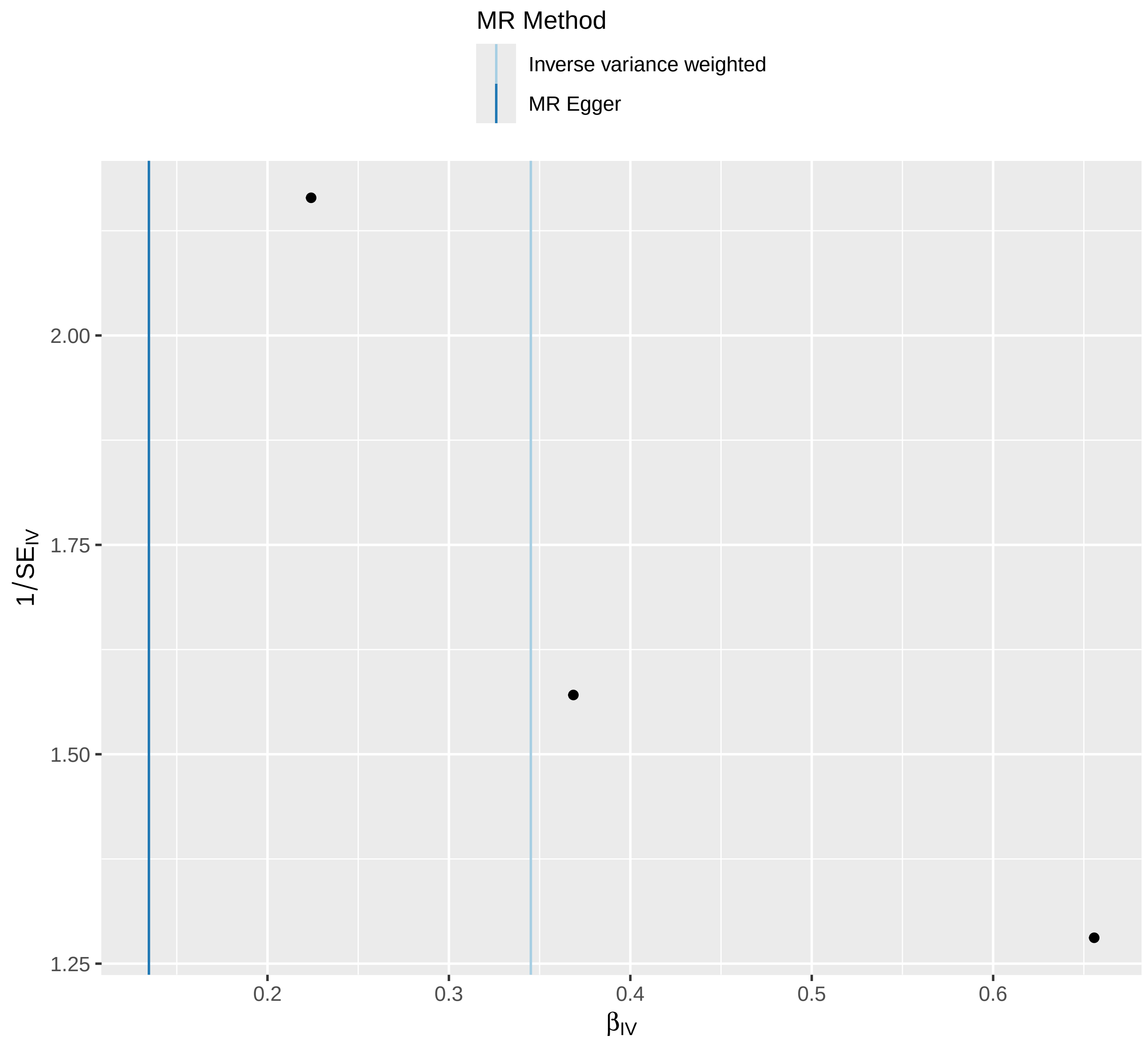

MR Method
Inverse variance weighted
MR Egger
2.00
1.75
1.50
1.25
0.2
0.3
0.4
0.5
0.6
$1/SE_{IV}$
β_{IV}

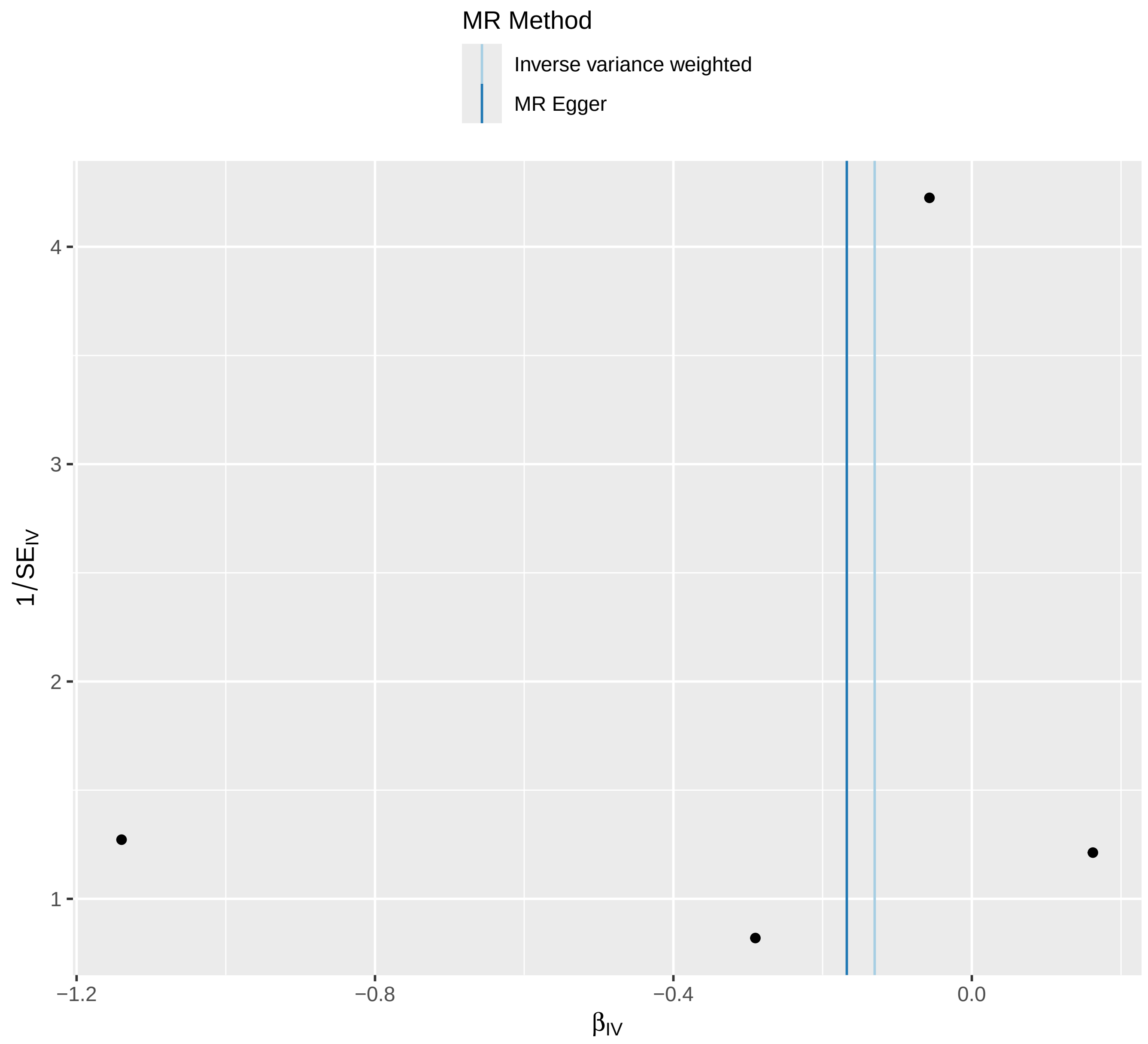

MR Method
Inverse variance weighted
MR Egger
4
3
2
1
1/SE_IV
−1.2
−0.8
−0.4
0.0
β_IV

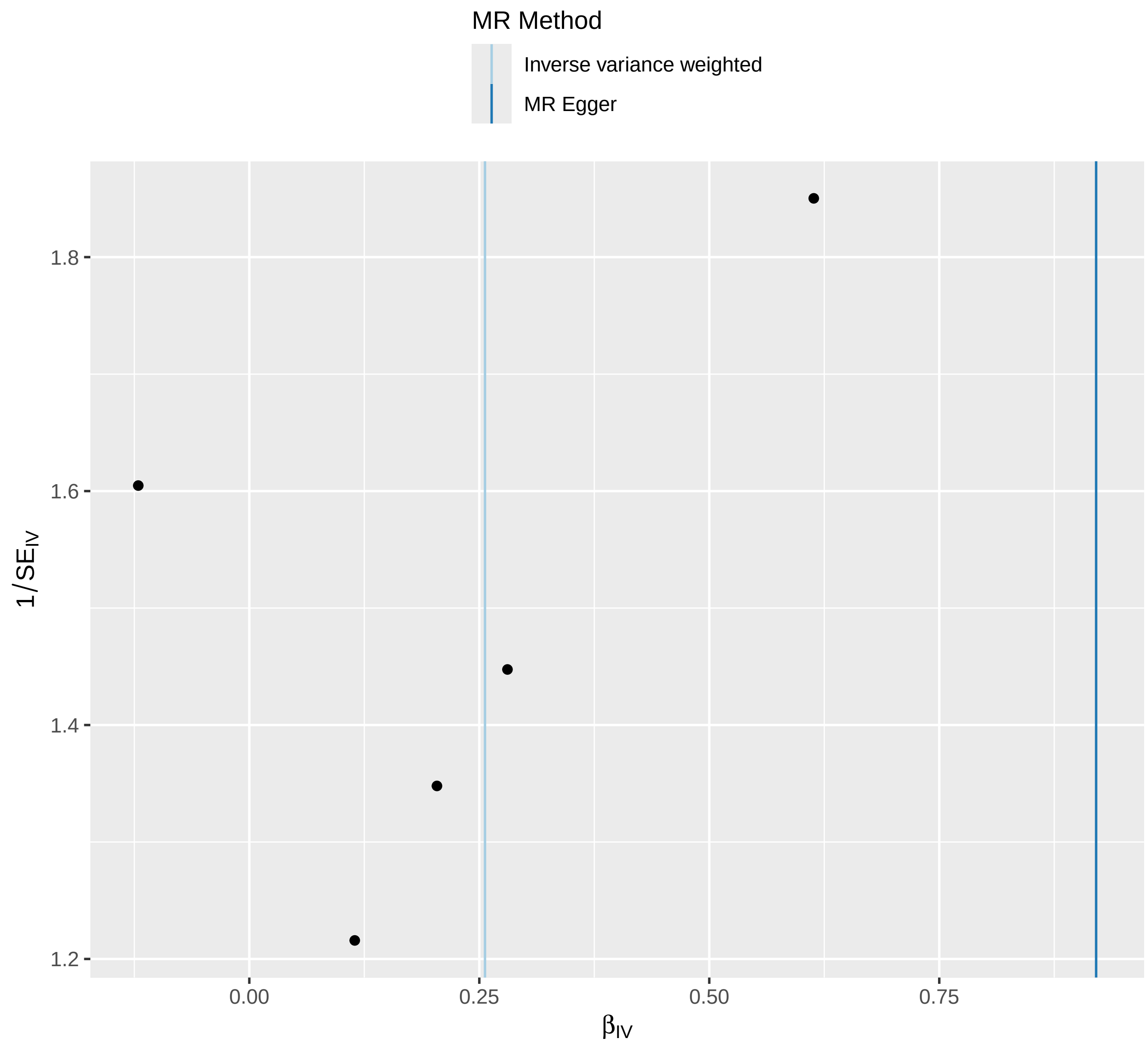
MR Method
Inverse variance weighted
MR Egger
1.8
1.6
1.4
1.2
$1/SE_{IV}$
0.00
0.25
0.50
0.75
β_{IV}

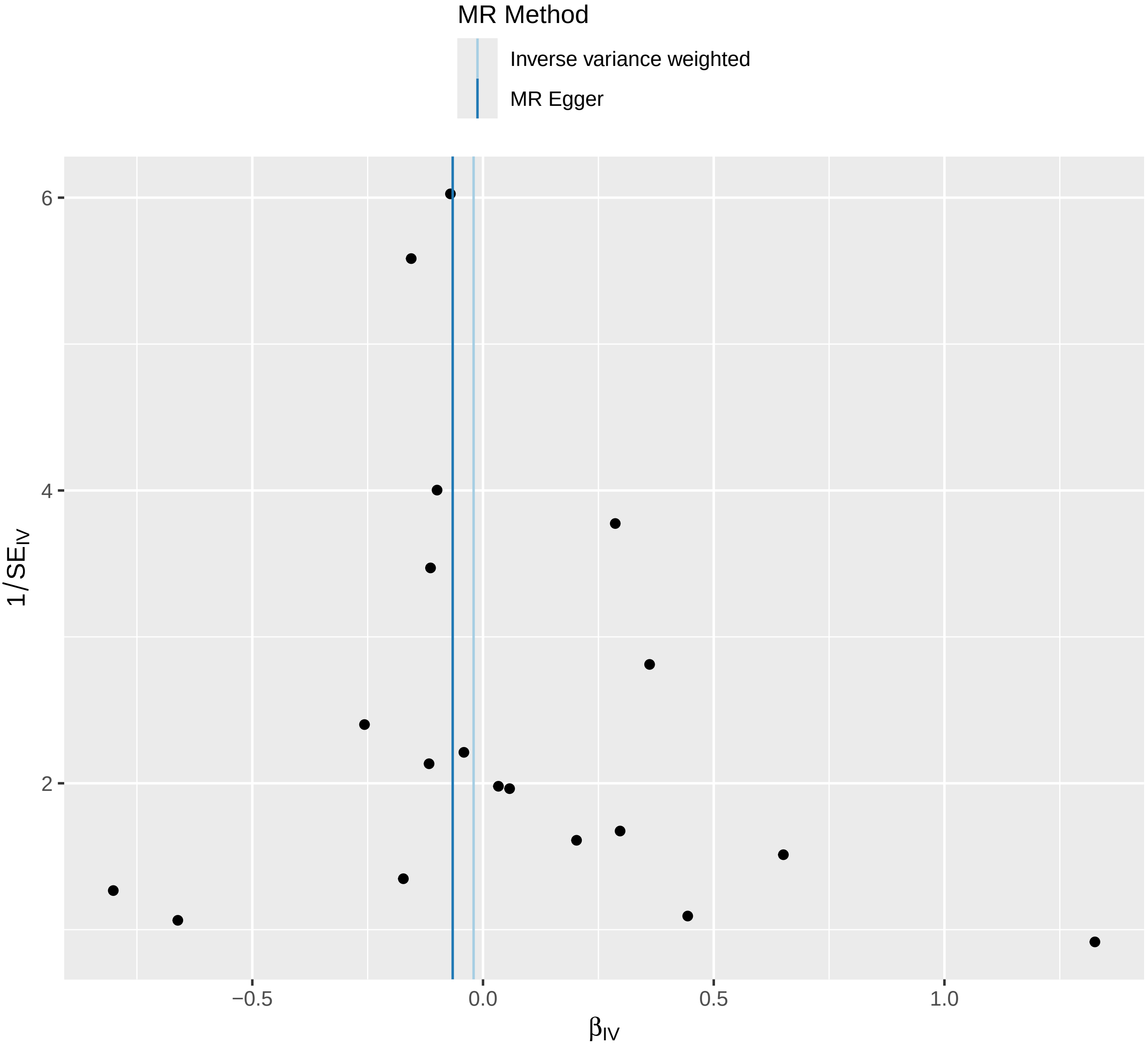
MR Method
Inverse variance weighted
MR Egger
6
4
2
1/SE_IV
−0.5
0.0
0.5
1.0
β_IV

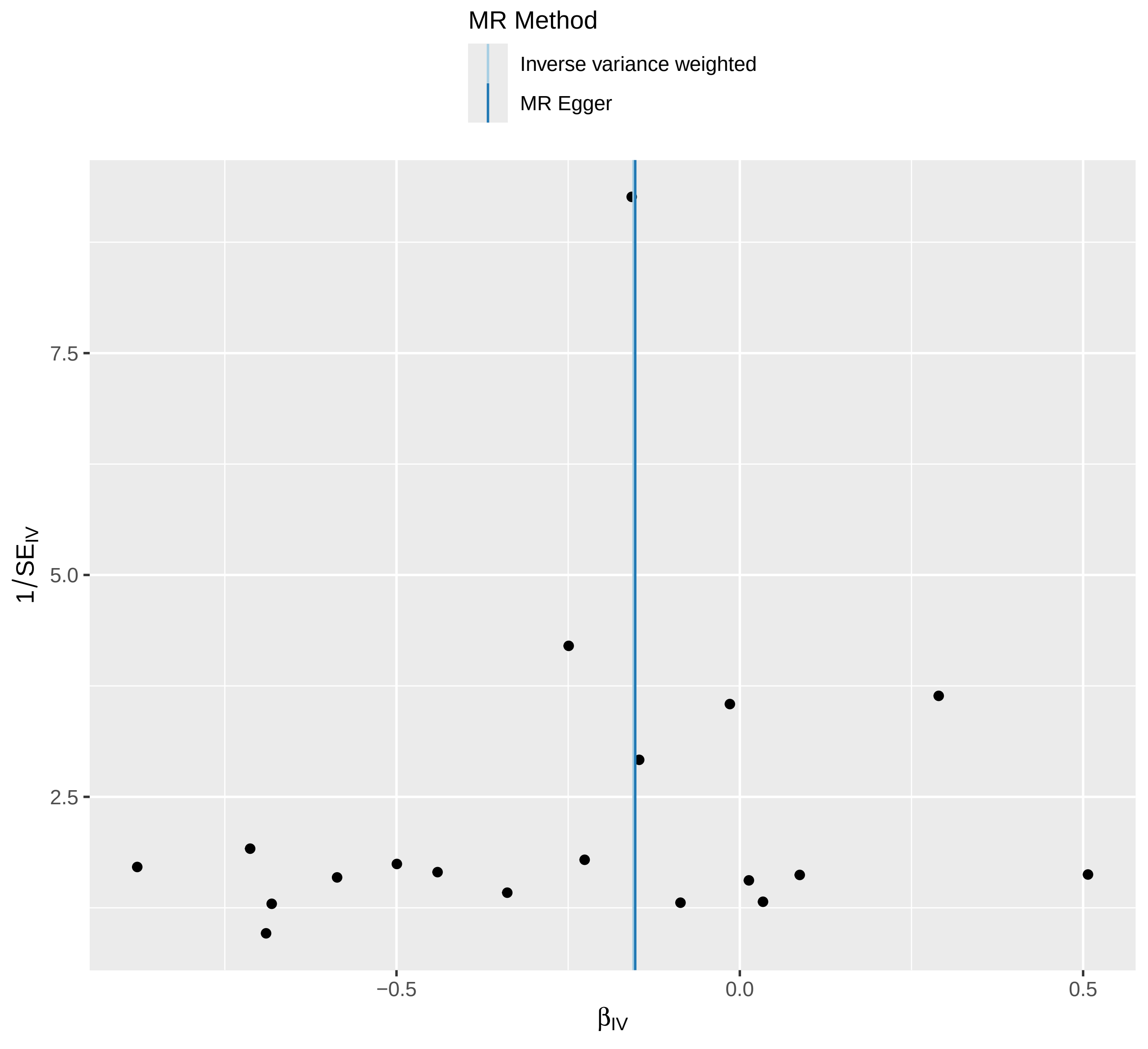
MR Method
Inverse variance weighted
MR Egger
7.5
5.0
2.5
1/SE_IV
−0.5
0.0
0.5
β_IV

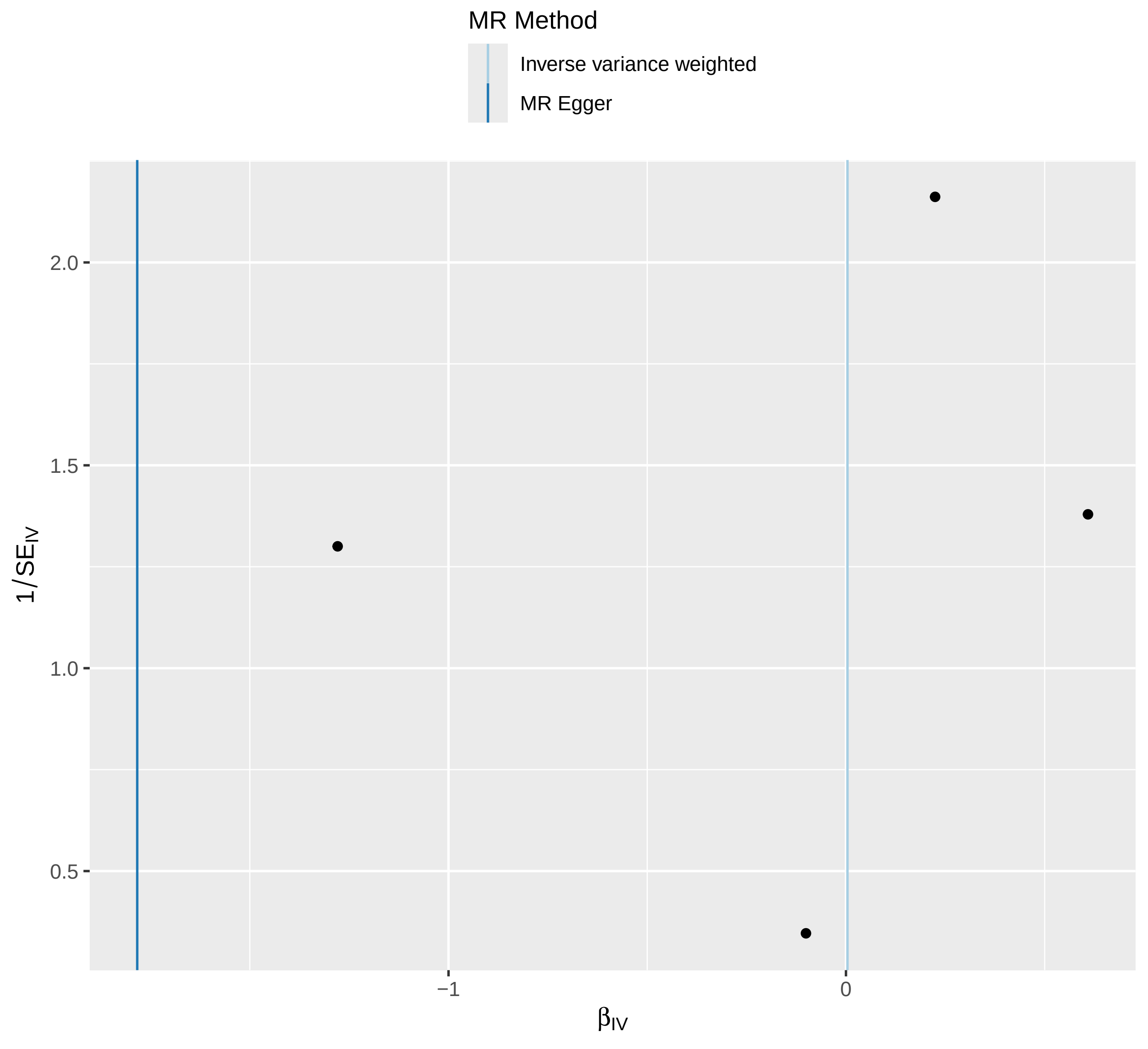
MR Method
Inverse variance weighted
MR Egger
2.0
1.5
1.0
0.5
1/SE$_{IV}$
−1
0
β$_{IV}$

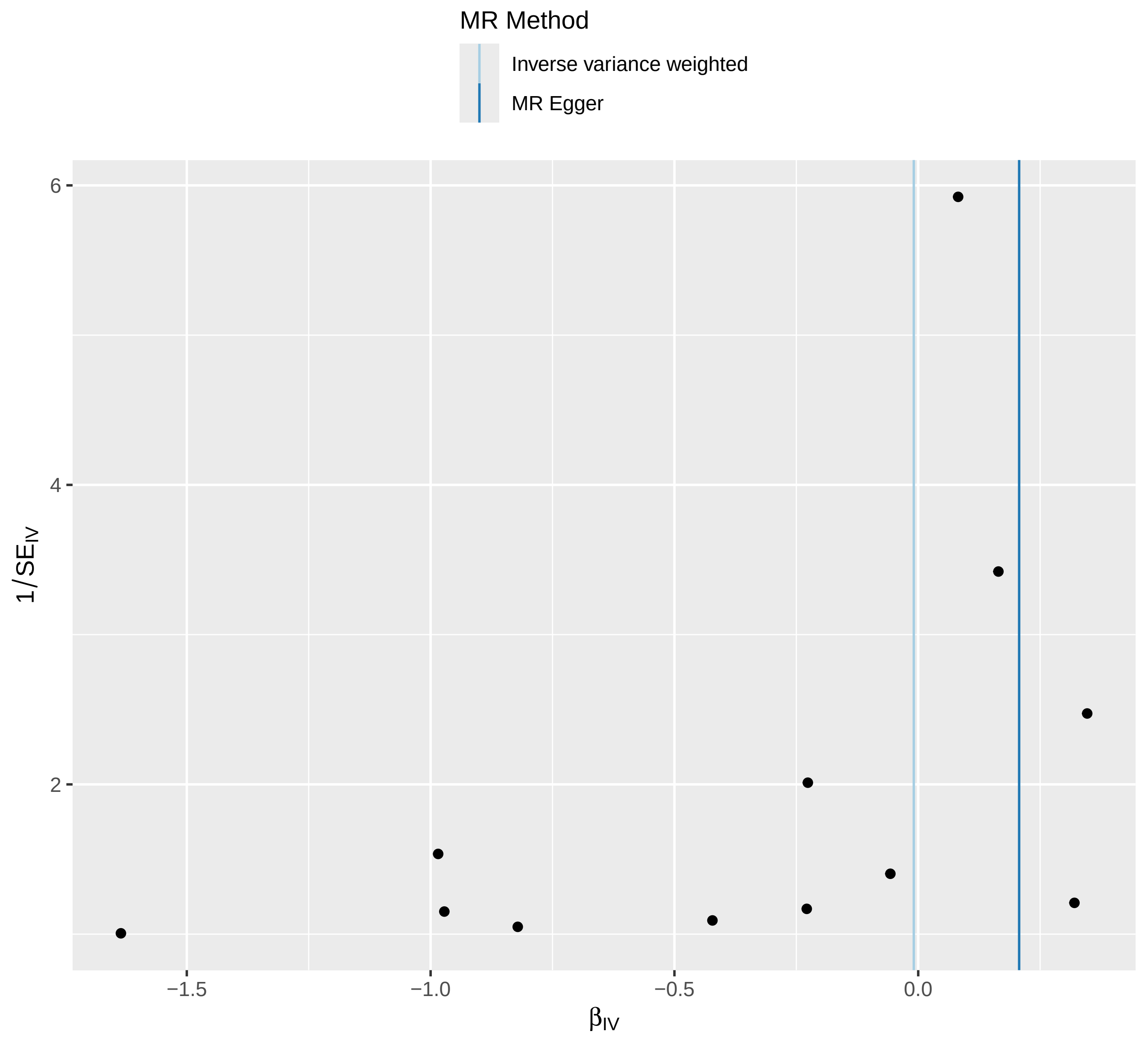
MR Method
Inverse variance weighted
MR Egger
6
4
2
1/SE$_{IV}$
−1.5
−1.0
−0.5
0.0
β$_{IV}$

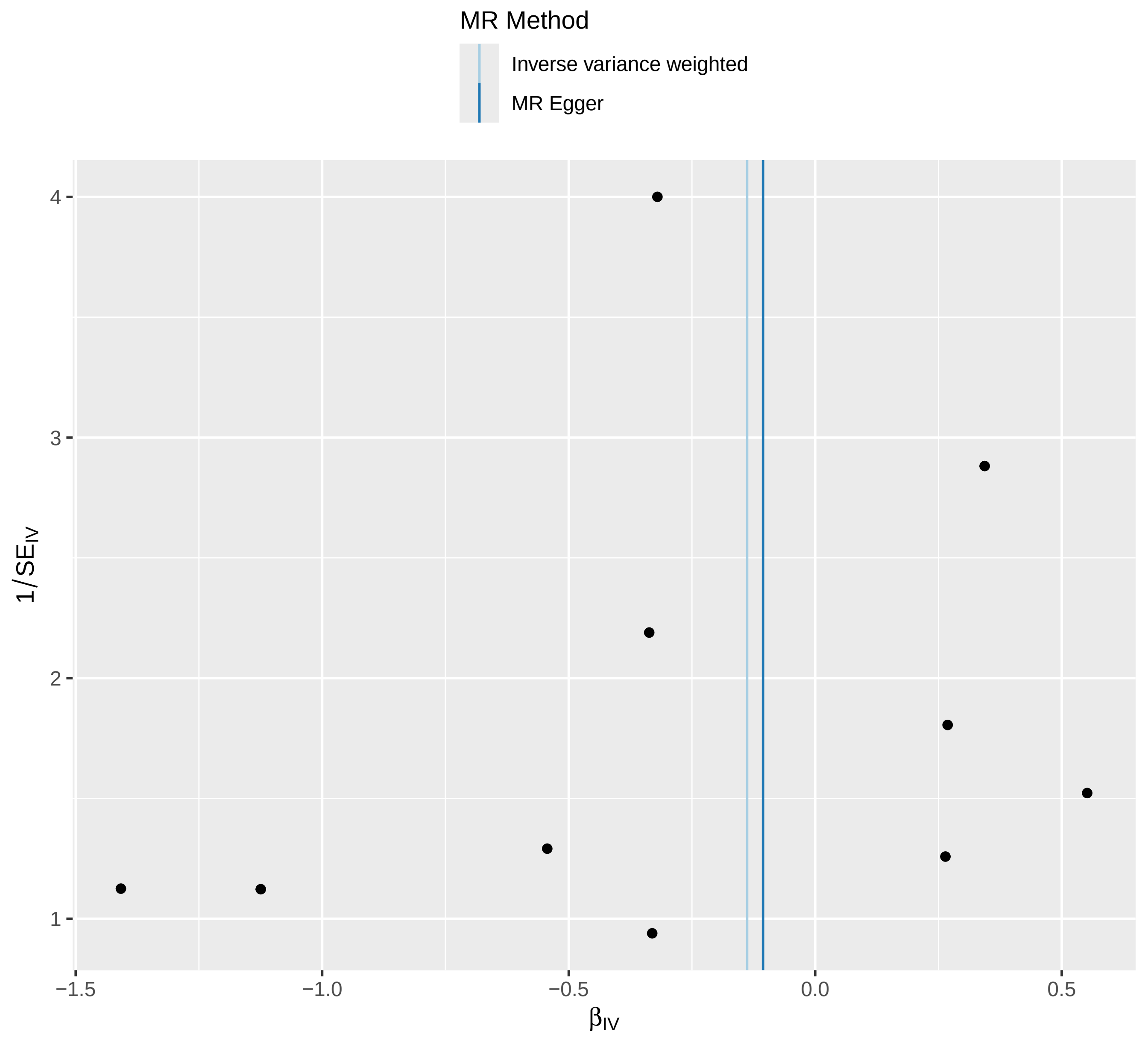
MR Method
Inverse variance weighted
MR Egger
4
3
2
1
−1.5
−1.0
−0.5
0.0
0.5
1/SE_IV
β_IV

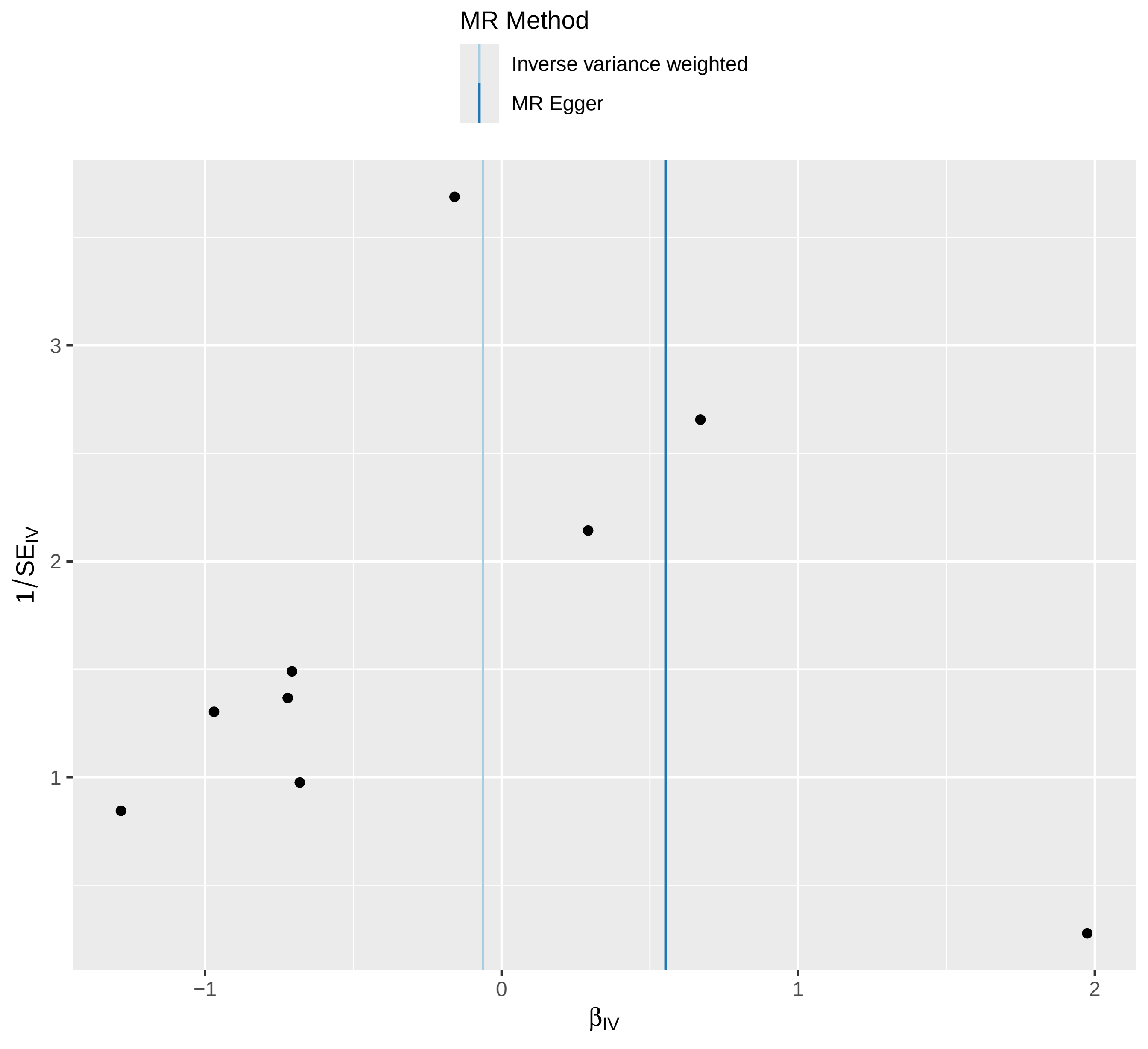
MR Method
Inverse variance weighted
MR Egger
3
2
1
1/SE_IV
−1
0
1
2
β_IV

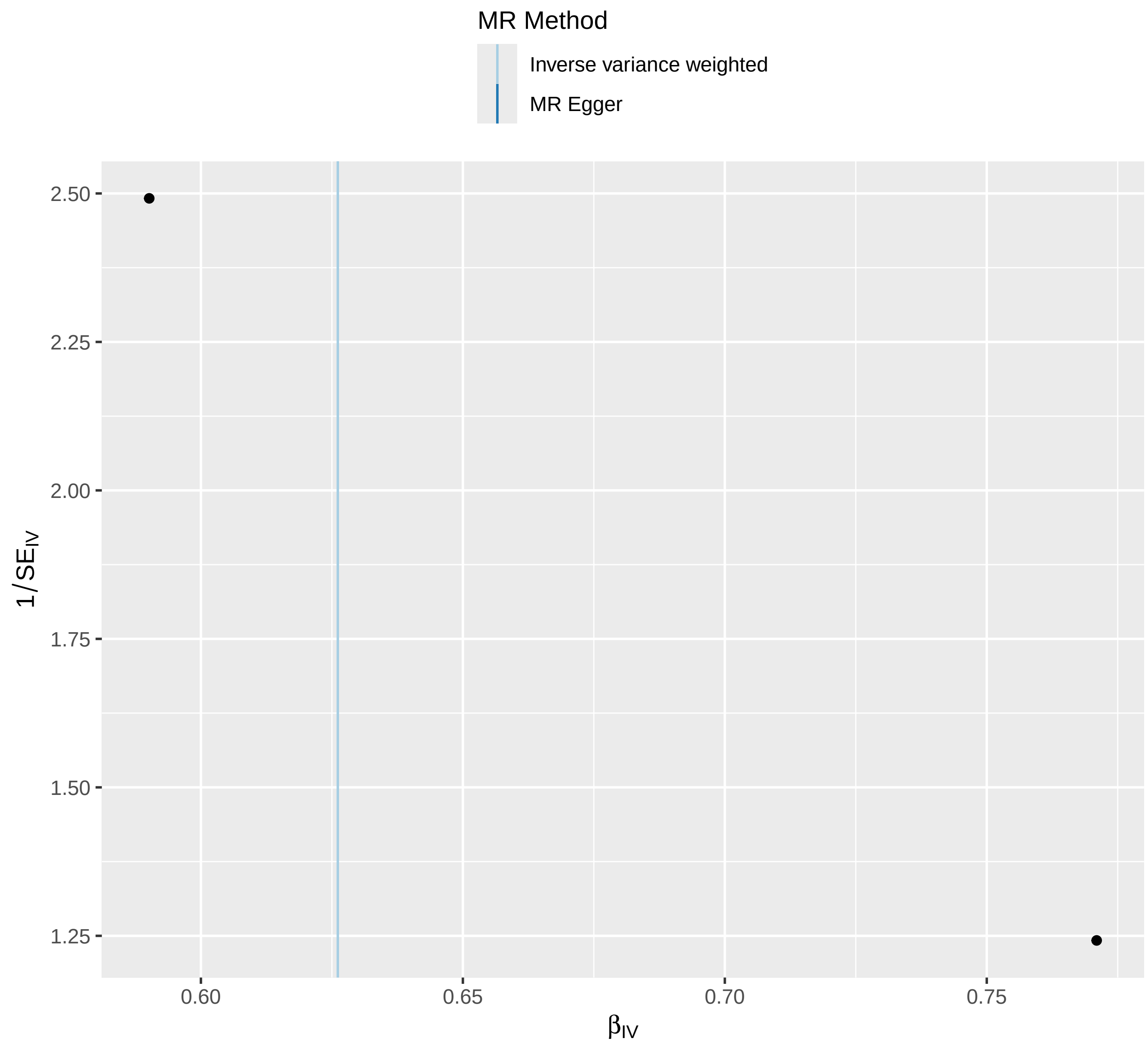
MR Method
Inverse variance weighted
MR Egger
2.50
2.25
2.00
1.75
1.50
1.25
$1/SE_{IV}$
0.60
0.65
0.70
0.75
β_{IV}

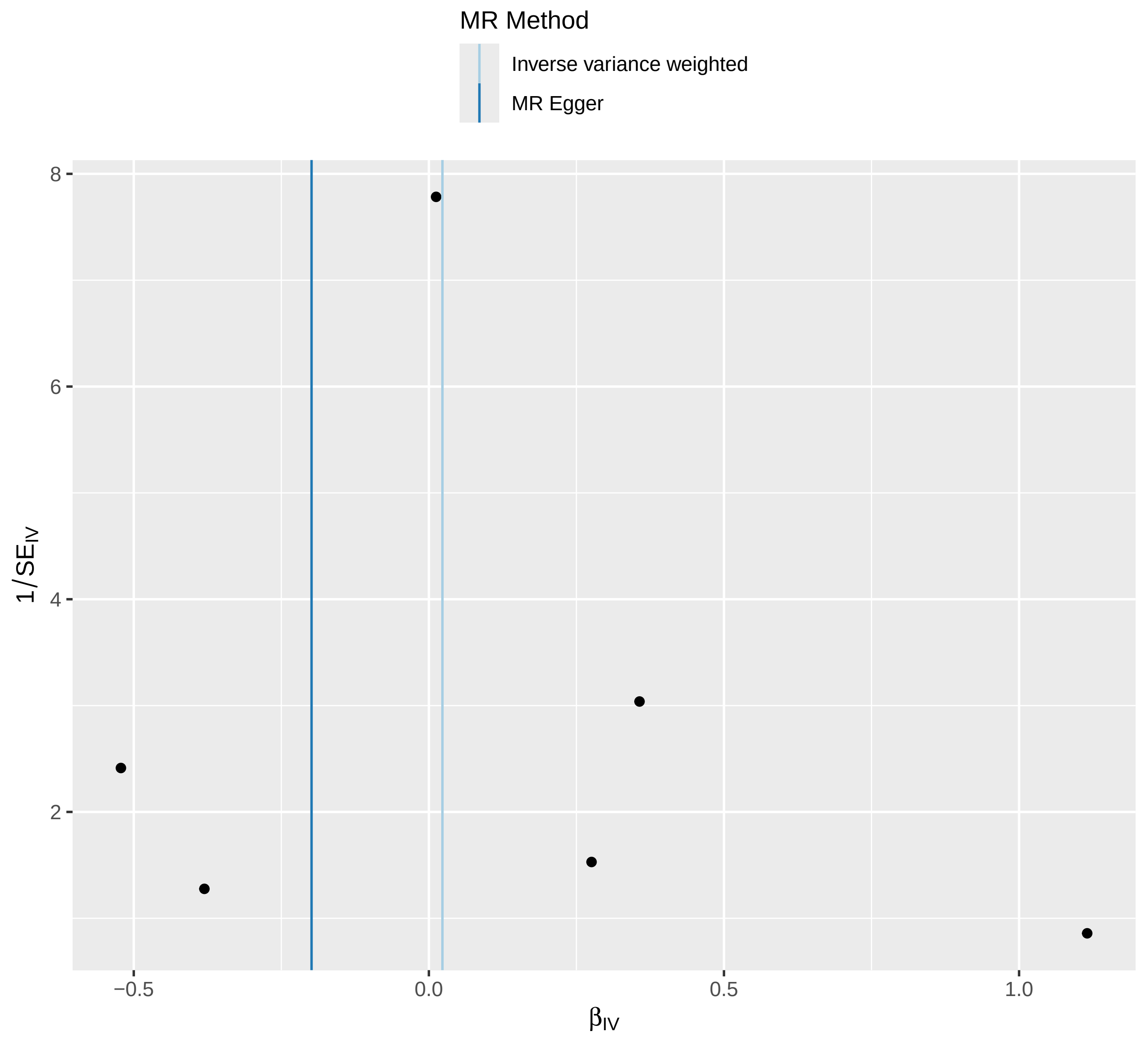

MR Method
Inverse variance weighted
MR Egger
8
6
4
2
1/SE_IV
−0.5
0.0
0.5
1.0
β_IV

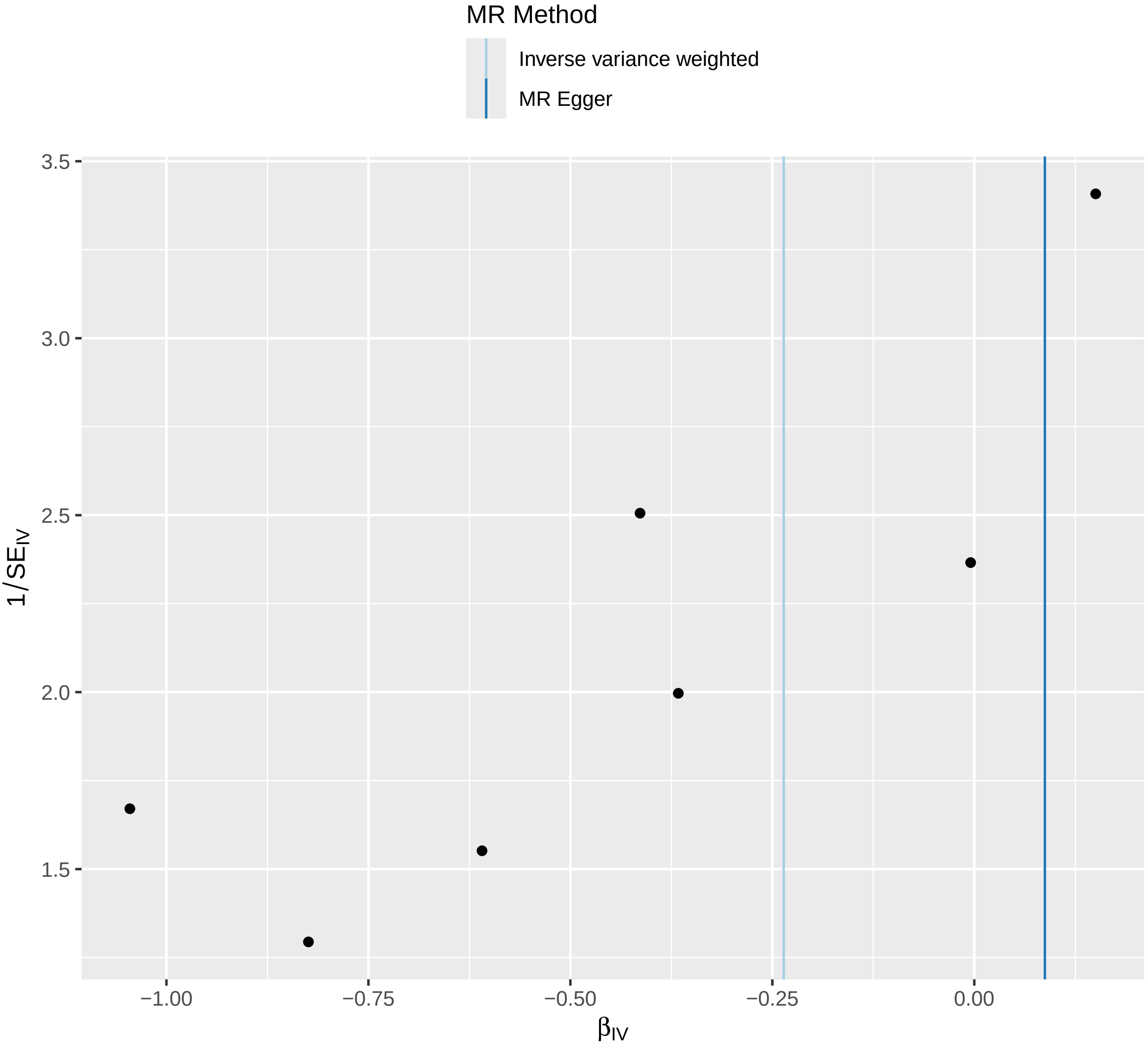
MR Method
Inverse variance weighted
MR Egger
3.5
3.0
2.5
2.0
1.5
1/SE$_{IV}$
−1.00
−0.75
−0.50
−0.25
0.00
β$_{IV}$

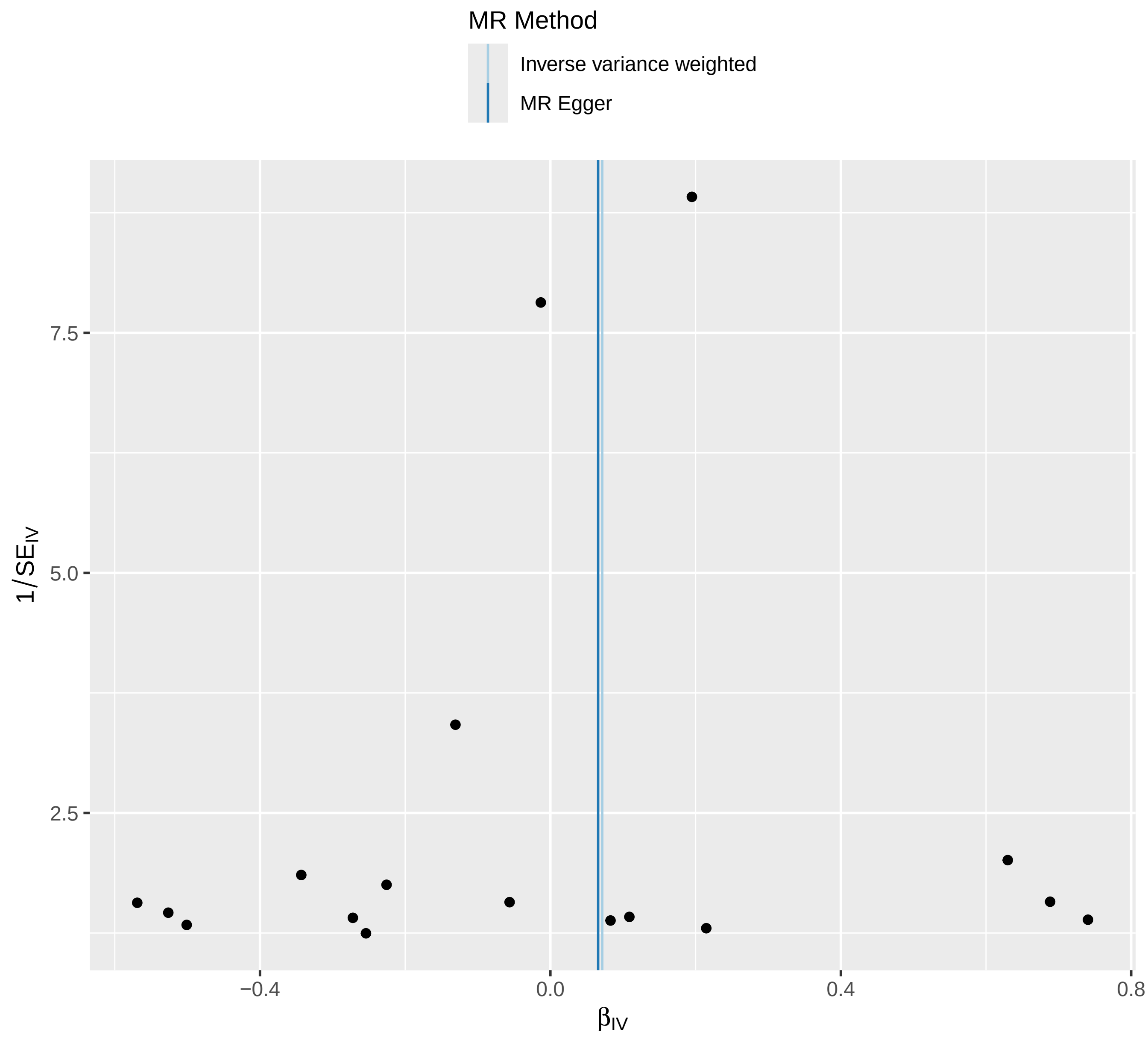

MR Method
Inverse variance weighted
MR Egger
7.5
5.0
2.5
1/SE$_{IV}$
−0.4
0.0
0.4
0.8
β$_{IV}$

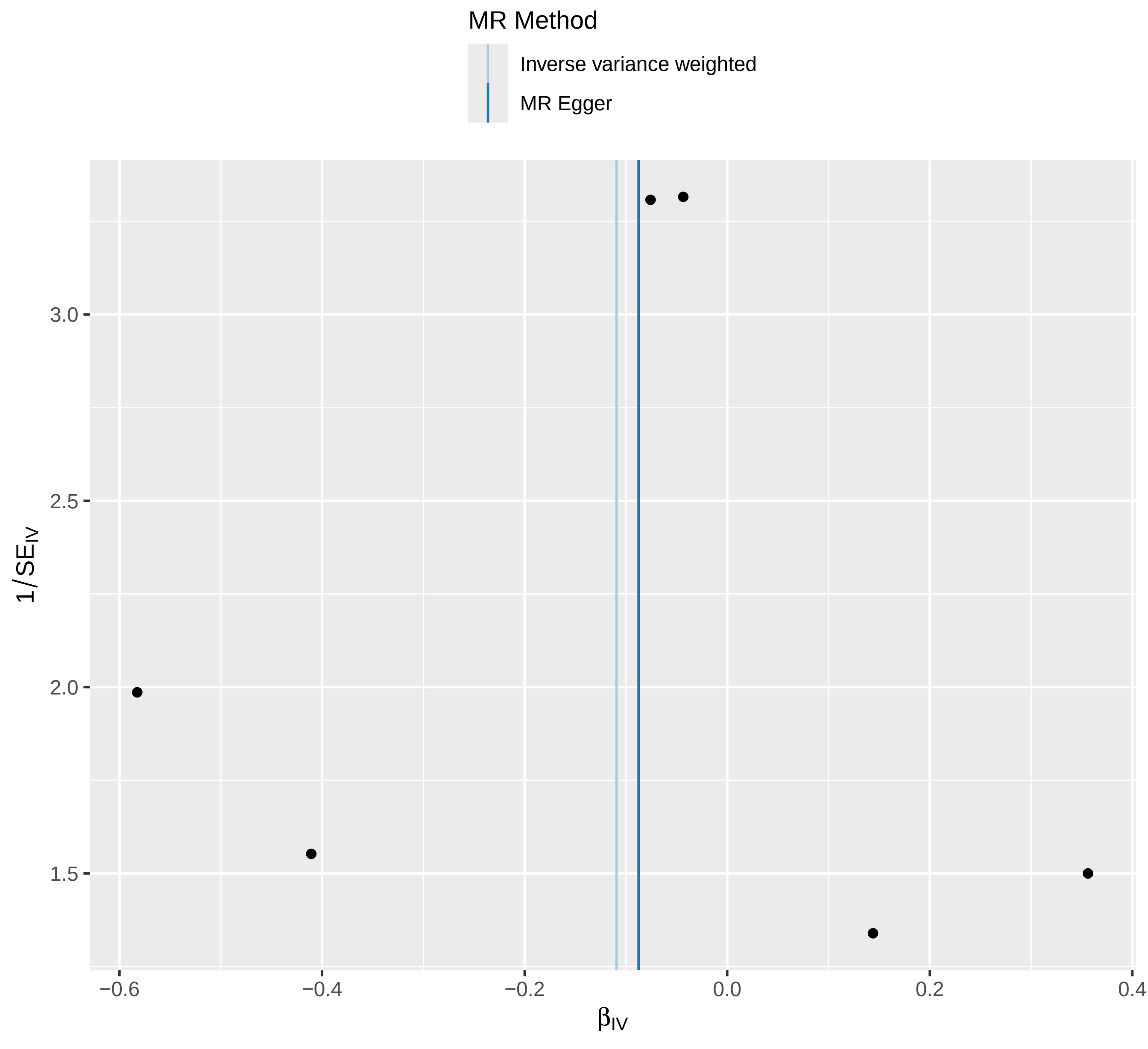
MR Method
Inverse variance weighted
MR Egger
3.0
2.5
2.0
1.5
1/SE_IV
−0.6
−0.4
−0.2
0.0
0.2
0.4
β_IV

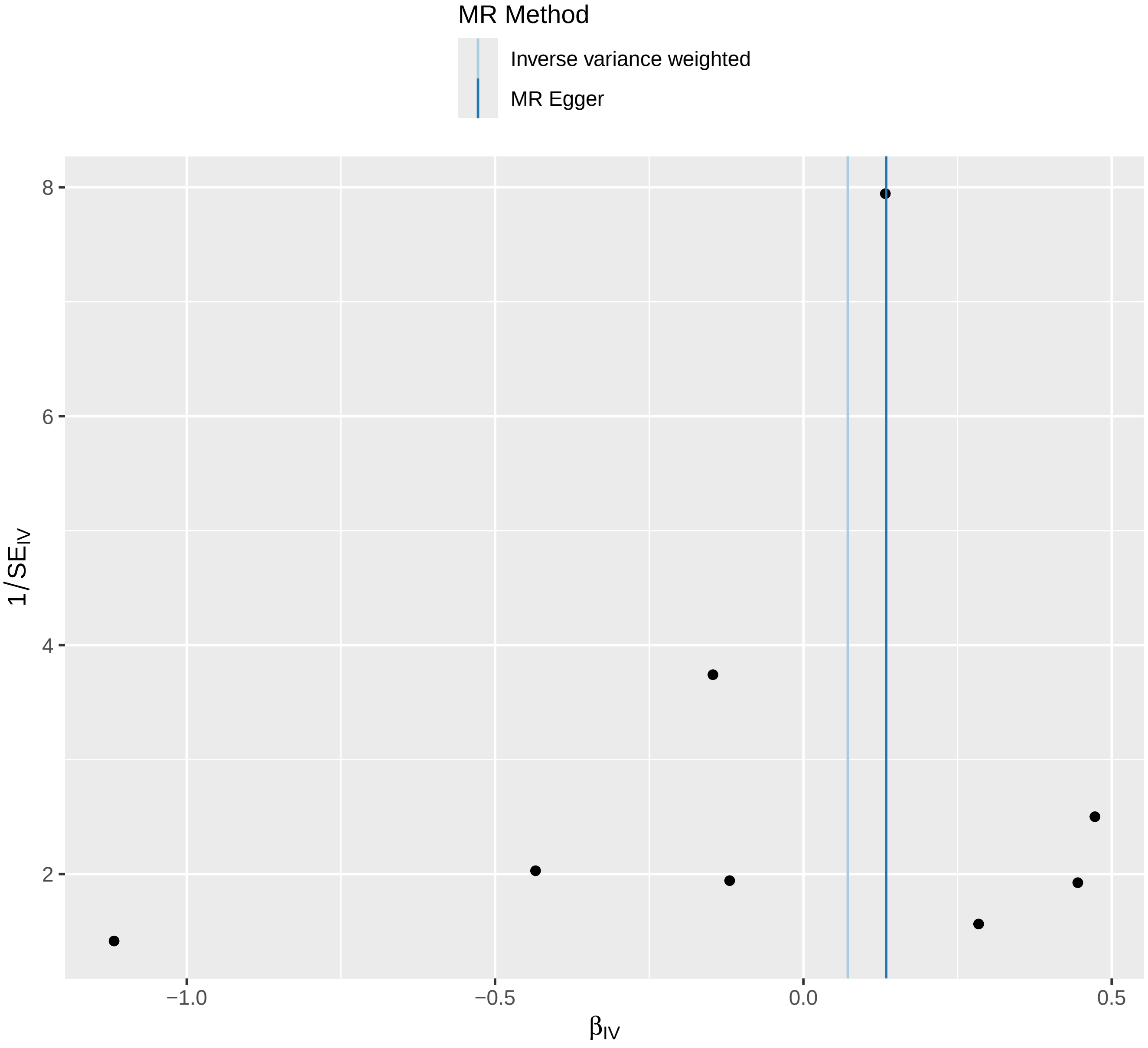
MR Method
Inverse variance weighted
MR Egger
8
6
4
2
1/SE_IV
−1.0
−0.5
0.0
0.5
β_IV

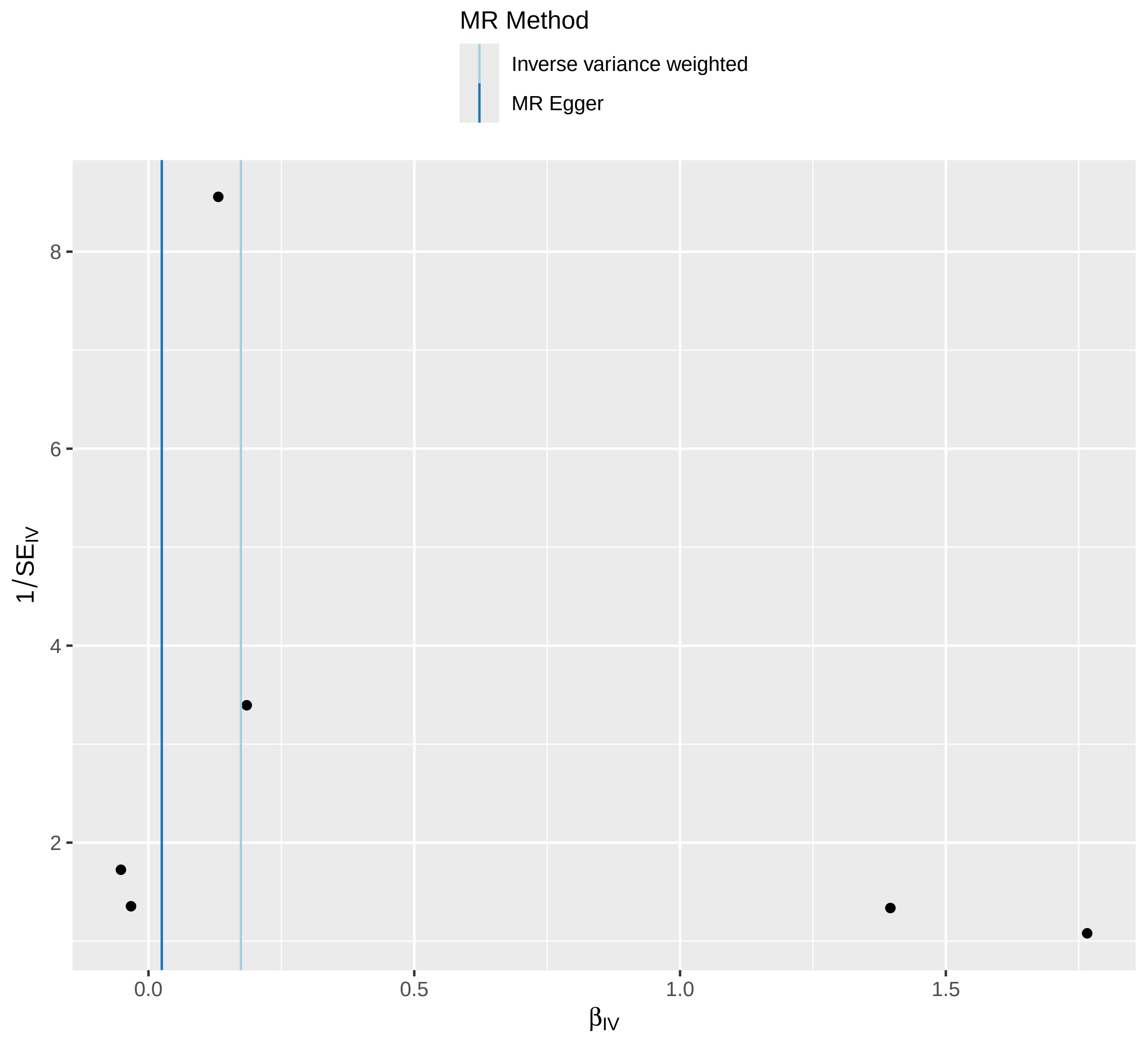
MR Method
Inverse variance weighted
MR Egger
1/SE_IV
8
6
4
2
0.0
0.5
1.0
1.5
β_IV

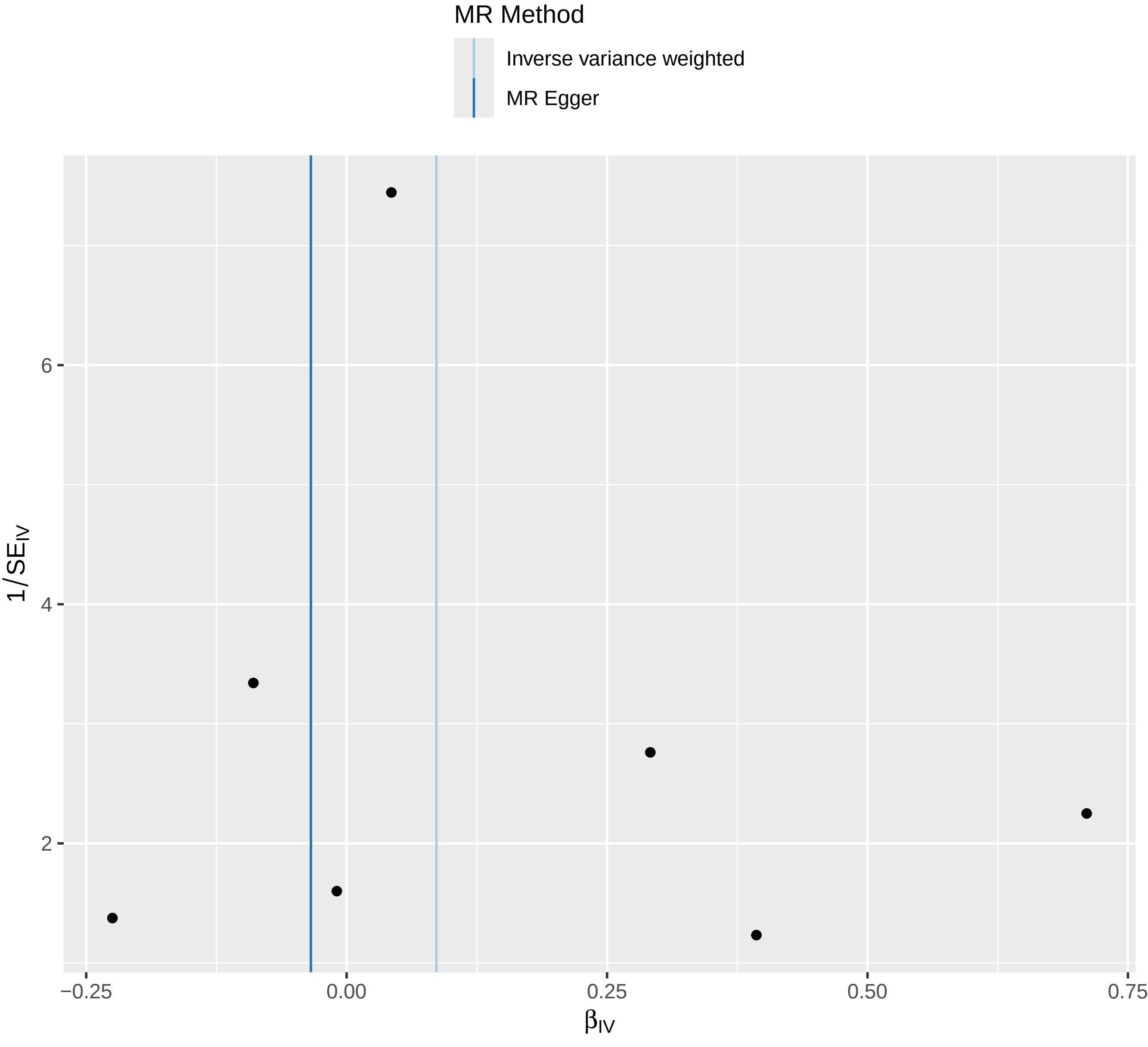
MR Method
Inverse variance weighted
MR Egger
1/SE_IV
6
4
2
−0.25
0.00
0.25
0.50
0.75
β_IV

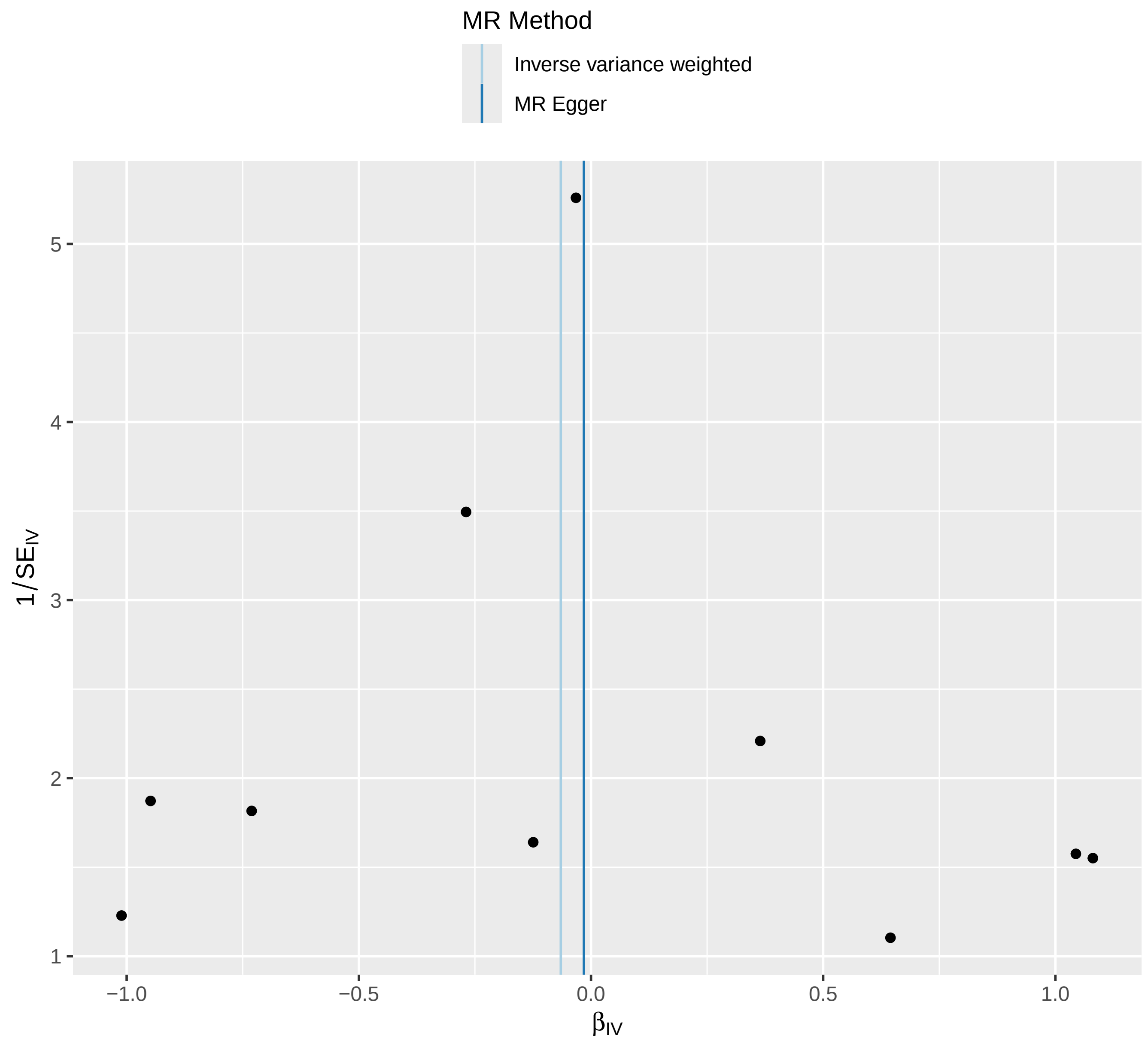

MR Method
Inverse variance weighted
MR Egger
1/SE_IV
5
4
3
2
1
−1.0
−0.5
0.0
0.5
1.0
β_IV

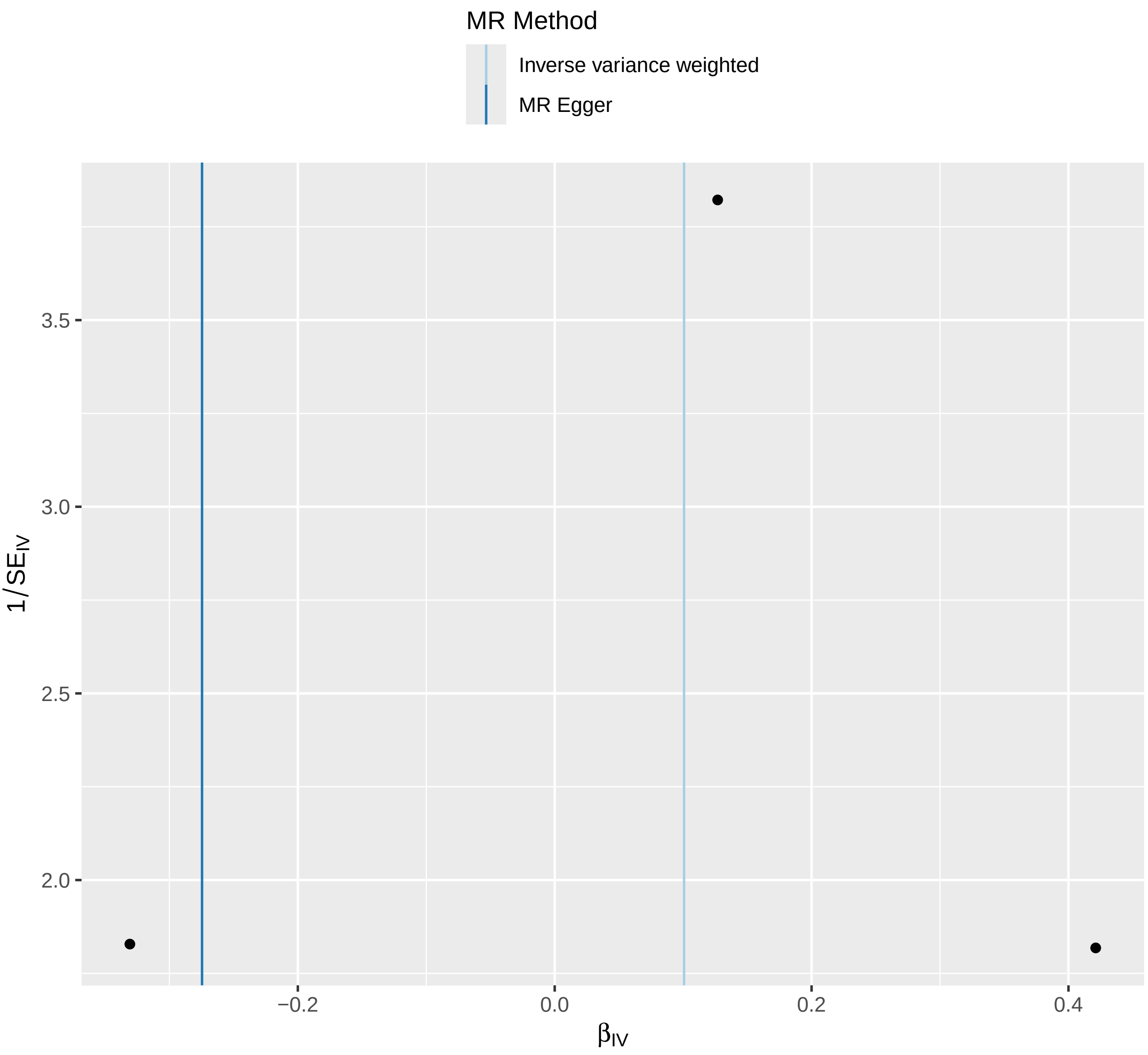
MR Method
Inverse variance weighted
MR Egger
3.5
3.0
2.5
2.0
1/SE_{IV}
−0.2
0.0
0.2
0.4
β_{IV}

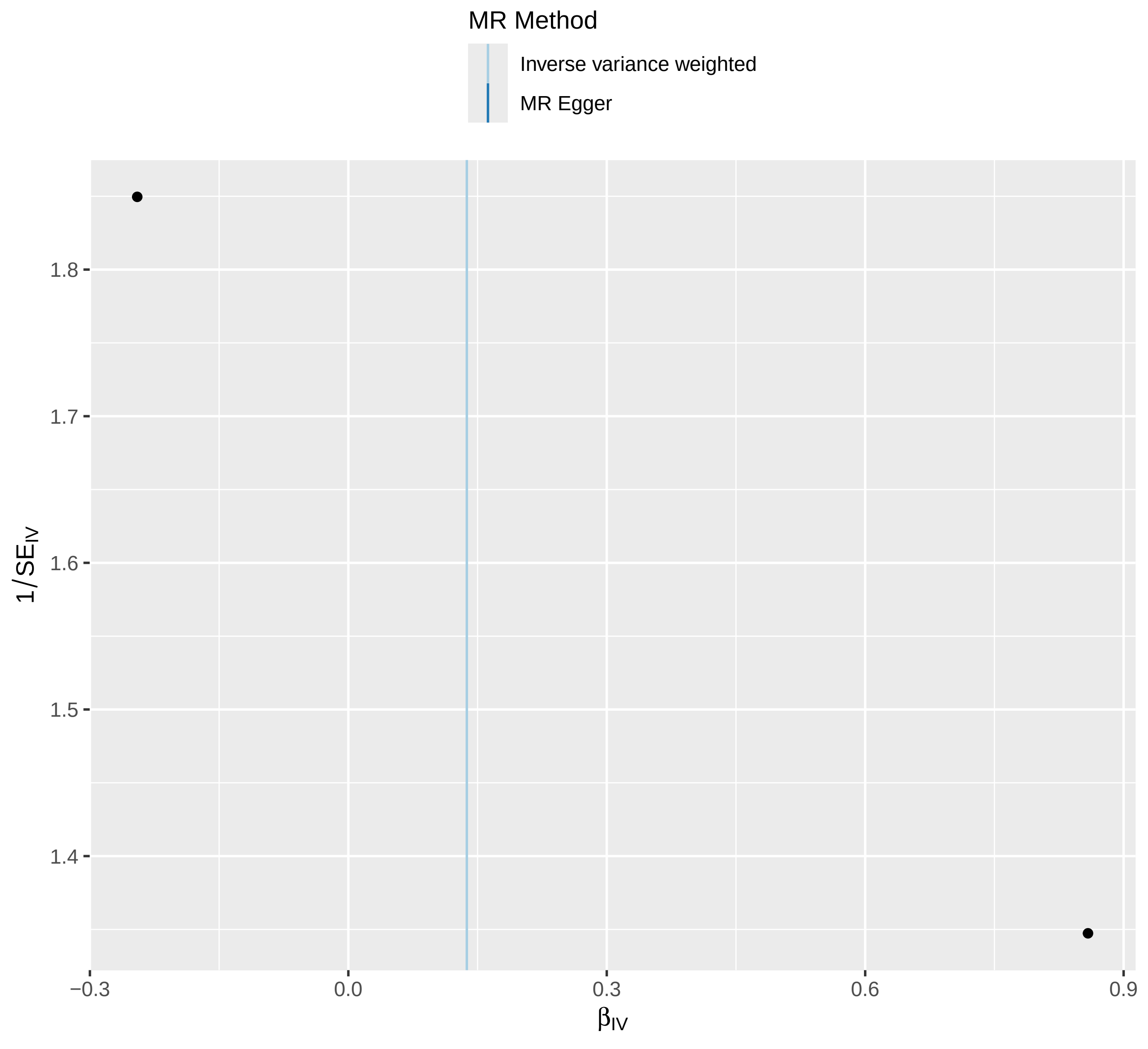

MR Method
Inverse variance weighted
MR Egger
1/SE_IV
1.8
1.7
1.6
1.5
1.4
−0.3
0.0
0.3
0.6
0.9
β_IV

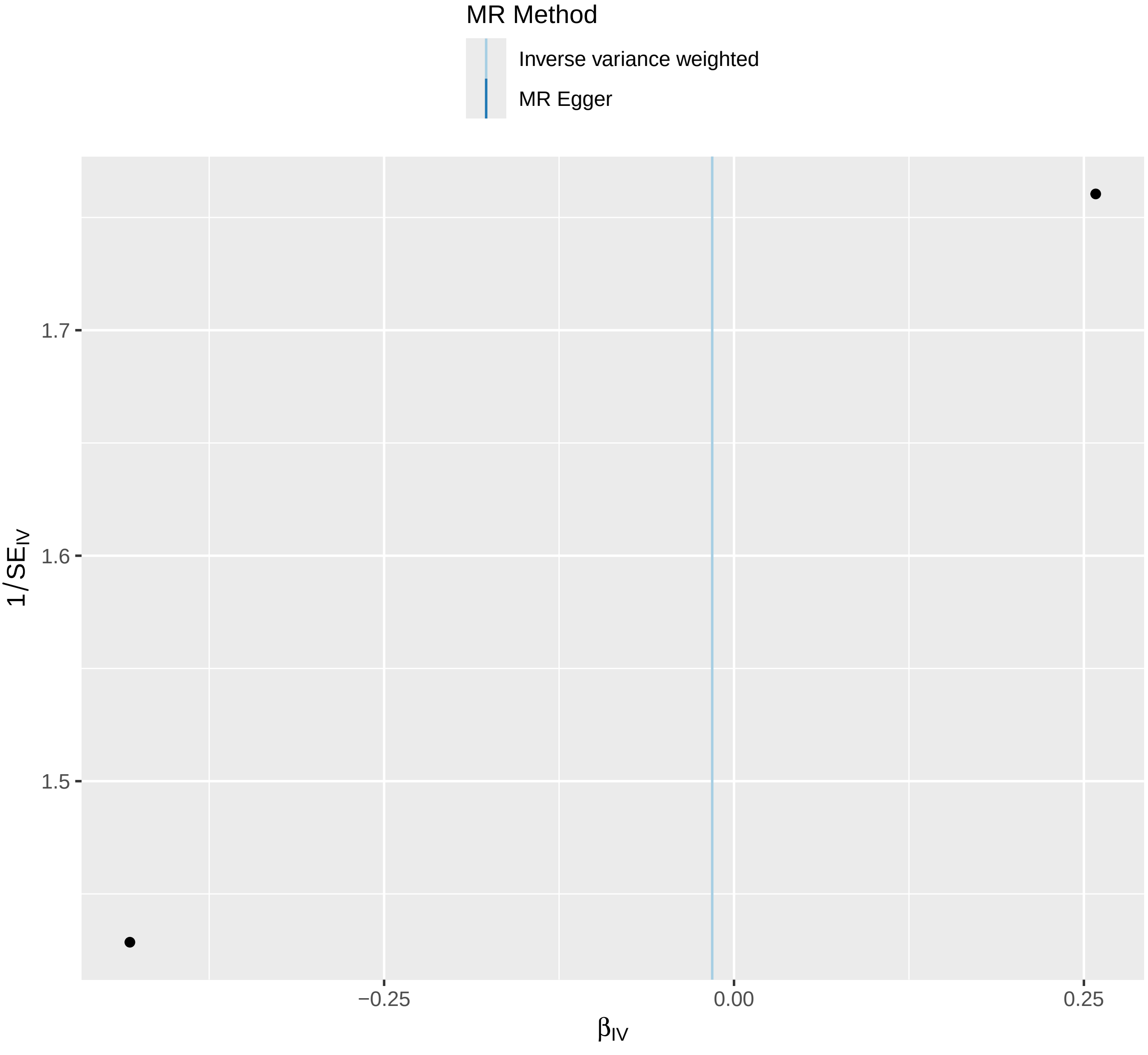
MR Method
Inverse variance weighted
MR Egger
1.7
1.6
1.5
1/SE_IV
−0.25
0.00
0.25
β_IV

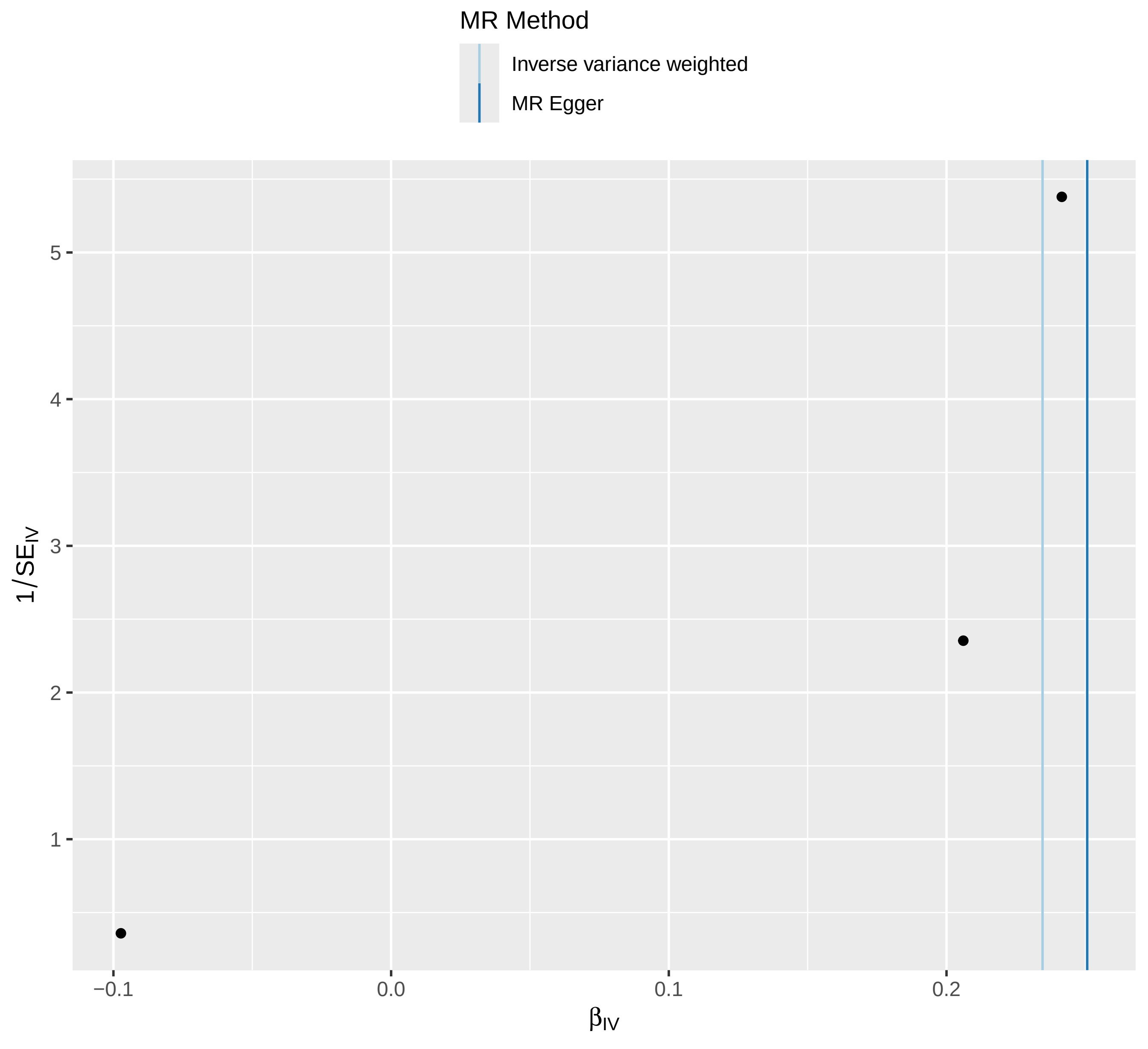

MR Method
Inverse variance weighted
MR Egger
5
4
3
2
1
1/SE_IV
−0.1
0.0
0.1
0.2
β_IV

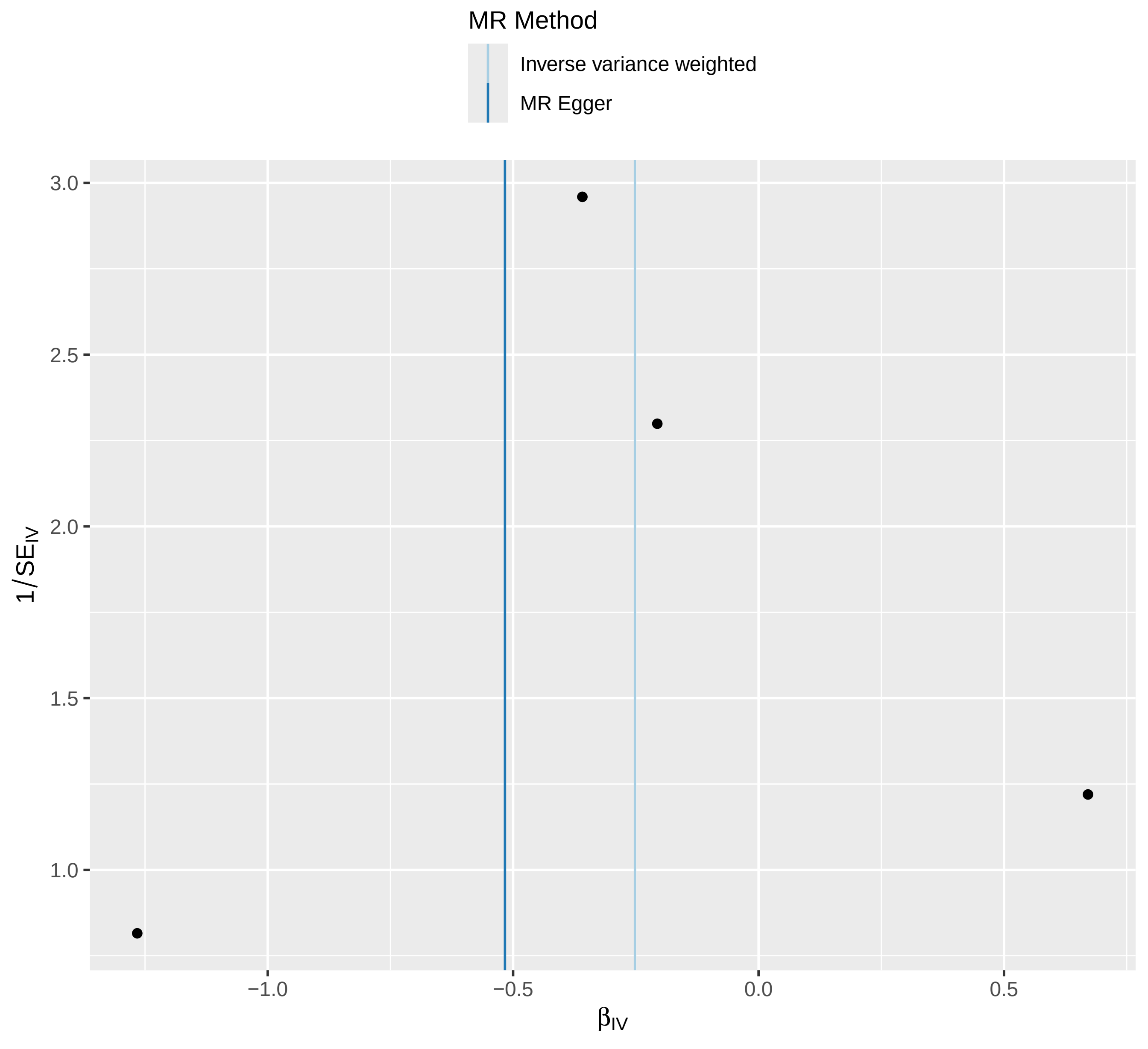

MR Method
Inverse variance weighted
MR Egger
3.0
2.5
2.0
1.5
1.0
$1/SE_{IV}$
−1.0
−0.5
0.0
0.5
β_{IV}

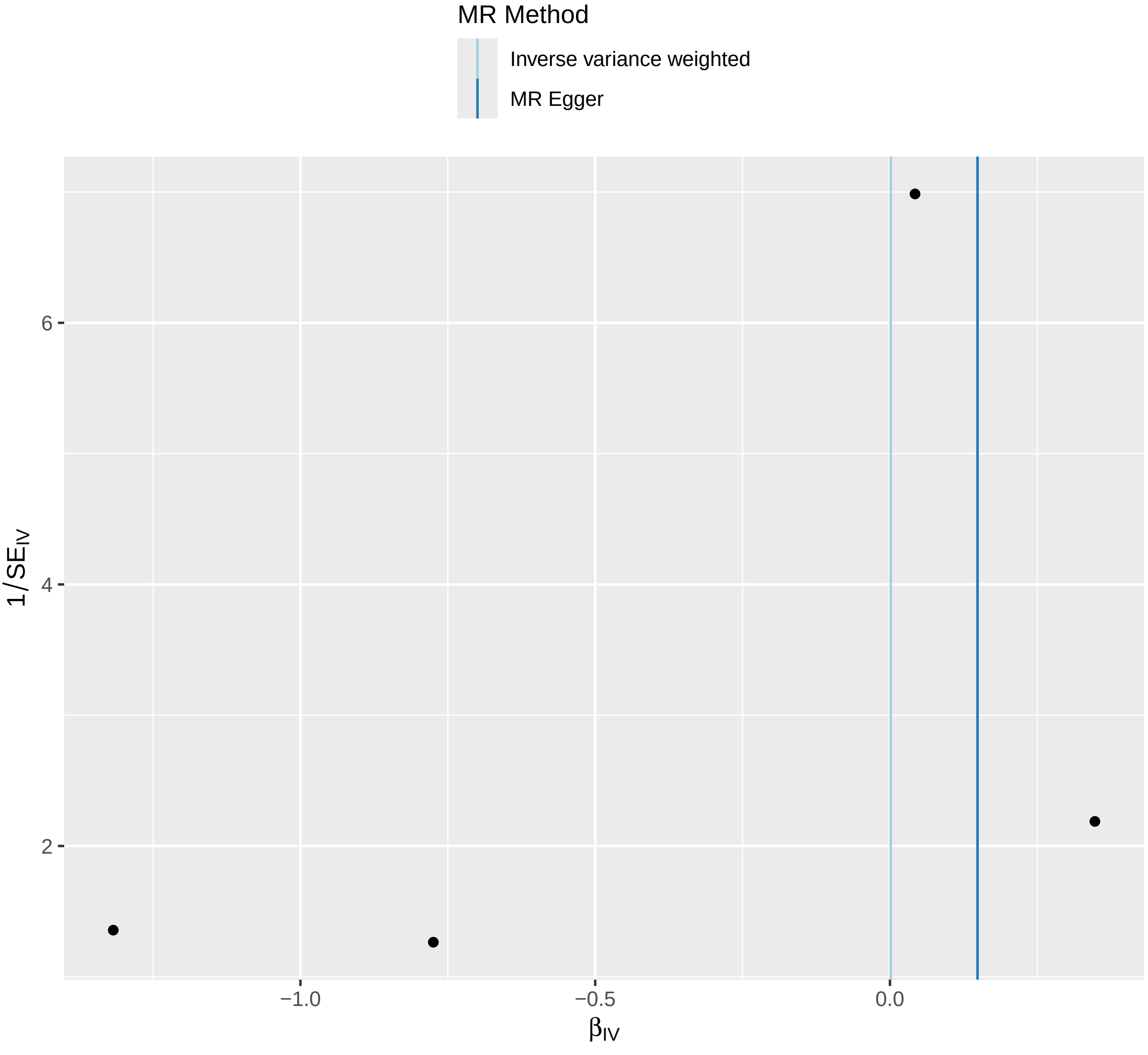

MR Method
Inverse variance weighted
MR Egger
6
4
2
1/SE_IV
−1.0
−0.5
0.0
β_IV

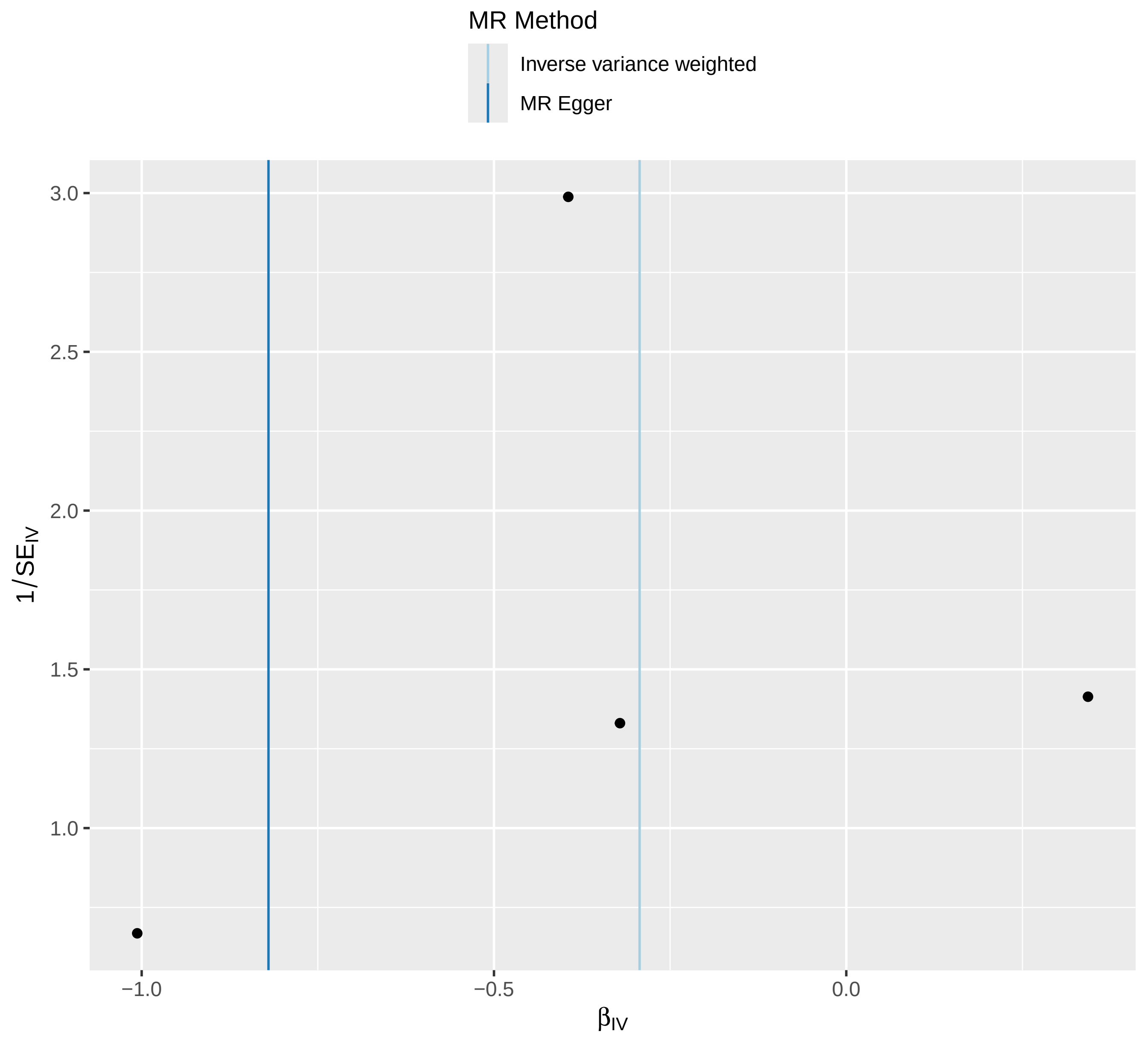
MR Method
Inverse variance weighted
MR Egger
3.0
2.5
2.0
1.5
1.0
1/SE_IV
−1.0
−0.5
0.0
β_IV

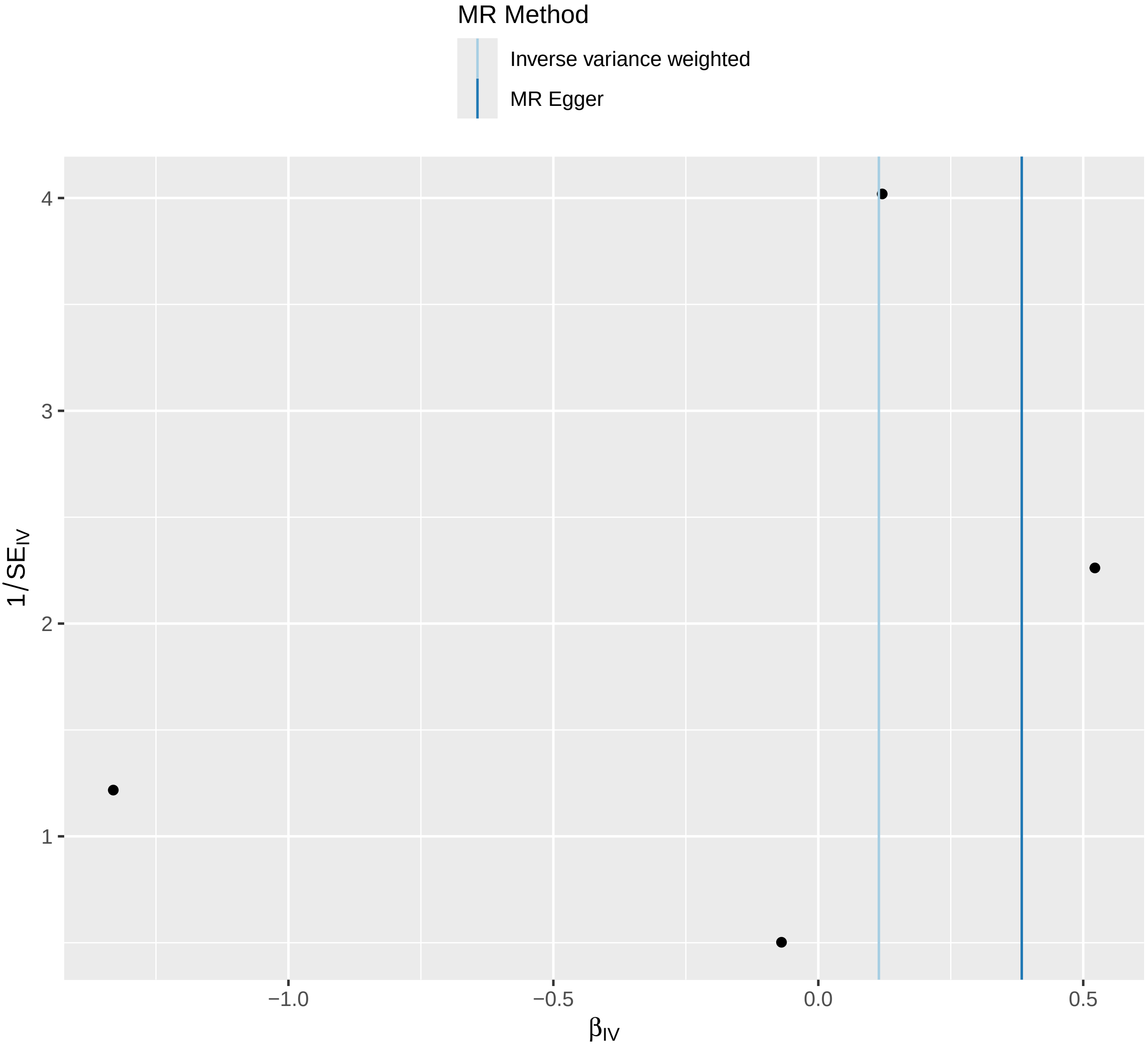
MR Method
Inverse variance weighted
MR Egger
4
3
2
1
1/SE_IV
−1.0
−0.5
0.0
0.5
β_IV

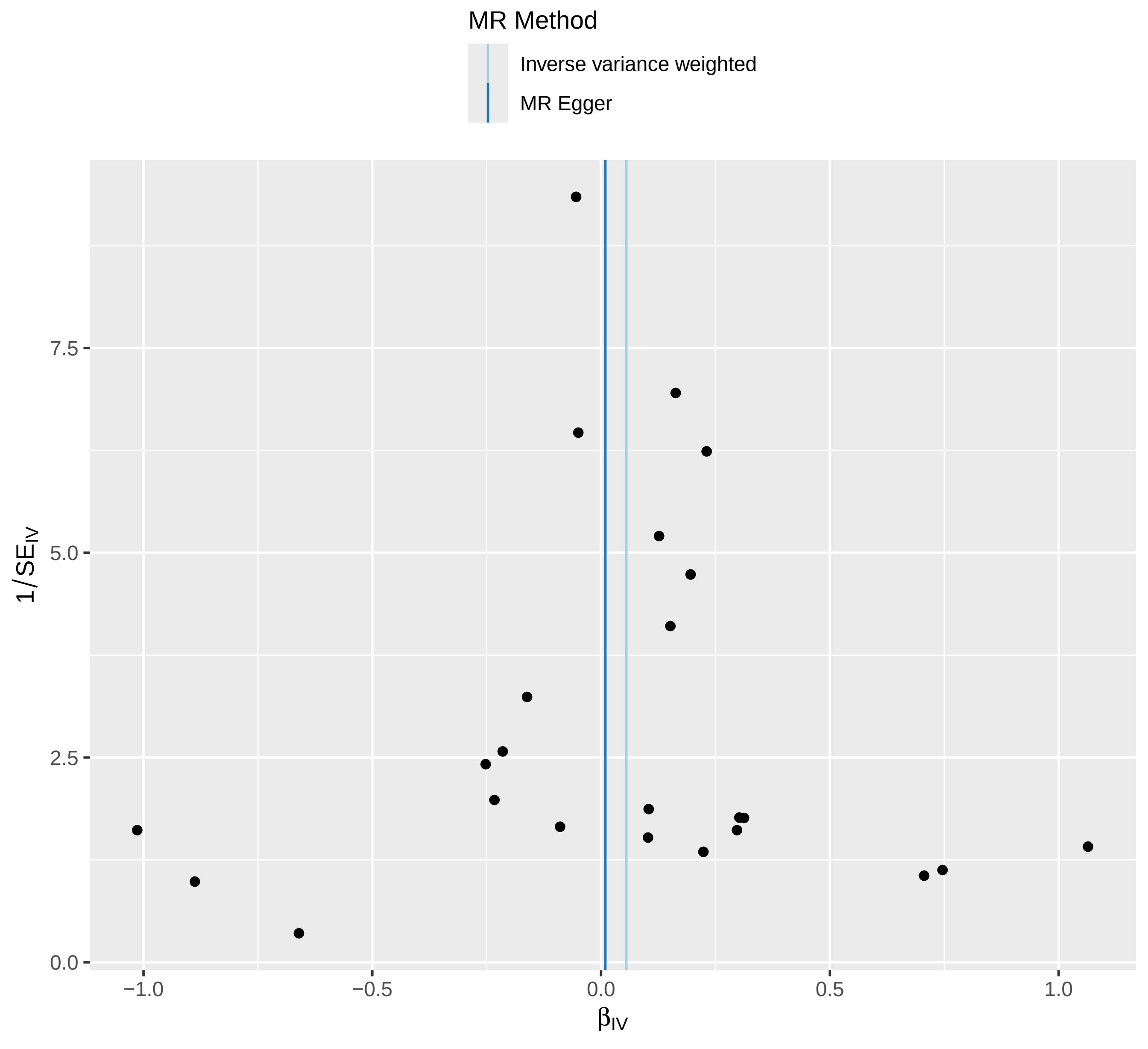
MR Method
Inverse variance weighted
MR Egger
1/SE_IV
7.5
5.0
2.5
0.0
−1.0
−0.5
0.0
0.5
1.0
β_IV

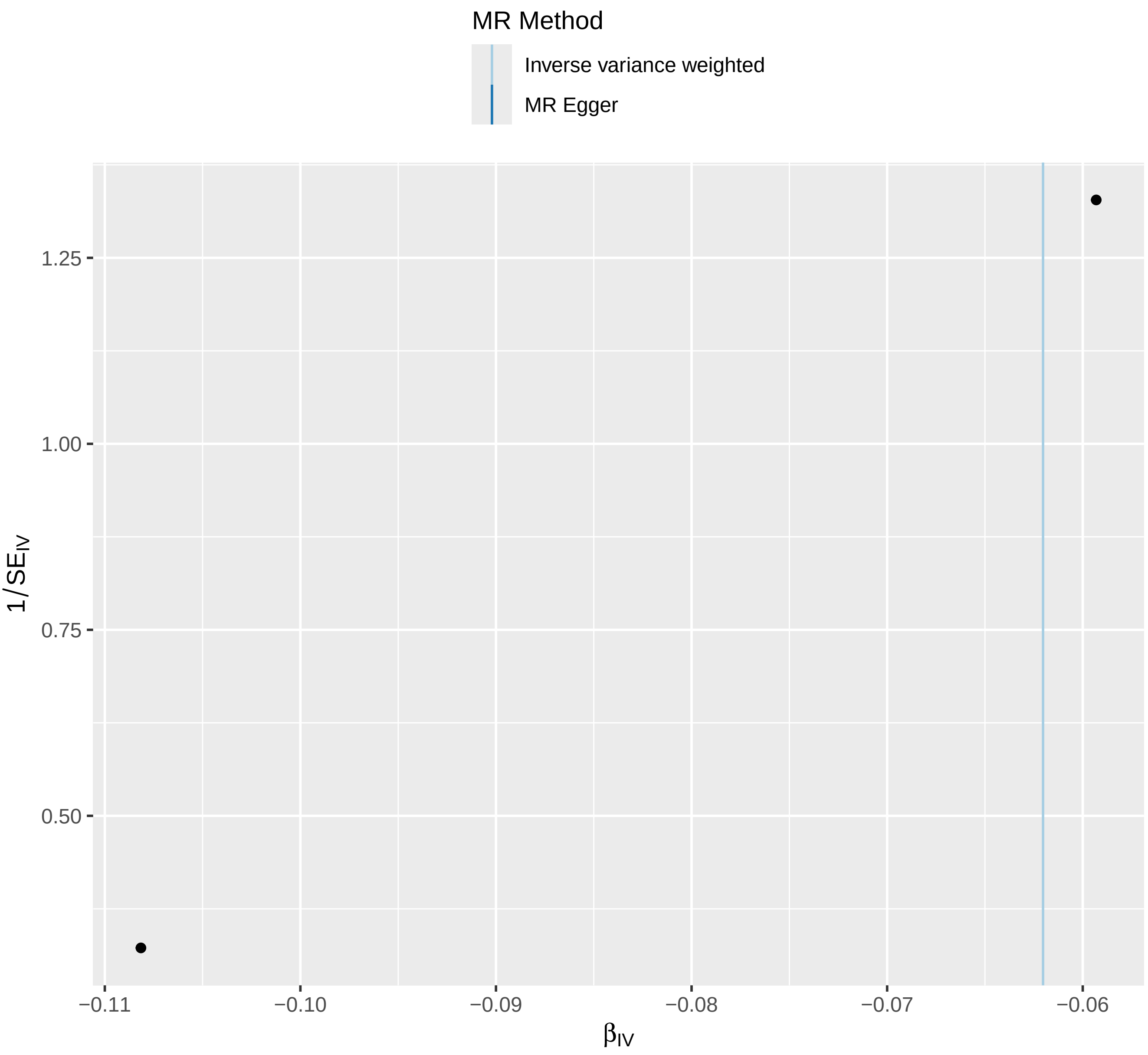
MR Method
Inverse variance weighted
MR Egger
1.25
1.00
0.75
0.50
$1/SE_{IV}$
−0.11
−0.10
−0.09
−0.08
−0.07
−0.06
β_{IV}

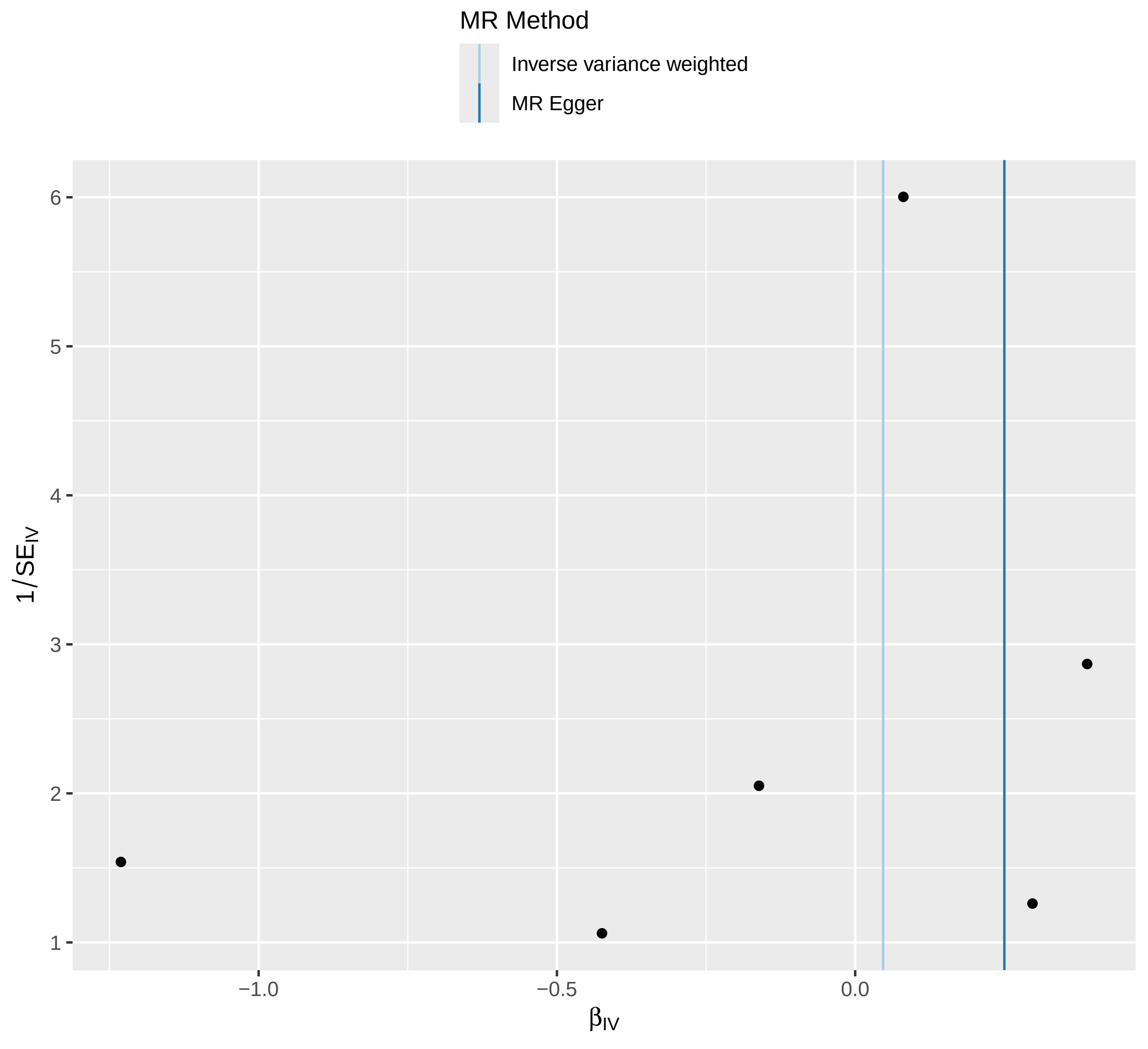
MR Method
Inverse variance weighted
MR Egger
1/SE_IV
6
5
4
3
2
1
−1.0
−0.5
0.0
β_IV

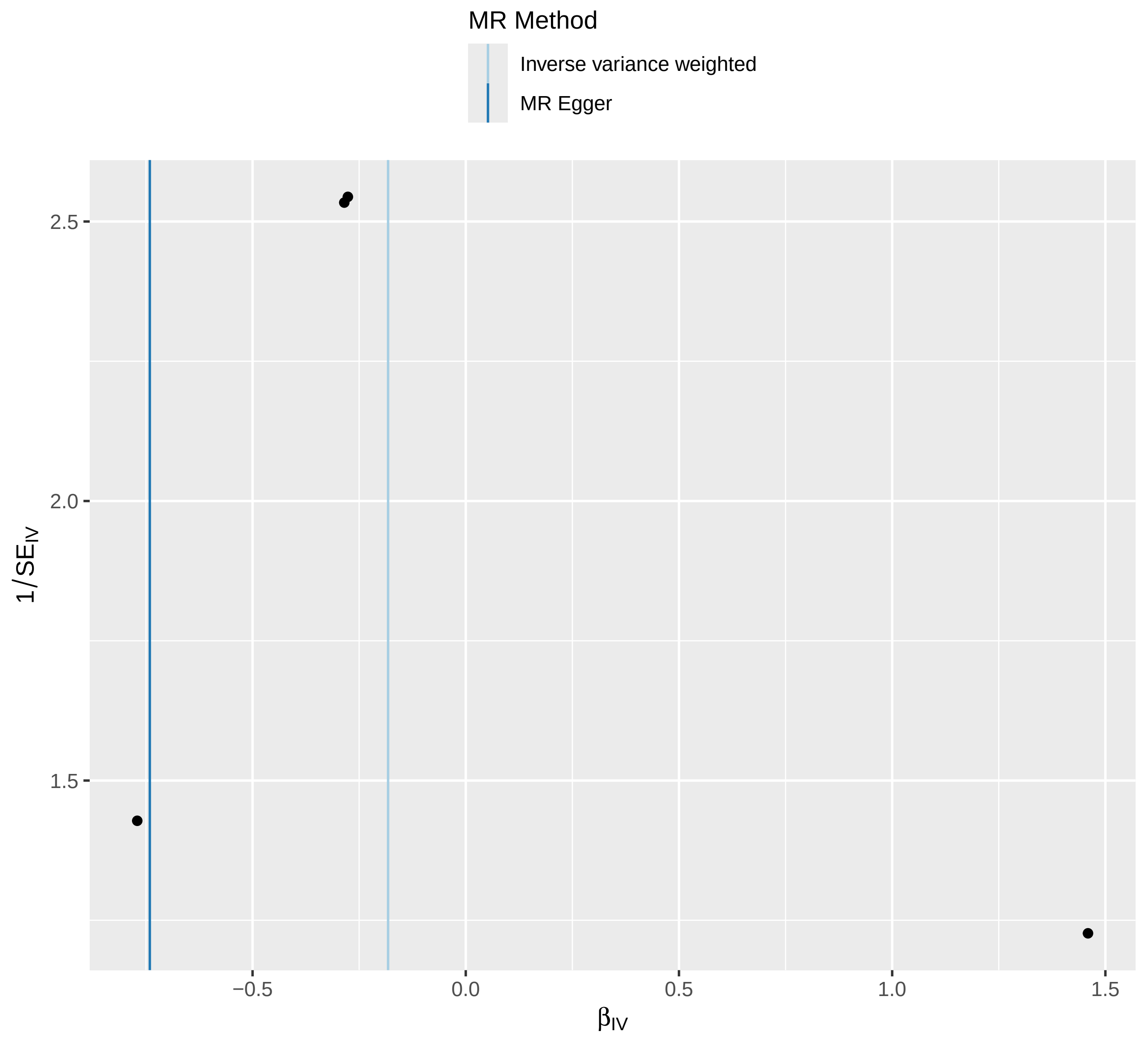
MR Method
Inverse variance weighted
MR Egger
2.5
2.0
1.5
1/SE_IV
−0.5
0.0
0.5
1.0
1.5
β_IV

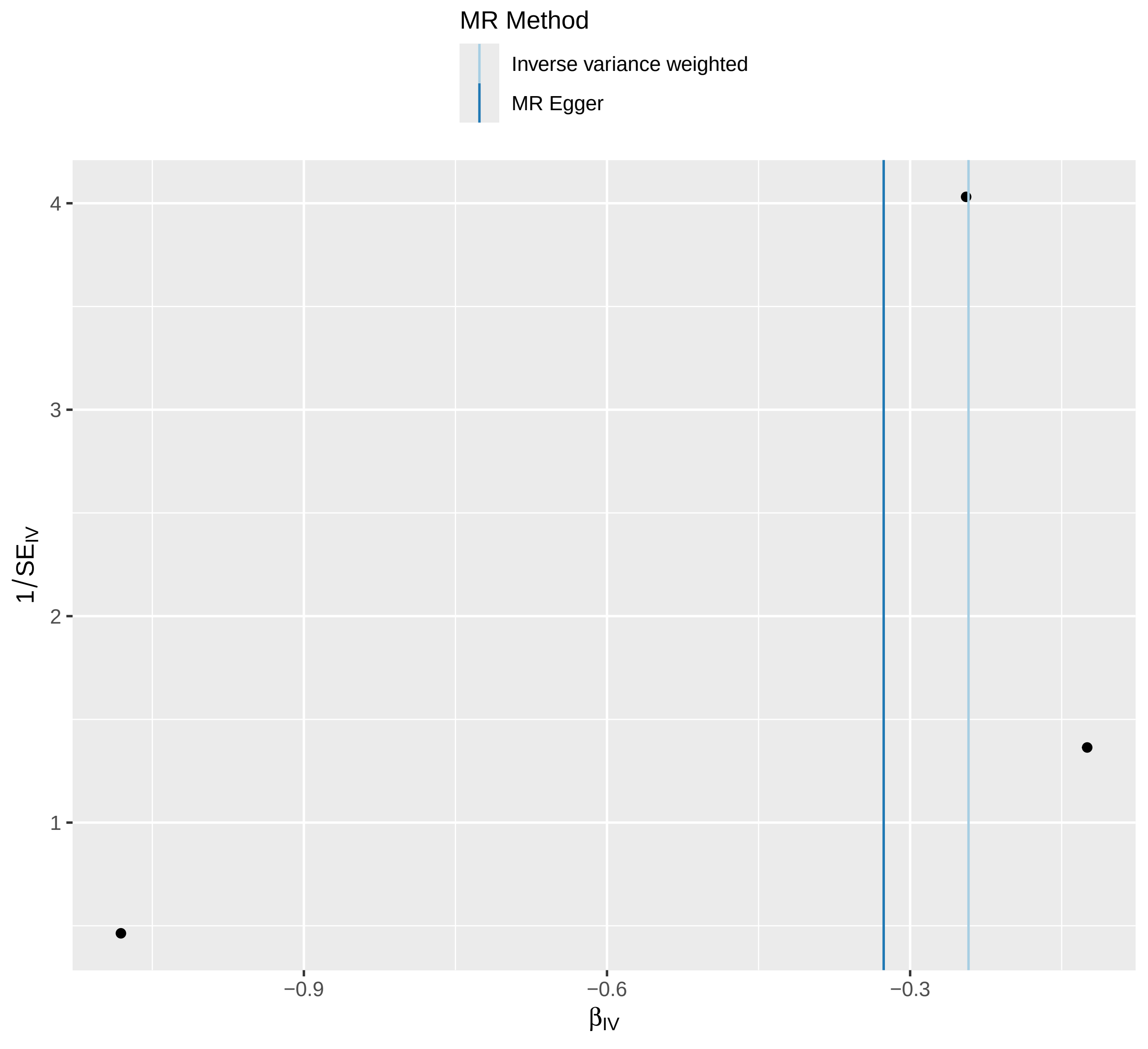

MR Method
Inverse variance weighted
MR Egger
4
3
2
1
1/SE_IV
−0.9
−0.6
−0.3
β_IV

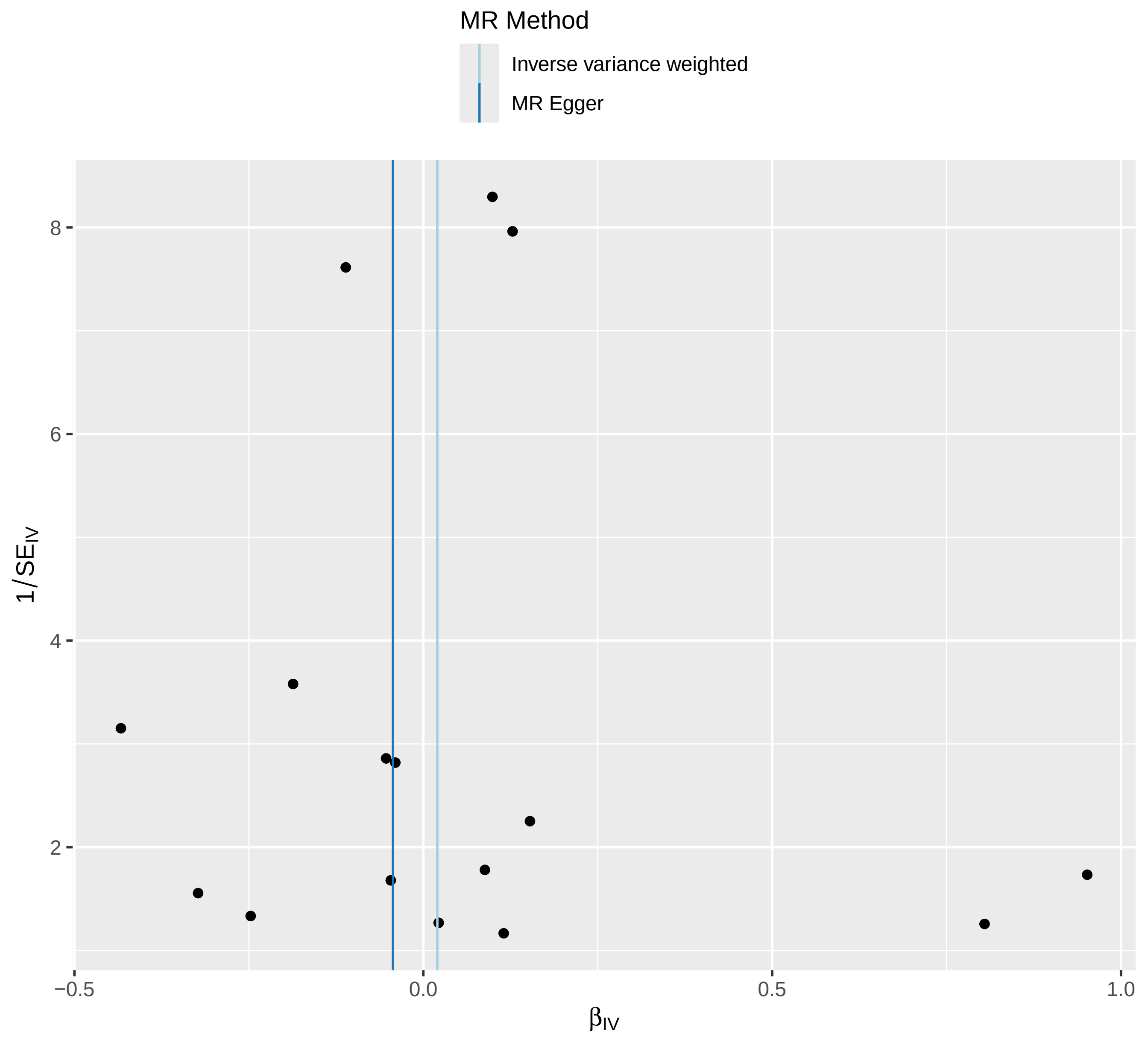
MR Method
Inverse variance weighted
MR Egger
1/SE_IV
8
6
4
2
−0.5
0.0
0.5
1.0
β_IV

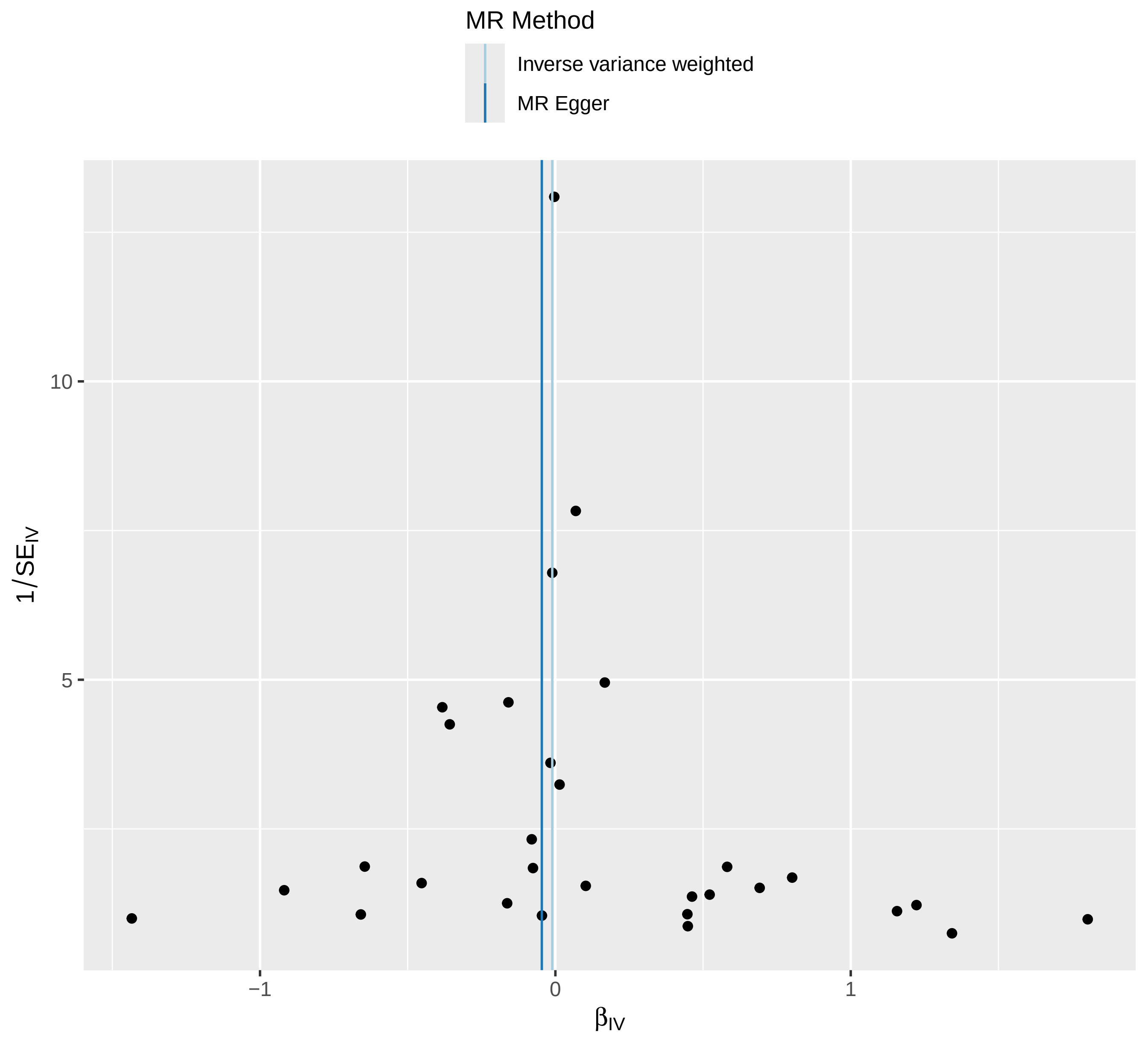

MR Method
Inverse variance weighted
MR Egger
10
5
1/SE_IV
−1
0
1
β_IV

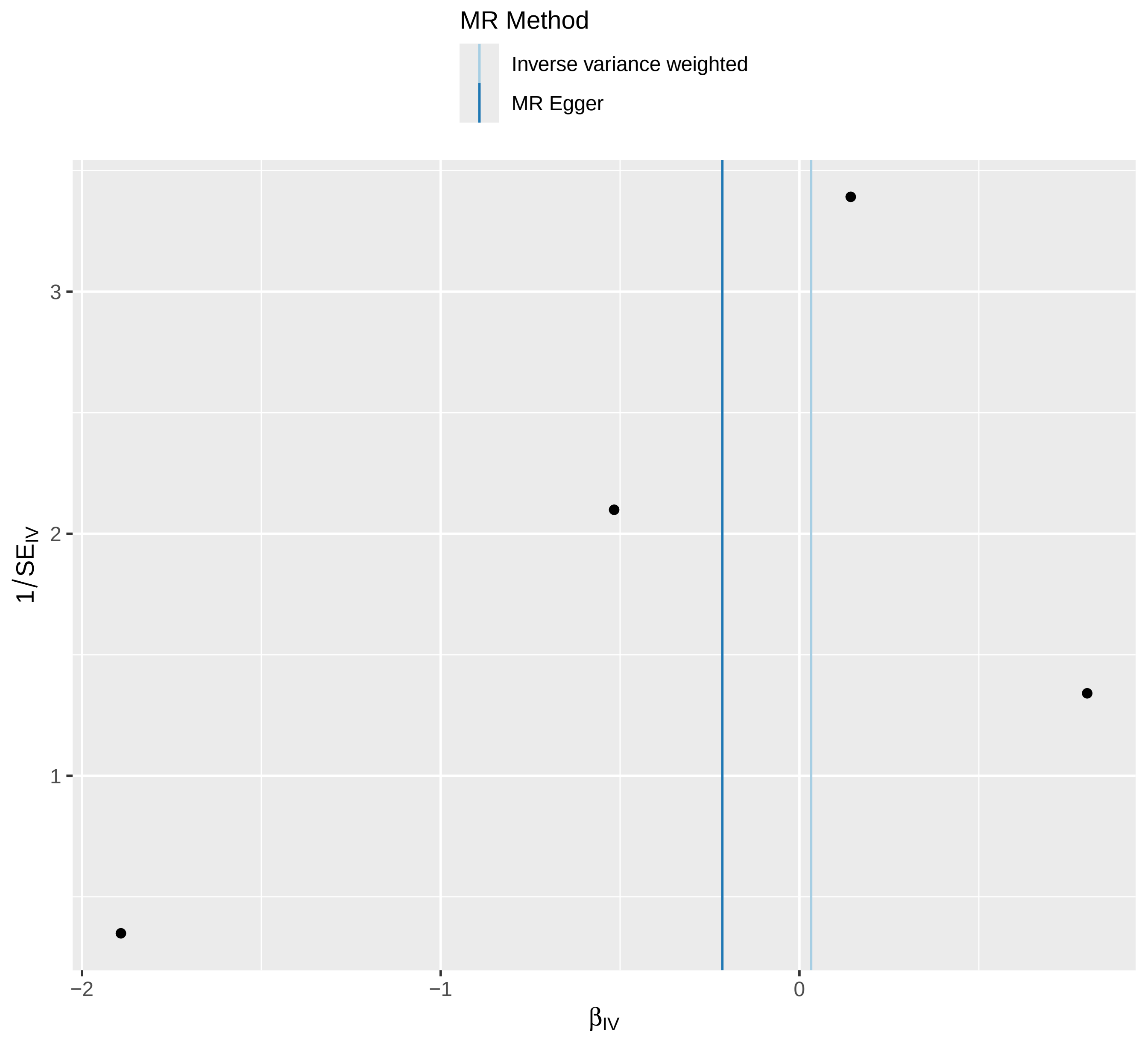

MR Method
Inverse variance weighted
MR Egger
3
2
1
1/SE_IV
−2
−1
0
β_IV

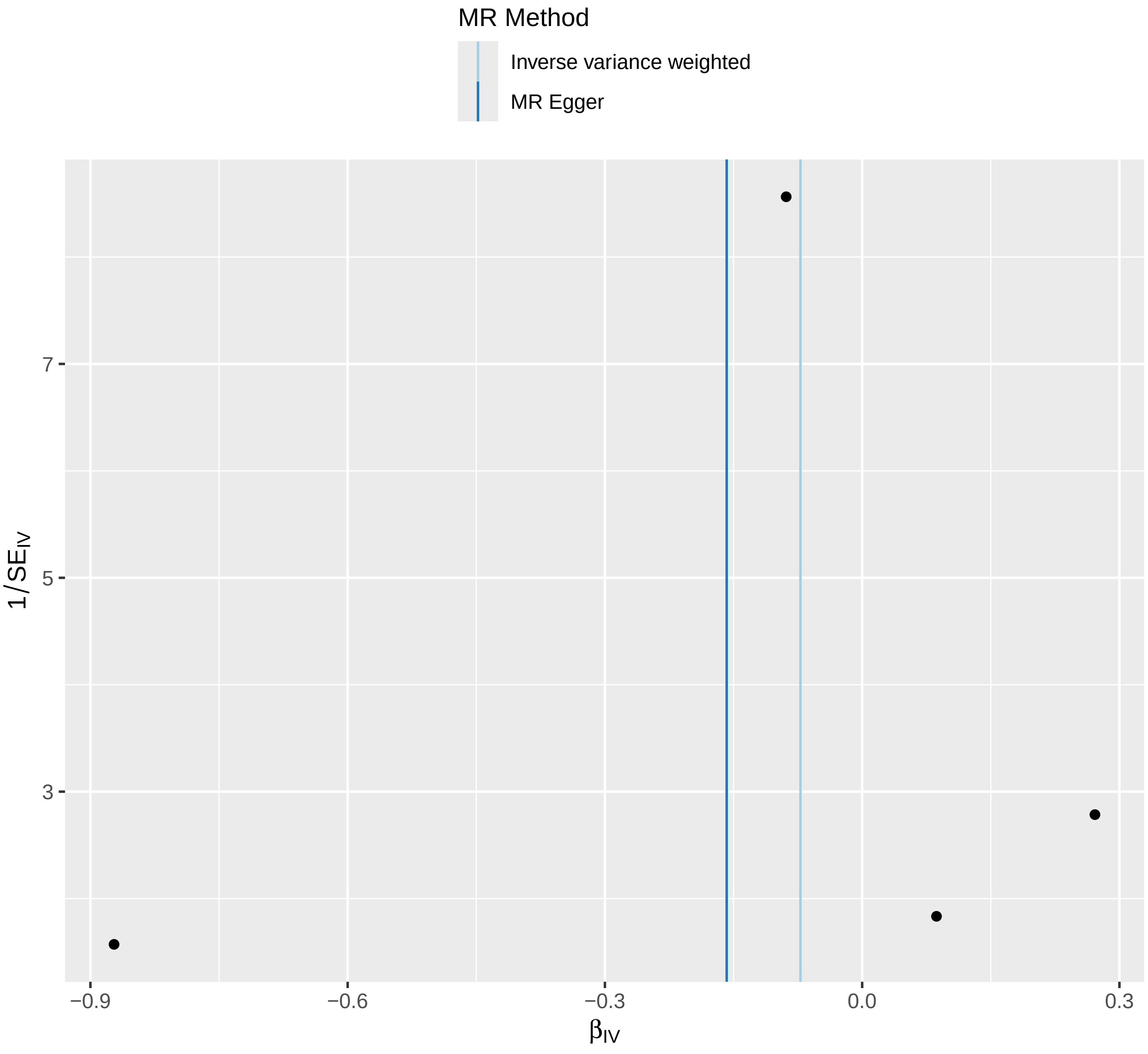
MR Method
Inverse variance weighted
MR Egger
1/SE_IV
7
5
3
−0.9
−0.6
−0.3
0.0
0.3
β_IV

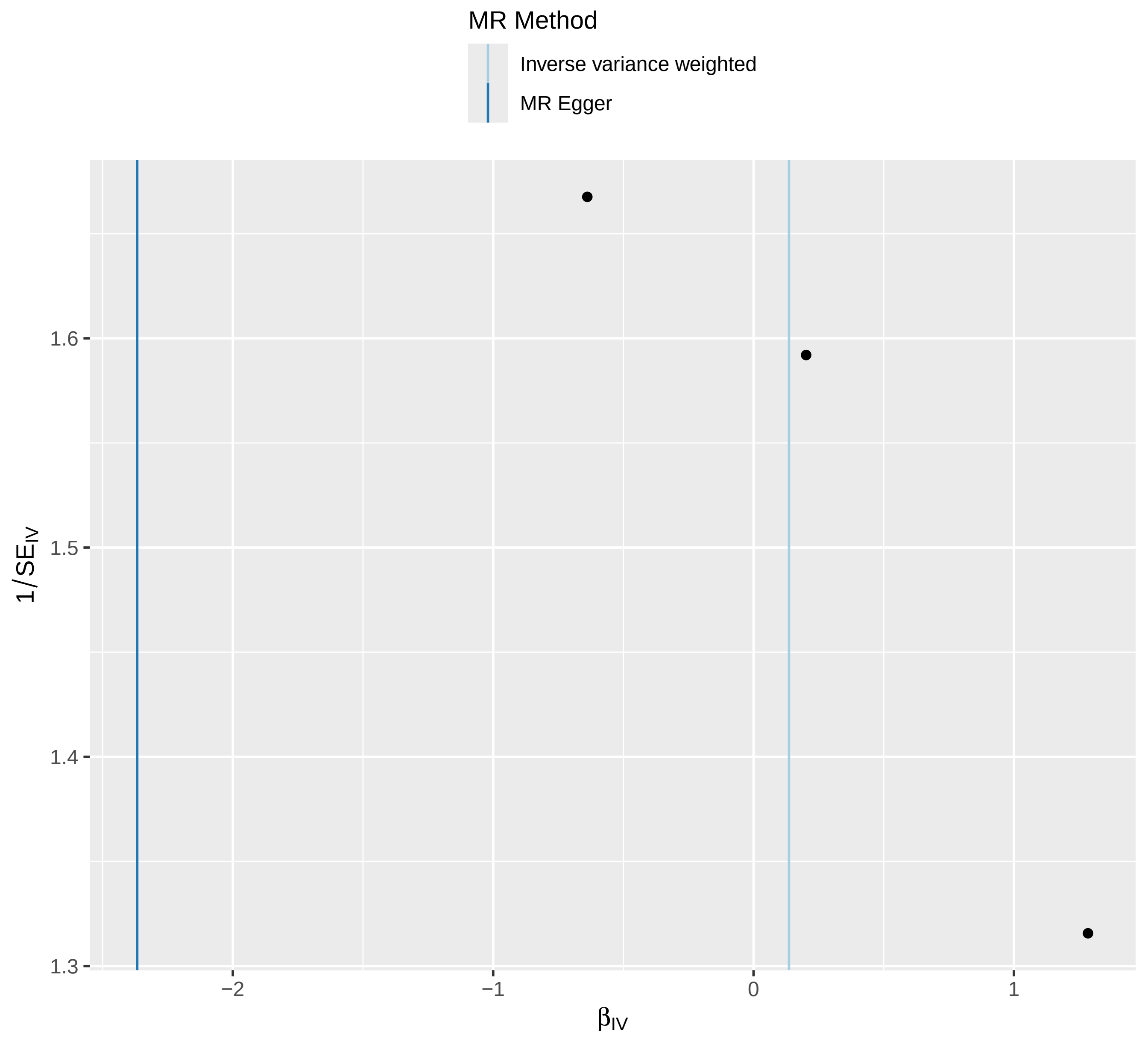
MR Method
Inverse variance weighted
MR Egger
1.6
1.5
1.4
1.3
1/SE_IV
−2
−1
0
1
β_IV

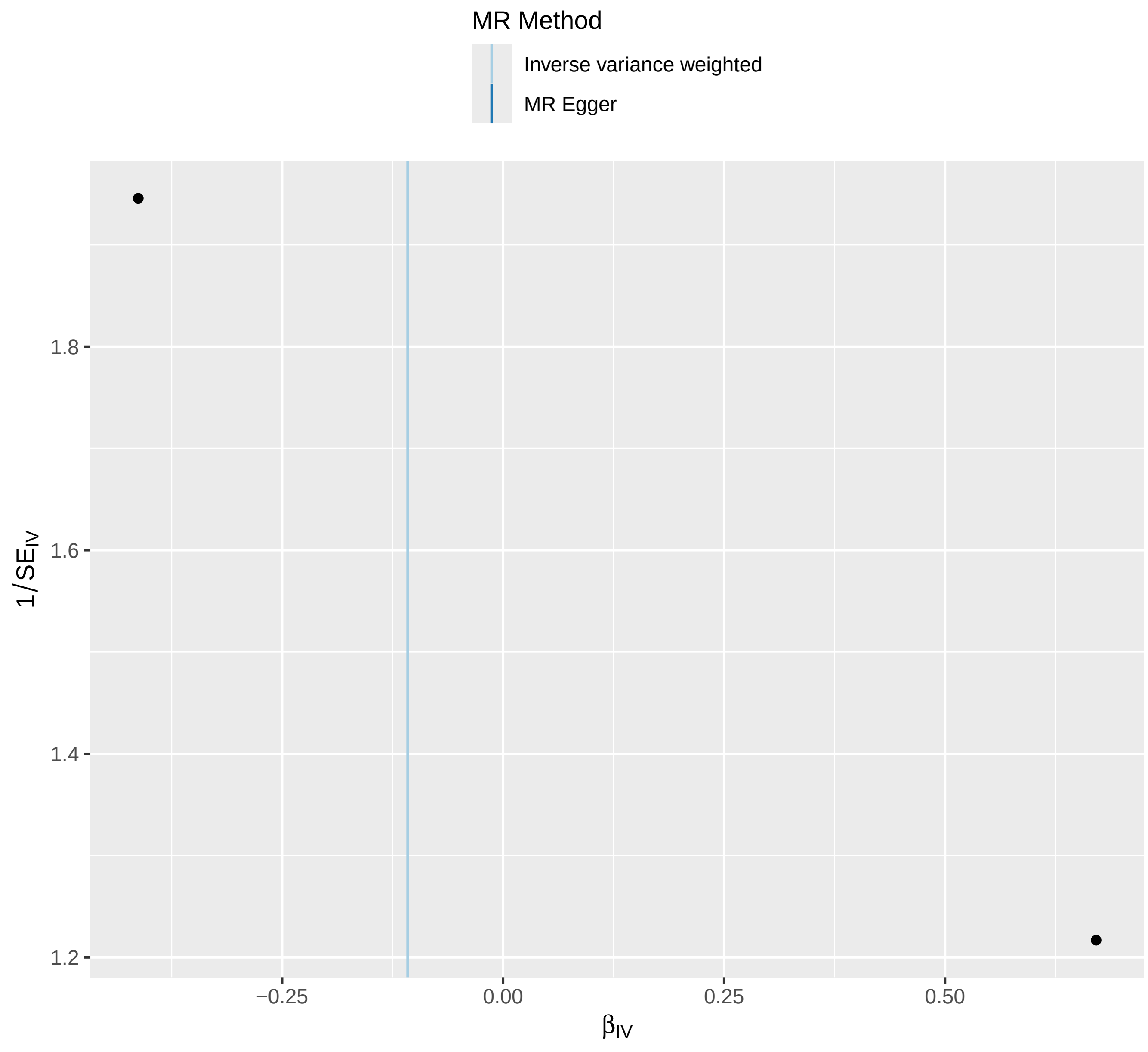
MR Method
Inverse variance weighted
MR Egger
1.8
1.6
1.4
1.2
1/SE_IV
−0.25
0.00
0.25
0.50
β_IV

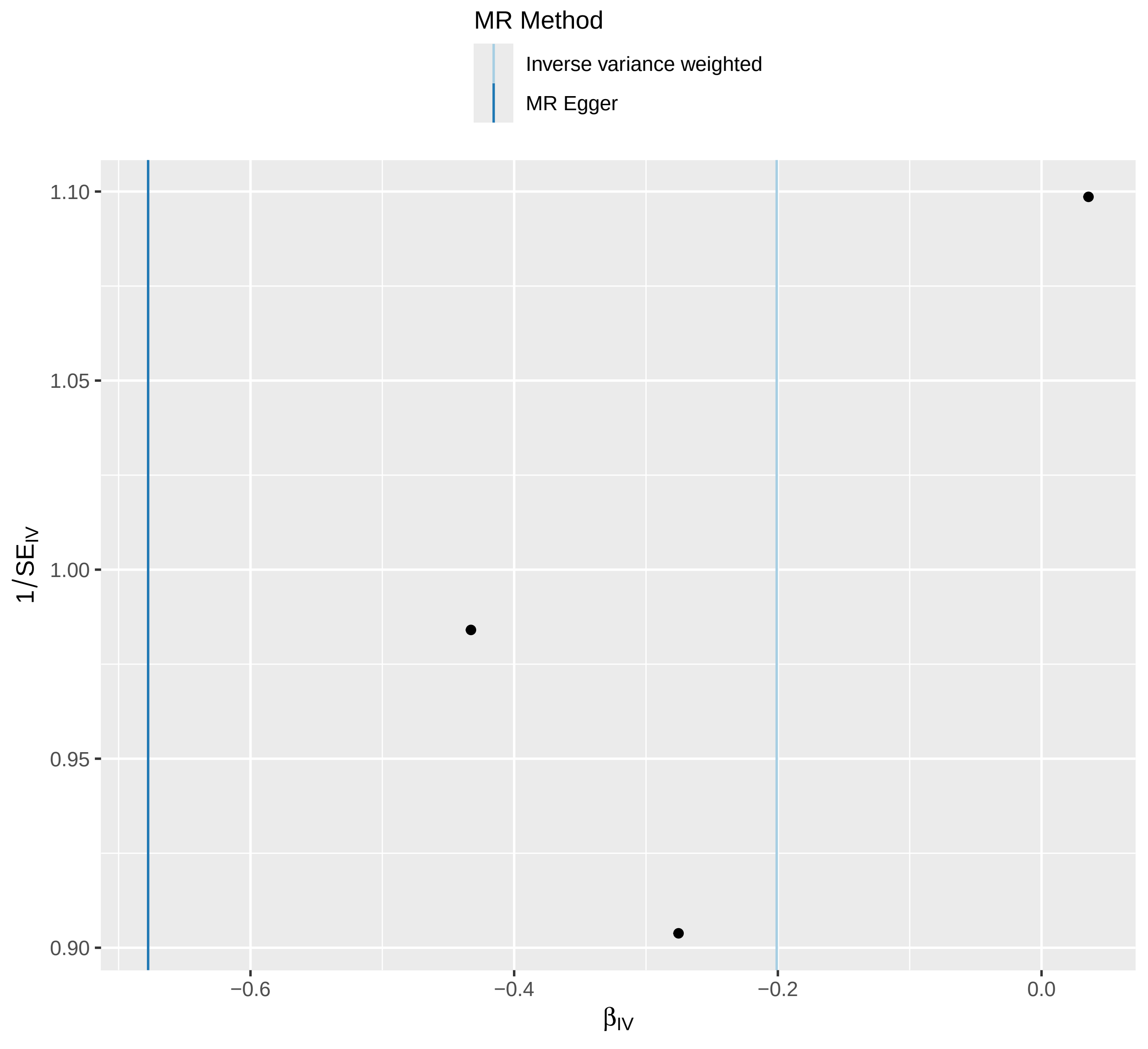

MR Method
Inverse variance weighted
MR Egger
1.10
1.05
1.00
0.95
0.90
1/SE$_{IV}$
−0.6
−0.4
−0.2
0.0
β$_{IV}$

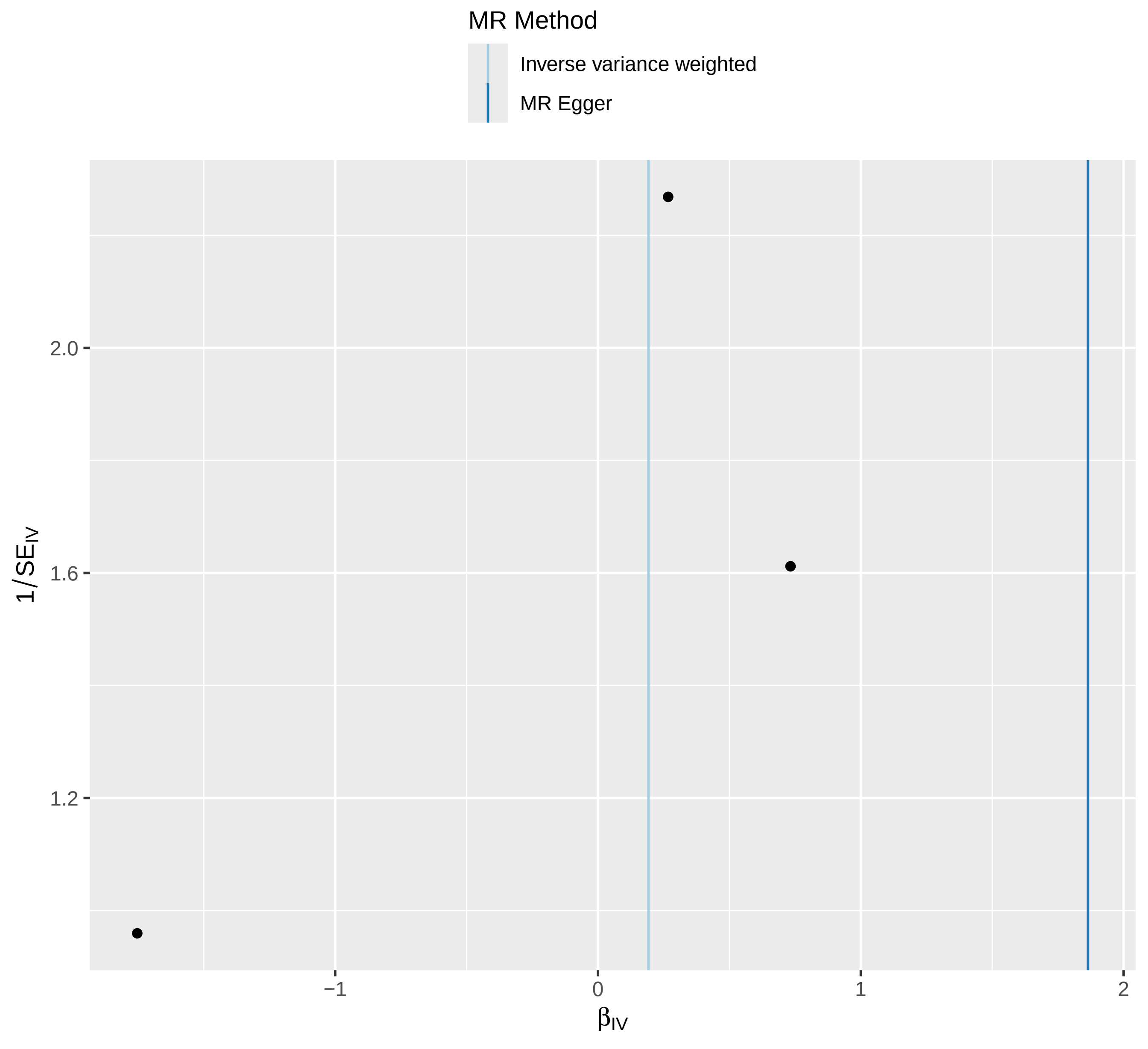

MR Method
Inverse variance weighted
MR Egger
2.0
1.6
1.2
$1/SE_{IV}$
−1
0
1
2
β_{IV}

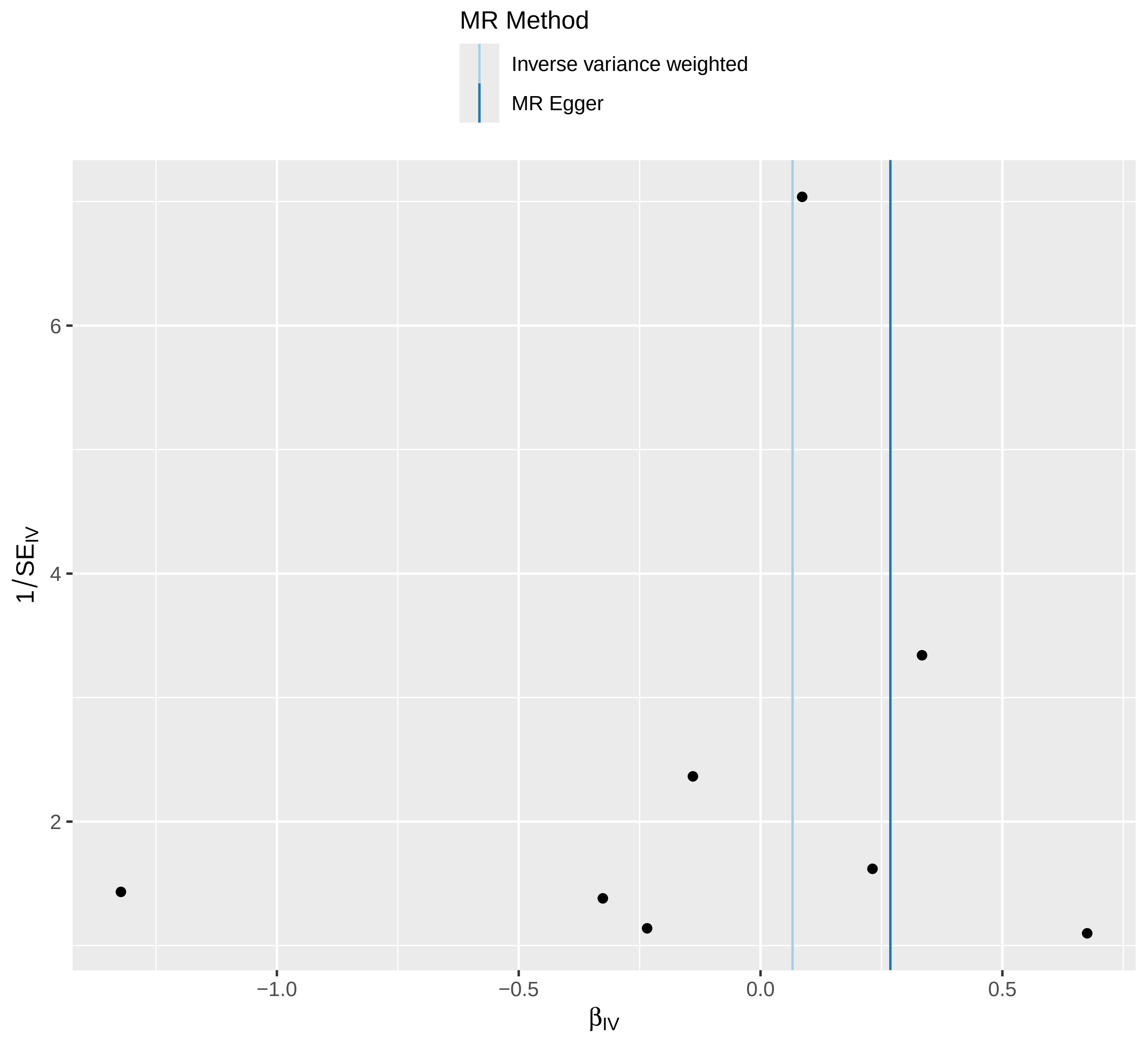
MR Method
Inverse variance weighted
MR Egger
6
4
2
1/SE$_{IV}$
−1.0
−0.5
0.0
0.5
β$_{IV}$

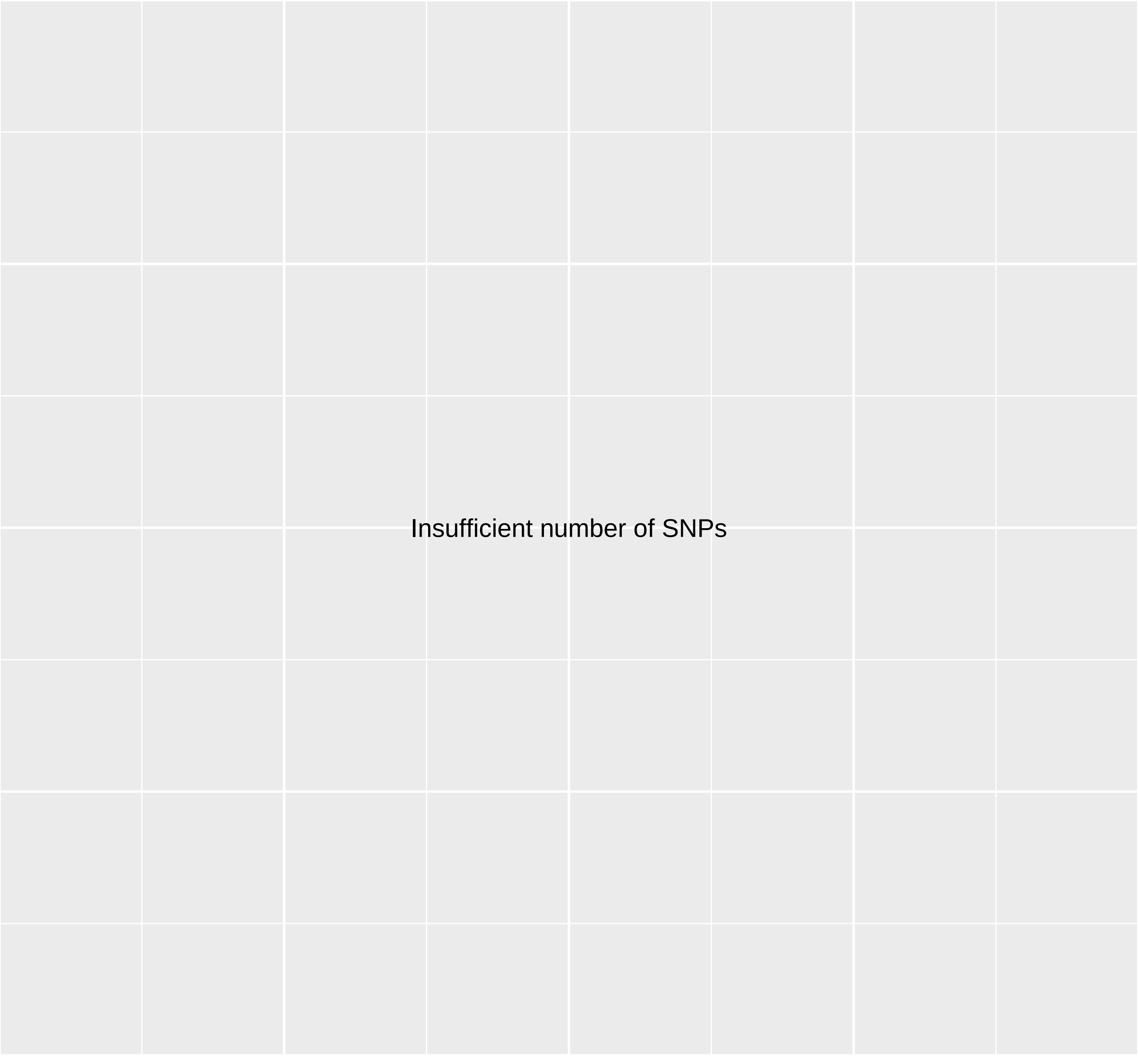
Insufficient number of SNPs

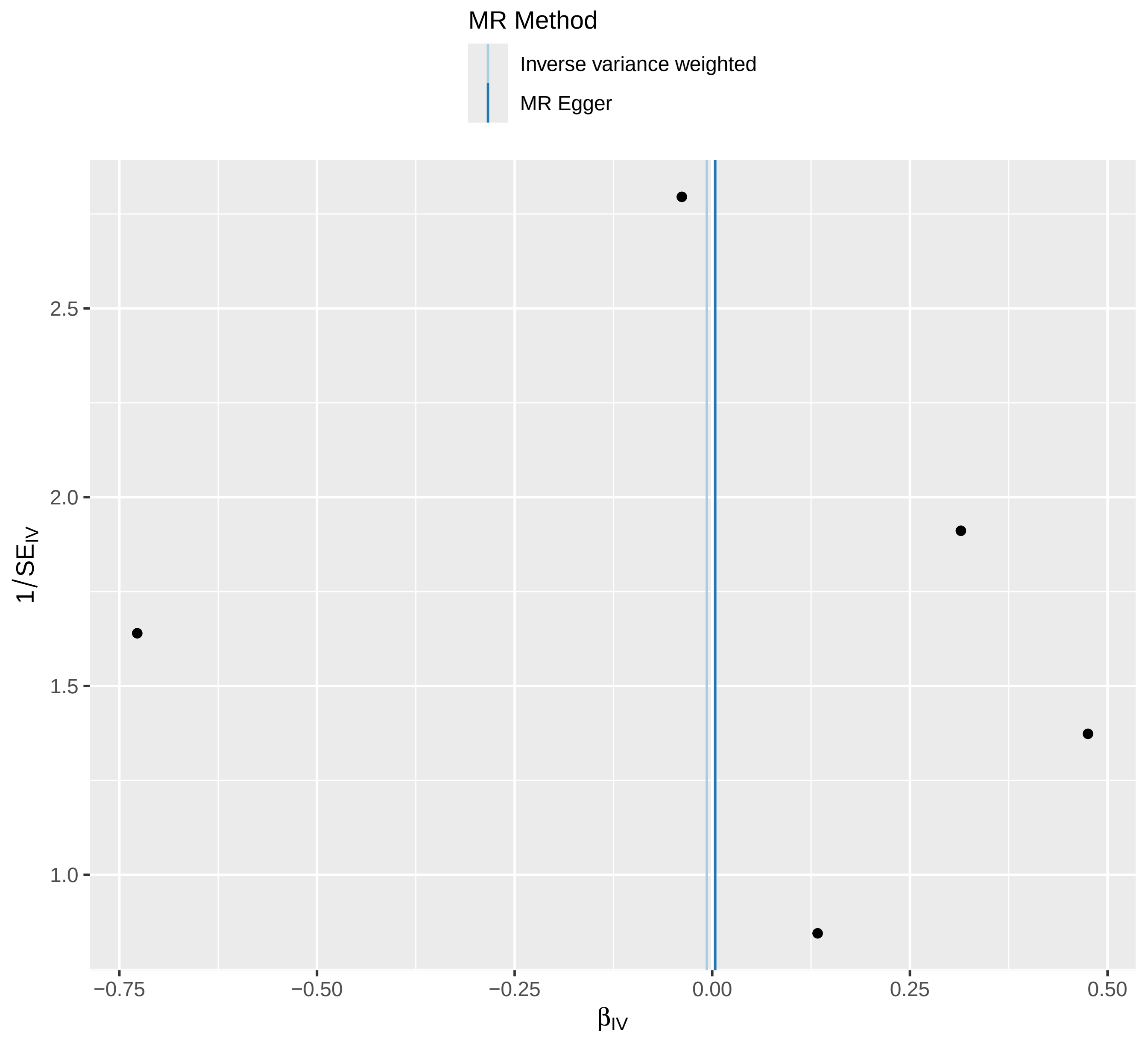
MR Method
Inverse variance weighted
MR Egger
1/SE_{IV}
2.5
2.0
1.5
1.0
−0.75
−0.50
−0.25
0.00
0.25
0.50
β_{IV}

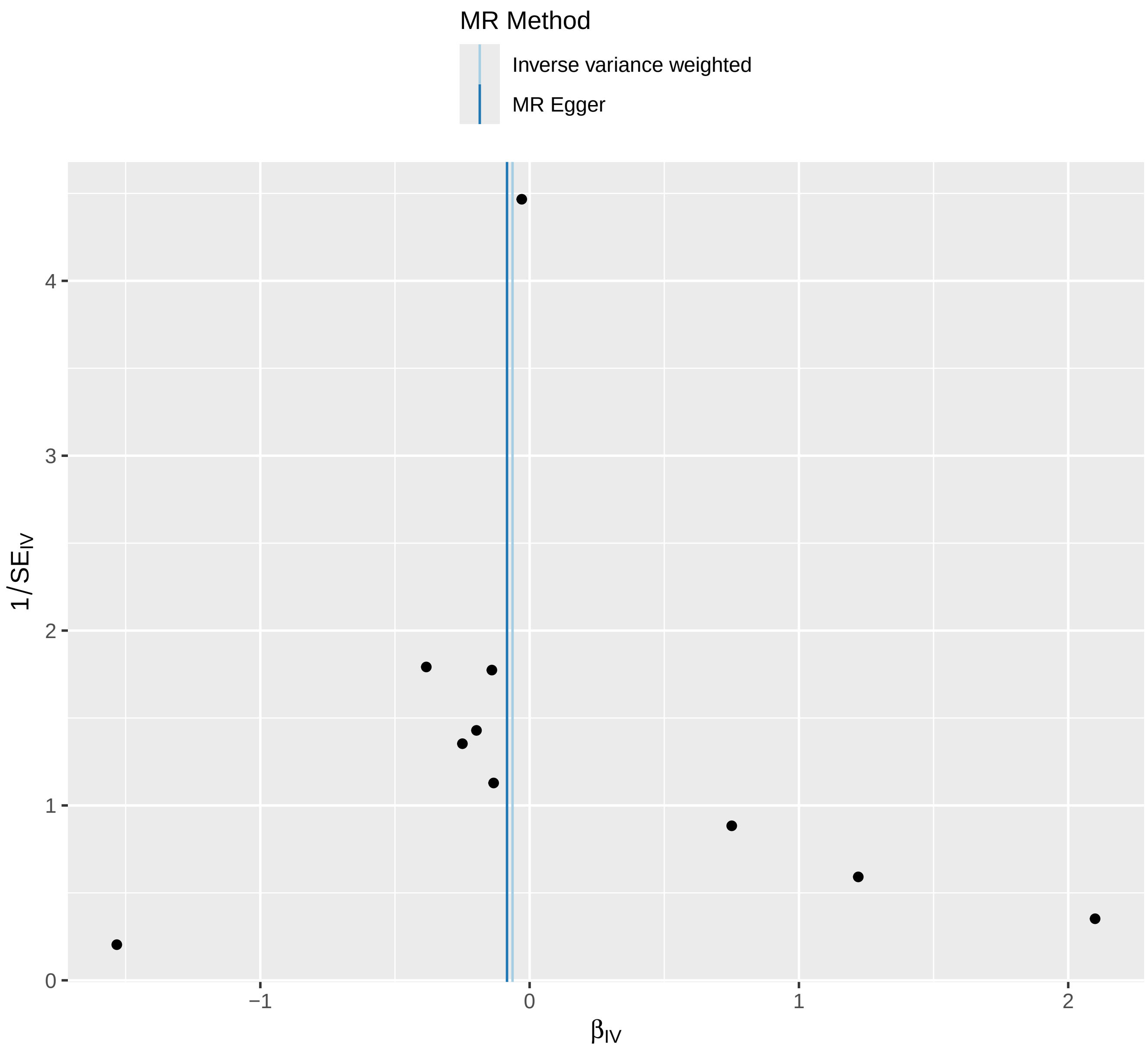
MR Method
Inverse variance weighted
MR Egger
4
3
2
1
0
−1
0
1
2
$1/SE_{IV}$
β_{IV}

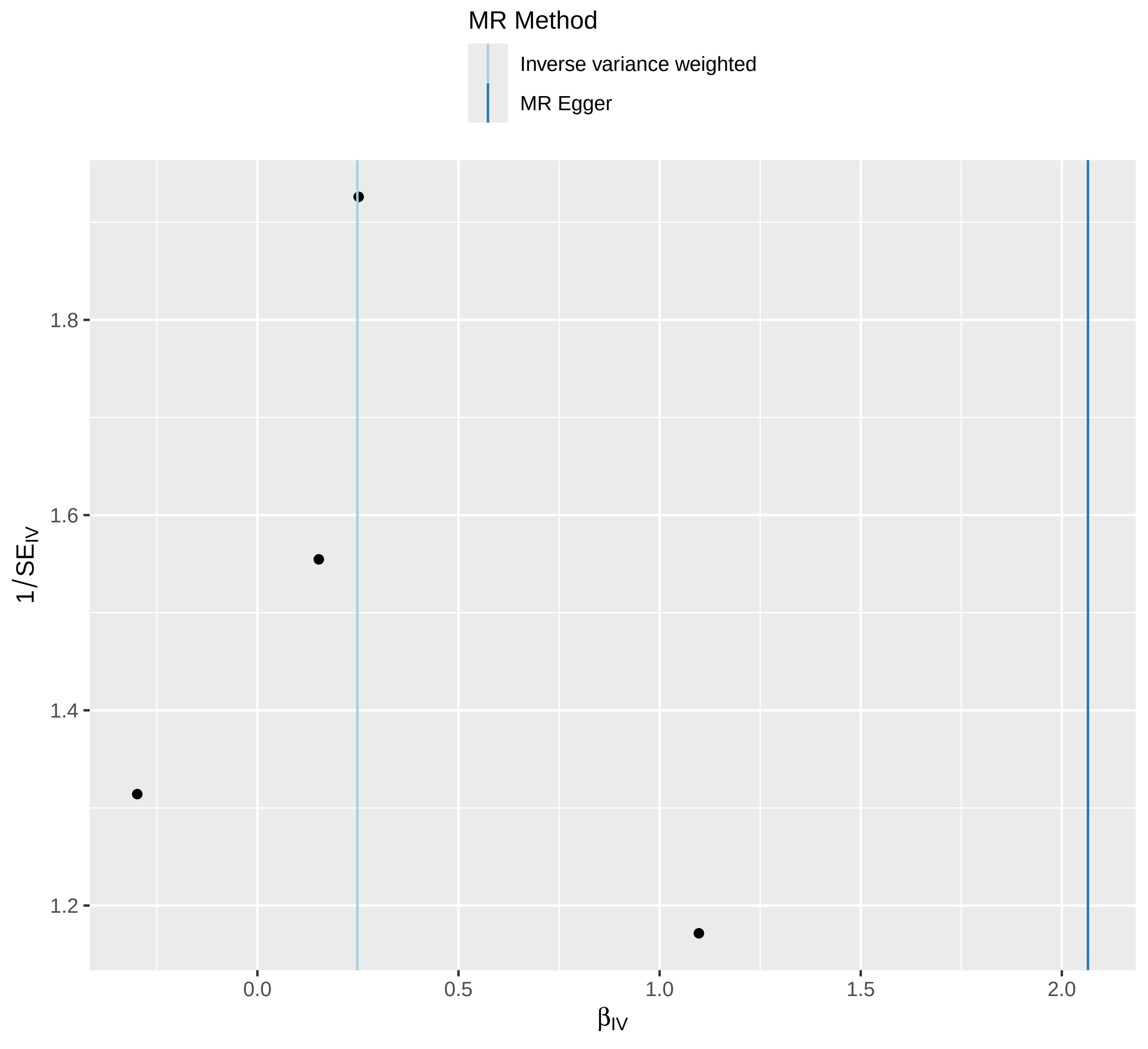
MR Method
Inverse variance weighted
MR Egger
1.8
1.6
1.4
1.2
1/SE_IV
0.0
0.5
1.0
1.5
2.0
β_IV

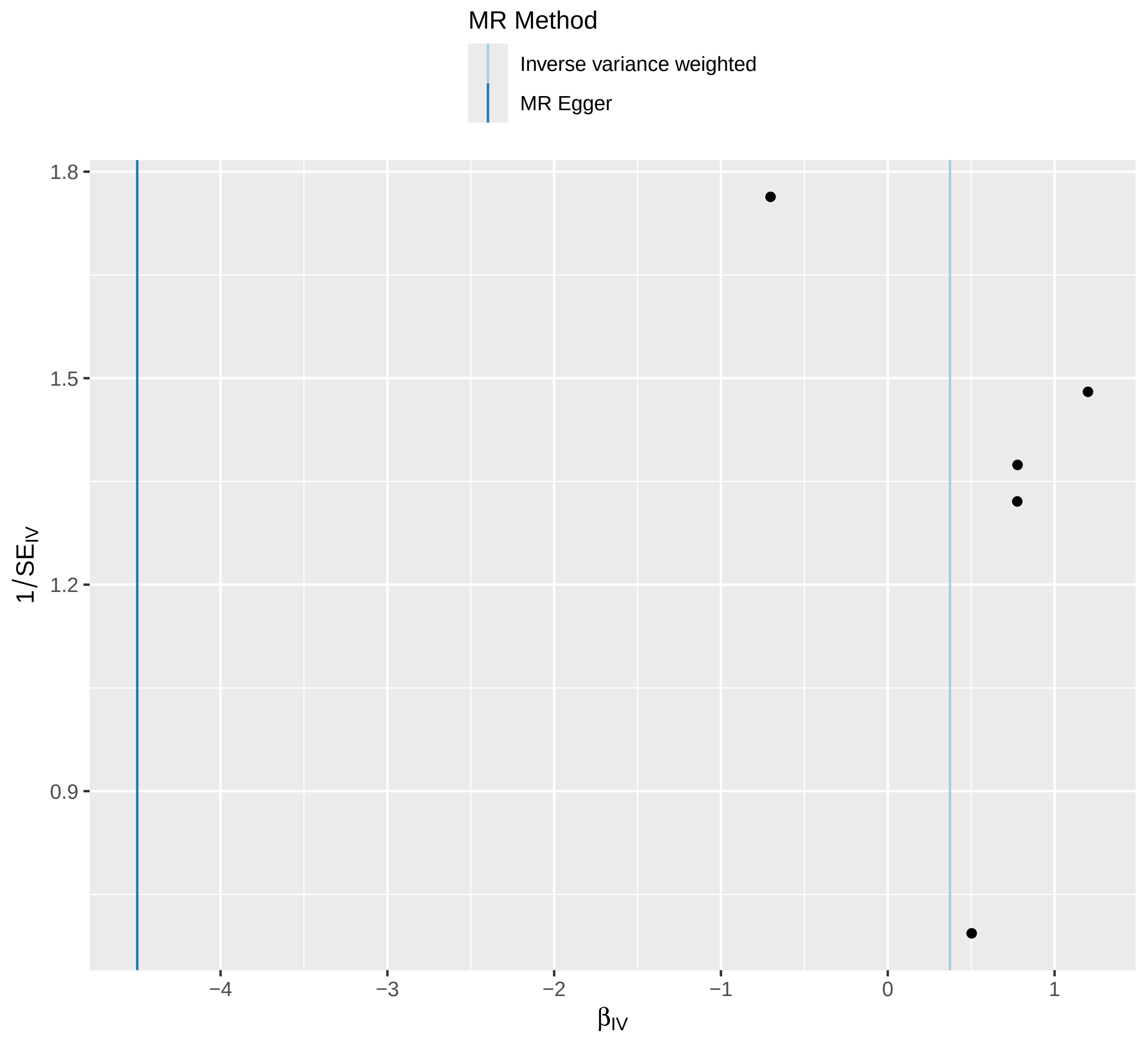

MR Method
Inverse variance weighted
MR Egger
1.8
1.5
1.2
0.9
$1/SE_{IV}$
−4
−3
−2
−1
0
1
β_{IV}

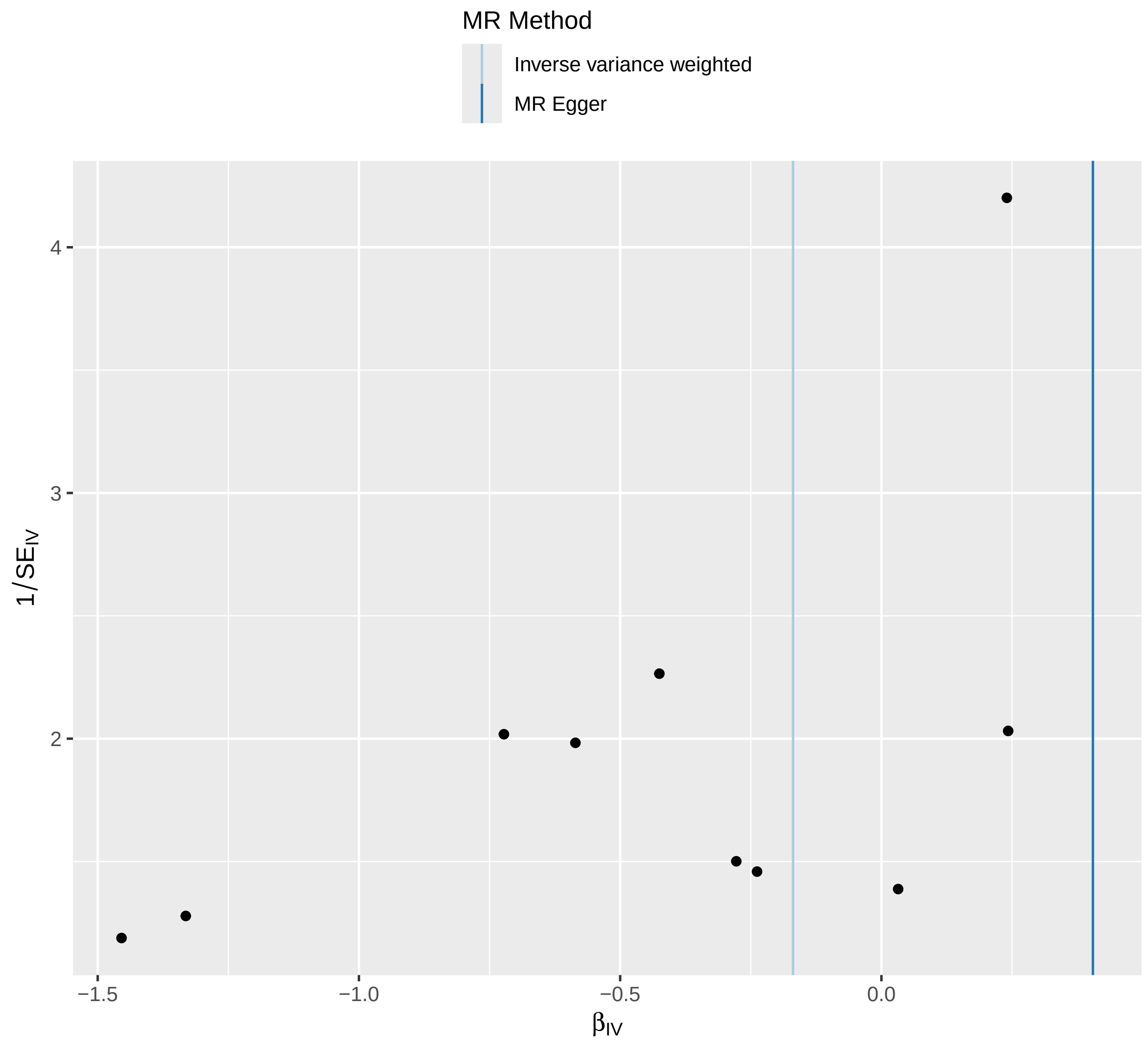
MR Method
Inverse variance weighted
MR Egger
4
3
2
$1/SE_{IV}$
−1.5
−1.0
−0.5
0.0
β_{IV}

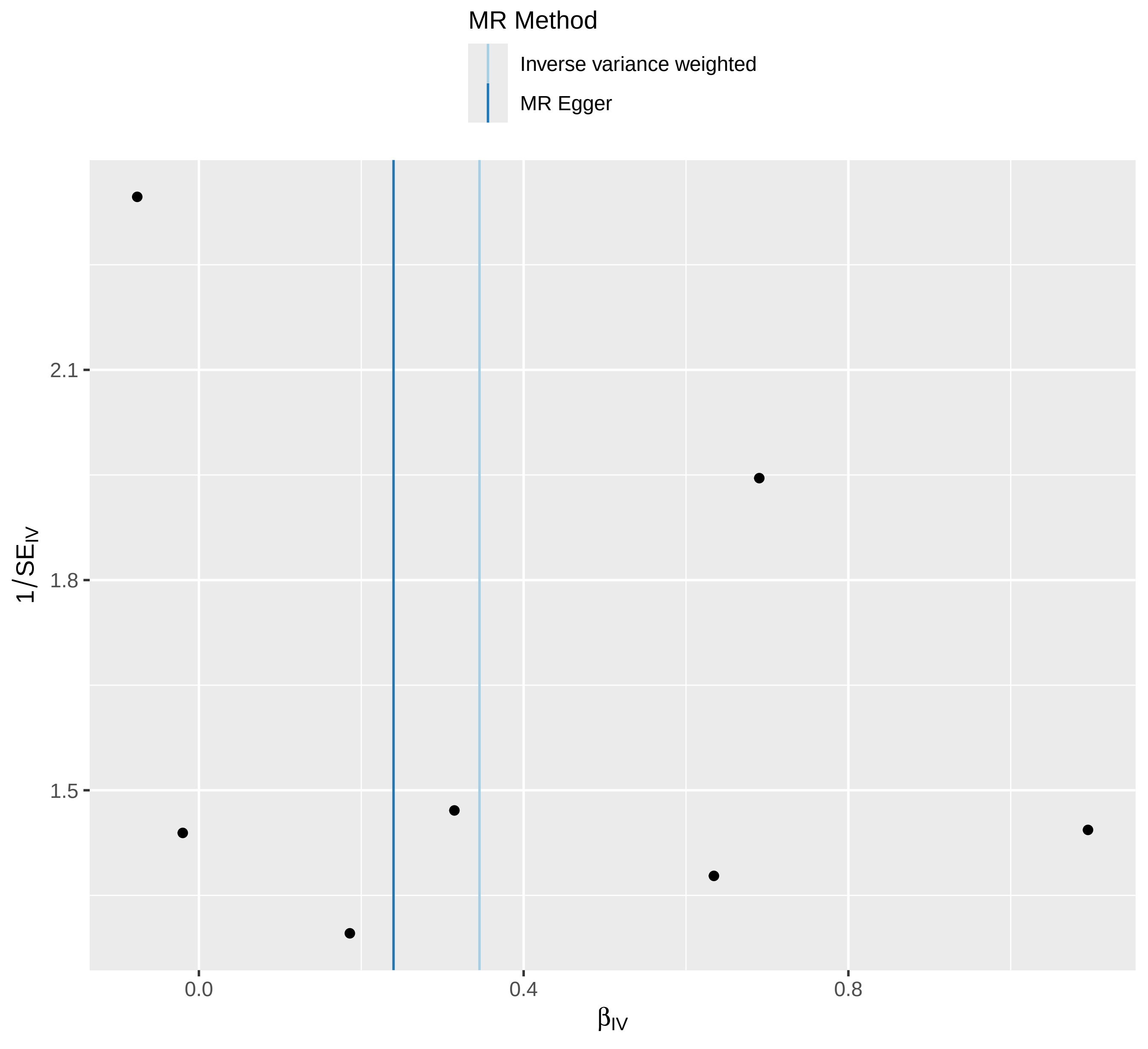

MR Method
Inverse variance weighted
MR Egger
2.1
1.8
1.5
1/SE_IV
0.0
0.4
0.8
β_IV

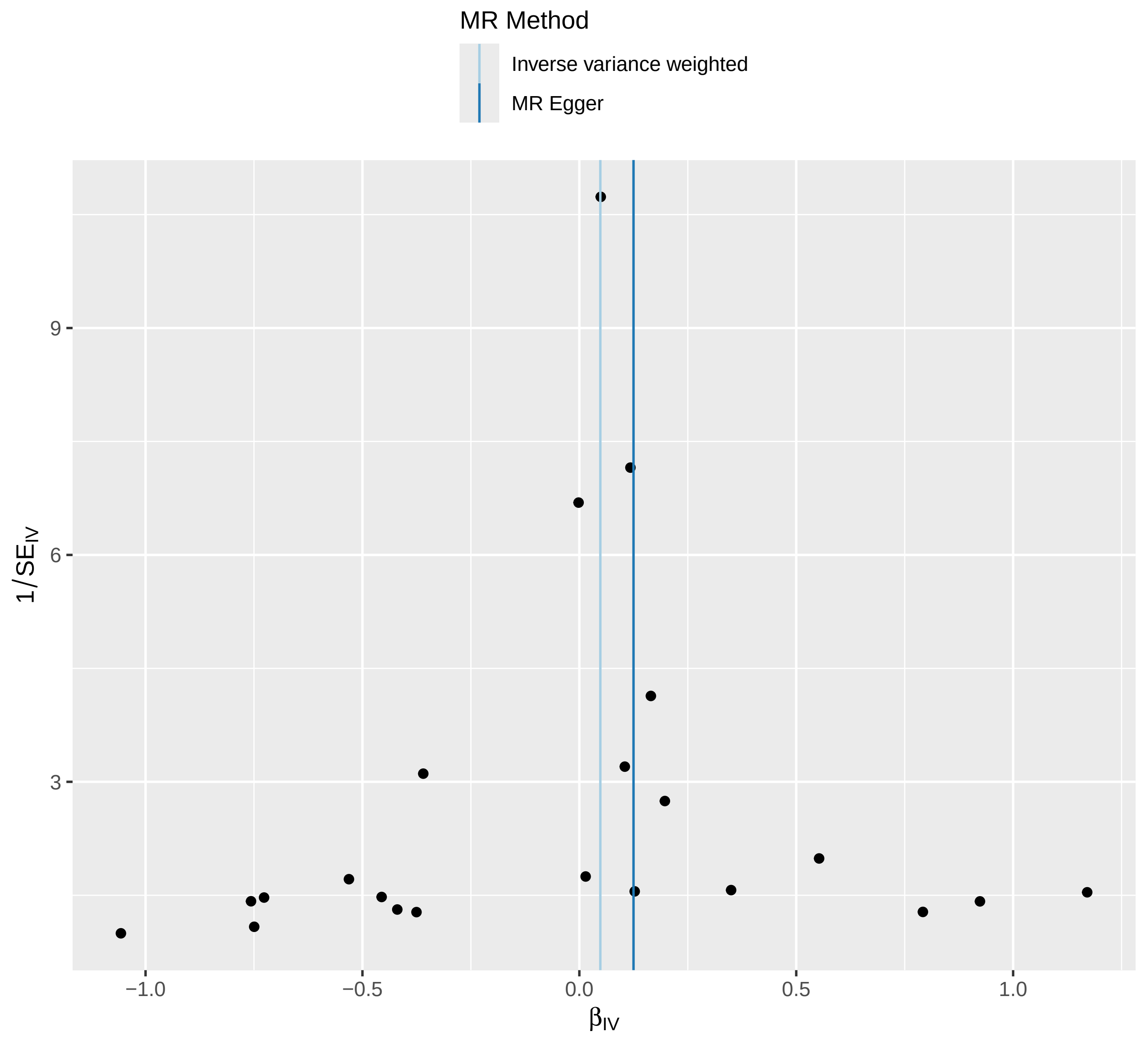
MR Method
Inverse variance weighted
MR Egger
1/SE_IV
9
6
3
−1.0
−0.5
0.0
0.5
1.0
β_IV

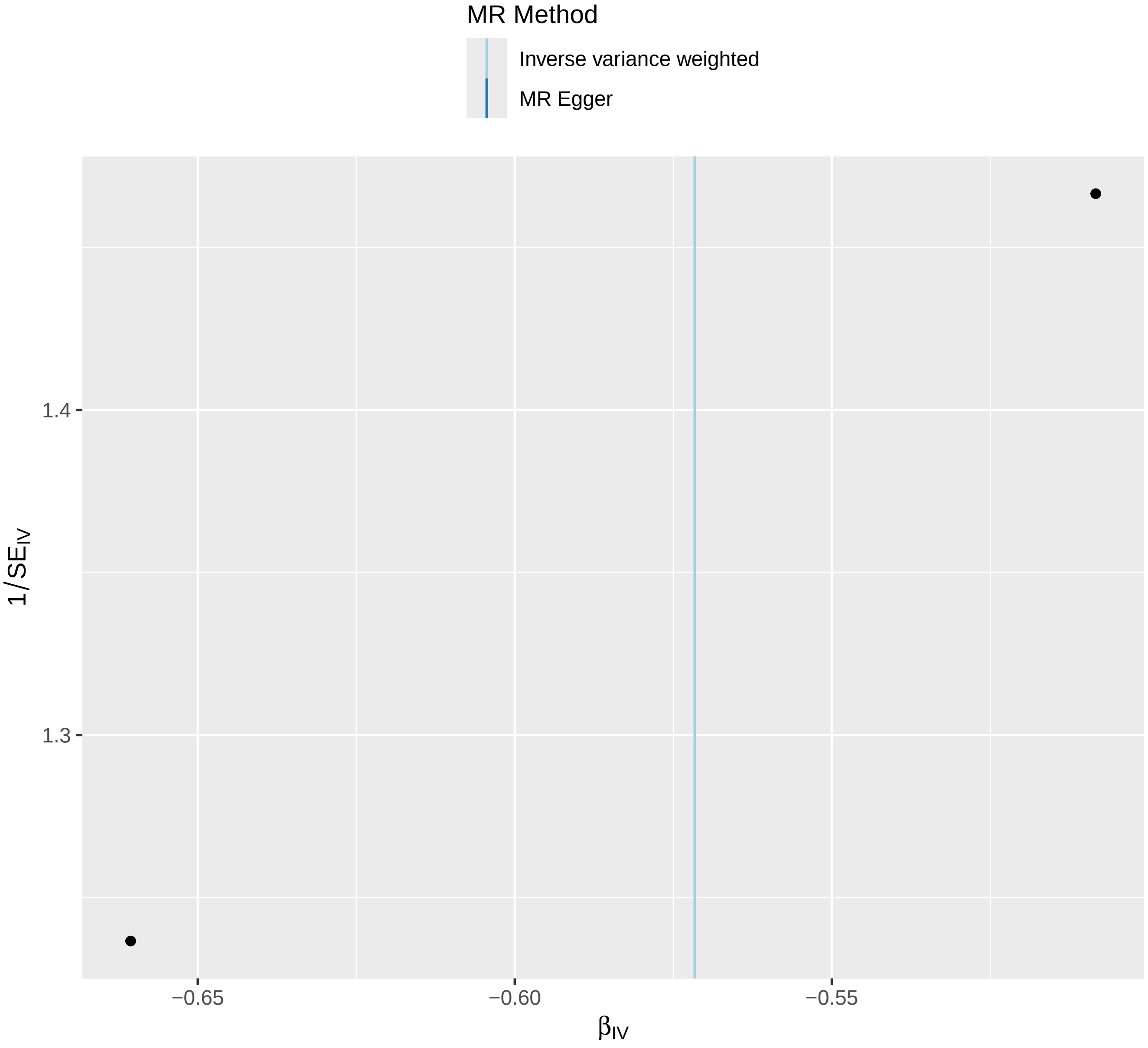

MR Method
Inverse variance weighted
MR Egger
1.4
1.3
1/SE_IV
−0.65
−0.60
−0.55
β_IV

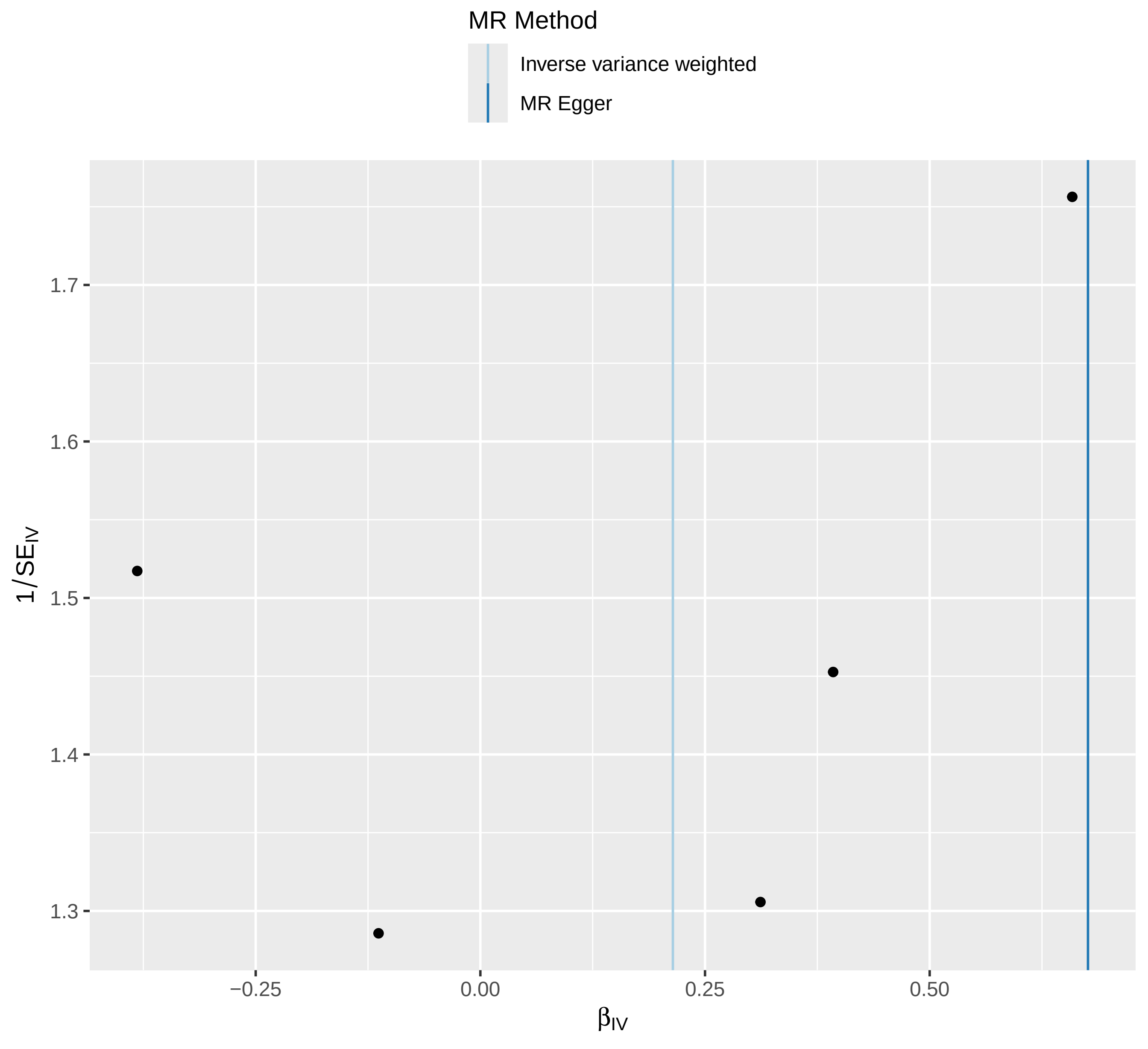
MR Method
Inverse variance weighted
MR Egger
1.7
1.6
1.5
1.4
1.3
1/SE$_{IV}$
−0.25
0.00
0.25
0.50
β$_{IV}$

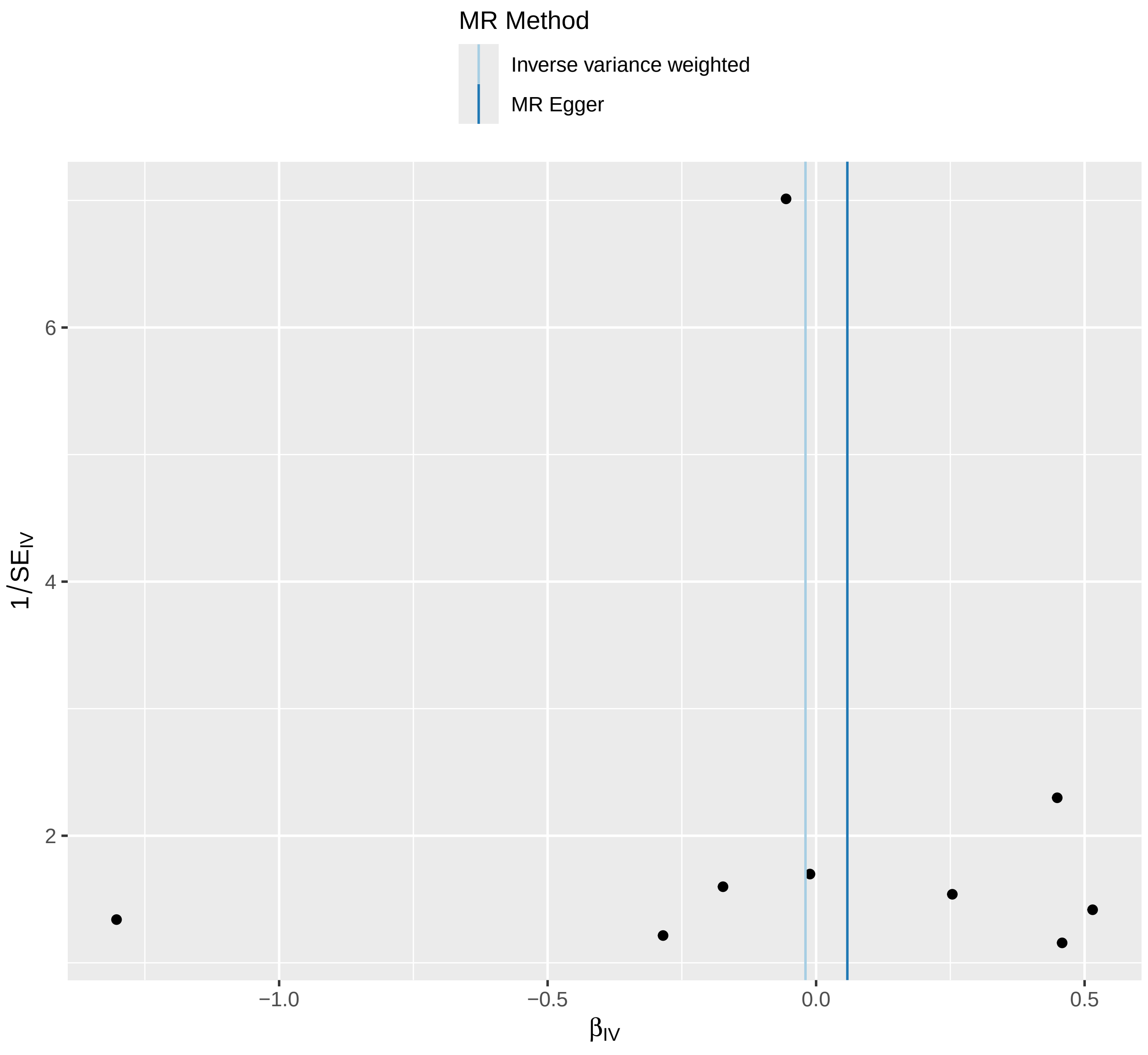
MR Method
Inverse variance weighted
MR Egger
1/SE$_{IV}$
6
4
2
−1.0
−0.5
0.0
0.5
β$_{IV}$

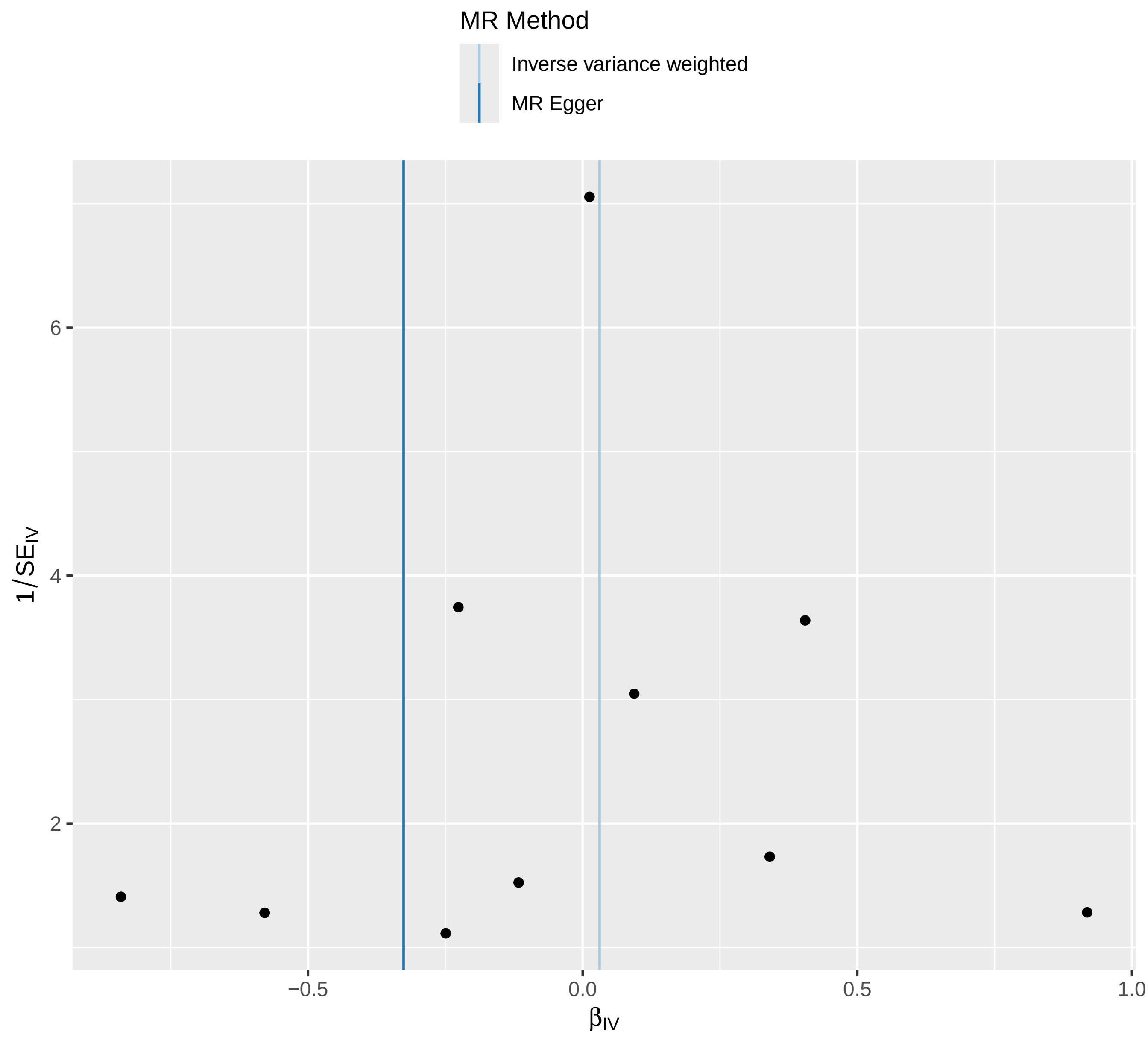

MR Method
Inverse variance weighted
MR Egger
6
4
2
1/SE_IV
−0.5
0.0
0.5
1.0
β_IV

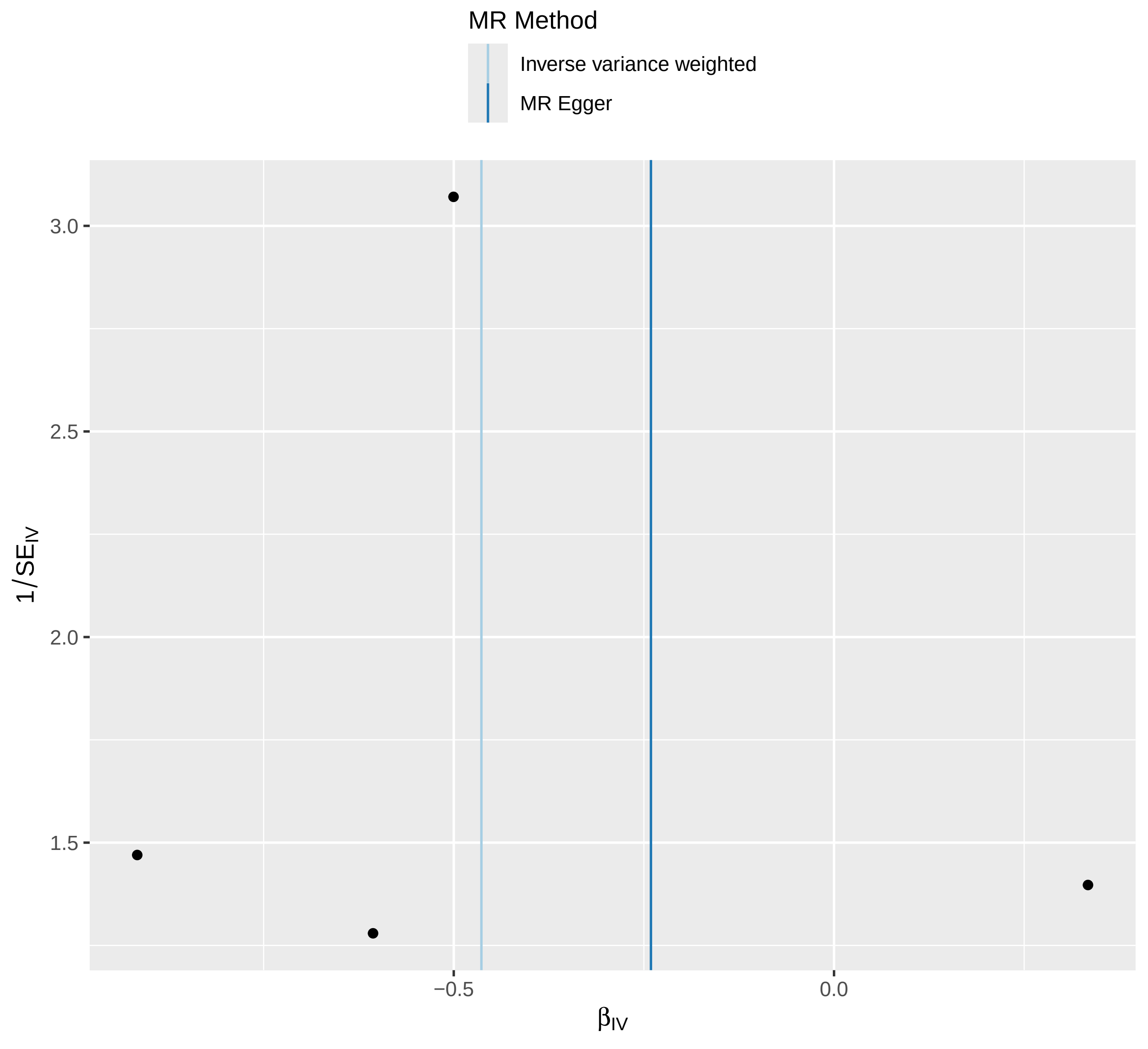
MR Method
Inverse variance weighted
MR Egger
3.0
2.5
2.0
1.5
1/SE_{IV}
−0.5
0.0
β_{IV}

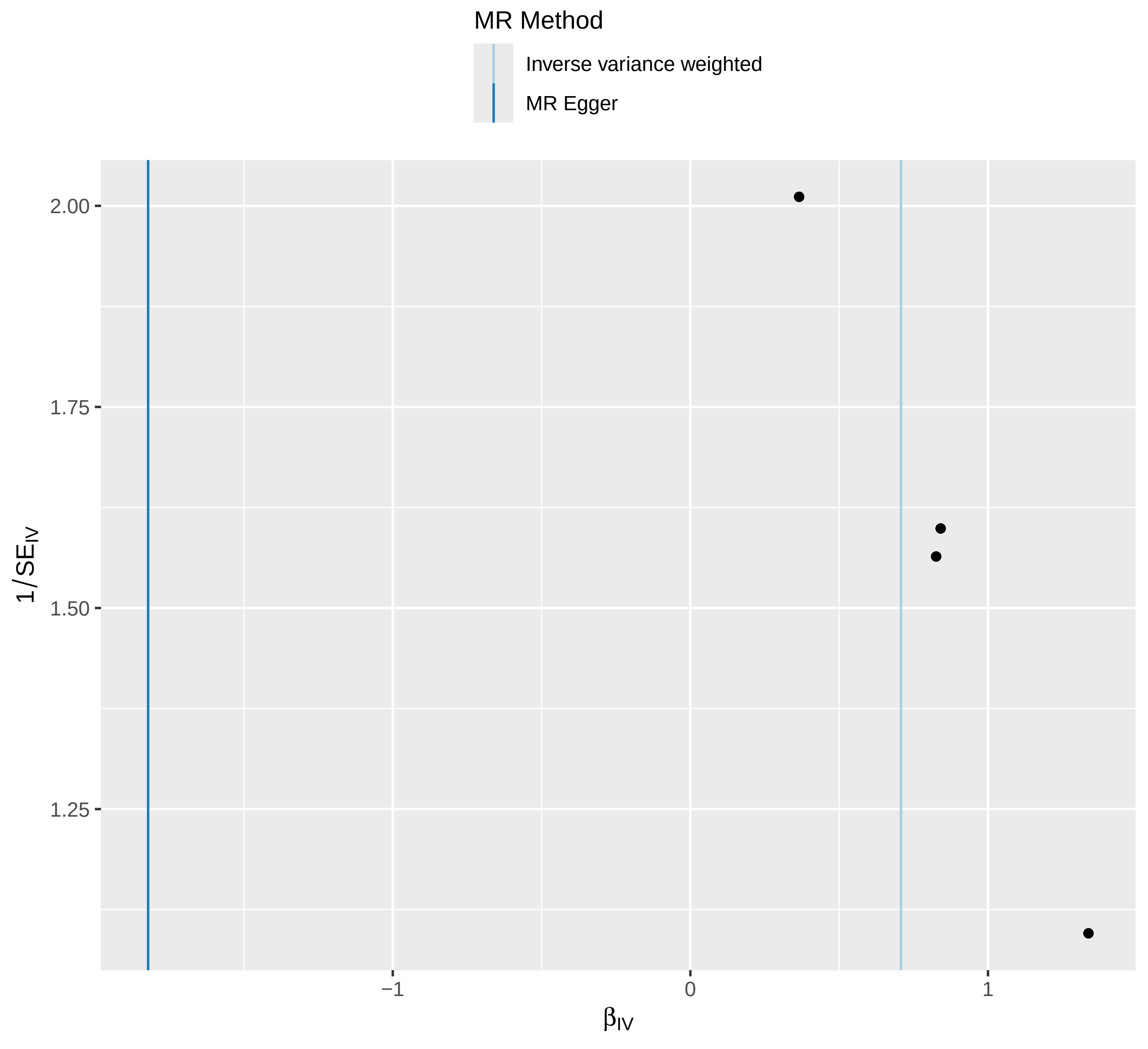
MR Method
Inverse variance weighted
MR Egger
2.00
1.75
1.50
1.25
1/SE_IV
−1
0
1
β_IV

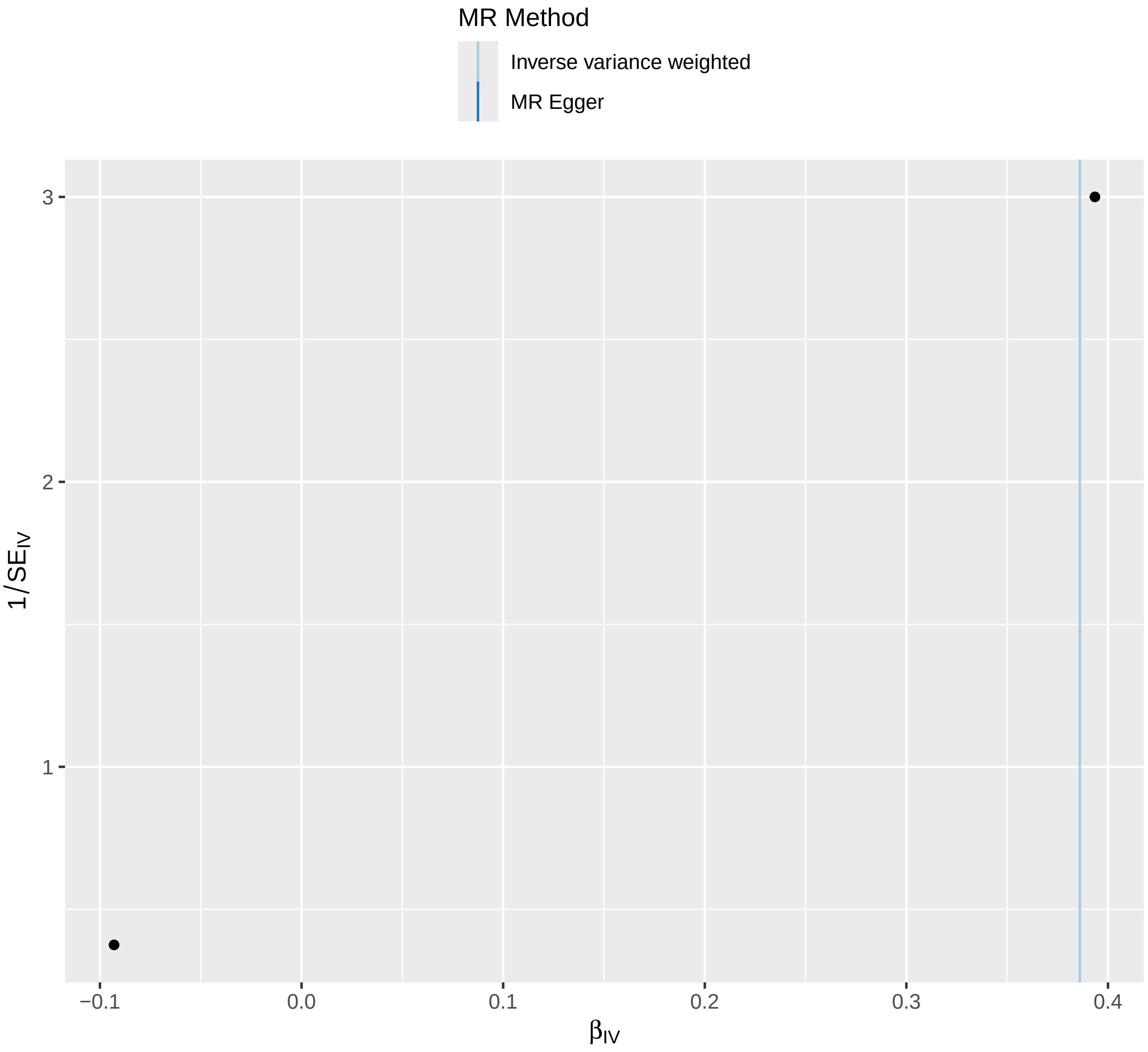
MR Method
Inverse variance weighted
MR Egger
3
2
1
1/SE_IV
−0.1
0.0
0.1
0.2
0.3
0.4
β_IV

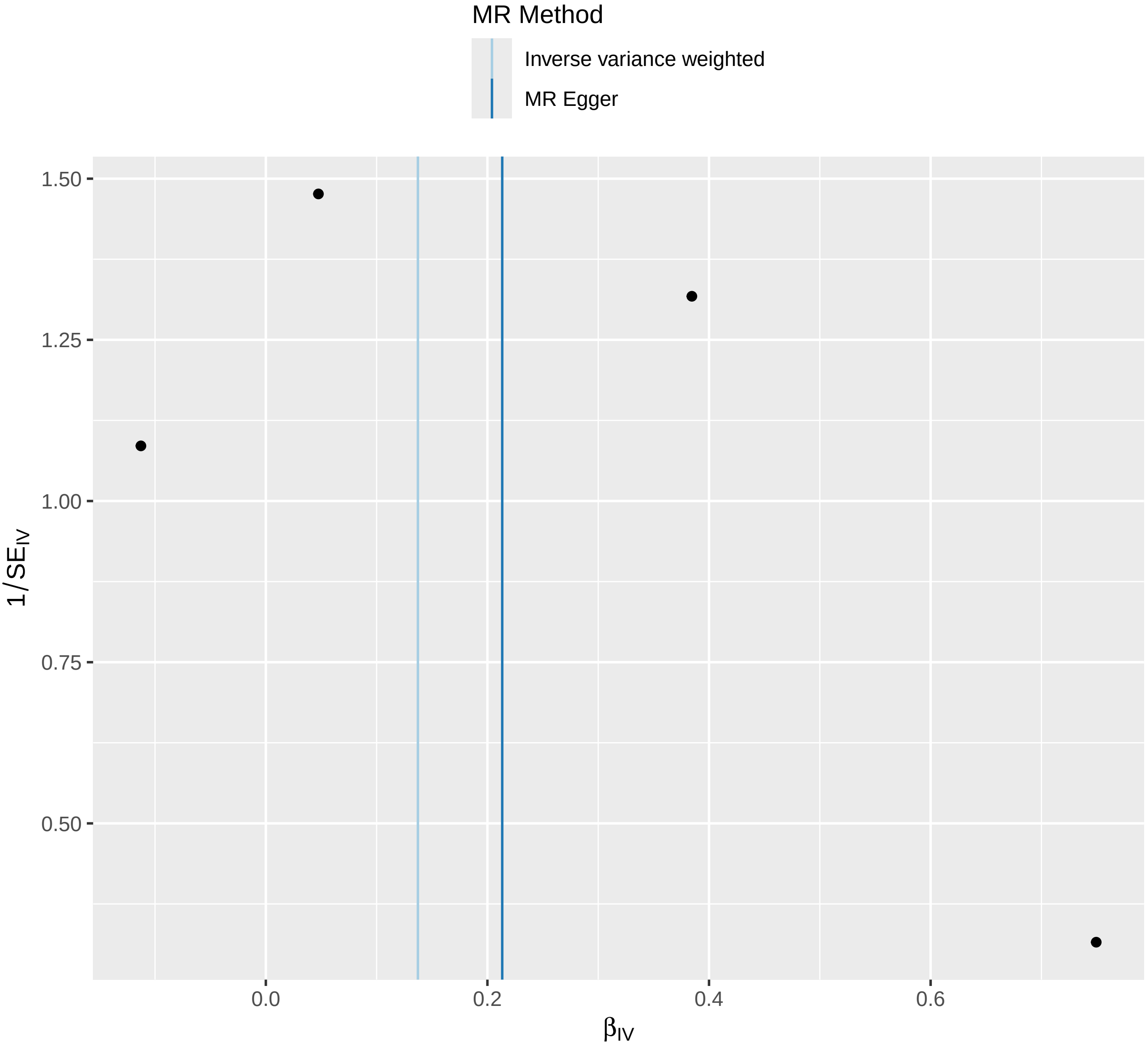

MR Method
Inverse variance weighted
MR Egger
1.50
1.25
1.00
0.75
0.50
0.0
0.2
0.4
0.6
1/SE_IV
β_IV

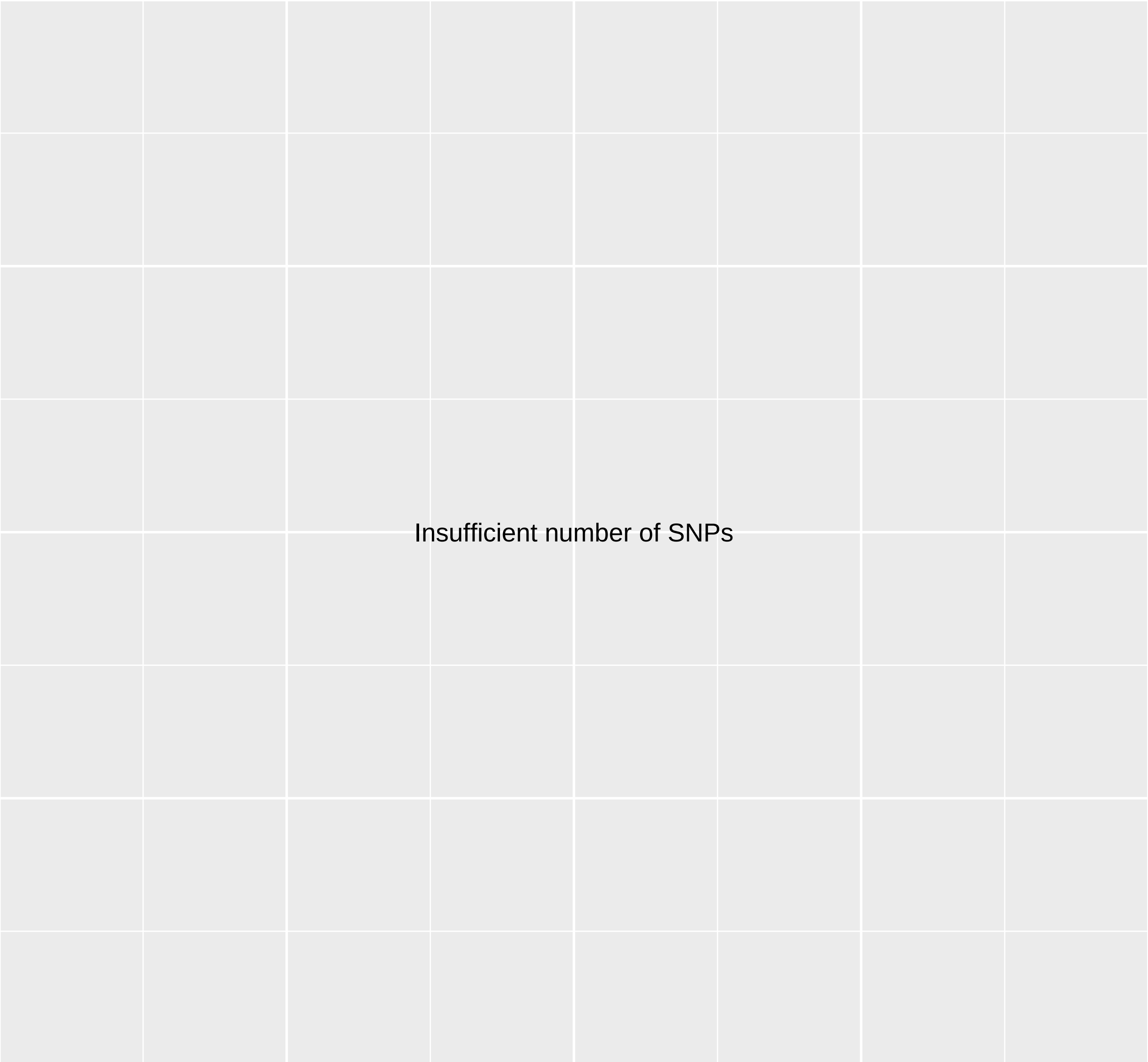
Insufficient number of SNPs

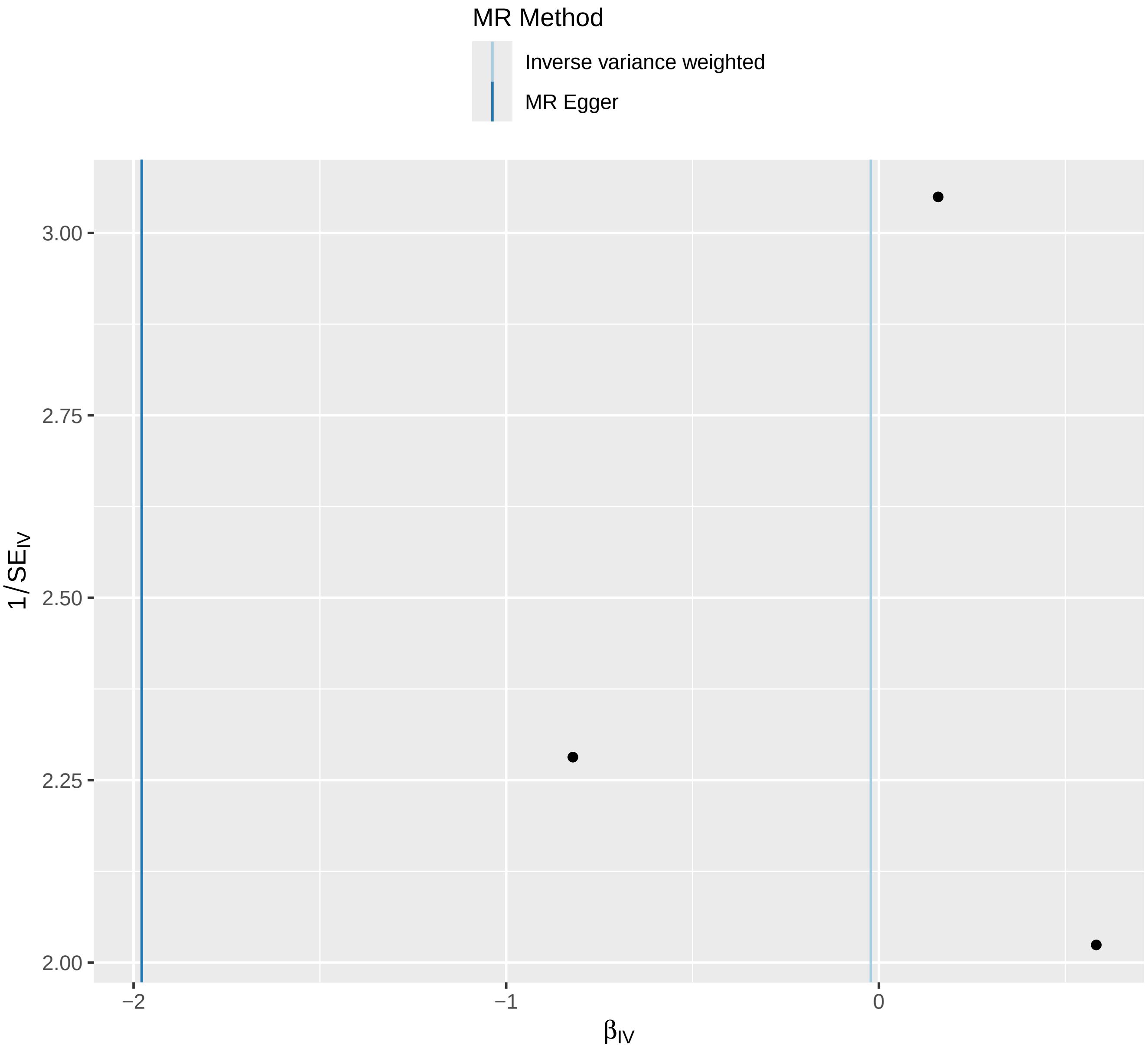
MR Method
Inverse variance weighted
MR Egger
3.00
2.75
2.50
2.25
2.00
1/SE_IV
−2
−1
0
β_IV

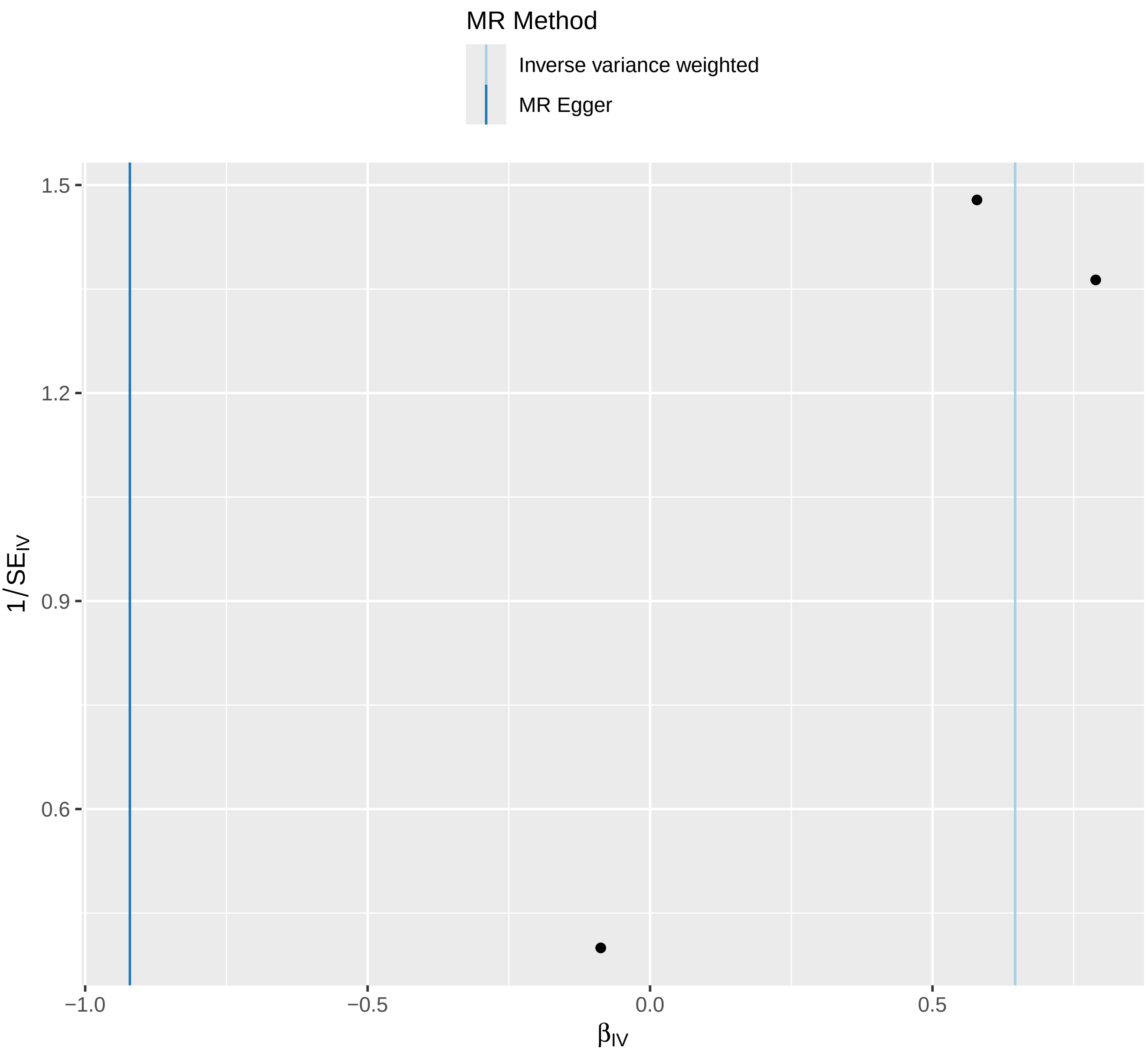
MR Method
Inverse variance weighted
MR Egger
1.5
1.2
0.9
0.6
1/SE$_{IV}$
−1.0
−0.5
0.0
0.5
β$_{IV}$

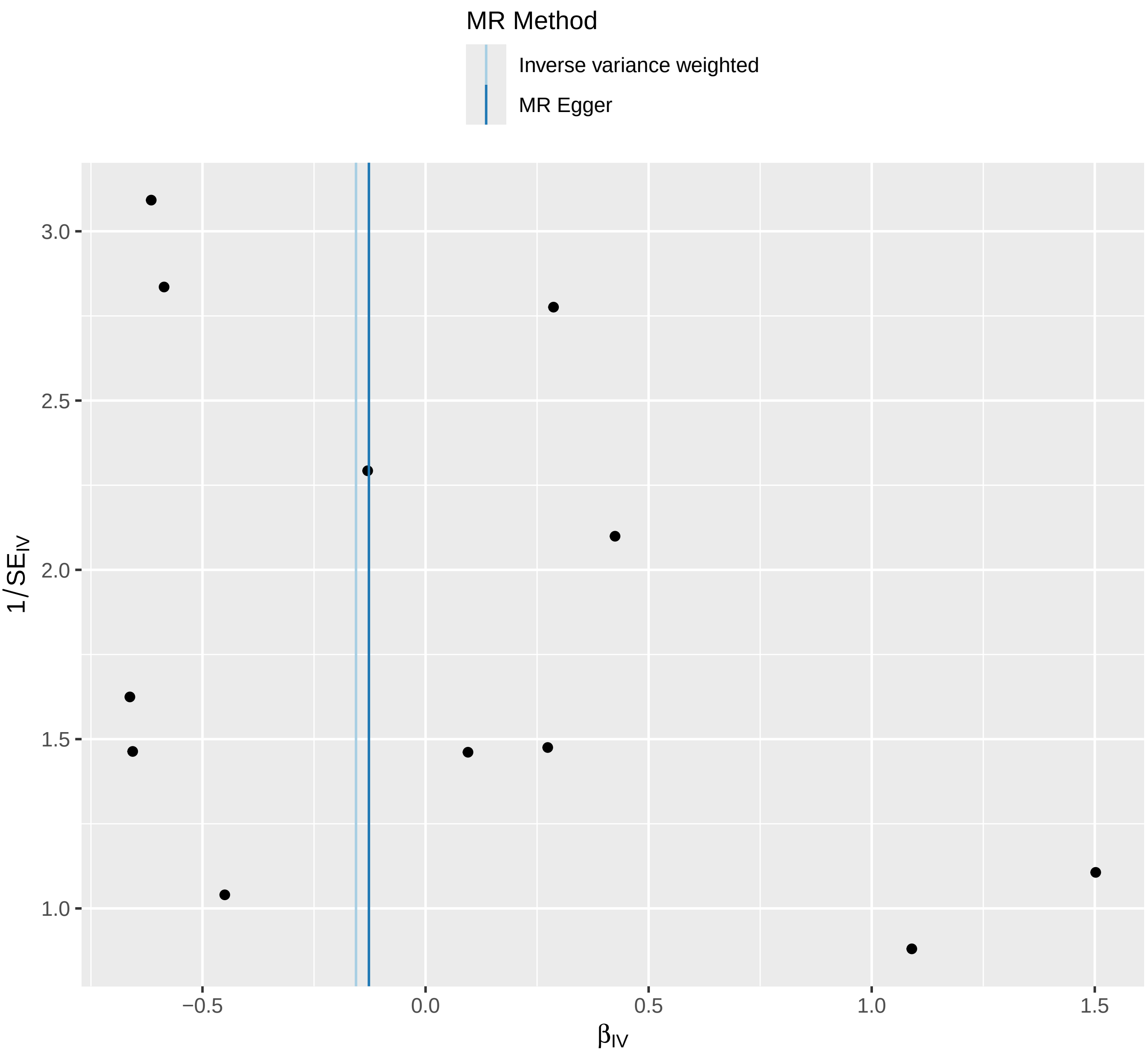
MR Method
Inverse variance weighted
MR Egger
3.0
2.5
2.0
1.5
1.0
$1/SE_{IV}$
−0.5
0.0
0.5
1.0
1.5
β_{IV}

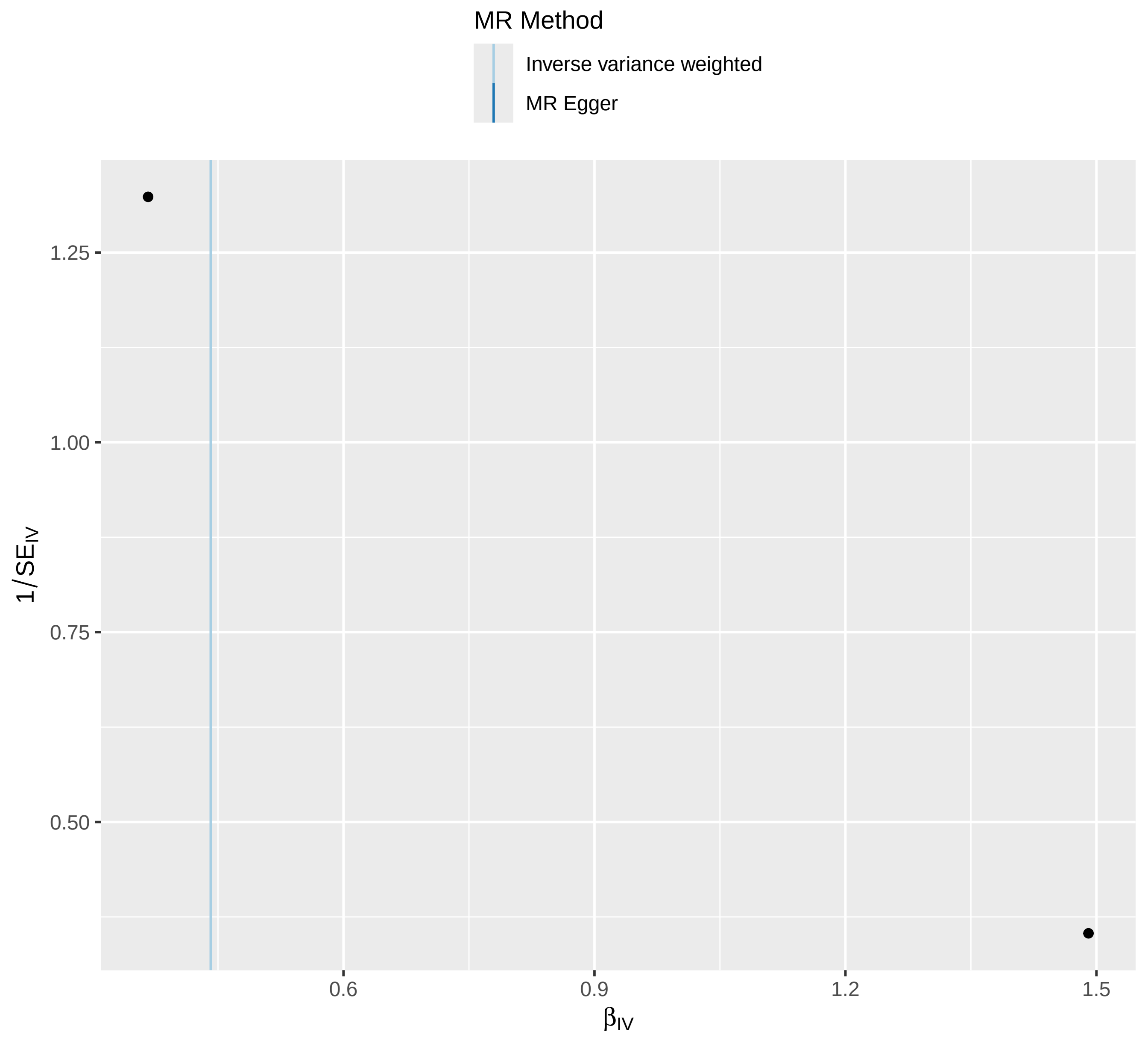
MR Method
Inverse variance weighted
MR Egger
1.25
1.00
0.75
0.50
0.6
0.9
1.2
1.5
$1/SE_{IV}$
β_{IV}

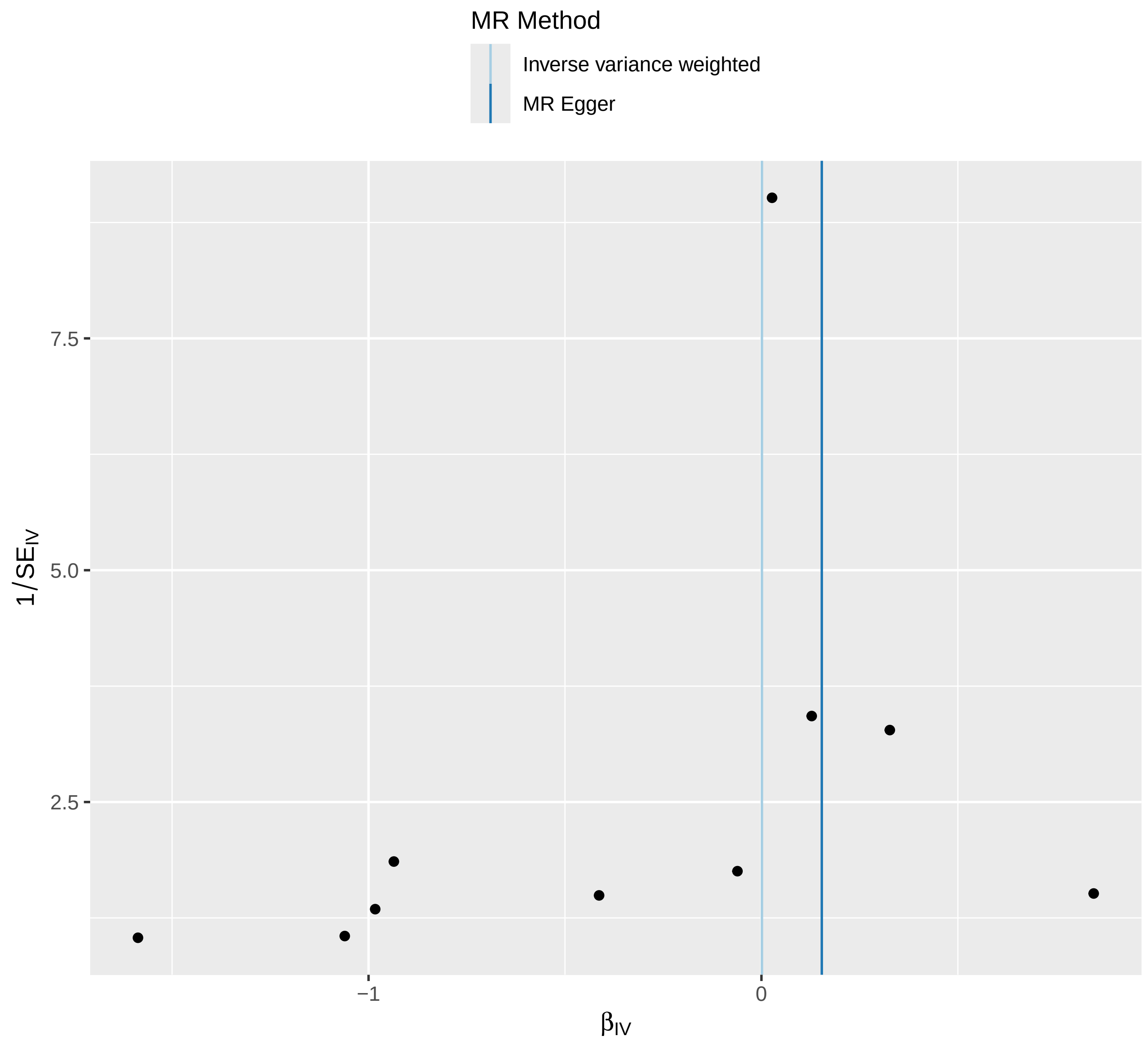
MR Method
Inverse variance weighted
MR Egger
7.5
5.0
2.5
1/SE$_{IV}$
−1
0
β$_{IV}$

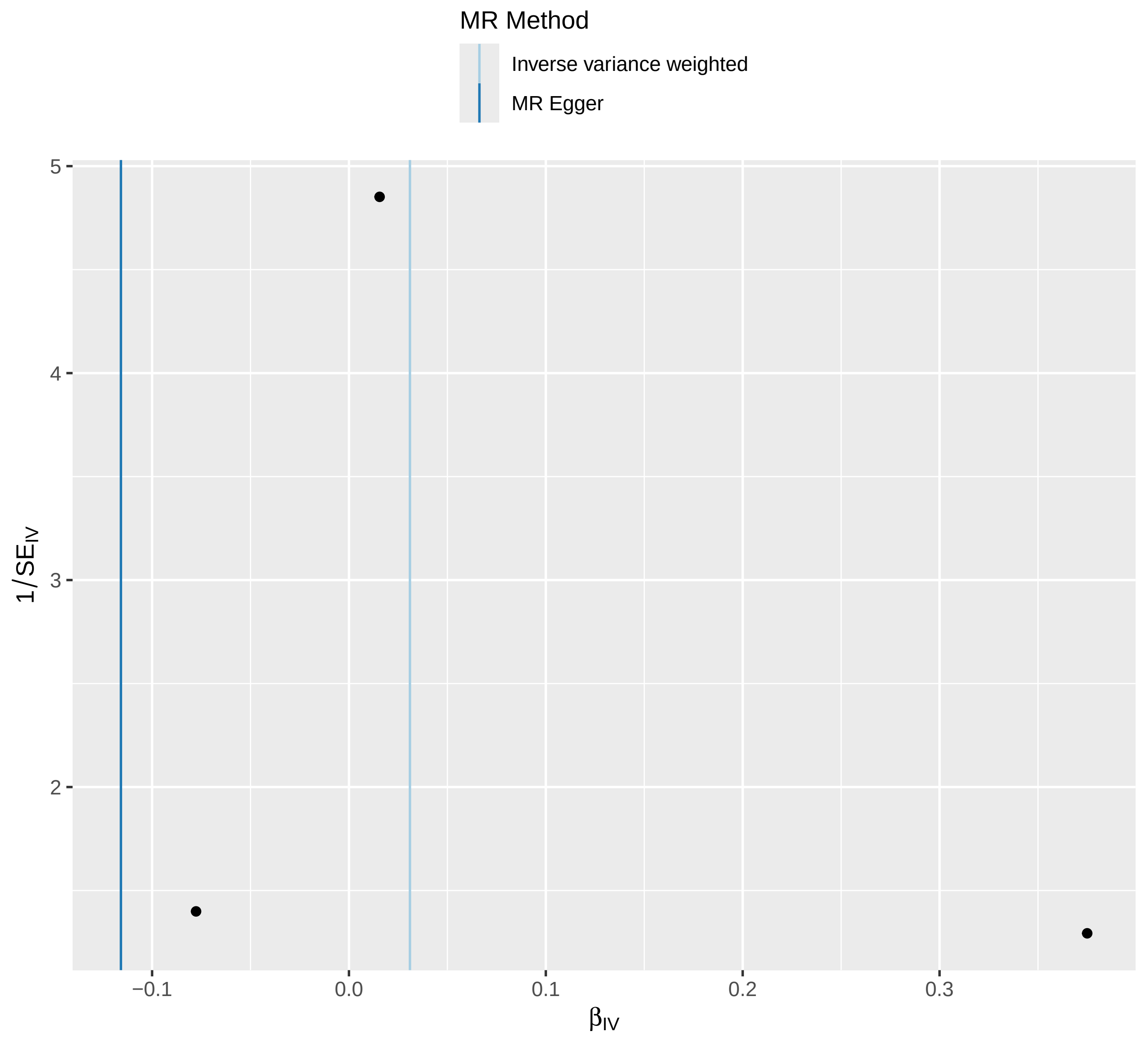
MR Method
Inverse variance weighted
MR Egger
1/SE$_{IV}$
5
4
3
2
−0.1
0.0
0.1
0.2
0.3
β$_{IV}$

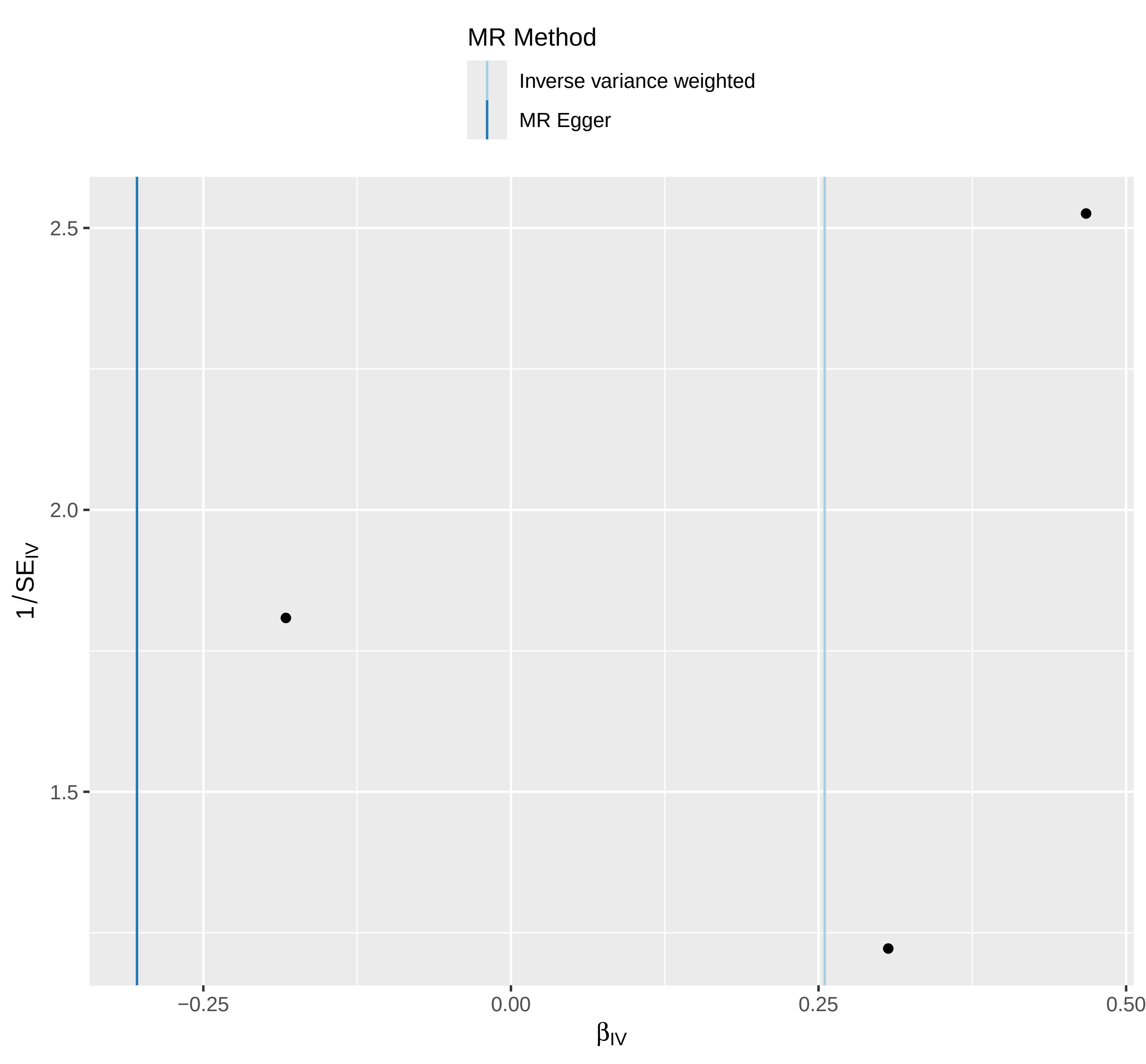

MR Method
Inverse variance weighted
MR Egger
2.5
2.0
1.5
1/SE_IV
−0.25
0.00
0.25
0.50
β_IV

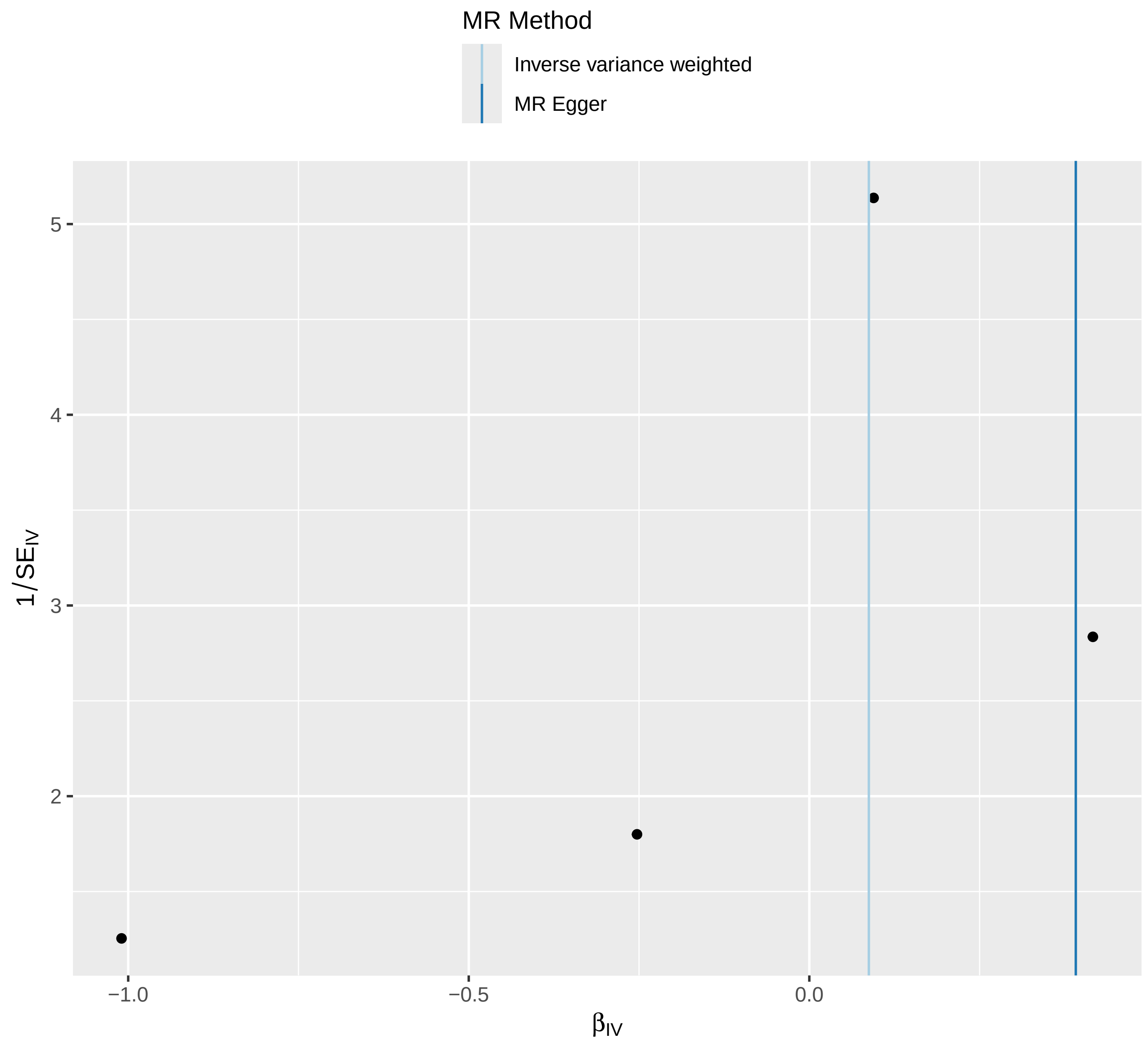

MR Method
Inverse variance weighted
MR Egger
5
4
3
2
1/SE_IV
−1.0
−0.5
0.0
β_IV

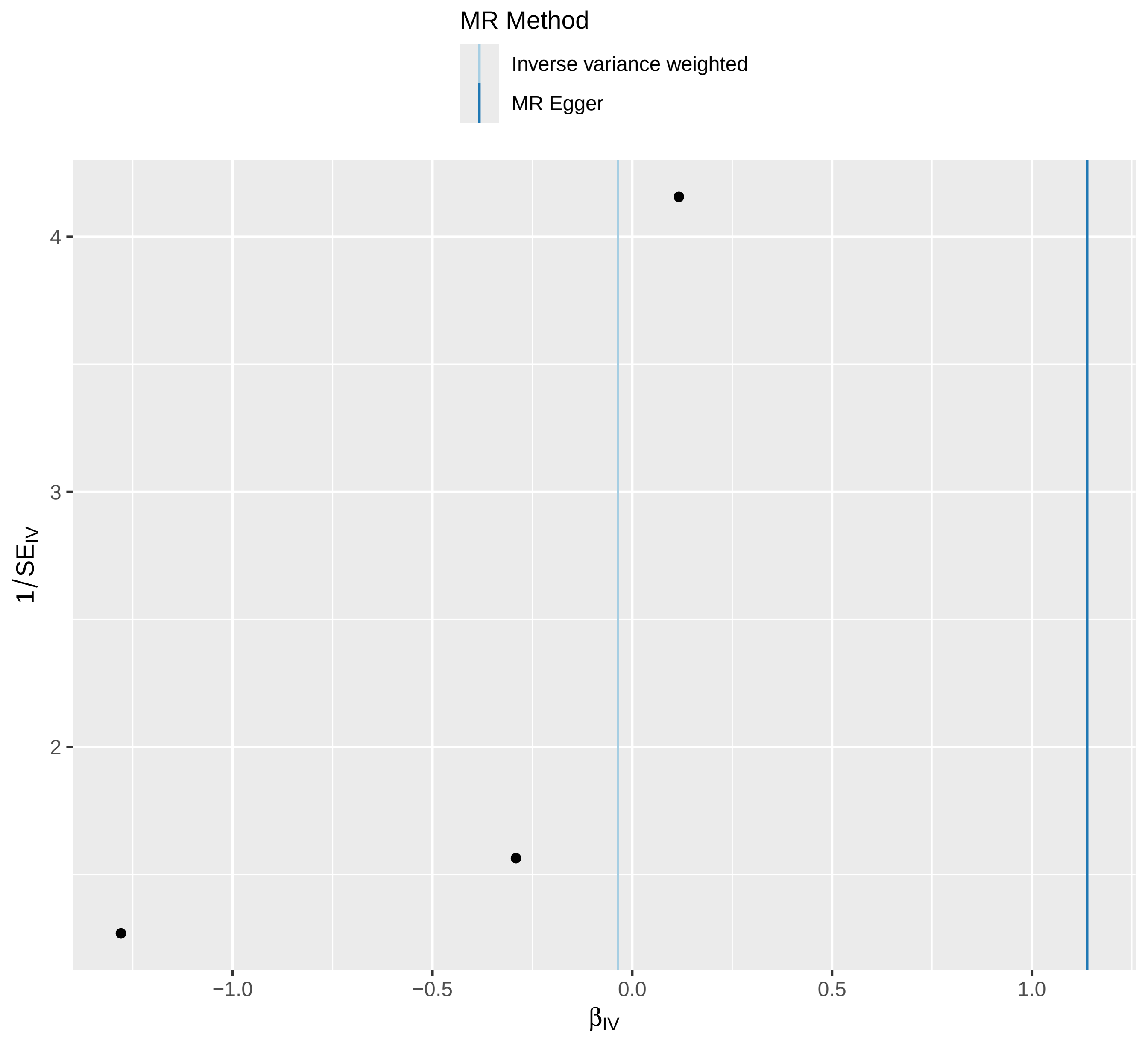

MR Method
Inverse variance weighted
MR Egger
4
3
2
1/SE_IV
−1.0
−0.5
0.0
0.5
1.0
β_IV

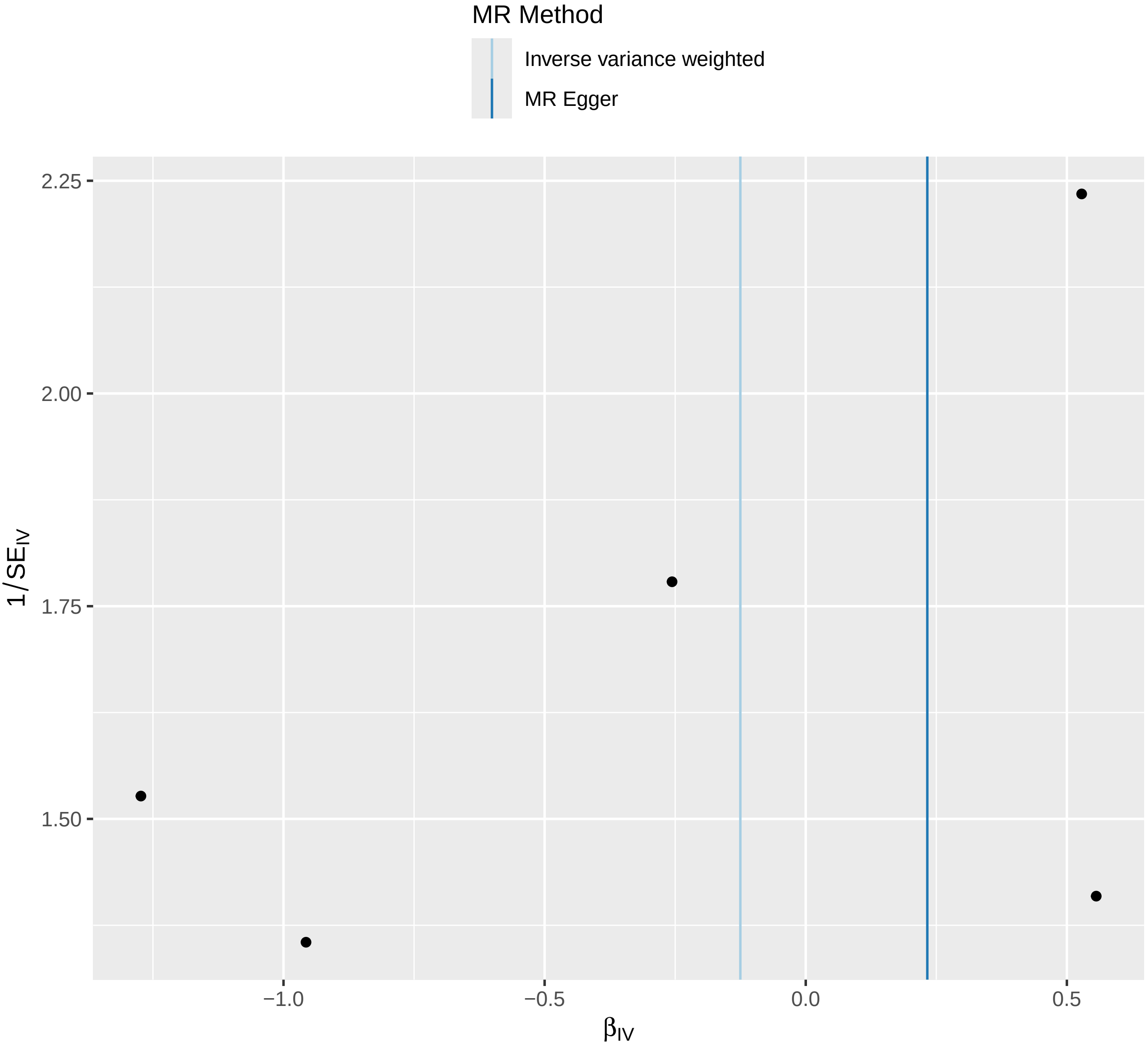
MR Method
Inverse variance weighted
MR Egger
2.25
2.00
1.75
1.50
$1/SE_{IV}$
−1.0
−0.5
0.0
0.5
β_{IV}

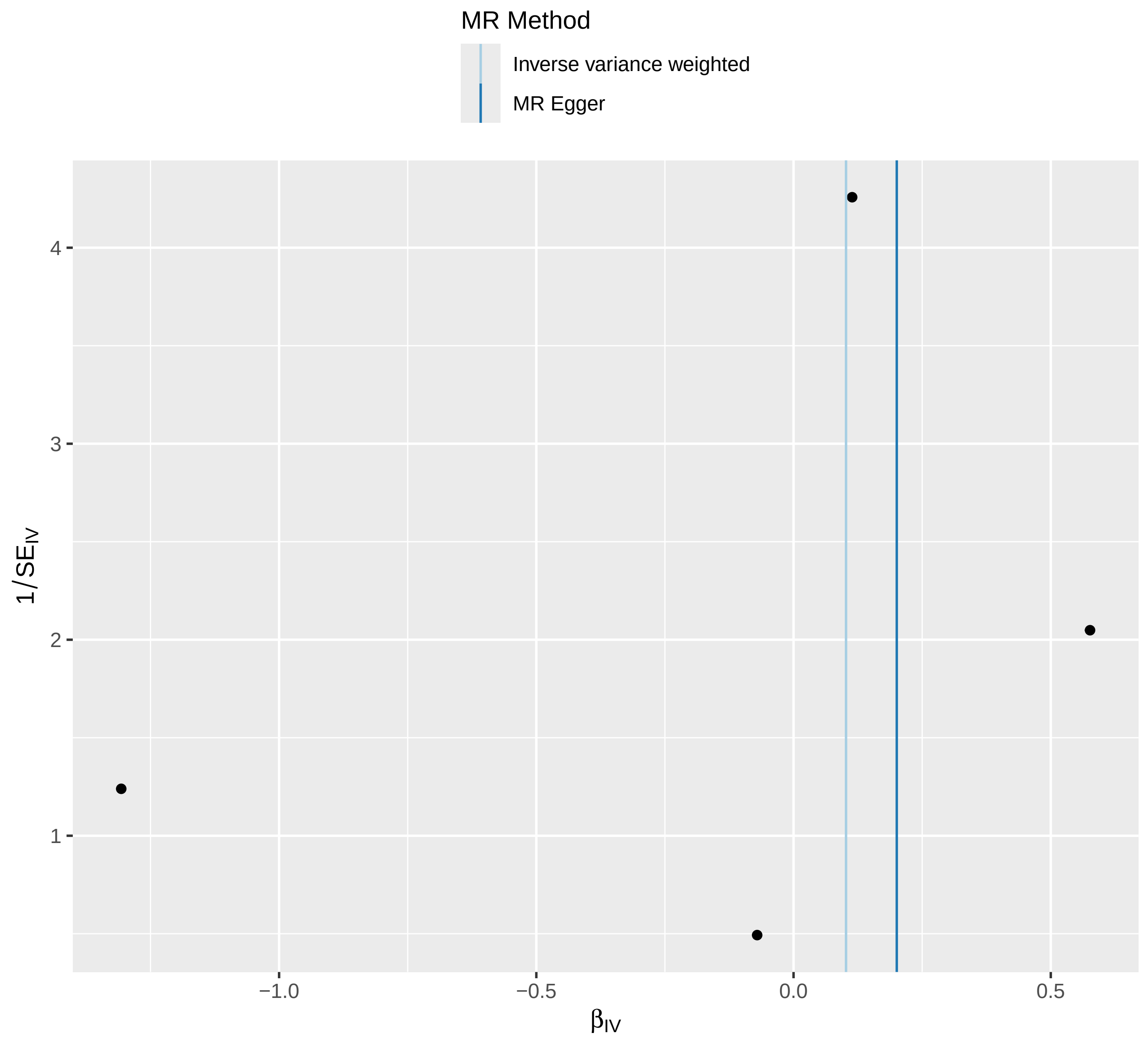

MR Method
Inverse variance weighted
MR Egger
4
3
2
1
$1/SE_{IV}$
−1.0
−0.5
0.0
0.5
β_{IV}

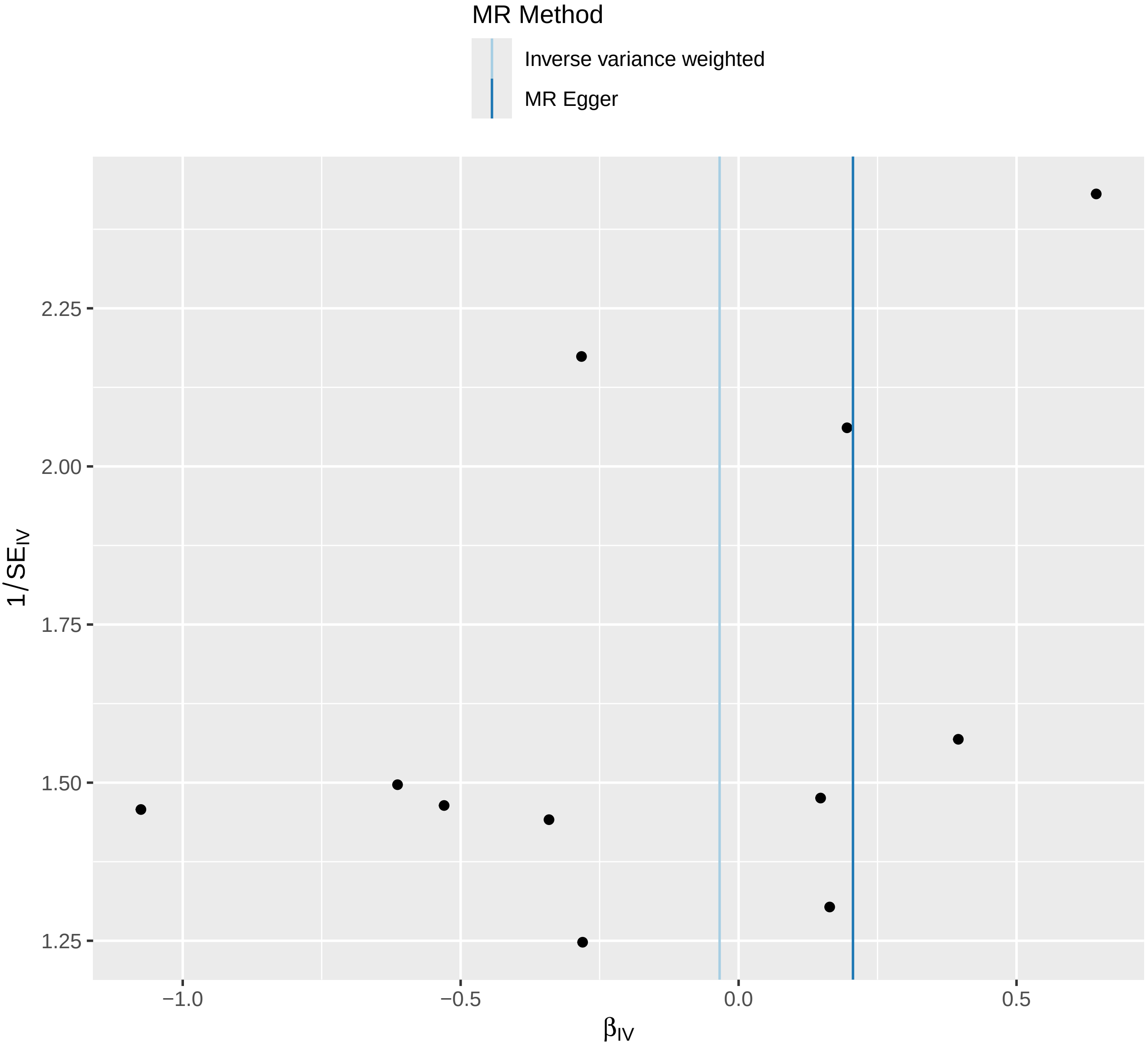
MR Method
Inverse variance weighted
MR Egger
2.25
2.00
1.75
1.50
1.25
1/SE$_{IV}$
−1.0
−0.5
0.0
0.5
β$_{IV}$

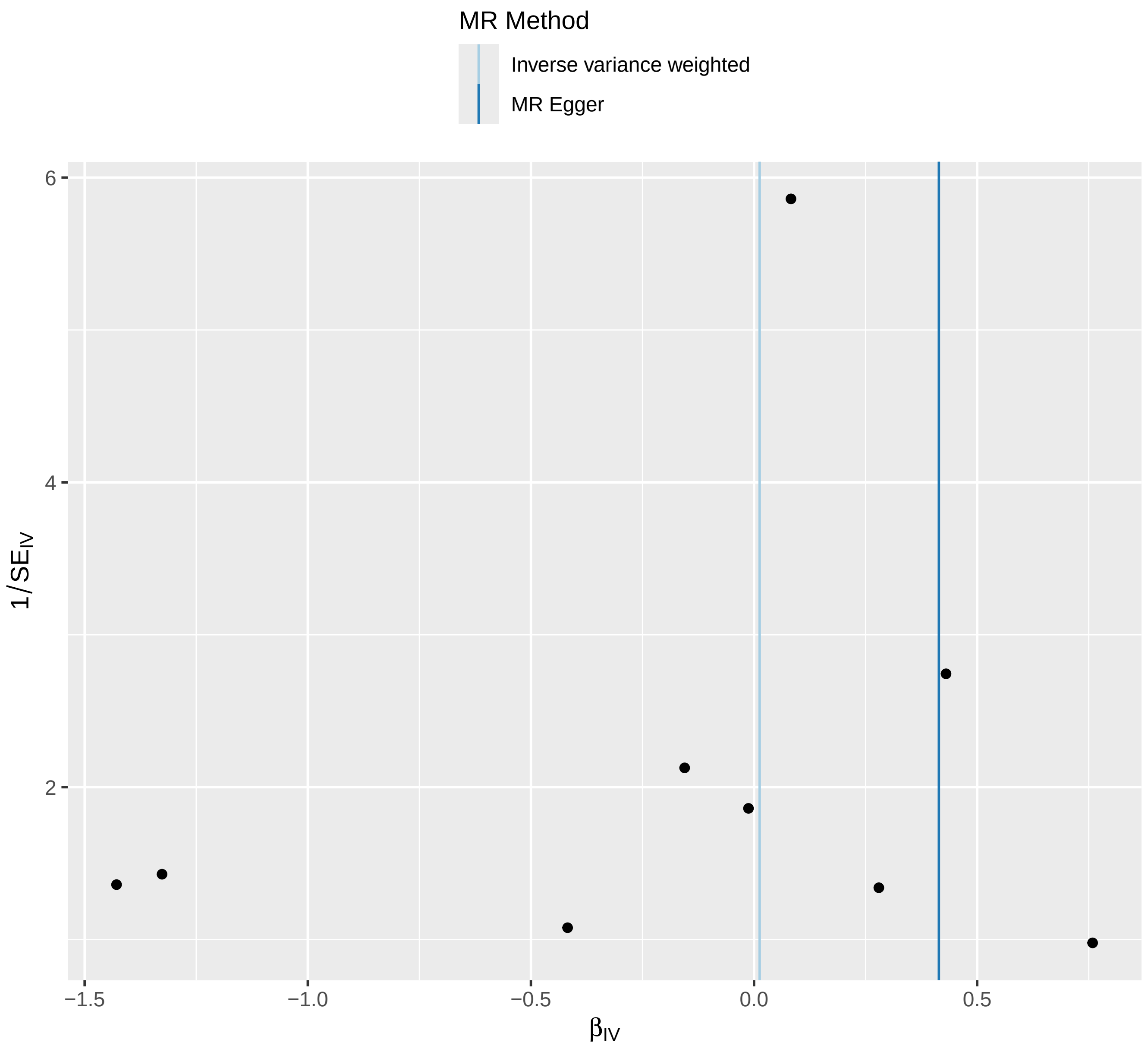

MR Method
Inverse variance weighted
MR Egger
6
4
2
$1/SE_{IV}$
−1.5
−1.0
−0.5
0.0
0.5
β_{IV}

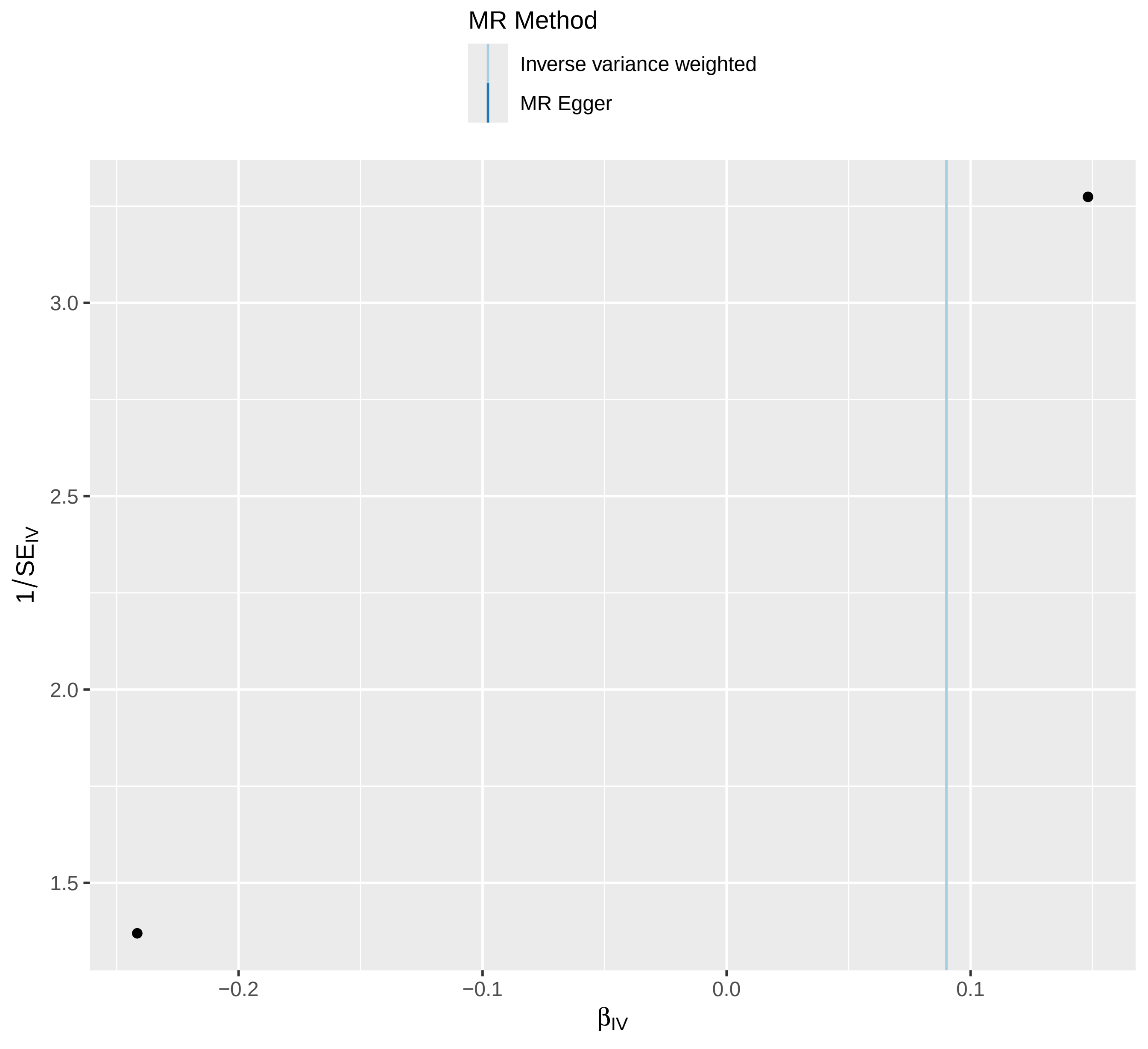
MR Method
Inverse variance weighted
MR Egger
1/SE_IV
3.0
2.5
2.0
1.5
−0.2
−0.1
0.0
0.1
β_IV

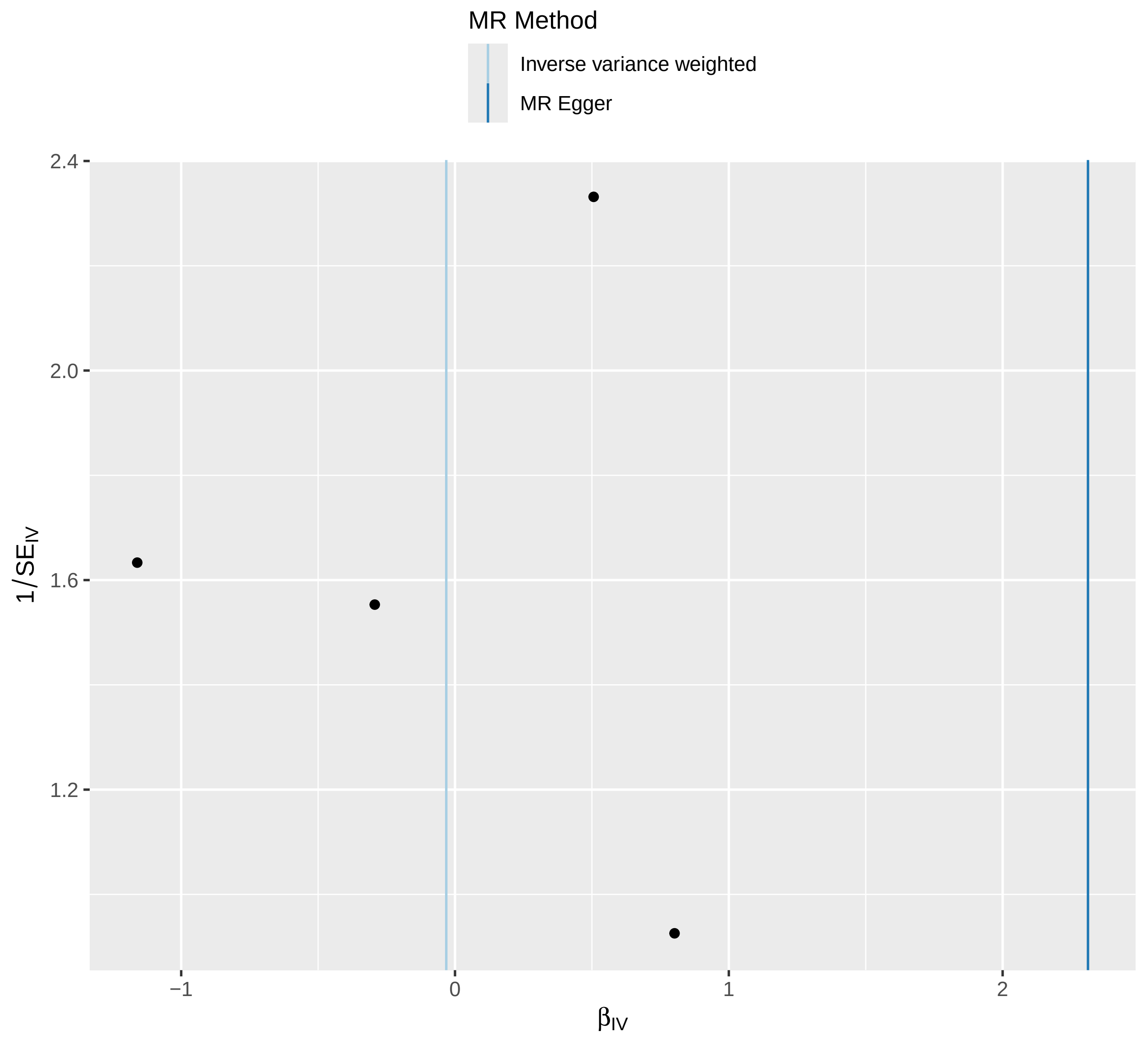
MR Method
Inverse variance weighted
MR Egger
2.4
2.0
1.6
1.2
1/SE_IV
−1
0
1
2
β_IV

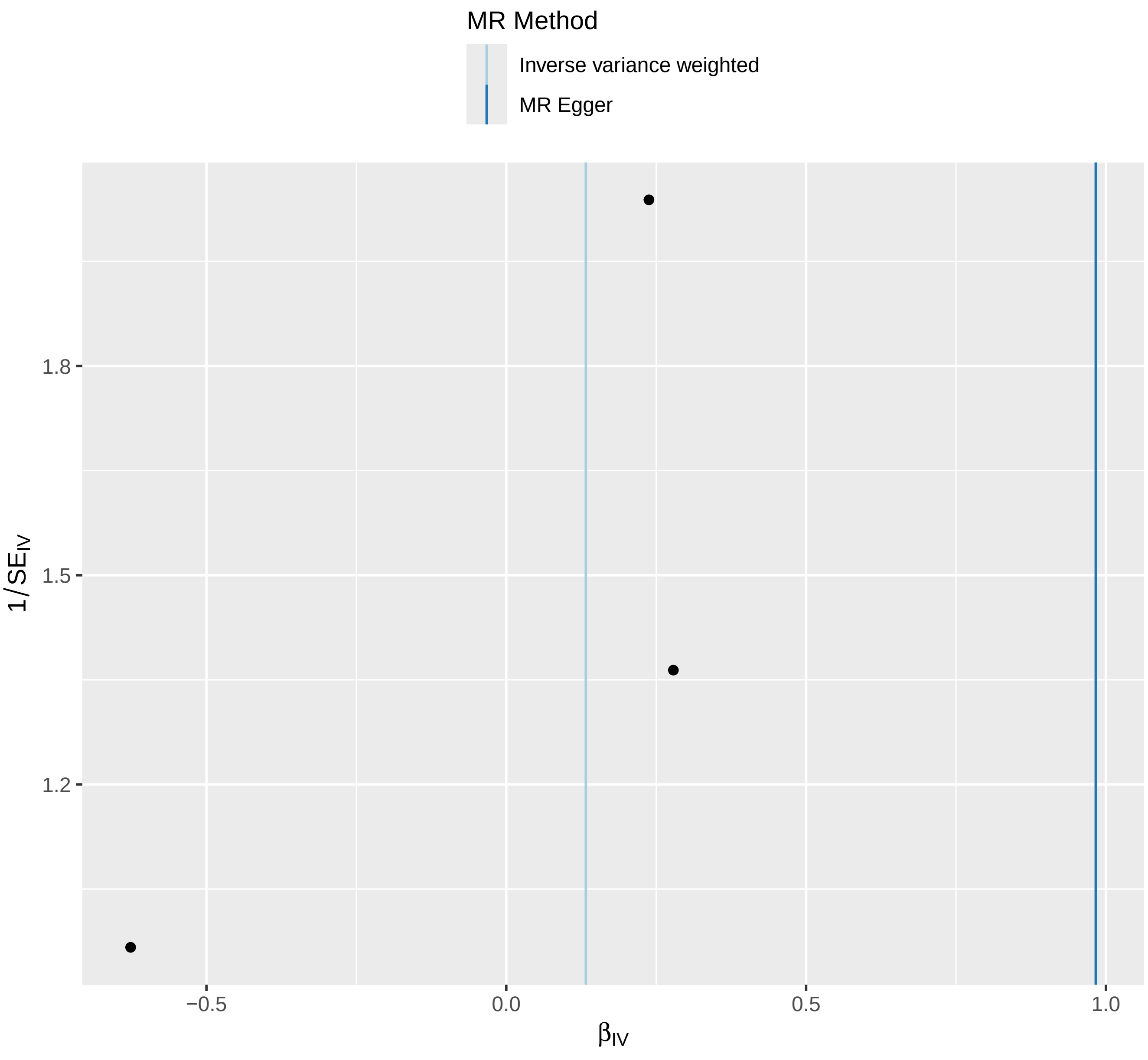

MR Method
Inverse variance weighted
MR Egger
1.8
1.5
1.2
1/SE$_{IV}$
−0.5
0.0
0.5
1.0
β$_{IV}$

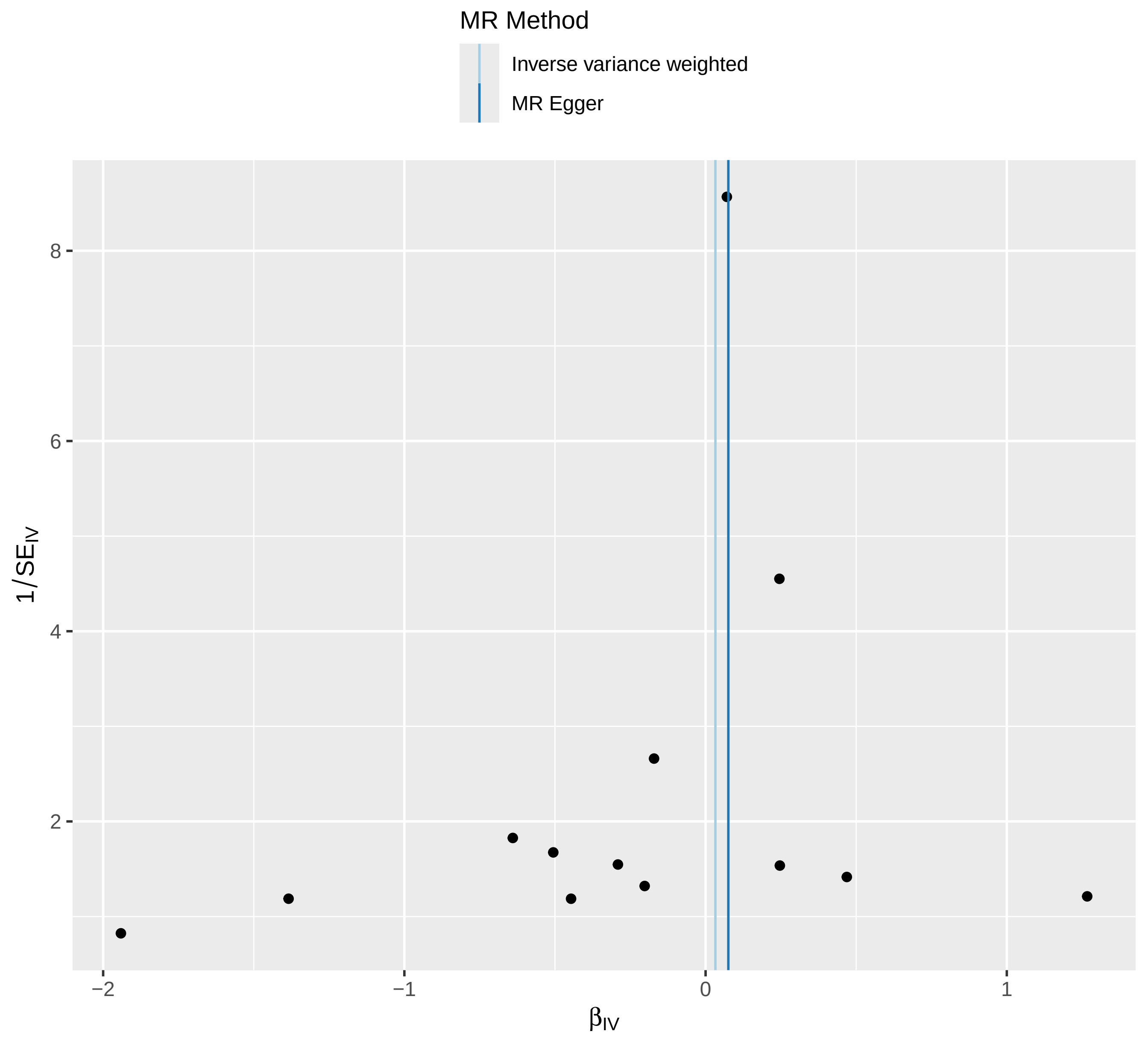

MR Method
Inverse variance weighted
MR Egger
8
6
4
2
$1/SE_{IV}$
−2
−1
0
1
β_{IV}

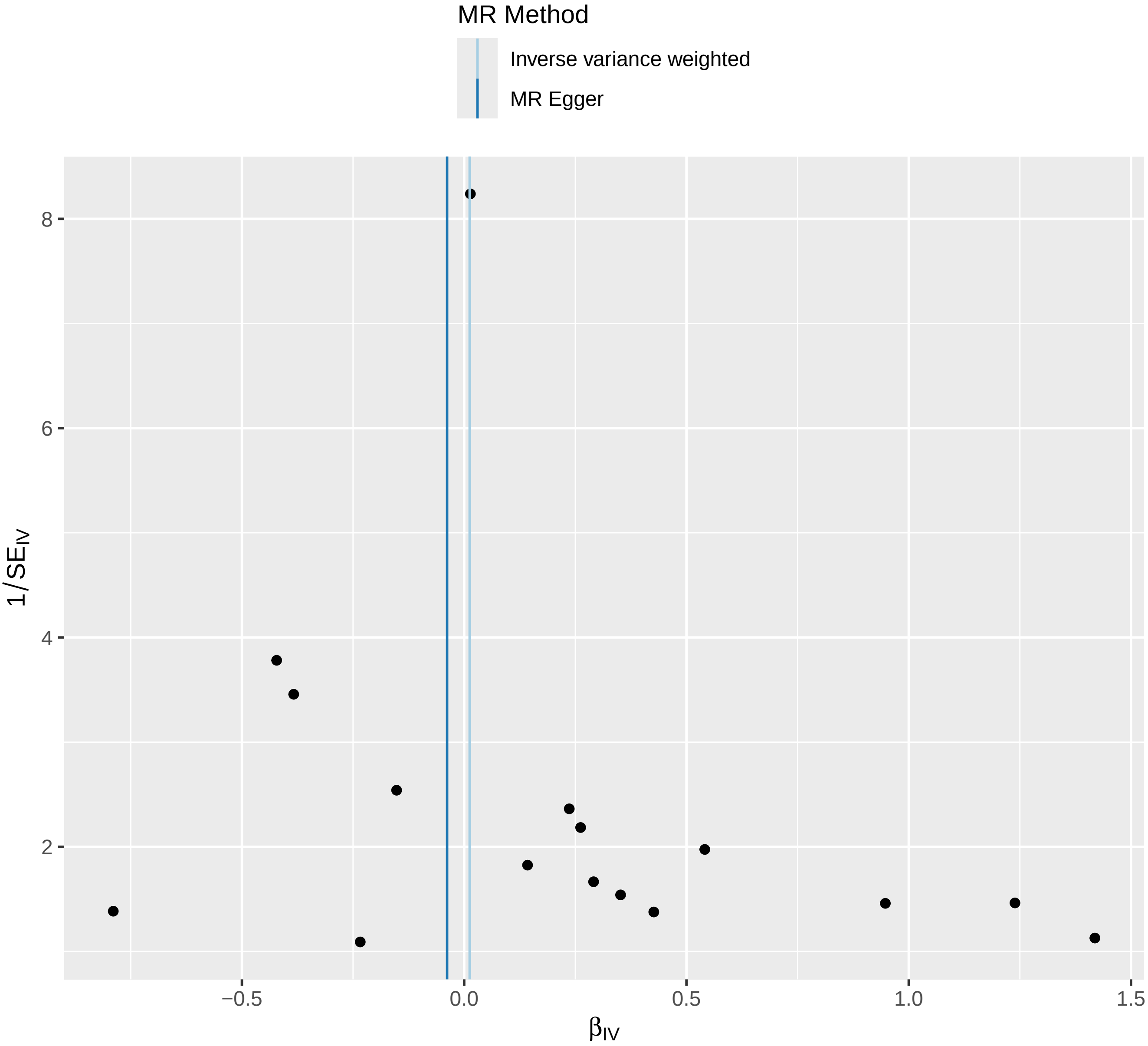
MR Method
Inverse variance weighted
MR Egger
1/SE_{IV}
8
6
4
2
−0.5
0.0
0.5
1.0
1.5
β_{IV}

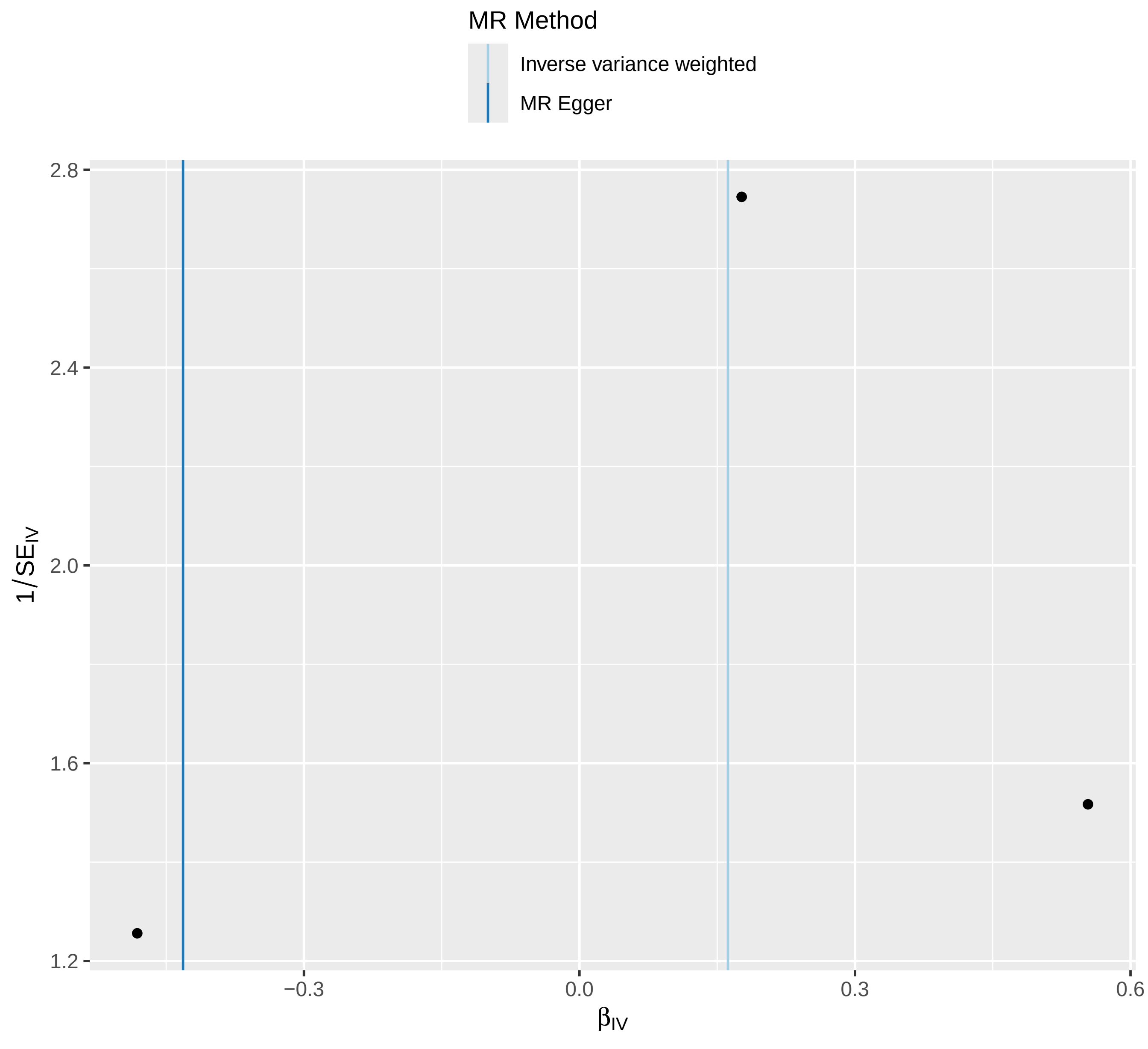

MR Method
Inverse variance weighted
MR Egger
2.8
2.4
2.0
1.6
1.2
$1/SE_{IV}$
−0.3
0.0
0.3
0.6
β_{IV}

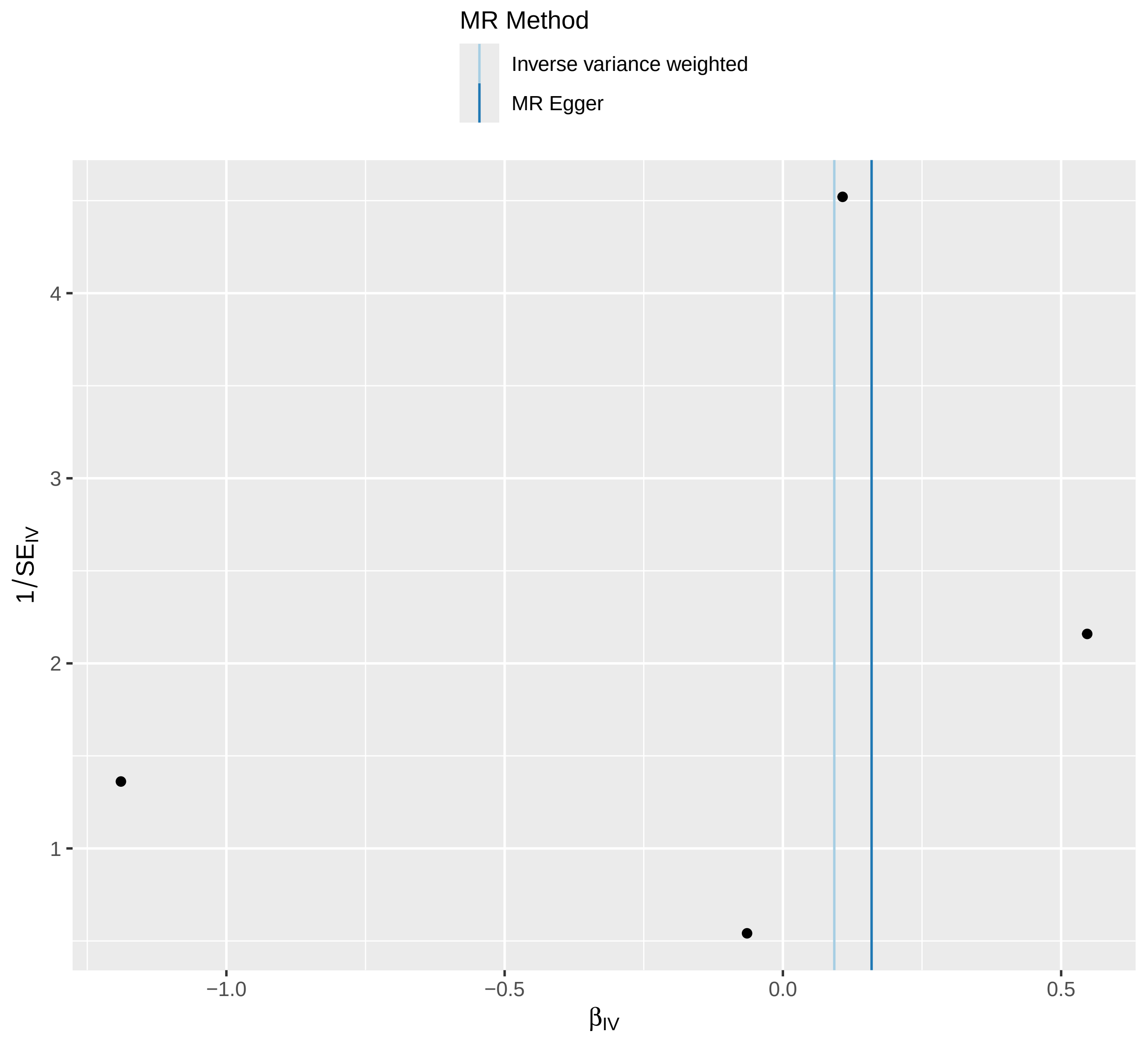

MR Method
Inverse variance weighted
MR Egger
4
3
2
1
1/SE_IV
−1.0
−0.5
0.0
0.5
β_IV

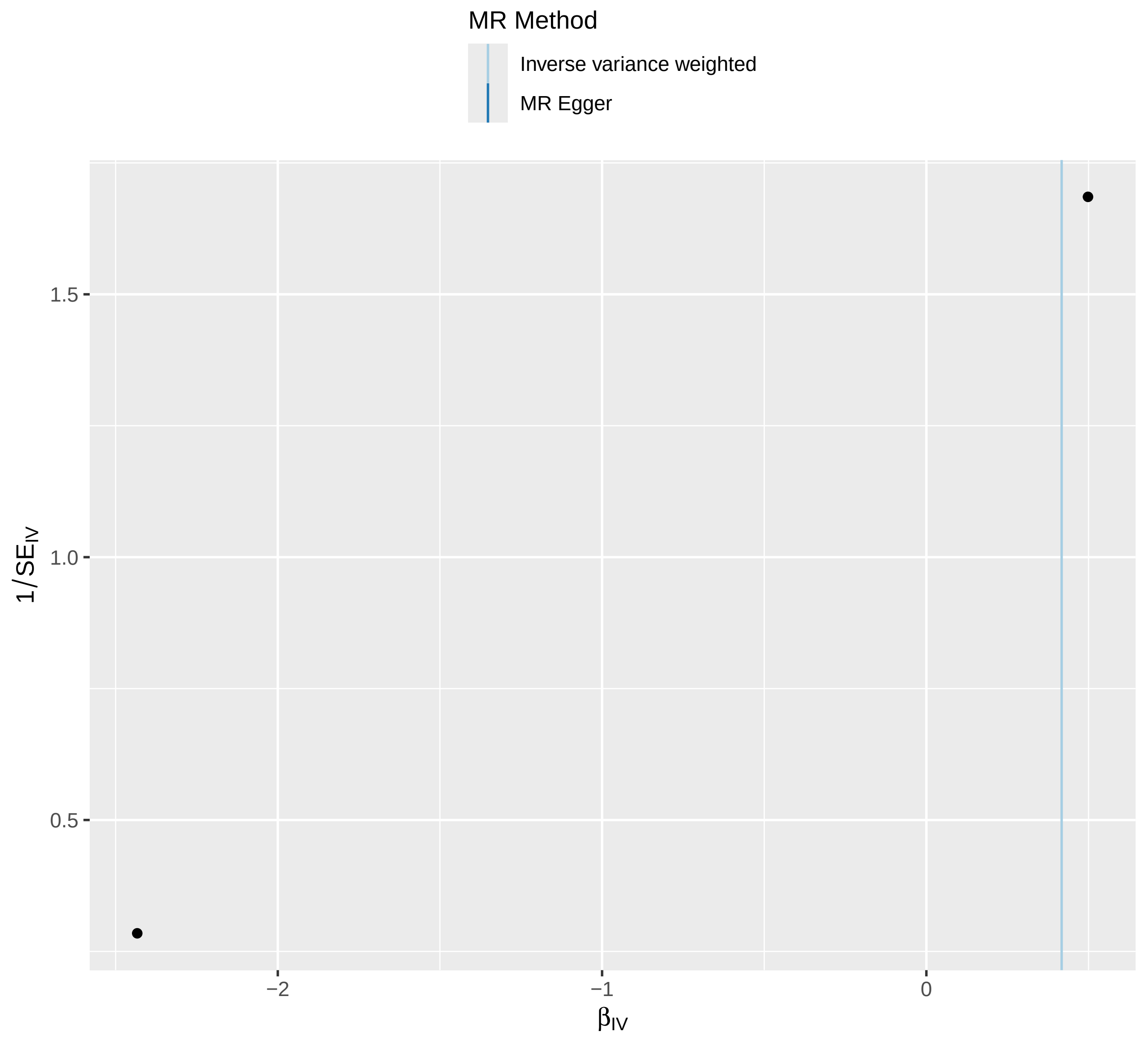

MR Method
Inverse variance weighted
MR Egger
1.5
1.0
0.5
$1/SE_{IV}$
−2
−1
0
β_{IV}

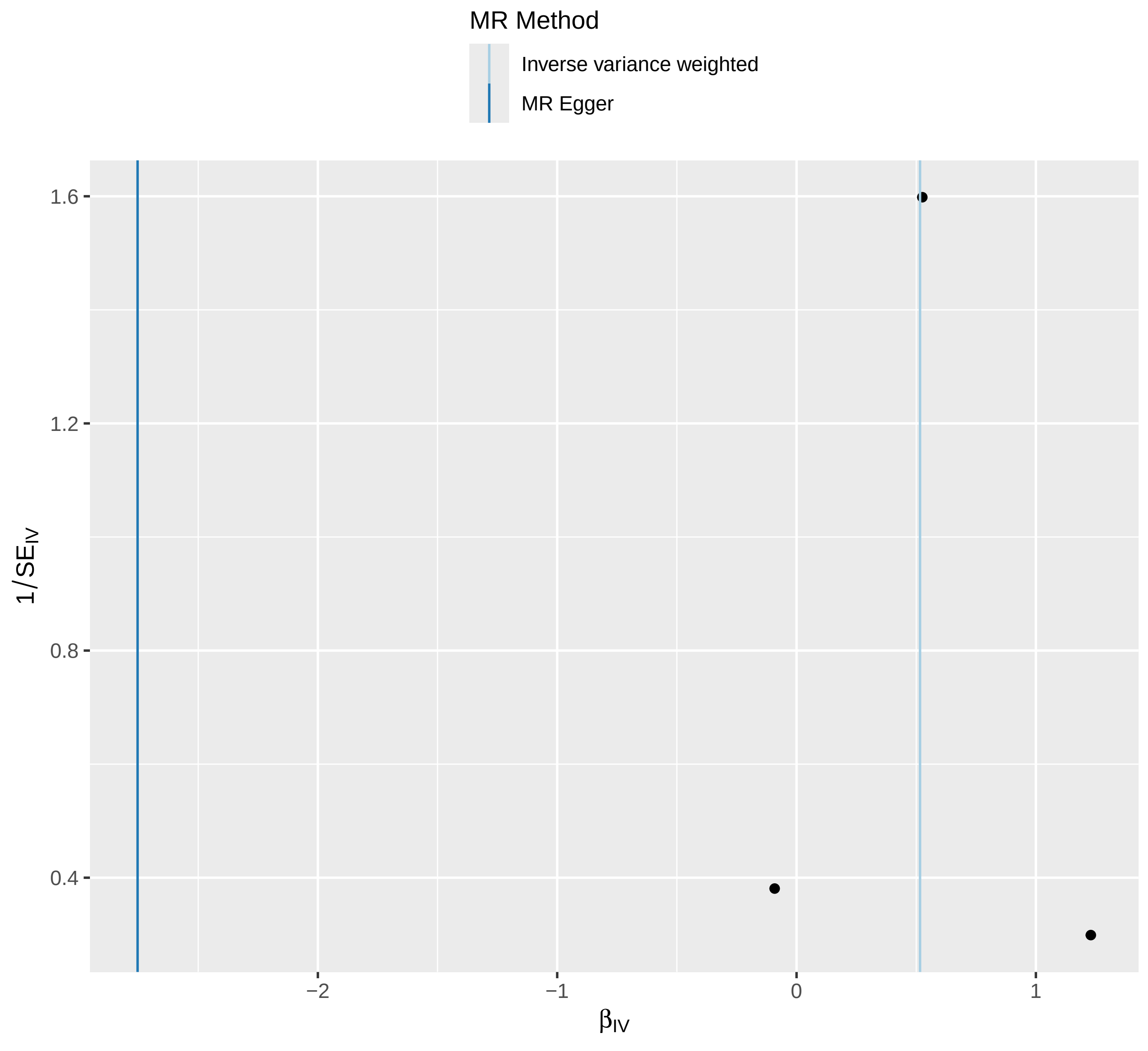
MR Method
Inverse variance weighted
MR Egger
1.6
1.2
0.8
0.4
1/SE_IV
−2
−1
0
1
β_IV

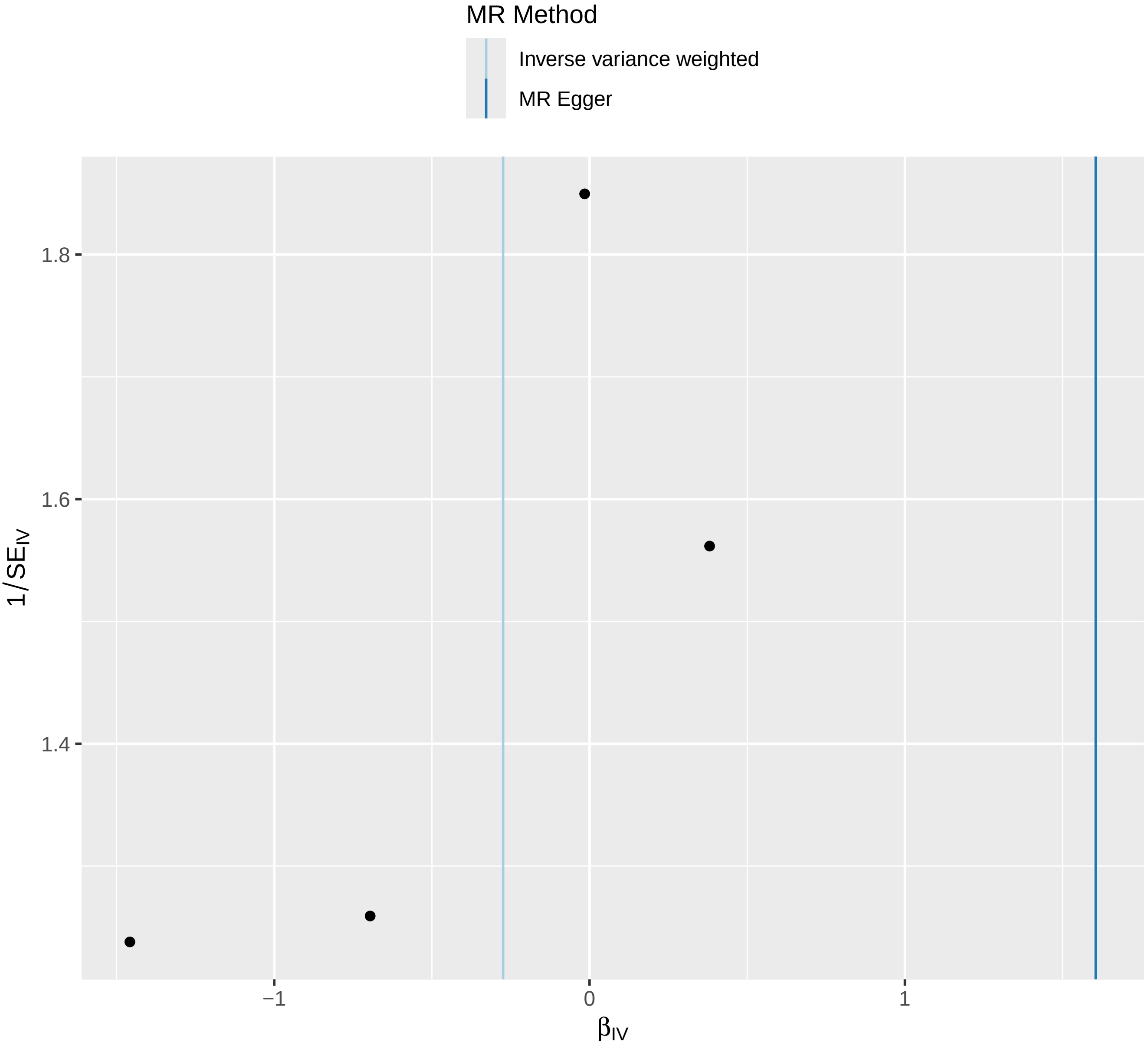
MR Method
Inverse variance weighted
MR Egger
1.8
1.6
1.4
1/SE$_{IV}$
−1
0
1
β$_{IV}$

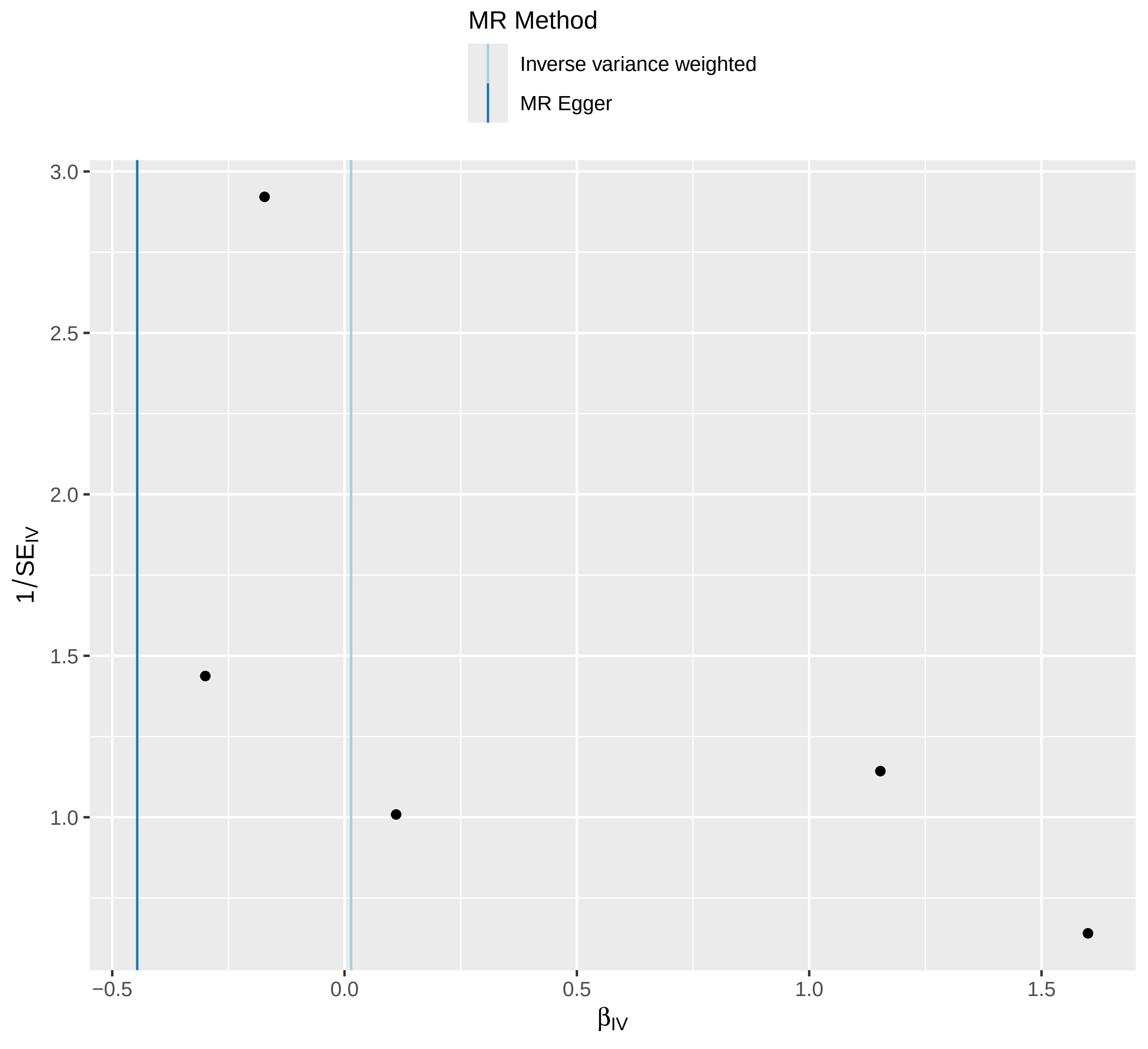

MR Method
Inverse variance weighted
MR Egger
3.0
2.5
2.0
1.5
1.0
1/SE$_{IV}$
−0.5
0.0
0.5
1.0
1.5
β$_{IV}$

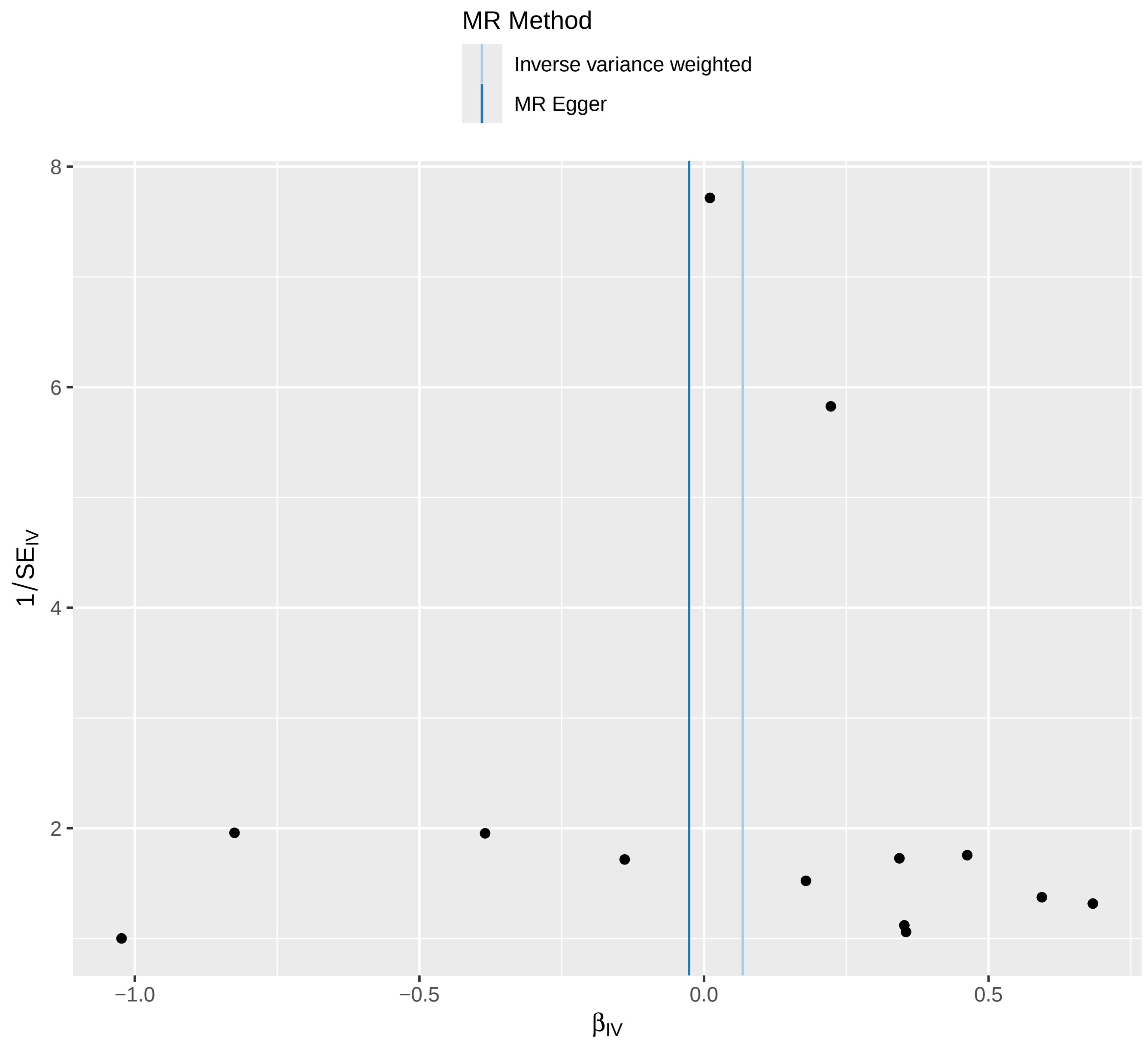
MR Method
Inverse variance weighted
MR Egger
8
6
4
2
1/SE$_{IV}$
−1.0
−0.5
0.0
0.5
β$_{IV}$

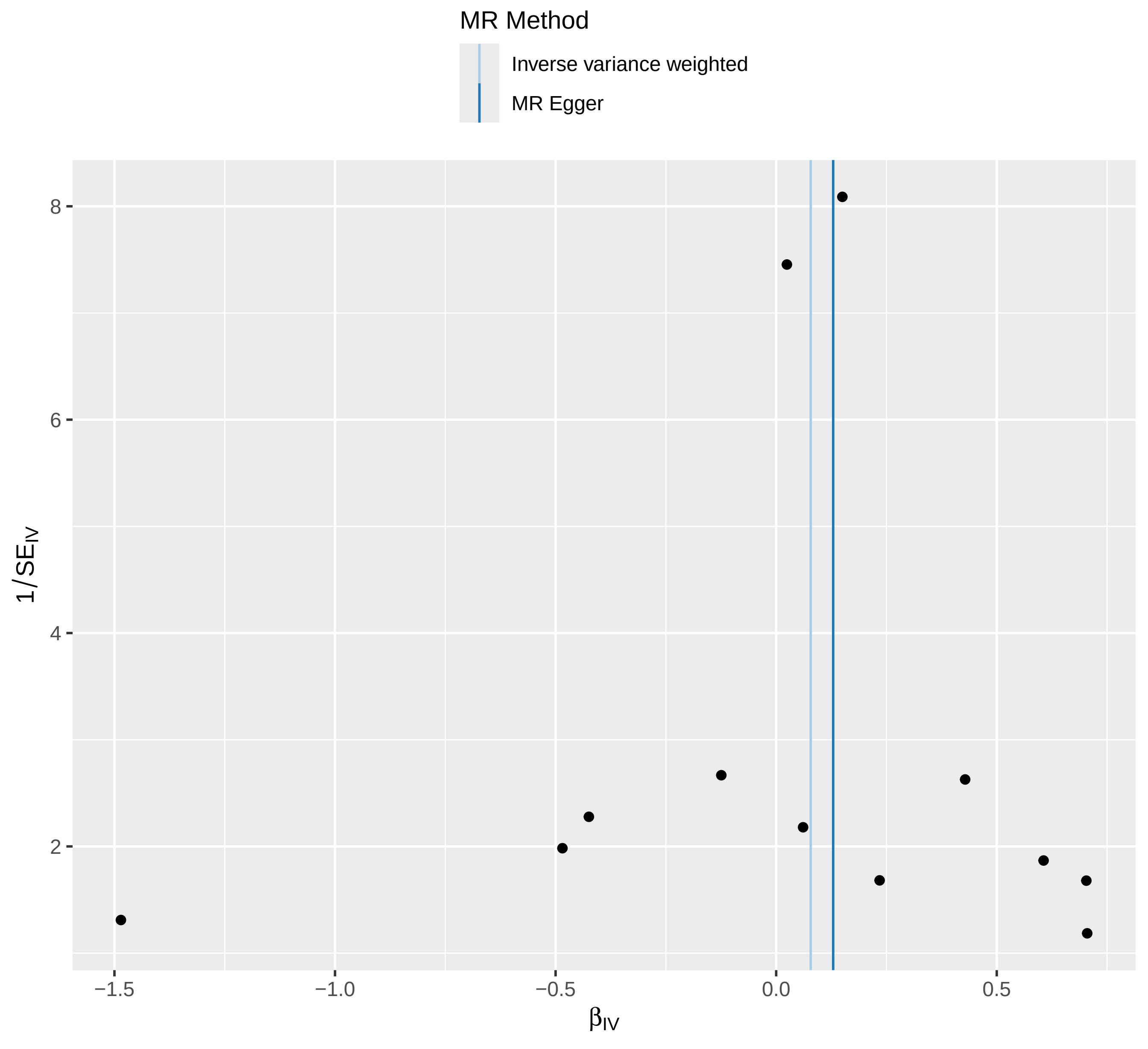

MR Method
Inverse variance weighted
MR Egger
8
6
4
2
1/SE_IV
−1.5
−1.0
−0.5
0.0
0.5
β_IV

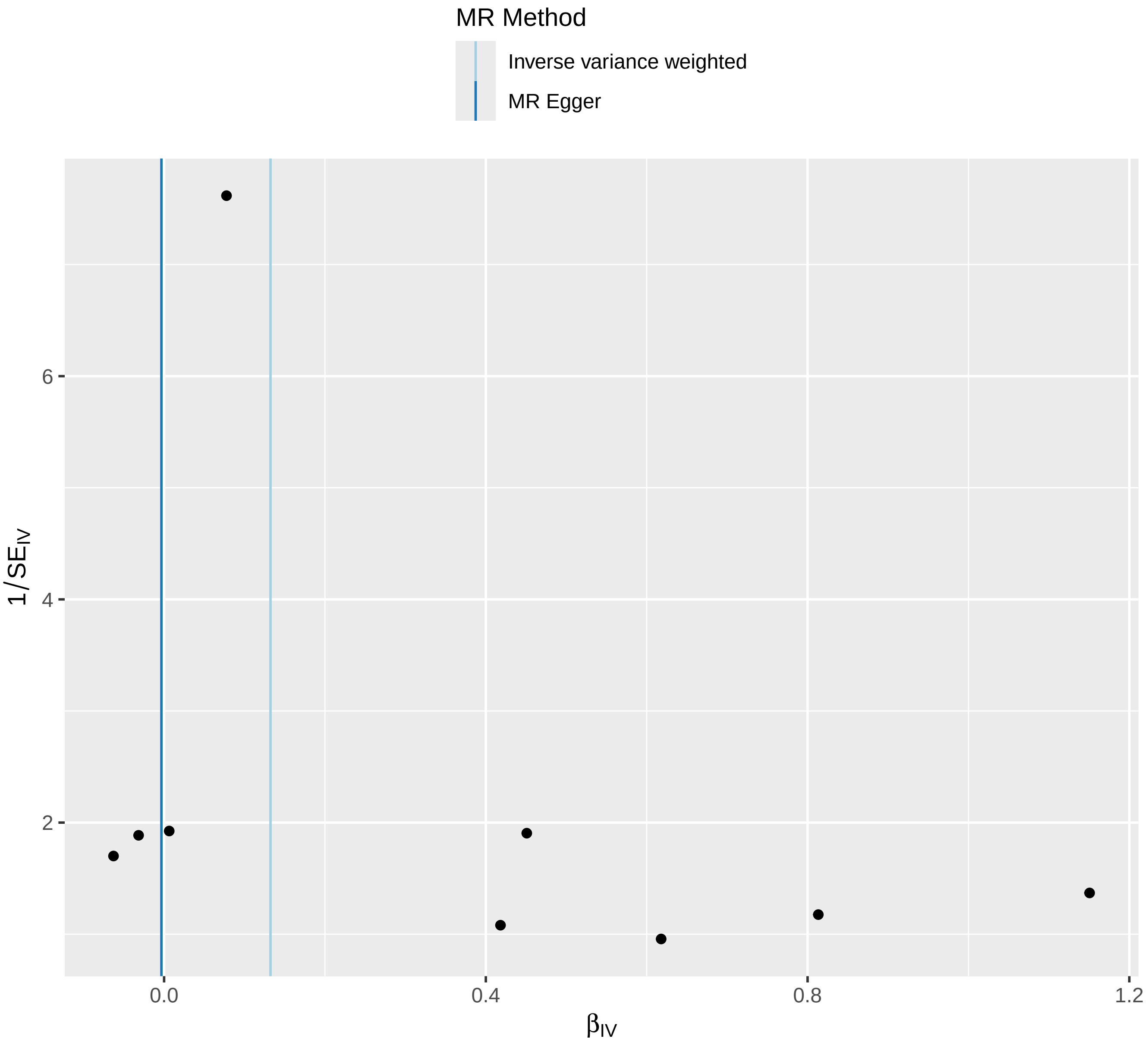

MR Method
Inverse variance weighted
MR Egger
6
4
2
$1/SE_{IV}$
0.0
0.4
0.8
1.2
β_{IV}

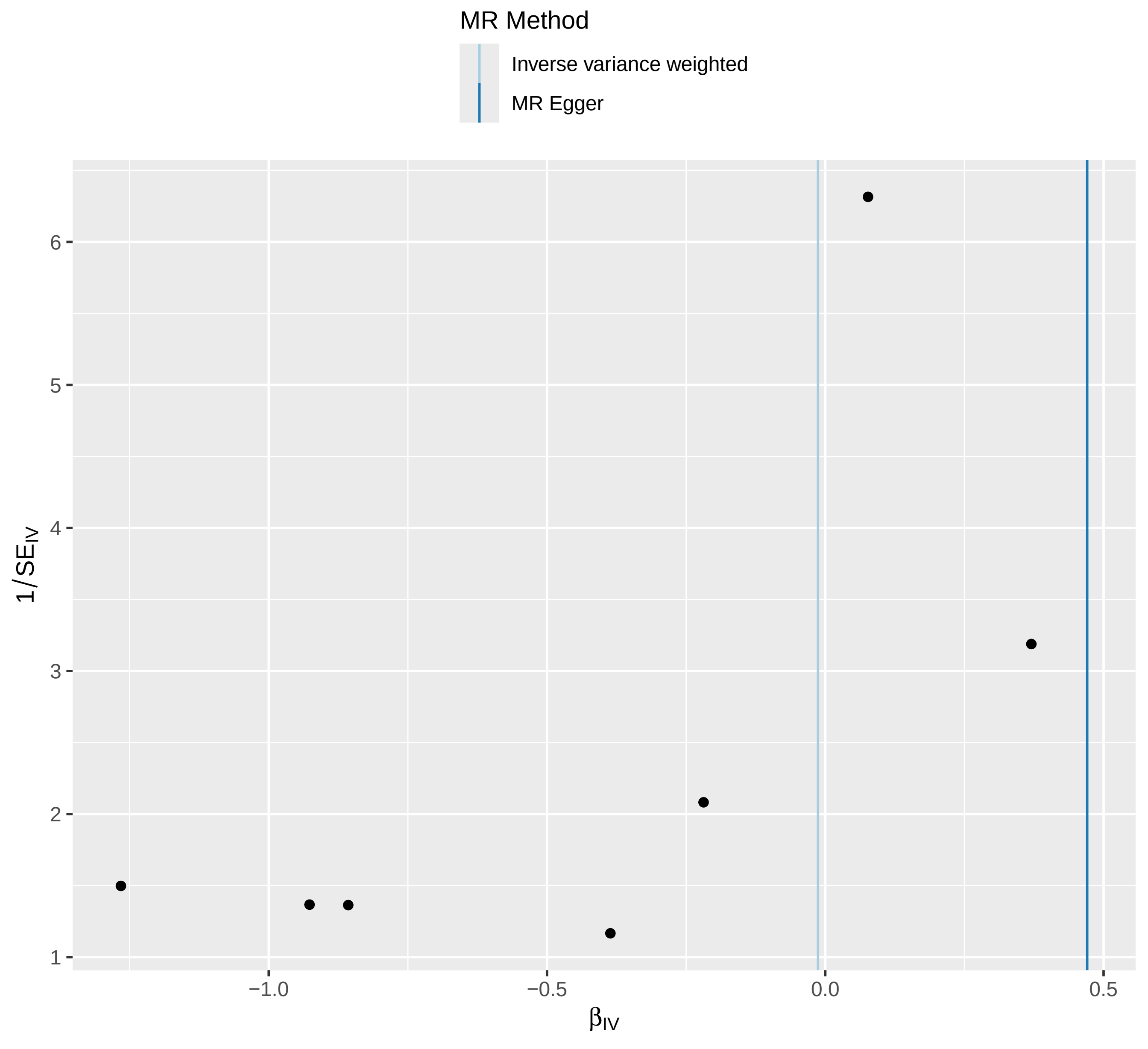
MR Method
Inverse variance weighted
MR Egger
6
5
4
3
2
1
1/SE_IV
−1.0
−0.5
0.0
0.5
β_IV

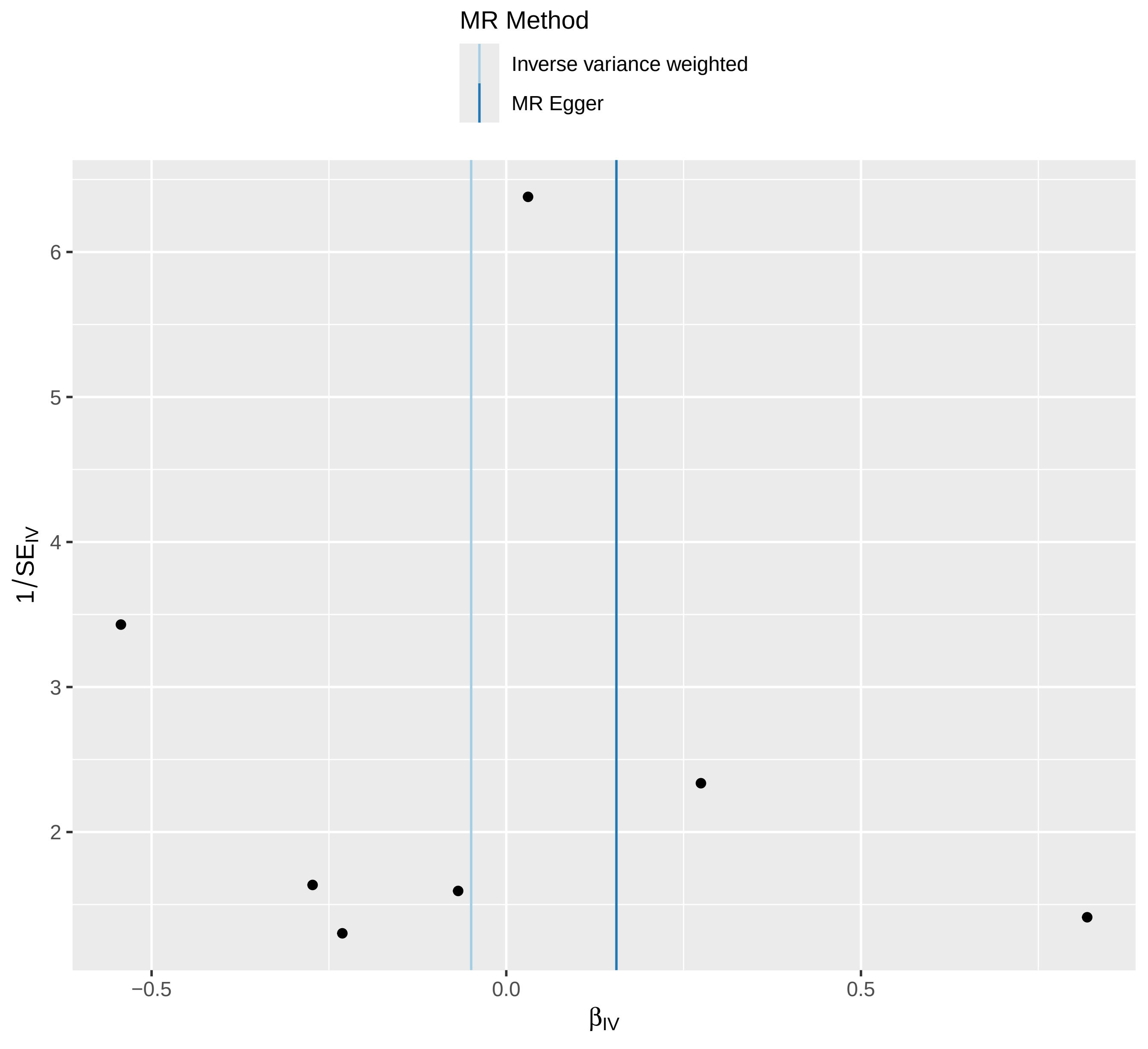
MR Method
Inverse variance weighted
MR Egger
6
5
4
3
2
−0.5
0.0
0.5
$1/SE_{IV}$
β_{IV}

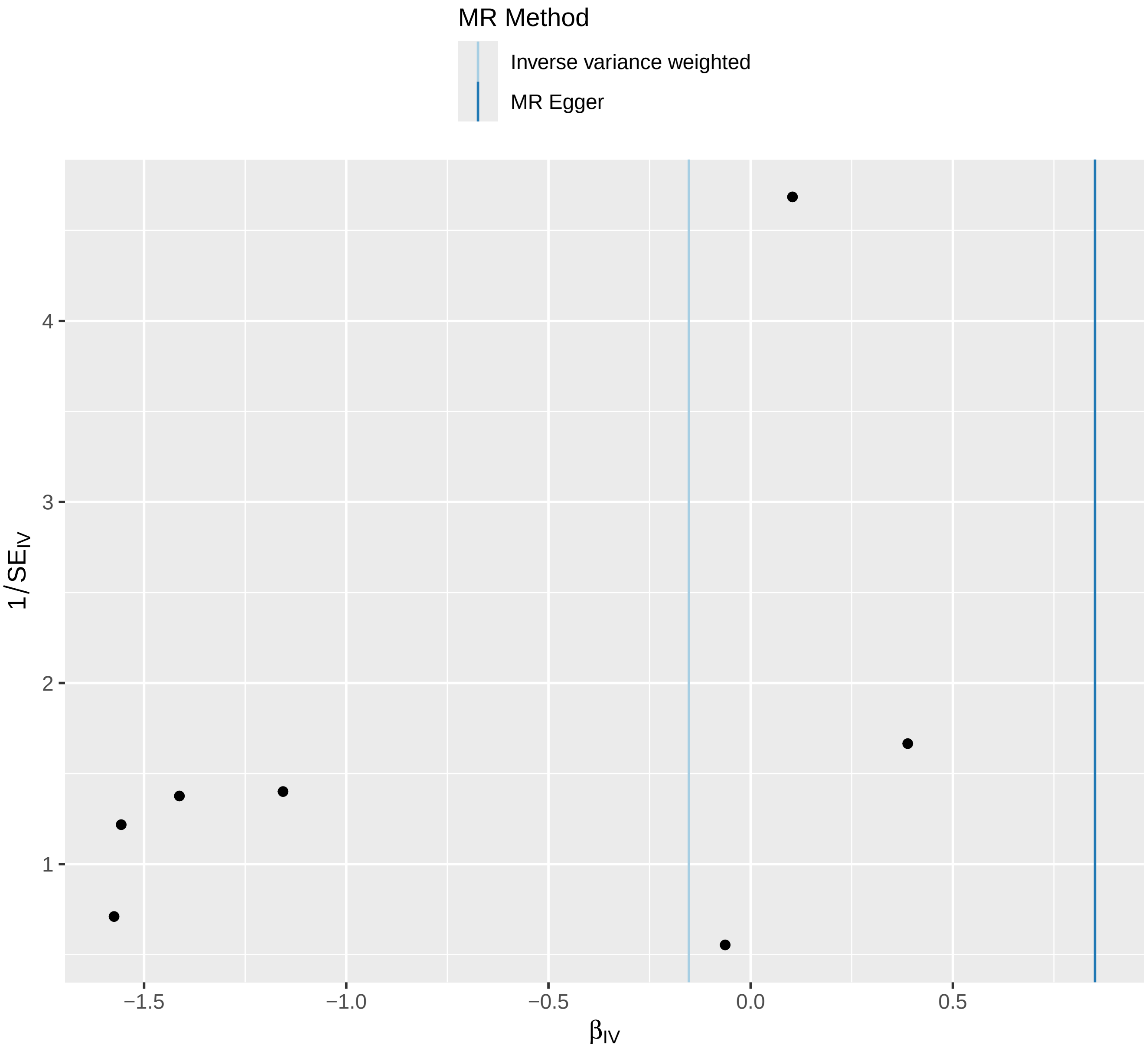
MR Method
Inverse variance weighted
MR Egger
1/SE$_{IV}$
4
3
2
1
−1.5
−1.0
−0.5
0.0
0.5
β$_{IV}$

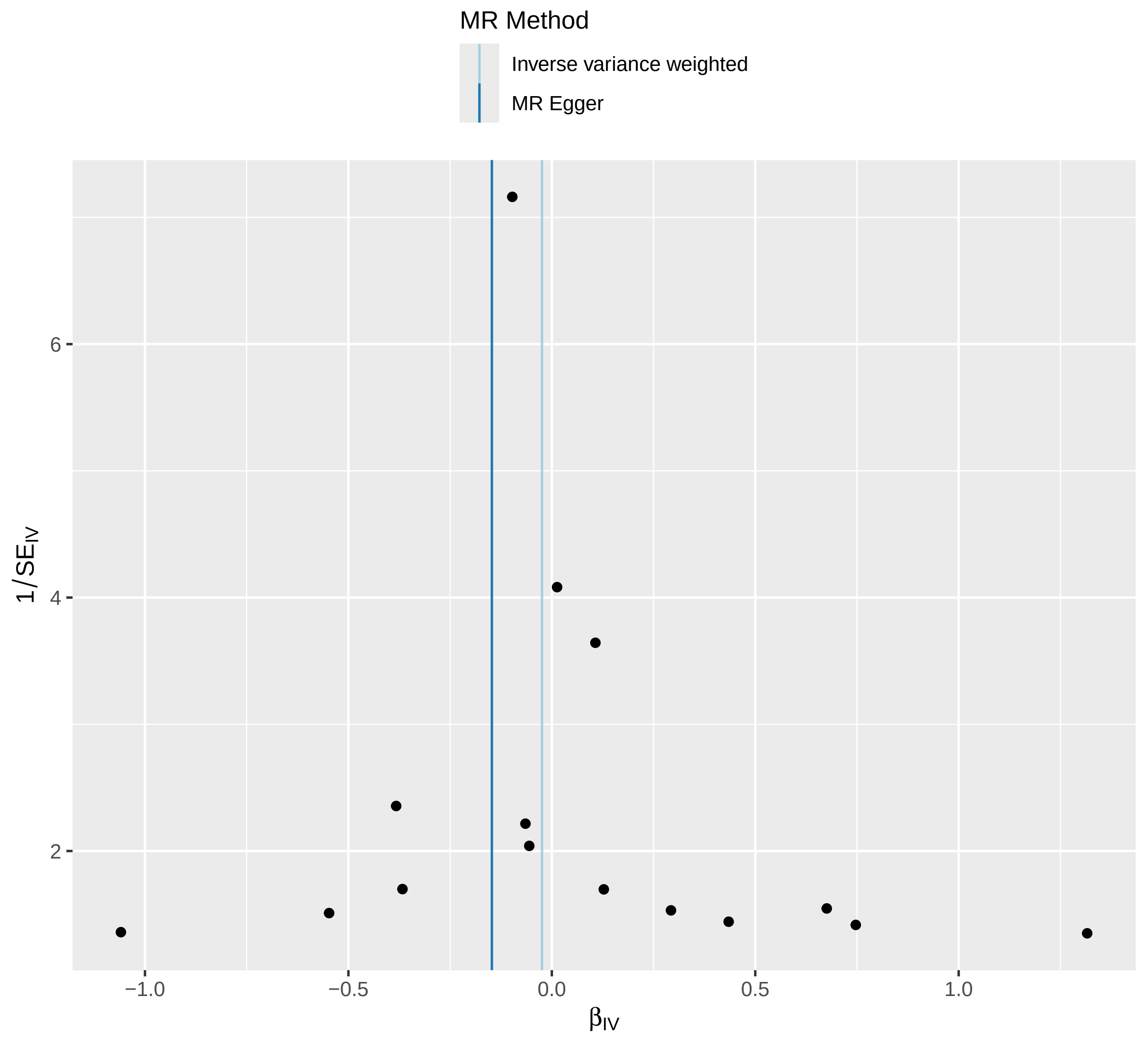

MR Method
Inverse variance weighted
MR Egger
1/SE_IV
6
4
2
−1.0
−0.5
0.0
0.5
1.0
β_IV

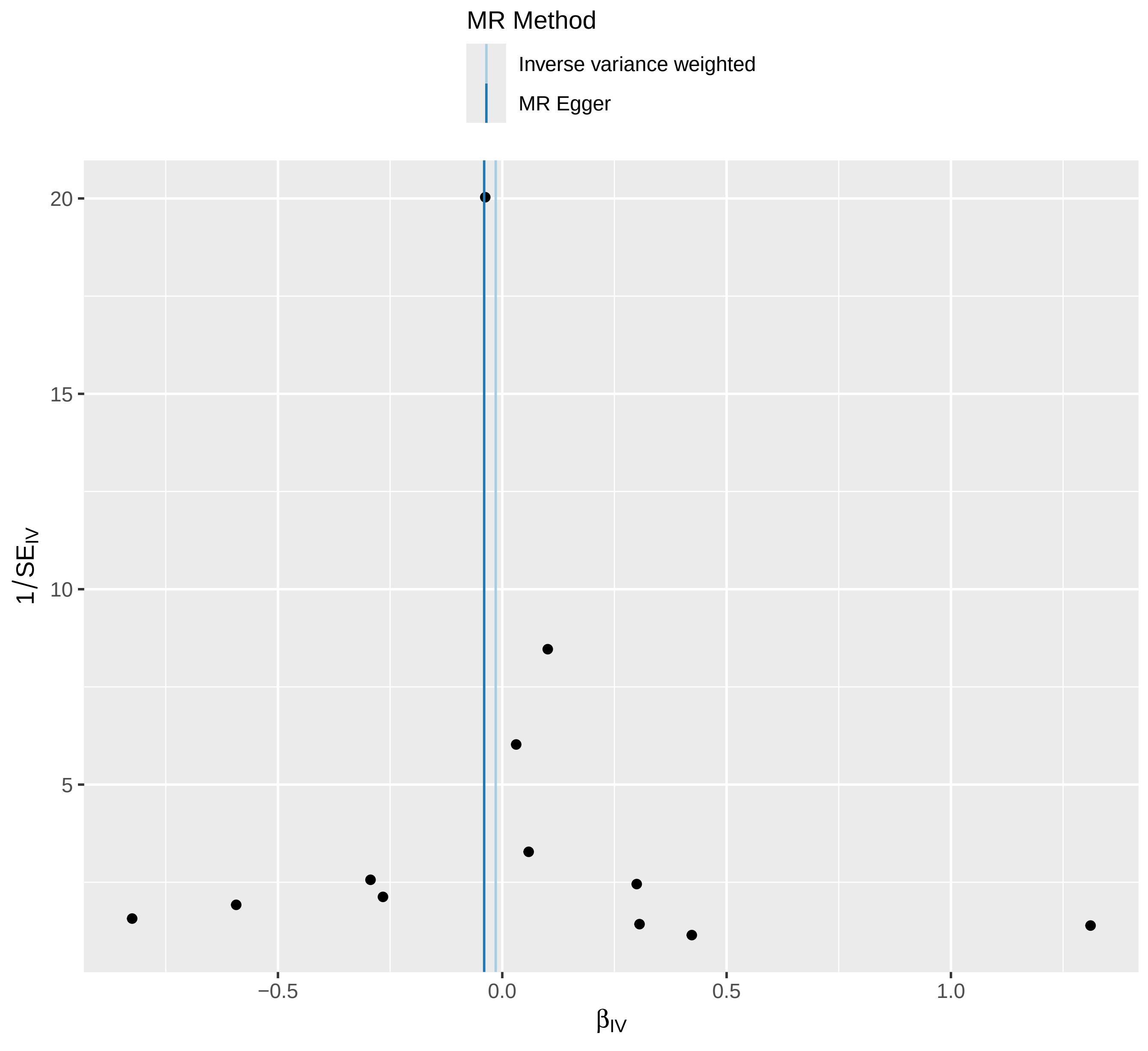
MR Method
Inverse variance weighted
MR Egger
20
15
10
5
1/SE_IV
−0.5
0.0
0.5
1.0
β_IV

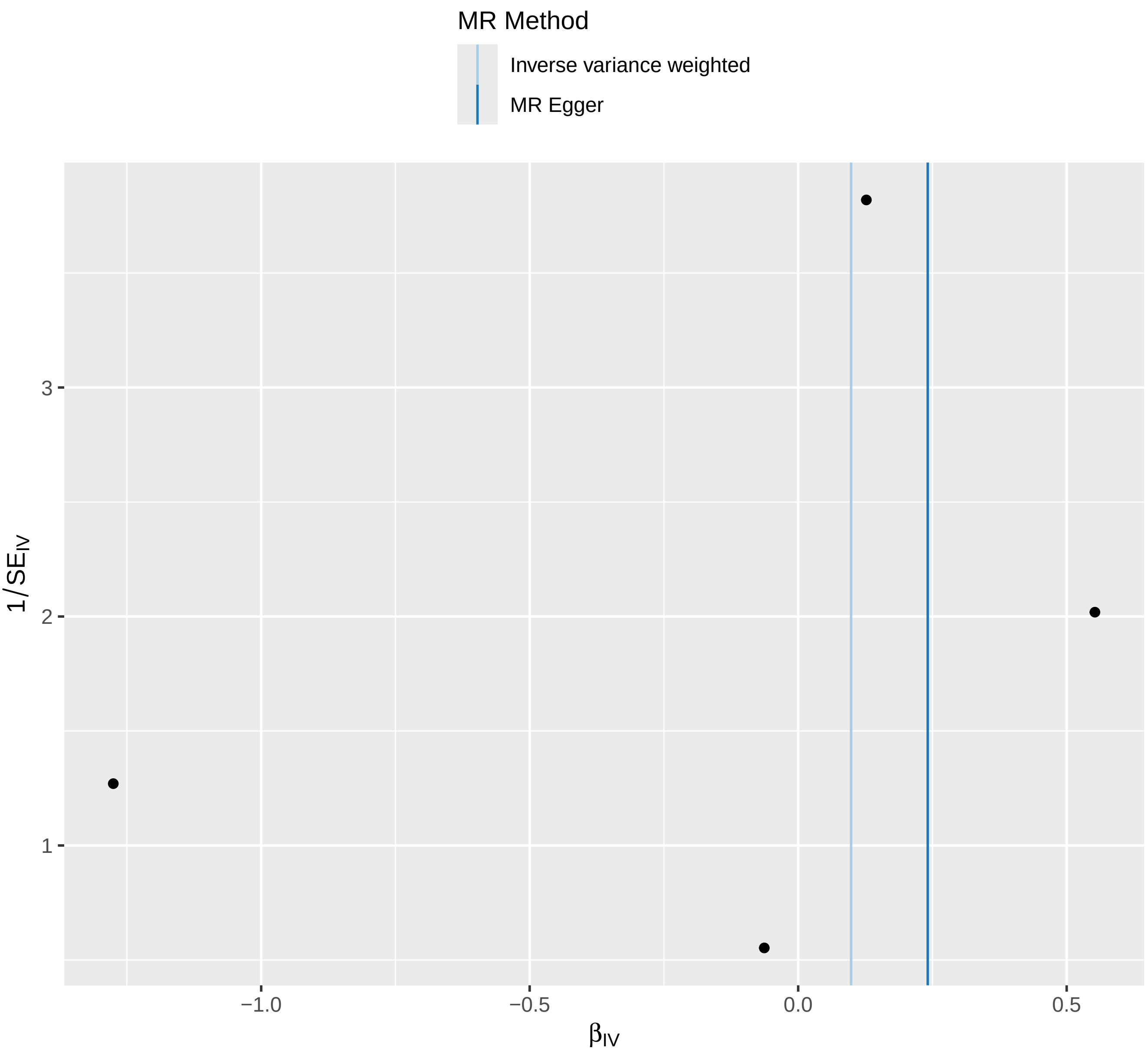
MR Method
Inverse variance weighted
MR Egger
1/SE_IV
3
2
1
−1.0
−0.5
0.0
0.5
β_IV

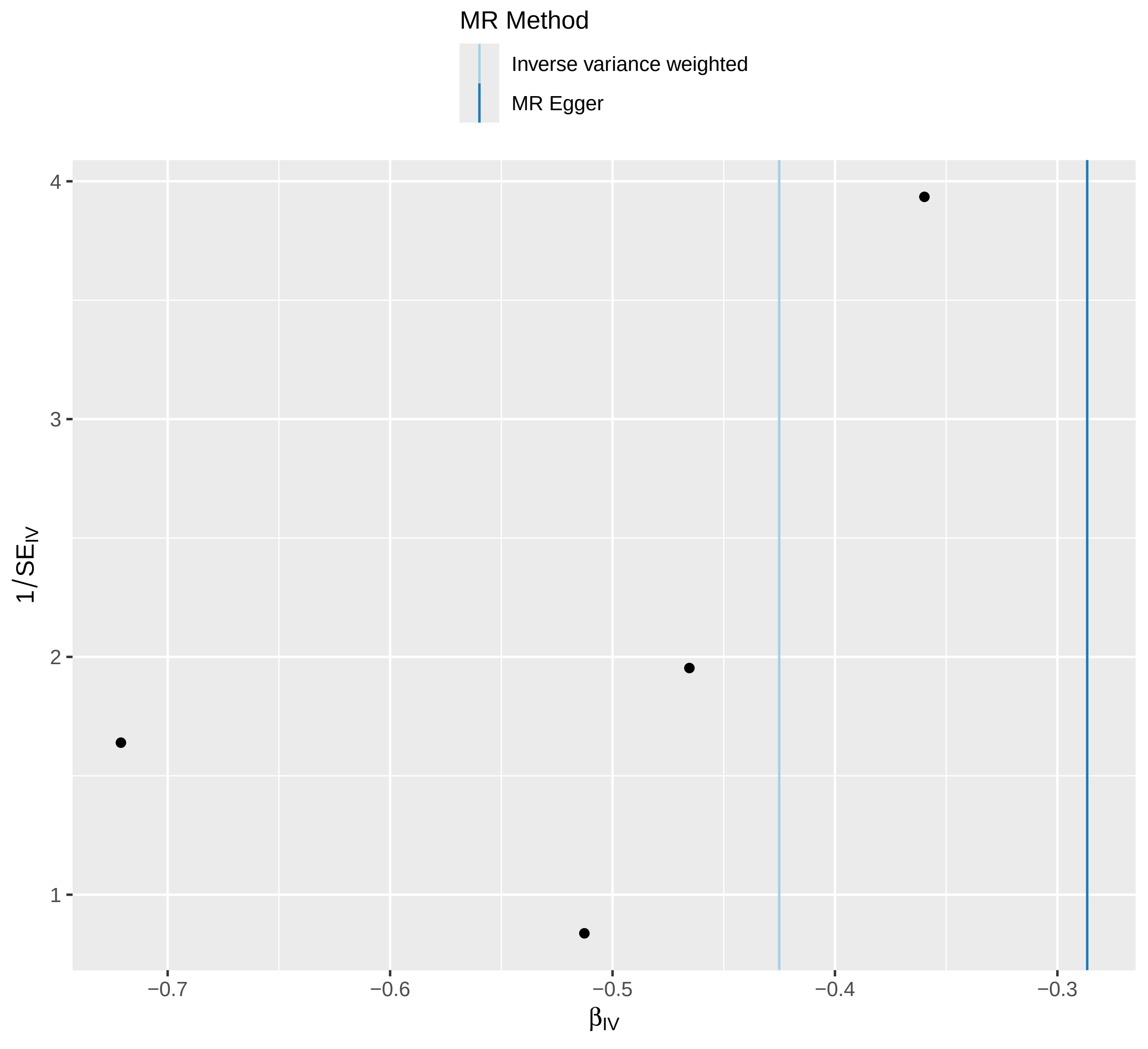
MR Method
Inverse variance weighted
MR Egger
4
3
2
1
1/SE_IV
−0.7
−0.6
−0.5
−0.4
−0.3
β_IV

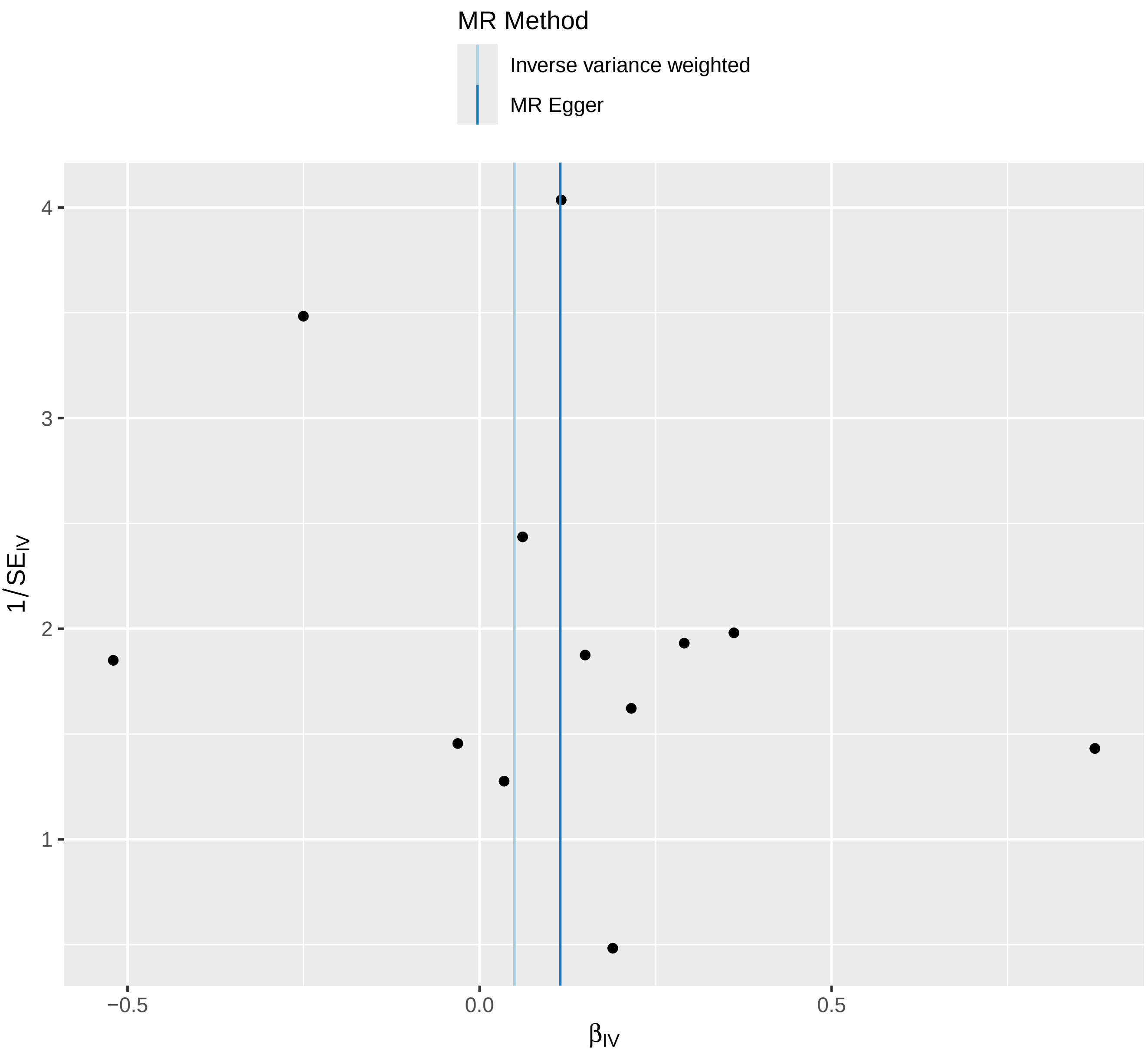
MR Method
Inverse variance weighted
MR Egger
1/SE_IV
4
3
2
1
−0.5
0.0
0.5
β_IV

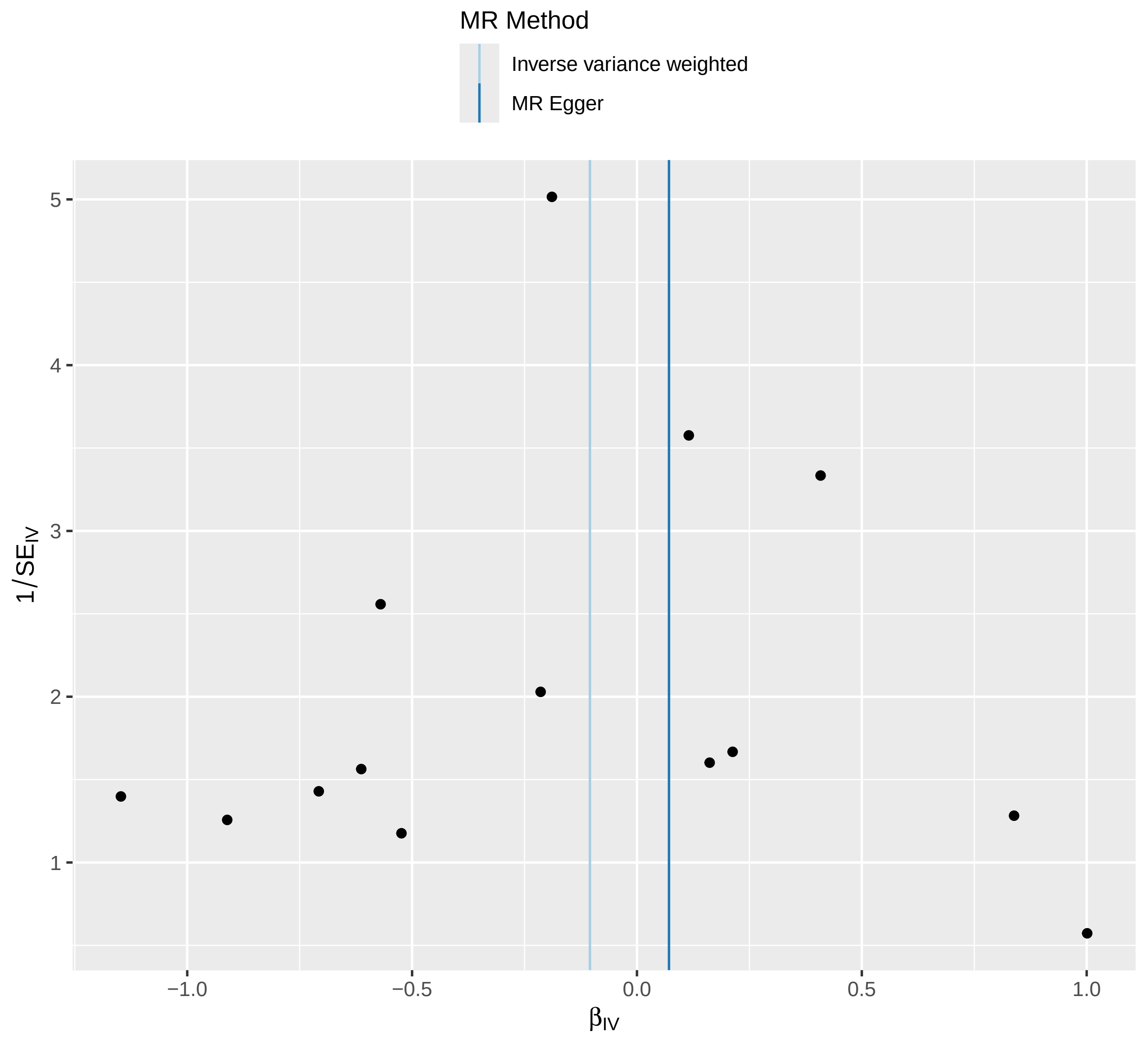
MR Method
Inverse variance weighted
MR Egger
1/SE_IV
β_IV
5
4
3
2
1
−1.0
−0.5
0.0
0.5
1.0

Insufficient number of SNPs

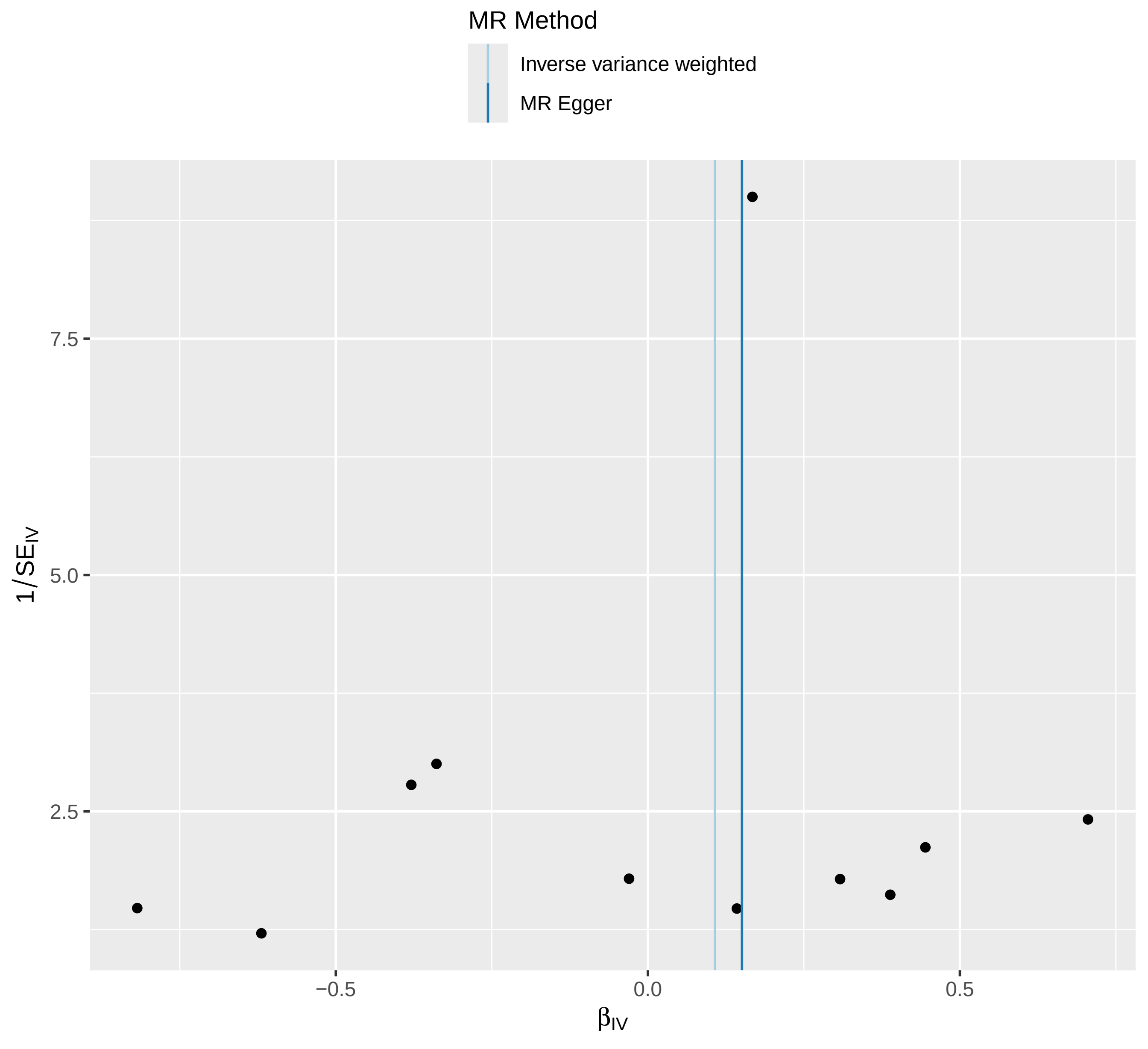
MR Method
Inverse variance weighted
MR Egger
7.5
5.0
2.5
1/SE_IV
−0.5
0.0
0.5
β_IV

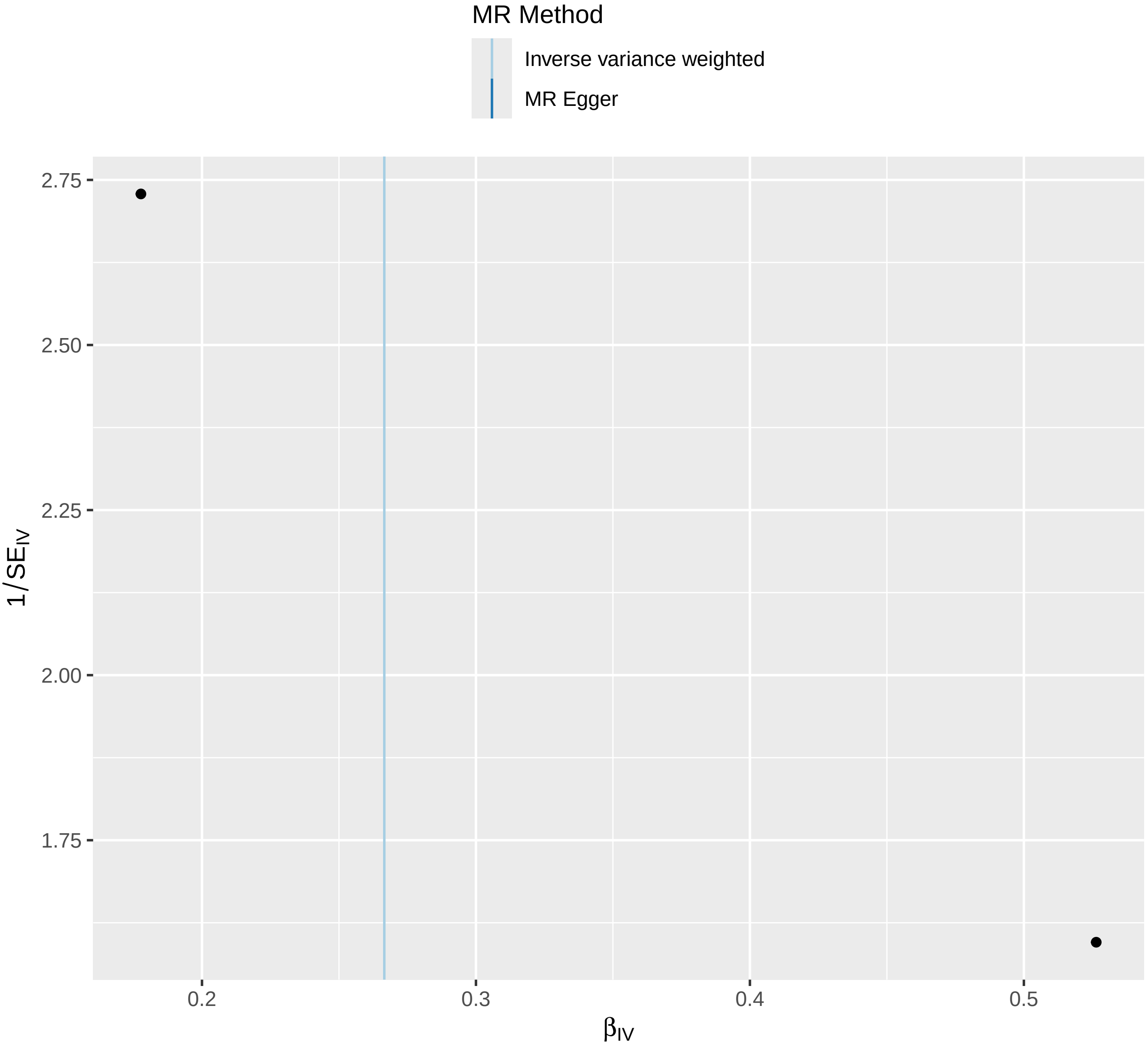
MR Method
Inverse variance weighted
MR Egger
2.75
2.50
2.25
2.00
1.75
$1/SE_{IV}$
0.2
0.3
0.4
0.5
β_{IV}

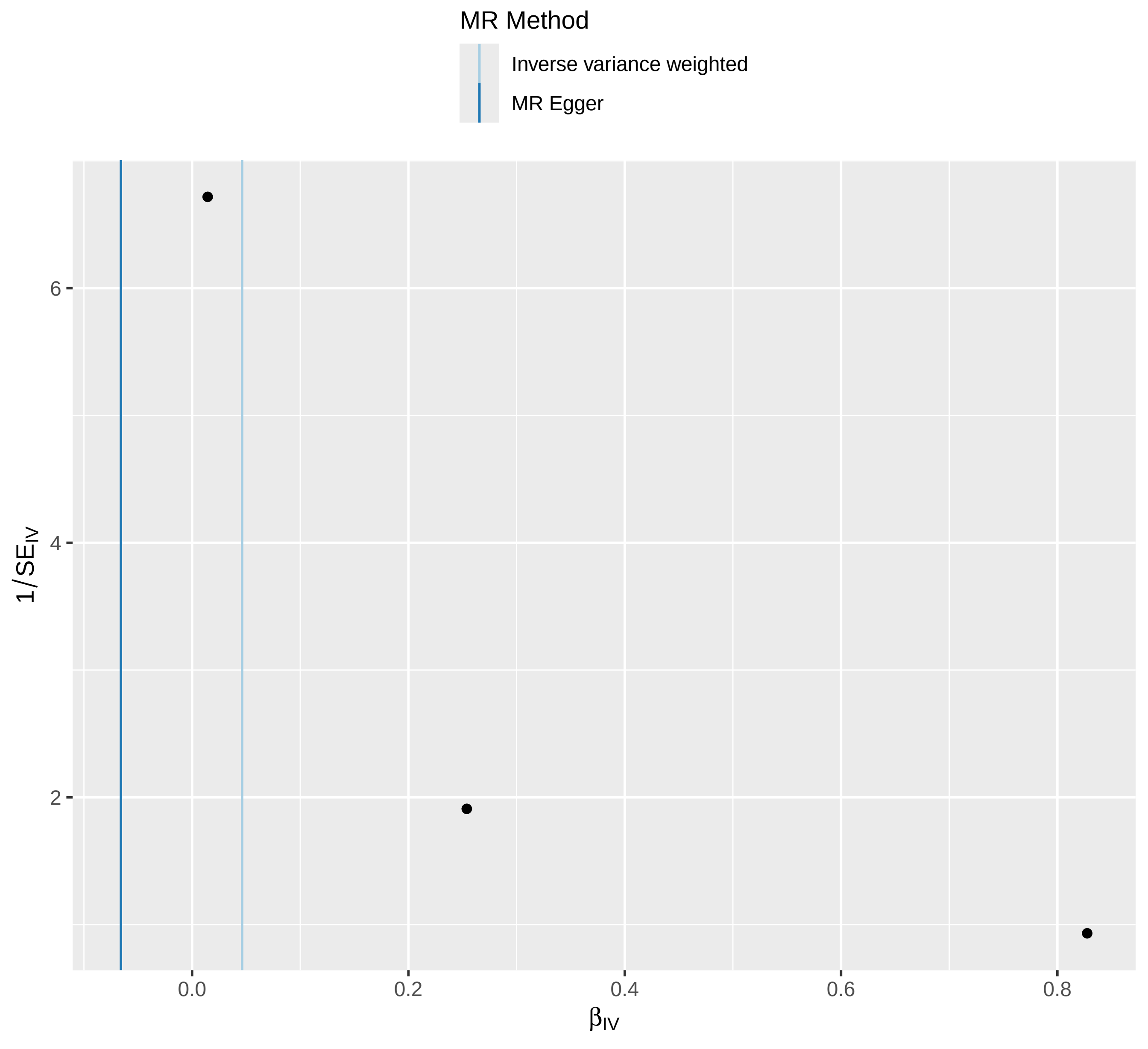

MR Method
Inverse variance weighted
MR Egger
6
4
2
0.0
0.2
0.4
0.6
0.8
$1/SE_{IV}$
β_{IV}

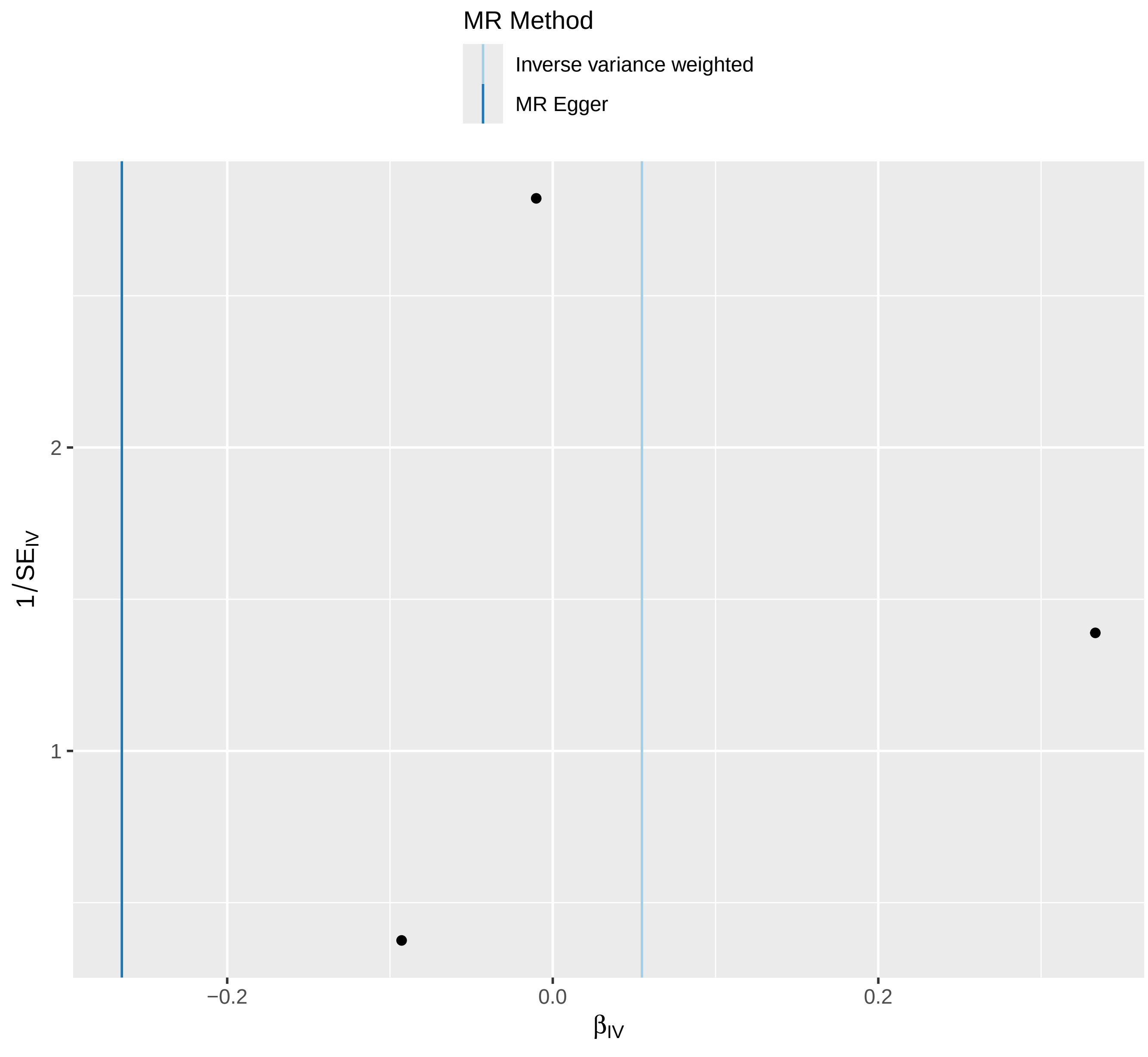

MR Method
Inverse variance weighted
MR Egger
2
1
1/SE_IV
−0.2
0.0
0.2
β_IV

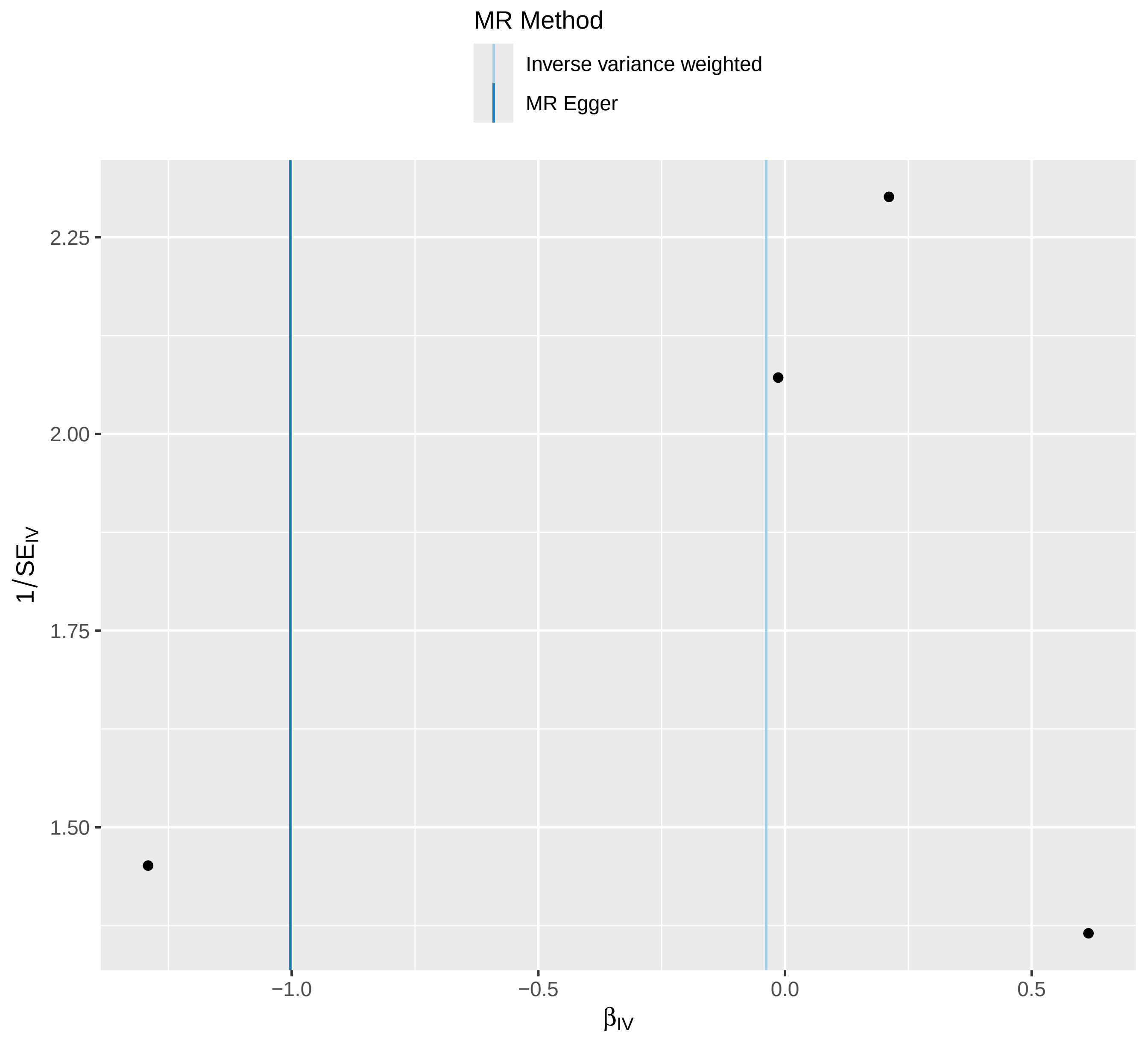

MR Method
Inverse variance weighted
MR Egger
2.25
2.00
1.75
1.50
1/SE$_{IV}$
−1.0
−0.5
0.0
0.5
β$_{IV}$

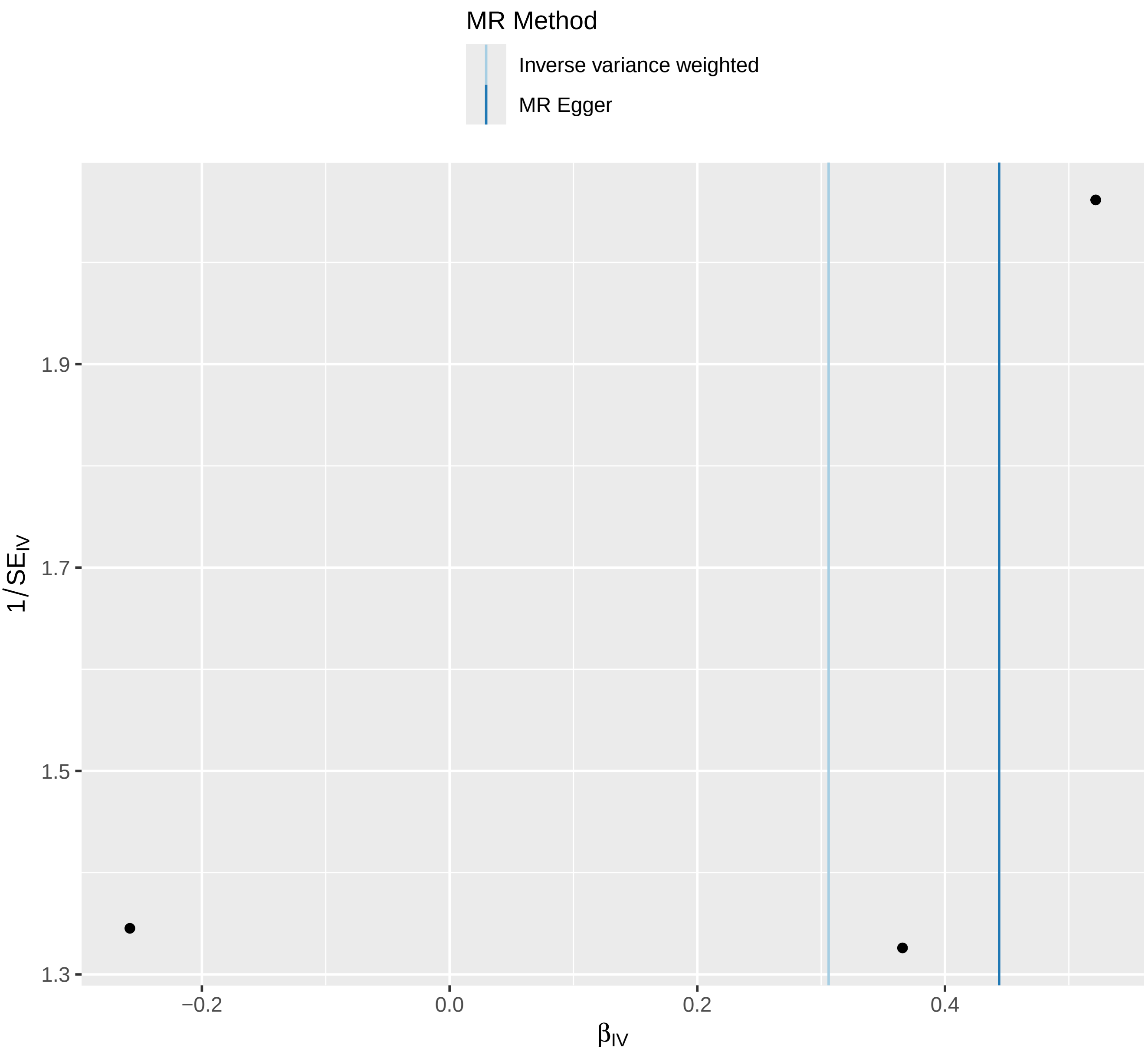
MR Method
Inverse variance weighted
MR Egger
1.9
1.7
1.5
1.3
1/SE_IV
−0.2
0.0
0.2
0.4
β_IV

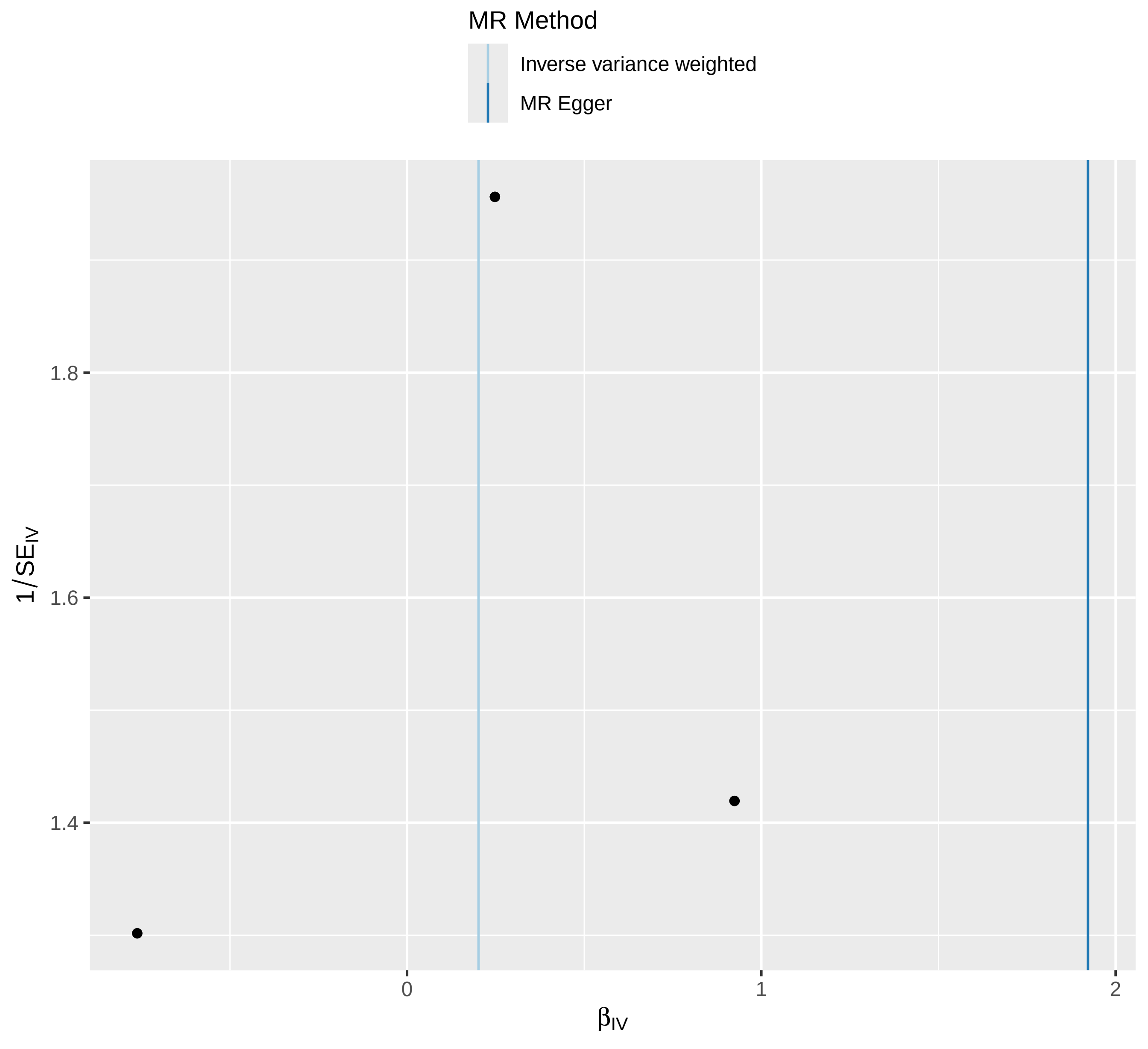
MR Method
Inverse variance weighted
MR Egger
1.8
1.6
1.4
1/SE_IV
0
1
2
β_IV

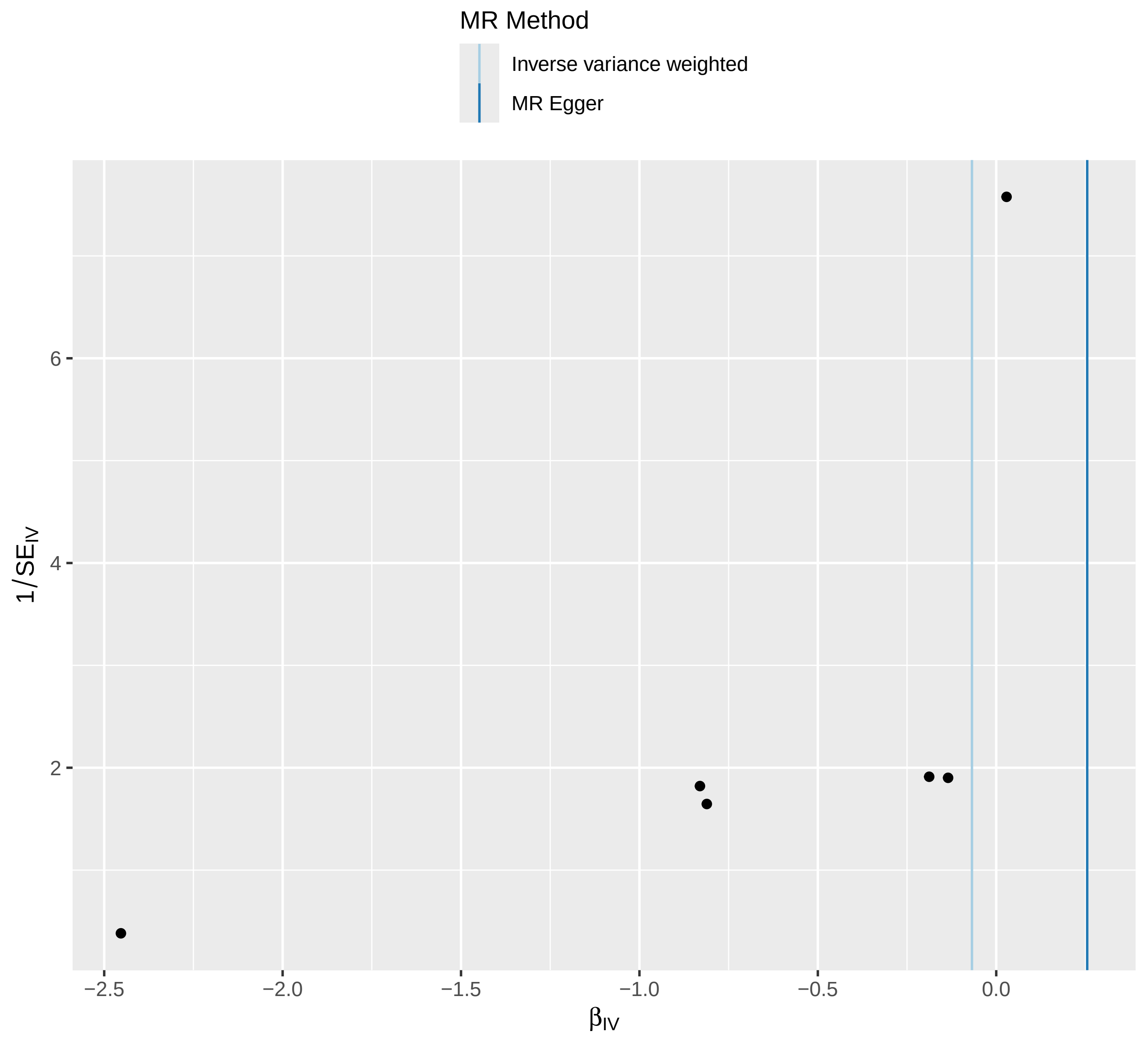

MR Method
Inverse variance weighted
MR Egger
1/SE_IV
6
4
2
−2.5
−2.0
−1.5
−1.0
−0.5
0.0
β_IV

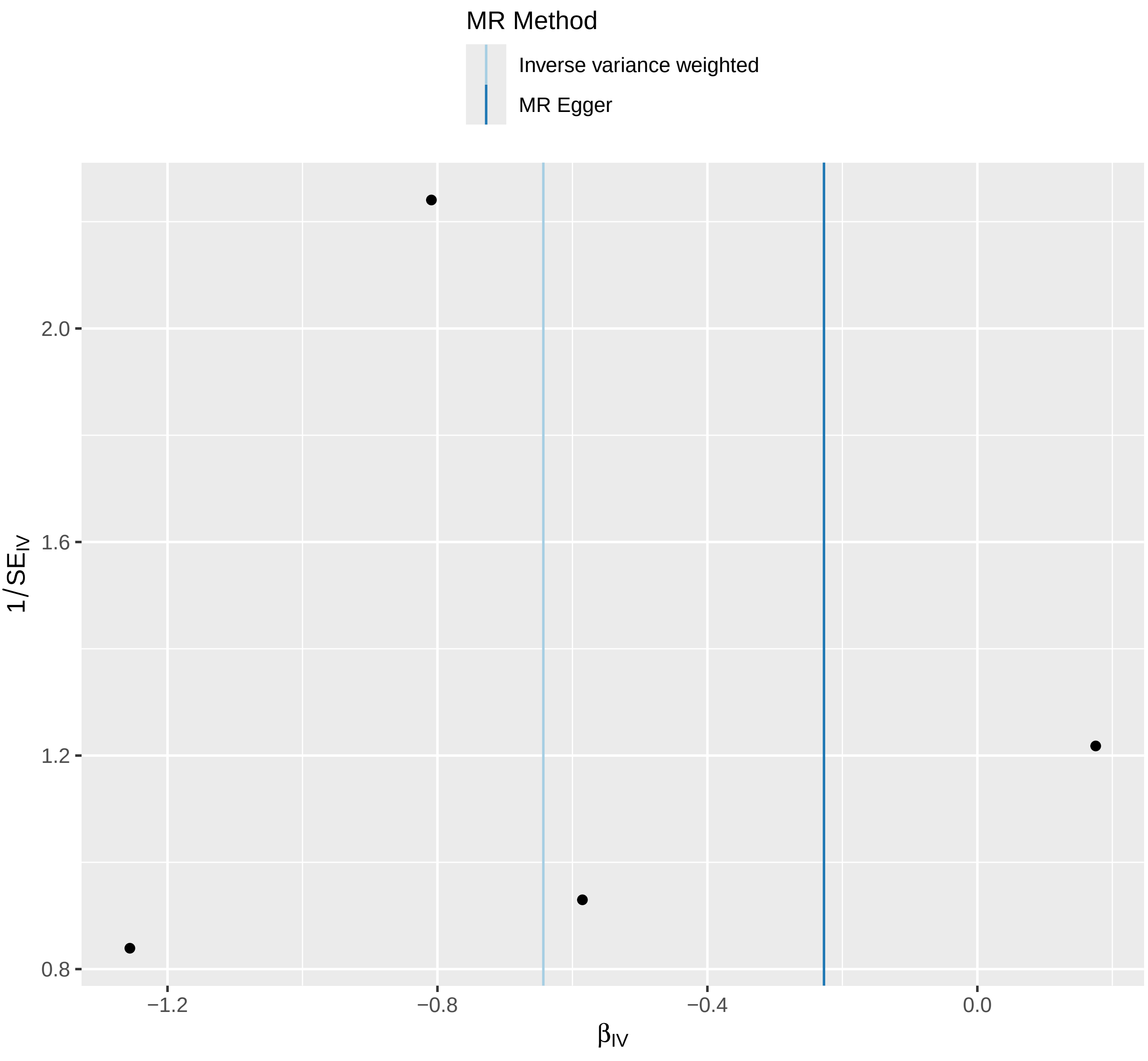

MR Method
Inverse variance weighted
MR Egger
2.0
1.6
1.2
0.8
1/SE$_{IV}$
−1.2
−0.8
−0.4
0.0
β_{IV}

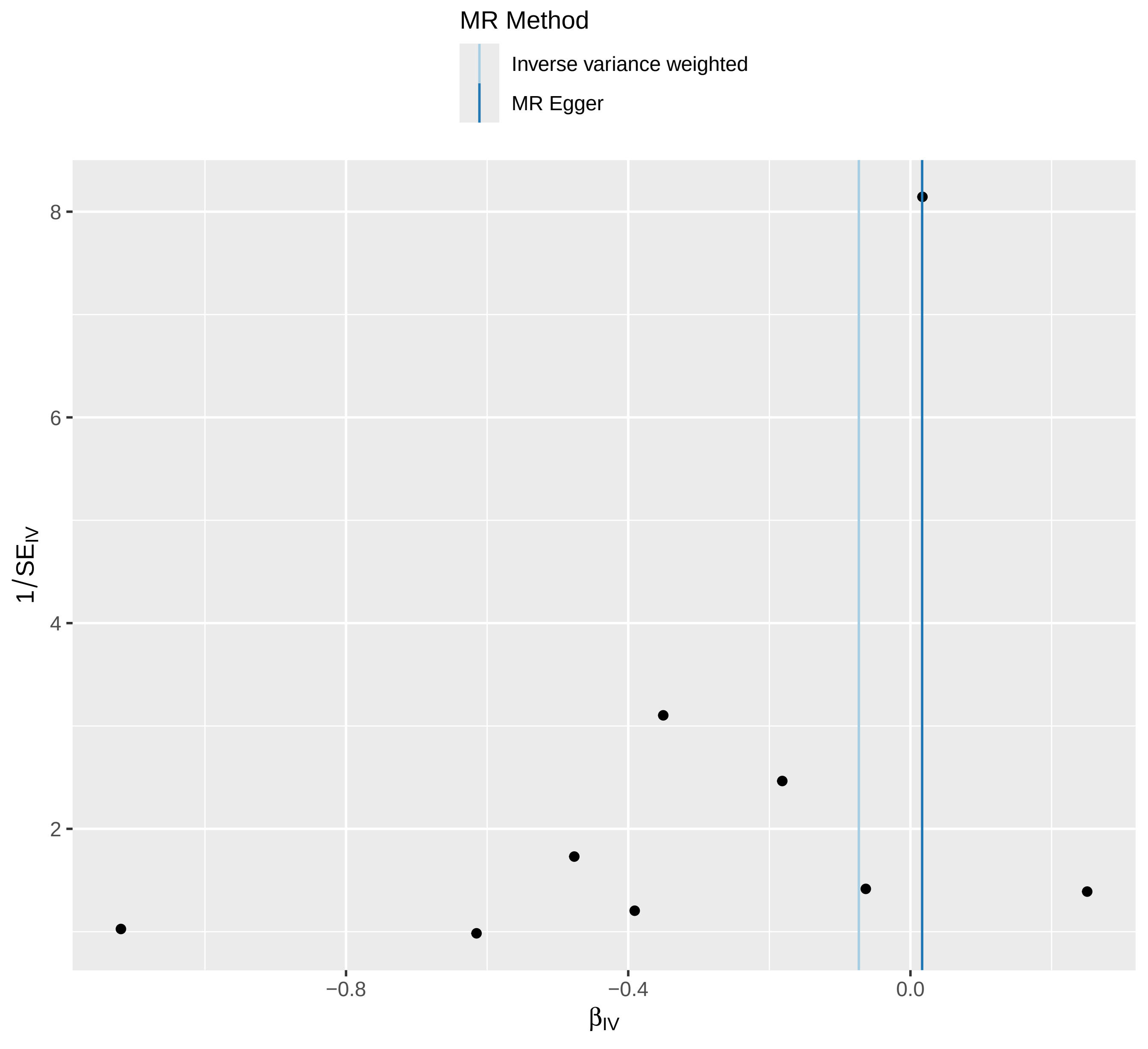
MR Method
Inverse variance weighted
MR Egger
8
6
4
2
1/SE_IV
−0.8
−0.4
0.0
β_IV

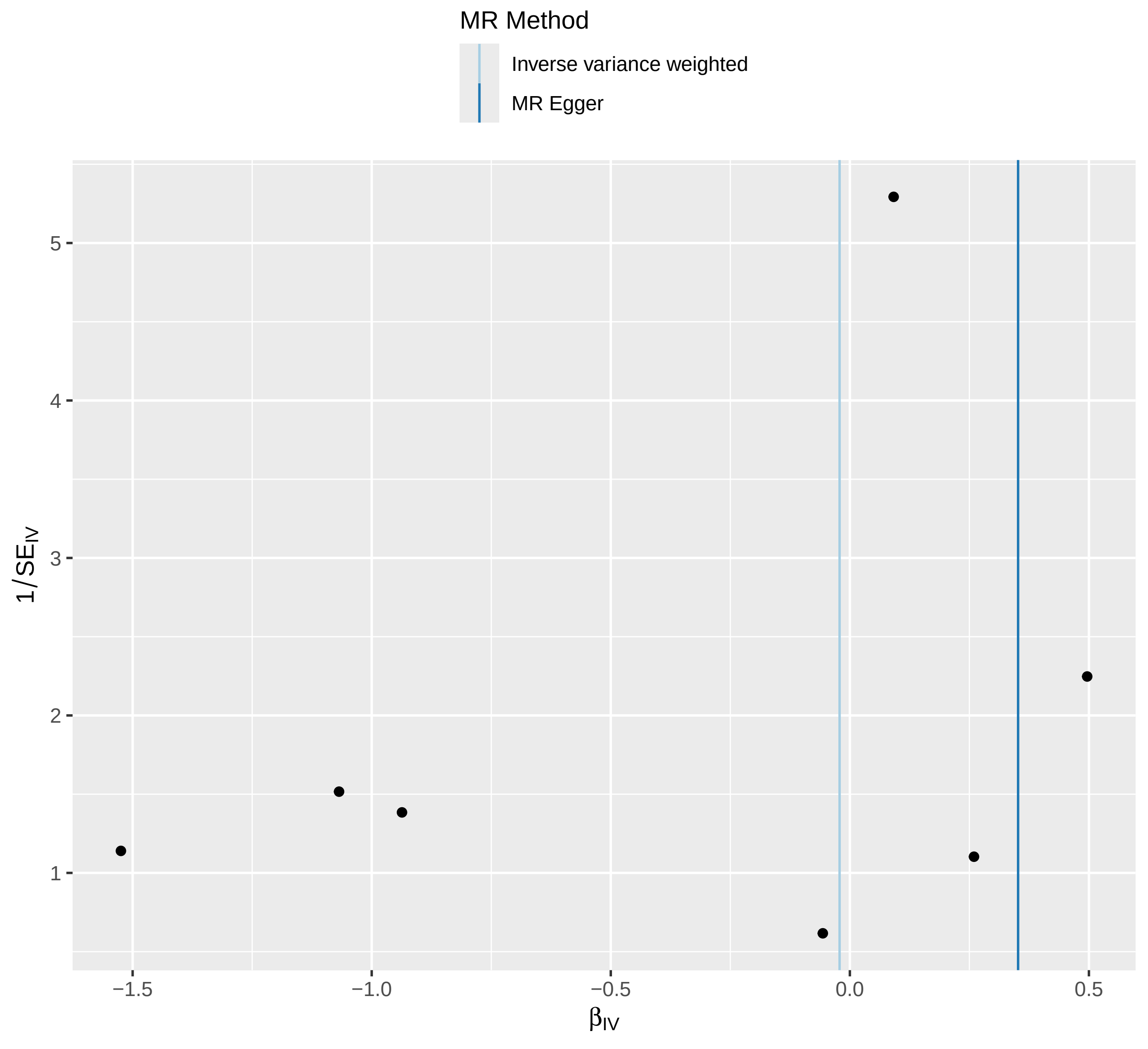
MR Method
Inverse variance weighted
MR Egger
1/SE_IV
5
4
3
2
1
−1.5
−1.0
−0.5
0.0
0.5
β_IV

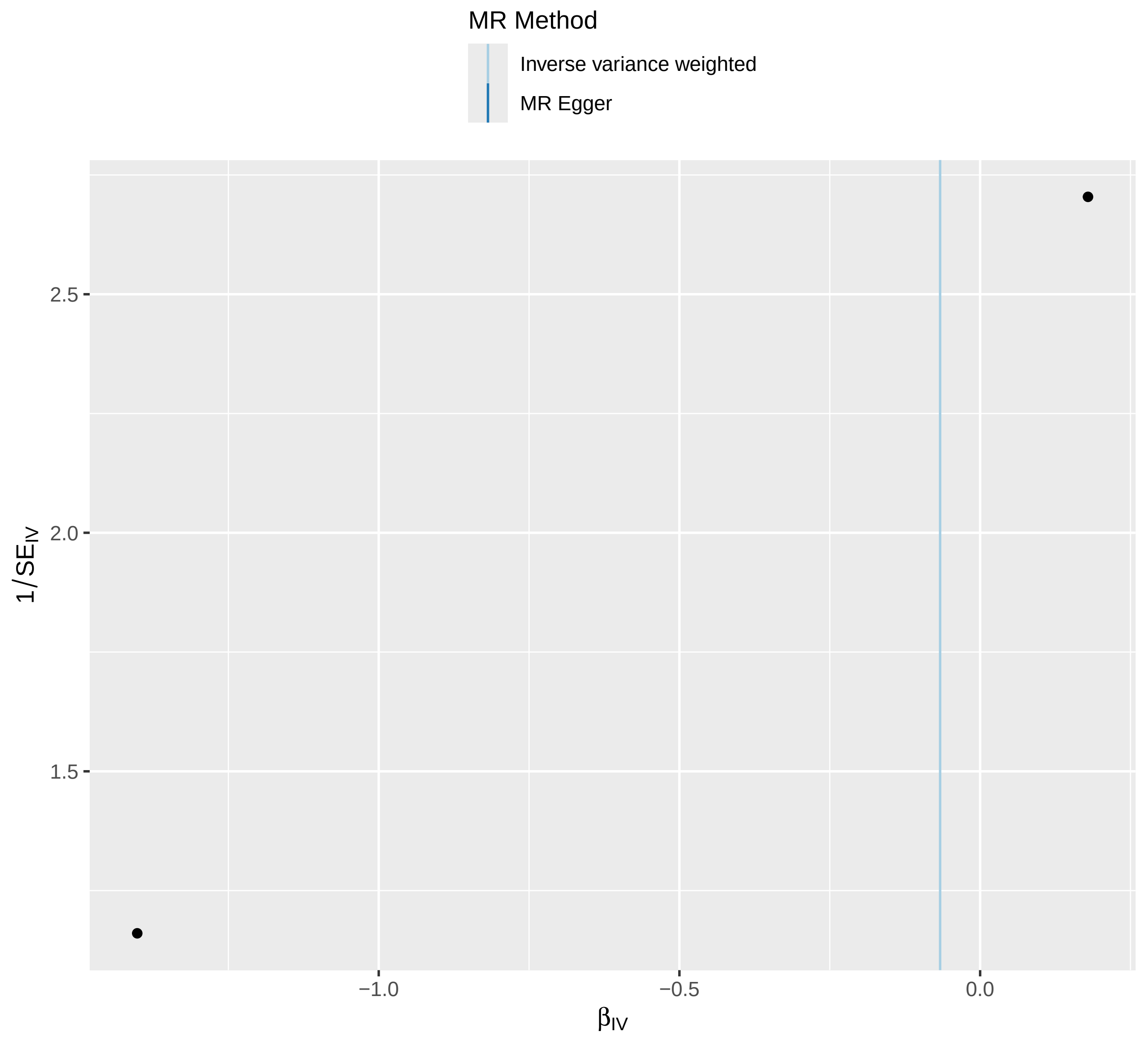

MR Method
Inverse variance weighted
MR Egger
2.5
2.0
1.5
1/SE_IV
−1.0
−0.5
0.0
β_IV

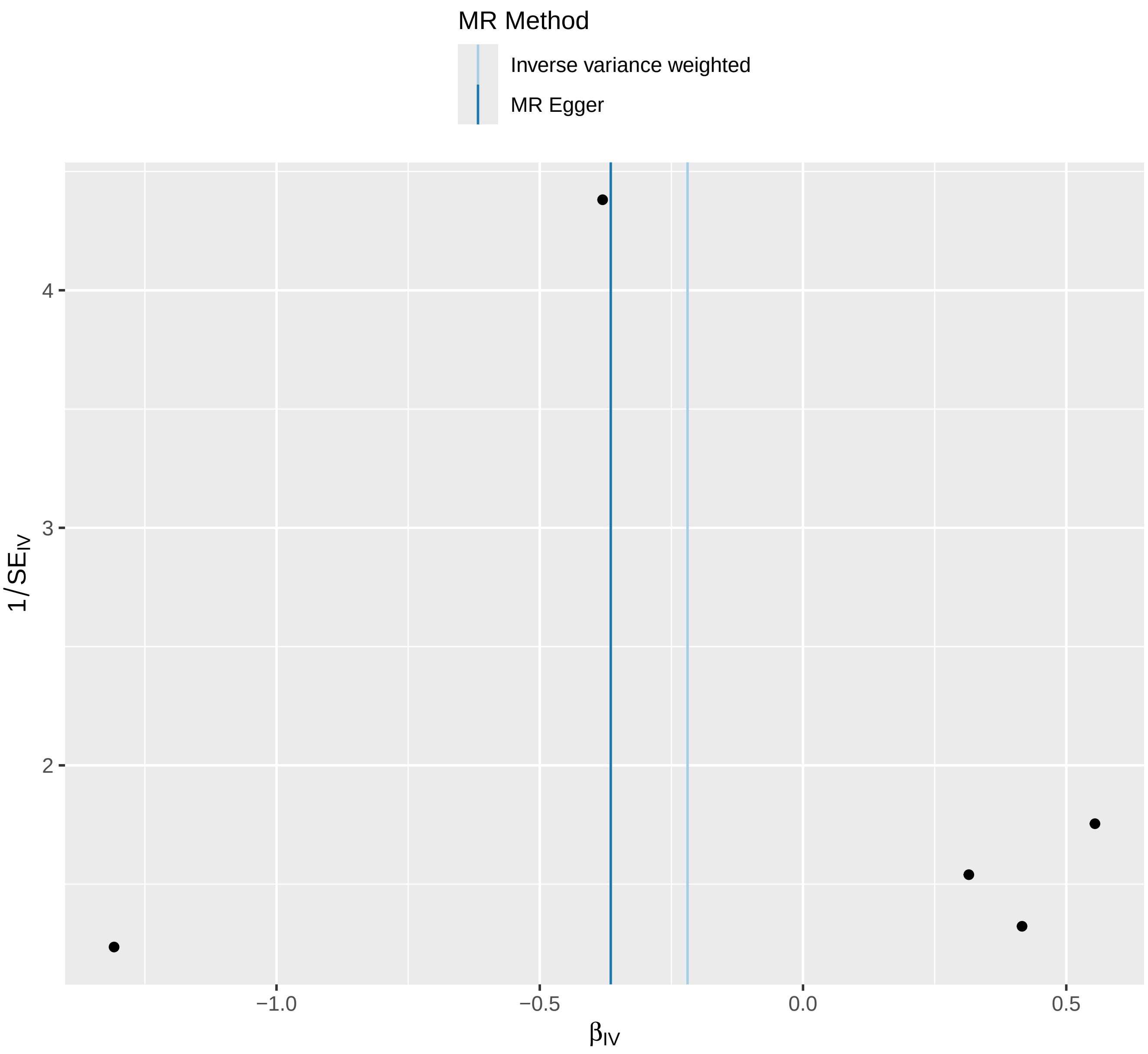
MR Method
Inverse variance weighted
MR Egger
4
3
2
1/SE_IV
−1.0
−0.5
0.0
0.5
β_IV

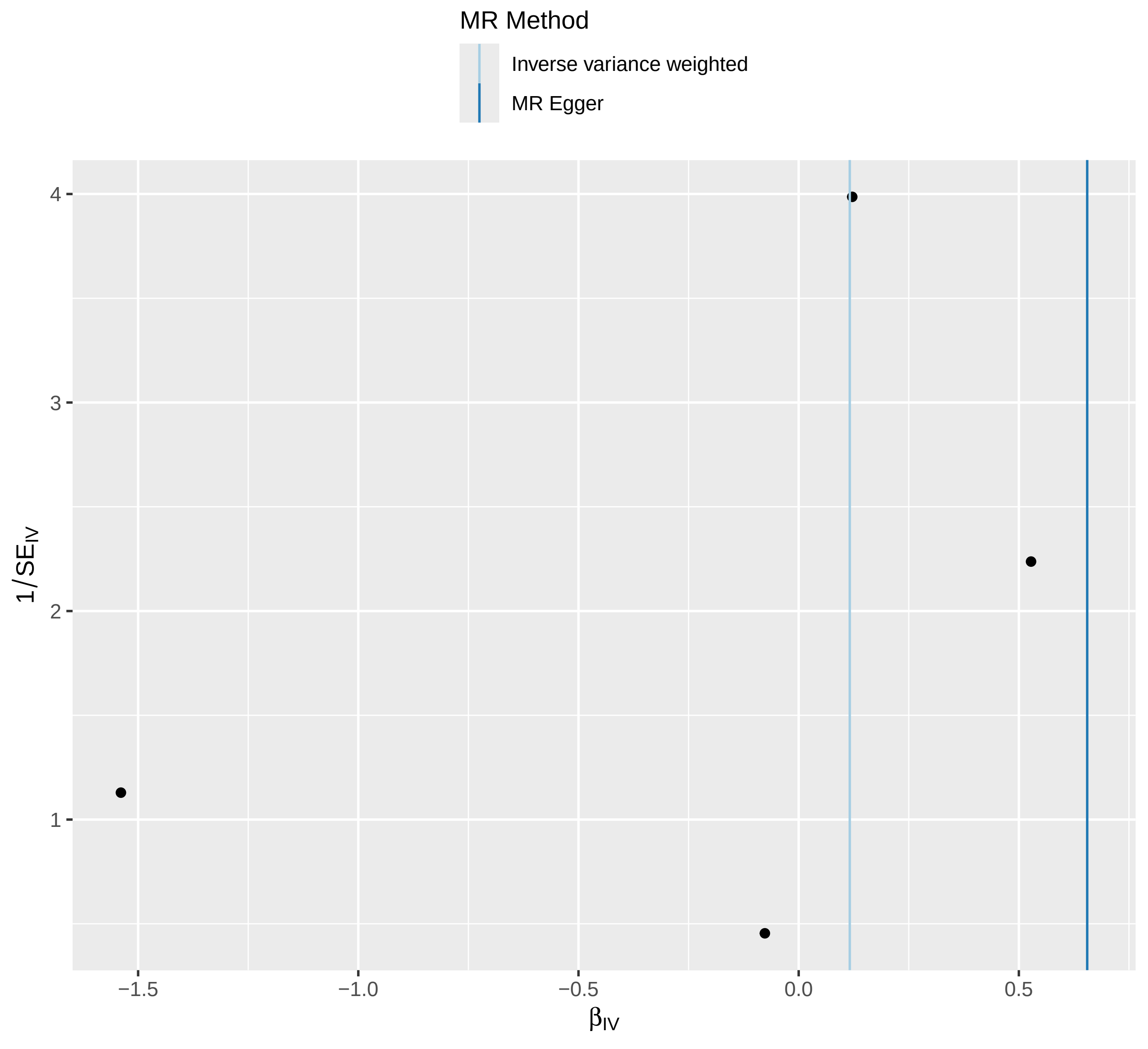
MR Method
Inverse variance weighted
MR Egger
4
3
2
1
−1.5
−1.0
−0.5
0.0
0.5
$1/SE_{IV}$
β_{IV}

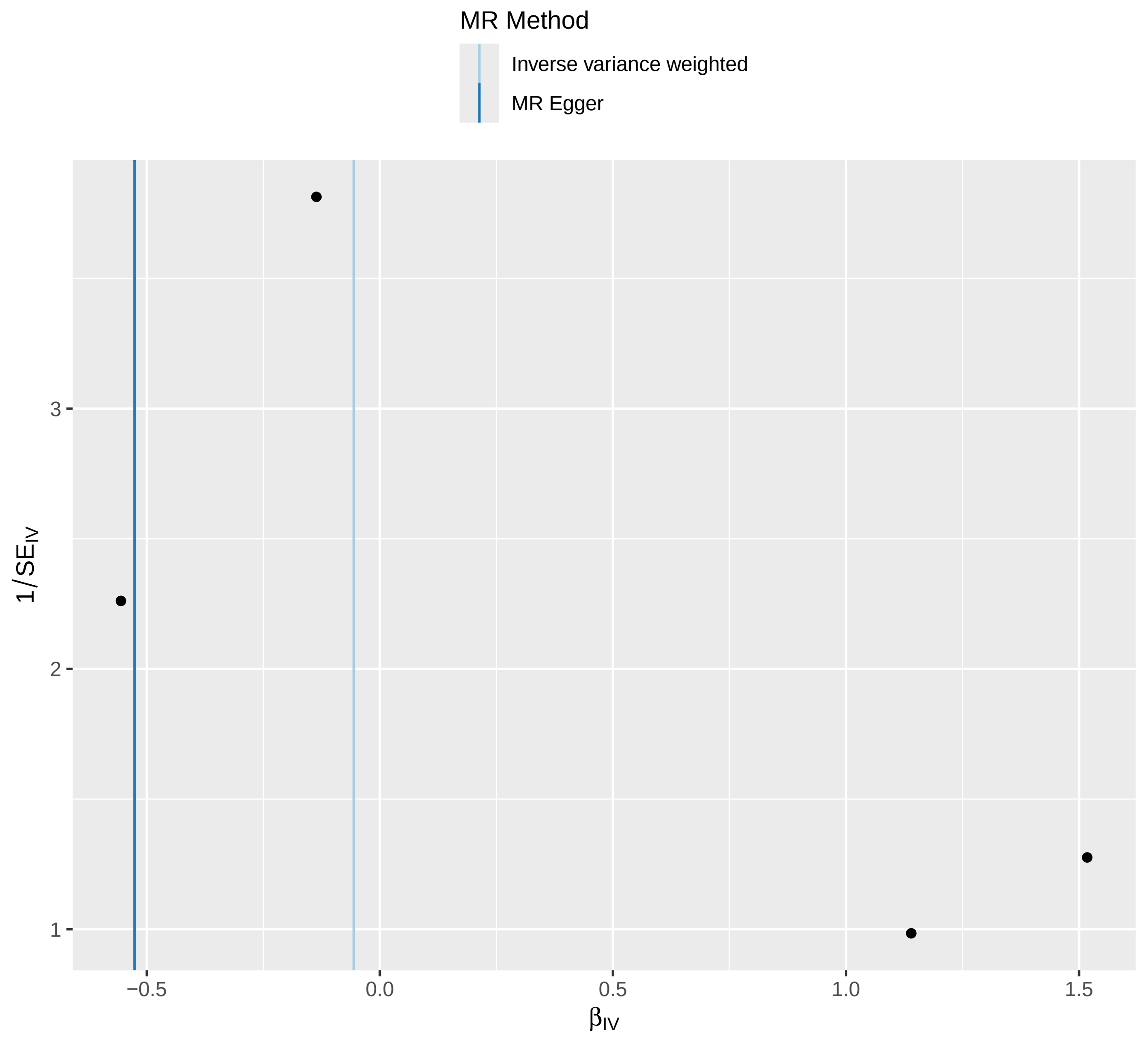

MR Method
Inverse variance weighted
MR Egger
1/SE_IV
3
2
1
−0.5
0.0
0.5
1.0
1.5
β_IV

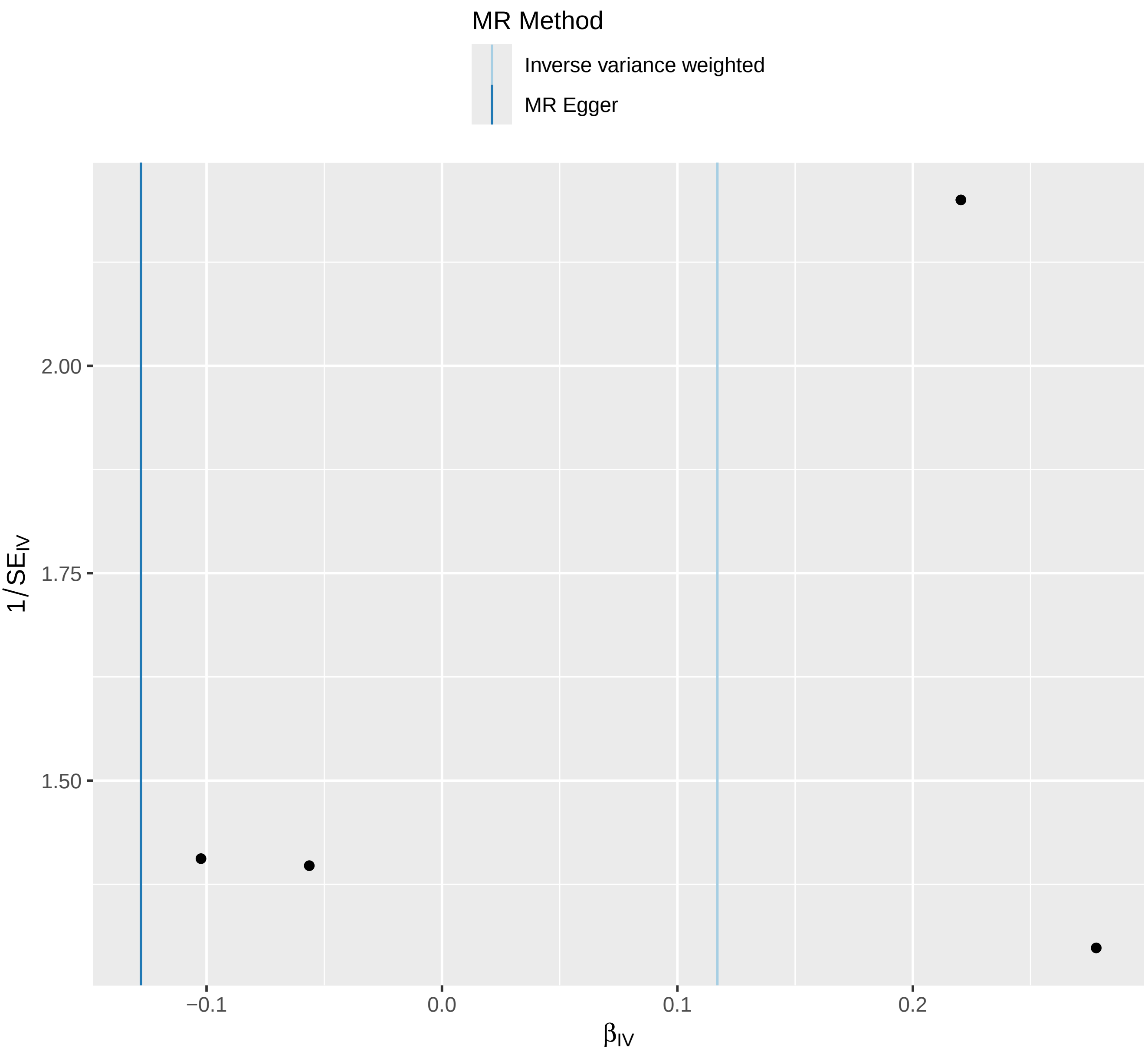
MR Method
Inverse variance weighted
MR Egger
2.00
1.75
1.50
$1/SE_{IV}$
−0.1
0.0
0.1
0.2
β_{IV}

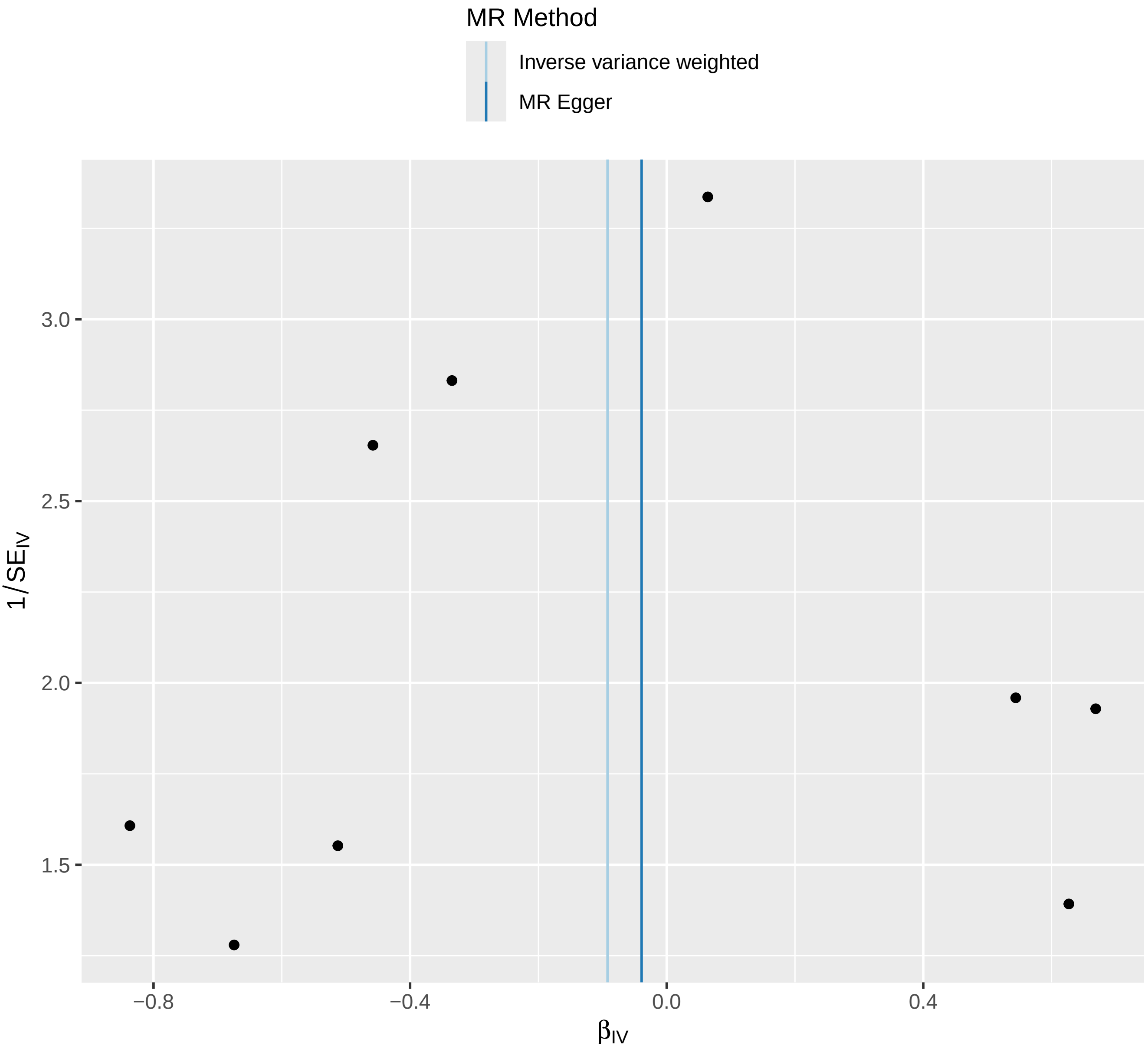
MR Method
Inverse variance weighted
MR Egger
1/SE_IV
3.0
2.5
2.0
1.5
−0.8
−0.4
0.0
0.4
β_IV

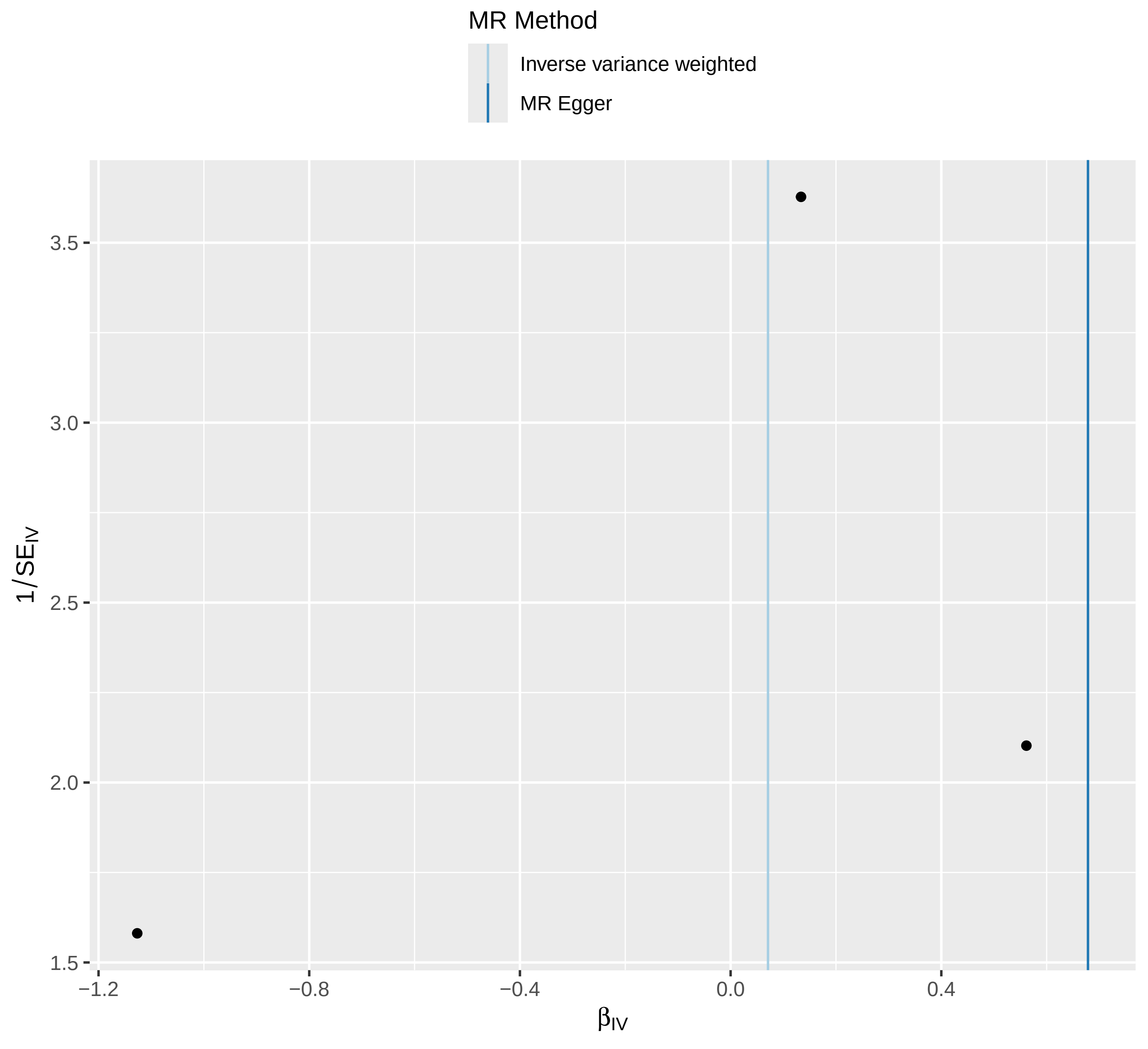
MR Method
Inverse variance weighted
MR Egger
3.5
3.0
2.5
2.0
1.5
1/SE_IV
−1.2
−0.8
−0.4
0.0
0.4
β_IV

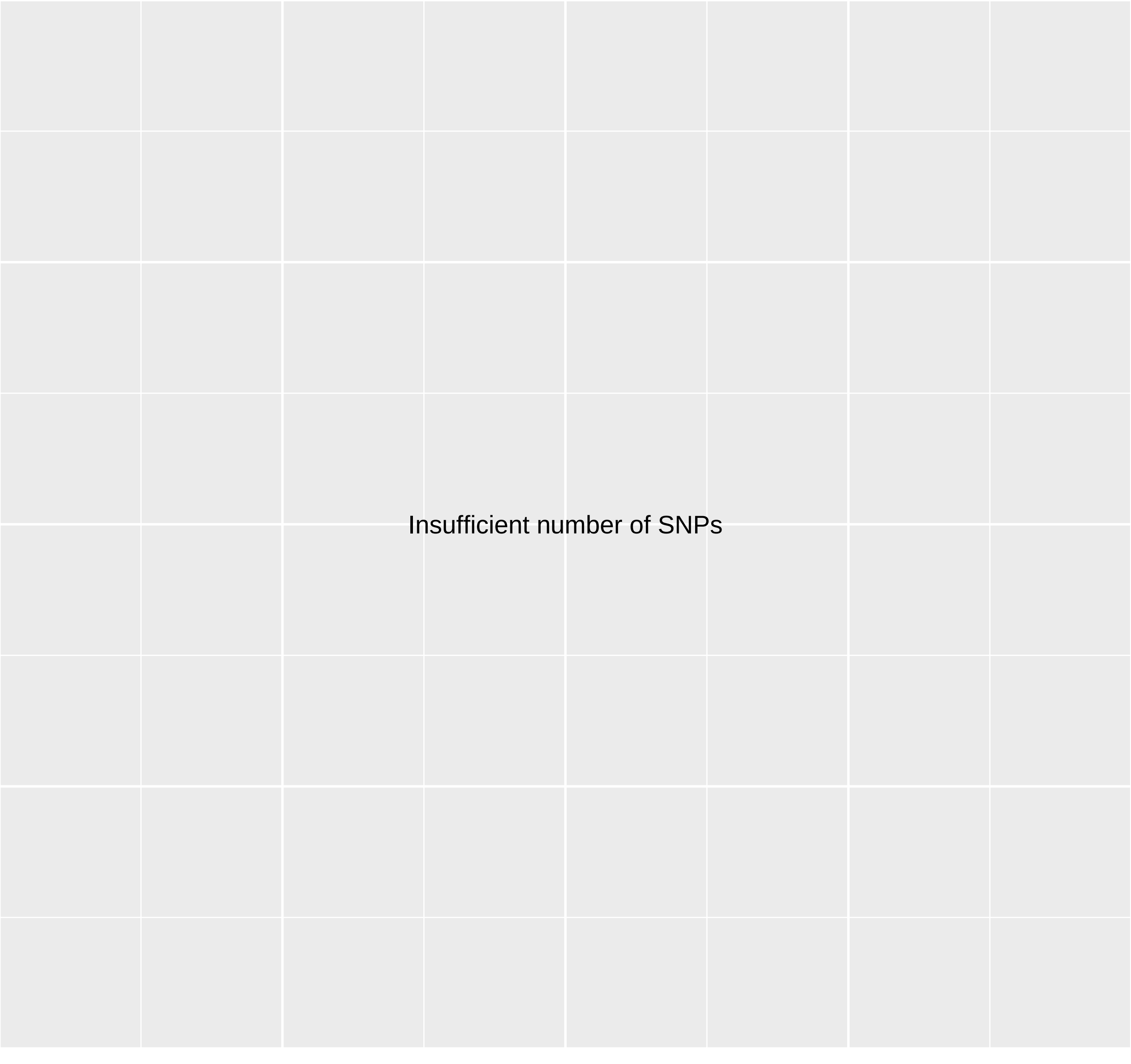
Insufficient number of SNPs

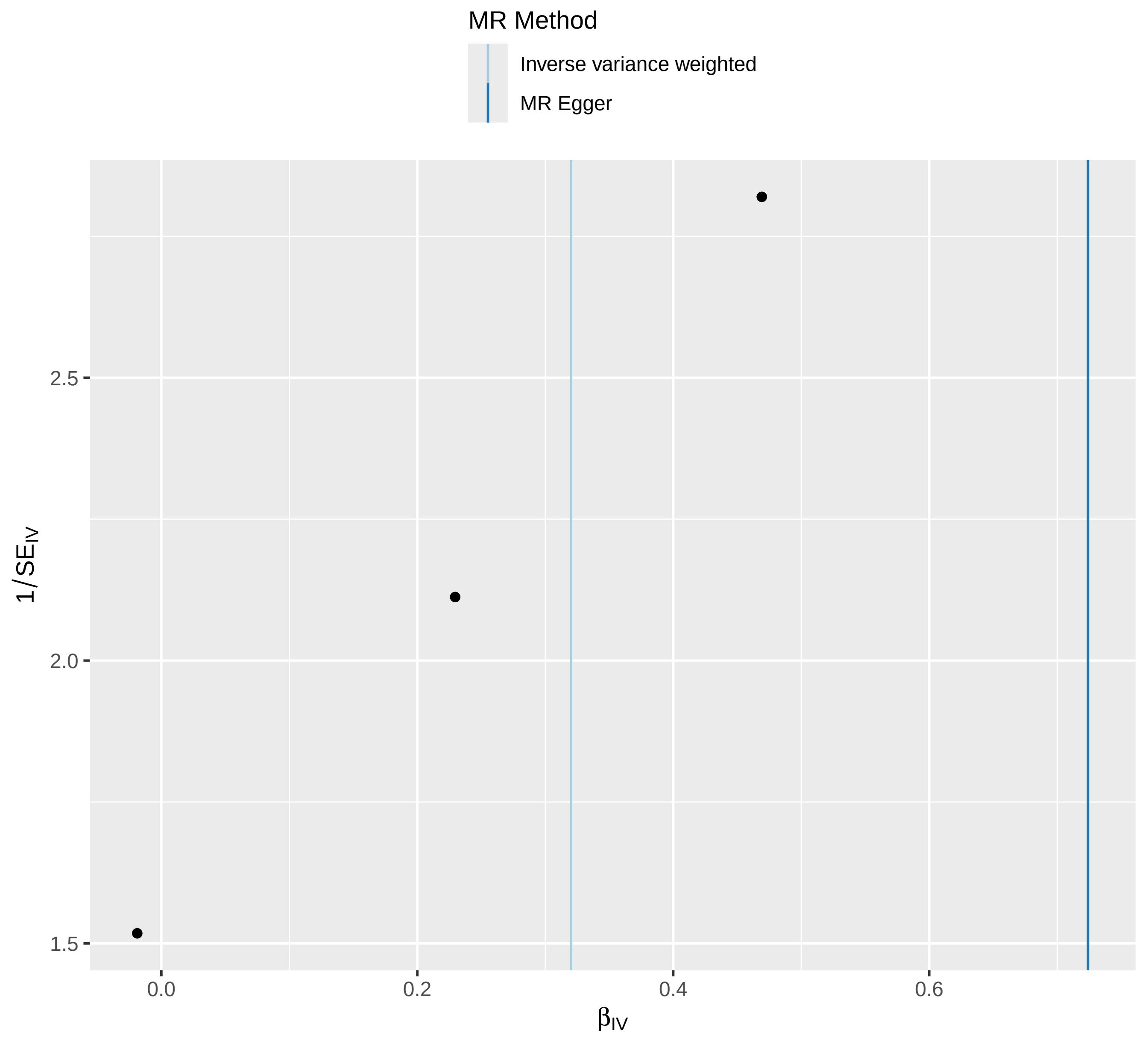
MR Method
Inverse variance weighted
MR Egger
1/SE_IV
β_IV
2.5
2.0
1.5
0.0
0.2
0.4
0.6

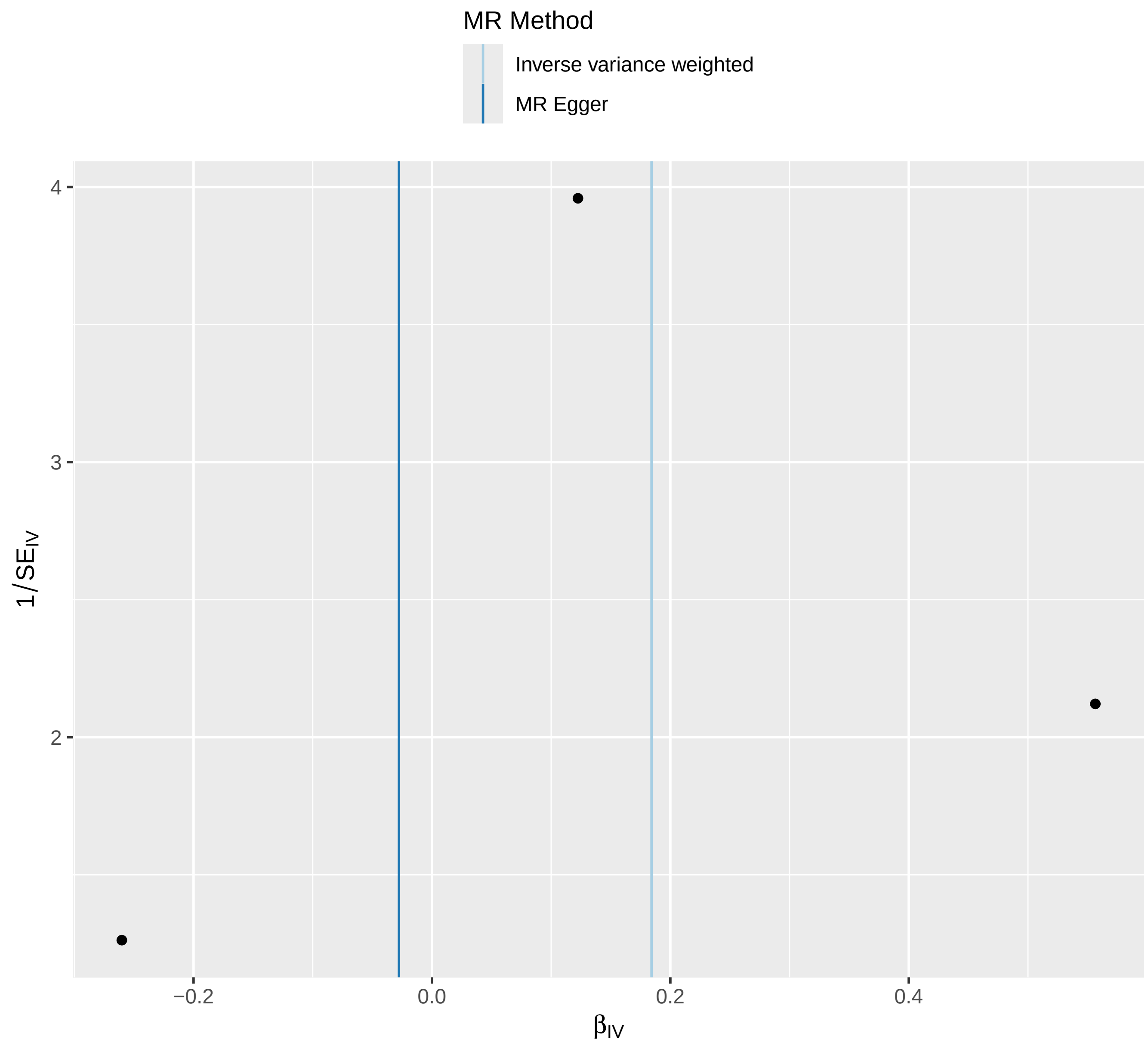
MR Method
Inverse variance weighted
MR Egger
4
3
2
1/SE$_{IV}$
−0.2
0.0
0.2
0.4
β_{IV}

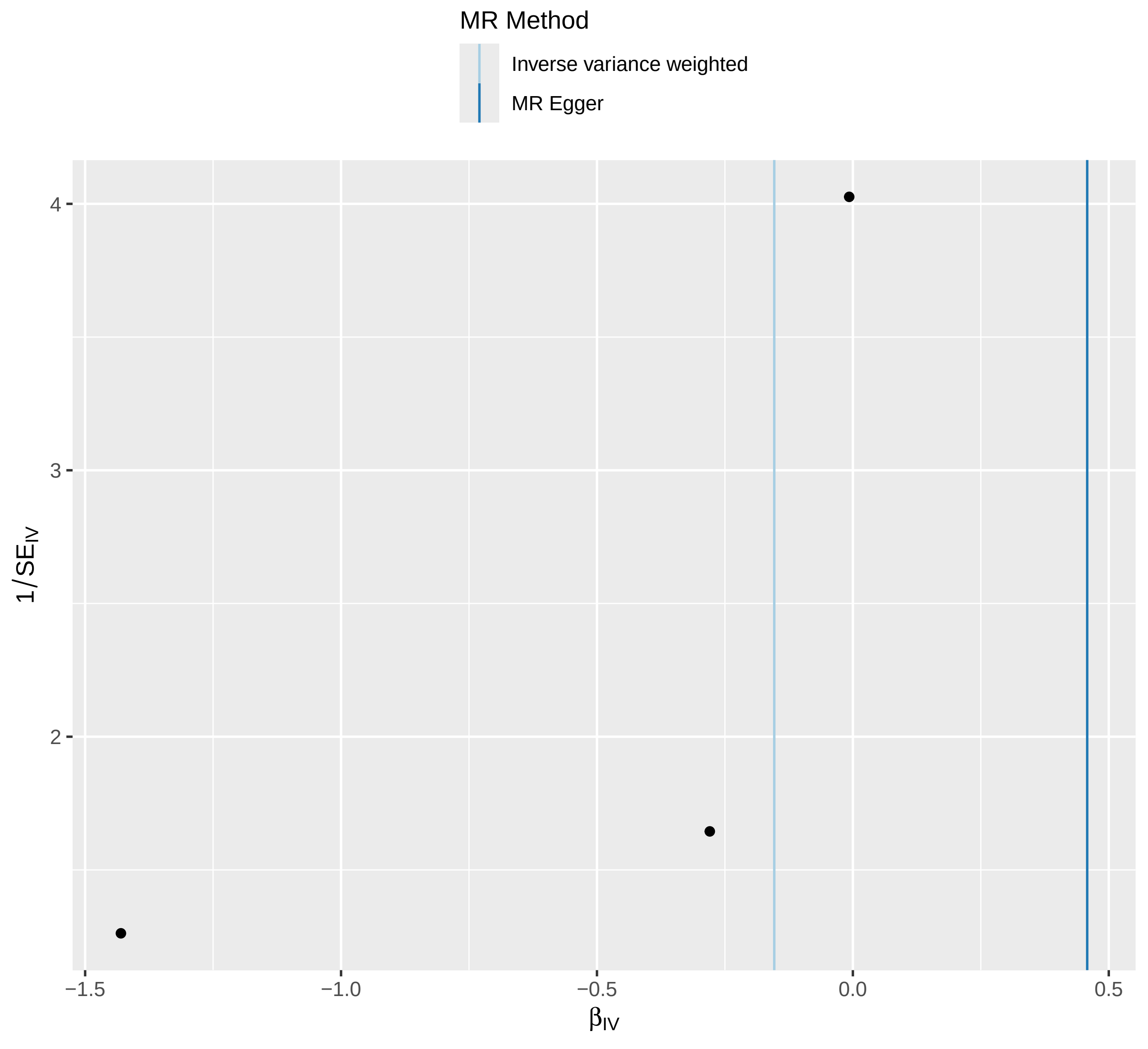
MR Method
Inverse variance weighted
MR Egger
4
3
2
1/SE_IV
−1.5
−1.0
−0.5
0.0
0.5
β_IV

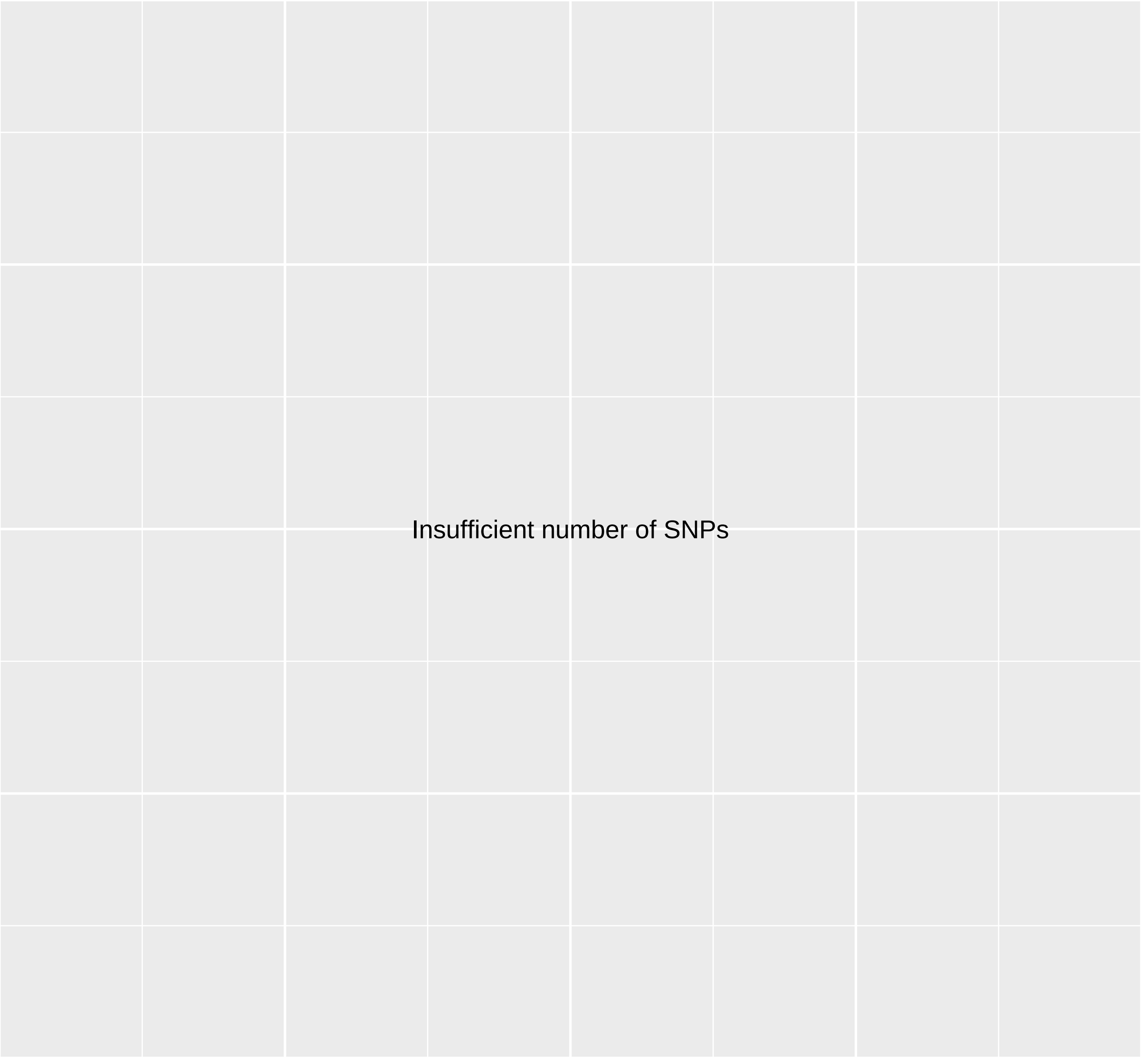
Insufficient number of SNPs

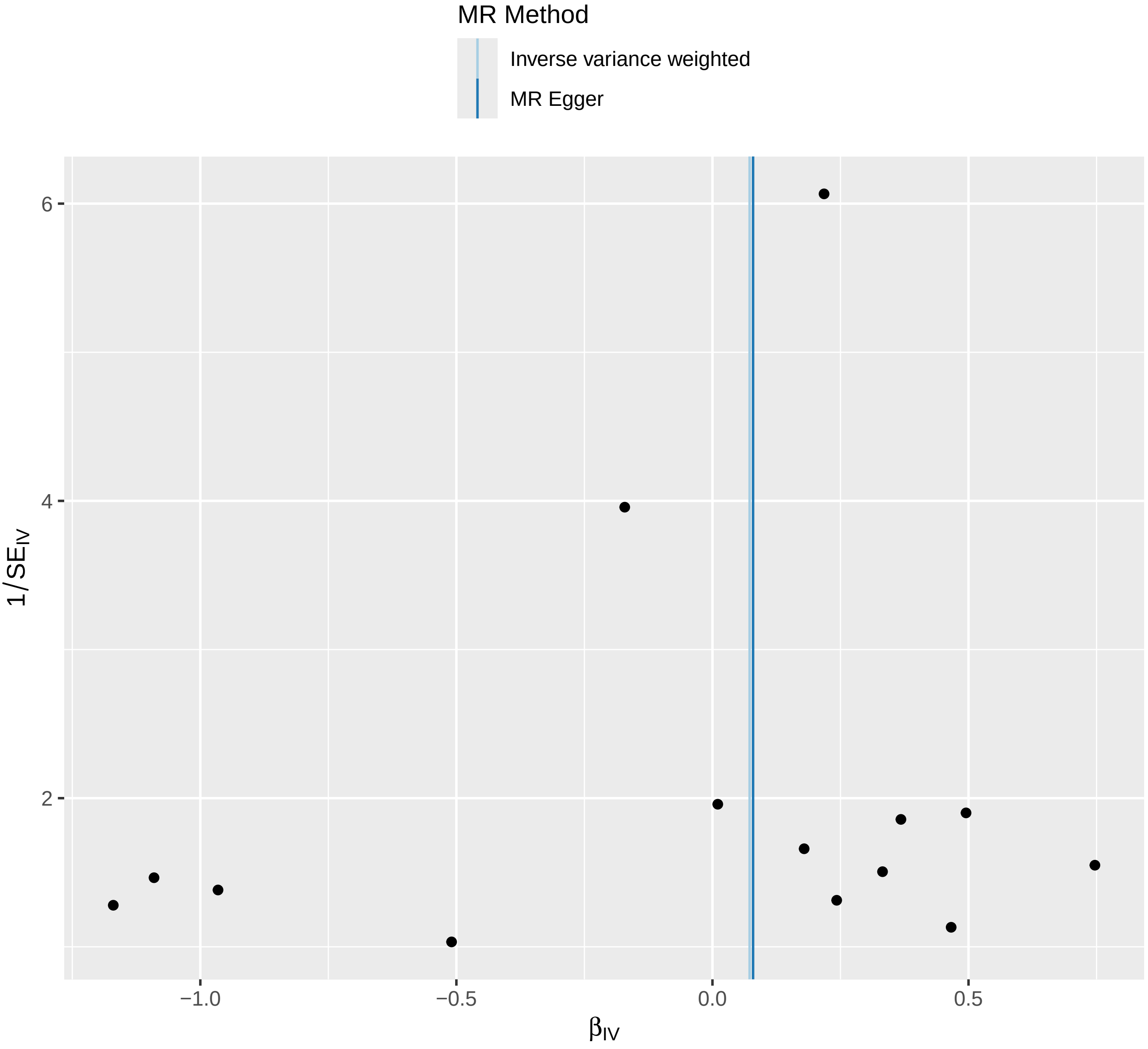

MR Method
Inverse variance weighted
MR Egger
6
4
2
1/SE_IV
−1.0
−0.5
0.0
0.5
β_IV

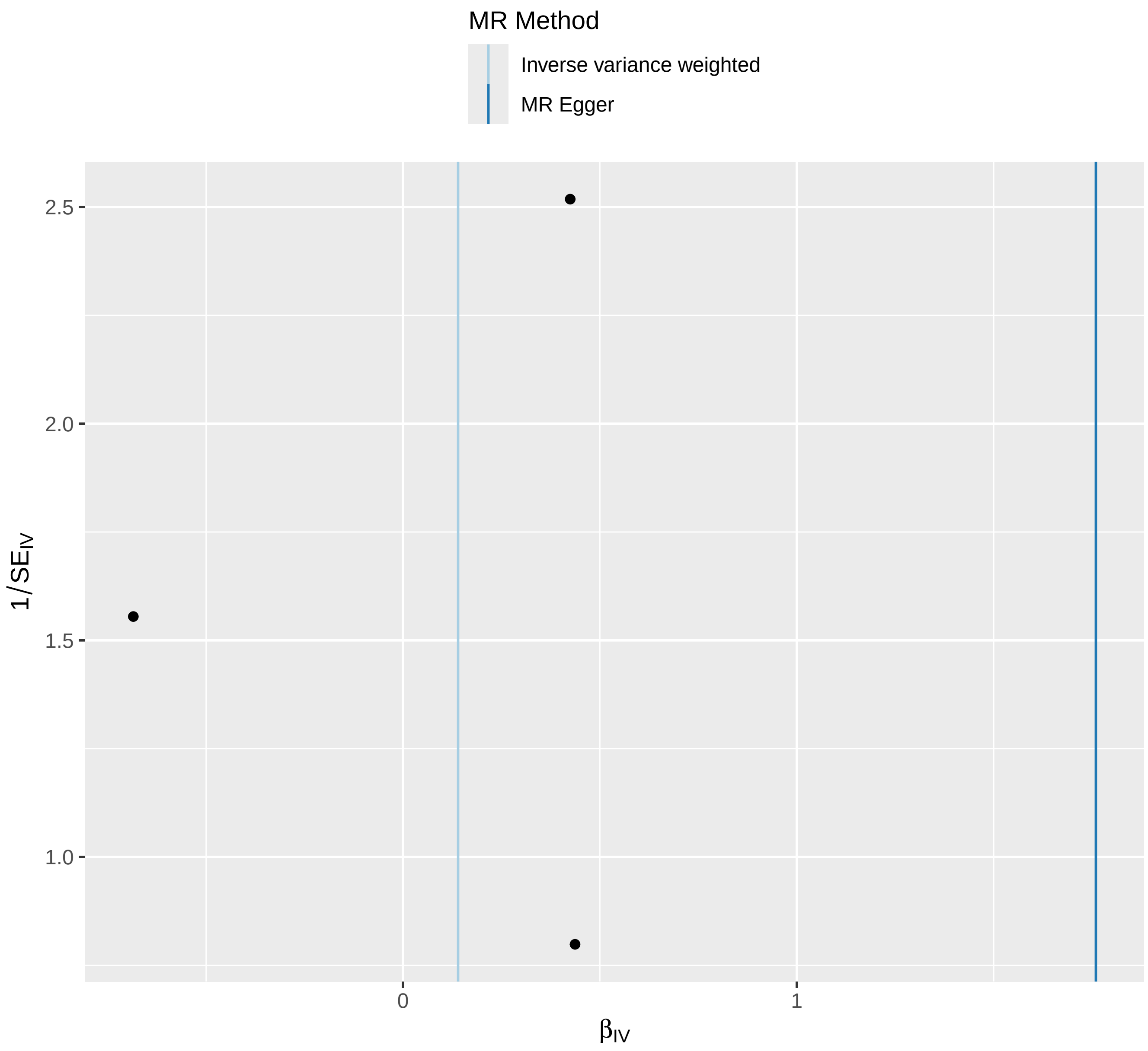
MR Method
Inverse variance weighted
MR Egger
2.5
2.0
1.5
1.0
1/SE_{IV}
0
1
β_{IV}

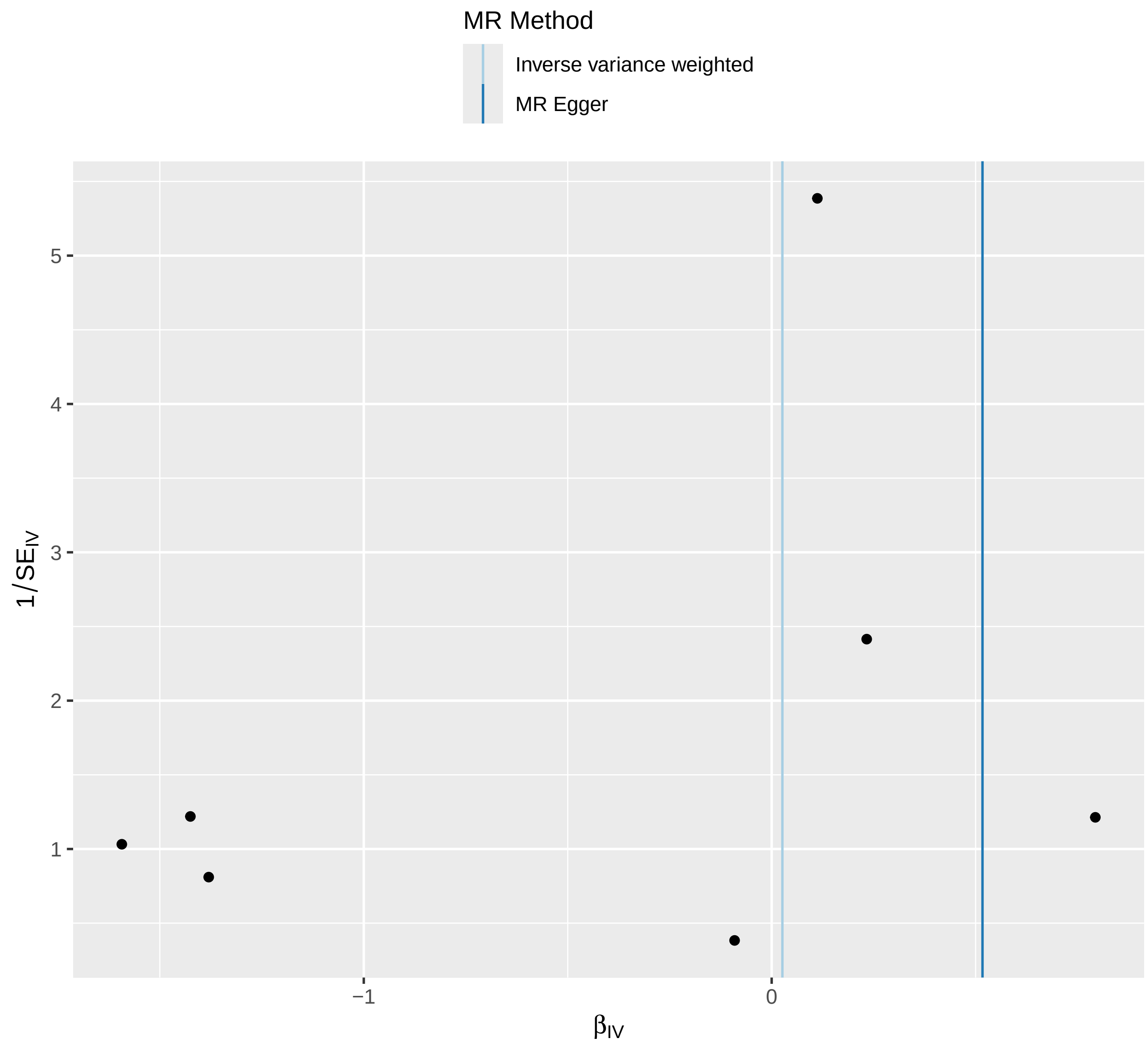
MR Method
Inverse variance weighted
MR Egger
1/SE_IV
5
4
3
2
1
−1
0
β_IV

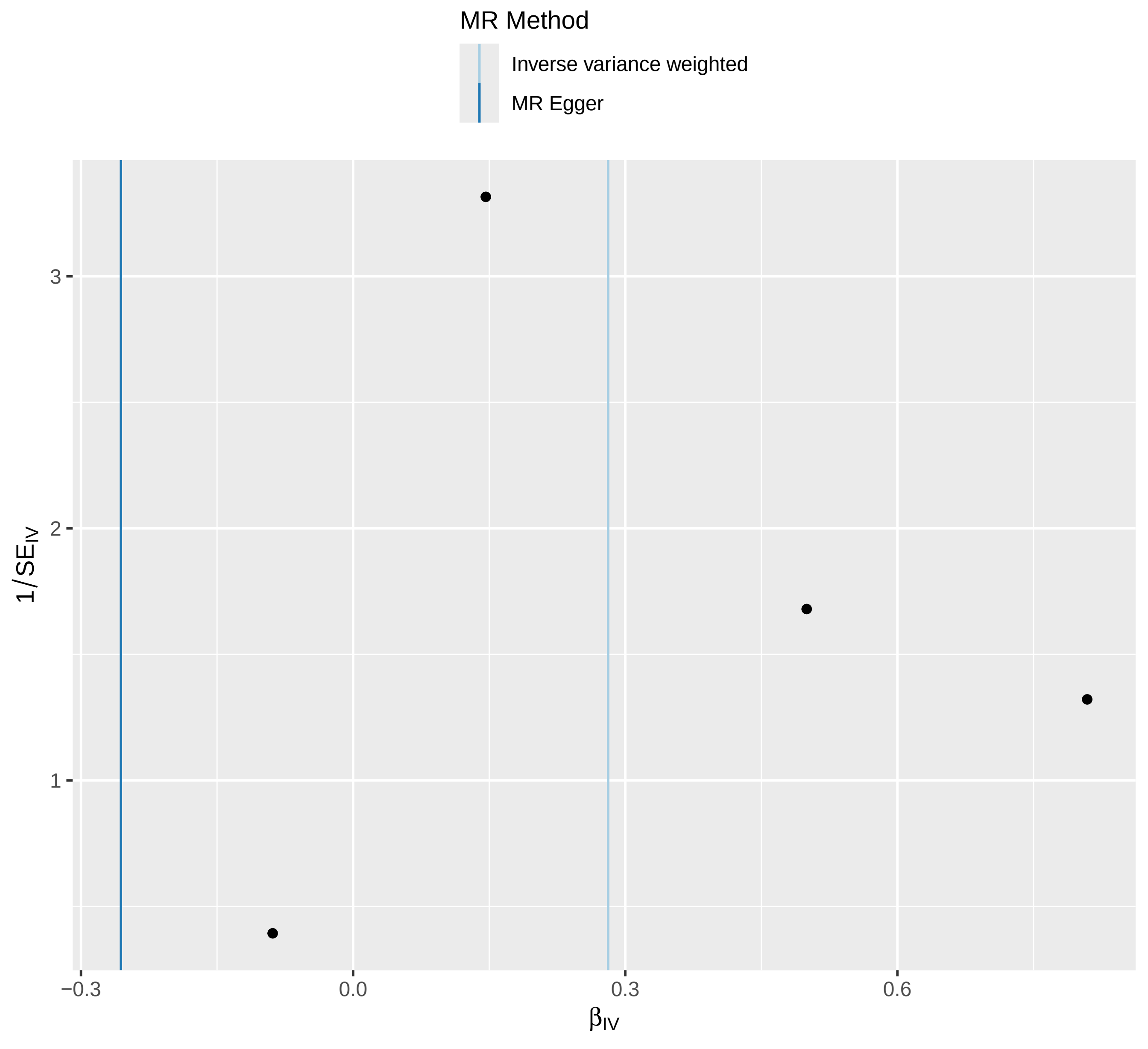
MR Method
Inverse variance weighted
MR Egger
3
2
1
1/SE$_{IV}$
−0.3
0.0
0.3
0.6
β$_{IV}$

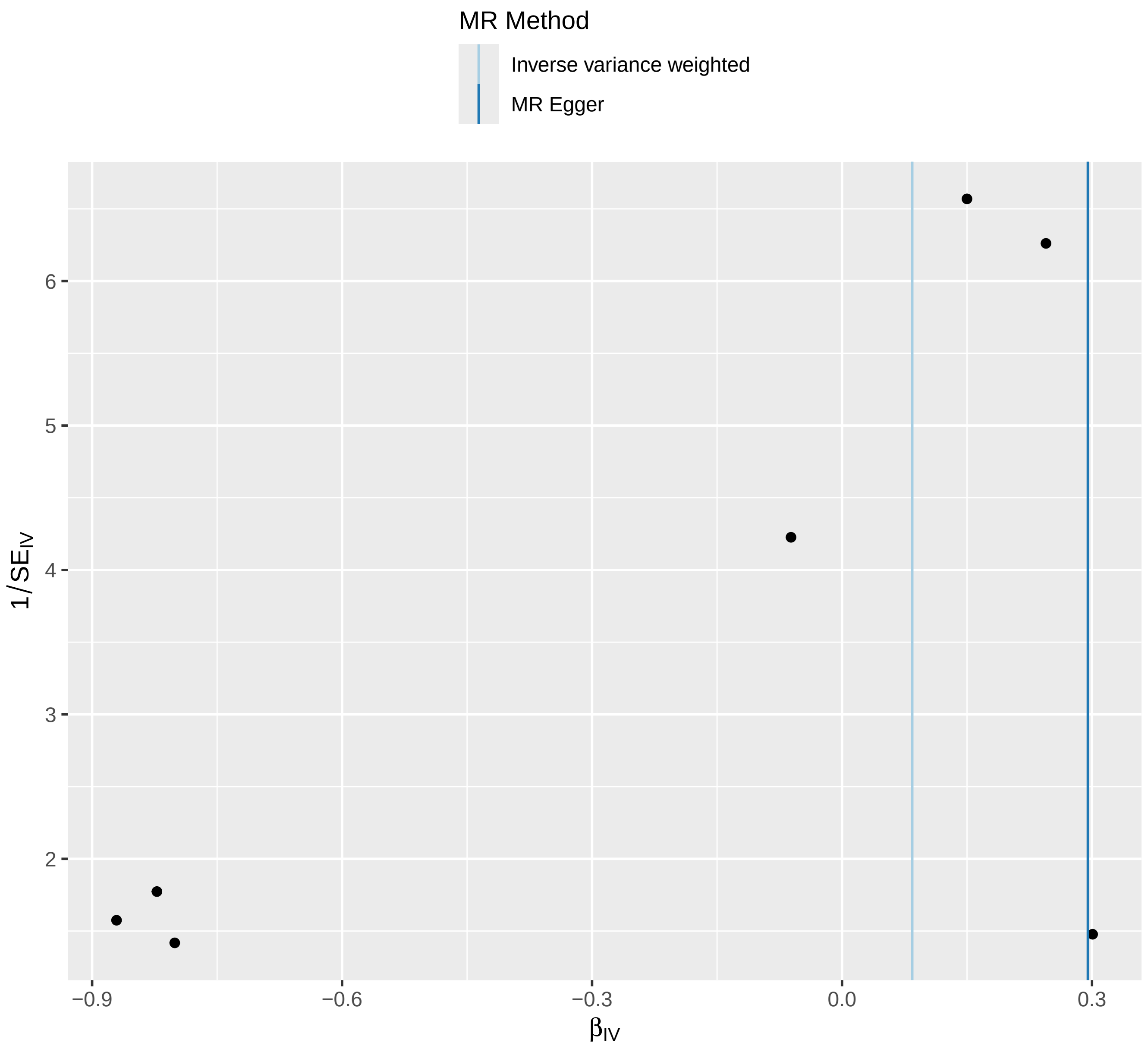
MR Method
Inverse variance weighted
MR Egger
6
5
4
3
2
1/SE$_{IV}$
−0.9
−0.6
−0.3
0.0
0.3
β$_{IV}$

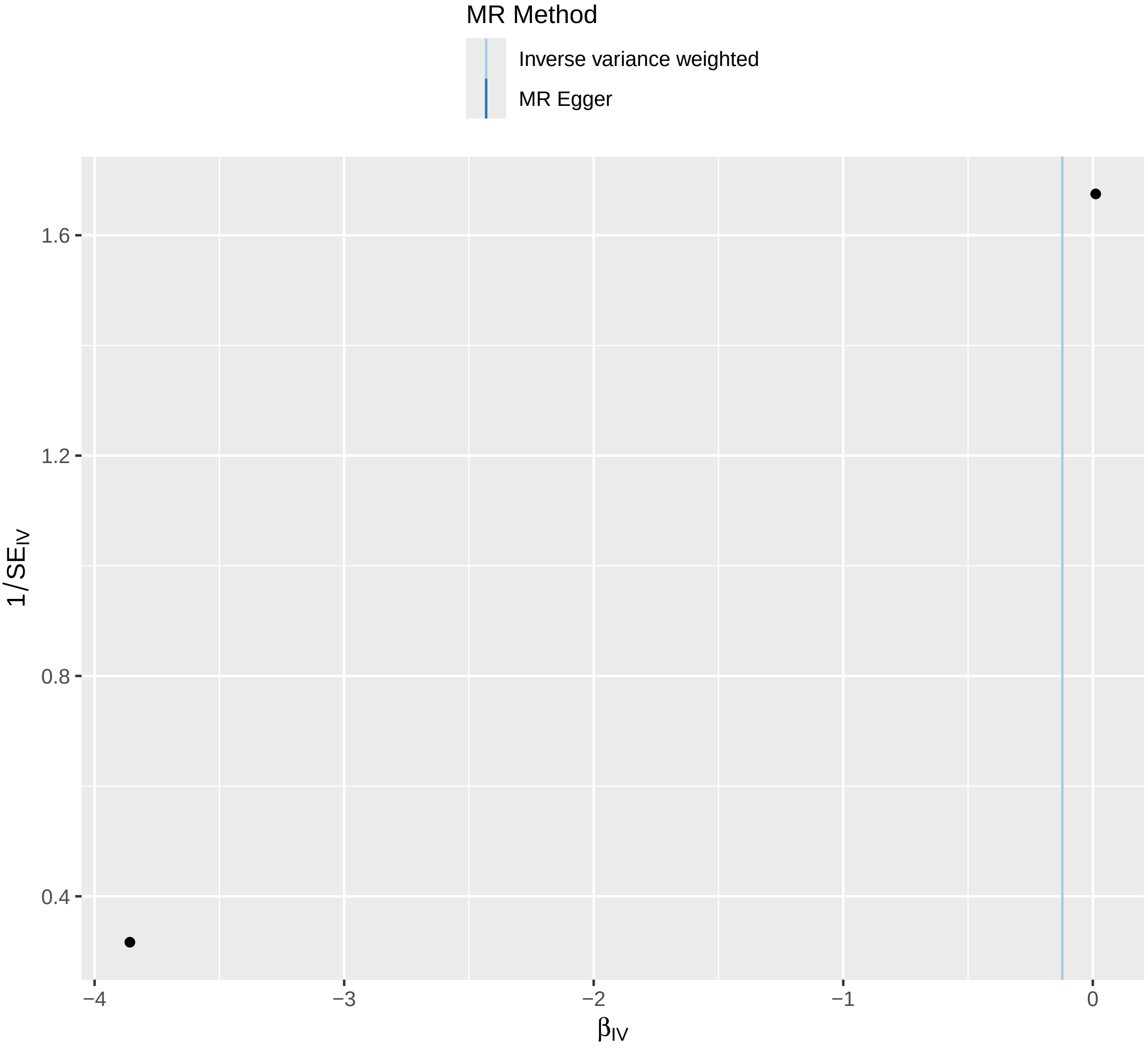
MR Method
Inverse variance weighted
MR Egger
1.6
1.2
0.8
0.4
1/SE_IV
−4
−3
−2
−1
0
β_IV

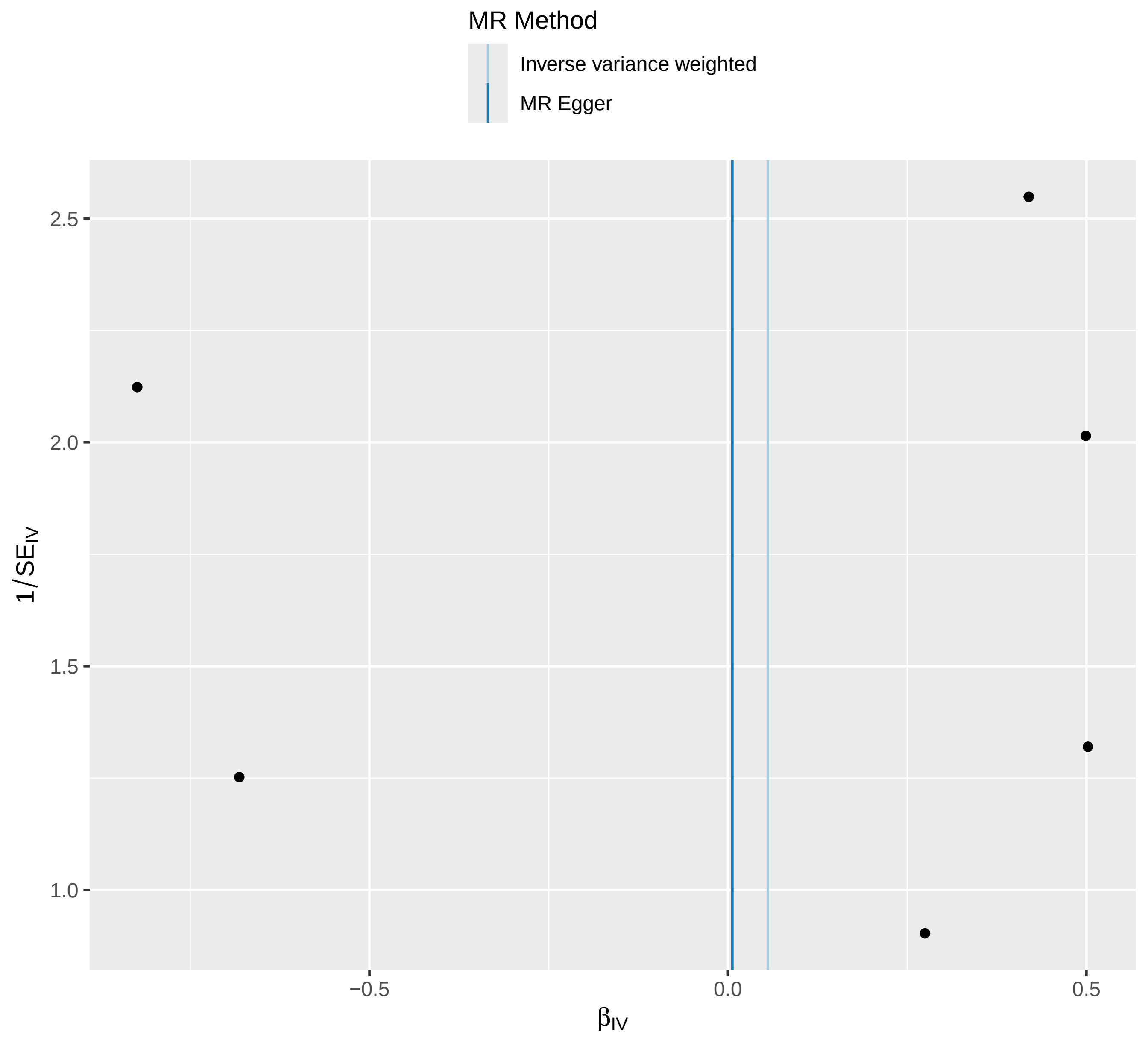

MR Method
Inverse variance weighted
MR Egger
2.5
2.0
1.5
1.0
1/SE_IV
−0.5
0.0
0.5
β_IV

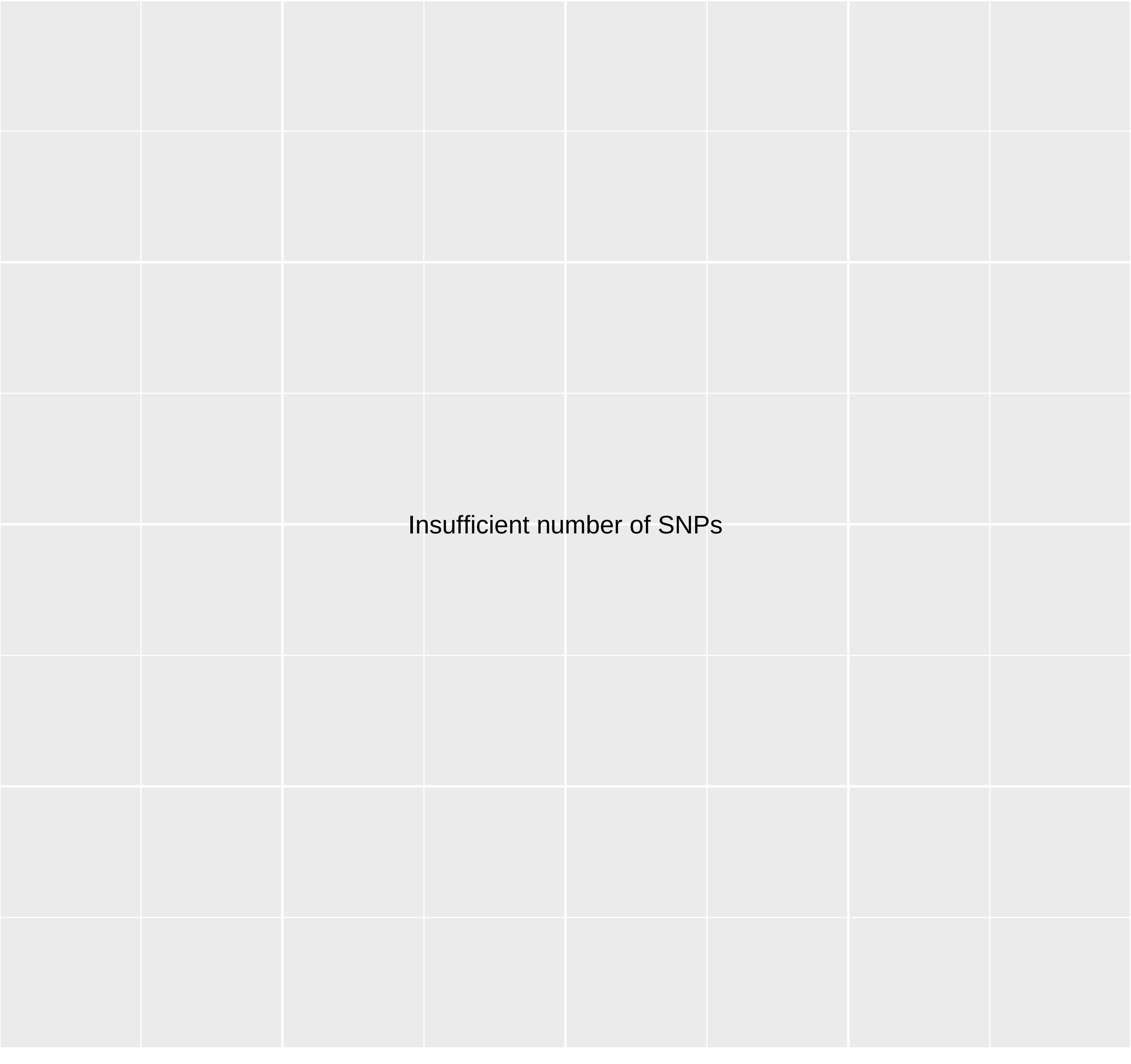
Insufficient number of SNPs

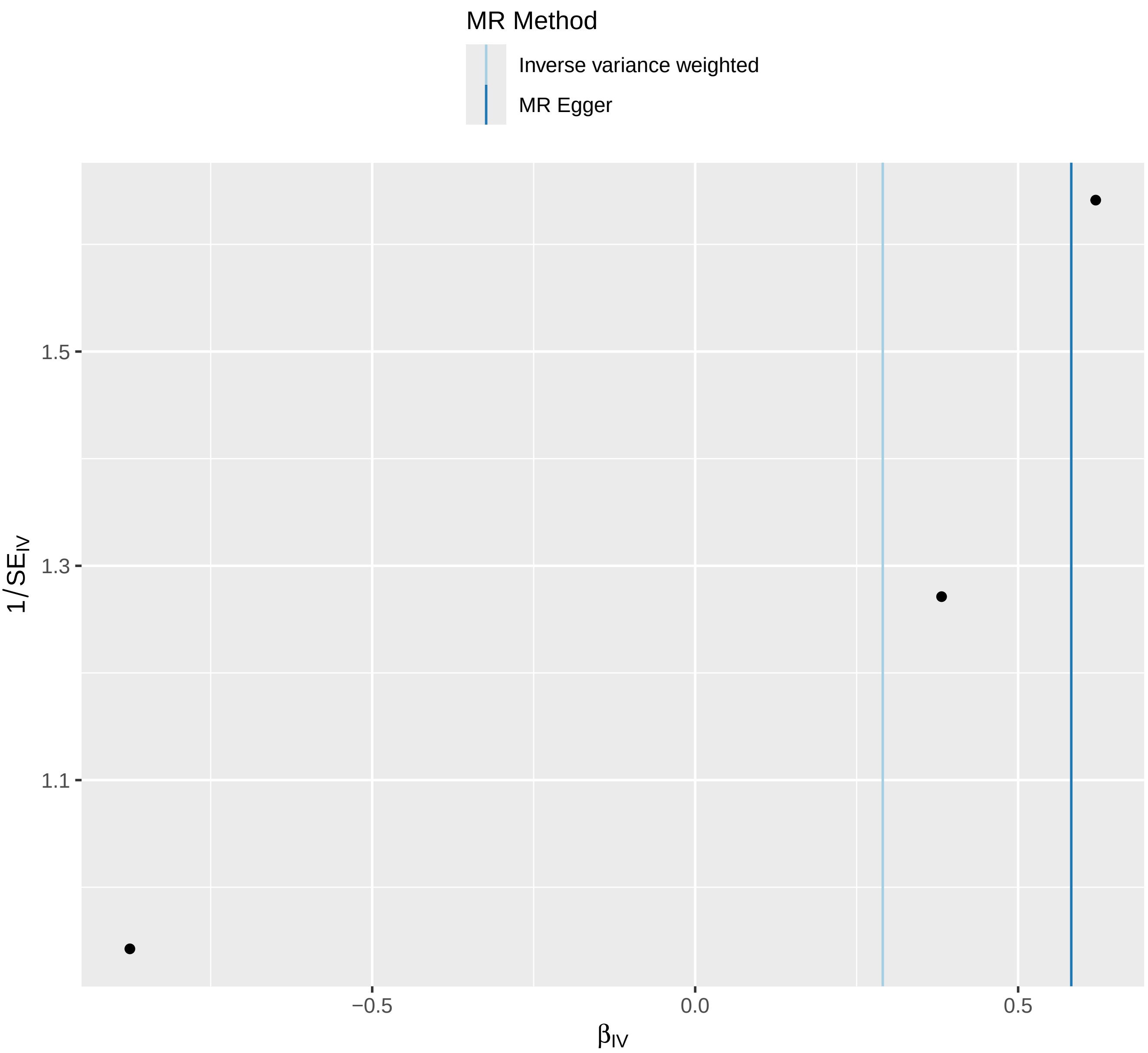

MR Method
Inverse variance weighted
MR Egger
1.5
1.3
1.1
1/SE_IV
−0.5
0.0
0.5
β_IV

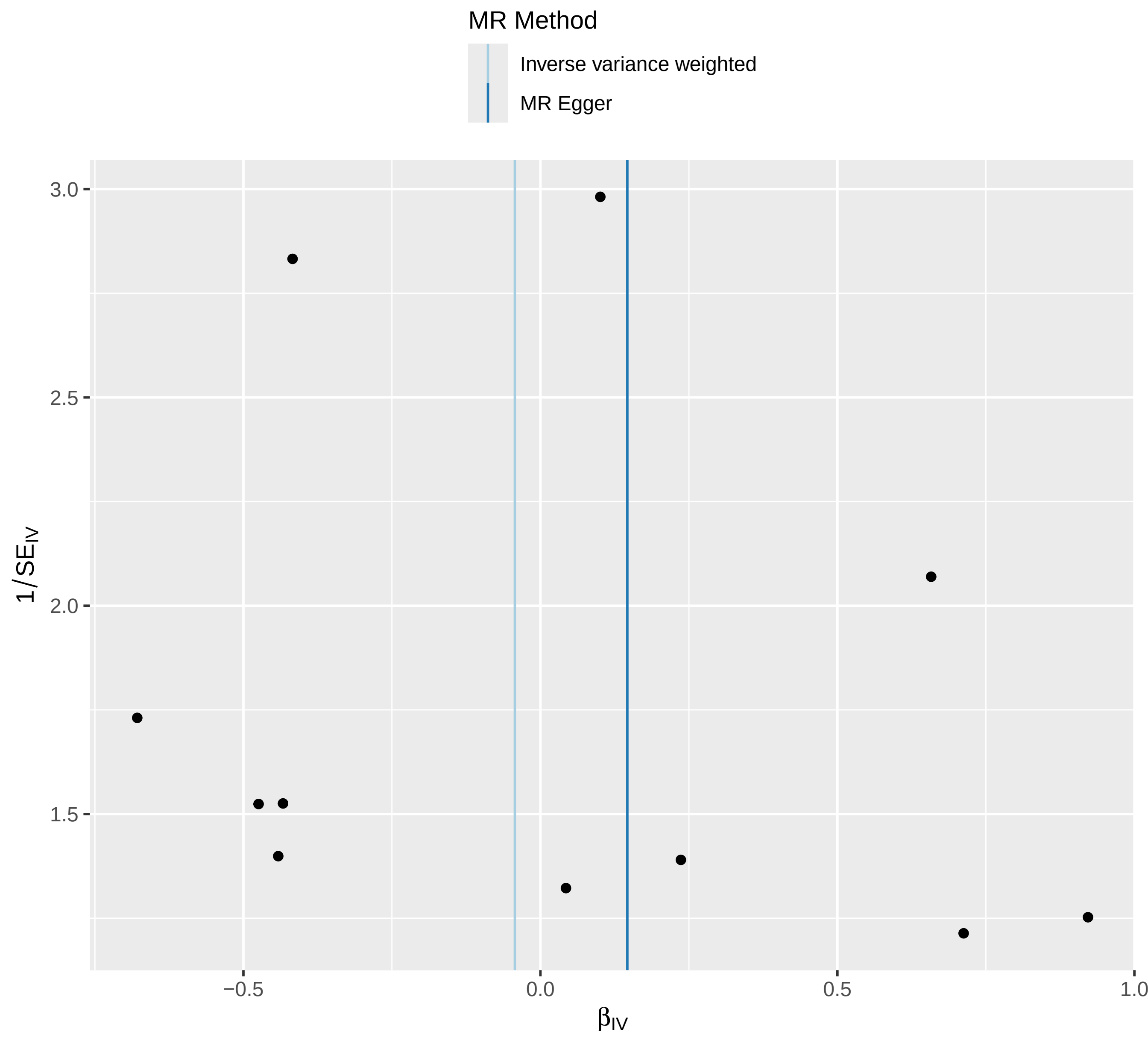

MR Method
Inverse variance weighted
MR Egger
3.0
2.5
2.0
1.5
1/SE_IV
−0.5
0.0
0.5
1.0
β_IV

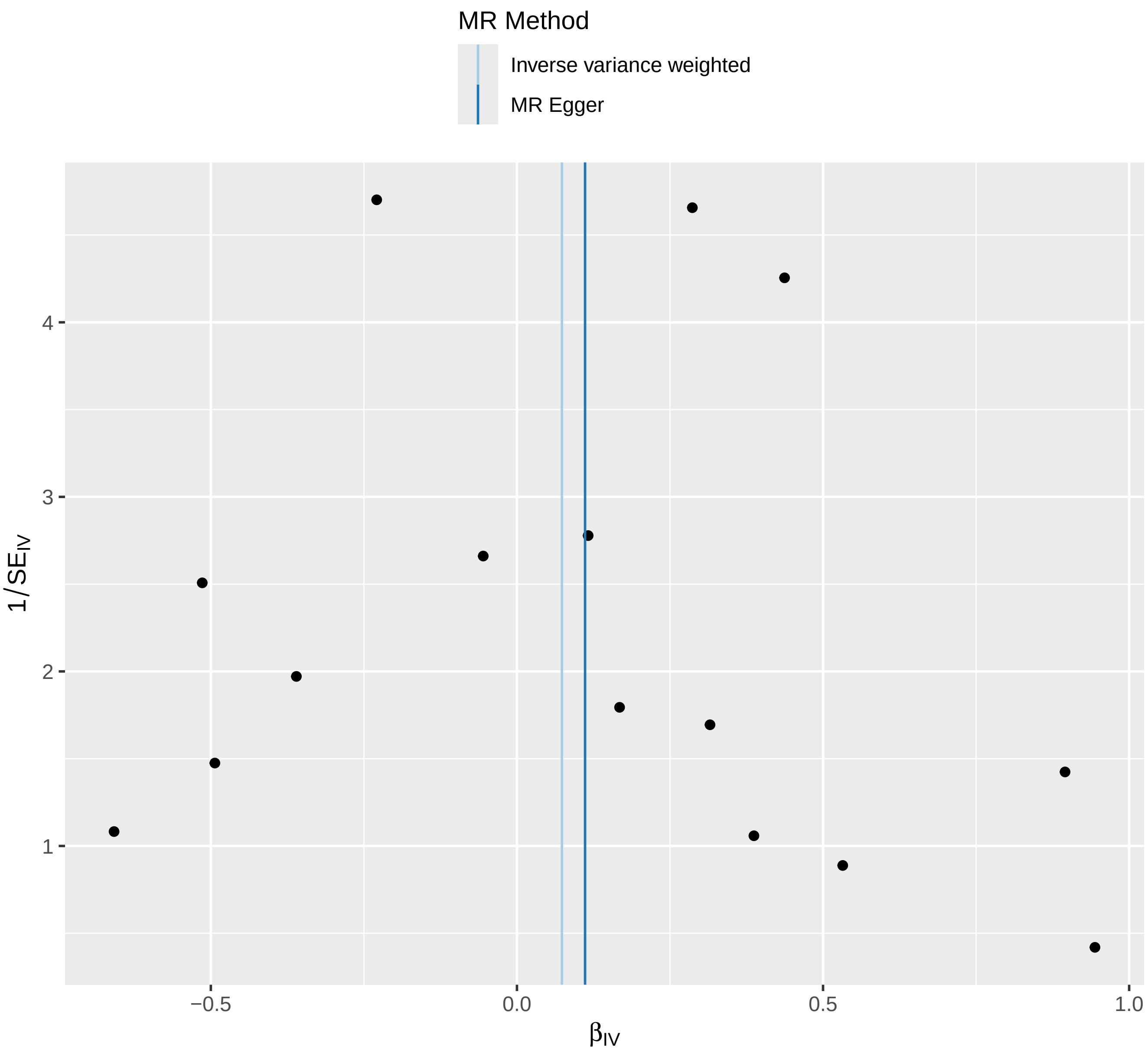
MR Method
Inverse variance weighted
MR Egger
1/SE$_{IV}$
4
3
2
1
−0.5
0.0
0.5
1.0
β$_{IV}$

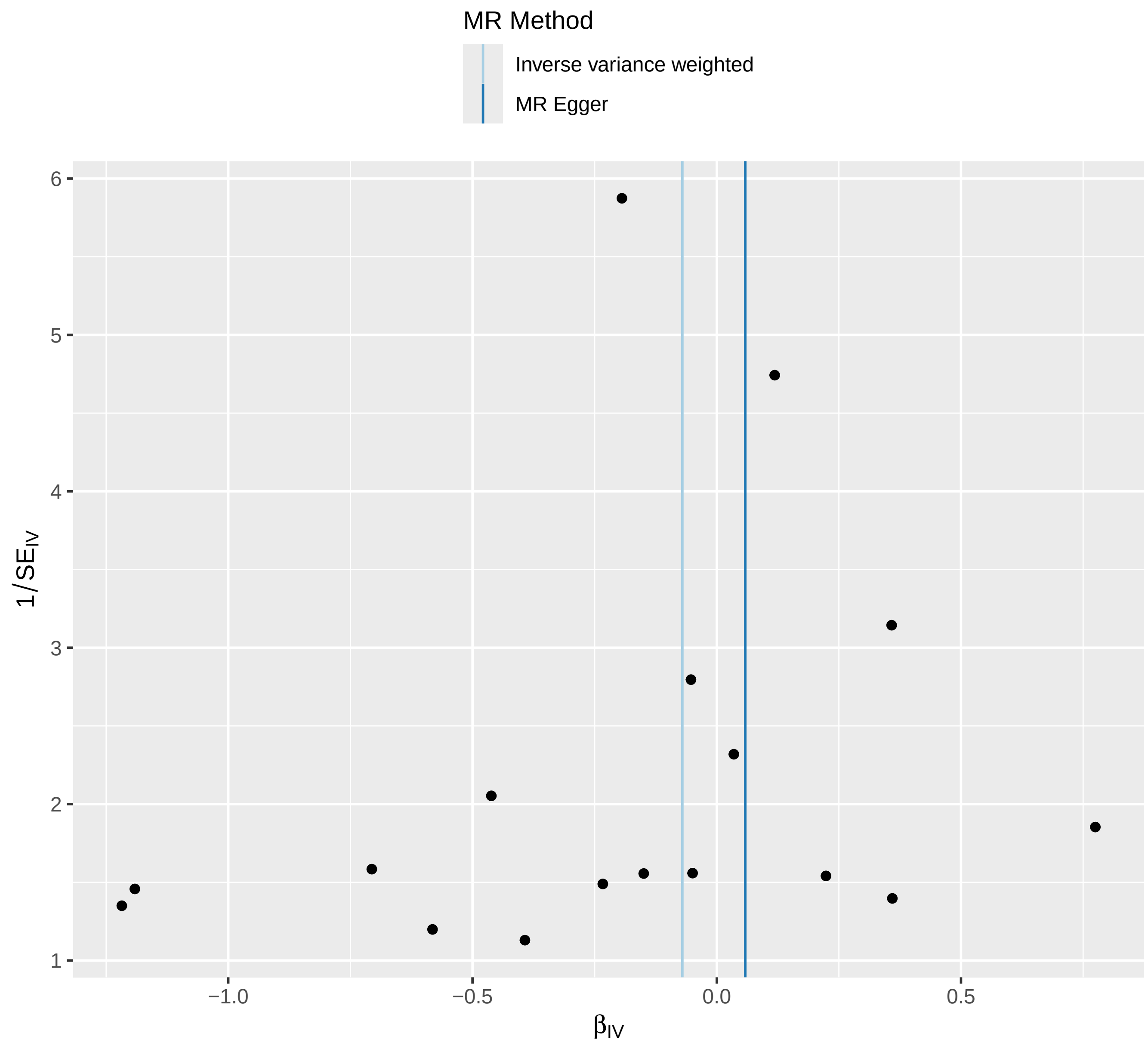

MR Method
Inverse variance weighted
MR Egger
1/SE_IV
β_IV
6
5
4
3
2
1
−1.0
−0.5
0.0
0.5

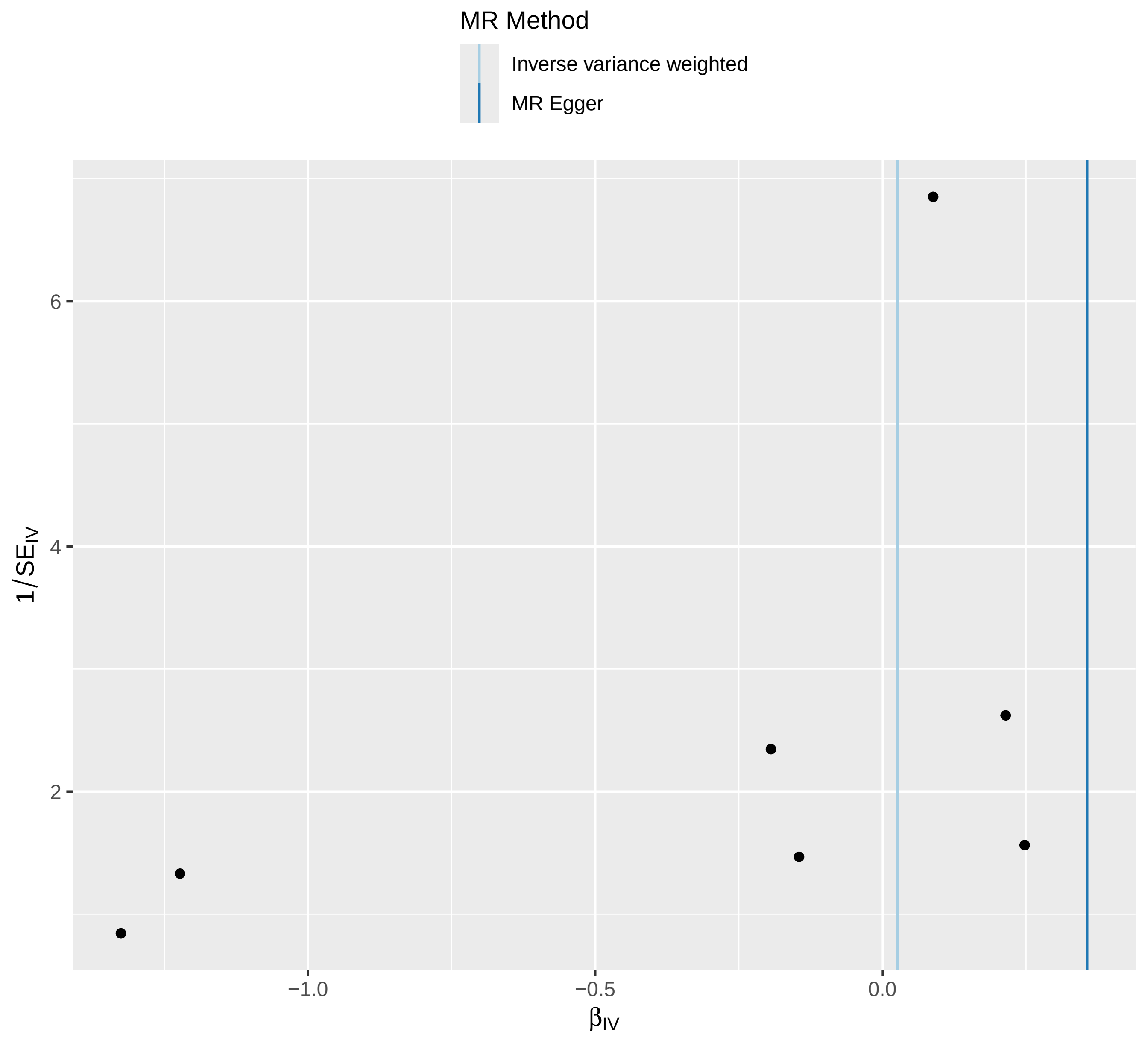

MR Method
Inverse variance weighted
MR Egger
6
4
2
1/SE$_{IV}$
−1.0
−0.5
0.0
β$_{IV}$

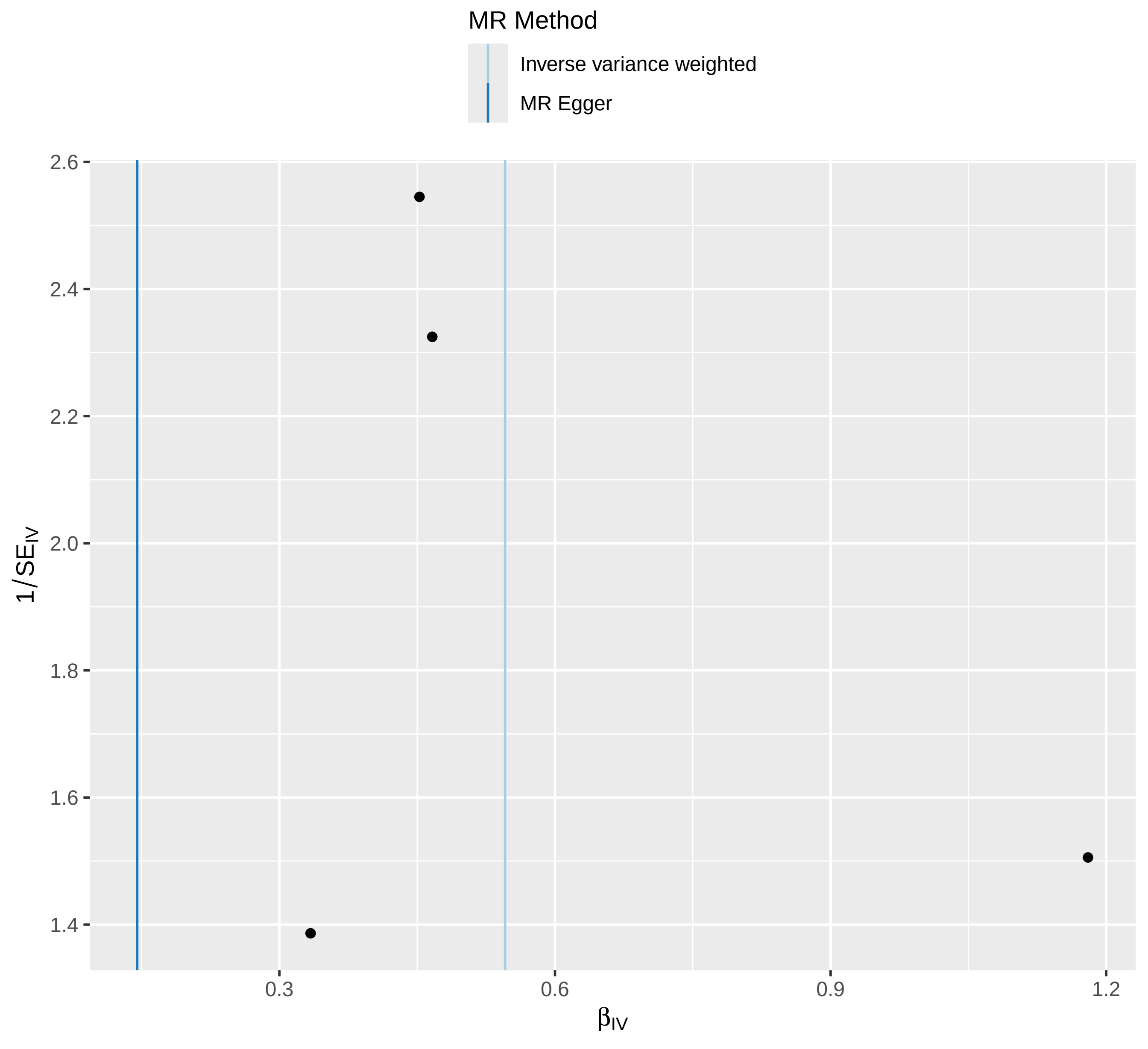

MR Method
Inverse variance weighted
MR Egger
2.6
2.4
2.2
2.0
1.8
1.6
1.4
1/SE_IV
0.3
0.6
0.9
1.2
β_IV

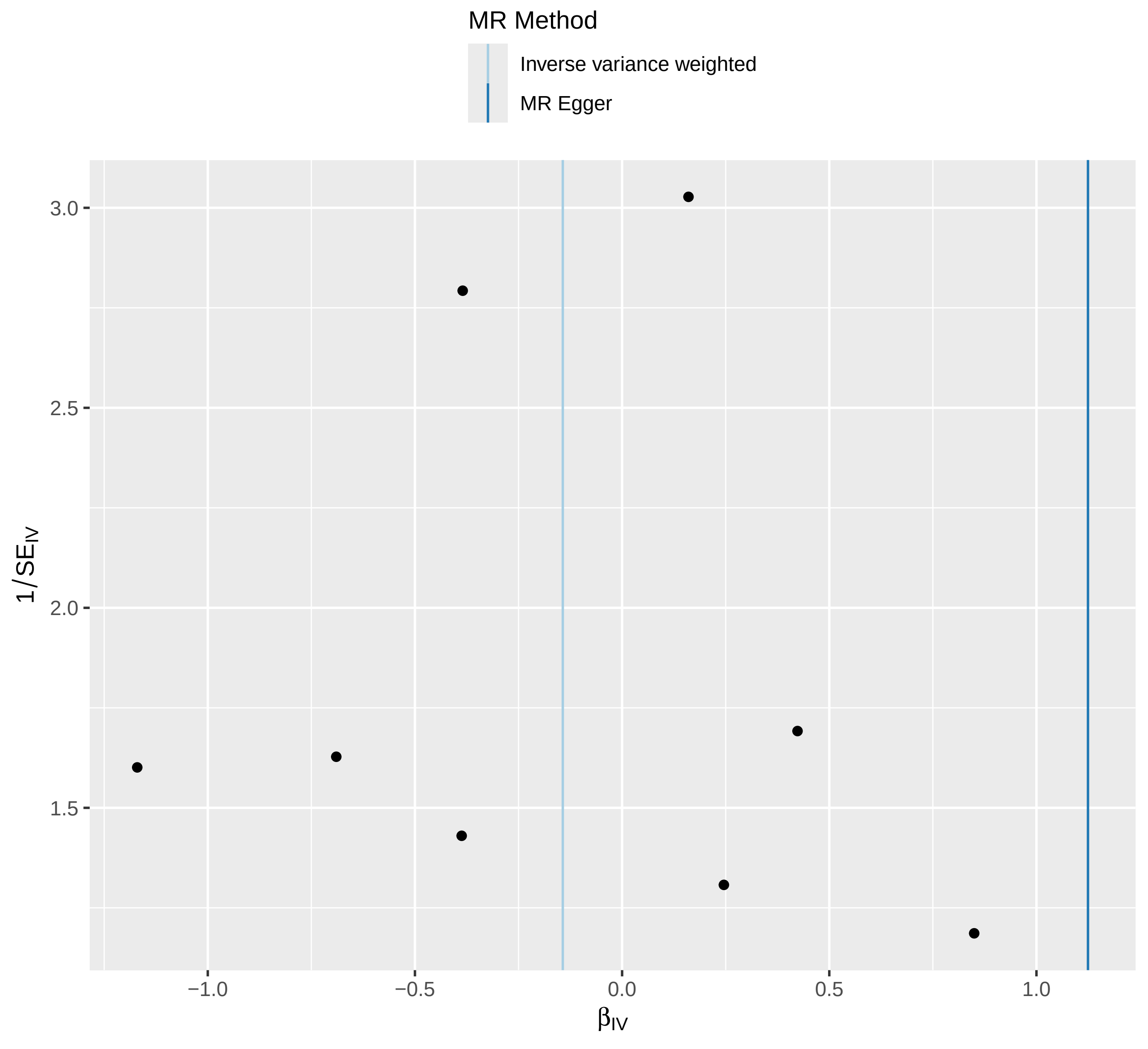

MR Method
Inverse variance weighted
MR Egger
3.0
2.5
2.0
1.5
1/SE_IV
−1.0
−0.5
0.0
0.5
1.0
β_IV

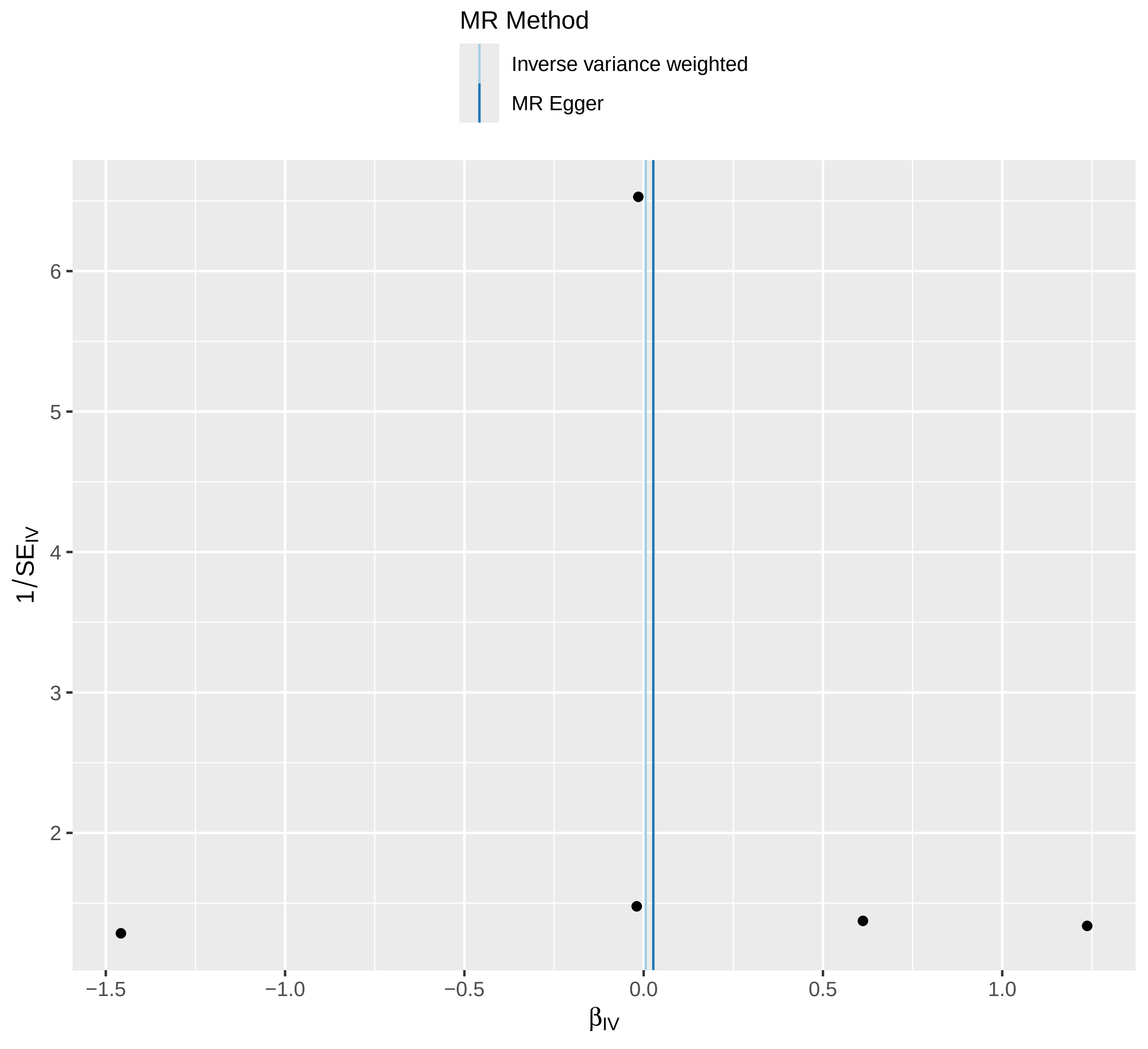
MR Method
Inverse variance weighted
MR Egger
6
5
4
3
2
1/SE_IV
−1.5
−1.0
−0.5
0.0
0.5
1.0
β_IV

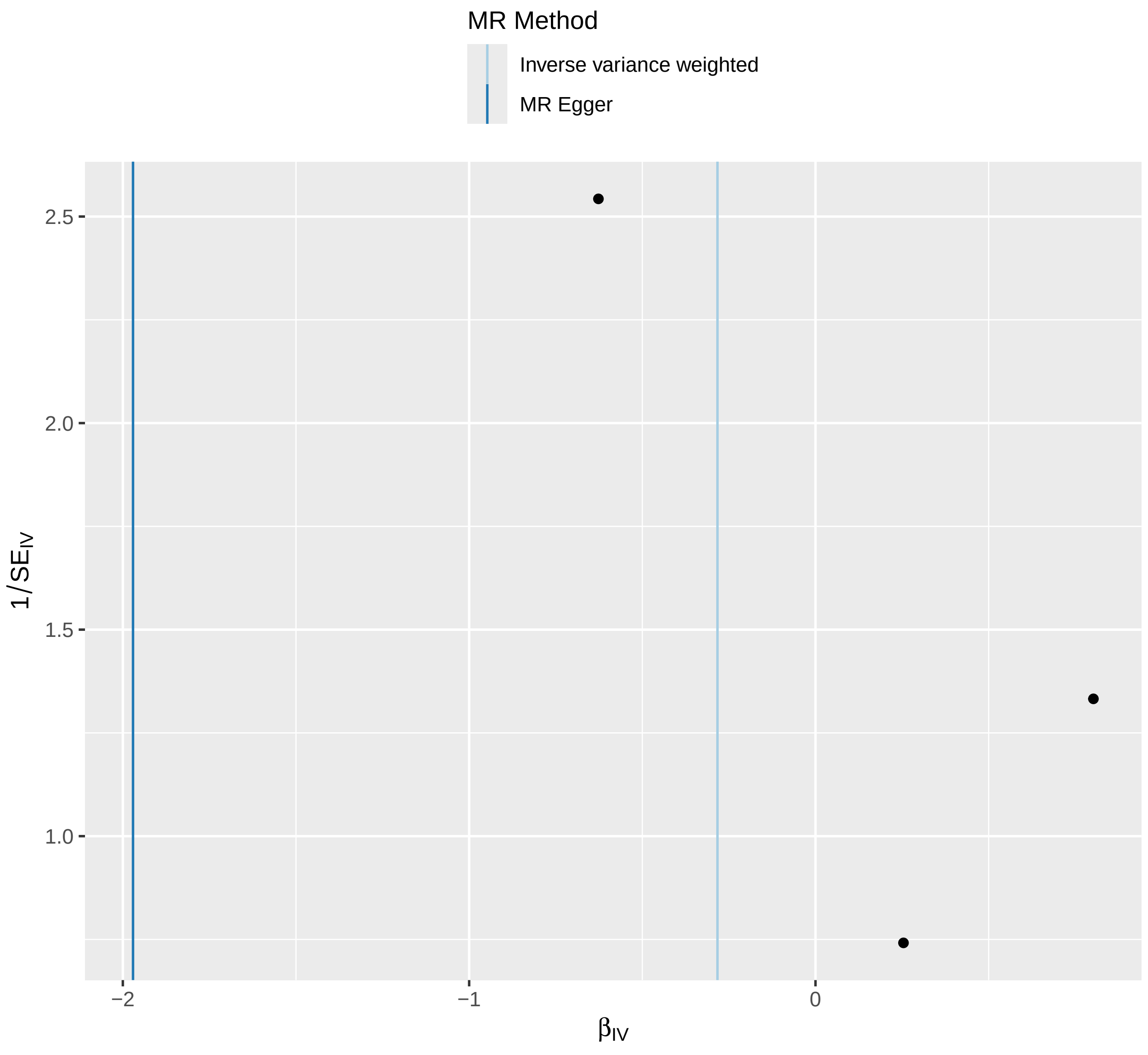
MR Method
Inverse variance weighted
MR Egger
2.5
2.0
1.5
1.0
1/SE_IV
−2
−1
0
β_IV

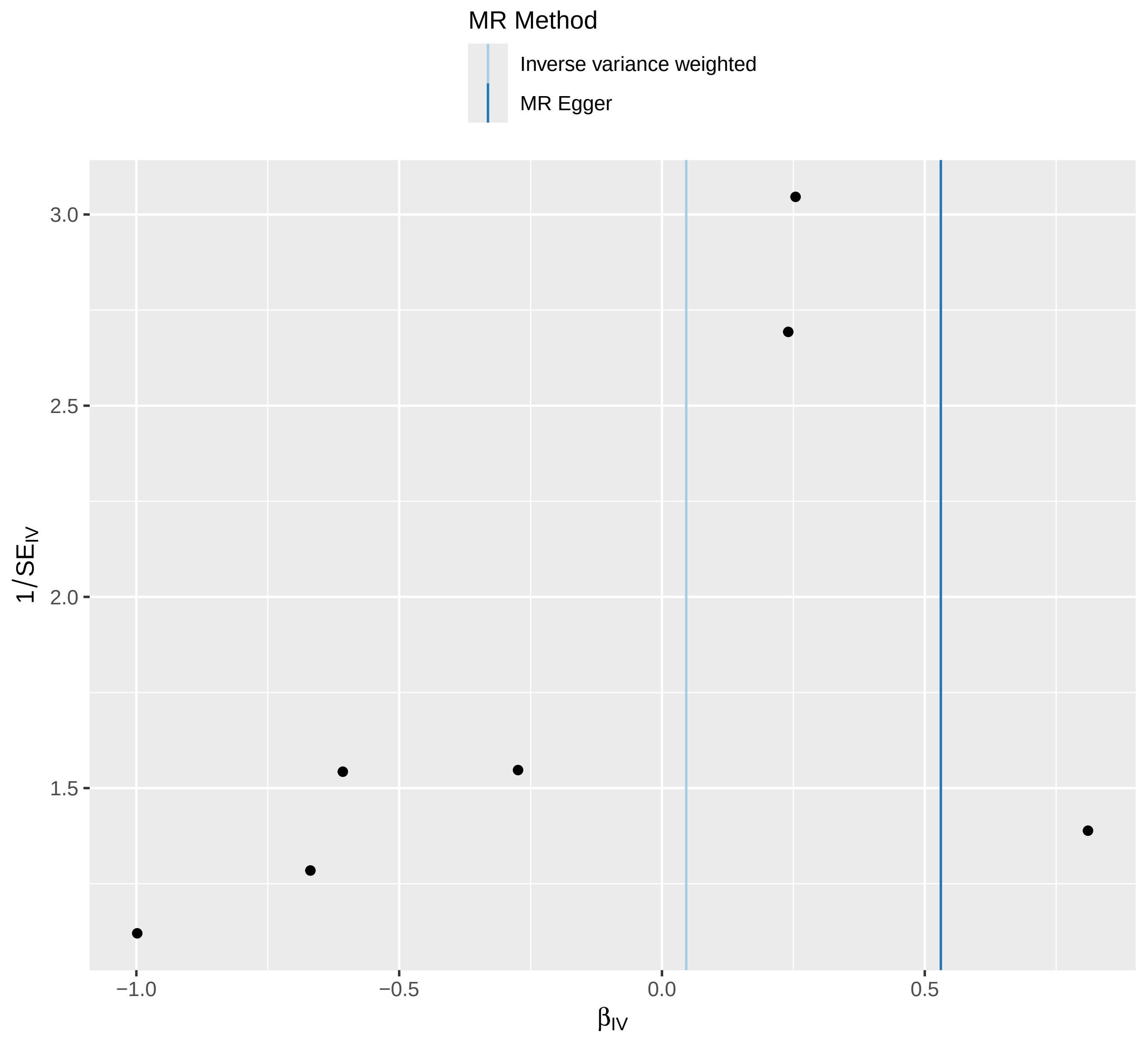
MR Method
Inverse variance weighted
MR Egger
3.0
2.5
2.0
1.5
$1/SE_{IV}$
−1.0
−0.5
0.0
0.5
β_{IV}

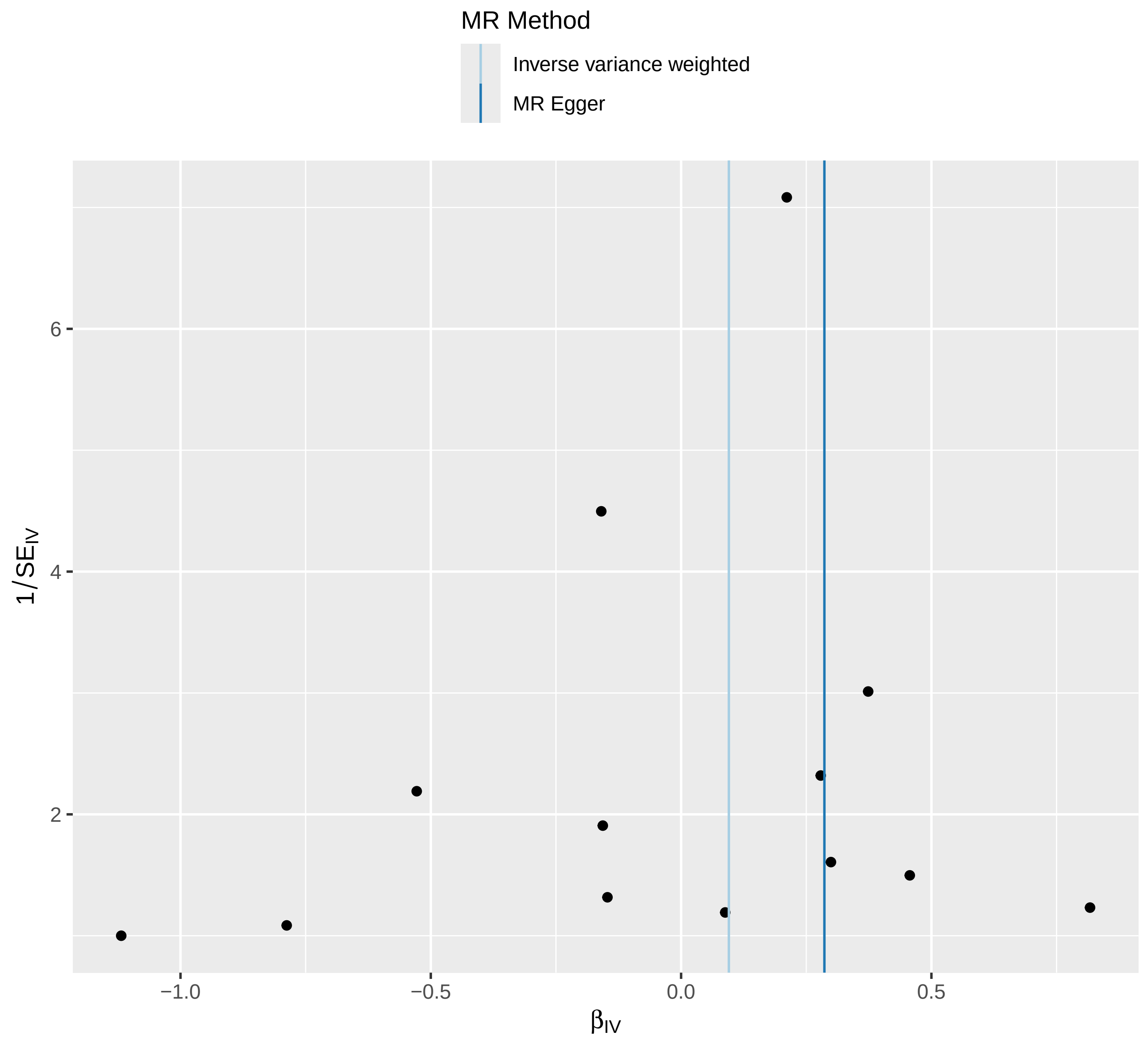
MR Method
Inverse variance weighted
MR Egger
1/SE_IV
6
4
2
−1.0
−0.5
0.0
0.5
β_IV

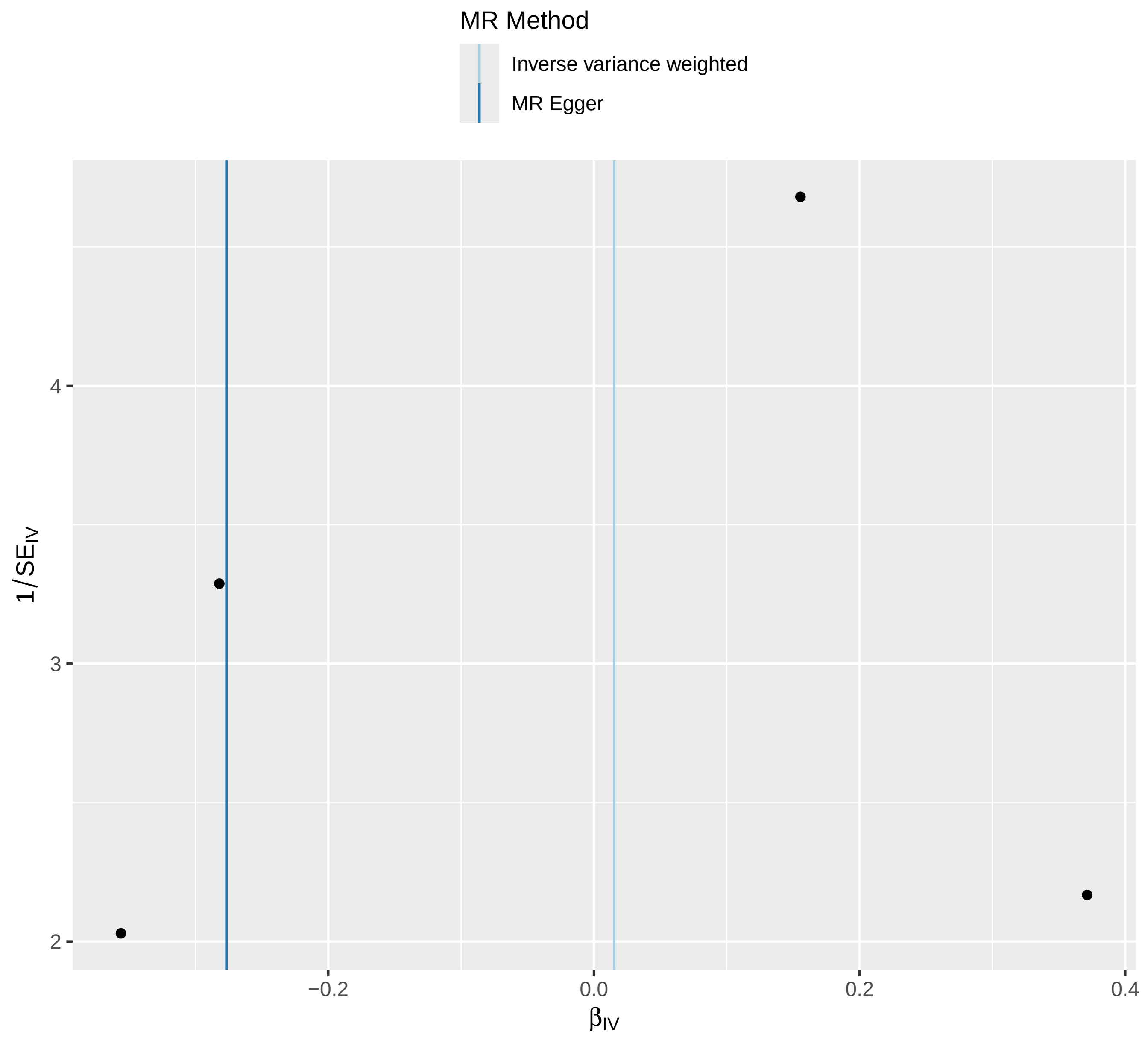

MR Method
Inverse variance weighted
MR Egger
4
3
2
1/SE$_{IV}$
−0.2
0.0
0.2
0.4
β$_{IV}$

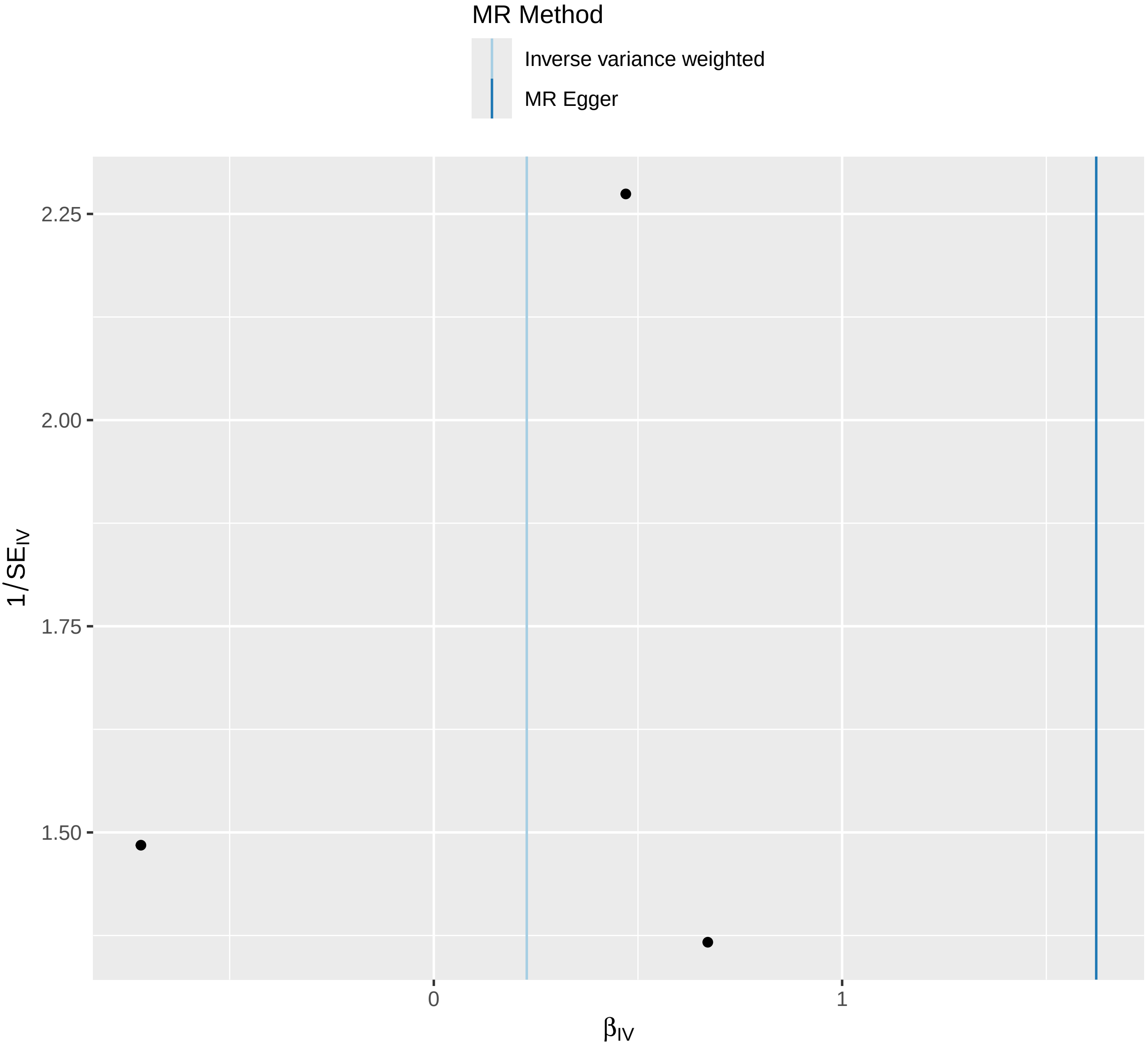
MR Method
Inverse variance weighted
MR Egger
2.25
2.00
1.75
1.50
1/SE_IV
0
1
β_IV

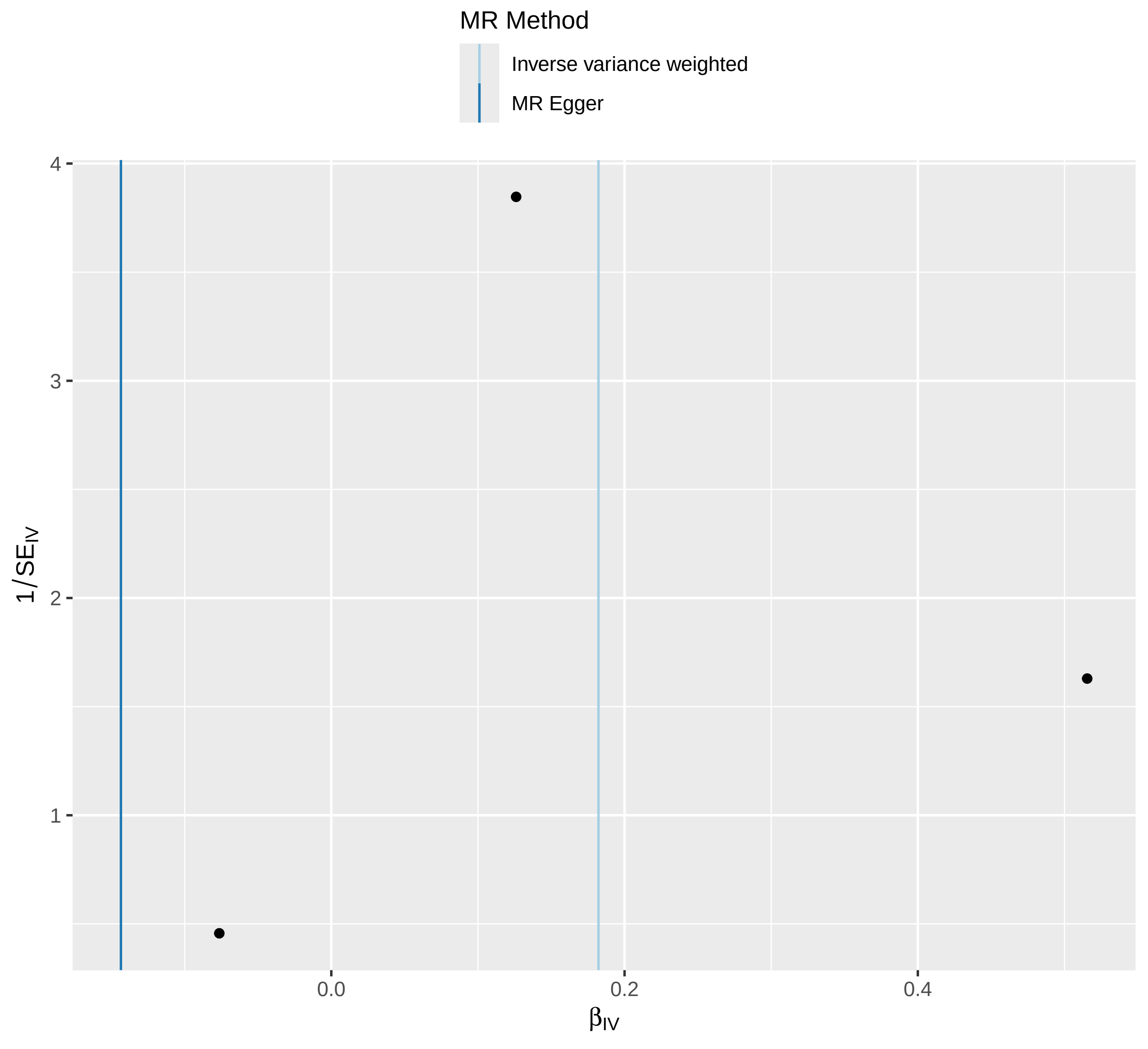

MR Method
Inverse variance weighted
MR Egger
4
3
2
1
1/SE$_{IV}$
0.0
0.2
0.4
β$_{IV}$

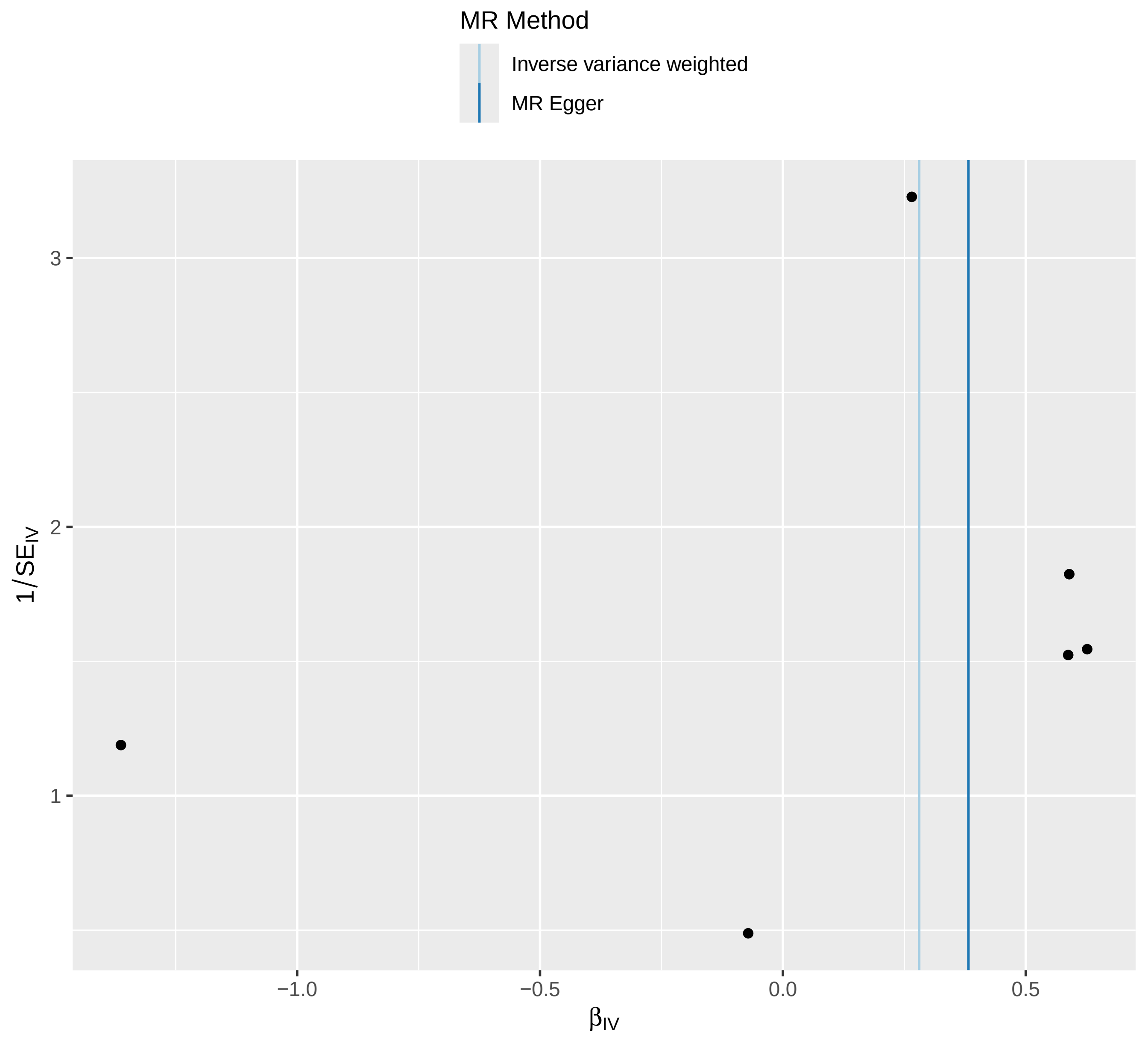
MR Method
Inverse variance weighted
MR Egger
3
2
1
1/SE_IV
−1.0
−0.5
0.0
0.5
β_IV

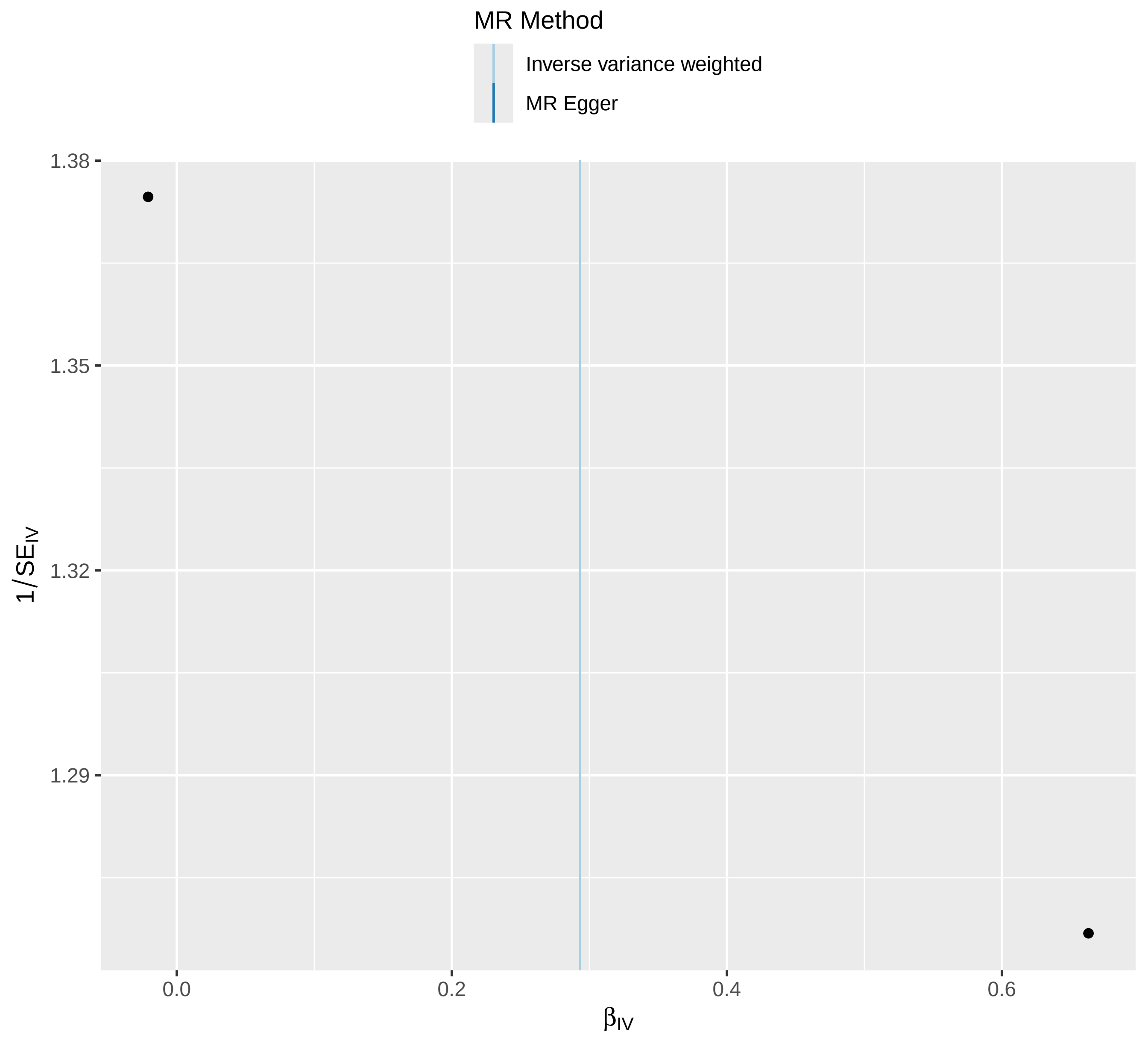
MR Method
Inverse variance weighted
MR Egger
1.38
1.35
1.32
1.29
$1/SE_{IV}$
0.0
0.2
0.4
0.6
β_{IV}

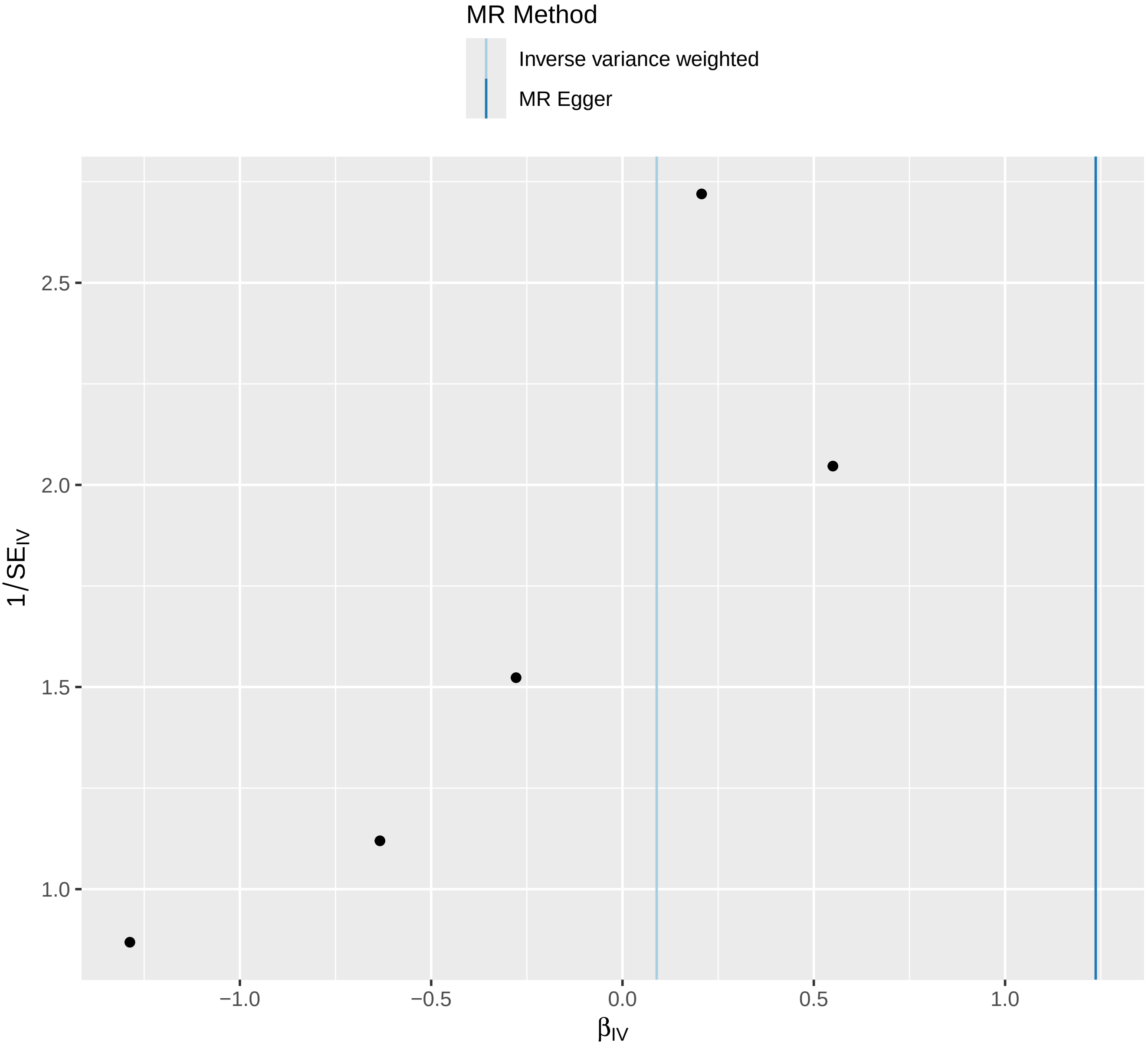

MR Method
Inverse variance weighted
MR Egger
2.5
2.0
1.5
1.0
1/SE_IV
−1.0
−0.5
0.0
0.5
1.0
β_IV

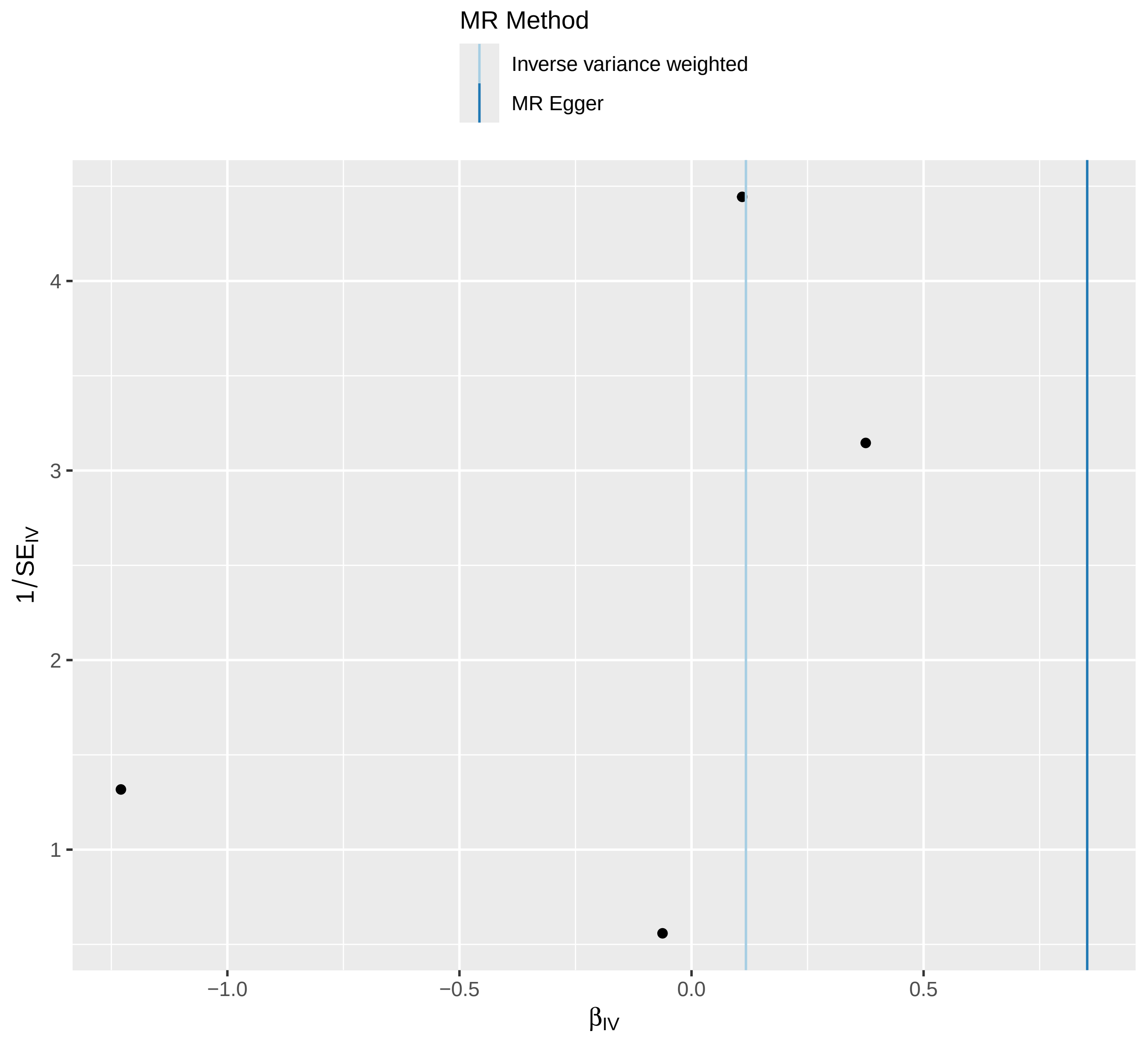
MR Method
Inverse variance weighted
MR Egger
4
3
2
1
1/SE$_{IV}$
−1.0
−0.5
0.0
0.5
β$_{IV}$

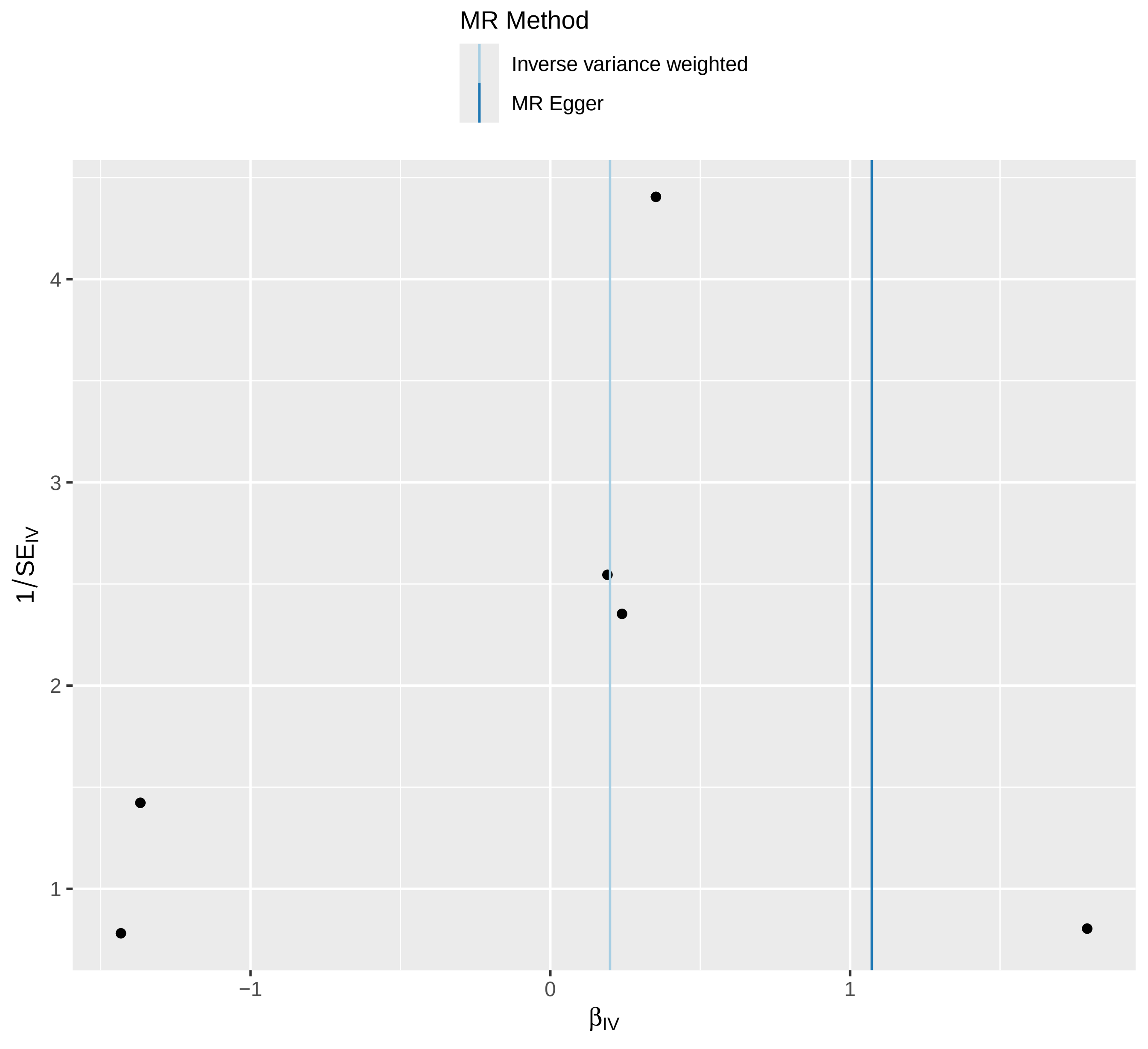

MR Method
Inverse variance weighted
MR Egger
$1/SE_{IV}$
4
3
2
1
−1
0
1
β_{IV}

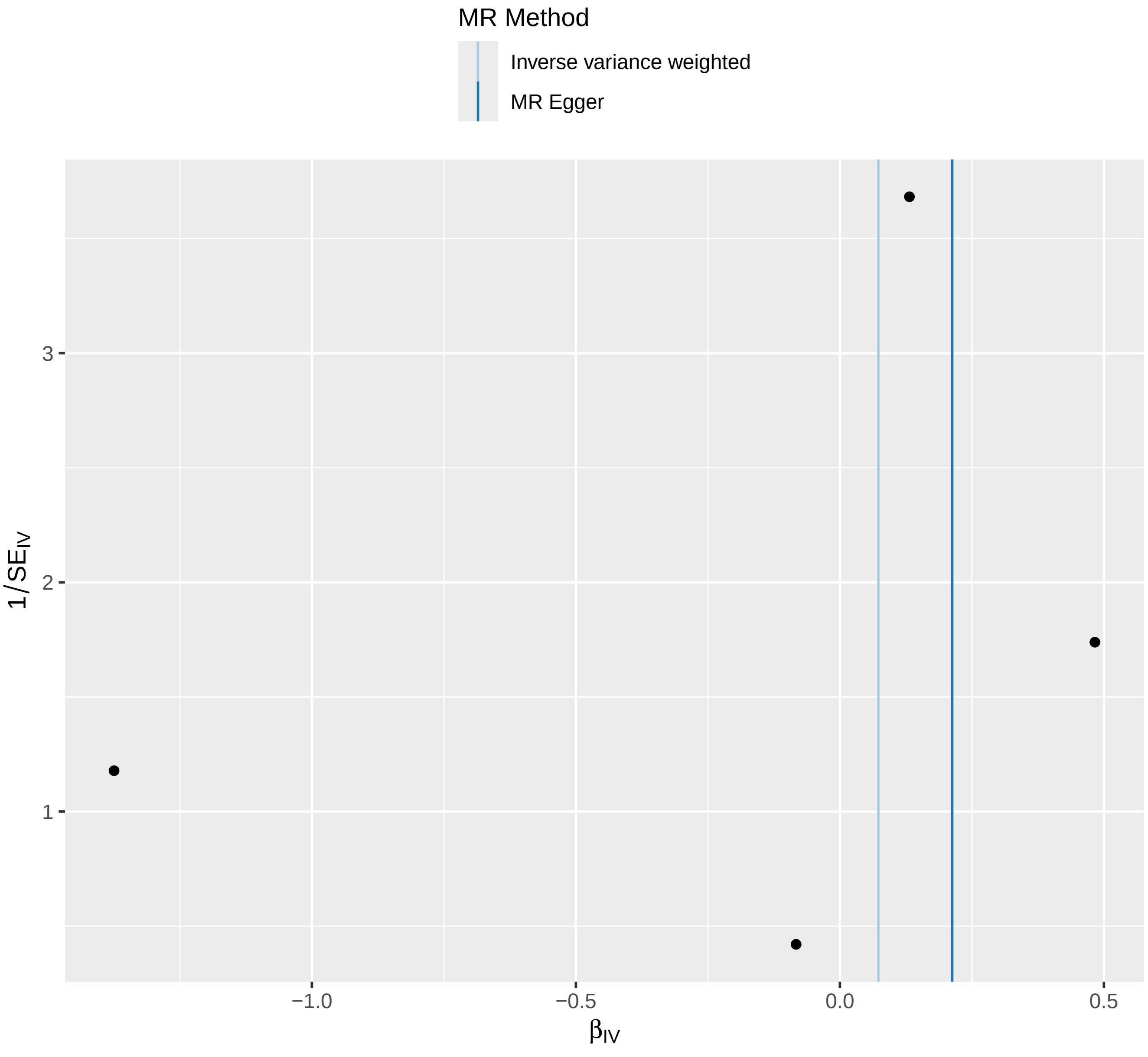
MR Method
Inverse variance weighted
MR Egger
1/SE_IV
3
2
1
−1.0
−0.5
0.0
0.5
β_IV

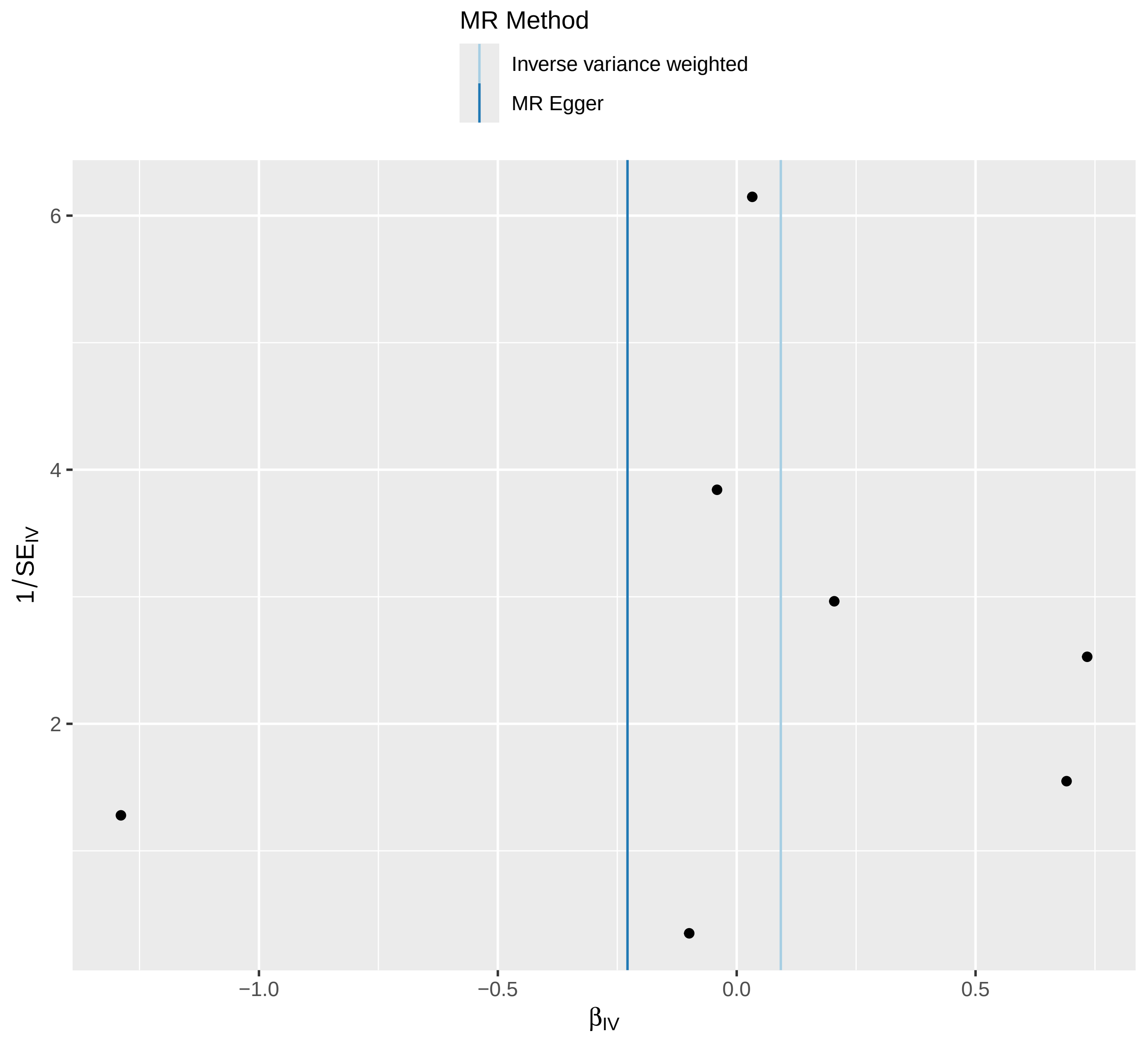

MR Method
Inverse variance weighted
MR Egger
6
4
2
1/SE_IV
−1.0
−0.5
0.0
0.5
β_IV

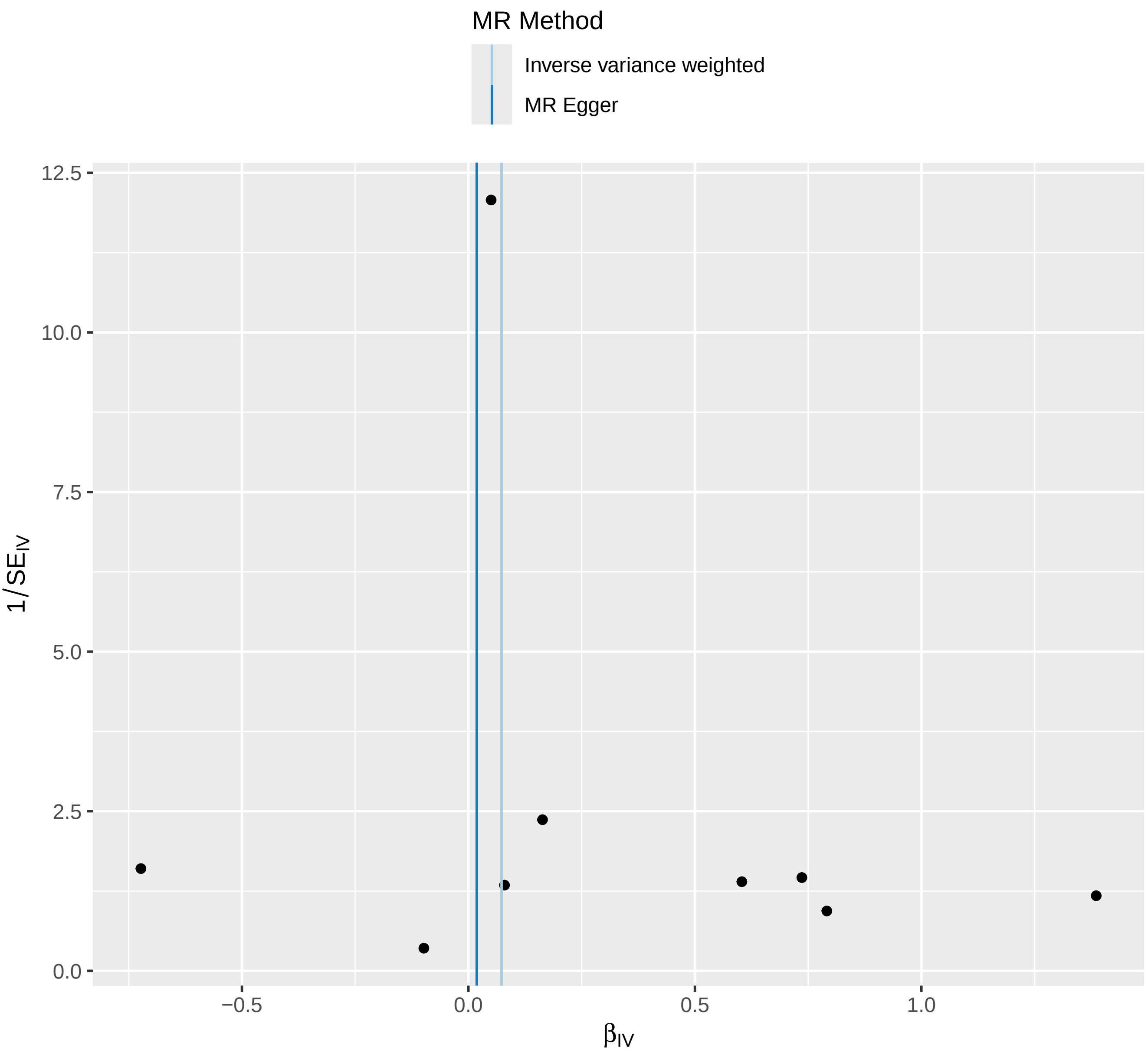
MR Method
Inverse variance weighted
MR Egger
12.5
10.0
7.5
5.0
2.5
0.0
1/SE_{IV}
−0.5
0.0
0.5
1.0
β_{IV}

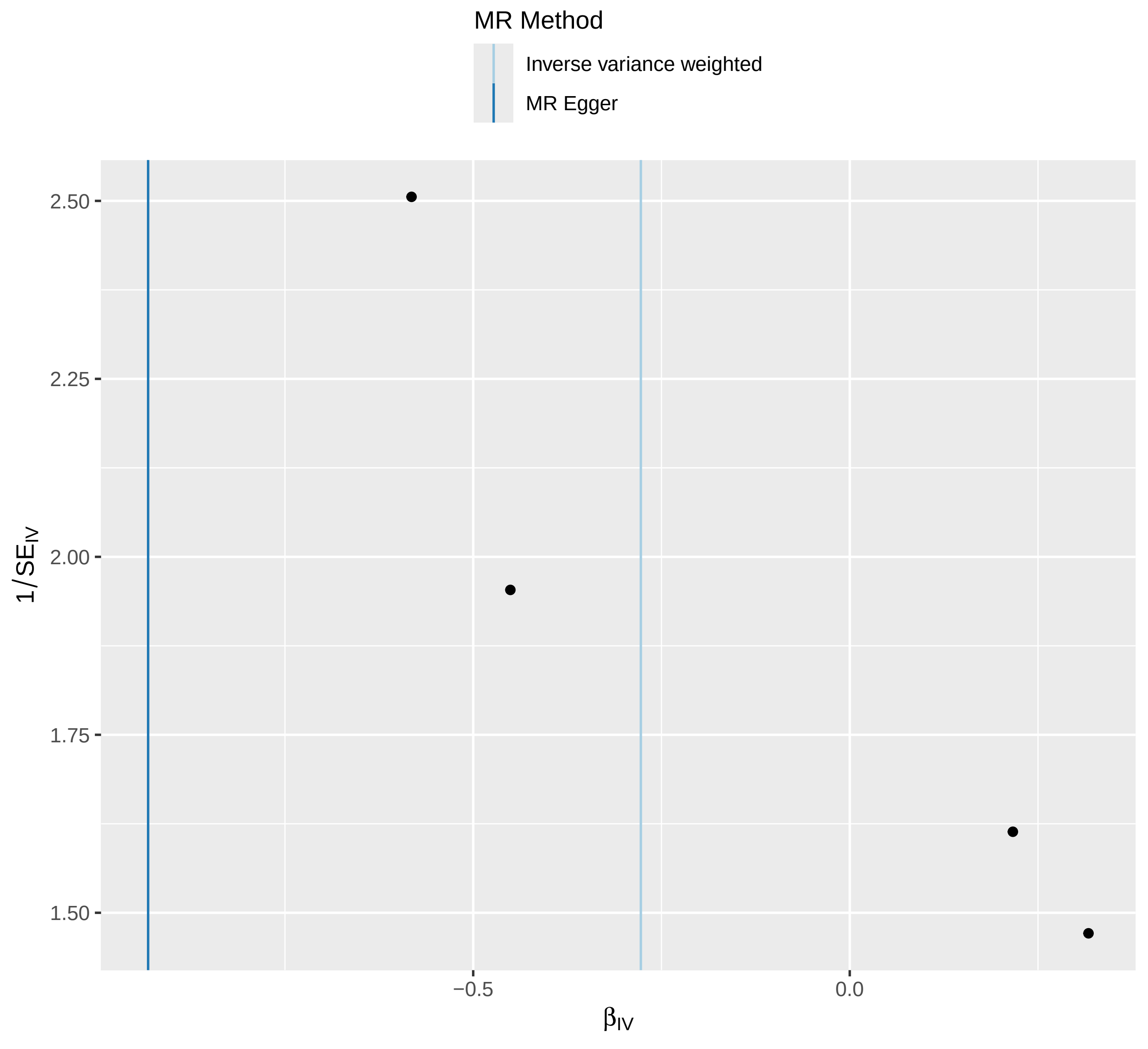
MR Method
Inverse variance weighted
MR Egger
2.50
2.25
2.00
1.75
1.50
$1/SE_{IV}$
−0.5
0.0
β_{IV}

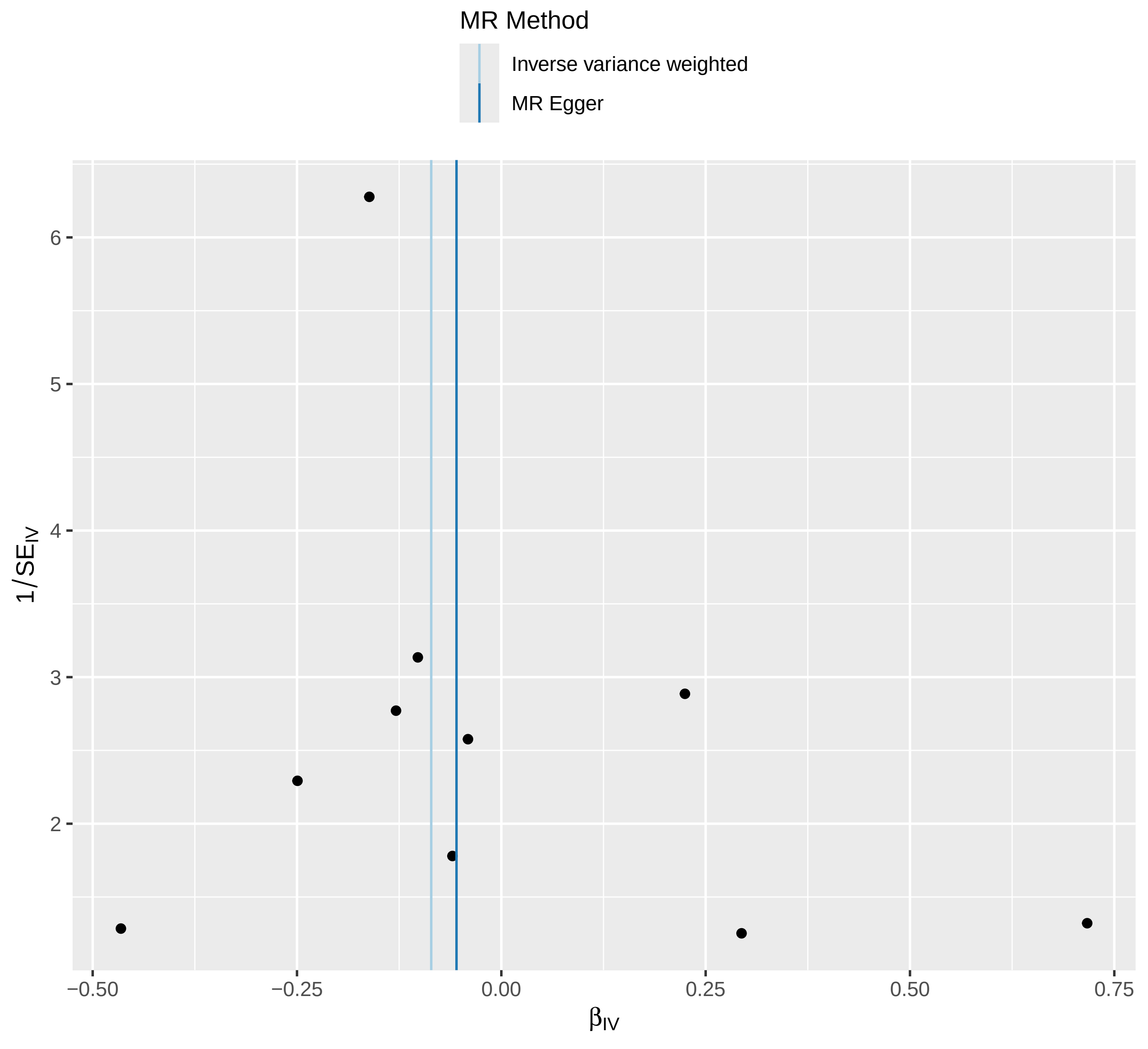

MR Method
Inverse variance weighted
MR Egger
6
5
4
3
2
1/SE$_{IV}$
−0.50
−0.25
0.00
0.25
0.50
0.75
β$_{IV}$

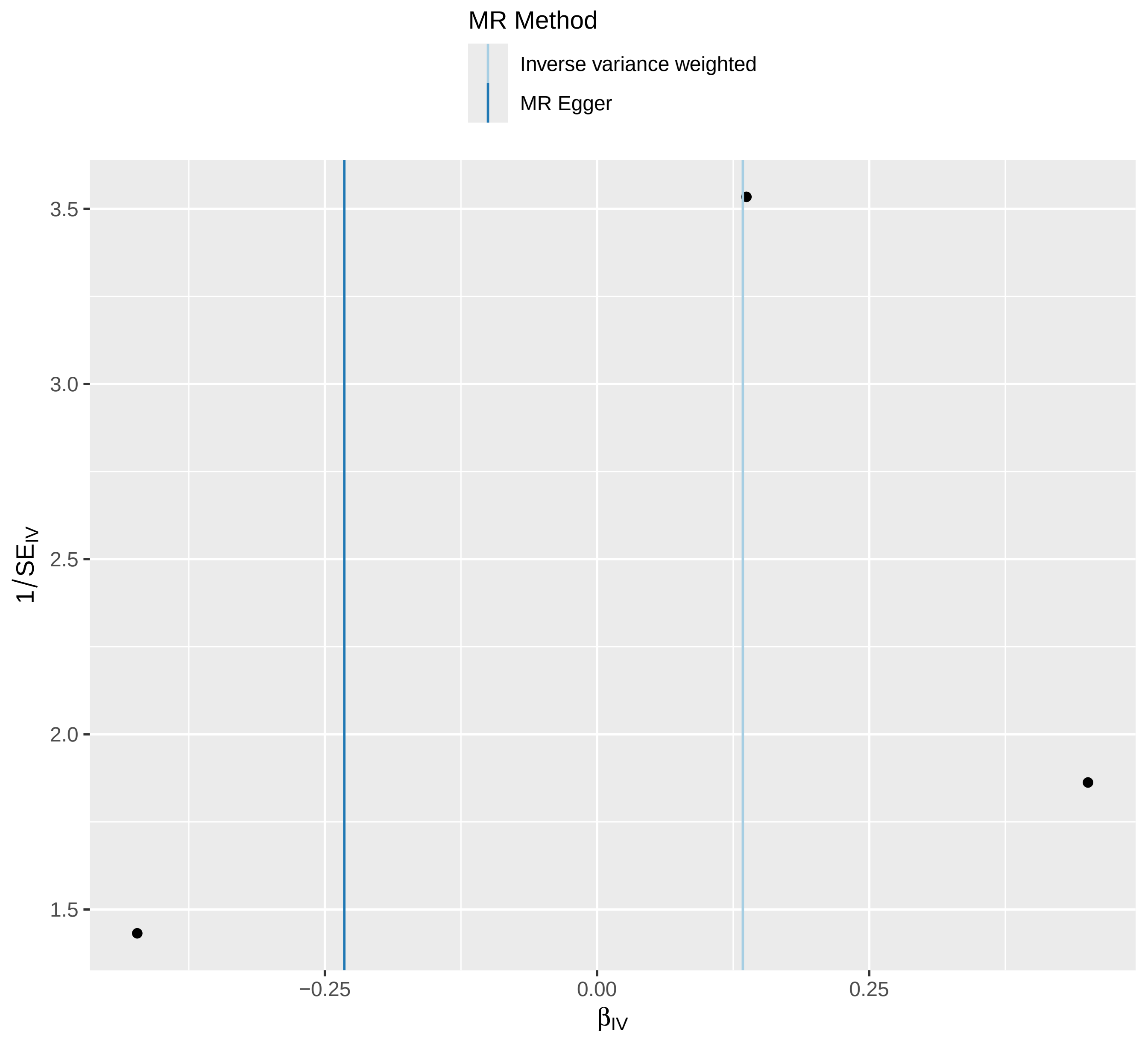
MR Method
Inverse variance weighted
MR Egger
3.5
3.0
2.5
2.0
1.5
1/SE_IV
−0.25
0.00
0.25
β_IV

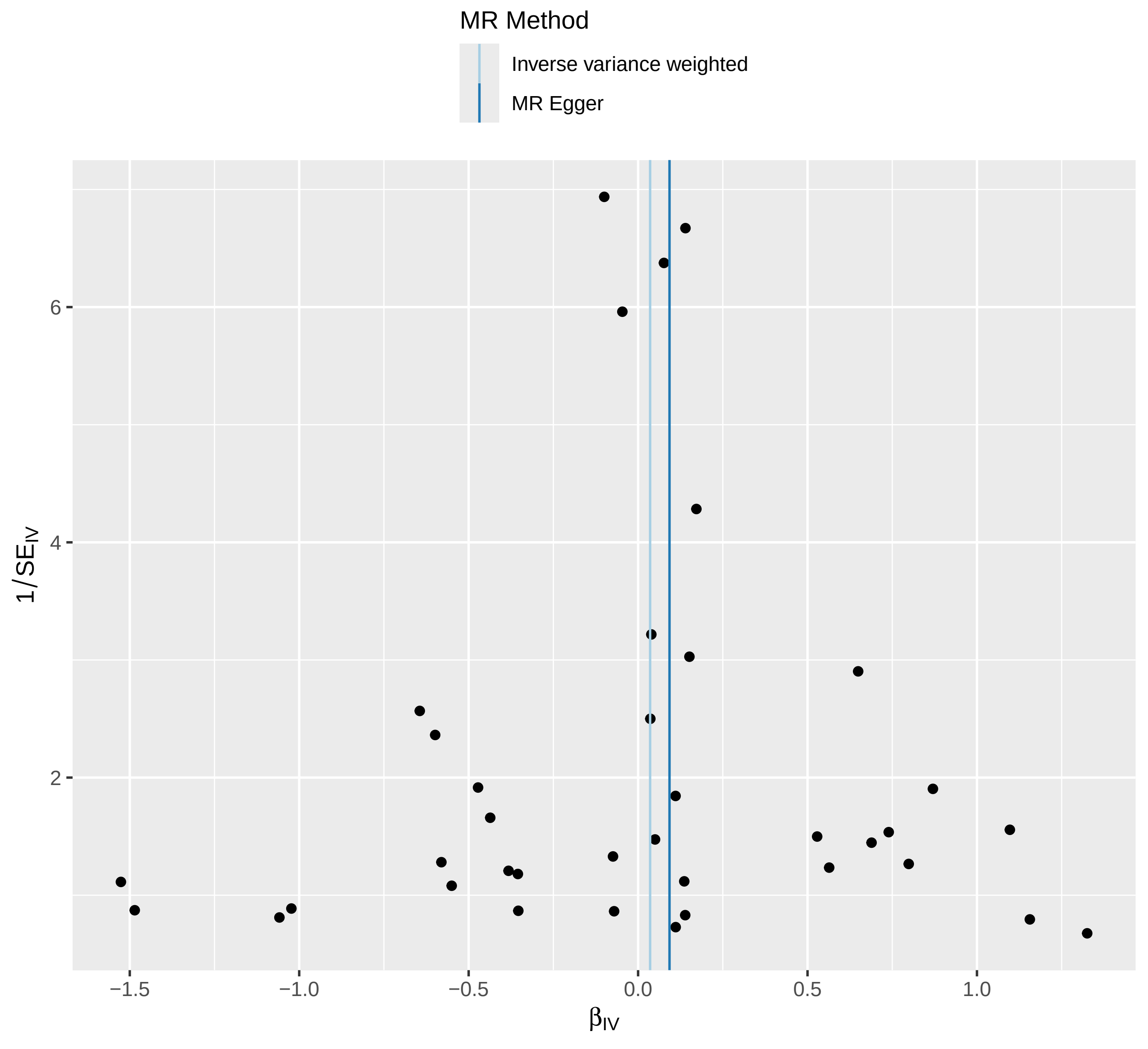
MR Method
Inverse variance weighted
MR Egger
1/SE_IV
6
4
2
−1.5
−1.0
−0.5
0.0
0.5
1.0
β_IV

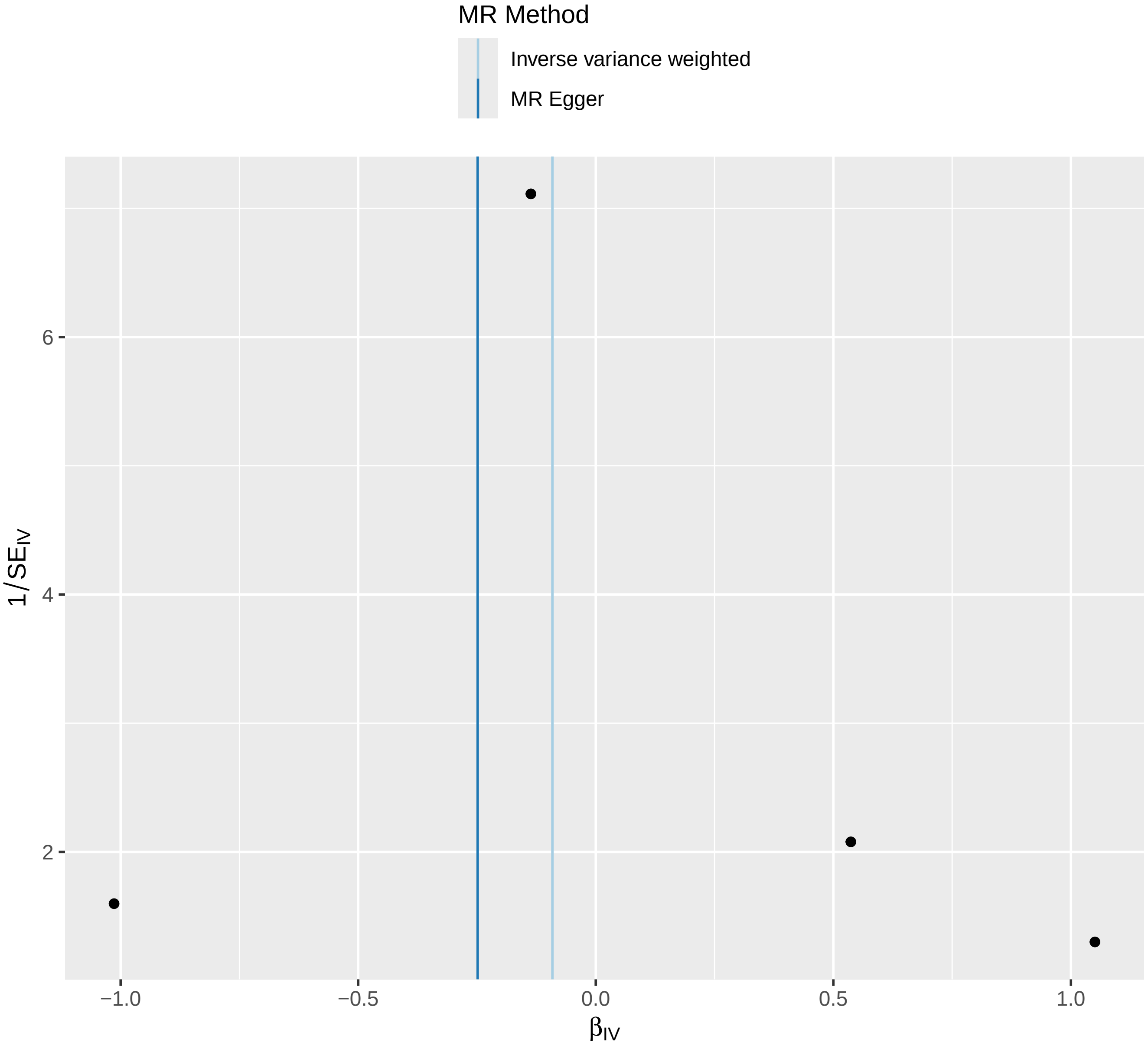
MR Method
Inverse variance weighted
MR Egger
6
4
2
1/SE$_{IV}$
−1.0
−0.5
0.0
0.5
1.0
β$_{IV}$

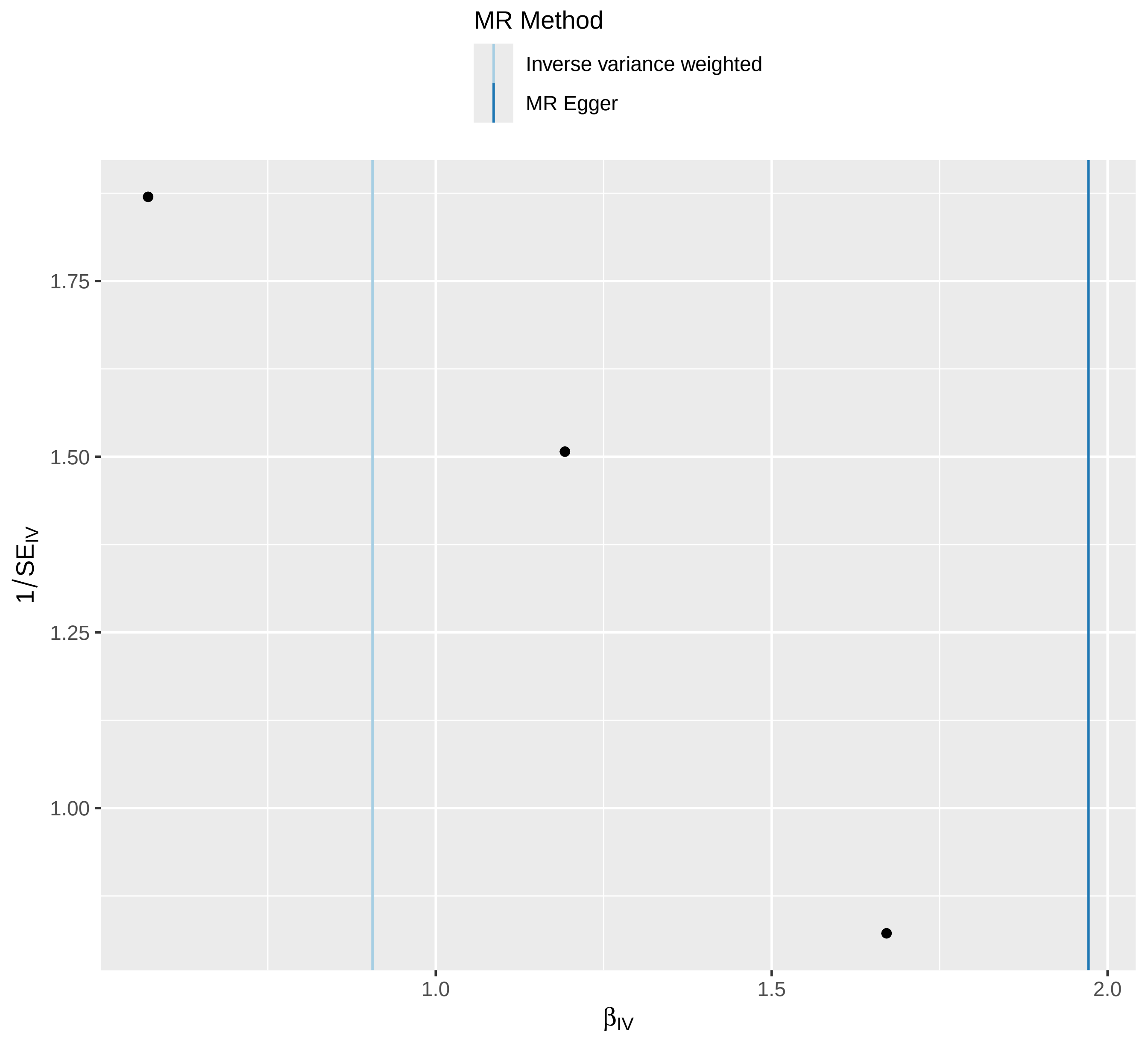
MR Method
Inverse variance weighted
MR Egger
1.75
1.50
1.25
1.00
1/SE$_{IV}$
1.0
1.5
2.0
β$_{IV}$

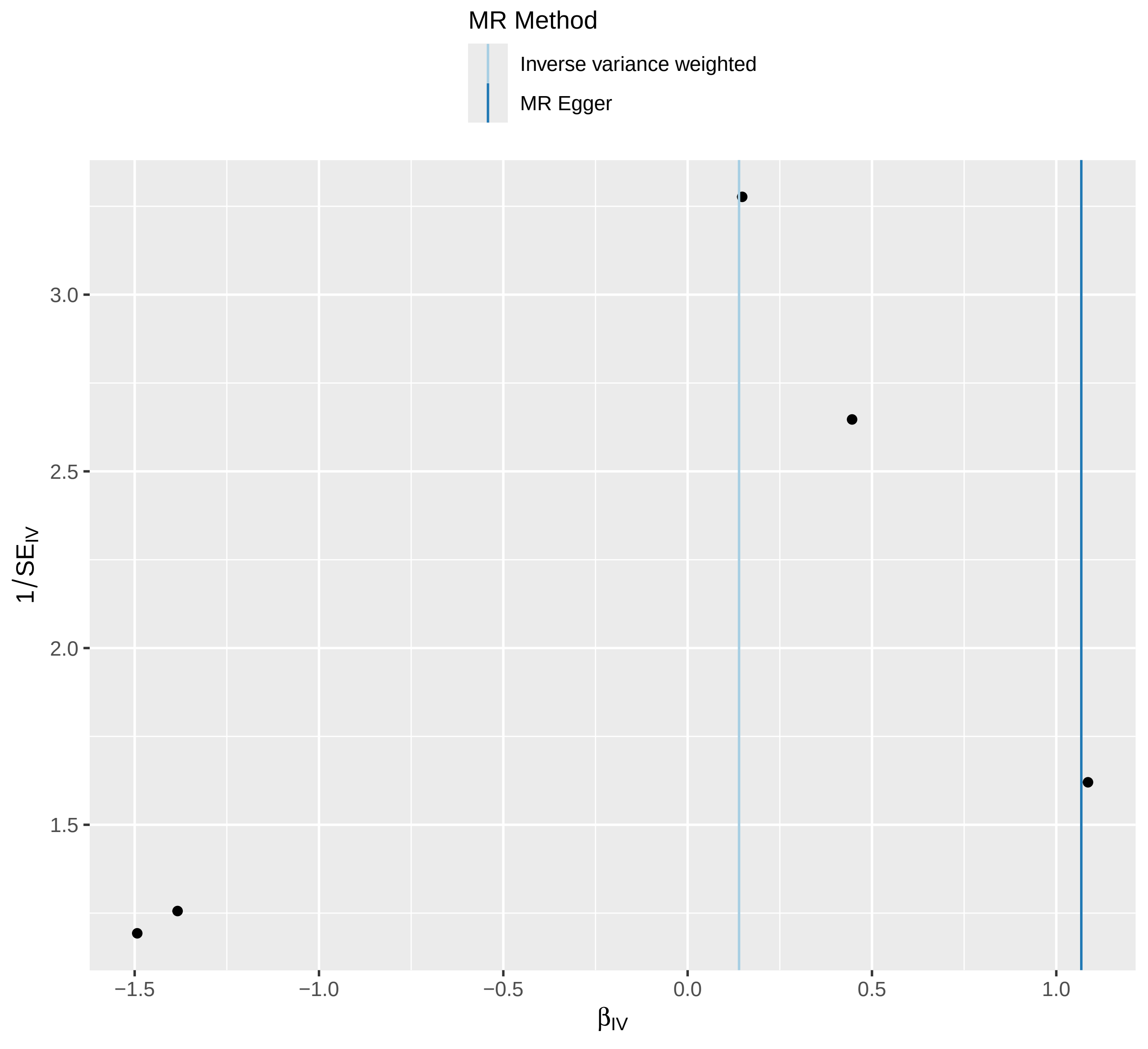

MR Method
Inverse variance weighted
MR Egger
3.0
2.5
2.0
1.5
1/SE$_{IV}$
−1.5
−1.0
−0.5
0.0
0.5
1.0
β$_{IV}$

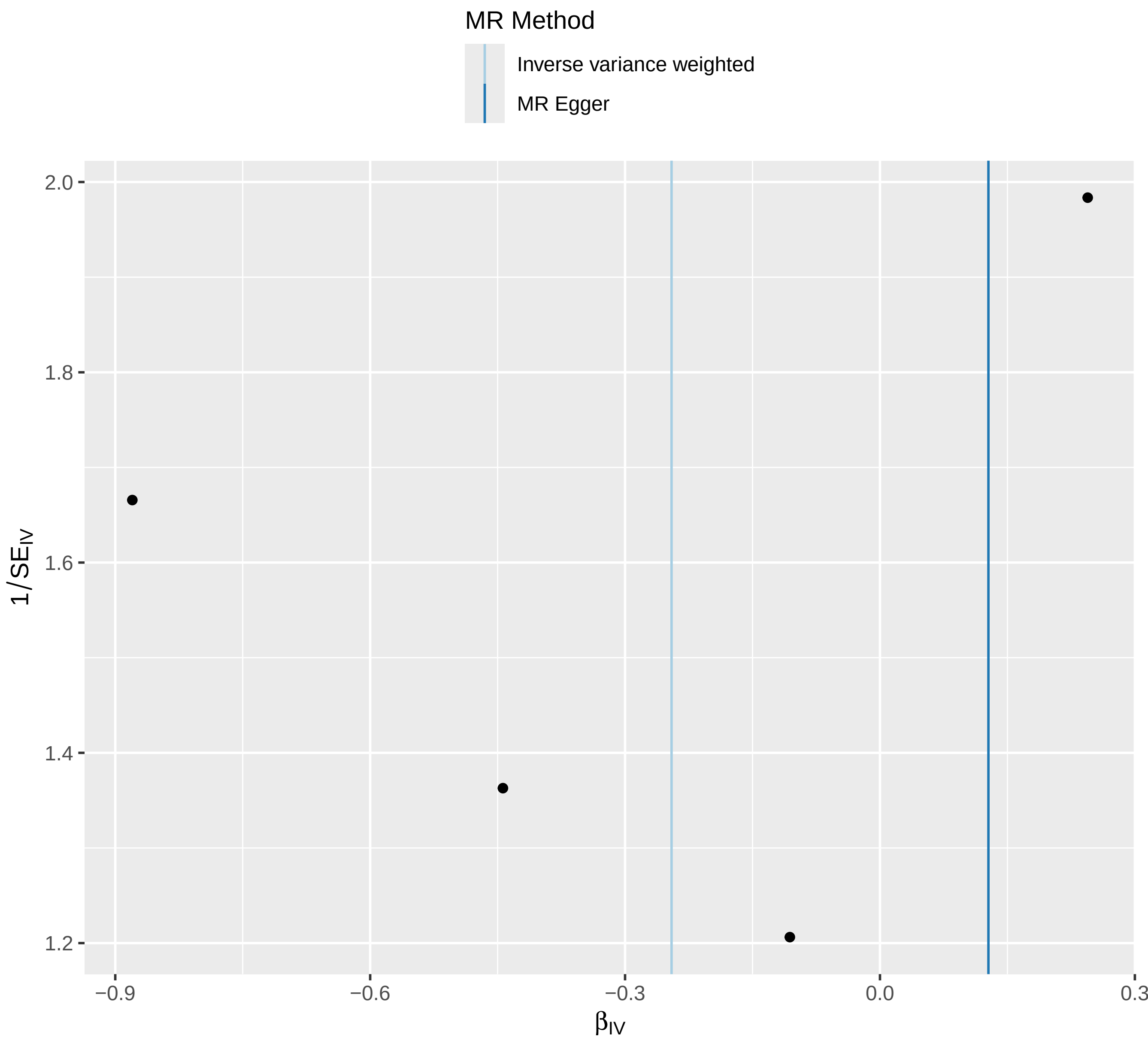
MR Method
Inverse variance weighted
MR Egger
2.0
1.8
1.6
1.4
1.2
1/SE_IV
−0.9
−0.6
−0.3
0.0
0.3
β_IV

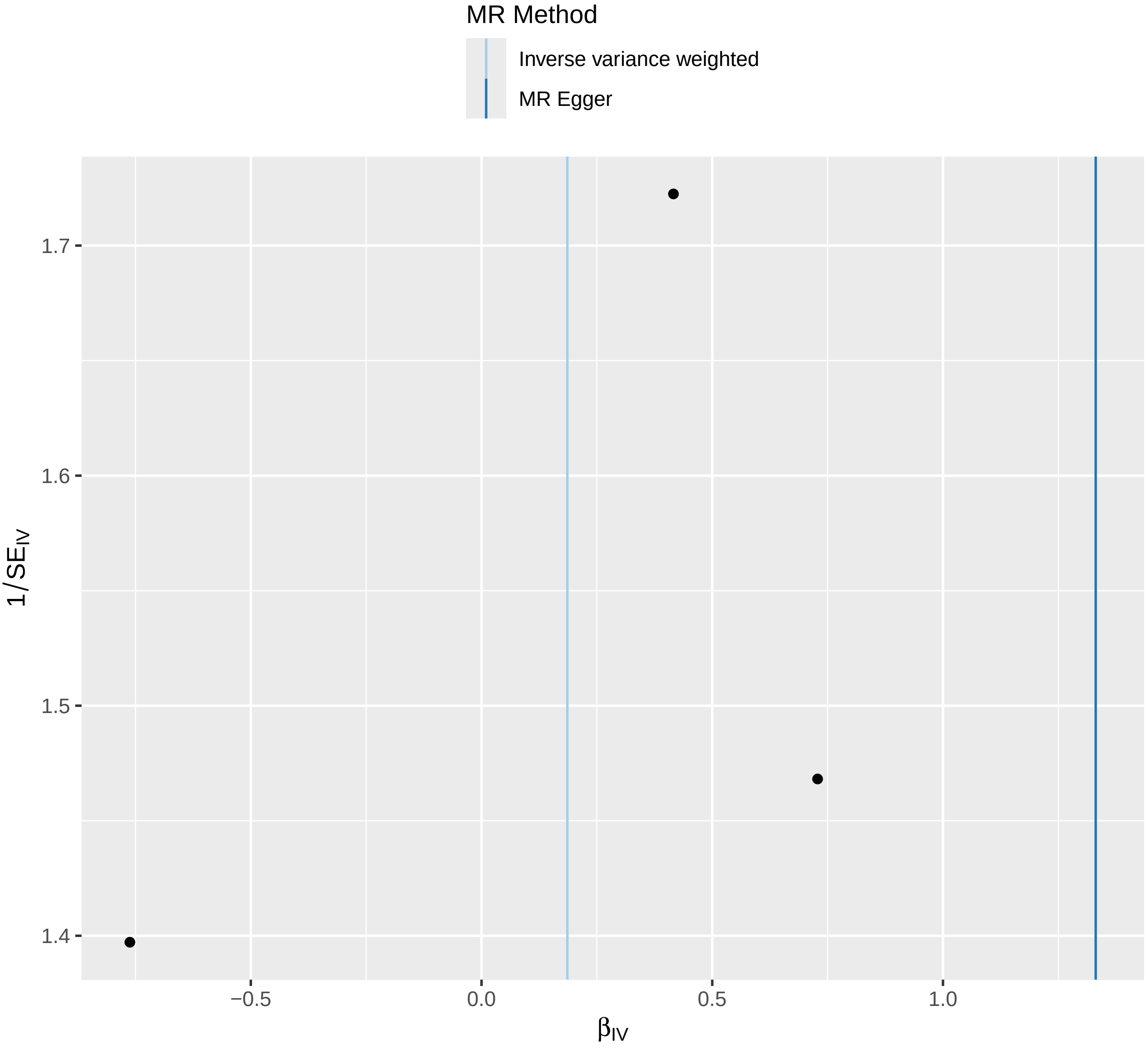
MR Method
Inverse variance weighted
MR Egger
1.7
1.6
1.5
1.4
1/SE$_{IV}$
−0.5
0.0
0.5
1.0
β$_{IV}$

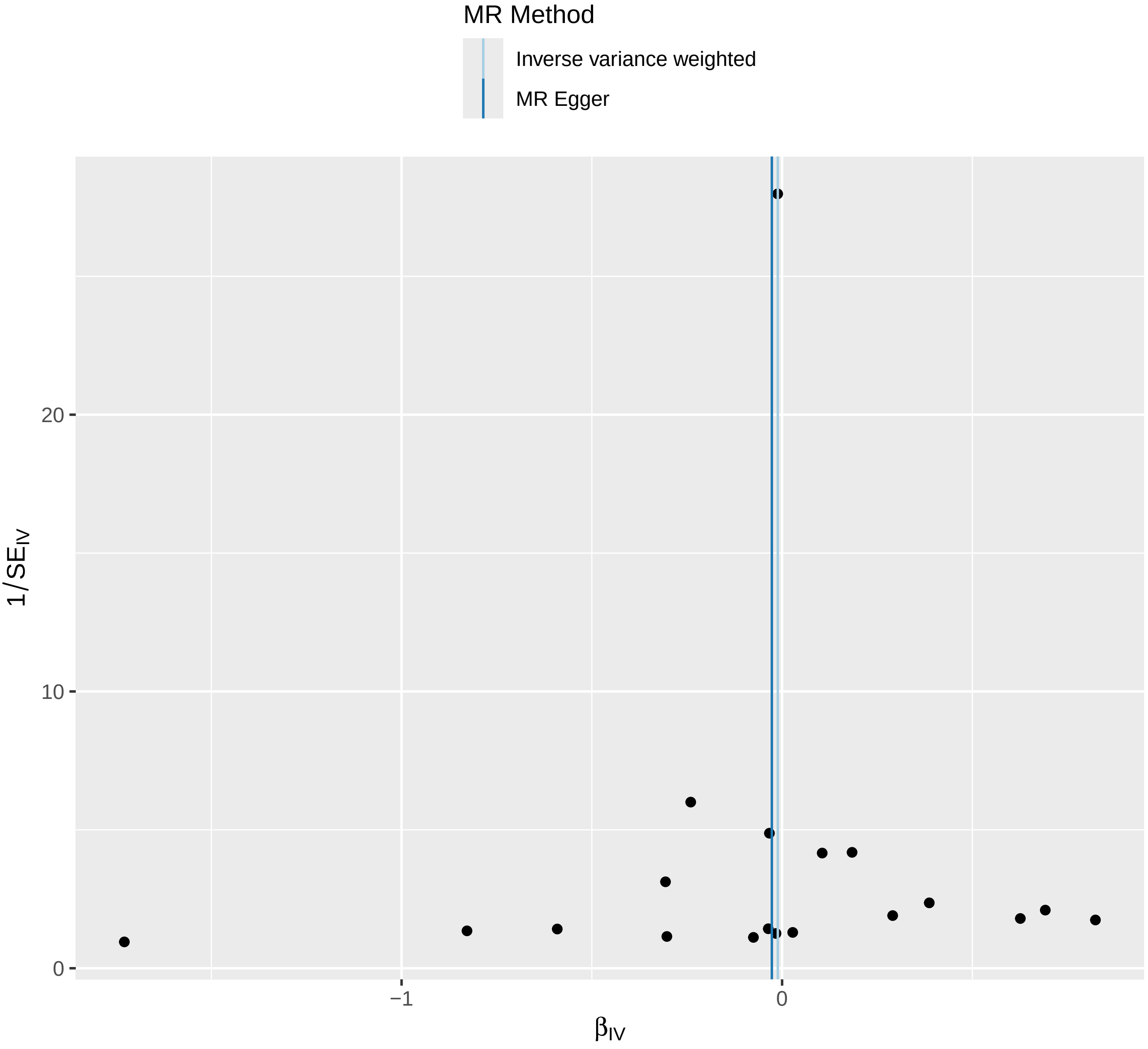
MR Method
Inverse variance weighted
MR Egger
20
10
0
−1
0
1/SE_IV
β_IV

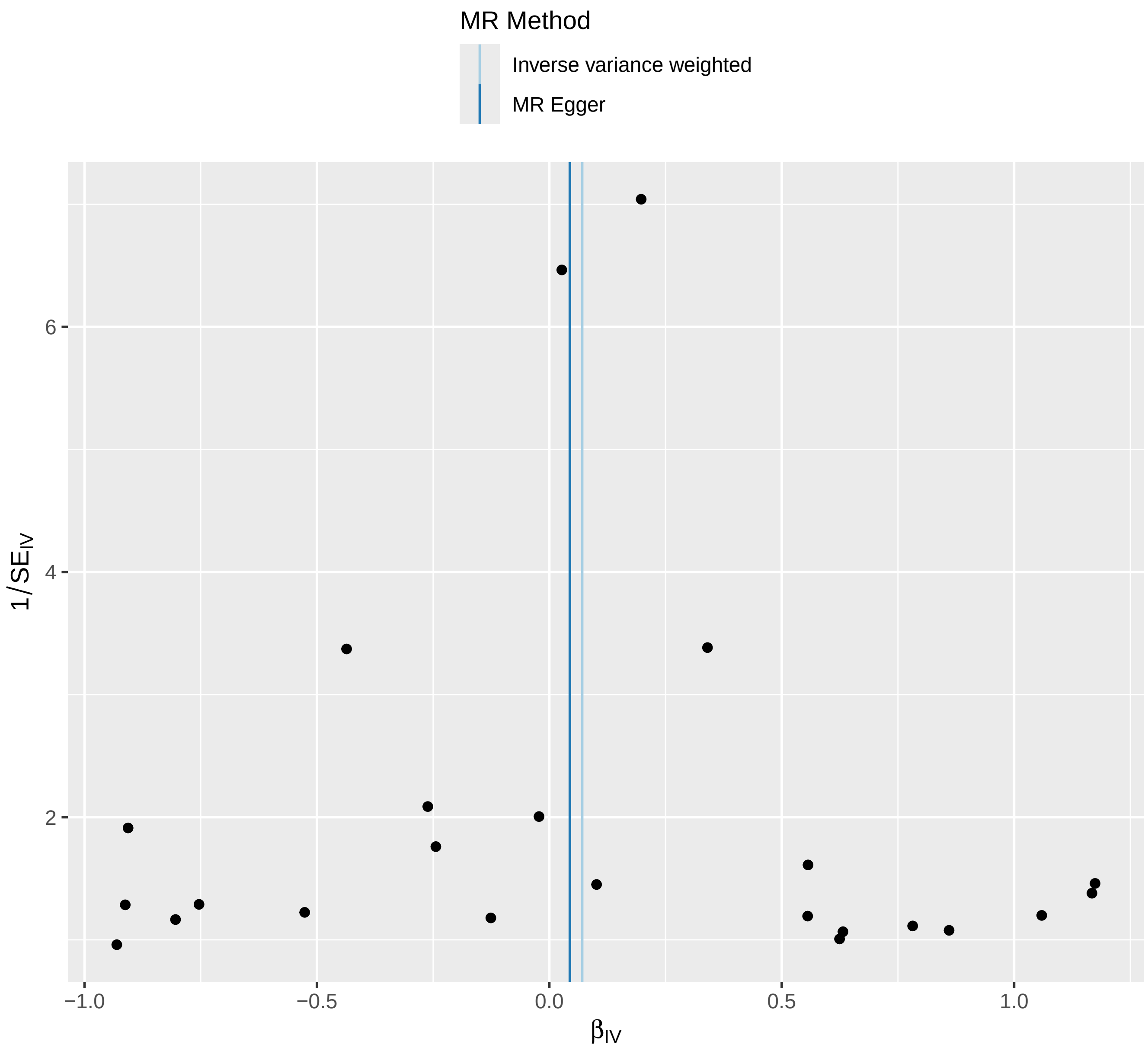

MR Method
Inverse variance weighted
MR Egger
6
4
2
1/SE$_{IV}$
−1.0
−0.5
0.0
0.5
1.0
β$_{IV}$

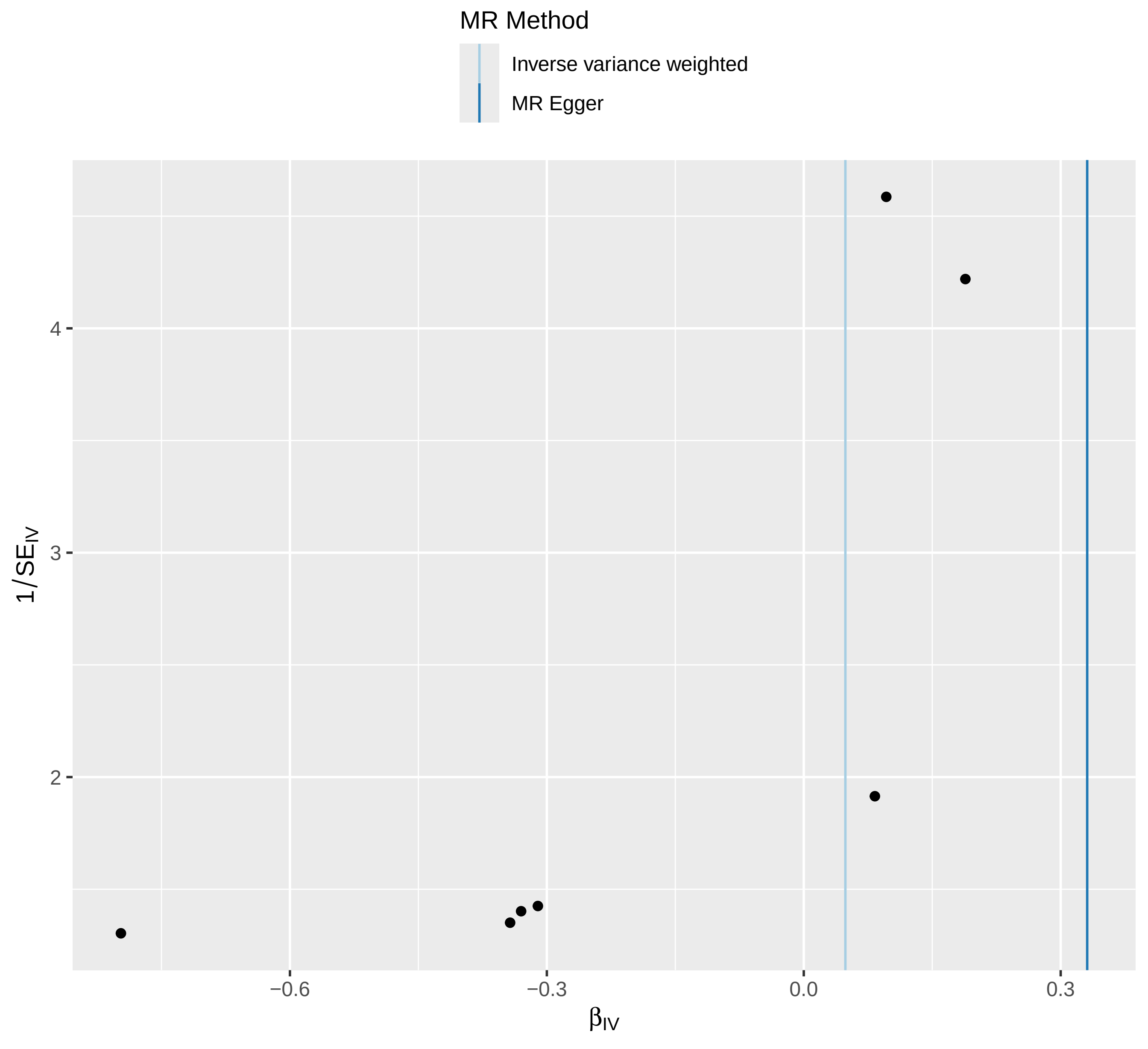
MR Method
Inverse variance weighted
MR Egger
4
3
2
1/SE_IV
−0.6
−0.3
0.0
0.3
β_IV

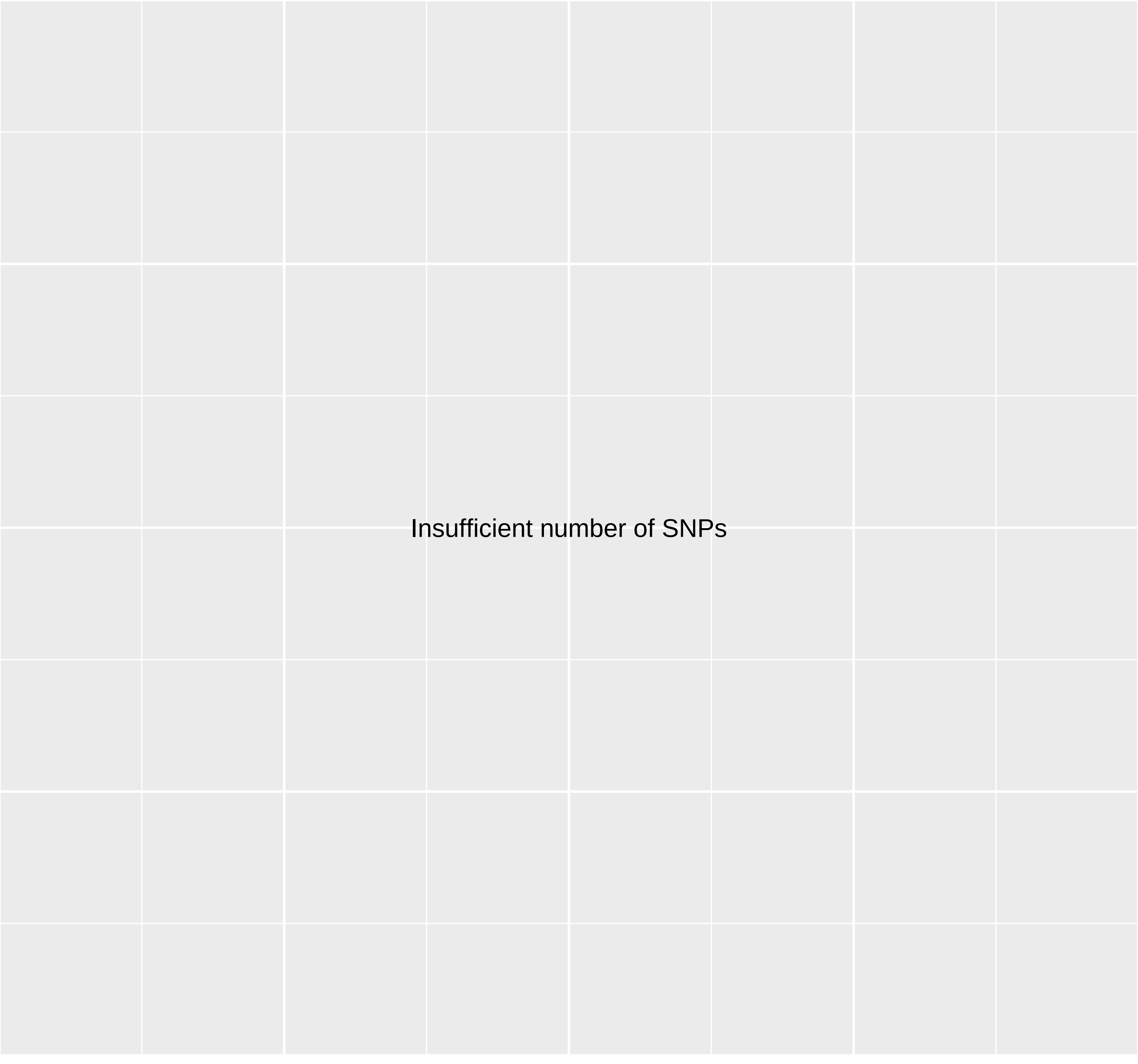
Insufficient number of SNPs

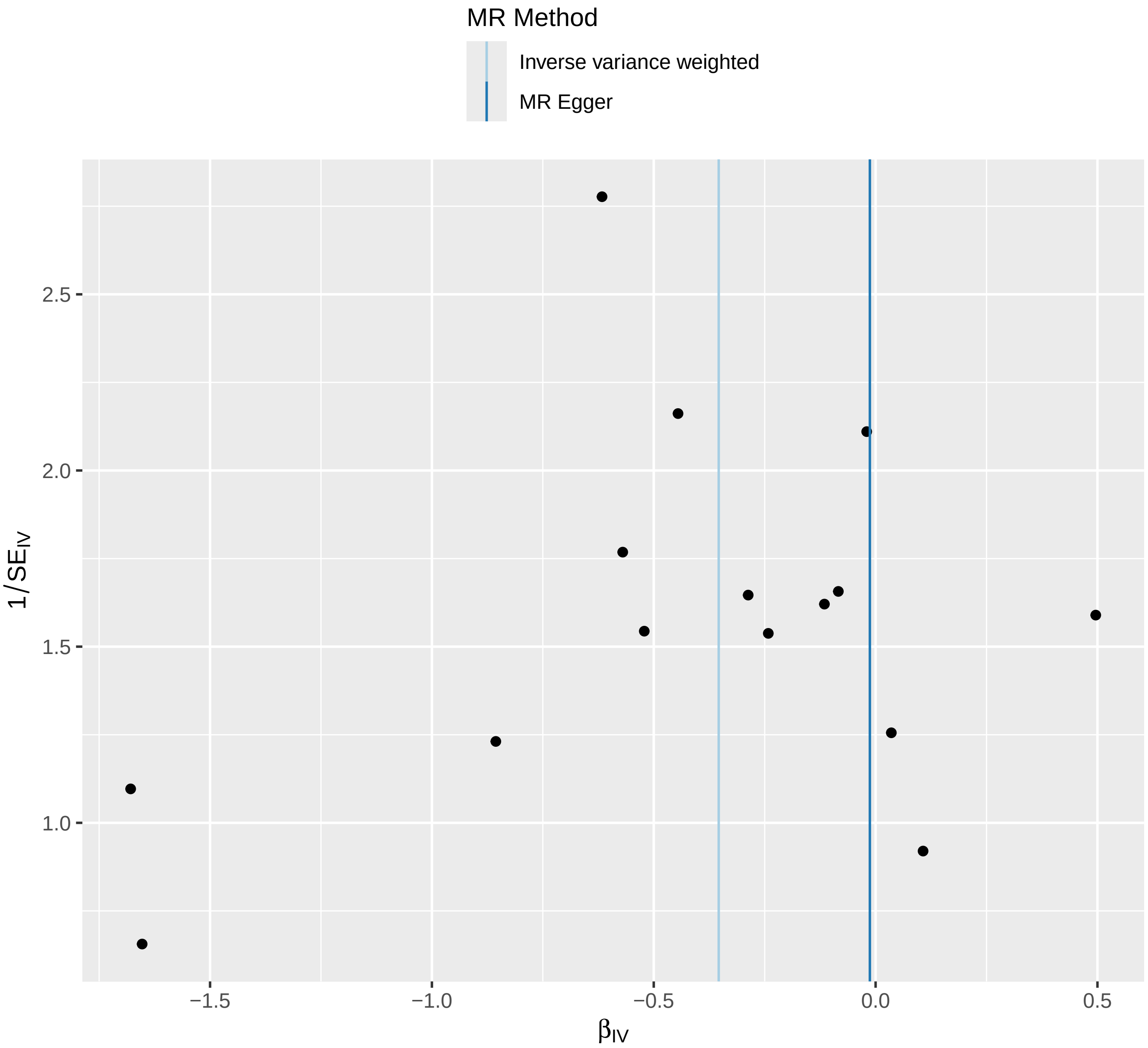

MR Method
Inverse variance weighted
MR Egger
2.5
2.0
1.5
1.0
1/SE_IV
−1.5
−1.0
−0.5
0.0
0.5
β_IV

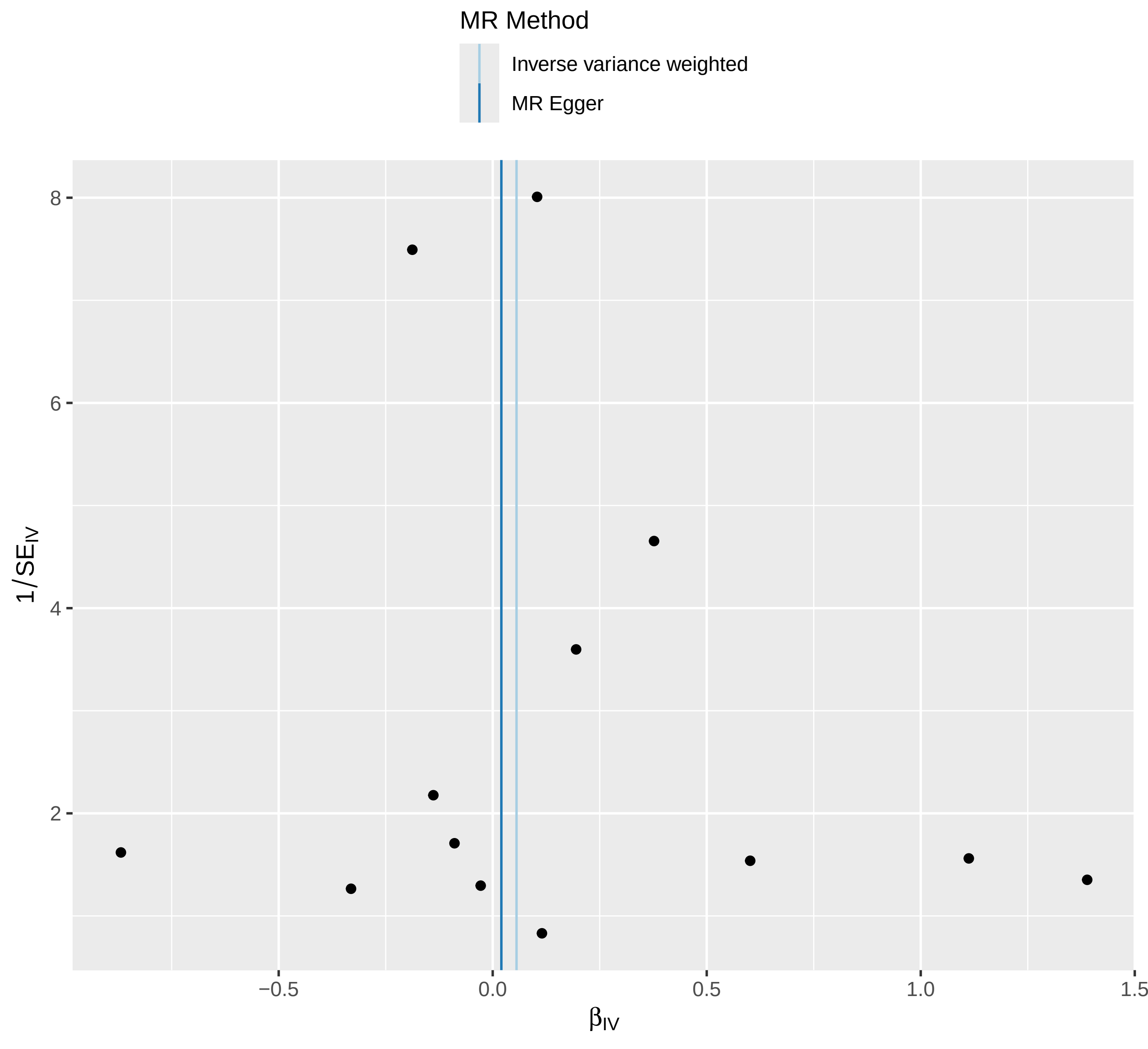

MR Method
Inverse variance weighted
MR Egger
8
6
4
2
1/SE$_{IV}$
−0.5
0.0
0.5
1.0
1.5
β$_{IV}$

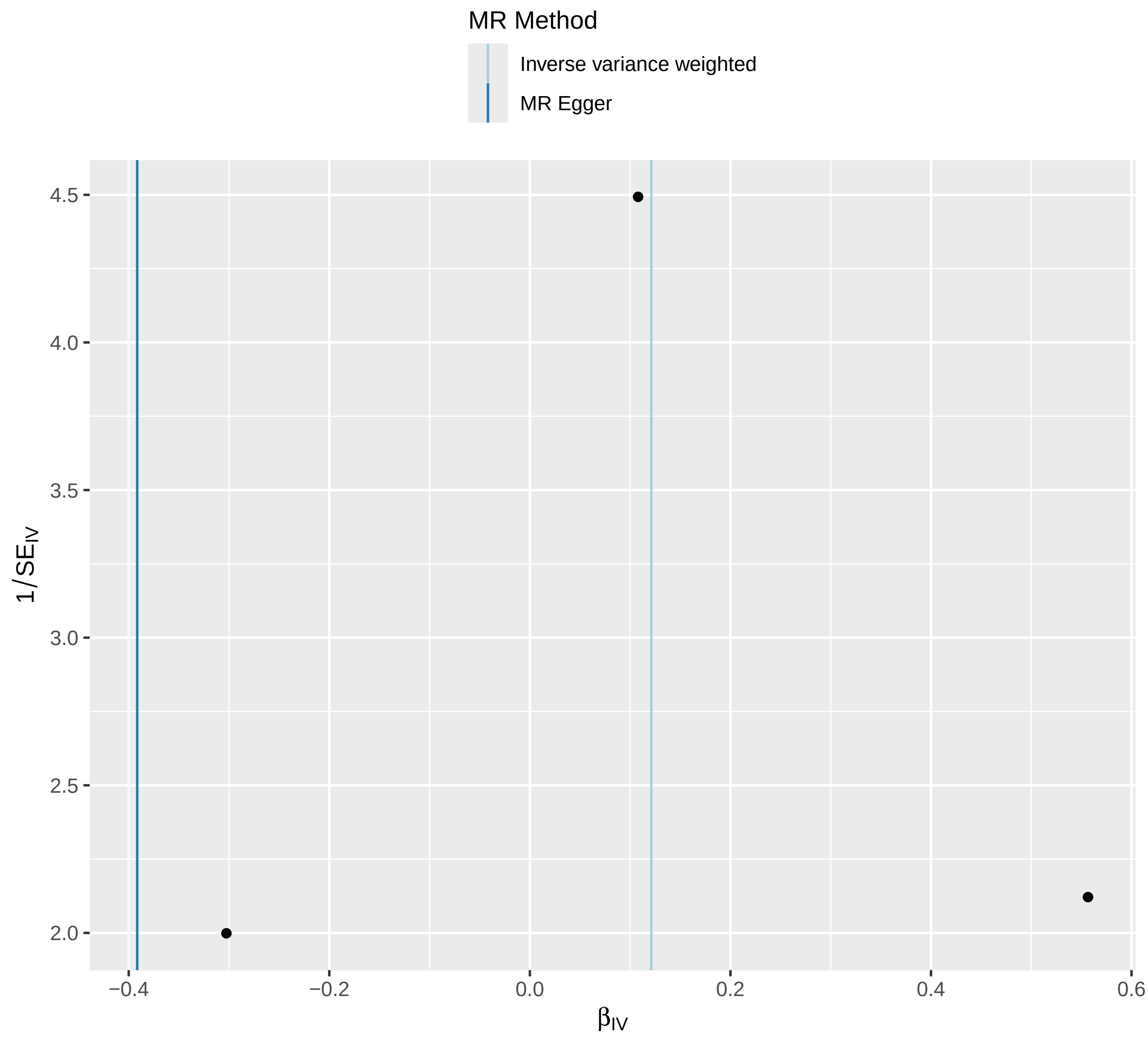
MR Method
Inverse variance weighted
MR Egger
4.5
4.0
3.5
3.0
2.5
2.0
1/SE_IV
−0.4
−0.2
0.0
0.2
0.4
0.6
β_IV

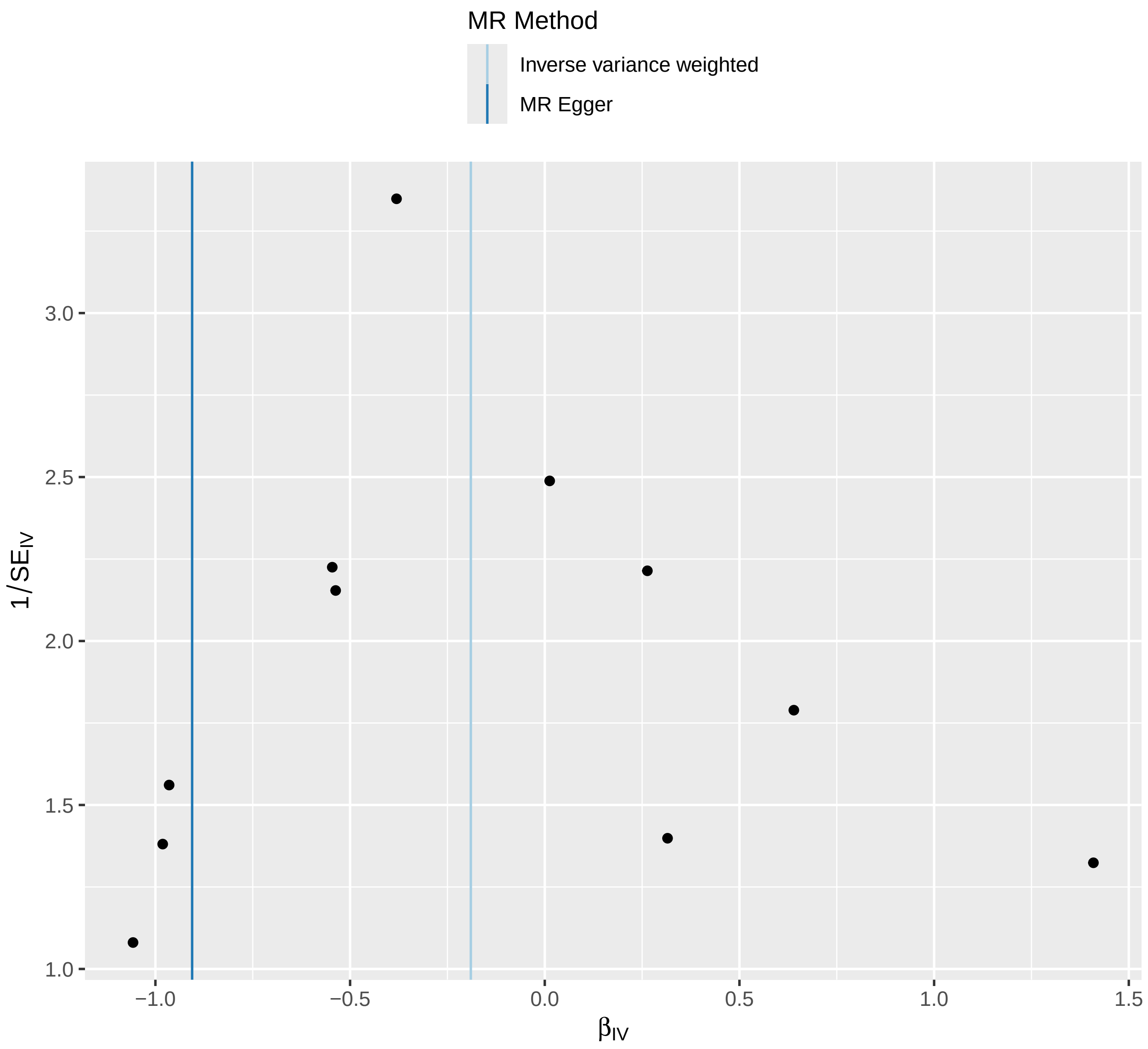
MR Method
Inverse variance weighted
MR Egger
1/SE_IV
β_IV
3.0
2.5
2.0
1.5
1.0
−1.0
−0.5
0.0
0.5
1.0
1.5

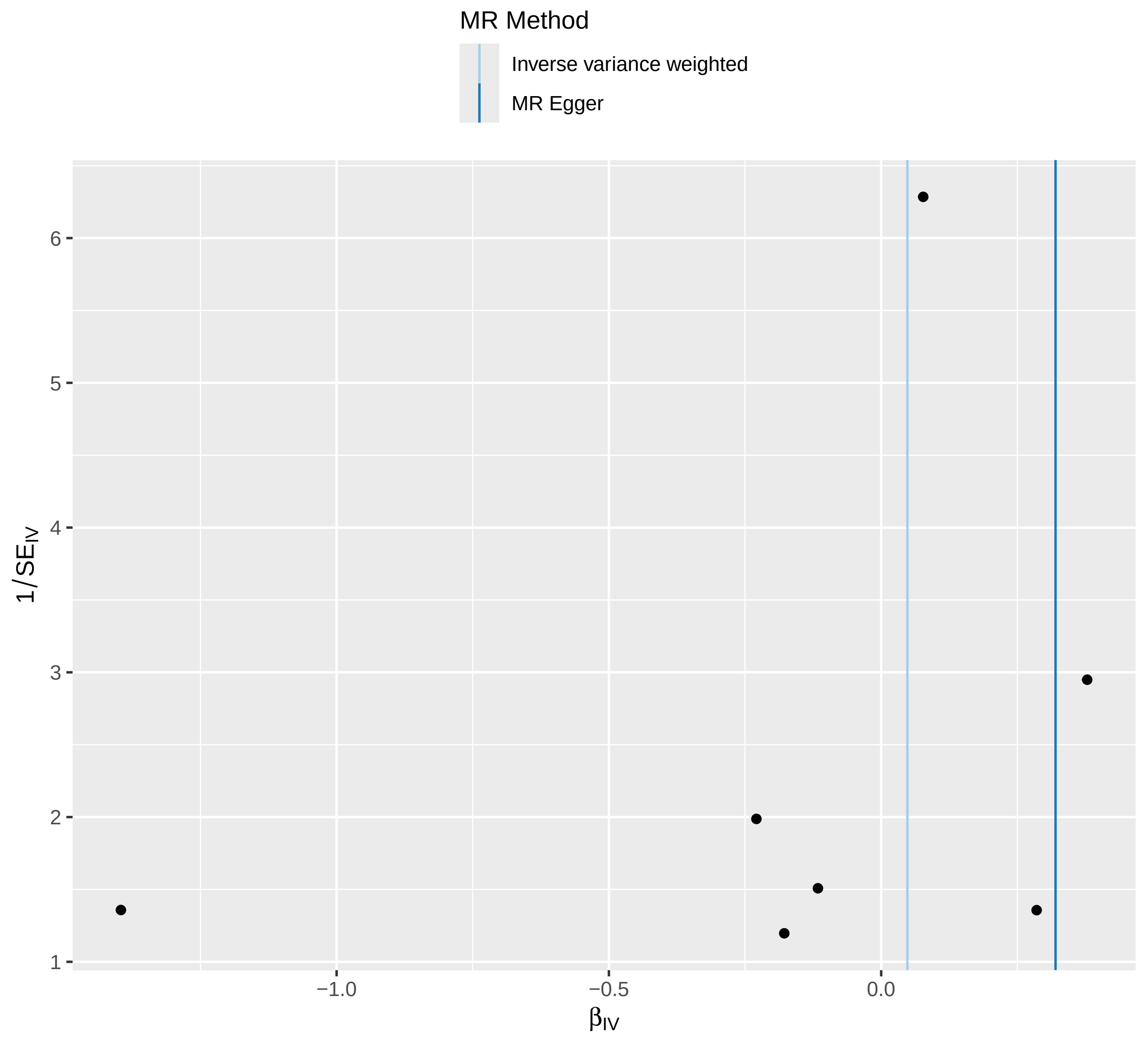

MR Method
Inverse variance weighted
MR Egger
6
5
4
3
2
1
1/SE_IV
−1.0
−0.5
0.0
β_IV

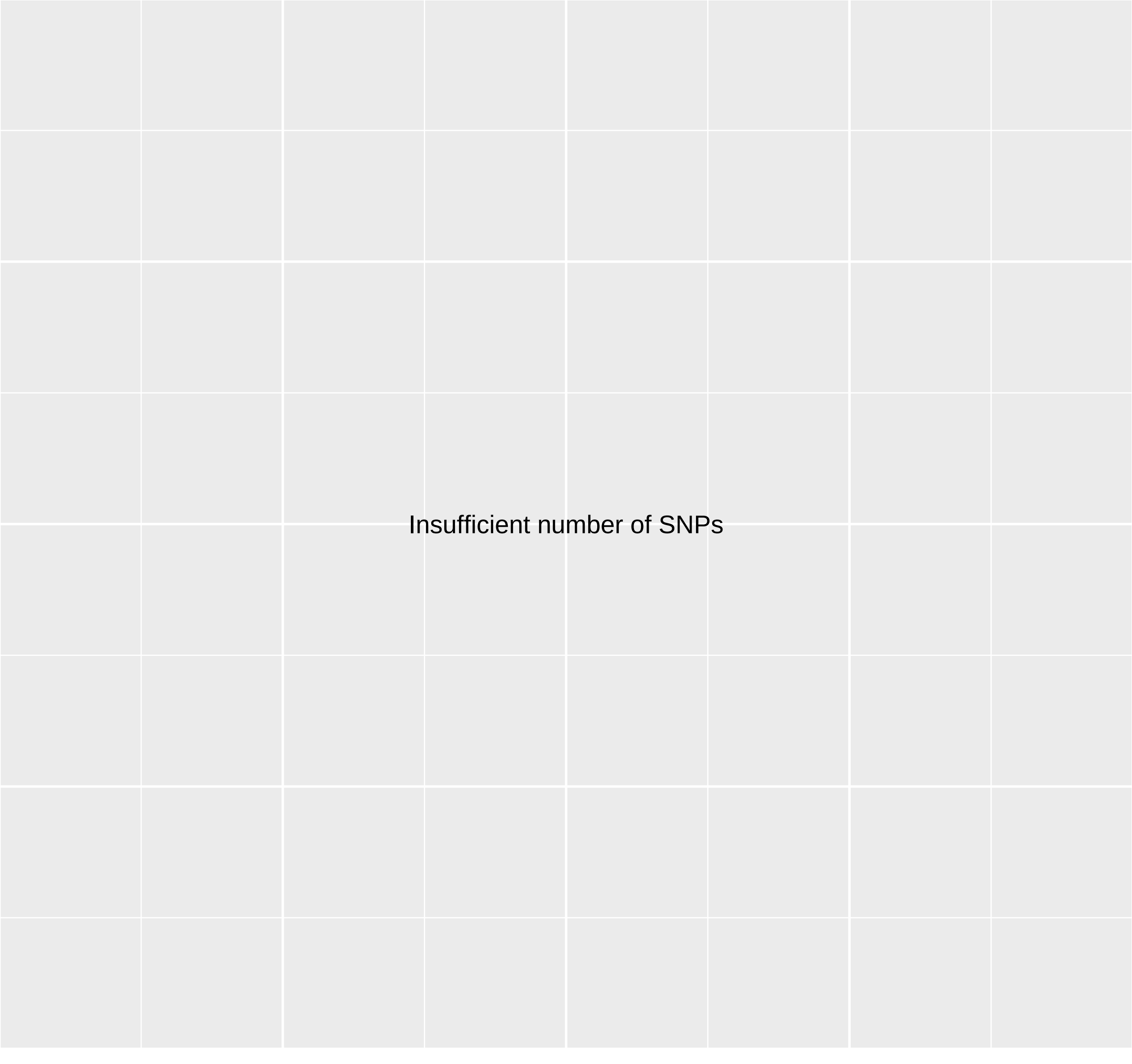
Insufficient number of SNPs

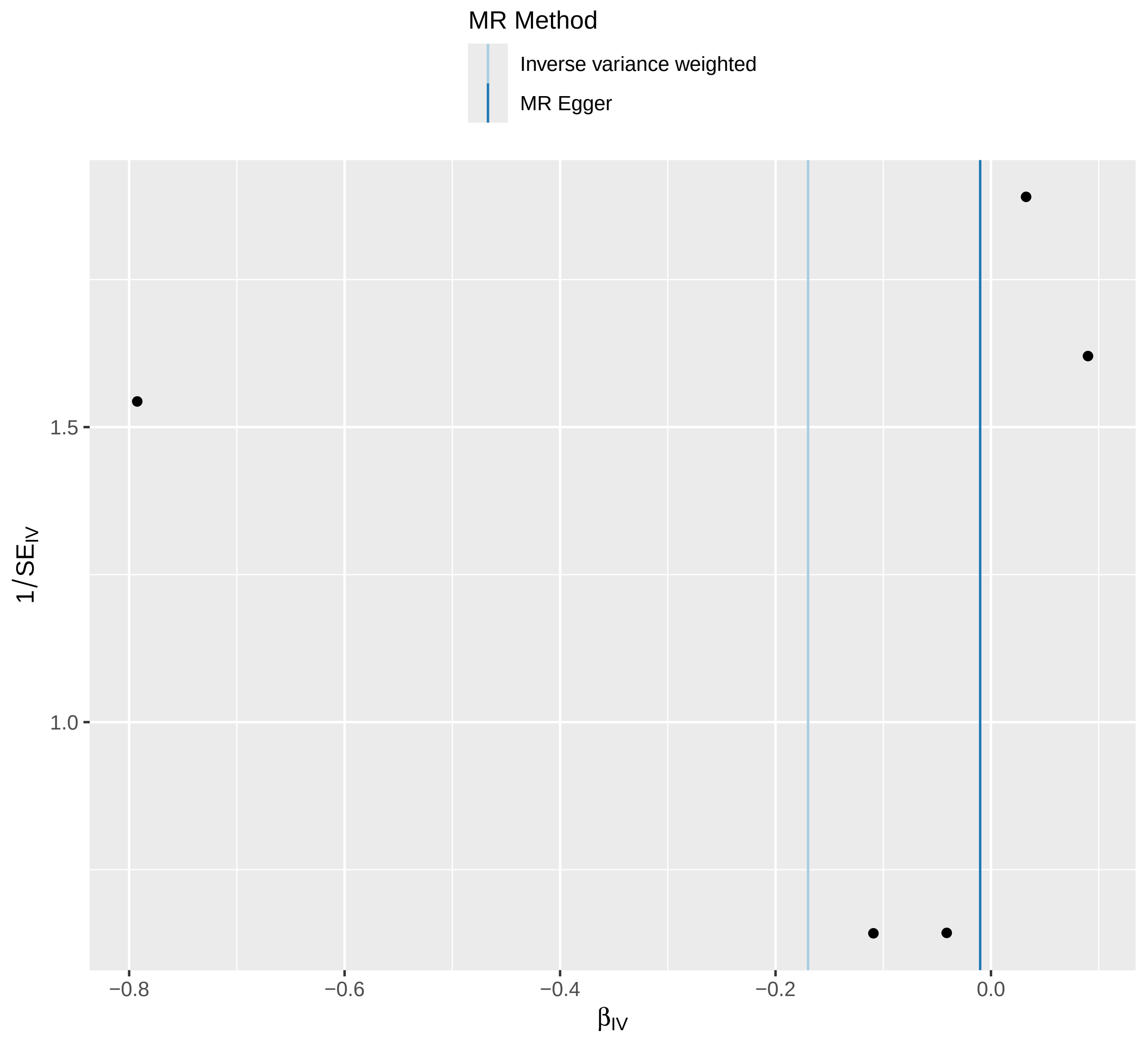
MR Method
Inverse variance weighted
MR Egger
1.5
1.0
1/SE_IV
−0.8
−0.6
−0.4
−0.2
0.0
β_IV

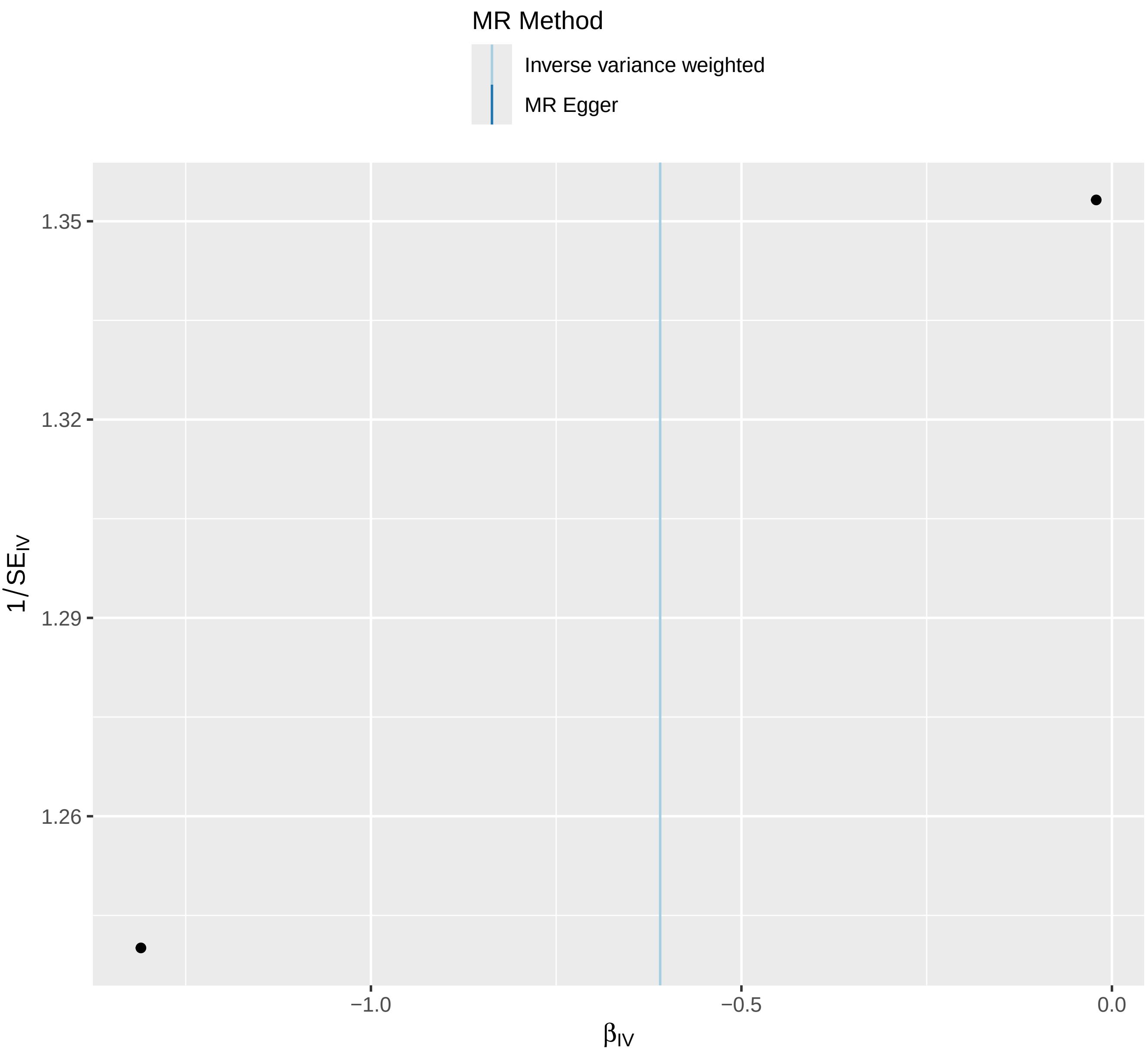
MR Method
Inverse variance weighted
MR Egger
1.35
1.32
1.29
1.26
1/SE$_{IV}$
−1.0
−0.5
0.0
β$_{IV}$

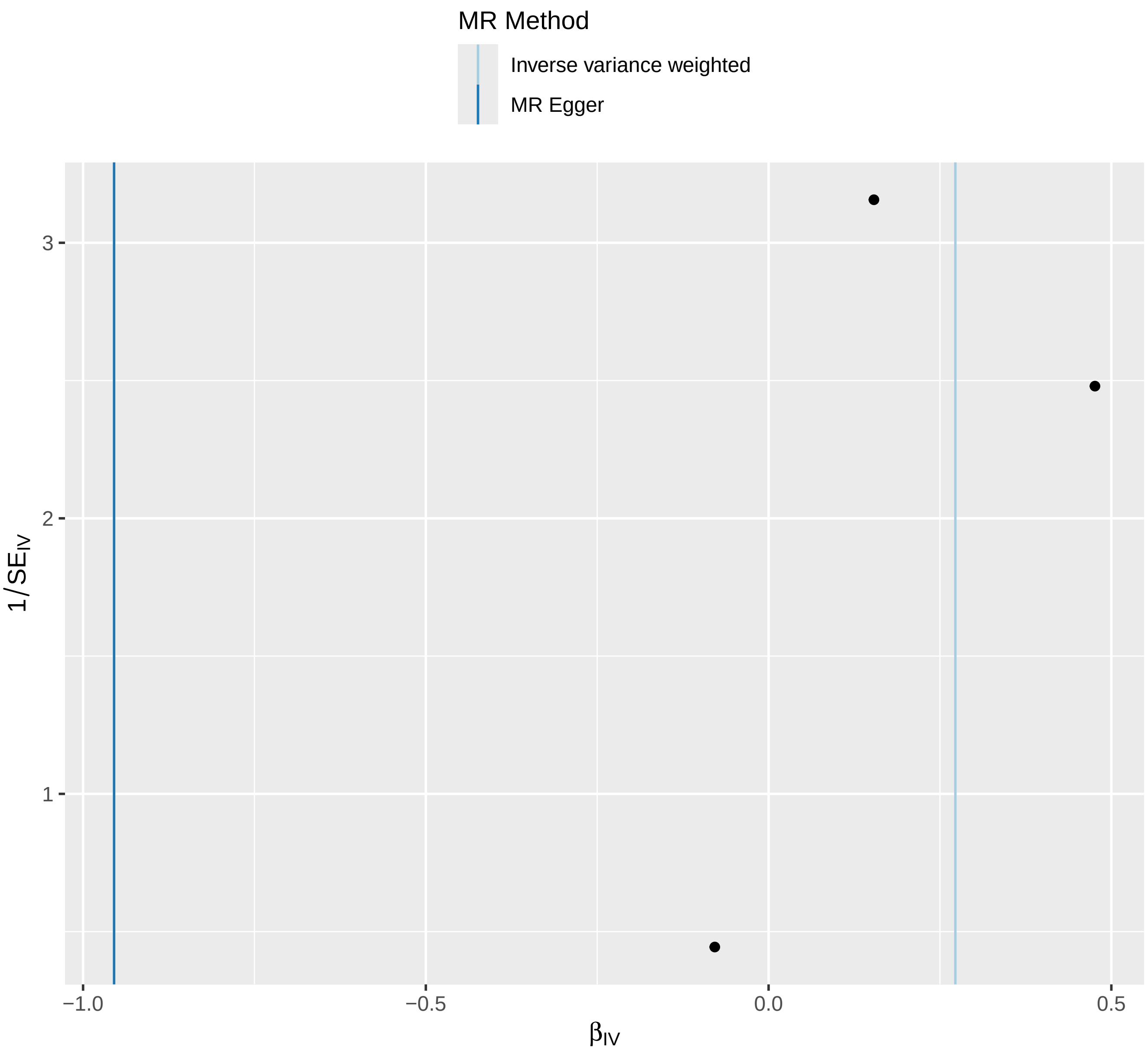
MR Method
Inverse variance weighted
MR Egger
3
2
1
1/SE$_{IV}$
−1.0
−0.5
0.0
0.5
β$_{IV}$

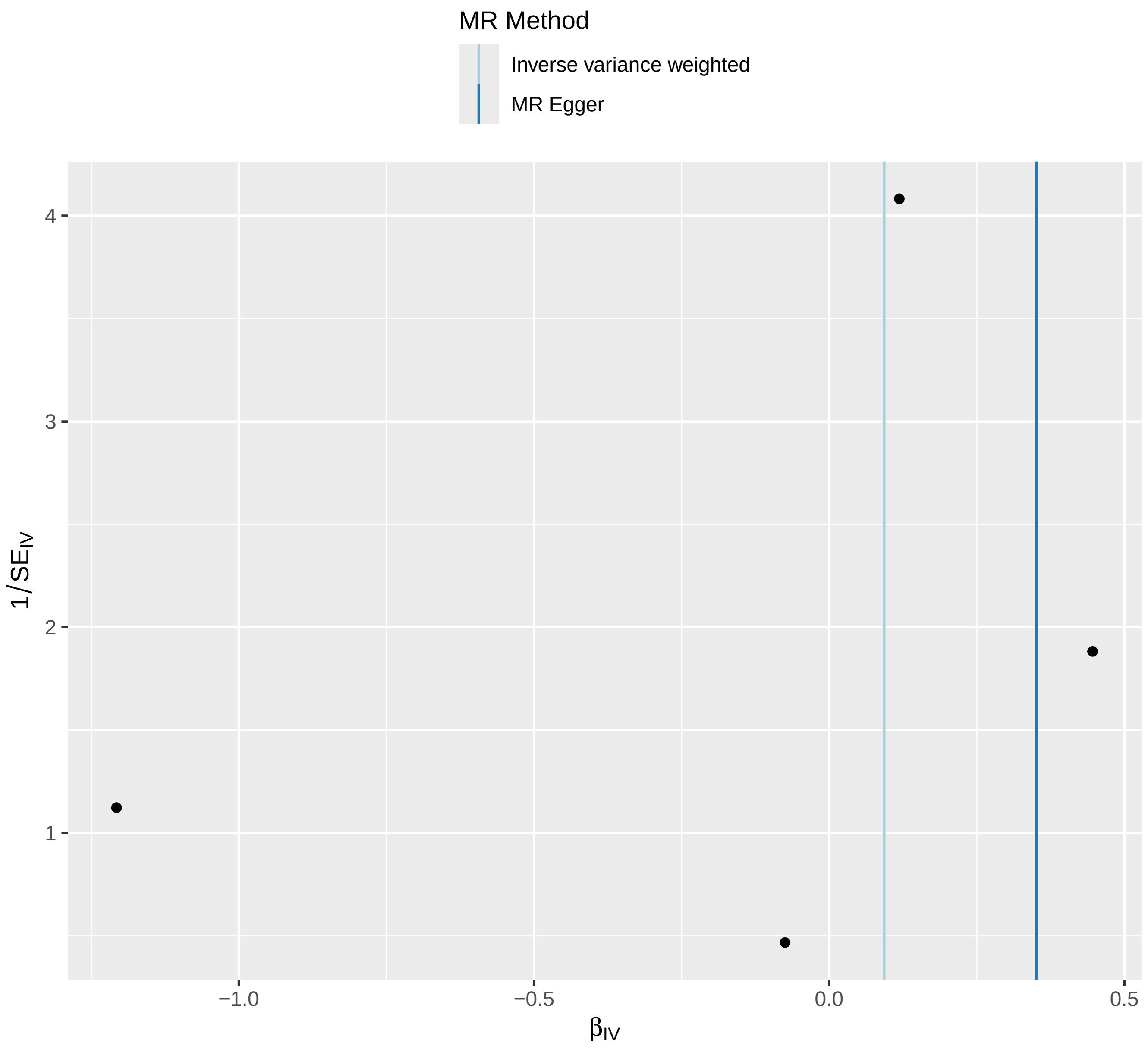

MR Method
Inverse variance weighted
MR Egger
4
3
2
1
1/SE_IV
−1.0
−0.5
0.0
0.5
β_IV

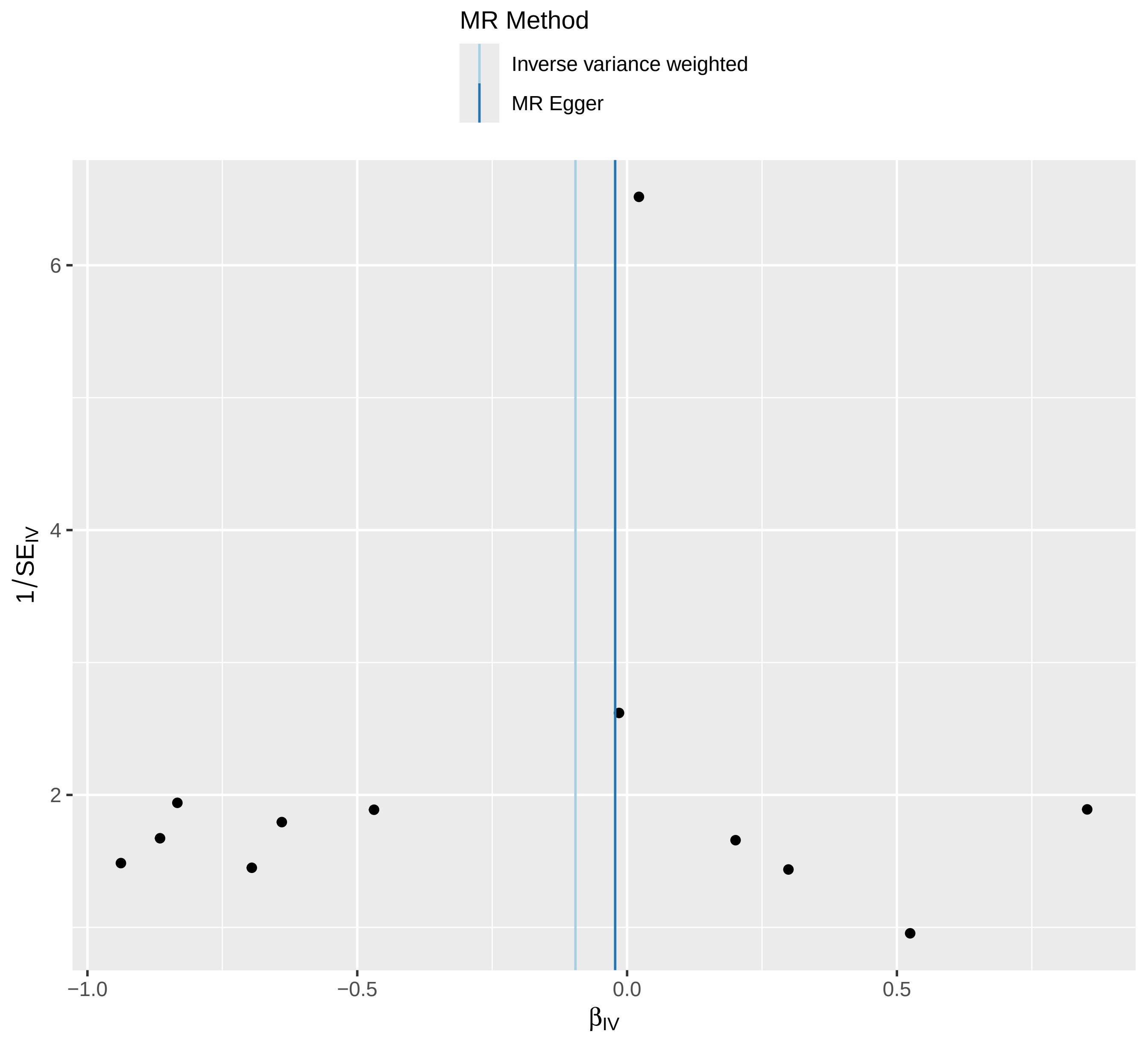

MR Method
Inverse variance weighted
MR Egger
1/SE_IV
6
4
2
−1.0
−0.5
0.0
0.5
β_IV

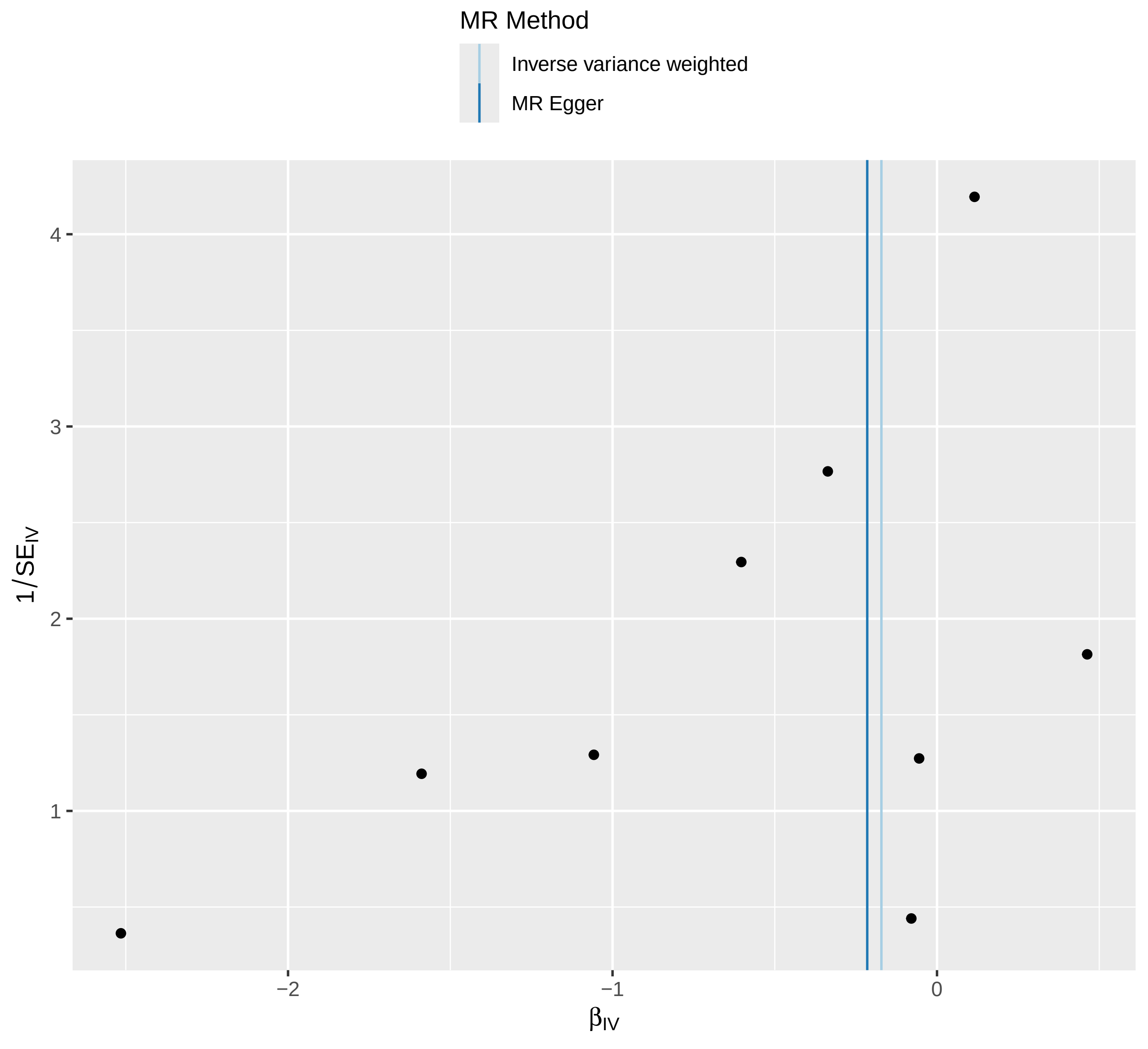
MR Method
Inverse variance weighted
MR Egger
4
3
2
1
−2
−1
0
$1/SE_{IV}$
β_{IV}

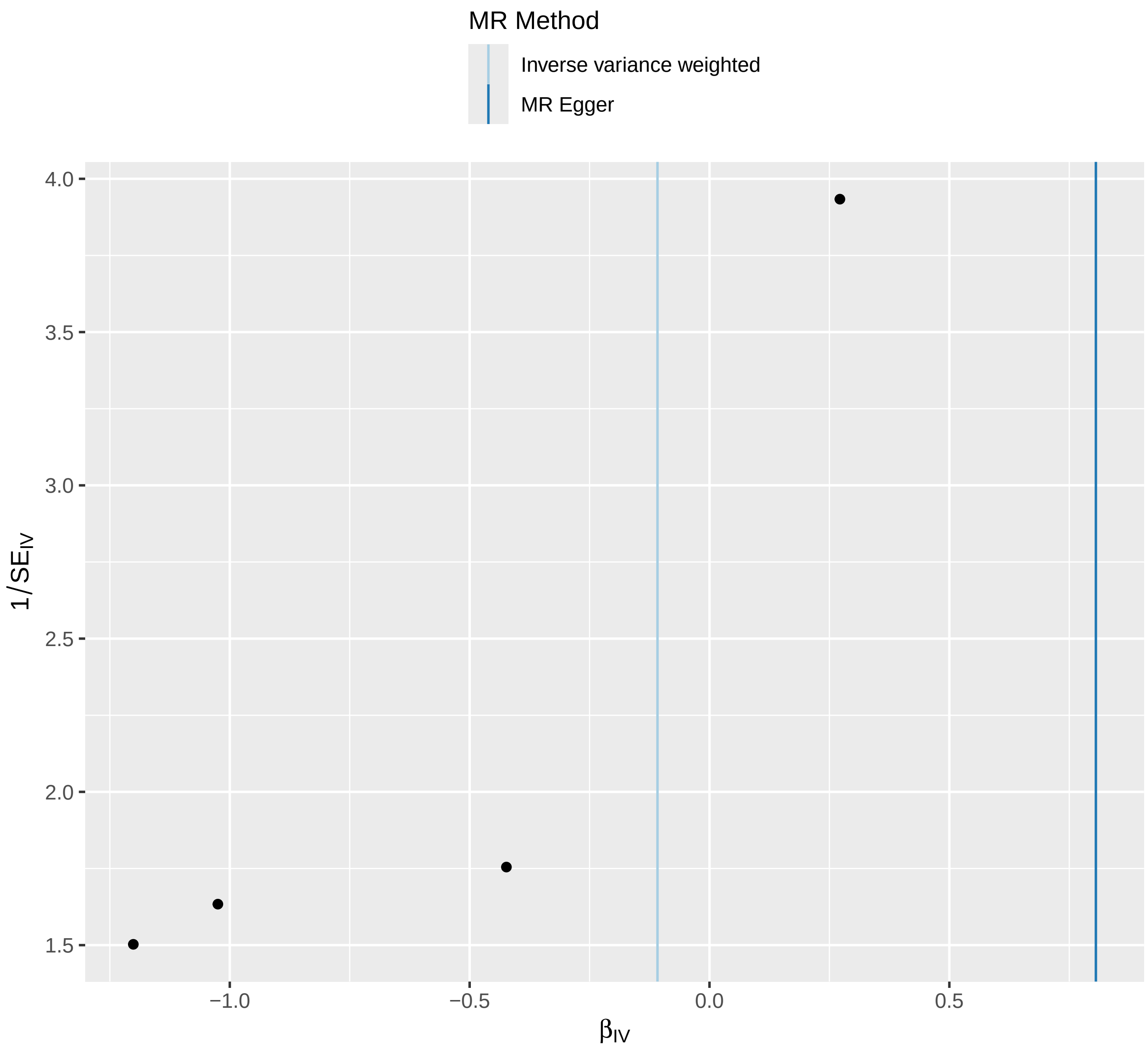
MR Method
Inverse variance weighted
MR Egger
4.0
3.5
3.0
2.5
2.0
1.5
1/SE$_{IV}$
−1.0
−0.5
0.0
0.5
β$_{IV}$

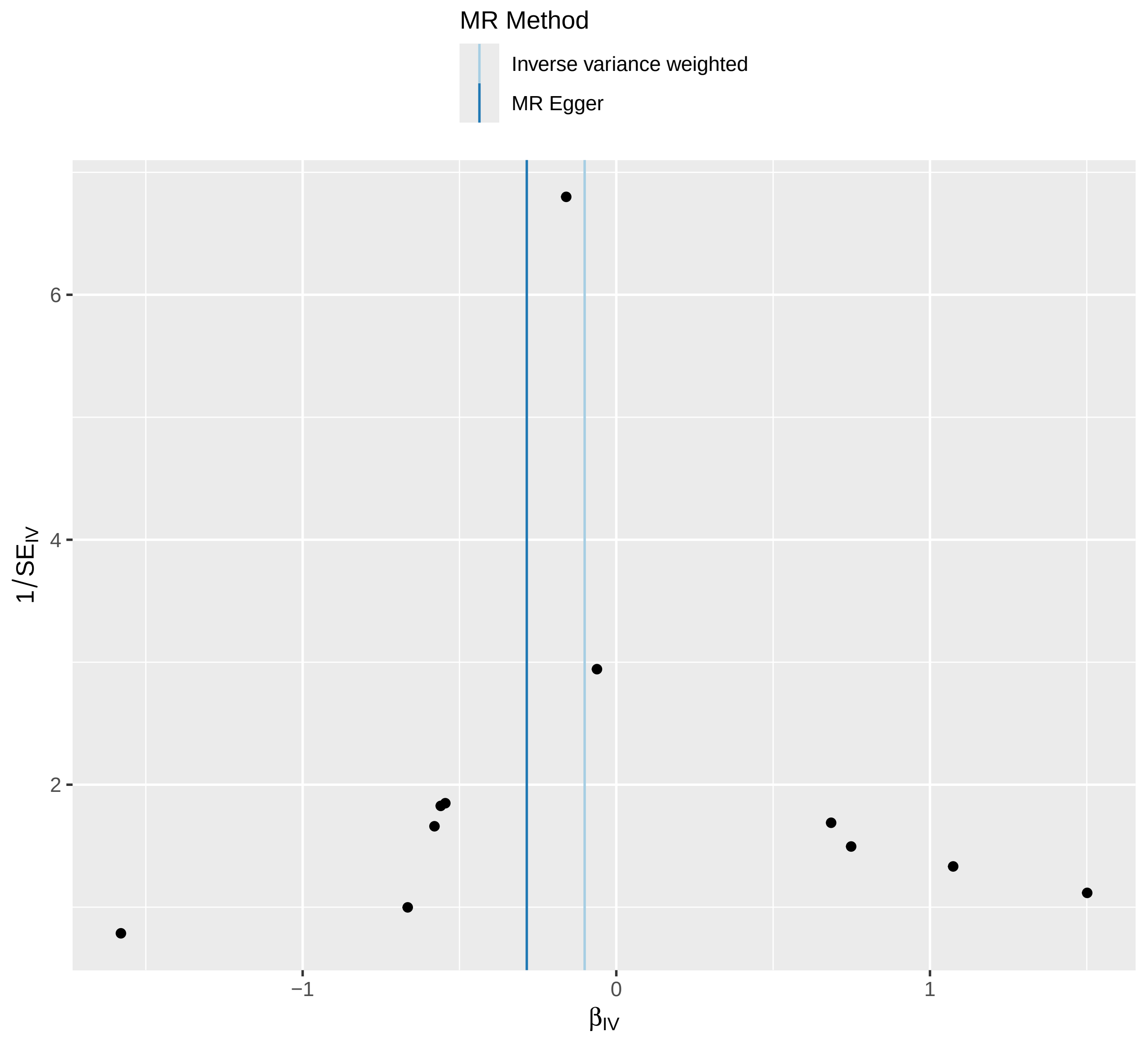

MR Method
Inverse variance weighted
MR Egger
6
4
2
1/SE$_{IV}$
−1
0
1
β$_{IV}$

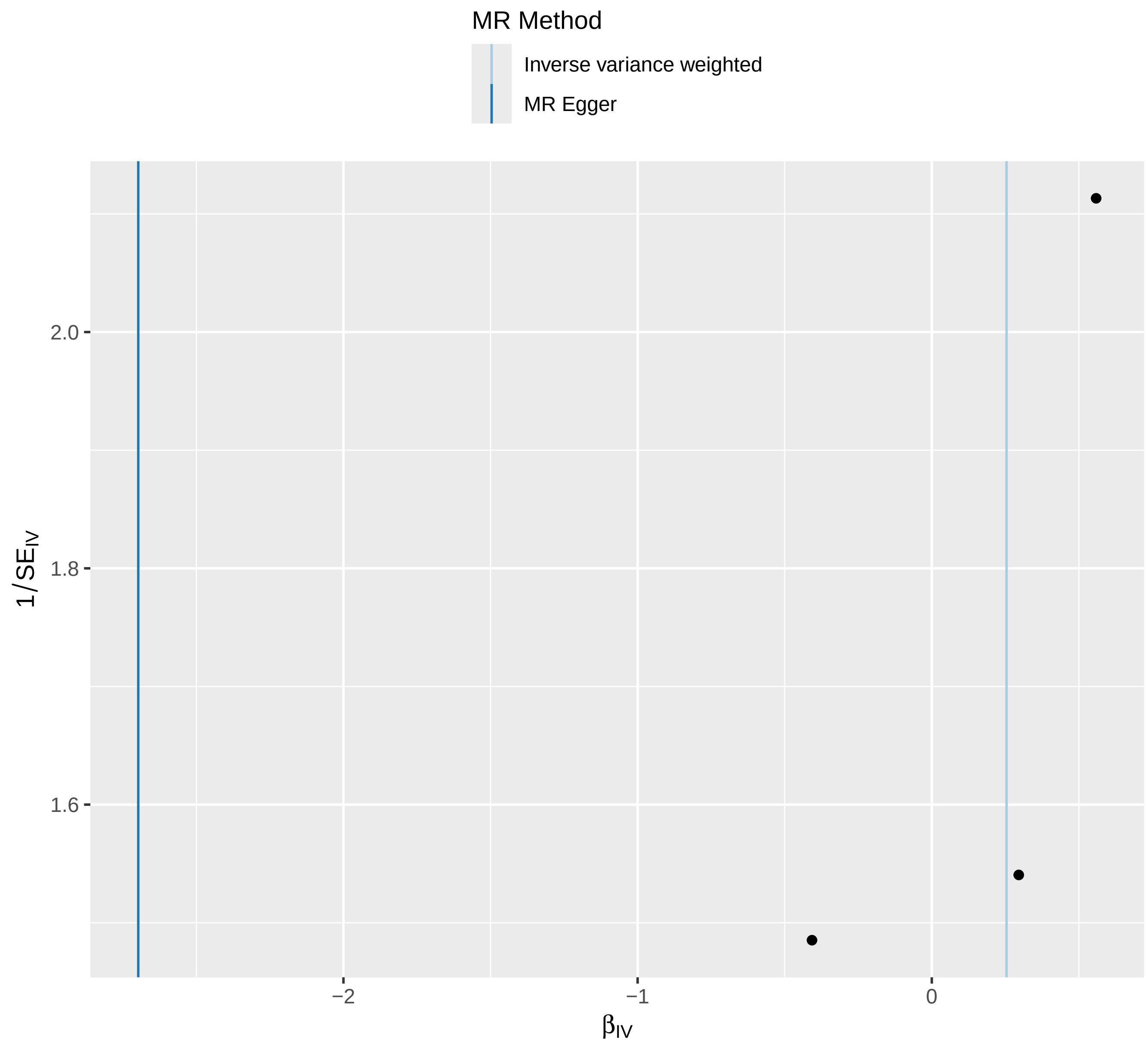

MR Method
Inverse variance weighted
MR Egger
2.0
1.8
1.6
1/SE_IV
−2
−1
0
β_IV

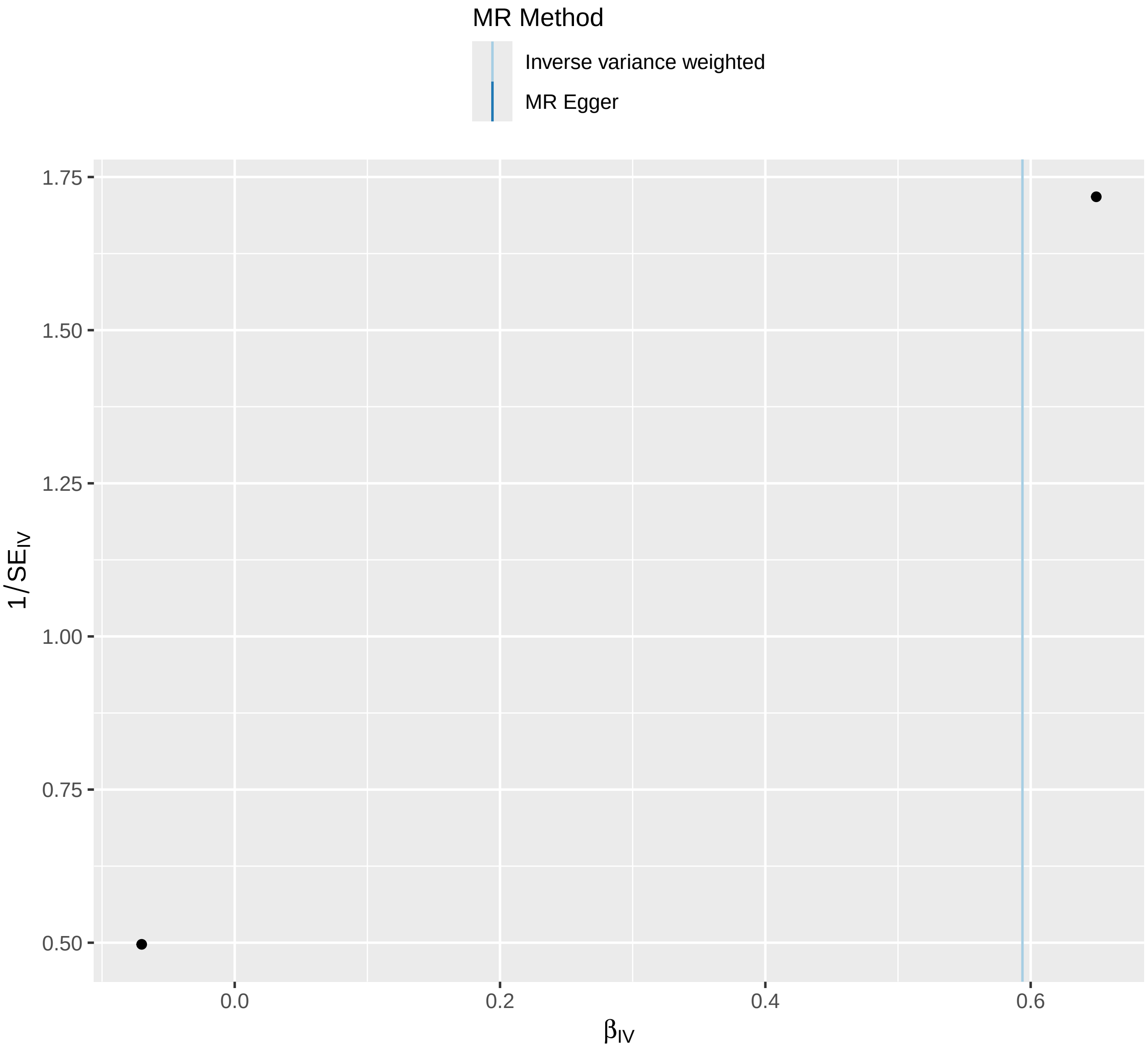
MR Method
Inverse variance weighted
MR Egger
1.75
1.50
1.25
1.00
0.75
0.50
$1/SE_{IV}$
0.0
0.2
0.4
0.6
β_{IV}

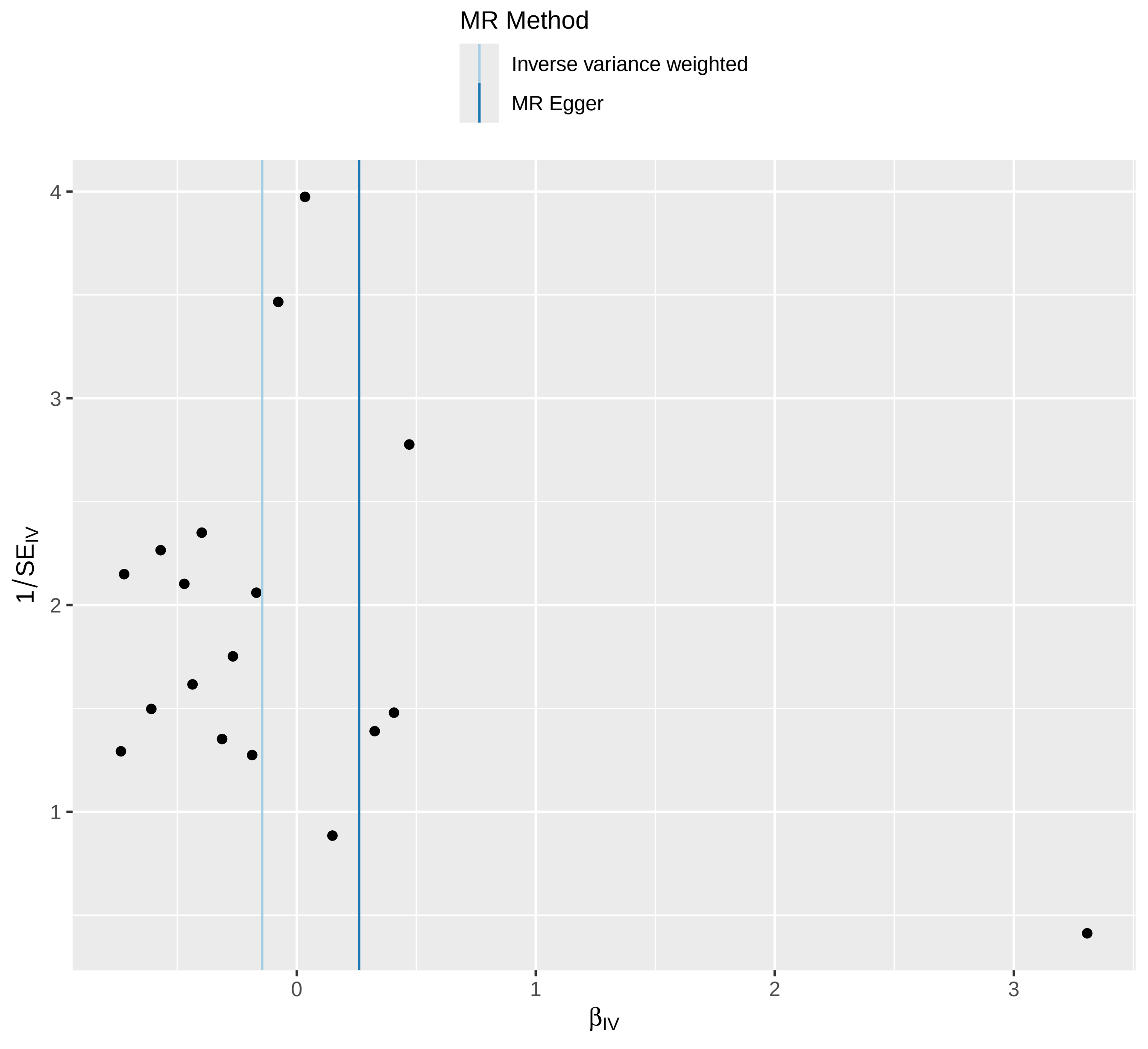

MR Method
Inverse variance weighted
MR Egger
1/SE$_{IV}$
4
3
2
1
0
1
2
3
β$_{IV}$

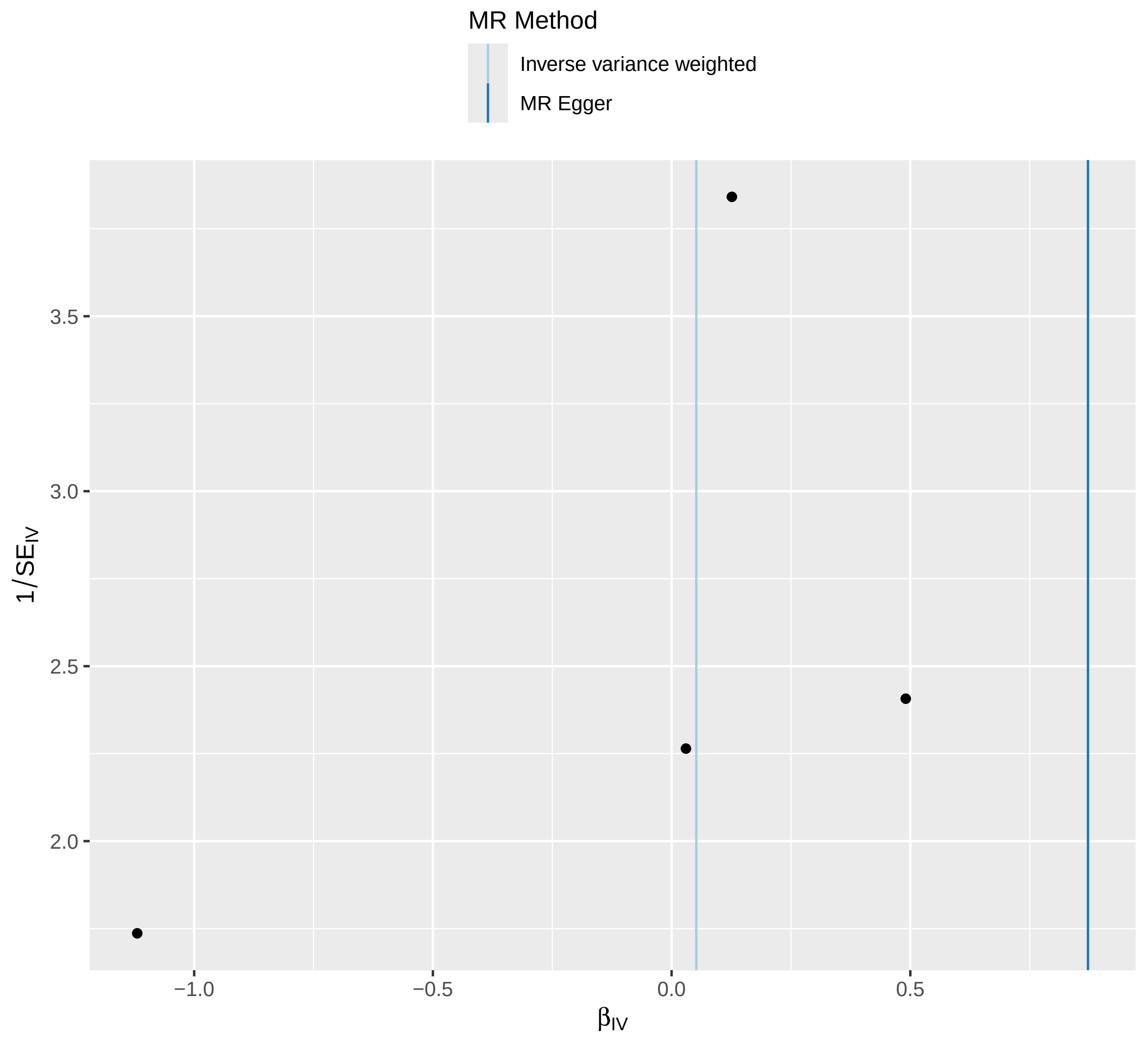

MR Method
Inverse variance weighted
MR Egger
3.5
3.0
2.5
2.0
1/SE_IV
−1.0
−0.5
0.0
0.5
β_IV

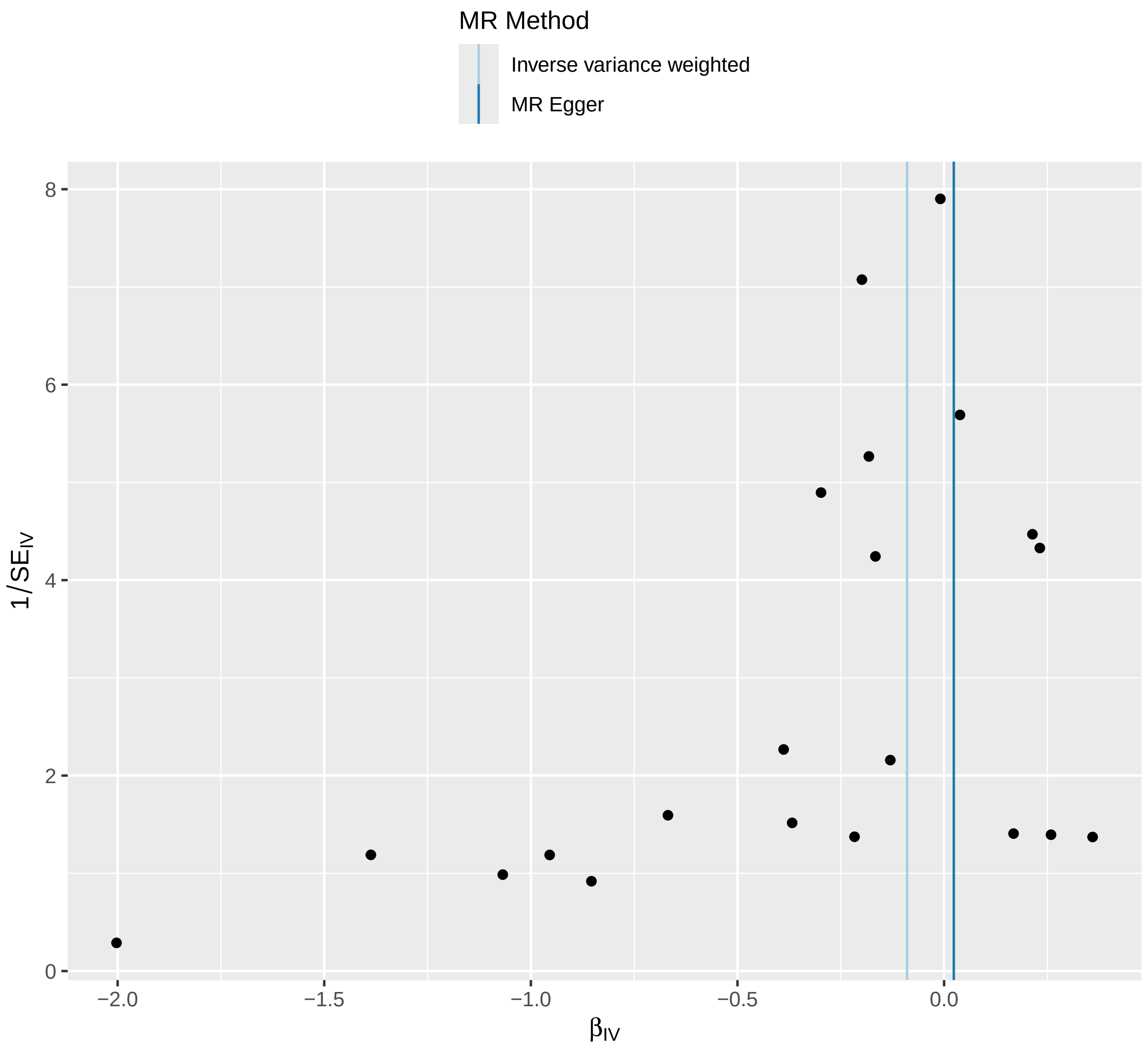
MR Method
Inverse variance weighted
MR Egger
8
6
4
2
0
1/SE_IV
−2.0
−1.5
−1.0
−0.5
0.0
β_IV

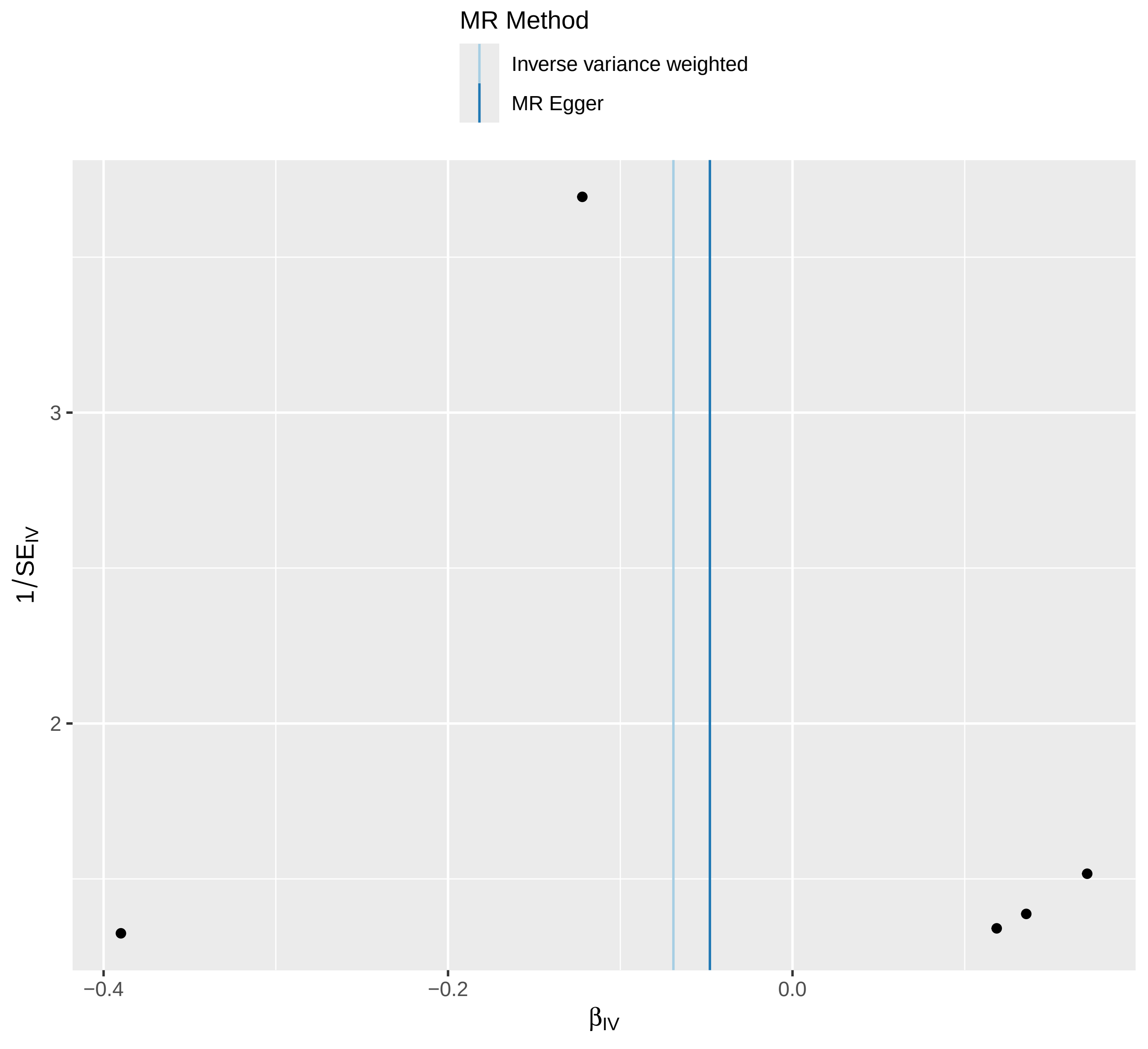

MR Method
Inverse variance weighted
MR Egger
3
2
1/SE_IV
−0.4
−0.2
0.0
β_IV

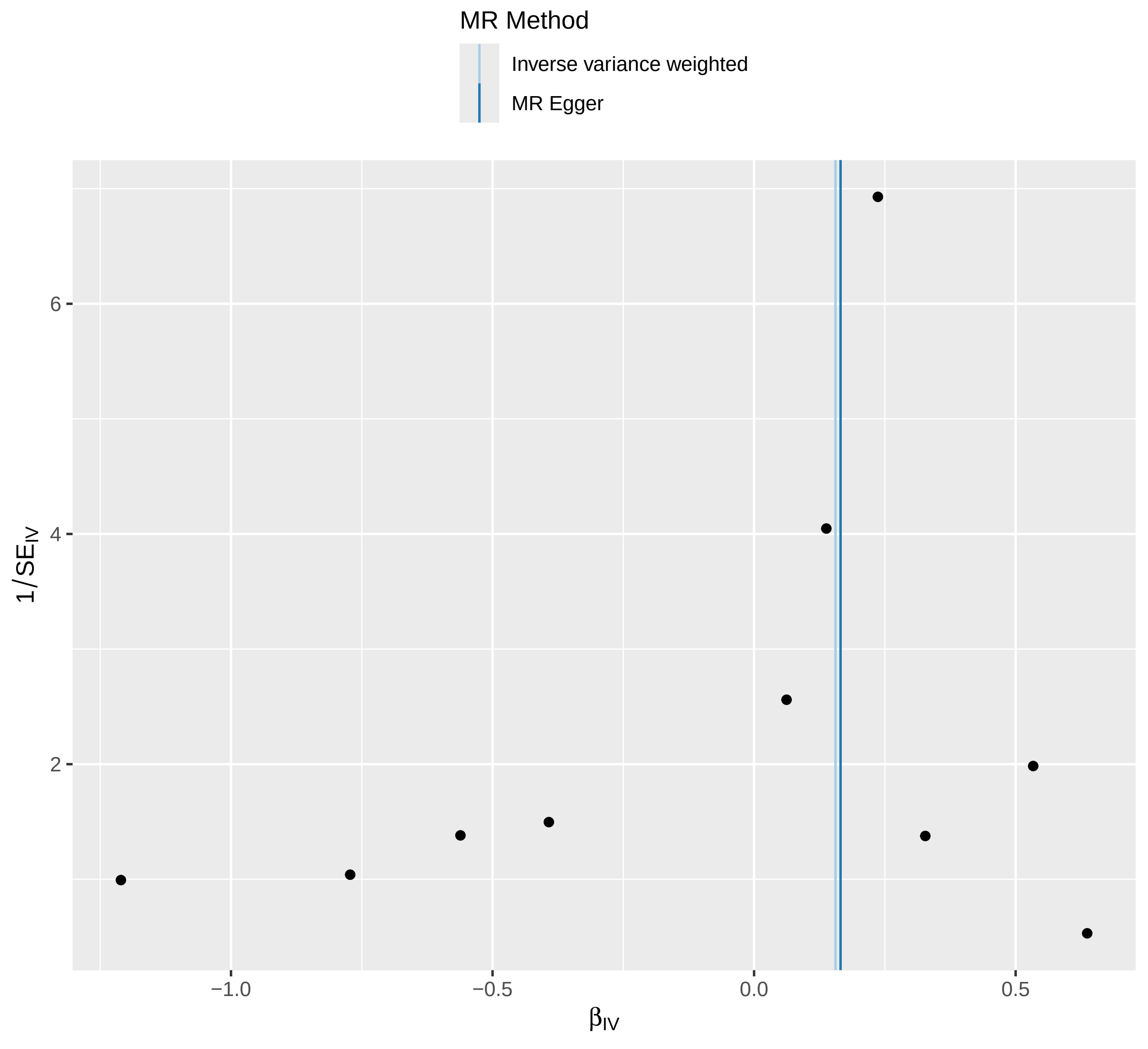

MR Method
Inverse variance weighted
MR Egger
6
4
2
1/SE$_{IV}$
−1.0
−0.5
0.0
0.5
β$_{IV}$

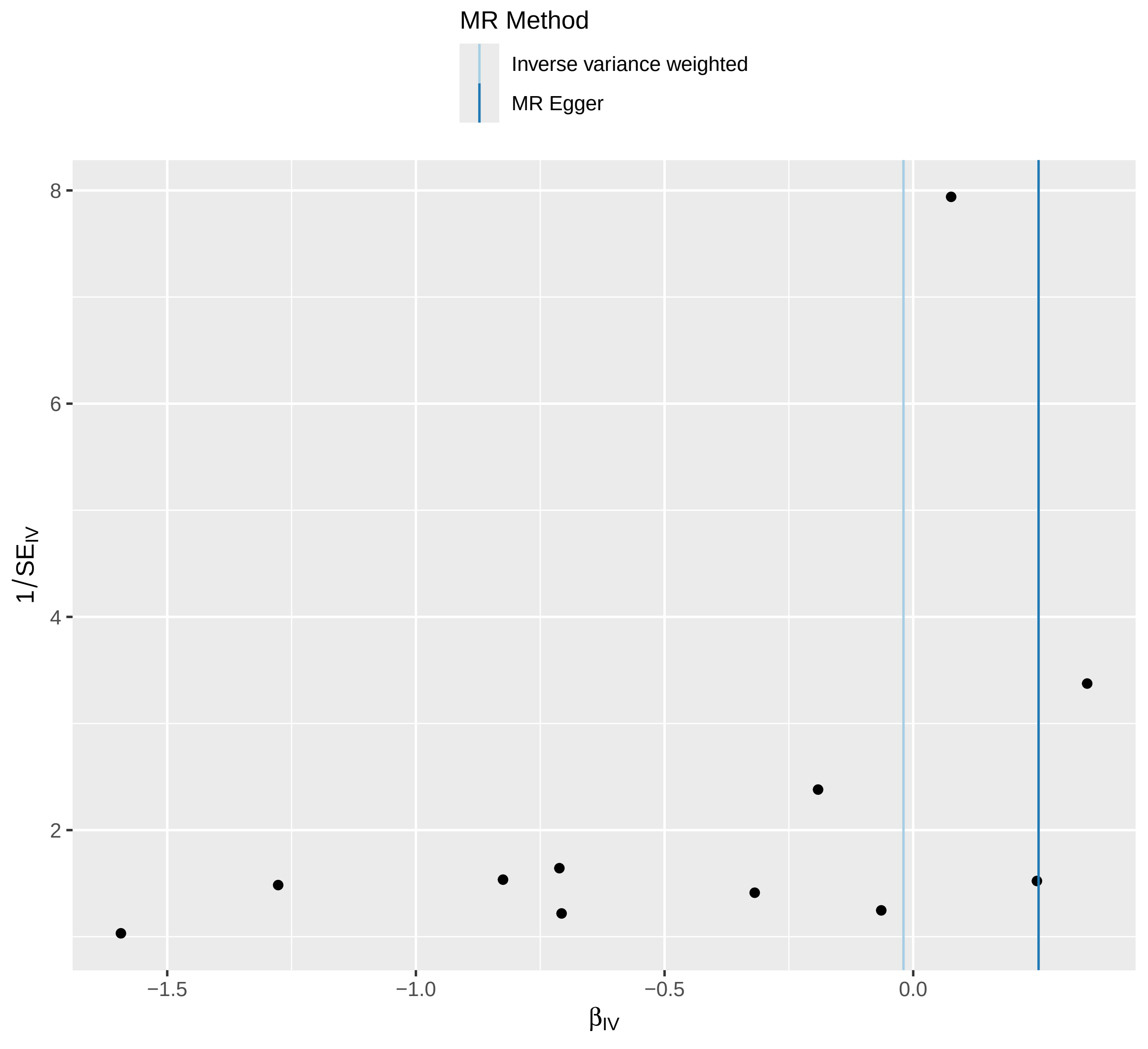
MR Method
Inverse variance weighted
MR Egger
8
6
4
2
1/SE_IV
−1.5
−1.0
−0.5
0.0
β_IV

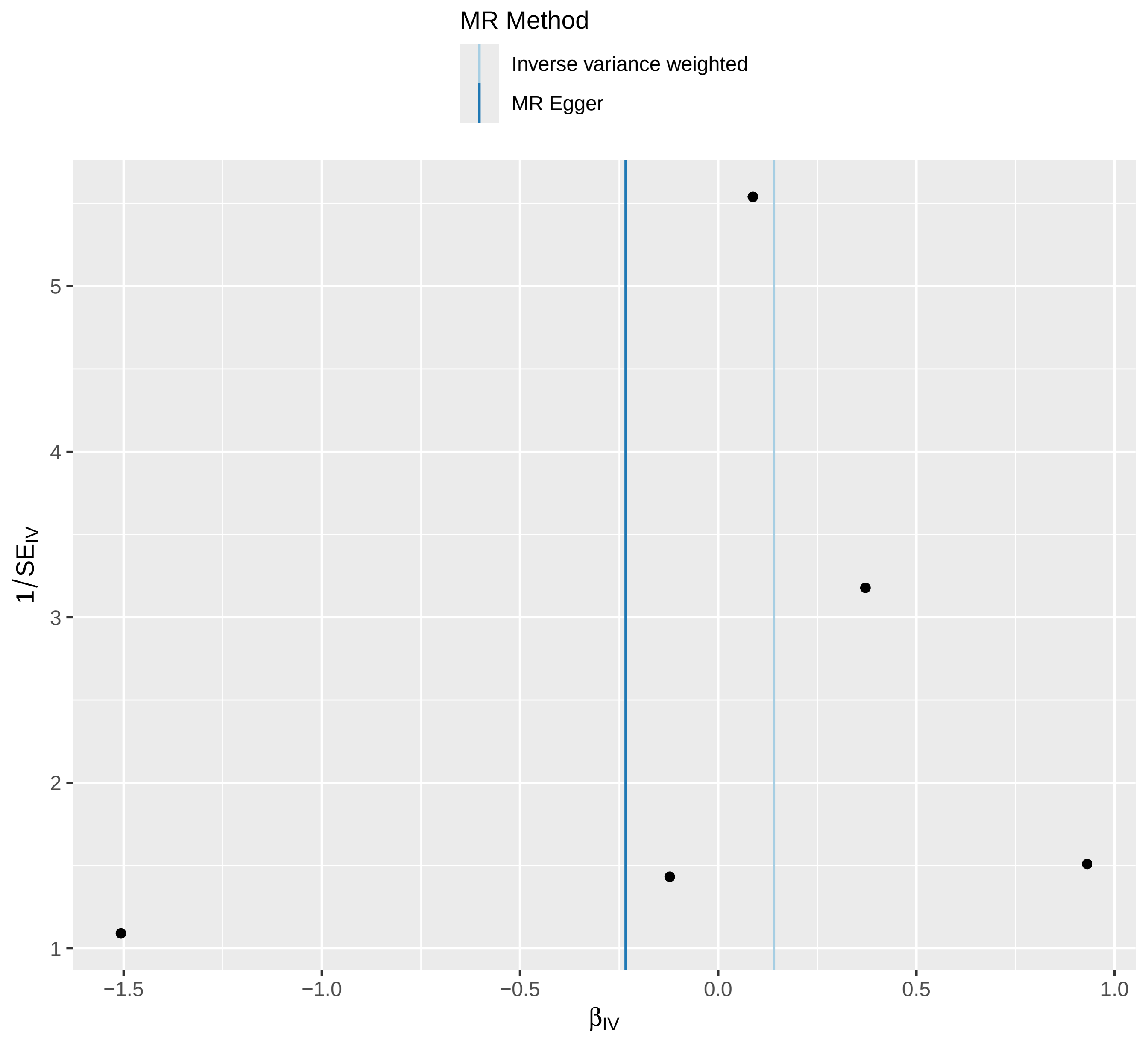

MR Method
Inverse variance weighted
MR Egger
5
4
3
2
1
1/SE_IV
−1.5
−1.0
−0.5
0.0
0.5
1.0
β_IV

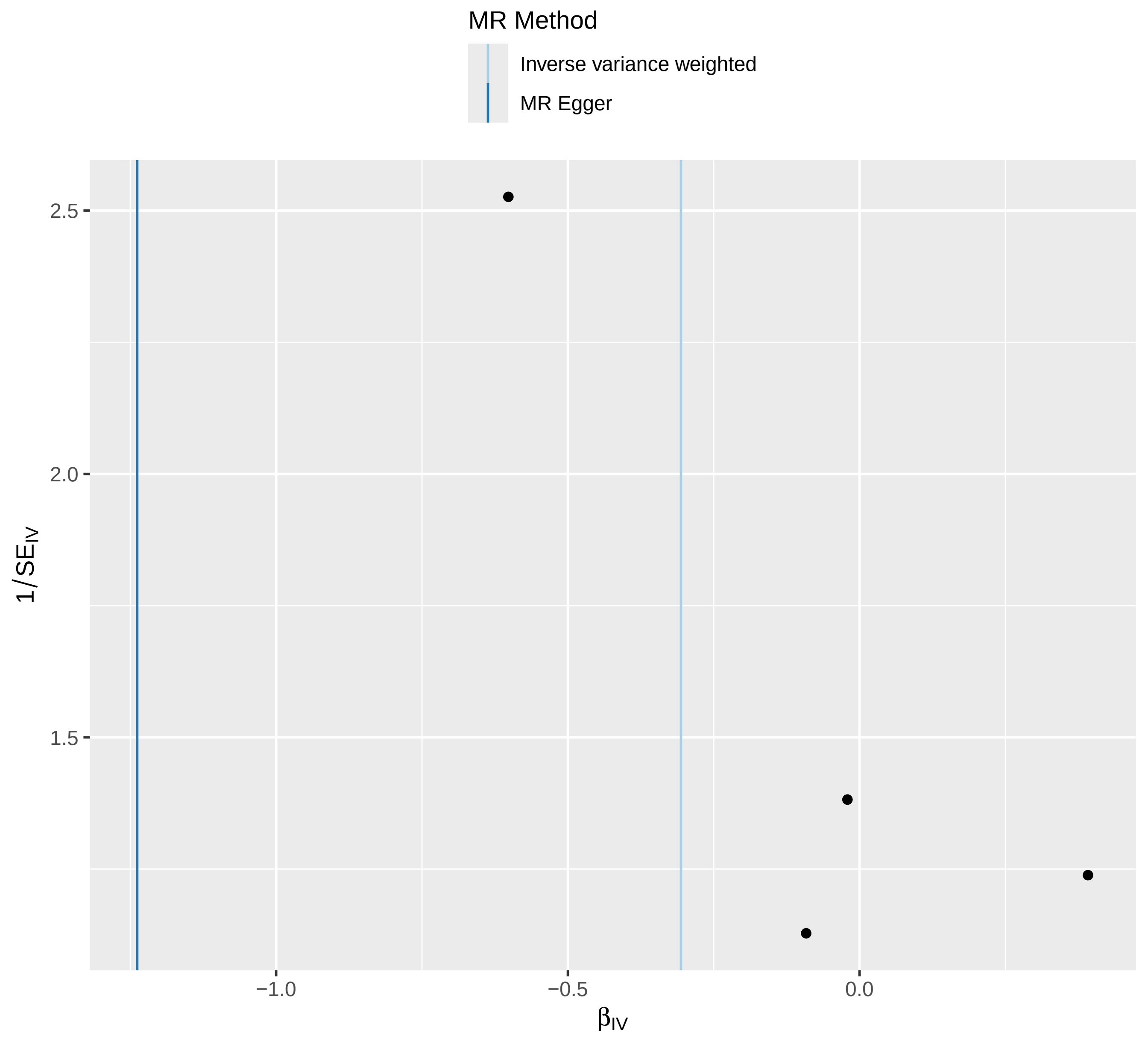
MR Method
Inverse variance weighted
MR Egger
2.5
2.0
1.5
1/SE_IV
−1.0
−0.5
0.0
β_IV

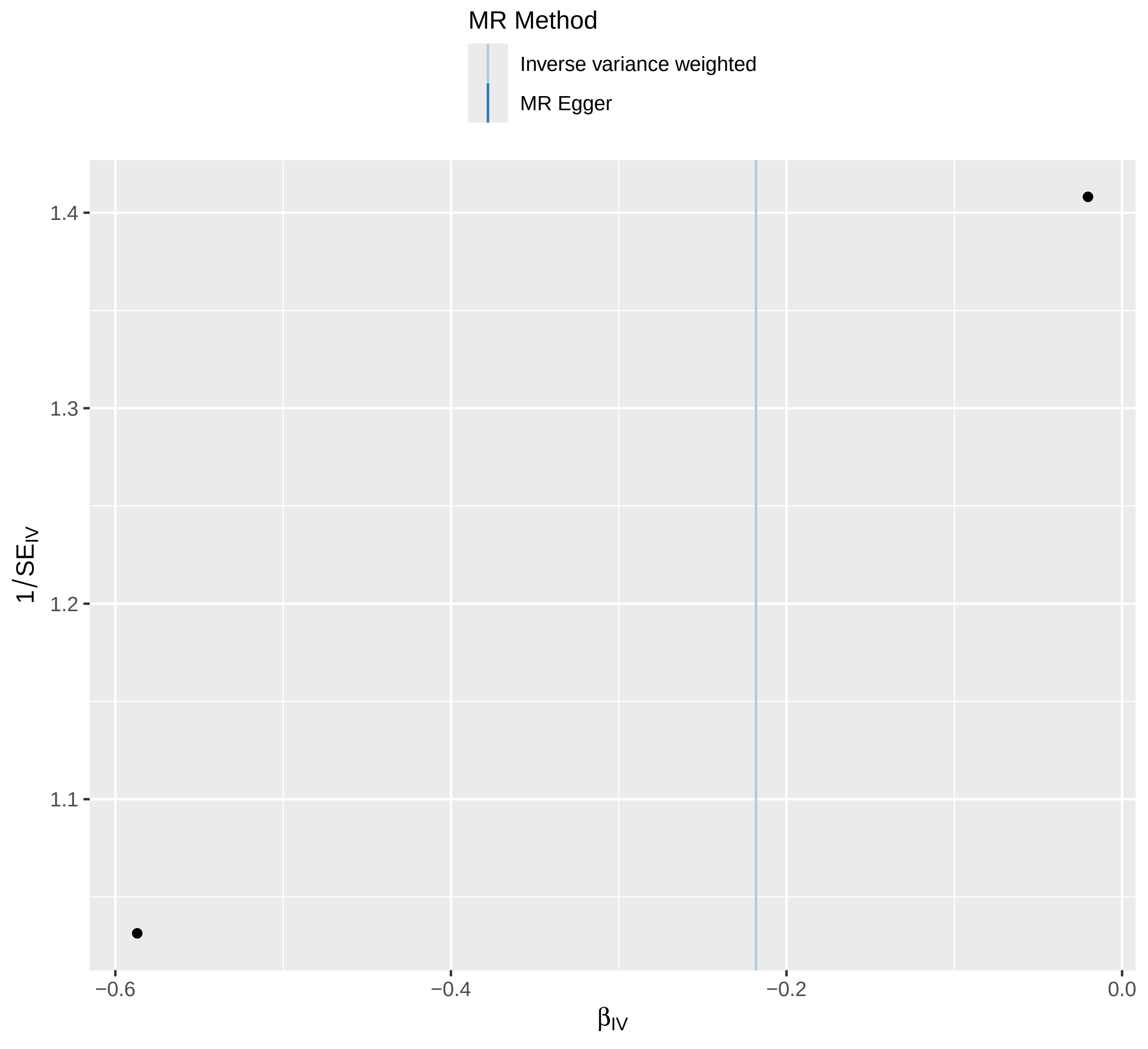

MR Method
Inverse variance weighted
MR Egger
1.4
1.3
1.2
1.1
$1/SE_{IV}$
−0.6
−0.4
−0.2
0.0
β_{IV}

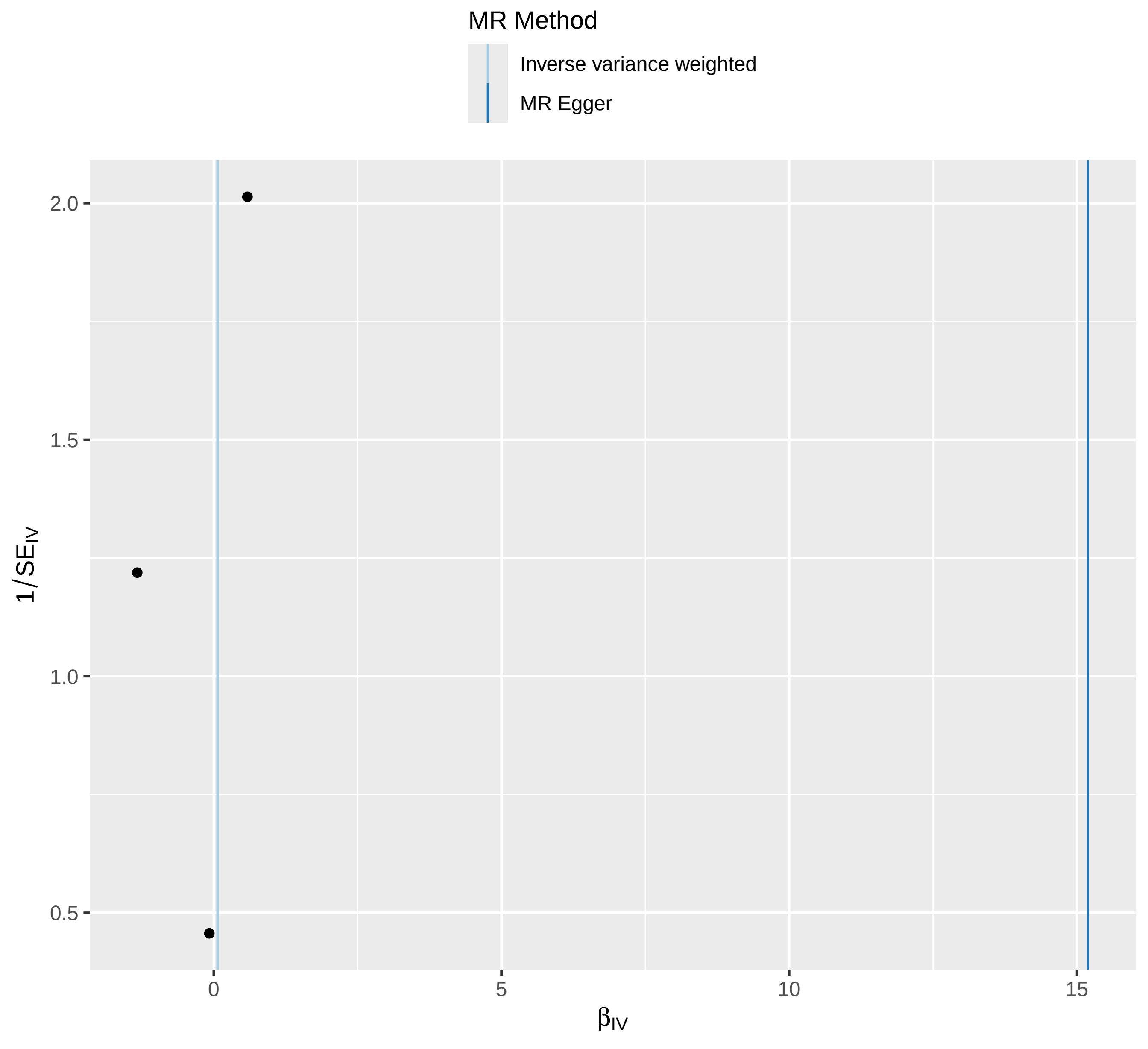
MR Method
Inverse variance weighted
MR Egger
2.0
1.5
1.0
0.5
1/SE_IV
0
5
10
15
β_IV

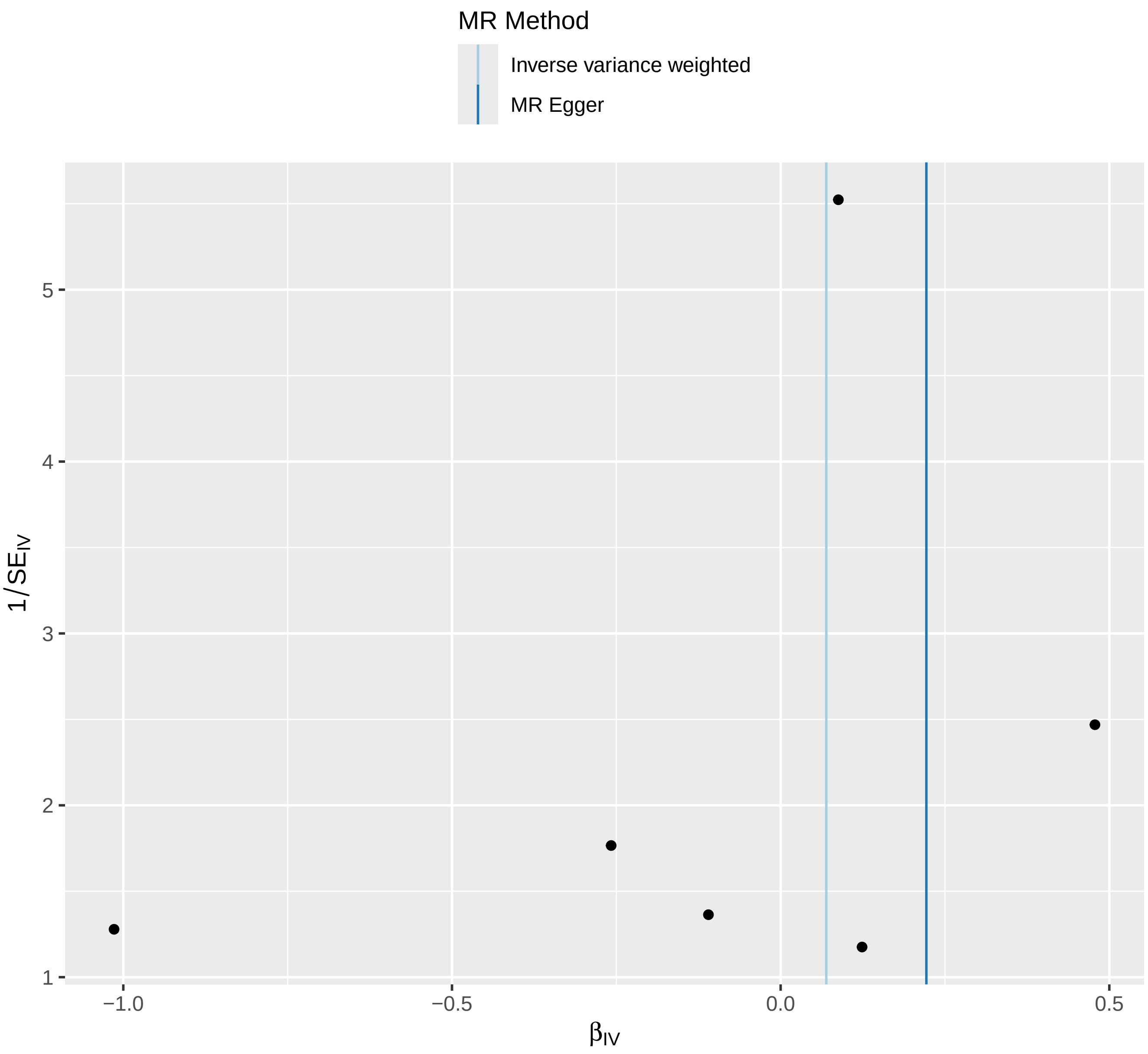
MR Method
Inverse variance weighted
MR Egger
1/SE_IV
5
4
3
2
1
−1.0
−0.5
0.0
0.5
β_IV

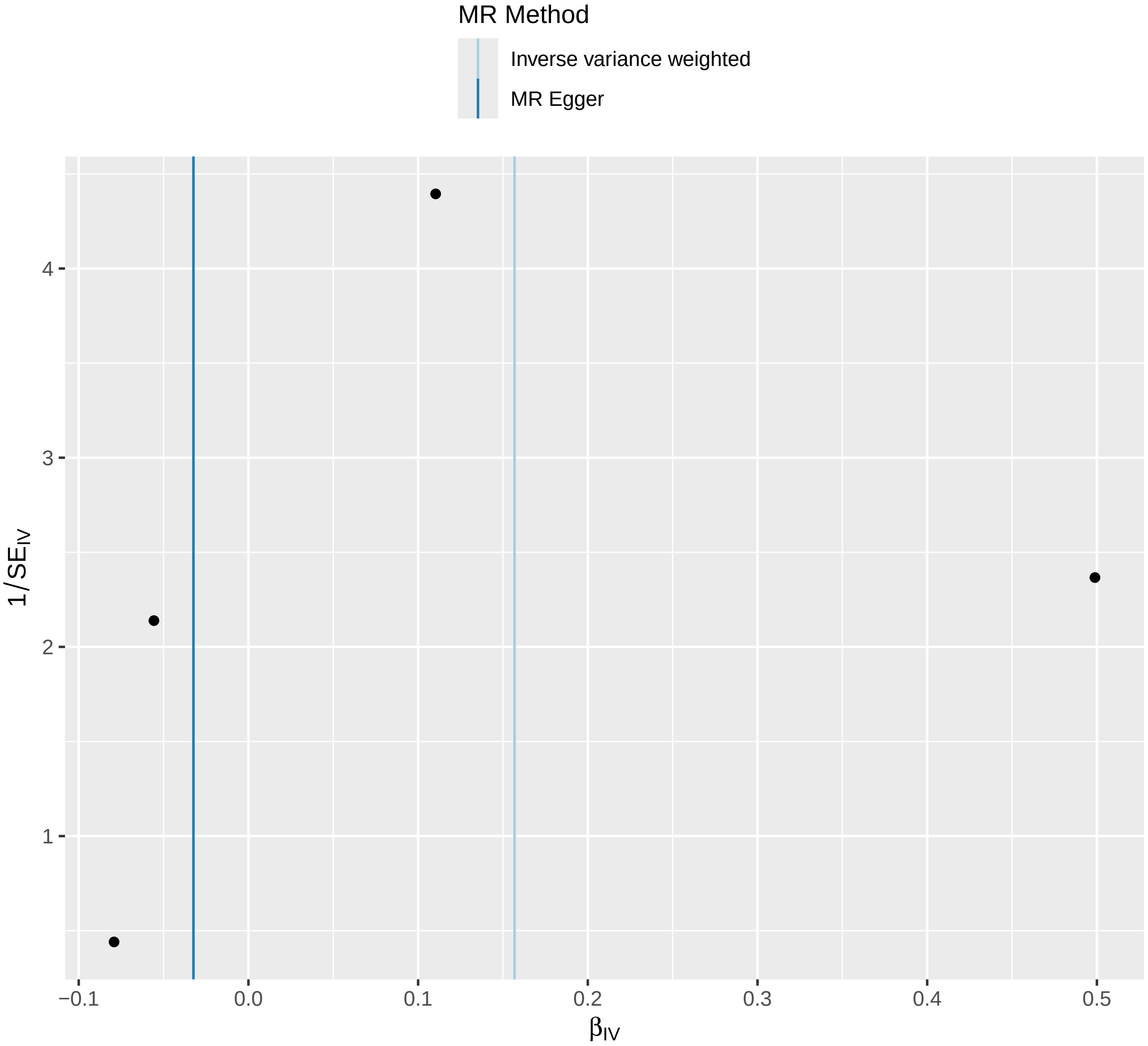
MR Method
Inverse variance weighted
MR Egger
4
3
2
1
1/SE$_{IV}$
−0.1
0.0
0.1
0.2
0.3
0.4
0.5
β_{IV}

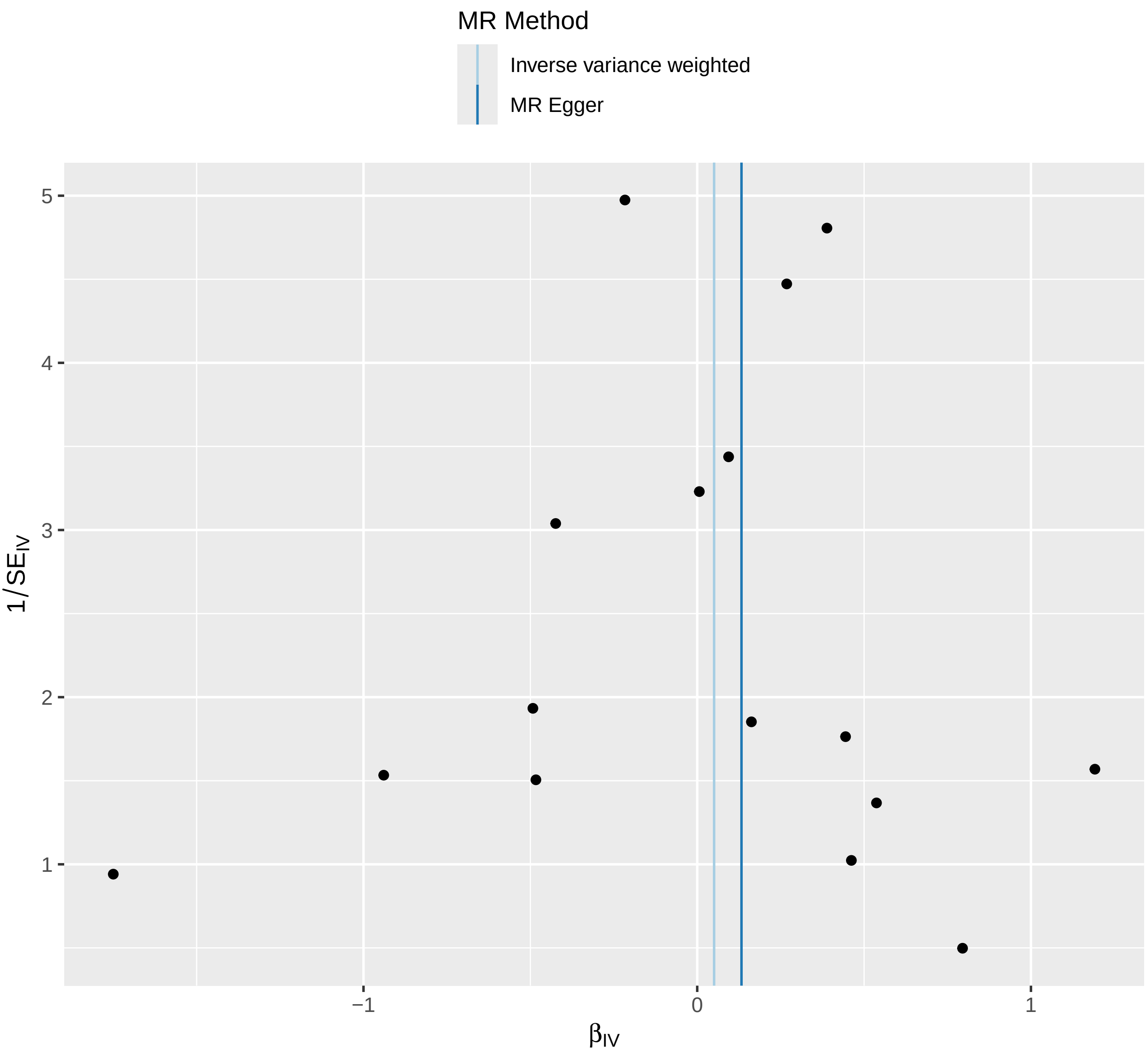
MR Method
Inverse variance weighted
MR Egger
1/SE_IV
5
4
3
2
1
−1
0
1
β_IV

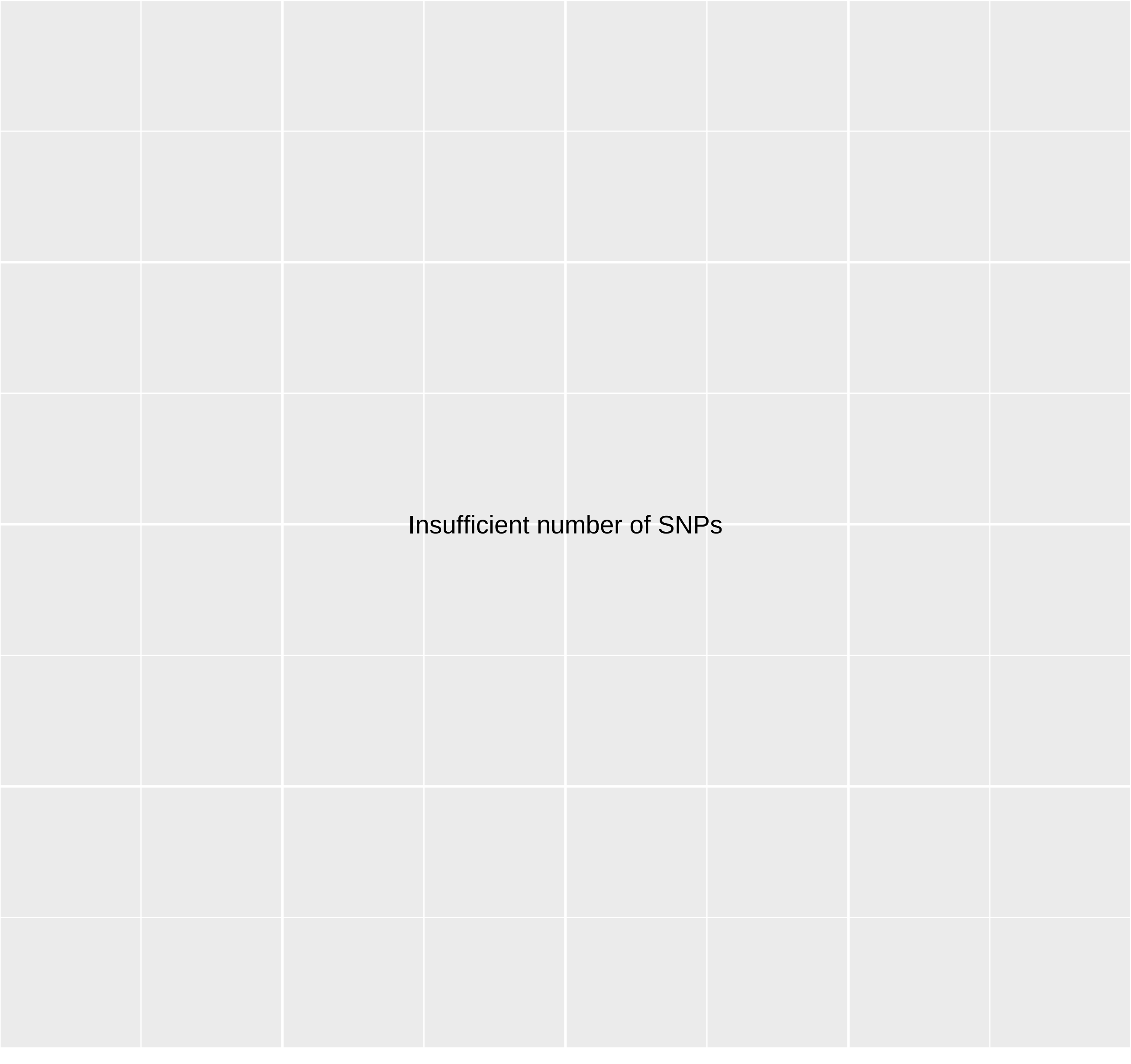
Insufficient number of SNPs

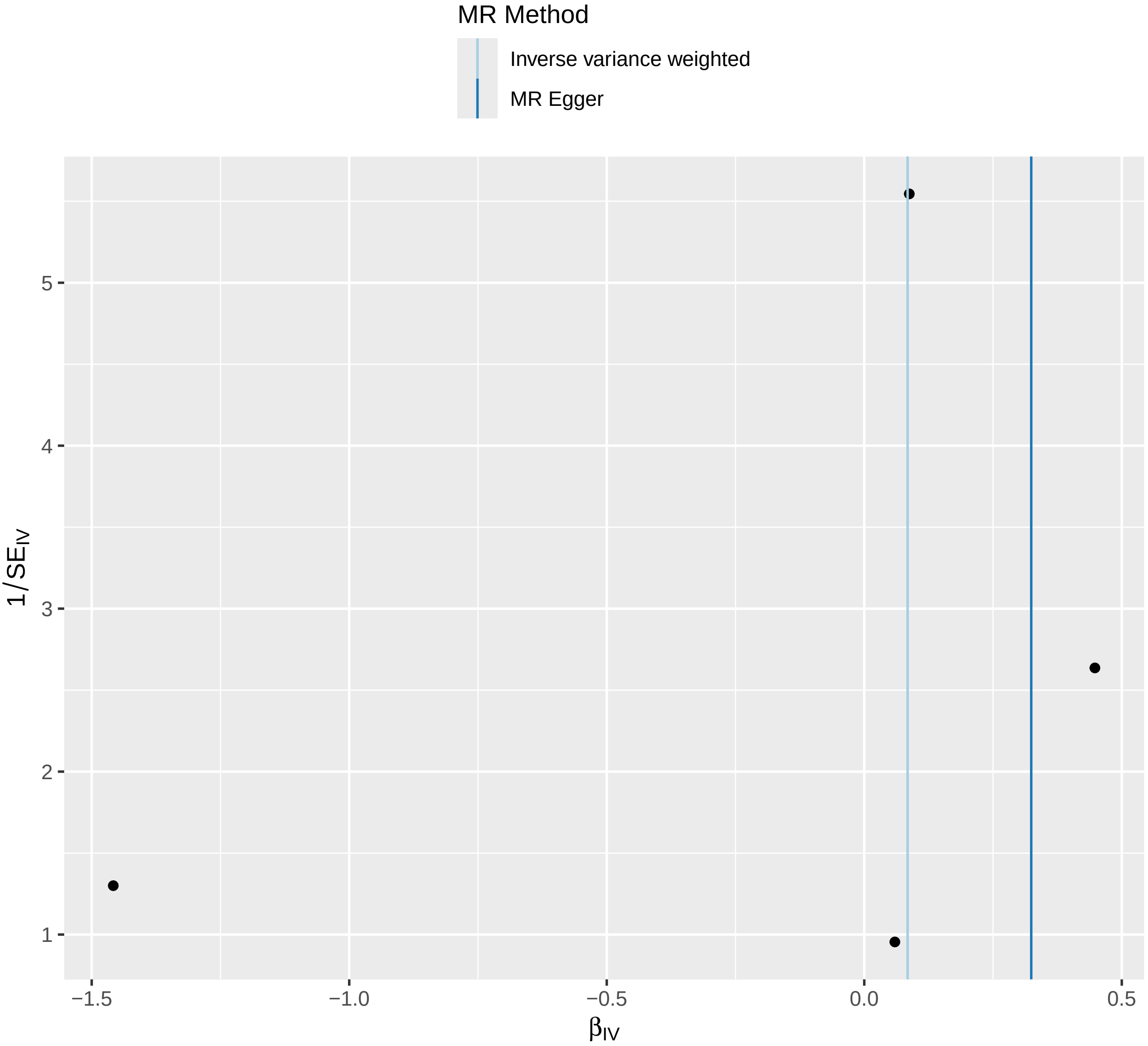
MR Method
Inverse variance weighted
MR Egger
1/SE_{IV}
5
4
3
2
1
−1.5
−1.0
−0.5
0.0
0.5
β_{IV}

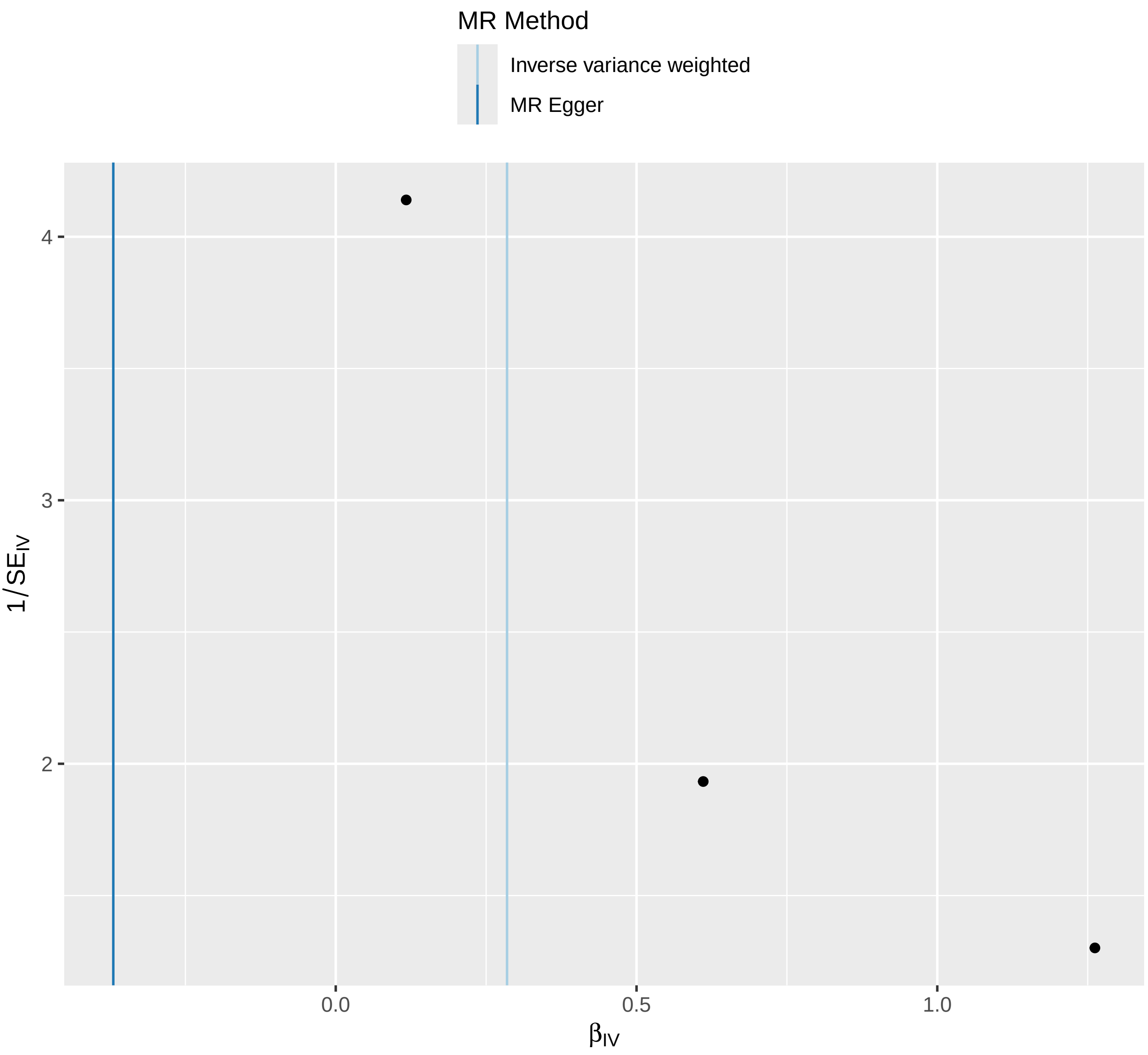

MR Method
Inverse variance weighted
MR Egger
4
3
2
1/SE_IV
0.0
0.5
1.0
β_IV

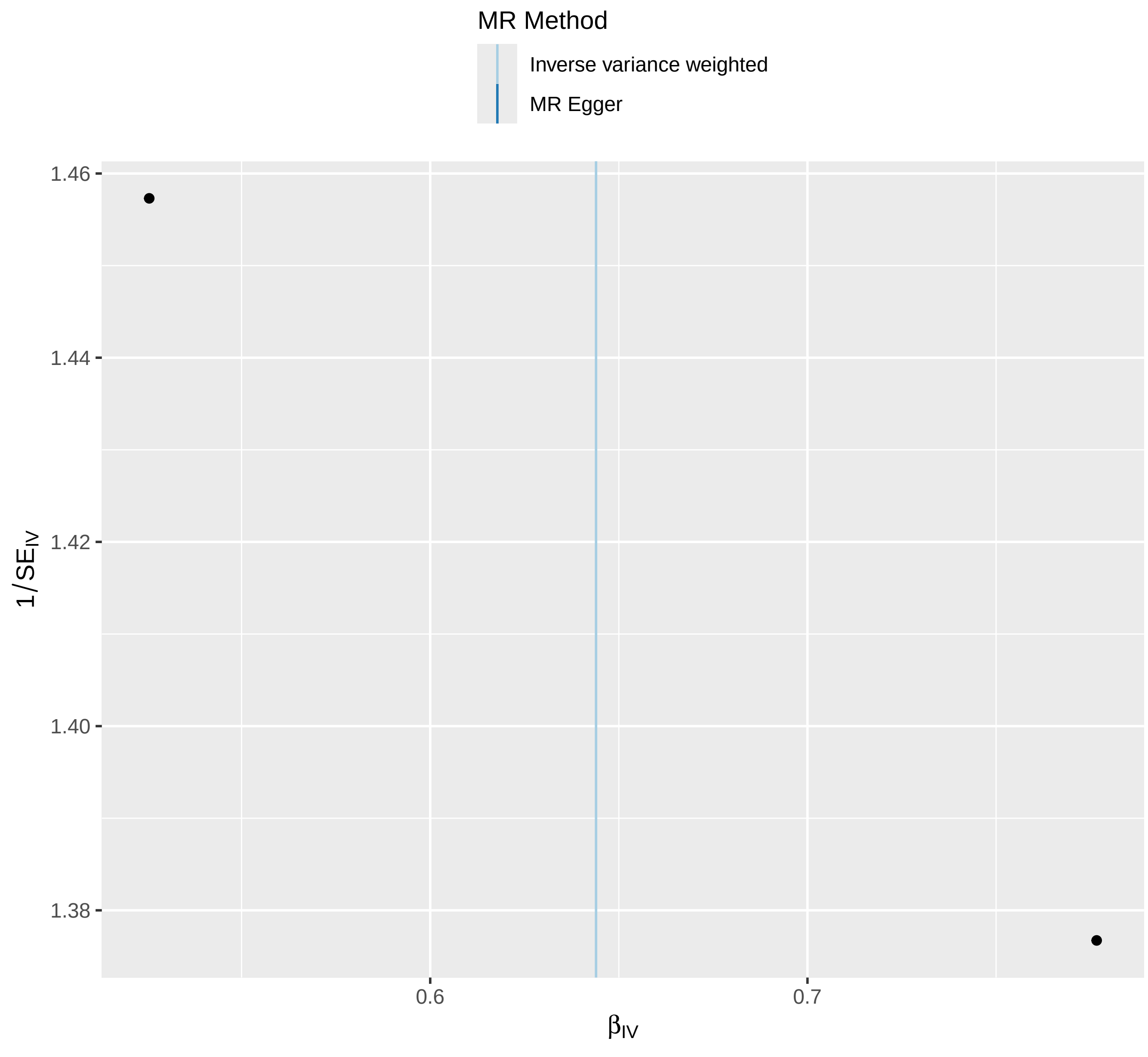
MR Method
Inverse variance weighted
MR Egger
1.46
1.44
1.42
1.40
1.38
$1/SE_{IV}$
0.6
0.7
β_{IV}

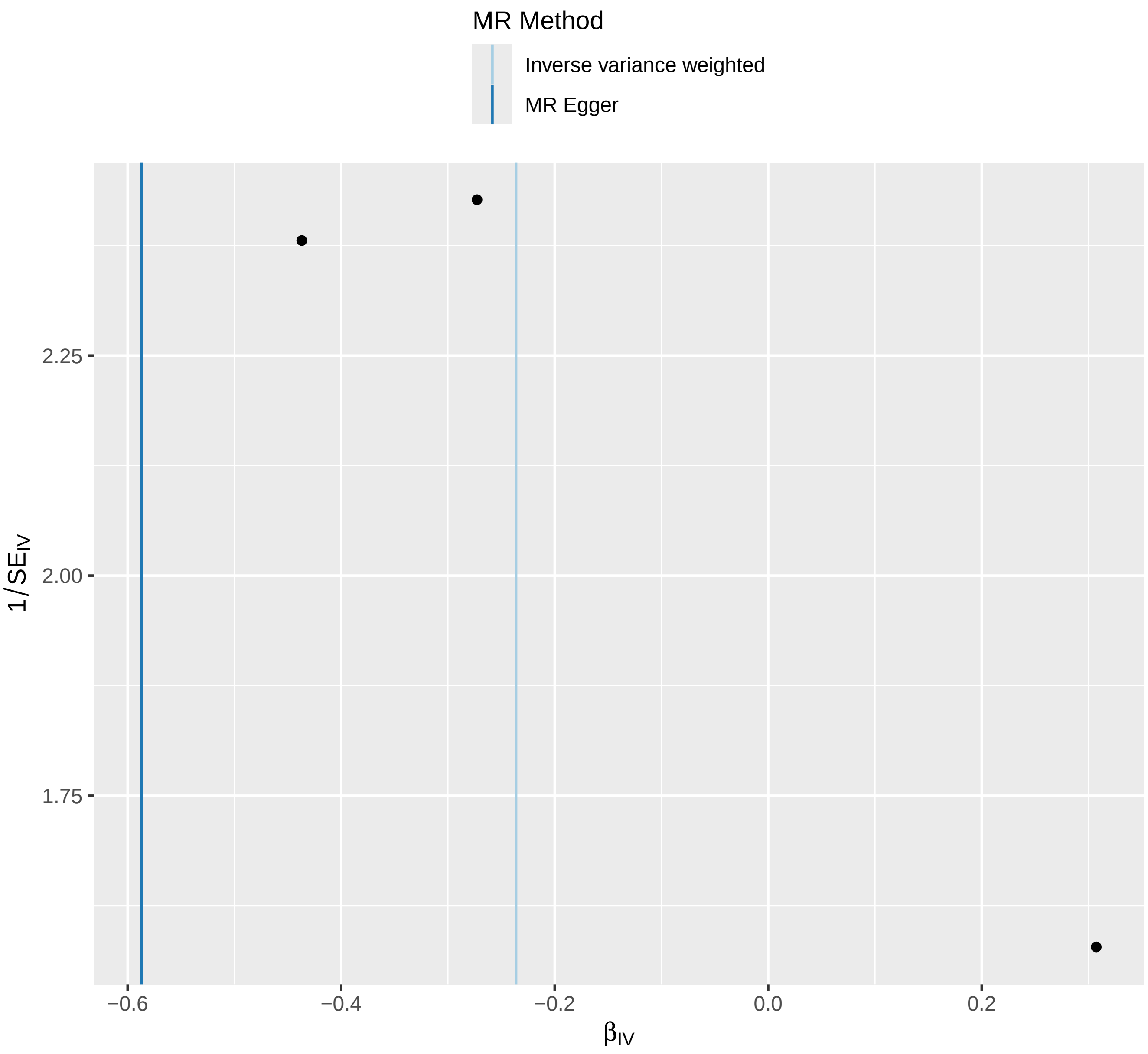
MR Method
Inverse variance weighted
MR Egger
2.25
2.00
1.75
1/SE_IV
−0.6
−0.4
−0.2
0.0
0.2
β_IV

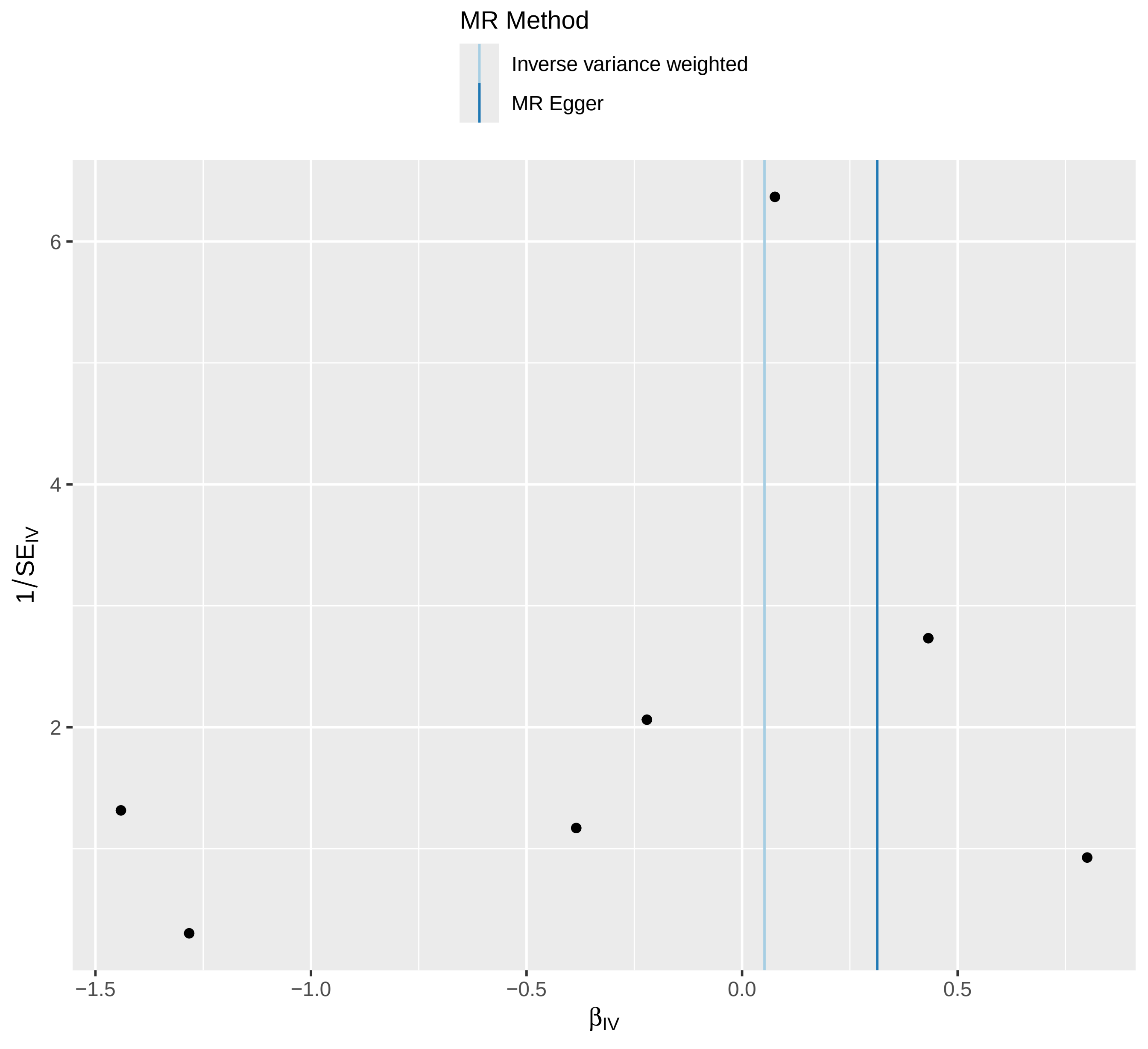

MR Method
Inverse variance weighted
MR Egger
6
4
2
1/SE_IV
−1.5
−1.0
−0.5
0.0
0.5
β_IV

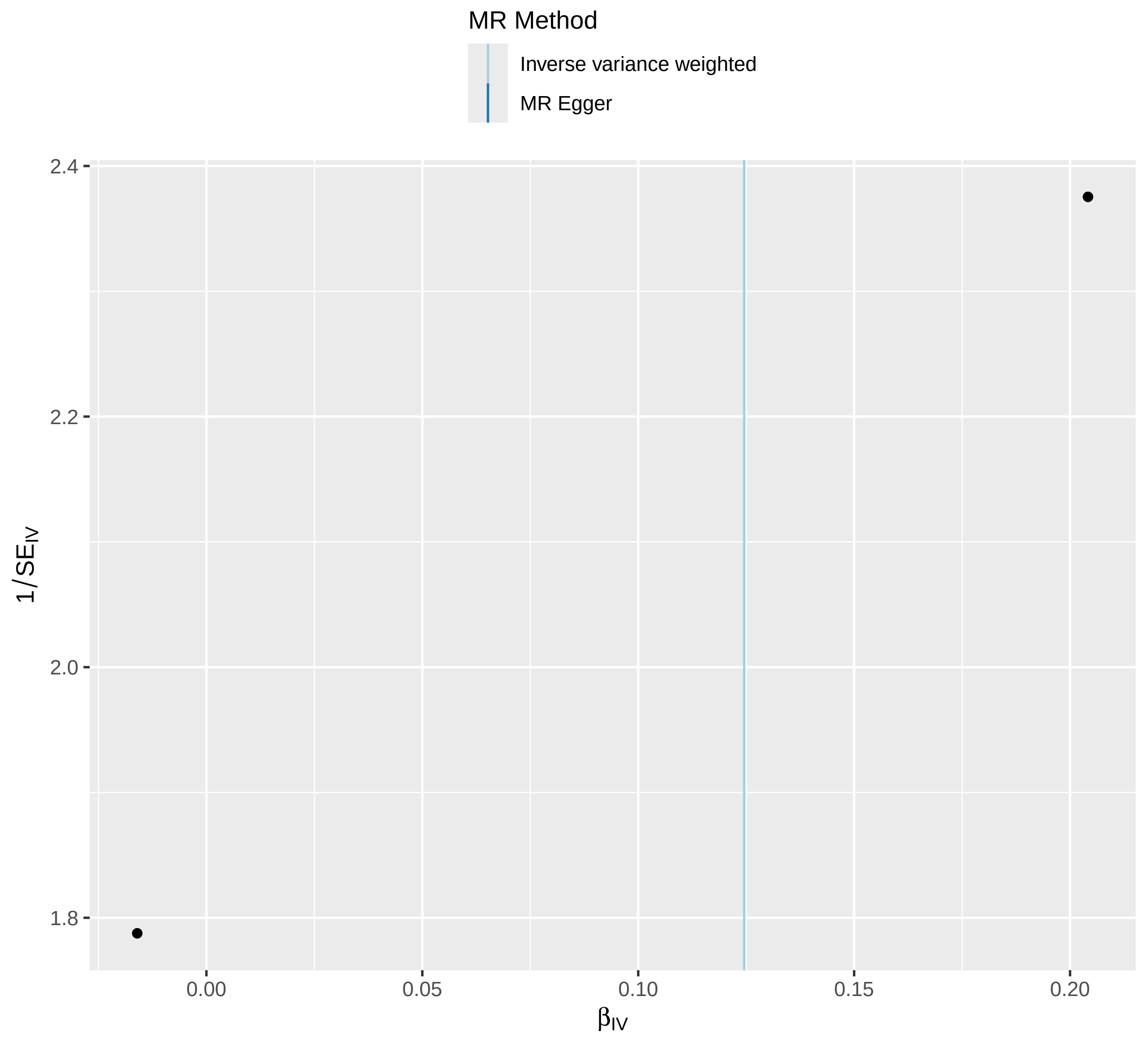

MR Method
Inverse variance weighted
MR Egger
2.4
2.2
2.0
1.8
$1/SE_{IV}$
0.00
0.05
0.10
0.15
0.20
β_{IV}

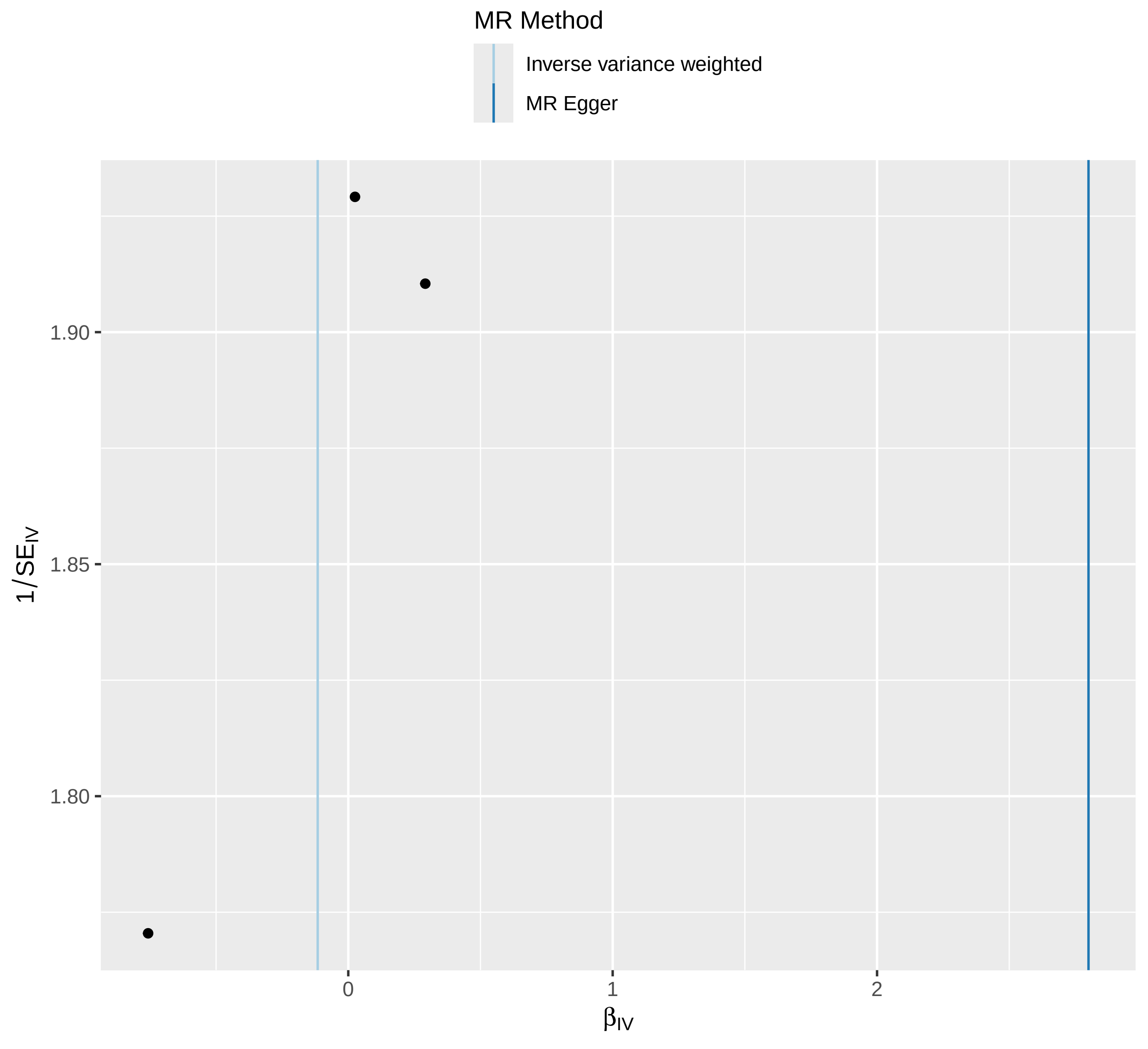

MR Method
Inverse variance weighted
MR Egger
1.90
1.85
1.80
1/SE_IV
0
1
2
β_IV

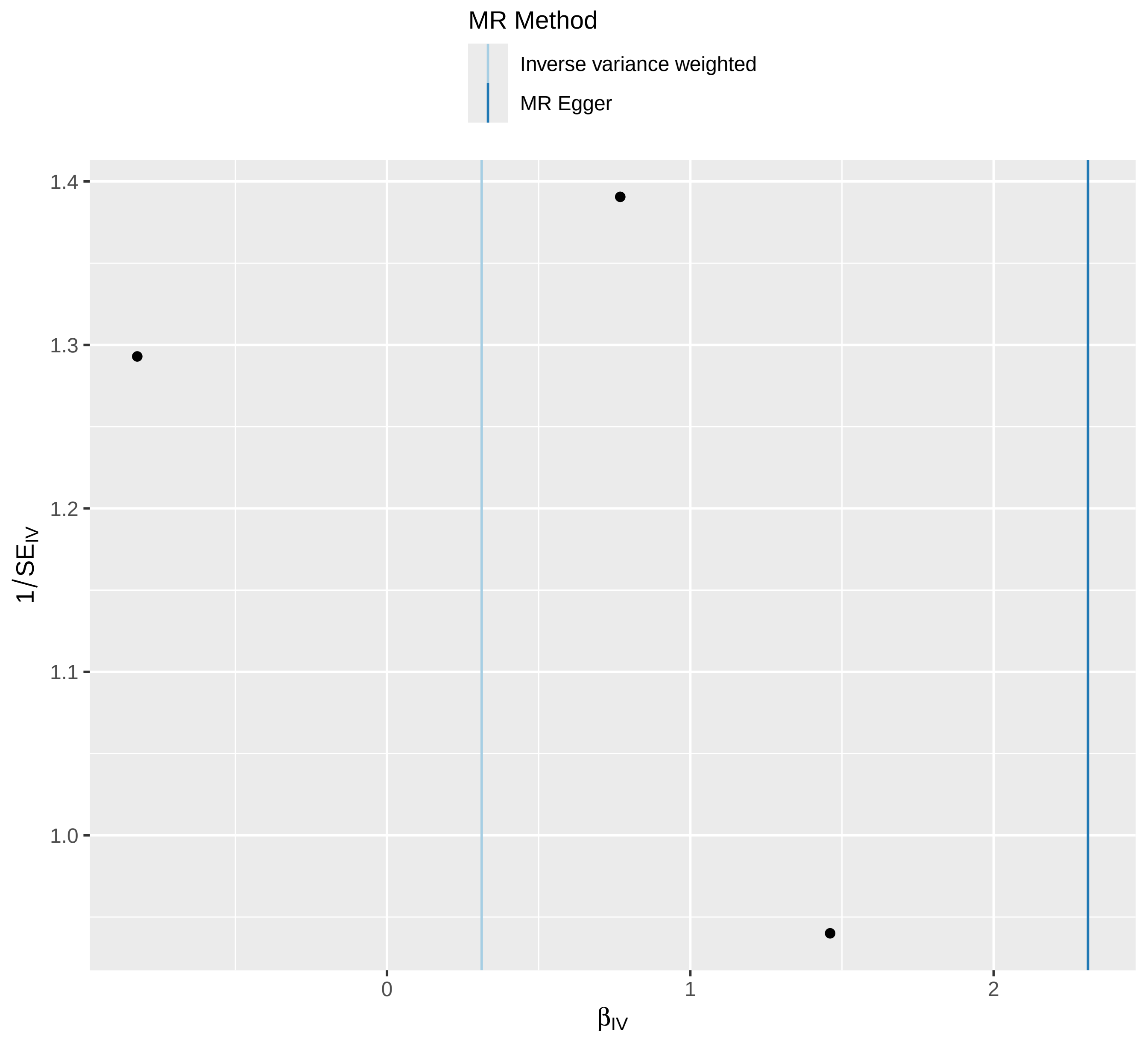
MR Method
Inverse variance weighted
MR Egger
1.4
1.3
1.2
1.1
1.0
1/SE_IV
0
1
2
β_IV

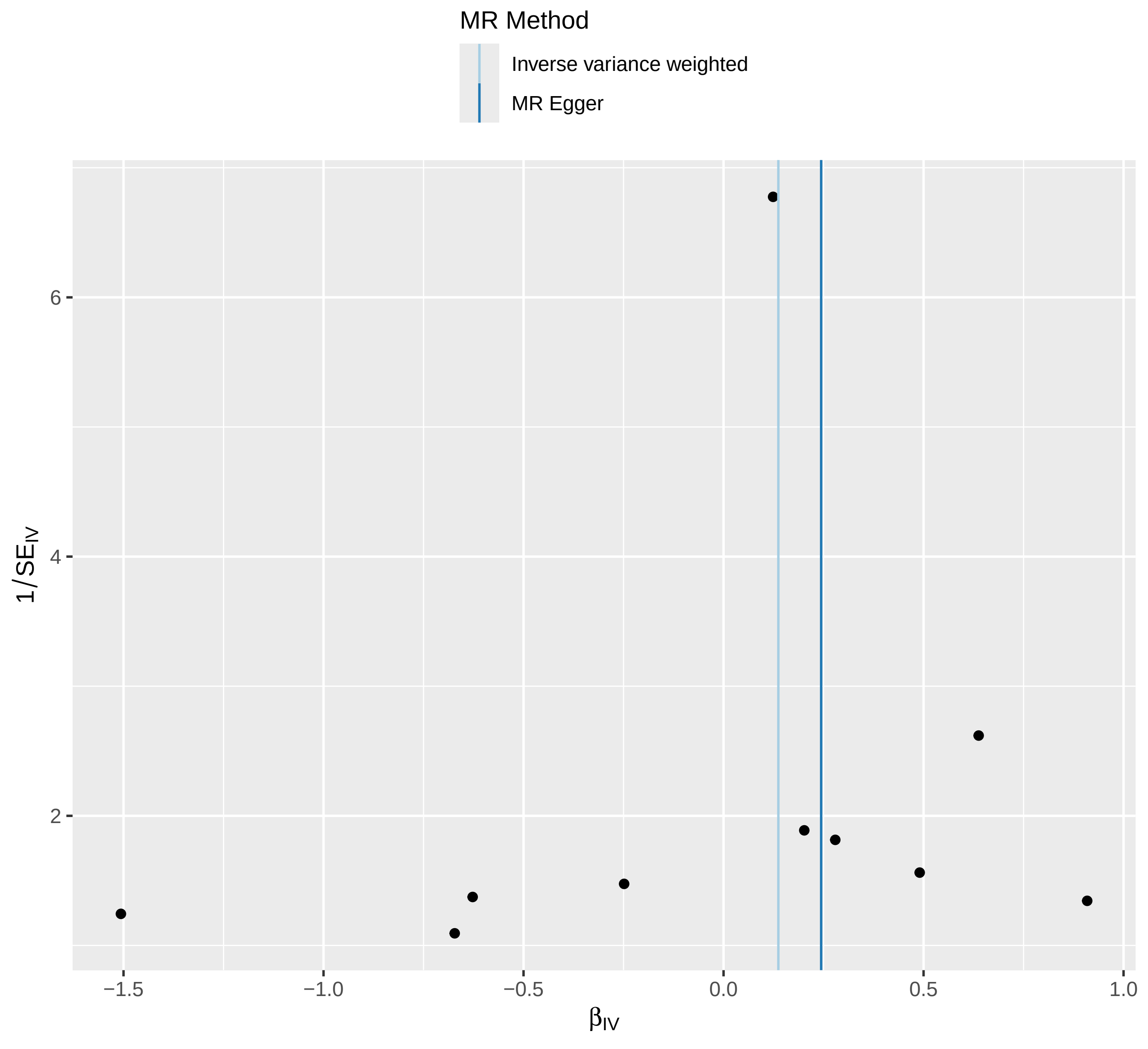
MR Method
Inverse variance weighted
MR Egger
1/SE_IV
6
4
2
−1.5
−1.0
−0.5
0.0
0.5
1.0
β_IV

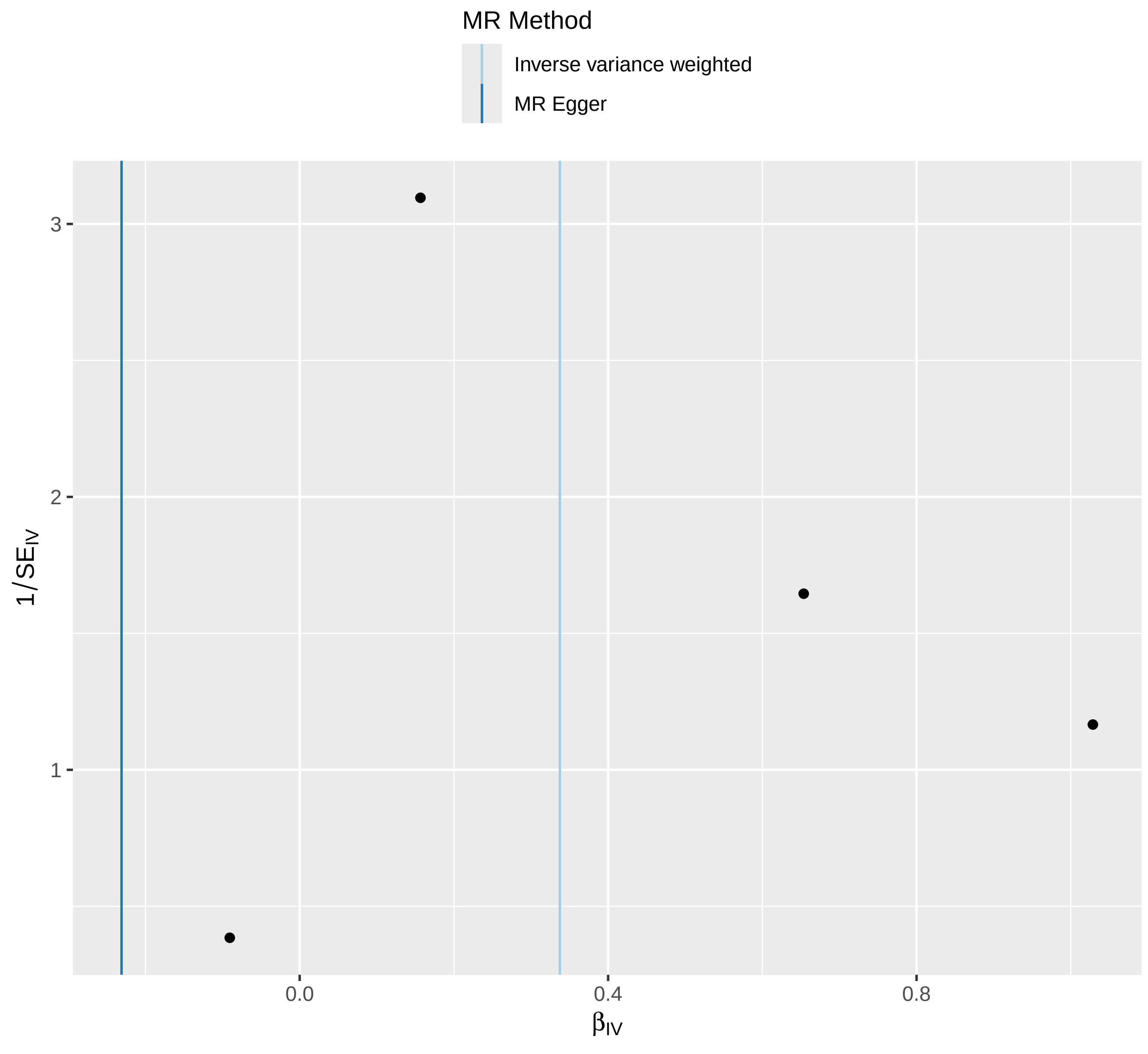
MR Method
Inverse variance weighted
MR Egger
3
2
1
0.0
0.4
0.8
$1/SE_{IV}$
β_{IV}

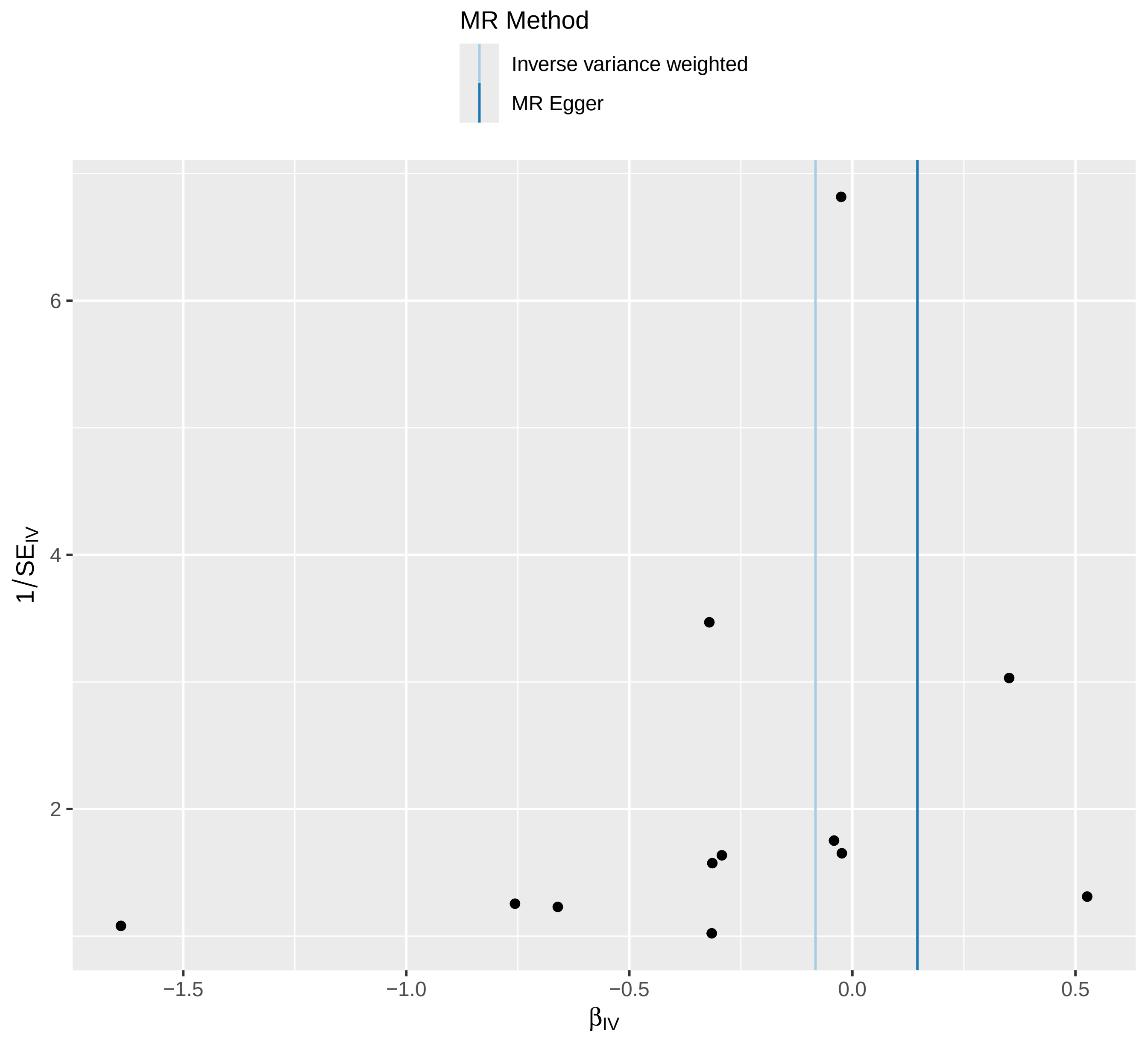

MR Method
Inverse variance weighted
MR Egger
6
4
2
1/SE_IV
−1.5
−1.0
−0.5
0.0
0.5
β_IV

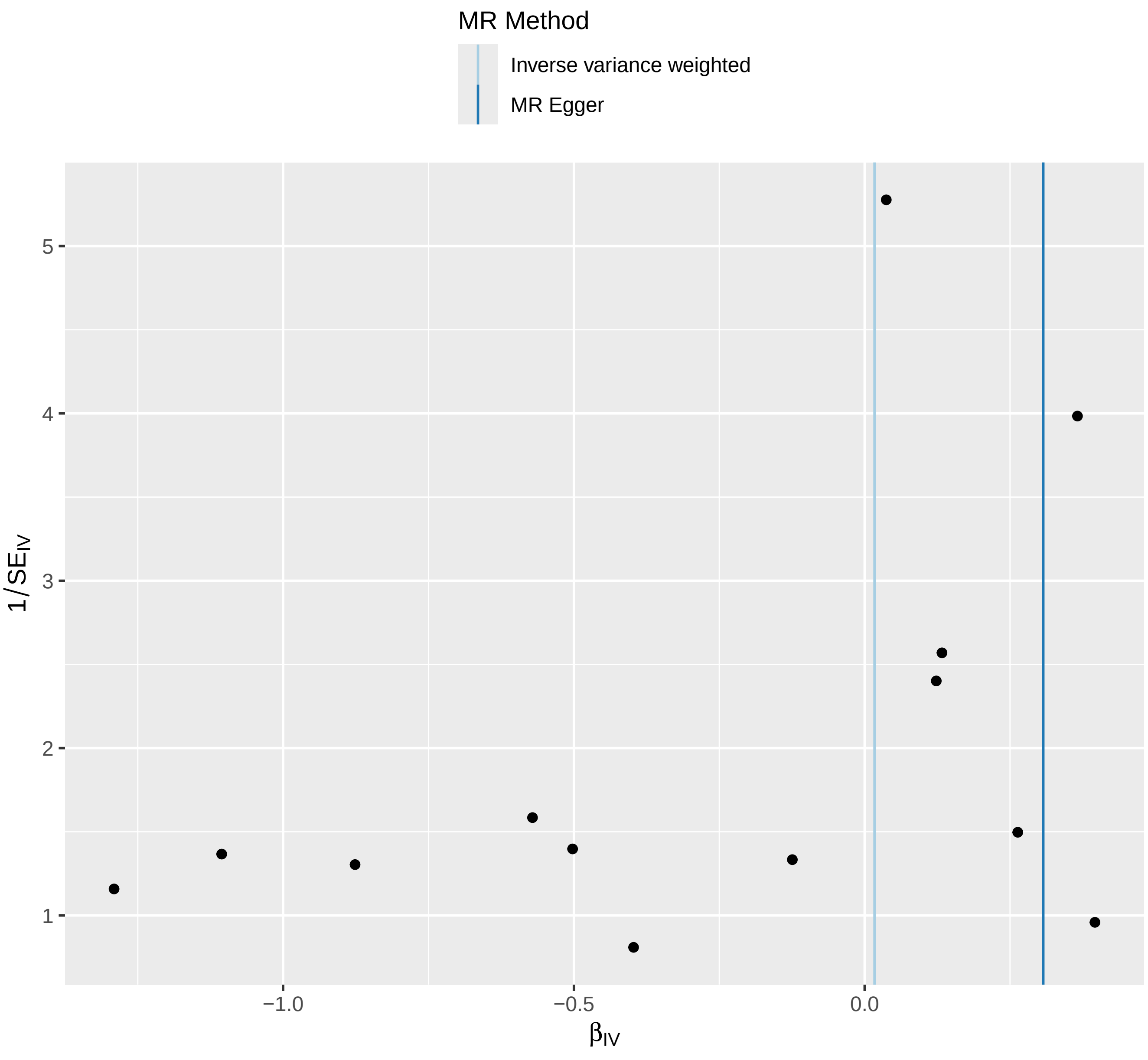
MR Method
Inverse variance weighted
MR Egger
5
4
3
2
1
1/SE_IV
−1.0
−0.5
0.0
β_IV

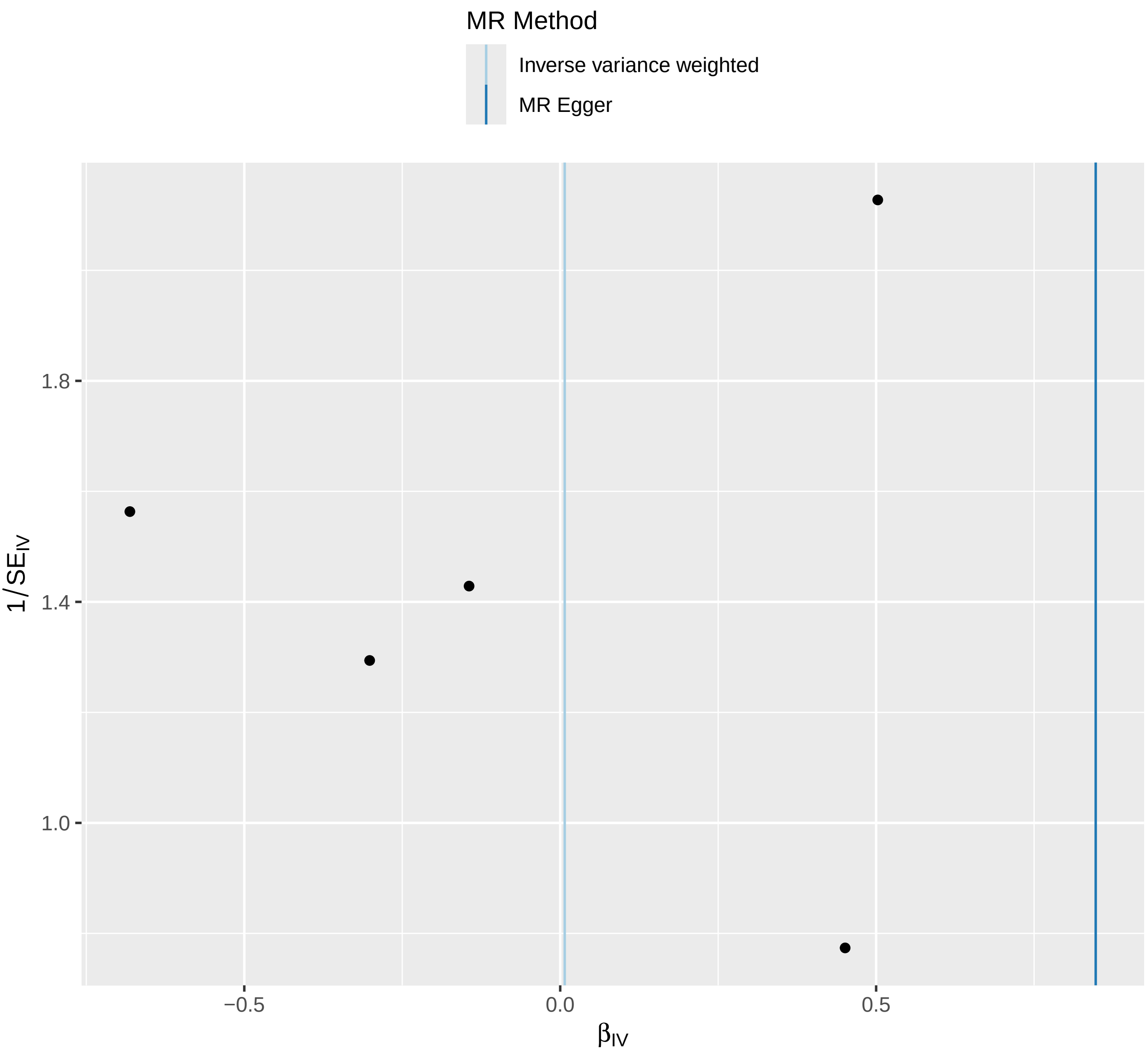
MR Method
Inverse variance weighted
MR Egger
1.8
1.4
1.0
1/SE$_{IV}$
−0.5
0.0
0.5
β$_{IV}$

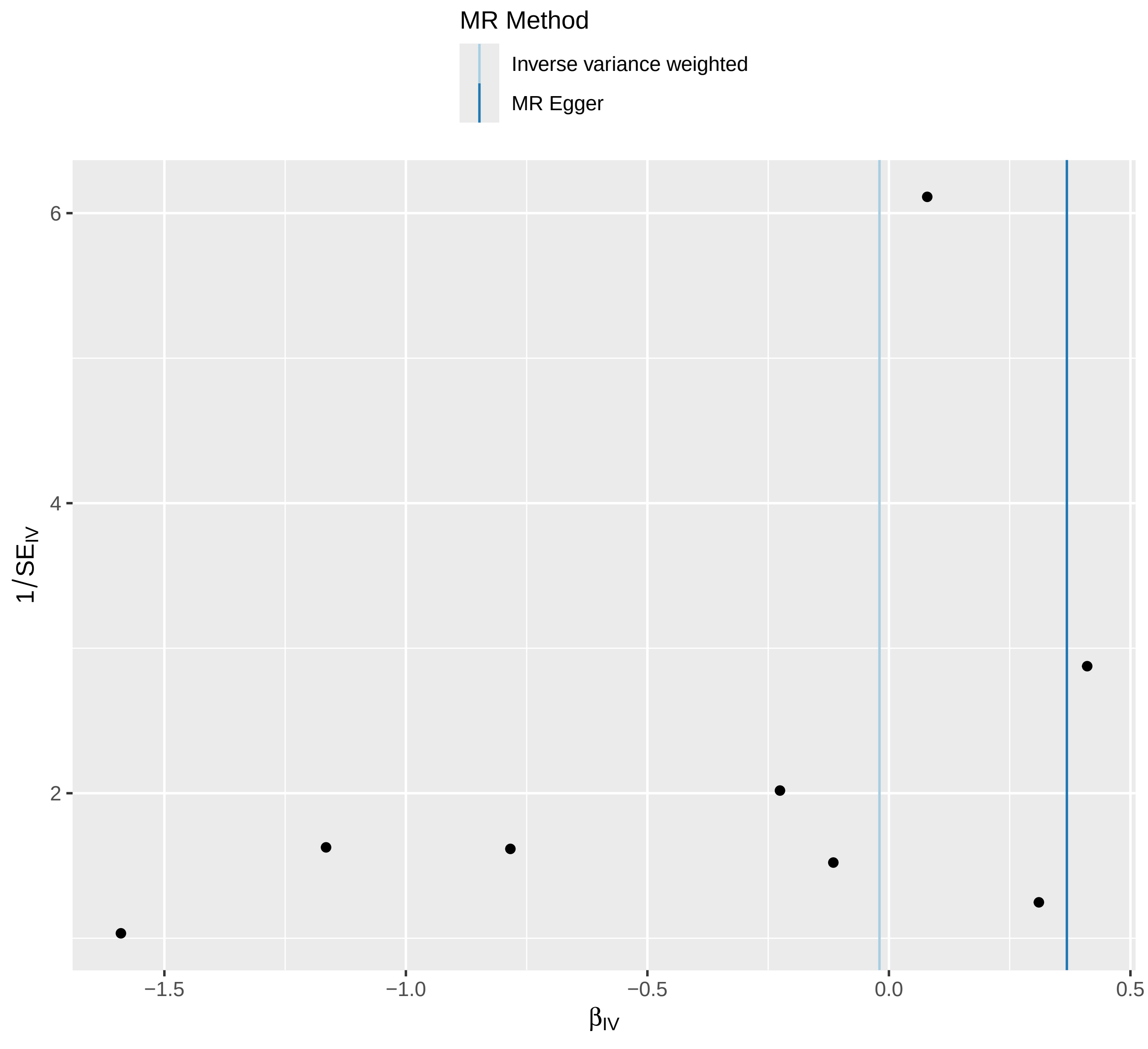
MR Method
Inverse variance weighted
MR Egger
6
4
2
$1/SE_{IV}$
−1.5
−1.0
−0.5
0.0
0.5
β_{IV}

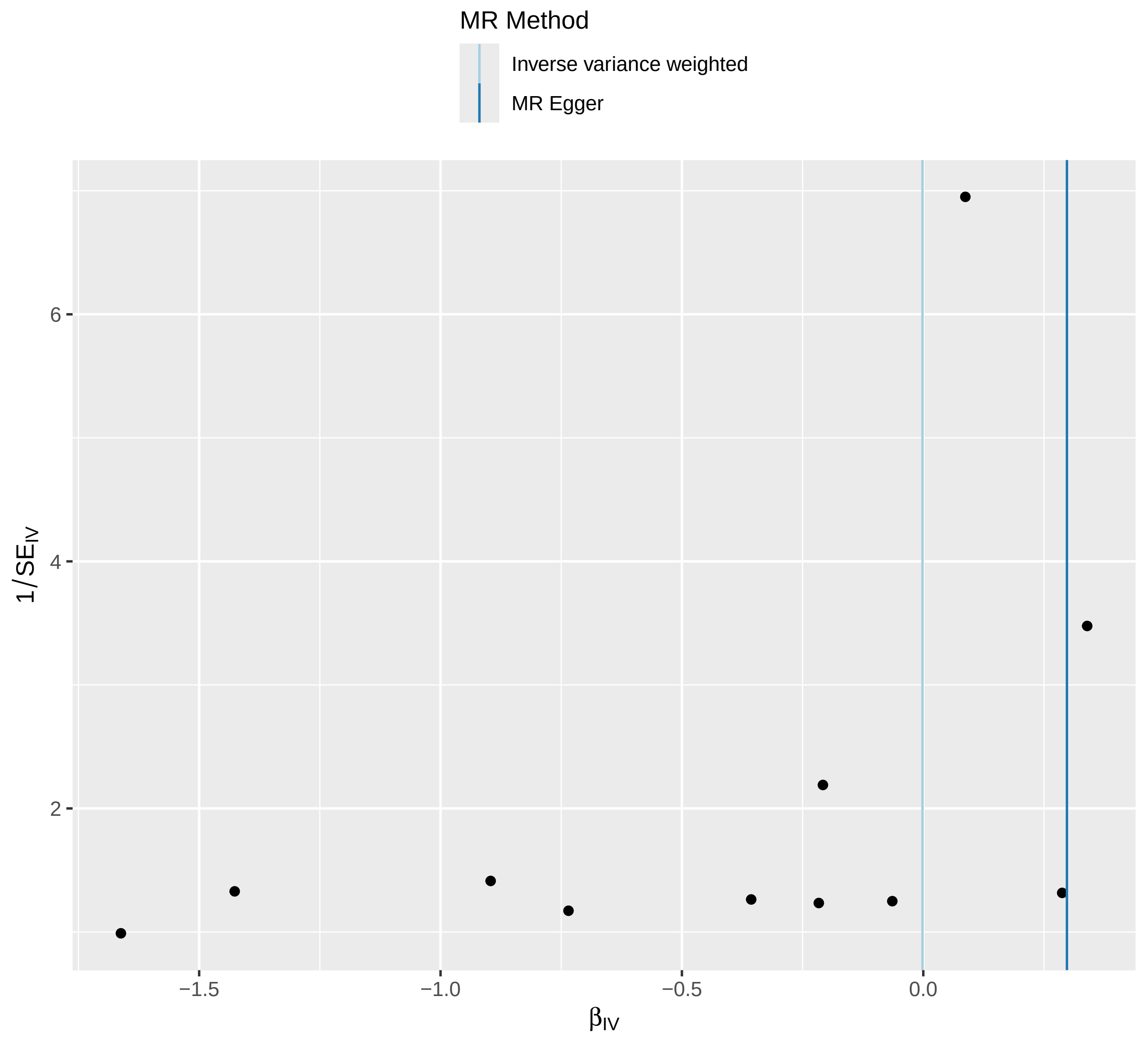
MR Method
Inverse variance weighted
MR Egger
6
4
2
1/SE_IV
−1.5
−1.0
−0.5
0.0
β_IV

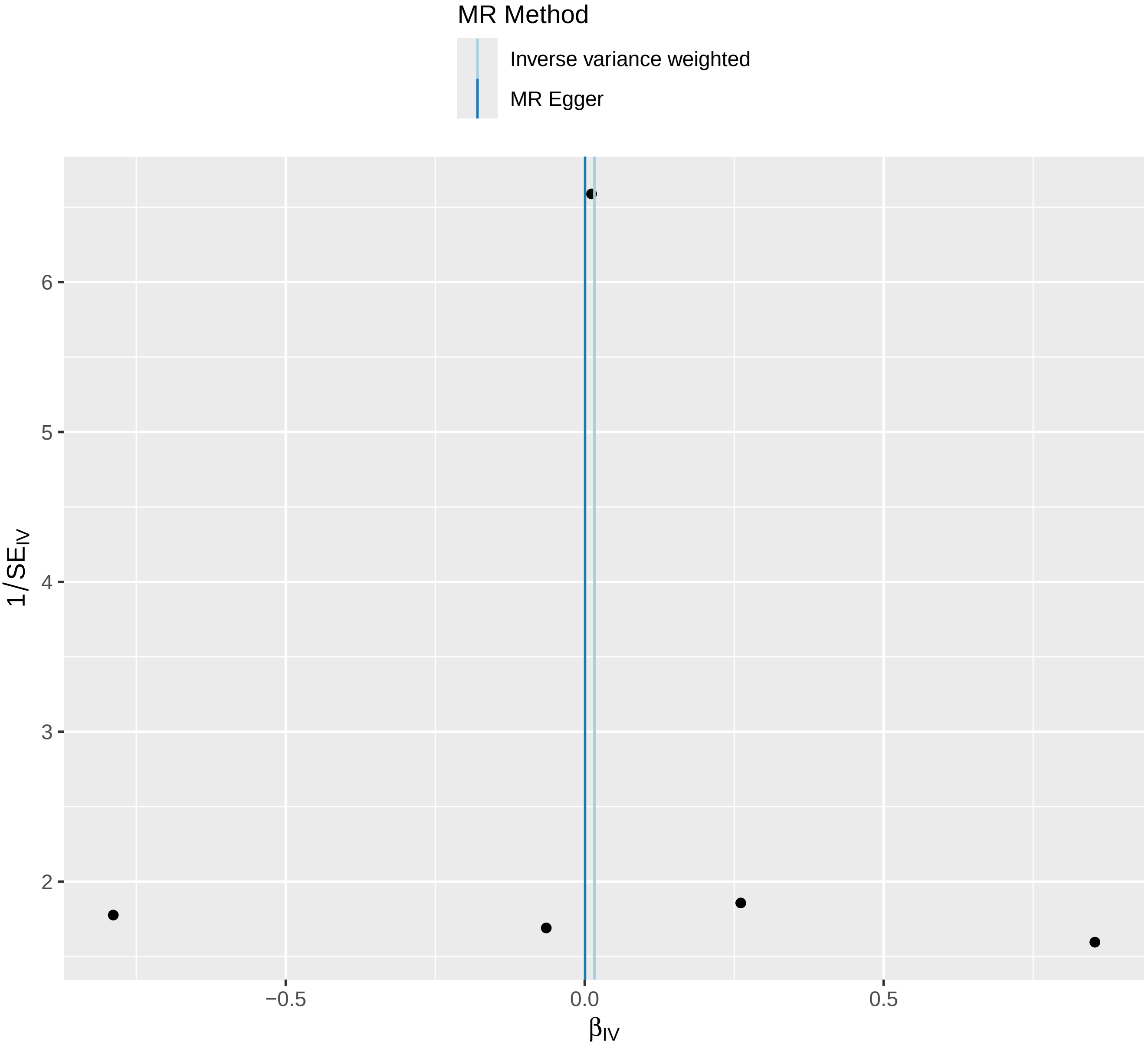
MR Method
Inverse variance weighted
MR Egger
1/SE_IV
6
5
4
3
2
−0.5
0.0
0.5
β_IV

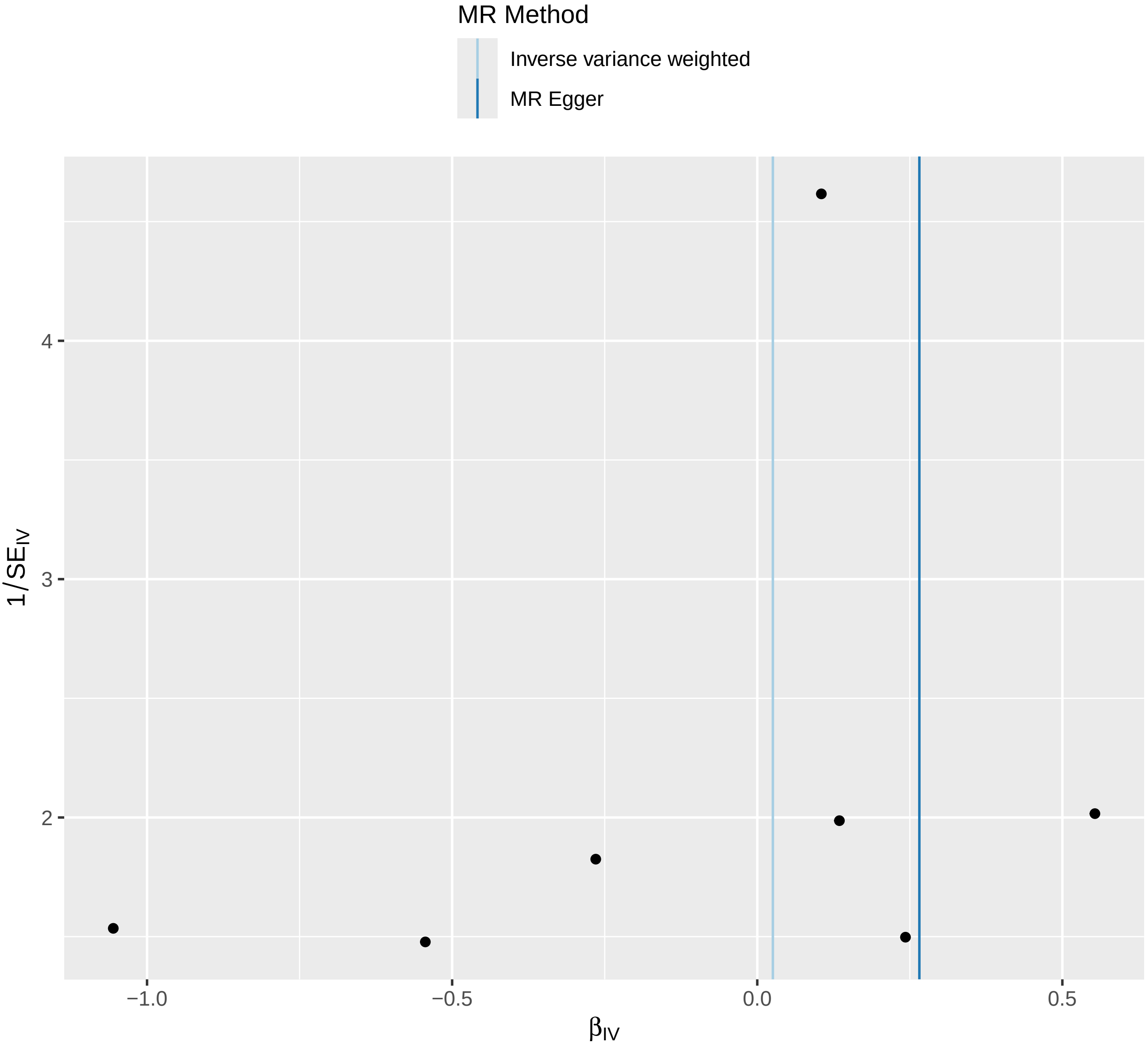
MR Method
Inverse variance weighted
MR Egger
1/SE_IV
4
3
2
−1.0
−0.5
0.0
0.5
β_IV

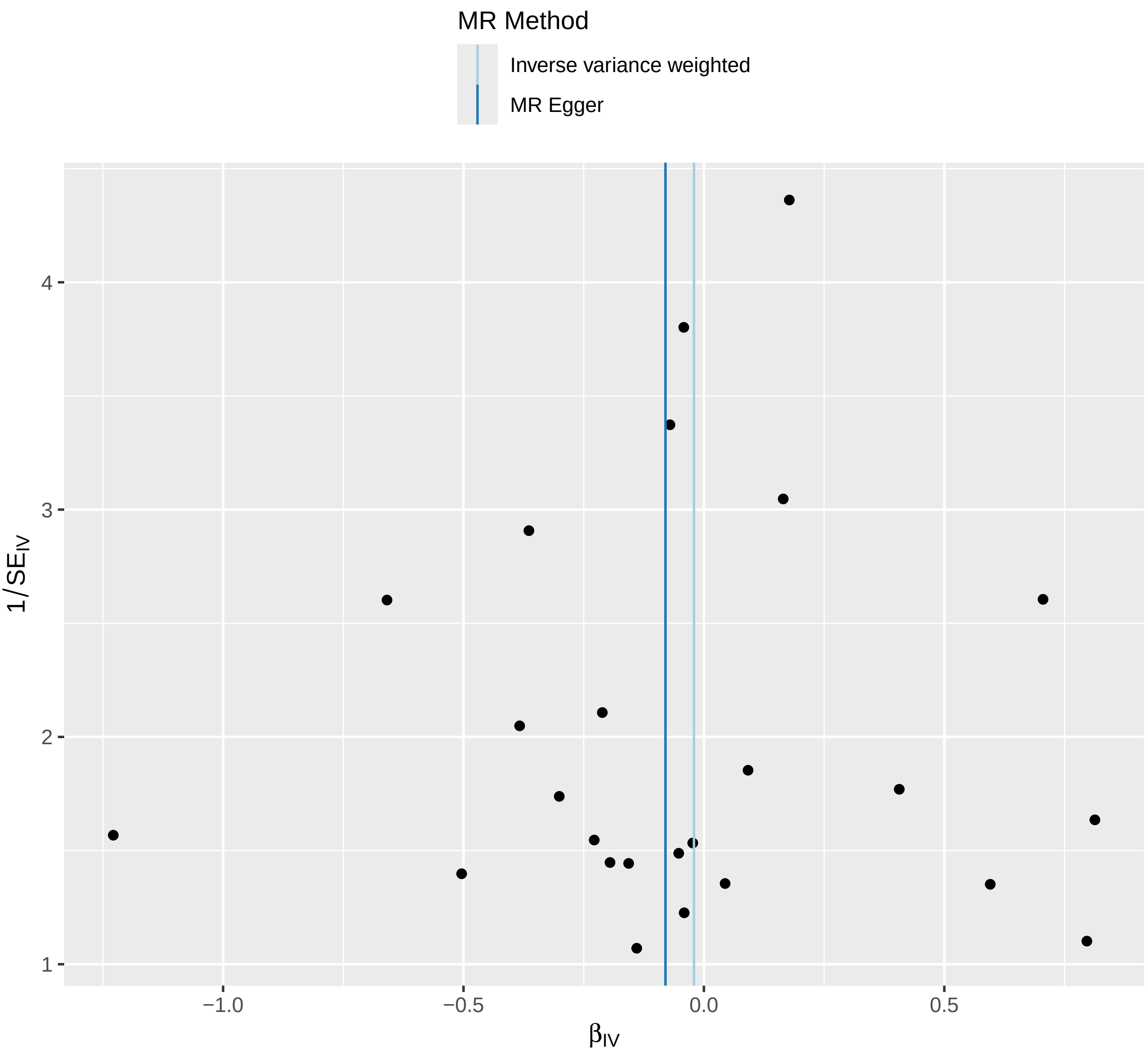
MR Method
Inverse variance weighted
MR Egger
1/SE_IV
β_IV
4
3
2
1
−1.0
−0.5
0.0
0.5

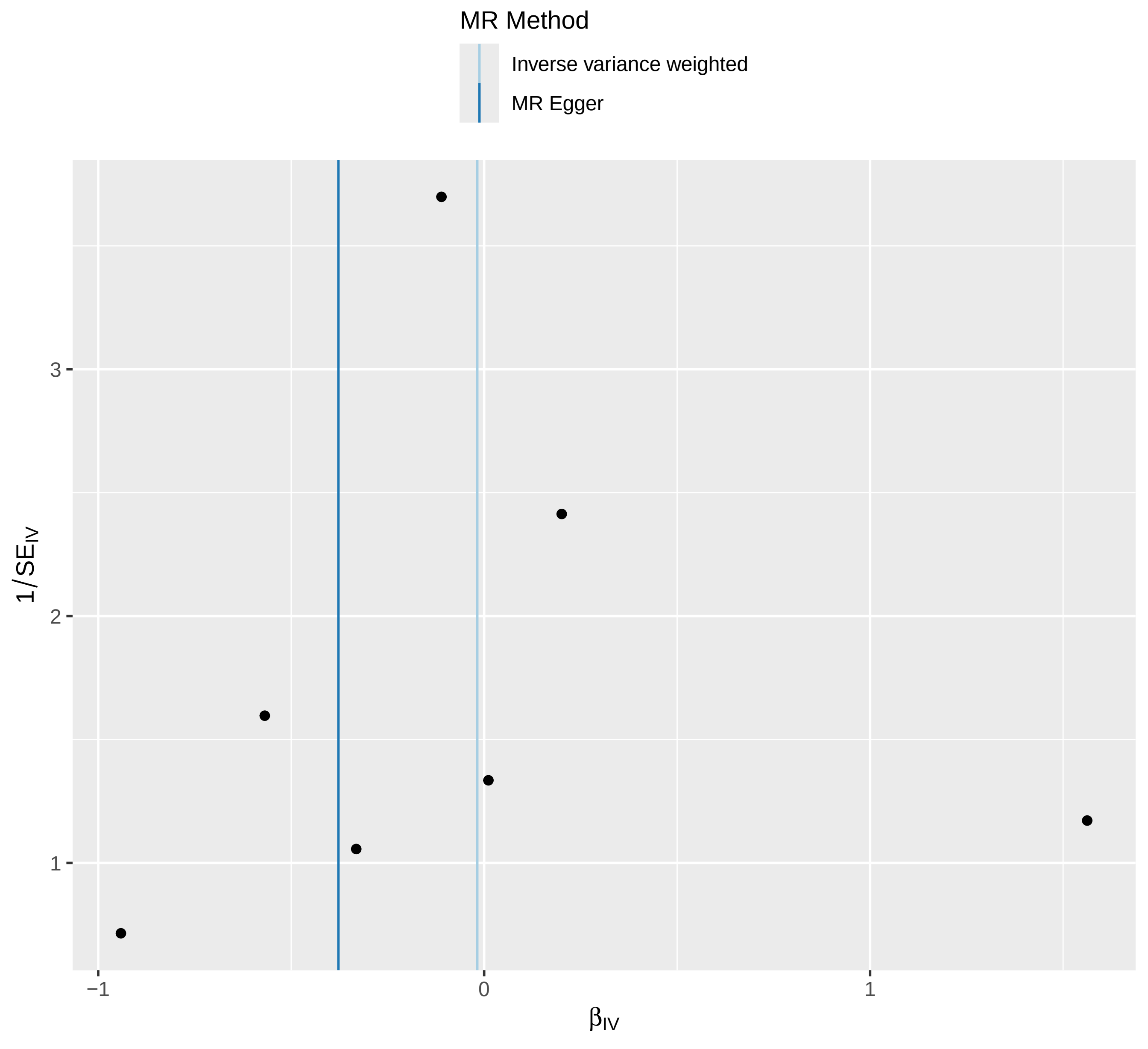

MR Method
Inverse variance weighted
MR Egger
3
2
1
1/SE_IV
−1
0
1
β_IV

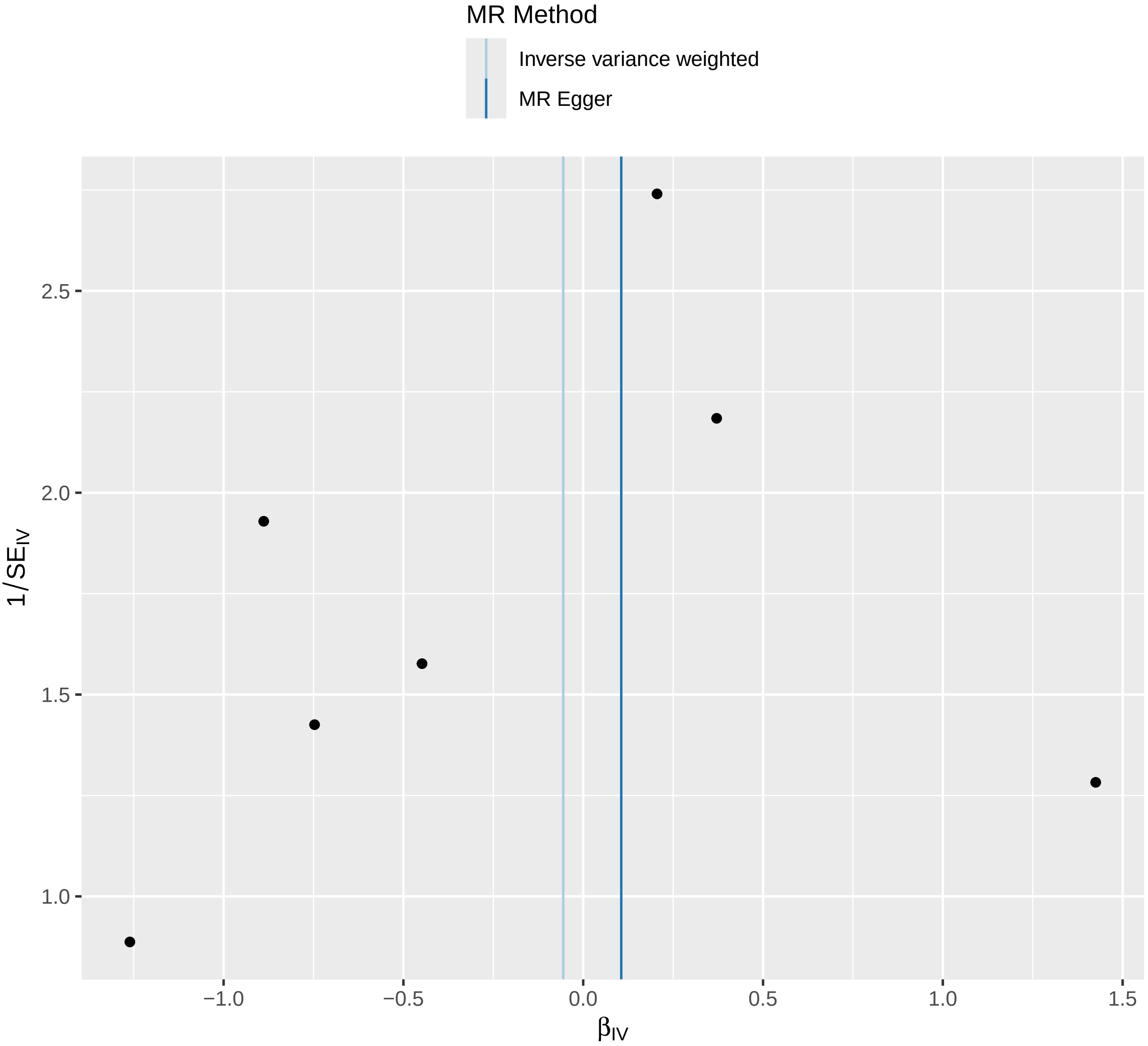

MR Method
Inverse variance weighted
MR Egger
2.5
2.0
1.5
1.0
1/SE_IV
−1.0
−0.5
0.0
0.5
1.0
1.5
β_IV

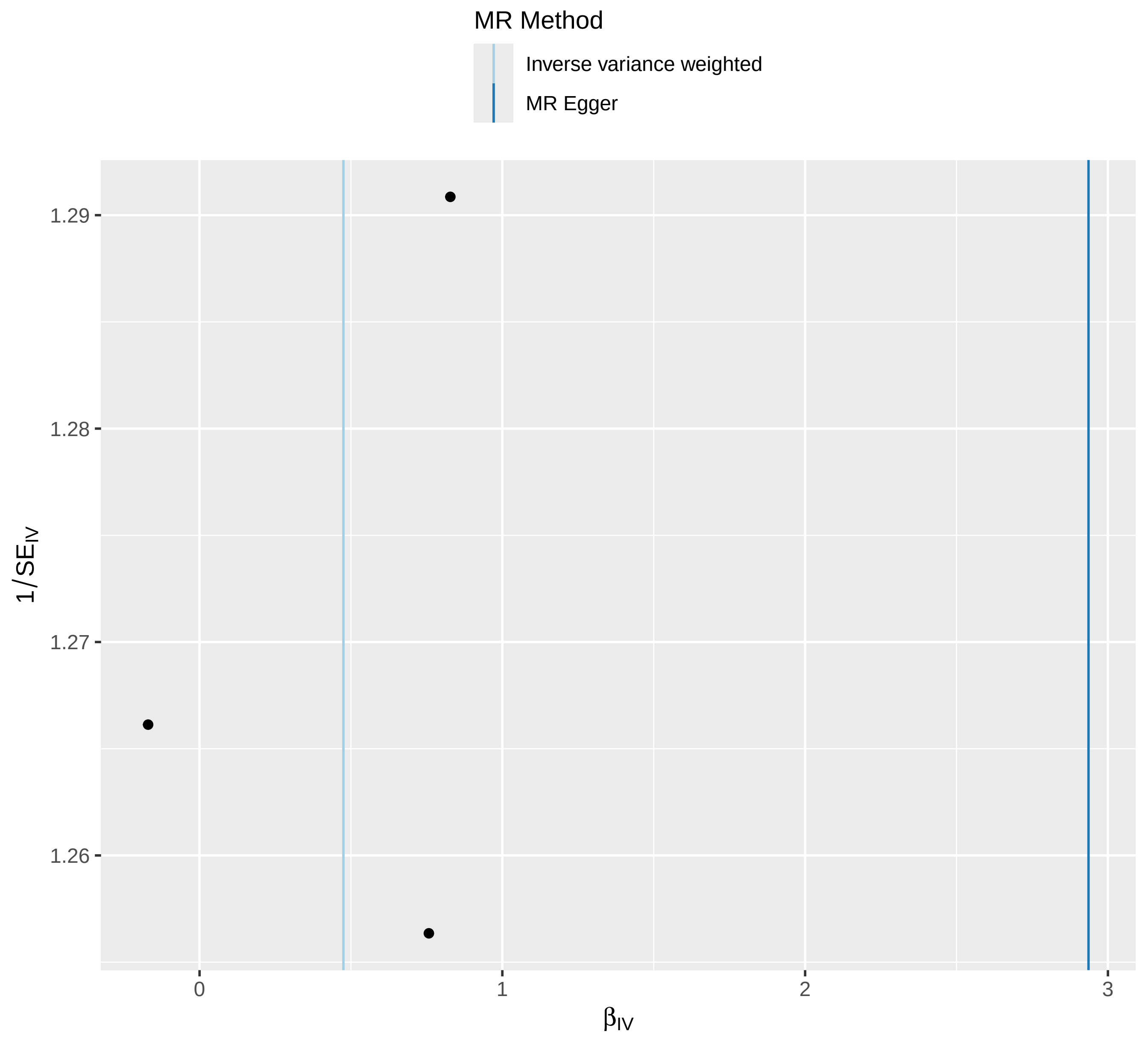
MR Method
Inverse variance weighted
MR Egger
1.29
1.28
1.27
1.26
$1/SE_{IV}$
0
1
2
3
β_{IV}

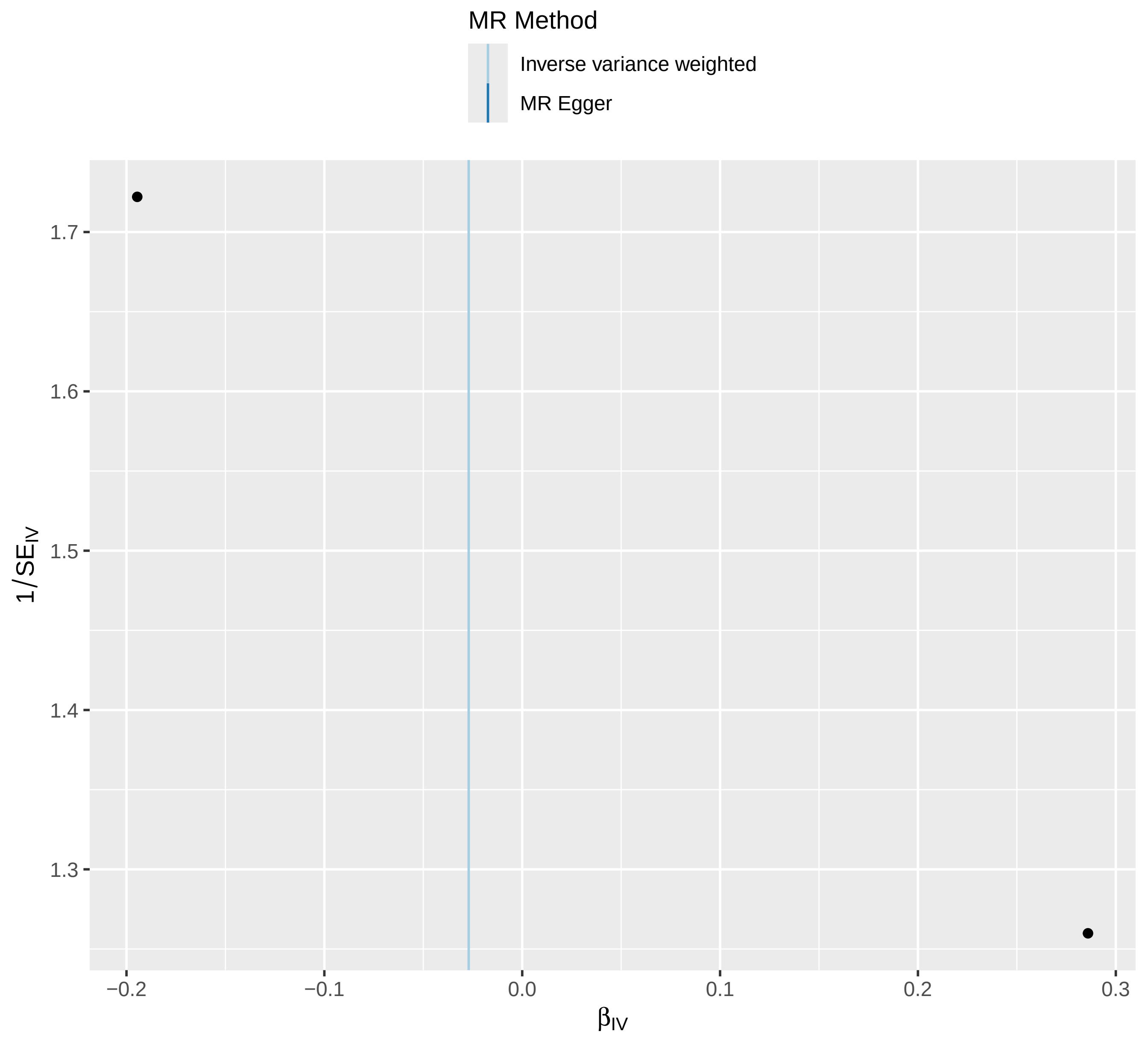
MR Method
Inverse variance weighted
MR Egger
1.7
1.6
1.5
1.4
1.3
$1/SE_{IV}$
−0.2
−0.1
0.0
0.1
0.2
0.3
β_{IV}

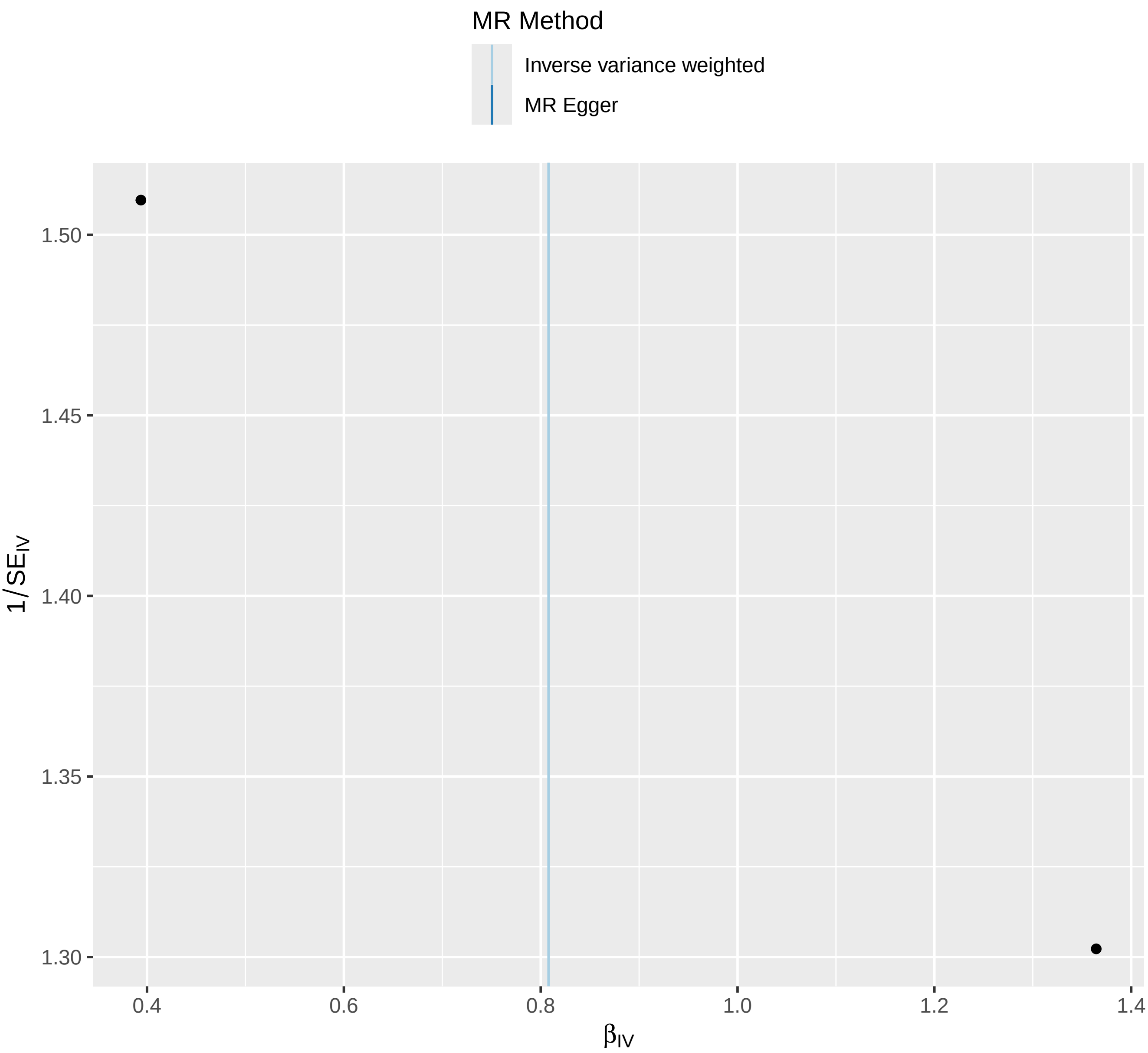
MR Method
Inverse variance weighted
MR Egger
1.50
1.45
1.40
1.35
1.30
$1/SE_{IV}$
0.4
0.6
0.8
1.0
1.2
1.4
β_{IV}

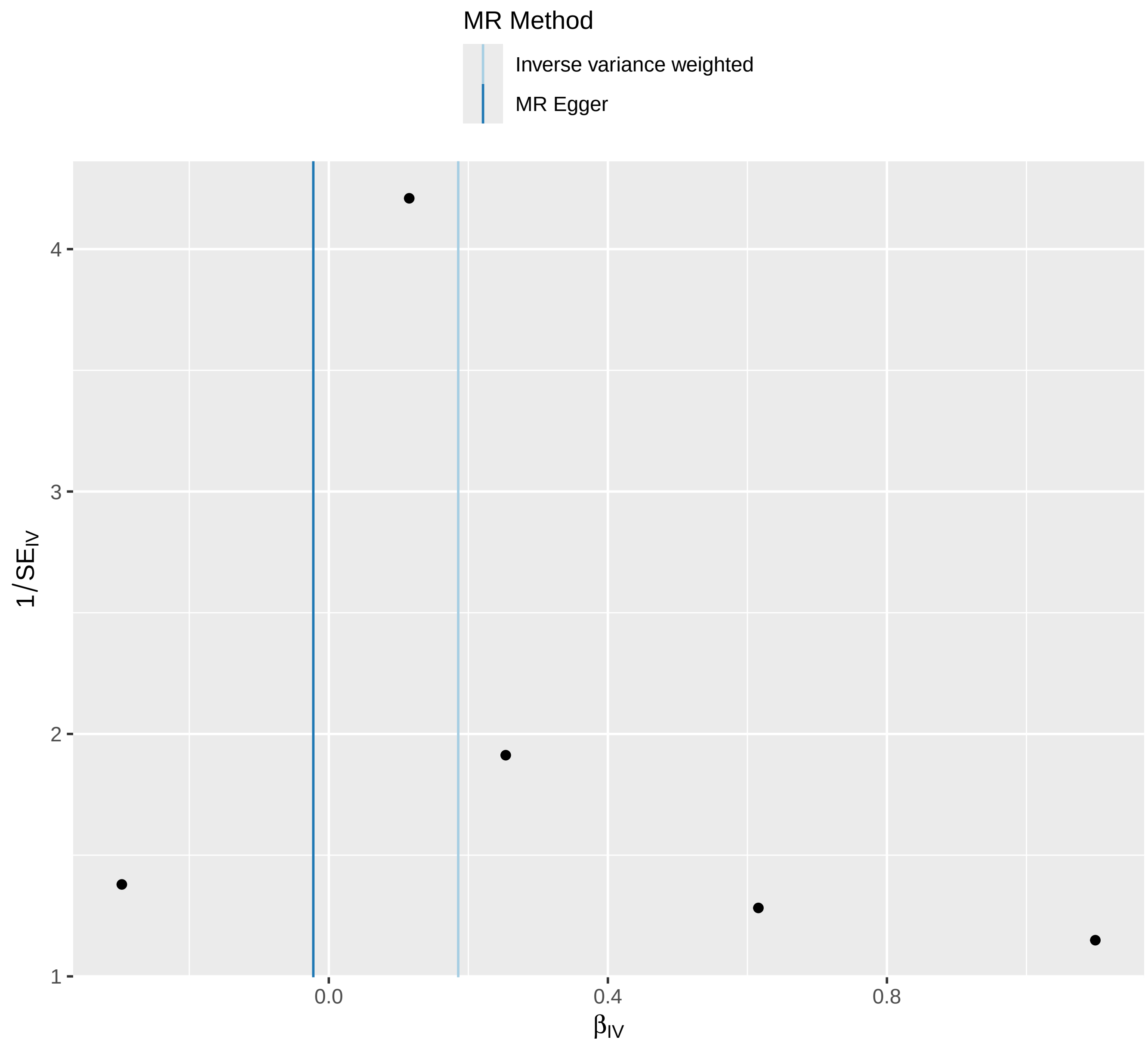

MR Method
Inverse variance weighted
MR Egger
4
3
2
1
1/SE_IV
0.0
0.4
0.8
β_IV

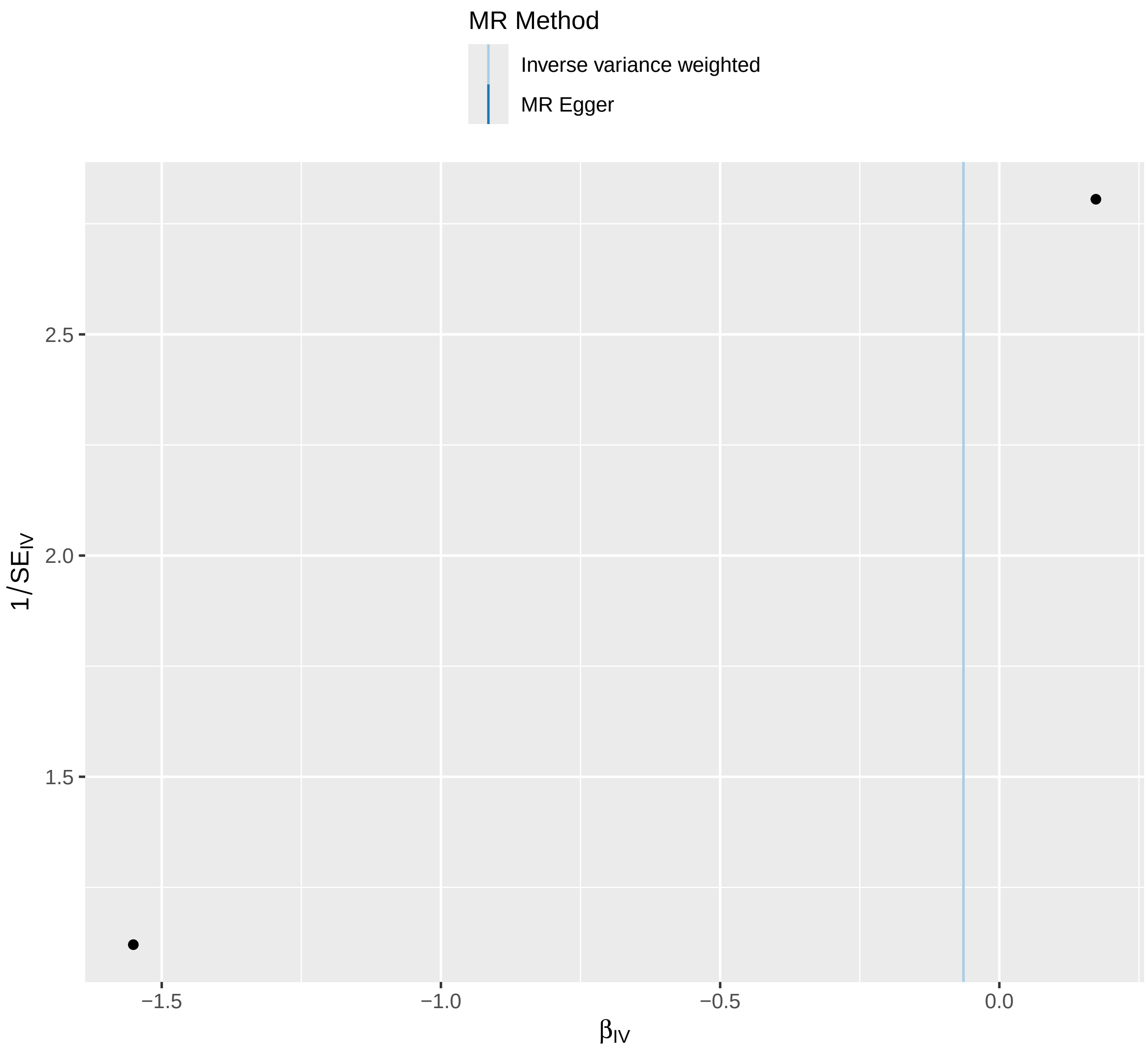
MR Method
Inverse variance weighted
MR Egger
2.5
2.0
1.5
1/SE$_{IV}$
−1.5
−1.0
−0.5
0.0
β$_{IV}$

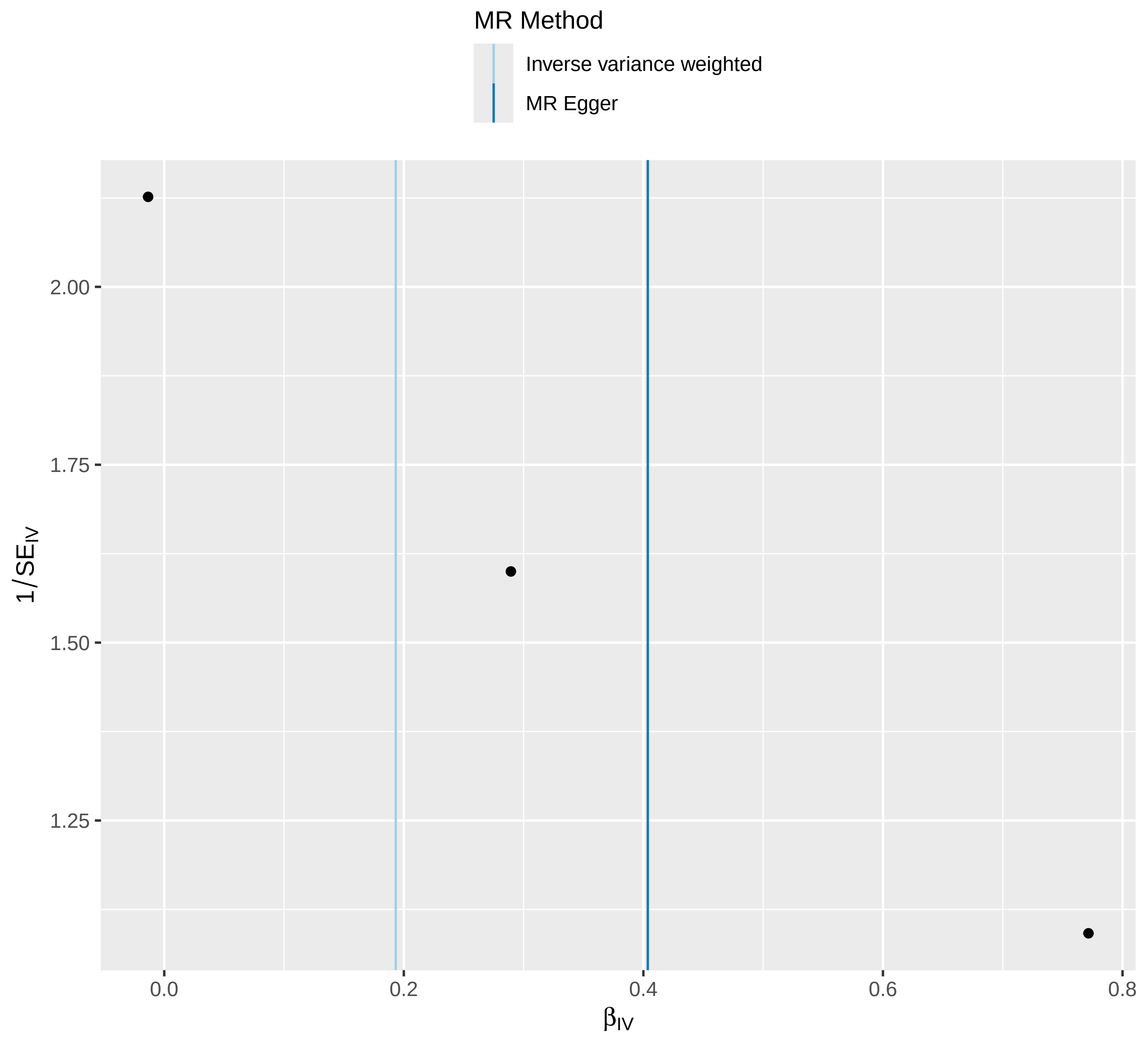
MR Method
Inverse variance weighted
MR Egger
2.00
1.75
1.50
1.25
0.0
0.2
0.4
0.6
0.8
$1/SE_{IV}$
β_{IV}

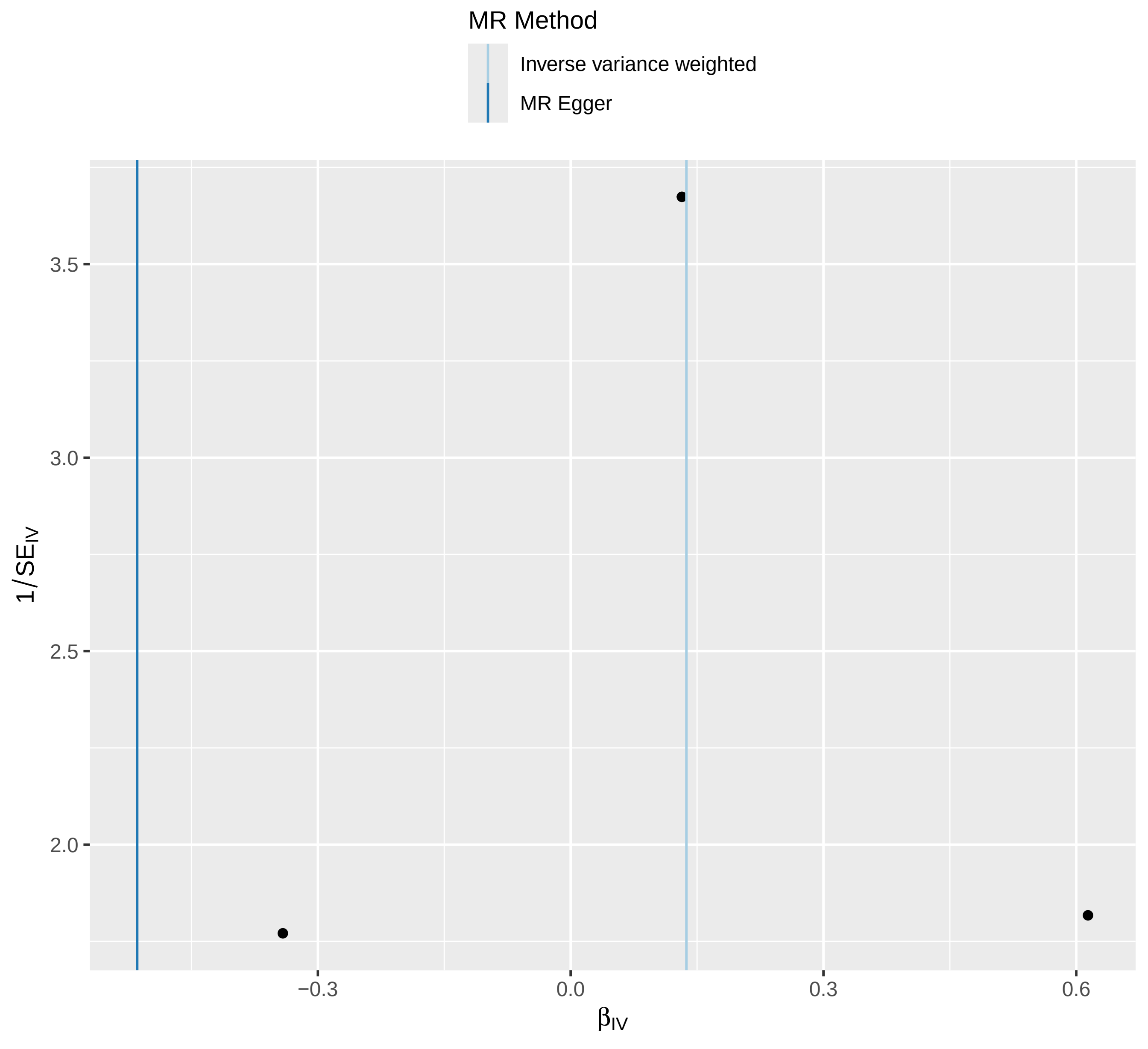

MR Method
Inverse variance weighted
MR Egger
3.5
3.0
2.5
2.0
1/SE_IV
−0.3
0.0
0.3
0.6
β_IV

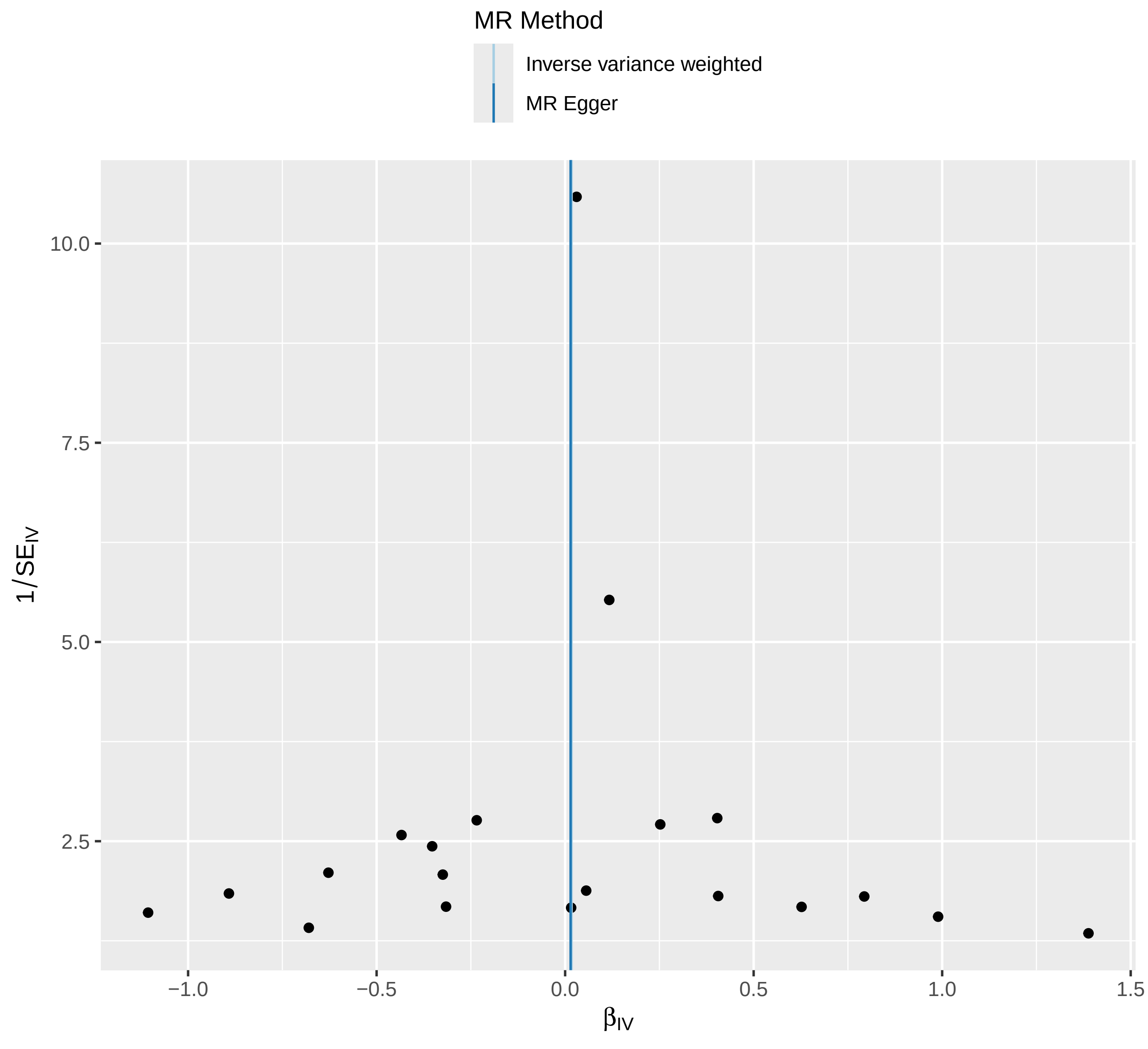
MR Method
Inverse variance weighted
MR Egger
10.0
7.5
5.0
2.5
1/SE$_{IV}$
−1.0
−0.5
0.0
0.5
1.0
1.5
β$_{IV}$

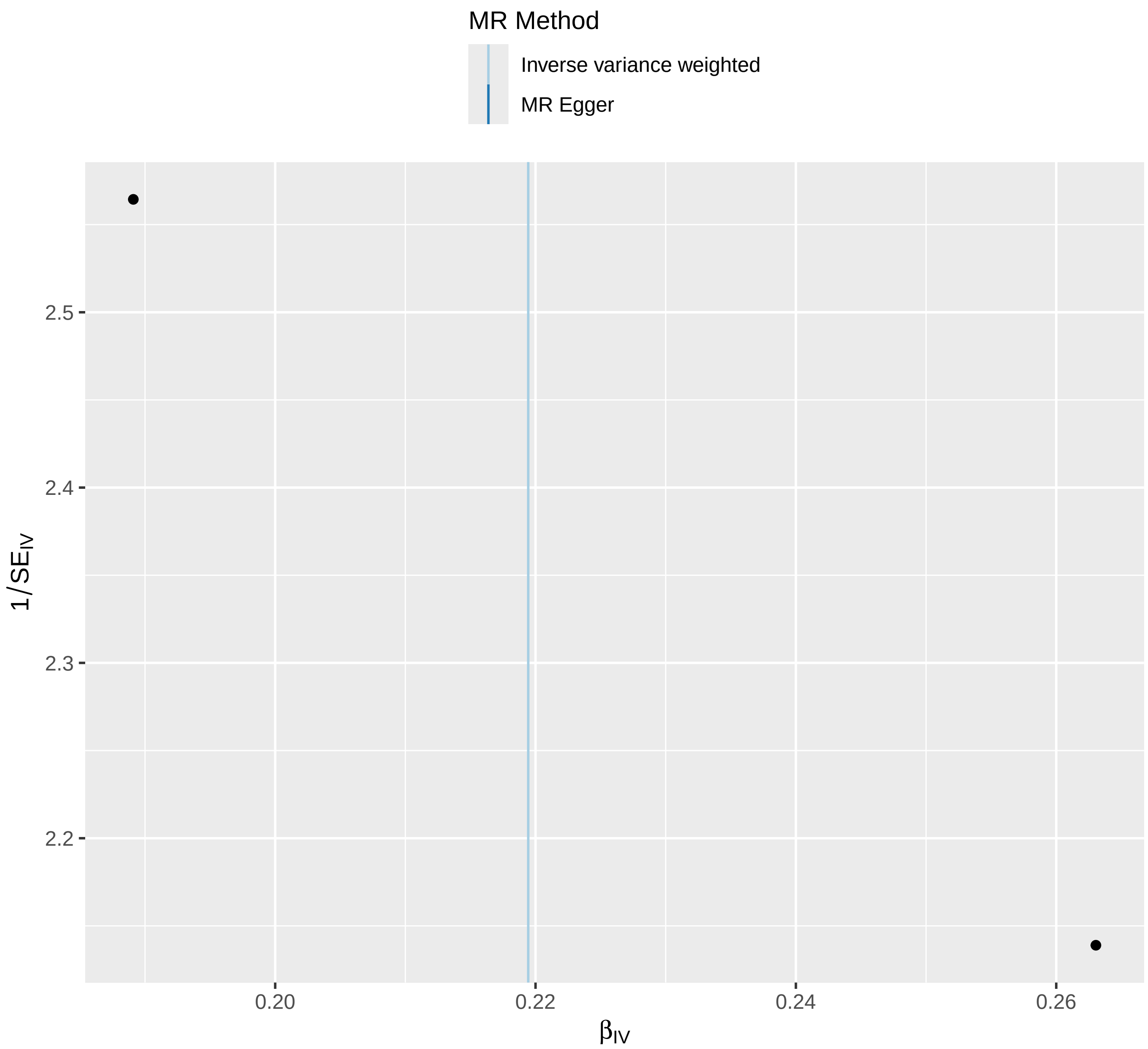
MR Method
Inverse variance weighted
MR Egger
2.5
2.4
2.3
2.2
1/SE$_{IV}$
0.20
0.22
0.24
0.26
β_{IV}

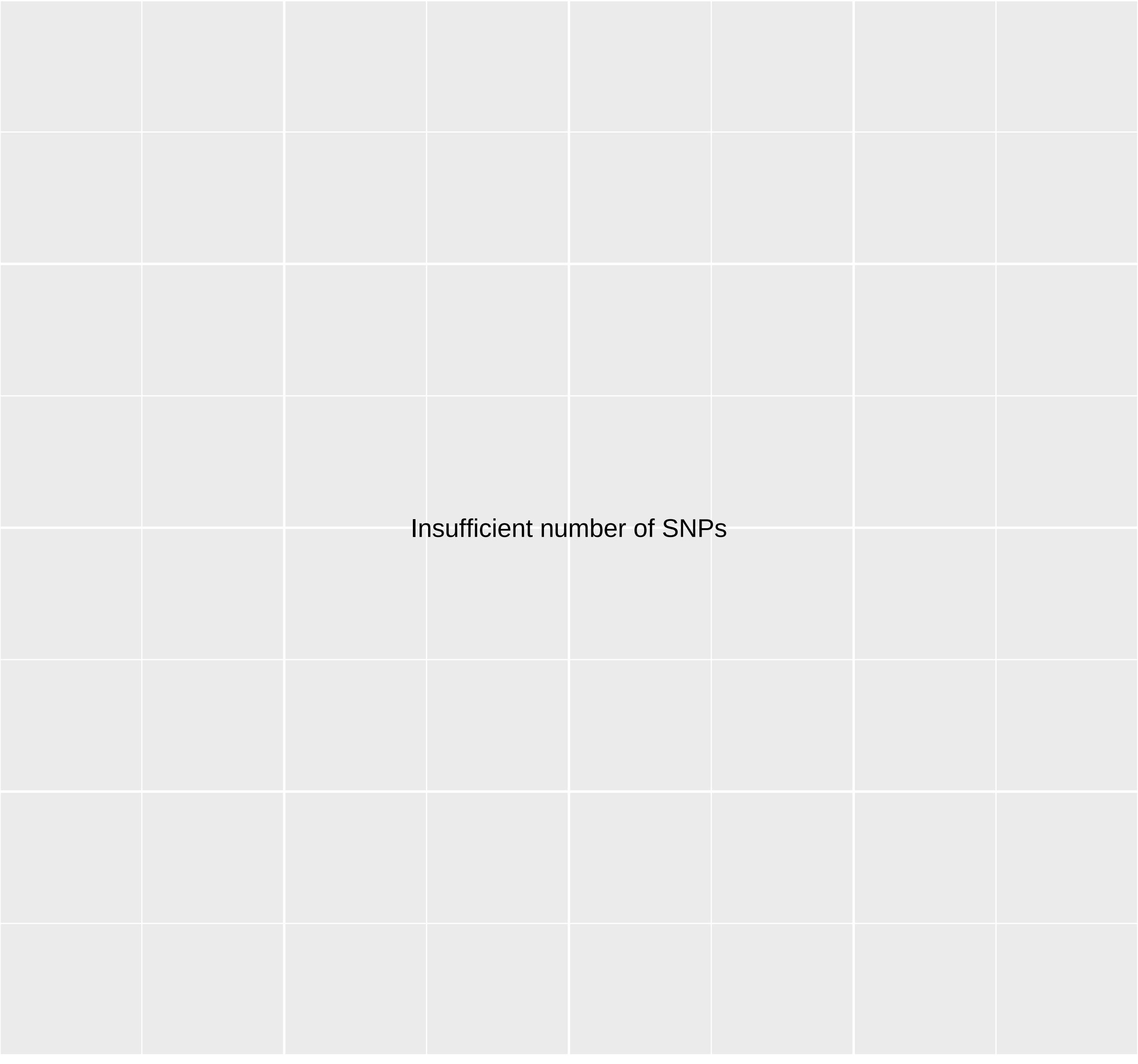
Insufficient number of SNPs

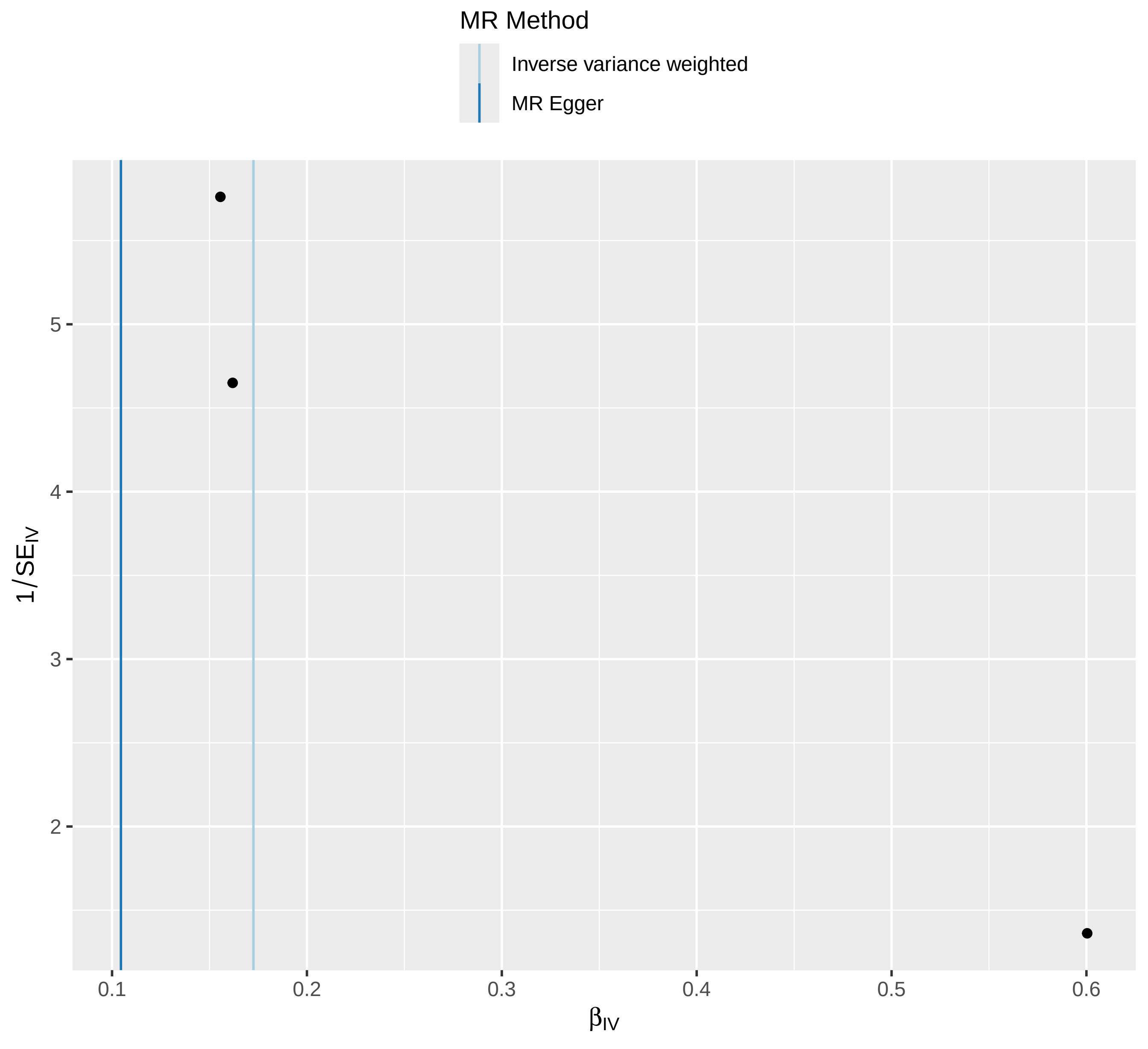

MR Method
Inverse variance weighted
MR Egger
5
4
3
2
1/SE_IV
0.1
0.2
0.3
0.4
0.5
0.6
β_IV

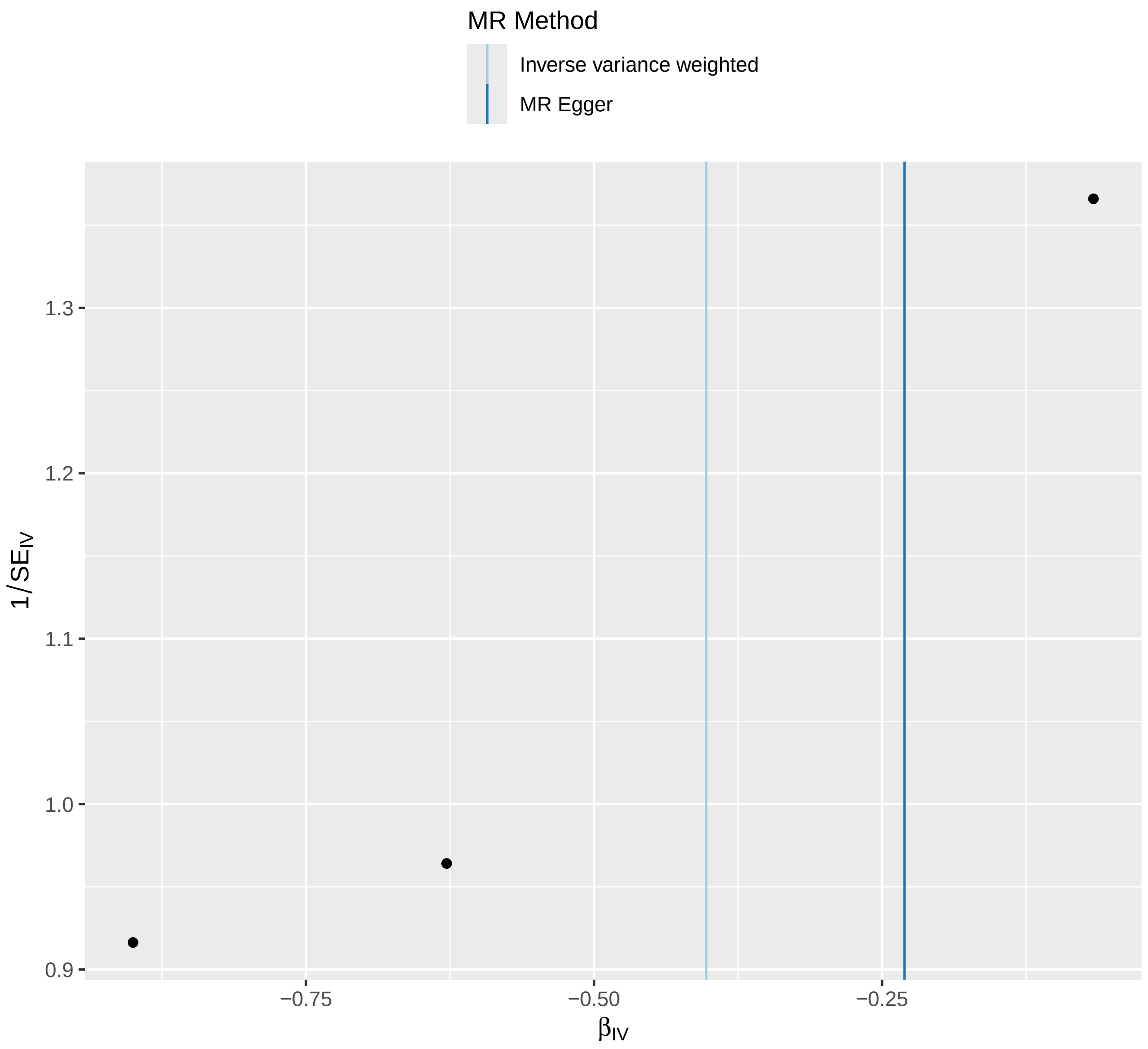

MR Method
Inverse variance weighted
MR Egger
1.3
1.2
1.1
1.0
0.9
1/SE_IV
−0.75
−0.50
−0.25
β_IV

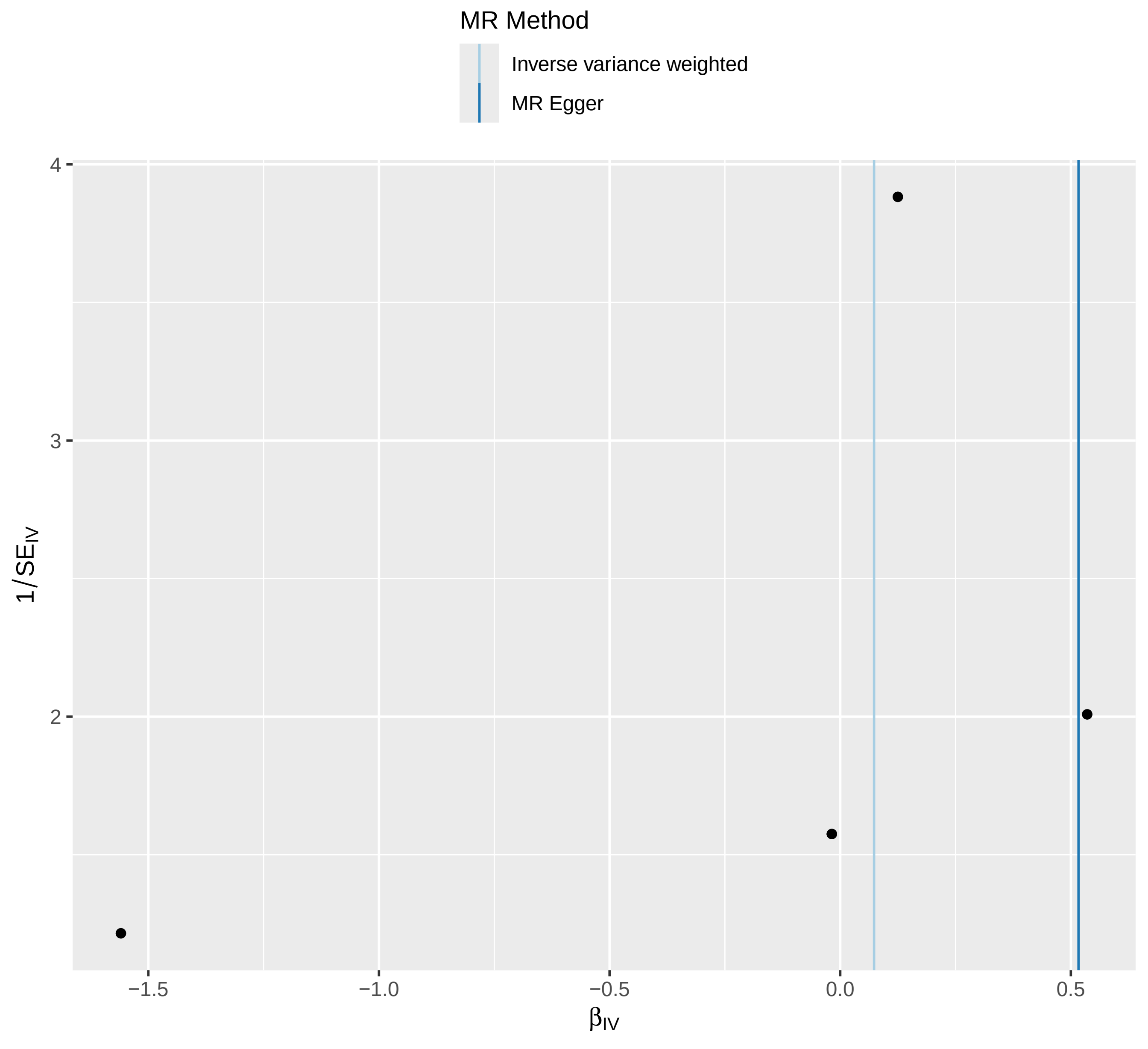
MR Method
Inverse variance weighted
MR Egger
4
3
2
1/SE$_{IV}$
−1.5
−1.0
−0.5
0.0
0.5
β$_{IV}$

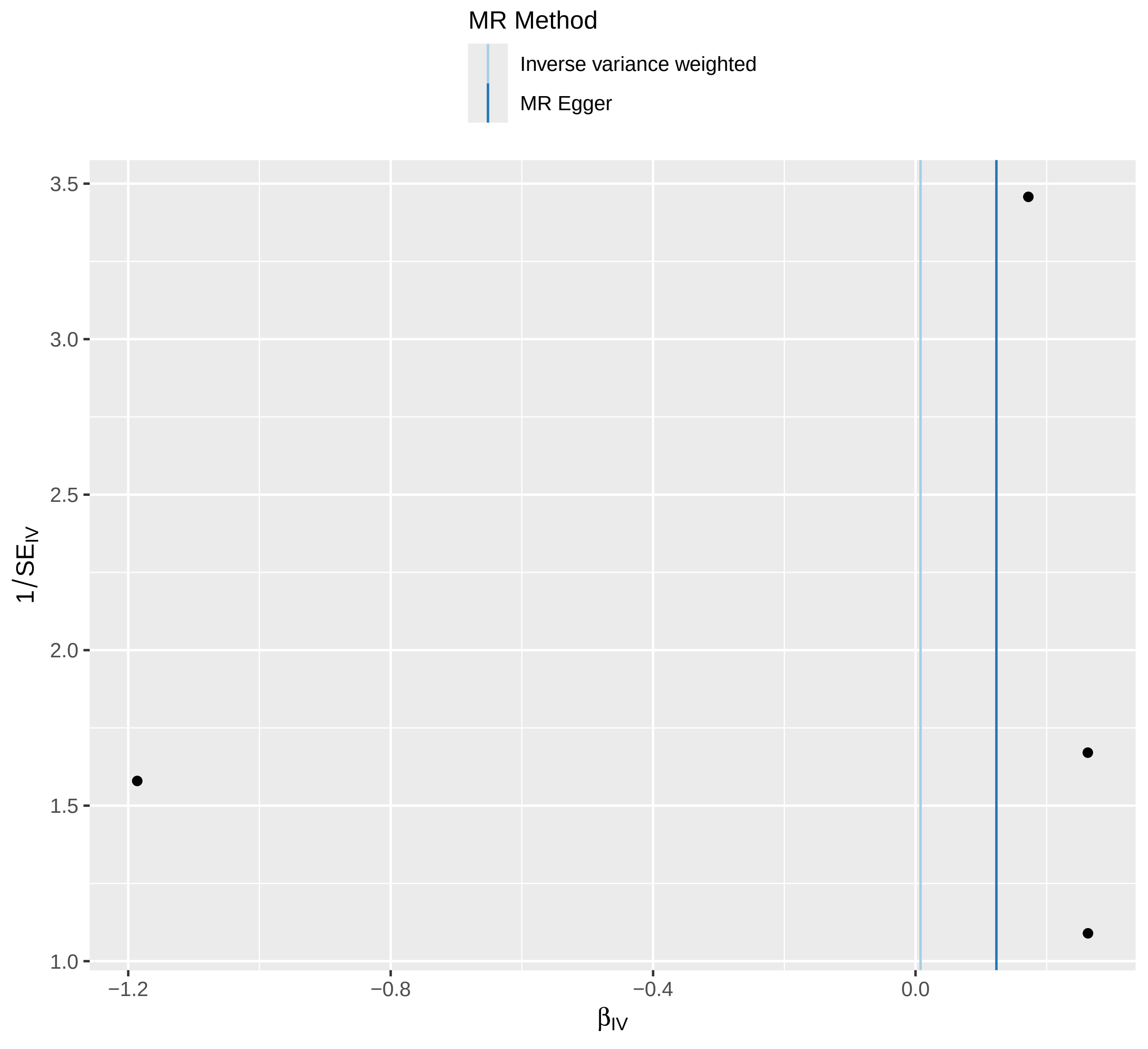

MR Method
Inverse variance weighted
MR Egger
3.5
3.0
2.5
2.0
1.5
1.0
$1/SE_{IV}$
−1.2
−0.8
−0.4
0.0
β_{IV}

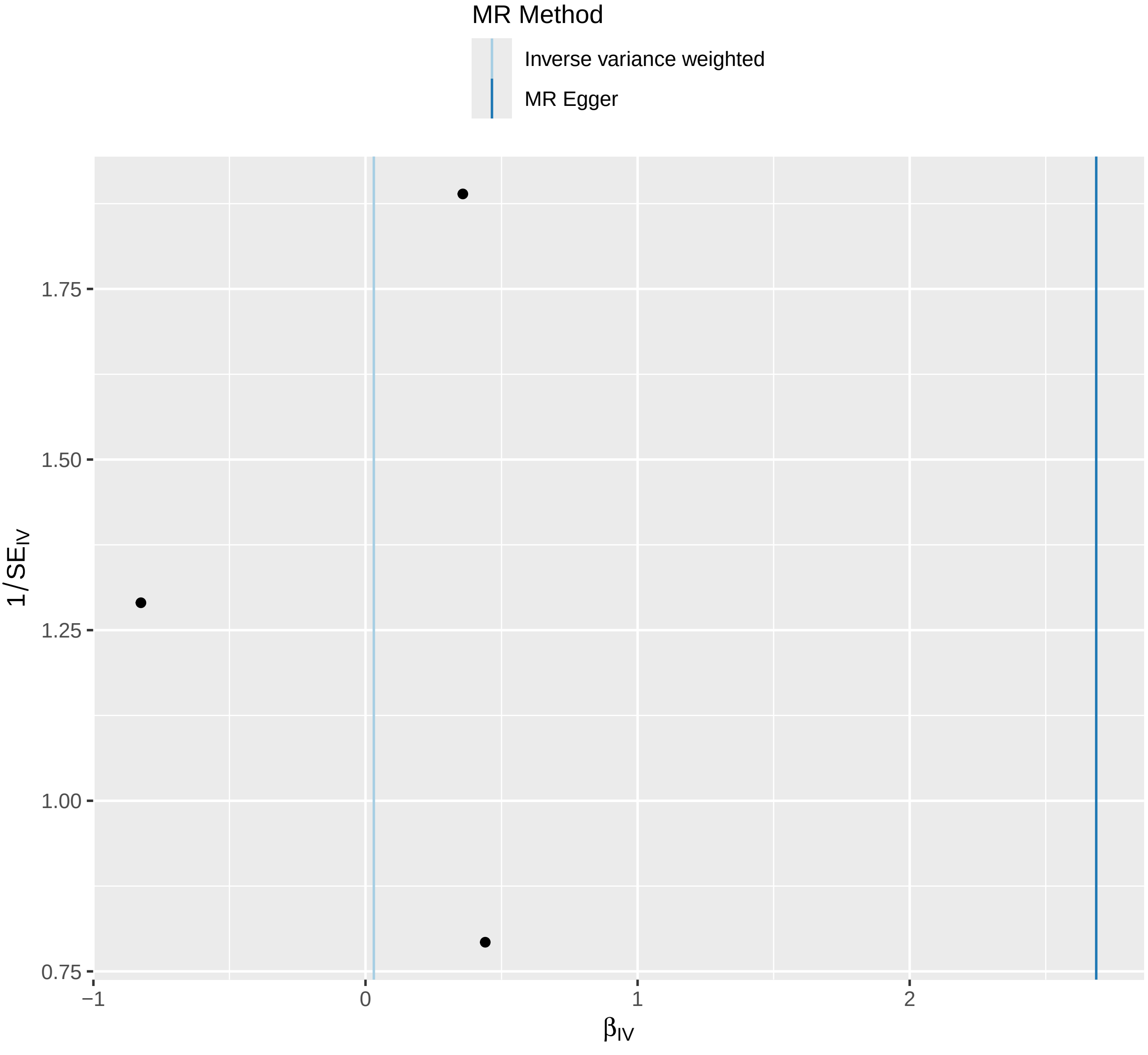

MR Method
Inverse variance weighted
MR Egger
1.75
1.50
1.25
1.00
0.75
1/SE$_{IV}$
−1
0
1
2
β$_{IV}$

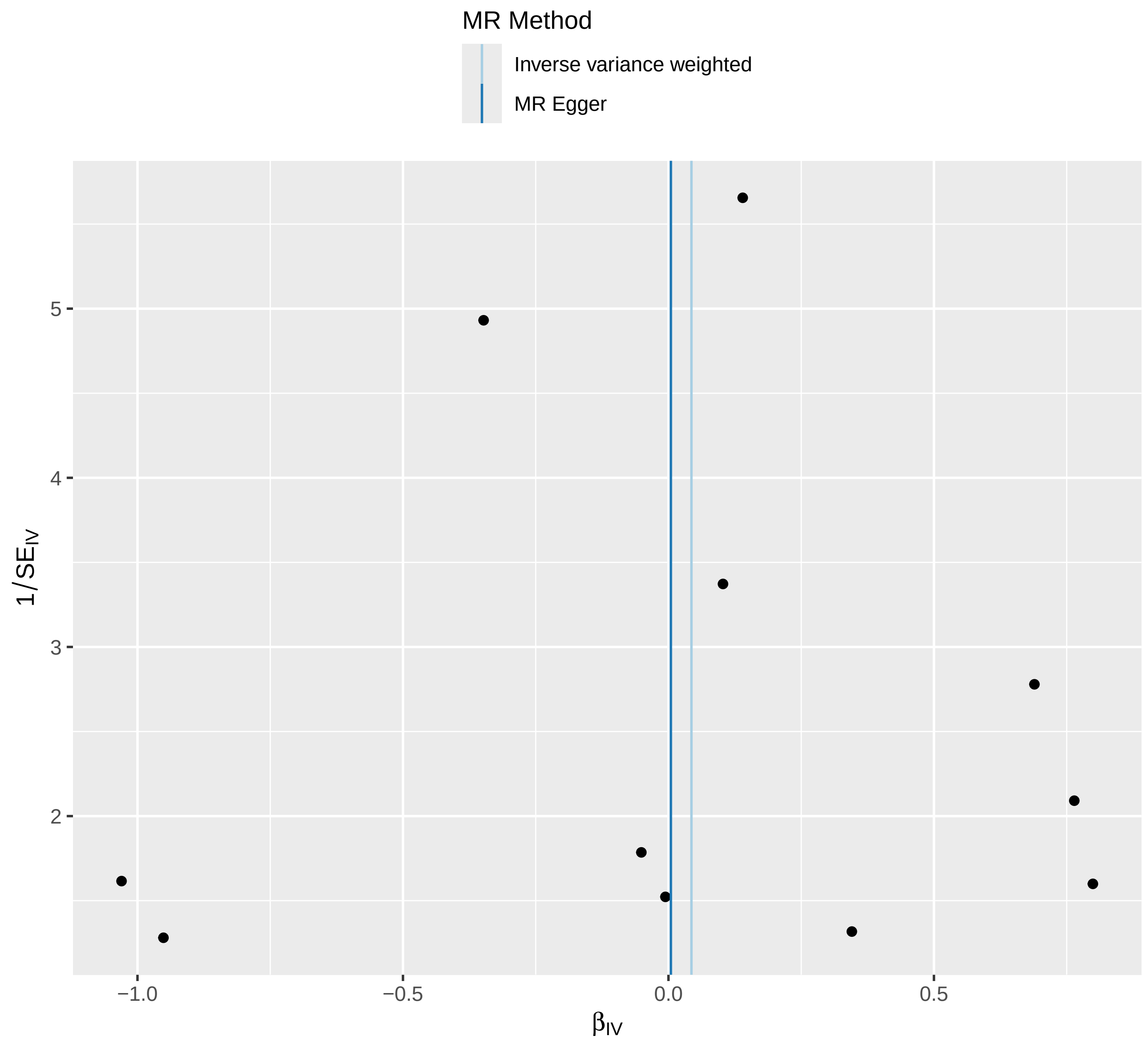
MR Method
Inverse variance weighted
MR Egger
5
4
3
2
1/SE$_{IV}$
−1.0
−0.5
0.0
0.5
β$_{IV}$

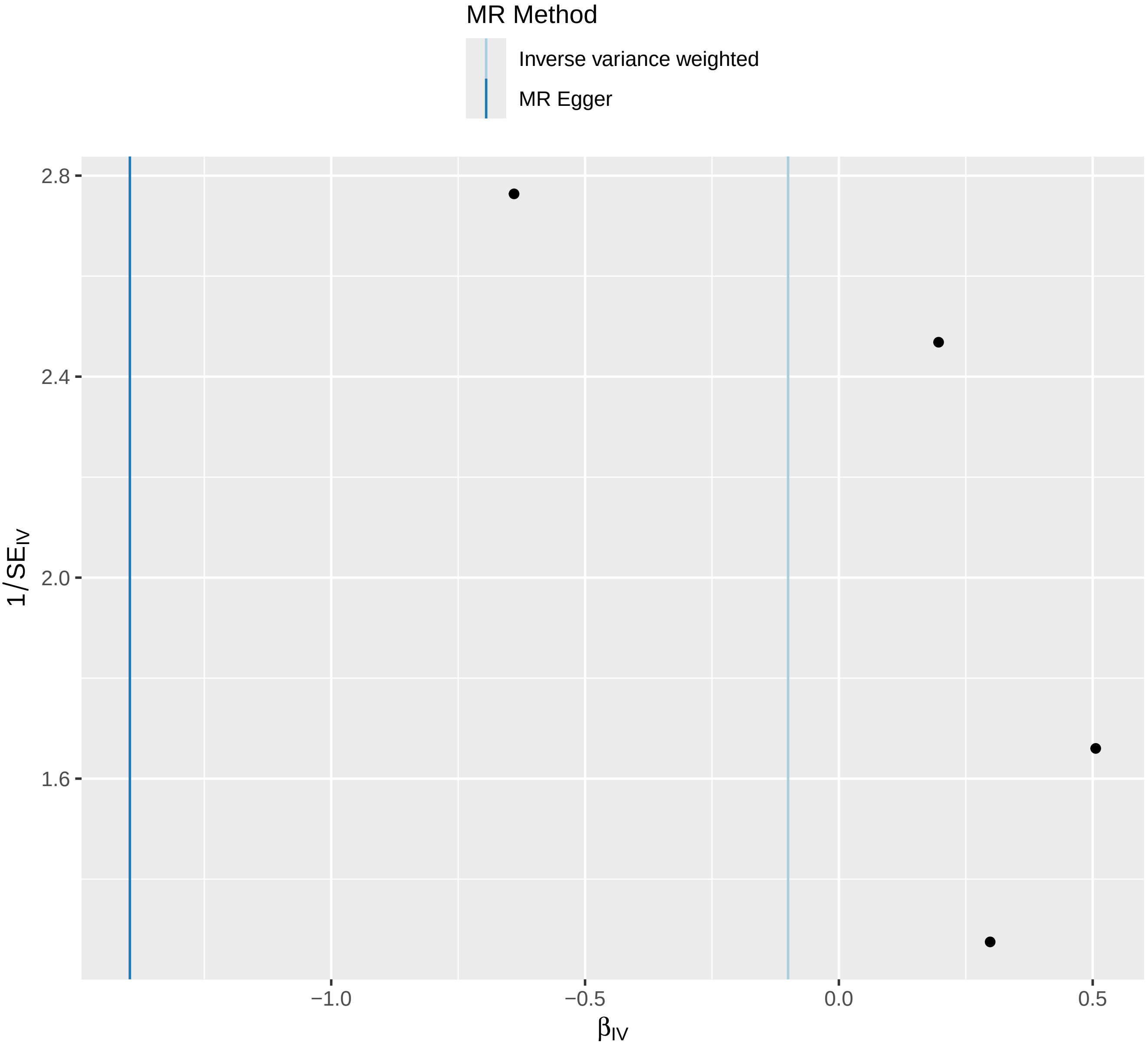
MR Method
Inverse variance weighted
MR Egger
2.8
2.4
2.0
1.6
$1/SE_{IV}$
−1.0
−0.5
0.0
0.5
β_{IV}

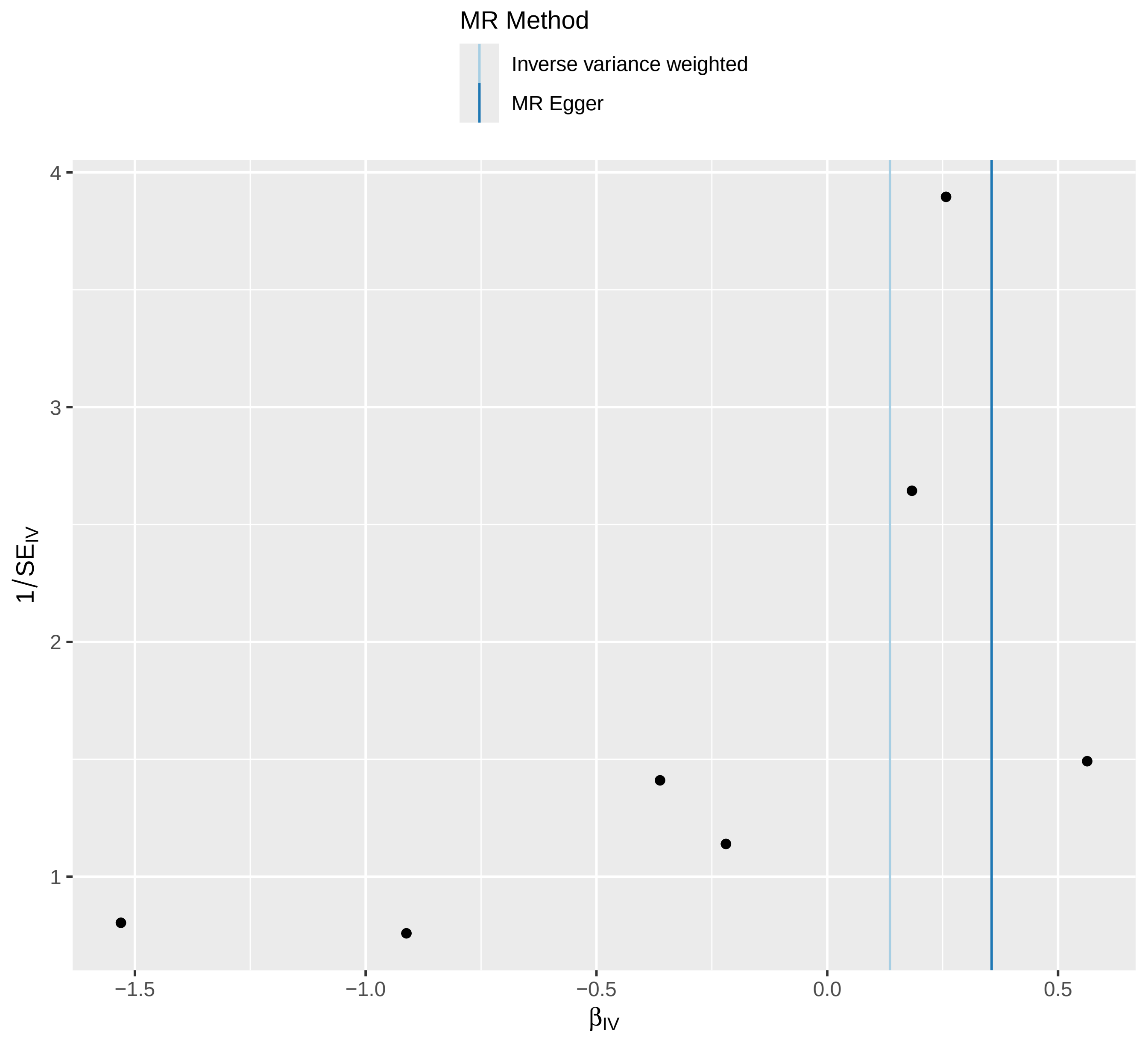

MR Method
Inverse variance weighted
MR Egger
4
3
2
1
1/SE$_{IV}$
−1.5
−1.0
−0.5
0.0
0.5
β$_{IV}$

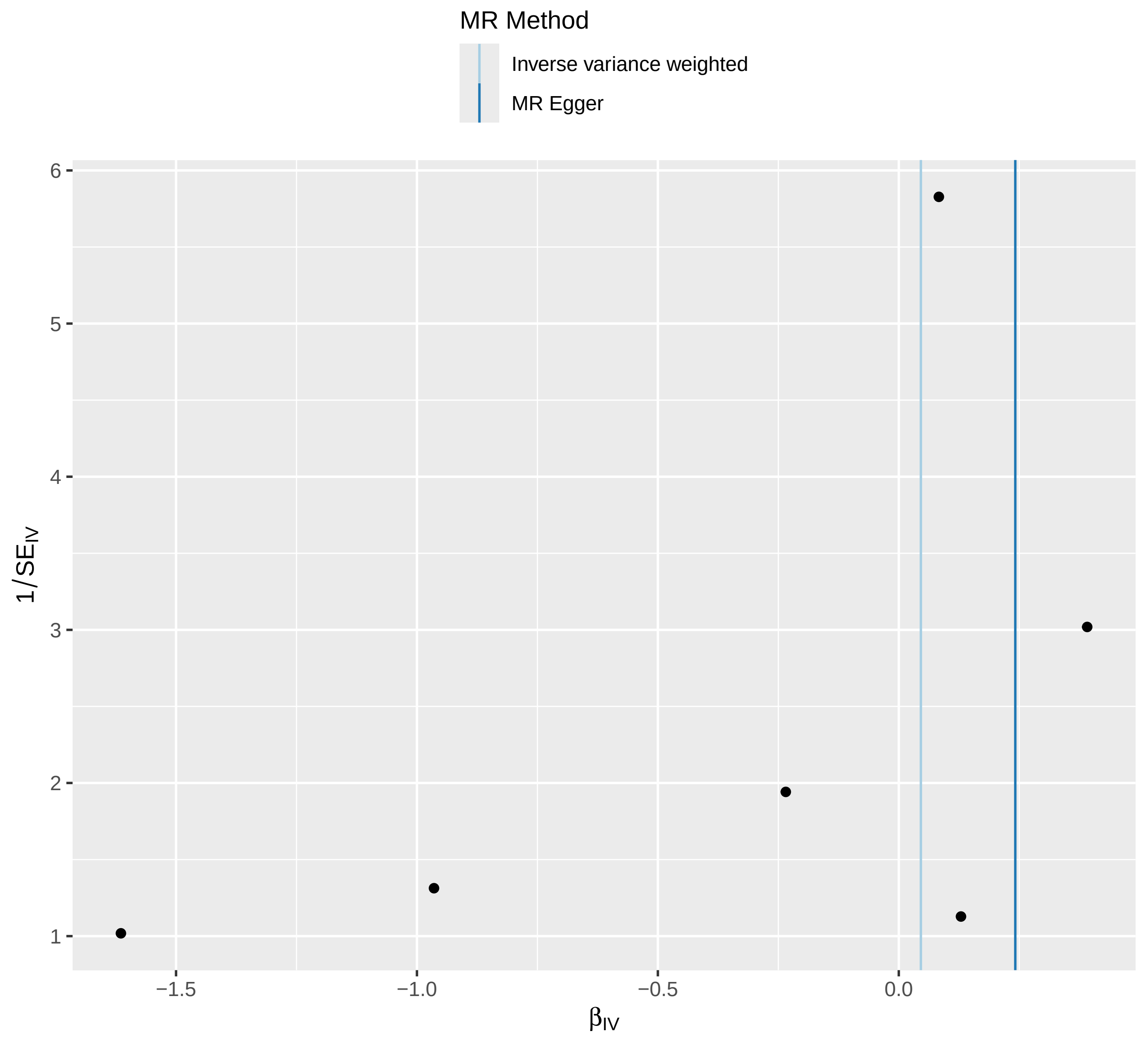

MR Method
Inverse variance weighted
MR Egger
6
5
4
3
2
1
1/SE_IV
−1.5
−1.0
−0.5
0.0
β_IV

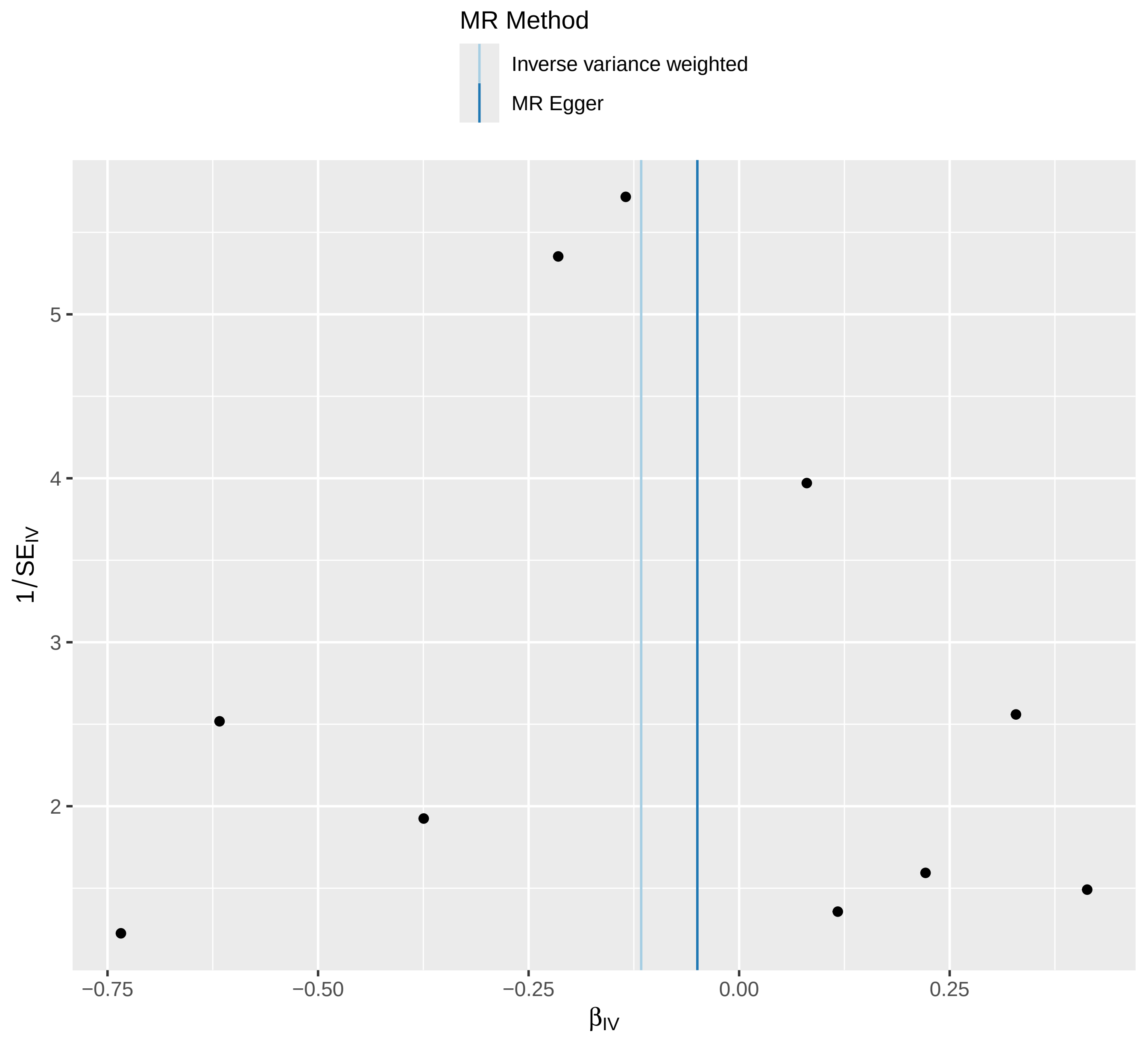
MR Method
Inverse variance weighted
MR Egger
5
4
3
2
1/SE_IV
−0.75
−0.50
−0.25
0.00
0.25
β_IV

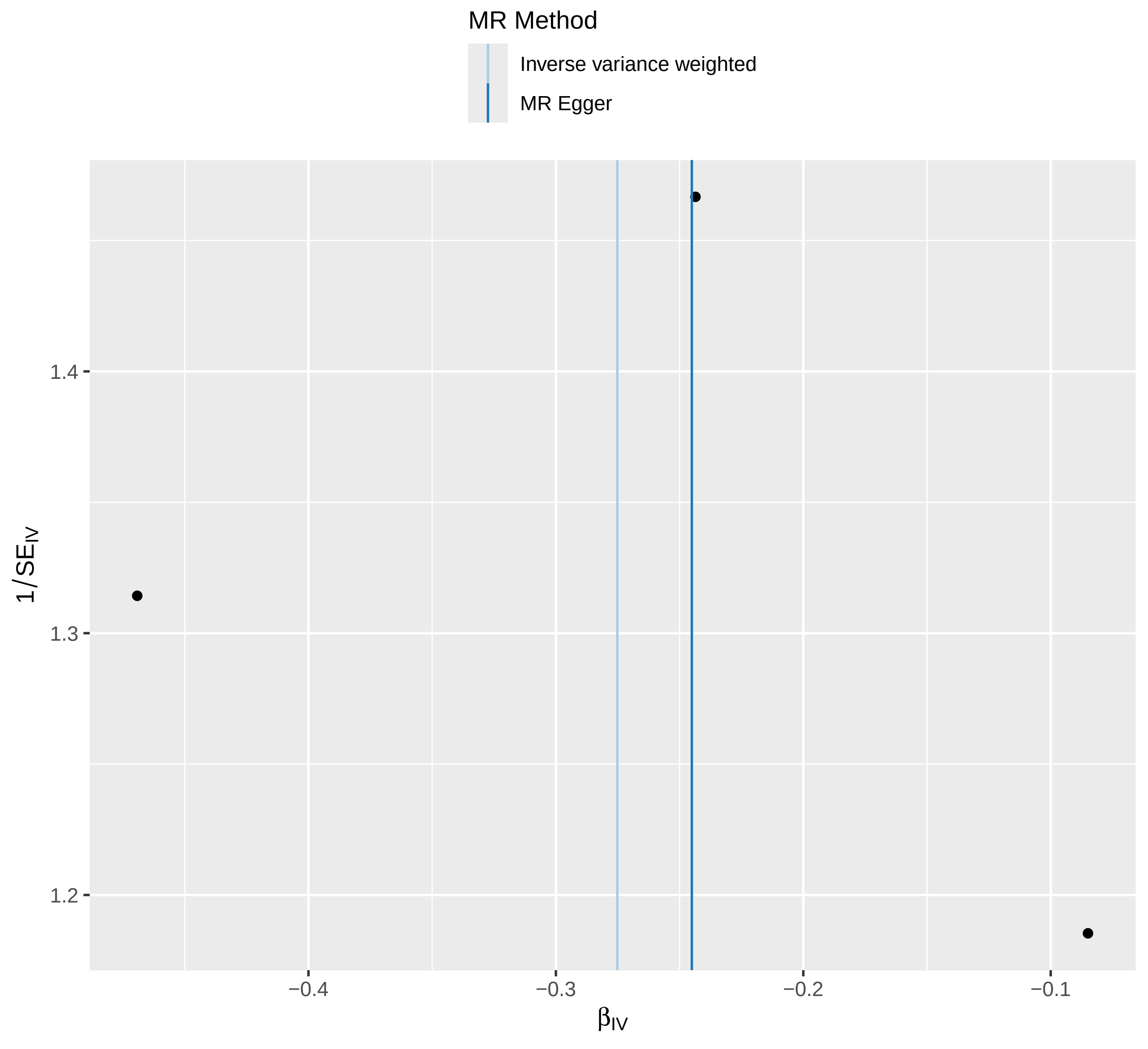
MR Method
Inverse variance weighted
MR Egger
1.4
1.3
1.2
1/SE_IV
−0.4
−0.3
−0.2
−0.1
β_IV

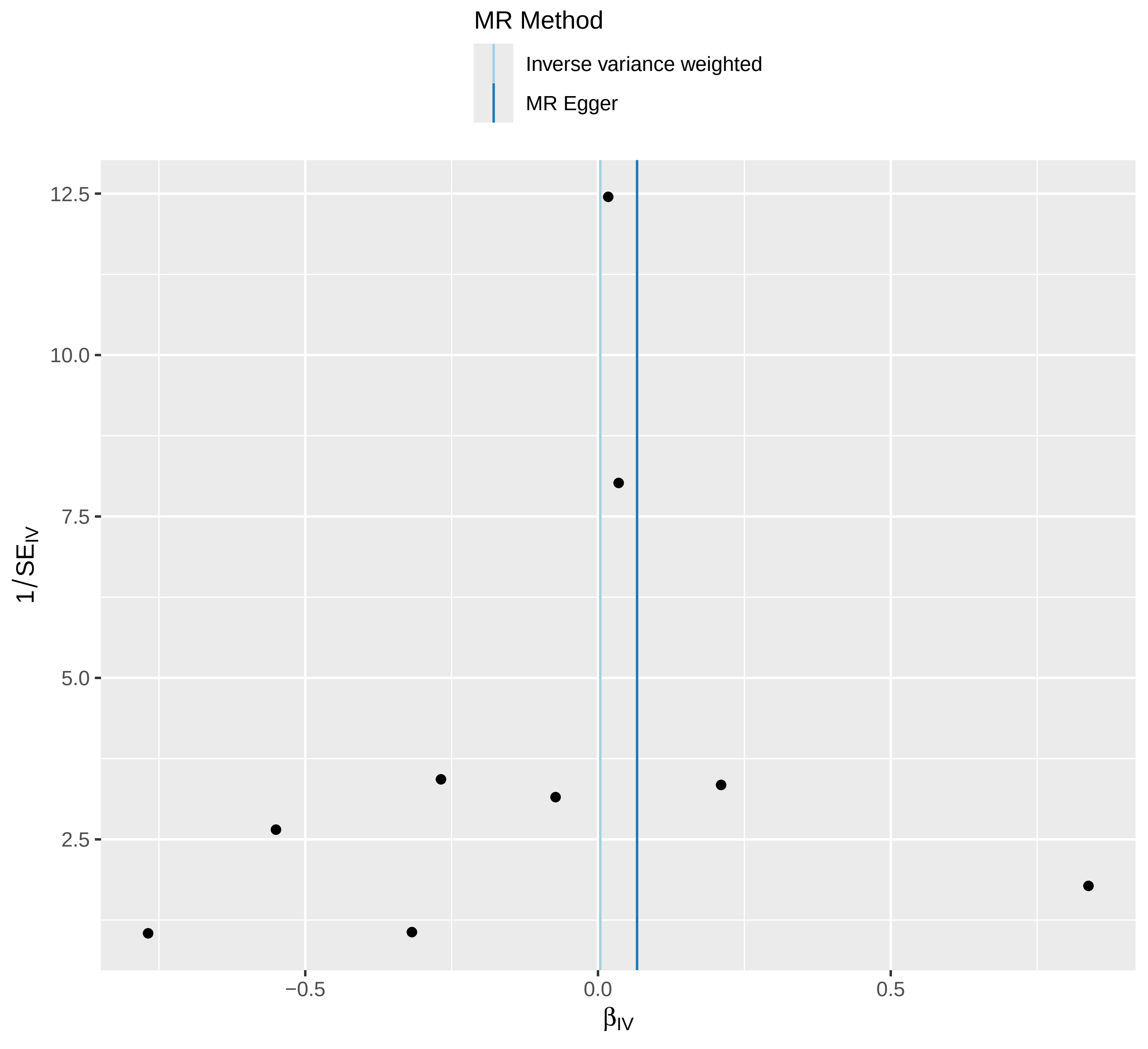
MR Method
Inverse variance weighted
MR Egger
12.5
10.0
7.5
5.0
2.5
1/SE_IV
−0.5
0.0
0.5
β_IV

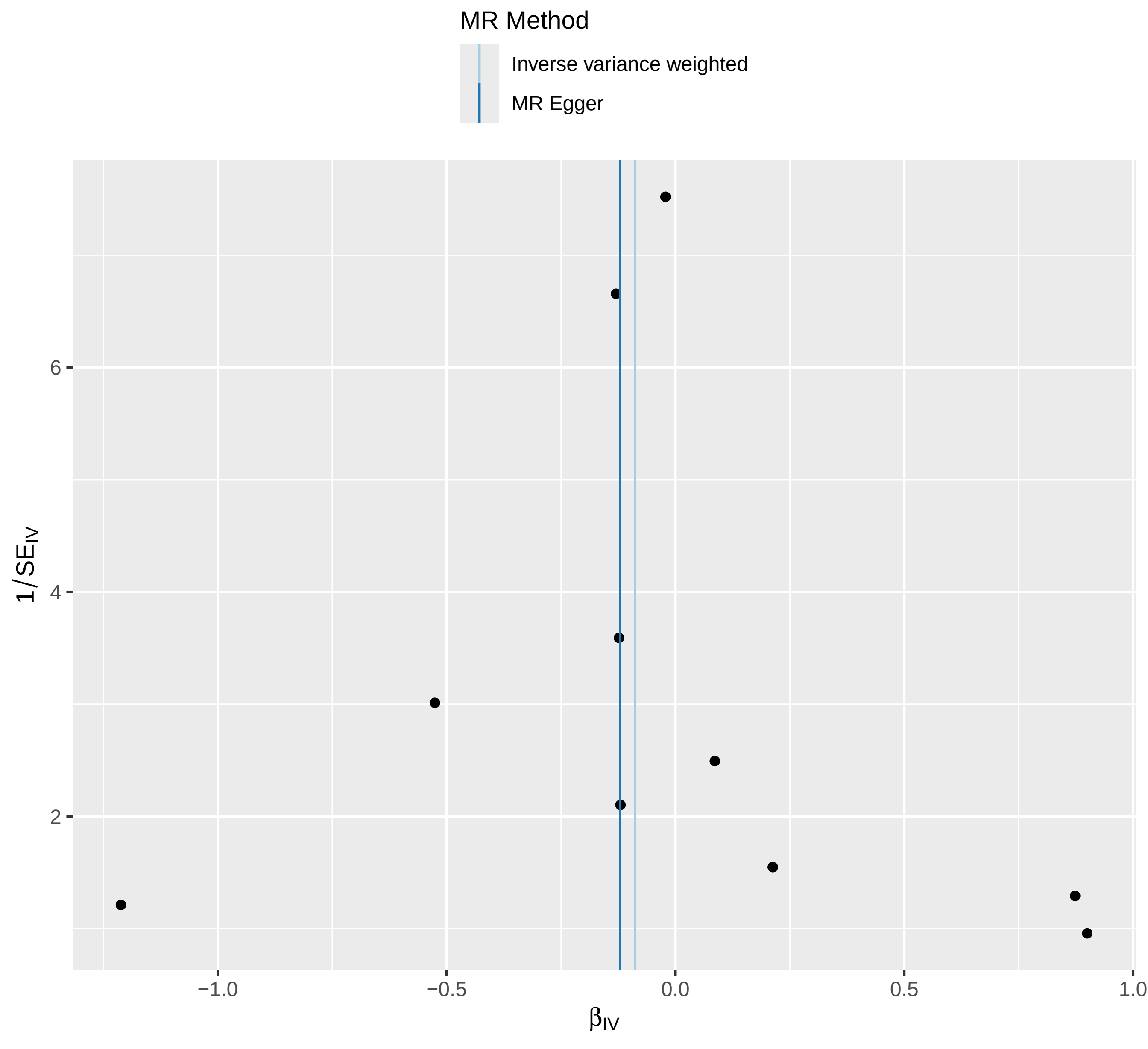
MR Method
Inverse variance weighted
MR Egger
1/SE_IV
6
4
2
−1.0
−0.5
0.0
0.5
1.0
β_IV

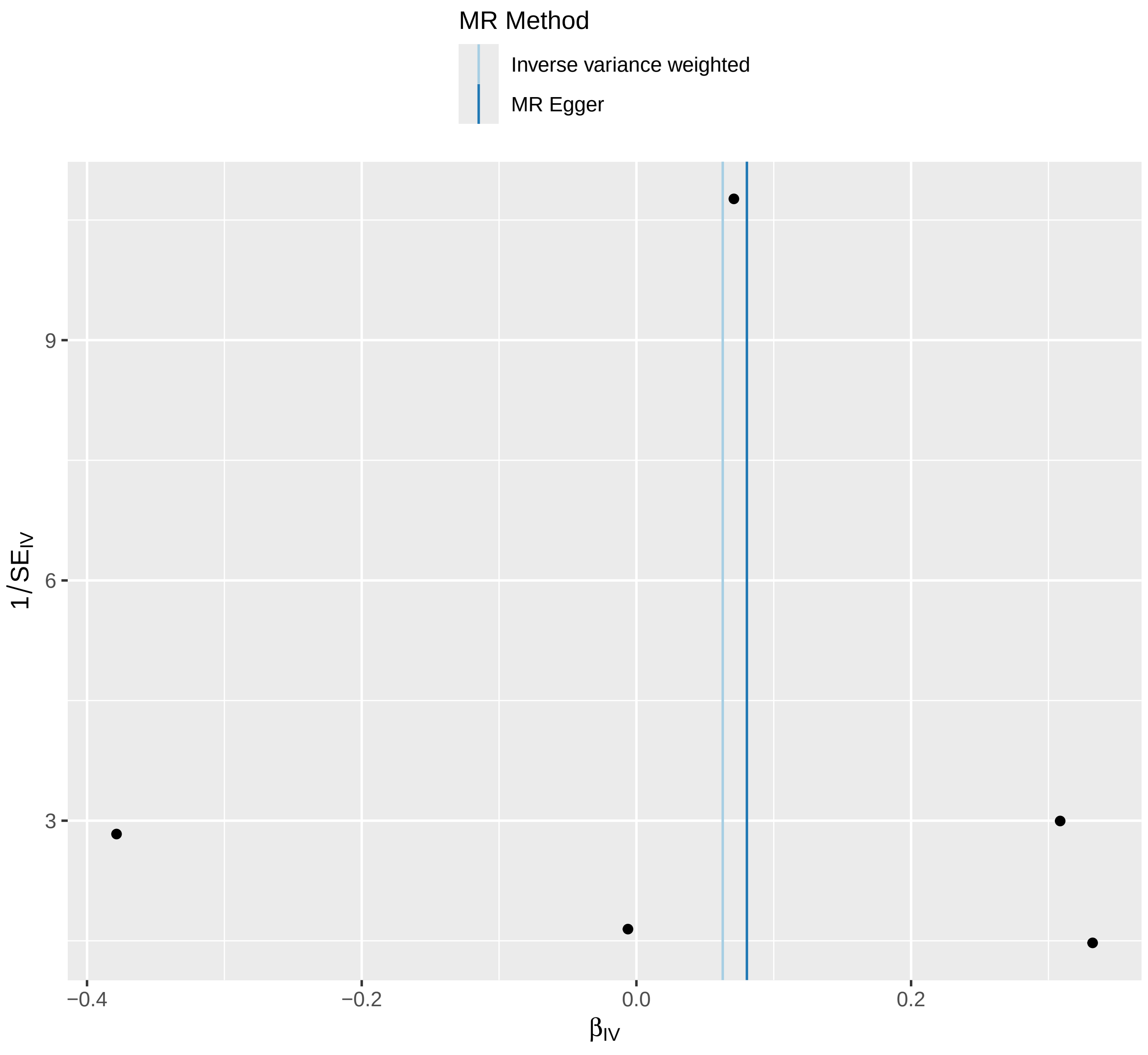
MR Method
Inverse variance weighted
MR Egger
9
6
3
$1/SE_{IV}$
−0.4
−0.2
0.0
0.2
β_{IV}

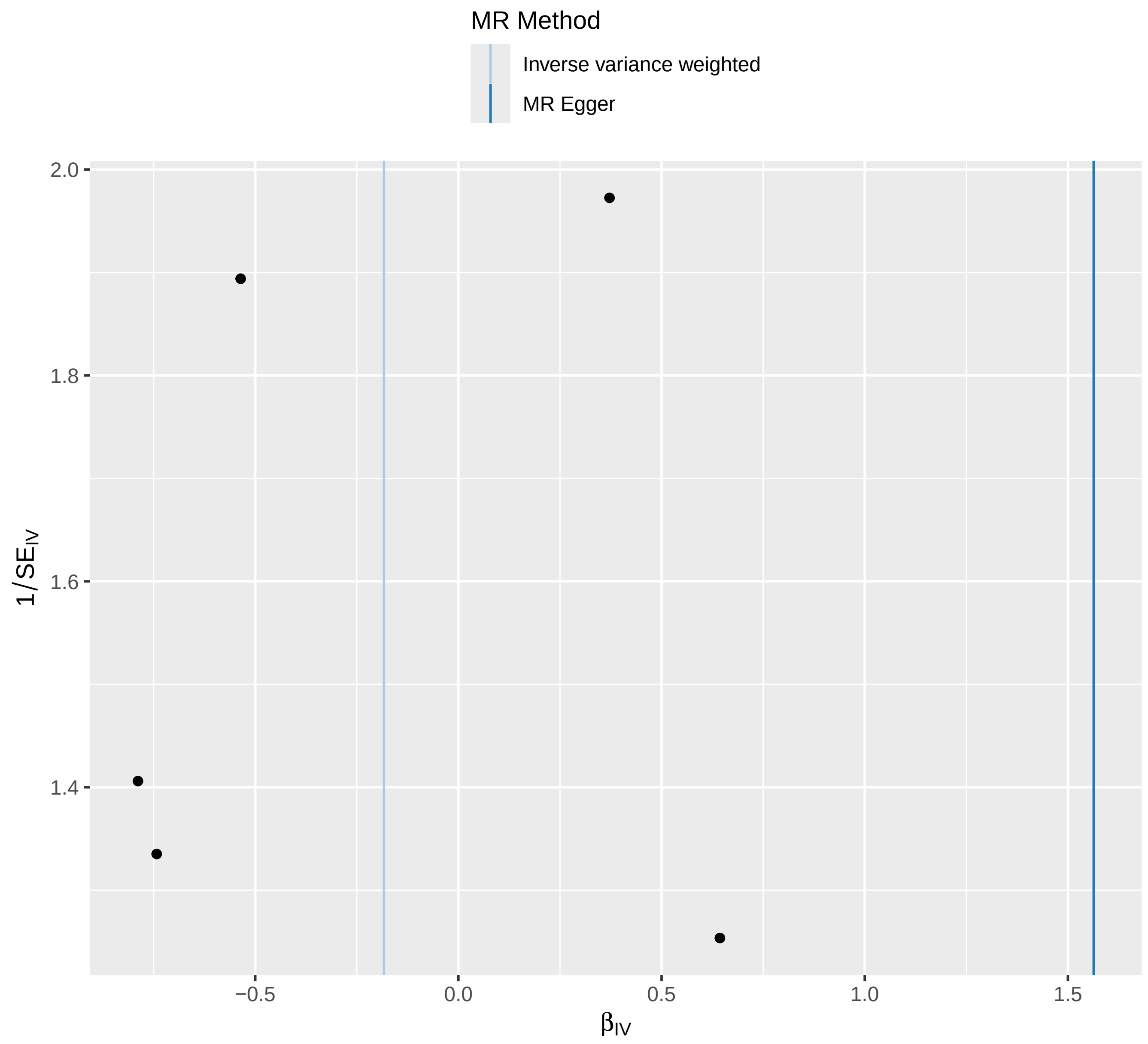
MR Method
Inverse variance weighted
MR Egger
2.0
1.8
1.6
1.4
1/SE$_{IV}$
−0.5
0.0
0.5
1.0
1.5
β$_{IV}$

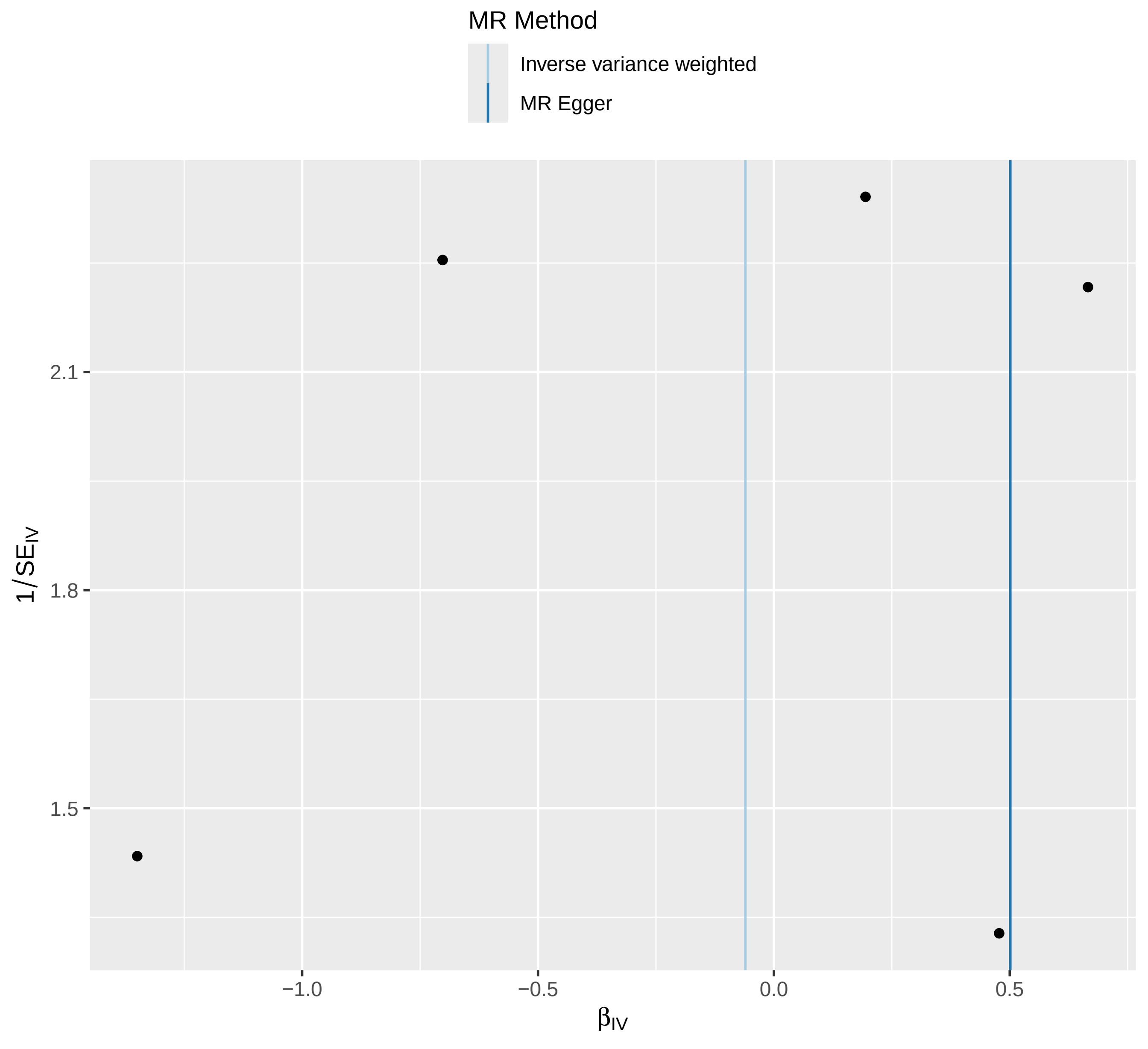
MR Method
Inverse variance weighted
MR Egger
2.1
1.8
1.5
1/SE$_{IV}$
−1.0
−0.5
0.0
0.5
β$_{IV}$

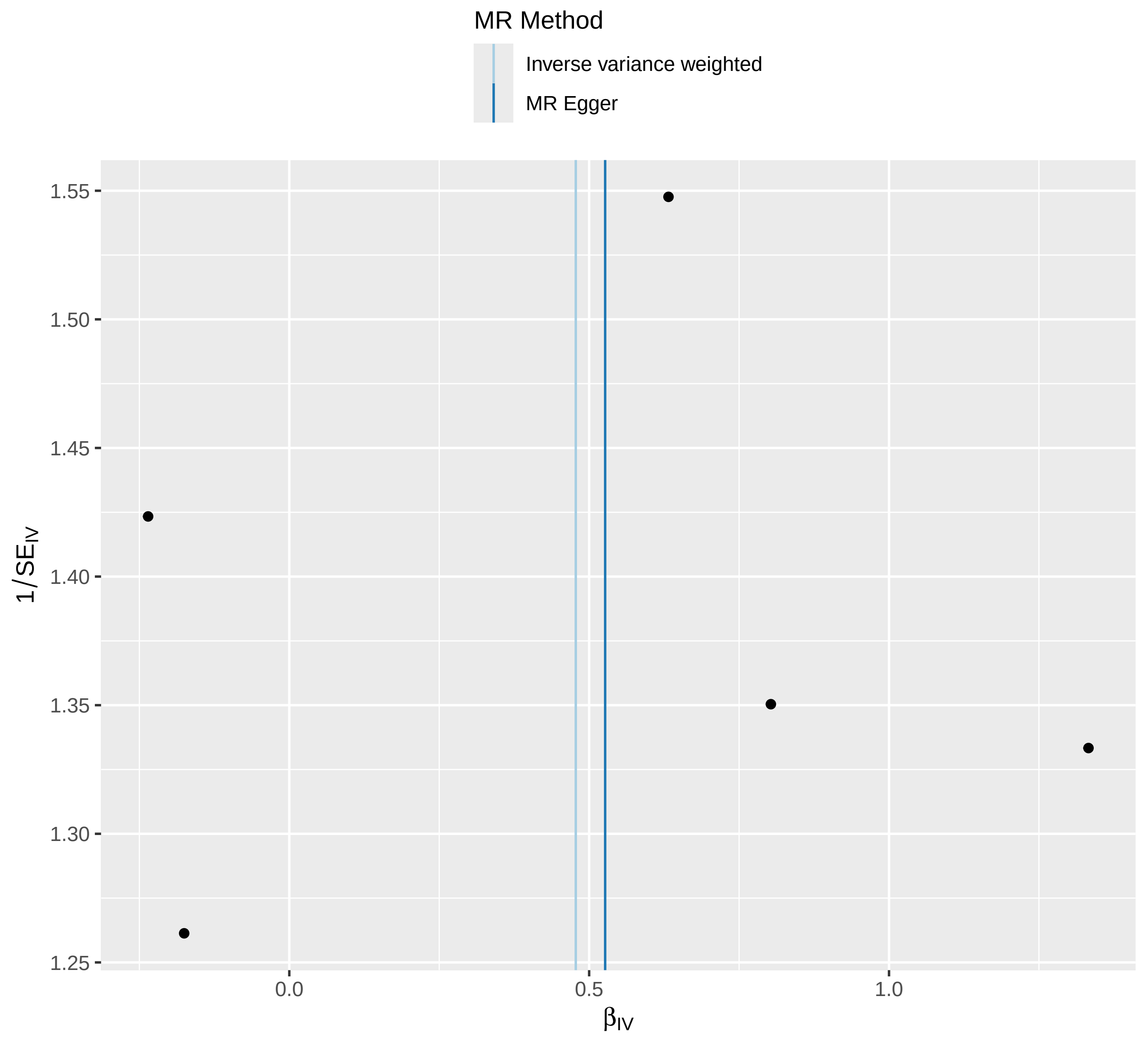

MR Method
Inverse variance weighted
MR Egger
1.55
1.50
1.45
1.40
1.35
1.30
1.25
$1/SE_{IV}$
0.0
0.5
1.0
β_{IV}

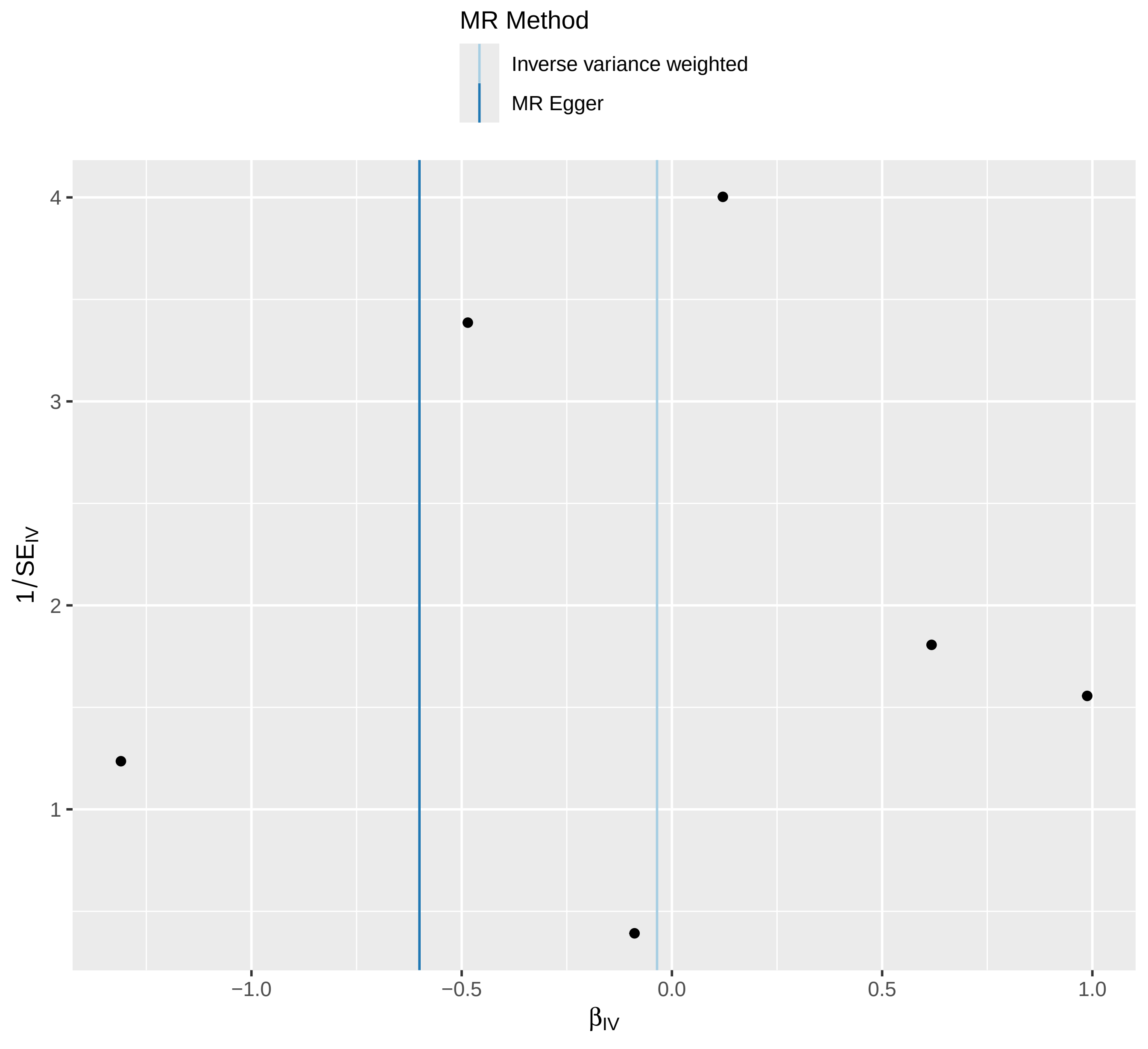

MR Method
Inverse variance weighted
MR Egger
4
3
2
1
1/SE$_{IV}$
−1.0
−0.5
0.0
0.5
1.0
β$_{IV}$

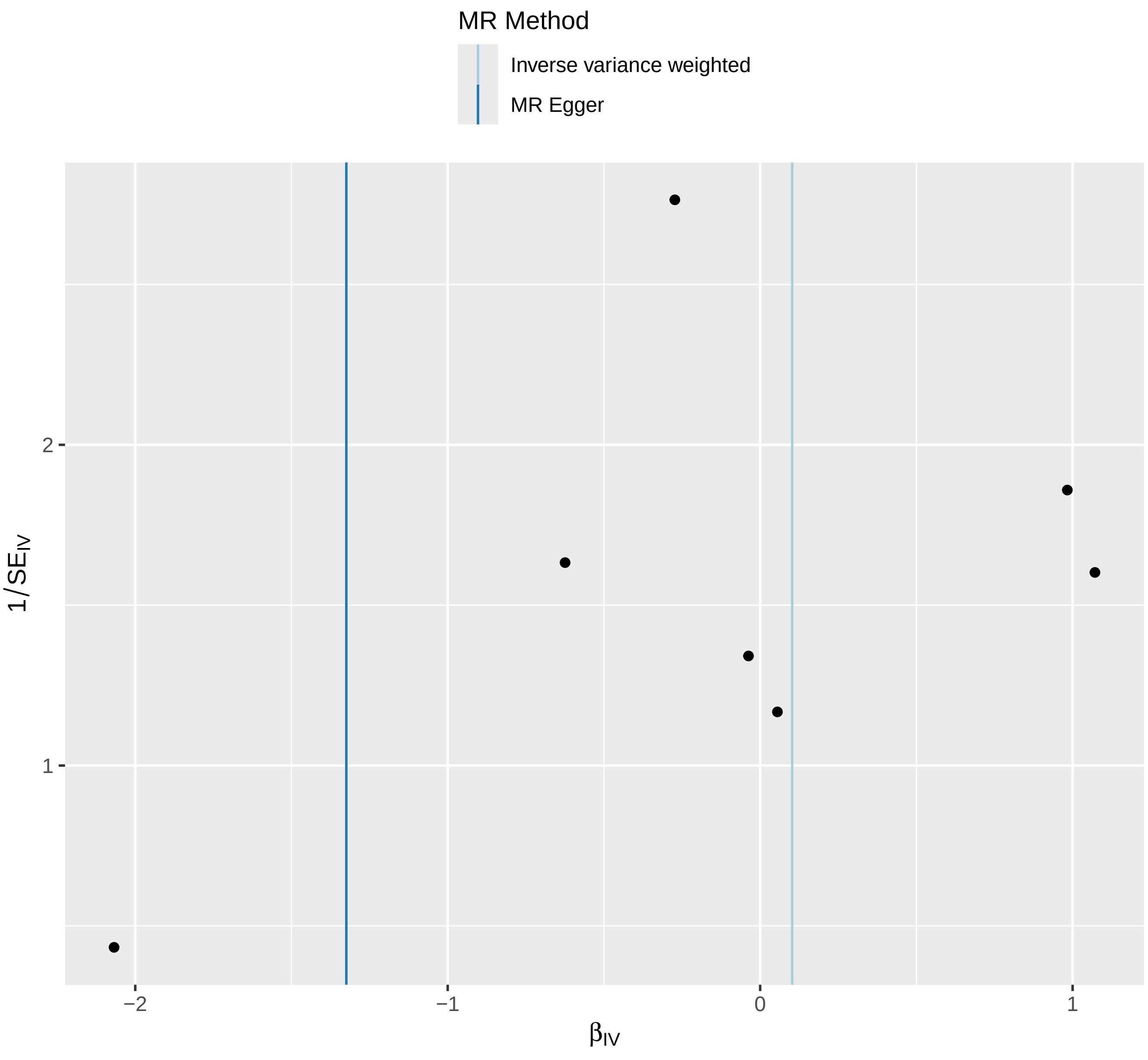

MR Method
Inverse variance weighted
MR Egger
2
1
1/SE_IV
−2
−1
0
1
β_IV

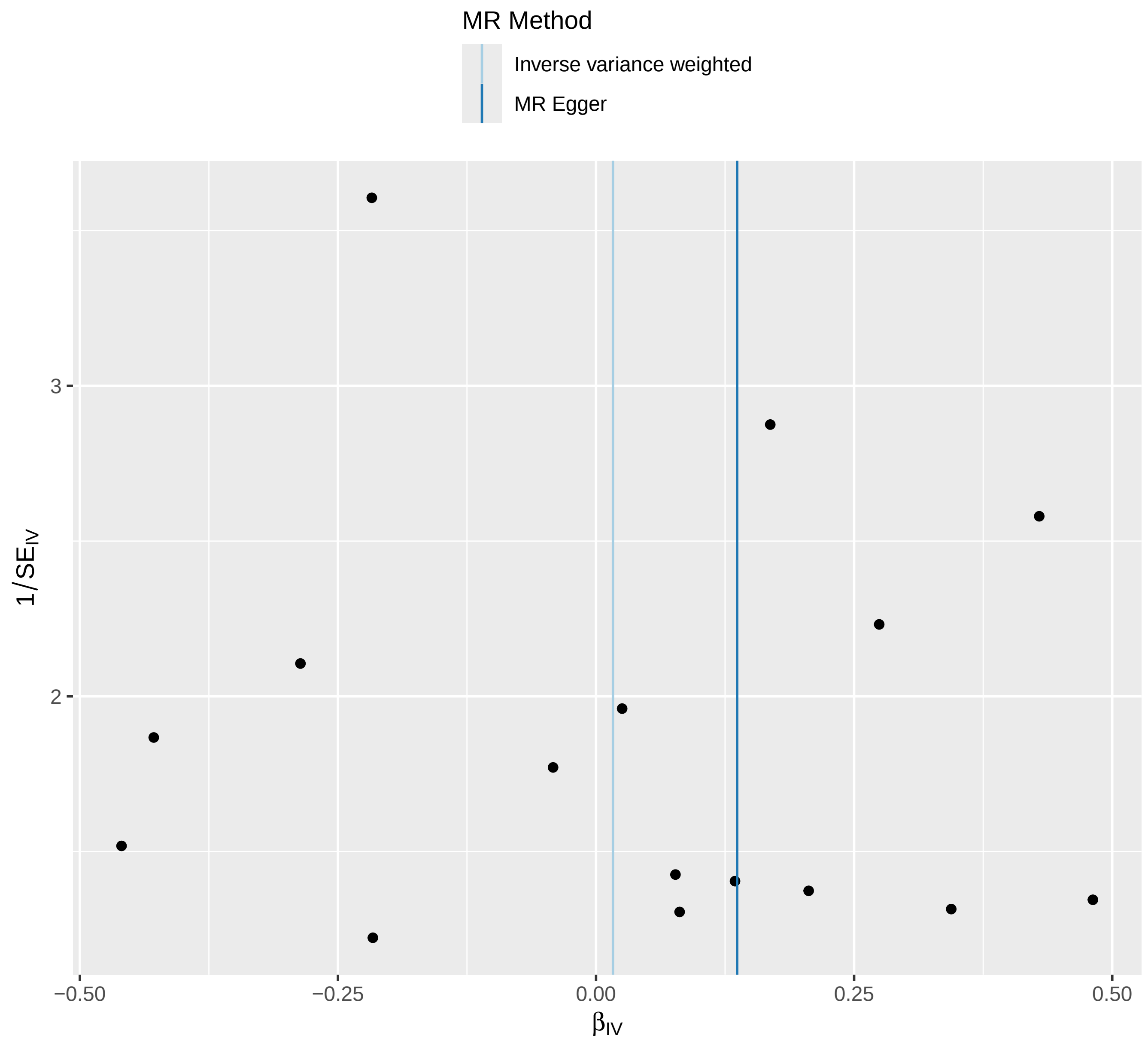
MR Method
Inverse variance weighted
MR Egger
3
2
1/SE_IV
−0.50
−0.25
0.00
0.25
0.50
β_IV

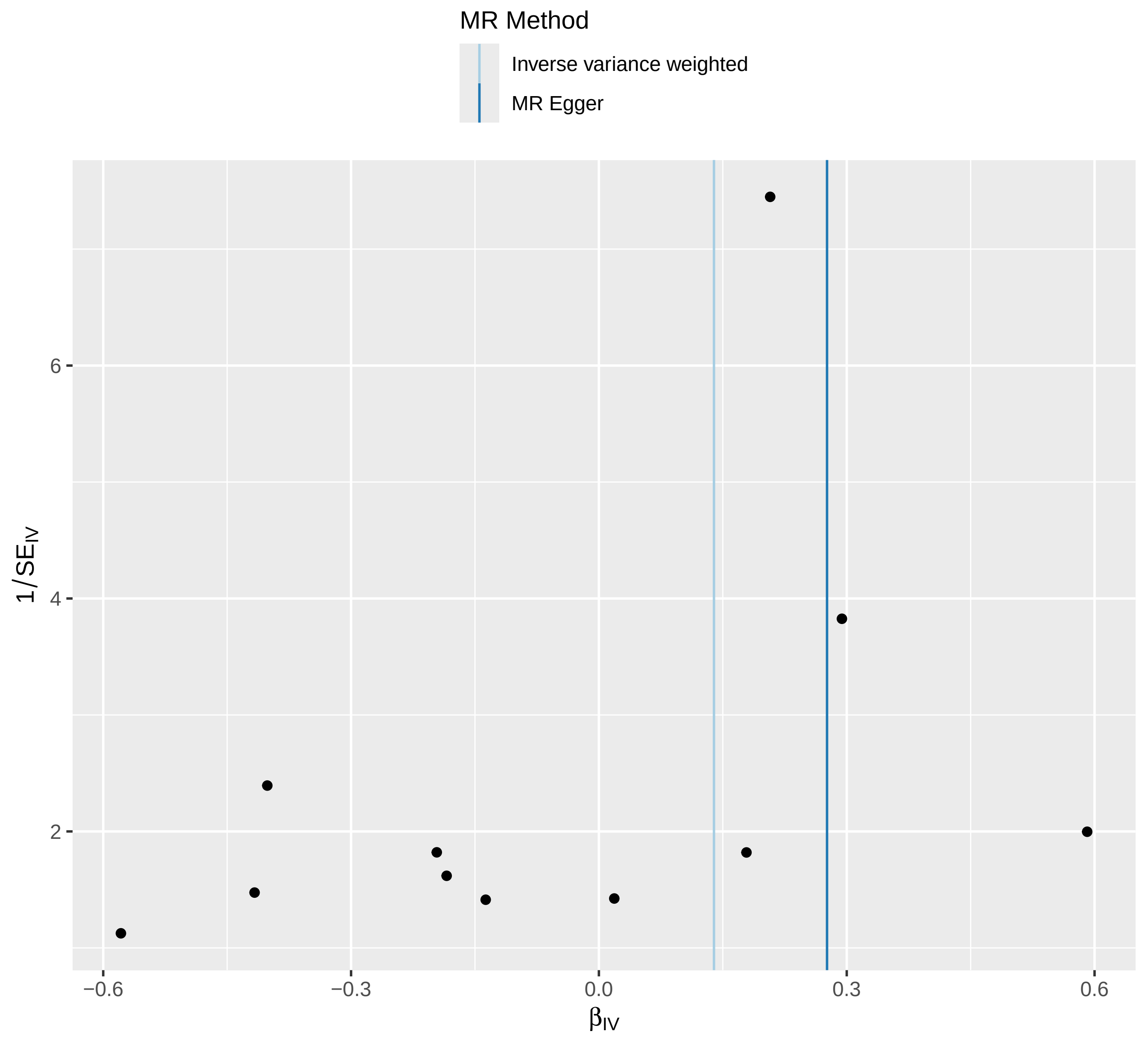

MR Method
Inverse variance weighted
MR Egger
1/SE_IV
6
4
2
−0.6
−0.3
0.0
0.3
0.6
β_IV

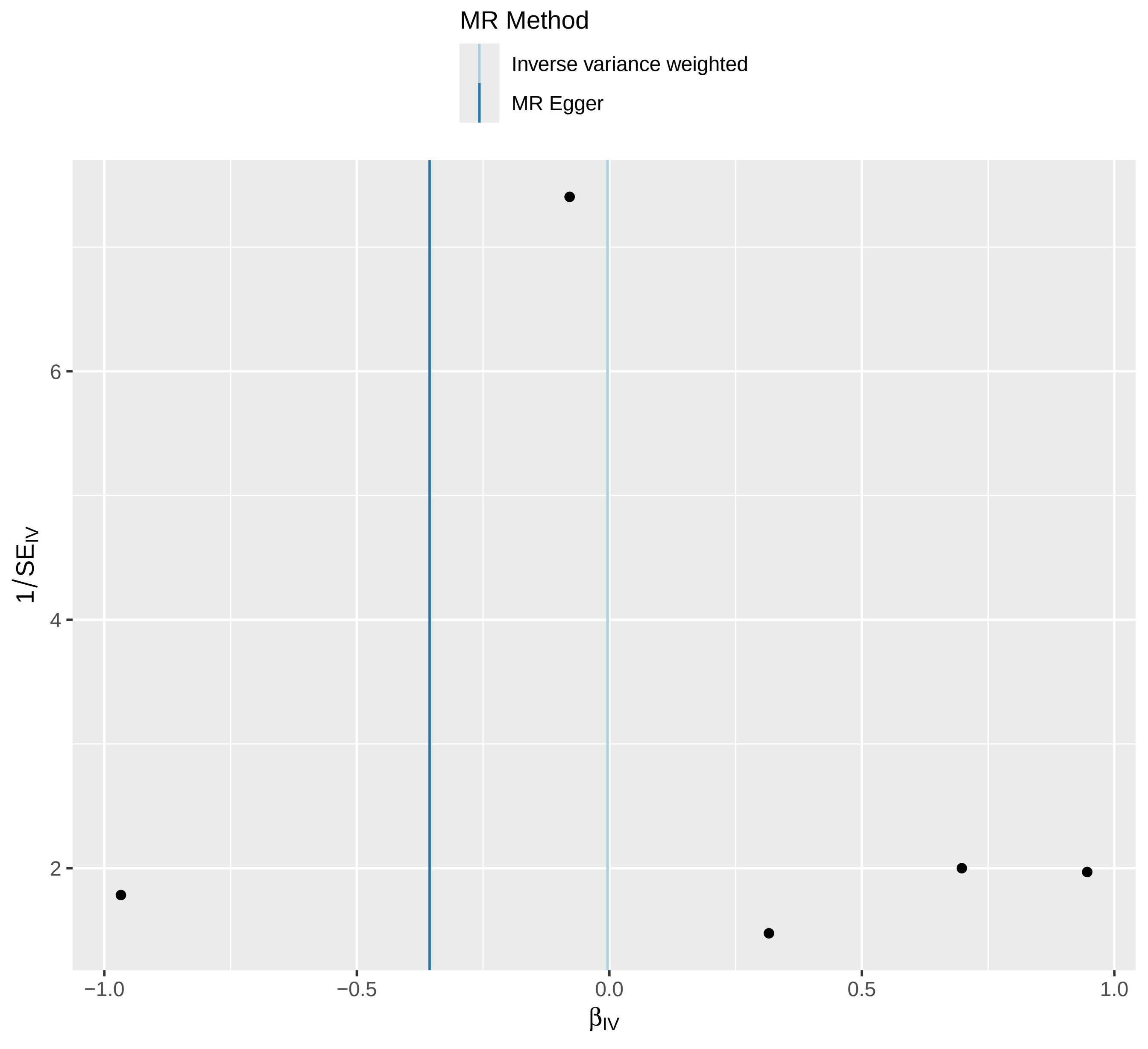

MR Method
Inverse variance weighted
MR Egger
6
4
2
$1/SE_{IV}$
−1.0
−0.5
0.0
0.5
1.0
β_{IV}

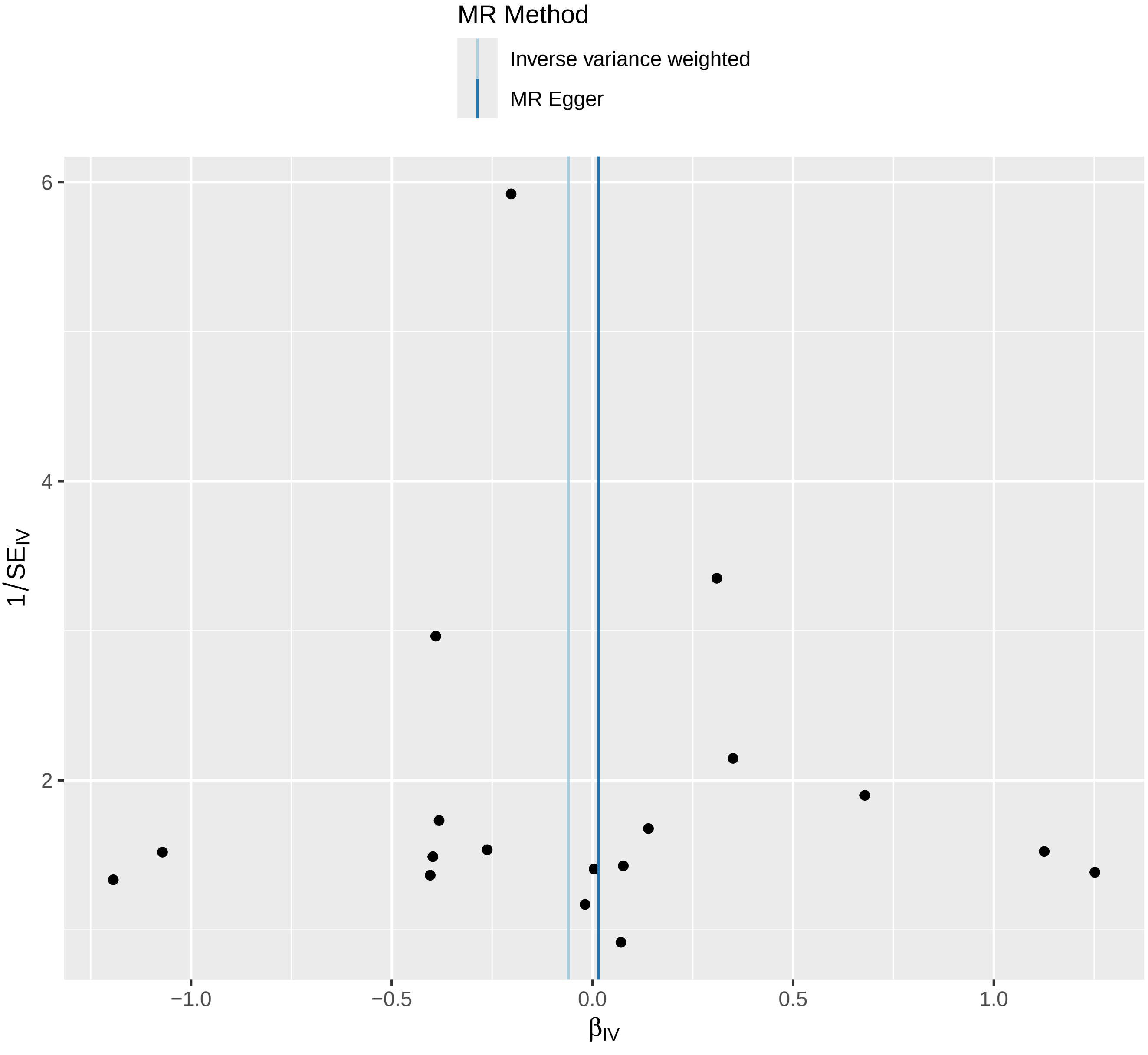

MR Method
Inverse variance weighted
MR Egger
6
4
2
$1/SE_{IV}$
−1.0
−0.5
0.0
0.5
1.0
β_{IV}

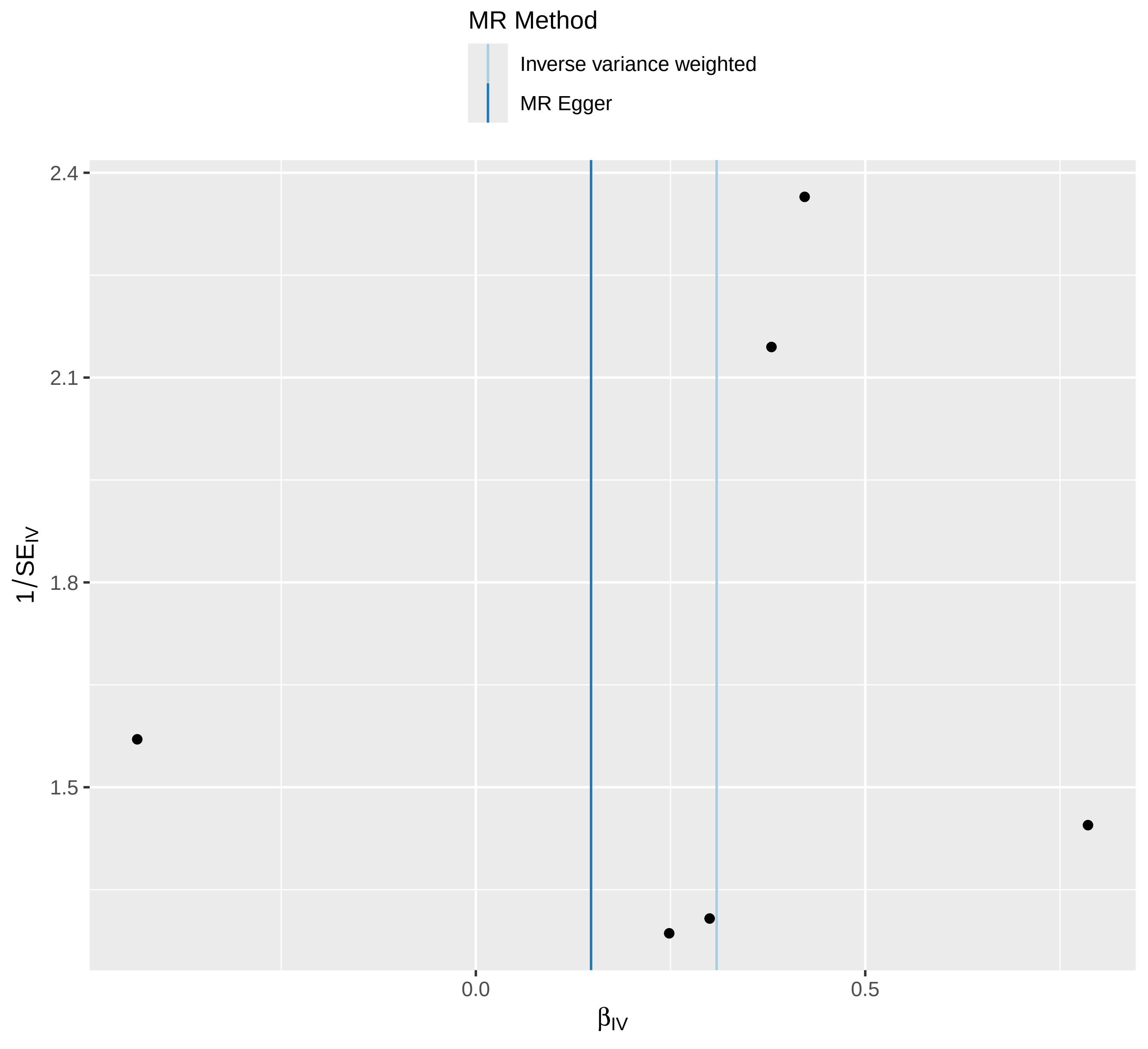

MR Method
Inverse variance weighted
MR Egger
2.4
2.1
1.8
1.5
1/SE$_{IV}$
0.0
0.5
β$_{IV}$

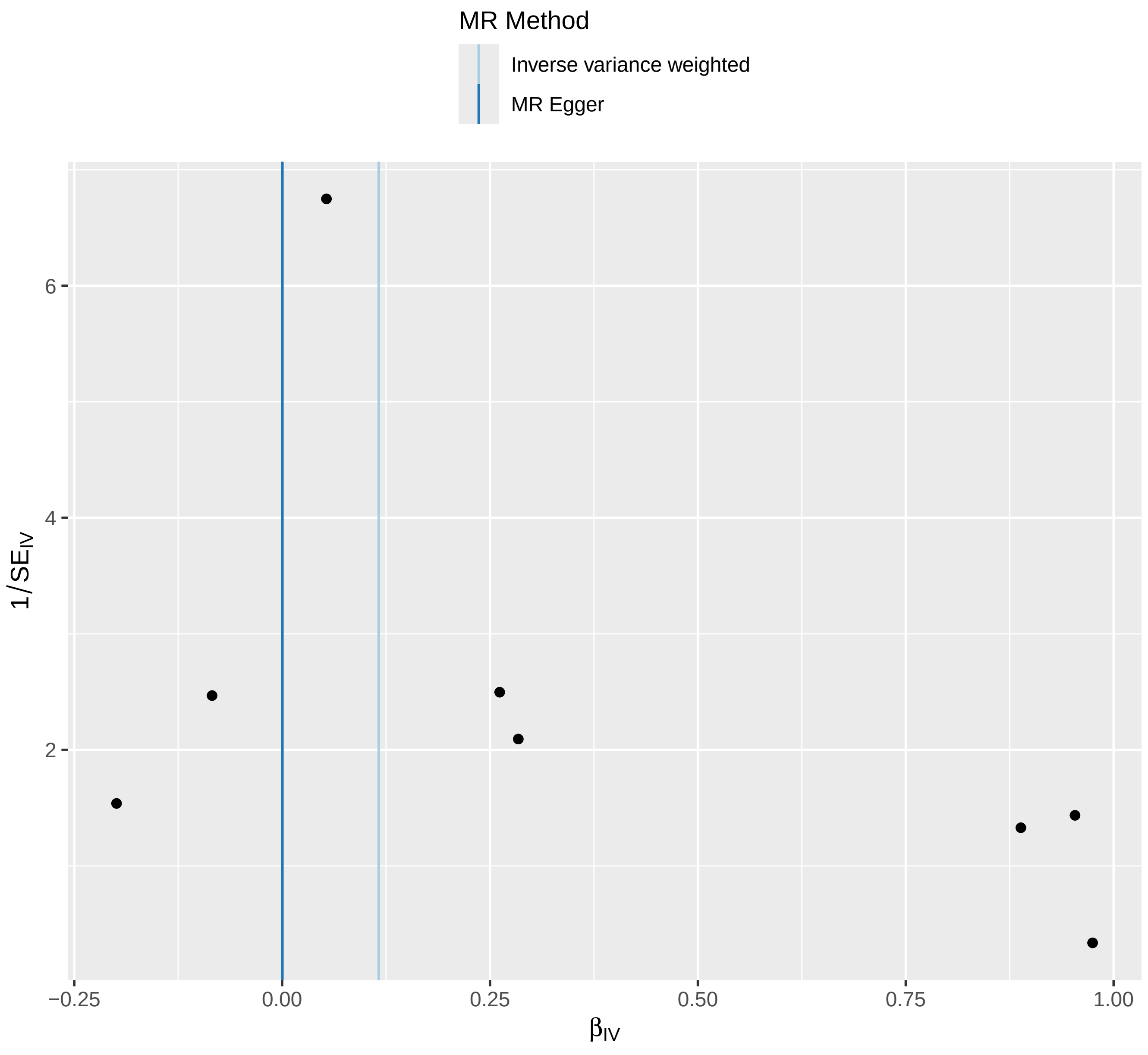
MR Method
Inverse variance weighted
MR Egger
6
4
2
1/SE_IV
−0.25
0.00
0.25
0.50
0.75
1.00
β_IV

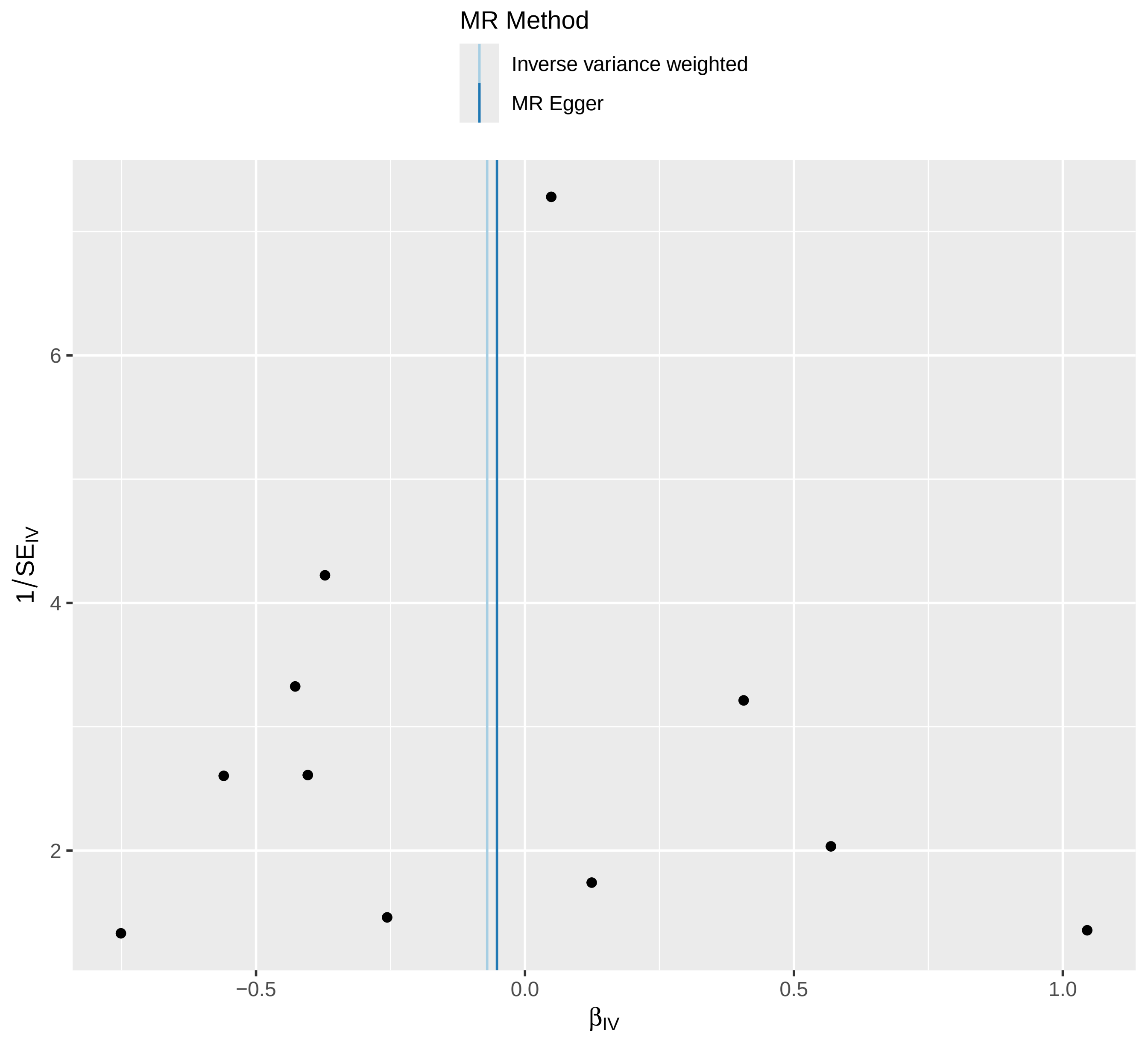
MR Method
Inverse variance weighted
MR Egger
6
4
2
1/SE_IV
−0.5
0.0
0.5
1.0
β_IV

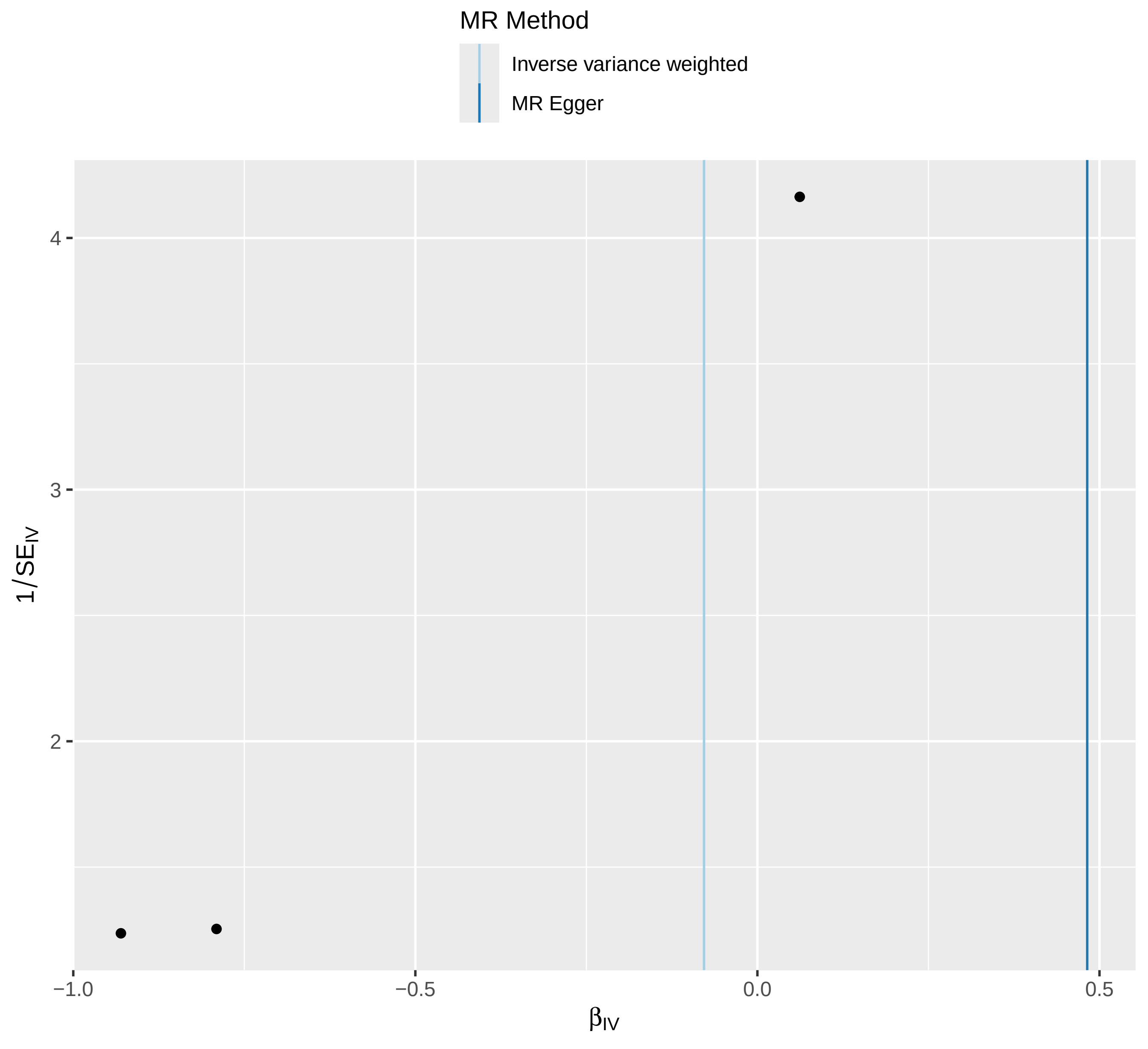

MR Method
Inverse variance weighted
MR Egger
4
3
2
1/SE_IV
−1.0
−0.5
0.0
0.5
β_IV

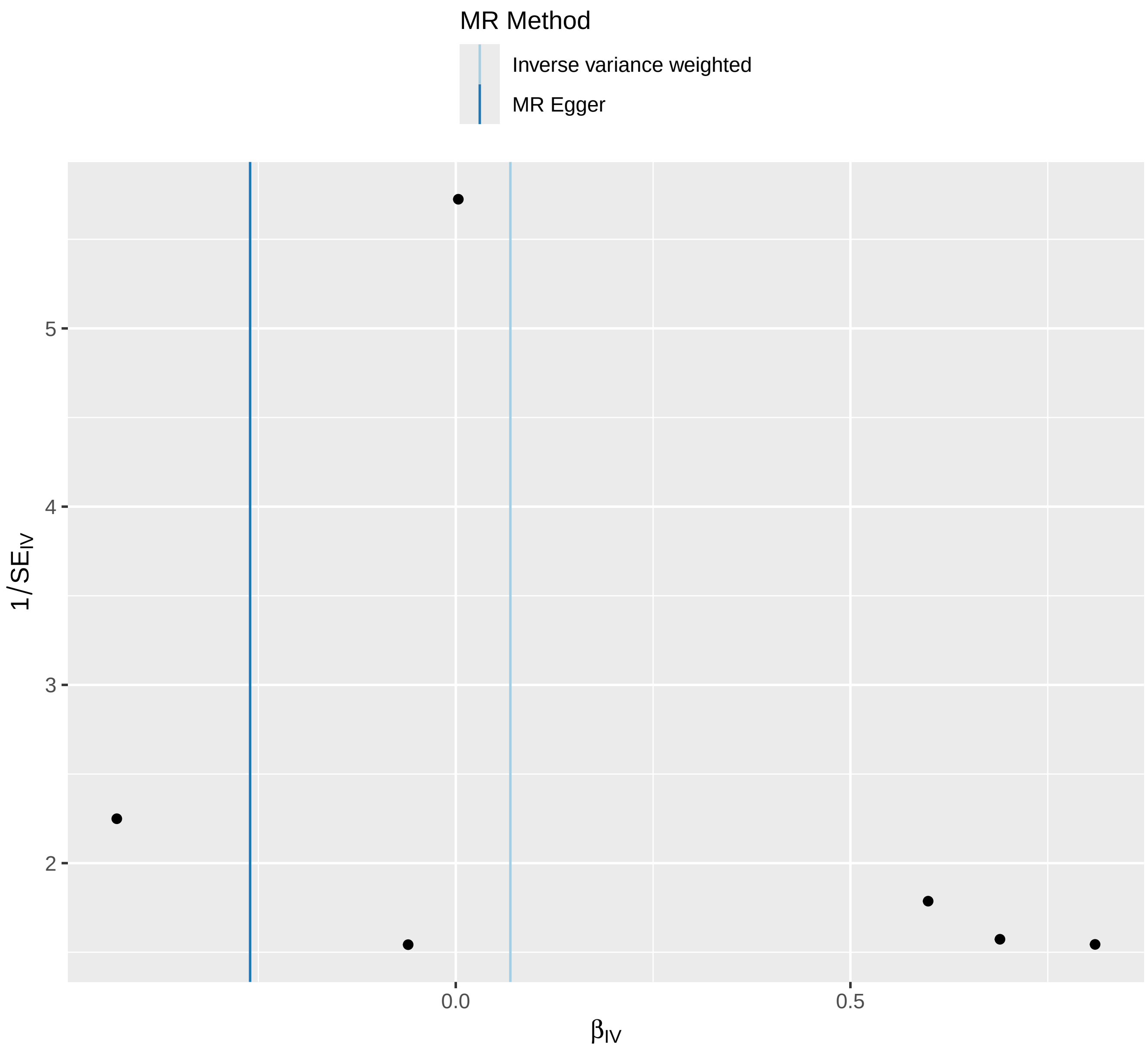

MR Method
Inverse variance weighted
MR Egger
5
4
3
2
1/SE$_{IV}$
0.0
0.5
β$_{IV}$

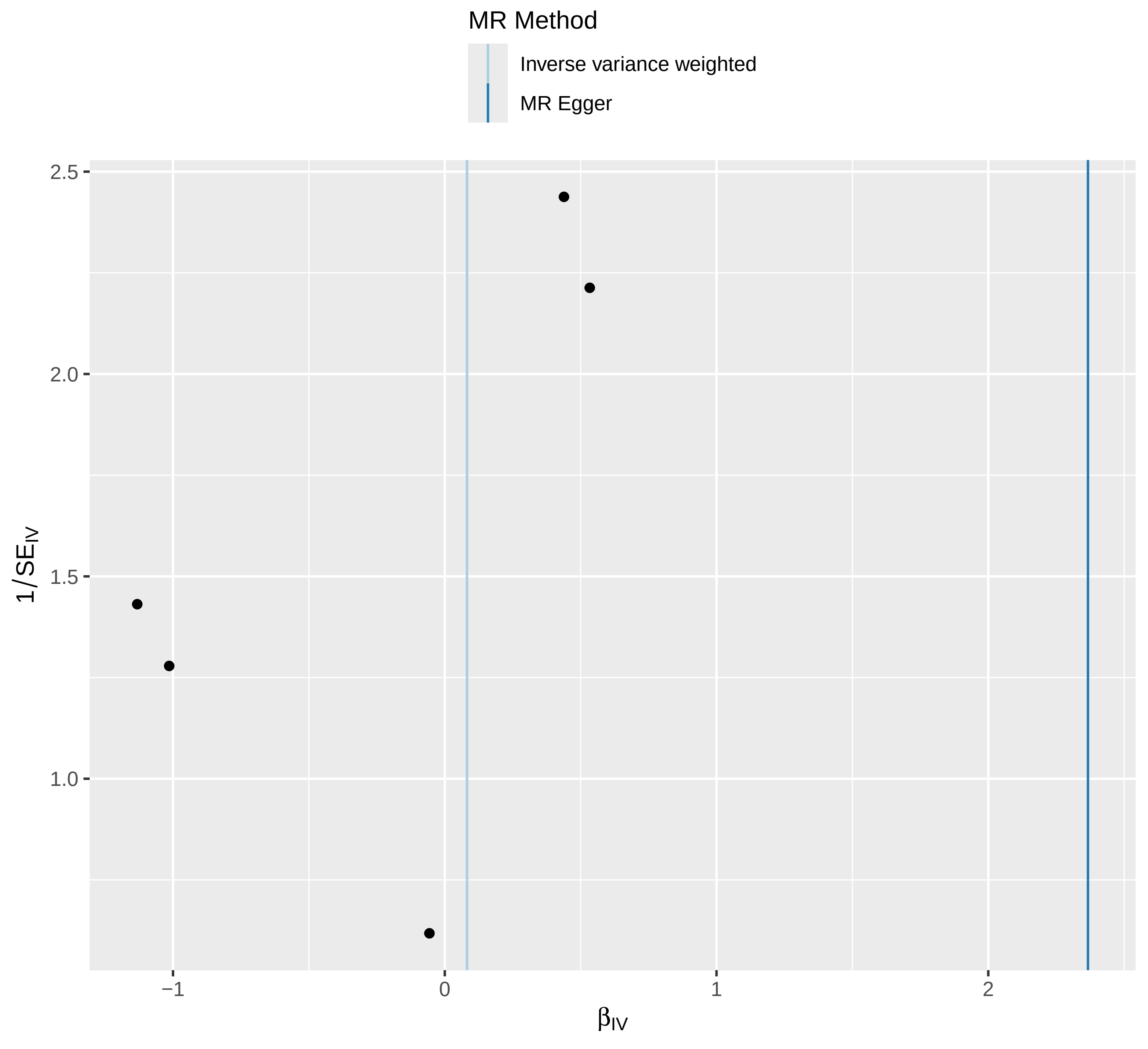

MR Method
Inverse variance weighted
MR Egger
2.5
2.0
1.5
1.0
1/SE_IV
−1
0
1
2
β_IV

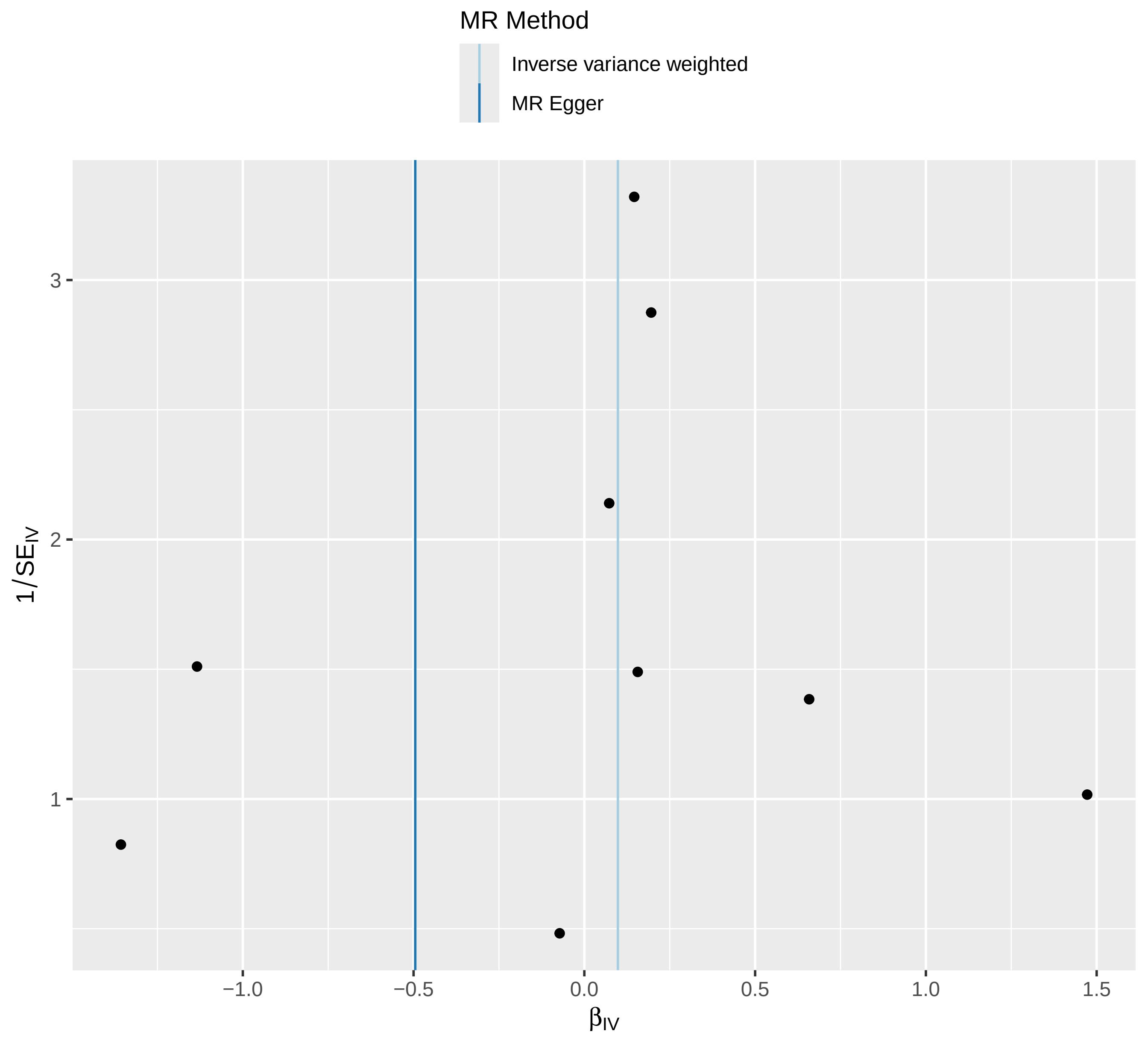

MR Method
Inverse variance weighted
MR Egger
3
2
1
1/SE$_{IV}$
−1.0
−0.5
0.0
0.5
1.0
1.5
β$_{IV}$

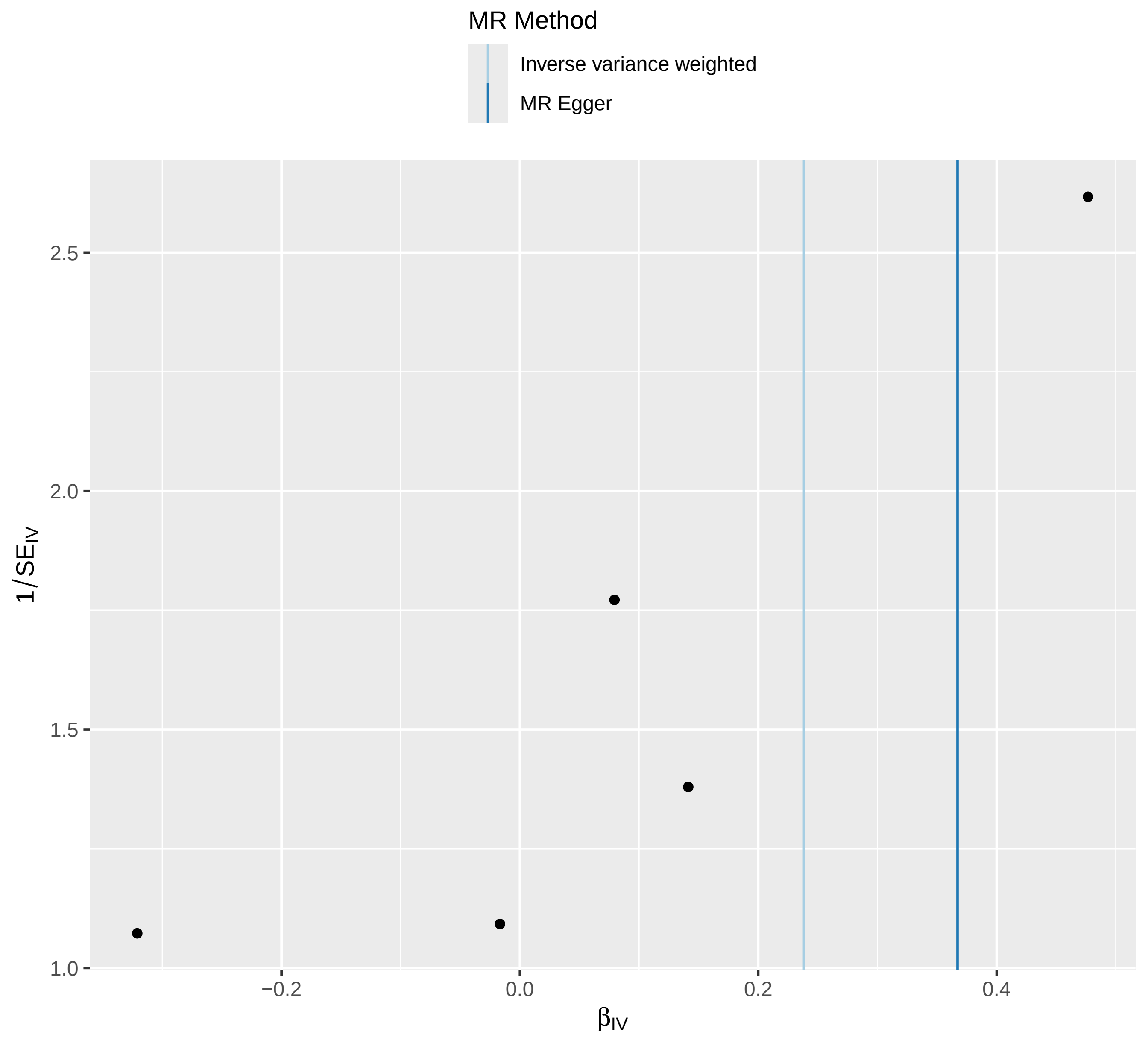

MR Method
Inverse variance weighted
MR Egger
2.5
2.0
1.5
1.0
1/SE_IV
−0.2
0.0
0.2
0.4
β_IV

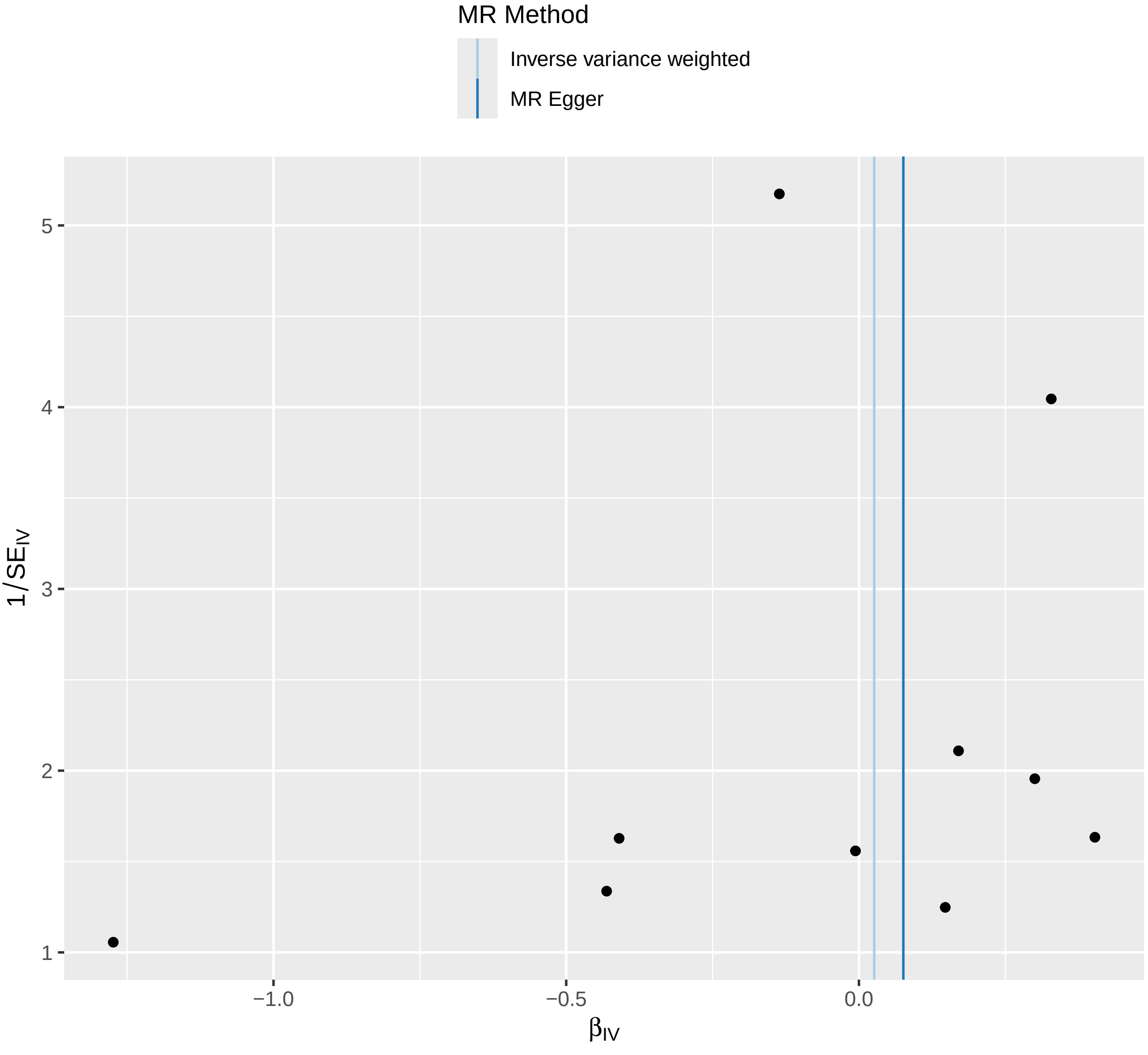
MR Method
Inverse variance weighted
MR Egger
1/SE$_{IV}$
5
4
3
2
1
−1.0
−0.5
0.0
β$_{IV}$

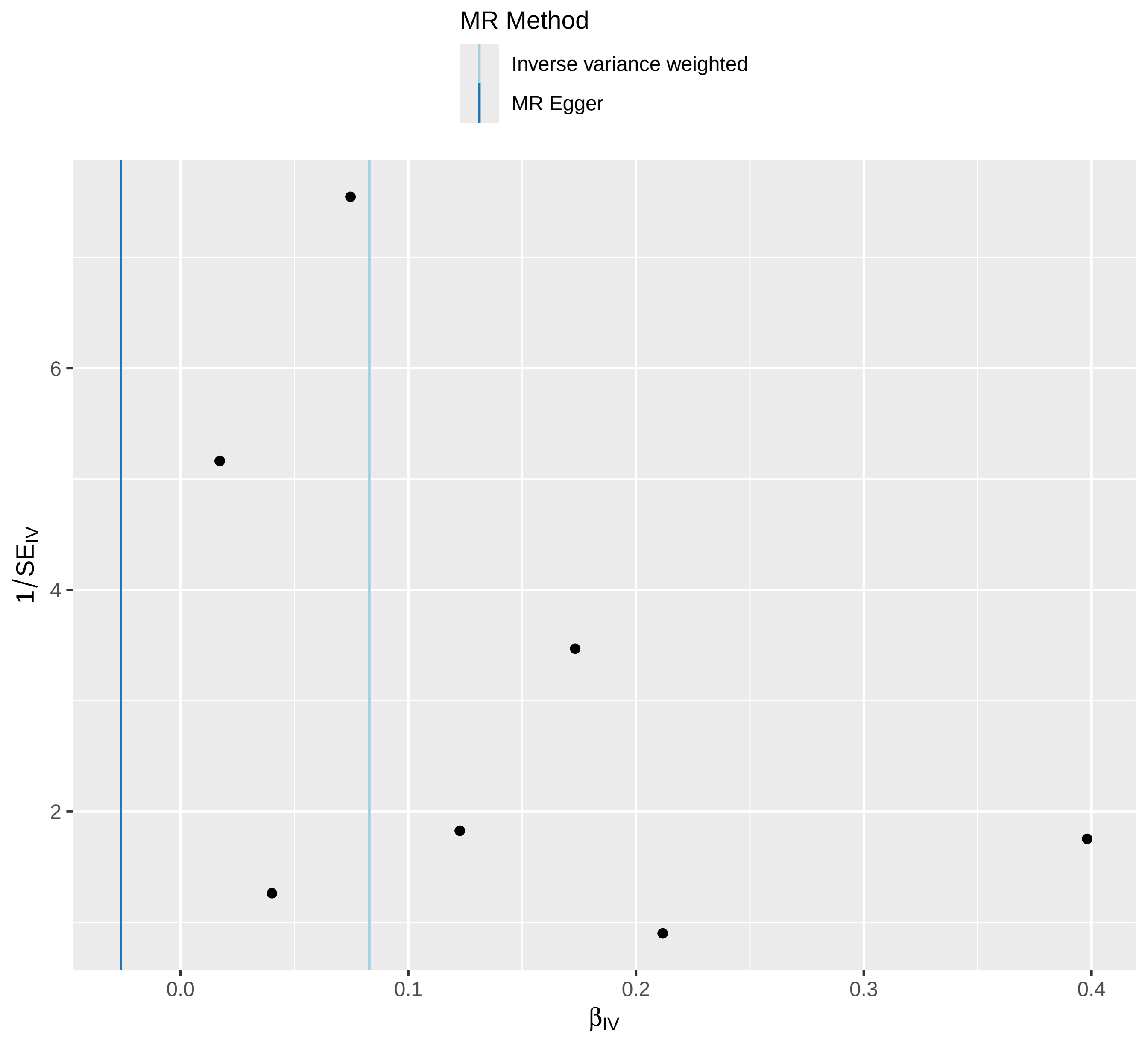

MR Method
Inverse variance weighted
MR Egger
6
4
2
1/SE_IV
0.0
0.1
0.2
0.3
0.4
β_IV

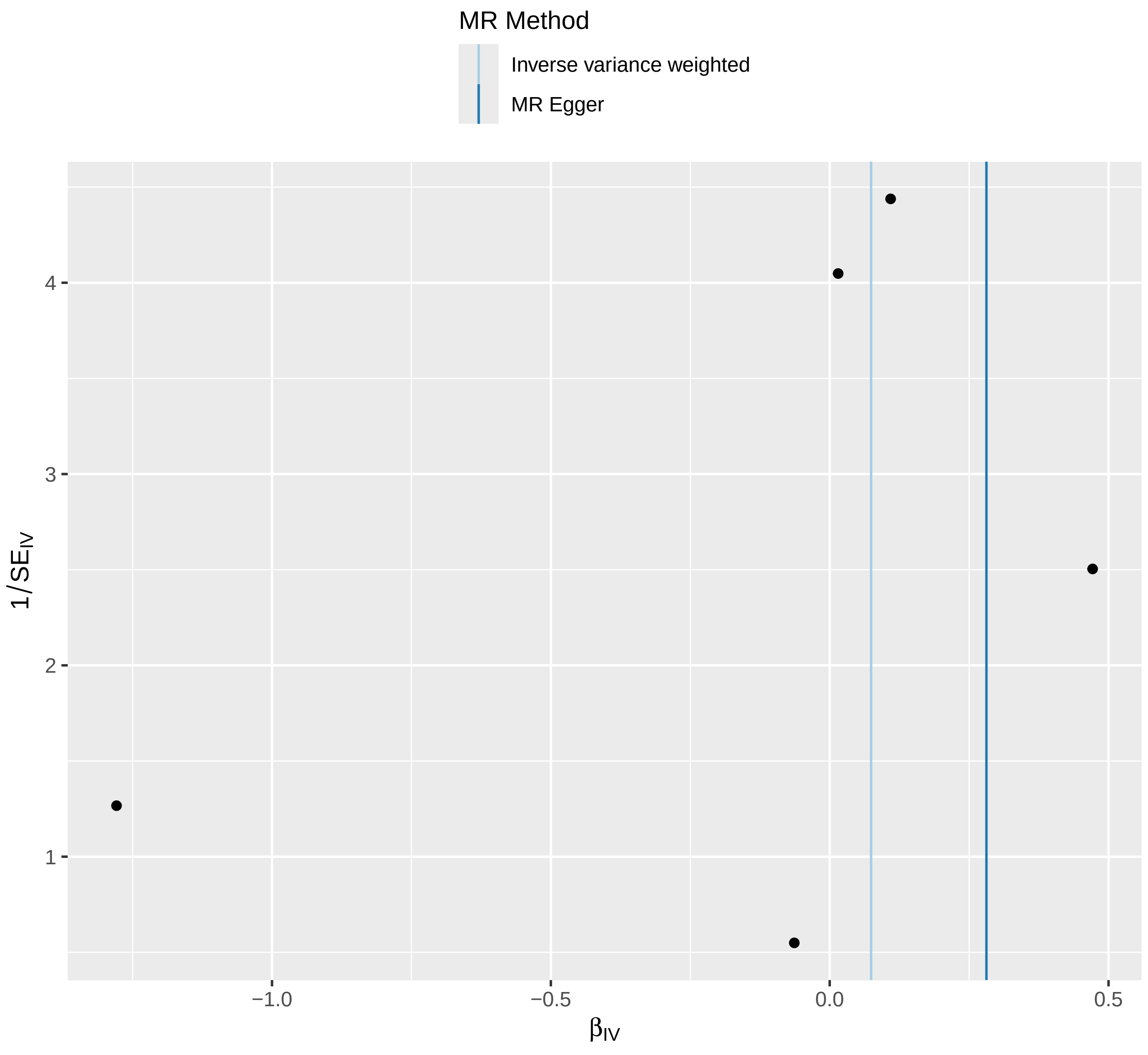

MR Method
Inverse variance weighted
MR Egger
4
3
2
1
1/SE$_{IV}$
−1.0
−0.5
0.0
0.5
β$_{IV}$

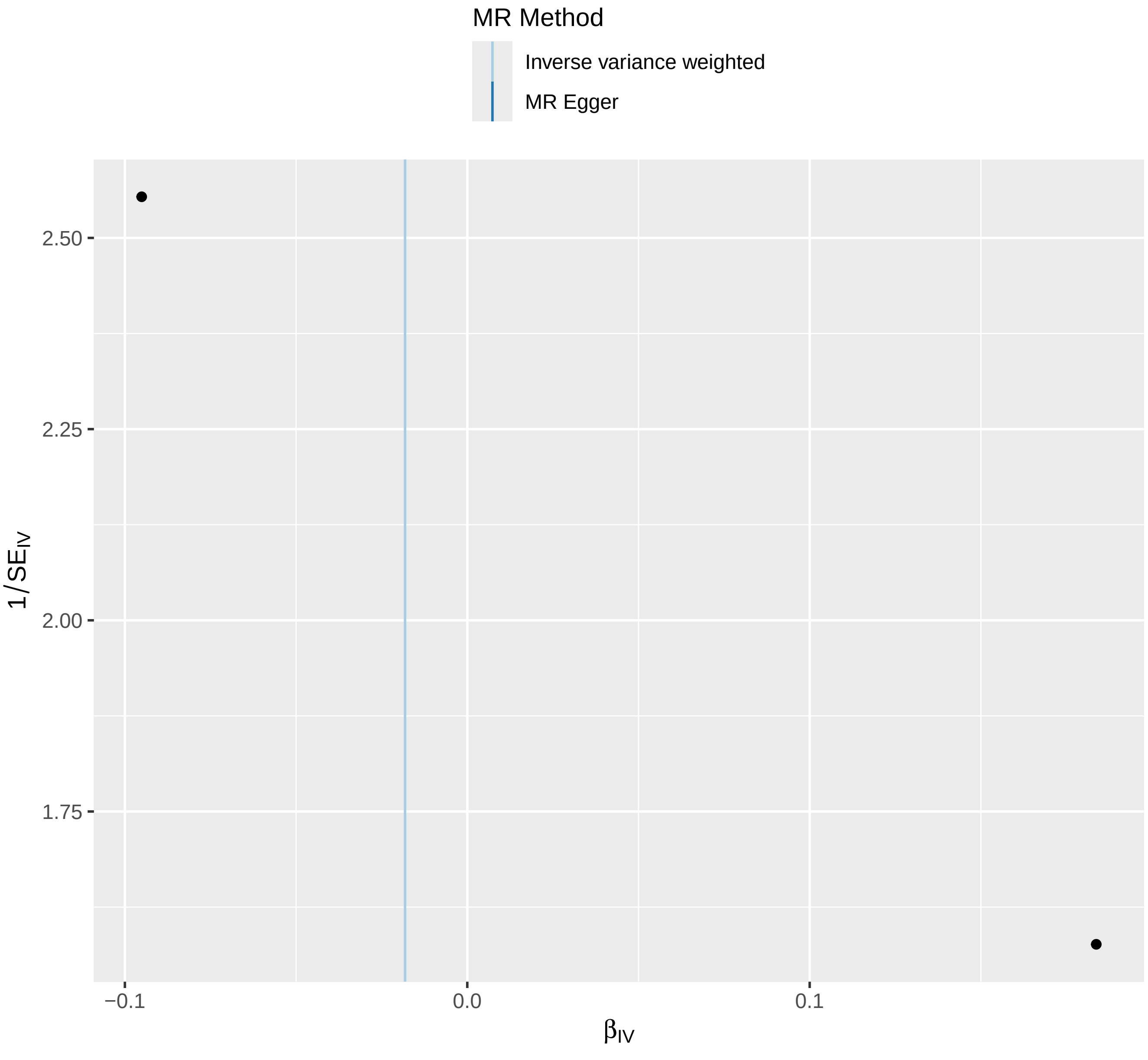

MR Method
Inverse variance weighted
MR Egger
2.50
2.25
2.00
1.75
1/SE_IV
−0.1
0.0
0.1
β_IV

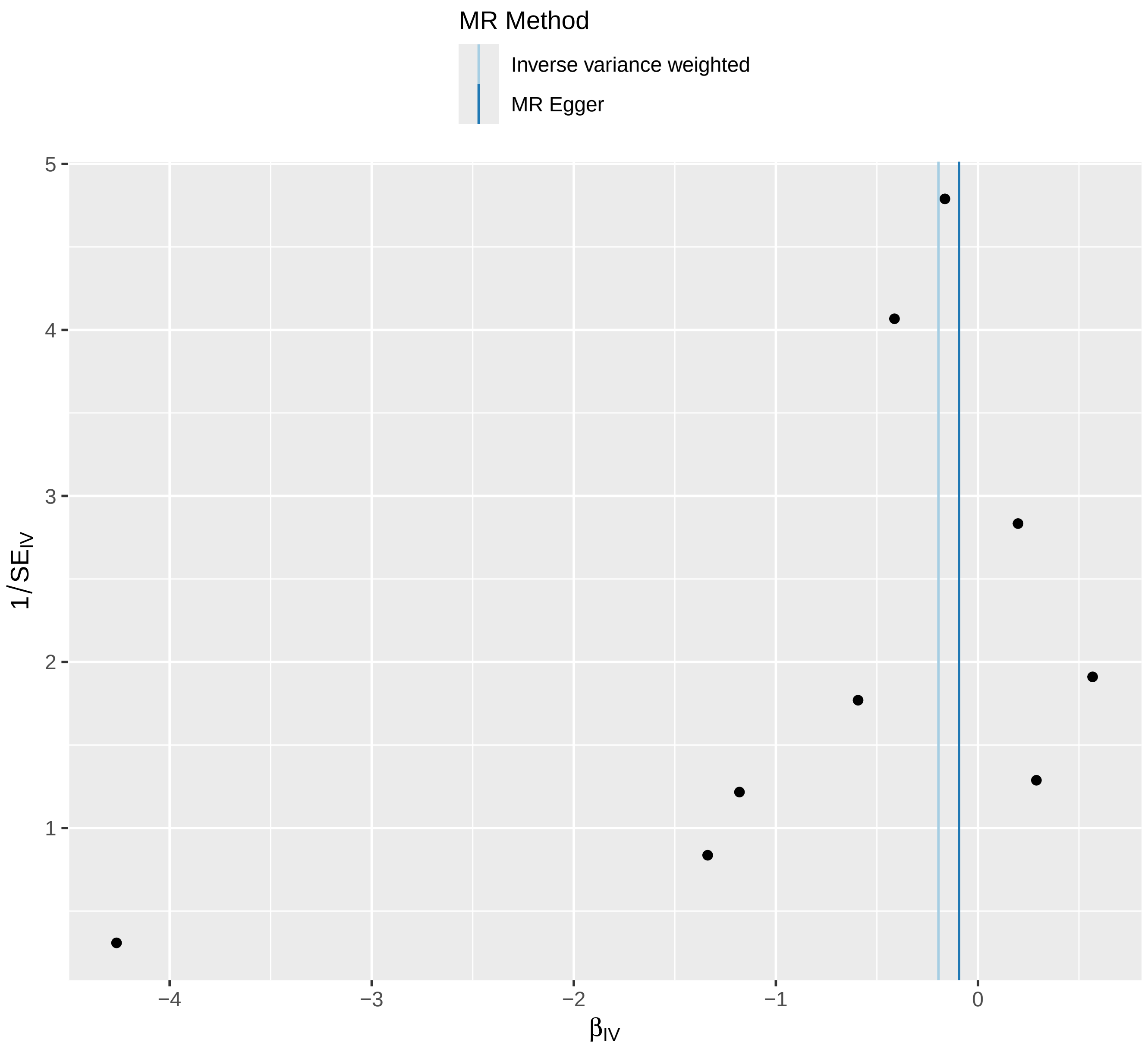
MR Method
Inverse variance weighted
MR Egger
5
4
3
2
1
−4
−3
−2
−1
0
$1/SE_{IV}$
β_{IV}

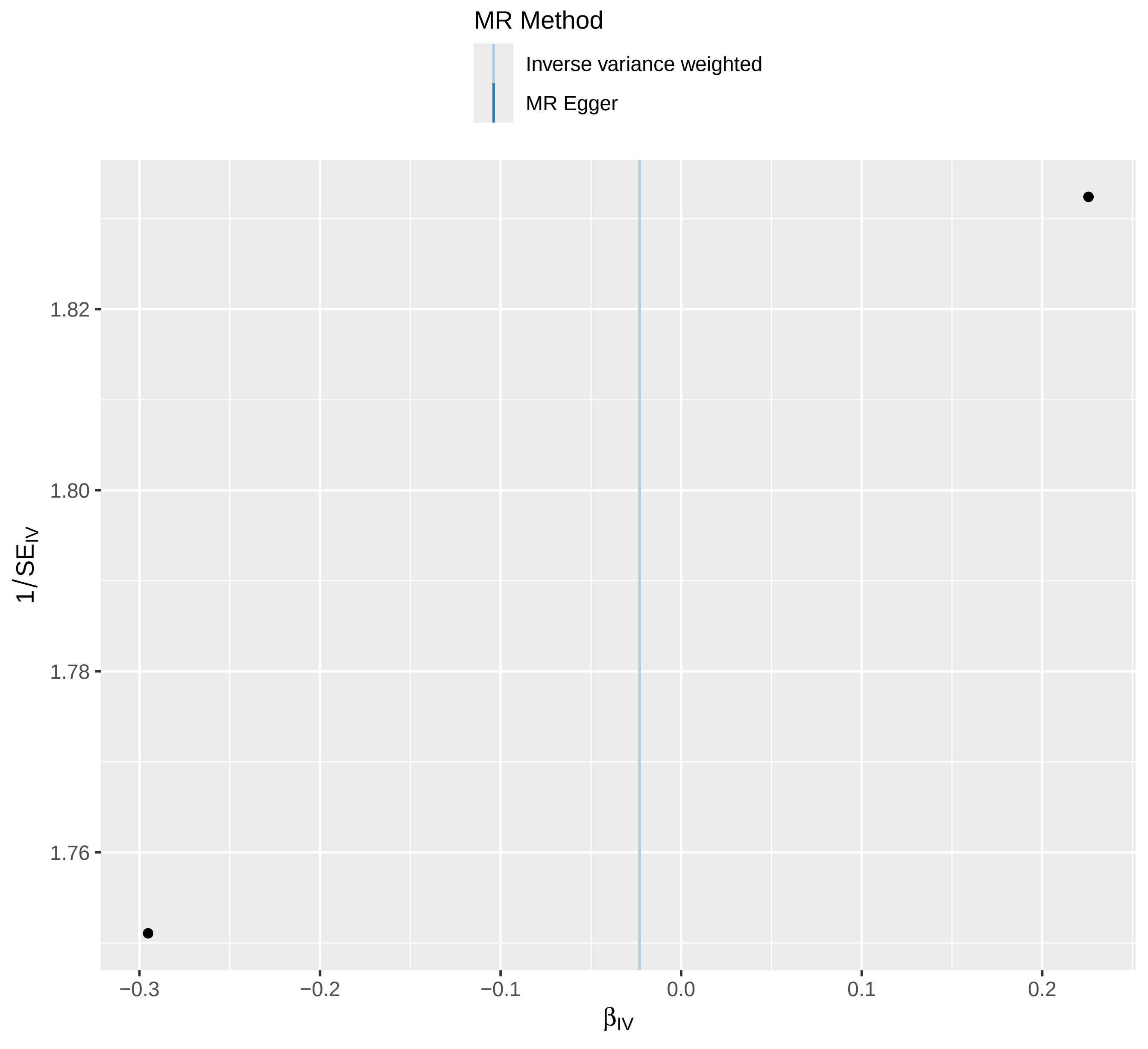
MR Method
Inverse variance weighted
MR Egger
1.82
1.80
1.78
1.76
1/SE_IV
−0.3
−0.2
−0.1
0.0
0.1
0.2
β_IV

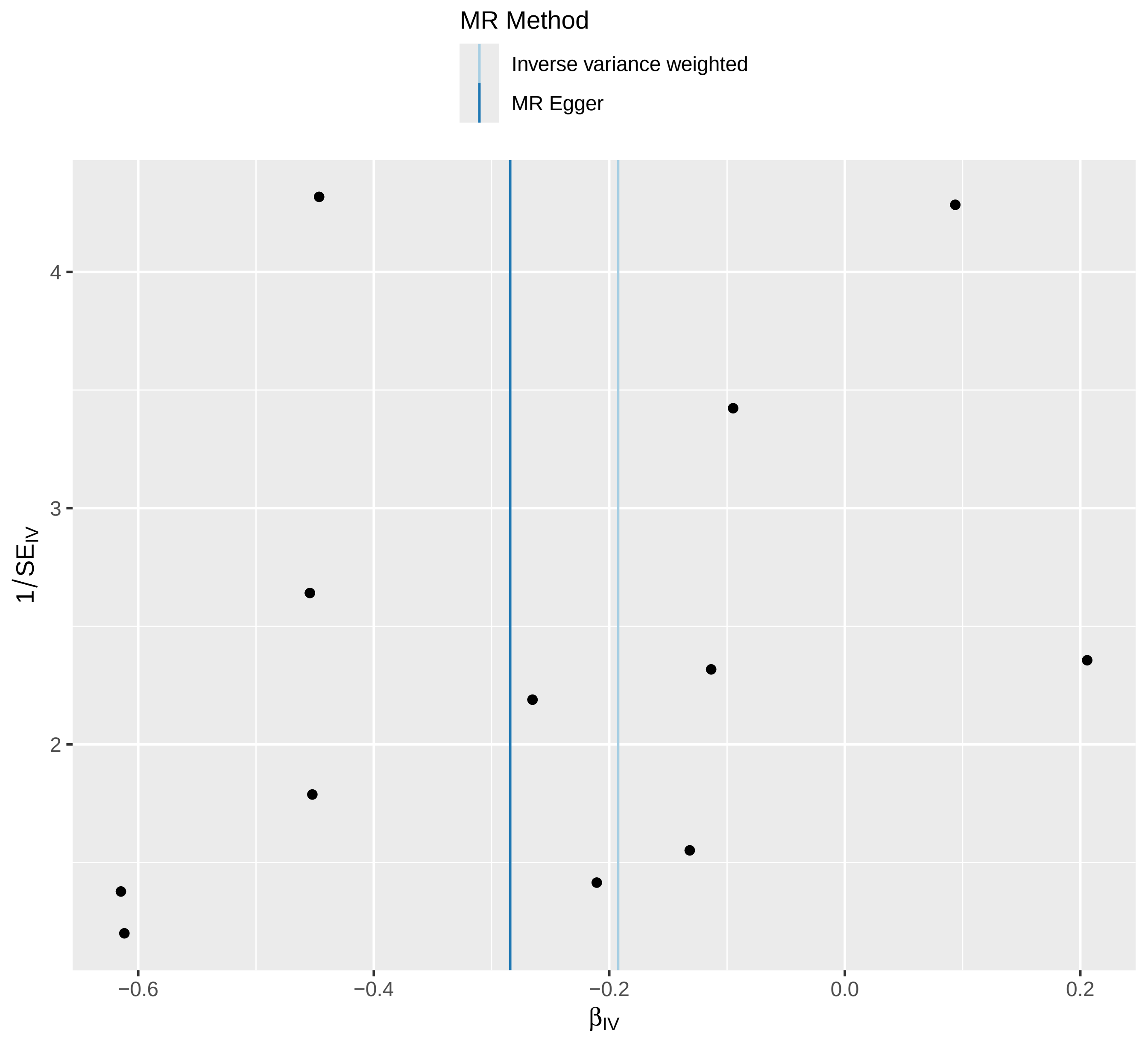
MR Method
Inverse variance weighted
MR Egger
4
3
2
1/SE$_{IV}$
−0.6
−0.4
−0.2
0.0
0.2
β$_{IV}$

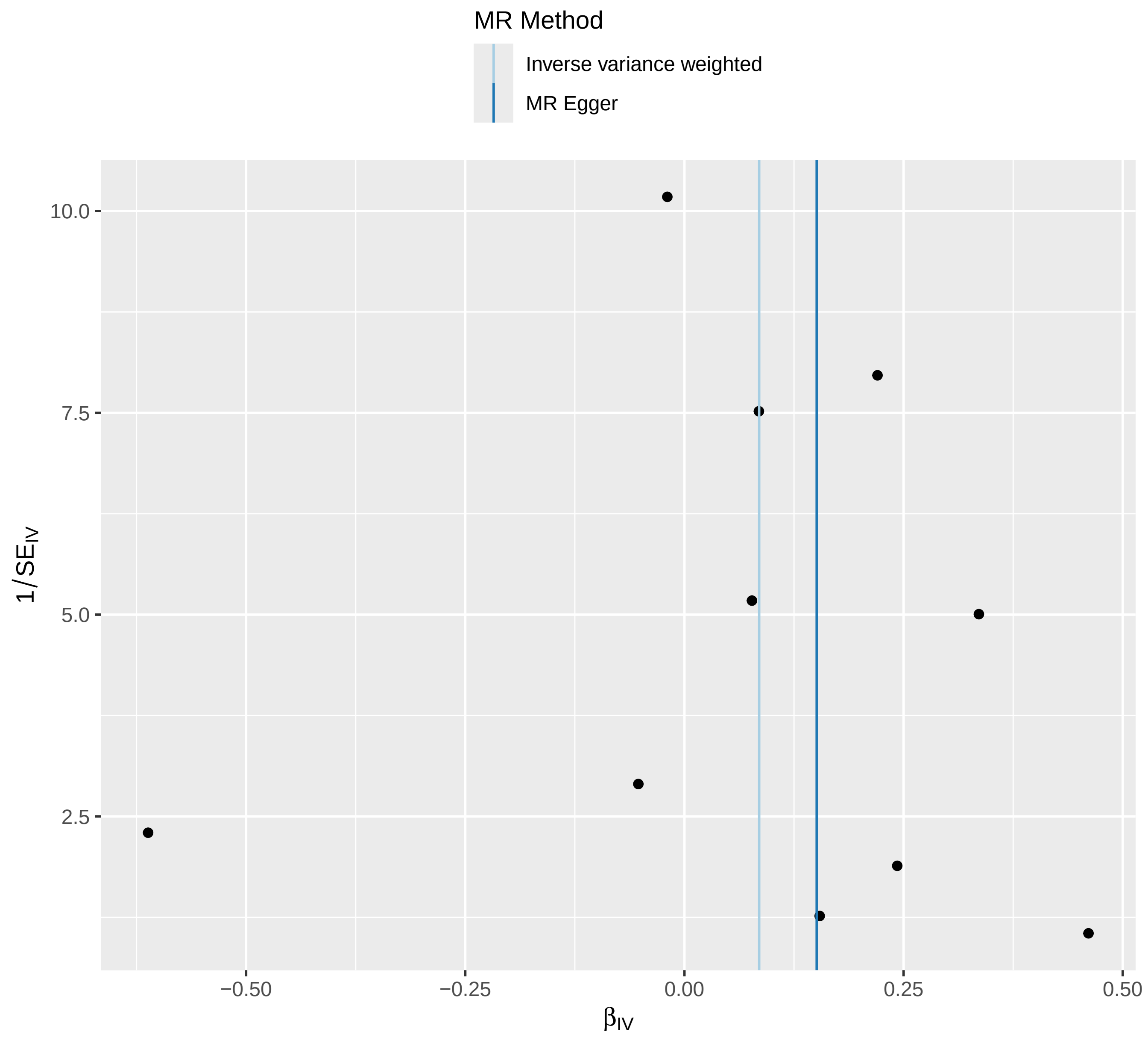
MR Method
Inverse variance weighted
MR Egger
10.0
7.5
5.0
2.5
1/SE_IV
−0.50
−0.25
0.00
0.25
0.50
β_IV

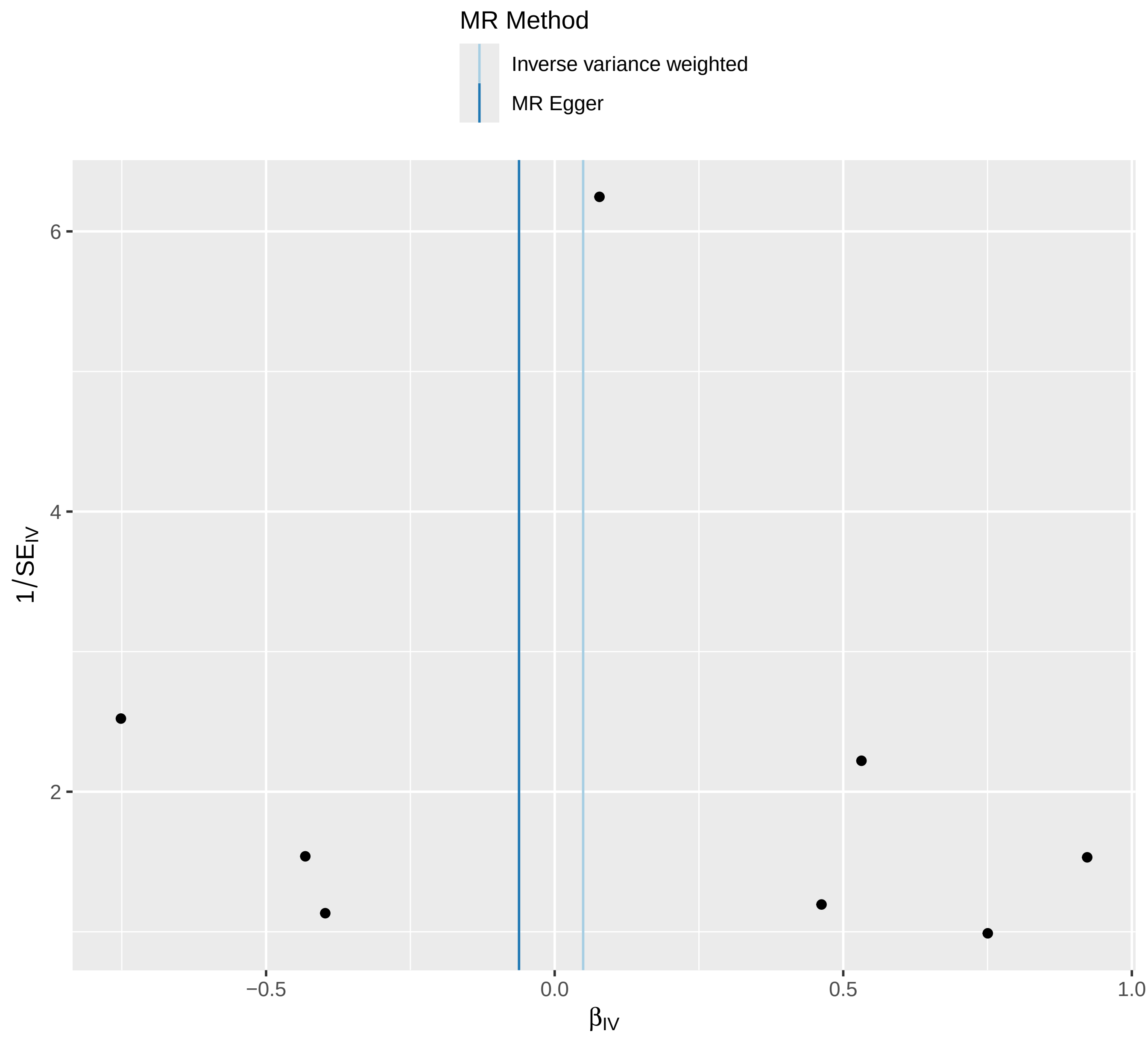
MR Method
Inverse variance weighted
MR Egger
6
4
2
1/SE_IV
−0.5
0.0
0.5
1.0
β_IV

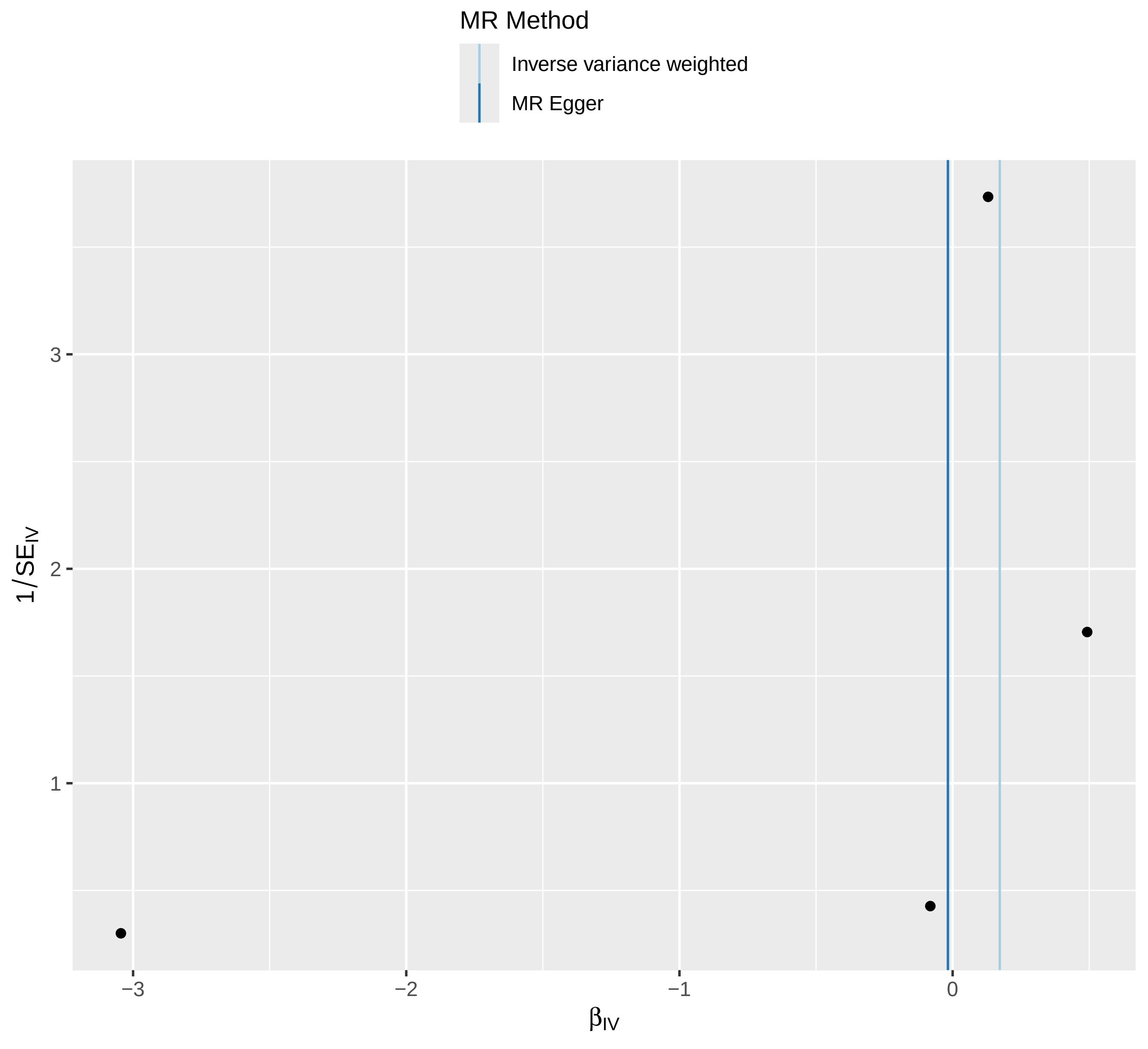
MR Method
Inverse variance weighted
MR Egger
3
2
1
1/SE_IV
−3
−2
−1
0
β_IV

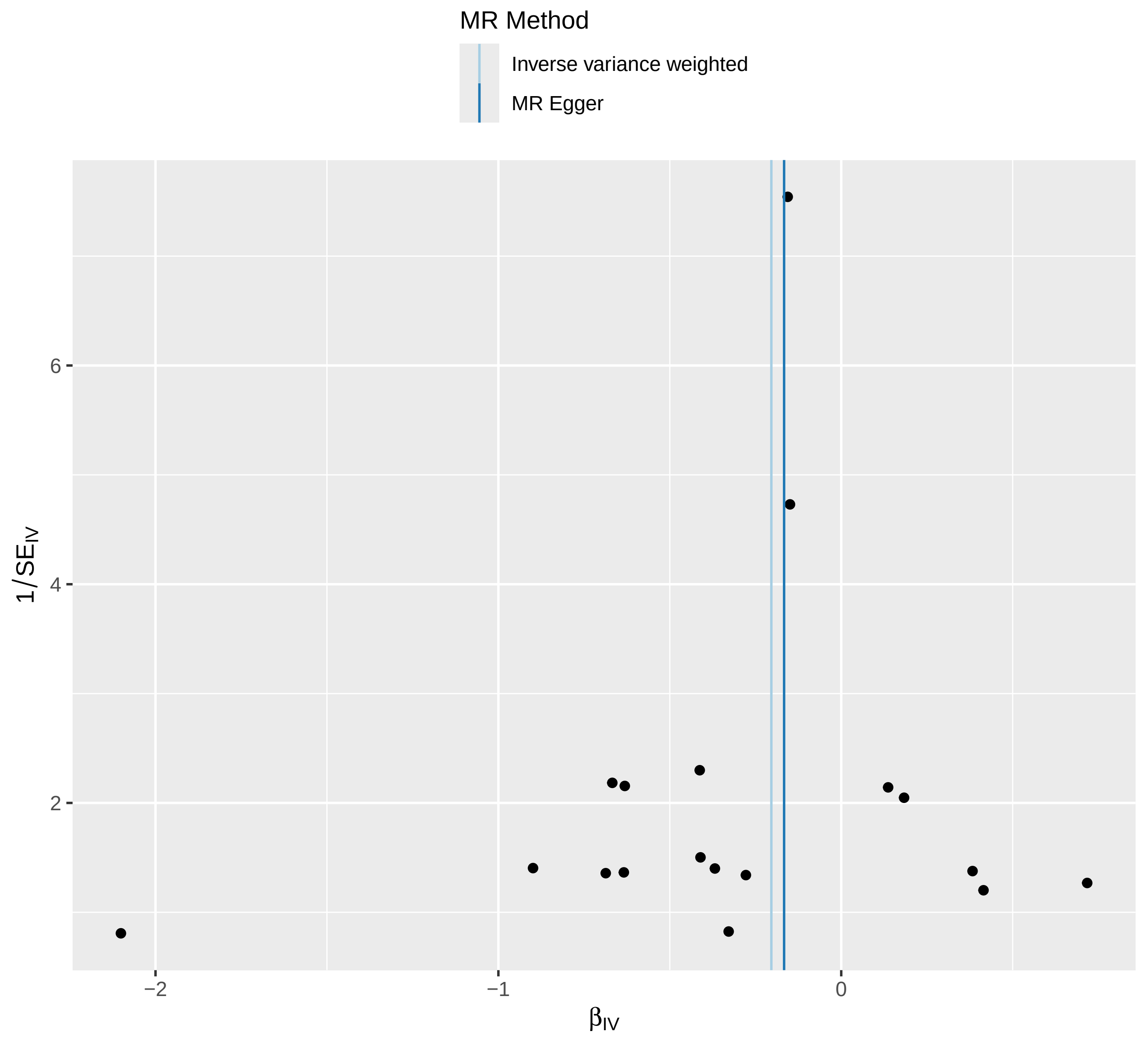

MR Method
Inverse variance weighted
MR Egger
6
4
2
1/SE_IV
−2
−1
0
β_IV

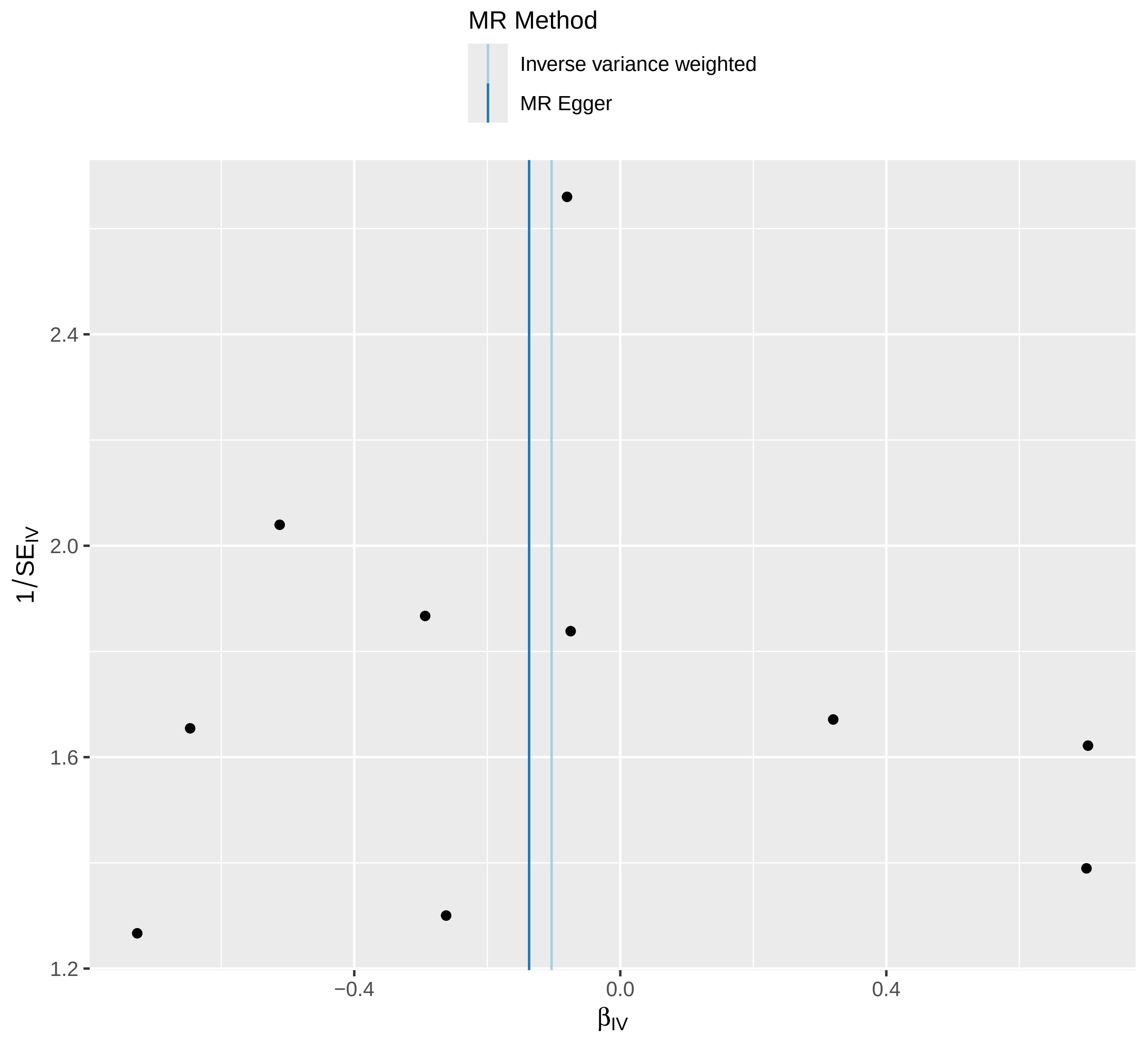

MR Method
Inverse variance weighted
MR Egger
2.4
2.0
1.6
1.2
1/SE$_{IV}$
−0.4
0.0
0.4
β$_{IV}$

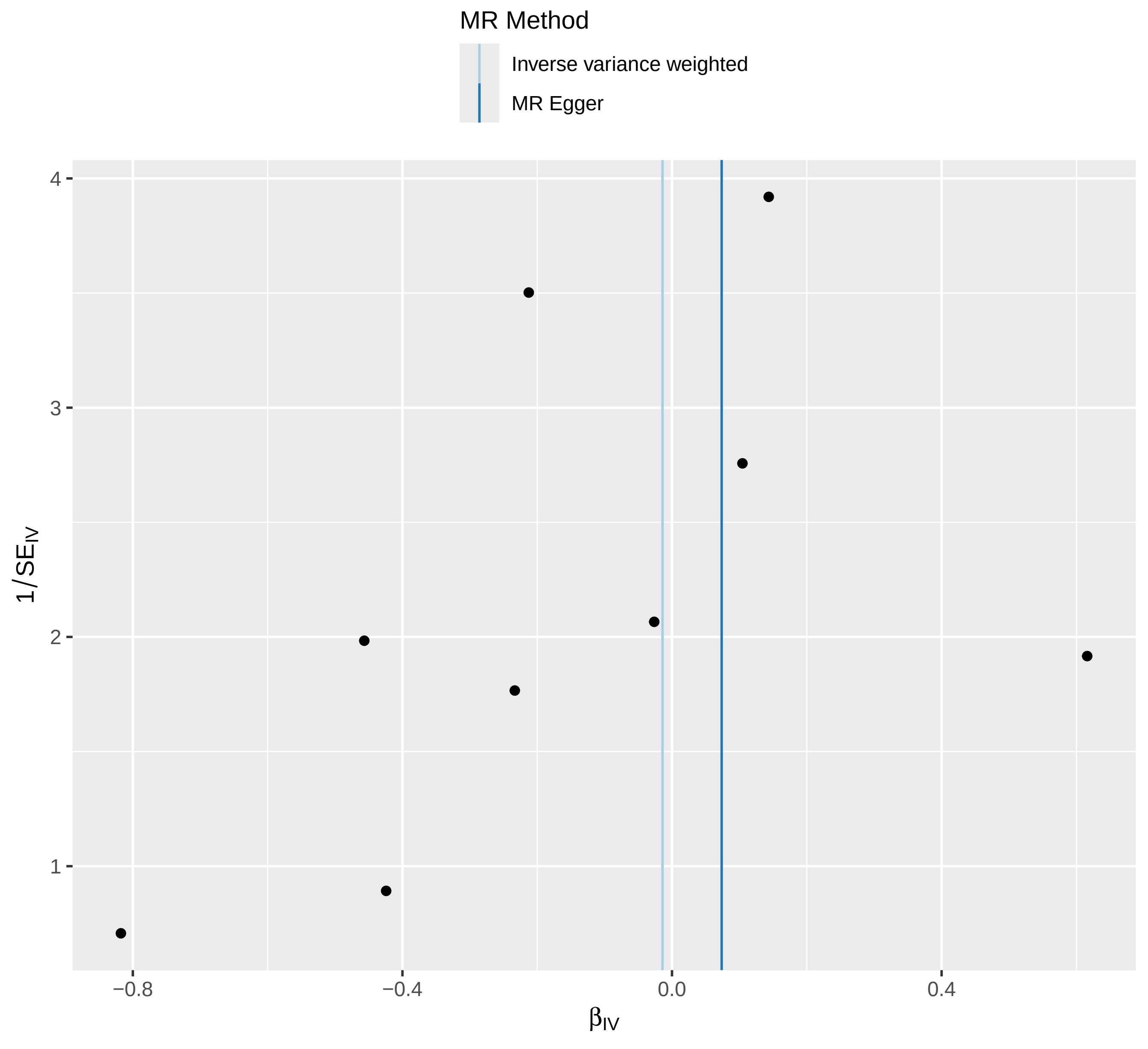
MR Method
Inverse variance weighted
MR Egger
1/SE$_{IV}$
4
3
2
1
−0.8
−0.4
0.0
0.4
β$_{IV}$

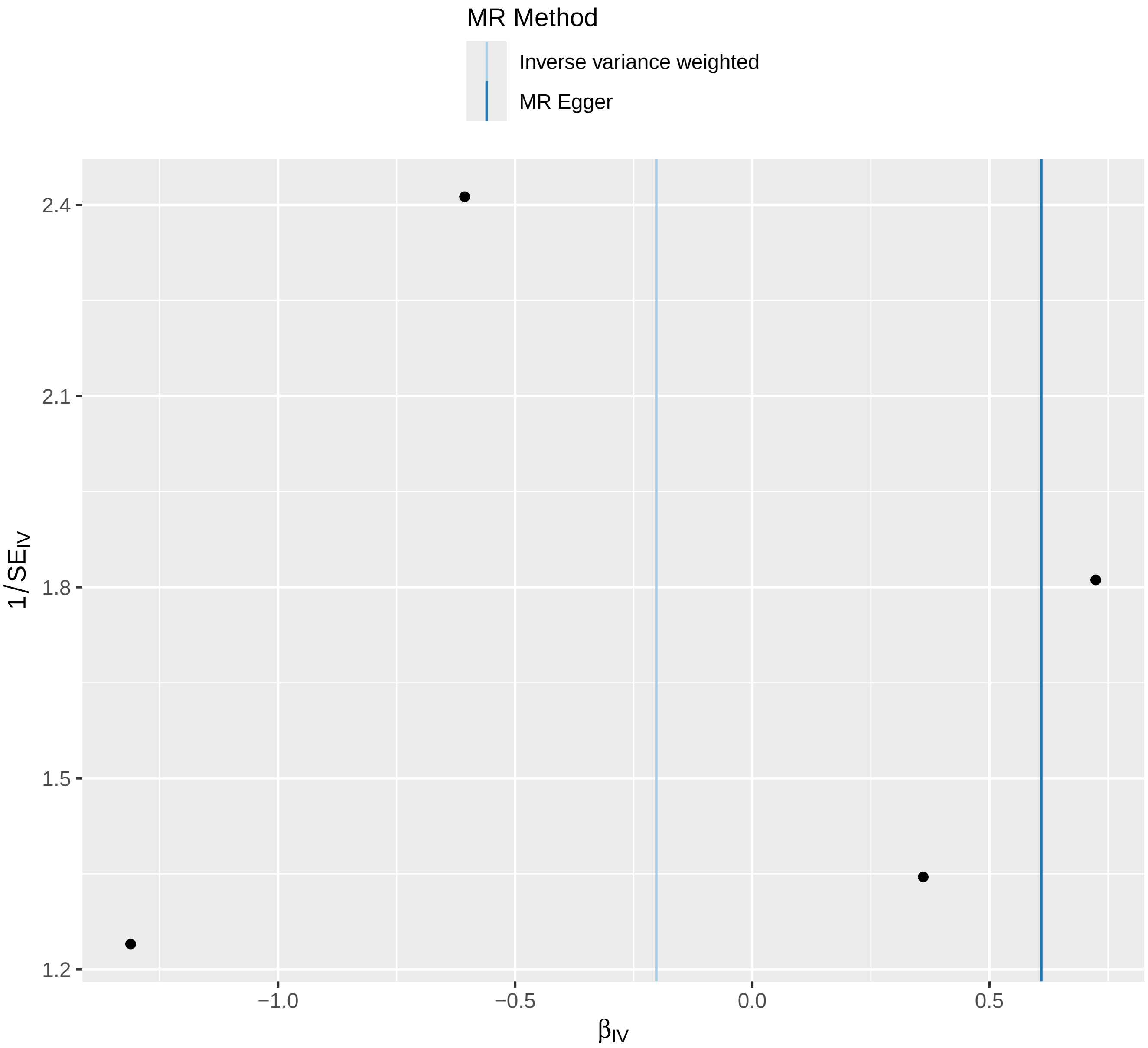
MR Method
Inverse variance weighted
MR Egger
2.4
2.1
1.8
1.5
1.2
1/SE_IV
−1.0
−0.5
0.0
0.5
β_IV

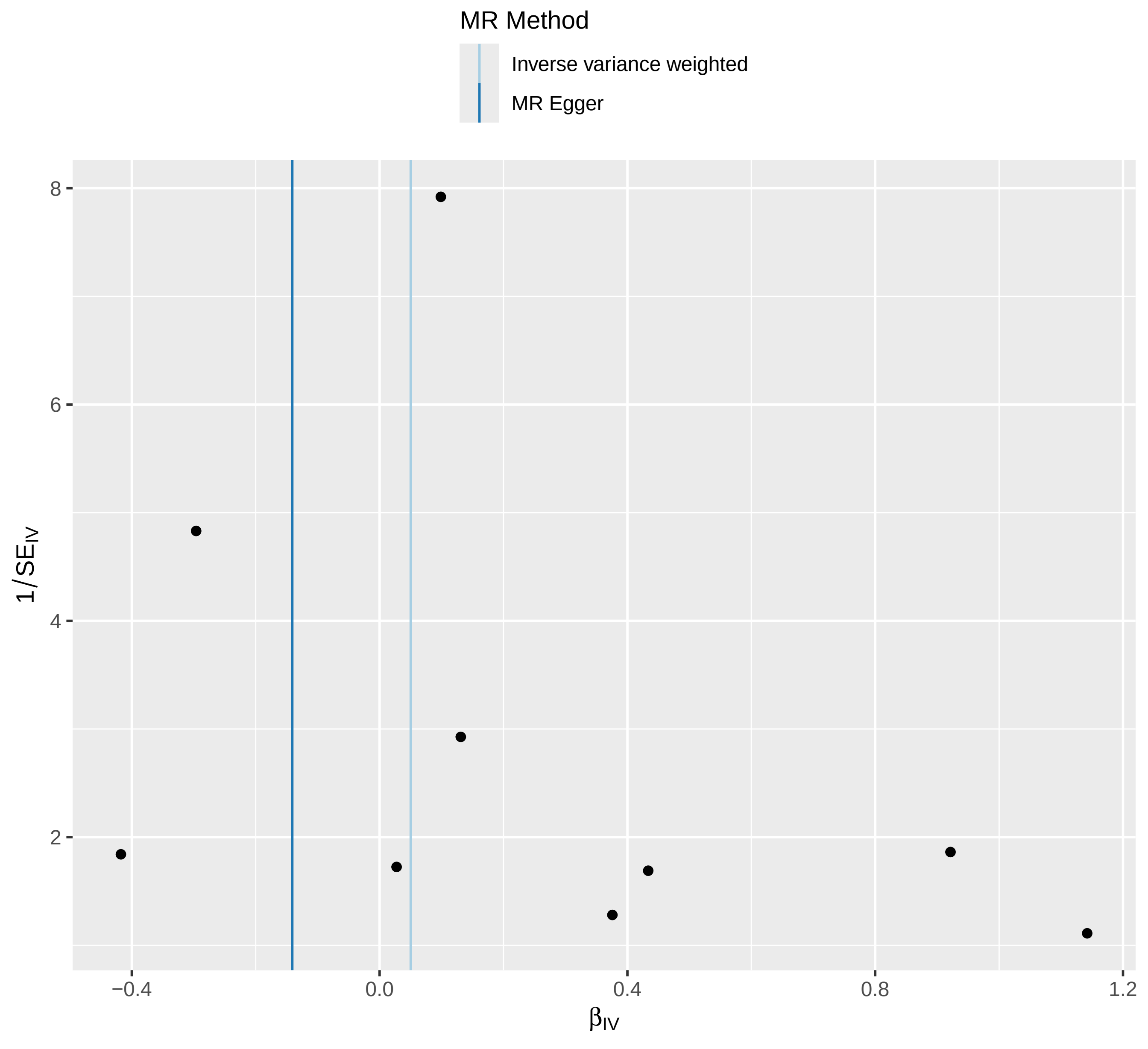

MR Method
Inverse variance weighted
MR Egger
8
6
4
2
1/SE_IV
−0.4
0.0
0.4
0.8
1.2
β_IV

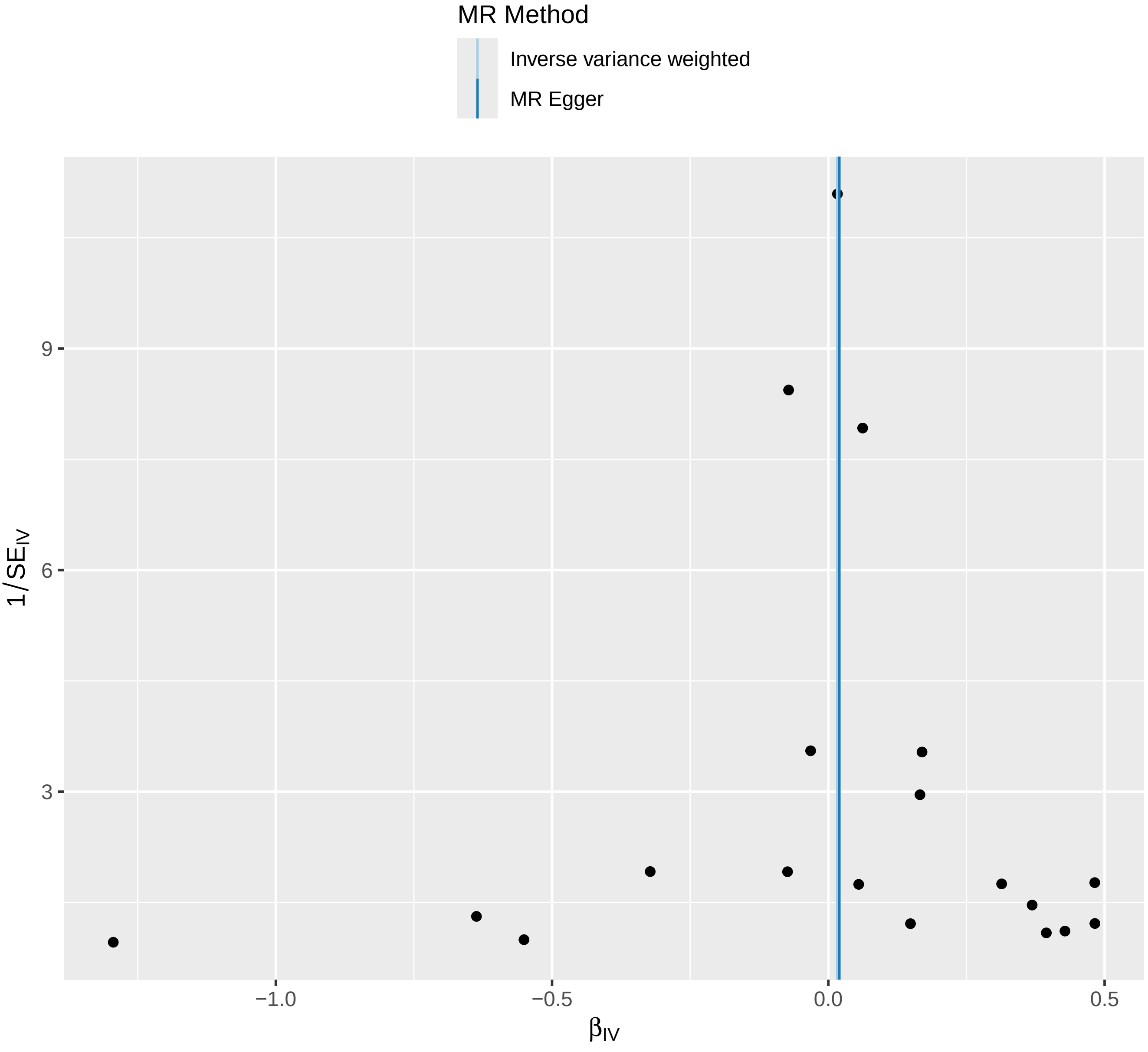
MR Method
Inverse variance weighted
MR Egger
9
6
3
1/SE_IV
−1.0
−0.5
0.0
0.5
β_IV

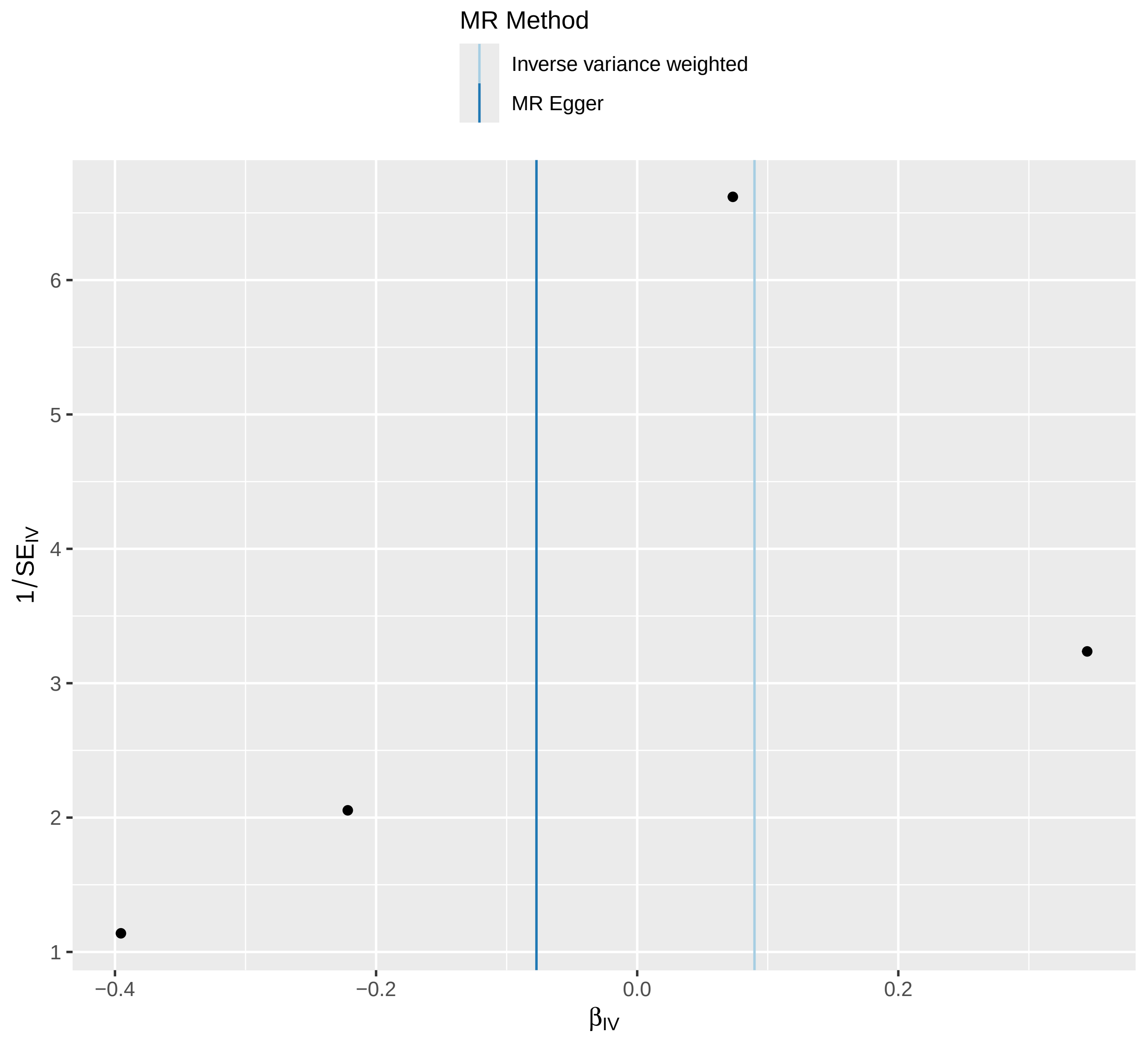
MR Method
Inverse variance weighted
MR Egger
1/SE_IV
6
5
4
3
2
1
−0.4
−0.2
0.0
0.2
β_IV

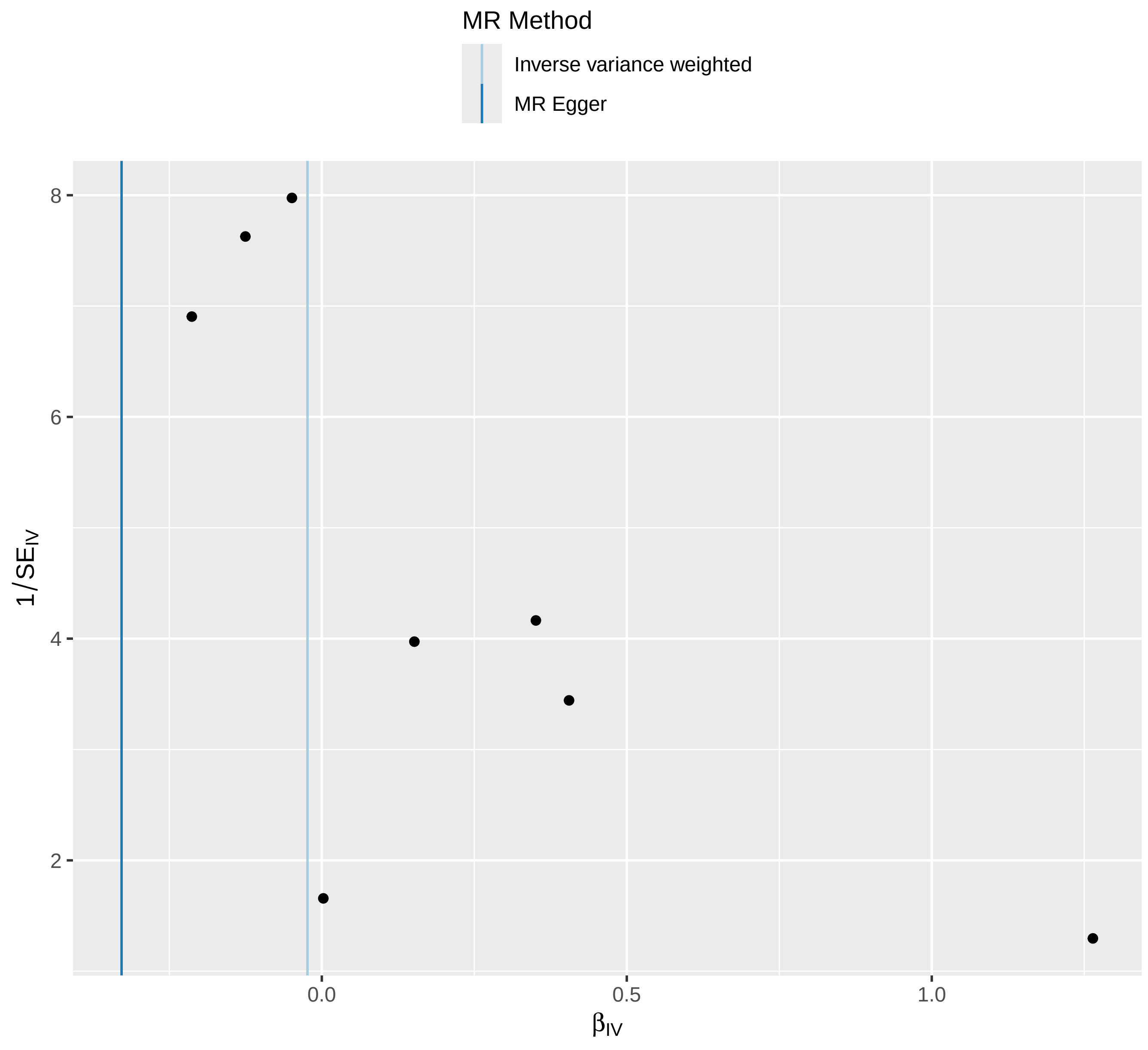
MR Method
Inverse variance weighted
MR Egger
8
6
4
2
1/SE_IV
0.0
0.5
1.0
β_IV

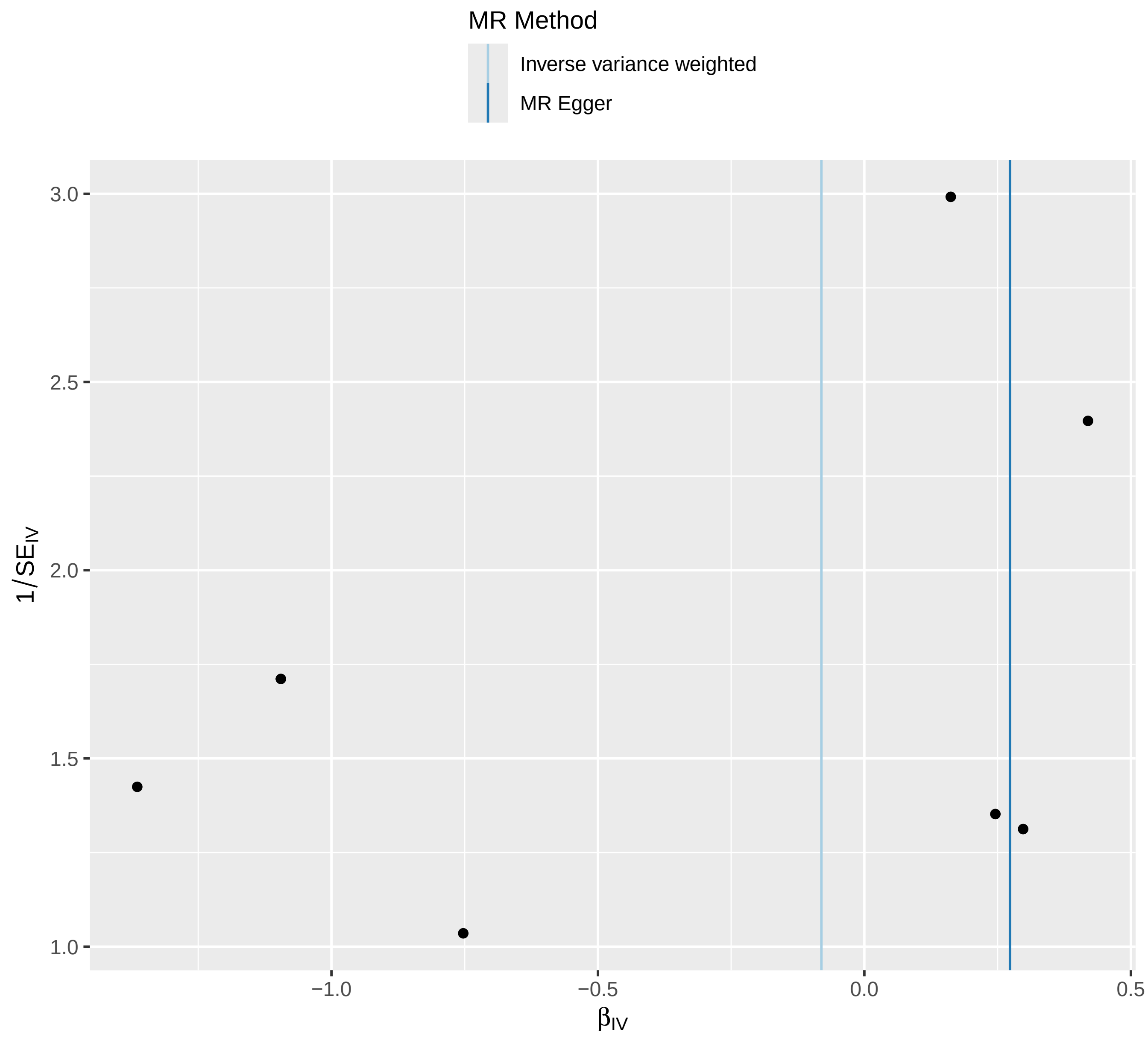
MR Method
Inverse variance weighted
MR Egger
3.0
2.5
2.0
1.5
1.0
1/SE_IV
−1.0
−0.5
0.0
0.5
β_IV

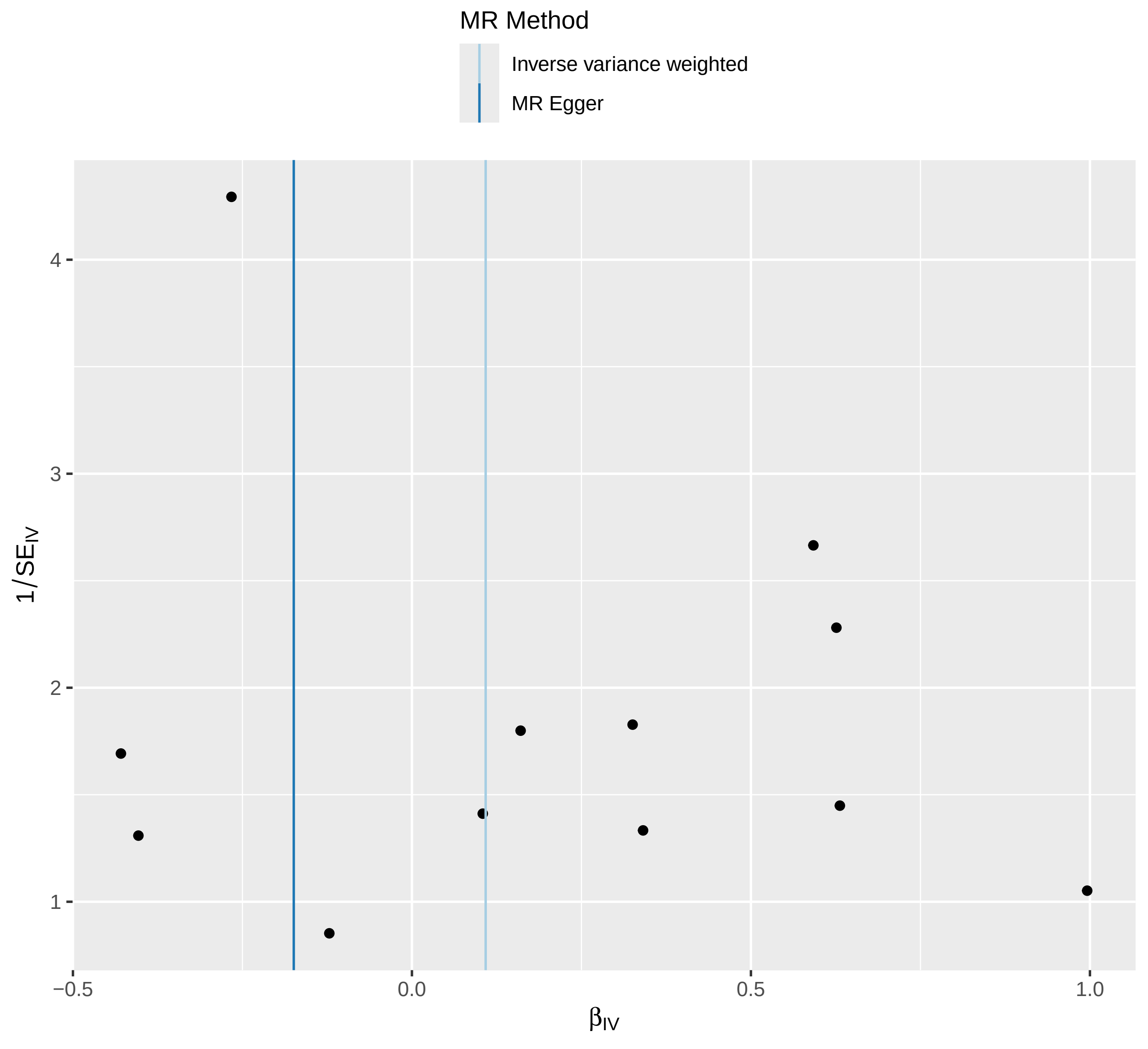
MR Method
Inverse variance weighted
MR Egger
4
3
2
1
1/SE$_{IV}$
−0.5
0.0
0.5
1.0
β$_{IV}$

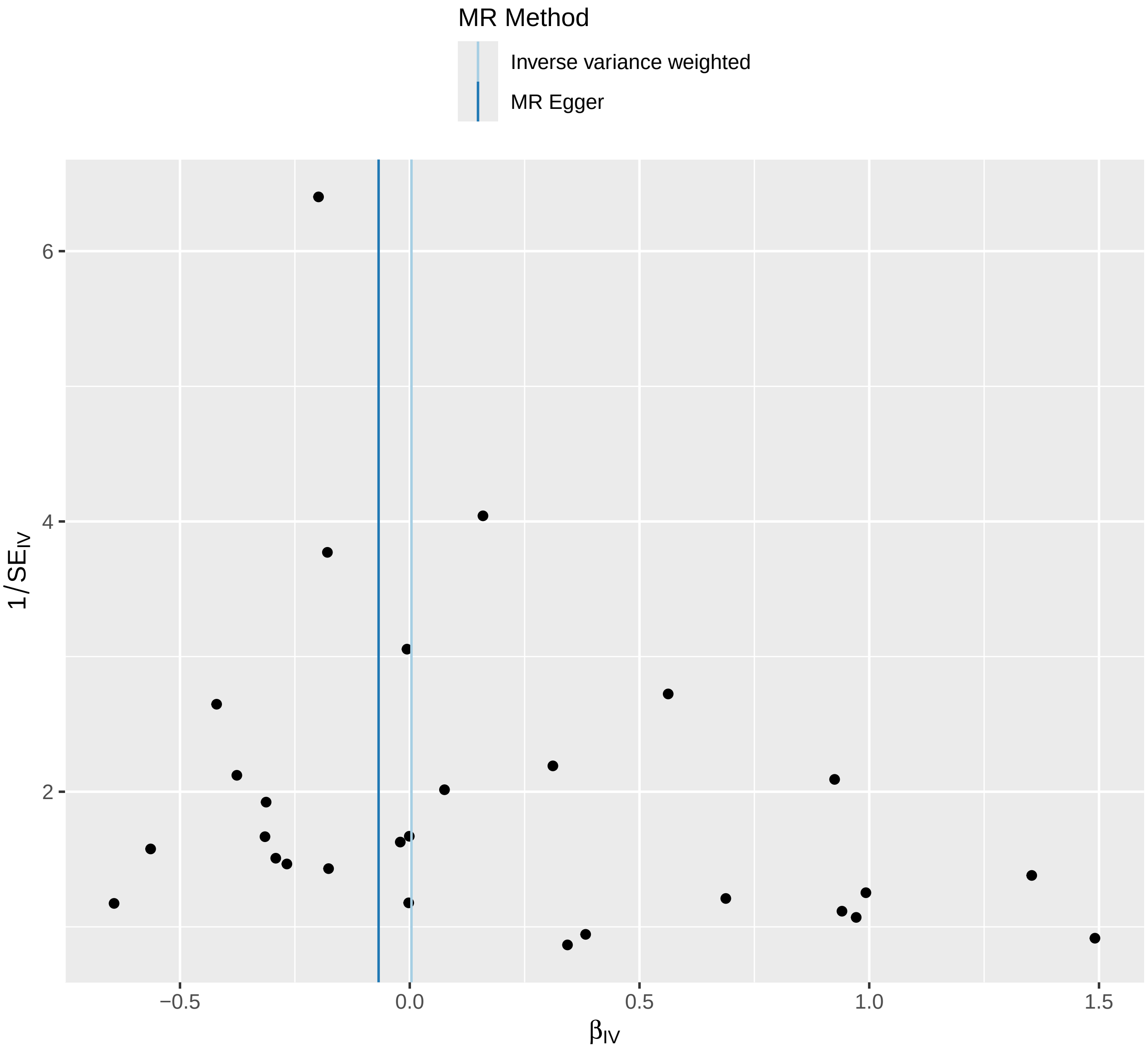

MR Method
Inverse variance weighted
MR Egger
6
4
2
1/SE_IV
−0.5
0.0
0.5
1.0
1.5
β_IV

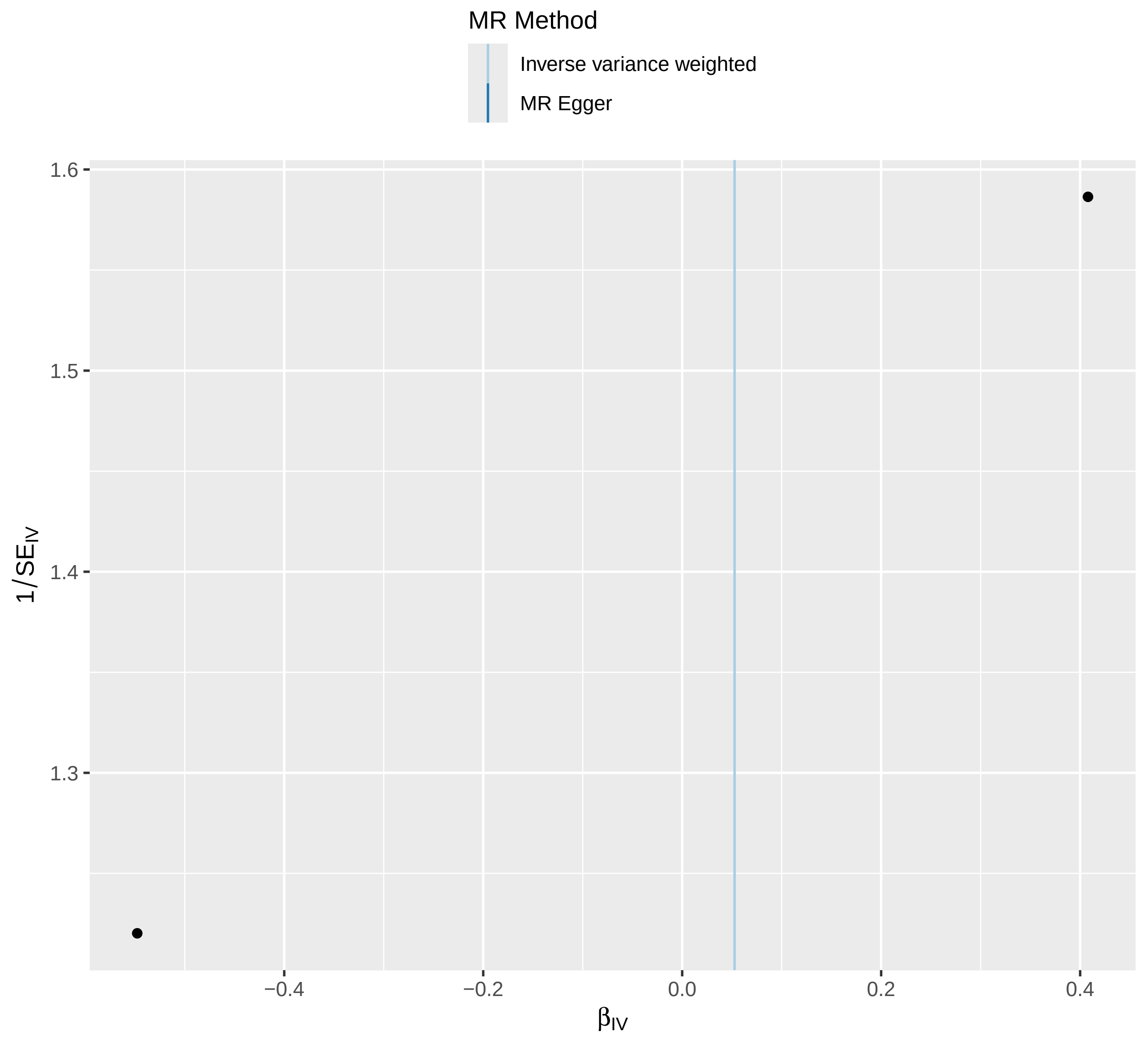
MR Method
Inverse variance weighted
MR Egger
1.6
1.5
1.4
1.3
1/SE_IV
−0.4
−0.2
0.0
0.2
0.4
β_IV

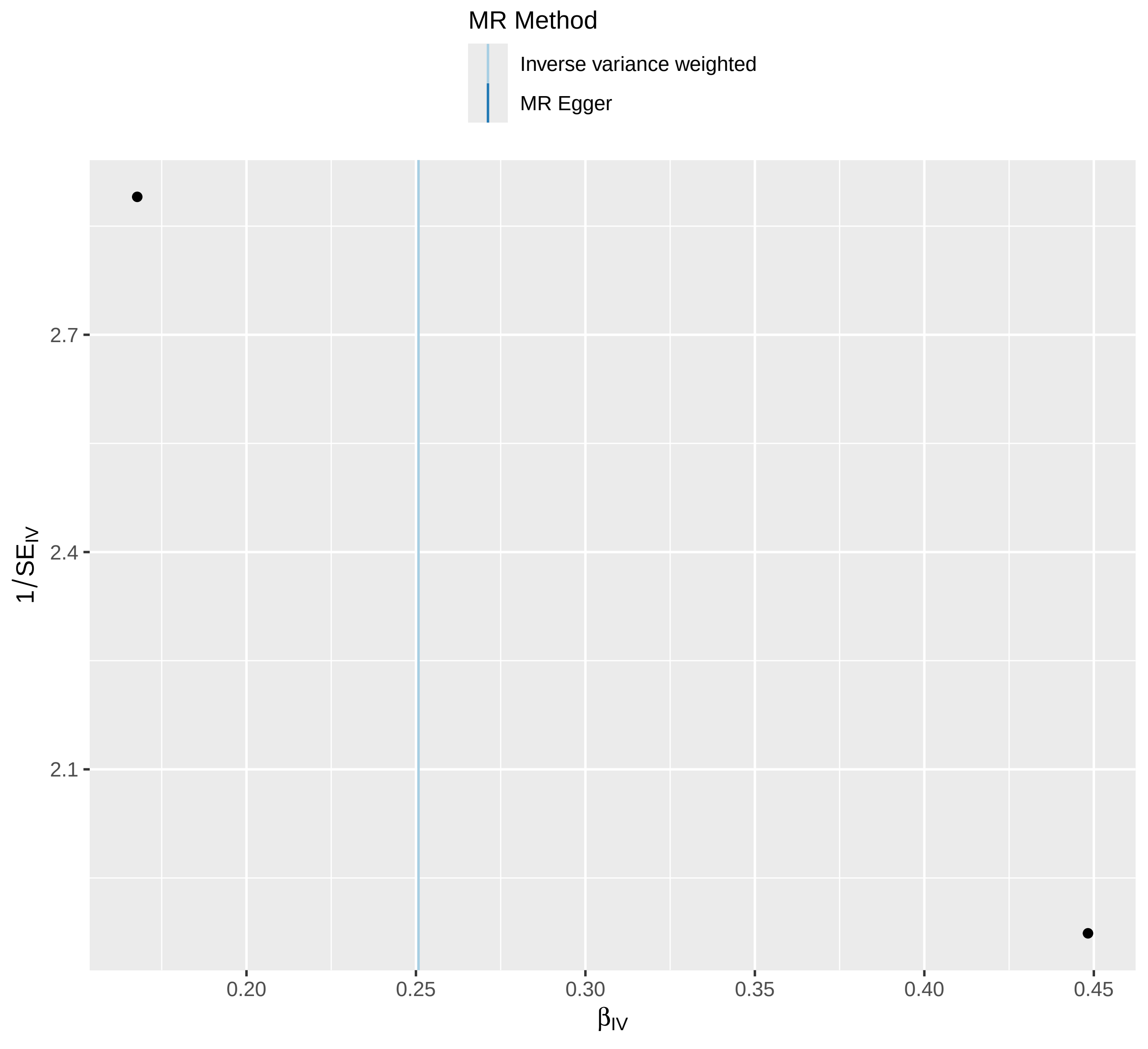

MR Method
Inverse variance weighted
MR Egger
2.7
2.4
2.1
$1/SE_{IV}$
0.20
0.25
0.30
0.35
0.40
0.45
β_{IV}

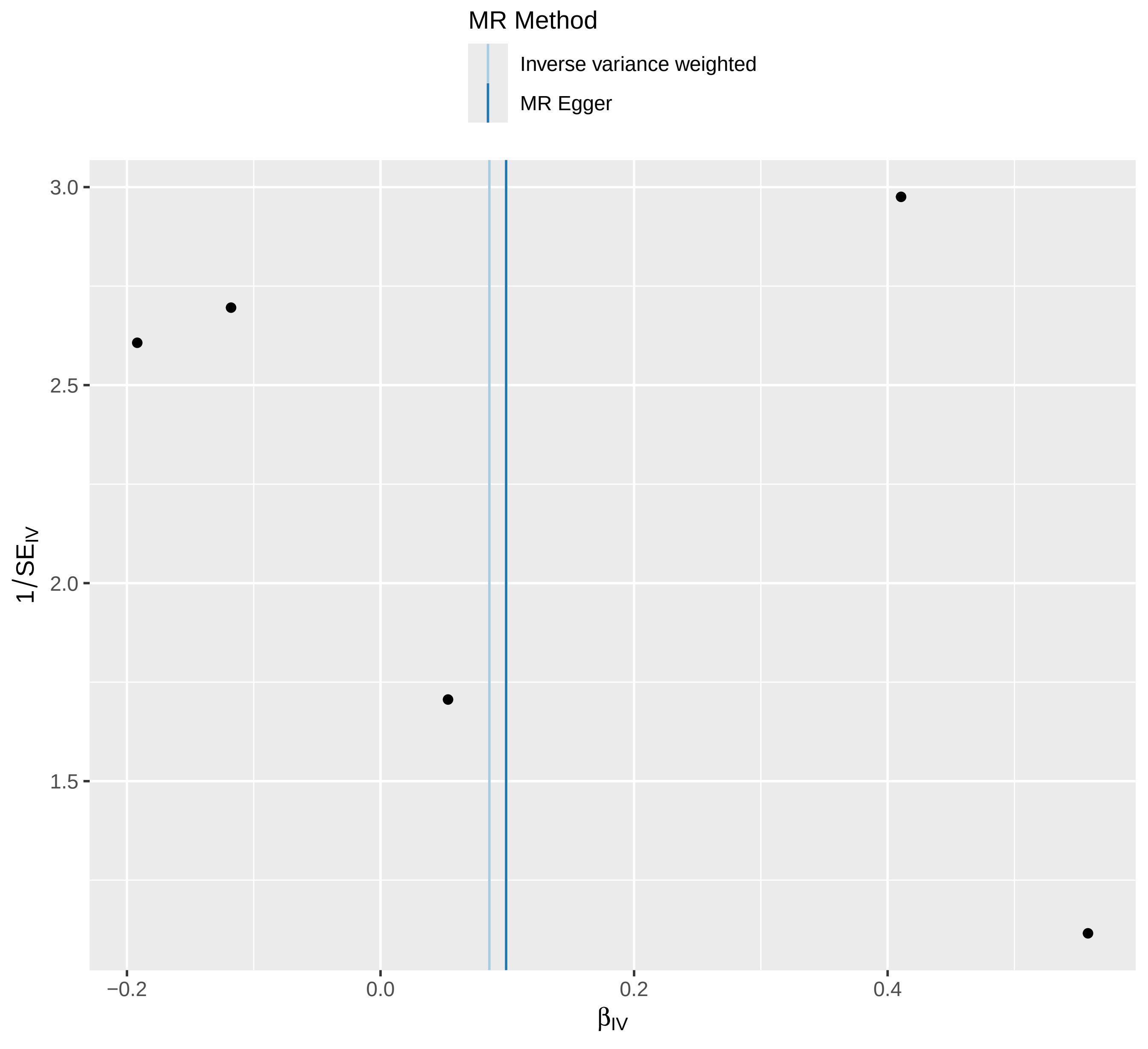

MR Method
Inverse variance weighted
MR Egger
3.0
2.5
2.0
1.5
1/SE$_{IV}$
−0.2
0.0
0.2
0.4
β$_{IV}$

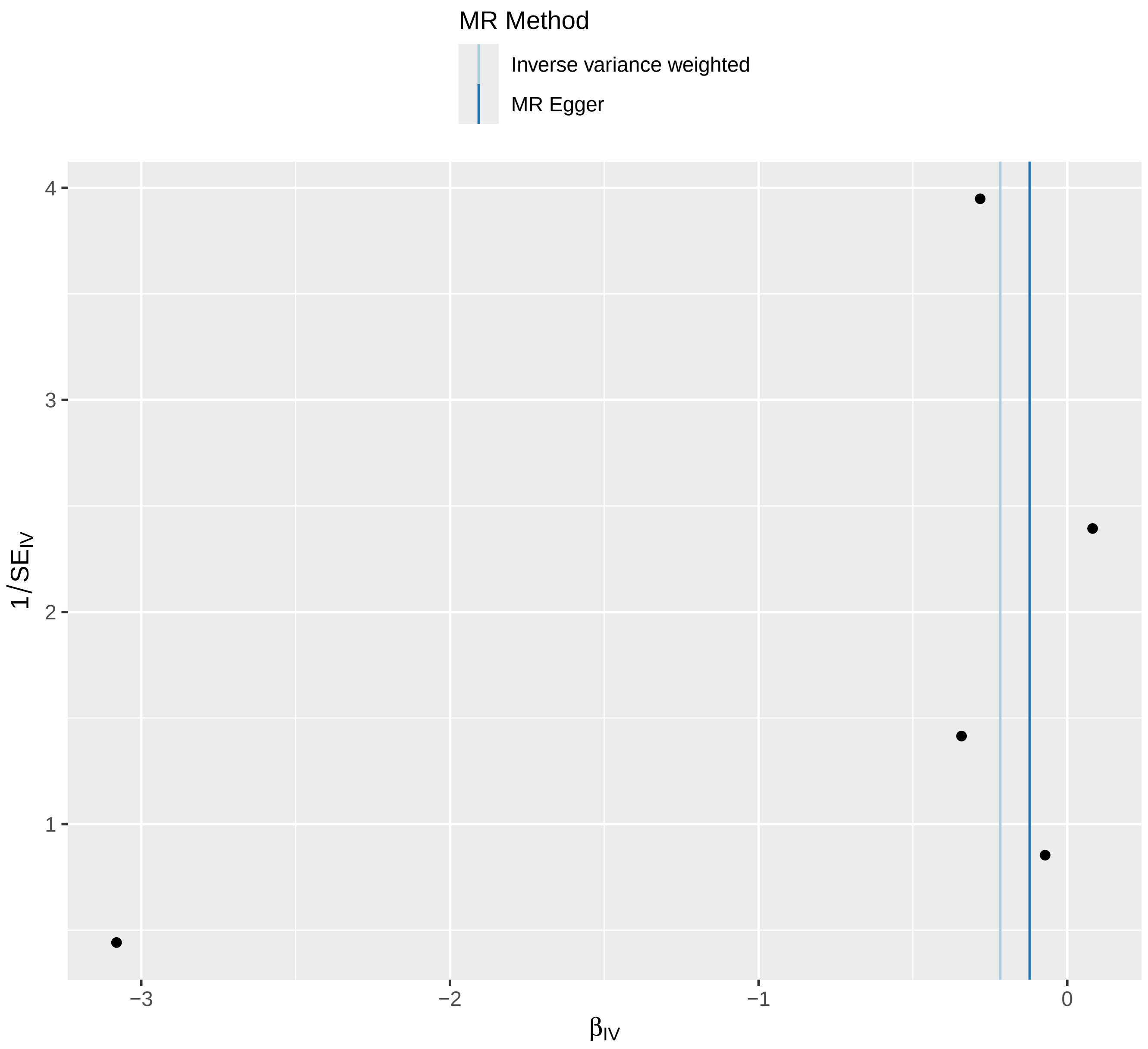
MR Method
Inverse variance weighted
MR Egger
1/SE_IV
β_IV
4
3
2
1
−3
−2
−1
0

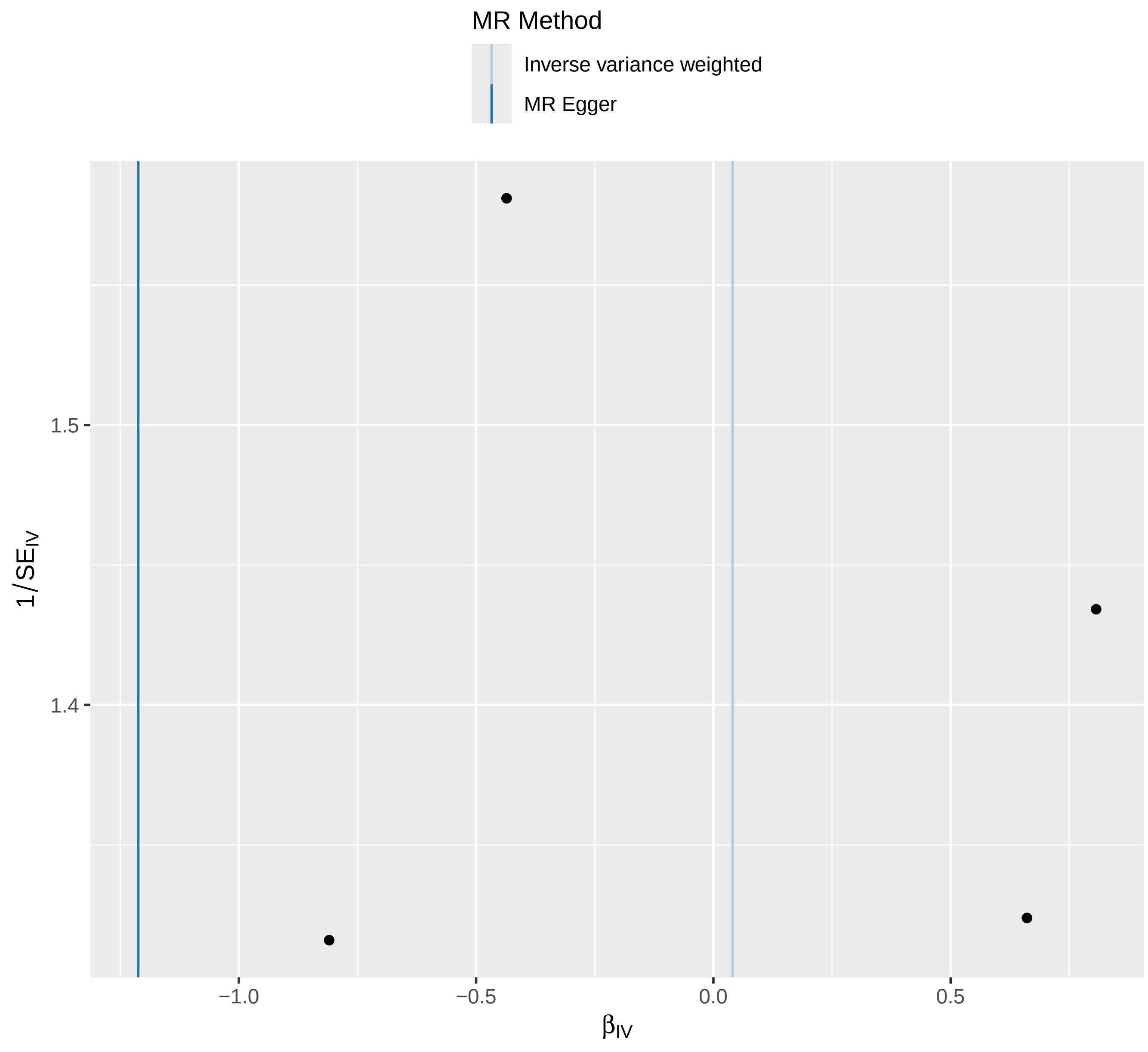

MR Method
Inverse variance weighted
MR Egger
1.5
1.4
1/SE$_{IV}$
−1.0
−0.5
0.0
0.5
β$_{IV}$

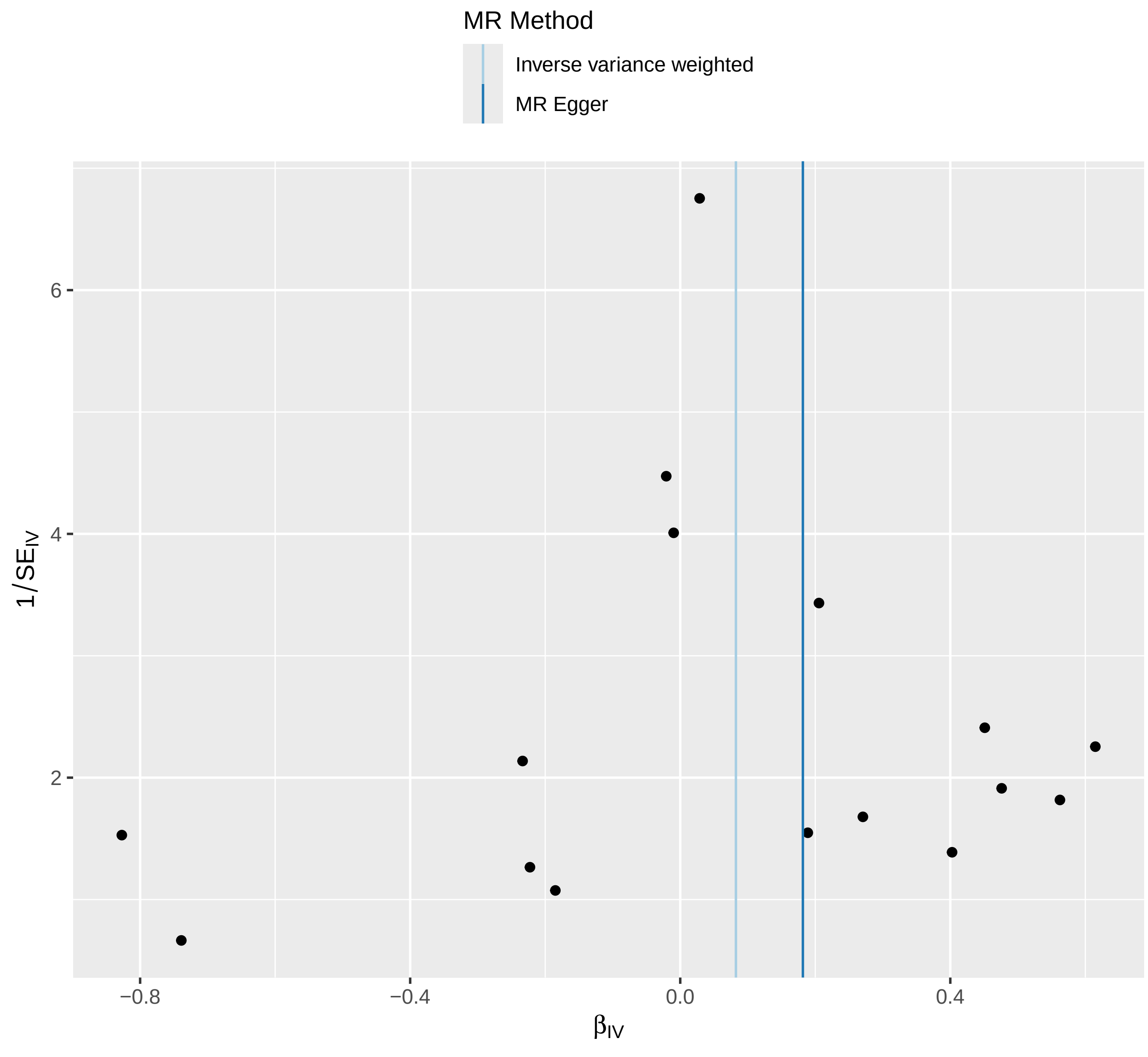

MR Method
Inverse variance weighted
MR Egger
6
4
2
1/SE_IV
−0.8
−0.4
0.0
0.4
β_IV

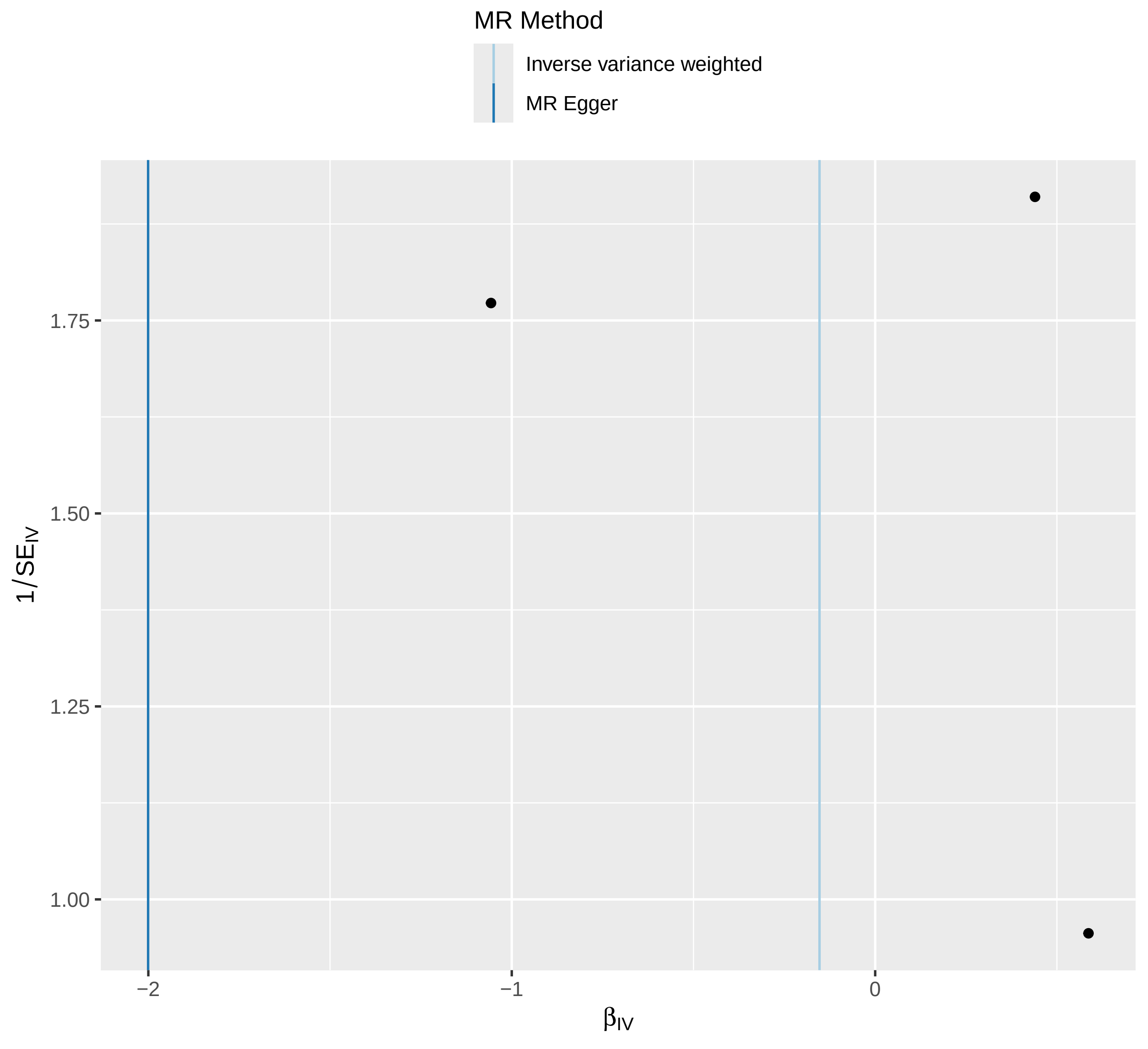

MR Method
Inverse variance weighted
MR Egger
1.75
1.50
1.25
1.00
1/SE_IV
−2
−1
0
β_IV

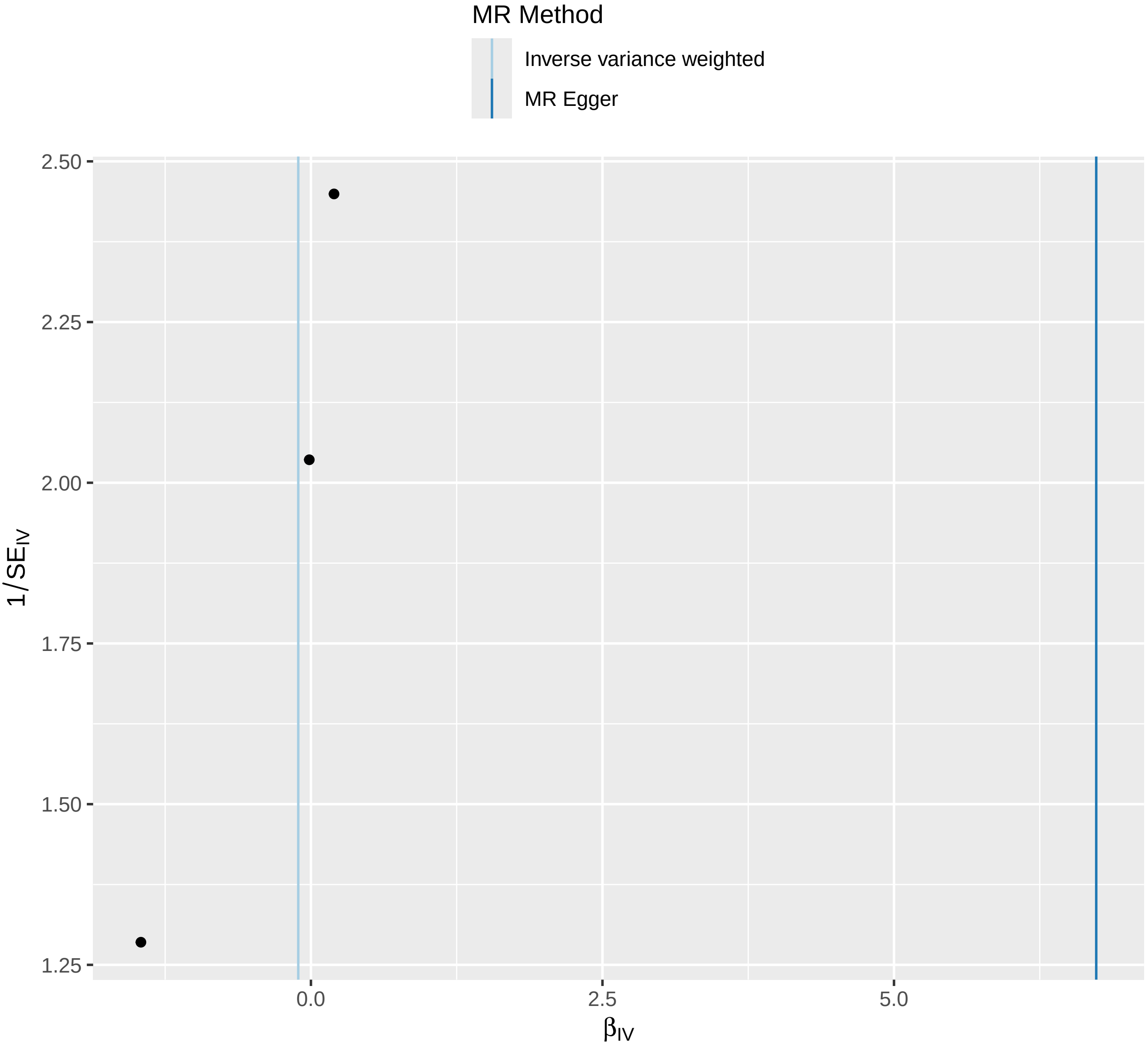

MR Method
Inverse variance weighted
MR Egger
2.50
2.25
2.00
1.75
1.50
1.25
1/SE$_{IV}$
0.0
2.5
5.0
β$_{IV}$

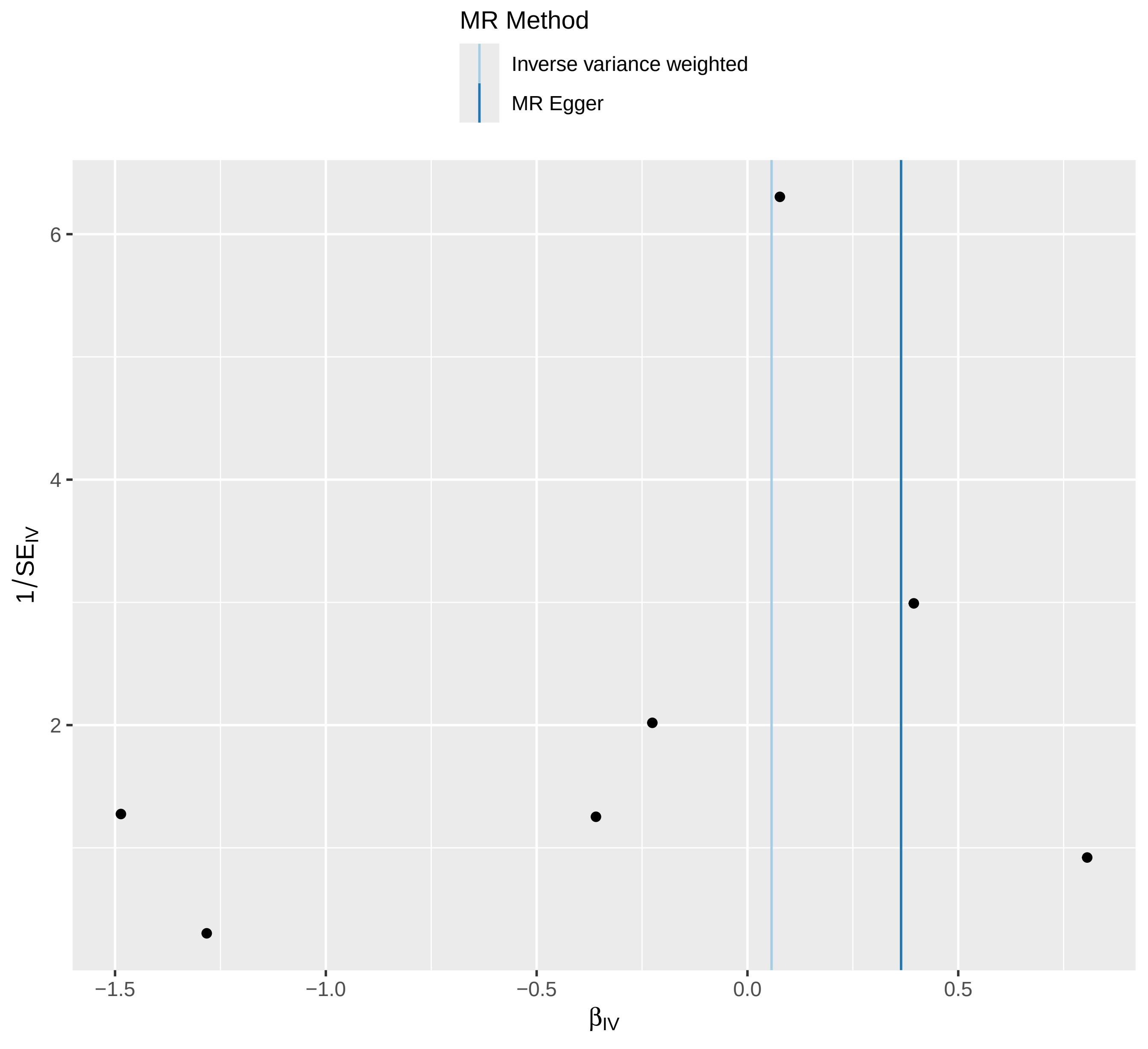
MR Method
Inverse variance weighted
MR Egger
6
4
2
$1/SE_{IV}$
−1.5
−1.0
−0.5
0.0
0.5
β_{IV}

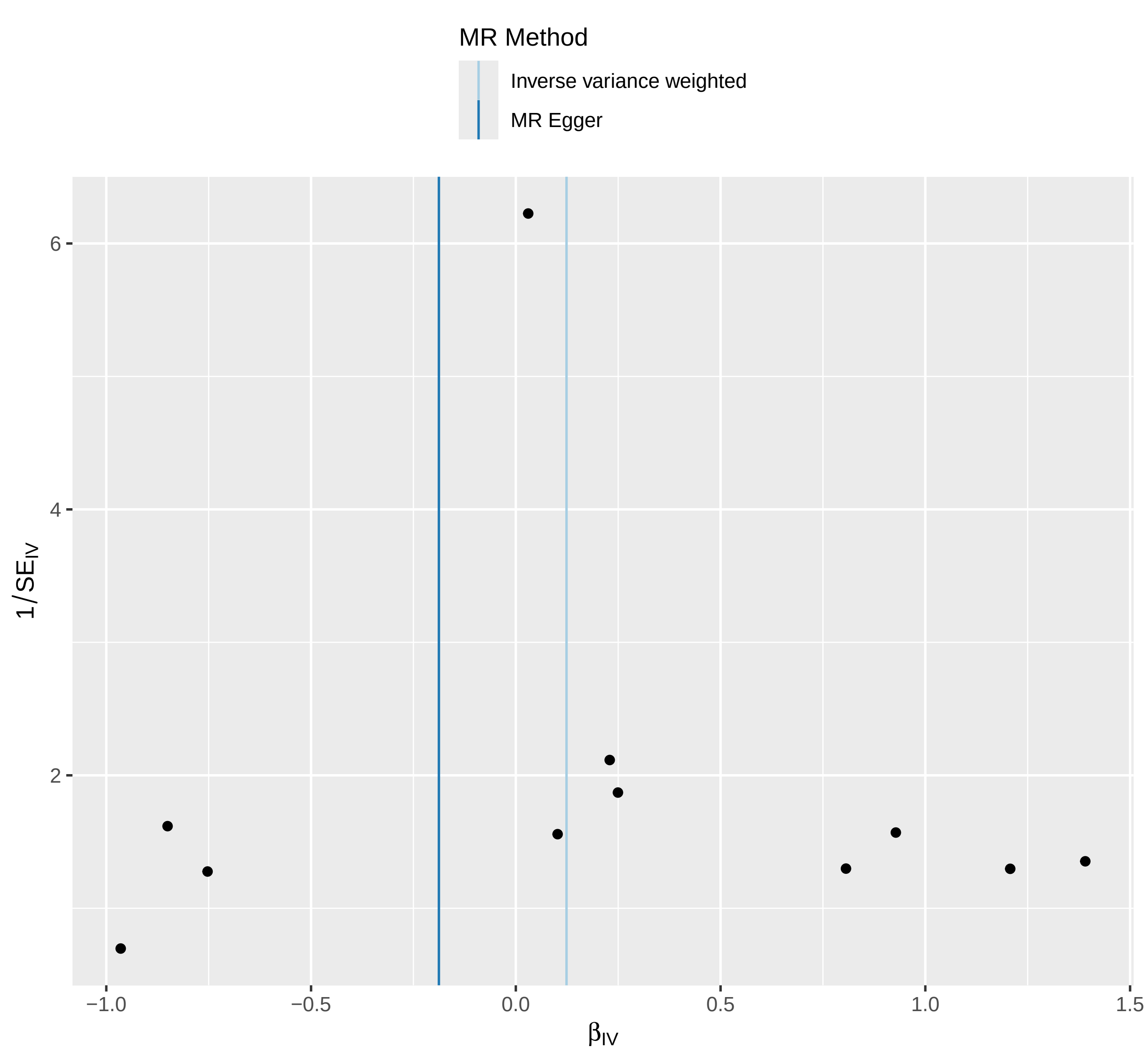

MR Method
Inverse variance weighted
MR Egger
1/SE_IV
β_IV
6
4
2
−1.0
−0.5
0.0
0.5
1.0
1.5

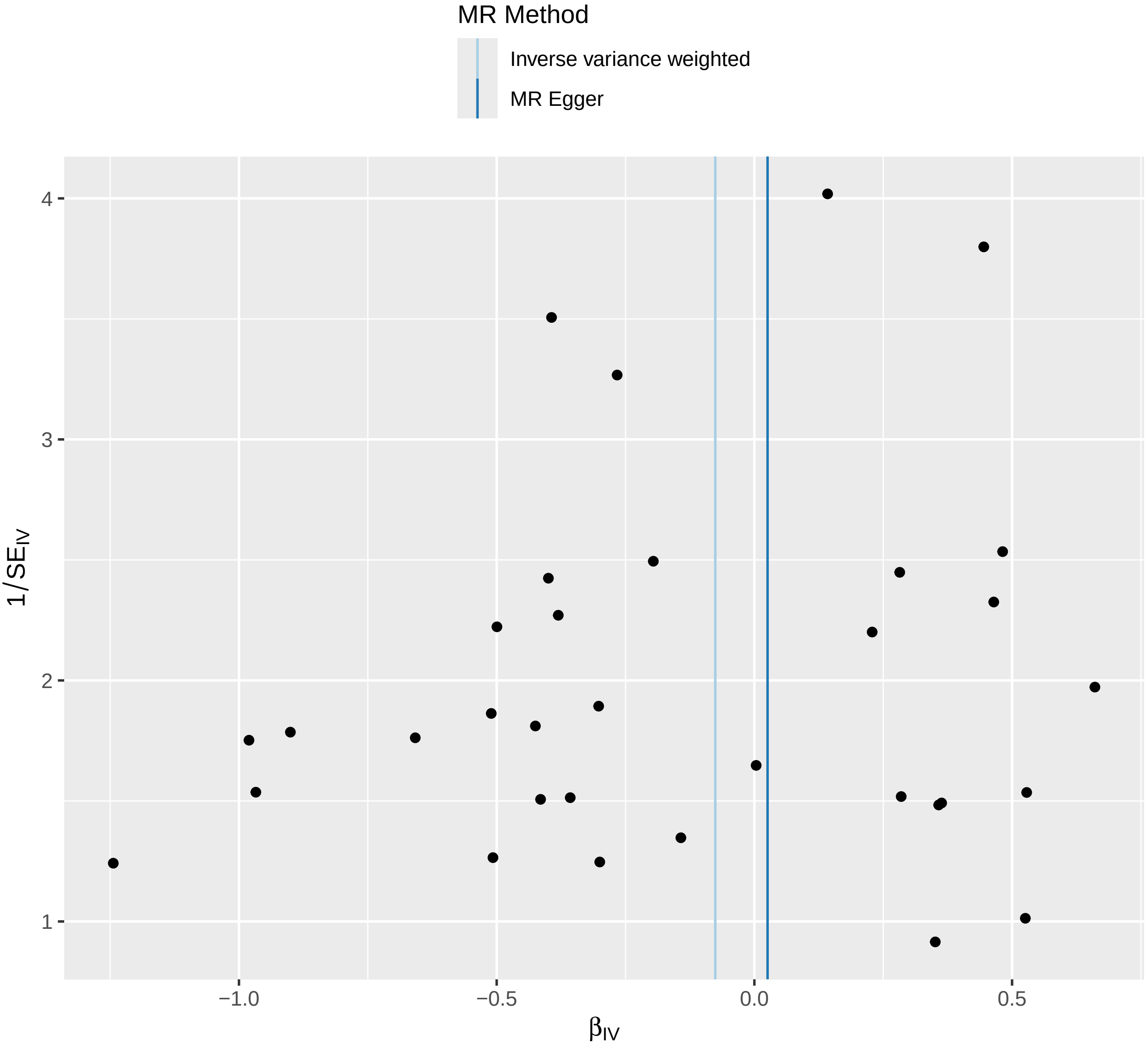

MR Method
Inverse variance weighted
MR Egger
4
3
2
1
1/SE$_{IV}$
−1.0
−0.5
0.0
0.5
β$_{IV}$

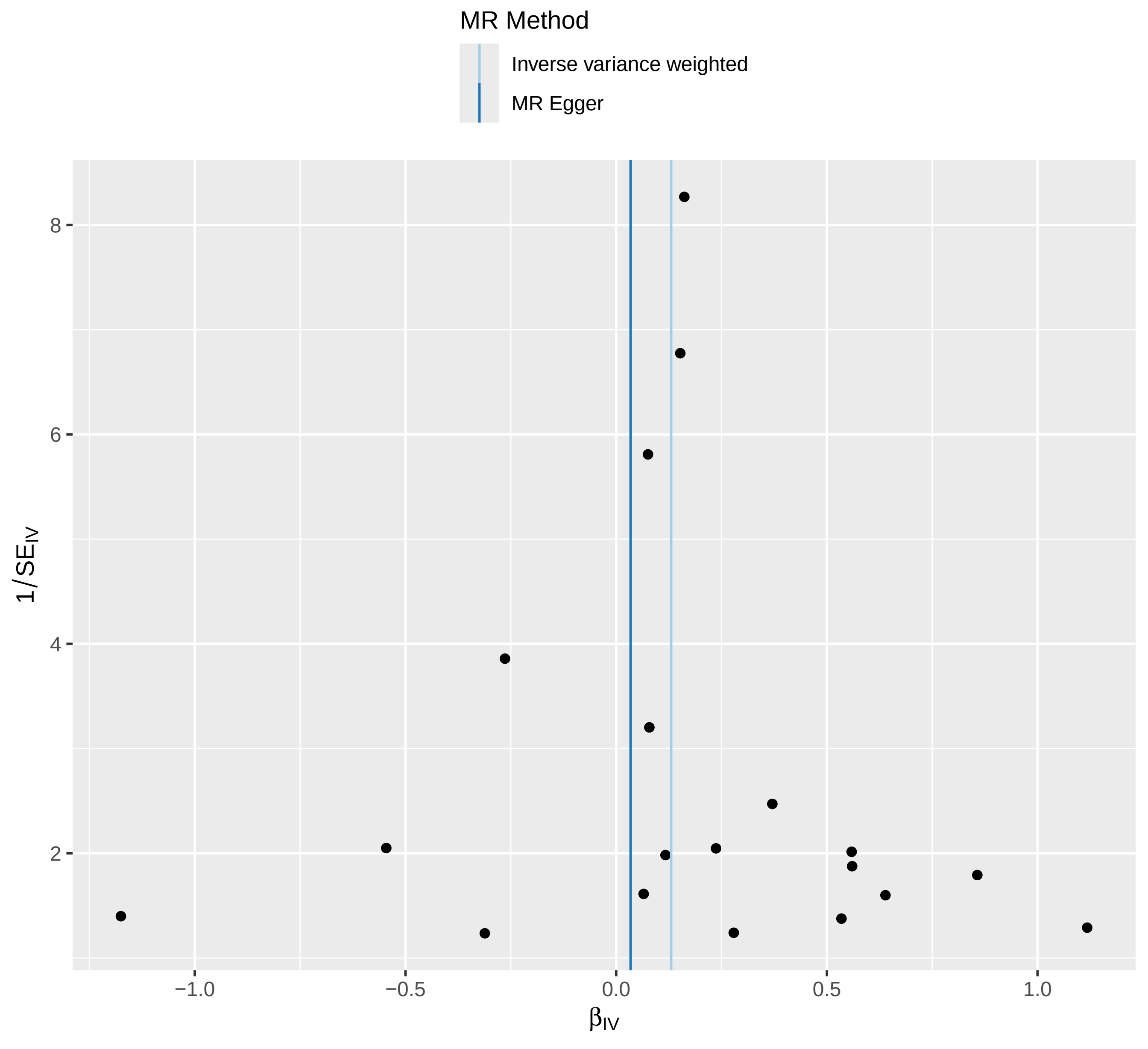
MR Method
Inverse variance weighted
MR Egger
1/SE_IV
8
6
4
2
−1.0
−0.5
0.0
0.5
1.0
β_IV

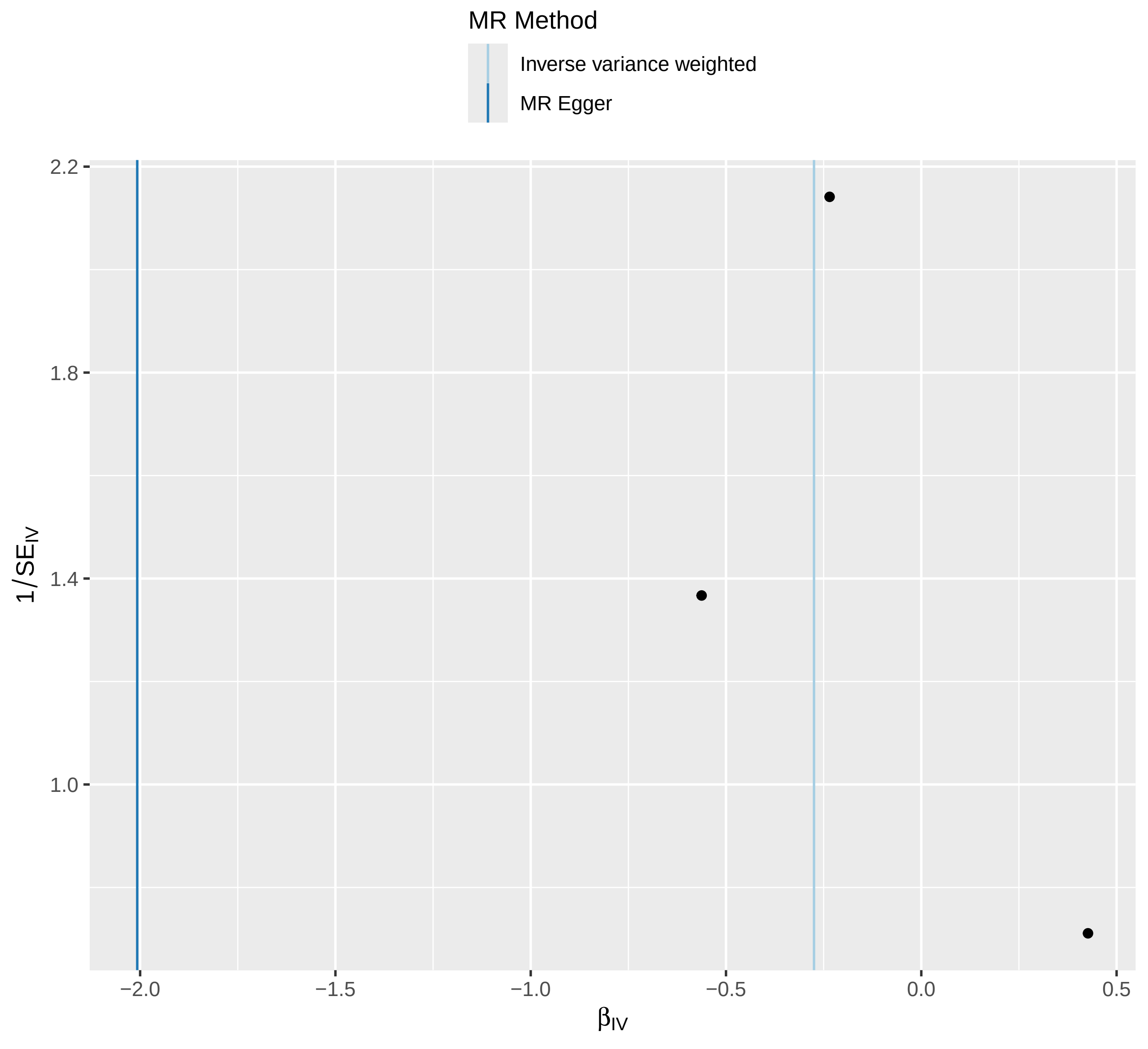
MR Method
Inverse variance weighted
MR Egger
2.2
1.8
1.4
1.0
1/SE_IV
−2.0
−1.5
−1.0
−0.5
0.0
0.5
β_IV

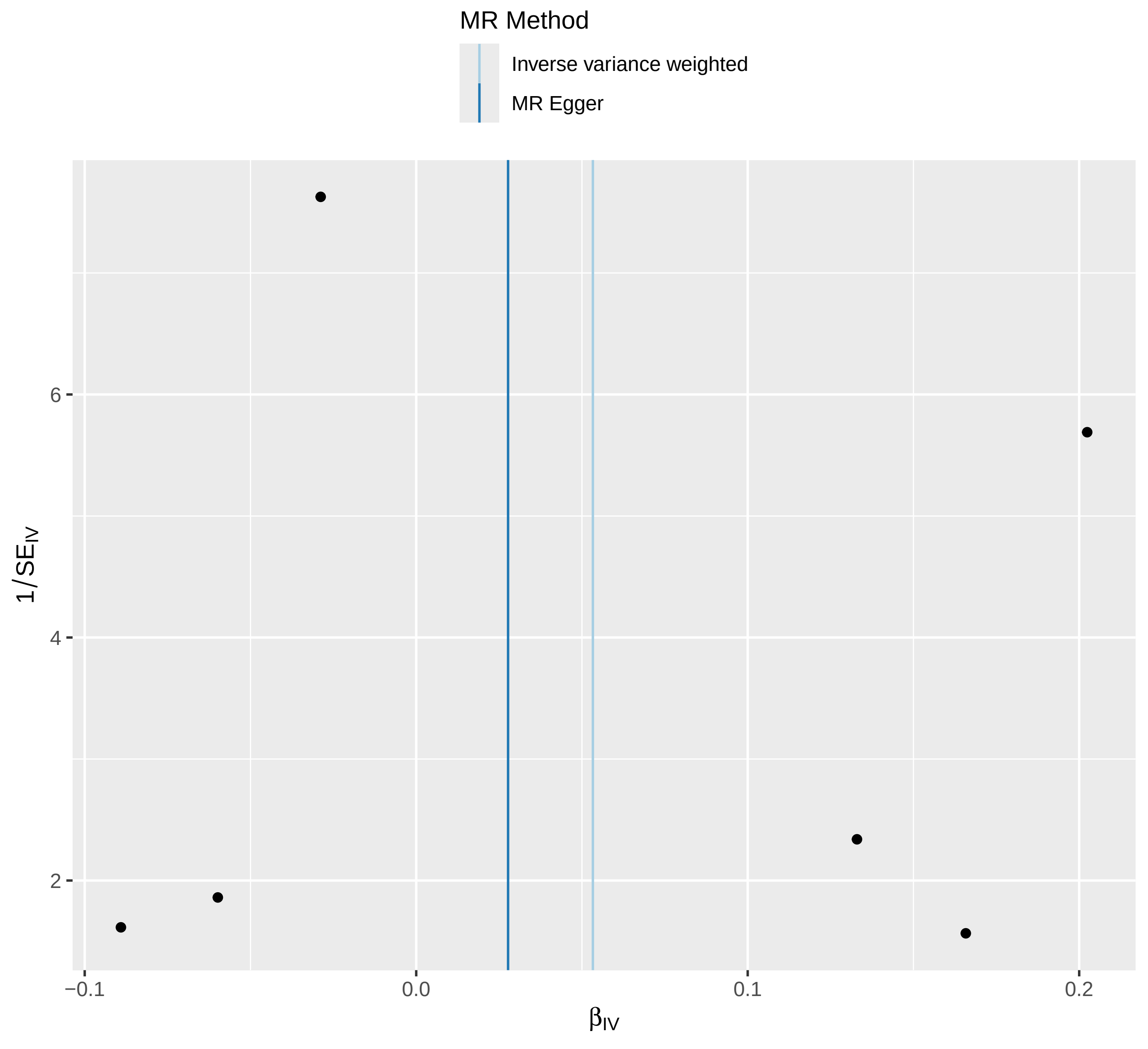
MR Method
Inverse variance weighted
MR Egger
1/SE$_{IV}$
6
4
2
−0.1
0.0
0.1
0.2
β_{IV}

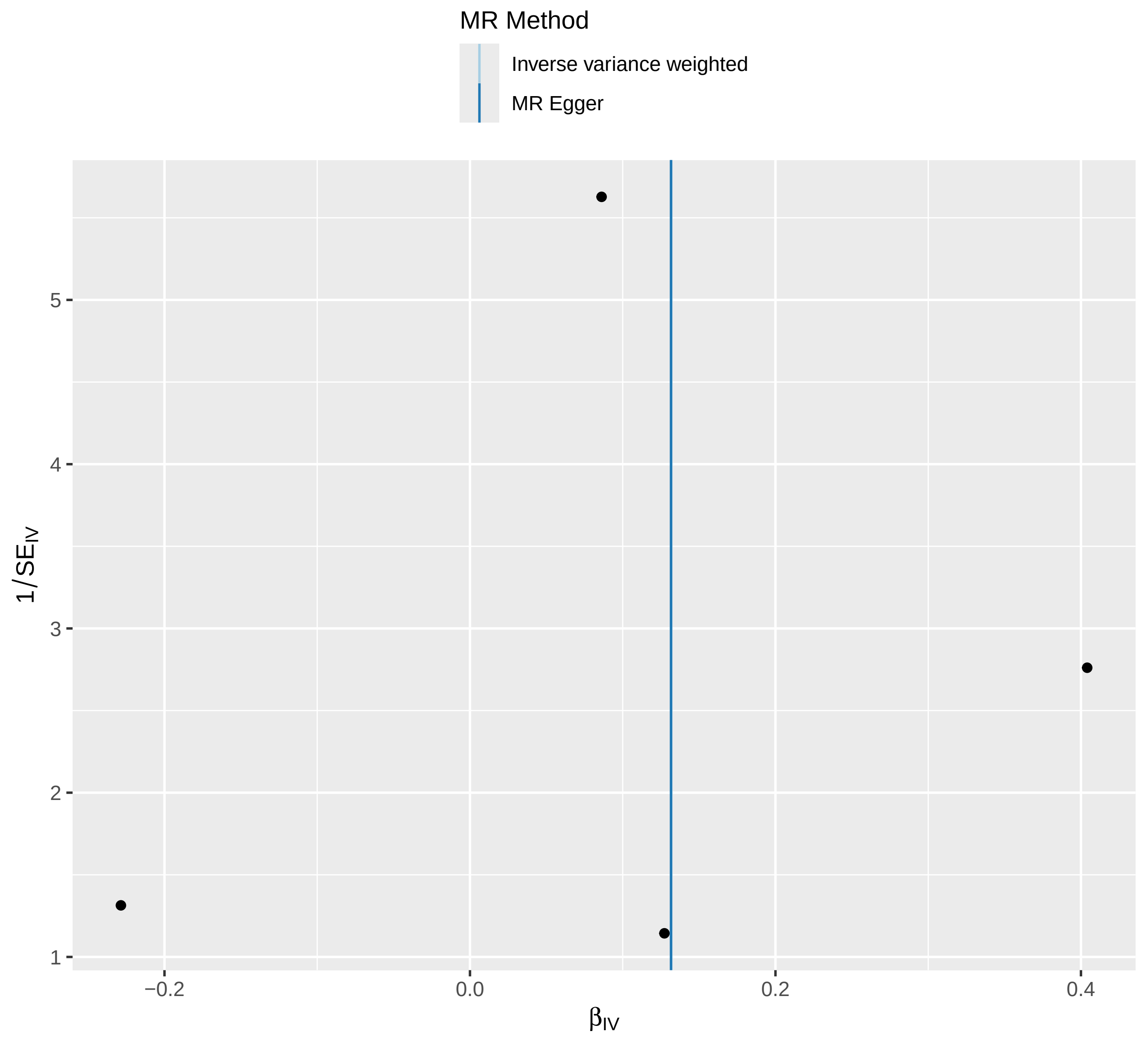

MR Method
Inverse variance weighted
MR Egger
5
4
3
2
1
1/SE_IV
−0.2
0.0
0.2
0.4
β_IV

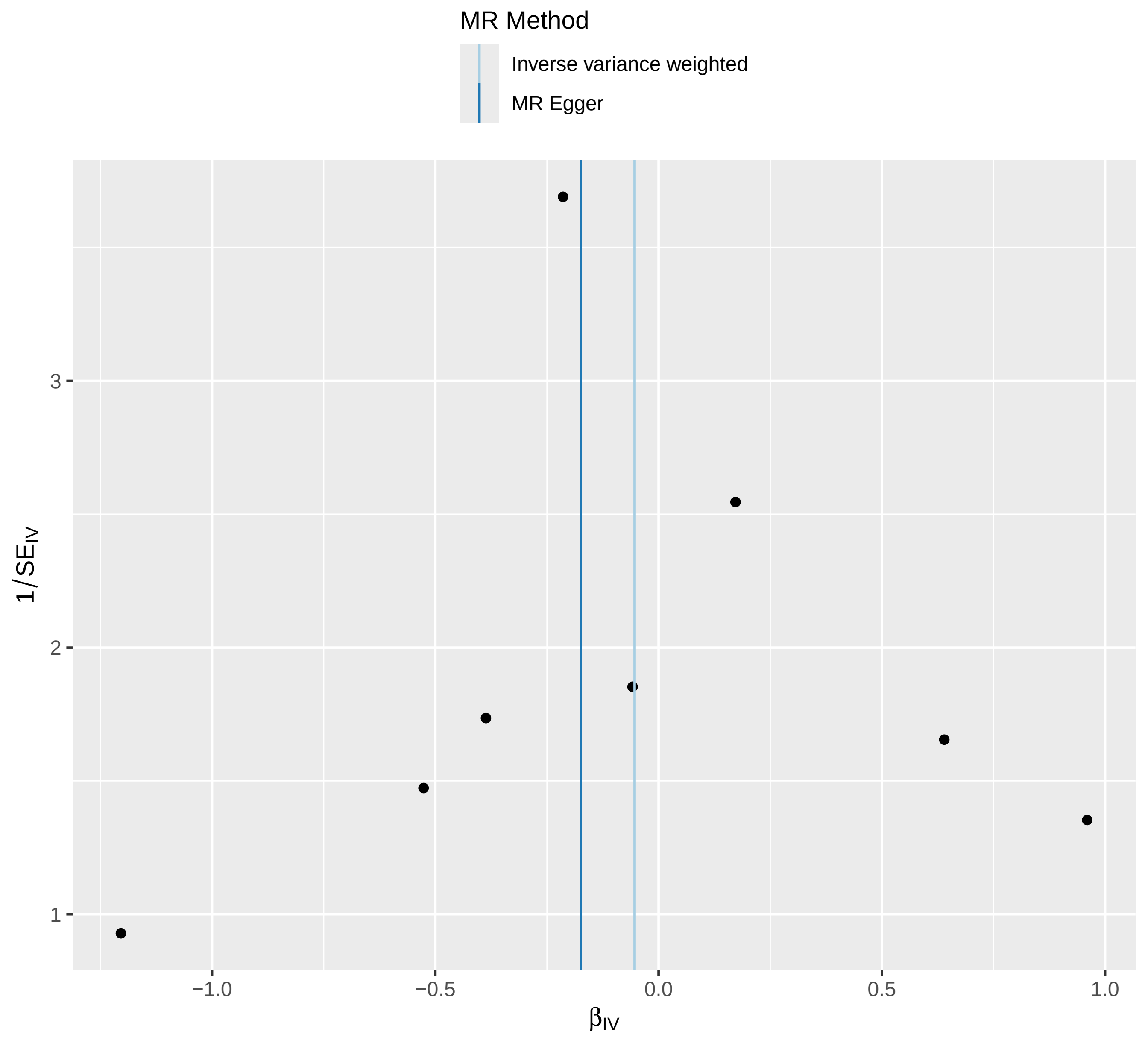

MR Method
Inverse variance weighted
MR Egger
1/SE_IV
3
2
1
−1.0
−0.5
0.0
0.5
1.0
β_IV

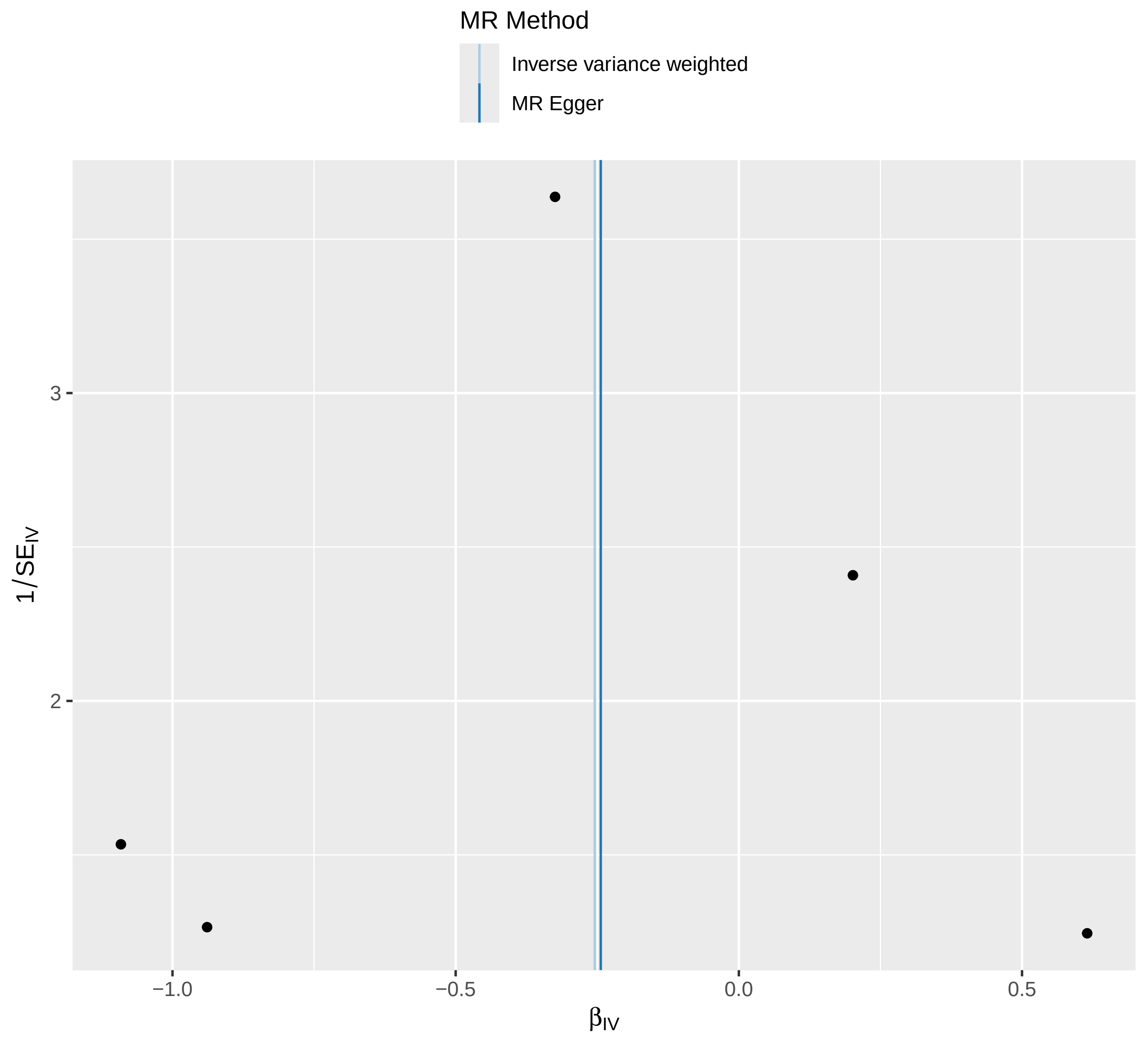
MR Method
Inverse variance weighted
MR Egger
3
2
1/SE$_{IV}$
−1.0
−0.5
0.0
0.5
β$_{IV}$

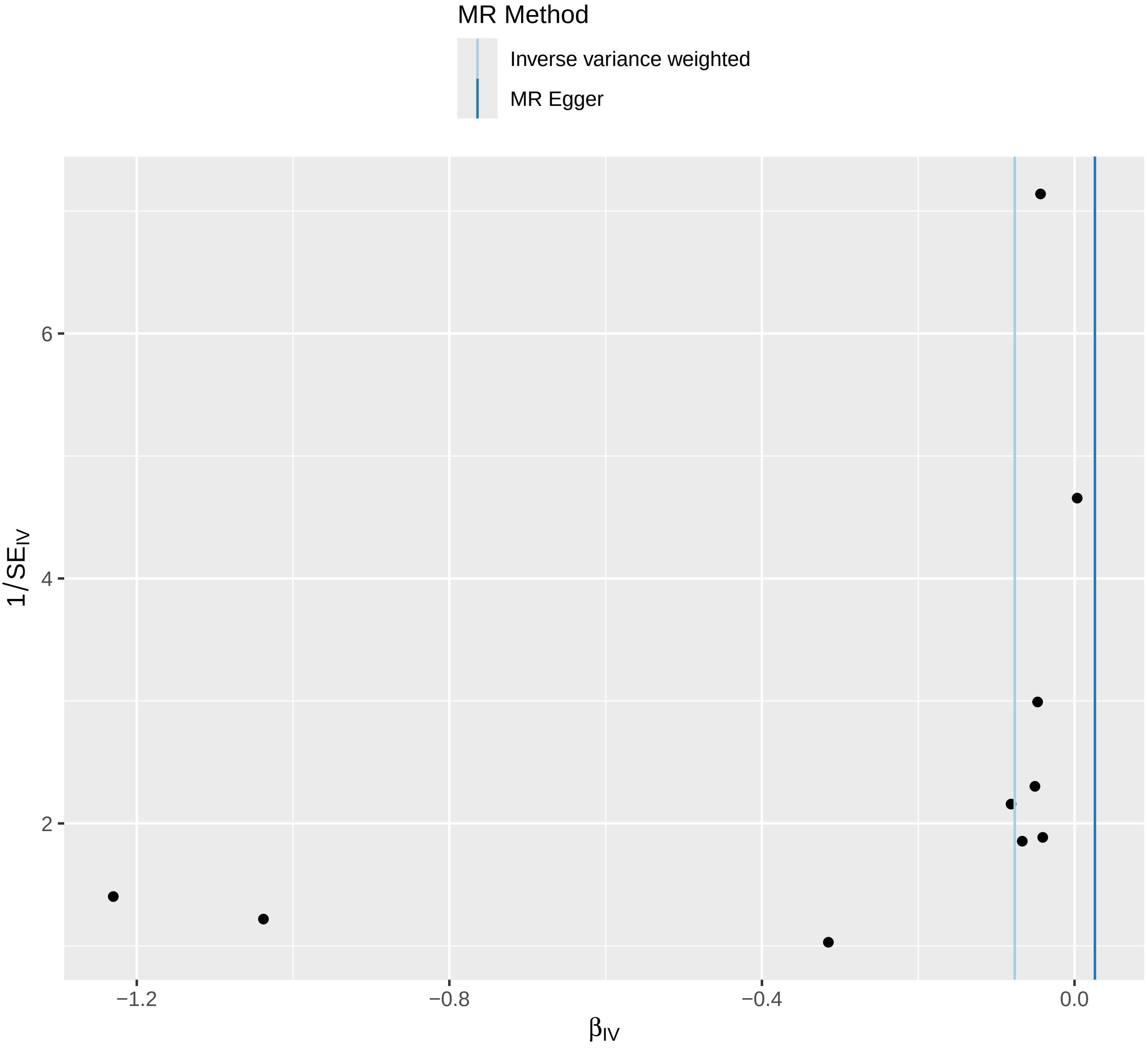

MR Method
Inverse variance weighted
MR Egger
1/SE$_{IV}$
6
4
2
−1.2
−0.8
−0.4
0.0
β$_{IV}$

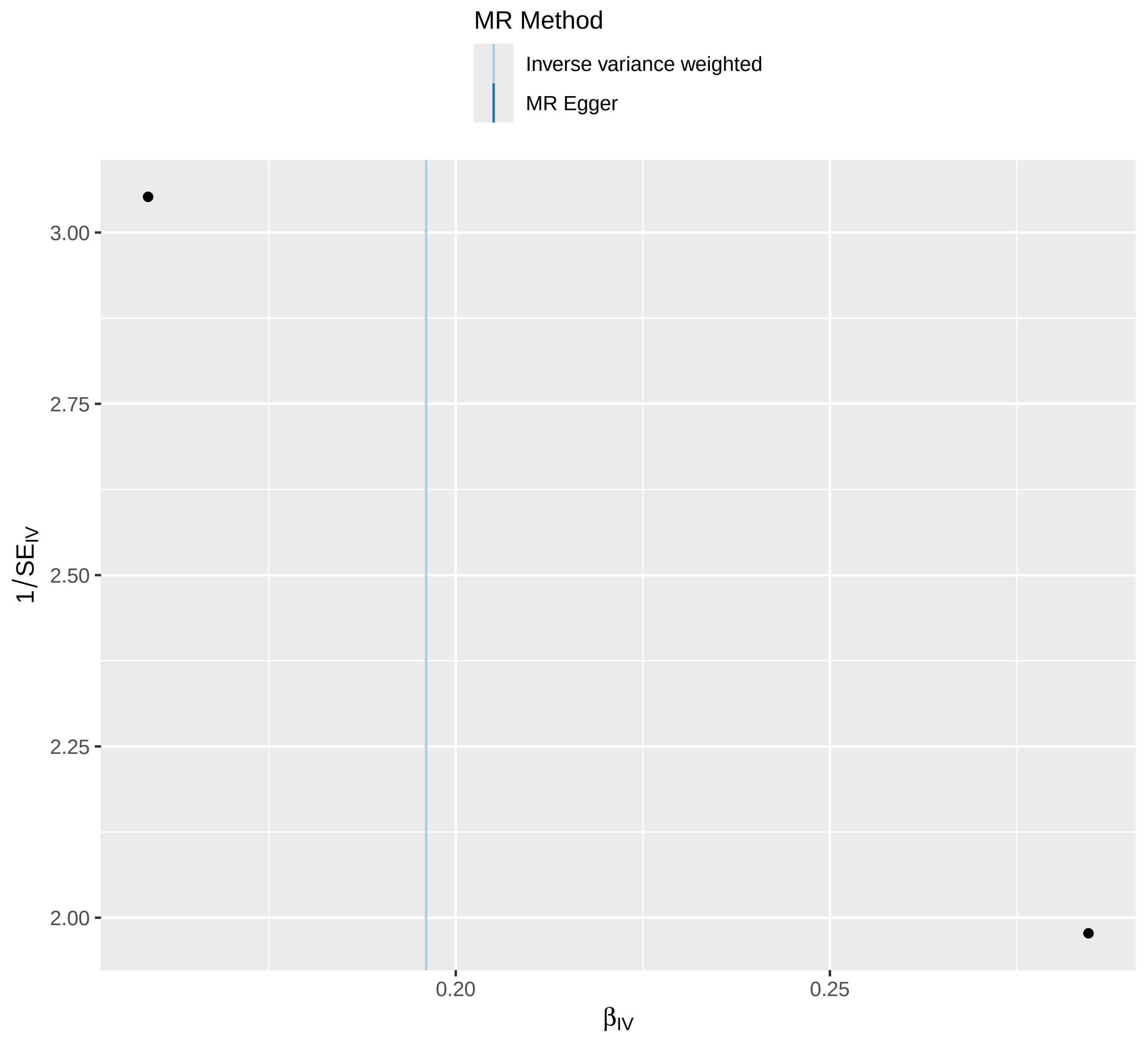

MR Method
Inverse variance weighted
MR Egger
3.00
2.75
2.50
2.25
2.00
1/SE_IV
0.20
0.25
β_IV

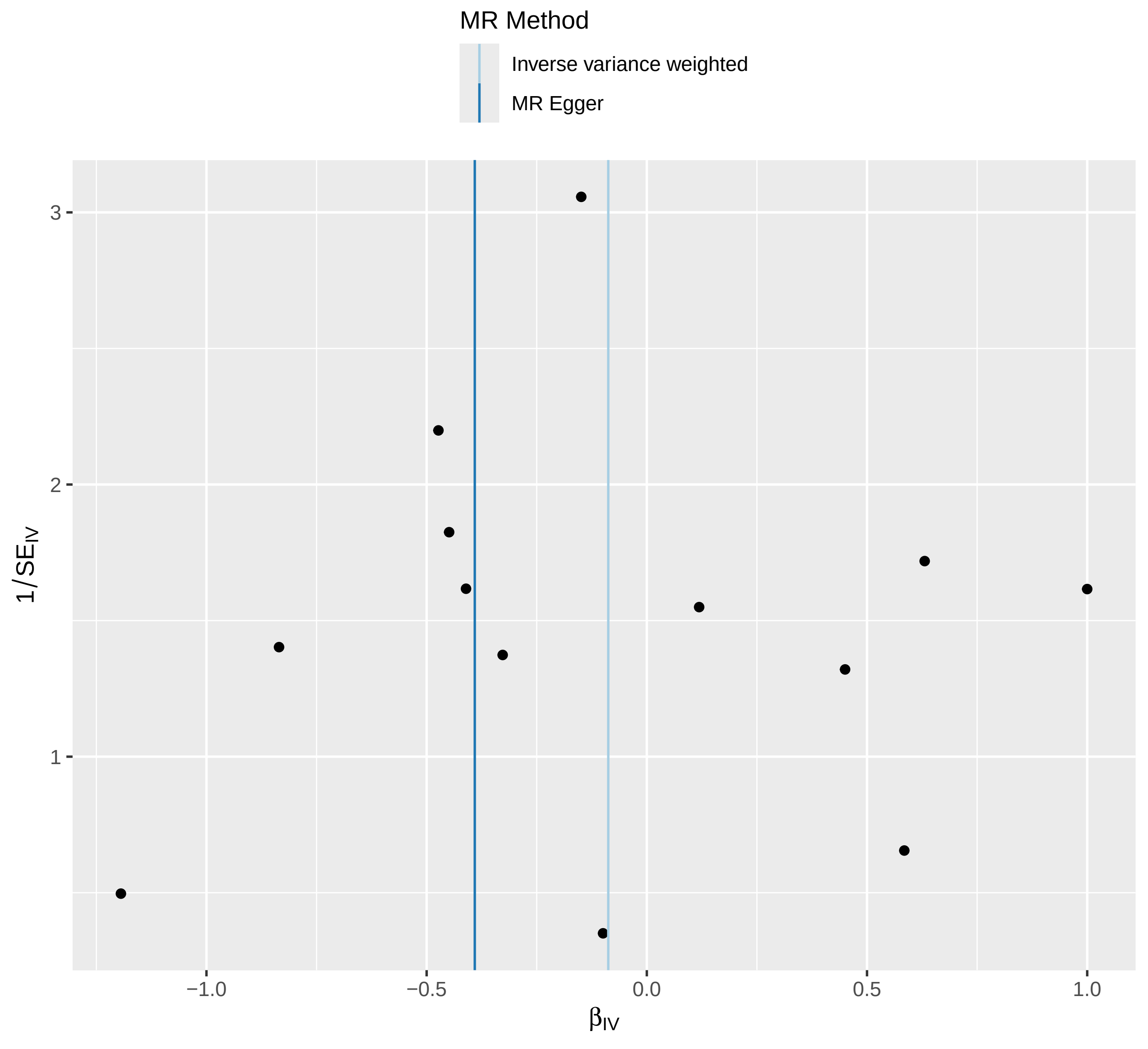
MR Method
Inverse variance weighted
MR Egger
3
2
1
$1/SE_{IV}$
−1.0
−0.5
0.0
0.5
1.0
β_{IV}

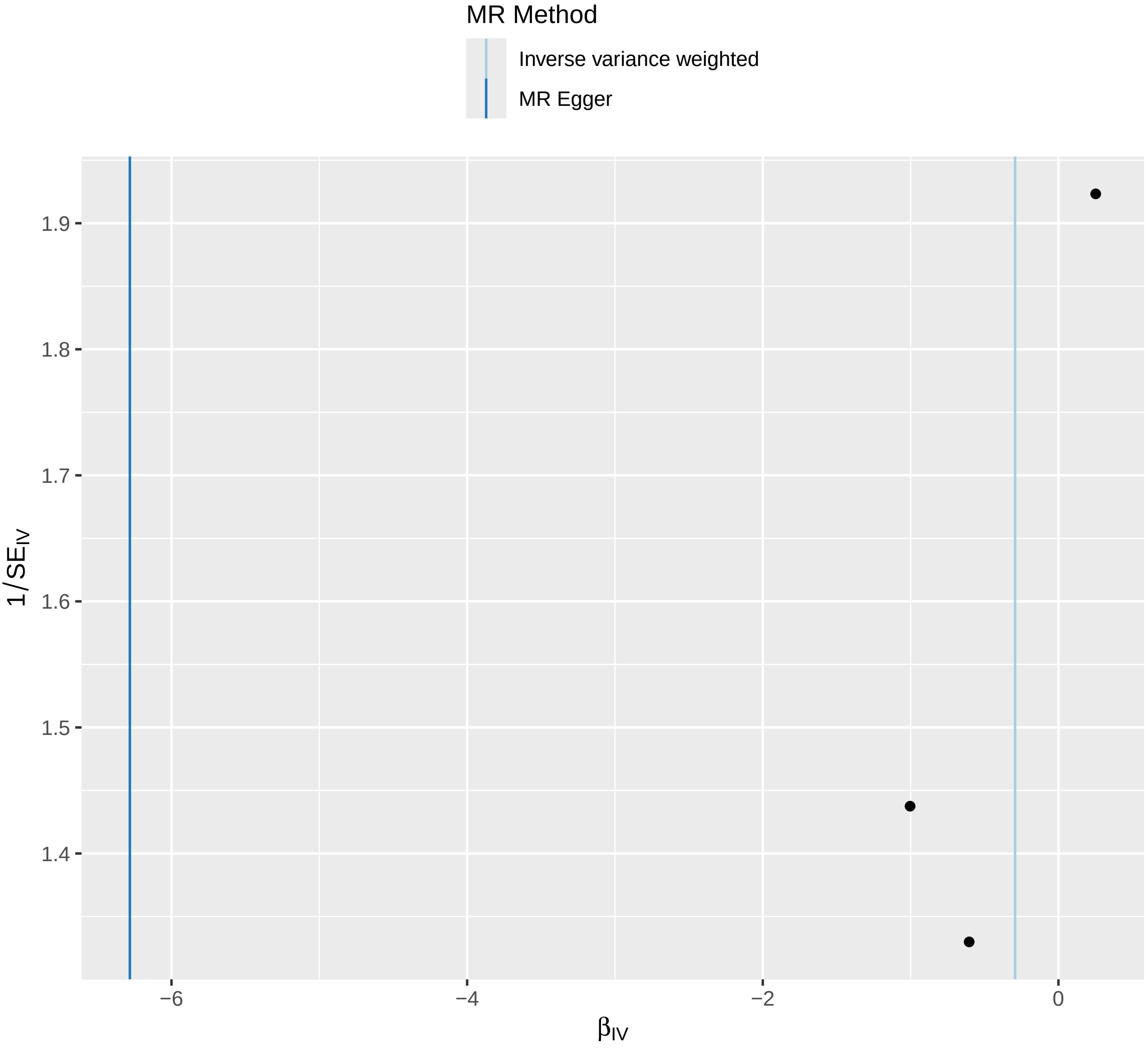
MR Method
Inverse variance weighted
MR Egger
1.9
1.8
1.7
1.6
1.5
1.4
$1/SE_{IV}$
−6
−4
−2
0
β_{IV}

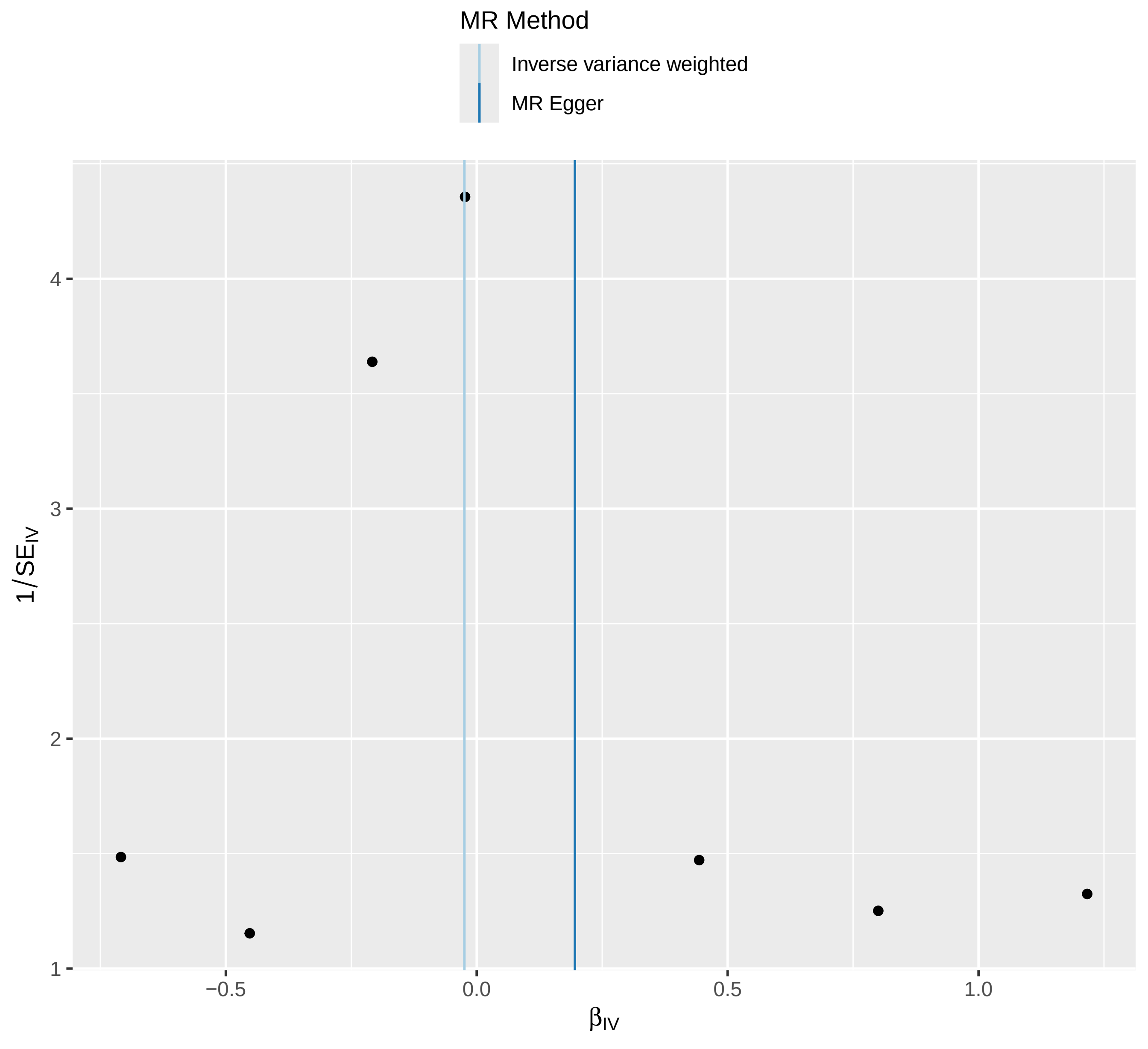

MR Method
Inverse variance weighted
MR Egger
4
3
2
1
1/SE_IV
−0.5
0.0
0.5
1.0
β_IV

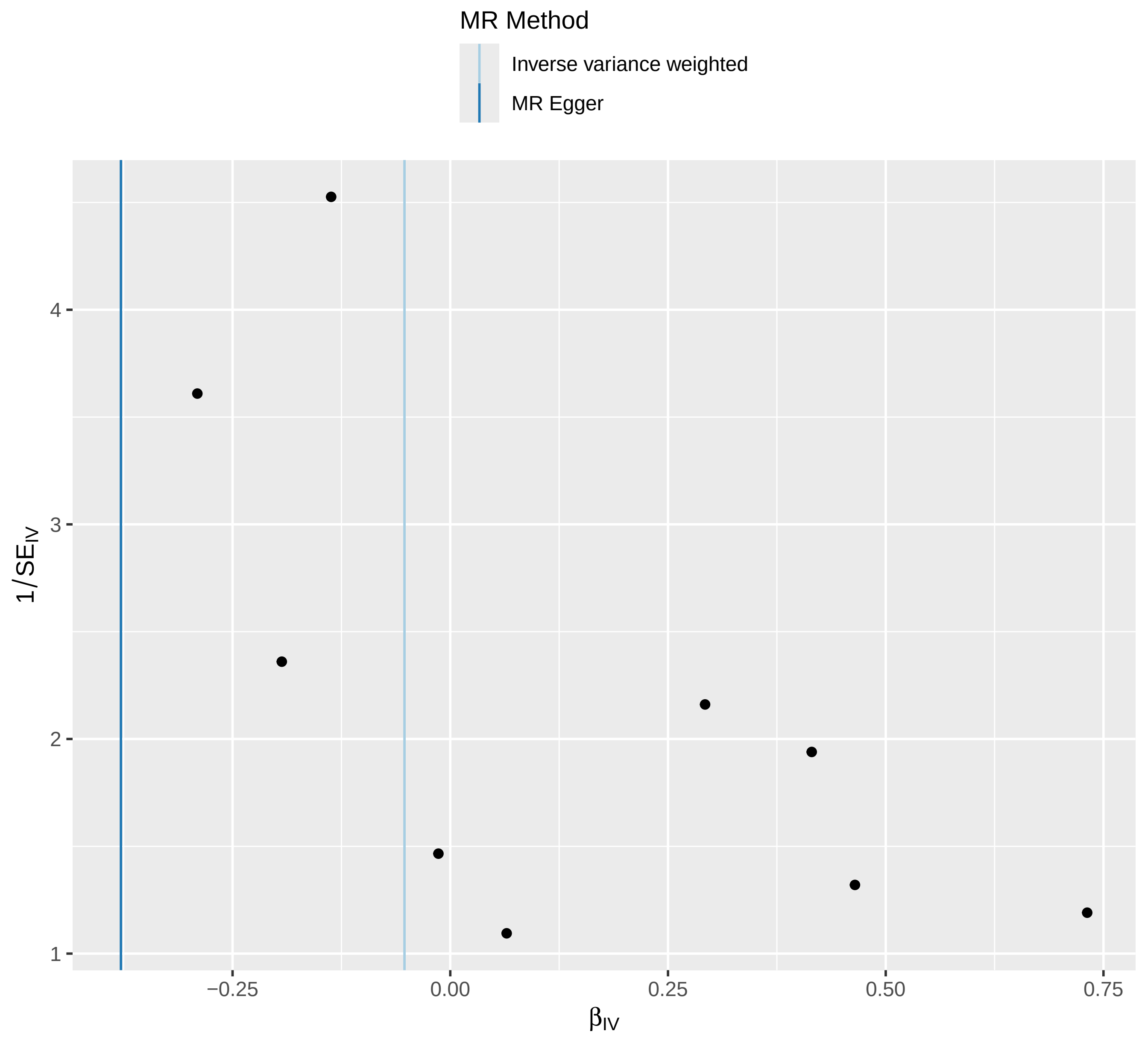
MR Method
Inverse variance weighted
MR Egger
1/SE_IV
4
3
2
1
−0.25
0.00
0.25
0.50
0.75
β_IV

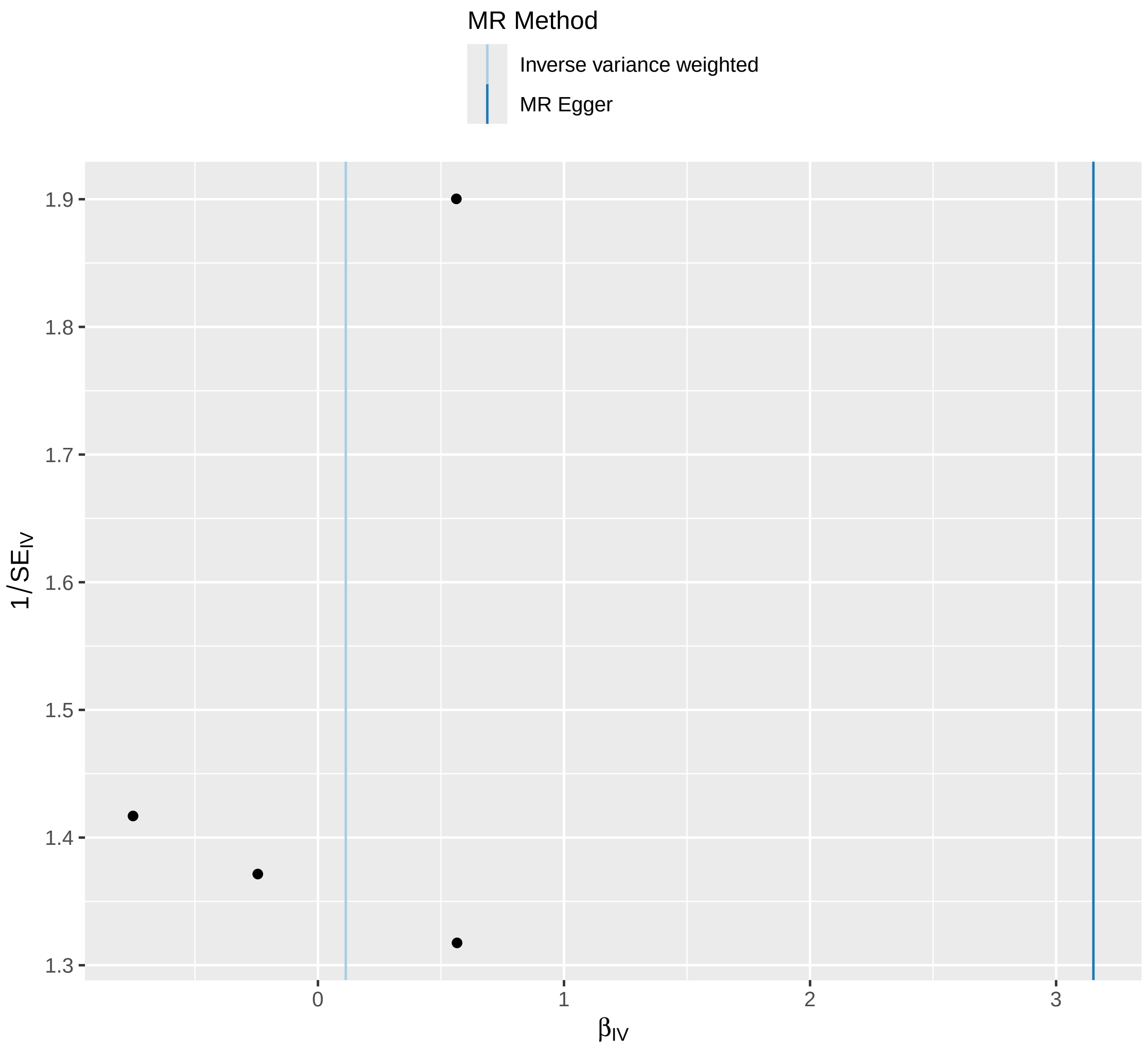

MR Method
Inverse variance weighted
MR Egger
1.9
1.8
1.7
1.6
1.5
1.4
1.3
$1/SE_{IV}$
0
1
2
3
β_{IV}

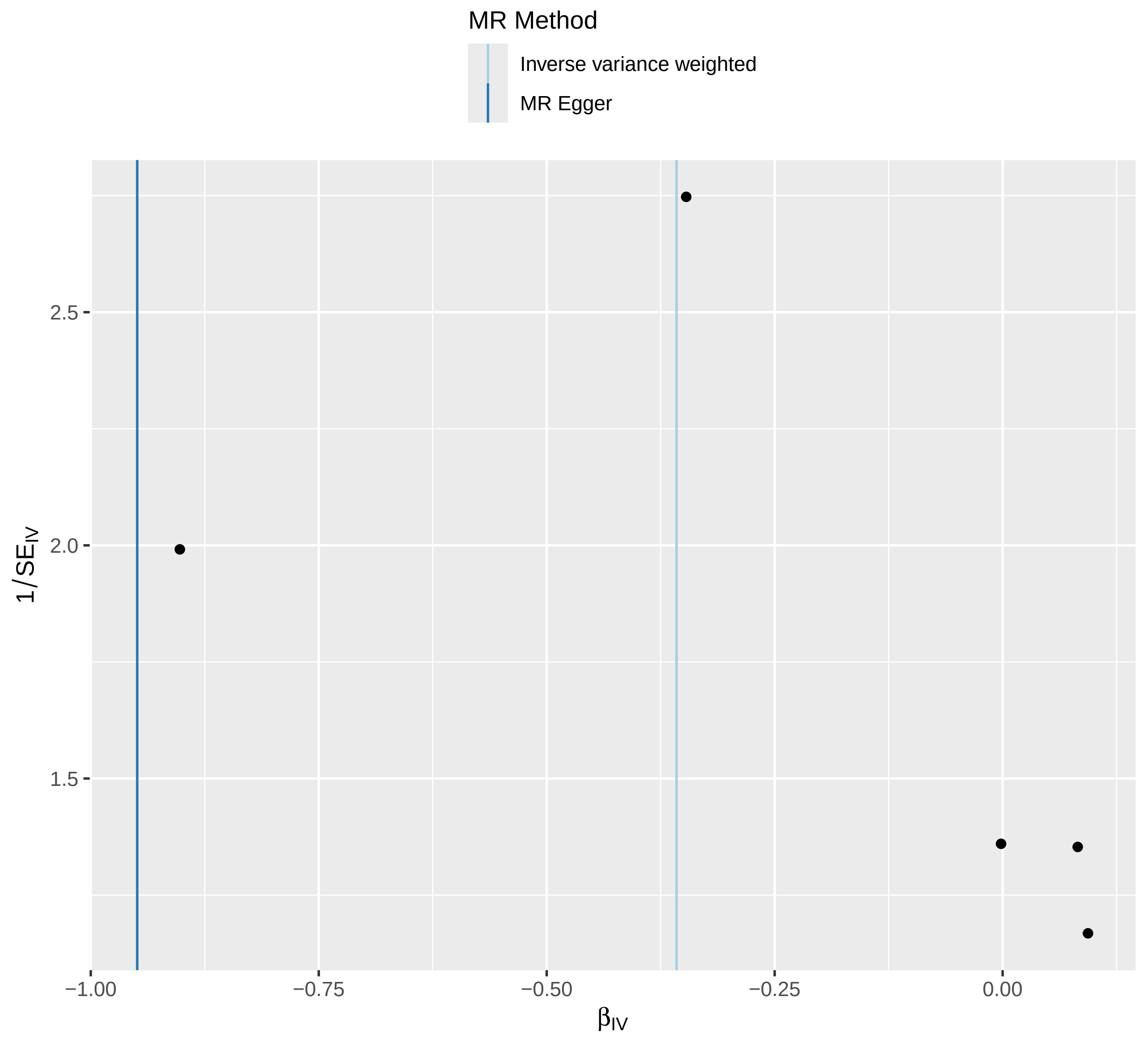
MR Method
Inverse variance weighted
MR Egger
2.5
2.0
1.5
$1/SE_{IV}$
−1.00
−0.75
−0.50
−0.25
0.00
β_{IV}

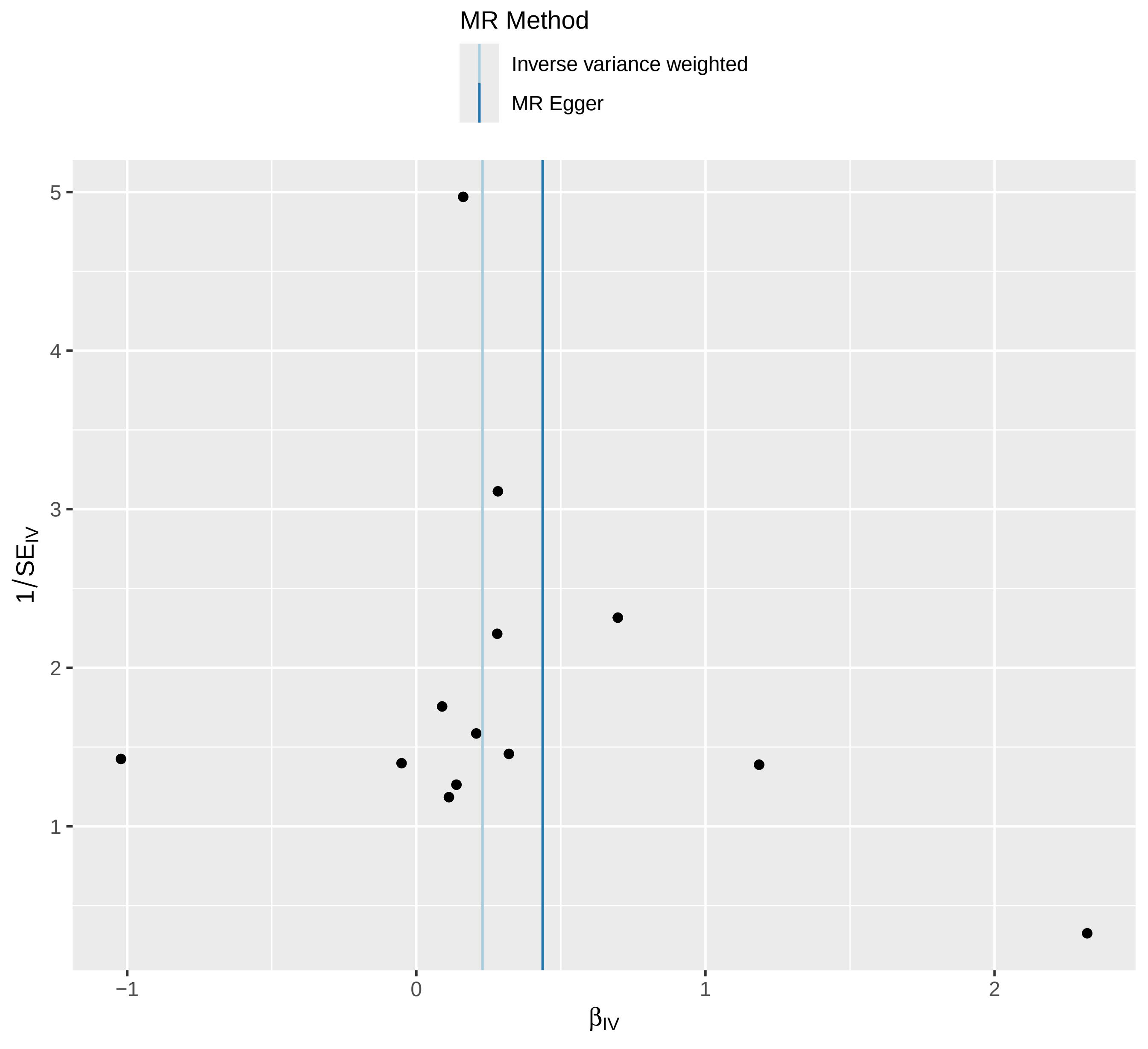

MR Method
Inverse variance weighted
MR Egger
5
4
3
2
1
1/SE_IV
−1
0
1
2
β_IV

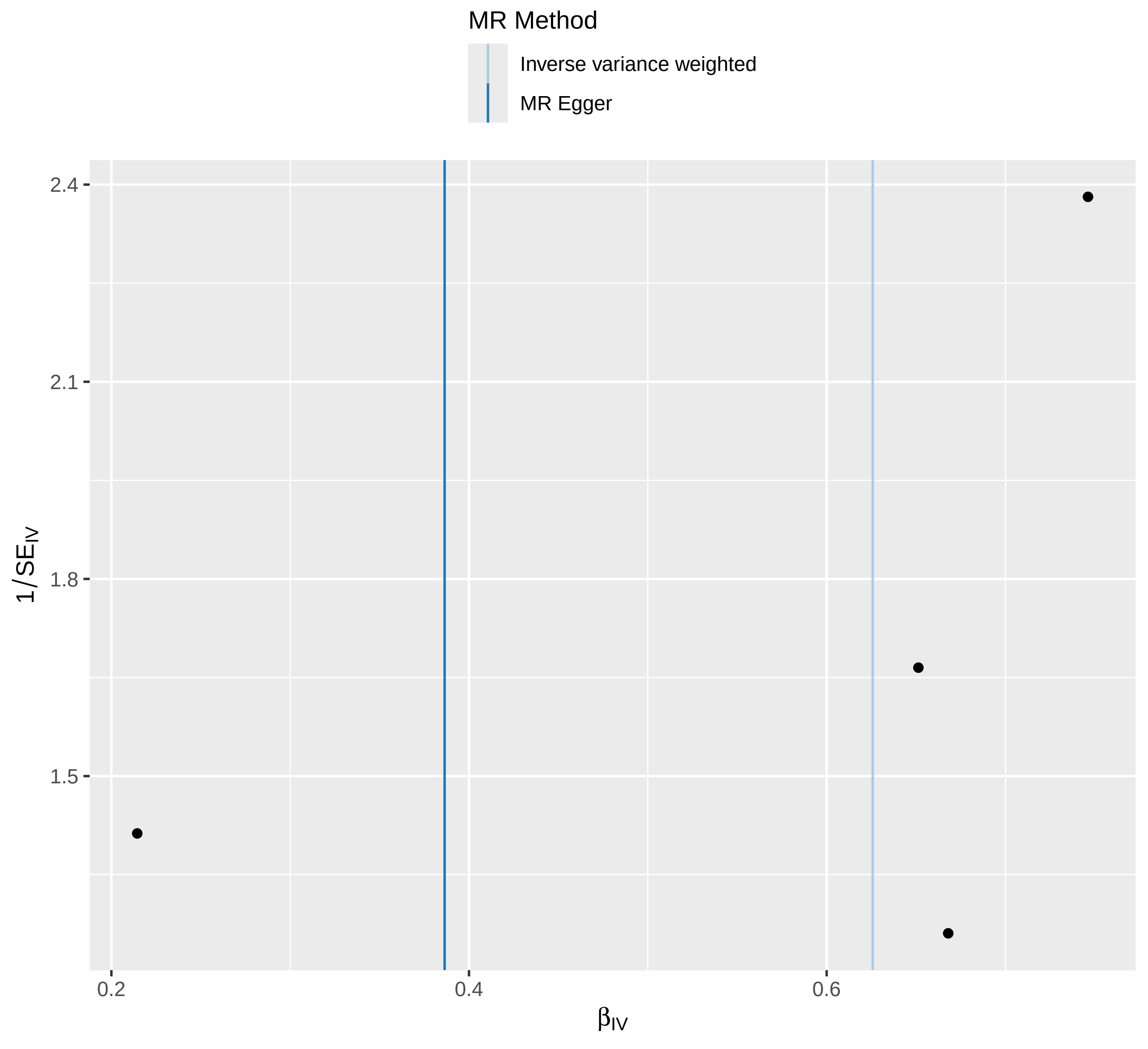

MR Method
Inverse variance weighted
MR Egger
2.4
2.1
1.8
1.5
1/SE_IV
0.2
0.4
0.6
β_IV

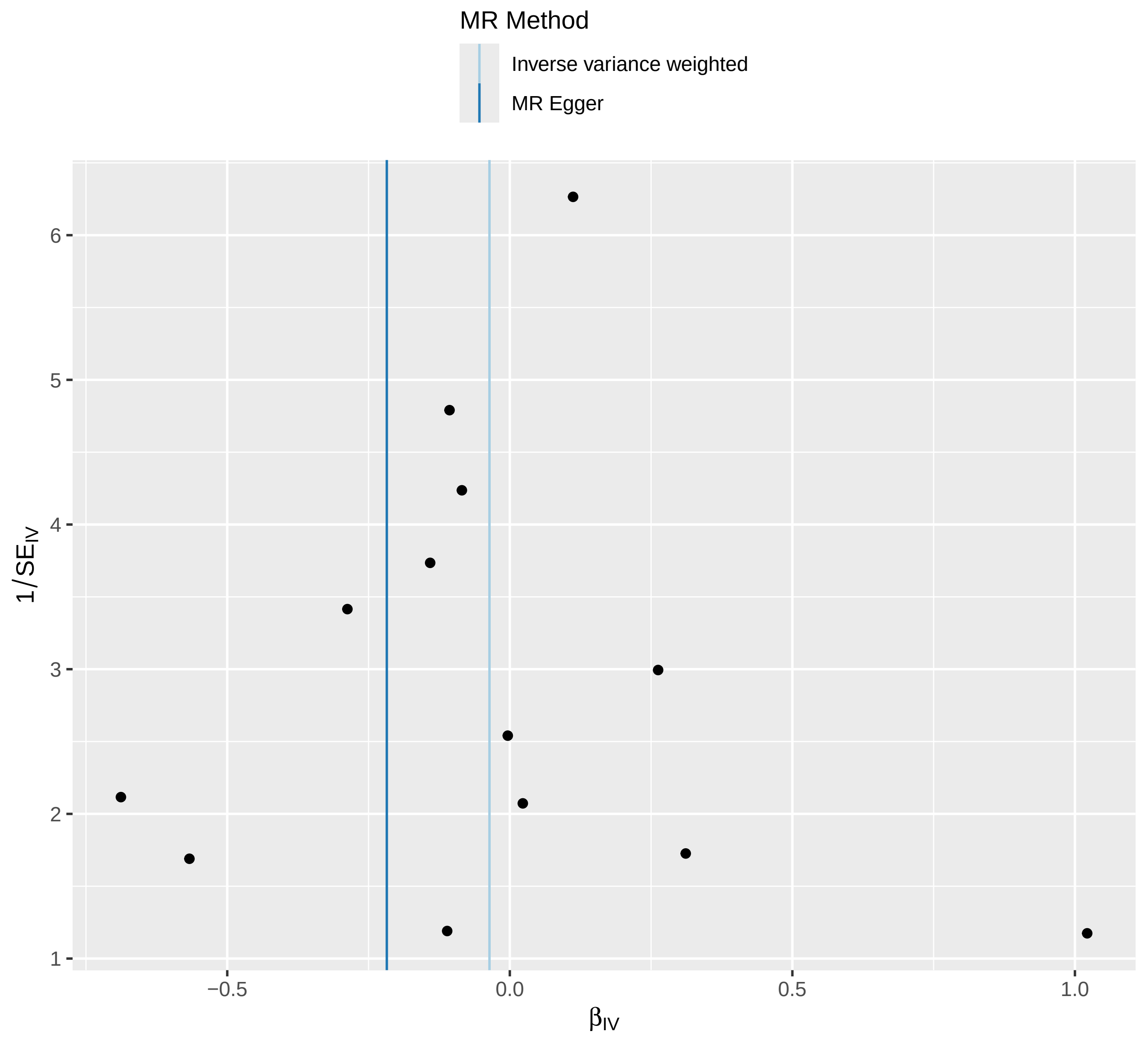

MR Method
Inverse variance weighted
MR Egger
1/SE_IV
β_IV
6
5
4
3
2
1
−0.5
0.0
0.5
1.0

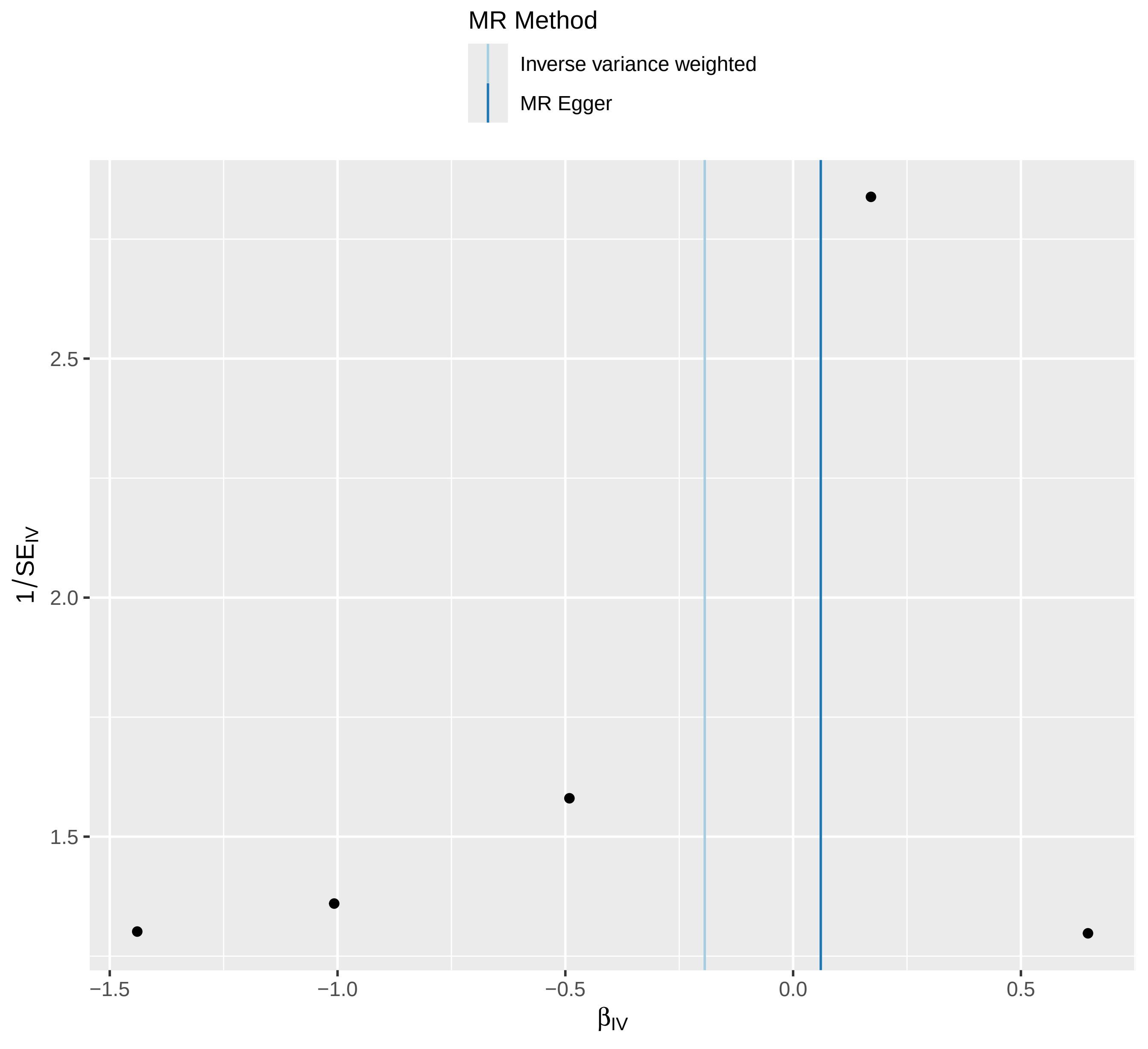

MR Method
Inverse variance weighted
MR Egger
1/SE$_{IV}$
2.5
2.0
1.5
−1.5
−1.0
−0.5
0.0
0.5
β$_{IV}$

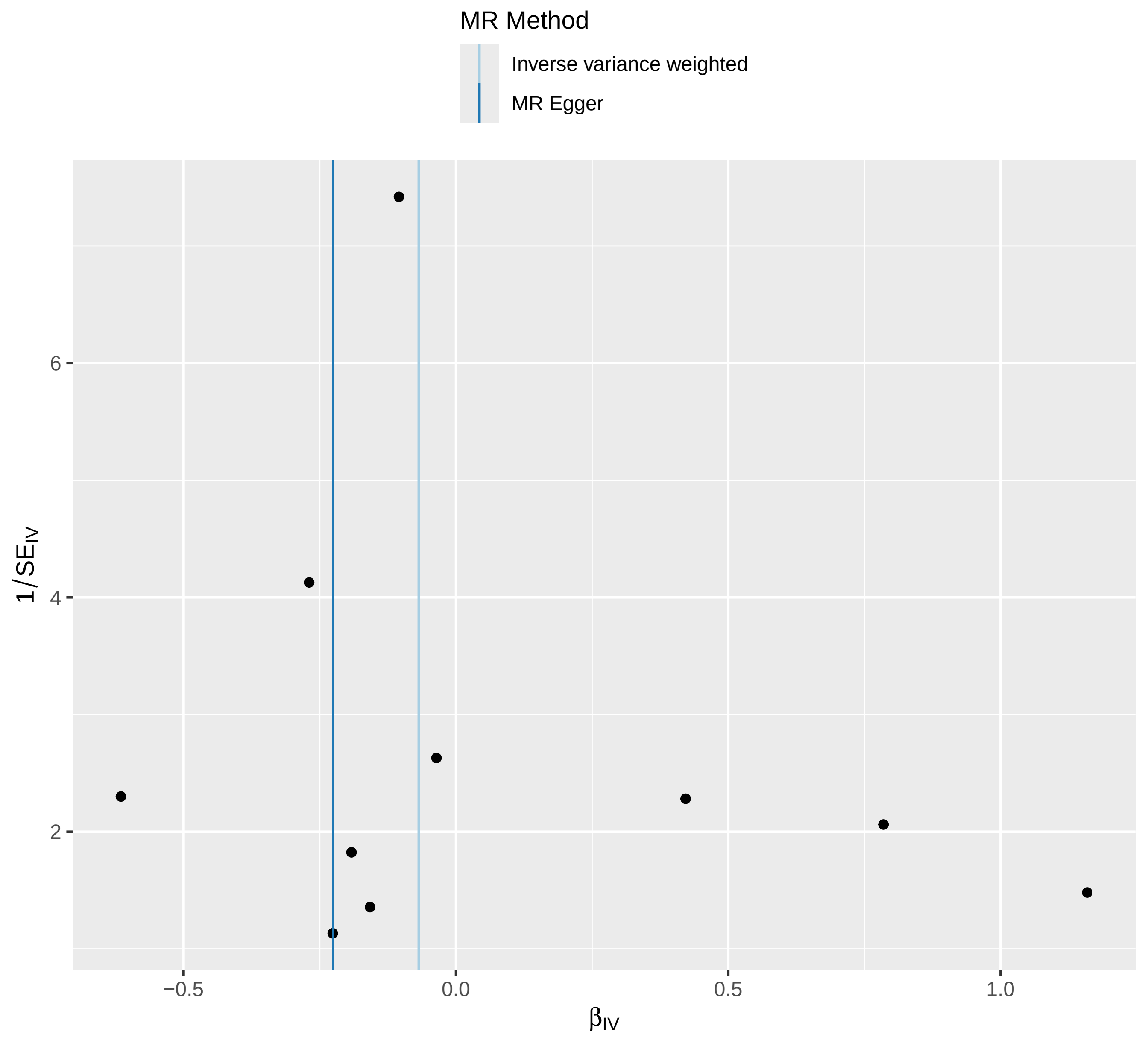
MR Method
Inverse variance weighted
MR Egger
1/SE$_{IV}$
6
4
2
−0.5
0.0
0.5
1.0
β$_{IV}$

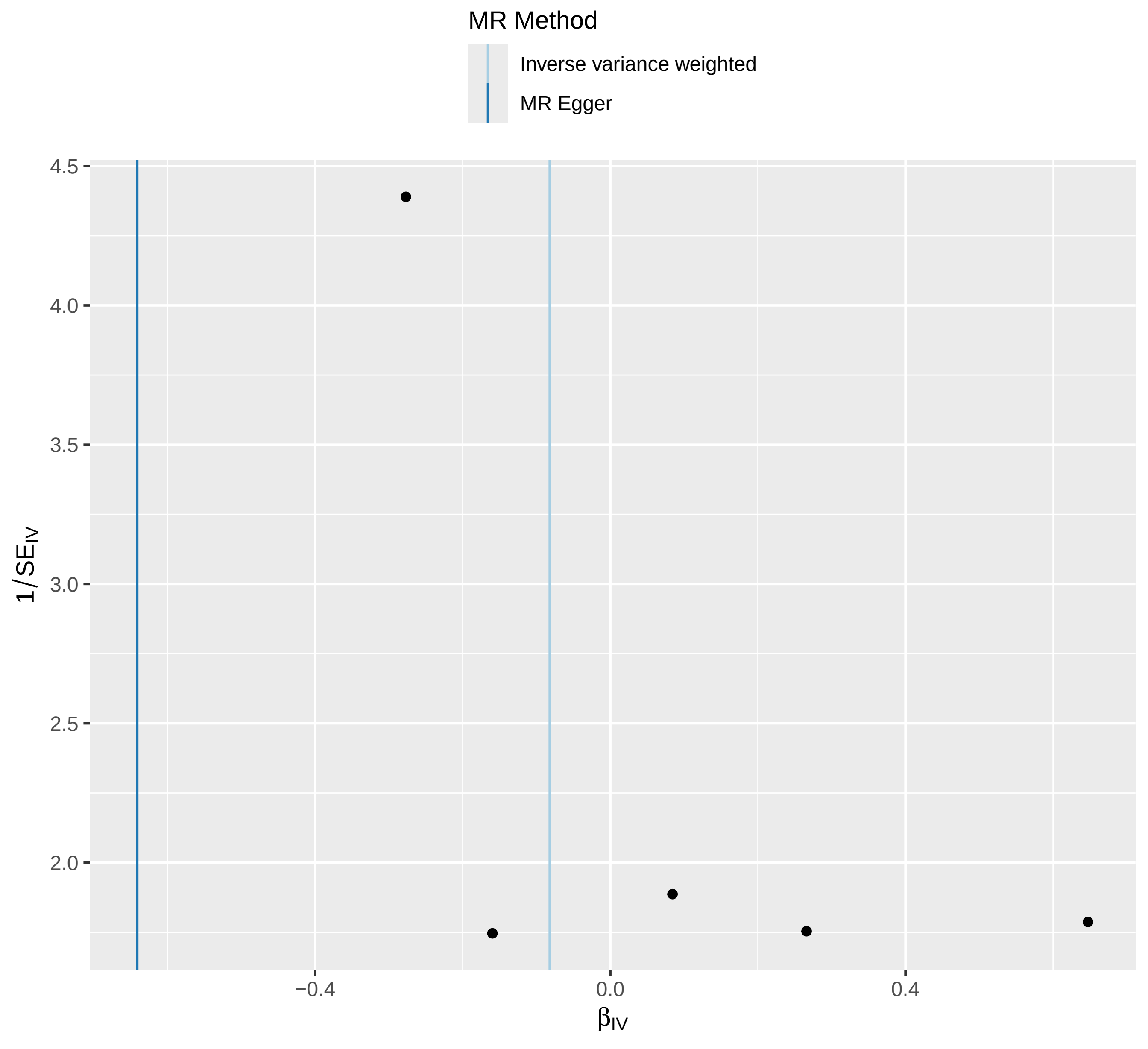

MR Method
Inverse variance weighted
MR Egger
4.5
4.0
3.5
3.0
2.5
2.0
1/SE_IV
−0.4
0.0
0.4
β_IV

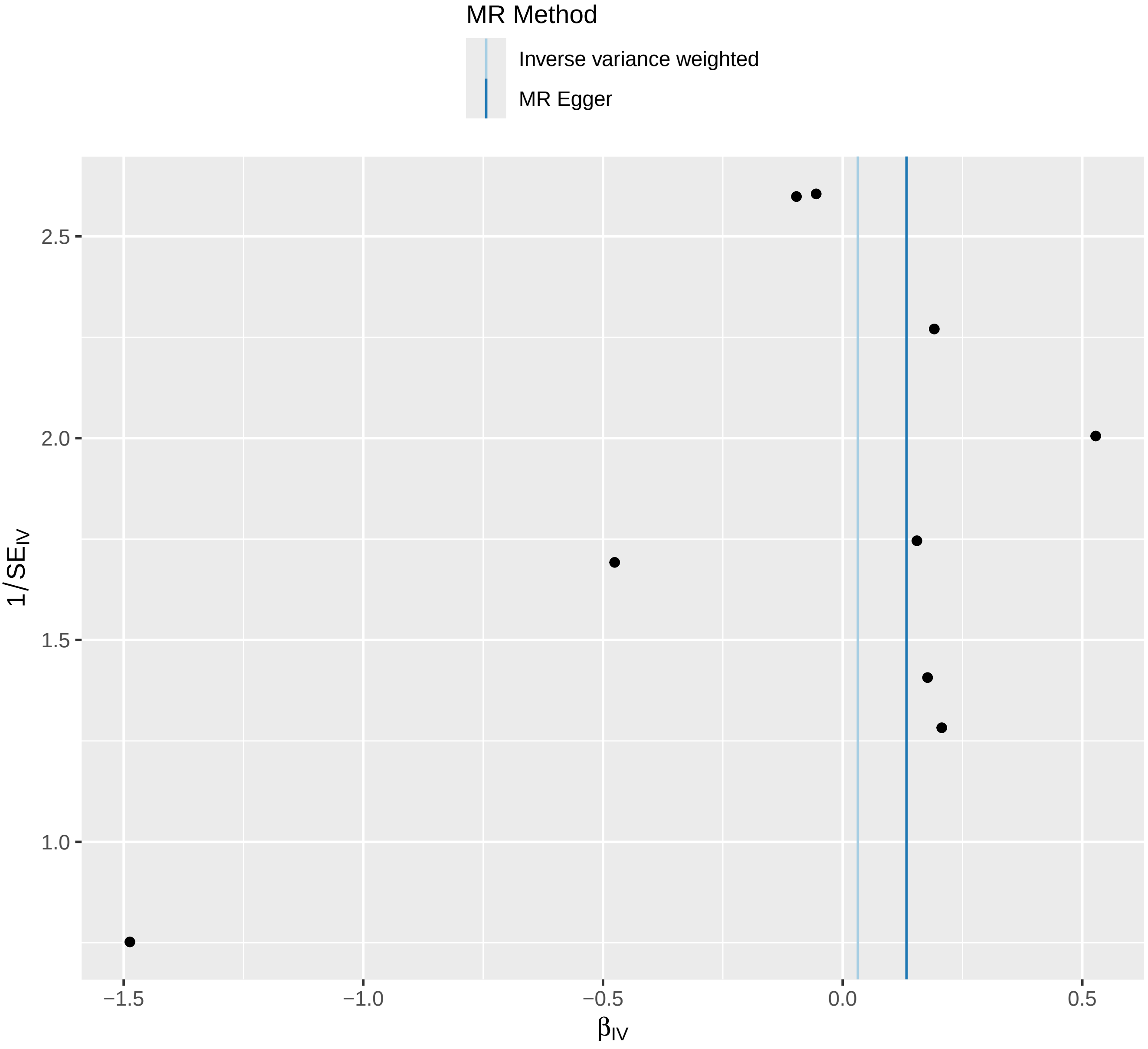
MR Method
Inverse variance weighted
MR Egger
1/SE_IV
2.5
2.0
1.5
1.0
−1.5
−1.0
−0.5
0.0
0.5
β_IV

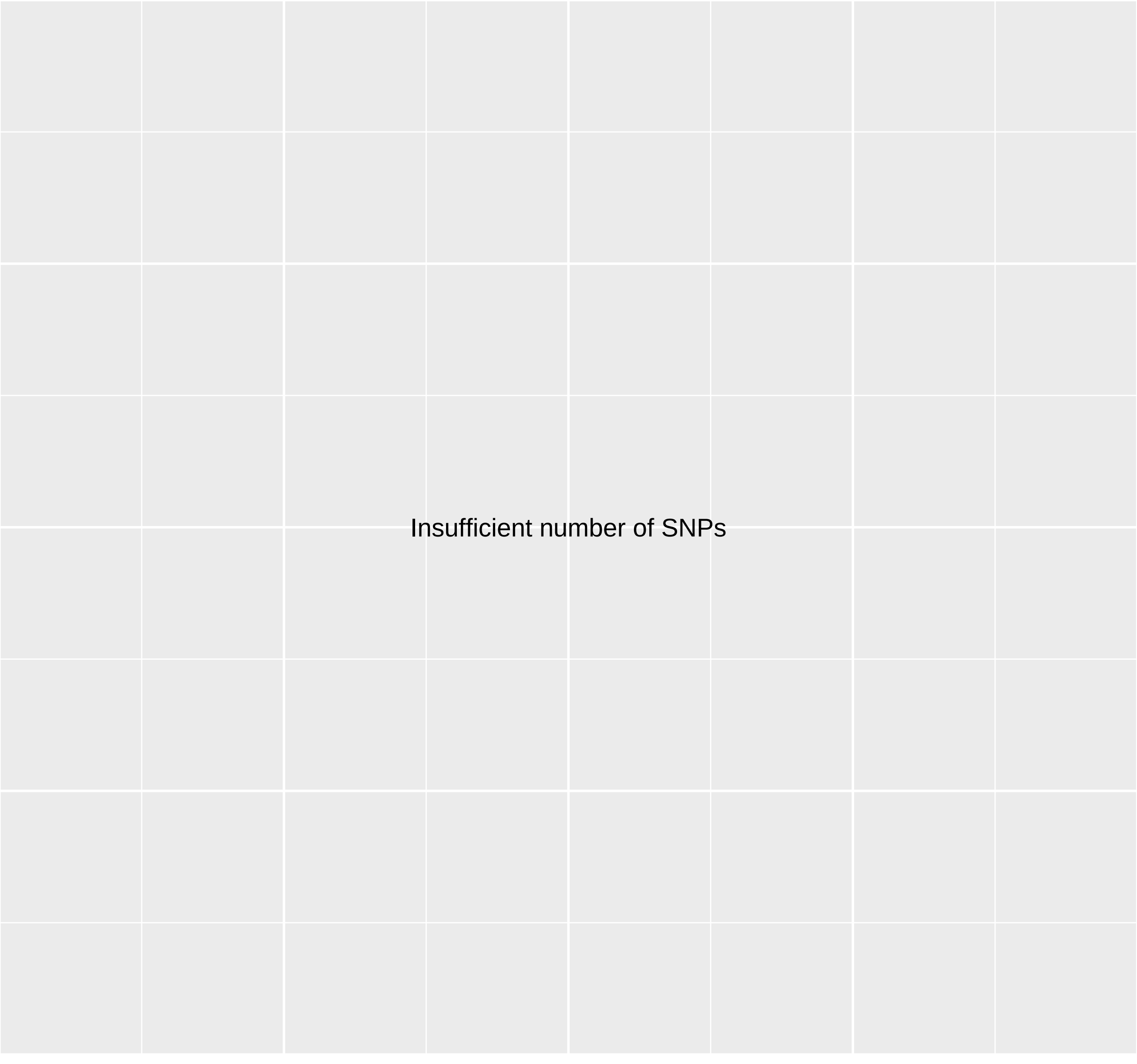
Insufficient number of SNPs

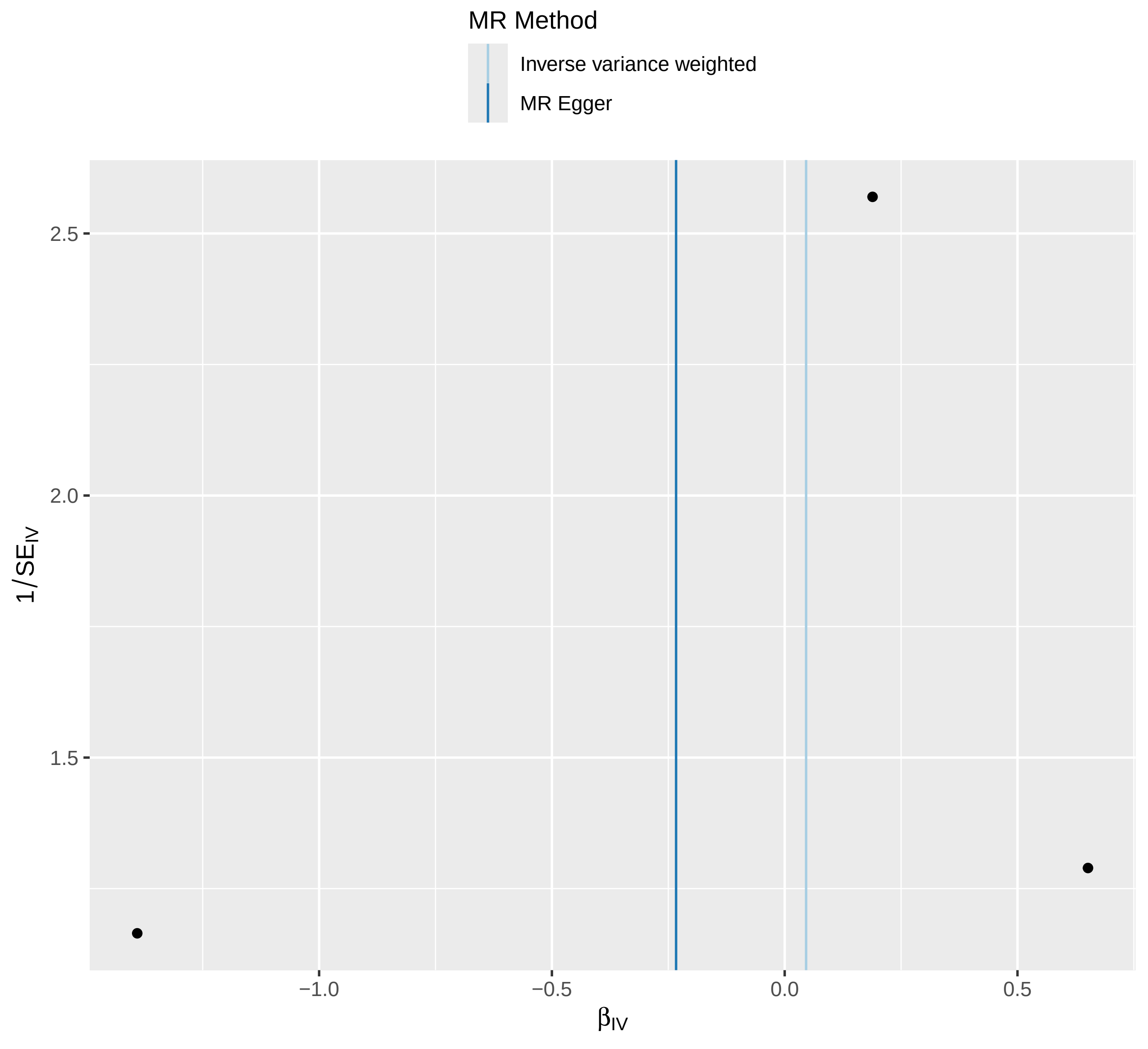
MR Method
Inverse variance weighted
MR Egger
2.5
2.0
1.5
1/SE_IV
−1.0
−0.5
0.0
0.5
β_IV

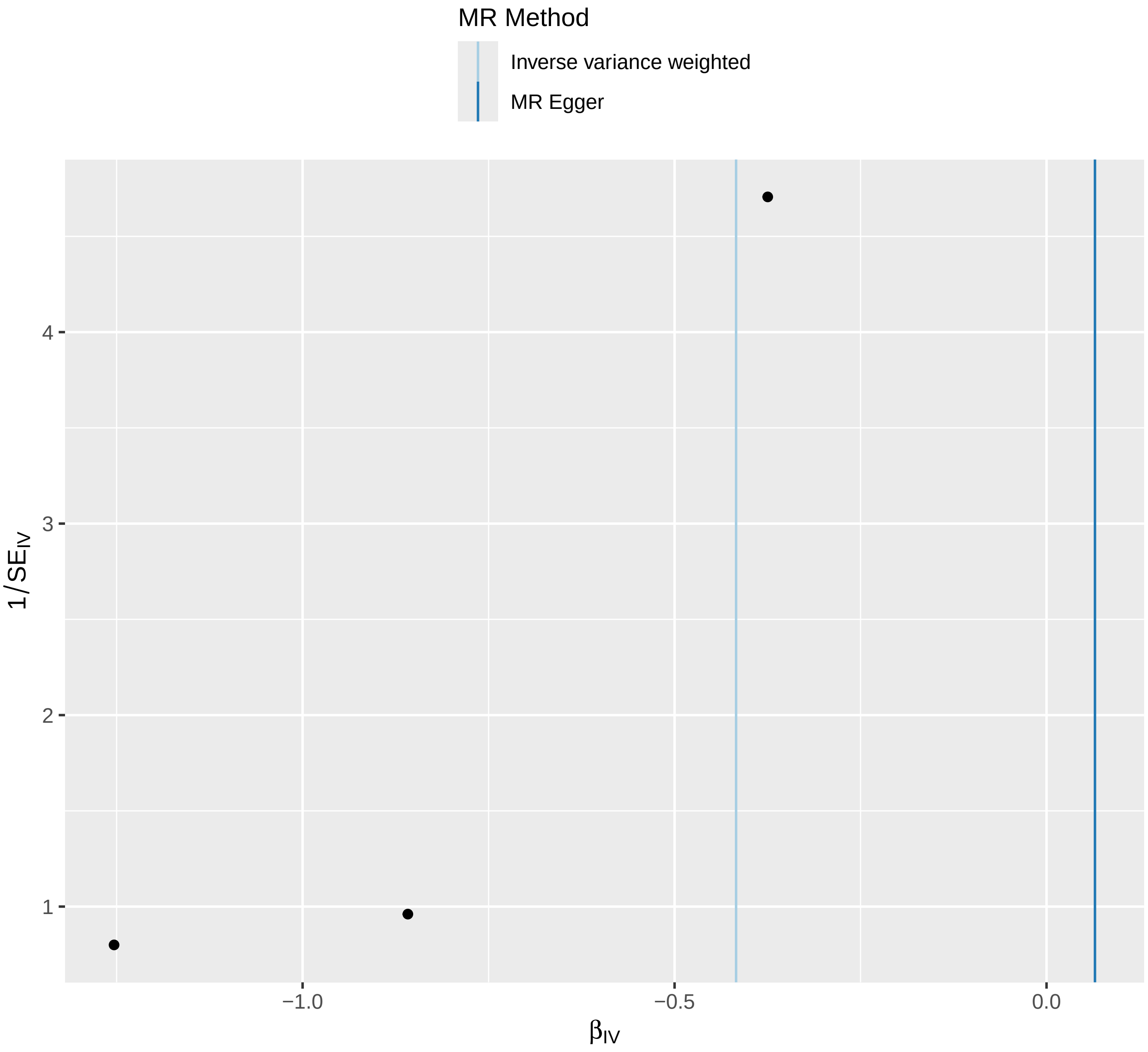

MR Method
Inverse variance weighted
MR Egger
4
3
2
1
$1/SE_{IV}$
−1.0
−0.5
0.0
β_{IV}

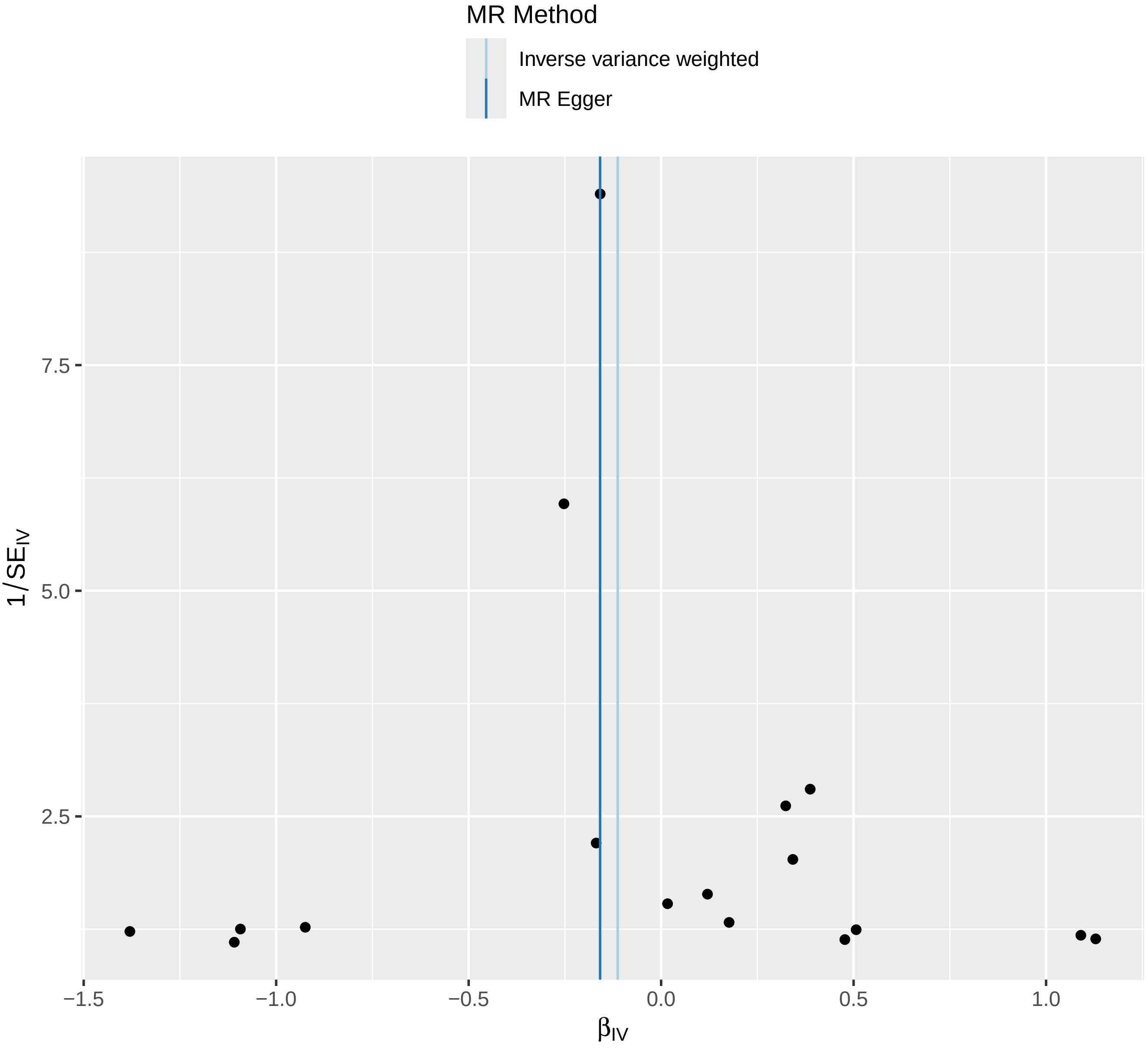

MR Method
Inverse variance weighted
MR Egger
7.5
5.0
2.5
1/SE$_{IV}$
−1.5
−1.0
−0.5
0.0
0.5
1.0
β$_{IV}$

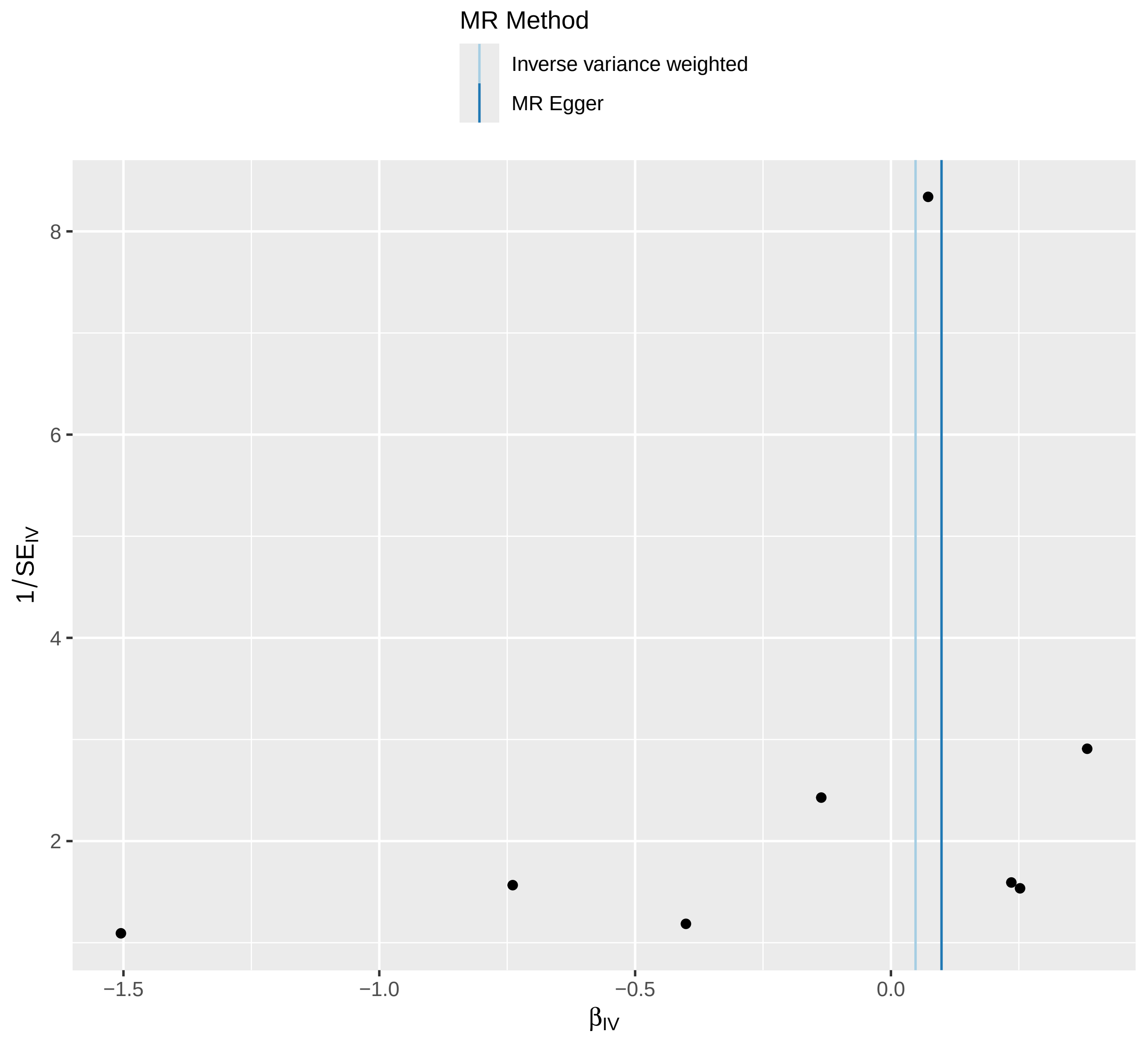
MR Method
Inverse variance weighted
MR Egger
8
6
4
2
1/SE_IV
−1.5
−1.0
−0.5
0.0
β_IV

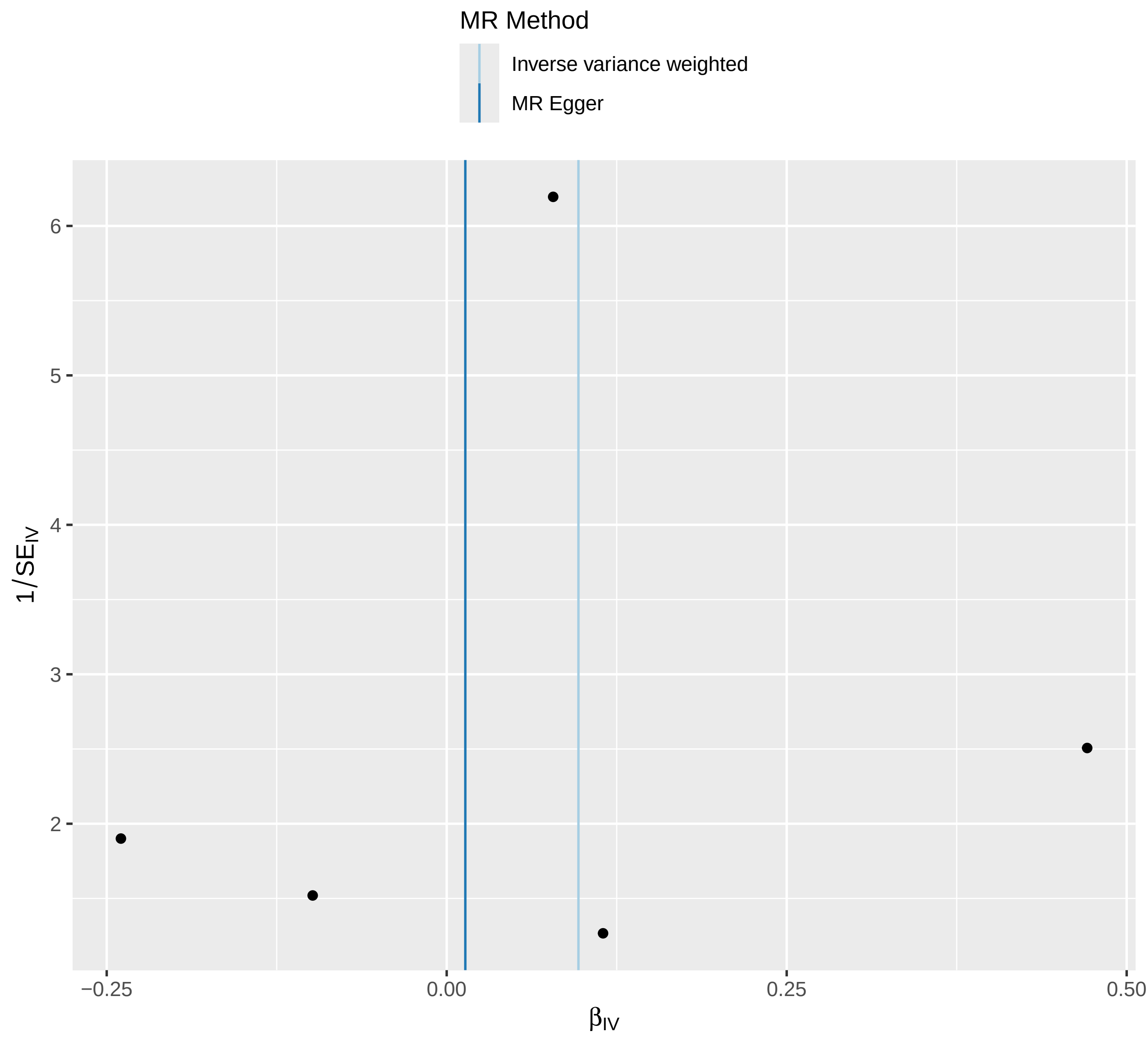
MR Method
Inverse variance weighted
MR Egger
$1/SE_{IV}$
β_{IV}
6
5
4
3
2
−0.25
0.00
0.25
0.50

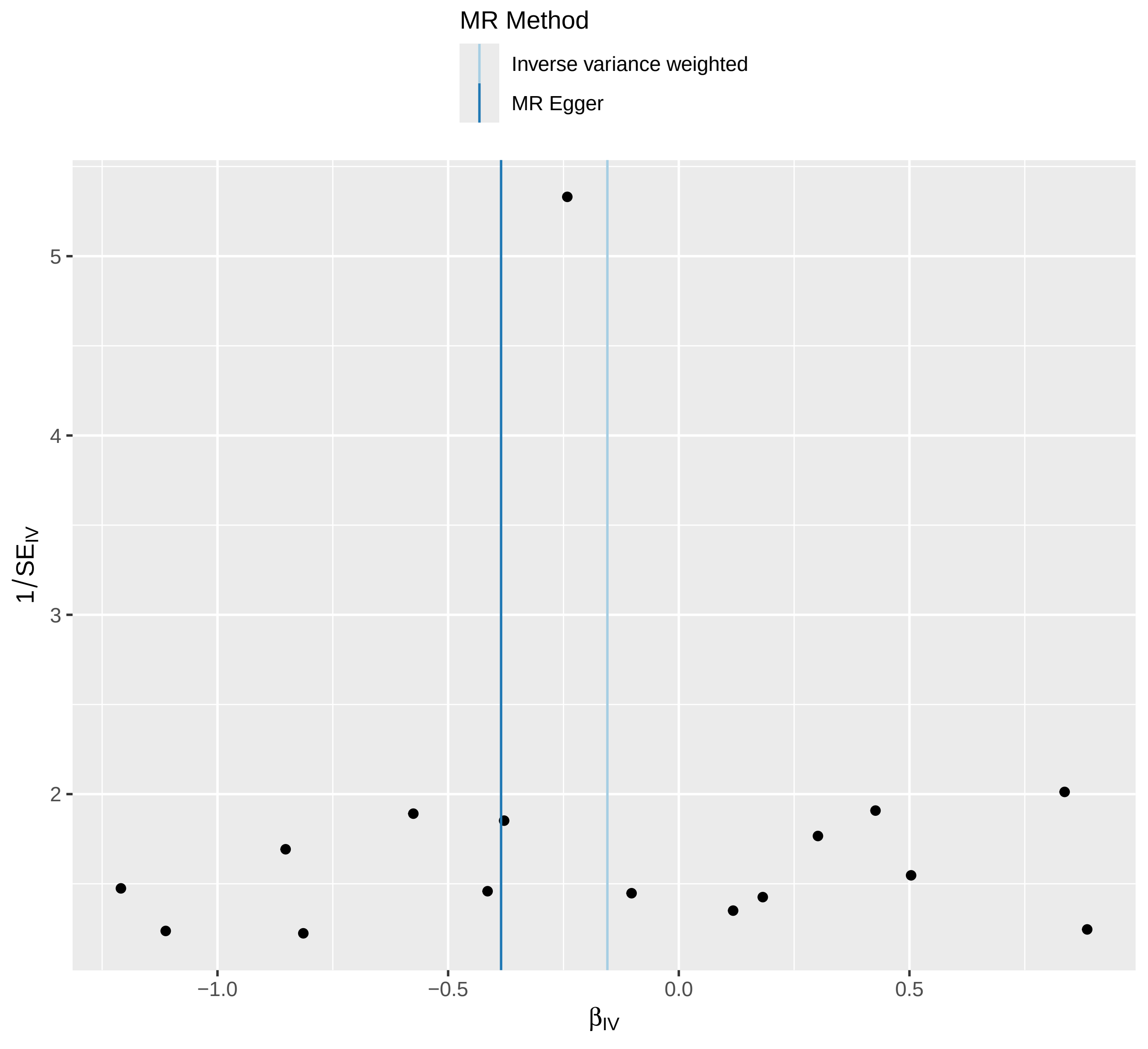

MR Method
Inverse variance weighted
MR Egger
$1/SE_{IV}$
5
4
3
2
−1.0
−0.5
0.0
0.5
β_{IV}

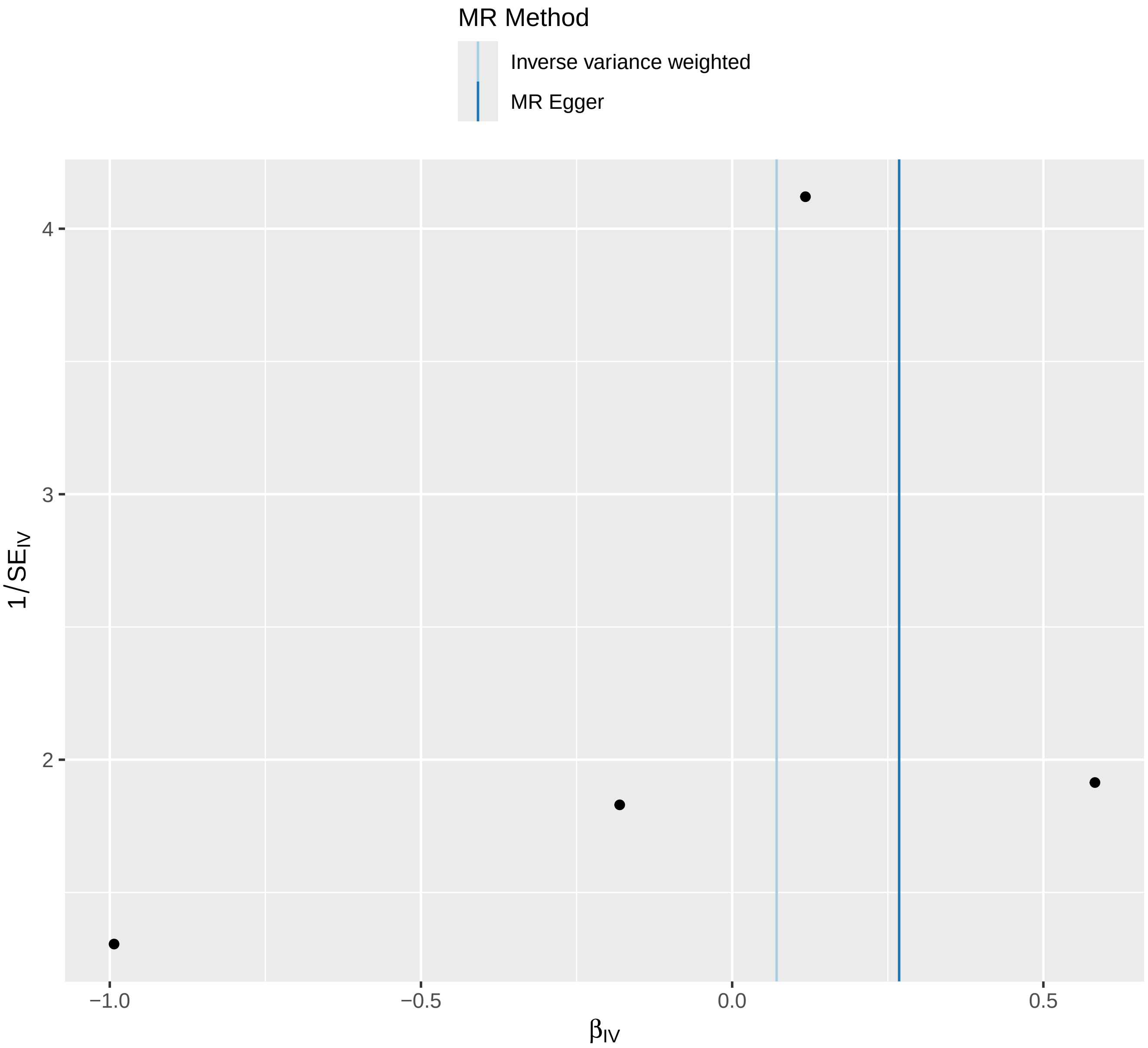
MR Method
Inverse variance weighted
MR Egger
4
3
2
$1/SE_{IV}$
−1.0
−0.5
0.0
0.5
β_{IV}

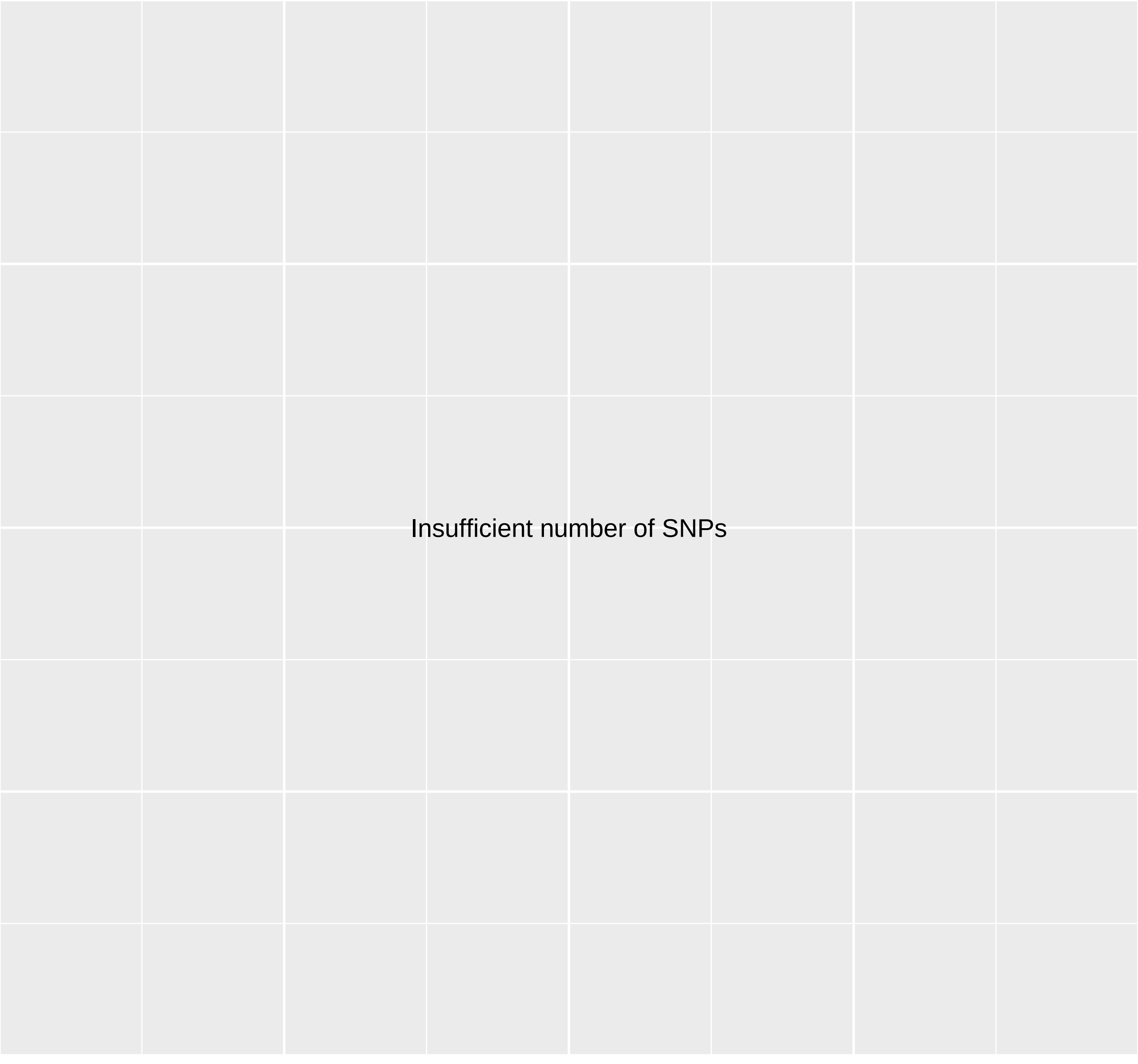
Insufficient number of SNPs

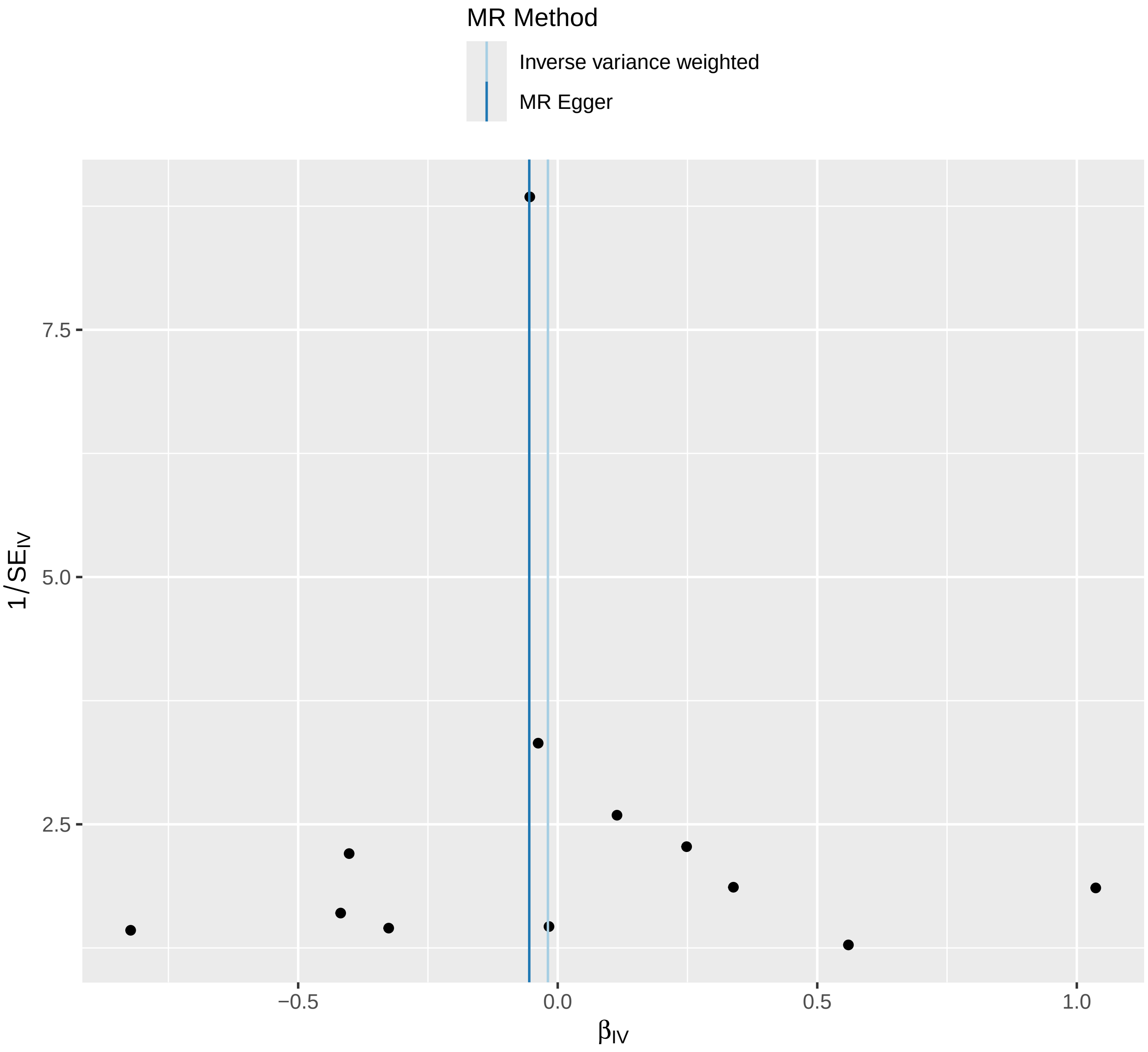

MR Method
Inverse variance weighted
MR Egger
7.5
5.0
2.5
1/SE$_{IV}$
−0.5
0.0
0.5
1.0
β$_{IV}$

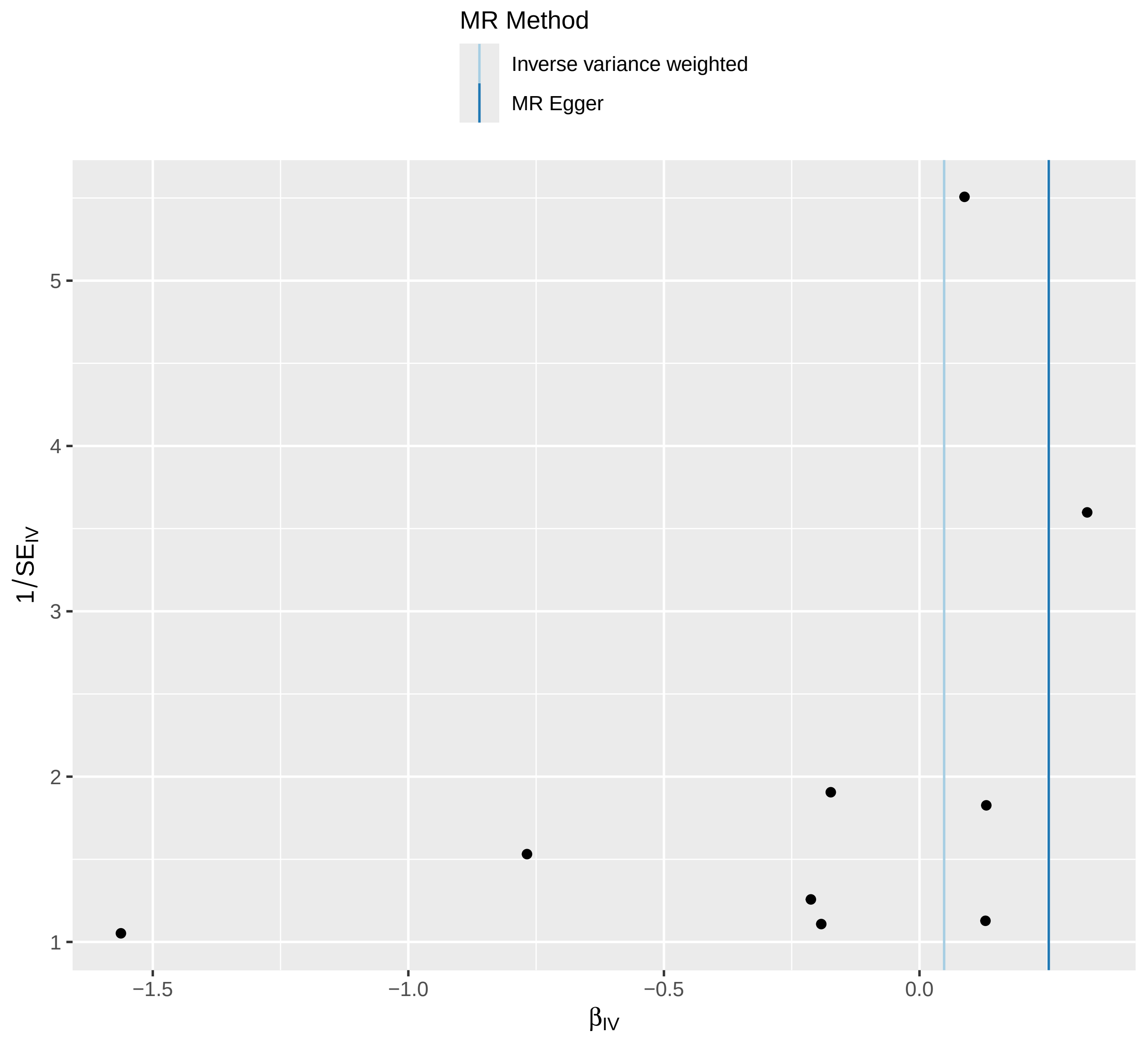

MR Method
Inverse variance weighted
MR Egger
1/SE$_{IV}$
5
4
3
2
1
−1.5
−1.0
−0.5
0.0
β$_{IV}$

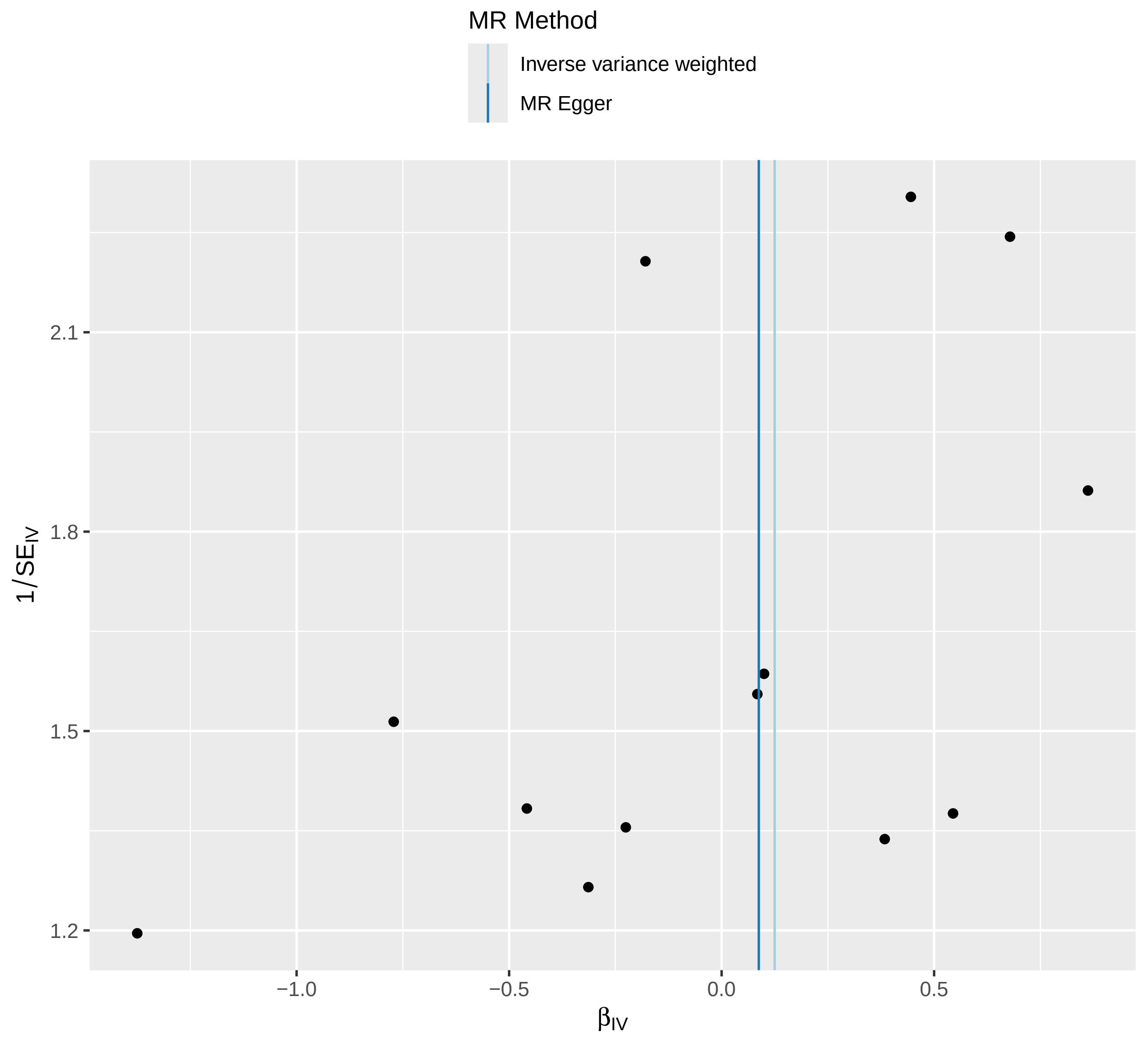

MR Method
Inverse variance weighted
MR Egger
2.1
1.8
1.5
1.2
$1/SE_{IV}$
−1.0
−0.5
0.0
0.5
β_{IV}

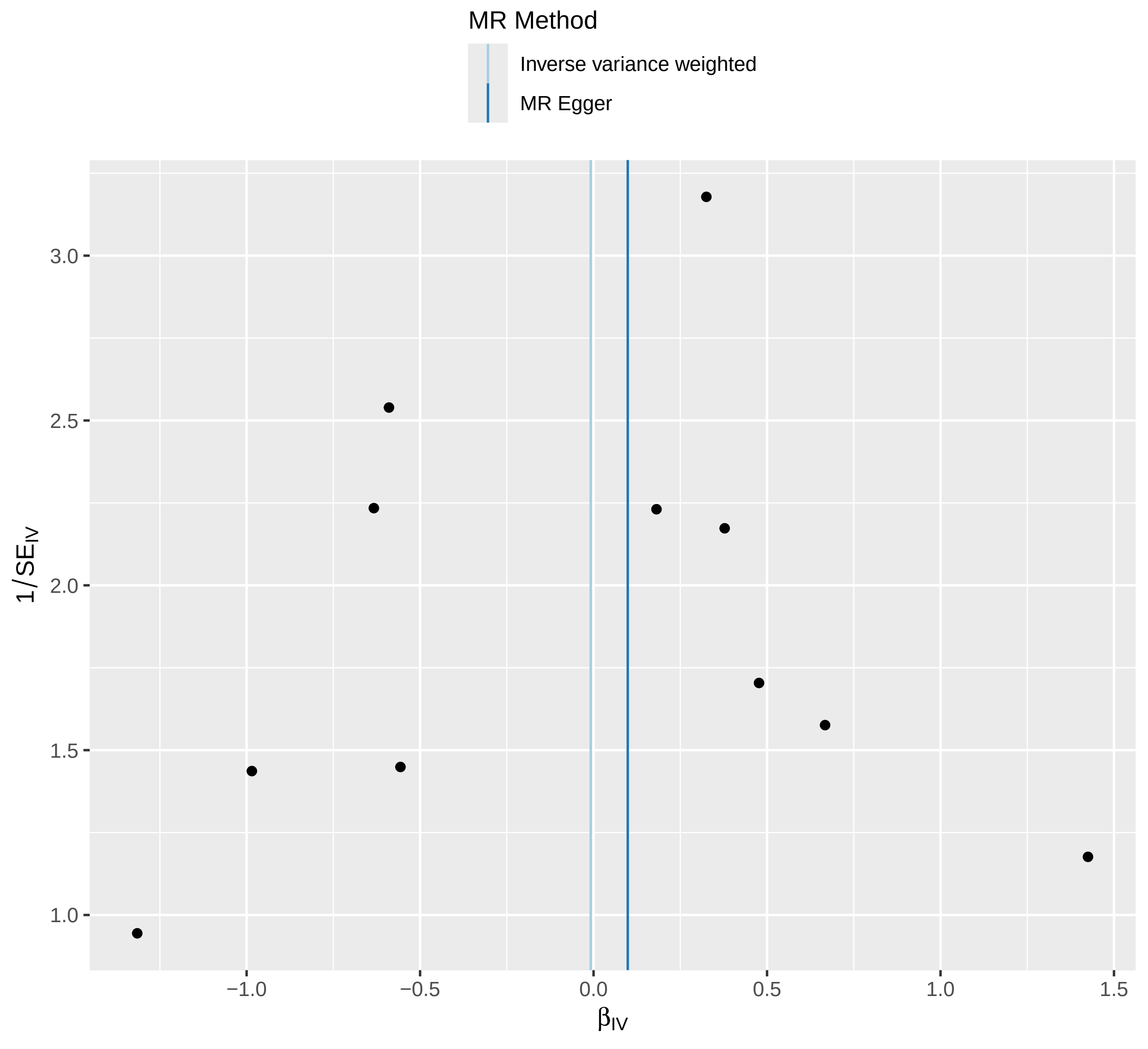
MR Method
Inverse variance weighted
MR Egger
3.0
2.5
2.0
1.5
1.0
1/SE_IV
−1.0
−0.5
0.0
0.5
1.0
1.5
β_IV

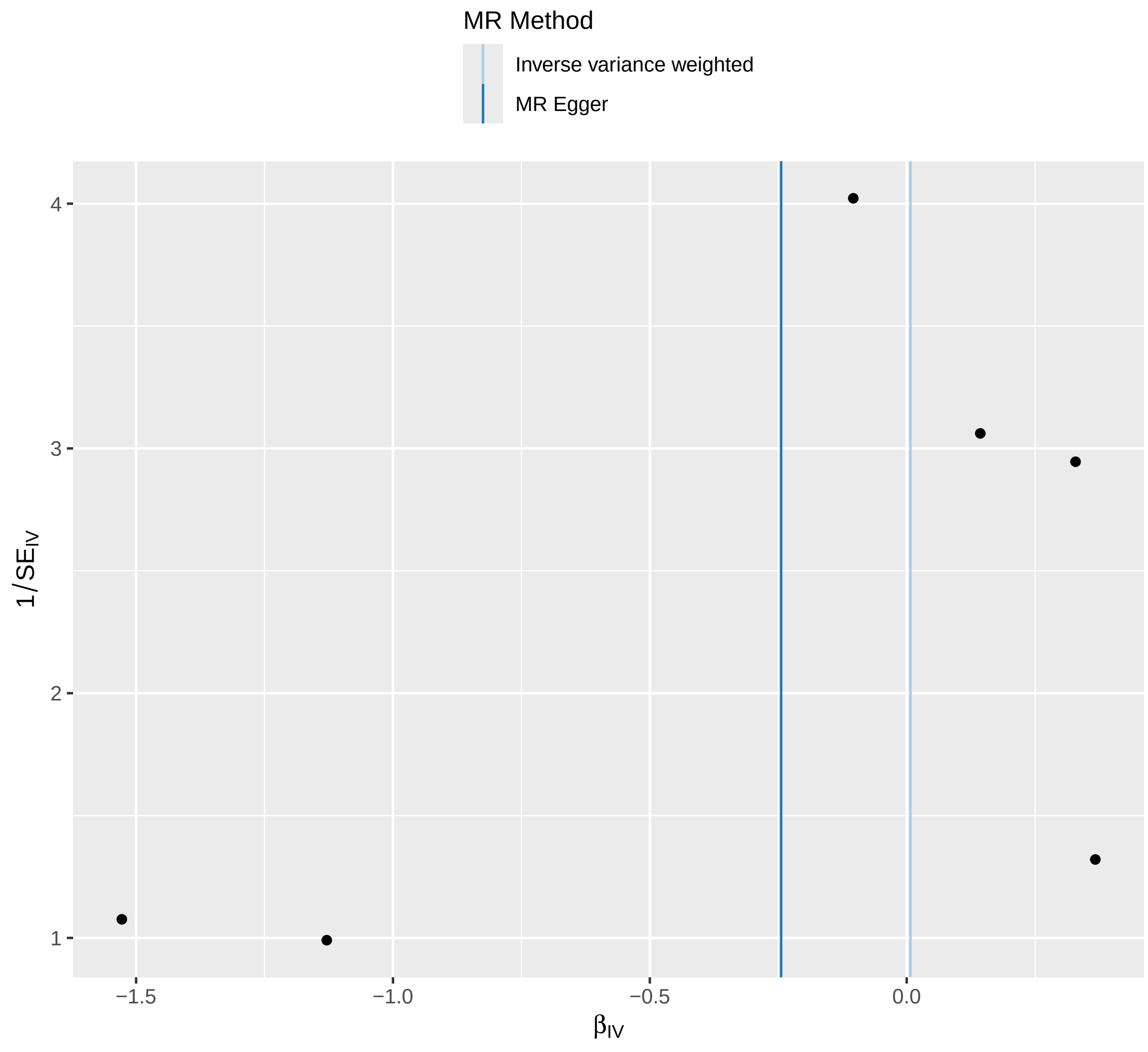

MR Method
Inverse variance weighted
MR Egger
4
3
2
1
$1/SE_{IV}$
−1.5
−1.0
−0.5
0.0
β_{IV}

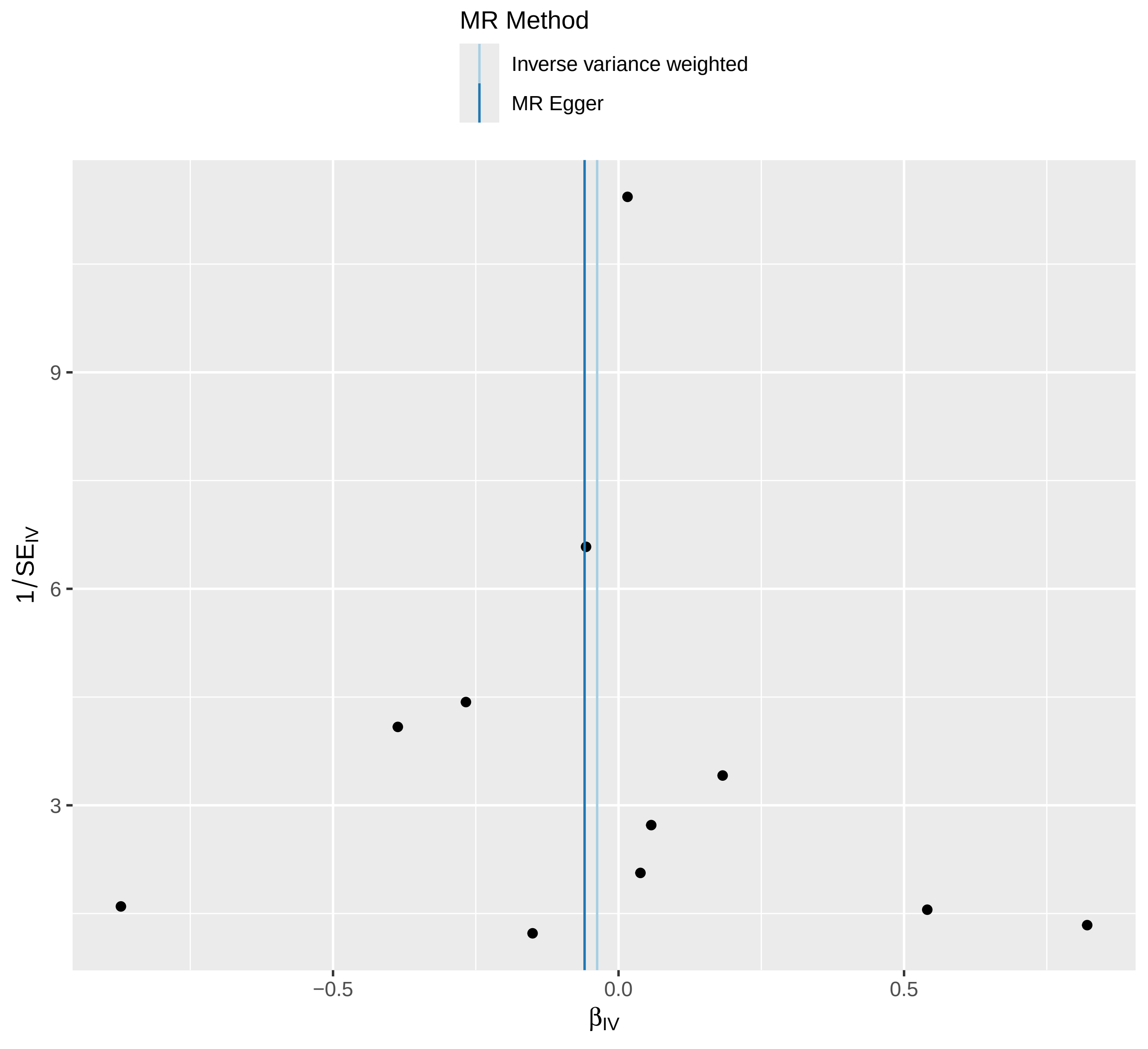

MR Method
Inverse variance weighted
MR Egger
9
6
3
1/SE_IV
−0.5
0.0
0.5
β_IV

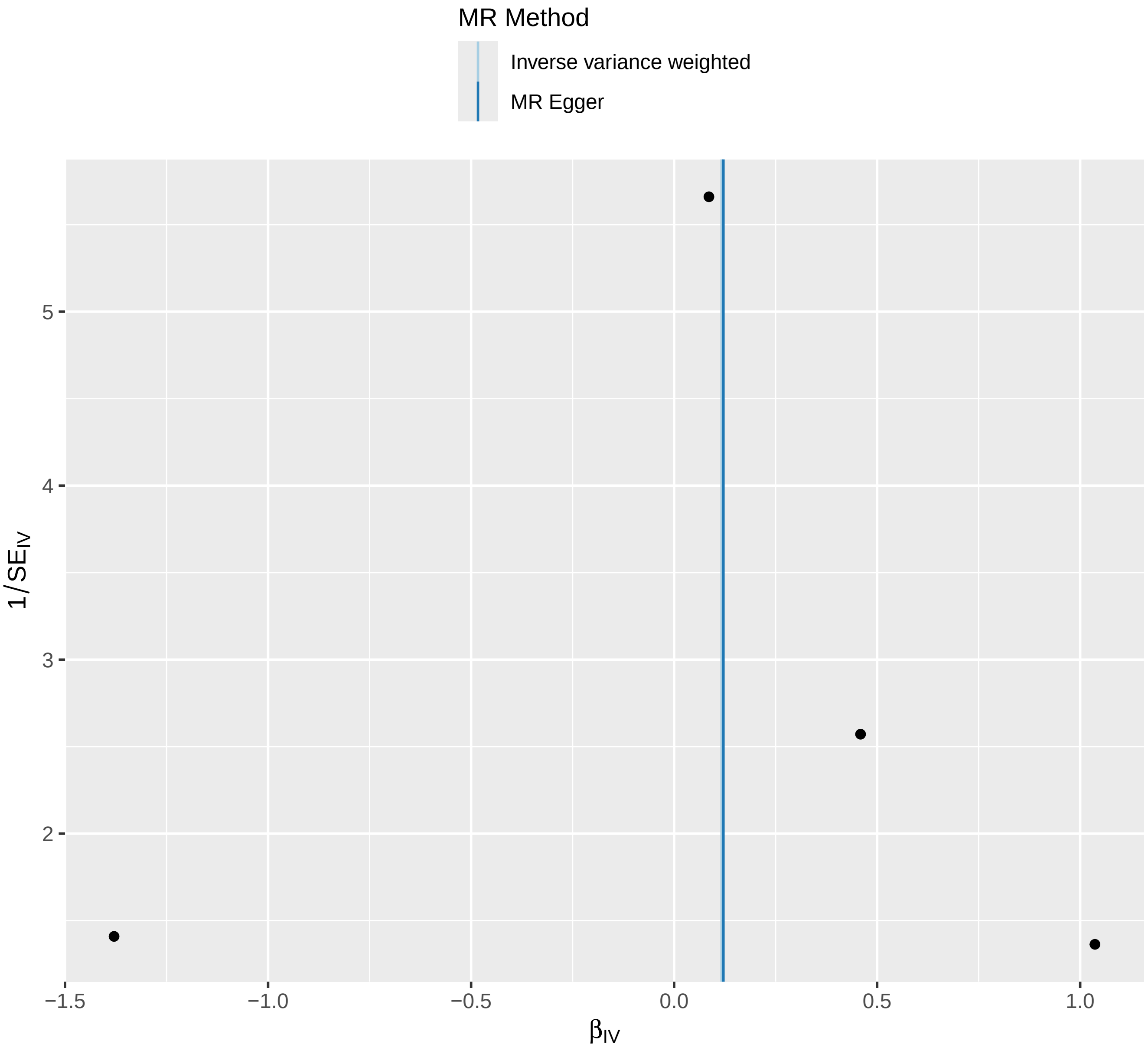
MR Method
Inverse variance weighted
MR Egger
1/SE$_{IV}$
5
4
3
2
−1.5
−1.0
−0.5
0.0
0.5
1.0
β$_{IV}$

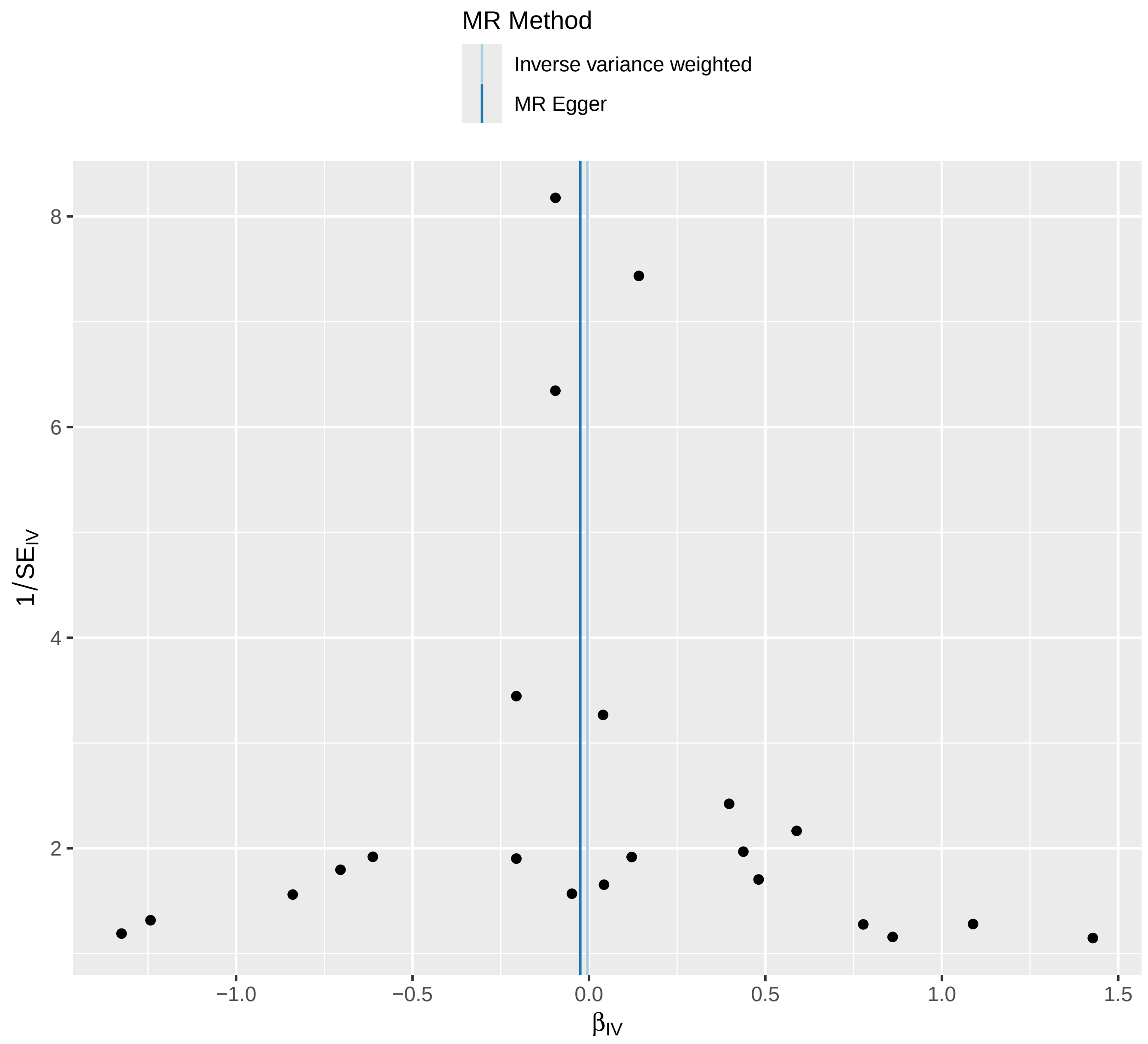
MR Method
Inverse variance weighted
MR Egger
8
6
4
2
1/SE_IV
−1.0
−0.5
0.0
0.5
1.0
1.5
β_IV

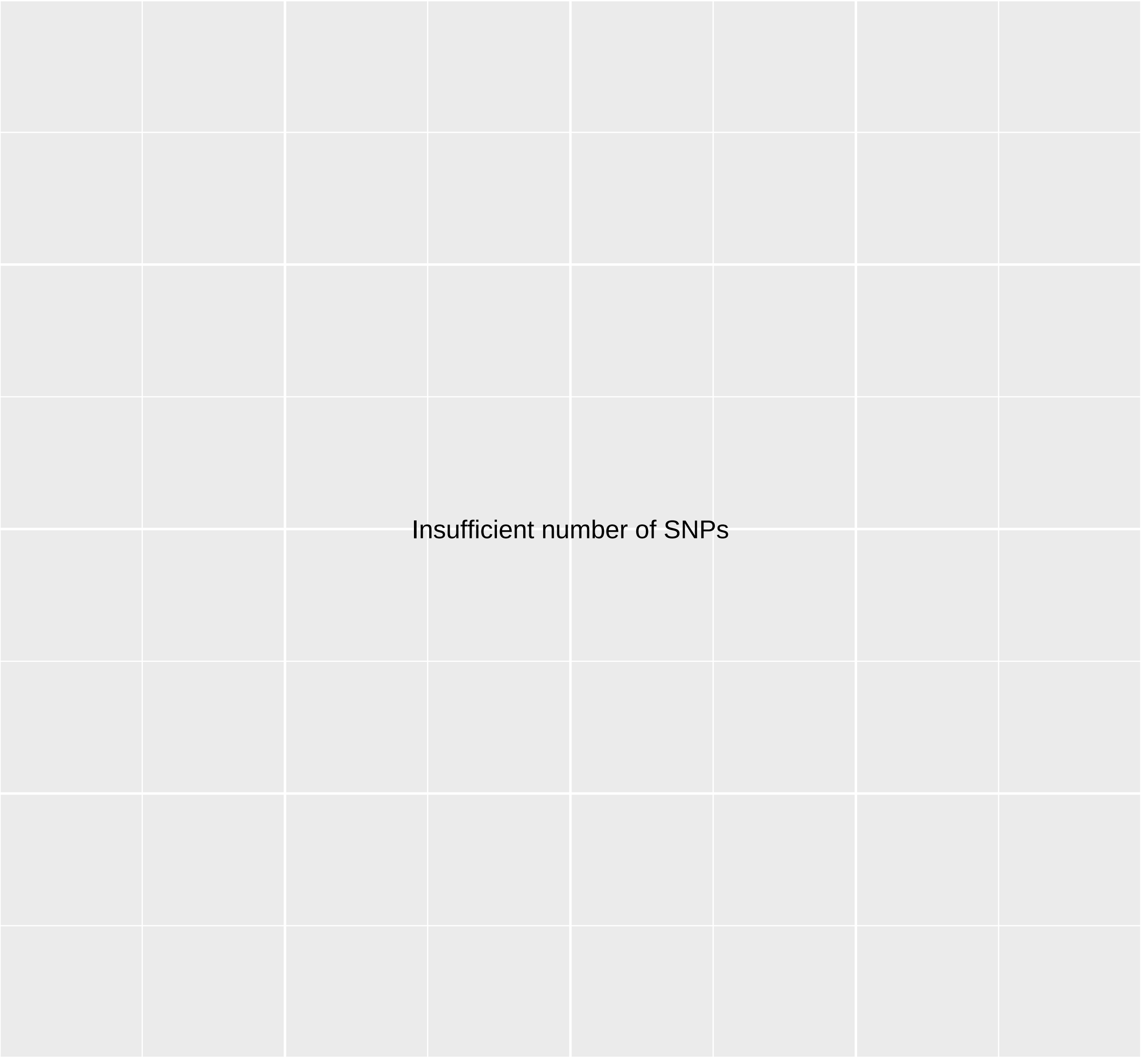
Insufficient number of SNPs

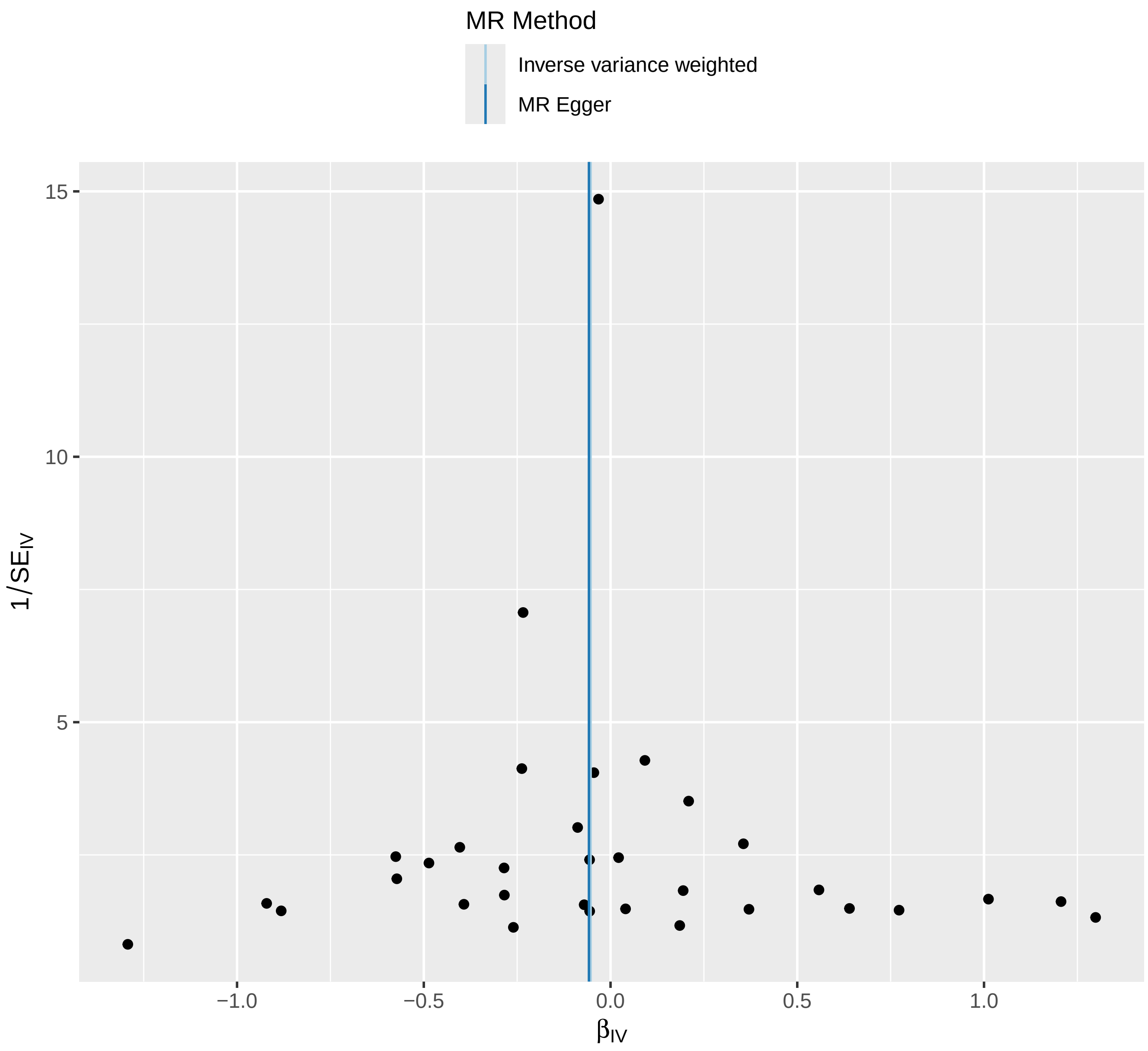
MR Method
Inverse variance weighted
MR Egger
15
10
5
1/SE$_{IV}$
−1.0
−0.5
0.0
0.5
1.0
β$_{IV}$

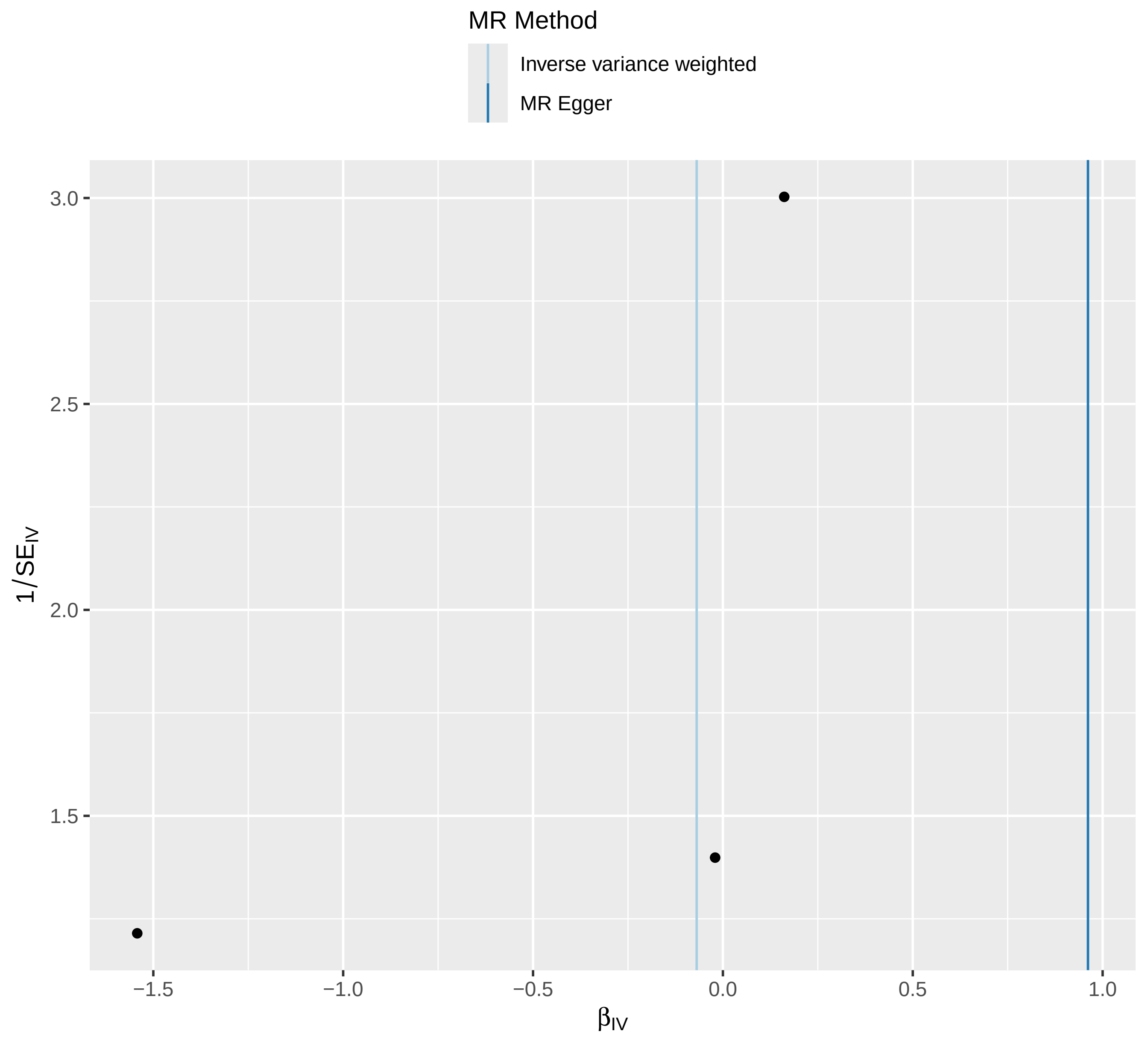
MR Method
Inverse variance weighted
MR Egger
3.0
2.5
2.0
1.5
1/SE_IV
−1.5
−1.0
−0.5
0.0
0.5
1.0
β_IV

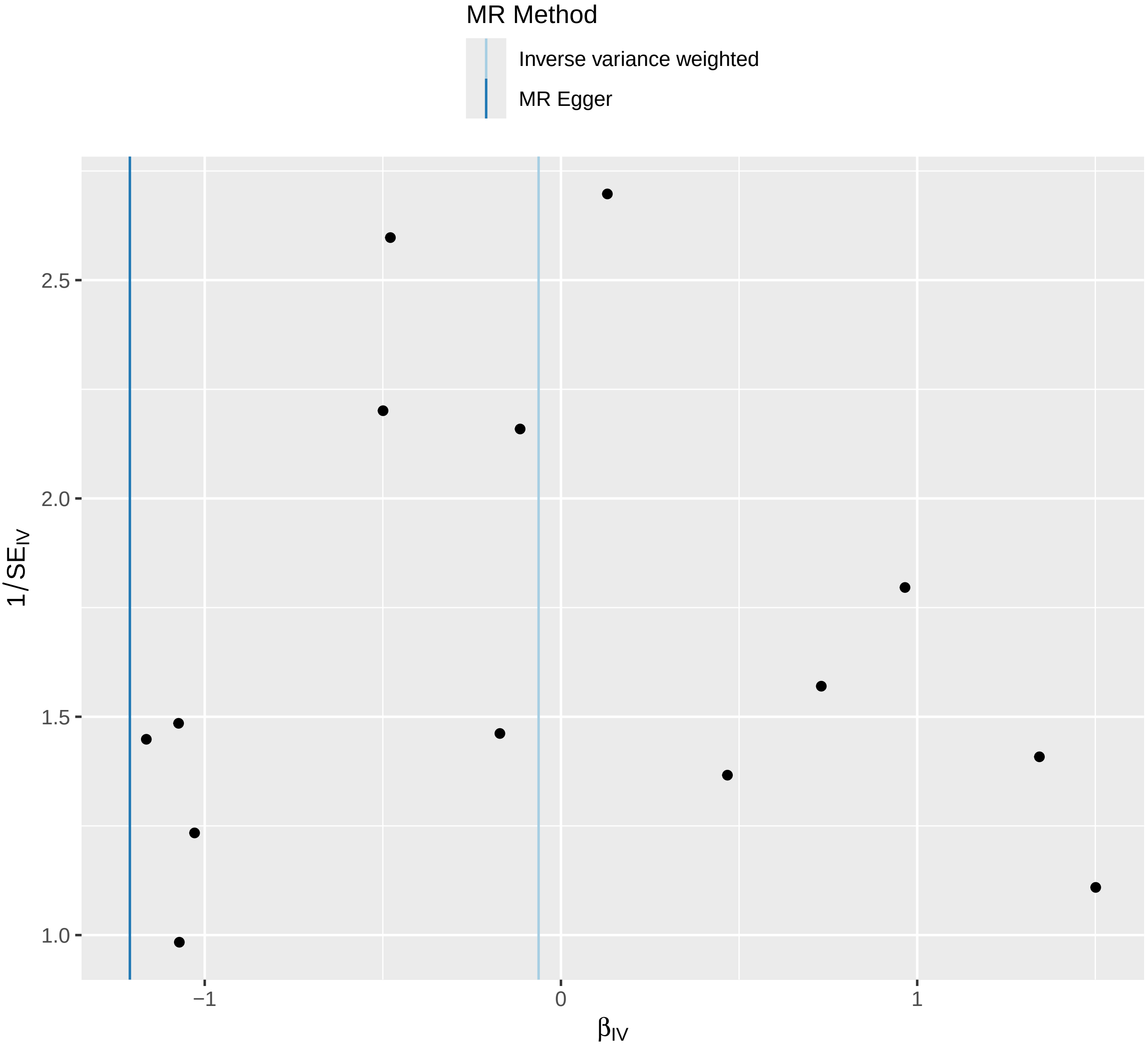
MR Method
Inverse variance weighted
MR Egger
2.5
2.0
1.5
1.0
1/SE_IV
−1
0
1
β_IV

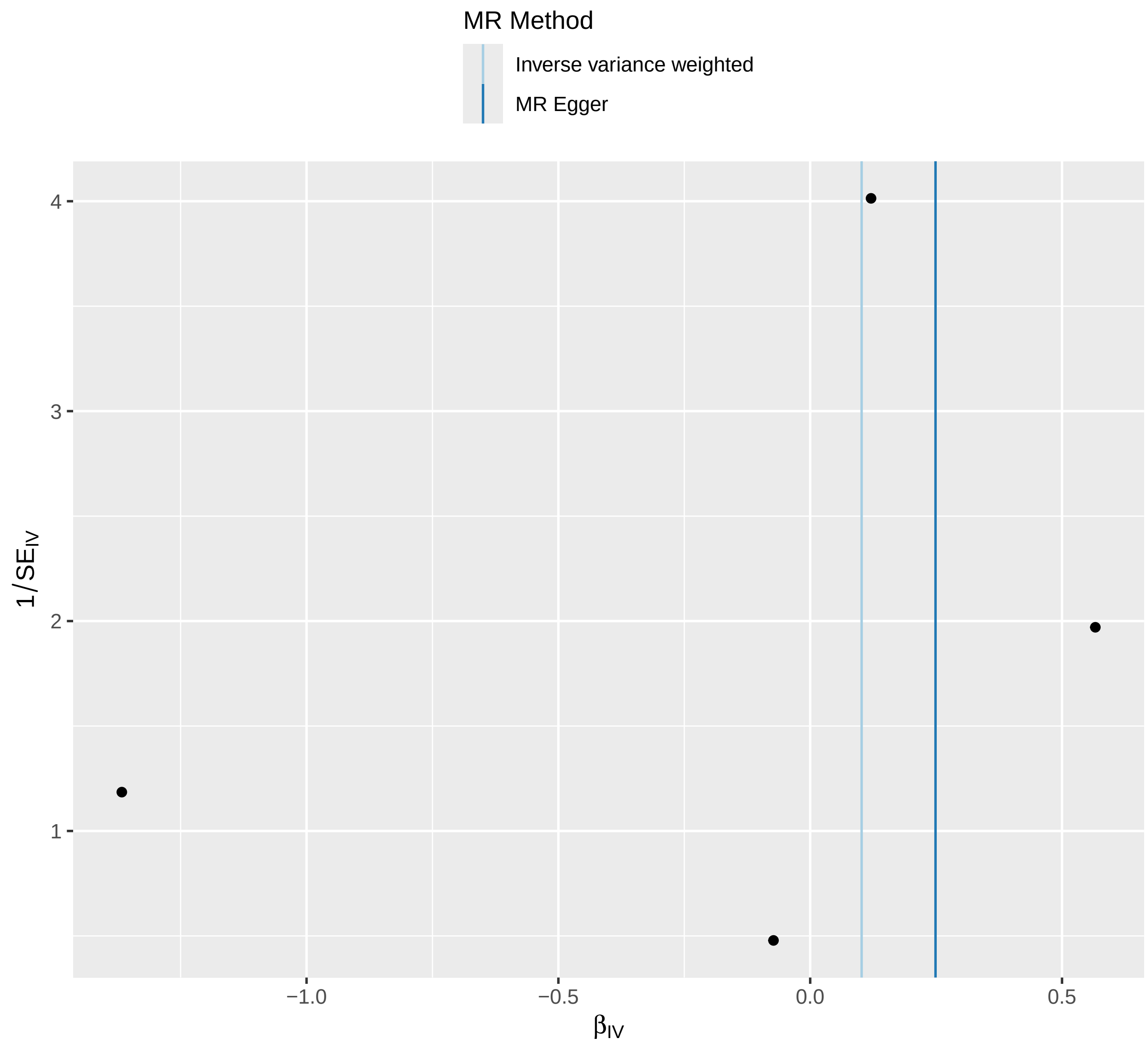
MR Method
Inverse variance weighted
MR Egger
4
3
2
1
1/SE_IV
−1.0
−0.5
0.0
0.5
β_IV

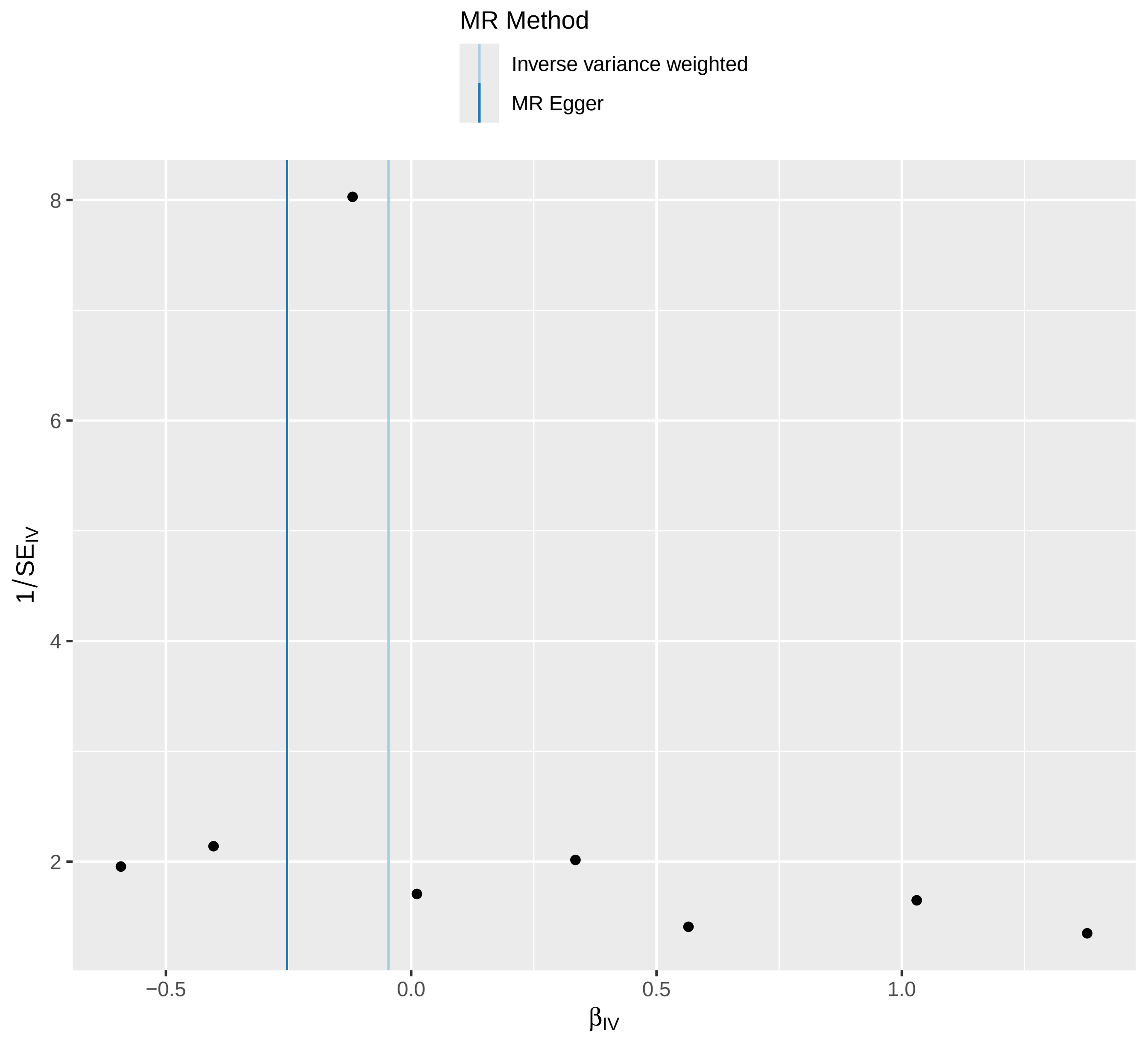

MR Method
Inverse variance weighted
MR Egger
8
6
4
2
1/SE$_{IV}$
−0.5
0.0
0.5
1.0
β$_{IV}$

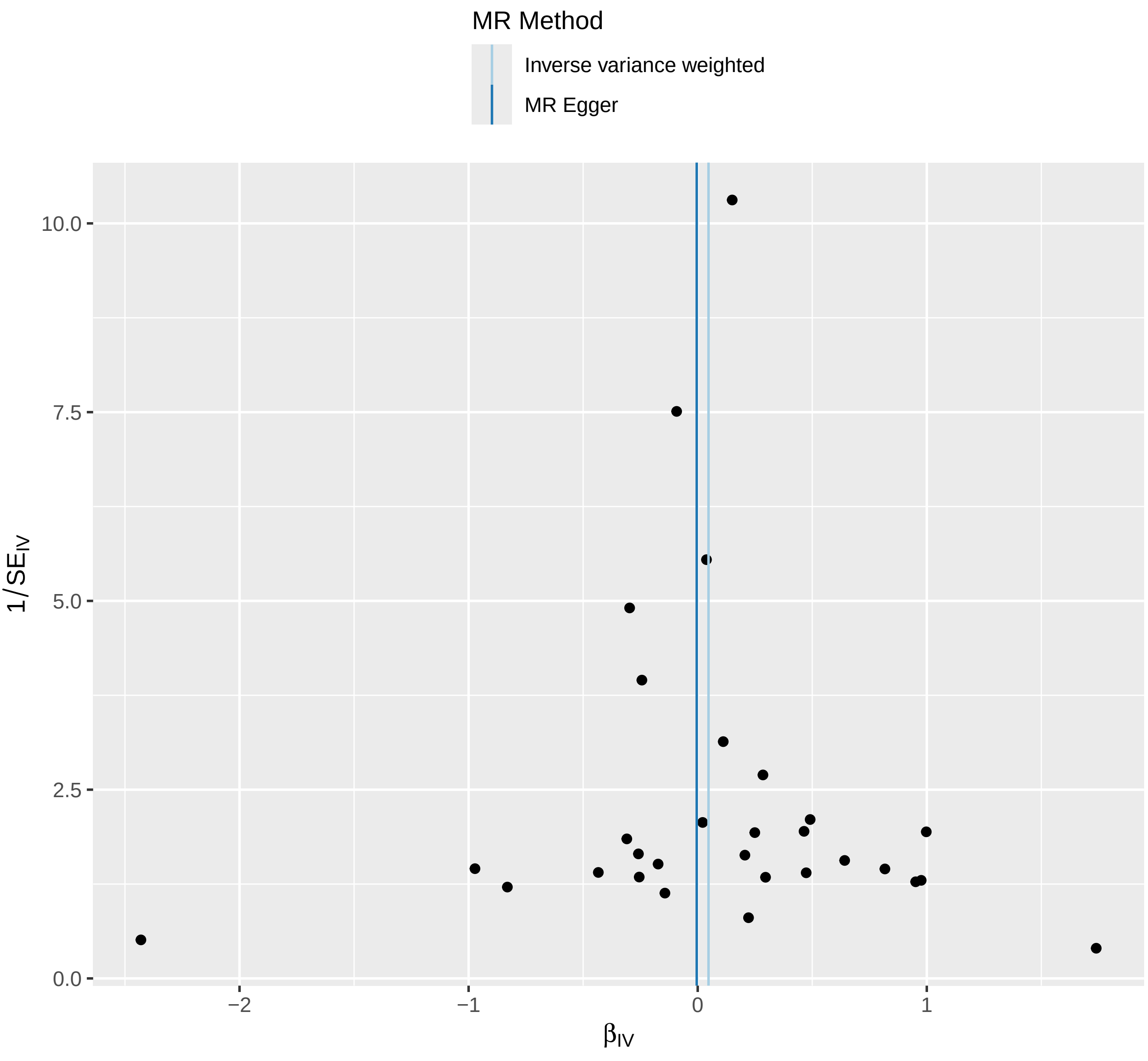
MR Method
Inverse variance weighted
MR Egger
10.0
7.5
5.0
2.5
0.0
1/SE$_{IV}$
−2
−1
0
1
β$_{IV}$

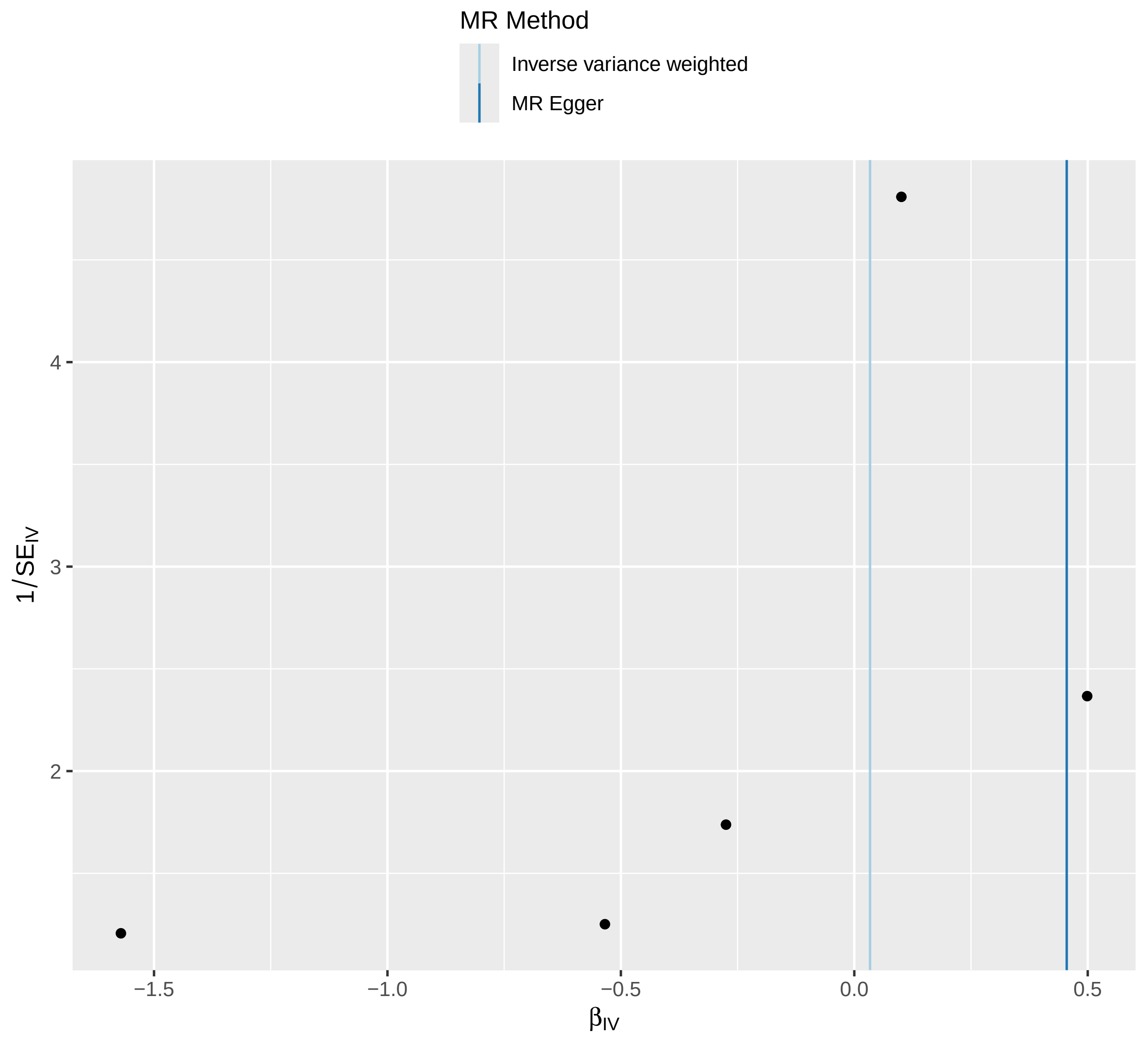

MR Method
Inverse variance weighted
MR Egger
4
3
2
1/SE_IV
−1.5
−1.0
−0.5
0.0
0.5
β_IV

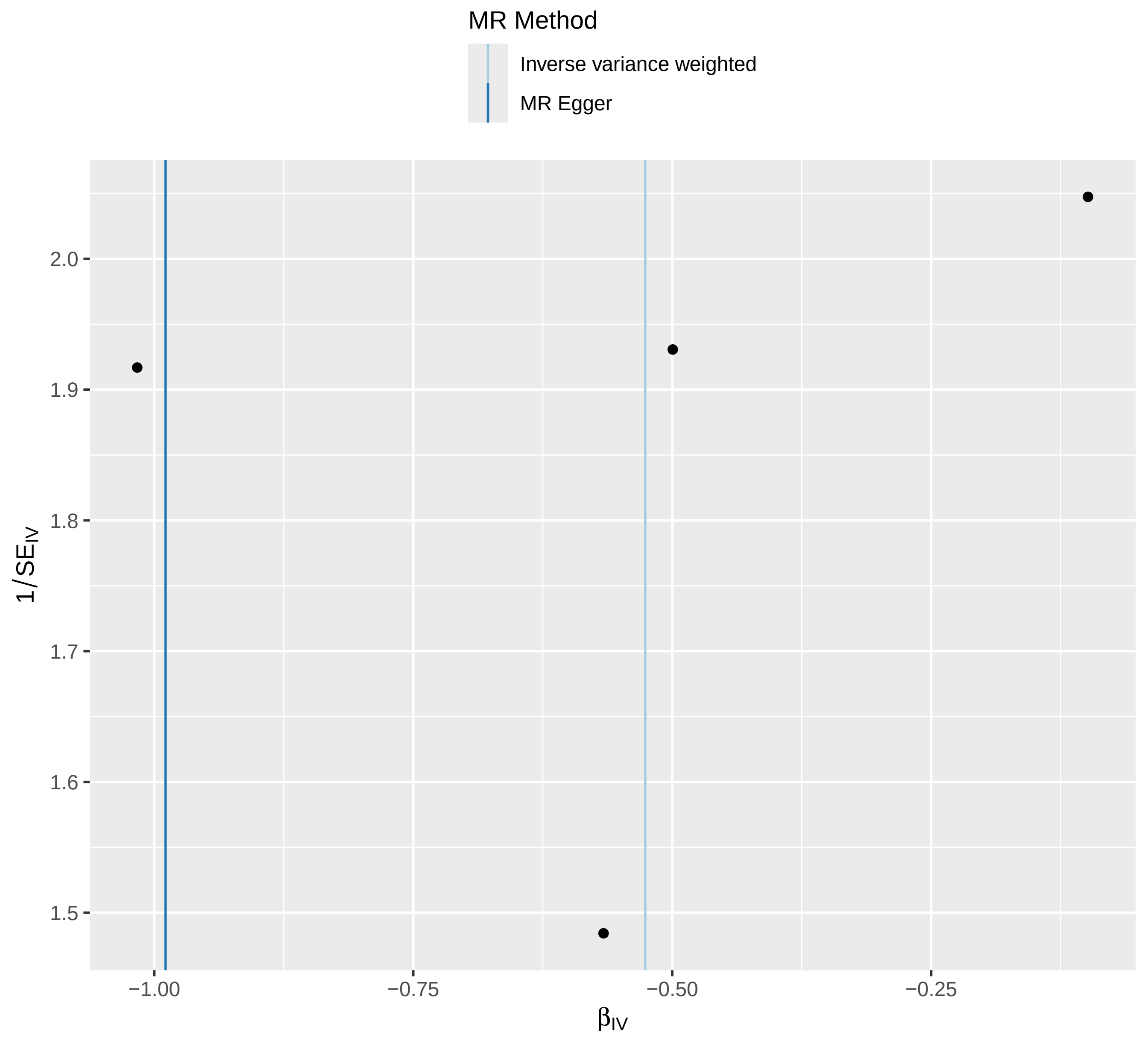
MR Method
Inverse variance weighted
MR Egger
2.0
1.9
1.8
1.7
1.6
1.5
1/SE_IV
−1.00
−0.75
−0.50
−0.25
β_IV

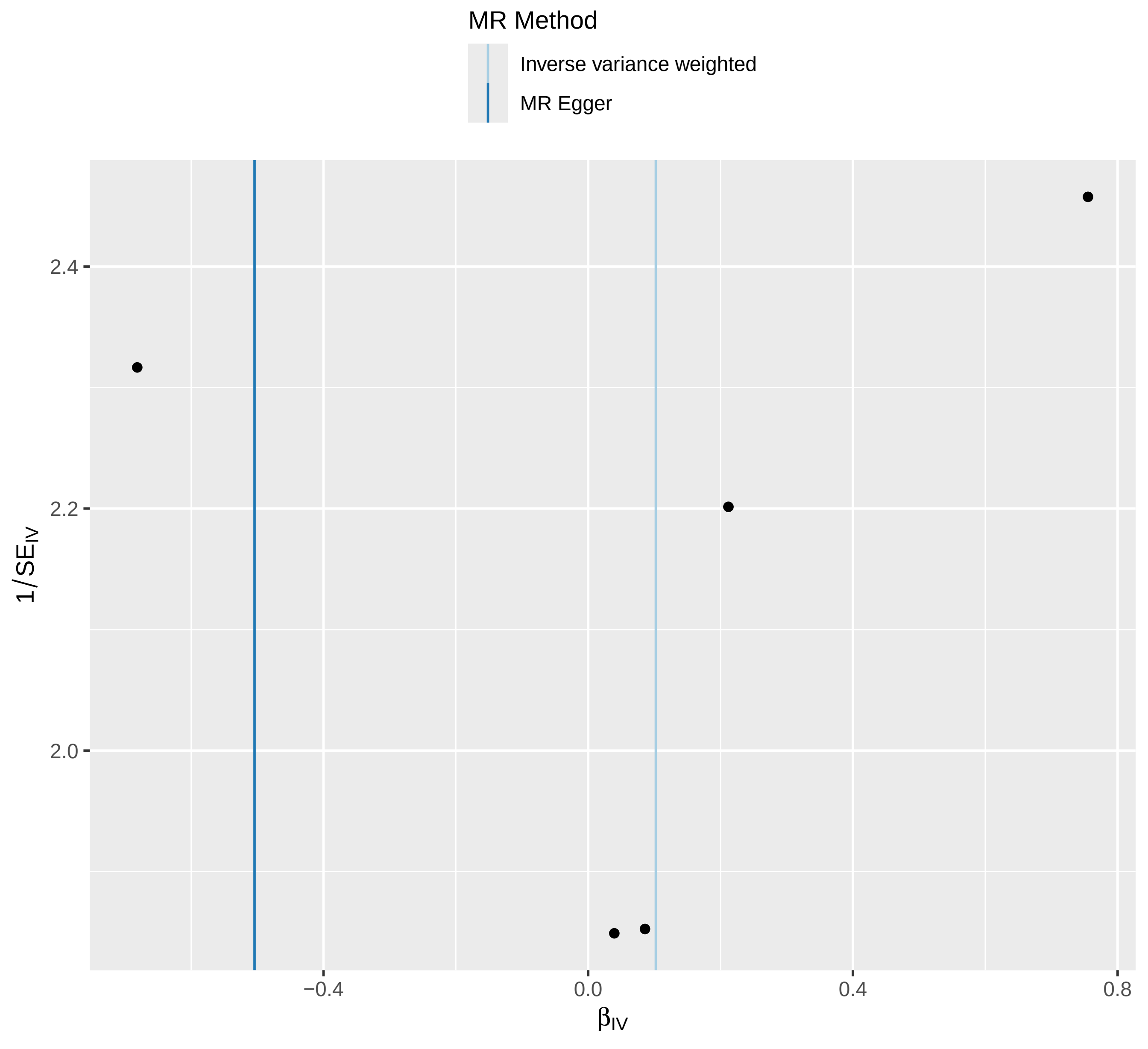

MR Method
Inverse variance weighted
MR Egger
2.4
2.2
2.0
$1/SE_{IV}$
−0.4
0.0
0.4
0.8
β_{IV}

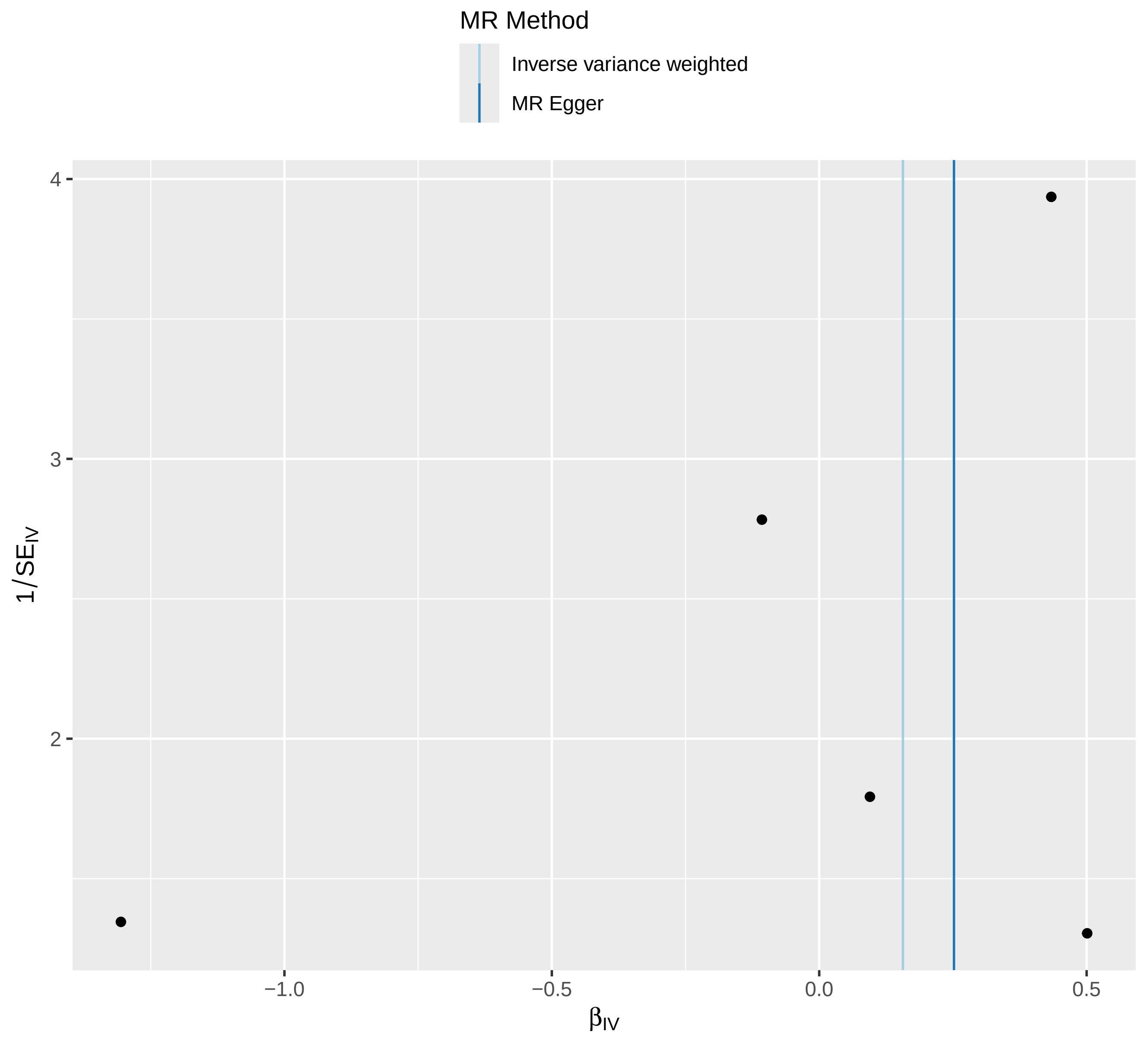
MR Method
Inverse variance weighted
MR Egger
4
3
2
1/SE_IV
−1.0
−0.5
0.0
0.5
β_IV

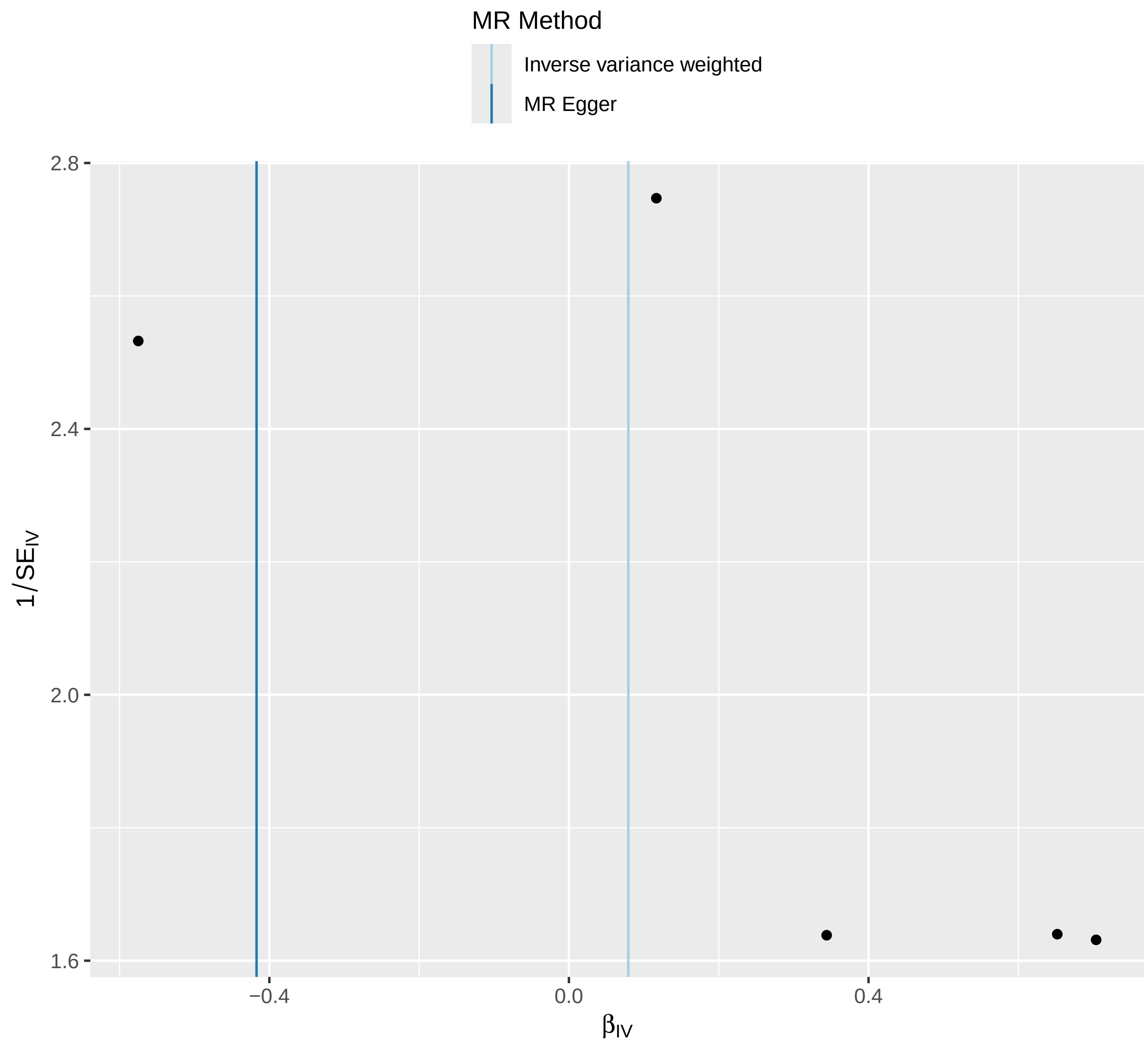
MR Method
Inverse variance weighted
MR Egger
2.8
2.4
2.0
1.6
1/SE_IV
−0.4
0.0
0.4
β_IV

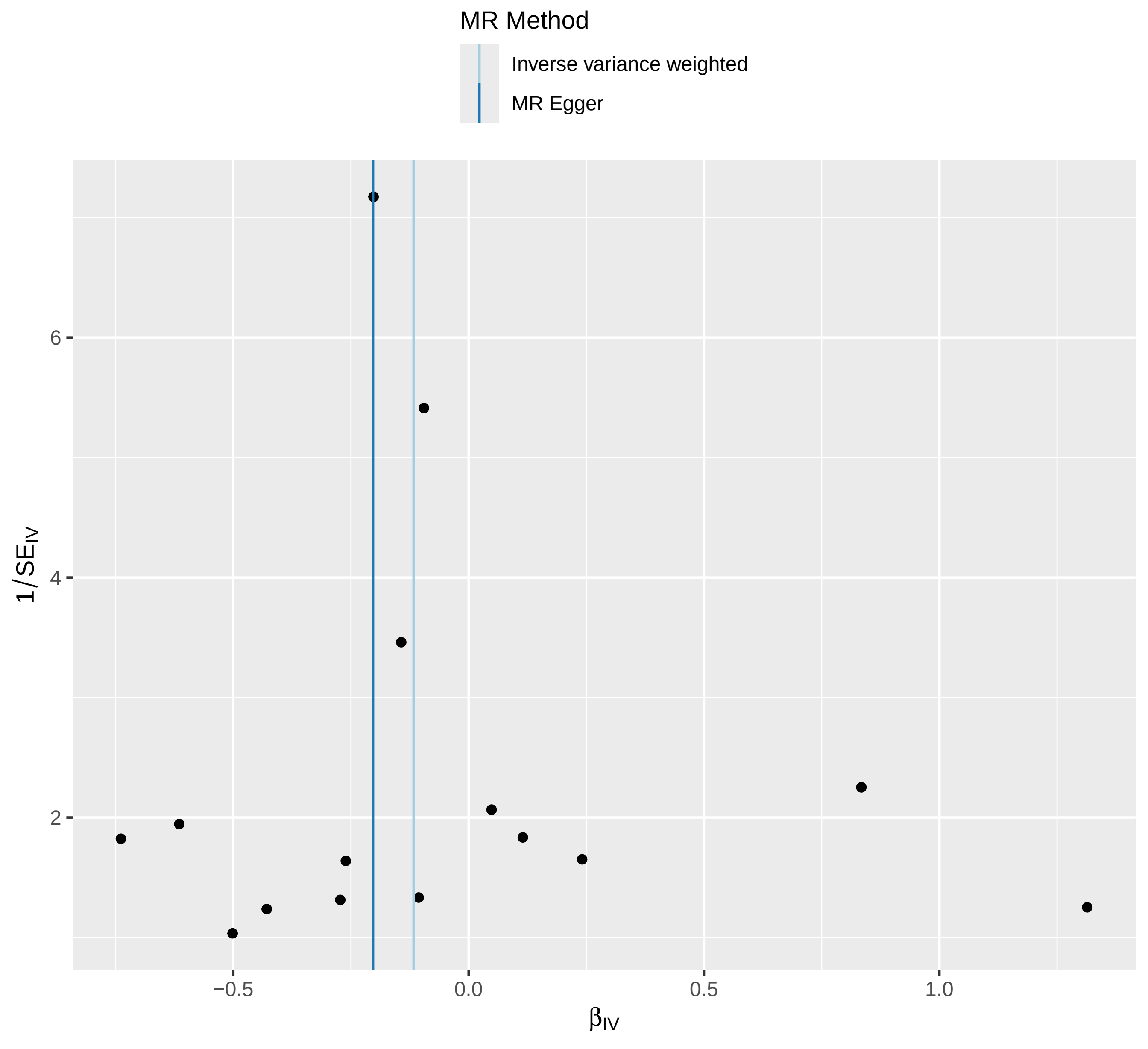

MR Method
Inverse variance weighted
MR Egger
6
4
2
1/SE$_{IV}$
−0.5
0.0
0.5
1.0
β$_{IV}$

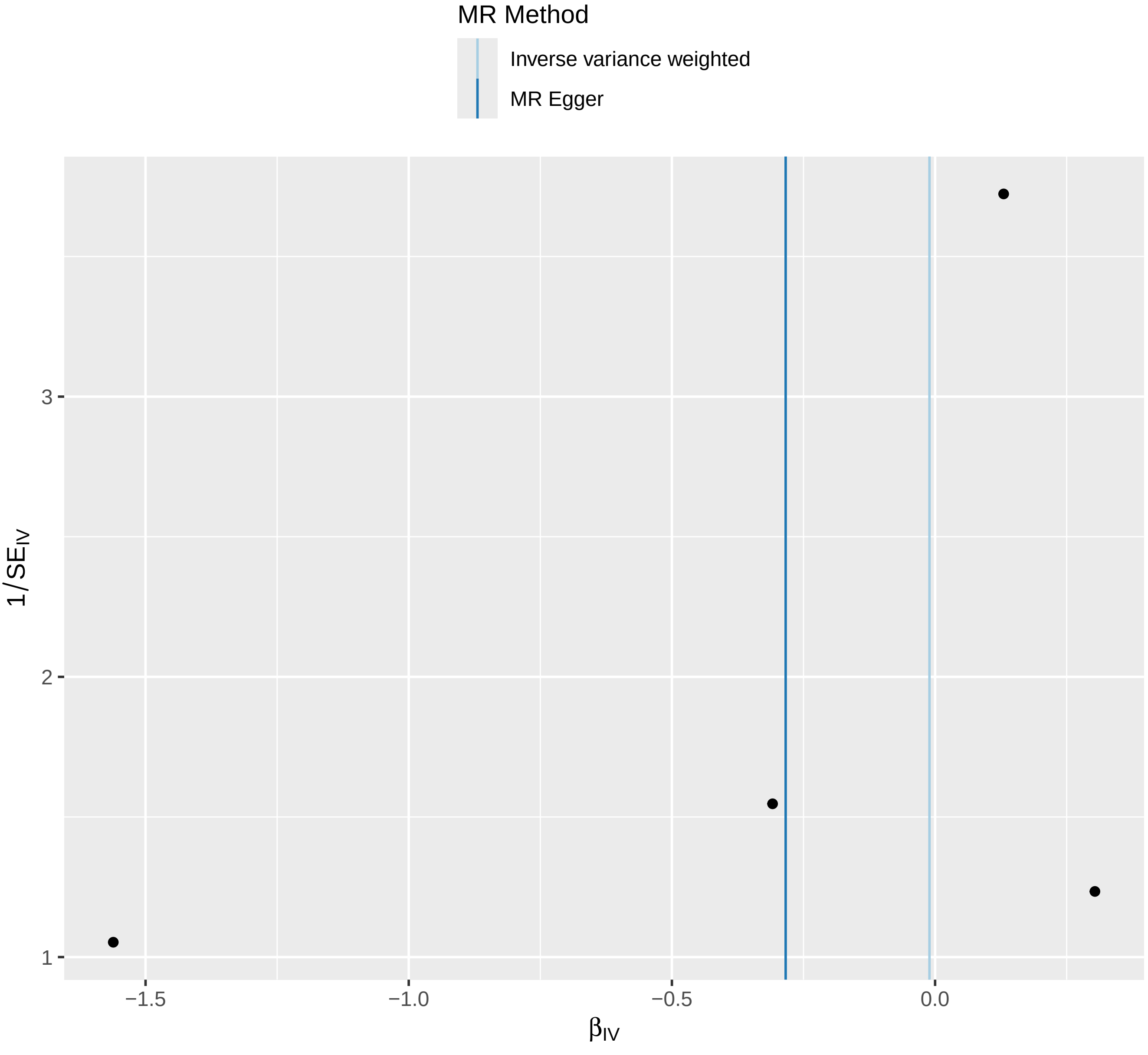

MR Method
Inverse variance weighted
MR Egger
1/SE_IV
3
2
1
−1.5
−1.0
−0.5
0.0
β_IV

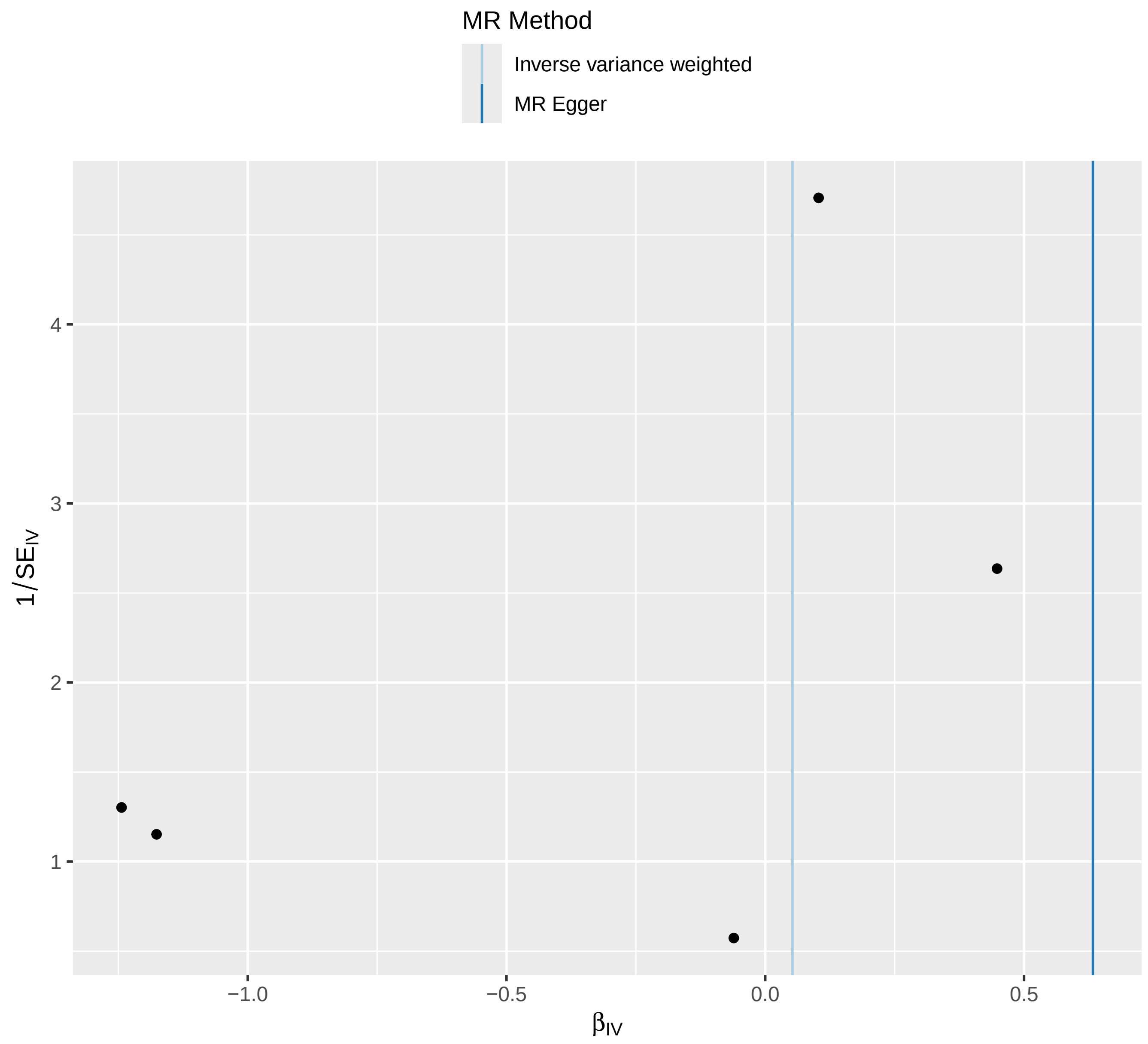

MR Method
Inverse variance weighted
MR Egger
4
3
2
1
1/SE_IV
−1.0
−0.5
0.0
0.5
β_IV

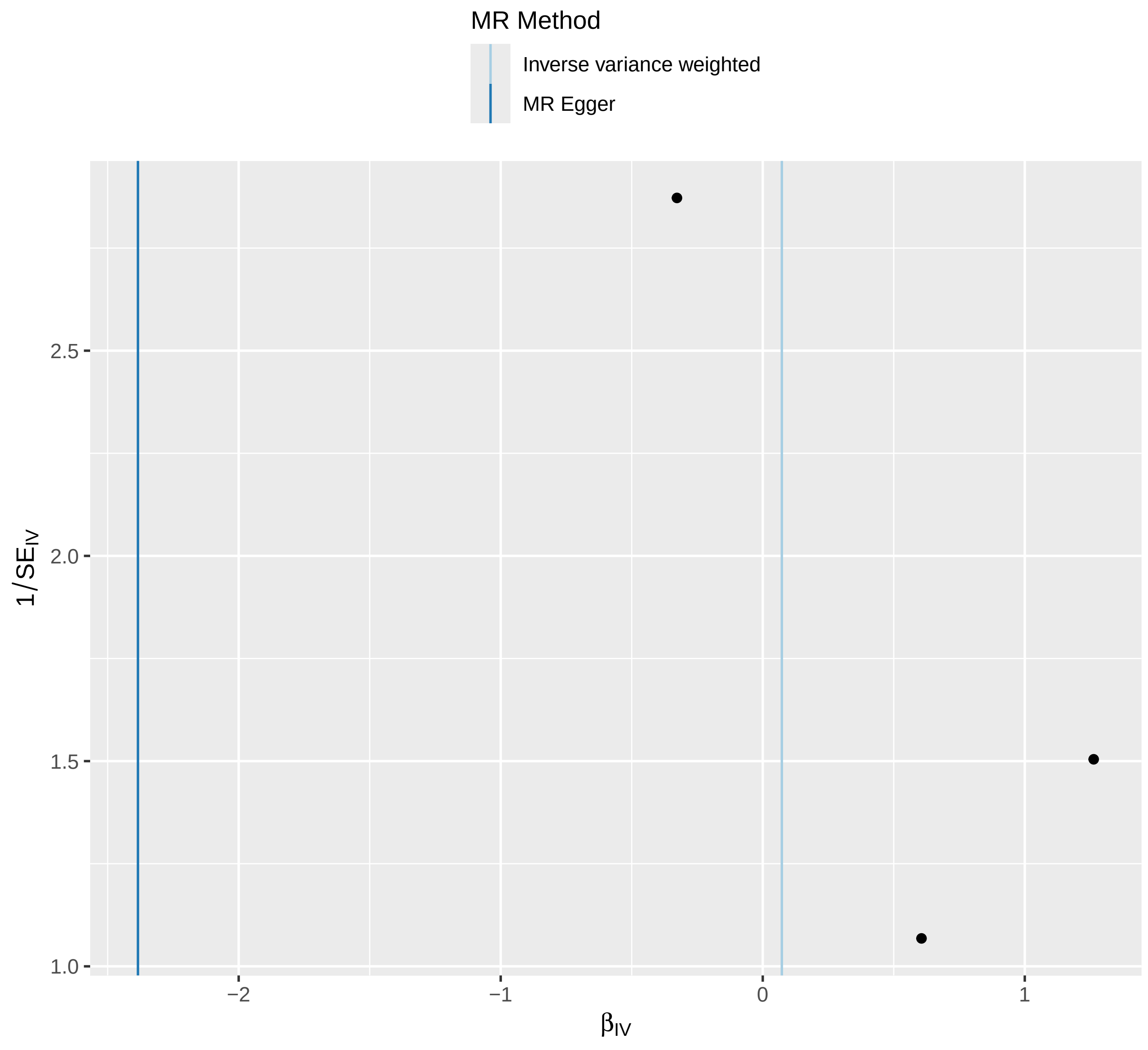
MR Method
Inverse variance weighted
MR Egger
1/SE$_{IV}$
2.5
2.0
1.5
1.0
−2
−1
0
1
β$_{IV}$

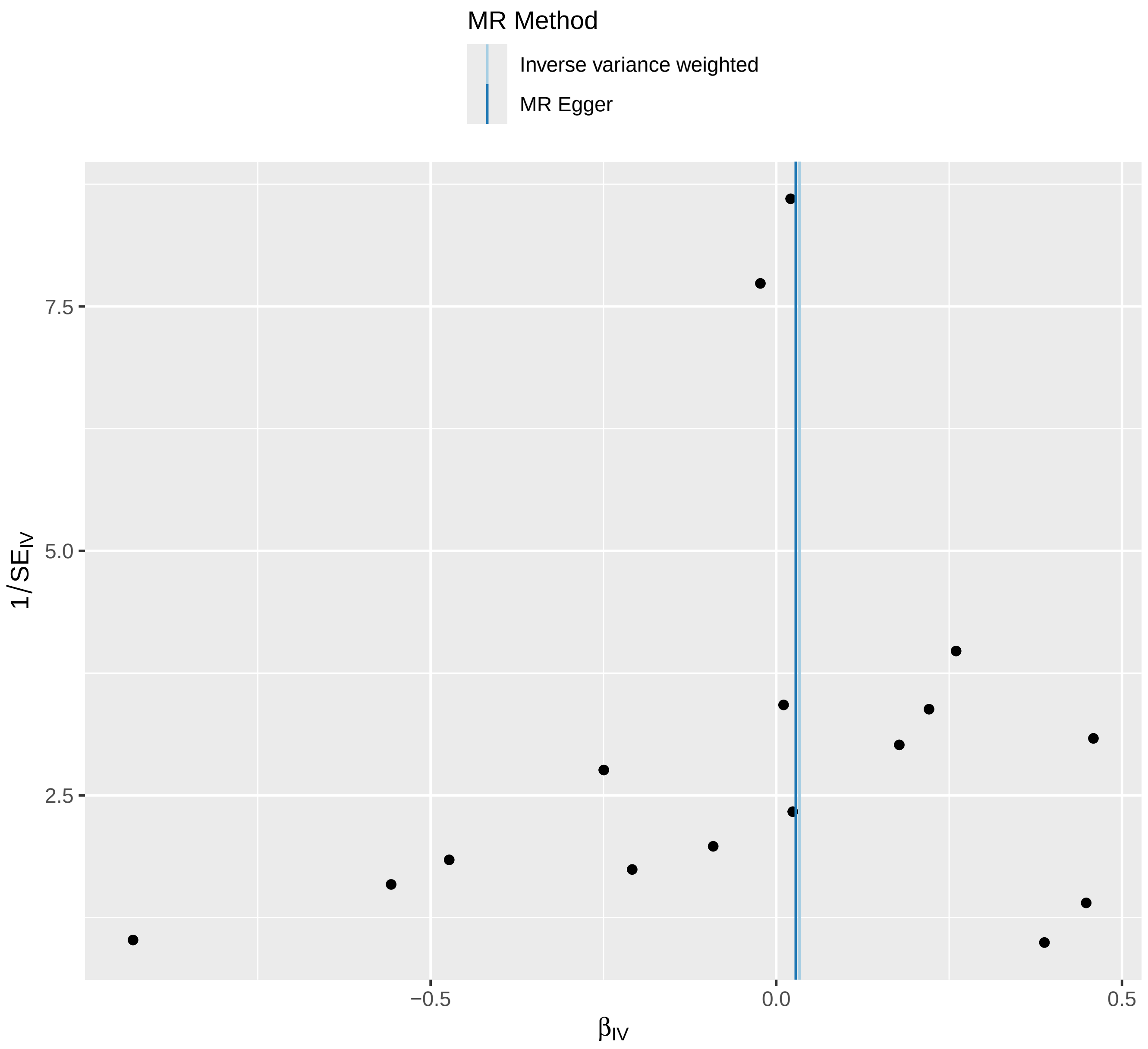
MR Method
Inverse variance weighted
MR Egger
7.5
5.0
2.5
1/SE_IV
−0.5
0.0
0.5
β_IV

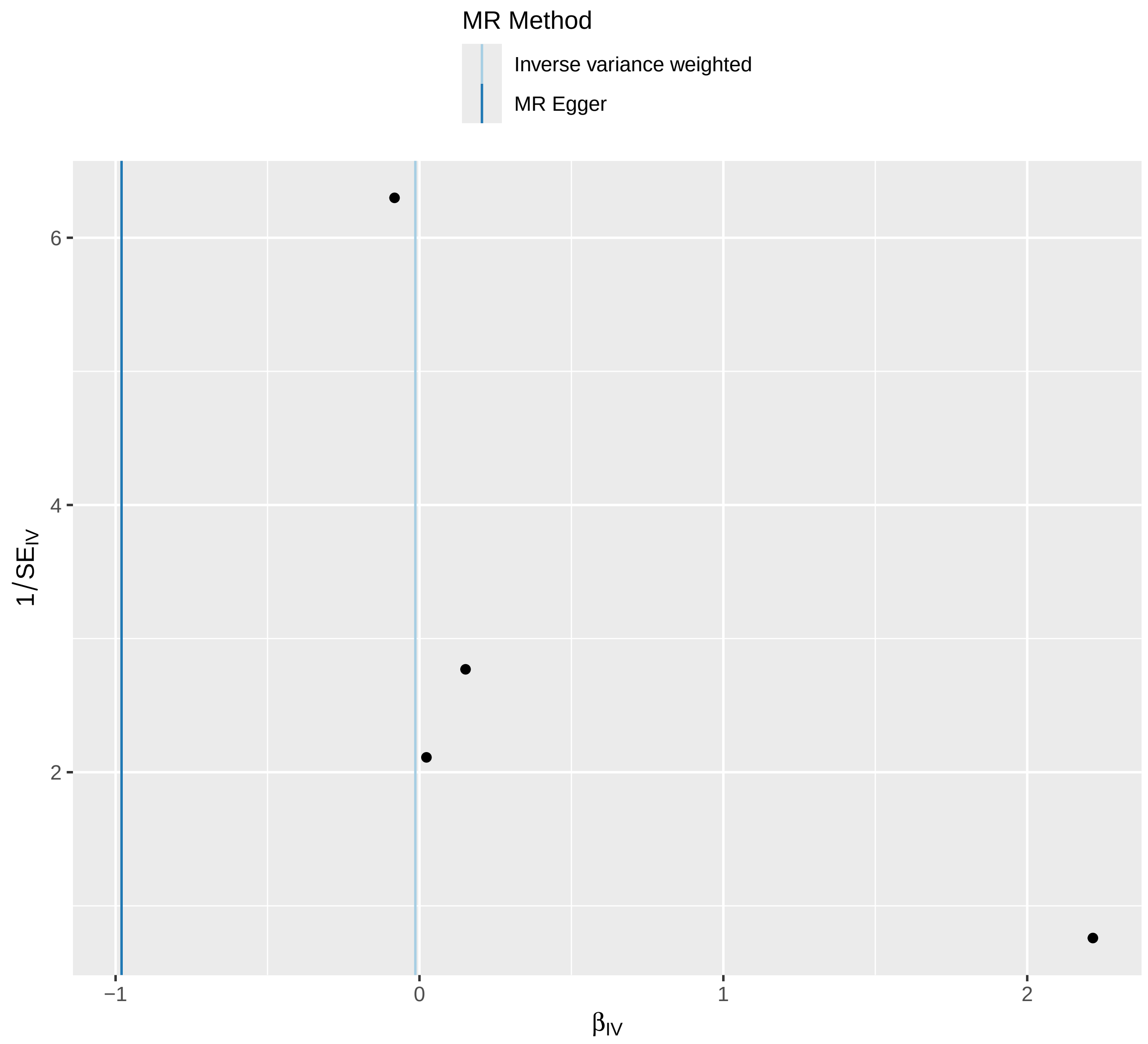
MR Method
Inverse variance weighted
MR Egger
6
4
2
1/SE_IV
−1
0
1
2
β_IV

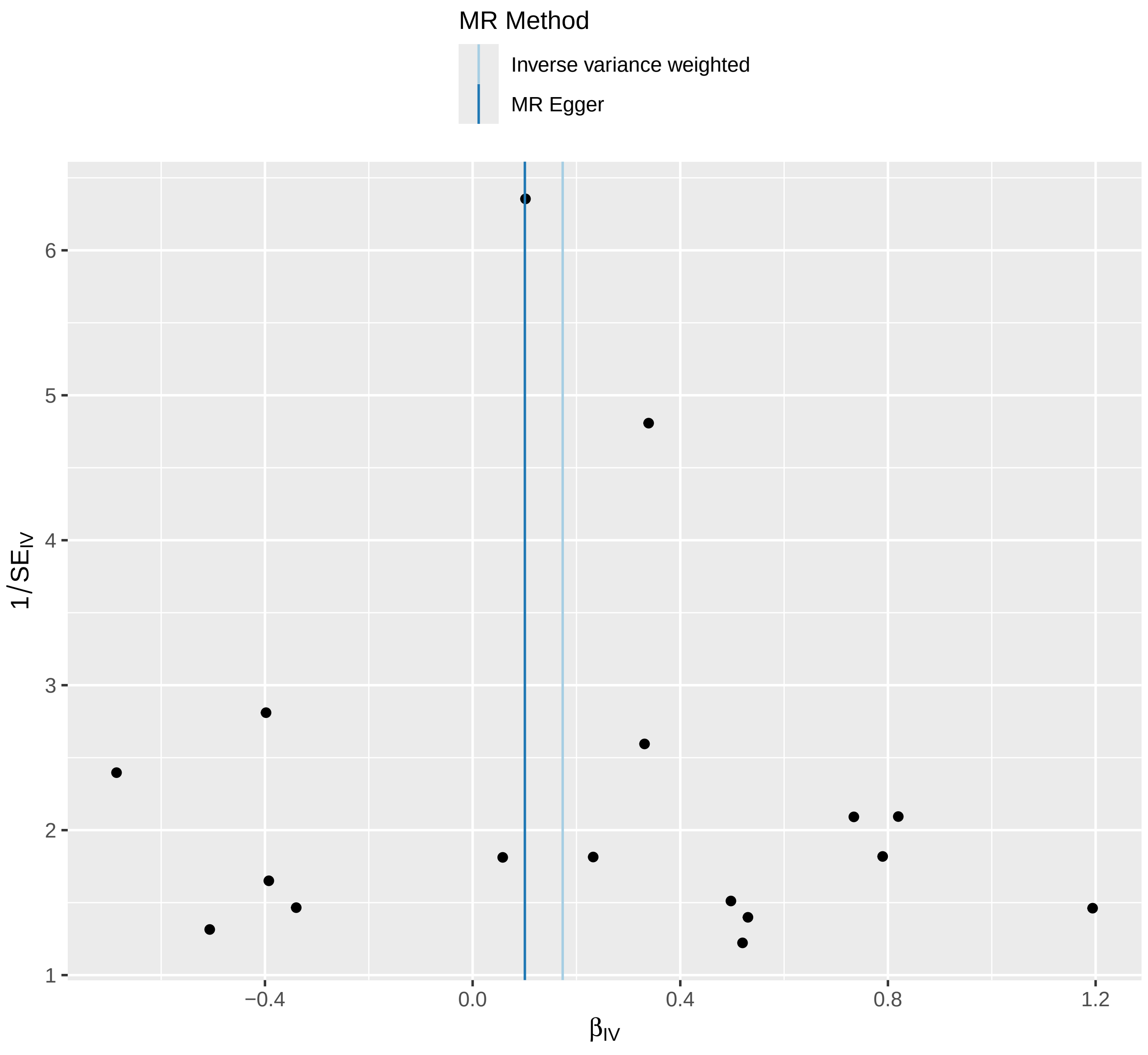

MR Method
Inverse variance weighted
MR Egger
6
5
4
3
2
1
1/SE_IV
−0.4
0.0
0.4
0.8
1.2
β_IV

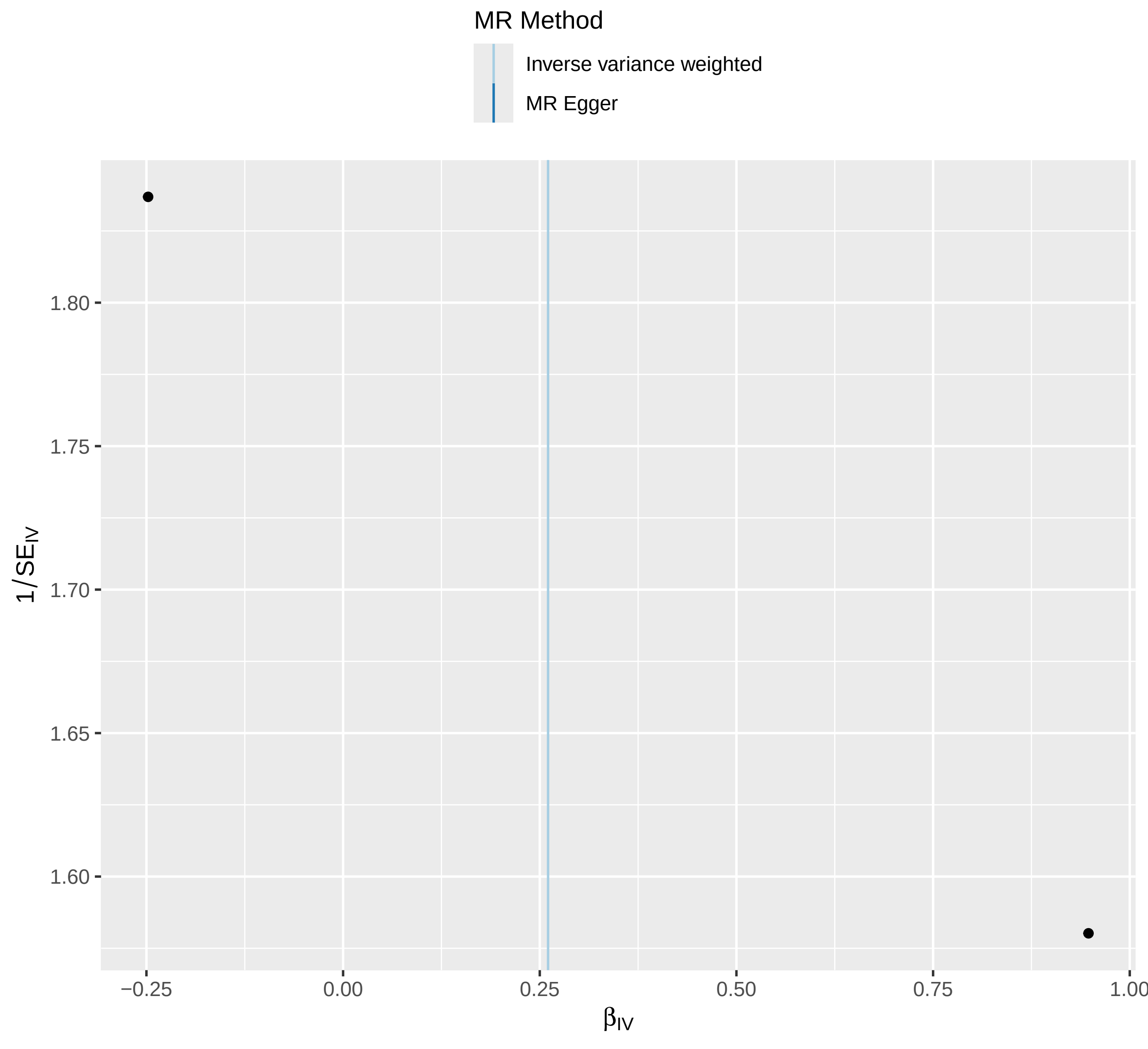

MR Method
Inverse variance weighted
MR Egger
1.80
1.75
1.70
1.65
1.60
1/SE_IV
−0.25
0.00
0.25
0.50
0.75
1.00
β_IV

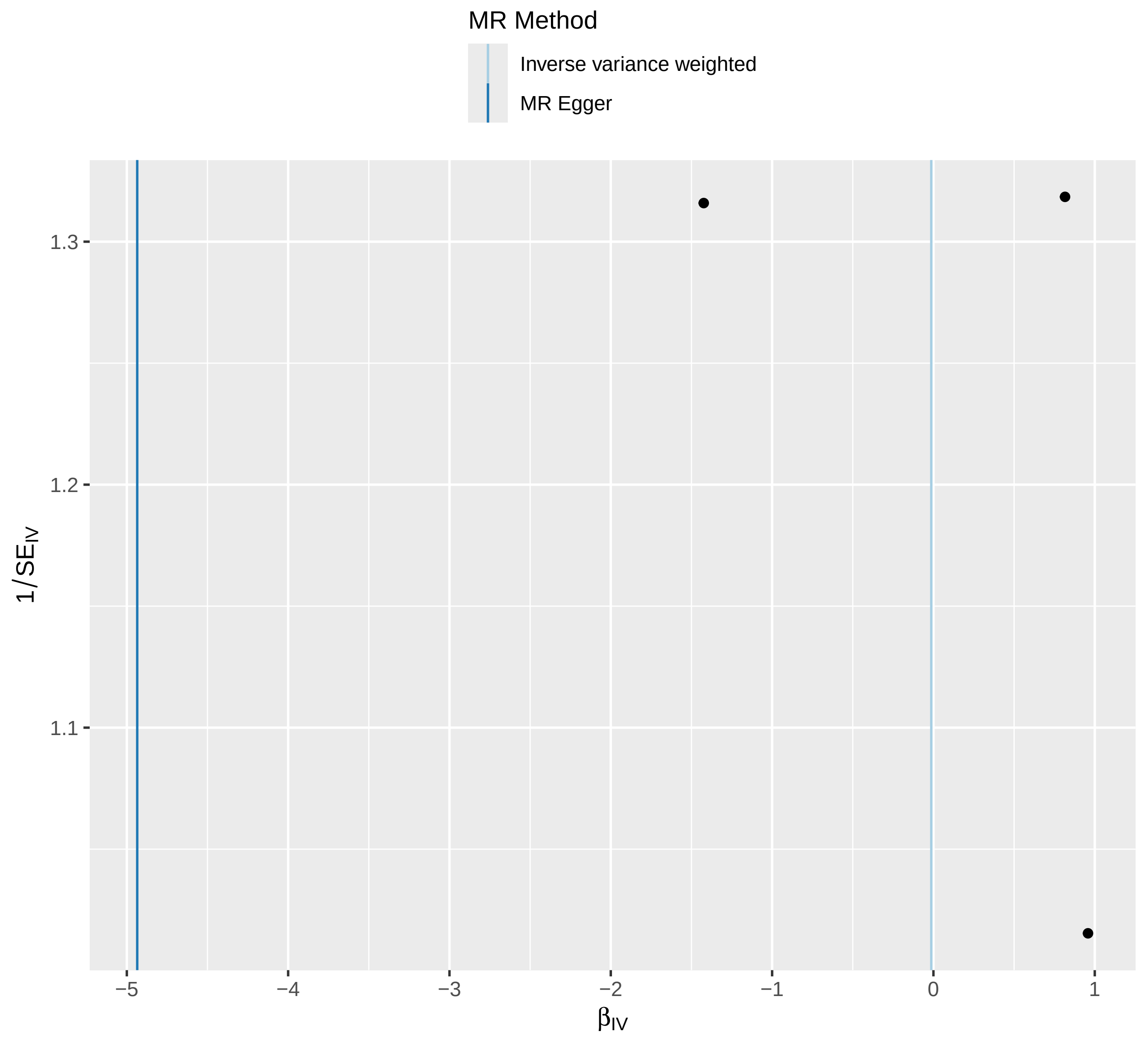

MR Method
Inverse variance weighted
MR Egger
1.3
1.2
1.1
1/SE_IV
−5
−4
−3
−2
−1
0
1
β_IV

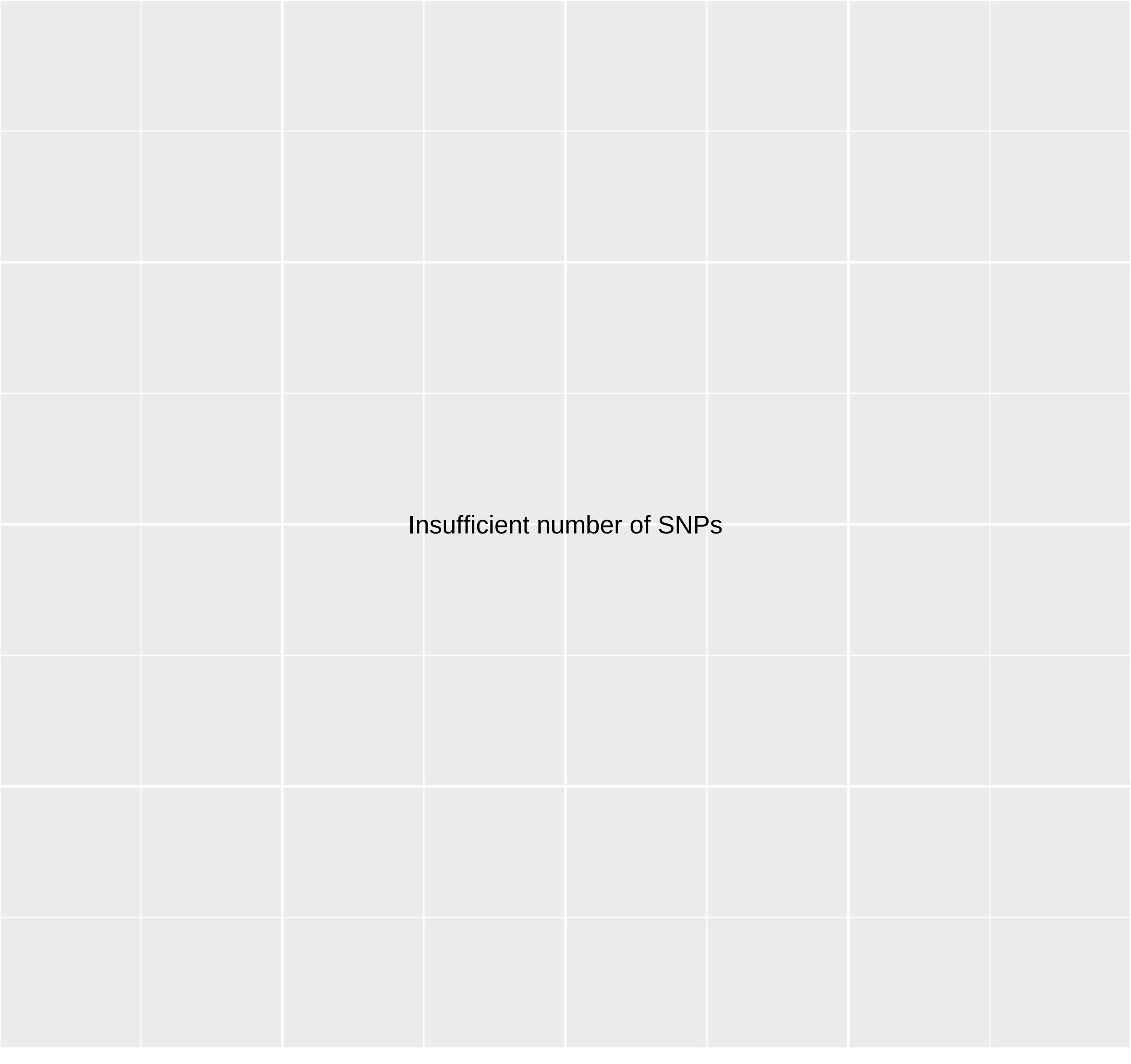
Insufficient number of SNPs

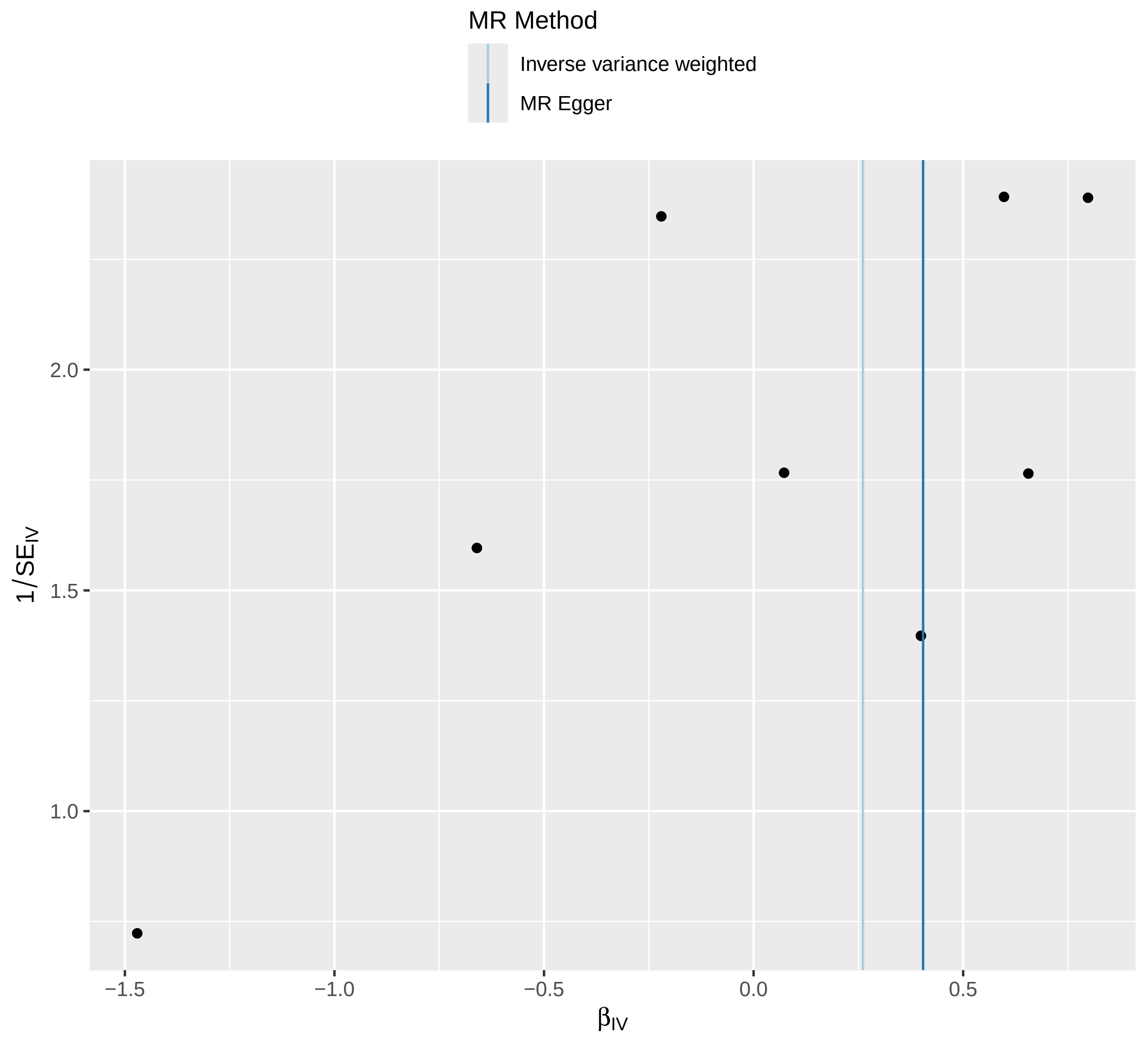

MR Method
Inverse variance weighted
MR Egger
2.0
1.5
1.0
1/SE_IV
−1.5
−1.0
−0.5
0.0
0.5
β_IV

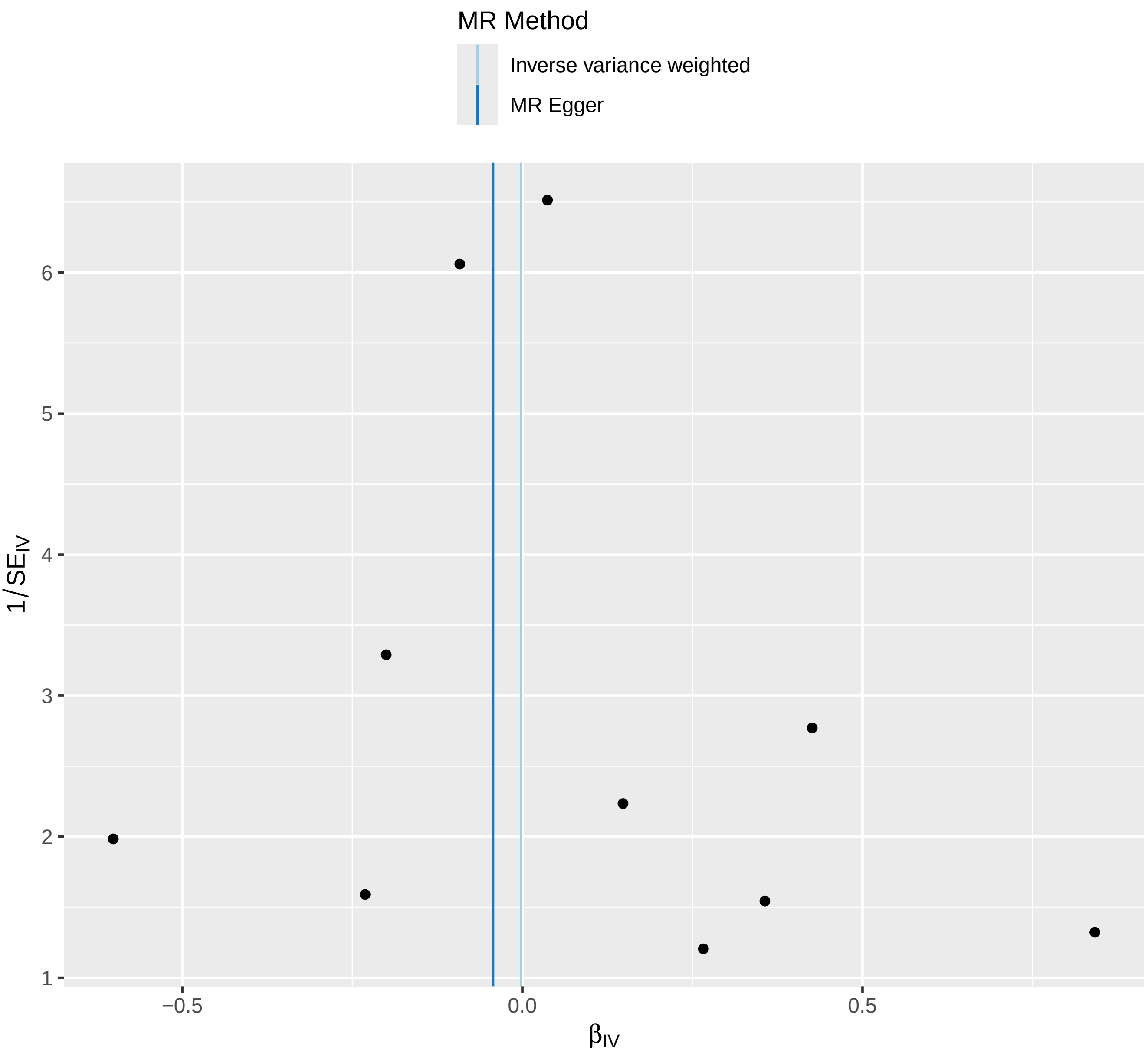
MR Method
Inverse variance weighted
MR Egger
6
5
4
3
2
1
1/SE_IV
−0.5
0.0
0.5
β_IV

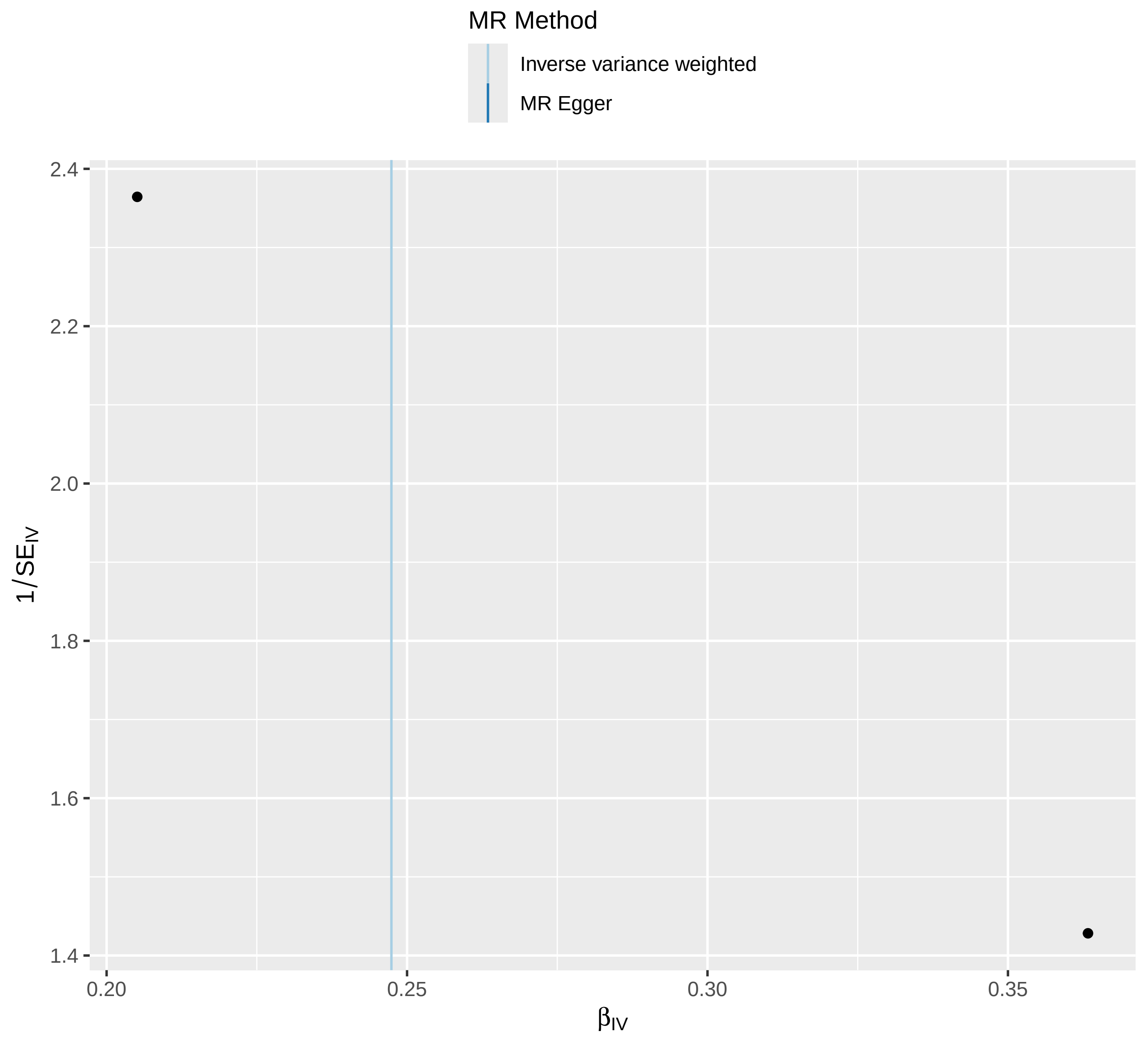
MR Method
Inverse variance weighted
MR Egger
2.4
2.2
2.0
1.8
1.6
1.4
1/SE_IV
0.20
0.25
0.30
0.35
β_IV

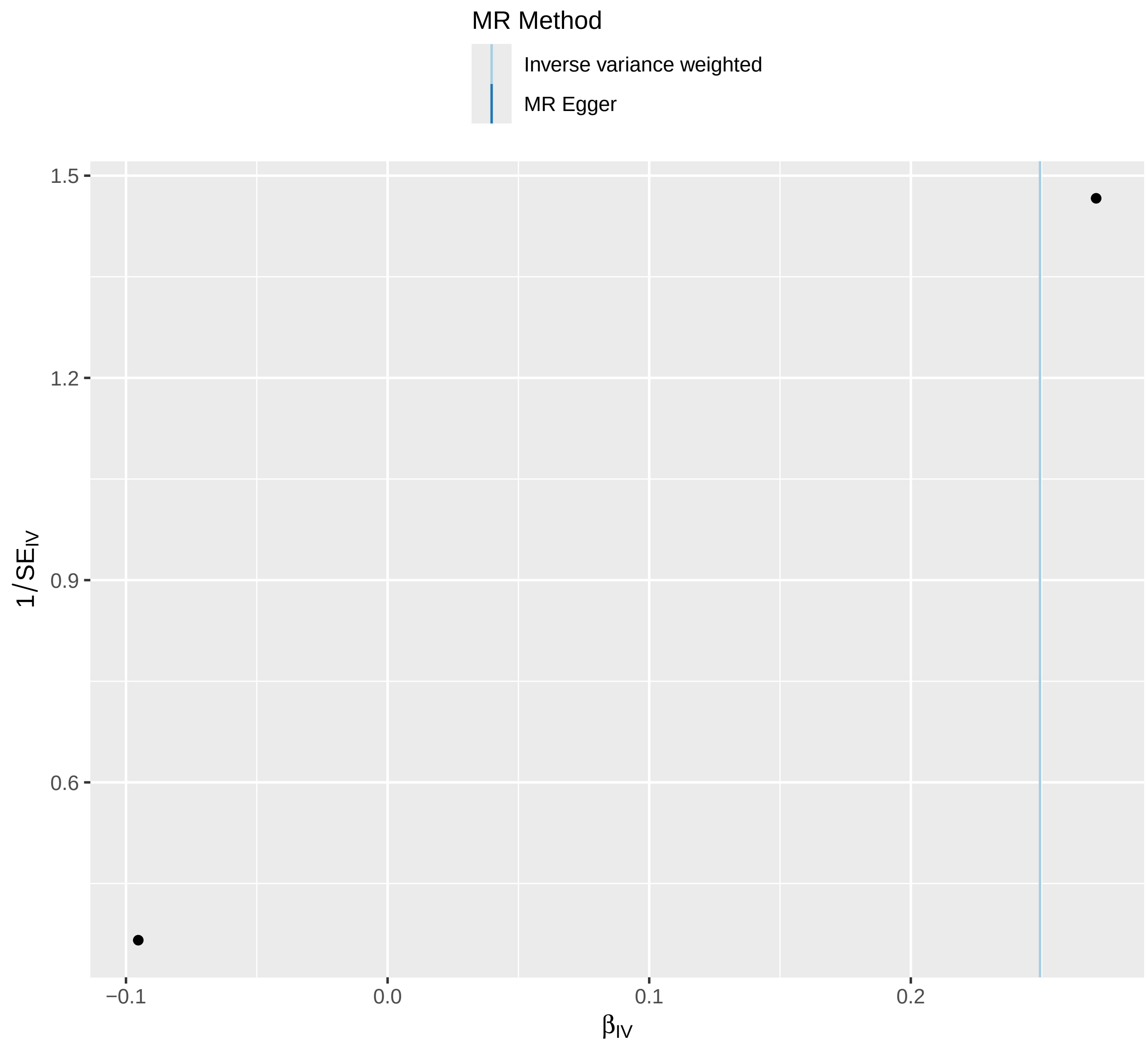

MR Method
Inverse variance weighted
MR Egger
1.5
1.2
0.9
0.6
1/SE_IV
−0.1
0.0
0.1
0.2
β_IV

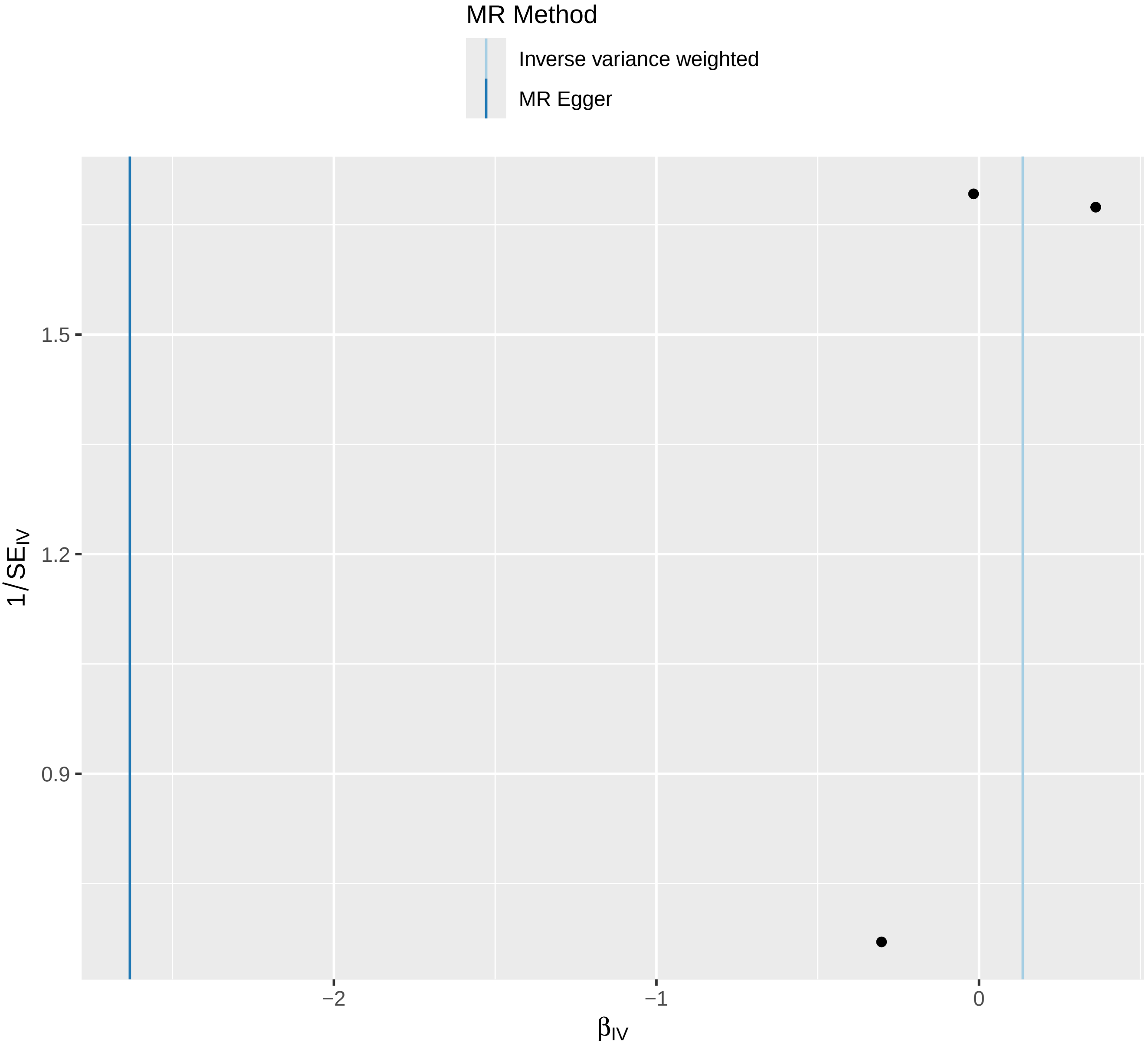
MR Method
Inverse variance weighted
MR Egger
1.5
1.2
0.9
1/SE$_{IV}$
−2
−1
0
β$_{IV}$

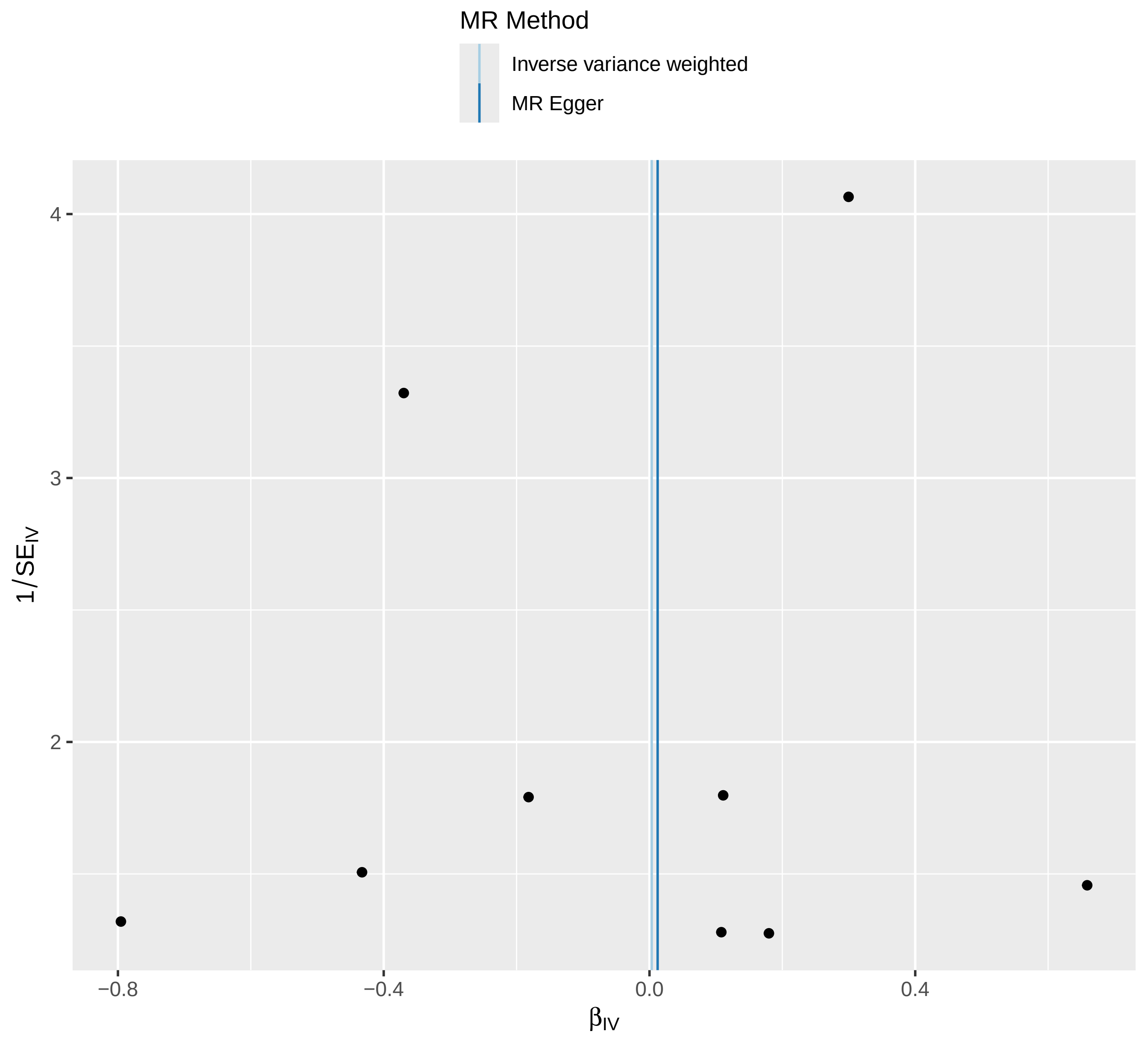
MR Method
Inverse variance weighted
MR Egger
4
3
2
1/SE$_{IV}$
−0.8
−0.4
0.0
0.4
β$_{IV}$

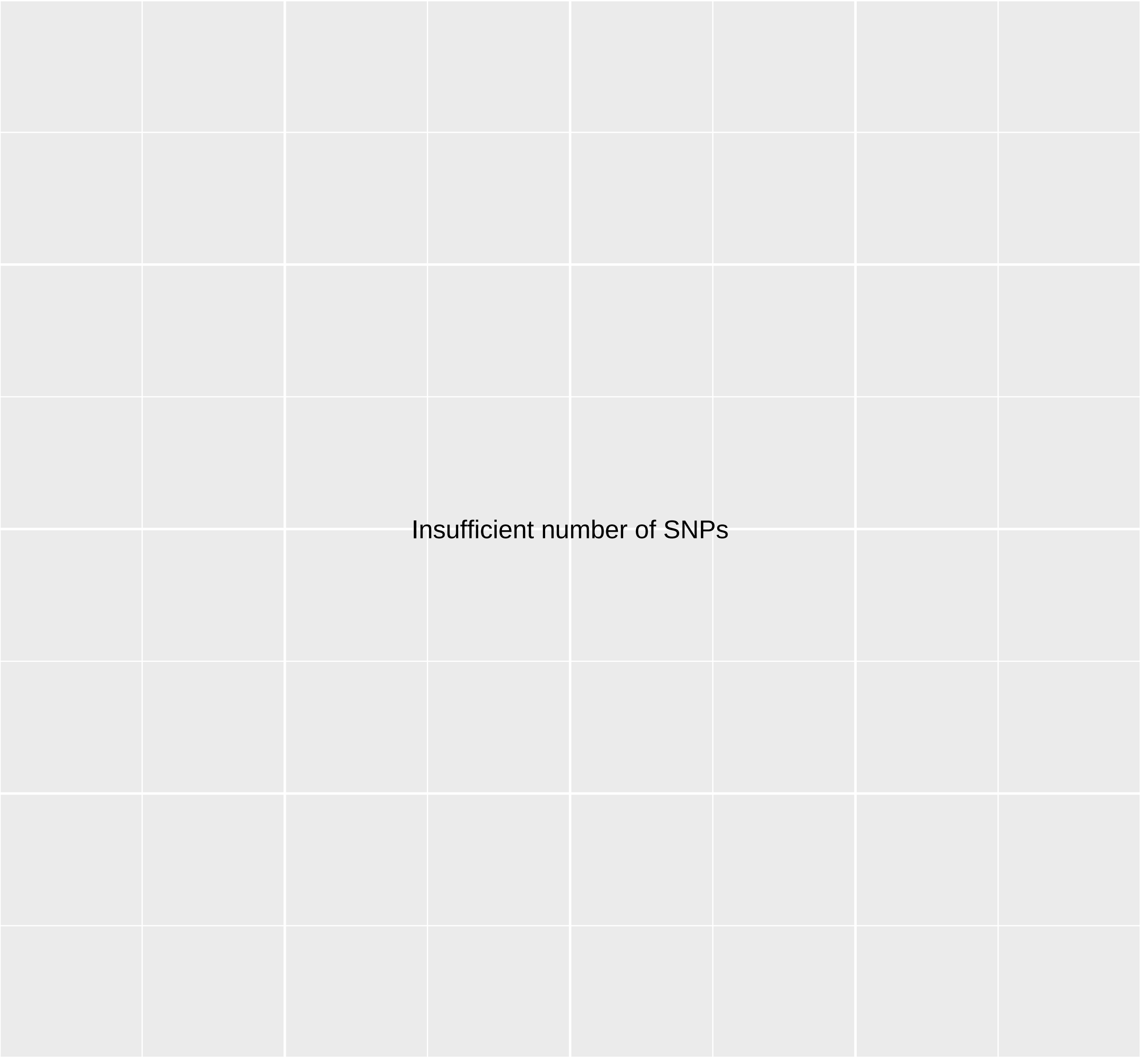
Insufficient number of SNPs

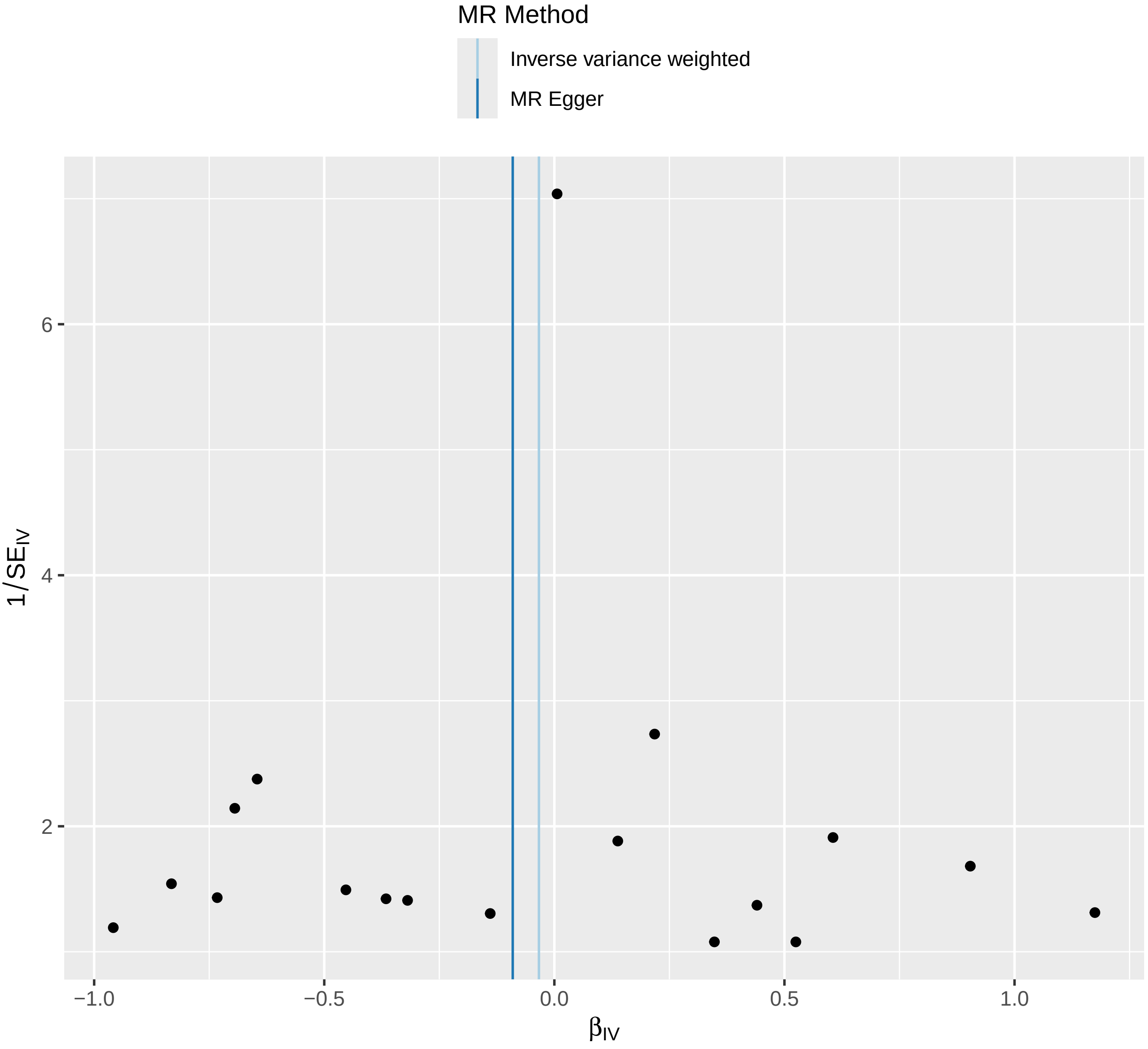
MR Method
Inverse variance weighted
MR Egger
$1/SE_{IV}$
6
4
2
−1.0
−0.5
0.0
0.5
1.0
β_{IV}

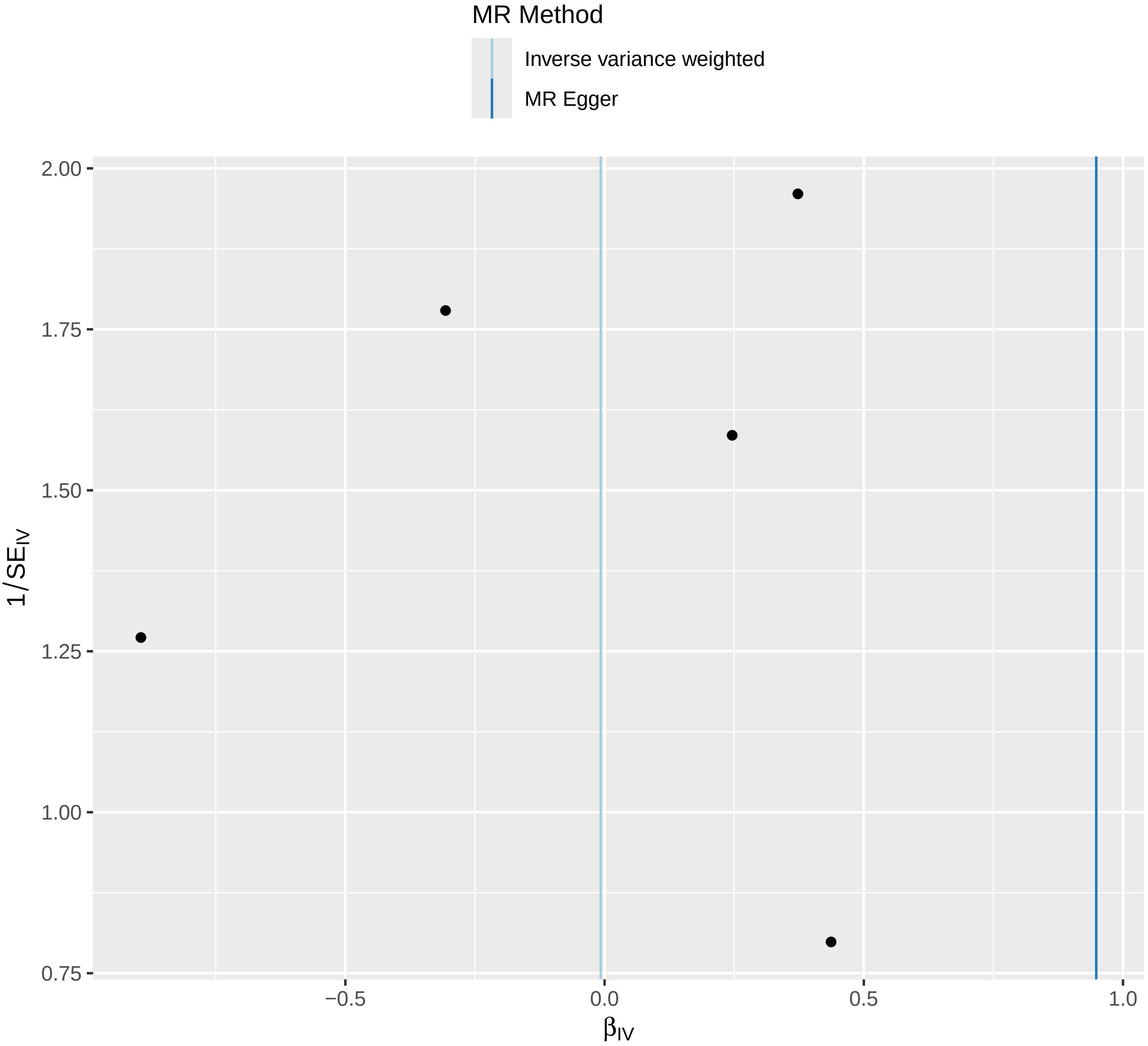

MR Method
Inverse variance weighted
MR Egger
2.00
1.75
1.50
1.25
1.00
0.75
1/SE_IV
−0.5
0.0
0.5
1.0
β_IV

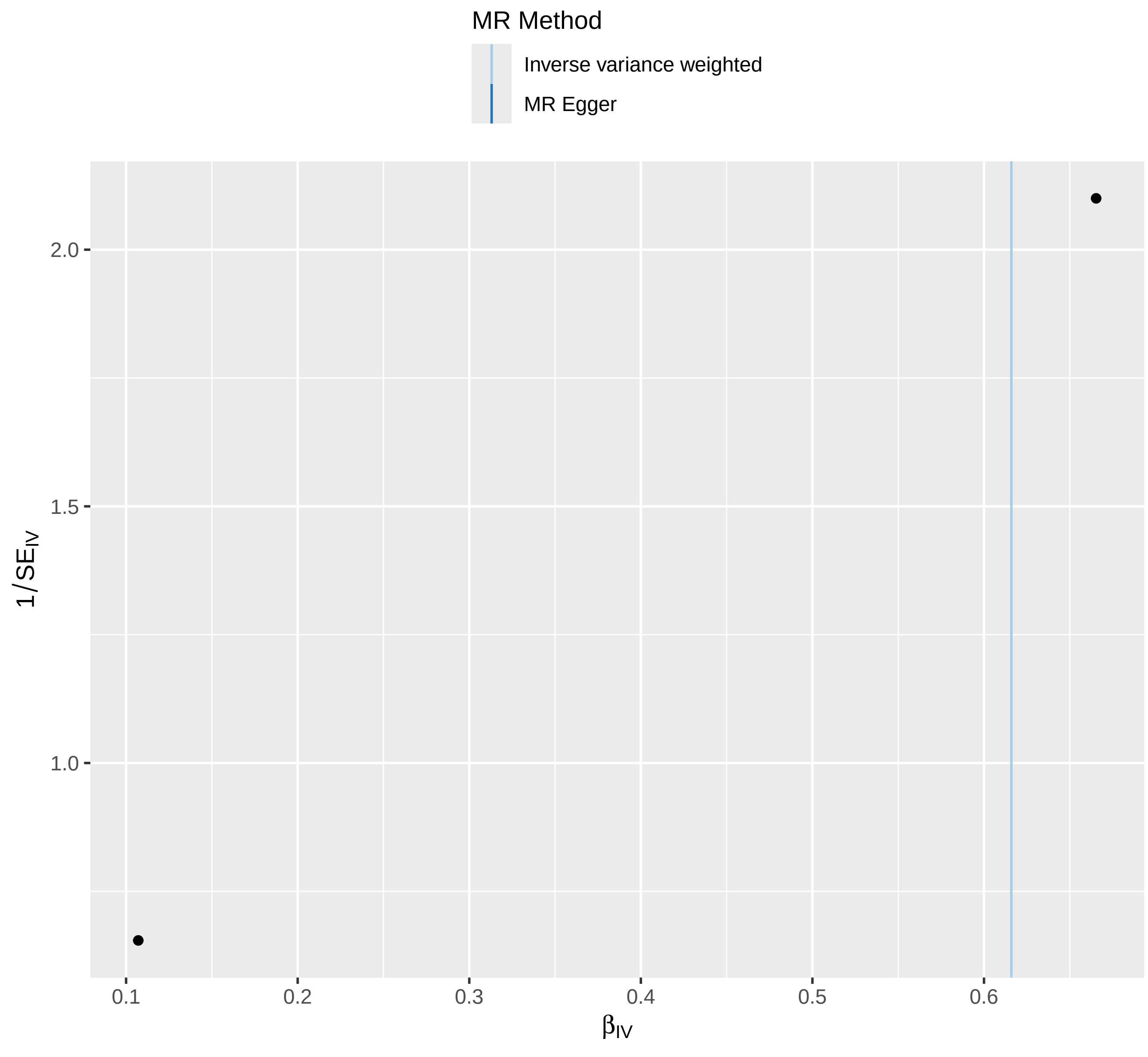
MR Method
Inverse variance weighted
MR Egger
2.0
1.5
1.0
1/SE_IV
0.1
0.2
0.3
0.4
0.5
0.6
β_IV

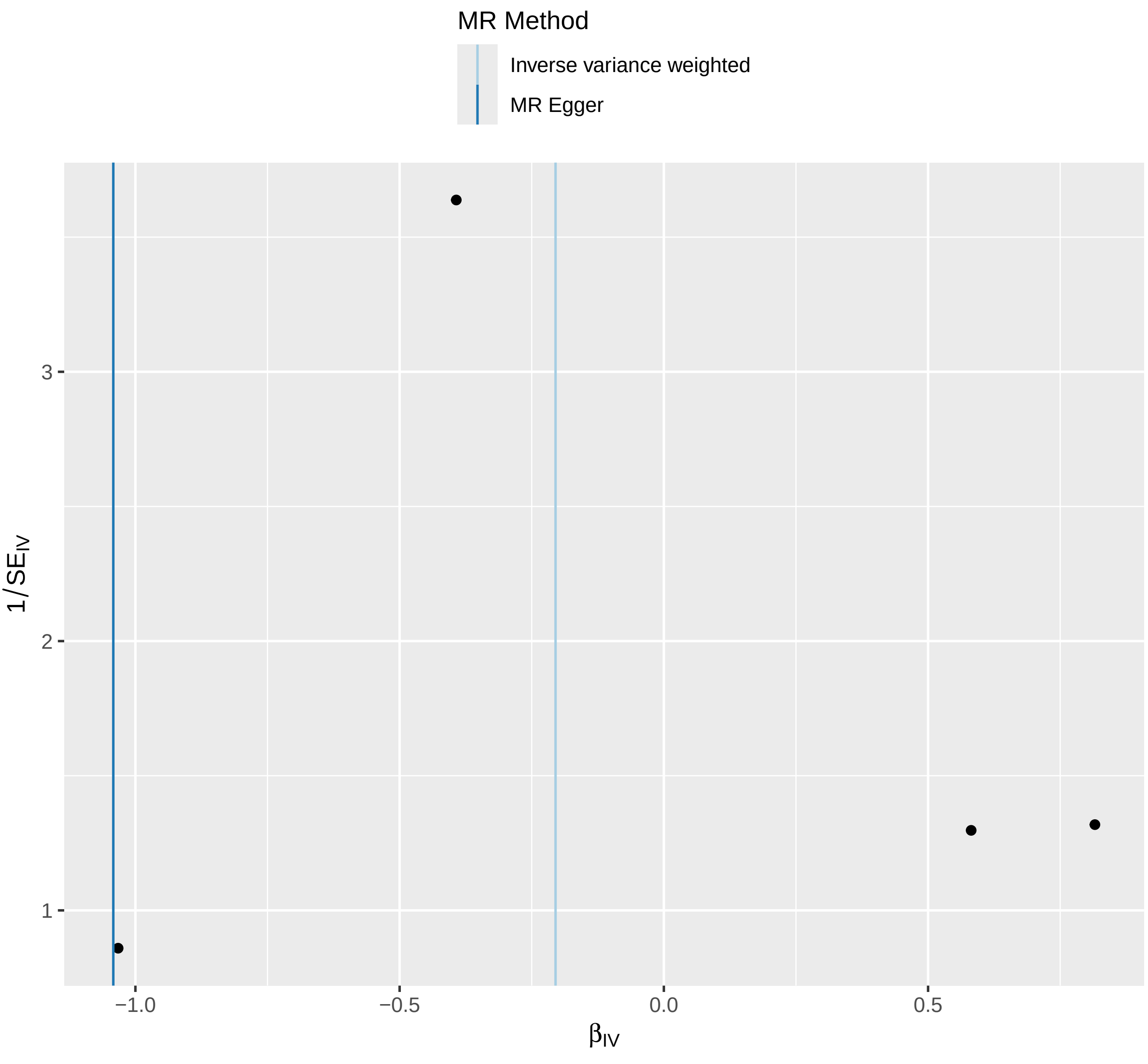
MR Method
Inverse variance weighted
MR Egger
1/SE_IV
3
2
1
−1.0
−0.5
0.0
0.5
β_IV

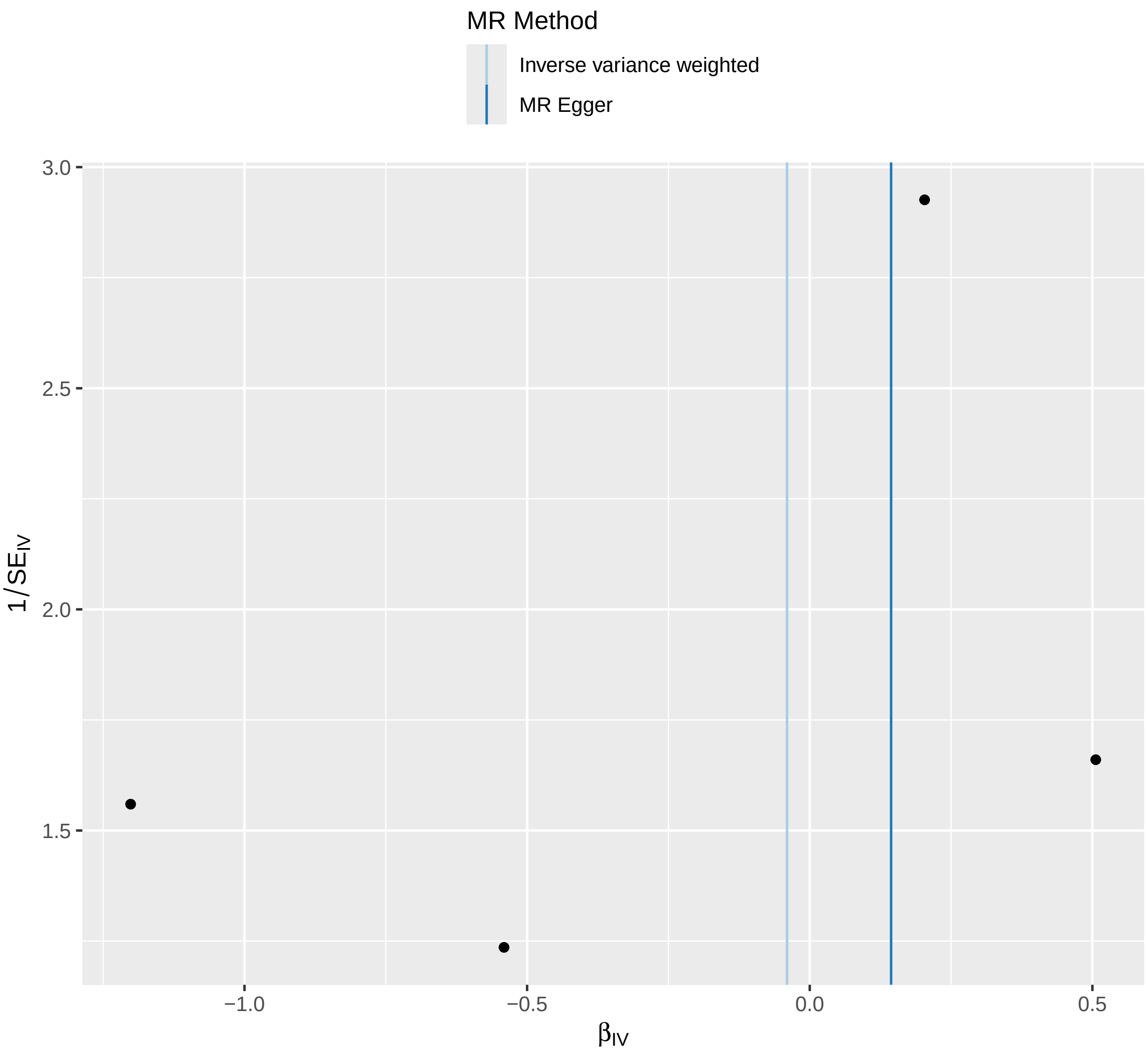

MR Method
Inverse variance weighted
MR Egger
3.0
2.5
2.0
1.5
1/SE_IV
−1.0
−0.5
0.0
0.5
β_IV

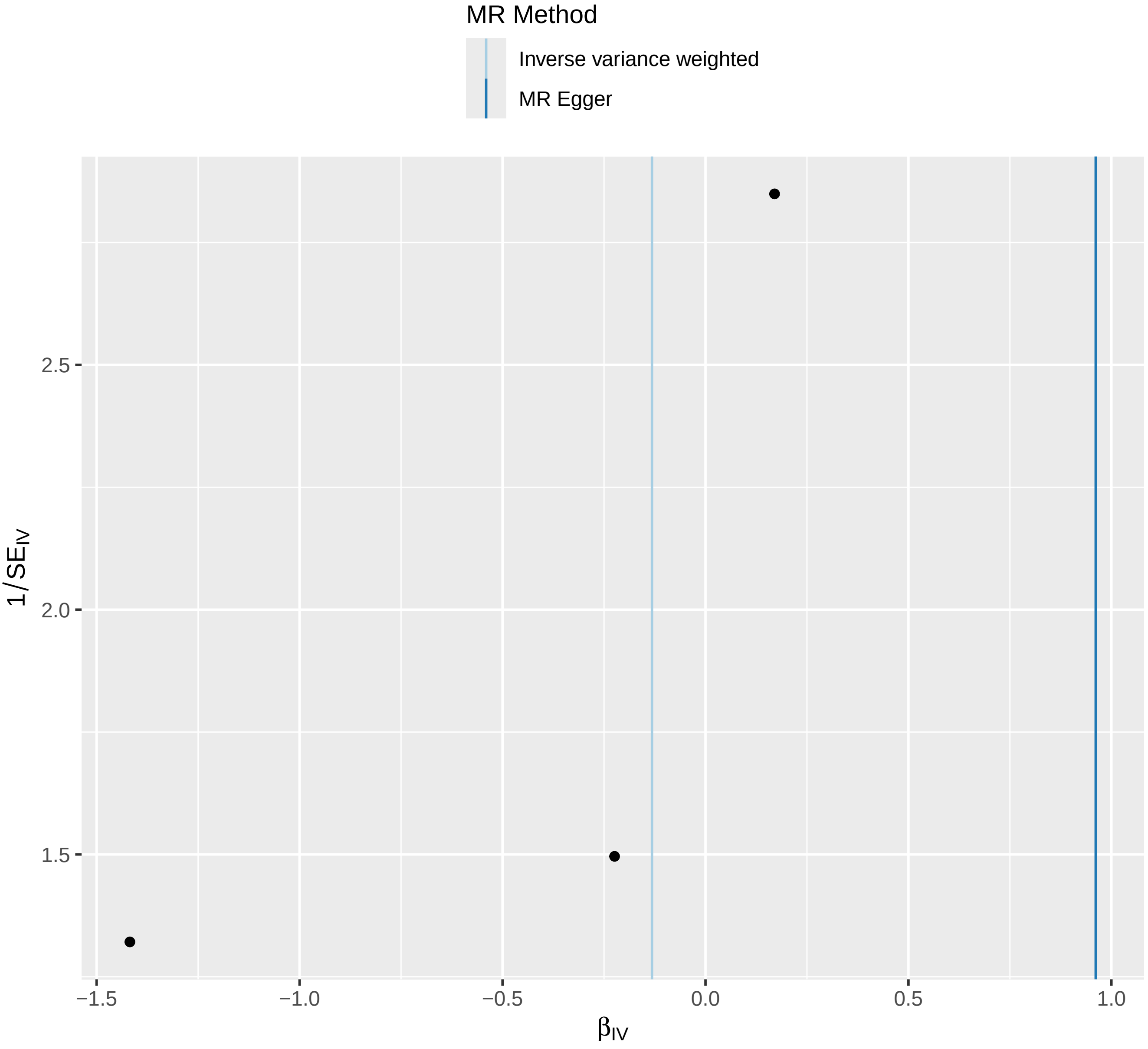
MR Method
Inverse variance weighted
MR Egger
2.5
2.0
1.5
1/SE$_{IV}$
−1.5
−1.0
−0.5
0.0
0.5
1.0
β$_{IV}$

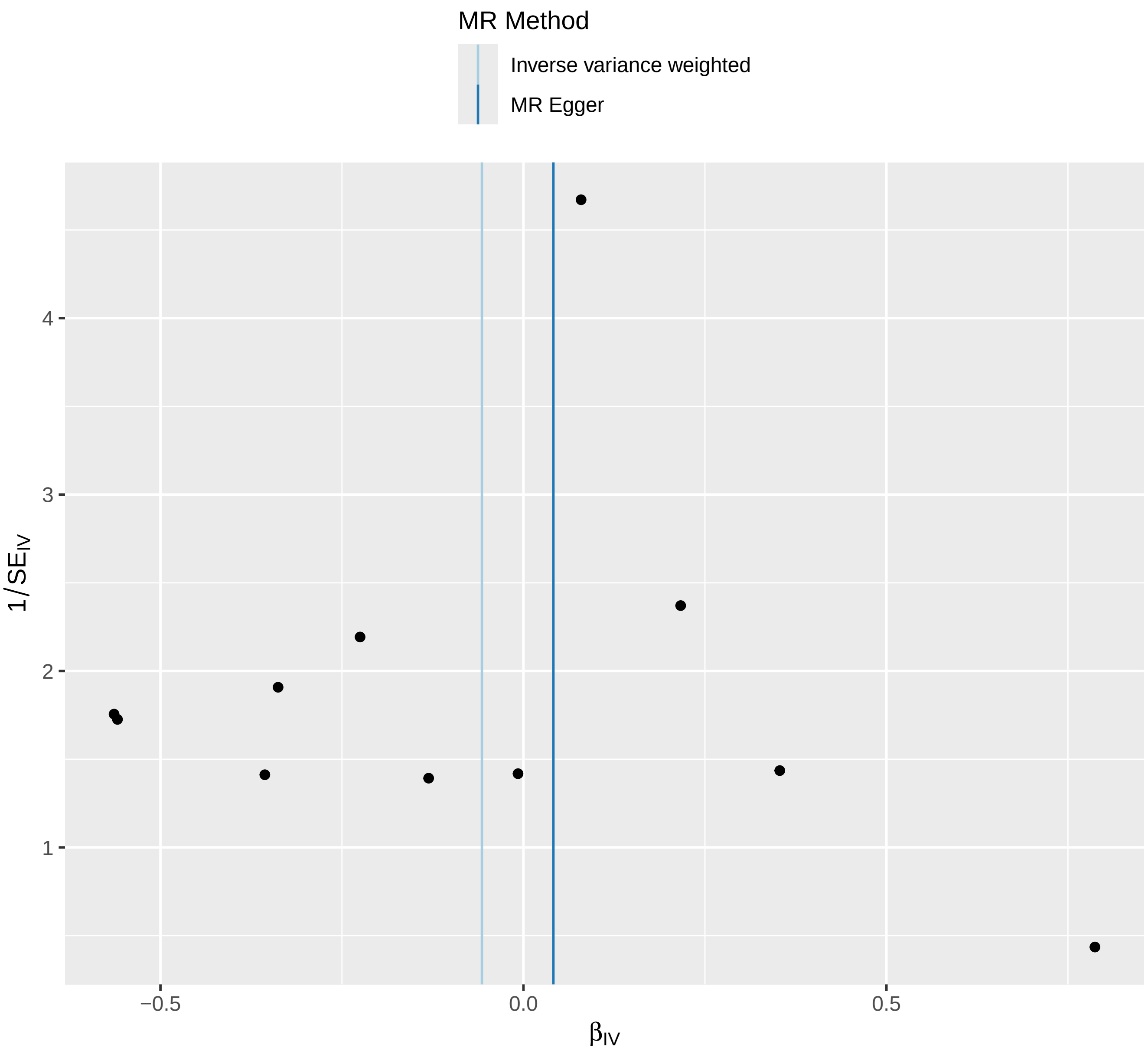
MR Method
Inverse variance weighted
MR Egger
4
3
2
1
1/SE$_{IV}$
−0.5
0.0
0.5
β$_{IV}$

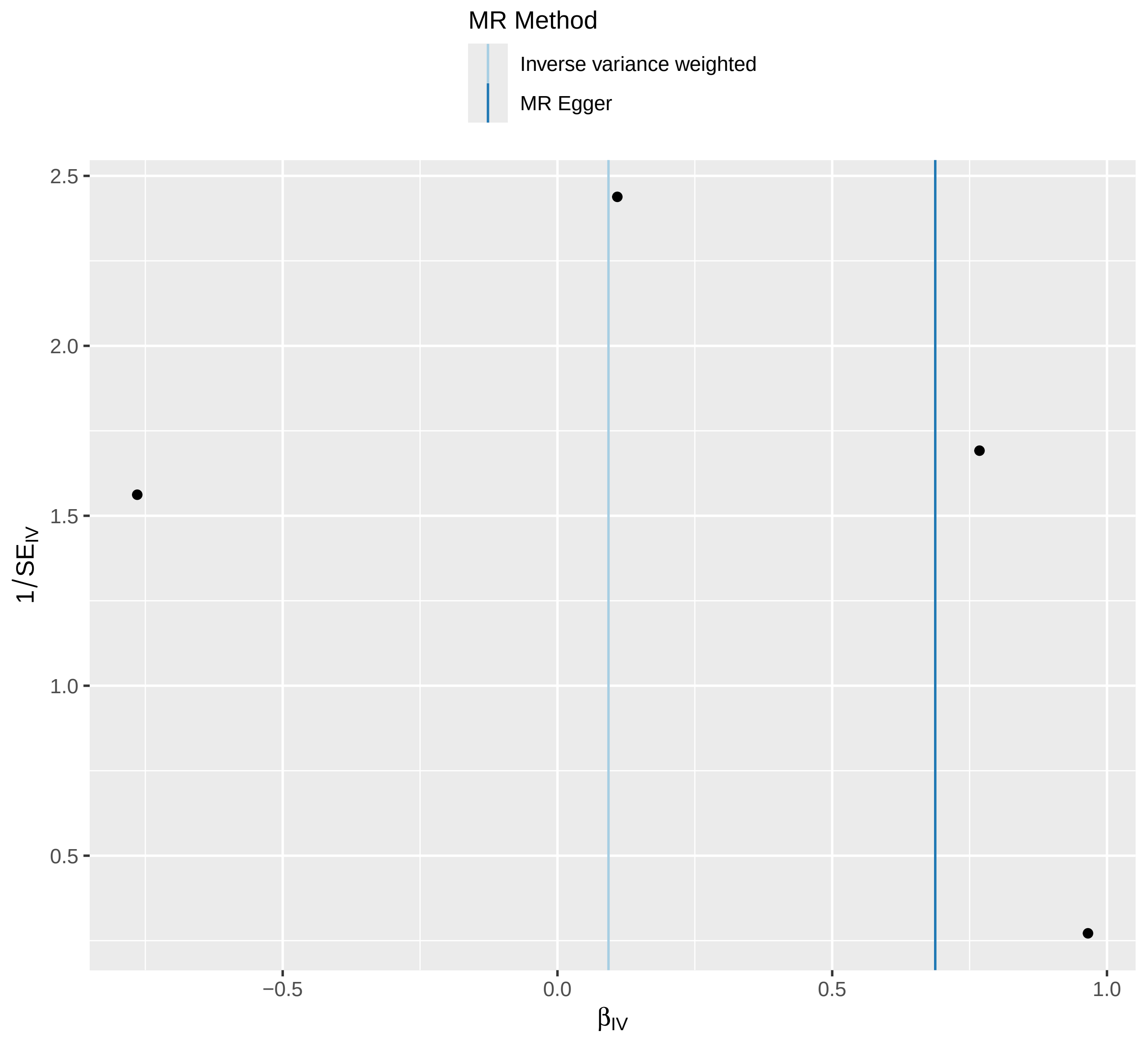
MR Method
Inverse variance weighted
MR Egger
2.5
2.0
1.5
1.0
0.5
1/SE$_{IV}$
−0.5
0.0
0.5
1.0
β_{IV}

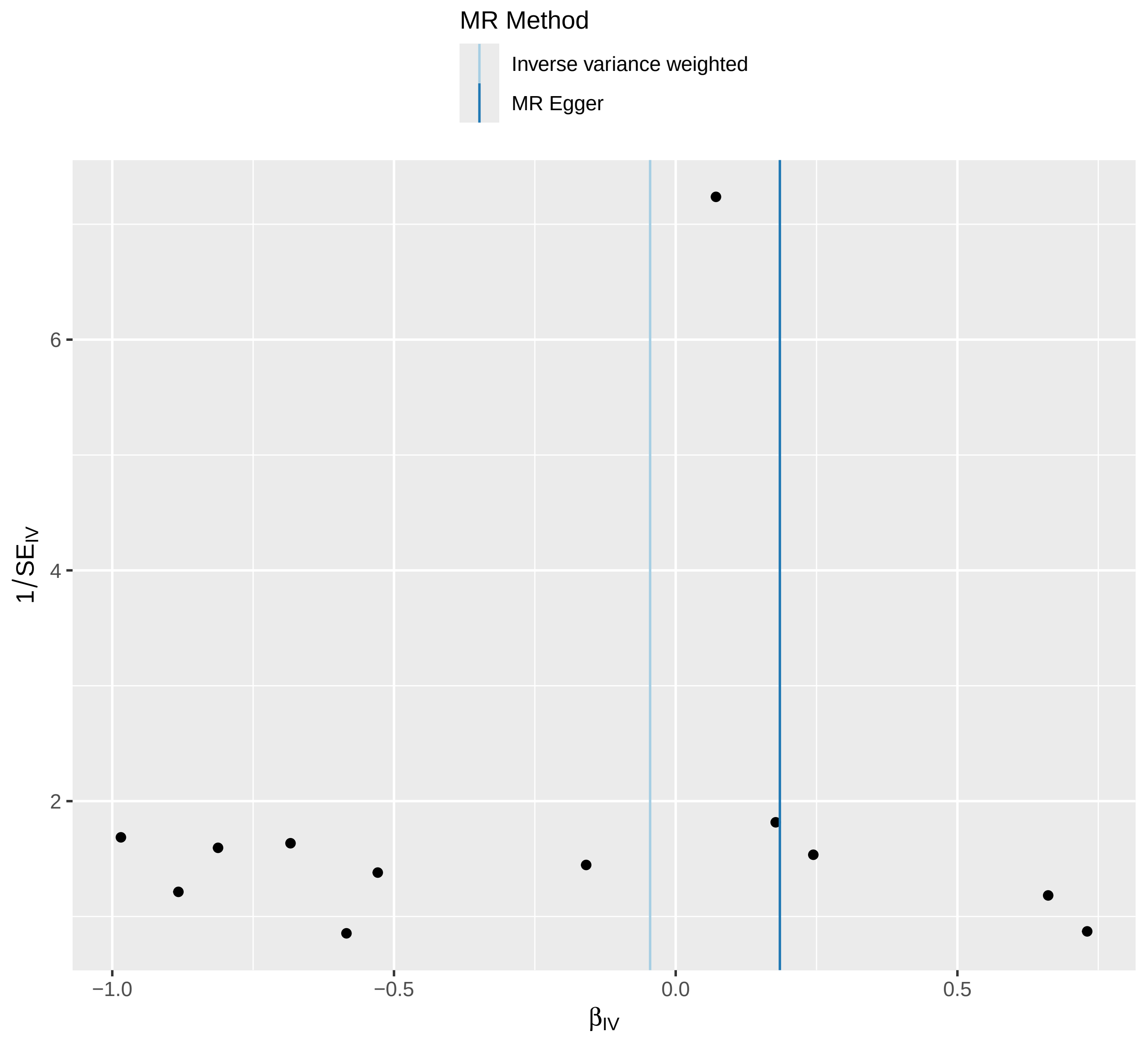

MR Method
Inverse variance weighted
MR Egger
6
4
2
1/SE_IV
−1.0
−0.5
0.0
0.5
β_IV

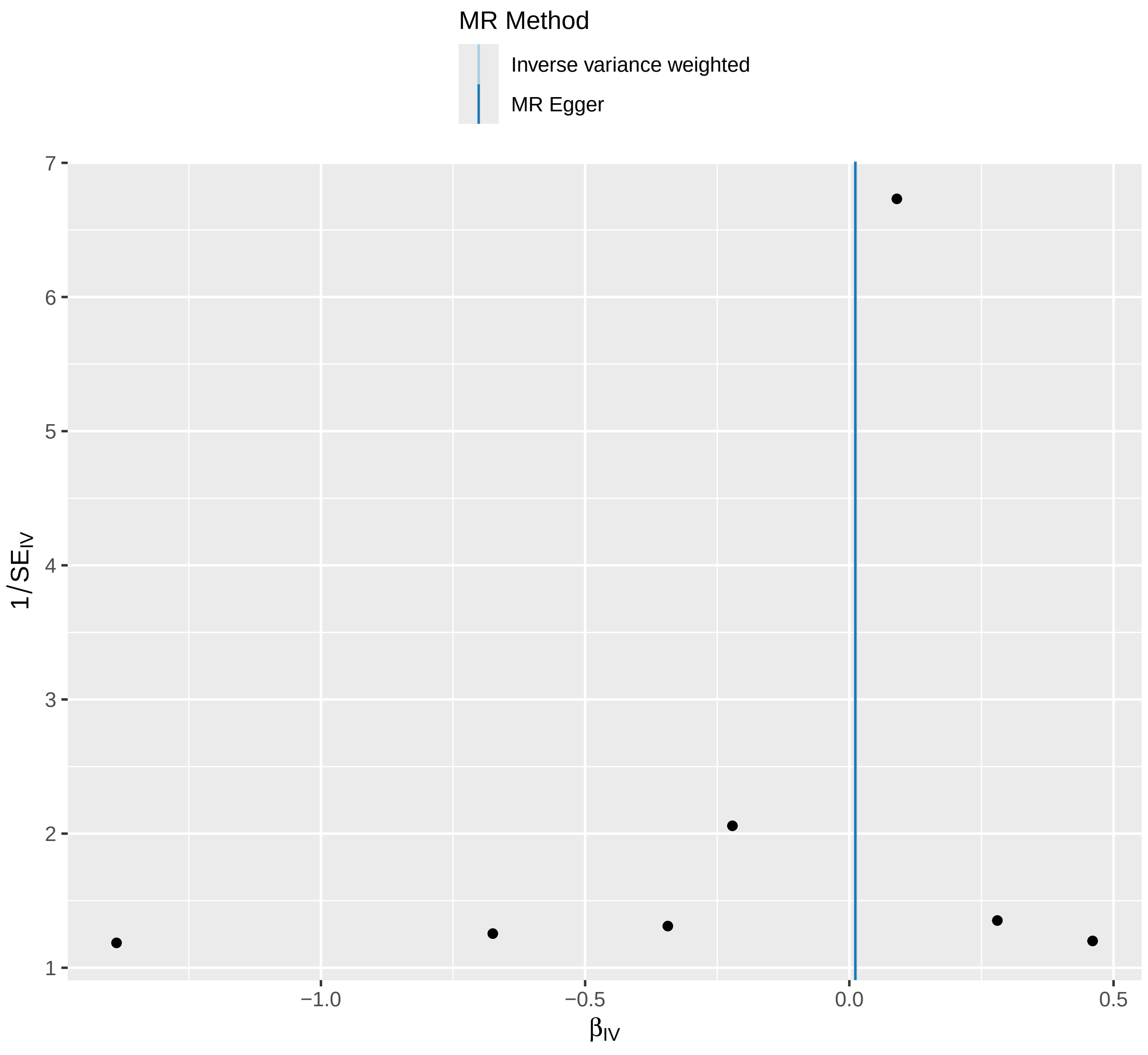
MR Method
Inverse variance weighted
MR Egger
7
6
5
4
3
2
1
1/SE_IV
−1.0
−0.5
0.0
0.5
β_IV

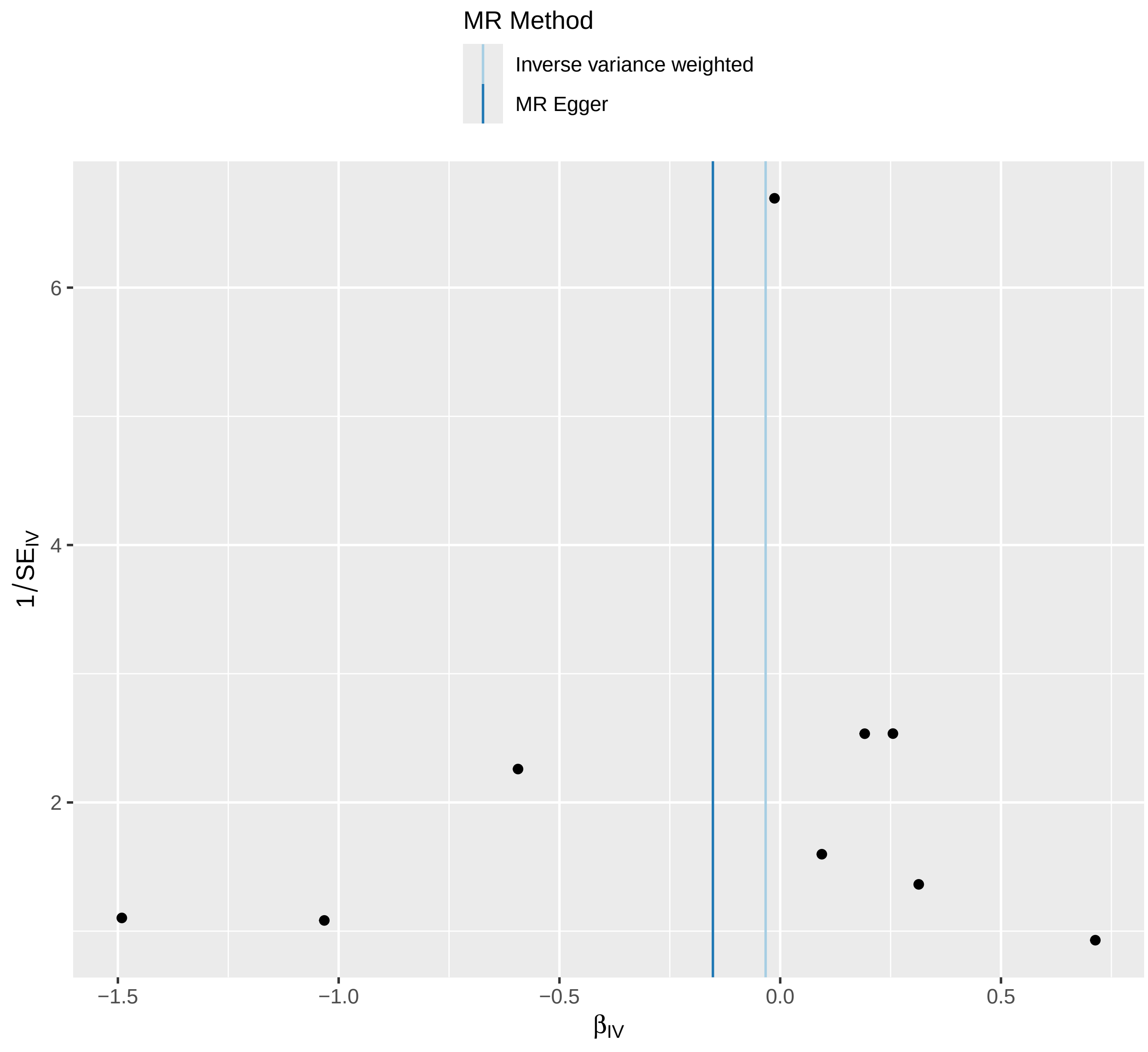

MR Method
Inverse variance weighted
MR Egger
6
4
2
1/SE_IV
−1.5
−1.0
−0.5
0.0
0.5
β_IV

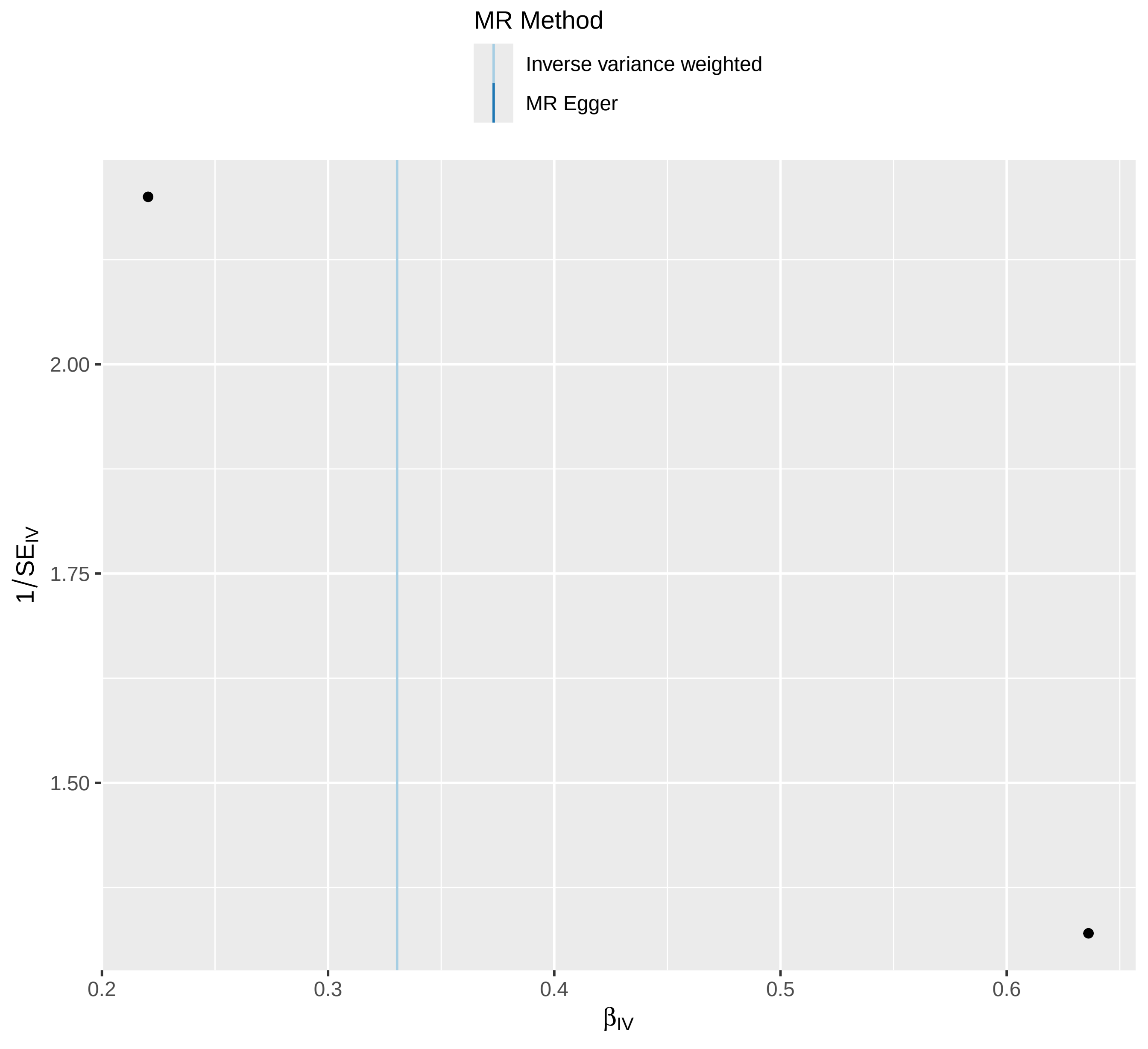
MR Method
Inverse variance weighted
MR Egger
1/SE$_{IV}$
2.00
1.75
1.50
0.2
0.3
0.4
0.5
0.6
β_{IV}

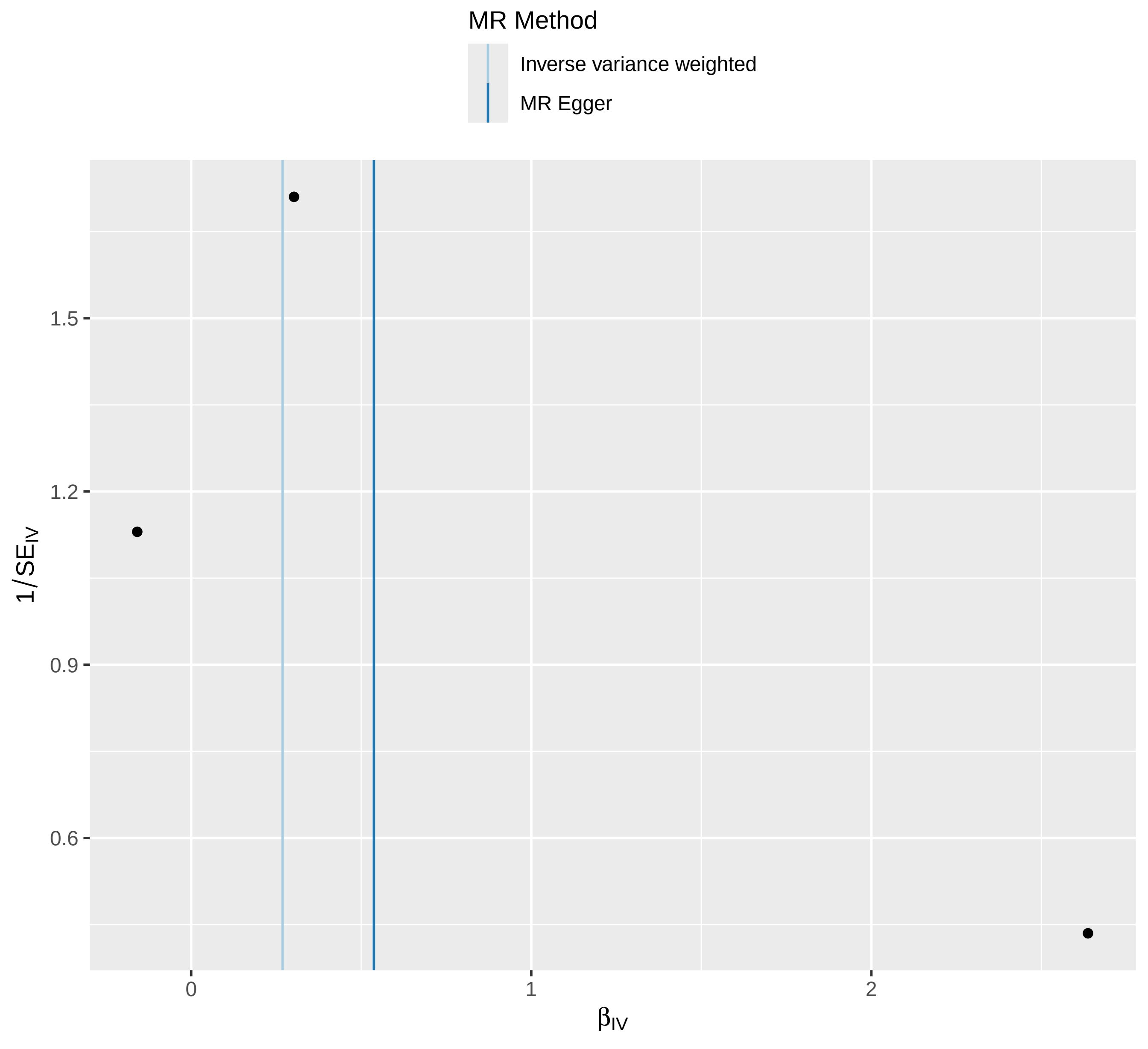
MR Method
Inverse variance weighted
MR Egger
1.5
1.2
0.9
0.6
$1/SE_{IV}$
0
1
2
β_{IV}

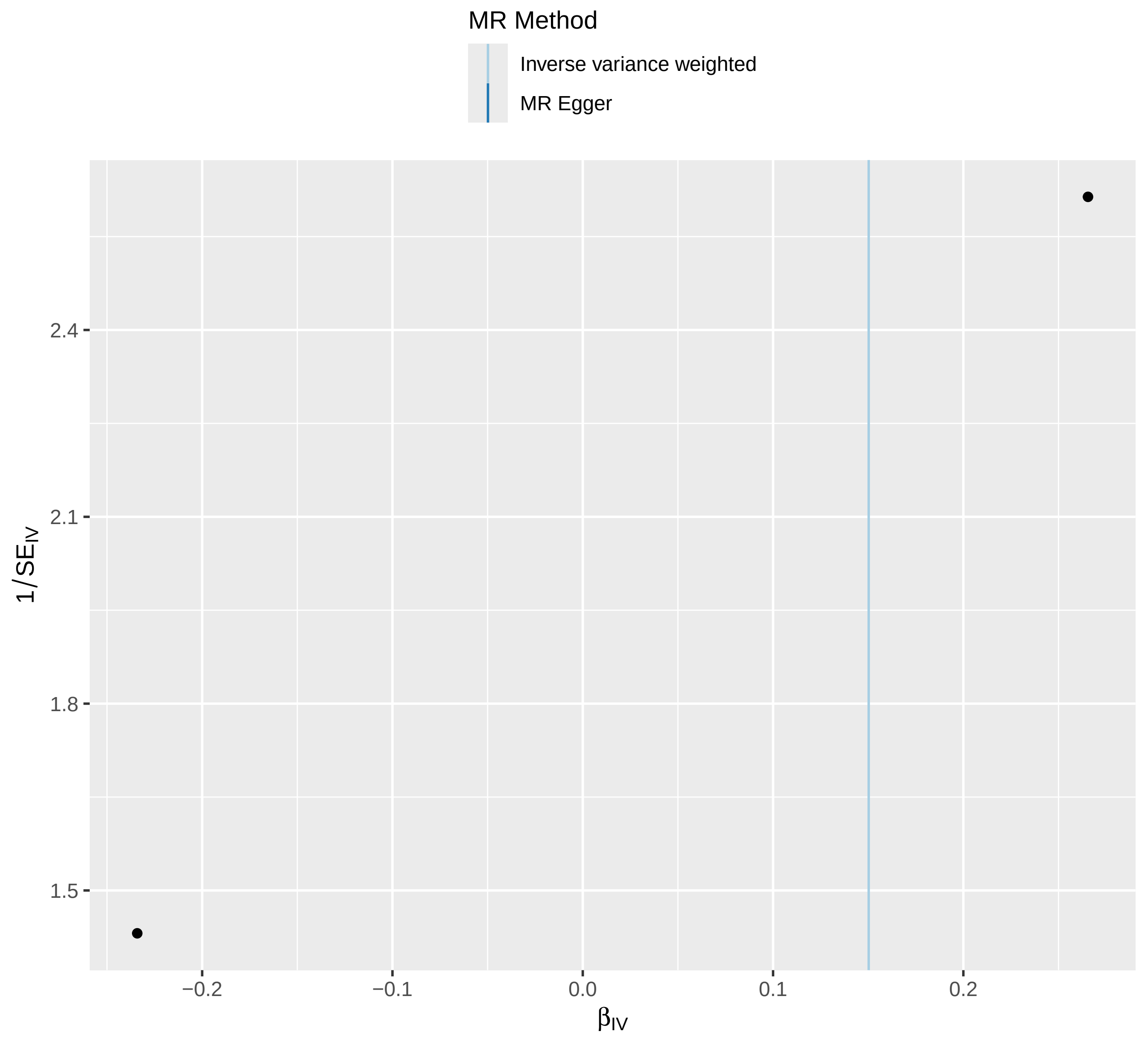
MR Method
Inverse variance weighted
MR Egger
2.4
2.1
1.8
1.5
$1/SE_{IV}$
−0.2
−0.1
0.0
0.1
0.2
β_{IV}

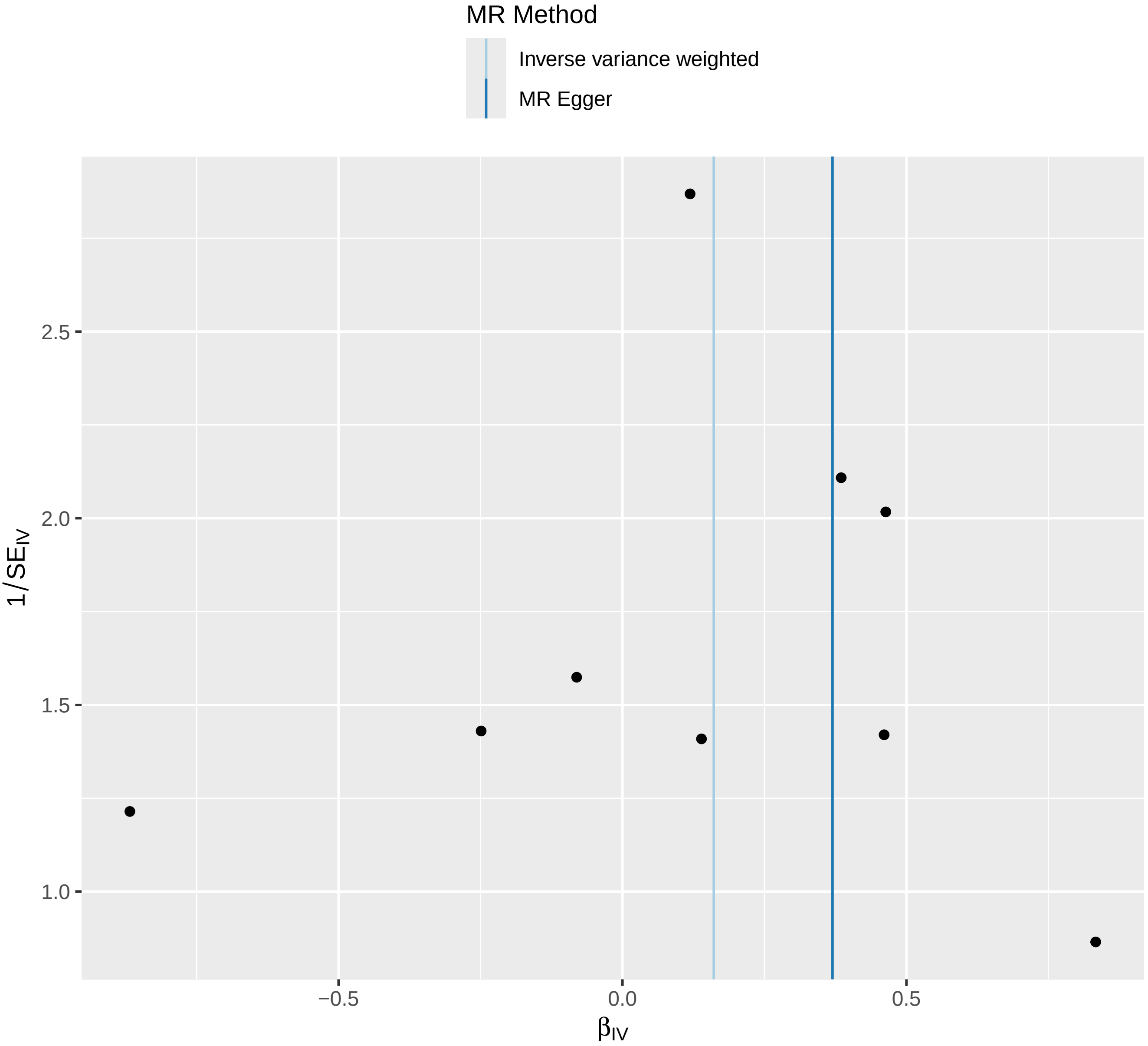

MR Method
Inverse variance weighted
MR Egger
2.5
2.0
1.5
1.0
1/SE_IV
−0.5
0.0
0.5
β_IV

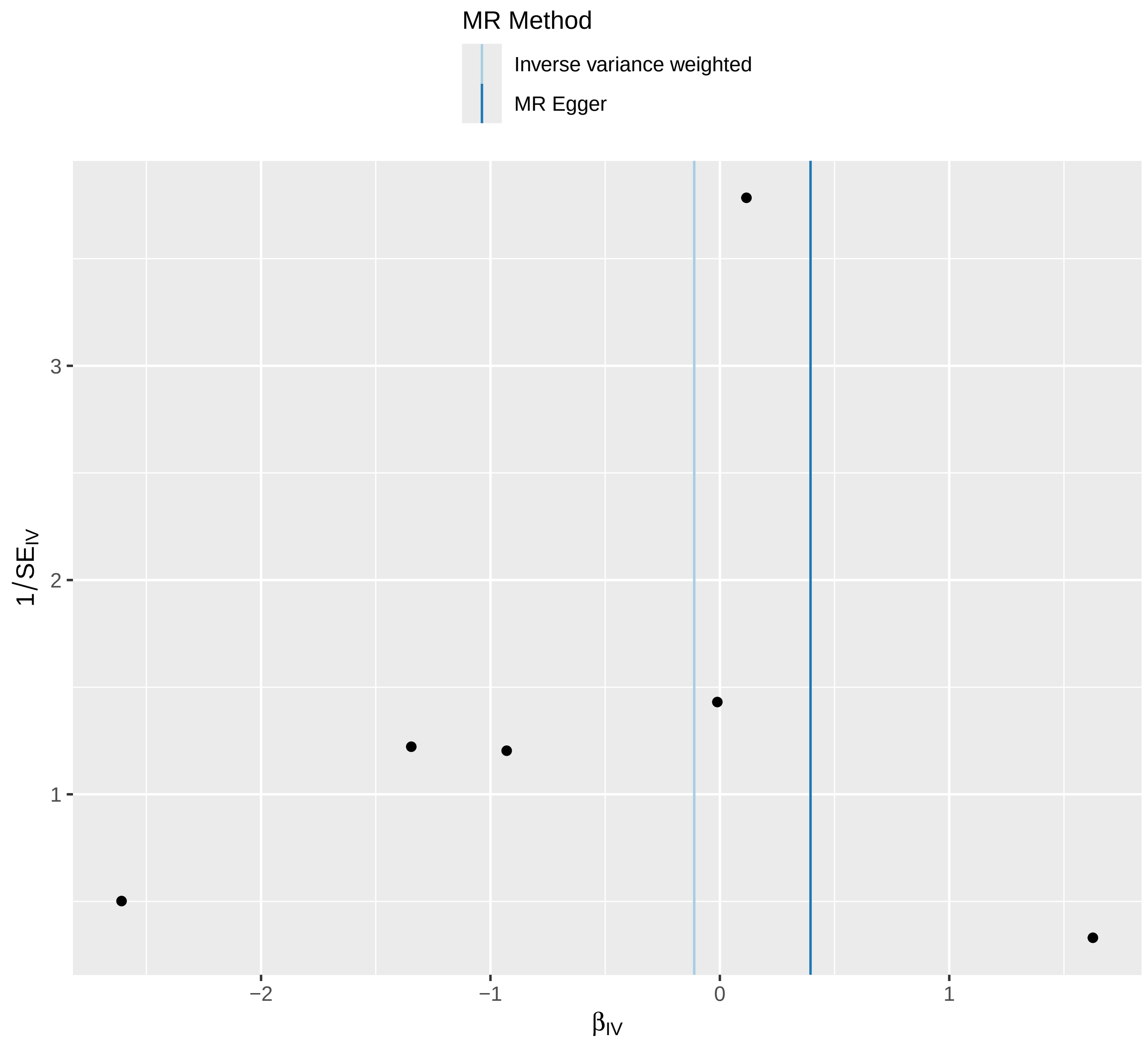

MR Method
Inverse variance weighted
MR Egger
3
2
1
1/SE_IV
−2
−1
0
1
β_IV

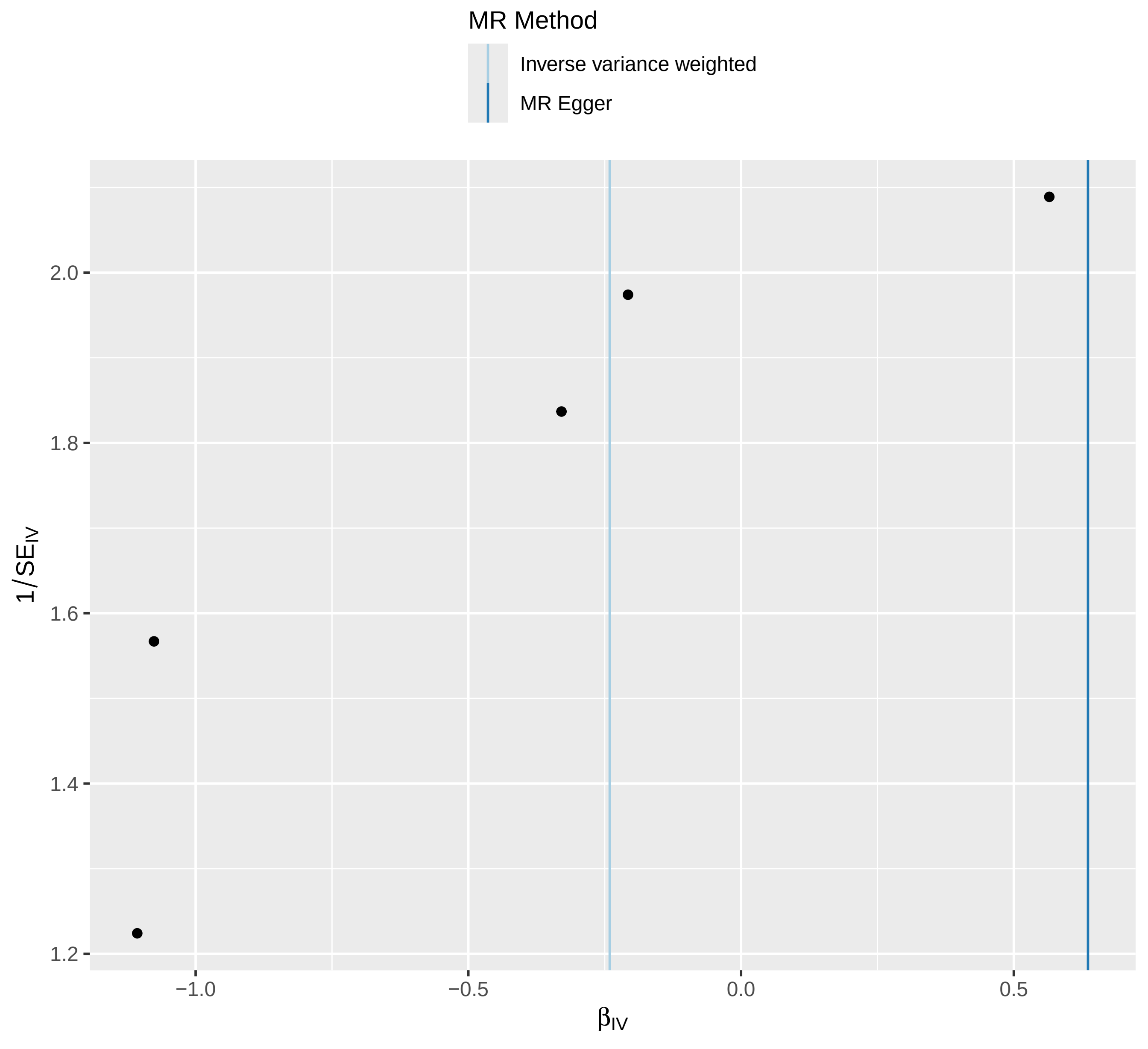
MR Method
Inverse variance weighted
MR Egger
2.0
1.8
1.6
1.4
1.2
1/SE_IV
−1.0
−0.5
0.0
0.5
β_IV

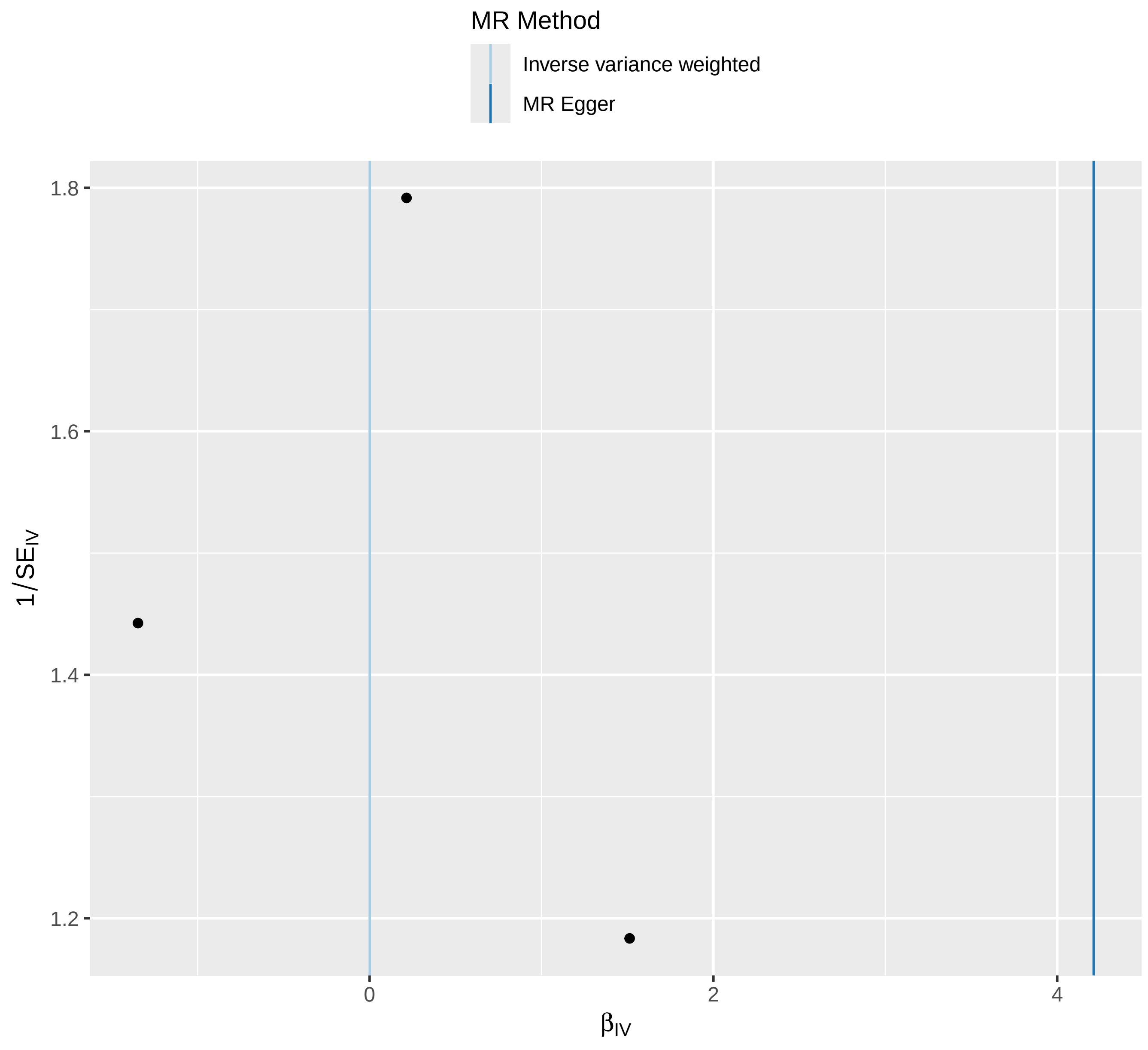

MR Method
Inverse variance weighted
MR Egger
1.8
1.6
1.4
1.2
$1/SE_{IV}$
0
2
4
β_{IV}

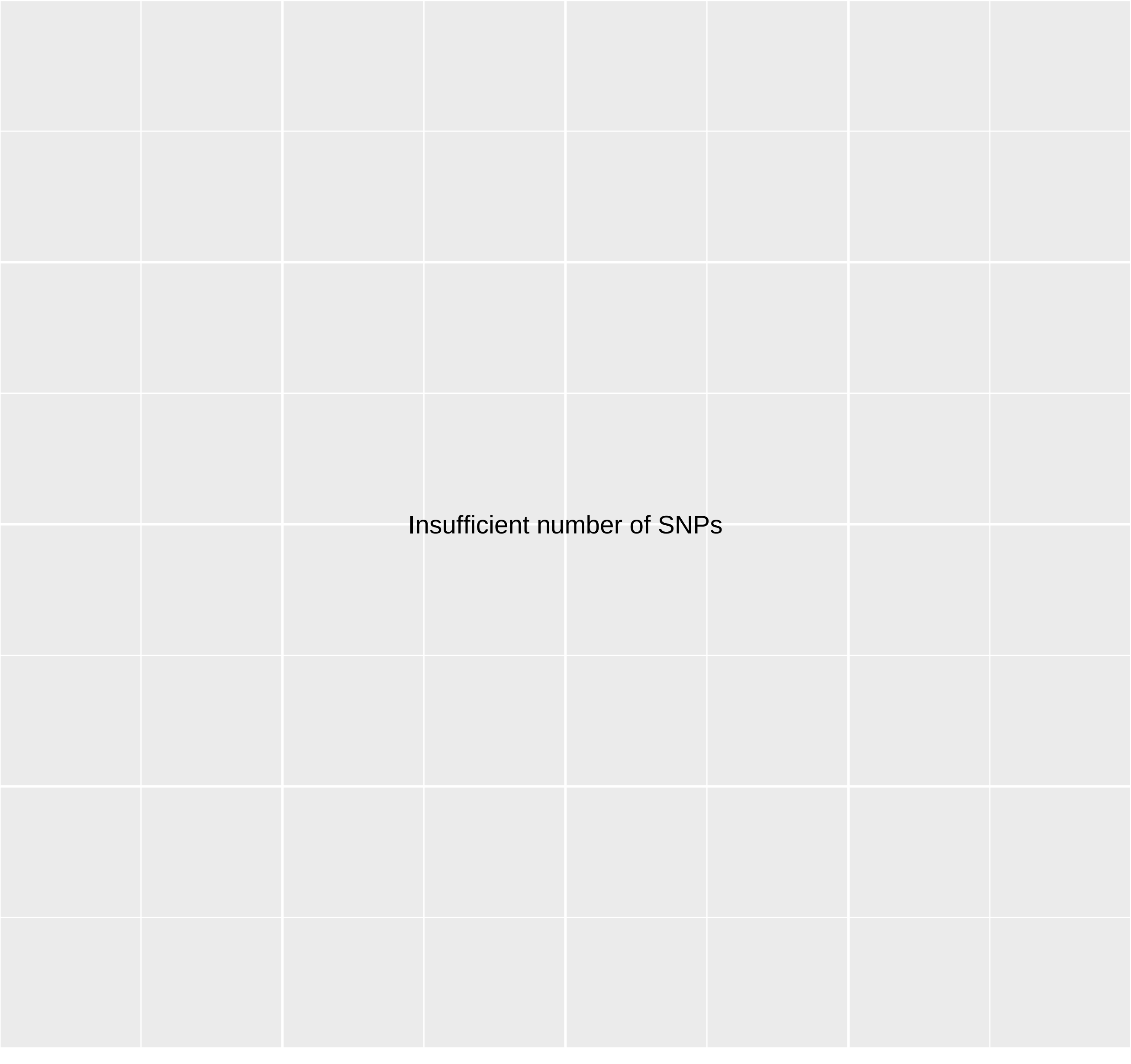
Insufficient number of SNPs

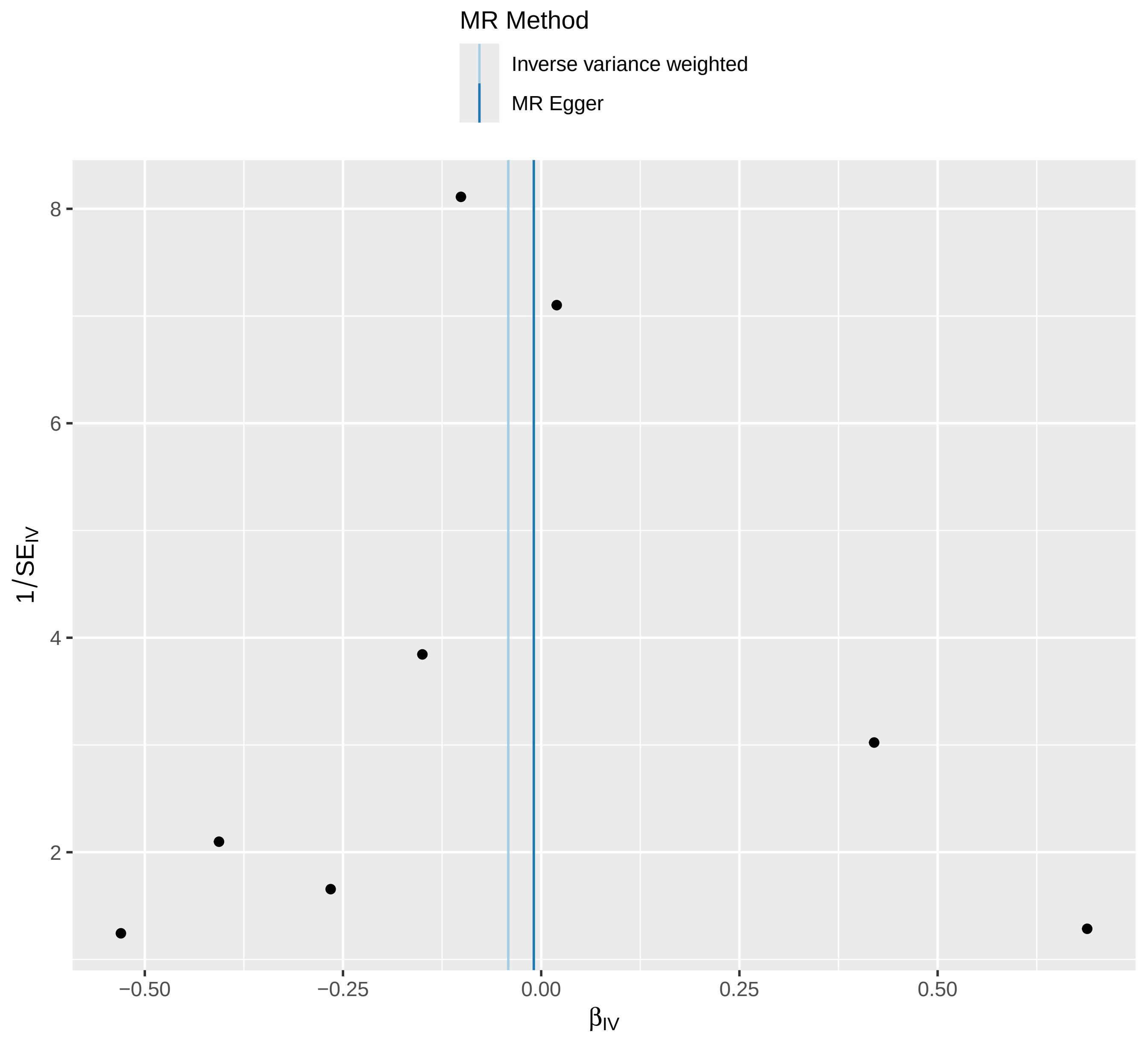

MR Method
Inverse variance weighted
MR Egger
1/SE_IV
8
6
4
2
−0.50
−0.25
0.00
0.25
0.50
β_IV

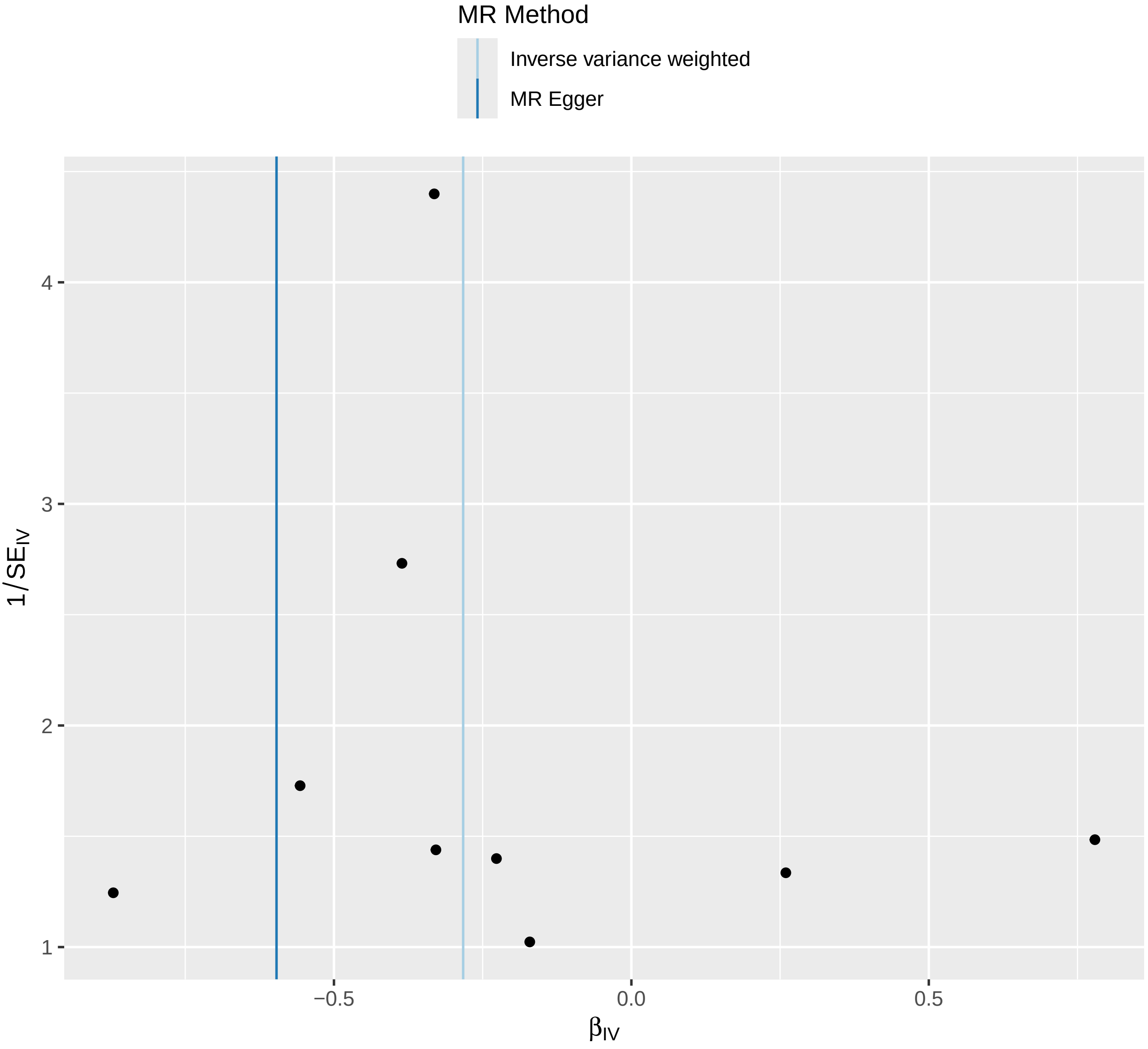
MR Method
Inverse variance weighted
MR Egger
1/SE$_{IV}$
4
3
2
1
−0.5
0.0
0.5
β$_{IV}$

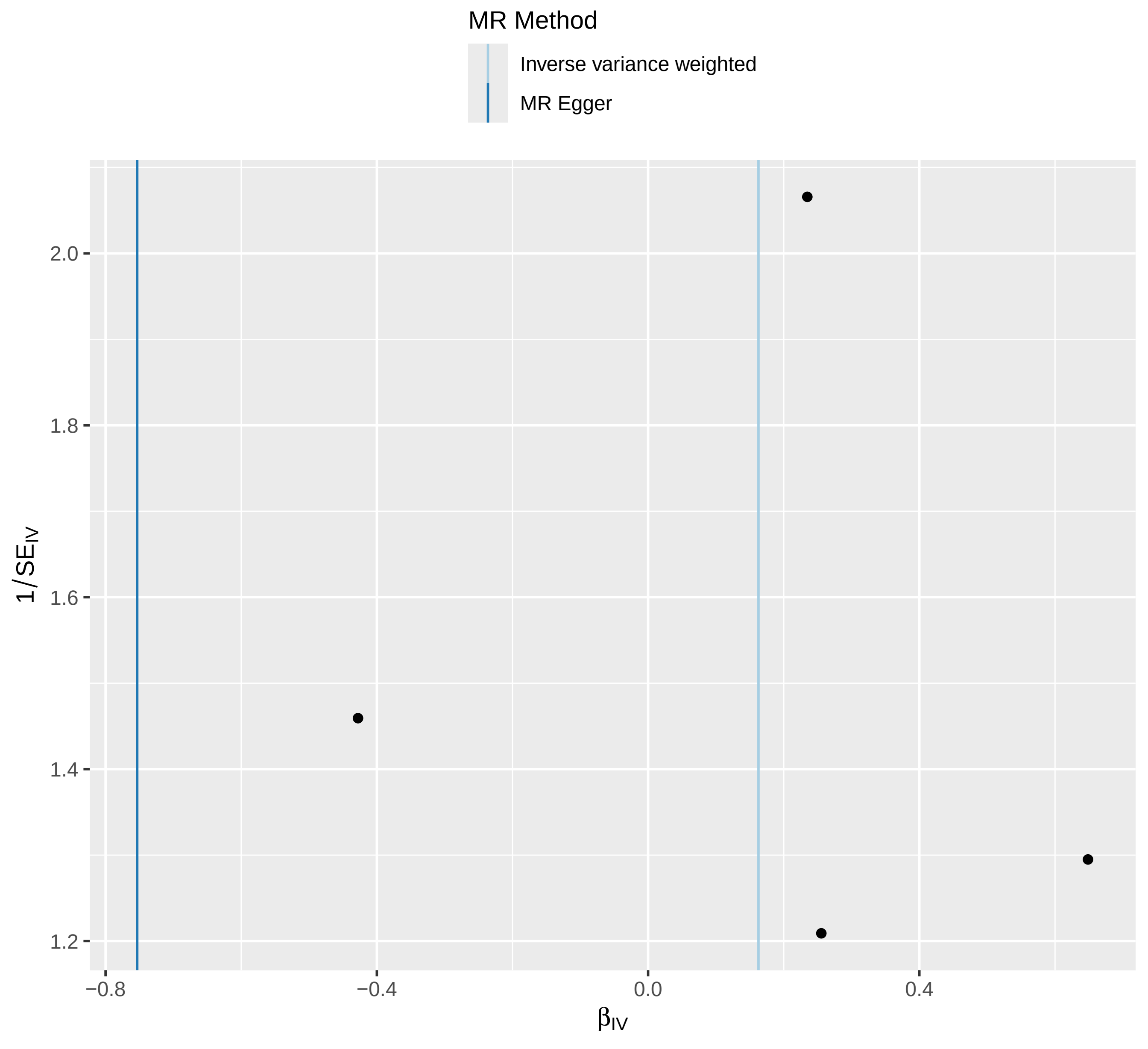

MR Method
Inverse variance weighted
MR Egger
2.0
1.8
1.6
1.4
1.2
$1/SE_{IV}$
−0.8
−0.4
0.0
0.4
β_{IV}

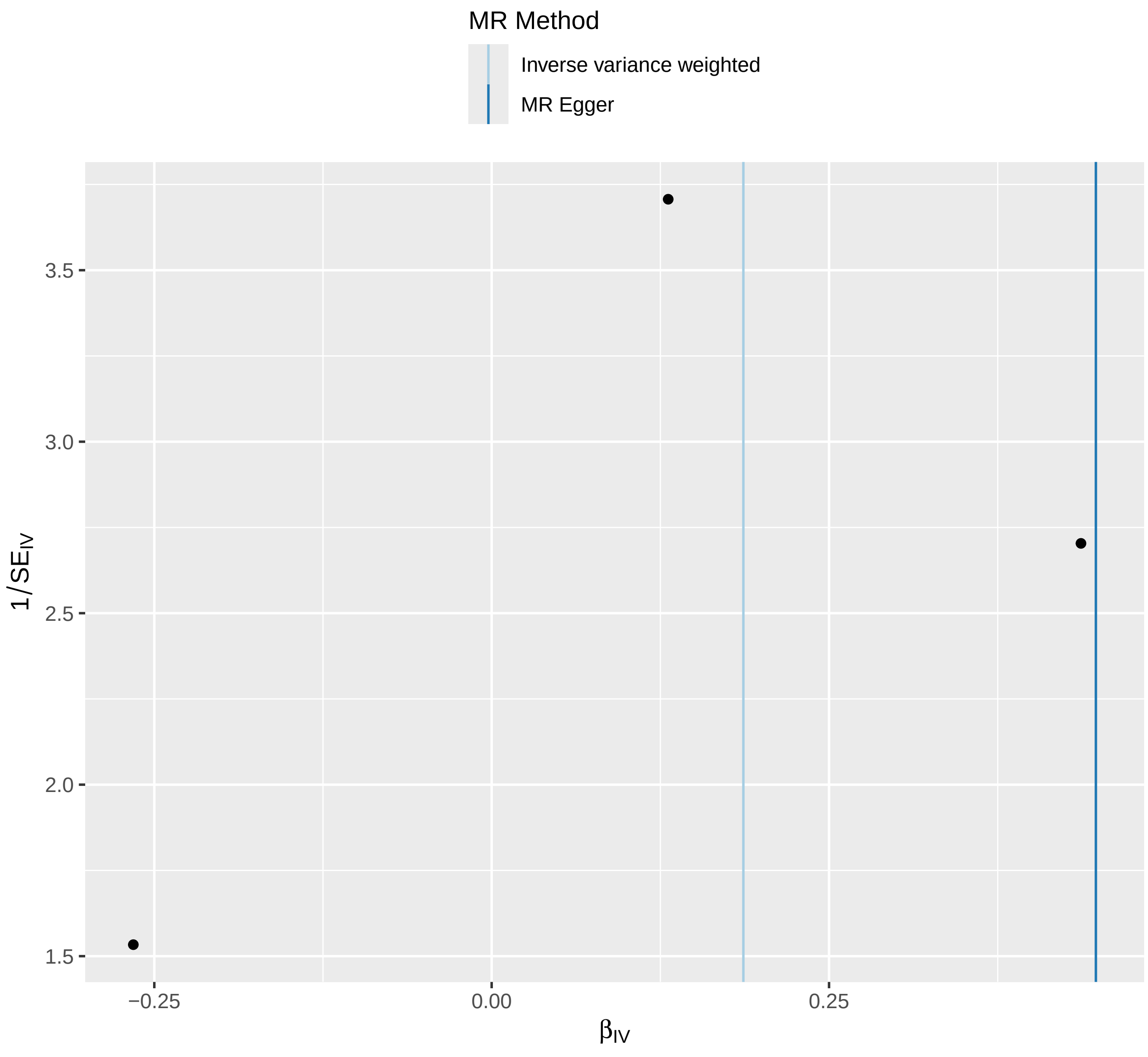
MR Method
Inverse variance weighted
MR Egger
1/SE$_{IV}$
3.5
3.0
2.5
2.0
1.5
−0.25
0.00
0.25
β_{IV}

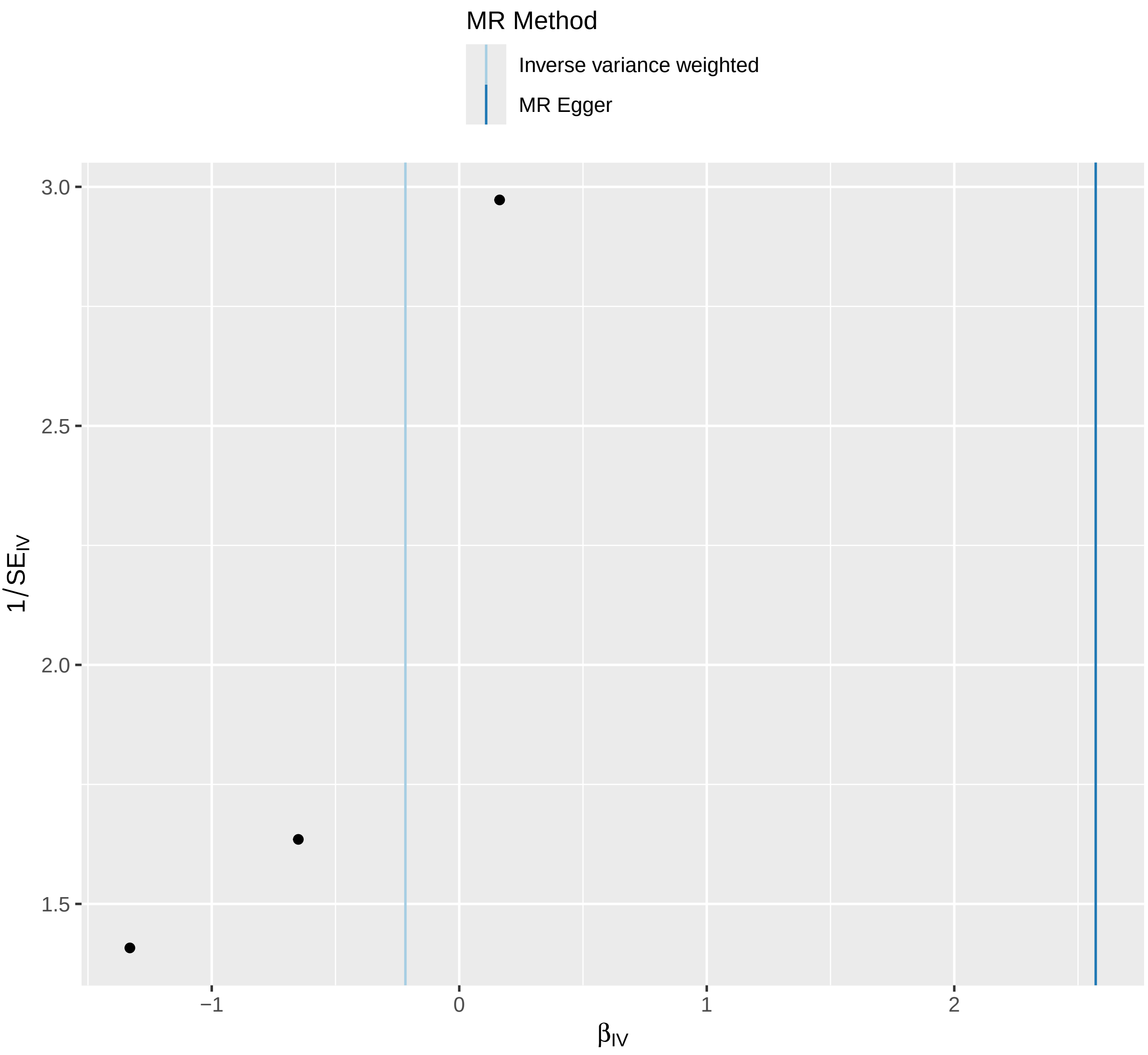
MR Method
Inverse variance weighted
MR Egger
3.0
2.5
2.0
1.5
1/SE_IV
−1
0
1
2
β_IV

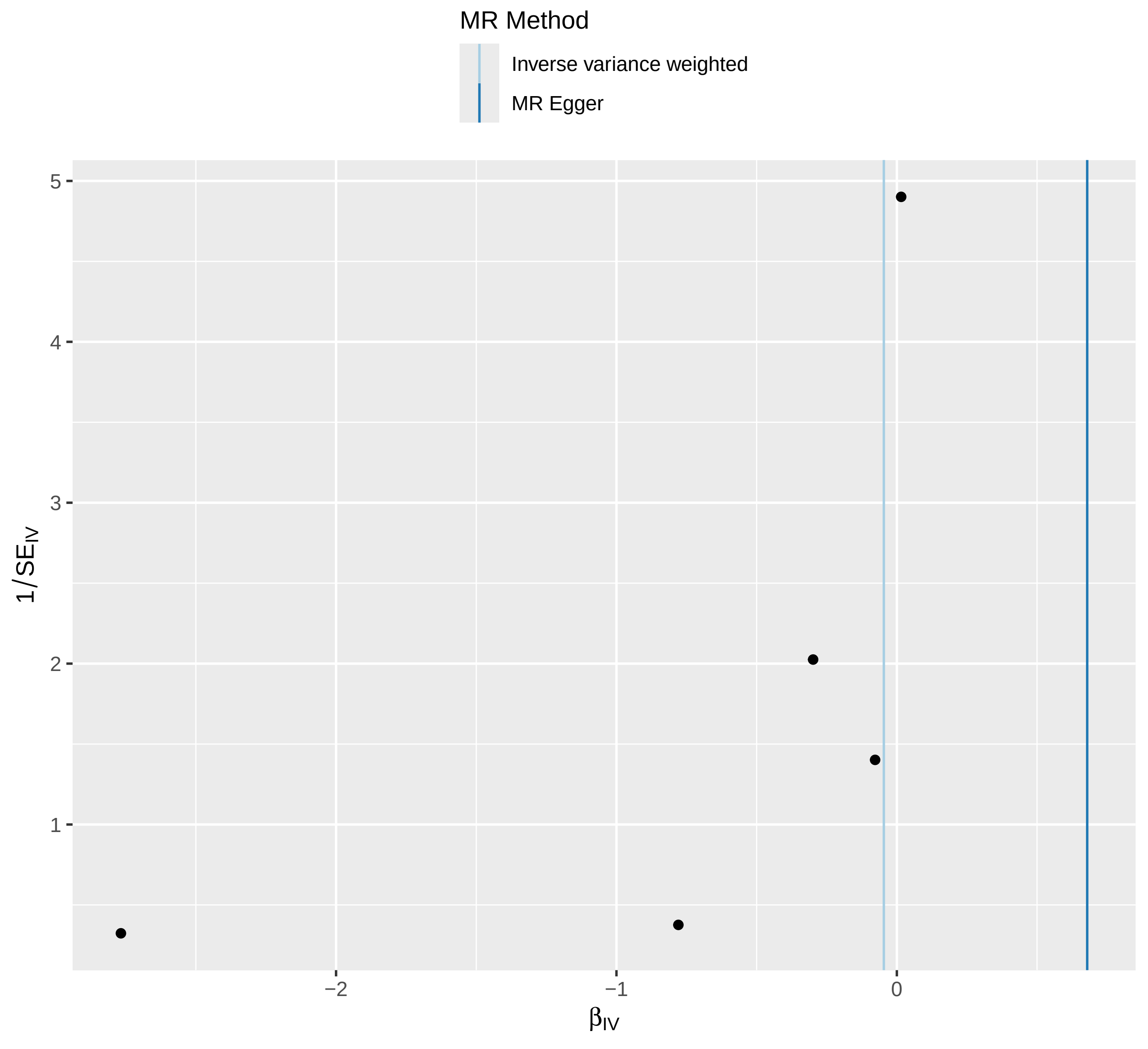

MR Method
Inverse variance weighted
MR Egger
5
4
3
2
1
1/SE$_{IV}$
−2
−1
0
β$_{IV}$

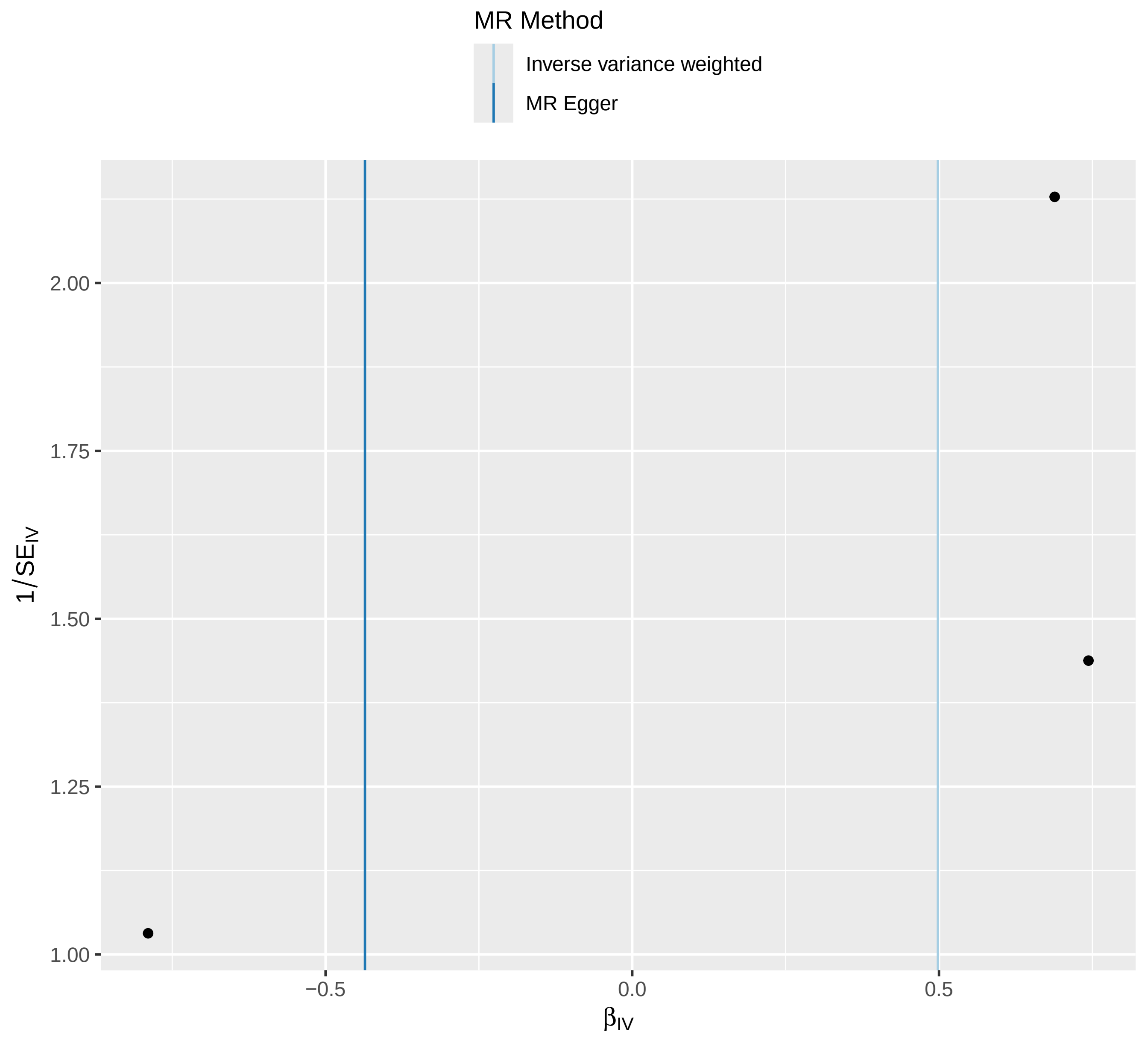
MR Method
Inverse variance weighted
MR Egger
2.00
1.75
1.50
1.25
1.00
1/SE_IV
−0.5
0.0
0.5
β_IV

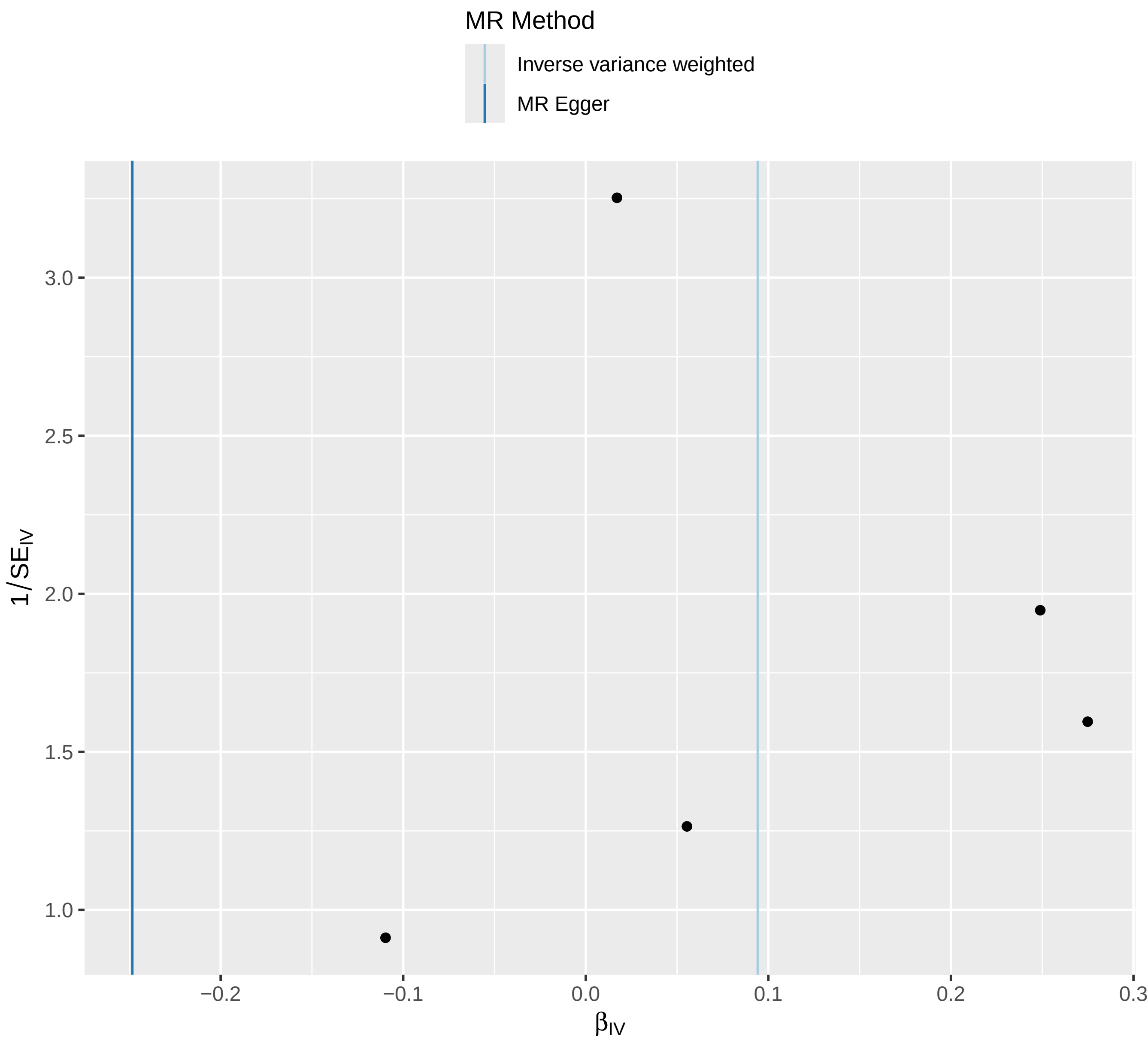
MR Method
Inverse variance weighted
MR Egger
1/SE_IV
3.0
2.5
2.0
1.5
1.0
−0.2
−0.1
0.0
0.1
0.2
0.3
β_IV

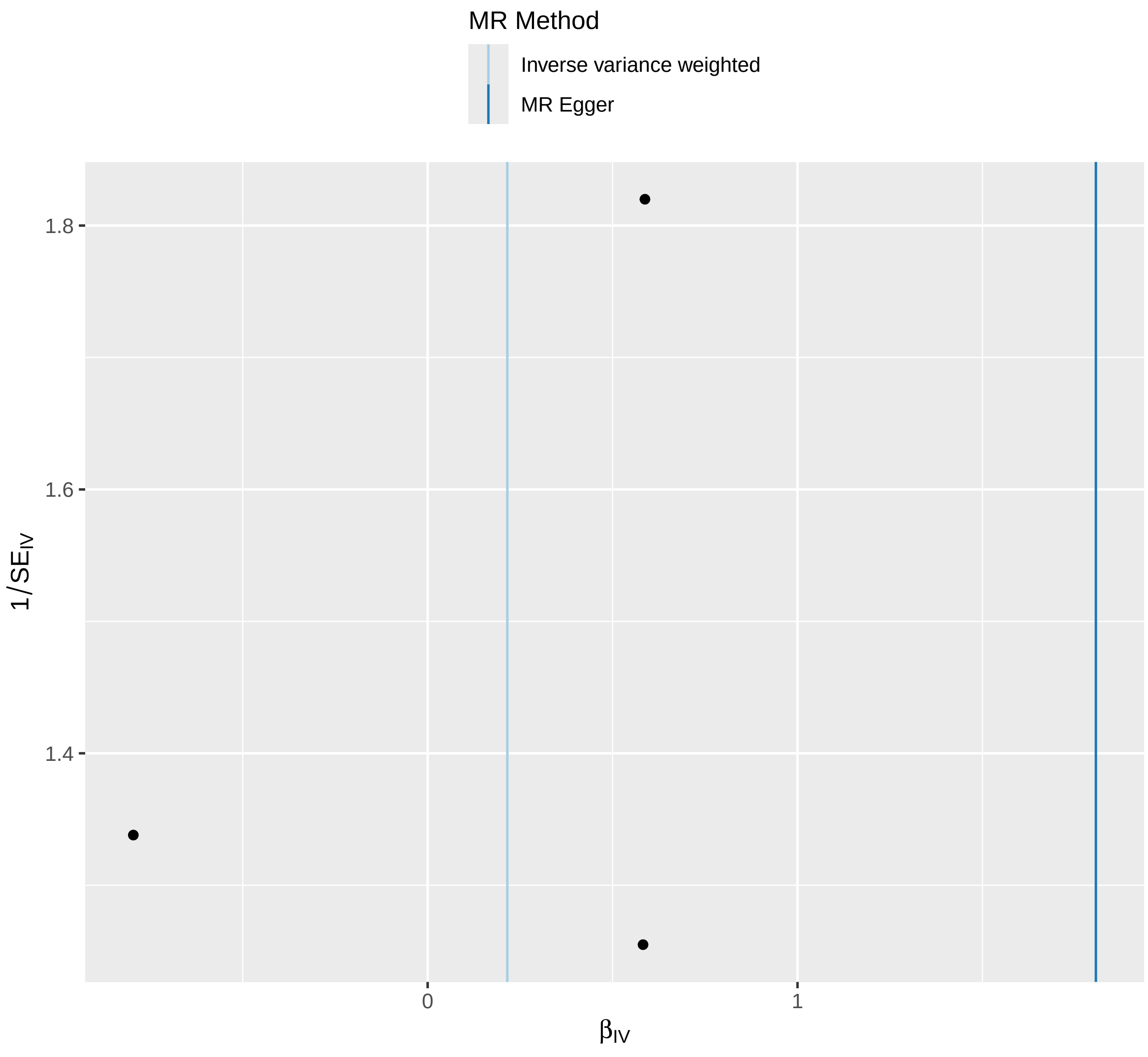
MR Method
Inverse variance weighted
MR Egger
1.8
1.6
1.4
1/SE_IV
0
1
β_IV

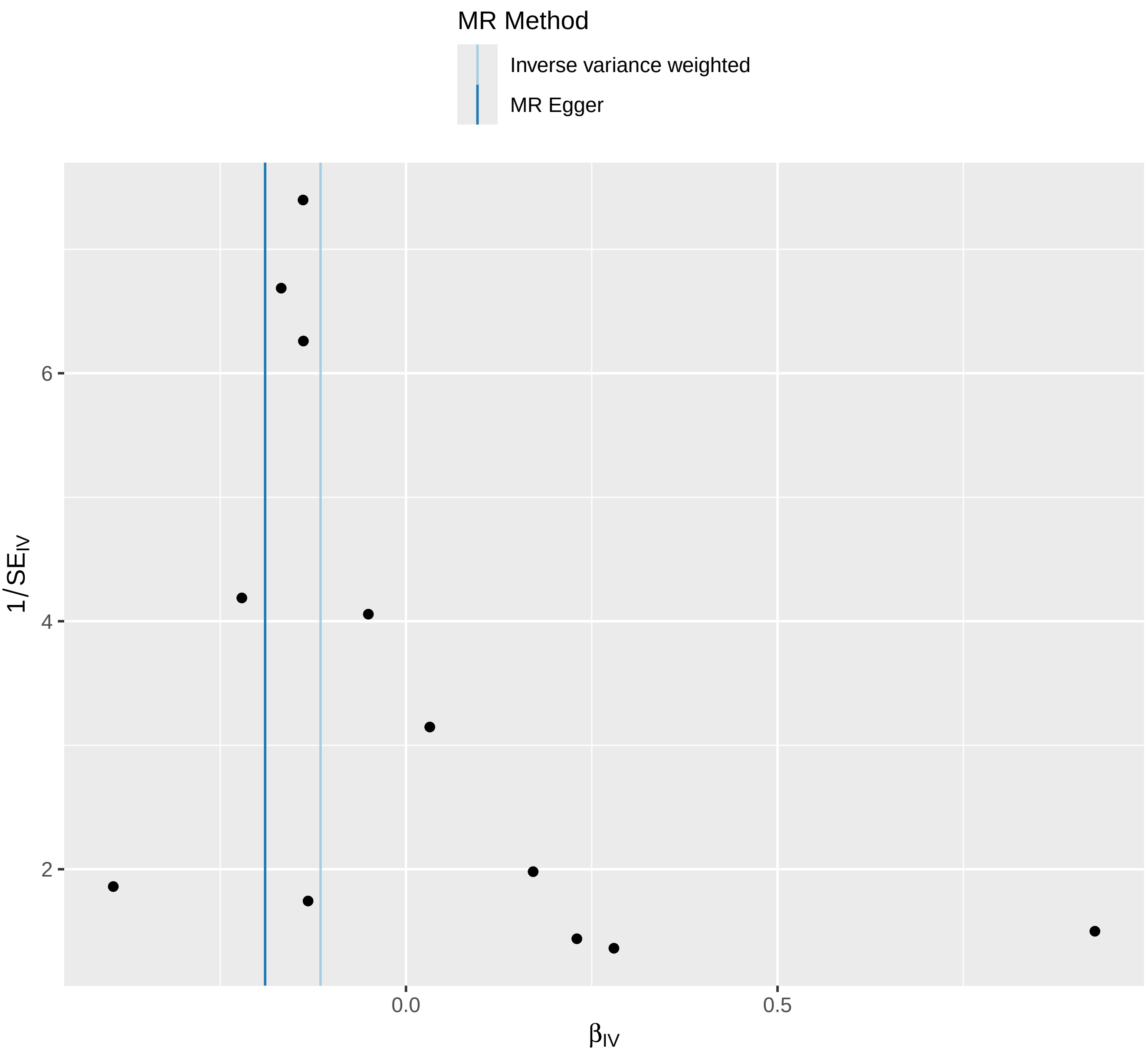
MR Method
Inverse variance weighted
MR Egger
1/SE_IV
6
4
2
0.0
0.5
β_IV

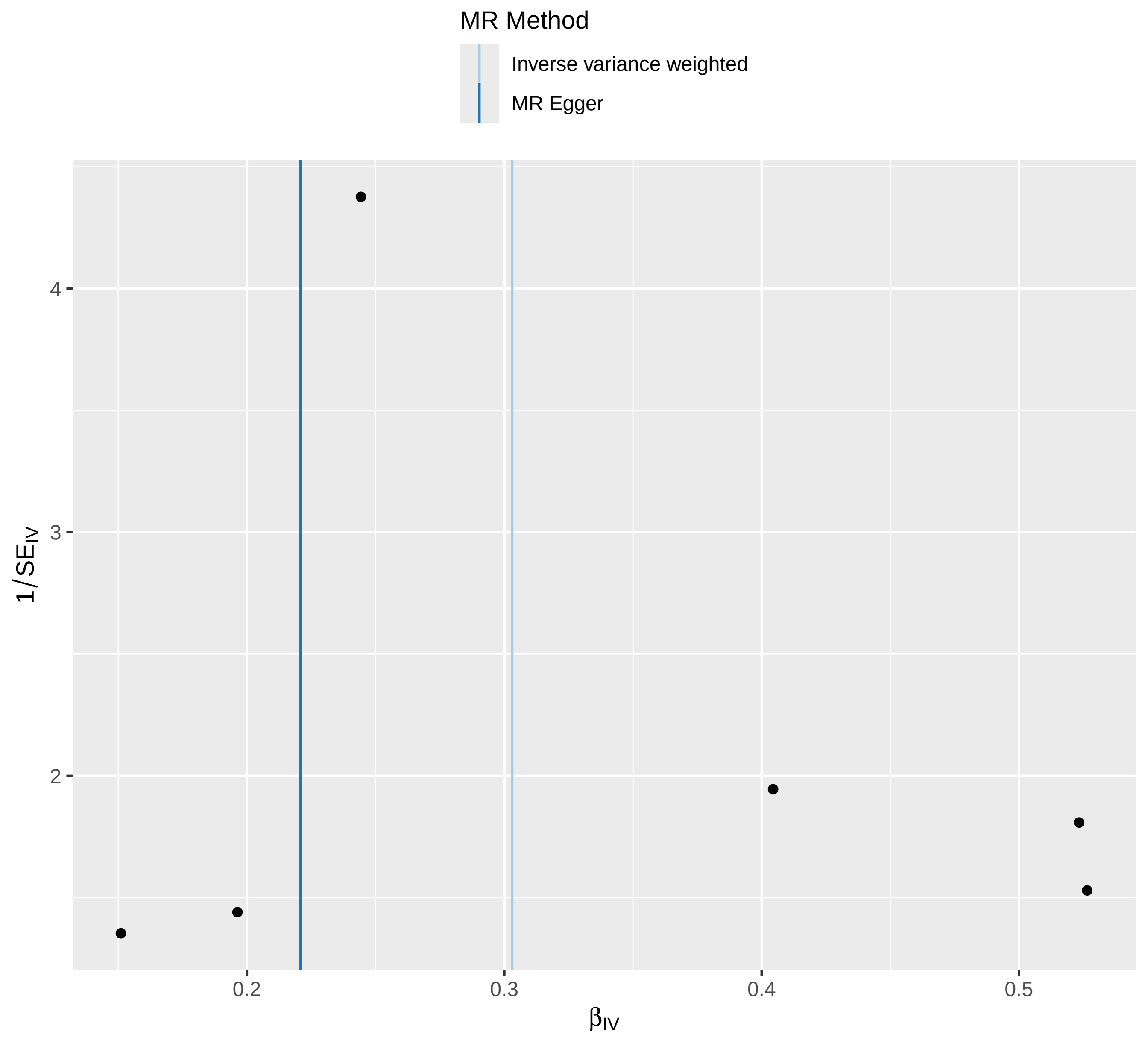
MR Method
Inverse variance weighted
MR Egger
1/SE$_{IV}$
4
3
2
0.2
0.3
0.4
0.5
β$_{IV}$

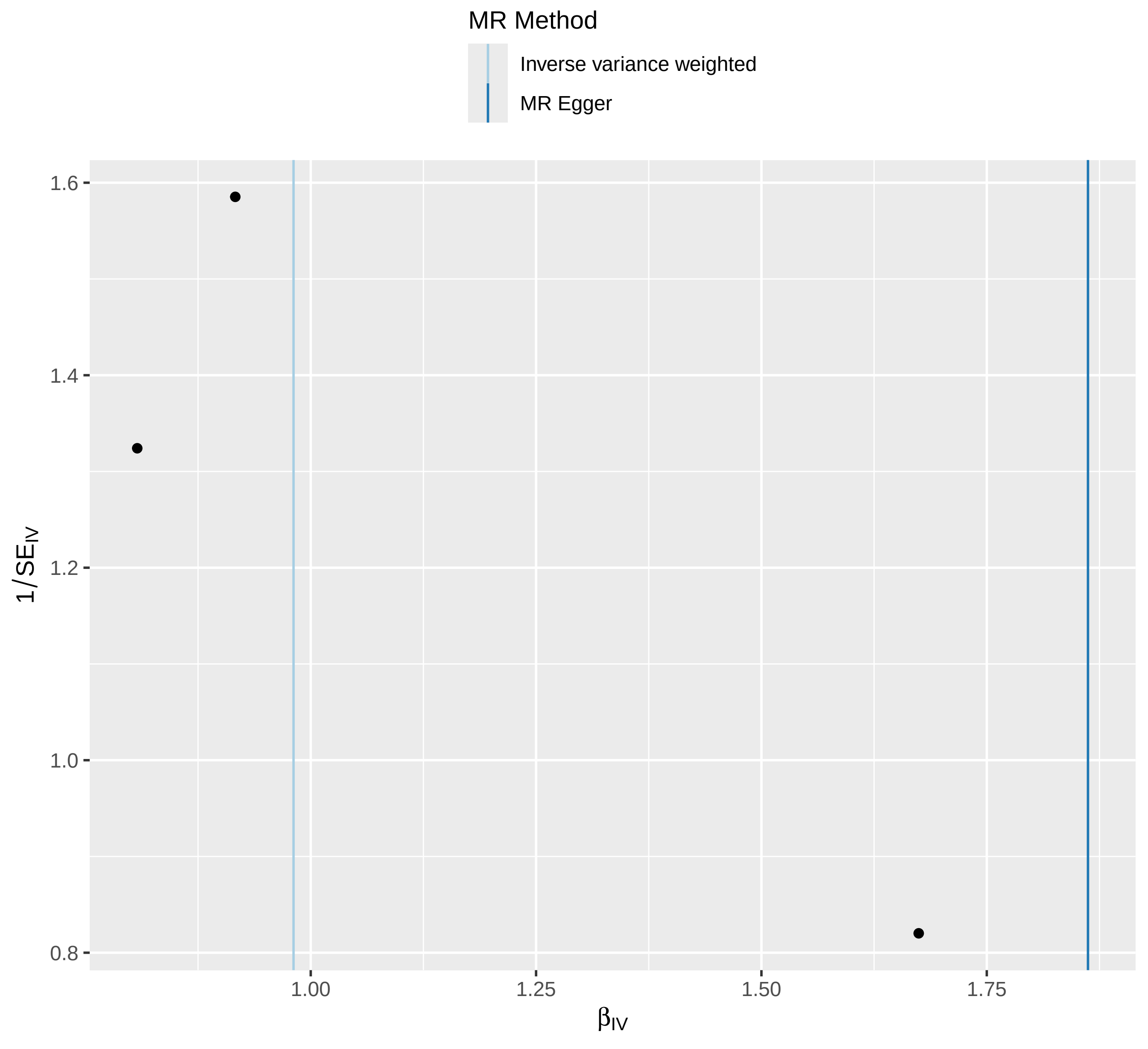
MR Method
Inverse variance weighted
MR Egger
1.6
1.4
1.2
1.0
0.8
1/SE$_{IV}$
1.00
1.25
1.50
1.75
β$_{IV}$

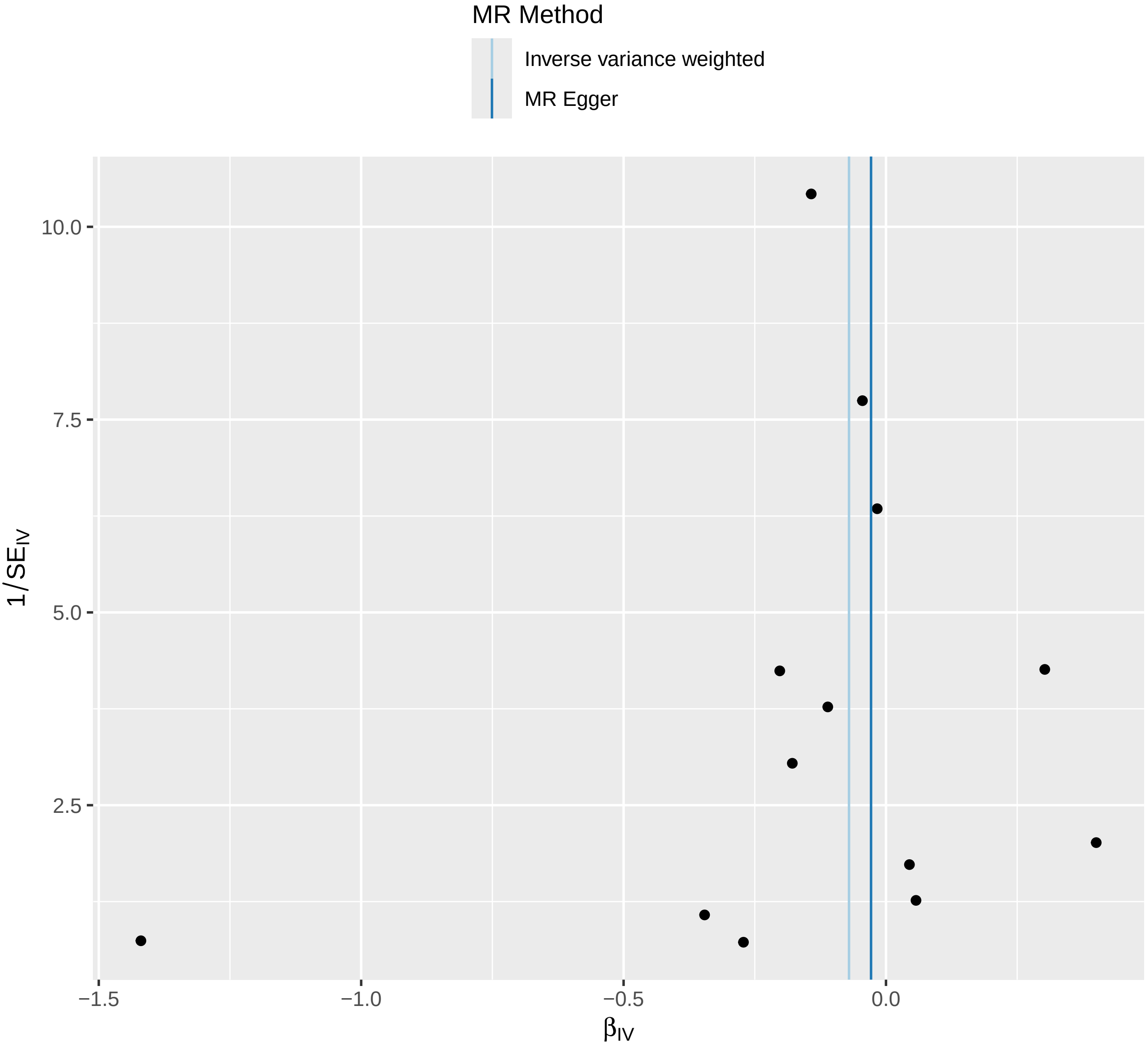

MR Method
Inverse variance weighted
MR Egger
10.0
7.5
5.0
2.5
1/SE$_{IV}$
−1.5
−1.0
−0.5
0.0
β$_{IV}$

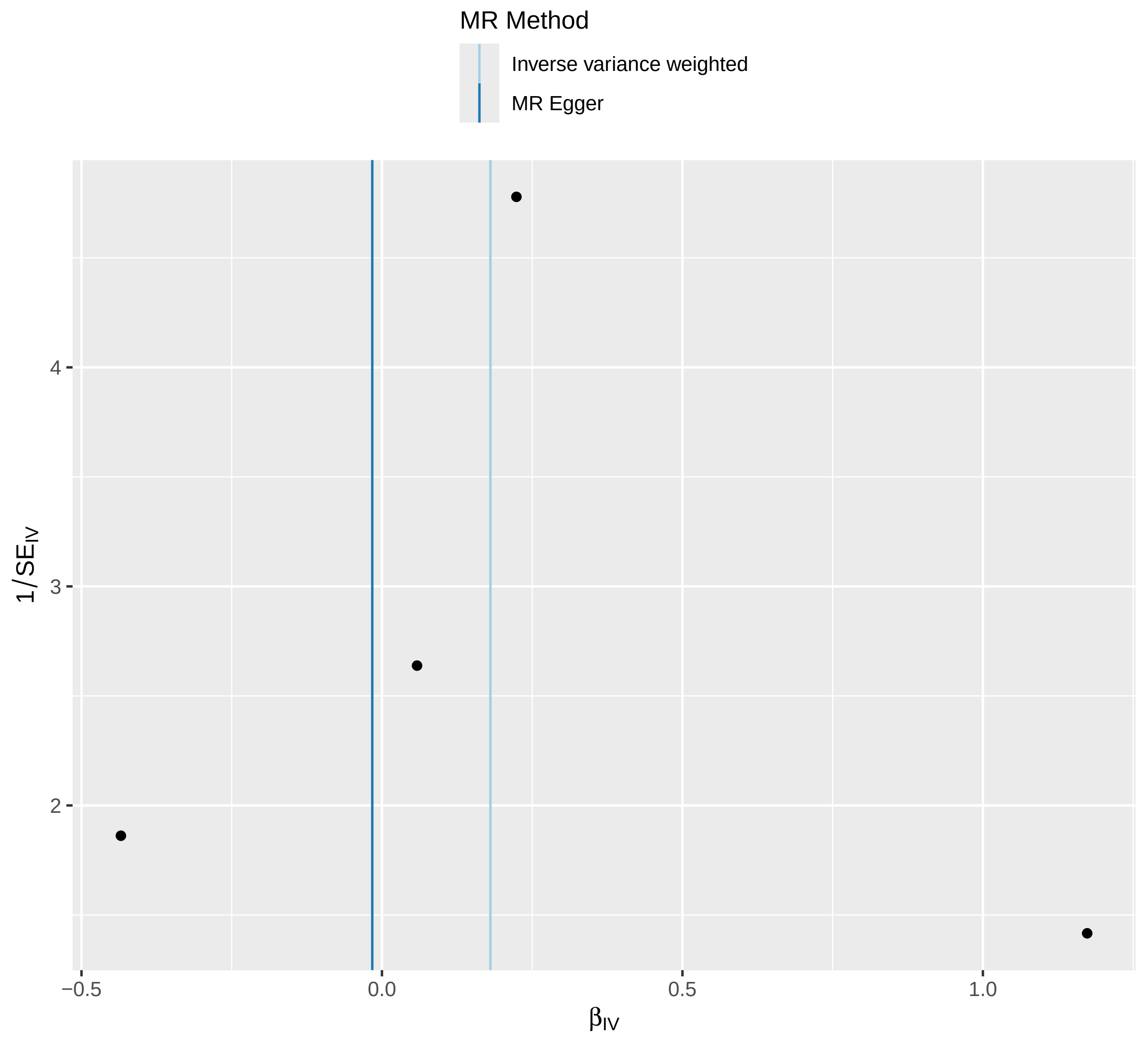

MR Method
Inverse variance weighted
MR Egger
1/SE_IV
4
3
2
−0.5
0.0
0.5
1.0
β_IV

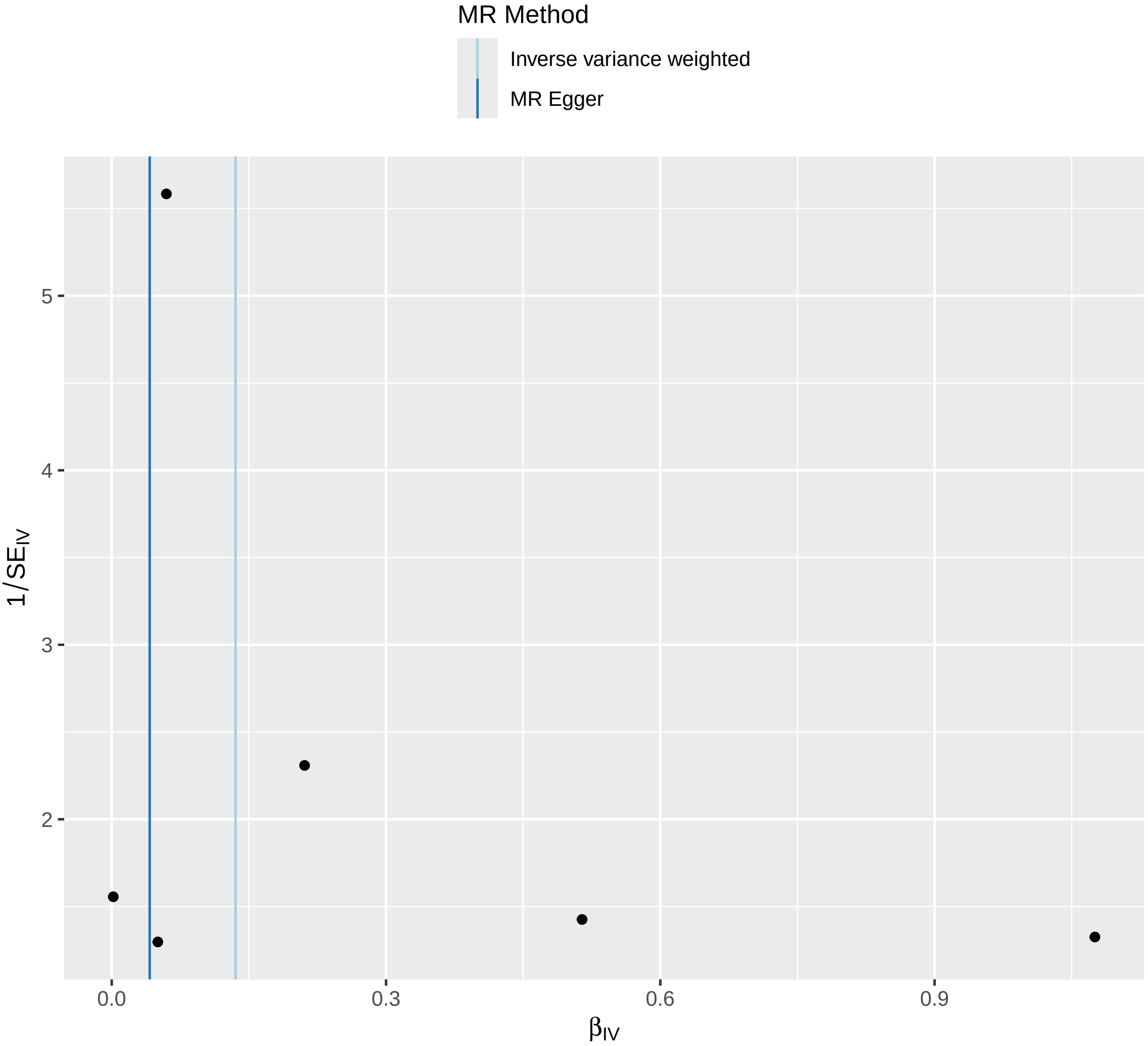

MR Method
Inverse variance weighted
MR Egger
5
4
3
2
1/SE$_{IV}$
0.0
0.3
0.6
0.9
β$_{IV}$

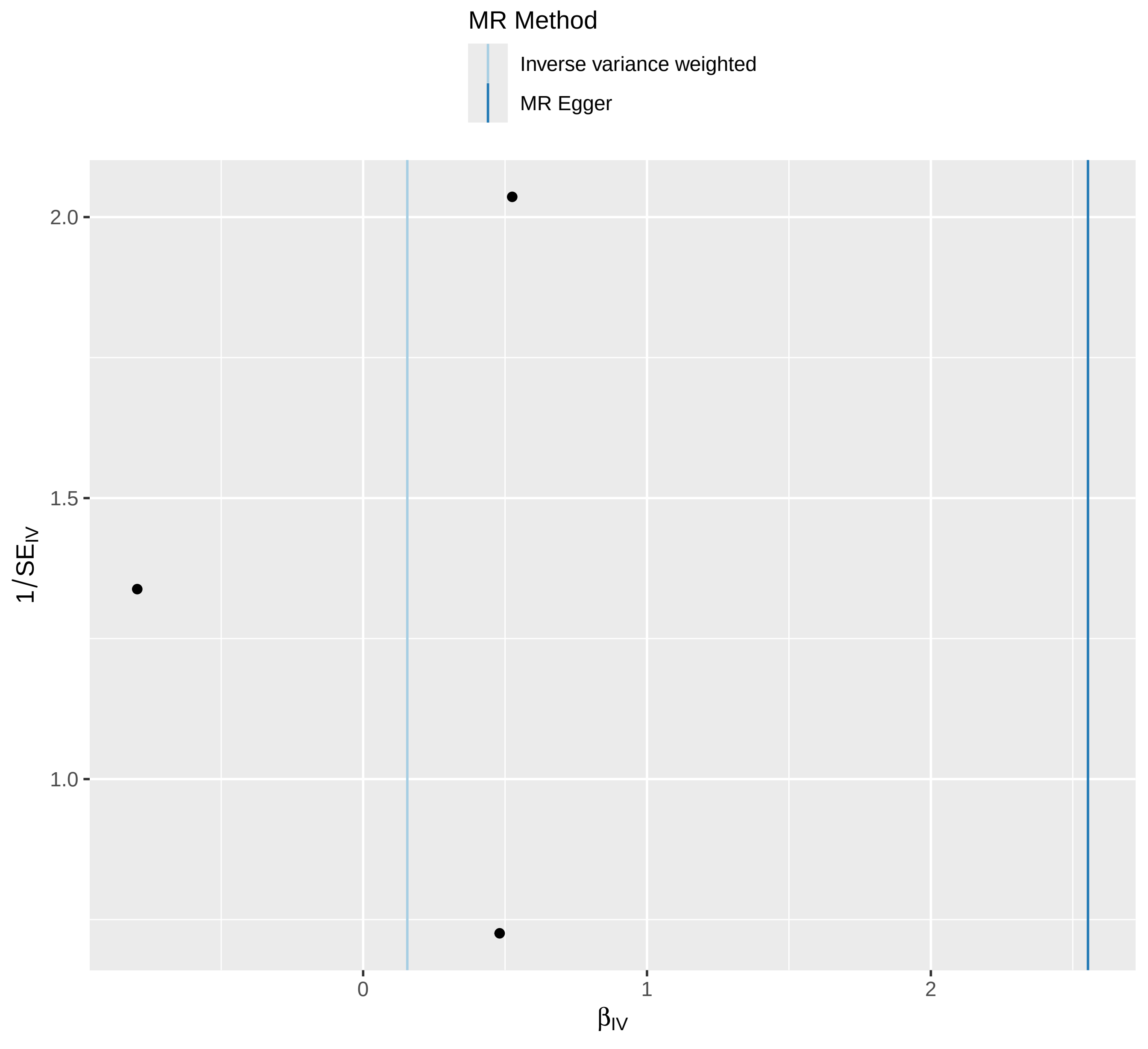

MR Method
Inverse variance weighted
MR Egger
2.0
1.5
1.0
0
1
2
$1/SE_{IV}$
β_{IV}

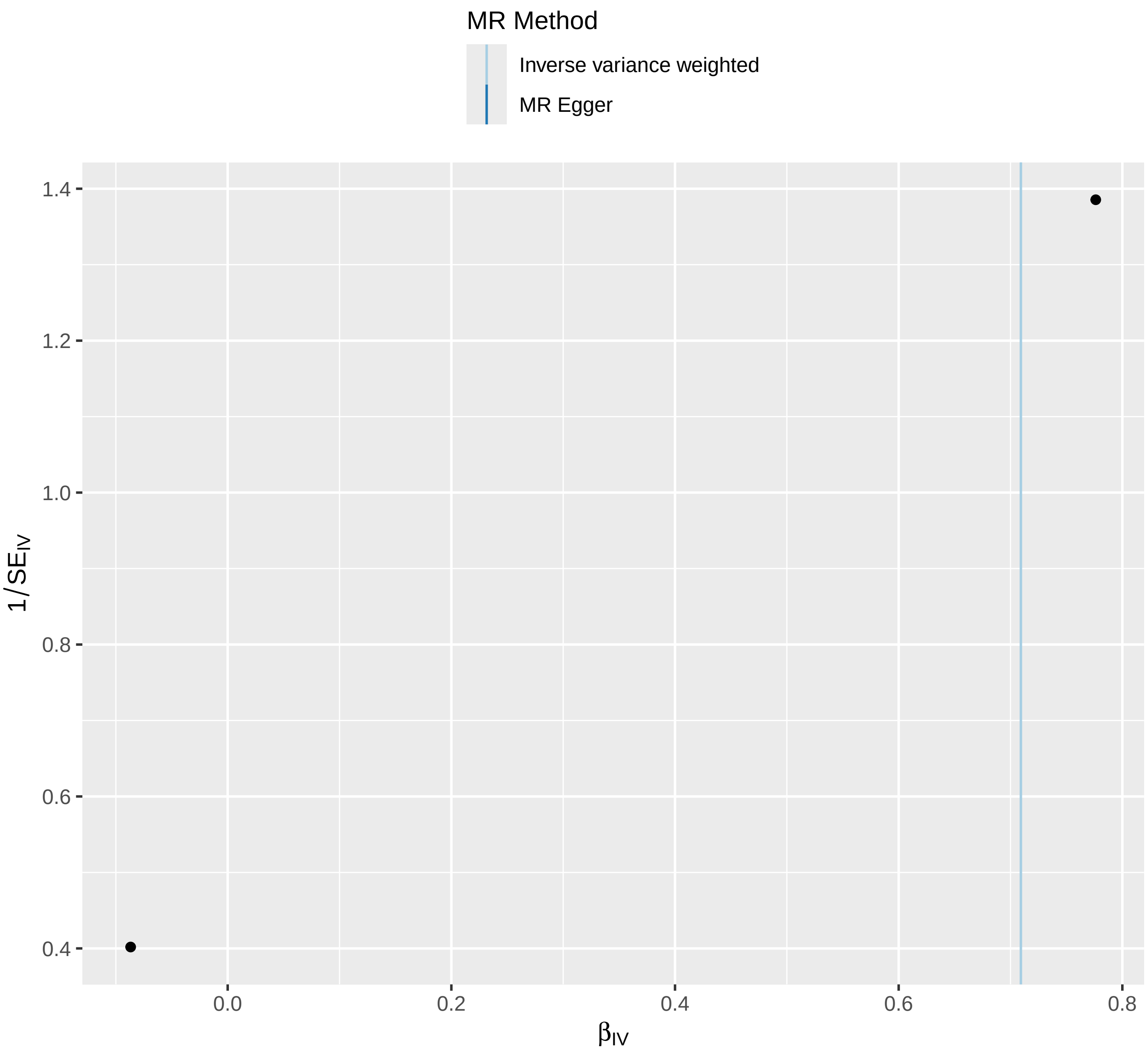

MR Method
Inverse variance weighted
MR Egger
1.4
1.2
1.0
0.8
0.6
0.4
$1/SE_{IV}$
0.0
0.2
0.4
0.6
0.8
β_{IV}

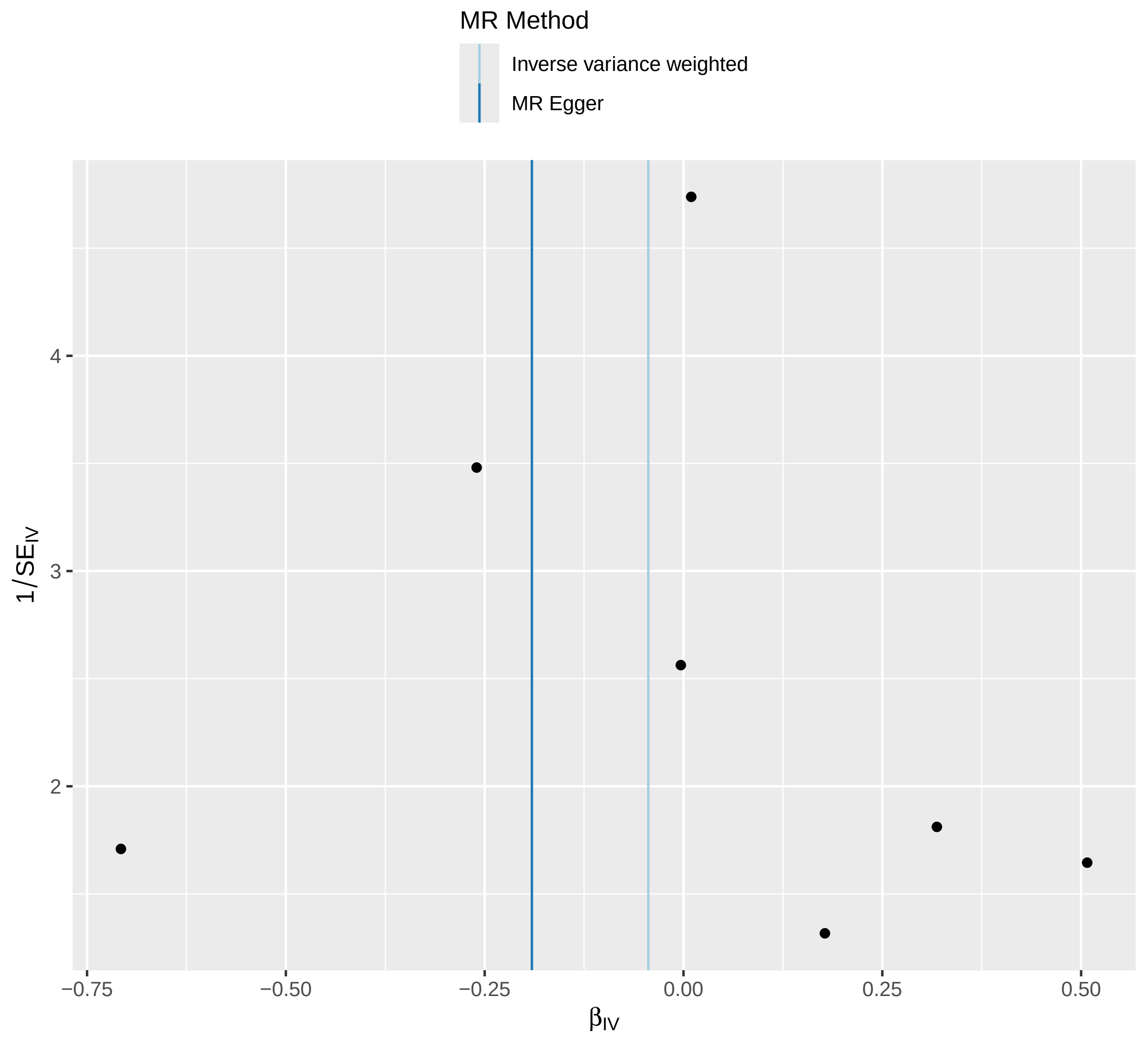

MR Method
Inverse variance weighted
MR Egger
4
3
2
$1/SE_{IV}$
−0.75
−0.50
−0.25
0.00
0.25
0.50
β_{IV}

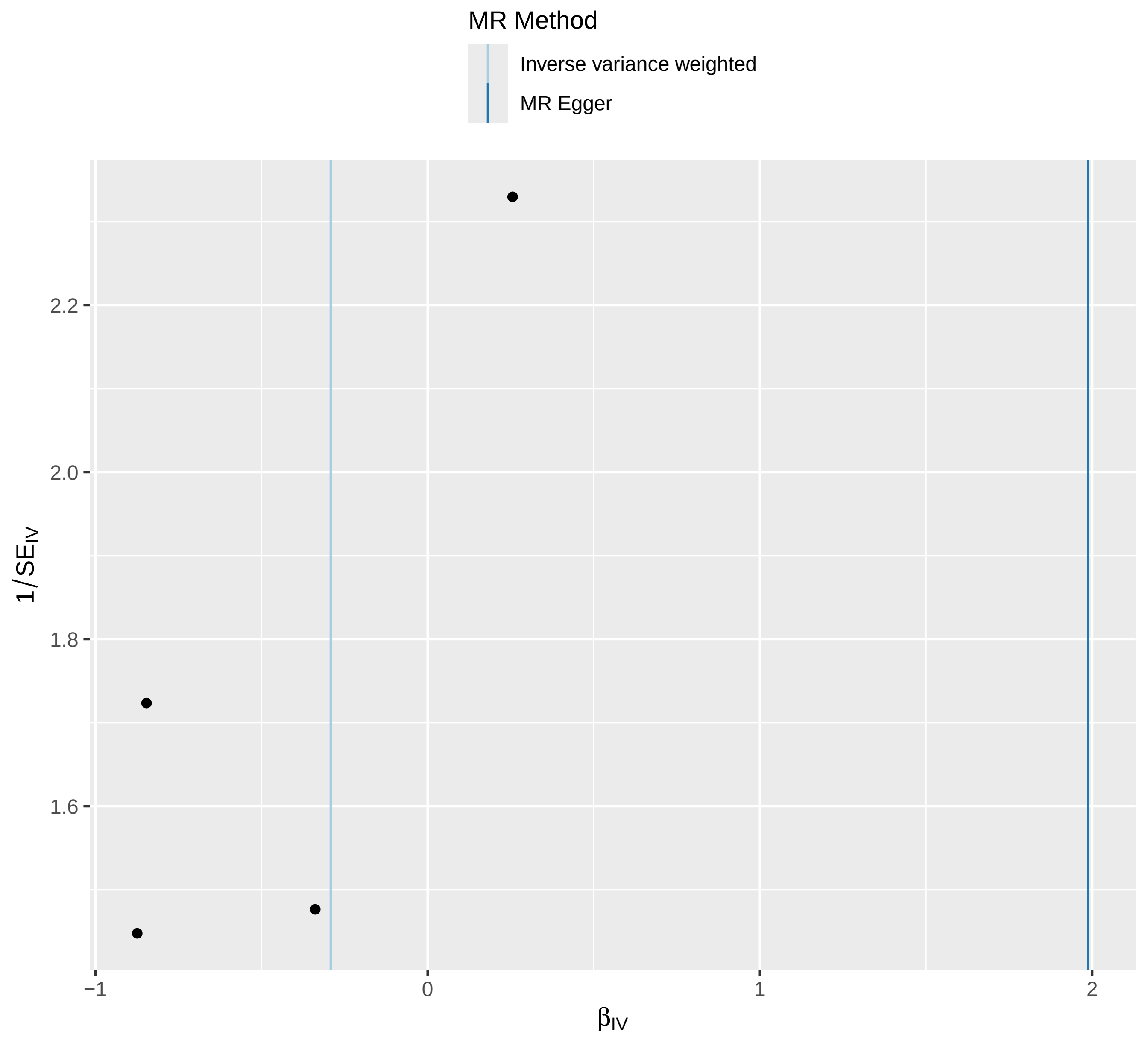
MR Method
Inverse variance weighted
MR Egger
2.2
2.0
1.8
1.6
1/SE_IV
−1
0
1
2
β_IV

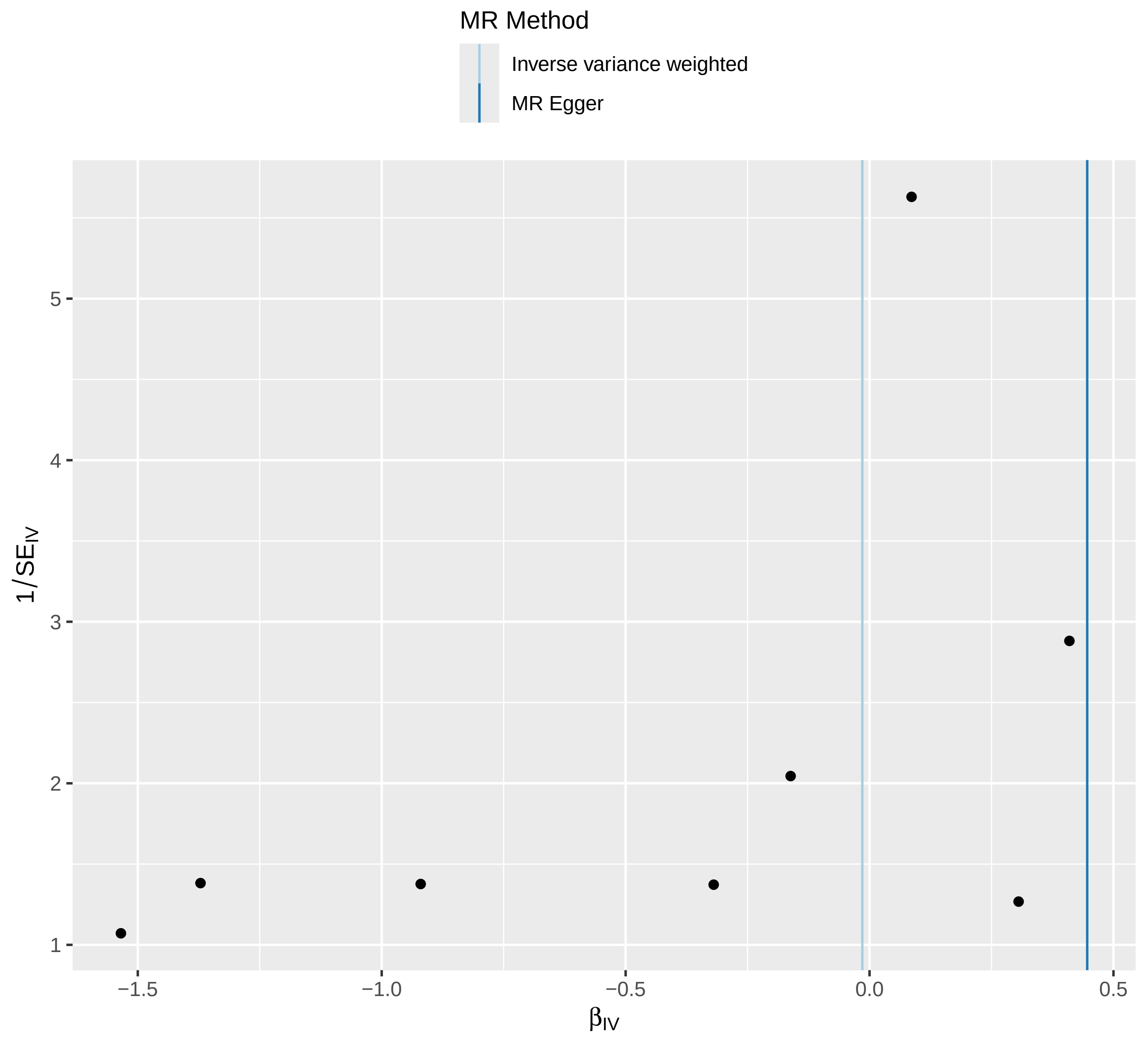
MR Method
Inverse variance weighted
MR Egger
1/SE_{IV}
5
4
3
2
1
−1.5
−1.0
−0.5
0.0
0.5
β_{IV}

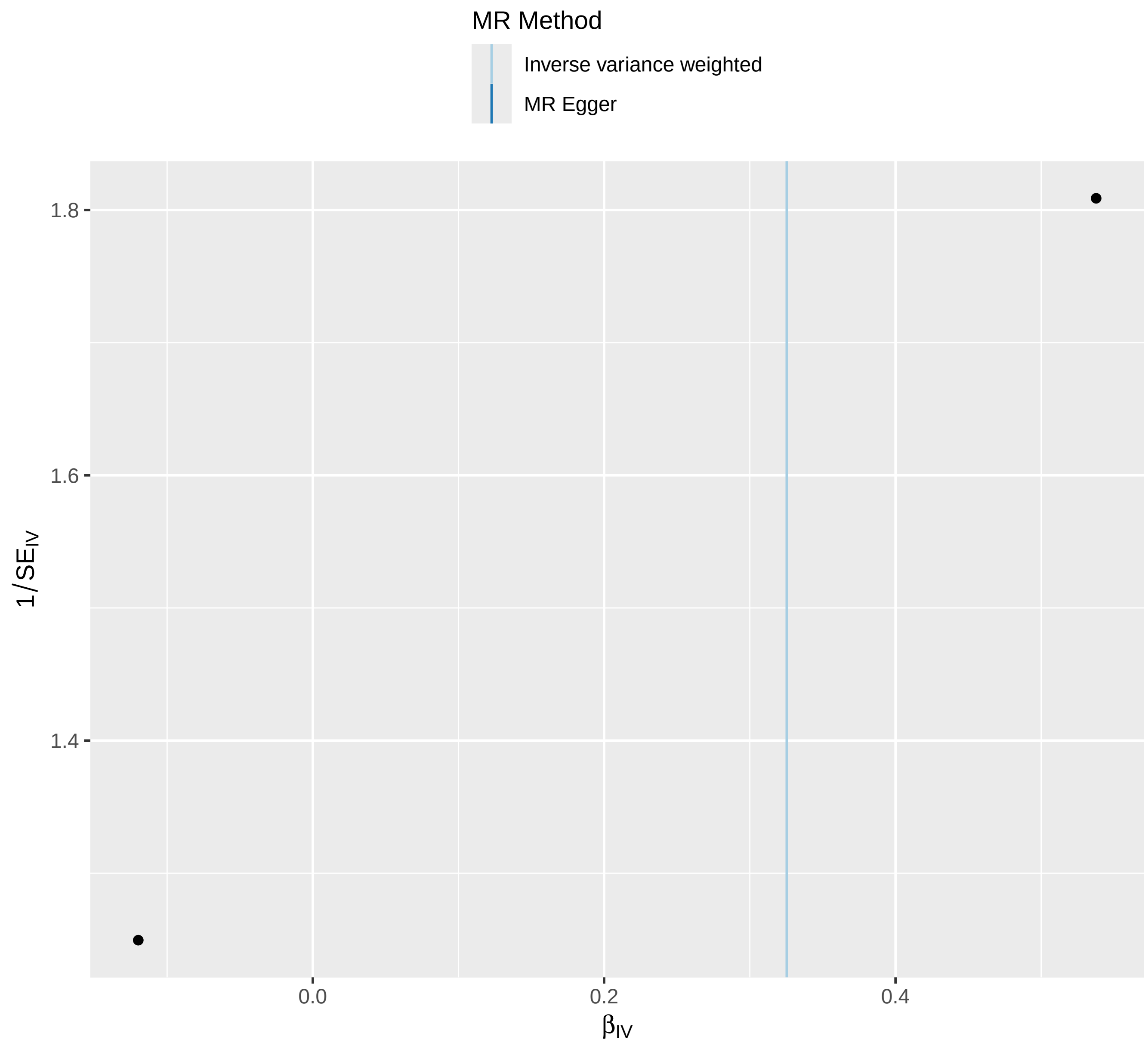
MR Method
Inverse variance weighted
MR Egger
1.8
1.6
1.4
$1/SE_{IV}$
0.0
0.2
0.4
β_{IV}

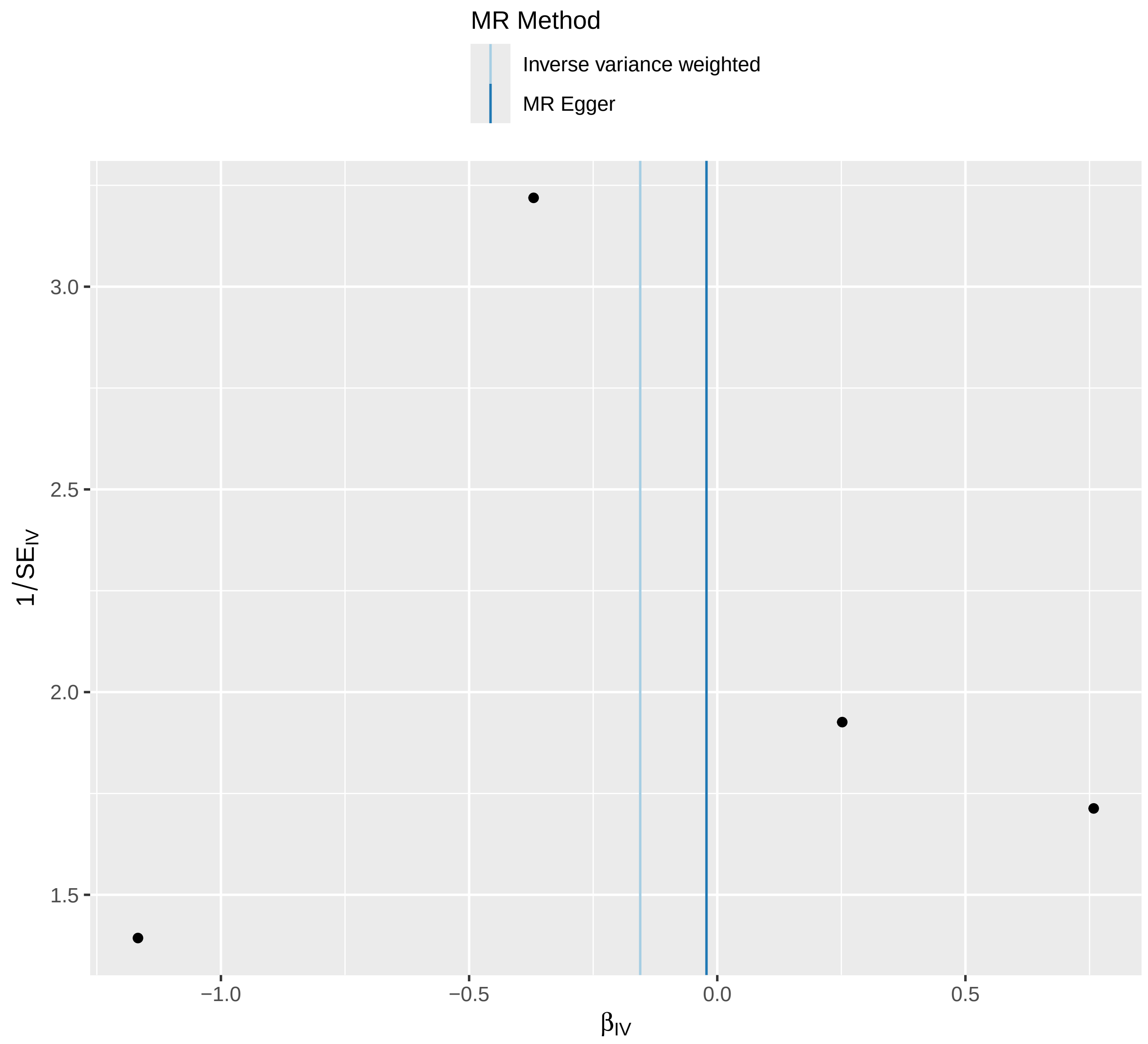

MR Method
Inverse variance weighted
MR Egger
3.0
2.5
2.0
1.5
1/SE_IV
−1.0
−0.5
0.0
0.5
β_IV

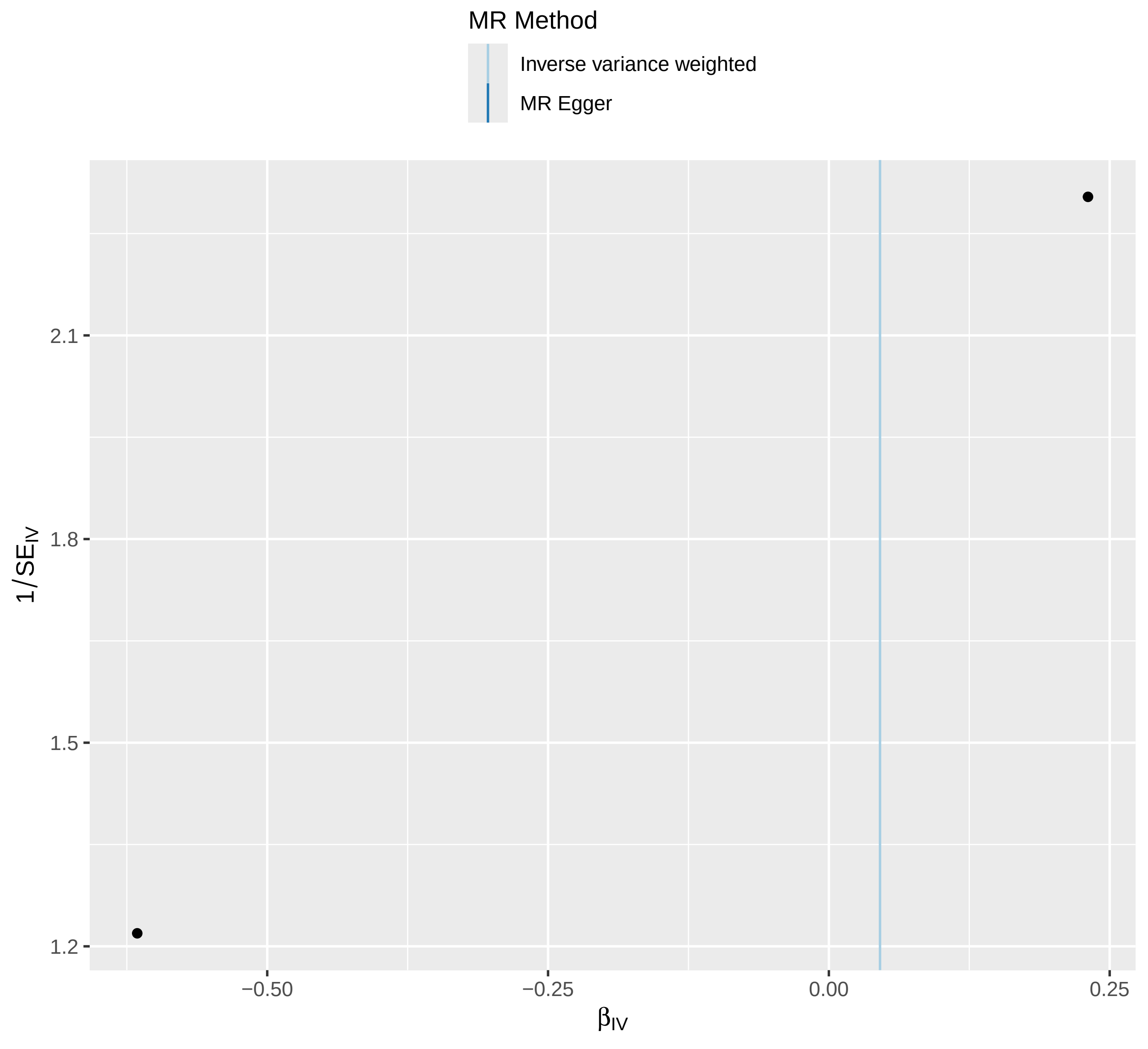
MR Method
Inverse variance weighted
MR Egger
2.1
1.8
1.5
1.2
$1/SE_{IV}$
−0.50
−0.25
0.00
0.25
β_{IV}

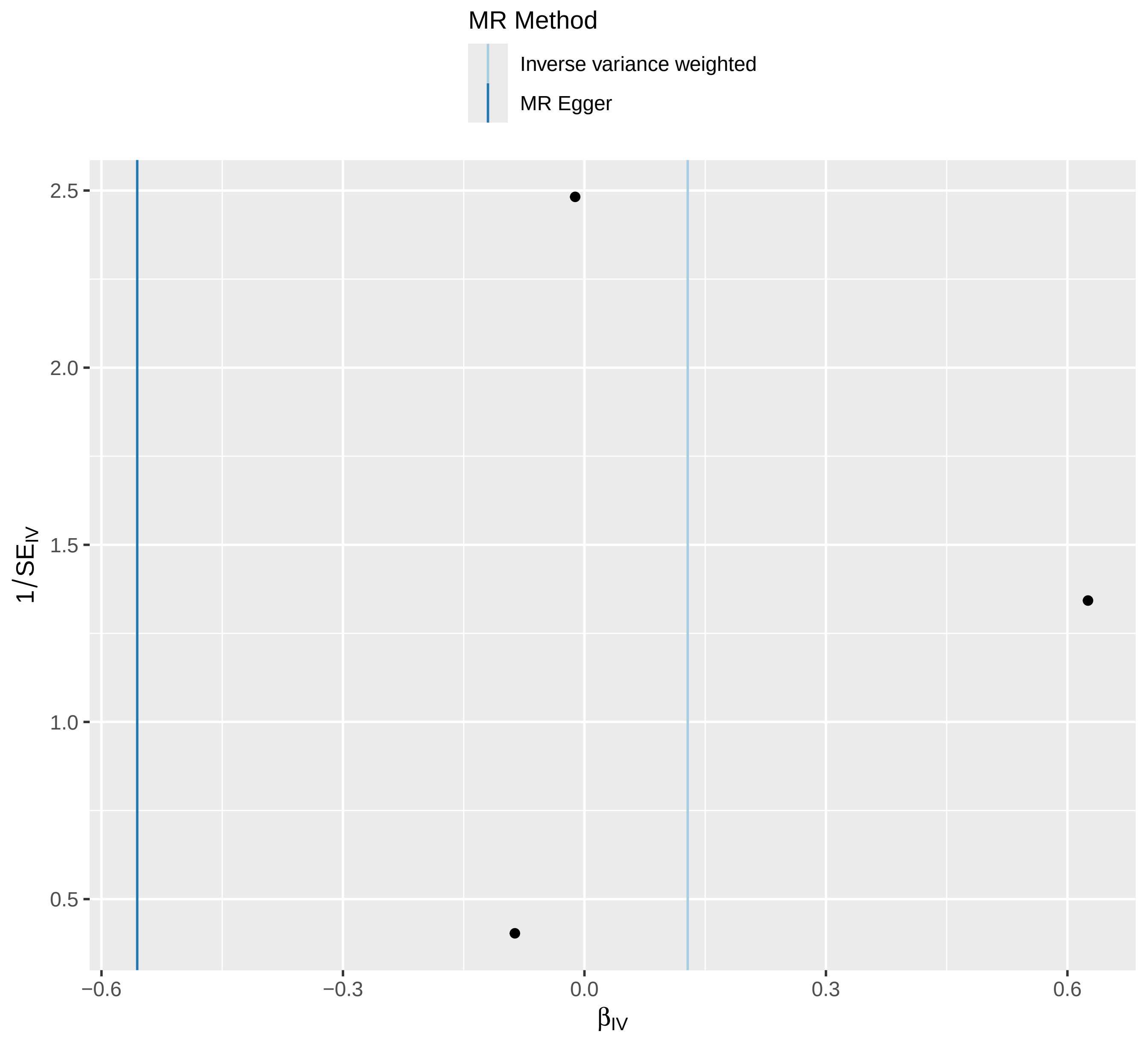
MR Method
Inverse variance weighted
MR Egger
2.5
2.0
1.5
1.0
0.5
$1/SE_{IV}$
−0.6
−0.3
0.0
0.3
0.6
β_{IV}

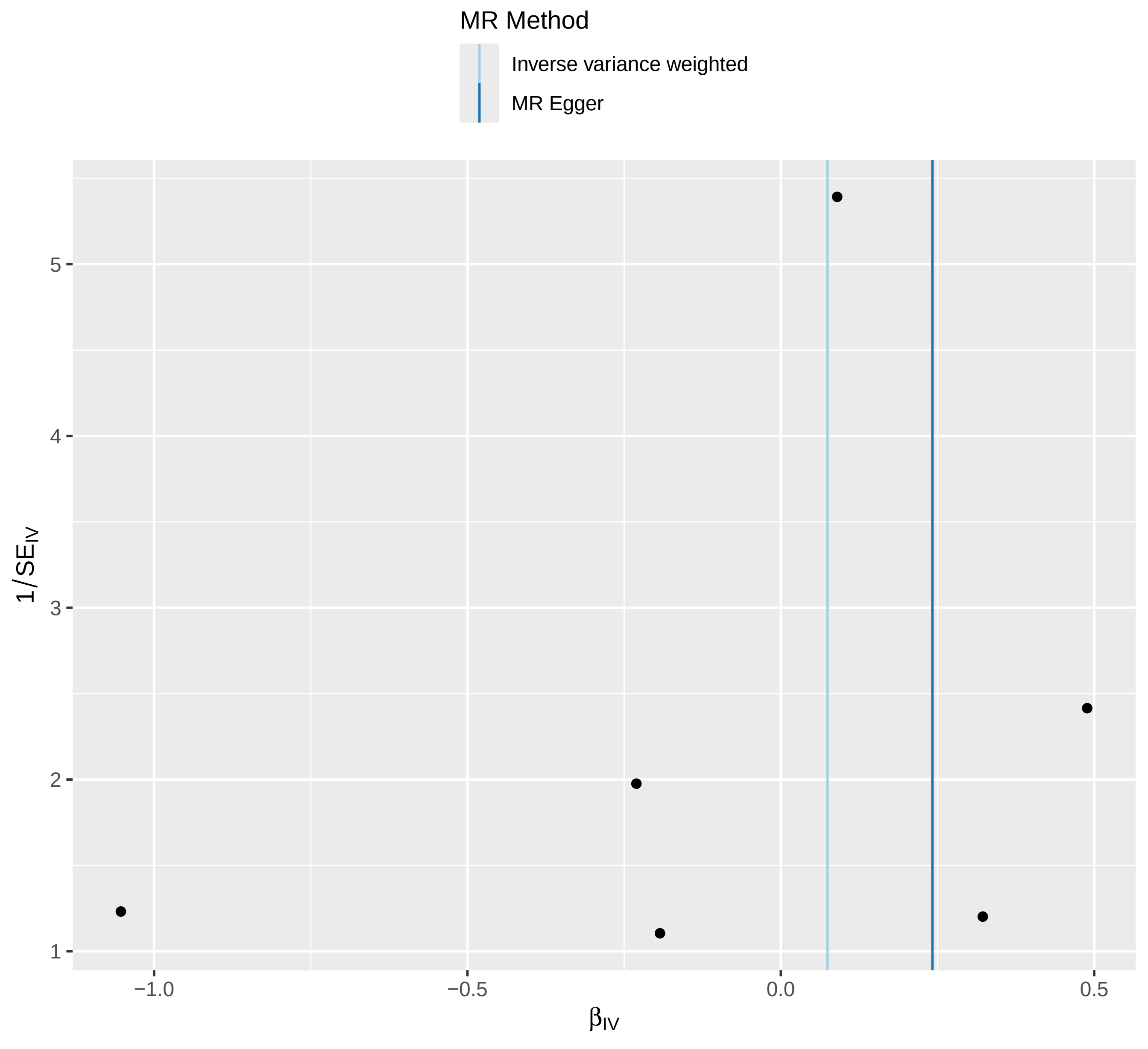
MR Method
Inverse variance weighted
MR Egger
1/SE_IV
β_IV
5
4
3
2
1
−1.0
−0.5
0.0
0.5

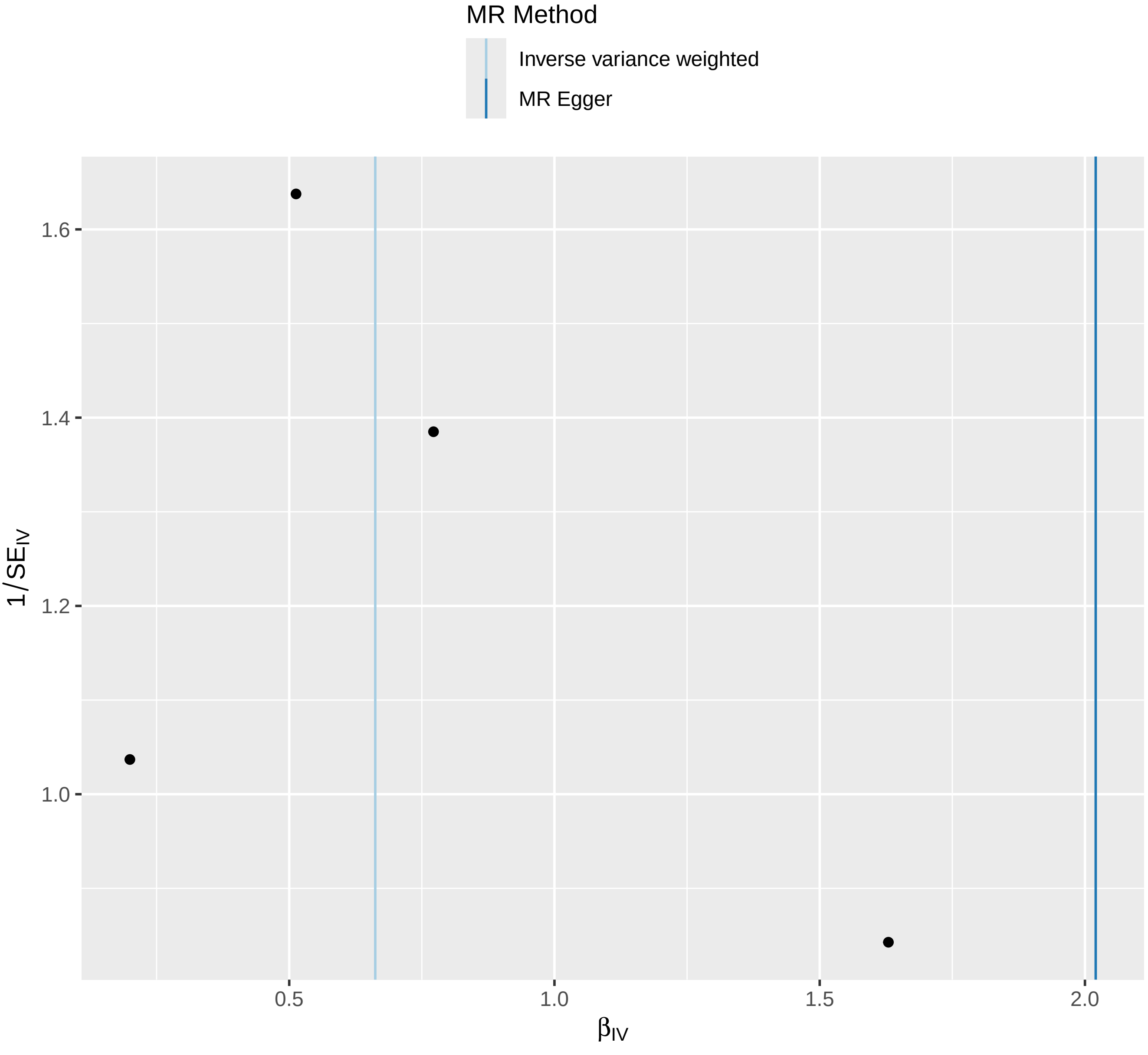
MR Method
Inverse variance weighted
MR Egger
1.6
1.4
1.2
1.0
0.5
1.0
1.5
2.0
$1/SE_{IV}$
β_{IV}

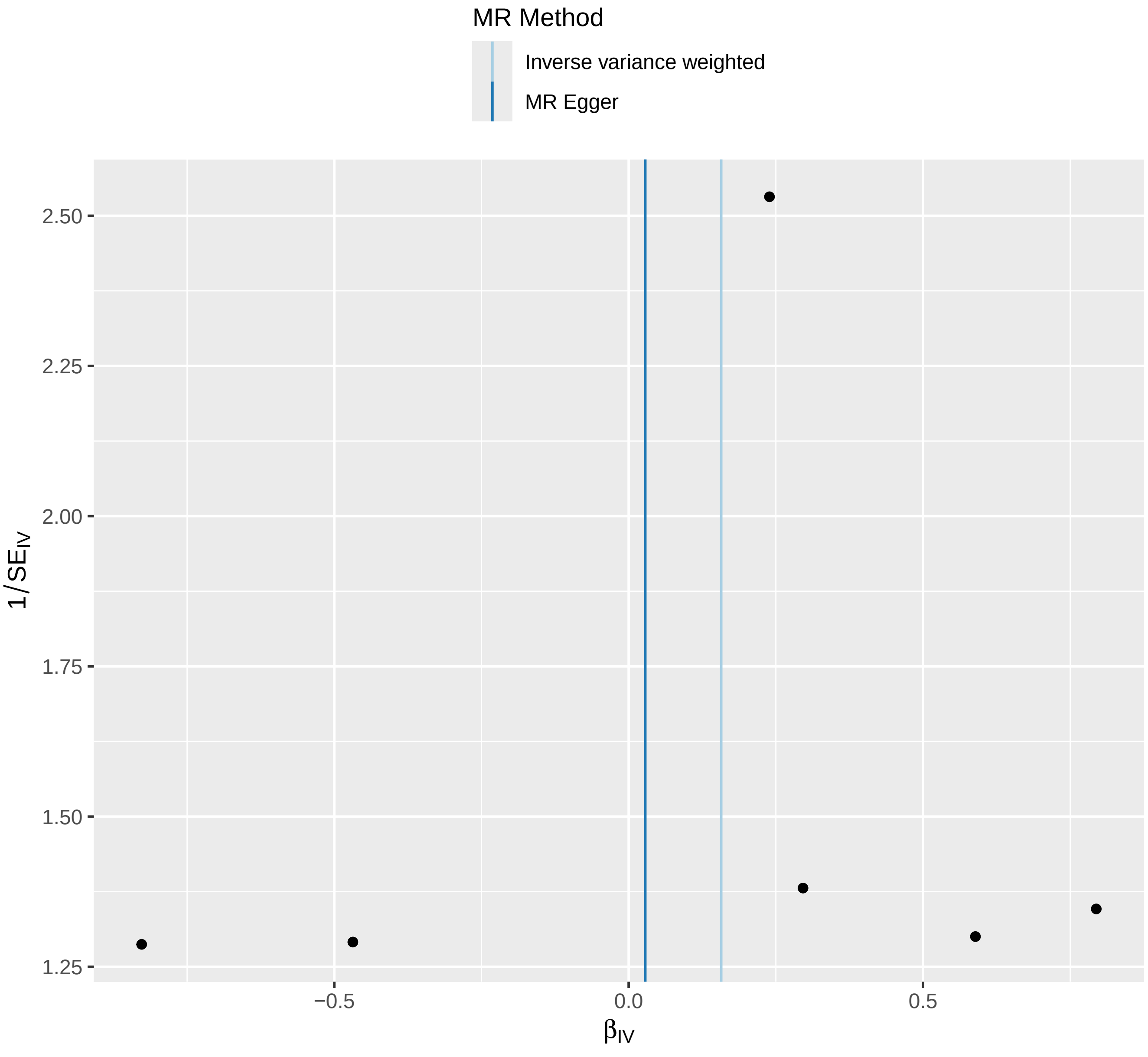
MR Method
Inverse variance weighted
MR Egger
2.50
2.25
2.00
1.75
1.50
1.25
$1/SE_{IV}$
−0.5
0.0
0.5
β_{IV}

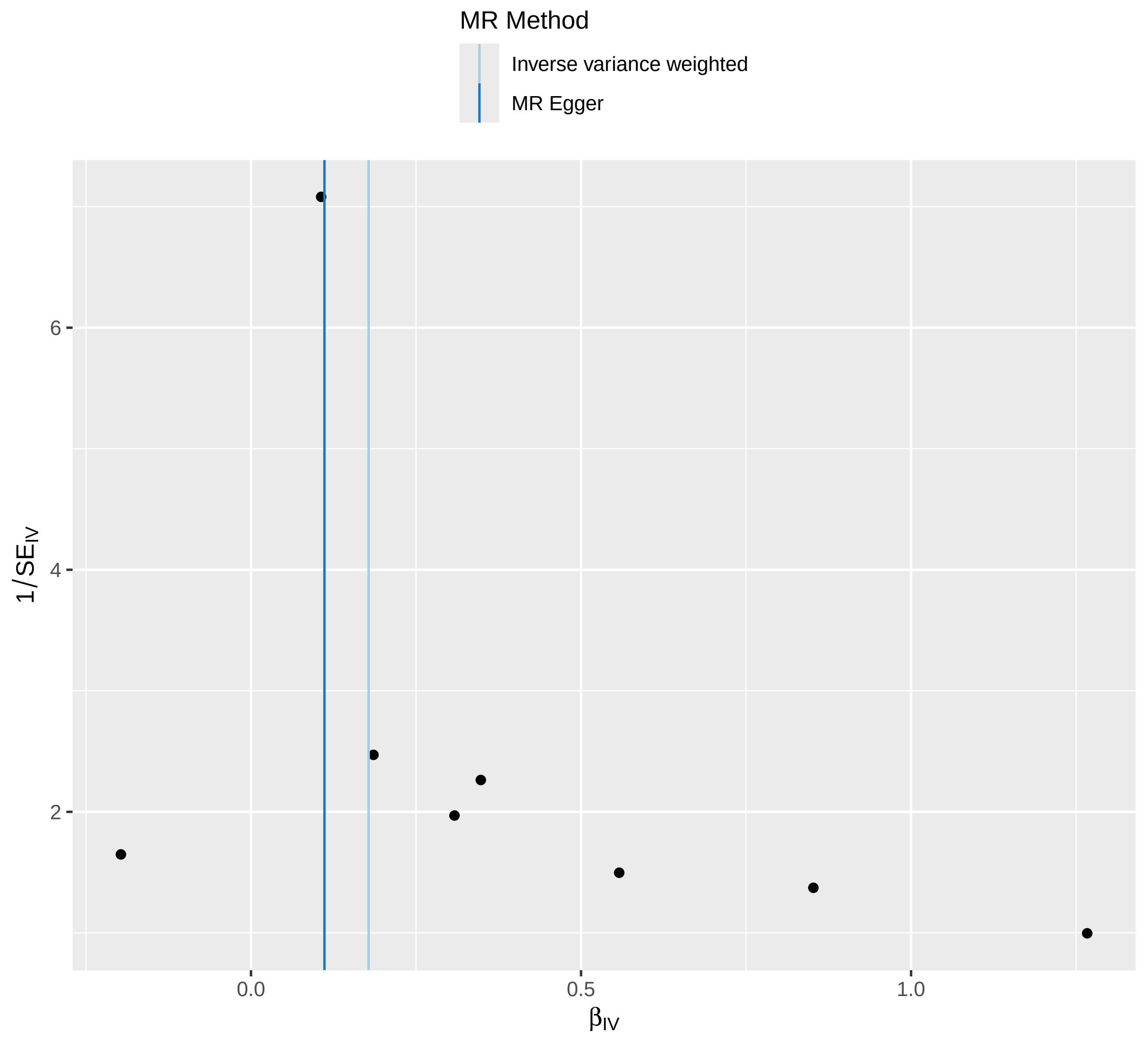
MR Method
Inverse variance weighted
MR Egger
6
4
2
1/SE_IV
0.0
0.5
1.0
β_IV

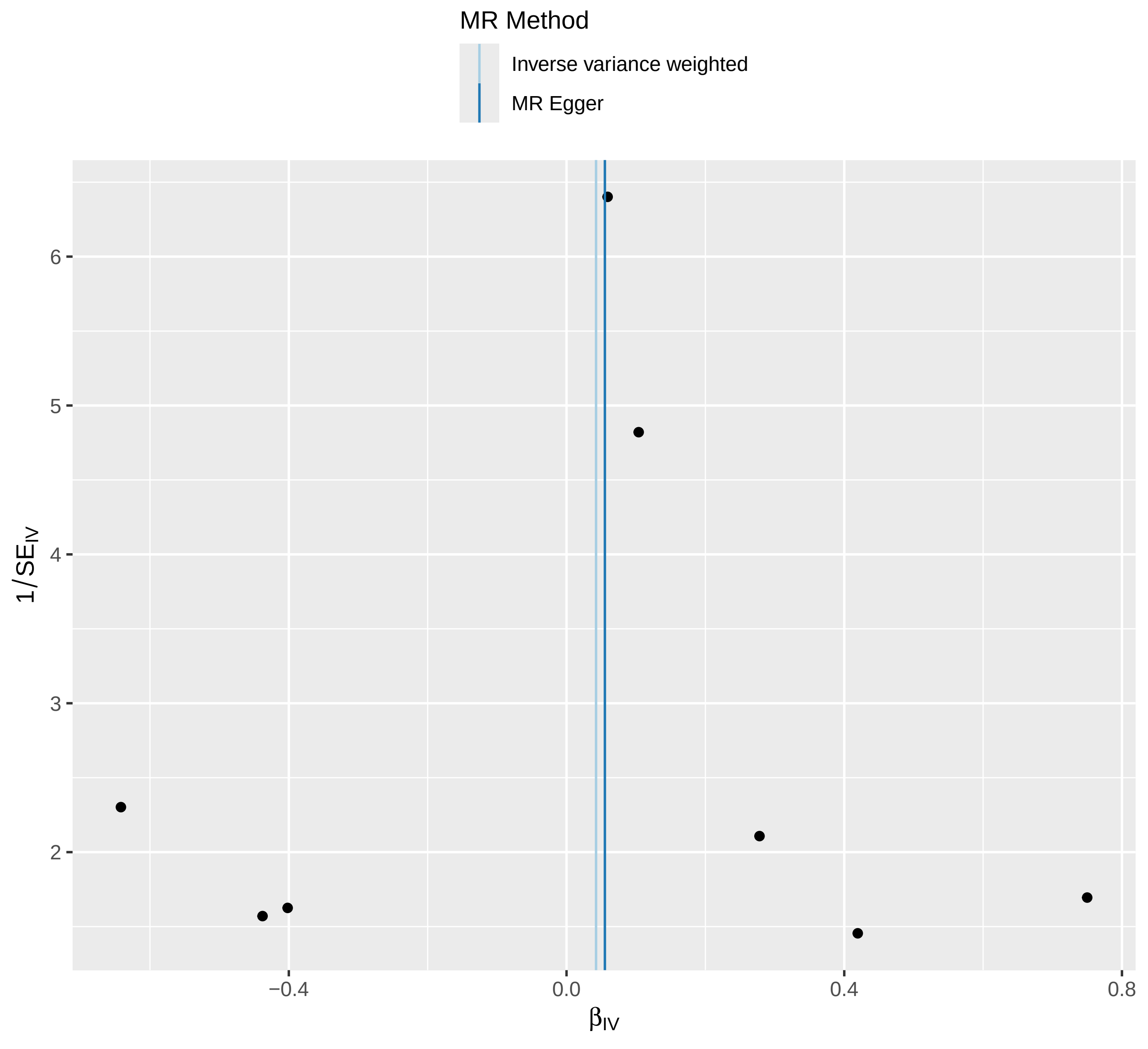

MR Method
Inverse variance weighted
MR Egger
1/SE_IV
6
5
4
3
2
−0.4
0.0
0.4
0.8
β_IV

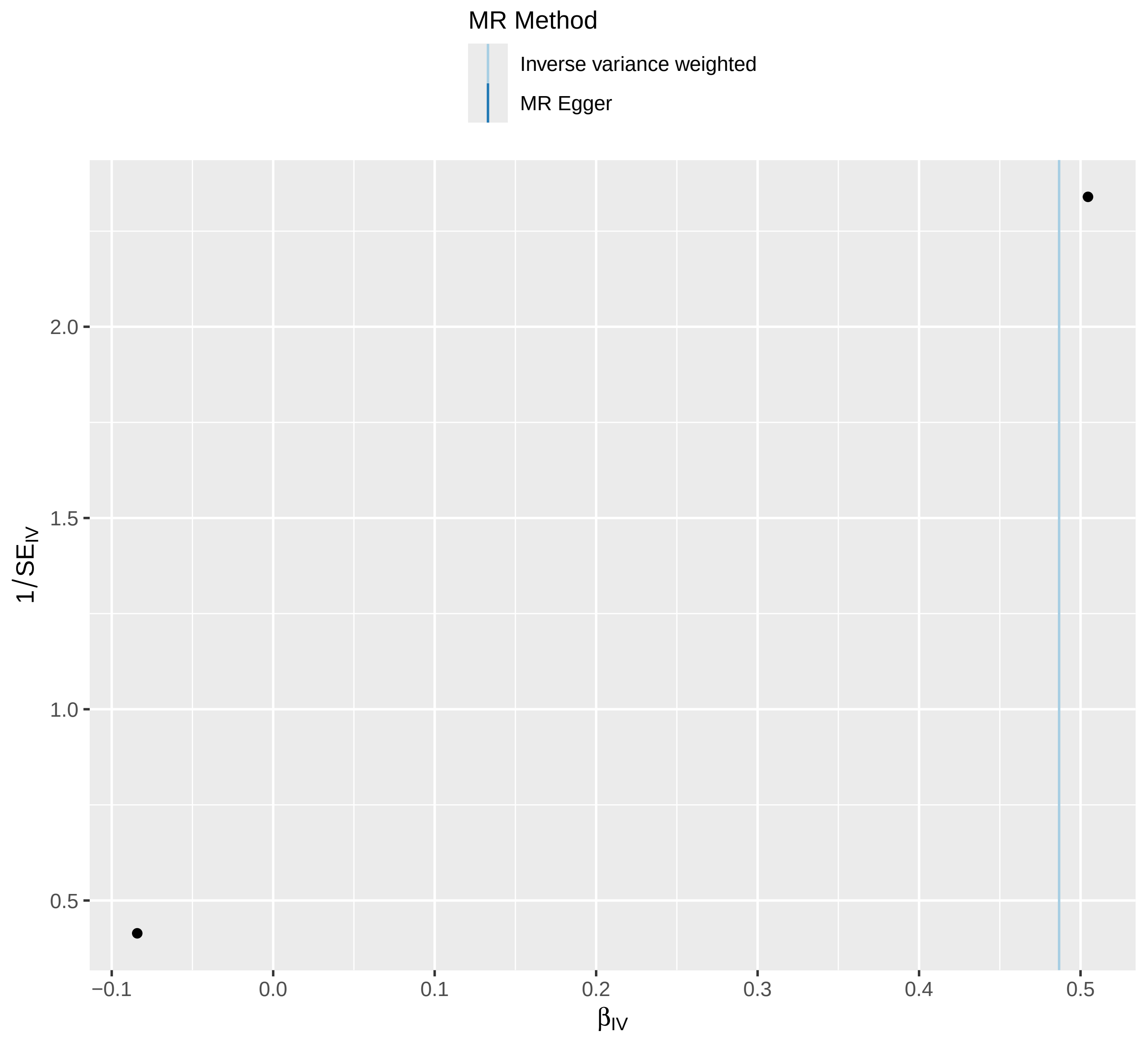
MR Method
Inverse variance weighted
MR Egger
2.0
1.5
1.0
0.5
1/SE_IV
−0.1
0.0
0.1
0.2
0.3
0.4
0.5
β_IV

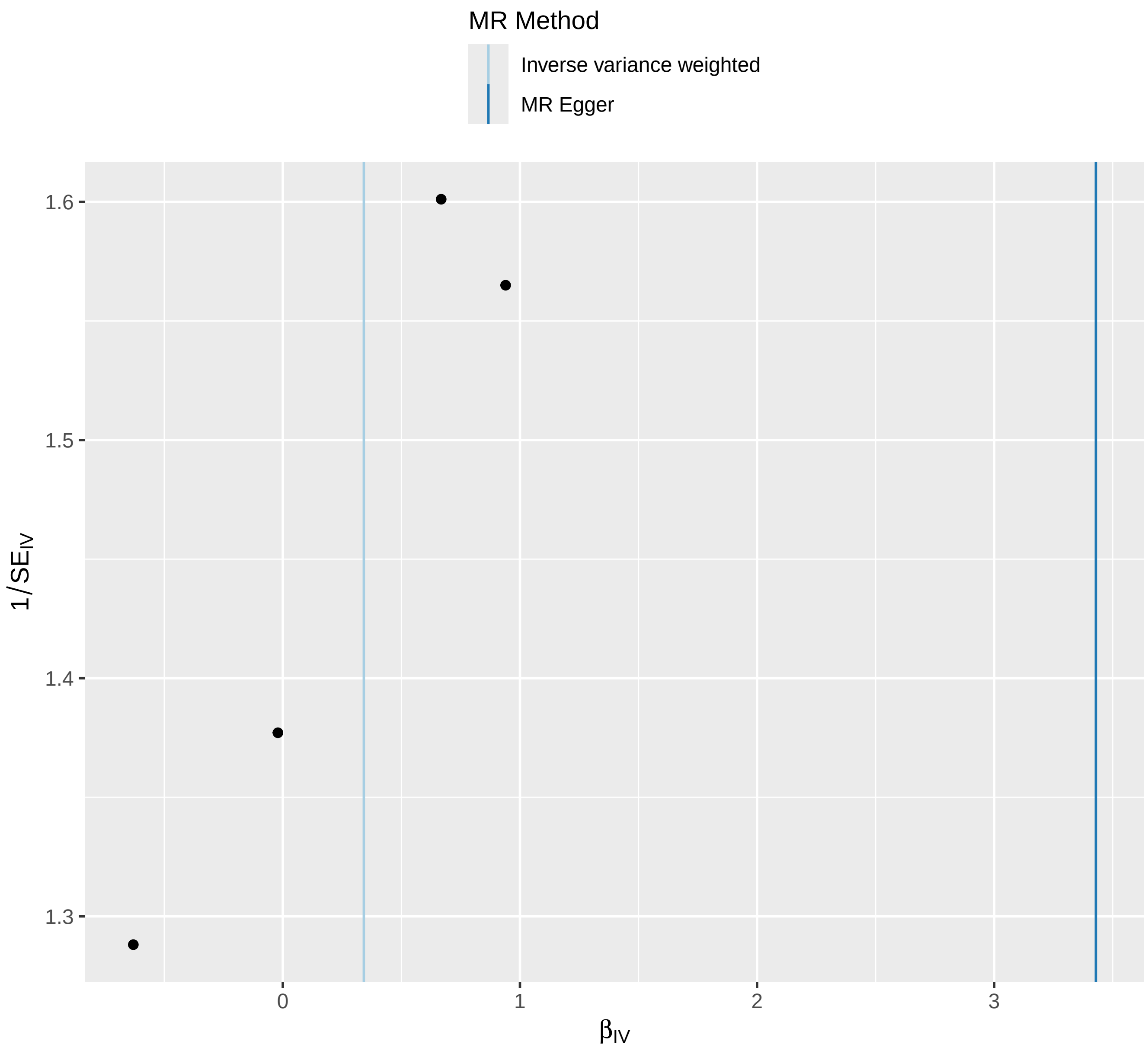
MR Method
Inverse variance weighted
MR Egger
1.6
1.5
1.4
1.3
1/SE$_{IV}$
0
1
2
3
β$_{IV}$

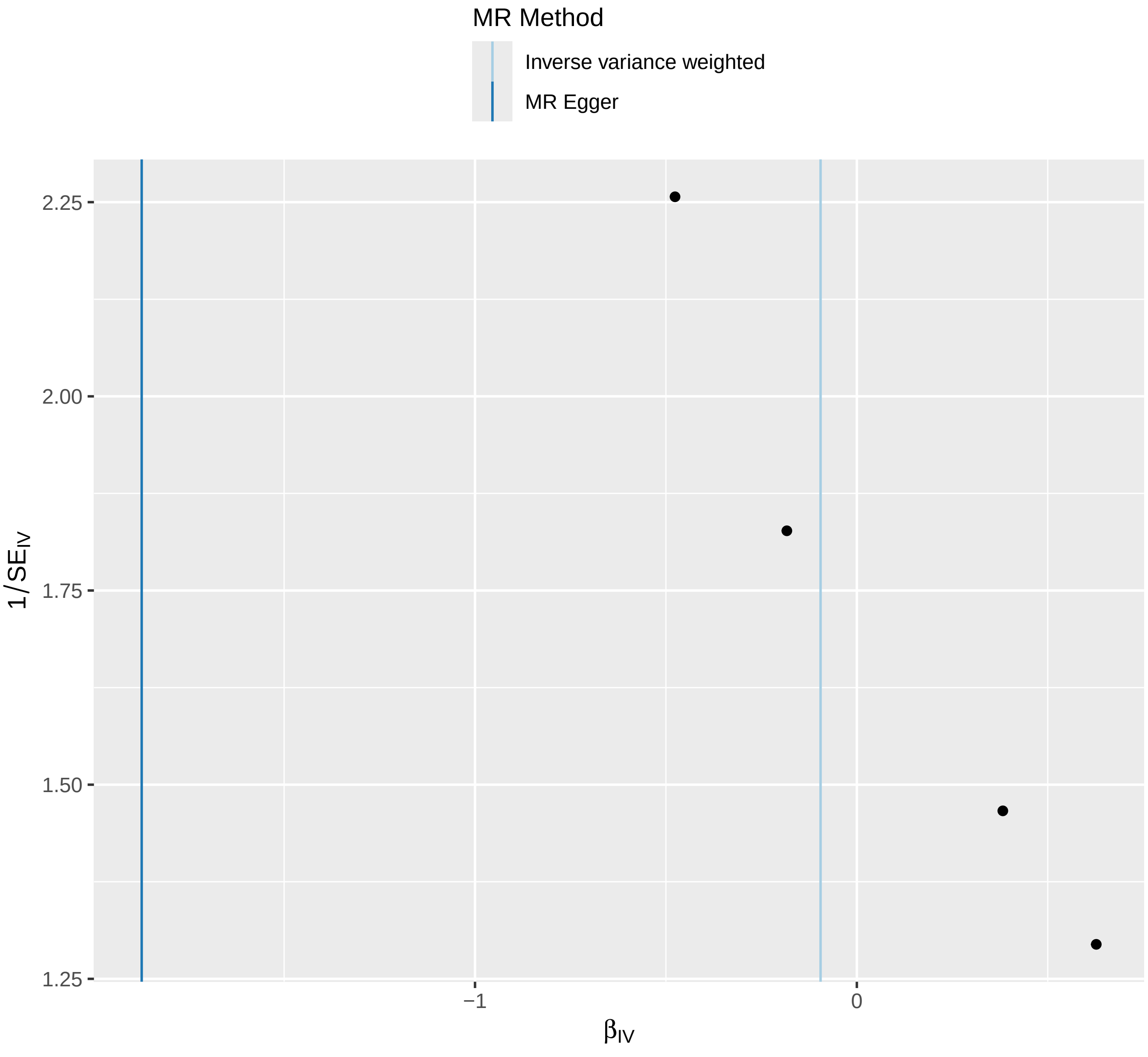
MR Method
Inverse variance weighted
MR Egger
2.25
2.00
1.75
1.50
1.25
1/SE$_{IV}$
−1
0
β$_{IV}$

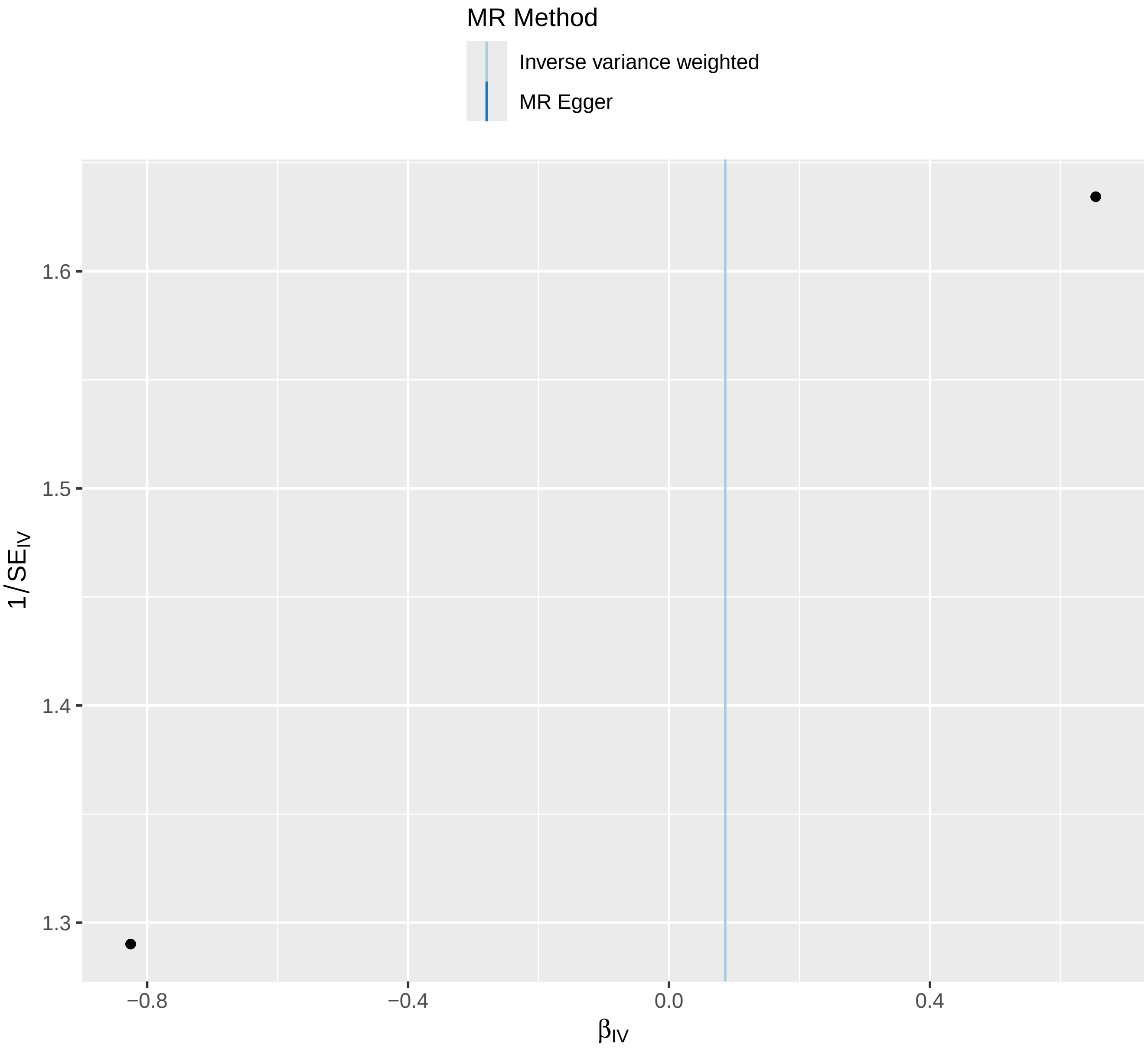

MR Method
Inverse variance weighted
MR Egger
1.6
1.5
1.4
1.3
1/SE_IV
−0.8
−0.4
0.0
0.4
β_IV

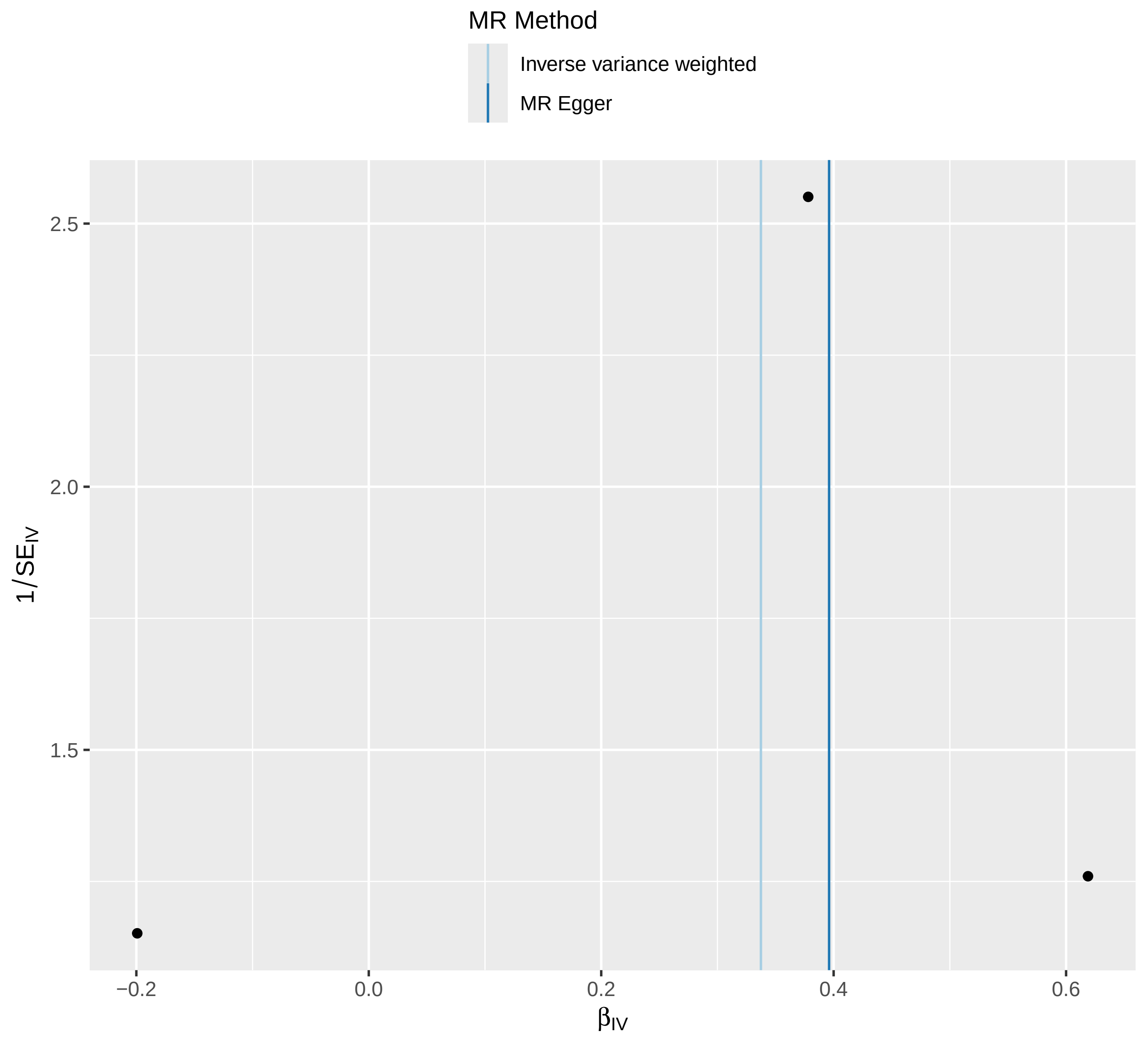

MR Method
Inverse variance weighted
MR Egger
2.5
2.0
1.5
1/SE_IV
−0.2
0.0
0.2
0.4
0.6
β_IV

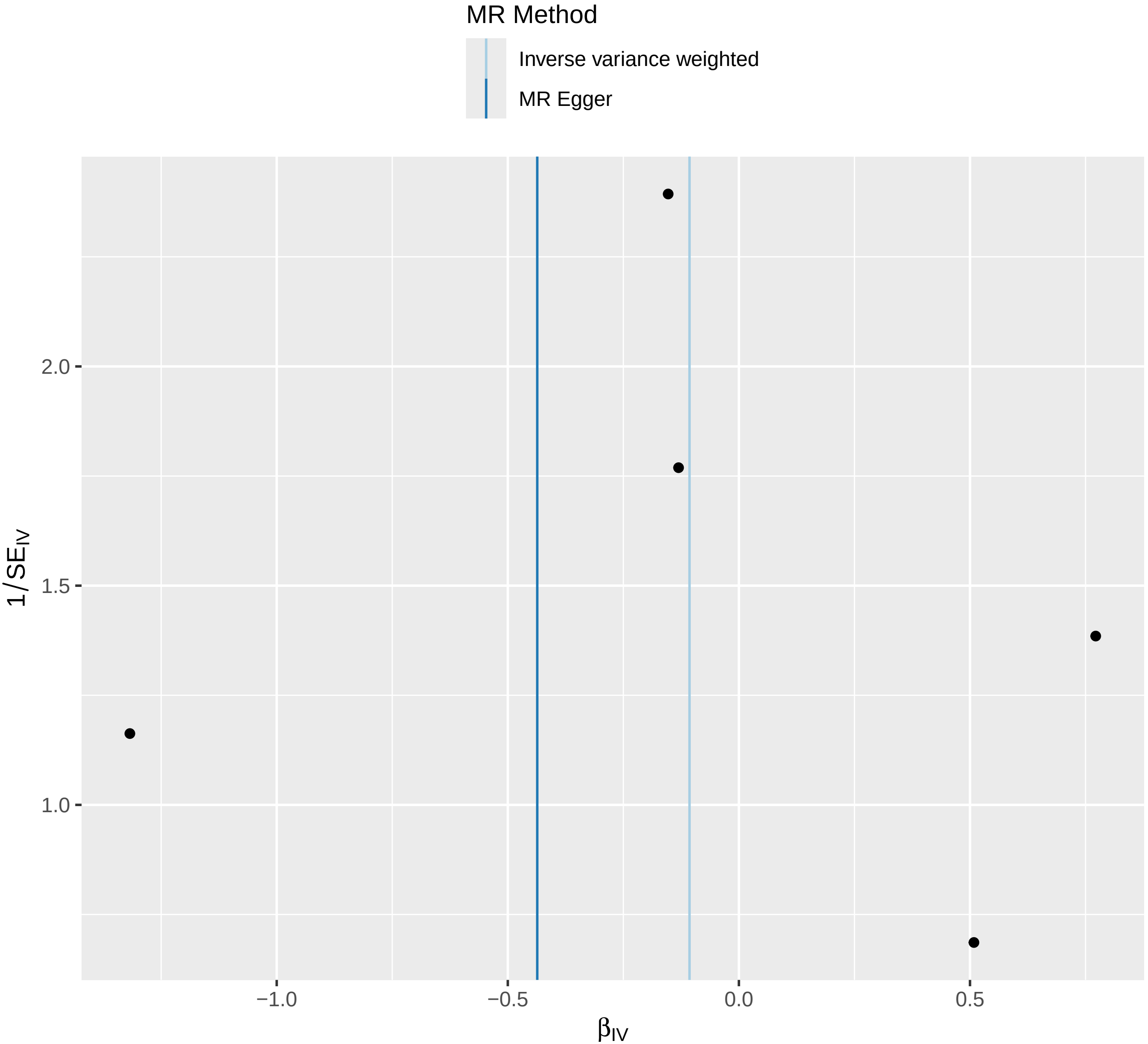
MR Method
Inverse variance weighted
MR Egger
2.0
1.5
1.0
1/SE$_{IV}$
−1.0
−0.5
0.0
0.5
β$_{IV}$

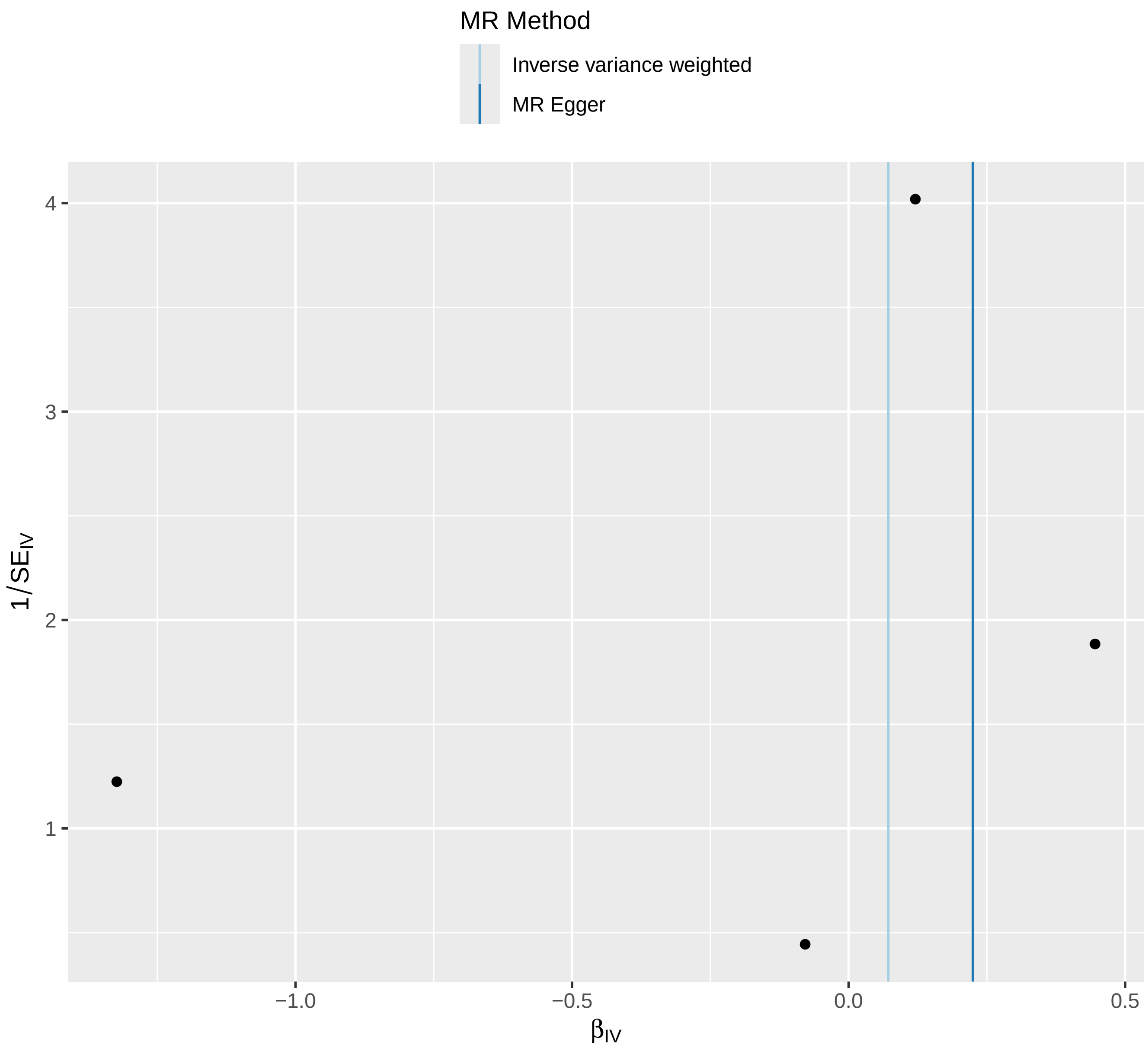

MR Method
Inverse variance weighted
MR Egger
4
3
2
1
1/SE$_{IV}$
−1.0
−0.5
0.0
0.5
β$_{IV}$

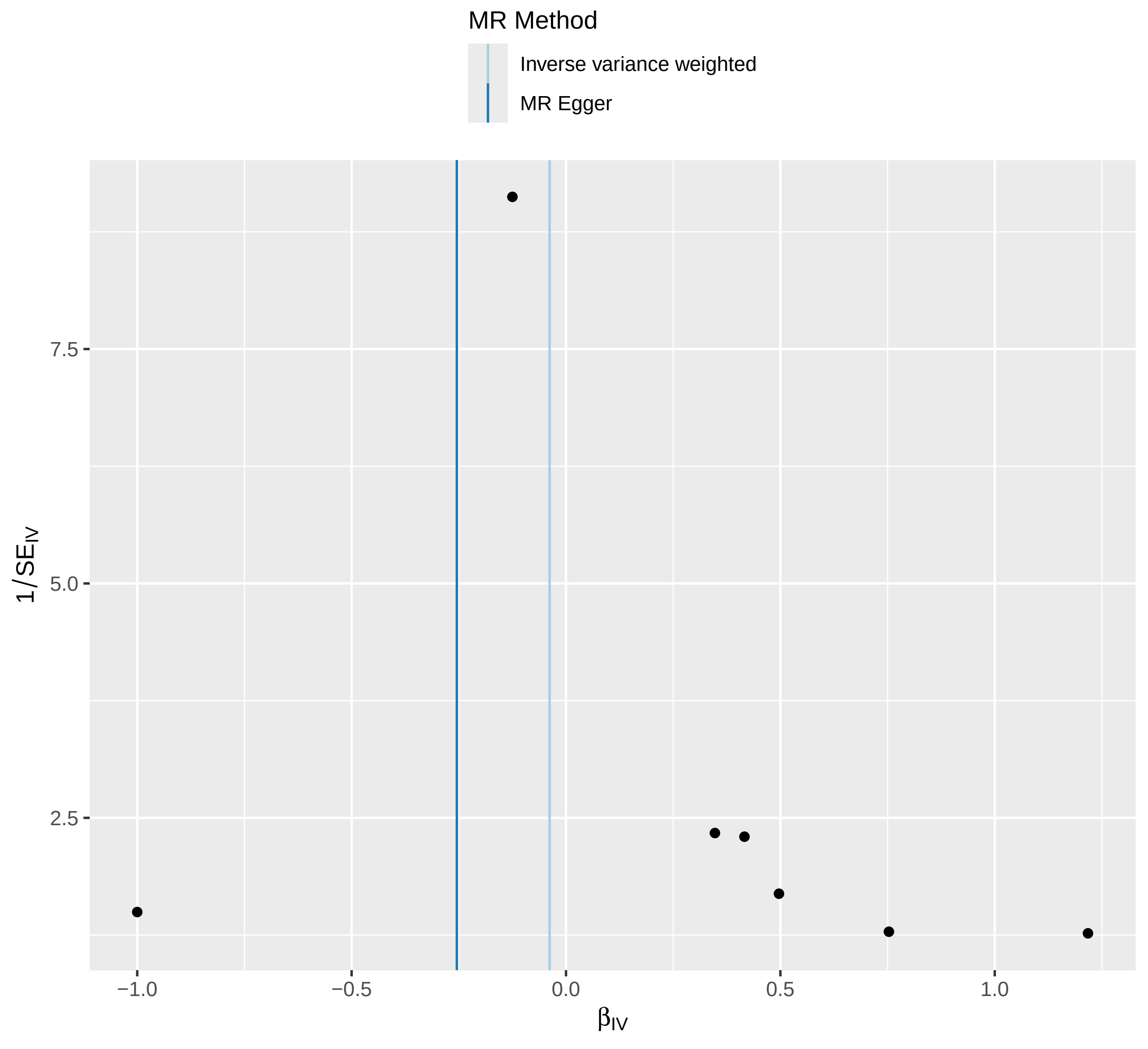
MR Method
Inverse variance weighted
MR Egger
7.5
5.0
2.5
1/SE_IV
−1.0
−0.5
0.0
0.5
1.0
β_IV

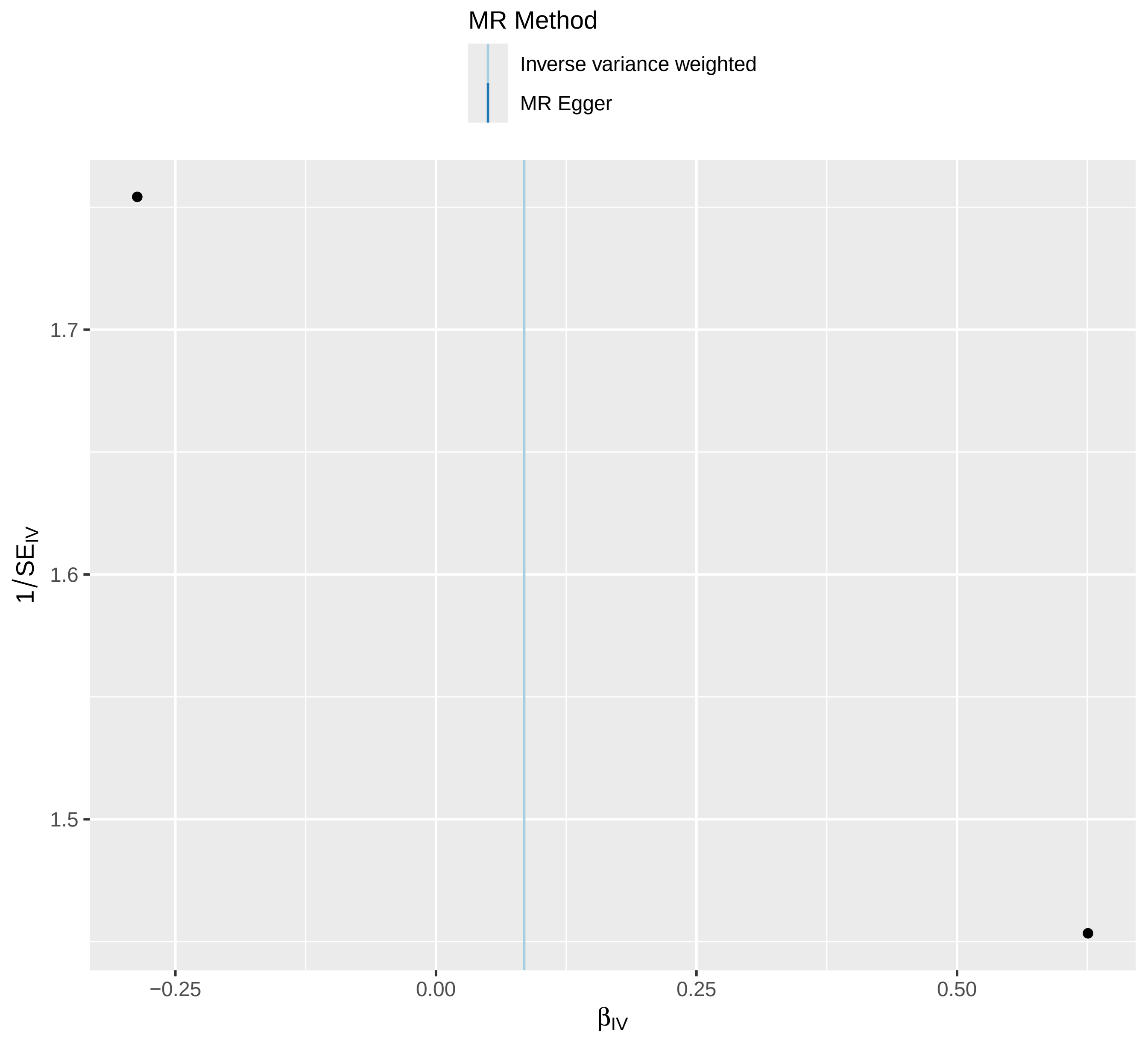
MR Method
Inverse variance weighted
MR Egger
1.7
1.6
1.5
$1/SE_{IV}$
−0.25
0.00
0.25
0.50
β_{IV}

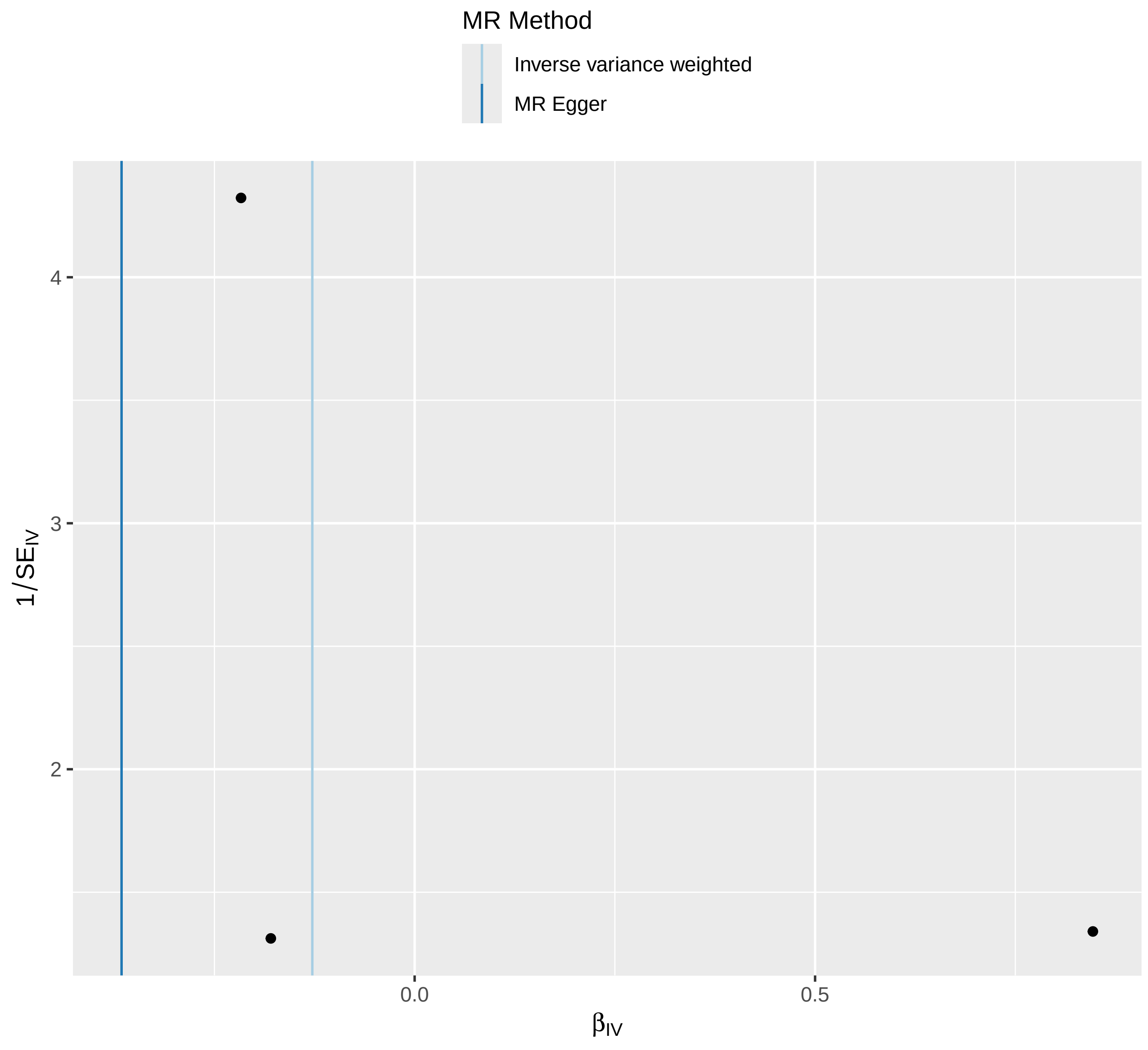

MR Method
Inverse variance weighted
MR Egger
4
3
2
$1/SE_{IV}$
0.0
0.5
β_{IV}

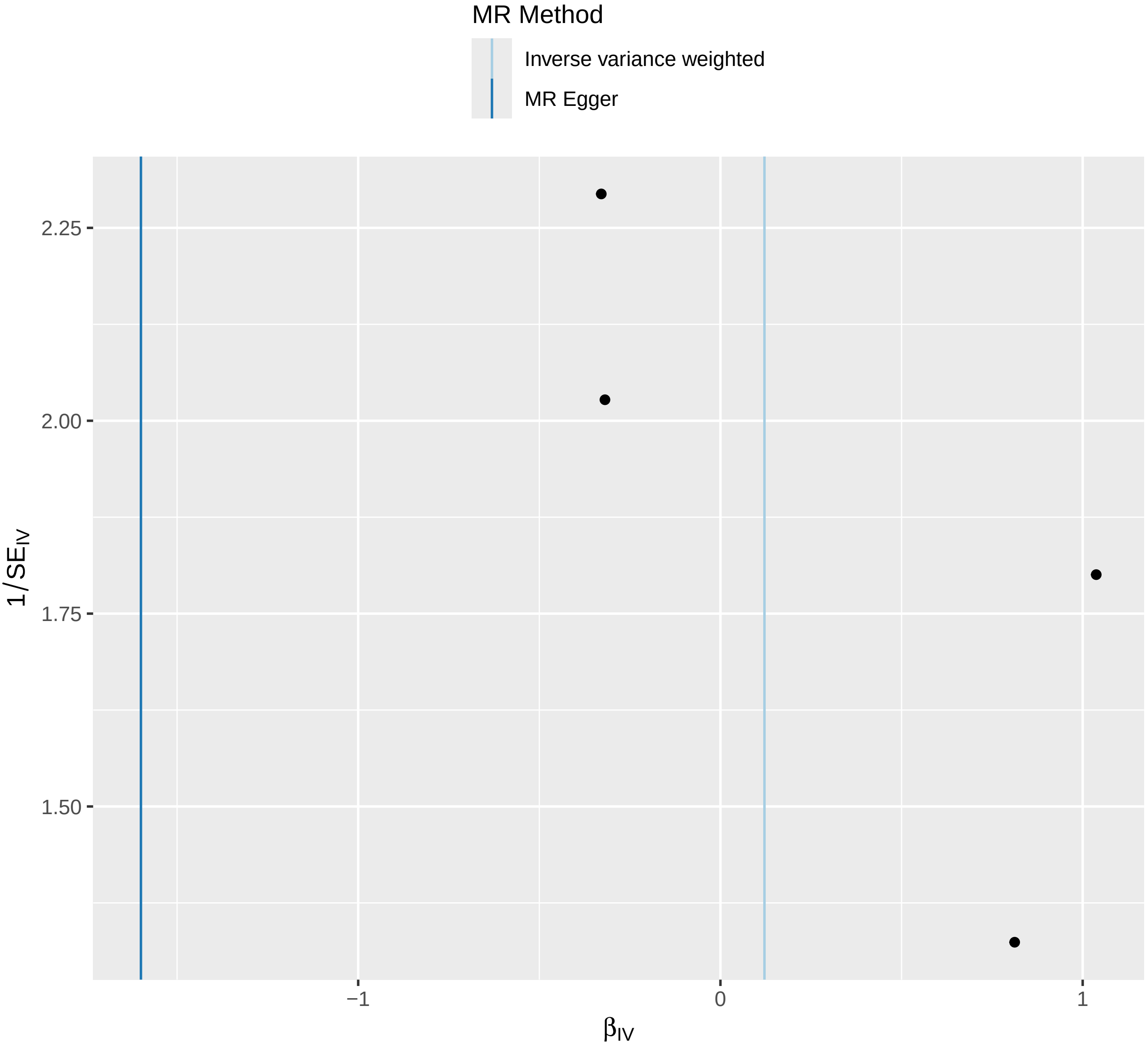
MR Method
Inverse variance weighted
MR Egger
2.25
2.00
1.75
1.50
1/SE_IV
−1
0
1
β_IV

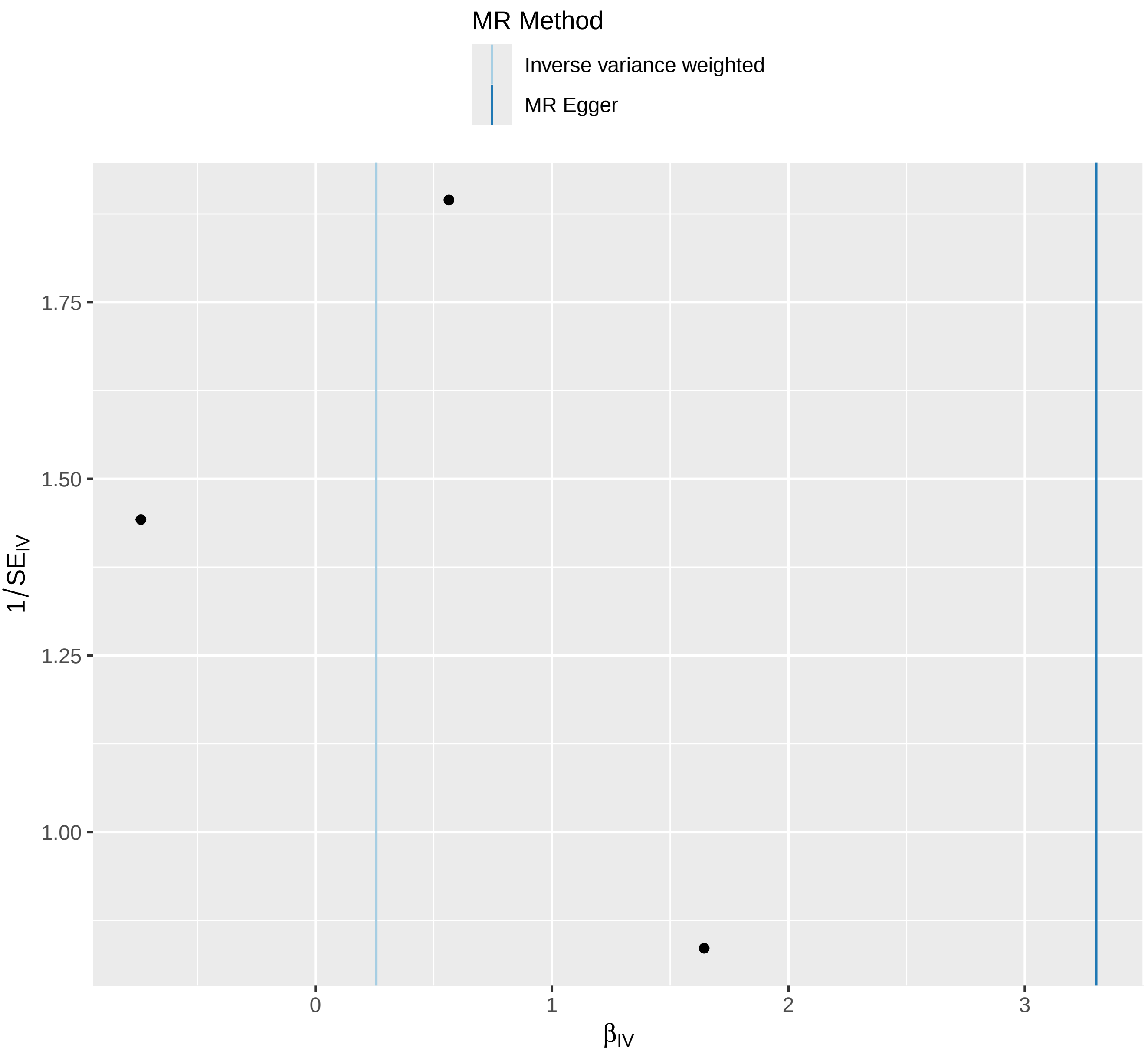
MR Method
Inverse variance weighted
MR Egger
1.75
1.50
1.25
1.00
$1/SE_{IV}$
0
1
2
3
β_{IV}

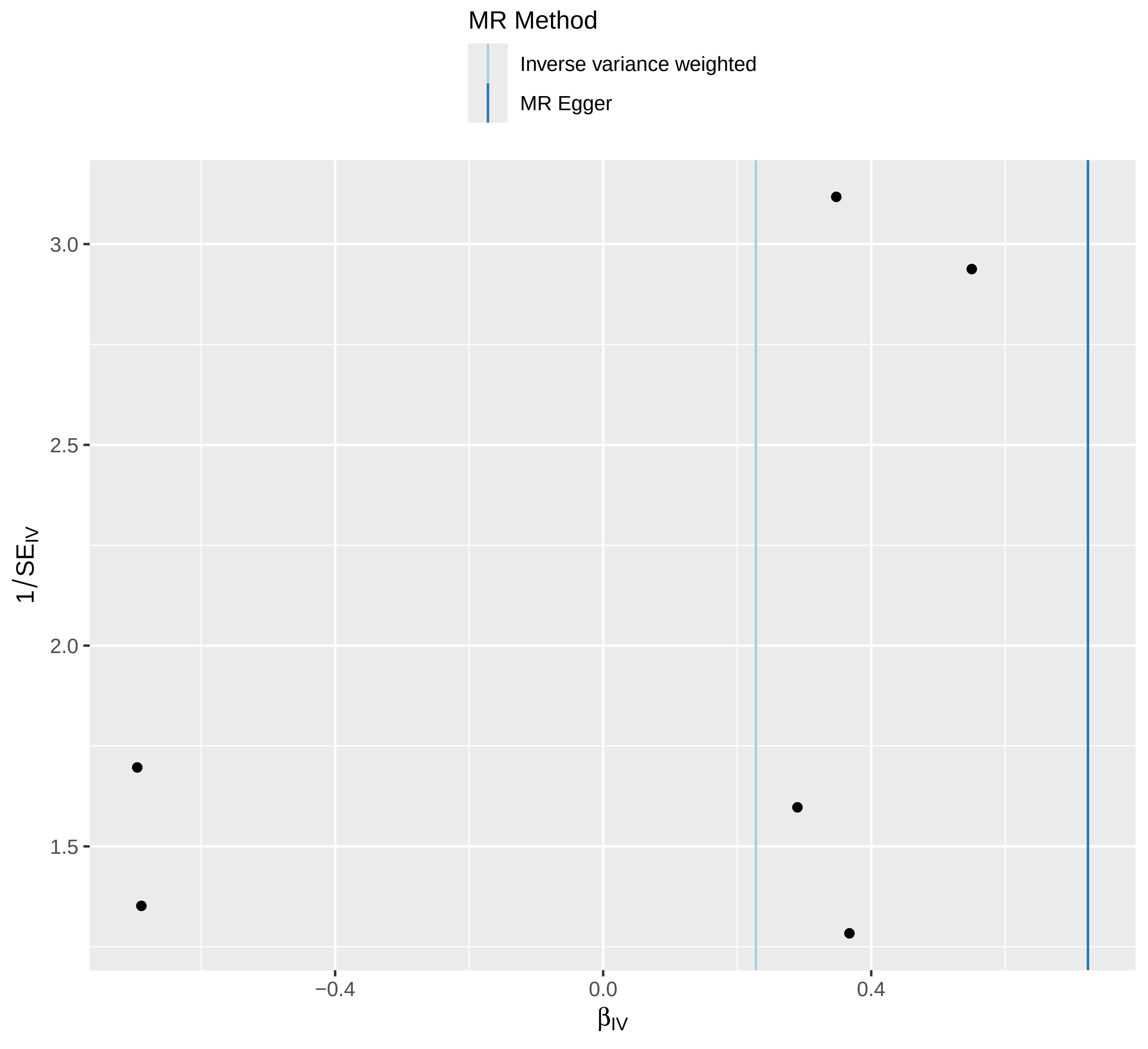

MR Method
Inverse variance weighted
MR Egger
3.0
2.5
2.0
1.5
$1/SE_{IV}$
−0.4
0.0
0.4
β_{IV}

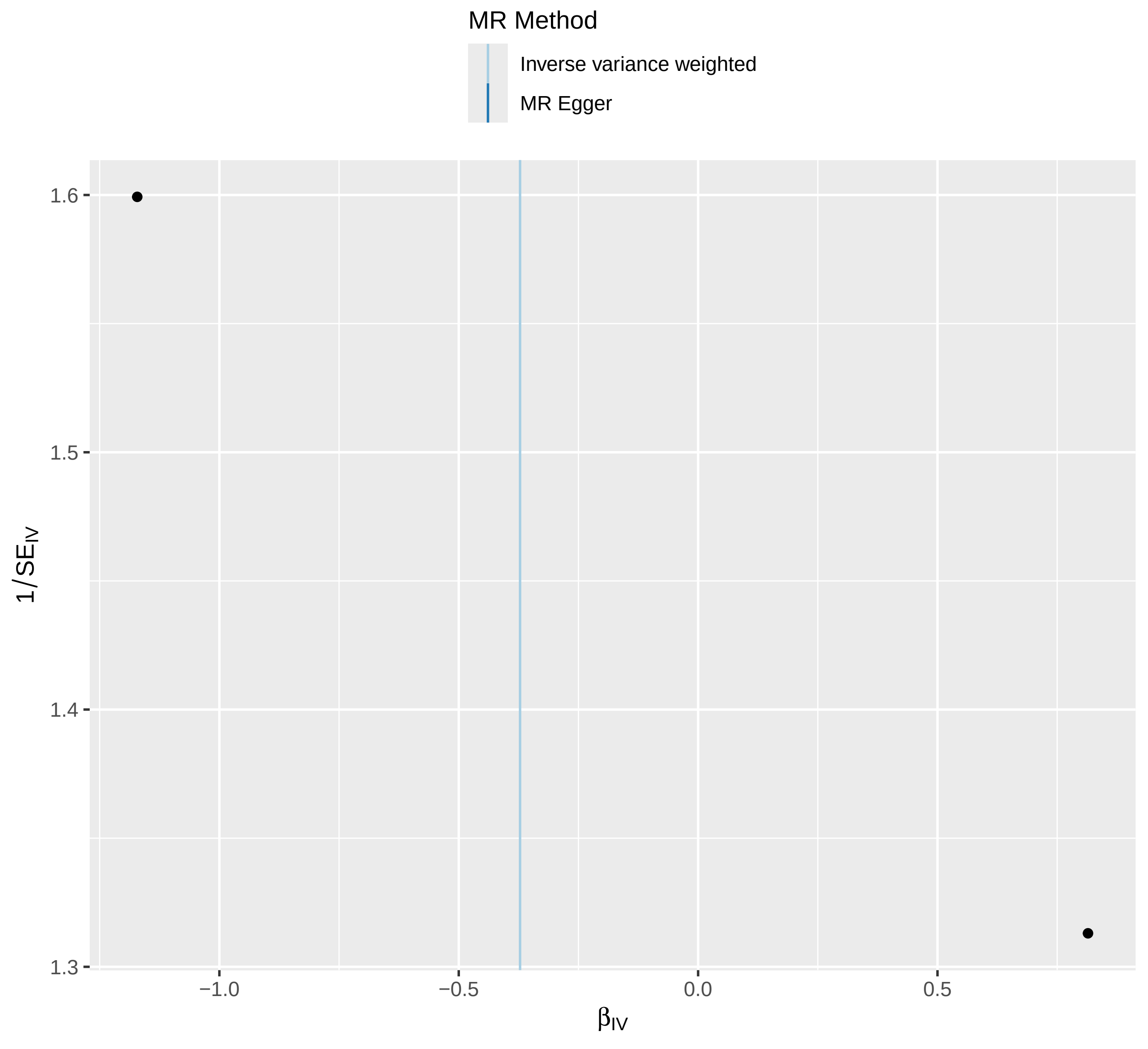

MR Method
Inverse variance weighted
MR Egger
1.6
1.5
1.4
1.3
1/SE_IV
−1.0
−0.5
0.0
0.5
β_IV

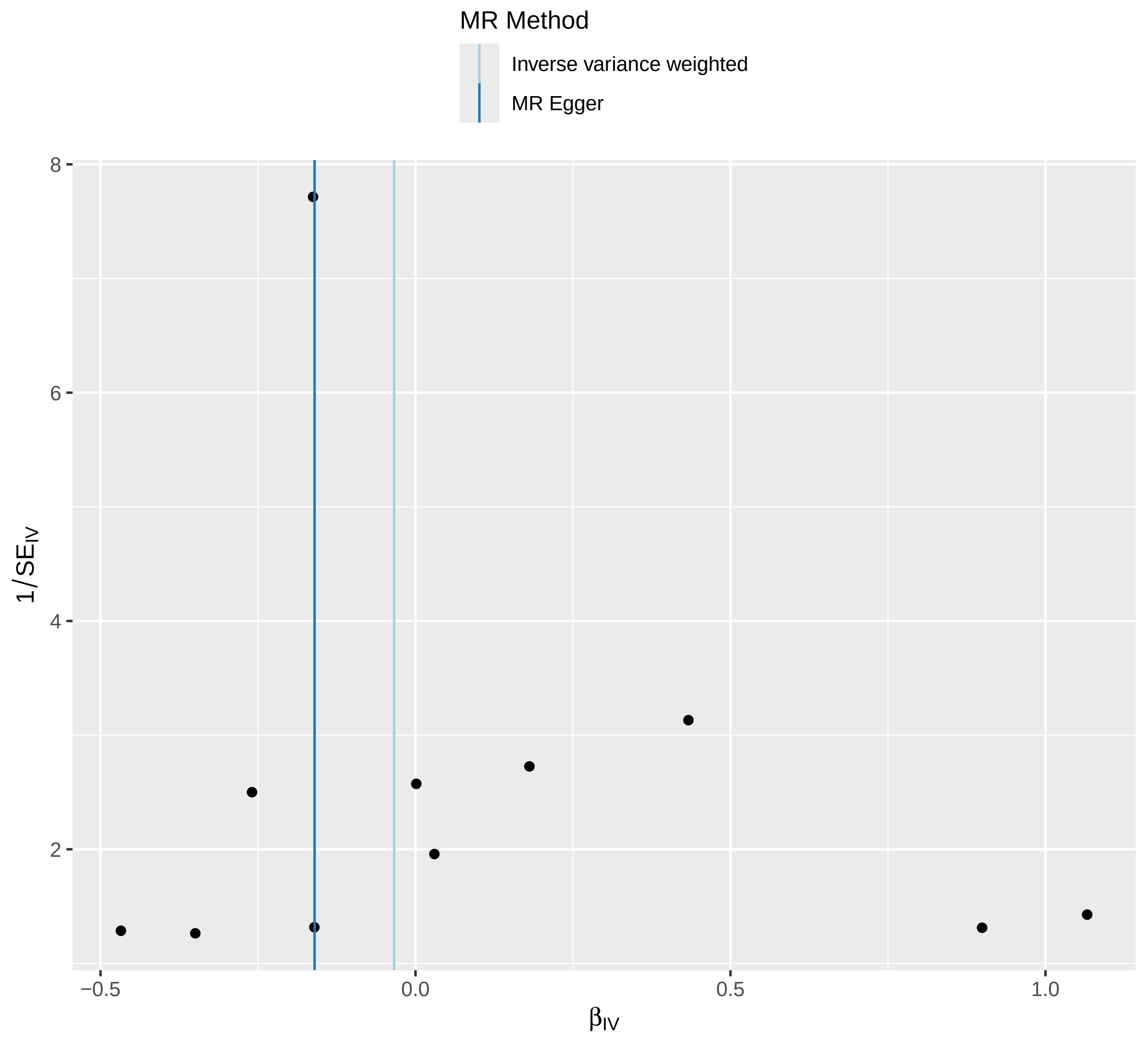

MR Method
Inverse variance weighted
MR Egger
8
6
4
2
1/SE_IV
−0.5
0.0
0.5
1.0
β_IV

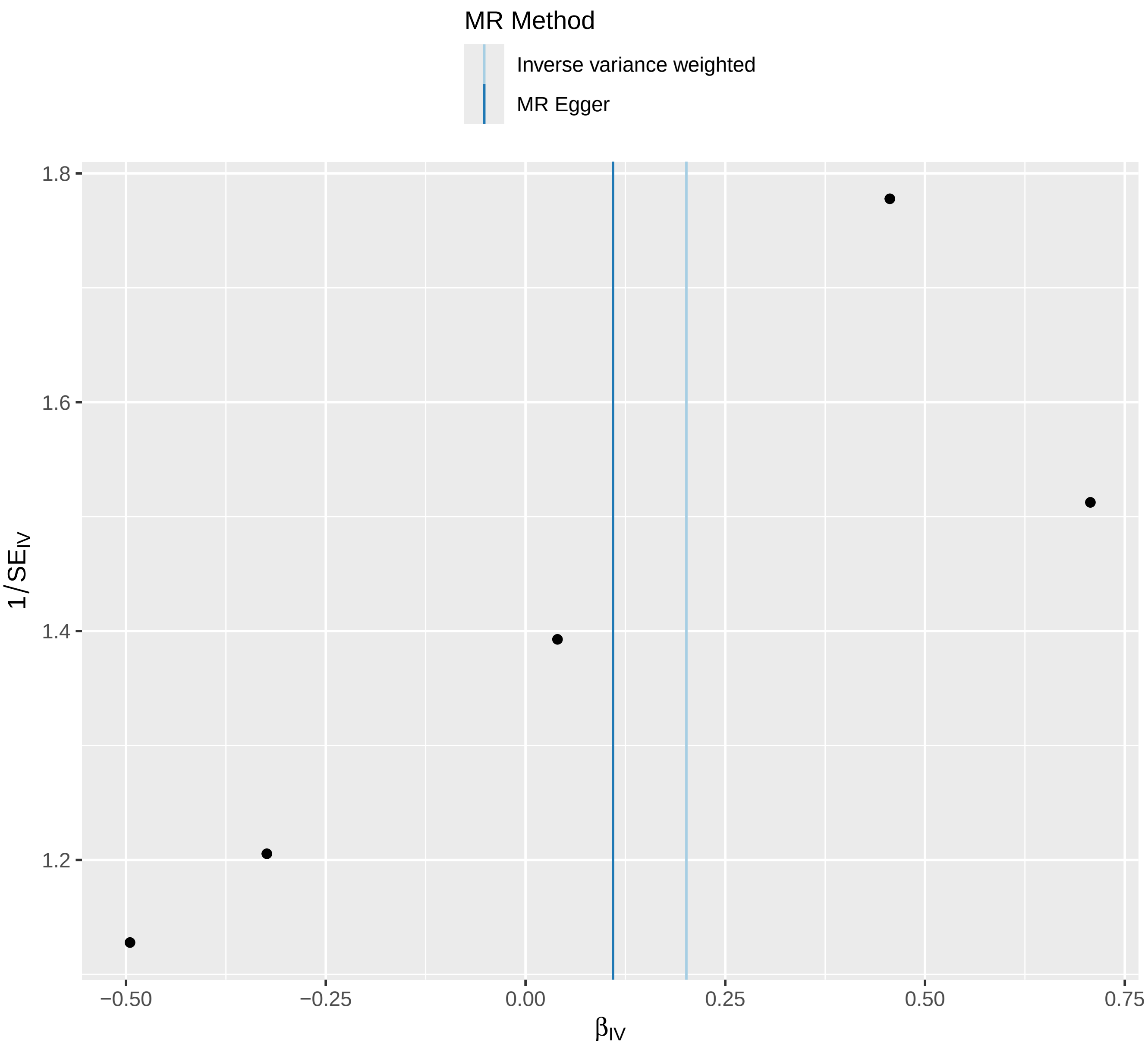
MR Method
Inverse variance weighted
MR Egger
1.8
1.6
1.4
1.2
1/SE_IV
−0.50
−0.25
0.00
0.25
0.50
0.75
β_IV

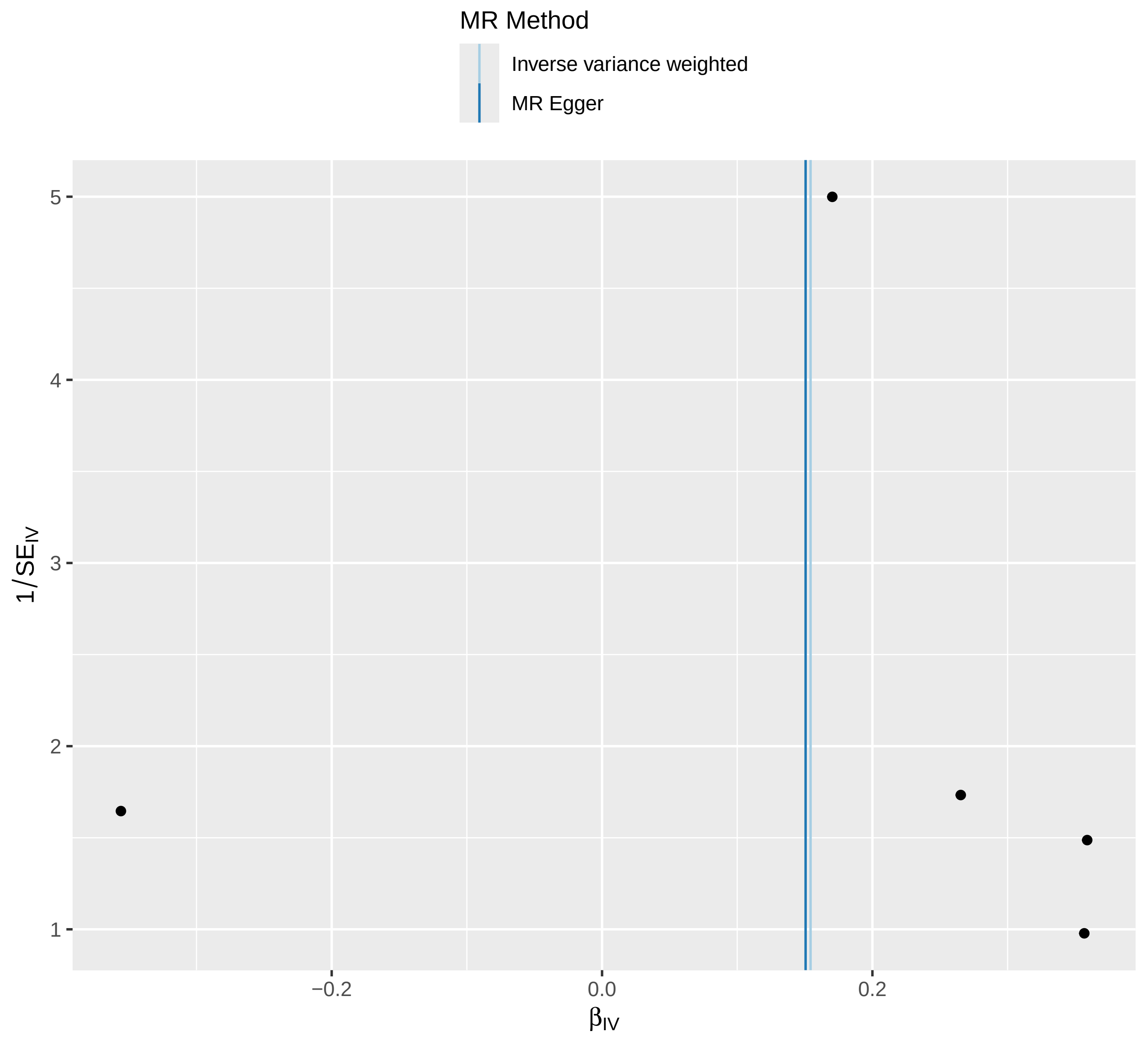
MR Method
Inverse variance weighted
MR Egger
1/SE_IV
5
4
3
2
1
−0.2
0.0
0.2
β_IV

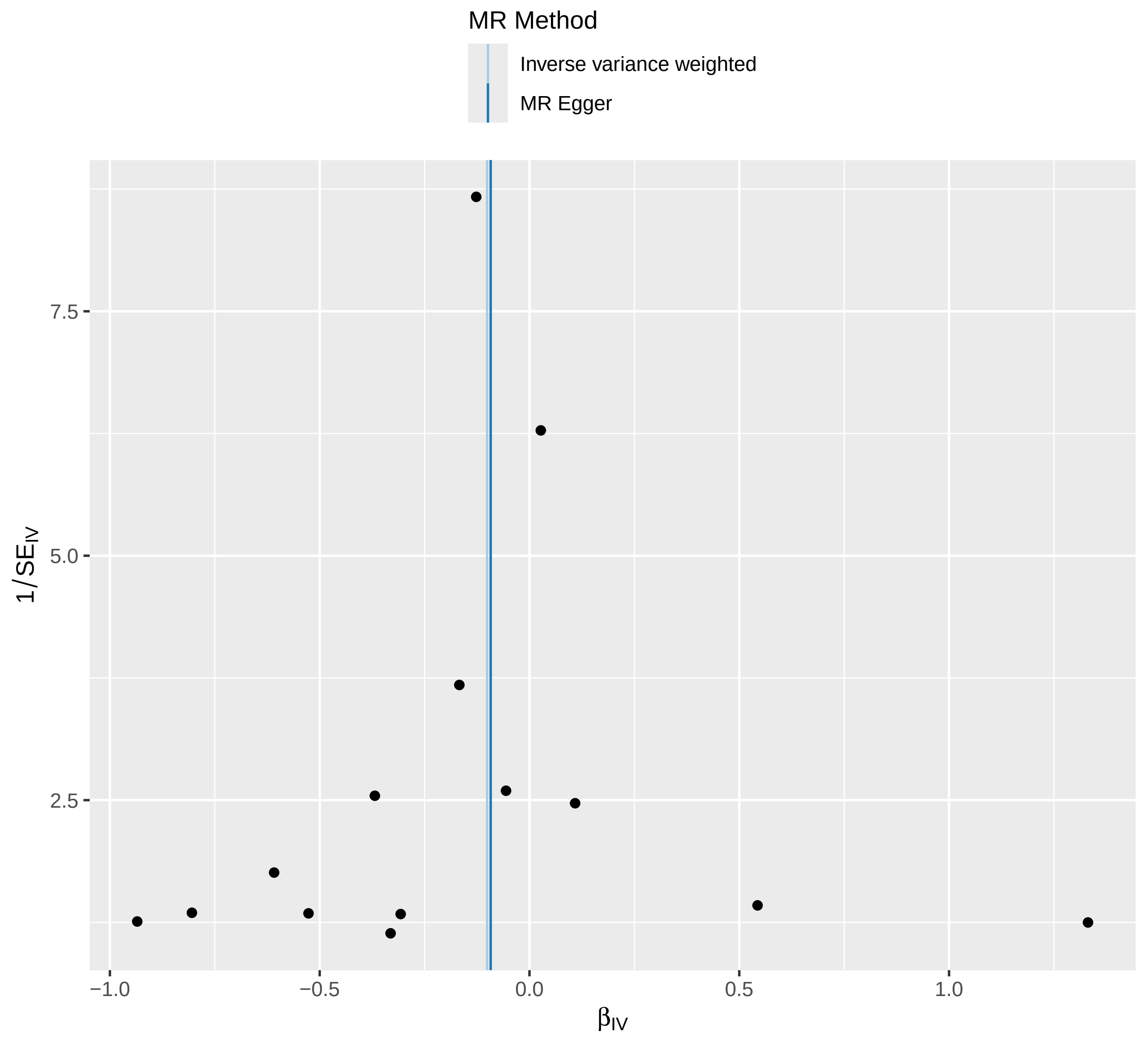

MR Method
Inverse variance weighted
MR Egger
7.5
5.0
2.5
1/SE_IV
−1.0
−0.5
0.0
0.5
1.0
β_IV

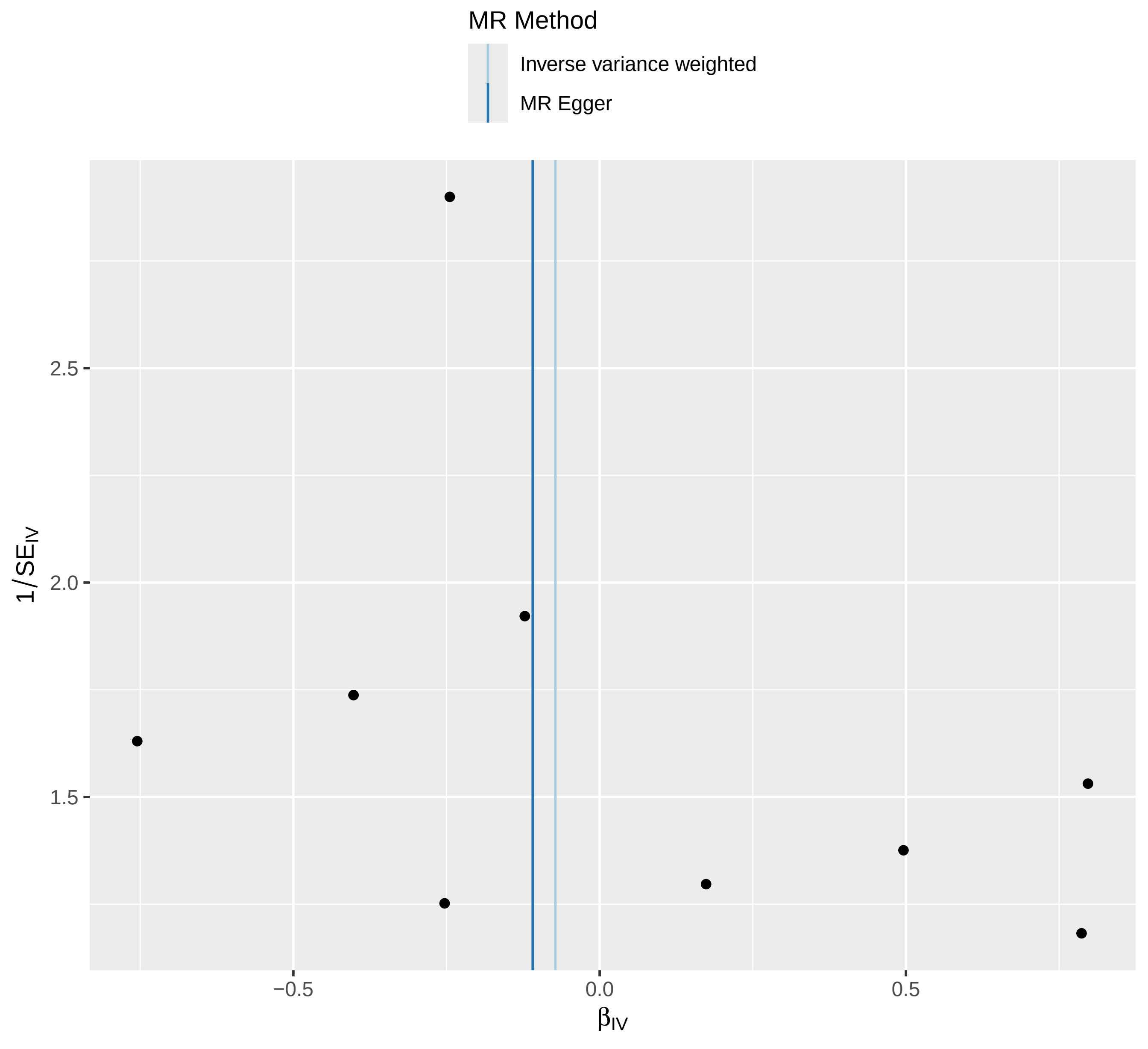

MR Method
Inverse variance weighted
MR Egger
2.5
2.0
1.5
1/SE$_{IV}$
−0.5
0.0
0.5
β$_{IV}$

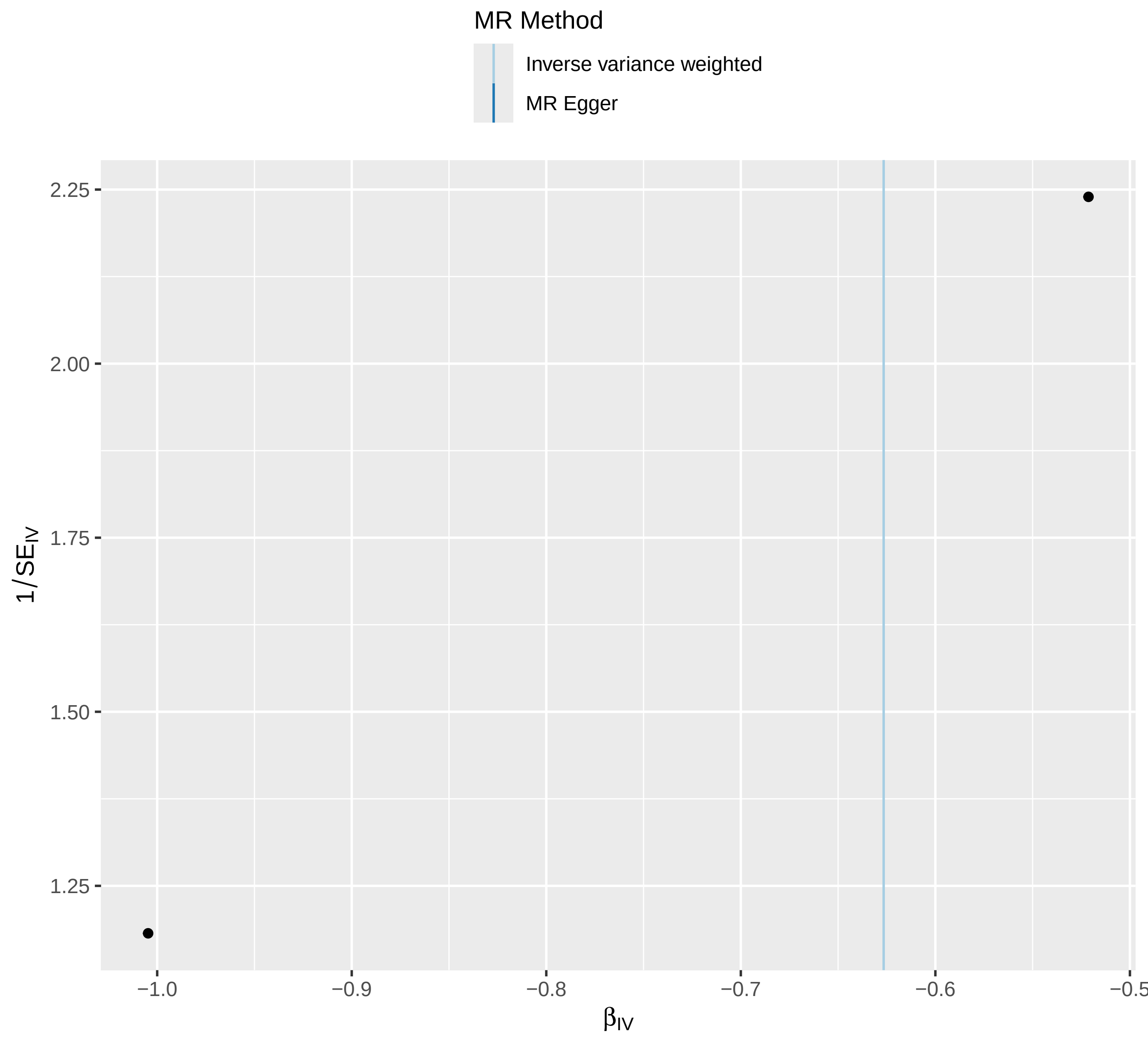

MR Method
Inverse variance weighted
MR Egger
2.25
2.00
1.75
1.50
1.25
1/SE_IV
−1.0
−0.9
−0.8
−0.7
−0.6
−0.5
β_IV

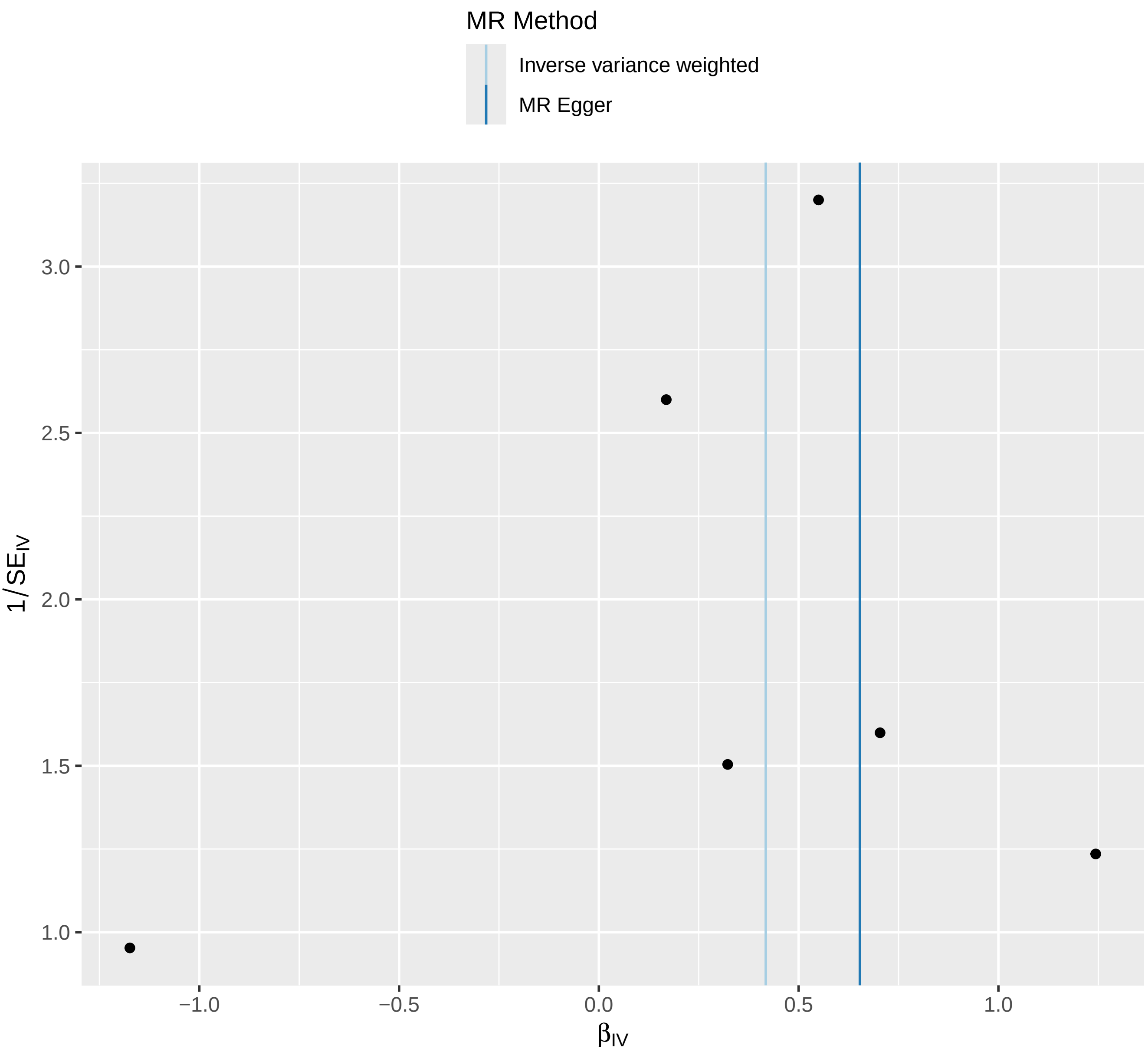

MR Method
Inverse variance weighted
MR Egger
1/SE_IV
3.0
2.5
2.0
1.5
1.0
−1.0
−0.5
0.0
0.5
1.0
β_IV

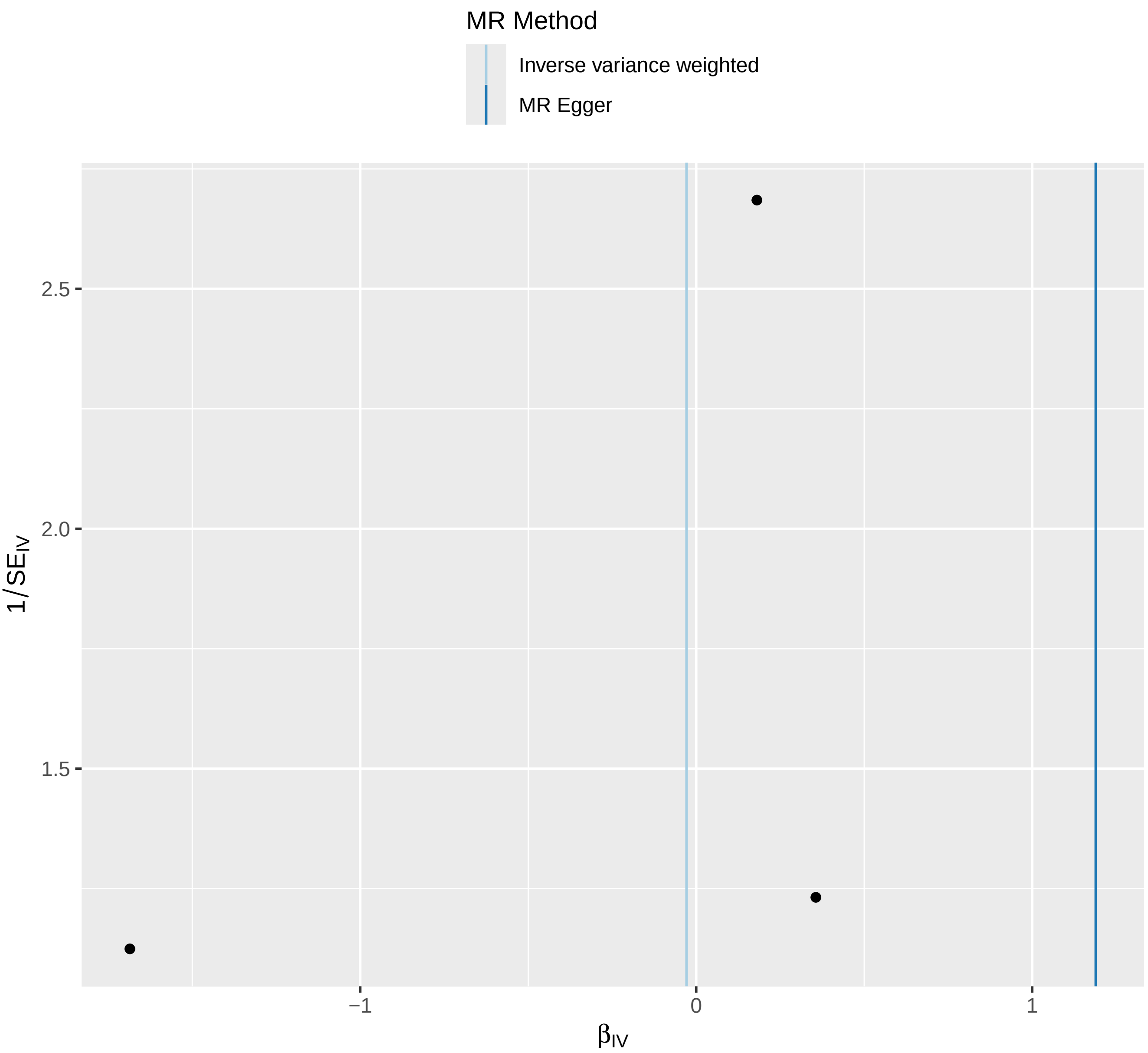
MR Method
Inverse variance weighted
MR Egger
2.5
2.0
1.5
1/SE$_{IV}$
−1
0
1
β_{IV}

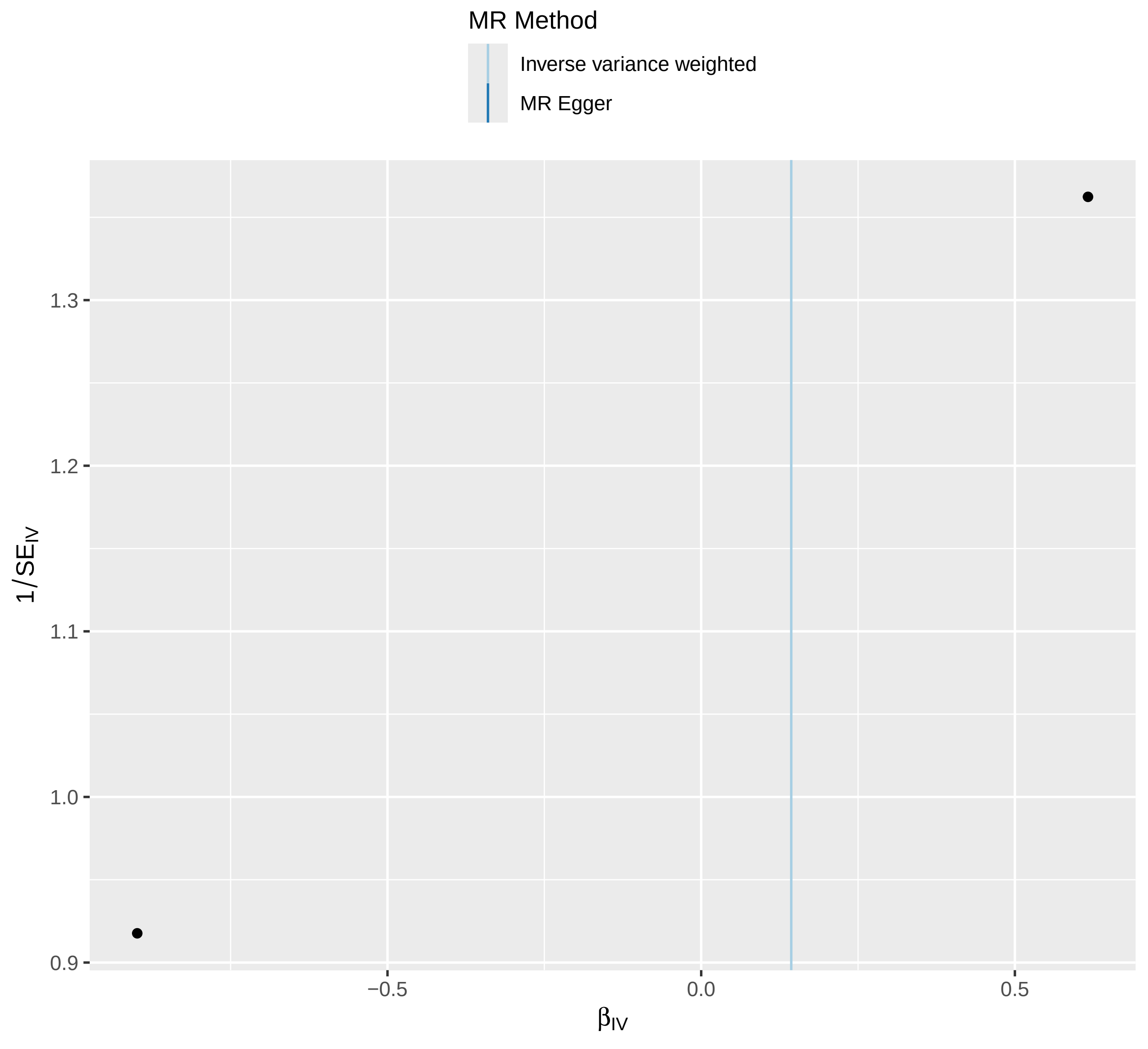
MR Method
Inverse variance weighted
MR Egger
1.3
1.2
1.1
1.0
0.9
1/SE_IV
−0.5
0.0
0.5
β_IV

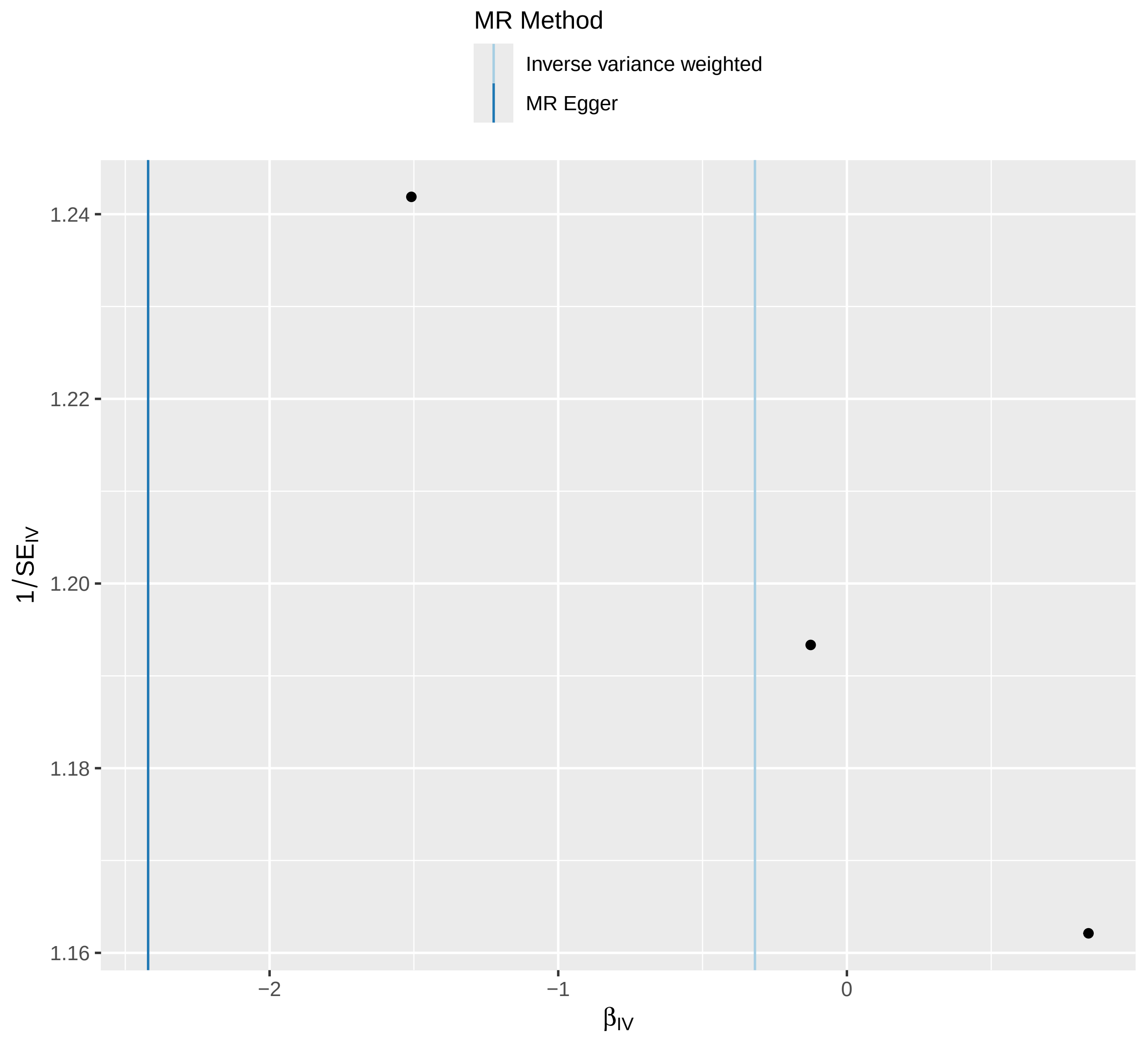

MR Method
Inverse variance weighted
MR Egger
1.24
1.22
1.20
1.18
1.16
1/SE_IV
−2
−1
0
β_IV

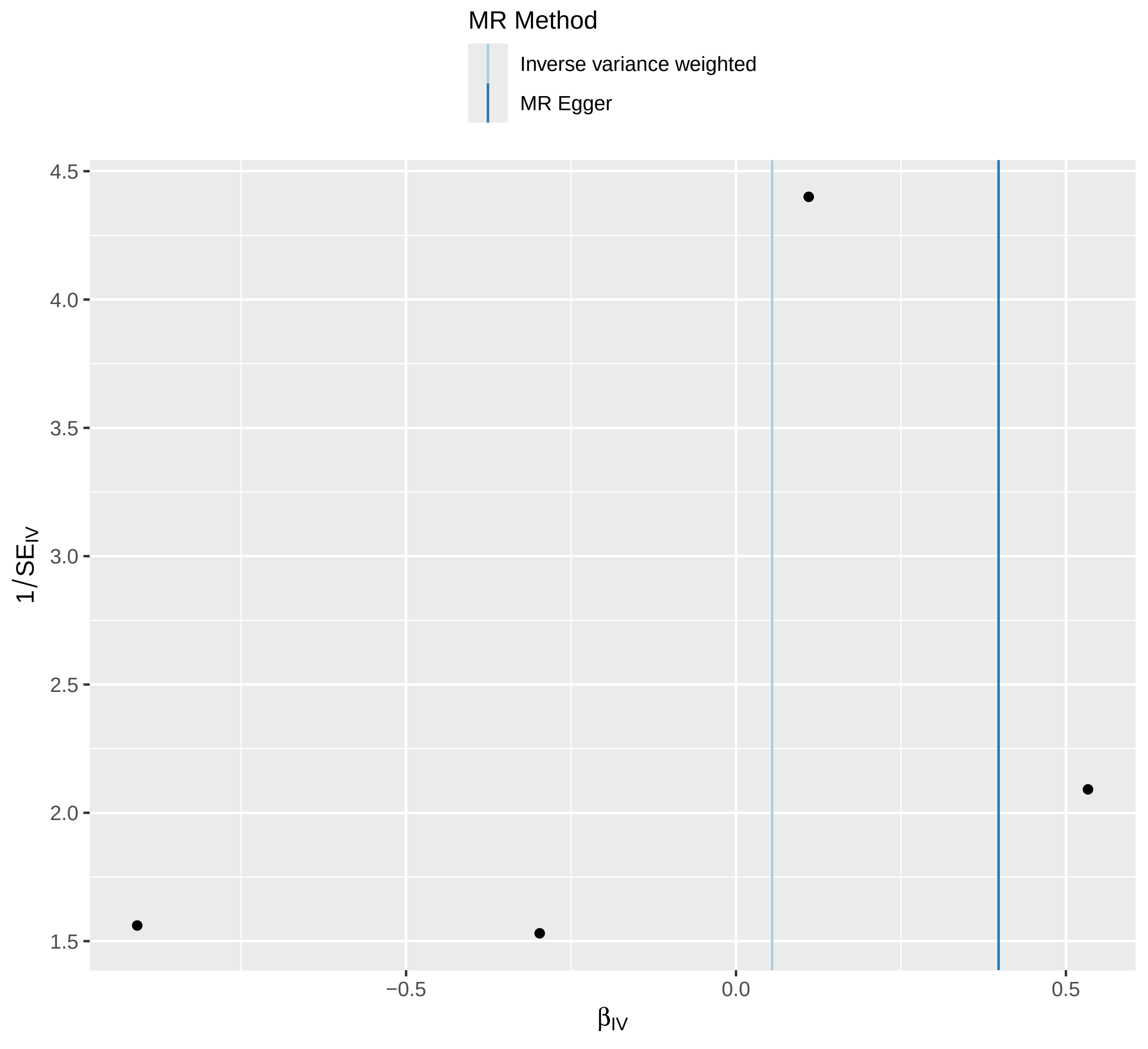
MR Method
Inverse variance weighted
MR Egger
4.5
4.0
3.5
3.0
2.5
2.0
1.5
1/SE_IV
−0.5
0.0
0.5
β_IV

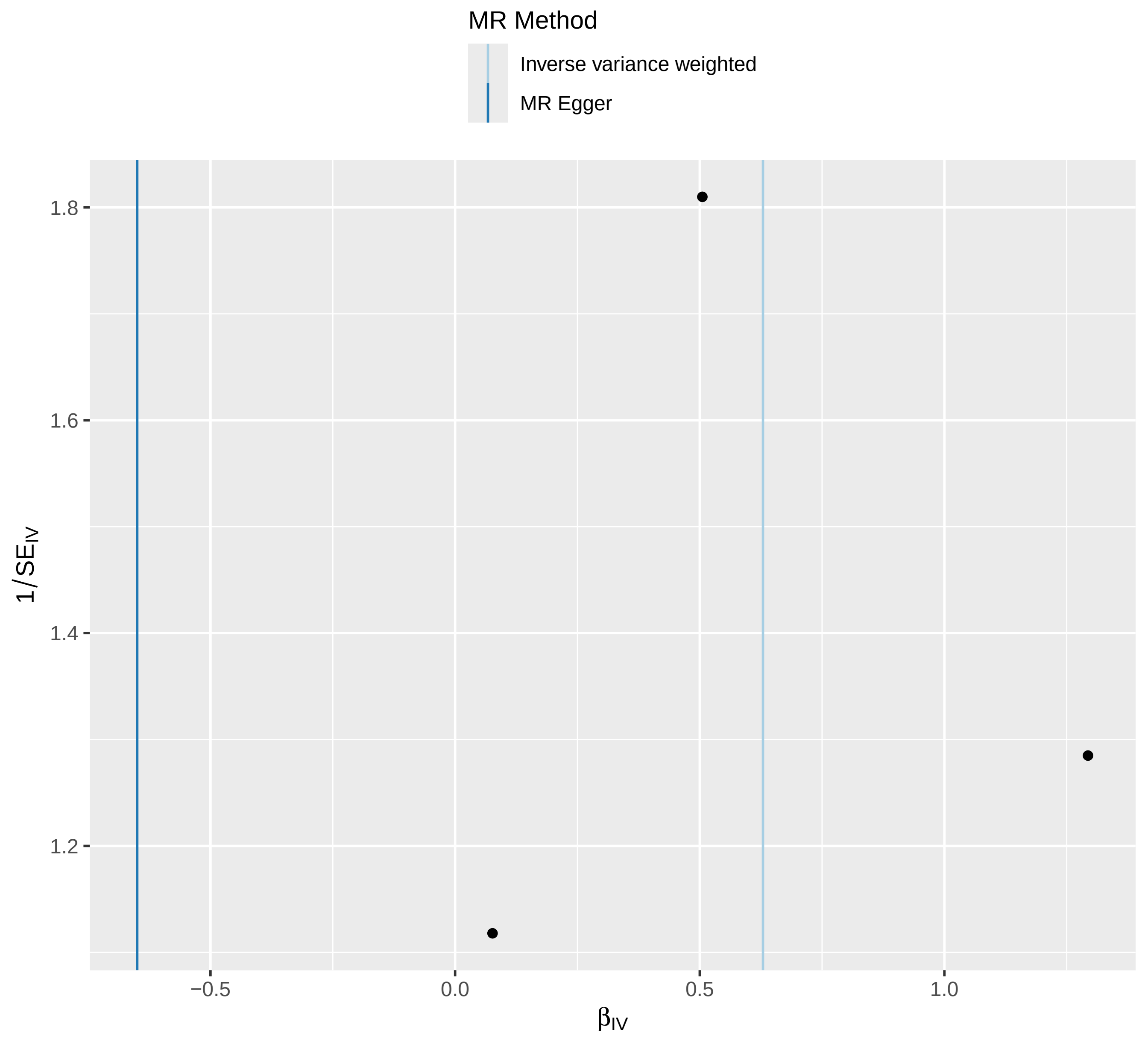
MR Method
Inverse variance weighted
MR Egger
1.8
1.6
1.4
1.2
1/SE_IV
−0.5
0.0
0.5
1.0
β_IV

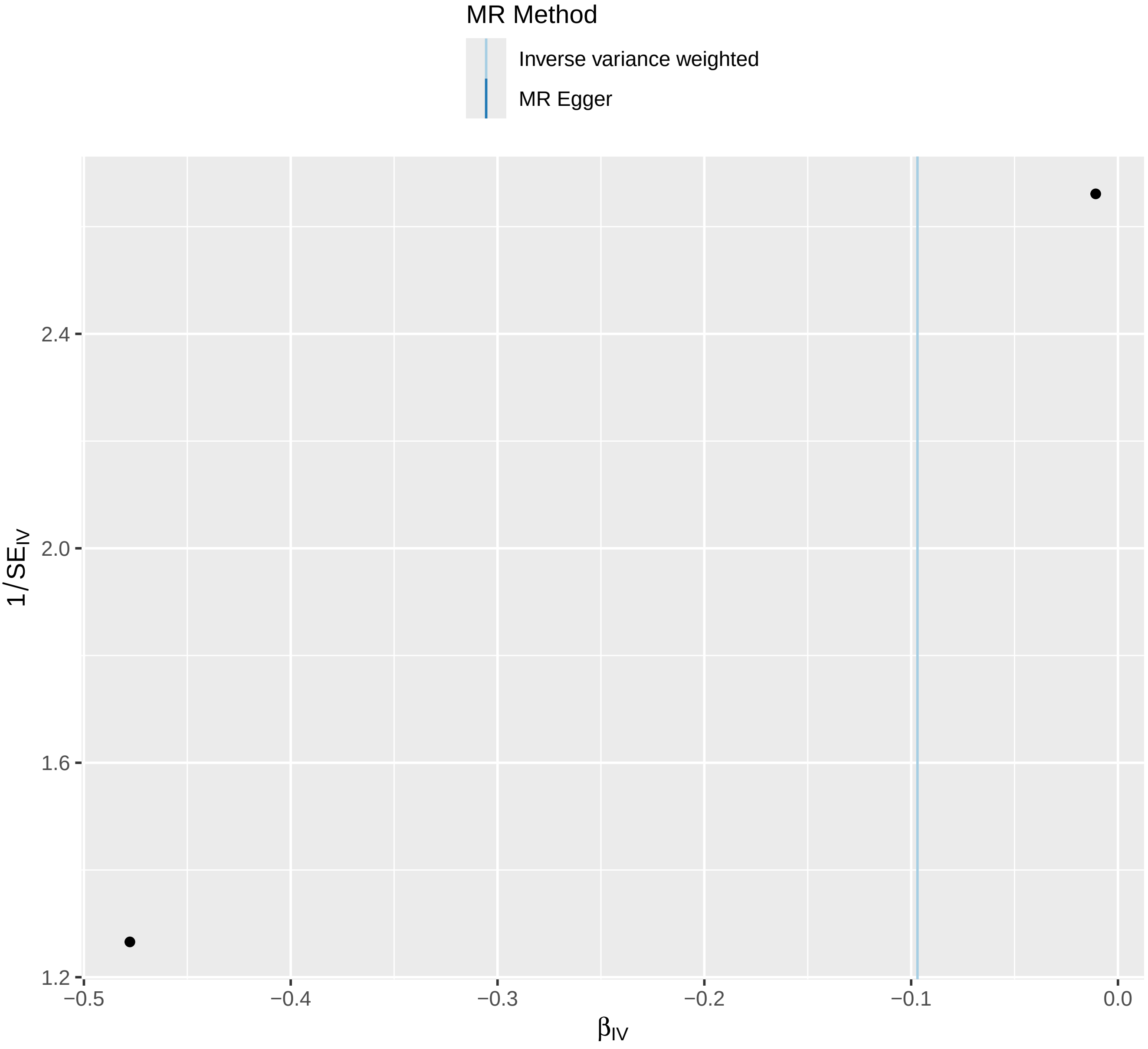

MR Method
Inverse variance weighted
MR Egger
2.4
2.0
1.6
1.2
1/SE$_{IV}$
−0.5
−0.4
−0.3
−0.2
−0.1
0.0
β$_{IV}$

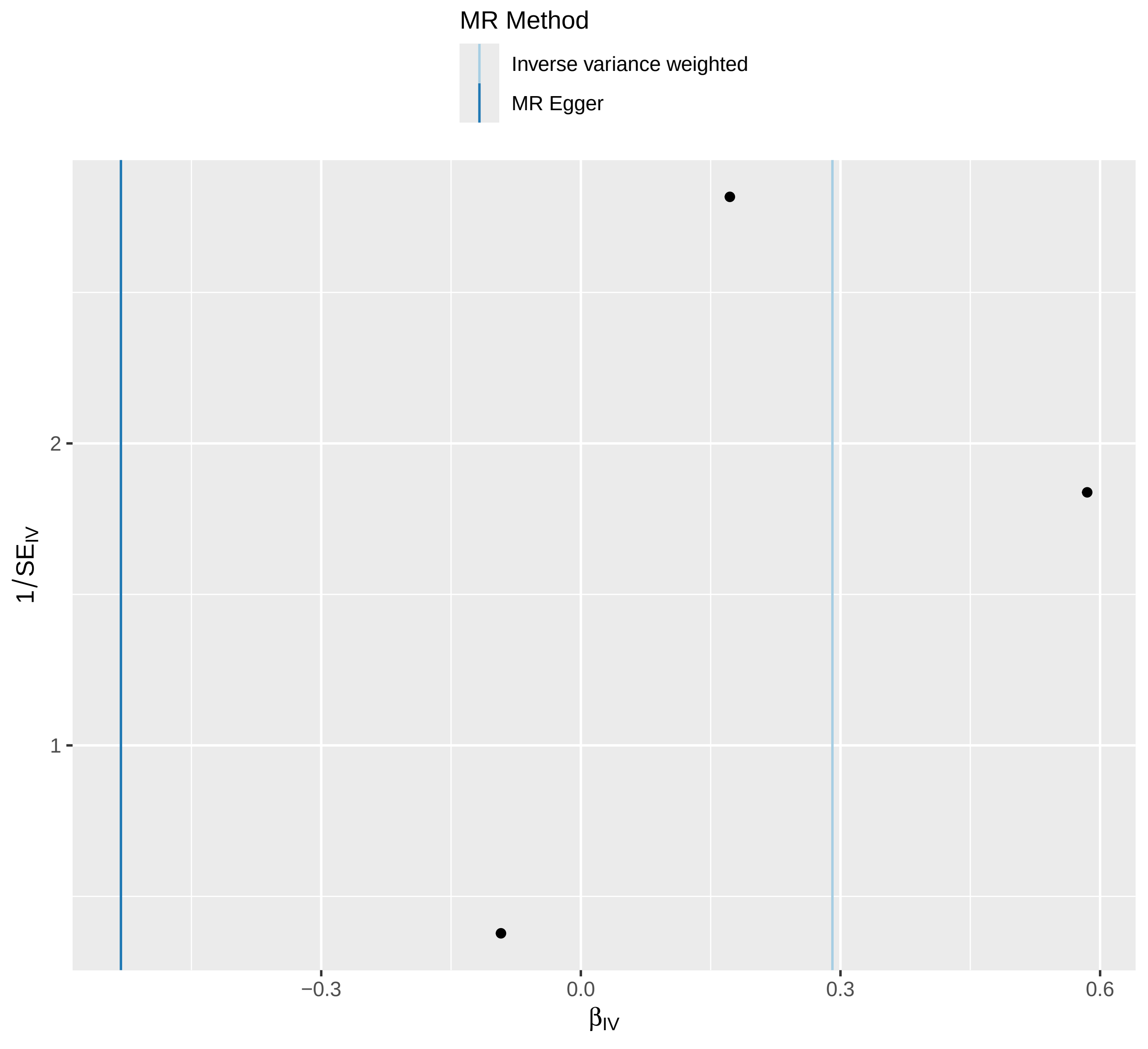
MR Method
Inverse variance weighted
MR Egger
2
1
1/SE_IV
−0.3
0.0
0.3
0.6
β_IV

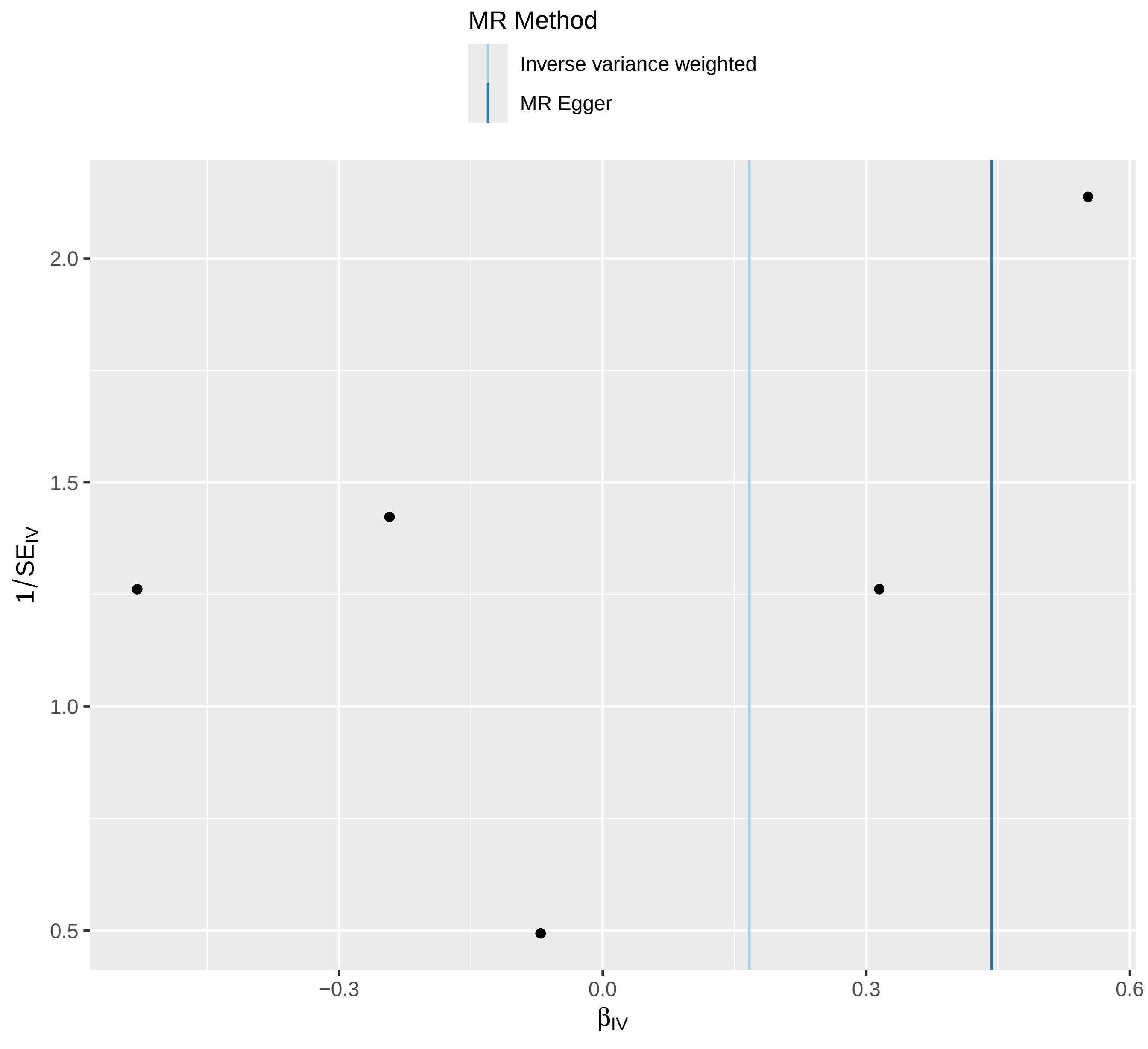

MR Method
Inverse variance weighted
MR Egger
2.0
1.5
1.0
0.5
1/SE_IV
−0.3
0.0
0.3
0.6
β_IV

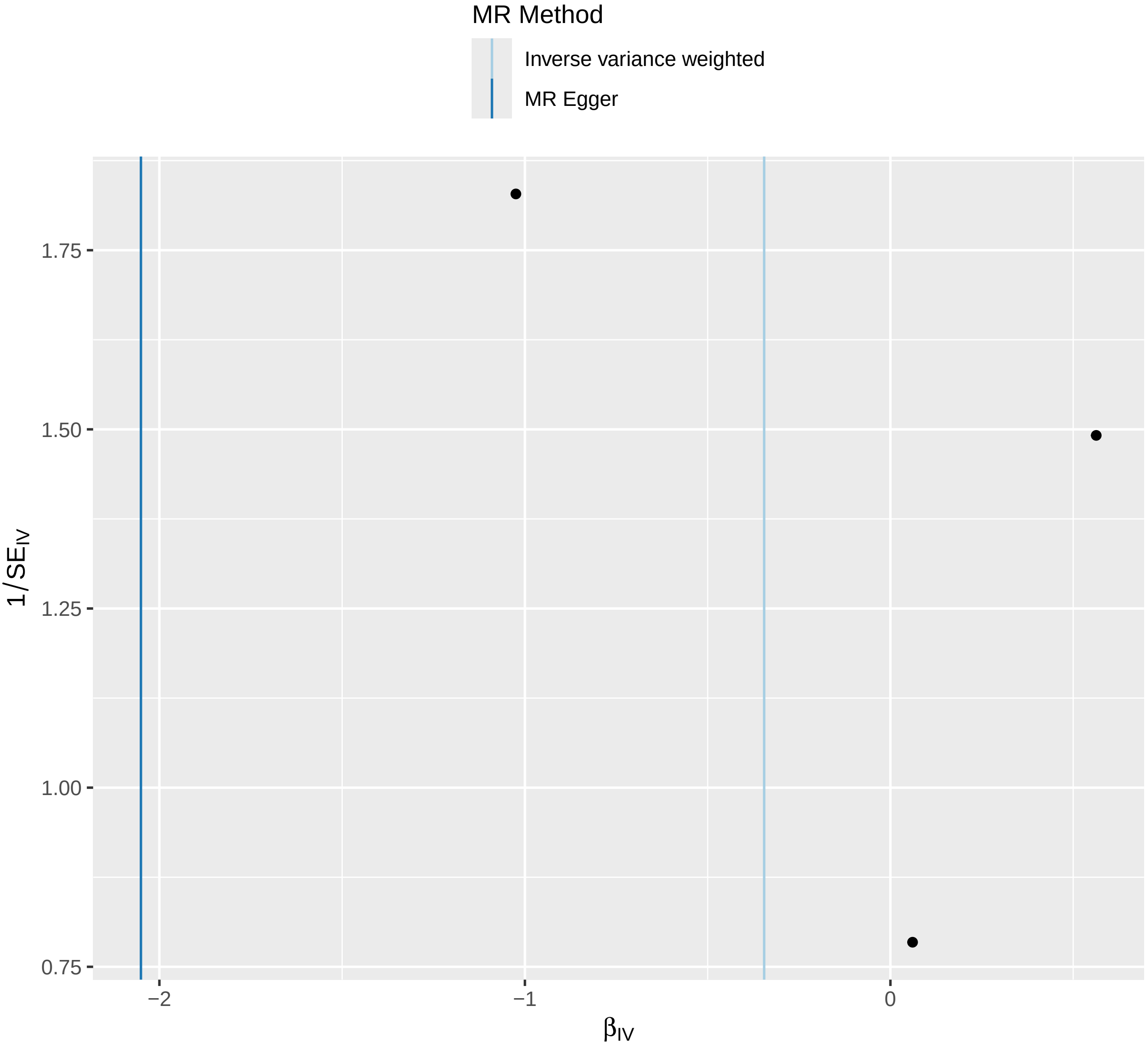
MR Method
Inverse variance weighted
MR Egger
1.75
1.50
1.25
1.00
0.75
$1/SE_{IV}$
−2
−1
0
β_{IV}

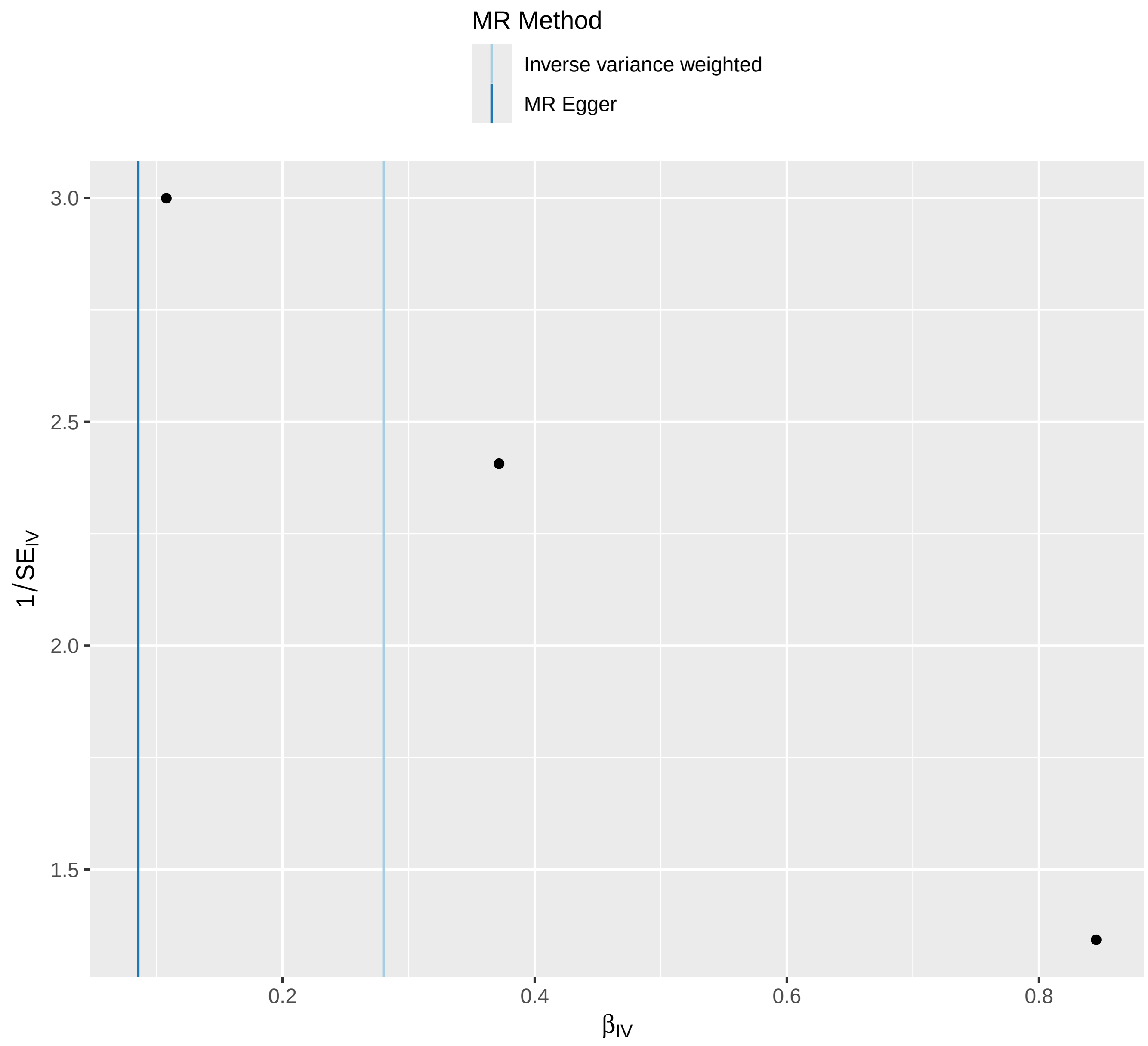

MR Method
Inverse variance weighted
MR Egger
3.0
2.5
2.0
1.5
1/SE$_{IV}$
0.2
0.4
0.6
0.8
β_{IV}

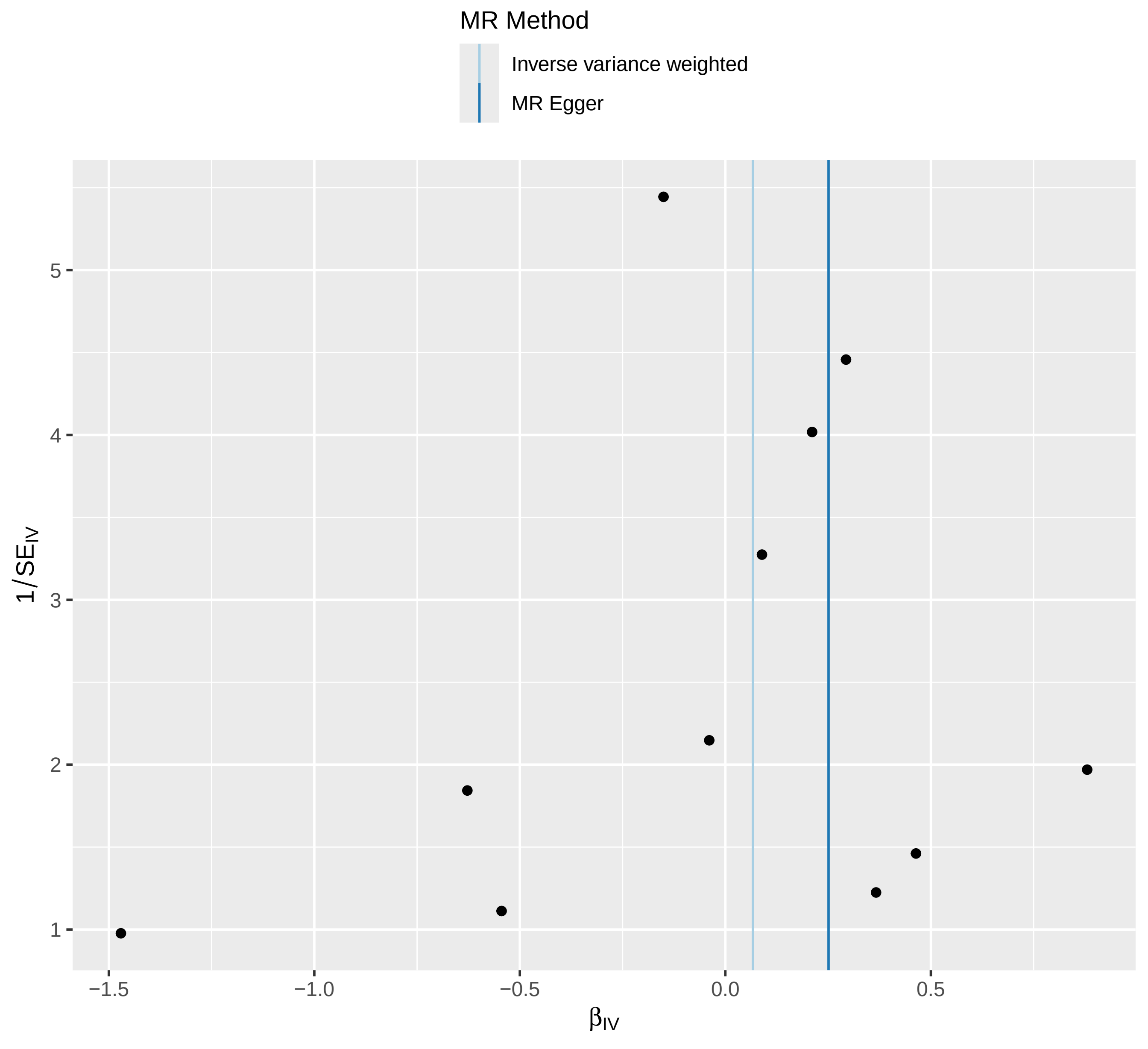
MR Method
Inverse variance weighted
MR Egger
1/SE_IV
5
4
3
2
1
−1.5
−1.0
−0.5
0.0
0.5
β_IV

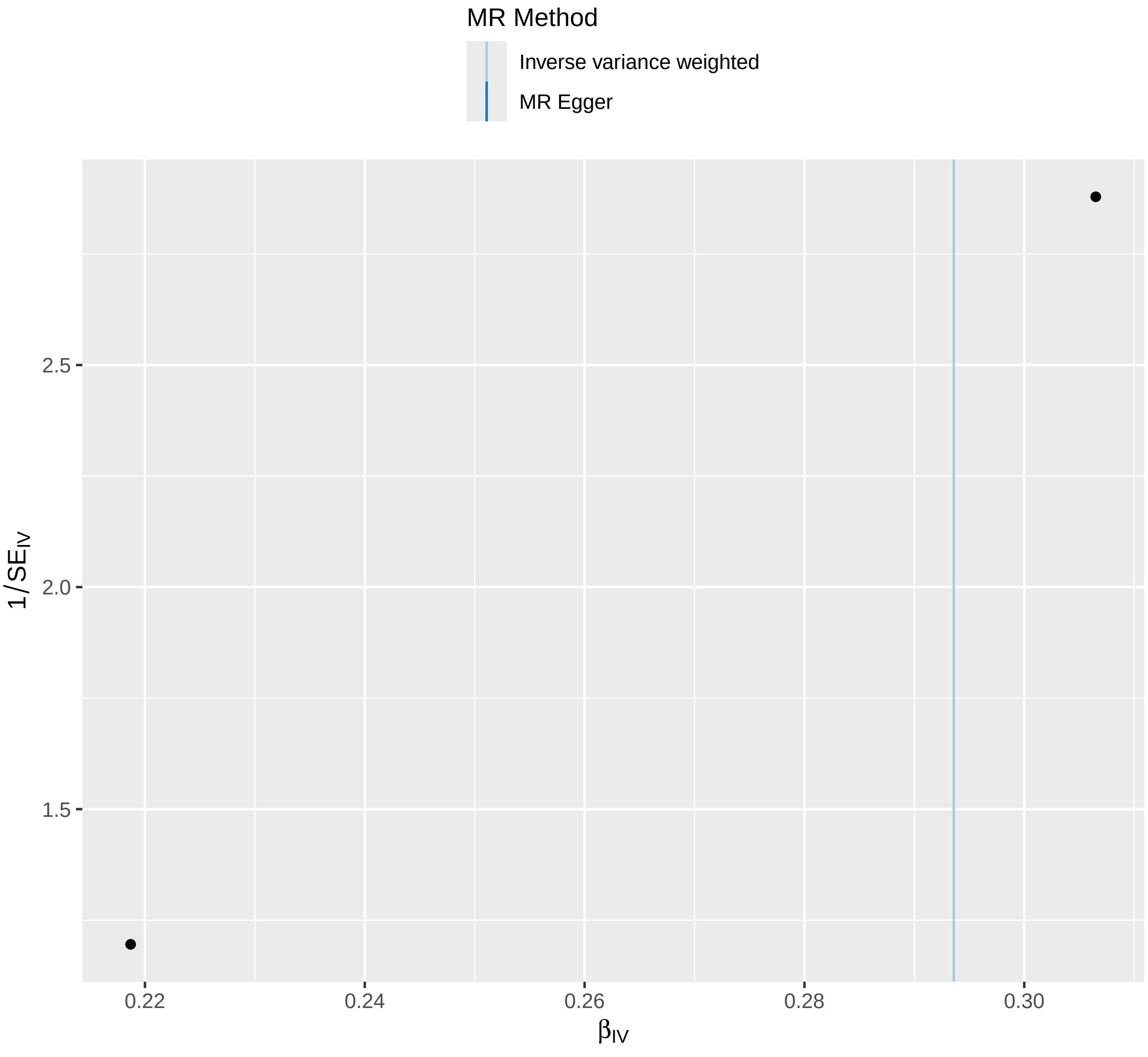

MR Method
Inverse variance weighted
MR Egger
2.5
2.0
1.5
1/SE$_{IV}$
0.22
0.24
0.26
0.28
0.30
β_{IV}

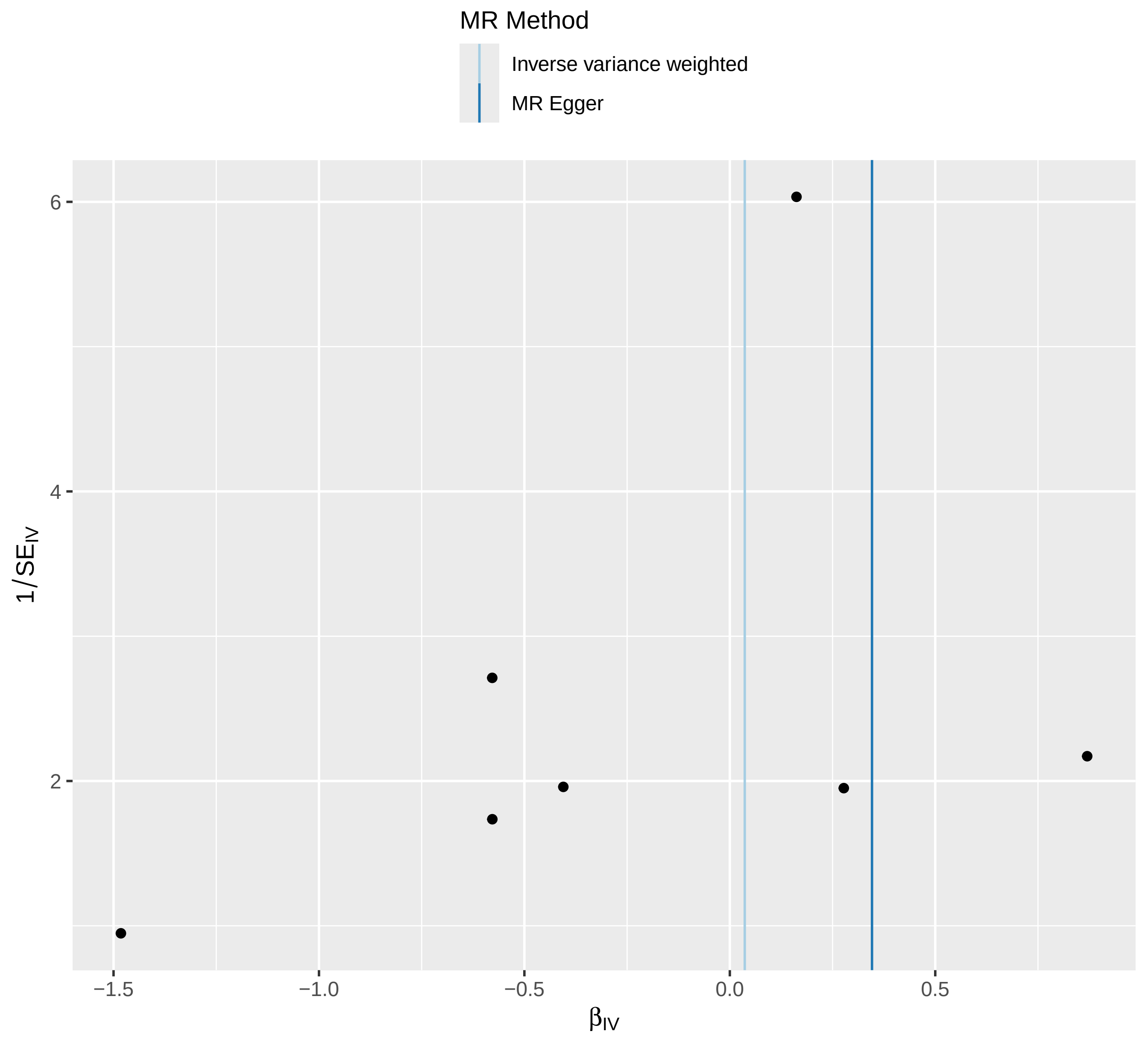
MR Method
Inverse variance weighted
MR Egger
6
4
2
$1/SE_{IV}$
−1.5
−1.0
−0.5
0.0
0.5
β_{IV}

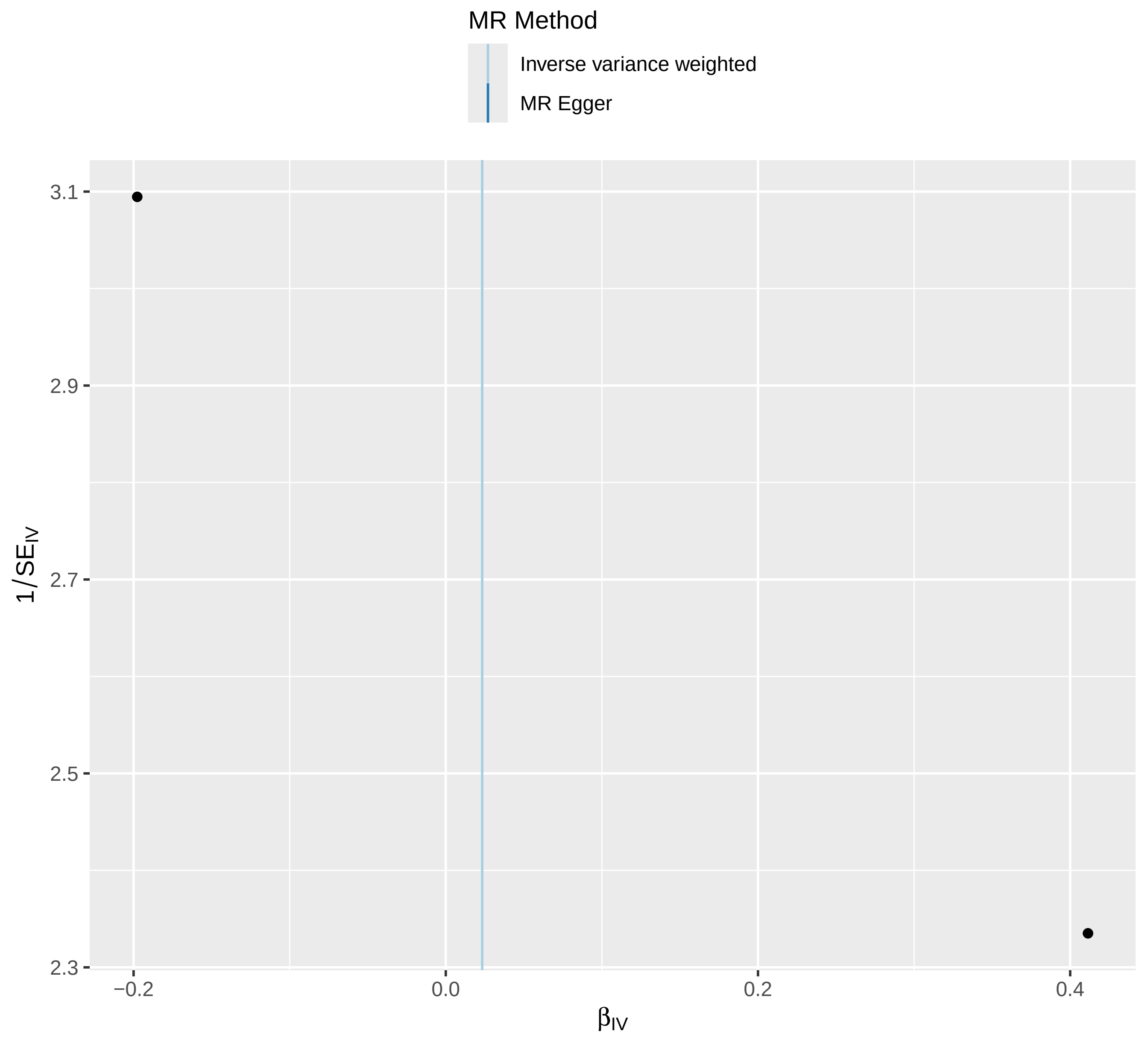

MR Method
Inverse variance weighted
MR Egger
3.1
2.9
2.7
2.5
2.3
1/SE_IV
−0.2
0.0
0.2
0.4
β_IV

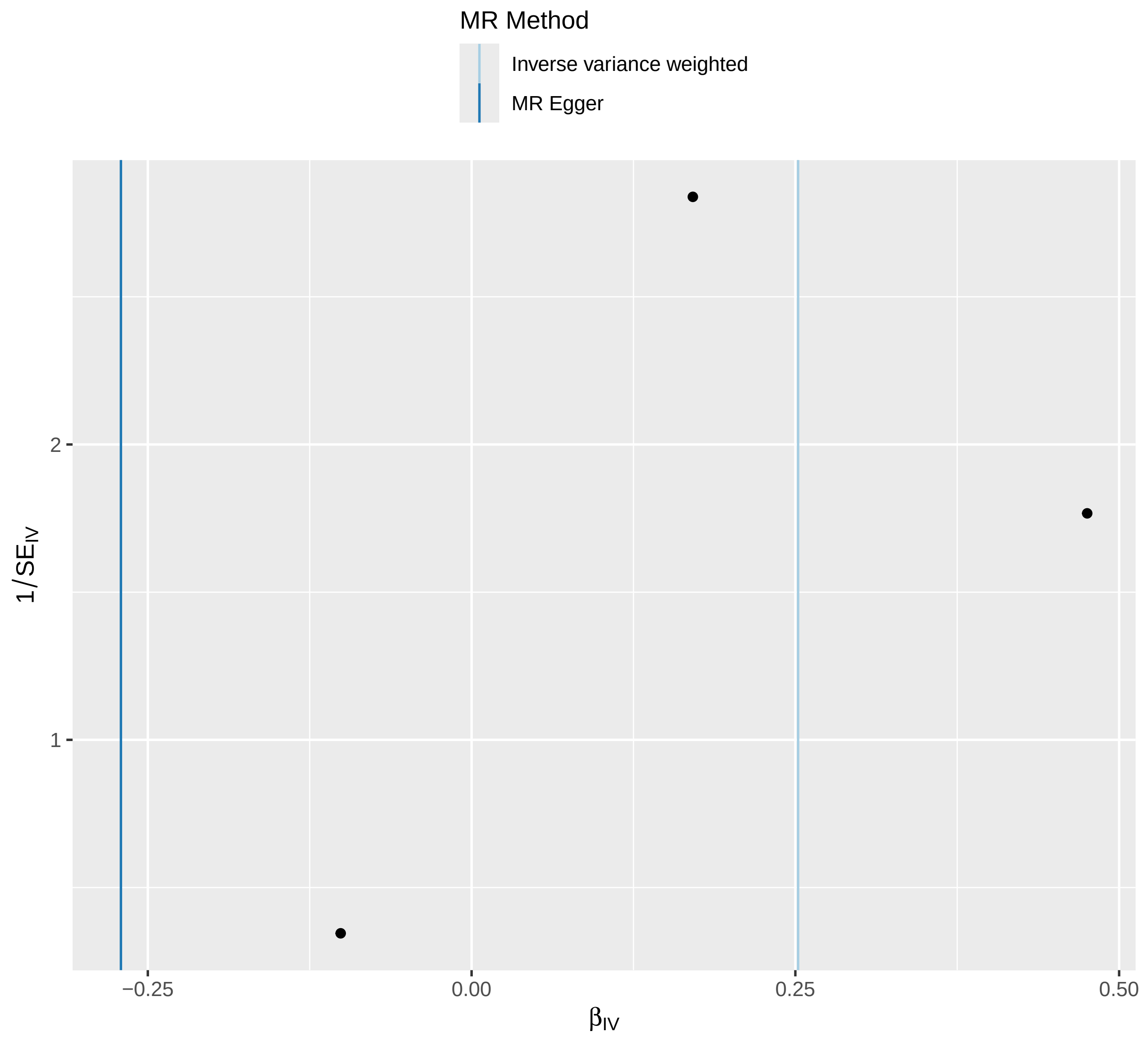

MR Method
Inverse variance weighted
MR Egger
2
1
1/SE_IV
−0.25
0.00
0.25
0.50
β_IV

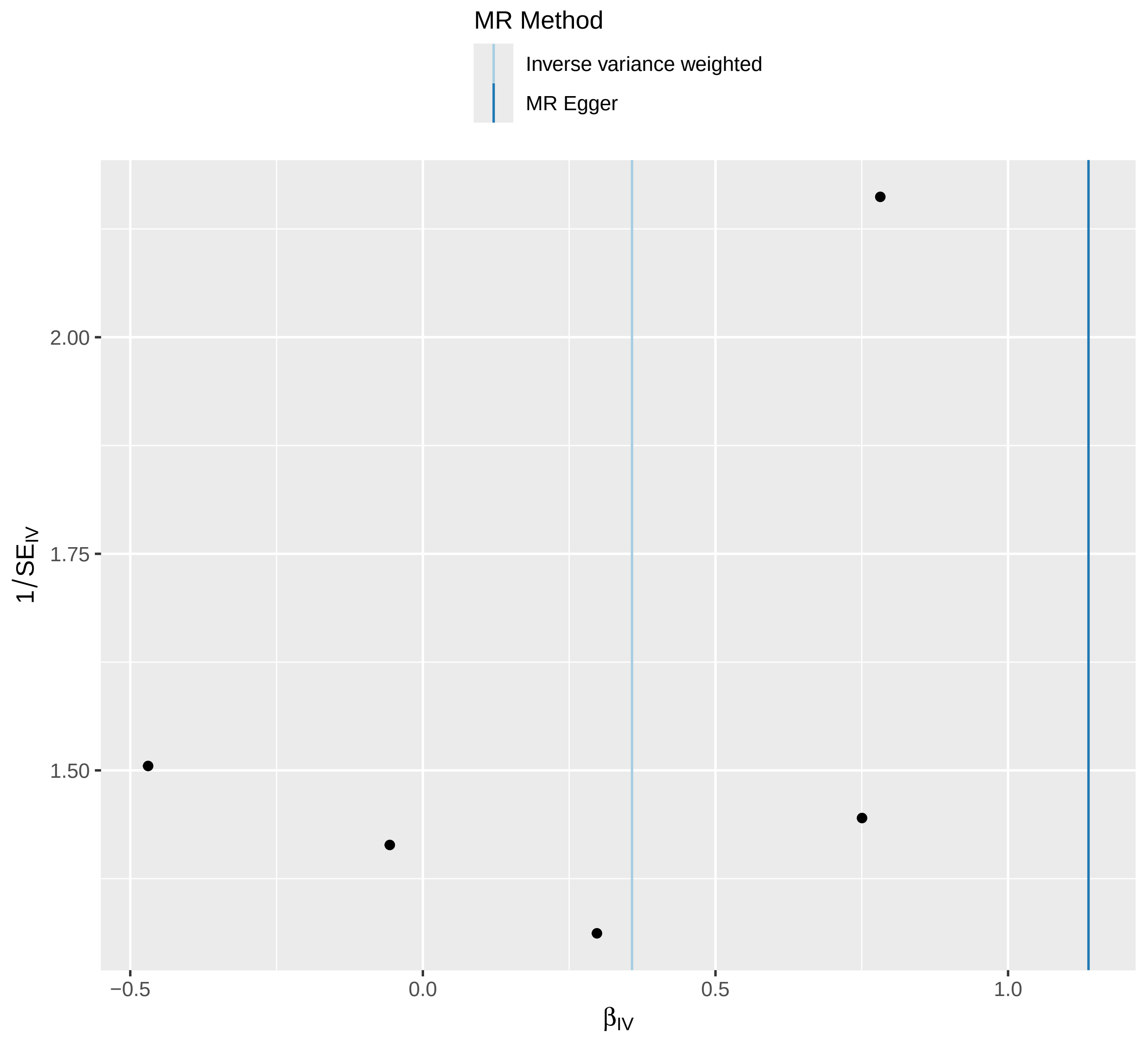
MR Method
Inverse variance weighted
MR Egger
2.00
1.75
1.50
1/SE_IV
−0.5
0.0
0.5
1.0
β_IV

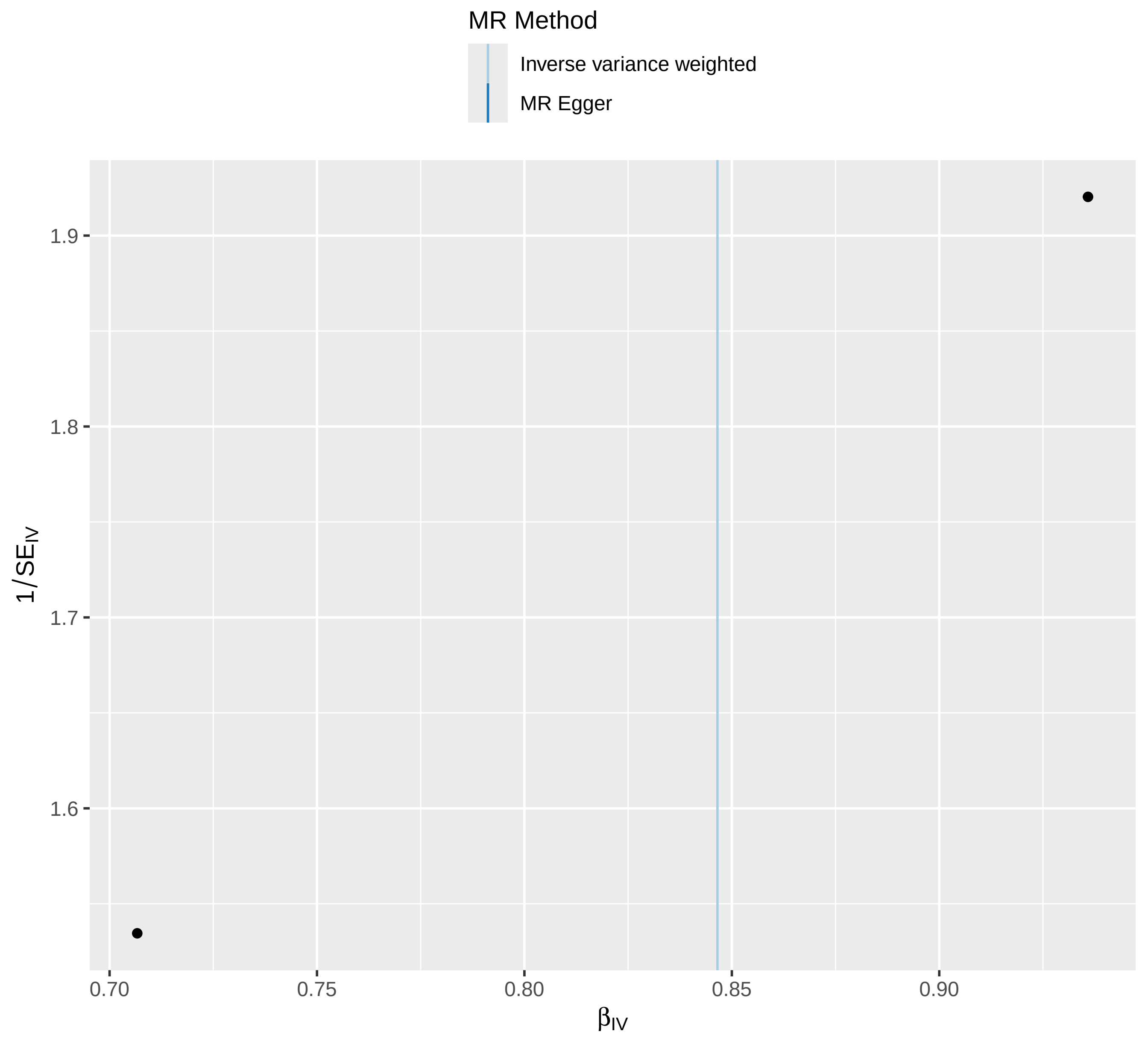

MR Method
Inverse variance weighted
MR Egger
1.9
1.8
1.7
1.6
0.70
0.75
0.80
0.85
0.90
$1/SE_{IV}$
β_{IV}

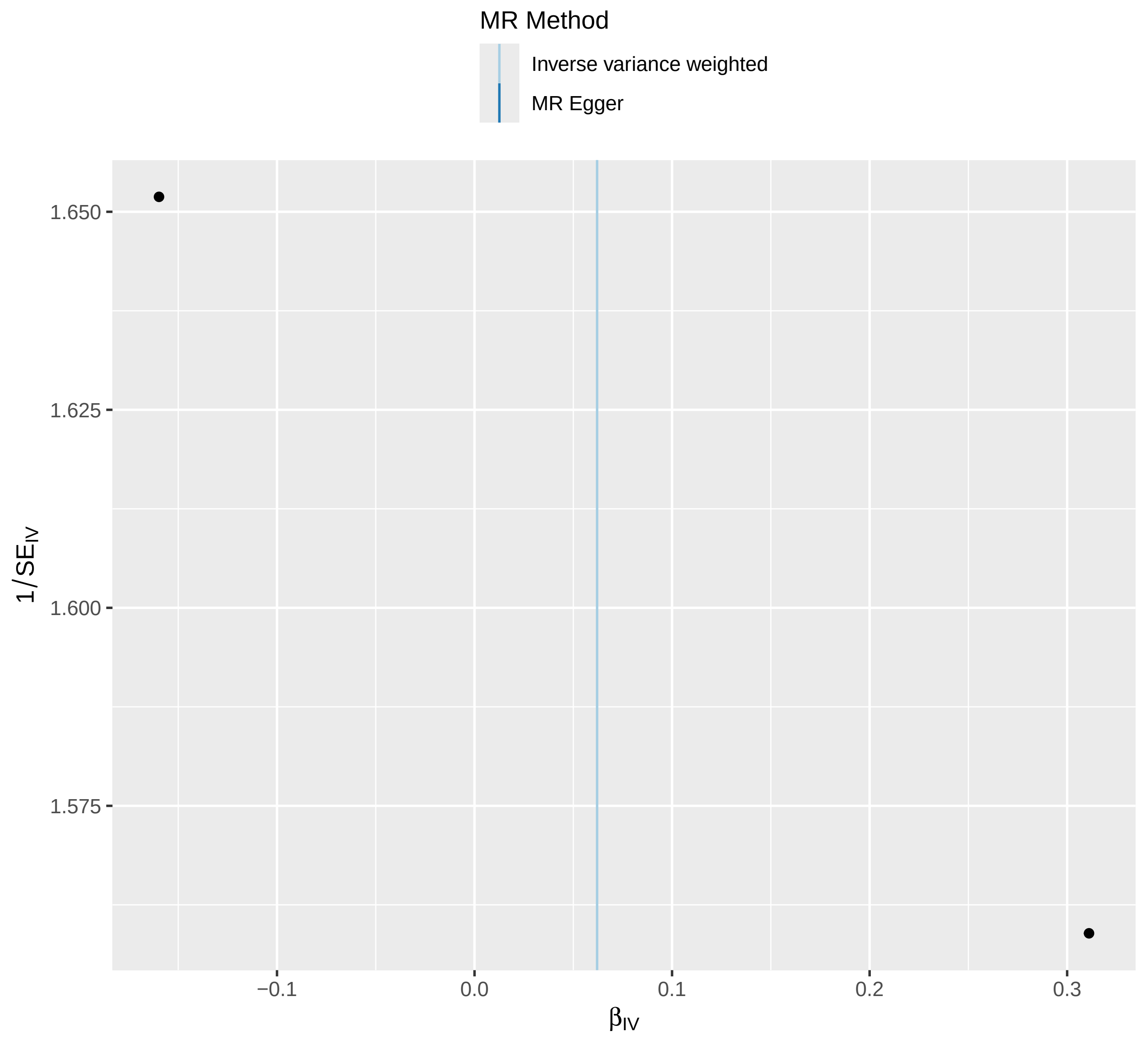
MR Method
Inverse variance weighted
MR Egger
1.650
1.625
1.600
1.575
1/SE_IV
−0.1
0.0
0.1
0.2
0.3
β_IV

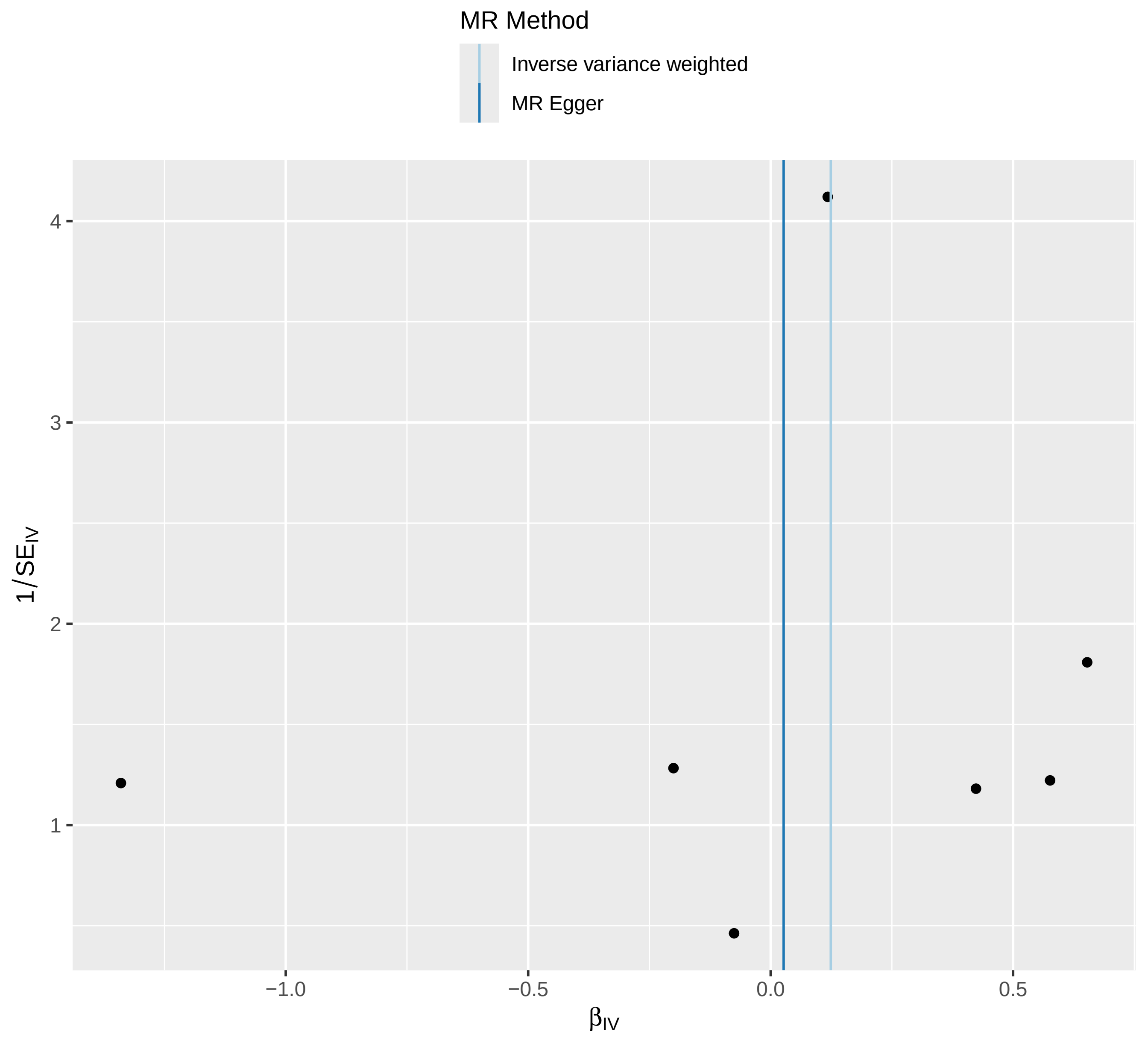

MR Method
Inverse variance weighted
MR Egger
$1/SE_{IV}$
4
3
2
1
−1.0
−0.5
0.0
0.5
β_{IV}

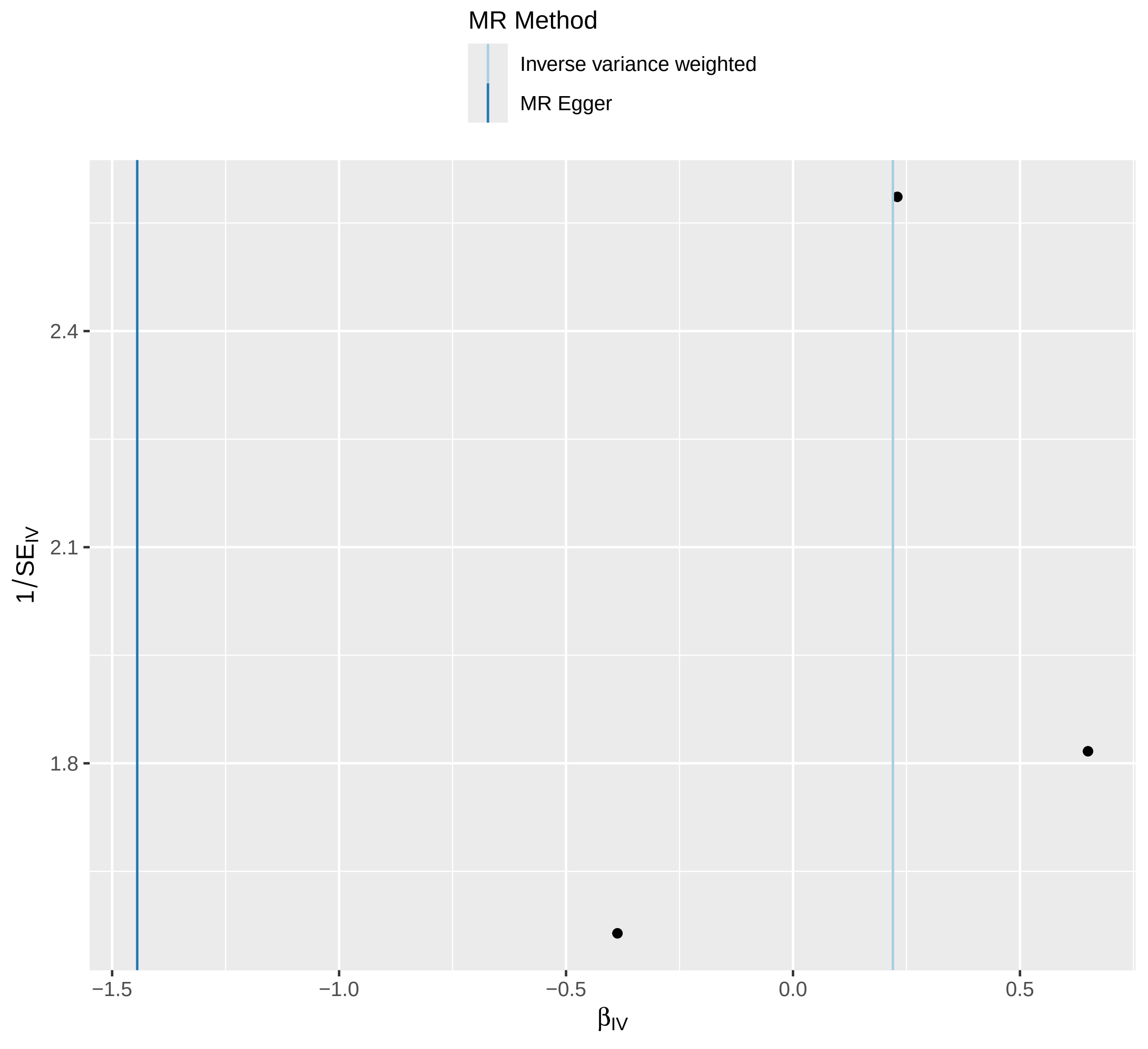
MR Method
Inverse variance weighted
MR Egger
2.4
2.1
1.8
1/SE$_{IV}$
−1.5
−1.0
−0.5
0.0
0.5
β$_{IV}$

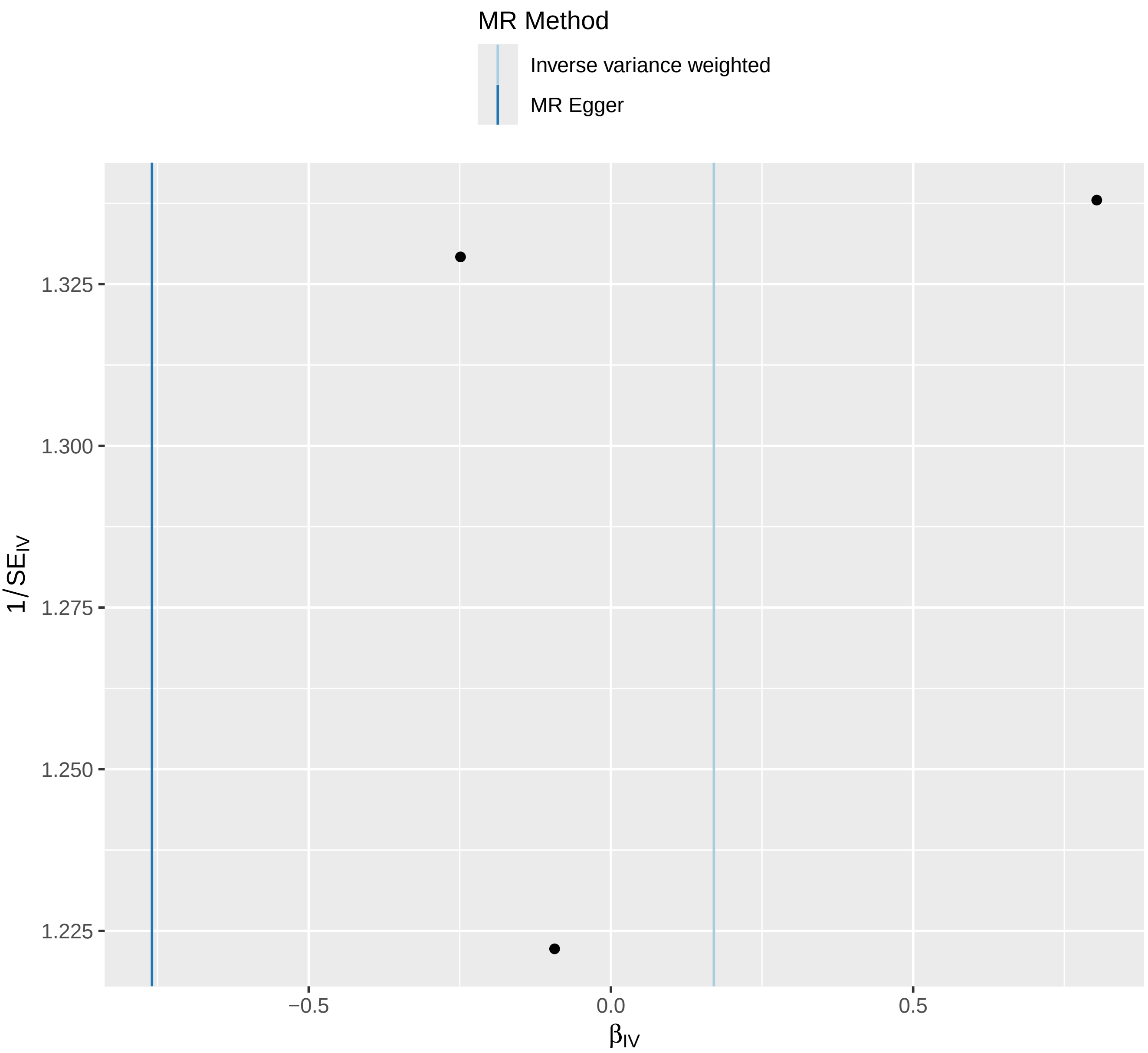
MR Method
Inverse variance weighted
MR Egger
1.325
1.300
1.275
1.250
1.225
1/SE_IV
−0.5
0.0
0.5
β_IV

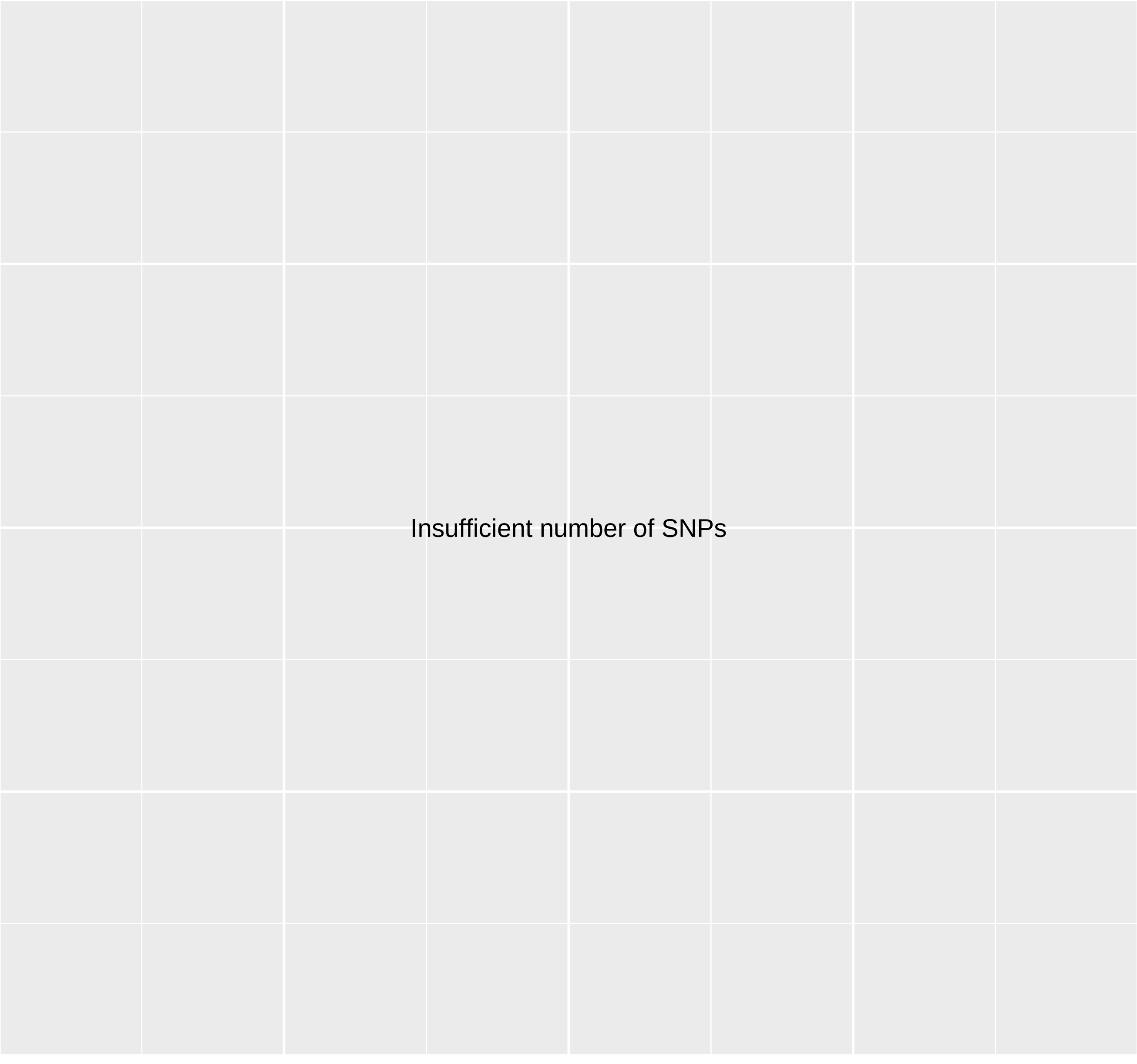
Insufficient number of SNPs

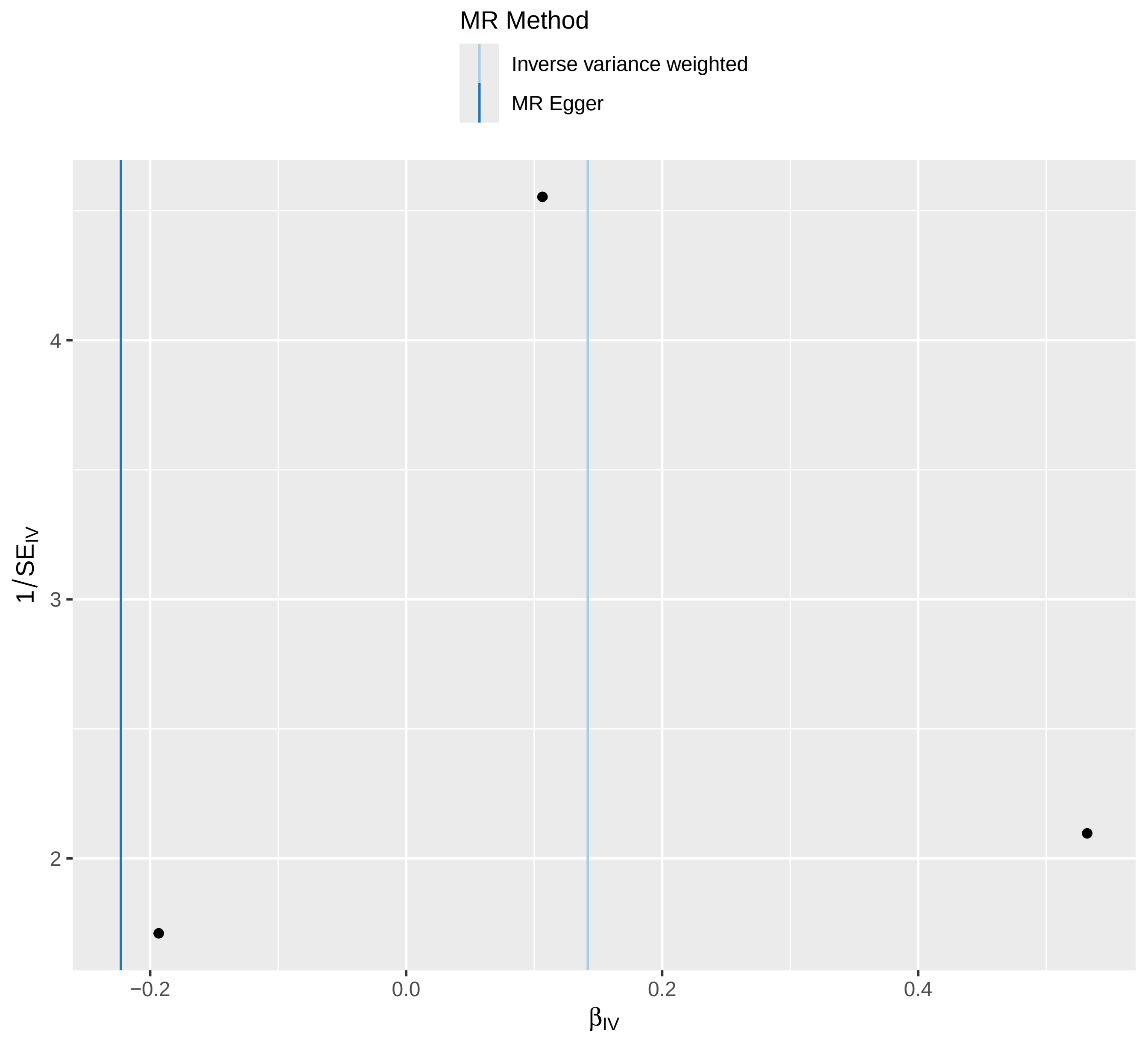

MR Method
Inverse variance weighted
MR Egger
4
3
2
−0.2
0.0
0.2
0.4
β_{IV}
$1/SE_{IV}$

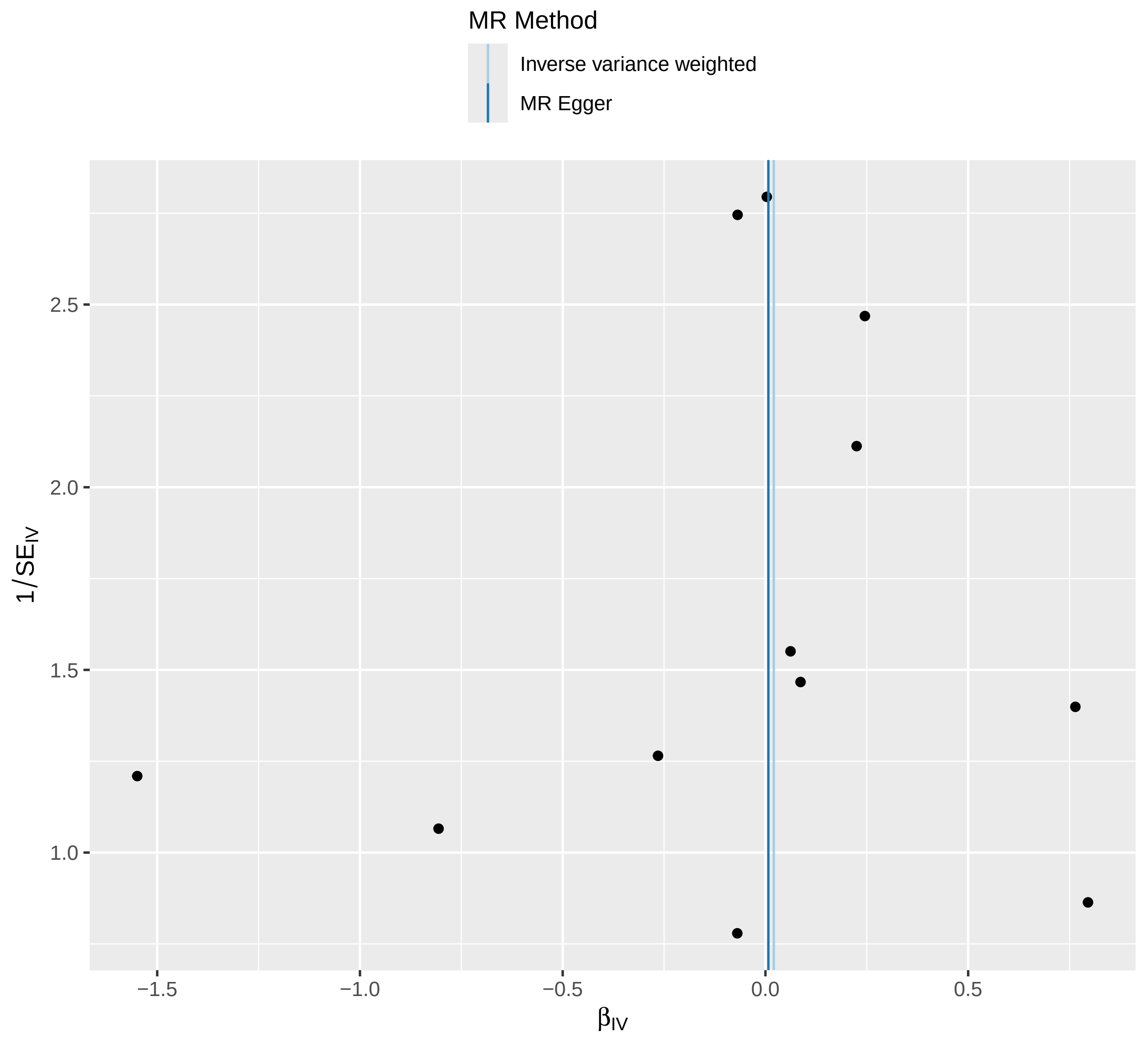
MR Method
Inverse variance weighted
MR Egger
2.5
2.0
1.5
1.0
1/SE_IV
−1.5
−1.0
−0.5
0.0
0.5
β_IV

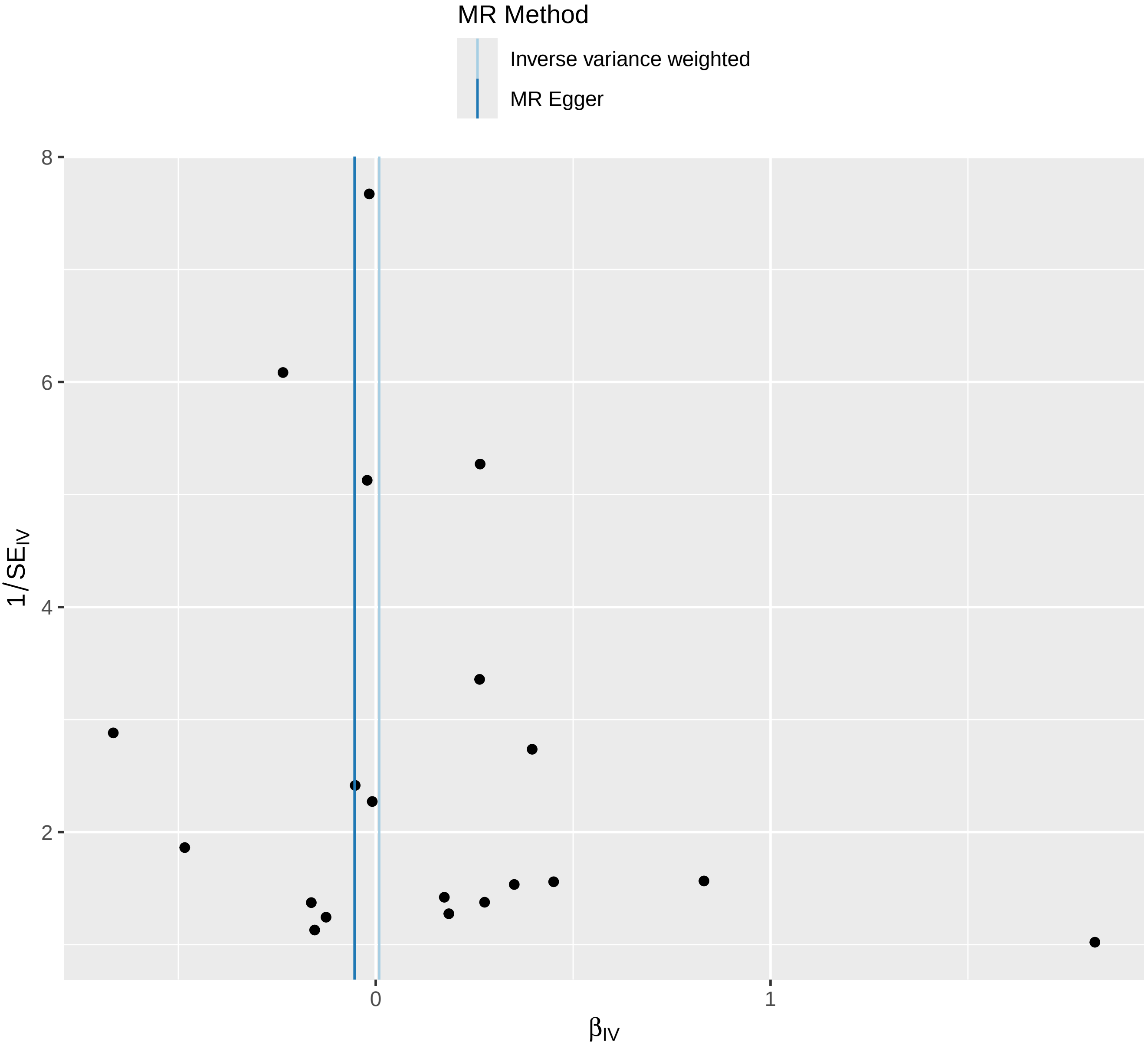

MR Method
Inverse variance weighted
MR Egger
8
6
4
2
1/SE_IV
0
1
β_IV

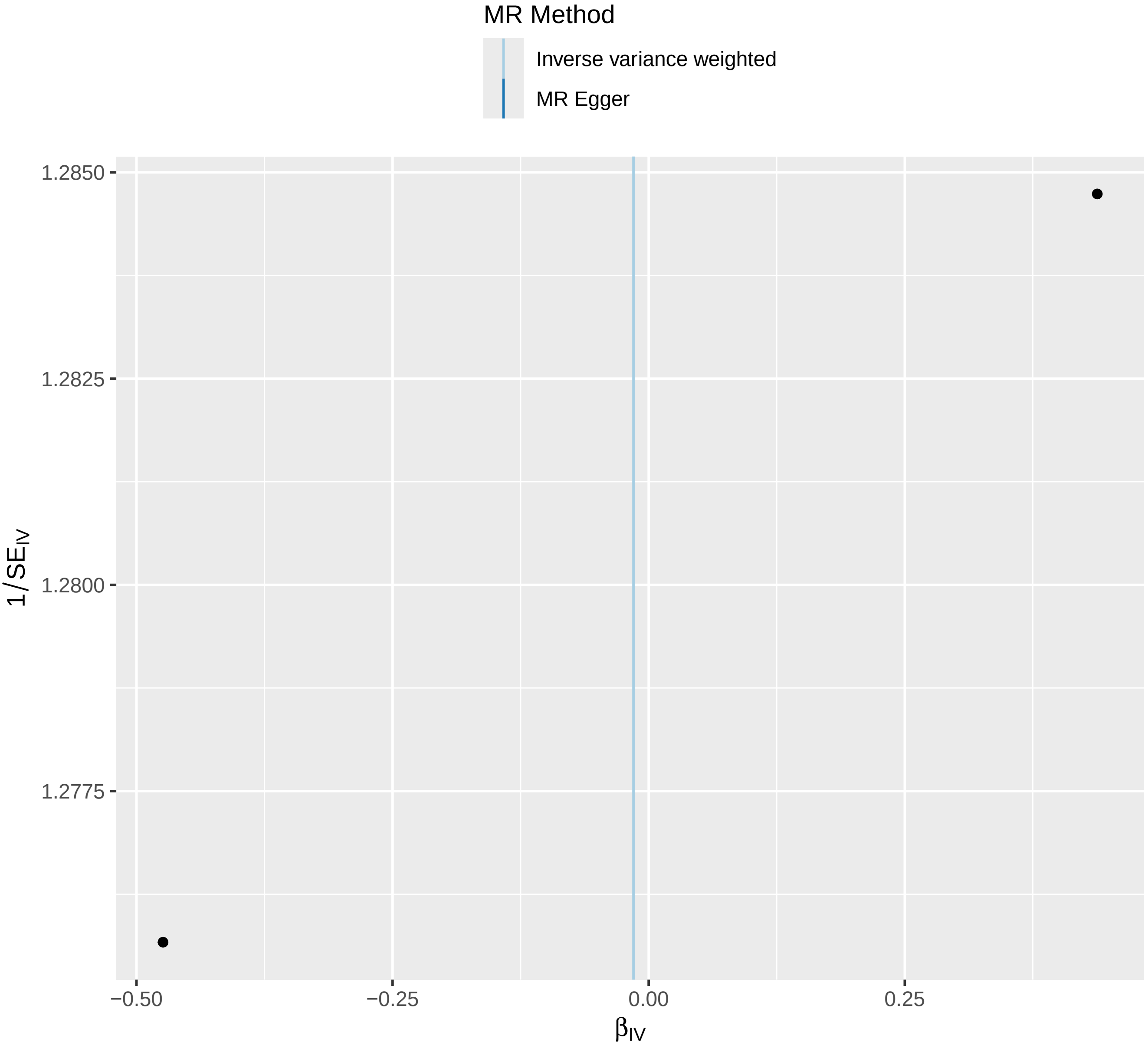
MR Method
Inverse variance weighted
MR Egger
1.2850
1.2825
1.2800
1.2775
1/SE_IV
−0.50
−0.25
0.00
0.25
β_IV

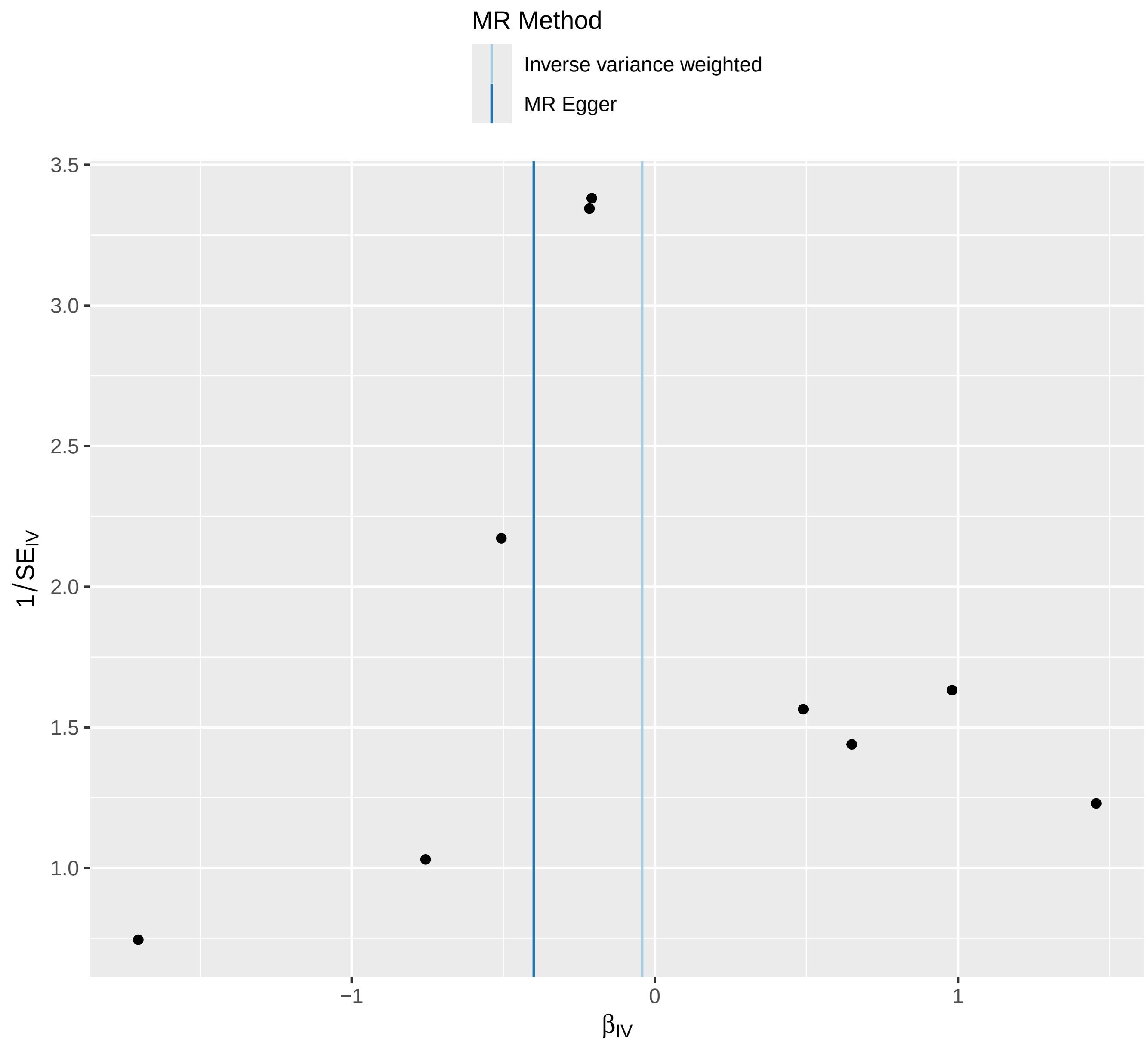

MR Method
Inverse variance weighted
MR Egger
3.5
3.0
2.5
2.0
1.5
1.0
1/SE$_{IV}$
−1
0
1
β$_{IV}$

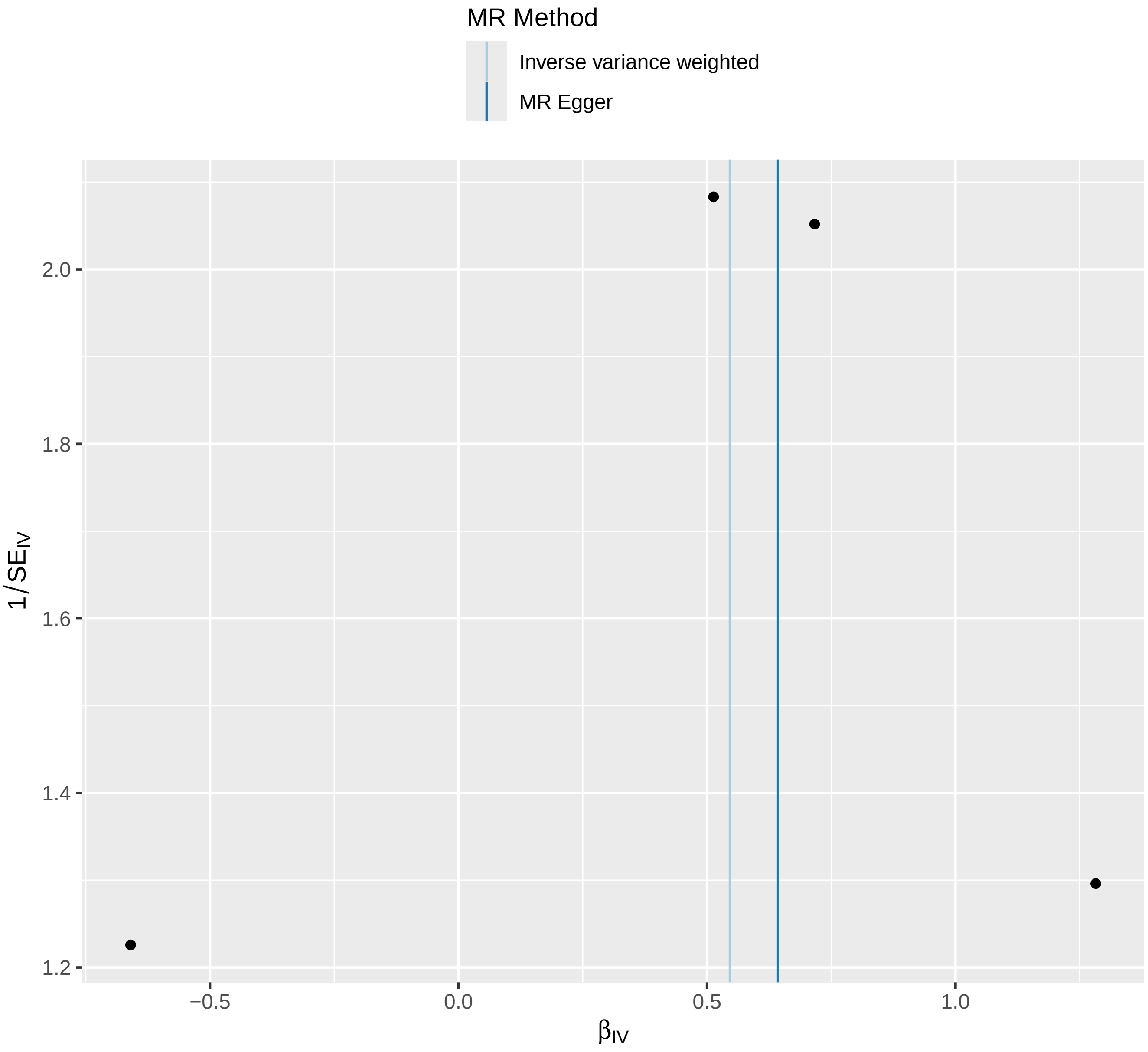

MR Method
Inverse variance weighted
MR Egger
1/SE_IV
2.0
1.8
1.6
1.4
1.2
−0.5
0.0
0.5
1.0
β_IV

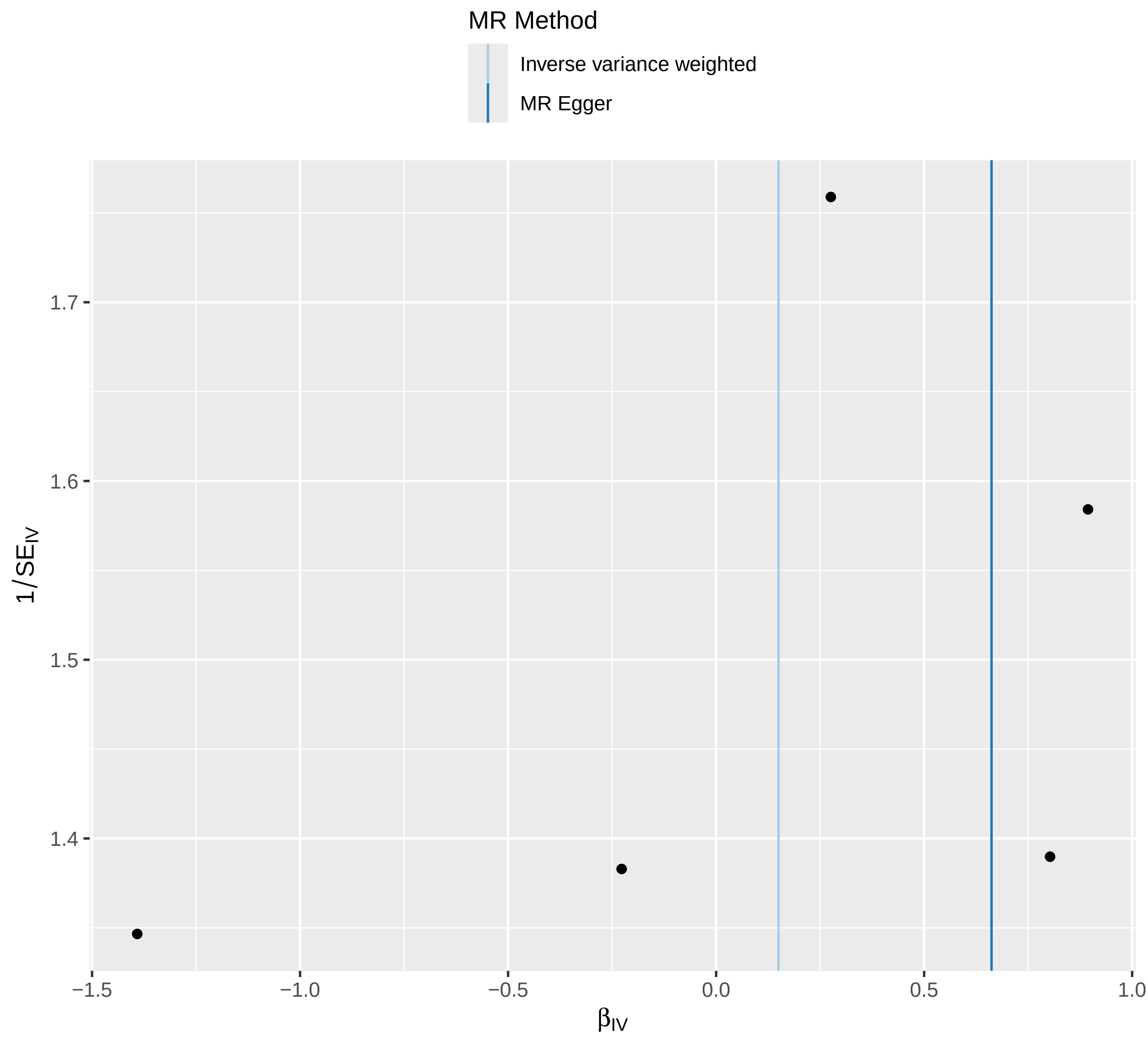

MR Method
Inverse variance weighted
MR Egger
1.7
1.6
1.5
1.4
1/SE_IV
−1.5
−1.0
−0.5
0.0
0.5
1.0
β_IV

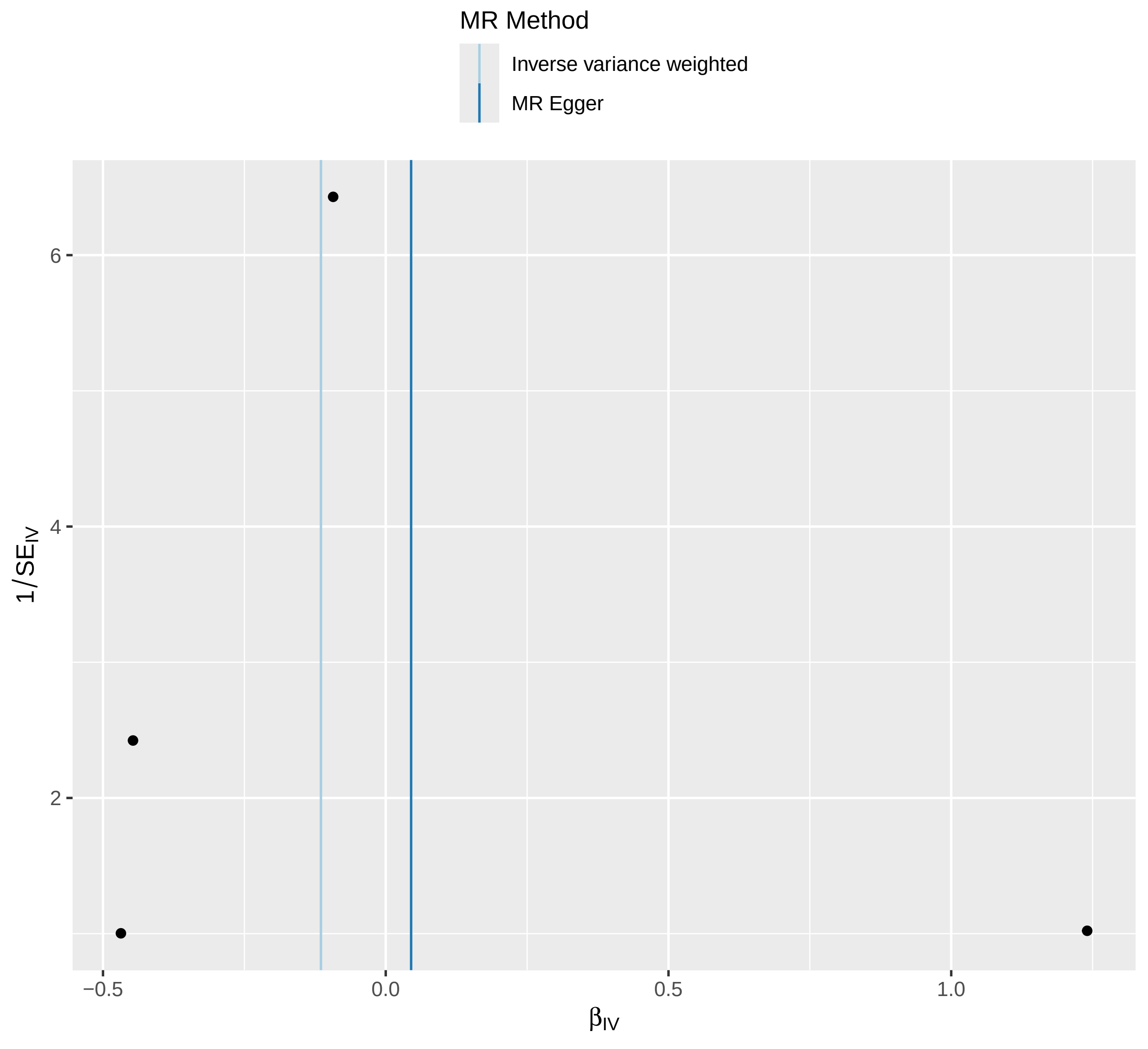
MR Method
Inverse variance weighted
MR Egger
6
4
2
1/SE_IV
−0.5
0.0
0.5
1.0
β_IV

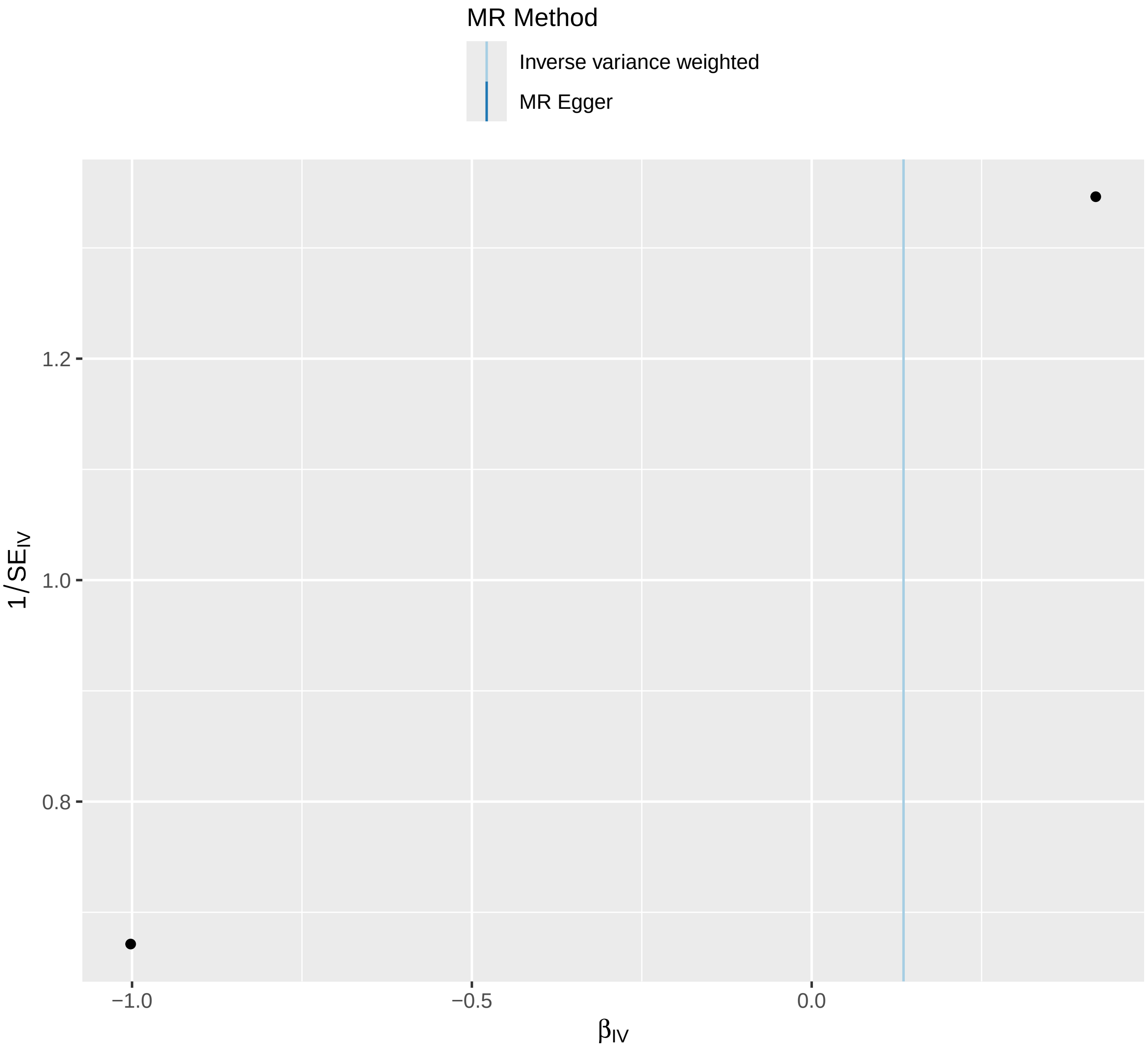

MR Method
Inverse variance weighted
MR Egger
1.2
1.0
0.8
1/SE$_{IV}$
−1.0
−0.5
0.0
β$_{IV}$

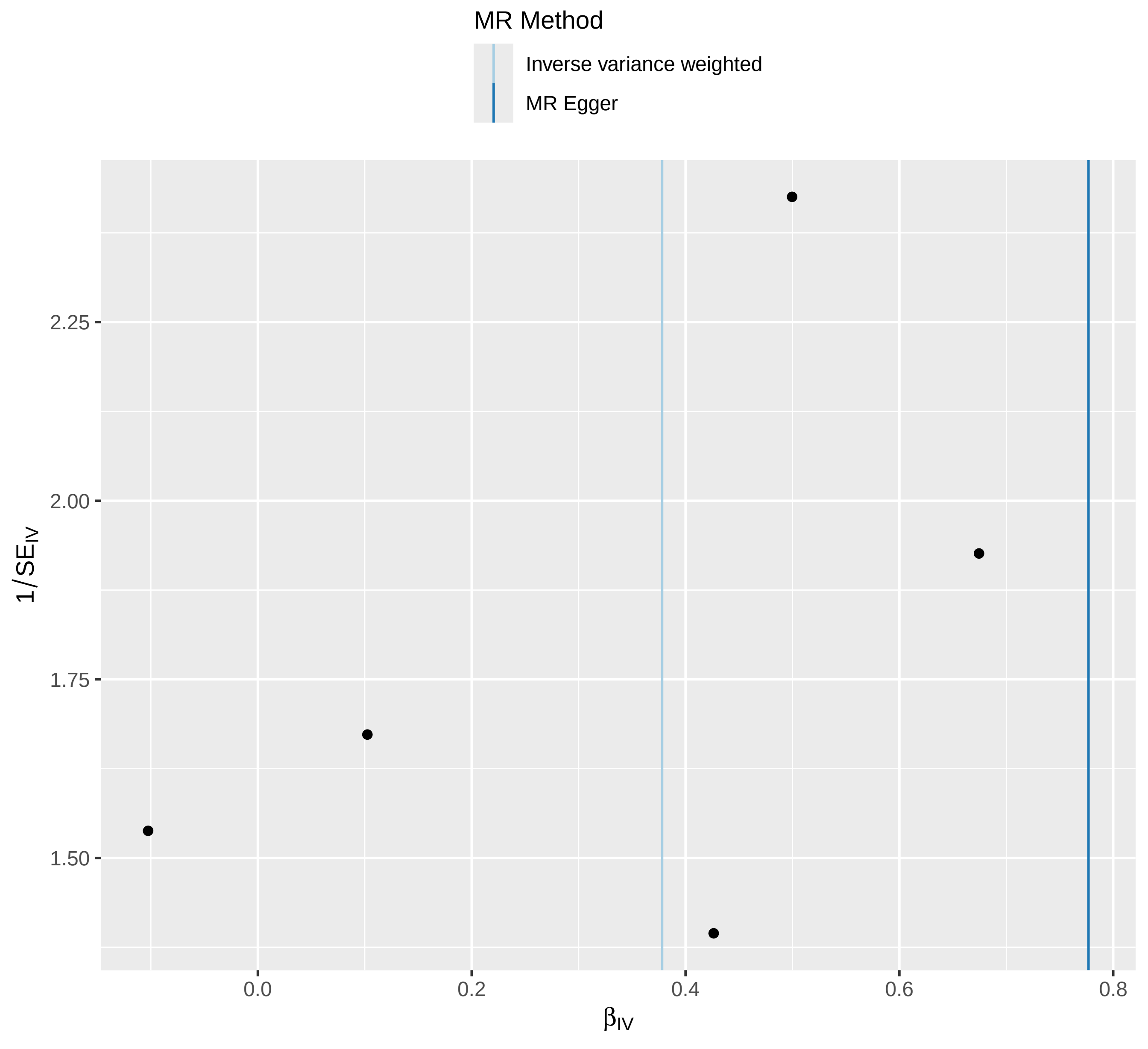
MR Method
Inverse variance weighted
MR Egger
2.25
2.00
1.75
1.50
1/SE_IV
0.0
0.2
0.4
0.6
0.8
β_IV

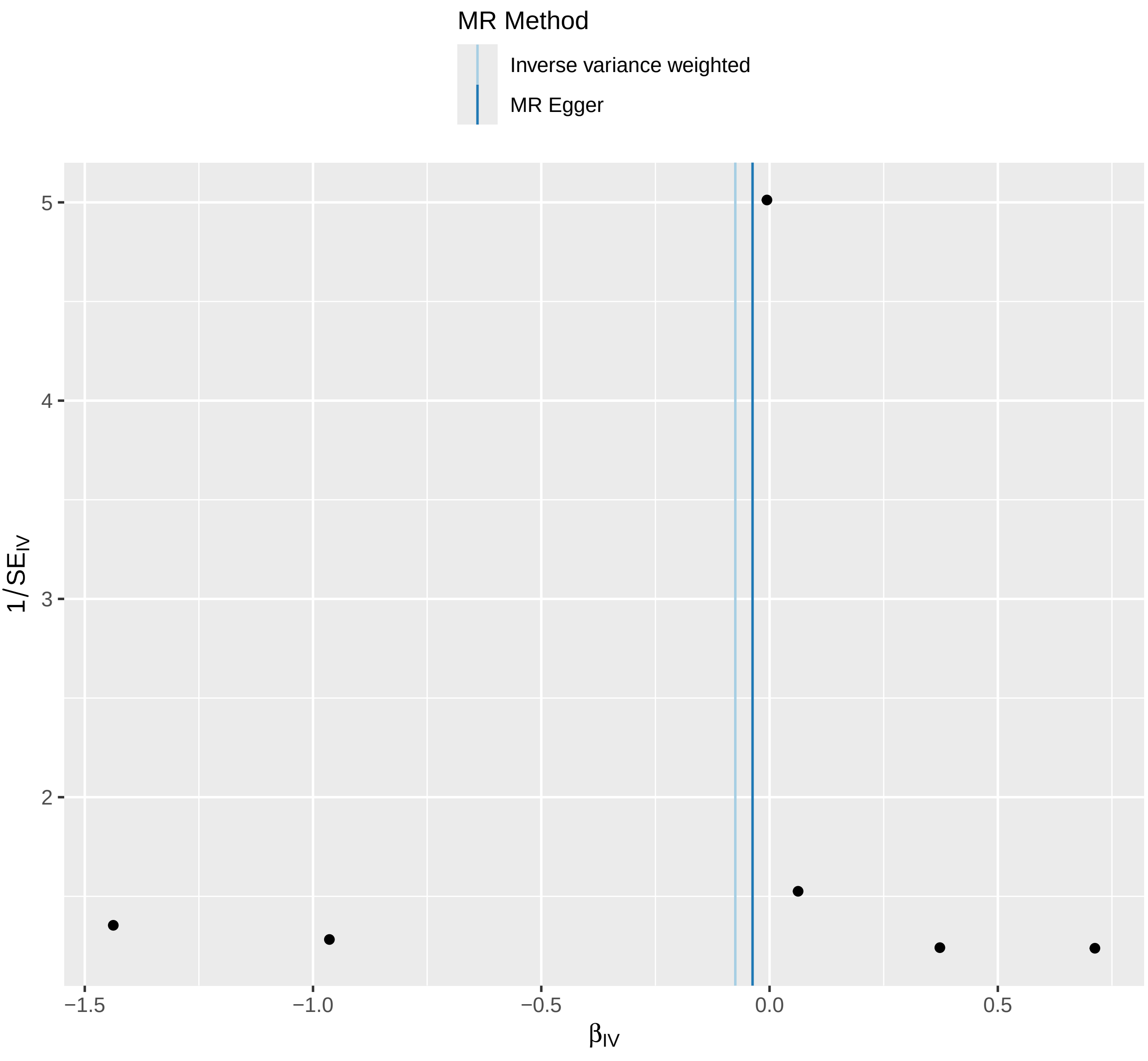
MR Method
Inverse variance weighted
MR Egger
5
4
3
2
1/SE$_{IV}$
−1.5
−1.0
−0.5
0.0
0.5
β$_{IV}$

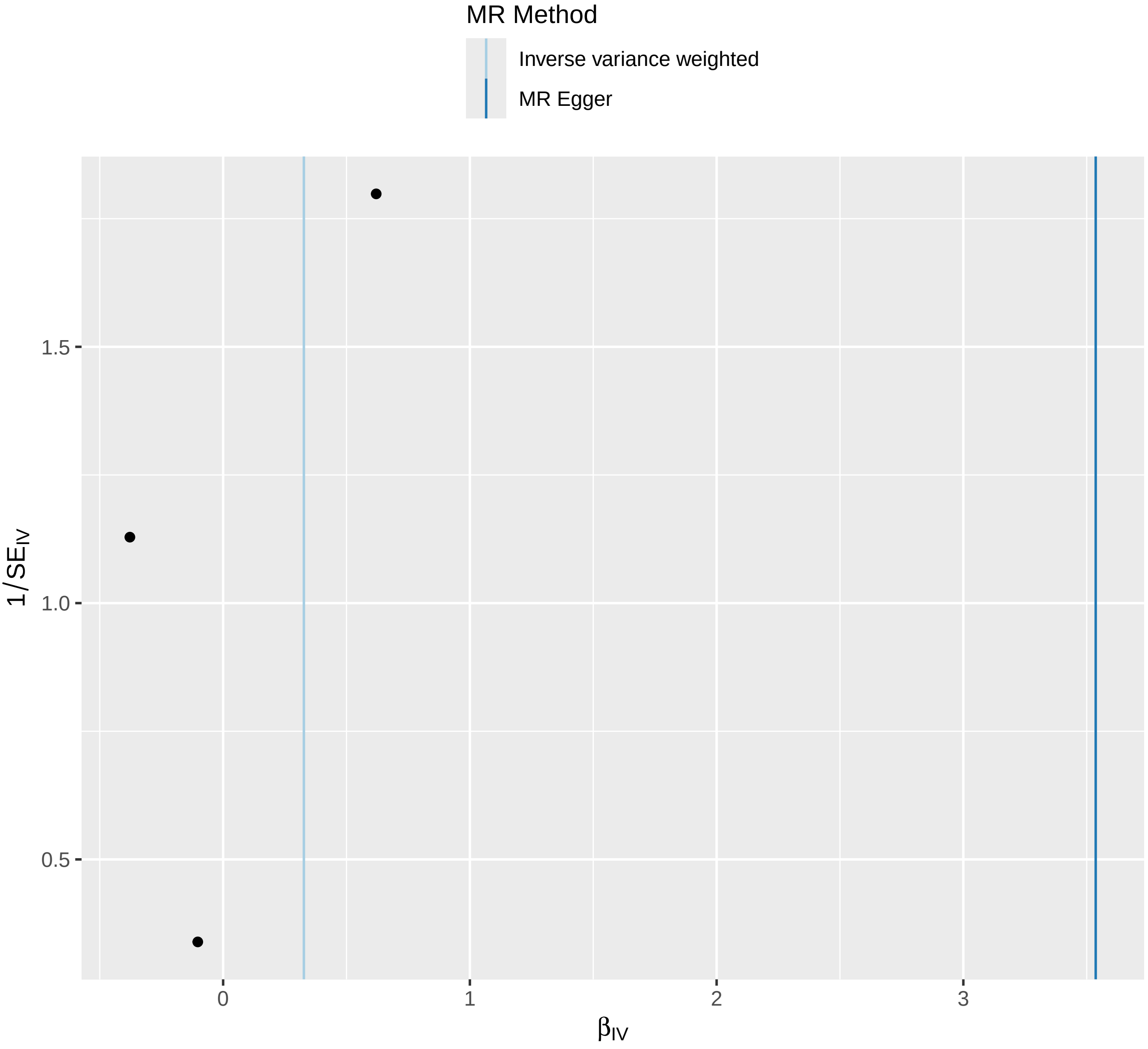
MR Method
Inverse variance weighted
MR Egger
1.5
1.0
0.5
1/SE_IV
0
1
2
3
β_IV

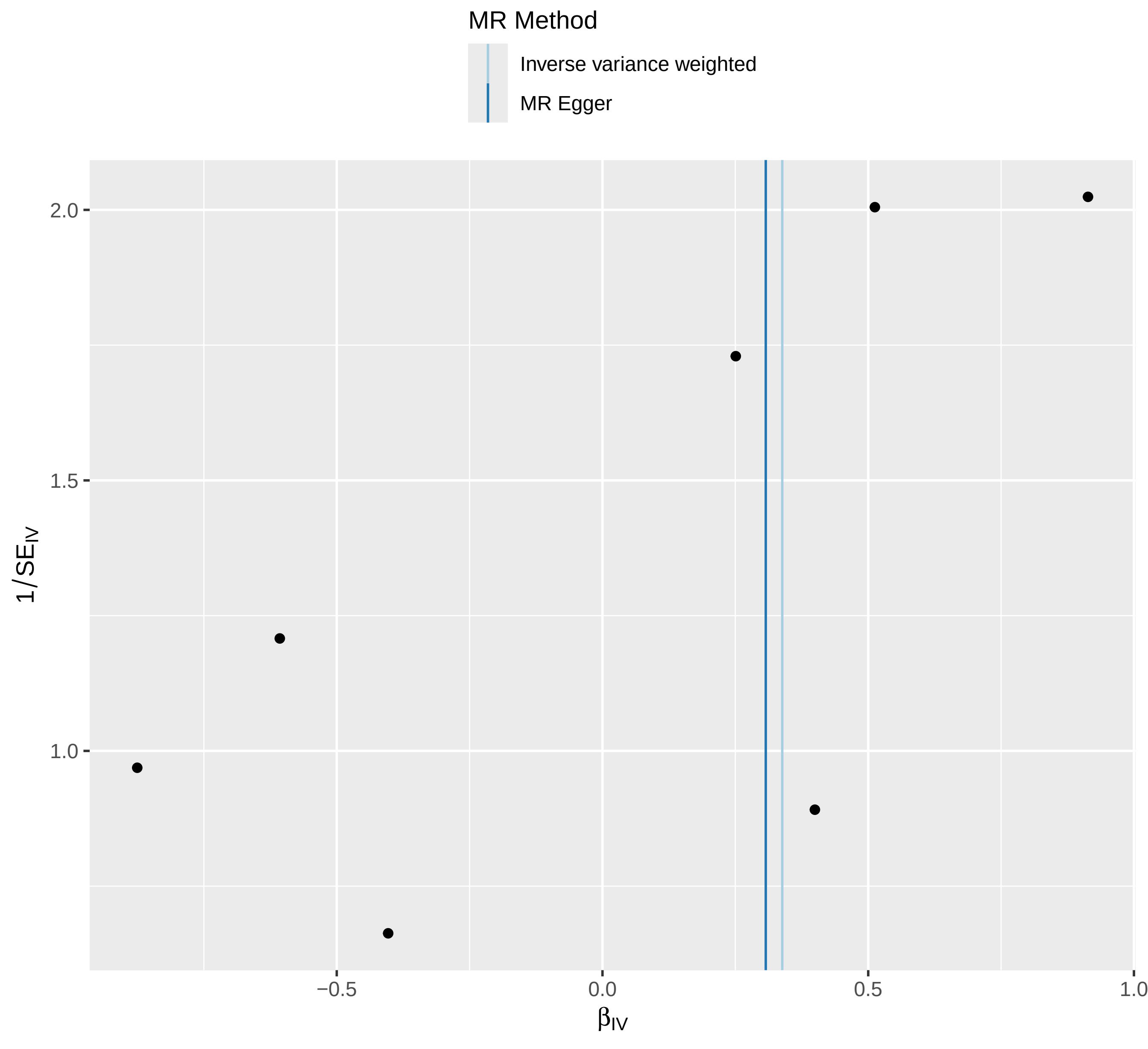
MR Method
Inverse variance weighted
MR Egger
2.0
1.5
1.0
1/SE_IV
−0.5
0.0
0.5
1.0
β_IV

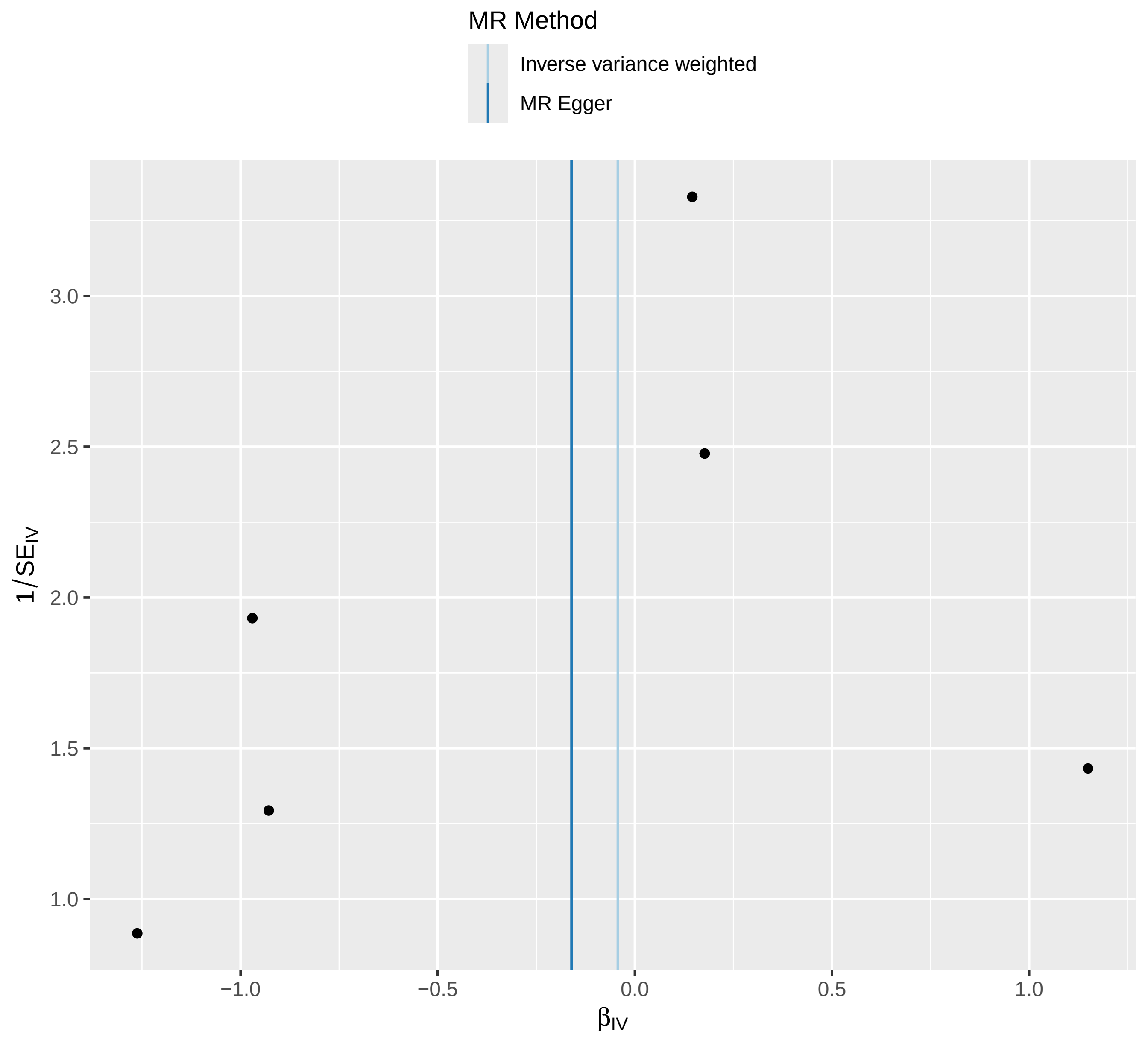
MR Method
Inverse variance weighted
MR Egger
3.0
2.5
2.0
1.5
1.0
1/SE_IV
−1.0
−0.5
0.0
0.5
1.0
β_IV

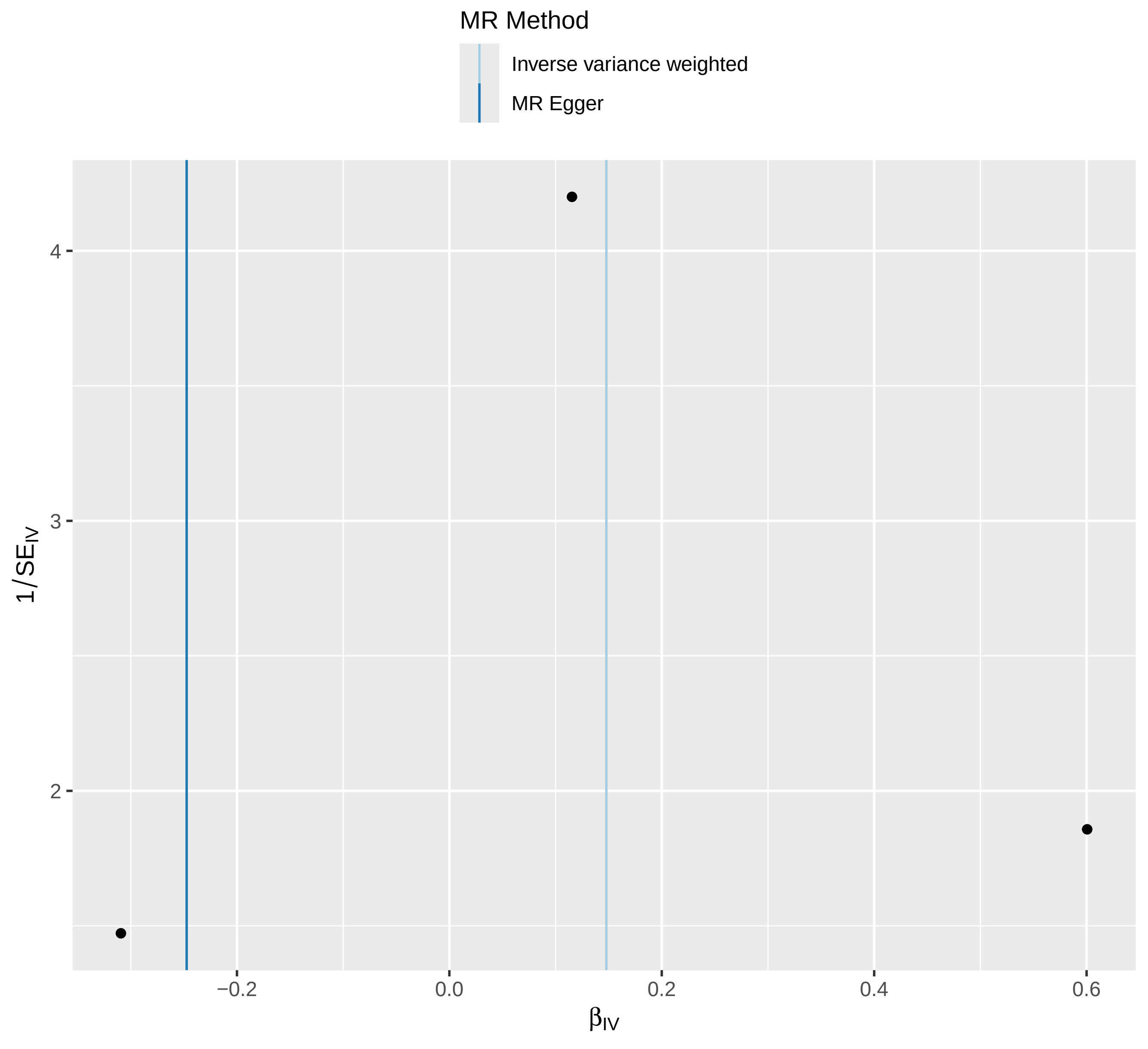
MR Method
Inverse variance weighted
MR Egger
4
3
2
1/SE_{IV}
−0.2
0.0
0.2
0.4
0.6
β_{IV}

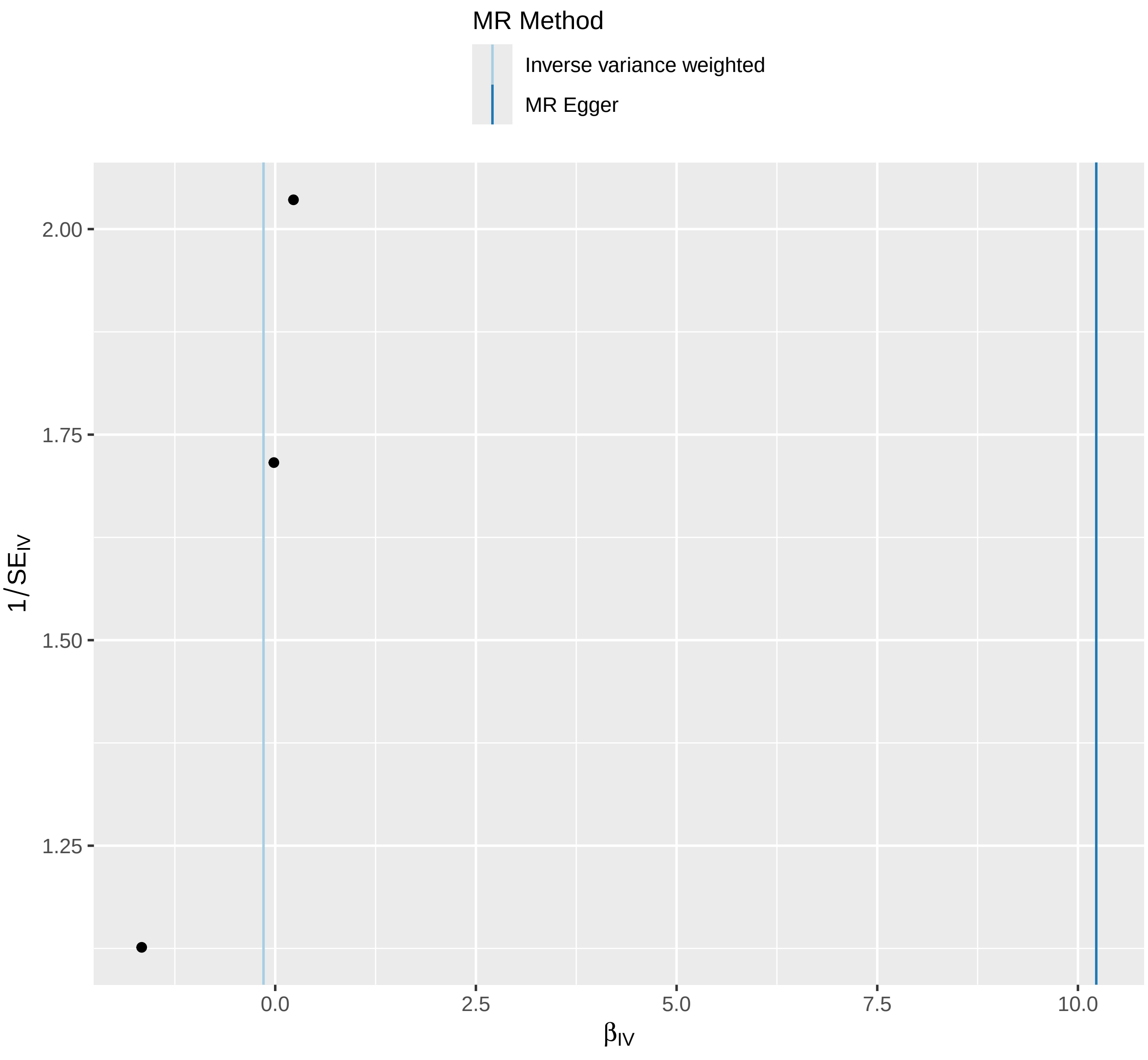
MR Method
Inverse variance weighted
MR Egger
2.00
1.75
1.50
1.25
1/SE$_{IV}$
0.0
2.5
5.0
7.5
10.0
β$_{IV}$

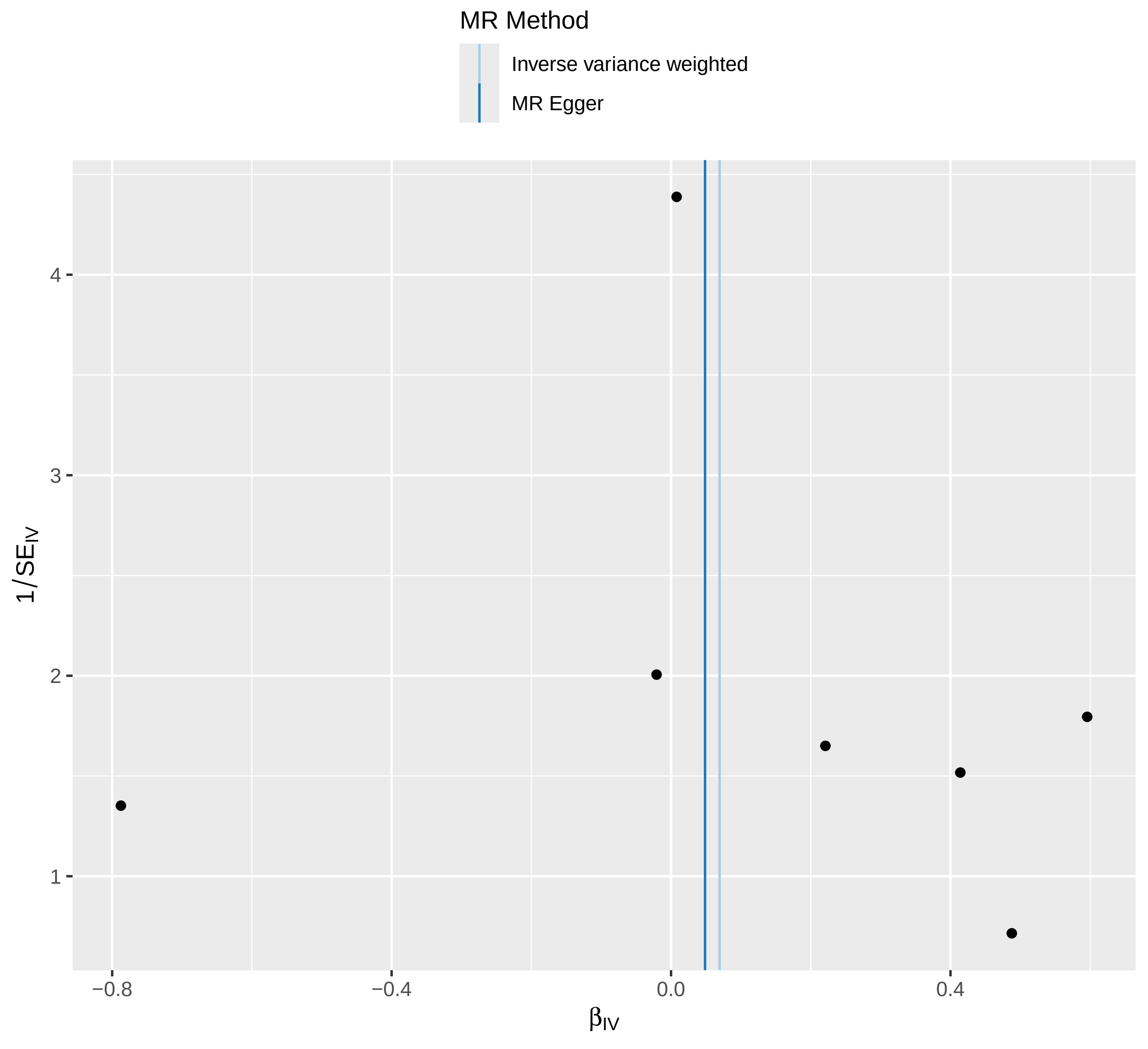

MR Method
Inverse variance weighted
MR Egger
4
3
2
1
1/SE_IV
−0.8
−0.4
0.0
0.4
β_IV

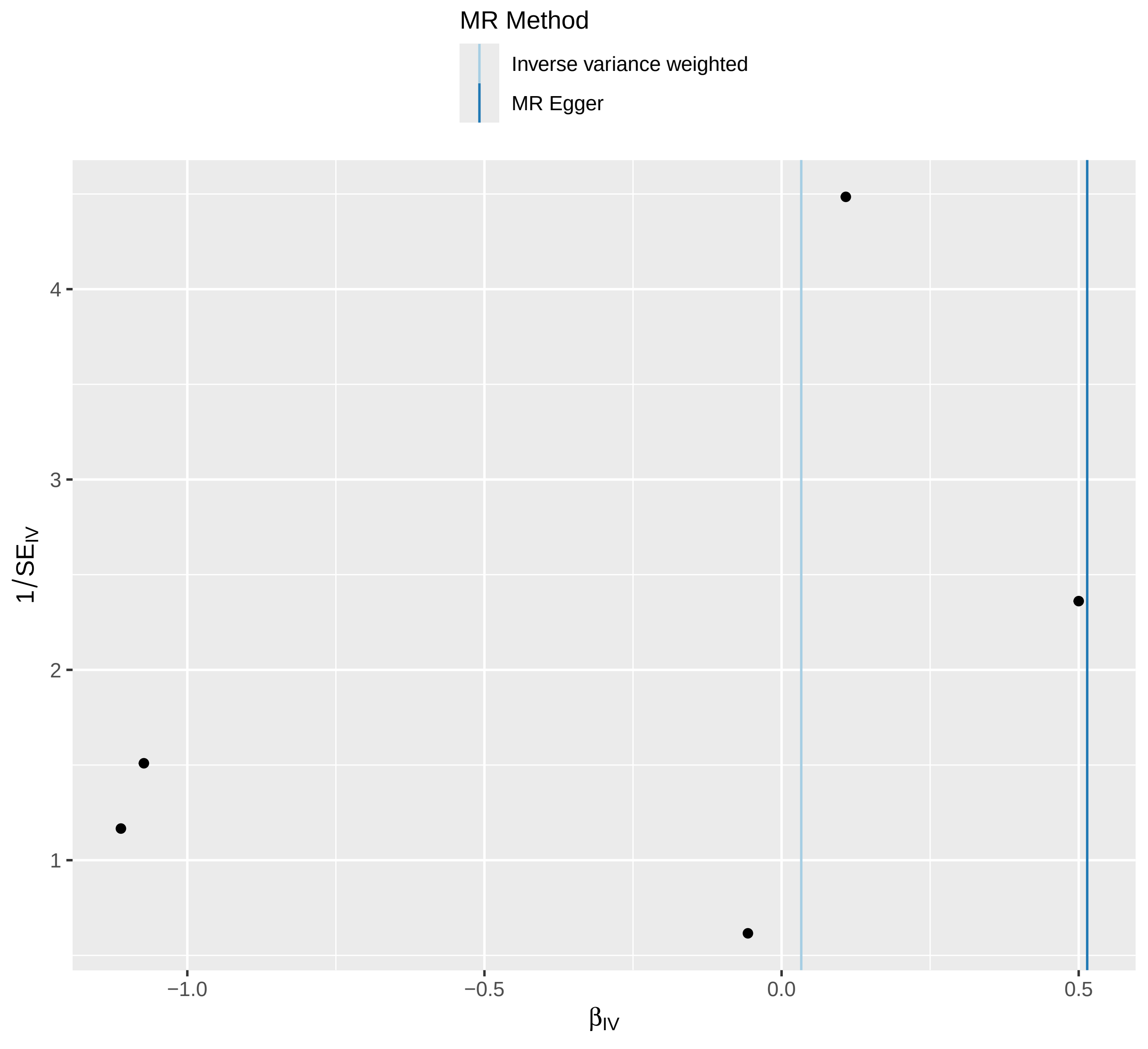
MR Method
Inverse variance weighted
MR Egger
4
3
2
1
$1/SE_{IV}$
−1.0
−0.5
0.0
0.5
β_{IV}

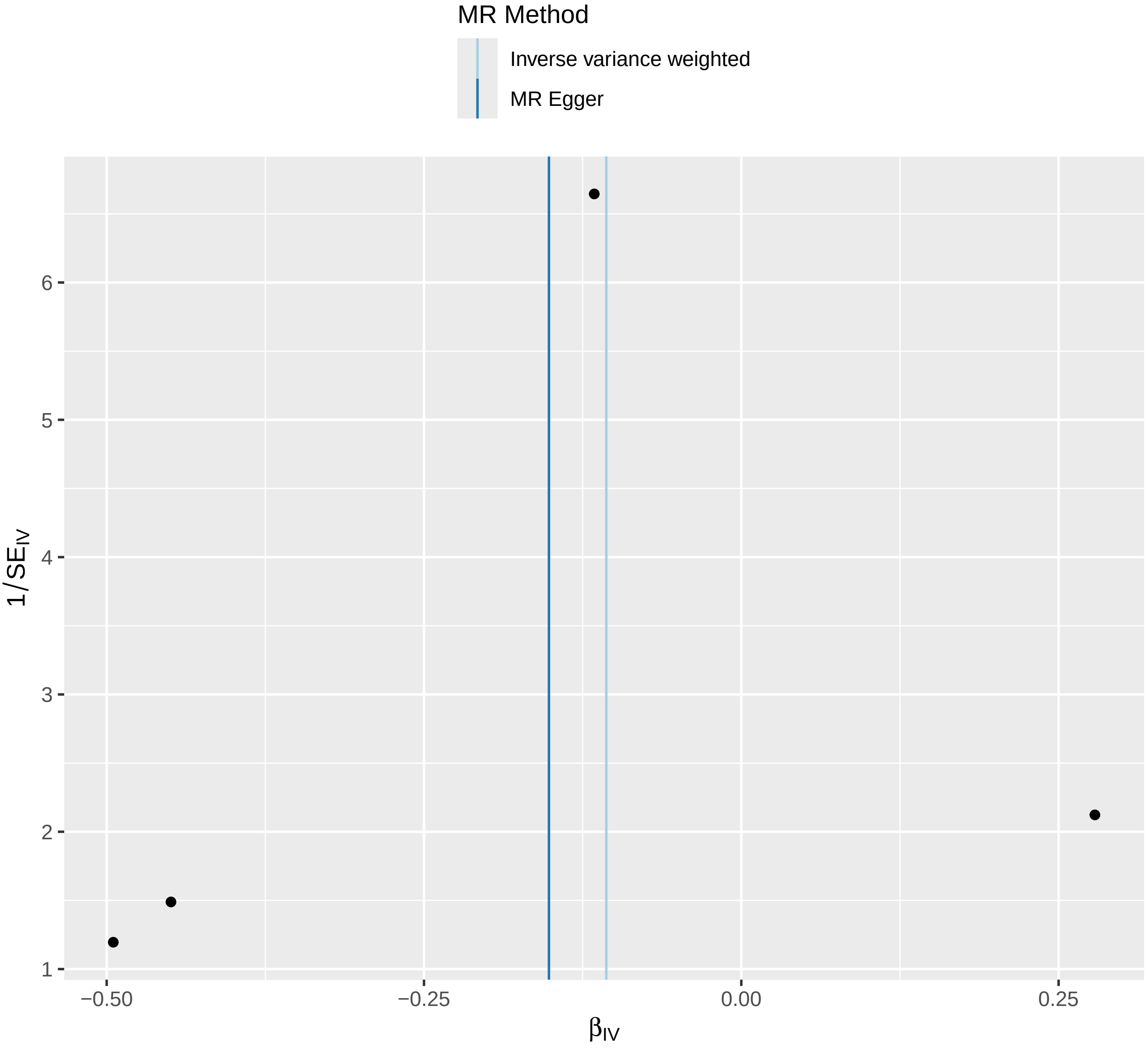
MR Method
Inverse variance weighted
MR Egger
1/SE$_{IV}$
6
5
4
3
2
1
−0.50
−0.25
0.00
0.25
β$_{IV}$

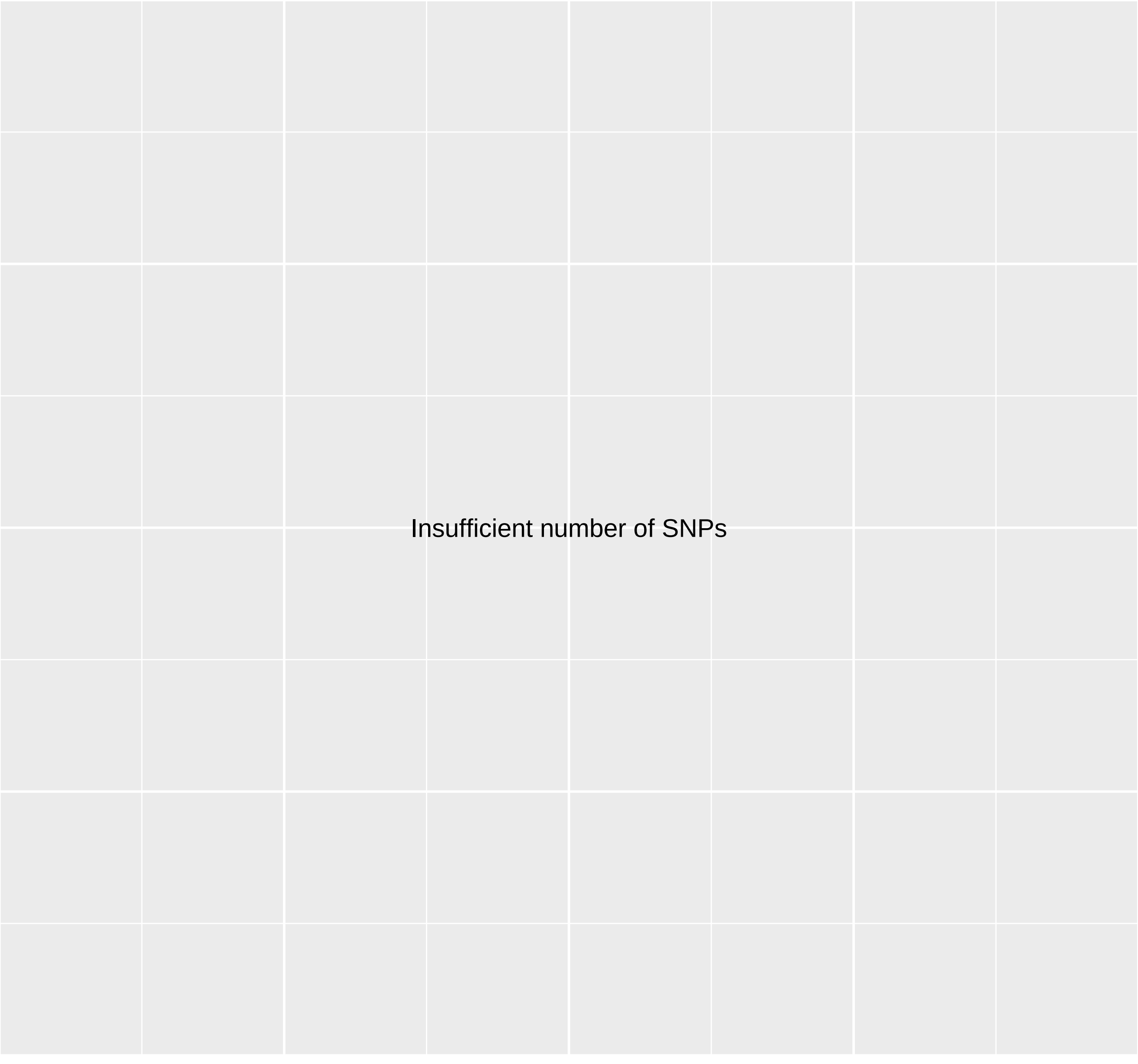
Insufficient number of SNPs

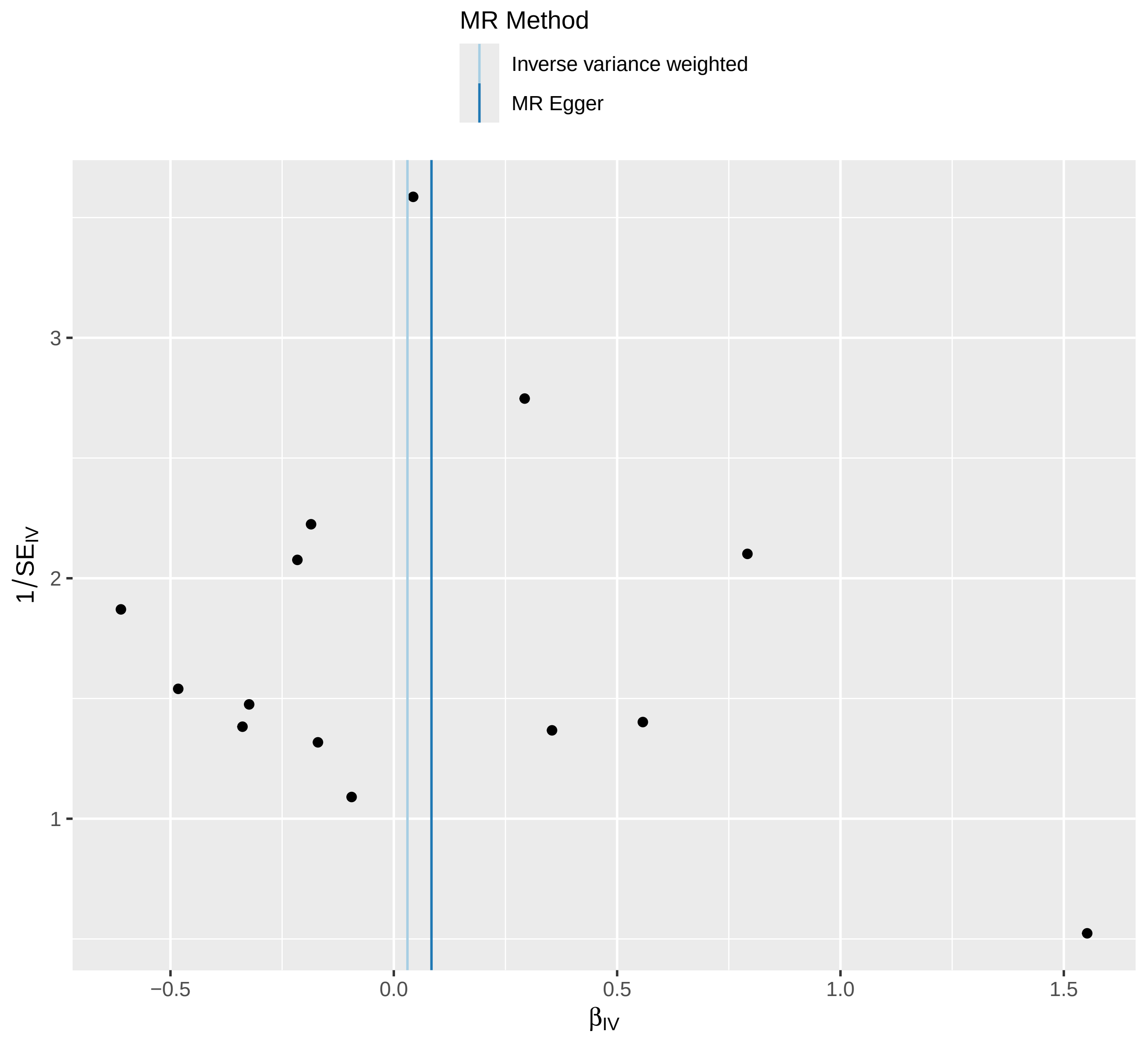
MR Method
Inverse variance weighted
MR Egger
3
2
1
1/SE_IV
−0.5
0.0
0.5
1.0
1.5
β_IV

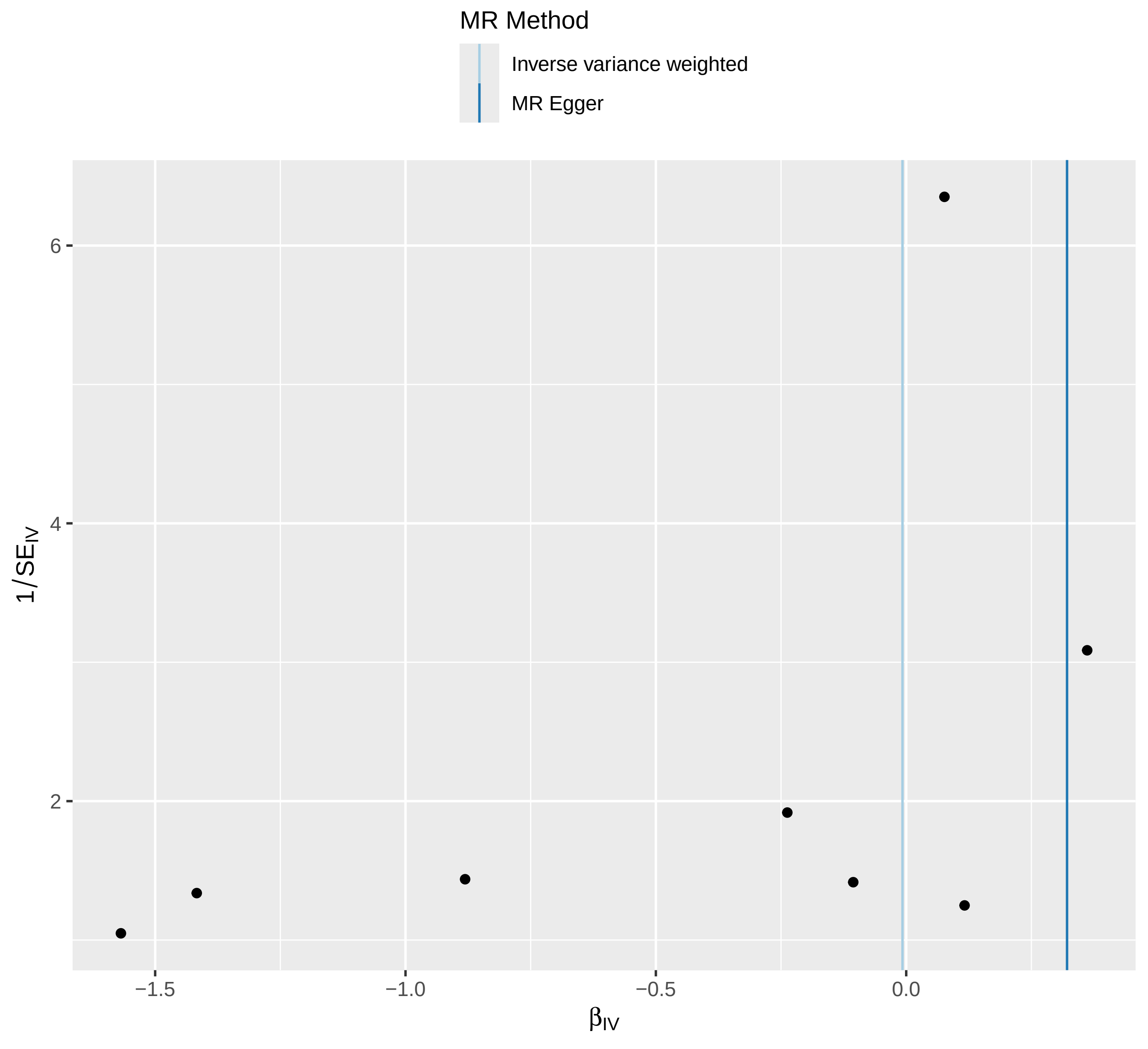
MR Method
Inverse variance weighted
MR Egger
6
4
2
1/SE_IV
−1.5
−1.0
−0.5
0.0
β_IV

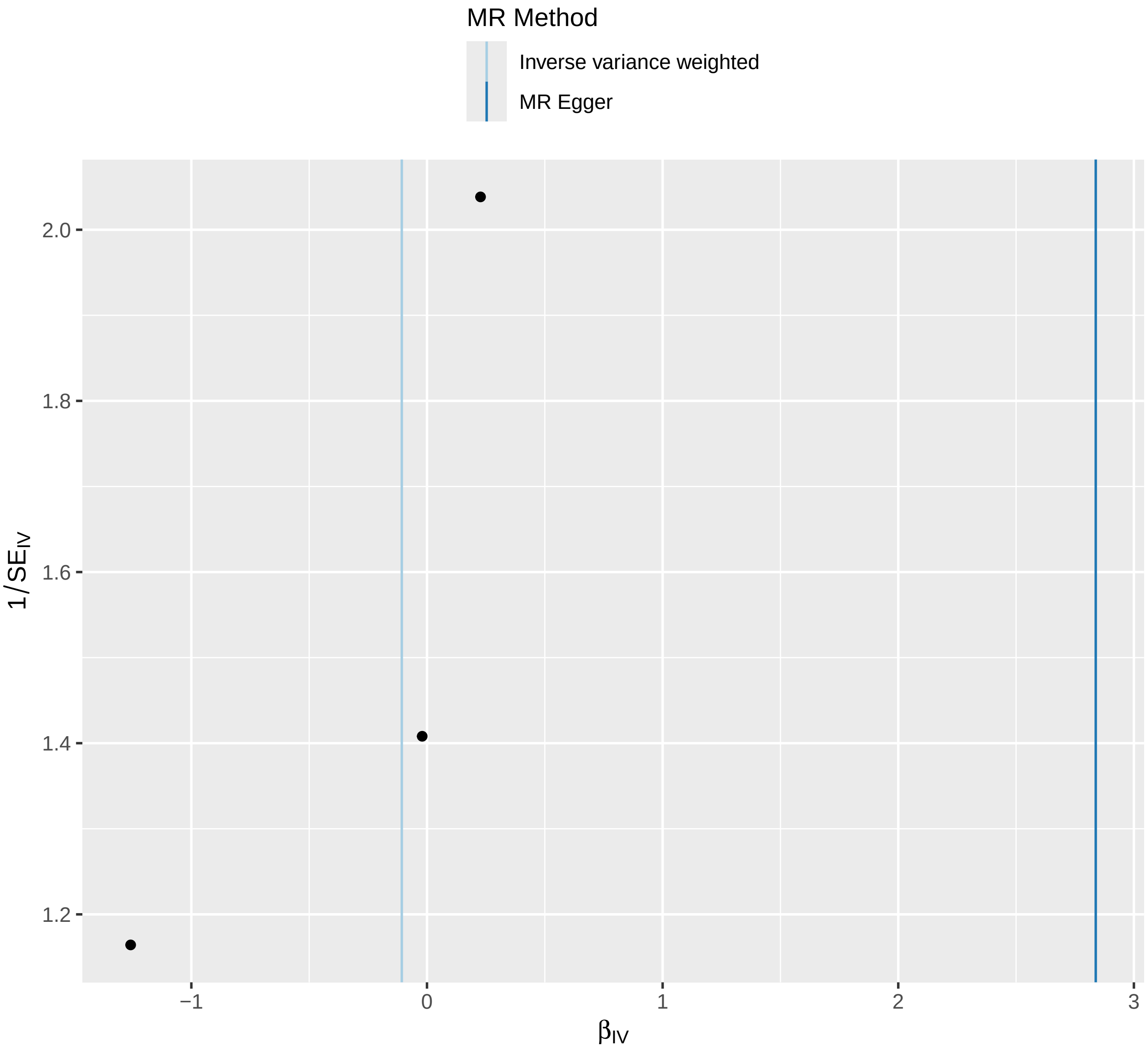

MR Method
Inverse variance weighted
MR Egger
2.0
1.8
1.6
1.4
1.2
1/SE$_{IV}$
−1
0
1
2
3
β$_{IV}$

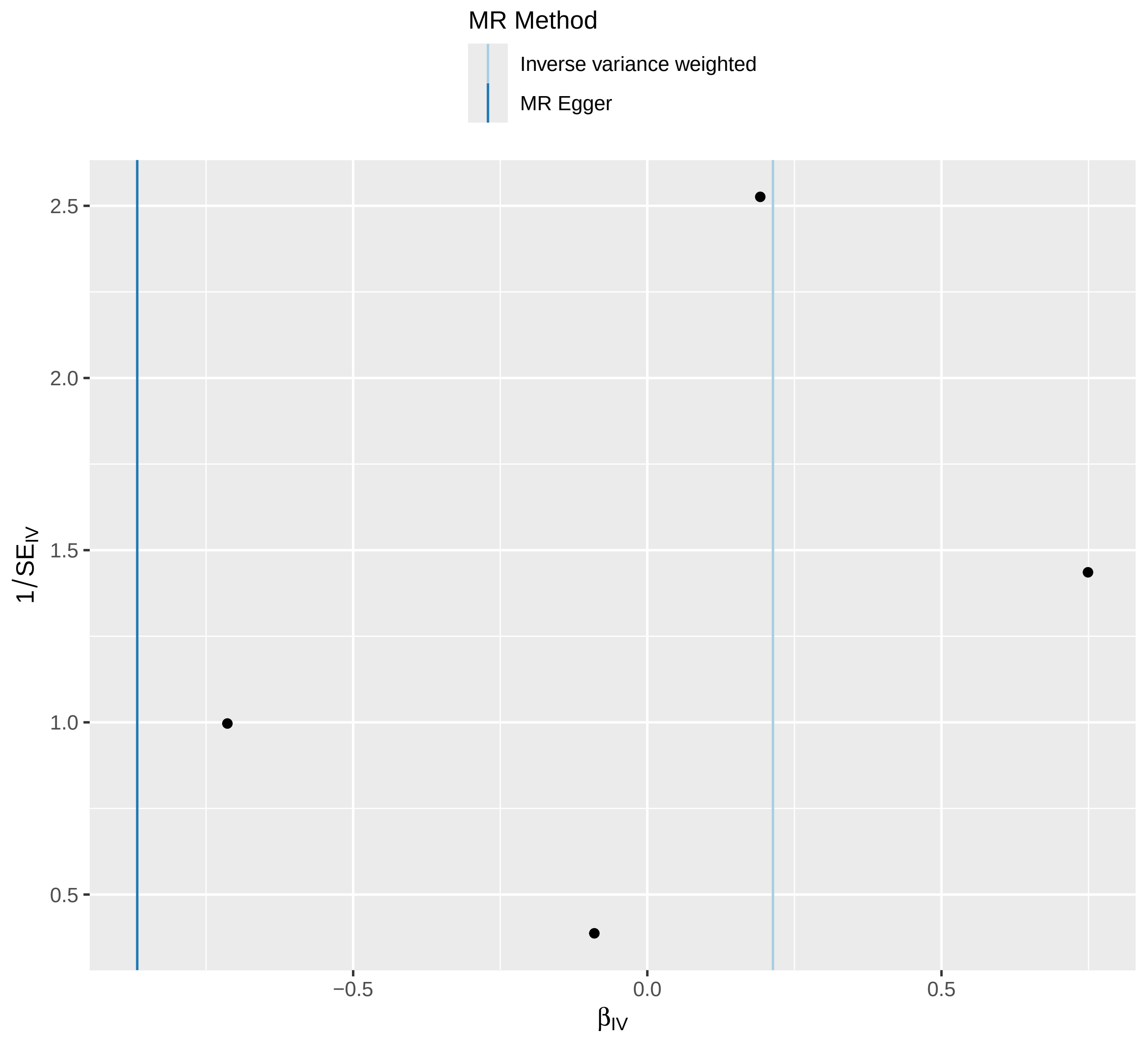
MR Method
Inverse variance weighted
MR Egger
2.5
2.0
1.5
1.0
0.5
1/SE_IV
−0.5
0.0
0.5
β_IV

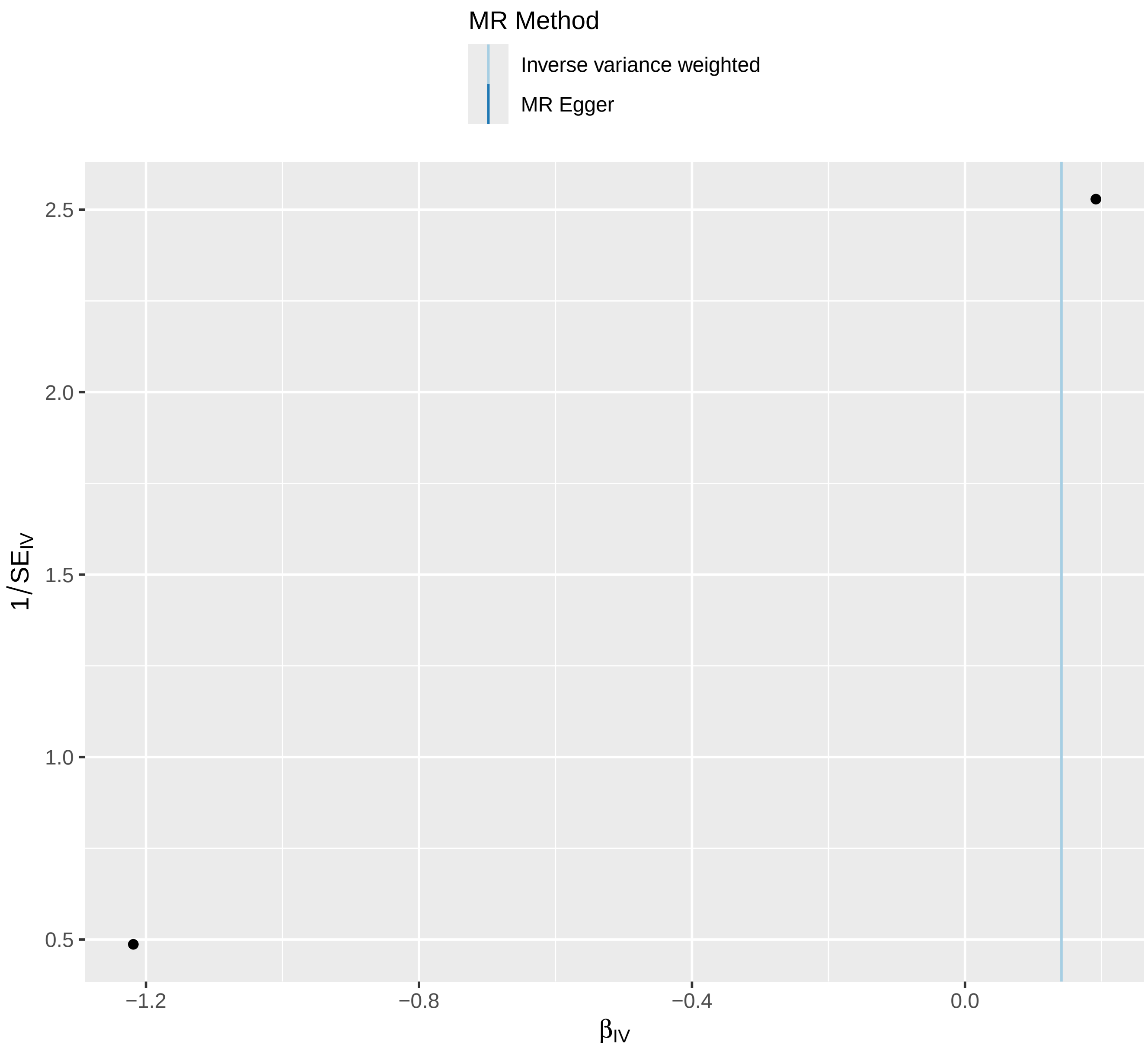

MR Method
Inverse variance weighted
MR Egger
2.5
2.0
1.5
1.0
0.5
1/SE_IV
−1.2
−0.8
−0.4
0.0
β_IV

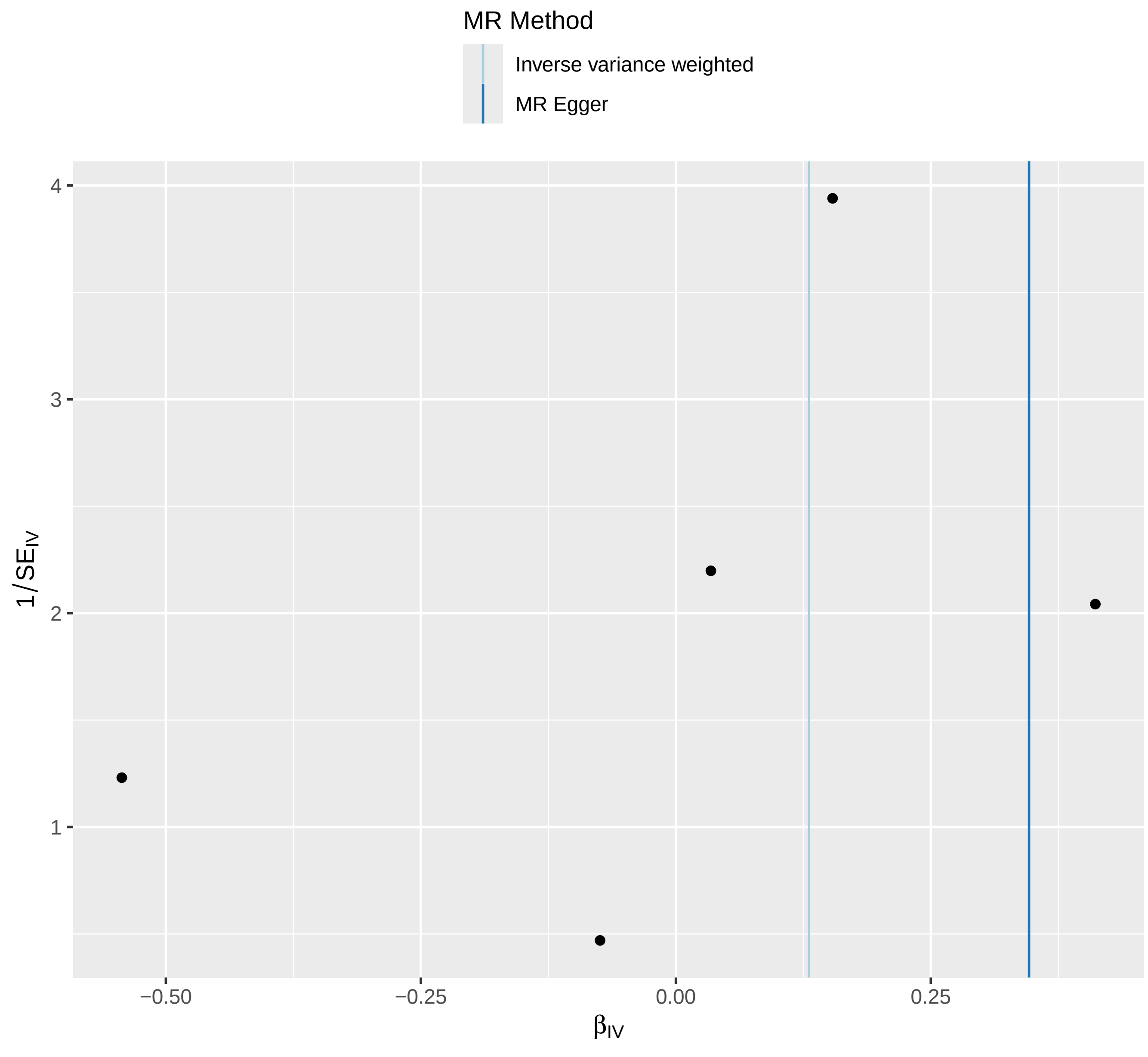
MR Method
Inverse variance weighted
MR Egger
4
3
2
1
1/SE_IV
−0.50
−0.25
0.00
0.25
β_IV

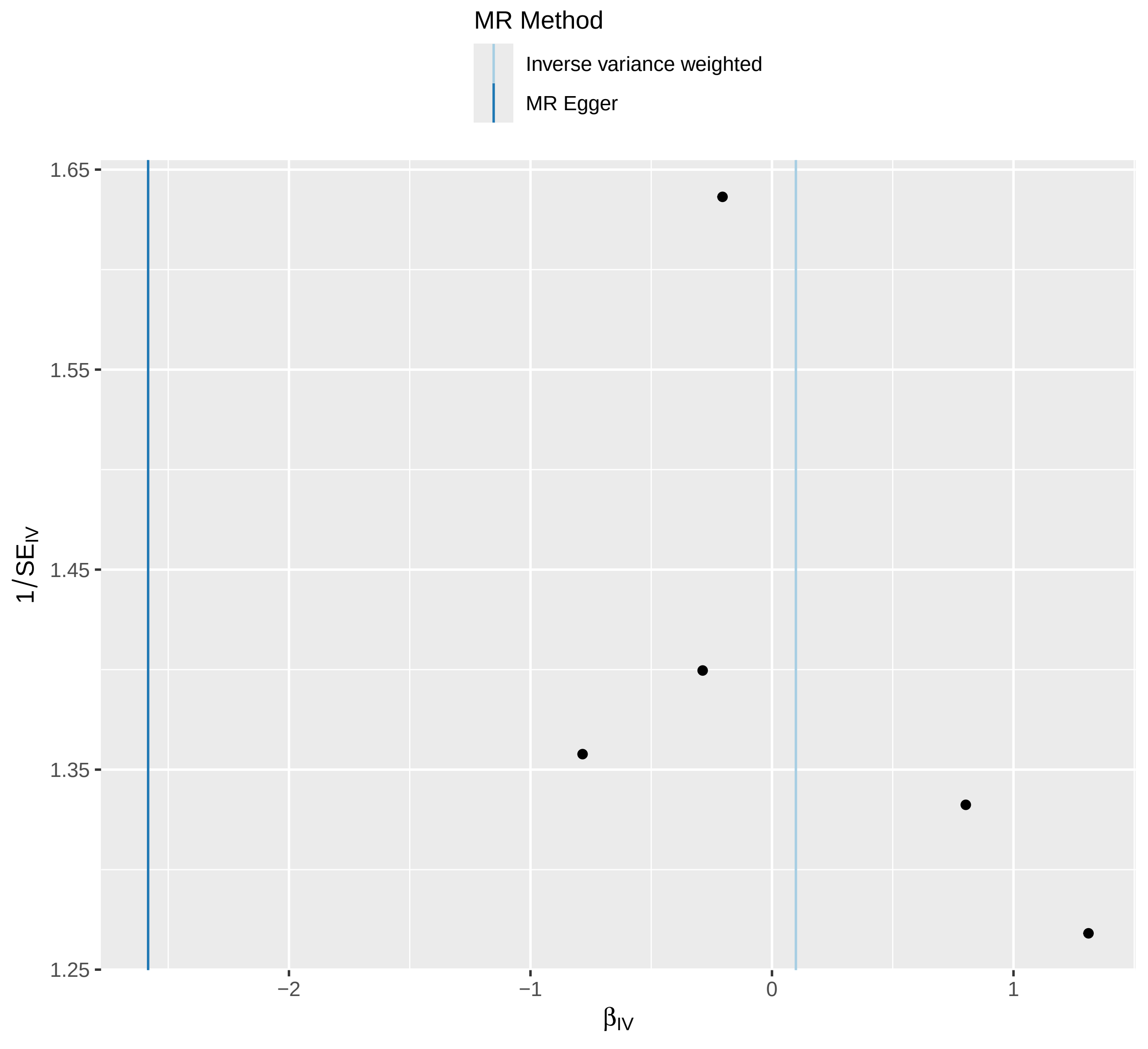

MR Method
Inverse variance weighted
MR Egger
1.65
1.55
1.45
1.35
1.25
1/SE_IV
−2
−1
0
1
β_IV

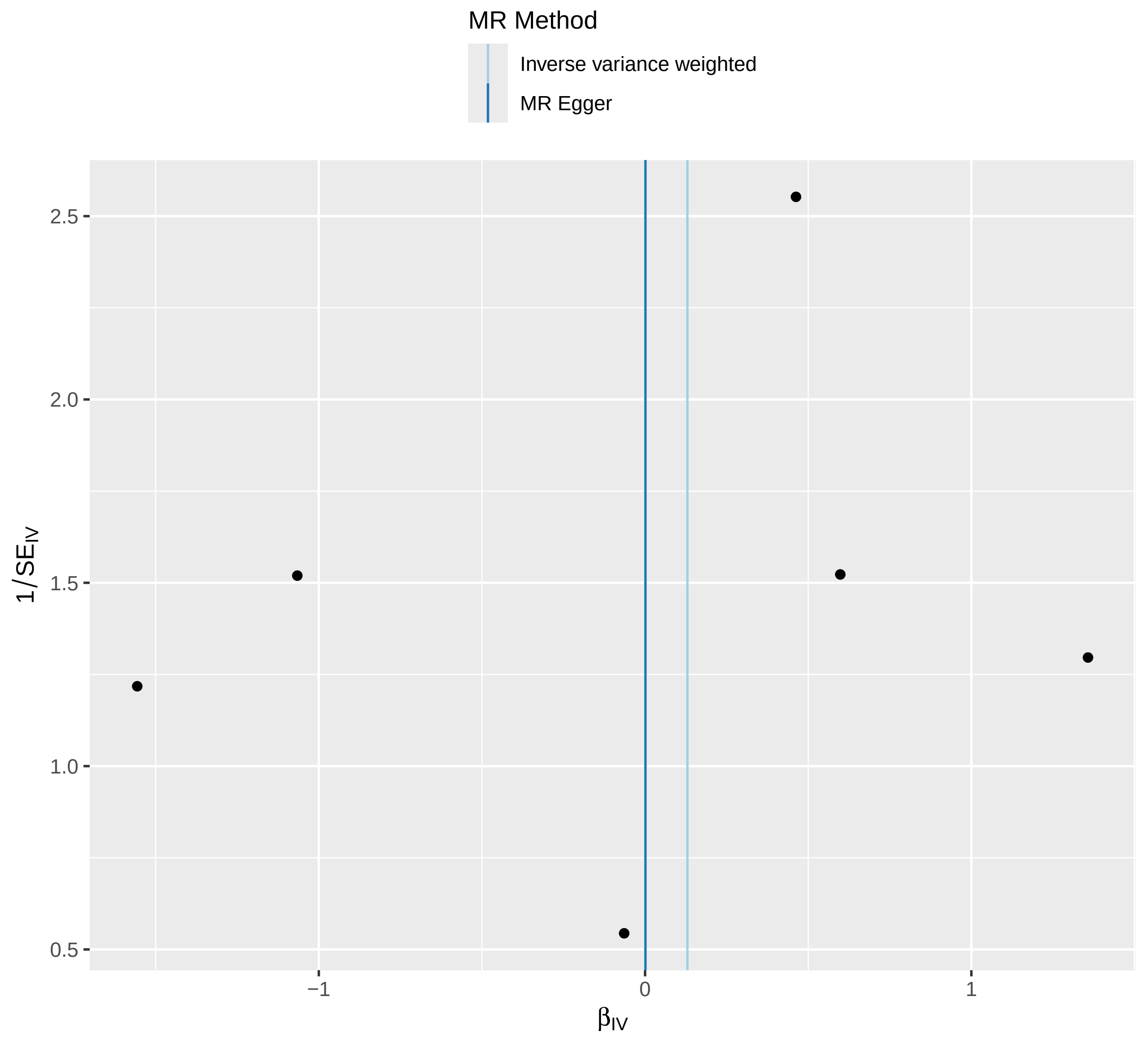

MR Method
Inverse variance weighted
MR Egger
2.5
2.0
1.5
1.0
0.5
1/SE_IV
−1
0
1
β_IV

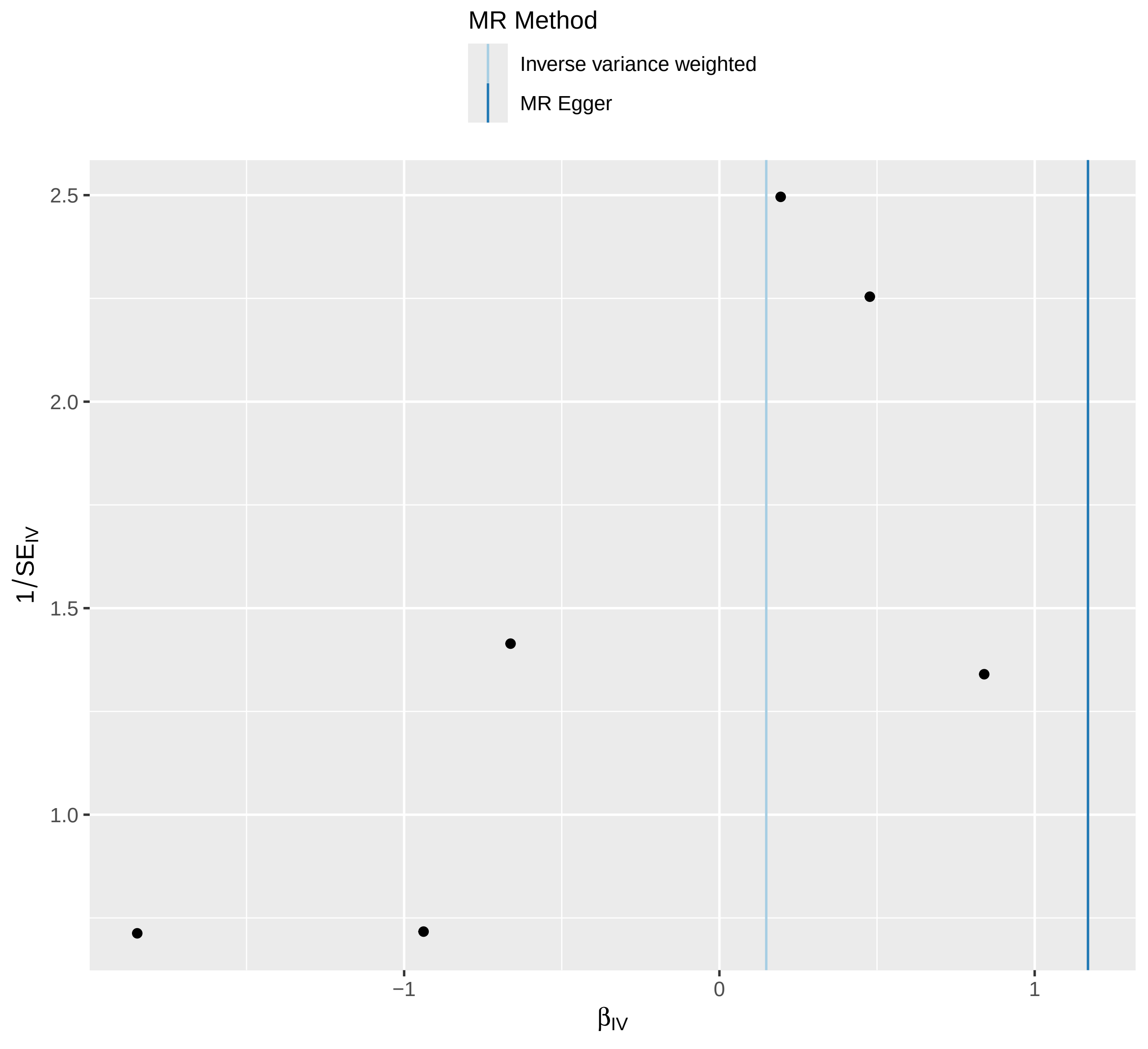
MR Method
Inverse variance weighted
MR Egger
2.5
2.0
1.5
1.0
1/SE_IV
−1
0
1
β_IV

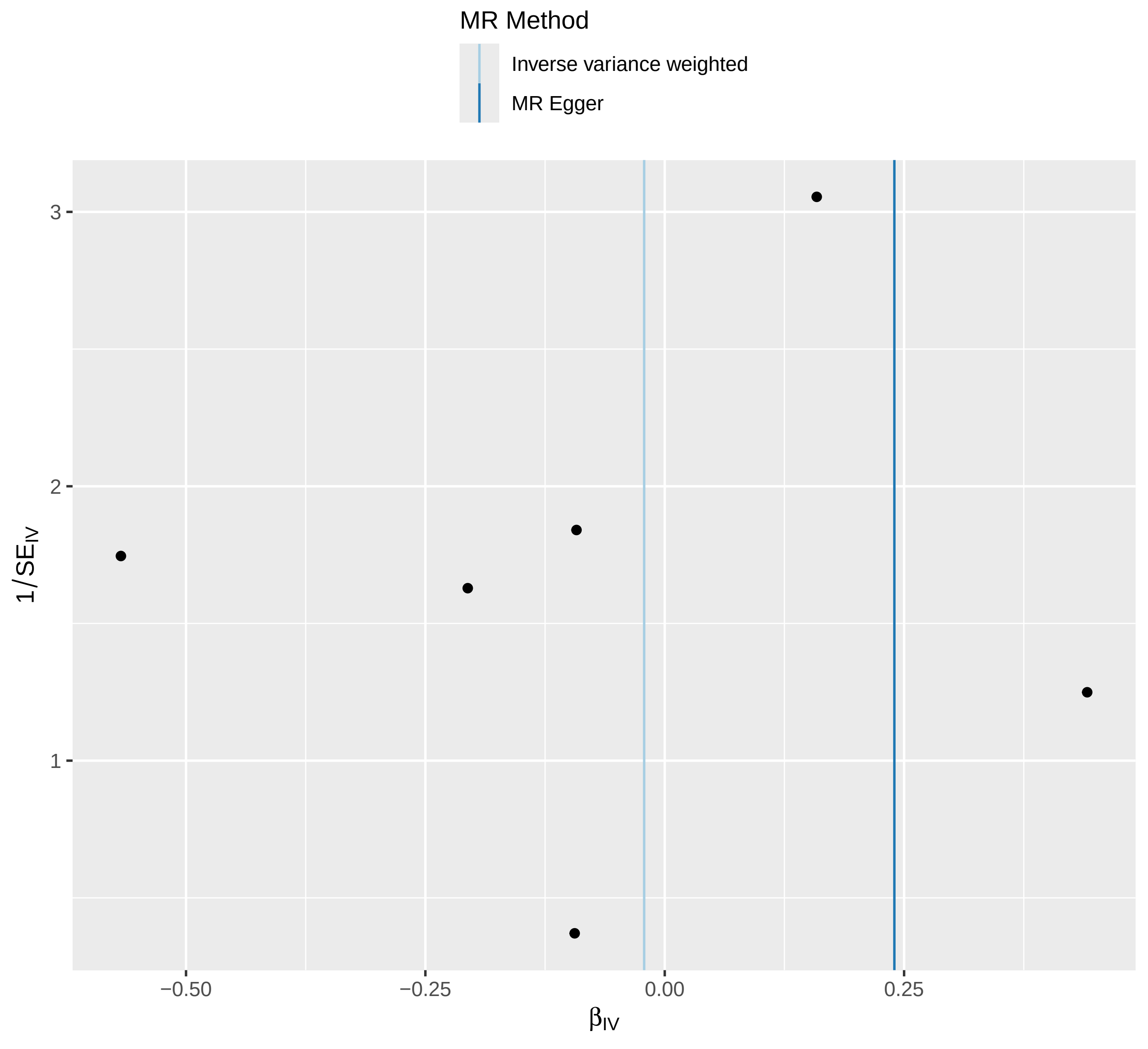
MR Method
Inverse variance weighted
MR Egger
1/SE_IV
3
2
1
−0.50
−0.25
0.00
0.25
β_IV

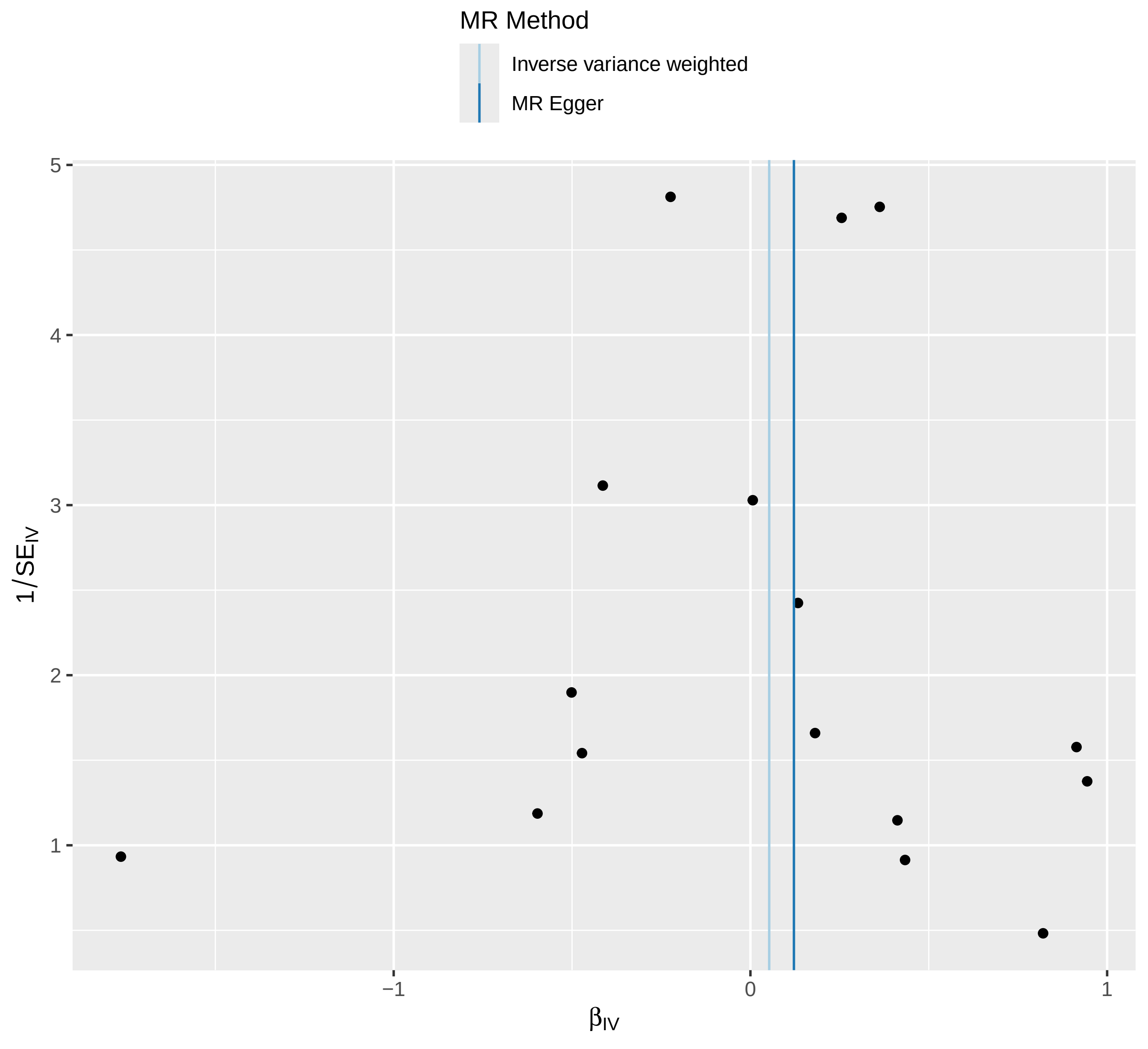

MR Method
Inverse variance weighted
MR Egger
5
4
3
2
1
1/SE$_{IV}$
−1
0
1
β$_{IV}$

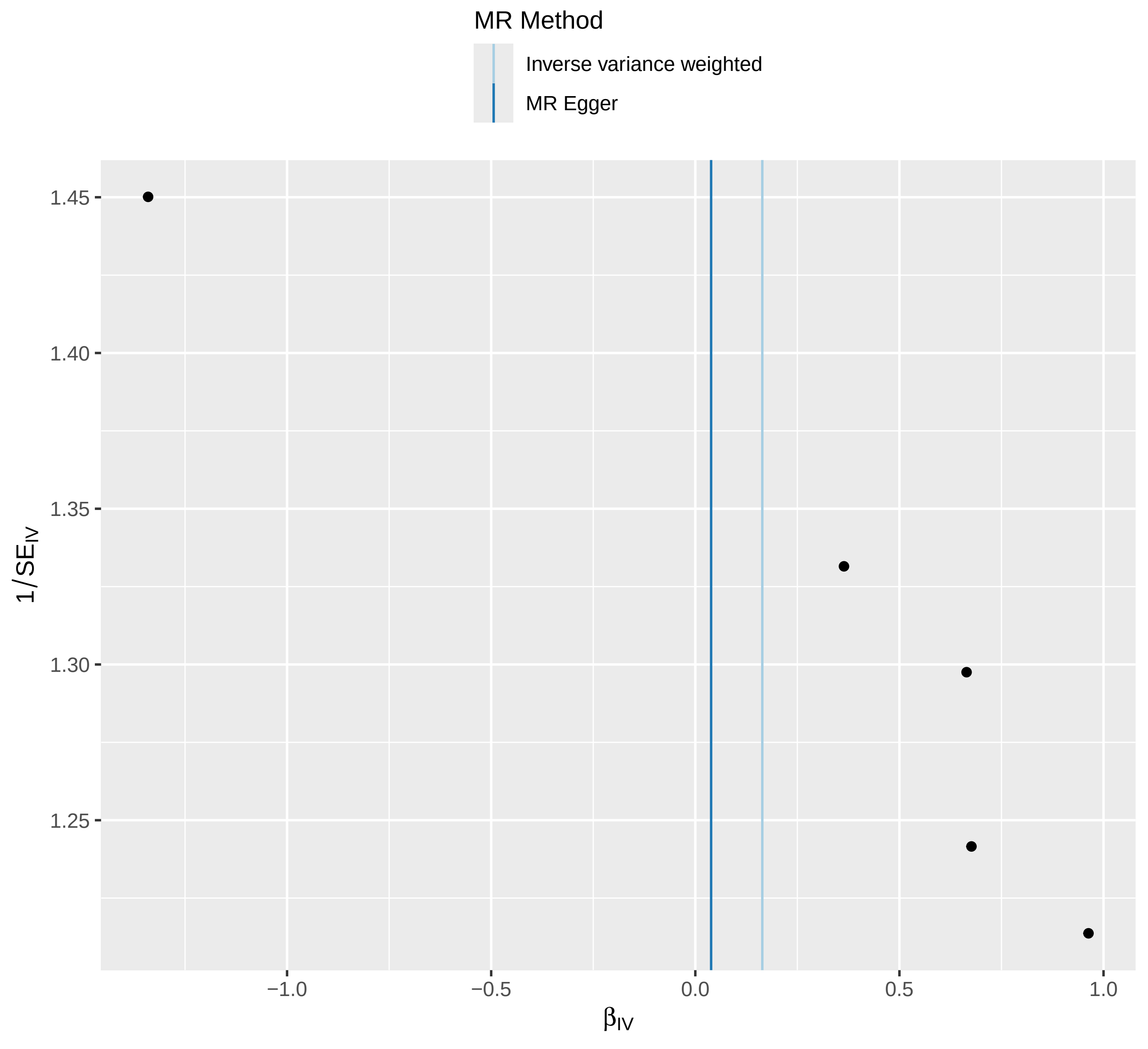

MR Method
Inverse variance weighted
MR Egger
1.45
1.40
1.35
1.30
1.25
1/SE_IV
−1.0
−0.5
0.0
0.5
1.0
β_IV

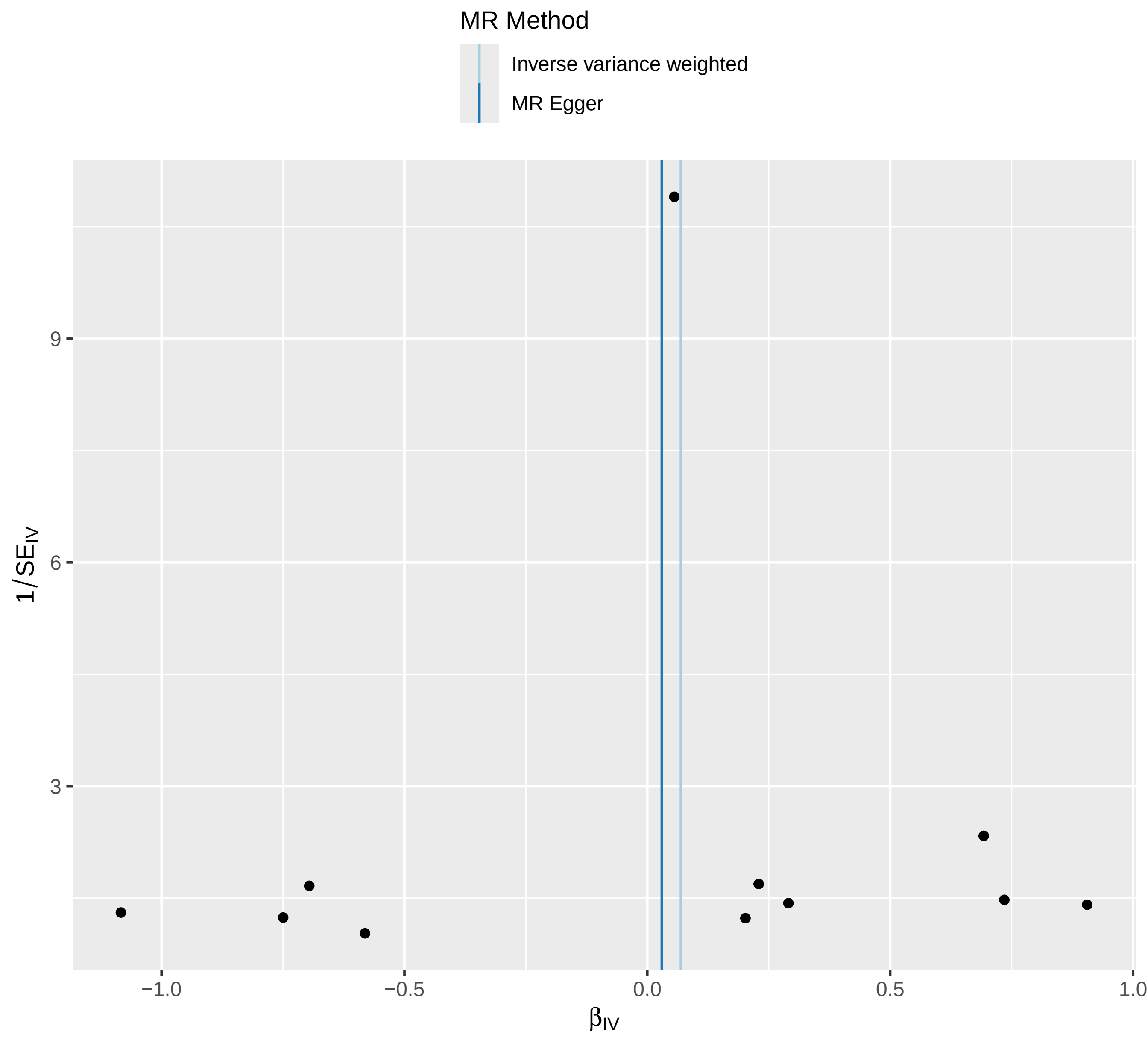
MR Method
Inverse variance weighted
MR Egger
9
6
3
1/SE$_{IV}$
−1.0
−0.5
0.0
0.5
1.0
β_{IV}

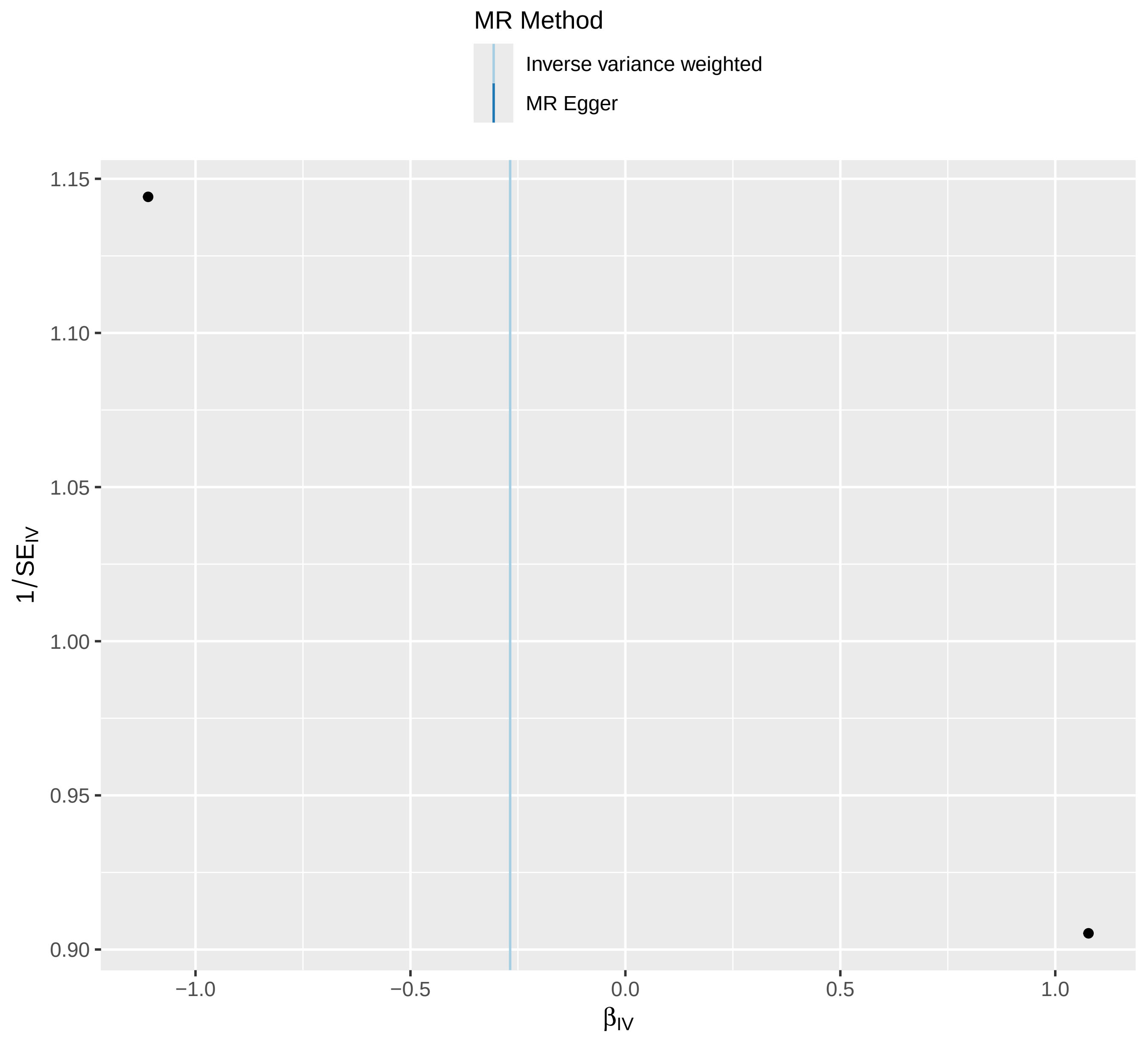

MR Method
Inverse variance weighted
MR Egger
1/SE_IV
1.15
1.10
1.05
1.00
0.95
0.90
−1.0
−0.5
0.0
0.5
1.0
β_IV

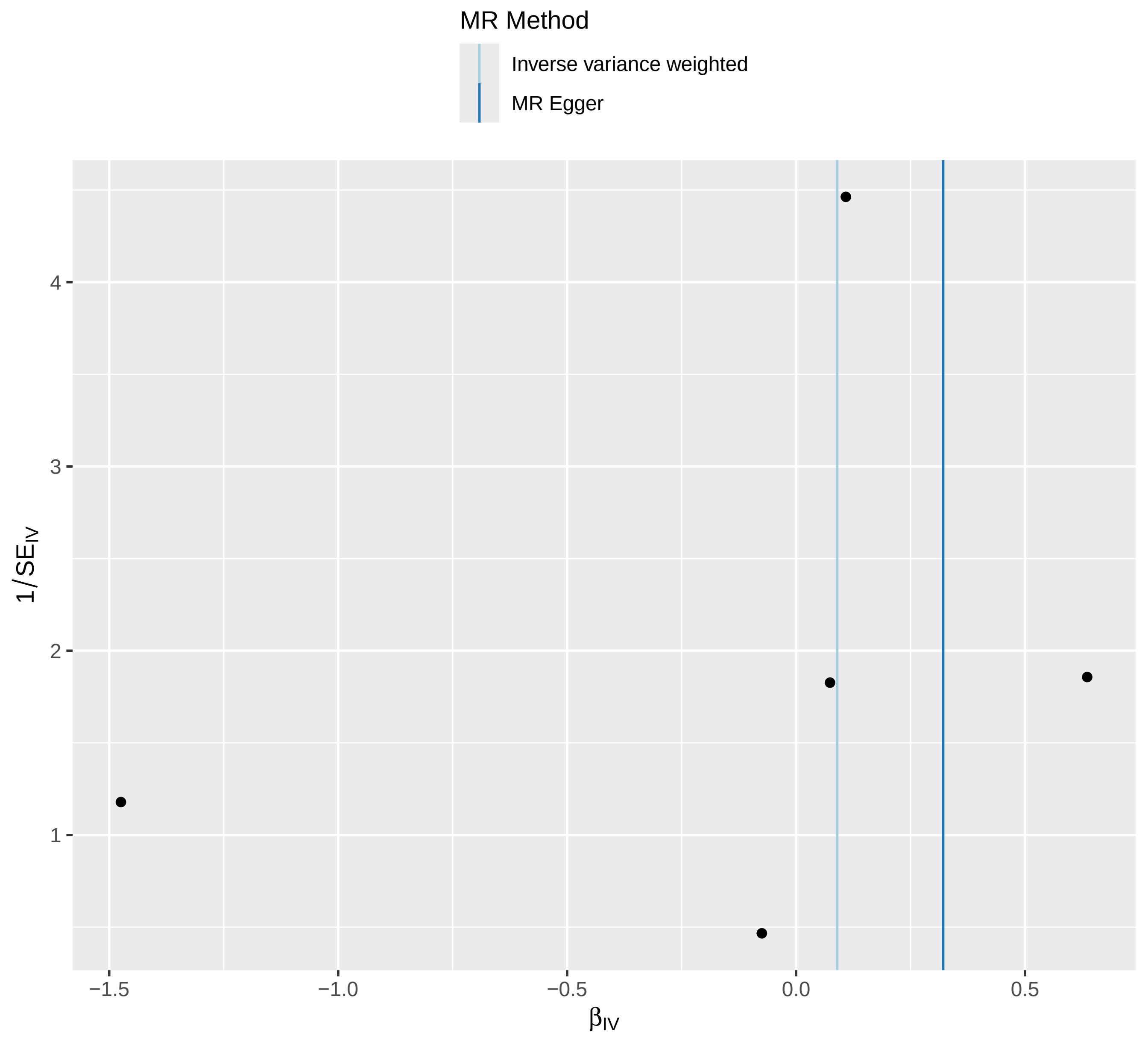
MR Method
Inverse variance weighted
MR Egger
4
3
2
1
1/SE_IV
−1.5
−1.0
−0.5
0.0
0.5
β_IV

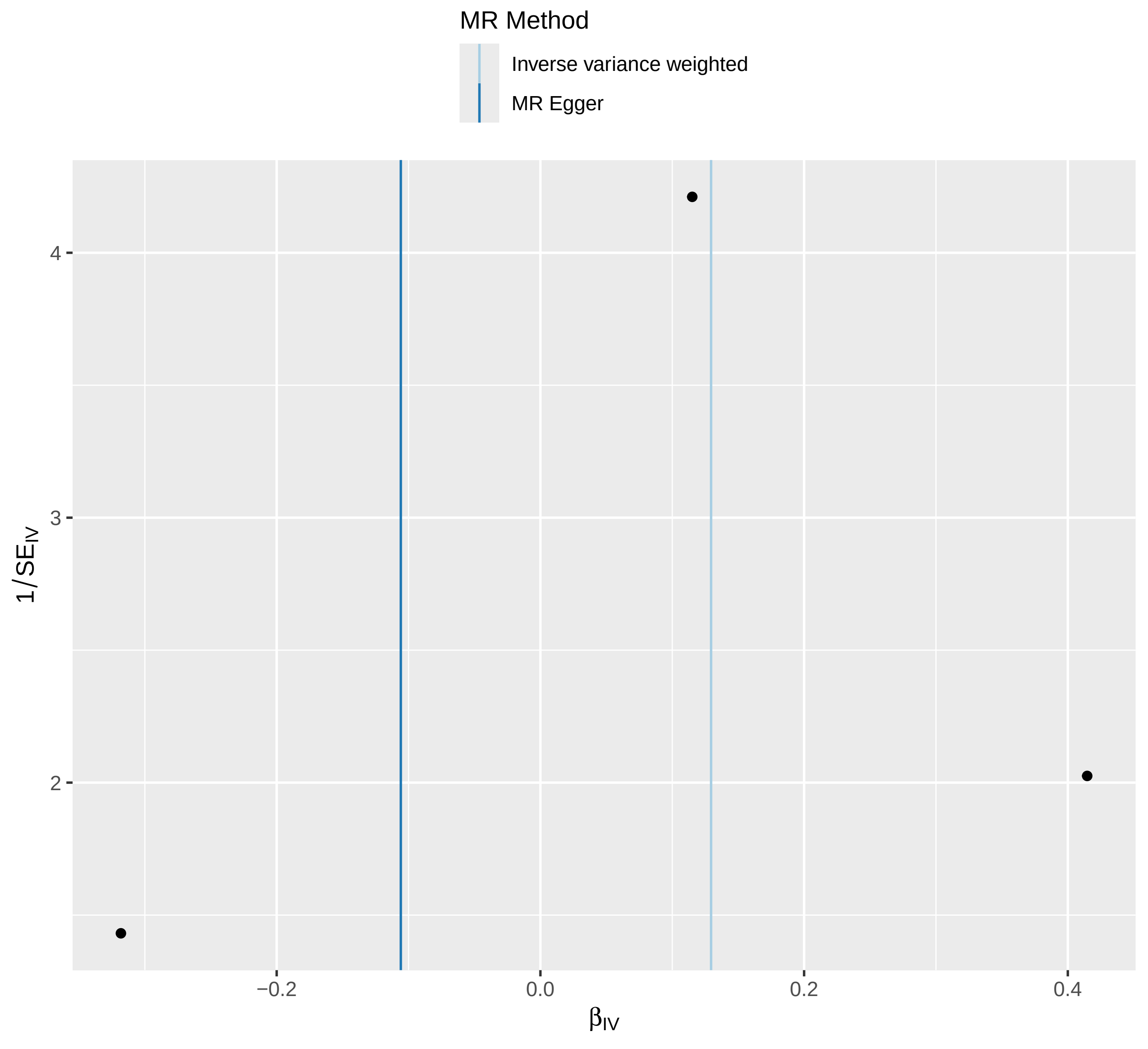

MR Method
Inverse variance weighted
MR Egger
4
3
2
1/SE_IV
−0.2
0.0
0.2
0.4
β_IV

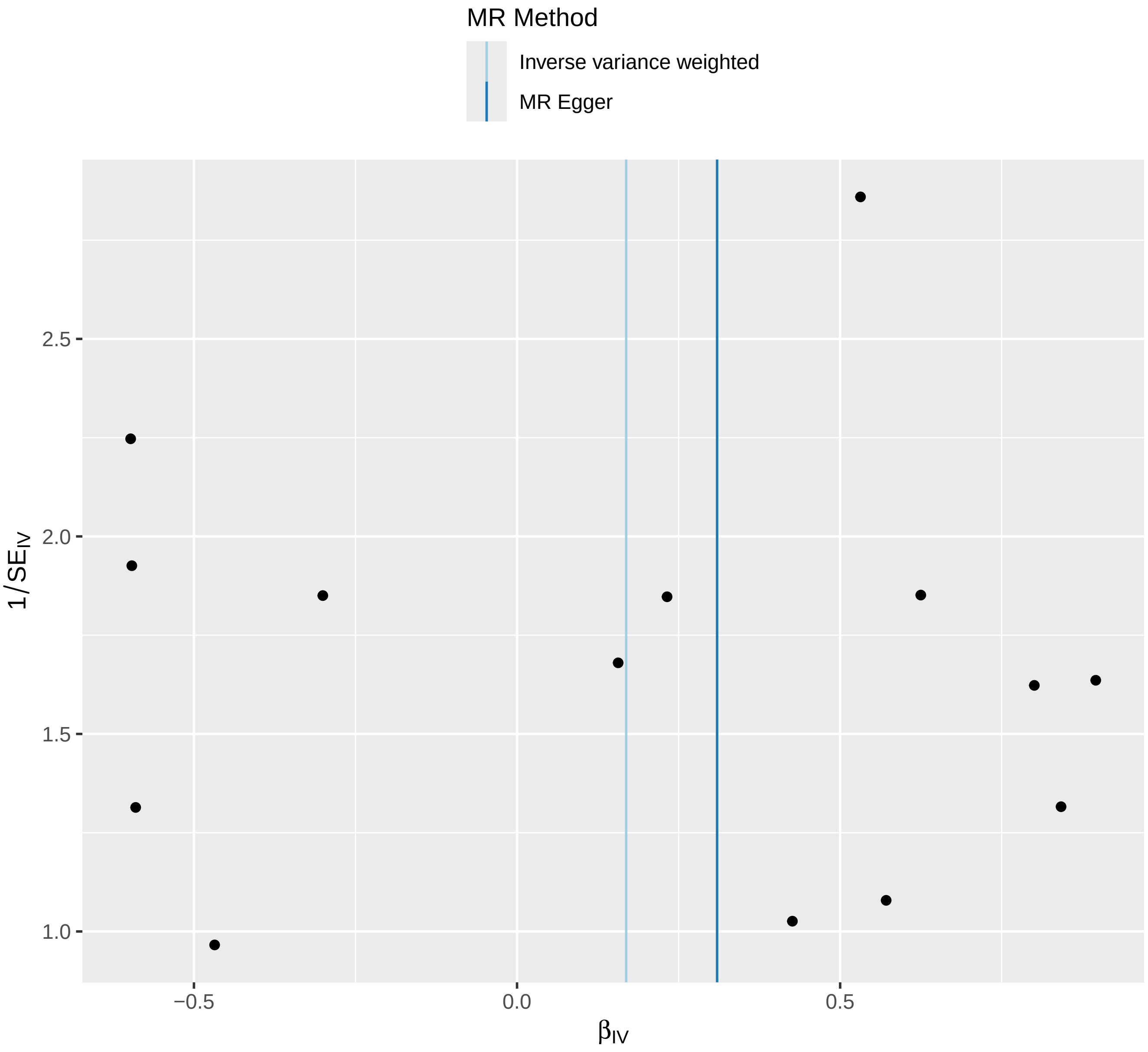

MR Method
Inverse variance weighted
MR Egger
2.5
2.0
1.5
1.0
1/SE_IV
−0.5
0.0
0.5
β_IV

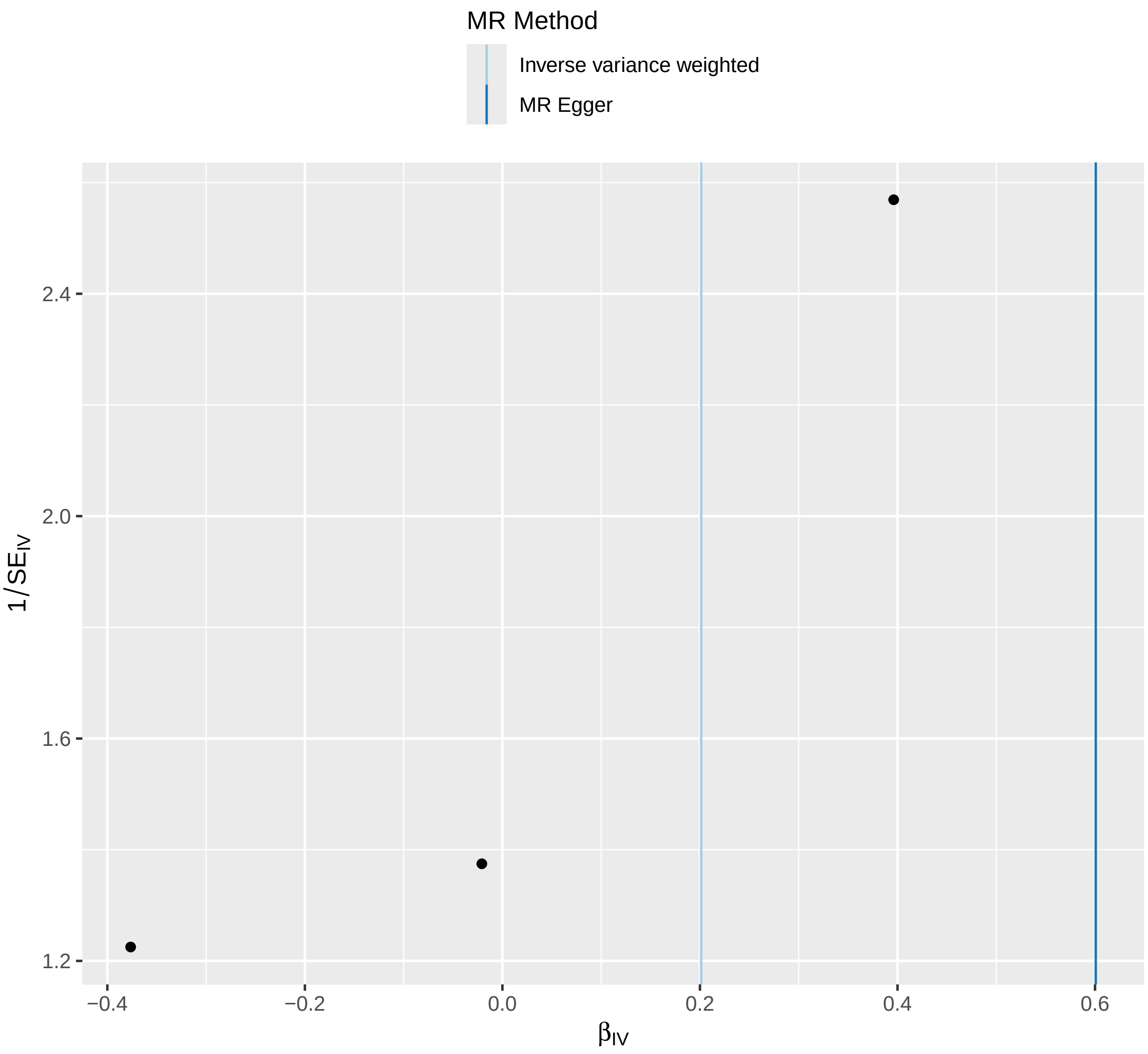

MR Method
Inverse variance weighted
MR Egger
2.4
2.0
1.6
1.2
1/SE_IV
−0.4
−0.2
0.0
0.2
0.4
0.6
β_IV

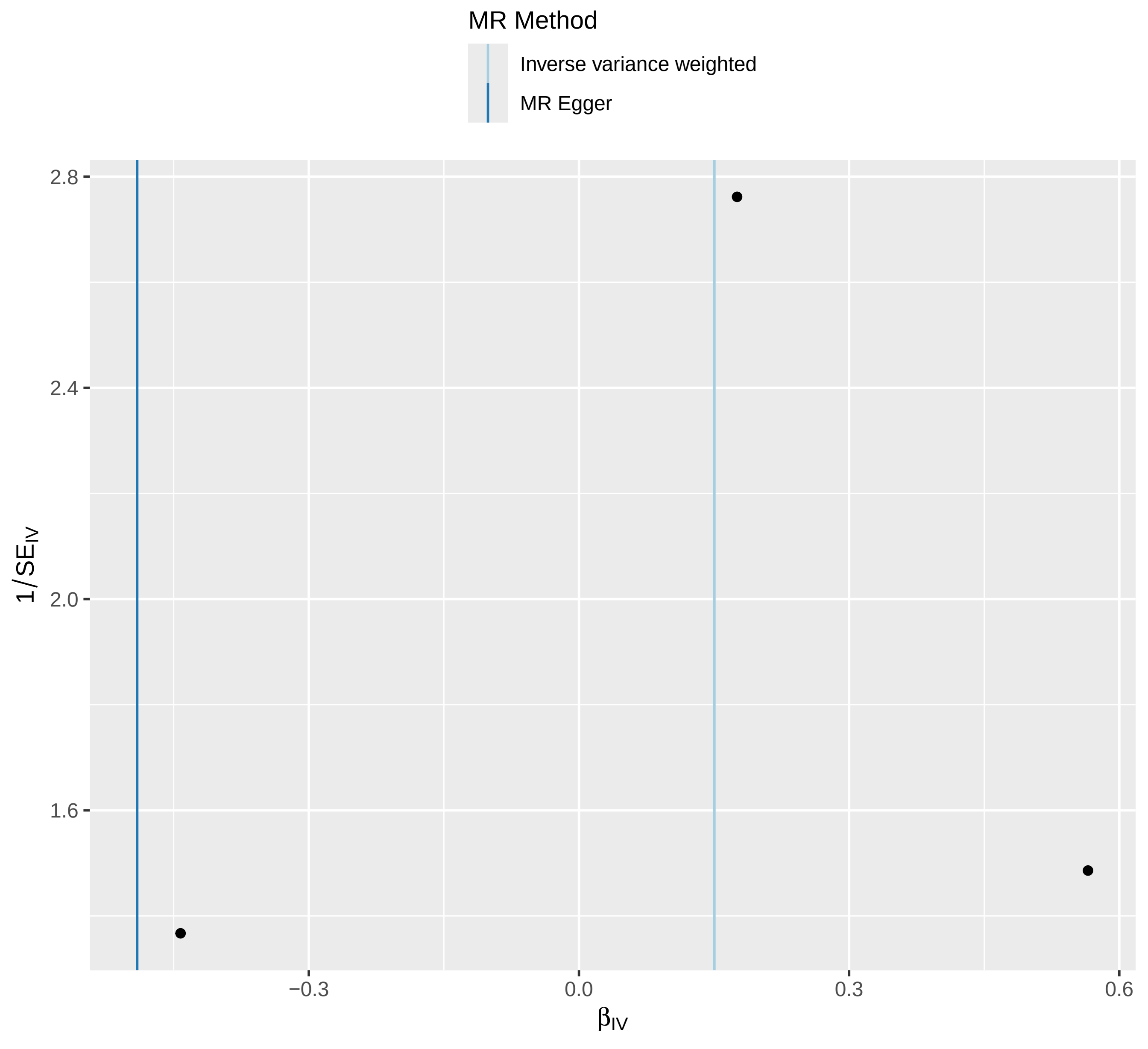

MR Method
Inverse variance weighted
MR Egger
2.8
2.4
2.0
1.6
1/SE_IV
−0.3
0.0
0.3
0.6
β_IV

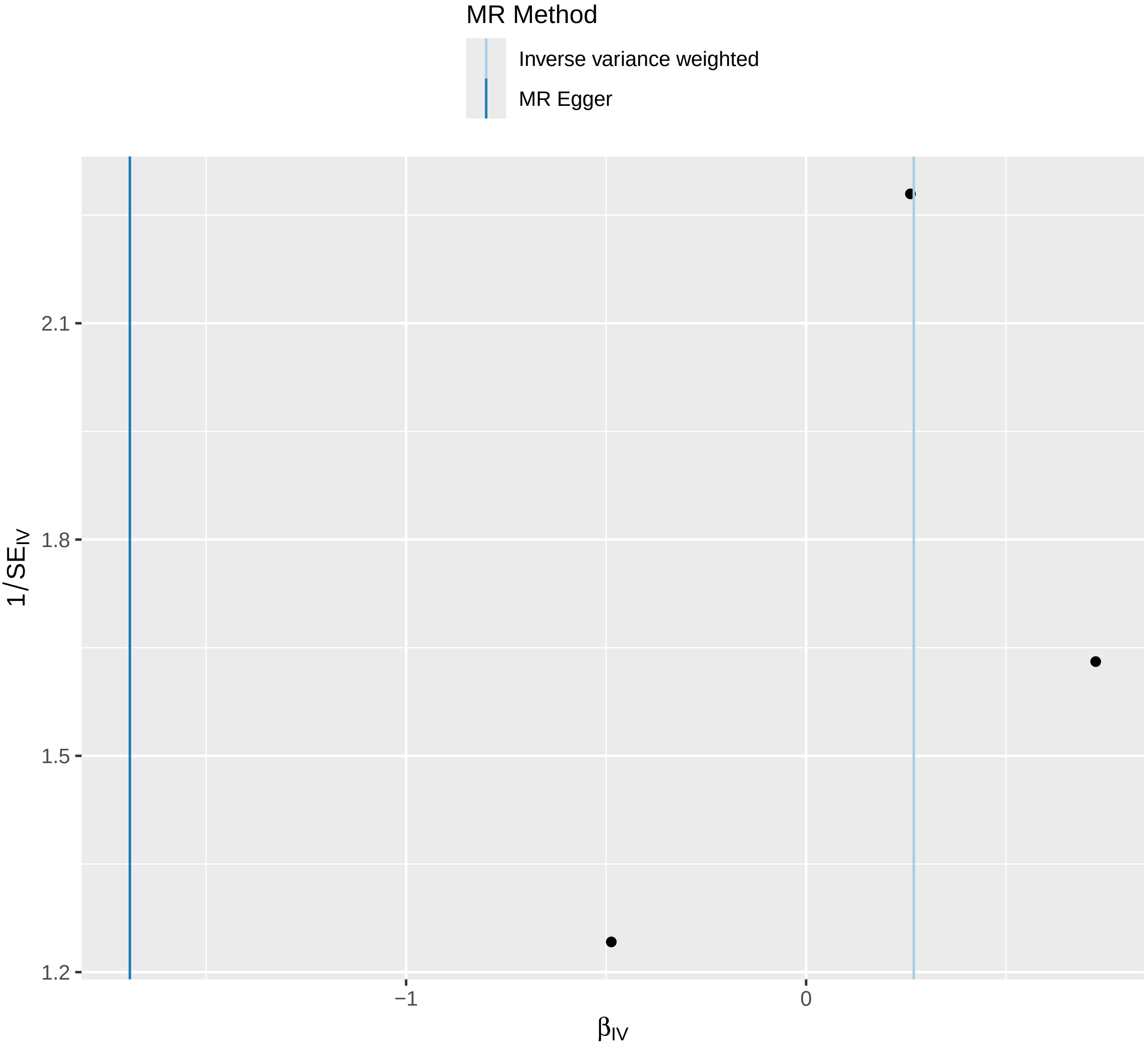
MR Method
Inverse variance weighted
MR Egger
2.1
1.8
1.5
1.2
−1
0
$1/SE_{IV}$
β_{IV}

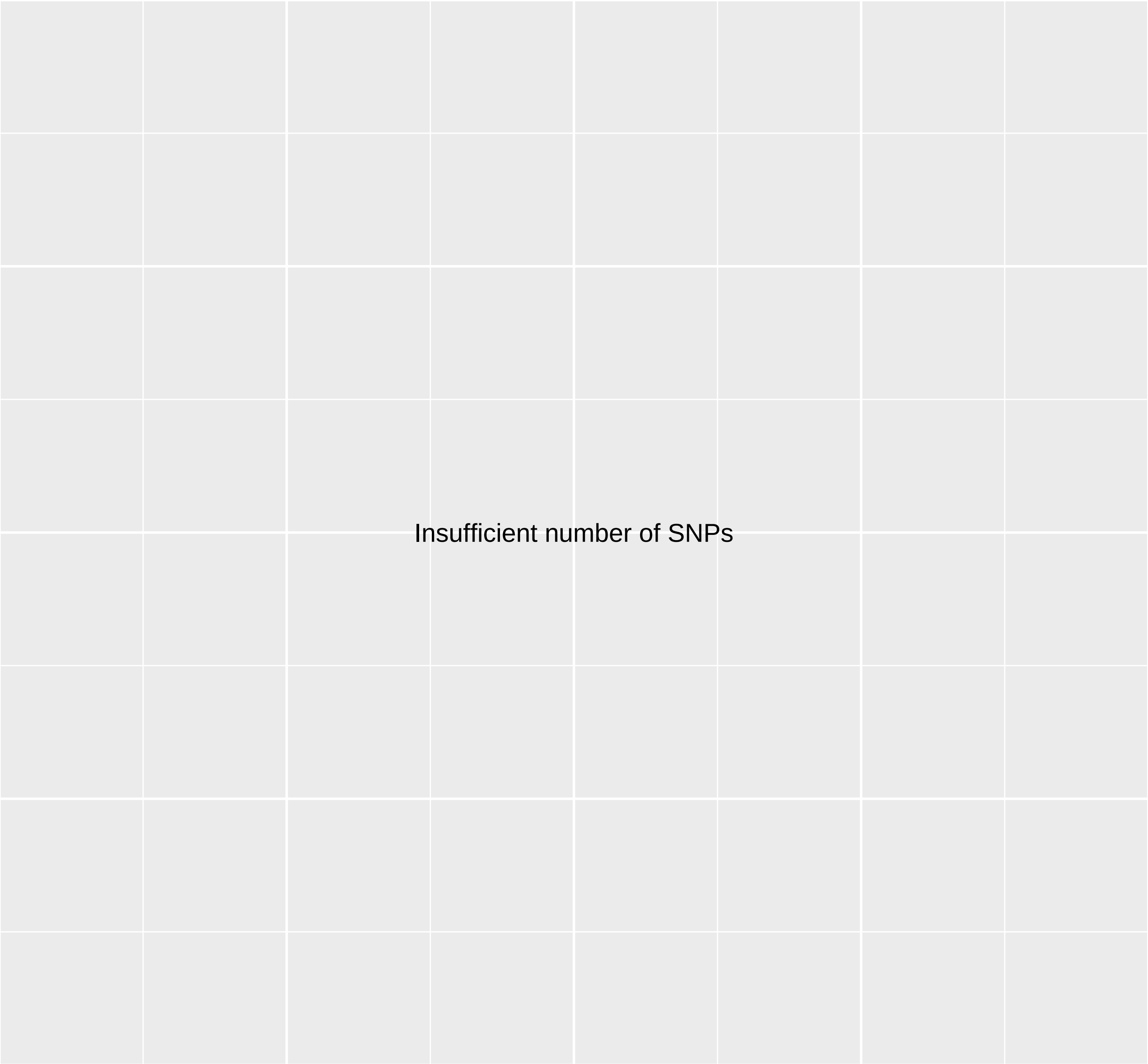
Insufficient number of SNPs
