# Supplementary material for: Assessing the Causal Relationship Between Plasma Proteins and Pulmonary Fibrosis: A Systematic Analysis Based on Mendelian Randomization
Source: Biology (Basel). 2025 Feb 14;14(2):200. doi: 10.3390/biology14020200 (PMC11852313; doi:10.3390/biology14020200)
Supplement: Supplementary file 1 [file Figure S3.pdf › Supplementary Material S1/Figure S3.pdf]

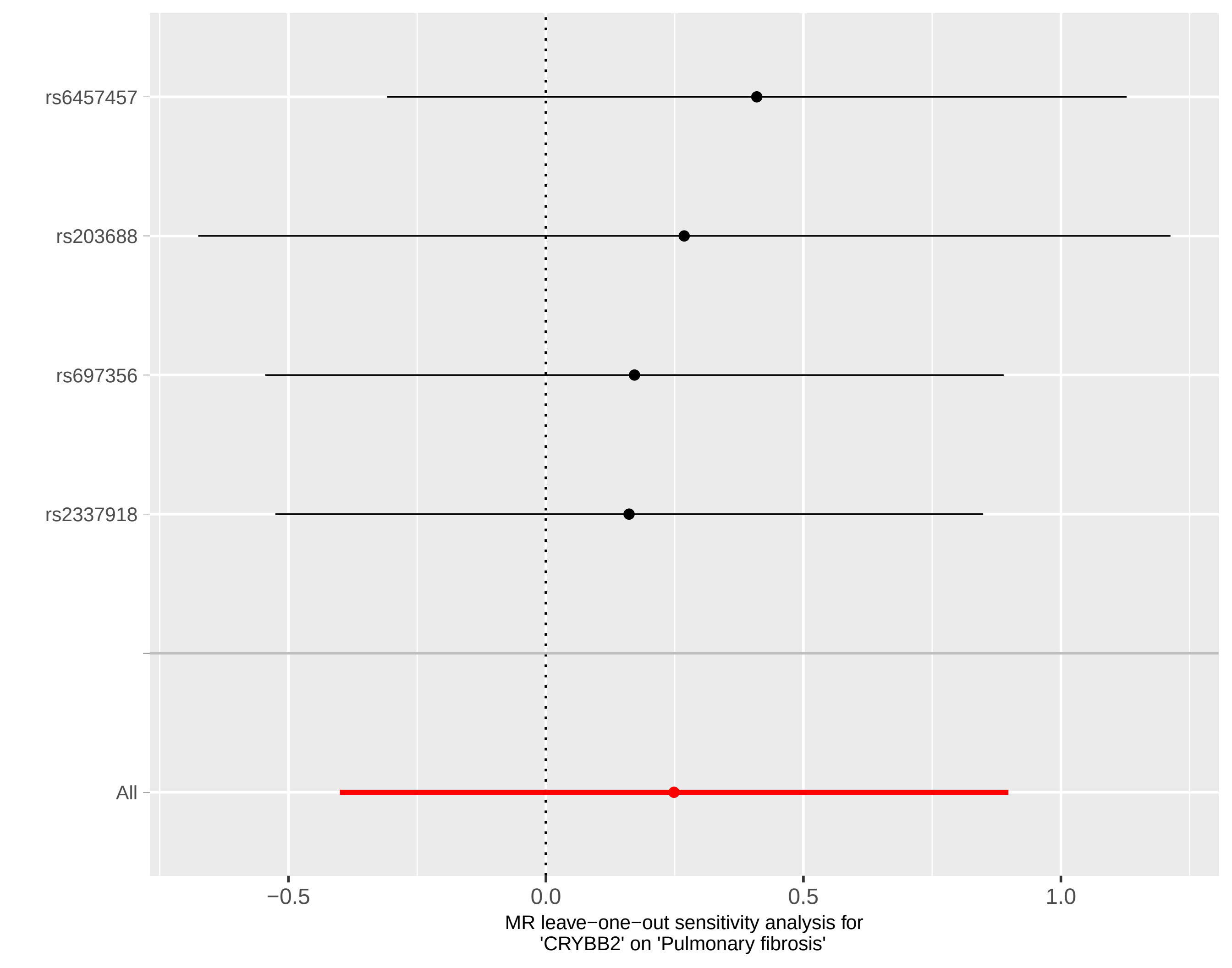

rs6457457
rs203688
rs697356
rs2337918
All
−0.5
0.0
0.5
1.0
MR leave−one−out sensitivity analysis for
'CRYBB2' on 'Pulmonary fibrosis'

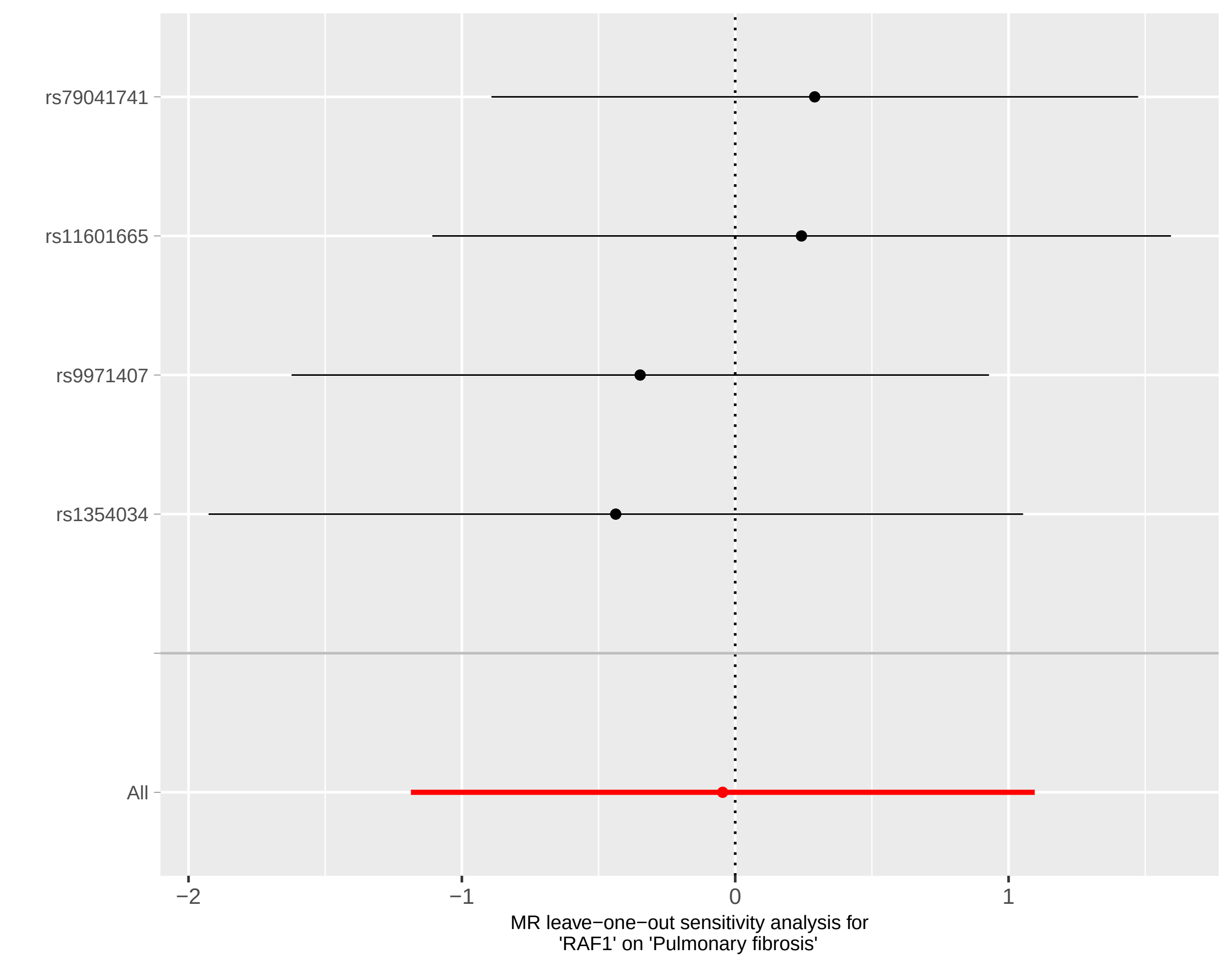

rs79041741
rs11601665
rs9971407
rs1354034
All
−2
−1
0
1
MR leave−one−out sensitivity analysis for
'RAF1' on 'Pulmonary fibrosis'

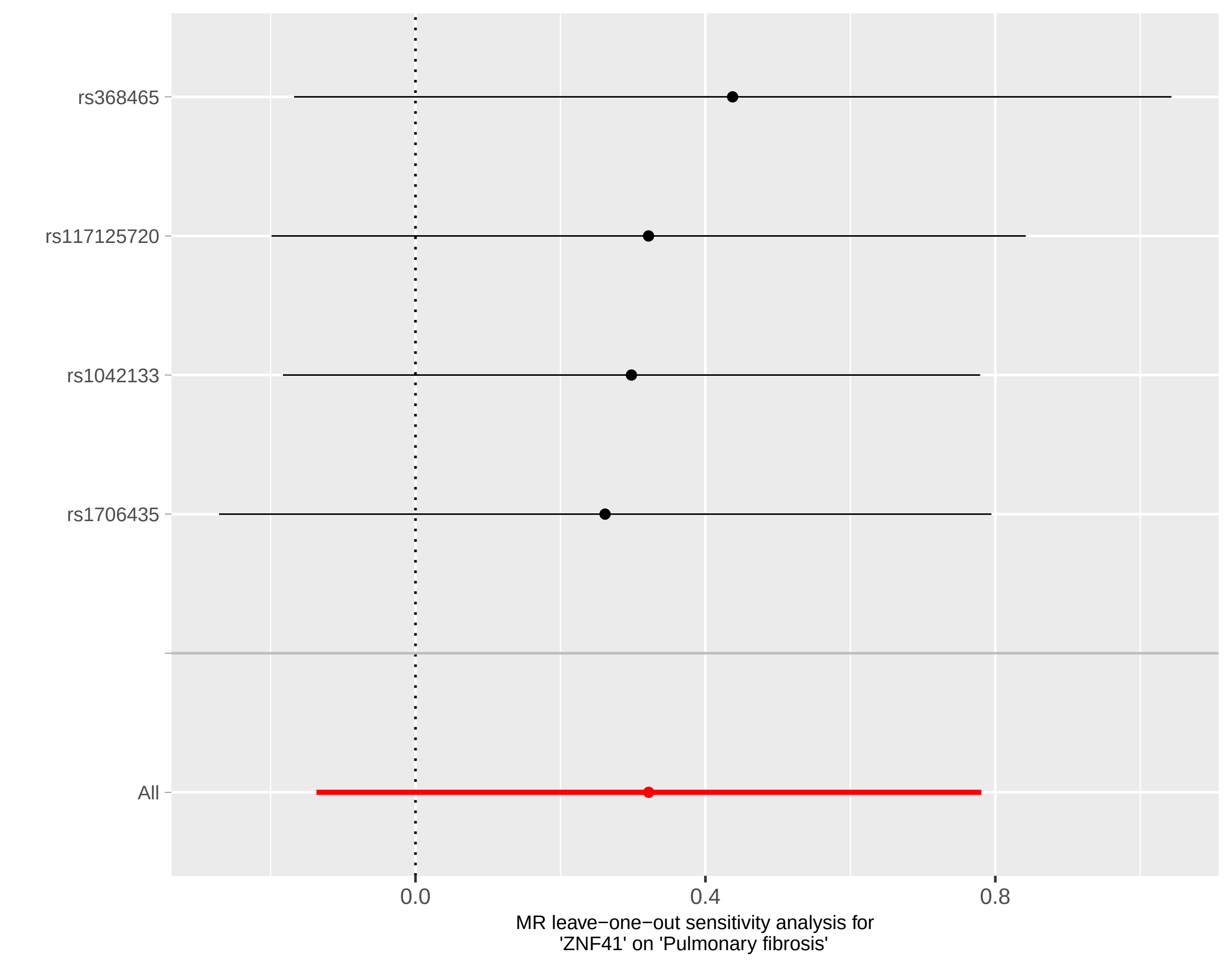
rs368465
rs117125720
rs1042133
rs1706435
All
0.0
0.4
0.8
MR leave−one−out sensitivity analysis for
'ZNF41' on 'Pulmonary fibrosis'

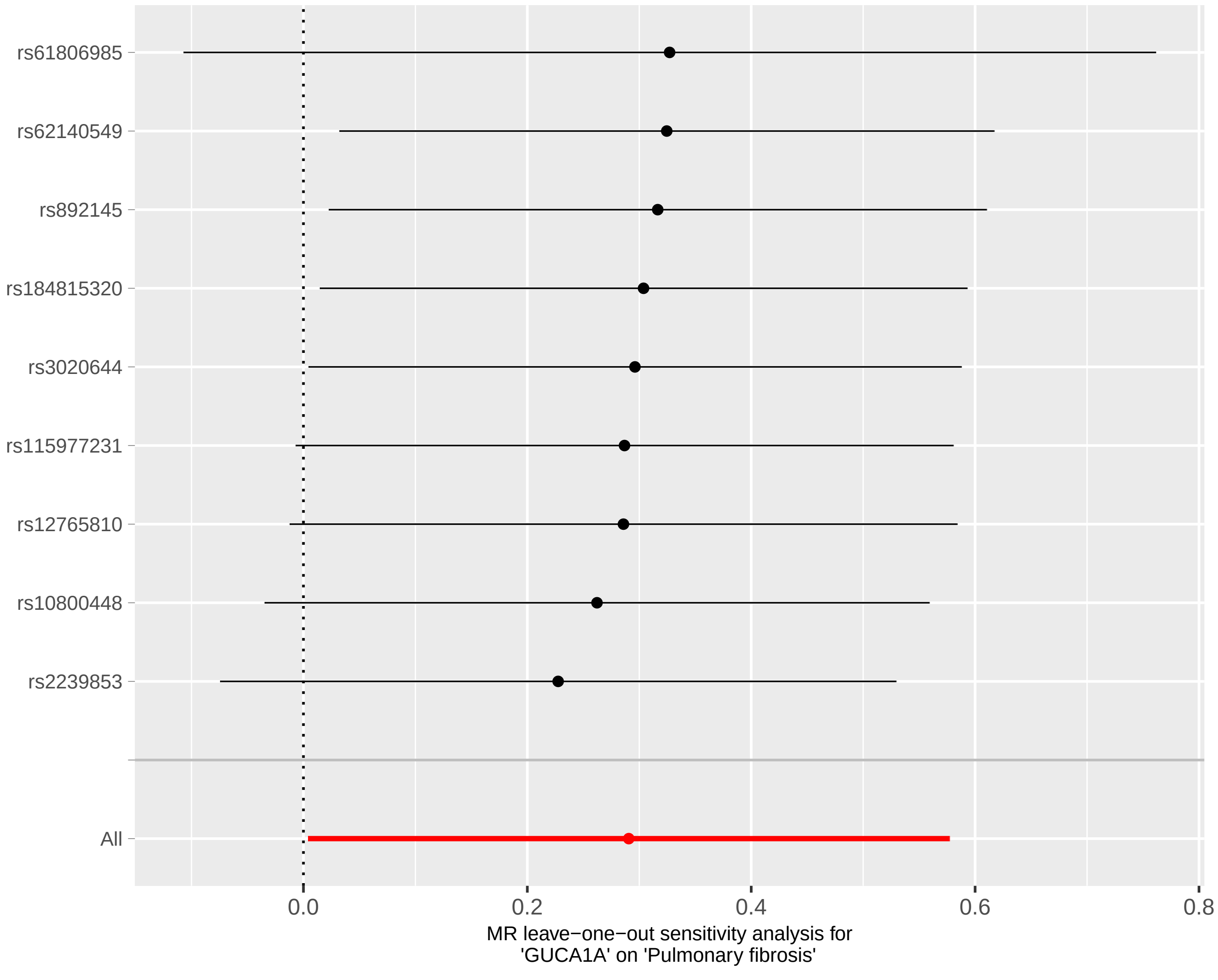

rs61806985
rs62140549
rs892145
rs184815320
rs3020644
rs115977231
rs12765810
rs10800448
rs2239853
All
0.0
0.2
0.4
0.6
0.8
MR leave−one−out sensitivity analysis for
'GUCA1A' on 'Pulmonary fibrosis'

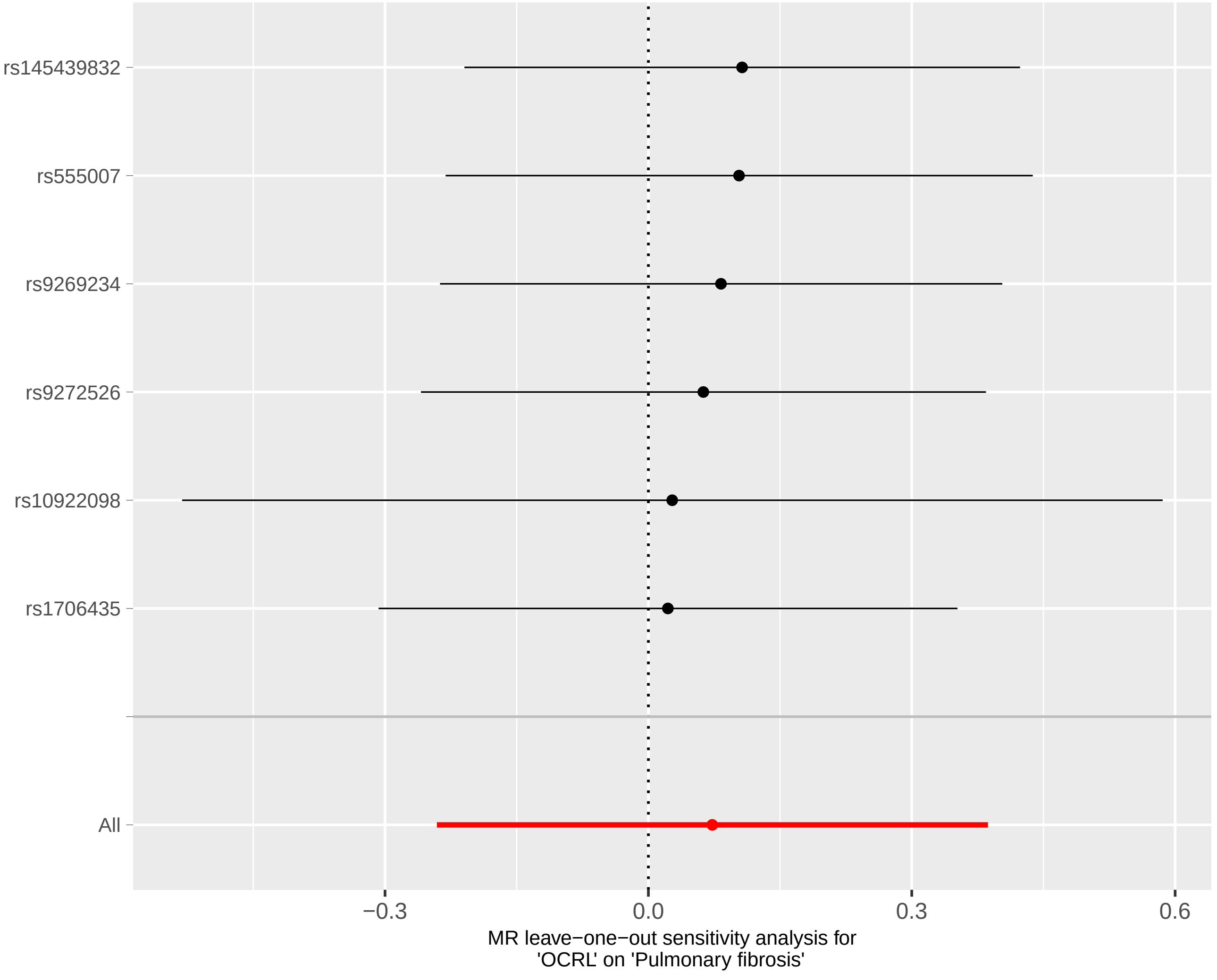
rs145439832
rs555007
rs9269234
rs9272526
rs10922098
rs1706435
All
−0.3
0.0
0.3
0.6
MR leave−one−out sensitivity analysis for
'OCRL' on 'Pulmonary fibrosis'

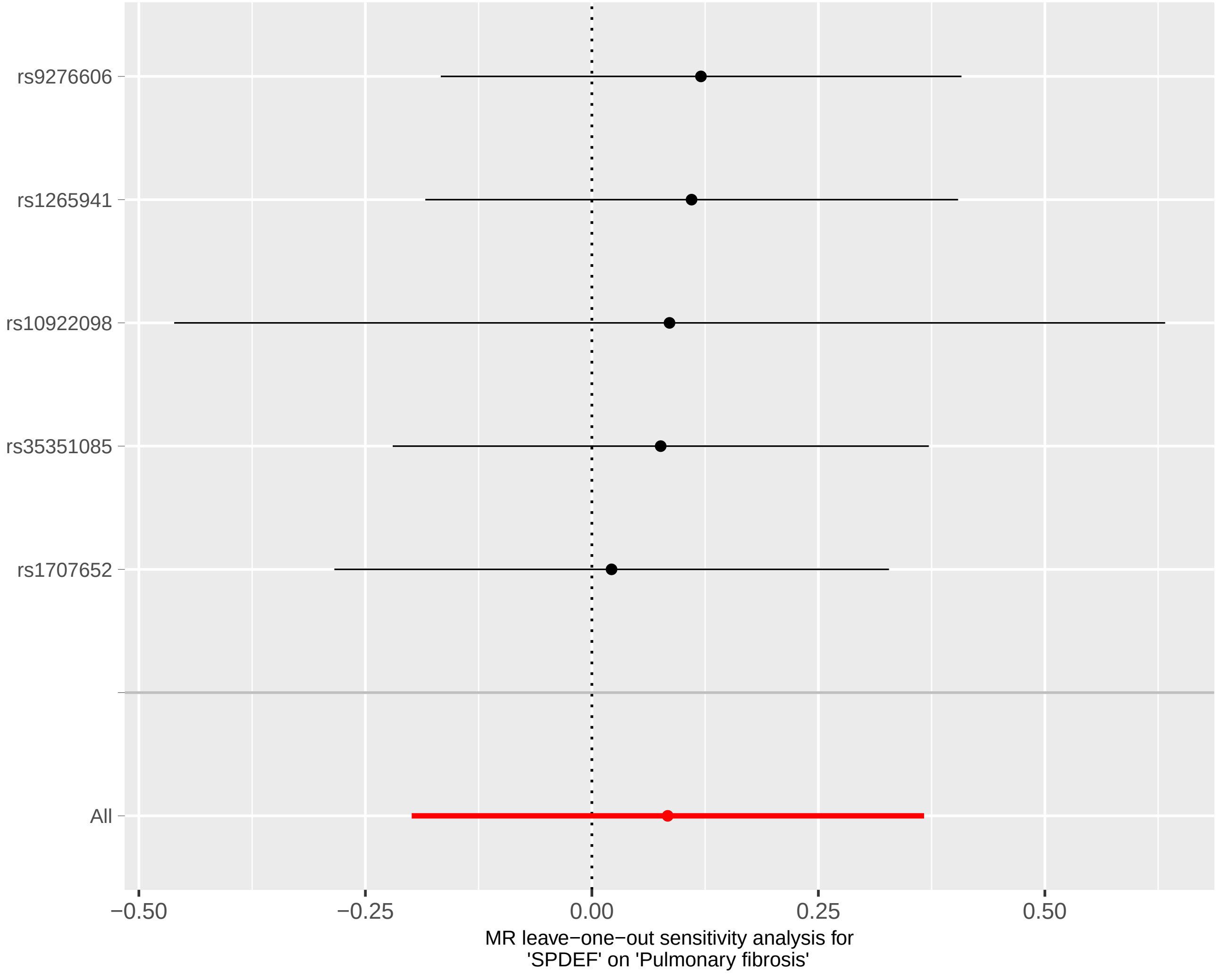
rs9276606
rs1265941
rs10922098
rs35351085
rs1707652
All
−0.50
−0.25
0.00
0.25
0.50
MR leave−one−out sensitivity analysis for
'SPDEF' on 'Pulmonary fibrosis'

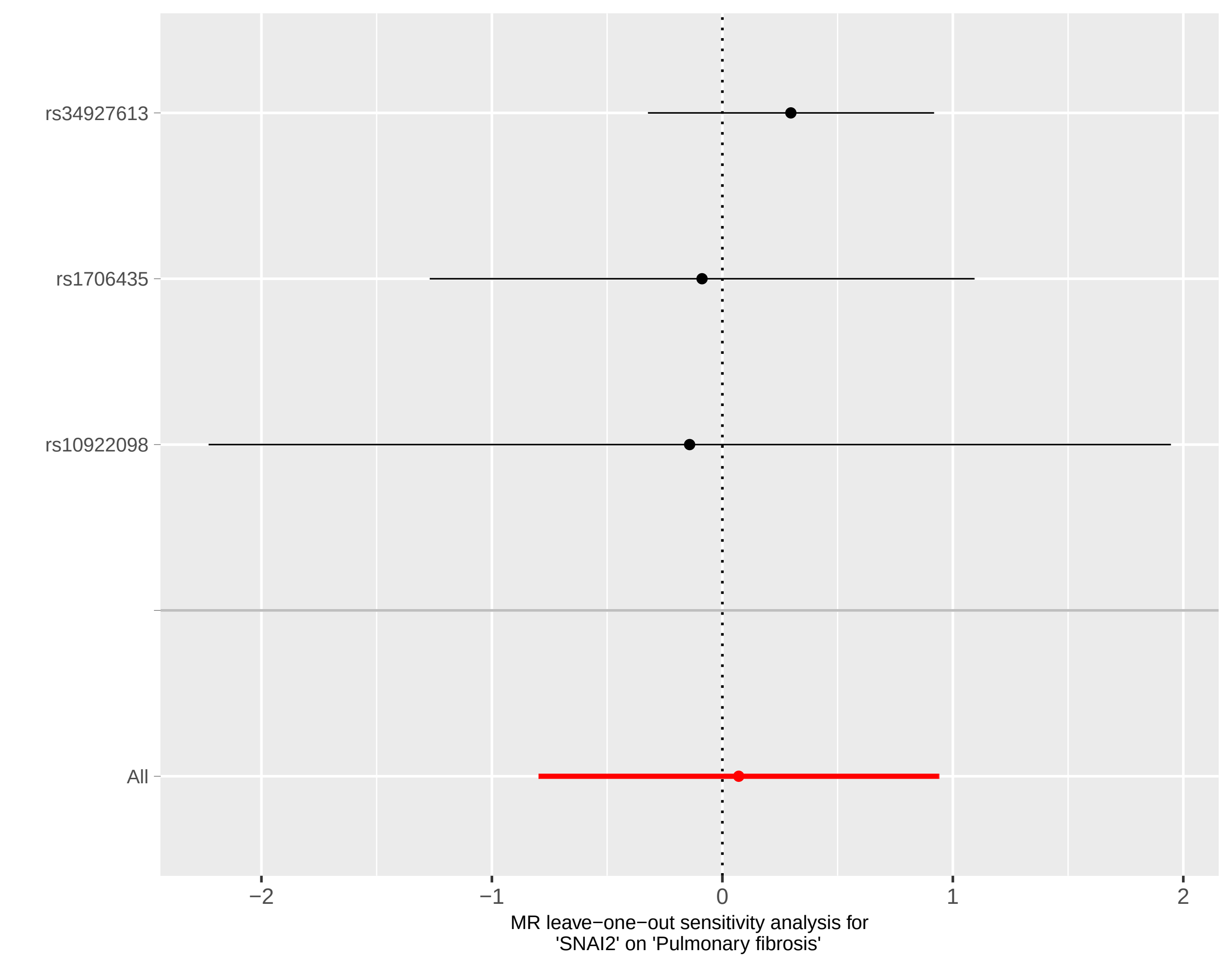
rs34927613
rs1706435
rs10922098
All
−2
−1
0
1
2
MR leave−one−out sensitivity analysis for
'SNAI2' on 'Pulmonary fibrosis'

Insufficient number of SNPs

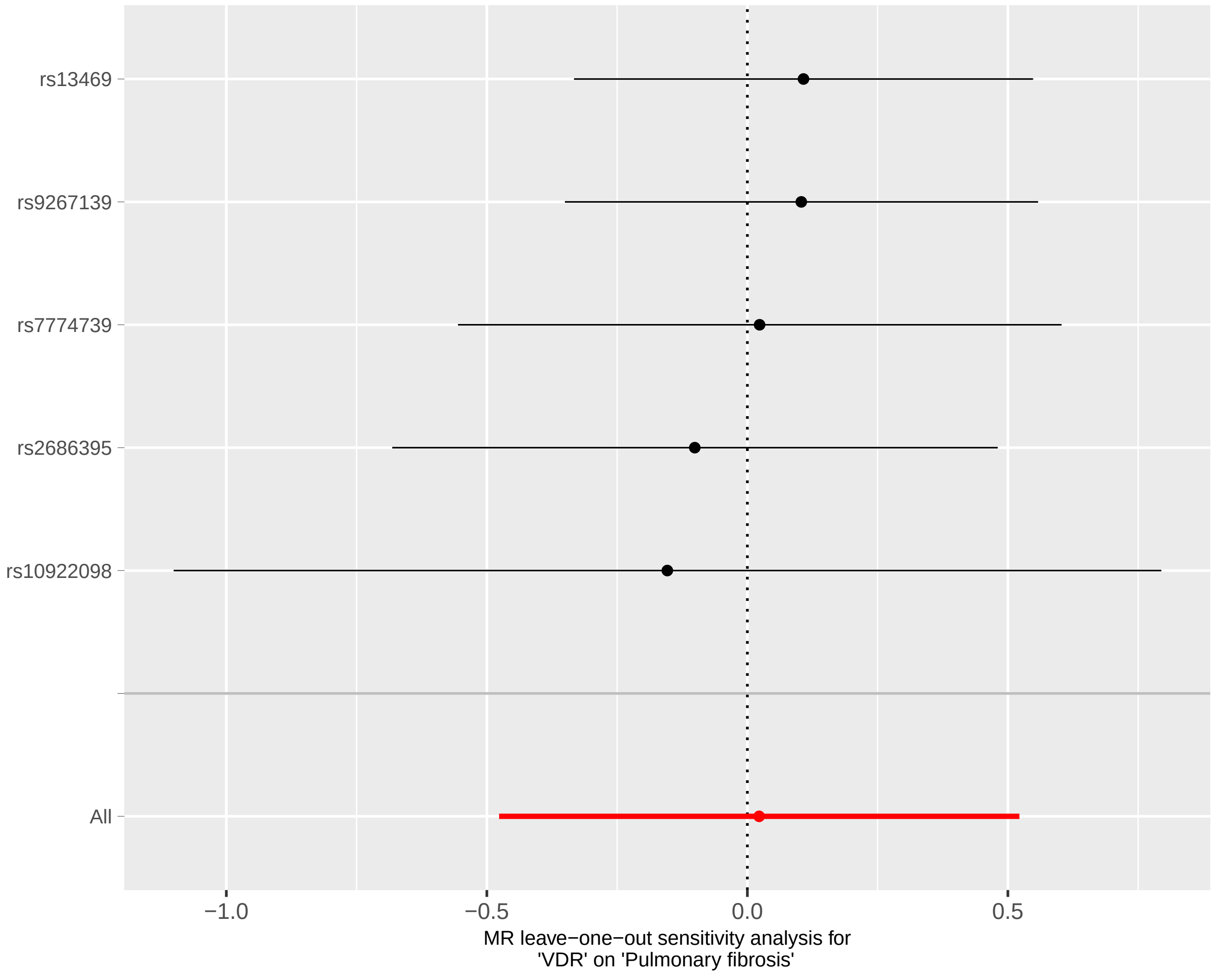
rs13469
rs9267139
rs7774739
rs2686395
rs10922098
All
−1.0
−0.5
0.0
0.5
MR leave−one−out sensitivity analysis for
'VDR' on 'Pulmonary fibrosis'

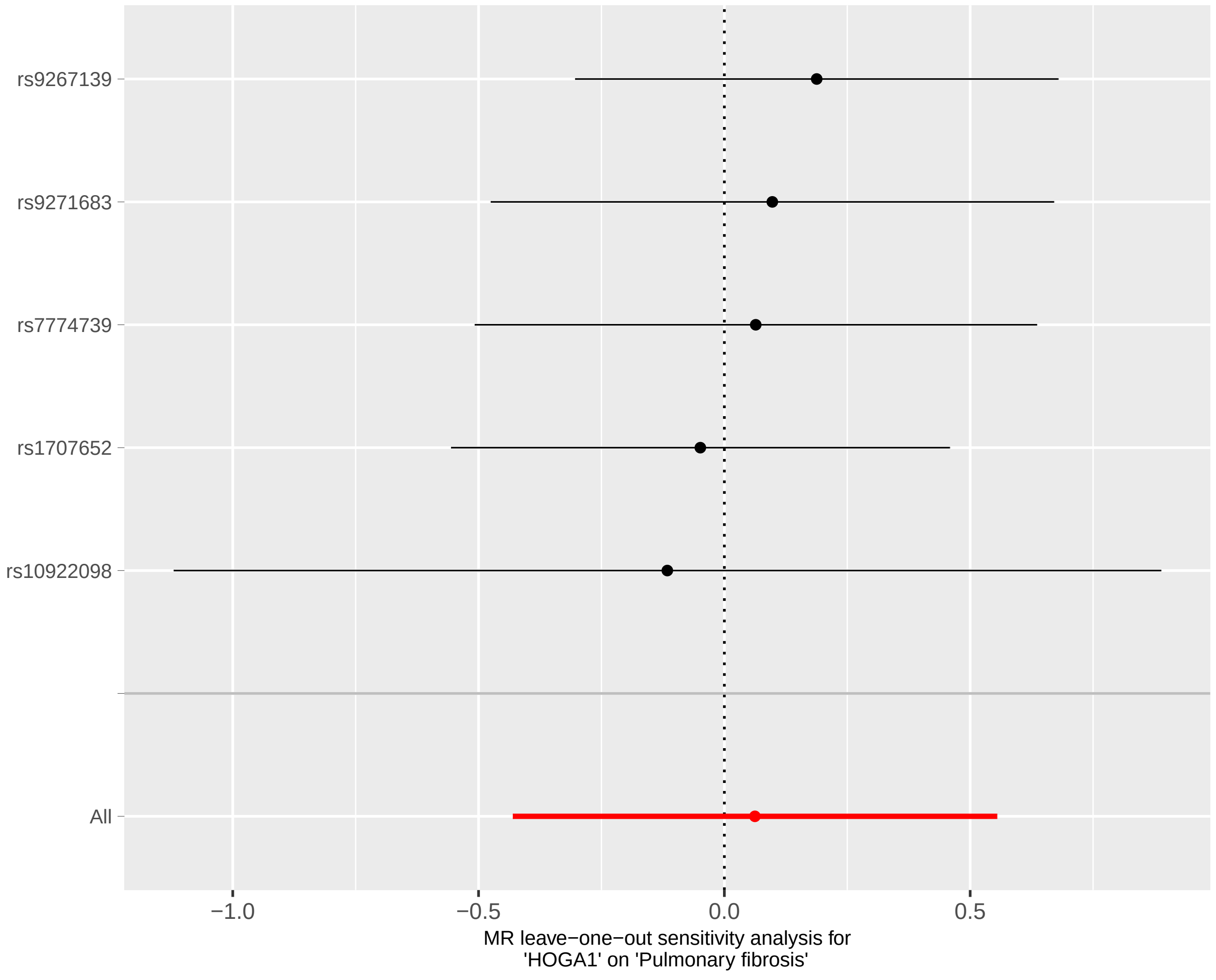
rs9267139
rs9271683
rs7774739
rs1707652
rs10922098
All
−1.0
−0.5
0.0
0.5
MR leave−one−out sensitivity analysis for
'HOGA1' on 'Pulmonary fibrosis'

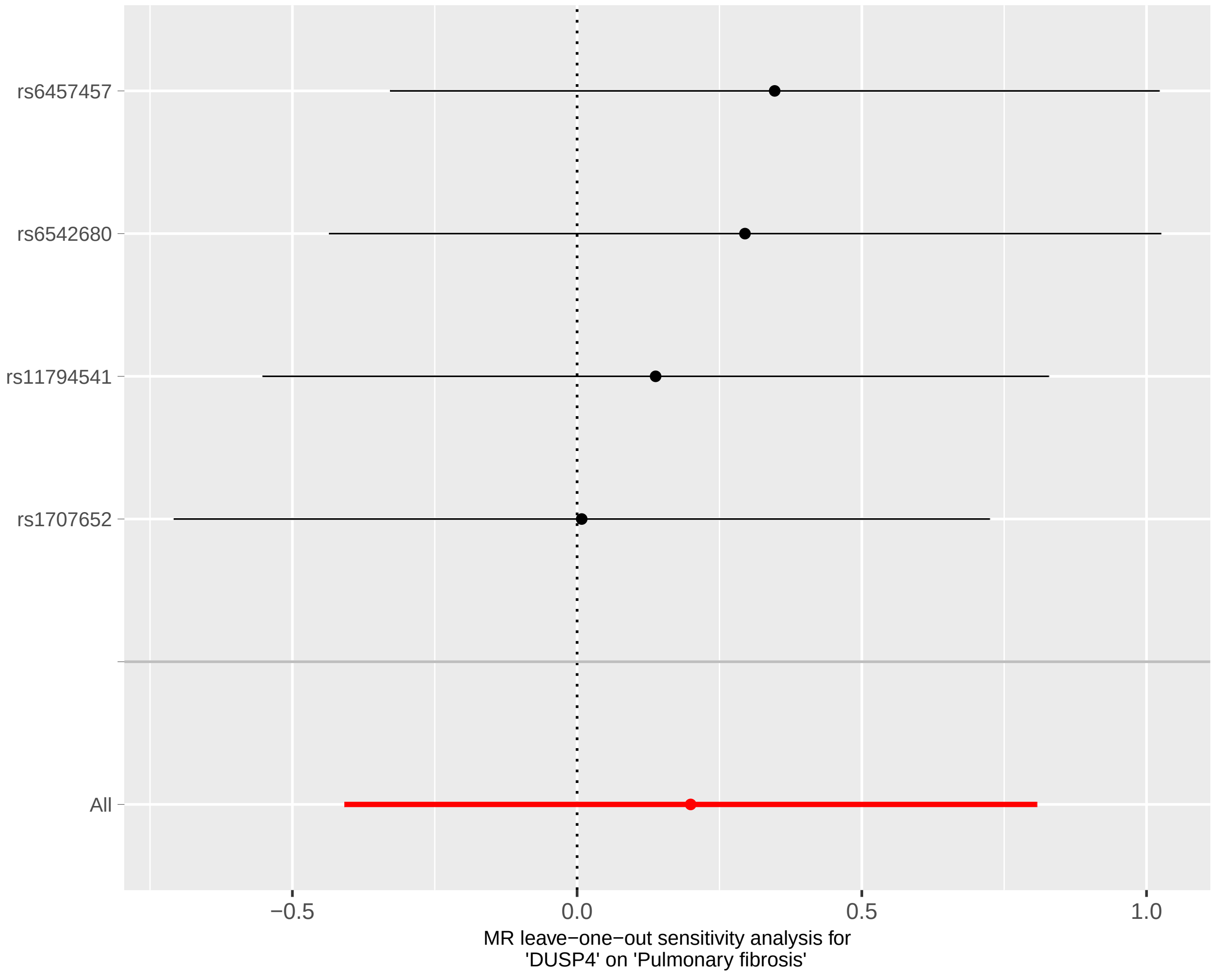
rs6457457
rs6542680
rs11794541
rs1707652
All
−0.5
0.0
0.5
1.0
MR leave−one−out sensitivity analysis for
'DUSP4' on 'Pulmonary fibrosis'

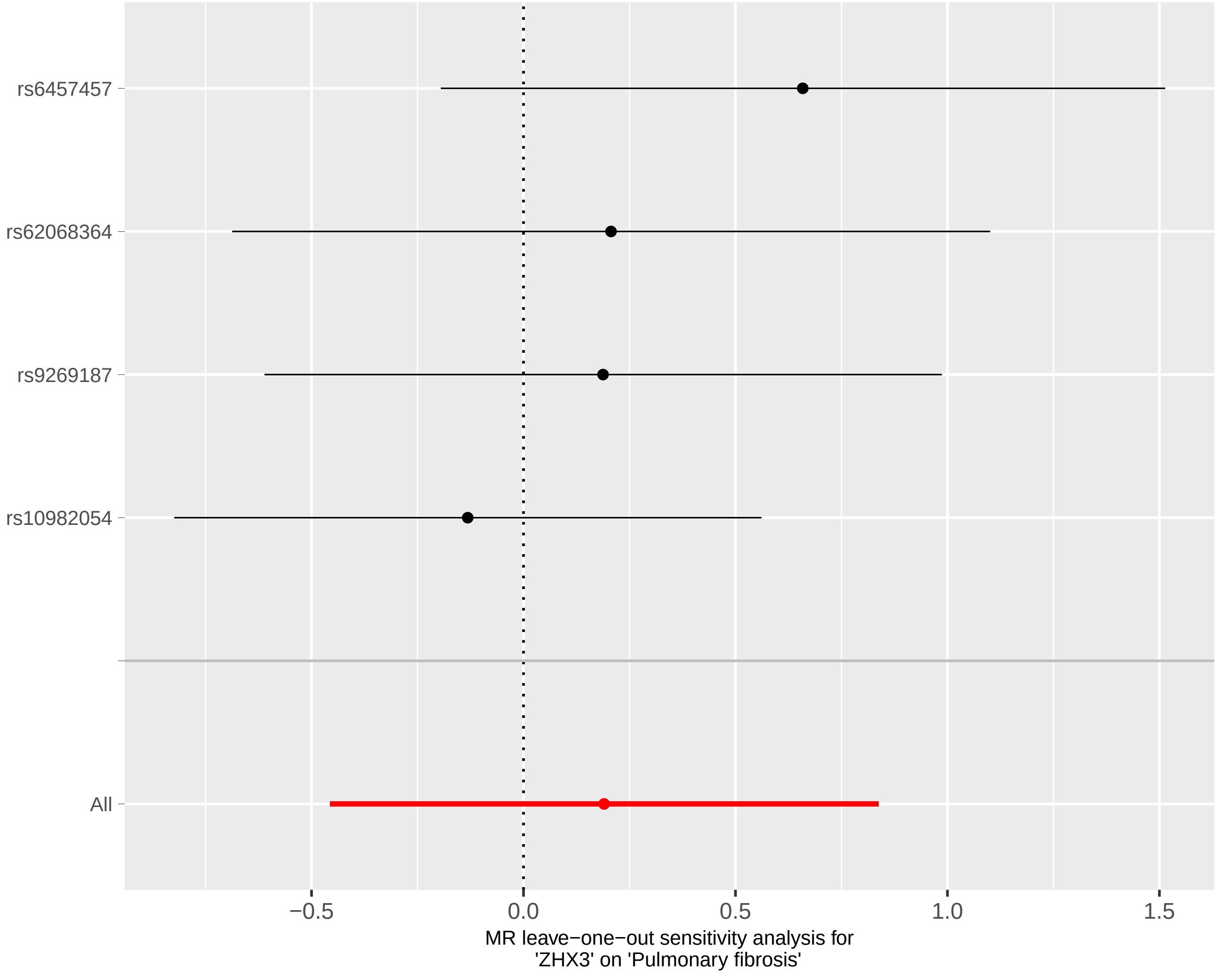

rs6457457
rs62068364
rs9269187
rs10982054
All
−0.5
0.0
0.5
1.0
1.5
MR leave−one−out sensitivity analysis for
'ZHX3' on 'Pulmonary fibrosis'

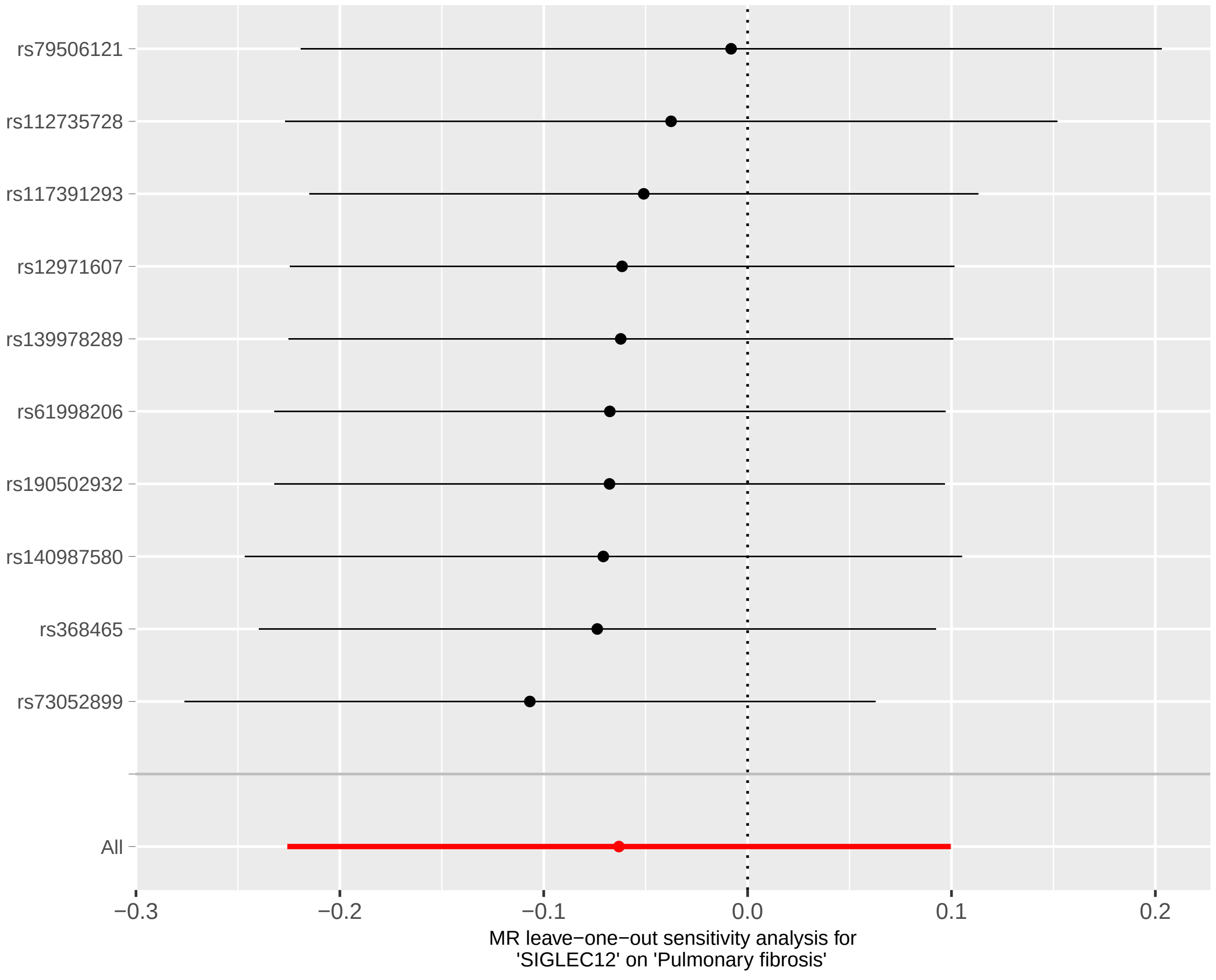
rs79506121
rs112735728
rs117391293
rs12971607
rs139978289
rs61998206
rs190502932
rs140987580
rs368465
rs73052899
All
−0.3
−0.2
−0.1
0.0
0.1
0.2
MR leave−one−out sensitivity analysis for
'SIGLEC12' on 'Pulmonary fibrosis'

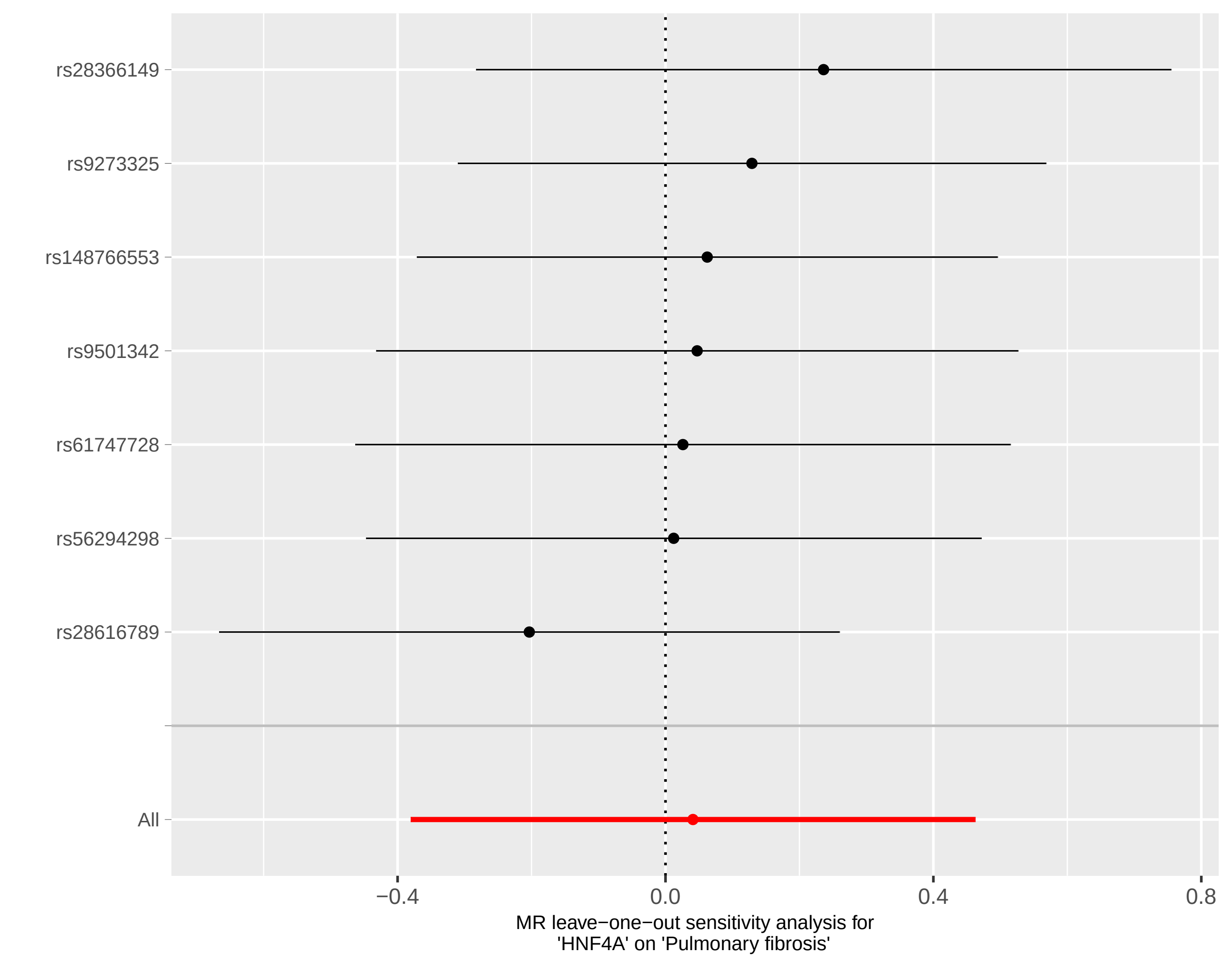
rs28366149
rs9273325
rs148766553
rs9501342
rs61747728
rs56294298
rs28616789
All
−0.4
0.0
0.4
0.8
MR leave−one−out sensitivity analysis for
'HNF4A' on 'Pulmonary fibrosis'

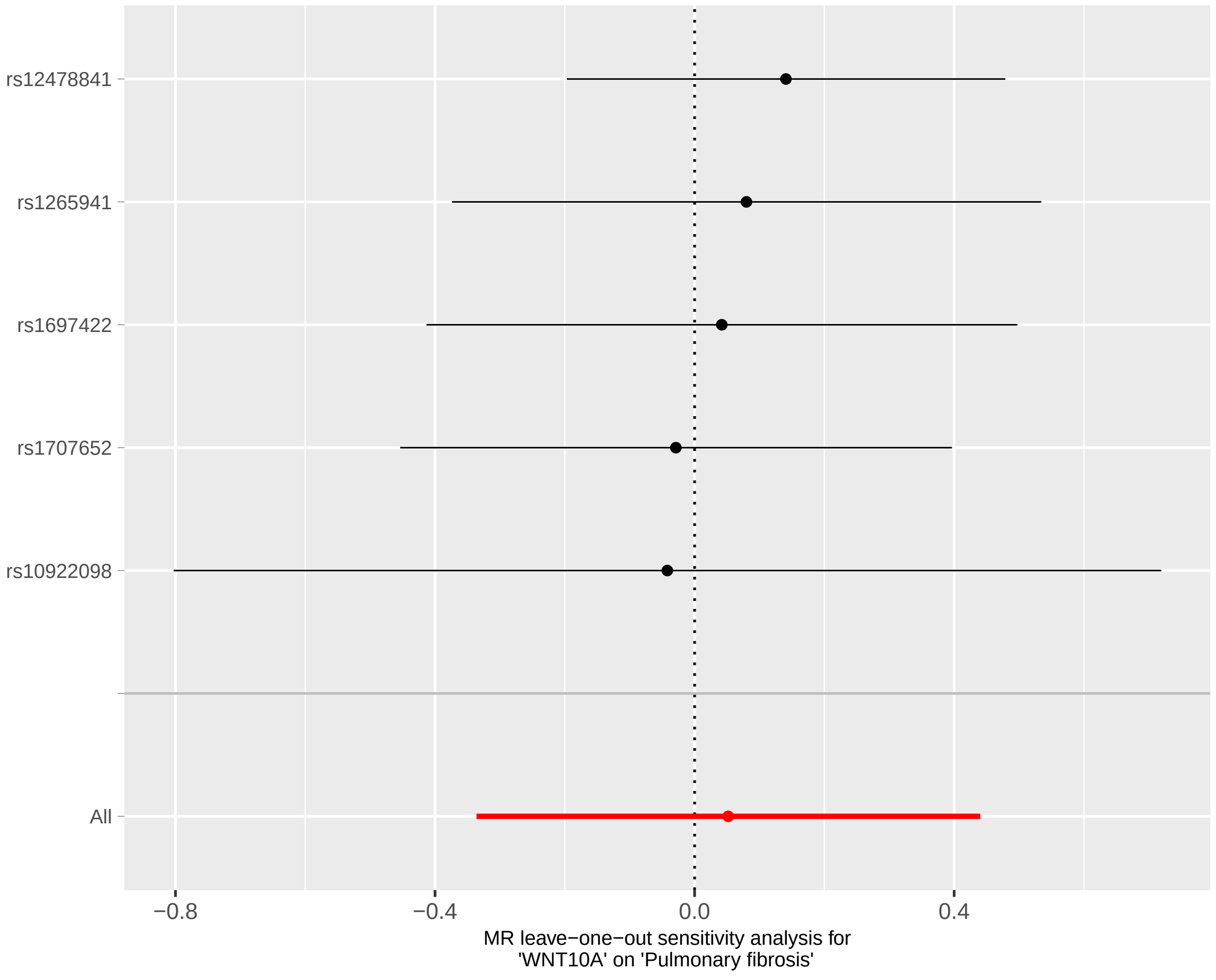

rs12478841
rs1265941
rs1697422
rs1707652
rs10922098
All
−0.8
−0.4
0.0
0.4
MR leave−one−out sensitivity analysis for
'WNT10A' on 'Pulmonary fibrosis'

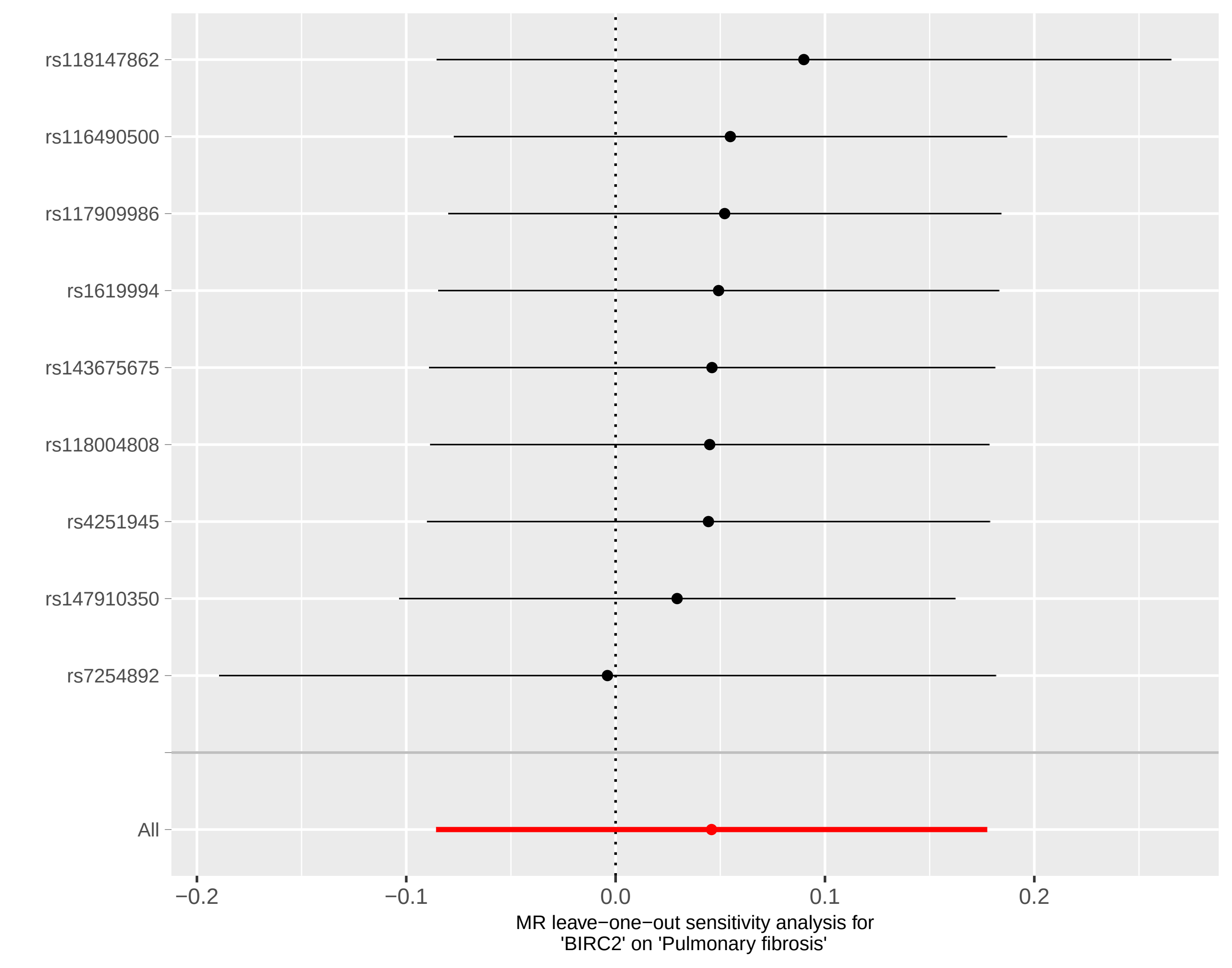
rs118147862
rs116490500
rs117909986
rs1619994
rs143675675
rs118004808
rs4251945
rs147910350
rs7254892
All
−0.2
−0.1
0.0
0.1
0.2
MR leave−one−out sensitivity analysis for
'BIRC2' on 'Pulmonary fibrosis'

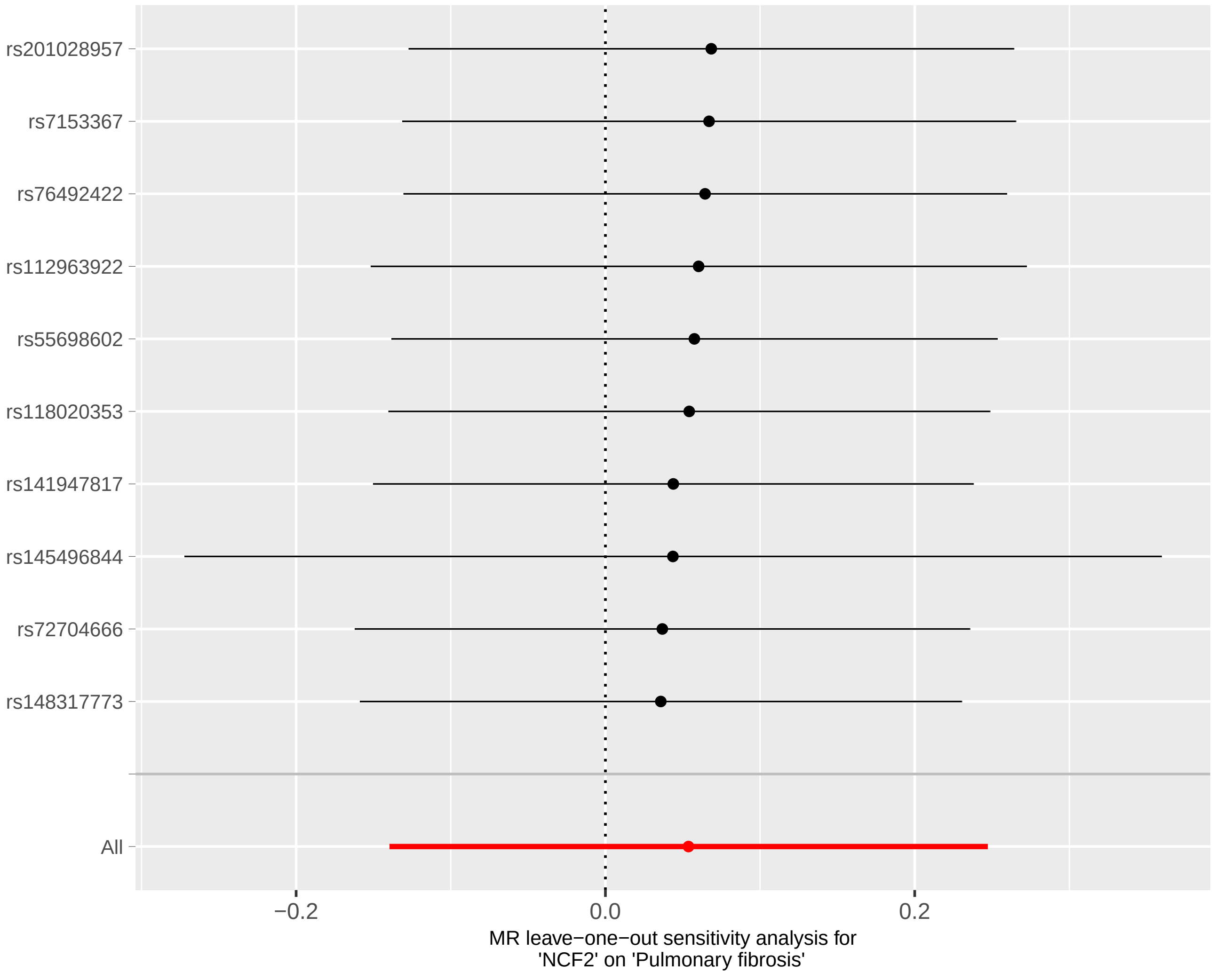
rs201028957
rs7153367
rs76492422
rs112963922
rs55698602
rs118020353
rs141947817
rs145496844
rs72704666
rs148317773
All
−0.2
0.0
0.2
MR leave−one−out sensitivity analysis for
'NCF2' on 'Pulmonary fibrosis'

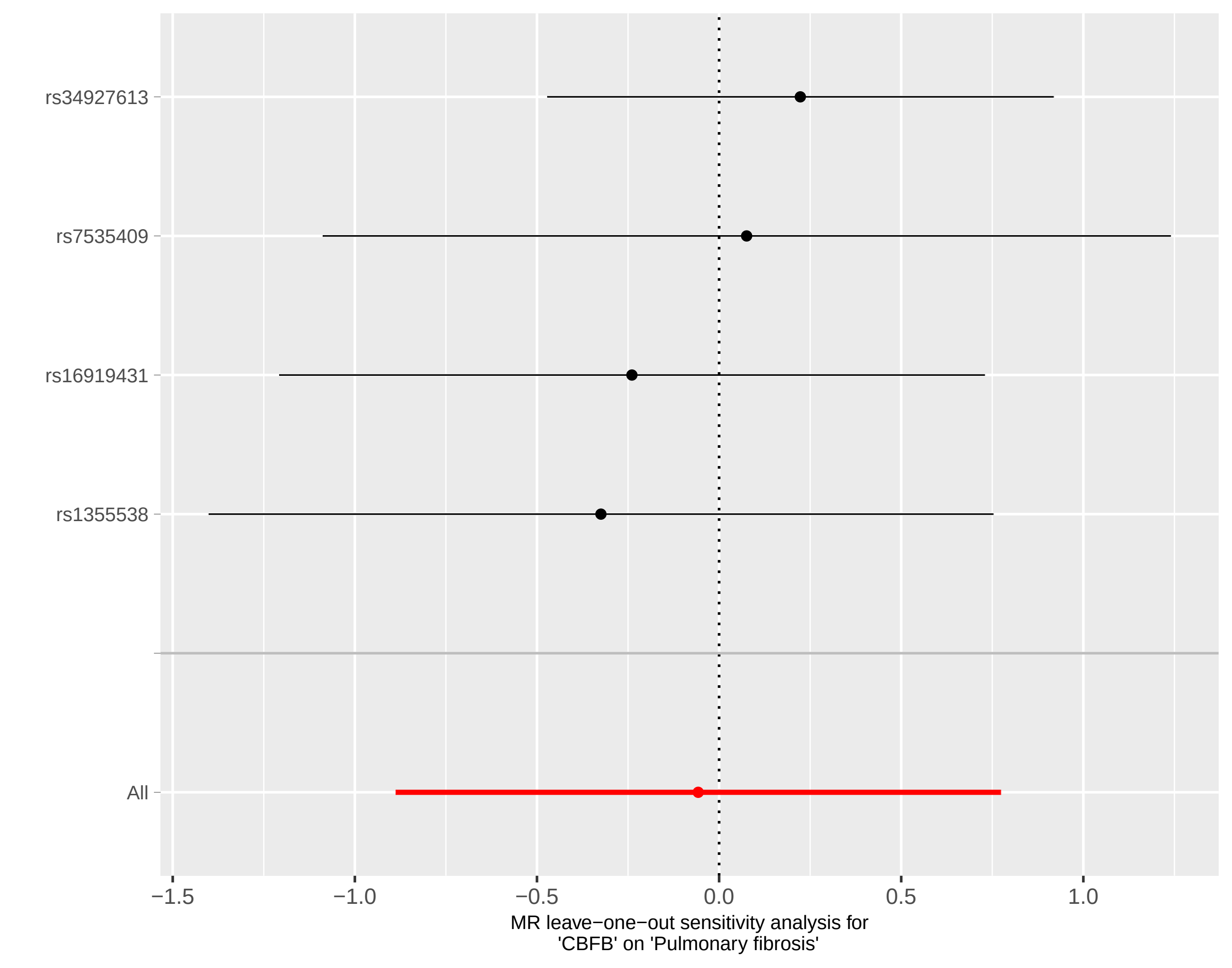
rs34927613
rs7535409
rs16919431
rs1355538
All
−1.5
−1.0
−0.5
0.0
0.5
1.0
MR leave−one−out sensitivity analysis for
'CBFB' on 'Pulmonary fibrosis'

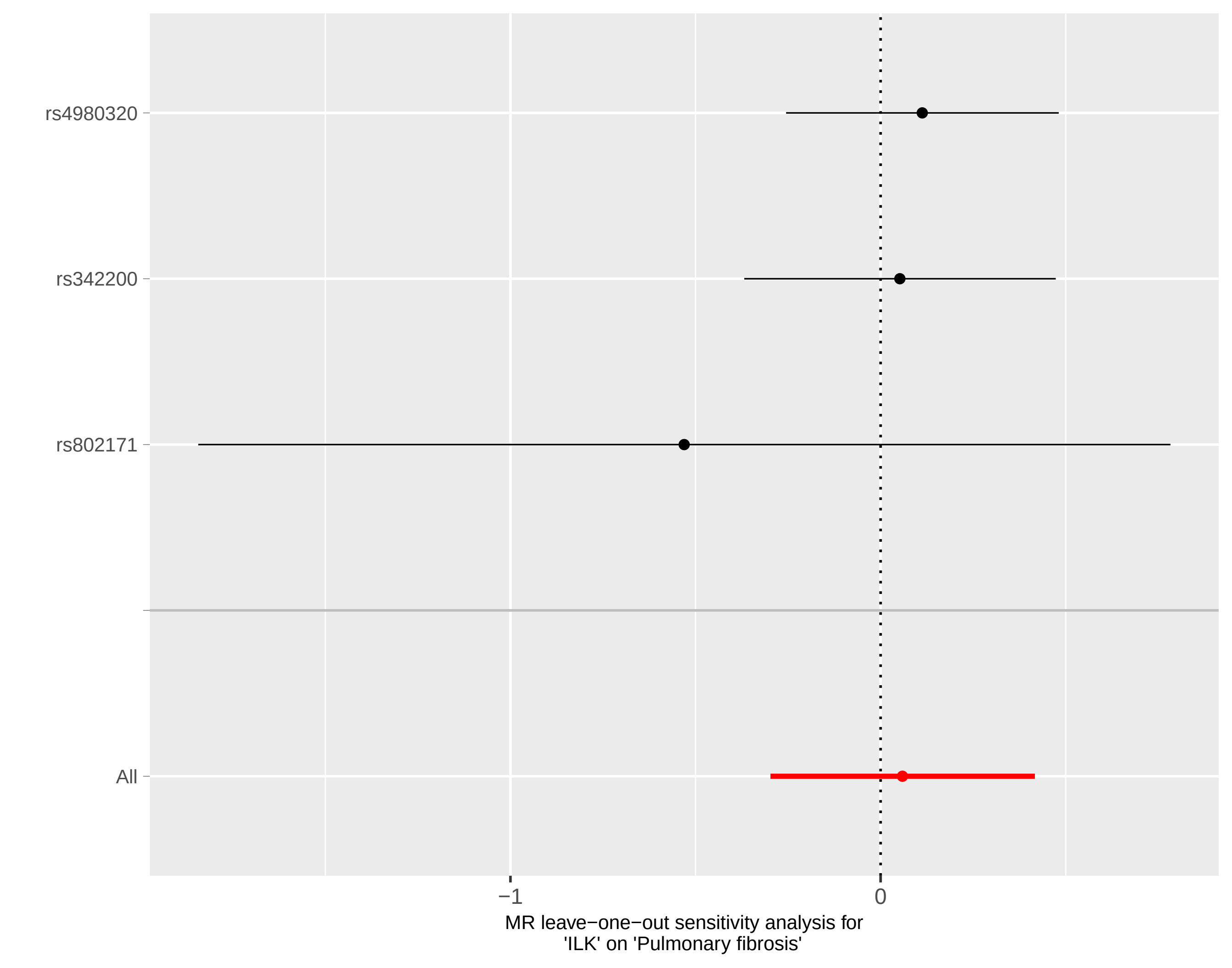
rs4980320
rs342200
rs802171
All
−1
0
MR leave−one−out sensitivity analysis for
'ILK' on 'Pulmonary fibrosis'

Insufficient number of SNPs

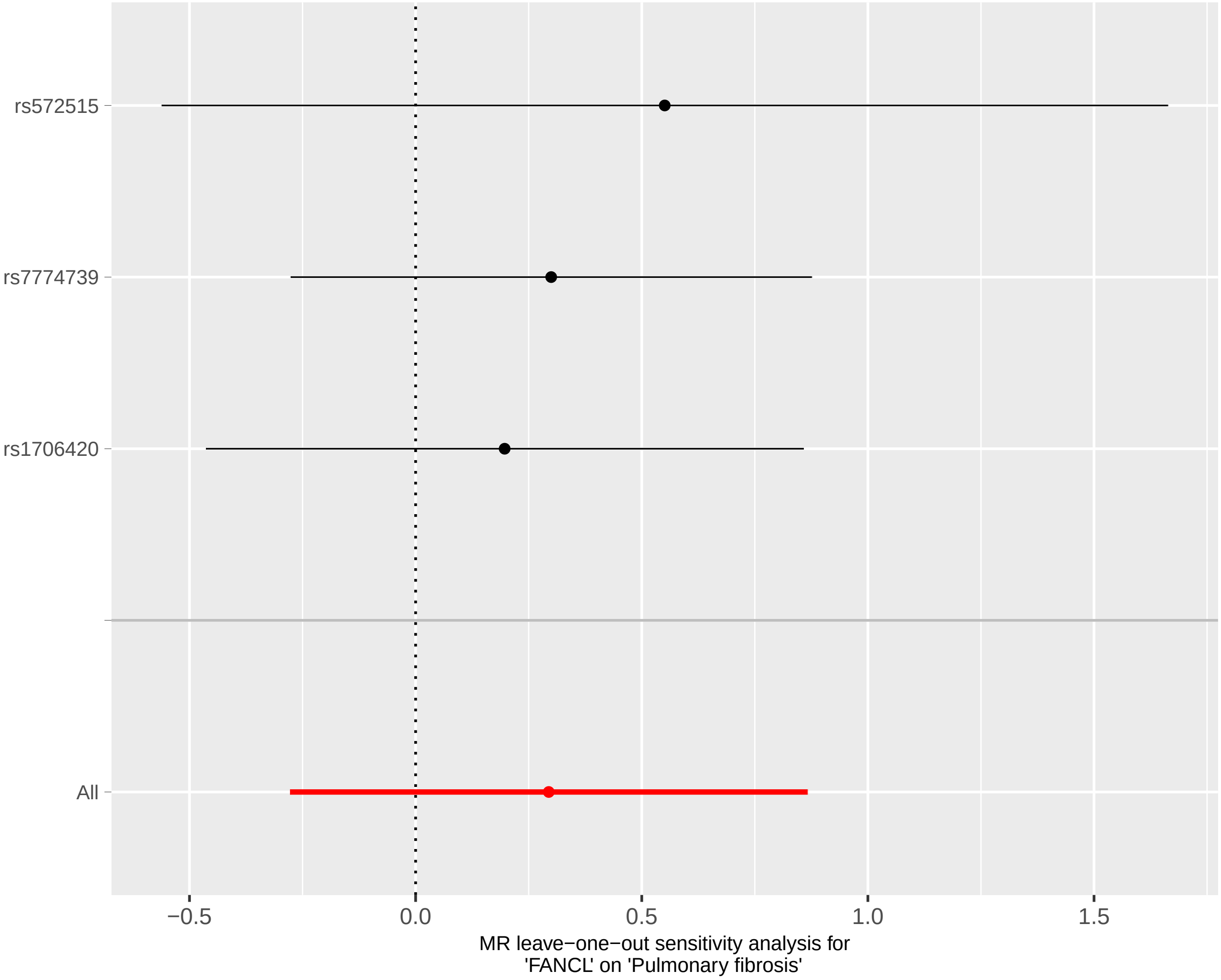
rs572515
rs7774739
rs1706420
All
−0.5
0.0
0.5
1.0
1.5
MR leave−one−out sensitivity analysis for
'FANCL' on 'Pulmonary fibrosis'

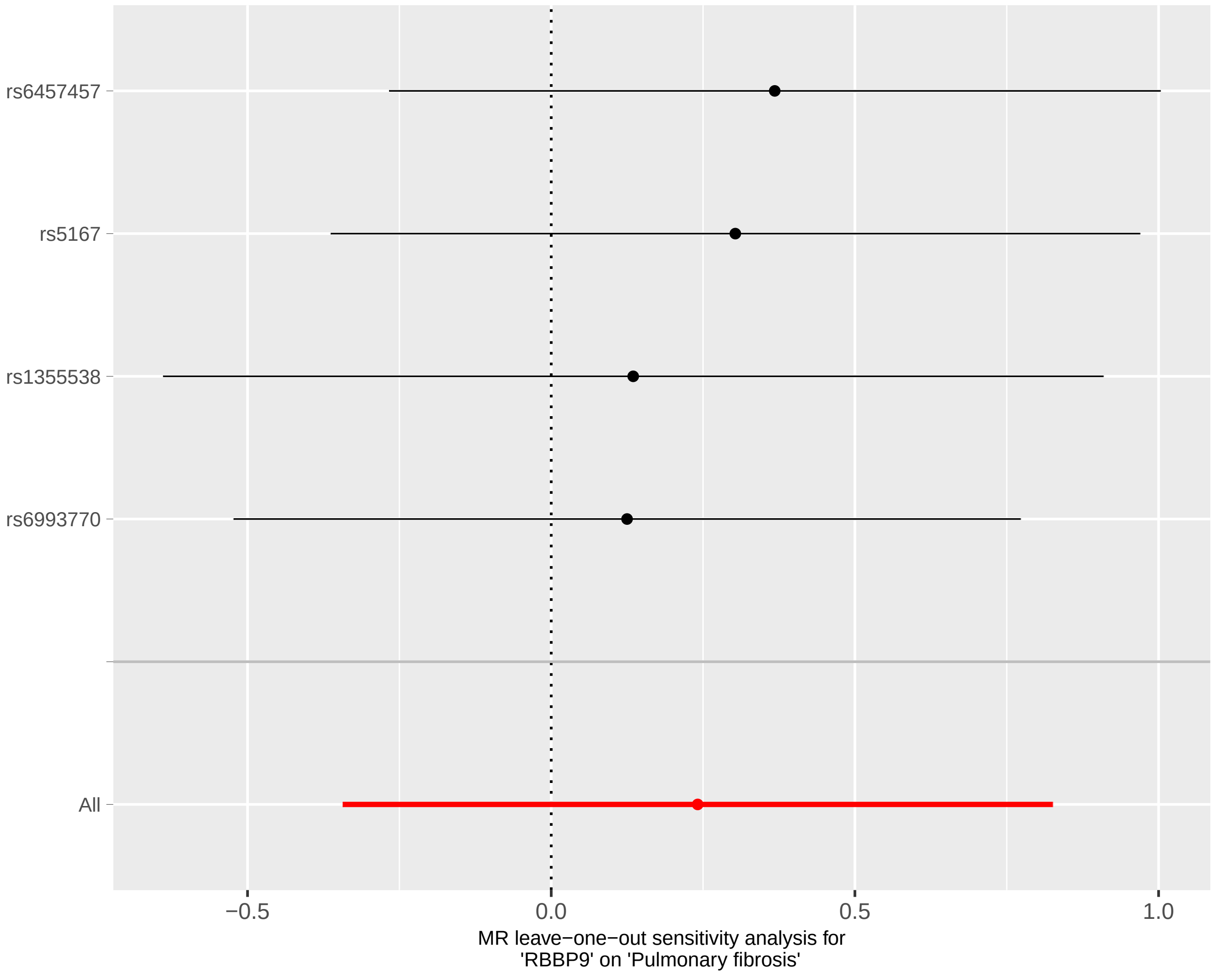

rs6457457
rs5167
rs1355538
rs6993770
All
−0.5
0.0
0.5
1.0
MR leave−one−out sensitivity analysis for
'RBBP9' on 'Pulmonary fibrosis'

Insufficient number of SNPs

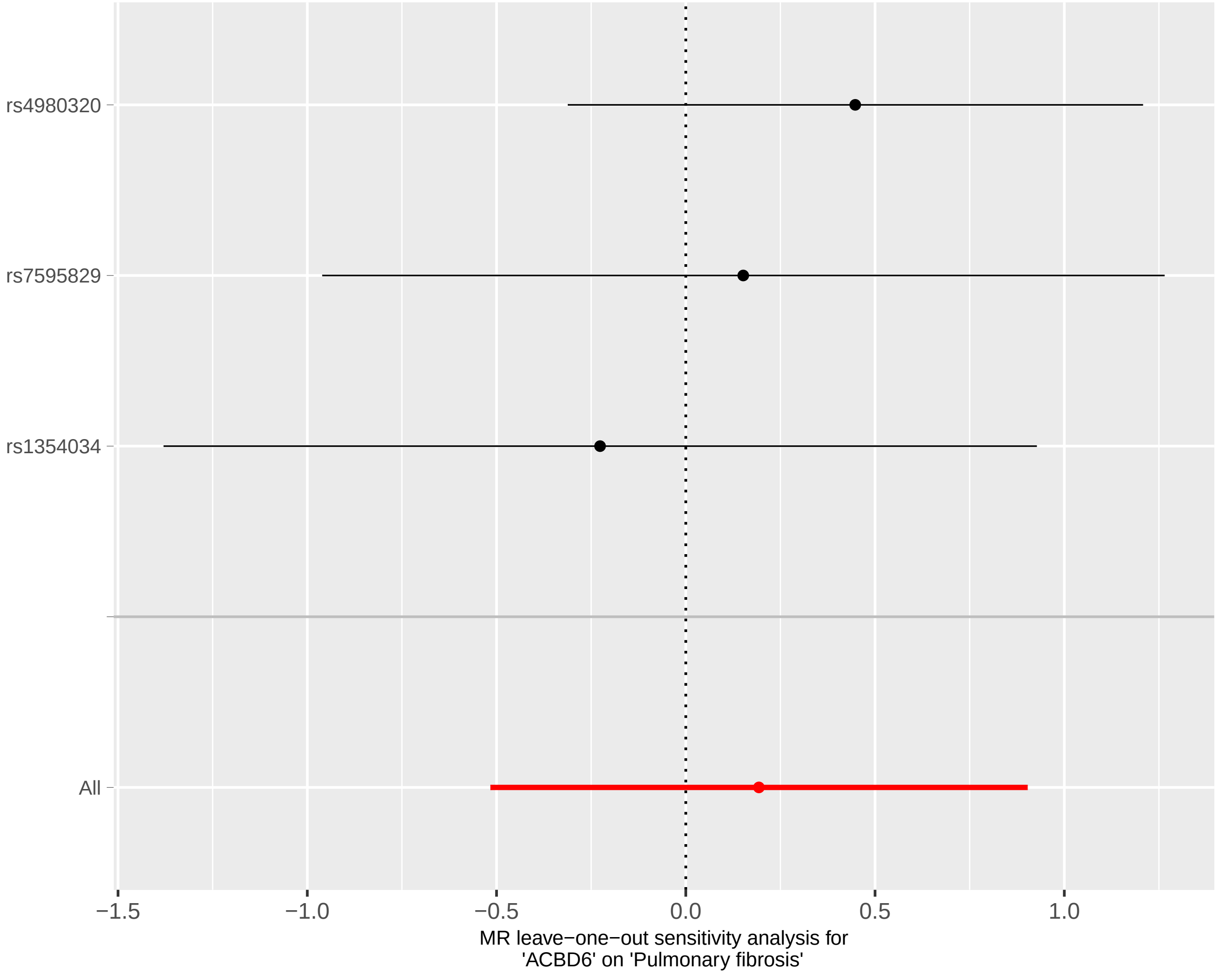
rs4980320
rs7595829
rs1354034
All
−1.5
−1.0
−0.5
0.0
0.5
1.0
MR leave−one−out sensitivity analysis for
'ACBD6' on 'Pulmonary fibrosis'

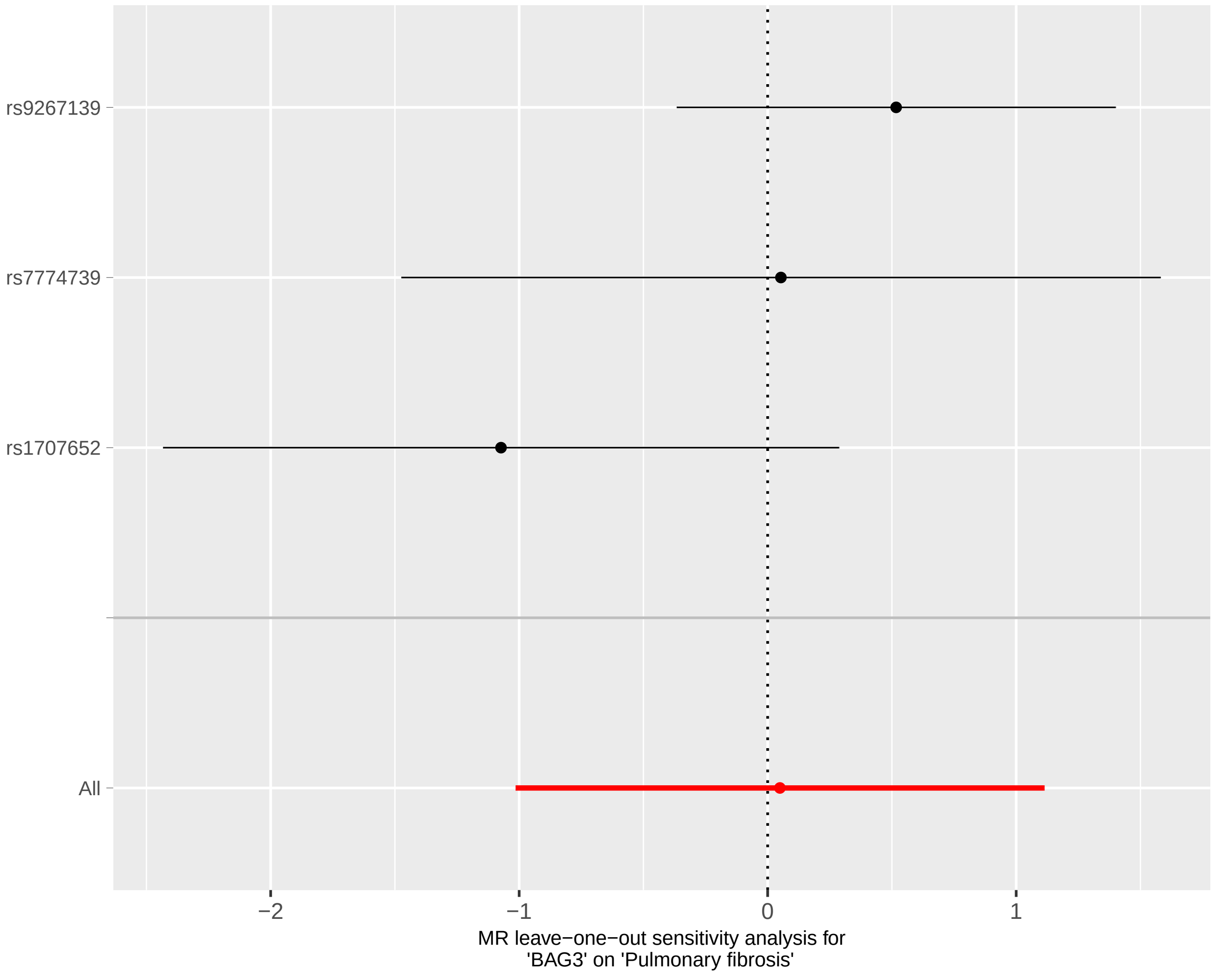
rs9267139
rs7774739
rs1707652
All
−2
−1
0
1
MR leave−one−out sensitivity analysis for
'BAG3' on 'Pulmonary fibrosis'

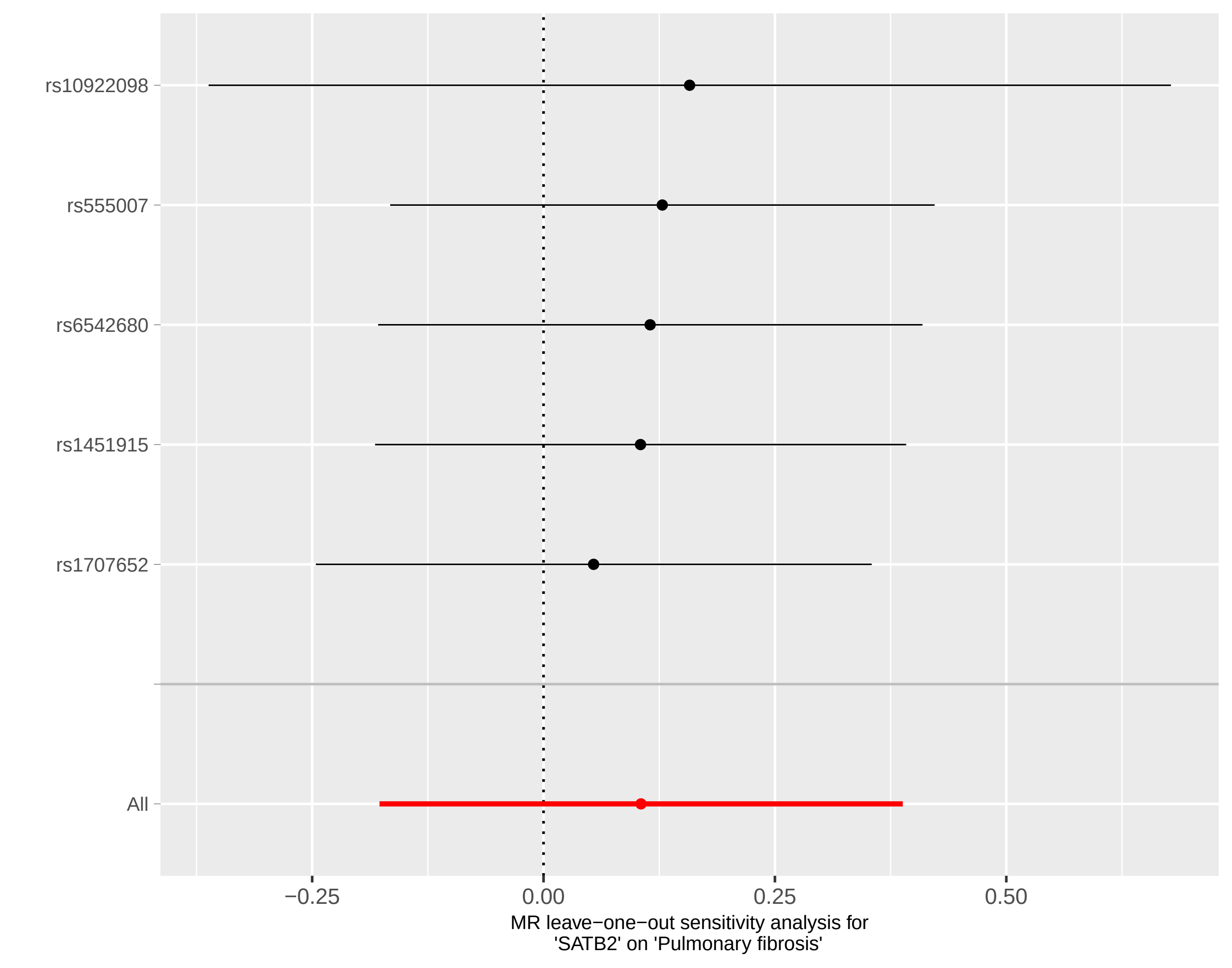
rs10922098
rs555007
rs6542680
rs1451915
rs1707652
All
−0.25
0.00
0.25
0.50
MR leave−one−out sensitivity analysis for
'SATB2' on 'Pulmonary fibrosis'

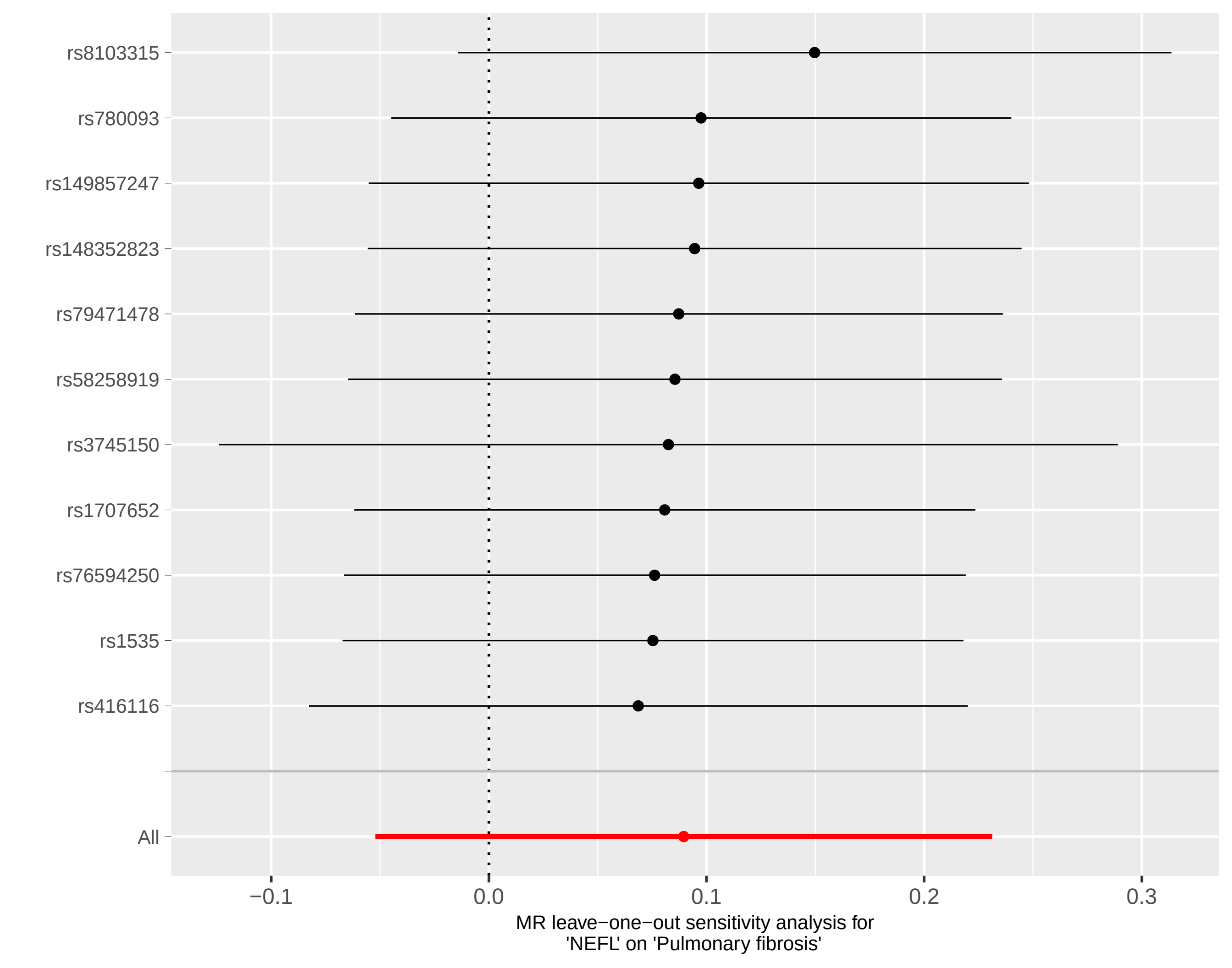
rs8103315
rs780093
rs149857247
rs148352823
rs79471478
rs58258919
rs3745150
rs1707652
rs76594250
rs1535
rs416116
All
−0.1
0.0
0.1
0.2
0.3
MR leave−one−out sensitivity analysis for
'NEFL' on 'Pulmonary fibrosis'

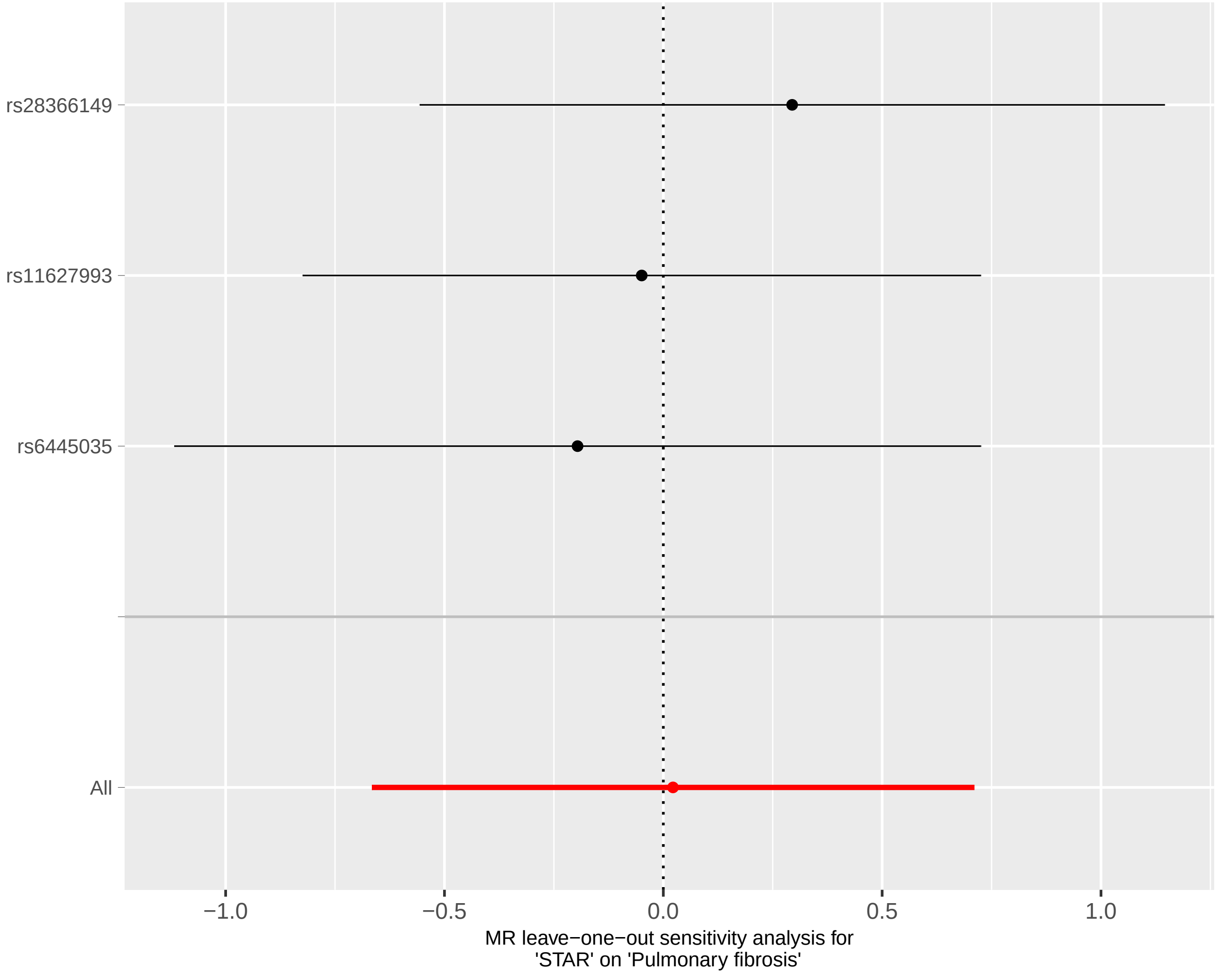
rs28366149
rs11627993
rs6445035
All
−1.0
−0.5
0.0
0.5
1.0
MR leave−one−out sensitivity analysis for
'STAR' on 'Pulmonary fibrosis'

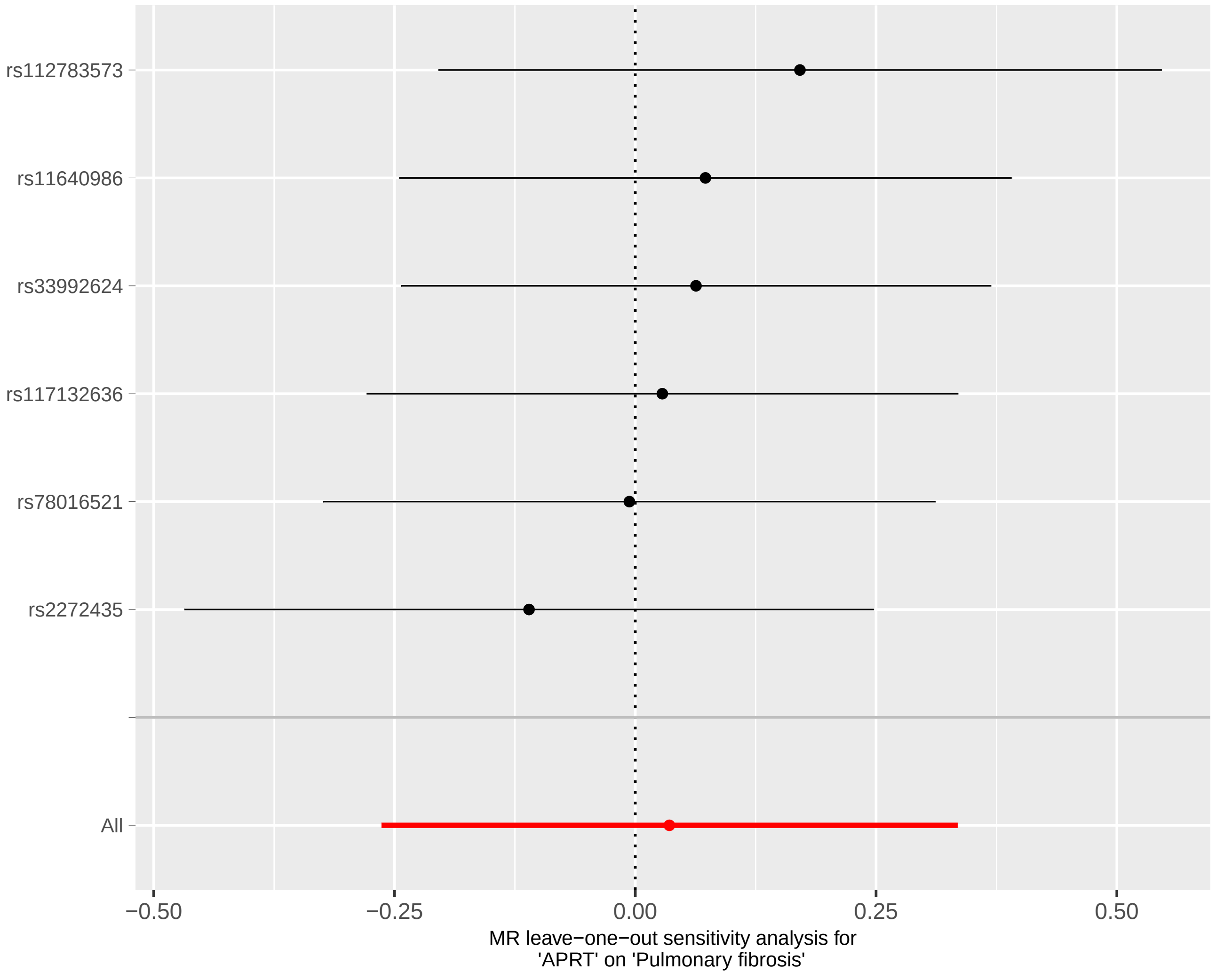
rs112783573
rs11640986
rs33992624
rs117132636
rs78016521
rs2272435
All
−0.50
−0.25
0.00
0.25
0.50
MR leave−one−out sensitivity analysis for
'APRT' on 'Pulmonary fibrosis'

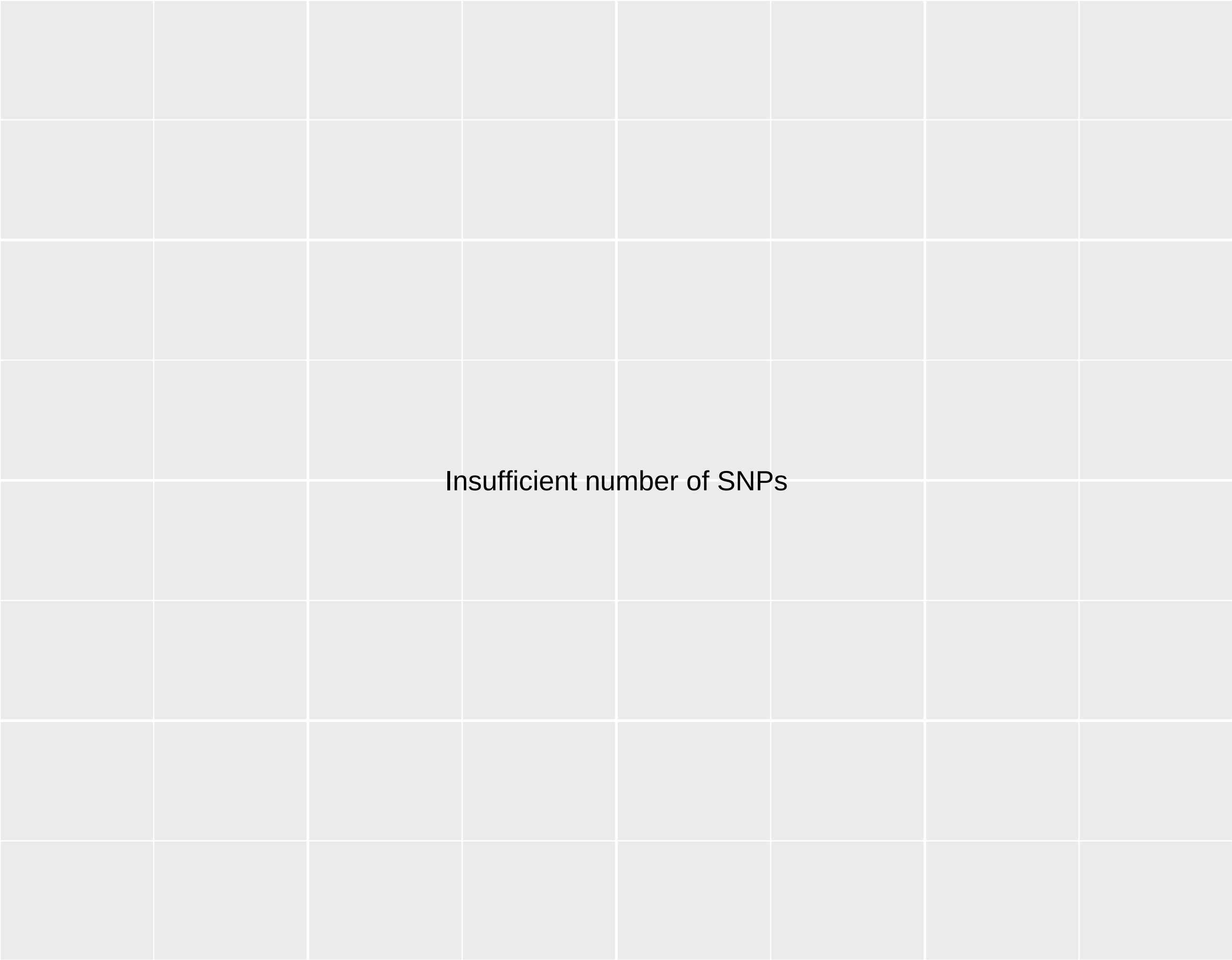
Insufficient number of SNPs

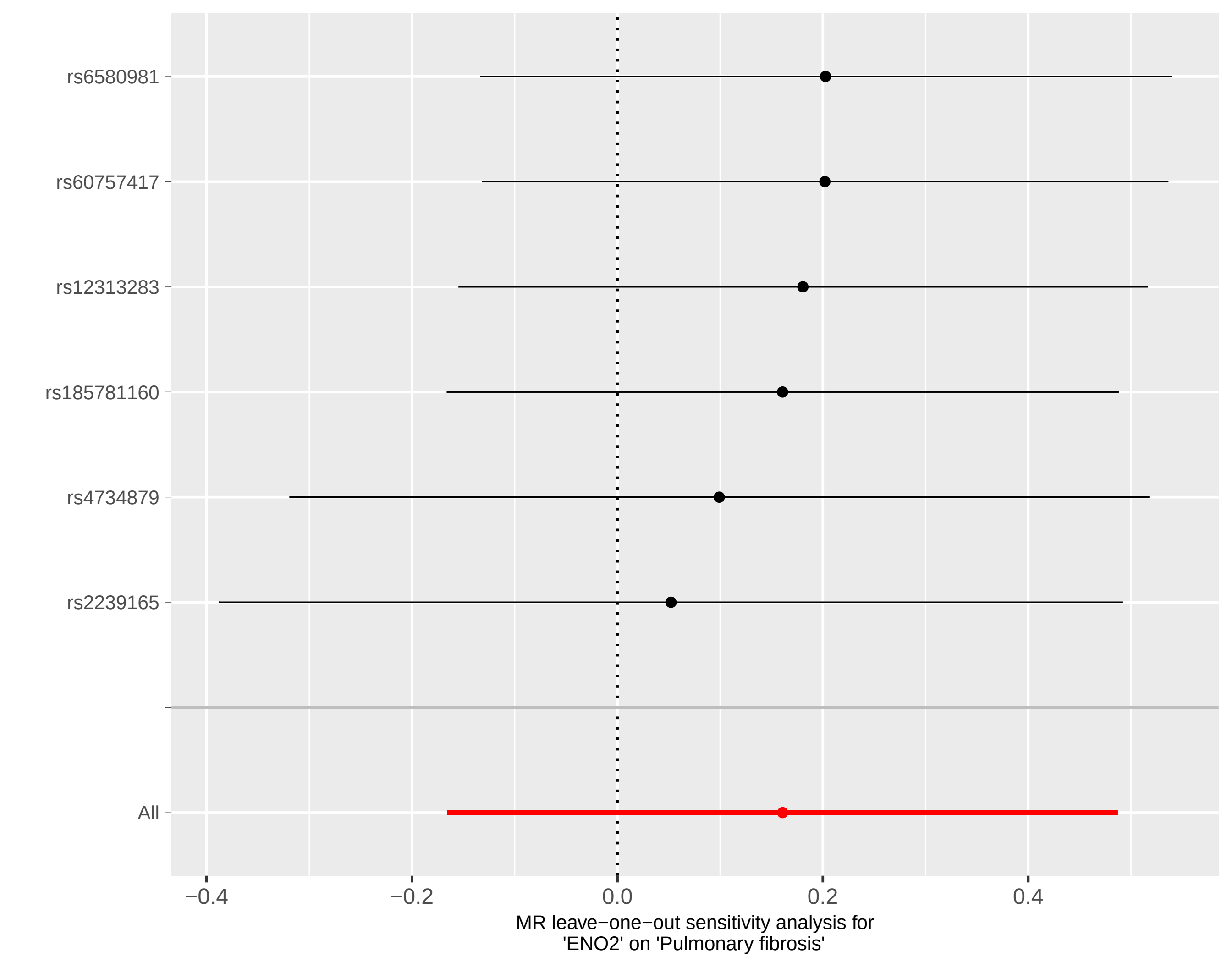
rs6580981
rs60757417
rs12313283
rs185781160
rs4734879
rs2239165
All
−0.4
−0.2
0.0
0.2
0.4
MR leave−one−out sensitivity analysis for
'ENO2' on 'Pulmonary fibrosis'

Insufficient number of SNPs

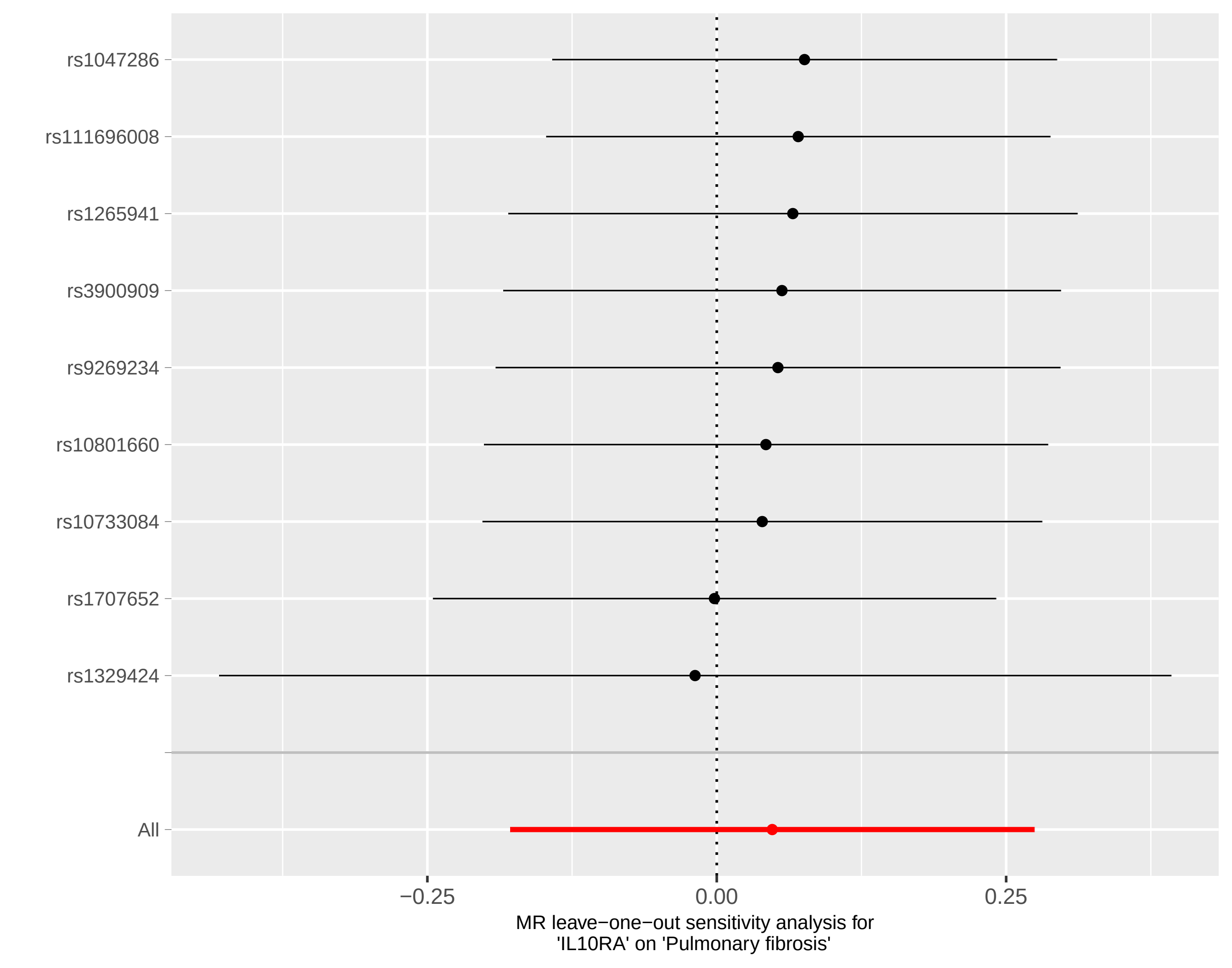
rs1047286
rs111696008
rs1265941
rs3900909
rs9269234
rs10801660
rs10733084
rs1707652
rs1329424
All
−0.25
0.00
0.25
MR leave−one−out sensitivity analysis for
'IL10RA' on 'Pulmonary fibrosis'

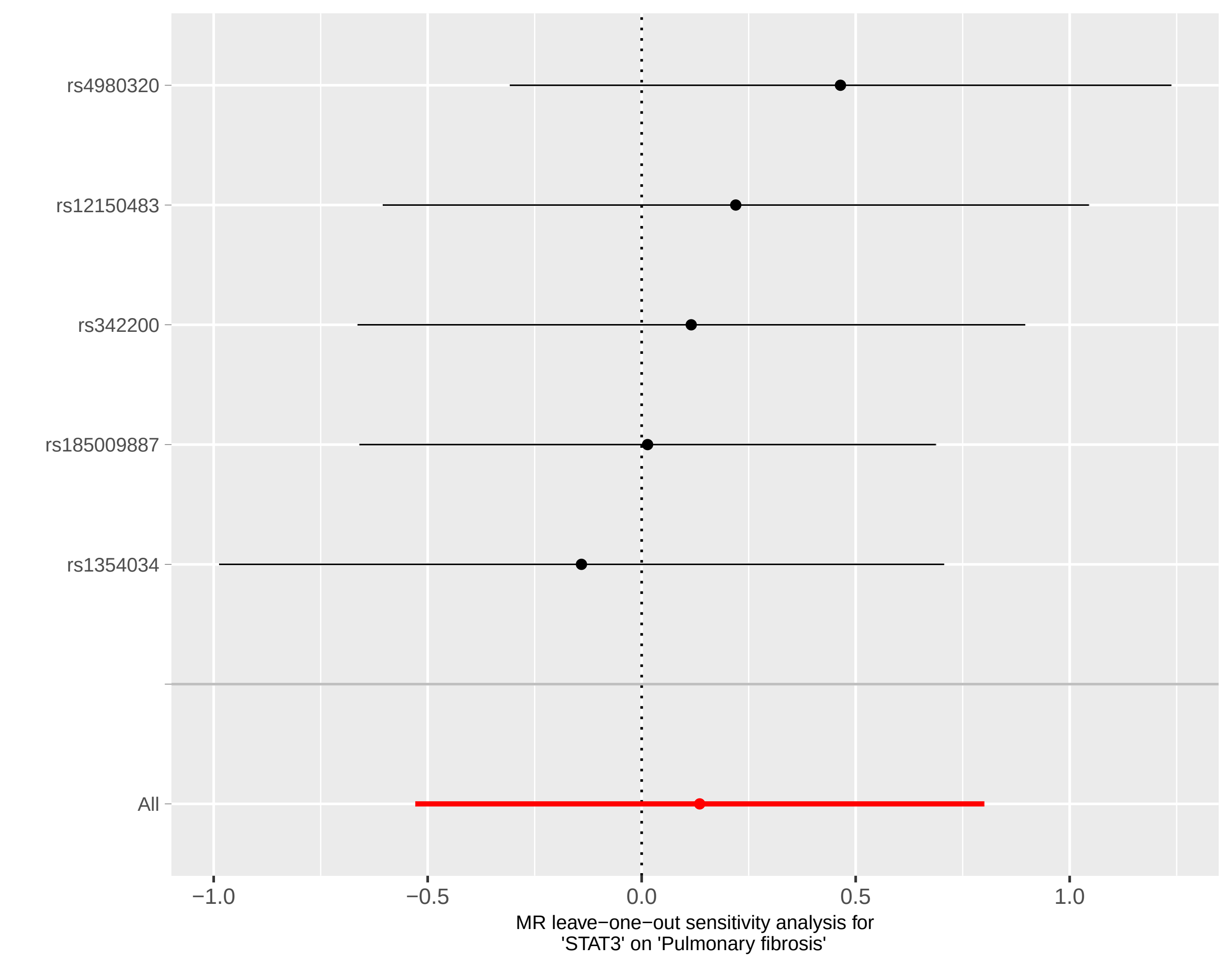
rs4980320
rs12150483
rs342200
rs185009887
rs1354034
All
−1.0
−0.5
0.0
0.5
1.0
MR leave−one−out sensitivity analysis for
'STAT3' on 'Pulmonary fibrosis'

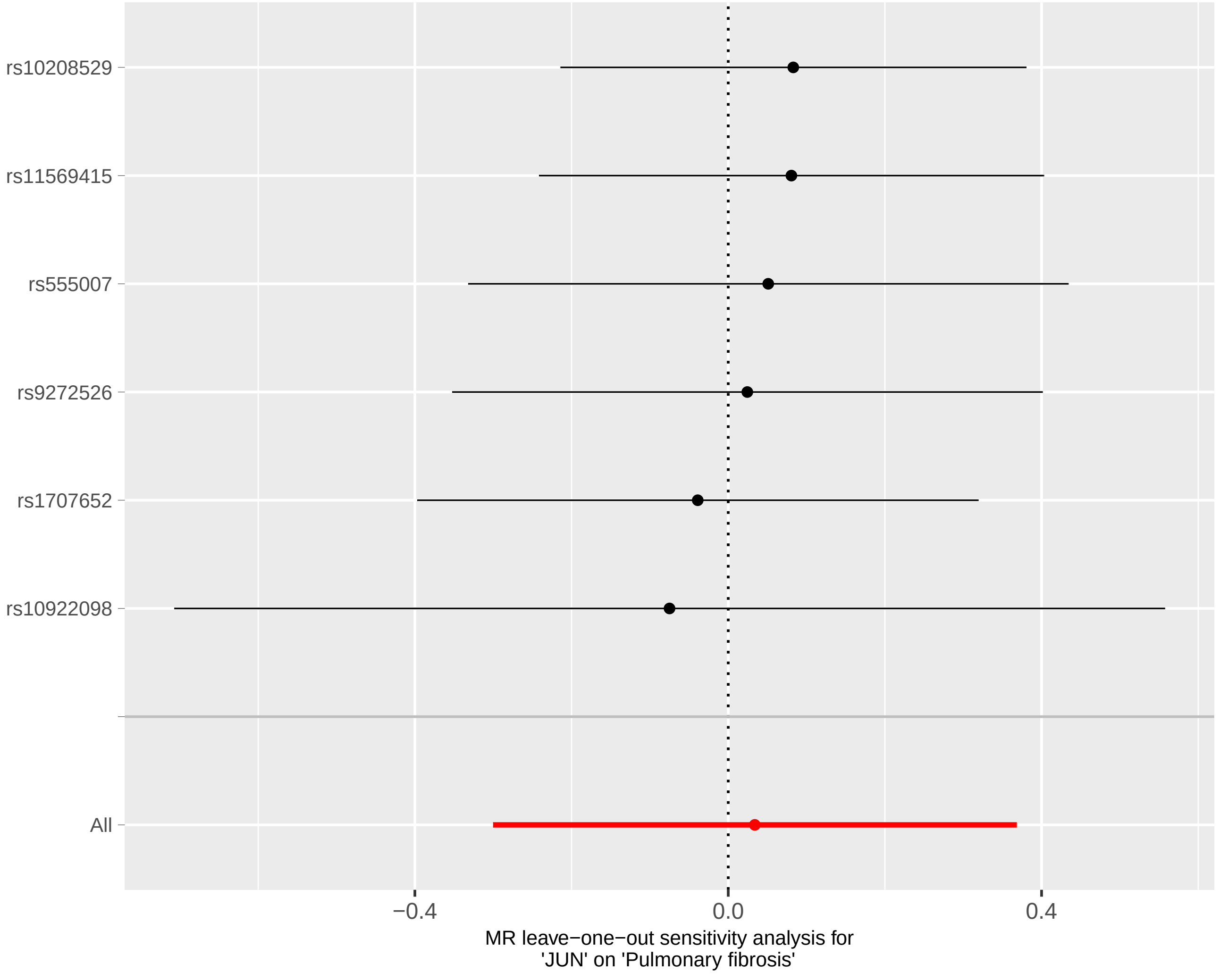

rs10208529
rs11569415
rs555007
rs9272526
rs1707652
rs10922098
All
−0.4
0.0
0.4
MR leave−one−out sensitivity analysis for
'JUN' on 'Pulmonary fibrosis'

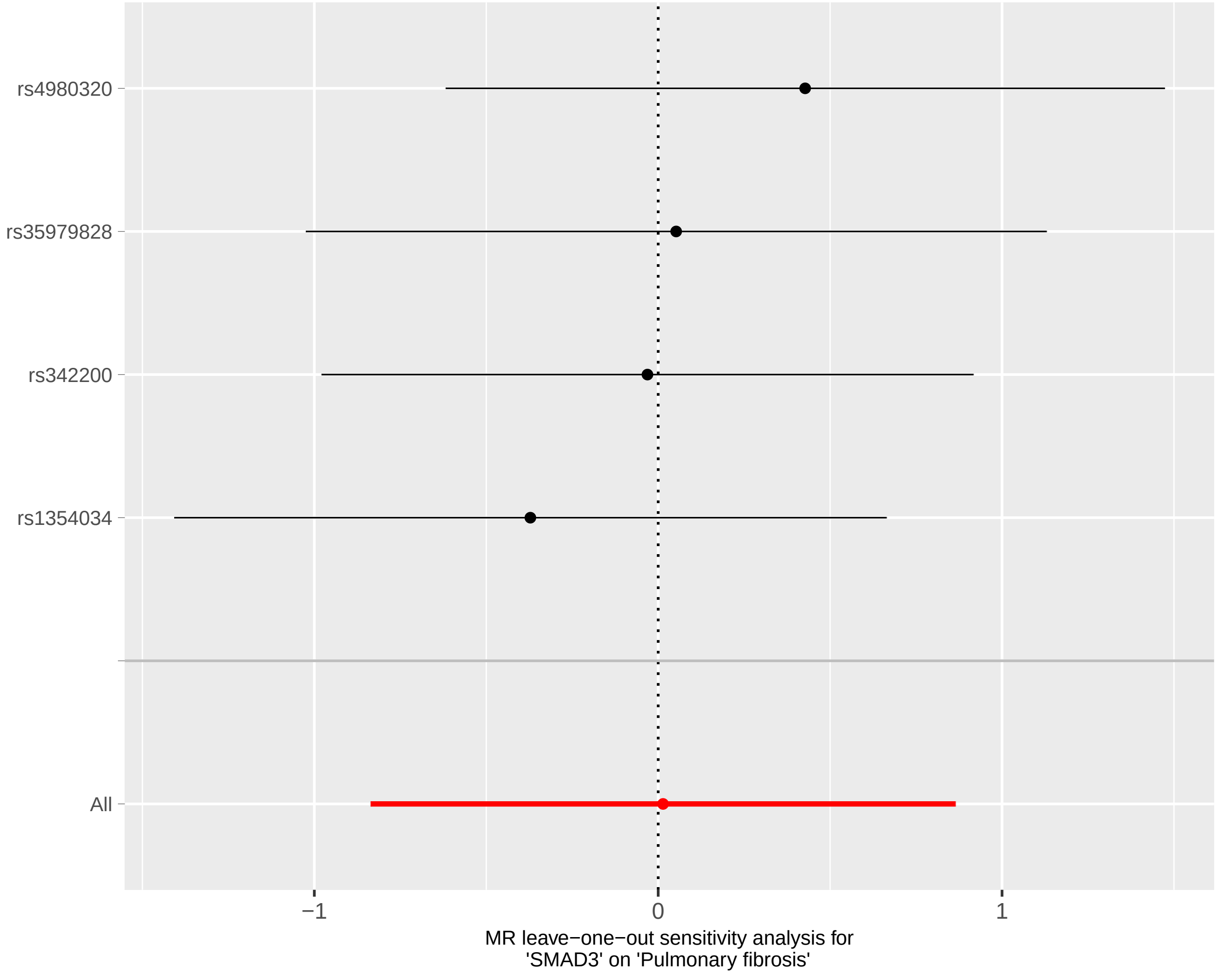
rs4980320
rs35979828
rs342200
rs1354034
All
−1
0
1
MR leave−one−out sensitivity analysis for
'SMAD3' on 'Pulmonary fibrosis'

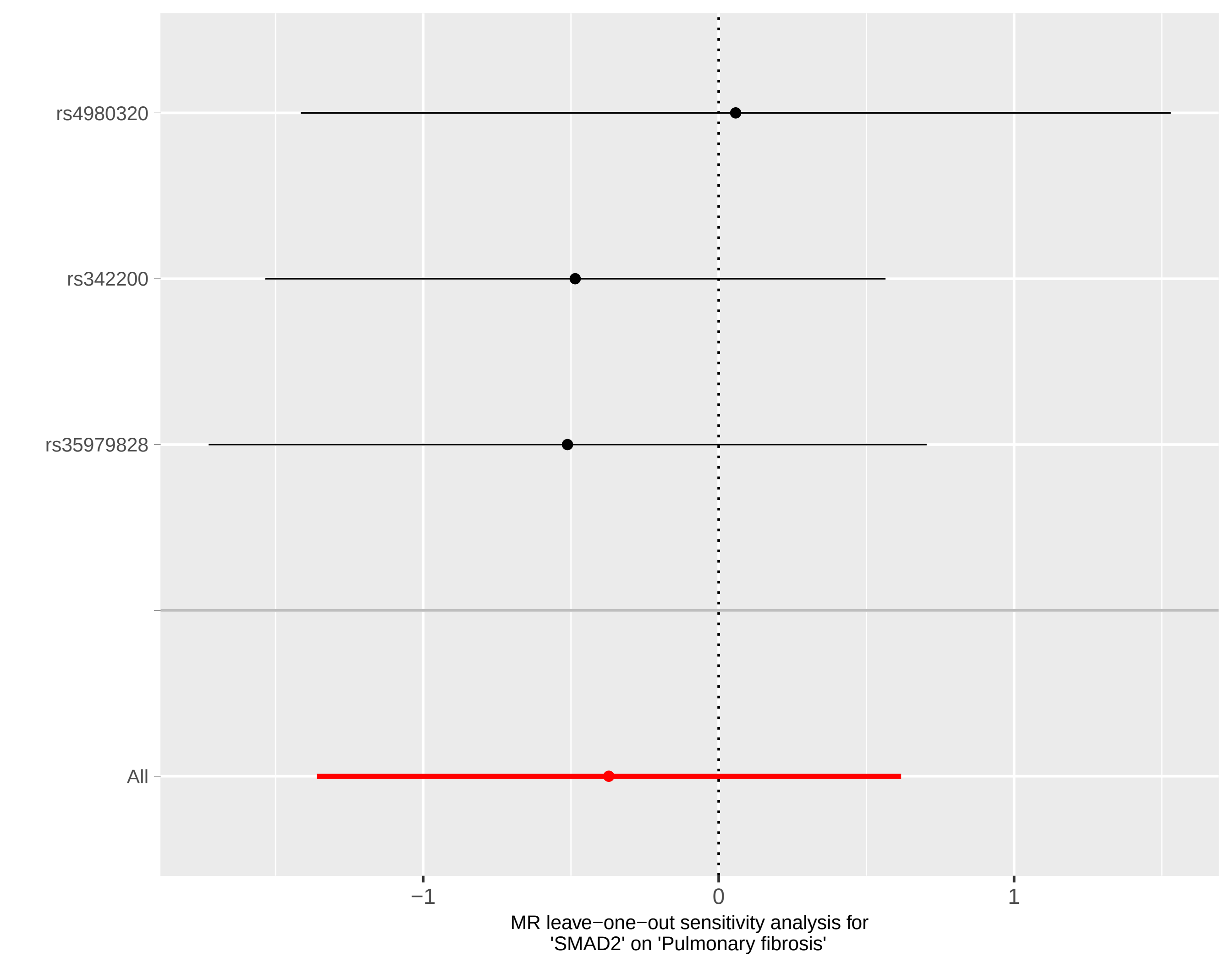
rs4980320
rs342200
rs35979828
All
−1
0
1
MR leave−one−out sensitivity analysis for
'SMAD2' on 'Pulmonary fibrosis'

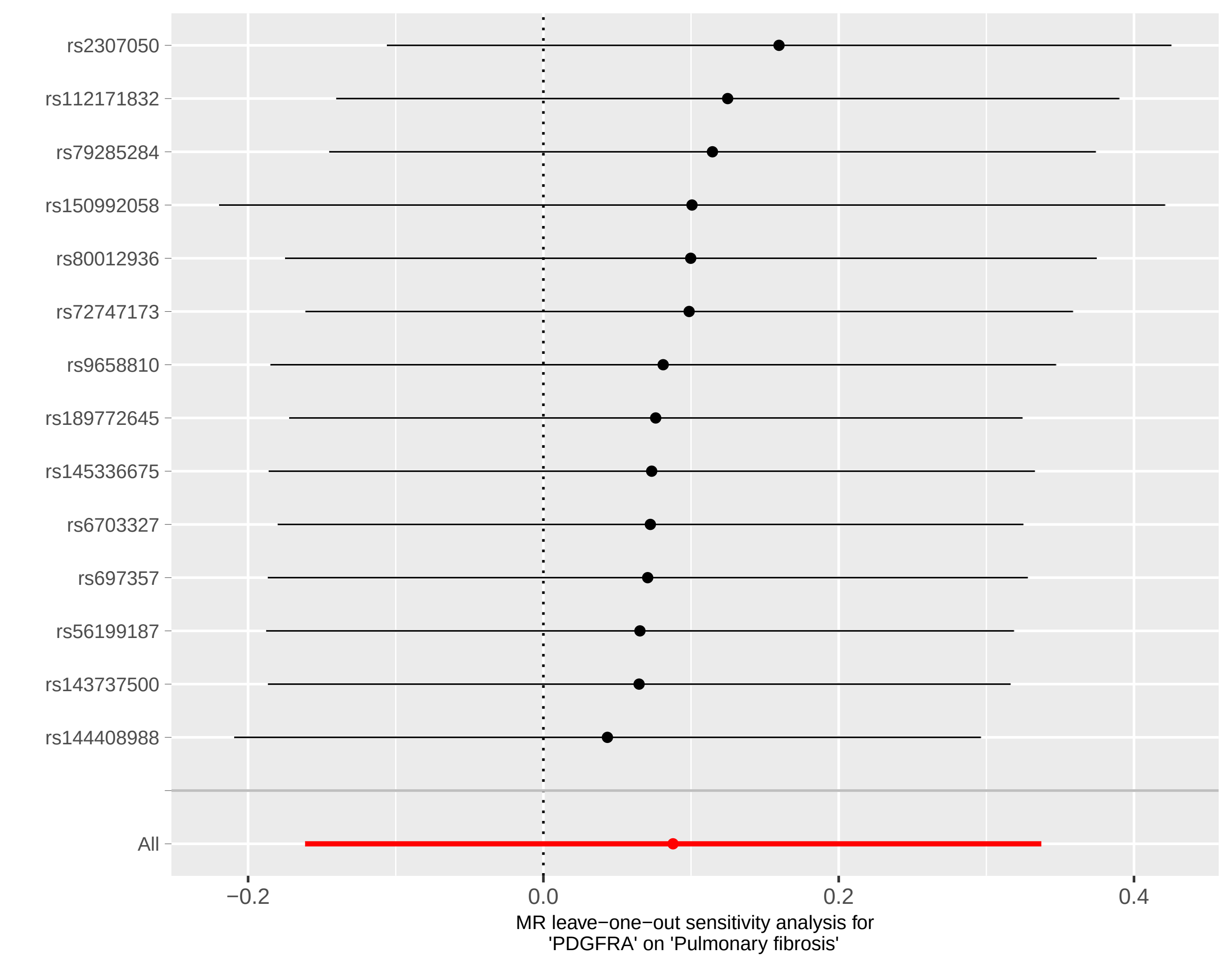
rs2307050
rs112171832
rs79285284
rs150992058
rs80012936
rs72747173
rs9658810
rs189772645
rs145336675
rs6703327
rs697357
rs56199187
rs143737500
rs144408988
All
−0.2
0.0
0.2
0.4
MR leave−one−out sensitivity analysis for
'PDGFRA' on 'Pulmonary fibrosis'

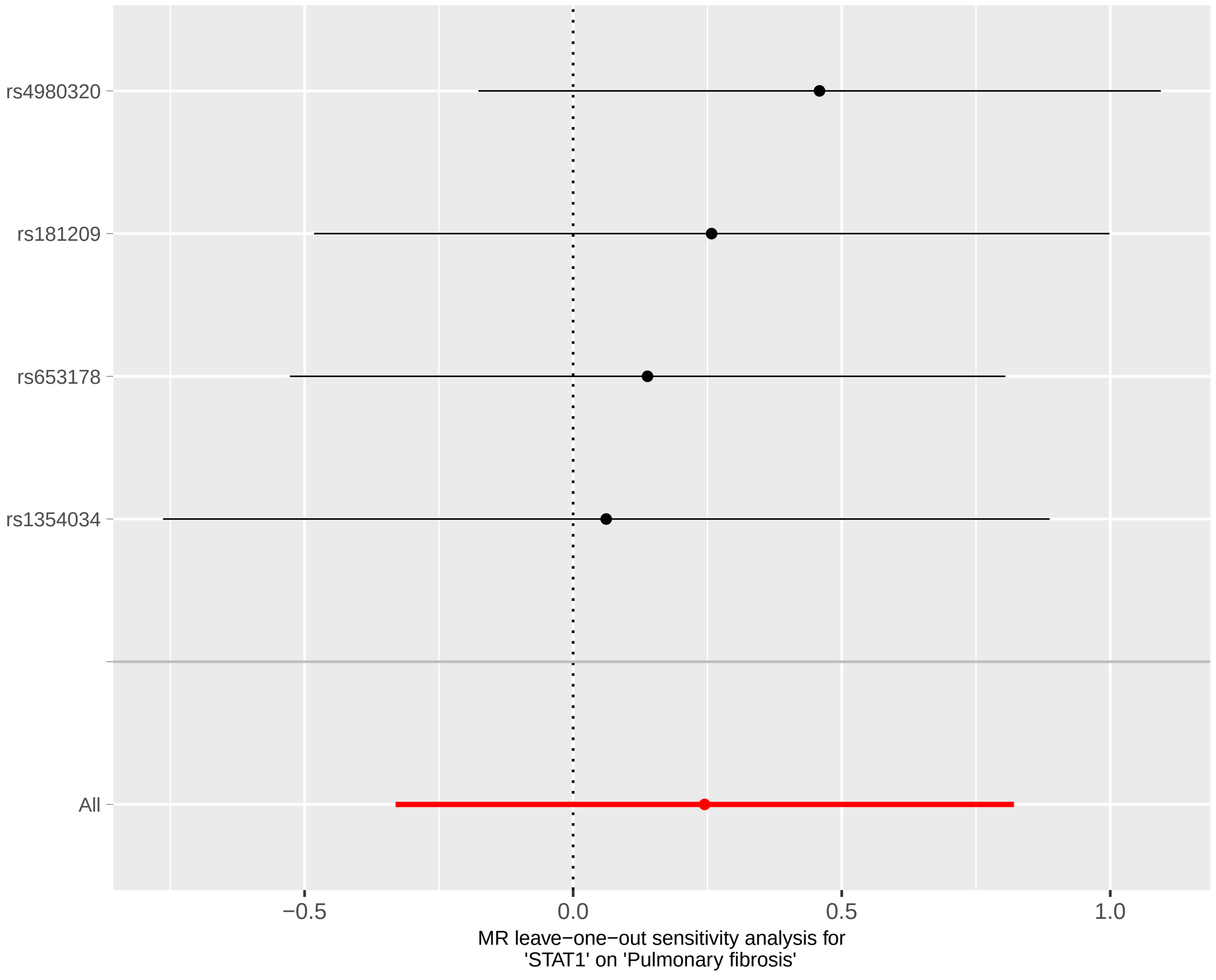

rs4980320
rs181209
rs653178
rs1354034
All
−0.5
0.0
0.5
1.0
MR leave−one−out sensitivity analysis for
'STAT1' on 'Pulmonary fibrosis'

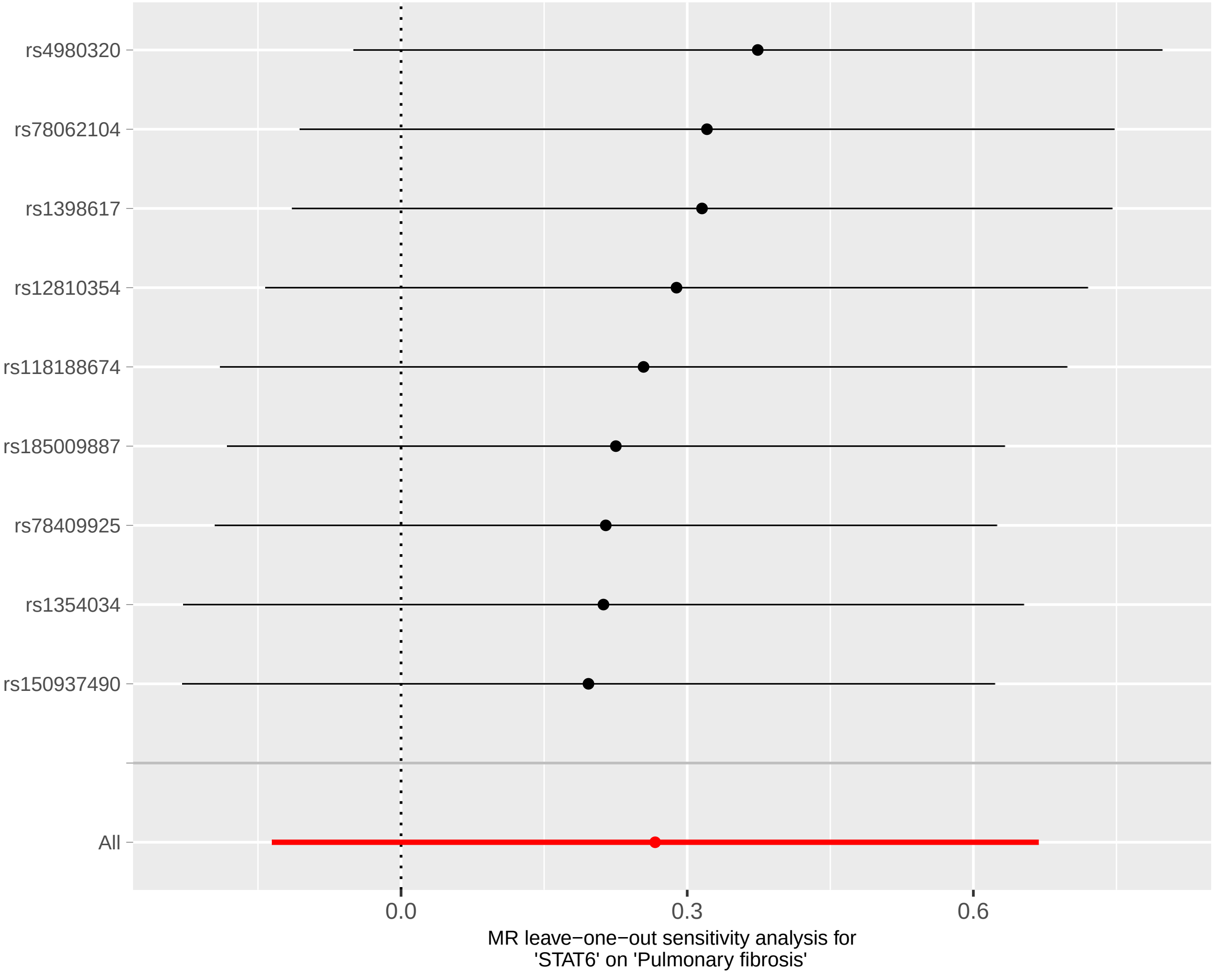
rs4980320
rs78062104
rs1398617
rs12810354
rs118188674
rs185009887
rs78409925
rs1354034
rs150937490
All
0.0
0.3
0.6
MR leave−one−out sensitivity analysis for
'STAT6' on 'Pulmonary fibrosis'

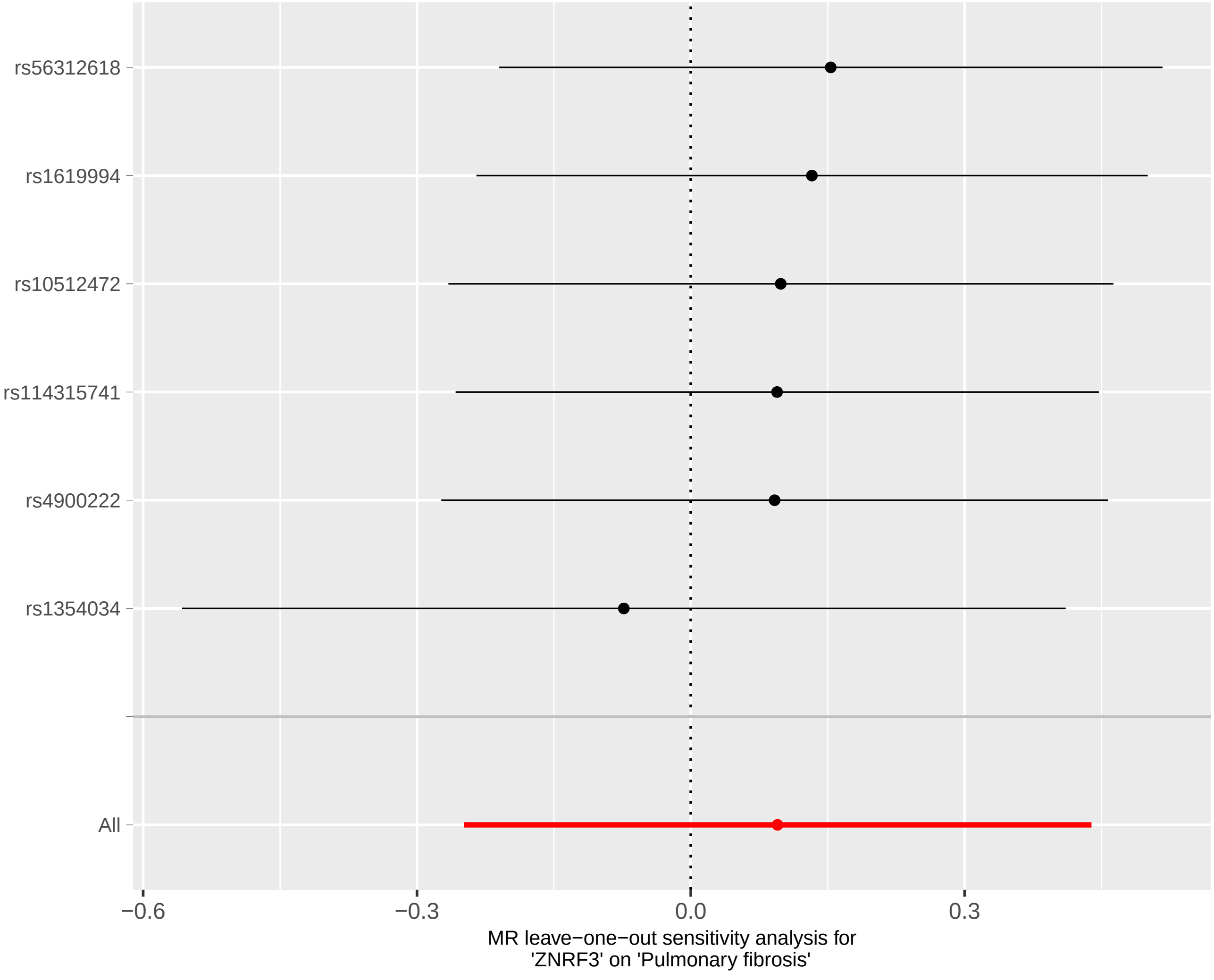
rs56312618
rs1619994
rs10512472
rs114315741
rs4900222
rs1354034
All
−0.6
−0.3
0.0
0.3
MR leave−one−out sensitivity analysis for
'ZNRF3' on 'Pulmonary fibrosis'

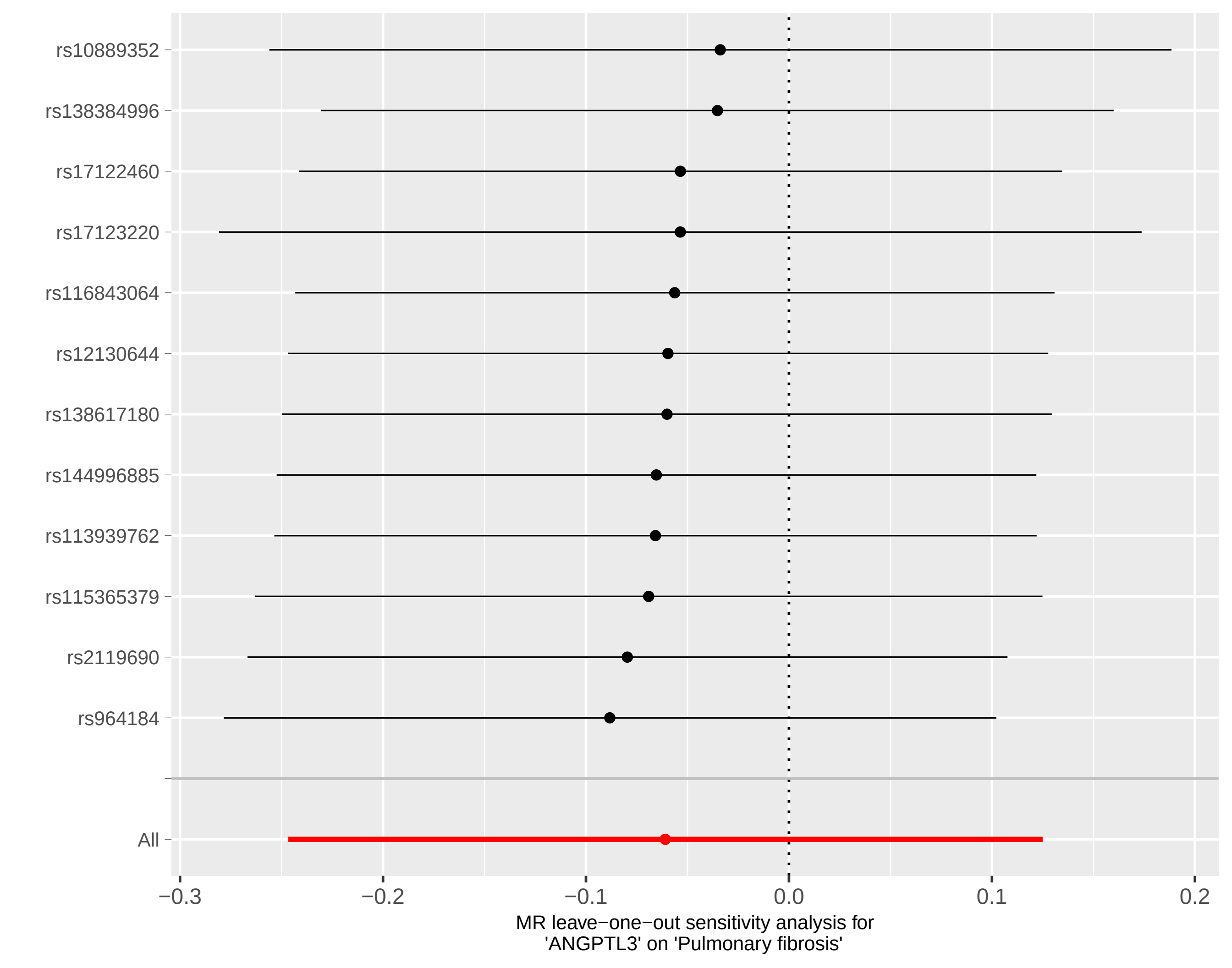
rs10889352
rs138384996
rs17122460
rs17123220
rs116843064
rs12130644
rs138617180
rs144996885
rs113939762
rs115365379
rs2119690
rs964184
All
−0.3
−0.2
−0.1
0.0
0.1
0.2
MR leave−one−out sensitivity analysis for
'ANGPTL3' on 'Pulmonary fibrosis'

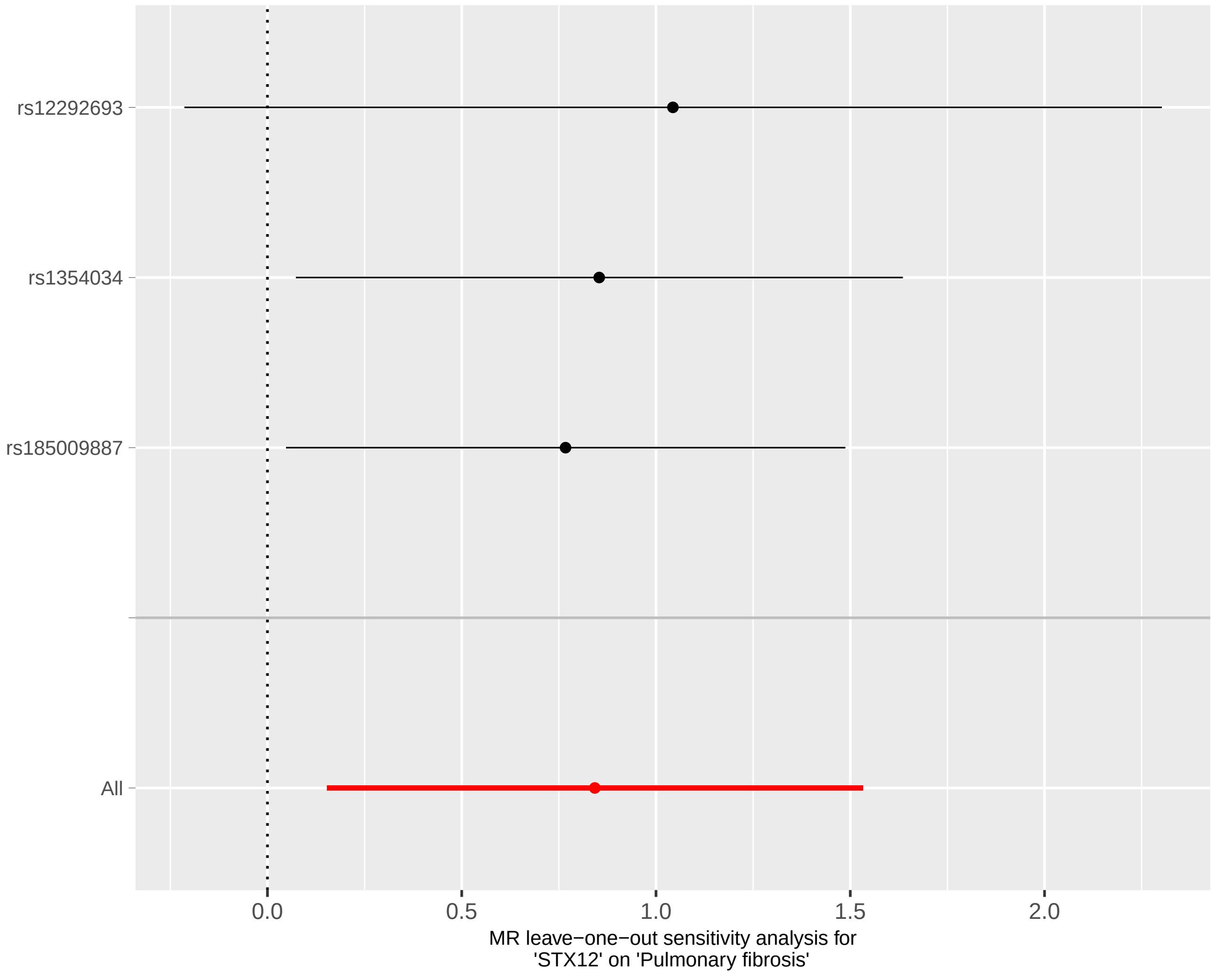
rs12292693
rs1354034
rs185009887
All
0.0
0.5
1.0
1.5
2.0
MR leave−one−out sensitivity analysis for
'STX12' on 'Pulmonary fibrosis'

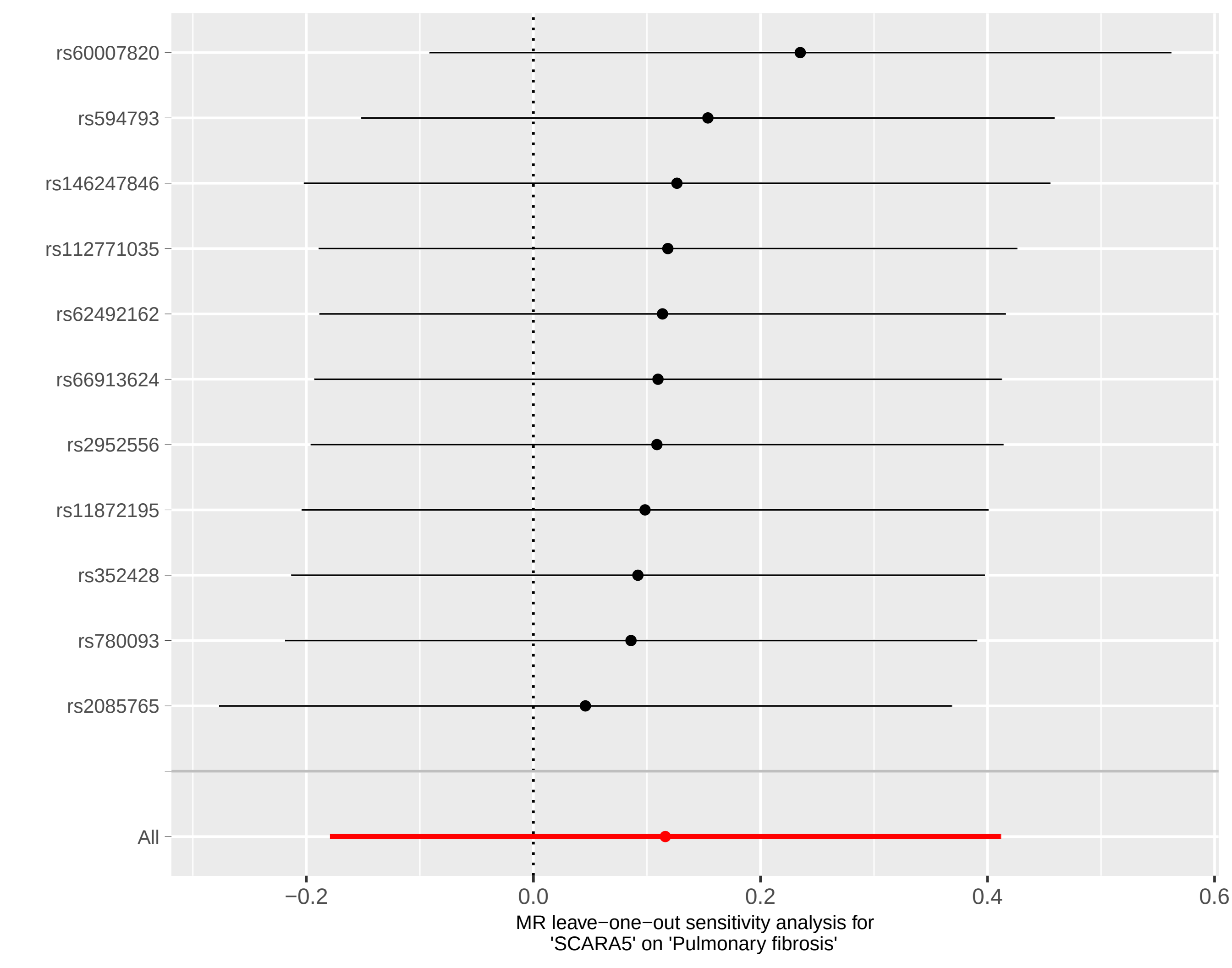
rs60007820
rs594793
rs146247846
rs112771035
rs62492162
rs66913624
rs2952556
rs11872195
rs352428
rs780093
rs2085765
All
−0.2
0.0
0.2
0.4
0.6
MR leave−one−out sensitivity analysis for
'SCARA5' on 'Pulmonary fibrosis'

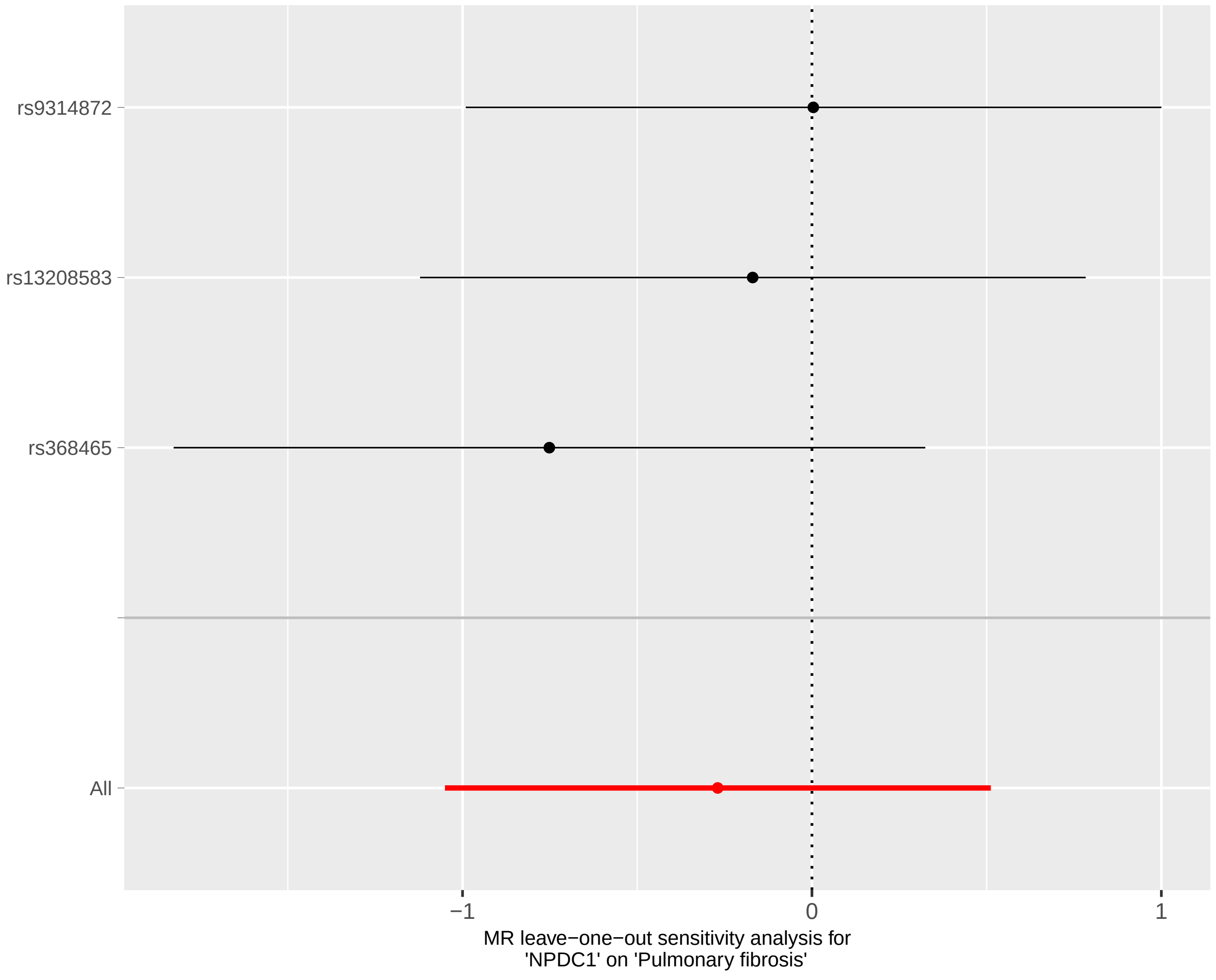
rs9314872
rs13208583
rs368465
All
−1
0
1
MR leave−one−out sensitivity analysis for
'NPDC1' on 'Pulmonary fibrosis'

Insufficient number of SNPs

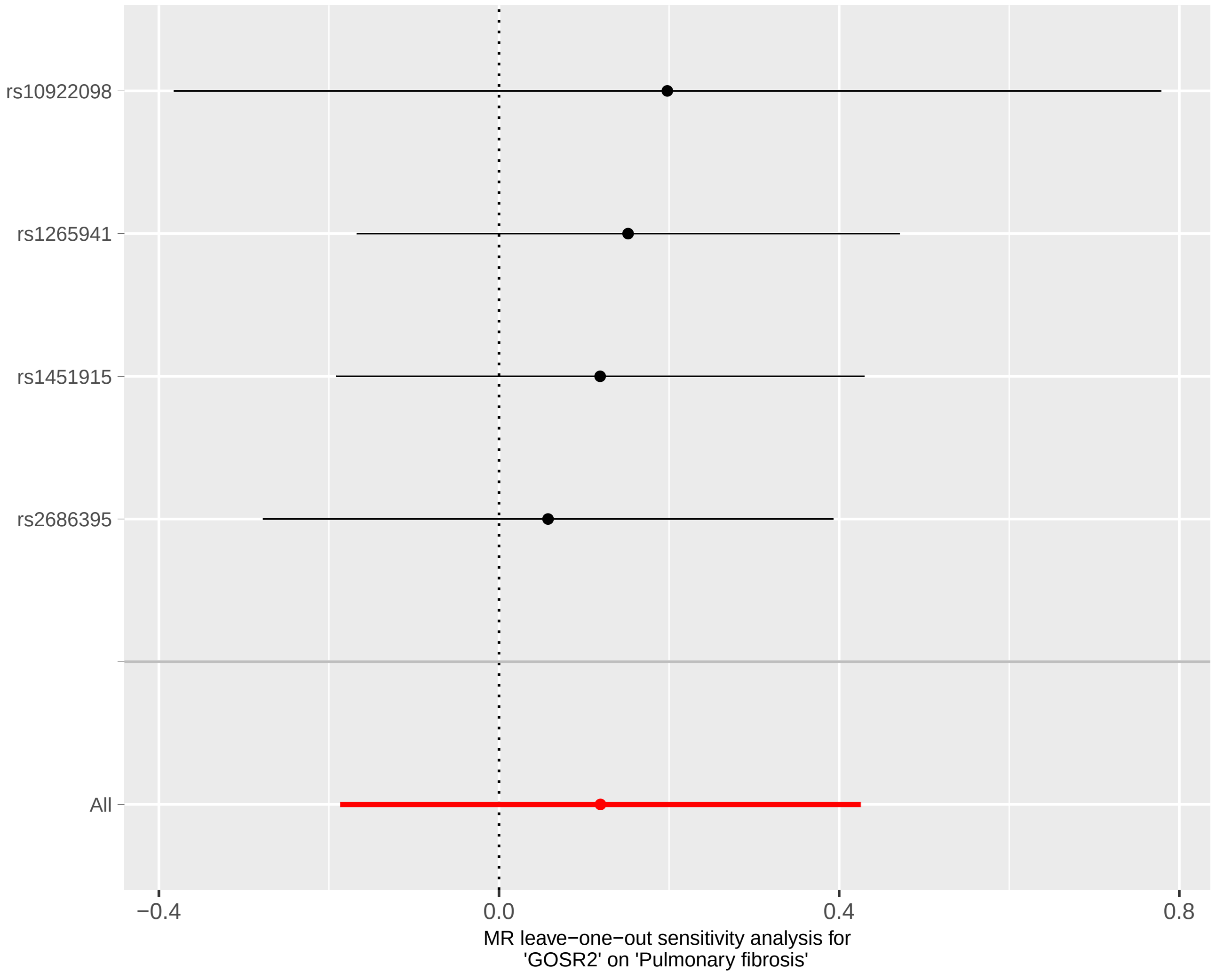
rs10922098
rs1265941
rs1451915
rs2686395
All
−0.4
0.0
0.4
0.8
MR leave−one−out sensitivity analysis for
'GOSR2' on 'Pulmonary fibrosis'

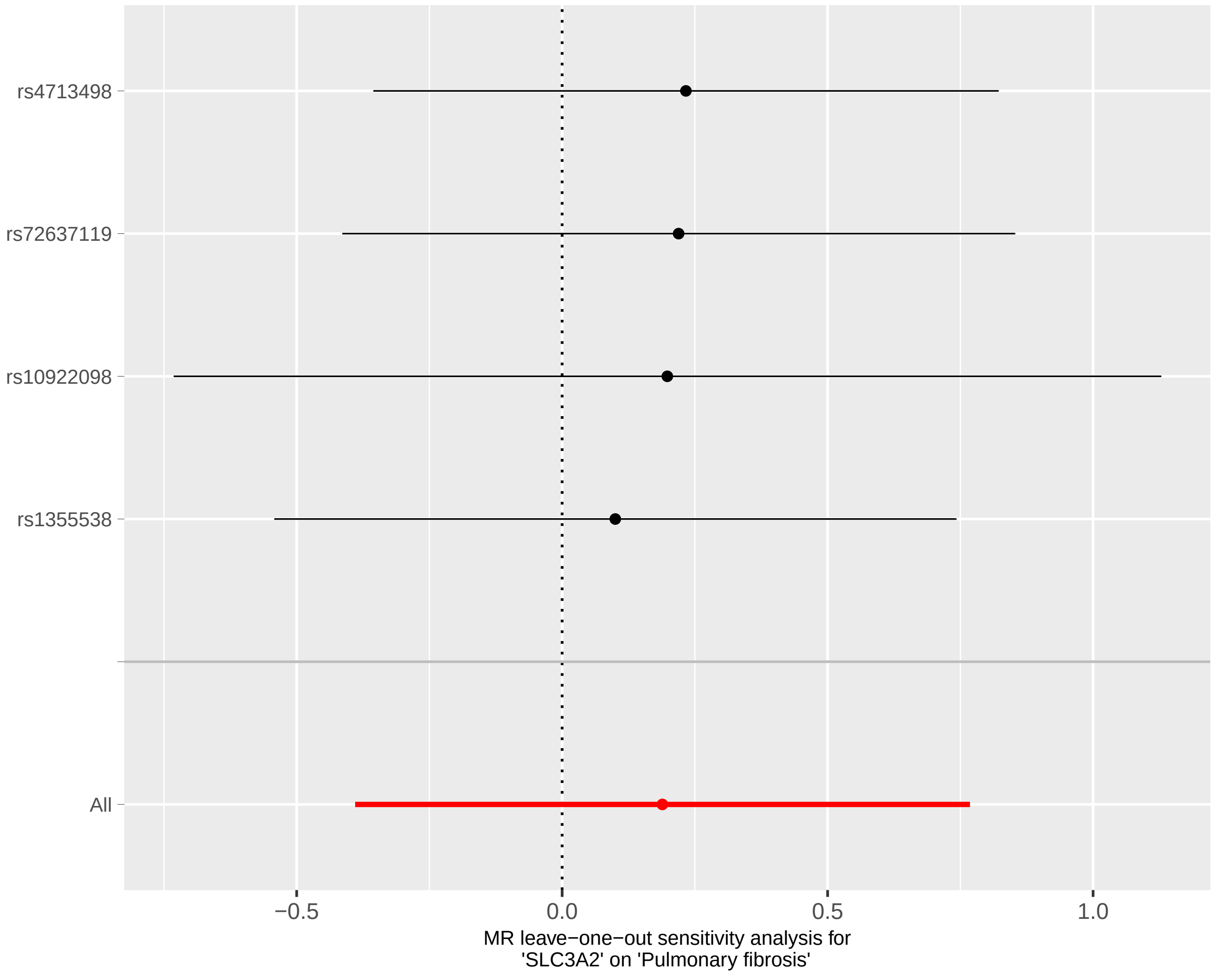
rs4713498
rs72637119
rs10922098
rs1355538
All
−0.5
0.0
0.5
1.0
MR leave−one−out sensitivity analysis for
'SLC3A2' on 'Pulmonary fibrosis'

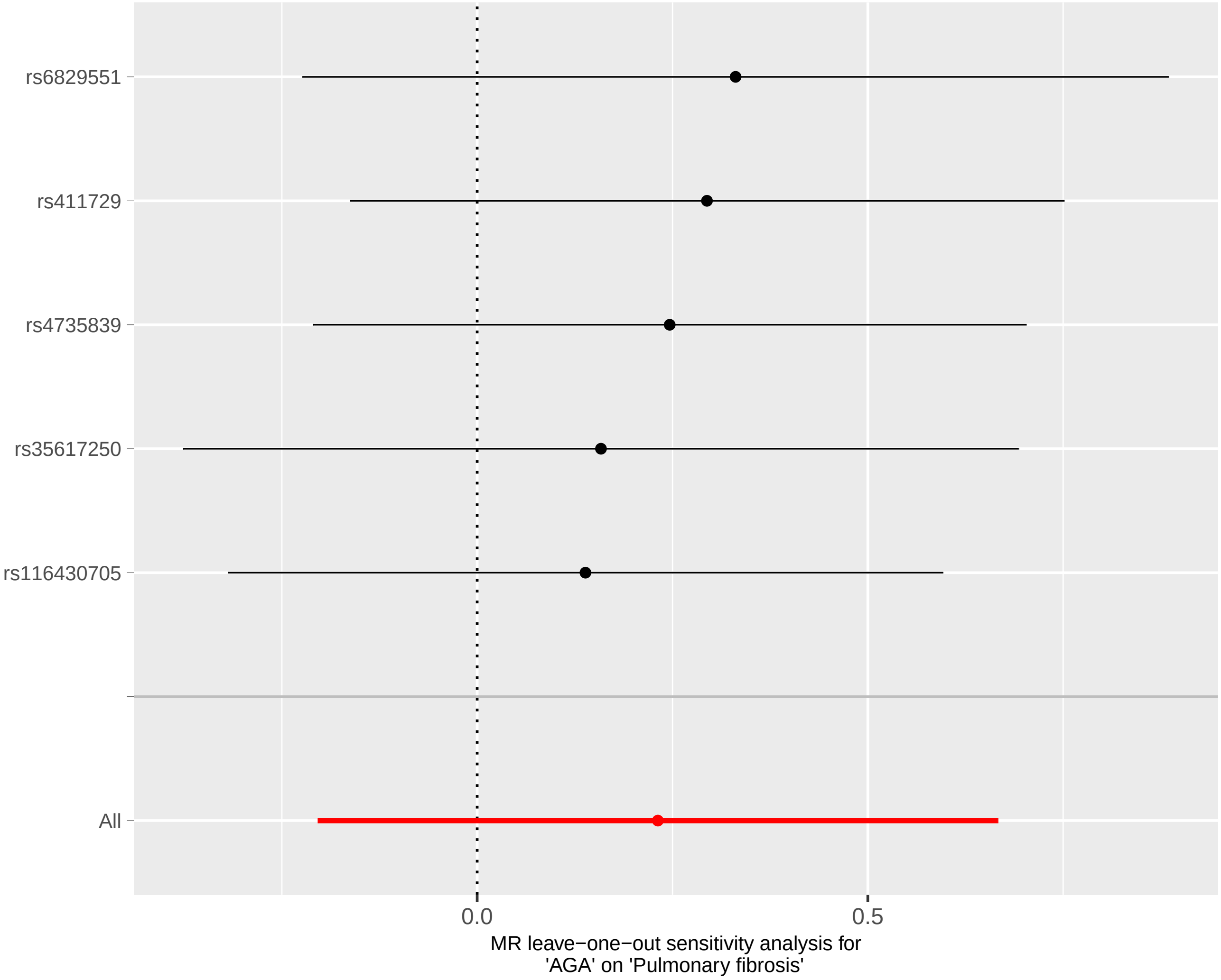

rs6829551
rs411729
rs4735839
rs35617250
rs116430705
All
0.0
0.5
MR leave−one−out sensitivity analysis for
'AGA' on 'Pulmonary fibrosis'

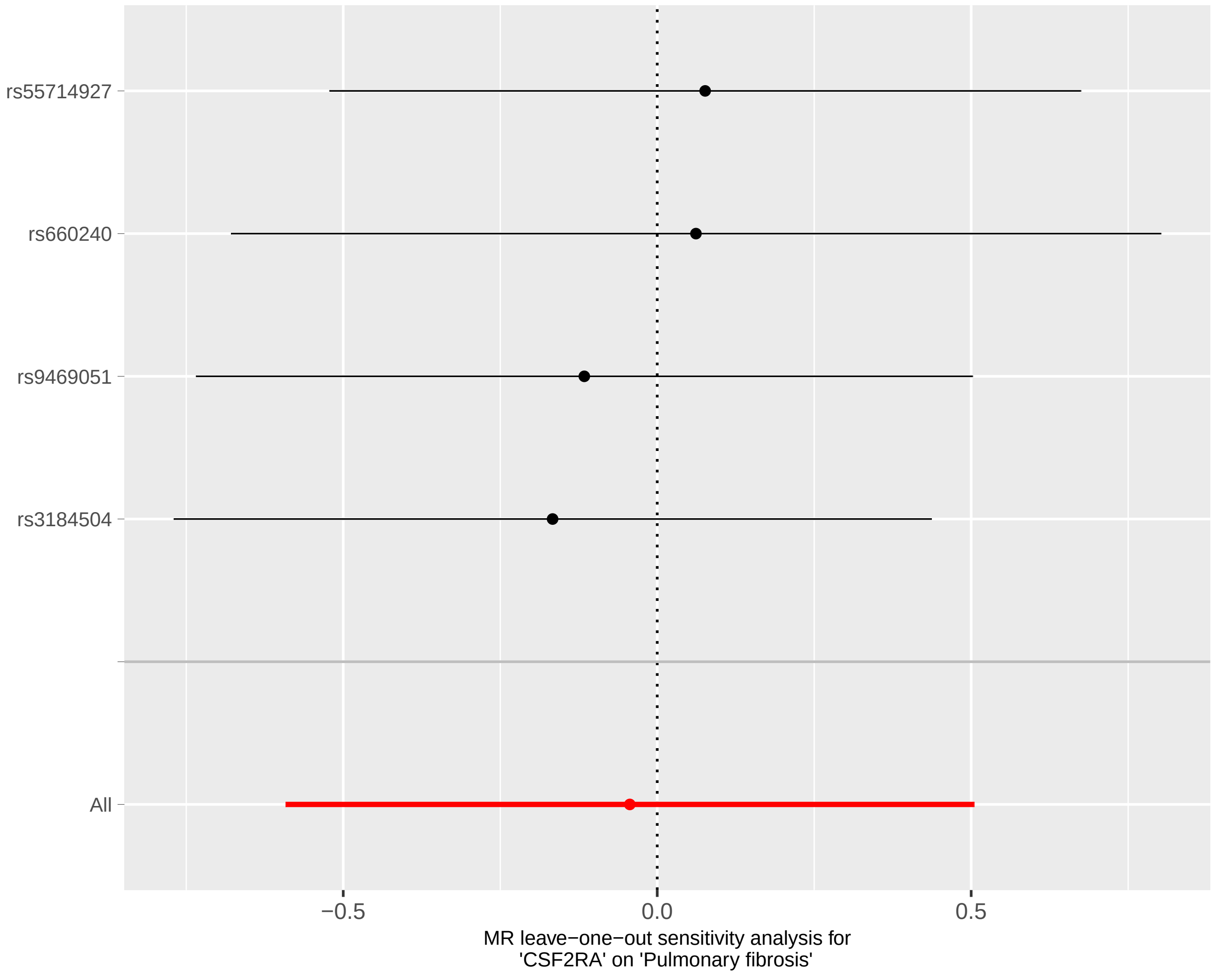
rs55714927
rs660240
rs9469051
rs3184504
All
−0.5
0.0
0.5
MR leave−one−out sensitivity analysis for
'CSF2RA' on 'Pulmonary fibrosis'

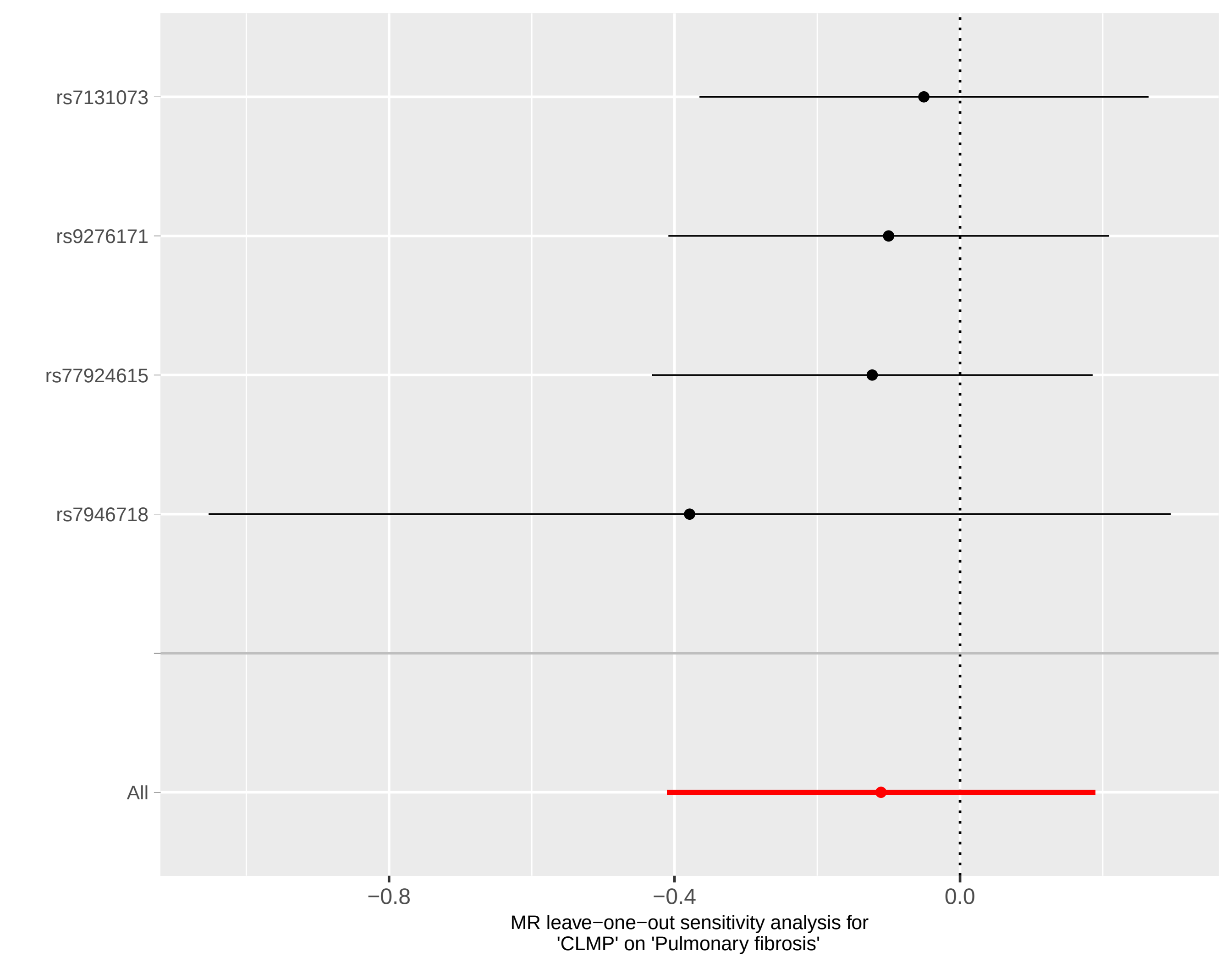
rs7131073
rs9276171
rs77924615
rs7946718
All
−0.8
−0.4
0.0
MR leave−one−out sensitivity analysis for
'CLMP' on 'Pulmonary fibrosis'

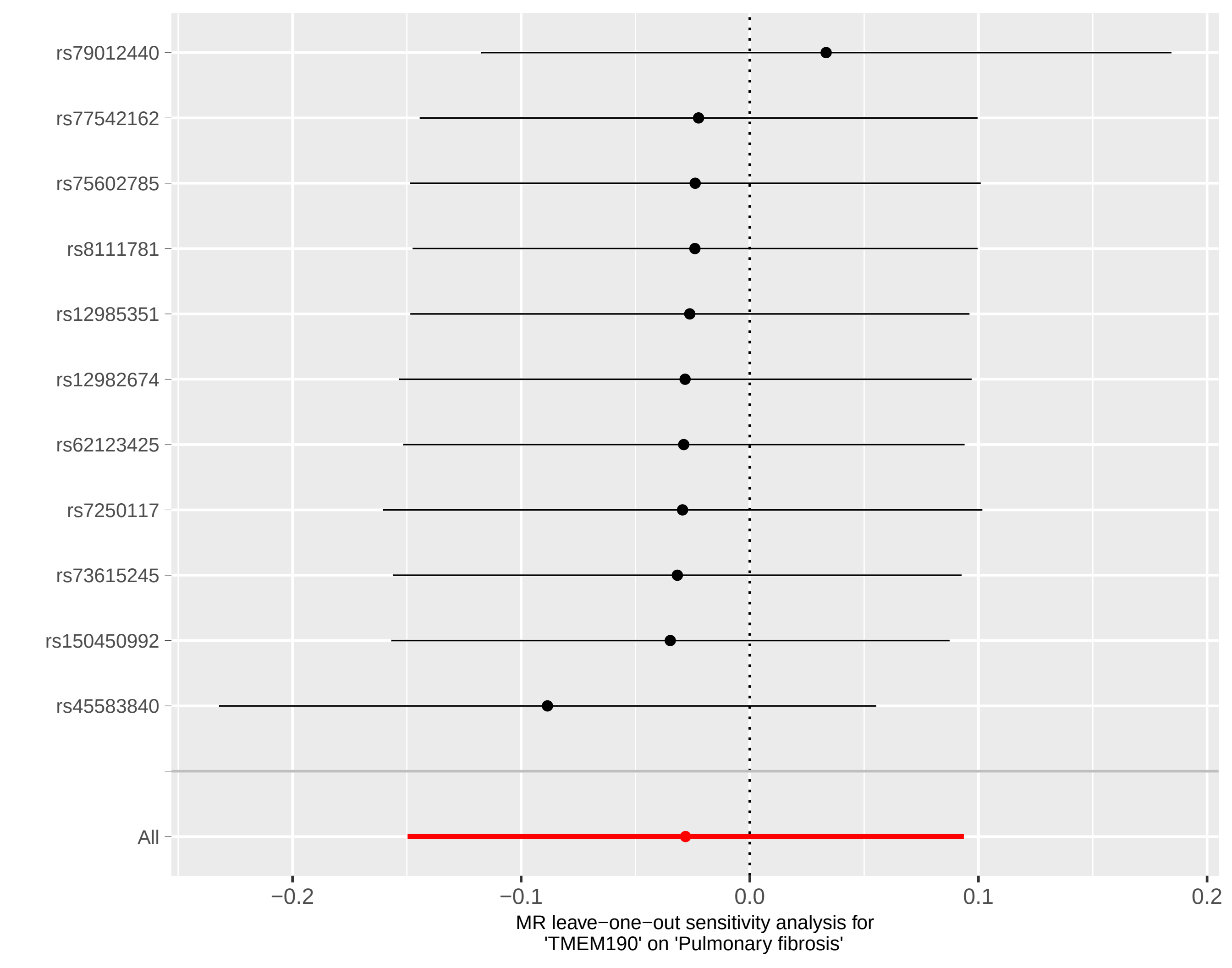
rs79012440
rs77542162
rs75602785
rs8111781
rs12985351
rs12982674
rs62123425
rs7250117
rs73615245
rs150450992
rs45583840
All
−0.2
−0.1
0.0
0.1
0.2
MR leave−one−out sensitivity analysis for
'TMEM190' on 'Pulmonary fibrosis'

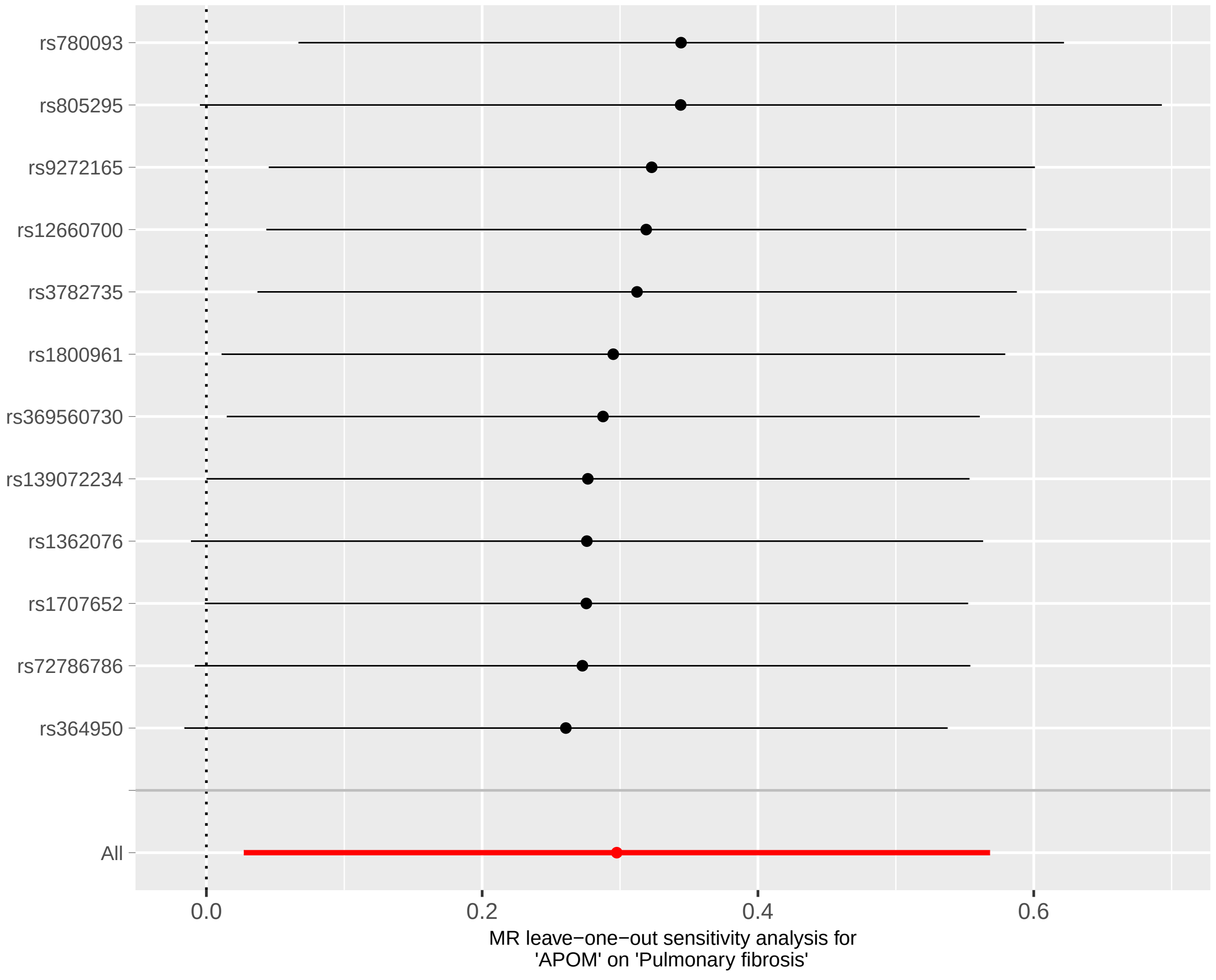
rs780093
rs805295
rs9272165
rs12660700
rs3782735
rs1800961
rs369560730
rs139072234
rs1362076
rs1707652
rs72786786
rs364950
All
0.0
0.2
0.4
0.6
MR leave−one−out sensitivity analysis for
'APOM' on 'Pulmonary fibrosis'

Insufficient number of SNPs

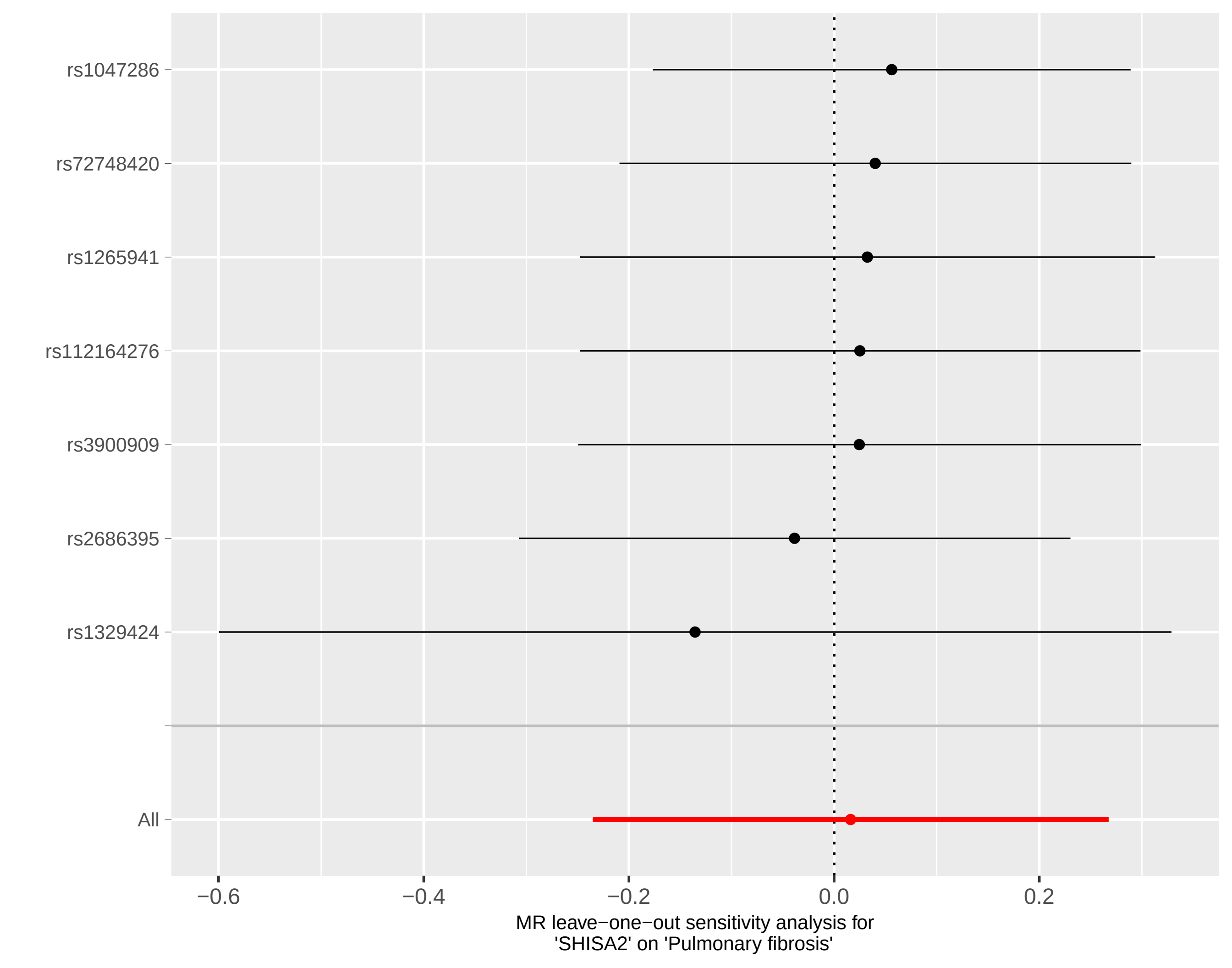

rs1047286
rs72748420
rs1265941
rs112164276
rs3900909
rs2686395
rs1329424
All
−0.6
−0.4
−0.2
0.0
0.2
MR leave−one−out sensitivity analysis for
'SHISA2' on 'Pulmonary fibrosis'

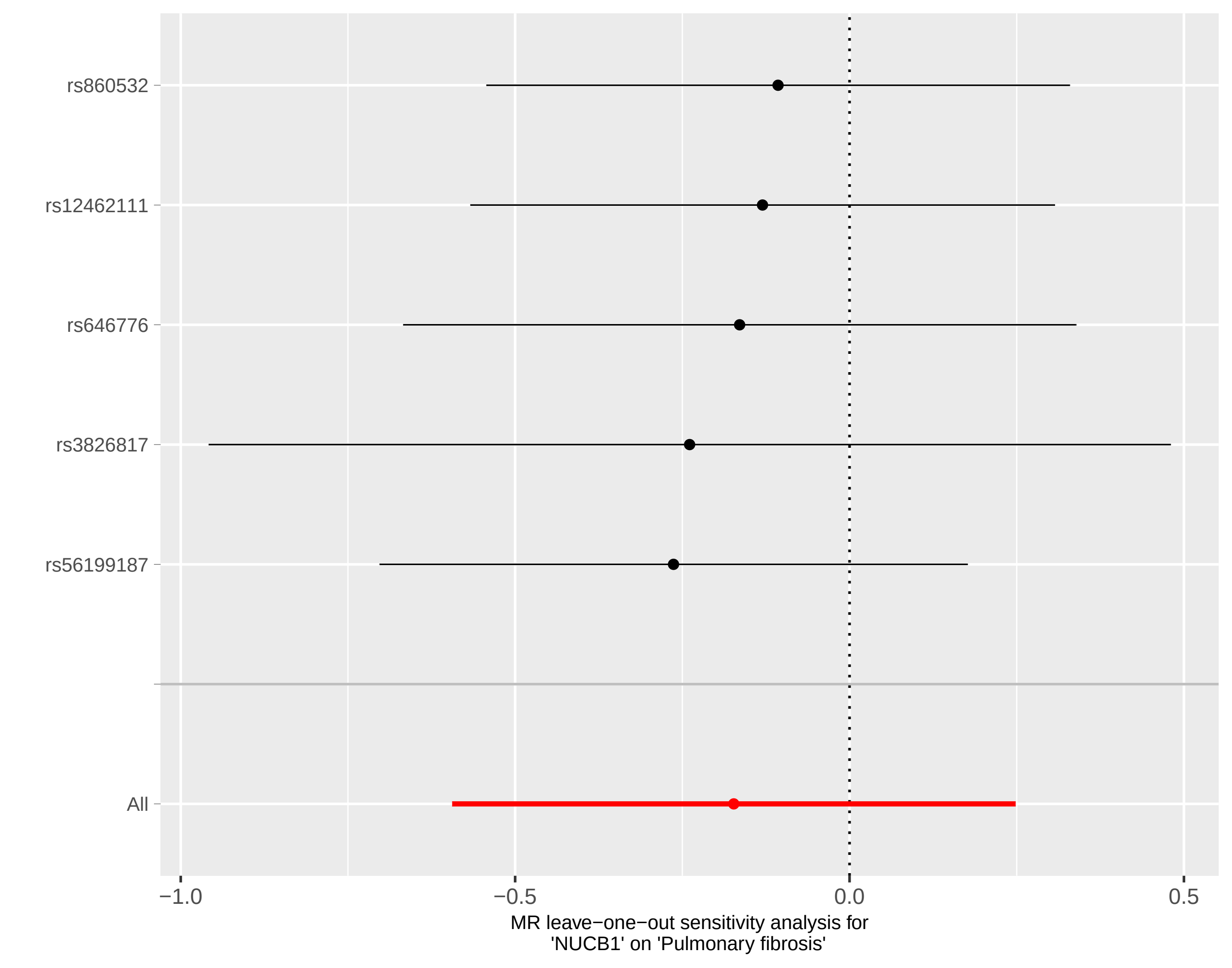
rs860532
rs12462111
rs646776
rs3826817
rs56199187
All
−1.0
−0.5
0.0
0.5
MR leave−one−out sensitivity analysis for
'NUCB1' on 'Pulmonary fibrosis'

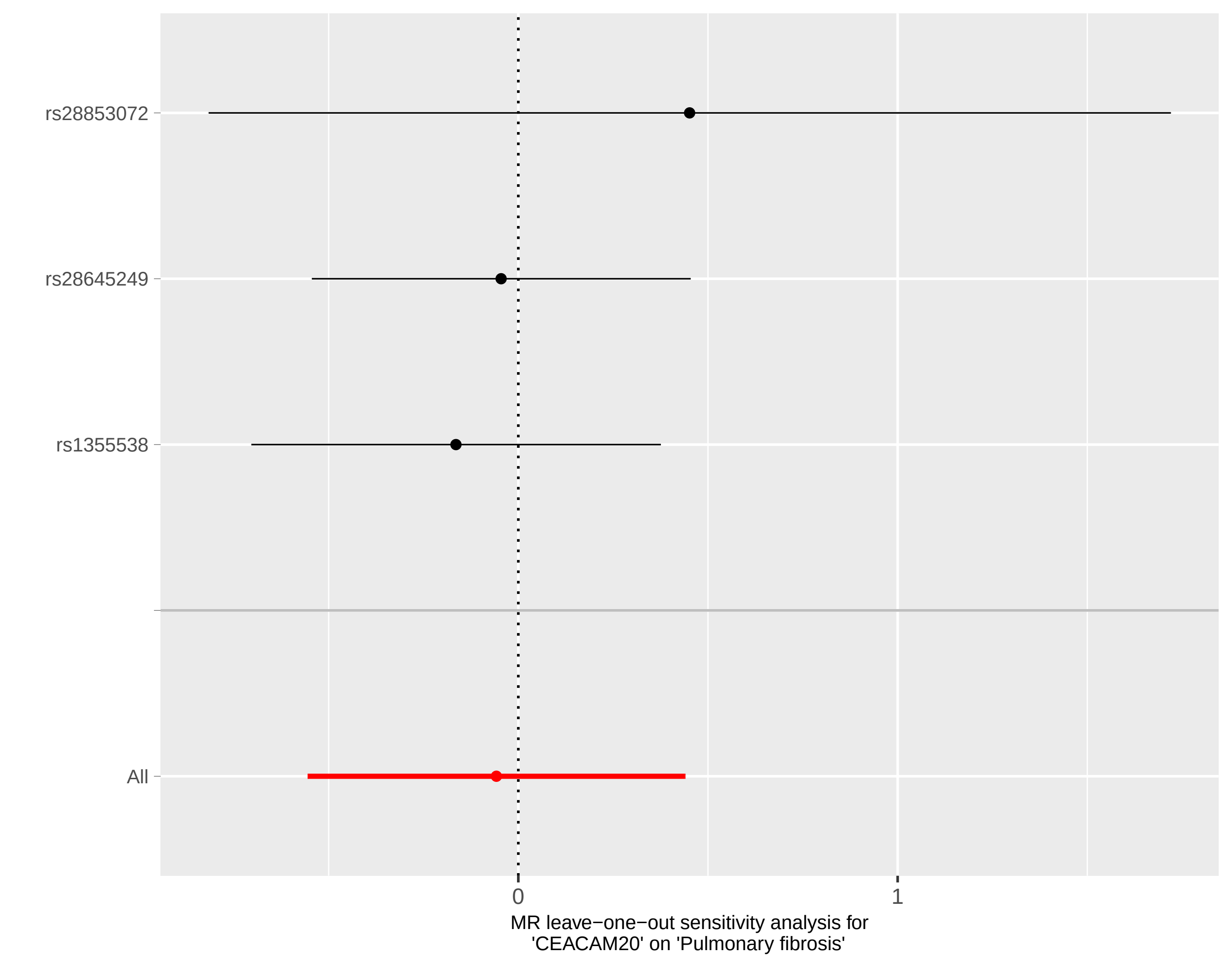
rs28853072
rs28645249
rs1355538
All
0
1
MR leave−one−out sensitivity analysis for
'CEACAM20' on 'Pulmonary fibrosis'

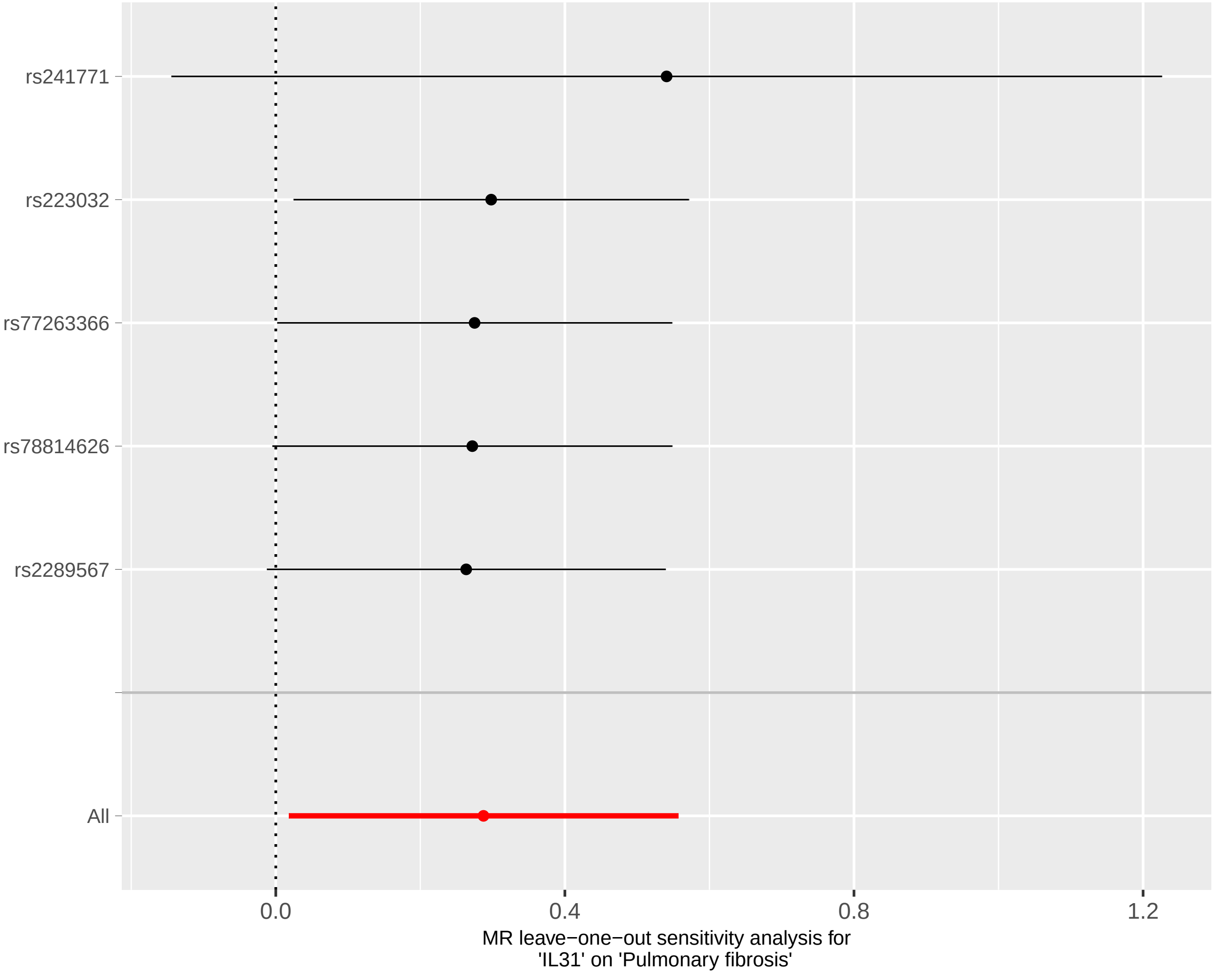

rs241771
rs223032
rs77263366
rs78814626
rs2289567
All
0.0
0.4
0.8
1.2
MR leave−one−out sensitivity analysis for
'IL31' on 'Pulmonary fibrosis'

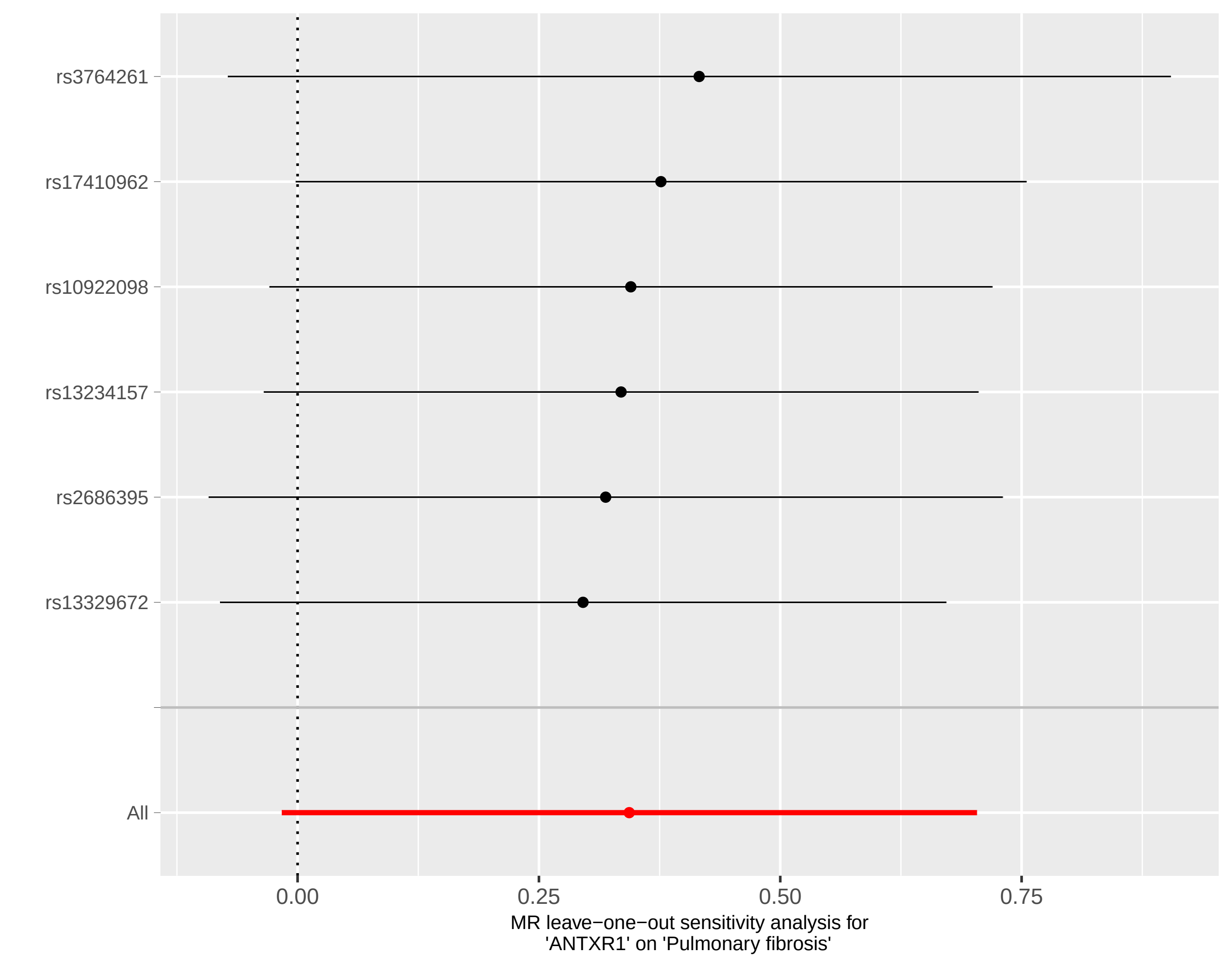

rs3764261
rs17410962
rs10922098
rs13234157
rs2686395
rs13329672
All
0.00
0.25
0.50
0.75
MR leave−one−out sensitivity analysis for
'ANTXR1' on 'Pulmonary fibrosis'

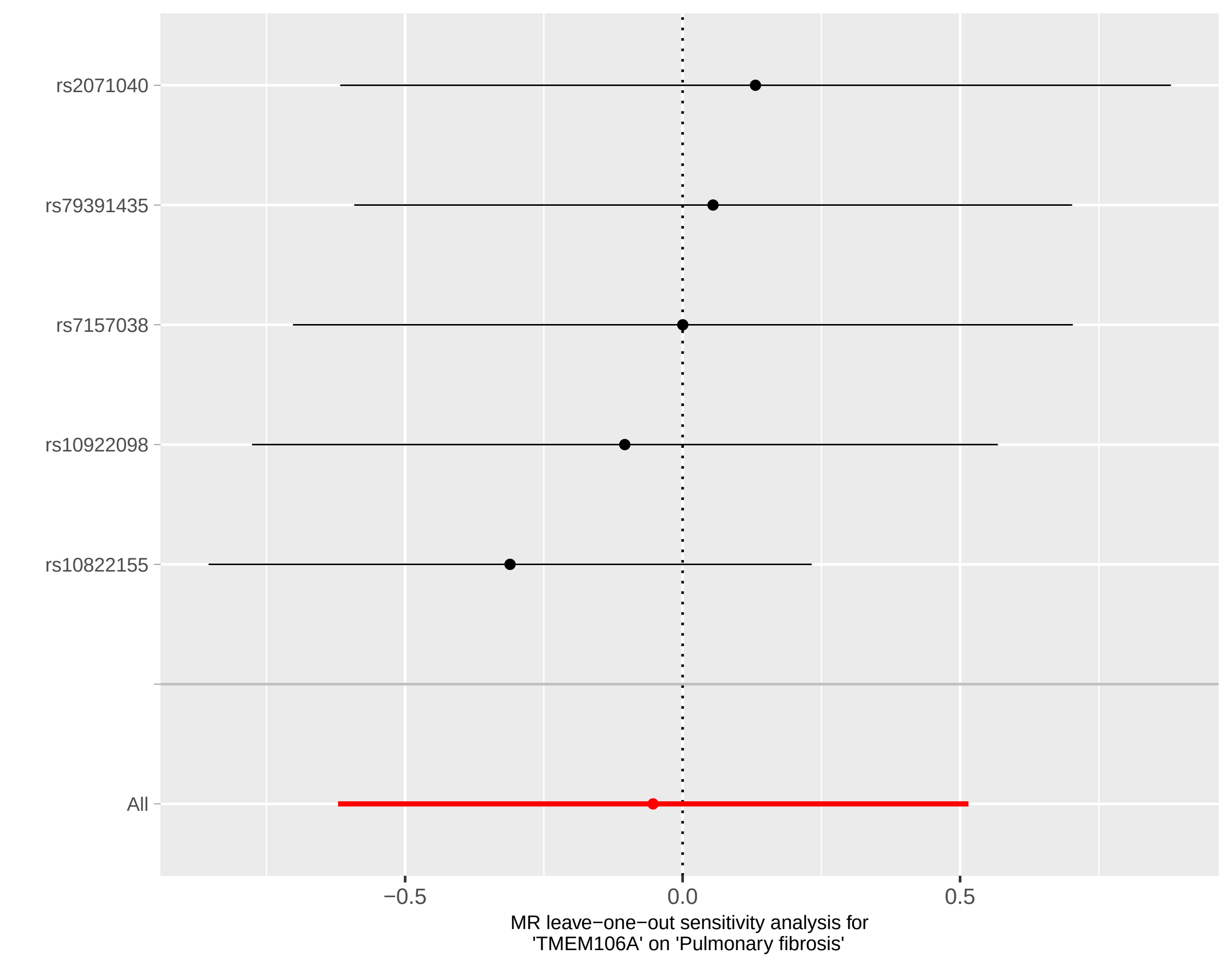
rs2071040
rs79391435
rs7157038
rs10922098
rs10822155
All
−0.5
0.0
0.5
MR leave−one−out sensitivity analysis for
'TMEM106A' on 'Pulmonary fibrosis'

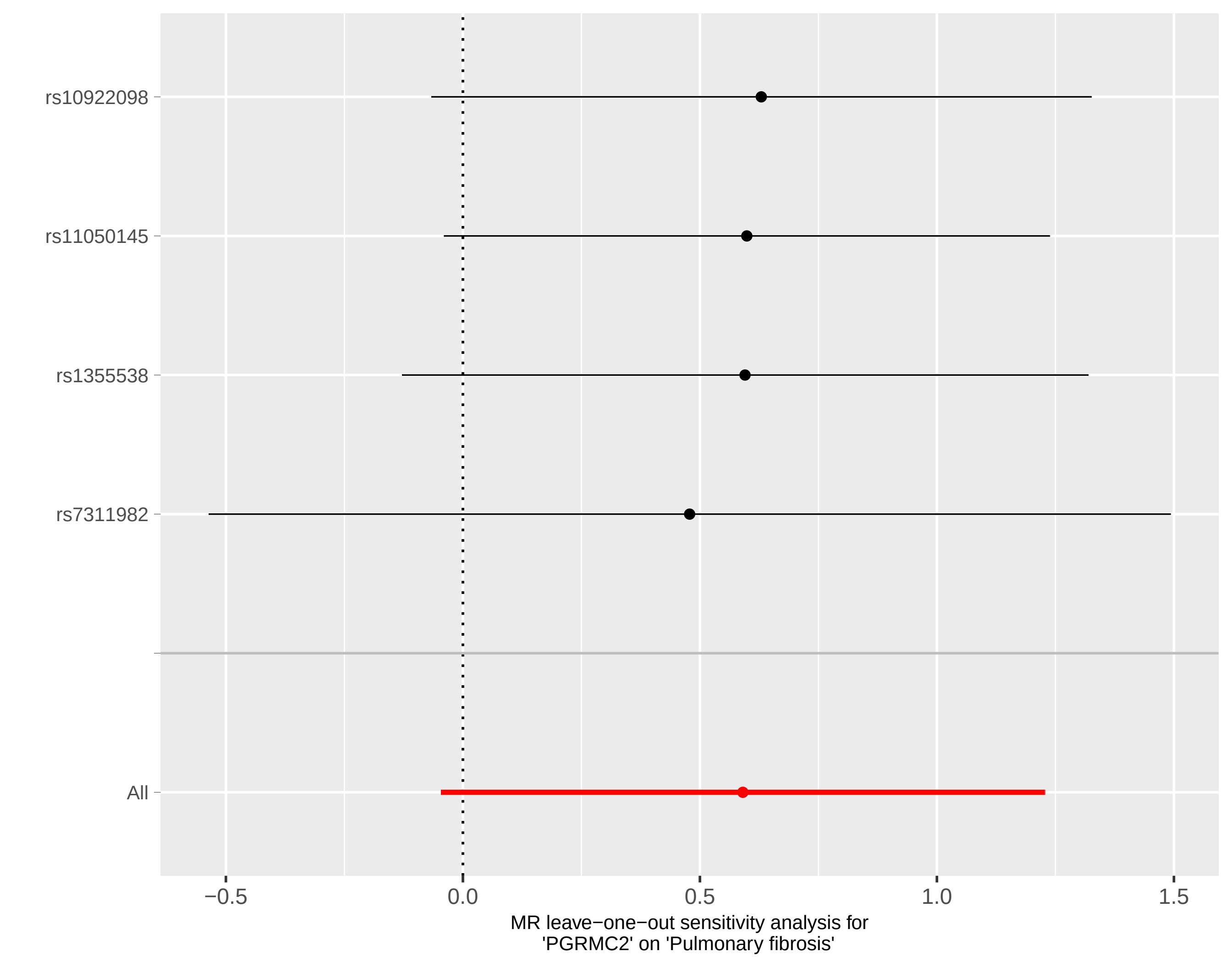

rs10922098
rs11050145
rs1355538
rs7311982
All
−0.5
0.0
0.5
1.0
1.5
MR leave−one−out sensitivity analysis for
'PGRMC2' on 'Pulmonary fibrosis'

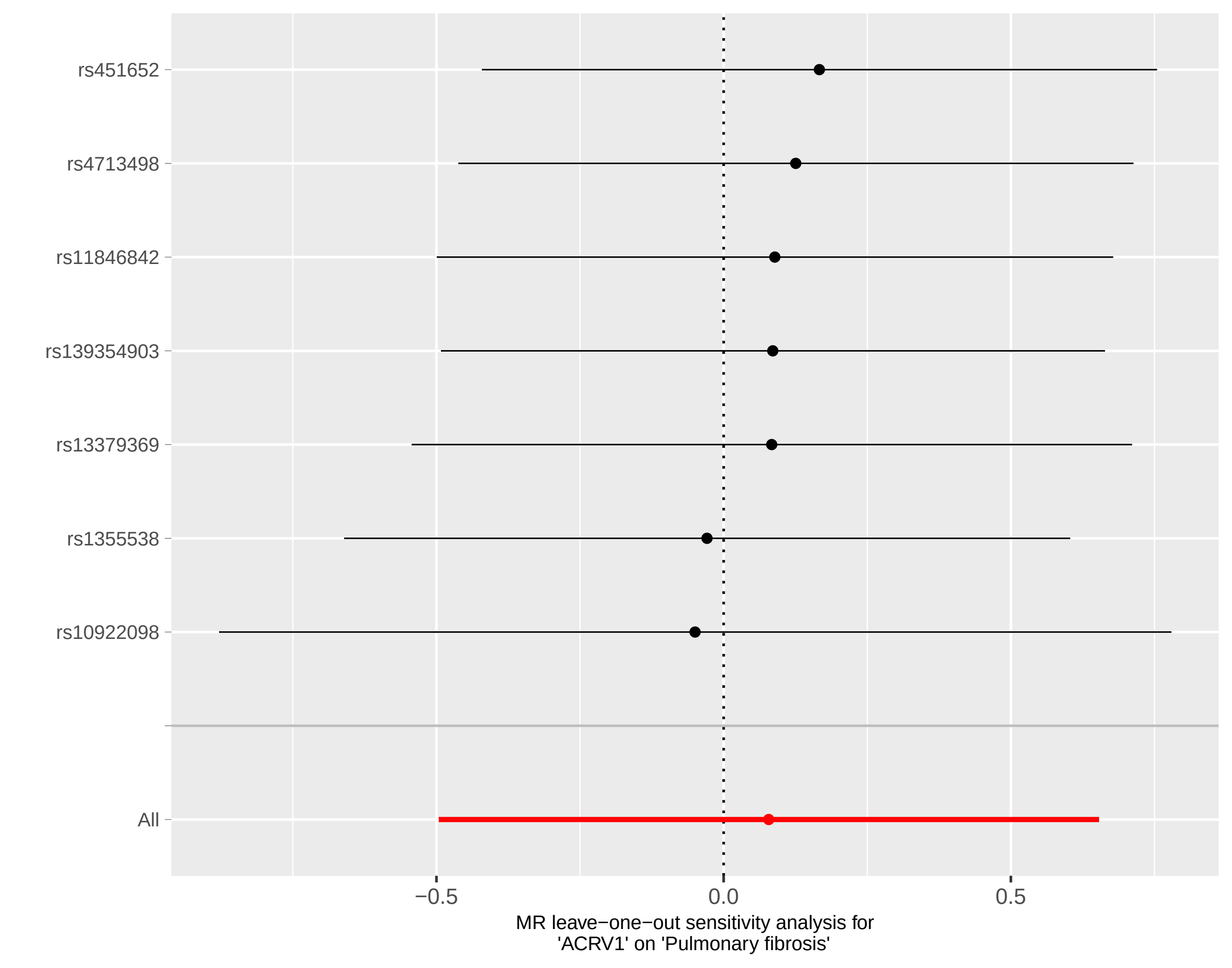
rs451652
rs4713498
rs11846842
rs139354903
rs13379369
rs1355538
rs10922098
All
−0.5
0.0
0.5
MR leave−one−out sensitivity analysis for
'ACRV1' on 'Pulmonary fibrosis'

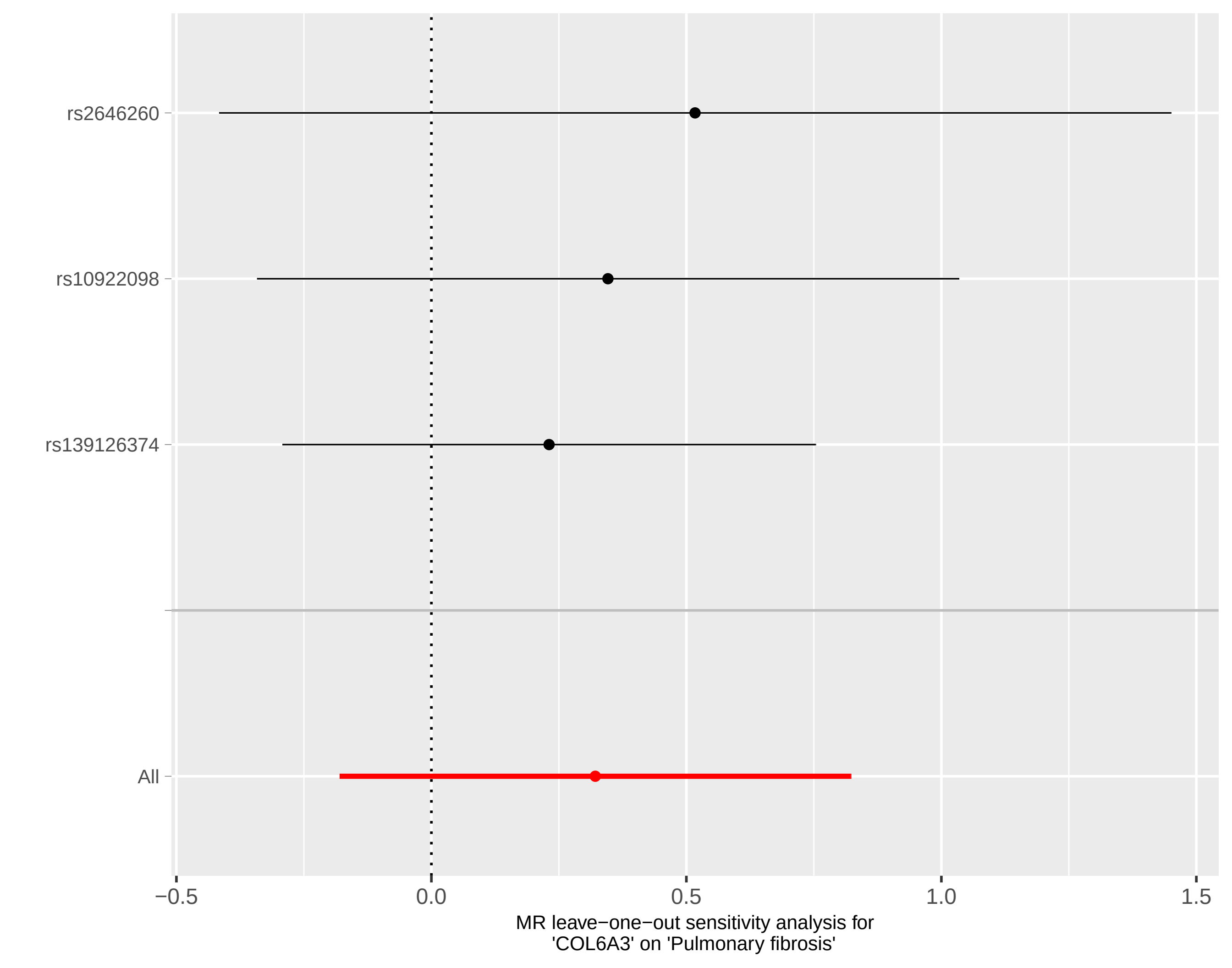
rs2646260
rs10922098
rs139126374
All
−0.5
0.0
0.5
1.0
1.5
MR leave−one−out sensitivity analysis for
'COL6A3' on 'Pulmonary fibrosis'

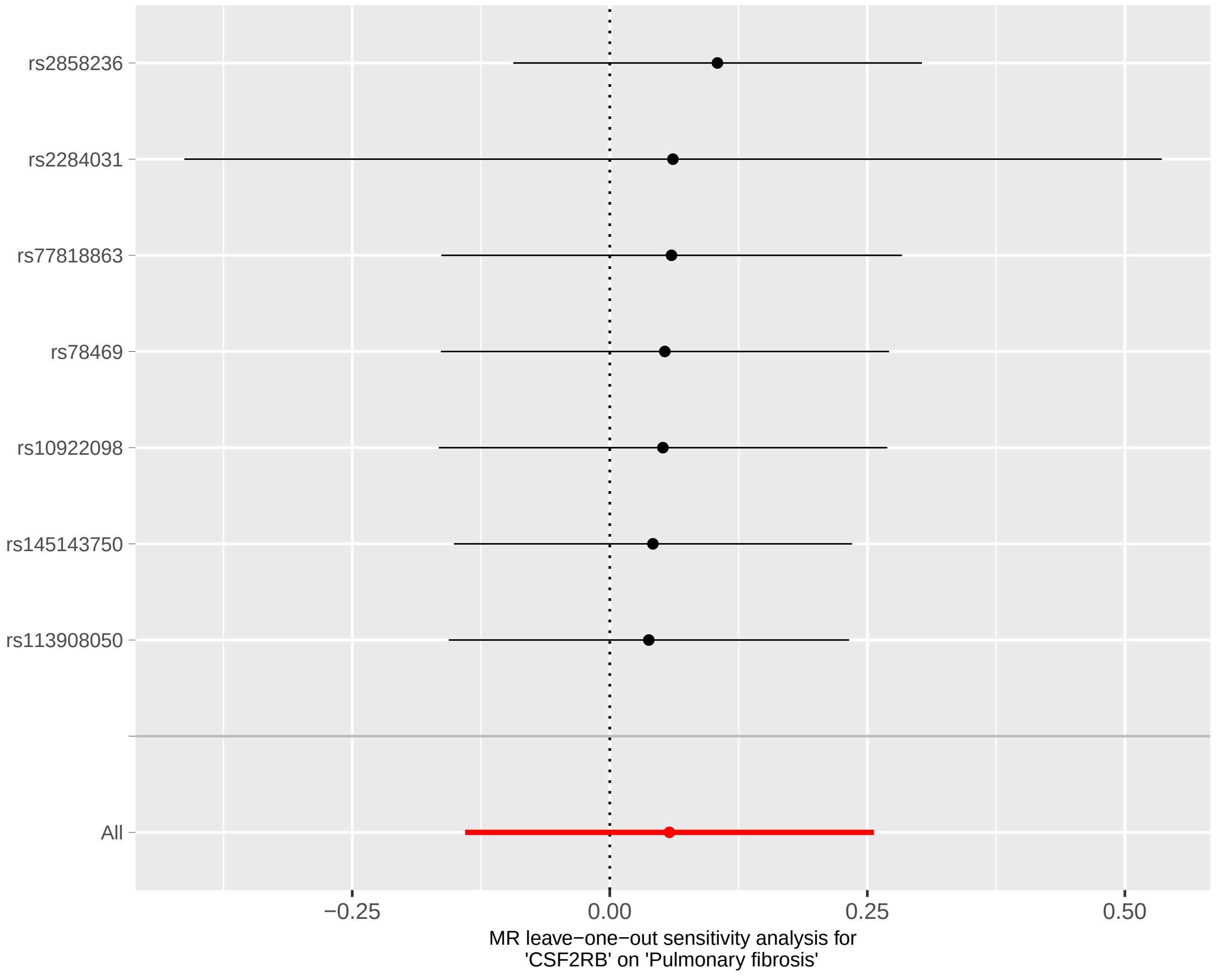
rs2858236
rs2284031
rs77818863
rs78469
rs10922098
rs145143750
rs113908050
All
−0.25
0.00
0.25
0.50
MR leave−one−out sensitivity analysis for
'CSF2RB' on 'Pulmonary fibrosis'

Insufficient number of SNPs

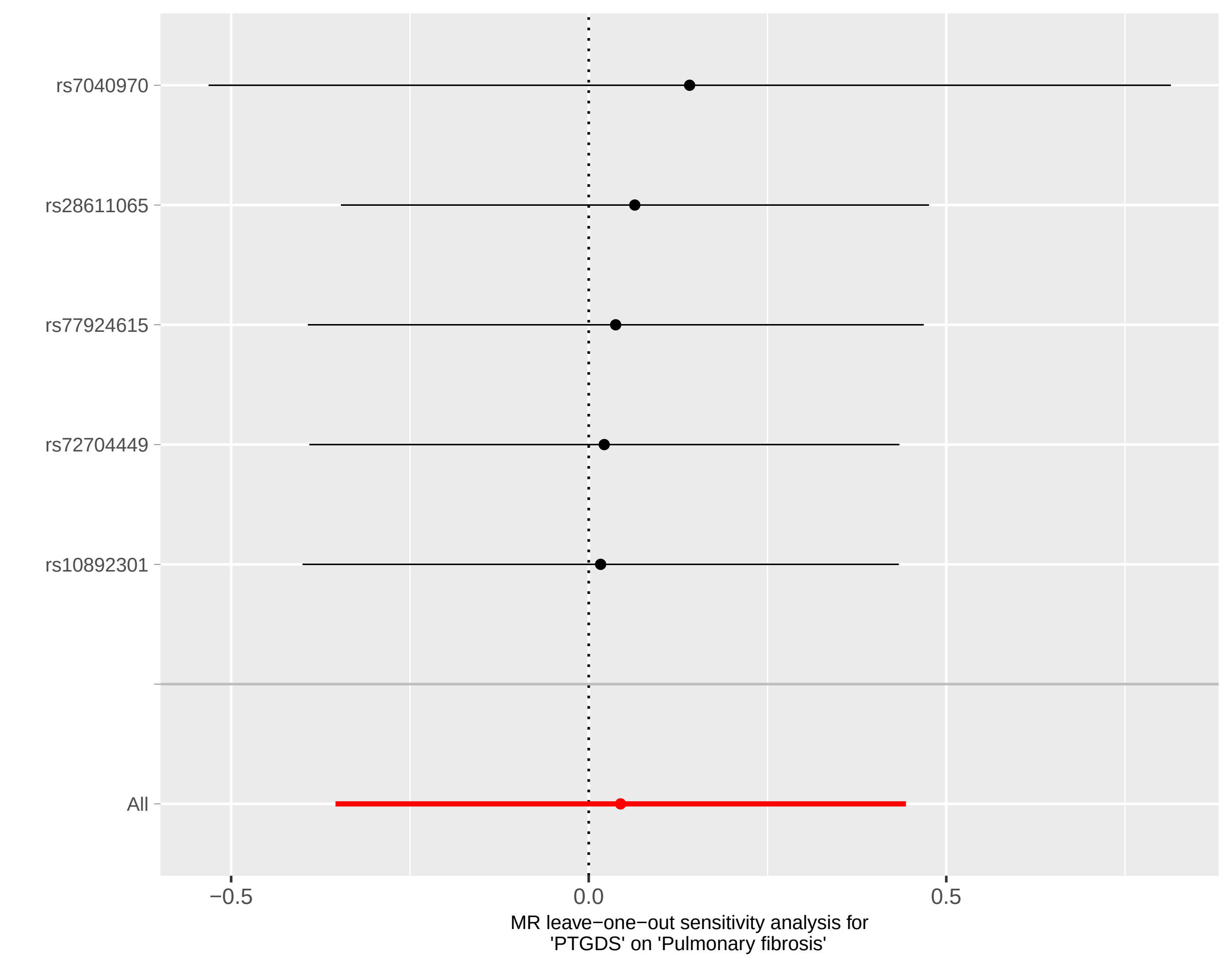
rs7040970
rs28611065
rs77924615
rs72704449
rs10892301
All
−0.5
0.0
0.5
MR leave−one−out sensitivity analysis for
'PTGDS' on 'Pulmonary fibrosis'

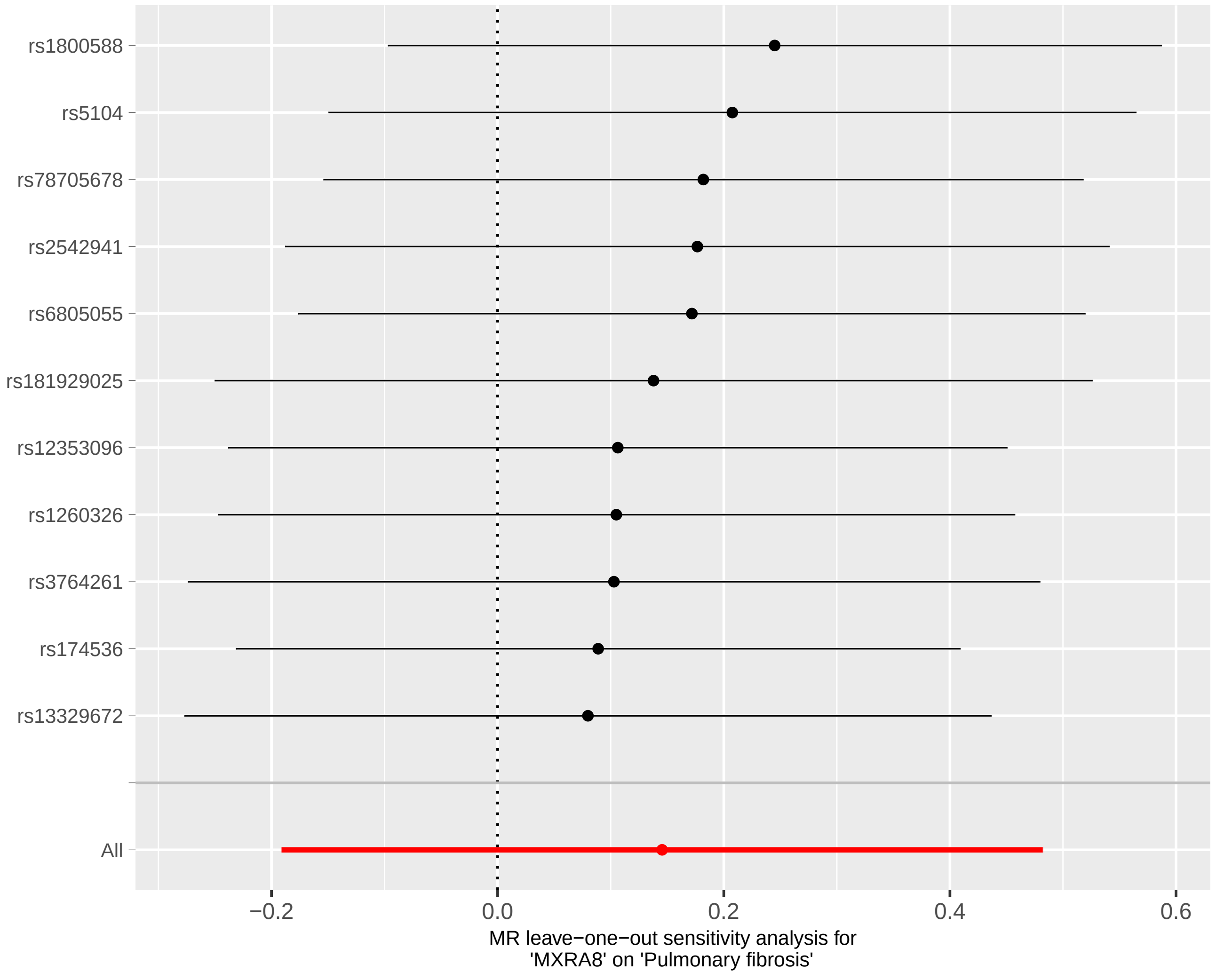

rs1800588
rs5104
rs78705678
rs2542941
rs6805055
rs181929025
rs12353096
rs1260326
rs3764261
rs174536
rs13329672
All
−0.2
0.0
0.2
0.4
0.6
MR leave−one−out sensitivity analysis for
'MXRA8' on 'Pulmonary fibrosis'

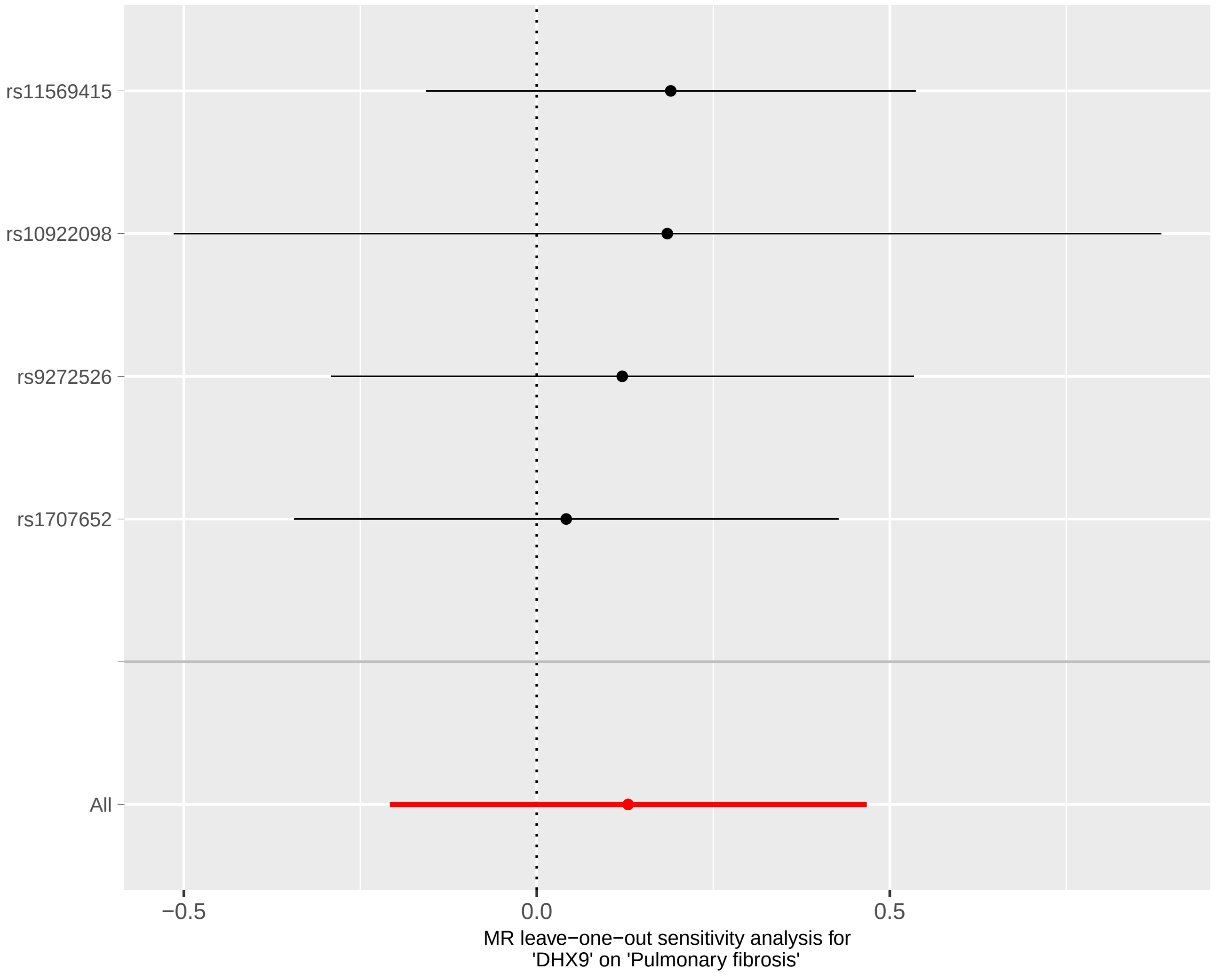
rs11569415
rs10922098
rs9272526
rs1707652
All
−0.5
0.0
0.5
MR leave−one−out sensitivity analysis for
'DHX9' on 'Pulmonary fibrosis'

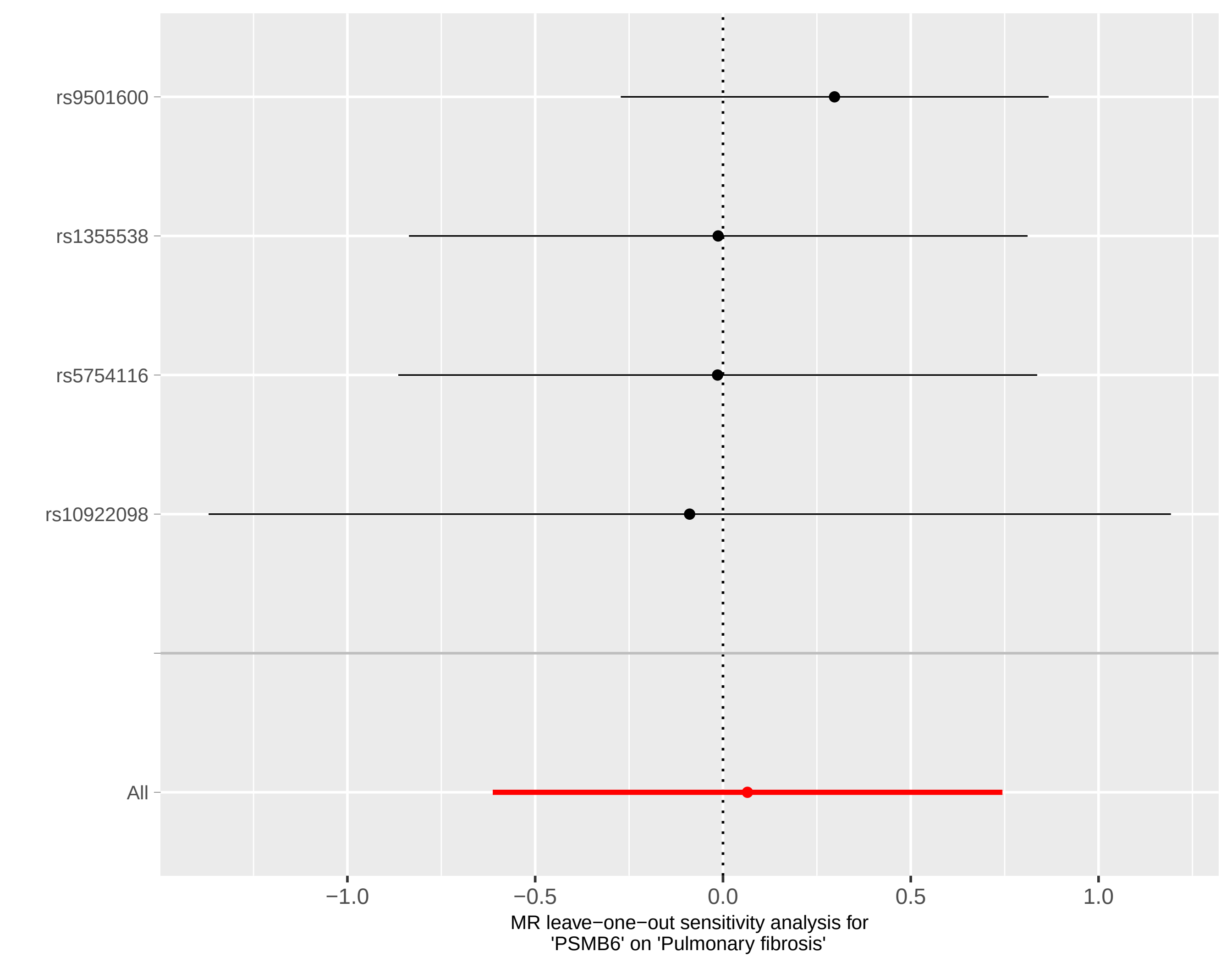
rs9501600
rs1355538
rs5754116
rs10922098
All
−1.0
−0.5
0.0
0.5
1.0
MR leave−one−out sensitivity analysis for
'PSMB6' on 'Pulmonary fibrosis'

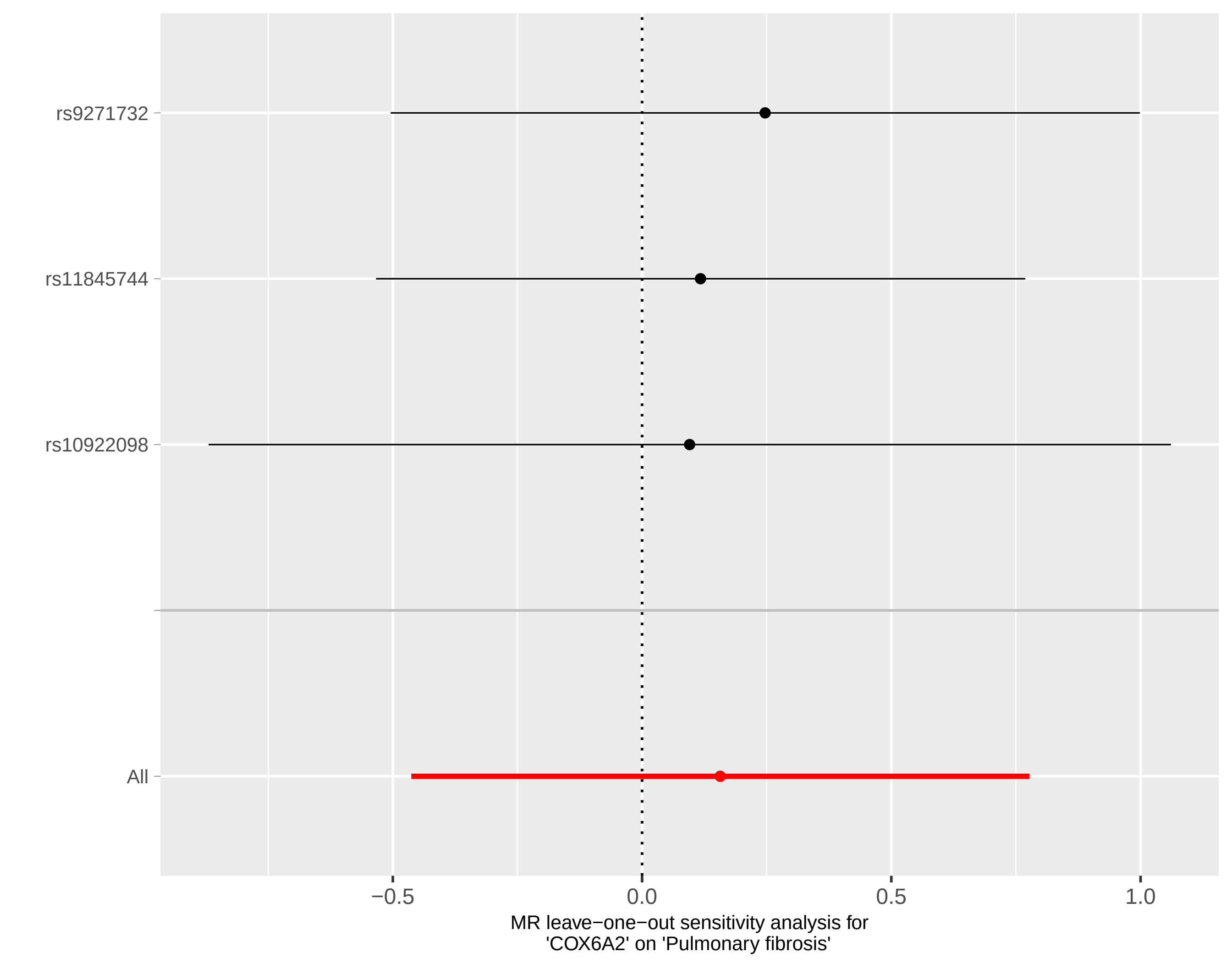

rs9271732
rs11845744
rs10922098
All
−0.5
0.0
0.5
1.0
MR leave−one−out sensitivity analysis for
'COX6A2' on 'Pulmonary fibrosis'

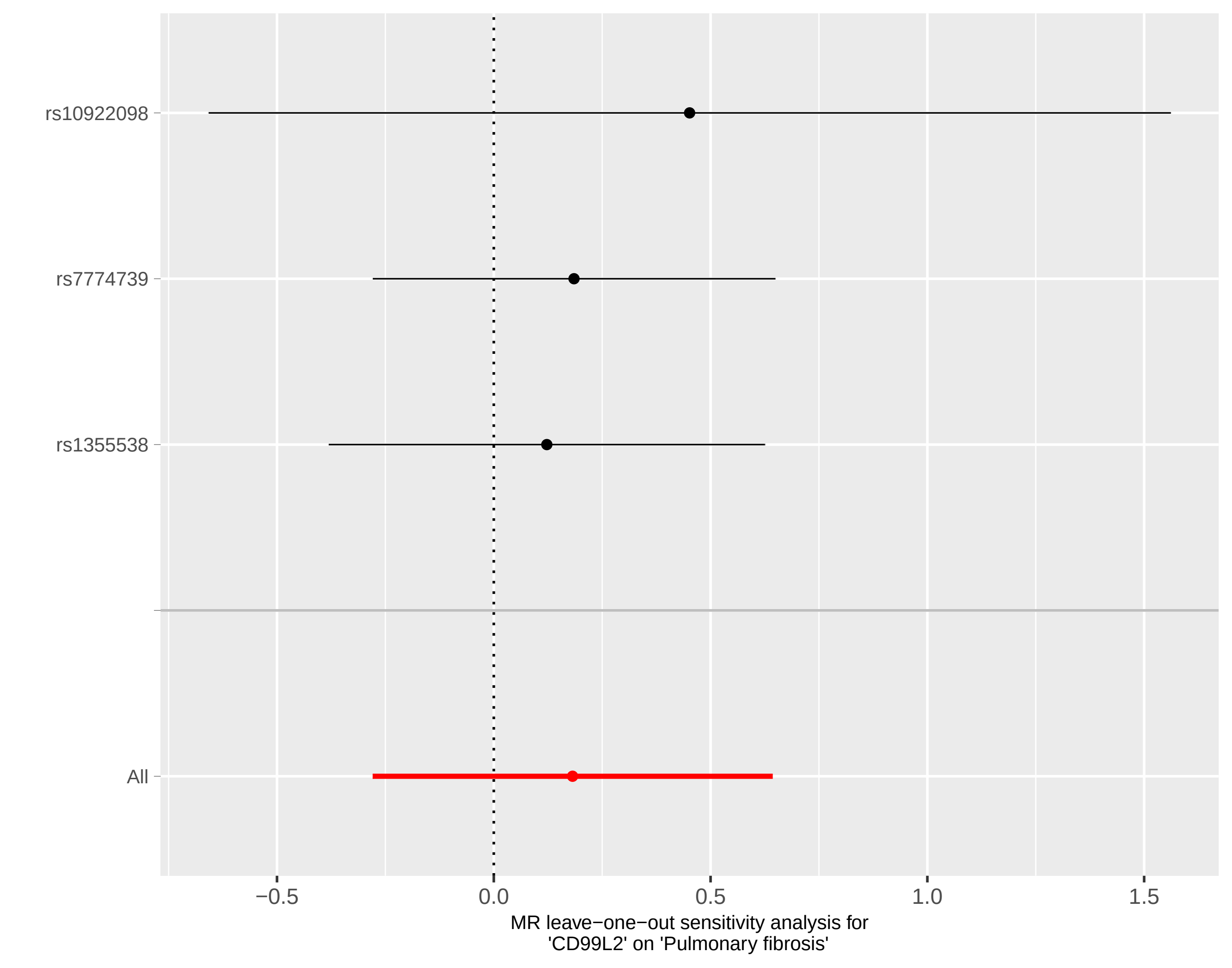

rs10922098
rs7774739
rs1355538
All
−0.5
0.0
0.5
1.0
1.5
MR leave−one−out sensitivity analysis for
'CD99L2' on 'Pulmonary fibrosis'

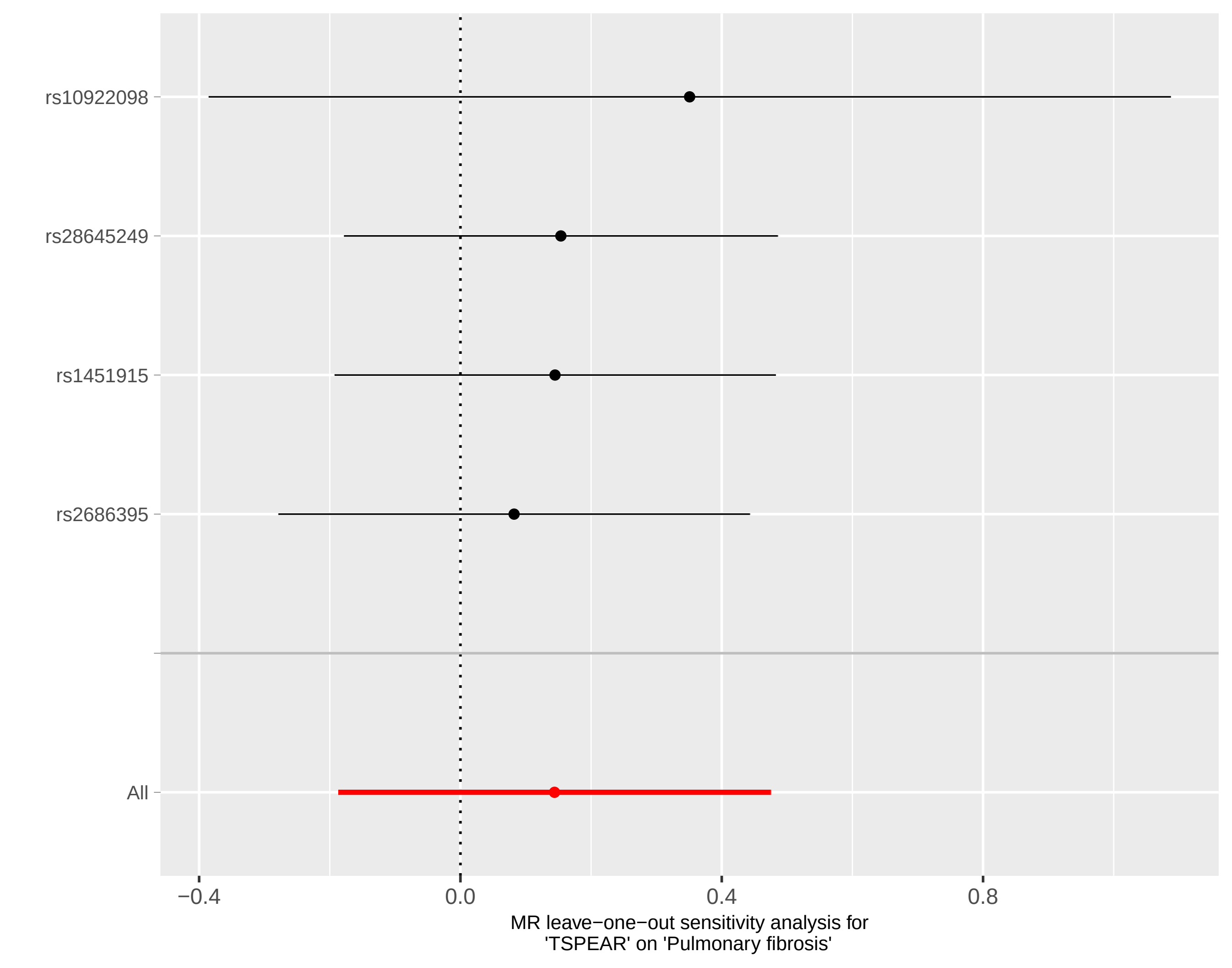

rs10922098
rs28645249
rs1451915
rs2686395
All
−0.4
0.0
0.4
0.8
MR leave−one−out sensitivity analysis for
'TSPEAR' on 'Pulmonary fibrosis'

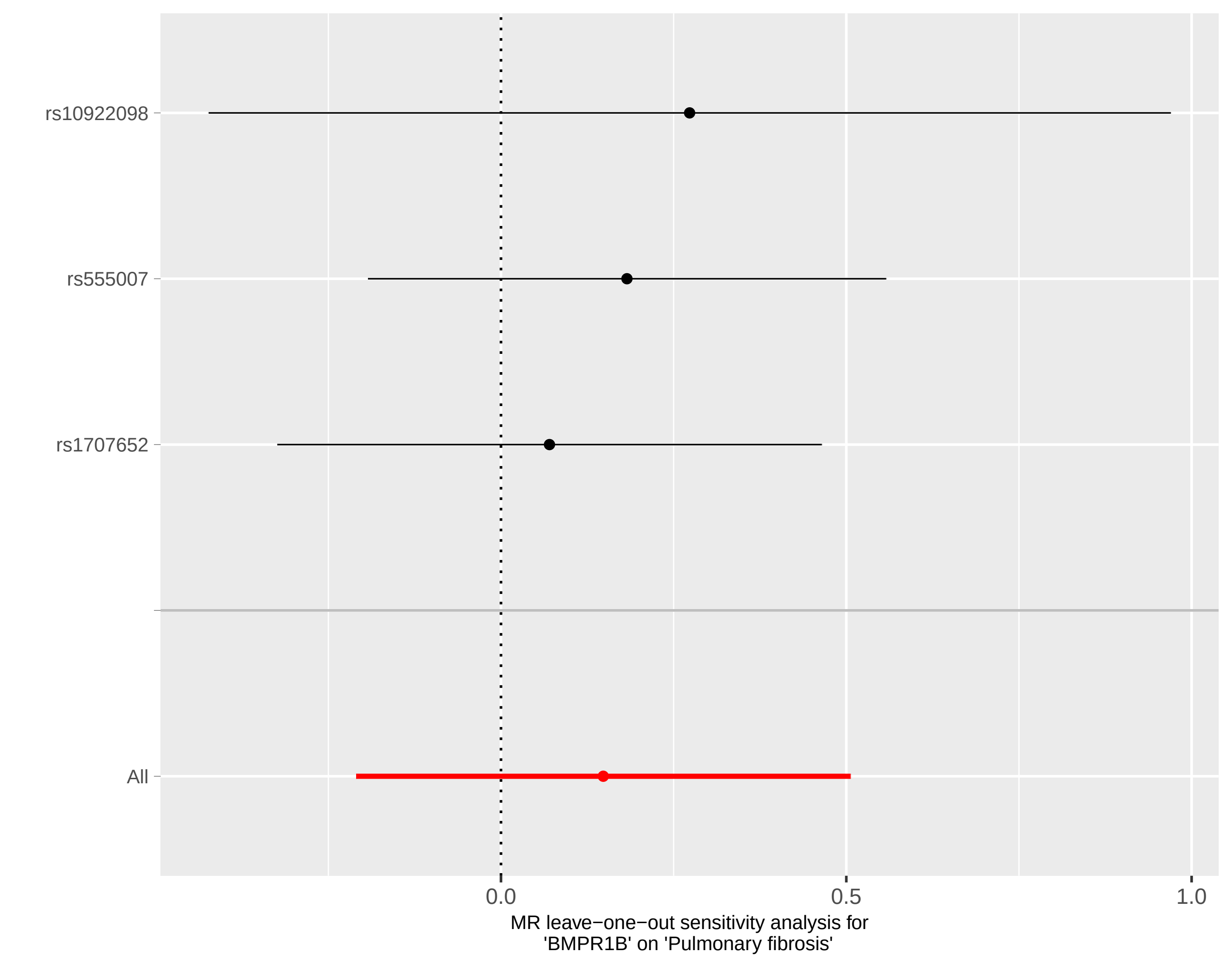
rs10922098
rs555007
rs1707652
All
0.0
0.5
1.0
MR leave−one−out sensitivity analysis for
'BMPR1B' on 'Pulmonary fibrosis'

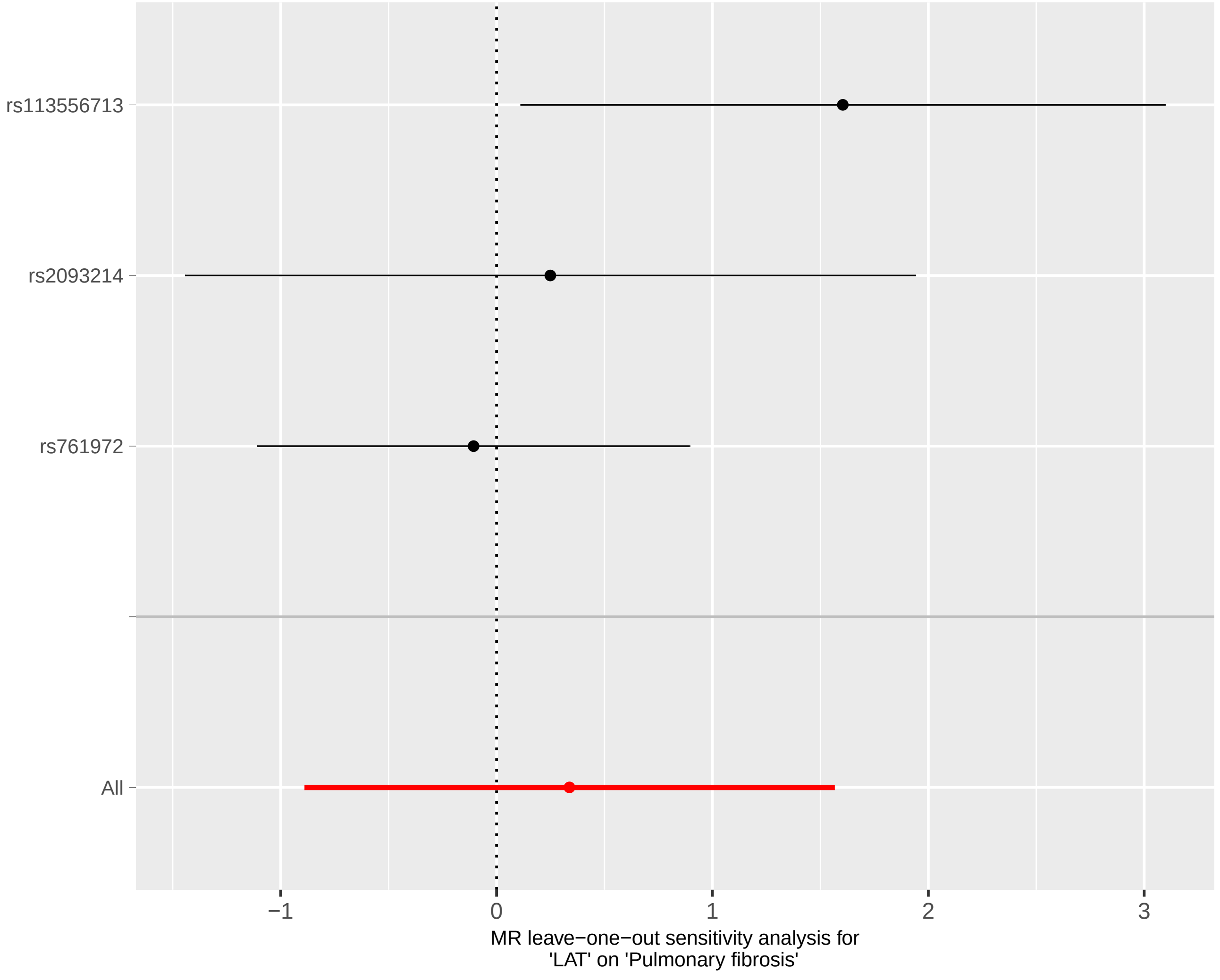
rs113556713
rs2093214
rs761972
All
−1
0
1
2
3
MR leave−one−out sensitivity analysis for
'LAT' on 'Pulmonary fibrosis'

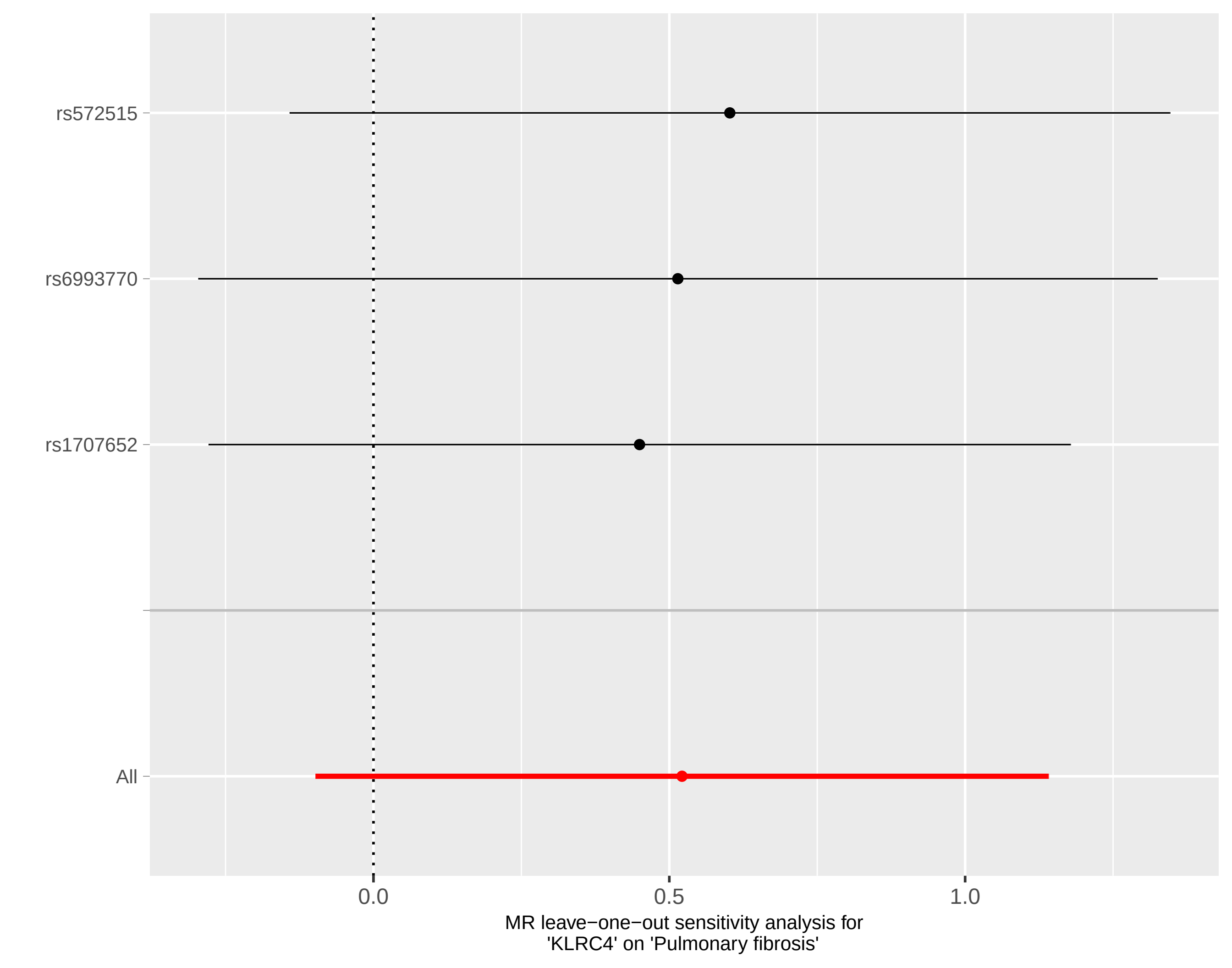
rs572515
rs6993770
rs1707652
All
0.0
0.5
1.0
MR leave−one−out sensitivity analysis for
'KLRC4' on 'Pulmonary fibrosis'

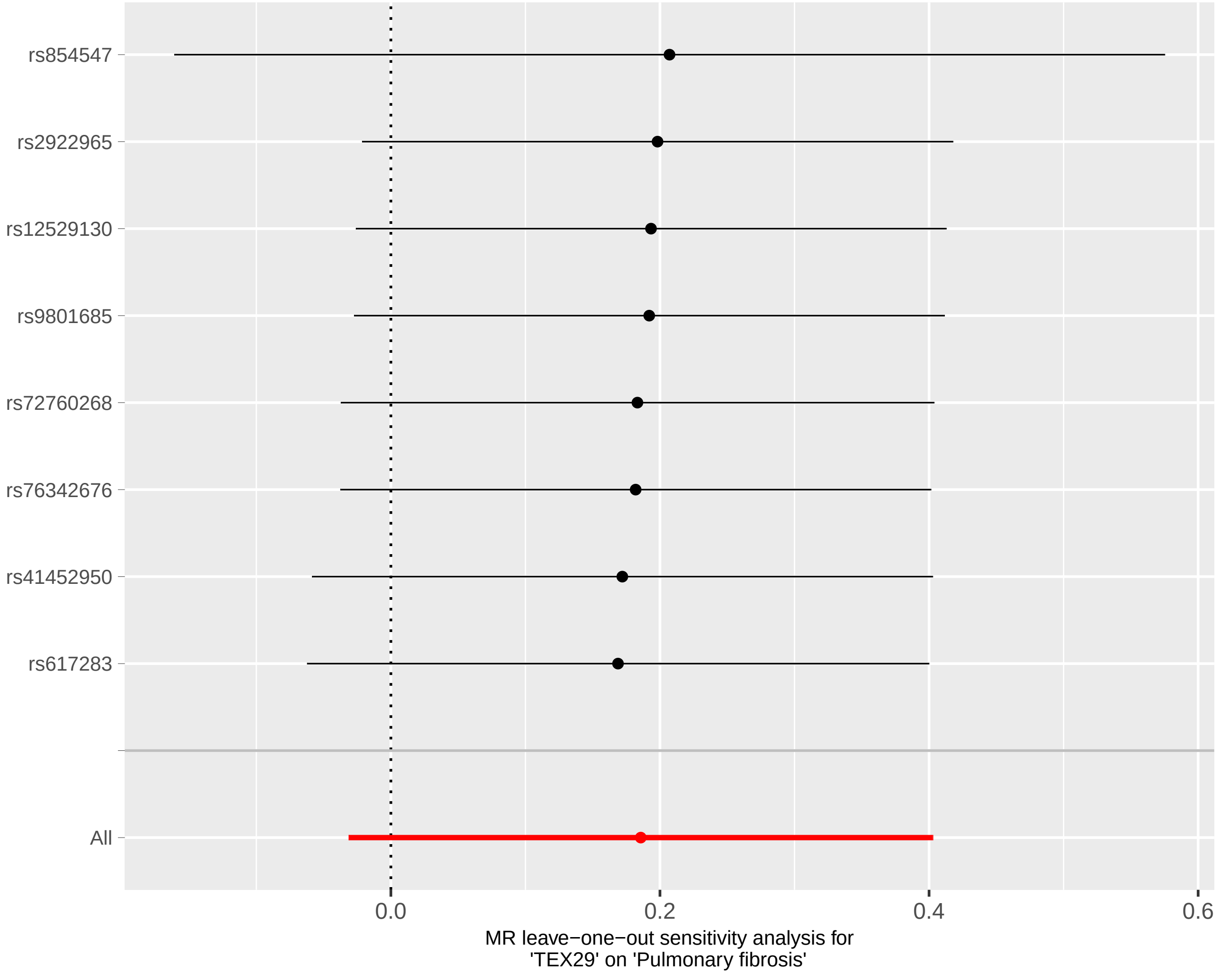

rs854547
rs2922965
rs12529130
rs9801685
rs72760268
rs76342676
rs41452950
rs617283
All
0.0
0.2
0.4
0.6
MR leave−one−out sensitivity analysis for
'TEX29' on 'Pulmonary fibrosis'

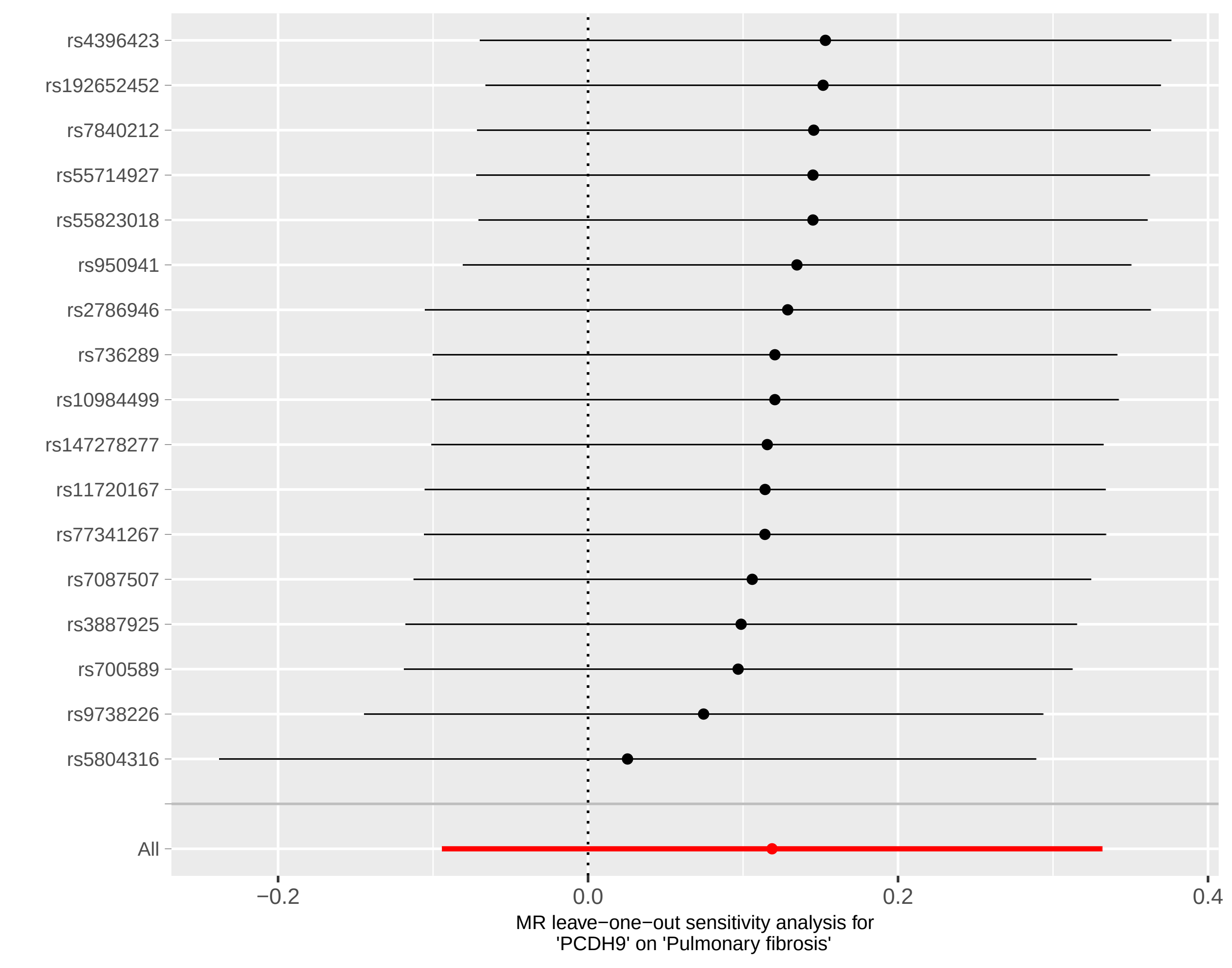
rs4396423
rs192652452
rs7840212
rs55714927
rs55823018
rs950941
rs2786946
rs736289
rs10984499
rs147278277
rs11720167
rs77341267
rs7087507
rs3887925
rs700589
rs9738226
rs5804316
All
−0.2
0.0
0.2
0.4
MR leave−one−out sensitivity analysis for
'PCDH9' on 'Pulmonary fibrosis'

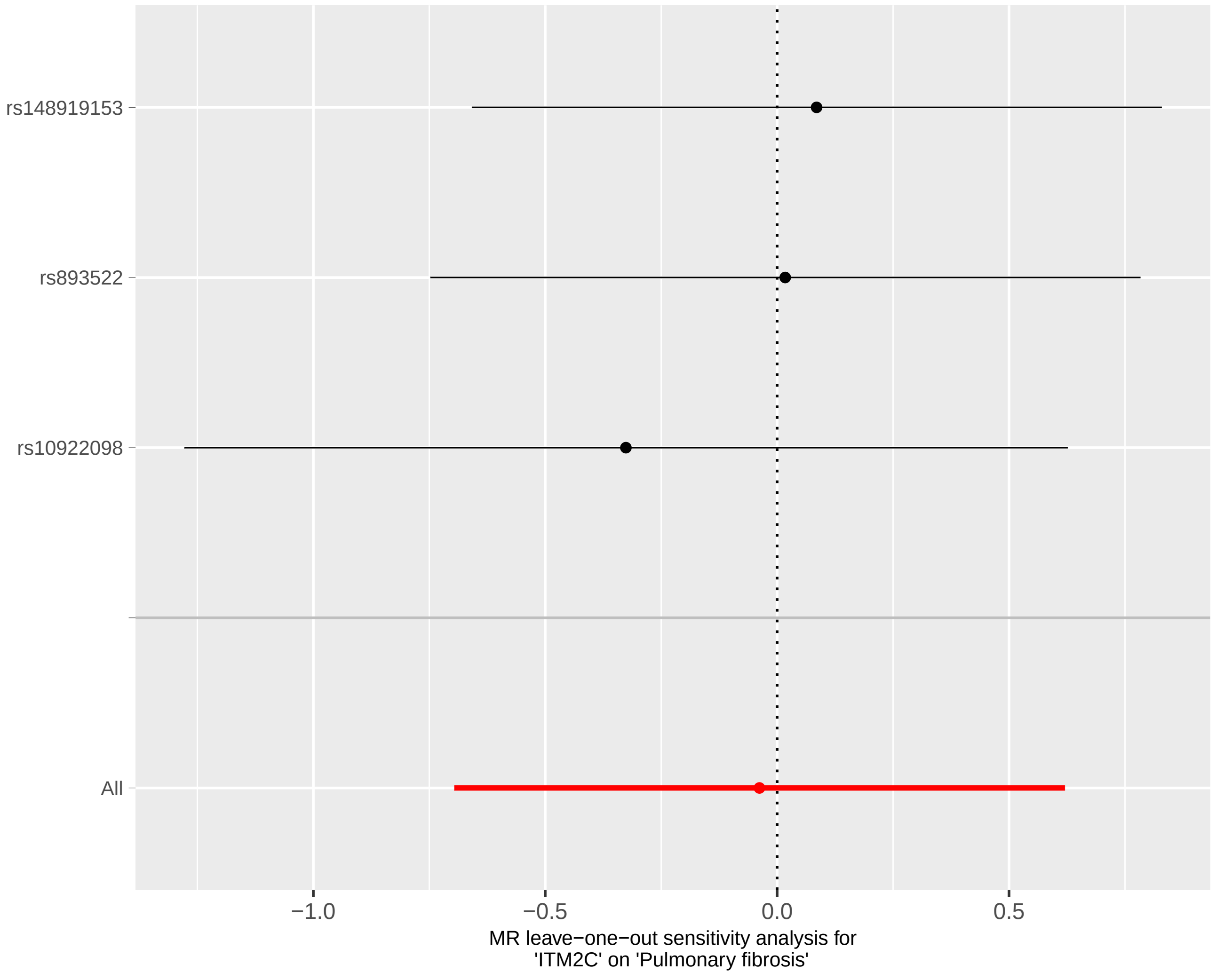
rs148919153
rs893522
rs10922098
All
−1.0
−0.5
0.0
0.5
MR leave−one−out sensitivity analysis for
'ITM2C' on 'Pulmonary fibrosis'

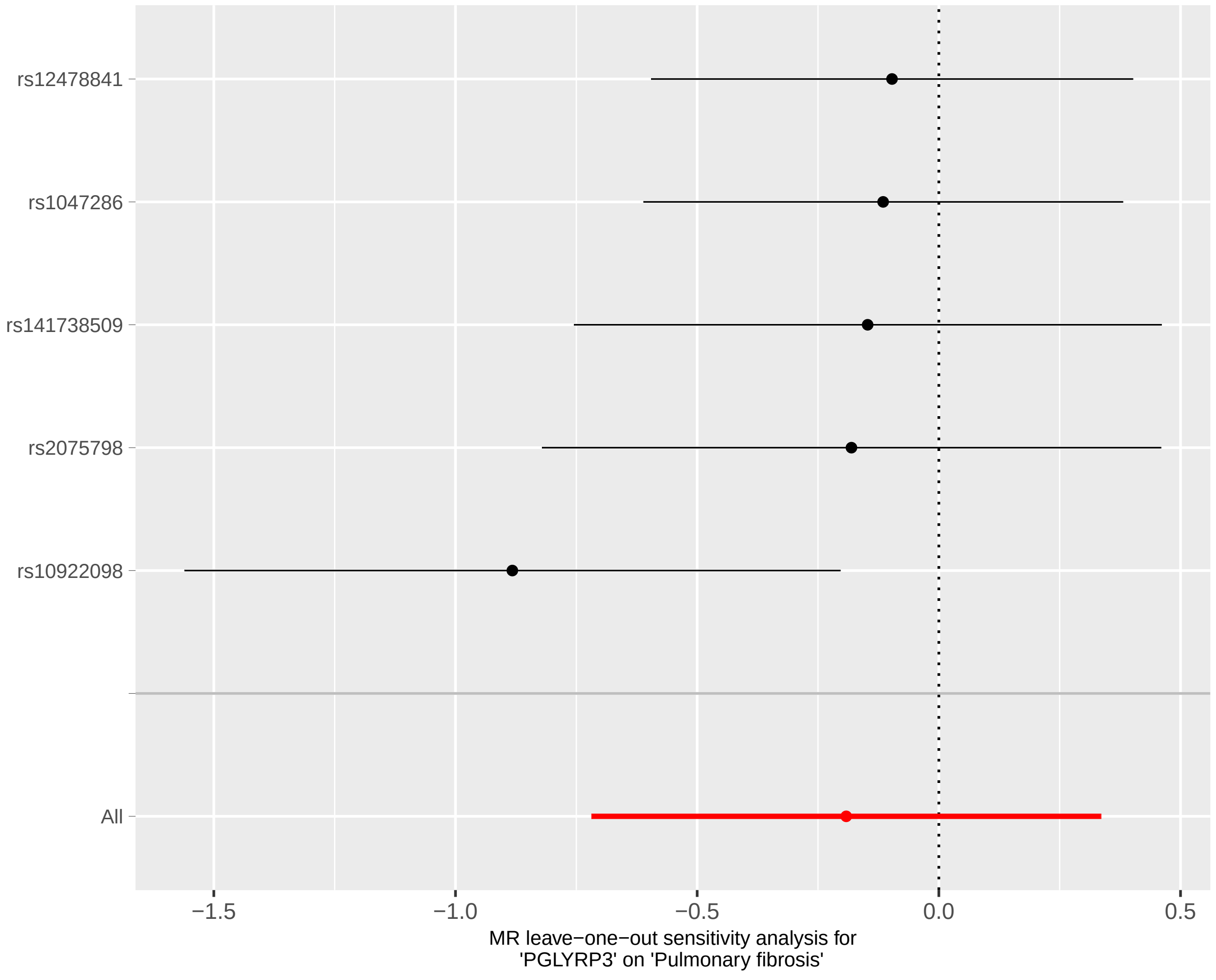

rs12478841
rs1047286
rs141738509
rs2075798
rs10922098
All
−1.5
−1.0
−0.5
0.0
0.5
MR leave−one−out sensitivity analysis for
'PGLYRP3' on 'Pulmonary fibrosis'

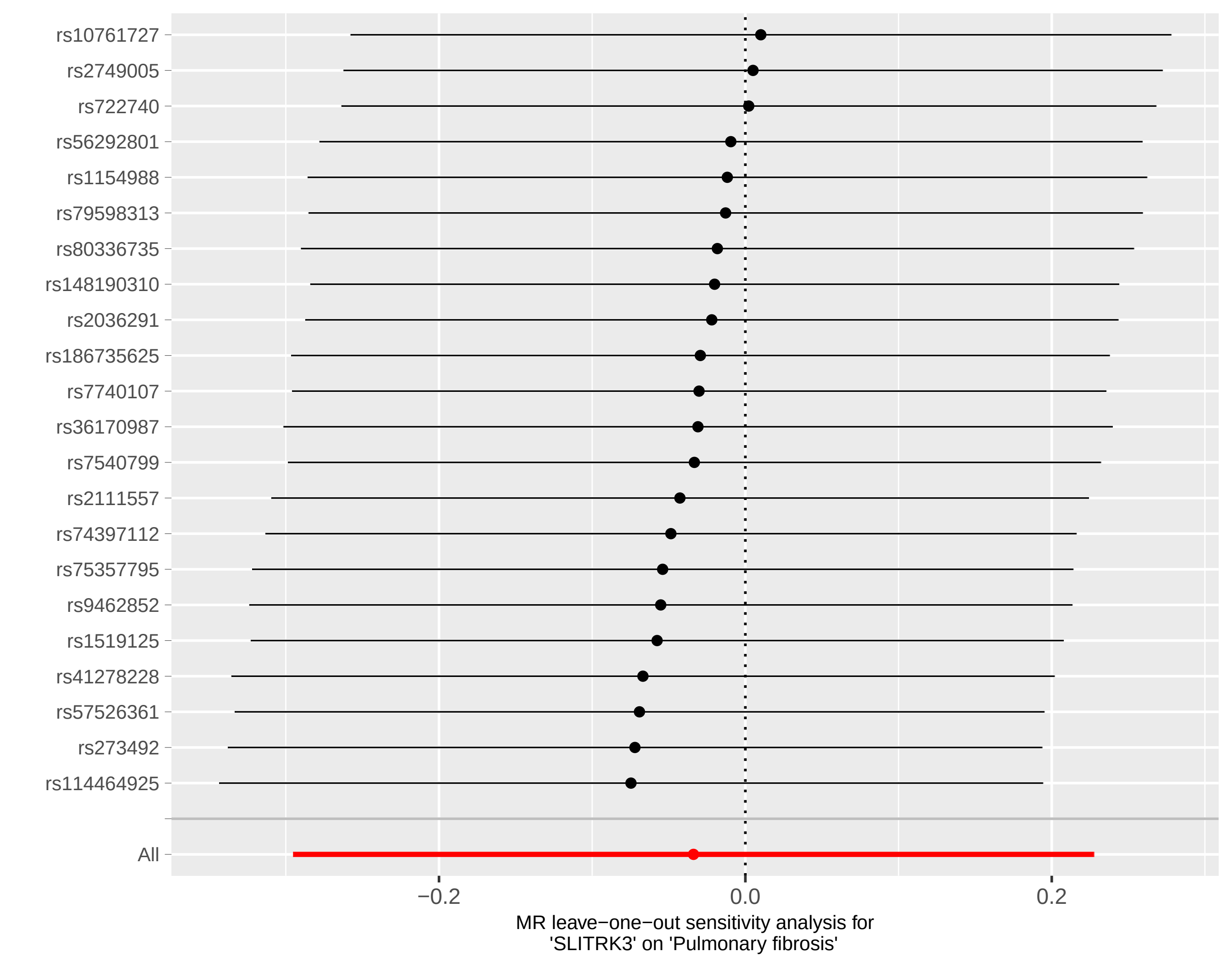
rs10761727
rs2749005
rs722740
rs56292801
rs1154988
rs79598313
rs80336735
rs148190310
rs2036291
rs186735625
rs7740107
rs36170987
rs7540799
rs2111557
rs74397112
rs75357795
rs9462852
rs1519125
rs41278228
rs57526361
rs273492
rs114464925
All
−0.2
0.0
0.2
MR leave−one−out sensitivity analysis for
'SLITRK3' on 'Pulmonary fibrosis'

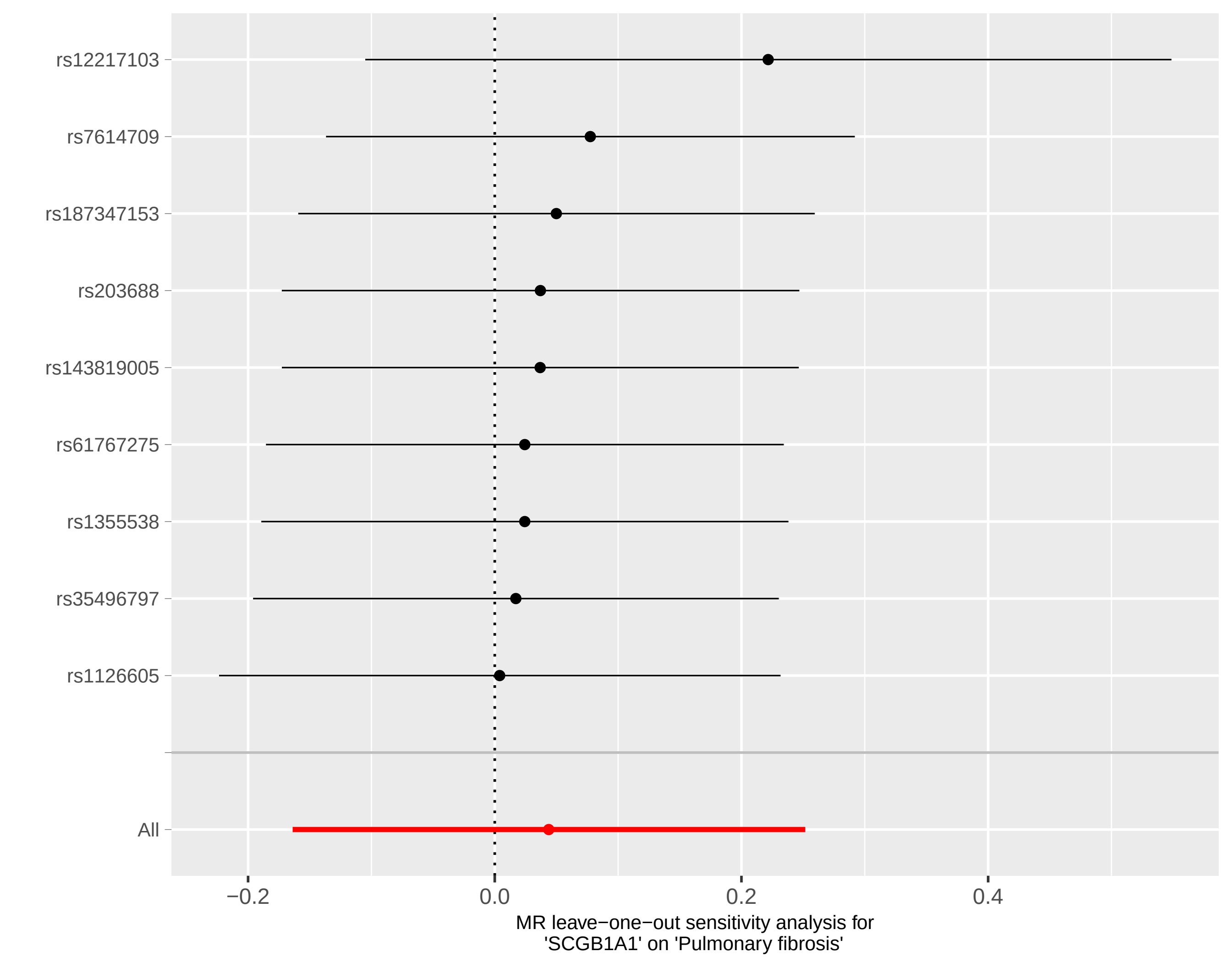
rs12217103
rs7614709
rs187347153
rs203688
rs143819005
rs61767275
rs1355538
rs35496797
rs1126605
All
−0.2
0.0
0.2
0.4
MR leave−one−out sensitivity analysis for
'SCGB1A1' on 'Pulmonary fibrosis'

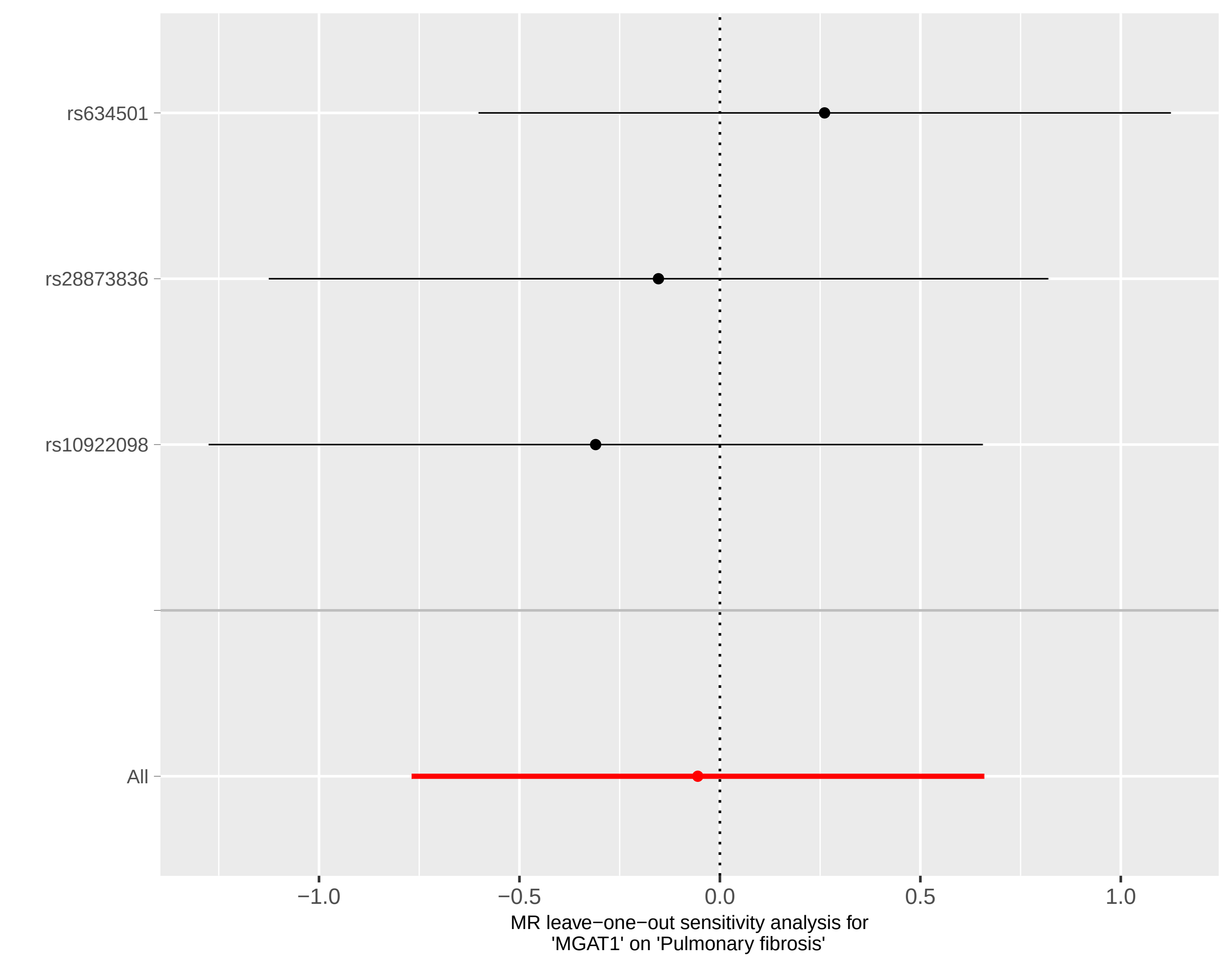
rs634501
rs28873836
rs10922098
All
−1.0
−0.5
0.0
0.5
1.0
MR leave−one−out sensitivity analysis for
'MGAT1' on 'Pulmonary fibrosis'

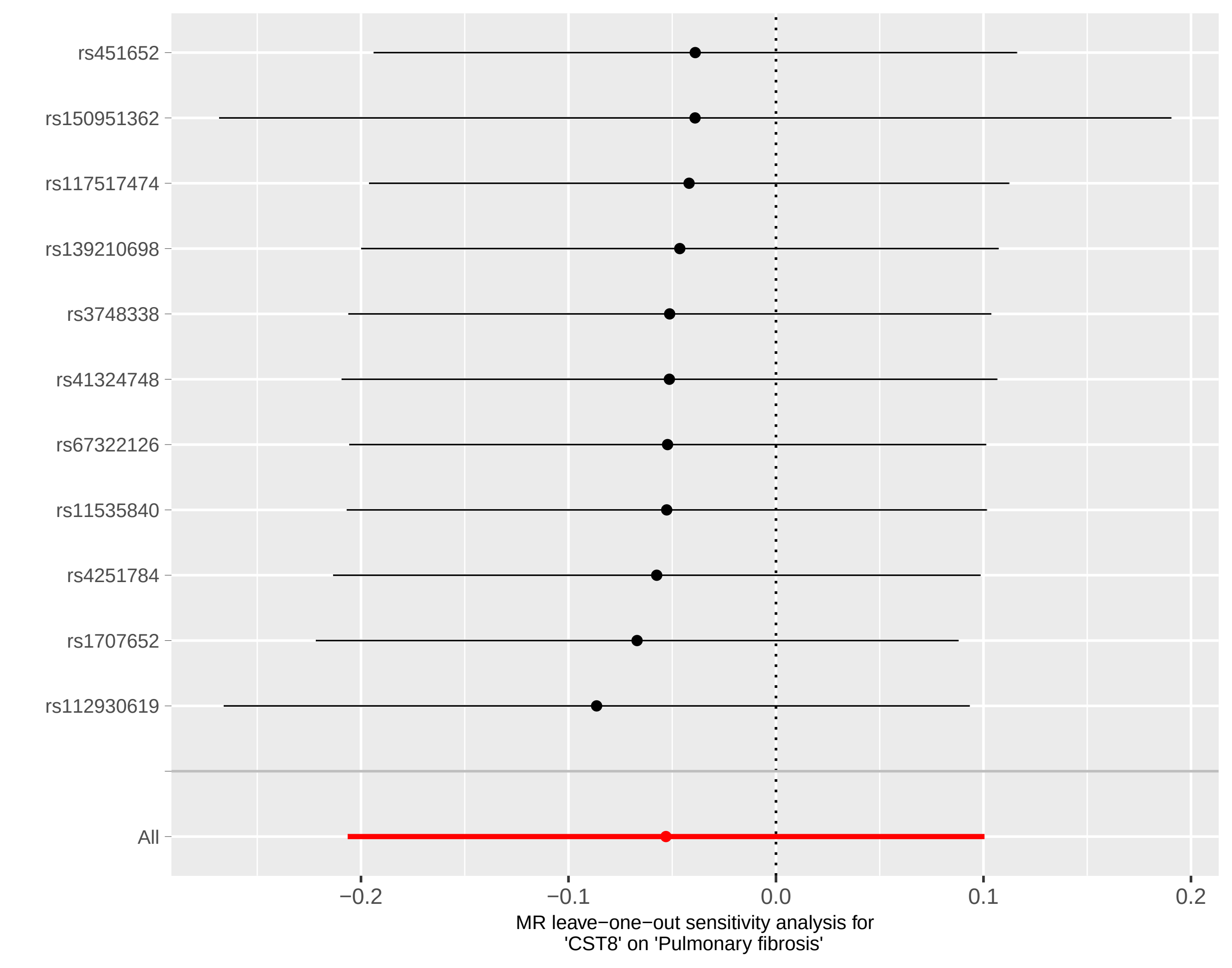
rs451652
rs150951362
rs117517474
rs139210698
rs3748338
rs41324748
rs67322126
rs11535840
rs4251784
rs1707652
rs112930619
All
−0.2
−0.1
0.0
0.1
0.2
MR leave−one−out sensitivity analysis for
'CST8' on 'Pulmonary fibrosis'

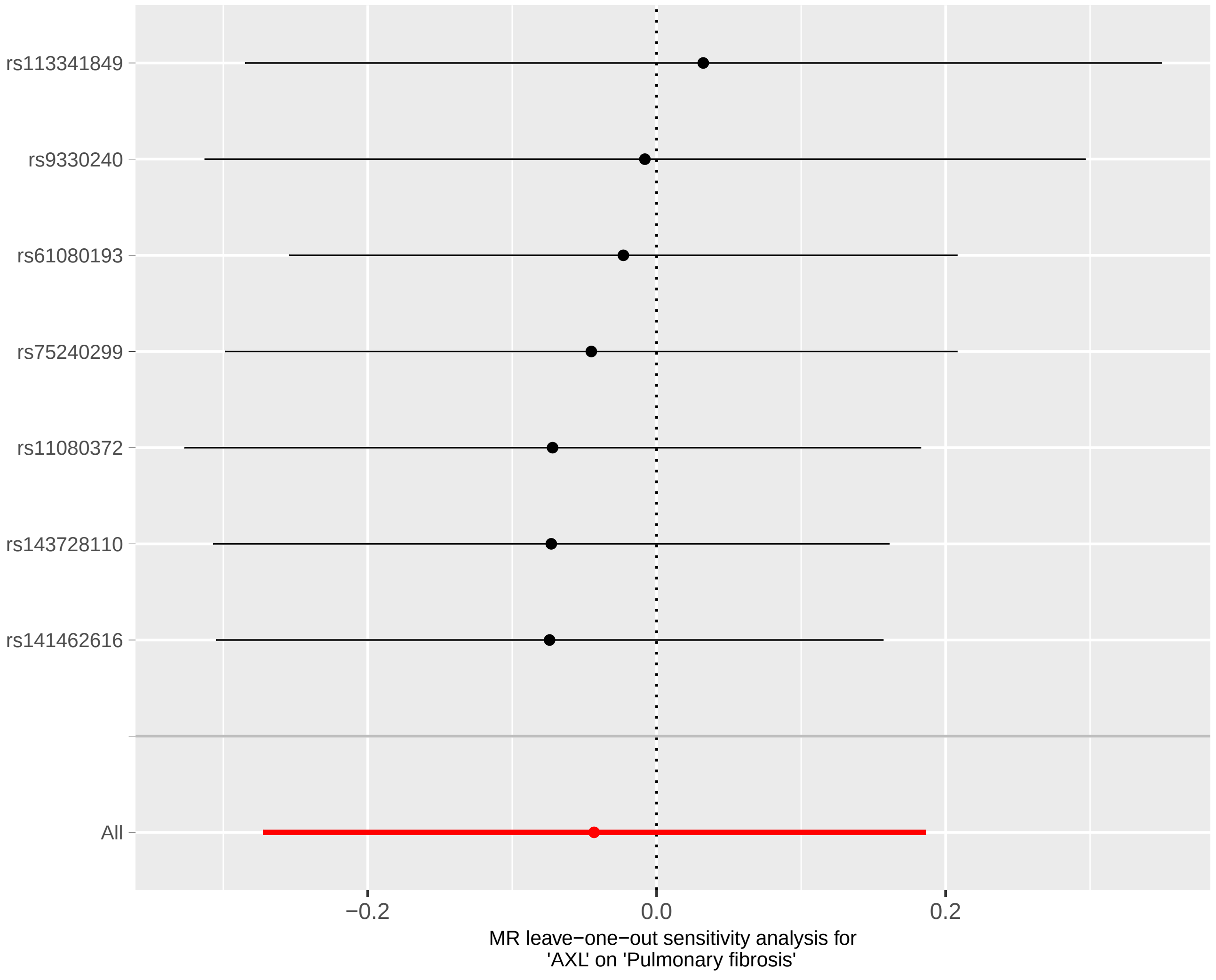
rs113341849
rs9330240
rs61080193
rs75240299
rs11080372
rs143728110
rs141462616
All
−0.2
0.0
0.2
MR leave−one−out sensitivity analysis for
'AXL' on 'Pulmonary fibrosis'

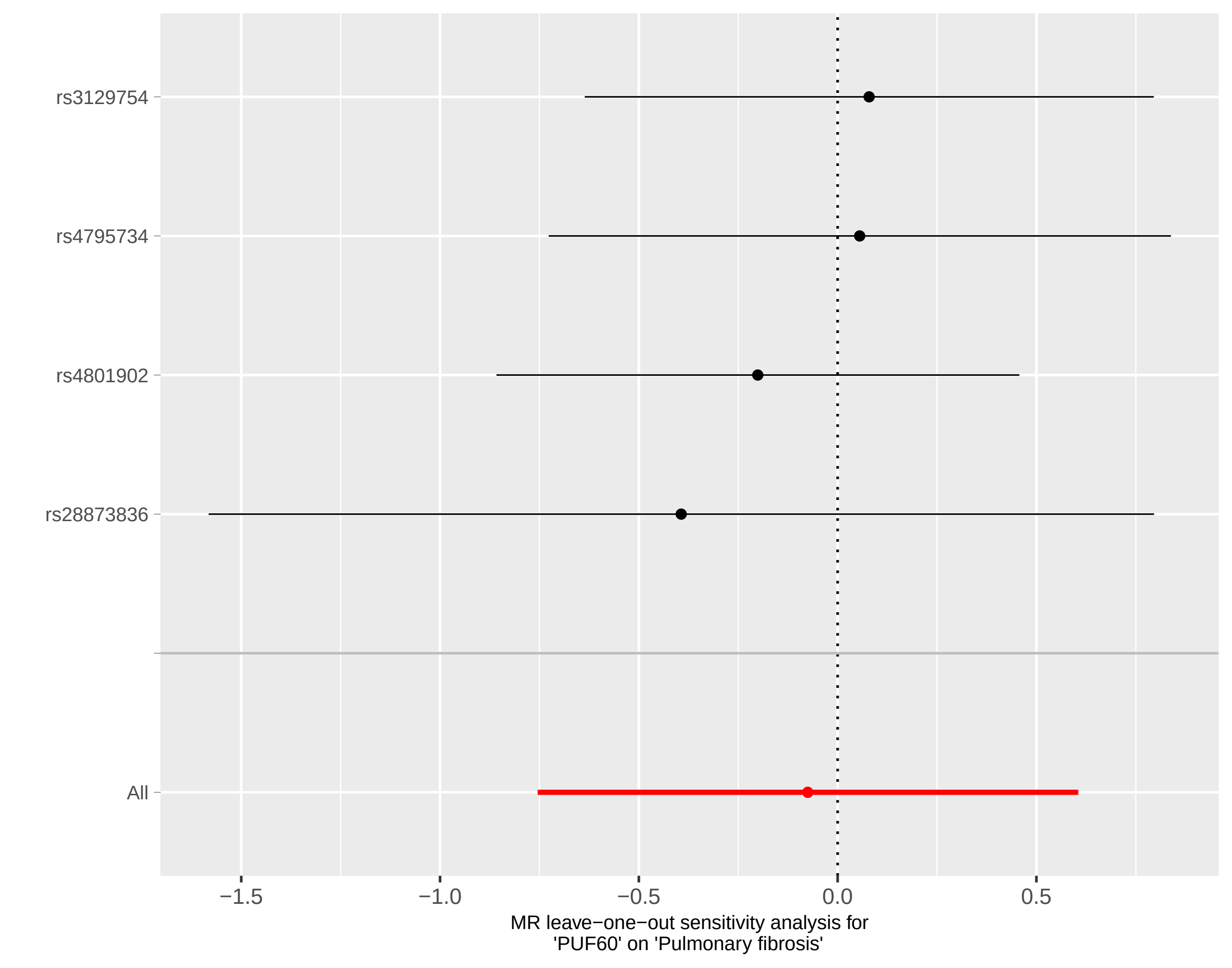

rs3129754
rs4795734
rs4801902
rs28873836
All
−1.5
−1.0
−0.5
0.0
0.5
MR leave−one−out sensitivity analysis for
'PUF60' on 'Pulmonary fibrosis'

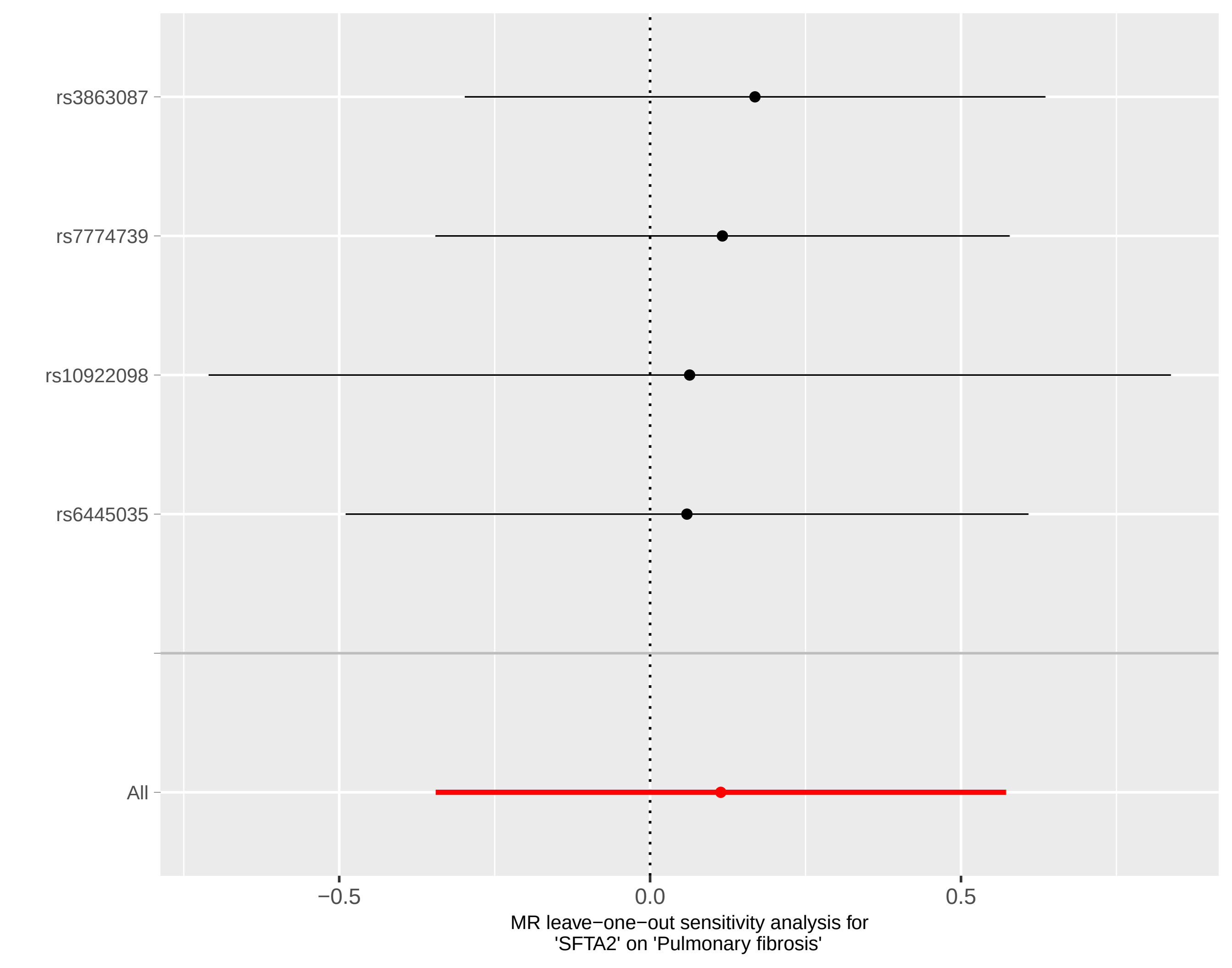
rs3863087
rs7774739
rs10922098
rs6445035
All
−0.5
0.0
0.5
MR leave−one−out sensitivity analysis for
'SFTA2' on 'Pulmonary fibrosis'

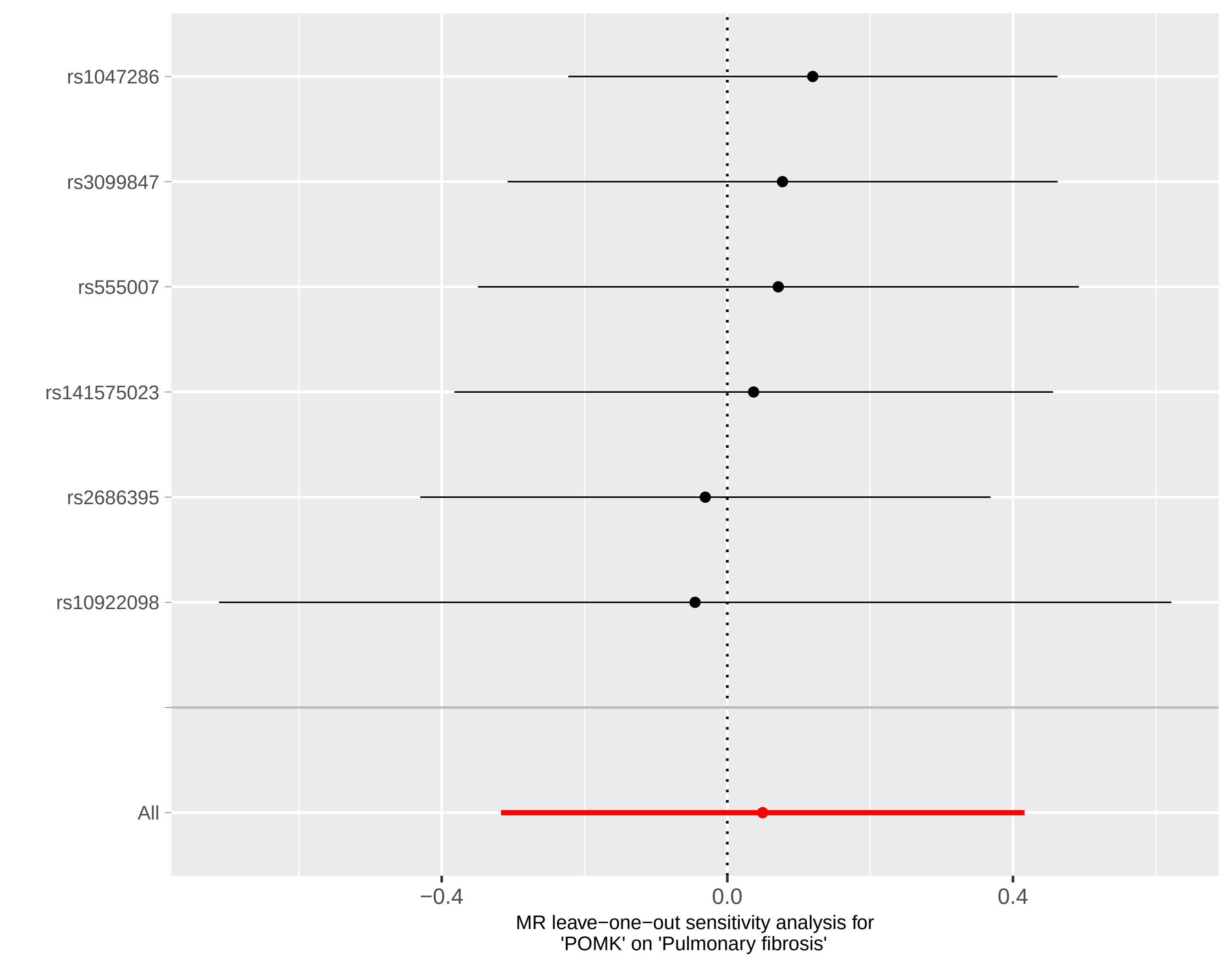
rs1047286
rs3099847
rs555007
rs141575023
rs2686395
rs10922098
All
−0.4
0.0
0.4
MR leave−one−out sensitivity analysis for
'POMK' on 'Pulmonary fibrosis'

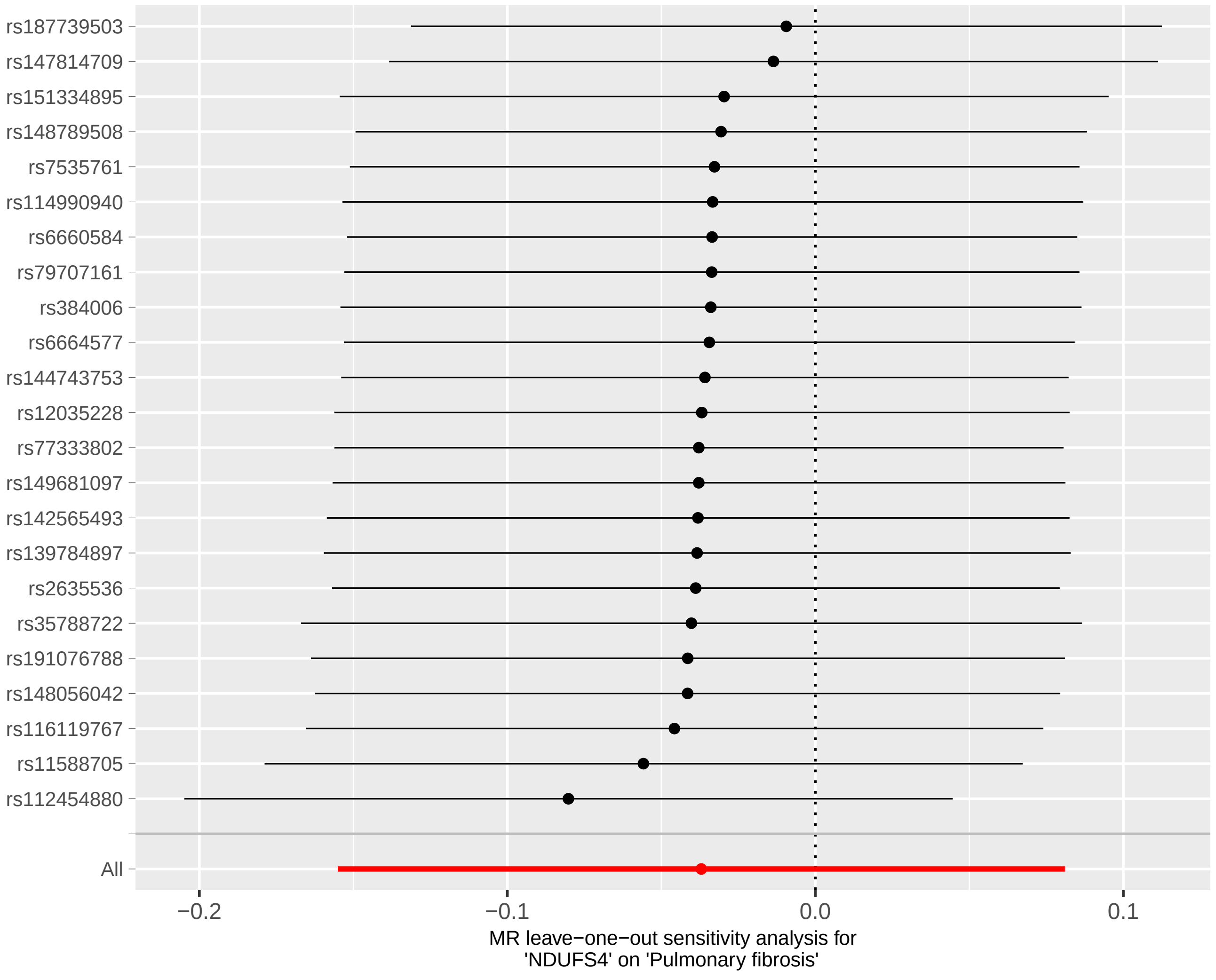
rs187739503
rs147814709
rs151334895
rs148789508
rs7535761
rs114990940
rs6660584
rs79707161
rs384006
rs6664577
rs144743753
rs12035228
rs77333802
rs149681097
rs142565493
rs139784897
rs2635536
rs35788722
rs191076788
rs148056042
rs116119767
rs11588705
rs112454880
All
−0.2
−0.1
0.0
0.1
MR leave−one−out sensitivity analysis for
'NDUFS4' on 'Pulmonary fibrosis'

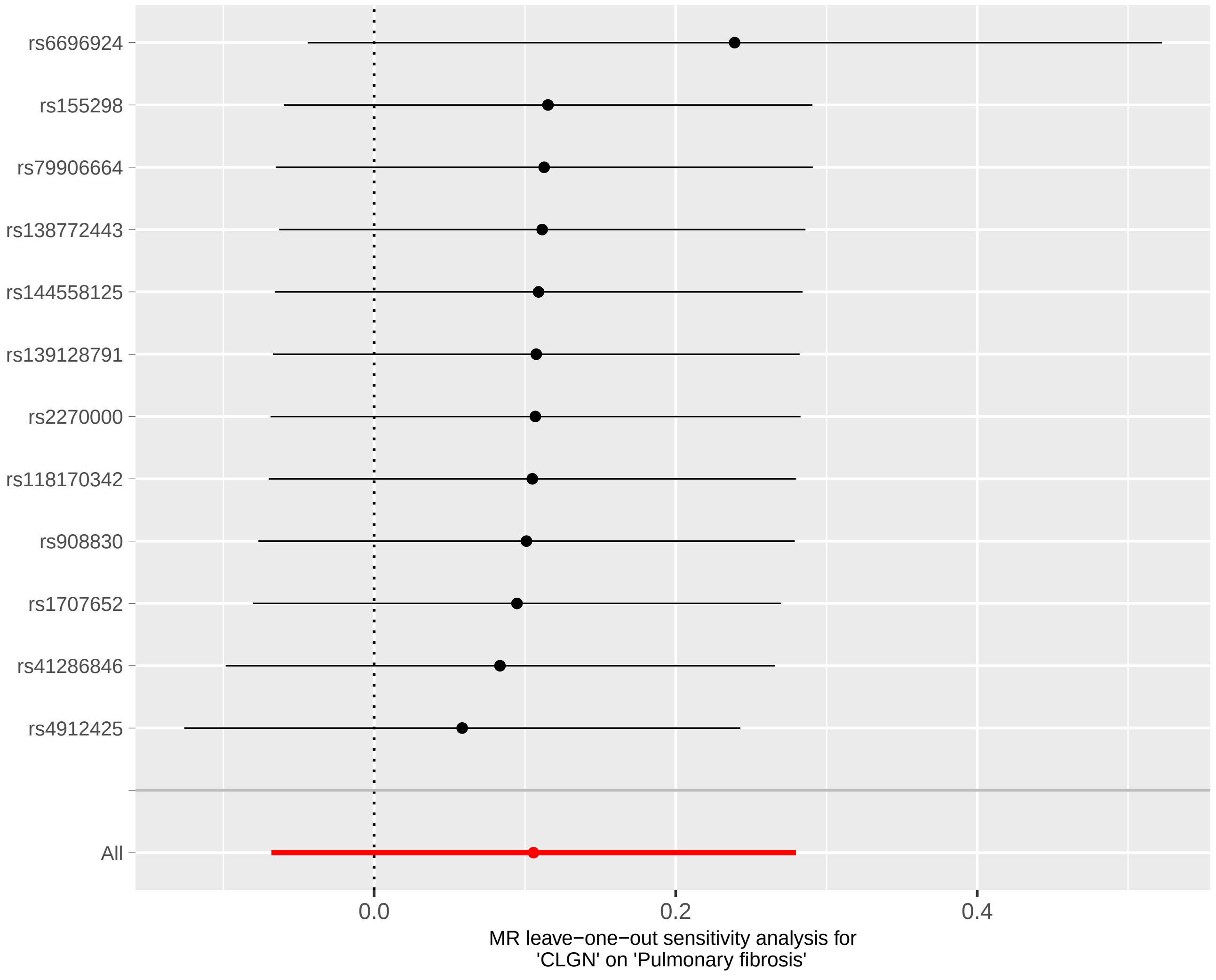
rs6696924
rs155298
rs79906664
rs138772443
rs144558125
rs139128791
rs2270000
rs118170342
rs908830
rs1707652
rs41286846
rs4912425
All
0.0
0.2
0.4
MR leave−one−out sensitivity analysis for
'CLGN' on 'Pulmonary fibrosis'

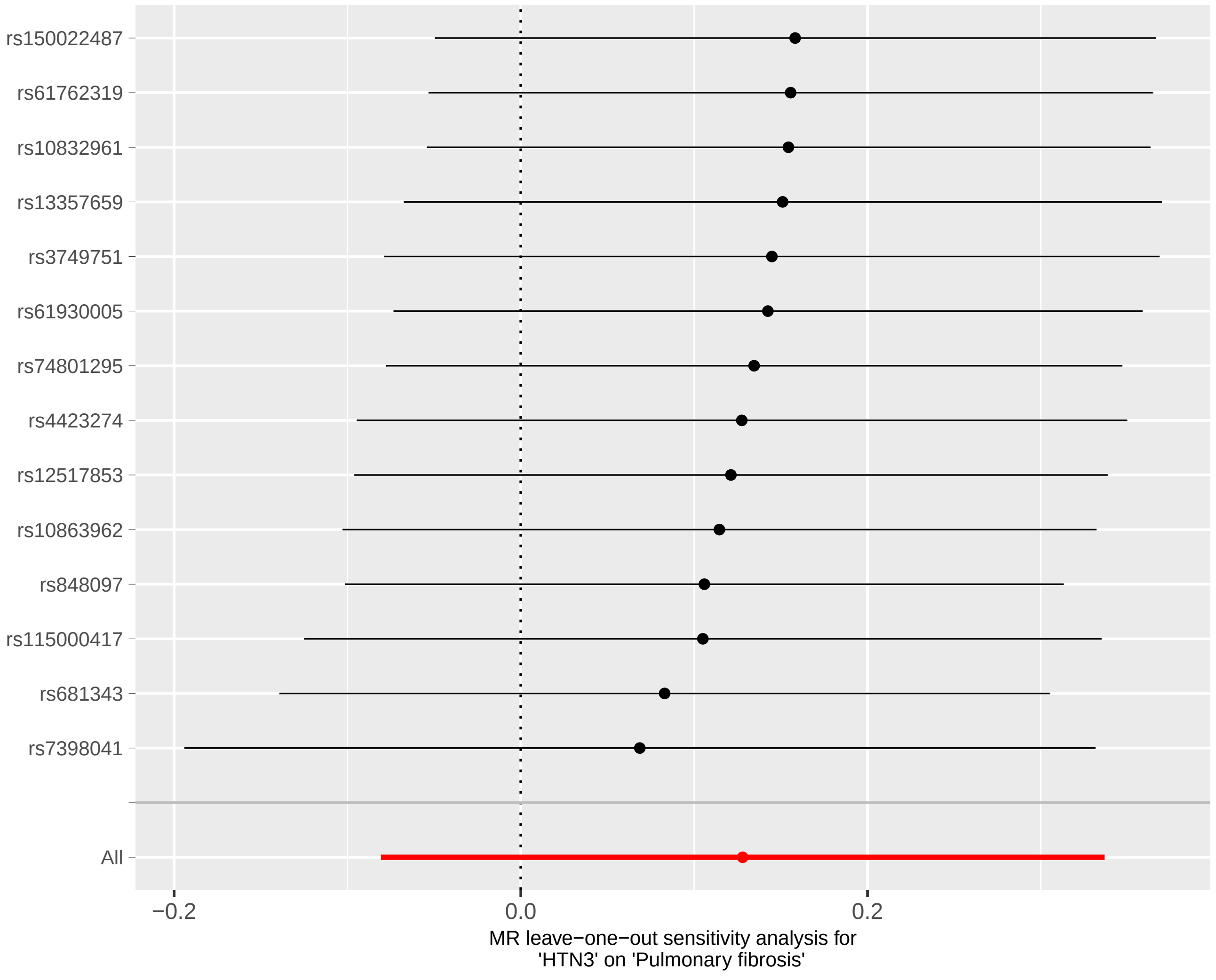
rs150022487
rs61762319
rs10832961
rs13357659
rs3749751
rs61930005
rs74801295
rs4423274
rs12517853
rs10863962
rs848097
rs115000417
rs681343
rs7398041
All
−0.2
0.0
0.2
MR leave−one−out sensitivity analysis for
'HTN3' on 'Pulmonary fibrosis'

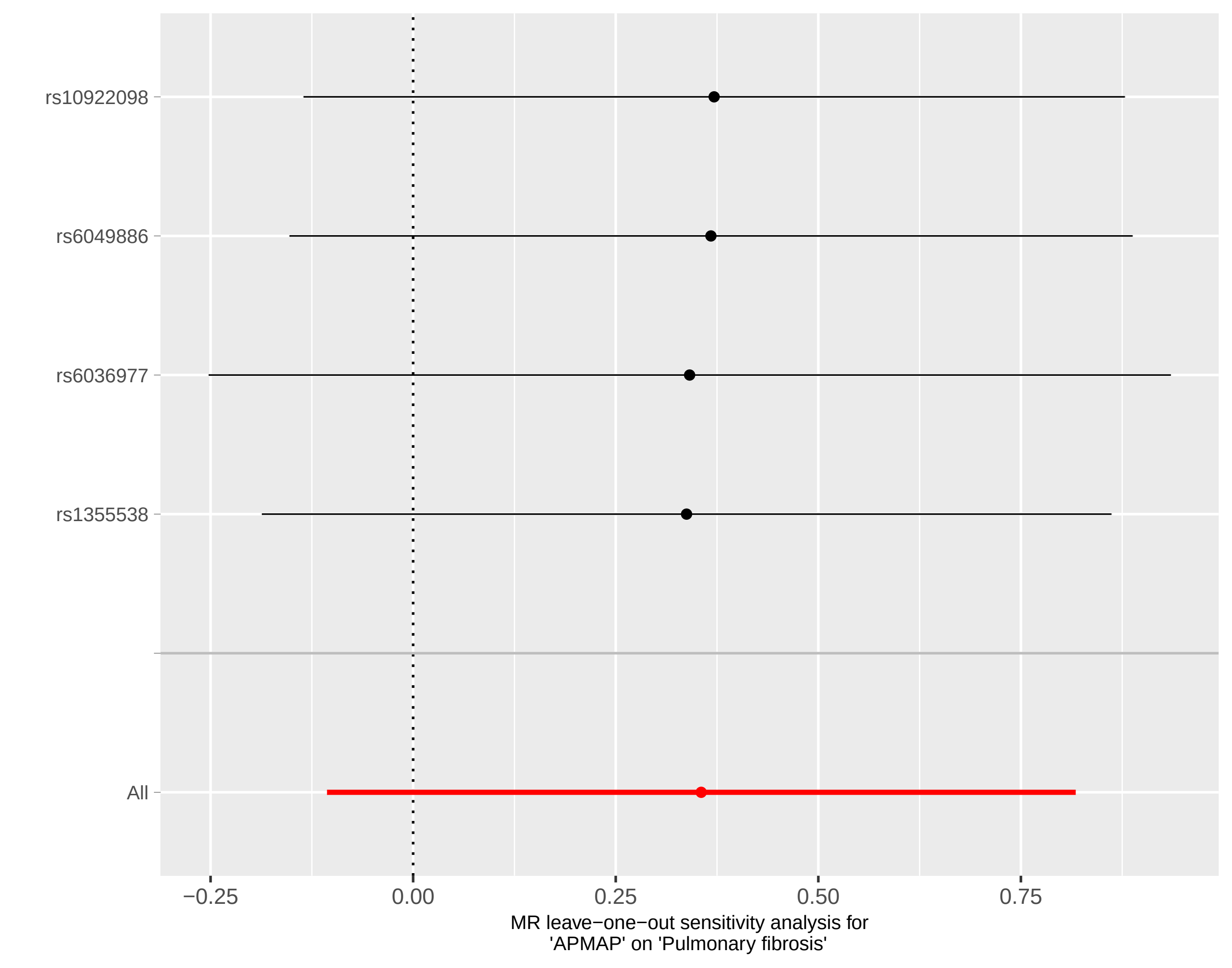
rs10922098
rs6049886
rs6036977
rs1355538
All
−0.25
0.00
0.25
0.50
0.75
MR leave−one−out sensitivity analysis for
'APMAP' on 'Pulmonary fibrosis'

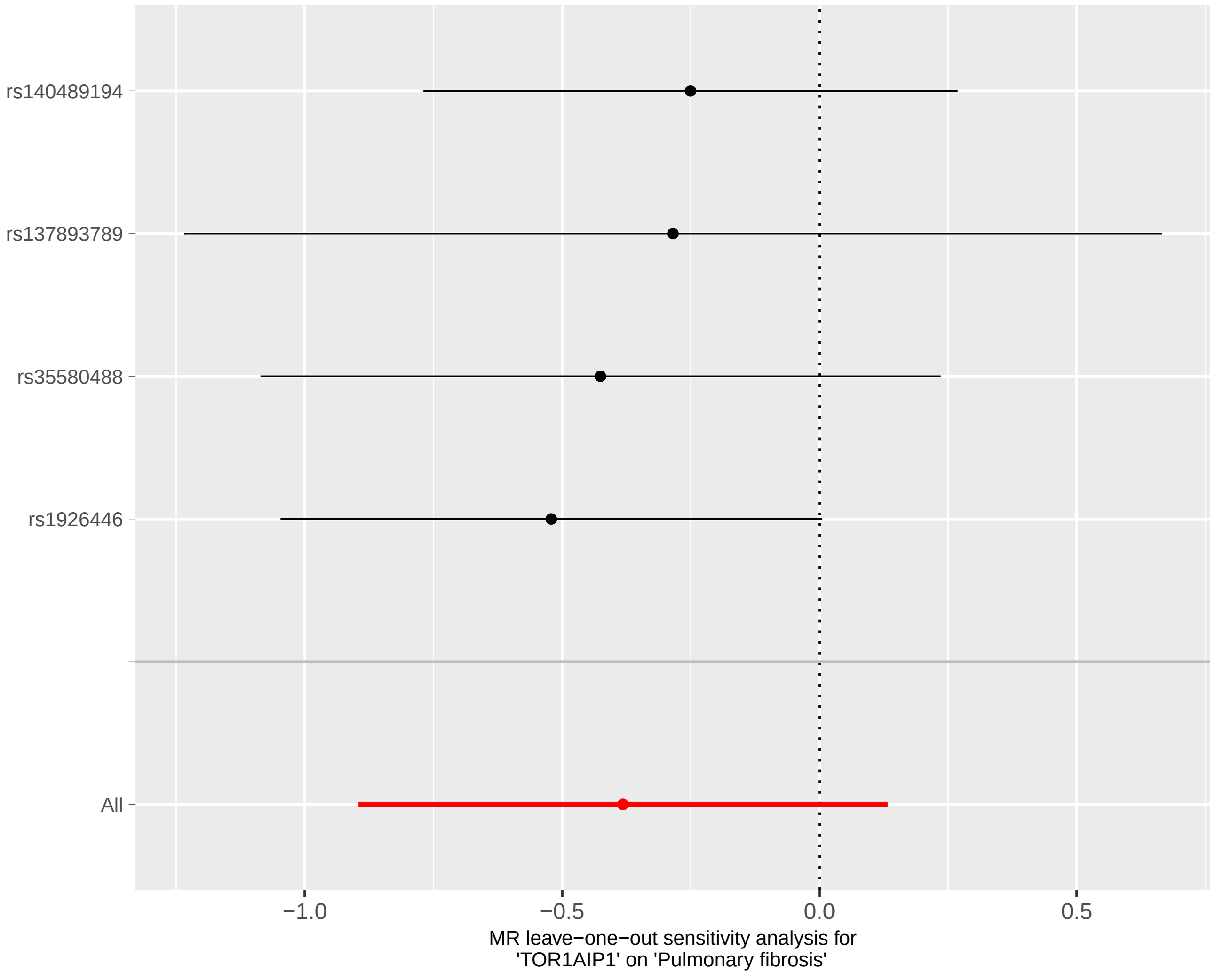
rs140489194
rs137893789
rs35580488
rs1926446
All
−1.0
−0.5
0.0
0.5
MR leave−one−out sensitivity analysis for
'TOR1AIP1' on 'Pulmonary fibrosis'

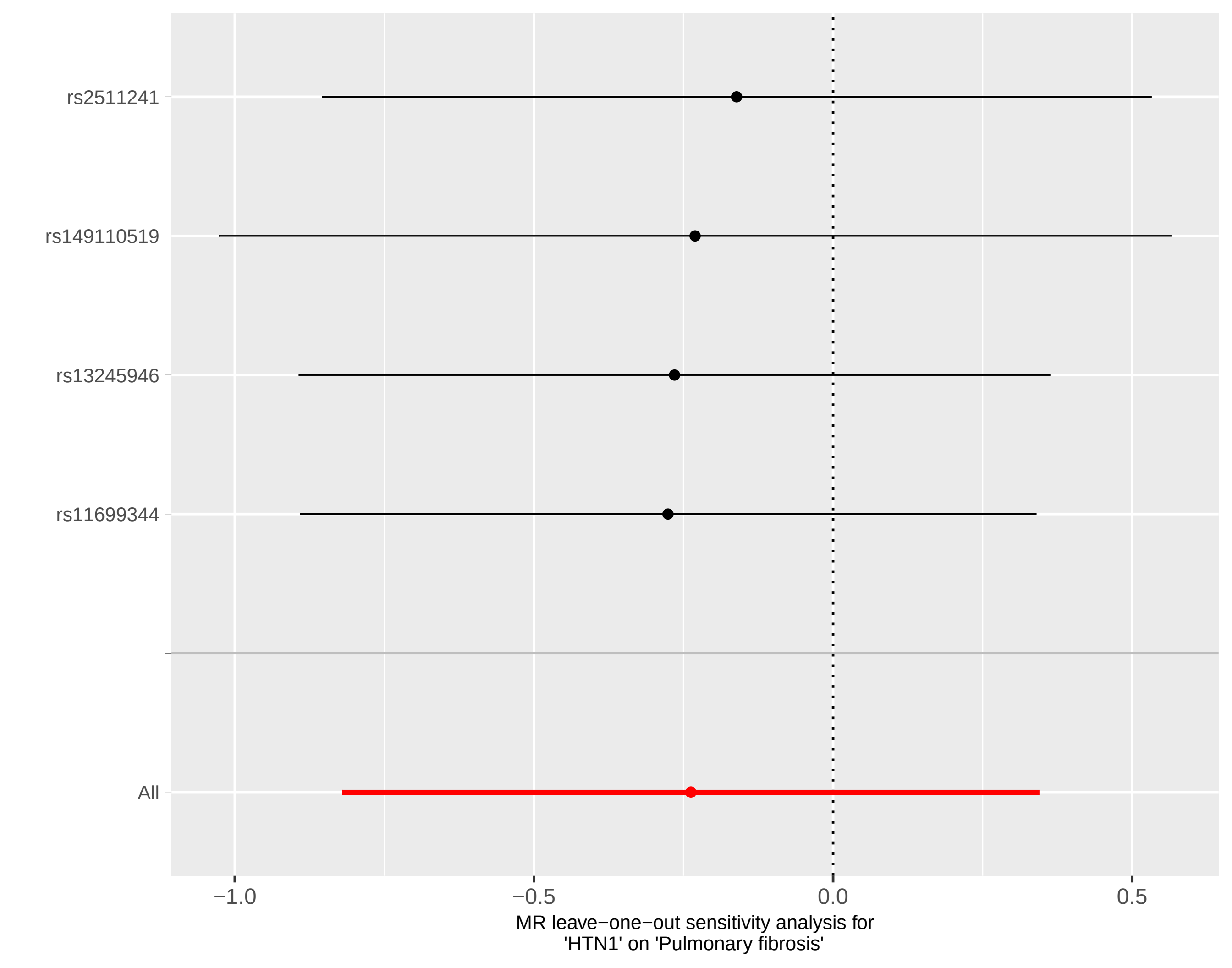
rs2511241
rs149110519
rs13245946
rs11699344
All
−1.0
−0.5
0.0
0.5
MR leave−one−out sensitivity analysis for
'HTN1' on 'Pulmonary fibrosis'

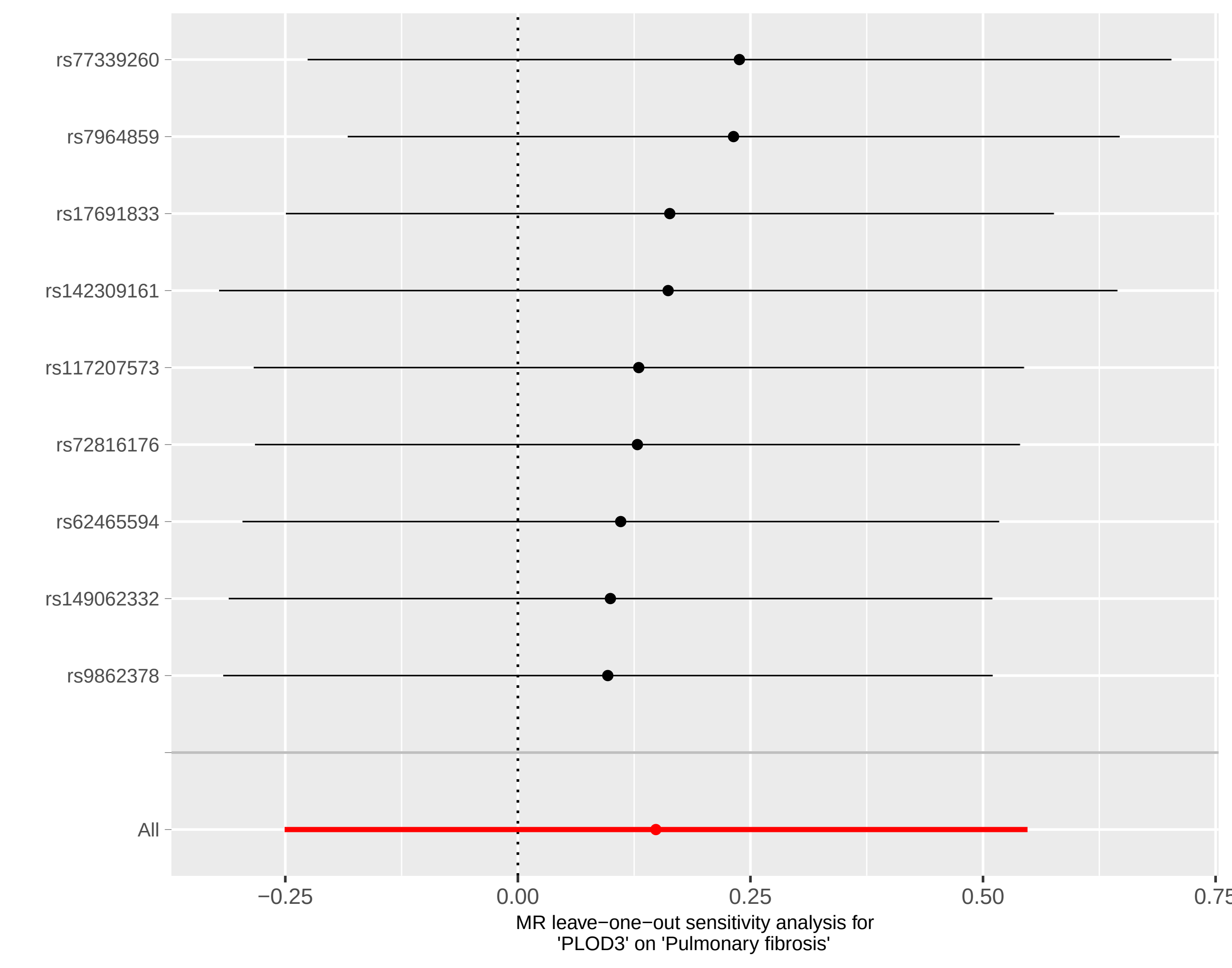

rs77339260
rs7964859
rs17691833
rs142309161
rs117207573
rs72816176
rs62465594
rs149062332
rs9862378
All
−0.25
0.00
0.25
0.50
0.75
MR leave−one−out sensitivity analysis for
'PLOD3' on 'Pulmonary fibrosis'

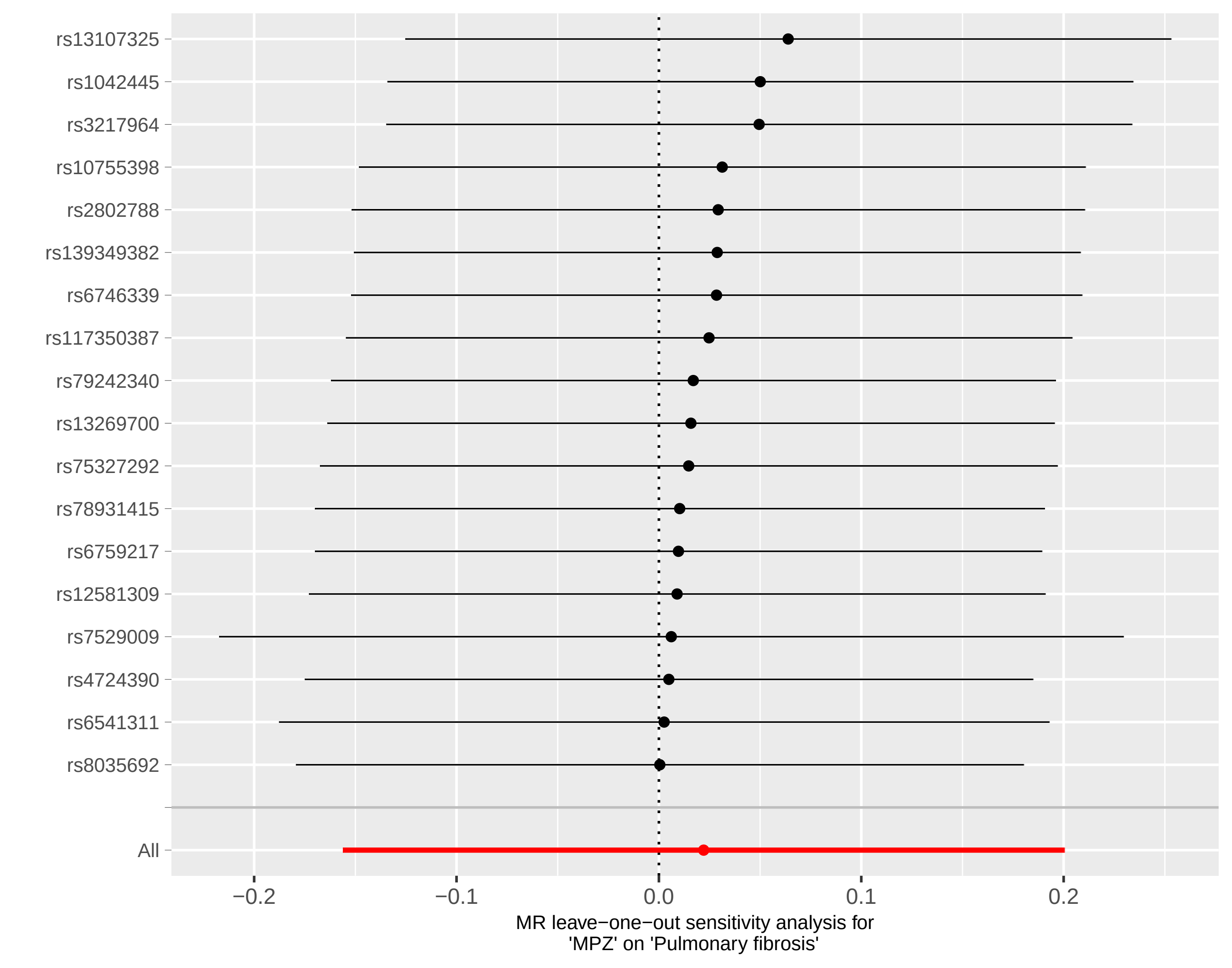
rs13107325
rs1042445
rs3217964
rs10755398
rs2802788
rs139349382
rs6746339
rs117350387
rs79242340
rs13269700
rs75327292
rs78931415
rs6759217
rs12581309
rs7529009
rs4724390
rs6541311
rs8035692
All
−0.2
−0.1
0.0
0.1
0.2
MR leave−one−out sensitivity analysis for
'MPZ' on 'Pulmonary fibrosis'

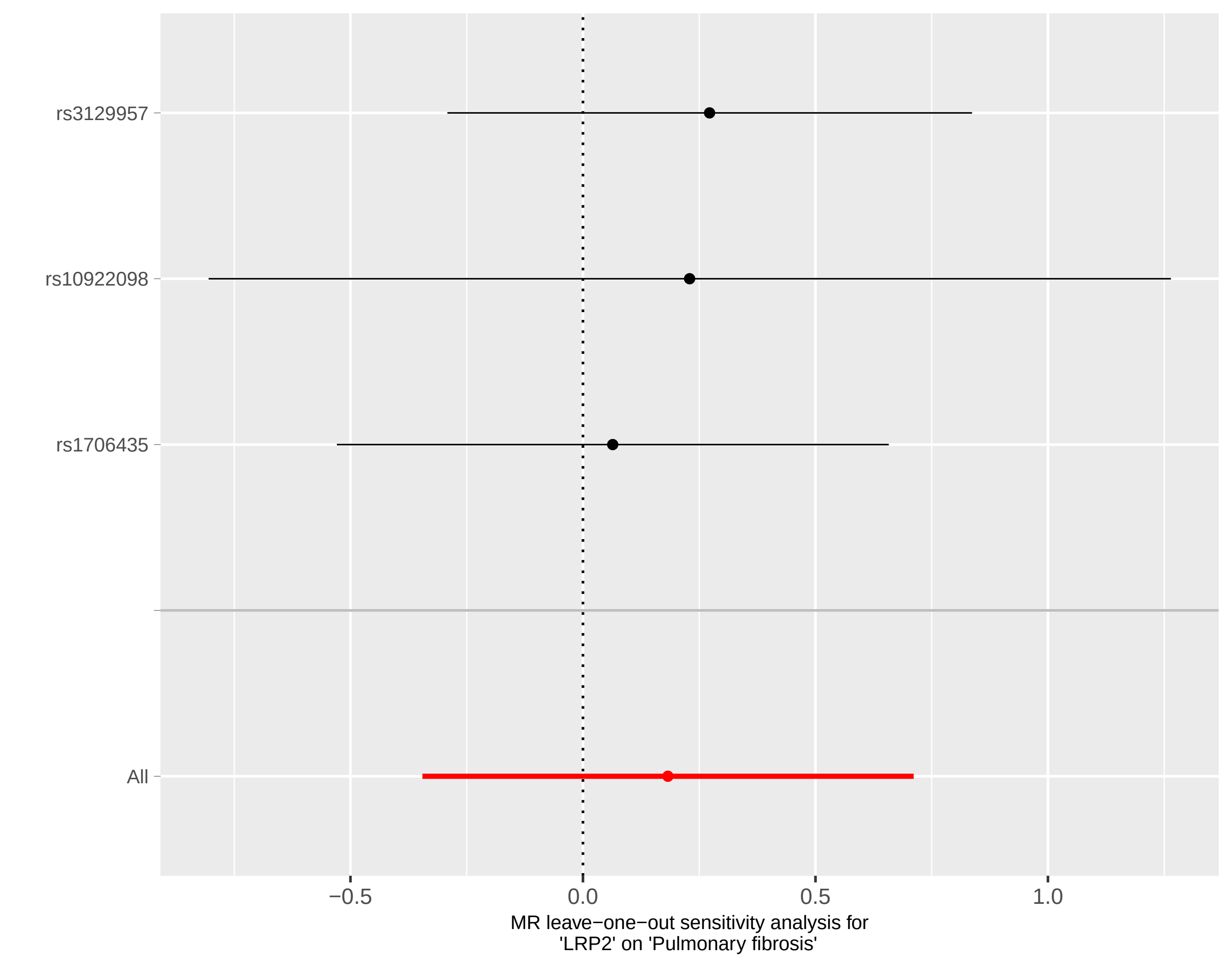

rs3129957
rs10922098
rs1706435
All
−0.5
0.0
0.5
1.0
MR leave−one−out sensitivity analysis for
'LRP2' on 'Pulmonary fibrosis'

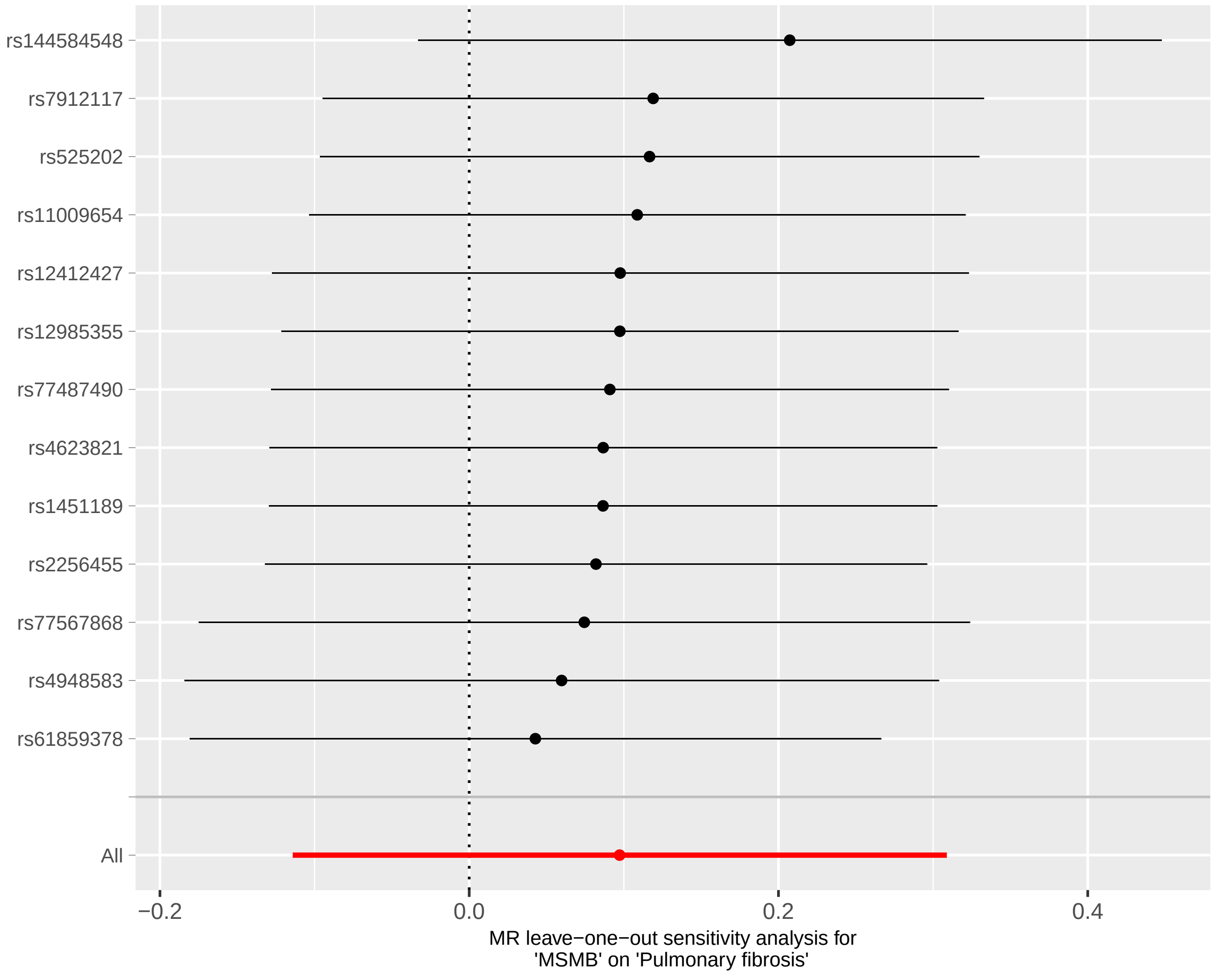
rs144584548
rs7912117
rs525202
rs11009654
rs12412427
rs12985355
rs77487490
rs4623821
rs1451189
rs2256455
rs77567868
rs4948583
rs61859378
All
−0.2
0.0
0.2
0.4
MR leave−one−out sensitivity analysis for
'MSMB' on 'Pulmonary fibrosis'

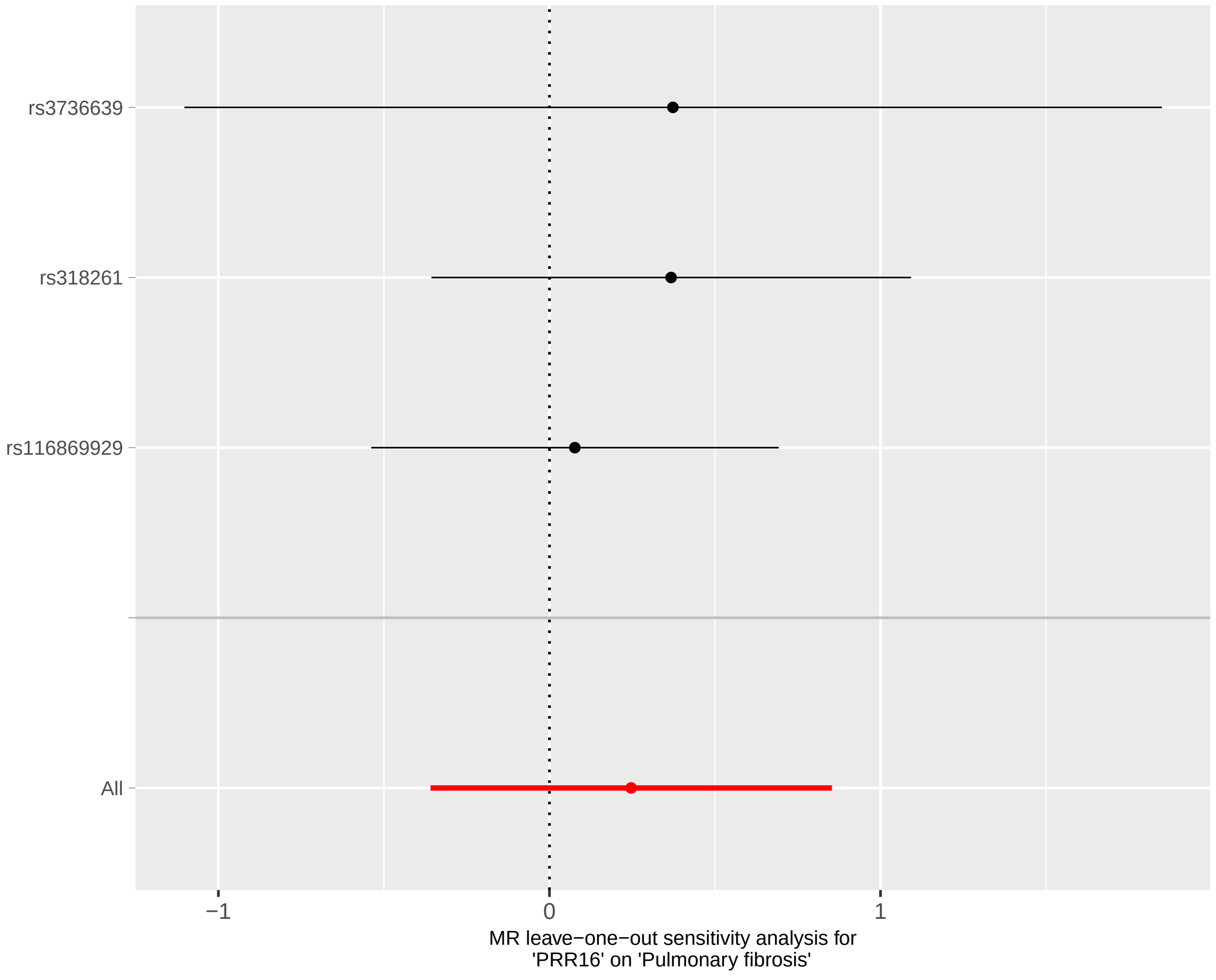
rs3736639
rs318261
rs116869929
All
−1
0
1
MR leave−one−out sensitivity analysis for
'PRR16' on 'Pulmonary fibrosis'

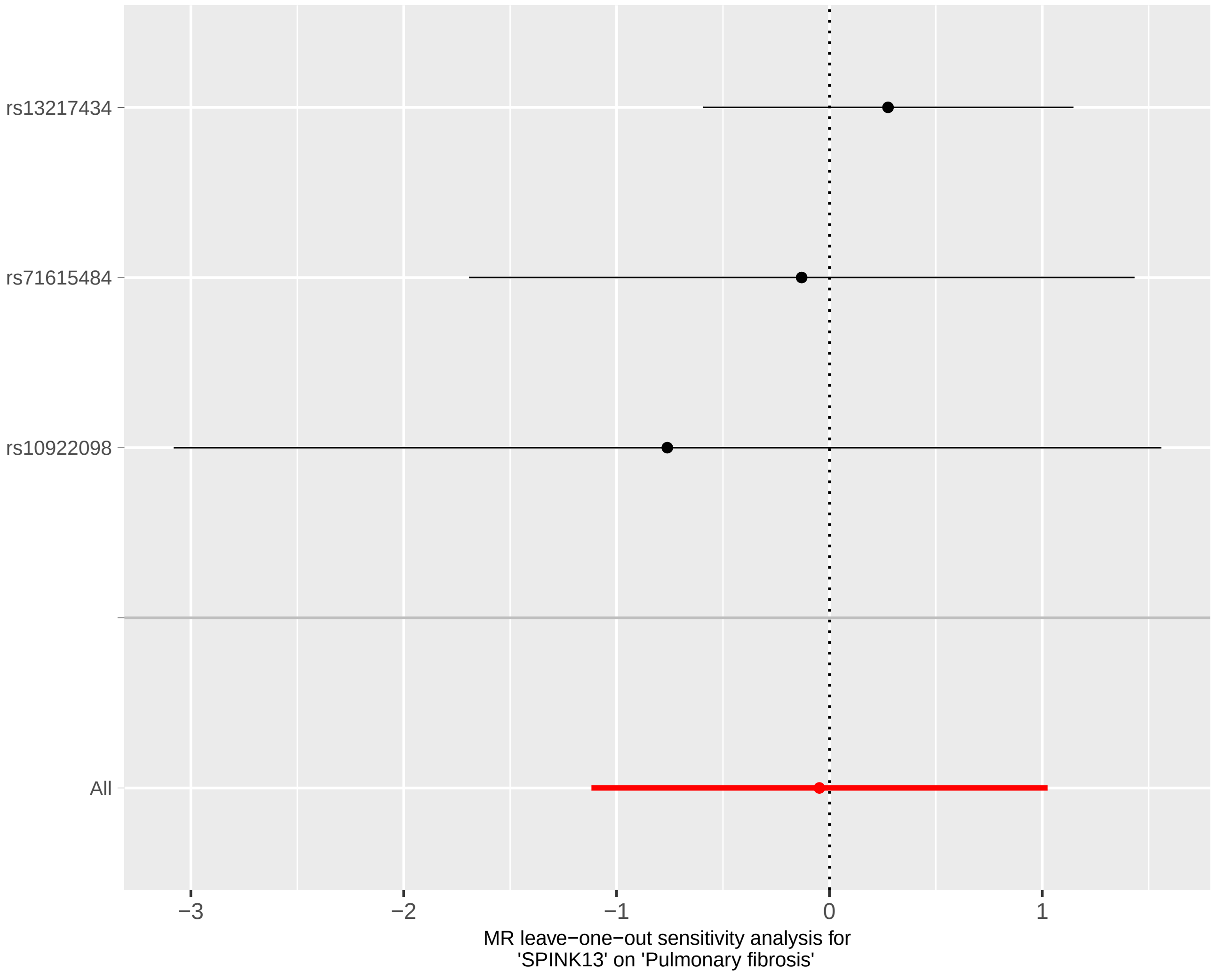
rs13217434
rs71615484
rs10922098
All
−3
−2
−1
0
1
MR leave−one−out sensitivity analysis for
'SPINK13' on 'Pulmonary fibrosis'

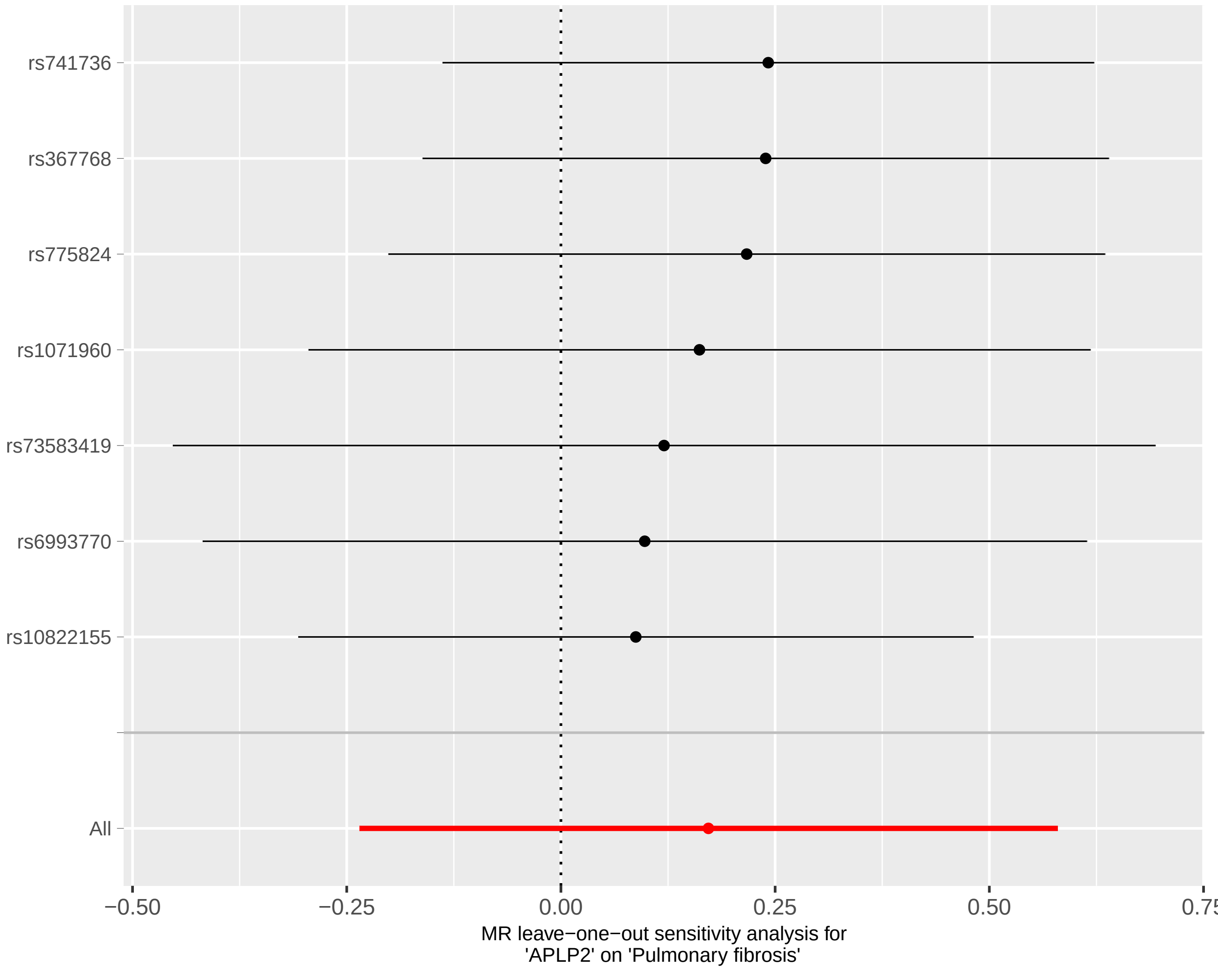
rs741736
rs367768
rs775824
rs1071960
rs73583419
rs6993770
rs10822155
All
−0.50
−0.25
0.00
0.25
0.50
MR leave−one−out sensitivity analysis for
'APLP2' on 'Pulmonary fibrosis'

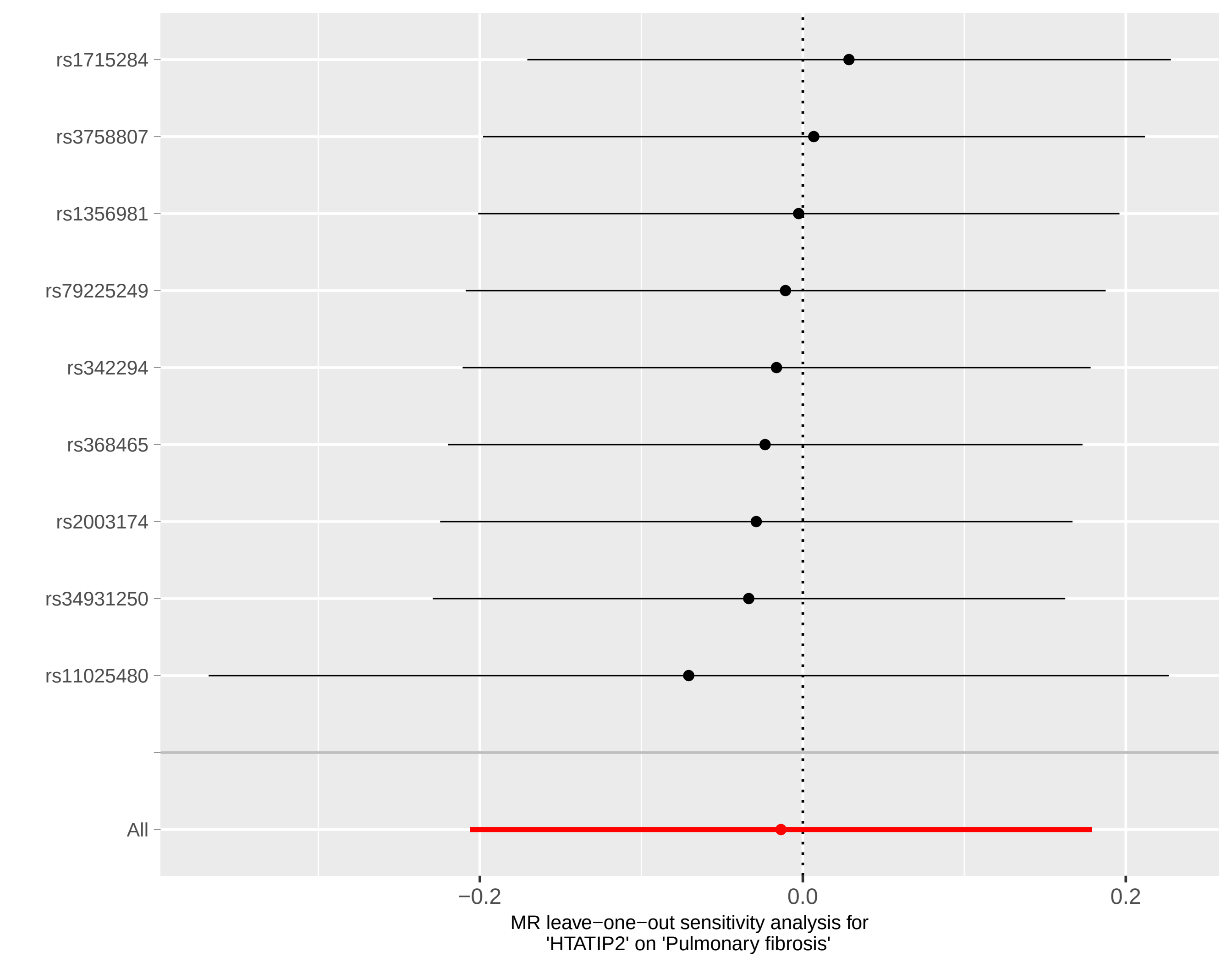
rs1715284
rs3758807
rs1356981
rs79225249
rs342294
rs368465
rs2003174
rs34931250
rs11025480
All
−0.2
0.0
0.2
MR leave−one−out sensitivity analysis for
'HTATIP2' on 'Pulmonary fibrosis'

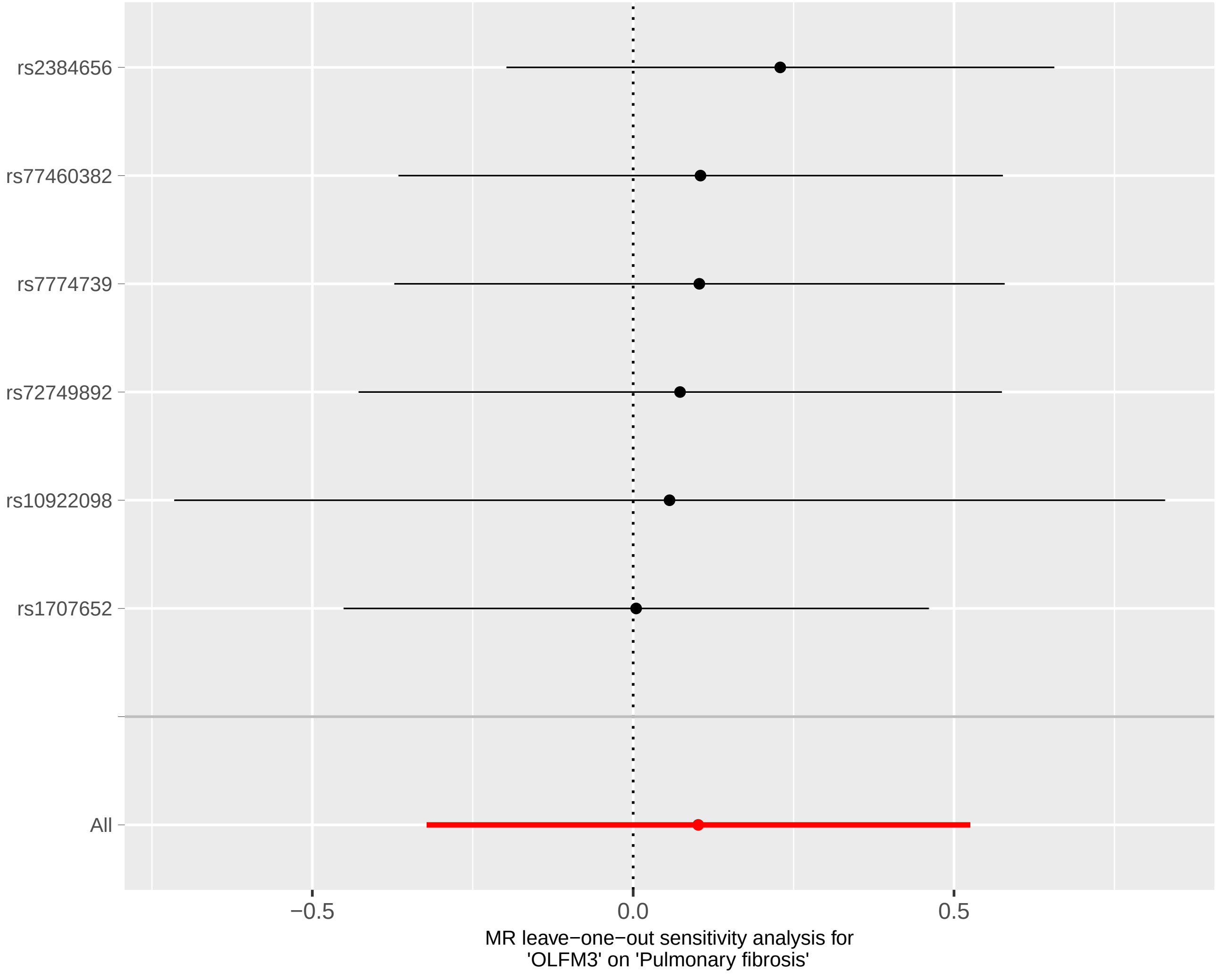

rs2384656
rs77460382
rs7774739
rs72749892
rs10922098
rs1707652
All
−0.5
0.0
0.5
MR leave−one−out sensitivity analysis for
'OLFM3' on 'Pulmonary fibrosis'

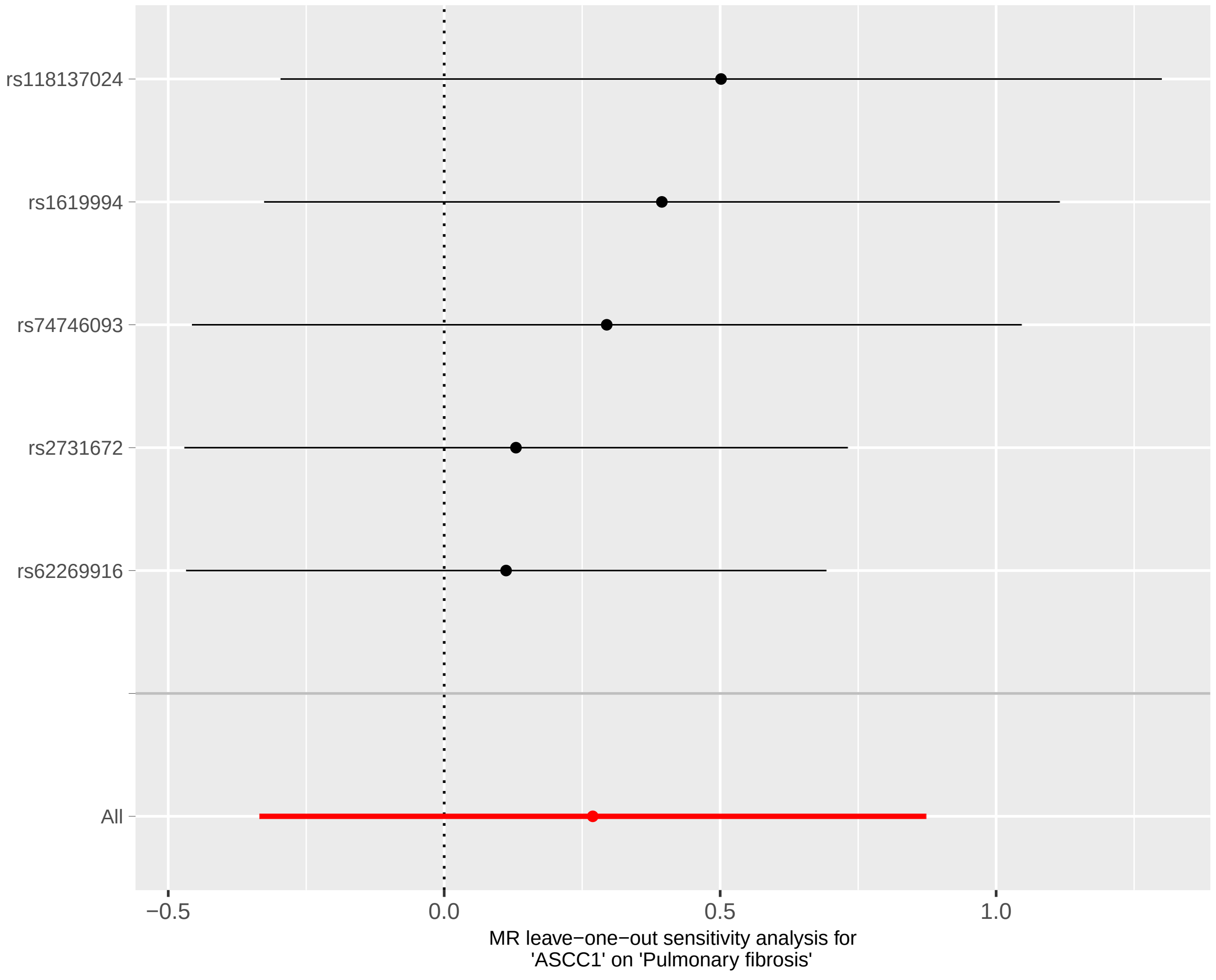

rs118137024
rs1619994
rs74746093
rs2731672
rs62269916
All
−0.5
0.0
0.5
1.0
MR leave−one−out sensitivity analysis for
'ASCC1' on 'Pulmonary fibrosis'

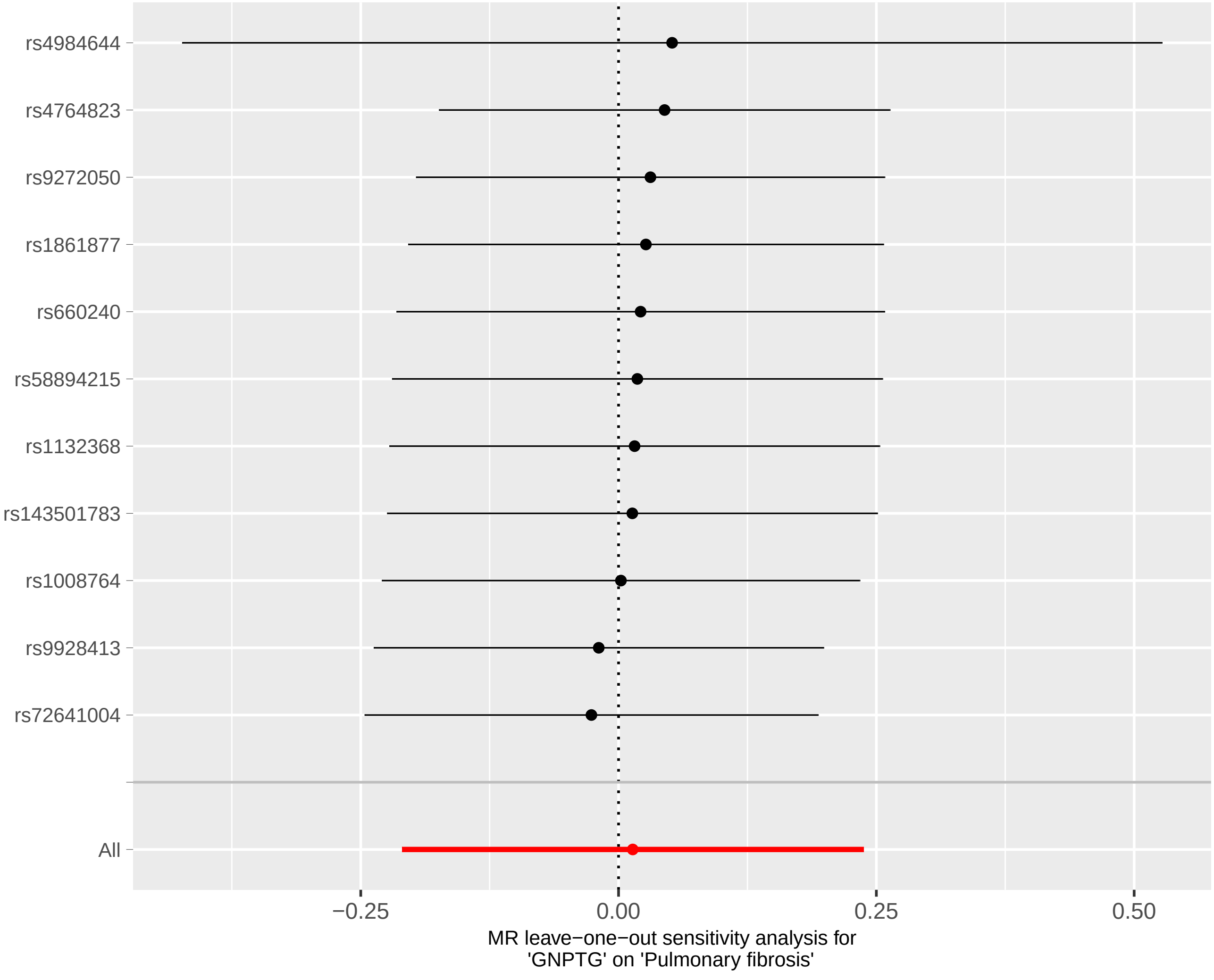
rs4984644
rs4764823
rs9272050
rs1861877
rs660240
rs58894215
rs1132368
rs143501783
rs1008764
rs9928413
rs72641004
All
−0.25
0.00
0.25
0.50
MR leave−one−out sensitivity analysis for
'GNPTG' on 'Pulmonary fibrosis'

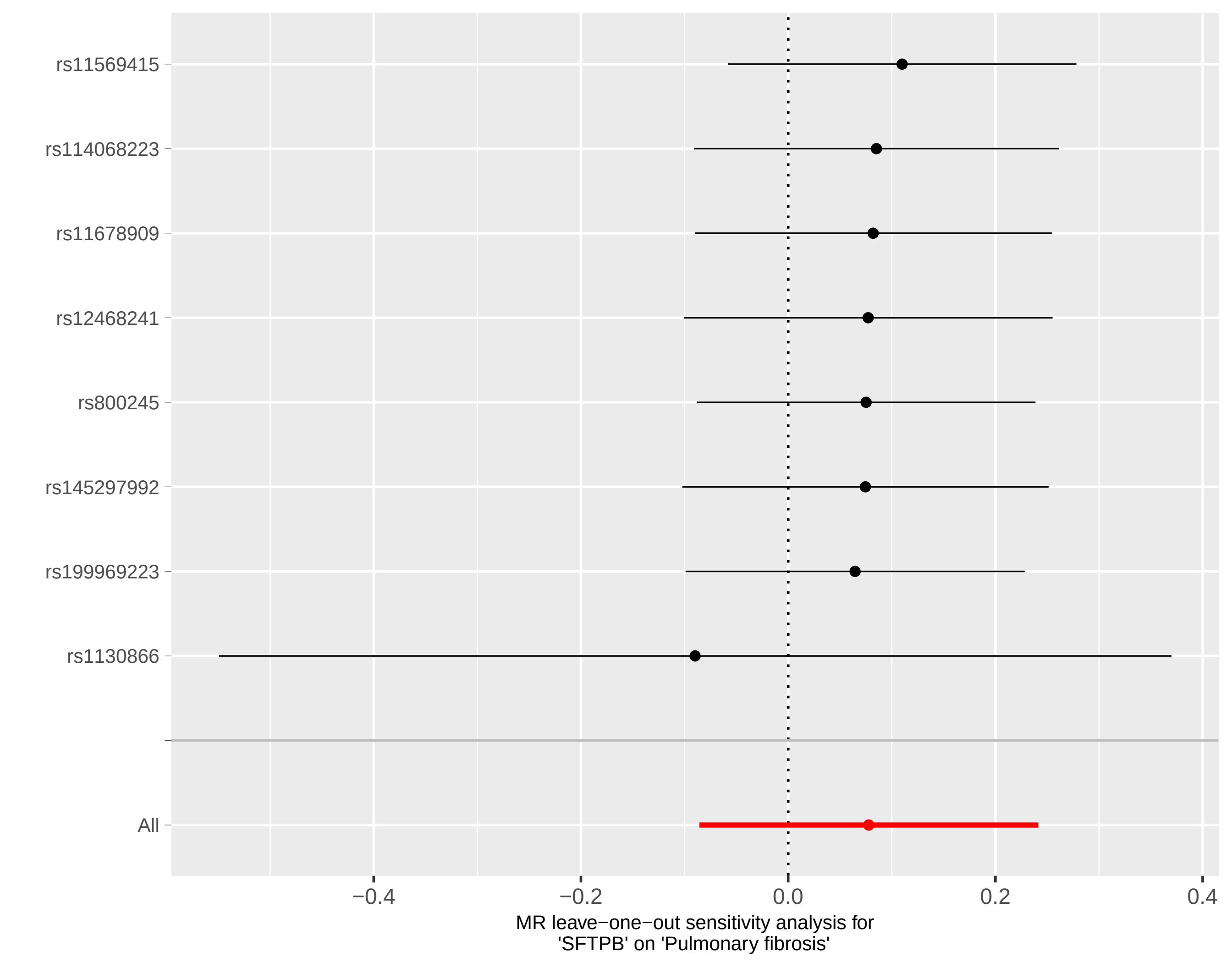
rs11569415
rs114068223
rs11678909
rs12468241
rs800245
rs145297992
rs199969223
rs1130866
All
−0.4
−0.2
0.0
0.2
0.4
MR leave−one−out sensitivity analysis for
'SFTPB' on 'Pulmonary fibrosis'

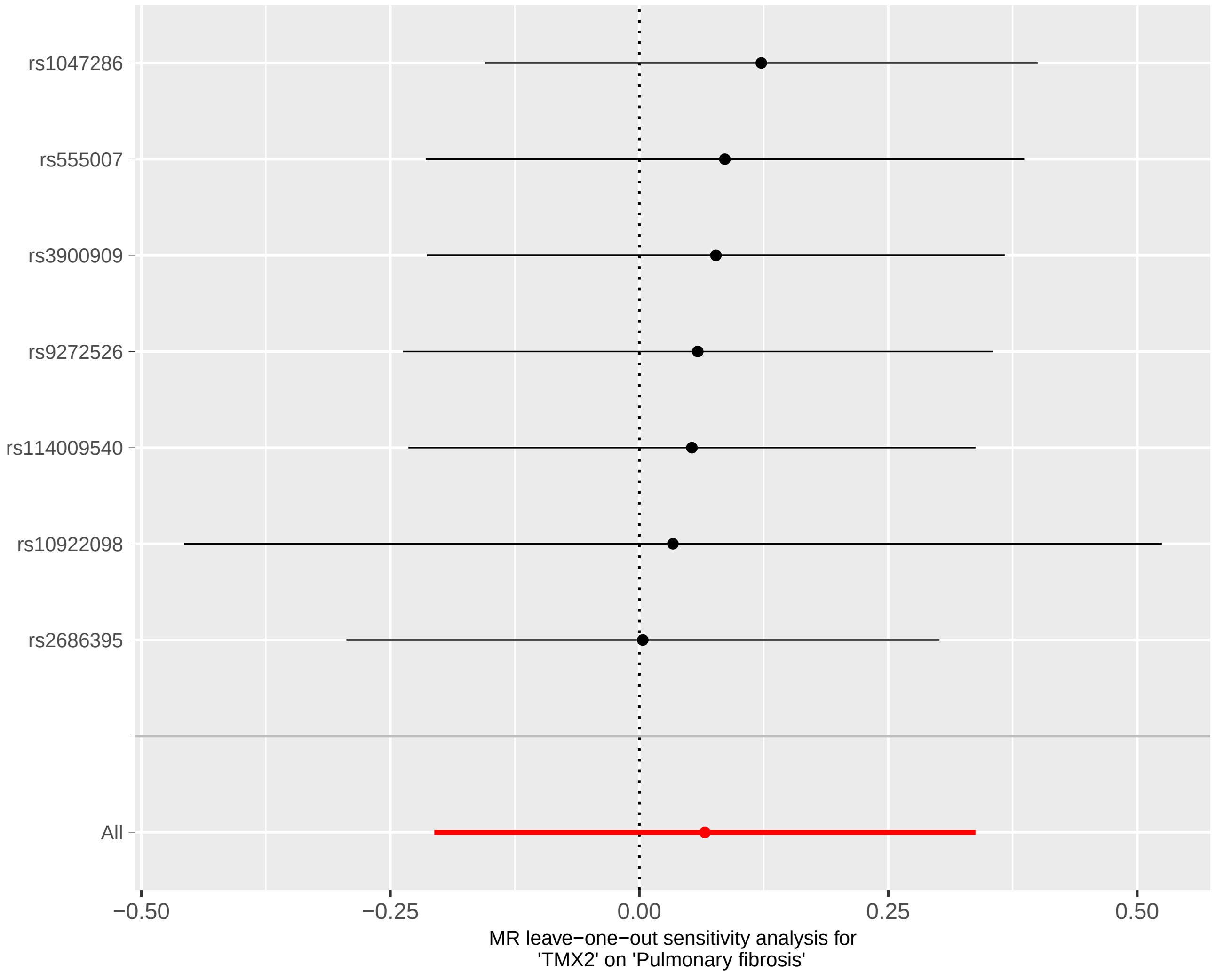
rs1047286
rs555007
rs3900909
rs9272526
rs114009540
rs10922098
rs2686395
All
−0.50
−0.25
0.00
0.25
0.50
MR leave−one−out sensitivity analysis for
'TMX2' on 'Pulmonary fibrosis'

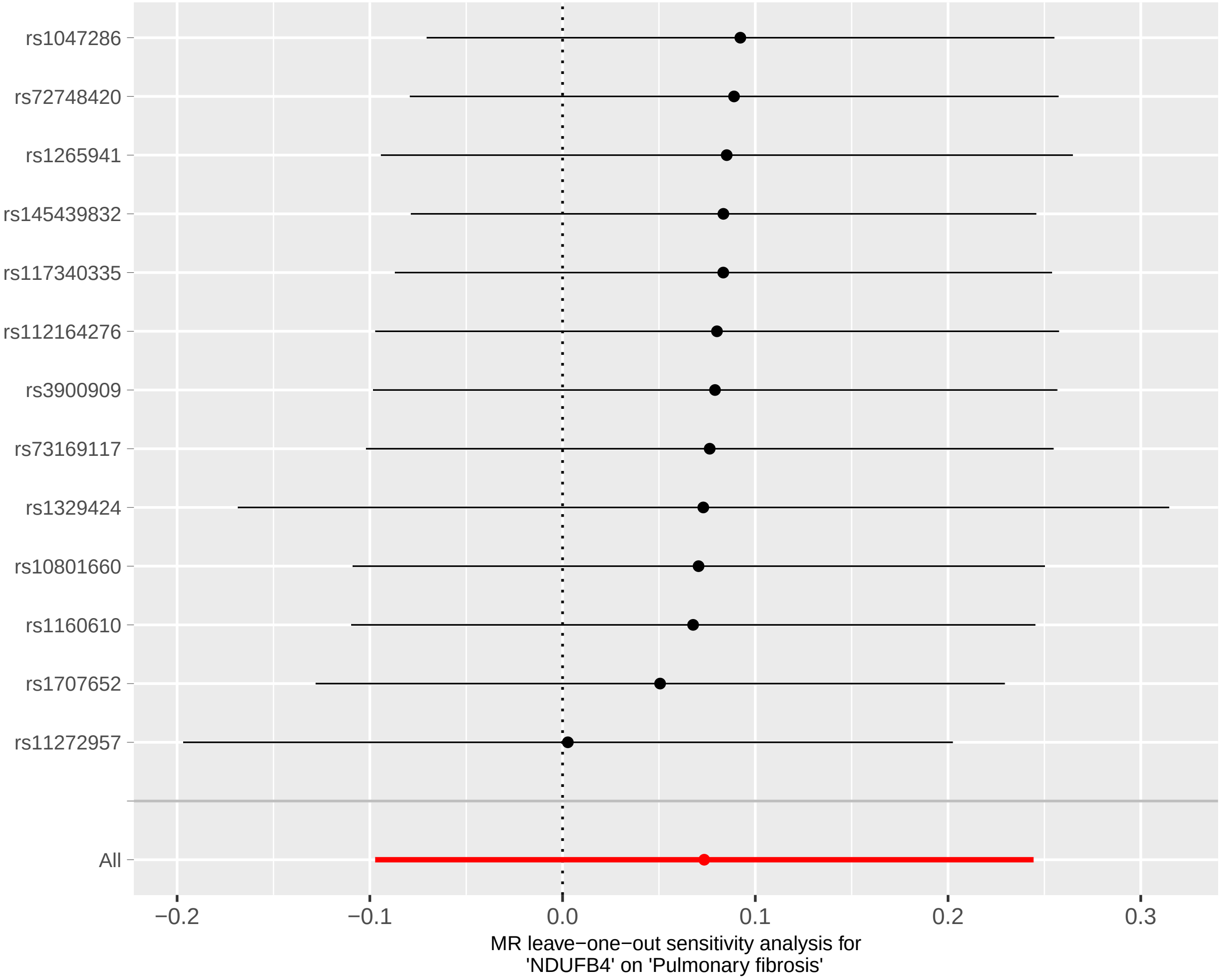
rs1047286
rs72748420
rs1265941
rs145439832
rs117340335
rs112164276
rs3900909
rs73169117
rs1329424
rs10801660
rs1160610
rs1707652
rs11272957
All
−0.2
−0.1
0.0
0.1
0.2
0.3
MR leave−one−out sensitivity analysis for
'NDUFB4' on 'Pulmonary fibrosis'

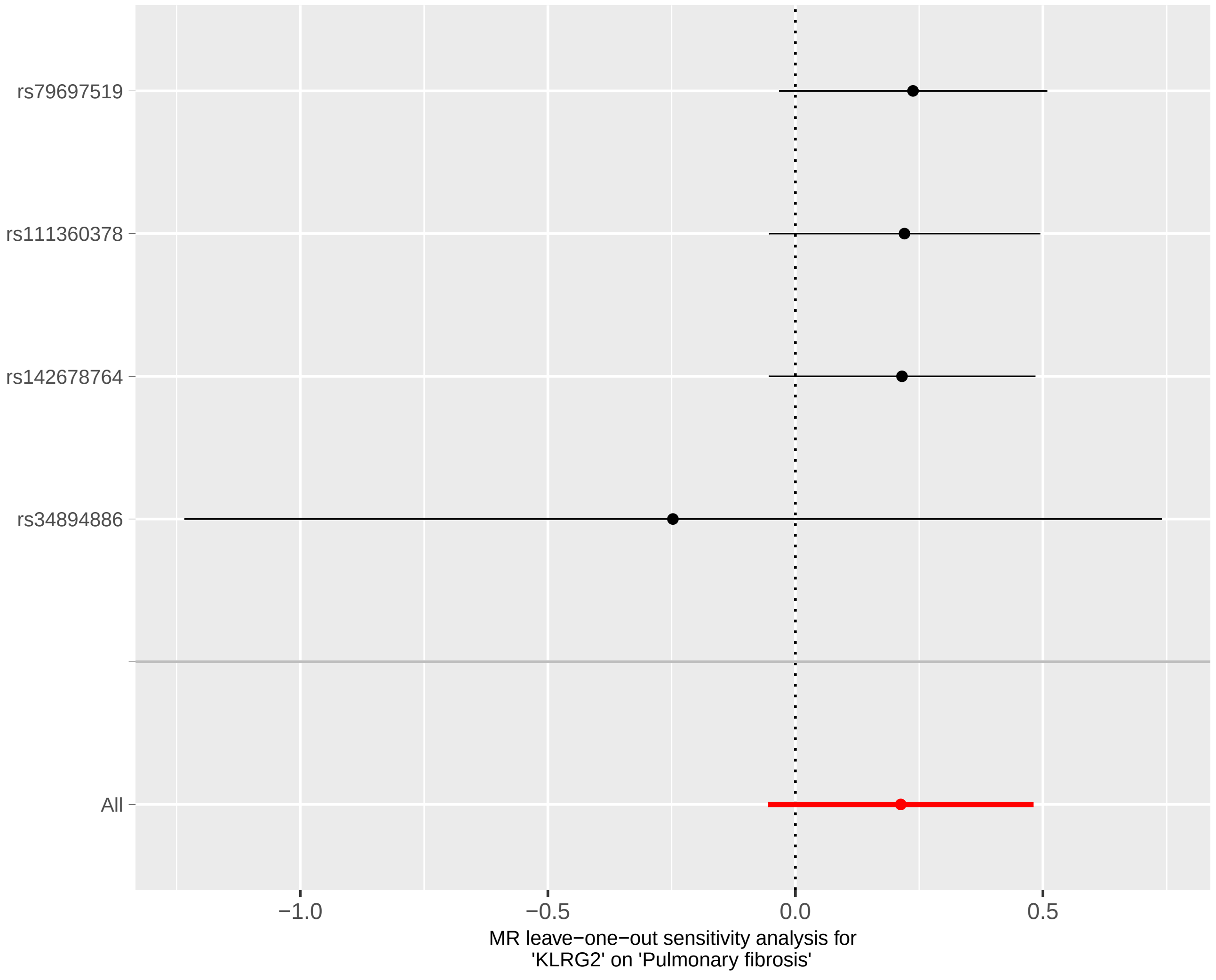
rs79697519
rs111360378
rs142678764
rs34894886
All
−1.0
−0.5
0.0
0.5
MR leave−one−out sensitivity analysis for
'KLRG2' on 'Pulmonary fibrosis'

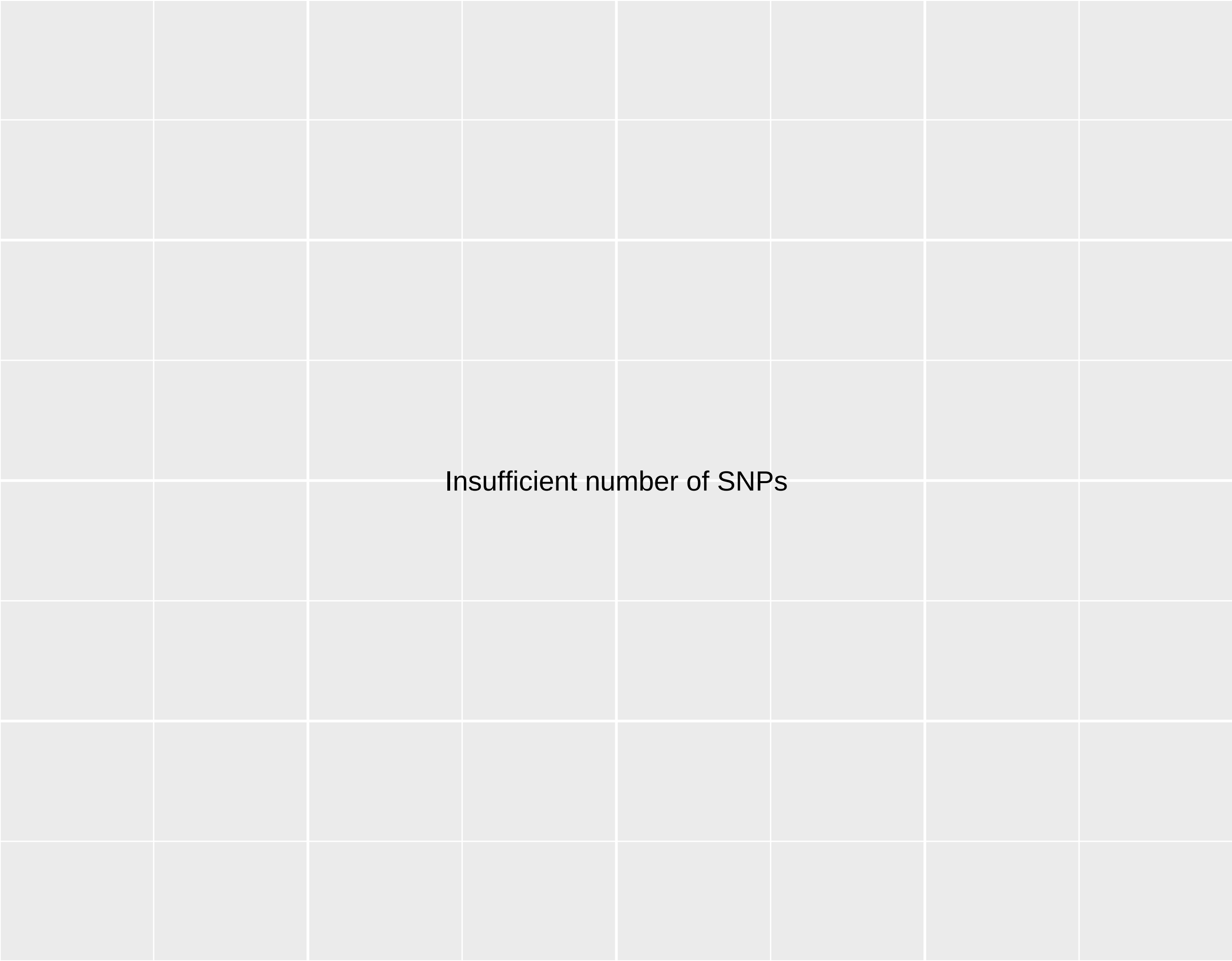
Insufficient number of SNPs

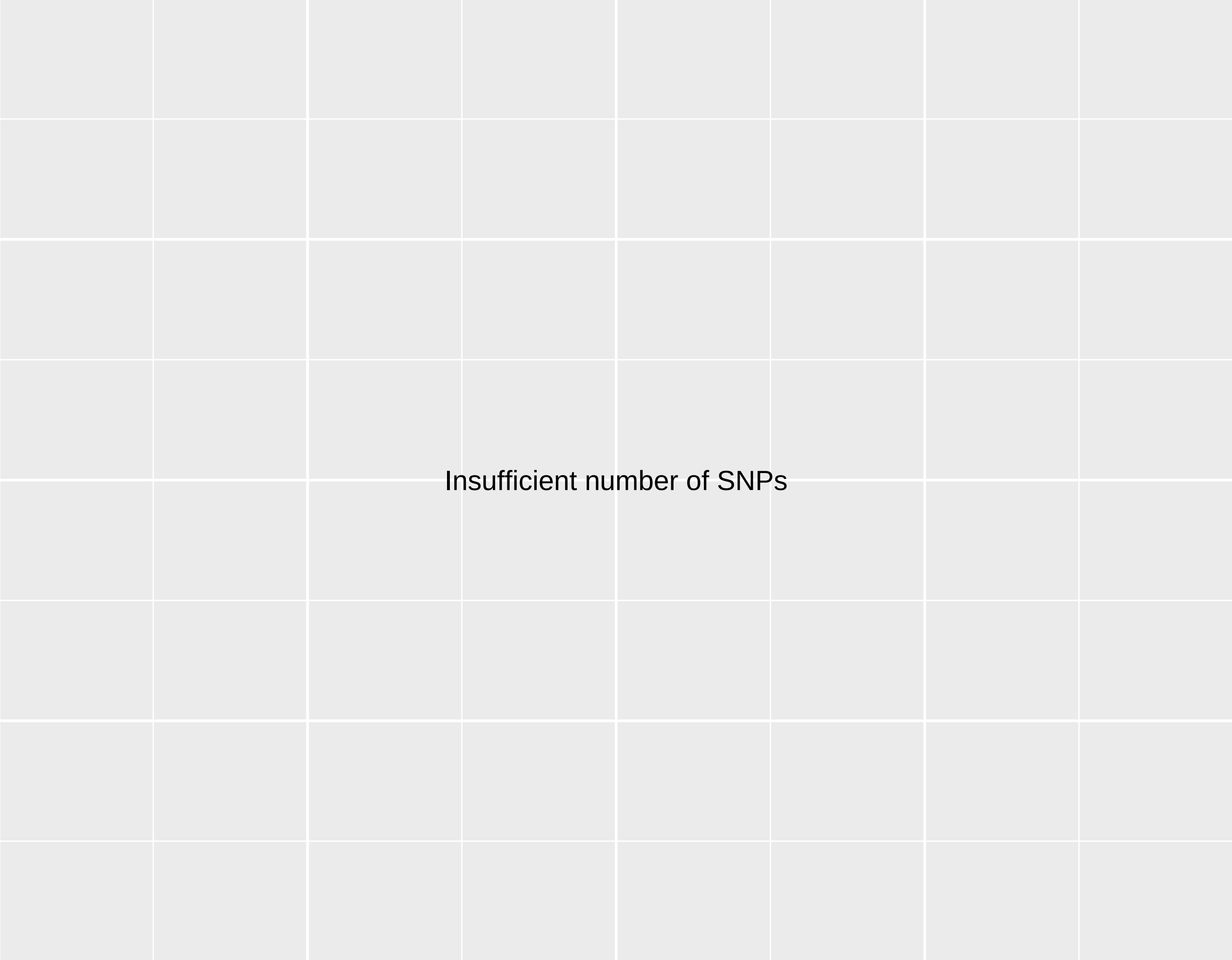
Insufficient number of SNPs

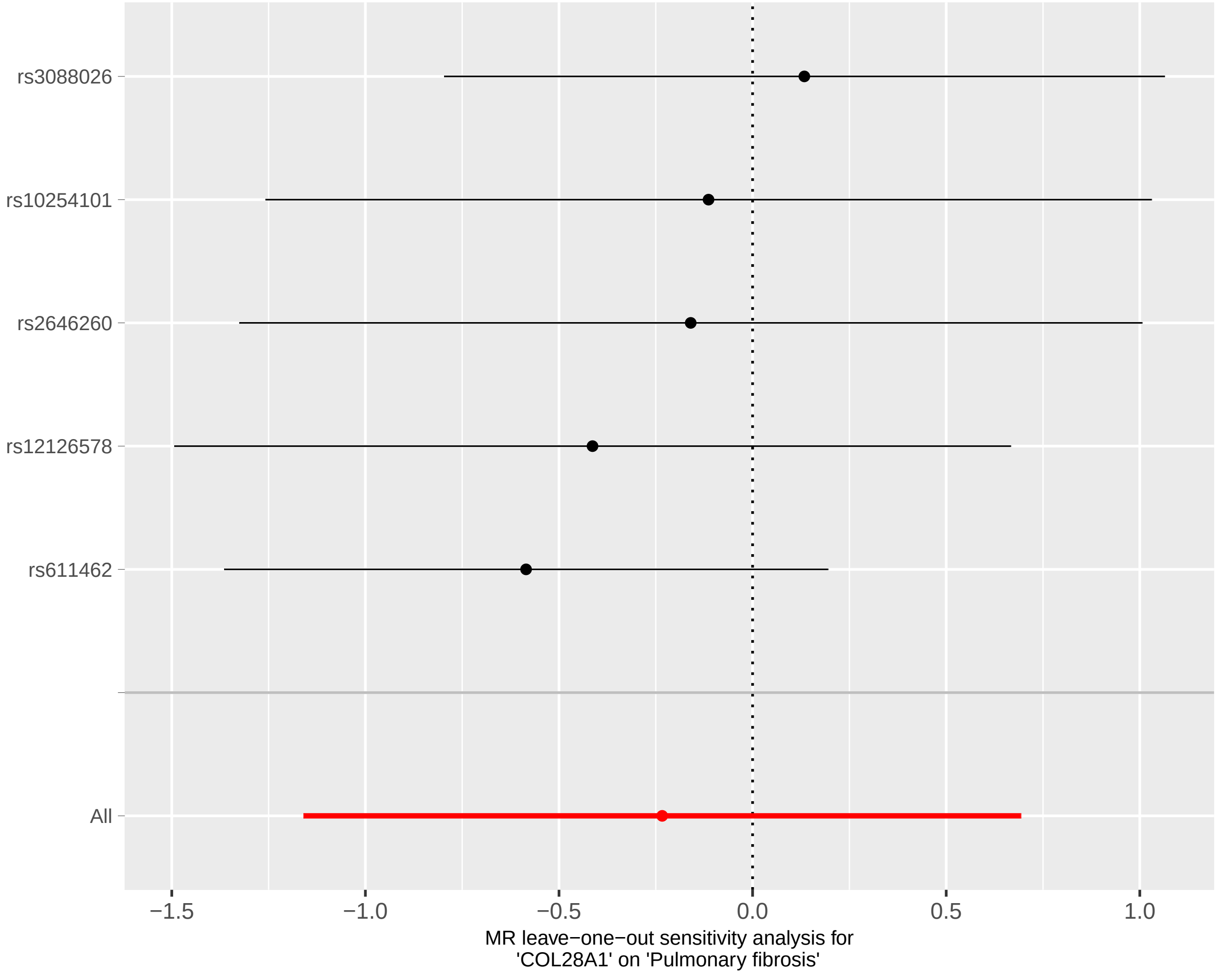

rs3088026
rs10254101
rs2646260
rs12126578
rs611462
All
−1.5
−1.0
−0.5
0.0
0.5
1.0
MR leave−one−out sensitivity analysis for
'COL28A1' on 'Pulmonary fibrosis'

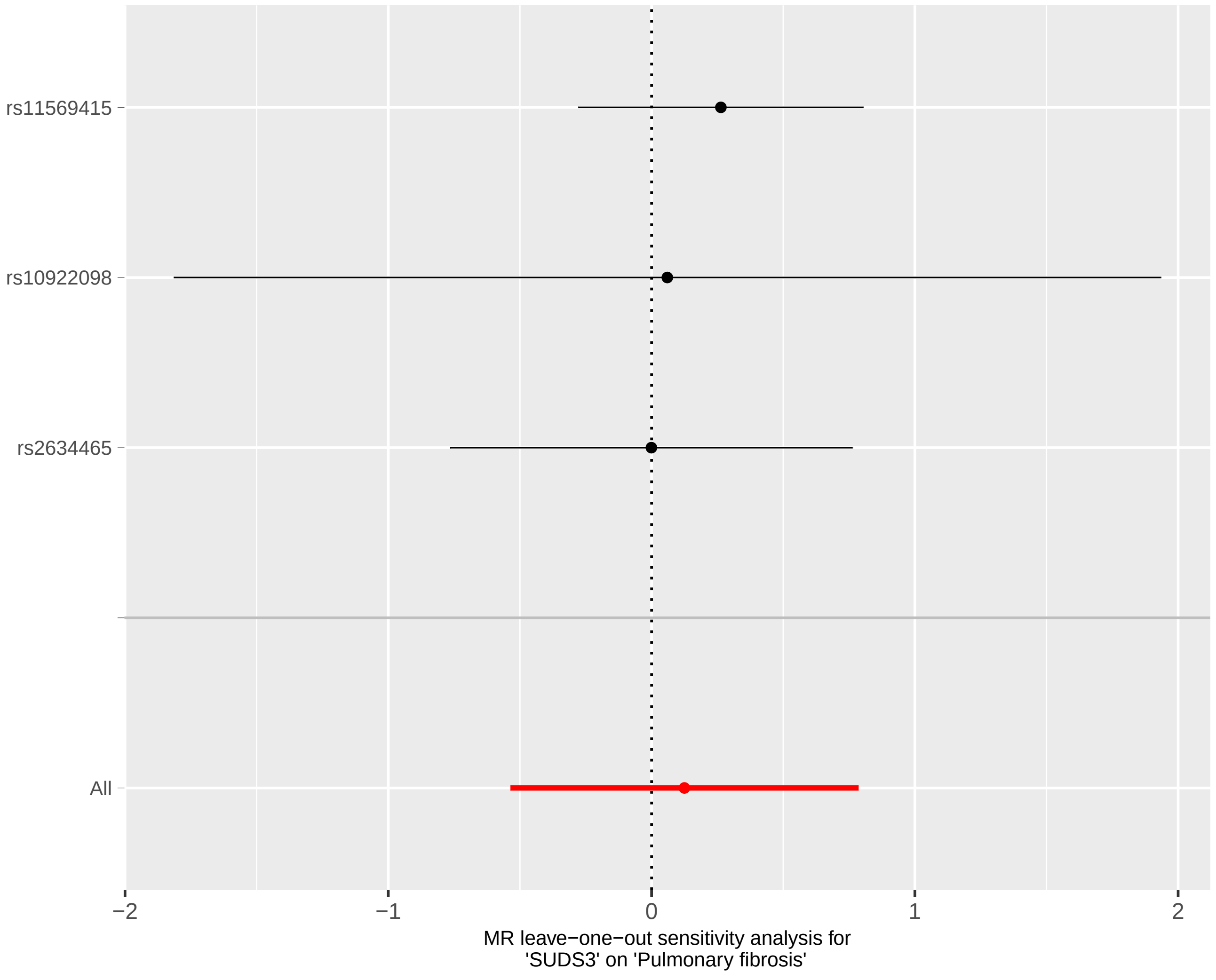
rs11569415
rs10922098
rs2634465
All
−2
−1
0
1
2
MR leave−one−out sensitivity analysis for
'SUDS3' on 'Pulmonary fibrosis'

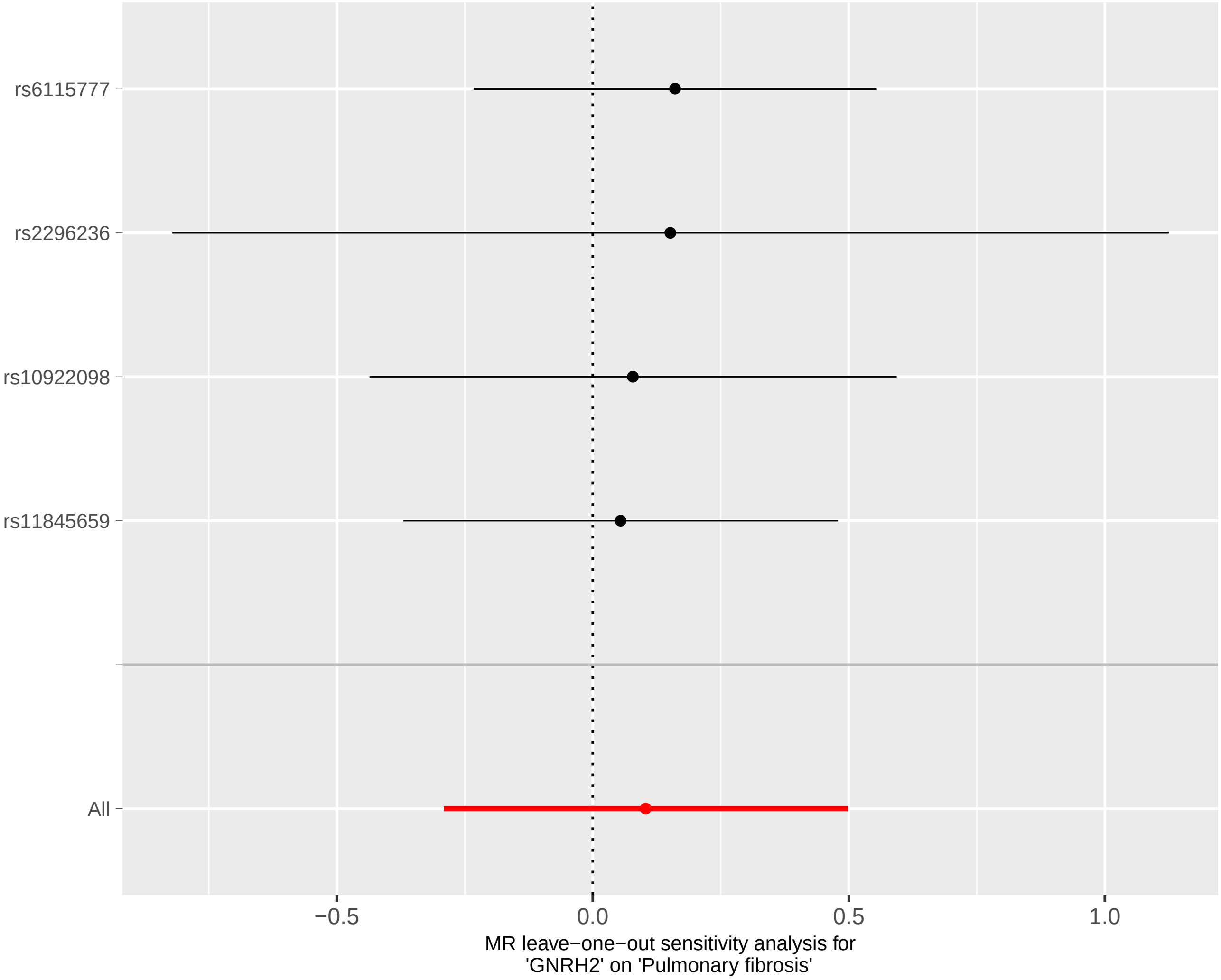
rs6115777
rs2296236
rs10922098
rs11845659
All
−0.5
0.0
0.5
1.0
MR leave−one−out sensitivity analysis for
'GNRH2' on 'Pulmonary fibrosis'

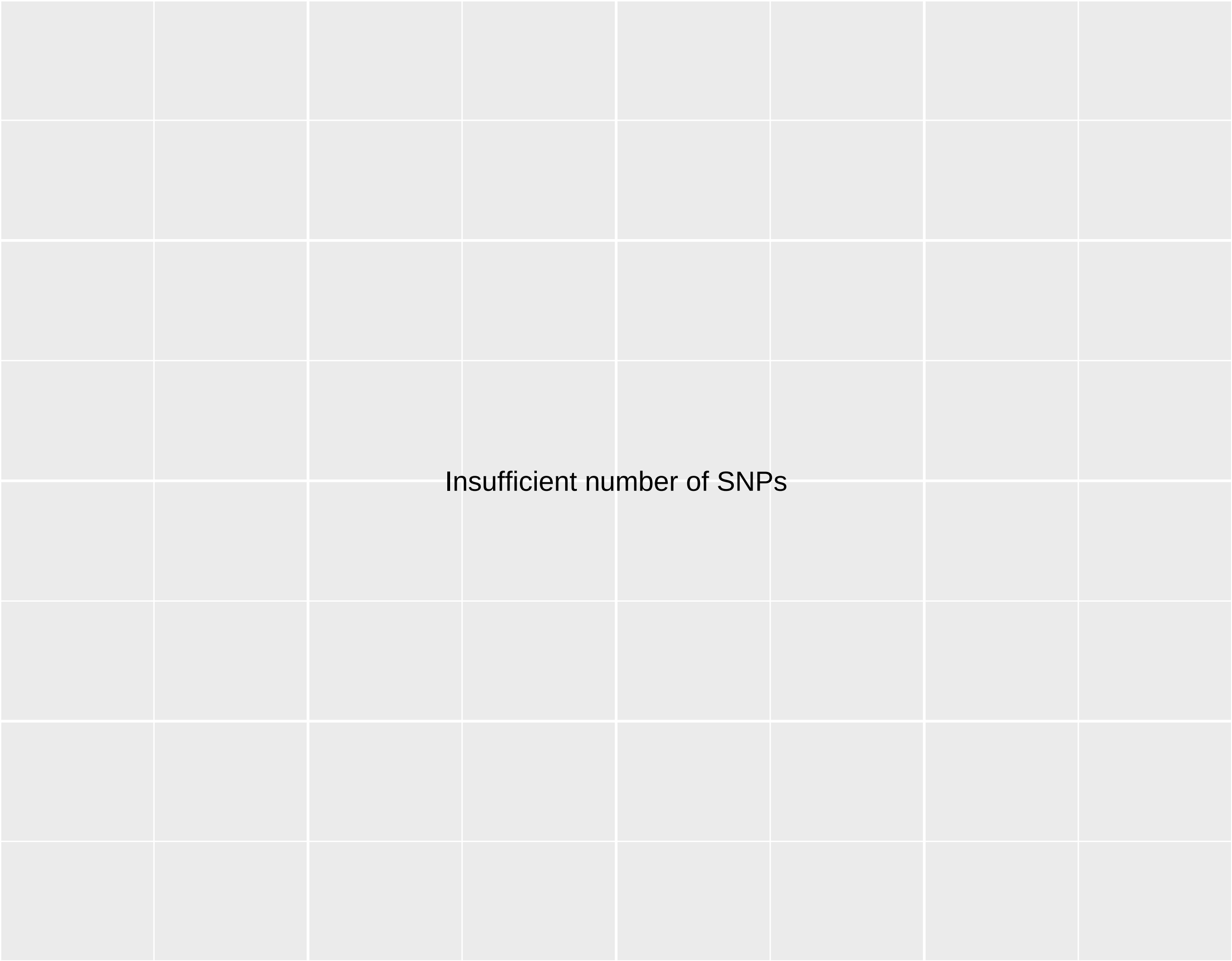
Insufficient number of SNPs

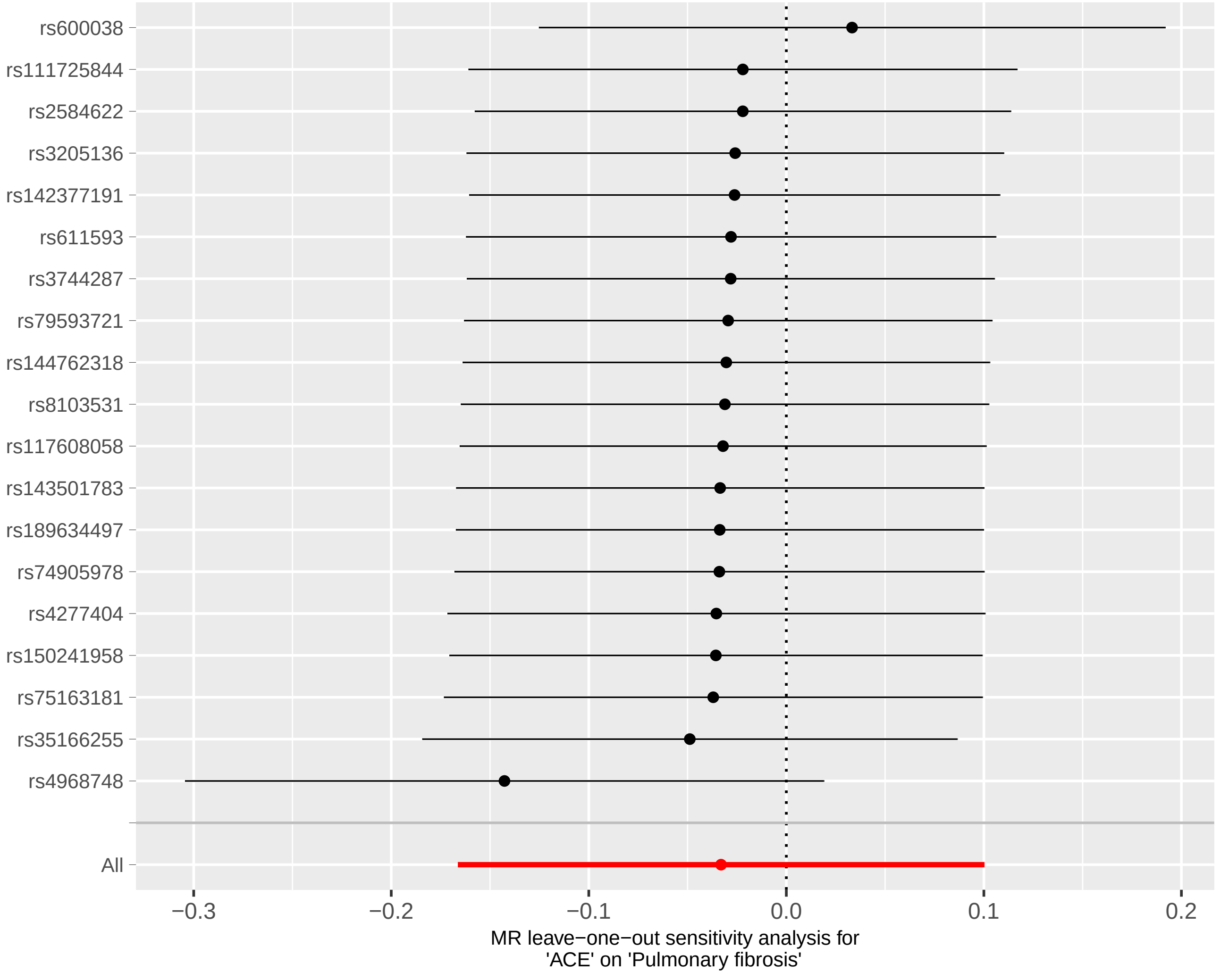

rs600038
rs111725844
rs2584622
rs3205136
rs142377191
rs611593
rs3744287
rs79593721
rs144762318
rs8103531
rs117608058
rs143501783
rs189634497
rs74905978
rs4277404
rs150241958
rs75163181
rs35166255
rs4968748
All
−0.3
−0.2
−0.1
0.0
0.1
0.2
MR leave−one−out sensitivity analysis for
'ACE' on 'Pulmonary fibrosis'

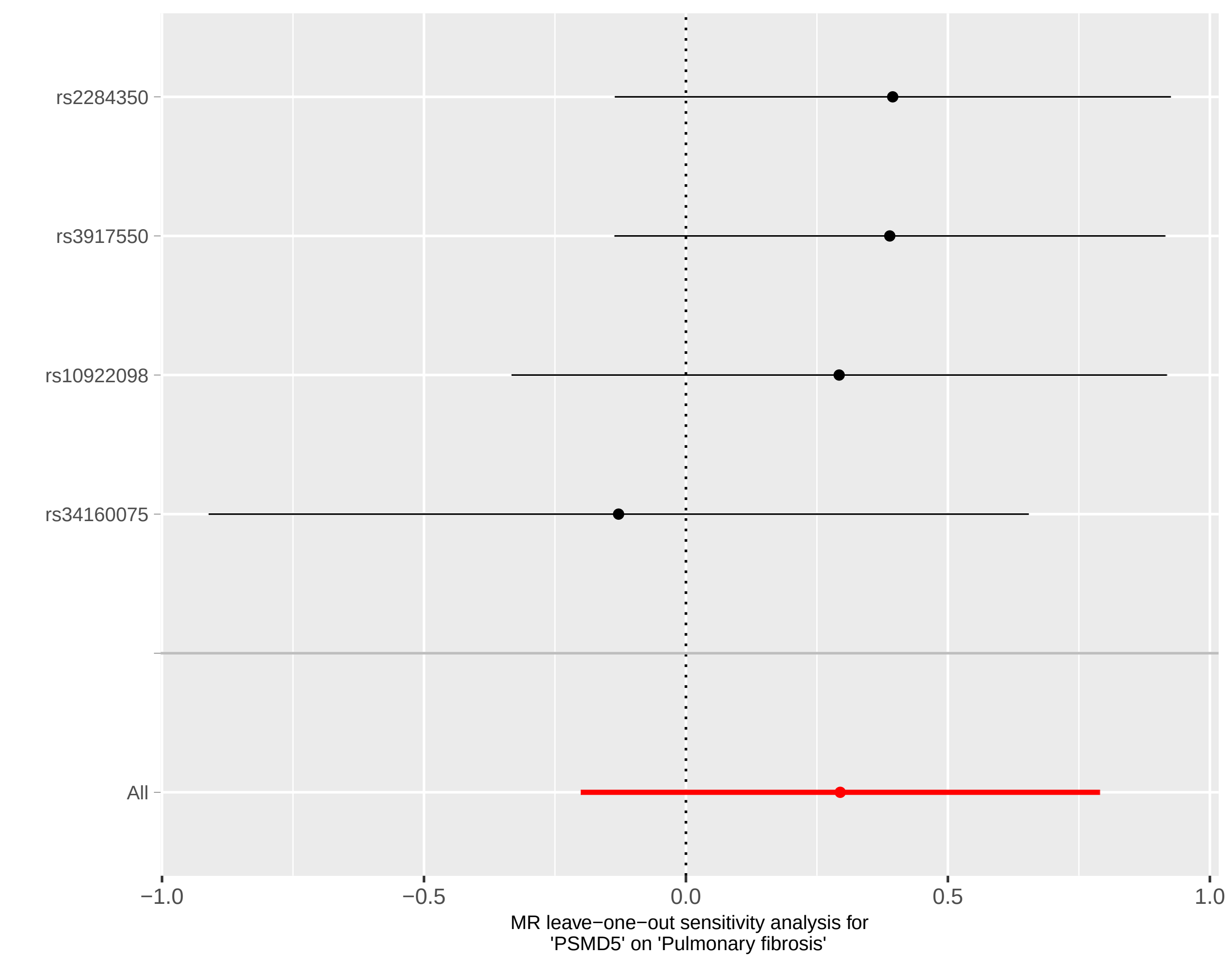

rs2284350
rs3917550
rs10922098
rs34160075
All
−1.0
−0.5
0.0
0.5
1.0
MR leave−one−out sensitivity analysis for
'PSMD5' on 'Pulmonary fibrosis'

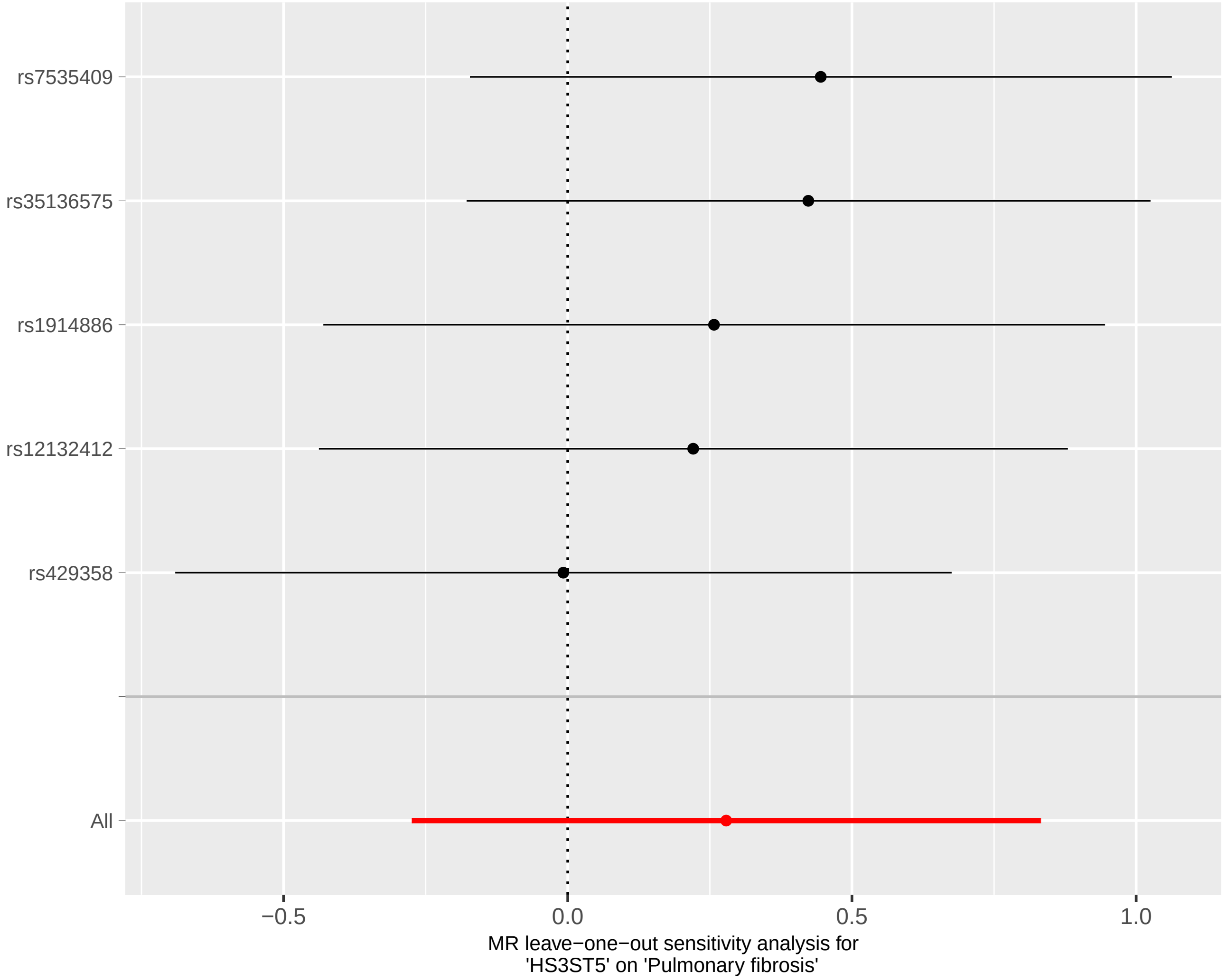

rs7535409
rs35136575
rs1914886
rs12132412
rs429358
All
−0.5
0.0
0.5
1.0
MR leave−one−out sensitivity analysis for
'HS3ST5' on 'Pulmonary fibrosis'

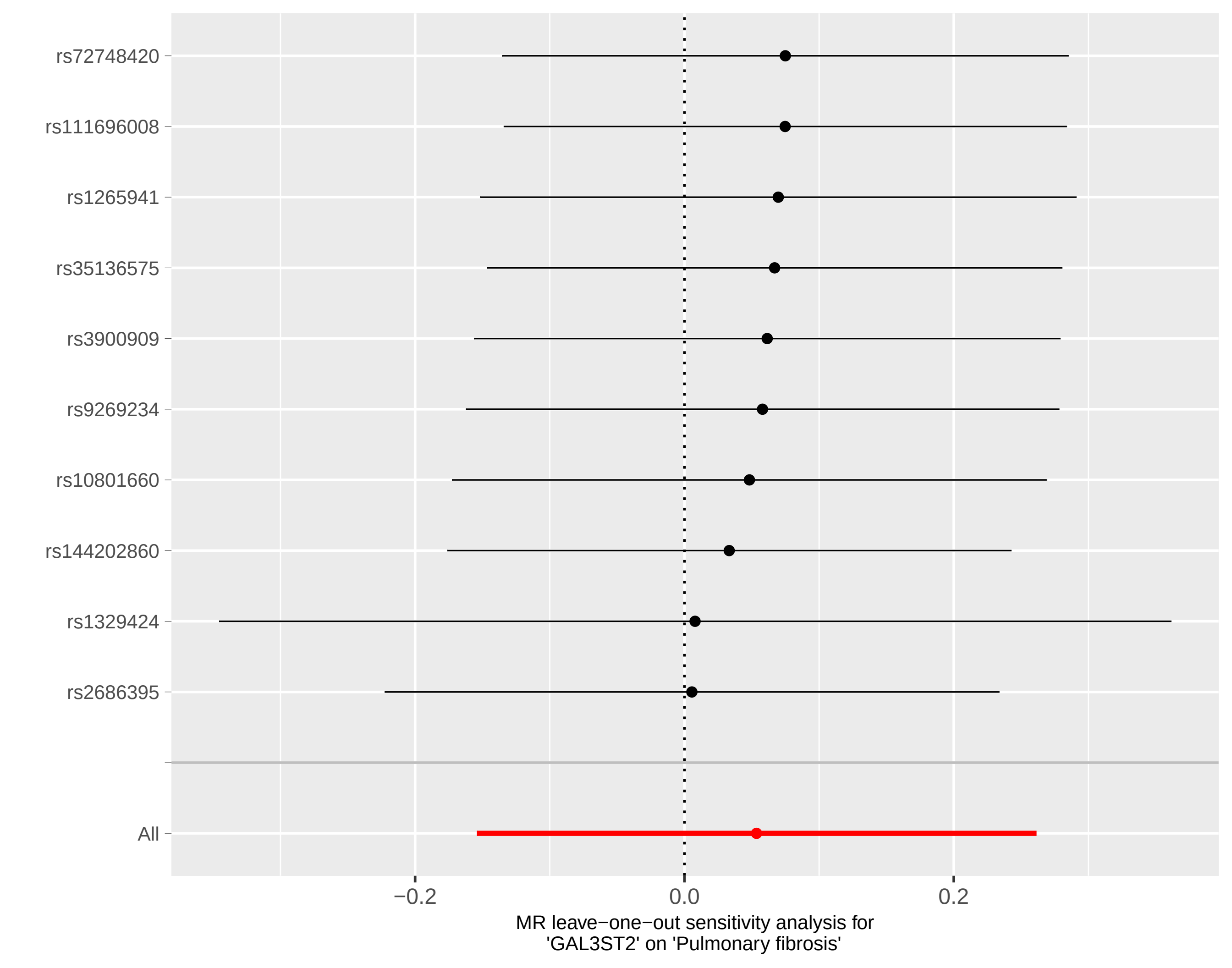

rs72748420
rs111696008
rs1265941
rs35136575
rs3900909
rs9269234
rs10801660
rs144202860
rs1329424
rs2686395
All
−0.2
0.0
0.2
MR leave−one−out sensitivity analysis for
'GAL3ST2' on 'Pulmonary fibrosis'

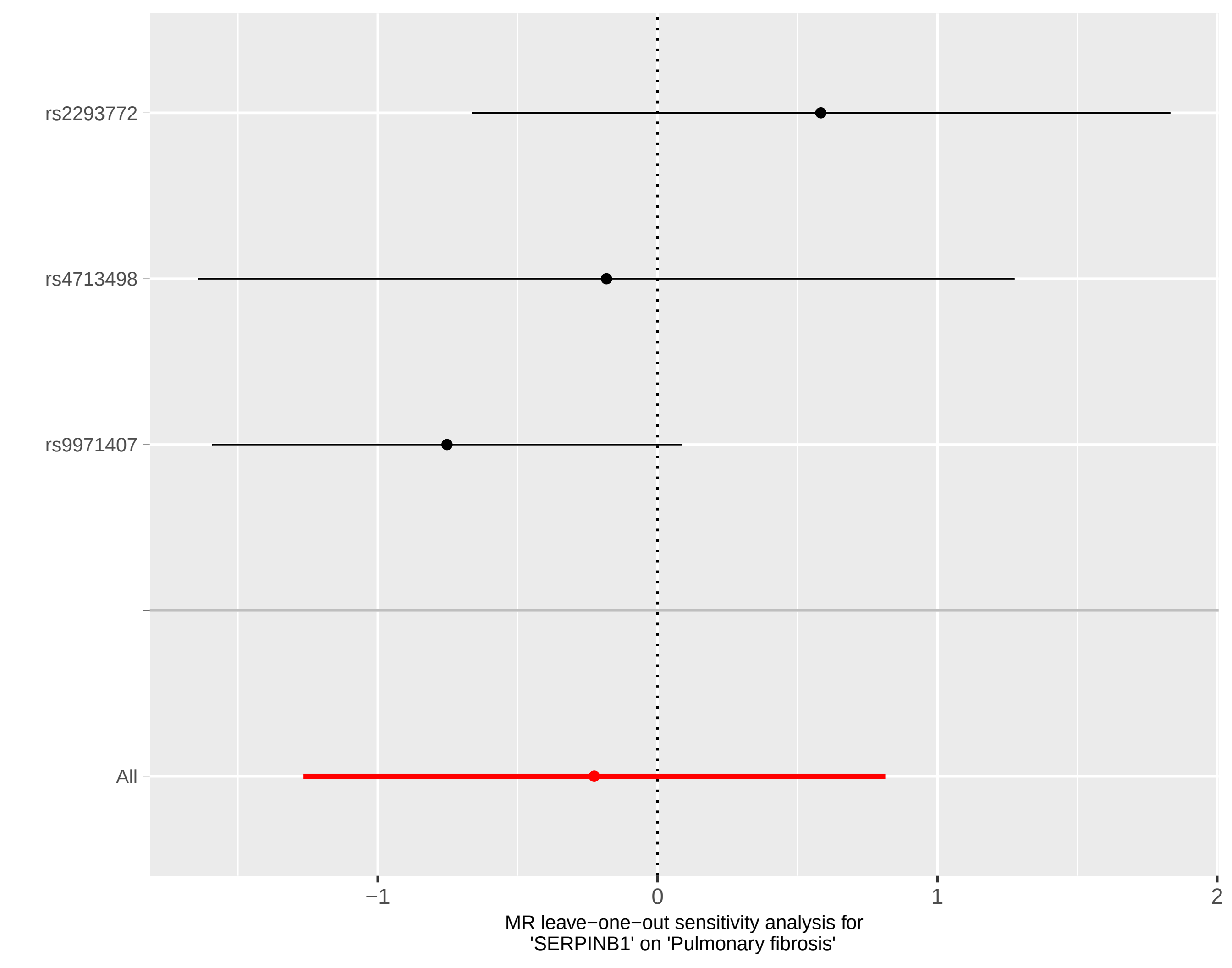
rs2293772
rs4713498
rs9971407
All
−1
0
1
2
MR leave−one−out sensitivity analysis for
'SERPINB1' on 'Pulmonary fibrosis'

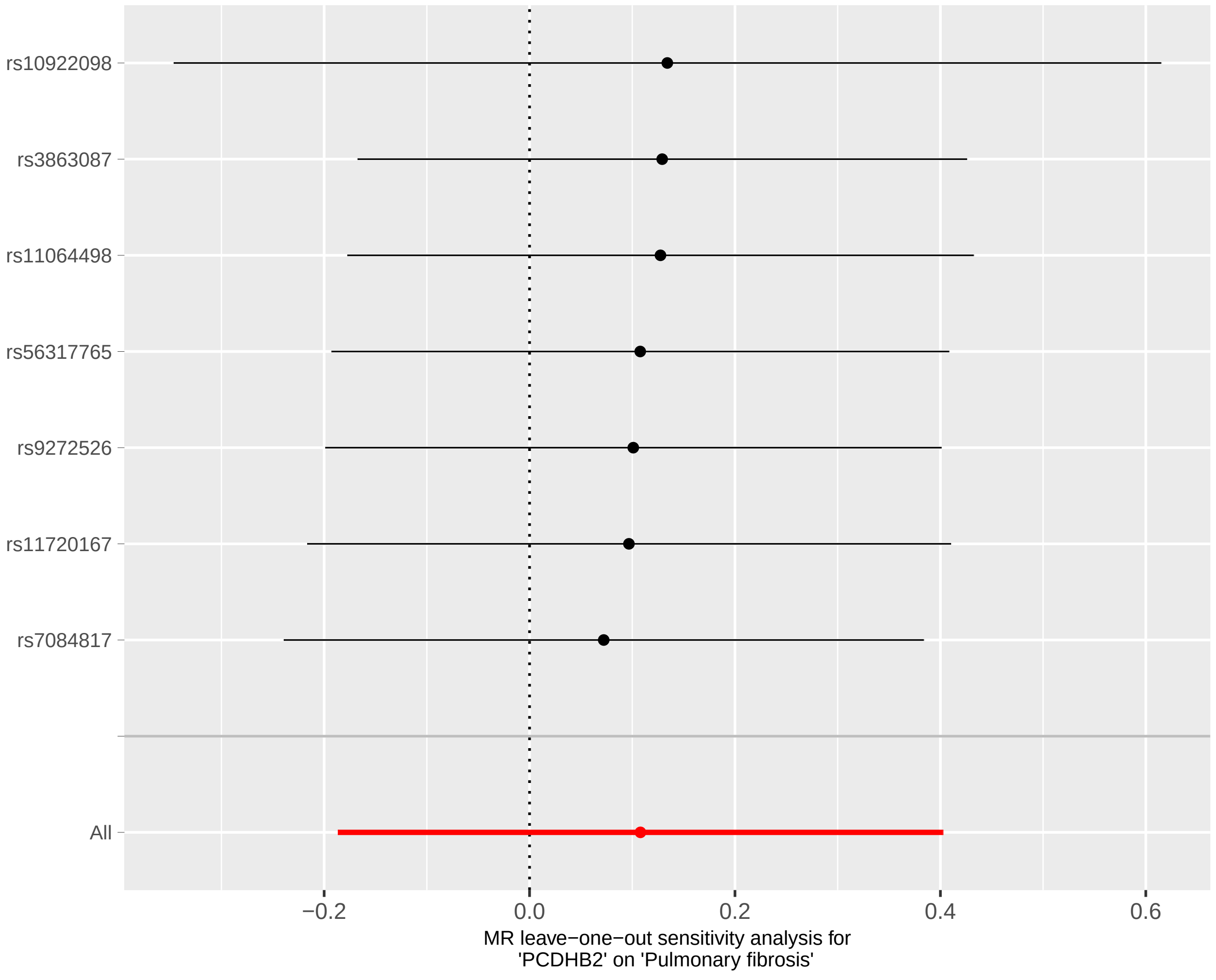
rs10922098
rs3863087
rs11064498
rs56317765
rs9272526
rs11720167
rs7084817
All
−0.2
0.0
0.2
0.4
0.6
MR leave−one−out sensitivity analysis for
'PCDHB2' on 'Pulmonary fibrosis'

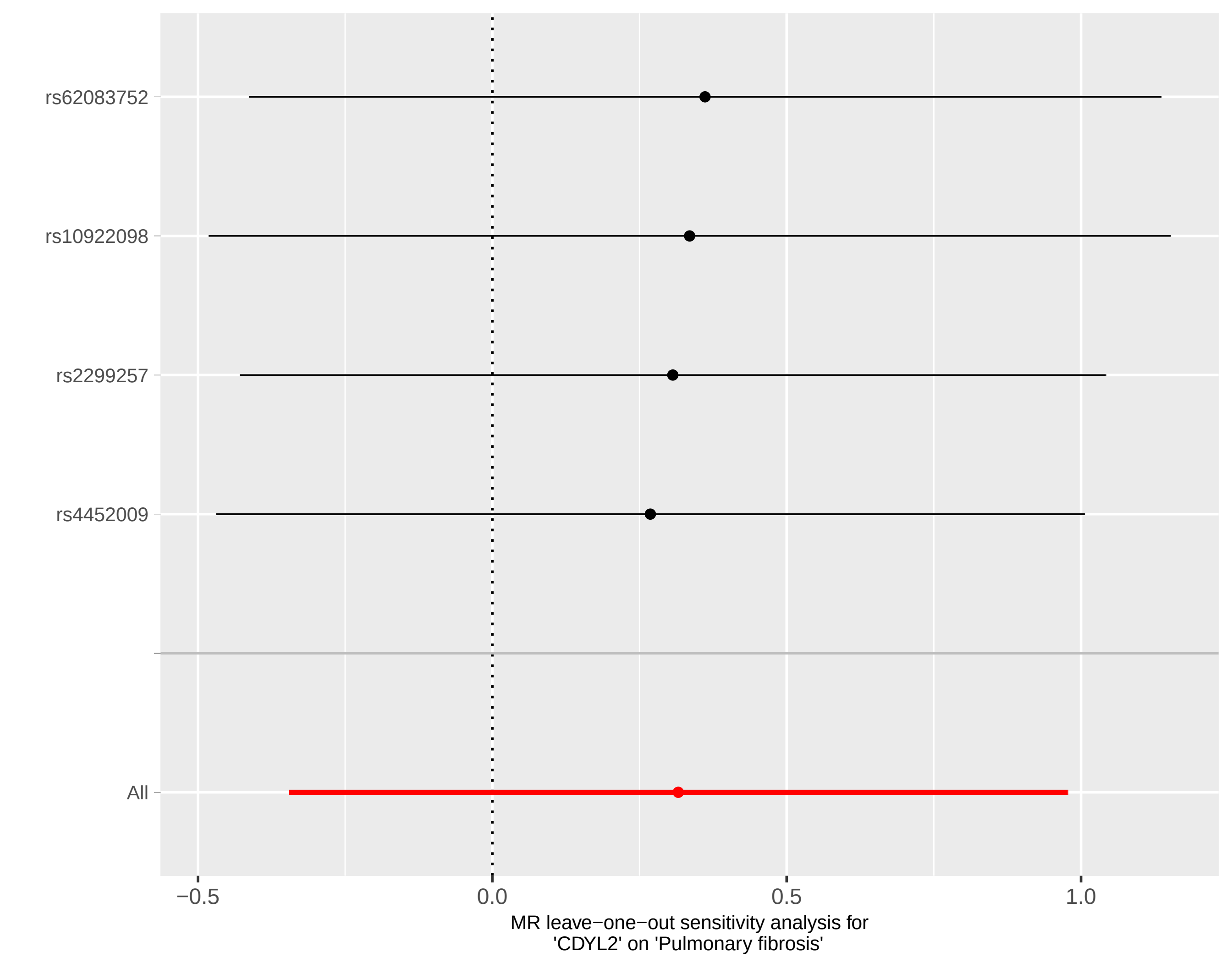
rs62083752
rs10922098
rs2299257
rs4452009
All
−0.5
0.0
0.5
1.0
MR leave−one−out sensitivity analysis for
'CDYL2' on 'Pulmonary fibrosis'

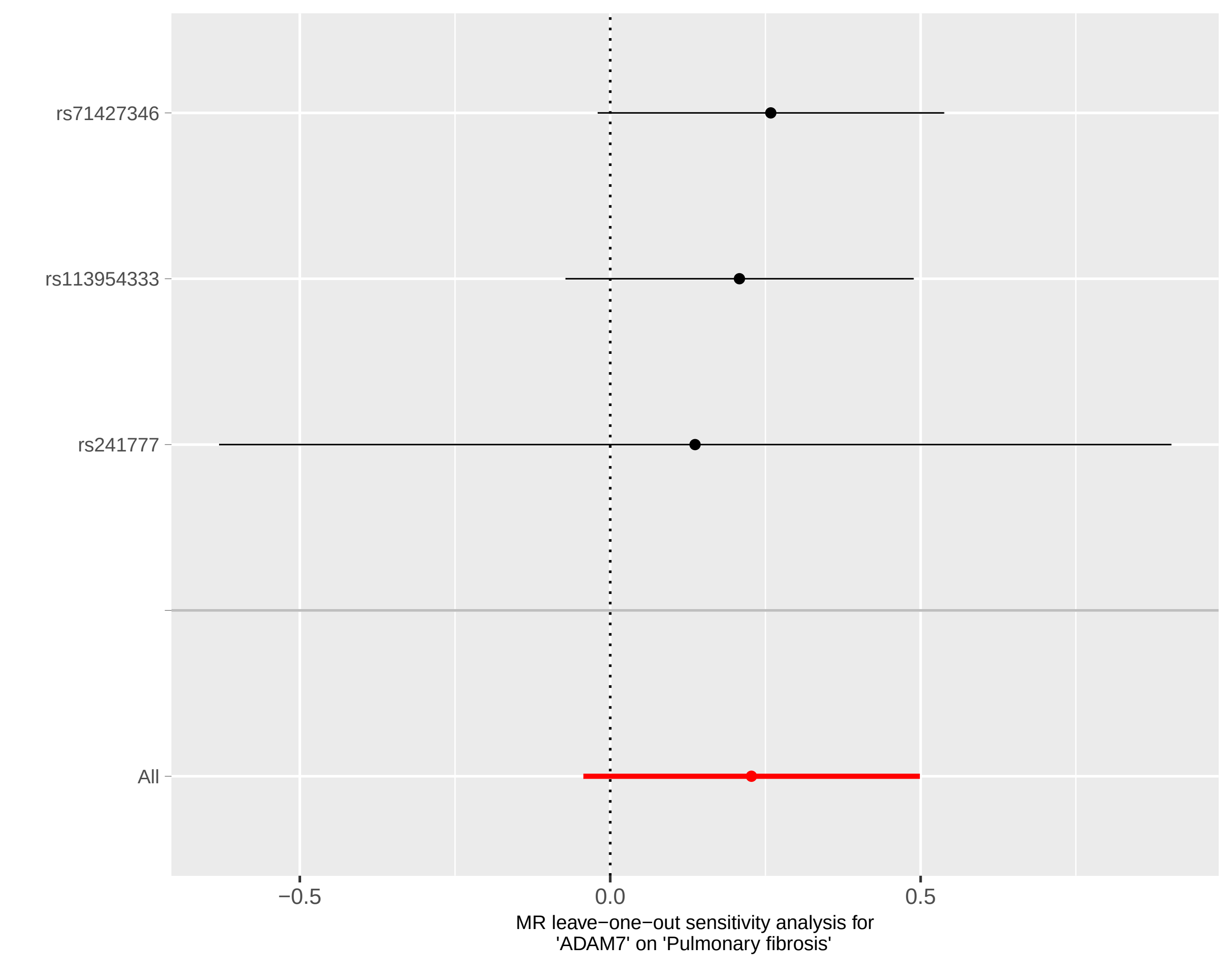
rs71427346
rs113954333
rs241777
All
−0.5
0.0
0.5
MR leave−one−out sensitivity analysis for
'ADAM7' on 'Pulmonary fibrosis'

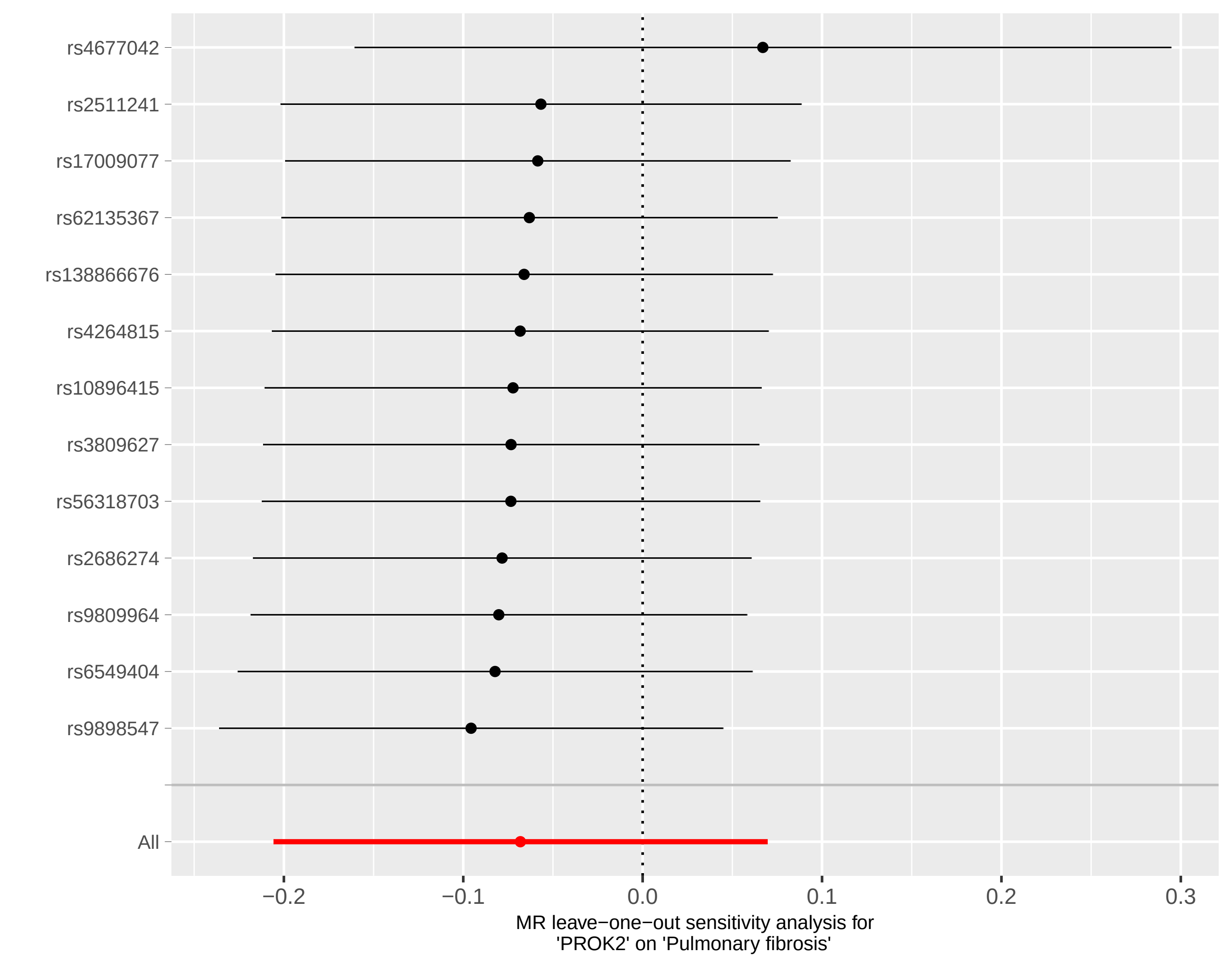
rs4677042
rs2511241
rs17009077
rs62135367
rs138866676
rs4264815
rs10896415
rs3809627
rs56318703
rs2686274
rs9809964
rs6549404
rs9898547
All
−0.2
−0.1
0.0
0.1
0.2
0.3
MR leave−one−out sensitivity analysis for
'PROK2' on 'Pulmonary fibrosis'

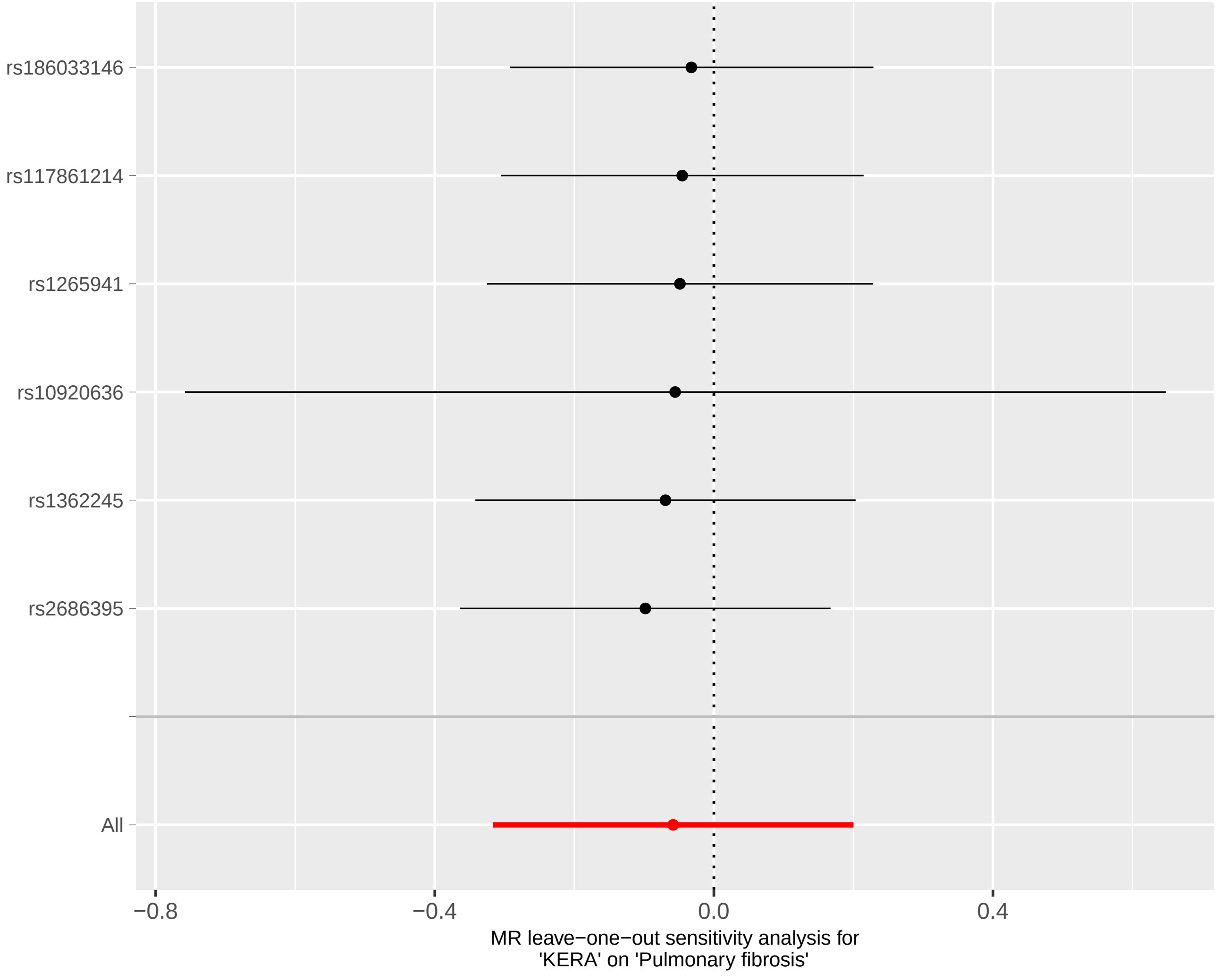
rs186033146
rs117861214
rs1265941
rs10920636
rs1362245
rs2686395
All
−0.8
−0.4
0.0
0.4
MR leave−one−out sensitivity analysis for
'KERA' on 'Pulmonary fibrosis'

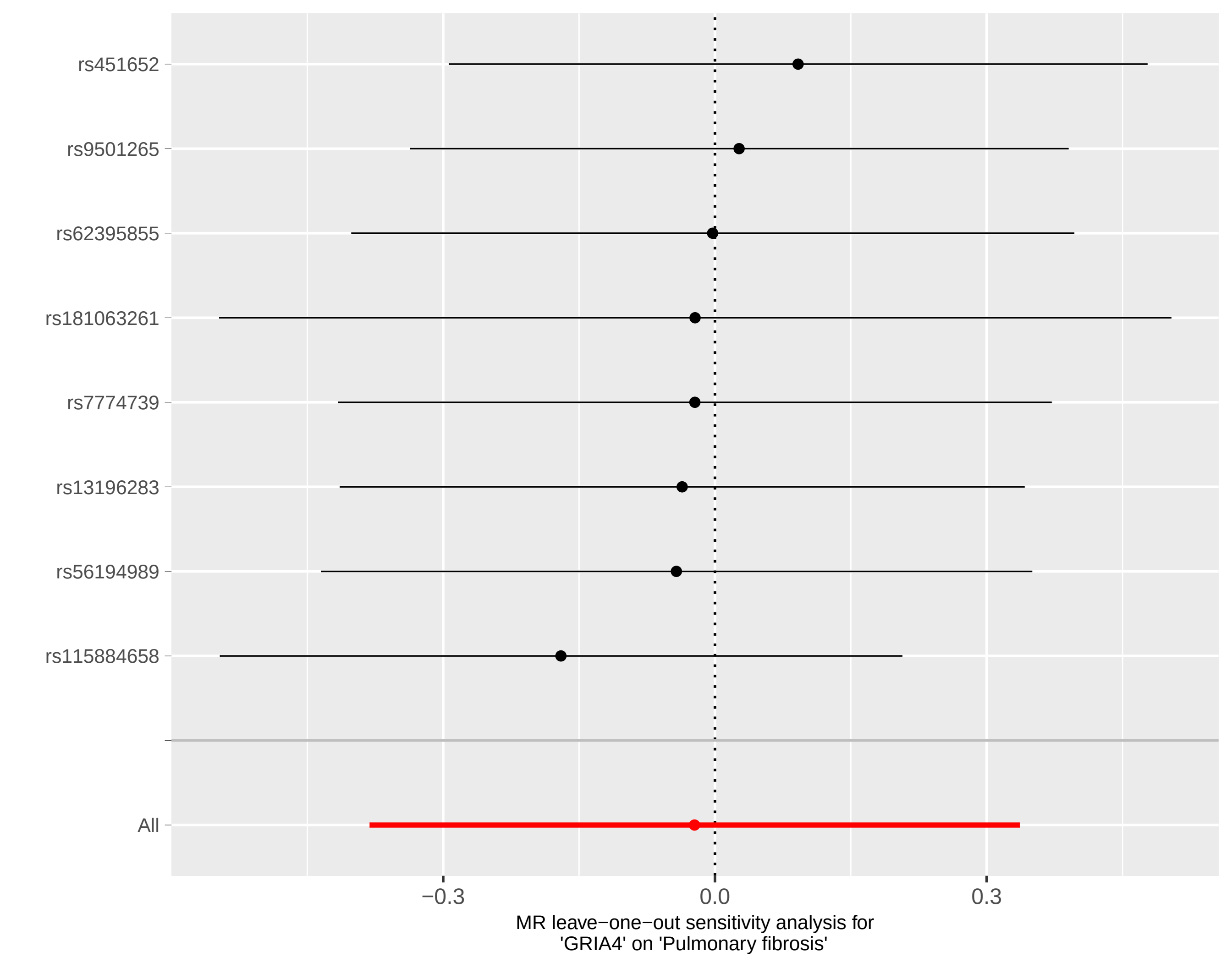

rs451652
rs9501265
rs62395855
rs181063261
rs7774739
rs13196283
rs56194989
rs115884658
All
−0.3
0.0
0.3
MR leave−one−out sensitivity analysis for
'GRIA4' on 'Pulmonary fibrosis'

Insufficient number of SNPs

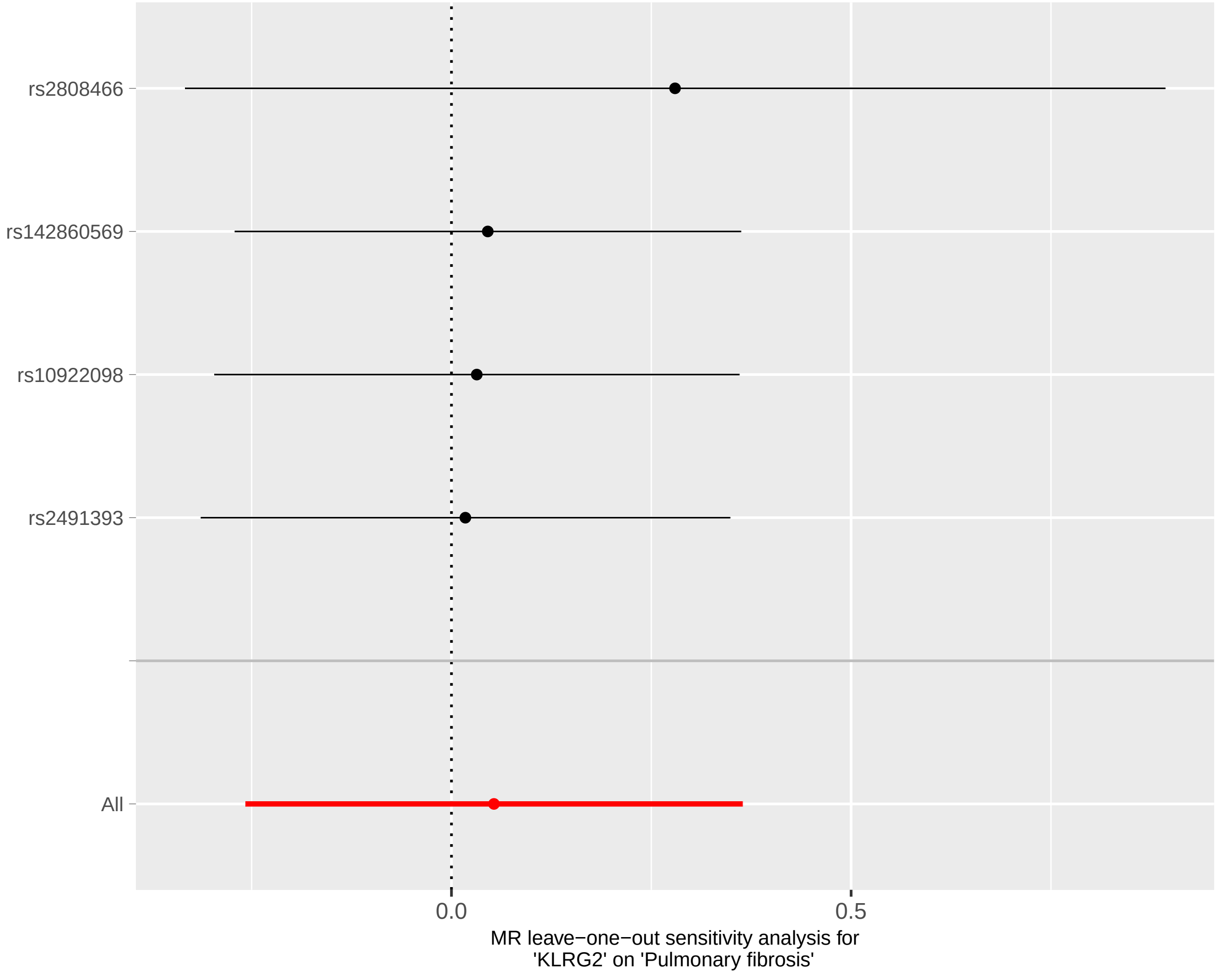

rs2808466
rs142860569
rs10922098
rs2491393
All
0.0
0.5
MR leave−one−out sensitivity analysis for
'KLRG2' on 'Pulmonary fibrosis'

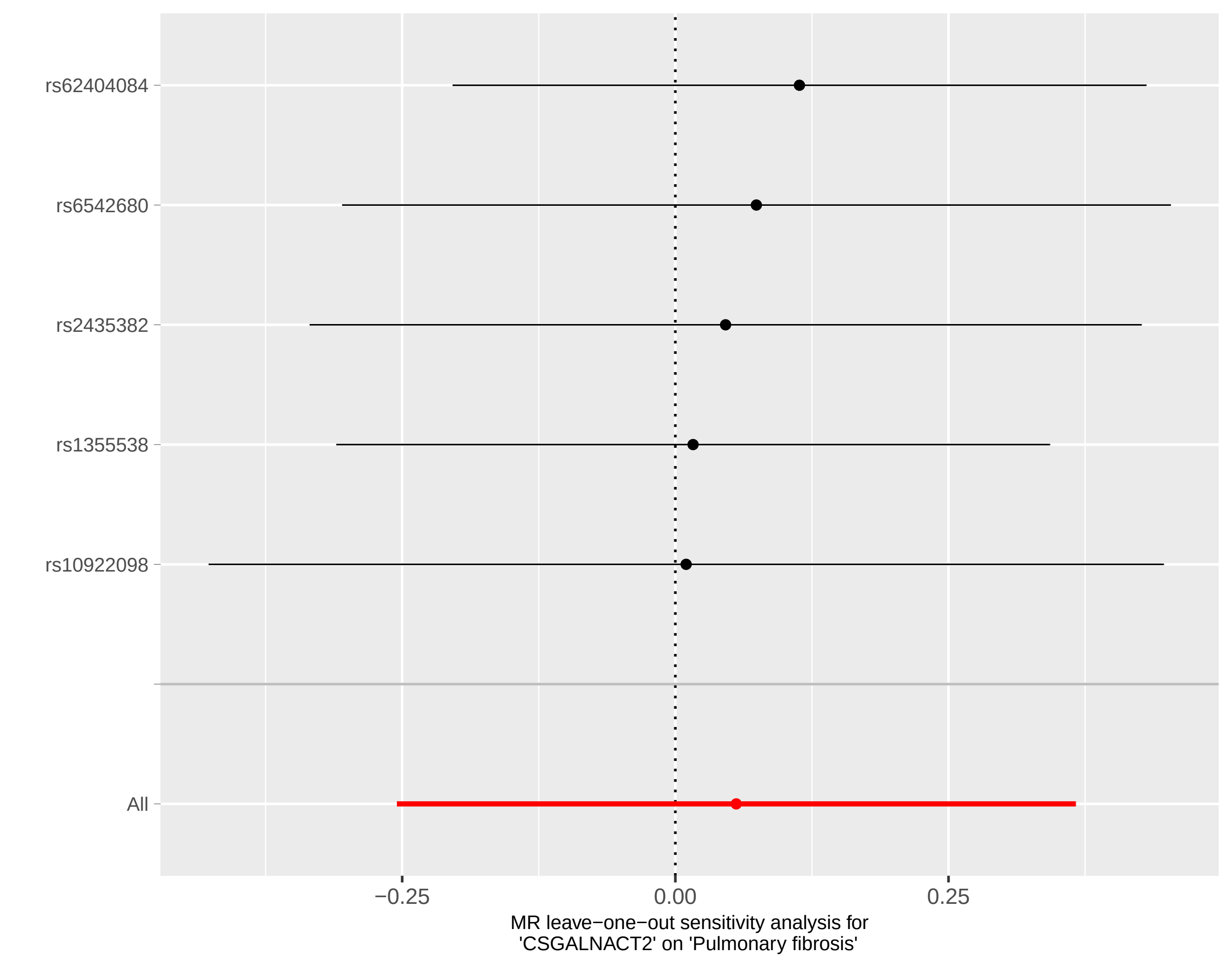

rs62404084
rs6542680
rs2435382
rs1355538
rs10922098
All
−0.25
0.00
0.25
MR leave−one−out sensitivity analysis for
'CSGALNACT2' on 'Pulmonary fibrosis'

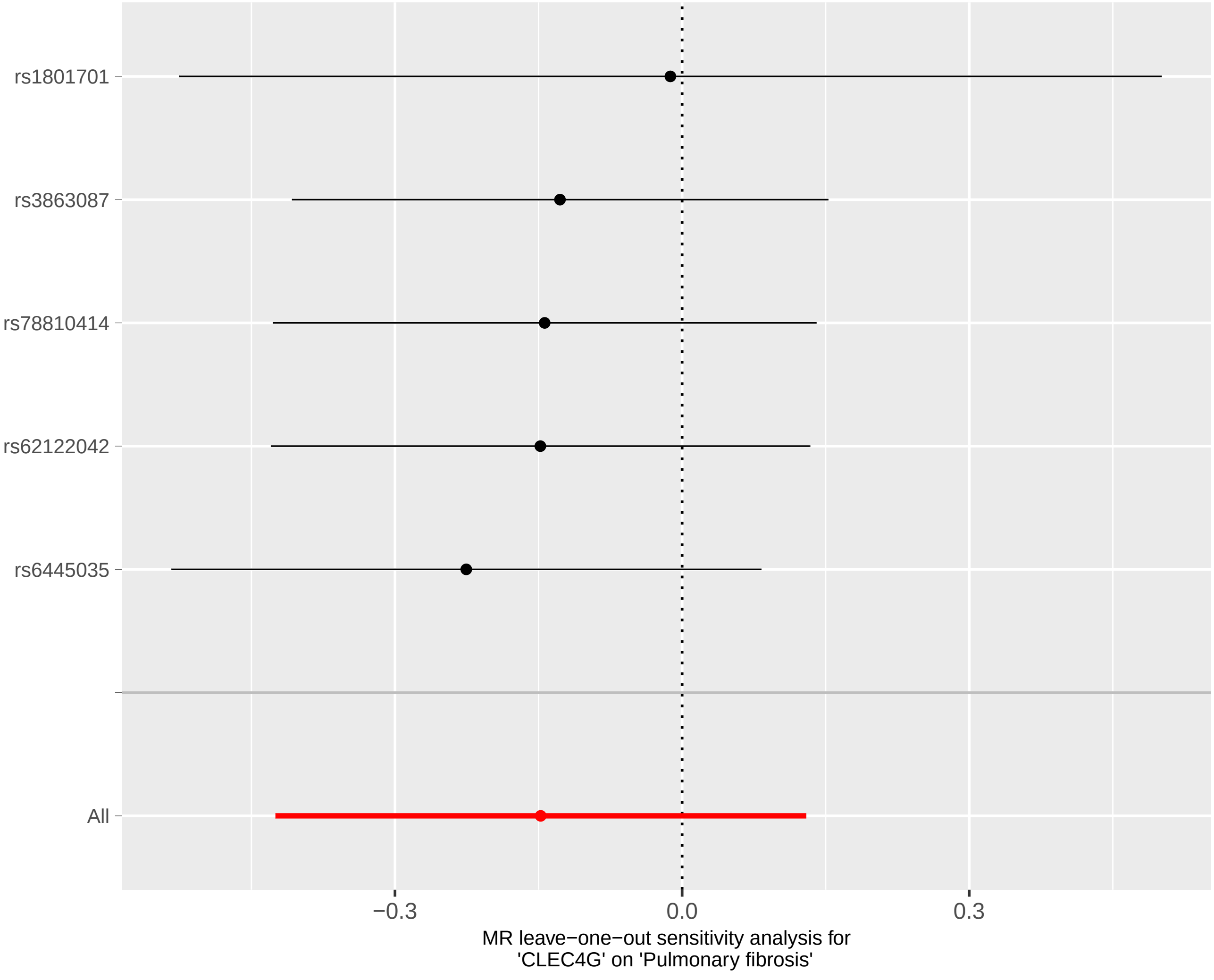
rs1801701
rs3863087
rs78810414
rs62122042
rs6445035
All
−0.3
0.0
0.3
MR leave−one−out sensitivity analysis for
'CLEC4G' on 'Pulmonary fibrosis'

Insufficient number of SNPs

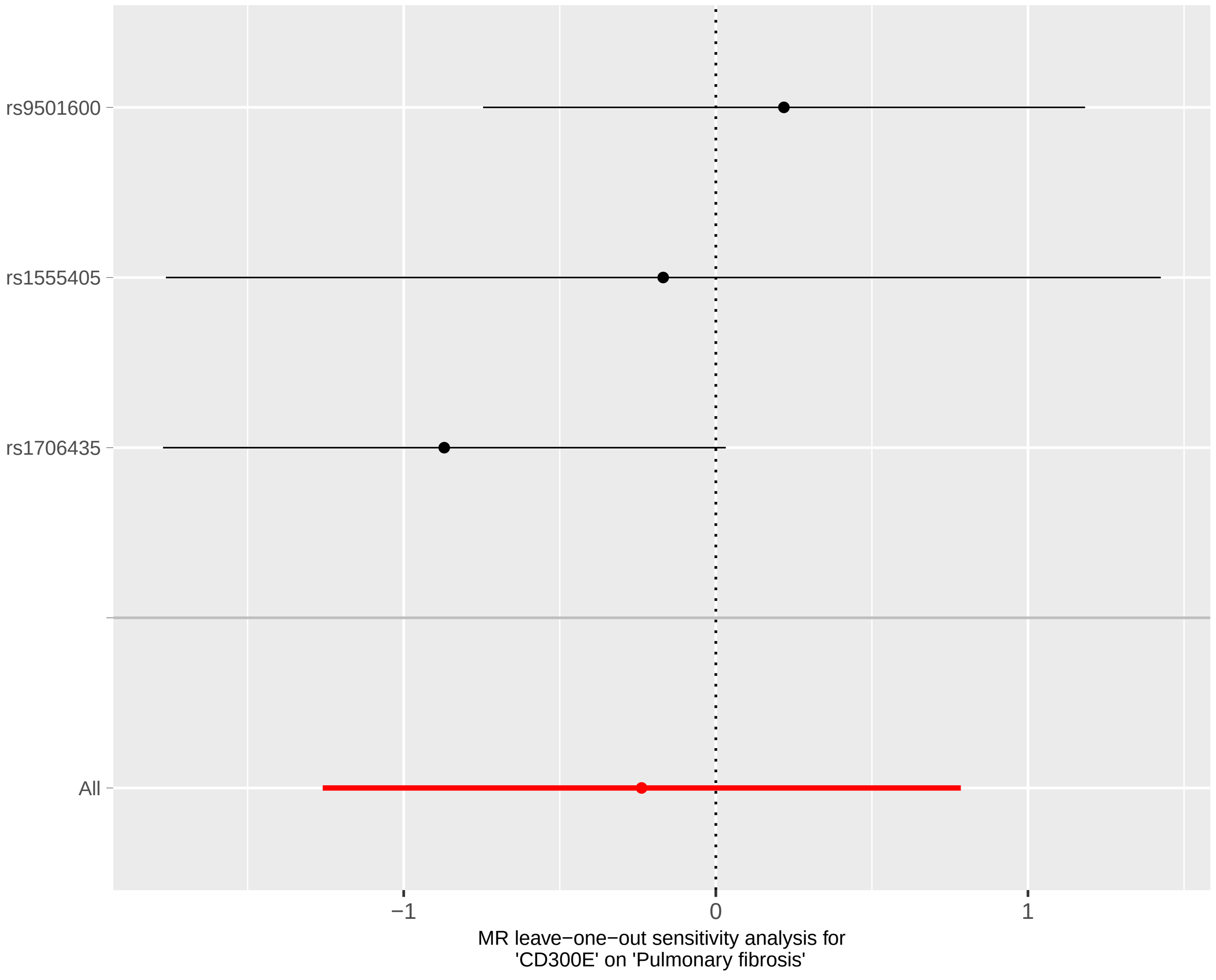
rs9501600
rs1555405
rs1706435
All
−1
0
1
MR leave−one−out sensitivity analysis for
'CD300E' on 'Pulmonary fibrosis'

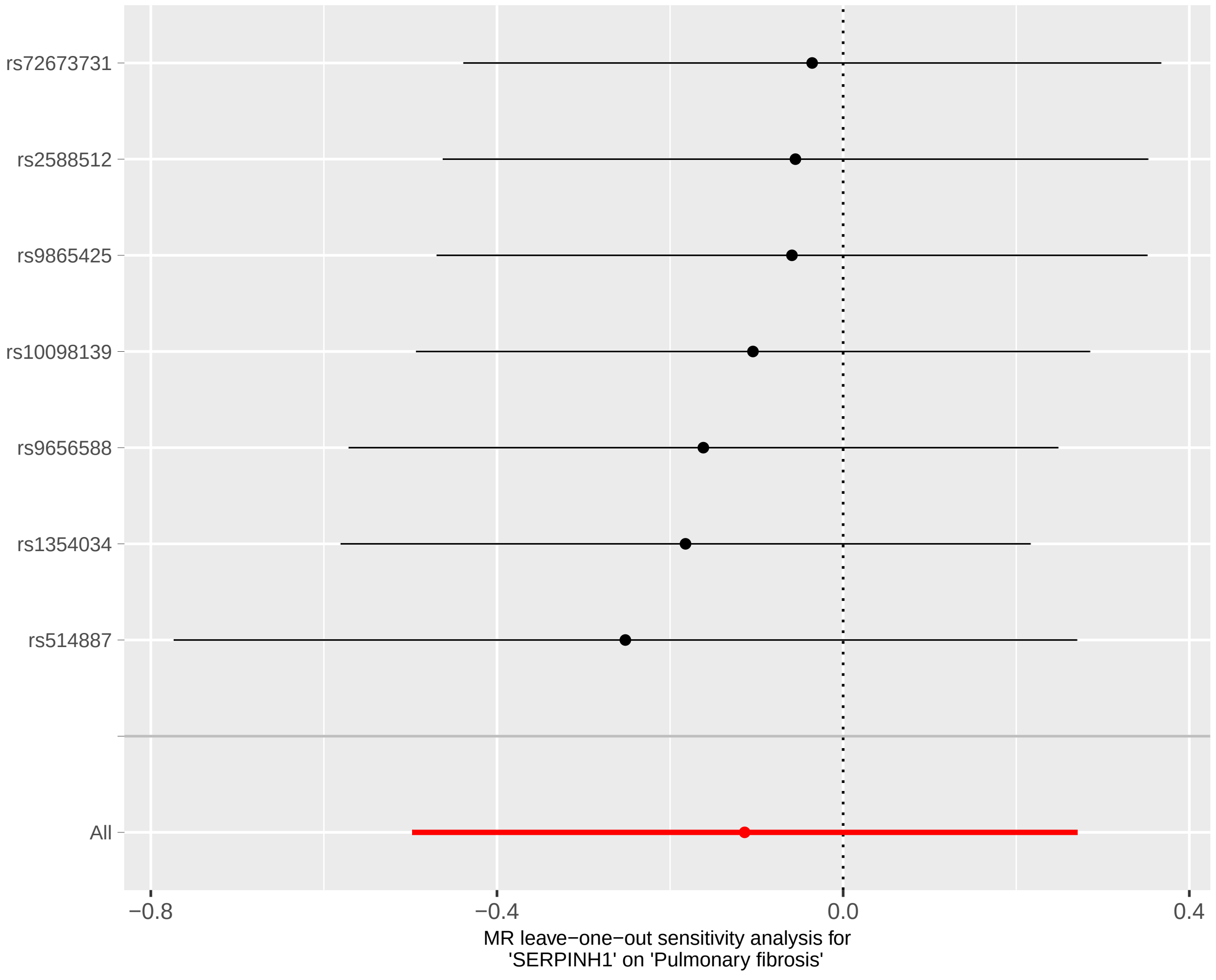

rs72673731
rs2588512
rs9865425
rs10098139
rs9656588
rs1354034
rs514887
All
−0.8
−0.4
0.0
0.4
MR leave−one−out sensitivity analysis for
'SERPINH1' on 'Pulmonary fibrosis'

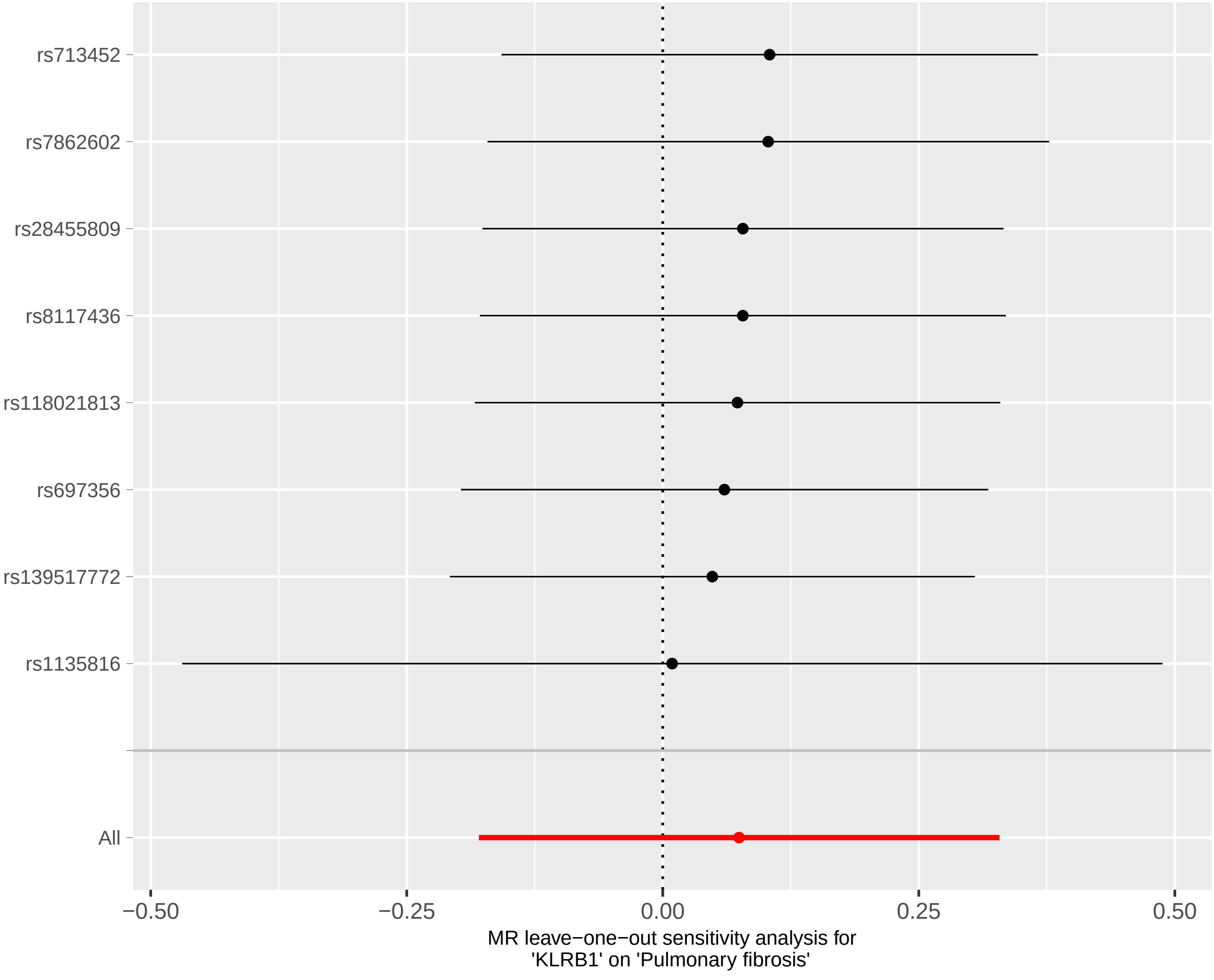
rs713452
rs7862602
rs28455809
rs8117436
rs118021813
rs697356
rs139517772
rs1135816
All
−0.50
−0.25
0.00
0.25
0.50
MR leave−one−out sensitivity analysis for
'KLRB1' on 'Pulmonary fibrosis'

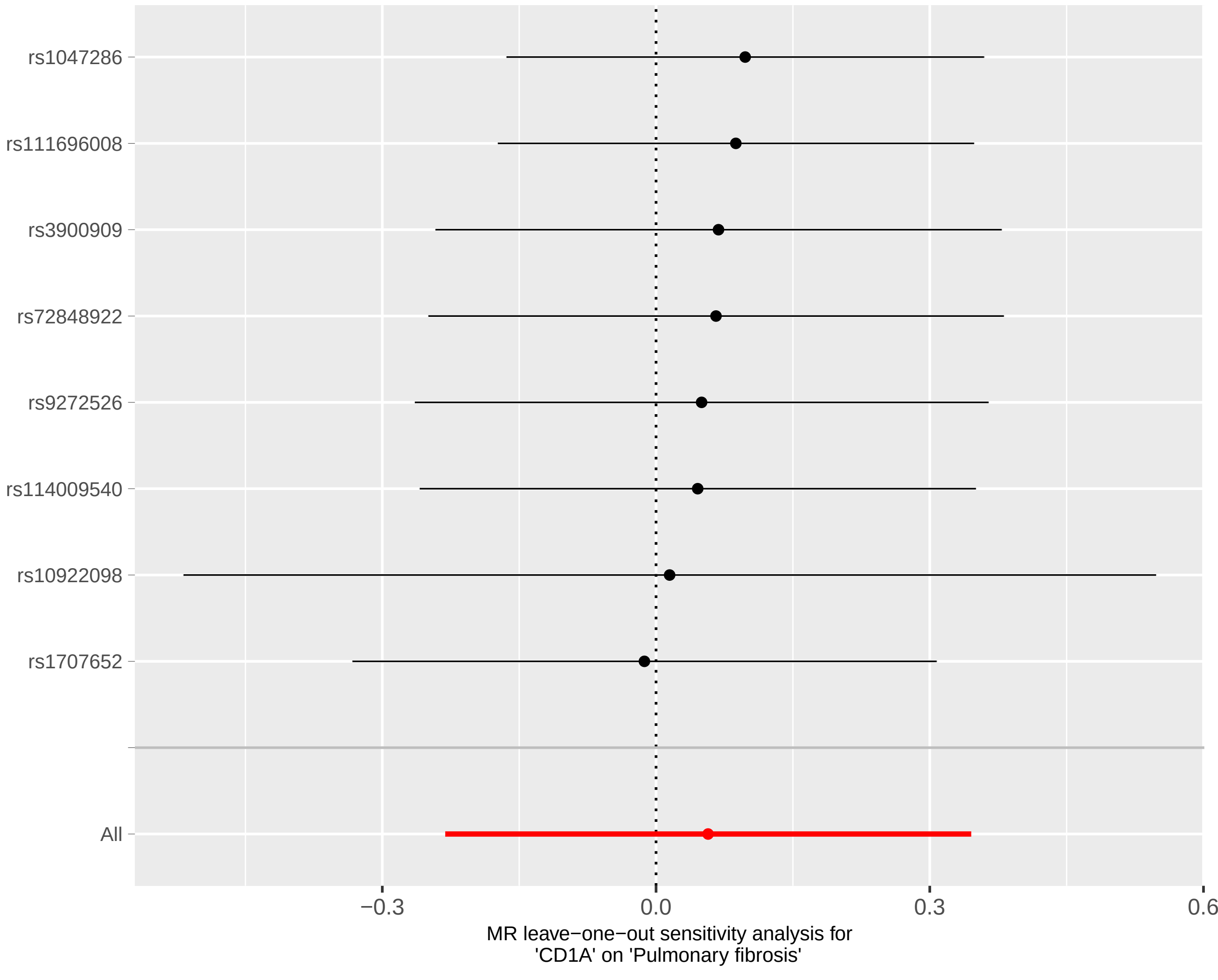
rs1047286
rs111696008
rs3900909
rs72848922
rs9272526
rs114009540
rs10922098
rs1707652
All
−0.3
0.0
0.3
0.6
MR leave−one−out sensitivity analysis for
'CD1A' on 'Pulmonary fibrosis'

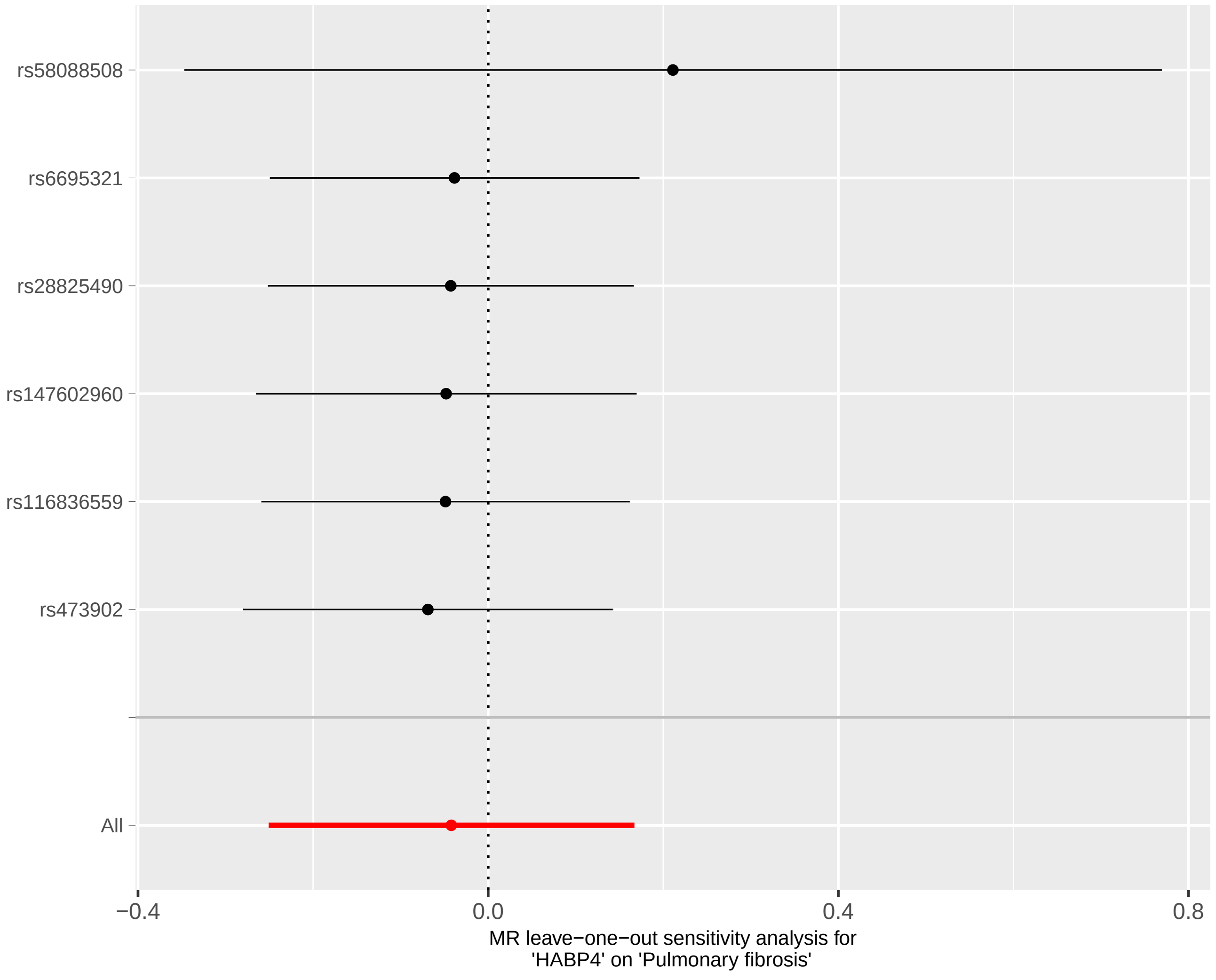
rs58088508
rs6695321
rs28825490
rs147602960
rs116836559
rs473902
All
−0.4
0.0
0.4
0.8
MR leave−one−out sensitivity analysis for
'HABP4' on 'Pulmonary fibrosis'

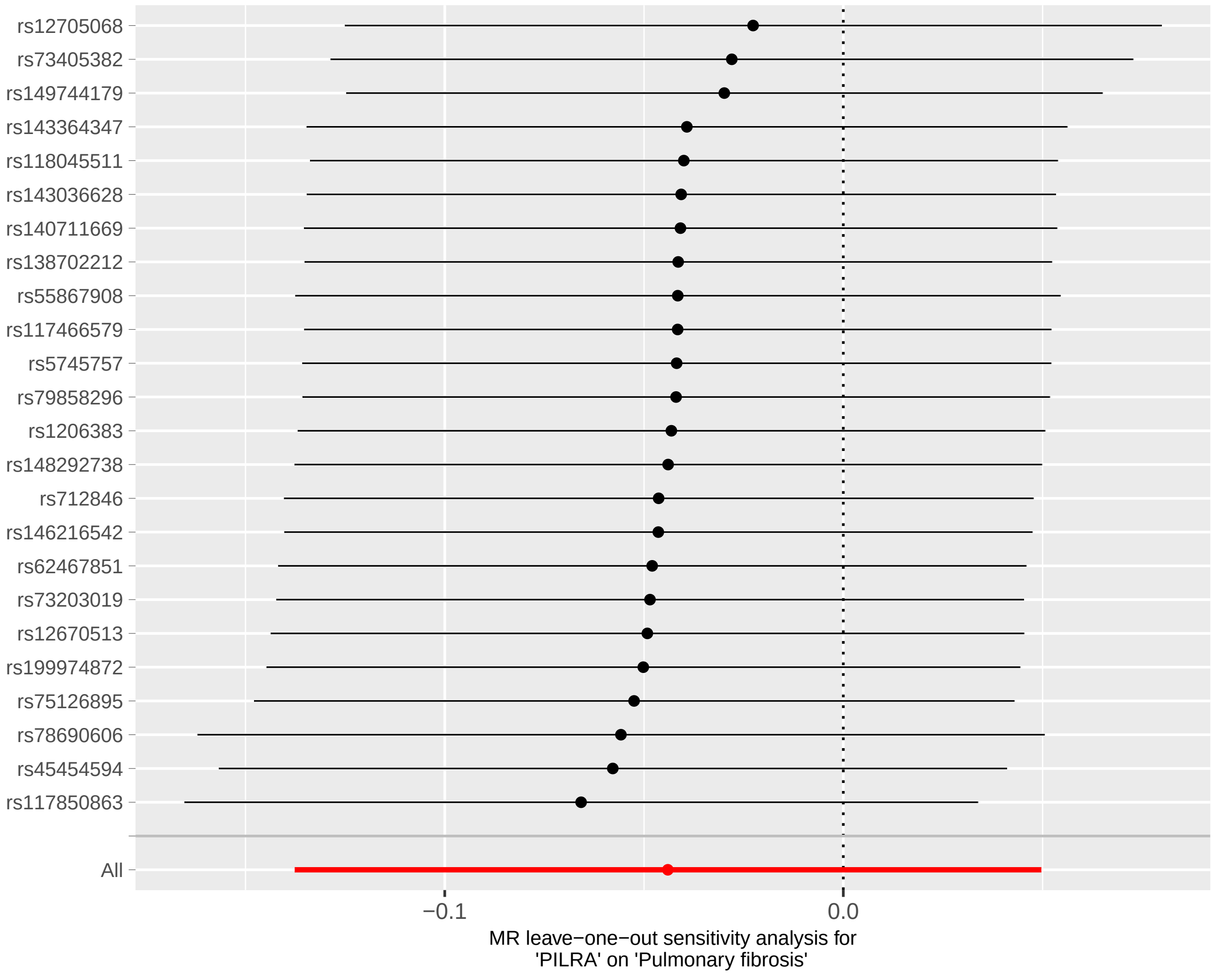
rs12705068
rs73405382
rs149744179
rs143364347
rs118045511
rs143036628
rs140711669
rs138702212
rs55867908
rs117466579
rs5745757
rs79858296
rs1206383
rs148292738
rs712846
rs146216542
rs62467851
rs73203019
rs12670513
rs199974872
rs75126895
rs78690606
rs45454594
rs117850863
All
−0.1
0.0
MR leave−one−out sensitivity analysis for
'PILRA' on 'Pulmonary fibrosis'

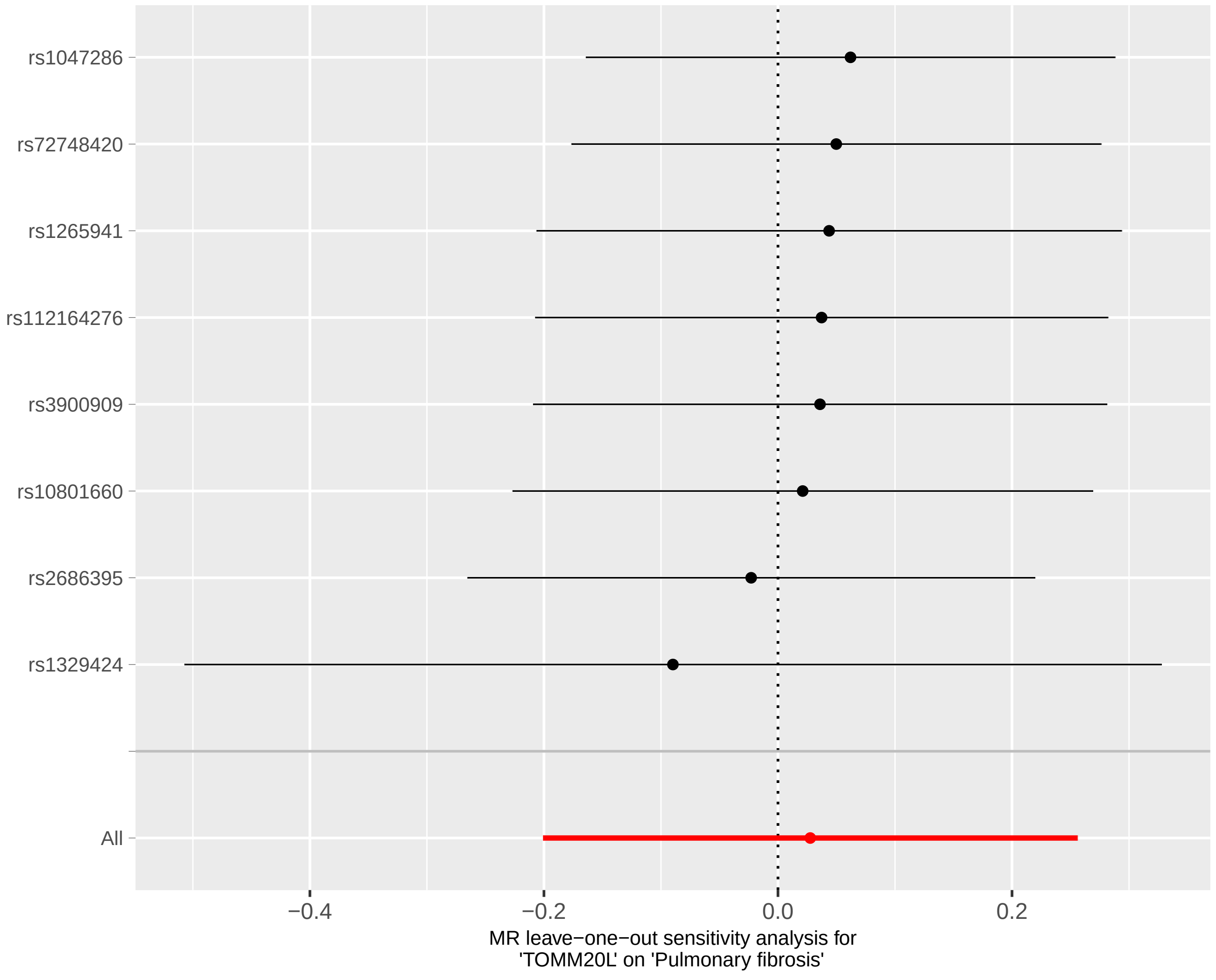
rs1047286
rs72748420
rs1265941
rs112164276
rs3900909
rs10801660
rs2686395
rs1329424
All
−0.4
−0.2
0.0
0.2
MR leave−one−out sensitivity analysis for
'TOMM20L' on 'Pulmonary fibrosis'

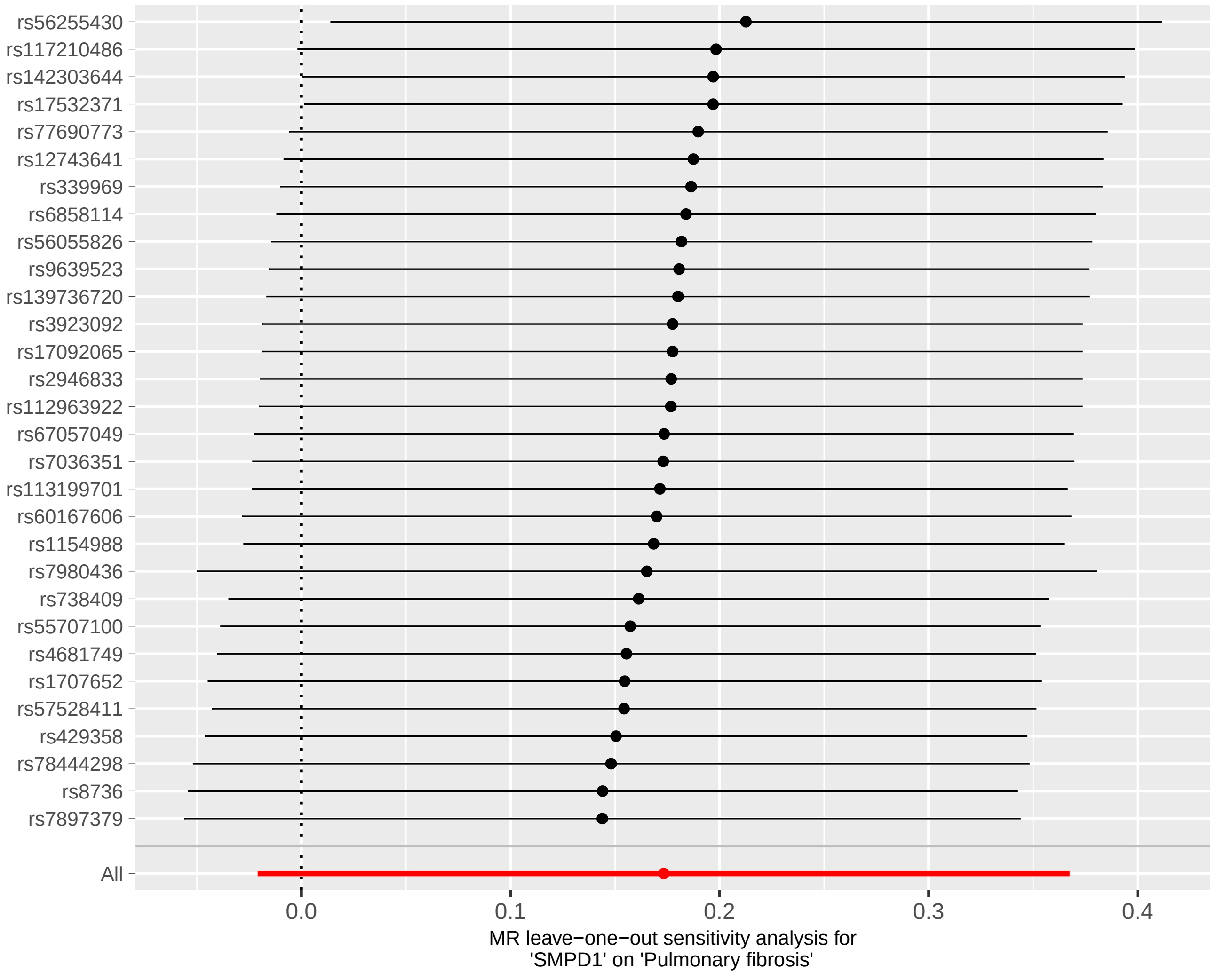

rs56255430
rs117210486
rs142303644
rs17532371
rs77690773
rs12743641
rs339969
rs6858114
rs56055826
rs9639523
rs139736720
rs3923092
rs17092065
rs2946833
rs112963922
rs67057049
rs7036351
rs113199701
rs60167606
rs1154988
rs7980436
rs738409
rs55707100
rs4681749
rs1707652
rs57528411
rs429358
rs78444298
rs8736
rs7897379
All
0.0
0.1
0.2
0.3
0.4
MR leave−one−out sensitivity analysis for
'SMPD1' on 'Pulmonary fibrosis'

Insufficient number of SNPs

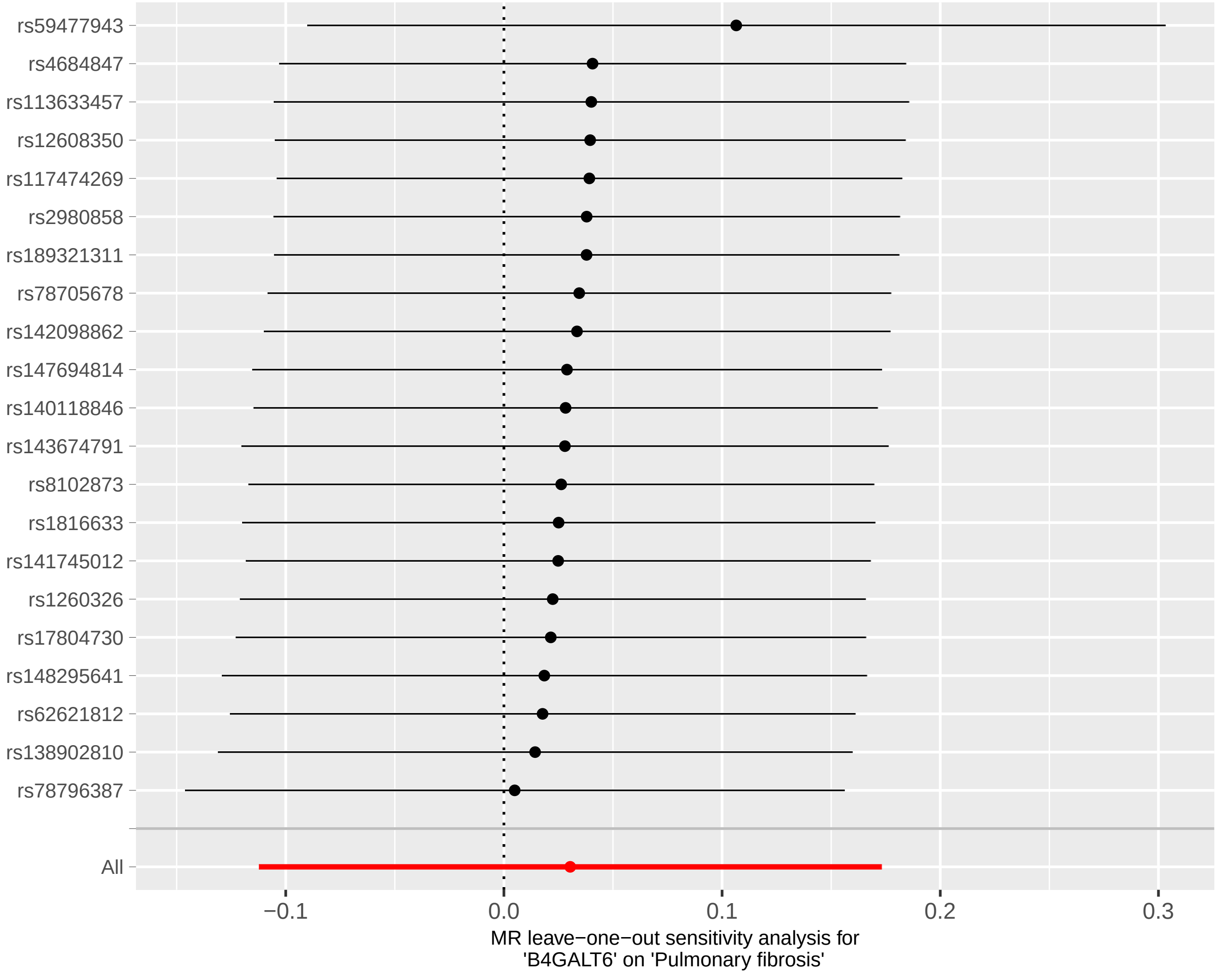
rs59477943
rs4684847
rs113633457
rs12608350
rs117474269
rs2980858
rs189321311
rs78705678
rs142098862
rs147694814
rs140118846
rs143674791
rs8102873
rs1816633
rs141745012
rs1260326
rs17804730
rs148295641
rs62621812
rs138902810
rs78796387
All
−0.1
0.0
0.1
0.2
0.3
MR leave−one−out sensitivity analysis for
'B4GALT6' on 'Pulmonary fibrosis'

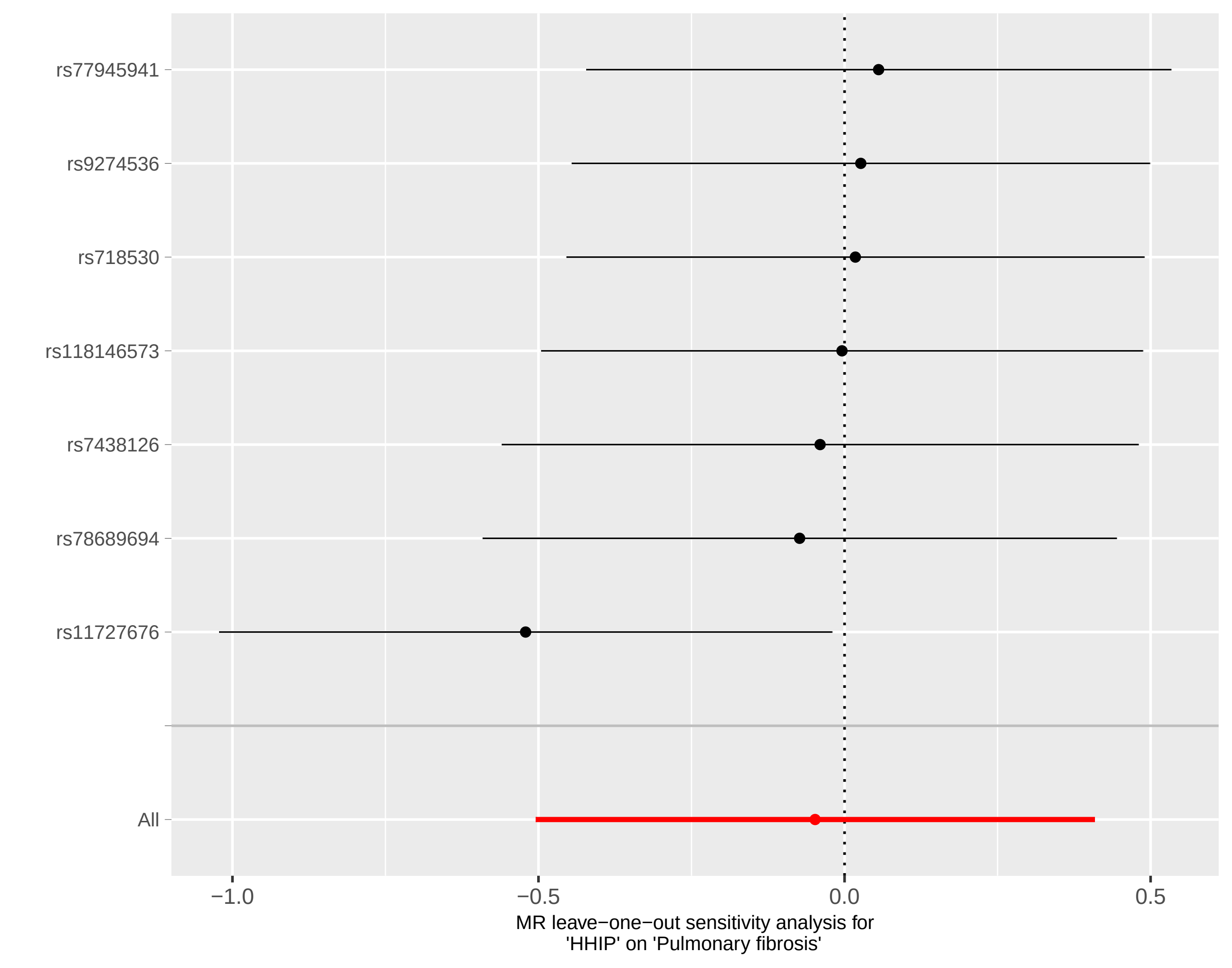

rs77945941
rs9274536
rs718530
rs118146573
rs7438126
rs78689694
rs11727676
All
−1.0
−0.5
0.0
0.5
MR leave−one−out sensitivity analysis for
'HHIP' on 'Pulmonary fibrosis'

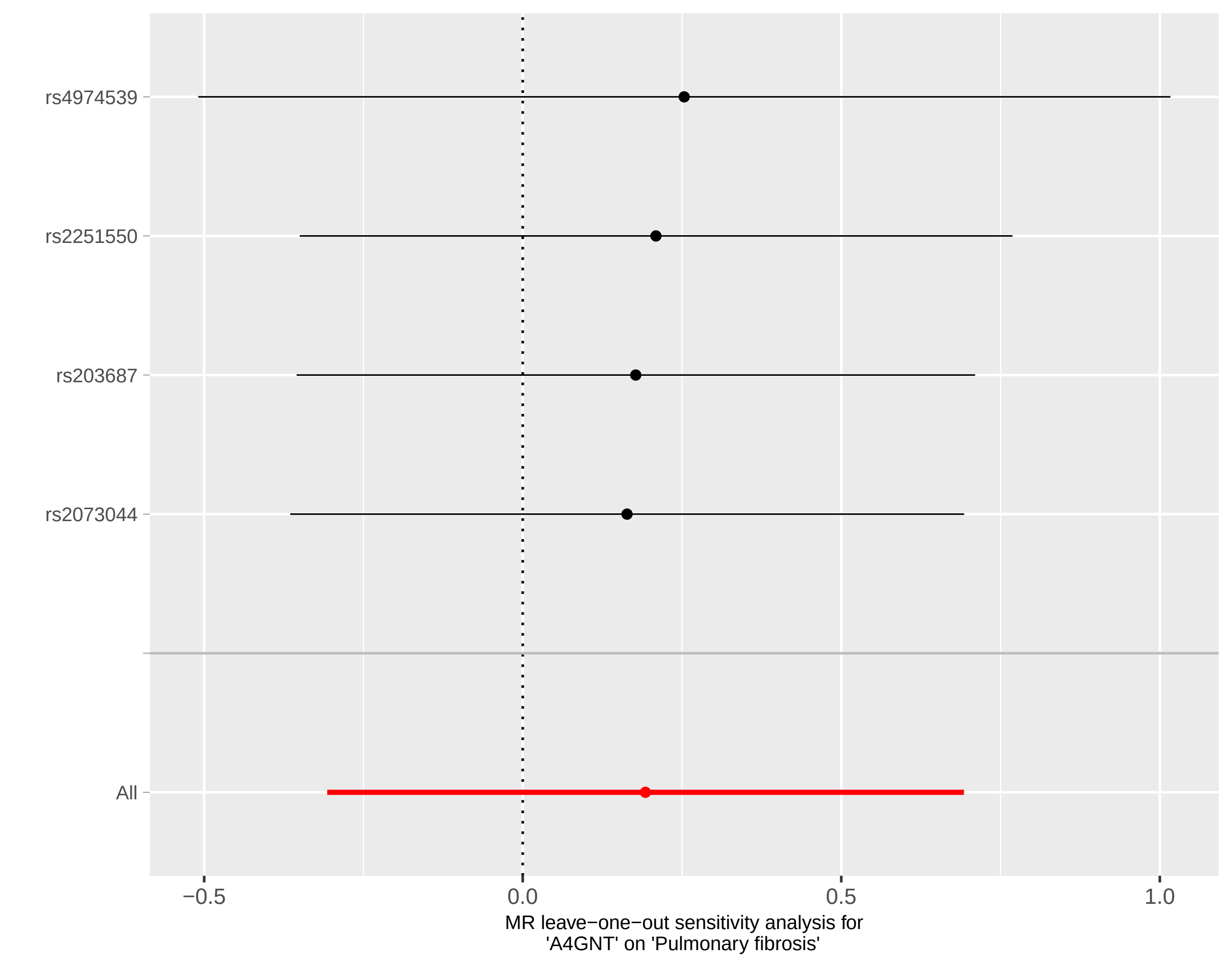
rs4974539
rs2251550
rs203687
rs2073044
All
−0.5
0.0
0.5
1.0
MR leave−one−out sensitivity analysis for
'A4GNT' on 'Pulmonary fibrosis'

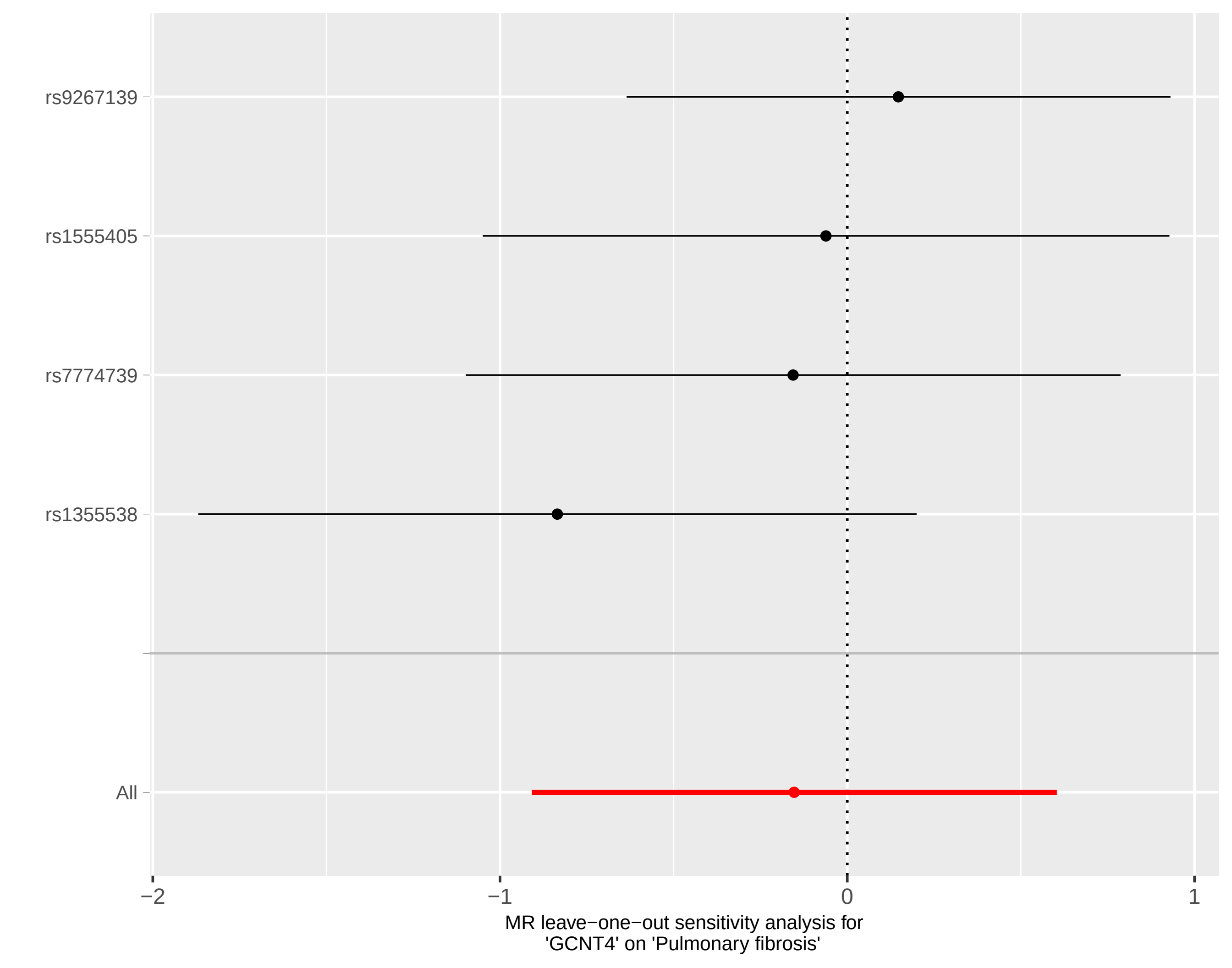
rs9267139
rs1555405
rs7774739
rs1355538
All
−2
−1
0
1
MR leave−one−out sensitivity analysis for
'GCNT4' on 'Pulmonary fibrosis'

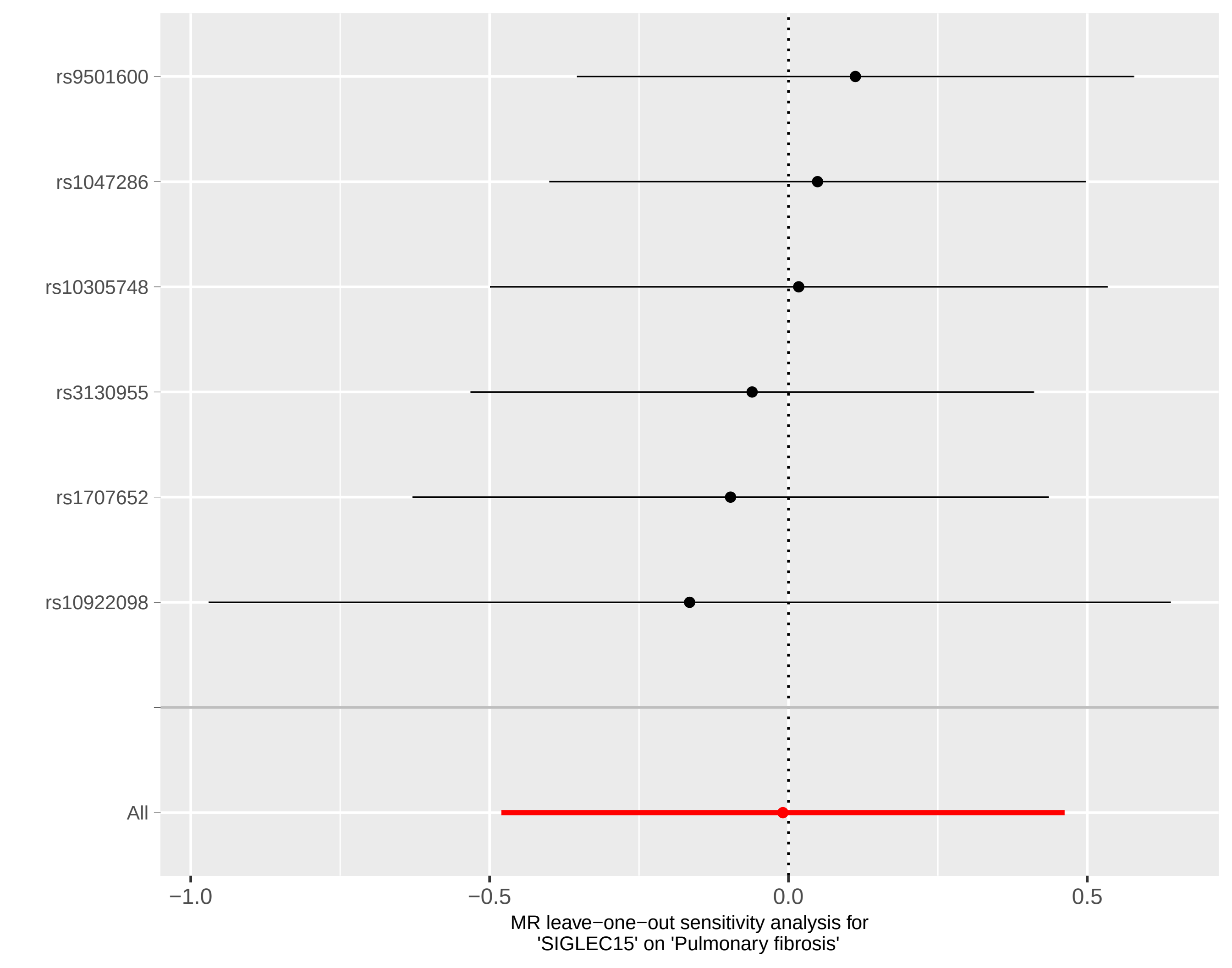

rs9501600
rs1047286
rs10305748
rs3130955
rs1707652
rs10922098
All
−1.0
−0.5
0.0
0.5
MR leave−one−out sensitivity analysis for
'SIGLEC15' on 'Pulmonary fibrosis'

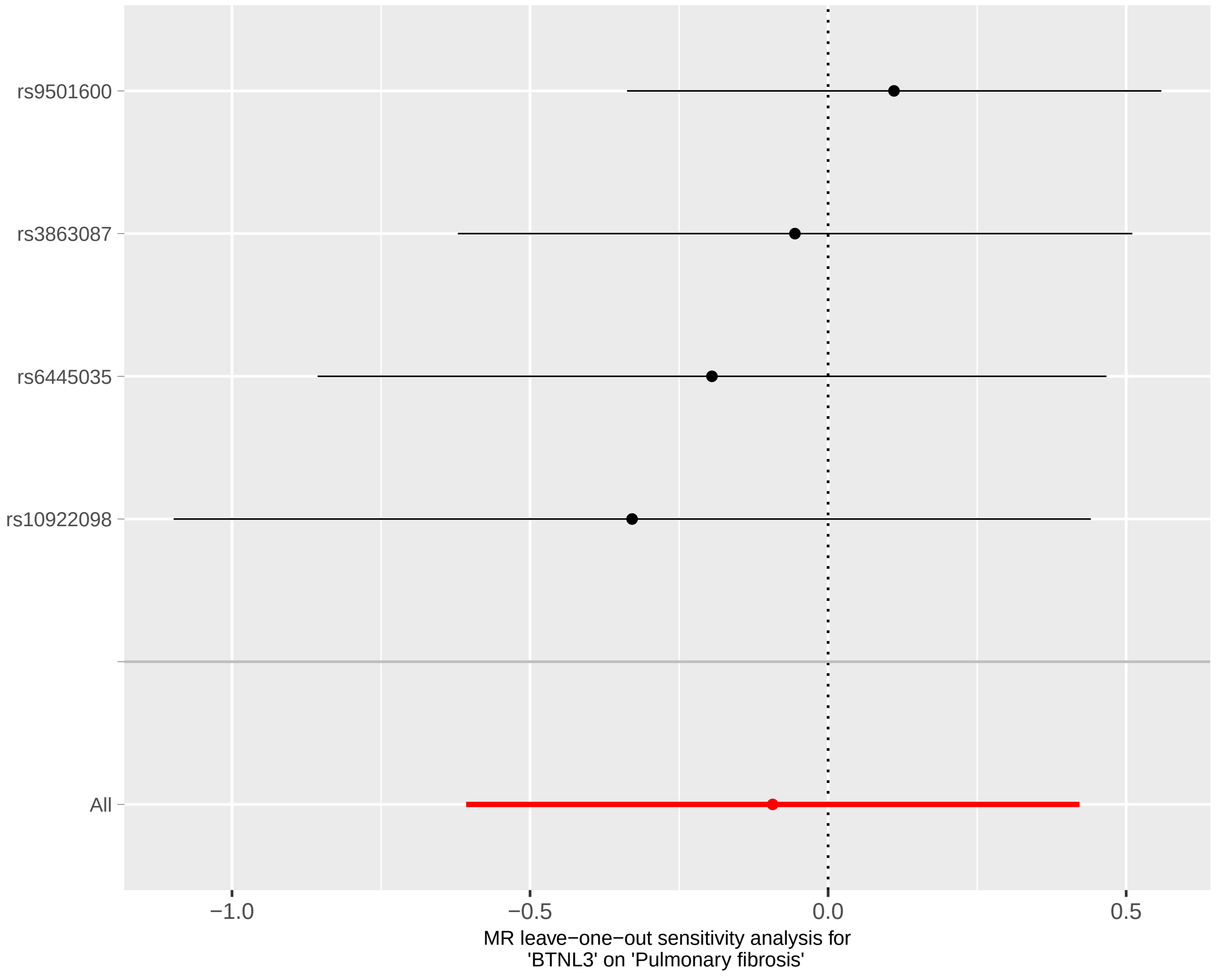
rs9501600
rs3863087
rs6445035
rs10922098
All
−1.0
−0.5
0.0
0.5
MR leave−one−out sensitivity analysis for
'BTNL3' on 'Pulmonary fibrosis'

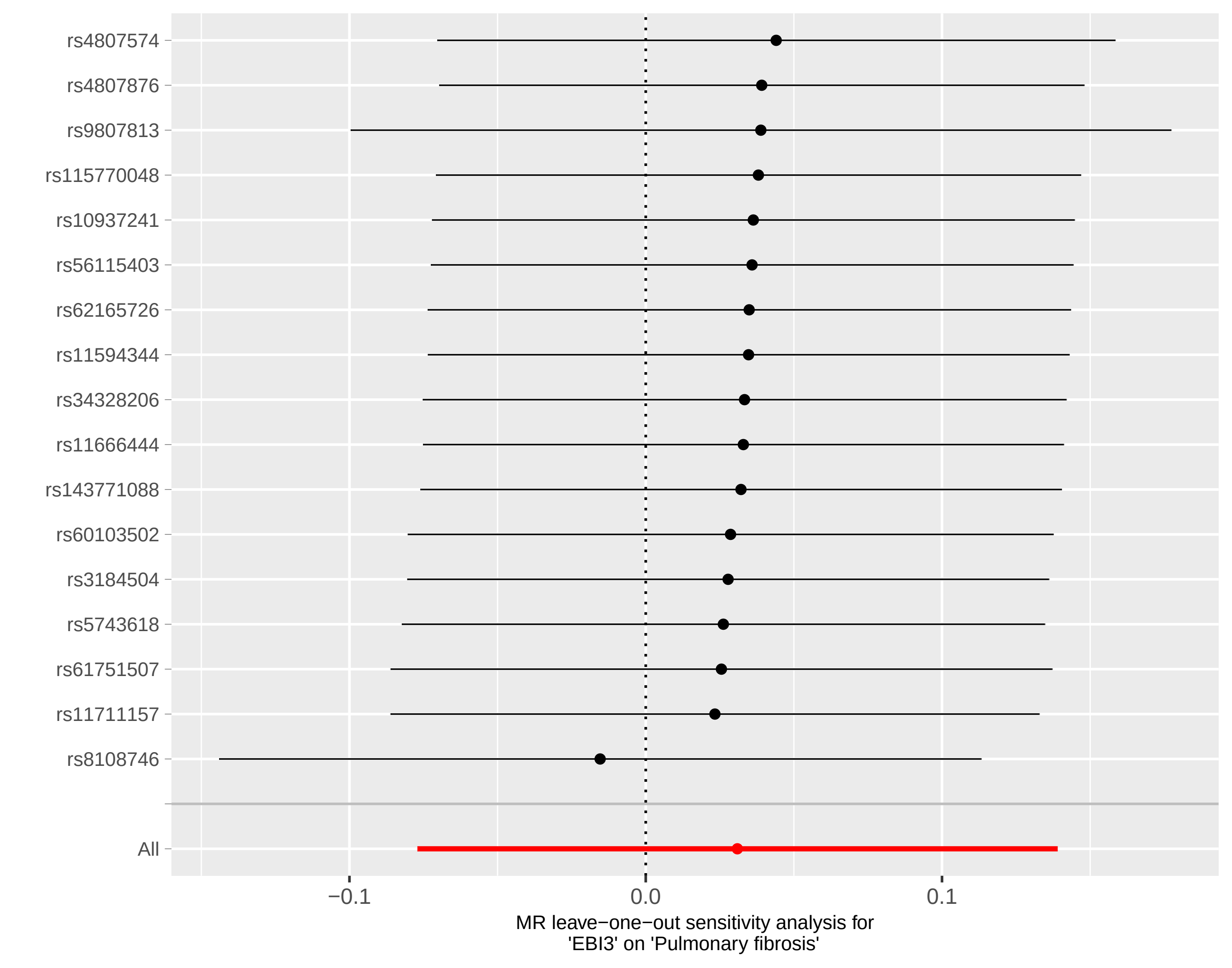

rs4807574
rs4807876
rs9807813
rs115770048
rs10937241
rs56115403
rs62165726
rs11594344
rs34328206
rs11666444
rs143771088
rs60103502
rs3184504
rs5743618
rs61751507
rs11711157
rs8108746
All
−0.1
0.0
0.1
MR leave−one−out sensitivity analysis for
'EBI3' on 'Pulmonary fibrosis'

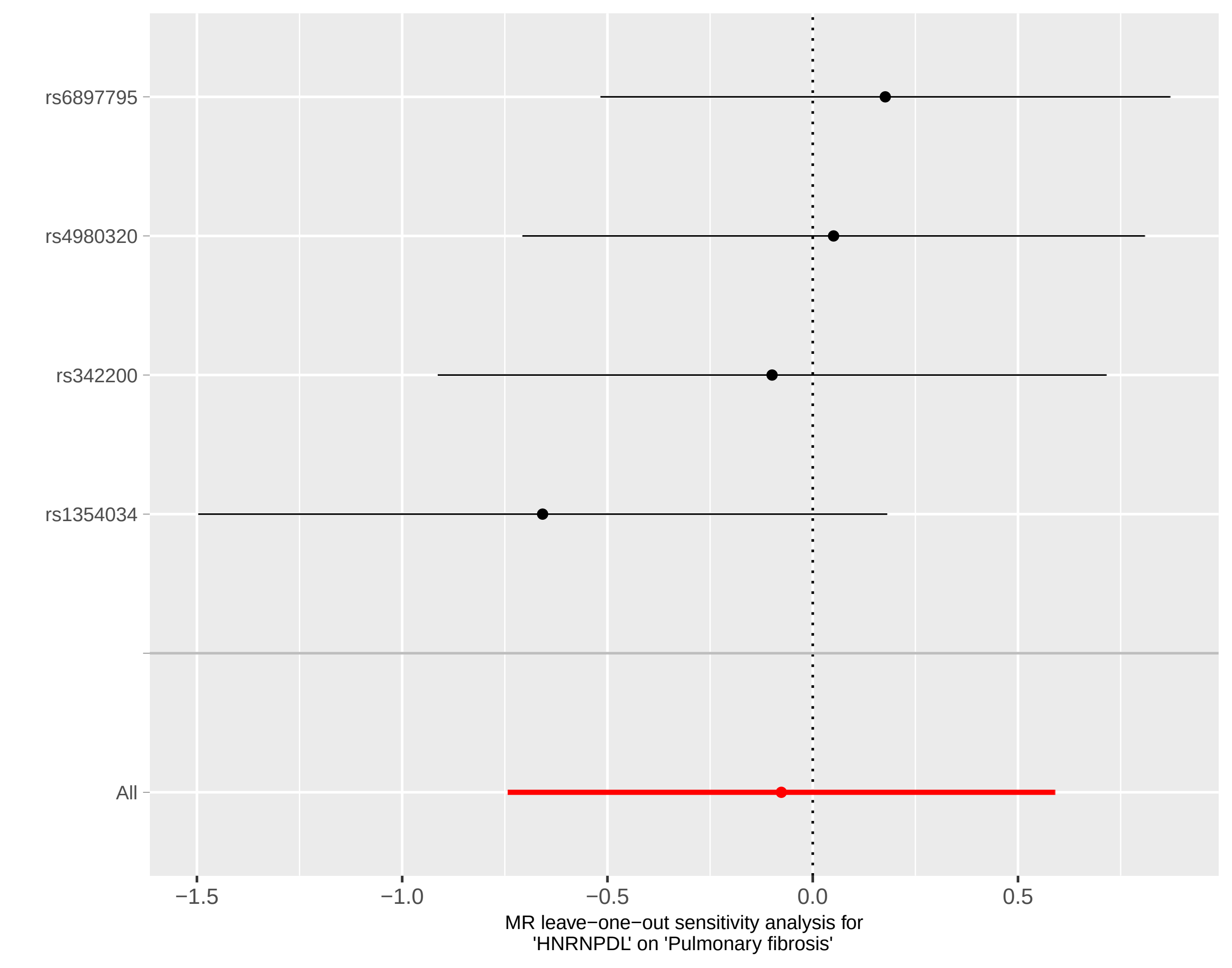
rs6897795
rs4980320
rs342200
rs1354034
All
−1.5
−1.0
−0.5
0.0
0.5
MR leave−one−out sensitivity analysis for
'HNRNPDL' on 'Pulmonary fibrosis'

Insufficient number of SNPs

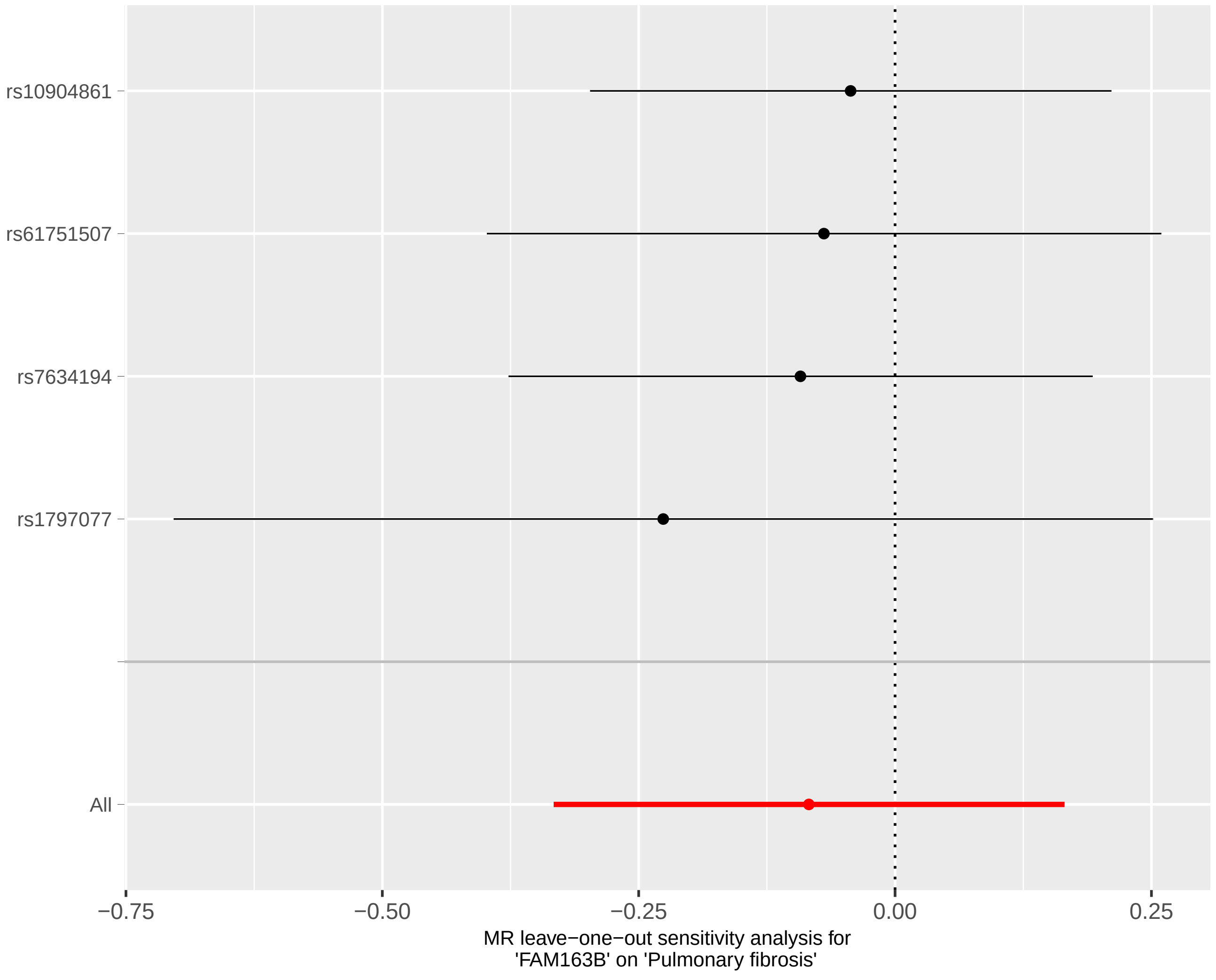
rs10904861
rs61751507
rs7634194
rs1797077
All
−0.75
−0.50
−0.25
0.00
0.25
MR leave−one−out sensitivity analysis for
'FAM163B' on 'Pulmonary fibrosis'

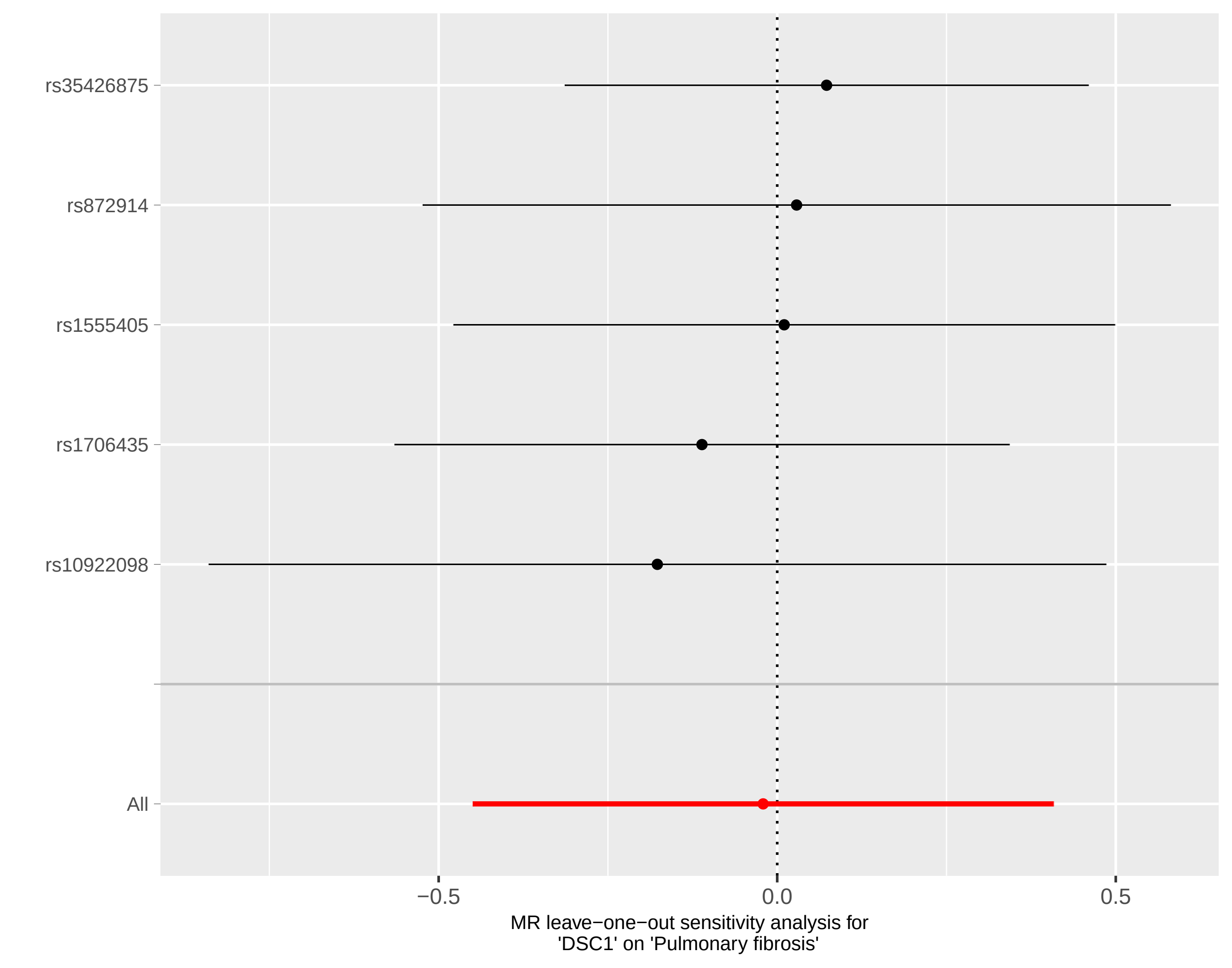

rs35426875
rs872914
rs1555405
rs1706435
rs10922098
All
−0.5
0.0
0.5
MR leave−one−out sensitivity analysis for
'DSC1' on 'Pulmonary fibrosis'

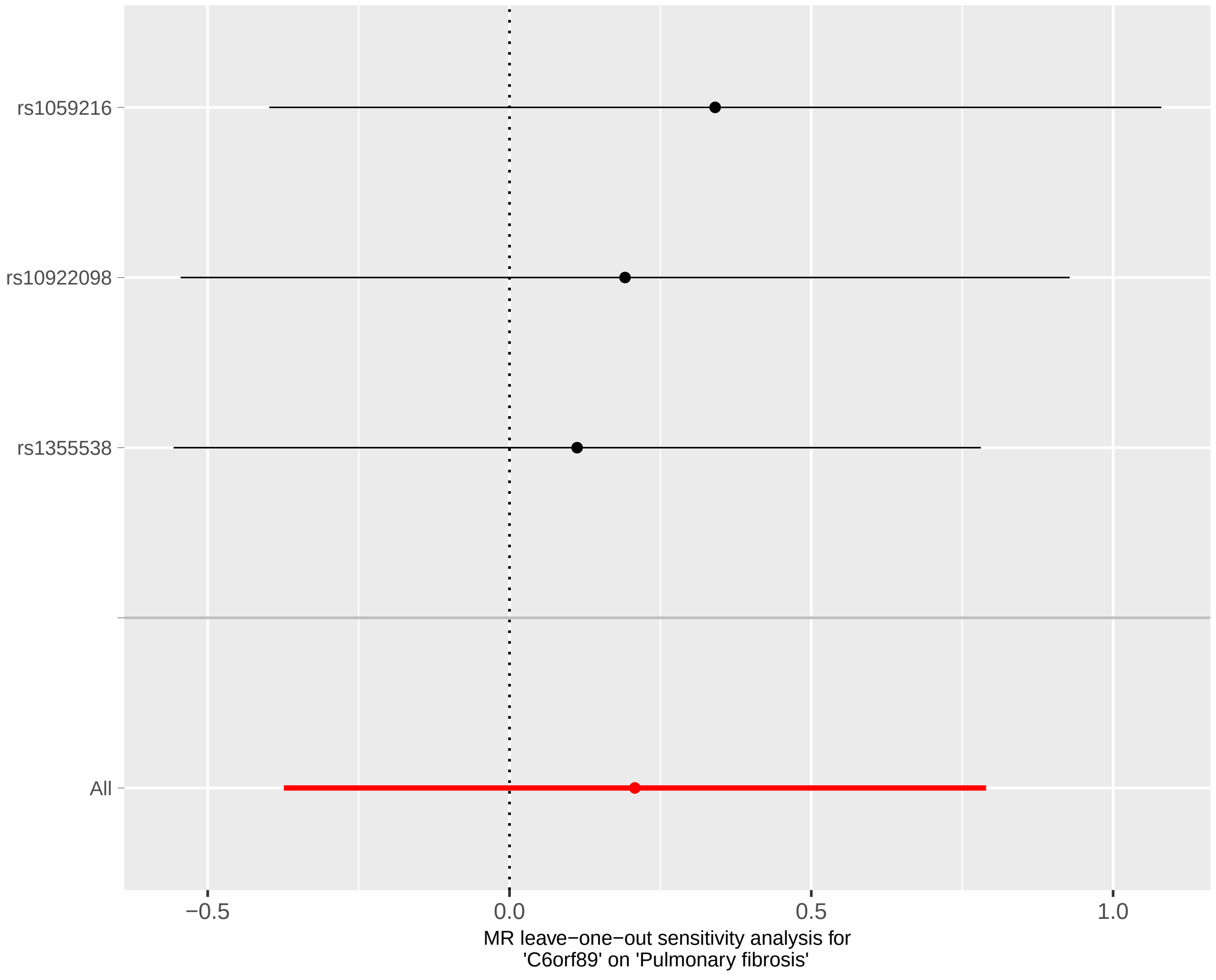
rs1059216
rs10922098
rs1355538
All
−0.5
0.0
0.5
1.0
MR leave−one−out sensitivity analysis for
'C6orf89' on 'Pulmonary fibrosis'

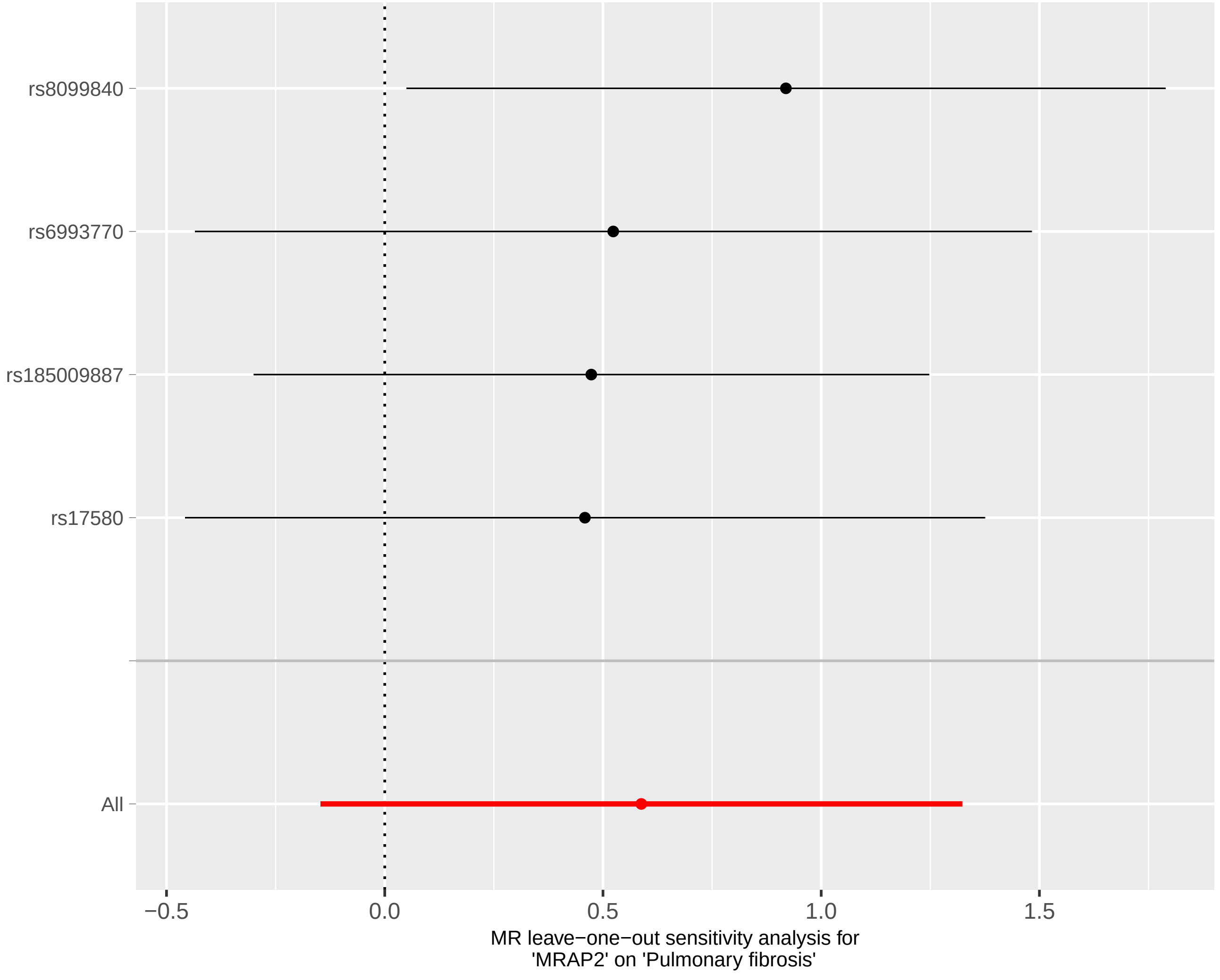
rs8099840
rs6993770
rs185009887
rs17580
All
−0.5
0.0
0.5
1.0
1.5
MR leave−one−out sensitivity analysis for
'MRAP2' on 'Pulmonary fibrosis'

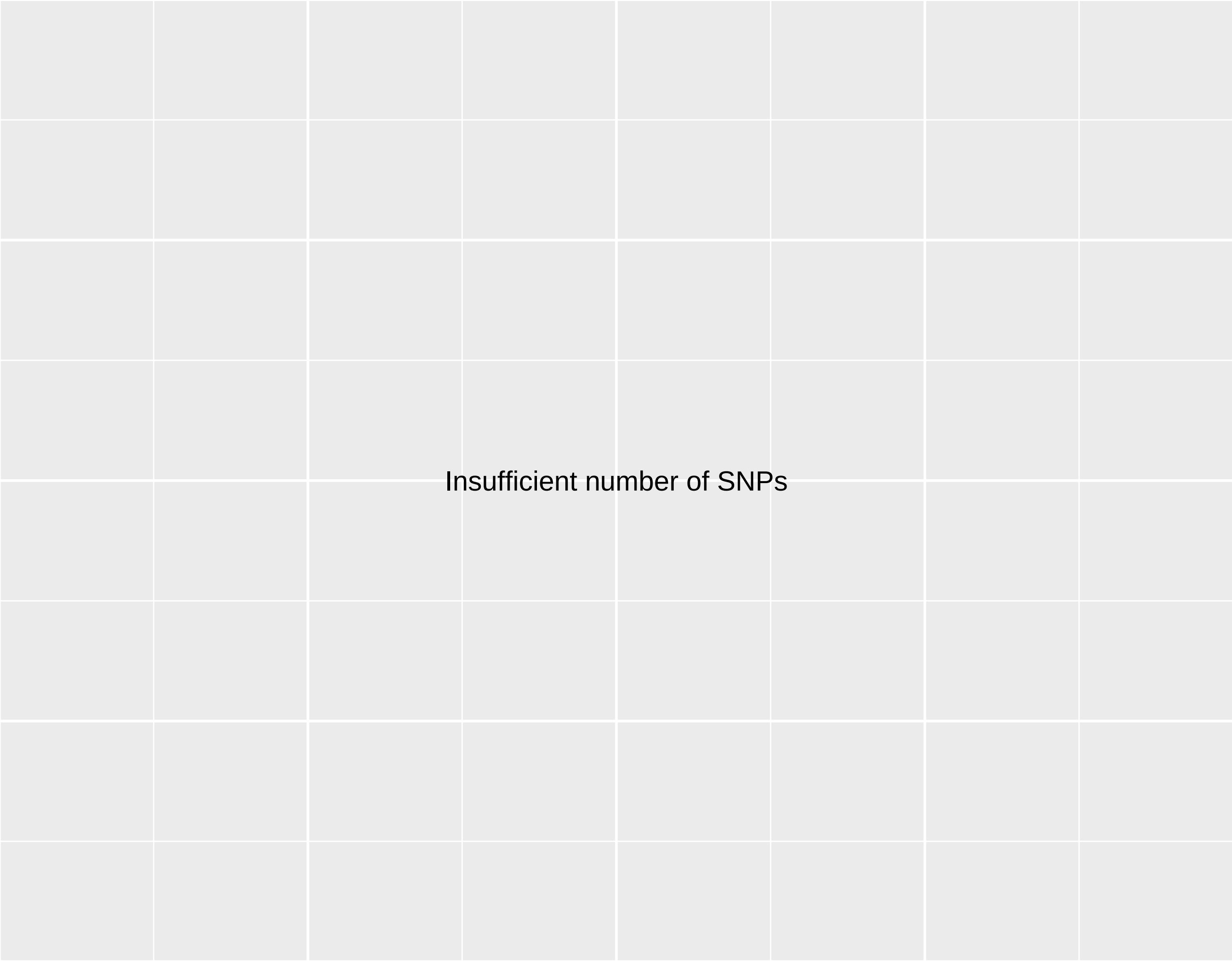
Insufficient number of SNPs

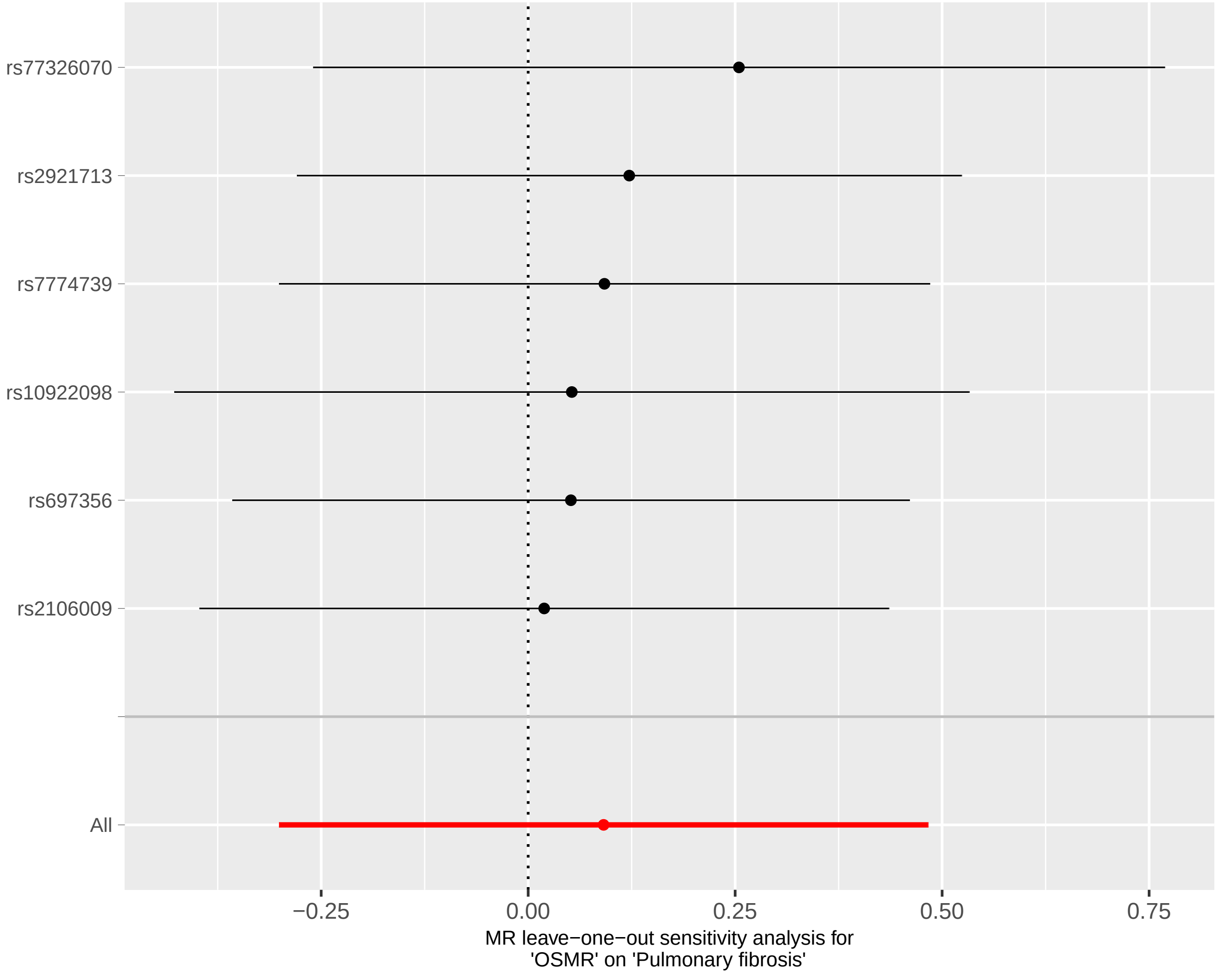
rs77326070
rs2921713
rs7774739
rs10922098
rs697356
rs2106009
All
−0.25
0.00
0.25
0.50
0.75
MR leave−one−out sensitivity analysis for
'OSMR' on 'Pulmonary fibrosis'

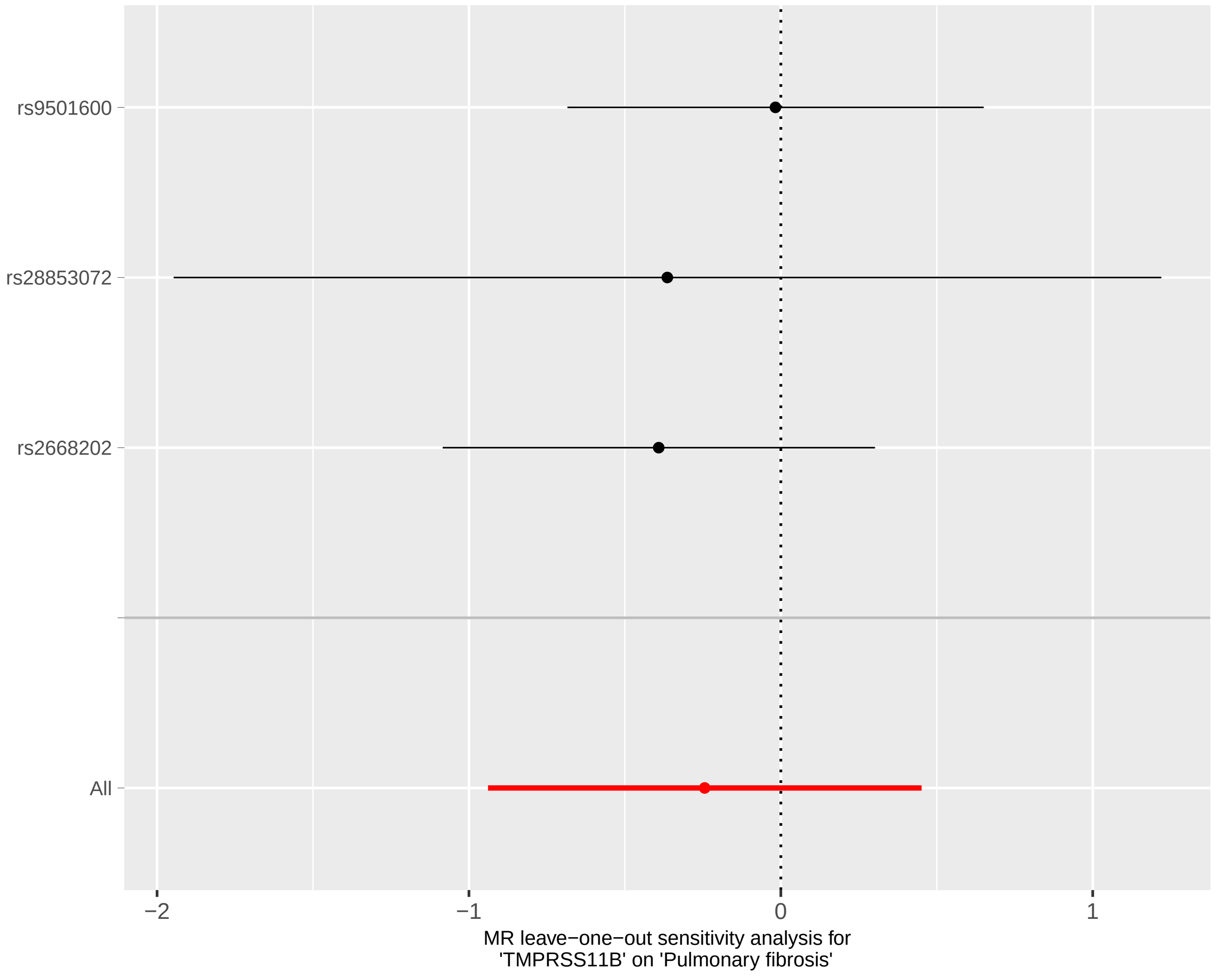
rs9501600
rs28853072
rs2668202
All
−2
−1
0
1
MR leave−one−out sensitivity analysis for
'TMPRSS11B' on 'Pulmonary fibrosis'

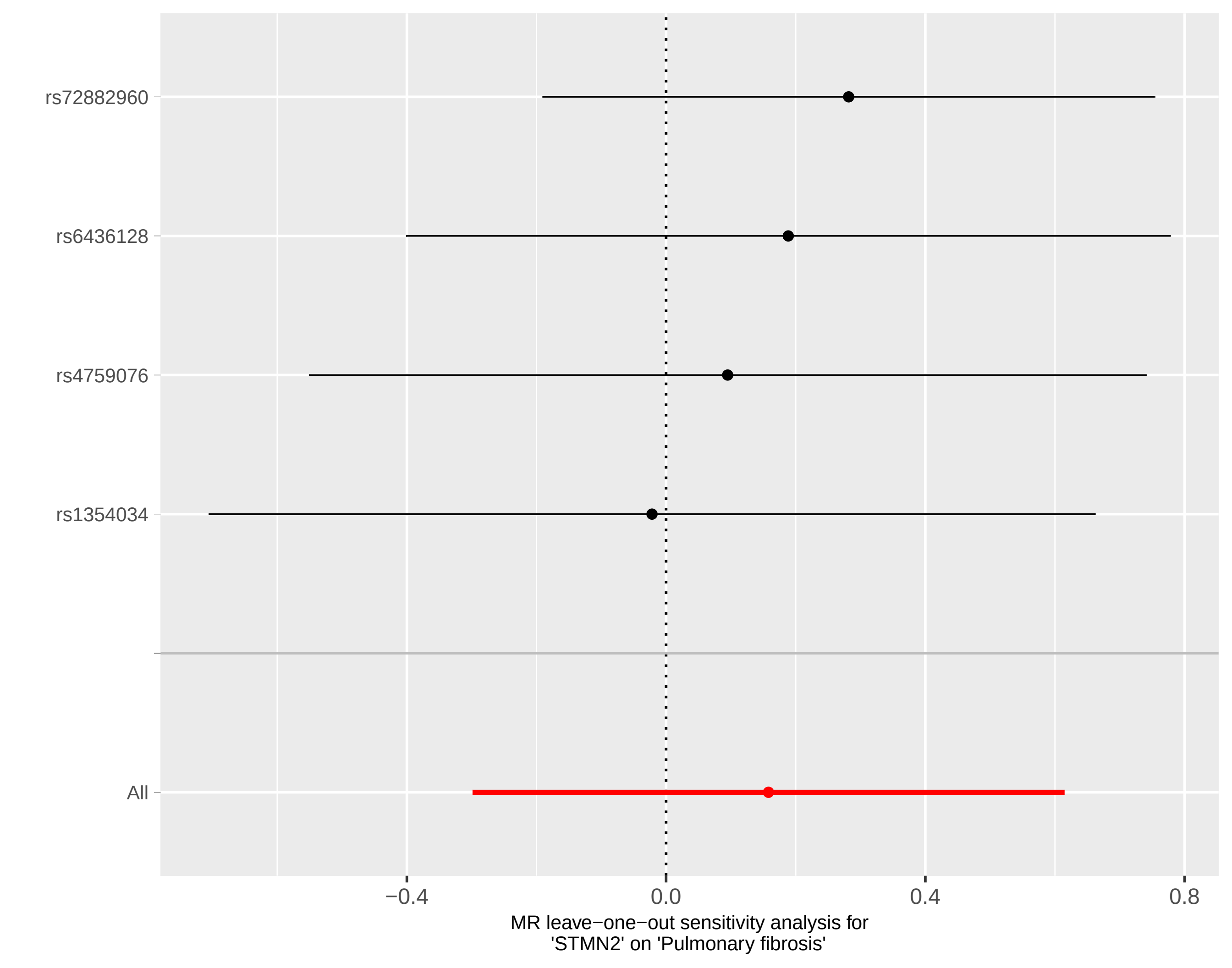

rs72882960
rs6436128
rs4759076
rs1354034
All
−0.4
0.0
0.4
0.8
MR leave−one−out sensitivity analysis for
'STMN2' on 'Pulmonary fibrosis'

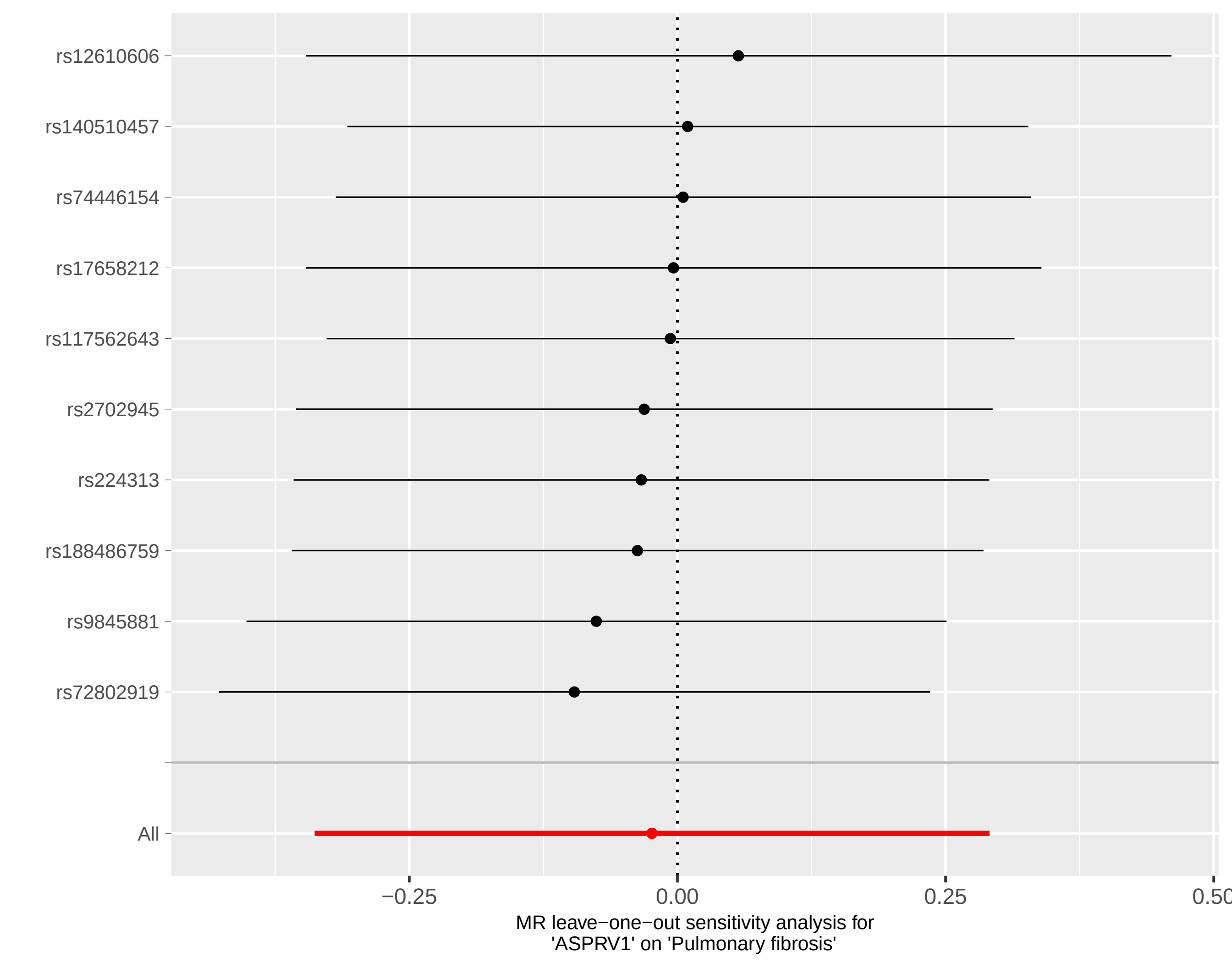
rs12610606
rs140510457
rs74446154
rs17658212
rs117562643
rs2702945
rs224313
rs188486759
rs9845881
rs72802919
All
−0.25
0.00
0.25
0.50
MR leave−one−out sensitivity analysis for
'ASPRV1' on 'Pulmonary fibrosis'

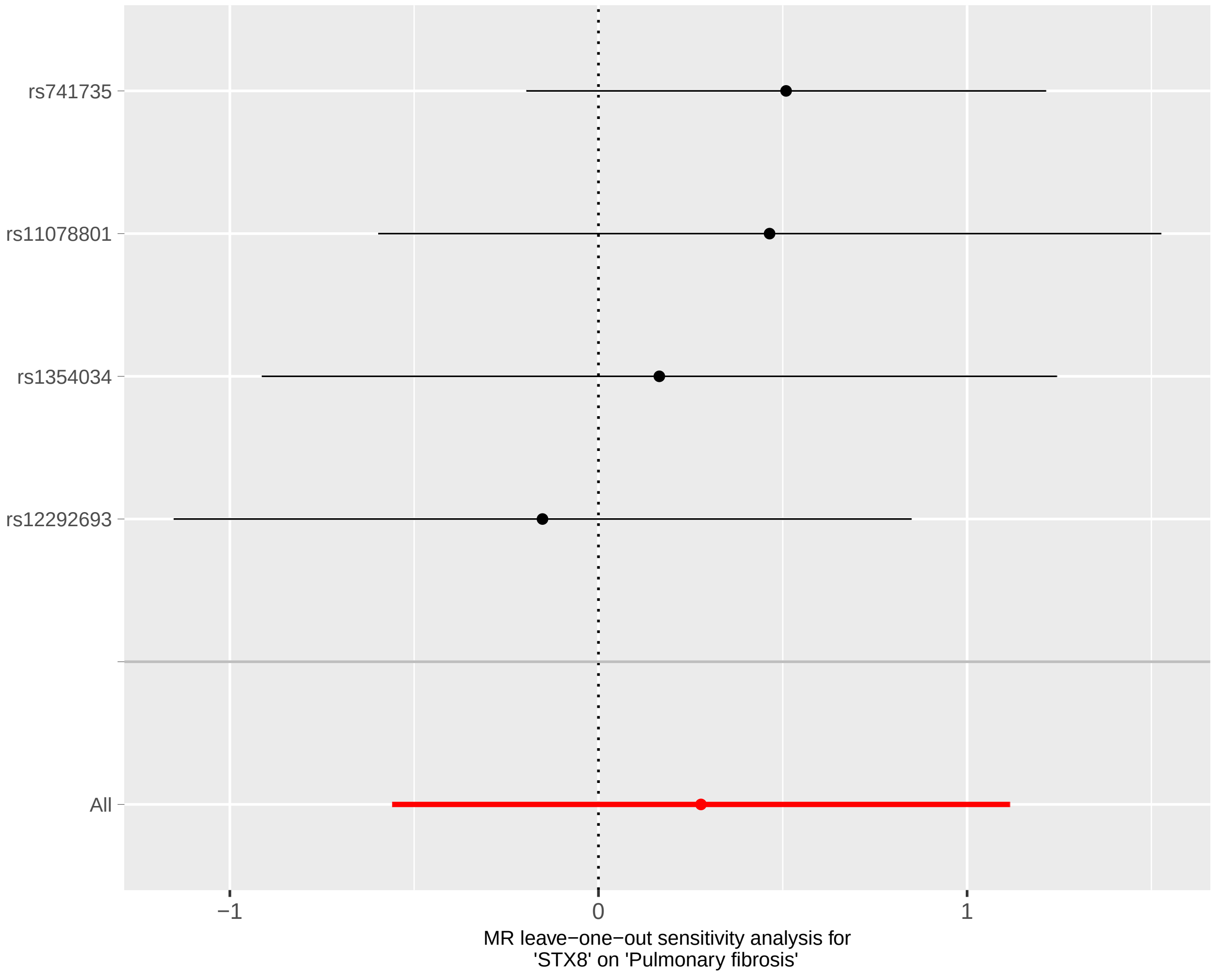

rs741735
rs11078801
rs1354034
rs12292693
All
−1
0
1
MR leave−one−out sensitivity analysis for
'STX8' on 'Pulmonary fibrosis'

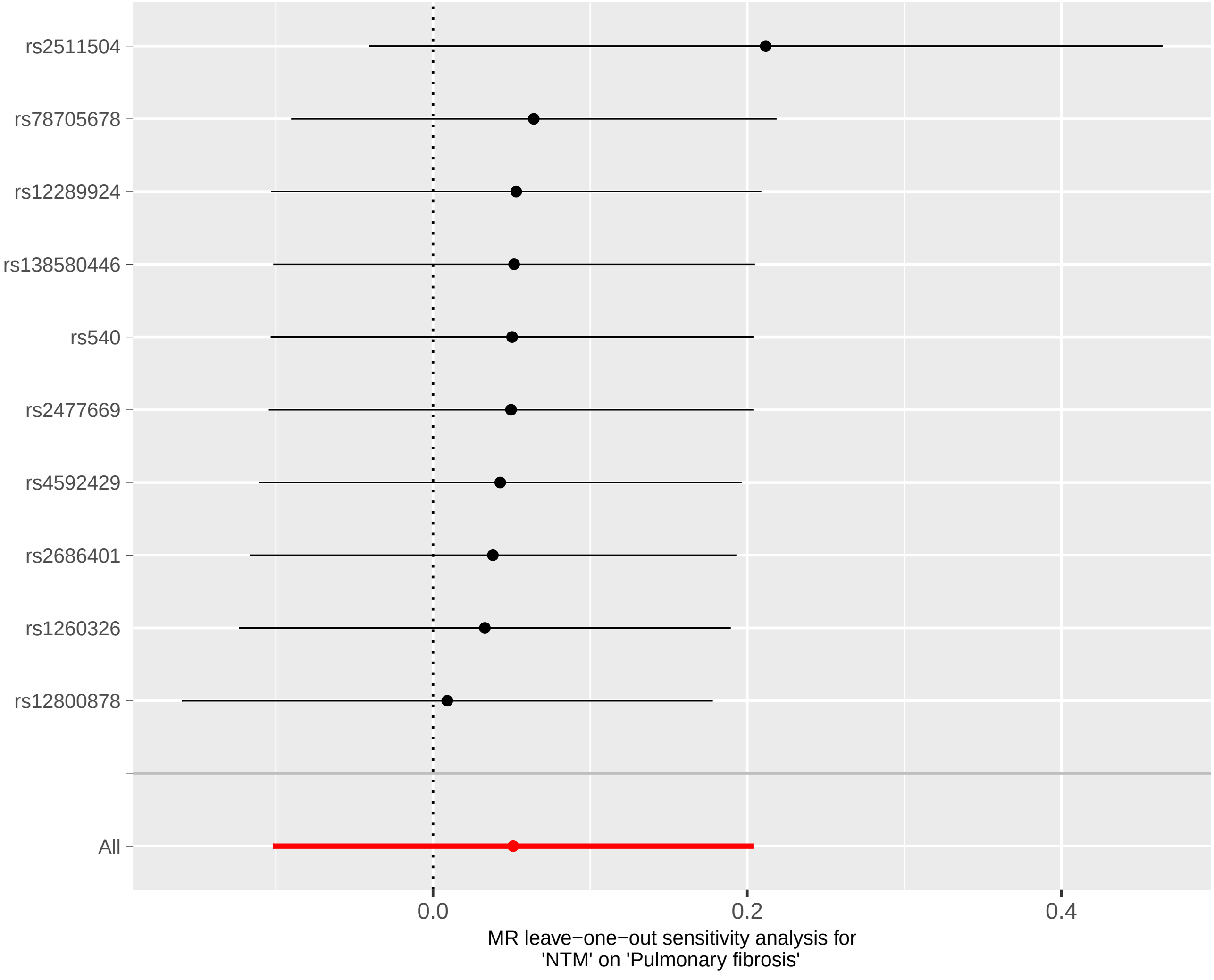

rs2511504
rs78705678
rs12289924
rs138580446
rs540
rs2477669
rs4592429
rs2686401
rs1260326
rs12800878
All
0.0
0.2
0.4
MR leave−one−out sensitivity analysis for
'NTM' on 'Pulmonary fibrosis'

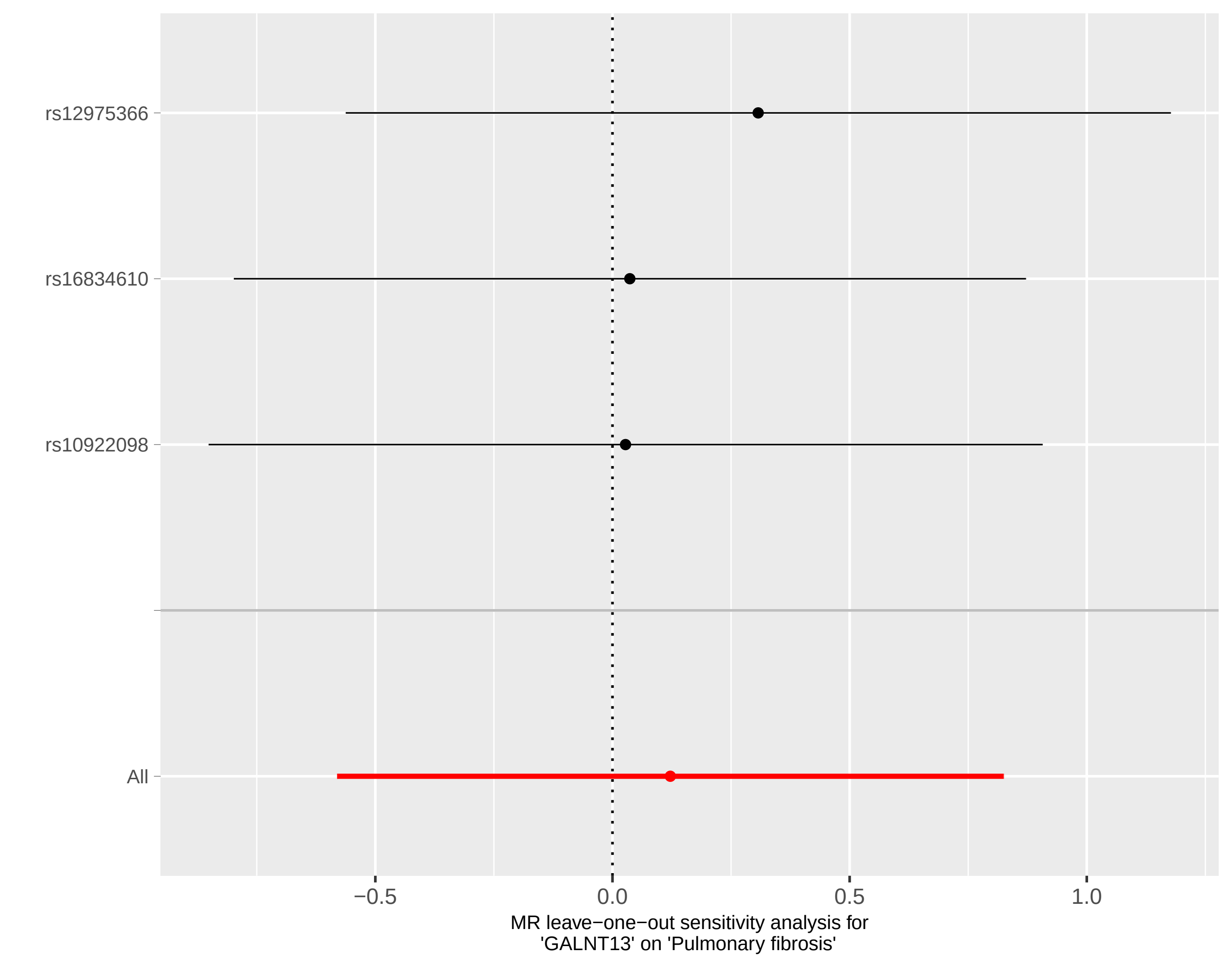
rs12975366
rs16834610
rs10922098
All
−0.5
0.0
0.5
1.0
MR leave−one−out sensitivity analysis for
'GALNT13' on 'Pulmonary fibrosis'

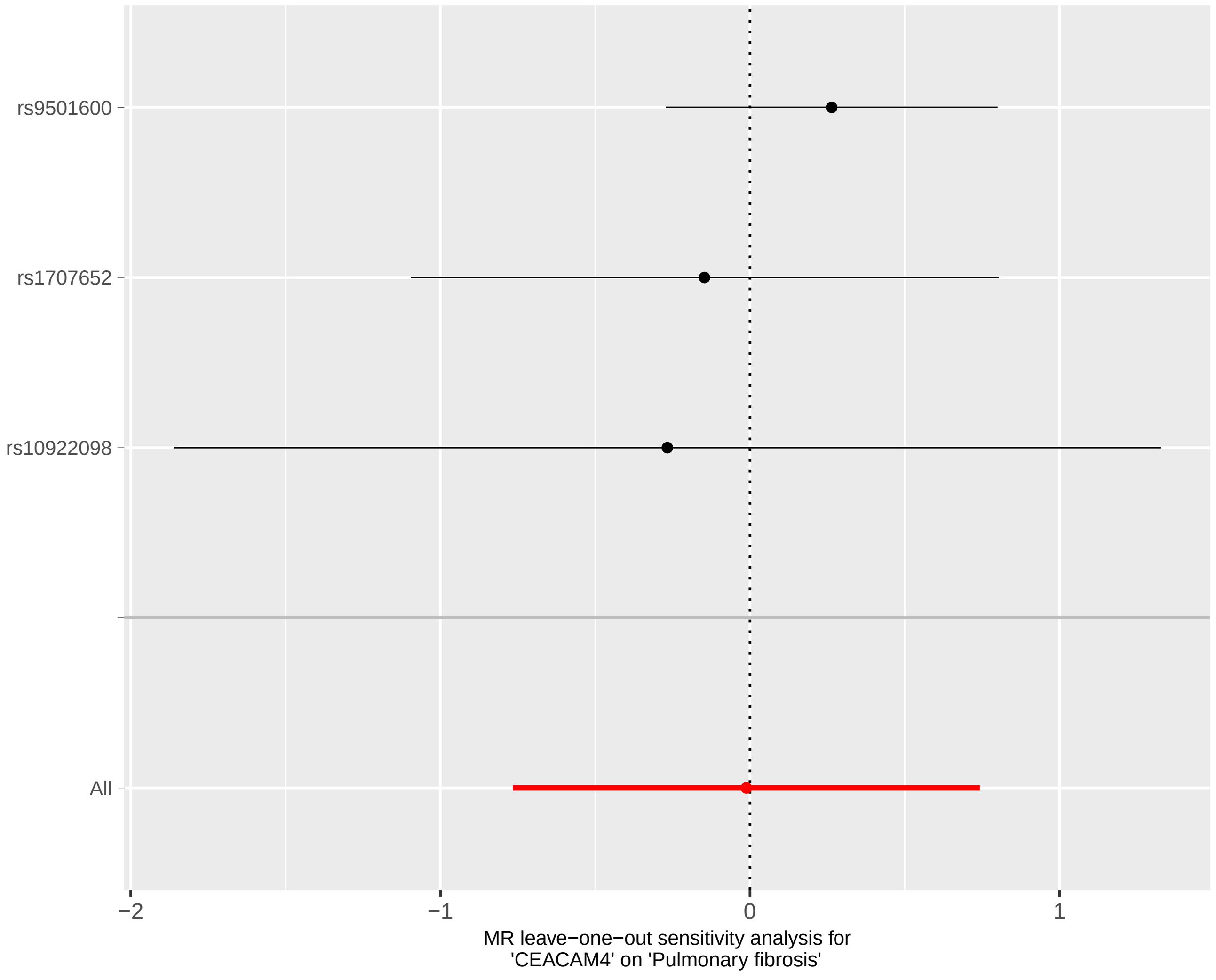
rs9501600
rs1707652
rs10922098
All
−2
−1
0
1
MR leave−one−out sensitivity analysis for
'CEACAM4' on 'Pulmonary fibrosis'

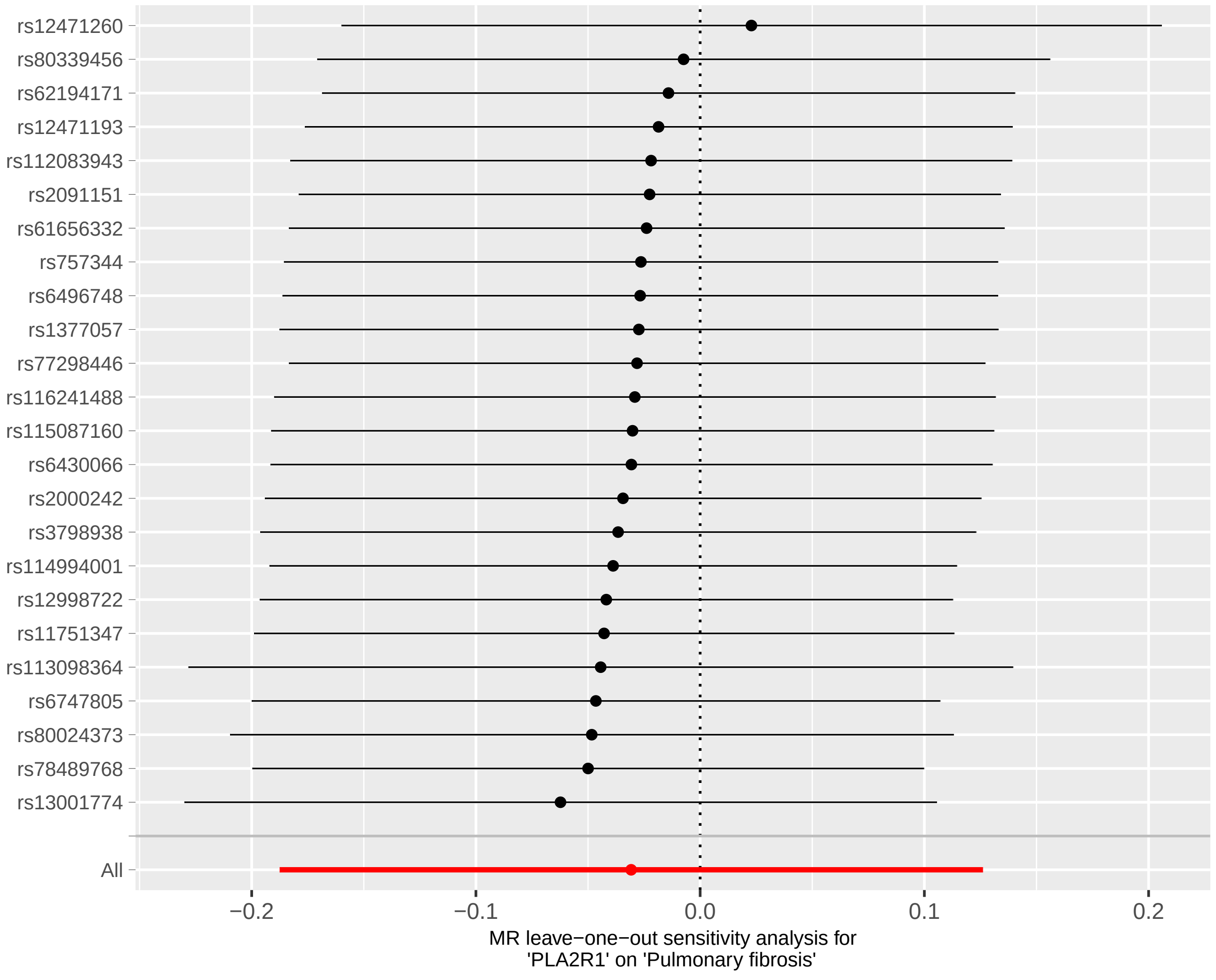
rs12471260
rs80339456
rs62194171
rs12471193
rs112083943
rs2091151
rs61656332
rs757344
rs6496748
rs1377057
rs77298446
rs116241488
rs115087160
rs6430066
rs2000242
rs3798938
rs114994001
rs12998722
rs11751347
rs113098364
rs6747805
rs80024373
rs78489768
rs13001774
All
−0.2
−0.1
0.0
0.1
0.2
MR leave−one−out sensitivity analysis for
'PLA2R1' on 'Pulmonary fibrosis'

Insufficient number of SNPs

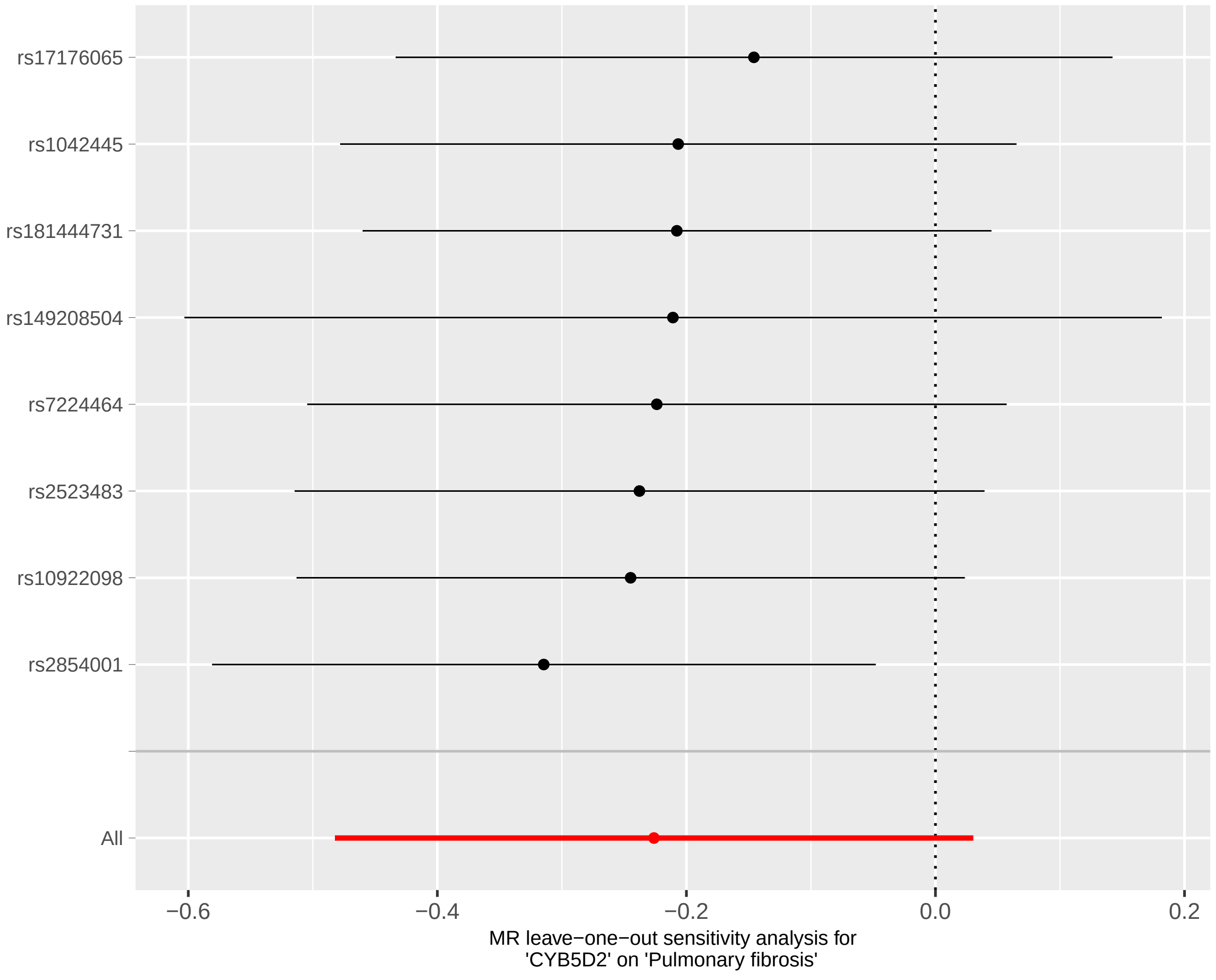
rs17176065
rs1042445
rs181444731
rs149208504
rs7224464
rs2523483
rs10922098
rs2854001
All
−0.6
−0.4
−0.2
0.0
0.2
MR leave−one−out sensitivity analysis for
'CYB5D2' on 'Pulmonary fibrosis'

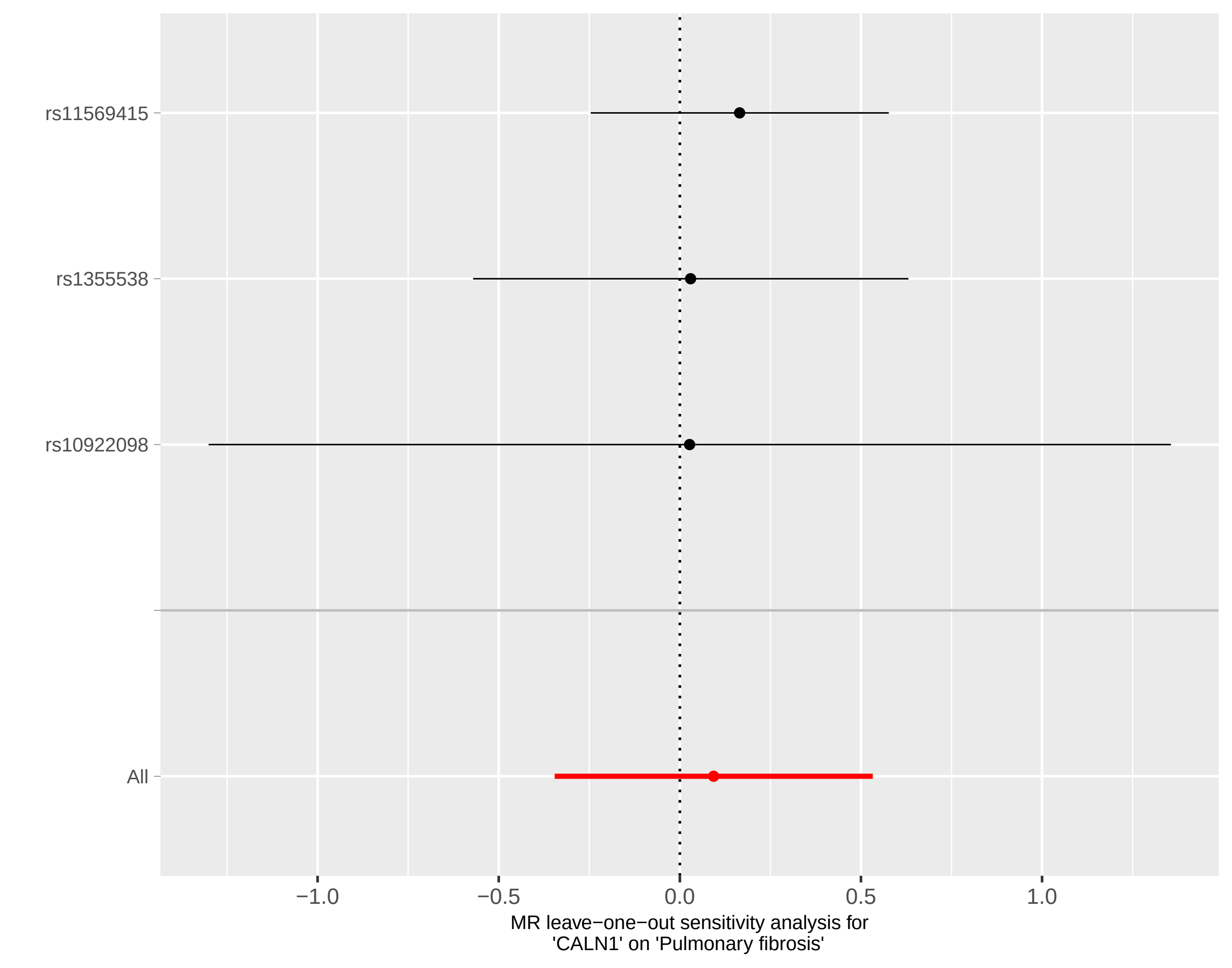
rs11569415
rs1355538
rs10922098
All
−1.0
−0.5
0.0
0.5
1.0
MR leave−one−out sensitivity analysis for
'CALN1' on 'Pulmonary fibrosis'

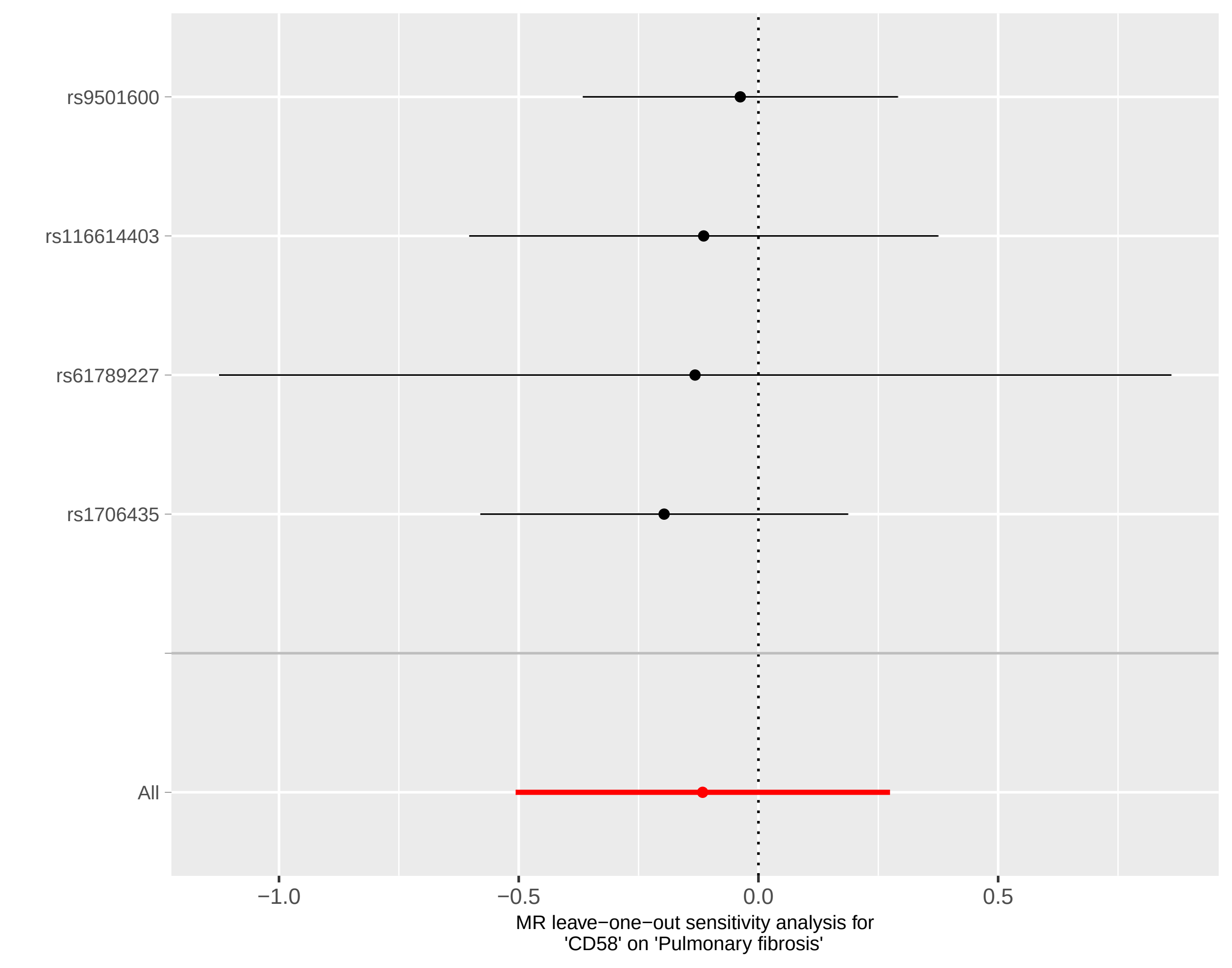
rs9501600
rs116614403
rs61789227
rs1706435
All
−1.0
−0.5
0.0
0.5
MR leave−one−out sensitivity analysis for
'CD58' on 'Pulmonary fibrosis'

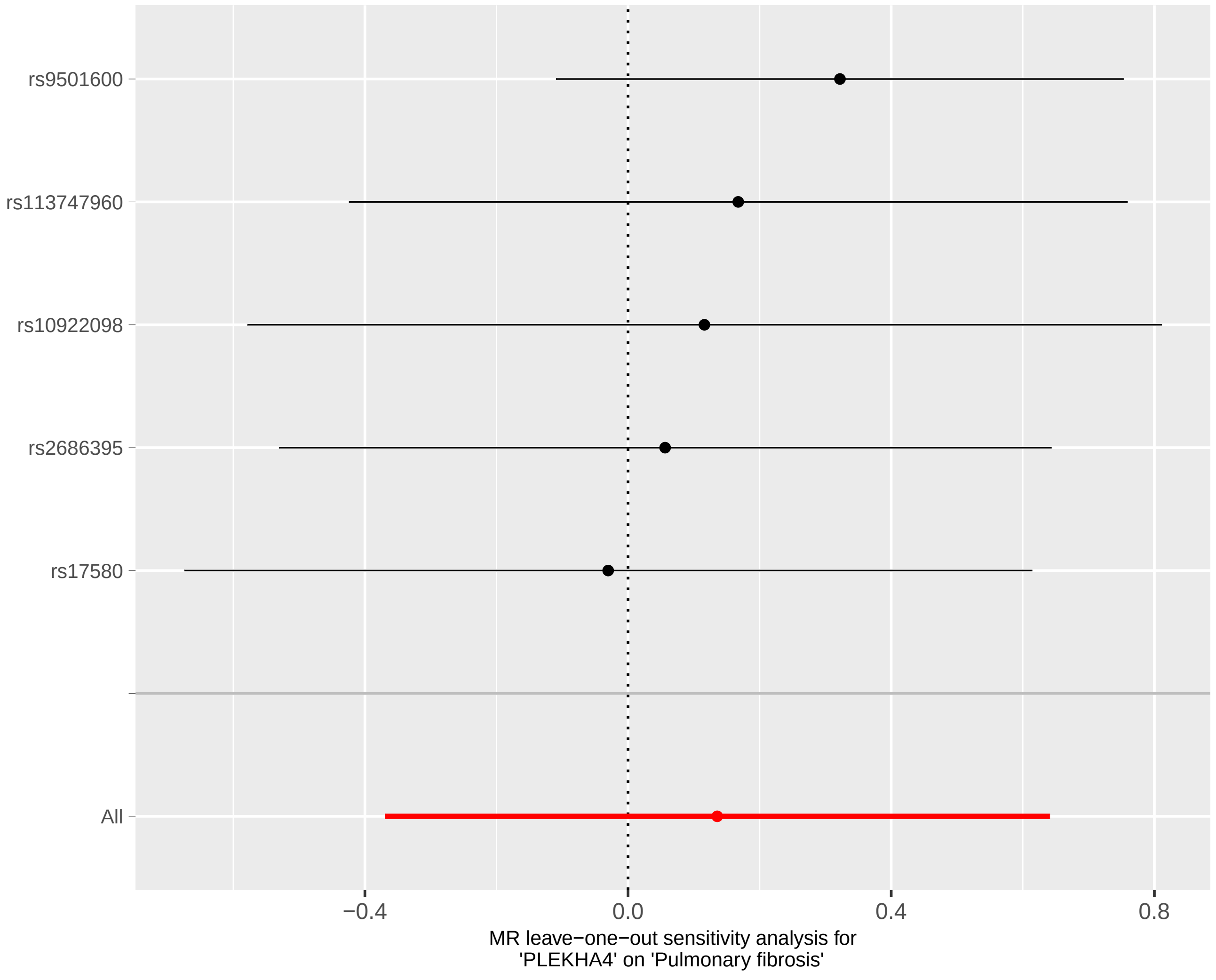
rs9501600
rs113747960
rs10922098
rs2686395
rs17580
All
−0.4
0.0
0.4
0.8
MR leave−one−out sensitivity analysis for
'PLEKHA4' on 'Pulmonary fibrosis'

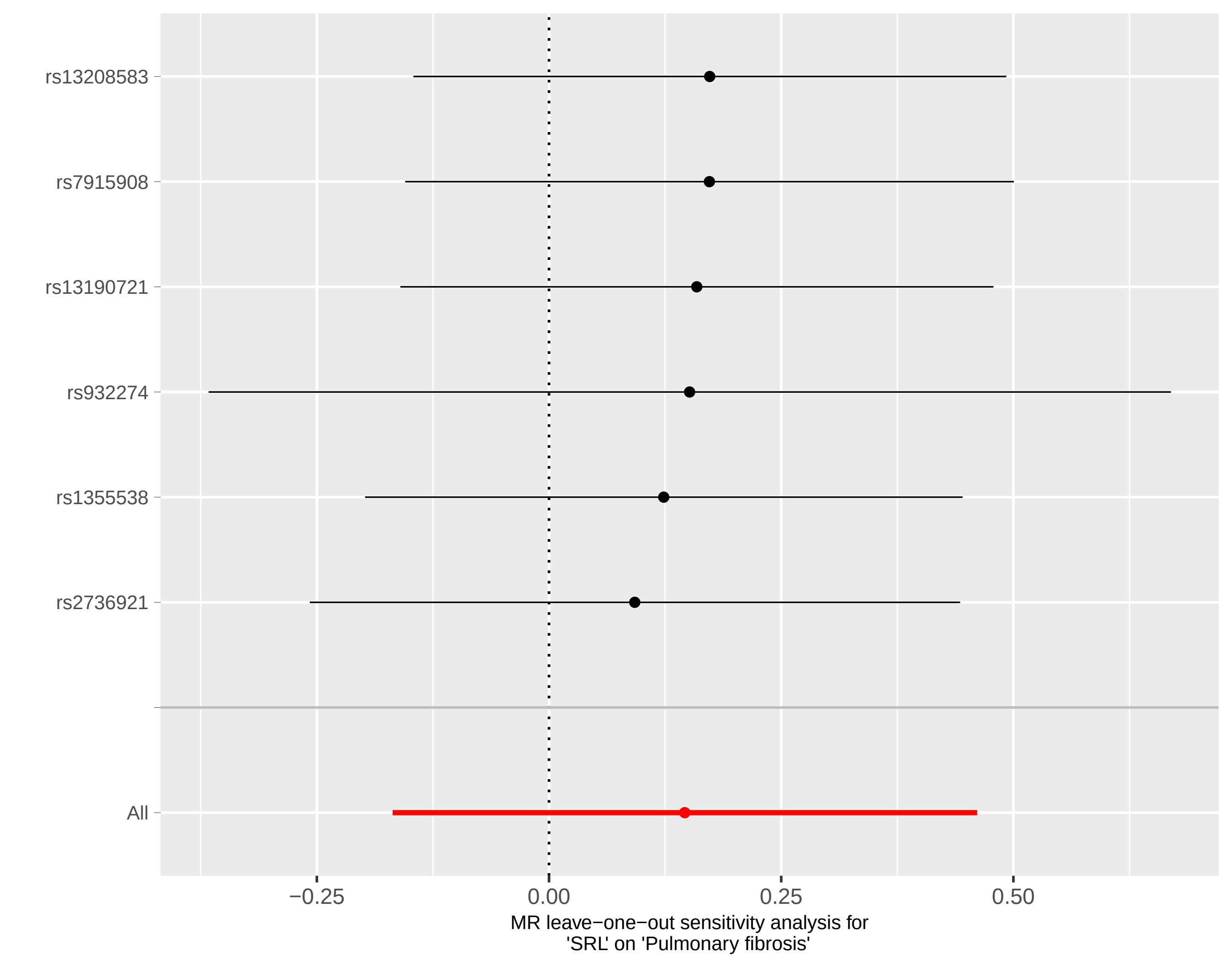
rs13208583
rs7915908
rs13190721
rs932274
rs1355538
rs2736921
All
−0.25
0.00
0.25
0.50
MR leave−one−out sensitivity analysis for
'SRL' on 'Pulmonary fibrosis'

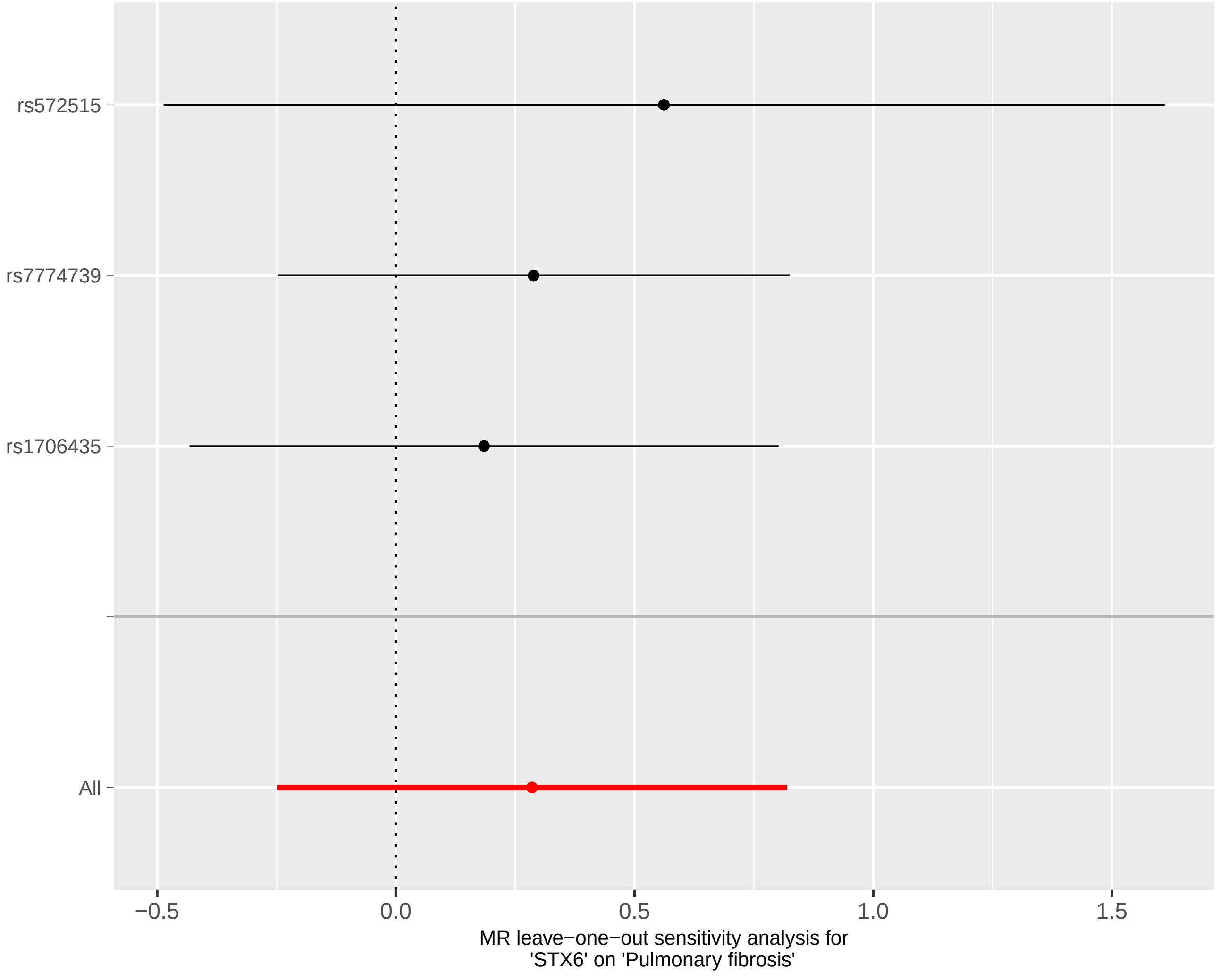

rs572515
rs7774739
rs1706435
All
−0.5
0.0
0.5
1.0
1.5
MR leave−one−out sensitivity analysis for
'STX6' on 'Pulmonary fibrosis'

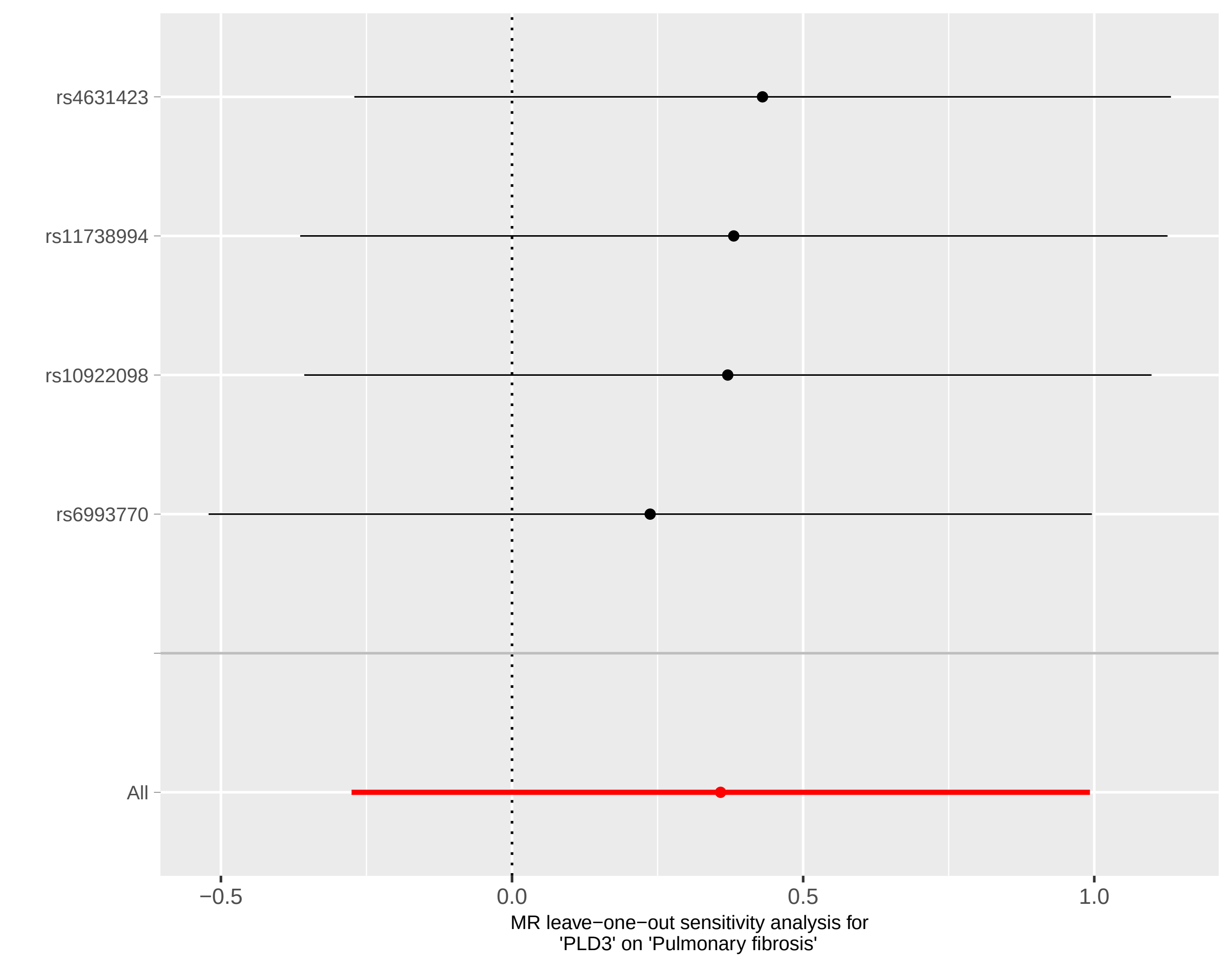
rs4631423
rs11738994
rs10922098
rs6993770
All
−0.5
0.0
0.5
1.0
MR leave−one−out sensitivity analysis for
'PLD3' on 'Pulmonary fibrosis'

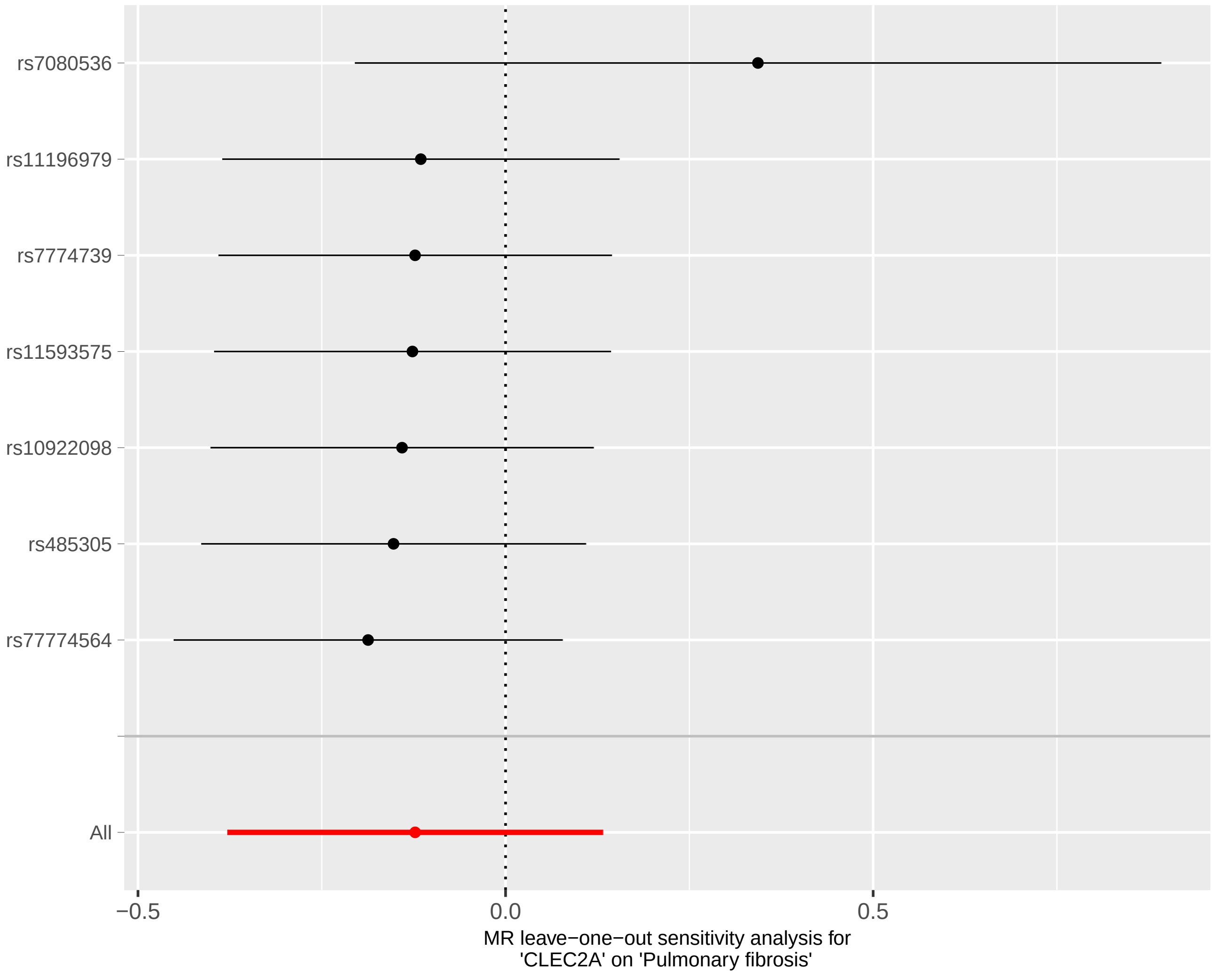
rs7080536
rs11196979
rs7774739
rs11593575
rs10922098
rs485305
rs77774564
All
−0.5
0.0
0.5
MR leave−one−out sensitivity analysis for
'CLEC2A' on 'Pulmonary fibrosis'

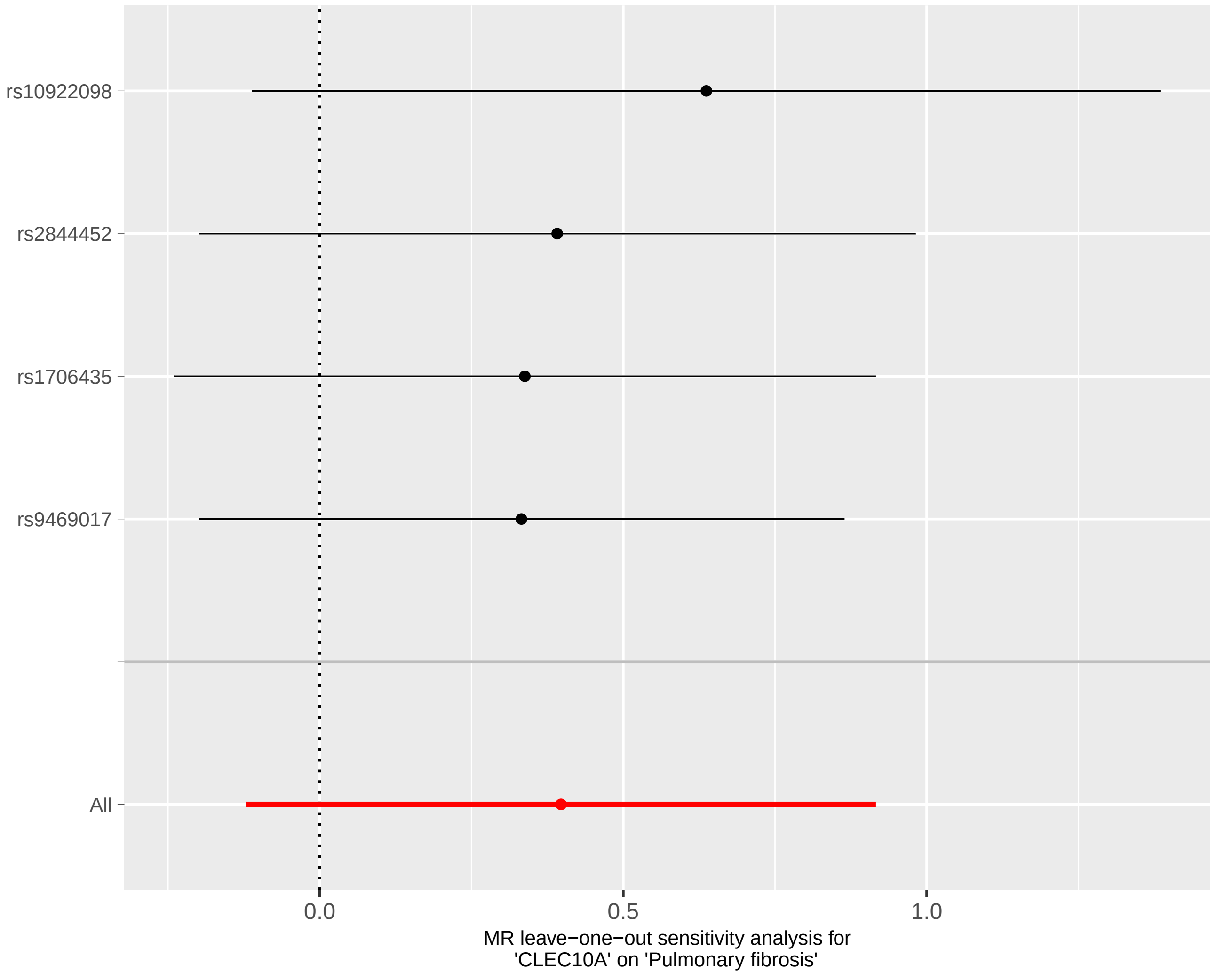
rs10922098
rs2844452
rs1706435
rs9469017
All
0.0
0.5
1.0
MR leave−one−out sensitivity analysis for
'CLEC10A' on 'Pulmonary fibrosis'

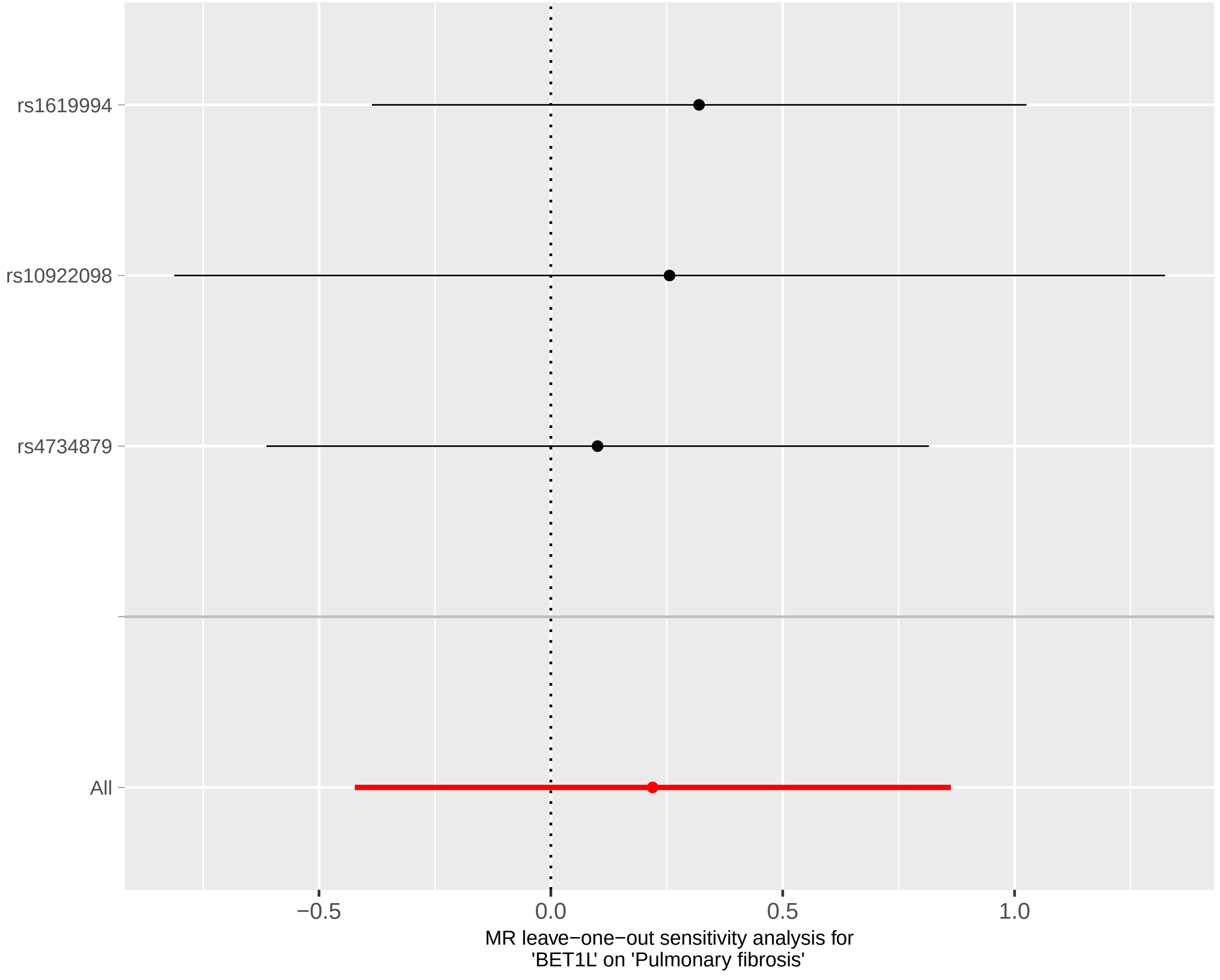
rs1619994
rs10922098
rs4734879
All
−0.5
0.0
0.5
1.0
MR leave−one−out sensitivity analysis for
'BET1L' on 'Pulmonary fibrosis'

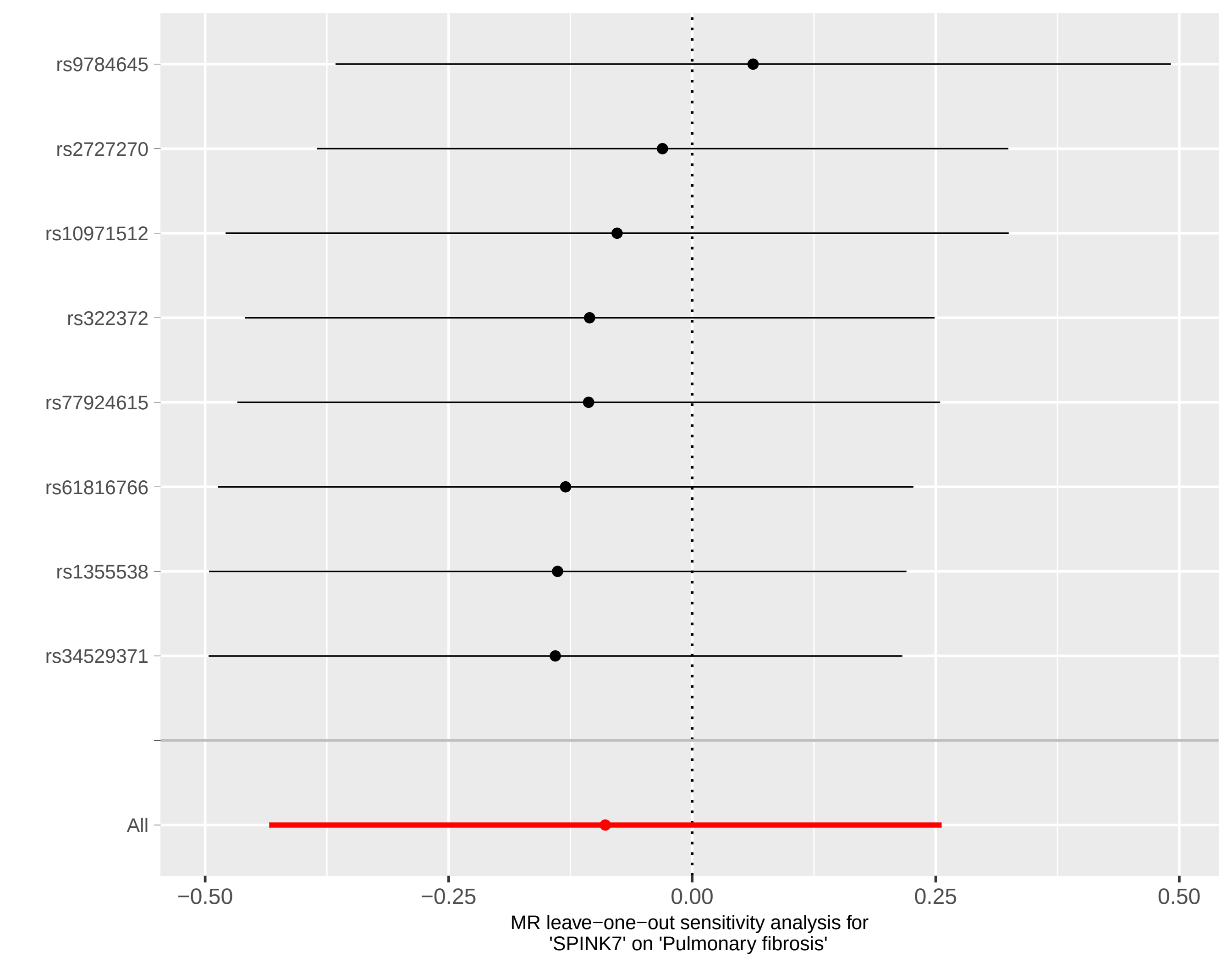
rs9784645
rs2727270
rs10971512
rs322372
rs77924615
rs61816766
rs1355538
rs34529371
All
−0.50
−0.25
0.00
0.25
0.50
MR leave−one−out sensitivity analysis for
'SPINK7' on 'Pulmonary fibrosis'

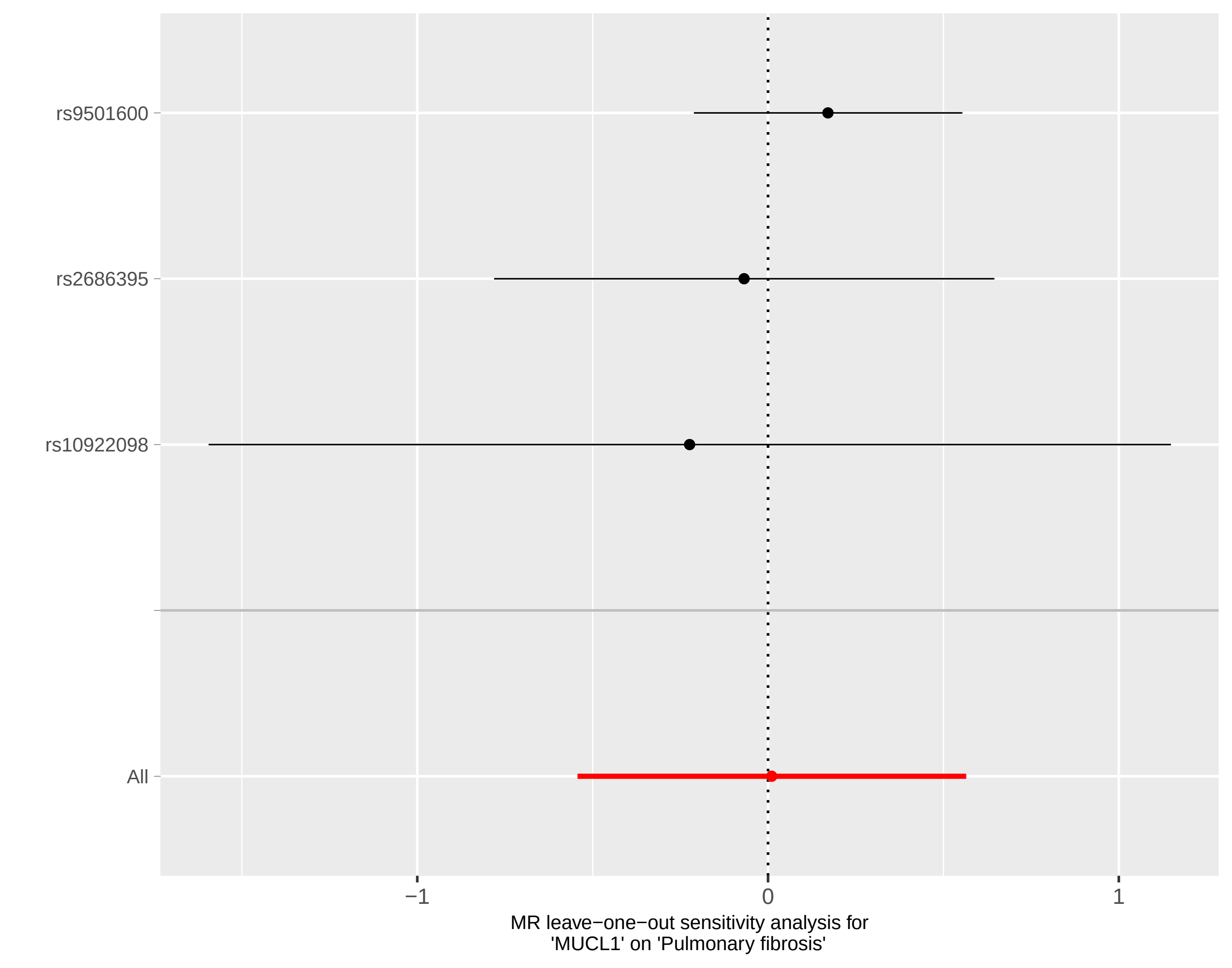
rs9501600
rs2686395
rs10922098
All
−1
0
1
MR leave−one−out sensitivity analysis for
'MUCL1' on 'Pulmonary fibrosis'

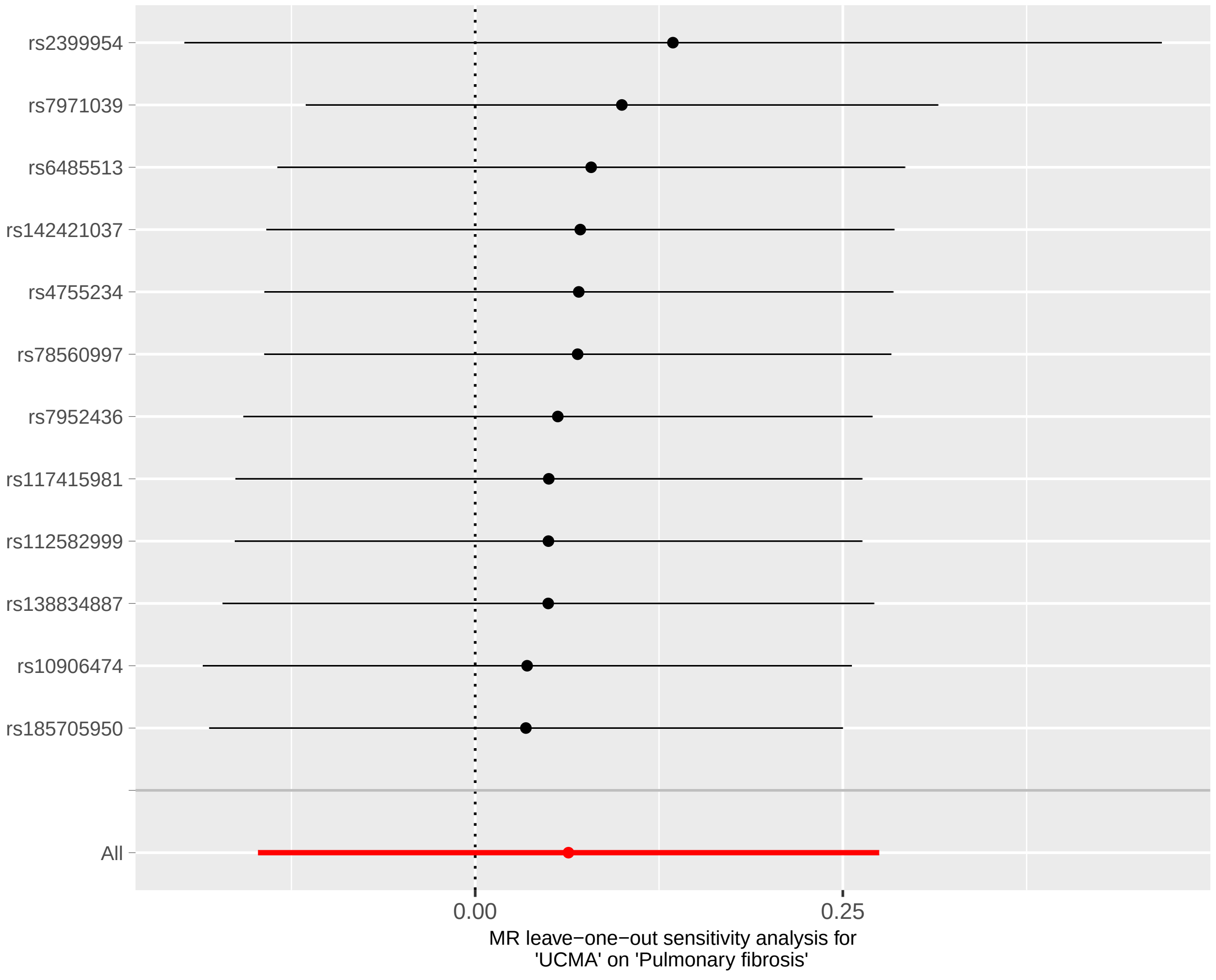

rs2399954
rs7971039
rs6485513
rs142421037
rs4755234
rs78560997
rs7952436
rs117415981
rs112582999
rs138834887
rs10906474
rs185705950
All
0.00
0.25
MR leave−one−out sensitivity analysis for
'UCMA' on 'Pulmonary fibrosis'

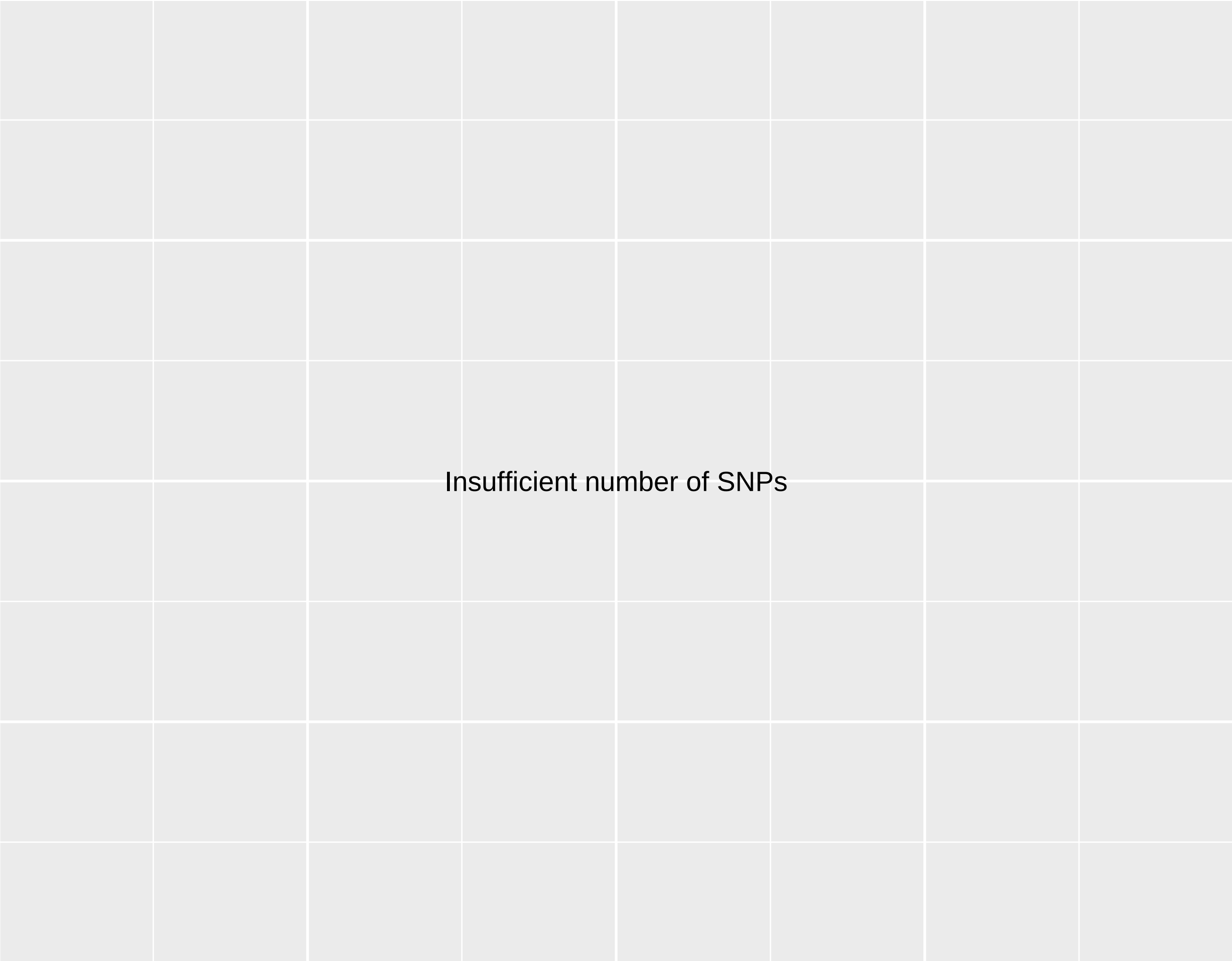
Insufficient number of SNPs

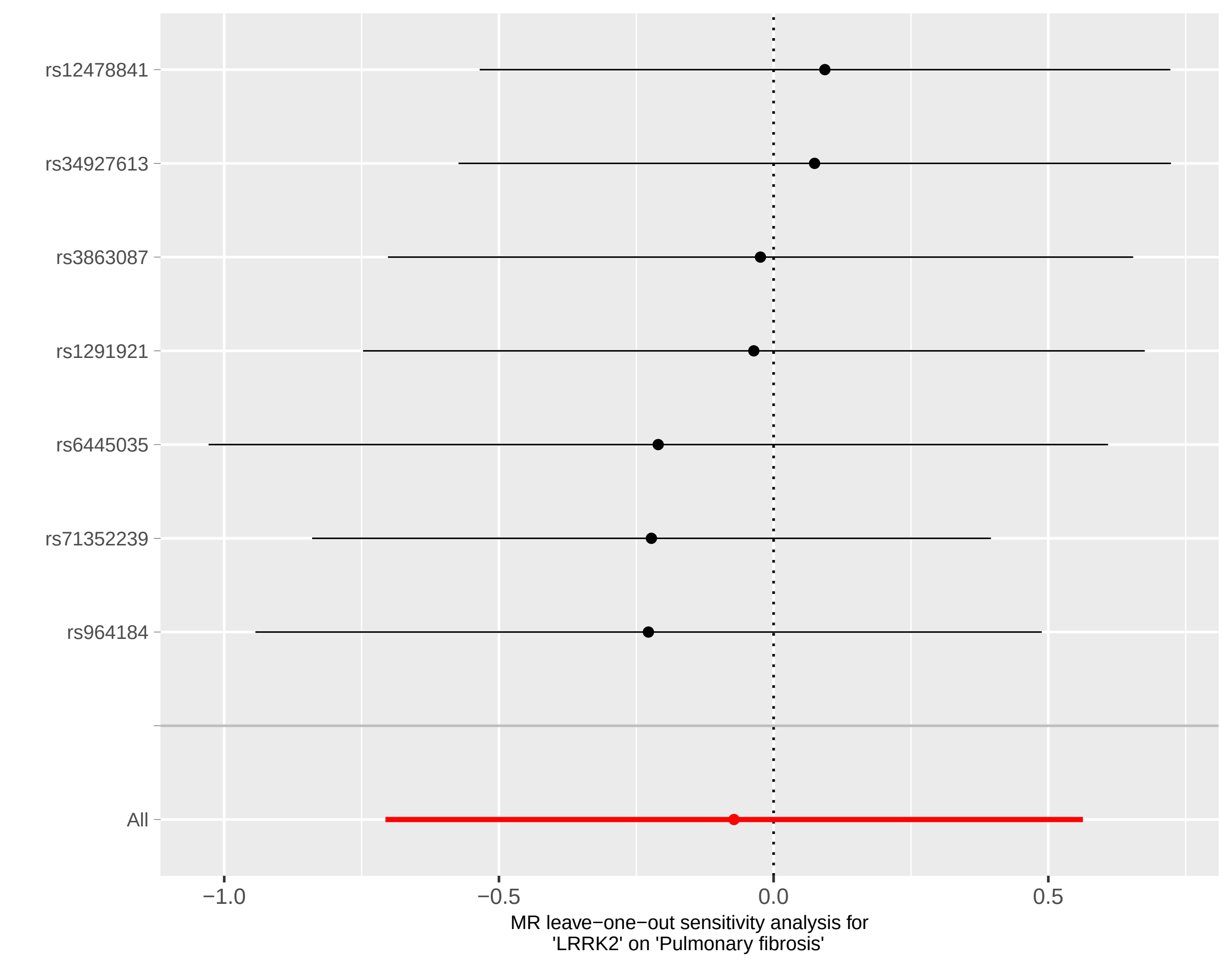
rs12478841
rs34927613
rs3863087
rs1291921
rs6445035
rs71352239
rs964184
All
−1.0
−0.5
0.0
0.5
MR leave−one−out sensitivity analysis for
'LRRK2' on 'Pulmonary fibrosis'

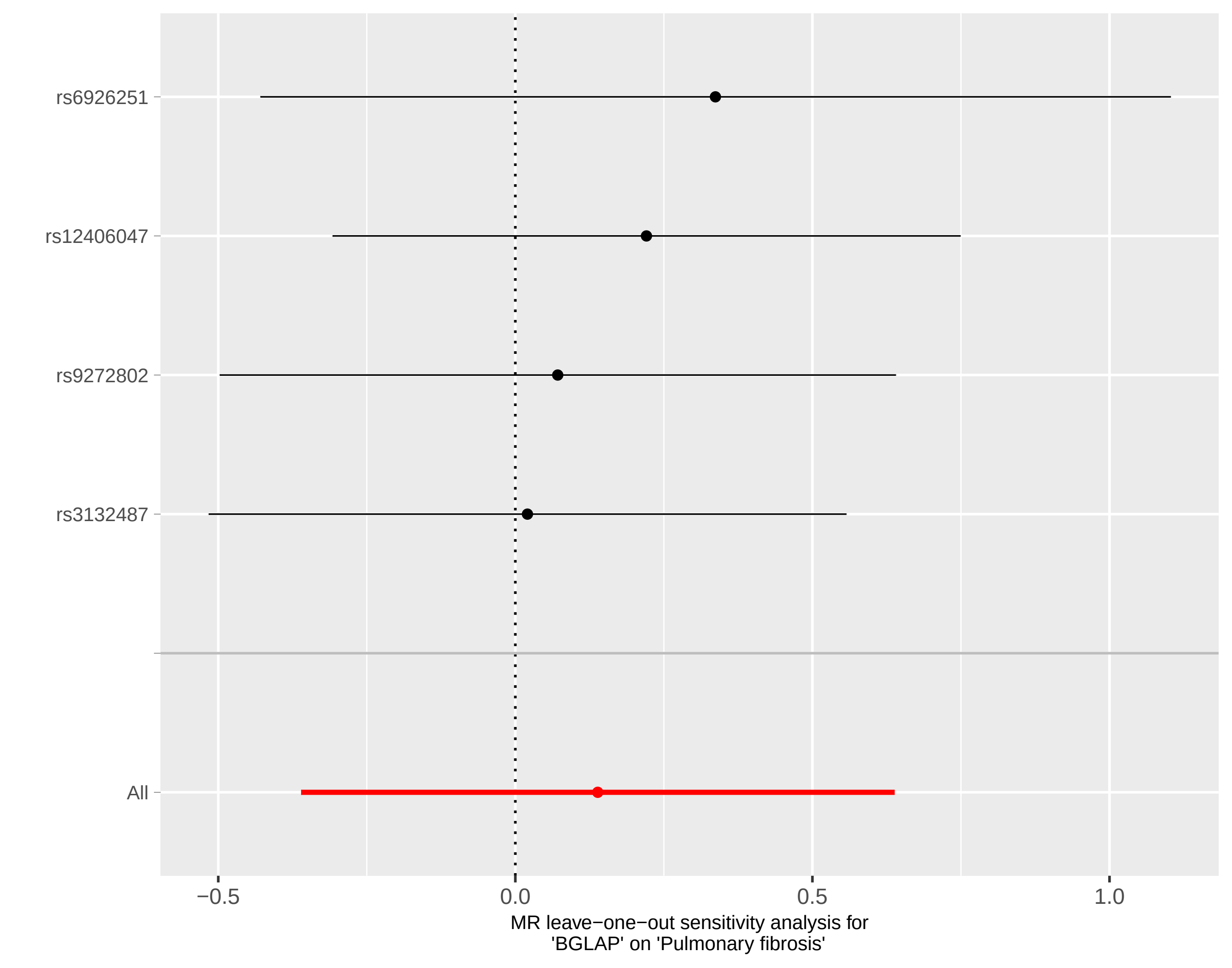

rs6926251
rs12406047
rs9272802
rs3132487
All
−0.5
0.0
0.5
1.0
MR leave−one−out sensitivity analysis for
'BGLAP' on 'Pulmonary fibrosis'

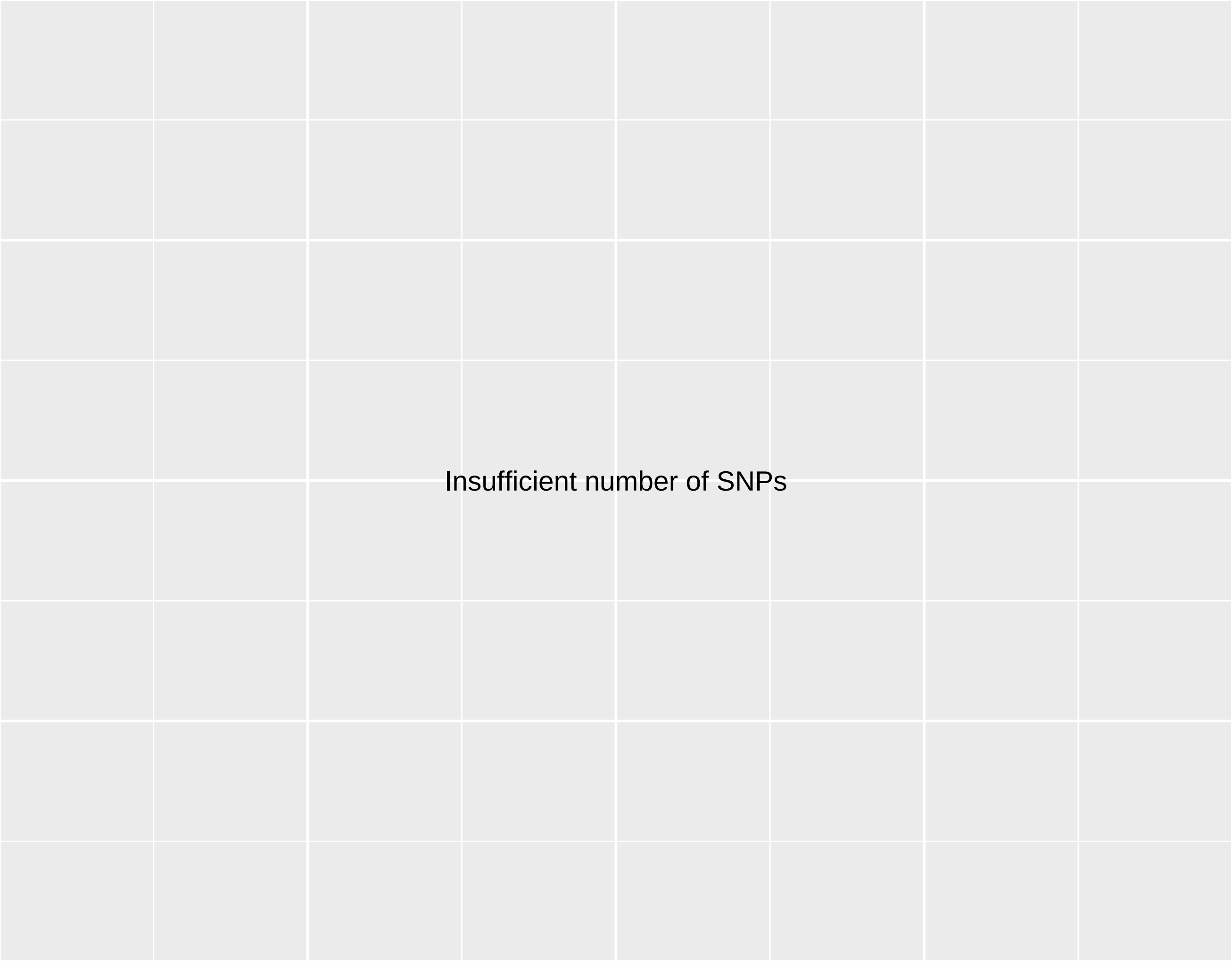
Insufficient number of SNPs

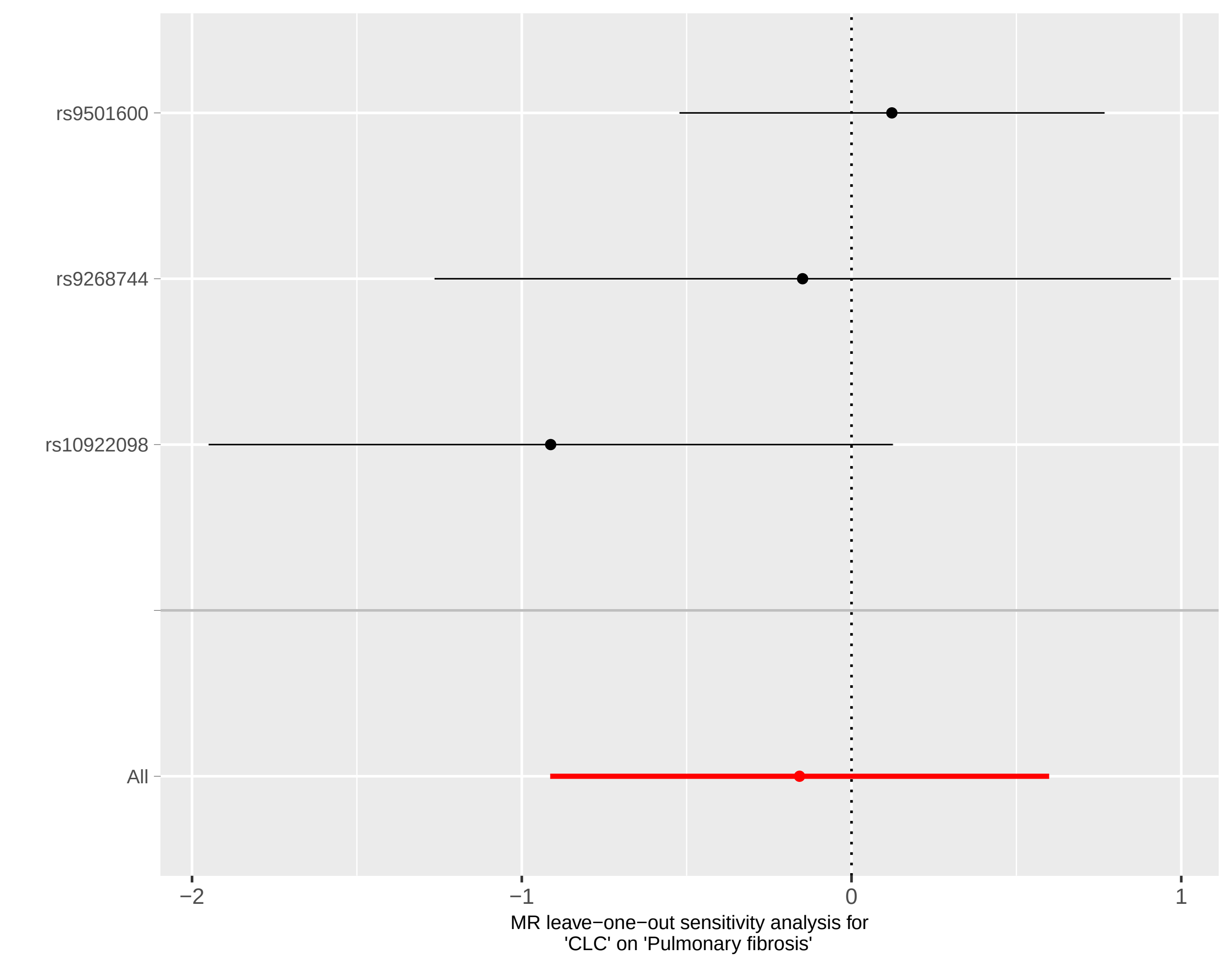
rs9501600
rs9268744
rs10922098
All
−2
−1
0
1
MR leave−one−out sensitivity analysis for
'CLC' on 'Pulmonary fibrosis'

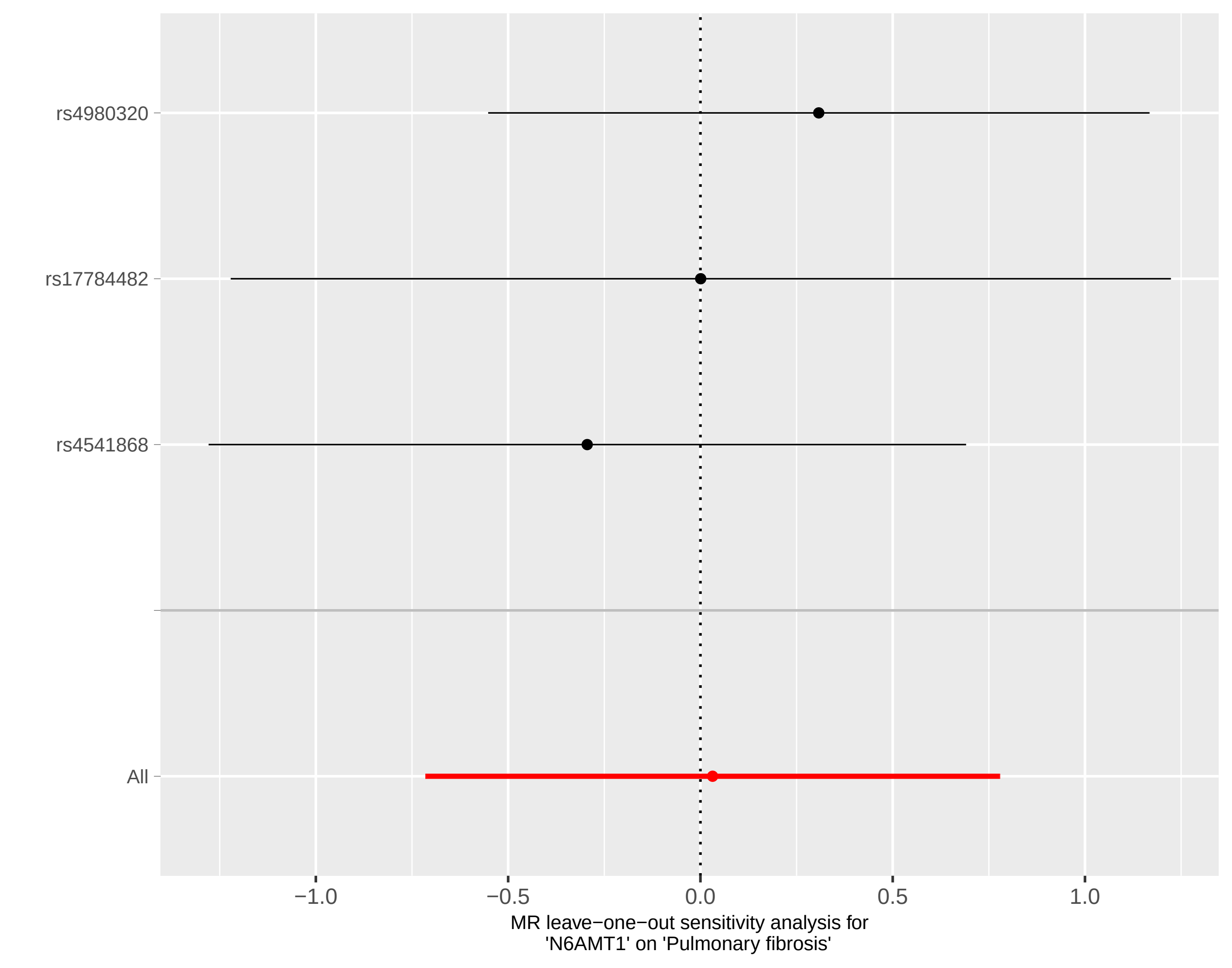
rs4980320
rs17784482
rs4541868
All
−1.0
−0.5
0.0
0.5
1.0
MR leave−one−out sensitivity analysis for
'N6AMT1' on 'Pulmonary fibrosis'

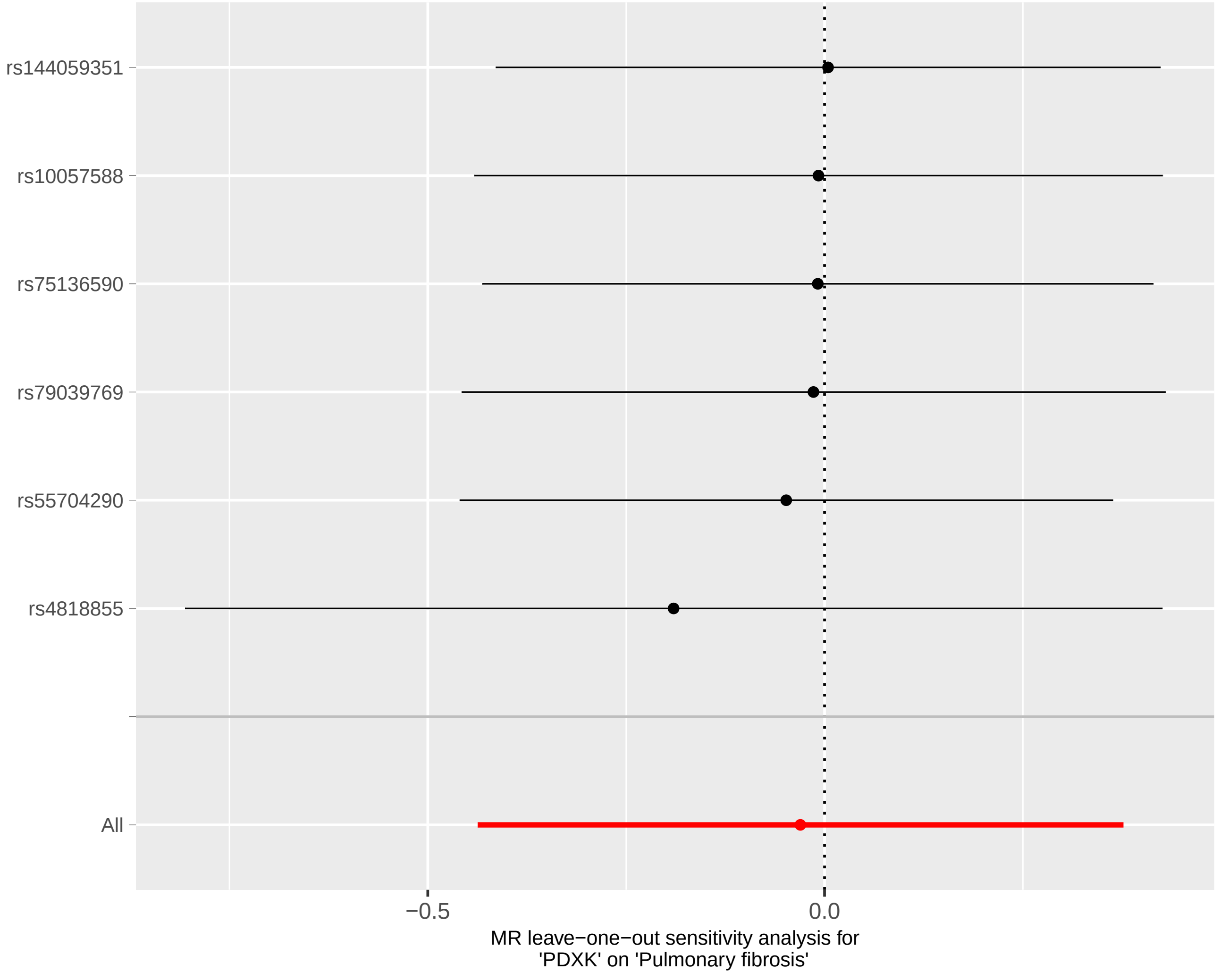
rs144059351
rs10057588
rs75136590
rs79039769
rs55704290
rs4818855
All
−0.5
0.0
MR leave−one−out sensitivity analysis for
'PDXK' on 'Pulmonary fibrosis'

Insufficient number of SNPs

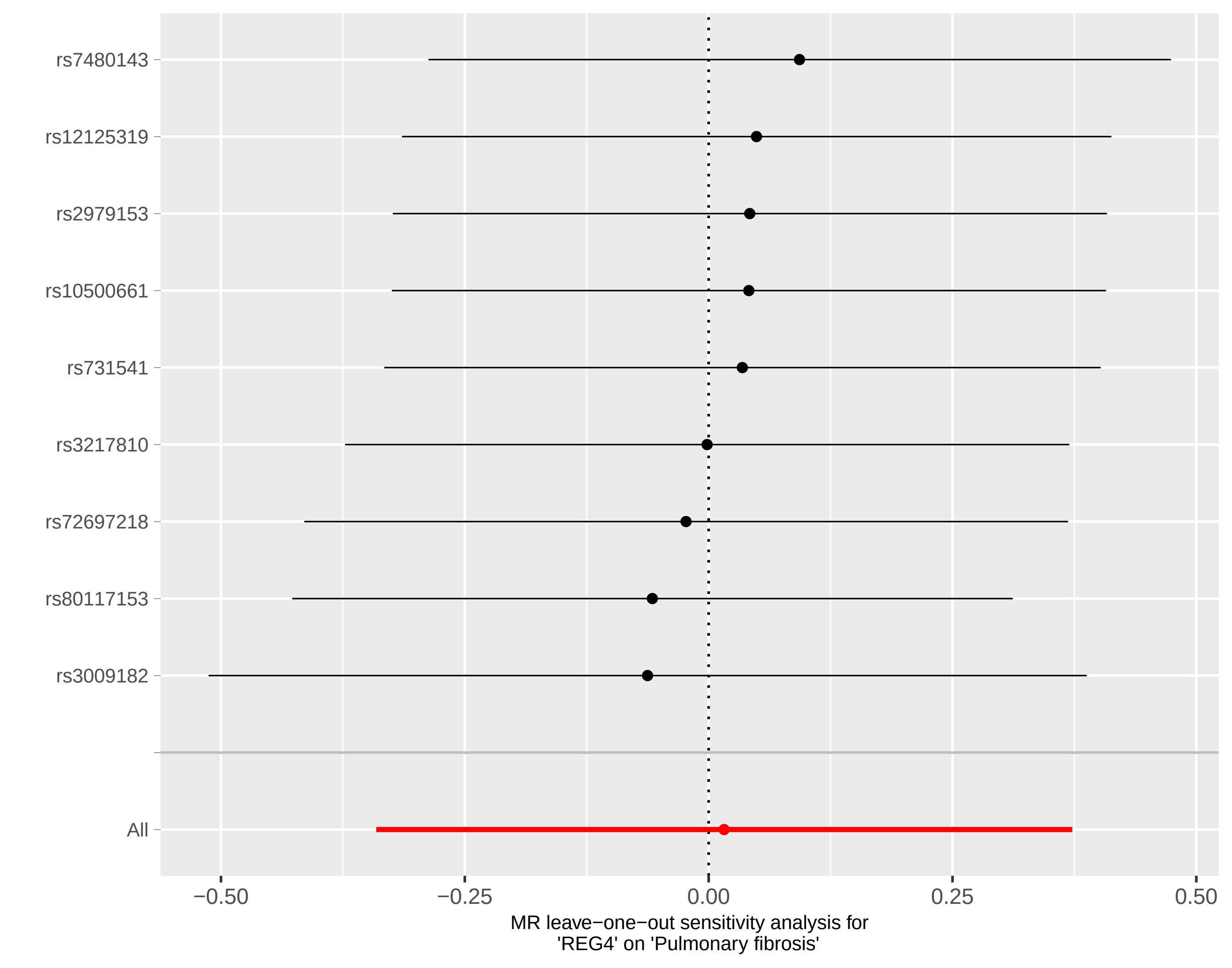
rs7480143
rs12125319
rs2979153
rs10500661
rs731541
rs3217810
rs72697218
rs80117153
rs3009182
All
−0.50
−0.25
0.00
0.25
0.50
MR leave−one−out sensitivity analysis for
'REG4' on 'Pulmonary fibrosis'

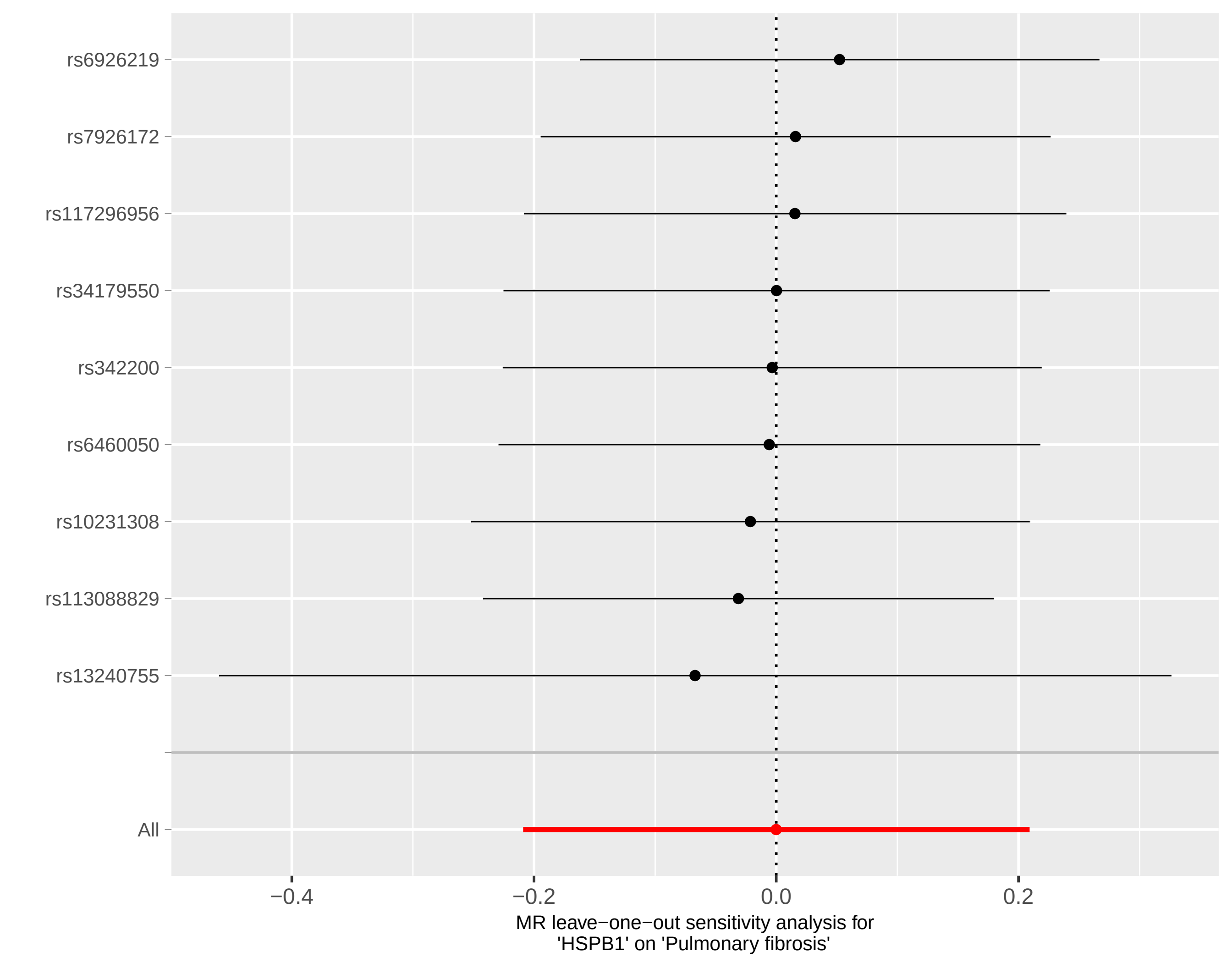
rs6926219
rs7926172
rs117296956
rs34179550
rs342200
rs6460050
rs10231308
rs113088829
rs13240755
All
−0.4
−0.2
0.0
0.2
MR leave−one−out sensitivity analysis for
'HSPB1' on 'Pulmonary fibrosis'

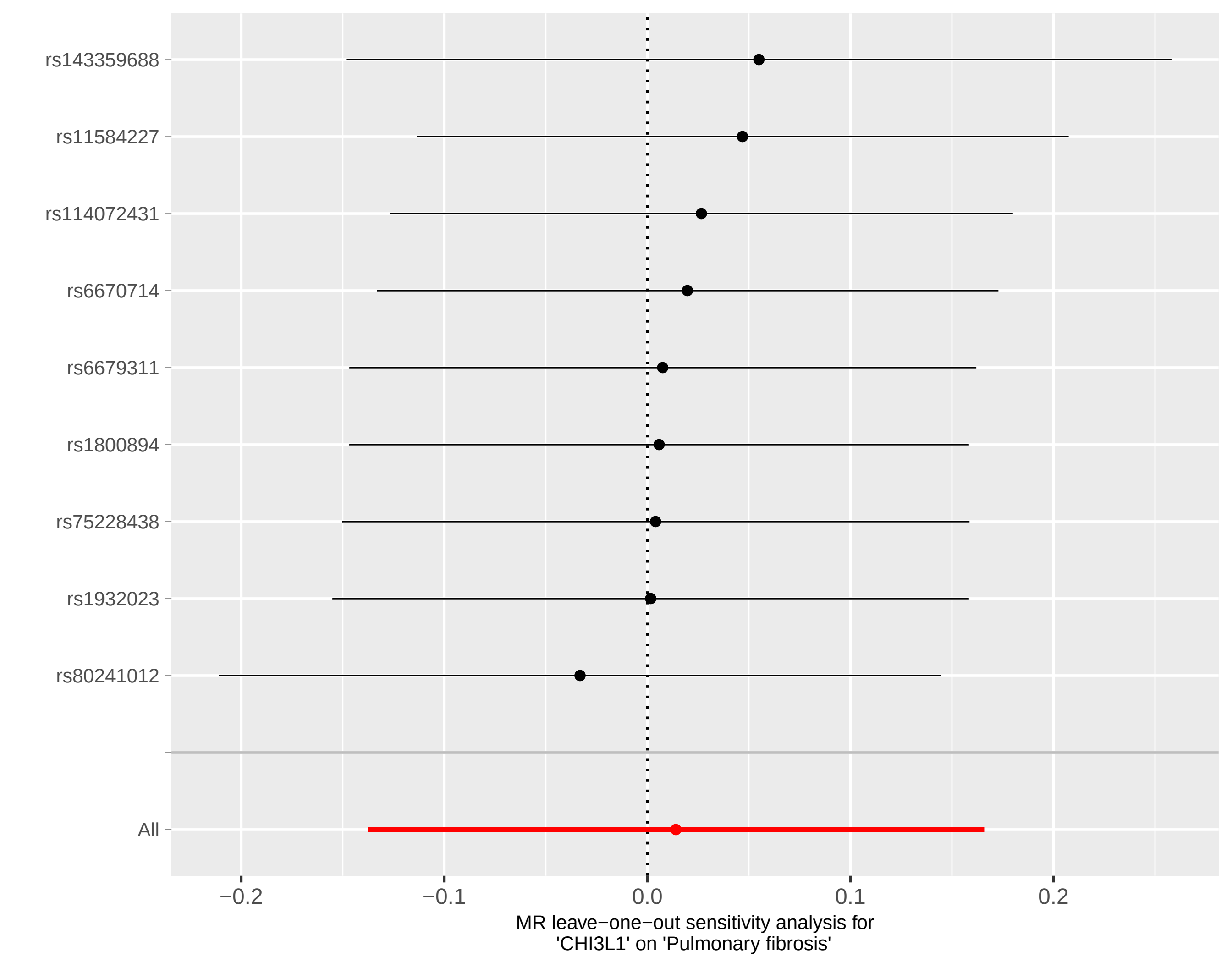
rs143359688
rs11584227
rs114072431
rs6670714
rs6679311
rs1800894
rs75228438
rs1932023
rs80241012
All
−0.2
−0.1
0.0
0.1
0.2
MR leave−one−out sensitivity analysis for
'CHI3L1' on 'Pulmonary fibrosis'

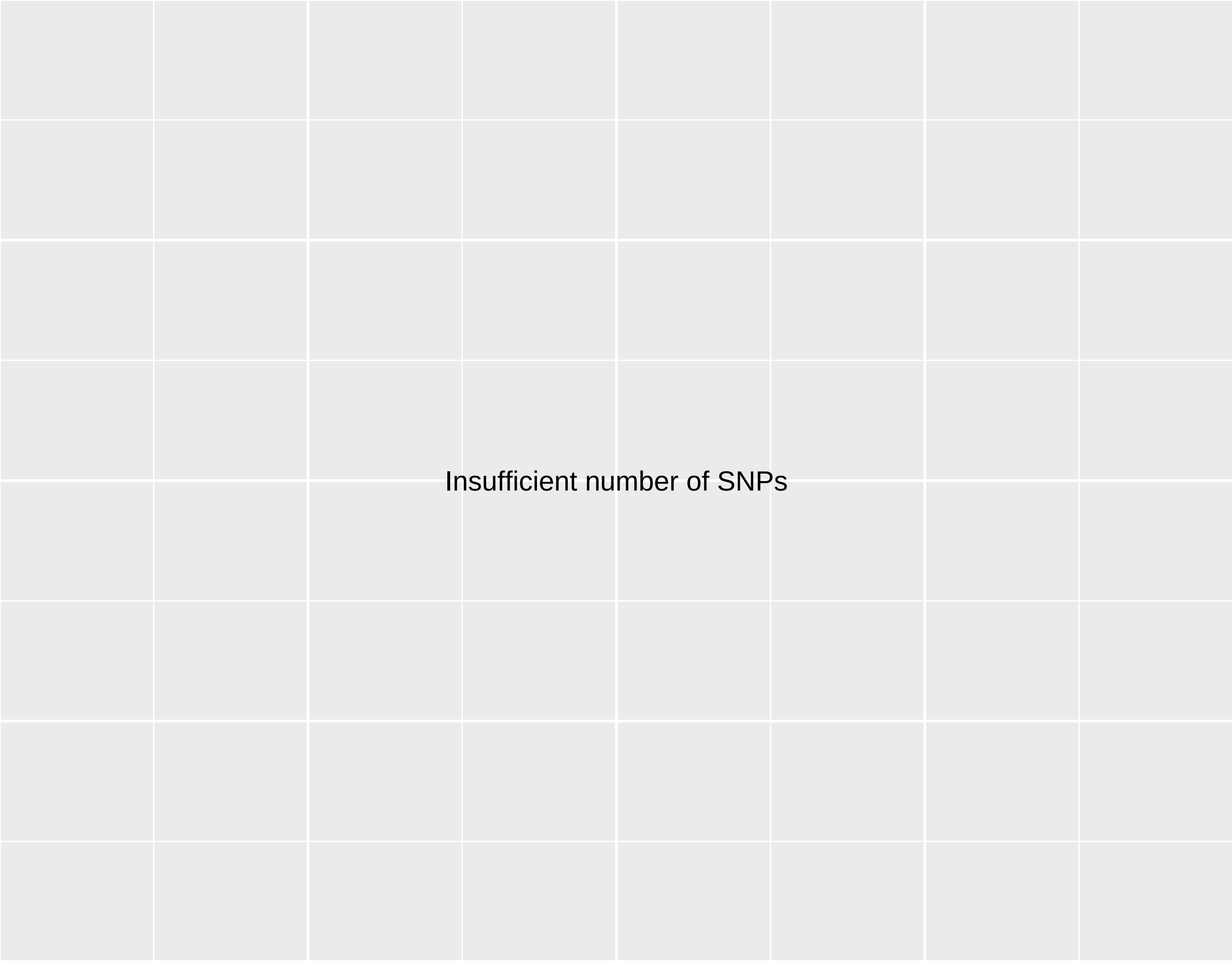
Insufficient number of SNPs

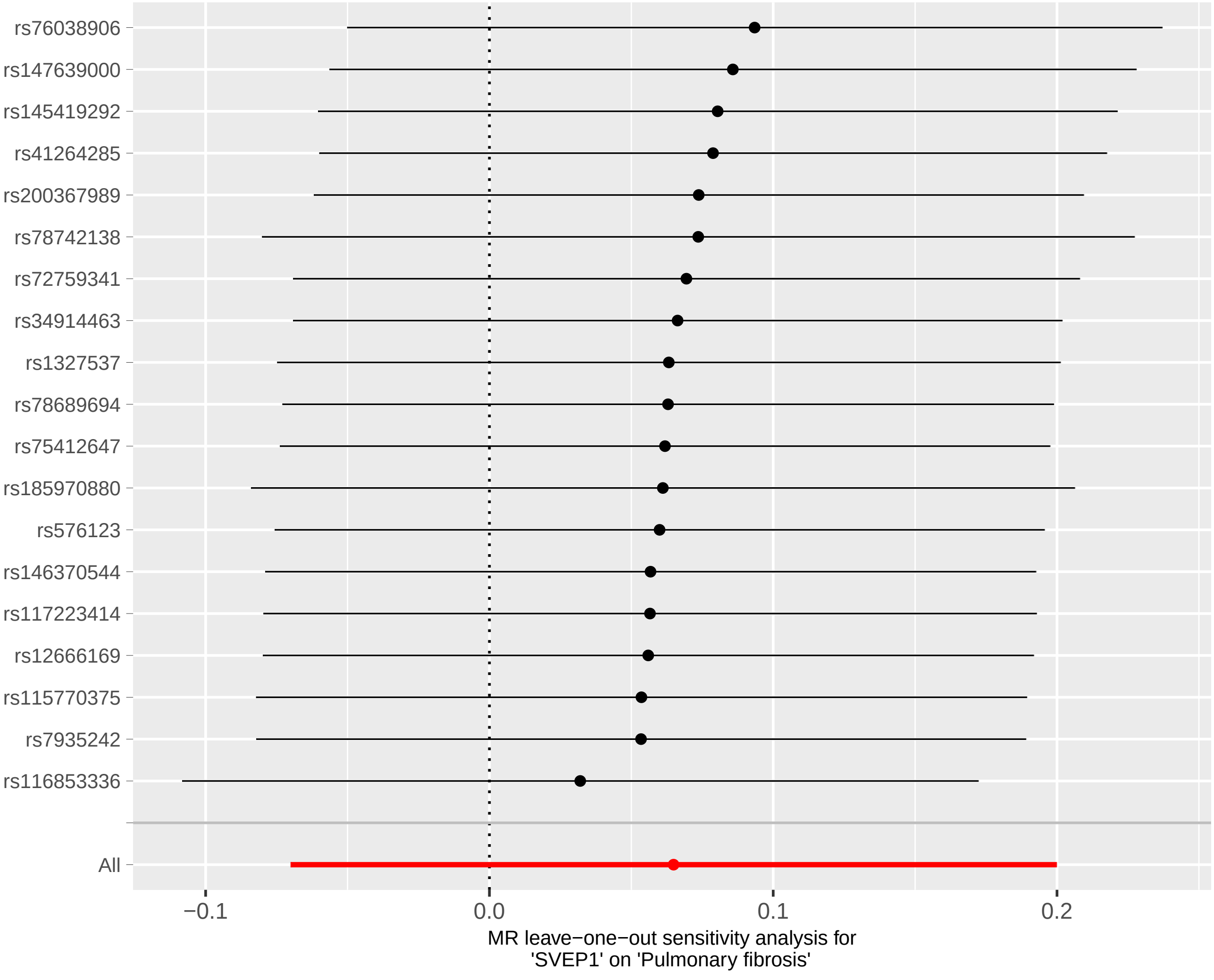
rs76038906
rs147639000
rs145419292
rs41264285
rs200367989
rs78742138
rs72759341
rs34914463
rs1327537
rs78689694
rs75412647
rs185970880
rs576123
rs146370544
rs117223414
rs12666169
rs115770375
rs7935242
rs116853336
All
−0.1
0.0
0.1
0.2
MR leave−one−out sensitivity analysis for
'SVEP1' on 'Pulmonary fibrosis'

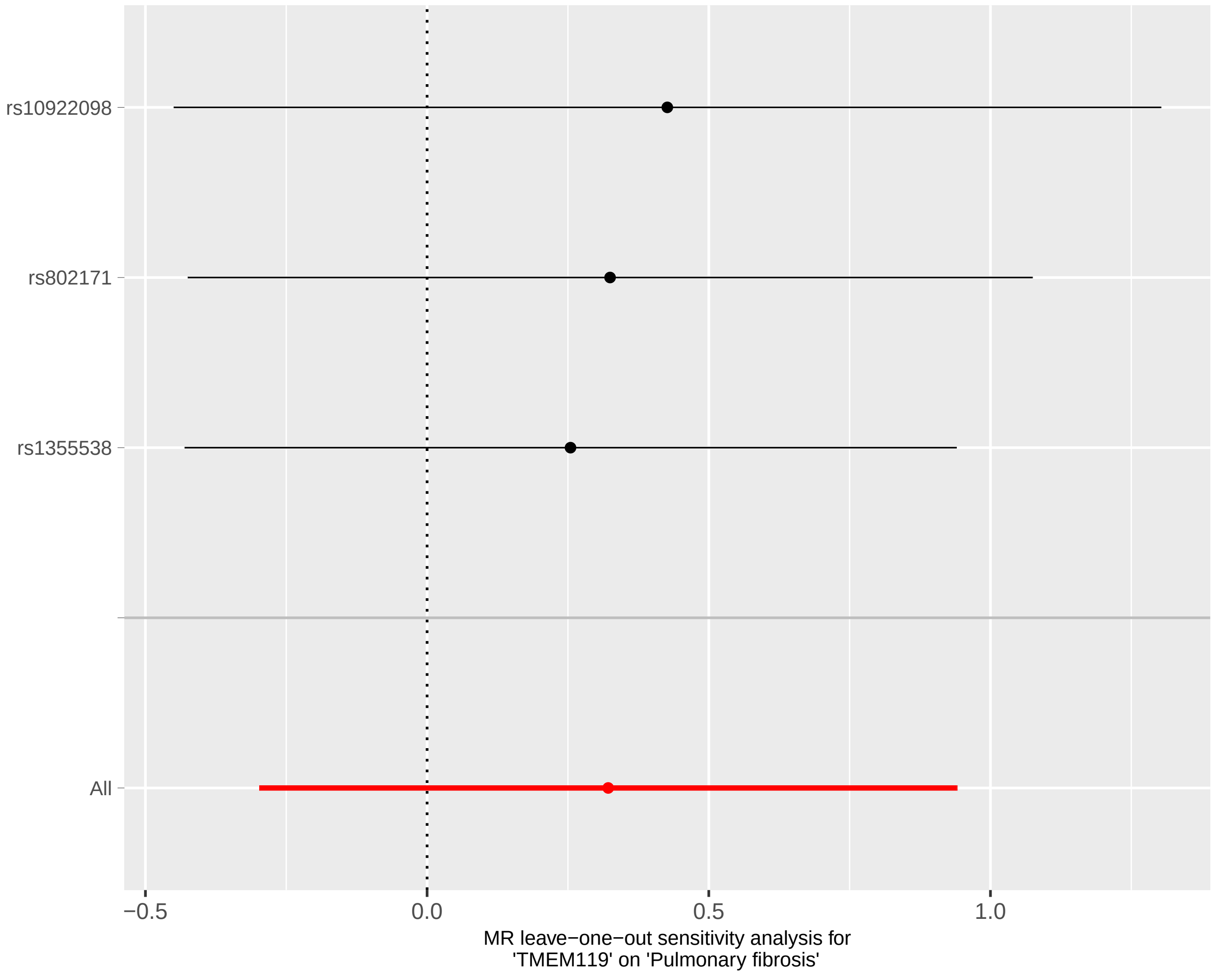
rs10922098
rs802171
rs1355538
All
−0.5
0.0
0.5
1.0
MR leave−one−out sensitivity analysis for
'TMEM119' on 'Pulmonary fibrosis'

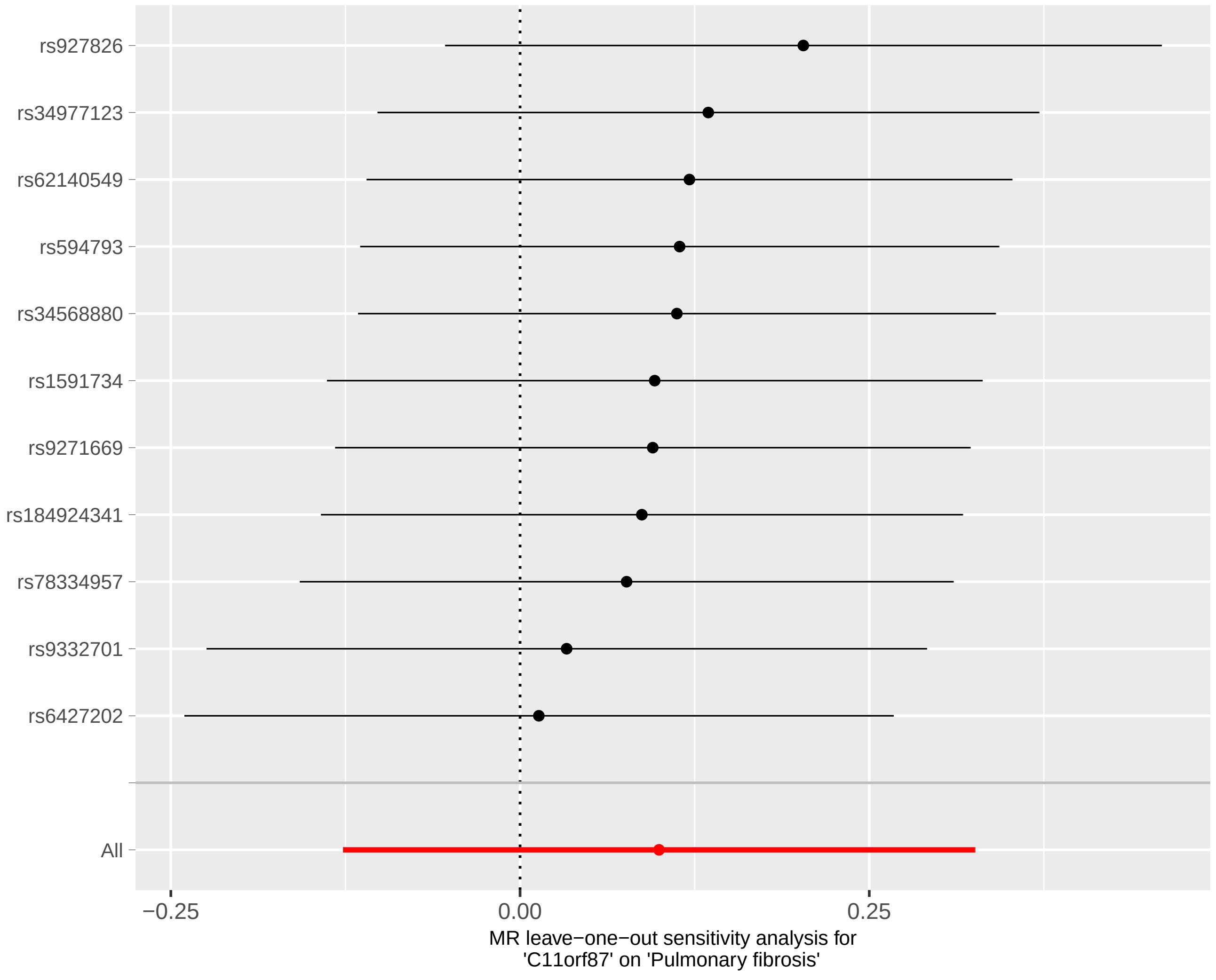
rs927826
rs34977123
rs62140549
rs594793
rs34568880
rs1591734
rs9271669
rs184924341
rs78334957
rs9332701
rs6427202
All
−0.25
0.00
0.25
MR leave−one−out sensitivity analysis for
'C11orf87' on 'Pulmonary fibrosis'

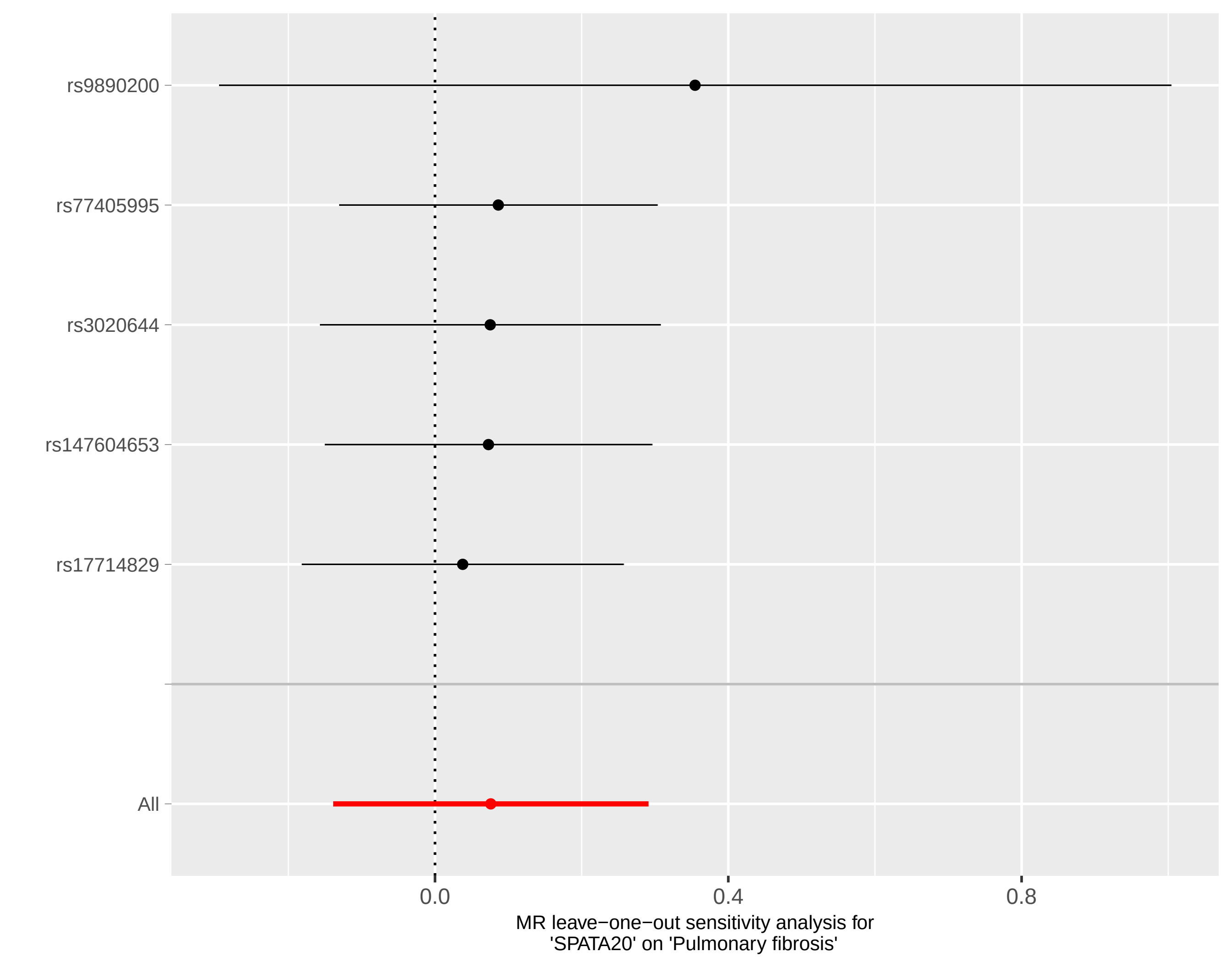
rs9890200
rs77405995
rs3020644
rs147604653
rs17714829
All
0.0
0.4
0.8
MR leave−one−out sensitivity analysis for
'SPATA20' on 'Pulmonary fibrosis'

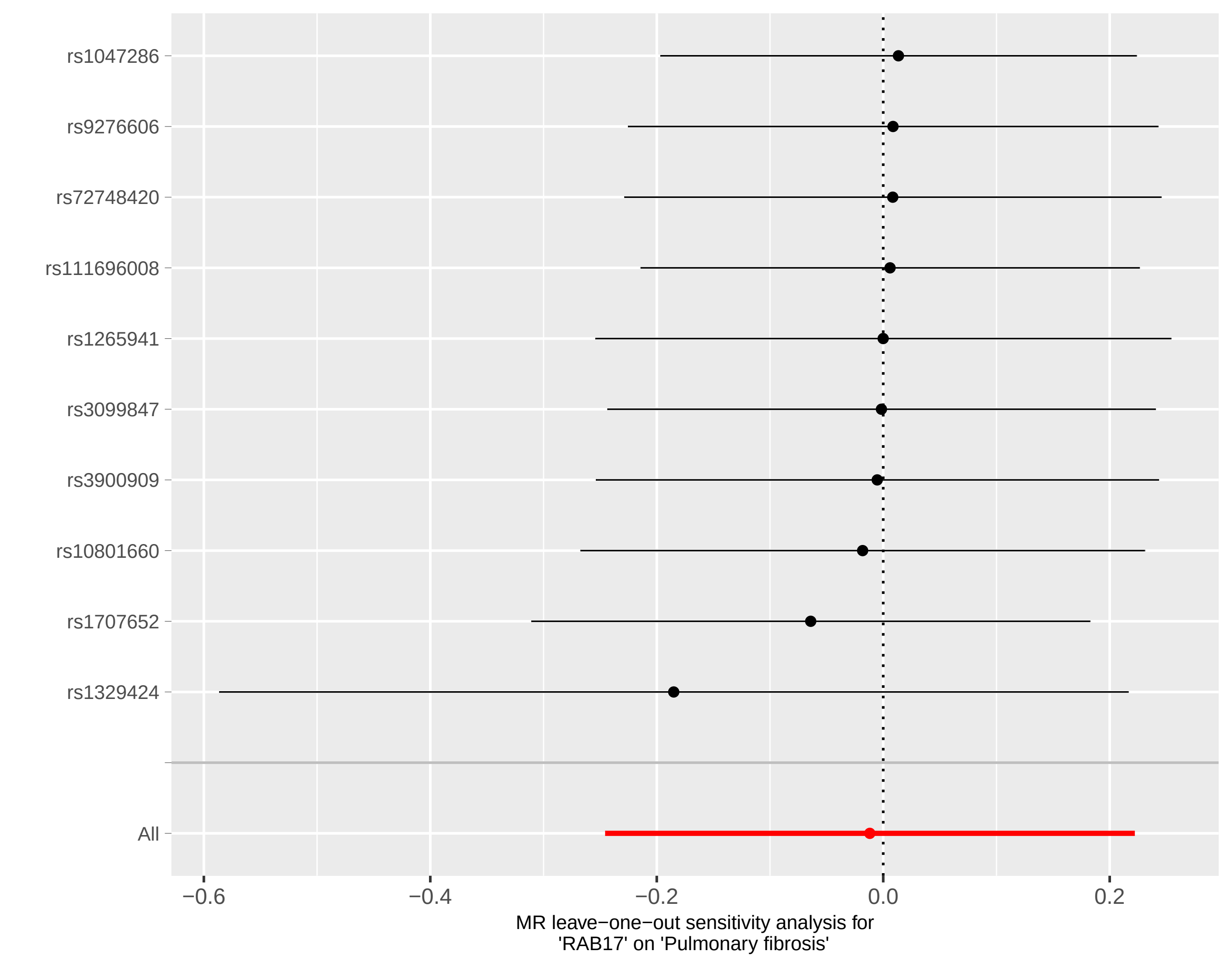

rs1047286
rs9276606
rs72748420
rs111696008
rs1265941
rs3099847
rs3900909
rs10801660
rs1707652
rs1329424
All
−0.6
−0.4
−0.2
0.0
0.2
MR leave−one−out sensitivity analysis for
'RAB17' on 'Pulmonary fibrosis'

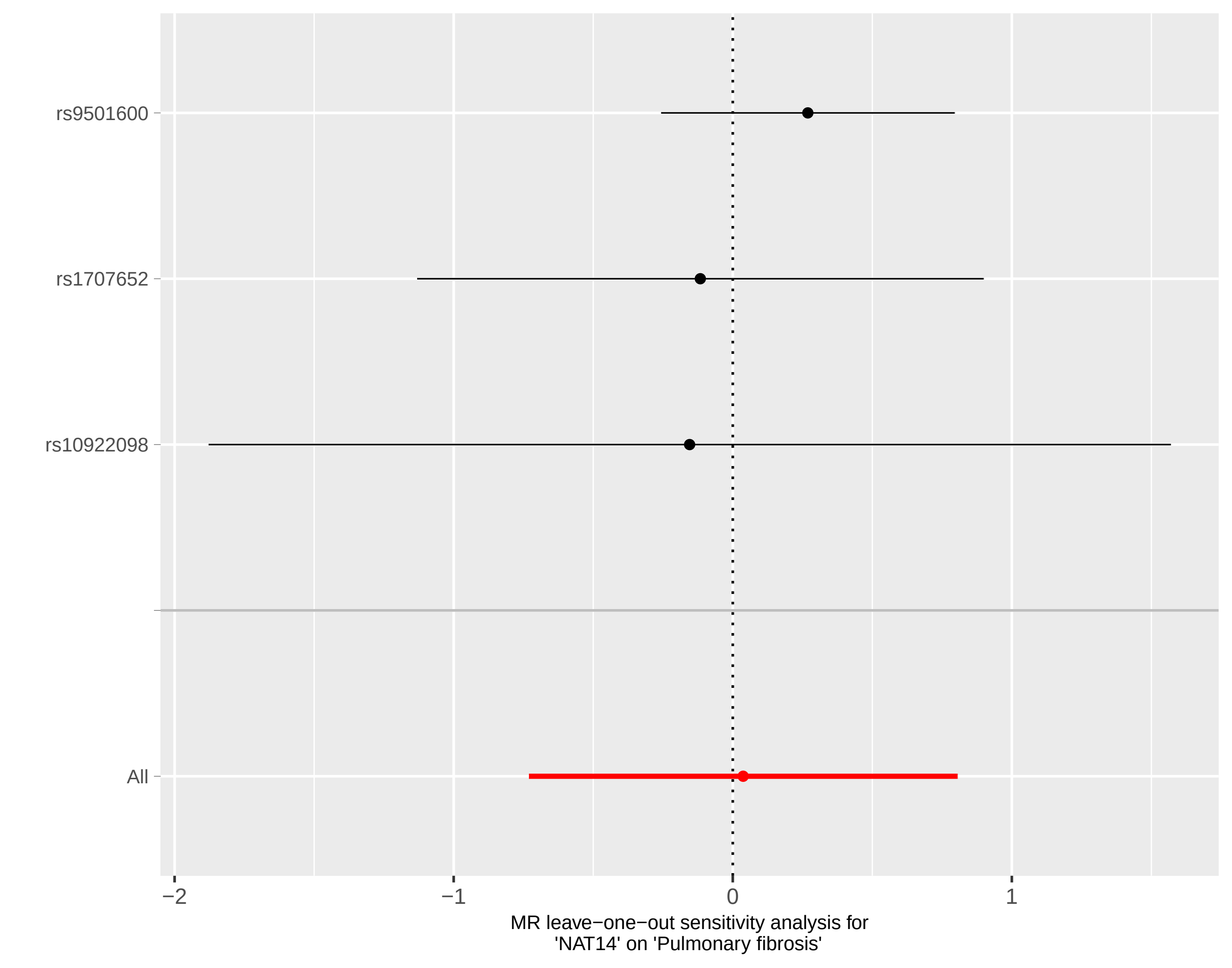
rs9501600
rs1707652
rs10922098
All
−2
−1
0
1
MR leave−one−out sensitivity analysis for
'NAT14' on 'Pulmonary fibrosis'

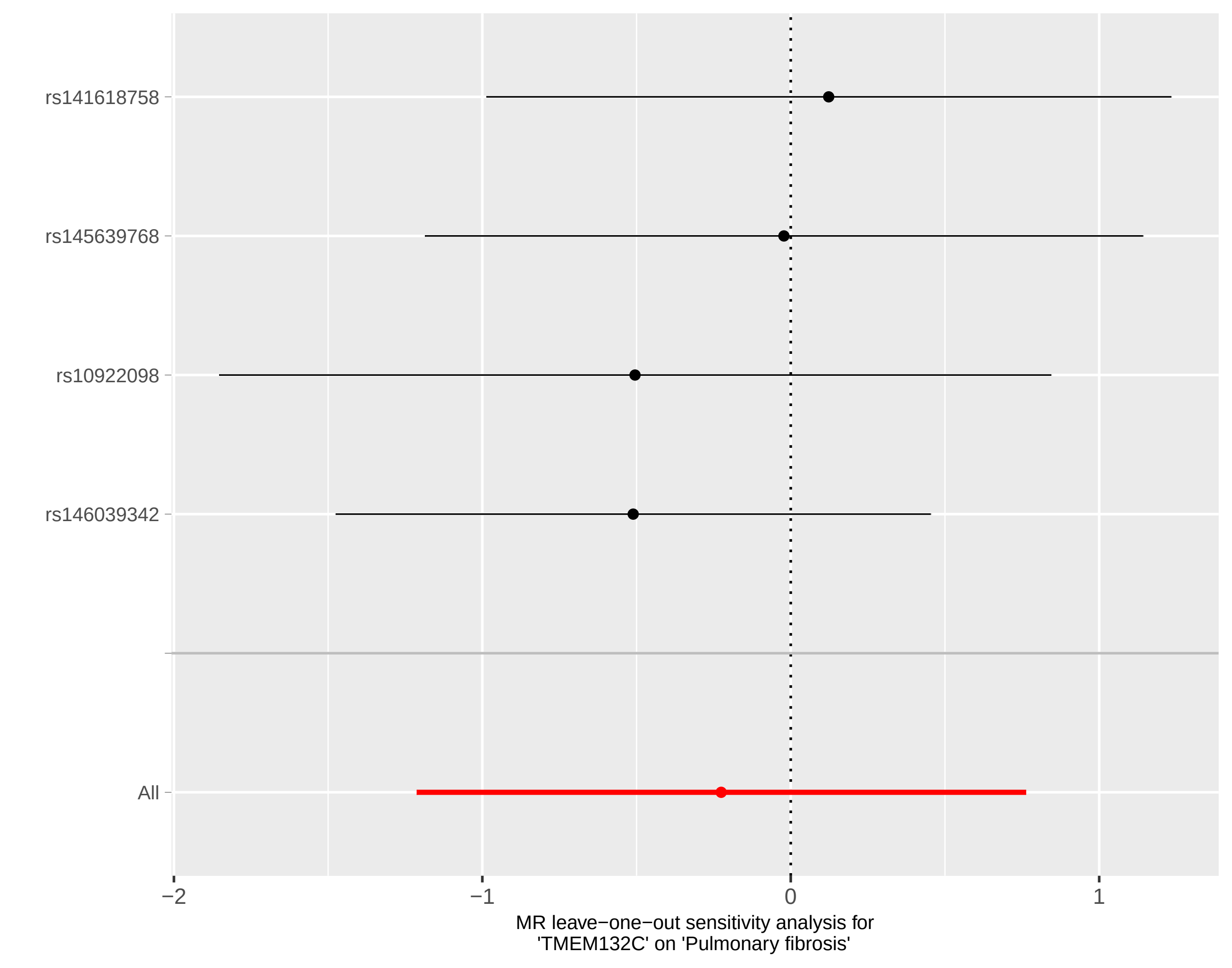

rs141618758
rs145639768
rs10922098
rs146039342
All
−2
−1
0
1
MR leave−one−out sensitivity analysis for
'TMEM132C' on 'Pulmonary fibrosis'

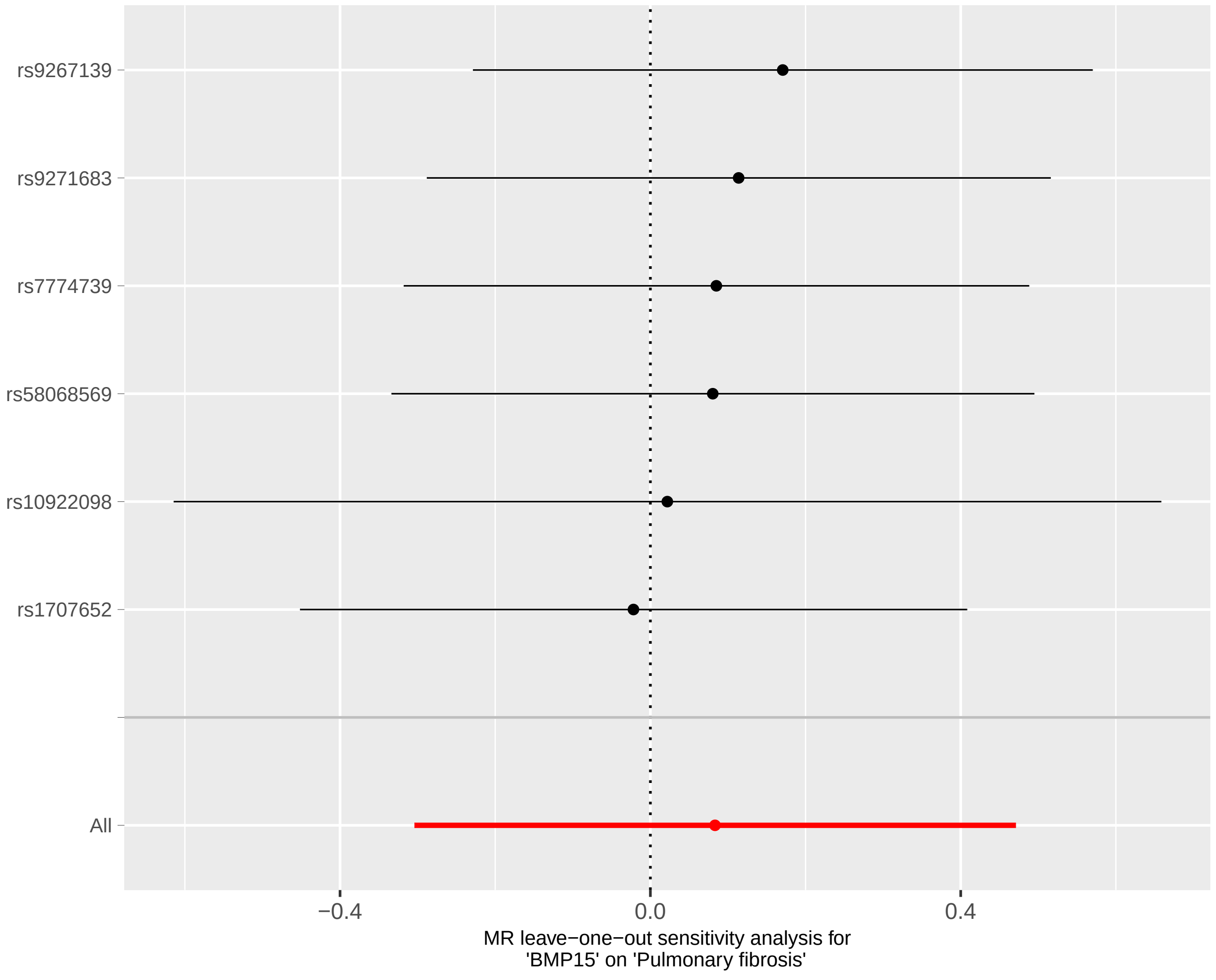
rs9267139
rs9271683
rs7774739
rs58068569
rs10922098
rs1707652
All
−0.4
0.0
0.4
MR leave−one−out sensitivity analysis for
'BMP15' on 'Pulmonary fibrosis'

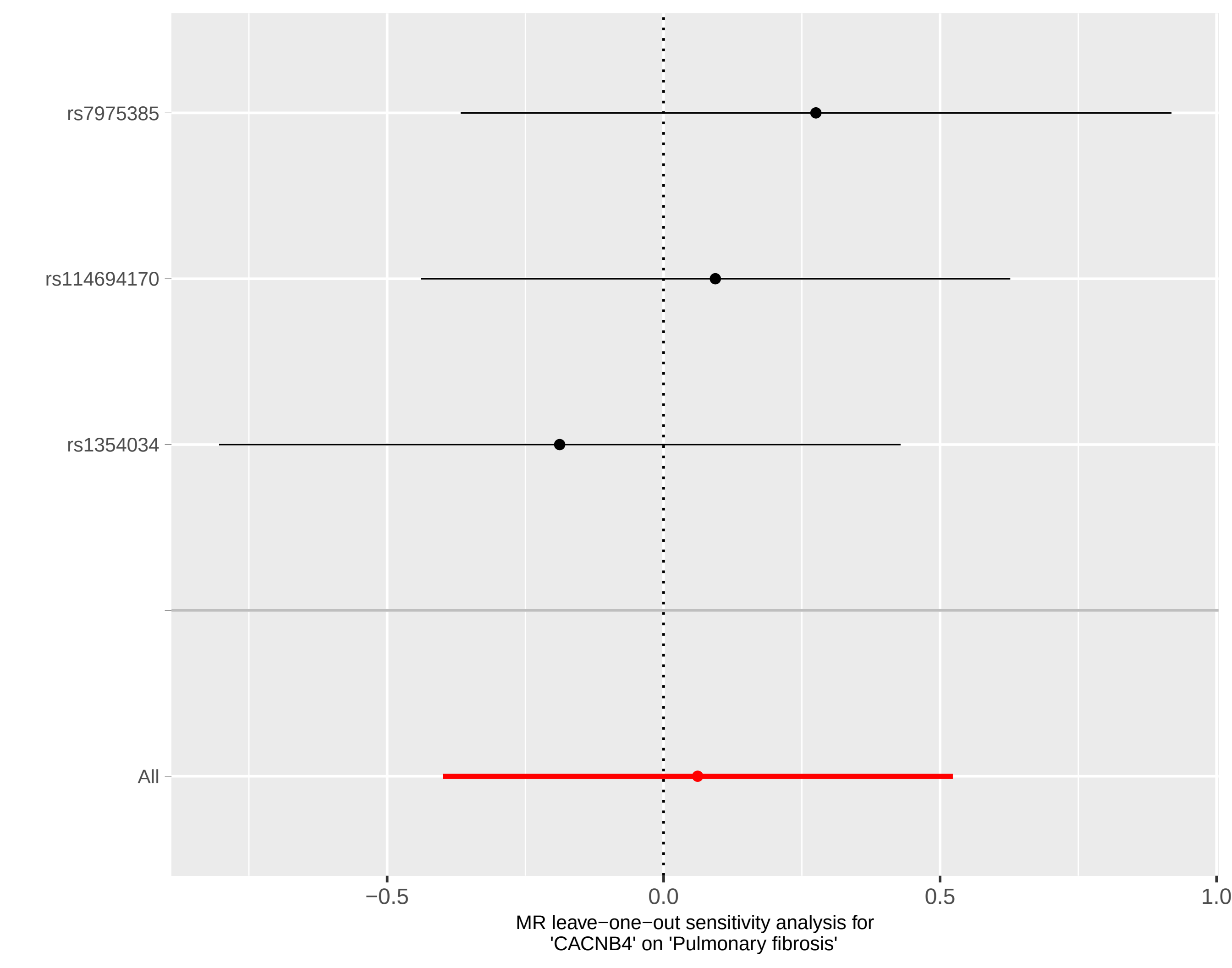

rs7975385
rs114694170
rs1354034
All
−0.5
0.0
0.5
1.0
MR leave−one−out sensitivity analysis for
'CACNB4' on 'Pulmonary fibrosis'

Insufficient number of SNPs

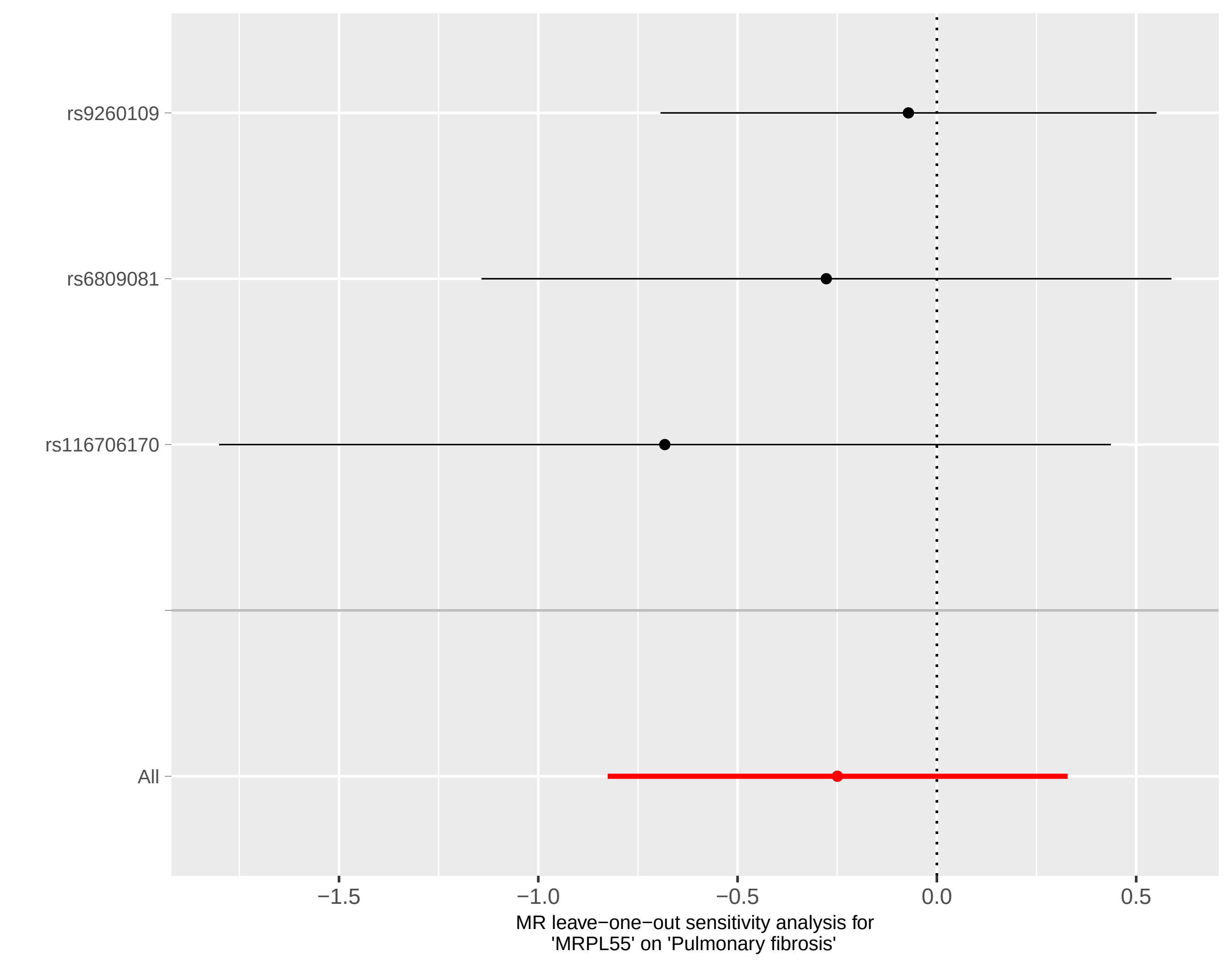

rs9260109
rs6809081
rs116706170
All
−1.5
−1.0
−0.5
0.0
0.5
MR leave−one−out sensitivity analysis for
'MRPL55' on 'Pulmonary fibrosis'

Insufficient number of SNPs

Insufficient number of SNPs

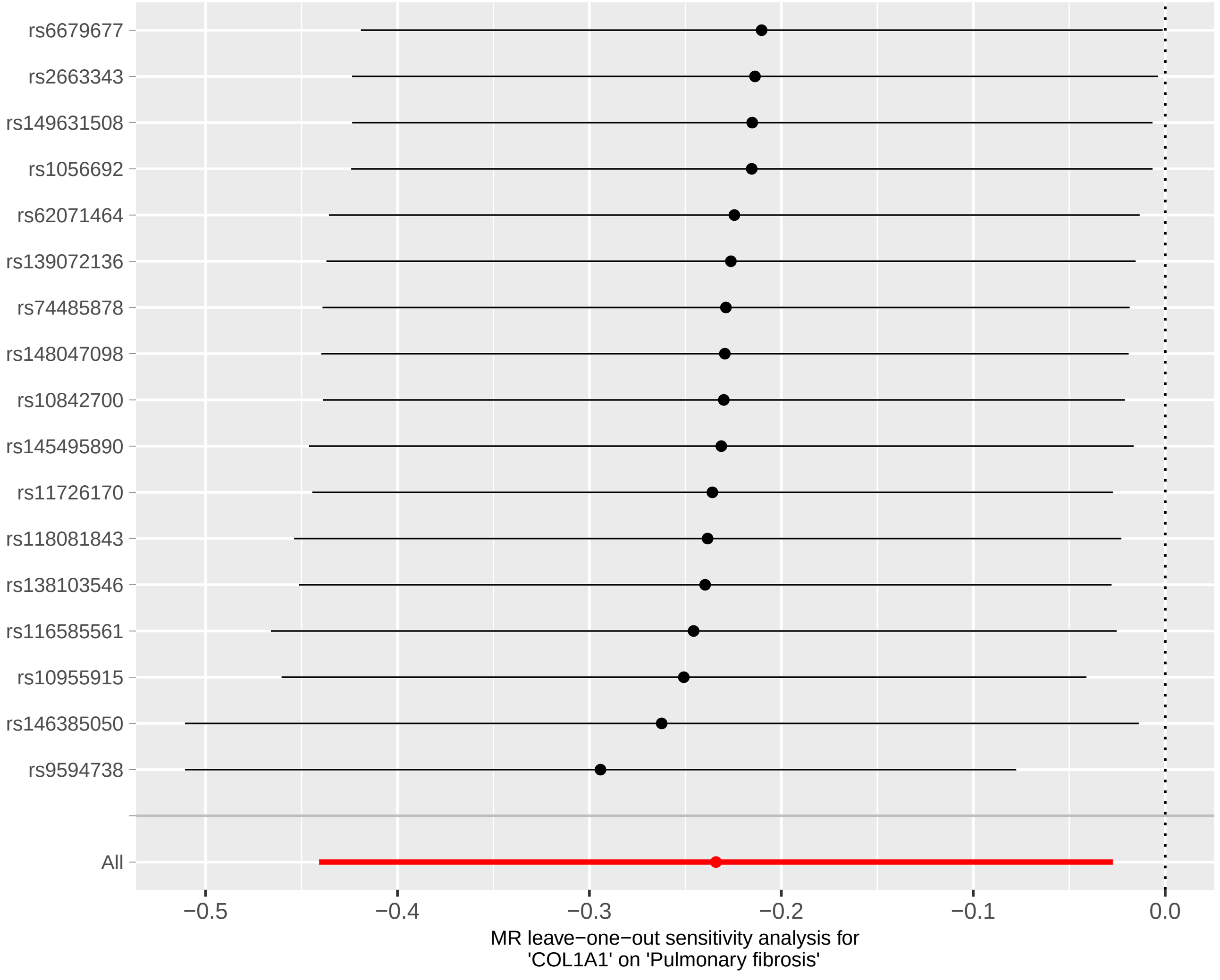

rs6679677
rs2663343
rs149631508
rs1056692
rs62071464
rs139072136
rs74485878
rs148047098
rs10842700
rs145495890
rs11726170
rs118081843
rs138103546
rs116585561
rs10955915
rs146385050
rs9594738
All
−0.5
−0.4
−0.3
−0.2
−0.1
0.0
MR leave−one−out sensitivity analysis for
'COL1A1' on 'Pulmonary fibrosis'

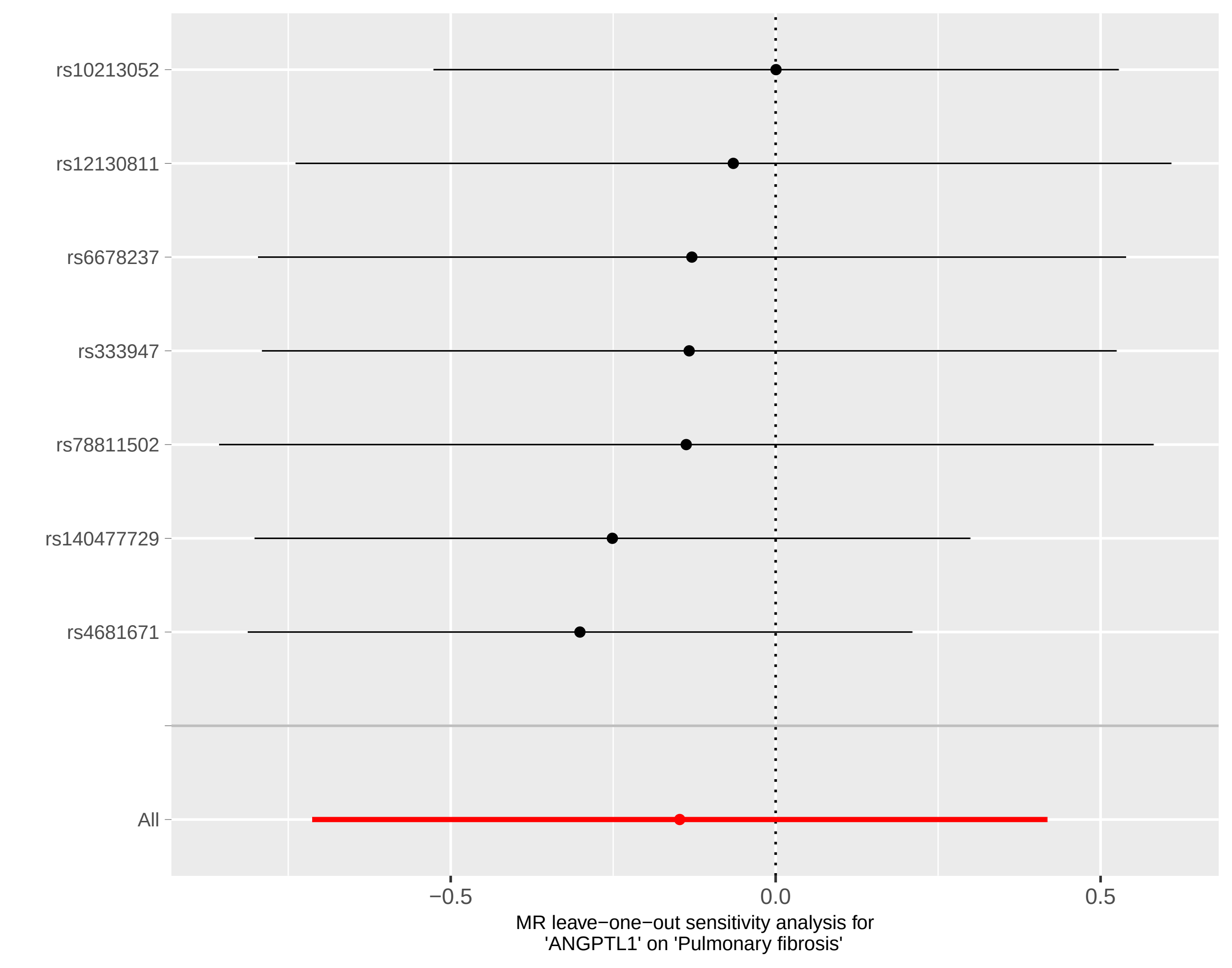

rs10213052
rs12130811
rs6678237
rs333947
rs78811502
rs140477729
rs4681671
All
−0.5
0.0
0.5
MR leave−one−out sensitivity analysis for
'ANGPTL1' on 'Pulmonary fibrosis'

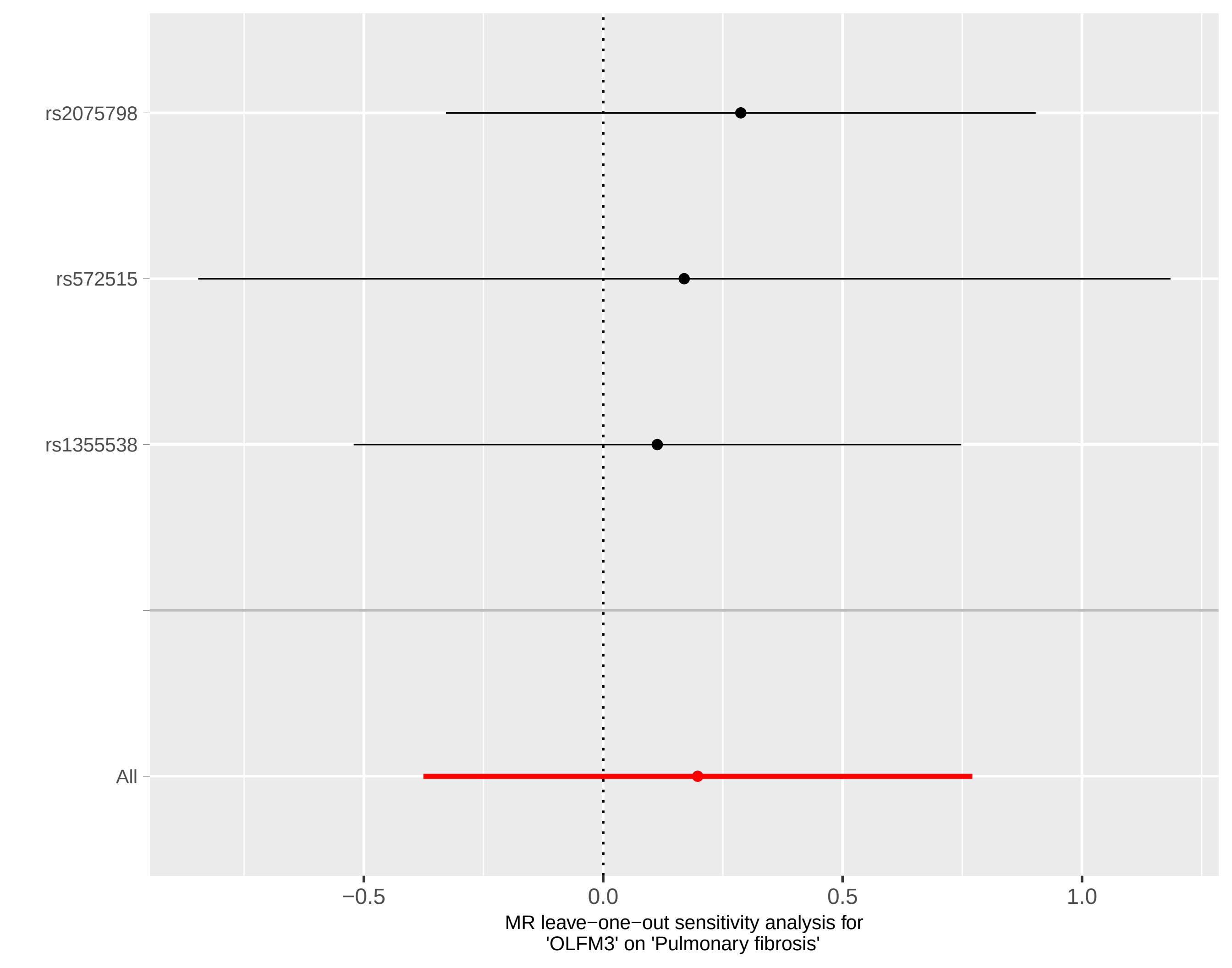
rs2075798
rs572515
rs1355538
All
−0.5
0.0
0.5
1.0
MR leave−one−out sensitivity analysis for
'OLFM3' on 'Pulmonary fibrosis'

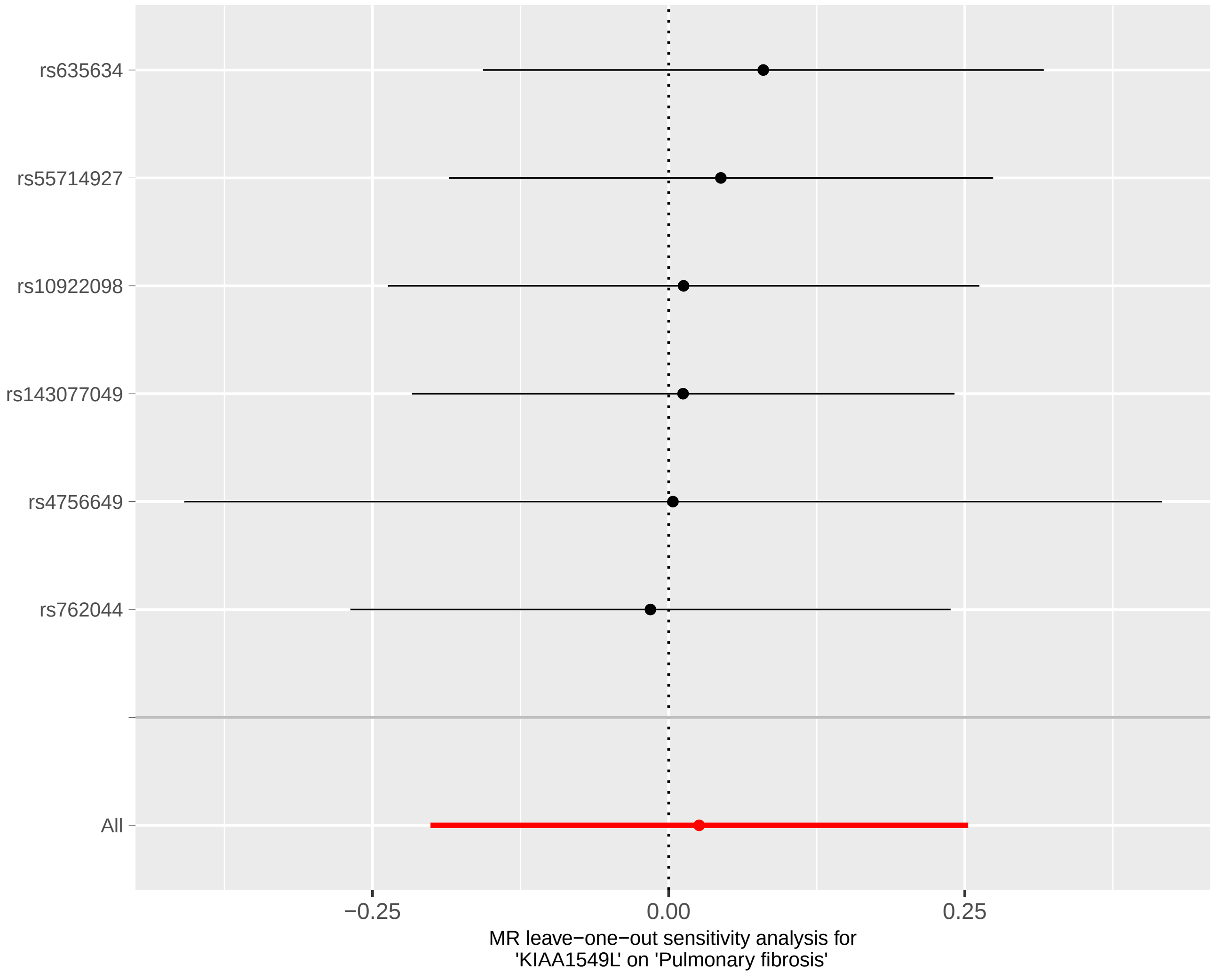
rs635634
rs55714927
rs10922098
rs143077049
rs4756649
rs762044
All
−0.25
0.00
0.25
MR leave−one−out sensitivity analysis for
'KIAA1549L' on 'Pulmonary fibrosis'

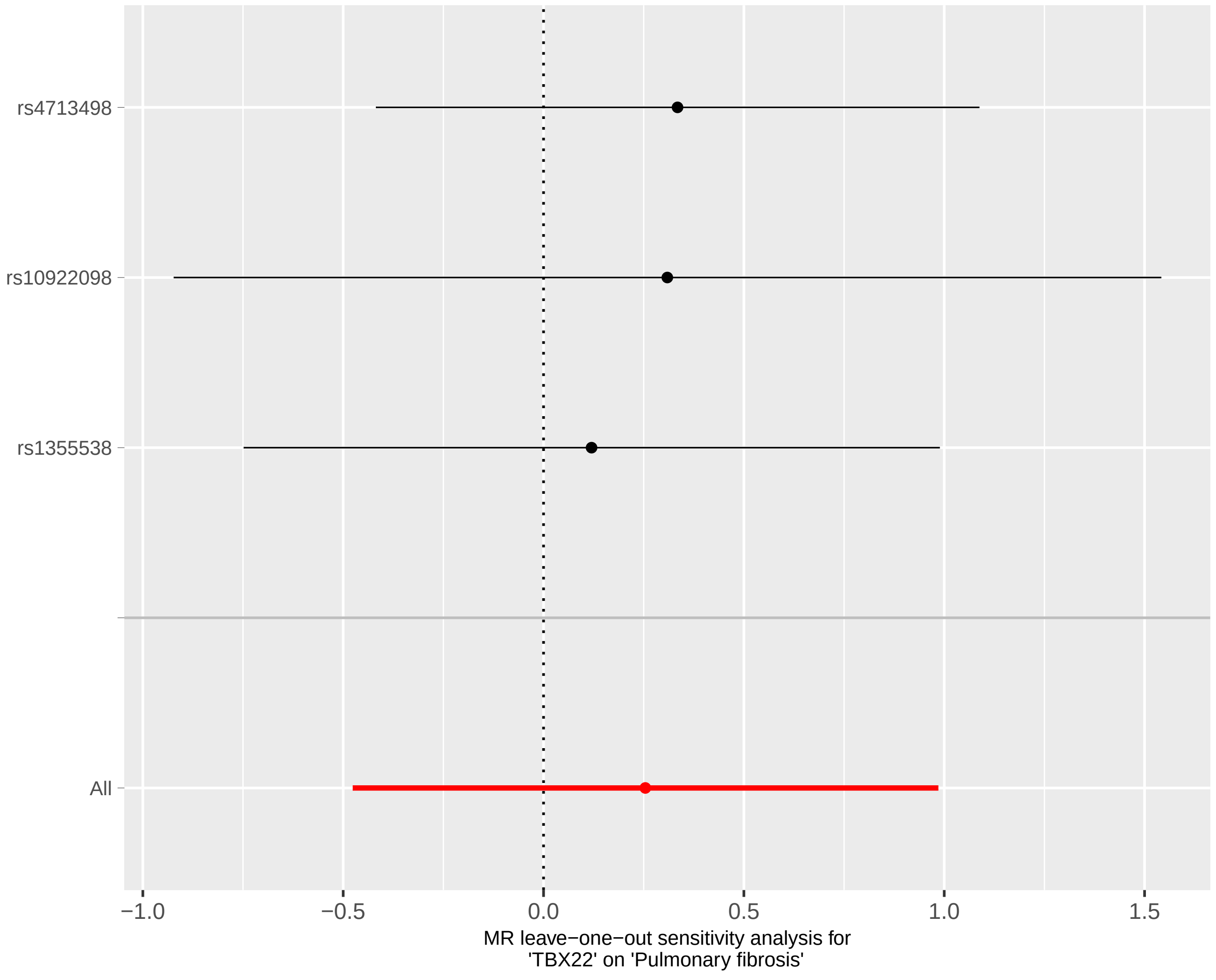
rs4713498
rs10922098
rs1355538
All
−1.0
−0.5
0.0
0.5
1.0
1.5
MR leave−one−out sensitivity analysis for
'TBX22' on 'Pulmonary fibrosis'

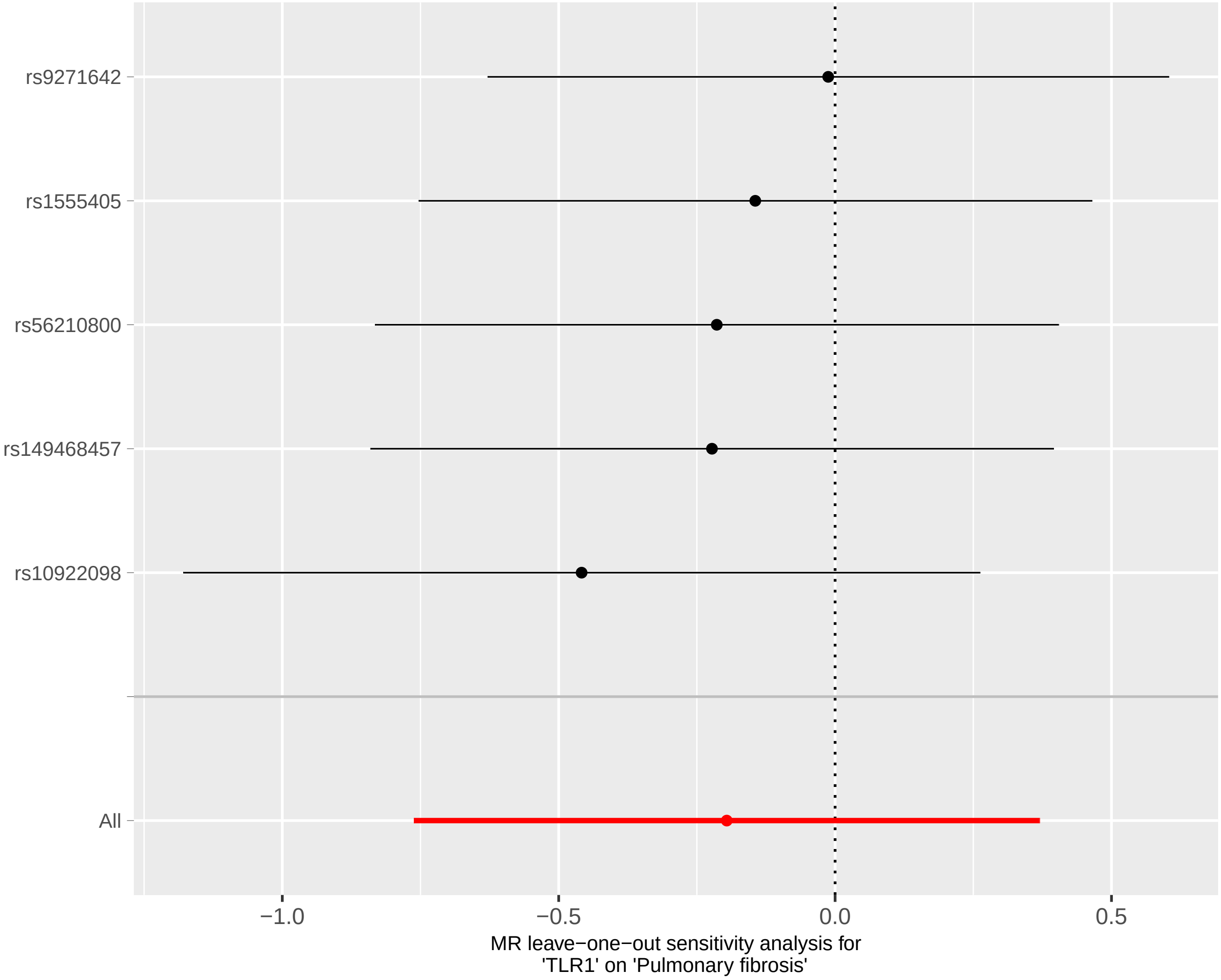

rs9271642
rs1555405
rs56210800
rs149468457
rs10922098
All
−1.0
−0.5
0.0
0.5
MR leave−one−out sensitivity analysis for
'TLR1' on 'Pulmonary fibrosis'

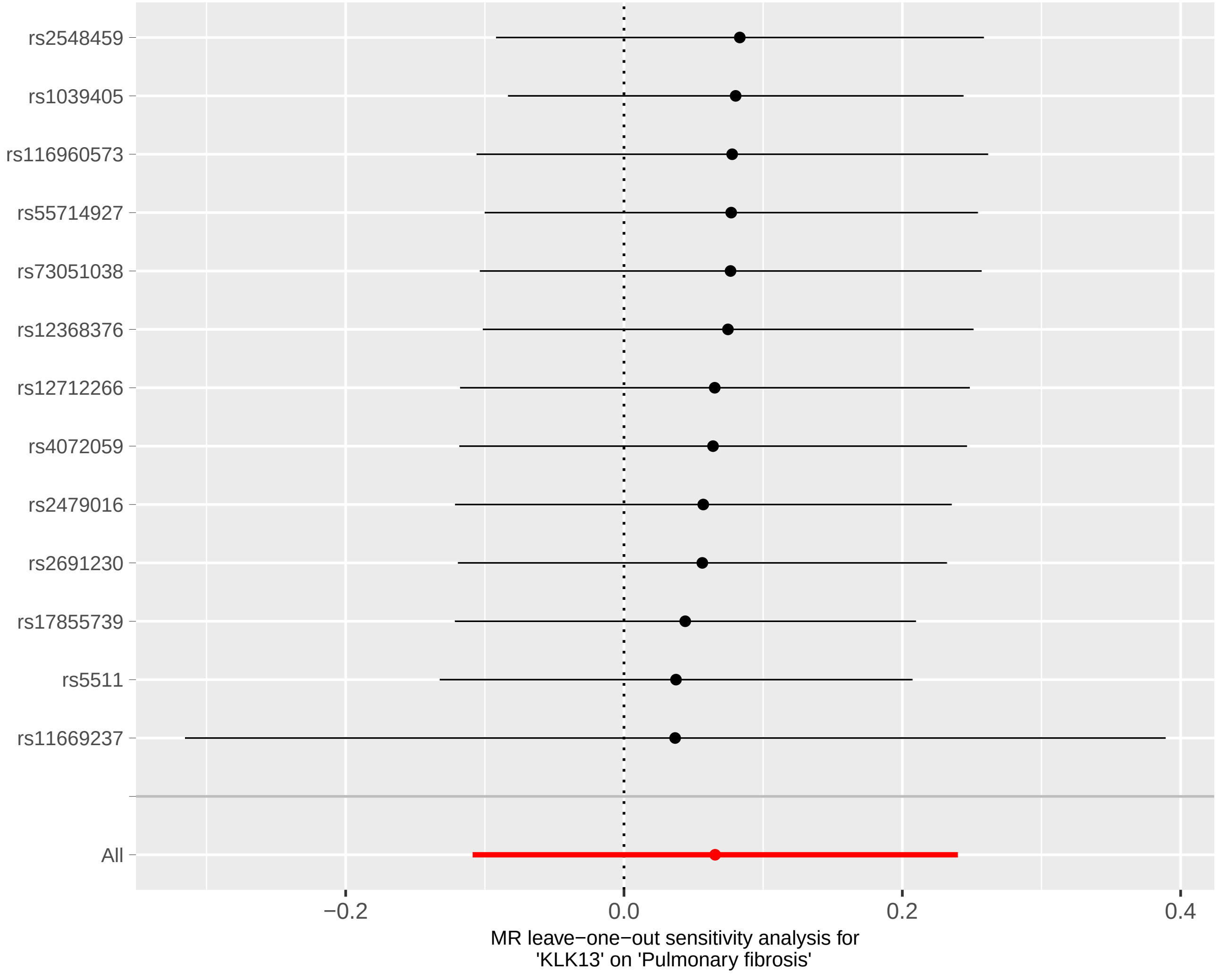
rs2548459
rs1039405
rs116960573
rs55714927
rs73051038
rs12368376
rs12712266
rs4072059
rs2479016
rs2691230
rs17855739
rs5511
rs11669237
All
−0.2
0.0
0.2
0.4
MR leave−one−out sensitivity analysis for
'KLK13' on 'Pulmonary fibrosis'

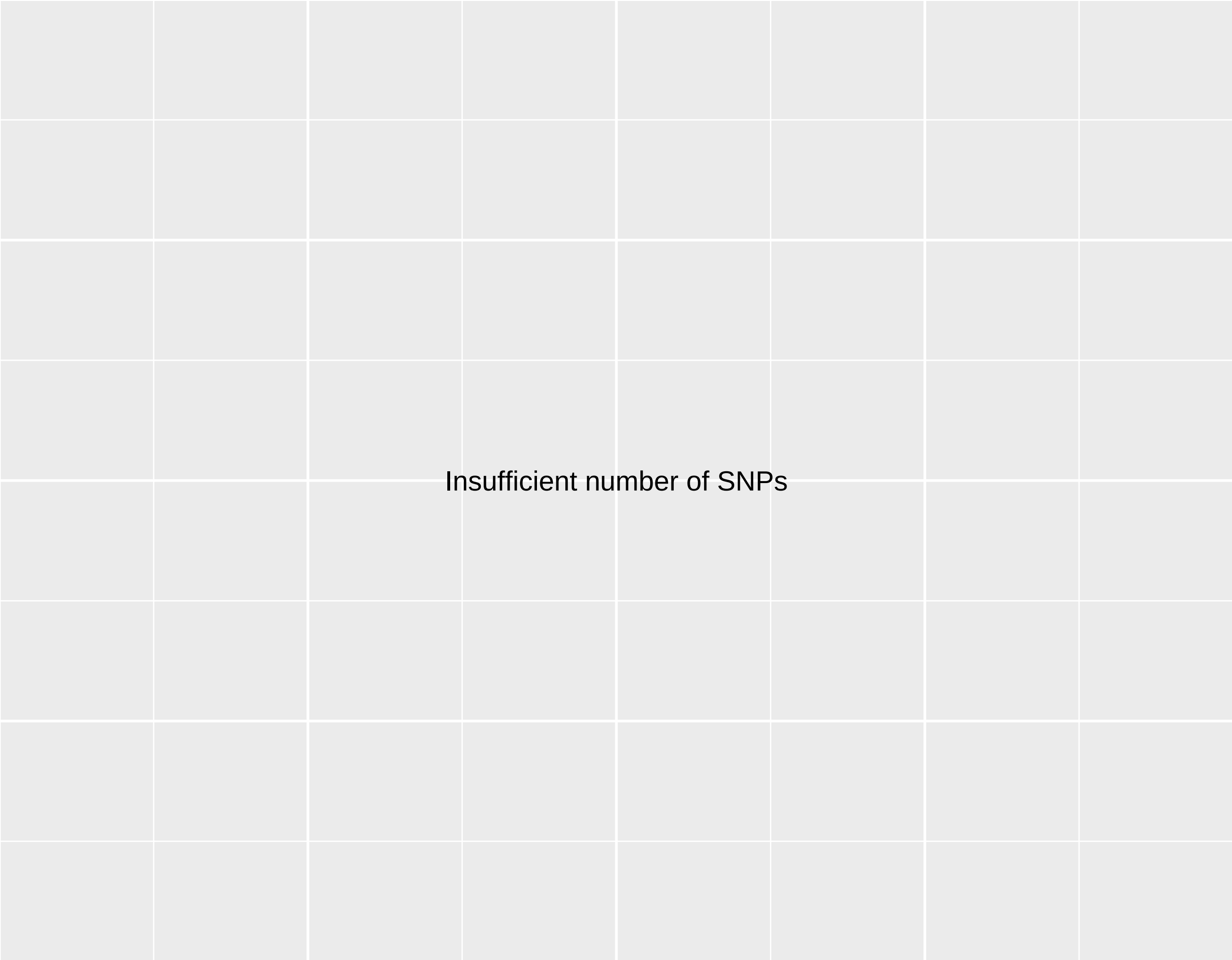
Insufficient number of SNPs

Insufficient number of SNPs

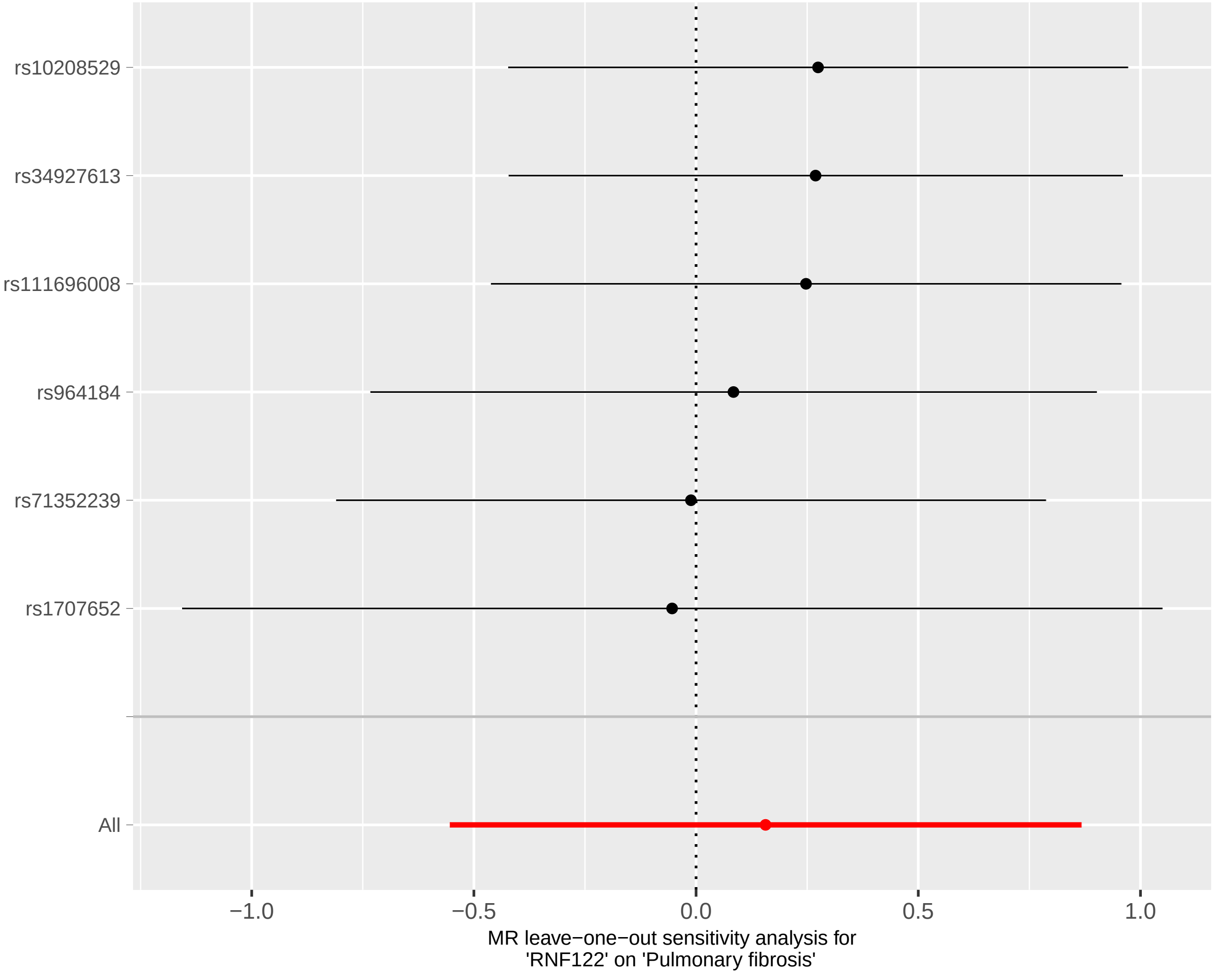

rs10208529
rs34927613
rs111696008
rs964184
rs71352239
rs1707652
All
−1.0
−0.5
0.0
0.5
1.0
MR leave−one−out sensitivity analysis for
'RNF122' on 'Pulmonary fibrosis'

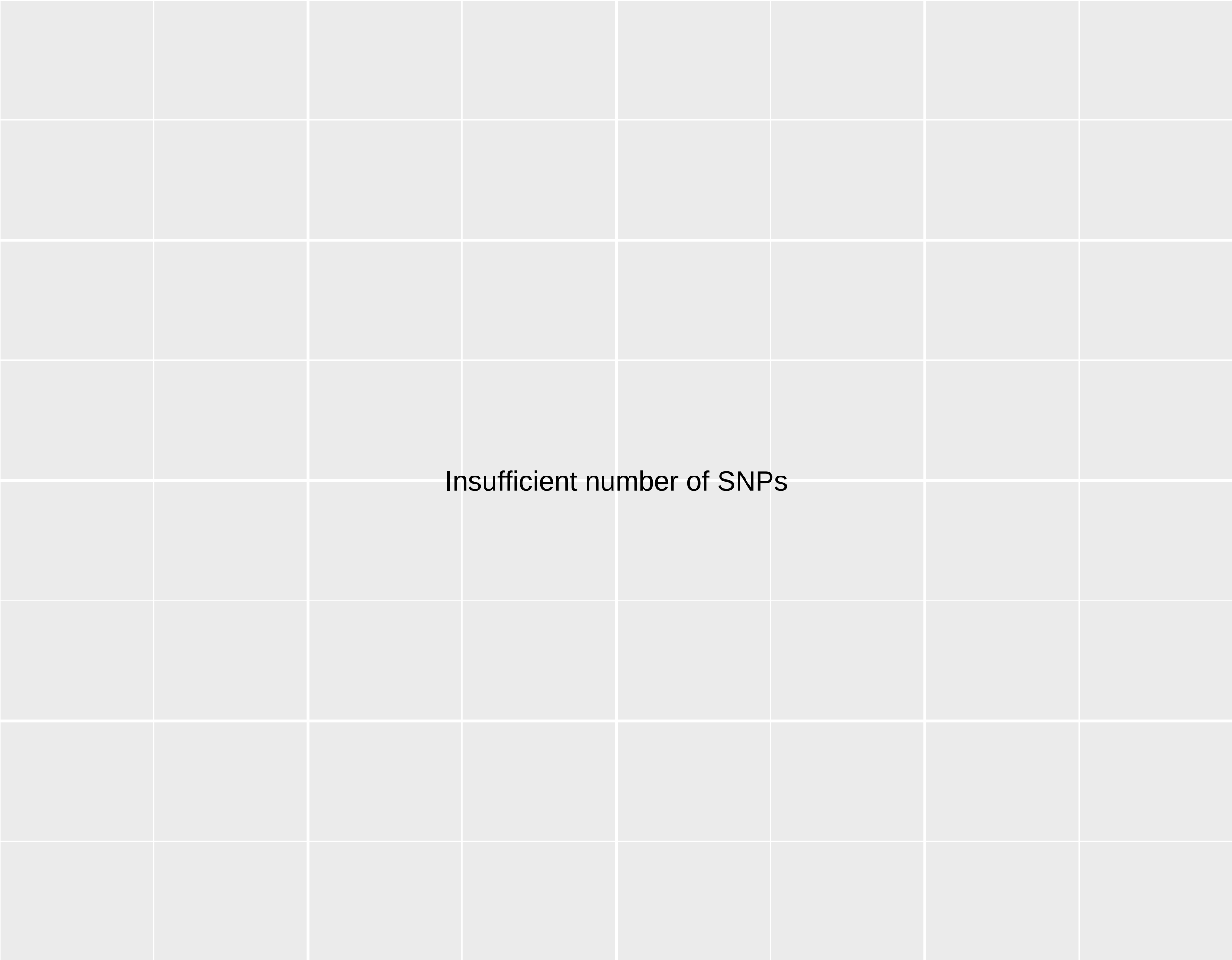
Insufficient number of SNPs

Insufficient number of SNPs

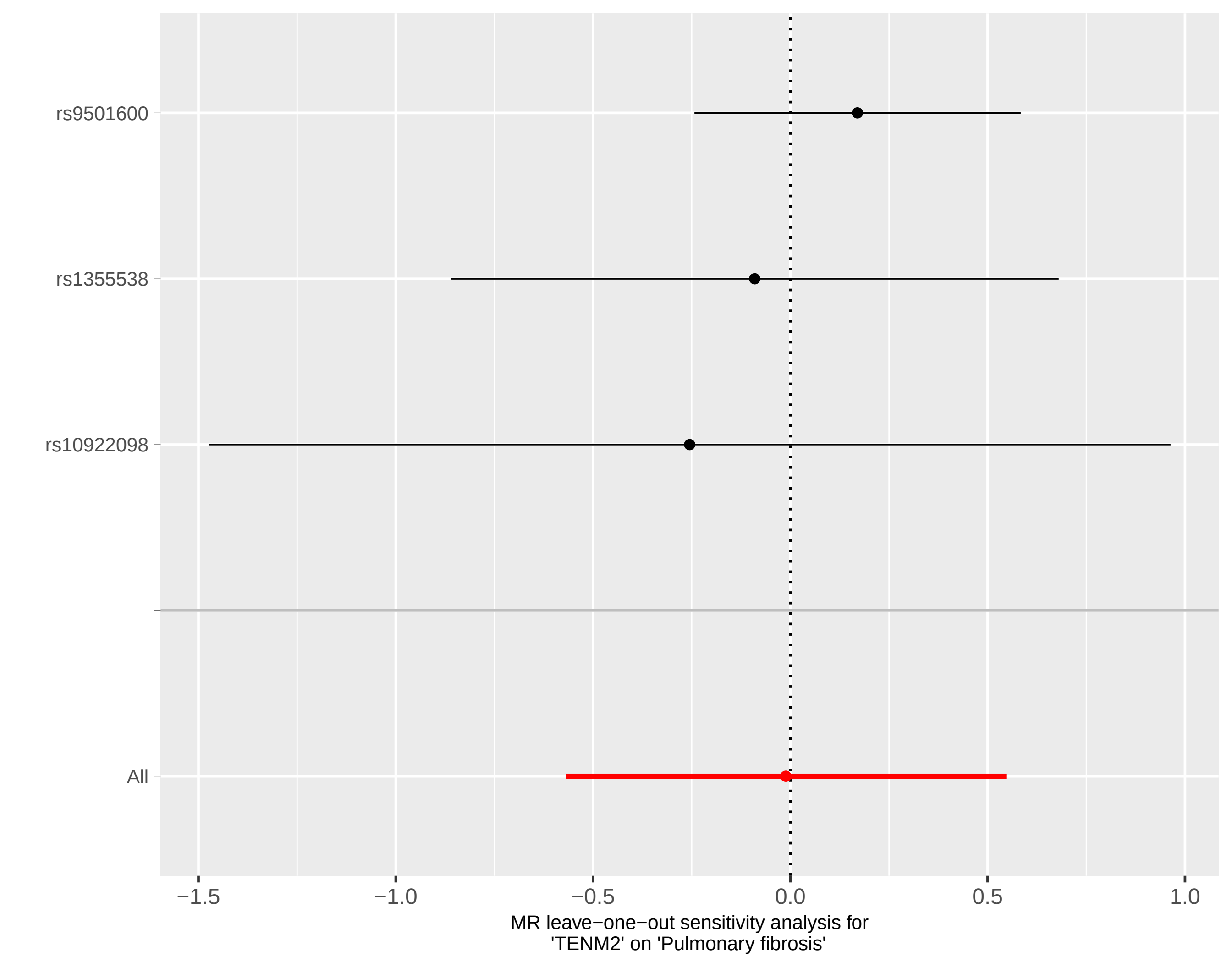
rs9501600
rs1355538
rs10922098
All
−1.5
−1.0
−0.5
0.0
0.5
1.0
MR leave−one−out sensitivity analysis for
'TENM2' on 'Pulmonary fibrosis'

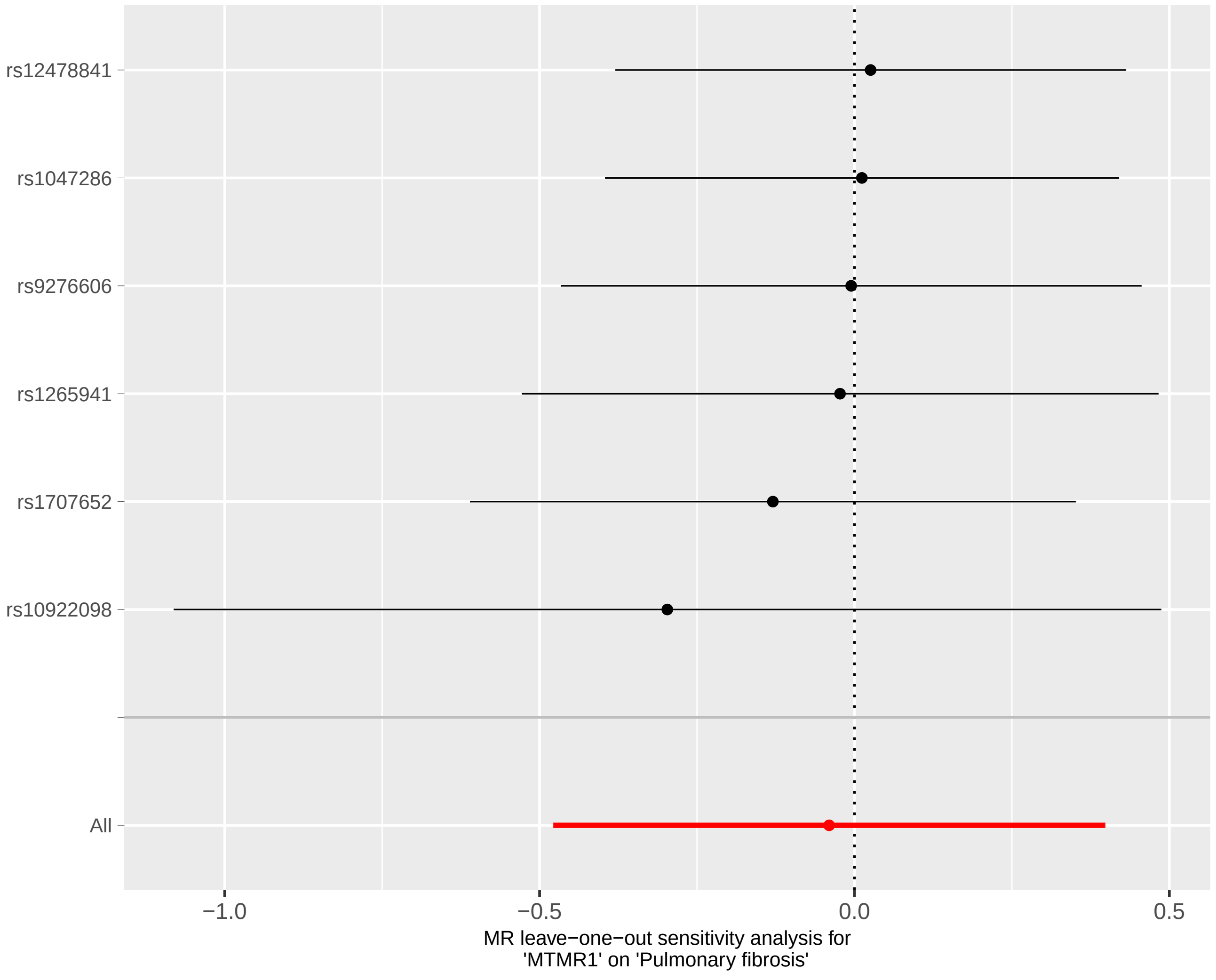
rs12478841
rs1047286
rs9276606
rs1265941
rs1707652
rs10922098
All
−1.0
−0.5
0.0
0.5
MR leave−one−out sensitivity analysis for
'MTMR1' on 'Pulmonary fibrosis'

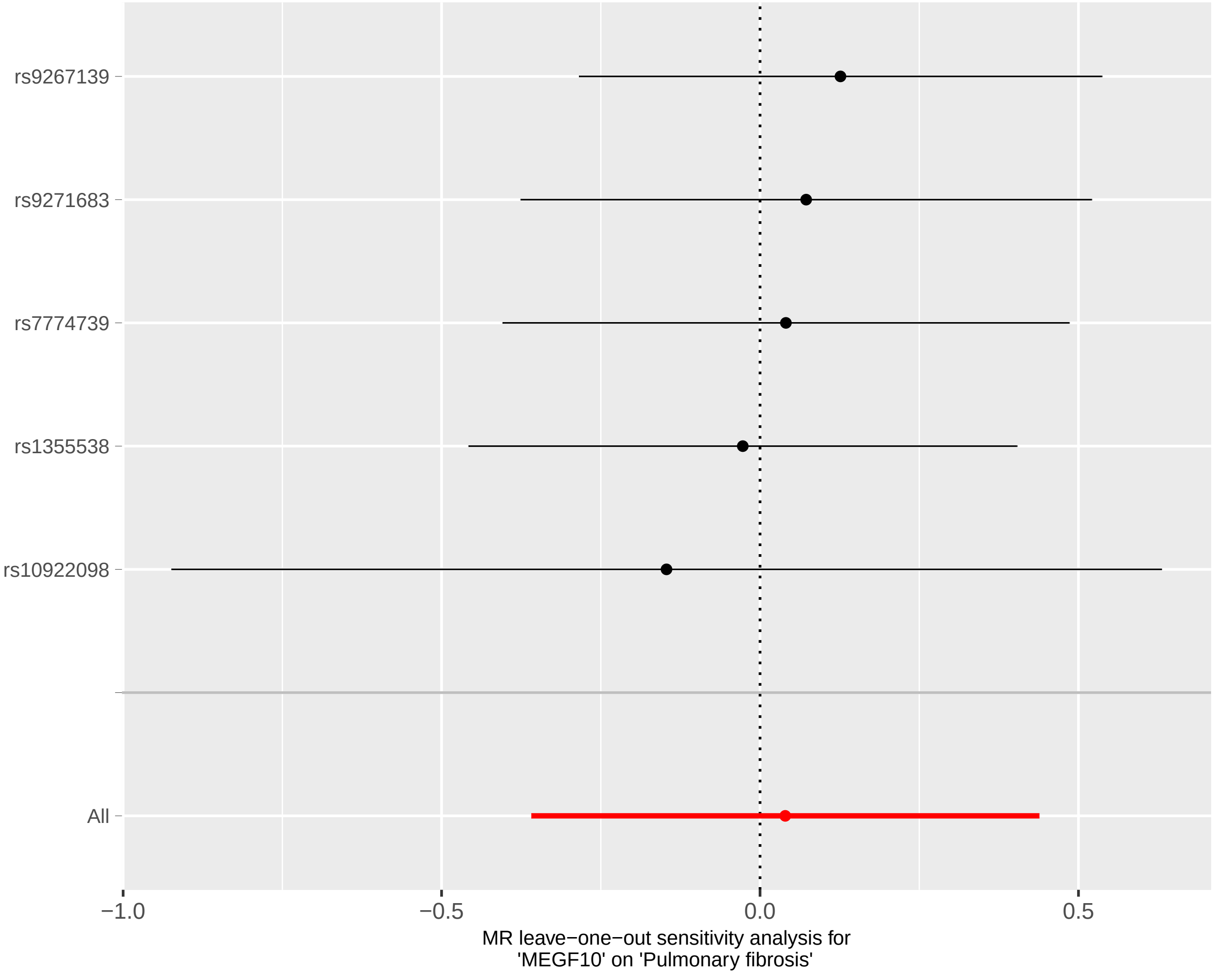
rs9267139
rs9271683
rs7774739
rs1355538
rs10922098
All
−1.0
−0.5
0.0
0.5
MR leave−one−out sensitivity analysis for
'MEGF10' on 'Pulmonary fibrosis'

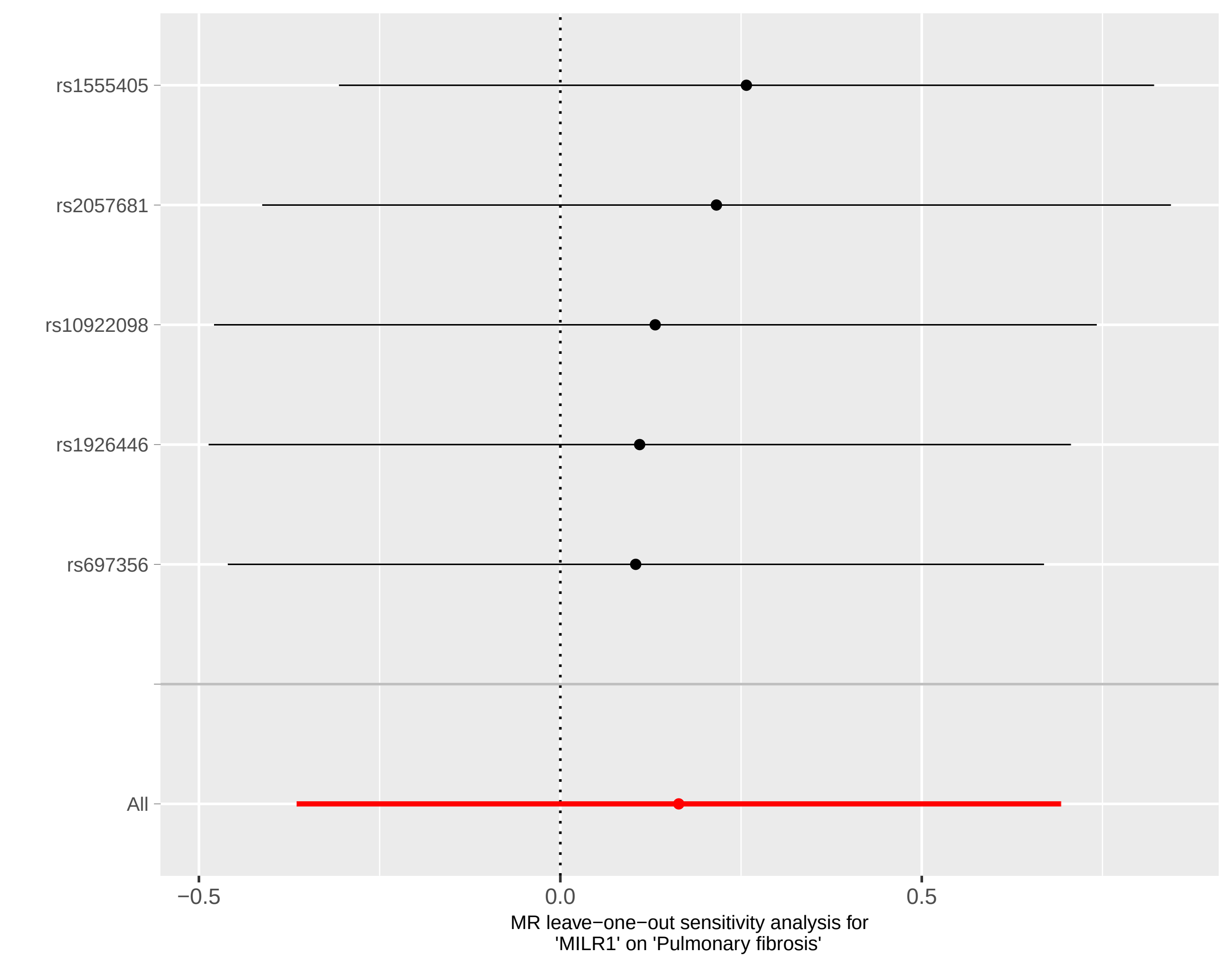

rs1555405
rs2057681
rs10922098
rs1926446
rs697356
All
−0.5
0.0
0.5
MR leave−one−out sensitivity analysis for
'MILR1' on 'Pulmonary fibrosis'

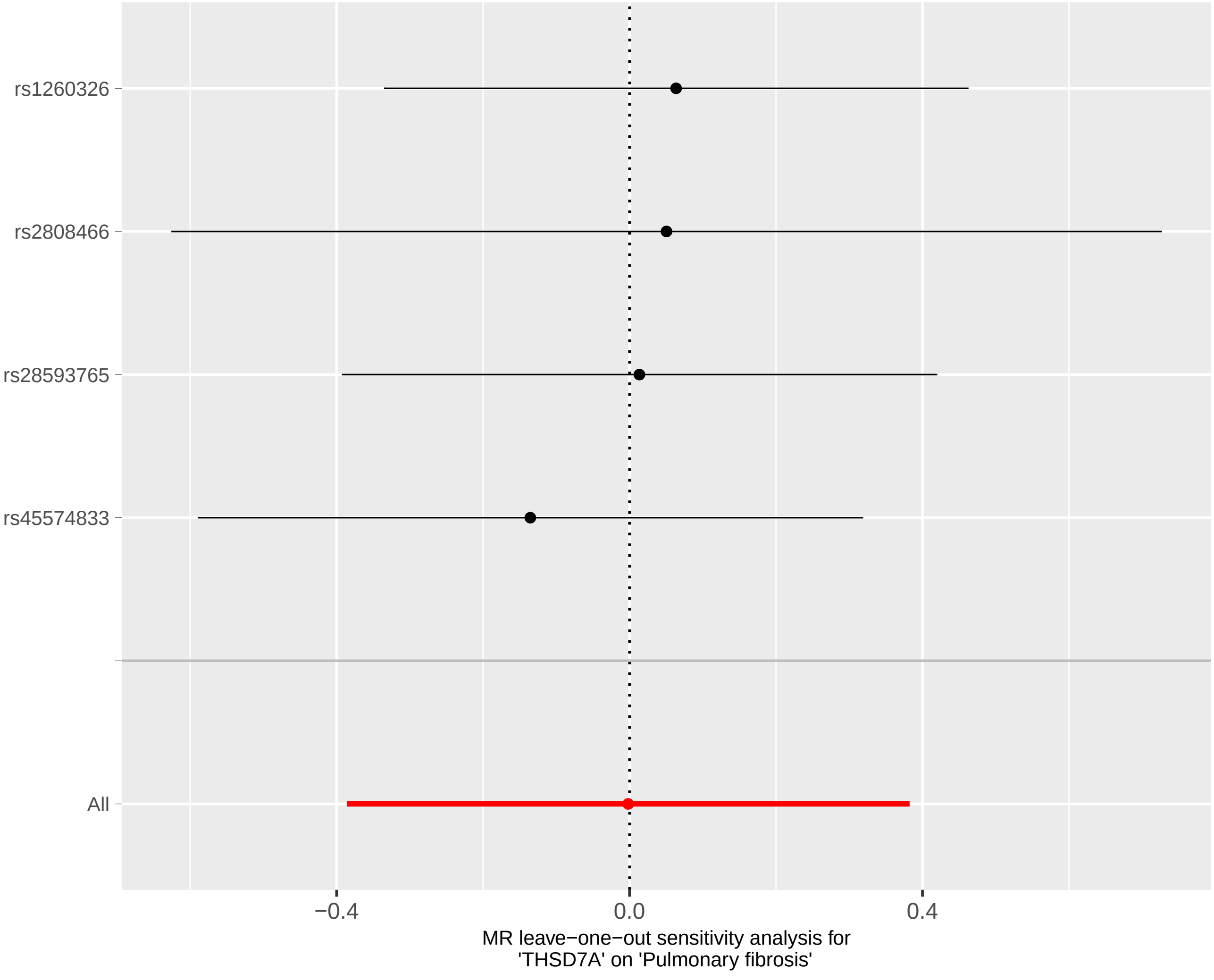

rs1260326
rs2808466
rs28593765
rs45574833
All
−0.4
0.0
0.4
MR leave−one−out sensitivity analysis for
'THSD7A' on 'Pulmonary fibrosis'

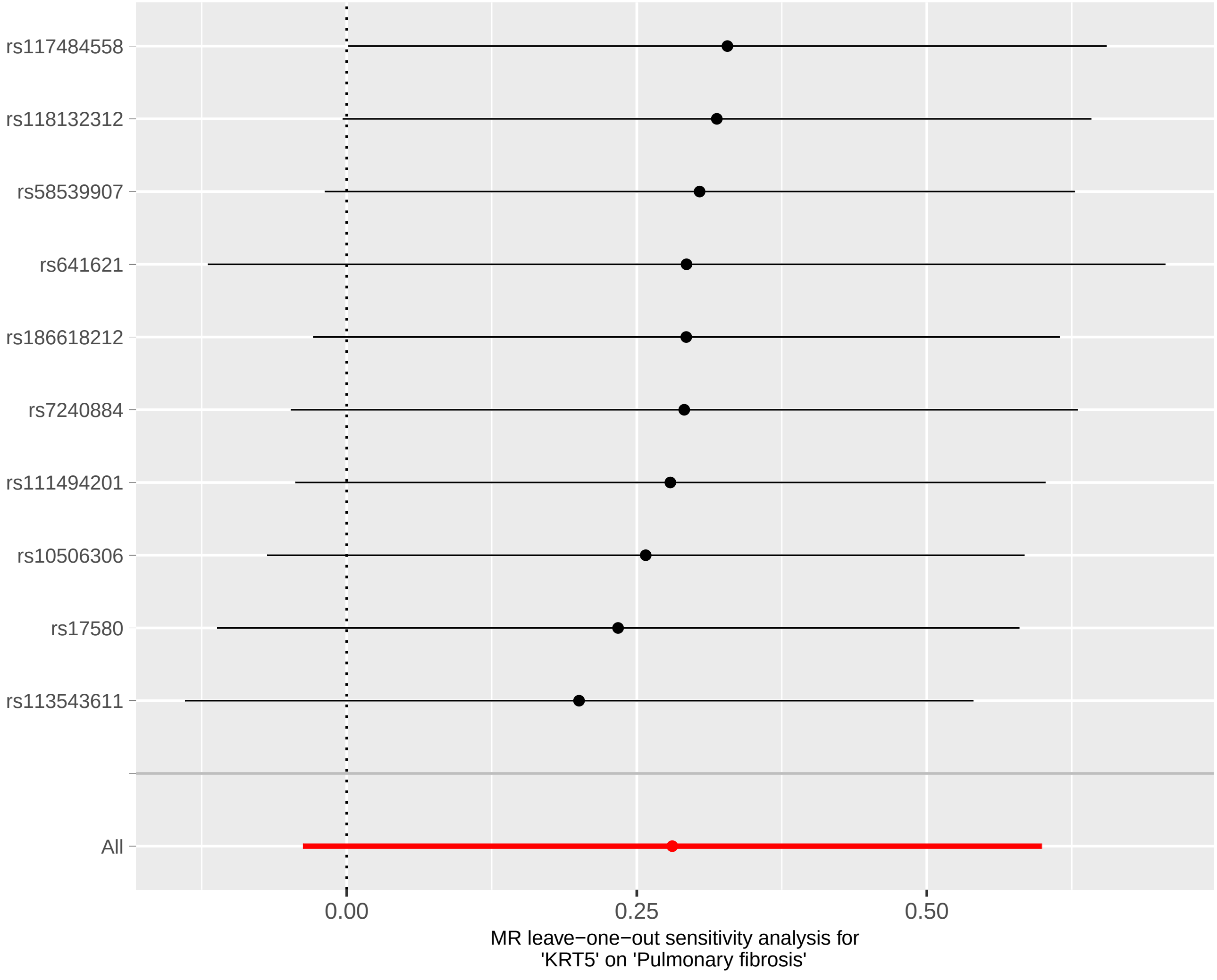

rs117484558
rs118132312
rs58539907
rs641621
rs186618212
rs7240884
rs111494201
rs10506306
rs17580
rs113543611
All
0.00
0.25
0.50
MR leave−one−out sensitivity analysis for
'KRT5' on 'Pulmonary fibrosis'

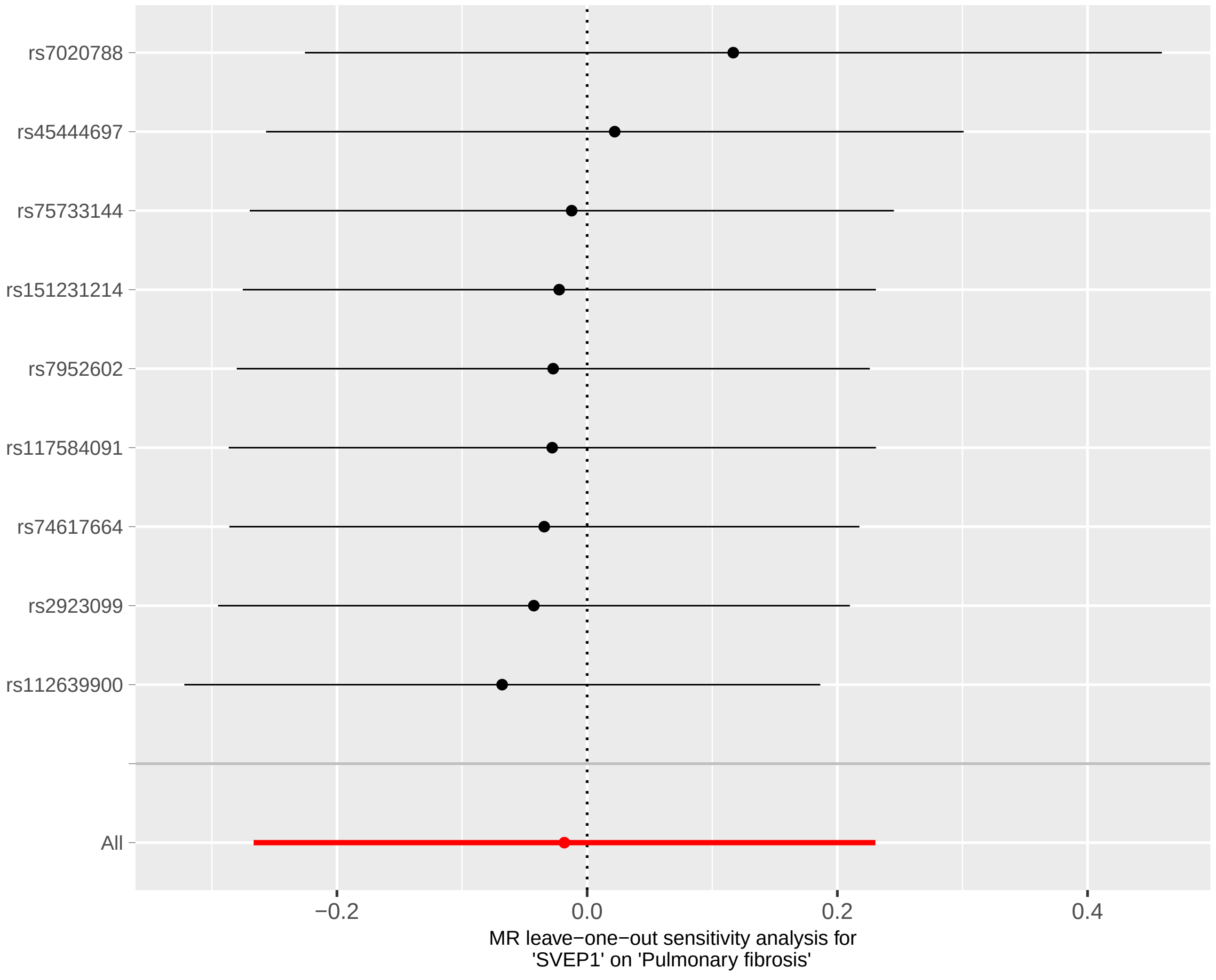

rs7020788
rs45444697
rs75733144
rs151231214
rs7952602
rs117584091
rs74617664
rs2923099
rs112639900
All
−0.2
0.0
0.2
0.4
MR leave−one−out sensitivity analysis for
'SVEP1' on 'Pulmonary fibrosis'

Insufficient number of SNPs

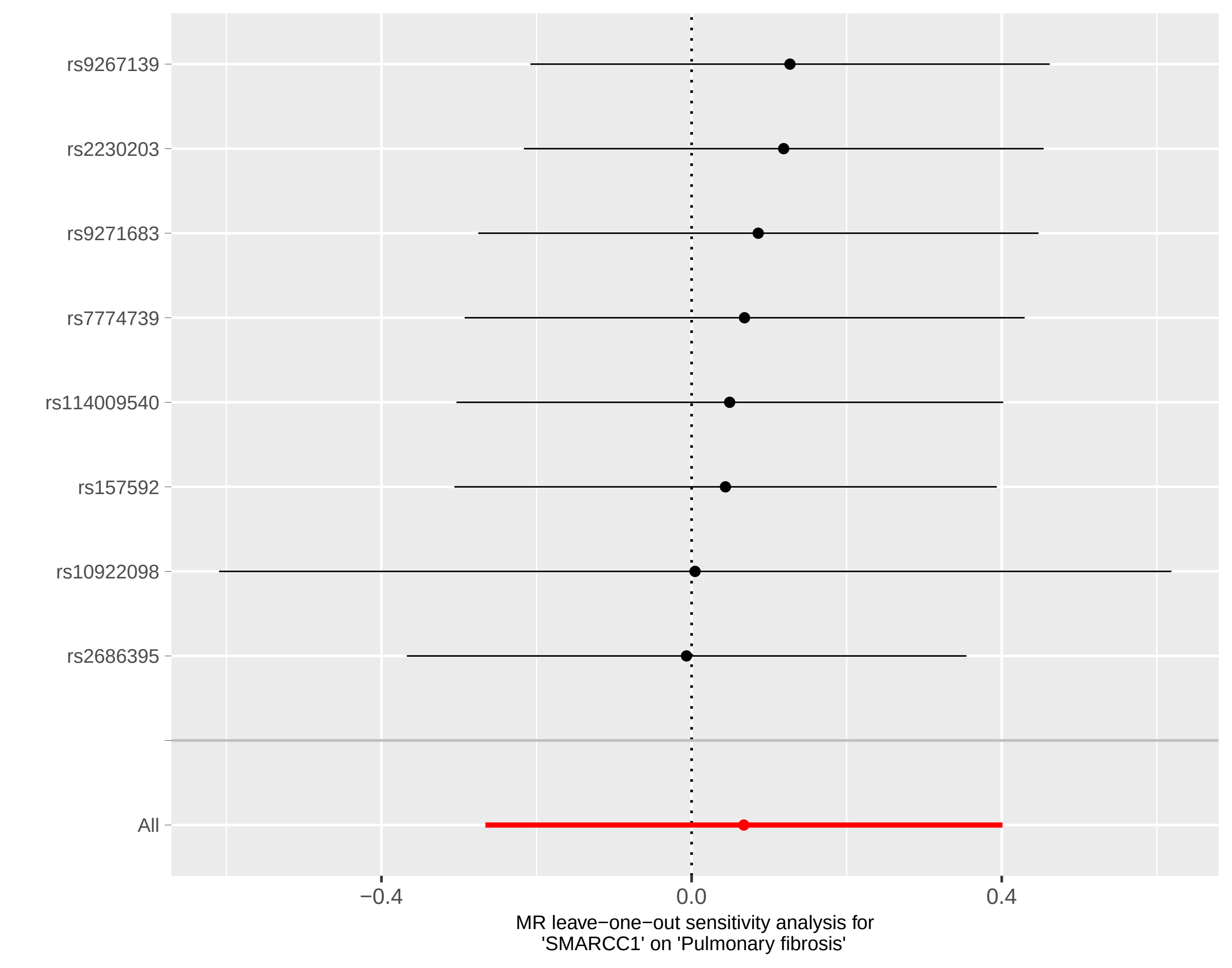
rs9267139
rs2230203
rs9271683
rs7774739
rs114009540
rs157592
rs10922098
rs2686395
All
−0.4
0.0
0.4
MR leave−one−out sensitivity analysis for
'SMARCC1' on 'Pulmonary fibrosis'

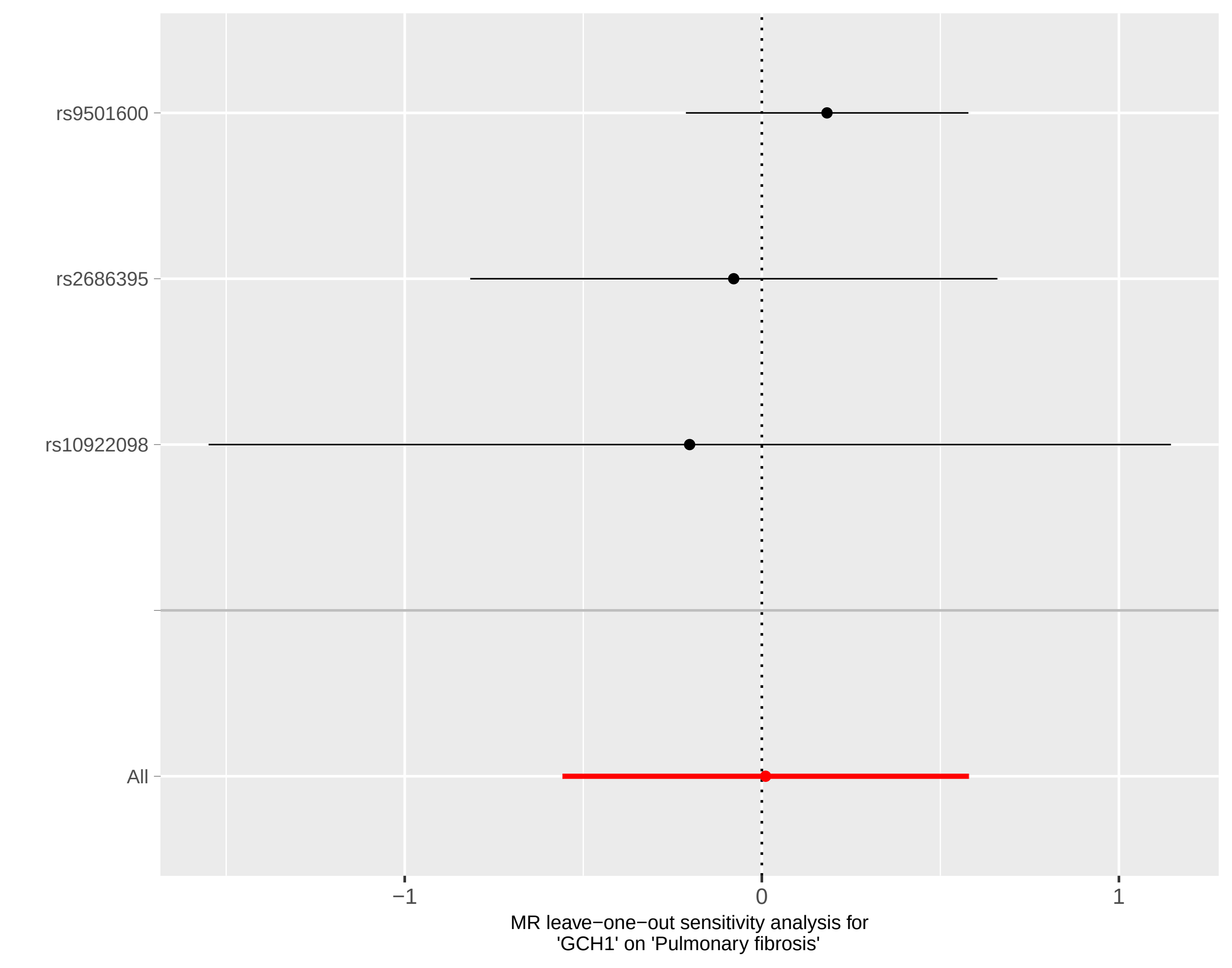

rs9501600
rs2686395
rs10922098
All
−1
0
1
MR leave−one−out sensitivity analysis for
'GCH1' on 'Pulmonary fibrosis'

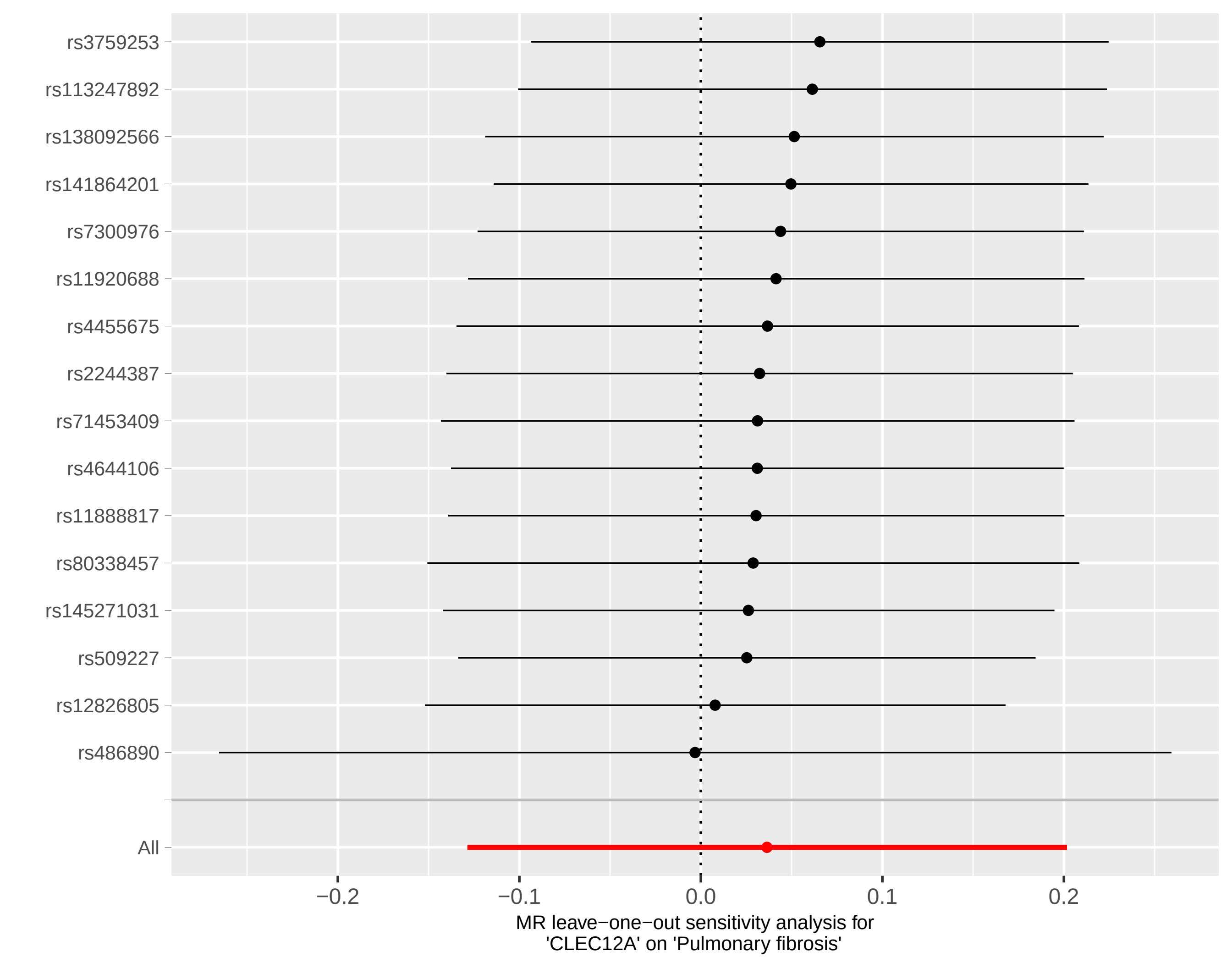
rs3759253
rs113247892
rs138092566
rs141864201
rs7300976
rs11920688
rs4455675
rs2244387
rs71453409
rs4644106
rs11888817
rs80338457
rs145271031
rs509227
rs12826805
rs486890
All
−0.2
−0.1
0.0
0.1
0.2
MR leave−one−out sensitivity analysis for
'CLEC12A' on 'Pulmonary fibrosis'

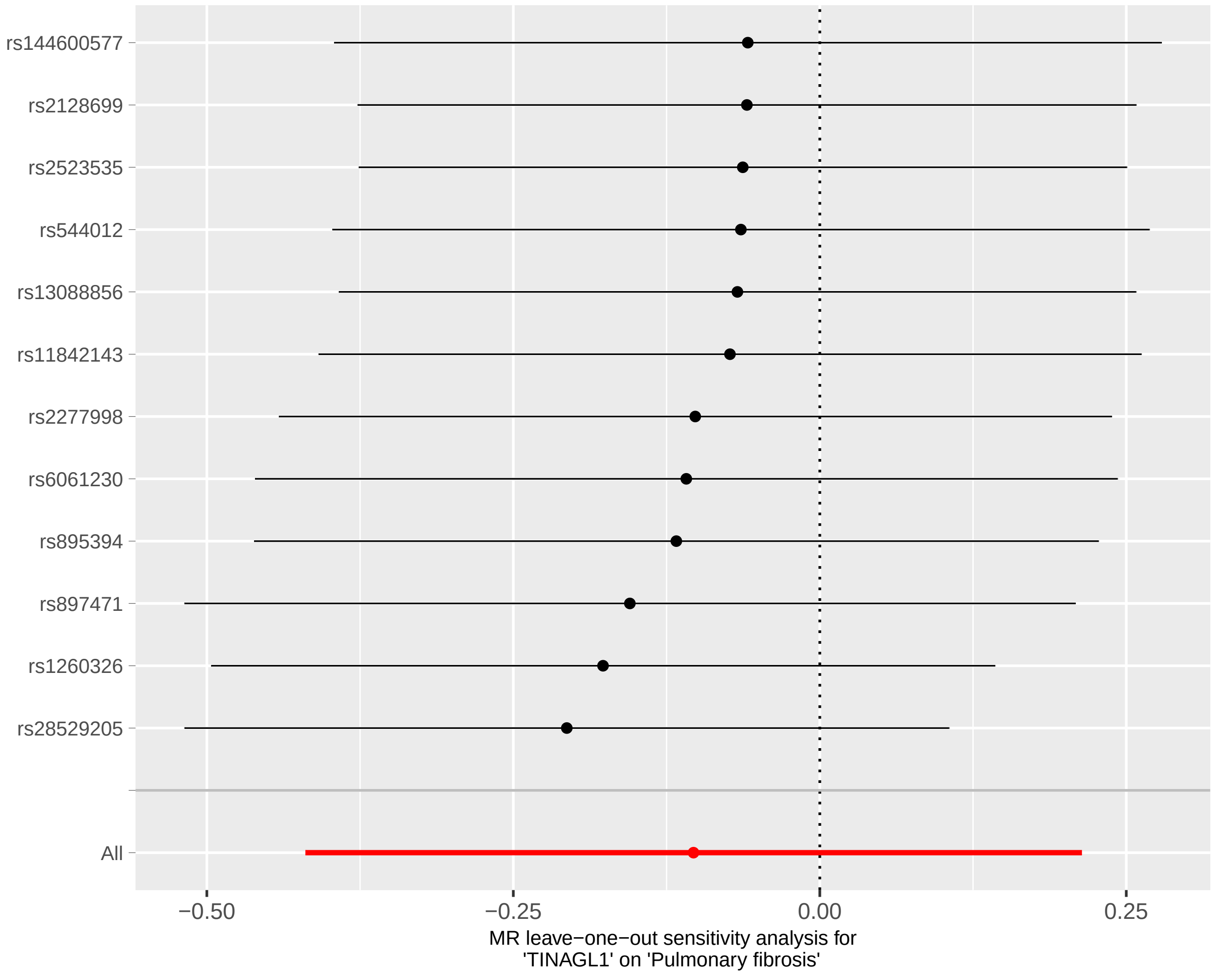
rs144600577
rs2128699
rs2523535
rs544012
rs13088856
rs11842143
rs2277998
rs6061230
rs895394
rs897471
rs1260326
rs28529205
All
−0.50
−0.25
0.00
0.25
MR leave−one−out sensitivity analysis for
'TINAGL1' on 'Pulmonary fibrosis'

Insufficient number of SNPs

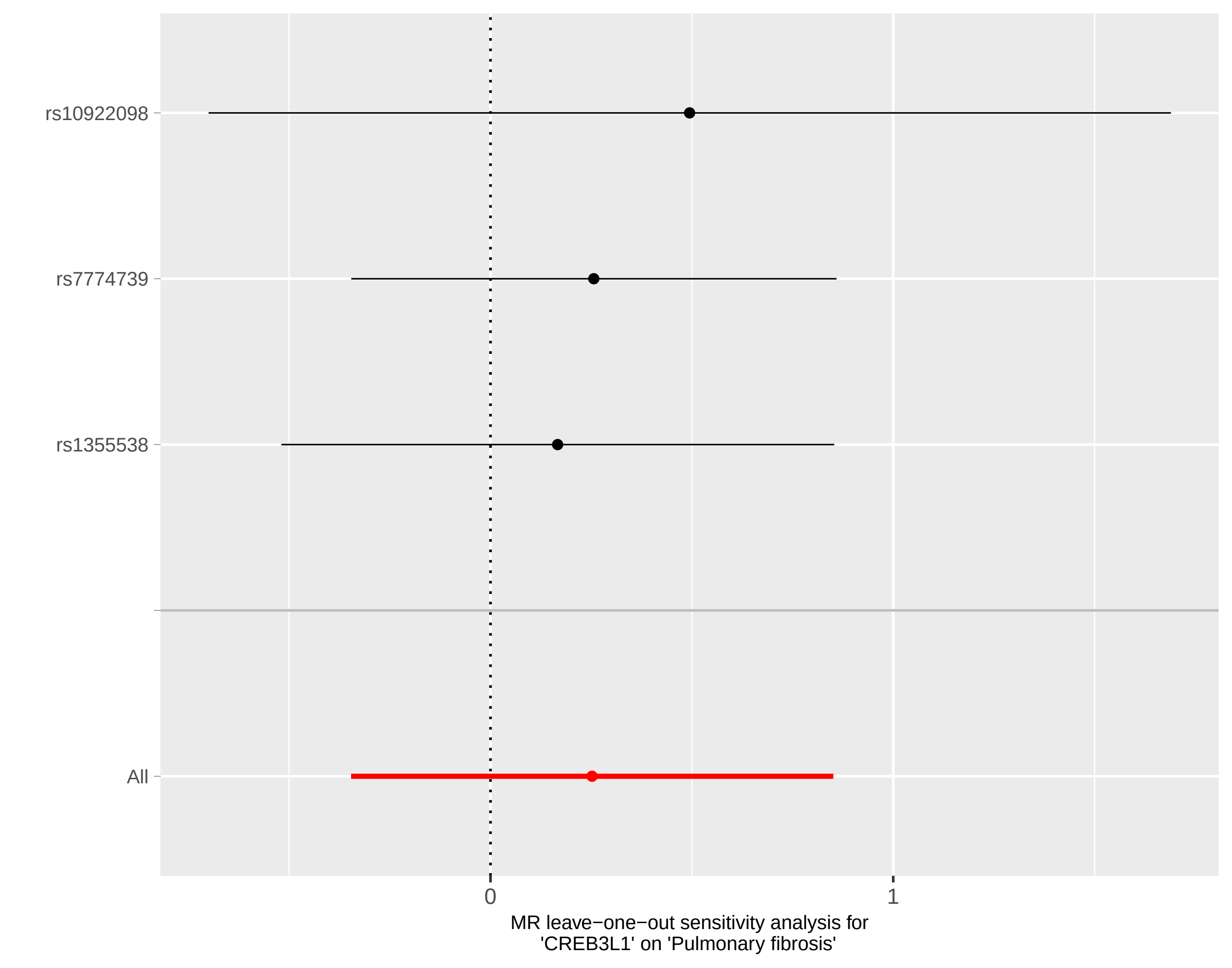

rs10922098
rs7774739
rs1355538
All
0
1
MR leave−one−out sensitivity analysis for
'CREB3L1' on 'Pulmonary fibrosis'

Insufficient number of SNPs

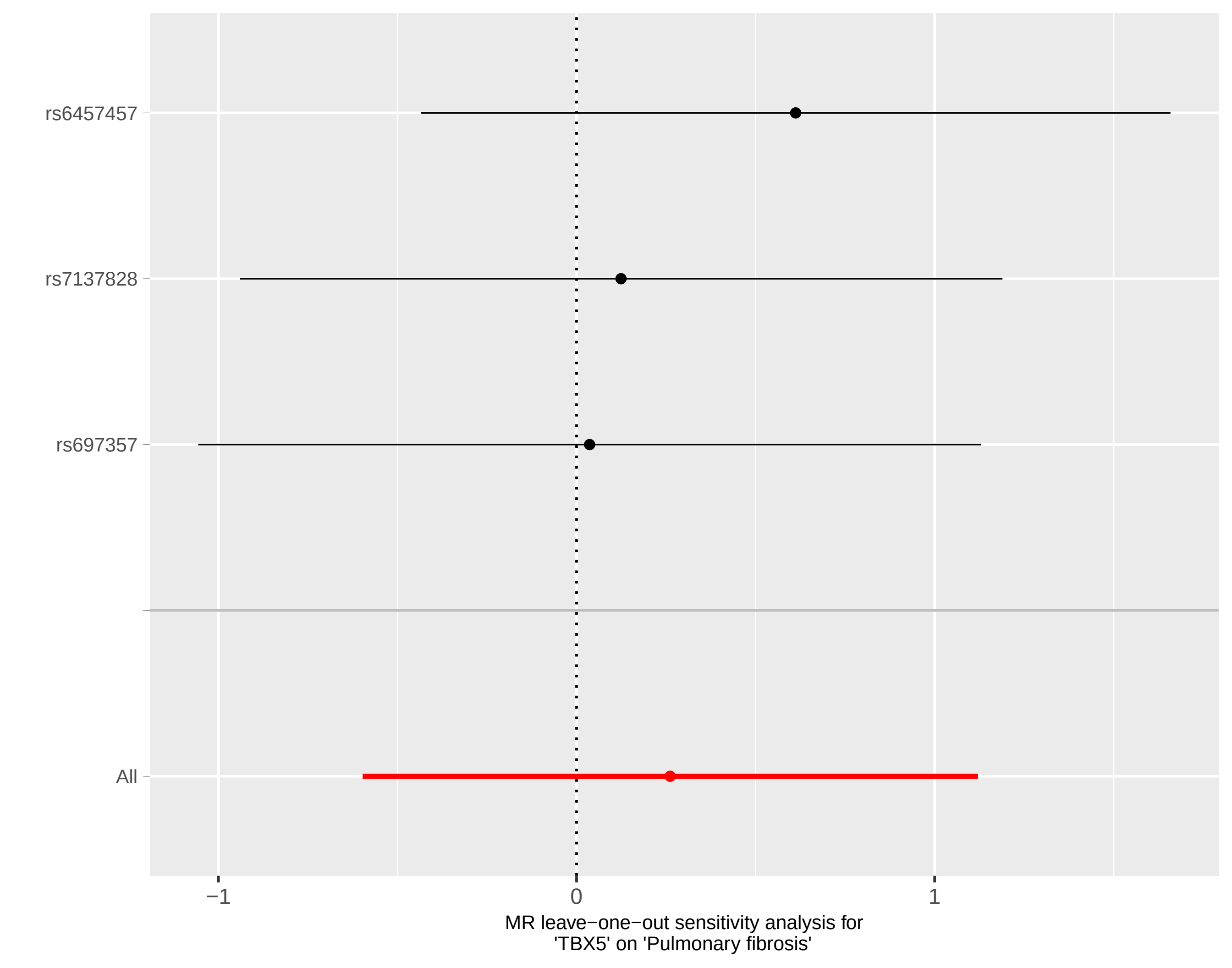
rs6457457
rs7137828
rs697357
All
−1
0
1
MR leave−one−out sensitivity analysis for
'TBX5' on 'Pulmonary fibrosis'

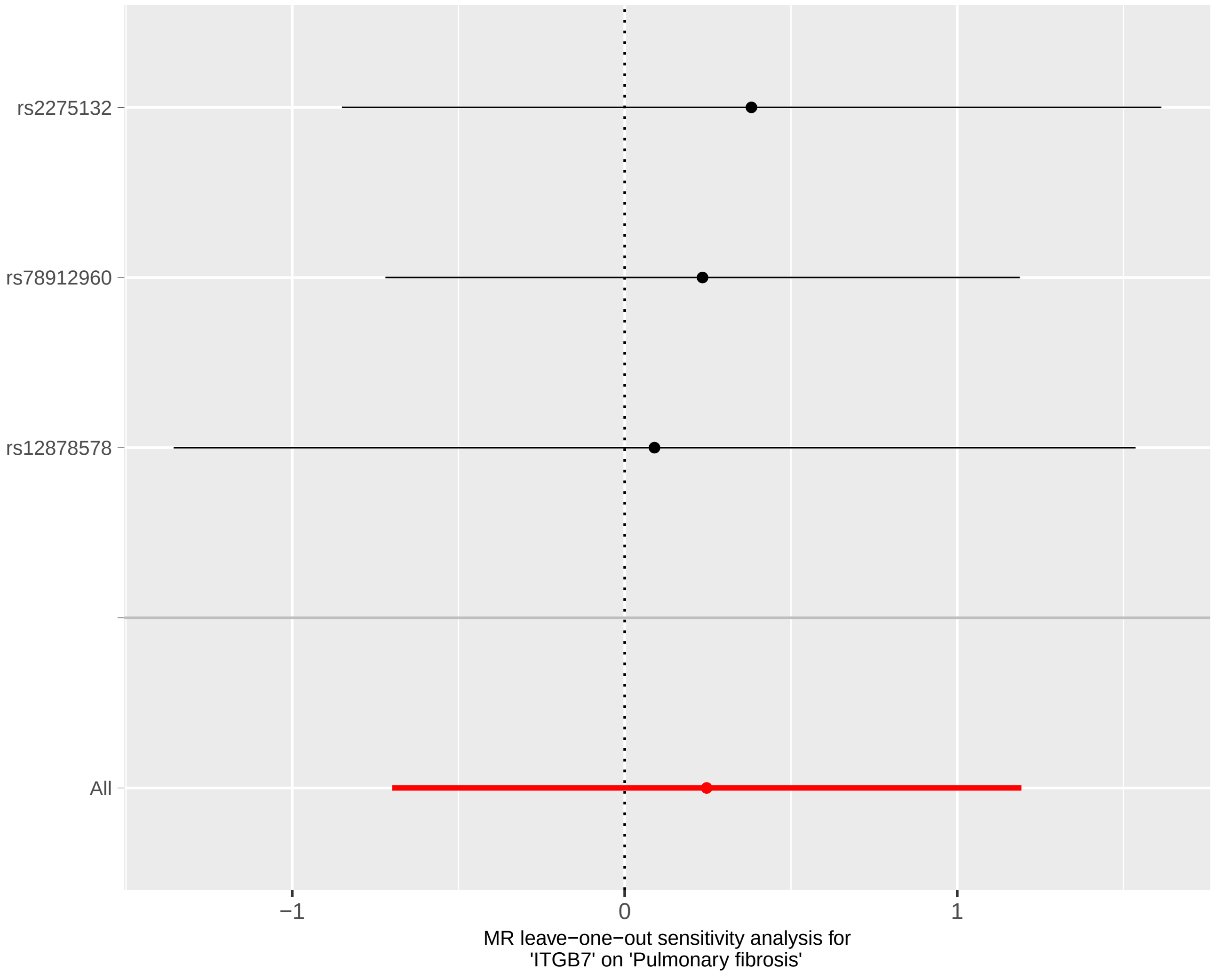

rs2275132
rs78912960
rs12878578
All
−1
0
1
MR leave−one−out sensitivity analysis for
'ITGB7' on 'Pulmonary fibrosis'

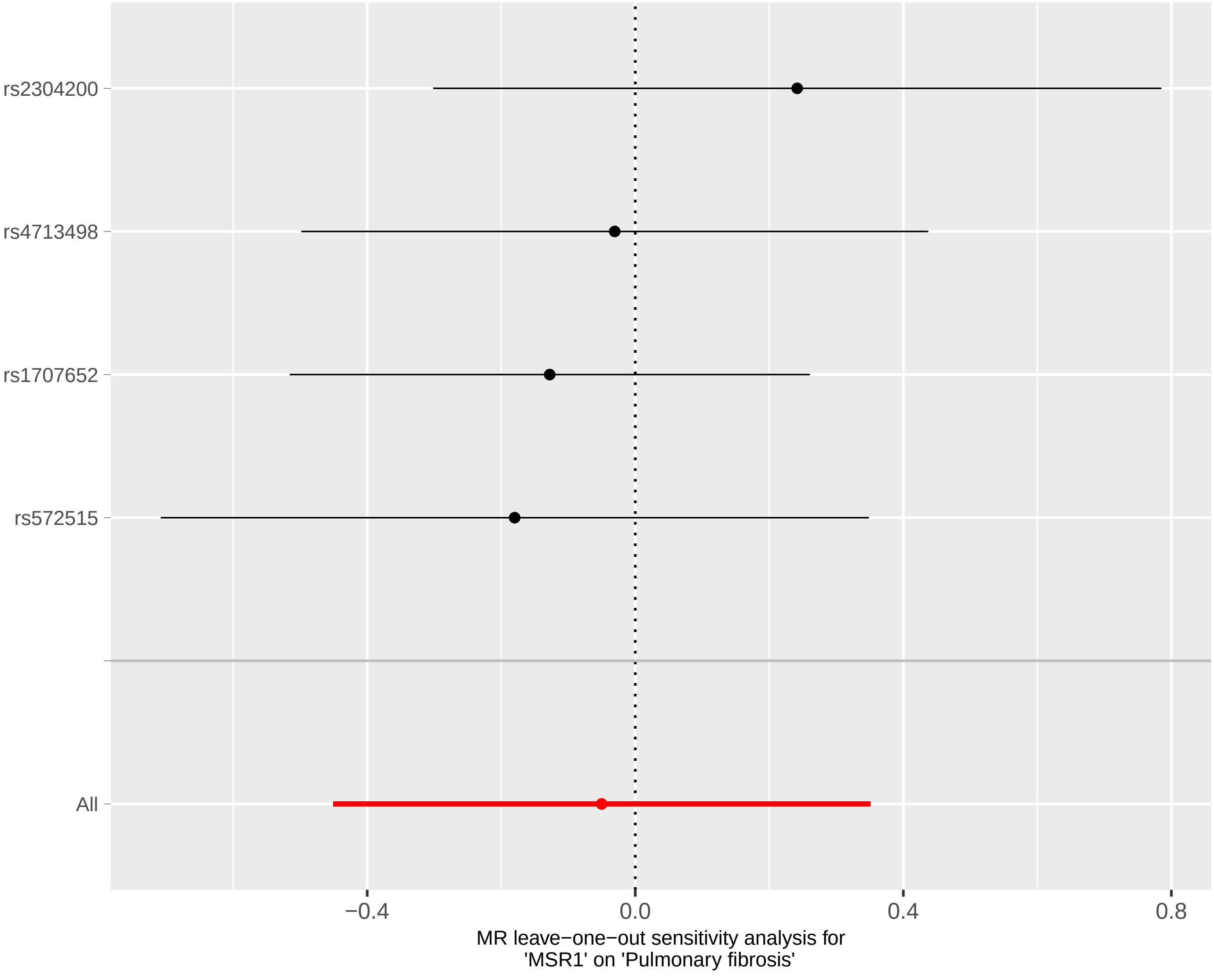

rs2304200
rs4713498
rs1707652
rs572515
All
−0.4
0.0
0.4
0.8
MR leave−one−out sensitivity analysis for
'MSR1' on 'Pulmonary fibrosis'

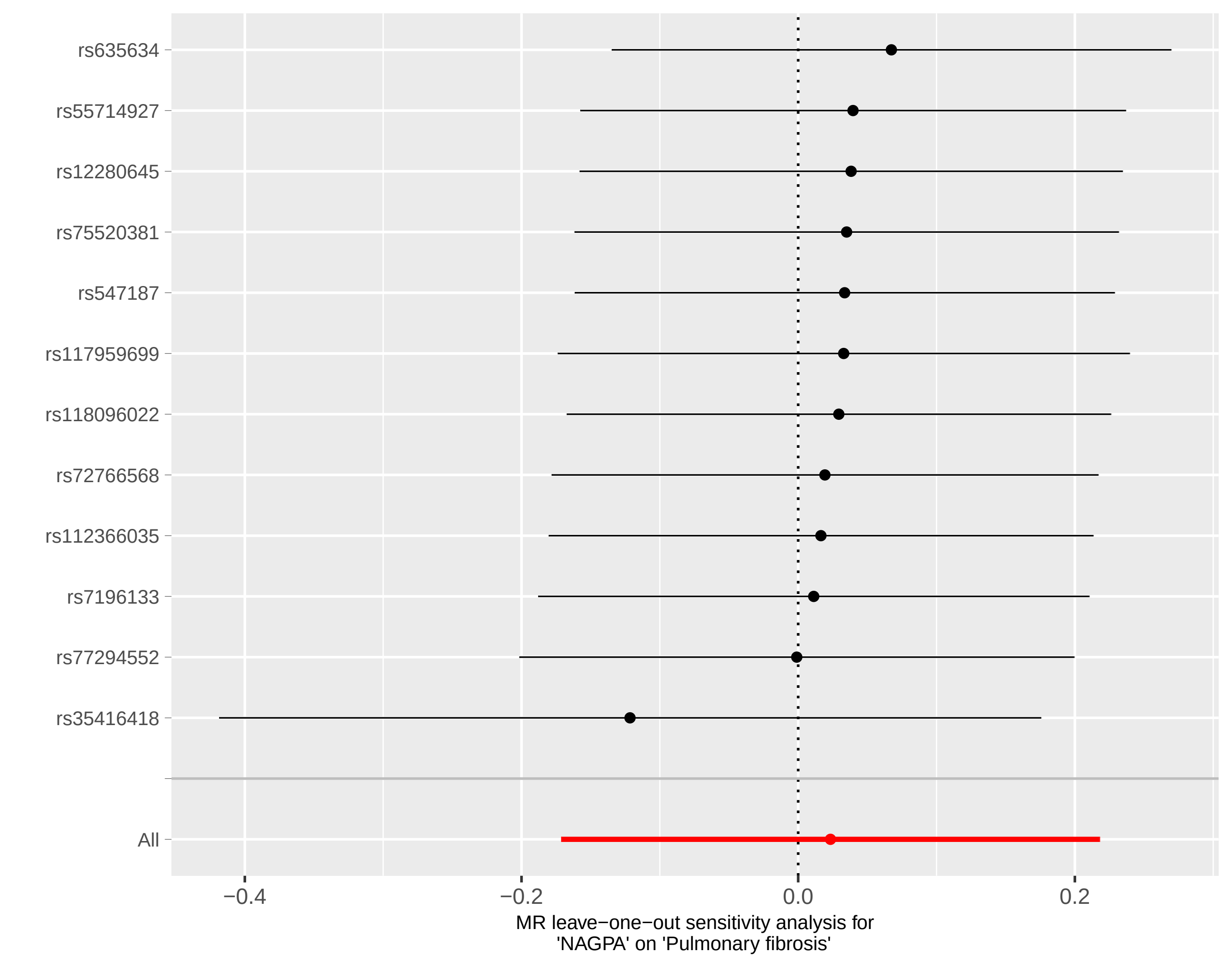
rs635634
rs55714927
rs12280645
rs75520381
rs547187
rs117959699
rs118096022
rs72766568
rs112366035
rs7196133
rs77294552
rs35416418
All
−0.4
−0.2
0.0
0.2
MR leave−one−out sensitivity analysis for
'NAGPA' on 'Pulmonary fibrosis'

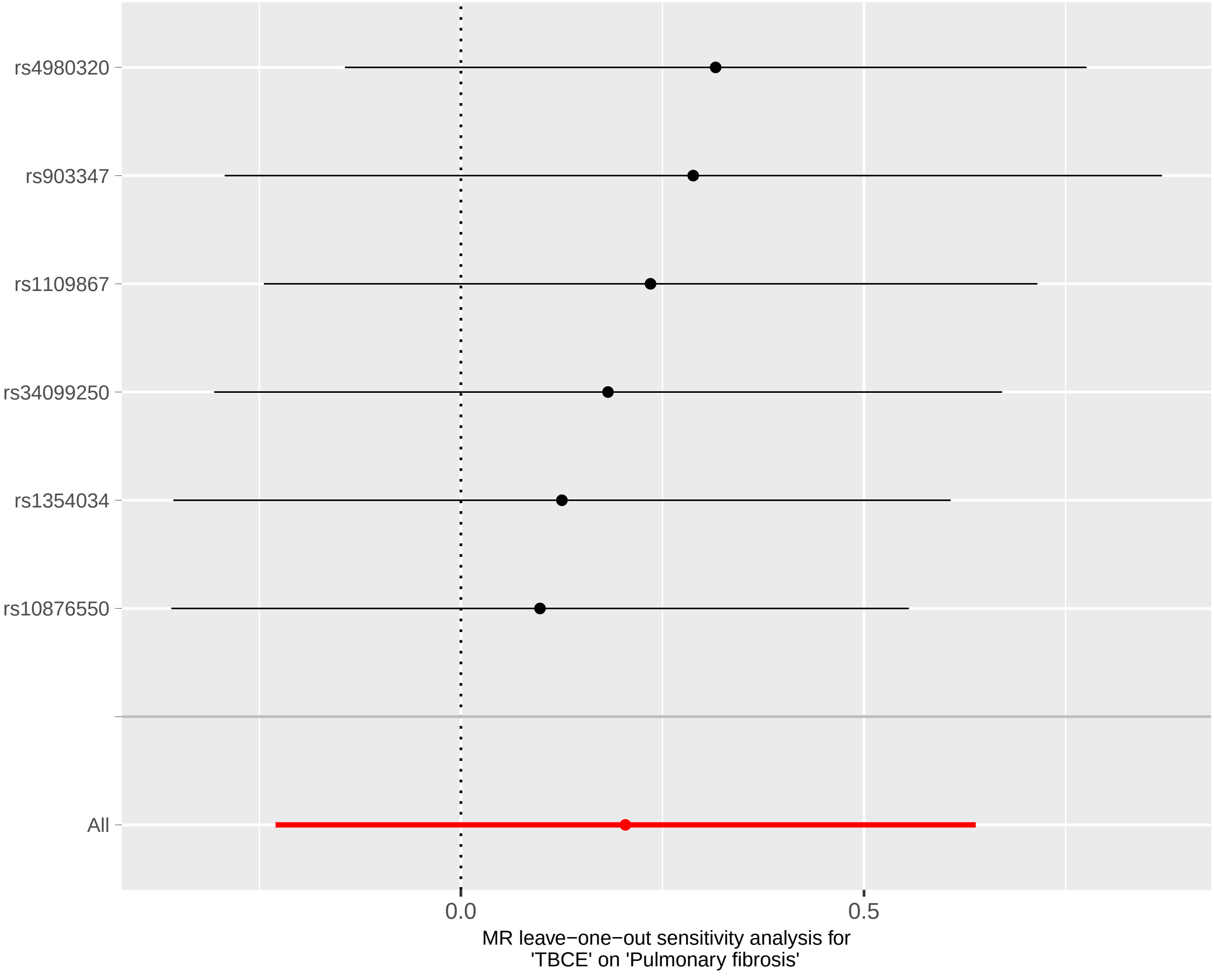
rs4980320
rs903347
rs1109867
rs34099250
rs1354034
rs10876550
All
0.0
0.5
MR leave−one−out sensitivity analysis for
'TBCE' on 'Pulmonary fibrosis'

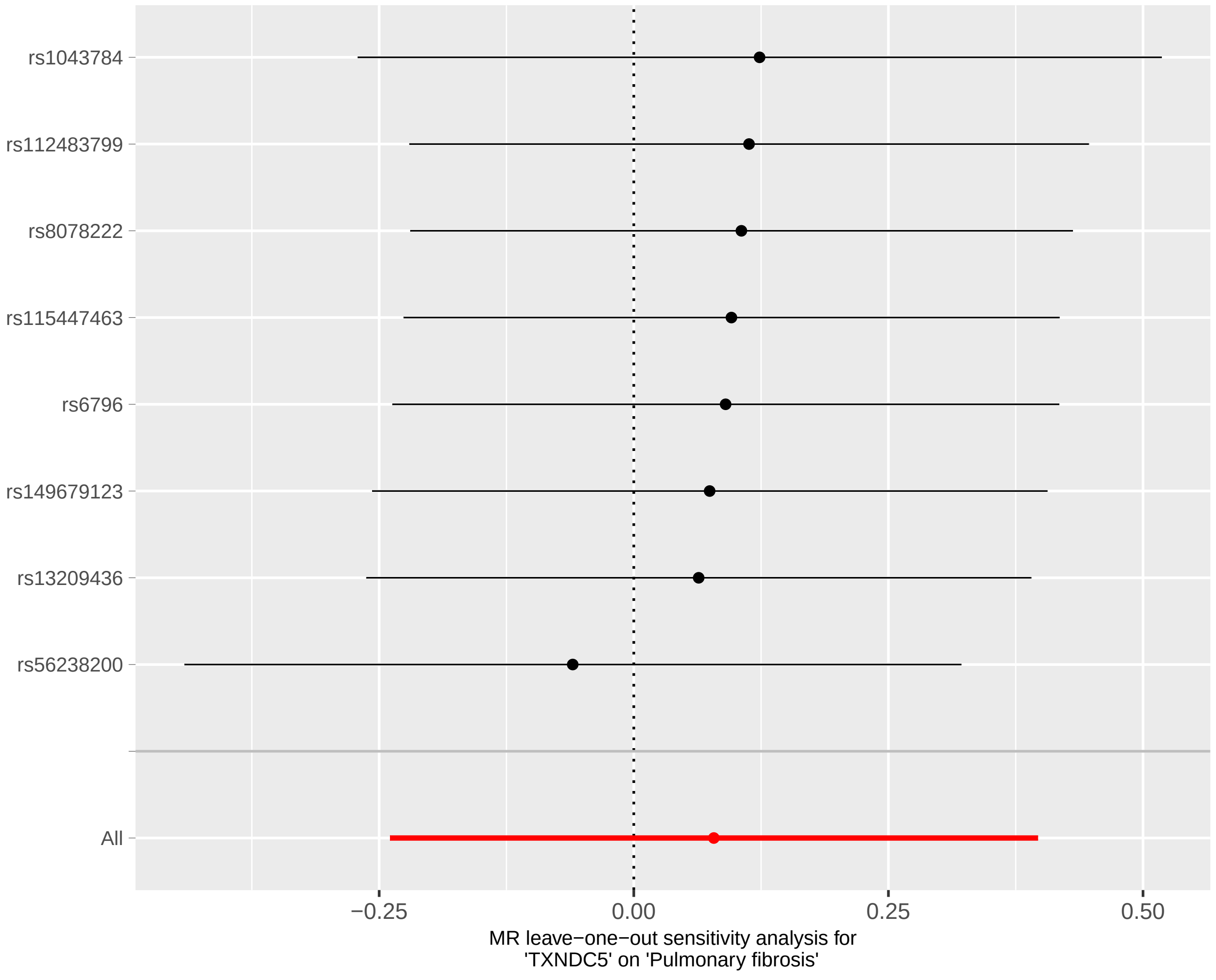
rs1043784
rs112483799
rs8078222
rs115447463
rs6796
rs149679123
rs13209436
rs56238200
All
−0.25
0.00
0.25
0.50
MR leave−one−out sensitivity analysis for
'TXNDC5' on 'Pulmonary fibrosis'

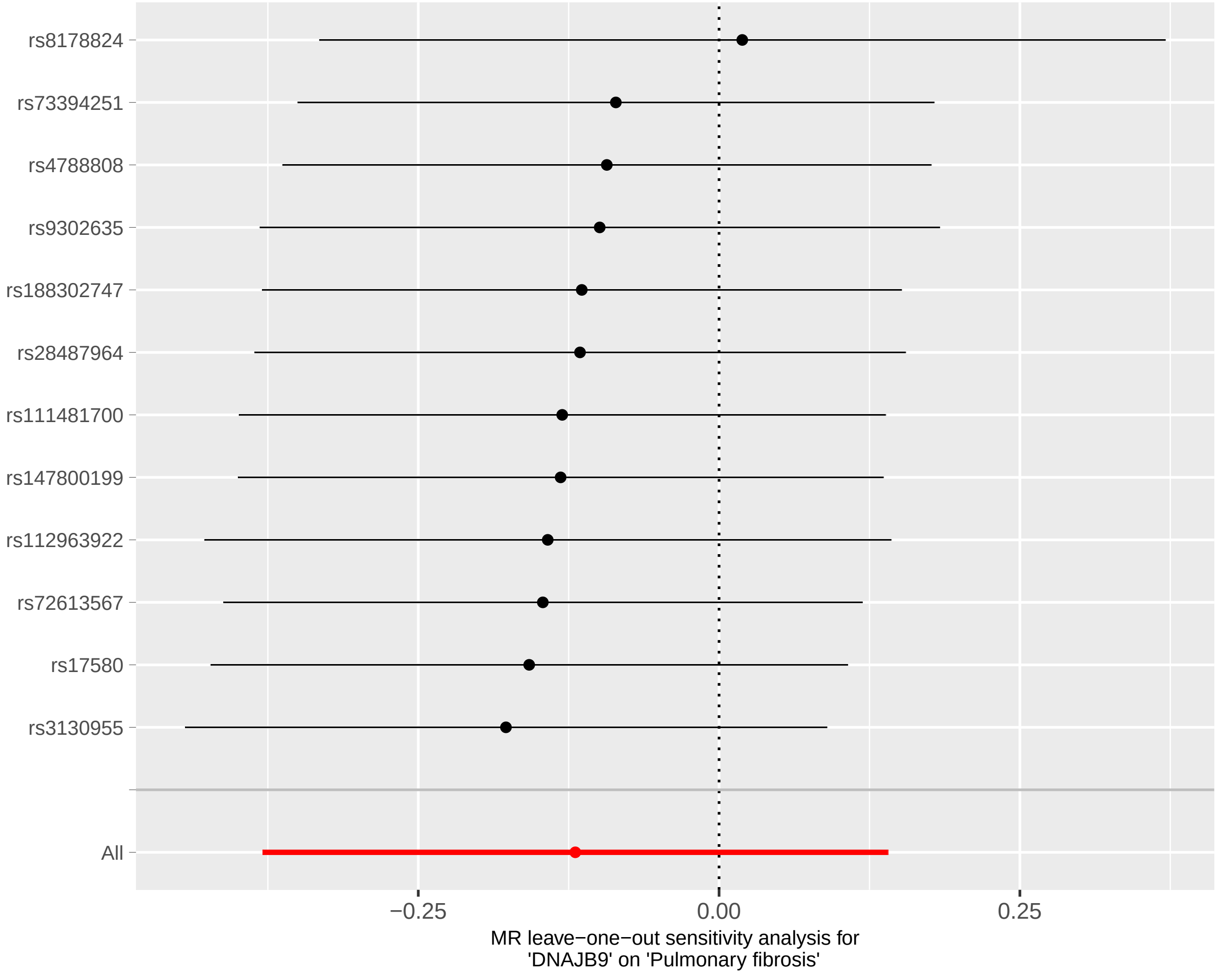
rs8178824
rs73394251
rs4788808
rs9302635
rs188302747
rs28487964
rs111481700
rs147800199
rs112963922
rs72613567
rs17580
rs3130955
All
−0.25
0.00
0.25
MR leave−one−out sensitivity analysis for
'DNAJB9' on 'Pulmonary fibrosis'

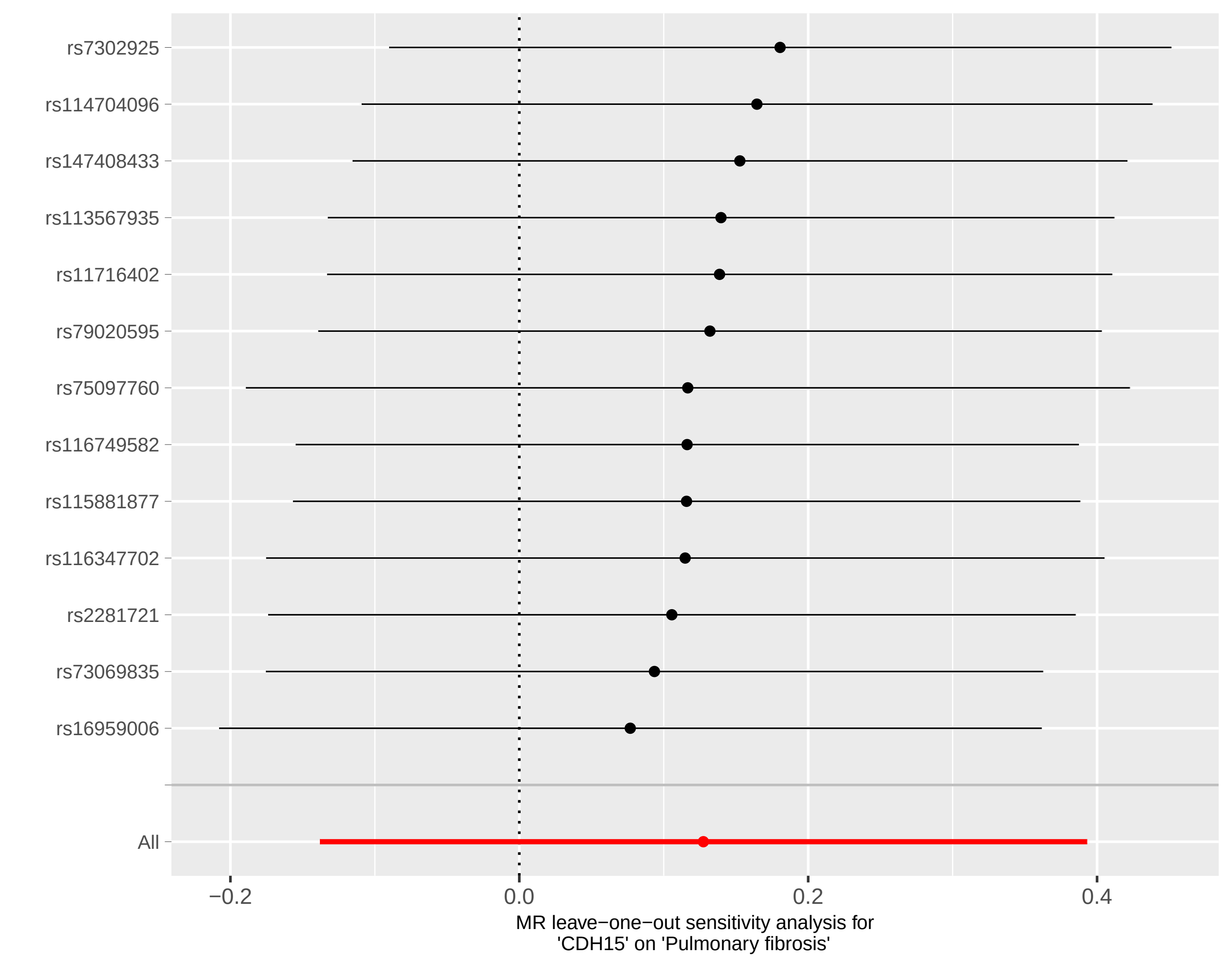

rs7302925
rs114704096
rs147408433
rs113567935
rs11716402
rs79020595
rs75097760
rs116749582
rs115881877
rs116347702
rs2281721
rs73069835
rs16959006
All
−0.2
0.0
0.2
0.4
MR leave−one−out sensitivity analysis for
'CDH15' on 'Pulmonary fibrosis'

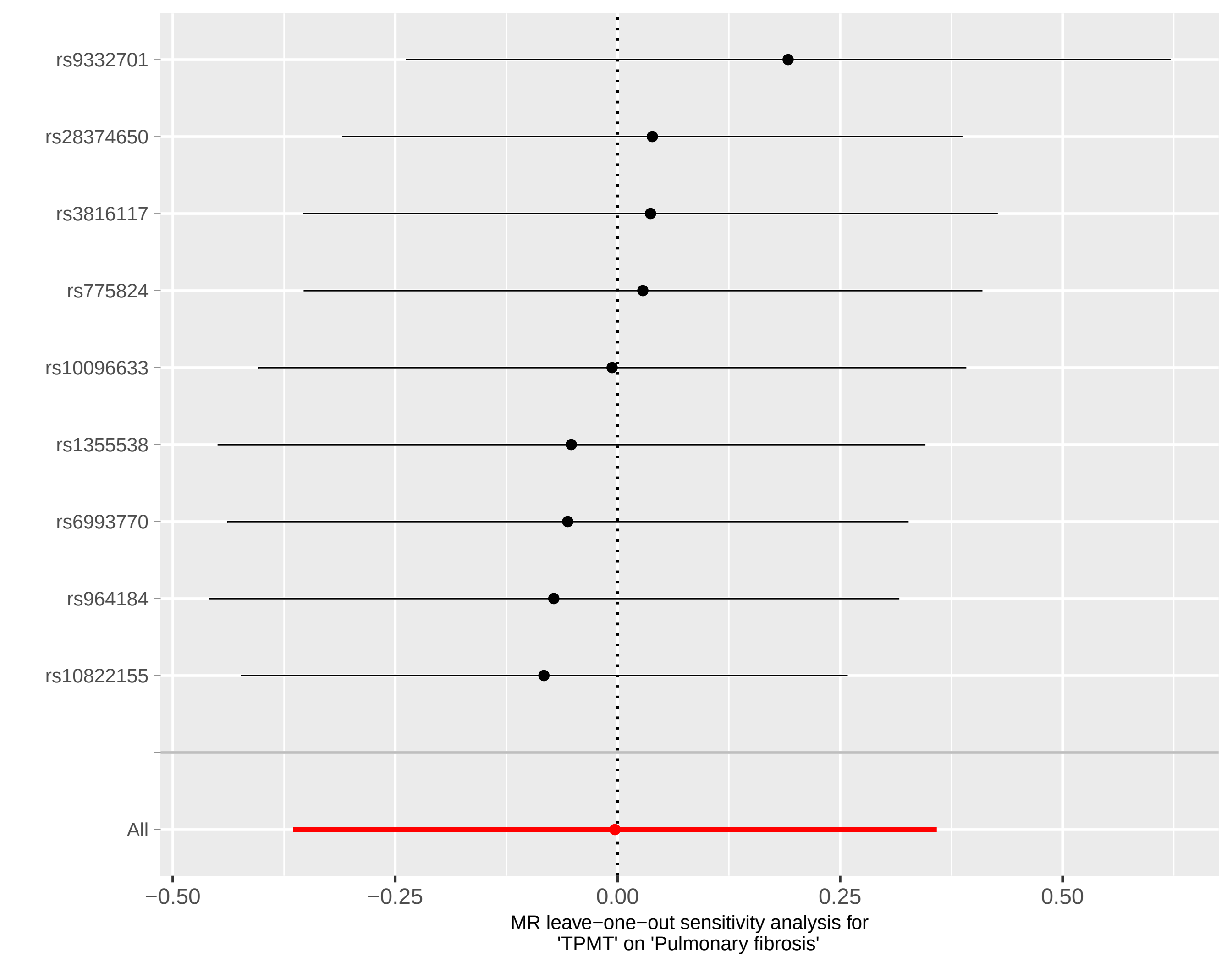
rs9332701
rs28374650
rs3816117
rs775824
rs10096633
rs1355538
rs6993770
rs964184
rs10822155
All
−0.50
−0.25
0.00
0.25
0.50
MR leave−one−out sensitivity analysis for
'TPMT' on 'Pulmonary fibrosis'

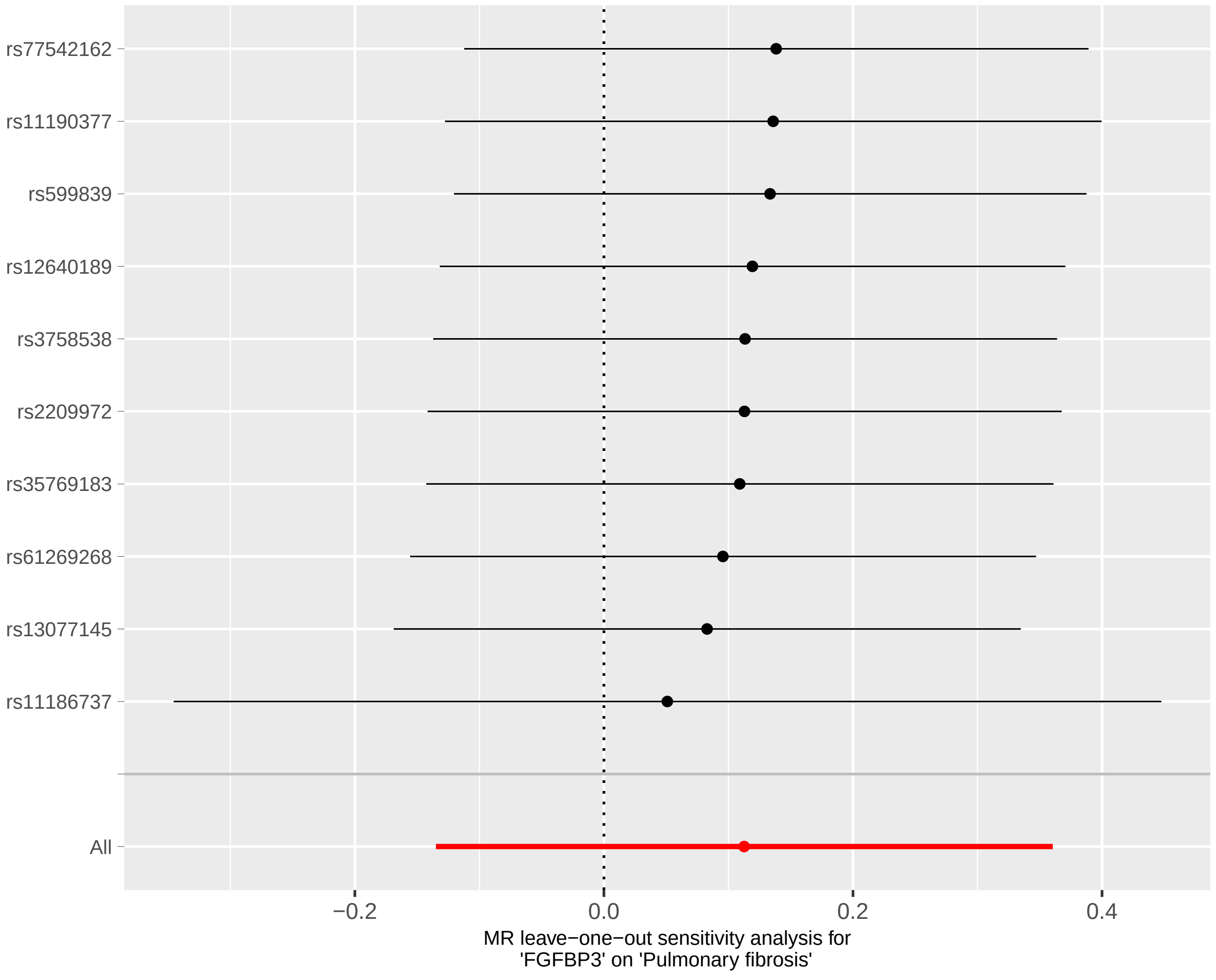
rs77542162
rs11190377
rs599839
rs12640189
rs3758538
rs2209972
rs35769183
rs61269268
rs13077145
rs11186737
All
−0.2
0.0
0.2
0.4
MR leave−one−out sensitivity analysis for
'FGFBP3' on 'Pulmonary fibrosis'

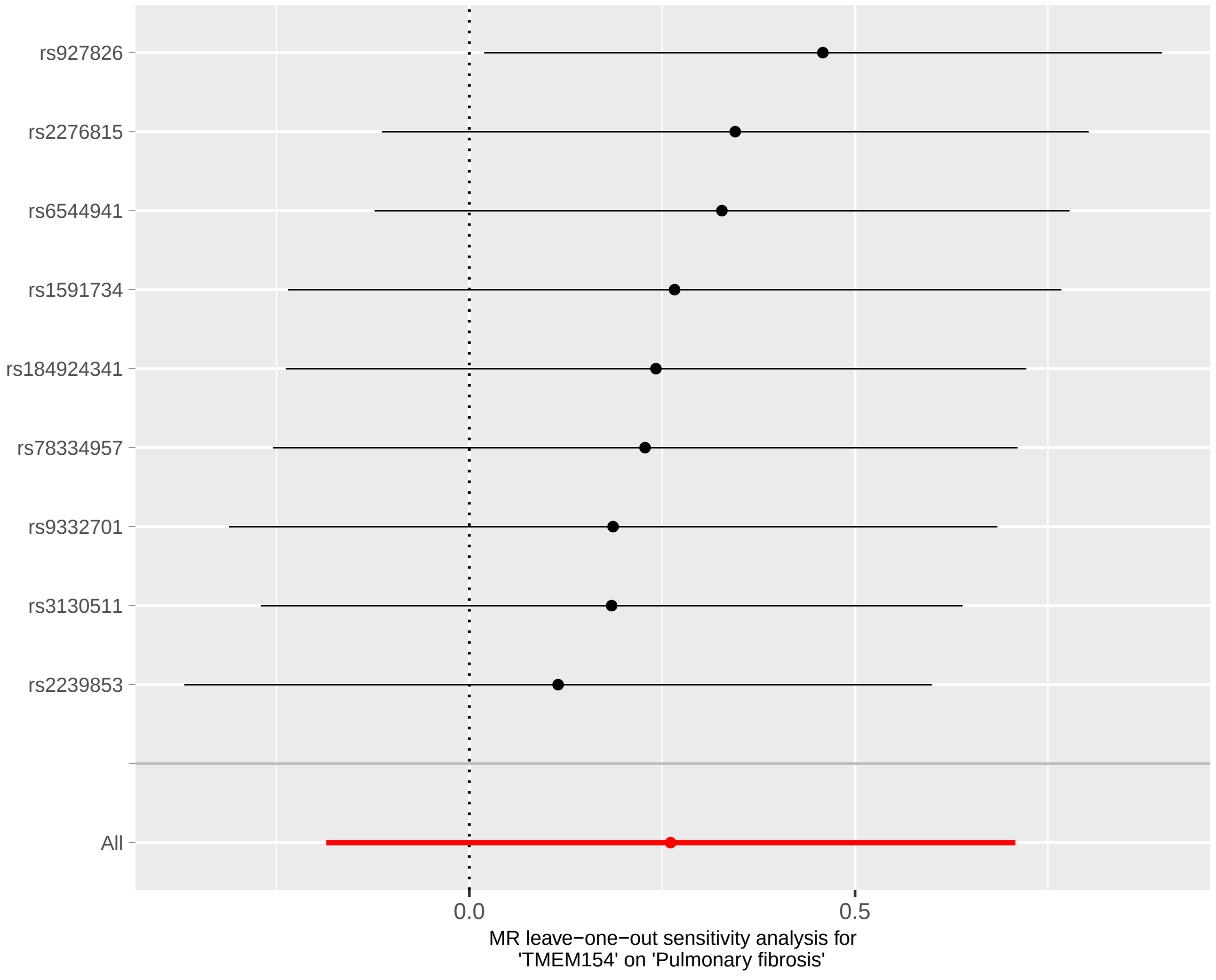
rs927826
rs2276815
rs6544941
rs1591734
rs184924341
rs78334957
rs9332701
rs3130511
rs2239853
All
0.0
0.5
MR leave−one−out sensitivity analysis for
'TMEM154' on 'Pulmonary fibrosis'

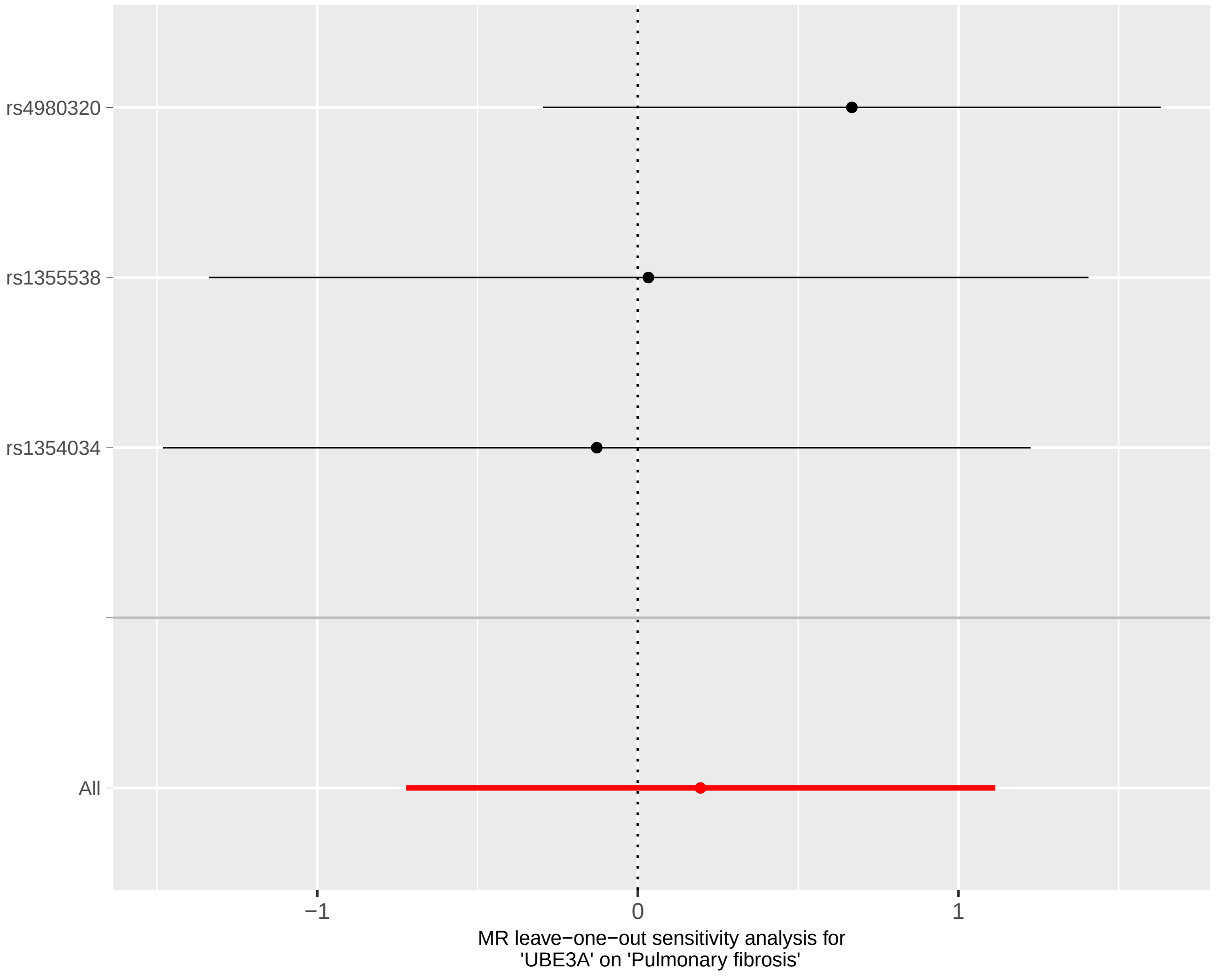
rs4980320
rs1355538
rs1354034
All
−1
0
1
MR leave−one−out sensitivity analysis for
'UBE3A' on 'Pulmonary fibrosis'

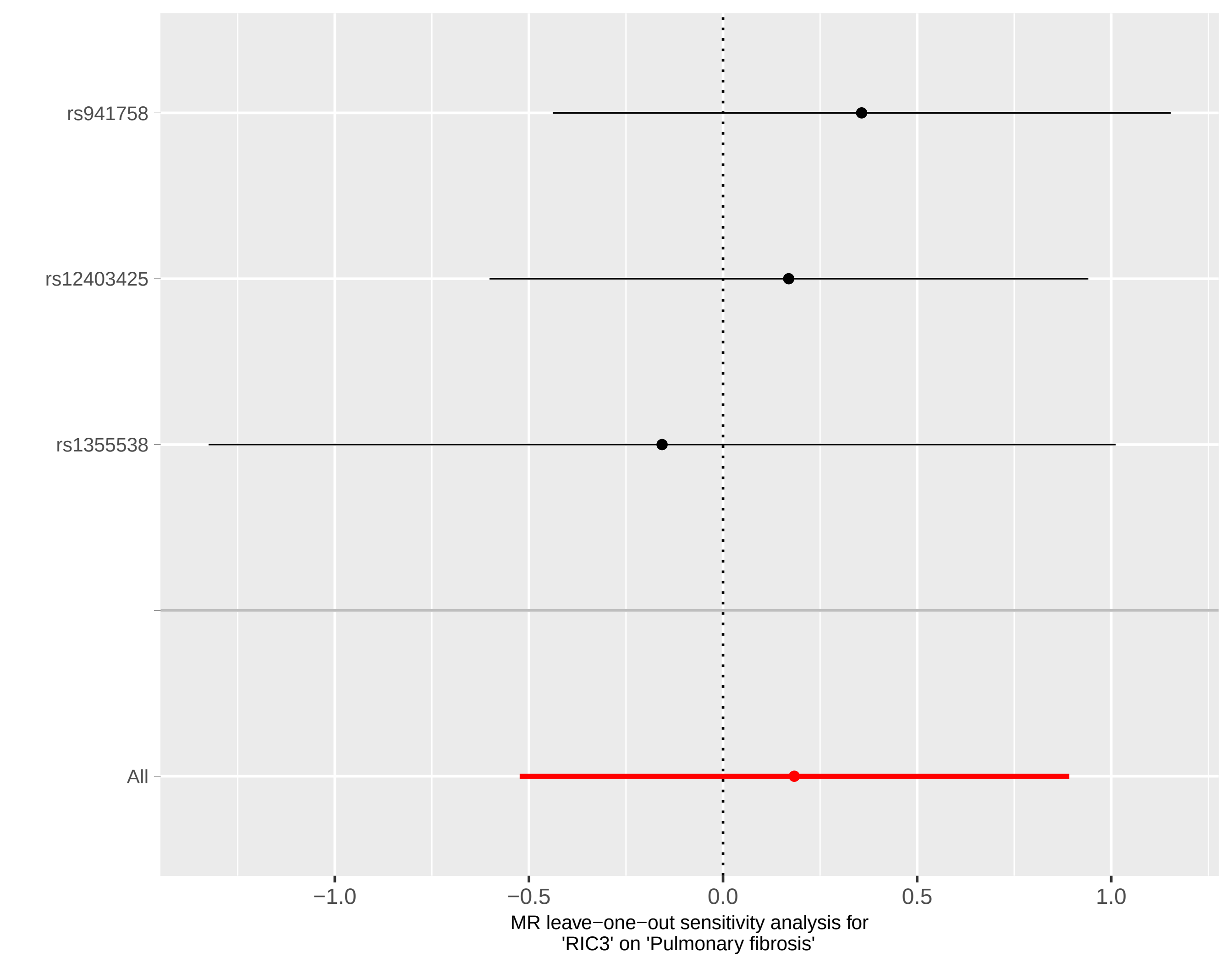
rs941758
rs12403425
rs1355538
All
−1.0
−0.5
0.0
0.5
1.0
MR leave−one−out sensitivity analysis for
'RIC3' on 'Pulmonary fibrosis'

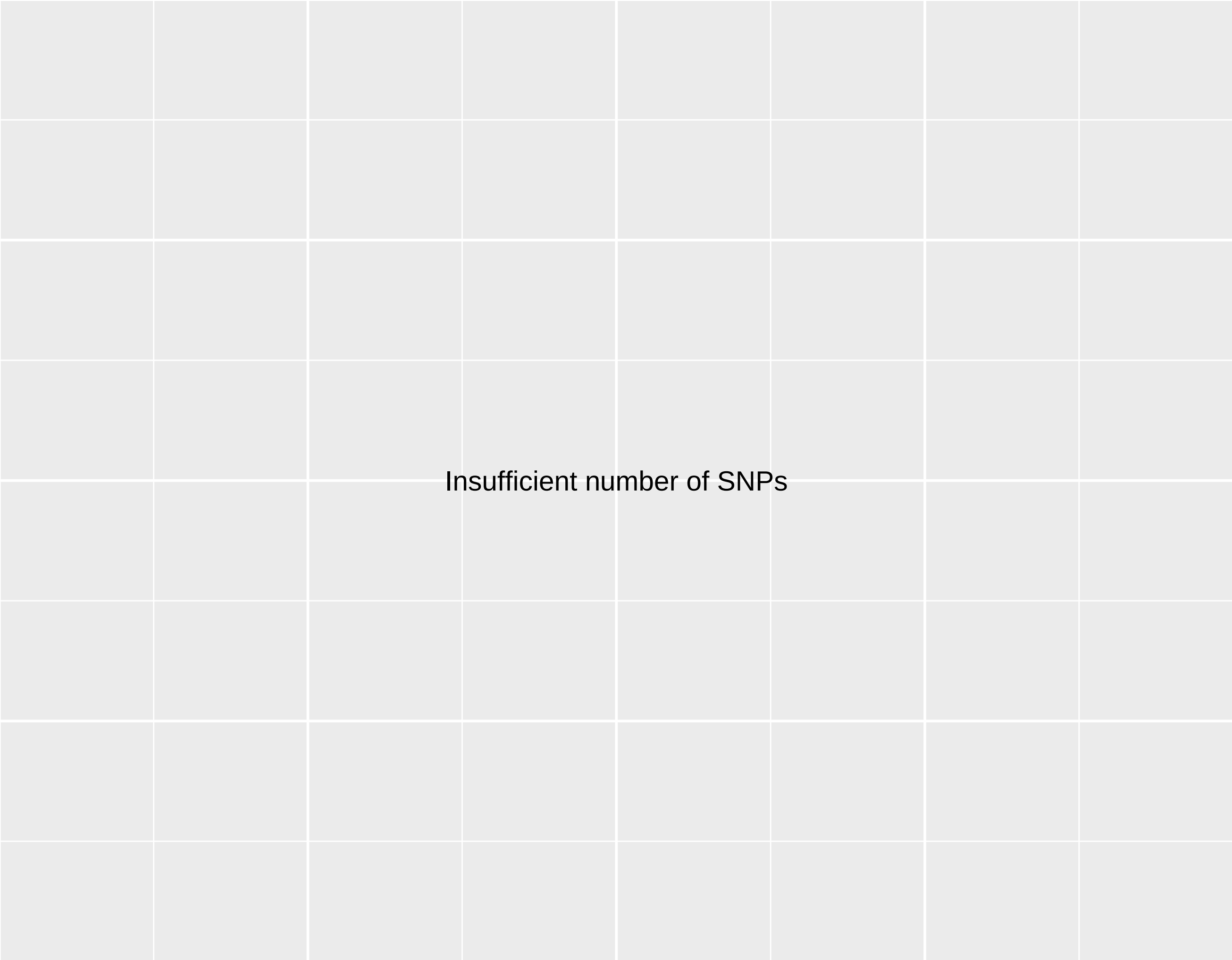
Insufficient number of SNPs

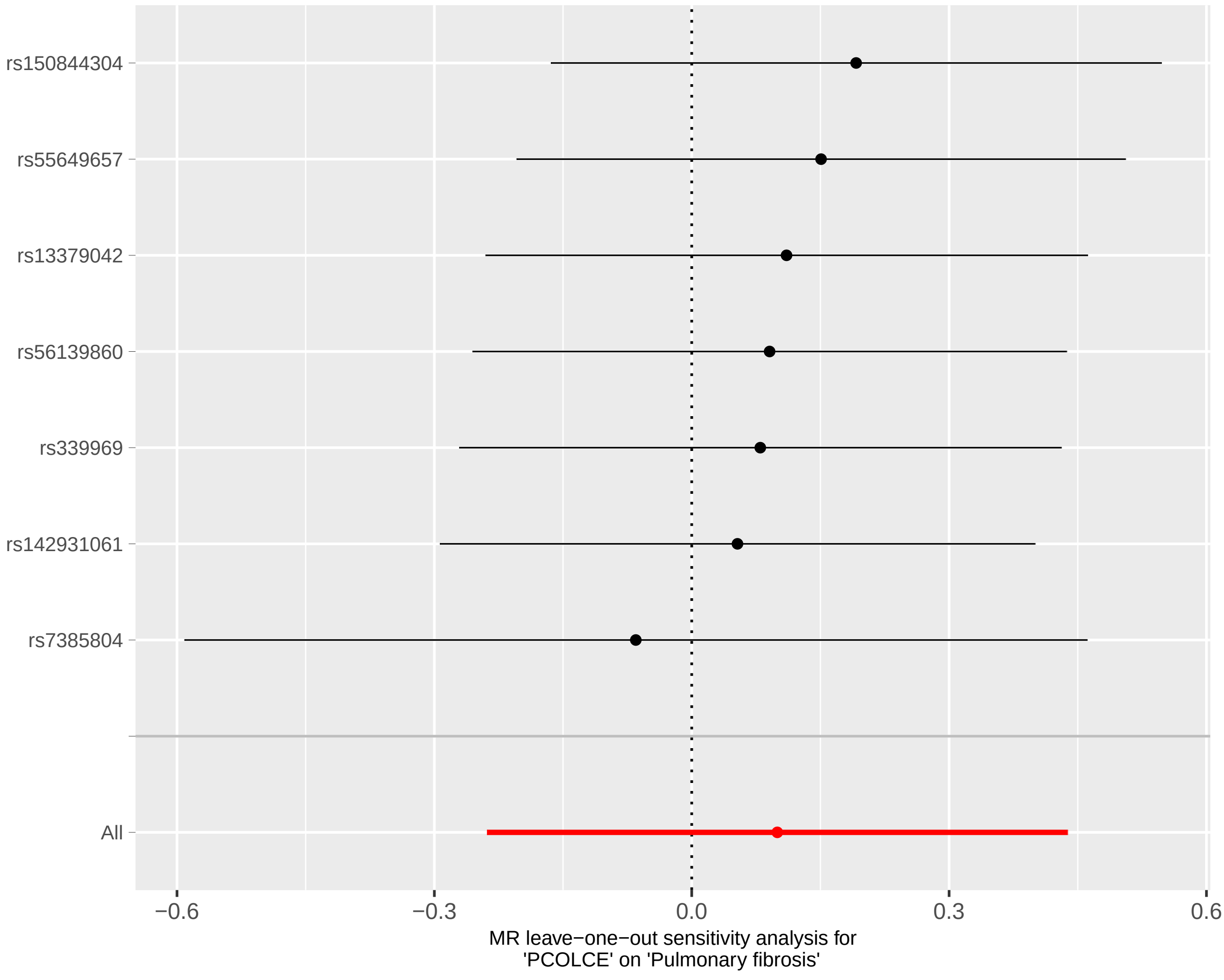

rs150844304
rs55649657
rs13379042
rs56139860
rs339969
rs142931061
rs7385804
All
−0.6
−0.3
0.0
0.3
0.6
MR leave−one−out sensitivity analysis for
'PCOLCE' on 'Pulmonary fibrosis'

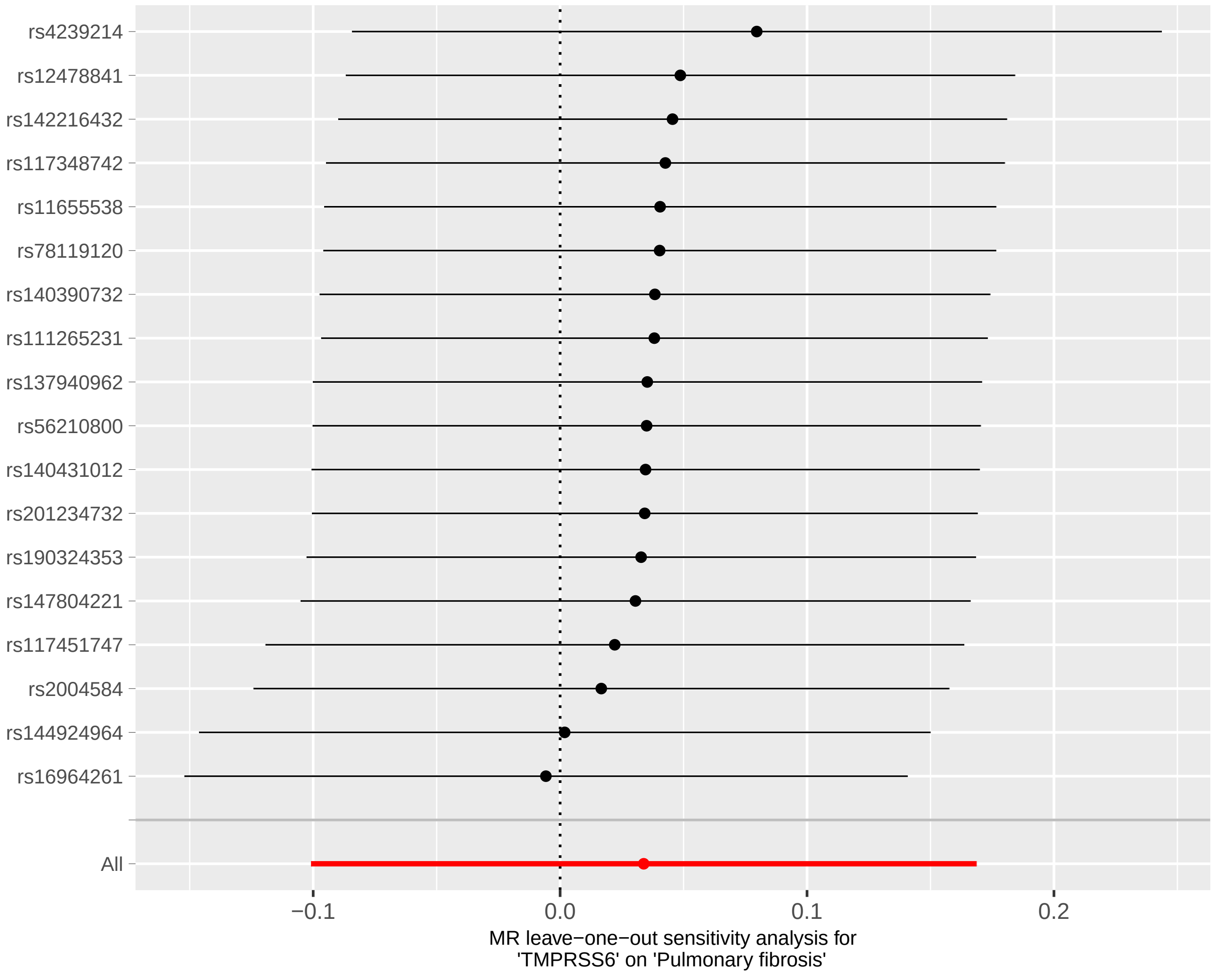
rs4239214
rs12478841
rs142216432
rs117348742
rs11655538
rs78119120
rs140390732
rs111265231
rs137940962
rs56210800
rs140431012
rs201234732
rs190324353
rs147804221
rs117451747
rs2004584
rs144924964
rs16964261
All
−0.1
0.0
0.1
0.2
MR leave−one−out sensitivity analysis for
'TMPRSS6' on 'Pulmonary fibrosis'

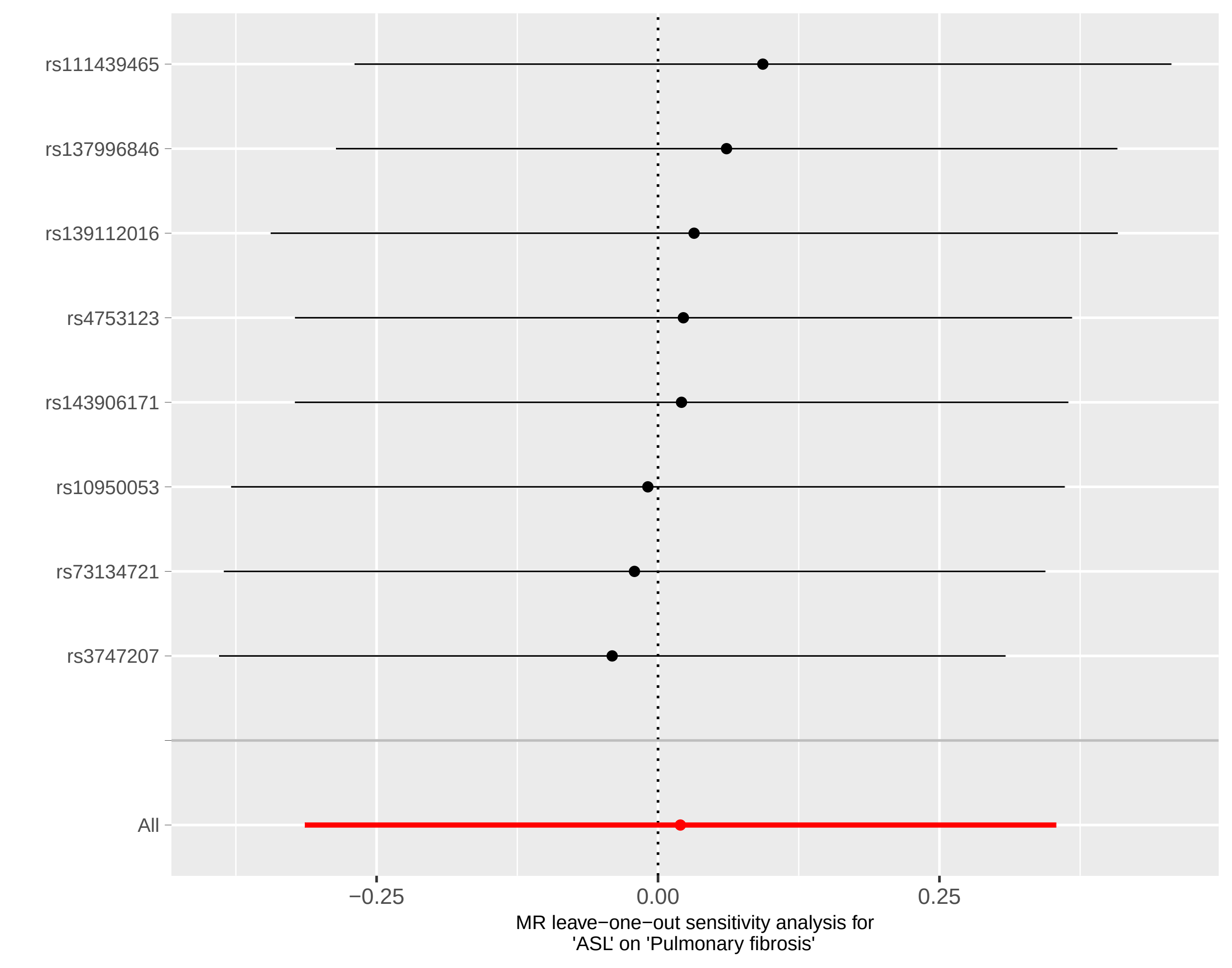

rs111439465
rs137996846
rs139112016
rs4753123
rs143906171
rs10950053
rs73134721
rs3747207
All
−0.25
0.00
0.25
MR leave−one−out sensitivity analysis for
'ASL' on 'Pulmonary fibrosis'

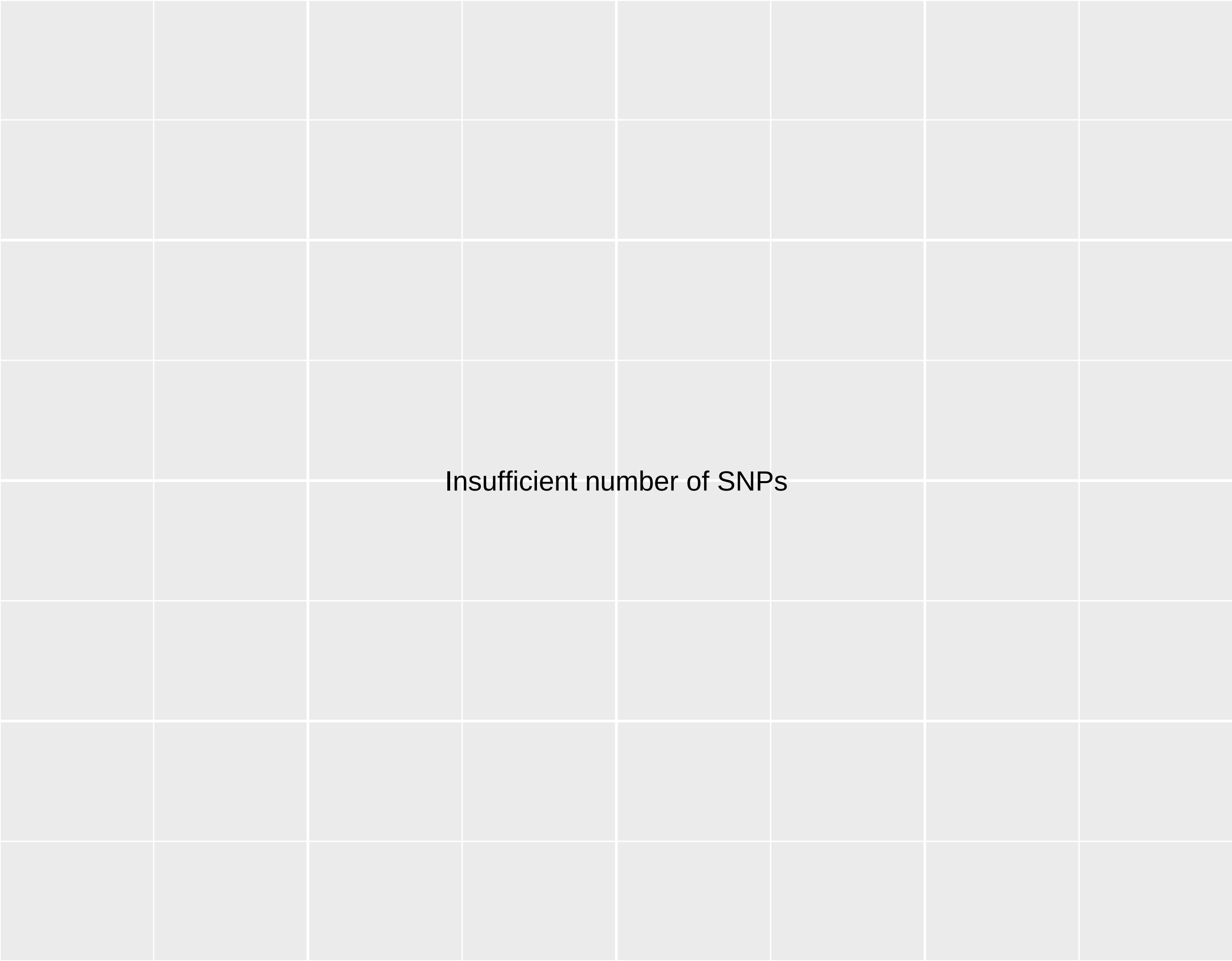
Insufficient number of SNPs

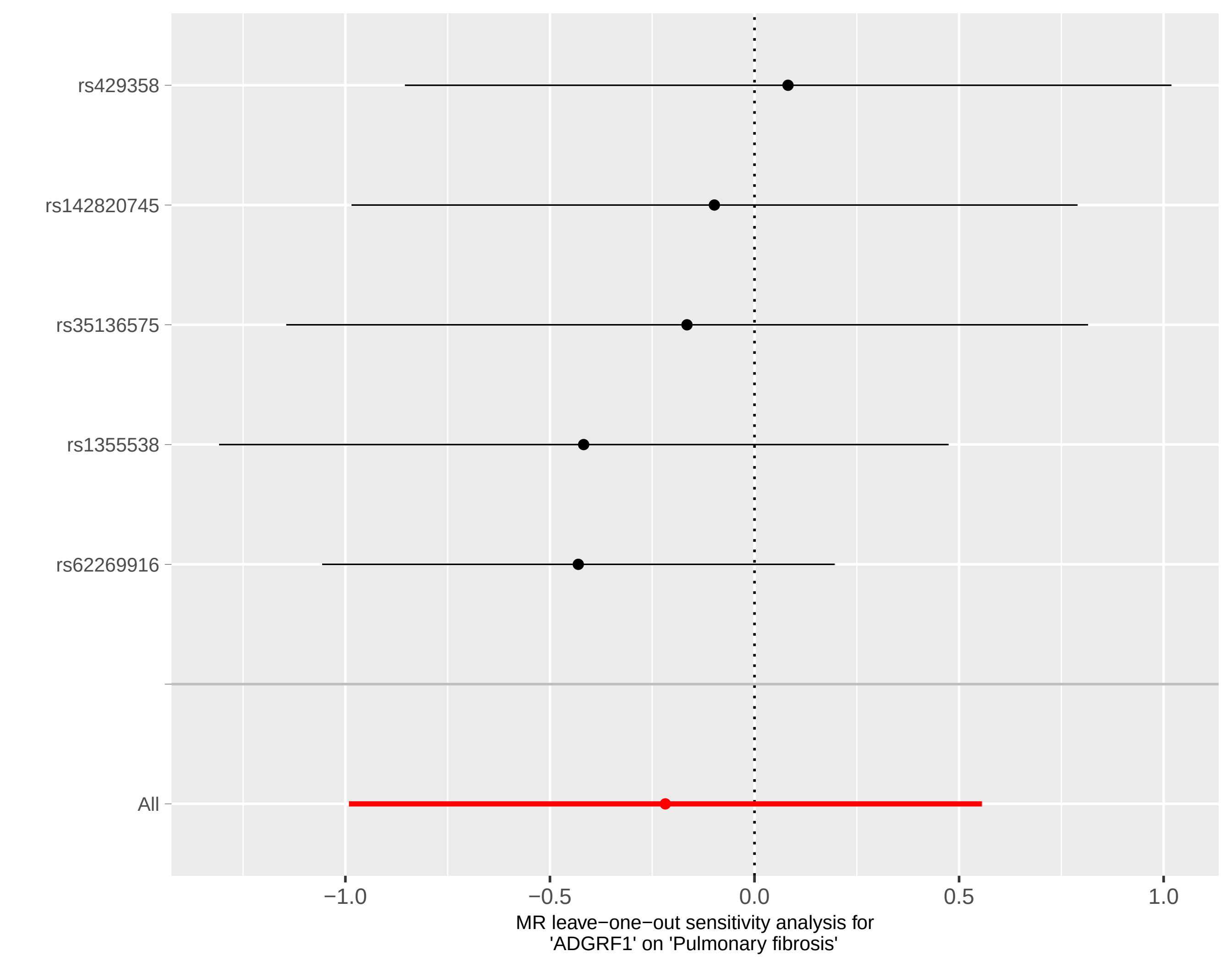
rs429358
rs142820745
rs35136575
rs1355538
rs62269916
All
−1.0
−0.5
0.0
0.5
1.0
MR leave−one−out sensitivity analysis for
'ADGRF1' on 'Pulmonary fibrosis'

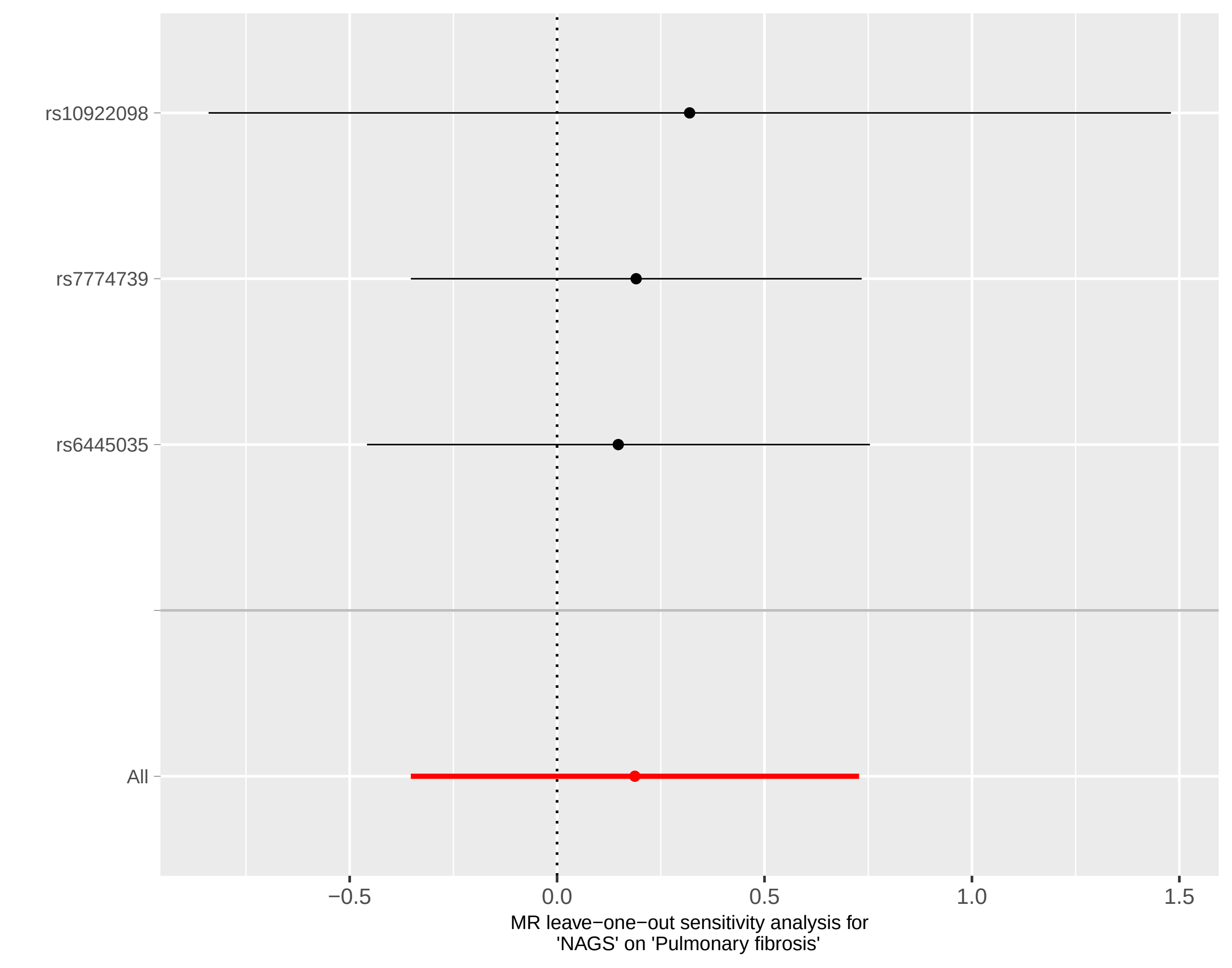
rs10922098
rs7774739
rs6445035
All
−0.5
0.0
0.5
1.0
1.5
MR leave−one−out sensitivity analysis for
'NAGS' on 'Pulmonary fibrosis'

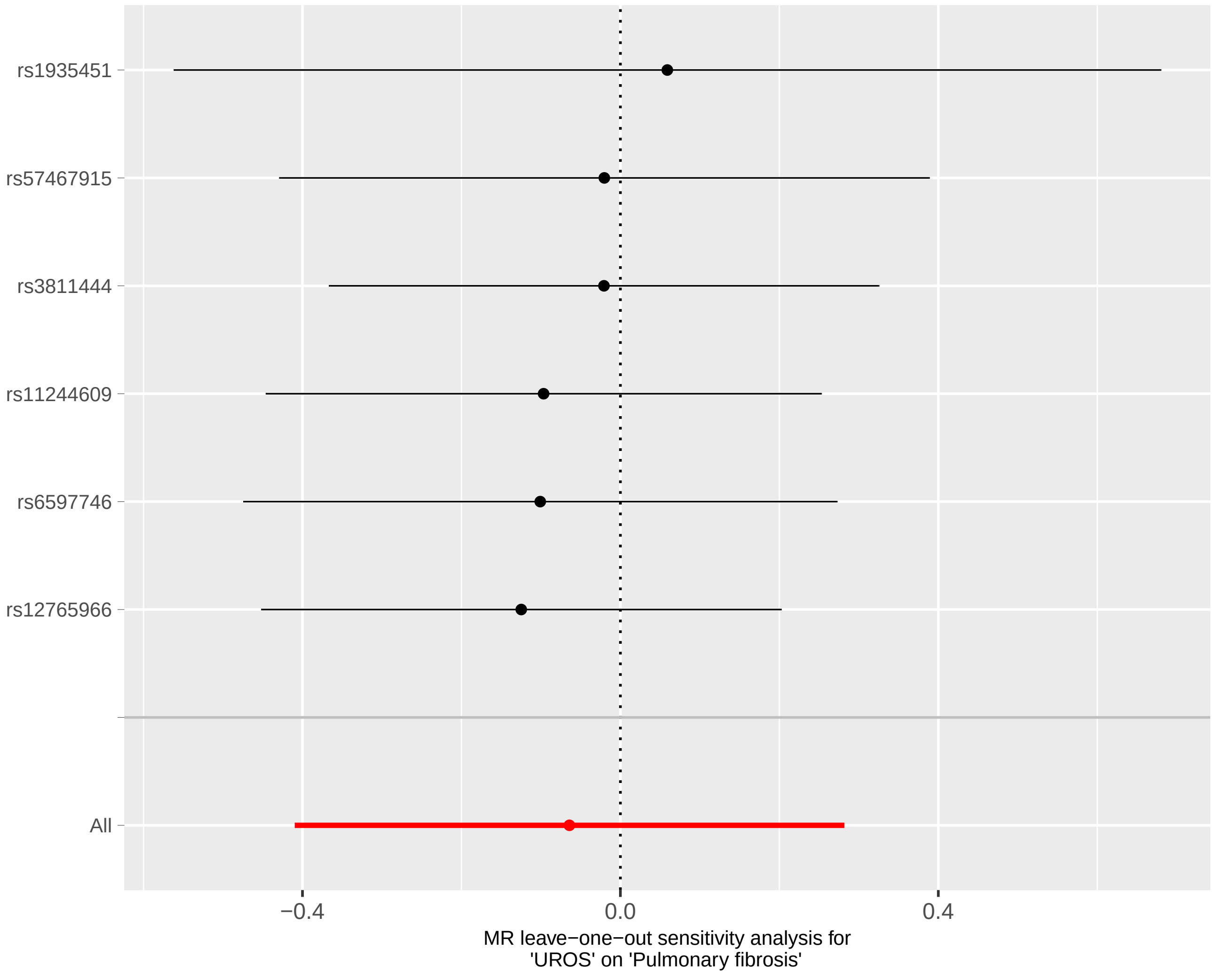
rs1935451
rs57467915
rs3811444
rs11244609
rs6597746
rs12765966
All
−0.4
0.0
0.4
MR leave−one−out sensitivity analysis for
'UROS' on 'Pulmonary fibrosis'

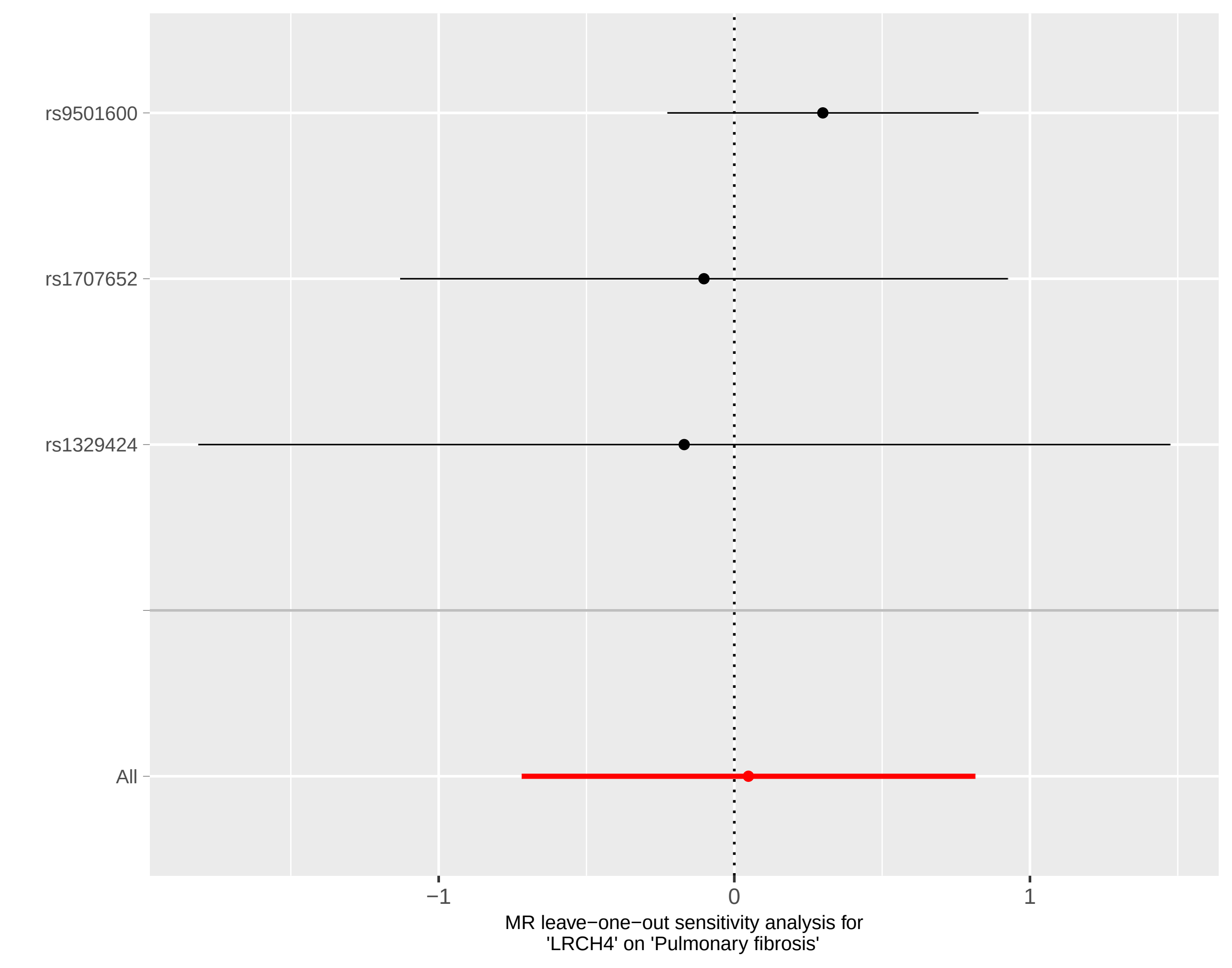
rs9501600
rs1707652
rs1329424
All
−1
0
1
MR leave−one−out sensitivity analysis for
'LRCH4' on 'Pulmonary fibrosis'

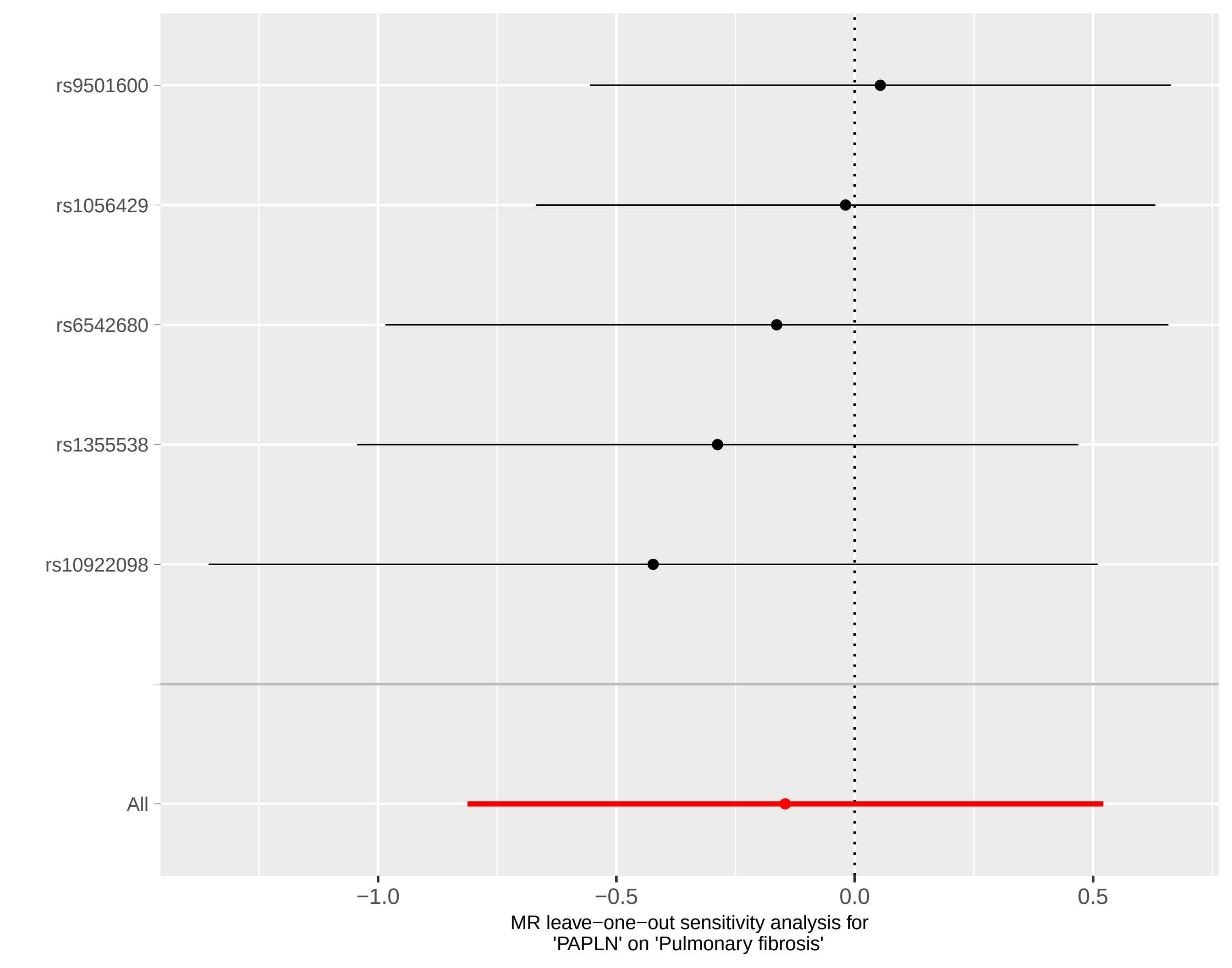
rs9501600
rs1056429
rs6542680
rs1355538
rs10922098
All
−1.0
−0.5
0.0
0.5
MR leave−one−out sensitivity analysis for
'PAPLN' on 'Pulmonary fibrosis'

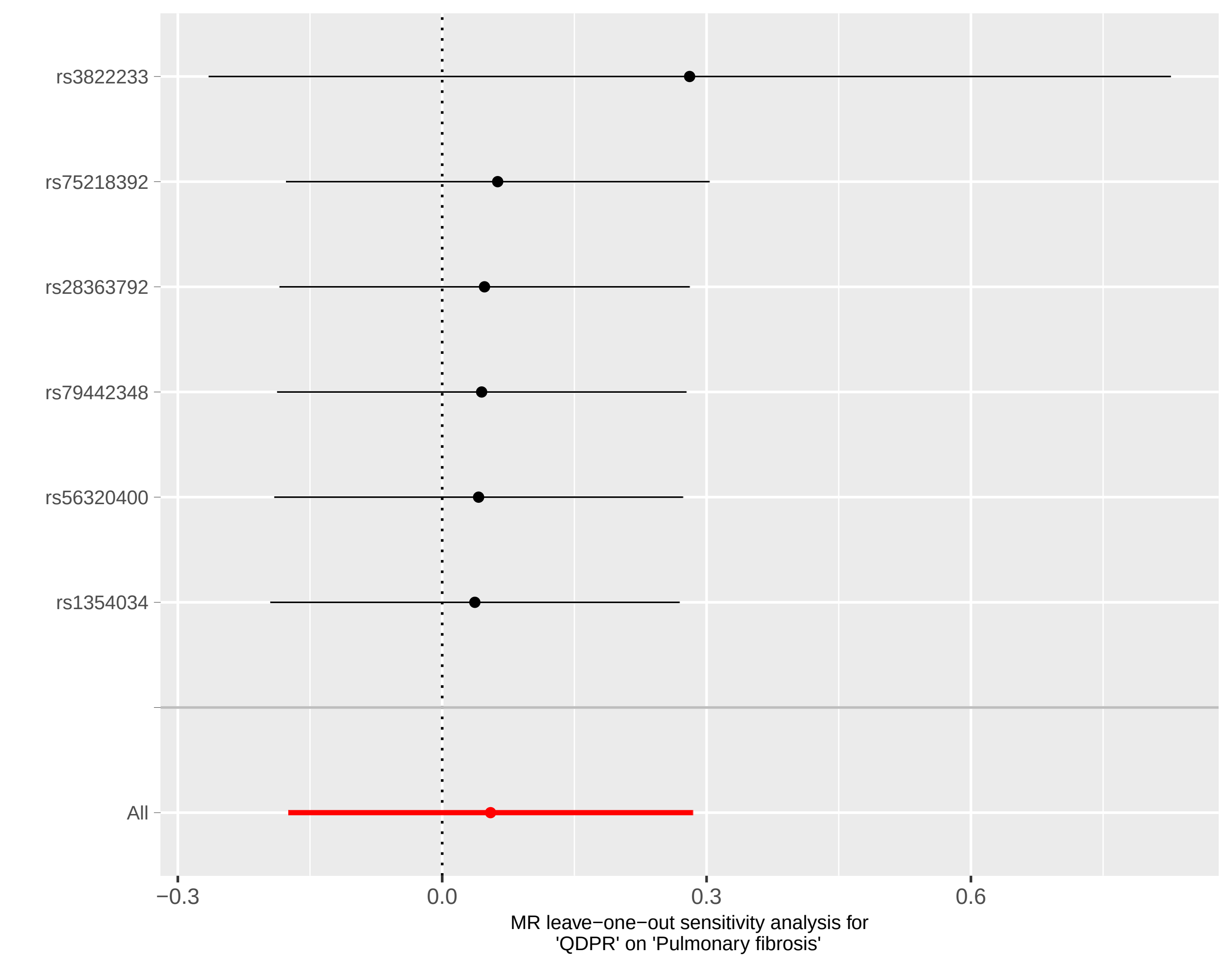
rs3822233
rs75218392
rs28363792
rs79442348
rs56320400
rs1354034
All
−0.3
0.0
0.3
0.6
MR leave−one−out sensitivity analysis for
'QDPR' on 'Pulmonary fibrosis'

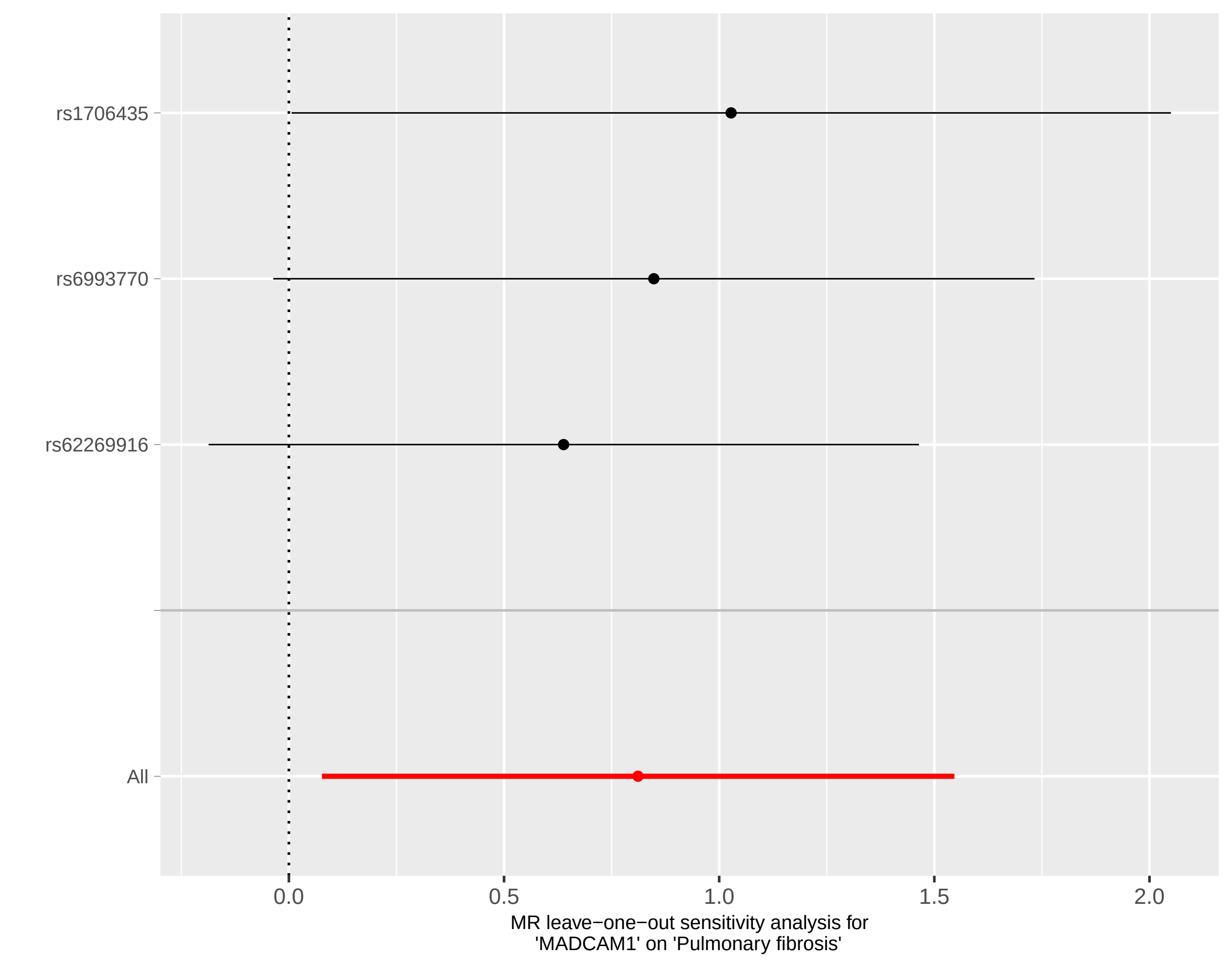
rs1706435
rs6993770
rs62269916
All
0.0
0.5
1.0
1.5
2.0
MR leave−one−out sensitivity analysis for
'MADCAM1' on 'Pulmonary fibrosis'

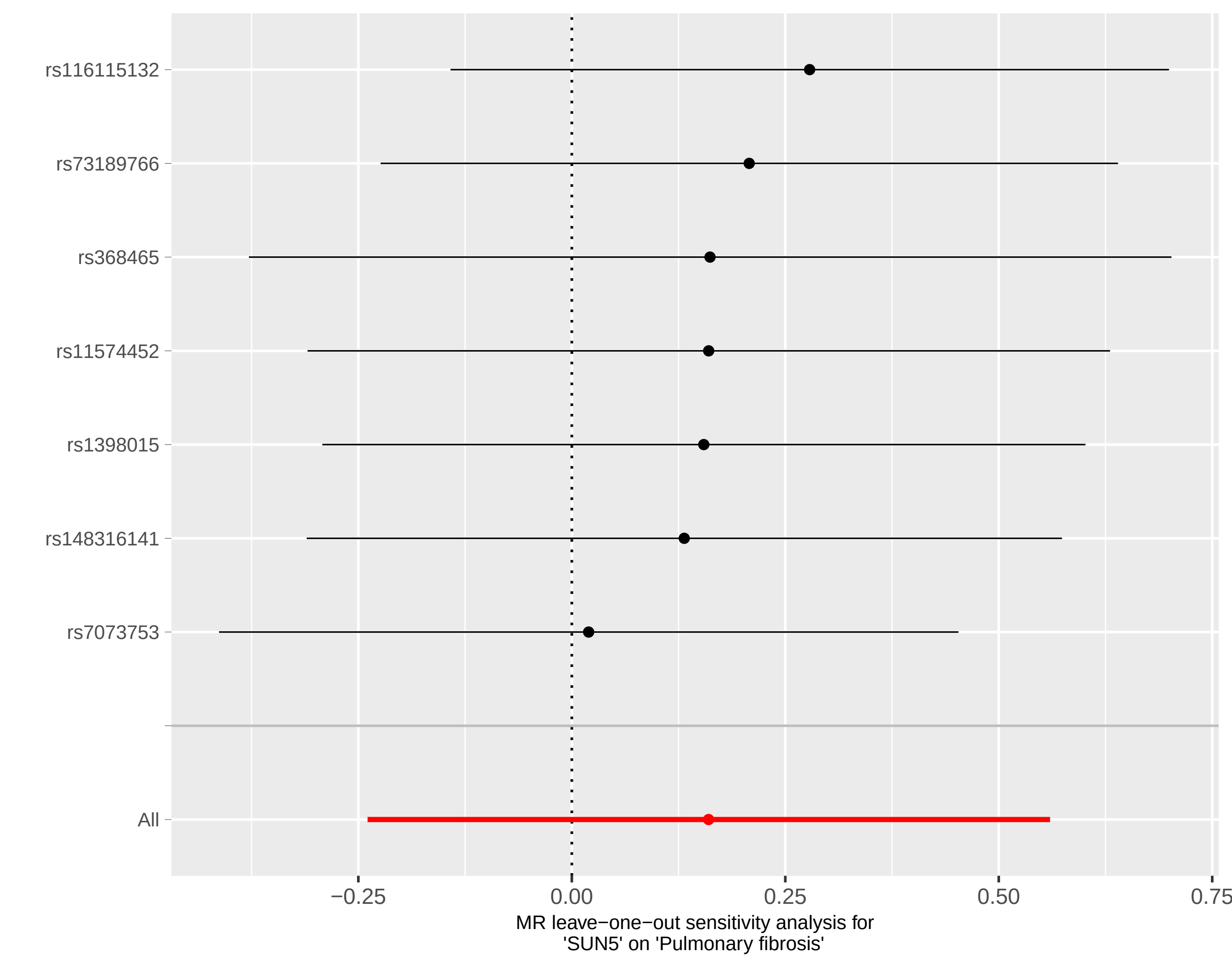
rs116115132
rs73189766
rs368465
rs11574452
rs1398015
rs148316141
rs7073753
All
−0.25
0.00
0.25
0.50
0.75
MR leave−one−out sensitivity analysis for
'SUN5' on 'Pulmonary fibrosis'

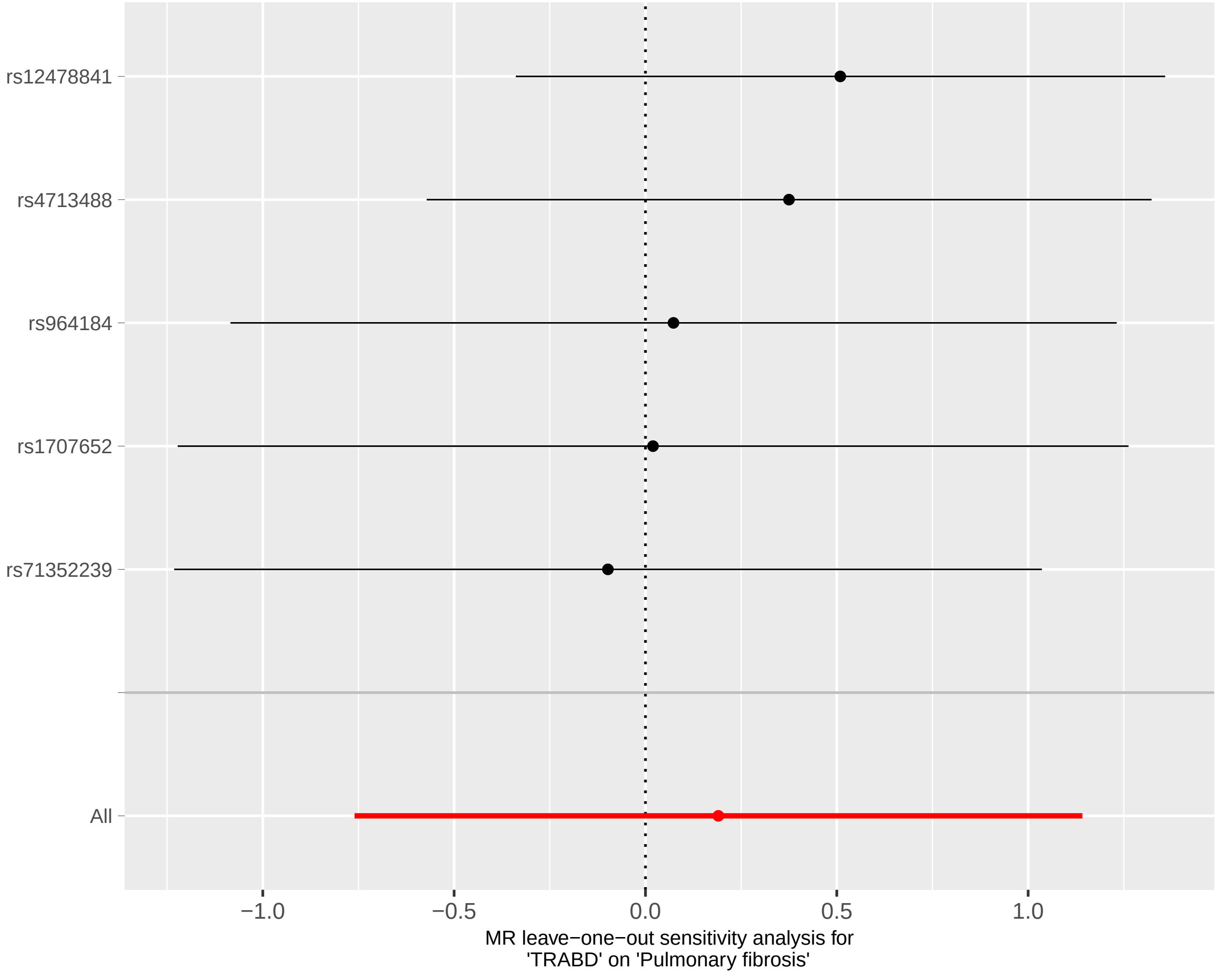
rs12478841
rs4713488
rs964184
rs1707652
rs71352239
All
−1.0
−0.5
0.0
0.5
1.0
MR leave−one−out sensitivity analysis for
'TRABD' on 'Pulmonary fibrosis'

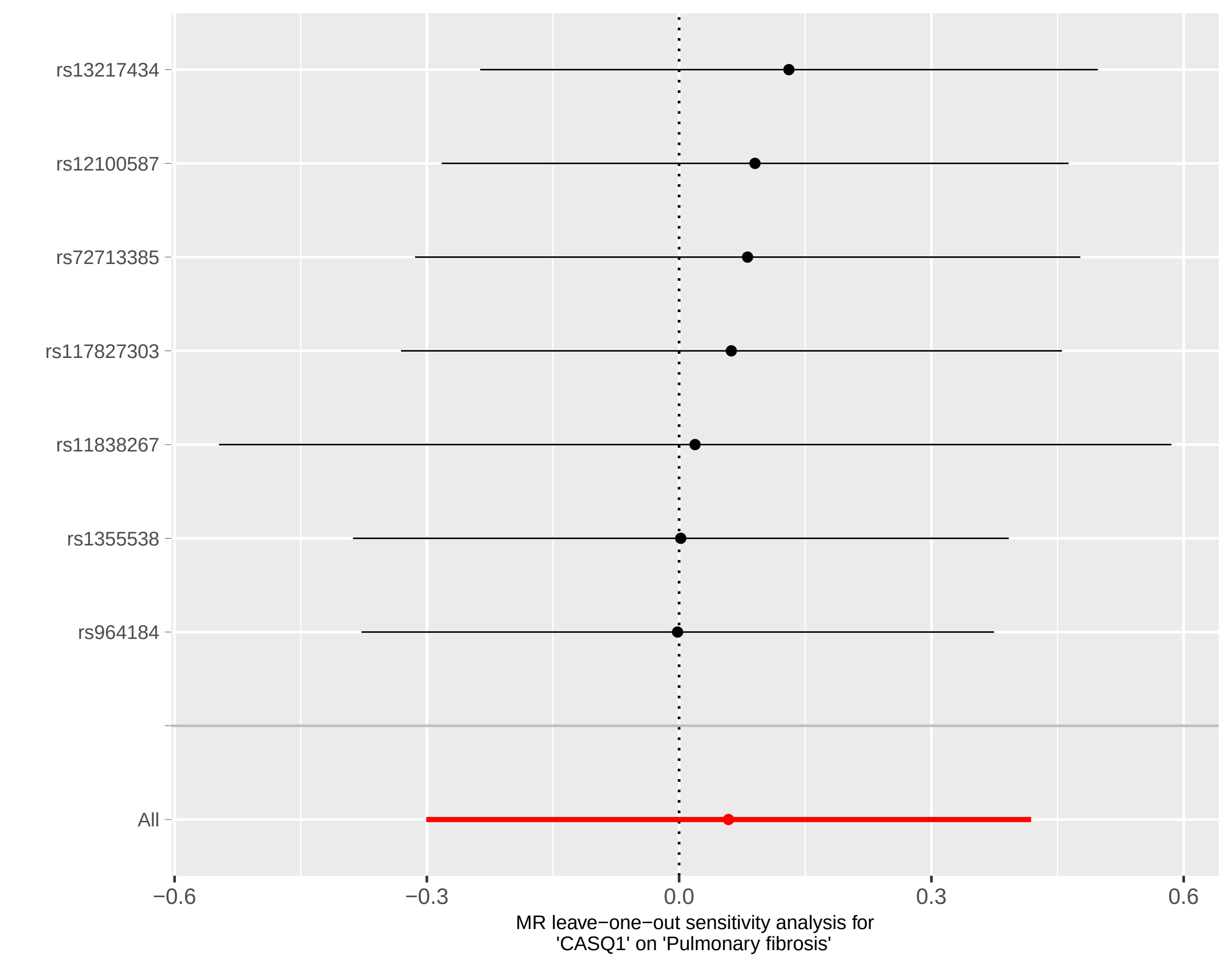
rs13217434
rs12100587
rs72713385
rs117827303
rs11838267
rs1355538
rs964184
All
−0.6
−0.3
0.0
0.3
0.6
MR leave−one−out sensitivity analysis for
'CASQ1' on 'Pulmonary fibrosis'

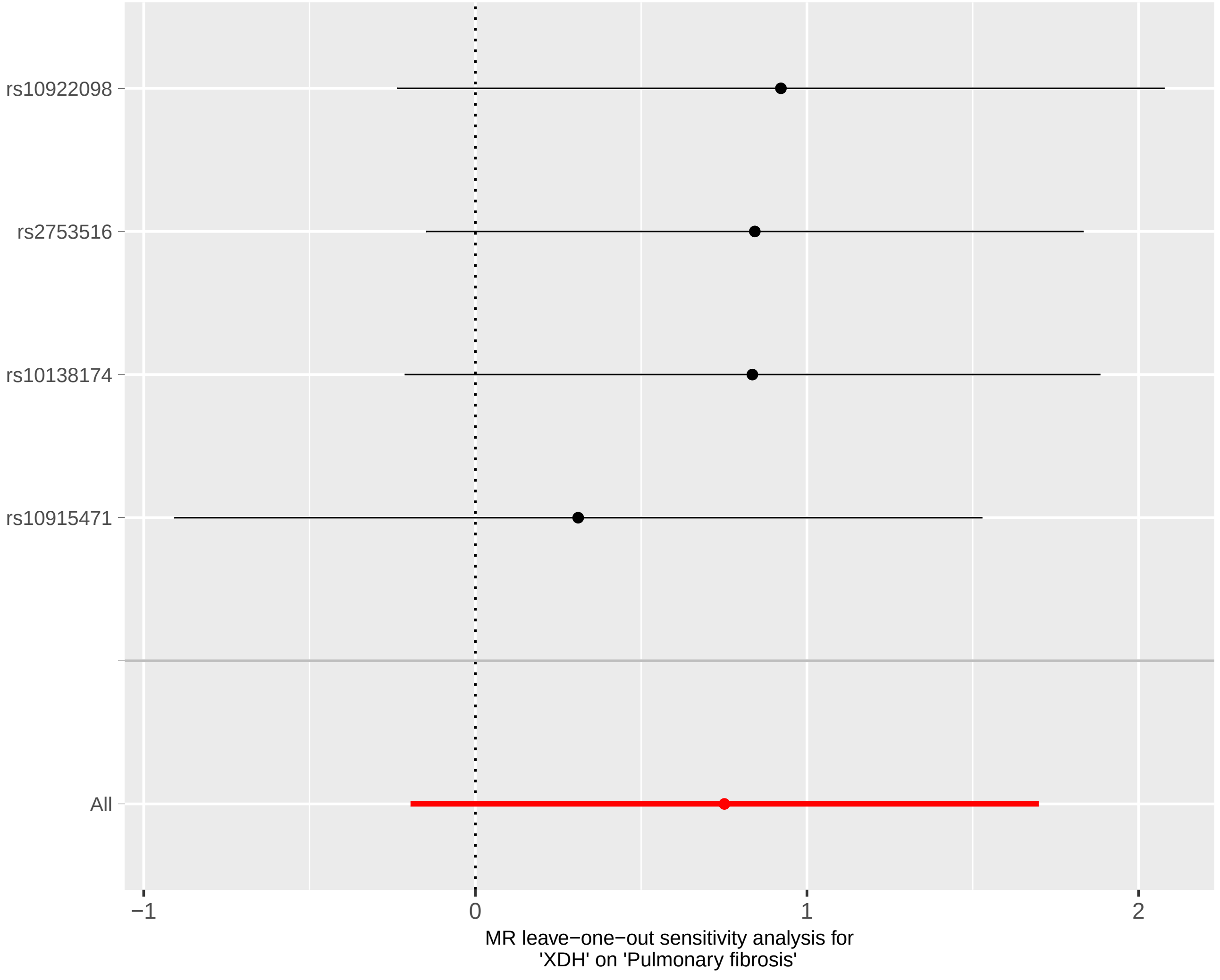
rs10922098
rs2753516
rs10138174
rs10915471
All
−1
0
1
2
MR leave−one−out sensitivity analysis for
'XDH' on 'Pulmonary fibrosis'

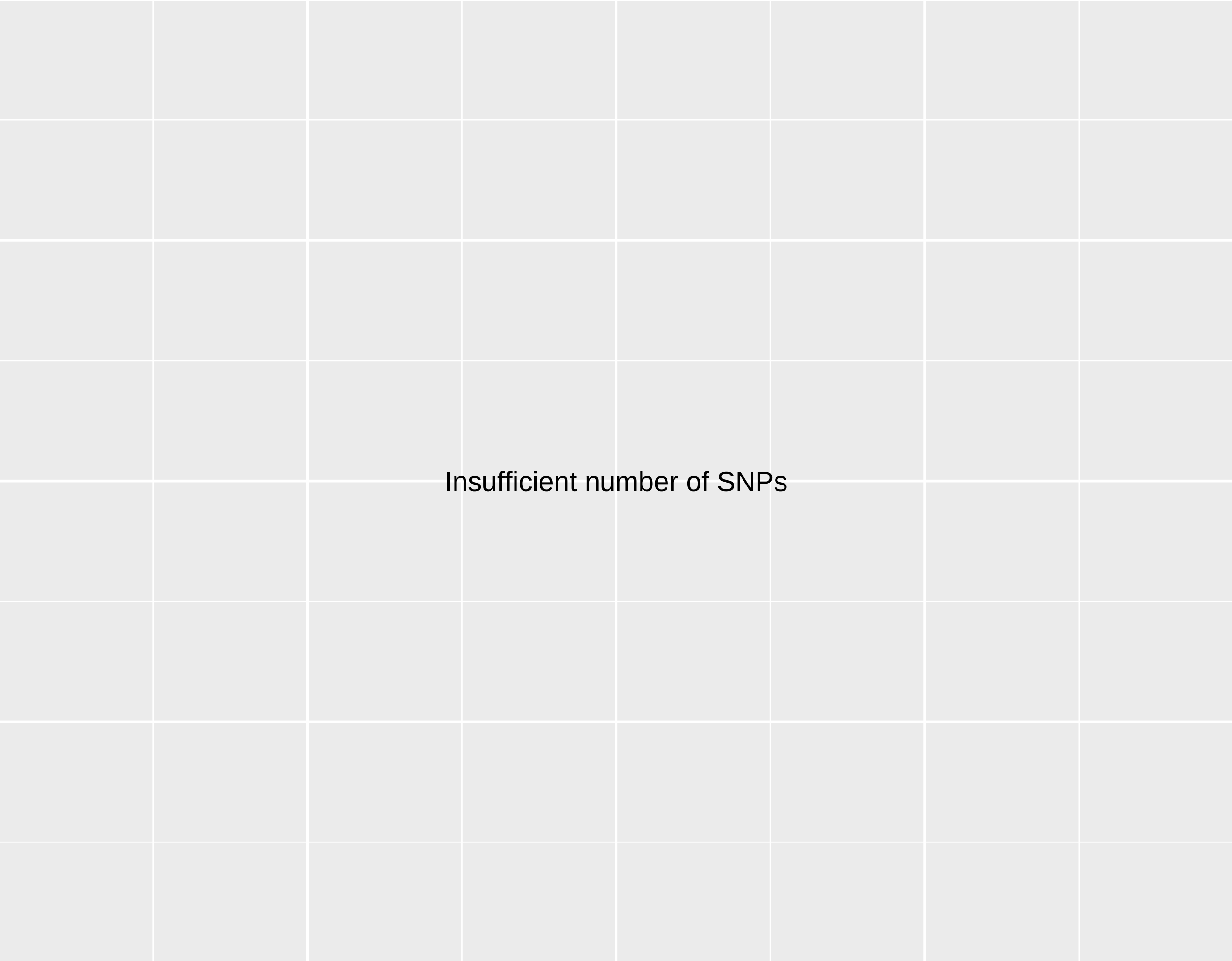
Insufficient number of SNPs

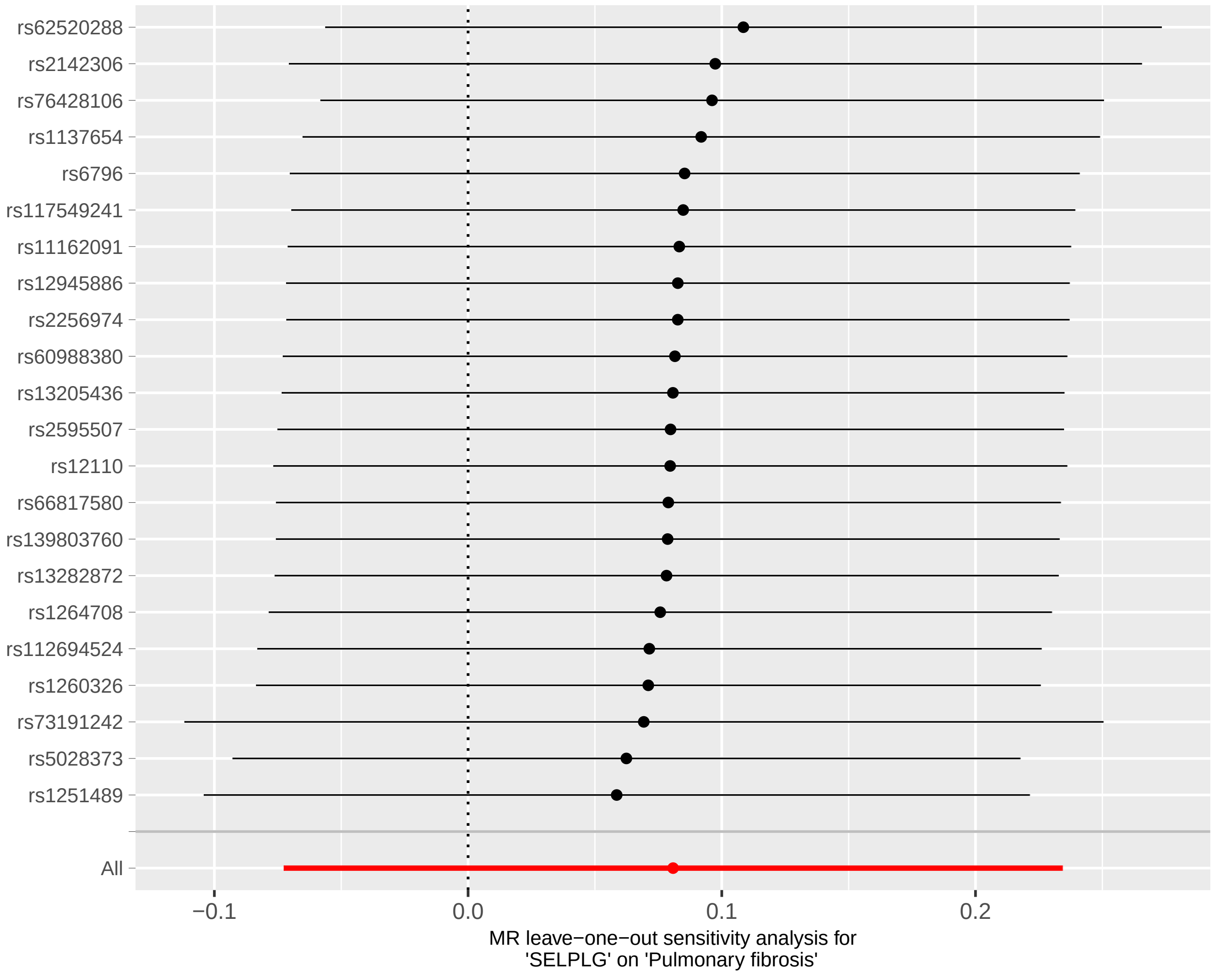
rs62520288
rs2142306
rs76428106
rs1137654
rs6796
rs117549241
rs11162091
rs12945886
rs2256974
rs60988380
rs13205436
rs2595507
rs12110
rs66817580
rs139803760
rs13282872
rs1264708
rs112694524
rs1260326
rs73191242
rs5028373
rs1251489
All
−0.1
0.0
0.1
0.2
MR leave−one−out sensitivity analysis for
'SELPLG' on 'Pulmonary fibrosis'

Insufficient number of SNPs

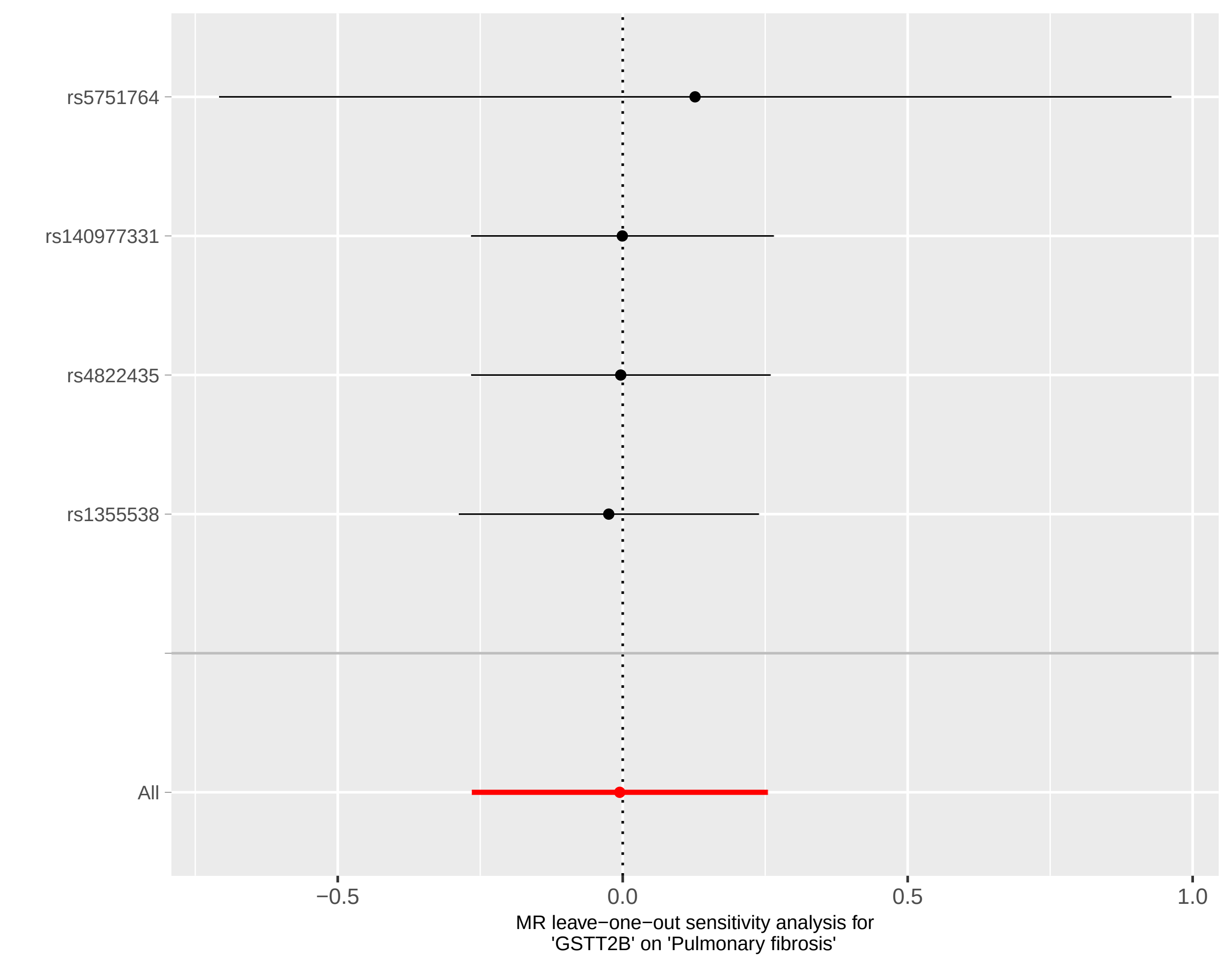
rs5751764
rs140977331
rs4822435
rs1355538
All
−0.5
0.0
0.5
1.0
MR leave−one−out sensitivity analysis for
'GSTT2B' on 'Pulmonary fibrosis'

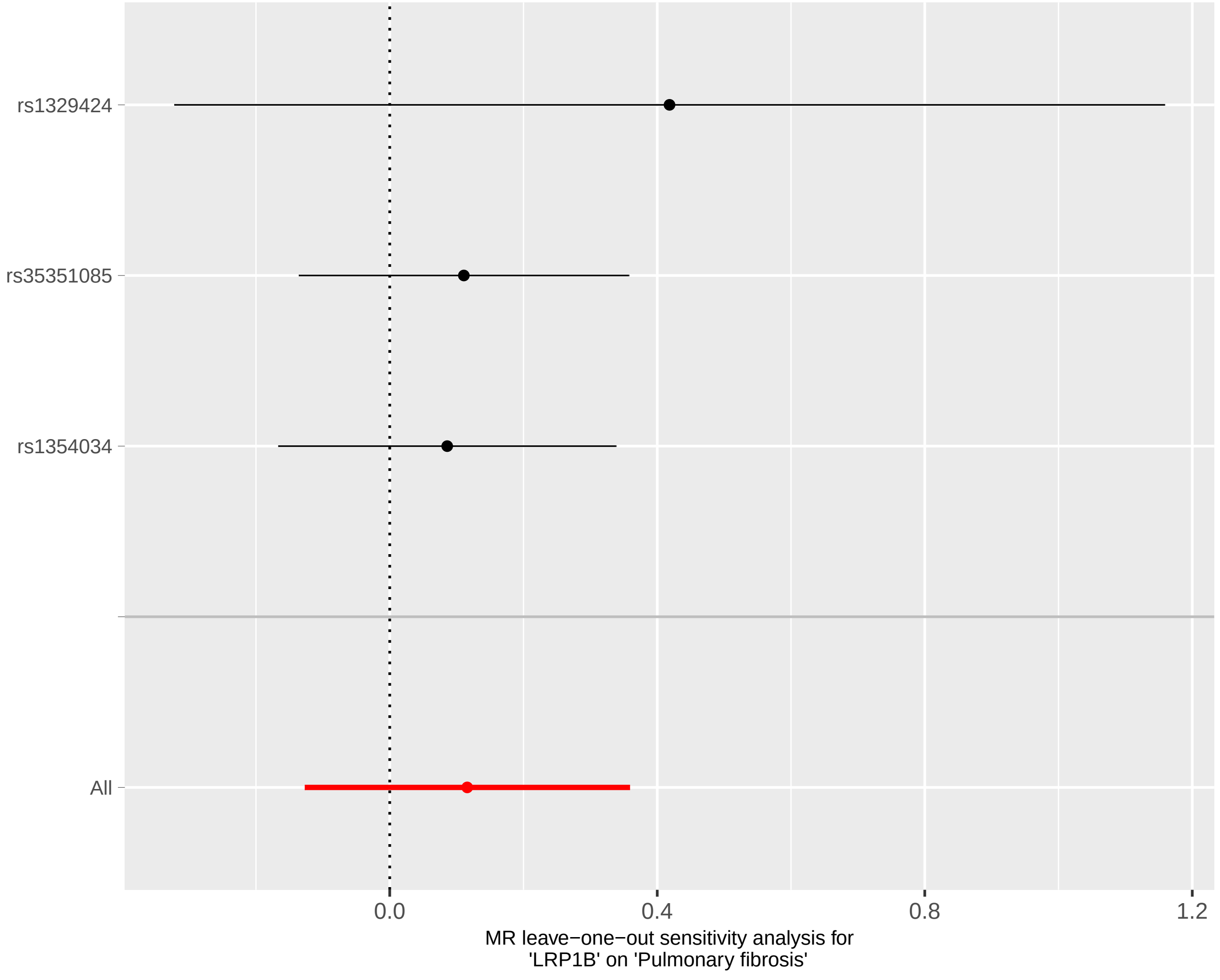
rs1329424
rs35351085
rs1354034
All
0.0
0.4
0.8
1.2
MR leave−one−out sensitivity analysis for
'LRP1B' on 'Pulmonary fibrosis'

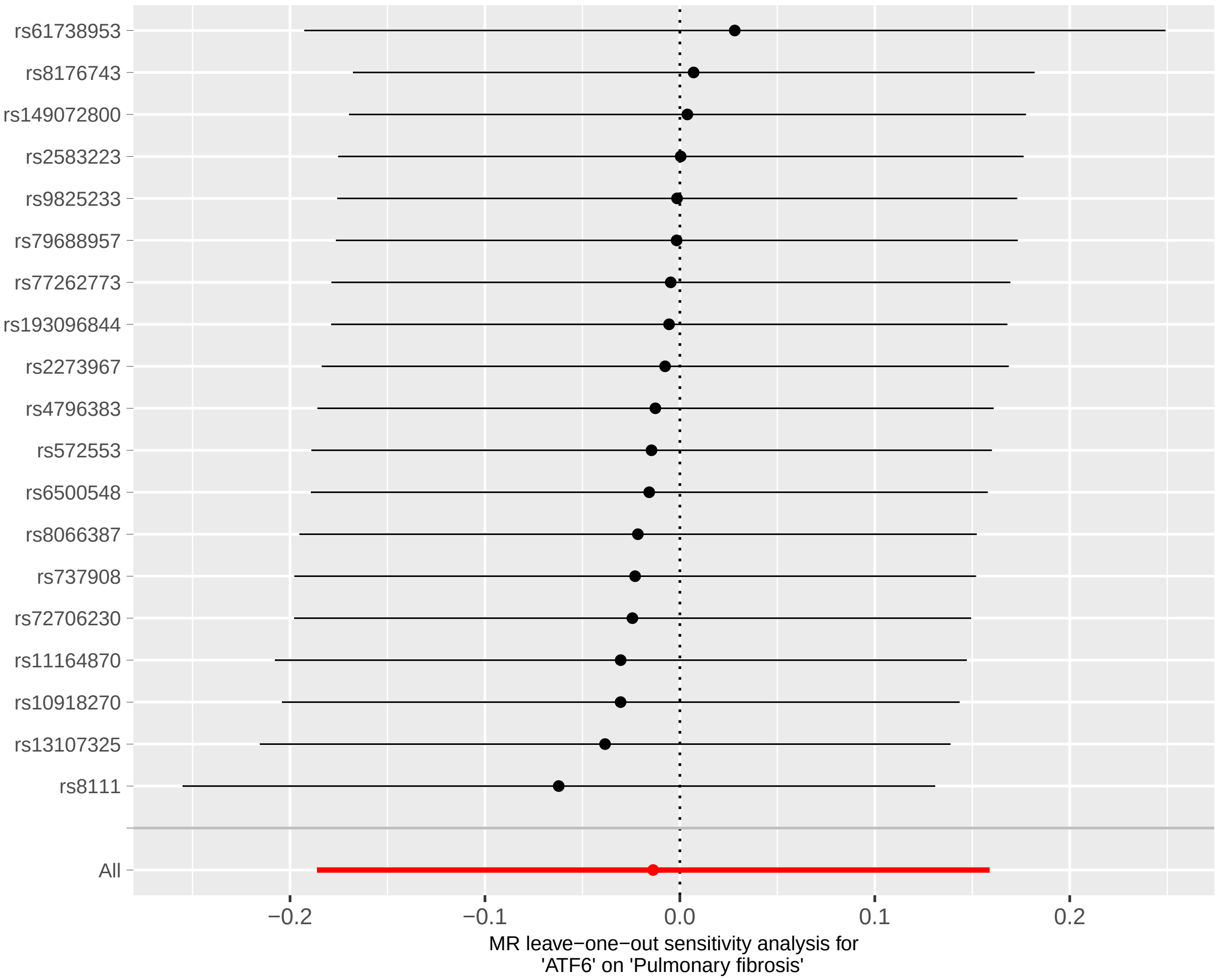
rs61738953
rs8176743
rs149072800
rs2583223
rs9825233
rs79688957
rs77262773
rs193096844
rs2273967
rs4796383
rs572553
rs6500548
rs8066387
rs737908
rs72706230
rs11164870
rs10918270
rs13107325
rs8111
All
−0.2
−0.1
0.0
0.1
0.2
MR leave−one−out sensitivity analysis for
'ATF6' on 'Pulmonary fibrosis'

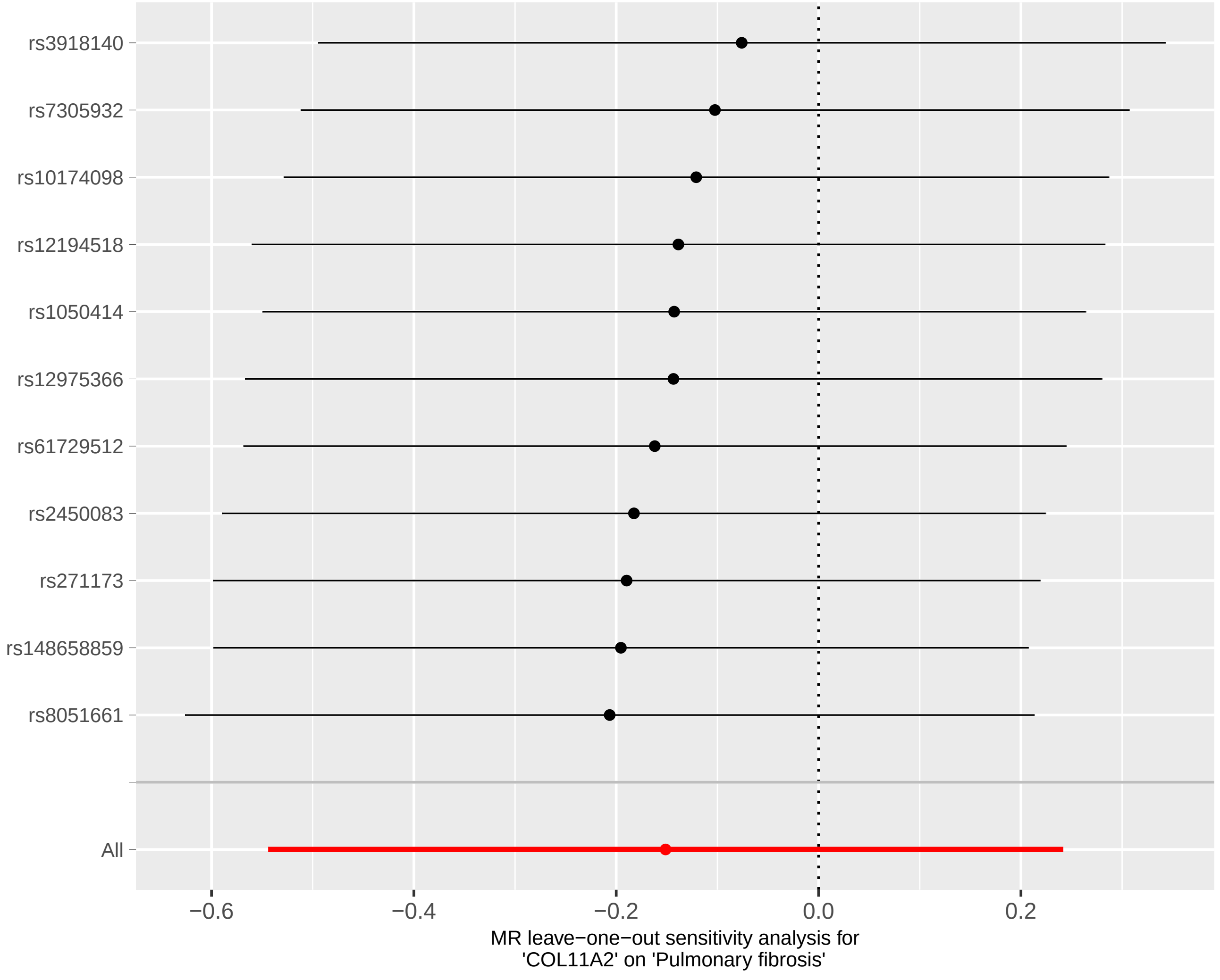
rs3918140
rs7305932
rs10174098
rs12194518
rs1050414
rs12975366
rs61729512
rs2450083
rs271173
rs148658859
rs8051661
All
−0.6
−0.4
−0.2
0.0
0.2
MR leave−one−out sensitivity analysis for
'COL11A2' on 'Pulmonary fibrosis'

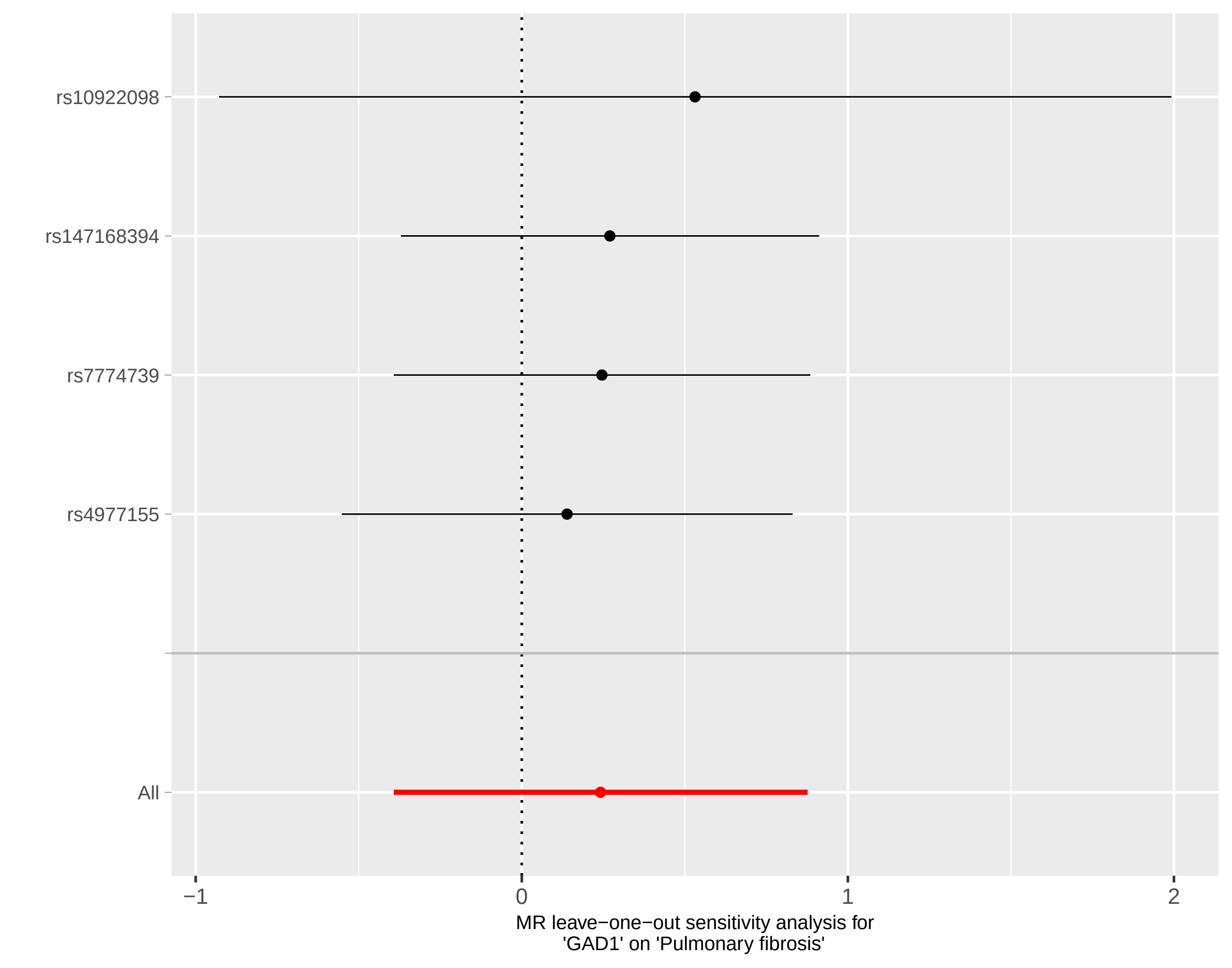
rs10922098
rs147168394
rs7774739
rs4977155
All
−1
0
1
2
MR leave−one−out sensitivity analysis for
'GAD1' on 'Pulmonary fibrosis'

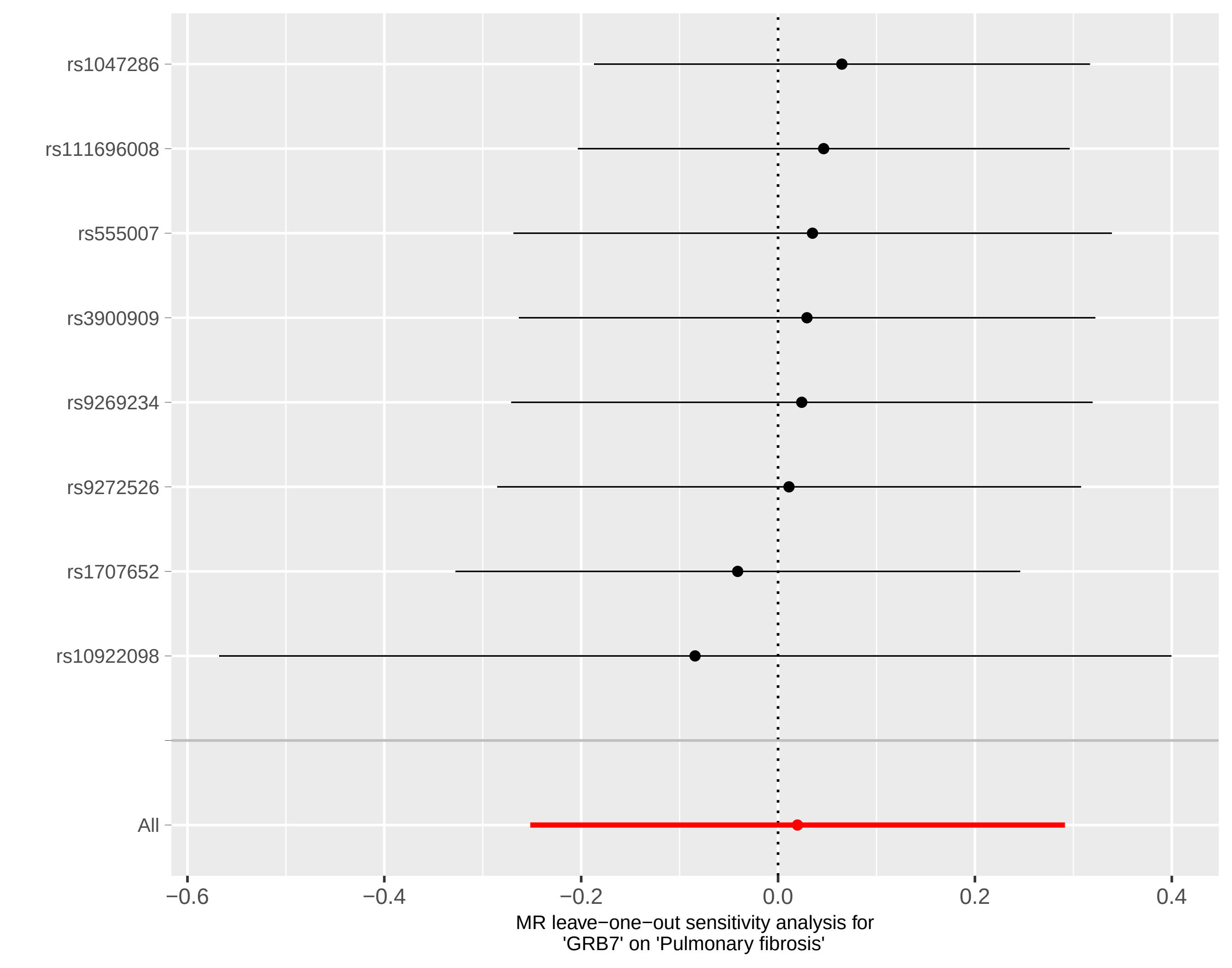
rs1047286
rs111696008
rs555007
rs3900909
rs9269234
rs9272526
rs1707652
rs10922098
All
−0.6
−0.4
−0.2
0.0
0.2
0.4
MR leave−one−out sensitivity analysis for
'GRB7' on 'Pulmonary fibrosis'

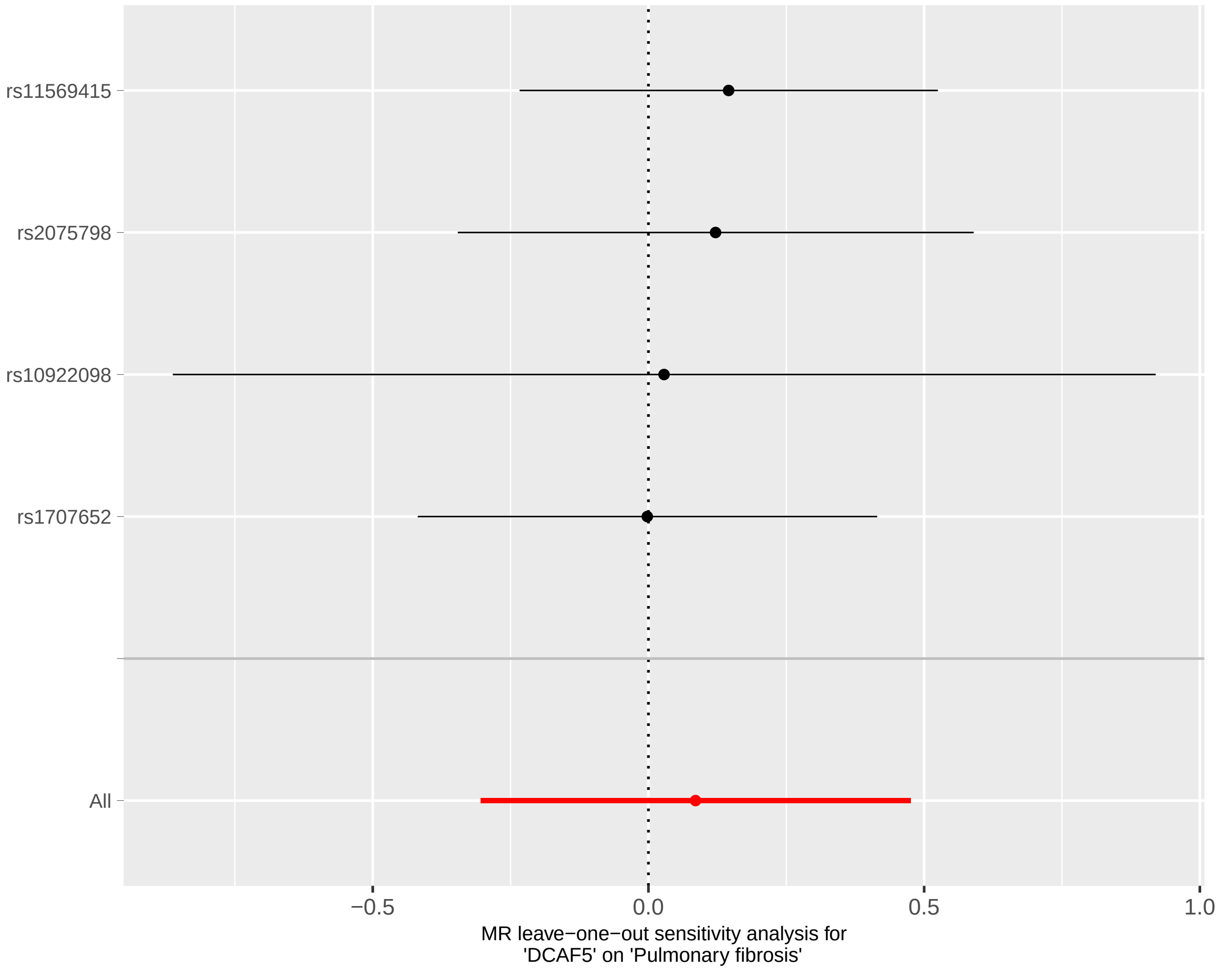
rs11569415
rs2075798
rs10922098
rs1707652
All
−0.5
0.0
0.5
1.0
MR leave−one−out sensitivity analysis for
'DCAF5' on 'Pulmonary fibrosis'

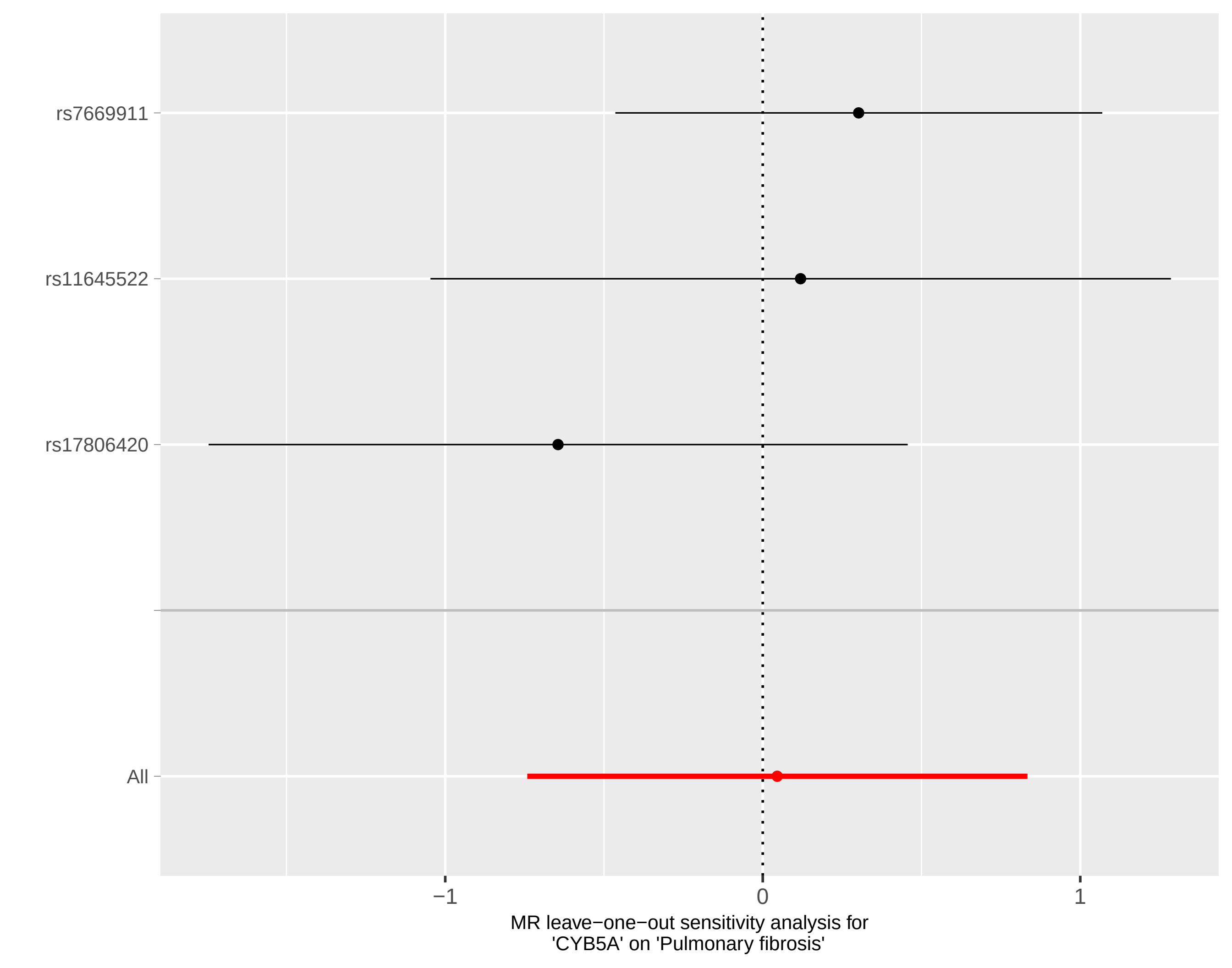

rs7669911
rs11645522
rs17806420
All
−1
0
1
MR leave−one−out sensitivity analysis for
'CYB5A' on 'Pulmonary fibrosis'

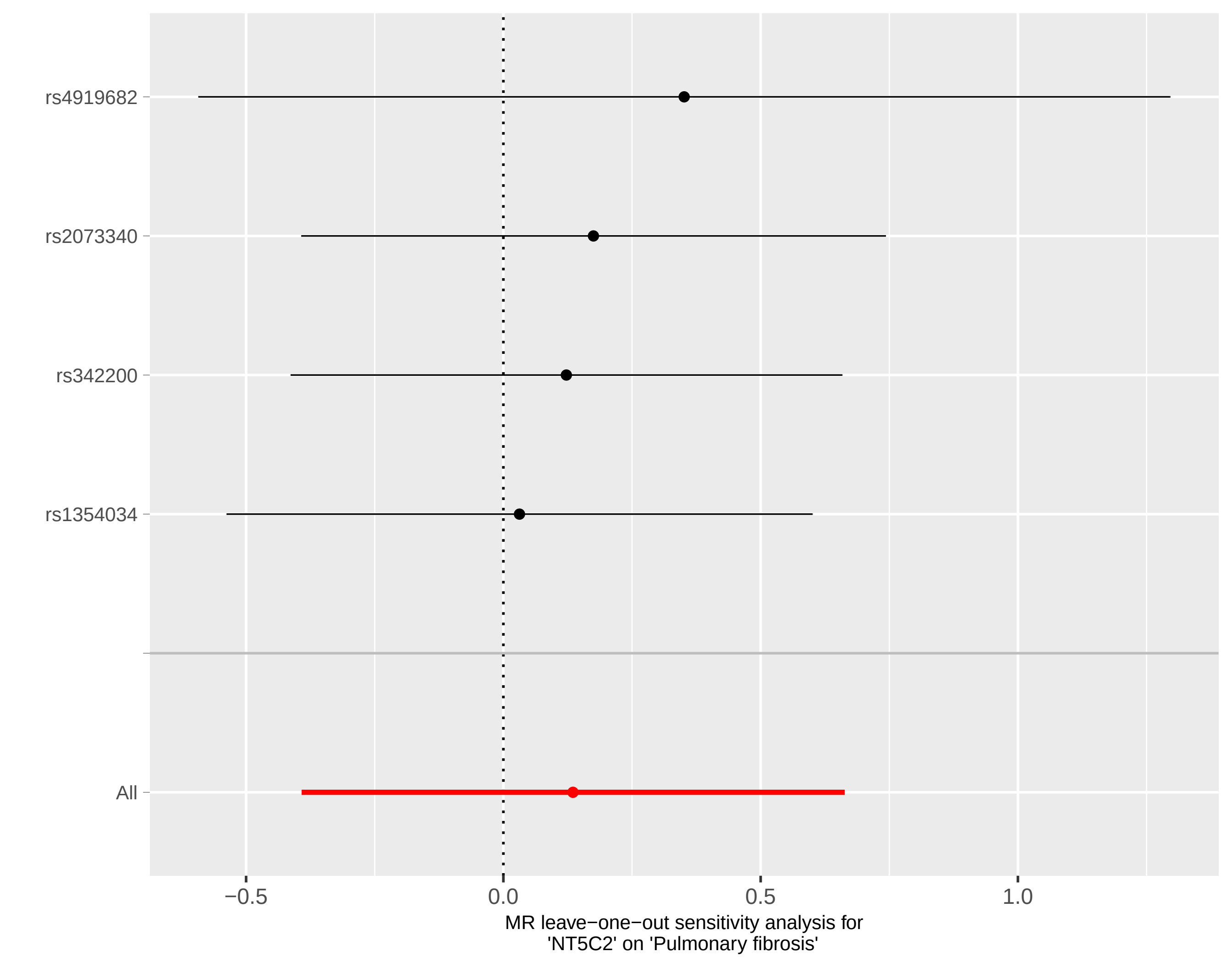

rs4919682
rs2073340
rs342200
rs1354034
All
−0.5
0.0
0.5
1.0
MR leave−one−out sensitivity analysis for
'NT5C2' on 'Pulmonary fibrosis'

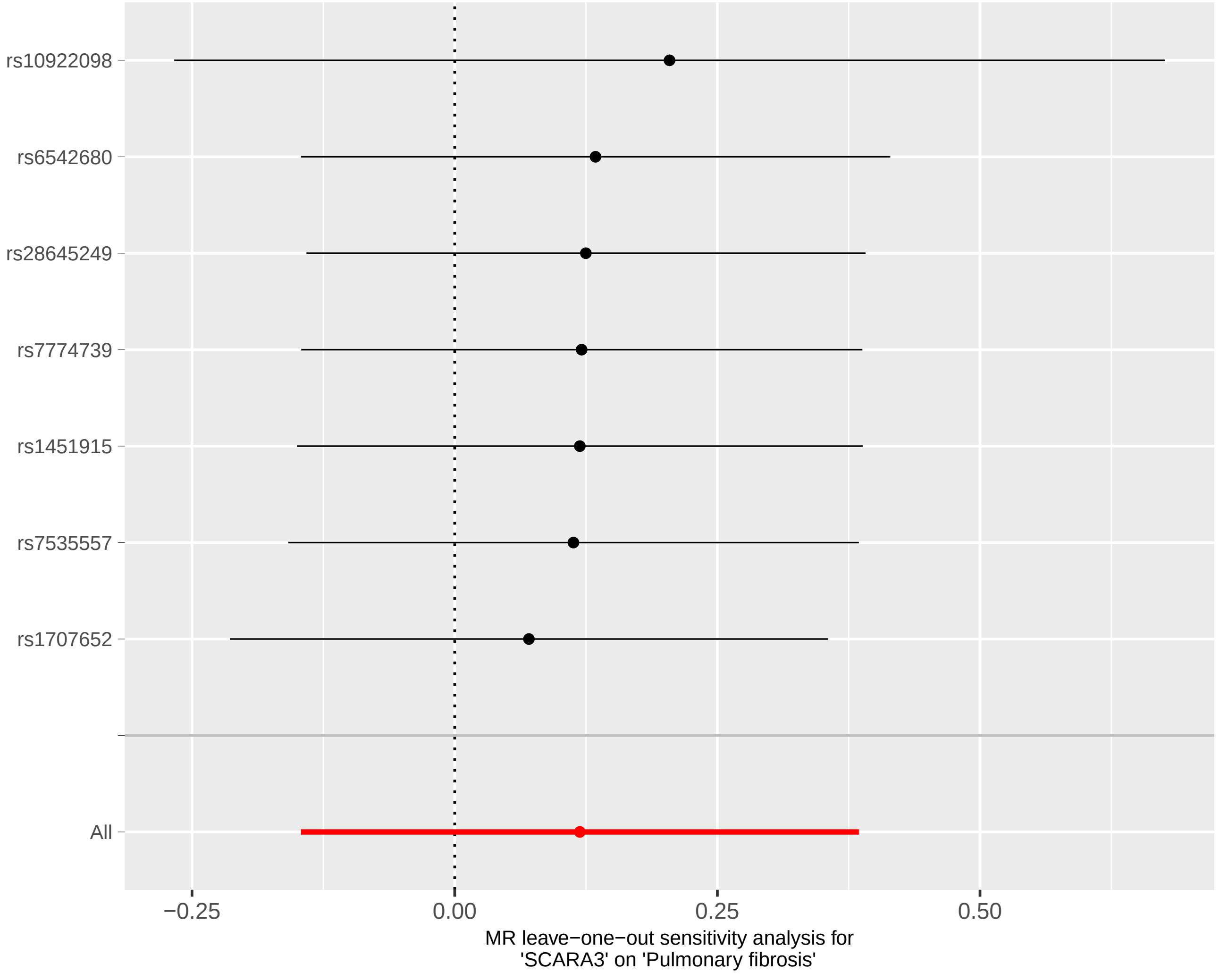
rs10922098
rs6542680
rs28645249
rs7774739
rs1451915
rs7535557
rs1707652
All
−0.25
0.00
0.25
0.50
MR leave−one−out sensitivity analysis for
'SCARA3' on 'Pulmonary fibrosis'

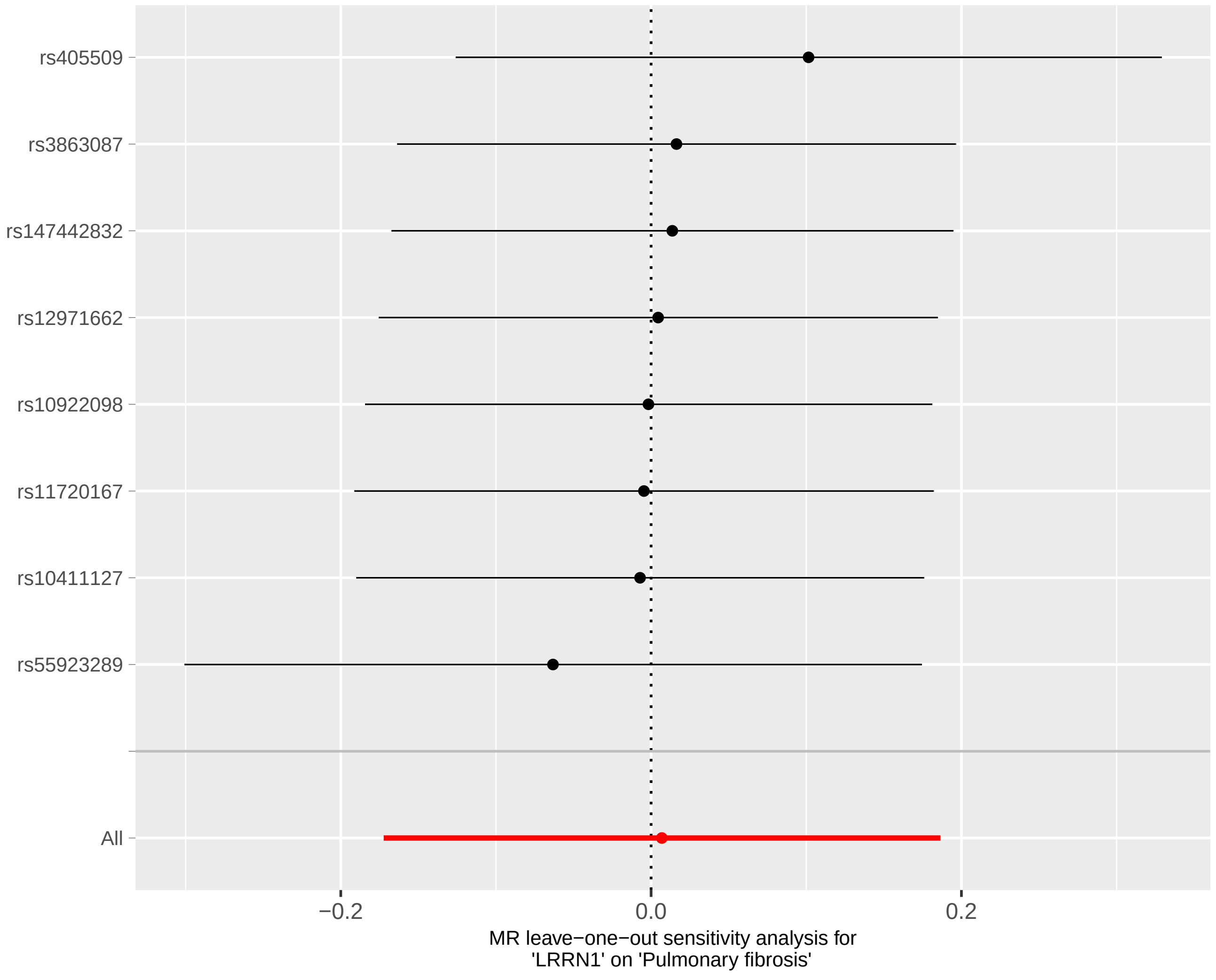

rs405509
rs3863087
rs147442832
rs12971662
rs10922098
rs11720167
rs10411127
rs55923289
All
−0.2
0.0
0.2
MR leave−one−out sensitivity analysis for
'LRRN1' on 'Pulmonary fibrosis'

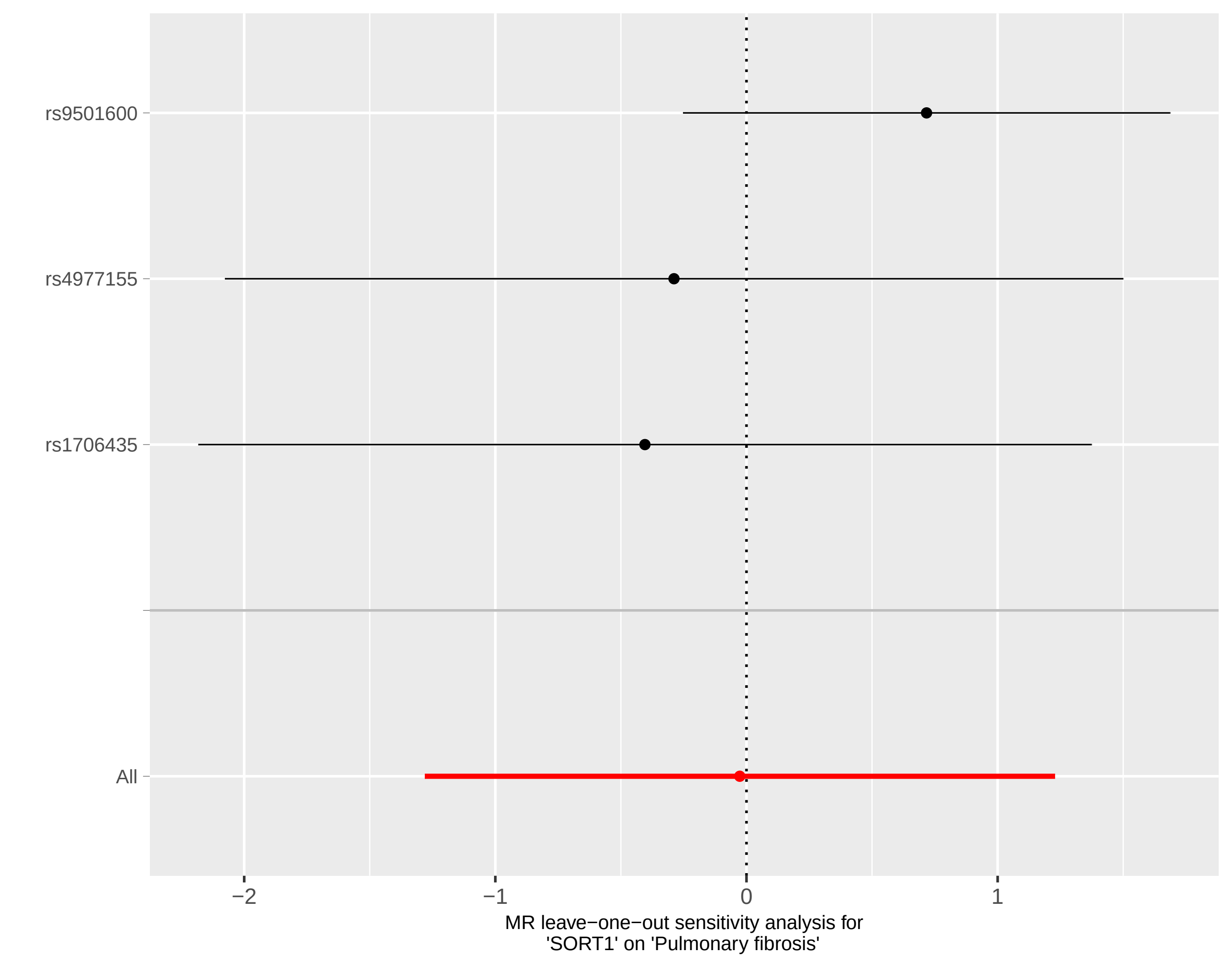
rs9501600
rs4977155
rs1706435
All
−2
−1
0
1
MR leave−one−out sensitivity analysis for
'SORT1' on 'Pulmonary fibrosis'

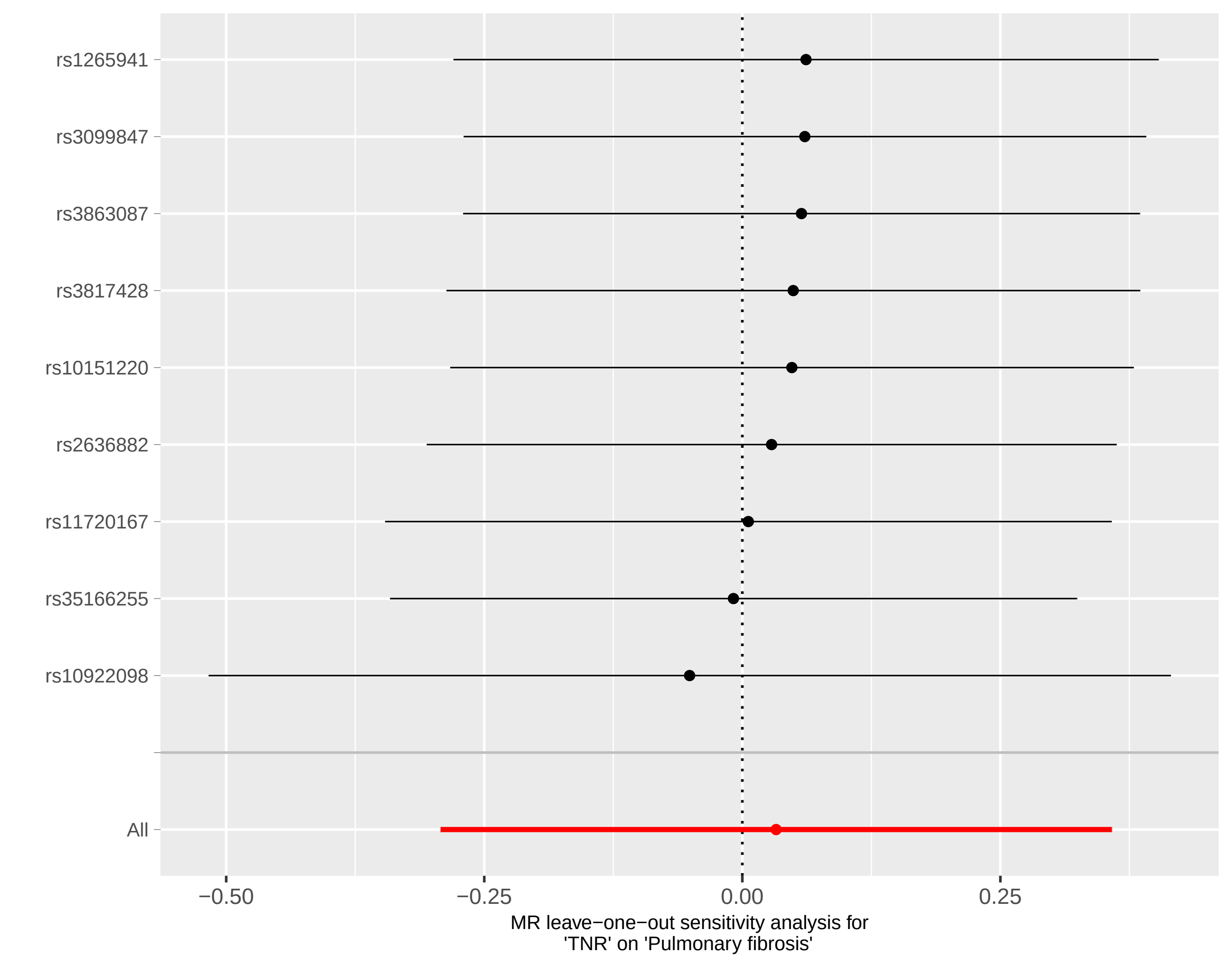
rs1265941
rs3099847
rs3863087
rs3817428
rs10151220
rs2636882
rs11720167
rs35166255
rs10922098
All
−0.50
−0.25
0.00
0.25
MR leave−one−out sensitivity analysis for
'TNR' on 'Pulmonary fibrosis'

Insufficient number of SNPs

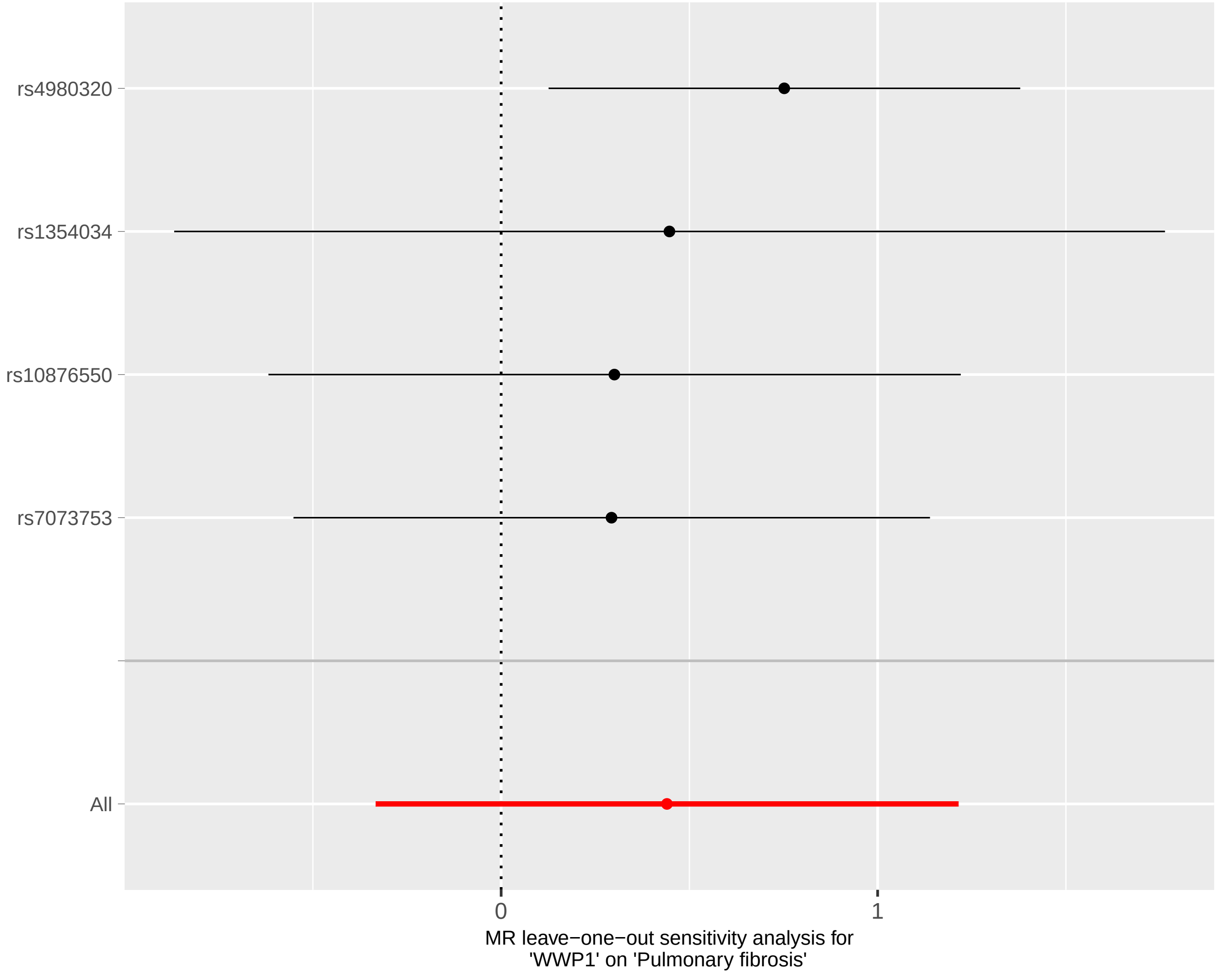
rs4980320
rs1354034
rs10876550
rs7073753
All
0
1
MR leave−one−out sensitivity analysis for
'WWP1' on 'Pulmonary fibrosis'

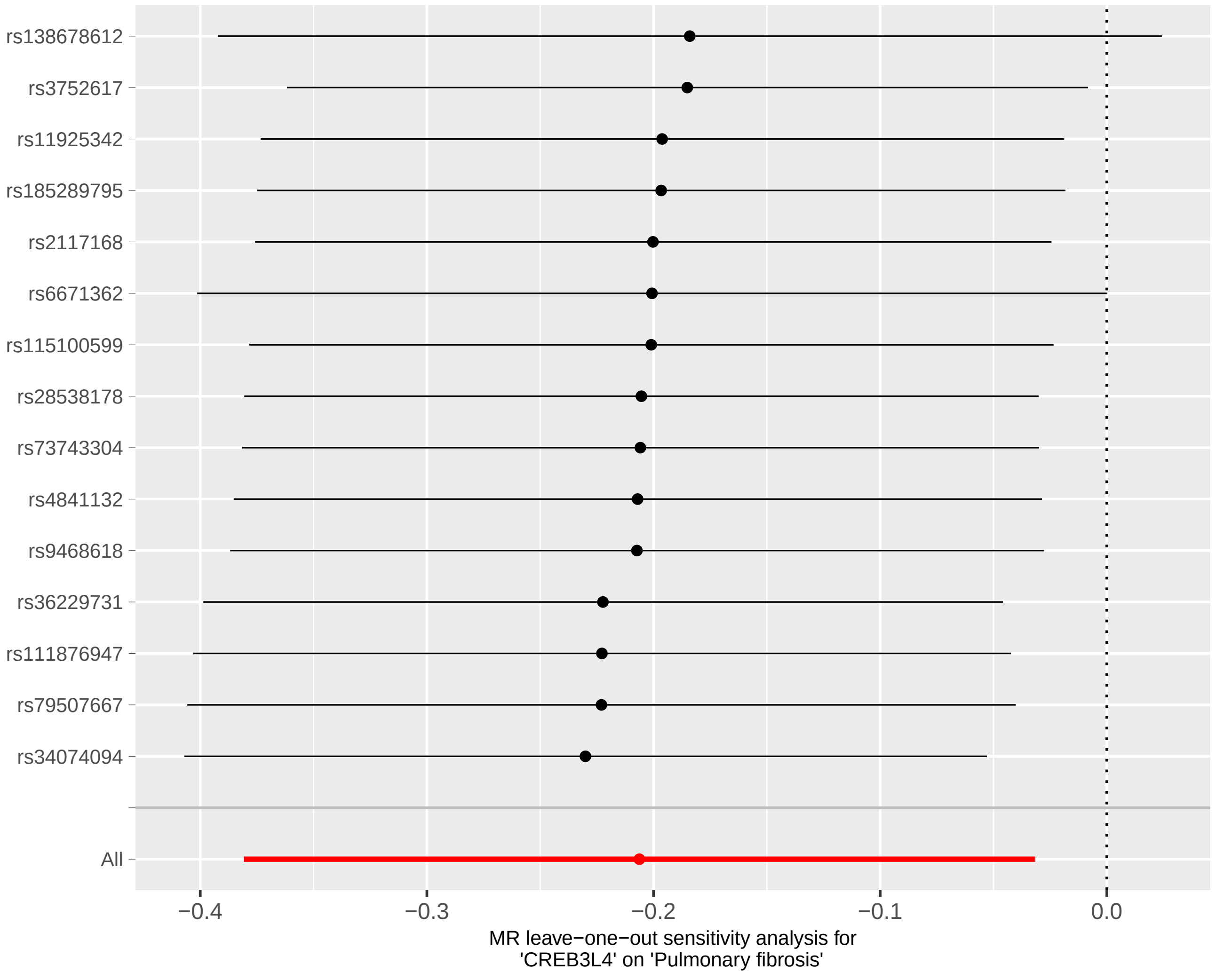
rs138678612
rs3752617
rs11925342
rs185289795
rs2117168
rs6671362
rs115100599
rs28538178
rs73743304
rs4841132
rs9468618
rs36229731
rs111876947
rs79507667
rs34074094
All
−0.4
−0.3
−0.2
−0.1
0.0
MR leave−one−out sensitivity analysis for
'CREB3L4' on 'Pulmonary fibrosis'

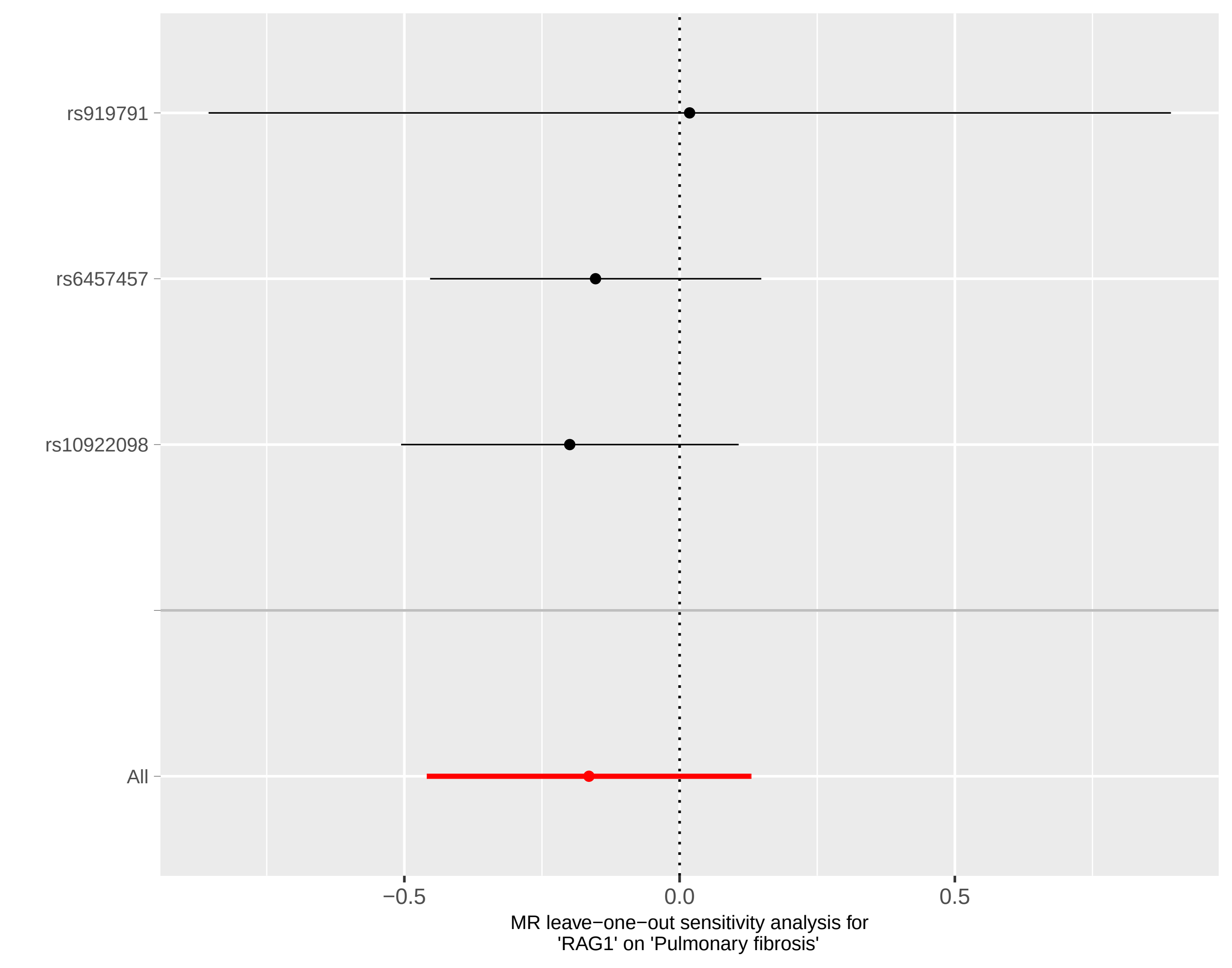
rs919791
rs6457457
rs10922098
All
−0.5
0.0
0.5
MR leave−one−out sensitivity analysis for
'RAG1' on 'Pulmonary fibrosis'

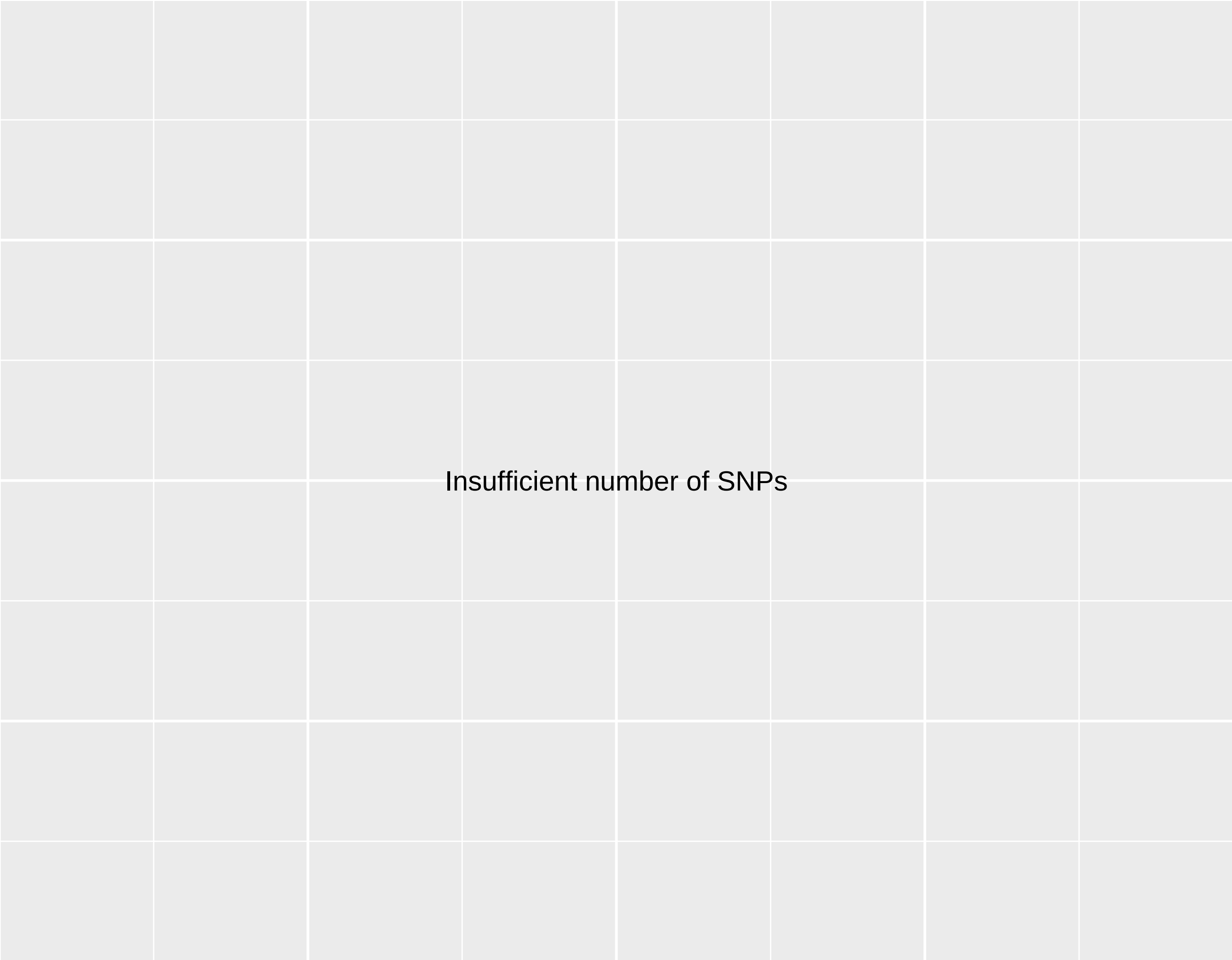
Insufficient number of SNPs

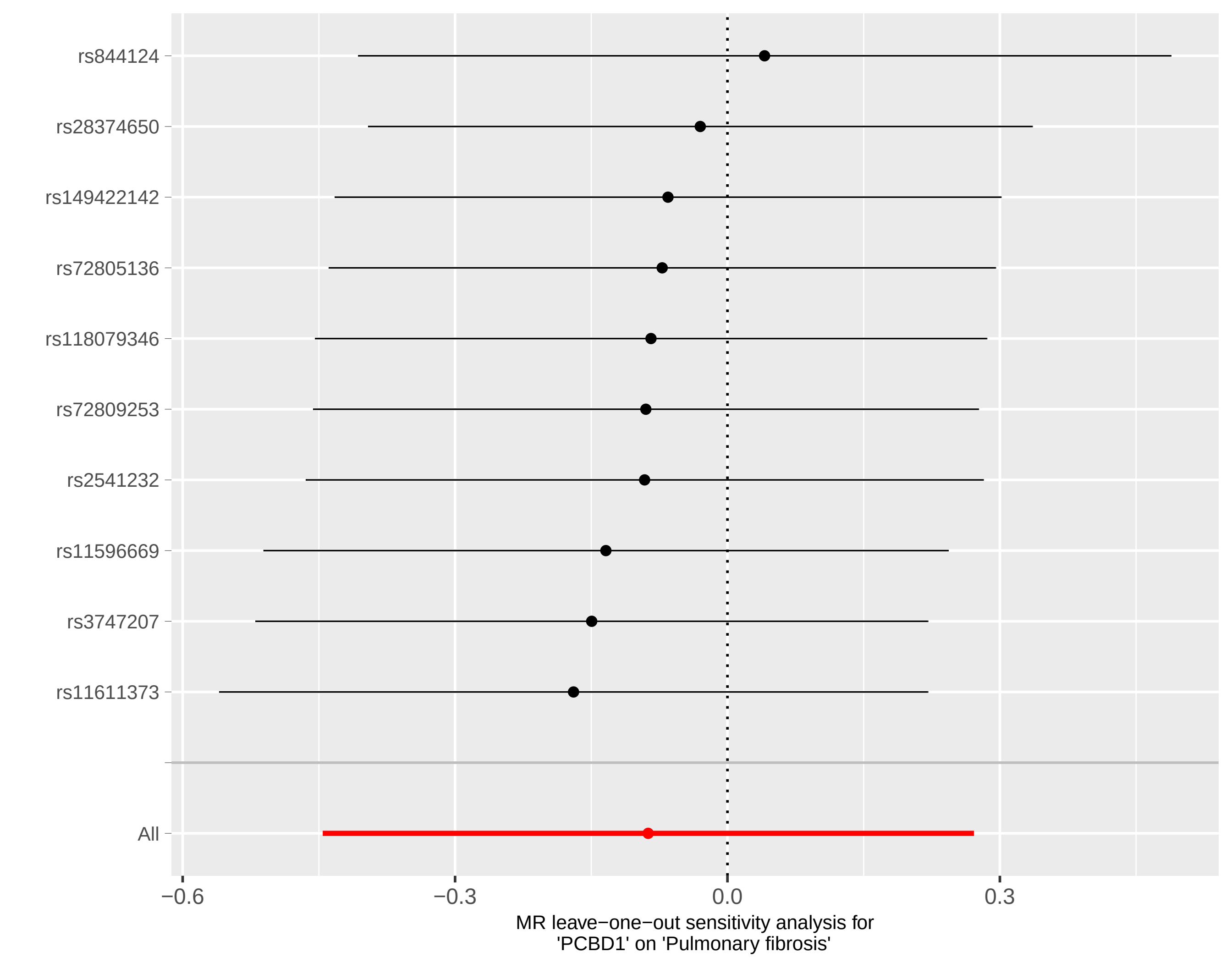

rs844124
rs28374650
rs149422142
rs72805136
rs118079346
rs72809253
rs2541232
rs11596669
rs3747207
rs11611373
All
−0.6
−0.3
0.0
0.3
MR leave−one−out sensitivity analysis for
'PCBD1' on 'Pulmonary fibrosis'

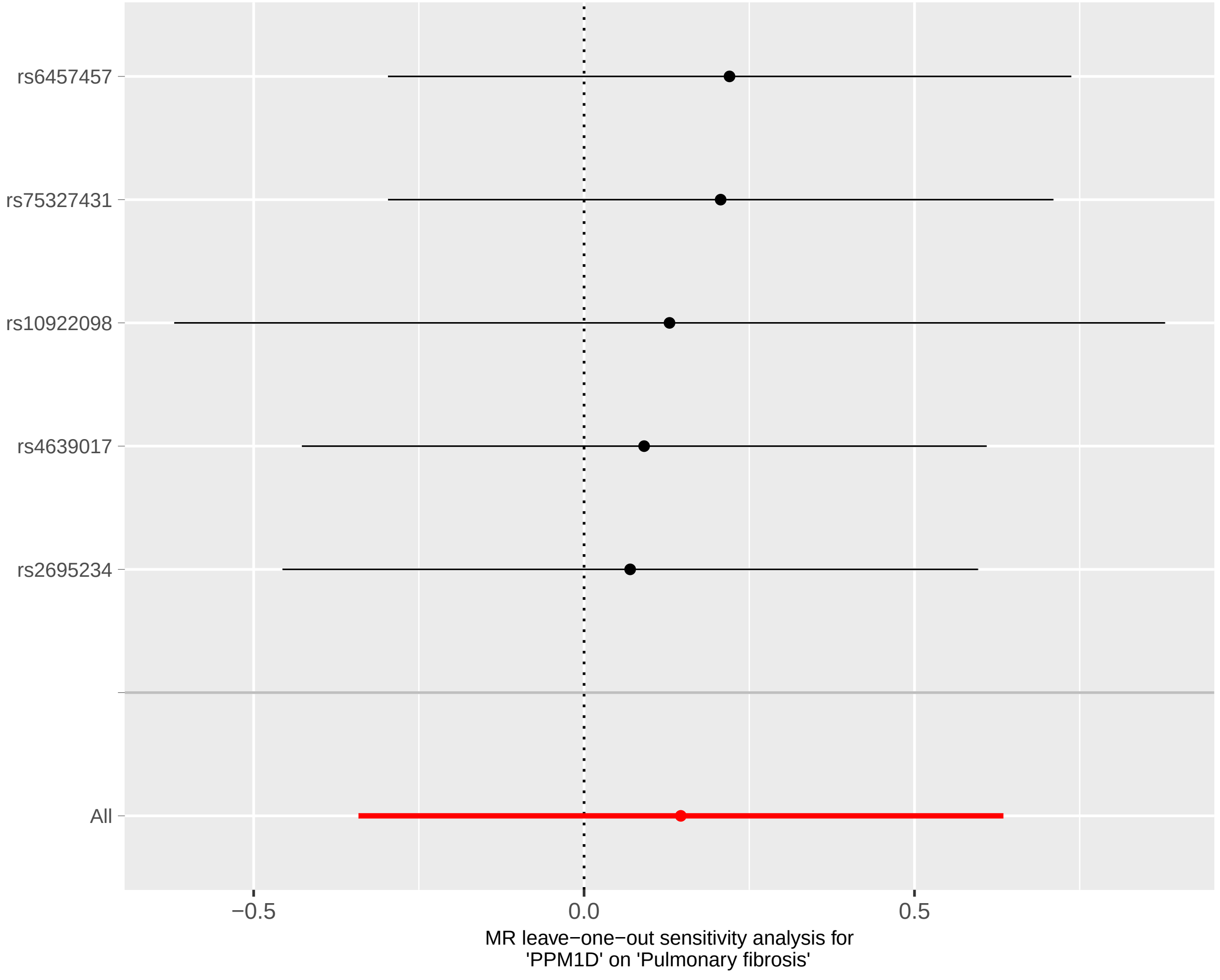

rs6457457
rs75327431
rs10922098
rs4639017
rs2695234
All
−0.5
0.0
0.5
MR leave−one−out sensitivity analysis for
'PPM1D' on 'Pulmonary fibrosis'

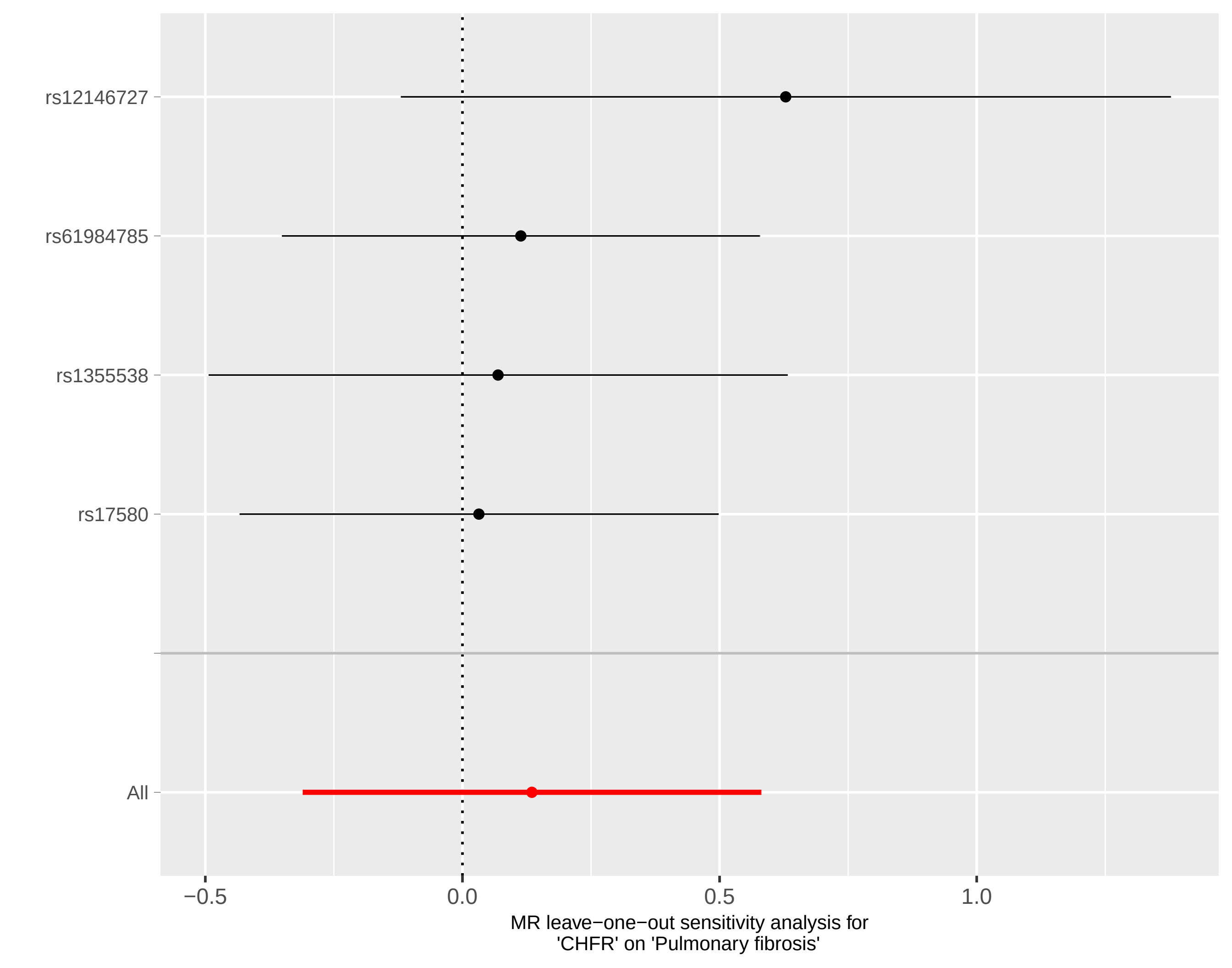
rs12146727
rs61984785
rs1355538
rs17580
All
−0.5
0.0
0.5
1.0
MR leave−one−out sensitivity analysis for
'CHFR' on 'Pulmonary fibrosis'

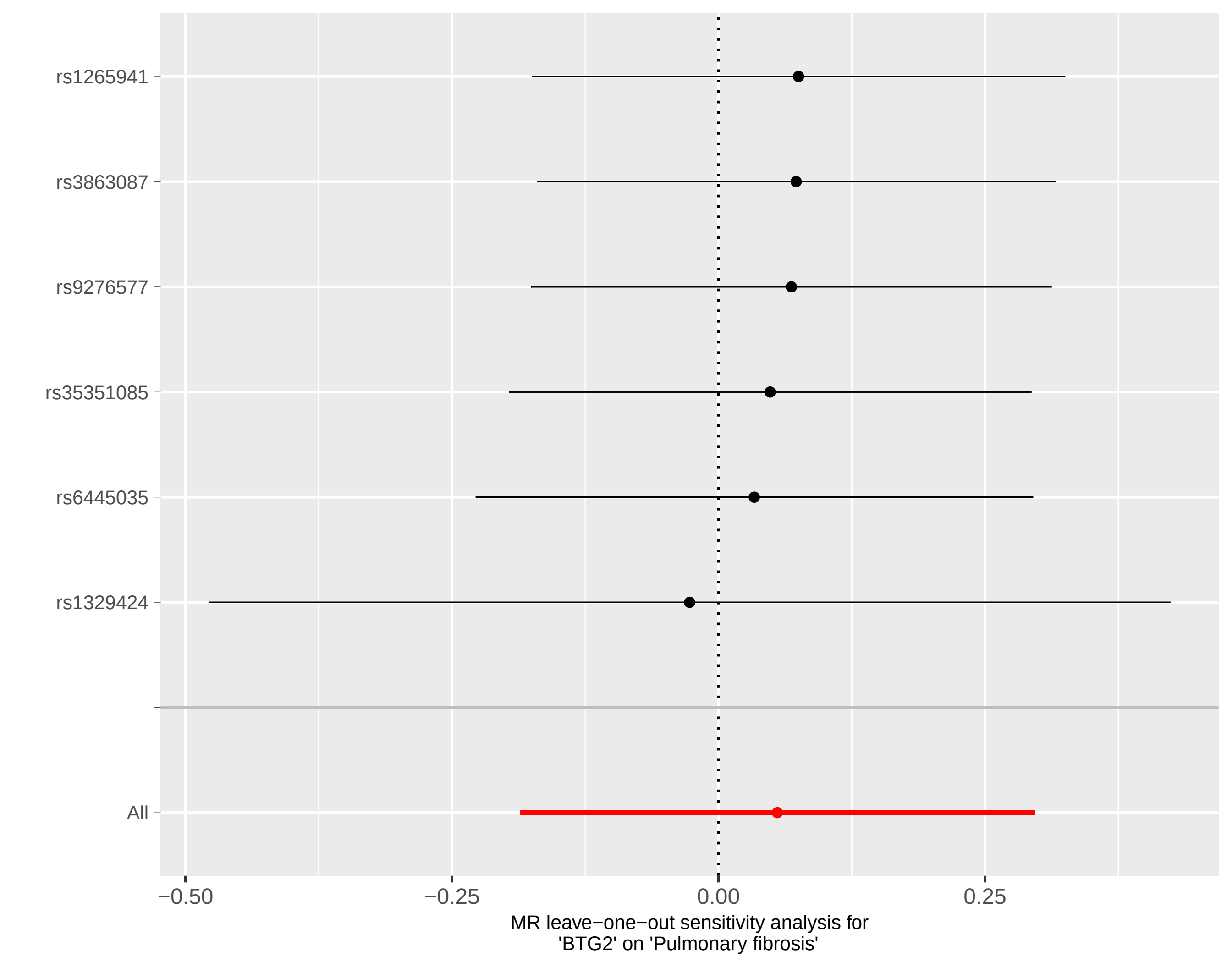

rs1265941
rs3863087
rs9276577
rs35351085
rs6445035
rs1329424
All
−0.50
−0.25
0.00
0.25
MR leave−one−out sensitivity analysis for
'BTG2' on 'Pulmonary fibrosis'

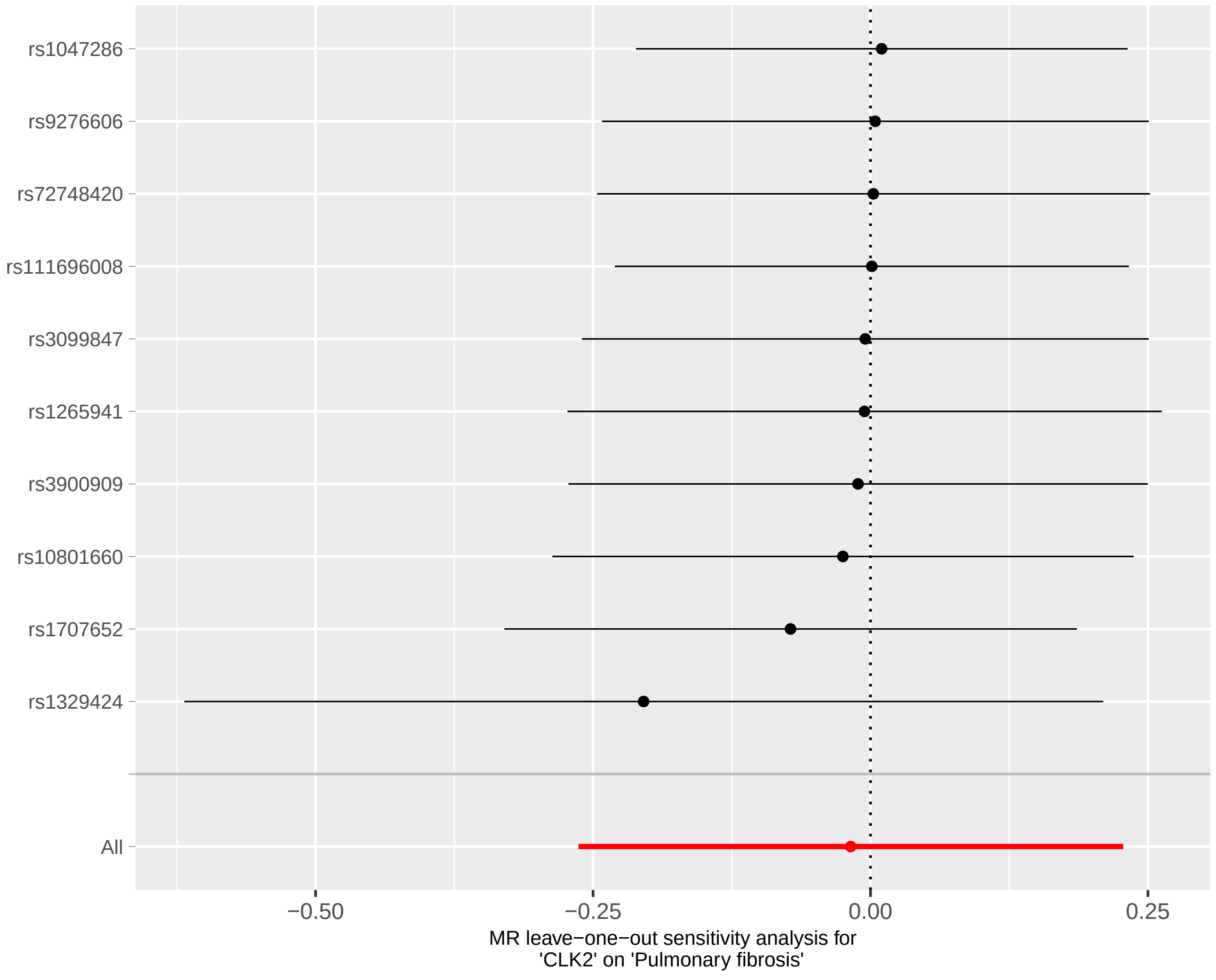

rs1047286
rs9276606
rs72748420
rs111696008
rs3099847
rs1265941
rs3900909
rs10801660
rs1707652
rs1329424
All
−0.50
−0.25
0.00
0.25
MR leave−one−out sensitivity analysis for
'CLK2' on 'Pulmonary fibrosis'

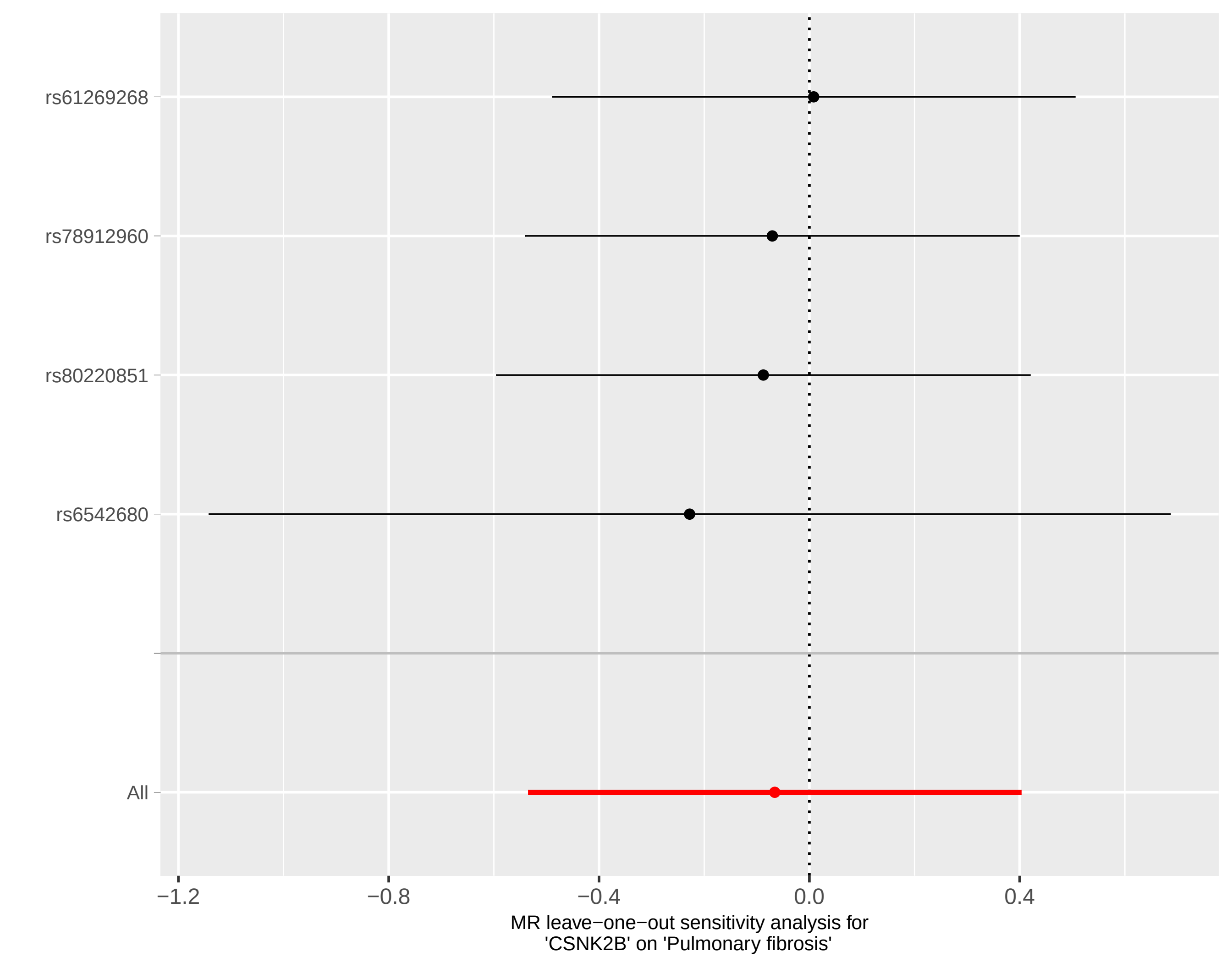
rs61269268
rs78912960
rs80220851
rs6542680
All
−1.2
−0.8
−0.4
0.0
0.4
MR leave−one−out sensitivity analysis for
'CSNK2B' on 'Pulmonary fibrosis'

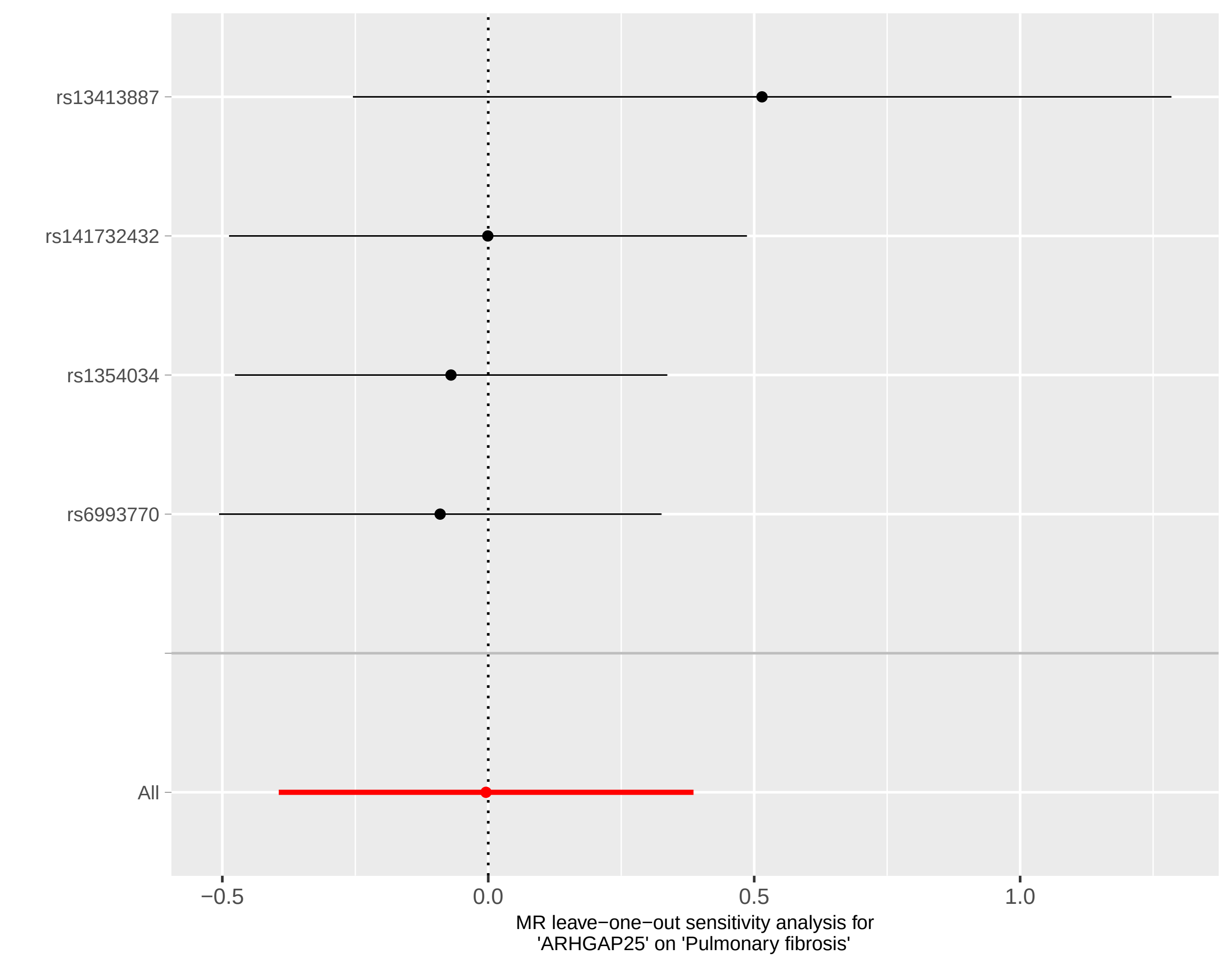

rs13413887
rs141732432
rs1354034
rs6993770
All
−0.5
0.0
0.5
1.0
MR leave−one−out sensitivity analysis for
'ARHGAP25' on 'Pulmonary fibrosis'

Insufficient number of SNPs

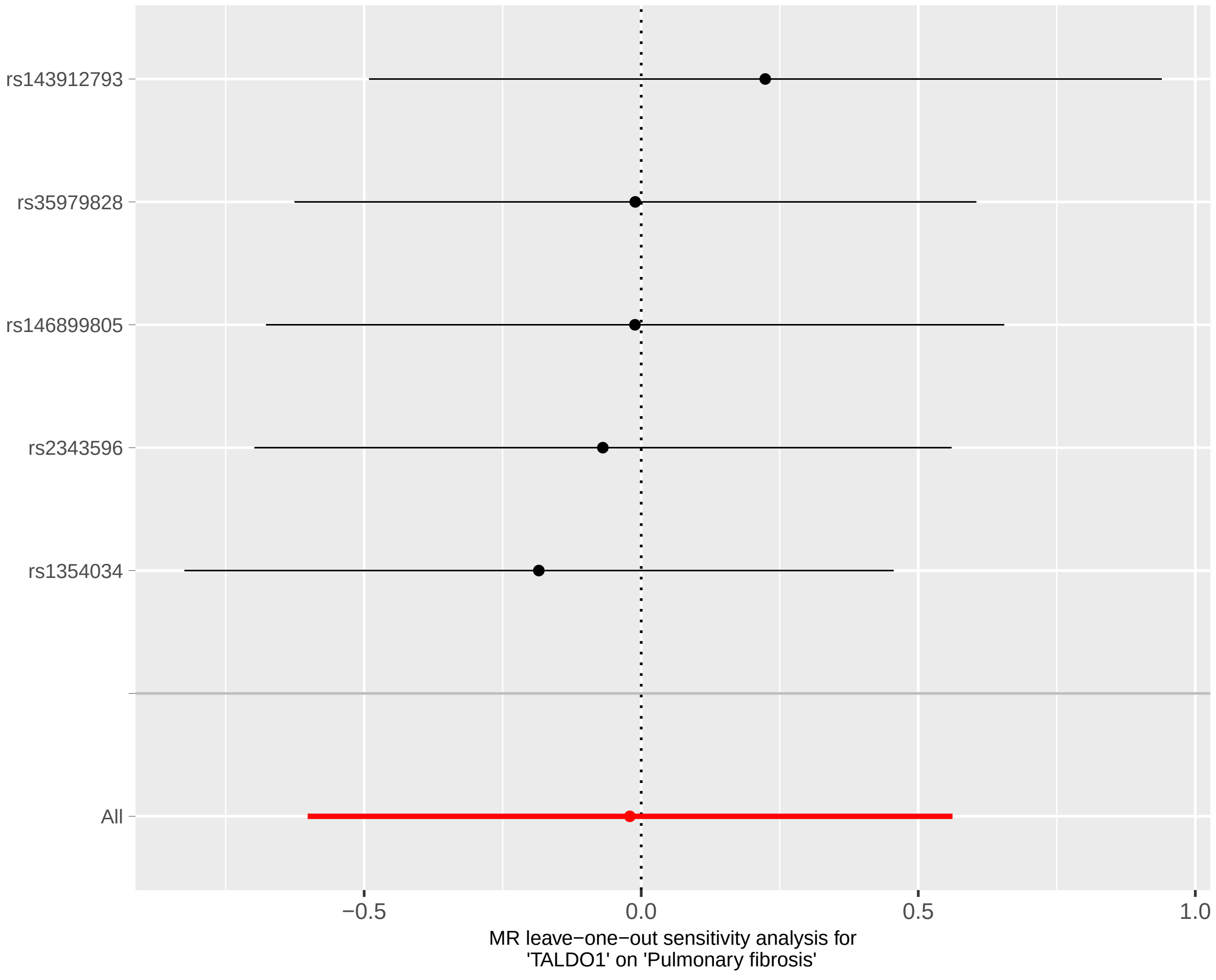

rs143912793
rs35979828
rs146899805
rs2343596
rs1354034
All
−0.5
0.0
0.5
1.0
MR leave−one−out sensitivity analysis for
'TALDO1' on 'Pulmonary fibrosis'

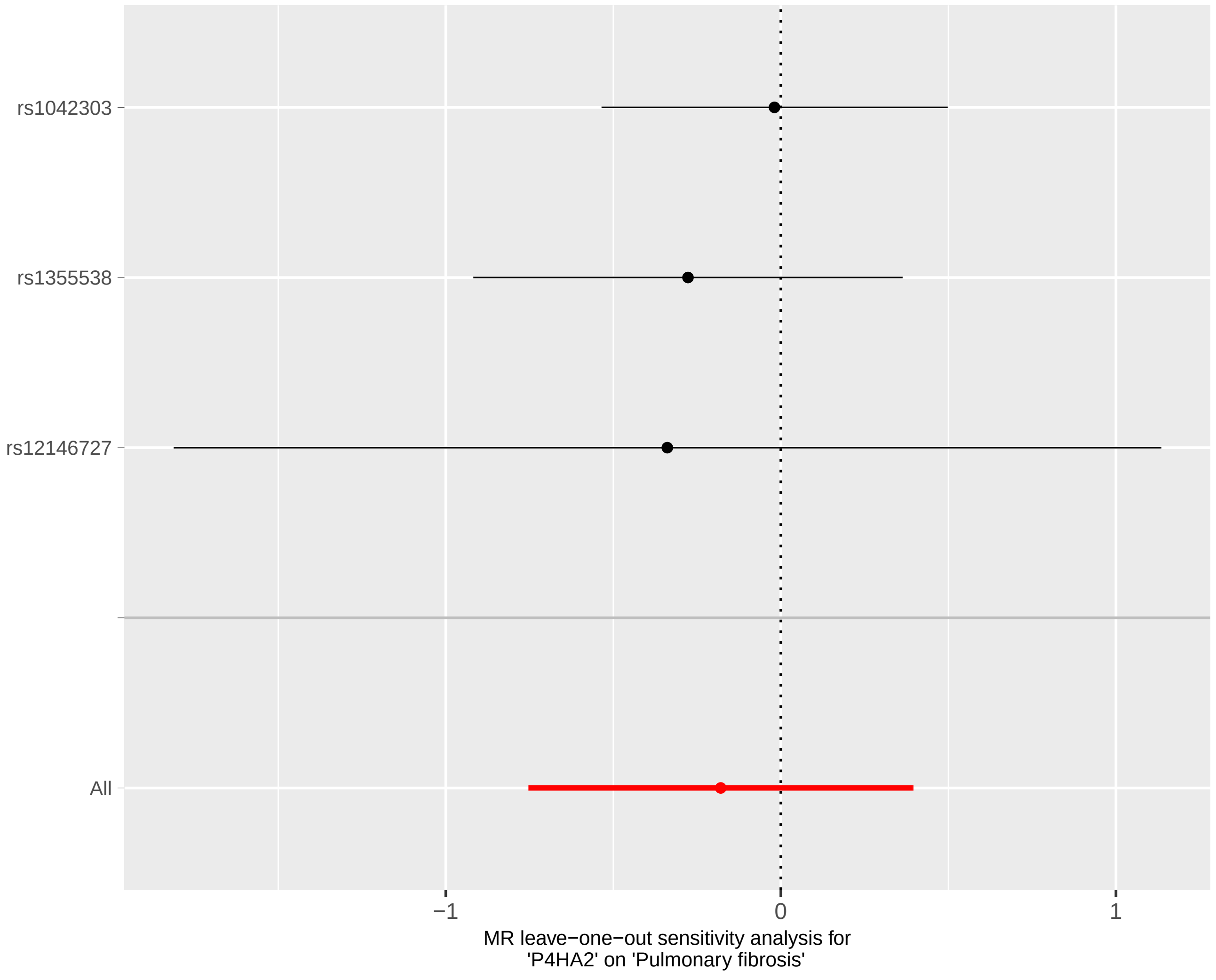

rs1042303
rs1355538
rs12146727
All
−1
0
1
MR leave−one−out sensitivity analysis for
'P4HA2' on 'Pulmonary fibrosis'

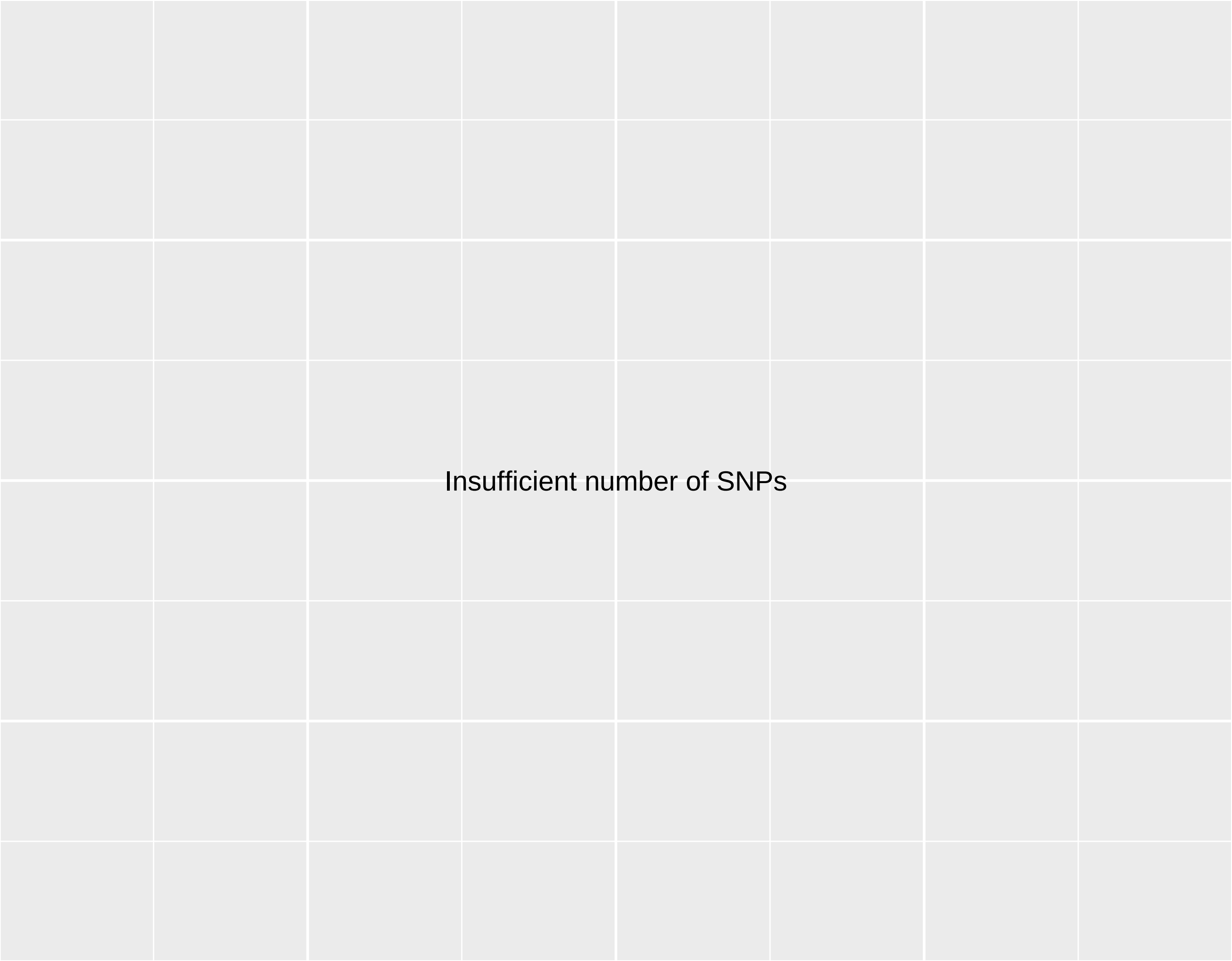
Insufficient number of SNPs

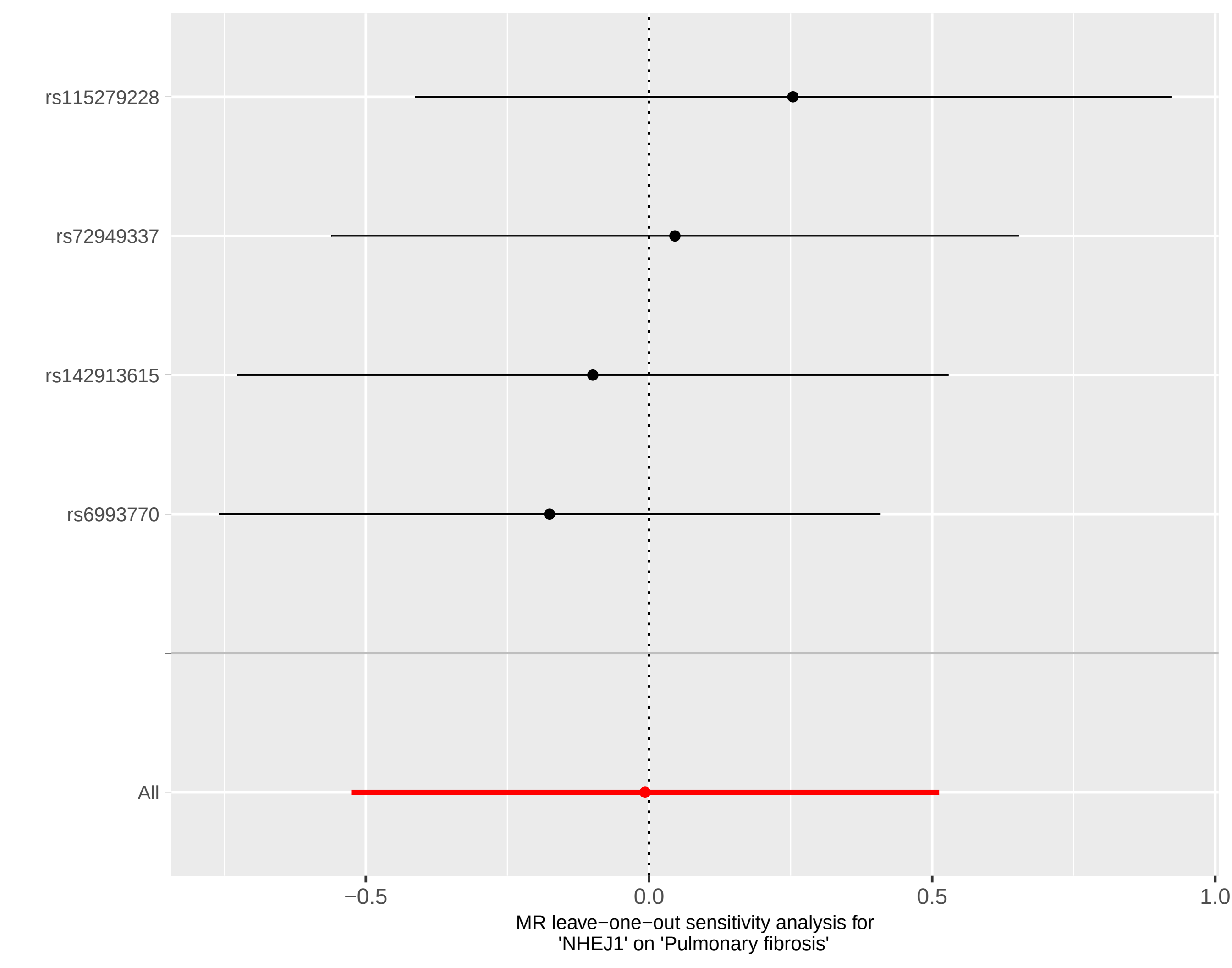

rs115279228
rs72949337
rs142913615
rs6993770
All
−0.5
0.0
0.5
1.0
MR leave−one−out sensitivity analysis for
'NHEJ1' on 'Pulmonary fibrosis'

Insufficient number of SNPs

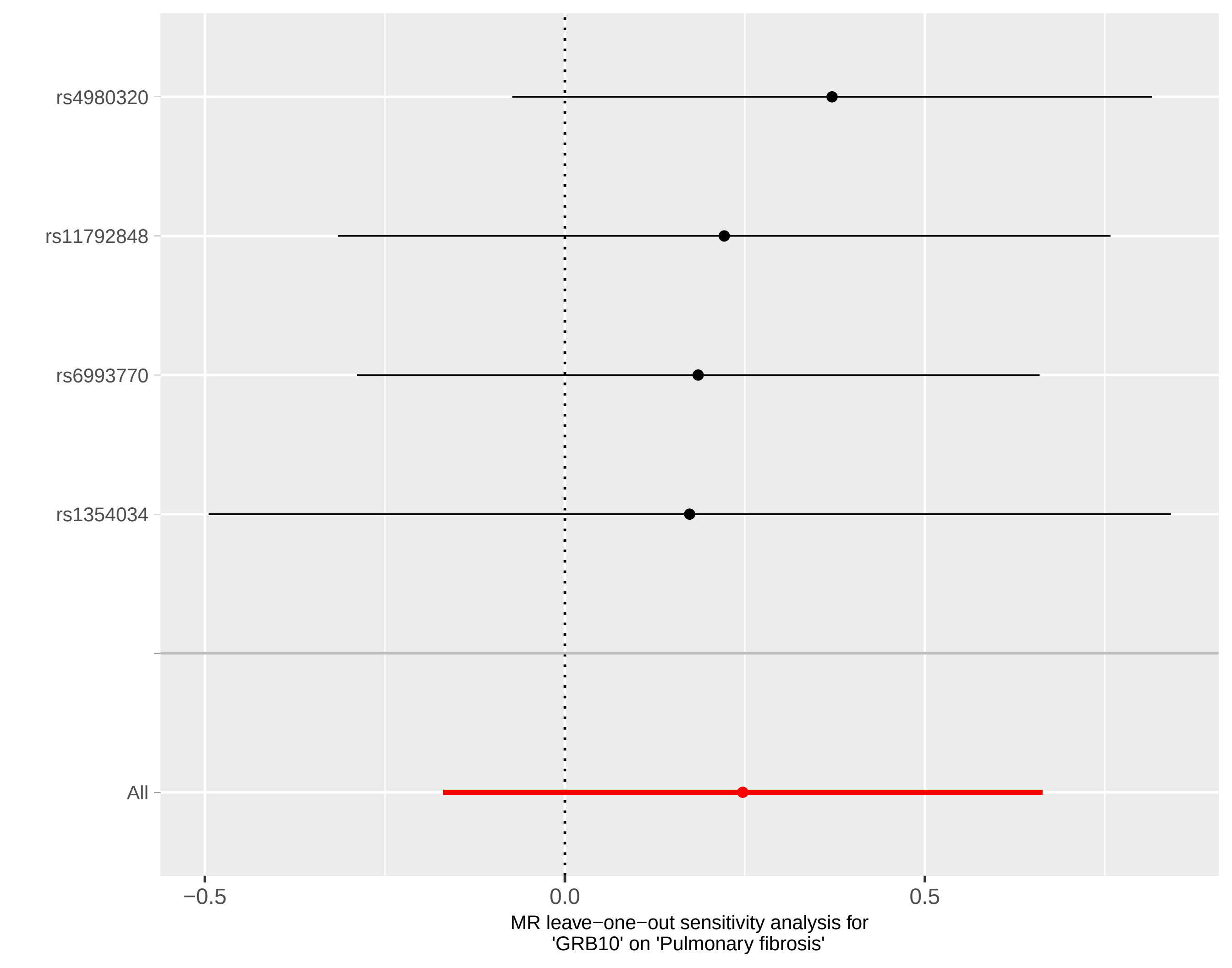

rs4980320
rs11792848
rs6993770
rs1354034
All
−0.5
0.0
0.5
MR leave−one−out sensitivity analysis for
'GRB10' on 'Pulmonary fibrosis'

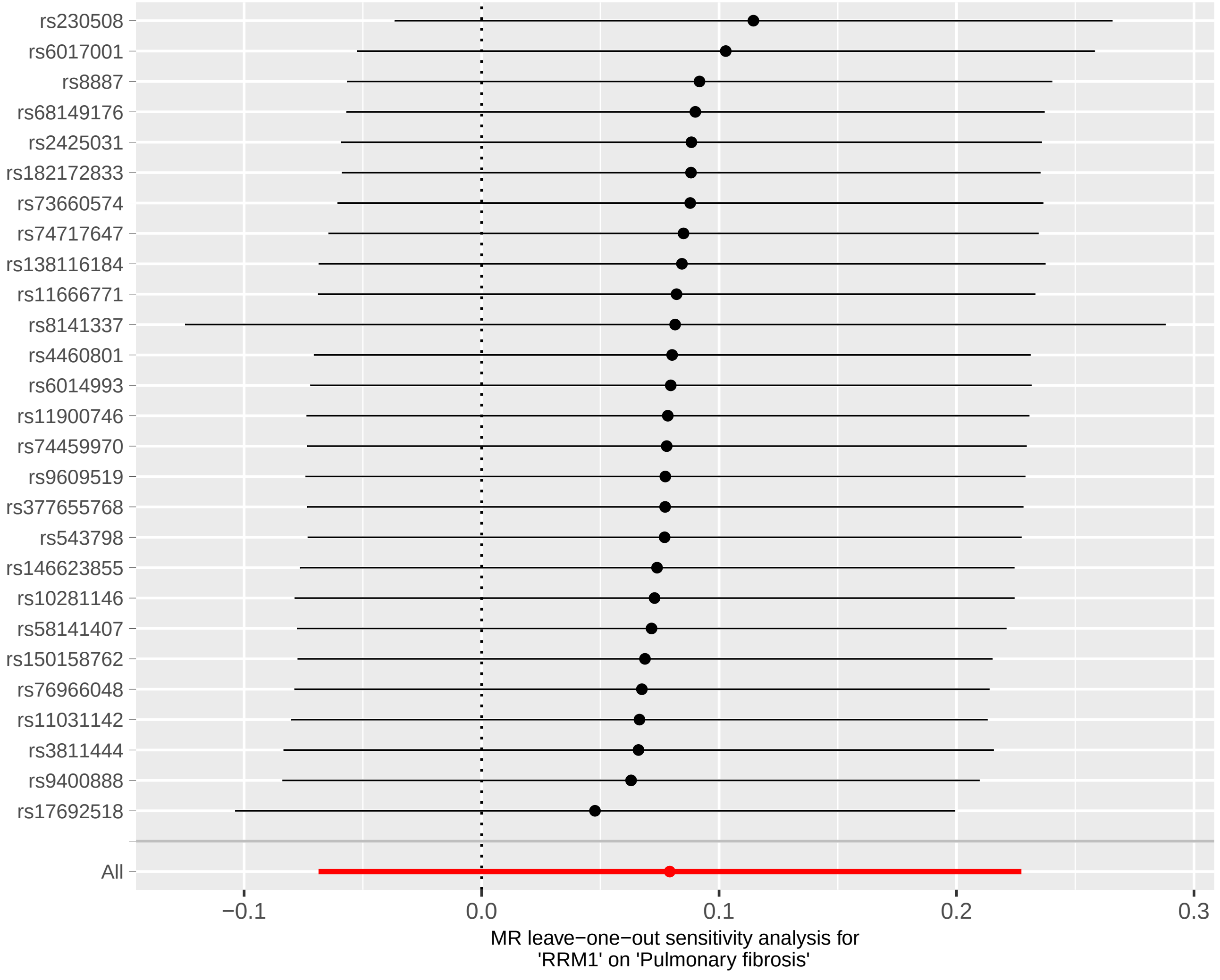
rs230508
rs6017001
rs8887
rs68149176
rs2425031
rs182172833
rs73660574
rs74717647
rs138116184
rs11666771
rs8141337
rs4460801
rs6014993
rs11900746
rs74459970
rs9609519
rs377655768
rs543798
rs146623855
rs10281146
rs58141407
rs150158762
rs76966048
rs11031142
rs3811444
rs9400888
rs17692518
All
−0.1
0.0
0.1
0.2
0.3
MR leave−one−out sensitivity analysis for
'RRM1' on 'Pulmonary fibrosis'

Insufficient number of SNPs

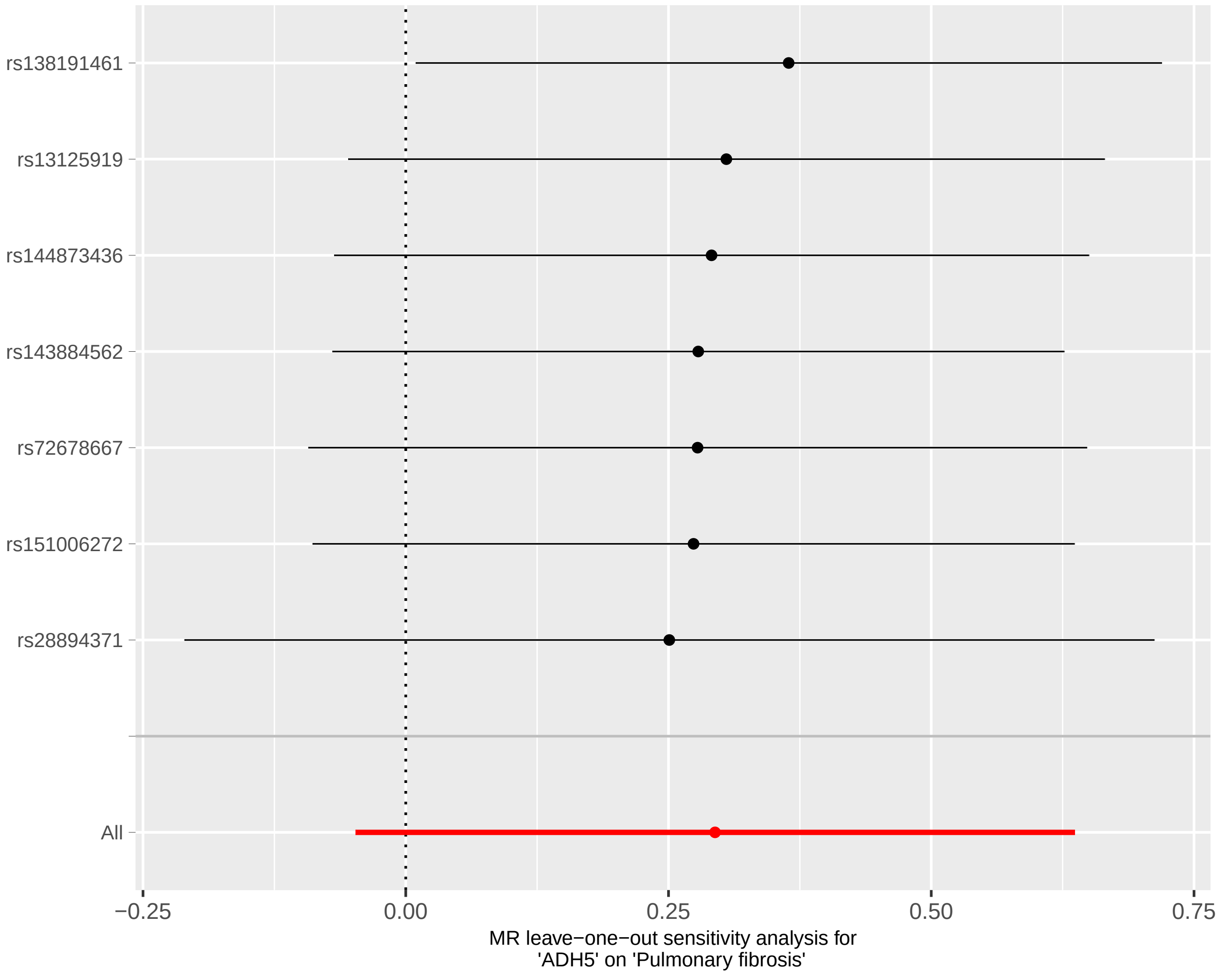

rs138191461
rs13125919
rs144873436
rs143884562
rs72678667
rs151006272
rs28894371
All
−0.25
0.00
0.25
0.50
0.75
MR leave−one−out sensitivity analysis for
'ADH5' on 'Pulmonary fibrosis'

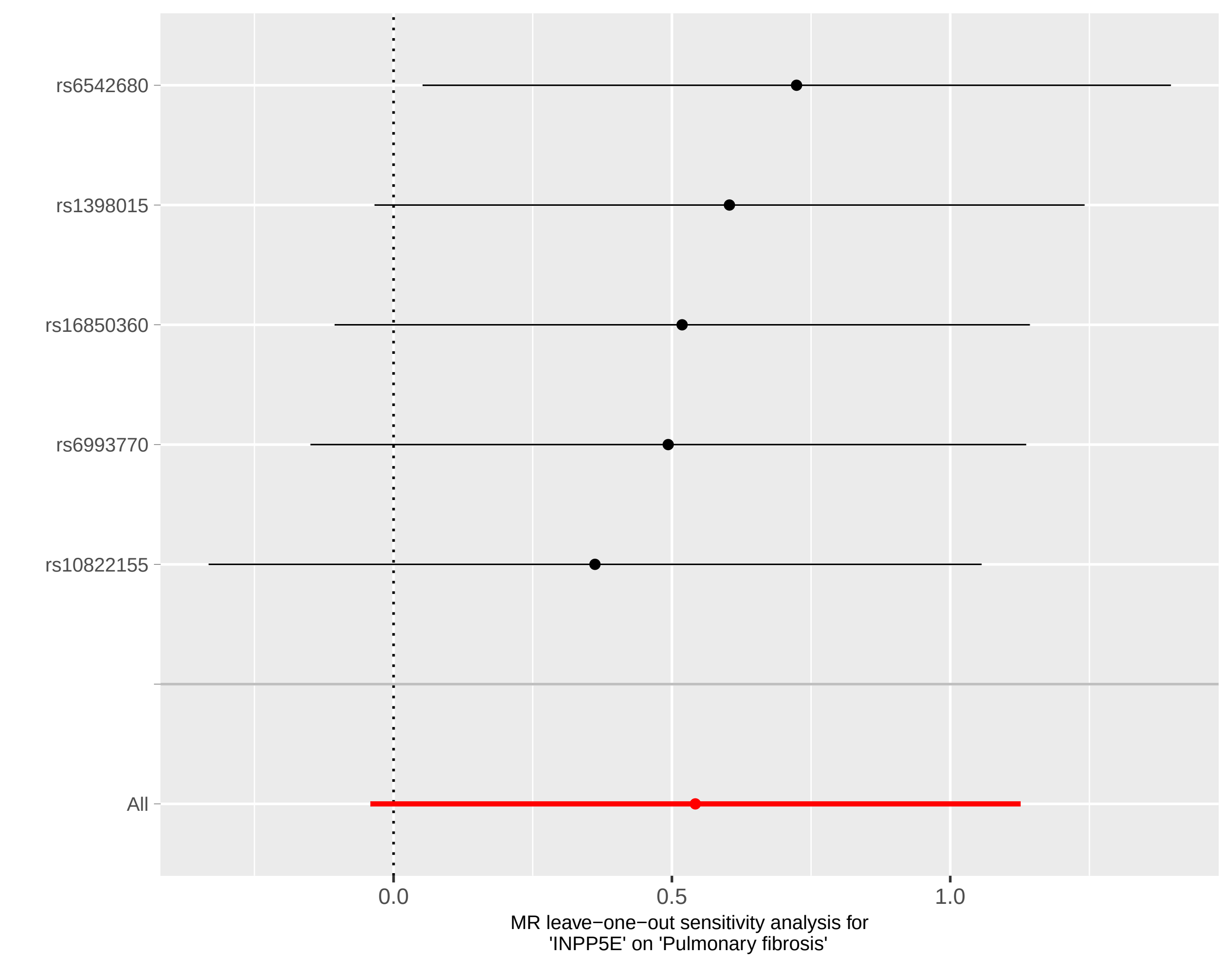
rs6542680
rs1398015
rs16850360
rs6993770
rs10822155
All
0.0
0.5
1.0
MR leave−one−out sensitivity analysis for
'INPP5E' on 'Pulmonary fibrosis'

Insufficient number of SNPs

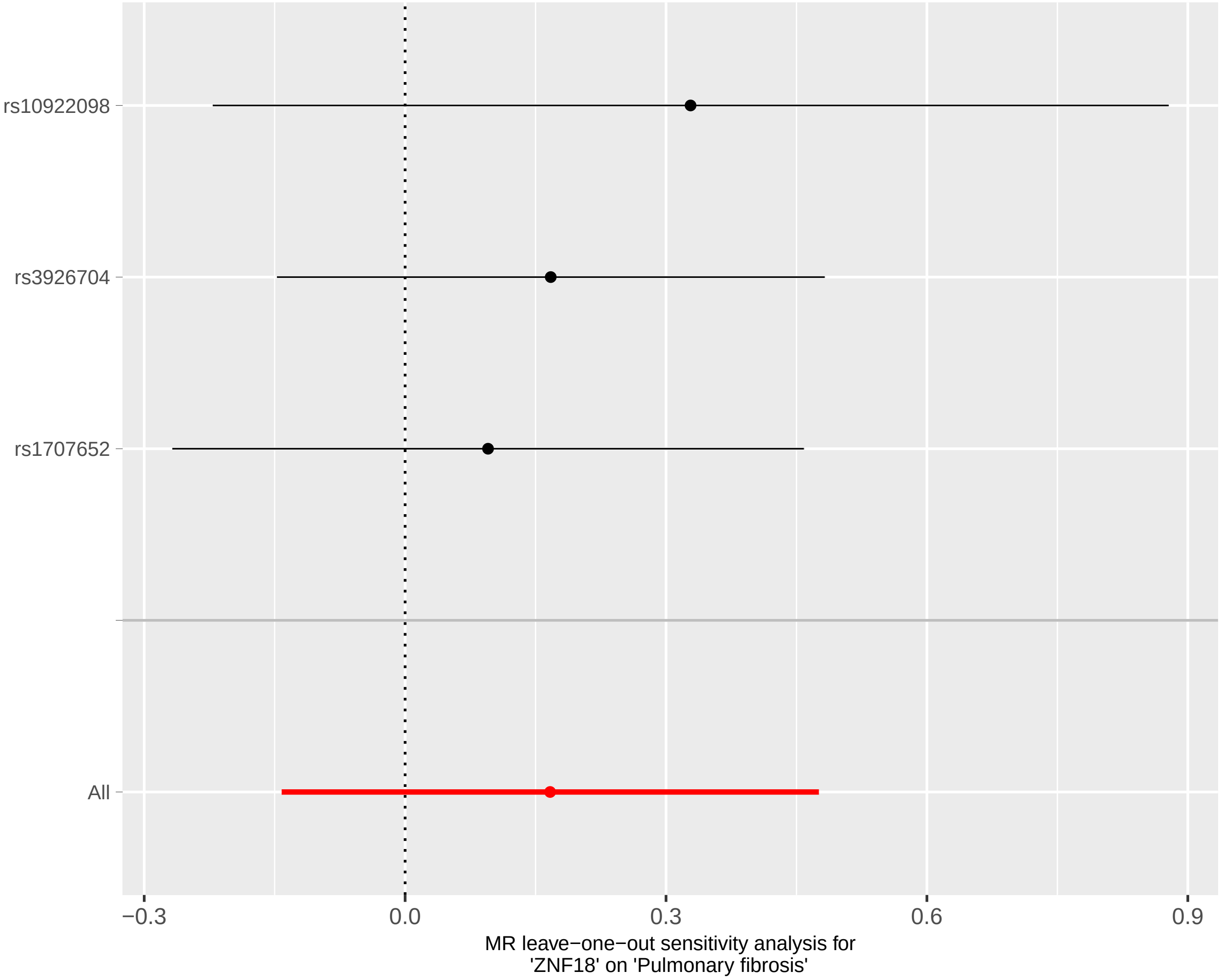

rs10922098
rs3926704
rs1707652
All
−0.3
0.0
0.3
0.6
0.9
MR leave−one−out sensitivity analysis for
'ZNF18' on 'Pulmonary fibrosis'

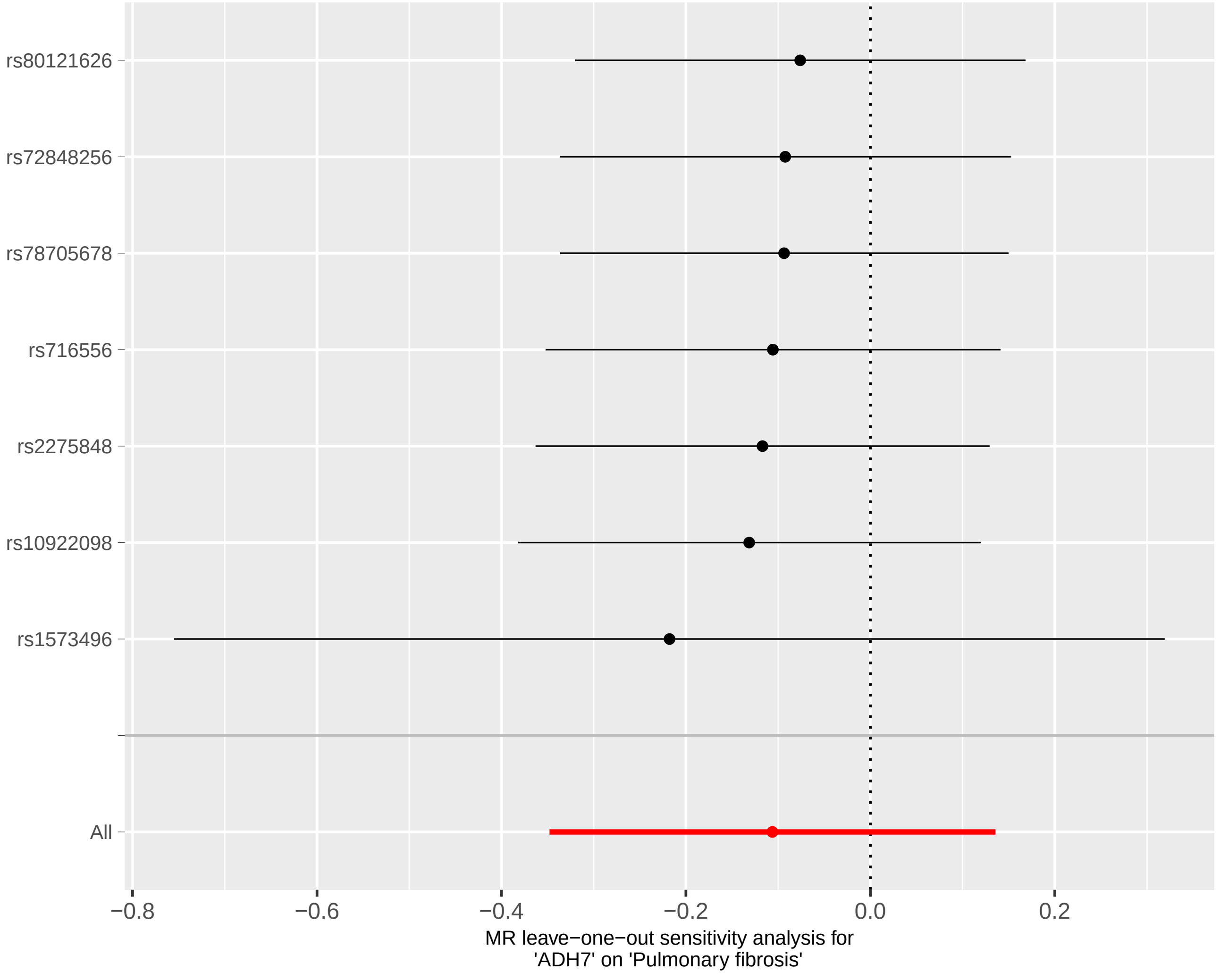

rs80121626
rs72848256
rs78705678
rs716556
rs2275848
rs10922098
rs1573496
All
−0.8
−0.6
−0.4
−0.2
0.0
0.2
MR leave−one−out sensitivity analysis for
'ADH7' on 'Pulmonary fibrosis'

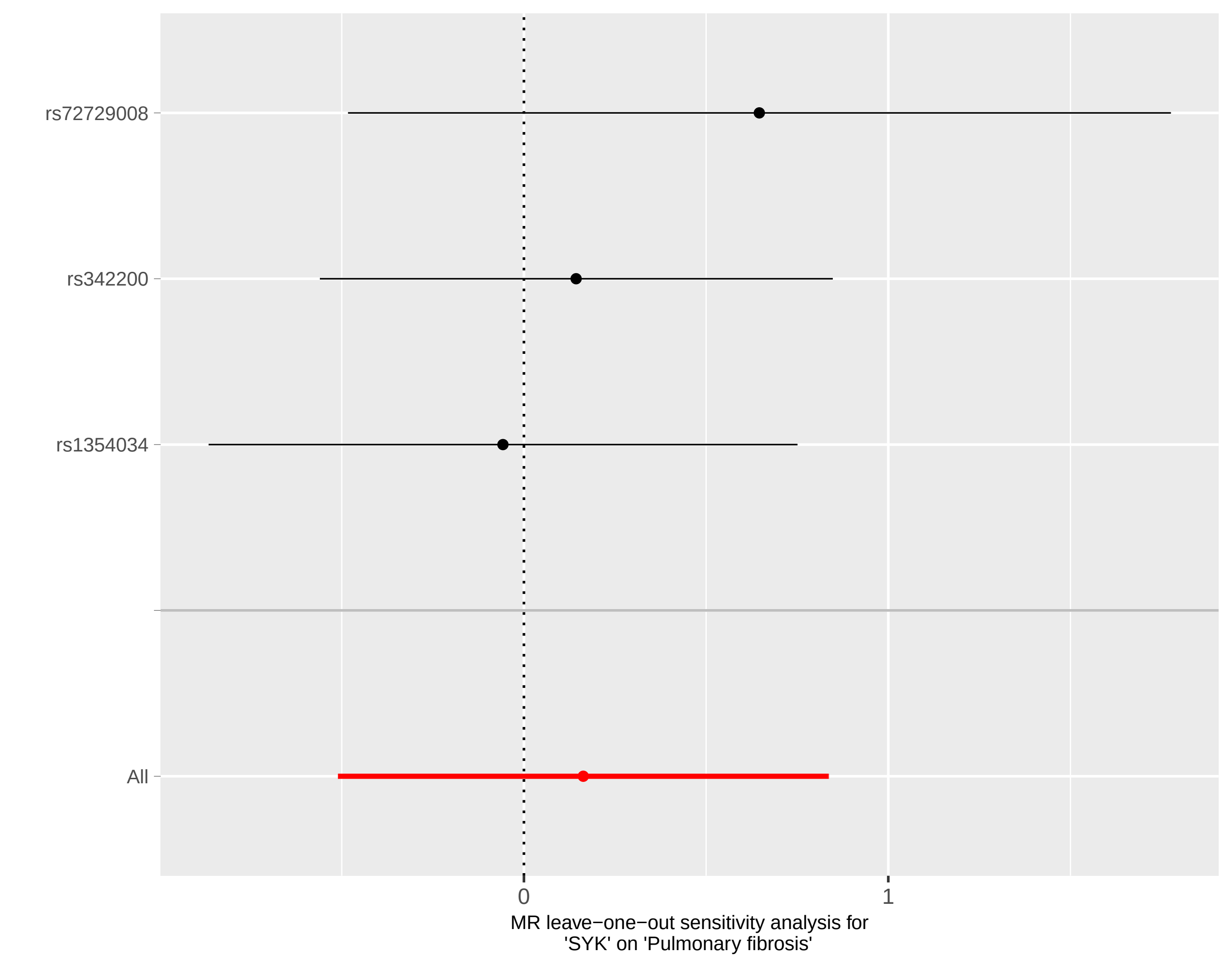
rs72729008
rs342200
rs1354034
All
0
1
MR leave−one−out sensitivity analysis for
'SYK' on 'Pulmonary fibrosis'

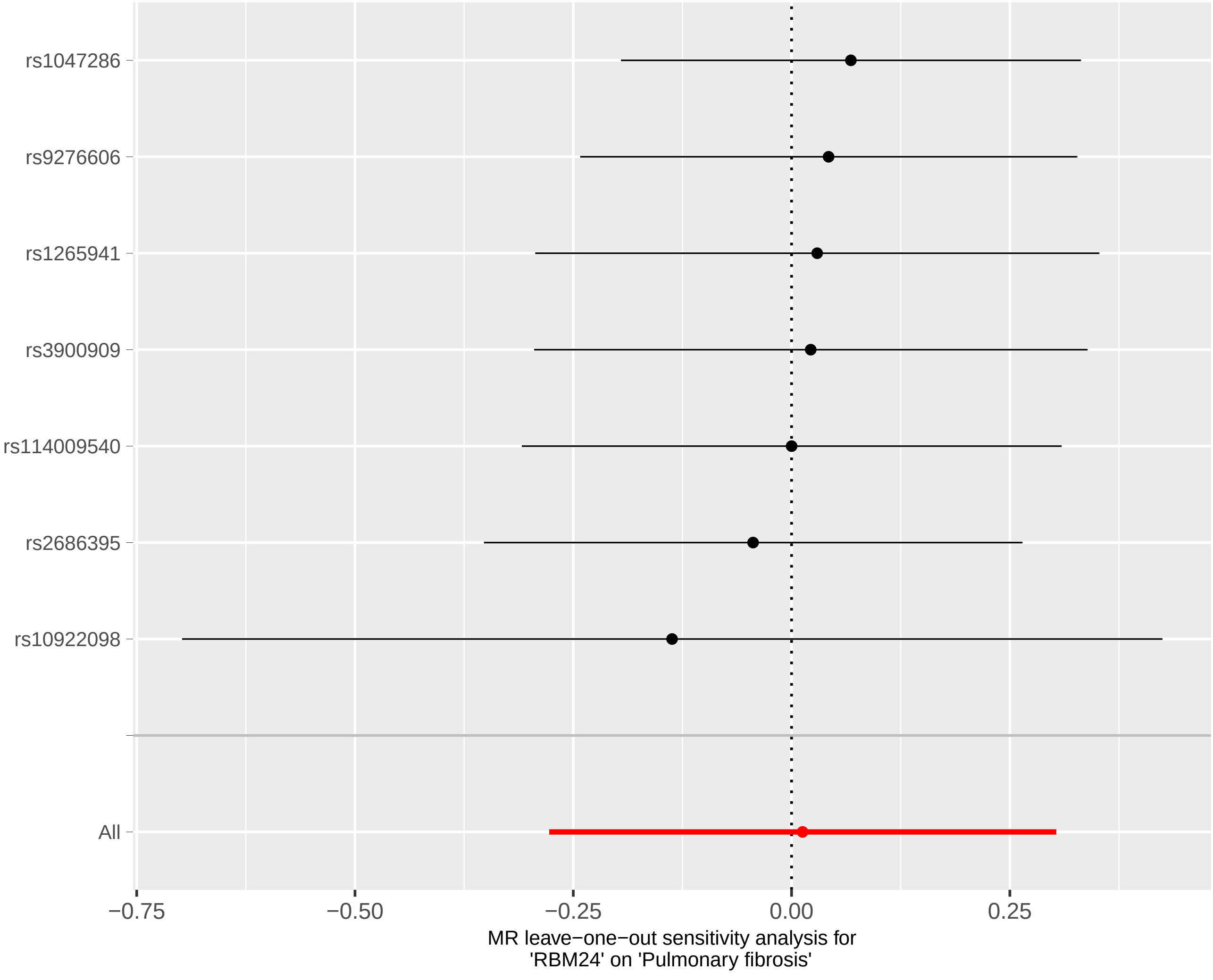
rs1047286
rs9276606
rs1265941
rs3900909
rs114009540
rs2686395
rs10922098
All
−0.75
−0.50
−0.25
0.00
0.25
MR leave−one−out sensitivity analysis for
'RBM24' on 'Pulmonary fibrosis'

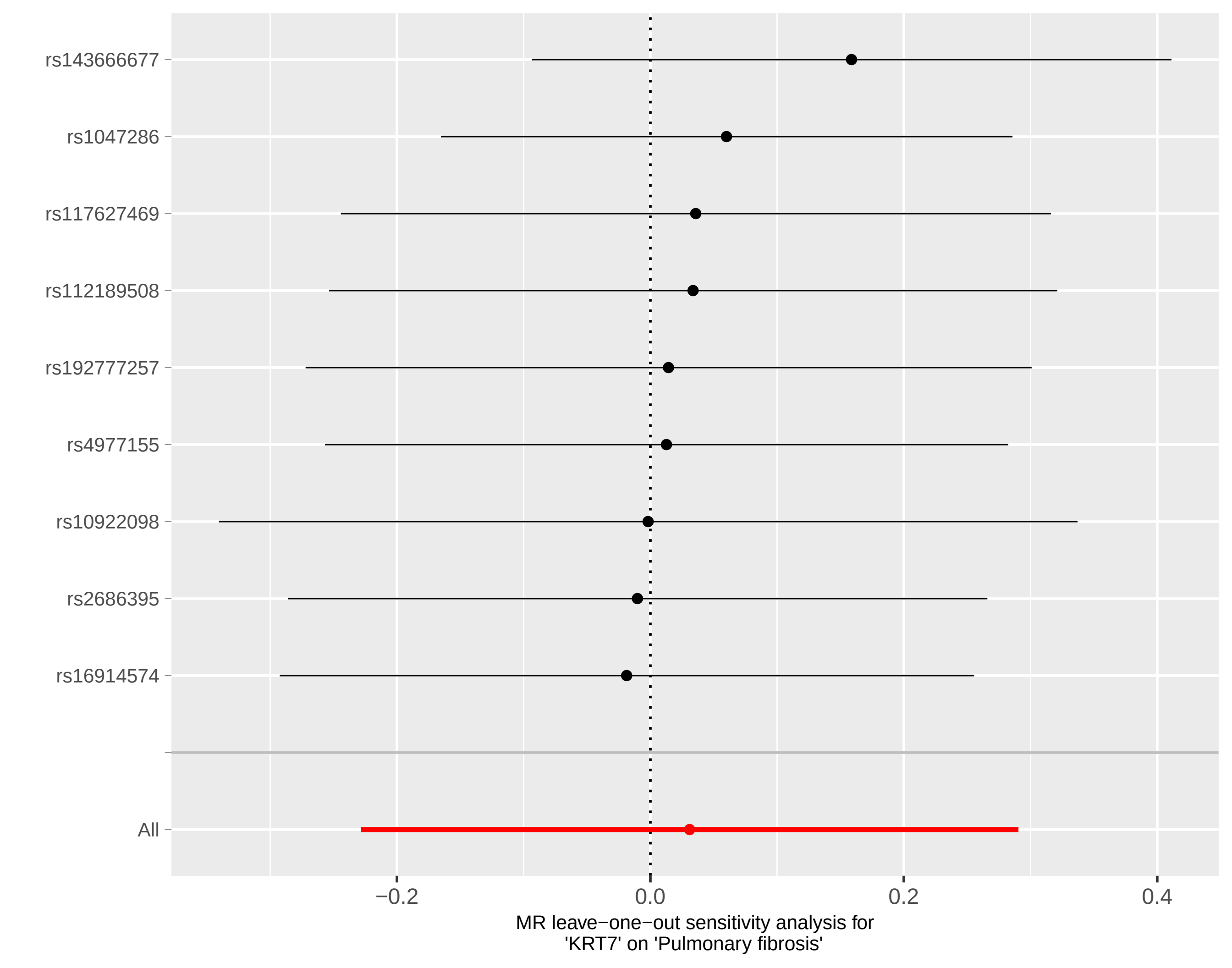

rs143666677
rs1047286
rs117627469
rs112189508
rs192777257
rs4977155
rs10922098
rs2686395
rs16914574
All
−0.2
0.0
0.2
0.4
MR leave−one−out sensitivity analysis for
'KRT7' on 'Pulmonary fibrosis'

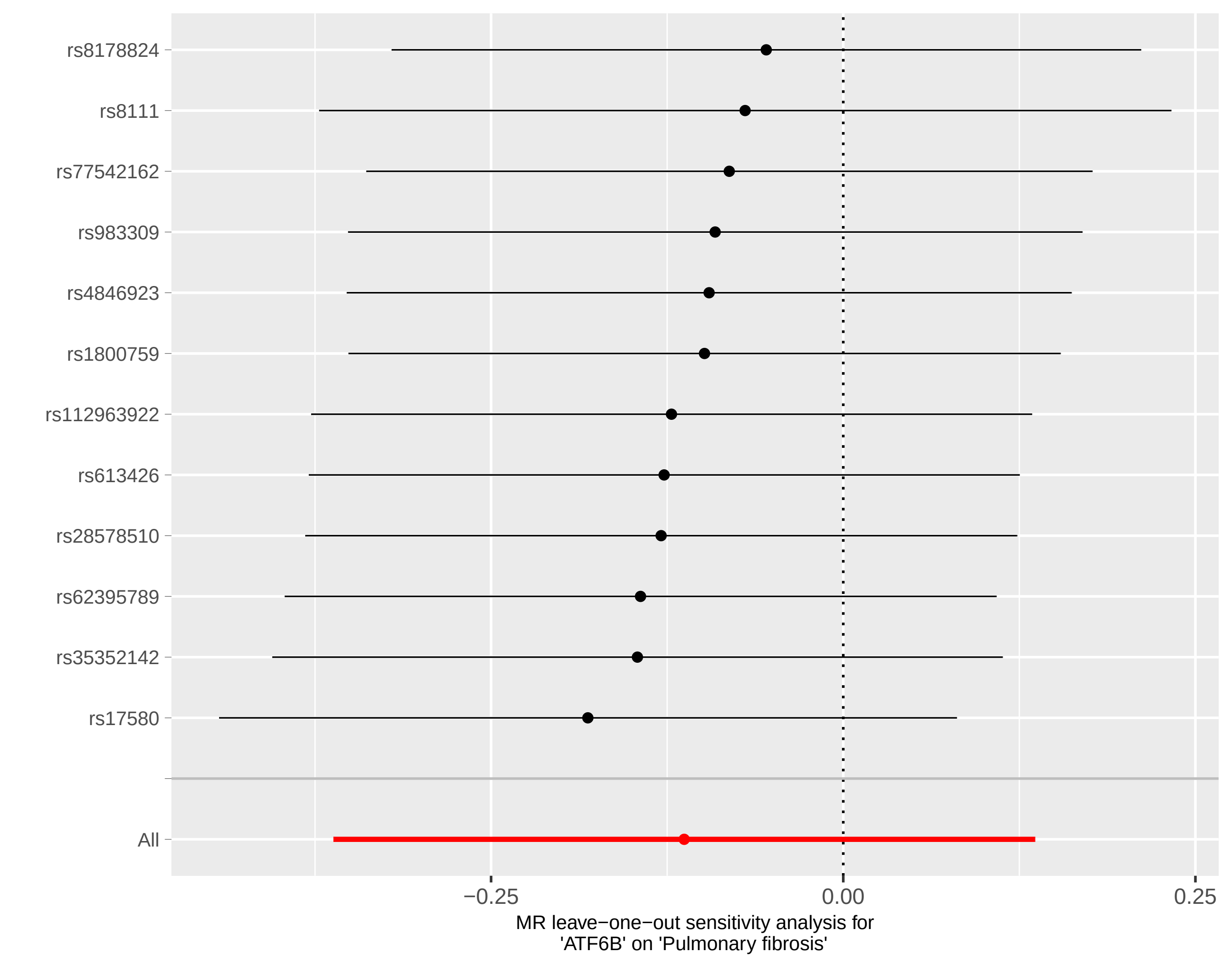

rs8178824
rs8111
rs77542162
rs983309
rs4846923
rs1800759
rs112963922
rs613426
rs28578510
rs62395789
rs35352142
rs17580
All
−0.25
0.00
0.25
MR leave−one−out sensitivity analysis for
'ATF6B' on 'Pulmonary fibrosis'

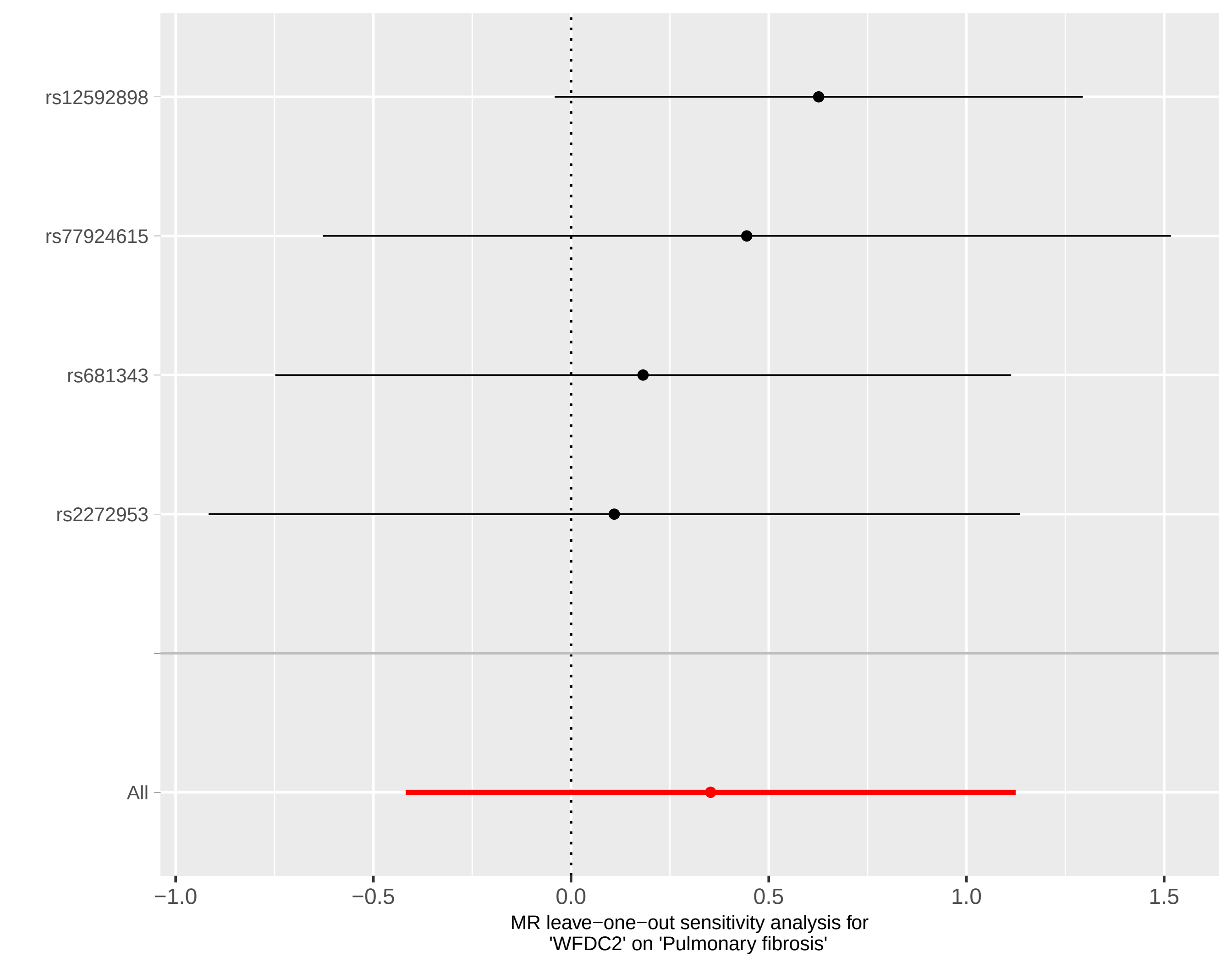
rs12592898
rs77924615
rs681343
rs2272953
All
−1.0
−0.5
0.0
0.5
1.0
1.5
MR leave−one−out sensitivity analysis for
'WFDC2' on 'Pulmonary fibrosis'

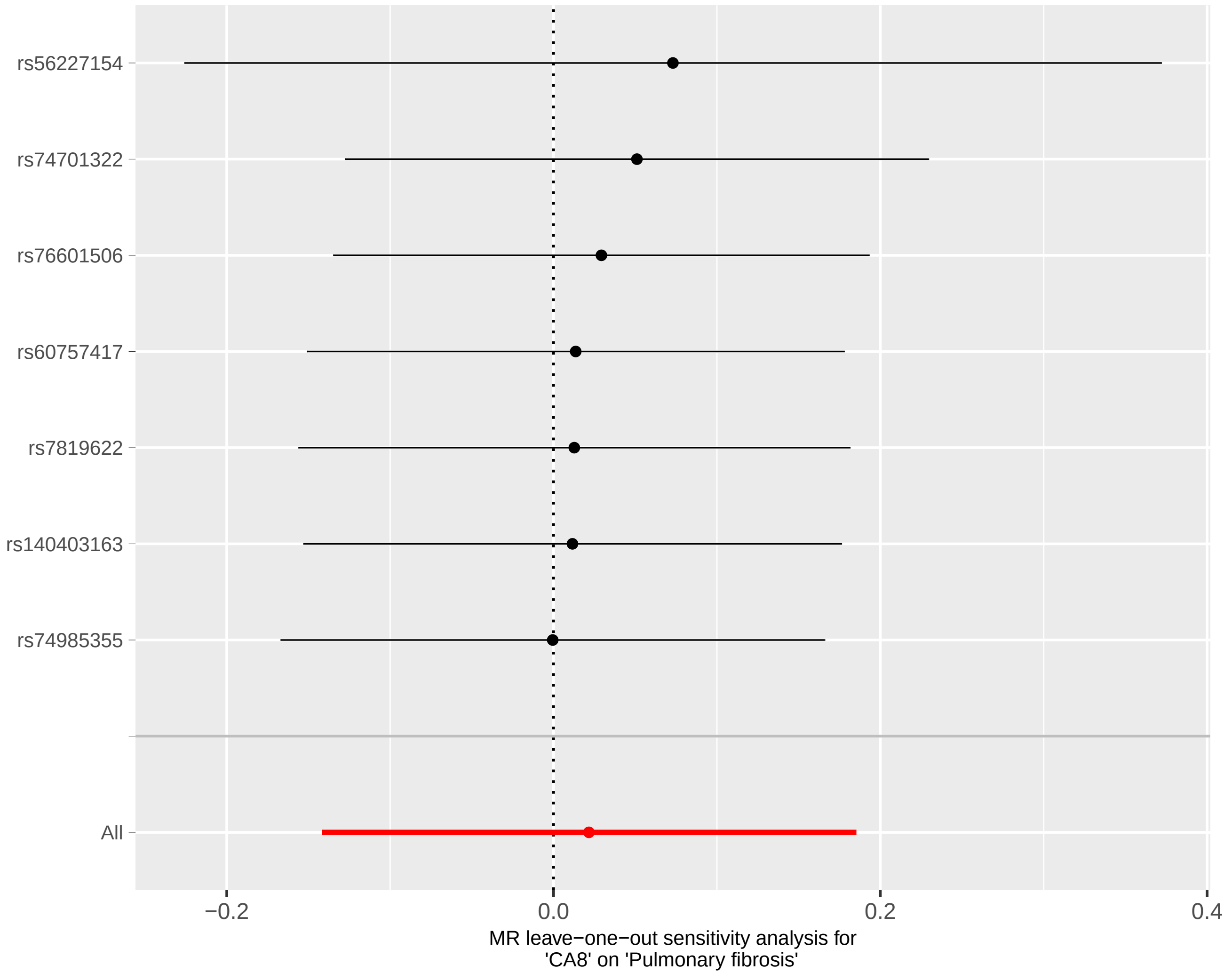
rs56227154
rs74701322
rs76601506
rs60757417
rs7819622
rs140403163
rs74985355
All
−0.2
0.0
0.2
0.4
MR leave−one−out sensitivity analysis for
'CA8' on 'Pulmonary fibrosis'

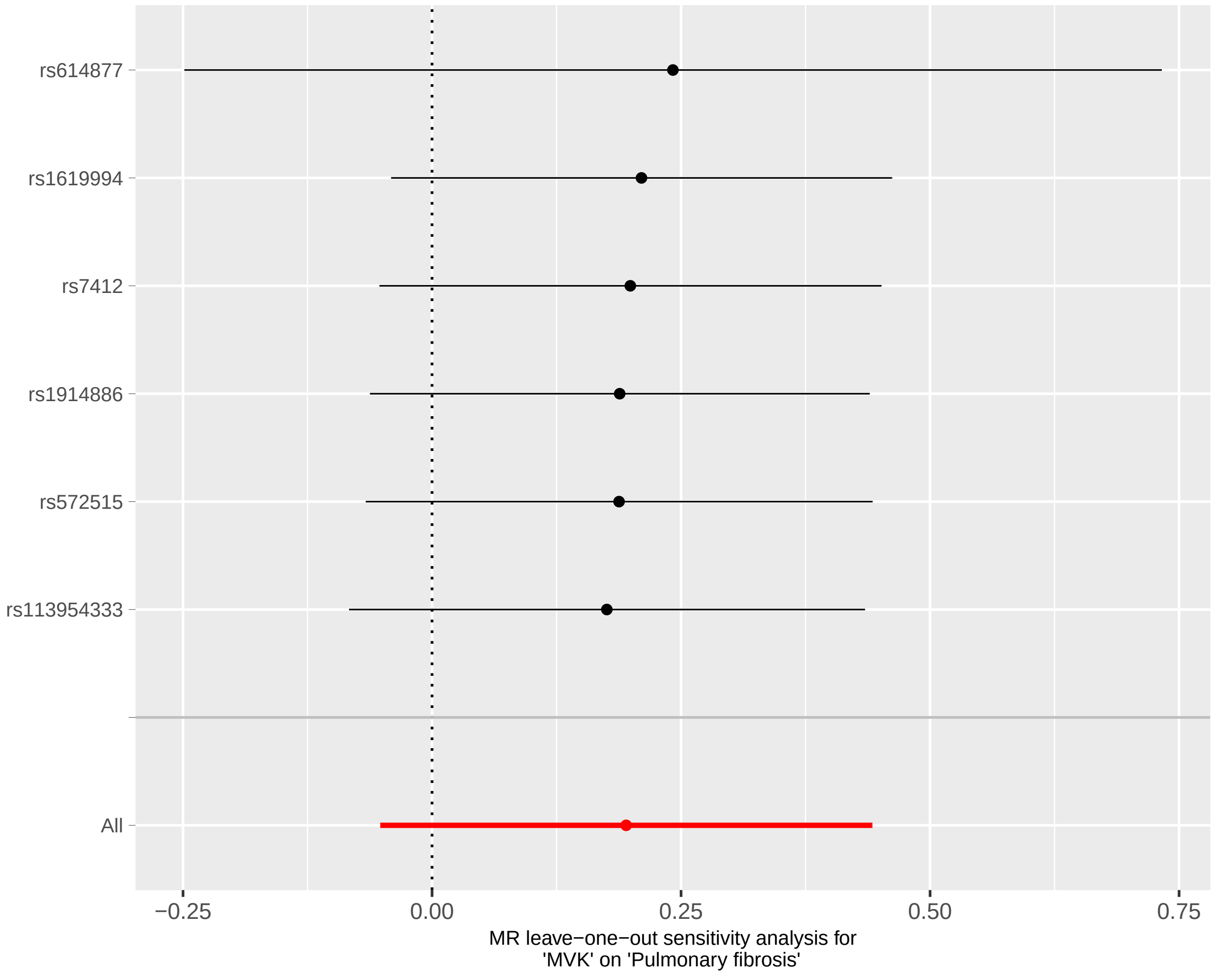

rs614877
rs1619994
rs7412
rs1914886
rs572515
rs113954333
All
−0.25
0.00
0.25
0.50
0.75
MR leave−one−out sensitivity analysis for
'MVK' on 'Pulmonary fibrosis'

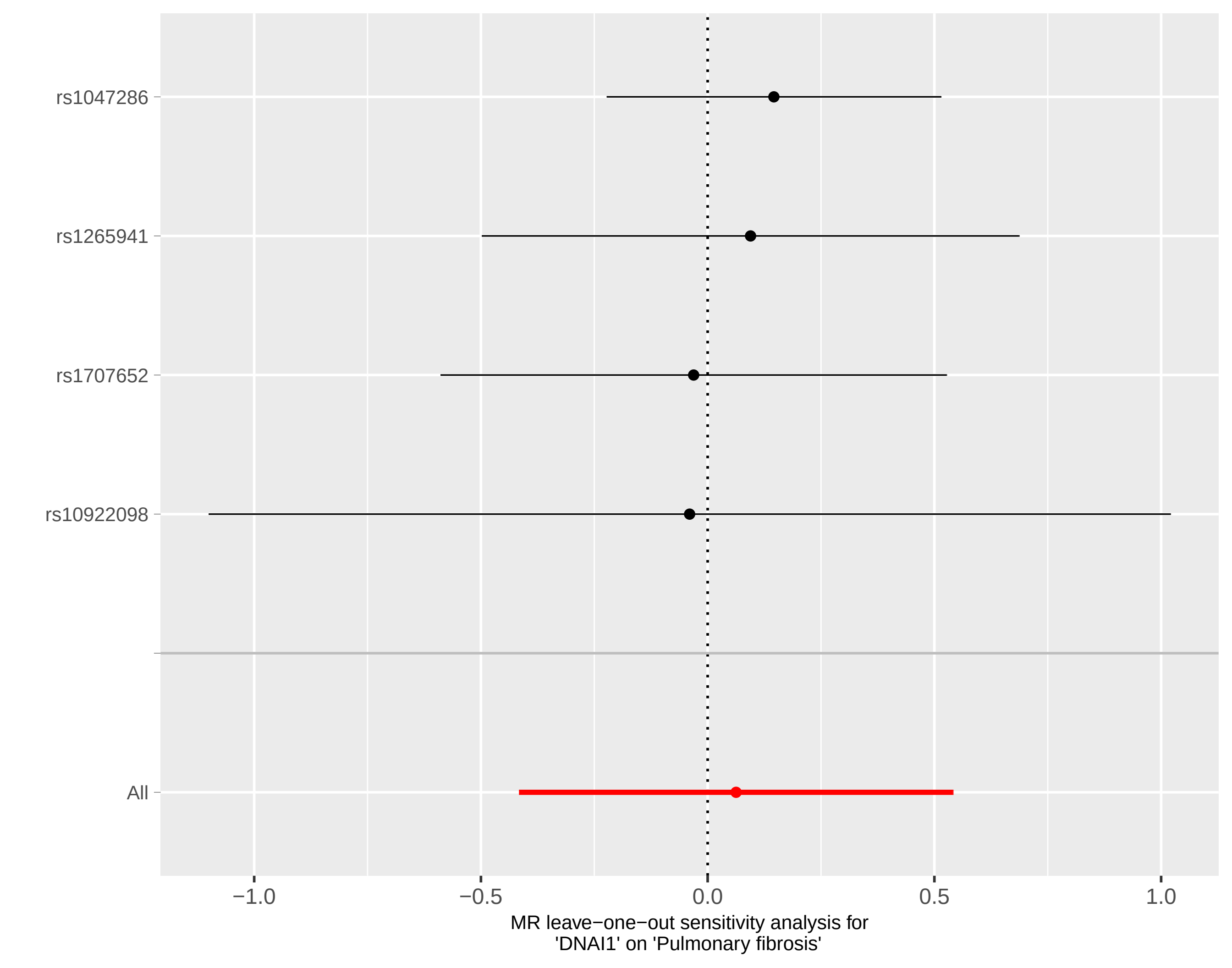
rs1047286
rs1265941
rs1707652
rs10922098
All
−1.0
−0.5
0.0
0.5
1.0
MR leave−one−out sensitivity analysis for
'DNAI1' on 'Pulmonary fibrosis'

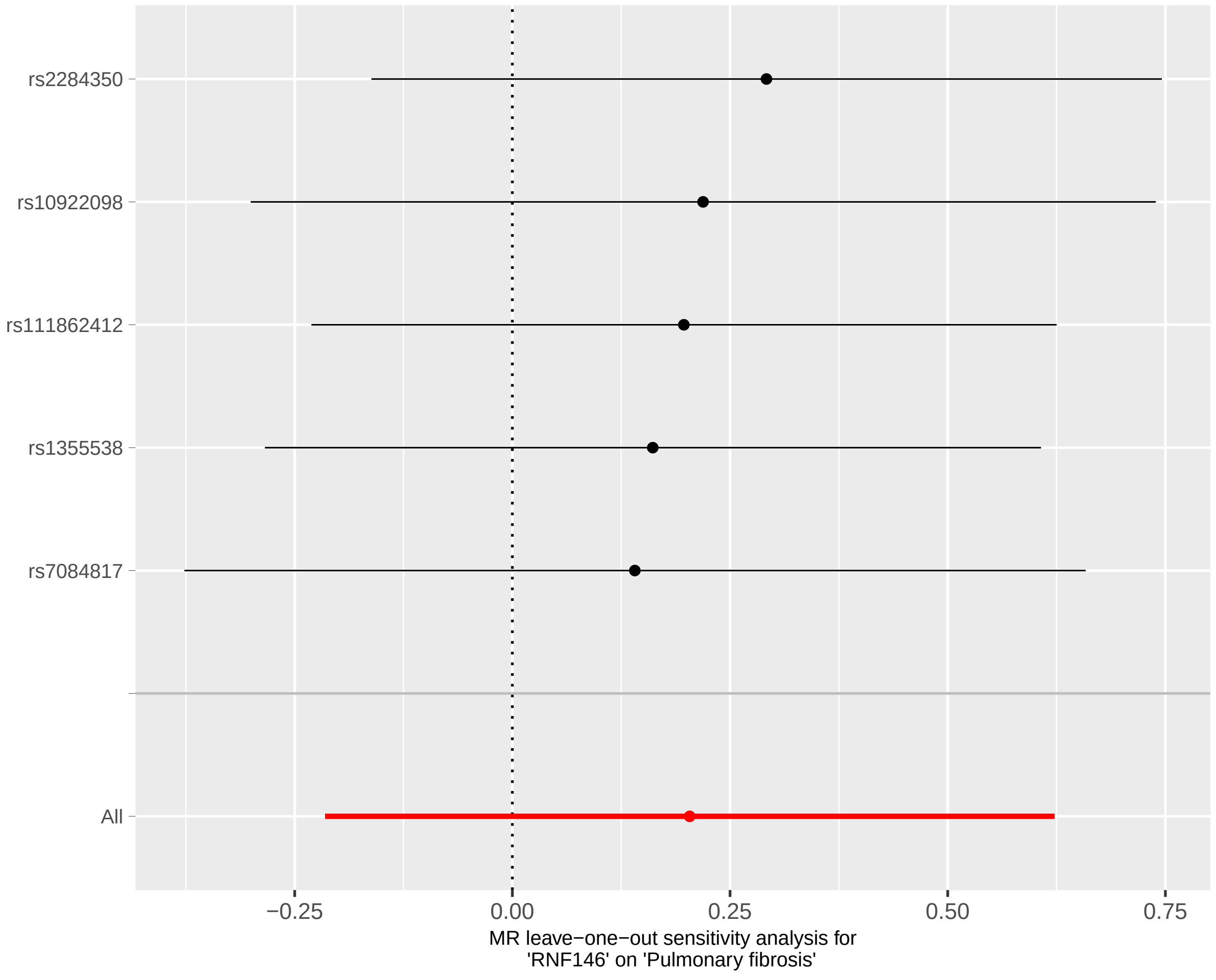
rs2284350
rs10922098
rs111862412
rs1355538
rs7084817
All
−0.25
0.00
0.25
0.50
0.75
MR leave−one−out sensitivity analysis for
'RNF146' on 'Pulmonary fibrosis'

Insufficient number of SNPs

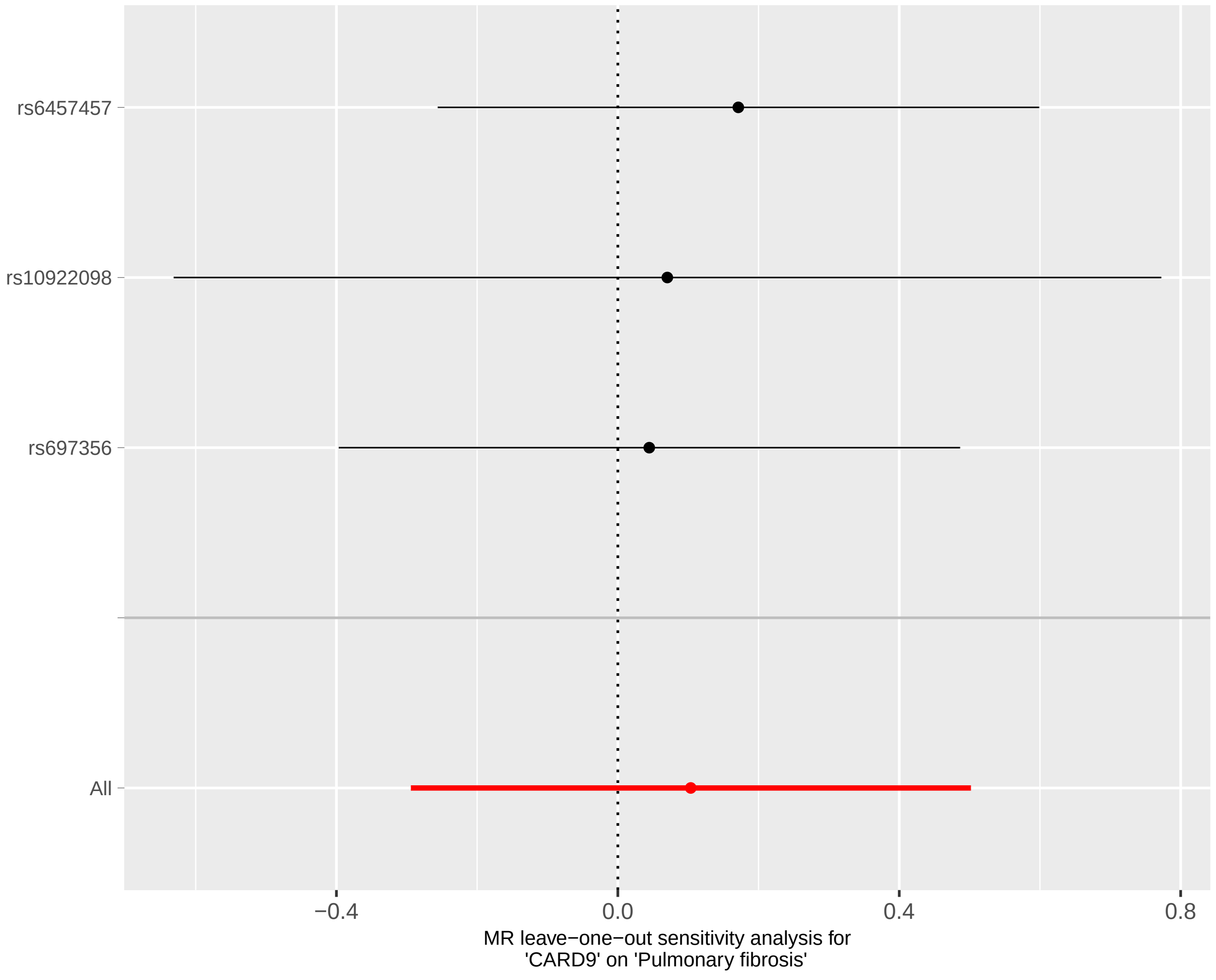
rs6457457
rs10922098
rs697356
All
−0.4
0.0
0.4
0.8
MR leave−one−out sensitivity analysis for
'CARD9' on 'Pulmonary fibrosis'

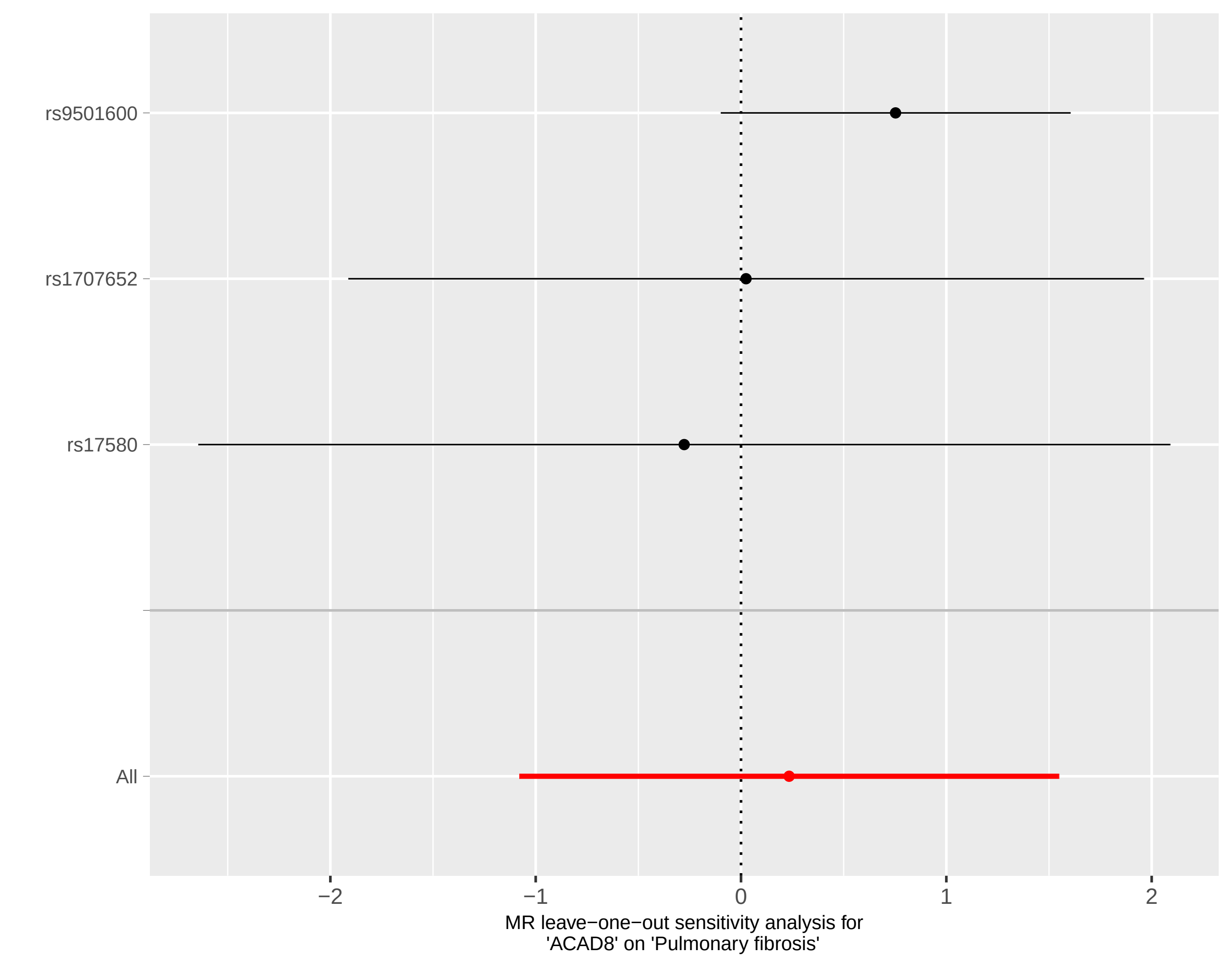
rs9501600
rs1707652
rs17580
All
−2
−1
0
1
2
MR leave−one−out sensitivity analysis for
'ACAD8' on 'Pulmonary fibrosis'

Insufficient number of SNPs

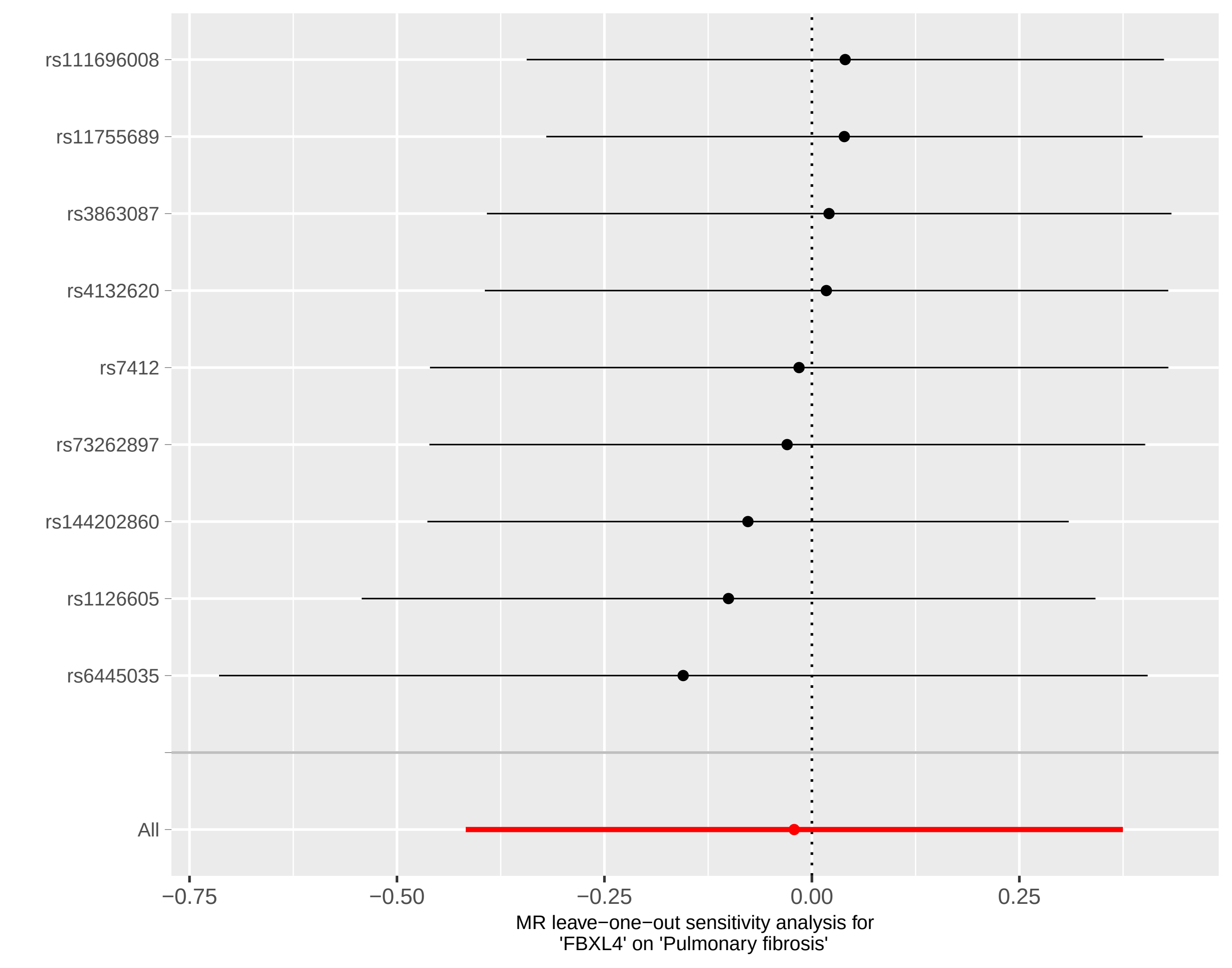
rs111696008
rs11755689
rs3863087
rs4132620
rs7412
rs73262897
rs144202860
rs1126605
rs6445035
All
−0.75
−0.50
−0.25
0.00
0.25
MR leave−one−out sensitivity analysis for
'FBXL4' on 'Pulmonary fibrosis'

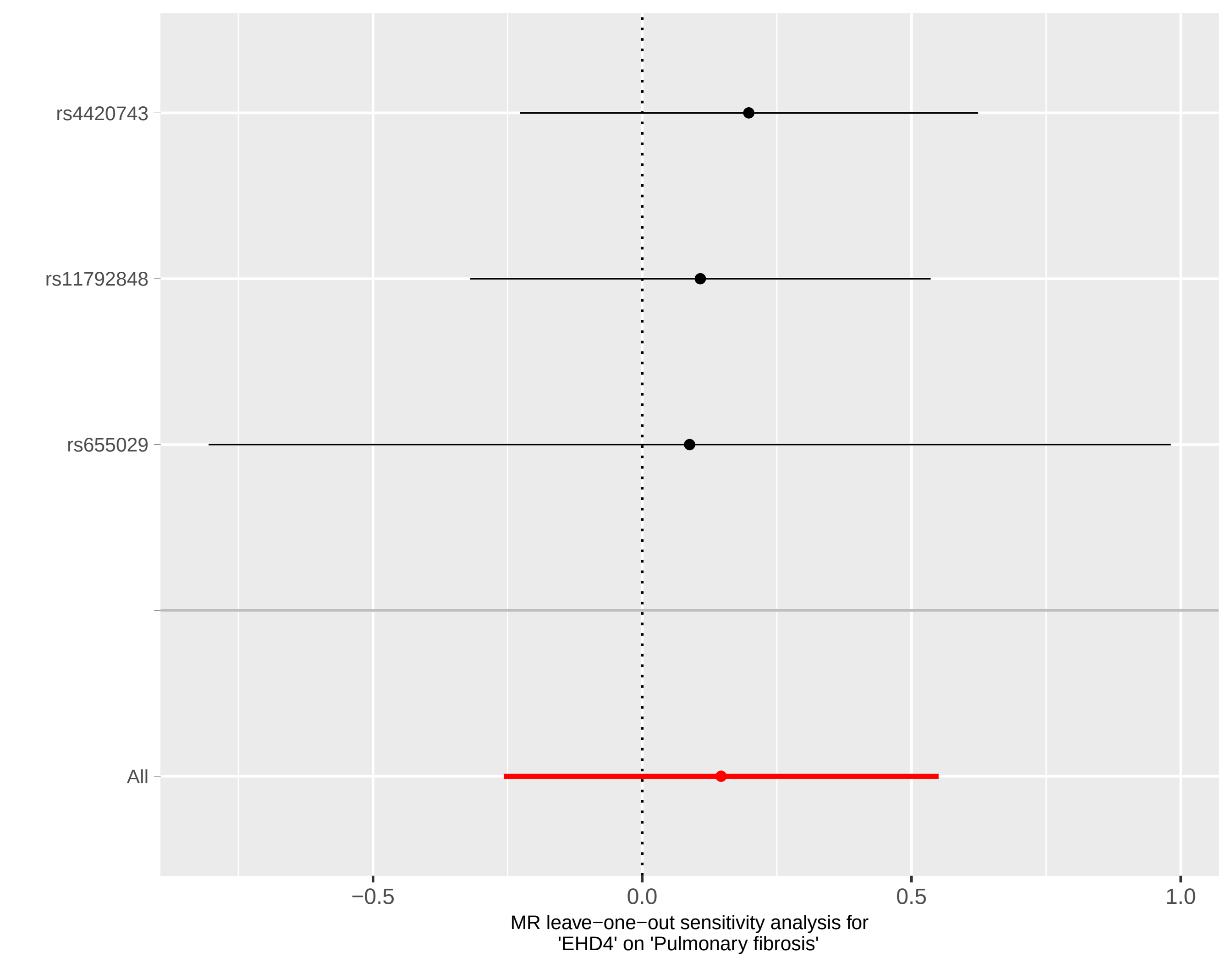

rs4420743
rs11792848
rs655029
All
−0.5
0.0
0.5
1.0
MR leave−one−out sensitivity analysis for
'EHD4' on 'Pulmonary fibrosis'

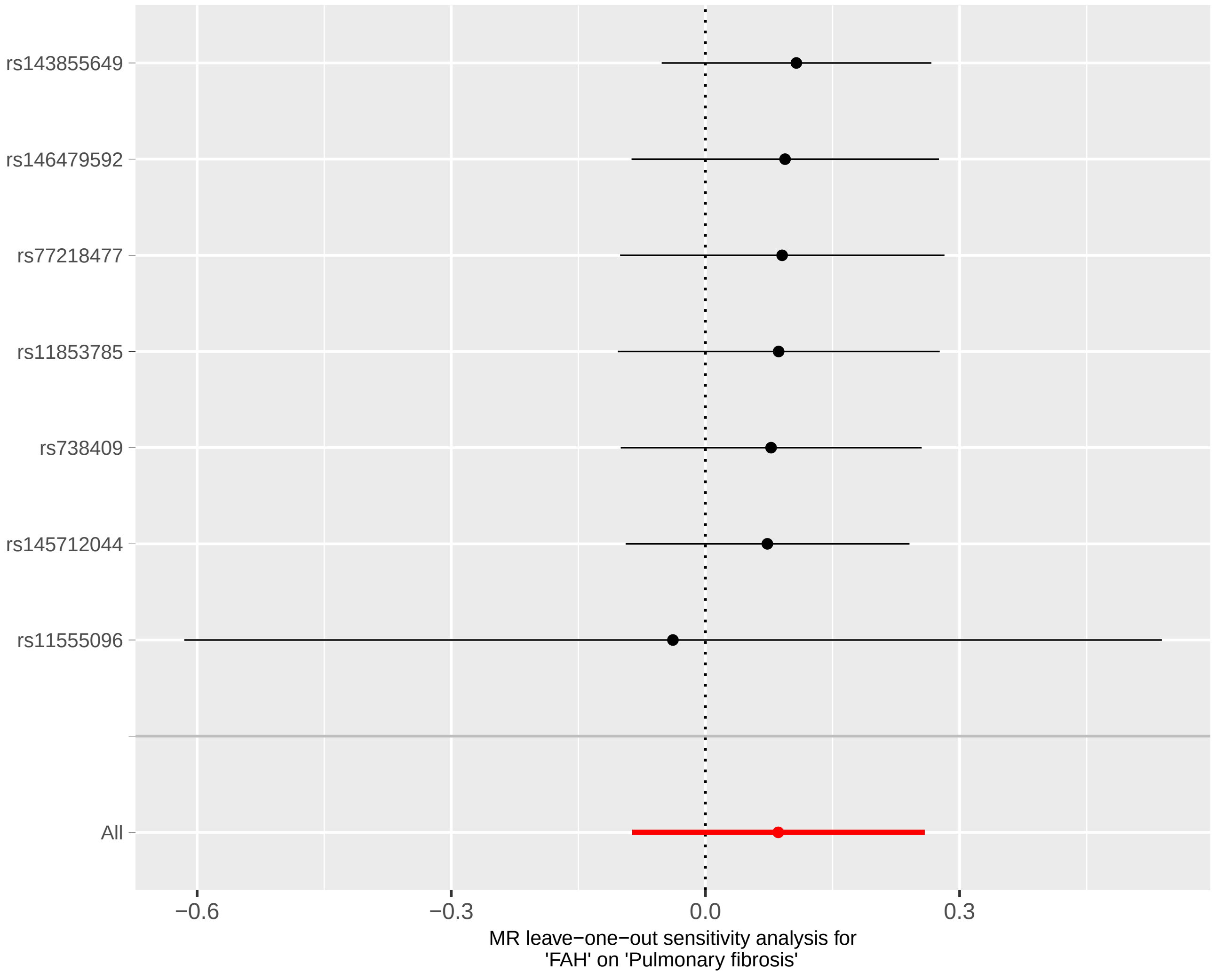
rs143855649
rs146479592
rs77218477
rs11853785
rs738409
rs145712044
rs11555096
All
−0.6
−0.3
0.0
0.3
MR leave−one−out sensitivity analysis for
'FAH' on 'Pulmonary fibrosis'

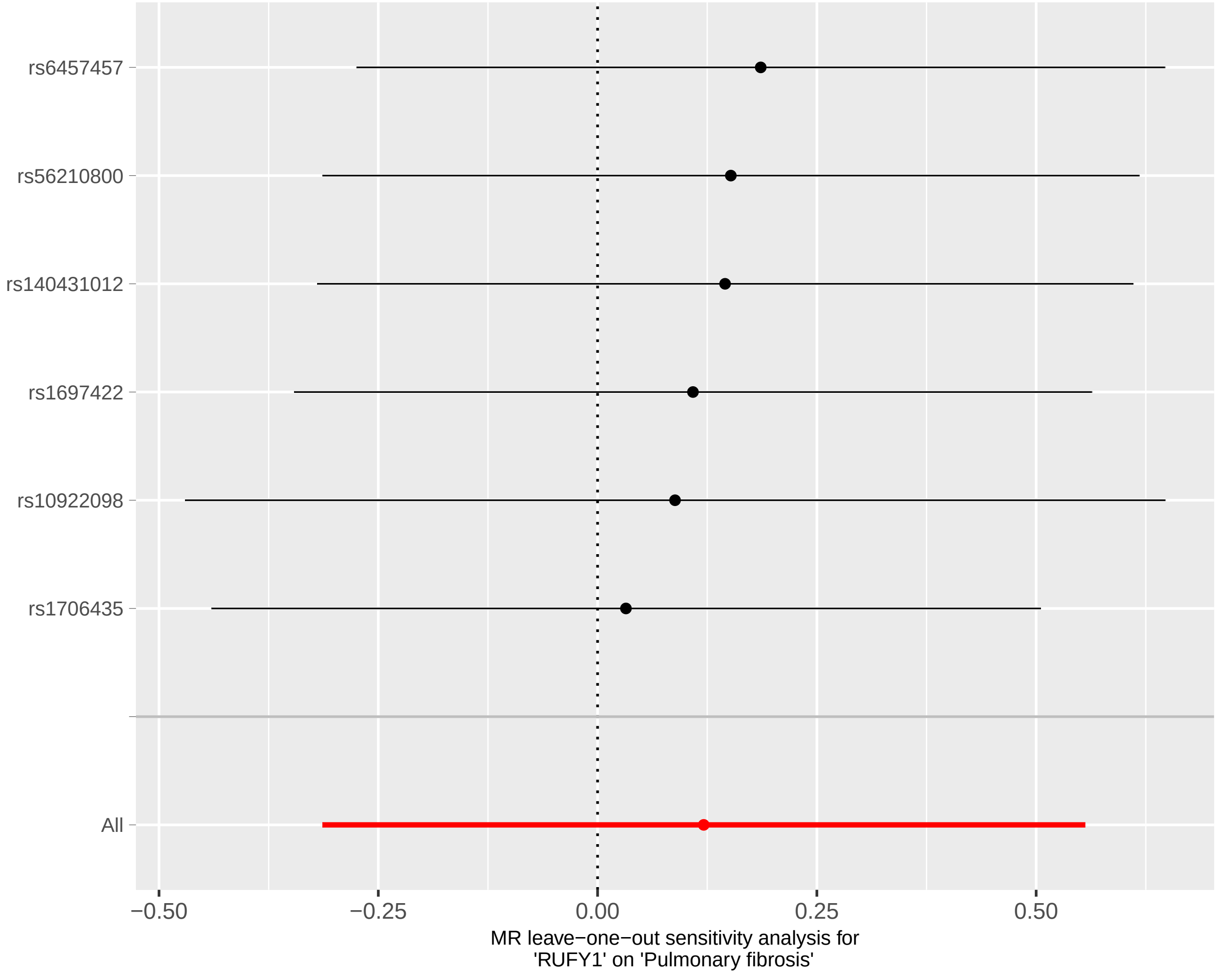

rs6457457
rs56210800
rs140431012
rs1697422
rs10922098
rs1706435
All
−0.50
−0.25
0.00
0.25
0.50
MR leave−one−out sensitivity analysis for
'RUFY1' on 'Pulmonary fibrosis'

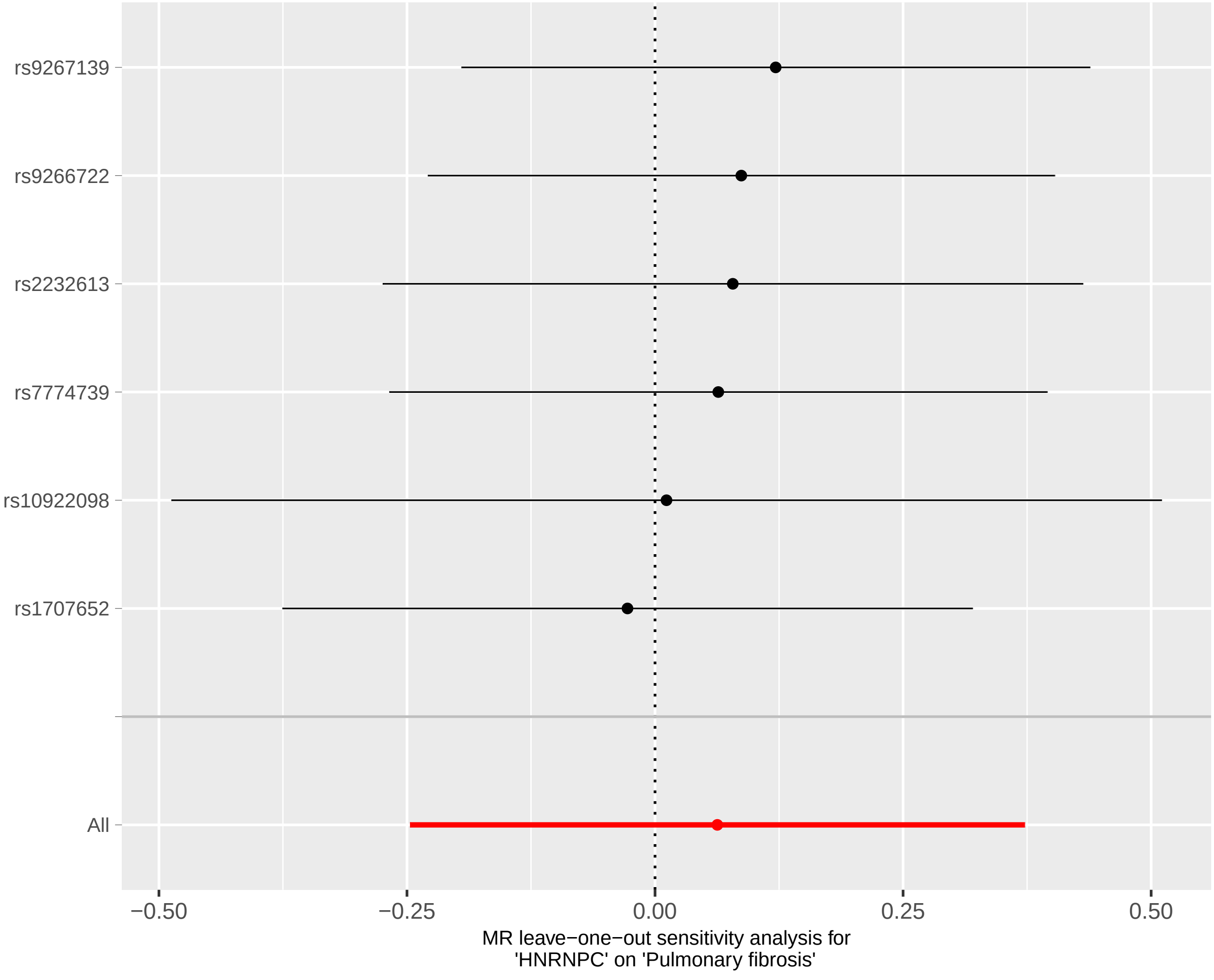

rs9267139
rs9266722
rs2232613
rs7774739
rs10922098
rs1707652
All
−0.50
−0.25
0.00
0.25
0.50
MR leave−one−out sensitivity analysis for
'HNRNPC' on 'Pulmonary fibrosis'

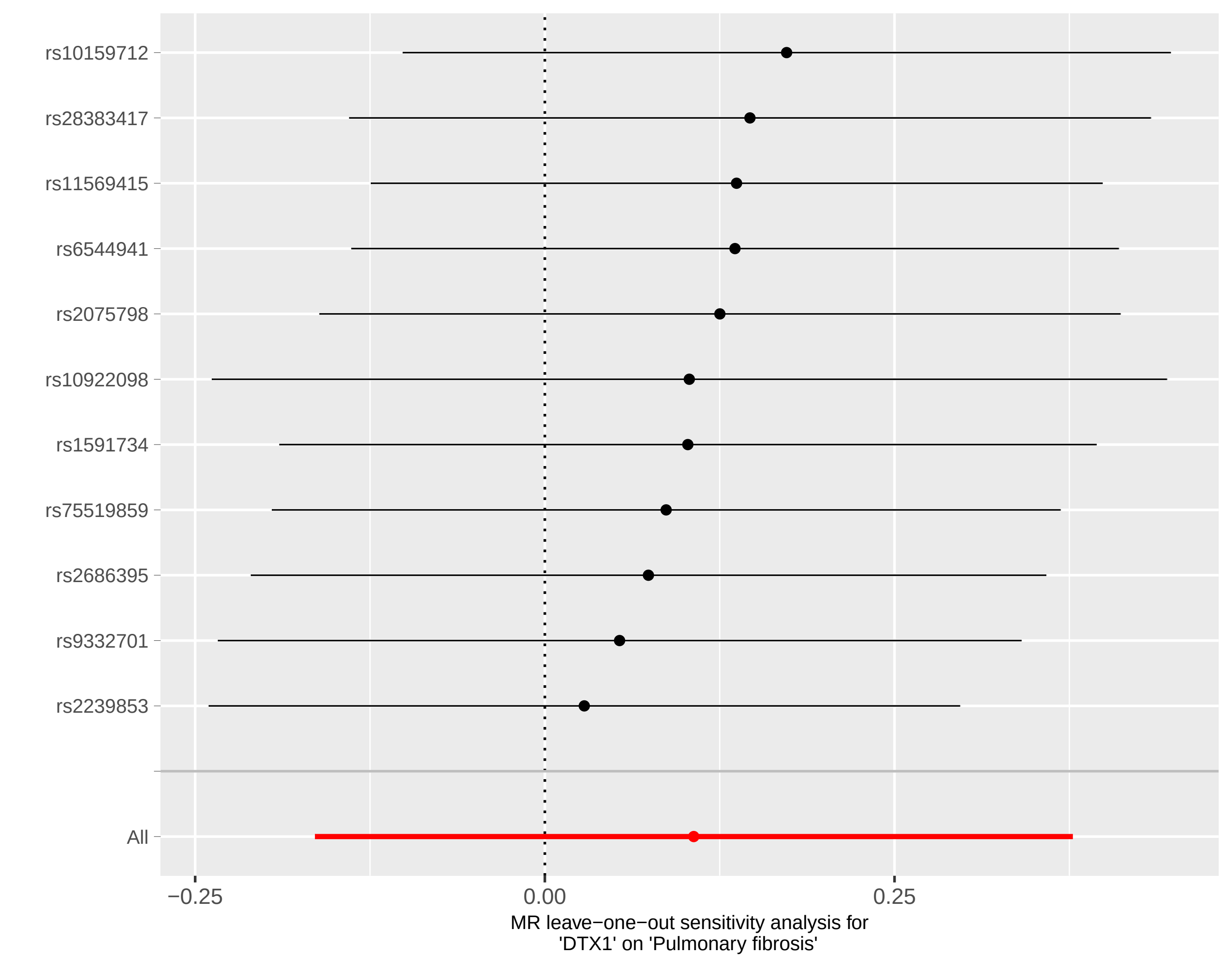
rs10159712
rs28383417
rs11569415
rs6544941
rs2075798
rs10922098
rs1591734
rs75519859
rs2686395
rs9332701
rs2239853
All
−0.25
0.00
0.25
MR leave−one−out sensitivity analysis for
'DTX1' on 'Pulmonary fibrosis'

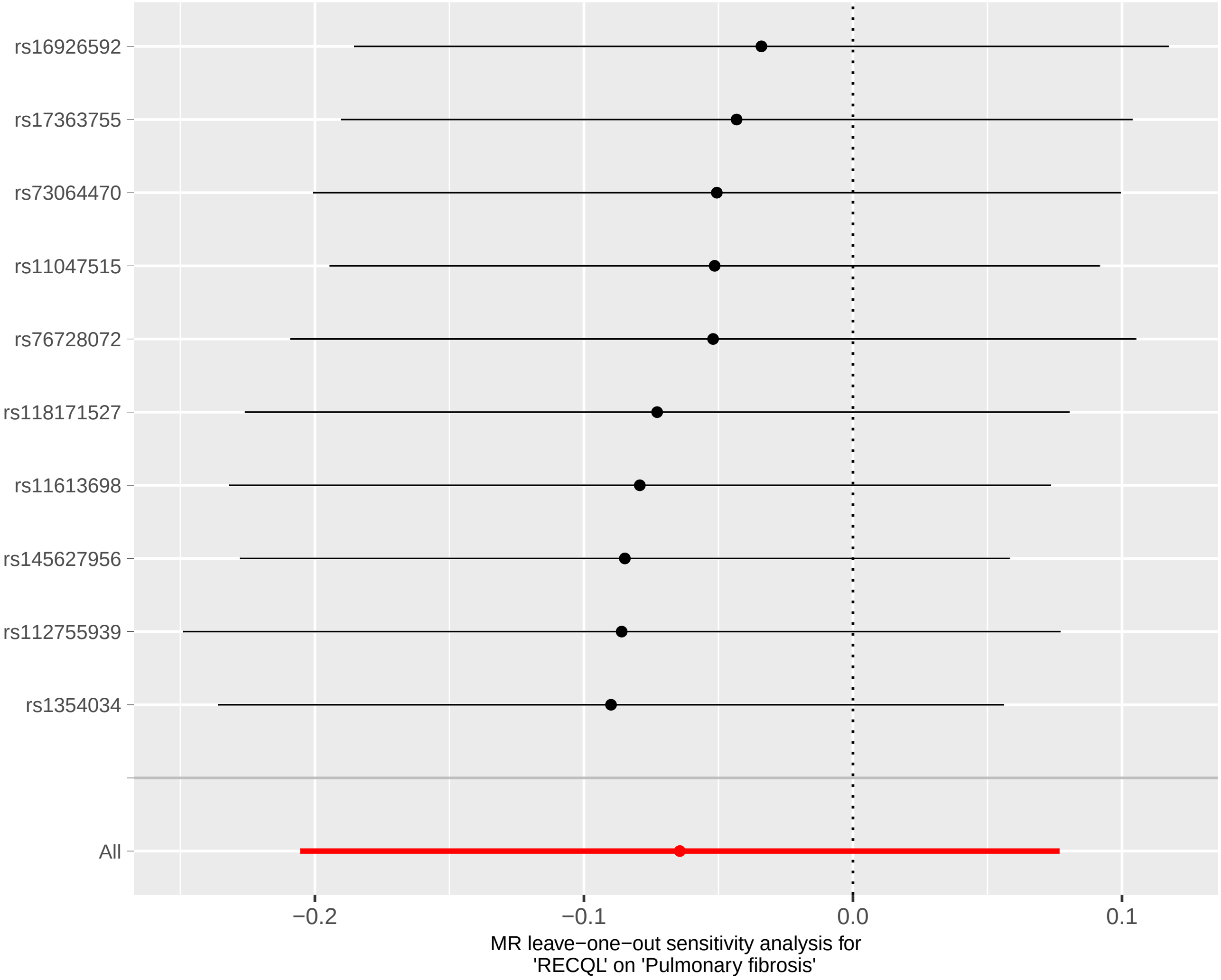
rs16926592
rs17363755
rs73064470
rs11047515
rs76728072
rs118171527
rs11613698
rs145627956
rs112755939
rs1354034
All
−0.2
−0.1
0.0
0.1
MR leave−one−out sensitivity analysis for
'RECQL' on 'Pulmonary fibrosis'

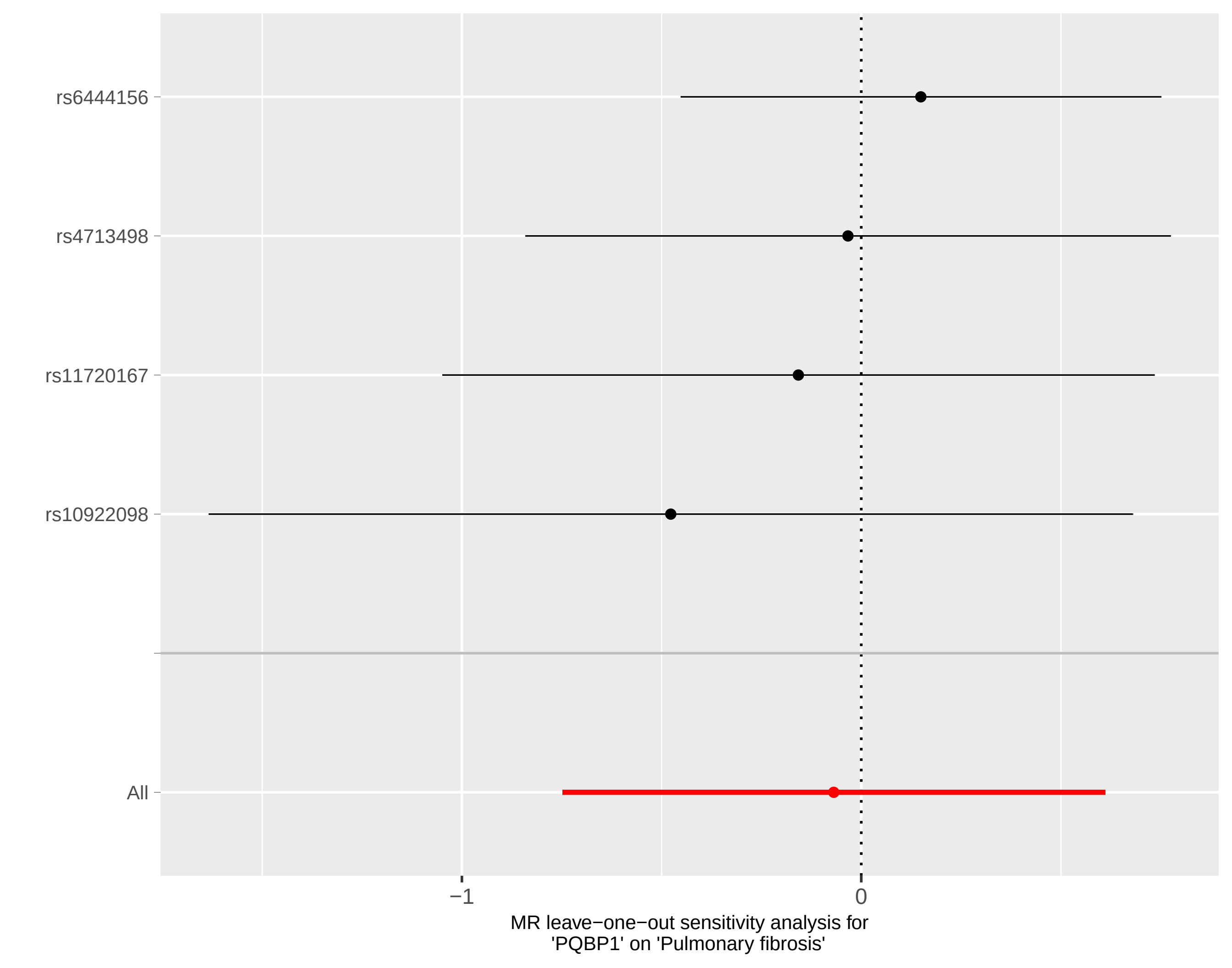
rs6444156
rs4713498
rs11720167
rs10922098
All
−1
0
MR leave−one−out sensitivity analysis for
'PQBP1' on 'Pulmonary fibrosis'

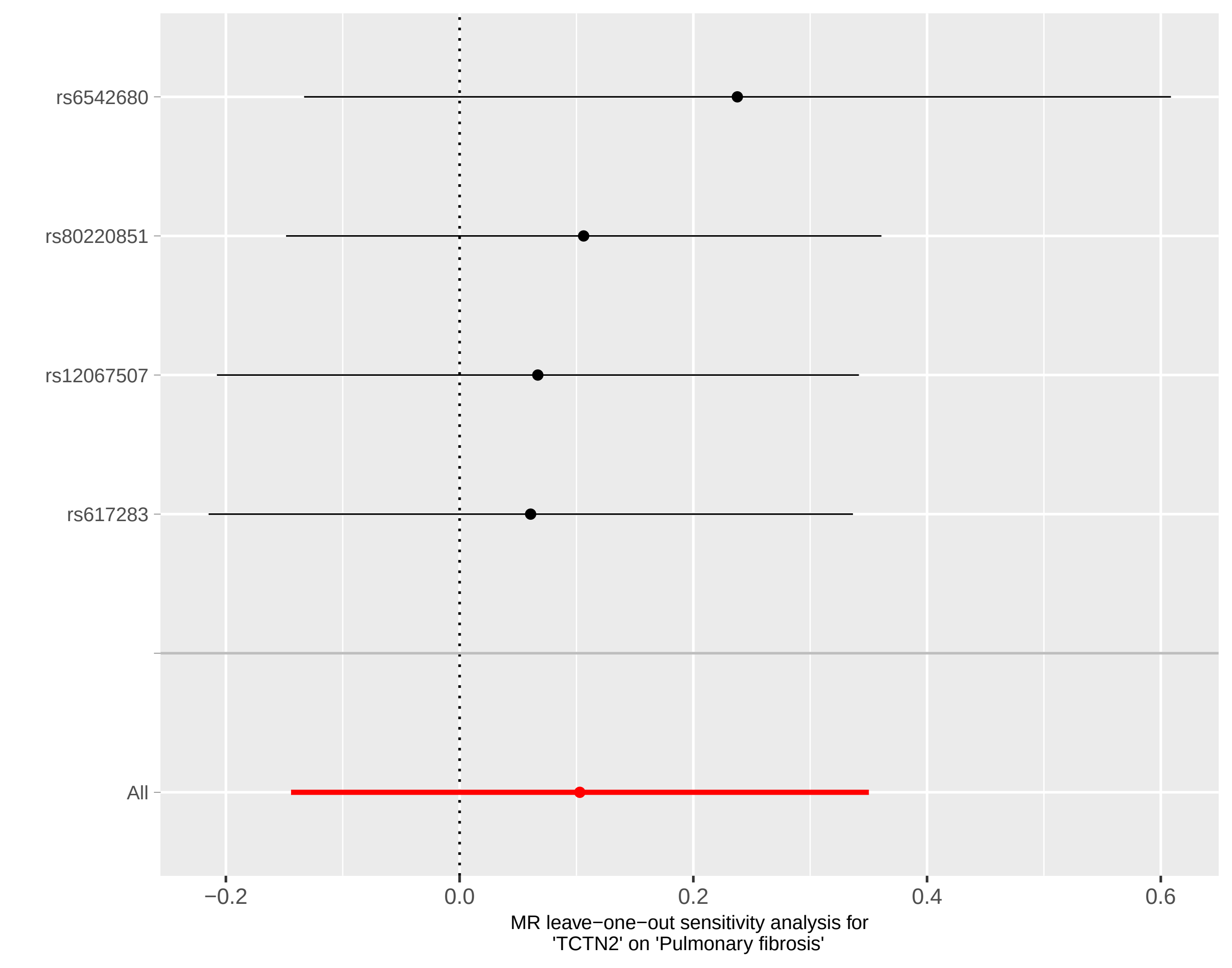
rs6542680
rs80220851
rs12067507
rs617283
All
−0.2
0.0
0.2
0.4
0.6
MR leave−one−out sensitivity analysis for
'TCTN2' on 'Pulmonary fibrosis'

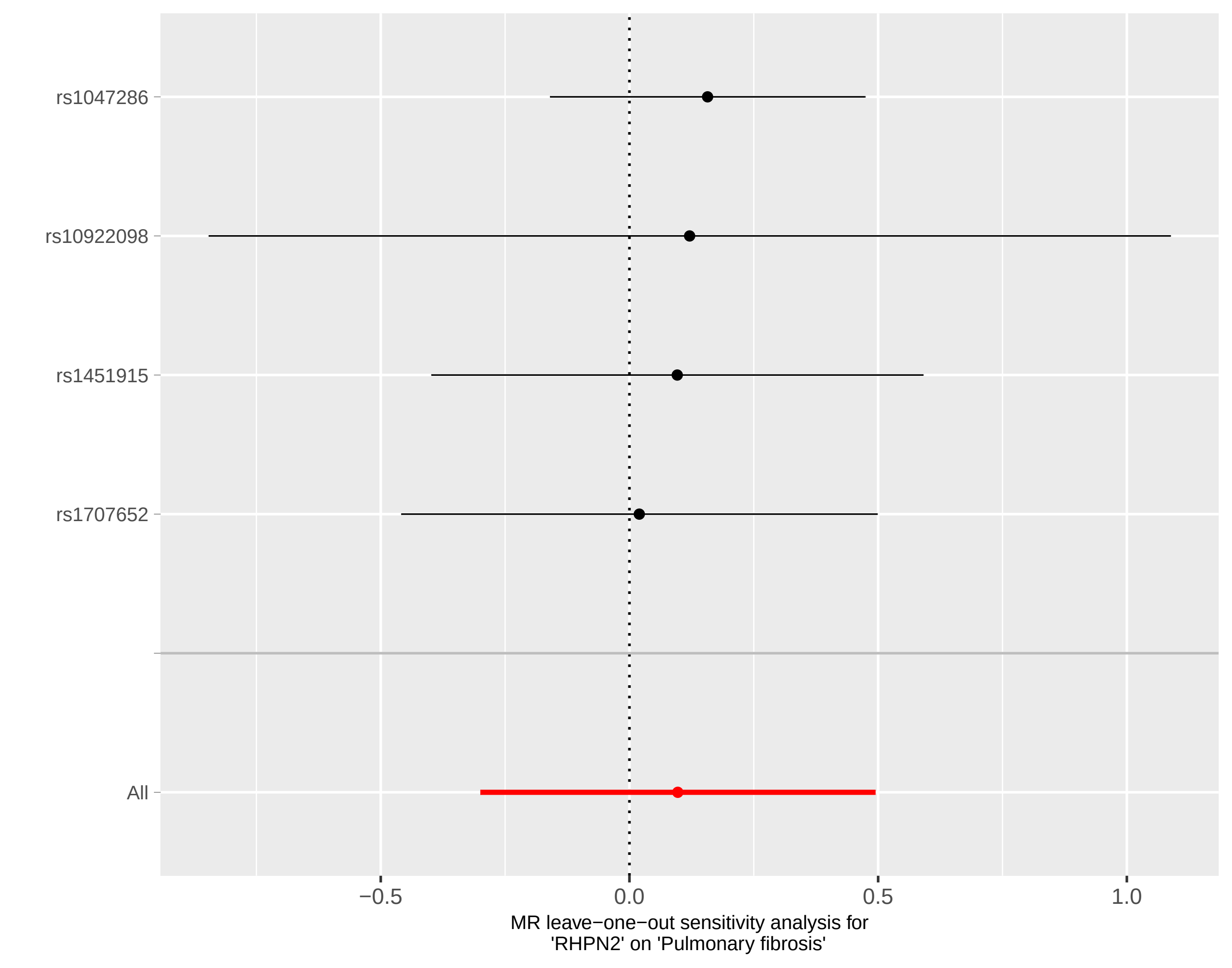
rs1047286
rs10922098
rs1451915
rs1707652
All
−0.5
0.0
0.5
1.0
MR leave−one−out sensitivity analysis for
'RHPN2' on 'Pulmonary fibrosis'

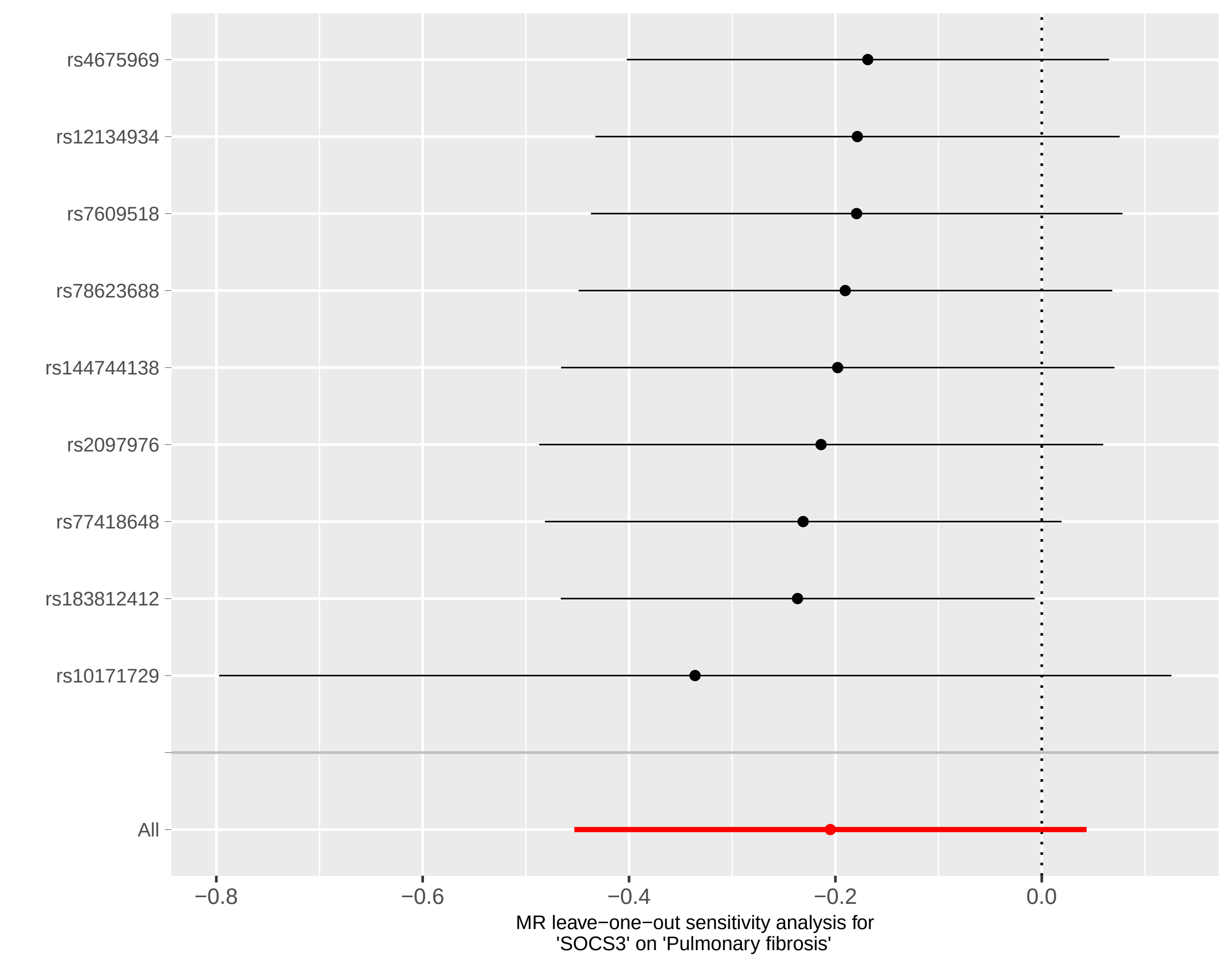
rs4675969
rs12134934
rs7609518
rs78623688
rs144744138
rs2097976
rs77418648
rs183812412
rs10171729
All
−0.8
−0.6
−0.4
−0.2
0.0
MR leave−one−out sensitivity analysis for
'SOCS3' on 'Pulmonary fibrosis'

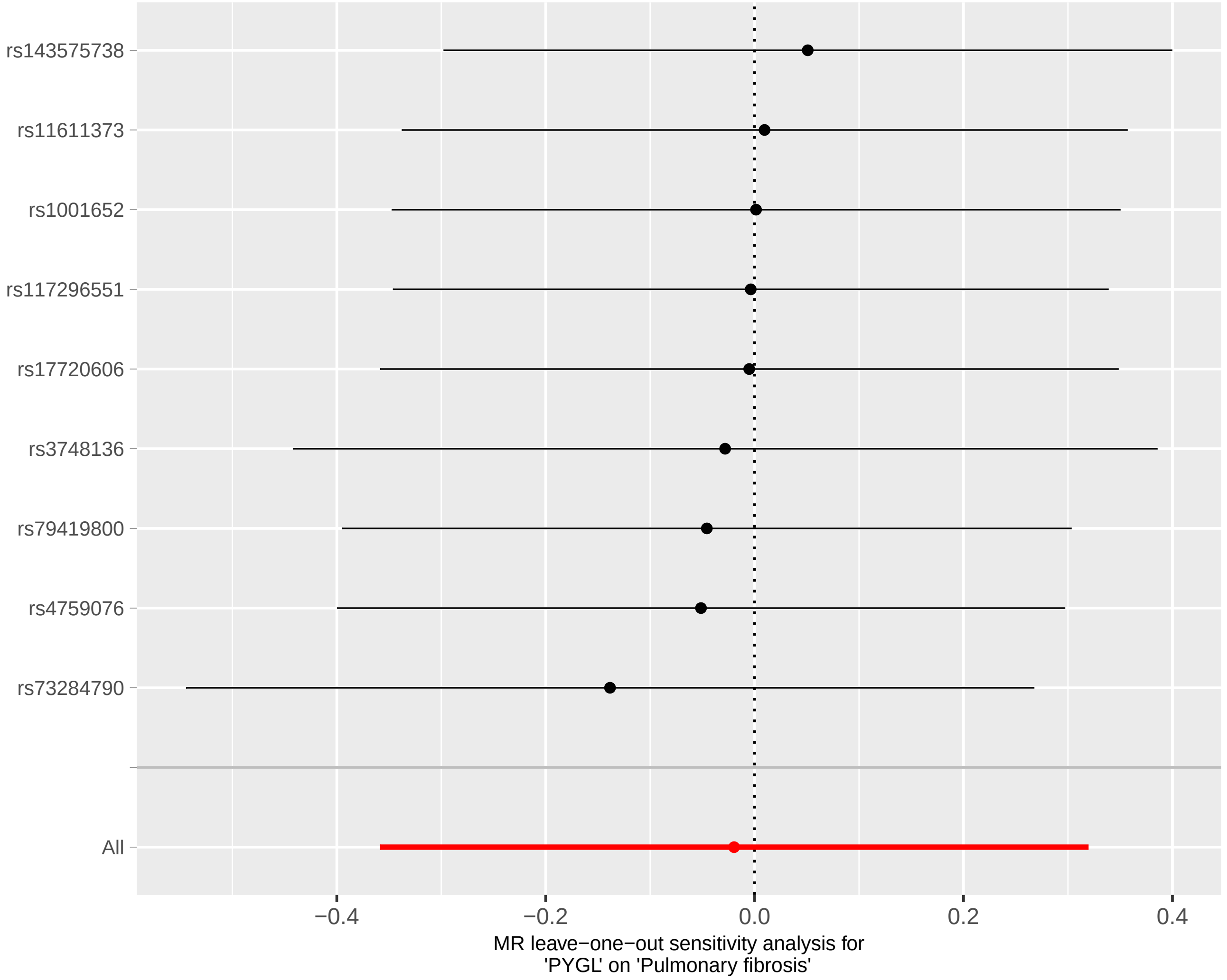

rs143575738
rs11611373
rs1001652
rs117296551
rs17720606
rs3748136
rs79419800
rs4759076
rs73284790
All
−0.4
−0.2
0.0
0.2
0.4
MR leave−one−out sensitivity analysis for
'PYGL' on 'Pulmonary fibrosis'

Insufficient number of SNPs

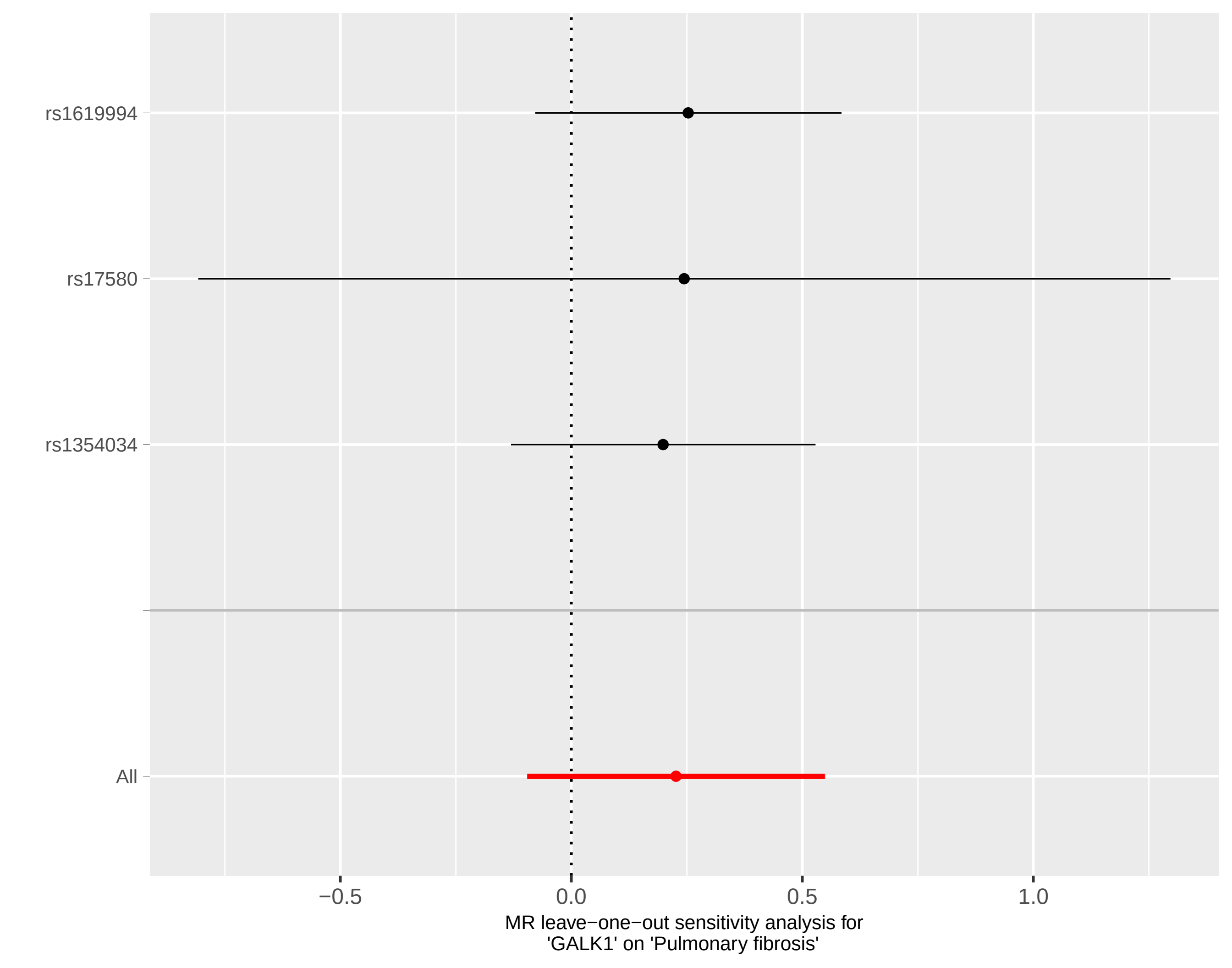
rs1619994
rs17580
rs1354034
All
−0.5
0.0
0.5
1.0
MR leave−one−out sensitivity analysis for
'GALK1' on 'Pulmonary fibrosis'

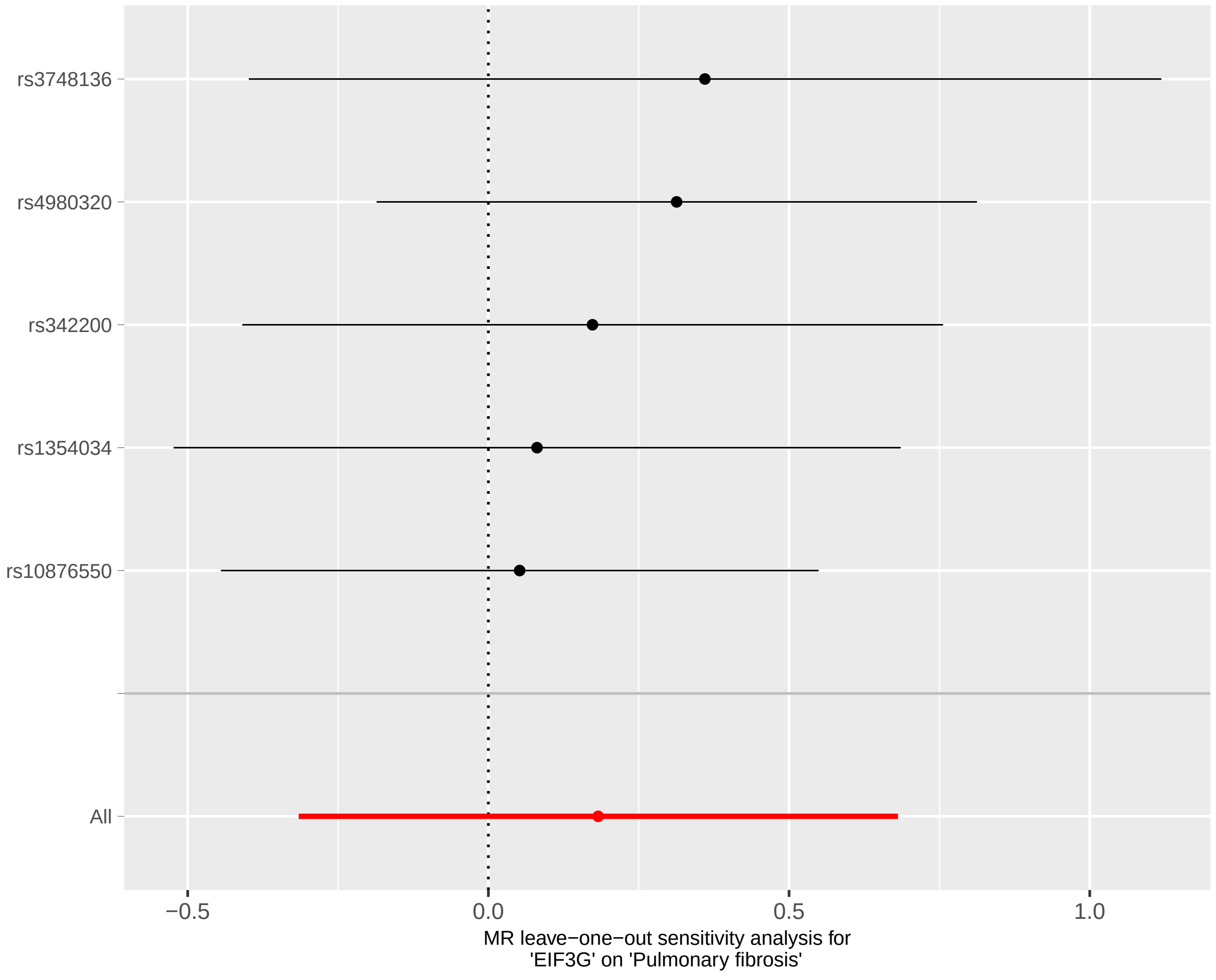
rs3748136
rs4980320
rs342200
rs1354034
rs10876550
All
−0.5
0.0
0.5
1.0
MR leave−one−out sensitivity analysis for
'EIF3G' on 'Pulmonary fibrosis'

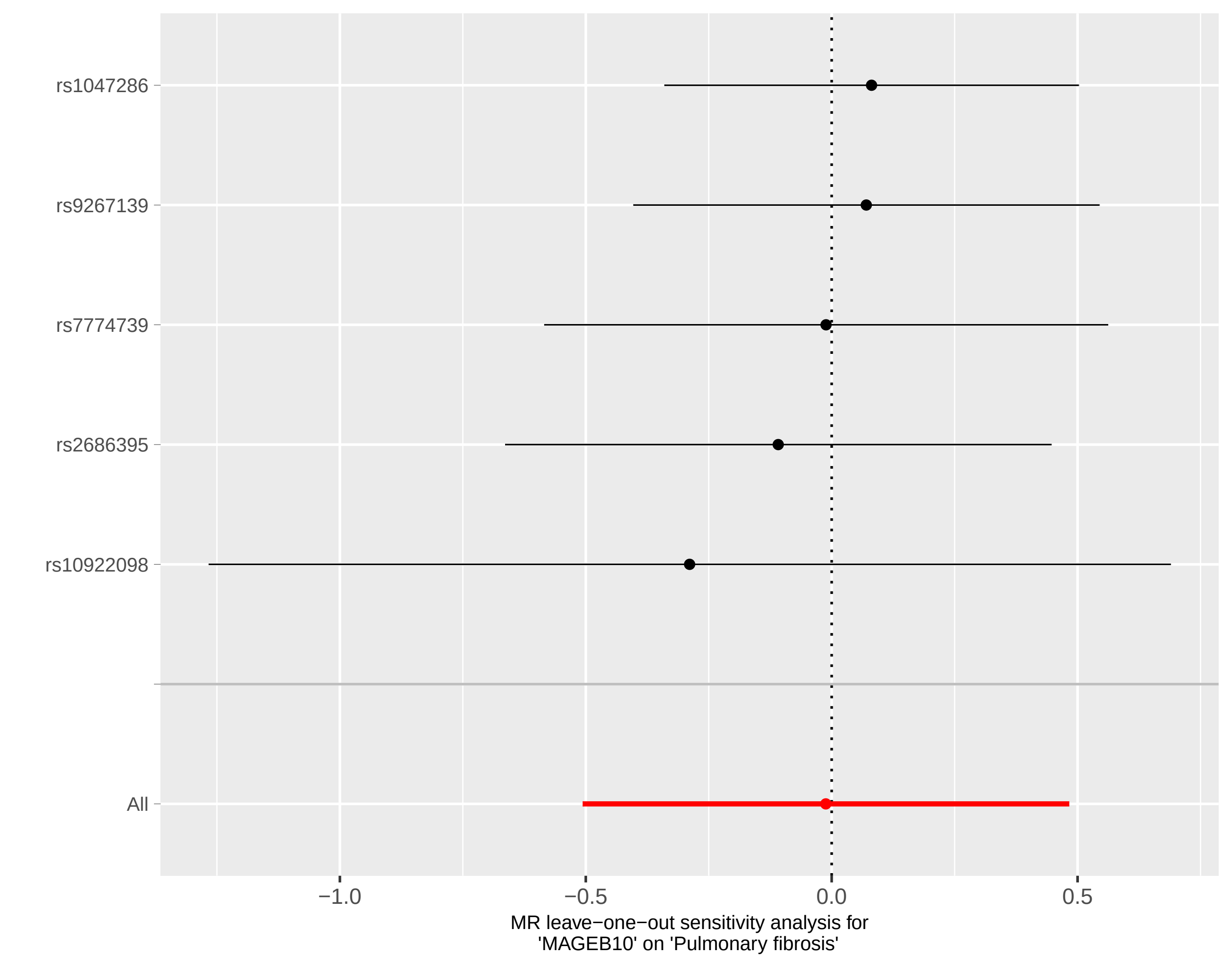

rs1047286
rs9267139
rs7774739
rs2686395
rs10922098
All
−1.0
−0.5
0.0
0.5
MR leave−one−out sensitivity analysis for
'MAGEB10' on 'Pulmonary fibrosis'

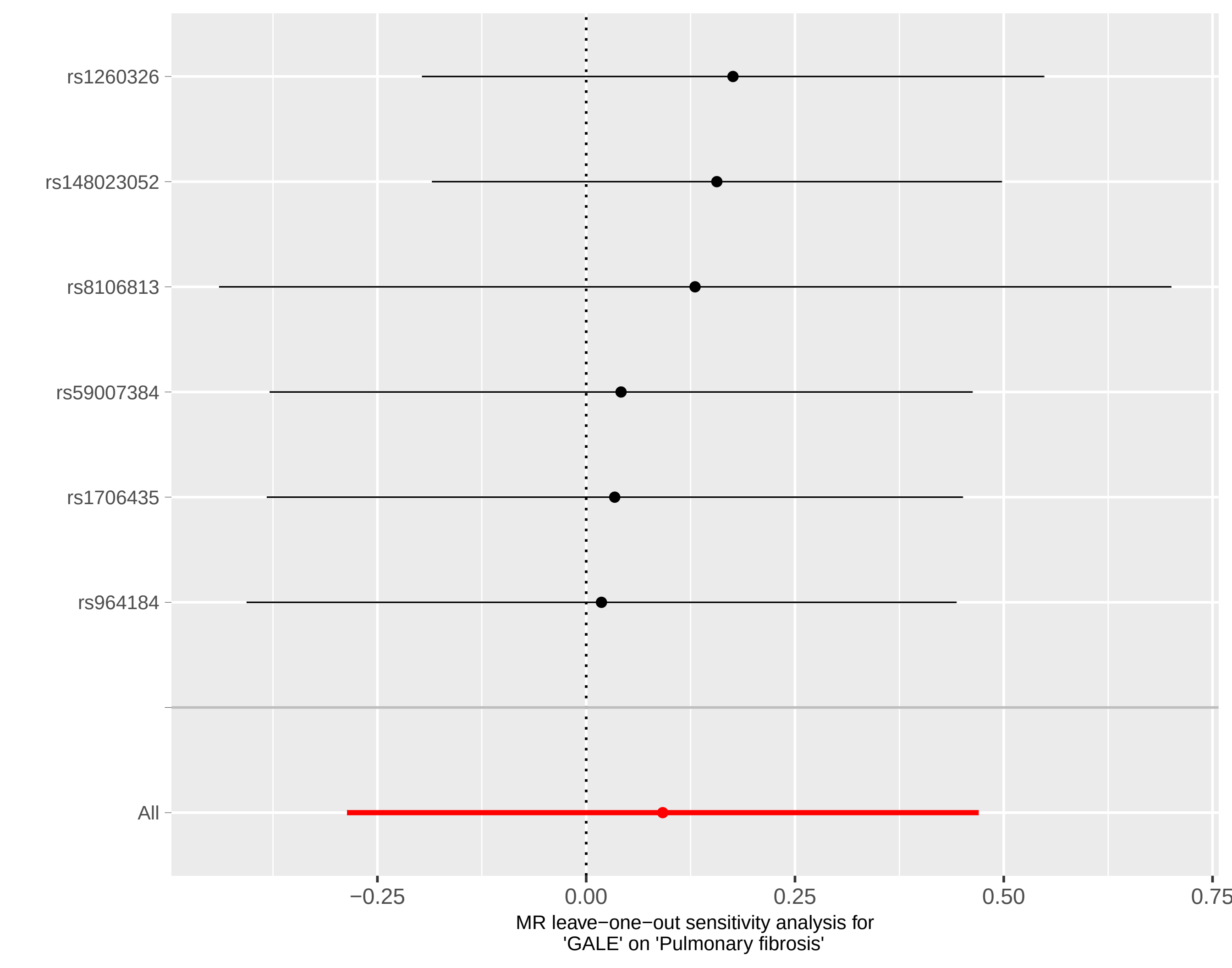
rs1260326
rs148023052
rs8106813
rs59007384
rs1706435
rs964184
All
−0.25
0.00
0.25
0.50
0.75
MR leave−one−out sensitivity analysis for
'GALE' on 'Pulmonary fibrosis'

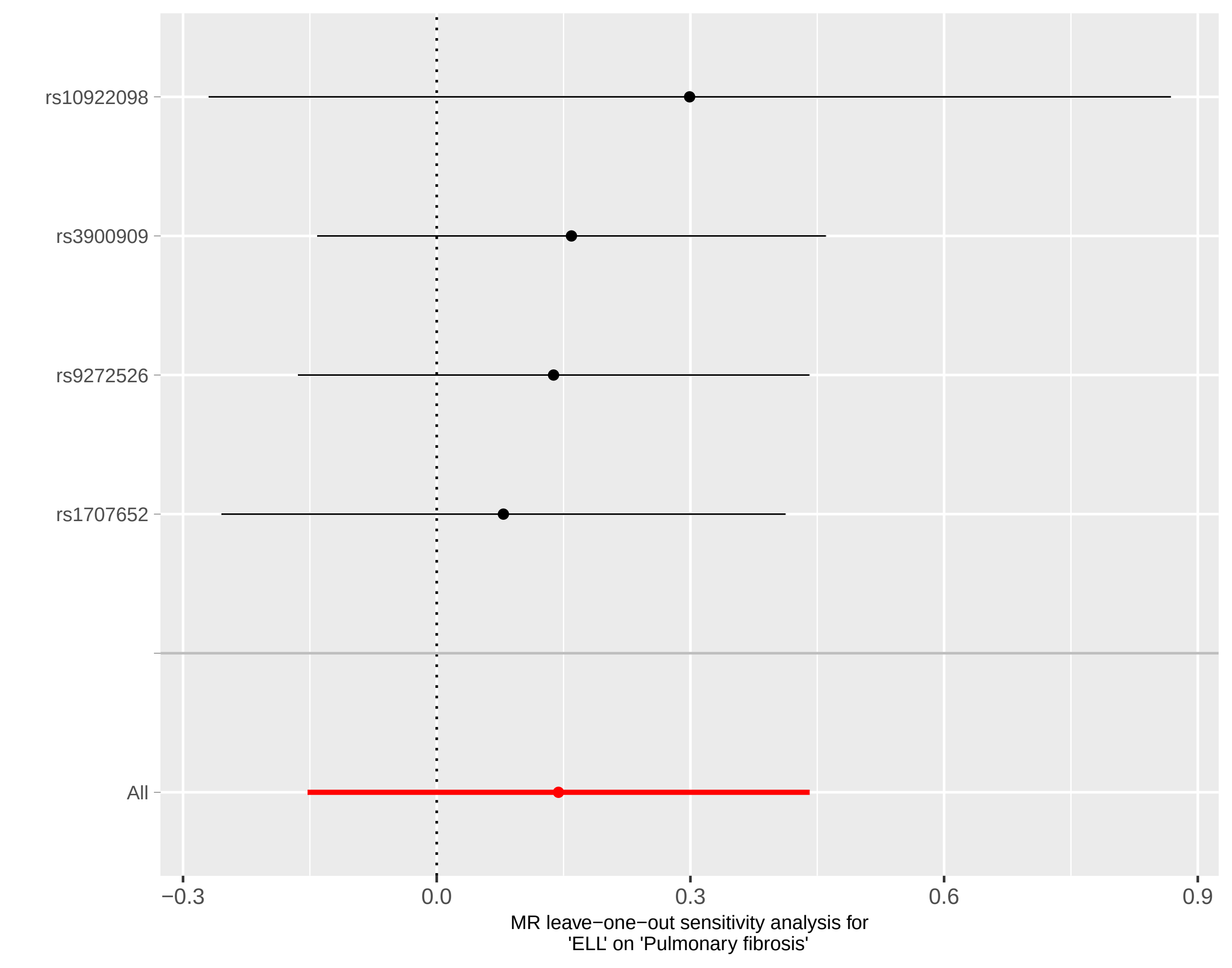

rs10922098
rs3900909
rs9272526
rs1707652
All
−0.3
0.0
0.3
0.6
0.9
MR leave−one−out sensitivity analysis for
'ELL' on 'Pulmonary fibrosis'

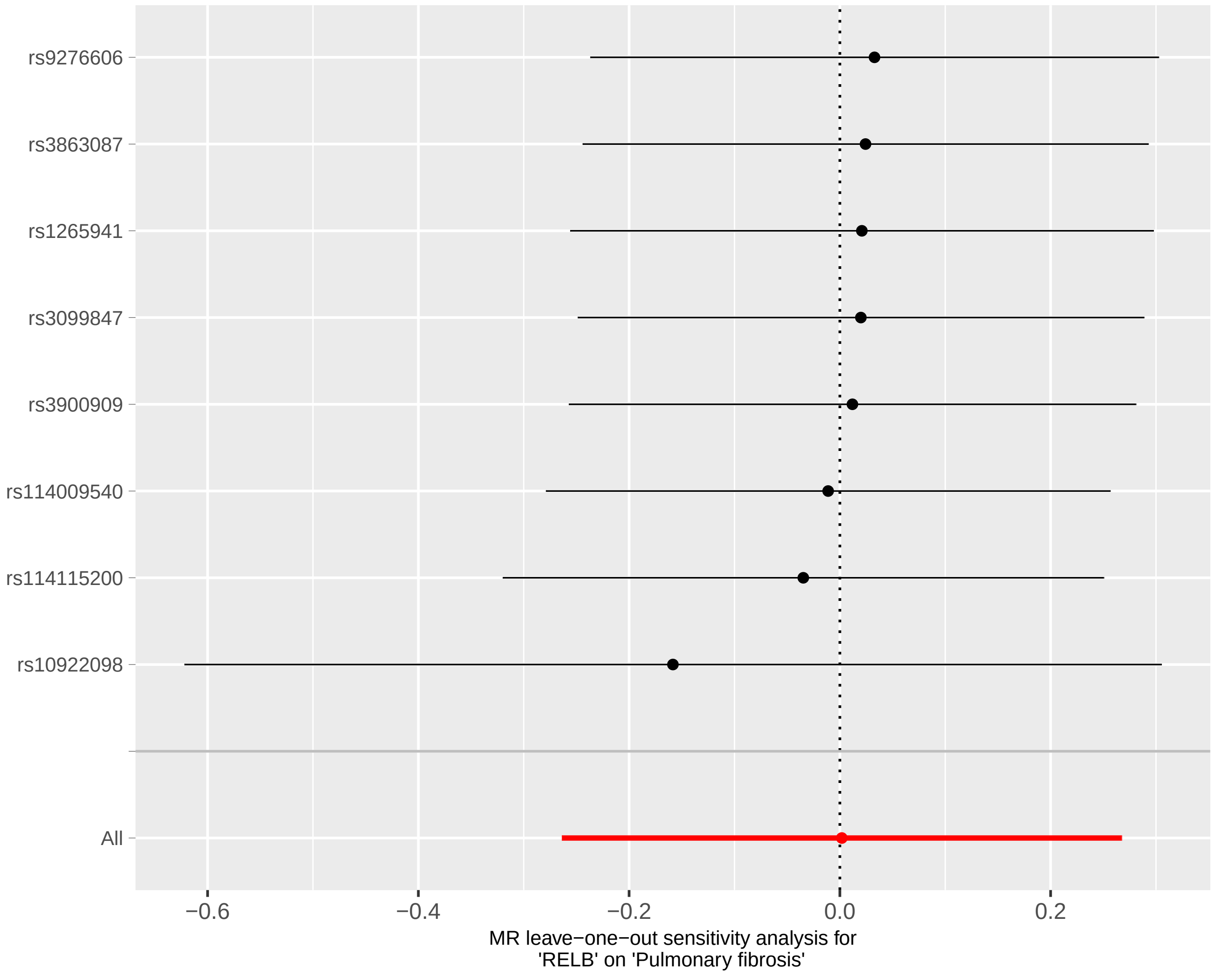
rs9276606
rs3863087
rs1265941
rs3099847
rs3900909
rs114009540
rs114115200
rs10922098
All
−0.6
−0.4
−0.2
0.0
0.2
MR leave−one−out sensitivity analysis for
'RELB' on 'Pulmonary fibrosis'

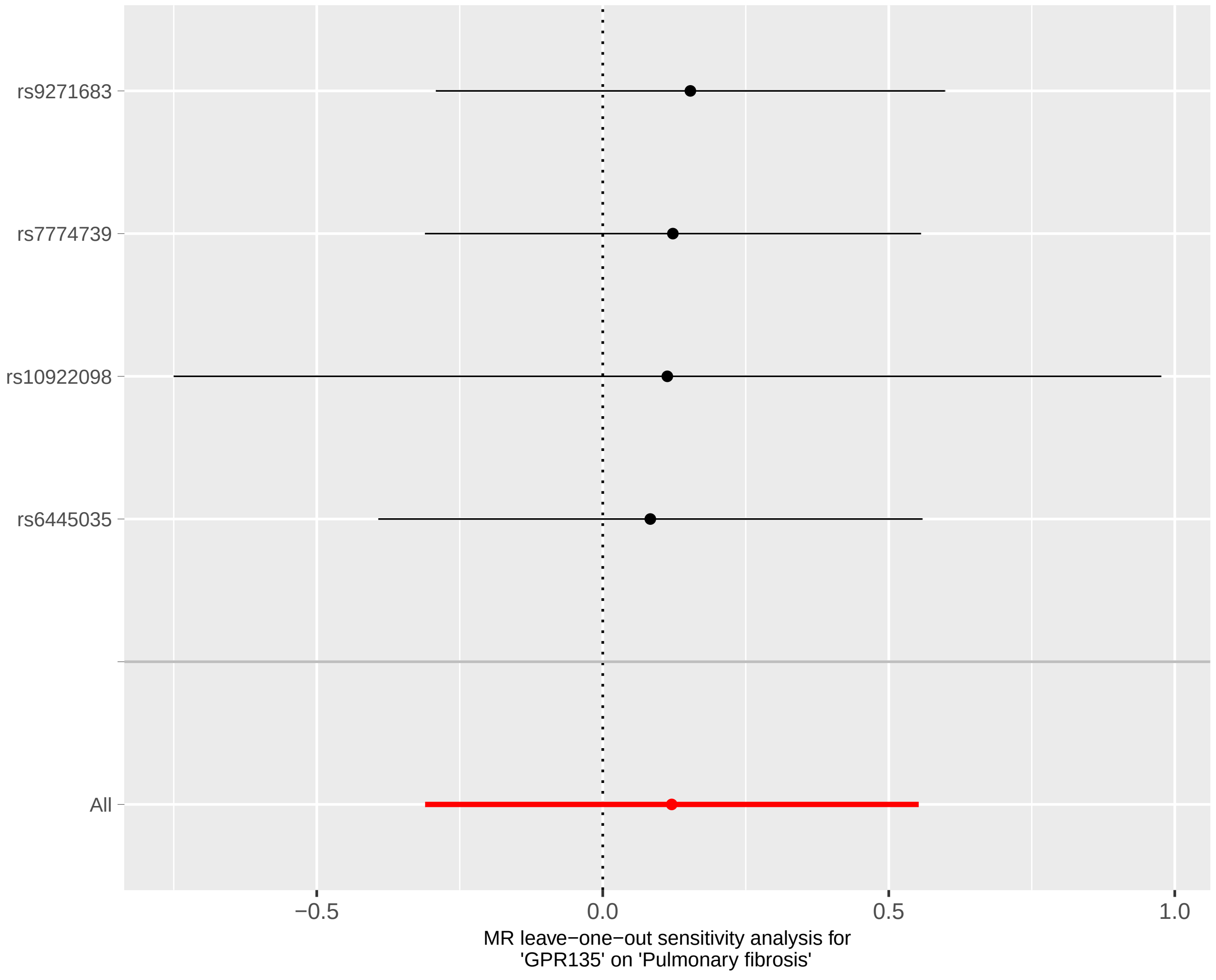
rs9271683
rs7774739
rs10922098
rs6445035
All
−0.5
0.0
0.5
1.0
MR leave−one−out sensitivity analysis for
'GPR135' on 'Pulmonary fibrosis'

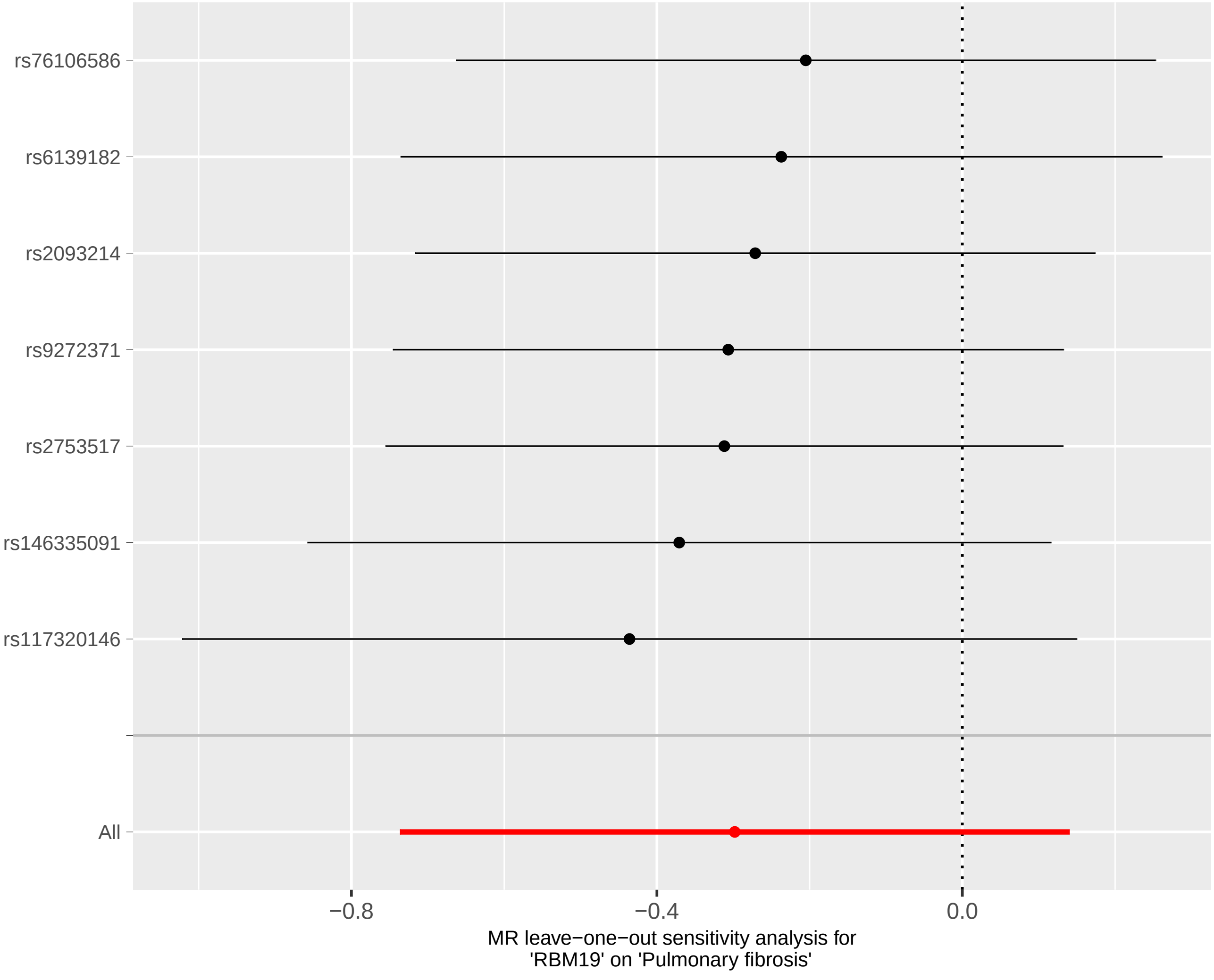

rs76106586
rs6139182
rs2093214
rs9272371
rs2753517
rs146335091
rs117320146
All
−0.8
−0.4
0.0
MR leave−one−out sensitivity analysis for
'RBM19' on 'Pulmonary fibrosis'

Insufficient number of SNPs

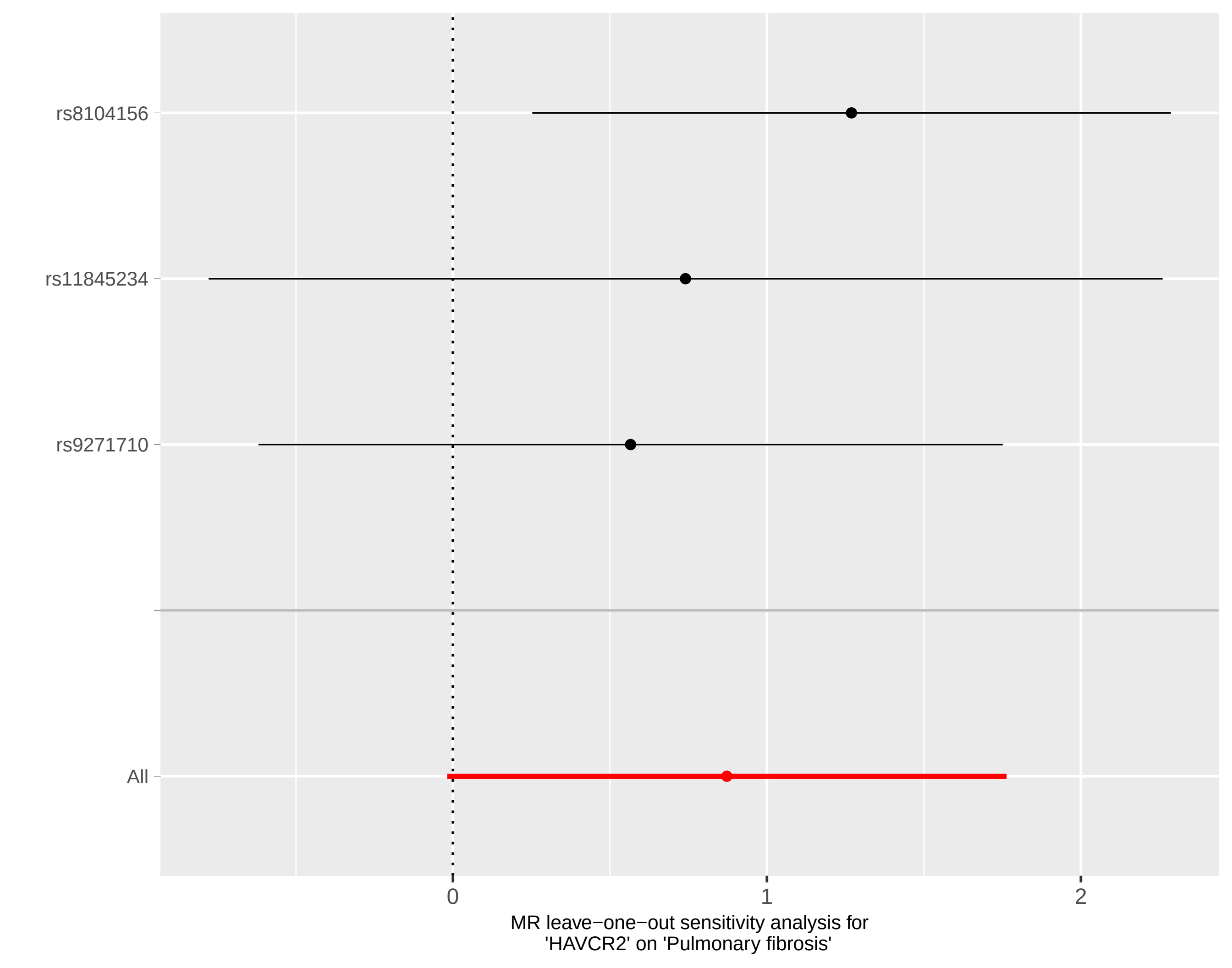
rs8104156
rs11845234
rs9271710
All
0
1
2
MR leave−one−out sensitivity analysis for
'HAVCR2' on 'Pulmonary fibrosis'

Insufficient number of SNPs

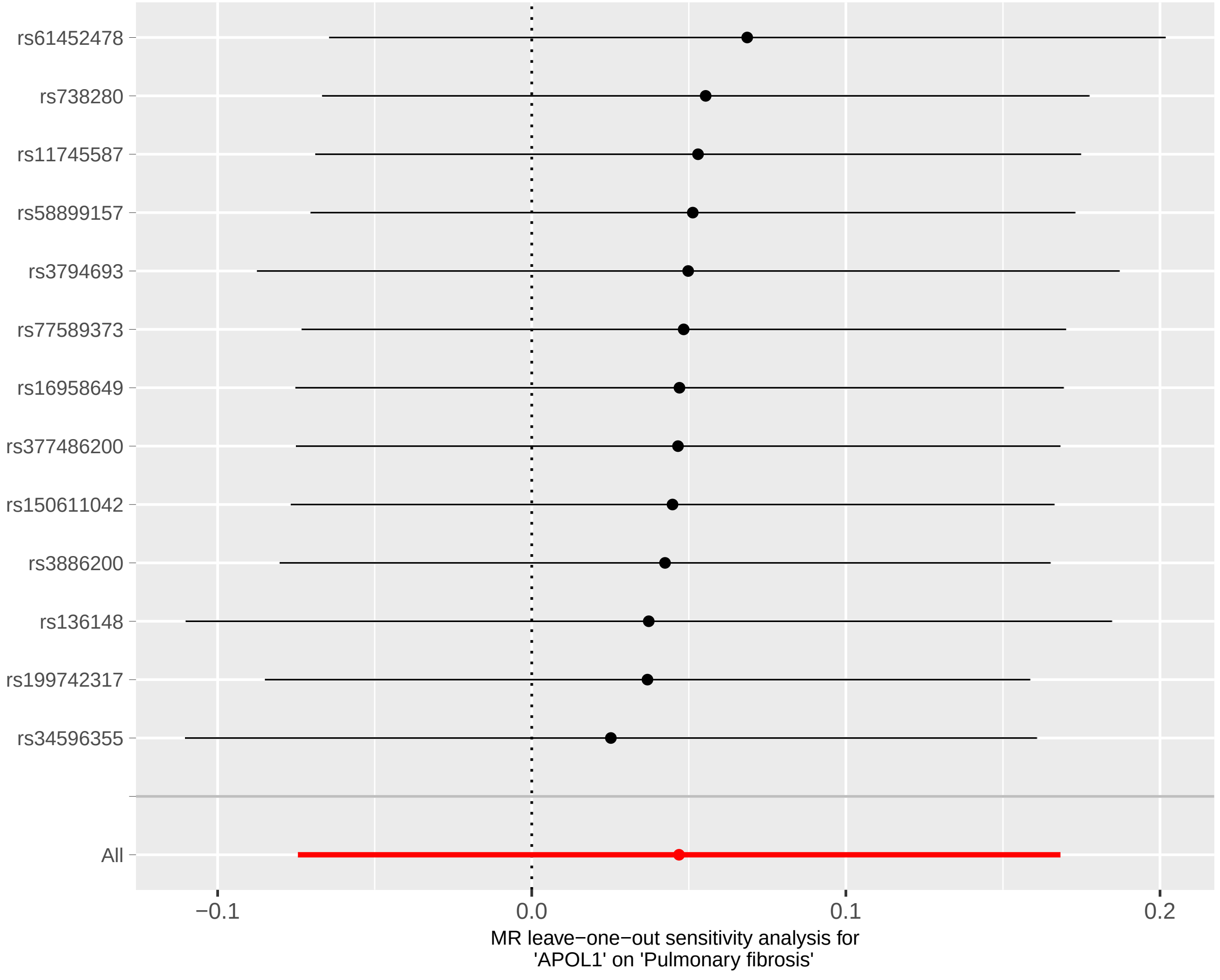
rs61452478
rs738280
rs11745587
rs58899157
rs3794693
rs77589373
rs16958649
rs377486200
rs150611042
rs3886200
rs136148
rs199742317
rs34596355
All
−0.1
0.0
0.1
0.2
MR leave−one−out sensitivity analysis for
'APOL1' on 'Pulmonary fibrosis'

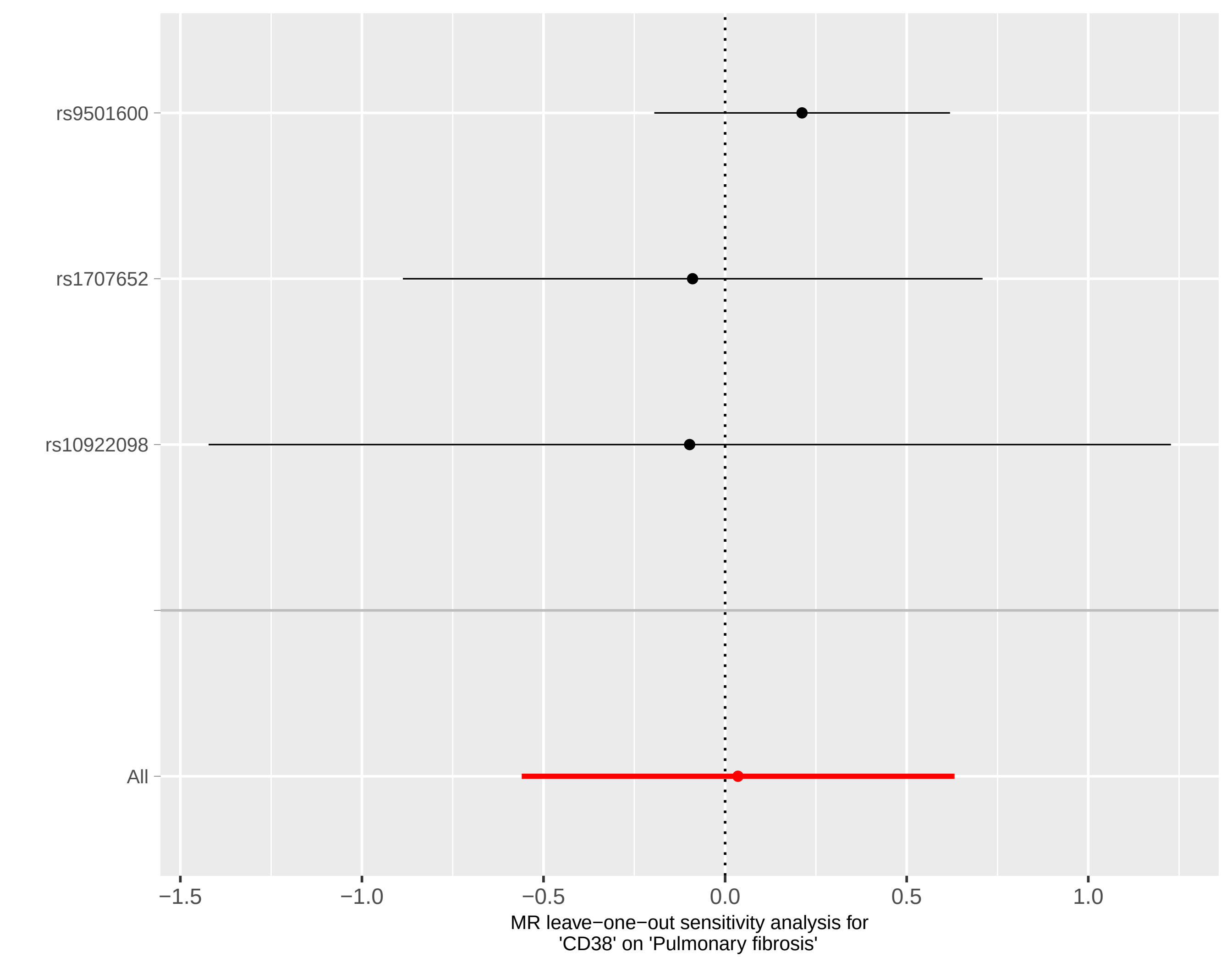

rs9501600
rs1707652
rs10922098
All
−1.5
−1.0
−0.5
0.0
0.5
1.0
MR leave−one−out sensitivity analysis for
'CD38' on 'Pulmonary fibrosis'

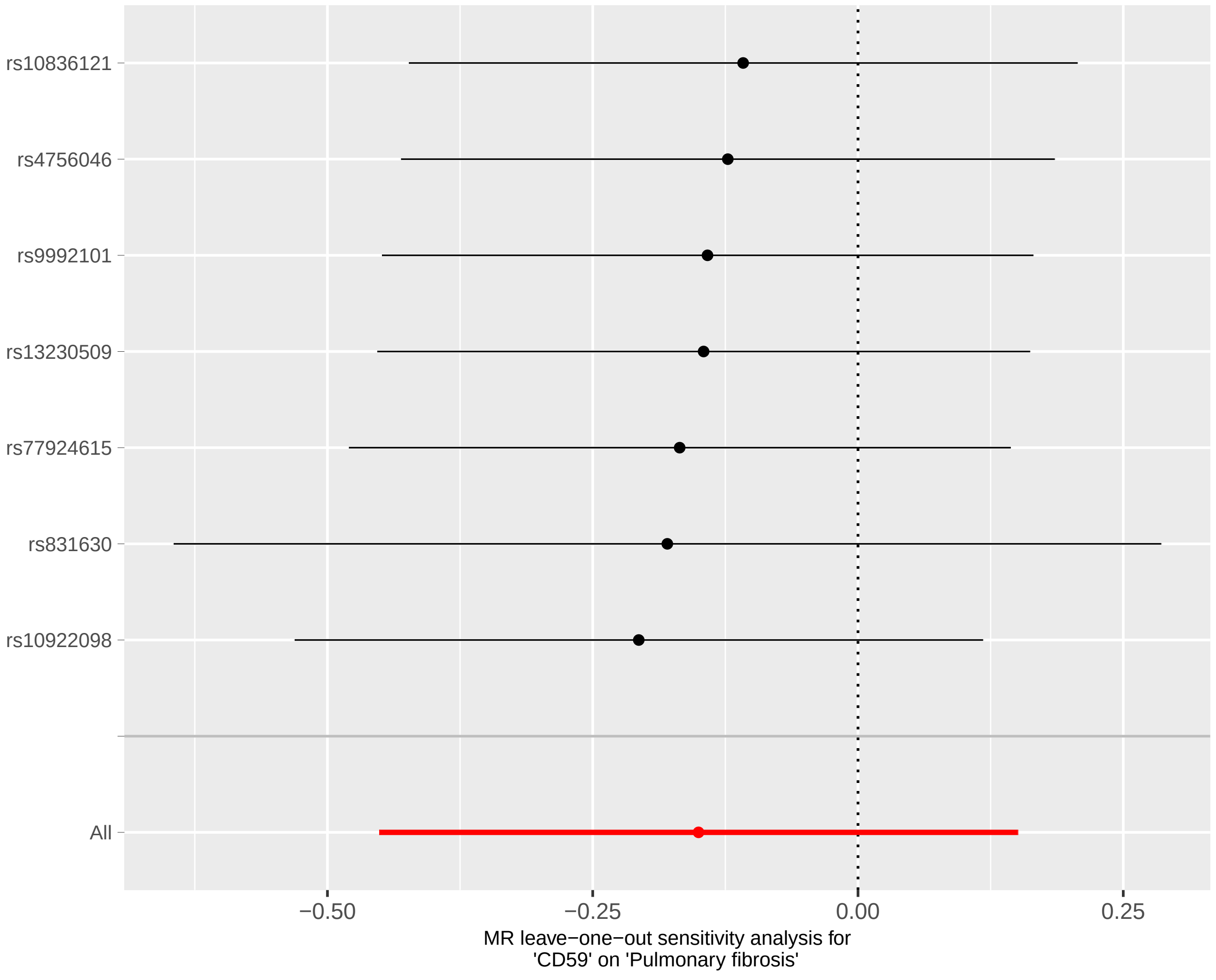

rs10836121
rs4756046
rs9992101
rs13230509
rs77924615
rs831630
rs10922098
All
−0.50
−0.25
0.00
0.25
MR leave−one−out sensitivity analysis for
'CD59' on 'Pulmonary fibrosis'

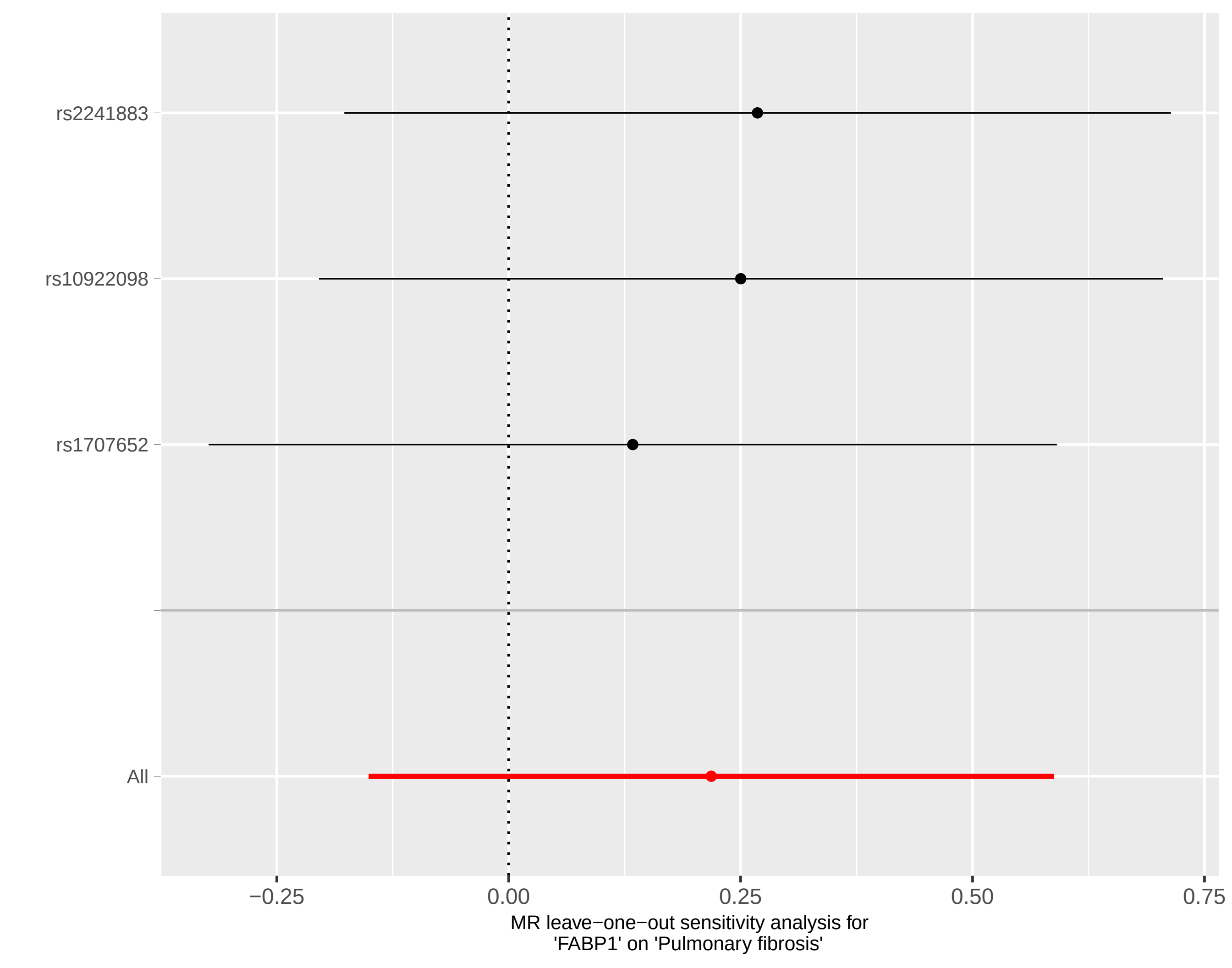
rs2241883
rs10922098
rs1707652
All
−0.25
0.00
0.25
0.50
0.75
MR leave−one−out sensitivity analysis for
'FABP1' on 'Pulmonary fibrosis'

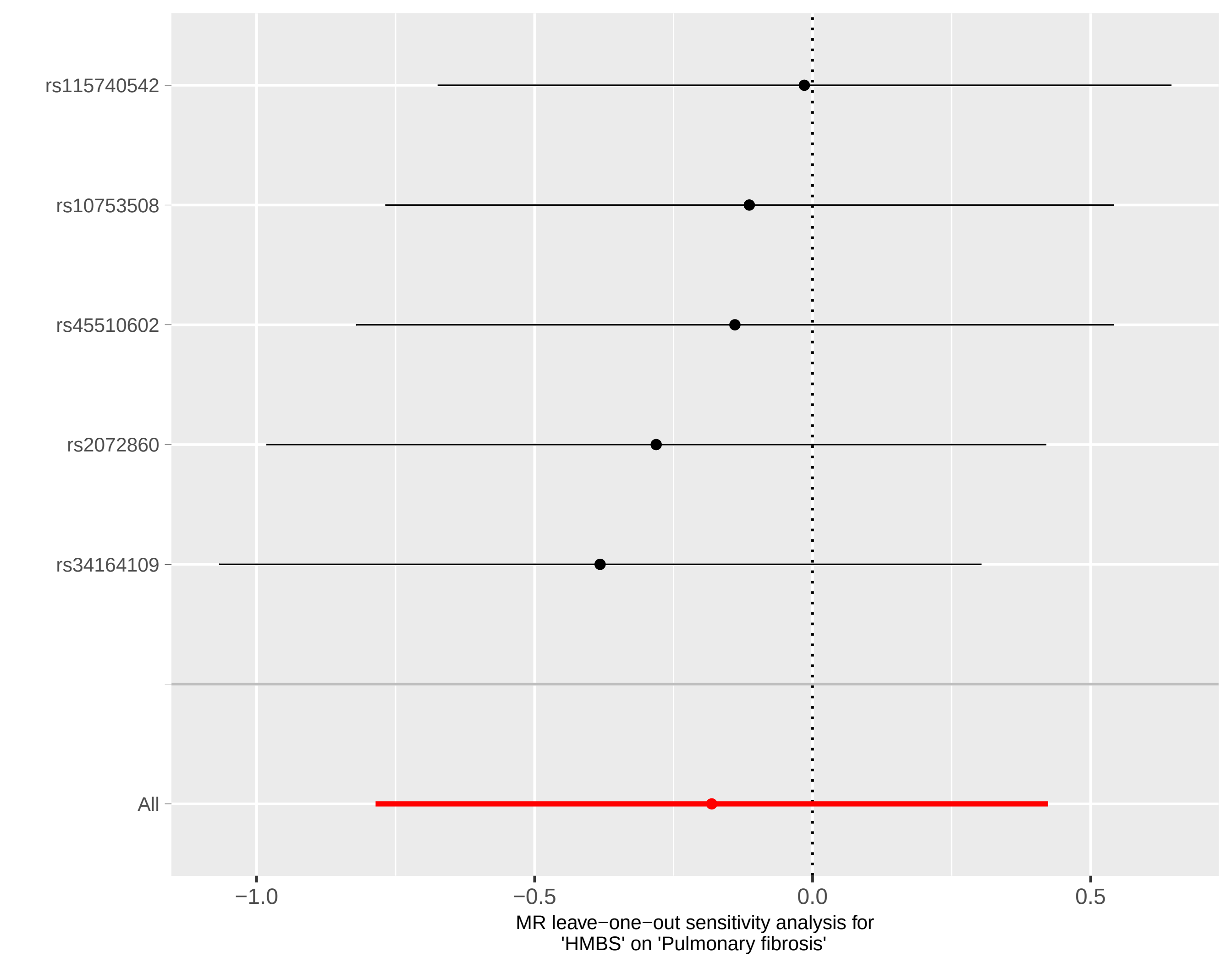
rs115740542
rs10753508
rs45510602
rs2072860
rs34164109
All
−1.0
−0.5
0.0
0.5
MR leave−one−out sensitivity analysis for
'HMBS' on 'Pulmonary fibrosis'

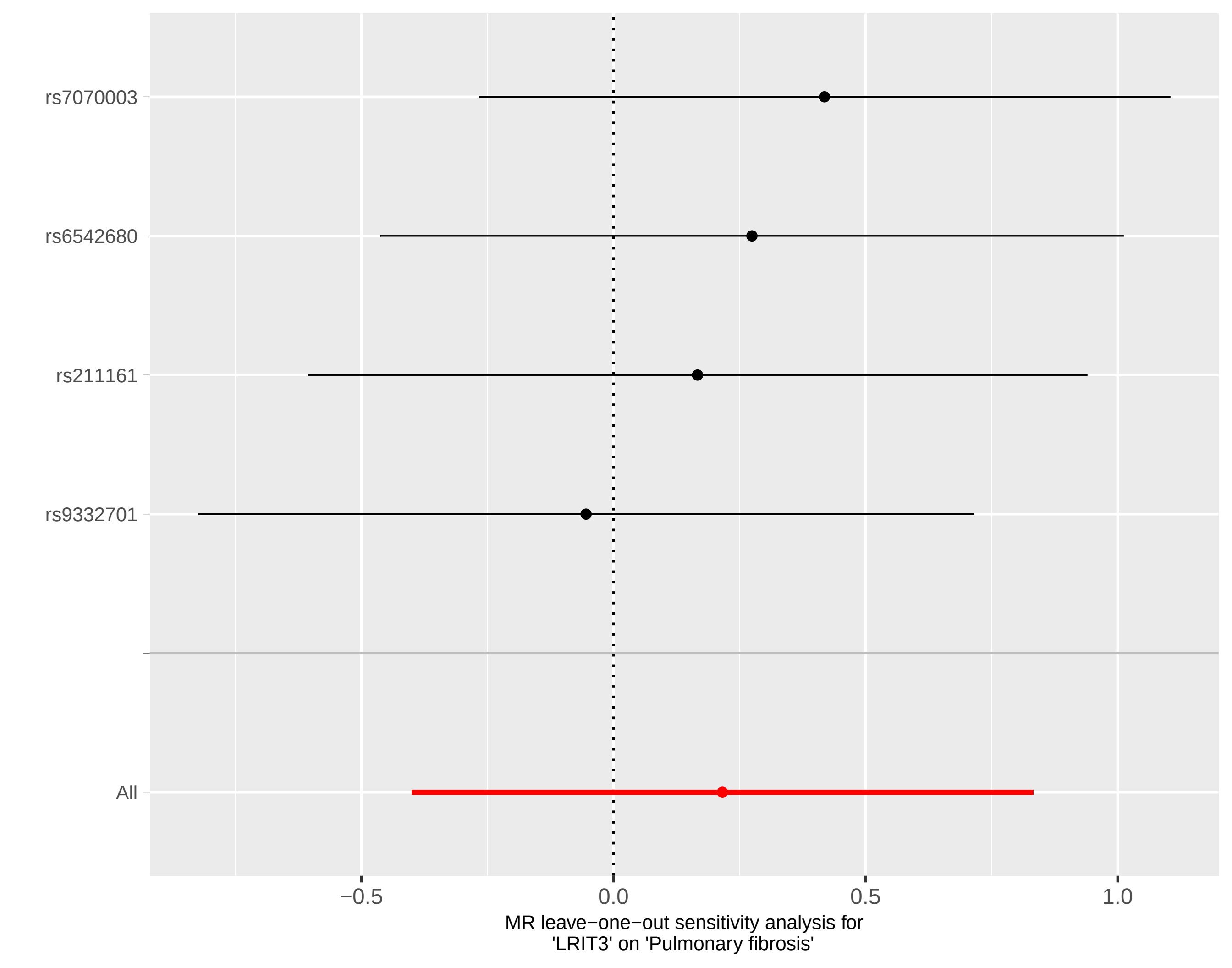

rs7070003
rs6542680
rs211161
rs9332701
All
−0.5
0.0
0.5
1.0
MR leave−one−out sensitivity analysis for
'LRIT3' on 'Pulmonary fibrosis'

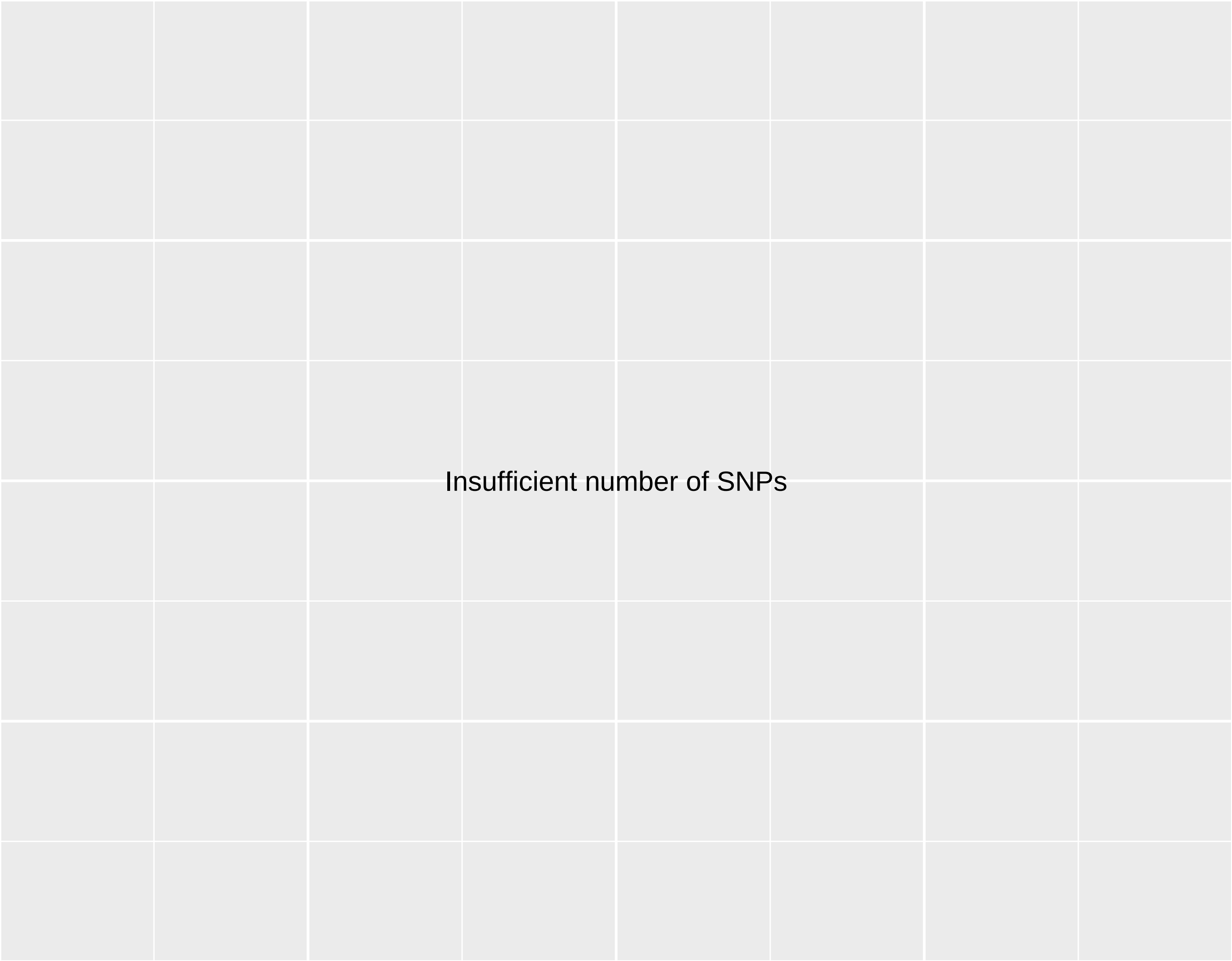
Insufficient number of SNPs

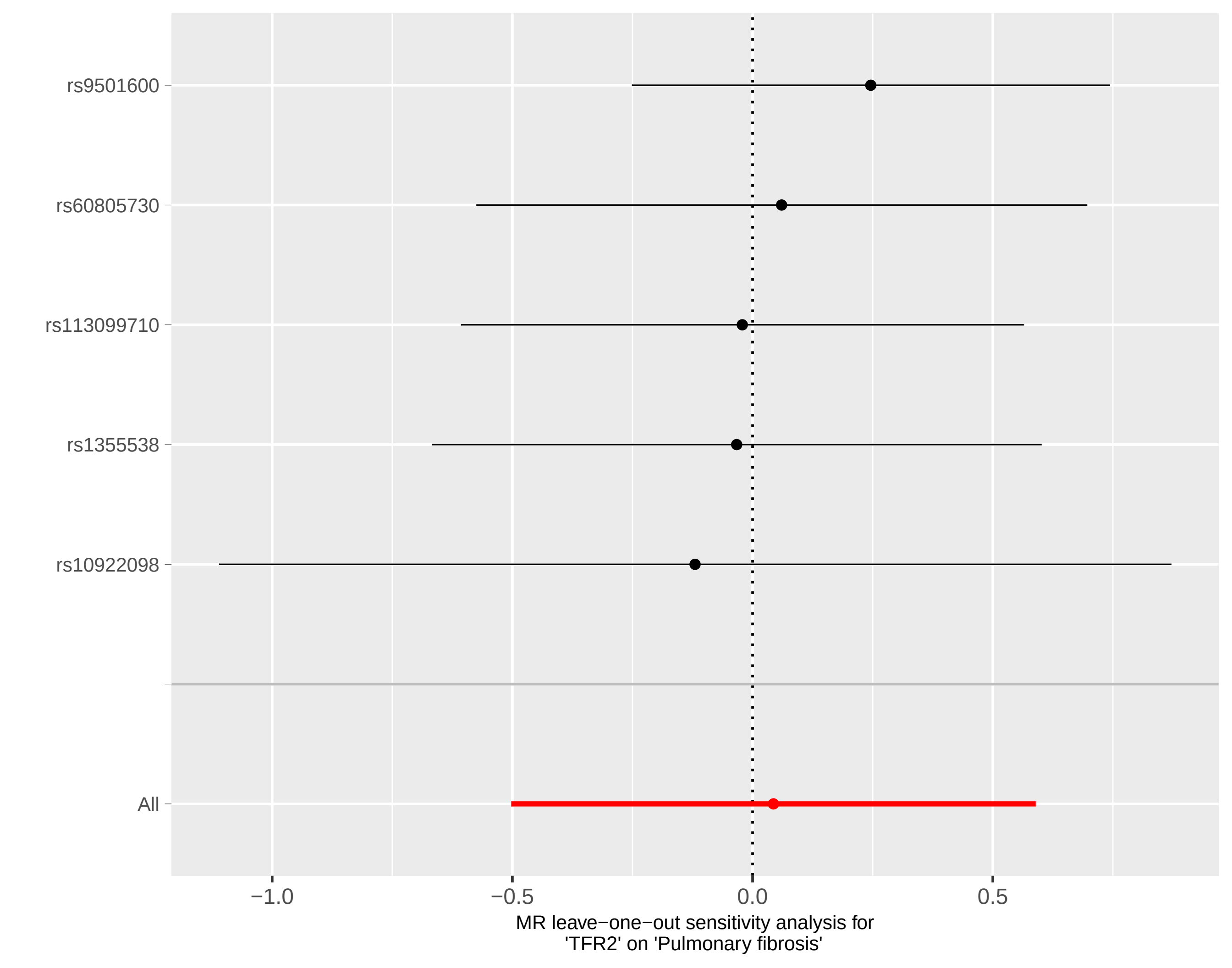
rs9501600
rs60805730
rs113099710
rs1355538
rs10922098
All
−1.0
−0.5
0.0
0.5
MR leave−one−out sensitivity analysis for
'TFR2' on 'Pulmonary fibrosis'

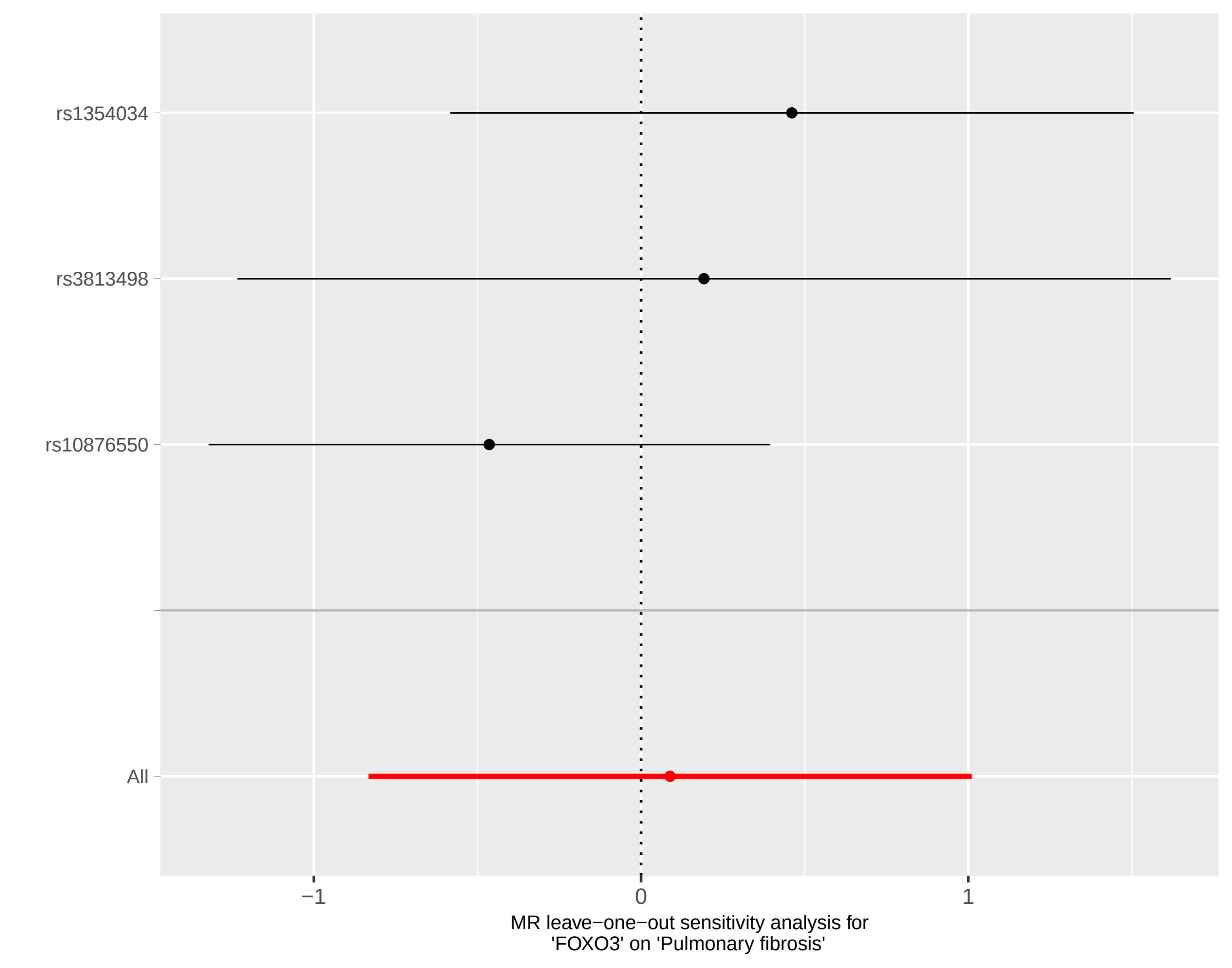
rs1354034
rs3813498
rs10876550
All
−1
0
1
MR leave−one−out sensitivity analysis for
'FOXO3' on 'Pulmonary fibrosis'

Insufficient number of SNPs

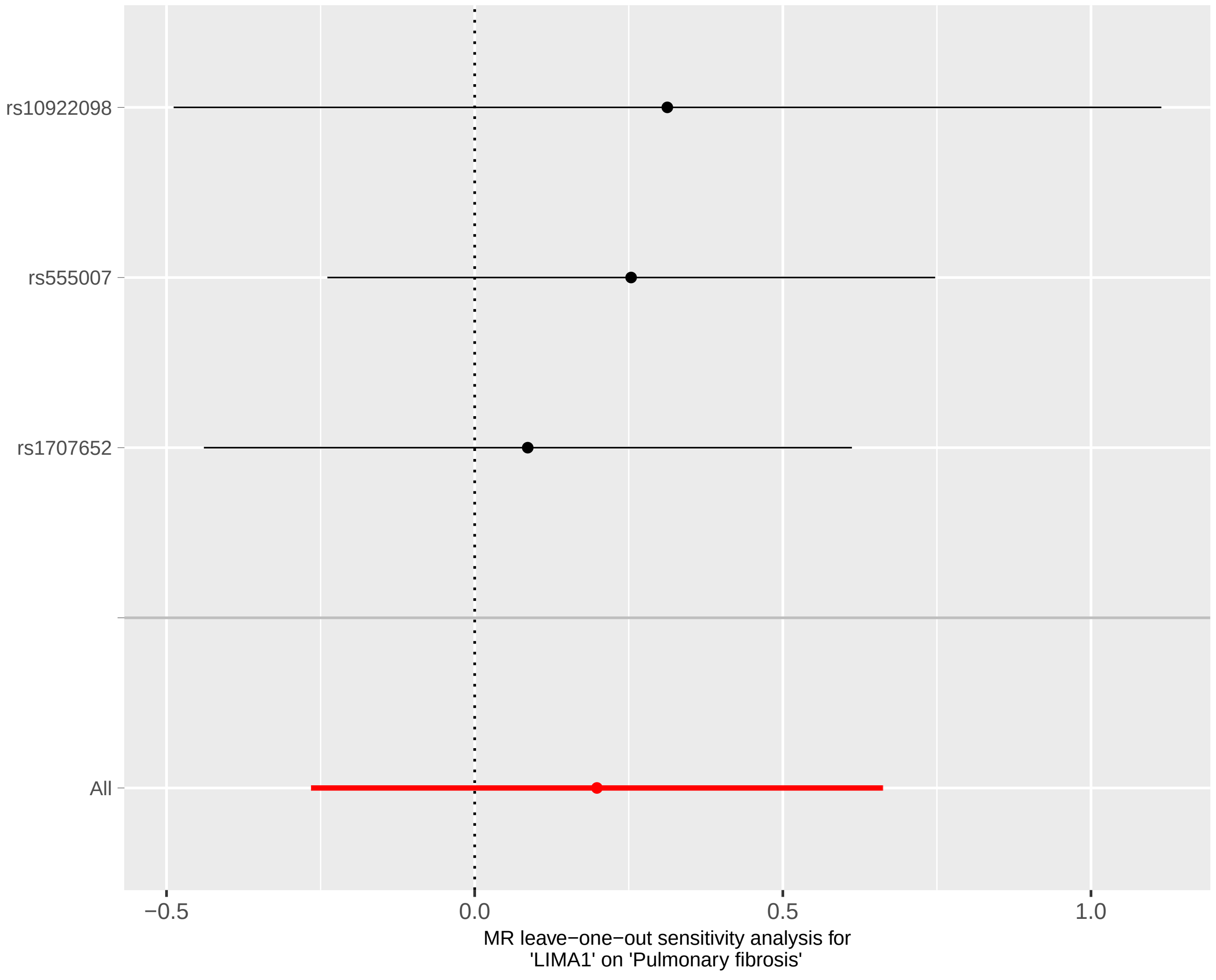

rs10922098
rs555007
rs1707652
All
−0.5
0.0
0.5
1.0
MR leave−one−out sensitivity analysis for
'LIMA1' on 'Pulmonary fibrosis'

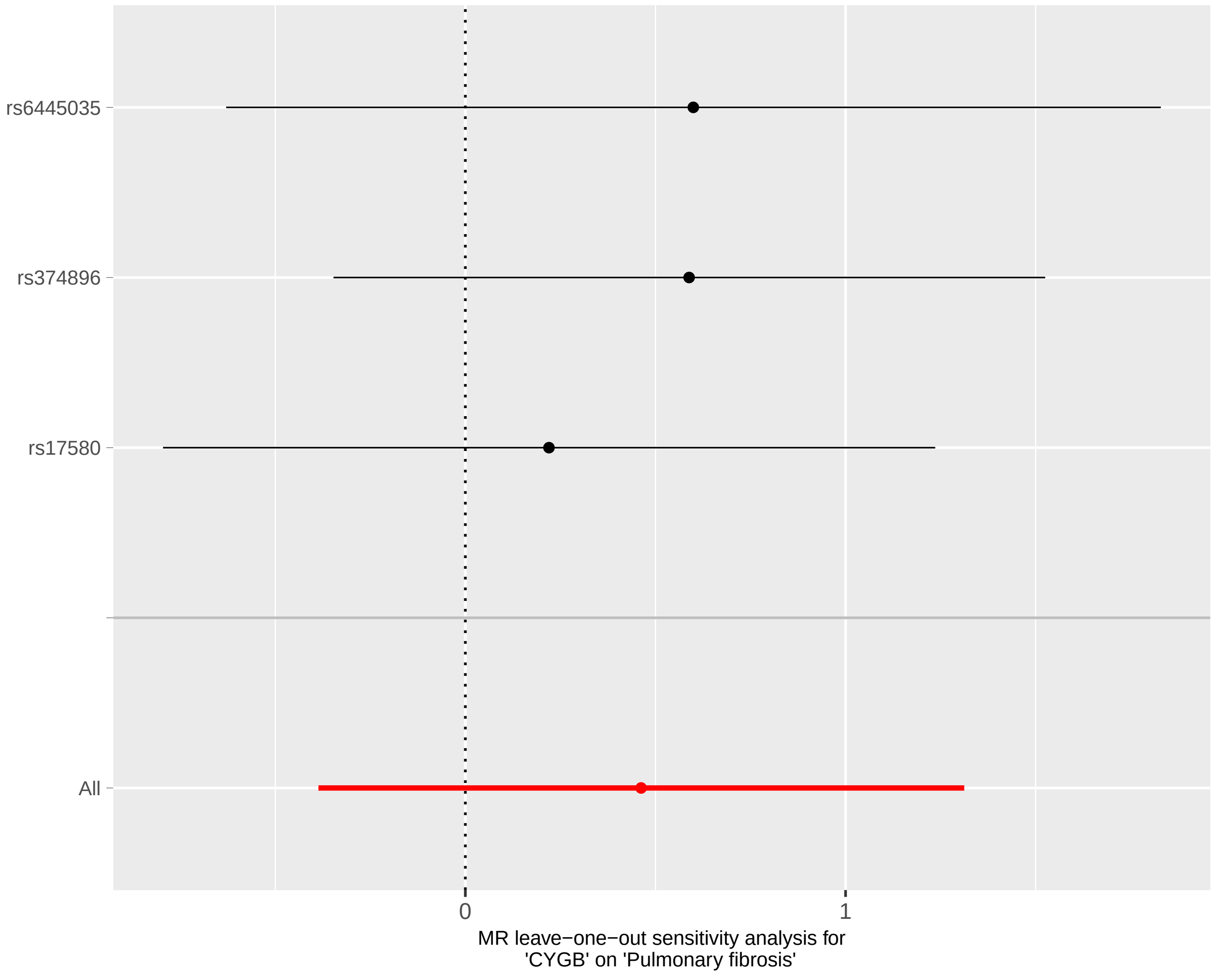
rs6445035
rs374896
rs17580
All
0
1
MR leave−one−out sensitivity analysis for
'CYGB' on 'Pulmonary fibrosis'

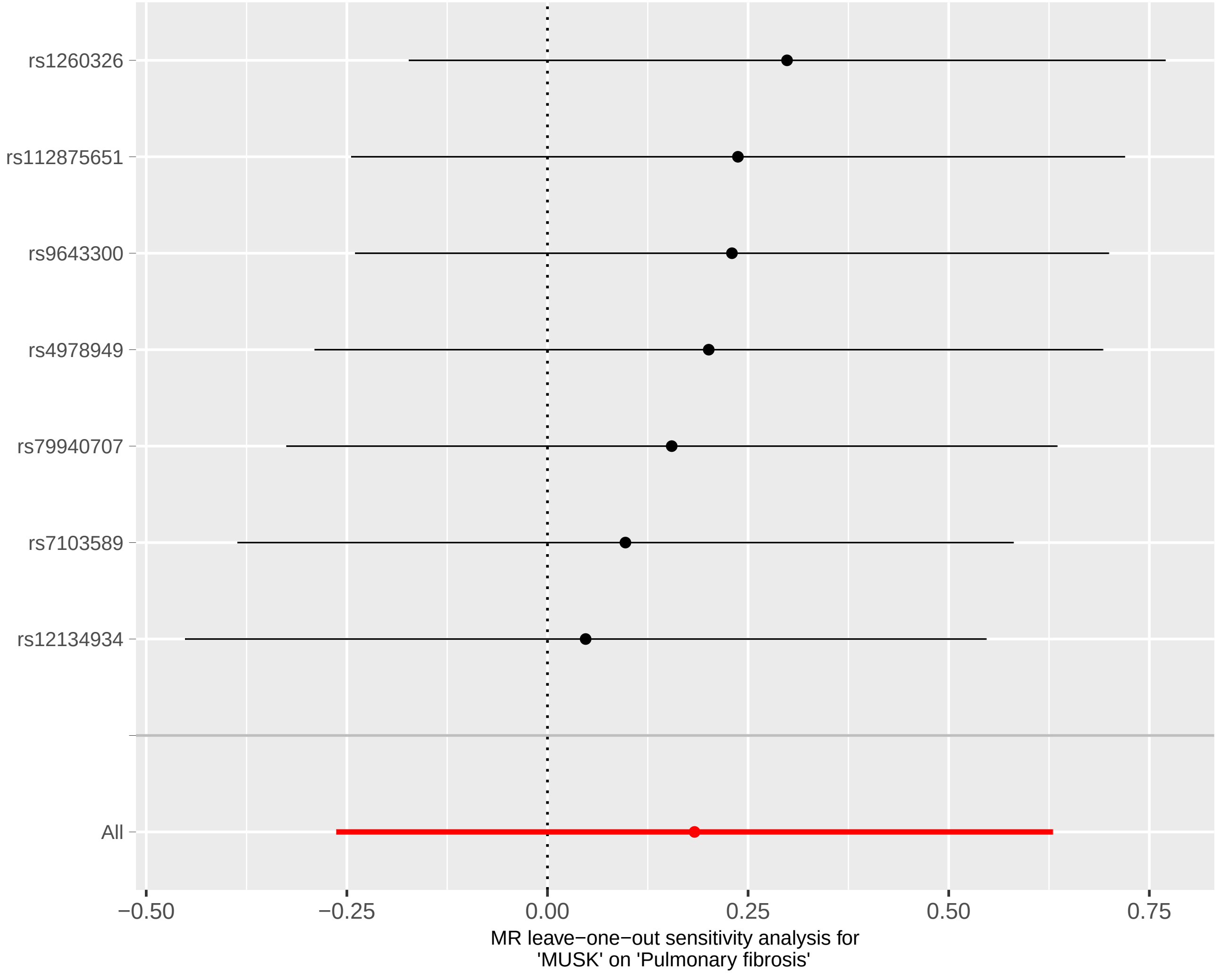
rs1260326
rs112875651
rs9643300
rs4978949
rs79940707
rs7103589
rs12134934
All
−0.50
−0.25
0.00
0.25
0.50
0.75
MR leave−one−out sensitivity analysis for
'MUSK' on 'Pulmonary fibrosis'

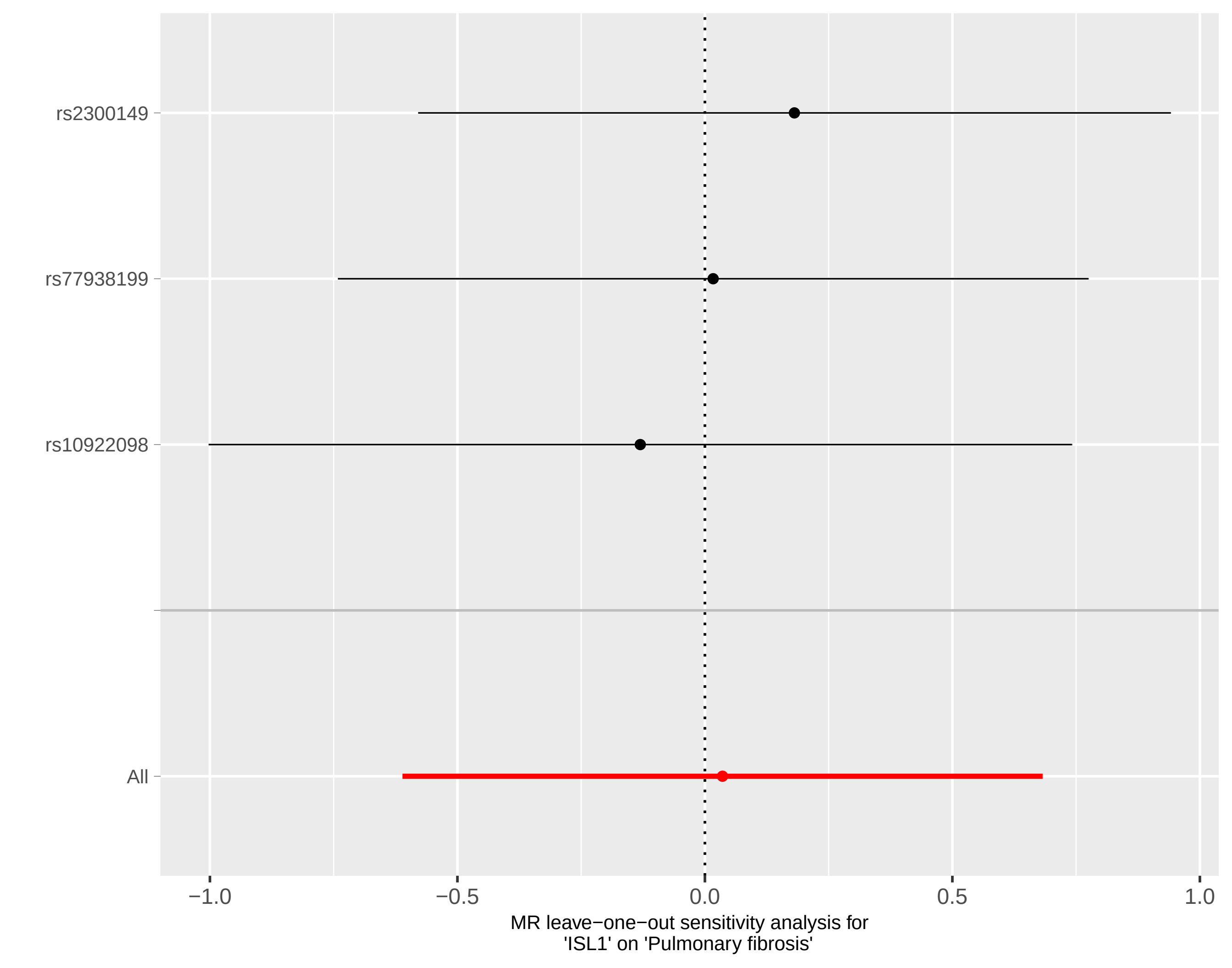
rs2300149
rs77938199
rs10922098
All
−1.0
−0.5
0.0
0.5
1.0
MR leave−one−out sensitivity analysis for
'ISL1' on 'Pulmonary fibrosis'

Insufficient number of SNPs

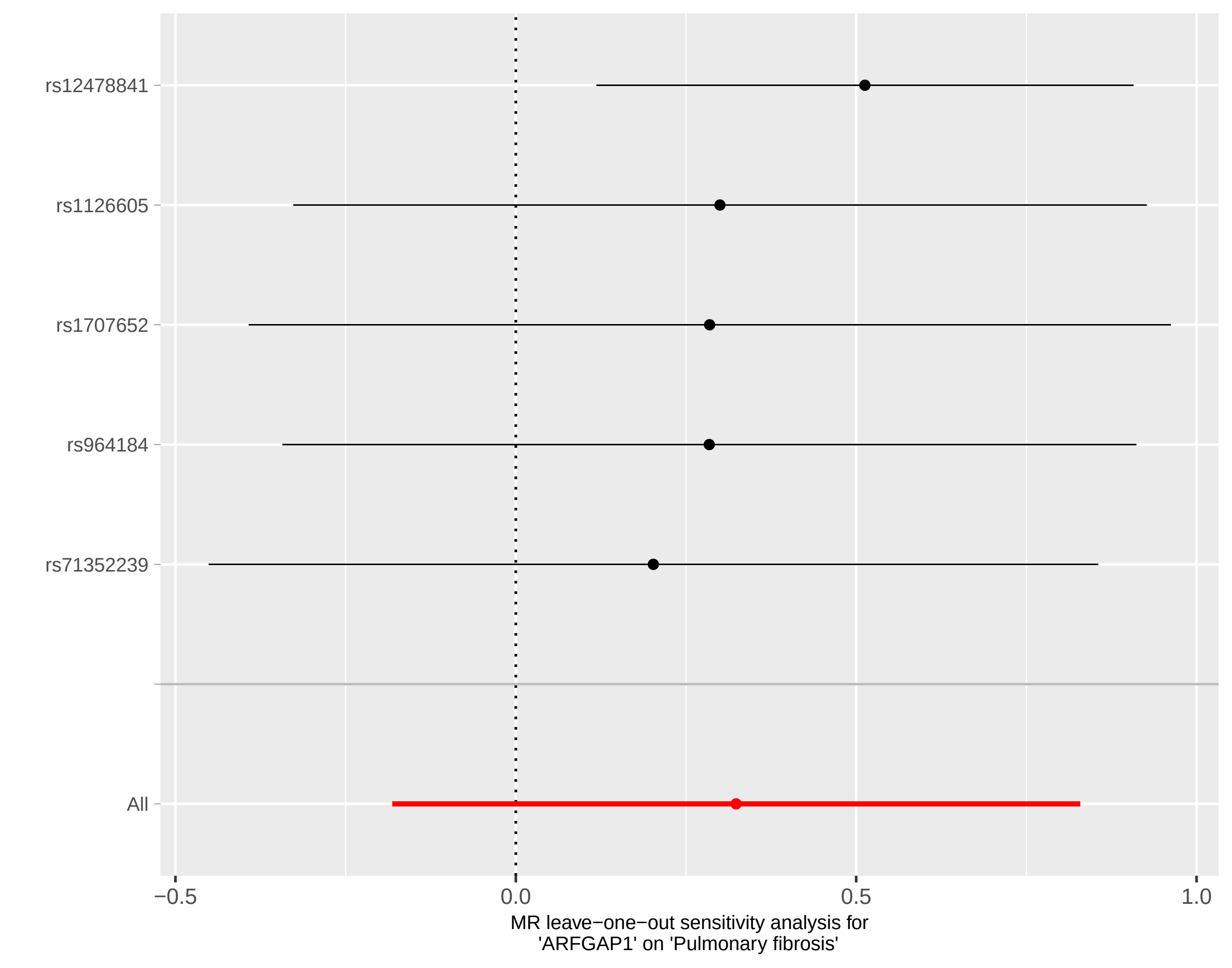

rs12478841
rs1126605
rs1707652
rs964184
rs71352239
All
−0.5
0.0
0.5
1.0
MR leave−one−out sensitivity analysis for
'ARFGAP1' on 'Pulmonary fibrosis'

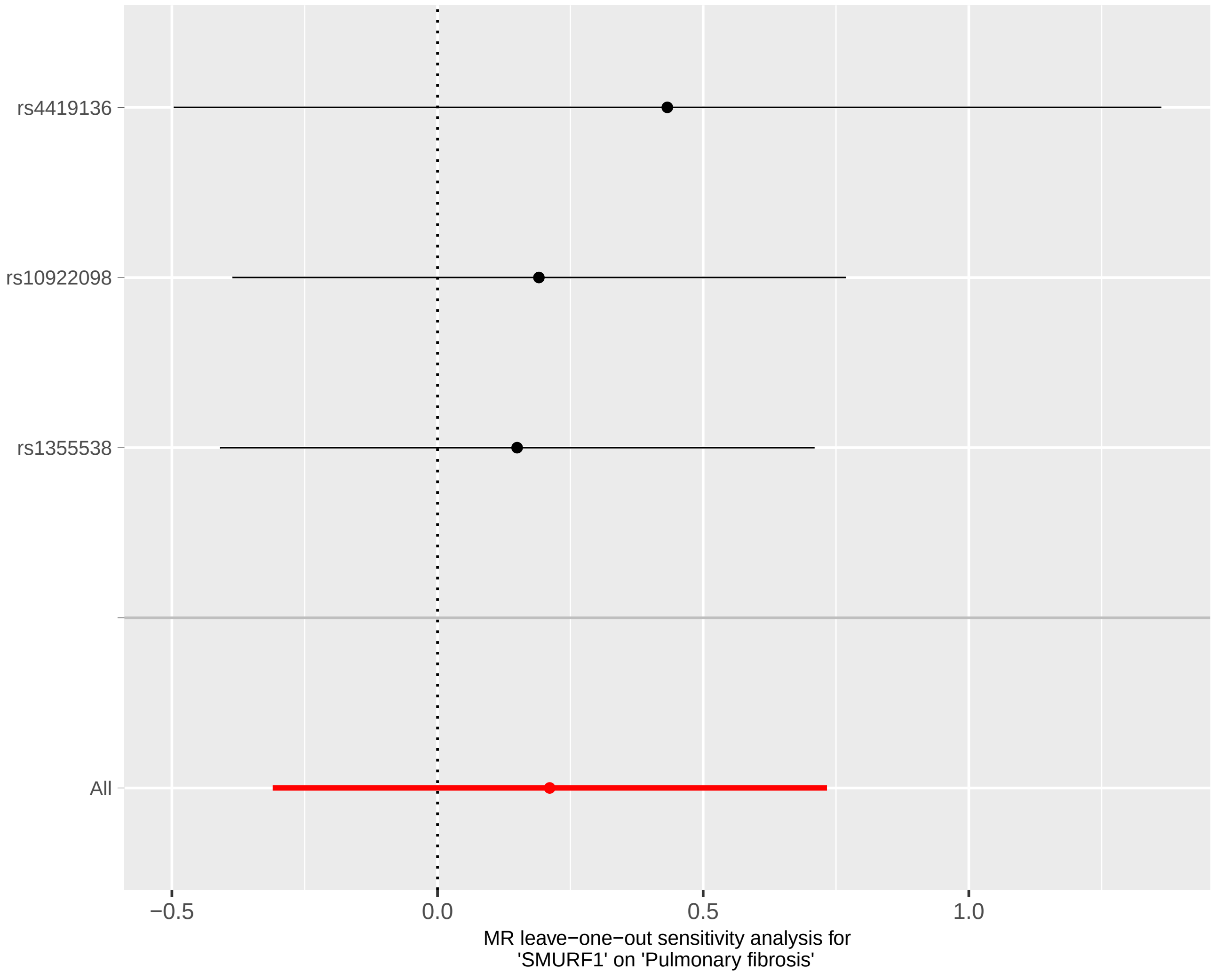
rs4419136
rs10922098
rs1355538
All
−0.5
0.0
0.5
1.0
MR leave−one−out sensitivity analysis for
'SMURF1' on 'Pulmonary fibrosis'

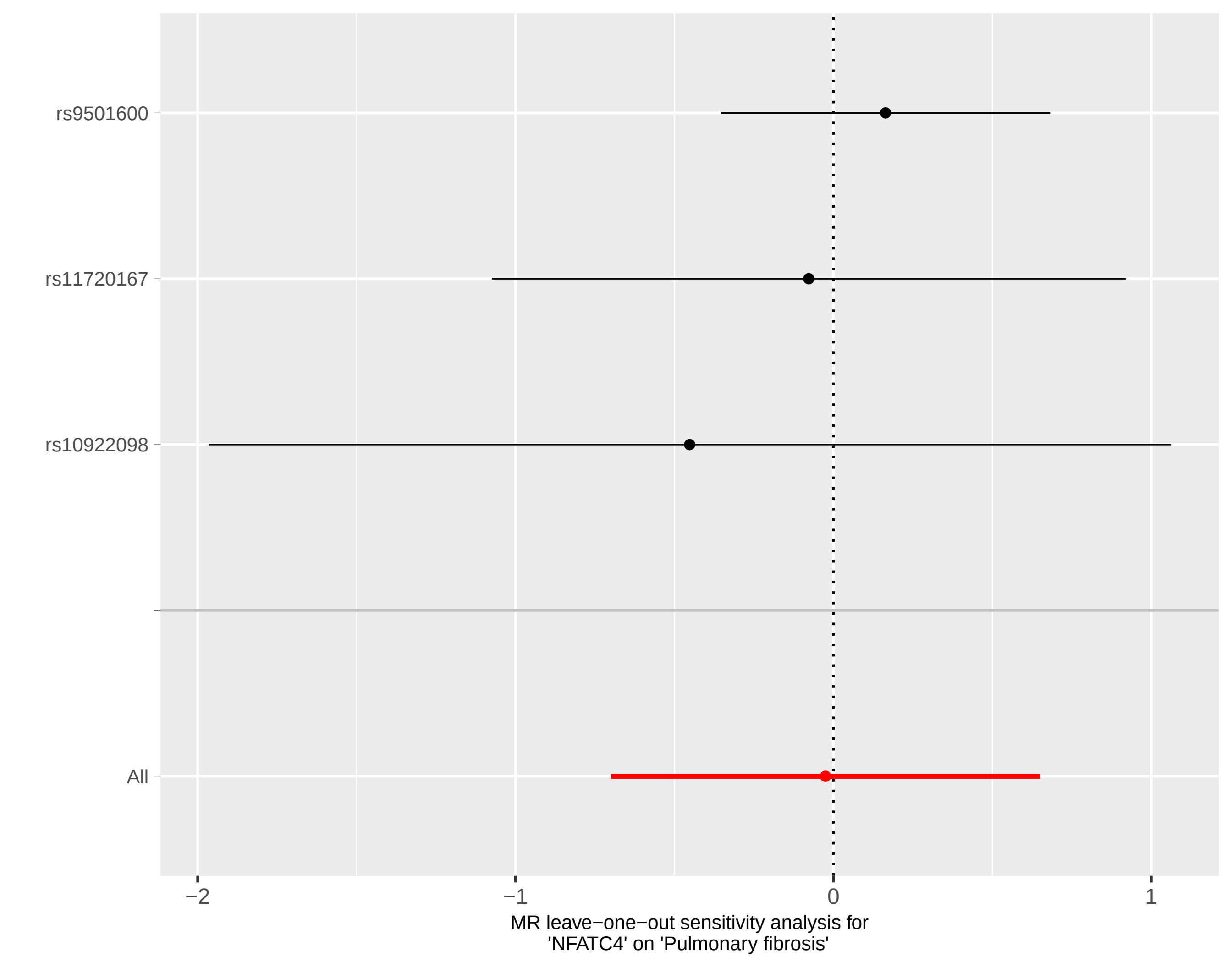

rs9501600
rs11720167
rs10922098
All
−2
−1
0
1
MR leave−one−out sensitivity analysis for
'NFATC4' on 'Pulmonary fibrosis'

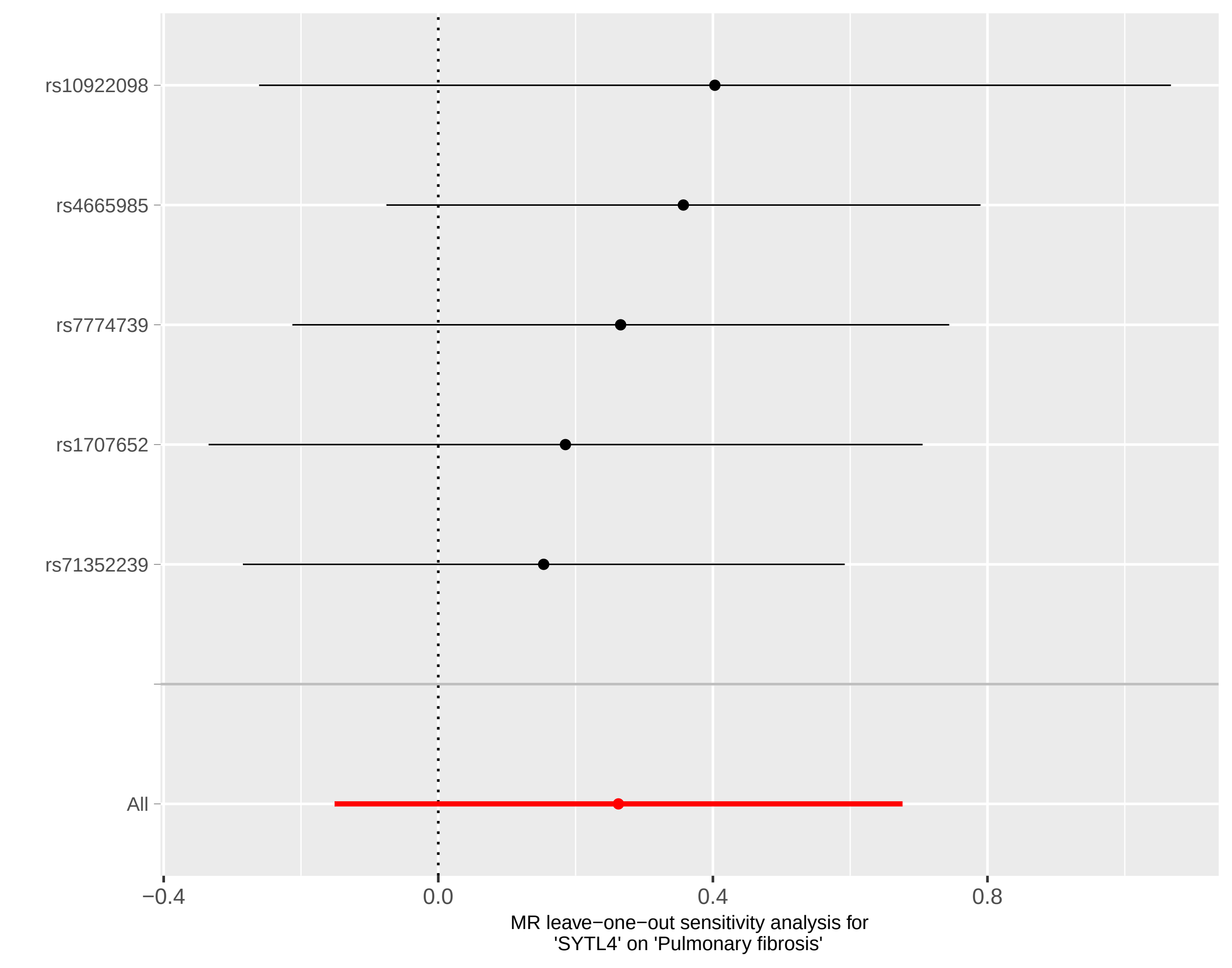

rs10922098
rs4665985
rs7774739
rs1707652
rs71352239
All
−0.4
0.0
0.4
0.8
MR leave−one−out sensitivity analysis for
'SYTL4' on 'Pulmonary fibrosis'

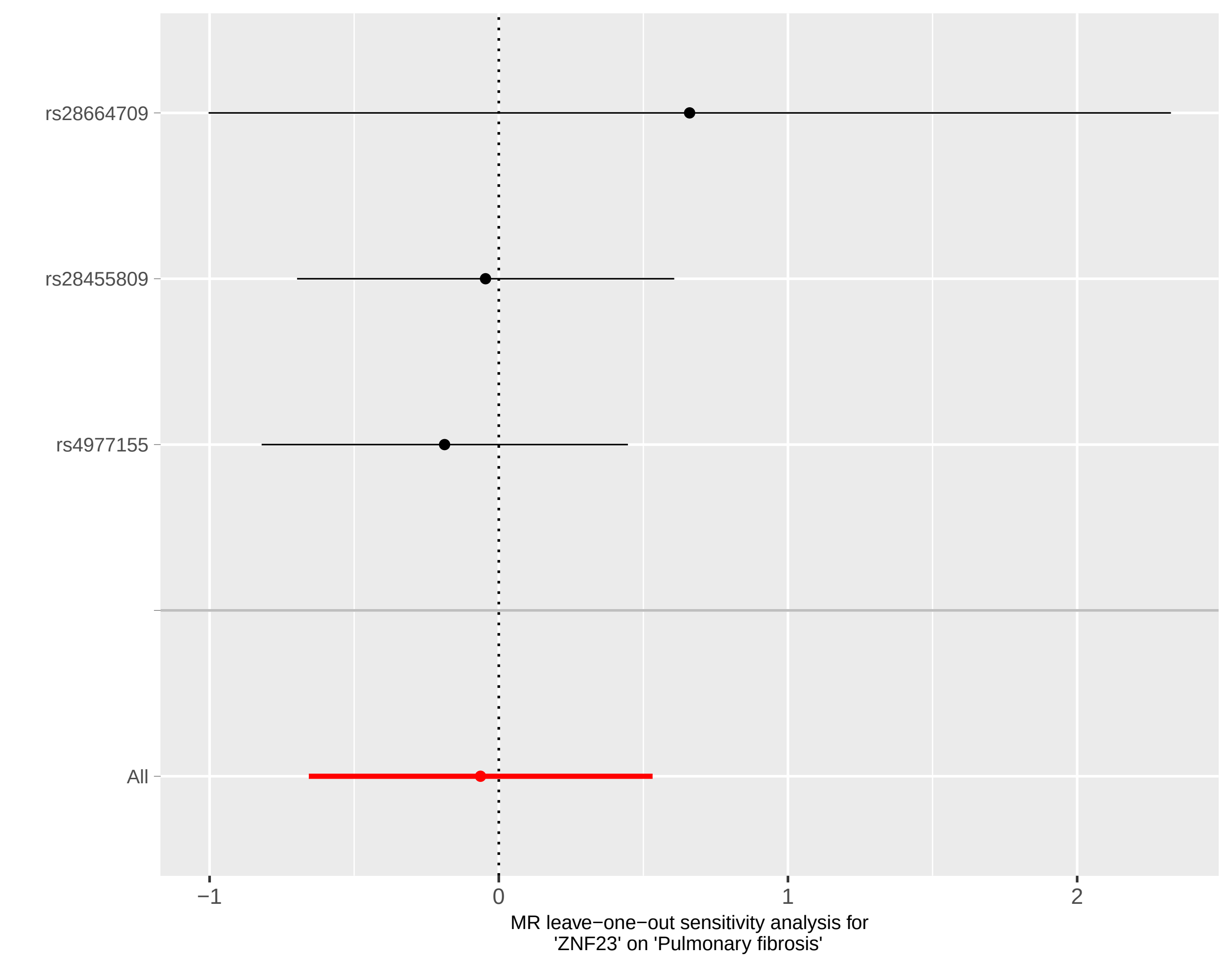
rs28664709
rs28455809
rs4977155
All
−1
0
1
2
MR leave−one−out sensitivity analysis for
'ZNF23' on 'Pulmonary fibrosis'

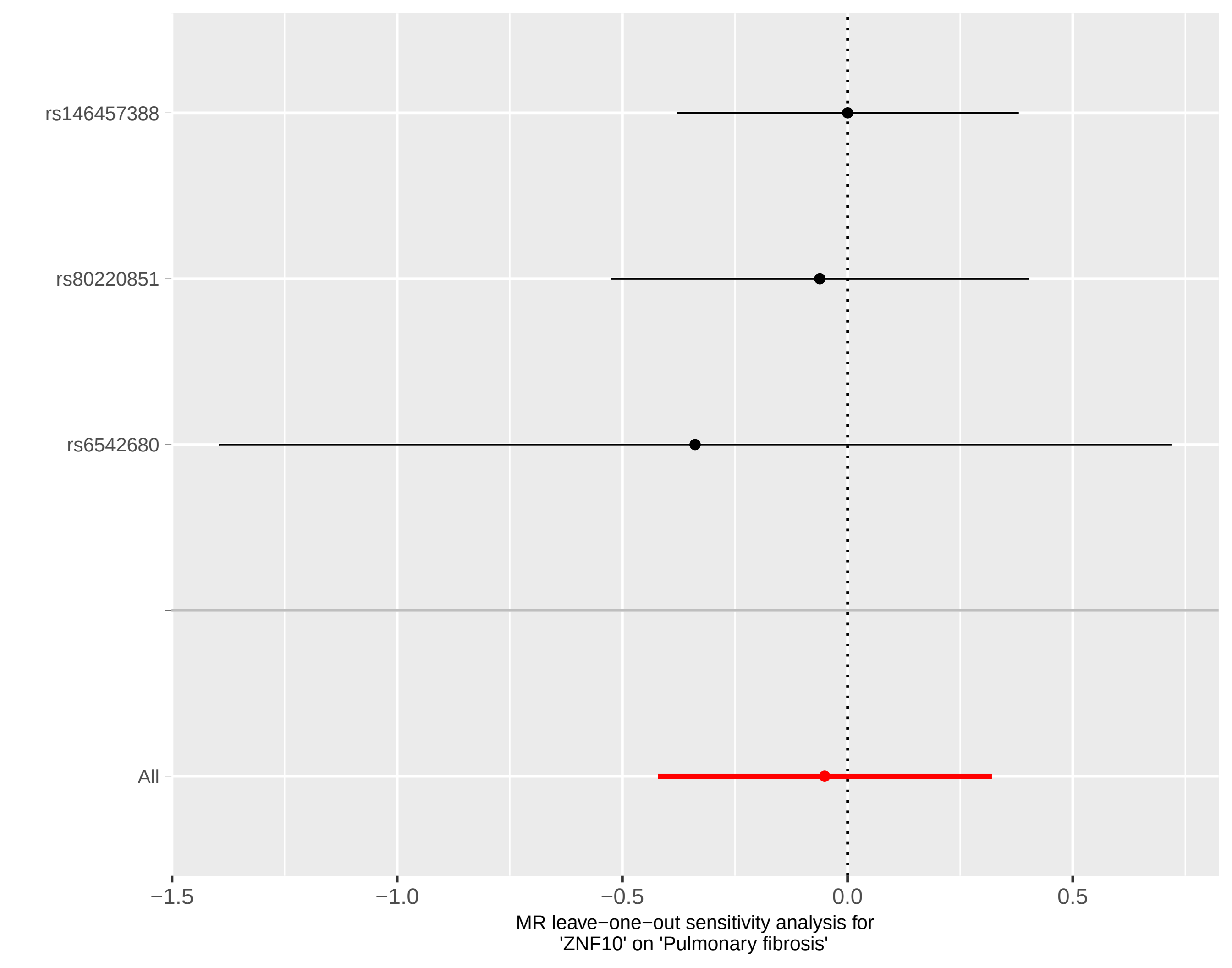
rs146457388
rs80220851
rs6542680
All
−1.5
−1.0
−0.5
0.0
0.5
MR leave−one−out sensitivity analysis for
'ZNF10' on 'Pulmonary fibrosis'

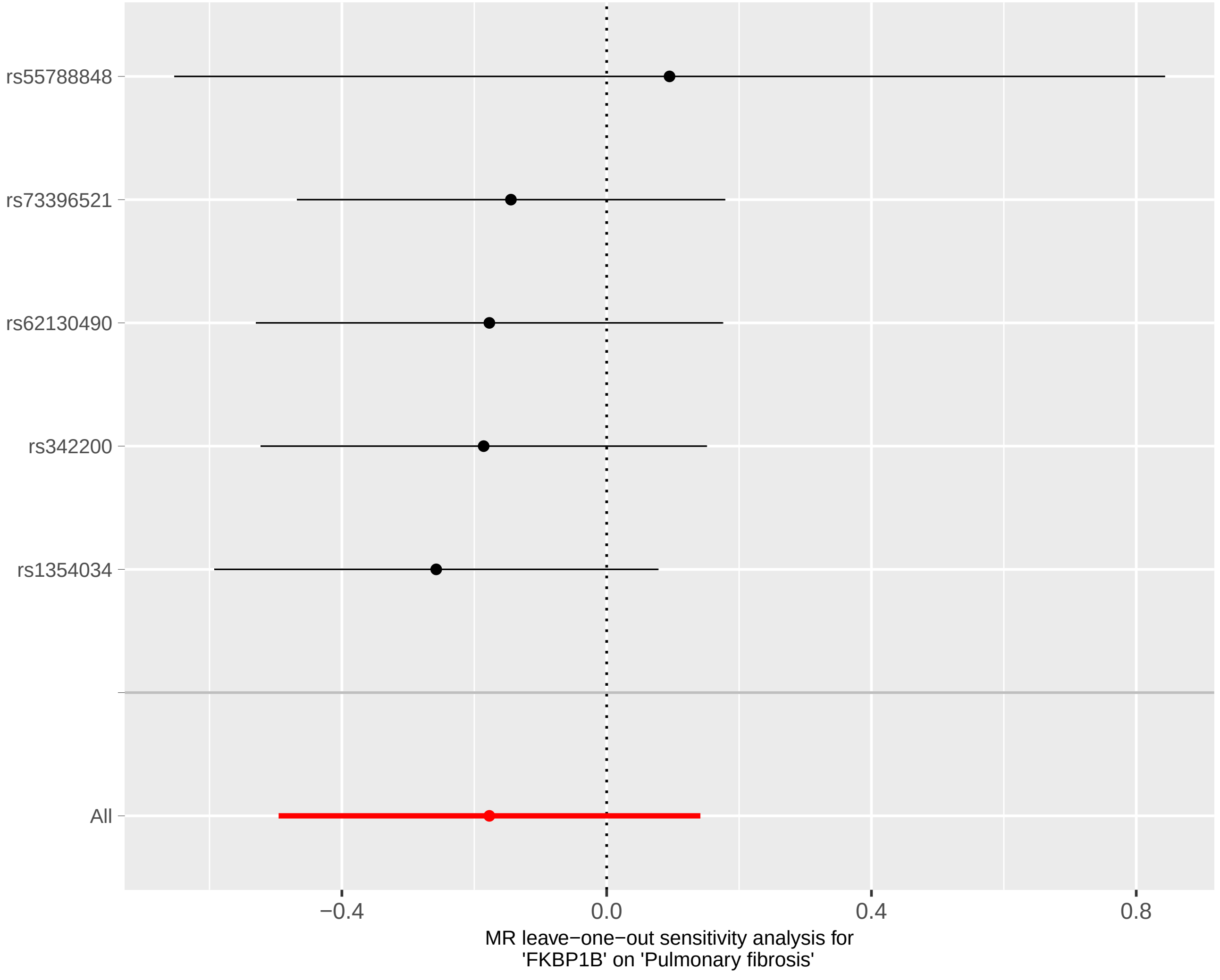
rs55788848
rs73396521
rs62130490
rs342200
rs1354034
All
−0.4
0.0
0.4
0.8
MR leave−one−out sensitivity analysis for
'FKBP1B' on 'Pulmonary fibrosis'

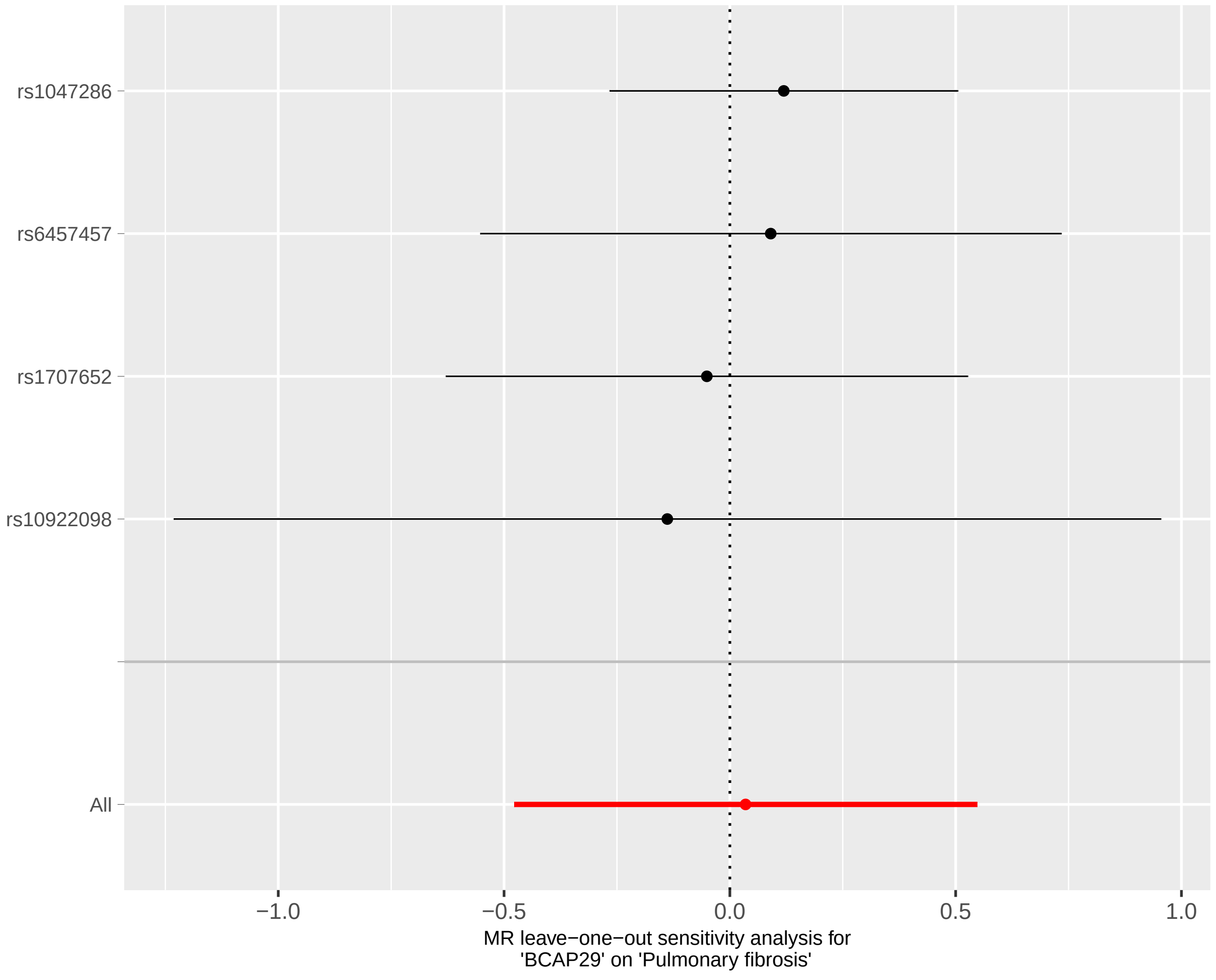
rs1047286
rs6457457
rs1707652
rs10922098
All
−1.0
−0.5
0.0
0.5
1.0
MR leave−one−out sensitivity analysis for
'BCAP29' on 'Pulmonary fibrosis'

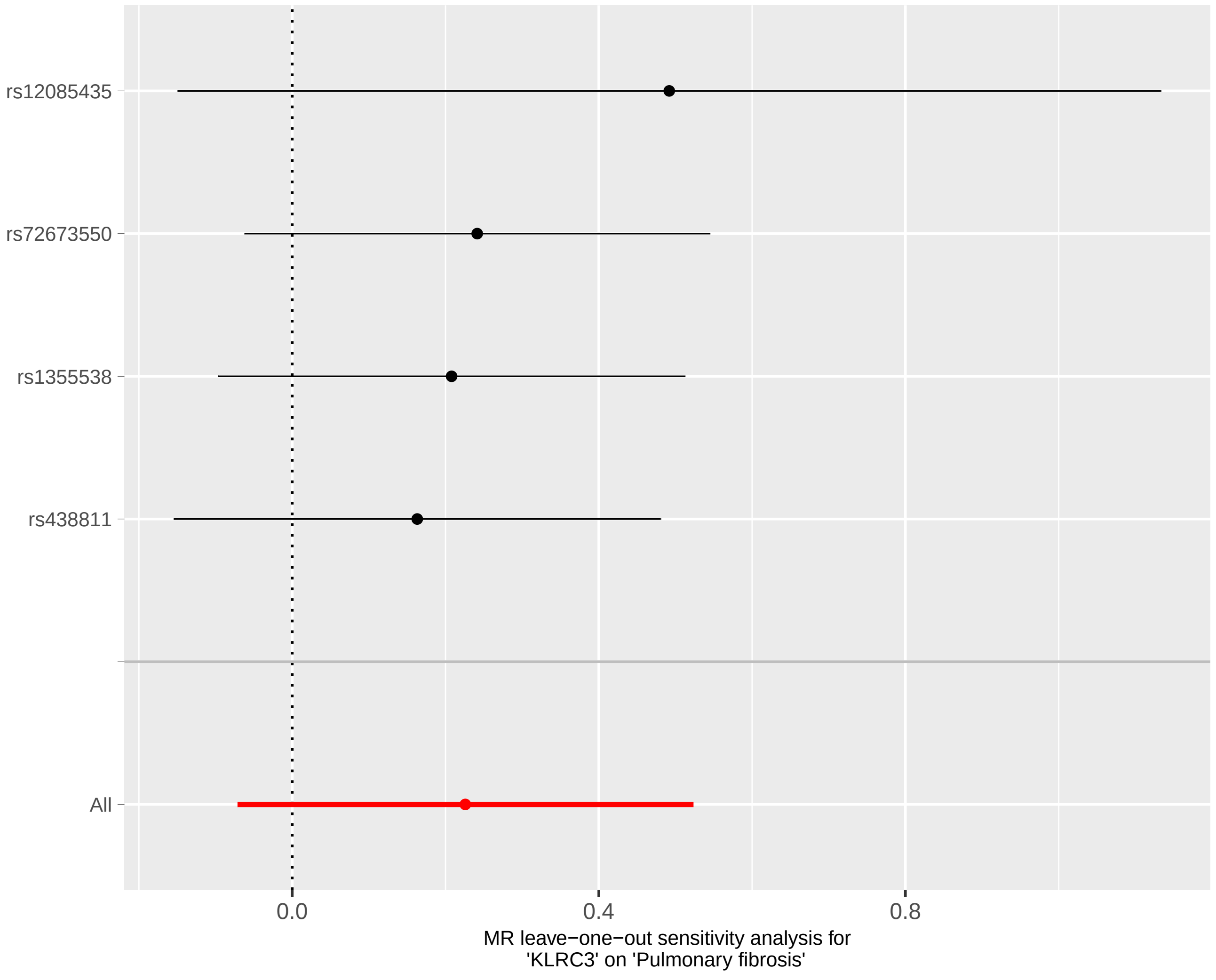
rs12085435
rs72673550
rs1355538
rs438811
All
0.0
0.4
0.8
MR leave−one−out sensitivity analysis for
'KLRC3' on 'Pulmonary fibrosis'

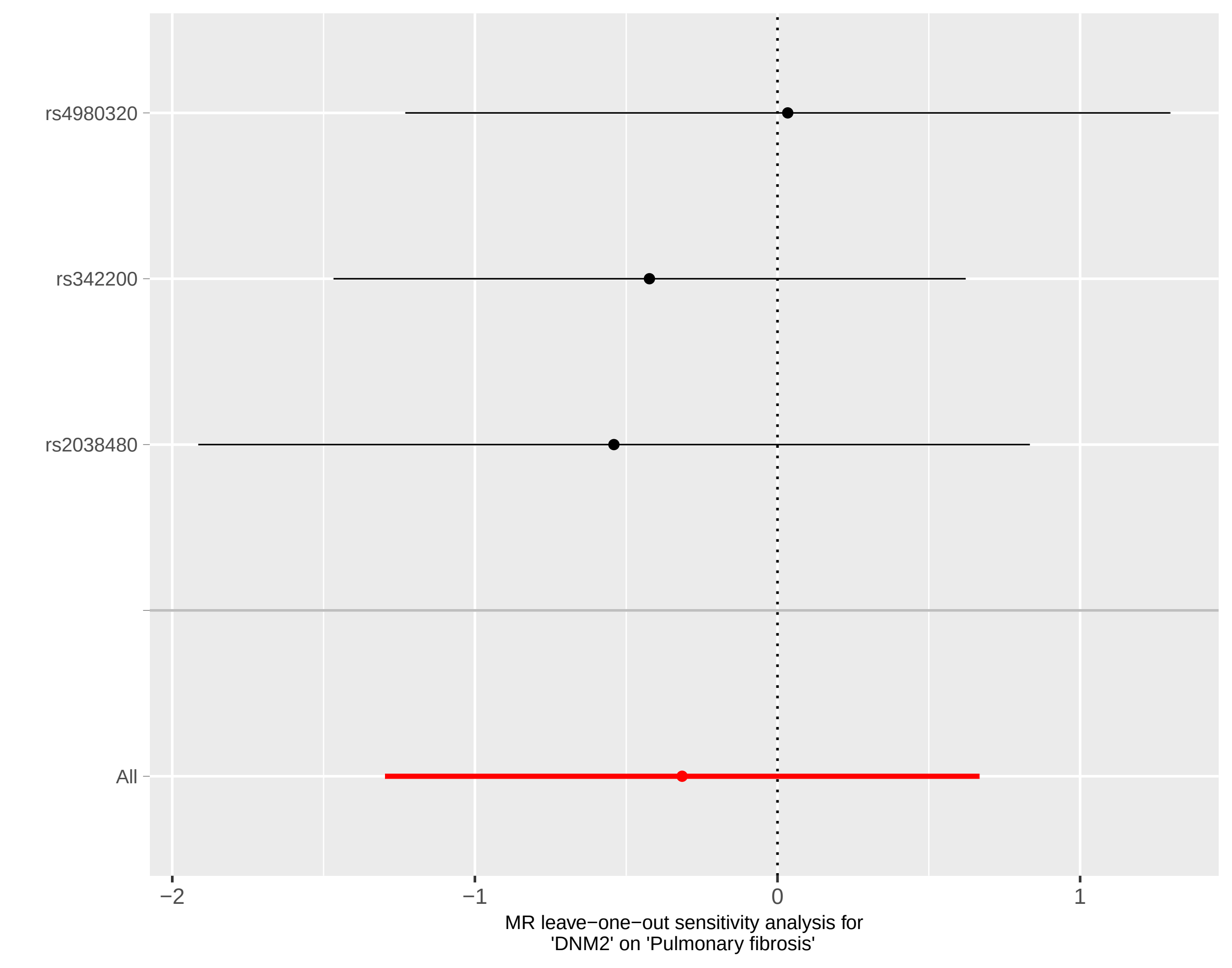
rs4980320
rs342200
rs2038480
All
−2
−1
0
1
MR leave−one−out sensitivity analysis for
'DNM2' on 'Pulmonary fibrosis'

Insufficient number of SNPs

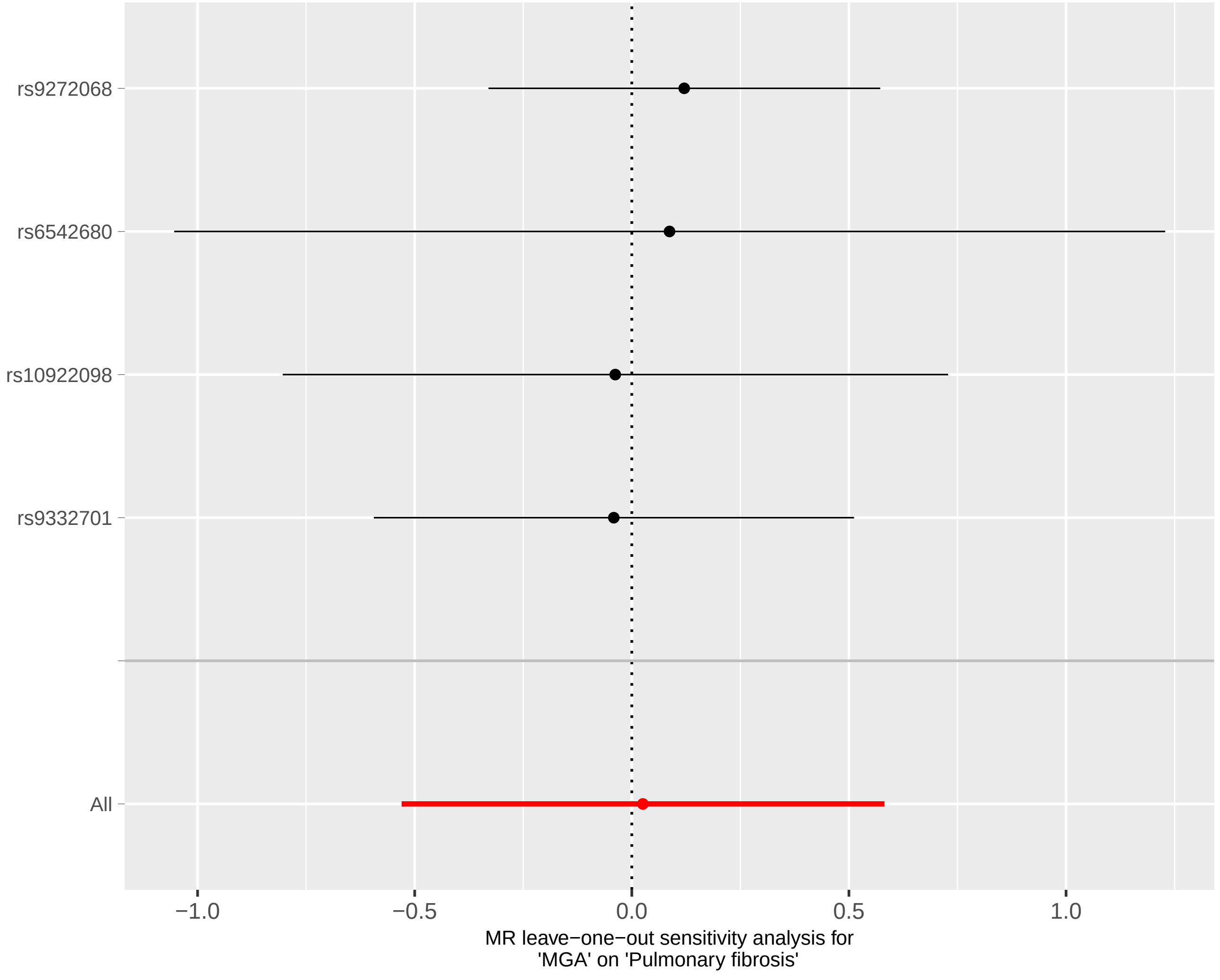

rs9272068
rs6542680
rs10922098
rs9332701
All
−1.0
−0.5
0.0
0.5
1.0
MR leave−one−out sensitivity analysis for
'MGA' on 'Pulmonary fibrosis'

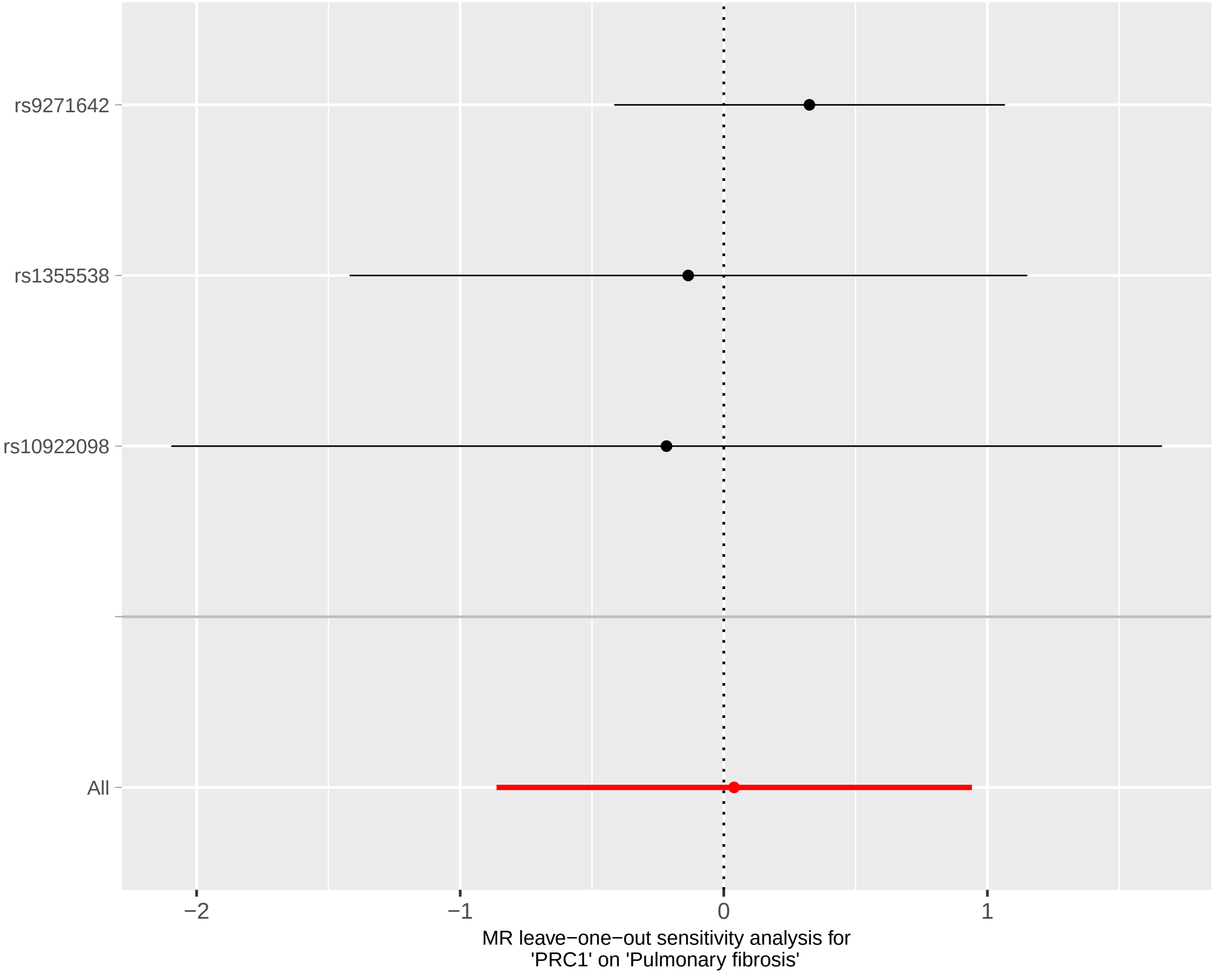

rs9271642
rs1355538
rs10922098
All
−2
−1
0
1
MR leave−one−out sensitivity analysis for
'PRC1' on 'Pulmonary fibrosis'

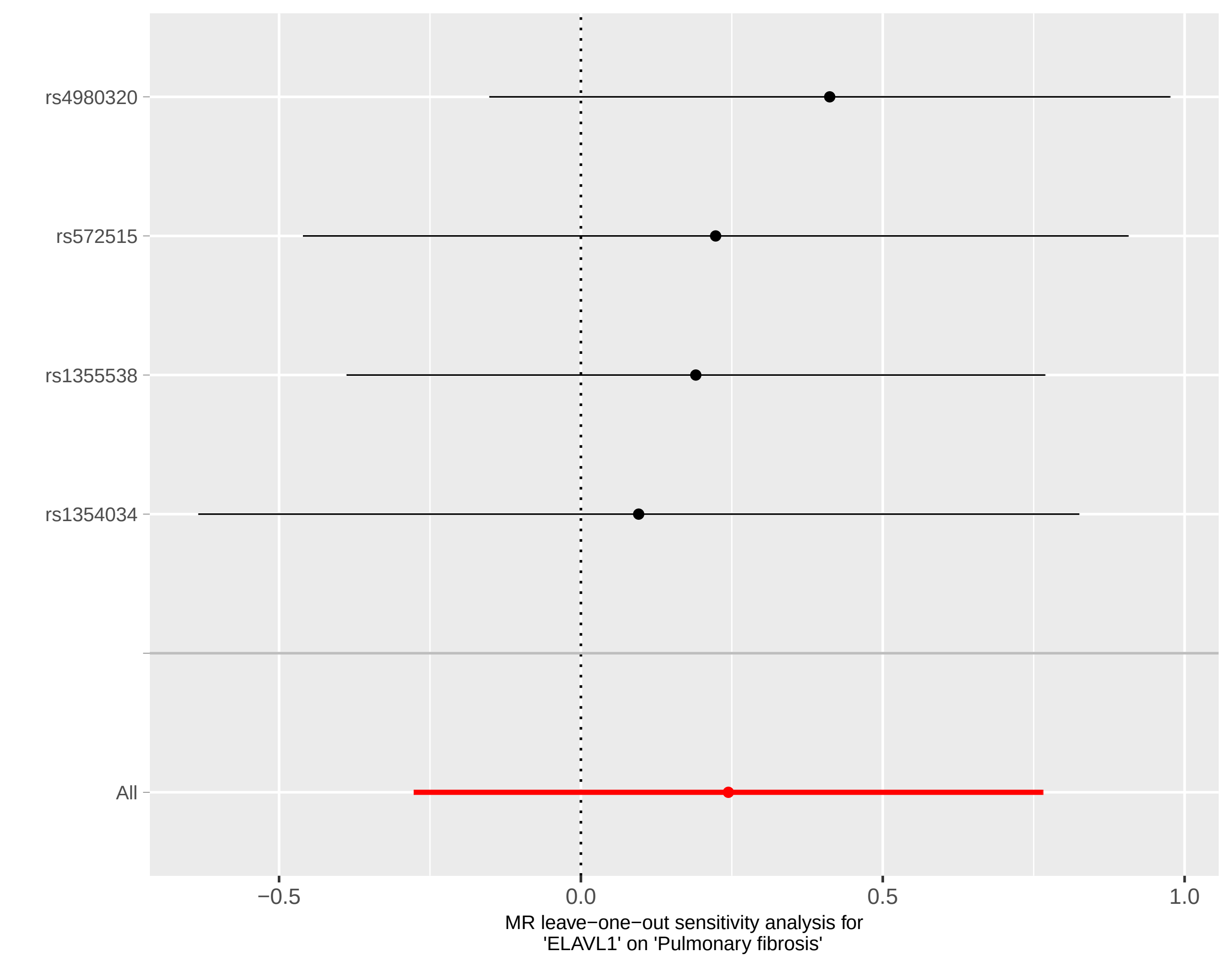
rs4980320
rs572515
rs1355538
rs1354034
All
−0.5
0.0
0.5
1.0
MR leave−one−out sensitivity analysis for
'ELAVL1' on 'Pulmonary fibrosis'

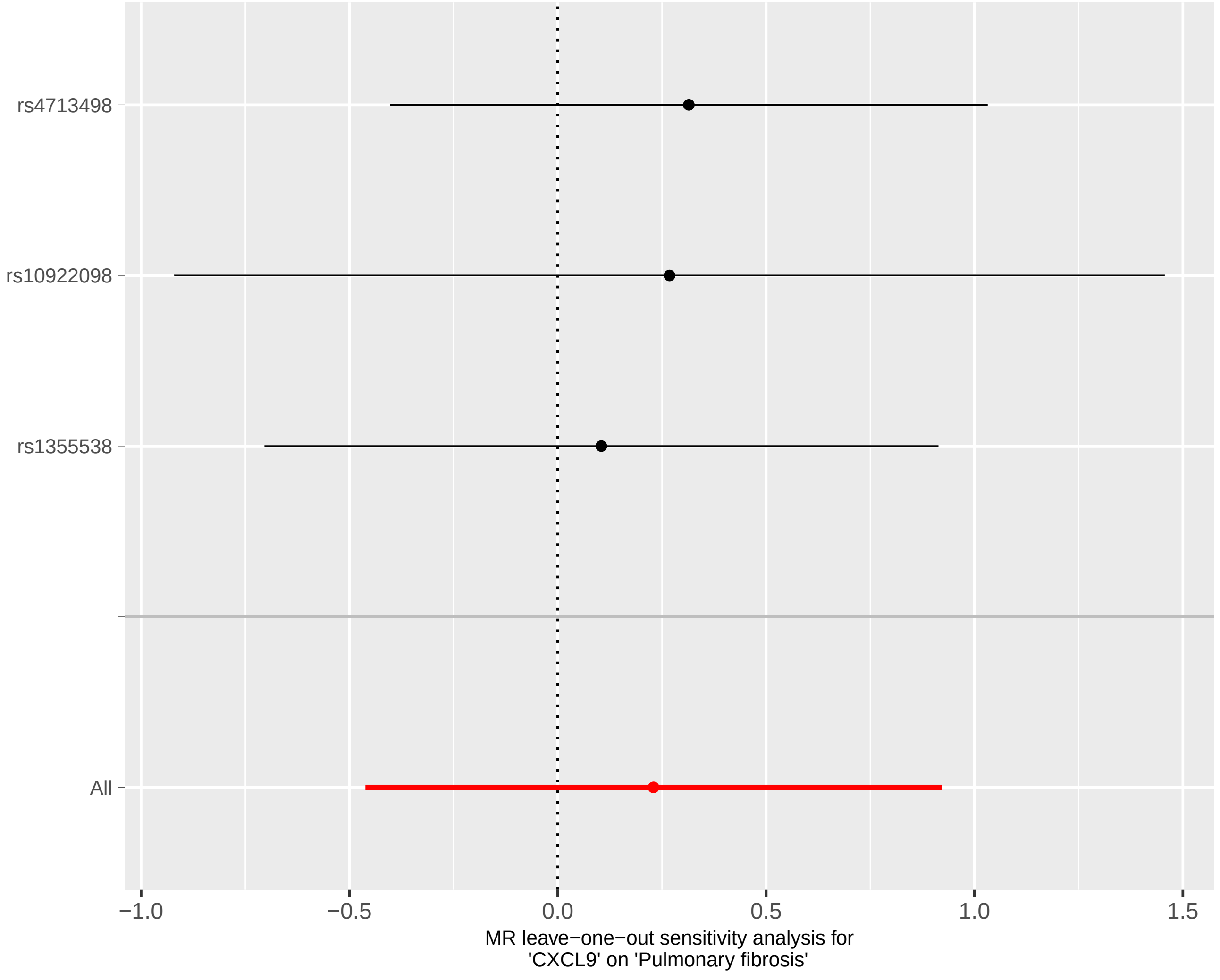

rs4713498
rs10922098
rs1355538
All
−1.0
−0.5
0.0
0.5
1.0
1.5
MR leave−one−out sensitivity analysis for
'CXCL9' on 'Pulmonary fibrosis'

Insufficient number of SNPs

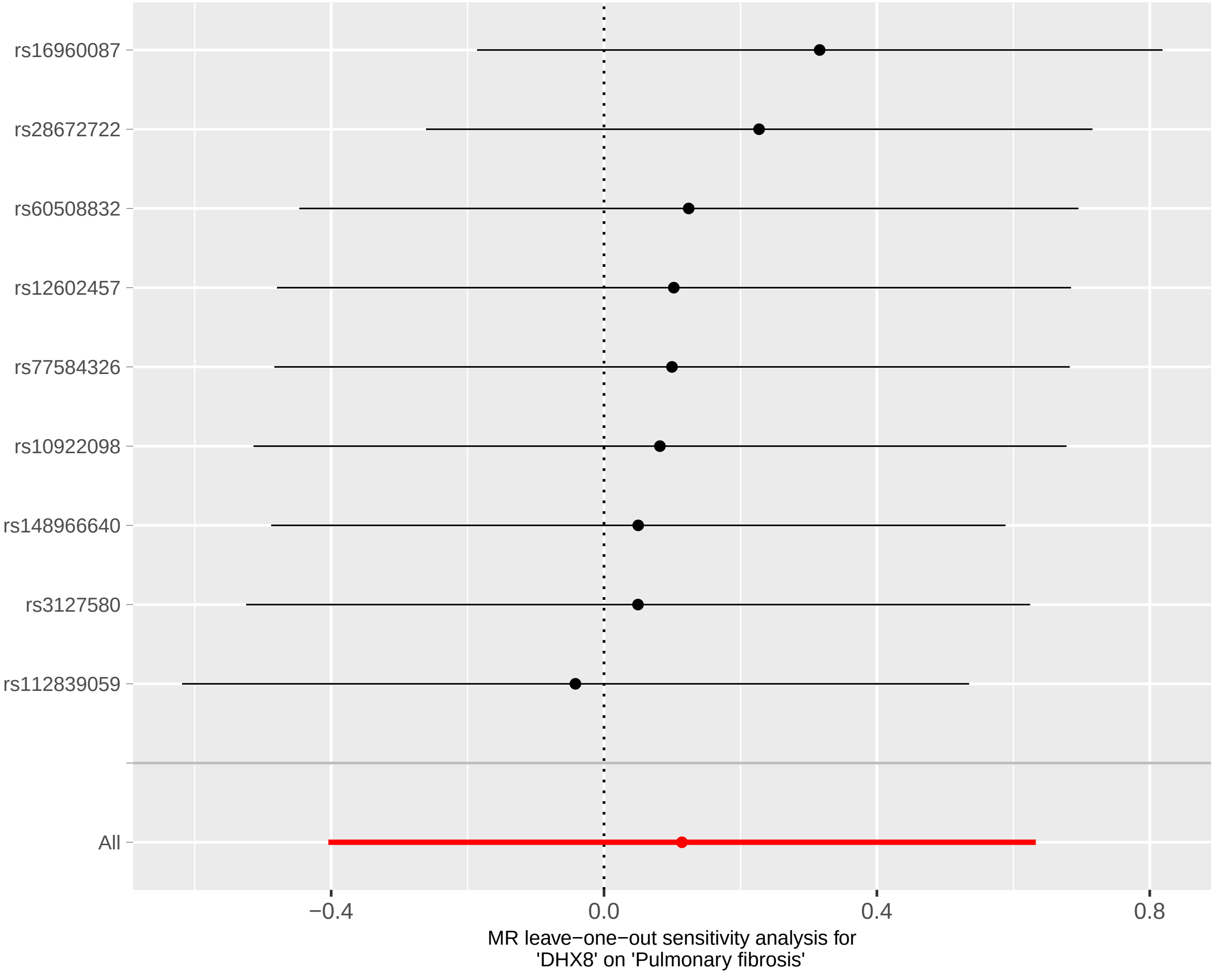

rs16960087
rs28672722
rs60508832
rs12602457
rs77584326
rs10922098
rs148966640
rs3127580
rs112839059
All
−0.4
0.0
0.4
0.8
MR leave−one−out sensitivity analysis for
'DHX8' on 'Pulmonary fibrosis'

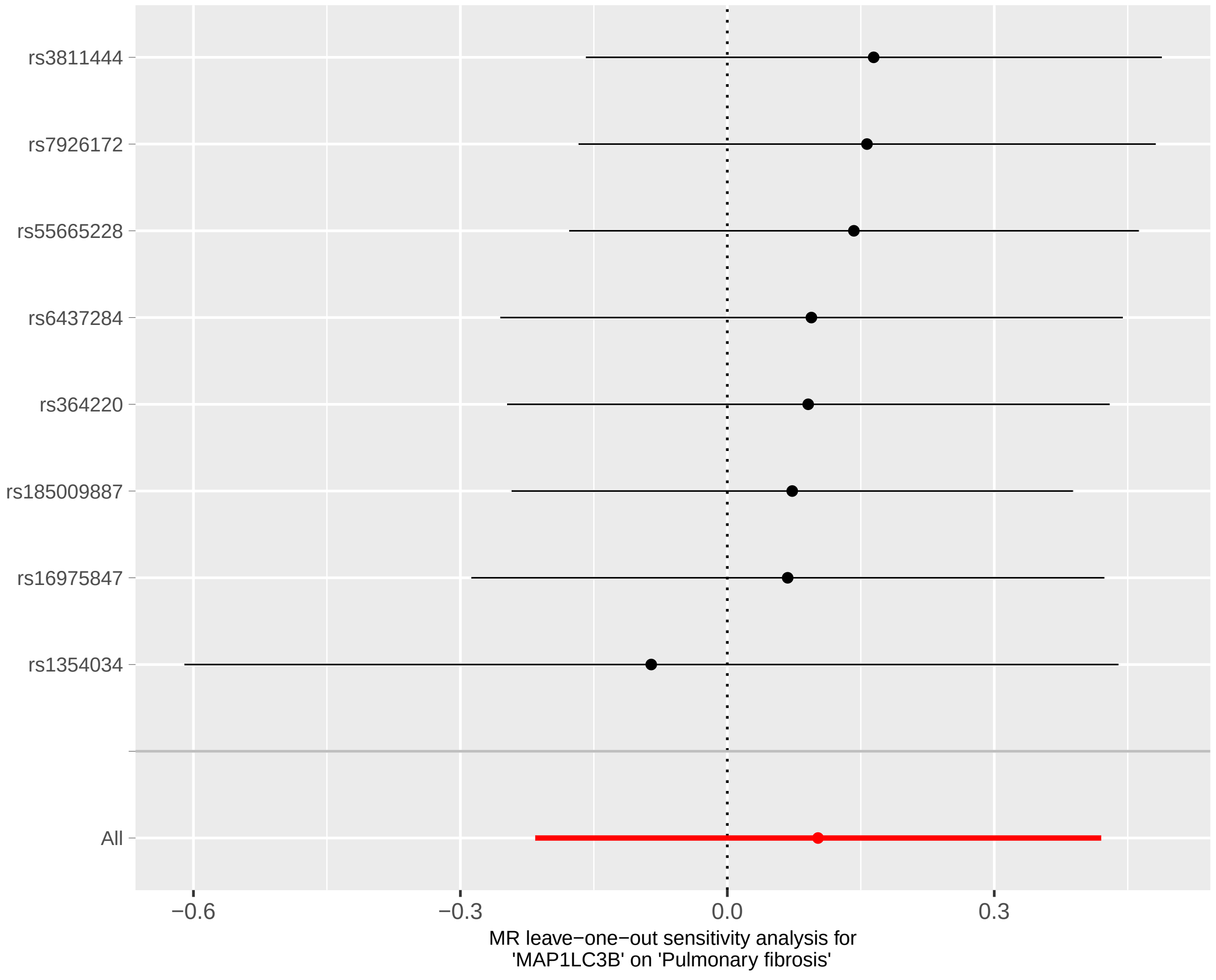
rs3811444
rs7926172
rs55665228
rs6437284
rs364220
rs185009887
rs16975847
rs1354034
All
−0.6
−0.3
0.0
0.3
MR leave−one−out sensitivity analysis for
'MAP1LC3B' on 'Pulmonary fibrosis'

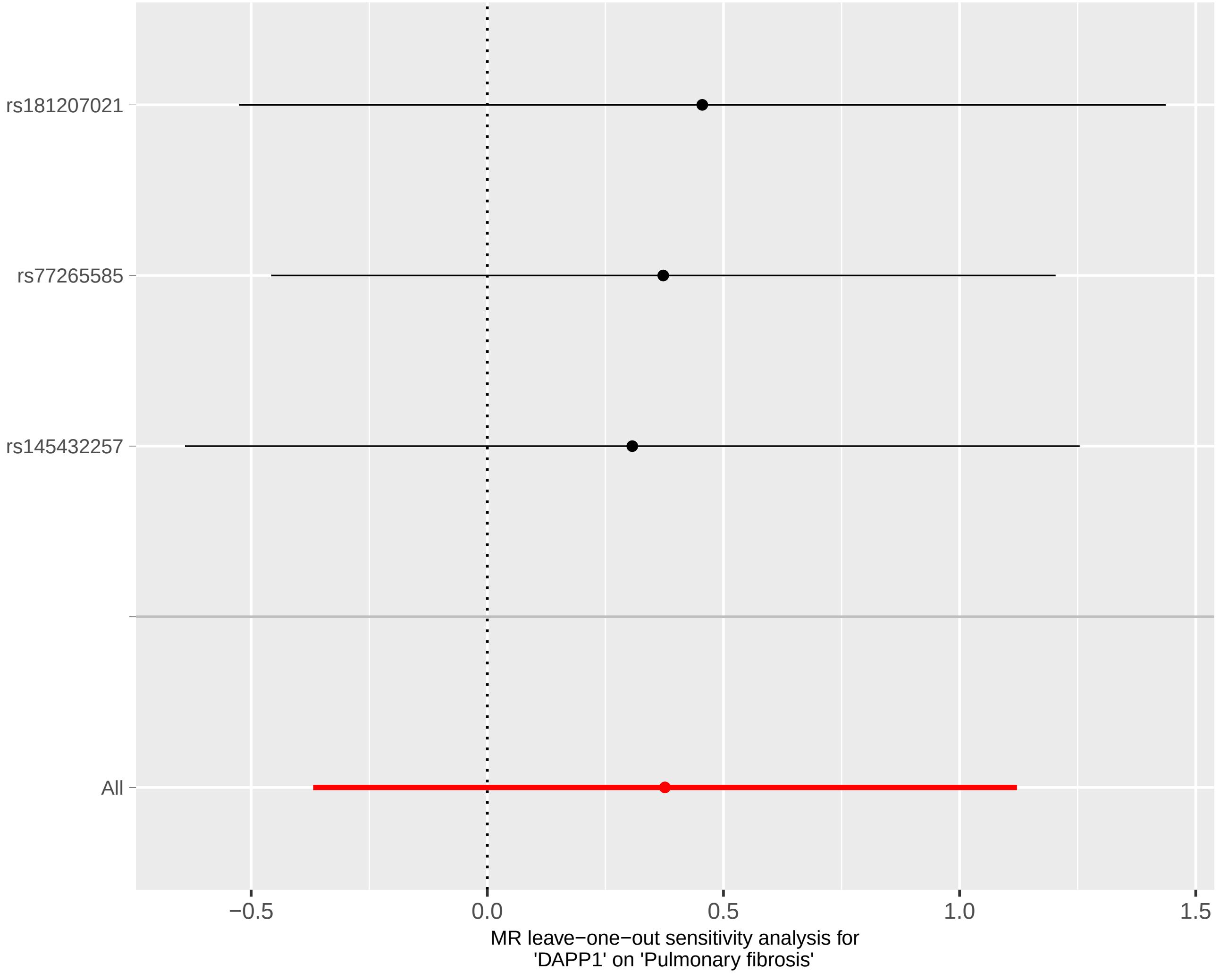
rs181207021
rs77265585
rs145432257
All
−0.5
0.0
0.5
1.0
1.5
MR leave−one−out sensitivity analysis for
'DAPP1' on 'Pulmonary fibrosis'

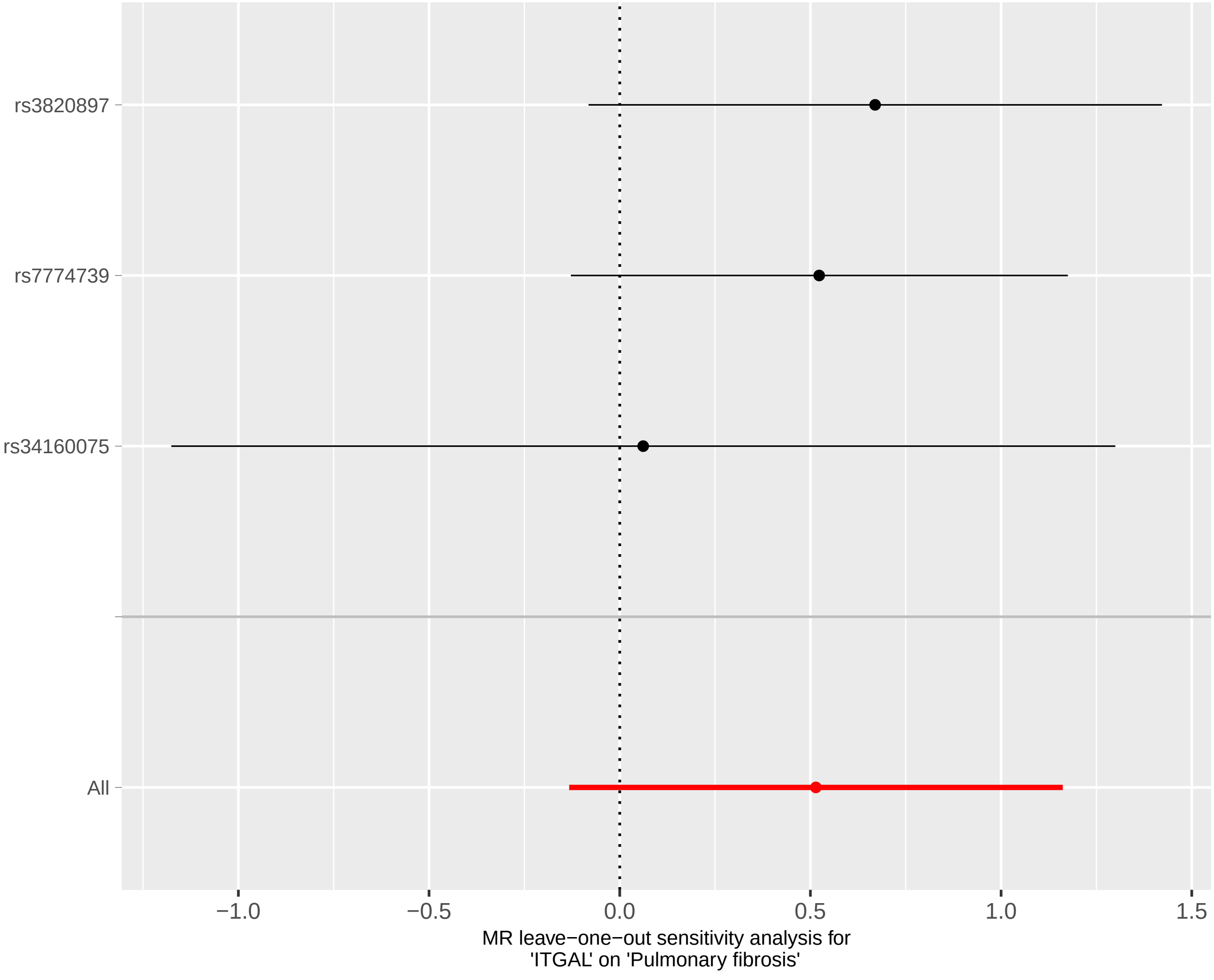
rs3820897
rs7774739
rs34160075
All
−1.0
−0.5
0.0
0.5
1.0
1.5
MR leave−one−out sensitivity analysis for
'ITGAL' on 'Pulmonary fibrosis'

Insufficient number of SNPs

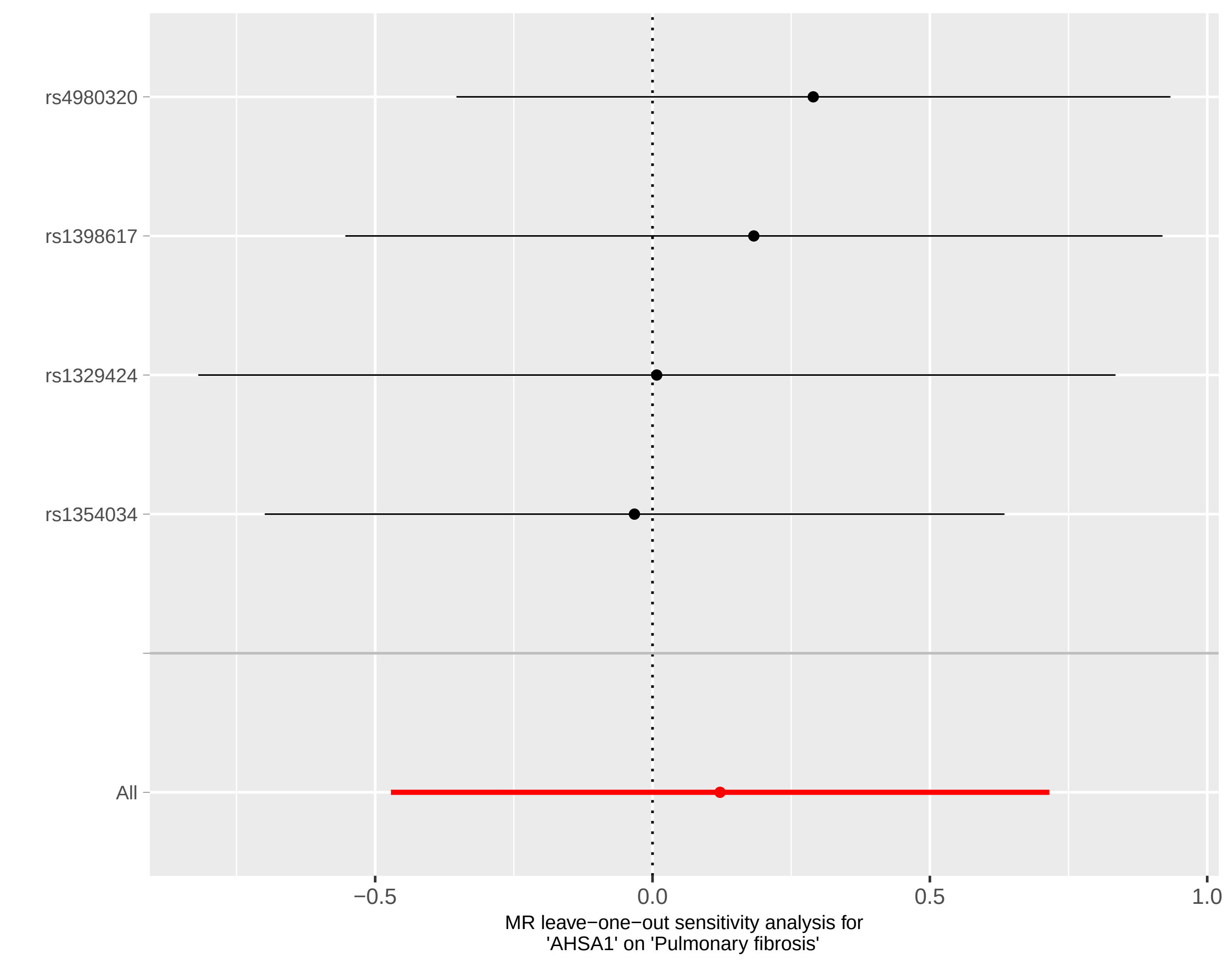
rs4980320
rs1398617
rs1329424
rs1354034
All
−0.5
0.0
0.5
1.0
MR leave−one−out sensitivity analysis for
'AHSA1' on 'Pulmonary fibrosis'

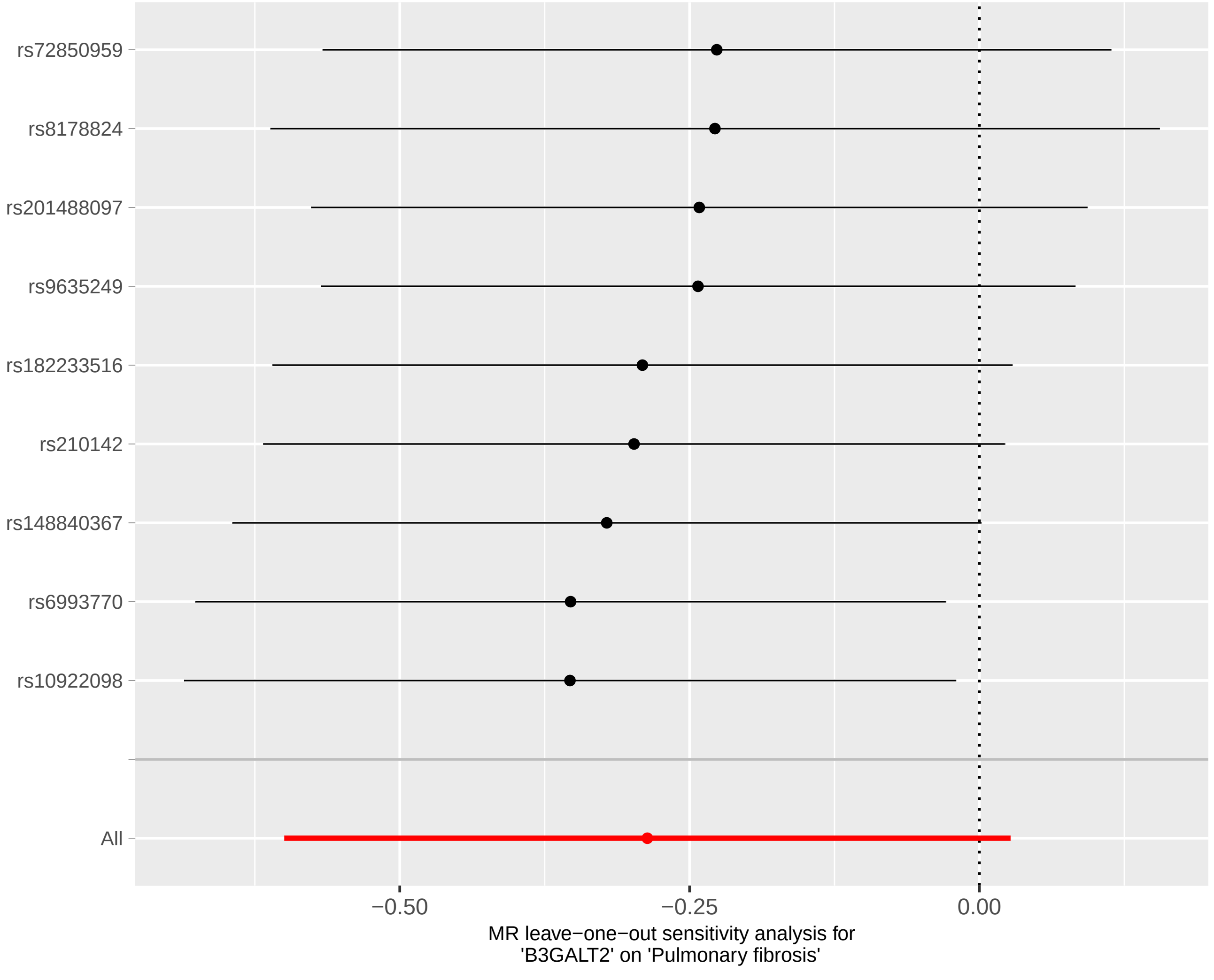

rs72850959
rs8178824
rs201488097
rs9635249
rs182233516
rs210142
rs148840367
rs6993770
rs10922098
All
−0.50
−0.25
0.00
MR leave−one−out sensitivity analysis for
'B3GALT2' on 'Pulmonary fibrosis'

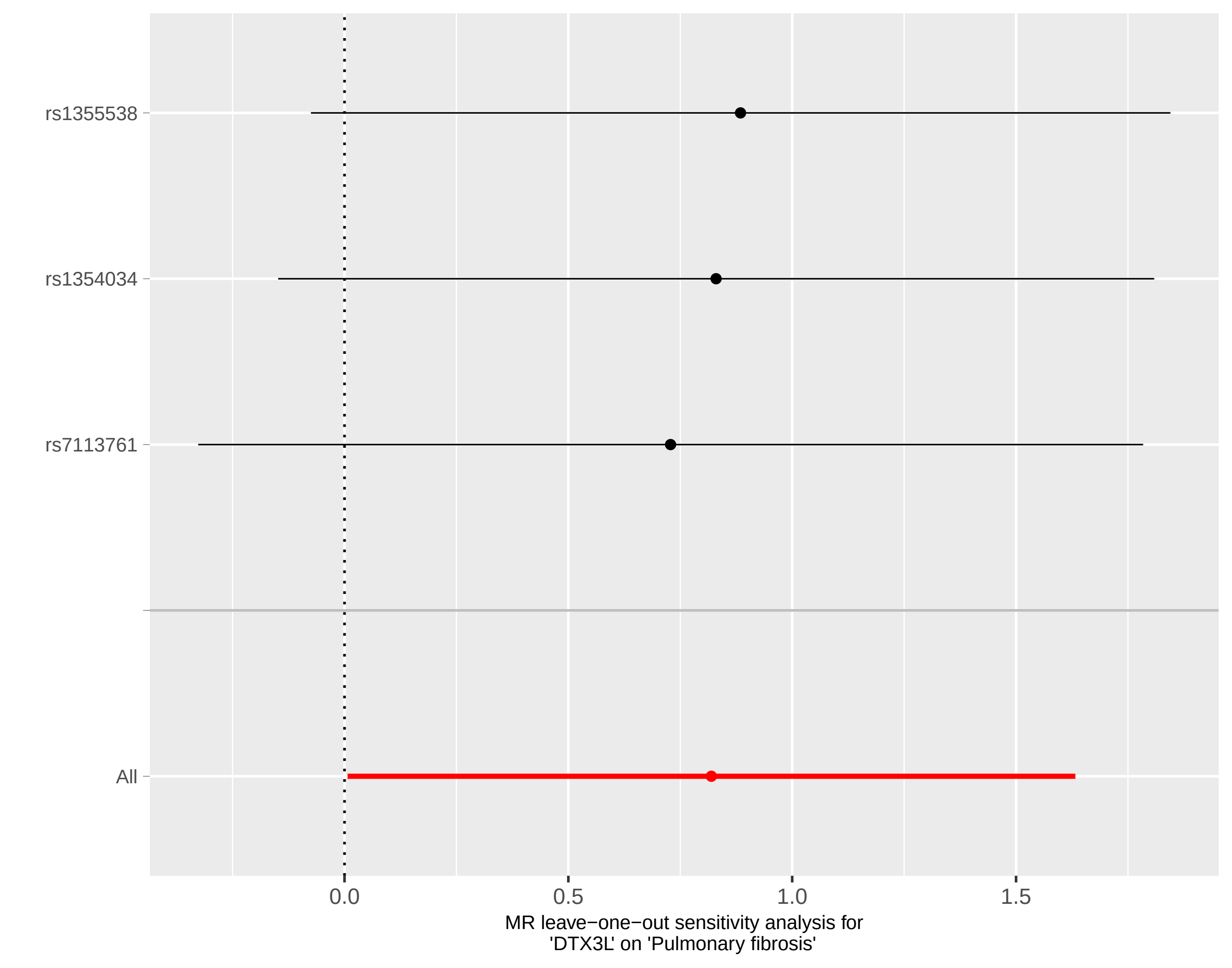
rs1355538
rs1354034
rs7113761
All
0.0
0.5
1.0
1.5
MR leave−one−out sensitivity analysis for
'DTX3L' on 'Pulmonary fibrosis'

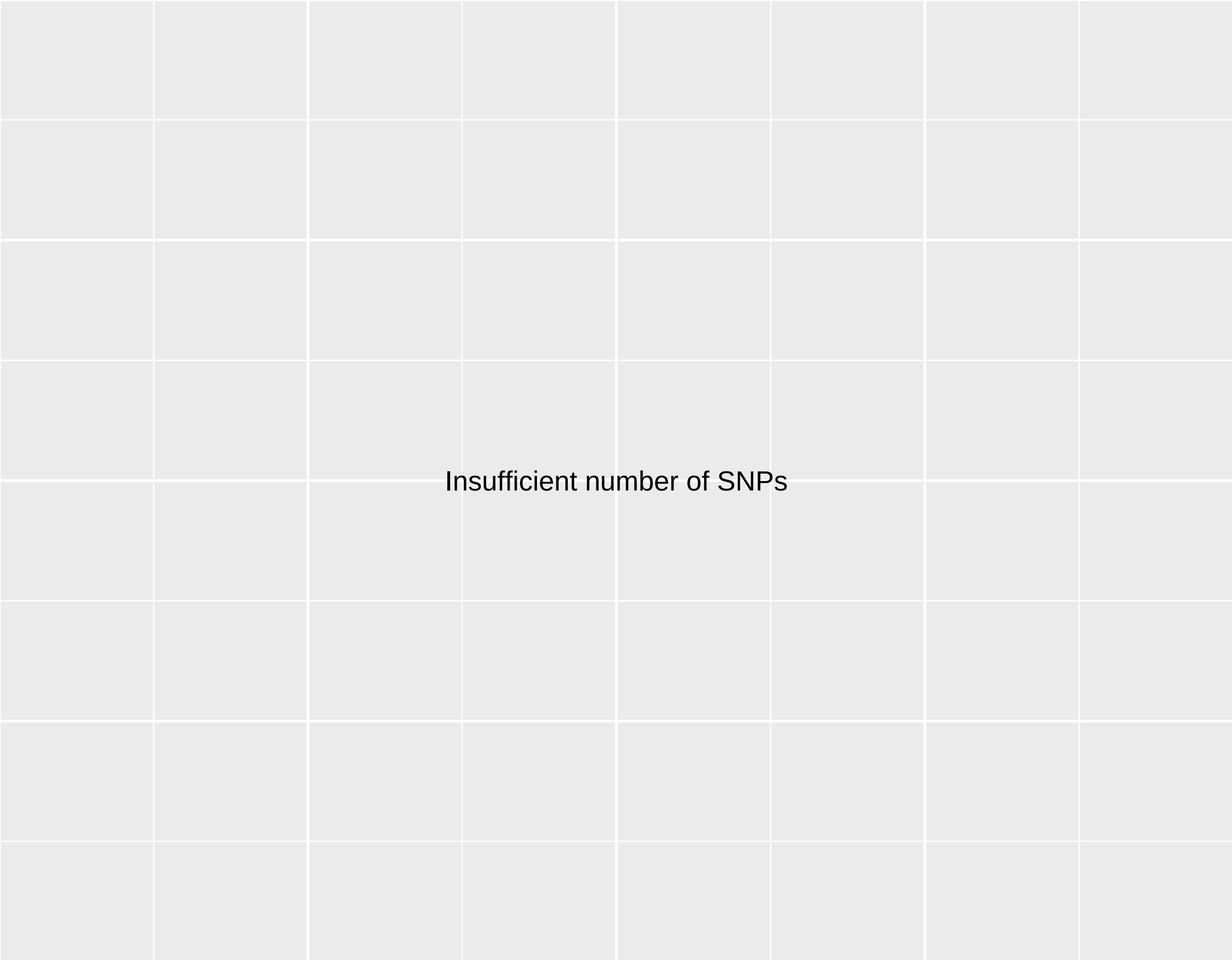
Insufficient number of SNPs

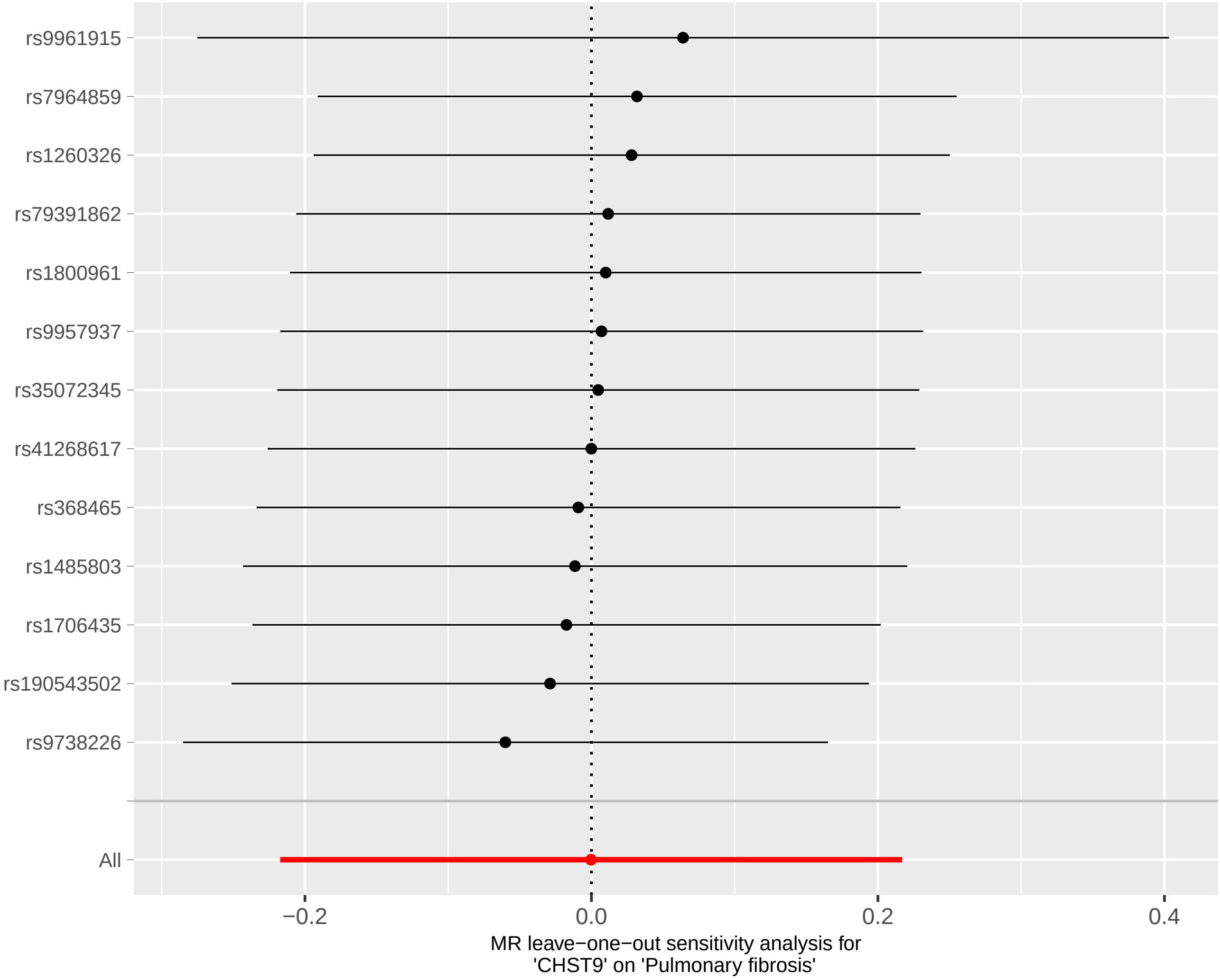
rs9961915
rs7964859
rs1260326
rs79391862
rs1800961
rs9957937
rs35072345
rs41268617
rs368465
rs1485803
rs1706435
rs190543502
rs9738226
All
−0.2
0.0
0.2
0.4
MR leave−one−out sensitivity analysis for
'CHST9' on 'Pulmonary fibrosis'

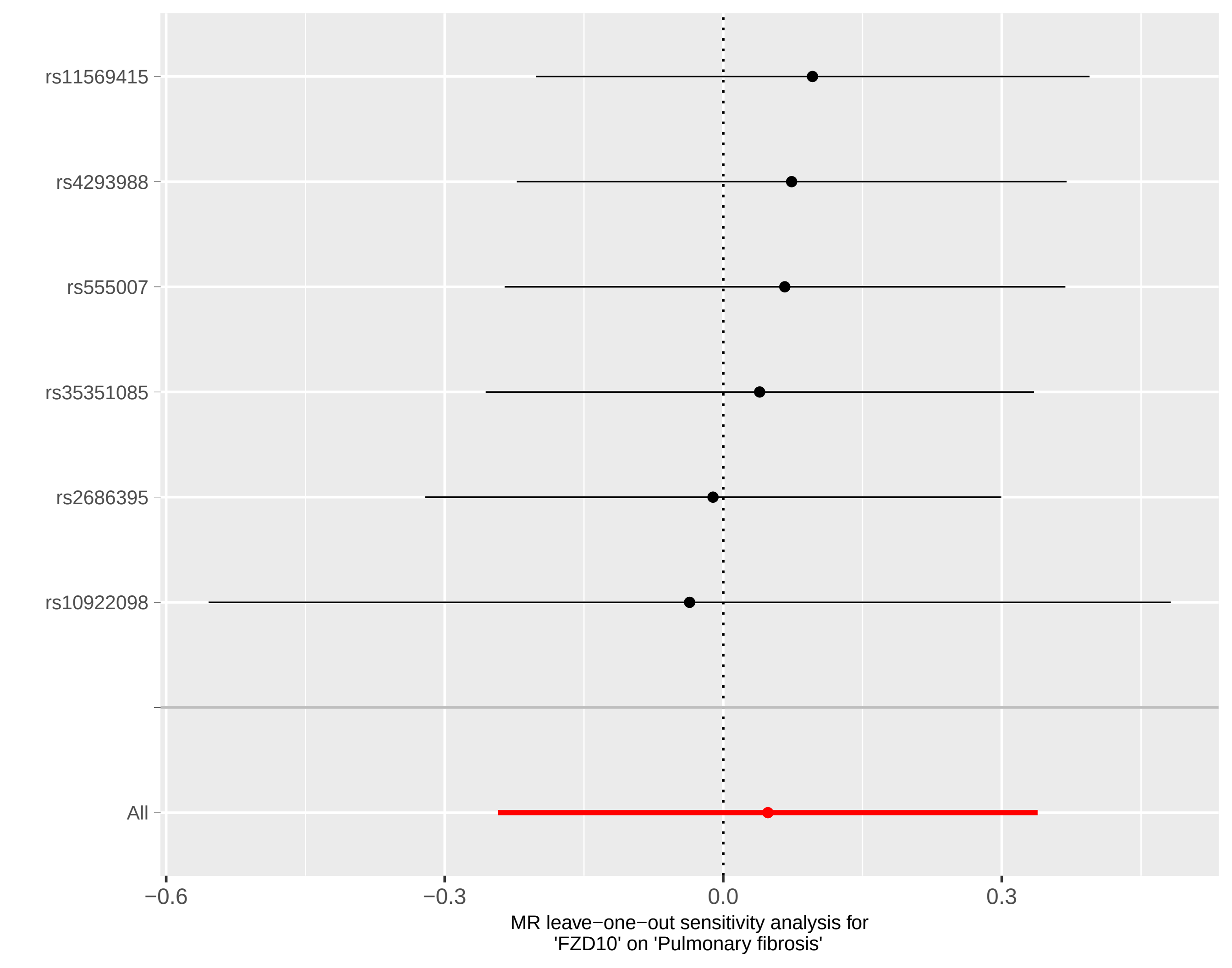
rs11569415
rs4293988
rs555007
rs35351085
rs2686395
rs10922098
All
−0.6
−0.3
0.0
0.3
MR leave−one−out sensitivity analysis for
'FZD10' on 'Pulmonary fibrosis'

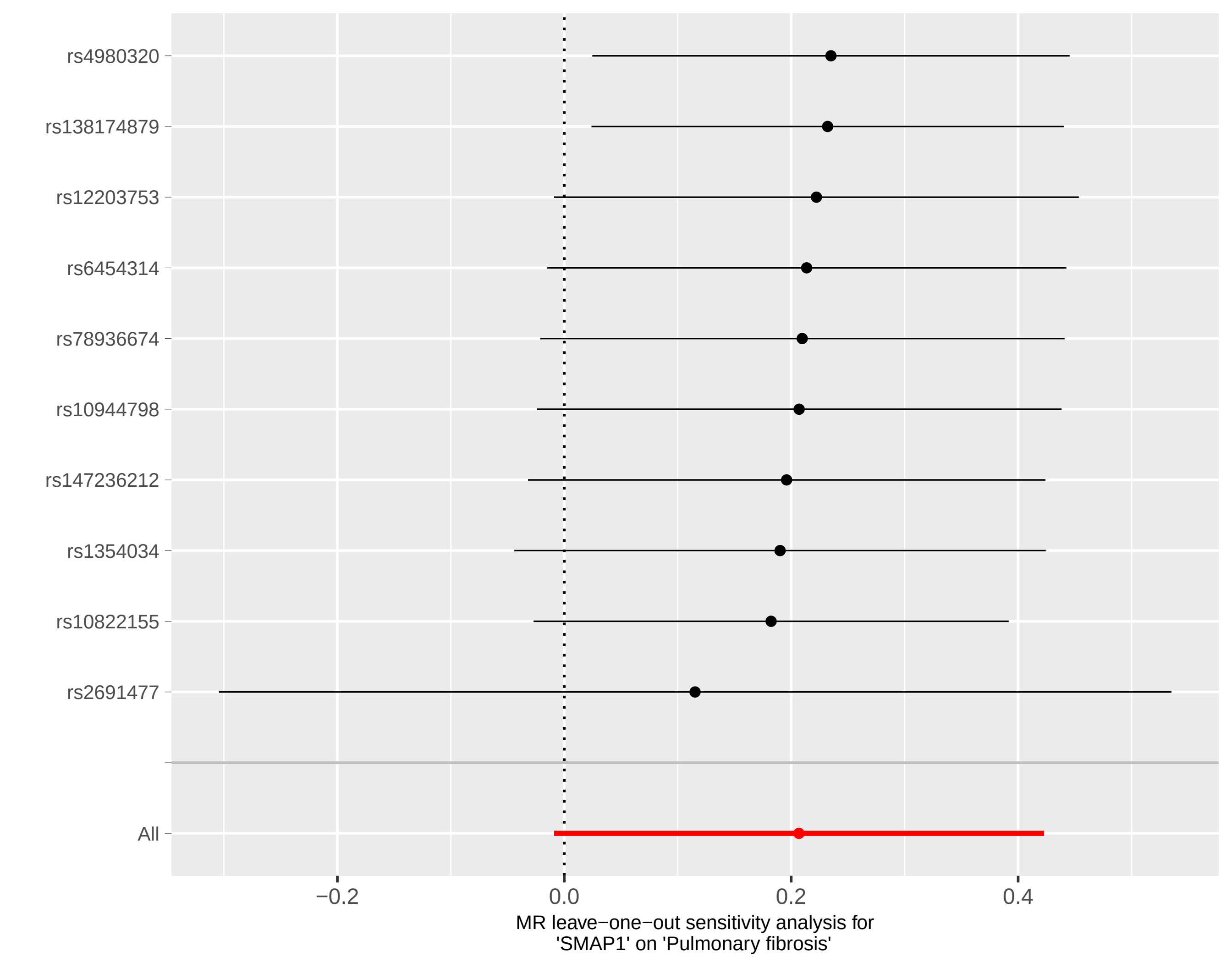
rs4980320
rs138174879
rs12203753
rs6454314
rs78936674
rs10944798
rs147236212
rs1354034
rs10822155
rs2691477
All
−0.2
0.0
0.2
0.4
MR leave−one−out sensitivity analysis for
'SMAP1' on 'Pulmonary fibrosis'

Insufficient number of SNPs

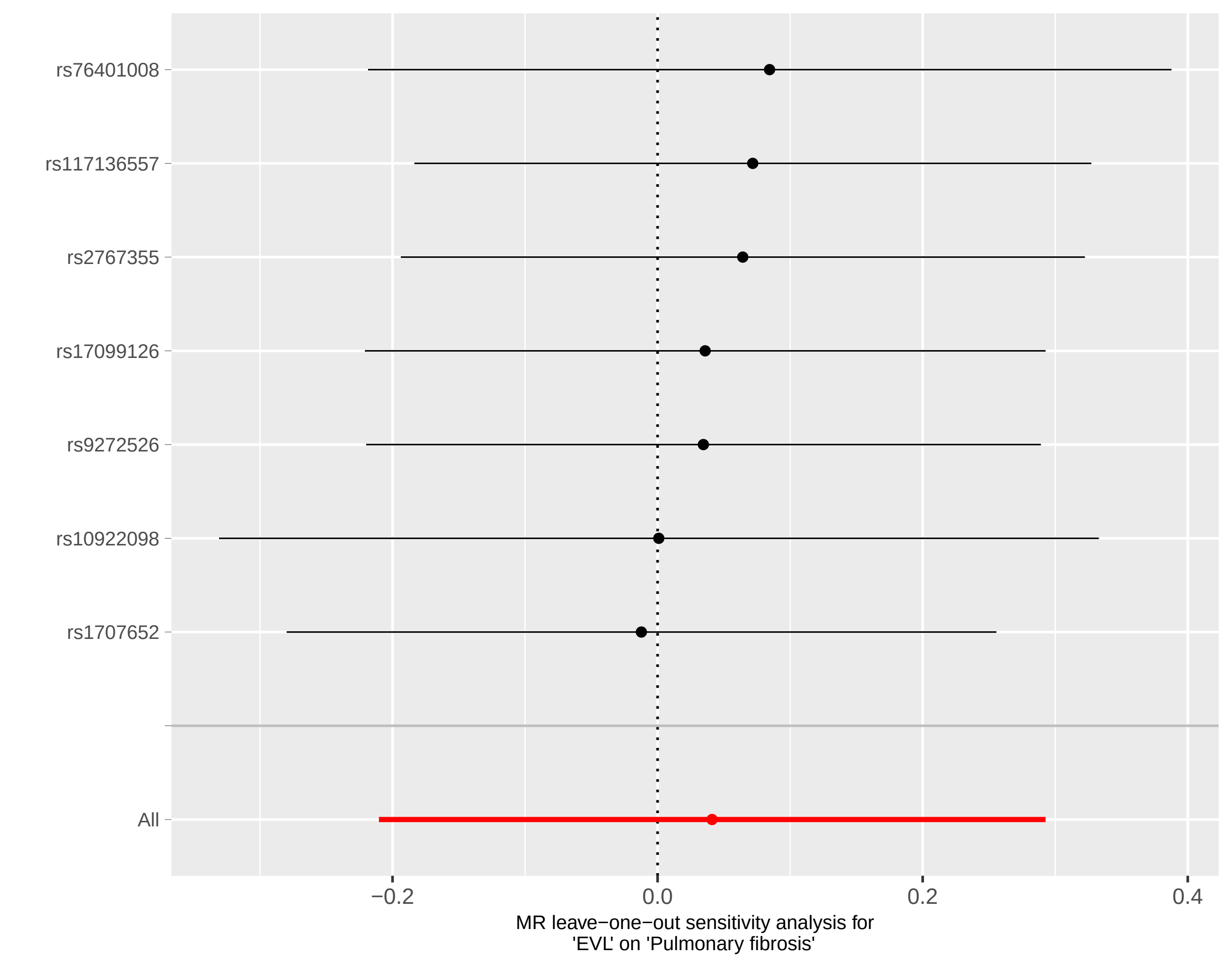
rs76401008
rs117136557
rs2767355
rs17099126
rs9272526
rs10922098
rs1707652
All
−0.2
0.0
0.2
0.4
MR leave−one−out sensitivity analysis for
'EVL' on 'Pulmonary fibrosis'

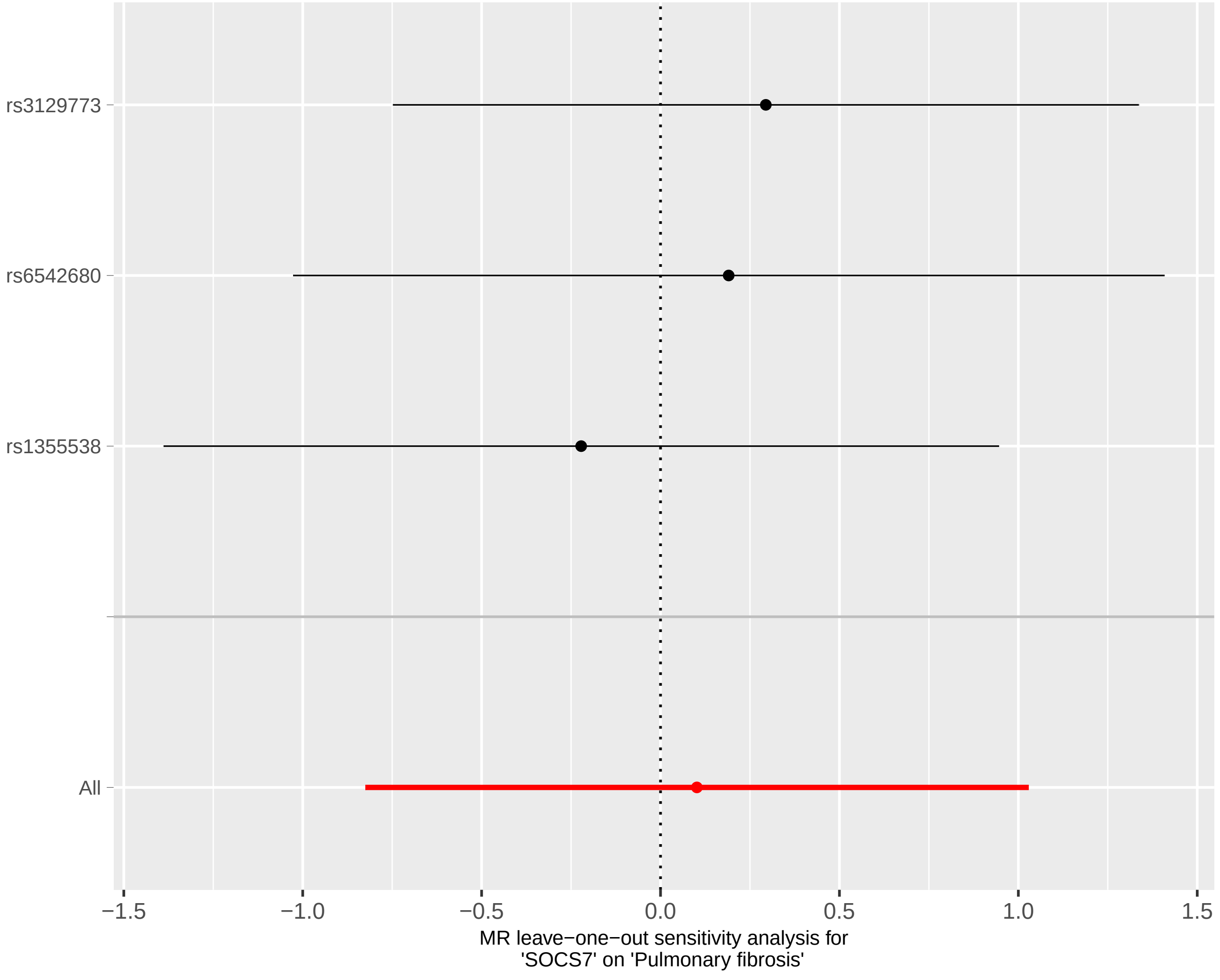
rs3129773
rs6542680
rs1355538
All
−1.5
−1.0
−0.5
0.0
0.5
1.0
1.5
MR leave−one−out sensitivity analysis for
'SOCS7' on 'Pulmonary fibrosis'

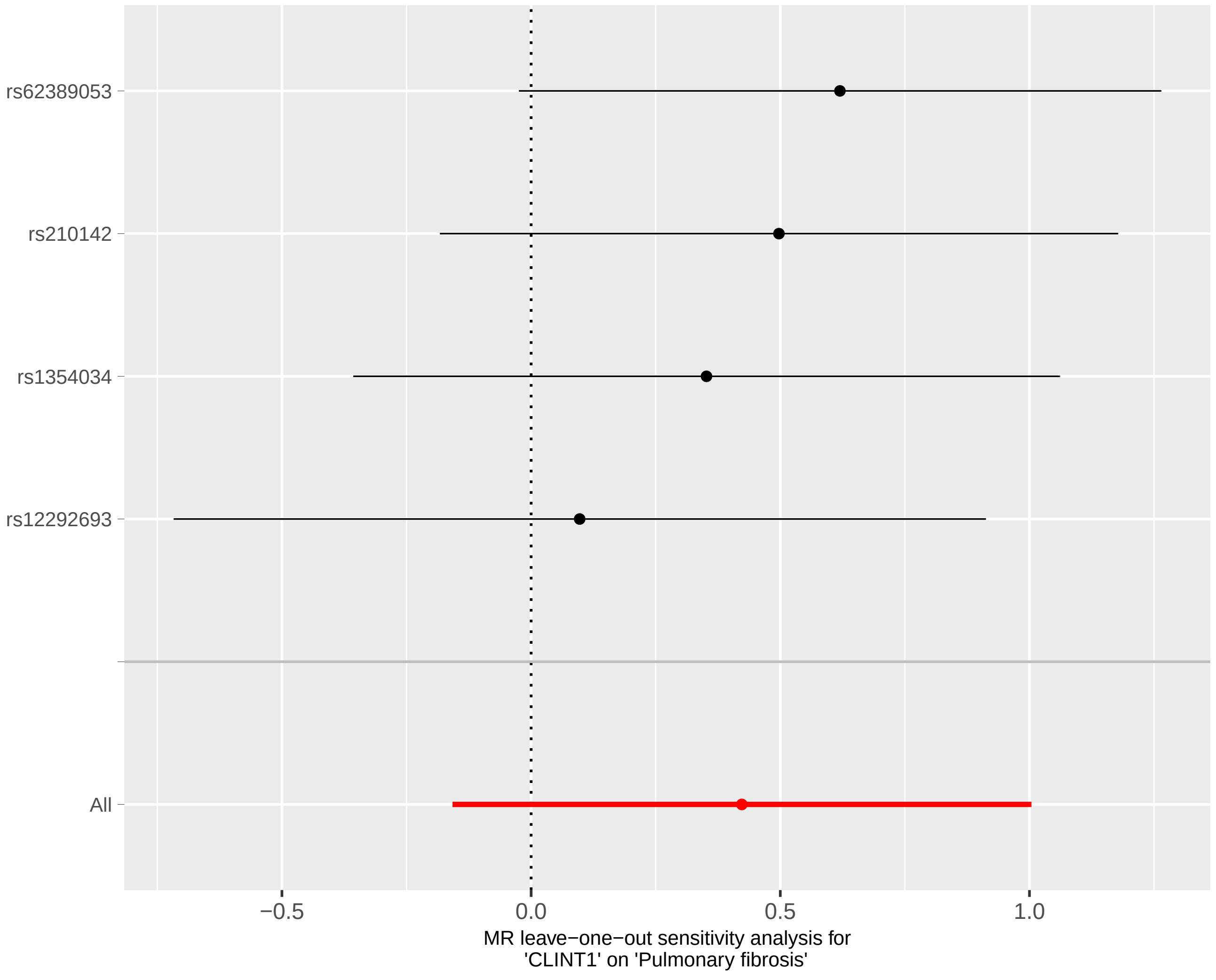

rs62389053
rs210142
rs1354034
rs12292693
All
−0.5
0.0
0.5
1.0
MR leave−one−out sensitivity analysis for
'CLINT1' on 'Pulmonary fibrosis'

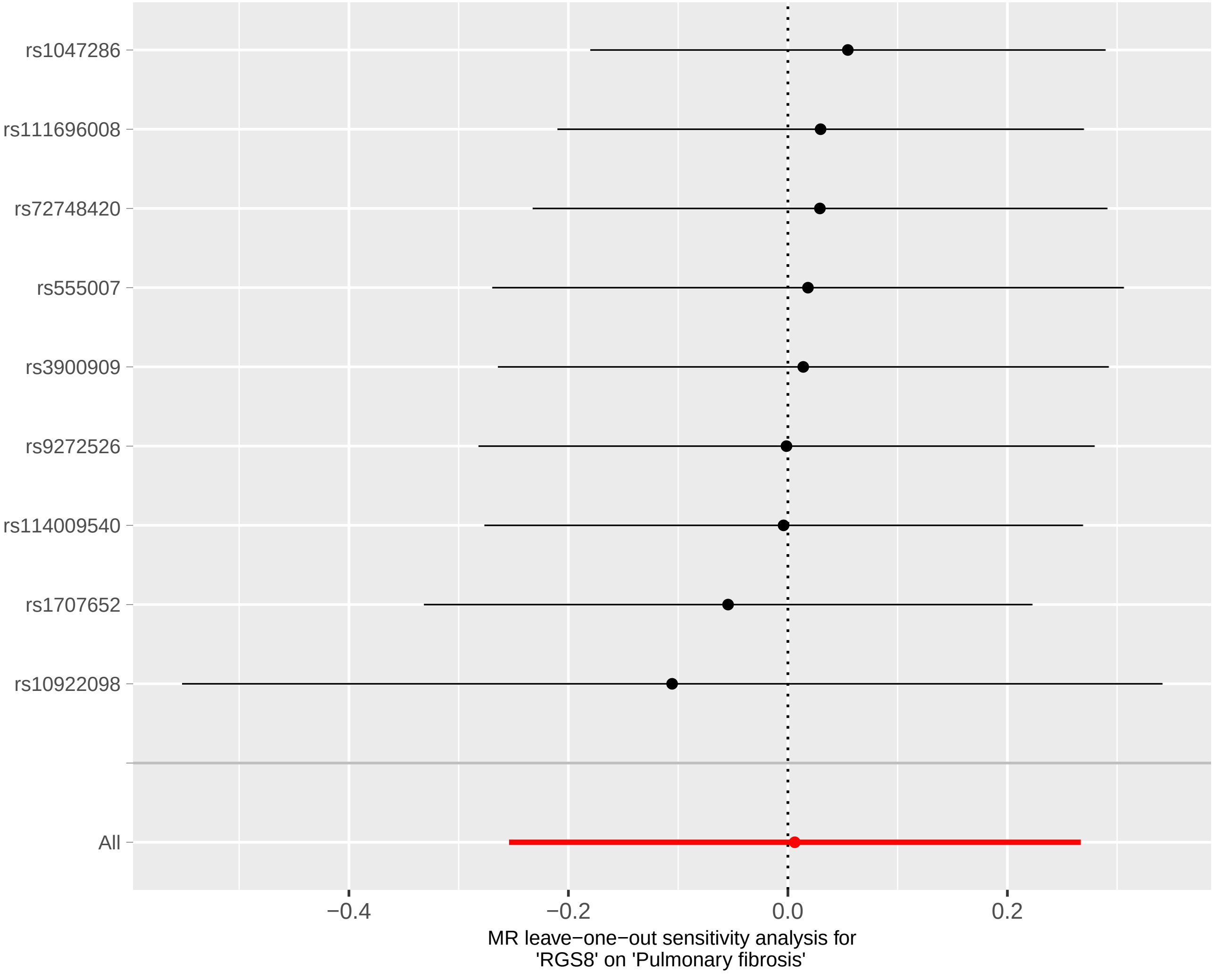
rs1047286
rs111696008
rs72748420
rs555007
rs3900909
rs9272526
rs114009540
rs1707652
rs10922098
All
−0.4
−0.2
0.0
0.2
MR leave−one−out sensitivity analysis for
'RGS8' on 'Pulmonary fibrosis'

Insufficient number of SNPs

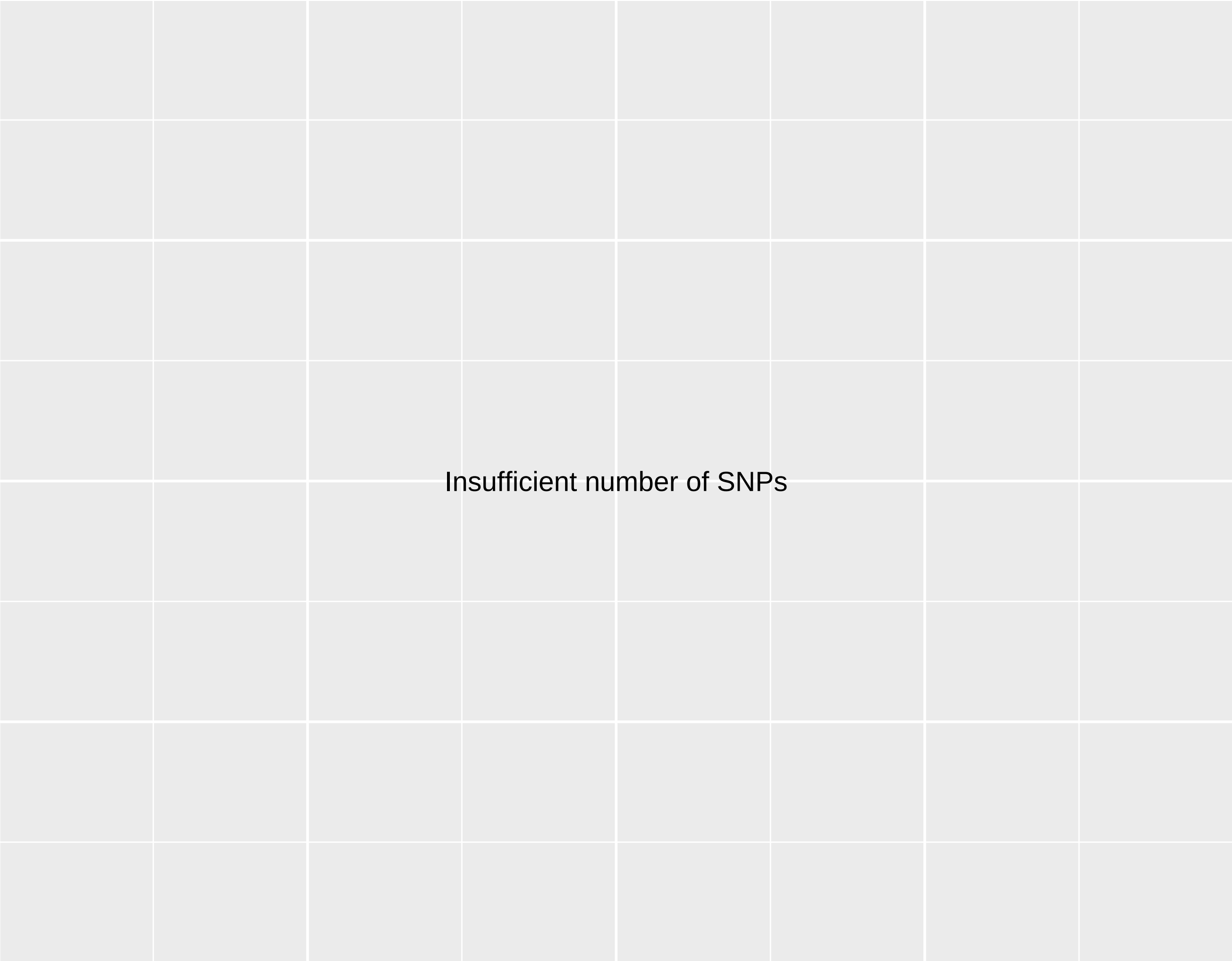
Insufficient number of SNPs

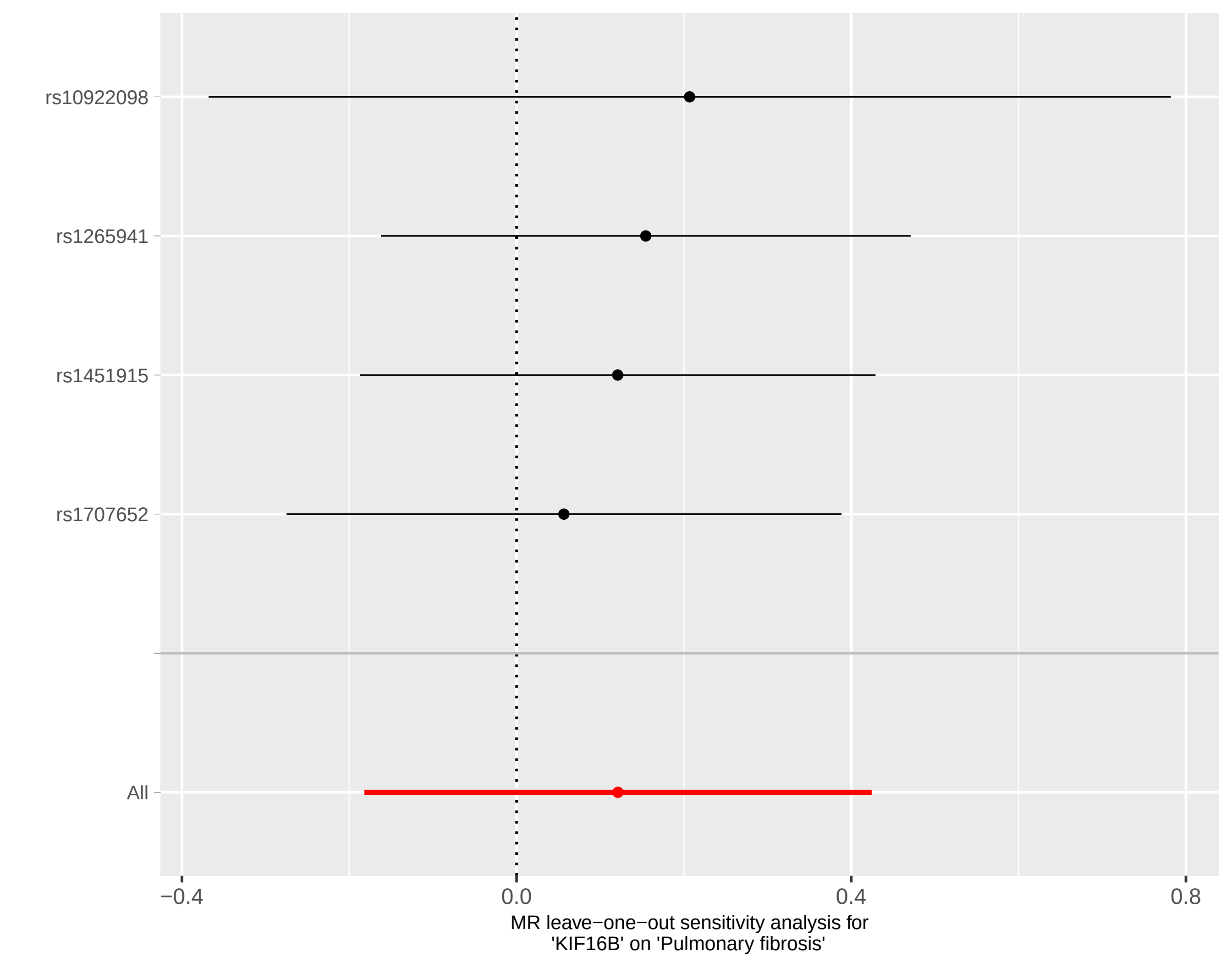
rs10922098
rs1265941
rs1451915
rs1707652
All
−0.4
0.0
0.4
0.8
MR leave−one−out sensitivity analysis for
'KIF16B' on 'Pulmonary fibrosis'

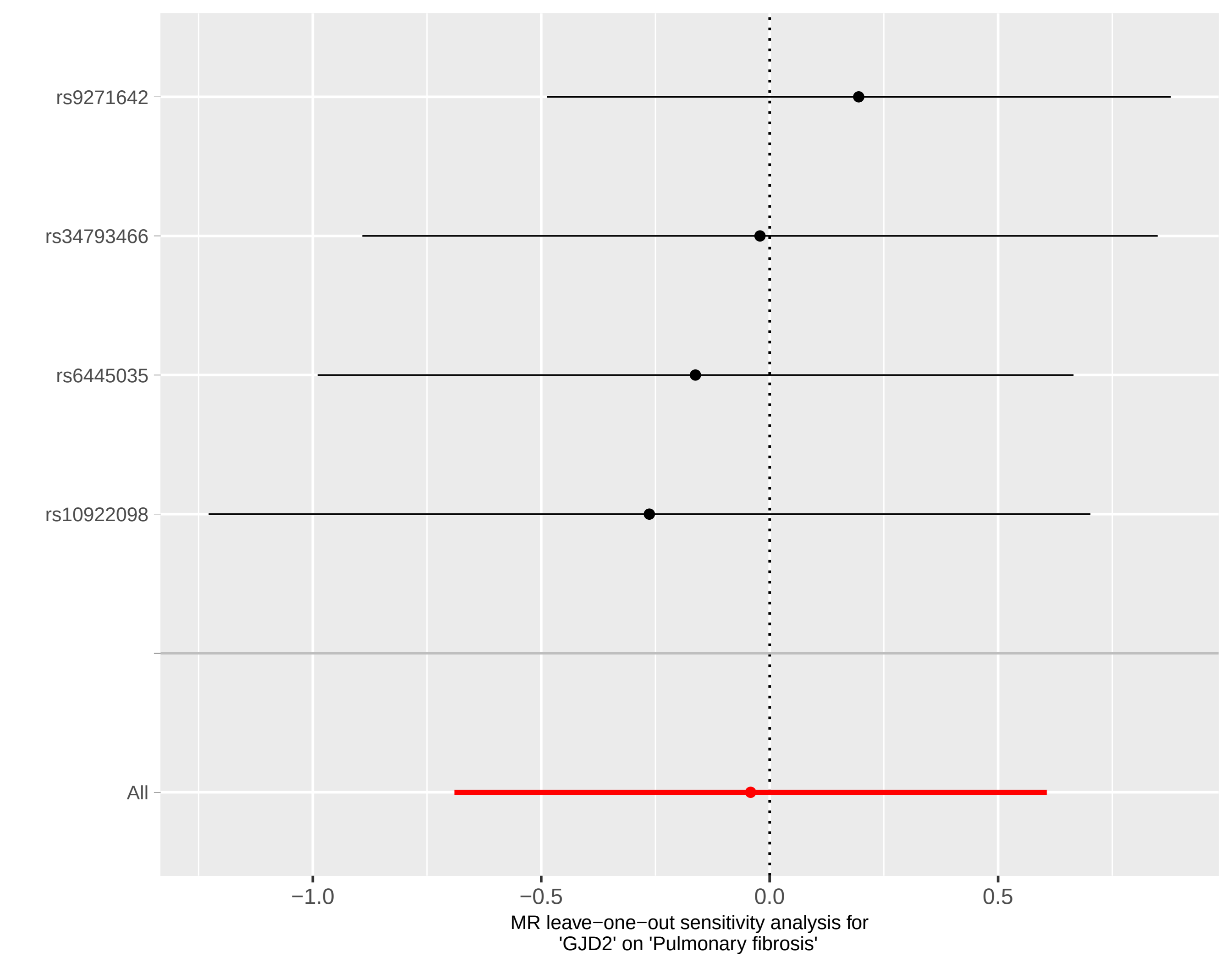

rs9271642
rs34793466
rs6445035
rs10922098
All
−1.0
−0.5
0.0
0.5
MR leave−one−out sensitivity analysis for
'GJD2' on 'Pulmonary fibrosis'

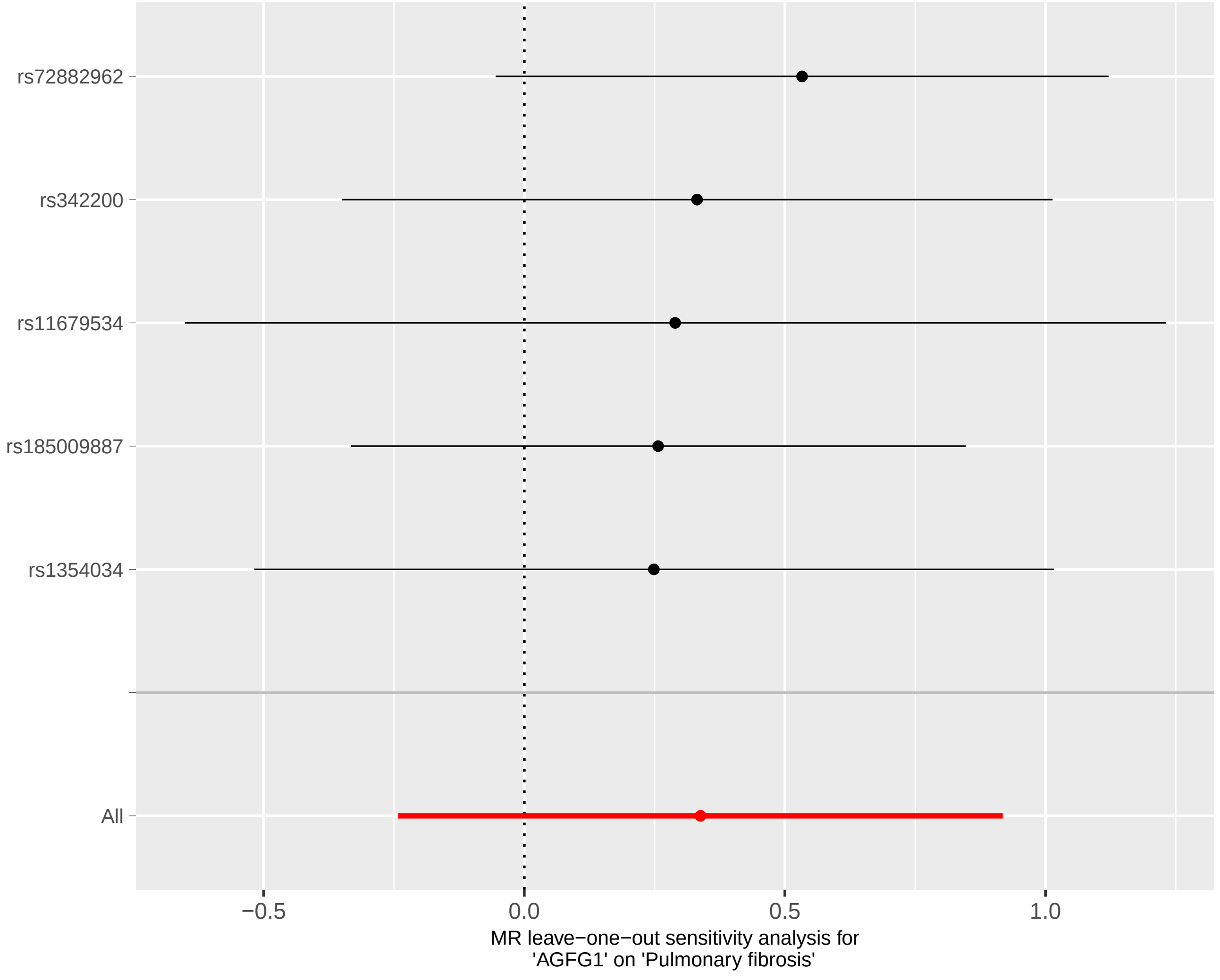
rs72882962
rs342200
rs11679534
rs185009887
rs1354034
All
−0.5
0.0
0.5
1.0
MR leave−one−out sensitivity analysis for
'AGFG1' on 'Pulmonary fibrosis'

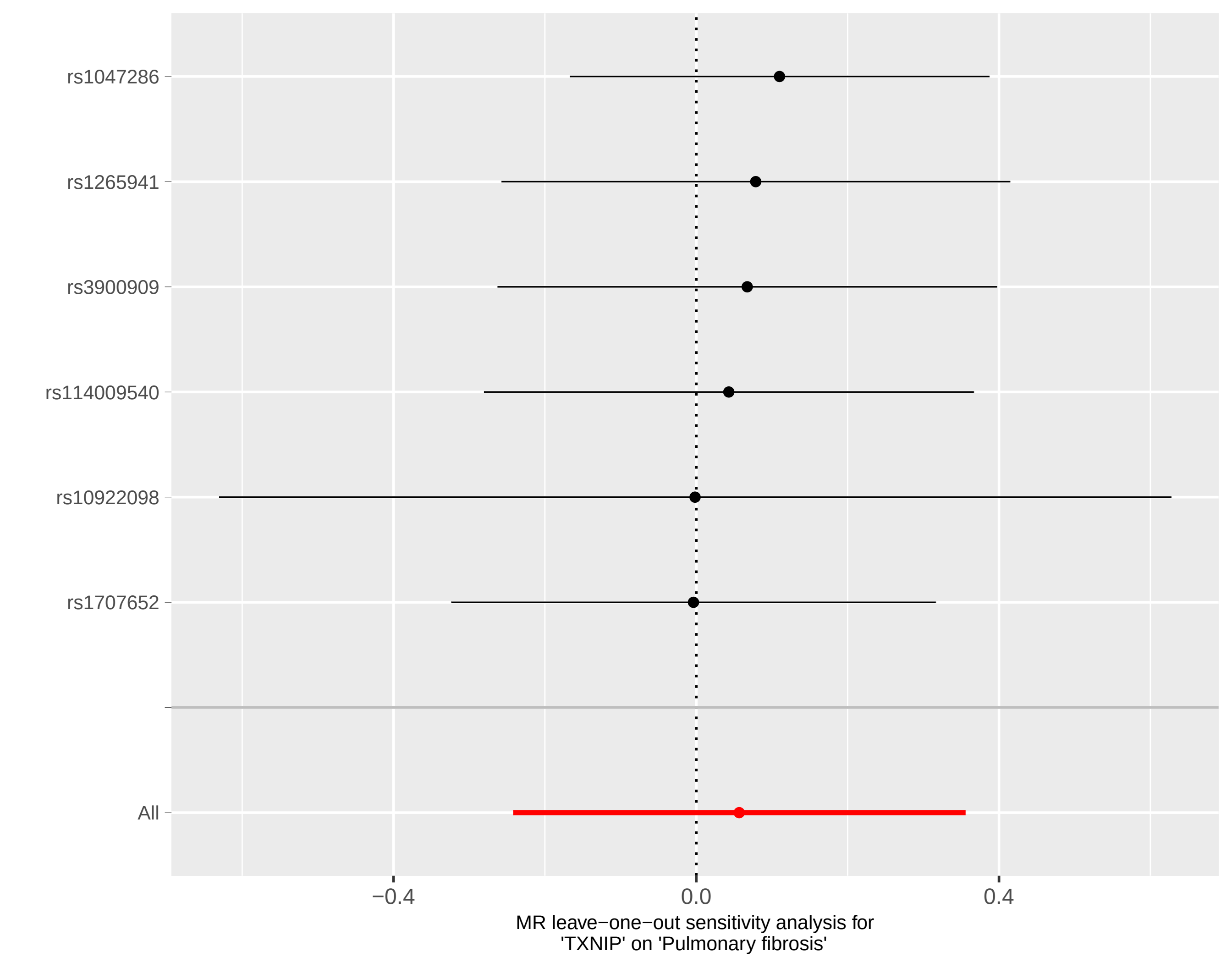

rs1047286
rs1265941
rs3900909
rs114009540
rs10922098
rs1707652
All
−0.4
0.0
0.4
MR leave−one−out sensitivity analysis for
'TXNIP' on 'Pulmonary fibrosis'

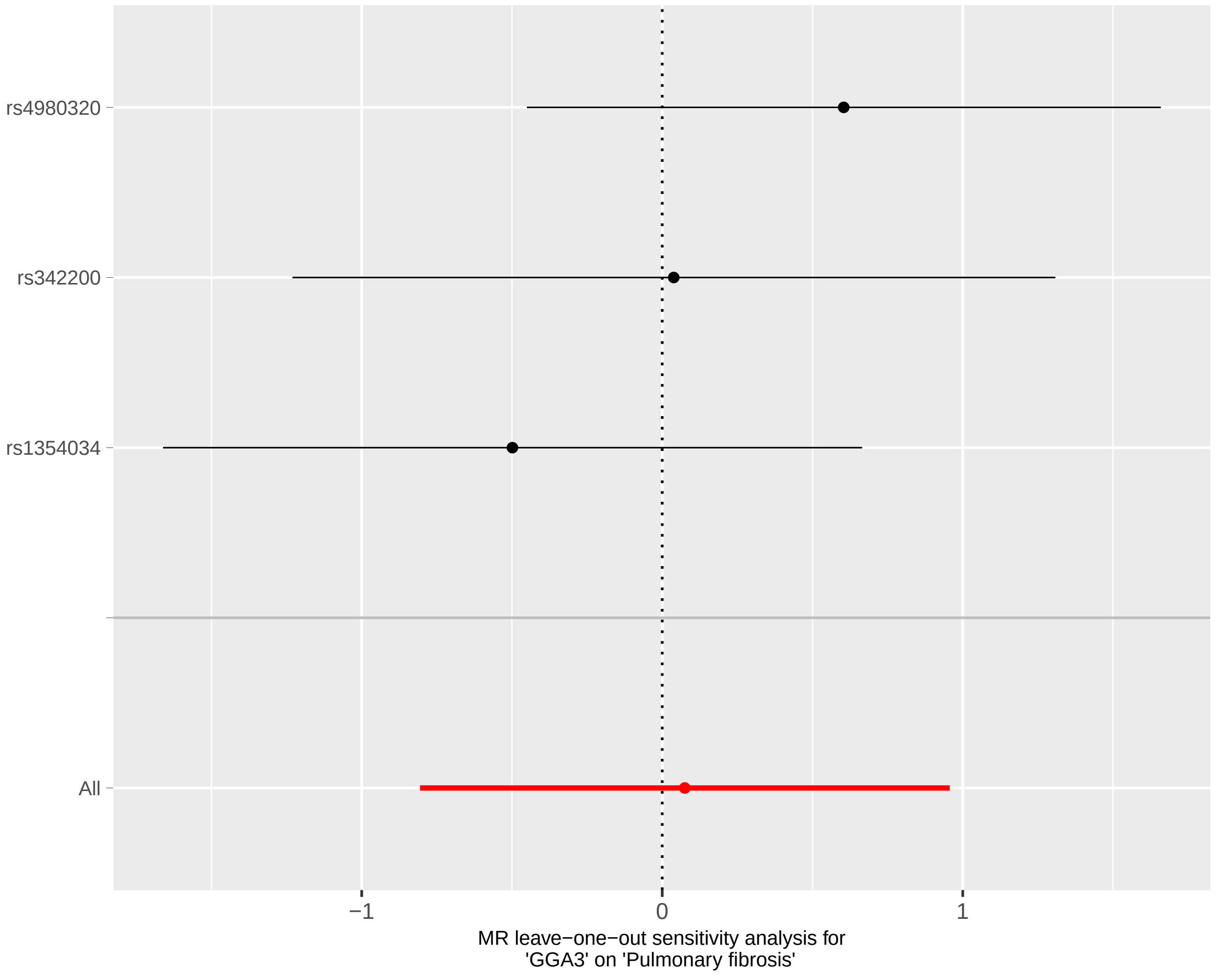
rs4980320
rs342200
rs1354034
All
−1
0
1
MR leave−one−out sensitivity analysis for
'GGA3' on 'Pulmonary fibrosis'

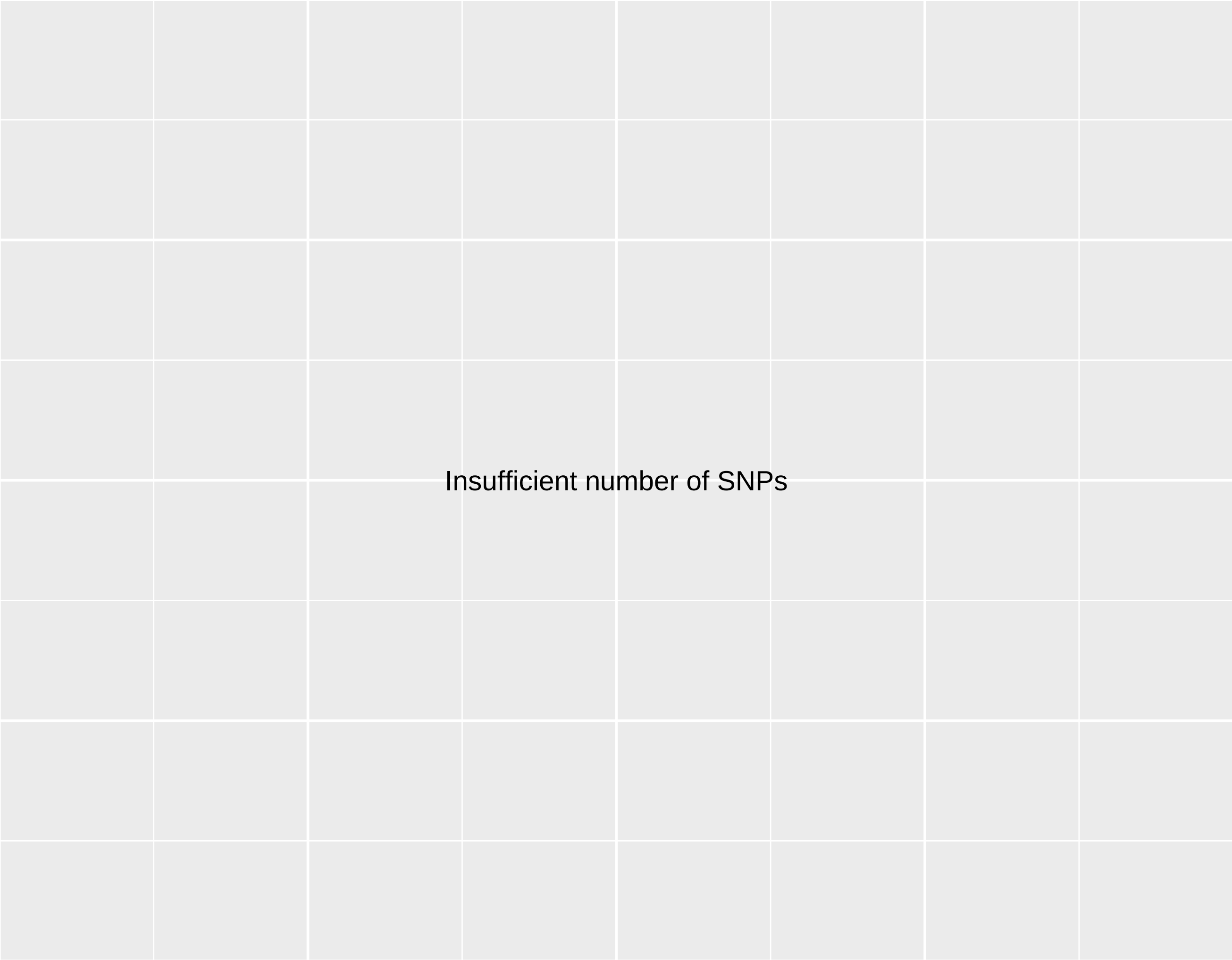
Insufficient number of SNPs

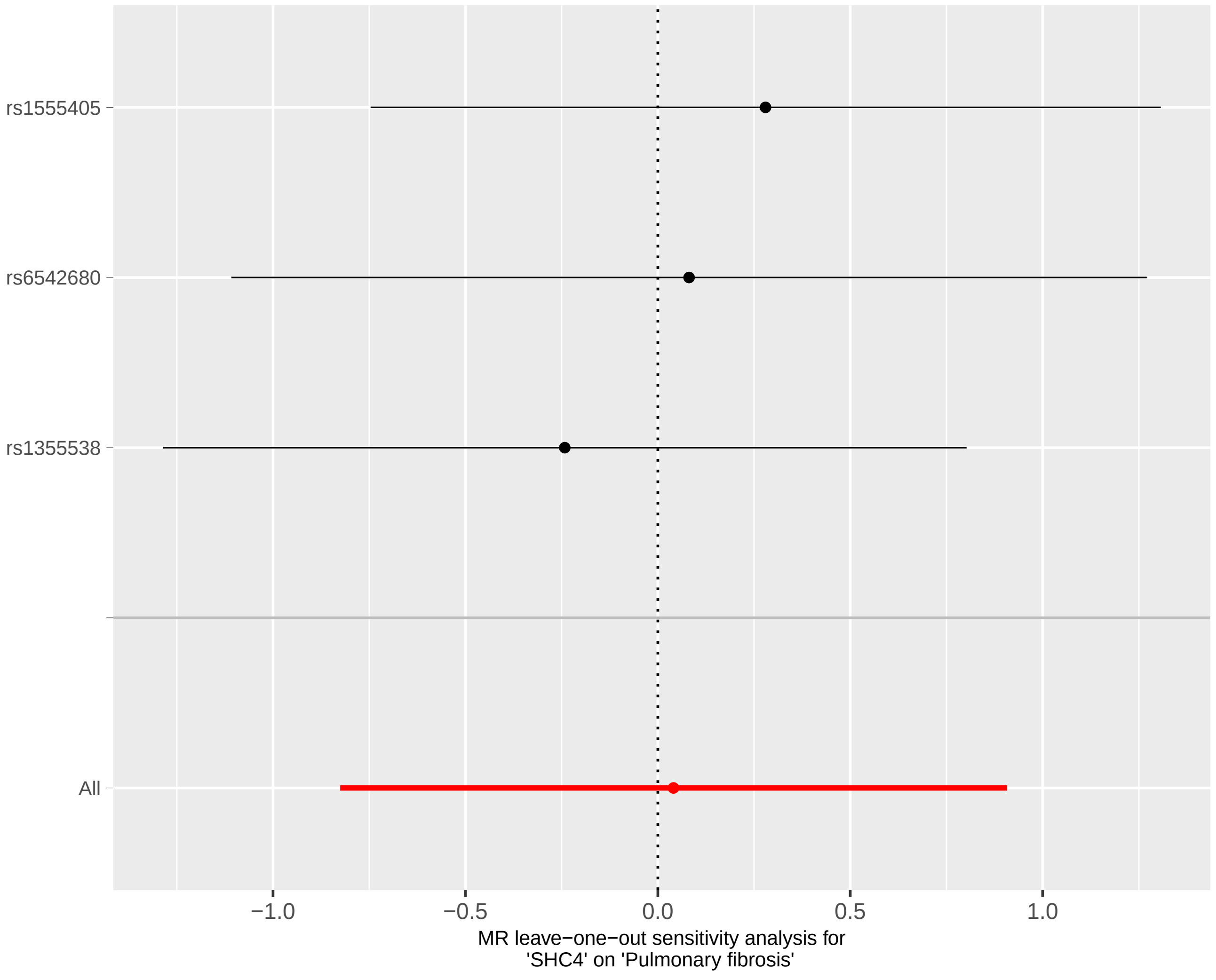
rs1555405
rs6542680
rs1355538
All
−1.0
−0.5
0.0
0.5
1.0
MR leave−one−out sensitivity analysis for
'SHC4' on 'Pulmonary fibrosis'

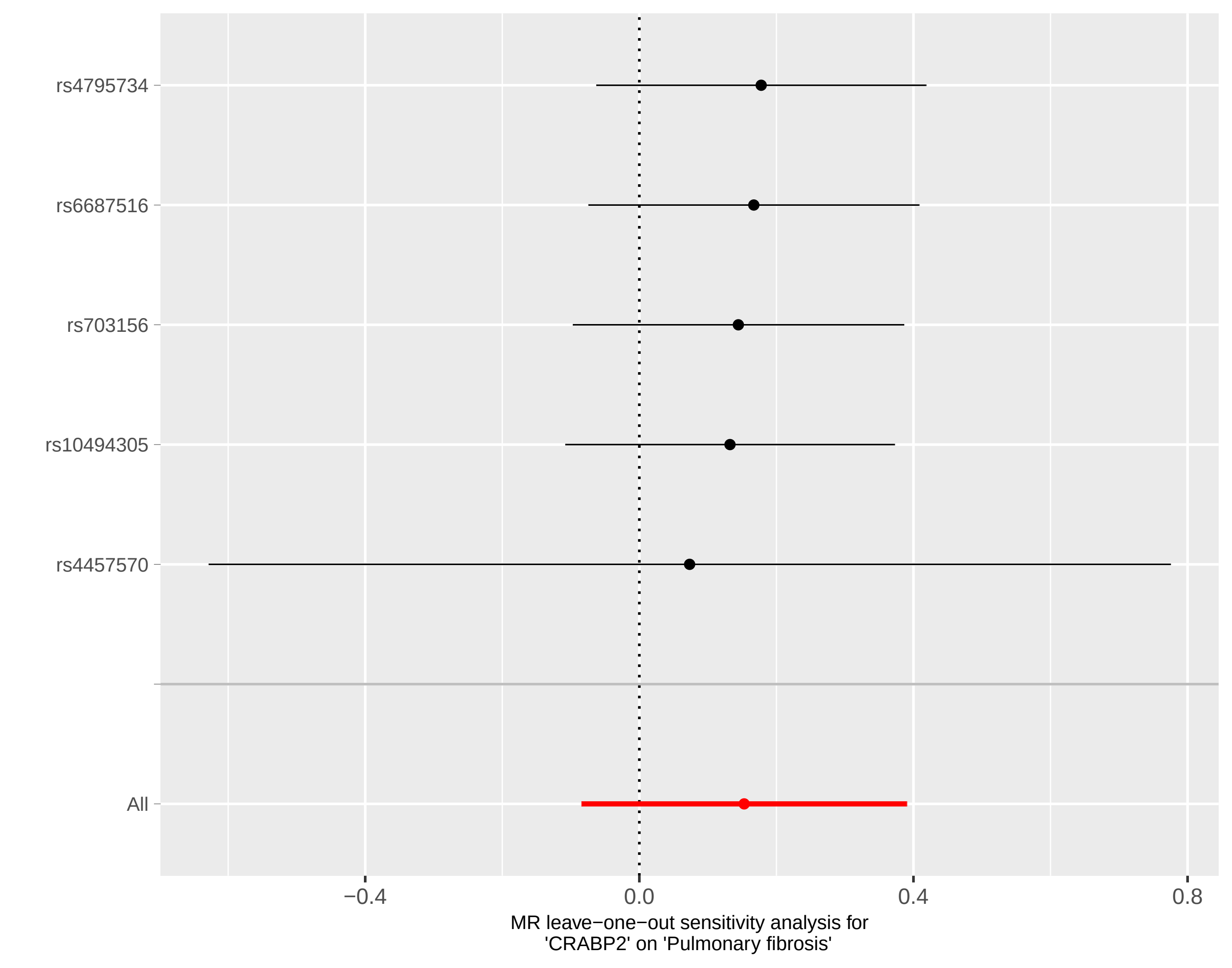

rs4795734
rs6687516
rs703156
rs10494305
rs4457570
All
−0.4
0.0
0.4
0.8
MR leave−one−out sensitivity analysis for
'CRABP2' on 'Pulmonary fibrosis'

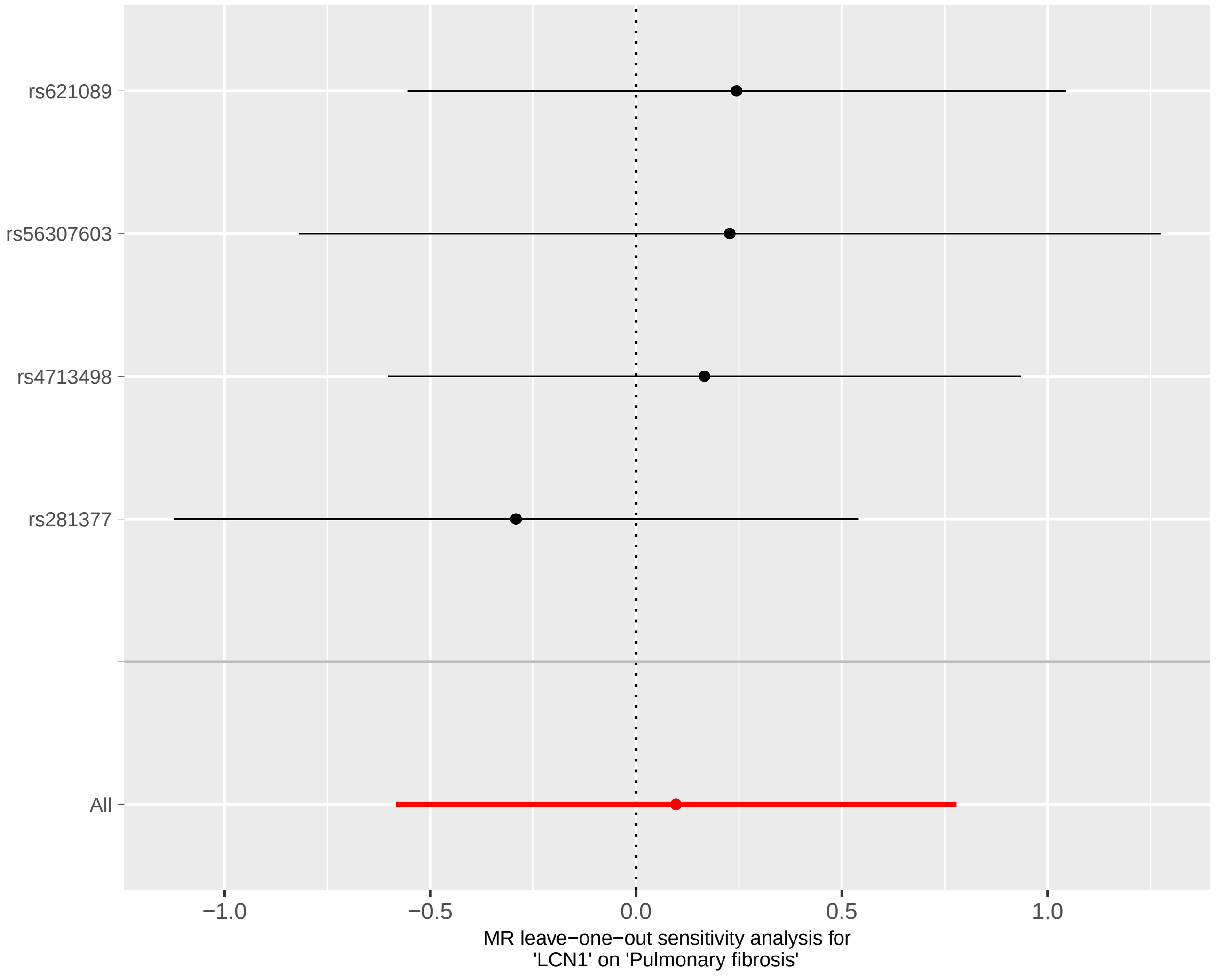
rs621089
rs56307603
rs4713498
rs281377
All
−1.0
−0.5
0.0
0.5
1.0
MR leave−one−out sensitivity analysis for
'LCN1' on 'Pulmonary fibrosis'

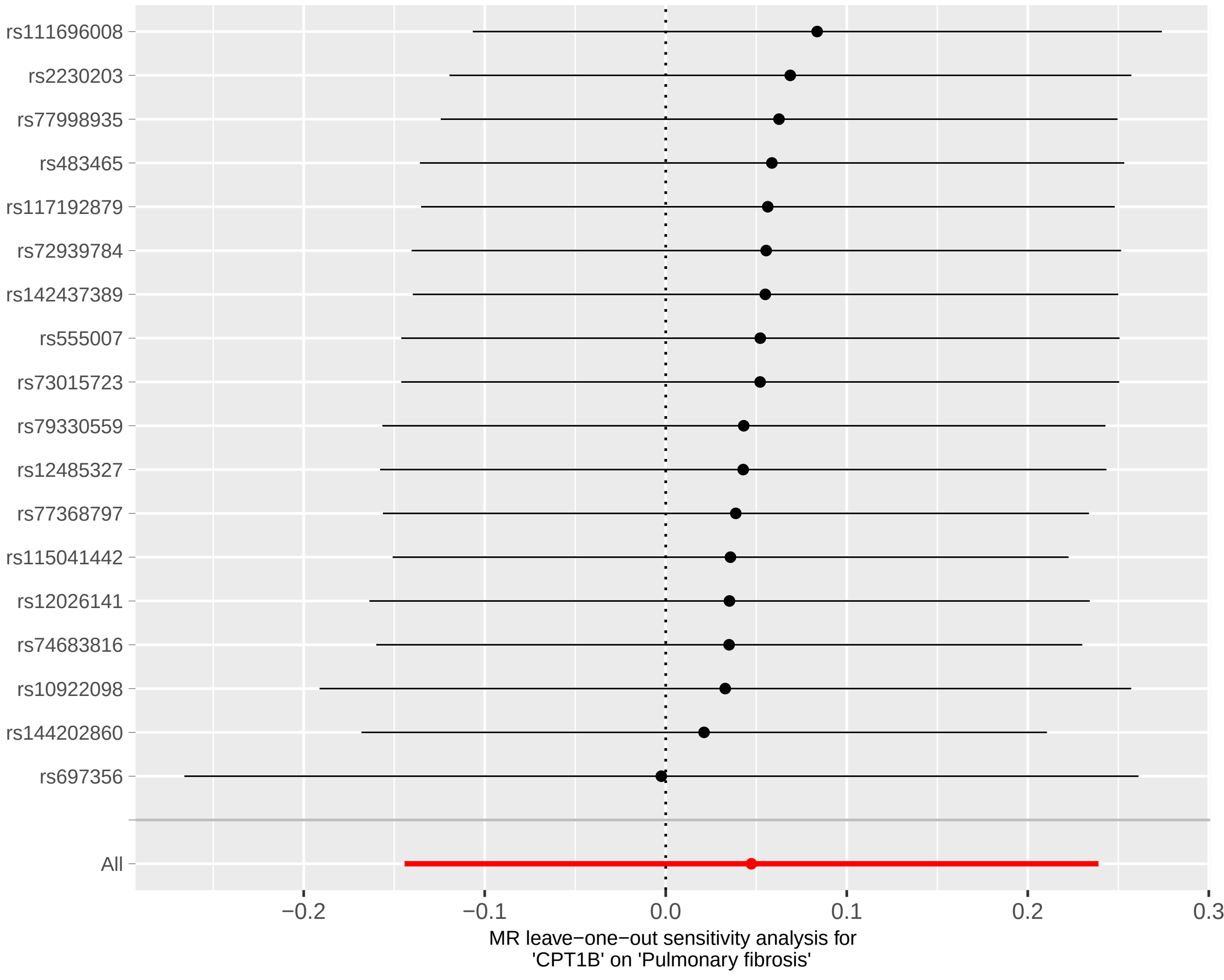

rs111696008
rs2230203
rs77998935
rs483465
rs117192879
rs72939784
rs142437389
rs555007
rs73015723
rs79330559
rs12485327
rs77368797
rs115041442
rs12026141
rs74683816
rs10922098
rs144202860
rs697356
All
−0.2
−0.1
0.0
0.1
0.2
0.3
MR leave−one−out sensitivity analysis for
'CPT1B' on 'Pulmonary fibrosis'

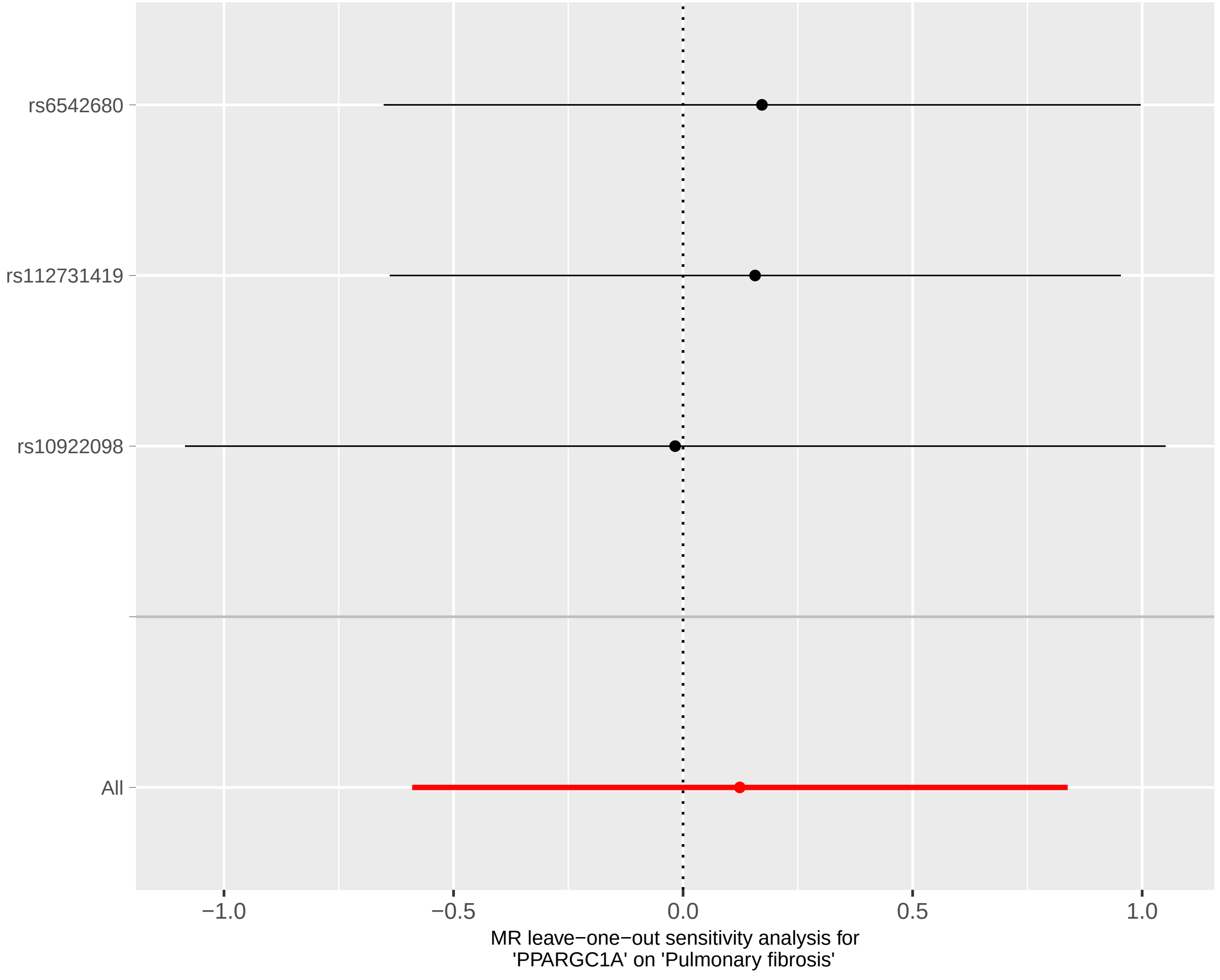

rs6542680
rs112731419
rs10922098
All
−1.0
−0.5
0.0
0.5
1.0
MR leave−one−out sensitivity analysis for
'PPARGC1A' on 'Pulmonary fibrosis'

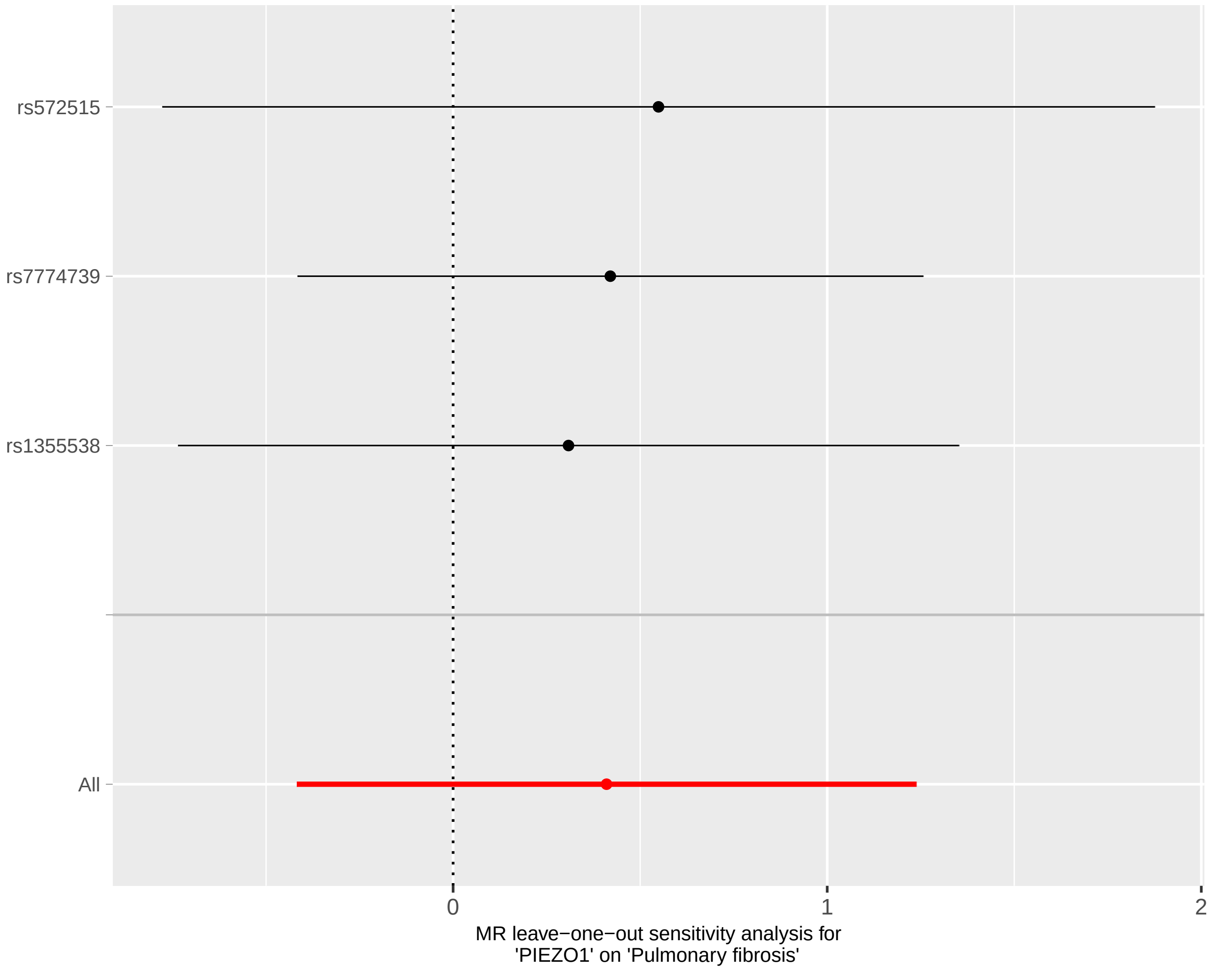

rs572515
rs7774739
rs1355538
All
0
1
2
MR leave−one−out sensitivity analysis for
'PIEZO1' on 'Pulmonary fibrosis'

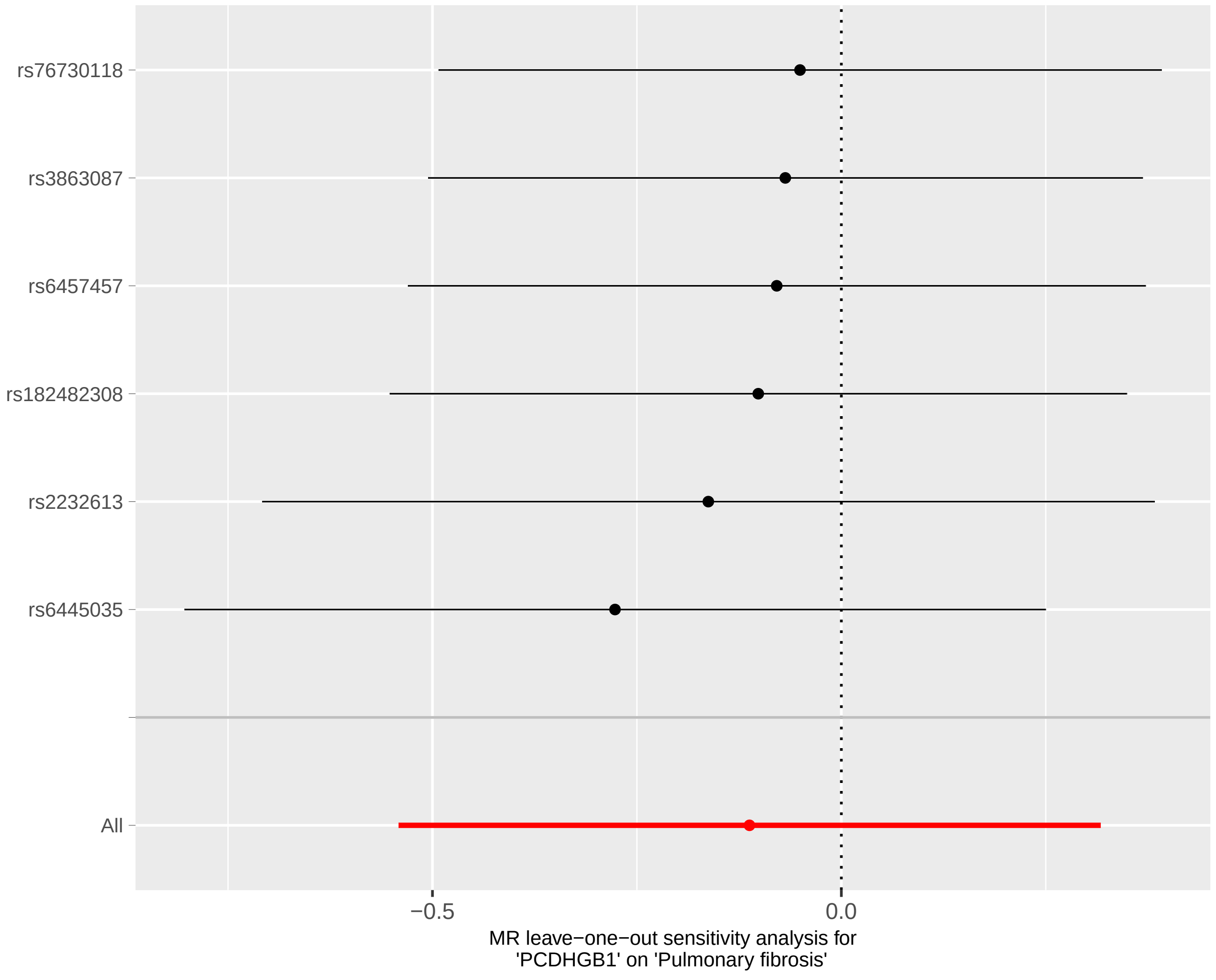
rs76730118
rs3863087
rs6457457
rs182482308
rs2232613
rs6445035
All
−0.5
0.0
MR leave−one−out sensitivity analysis for
'PCDHGB1' on 'Pulmonary fibrosis'

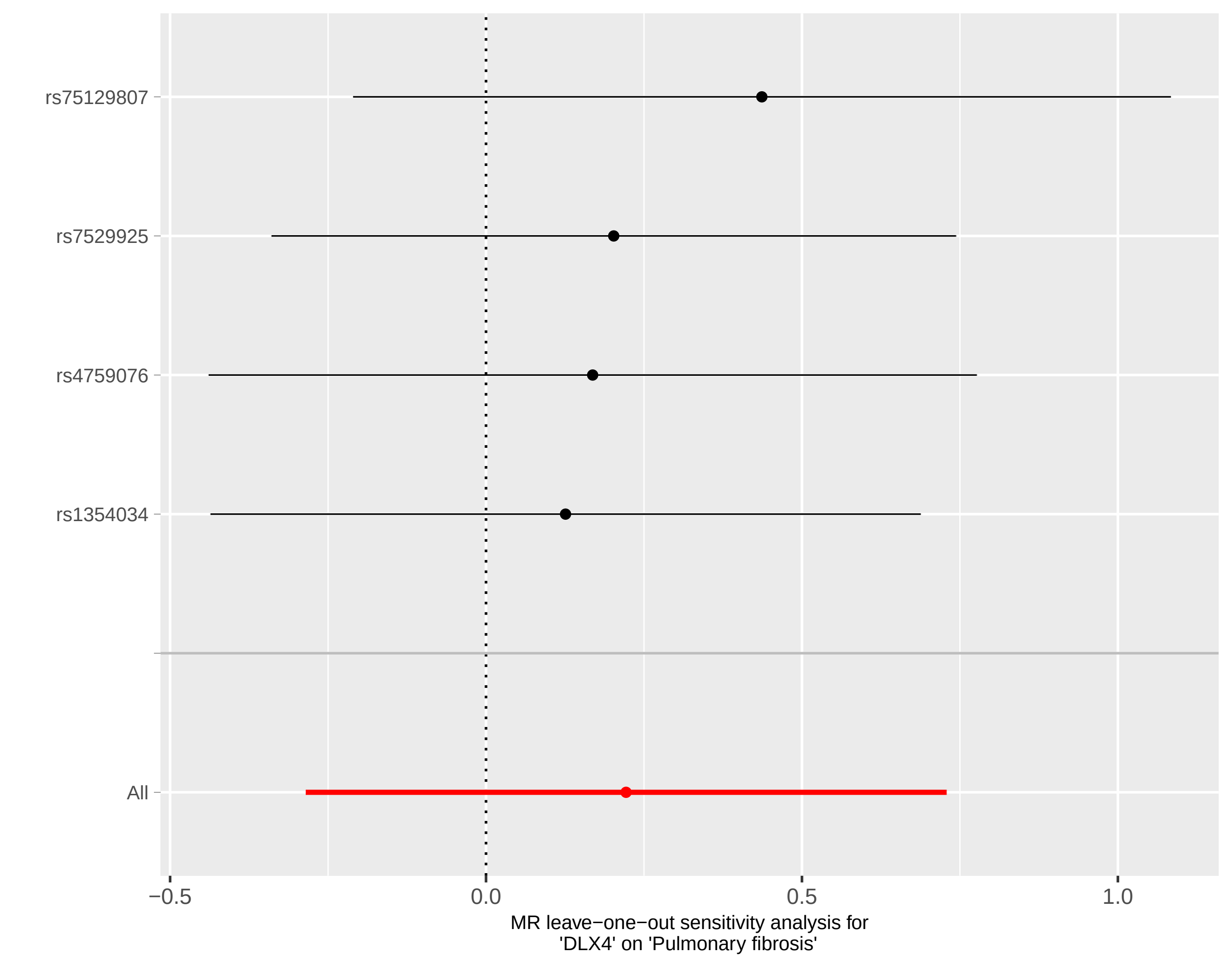
rs75129807
rs7529925
rs4759076
rs1354034
All
−0.5
0.0
0.5
1.0
MR leave−one−out sensitivity analysis for
'DLX4' on 'Pulmonary fibrosis'

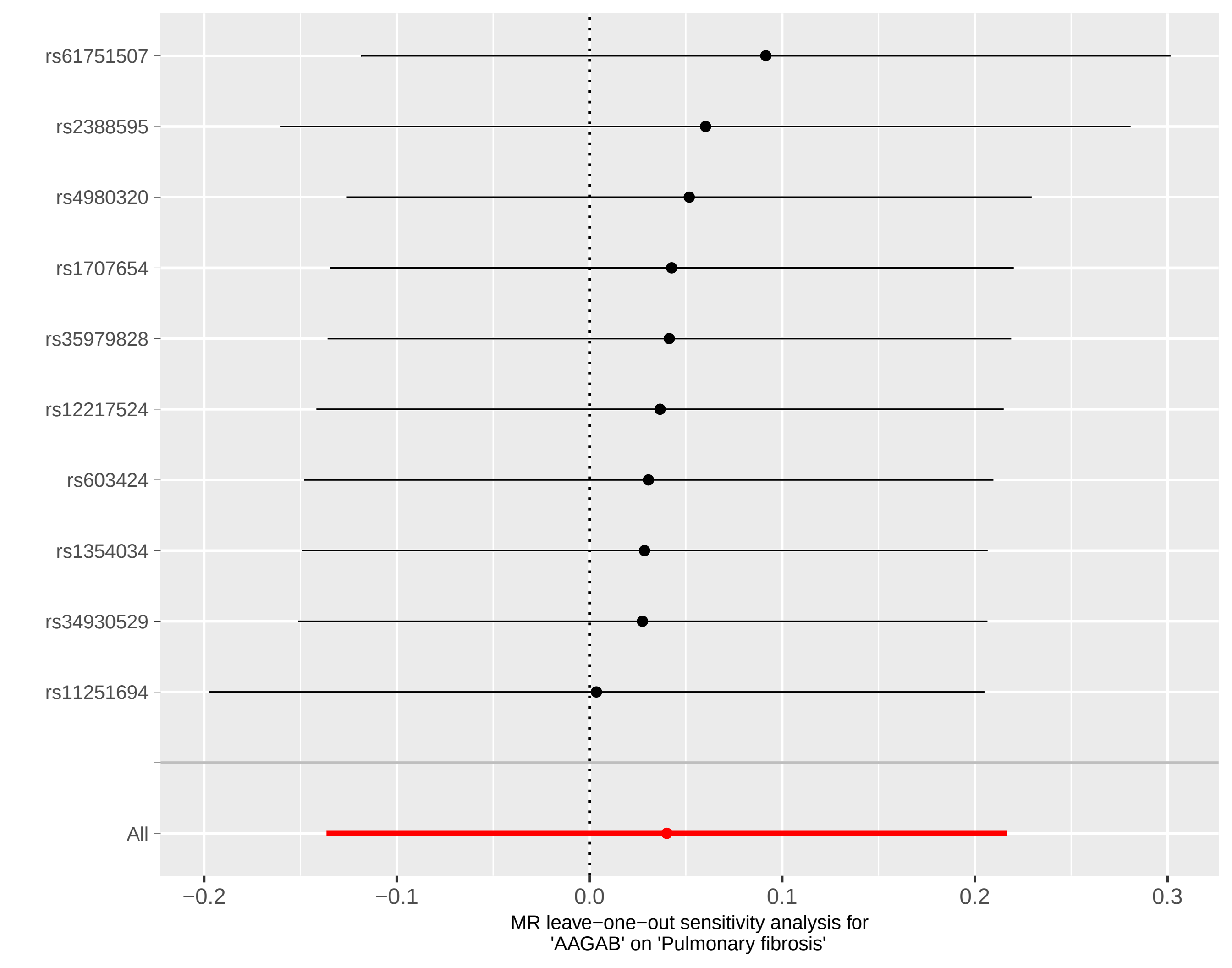
rs61751507
rs2388595
rs4980320
rs1707654
rs35979828
rs12217524
rs603424
rs1354034
rs34930529
rs11251694
All
−0.2
−0.1
0.0
0.1
0.2
0.3
MR leave−one−out sensitivity analysis for
'AAGAB' on 'Pulmonary fibrosis'

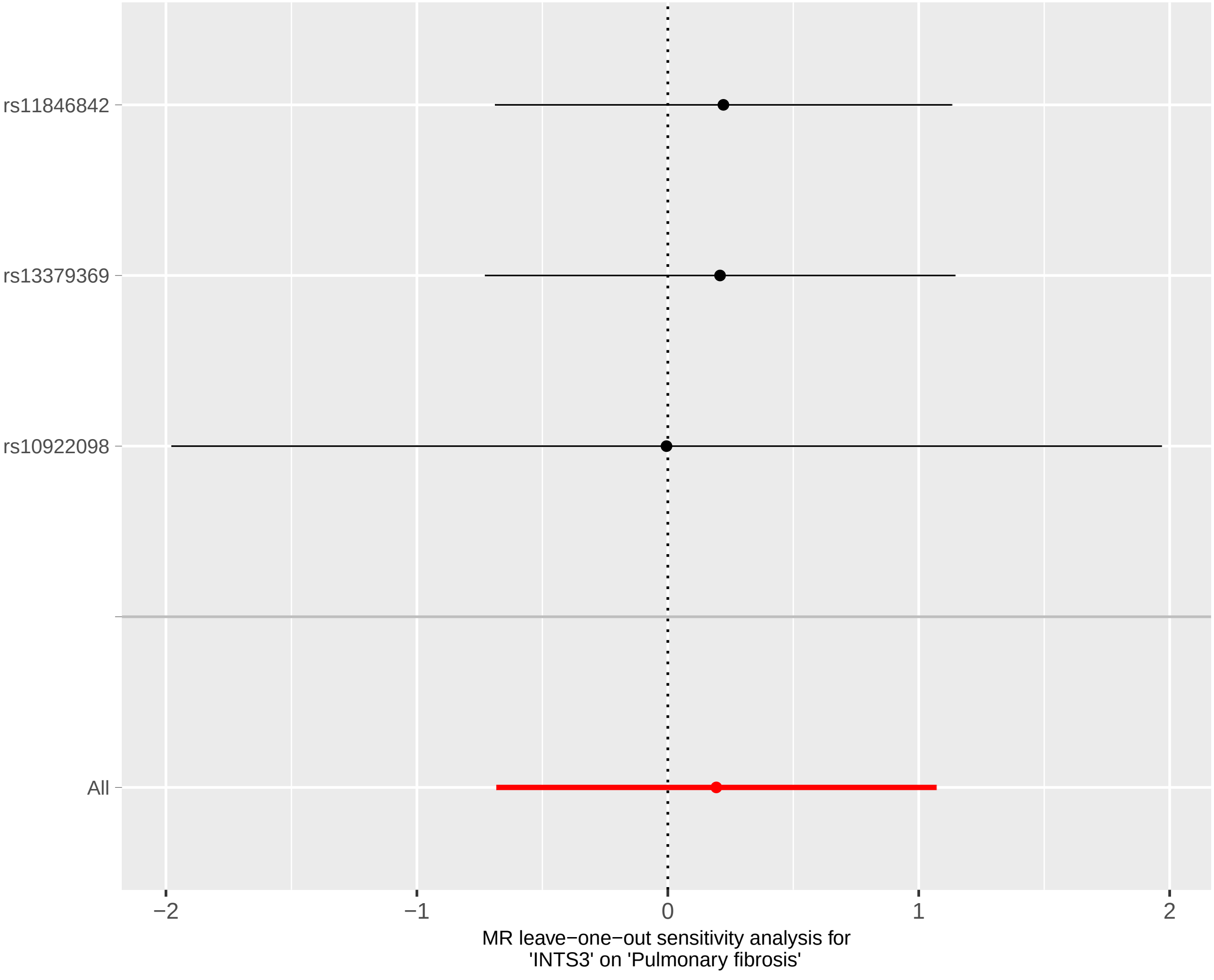

rs11846842
rs13379369
rs10922098
All
−2
−1
0
1
2
MR leave−one−out sensitivity analysis for
'INTS3' on 'Pulmonary fibrosis'

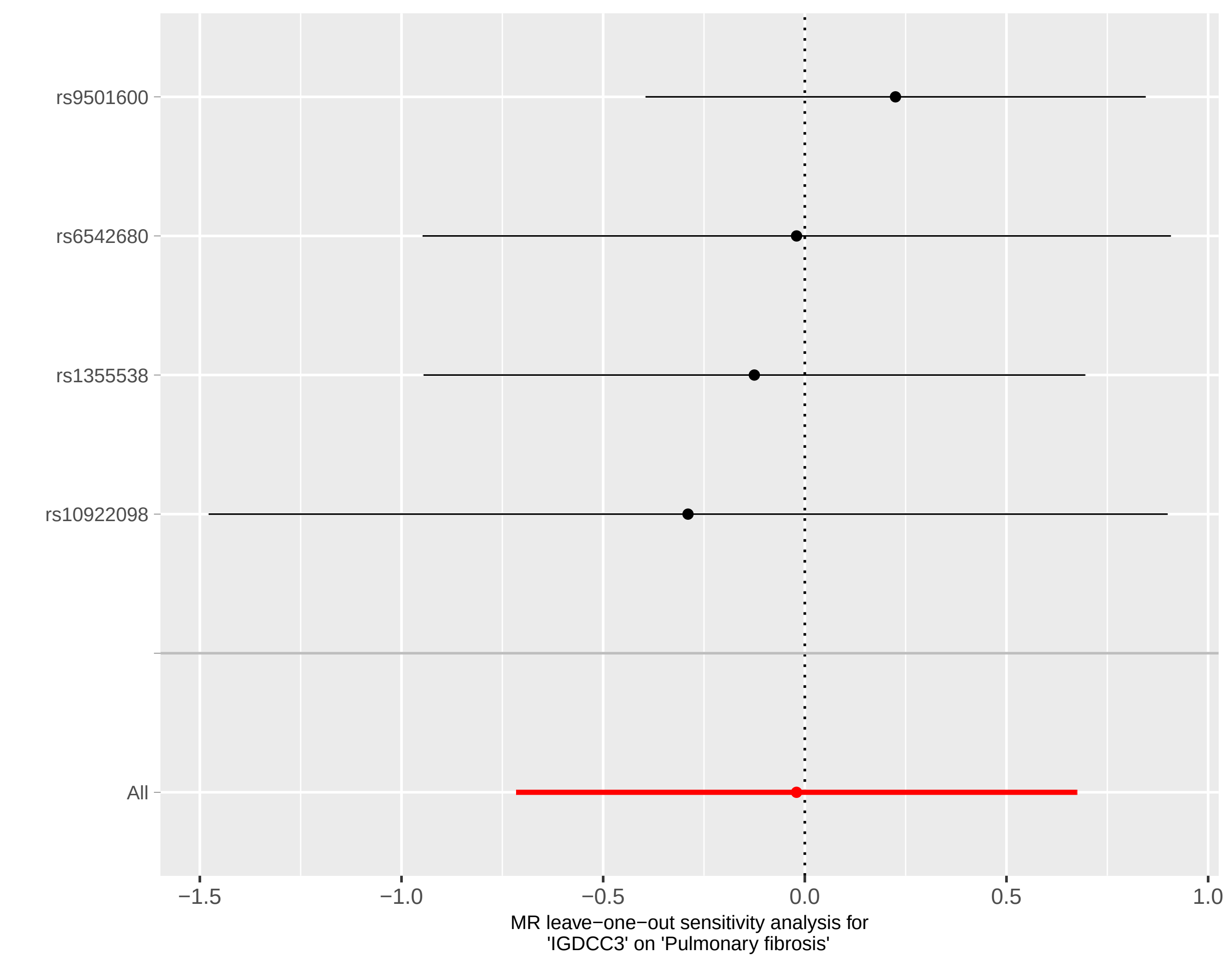
rs9501600
rs6542680
rs1355538
rs10922098
All
−1.5
−1.0
−0.5
0.0
0.5
1.0
MR leave−one−out sensitivity analysis for
'IGDCC3' on 'Pulmonary fibrosis'

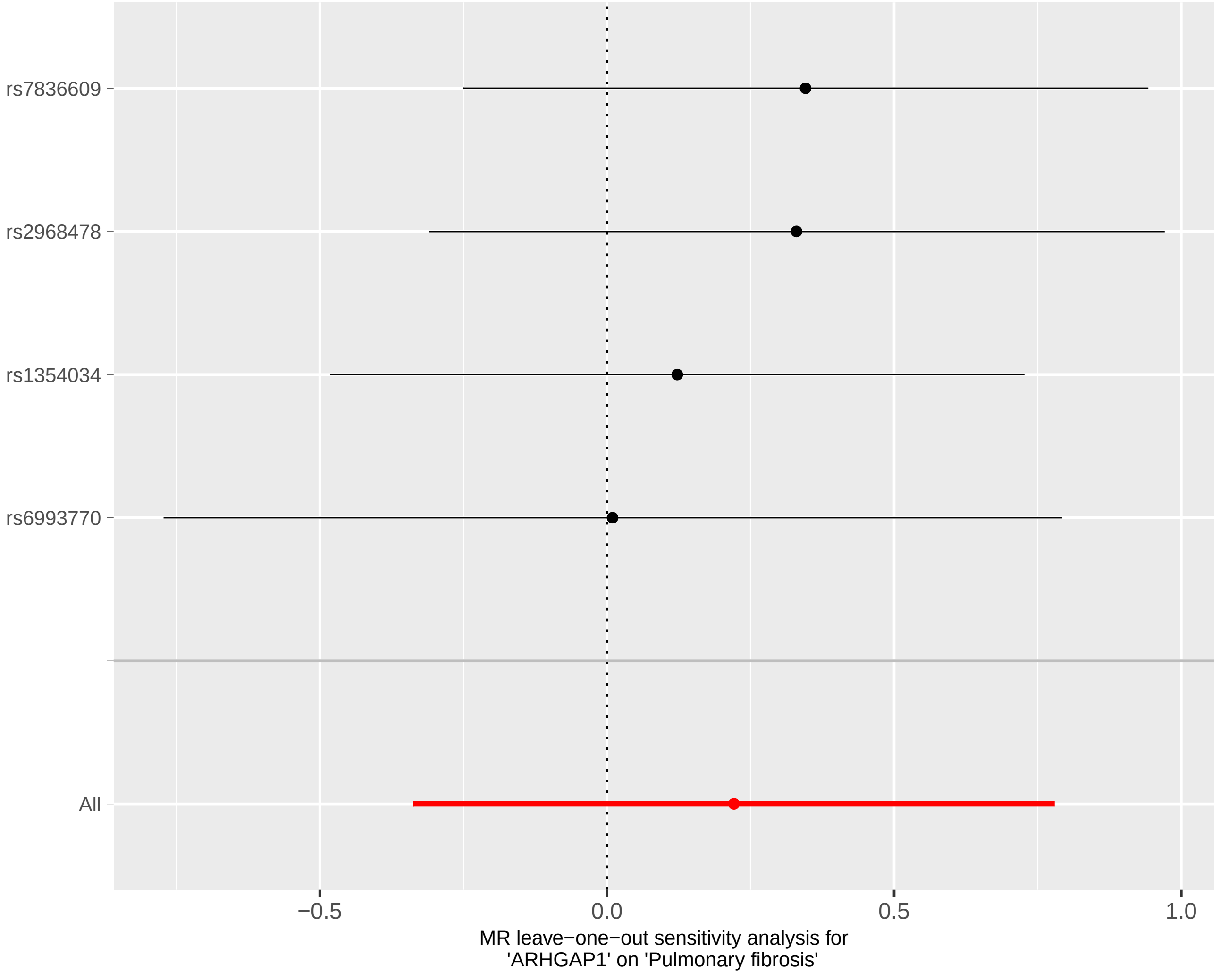

rs7836609
rs2968478
rs1354034
rs6993770
All
−0.5
0.0
0.5
1.0
MR leave−one−out sensitivity analysis for
'ARHGAP1' on 'Pulmonary fibrosis'

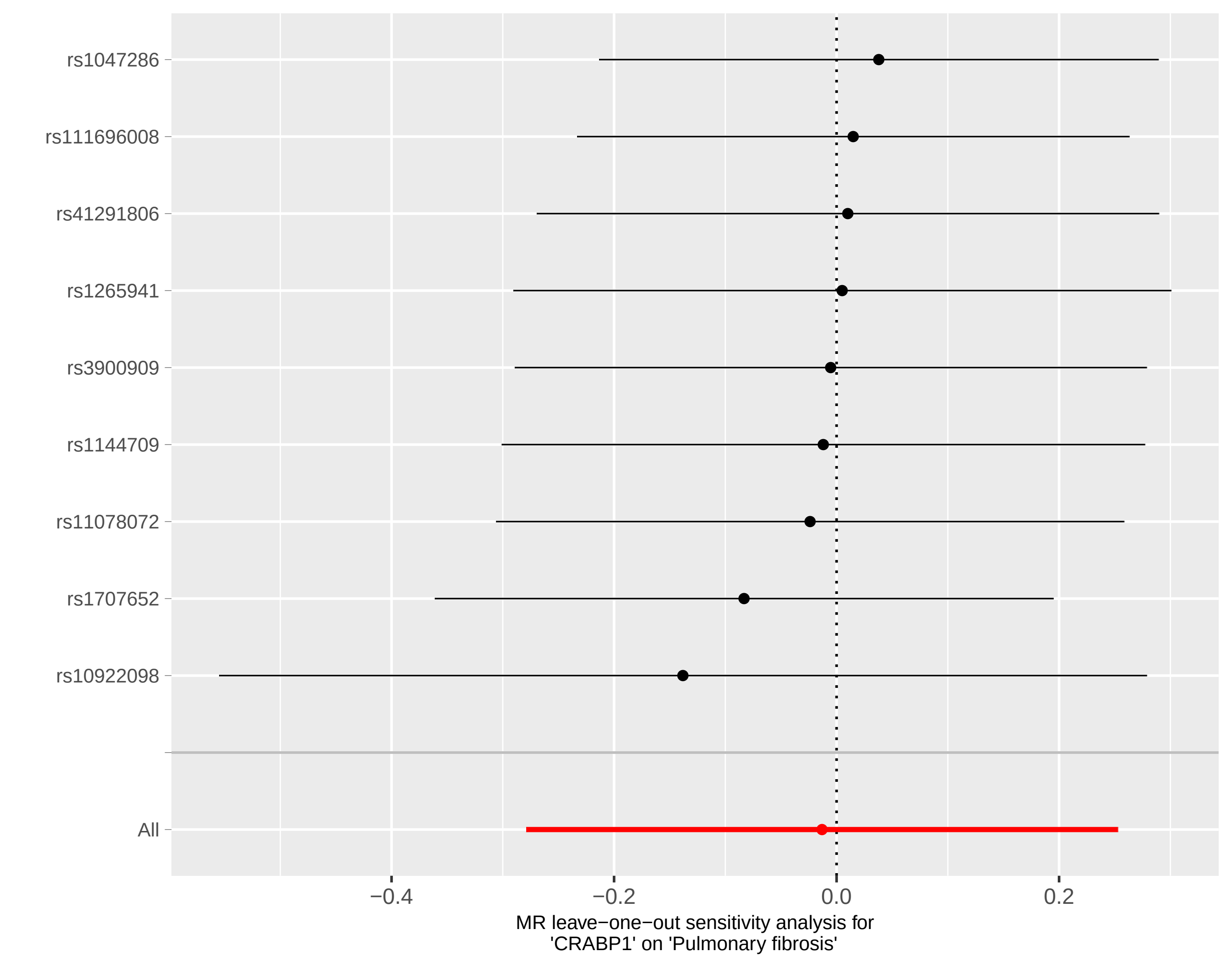
rs1047286
rs111696008
rs41291806
rs1265941
rs3900909
rs1144709
rs11078072
rs1707652
rs10922098
All
−0.4
−0.2
0.0
0.2
MR leave−one−out sensitivity analysis for
'CRABP1' on 'Pulmonary fibrosis'

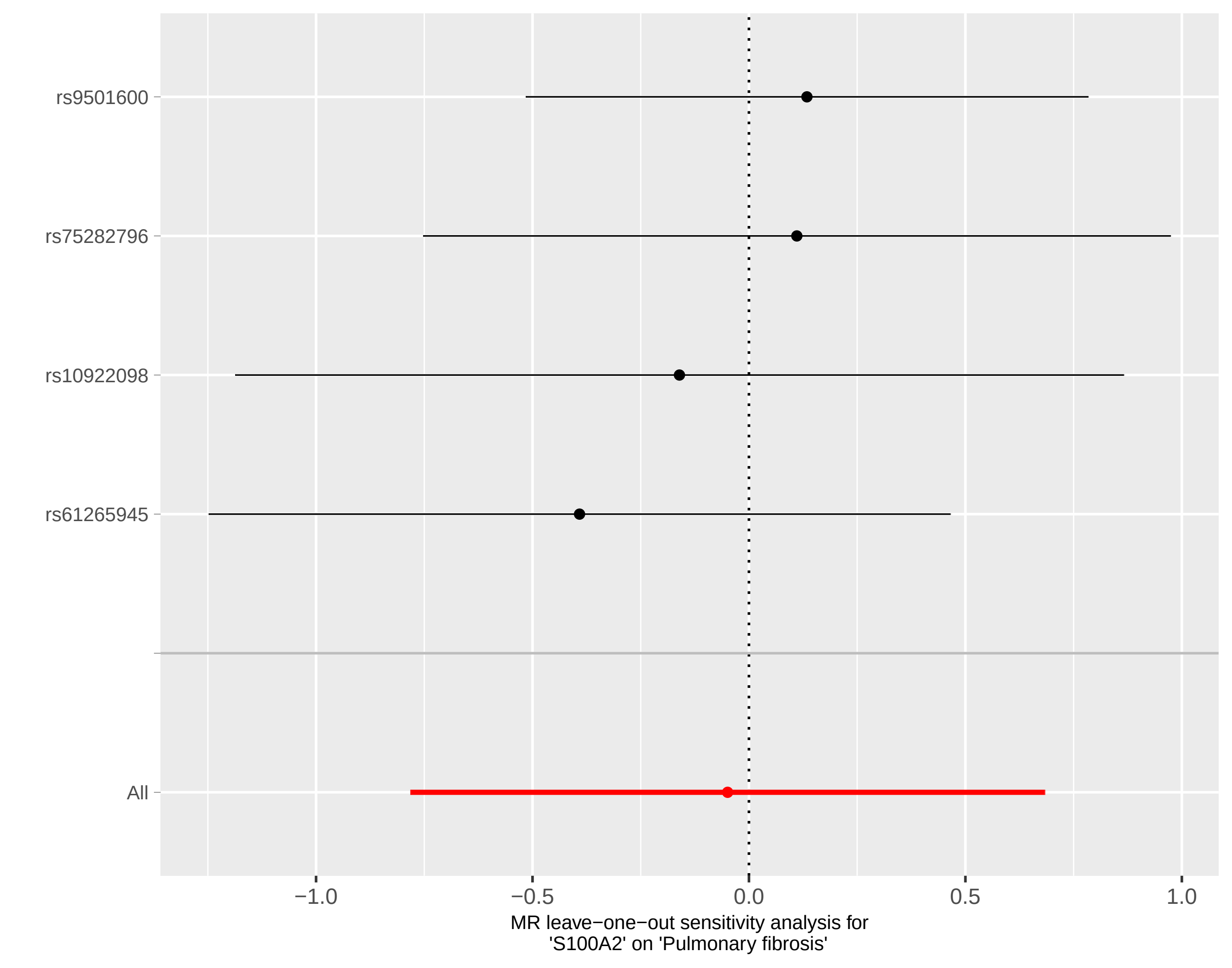
rs9501600
rs75282796
rs10922098
rs61265945
All
−1.0
−0.5
0.0
0.5
1.0
MR leave−one−out sensitivity analysis for
'S100A2' on 'Pulmonary fibrosis'

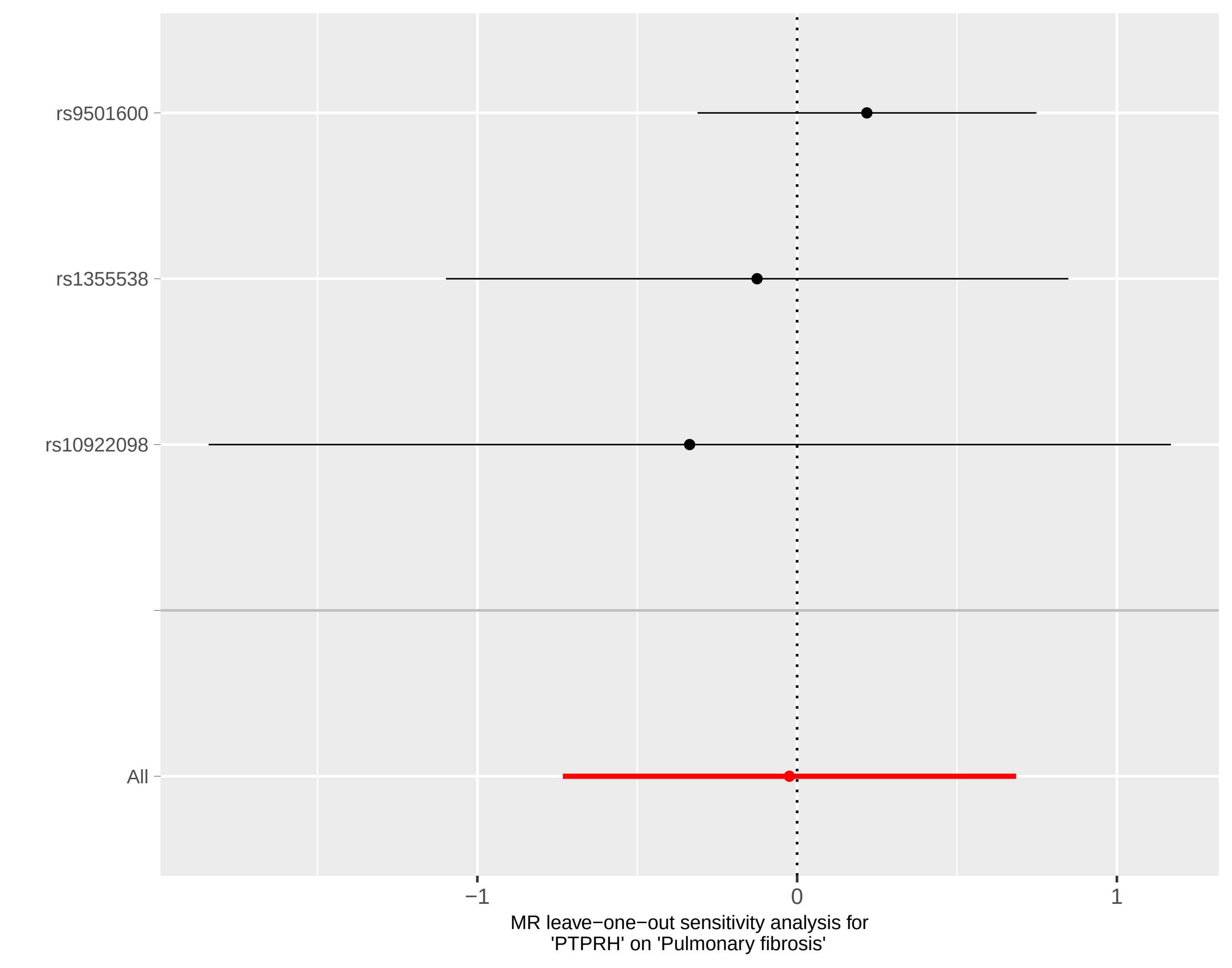
rs9501600
rs1355538
rs10922098
All
−1
0
1
MR leave−one−out sensitivity analysis for
'PTPRH' on 'Pulmonary fibrosis'

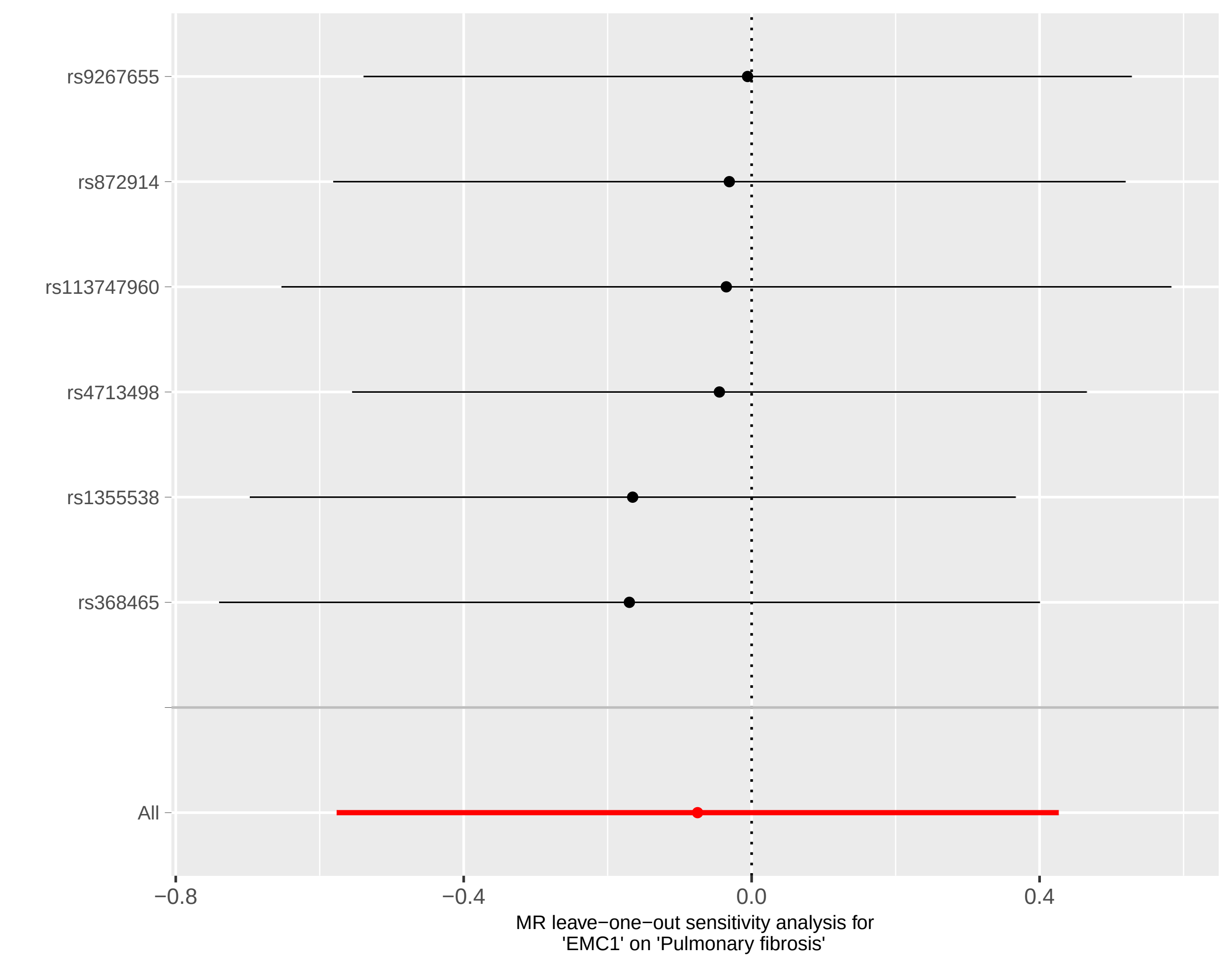
rs9267655
rs872914
rs113747960
rs4713498
rs1355538
rs368465
All
−0.8
−0.4
0.0
0.4
MR leave−one−out sensitivity analysis for
'EMC1' on 'Pulmonary fibrosis'

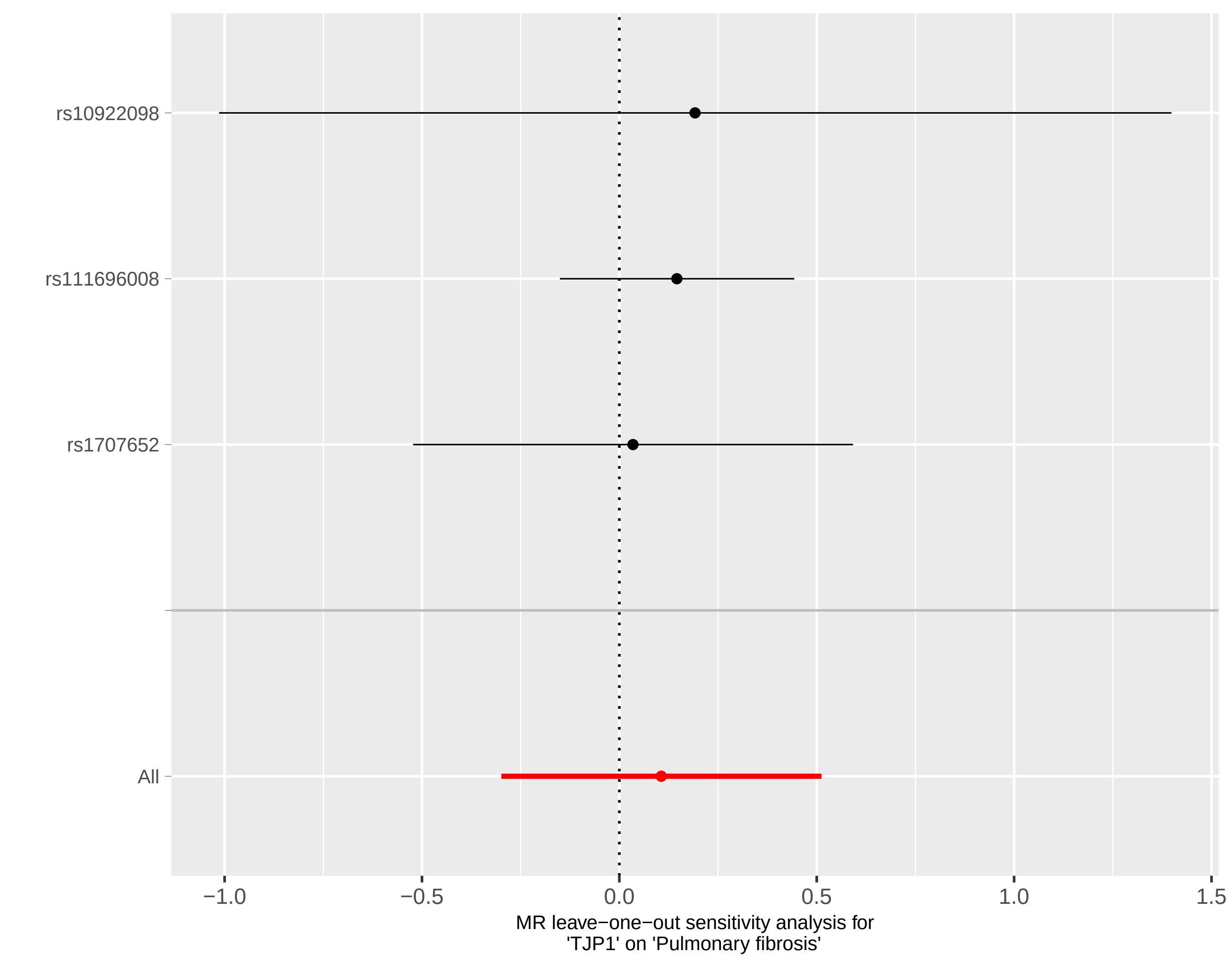
rs10922098
rs111696008
rs1707652
All
−1.0
−0.5
0.0
0.5
1.0
1.5
MR leave−one−out sensitivity analysis for
'TJP1' on 'Pulmonary fibrosis'

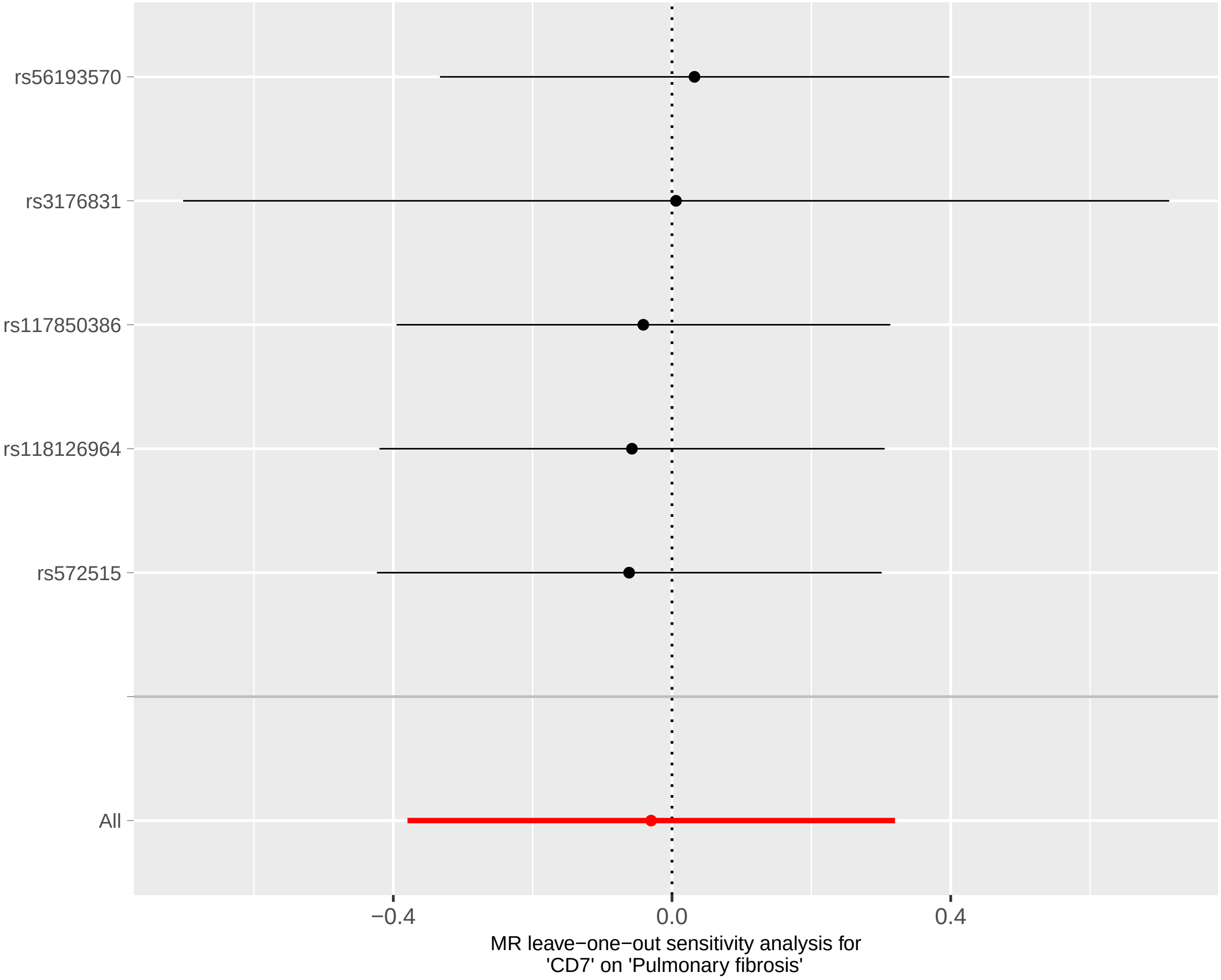

rs56193570
rs3176831
rs117850386
rs118126964
rs572515
All
−0.4
0.0
0.4
MR leave−one−out sensitivity analysis for
'CD7' on 'Pulmonary fibrosis'

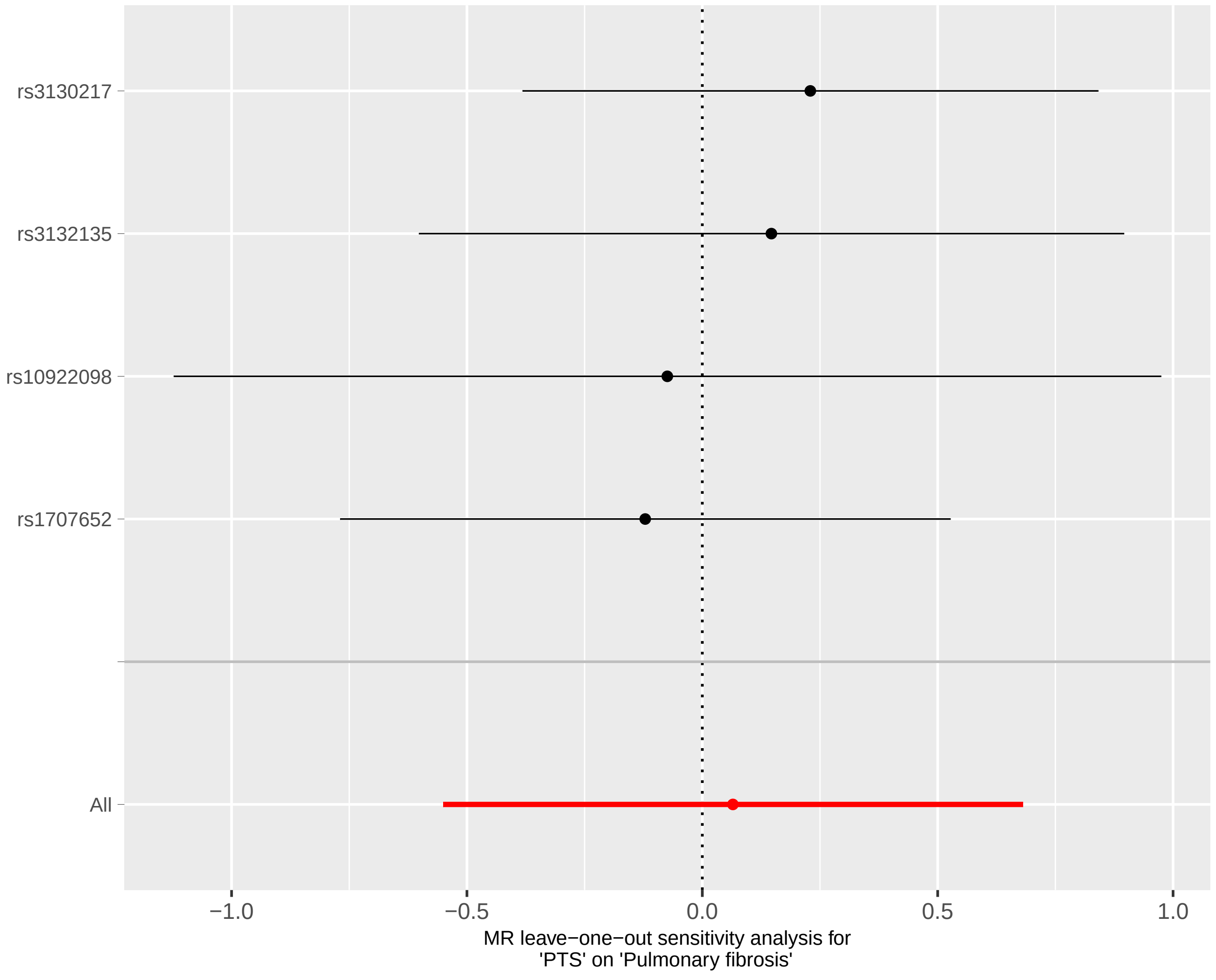
rs3130217
rs3132135
rs10922098
rs1707652
All
−1.0
−0.5
0.0
0.5
1.0
MR leave−one−out sensitivity analysis for
'PTS' on 'Pulmonary fibrosis'

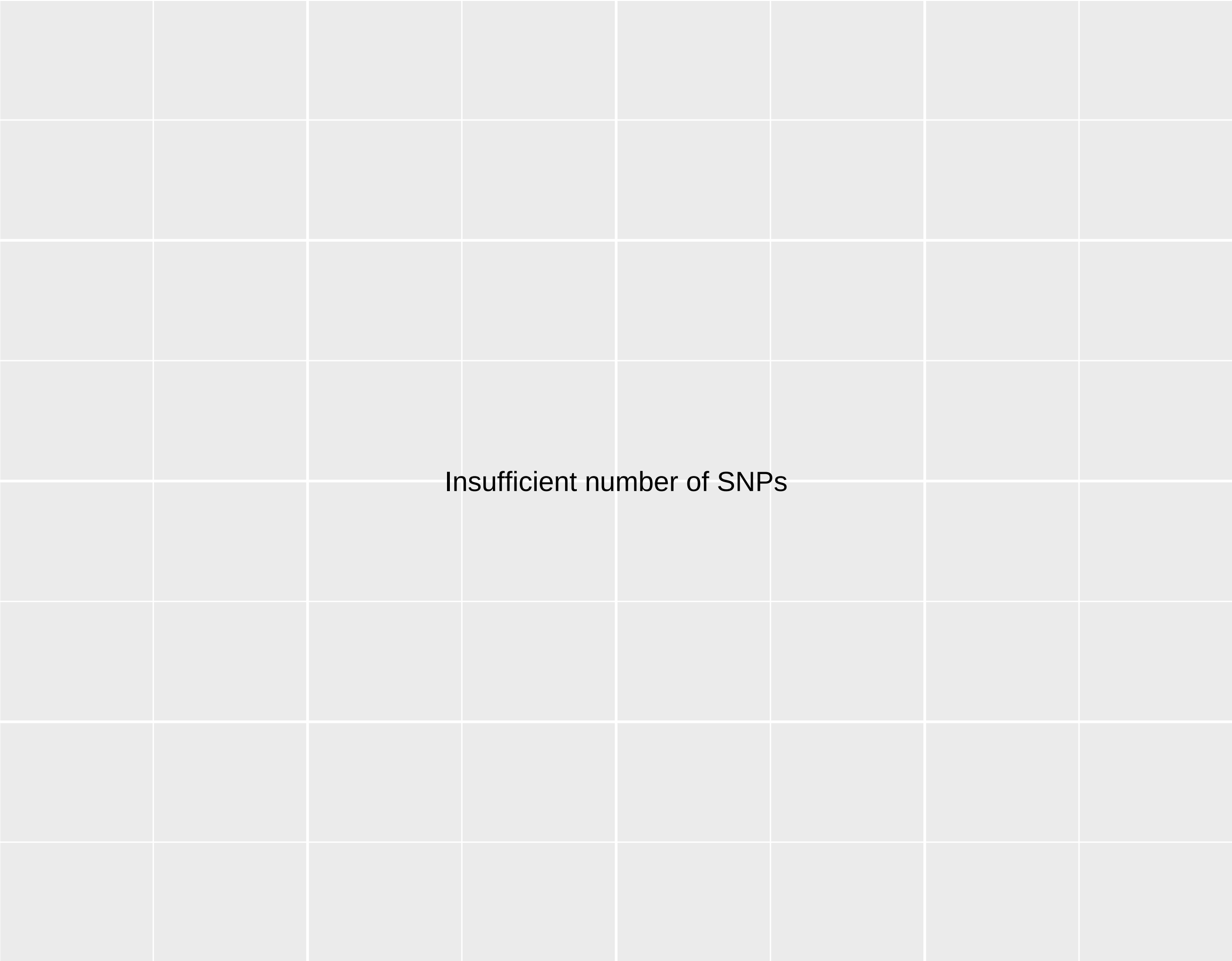
Insufficient number of SNPs

Insufficient number of SNPs

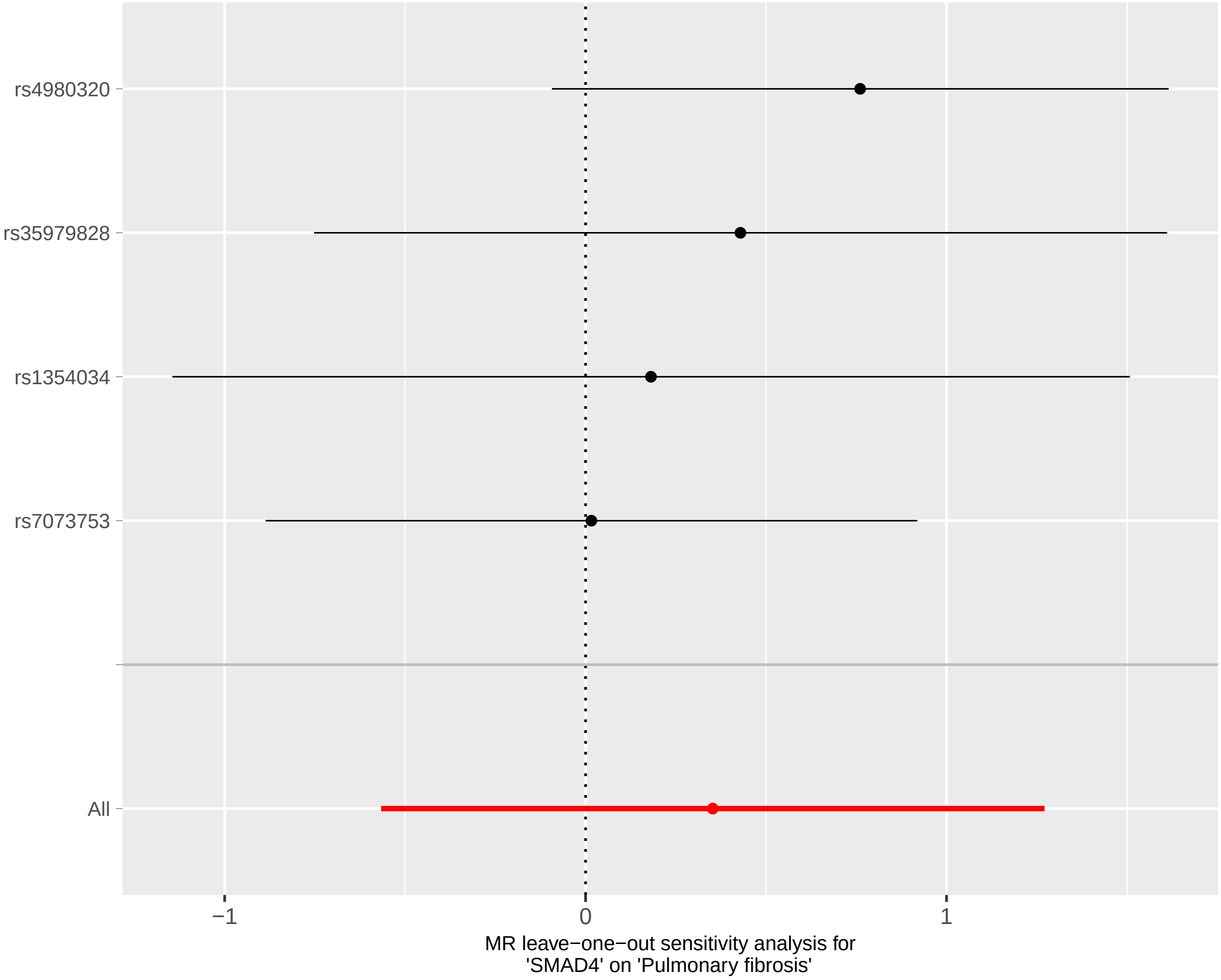

rs4980320
rs35979828
rs1354034
rs7073753
All
−1
0
1
MR leave−one−out sensitivity analysis for
'SMAD4' on 'Pulmonary fibrosis'

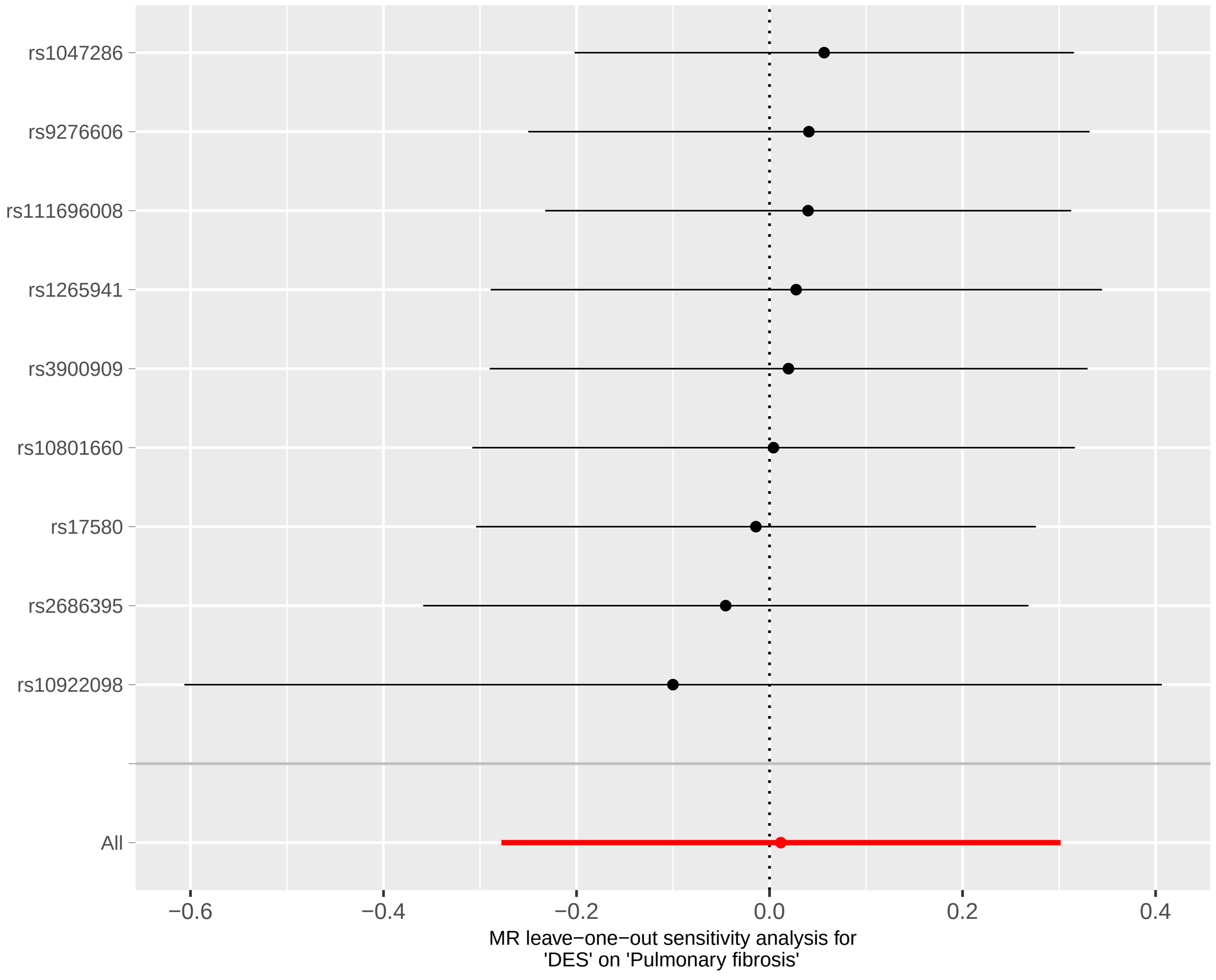
rs1047286
rs9276606
rs111696008
rs1265941
rs3900909
rs10801660
rs17580
rs2686395
rs10922098
All
−0.6
−0.4
−0.2
0.0
0.2
0.4
MR leave−one−out sensitivity analysis for
'DES' on 'Pulmonary fibrosis'

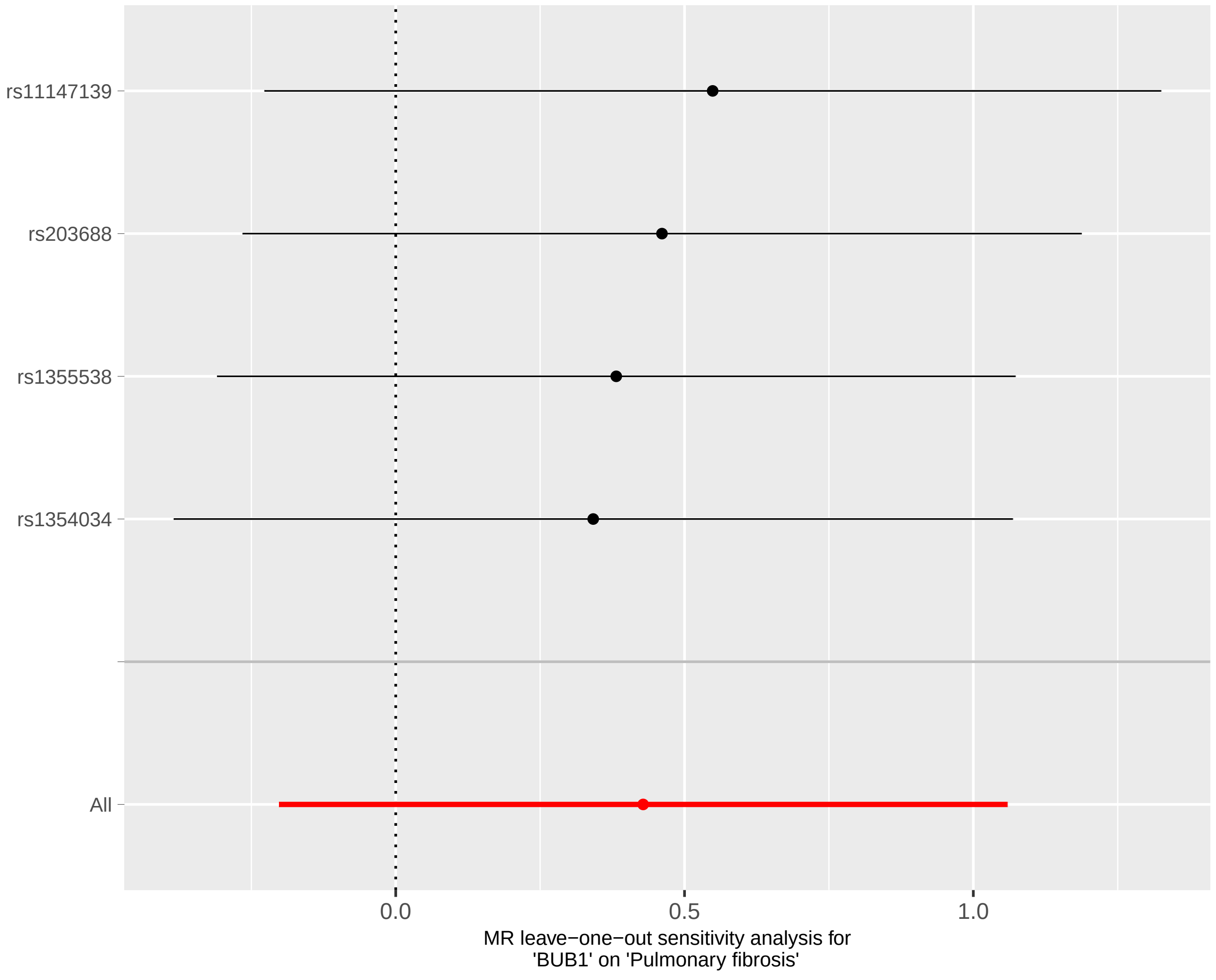

rs11147139
rs203688
rs1355538
rs1354034
All
0.0
0.5
1.0
MR leave−one−out sensitivity analysis for
'BUB1' on 'Pulmonary fibrosis'

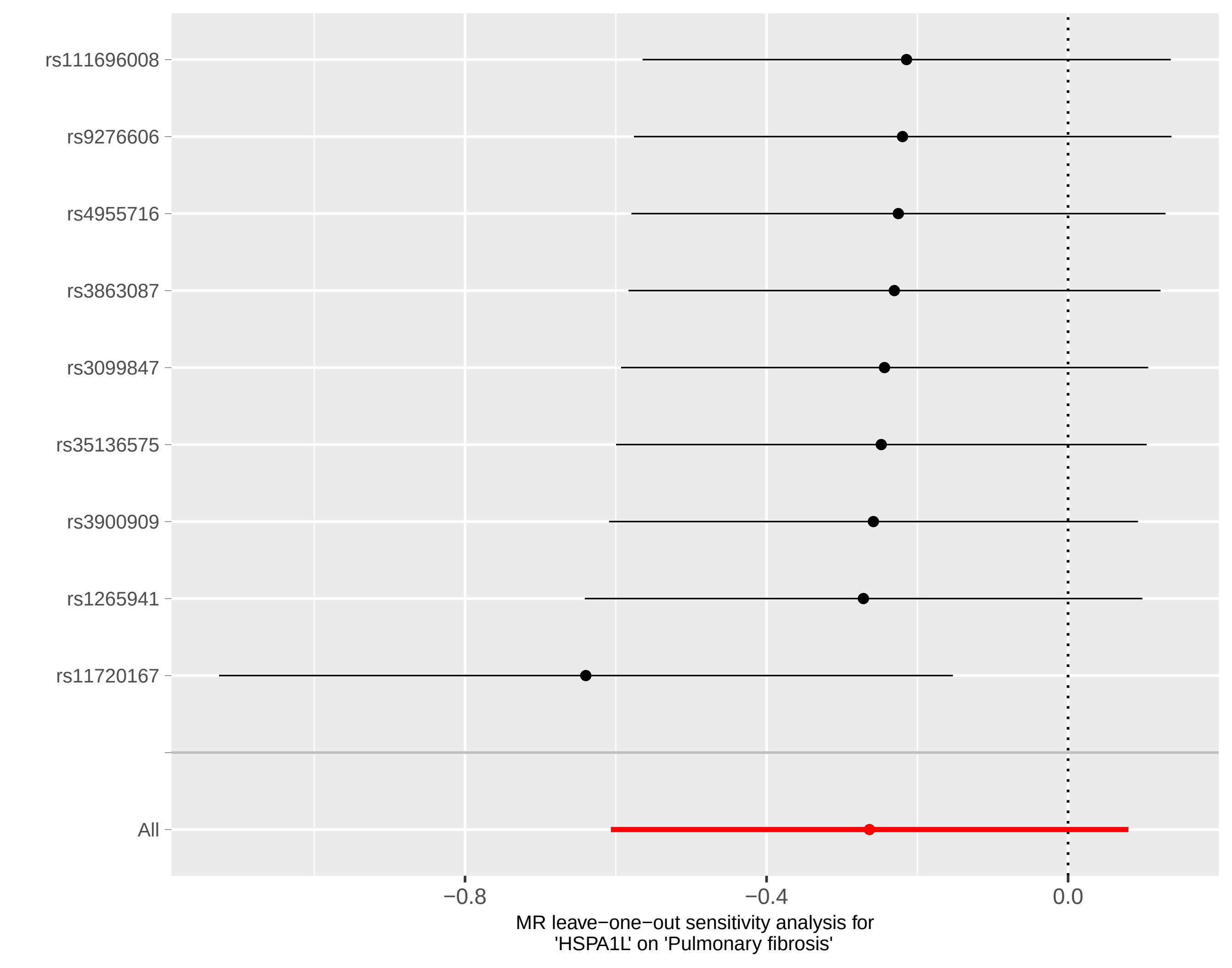

rs111696008
rs9276606
rs4955716
rs3863087
rs3099847
rs35136575
rs3900909
rs1265941
rs11720167
All
−0.8
−0.4
0.0
MR leave−one−out sensitivity analysis for
'HSPA1L' on 'Pulmonary fibrosis'

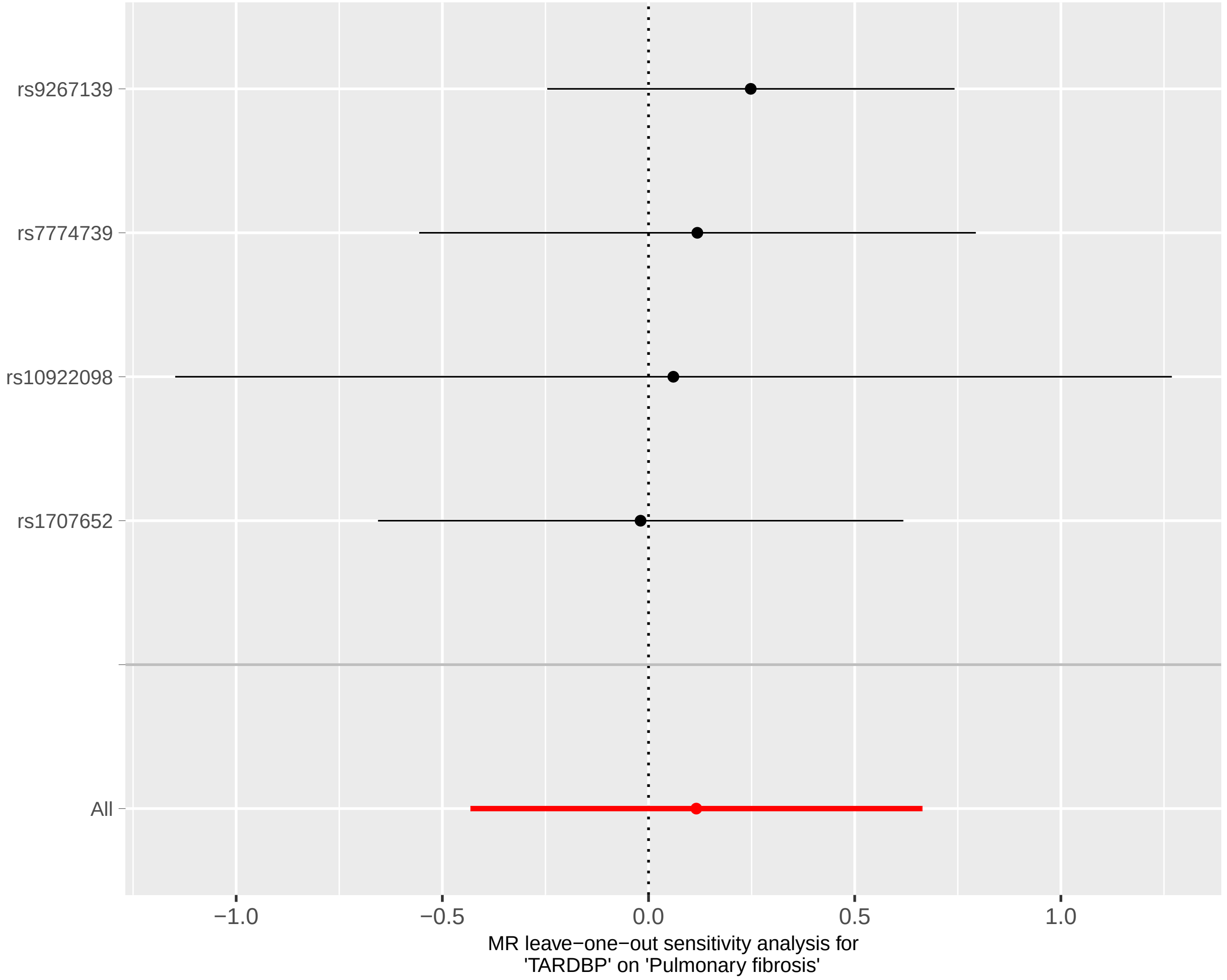
rs9267139
rs7774739
rs10922098
rs1707652
All
−1.0
−0.5
0.0
0.5
1.0
MR leave−one−out sensitivity analysis for
'TARDBP' on 'Pulmonary fibrosis'

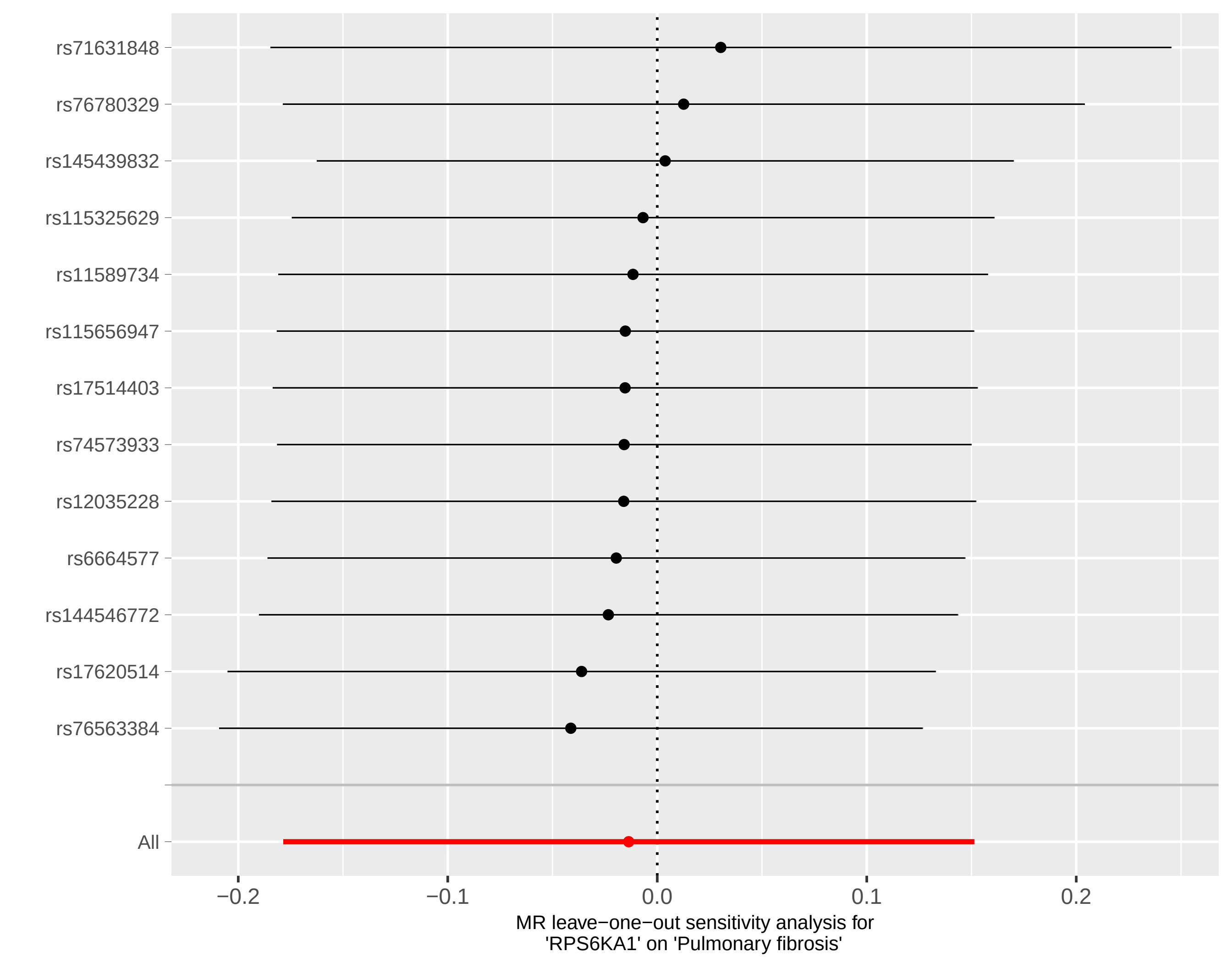
rs71631848
rs76780329
rs145439832
rs115325629
rs11589734
rs115656947
rs17514403
rs74573933
rs12035228
rs6664577
rs144546772
rs17620514
rs76563384
All
−0.2
−0.1
0.0
0.1
0.2
MR leave−one−out sensitivity analysis for
'RPS6KA1' on 'Pulmonary fibrosis'

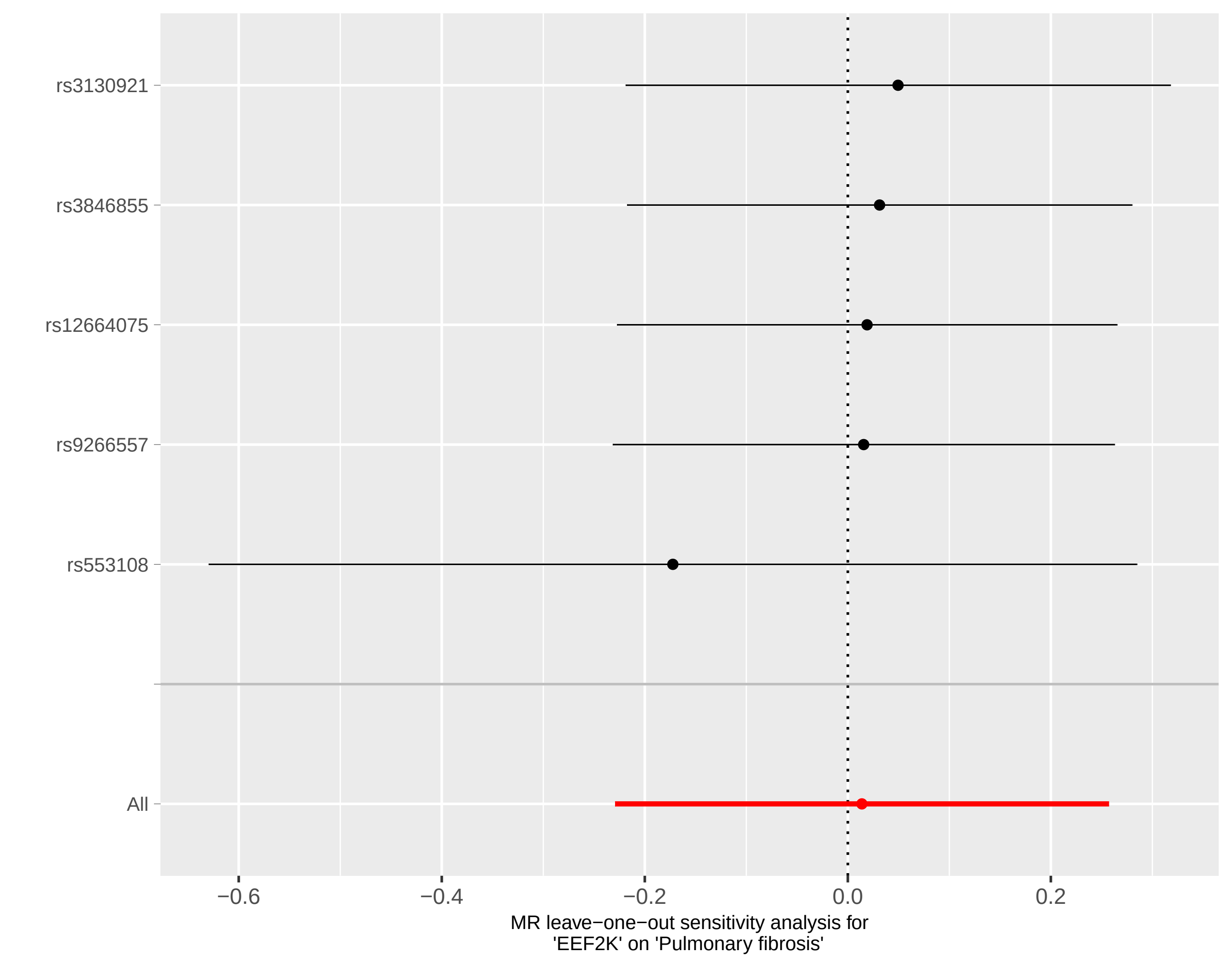

rs3130921
rs3846855
rs12664075
rs9266557
rs553108
All
−0.6
−0.4
−0.2
0.0
0.2
MR leave−one−out sensitivity analysis for
'EEF2K' on 'Pulmonary fibrosis'

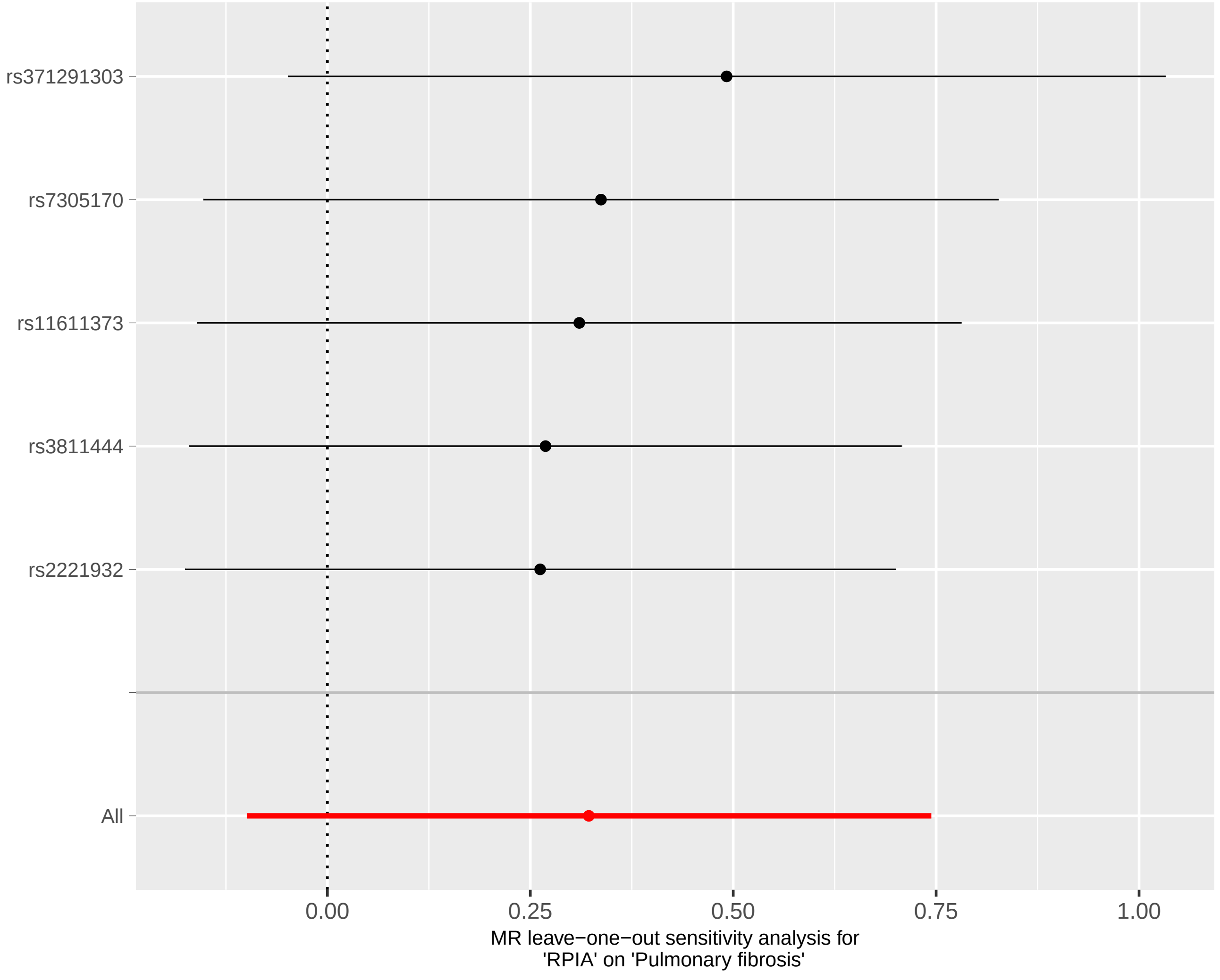
rs371291303
rs7305170
rs11611373
rs3811444
rs2221932
All
0.00
0.25
0.50
0.75
1.00
MR leave−one−out sensitivity analysis for
'RPIA' on 'Pulmonary fibrosis'

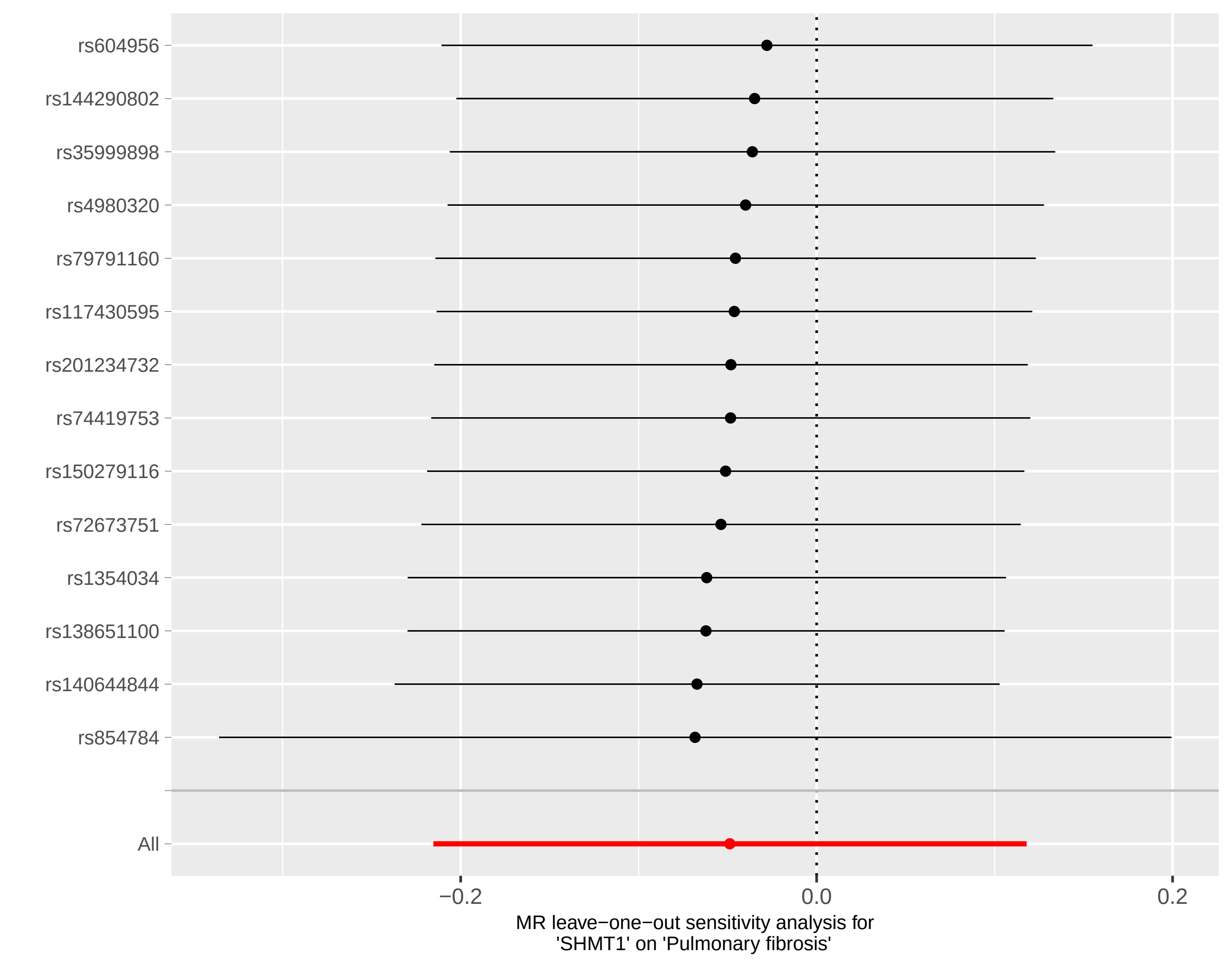
rs604956
rs144290802
rs35999898
rs4980320
rs79791160
rs117430595
rs201234732
rs74419753
rs150279116
rs72673751
rs1354034
rs138651100
rs140644844
rs854784
All
−0.2
0.0
0.2
MR leave−one−out sensitivity analysis for
'SHMT1' on 'Pulmonary fibrosis'

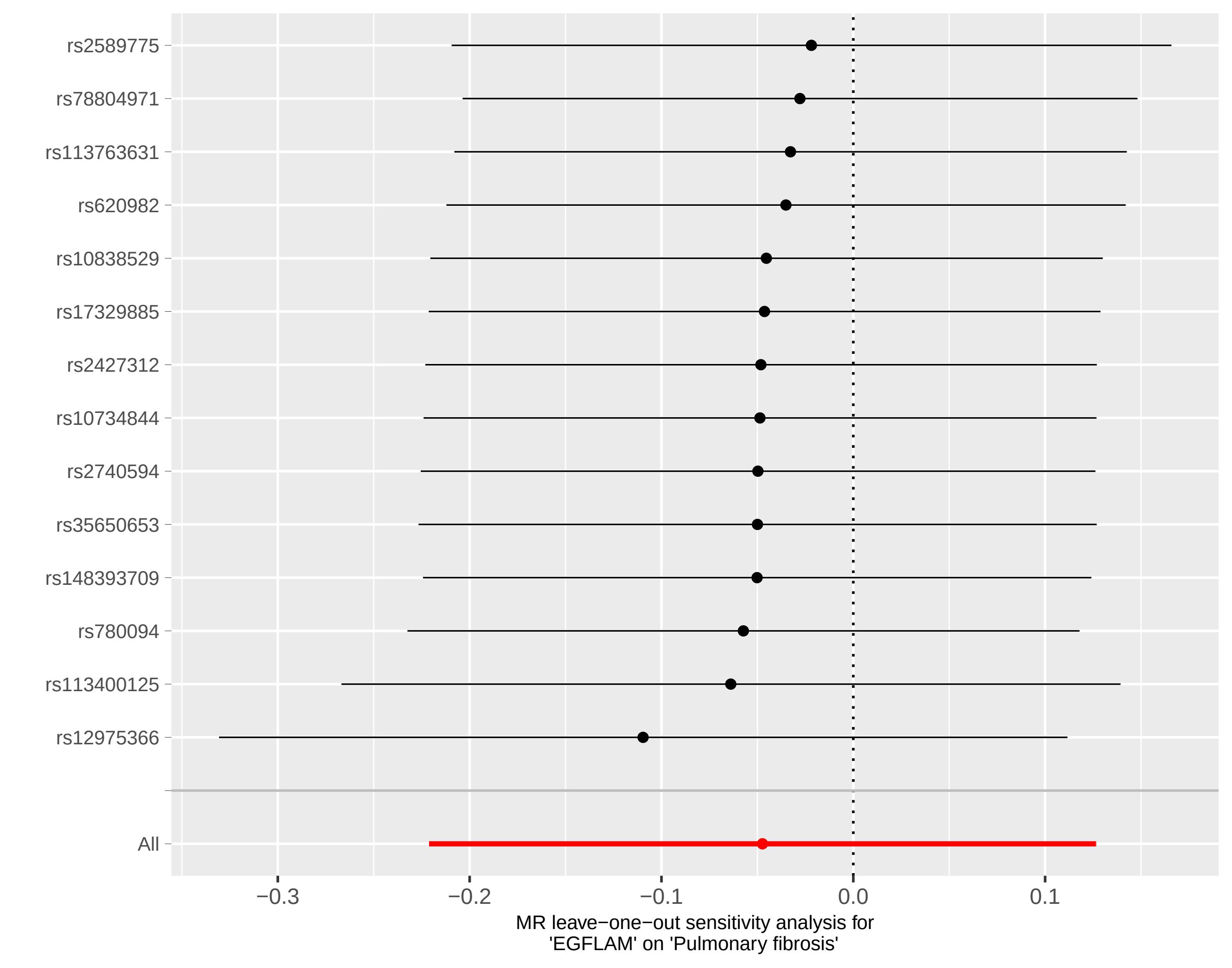
rs2589775
rs78804971
rs113763631
rs620982
rs10838529
rs17329885
rs2427312
rs10734844
rs2740594
rs35650653
rs148393709
rs780094
rs113400125
rs12975366
All
−0.3
−0.2
−0.1
0.0
0.1
MR leave−one−out sensitivity analysis for
'EGFLAM' on 'Pulmonary fibrosis'

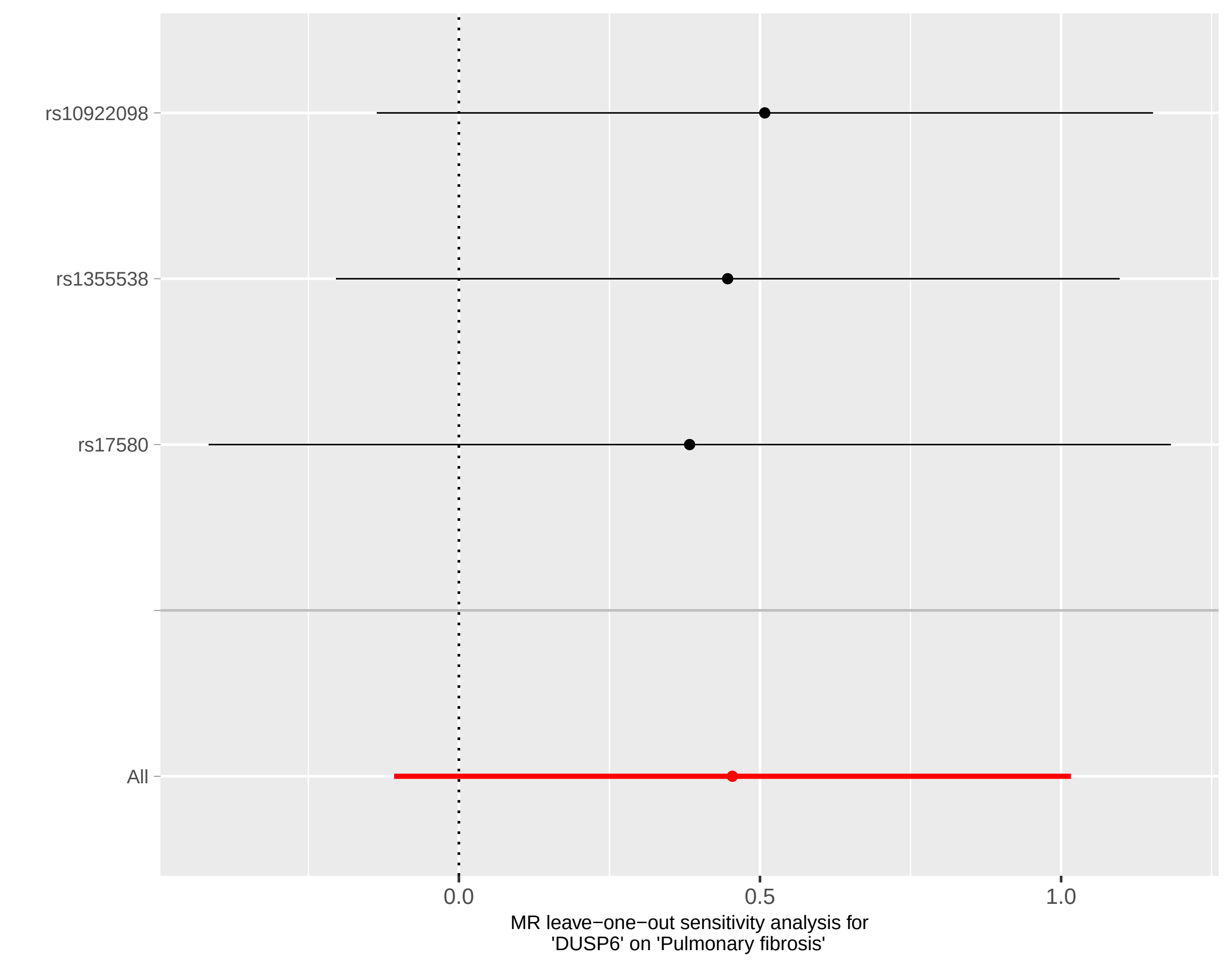
rs10922098
rs1355538
rs17580
All
0.0
0.5
1.0
MR leave−one−out sensitivity analysis for
'DUSP6' on 'Pulmonary fibrosis'

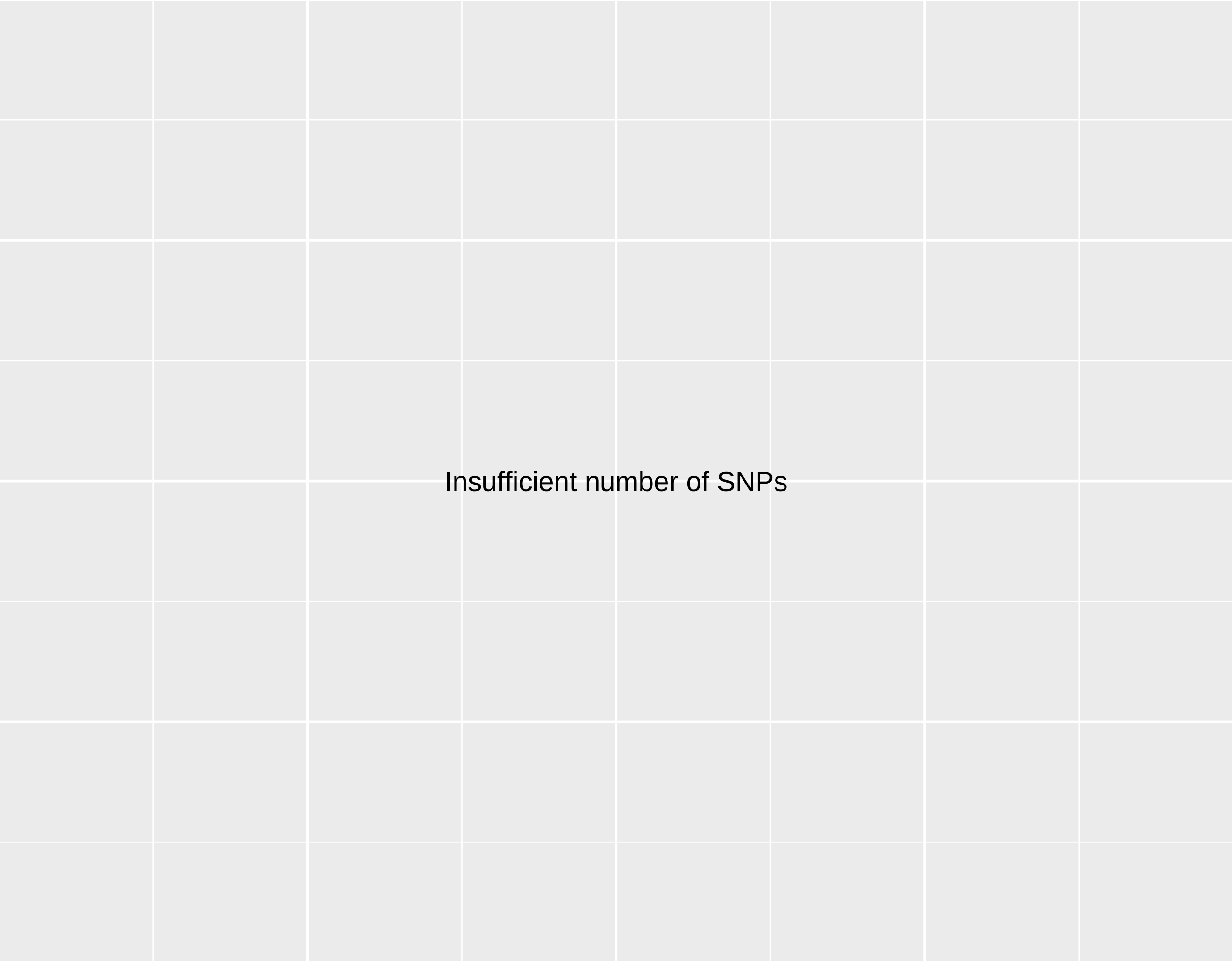
Insufficient number of SNPs

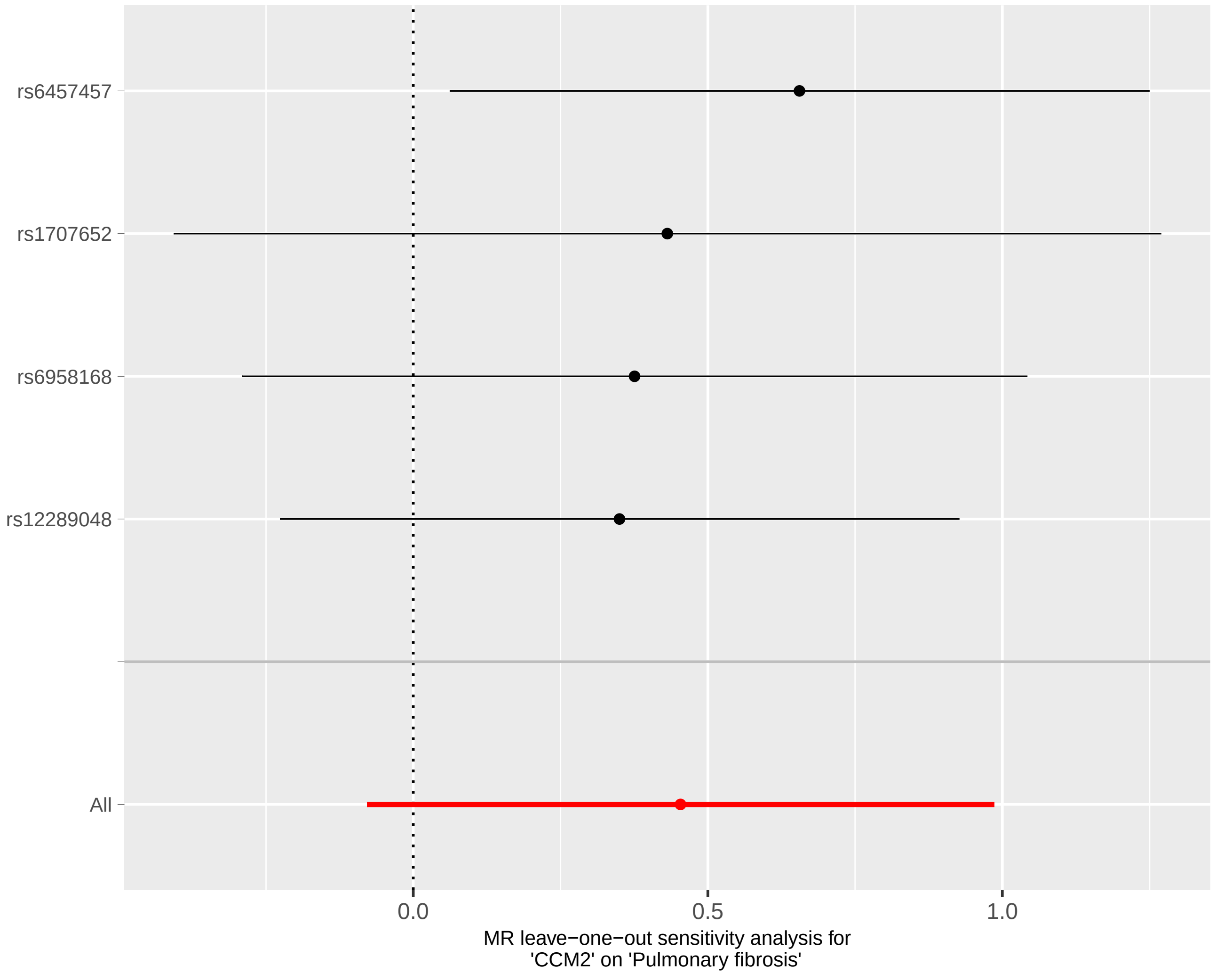
rs6457457
rs1707652
rs6958168
rs12289048
All
0.0
0.5
1.0
MR leave−one−out sensitivity analysis for
'CCM2' on 'Pulmonary fibrosis'

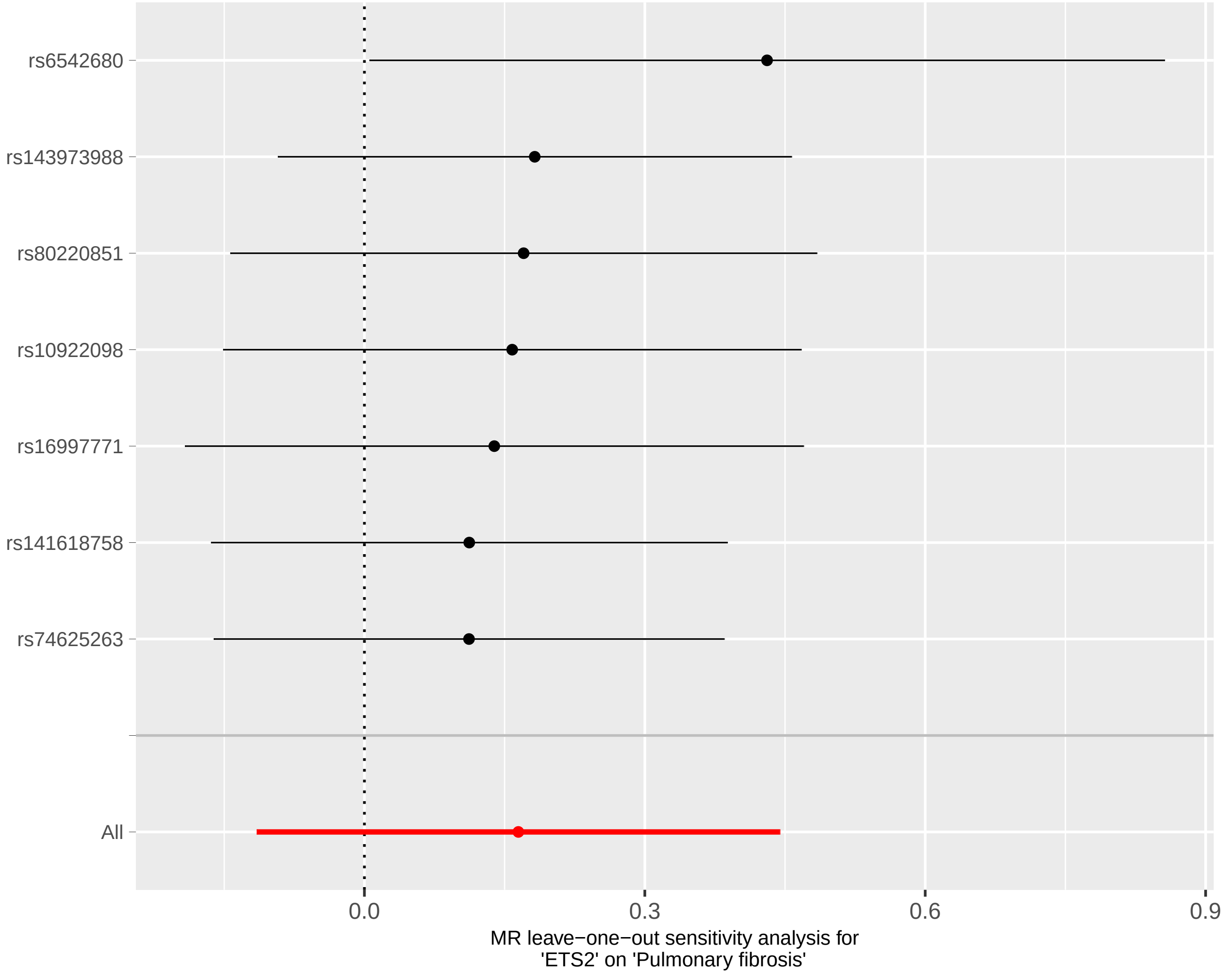
rs6542680
rs143973988
rs80220851
rs10922098
rs16997771
rs141618758
rs74625263
All
0.0
0.3
0.6
0.9
MR leave−one−out sensitivity analysis for
'ETS2' on 'Pulmonary fibrosis'

Insufficient number of SNPs

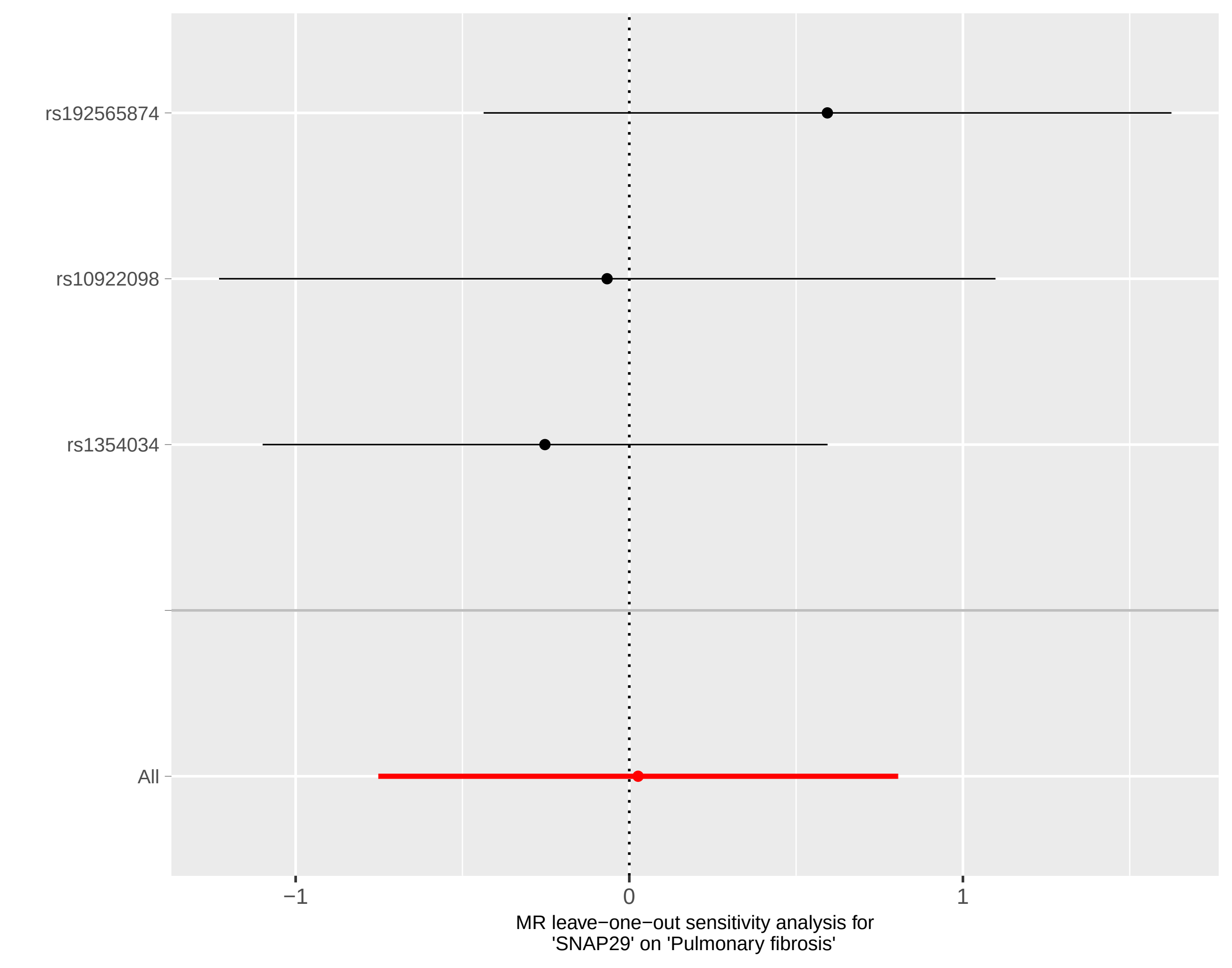

rs192565874
rs10922098
rs1354034
All
−1
0
1
MR leave−one−out sensitivity analysis for
'SNAP29' on 'Pulmonary fibrosis'

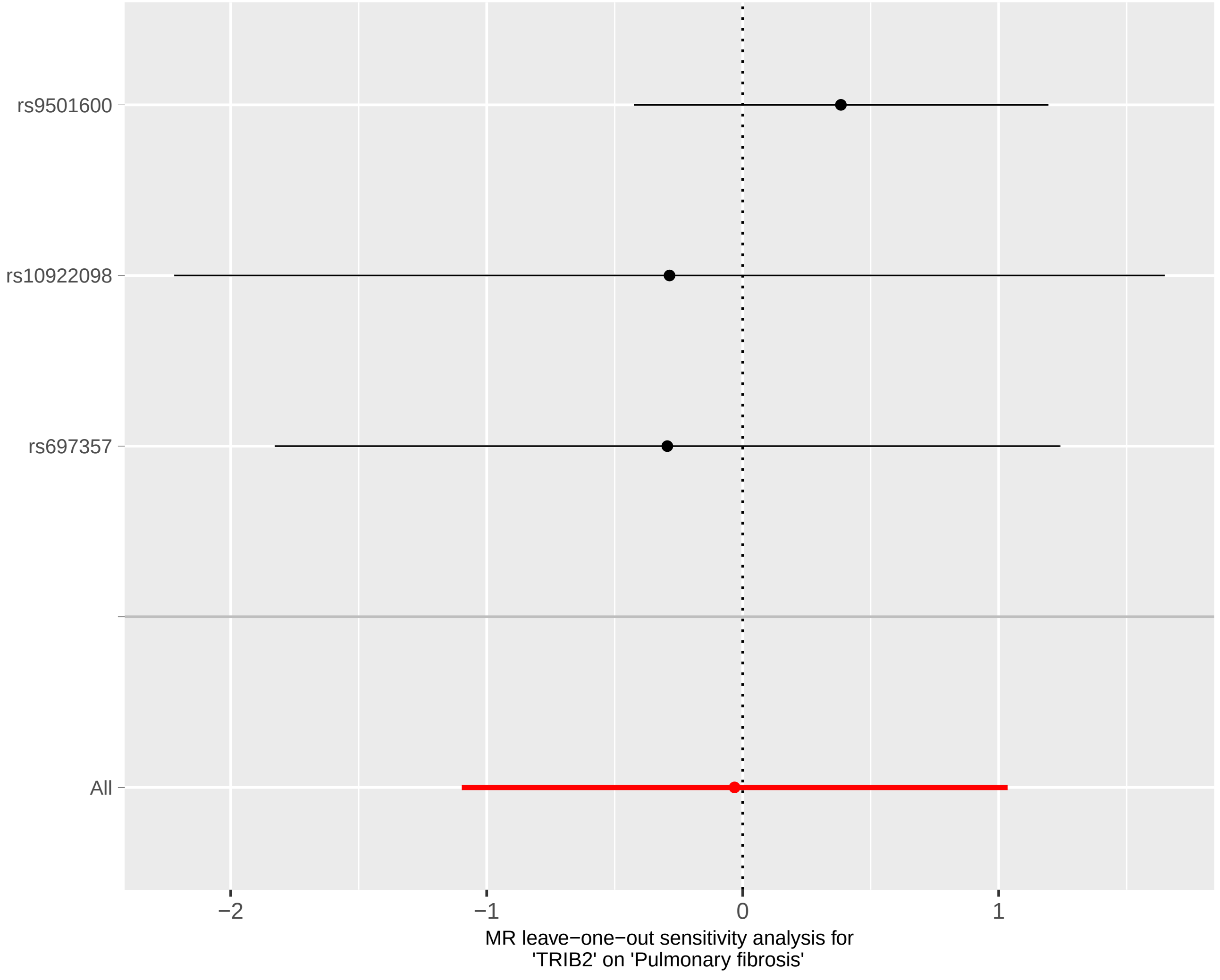
rs9501600
rs10922098
rs697357
All
−2
−1
0
1
MR leave−one−out sensitivity analysis for
'TRIB2' on 'Pulmonary fibrosis'

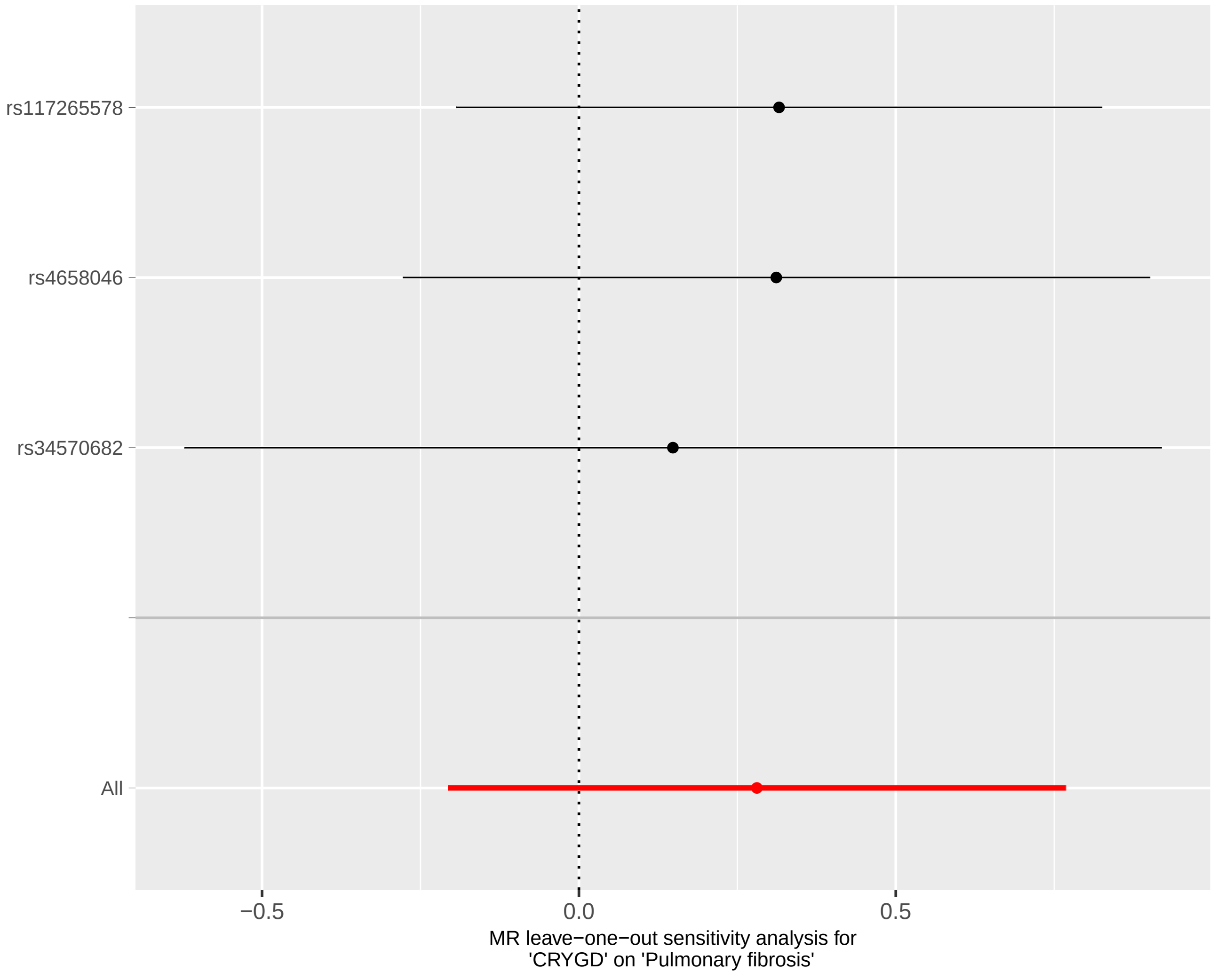

rs117265578
rs4658046
rs34570682
All
−0.5
0.0
0.5
MR leave−one−out sensitivity analysis for
'CRYGD' on 'Pulmonary fibrosis'

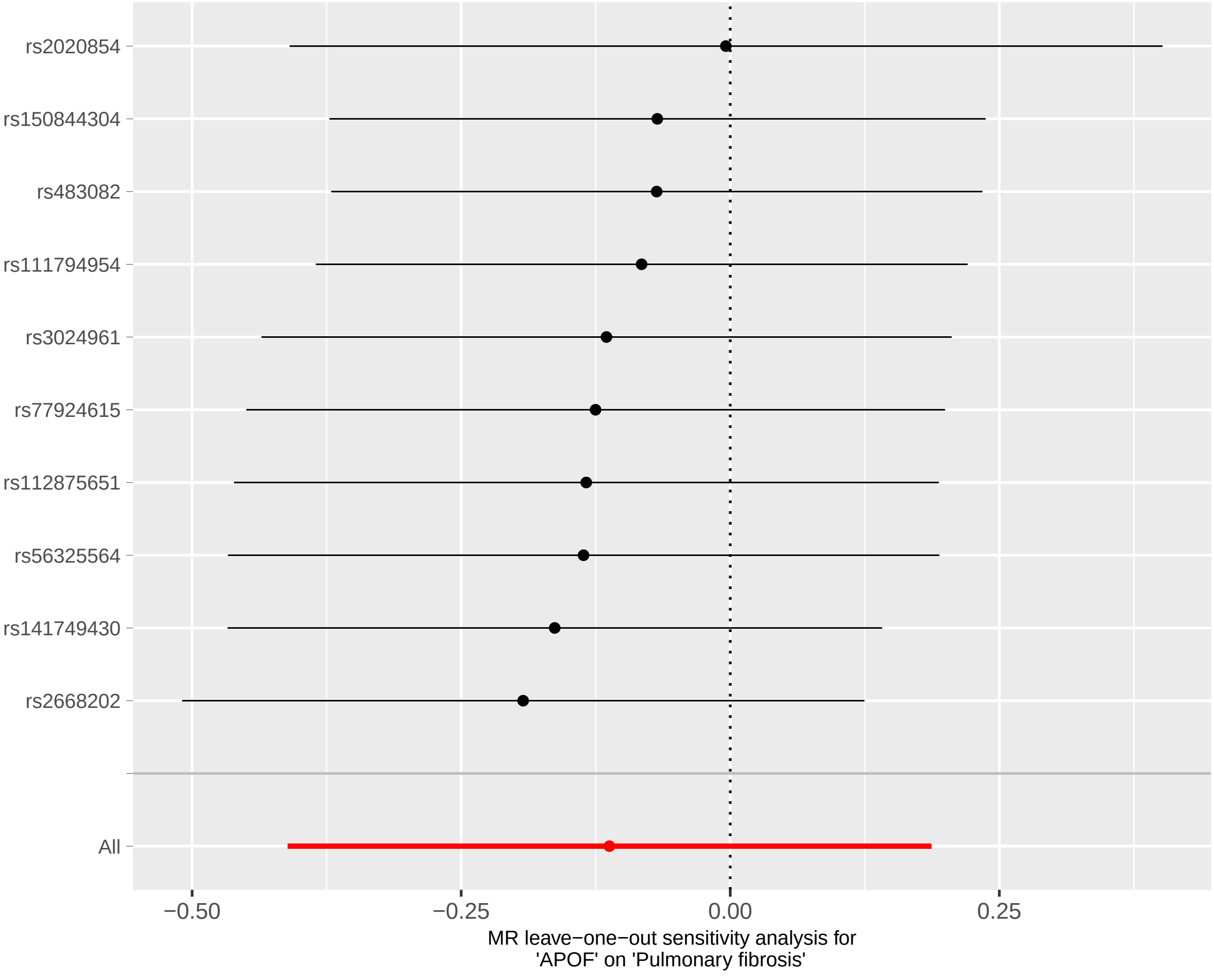
rs2020854
rs150844304
rs483082
rs111794954
rs3024961
rs77924615
rs112875651
rs56325564
rs141749430
rs2668202
All
−0.50
−0.25
0.00
0.25
MR leave−one−out sensitivity analysis for
'APOF' on 'Pulmonary fibrosis'

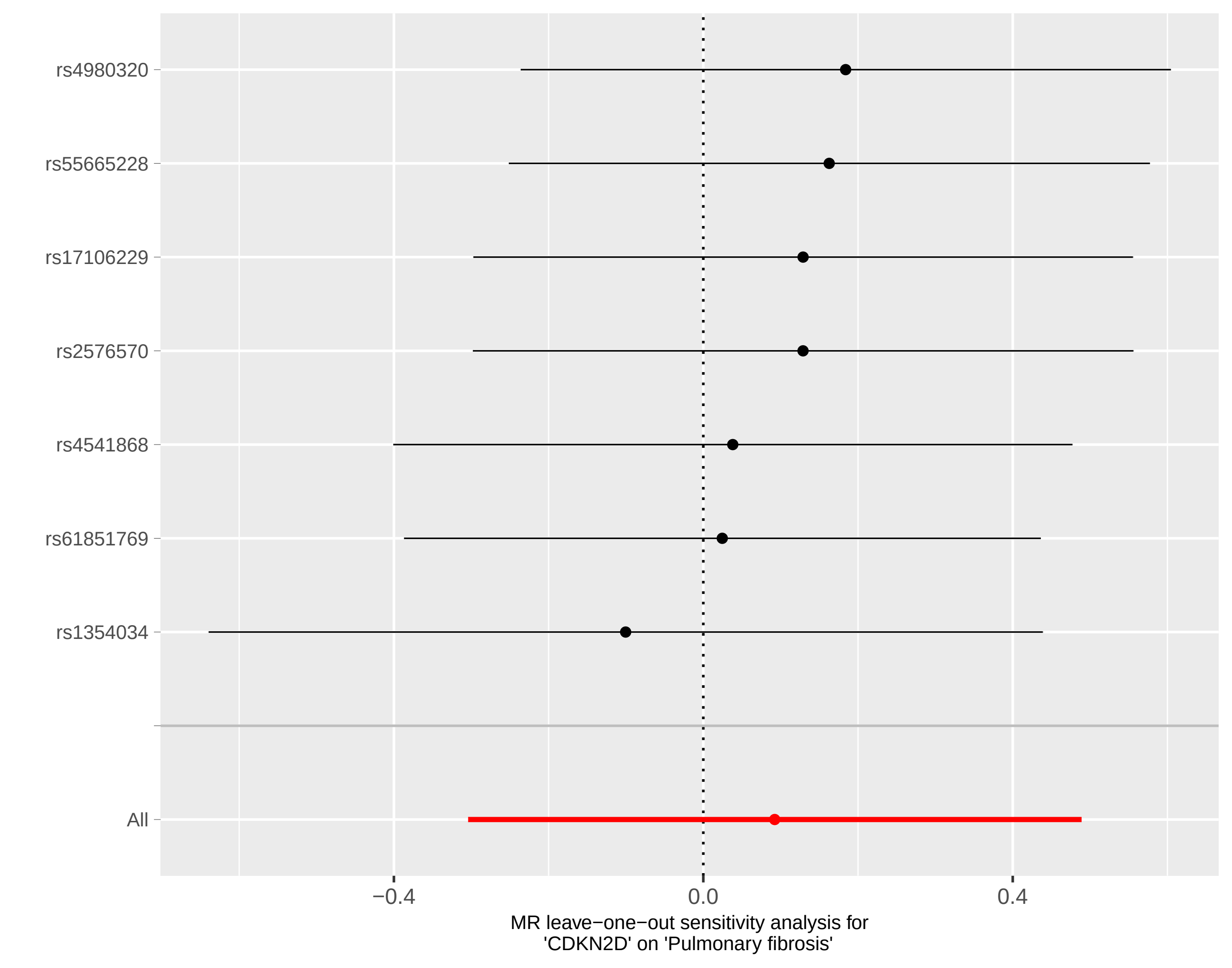
rs4980320
rs55665228
rs17106229
rs2576570
rs4541868
rs61851769
rs1354034
All
−0.4
0.0
0.4
MR leave−one−out sensitivity analysis for
'CDKN2D' on 'Pulmonary fibrosis'

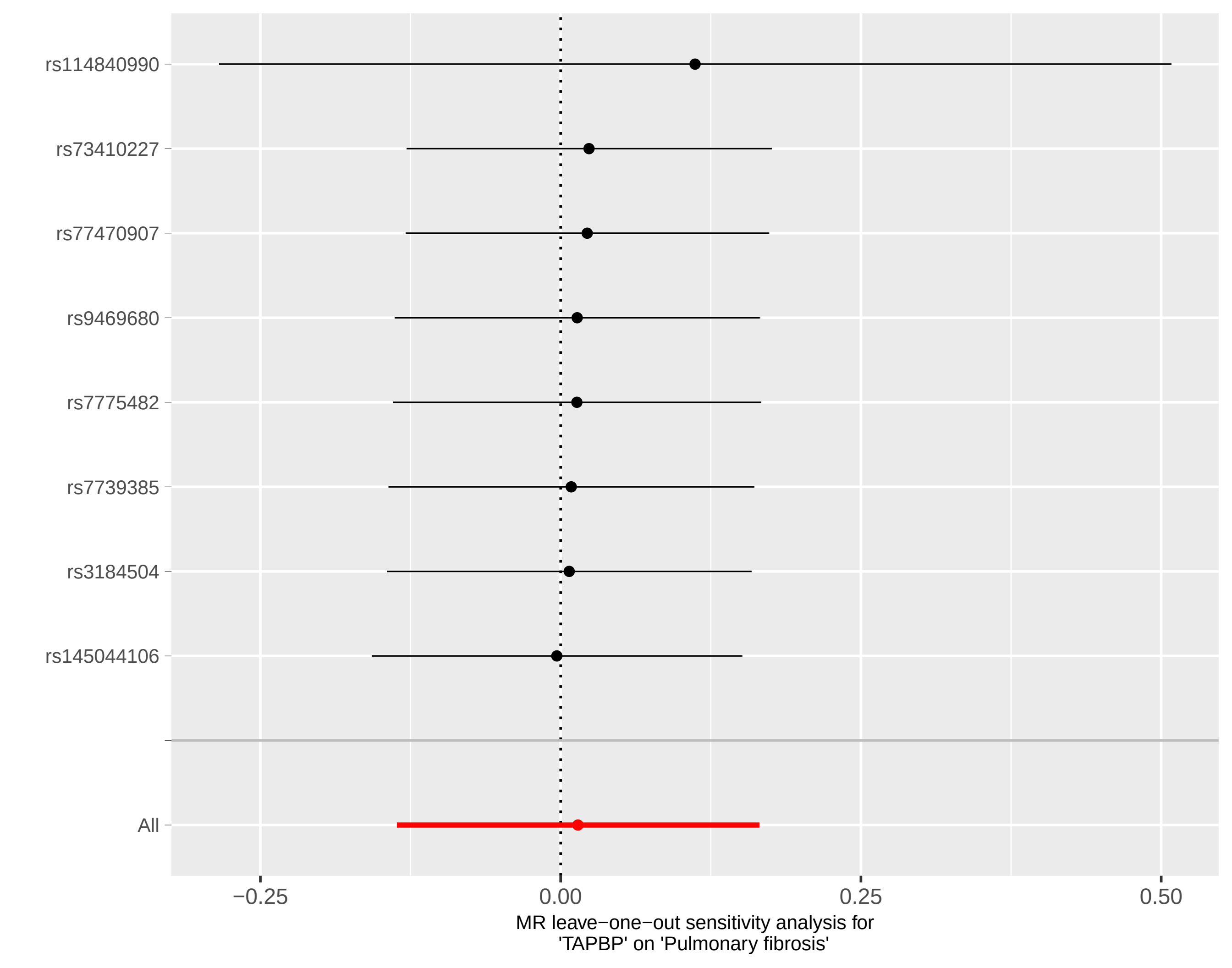
rs114840990
rs73410227
rs77470907
rs9469680
rs7775482
rs7739385
rs3184504
rs145044106
All
−0.25
0.00
0.25
0.50
MR leave−one−out sensitivity analysis for
'TAPBP' on 'Pulmonary fibrosis'

Insufficient number of SNPs

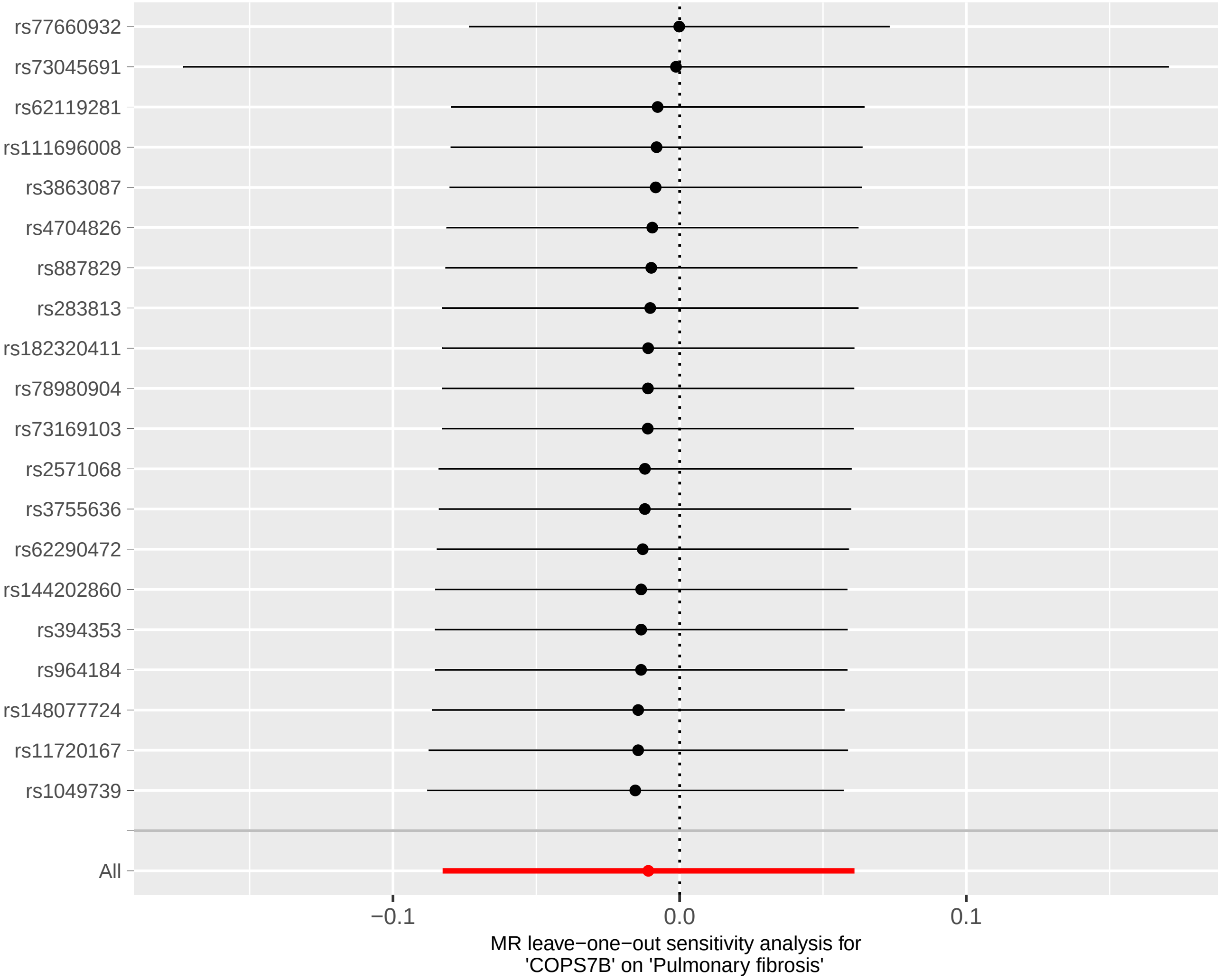
rs77660932
rs73045691
rs62119281
rs111696008
rs3863087
rs4704826
rs887829
rs283813
rs182320411
rs78980904
rs73169103
rs2571068
rs3755636
rs62290472
rs144202860
rs394353
rs964184
rs148077724
rs11720167
rs1049739
All
−0.1
0.0
0.1
MR leave−one−out sensitivity analysis for
'COPS7B' on 'Pulmonary fibrosis'

Insufficient number of SNPs

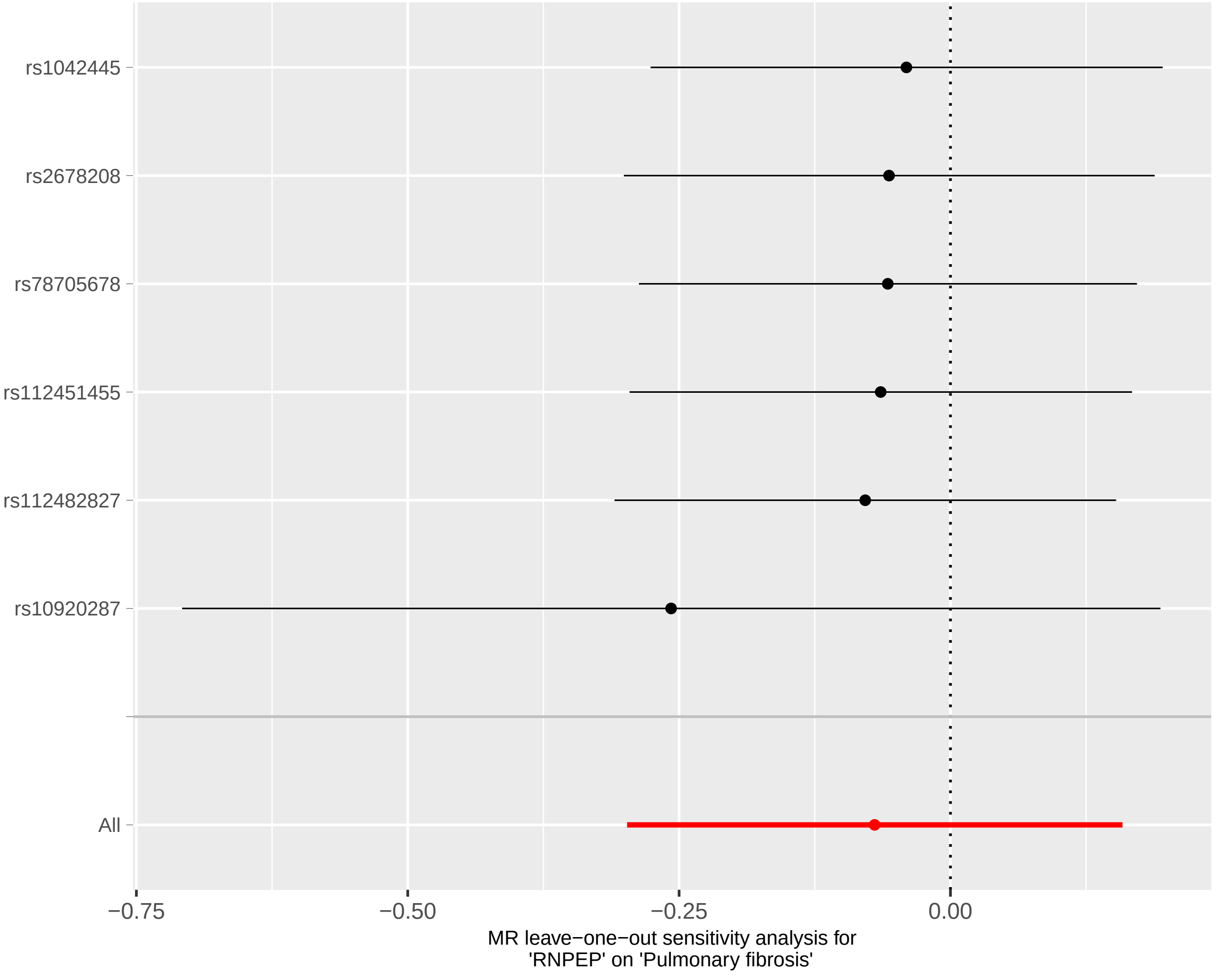
rs1042445
rs2678208
rs78705678
rs112451455
rs112482827
rs10920287
All
−0.75
−0.50
−0.25
0.00
MR leave−one−out sensitivity analysis for
'RNPEP' on 'Pulmonary fibrosis'

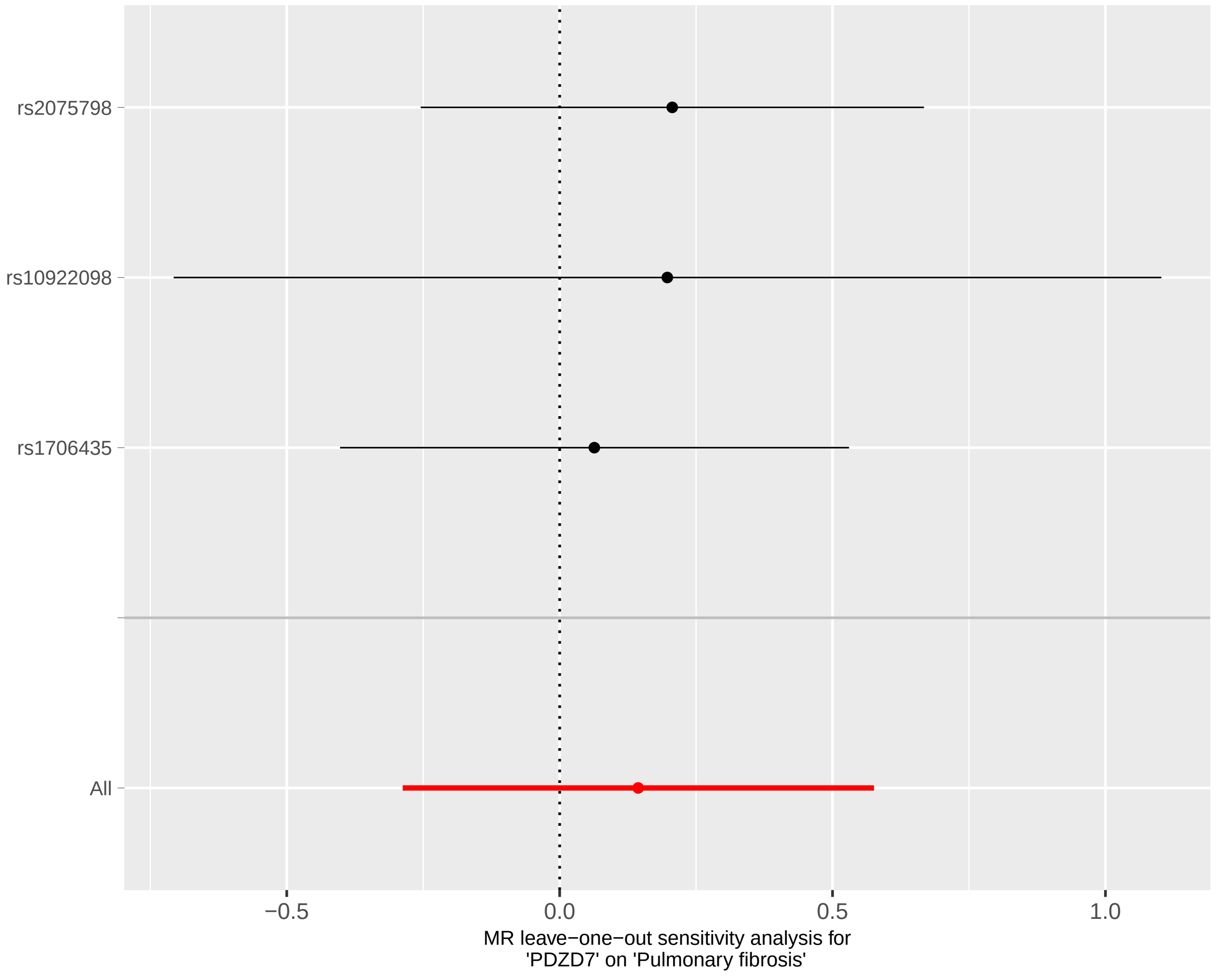
rs2075798
rs10922098
rs1706435
All
−0.5
0.0
0.5
1.0
MR leave−one−out sensitivity analysis for
'PDZD7' on 'Pulmonary fibrosis'

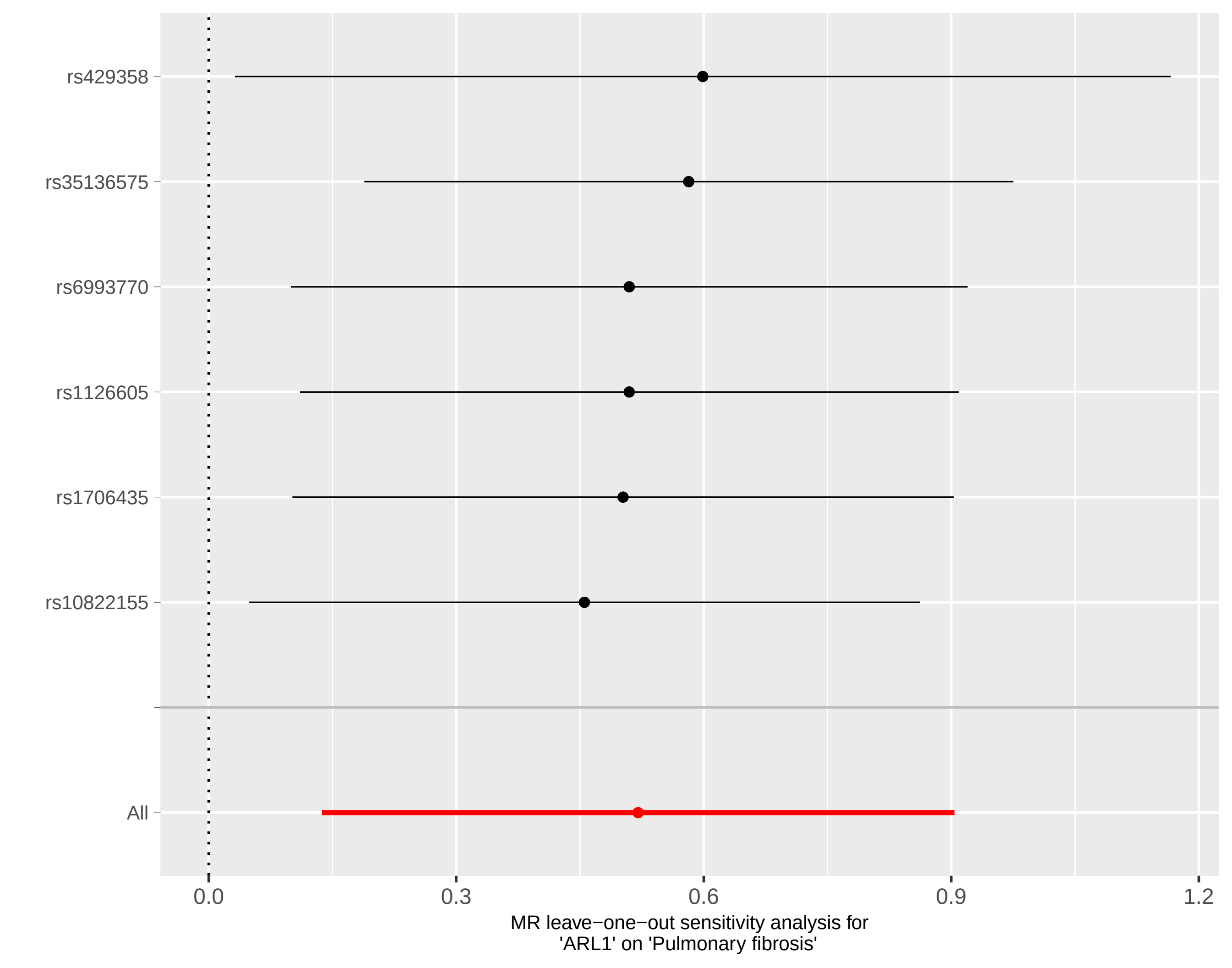

rs429358
rs35136575
rs6993770
rs1126605
rs1706435
rs10822155
All
0.0
0.3
0.6
0.9
1.2
MR leave−one−out sensitivity analysis for
'ARL1' on 'Pulmonary fibrosis'

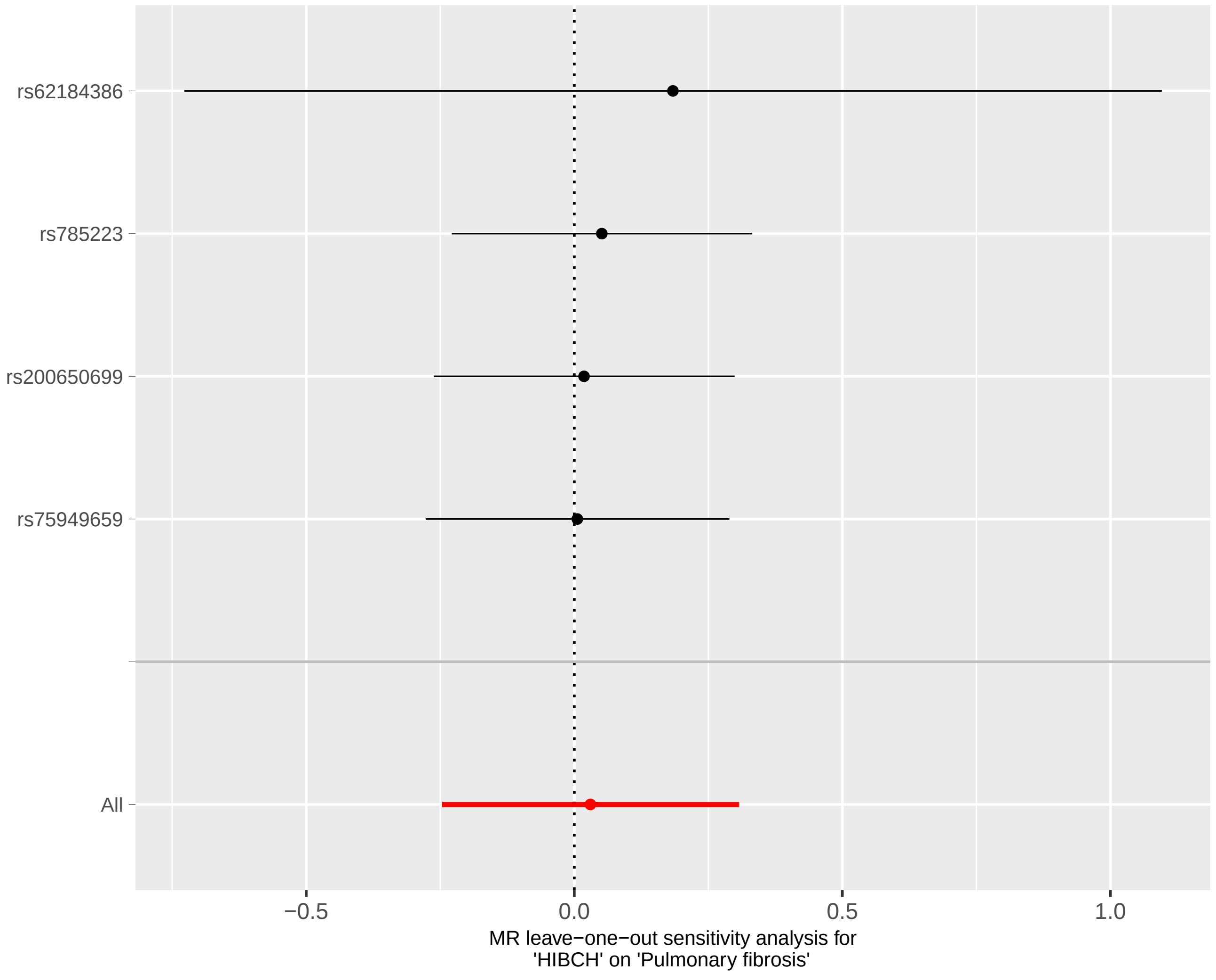
rs62184386
rs785223
rs200650699
rs75949659
All
−0.5
0.0
0.5
1.0
MR leave−one−out sensitivity analysis for
'HIBCH' on 'Pulmonary fibrosis'

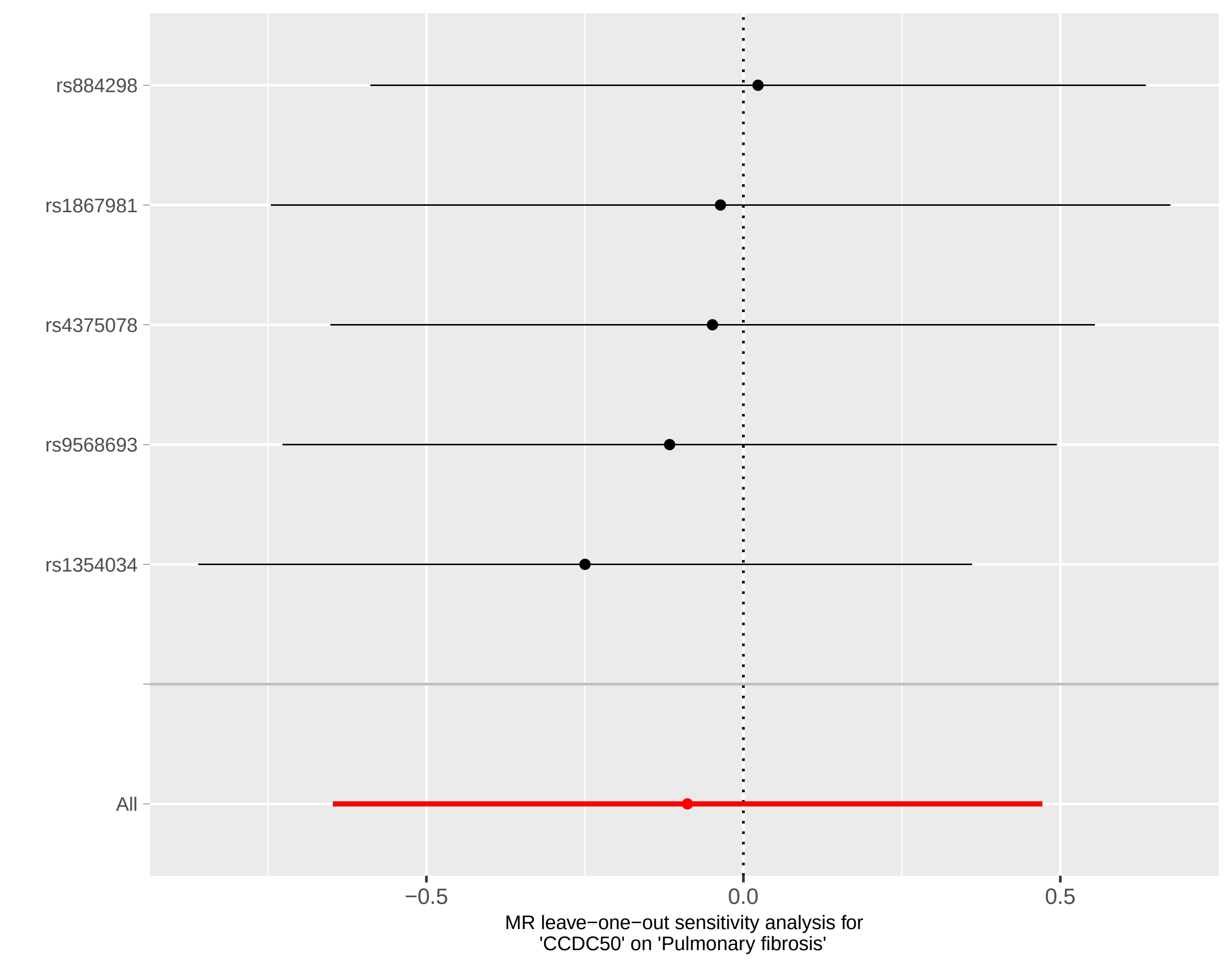
rs884298
rs1867981
rs4375078
rs9568693
rs1354034
All
−0.5
0.0
0.5
MR leave−one−out sensitivity analysis for
'CCDC50' on 'Pulmonary fibrosis'

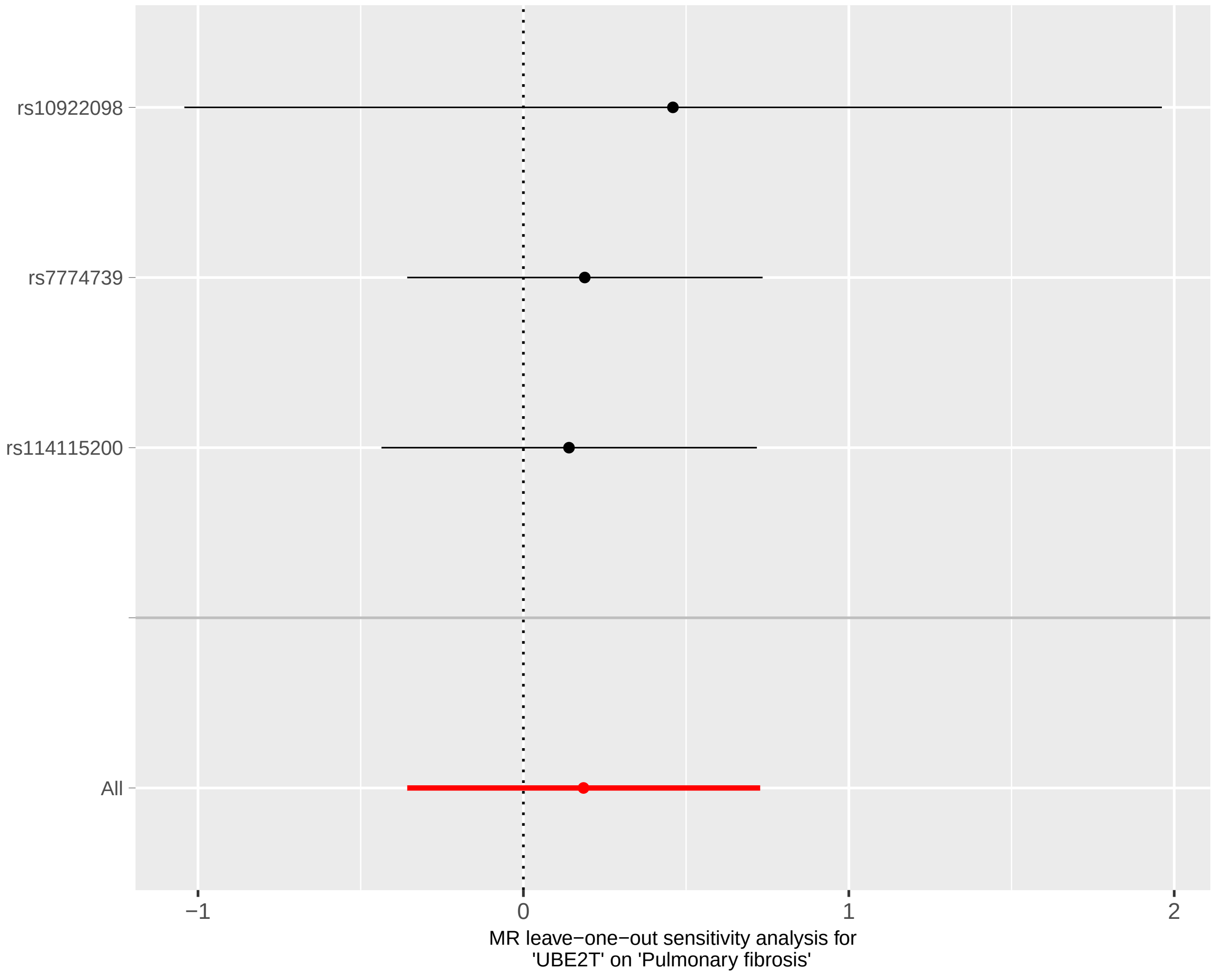
rs10922098
rs7774739
rs114115200
All
−1
0
1
2
MR leave−one−out sensitivity analysis for
'UBE2T' on 'Pulmonary fibrosis'

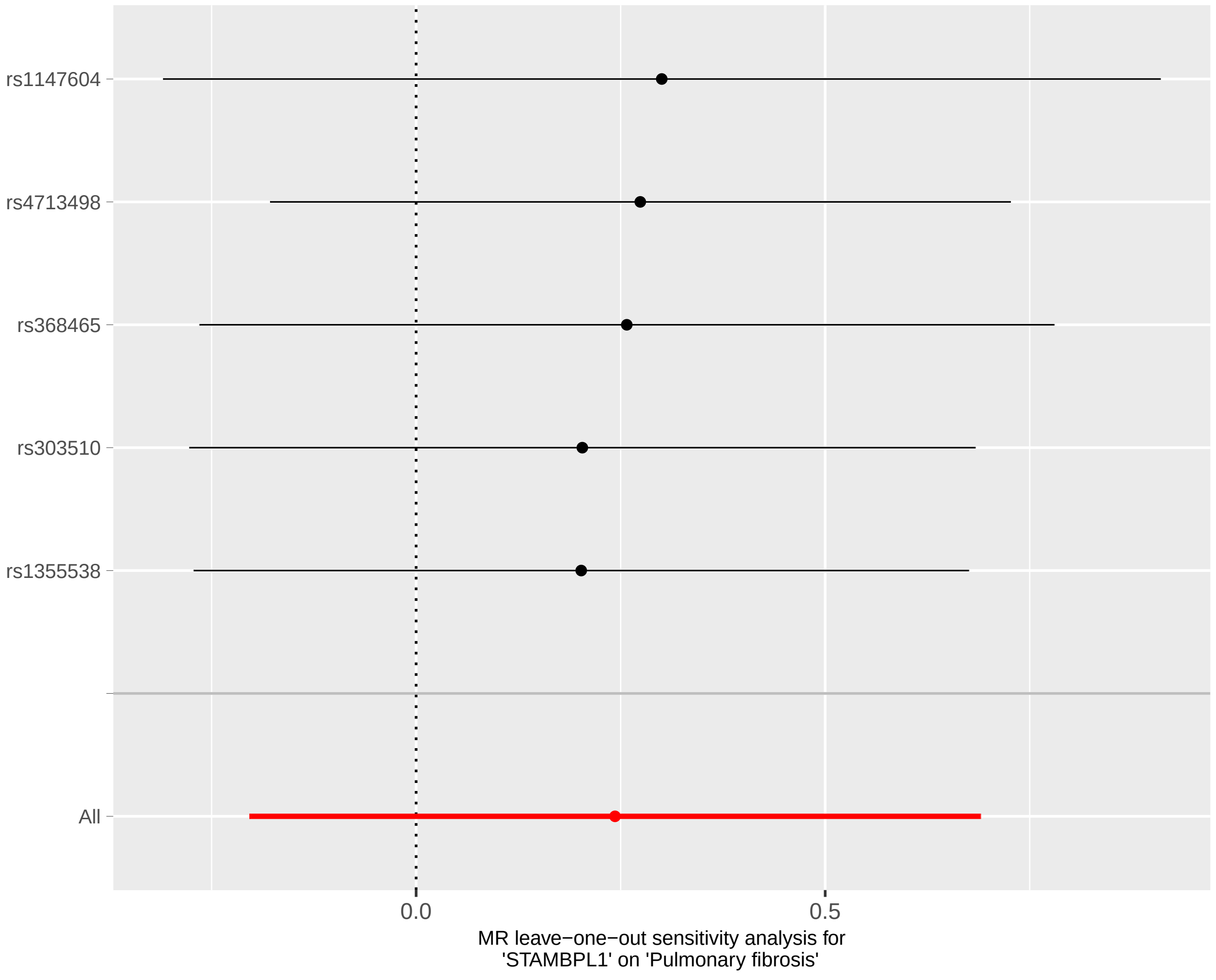

rs1147604
rs4713498
rs368465
rs303510
rs1355538
All
0.0
0.5
MR leave−one−out sensitivity analysis for
'STAMBPL1' on 'Pulmonary fibrosis'

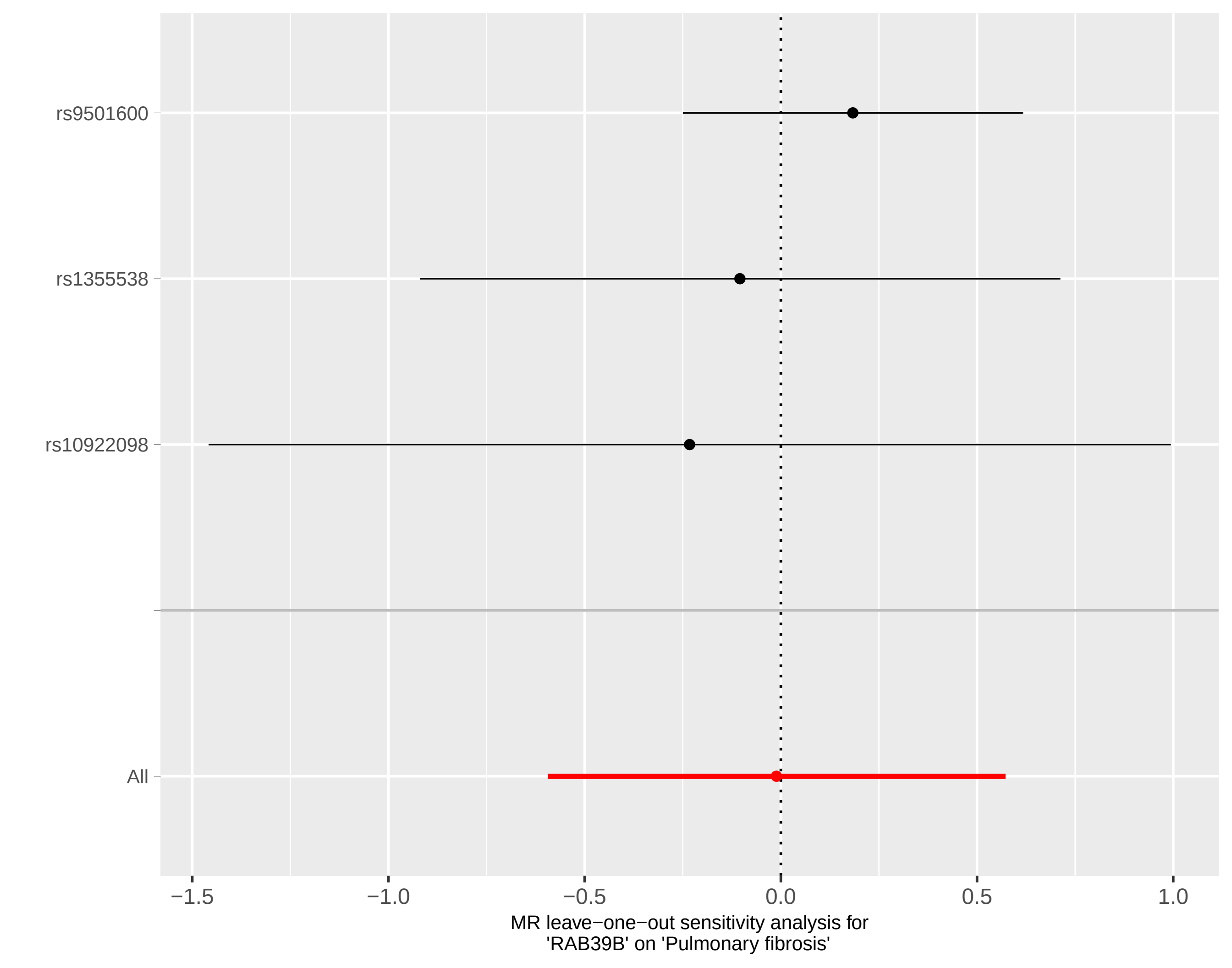
rs9501600
rs1355538
rs10922098
All
−1.5
−1.0
−0.5
0.0
0.5
1.0
MR leave−one−out sensitivity analysis for
'RAB39B' on 'Pulmonary fibrosis'

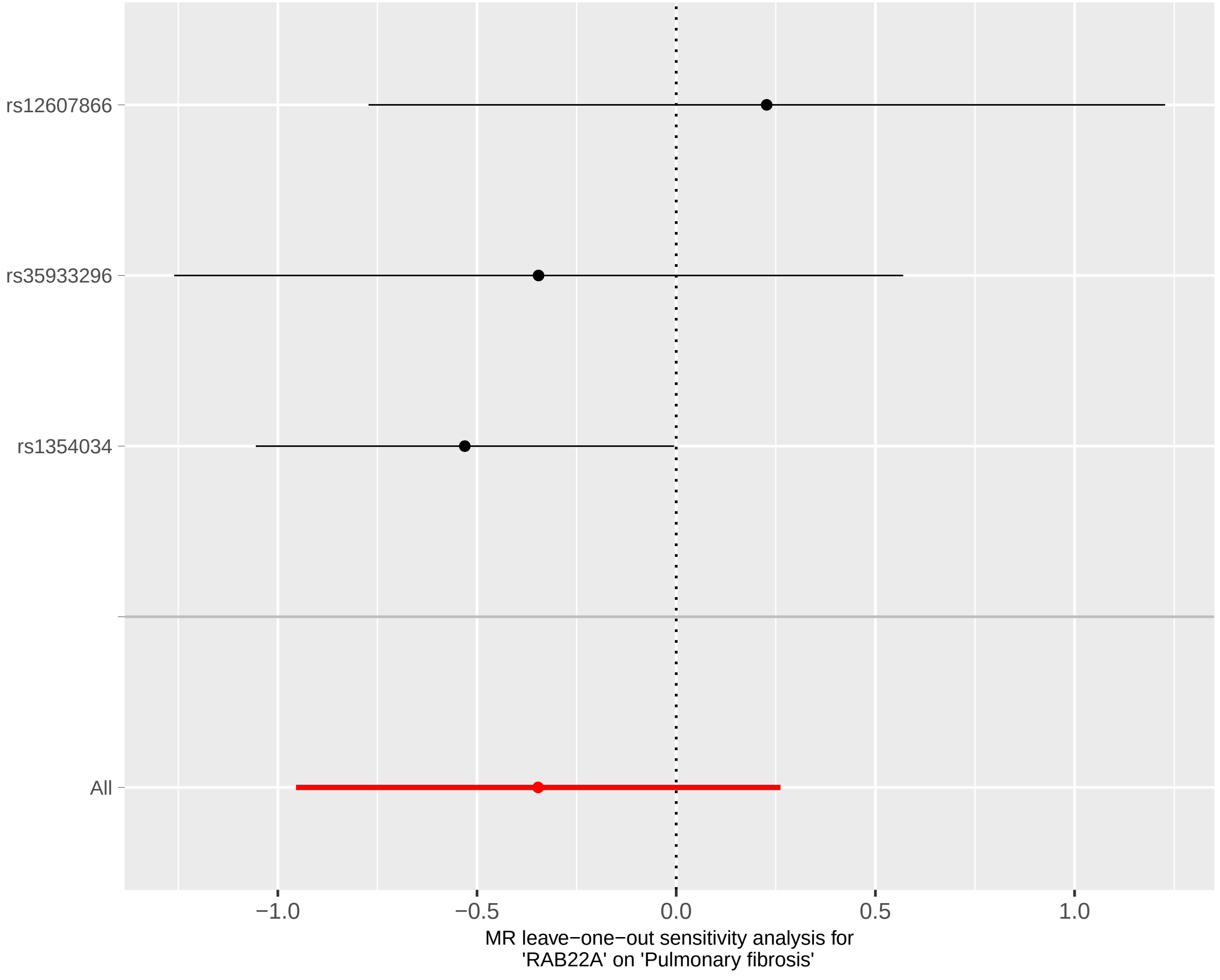

rs12607866
rs35933296
rs1354034
All
−1.0
−0.5
0.0
0.5
1.0
MR leave−one−out sensitivity analysis for
'RAB22A' on 'Pulmonary fibrosis'

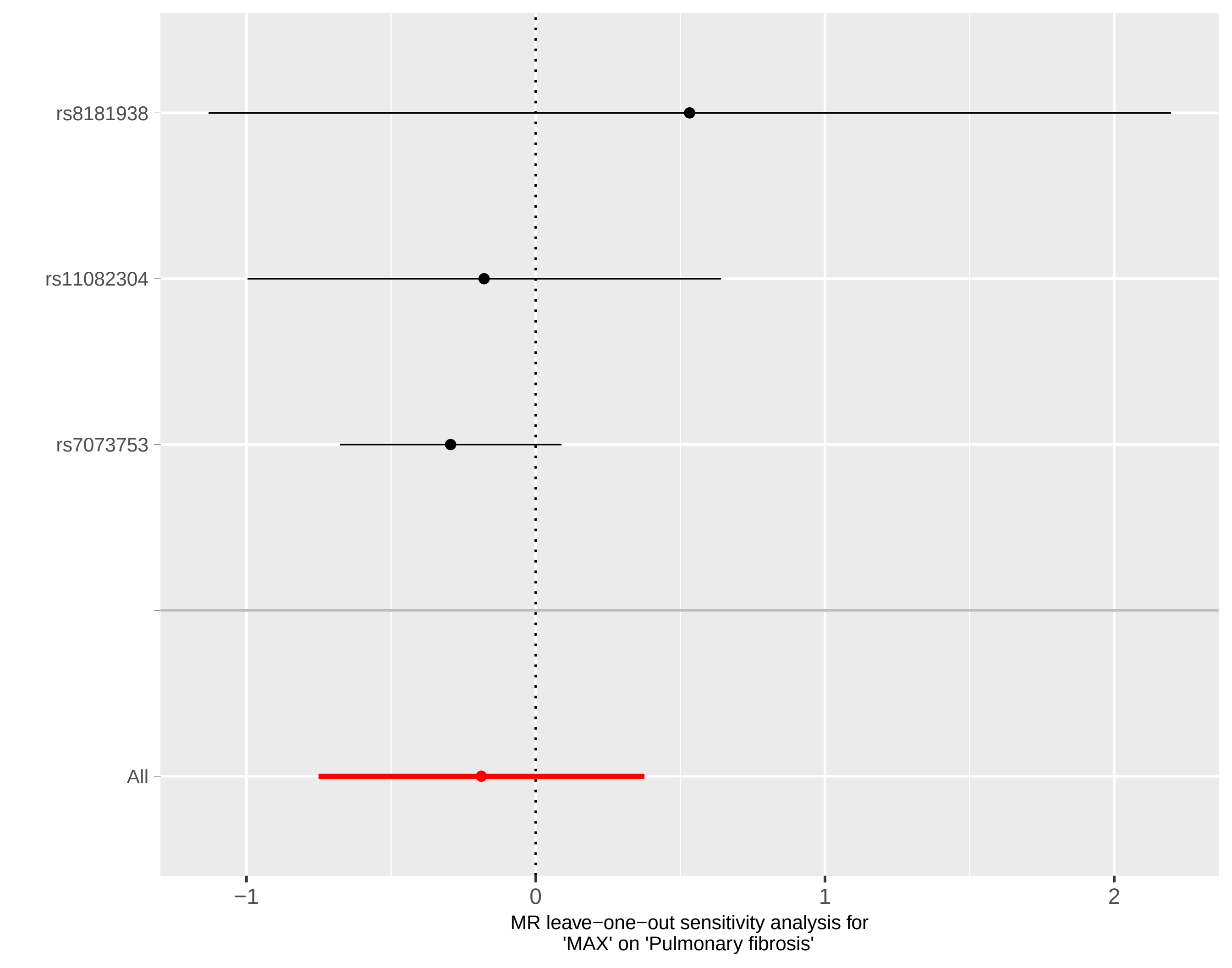
rs8181938
rs11082304
rs7073753
All
−1
0
1
2
MR leave−one−out sensitivity analysis for
'MAX' on 'Pulmonary fibrosis'

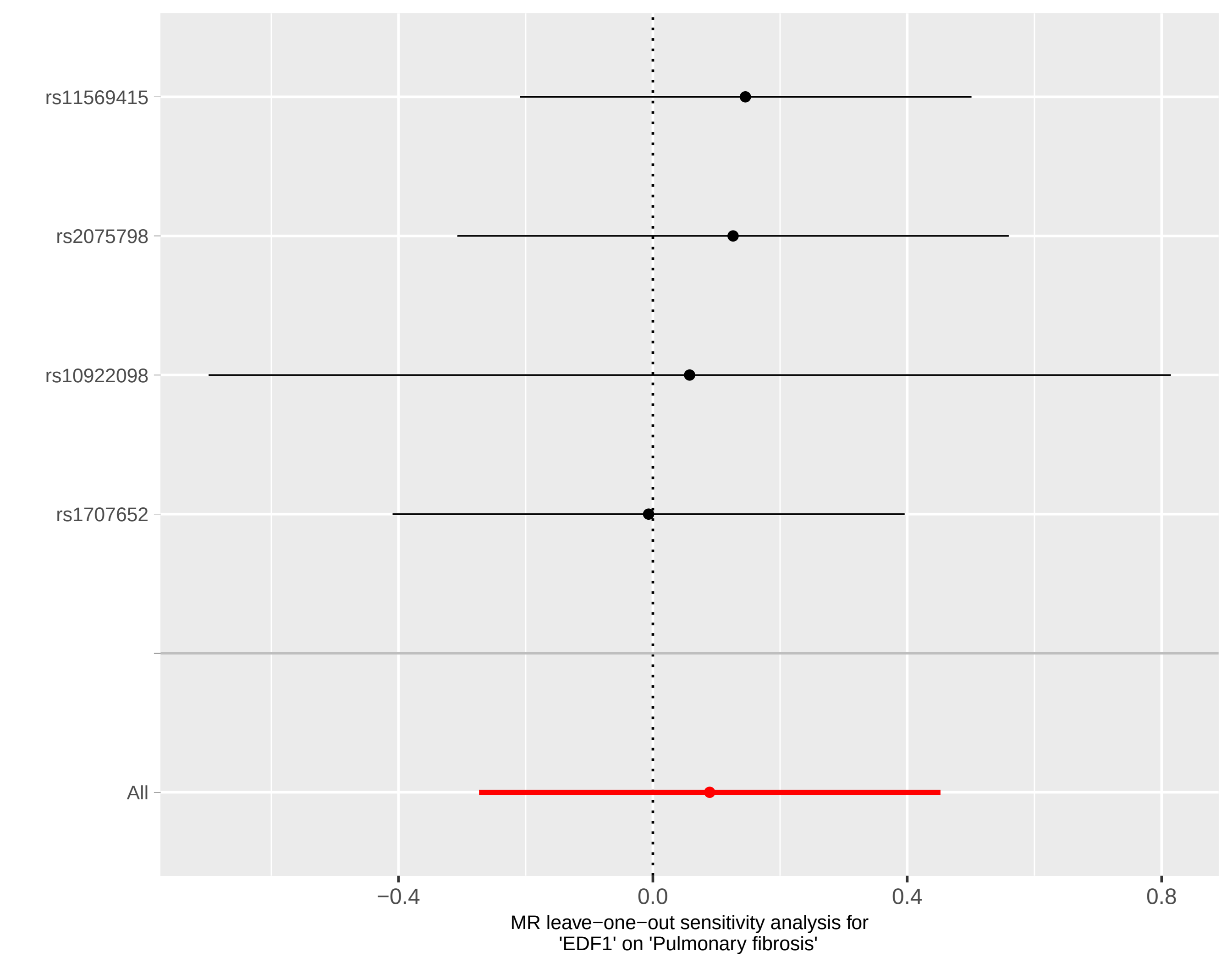
rs11569415
rs2075798
rs10922098
rs1707652
All
−0.4
0.0
0.4
0.8
MR leave−one−out sensitivity analysis for
'EDF1' on 'Pulmonary fibrosis'

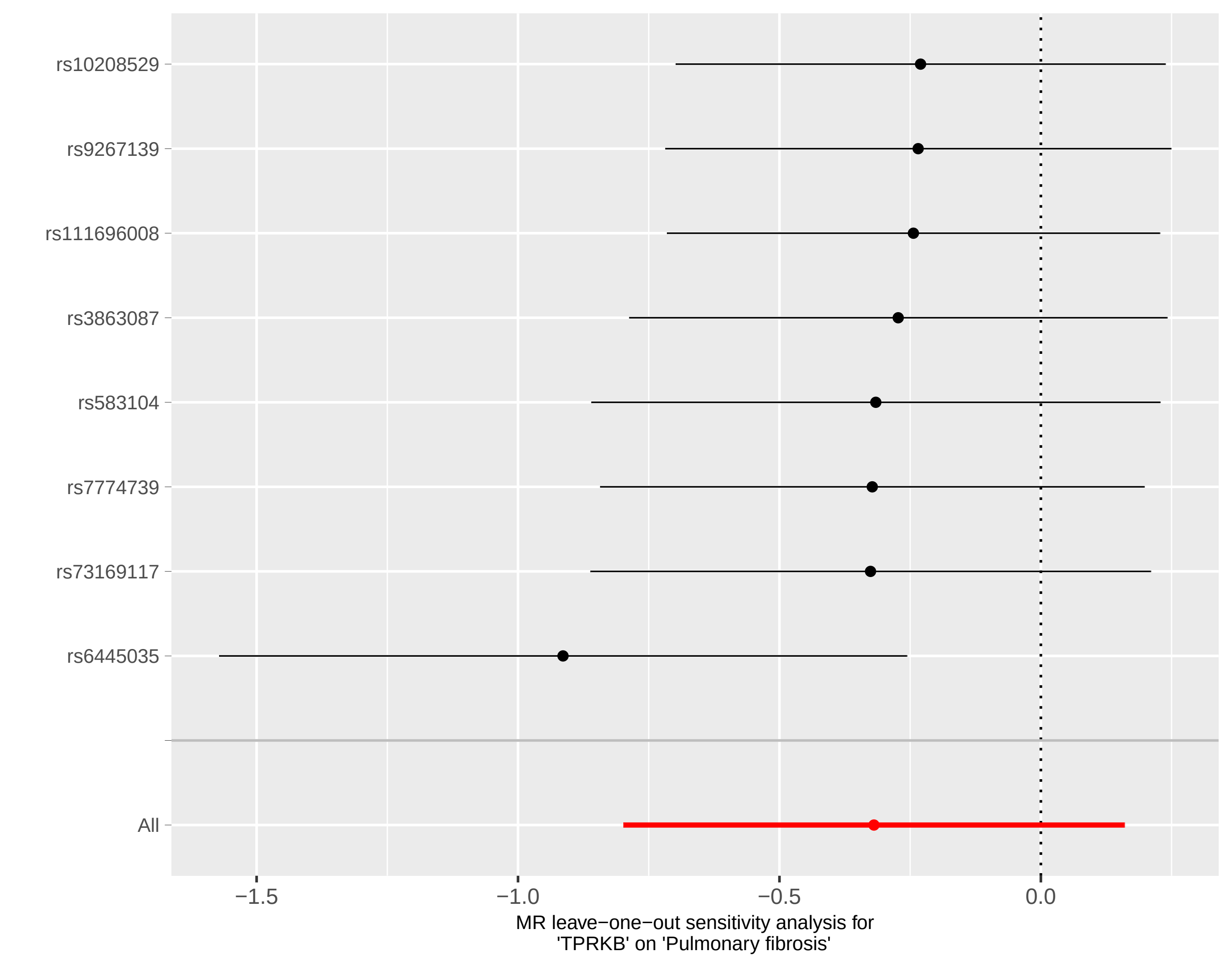
rs10208529
rs9267139
rs111696008
rs3863087
rs583104
rs7774739
rs73169117
rs6445035
All
−1.5
−1.0
−0.5
0.0
MR leave−one−out sensitivity analysis for
'TPRKB' on 'Pulmonary fibrosis'

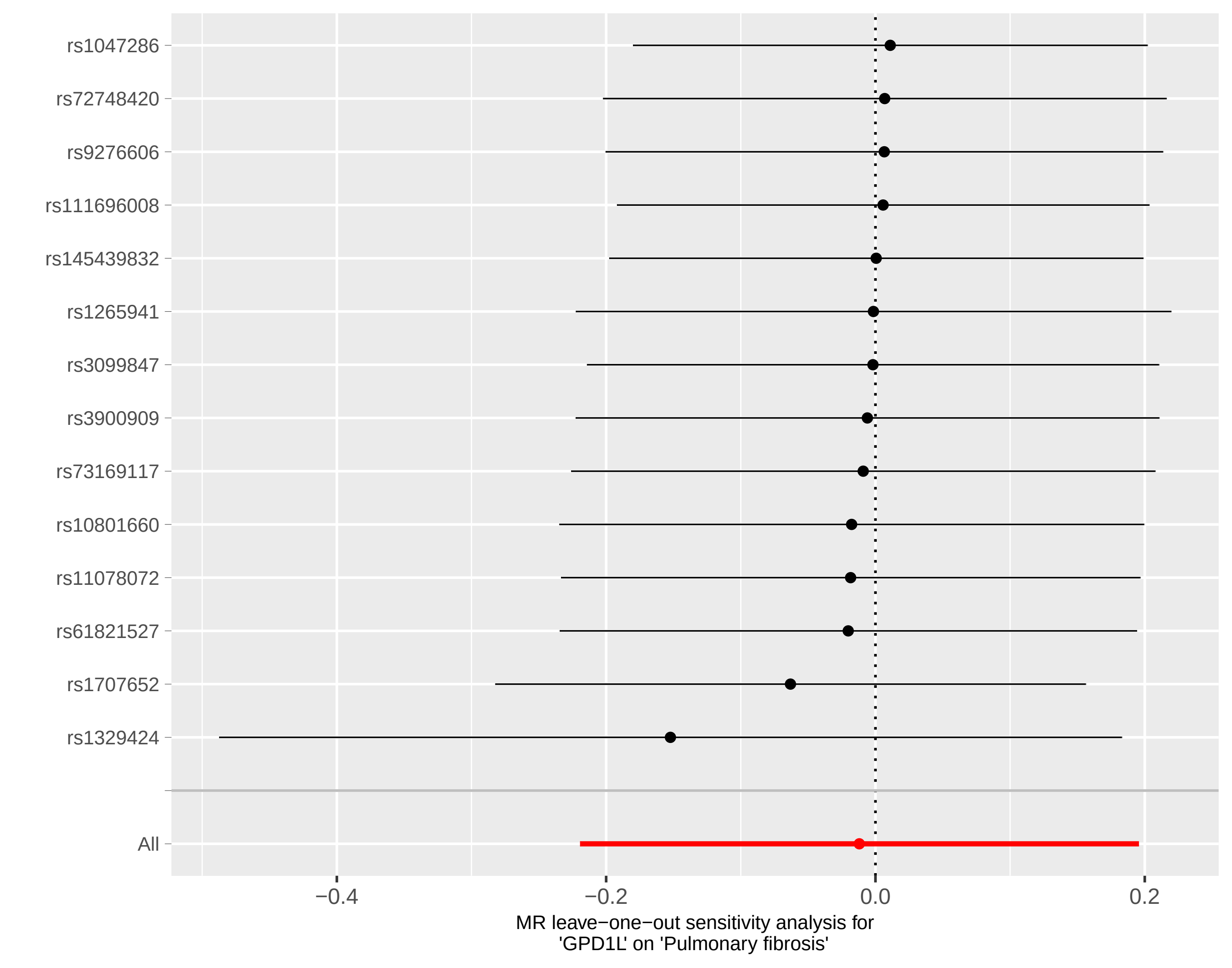
rs1047286
rs72748420
rs9276606
rs111696008
rs145439832
rs1265941
rs3099847
rs3900909
rs73169117
rs10801660
rs11078072
rs61821527
rs1707652
rs1329424
All
−0.4
−0.2
0.0
0.2
MR leave−one−out sensitivity analysis for
'GPD1L' on 'Pulmonary fibrosis'

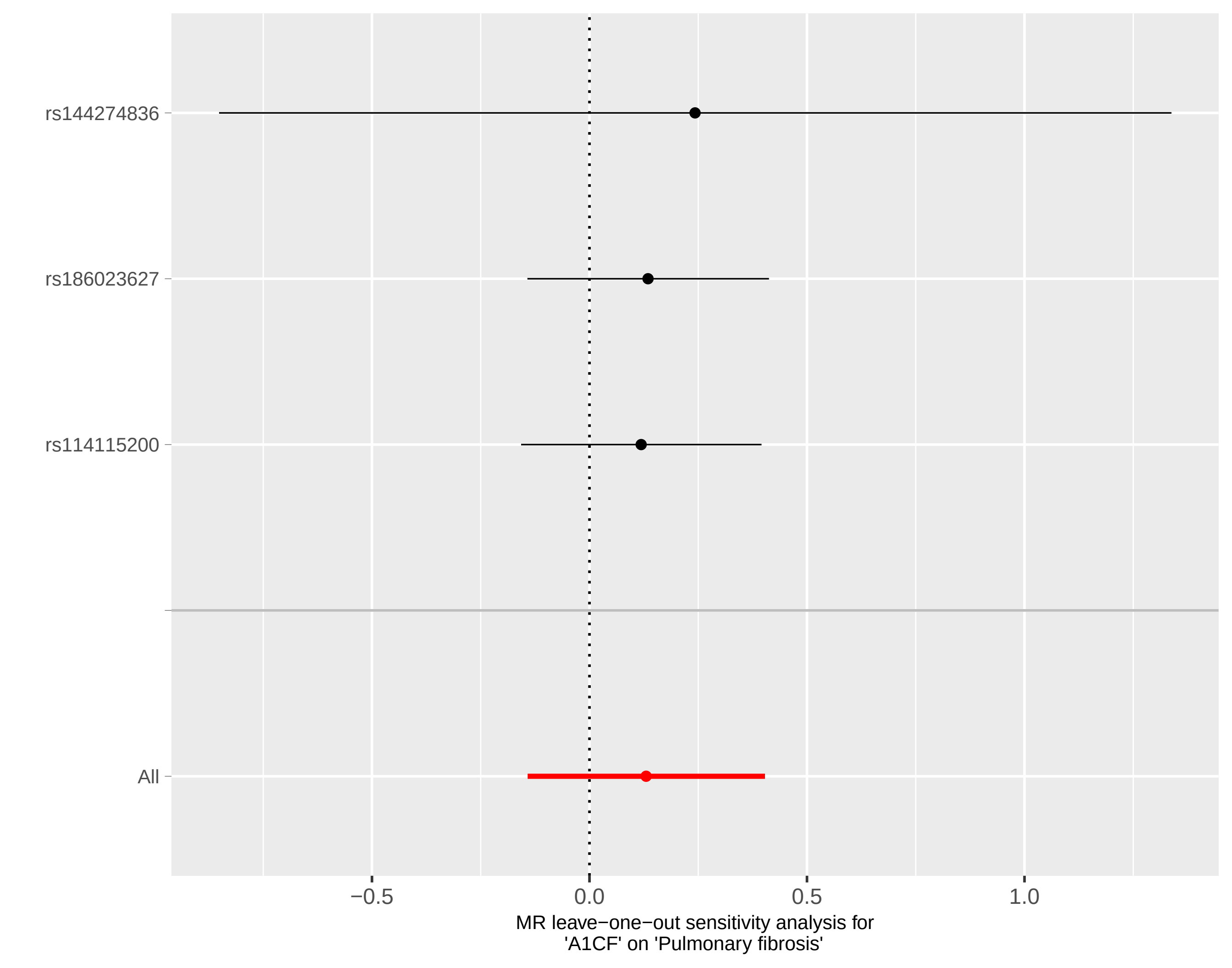
rs144274836
rs186023627
rs114115200
All
−0.5
0.0
0.5
1.0
MR leave−one−out sensitivity analysis for
'A1CF' on 'Pulmonary fibrosis'

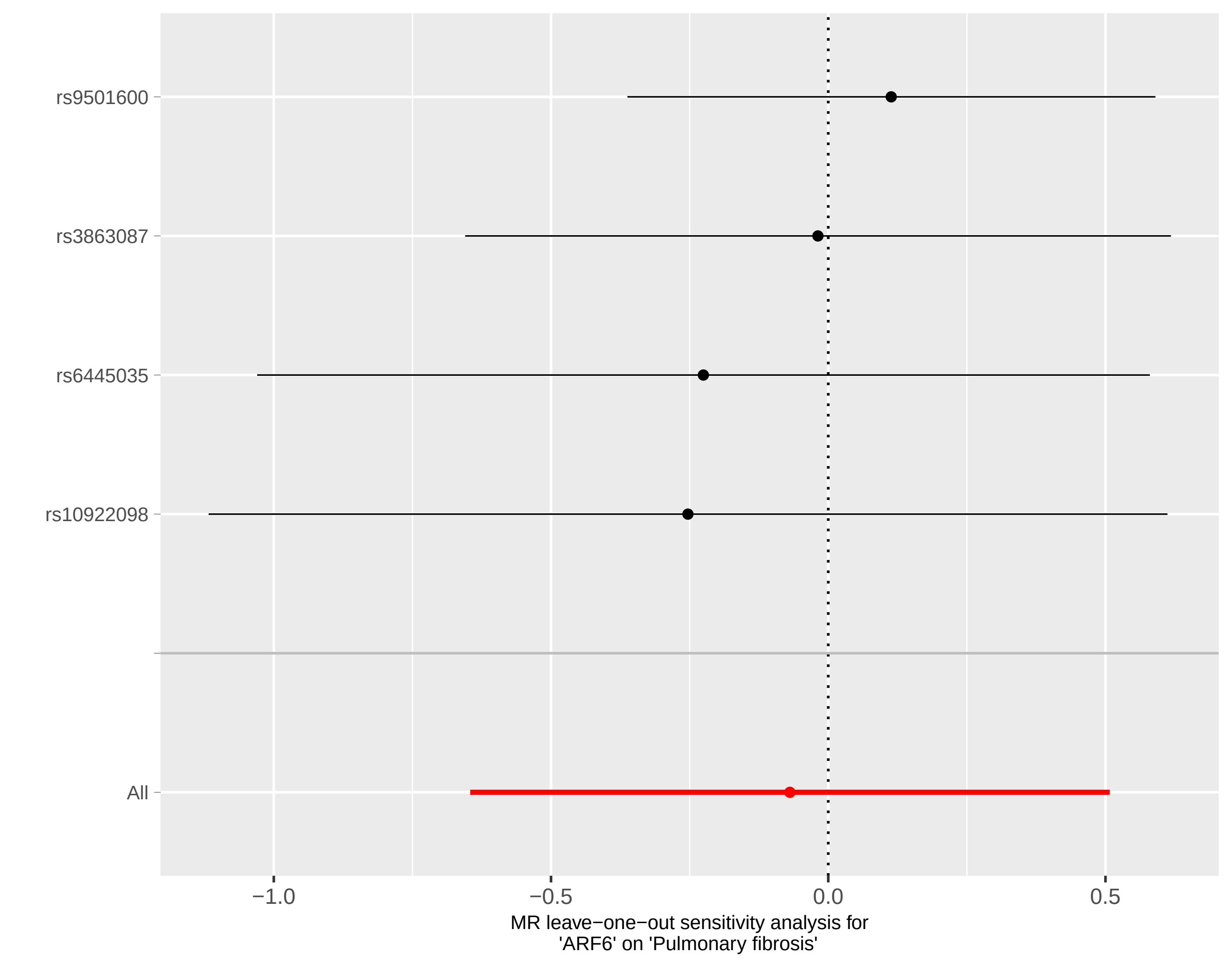

rs9501600
rs3863087
rs6445035
rs10922098
All
−1.0
−0.5
0.0
0.5
MR leave−one−out sensitivity analysis for
'ARF6' on 'Pulmonary fibrosis'

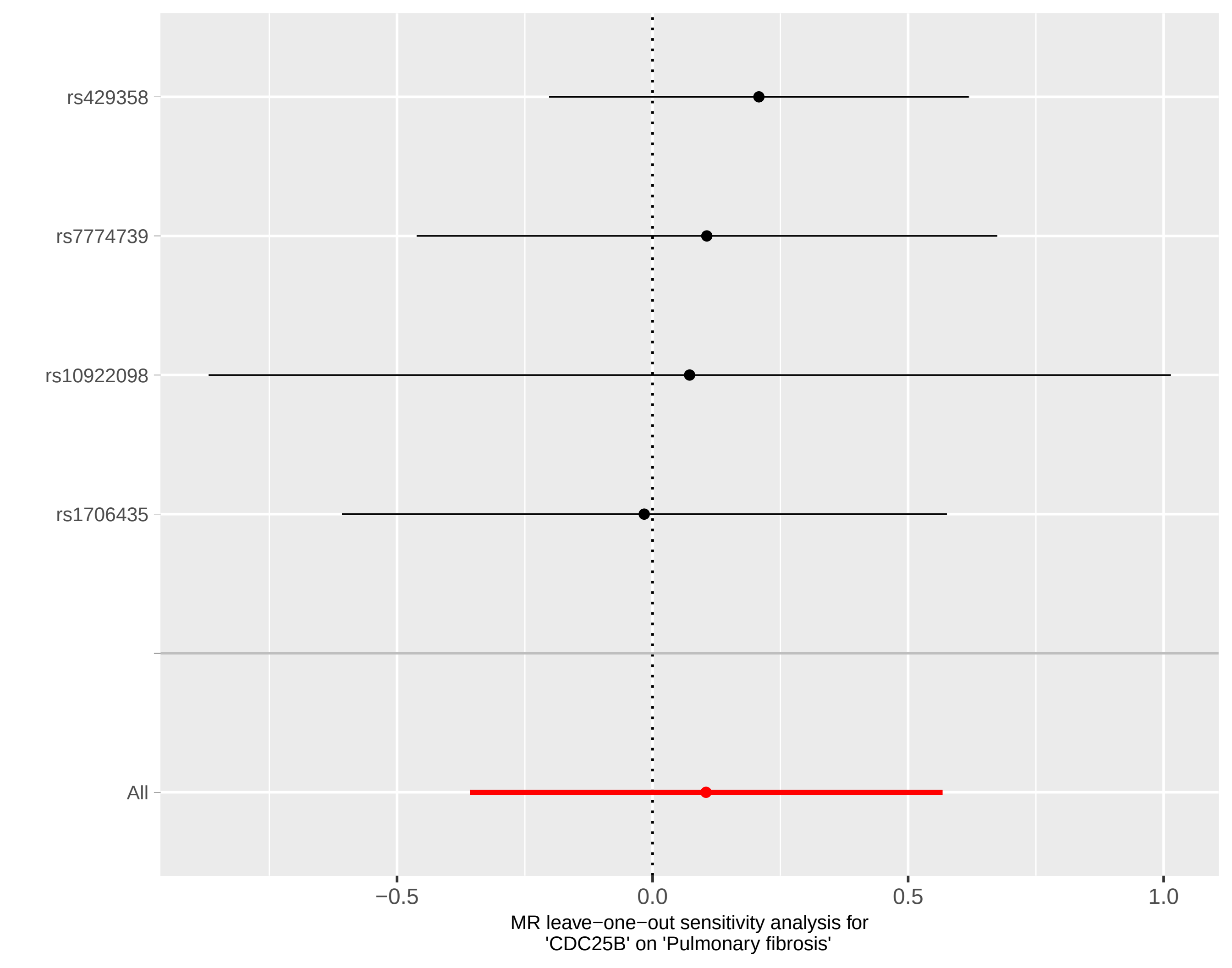
rs429358
rs7774739
rs10922098
rs1706435
All
−0.5
0.0
0.5
1.0
MR leave−one−out sensitivity analysis for
'CDC25B' on 'Pulmonary fibrosis'

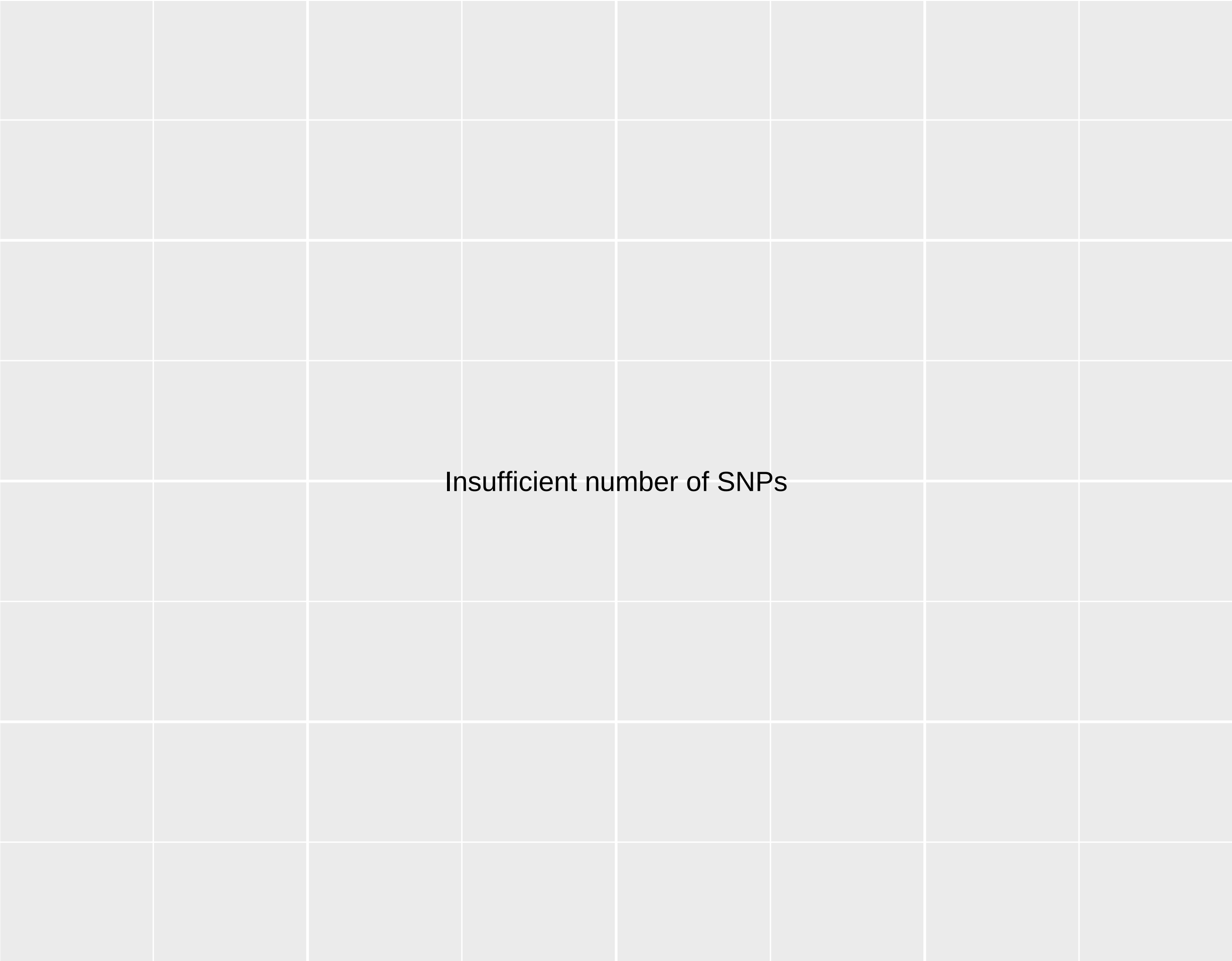
Insufficient number of SNPs

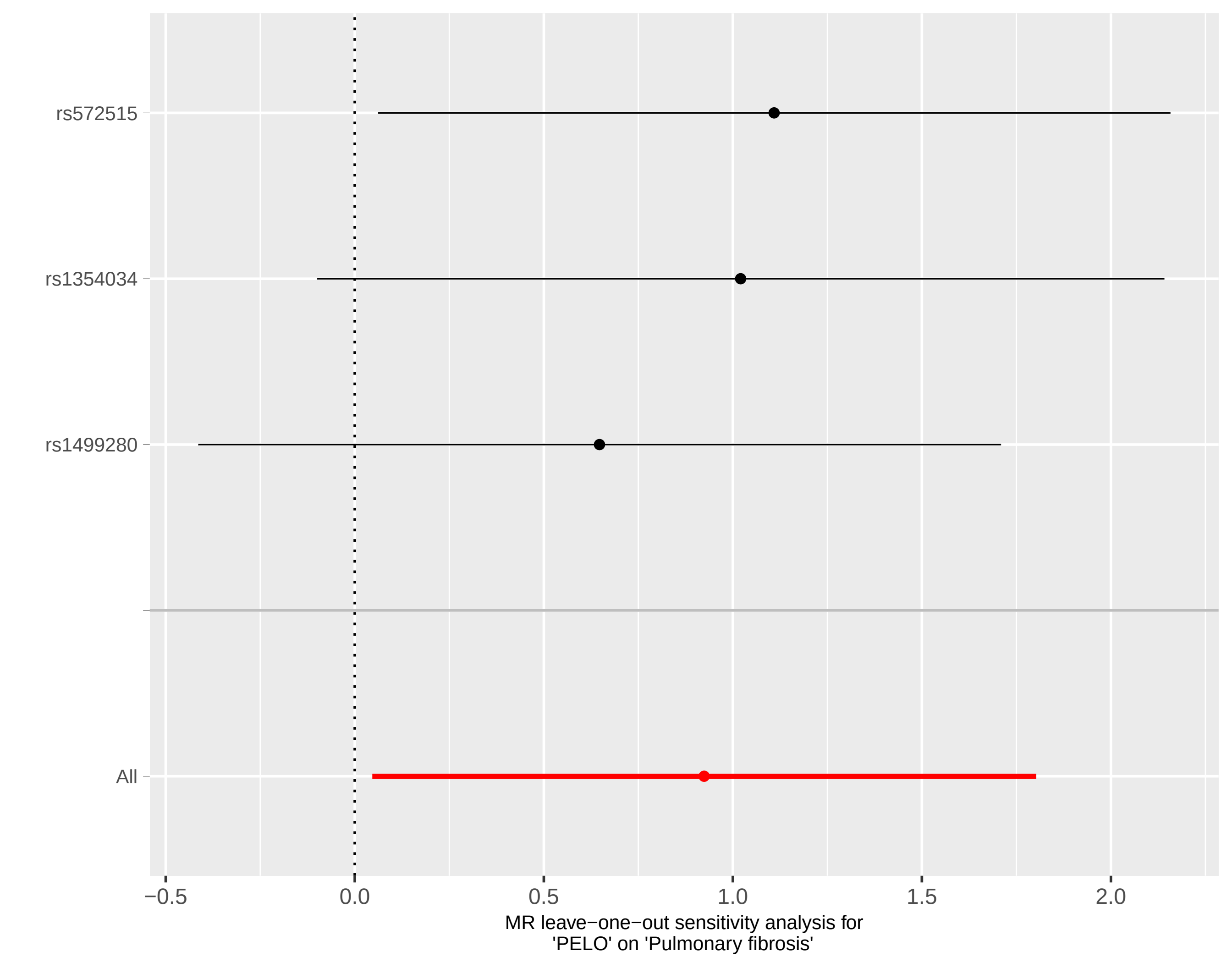

rs572515
rs1354034
rs1499280
All
−0.5
0.0
0.5
1.0
1.5
2.0
MR leave−one−out sensitivity analysis for
'PELO' on 'Pulmonary fibrosis'

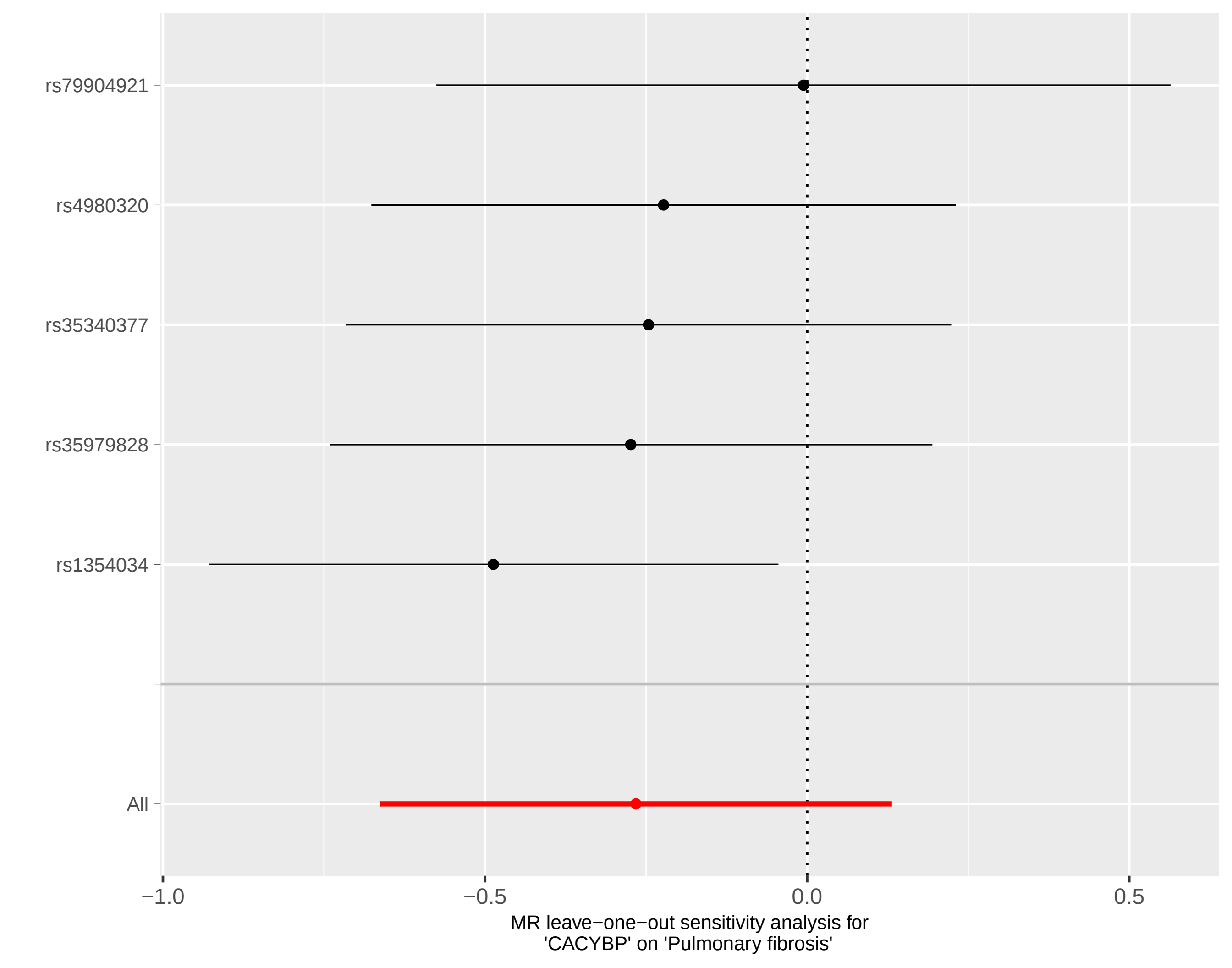
rs79904921
rs4980320
rs35340377
rs35979828
rs1354034
All
−1.0
−0.5
0.0
0.5
MR leave−one−out sensitivity analysis for
'CACYBP' on 'Pulmonary fibrosis'

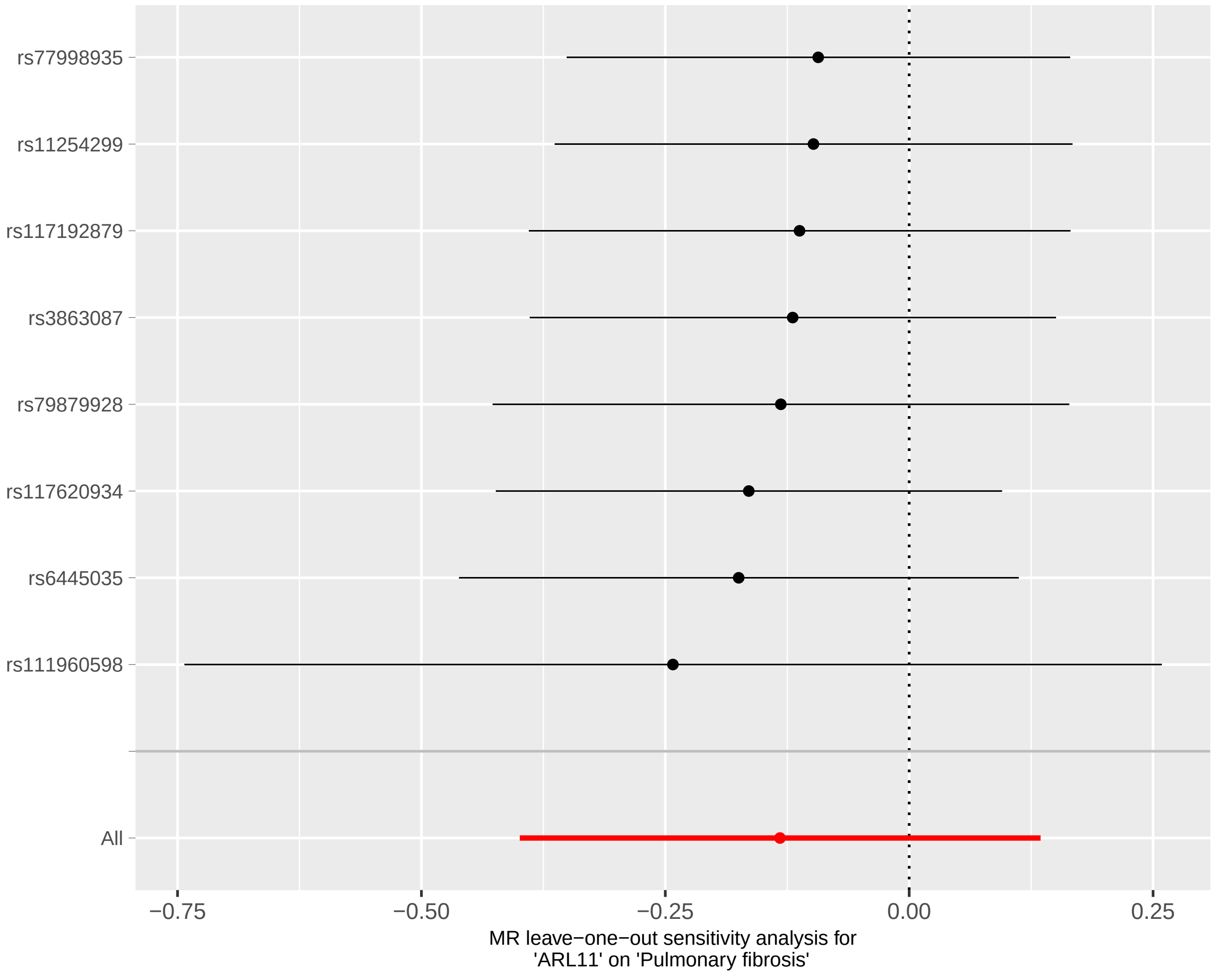

rs77998935
rs11254299
rs117192879
rs3863087
rs79879928
rs117620934
rs6445035
rs111960598
All
−0.75
−0.50
−0.25
0.00
0.25
MR leave−one−out sensitivity analysis for
'ARL11' on 'Pulmonary fibrosis'

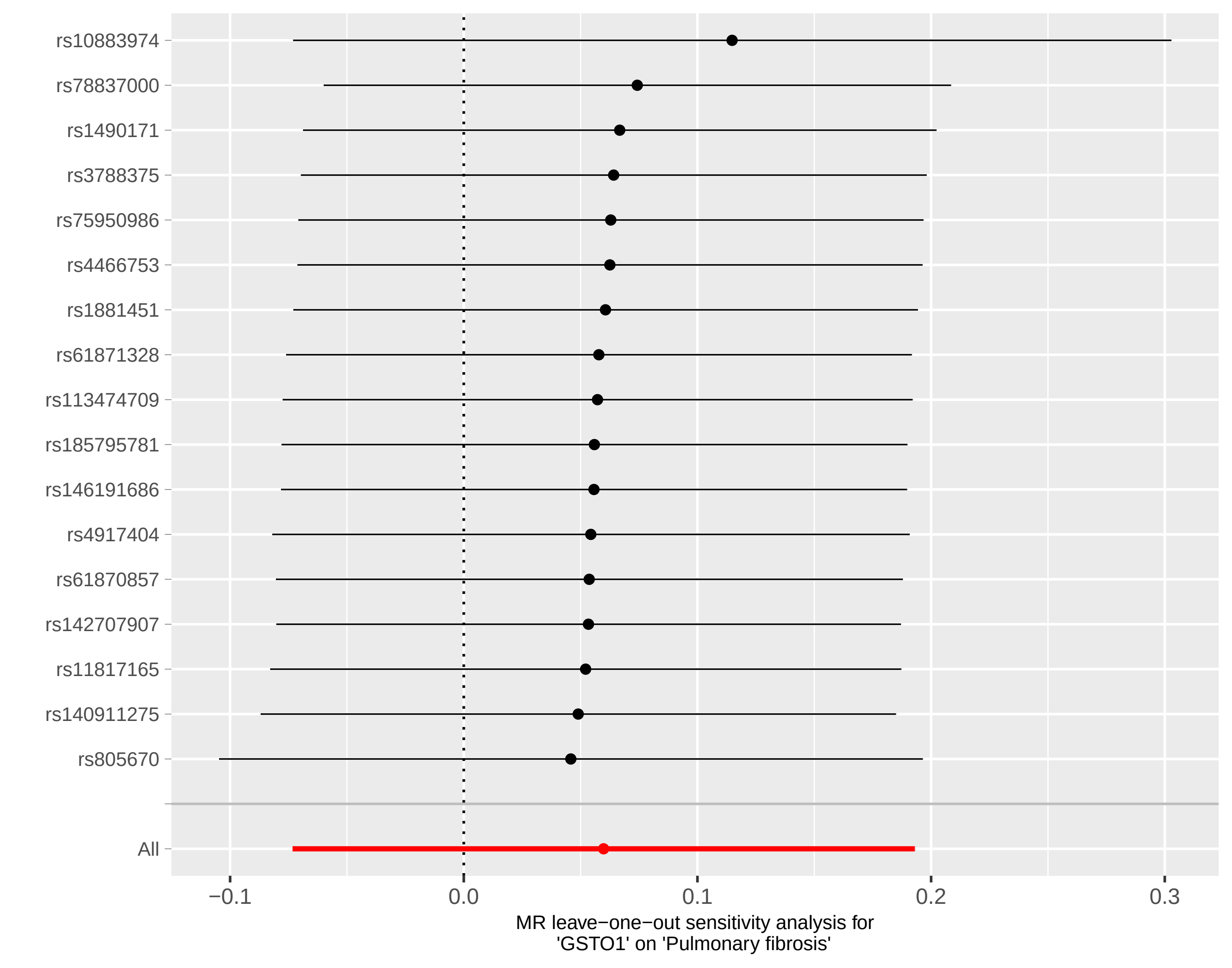
rs10883974
rs78837000
rs1490171
rs3788375
rs75950986
rs4466753
rs1881451
rs61871328
rs113474709
rs185795781
rs146191686
rs4917404
rs61870857
rs142707907
rs11817165
rs140911275
rs805670
All
−0.1
0.0
0.1
0.2
0.3
MR leave−one−out sensitivity analysis for
'GSTO1' on 'Pulmonary fibrosis'

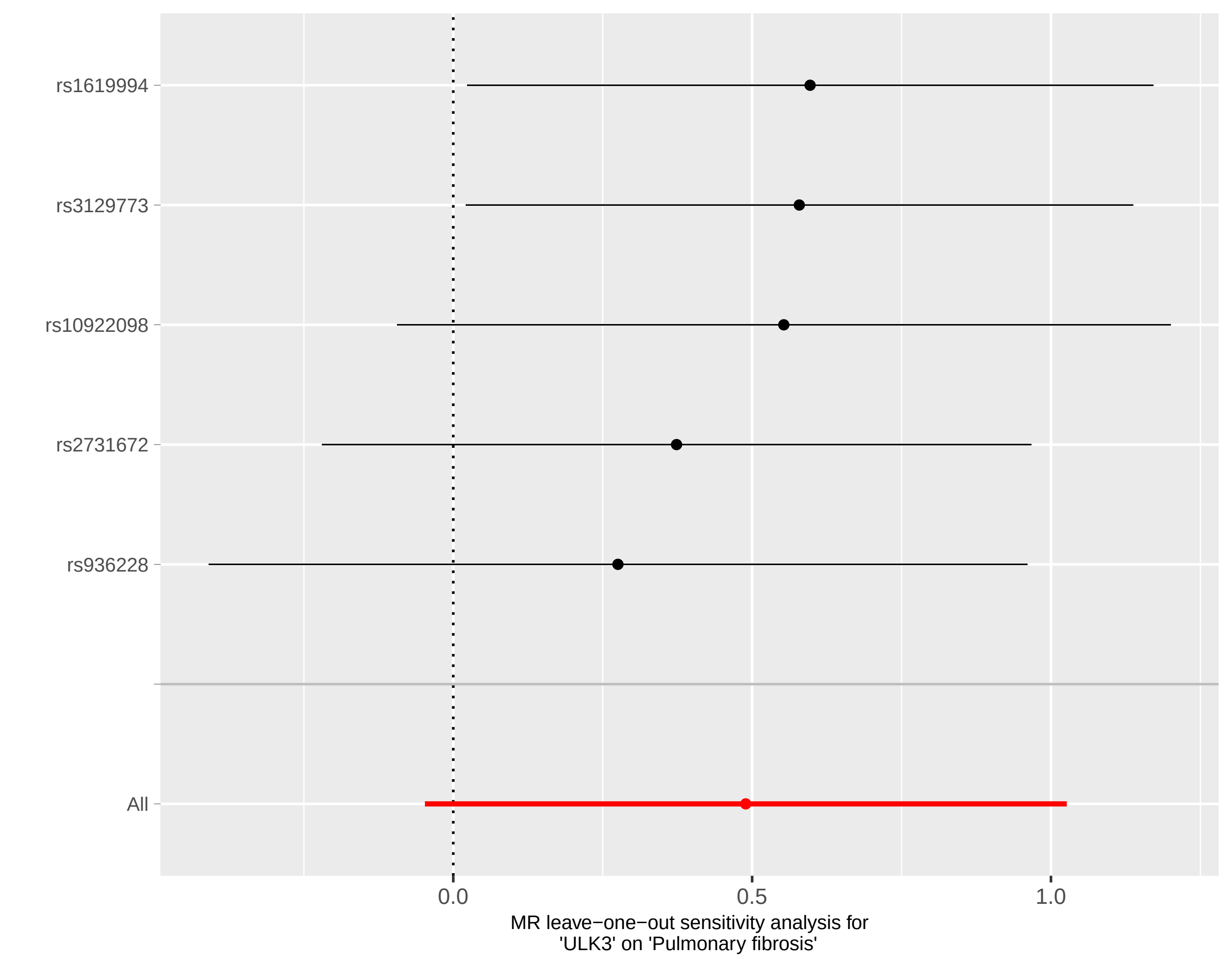
rs1619994
rs3129773
rs10922098
rs2731672
rs936228
All
0.0
0.5
1.0
MR leave−one−out sensitivity analysis for
'ULK3' on 'Pulmonary fibrosis'

Insufficient number of SNPs

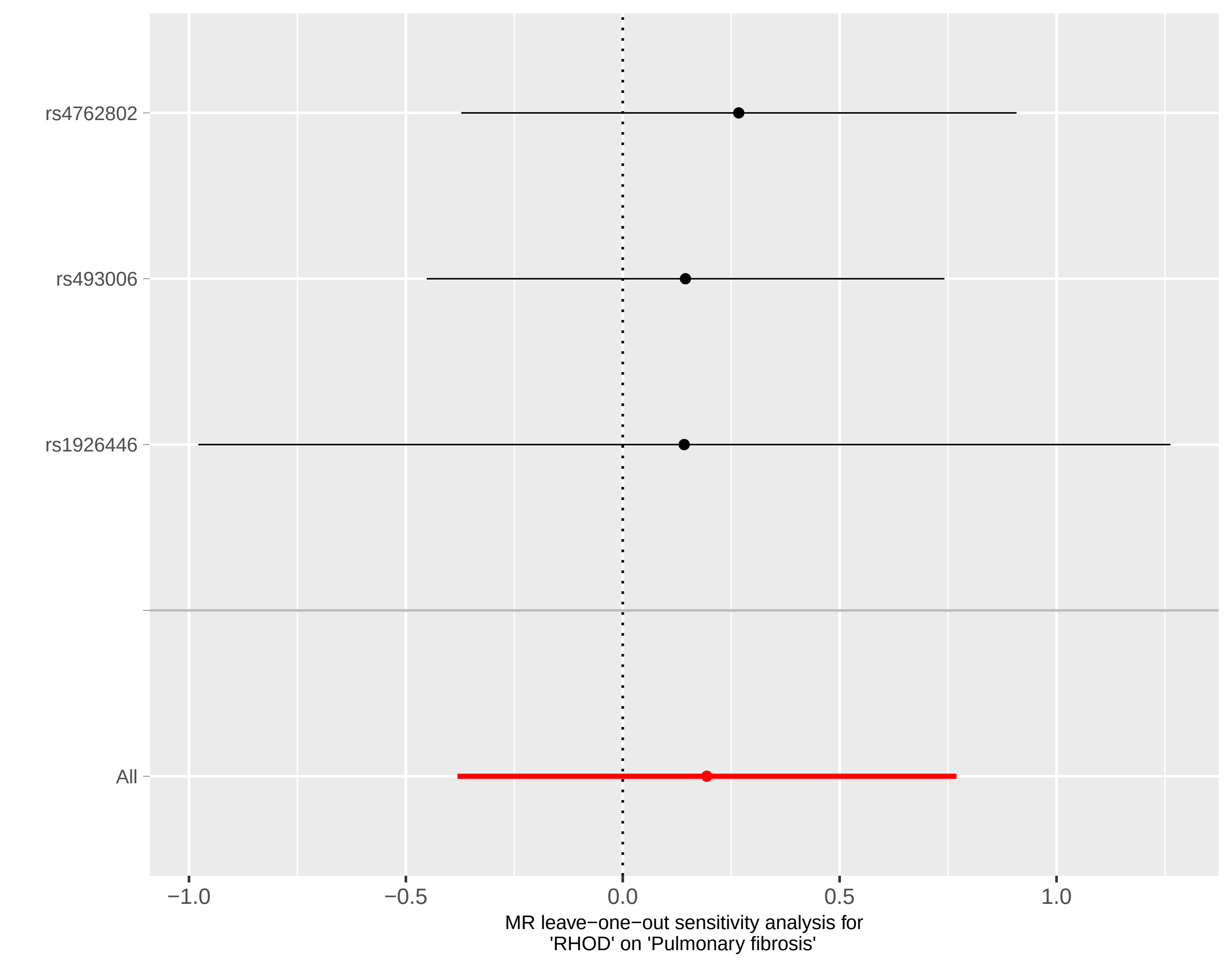
rs4762802
rs493006
rs1926446
All
−1.0
−0.5
0.0
0.5
1.0
MR leave−one−out sensitivity analysis for
'RHOD' on 'Pulmonary fibrosis'

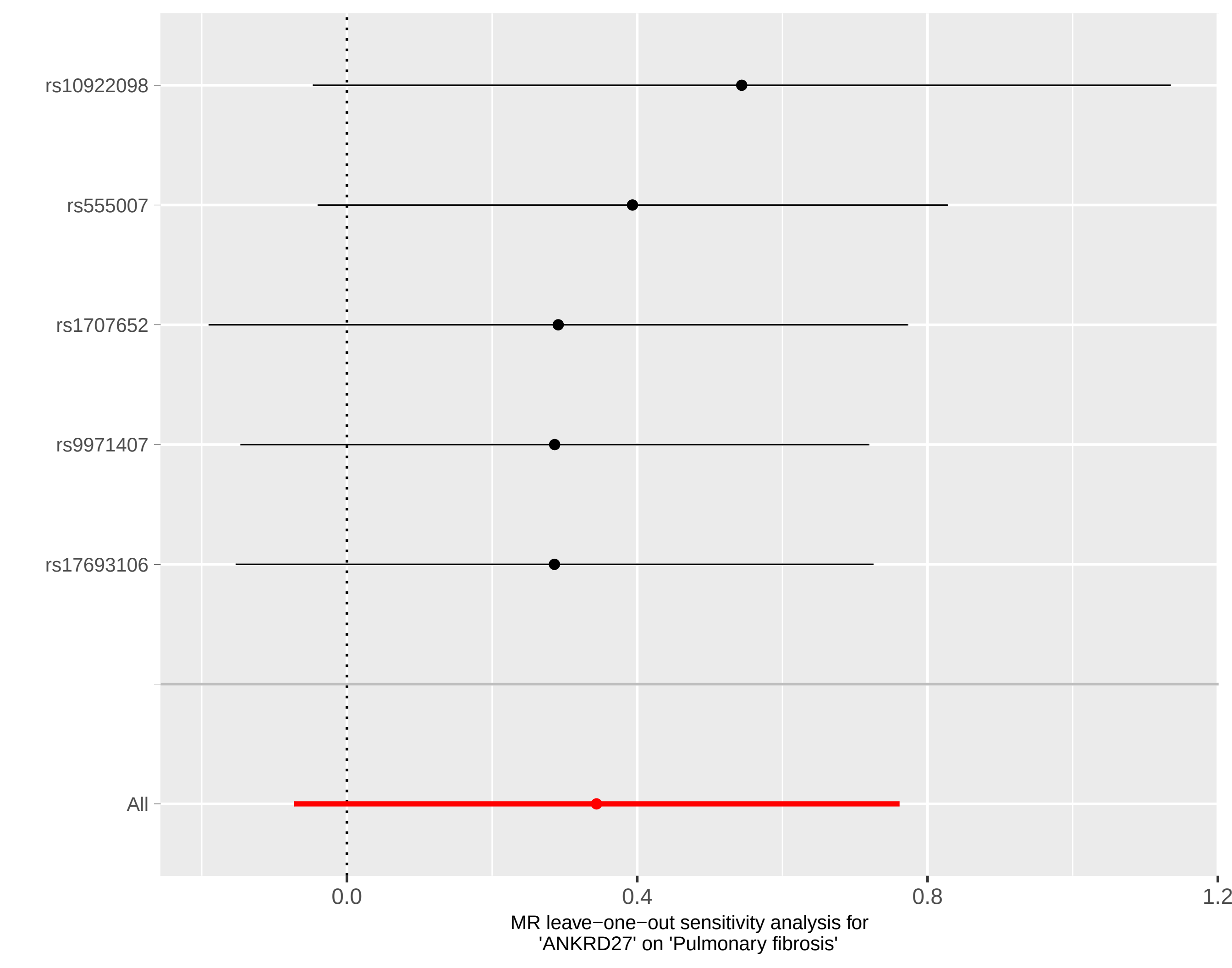
rs10922098
rs555007
rs1707652
rs9971407
rs17693106
All
0.0
0.4
0.8
1.2
MR leave−one−out sensitivity analysis for
'ANKRD27' on 'Pulmonary fibrosis'

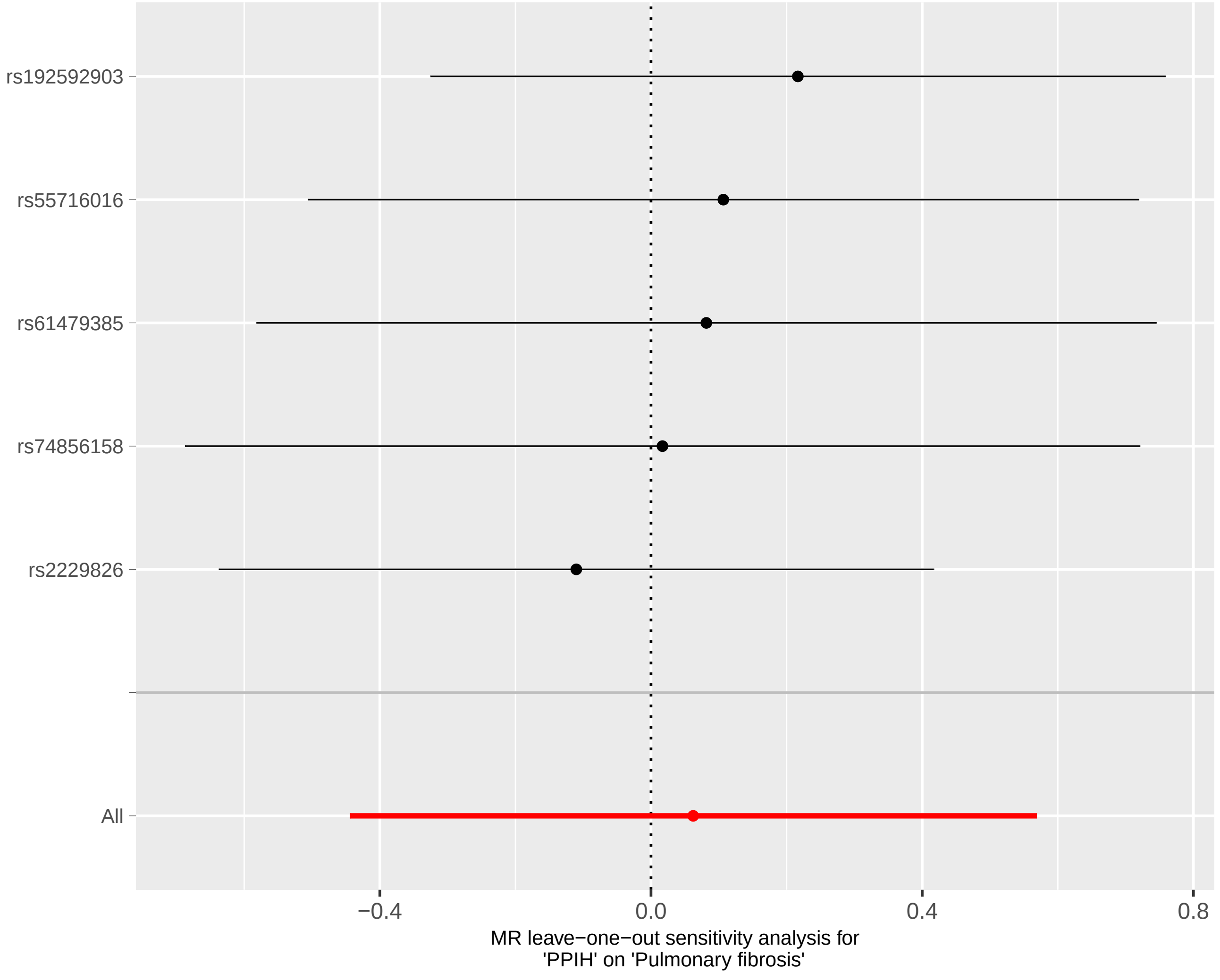
rs192592903
rs55716016
rs61479385
rs74856158
rs2229826
All
−0.4
0.0
0.4
0.8
MR leave−one−out sensitivity analysis for
'PPIH' on 'Pulmonary fibrosis'

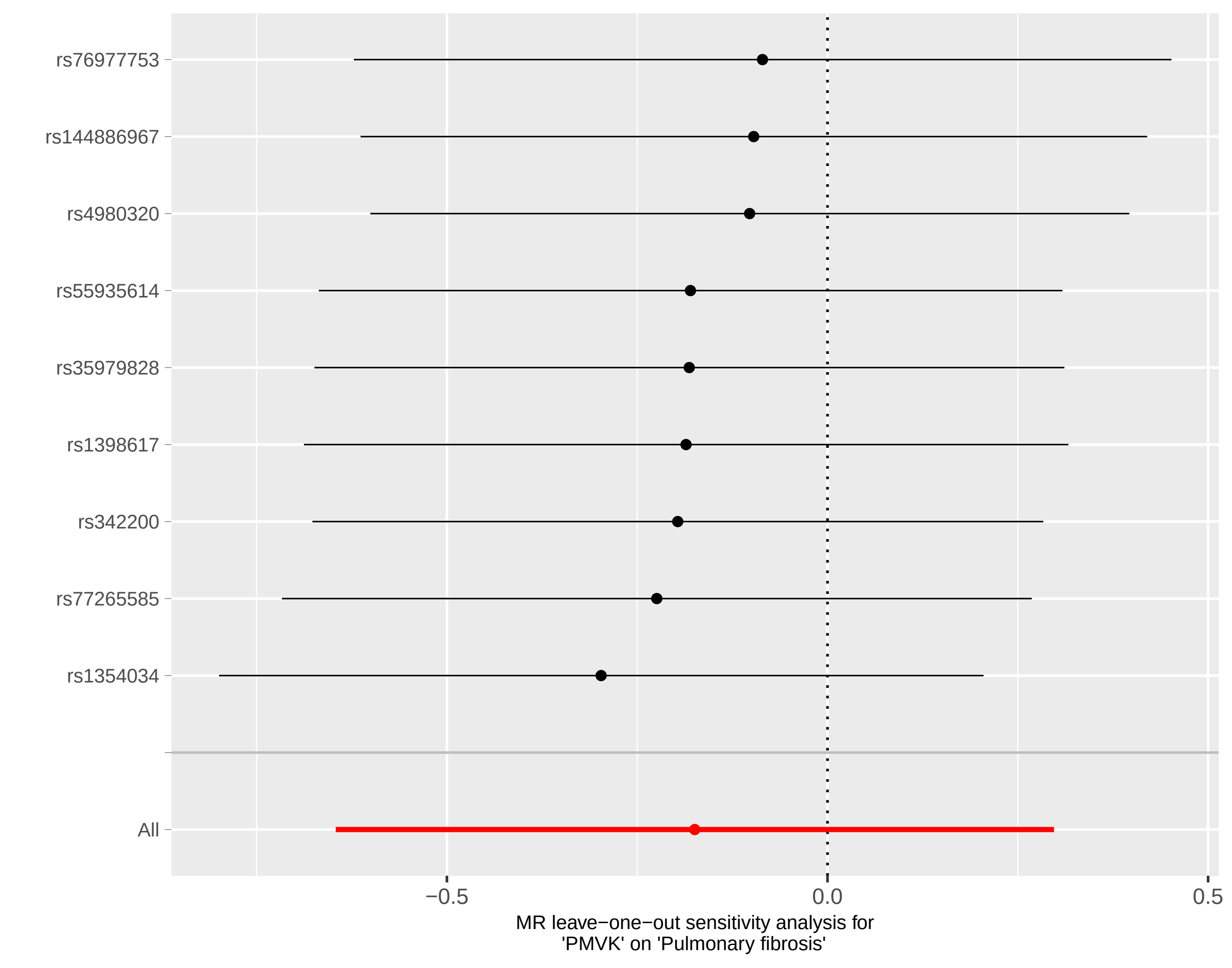
rs76977753
rs144886967
rs4980320
rs55935614
rs35979828
rs1398617
rs342200
rs77265585
rs1354034
All
−0.5
0.0
0.5
MR leave−one−out sensitivity analysis for
'PMVK' on 'Pulmonary fibrosis'

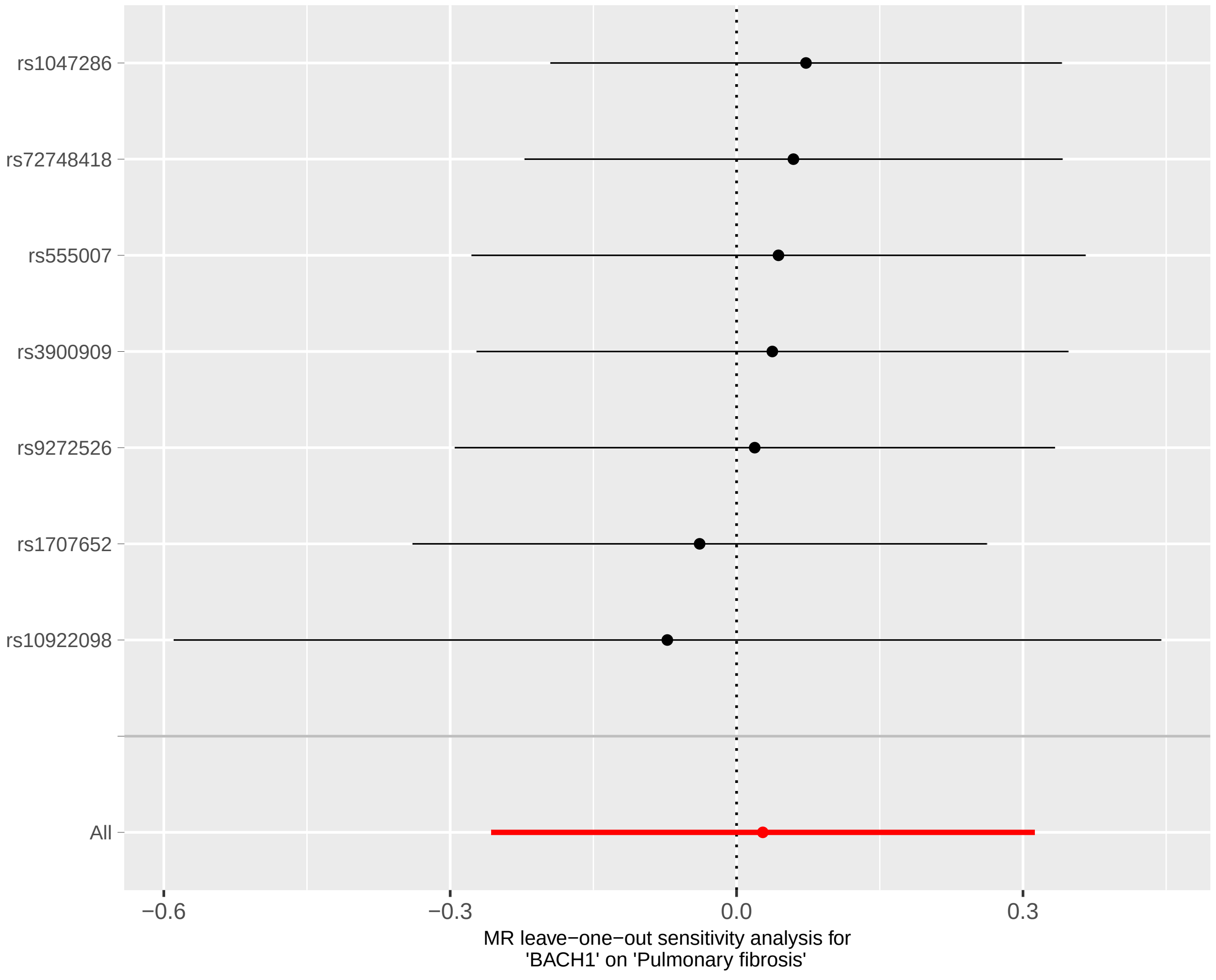
rs1047286
rs72748418
rs555007
rs3900909
rs9272526
rs1707652
rs10922098
All
−0.6
−0.3
0.0
0.3
MR leave−one−out sensitivity analysis for
'BACH1' on 'Pulmonary fibrosis'

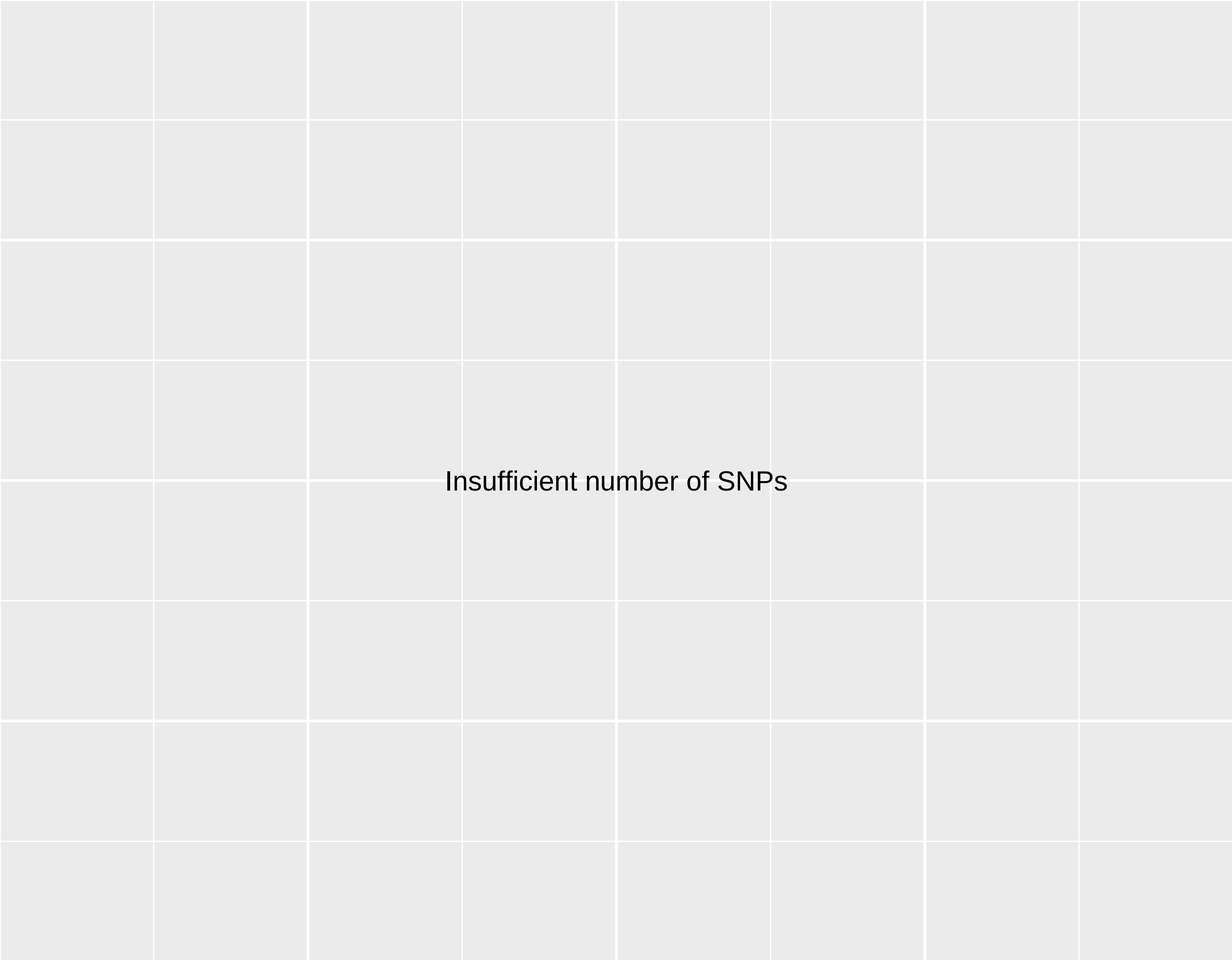
Insufficient number of SNPs

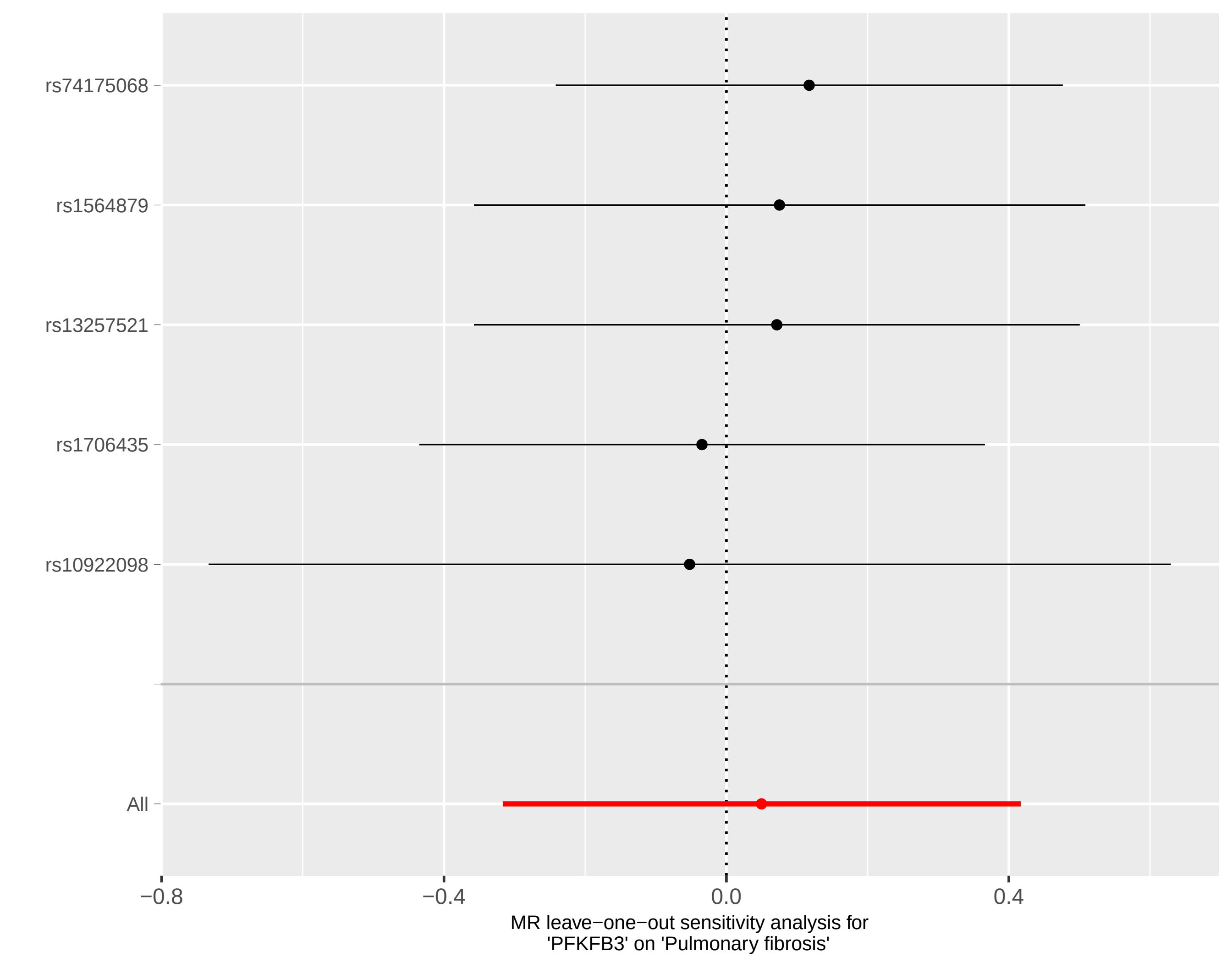

rs74175068
rs1564879
rs13257521
rs1706435
rs10922098
All
−0.8
−0.4
0.0
0.4
MR leave−one−out sensitivity analysis for
'PFKFB3' on 'Pulmonary fibrosis'

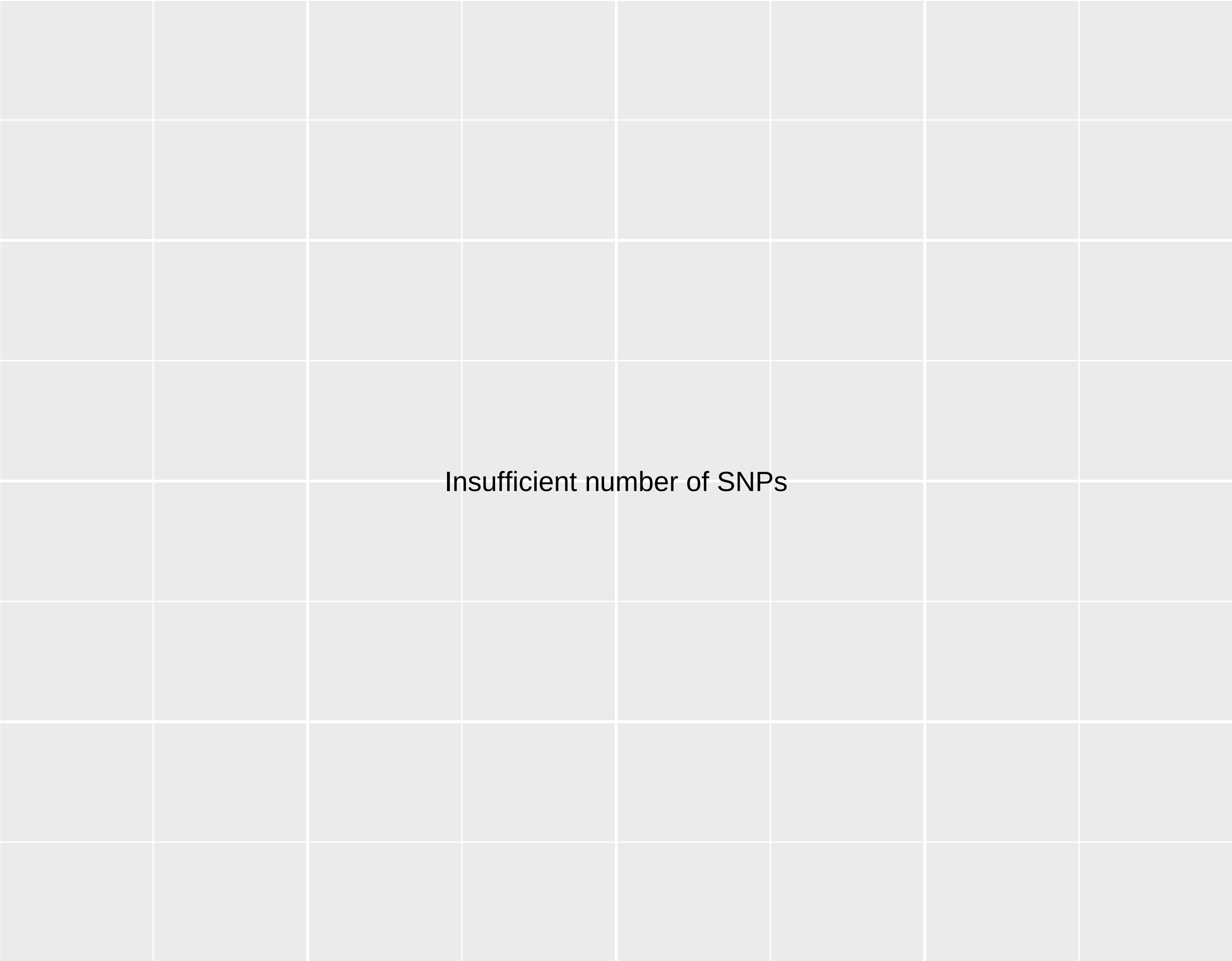
Insufficient number of SNPs

Insufficient number of SNPs

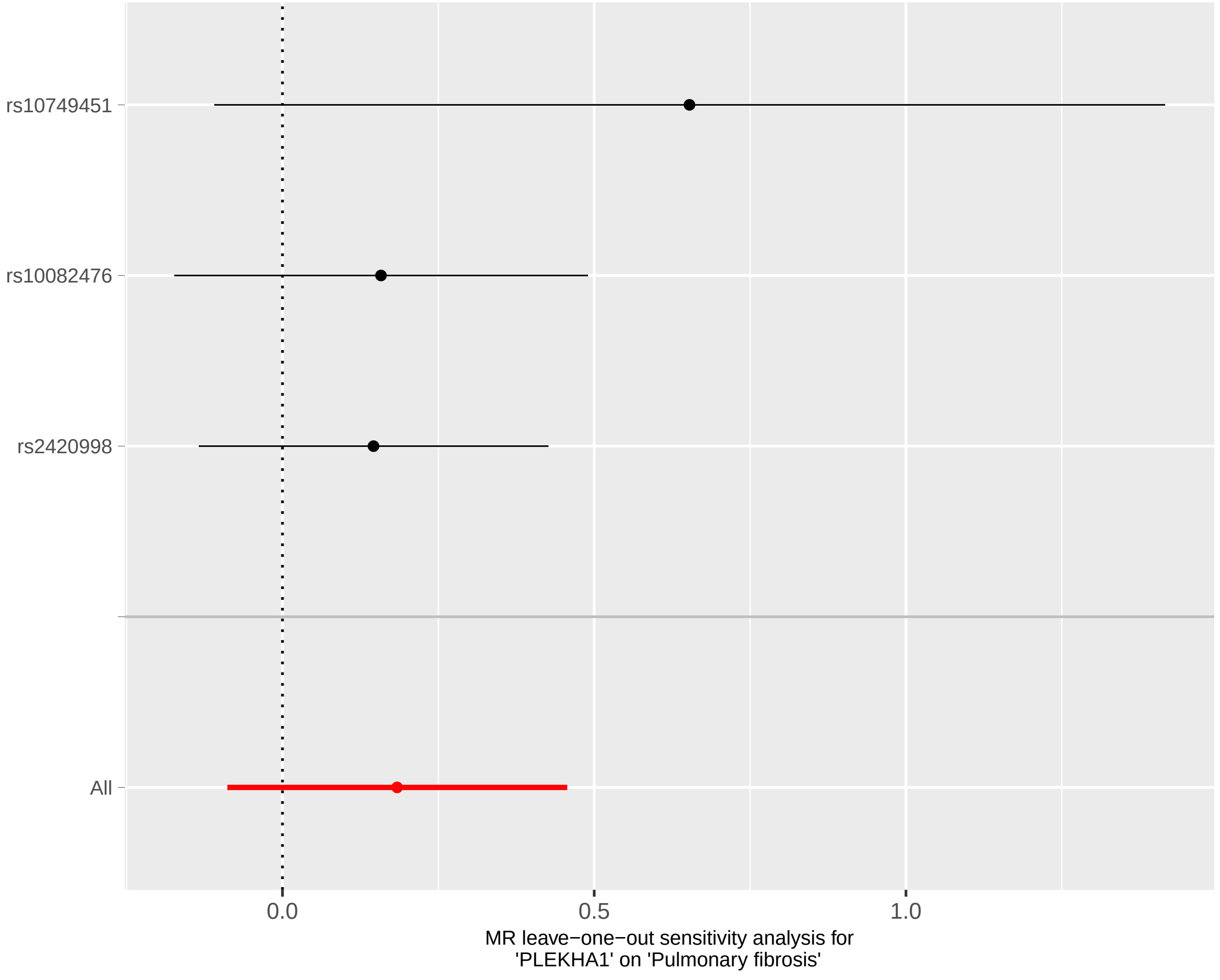
rs10749451
rs10082476
rs2420998
All
0.0
0.5
1.0
MR leave−one−out sensitivity analysis for
'PLEKHA1' on 'Pulmonary fibrosis'

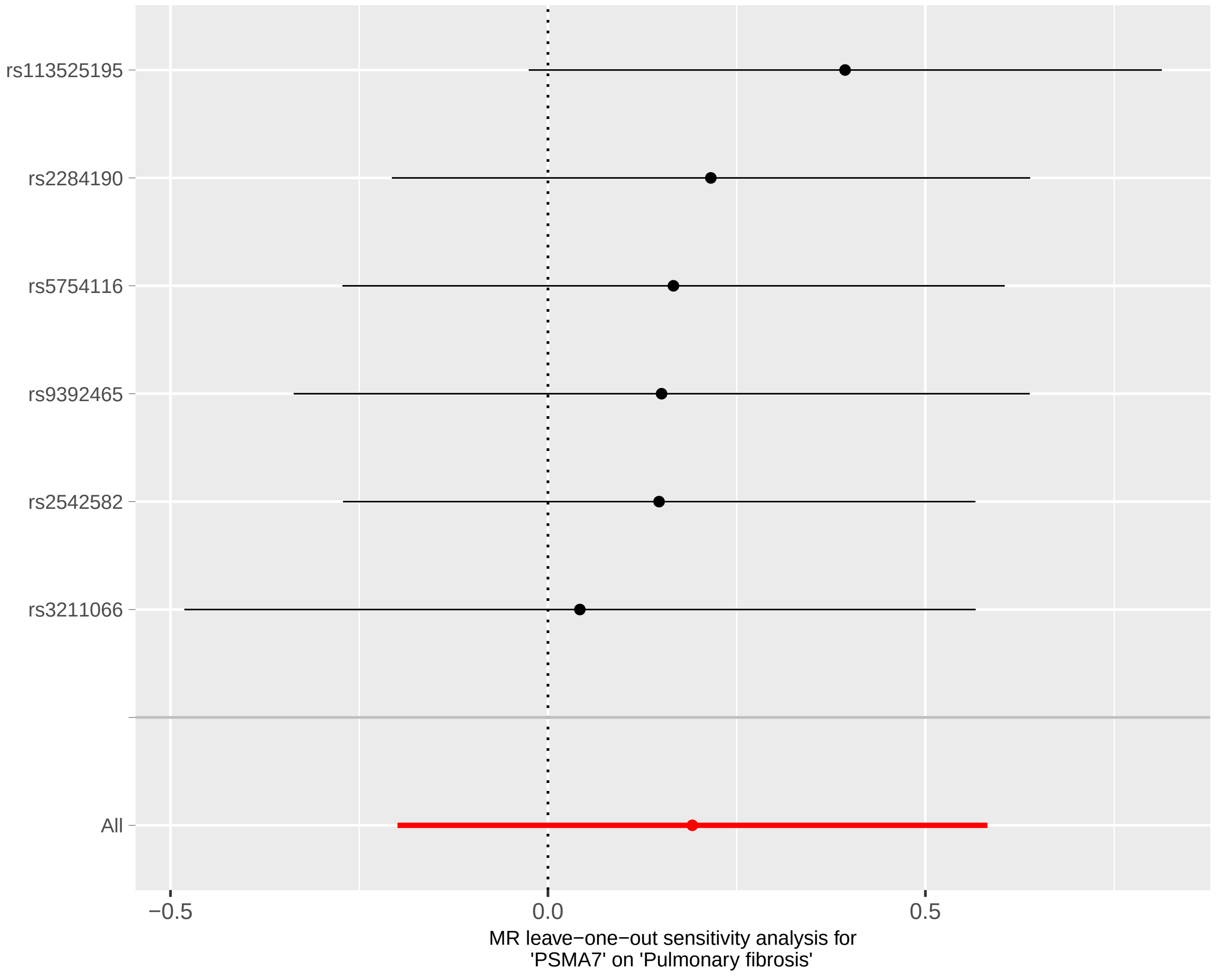
rs113525195
rs2284190
rs5754116
rs9392465
rs2542582
rs3211066
All
−0.5
0.0
0.5
MR leave−one−out sensitivity analysis for
'PSMA7' on 'Pulmonary fibrosis'

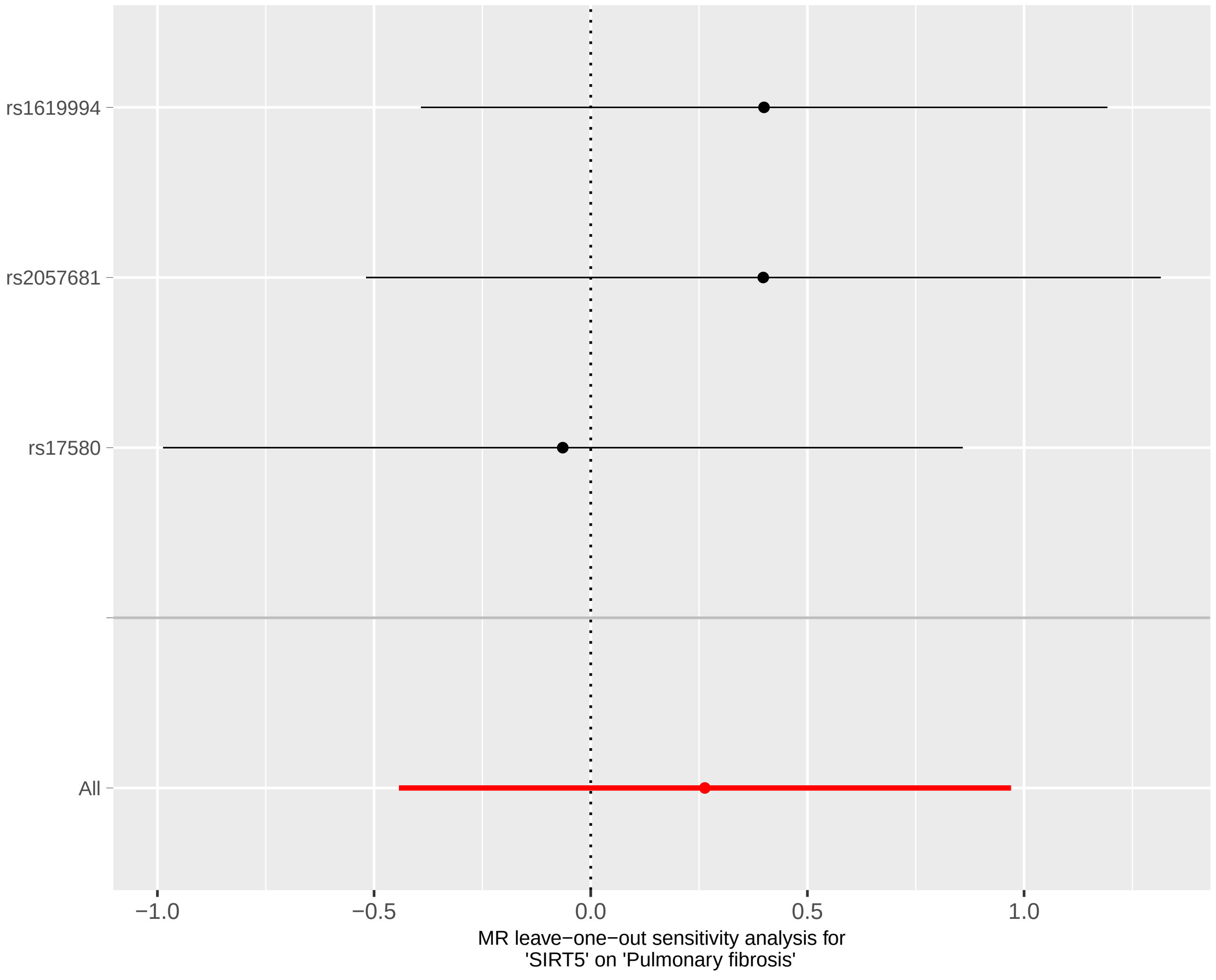
rs1619994
rs2057681
rs17580
All
−1.0
−0.5
0.0
0.5
1.0
MR leave−one−out sensitivity analysis for
'SIRT5' on 'Pulmonary fibrosis'

Insufficient number of SNPs

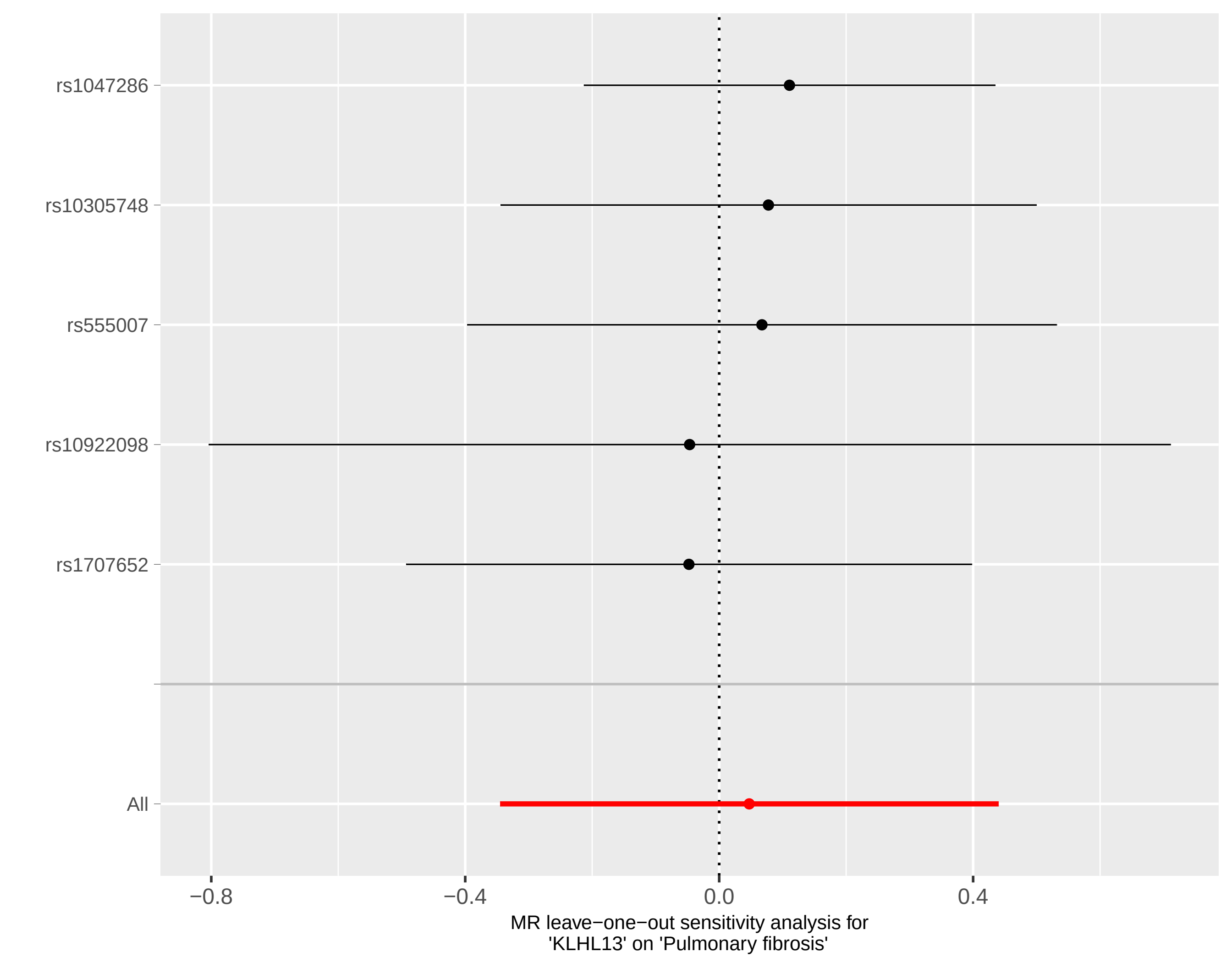
rs1047286
rs10305748
rs555007
rs10922098
rs1707652
All
−0.8
−0.4
0.0
0.4
MR leave−one−out sensitivity analysis for
'KLHL13' on 'Pulmonary fibrosis'

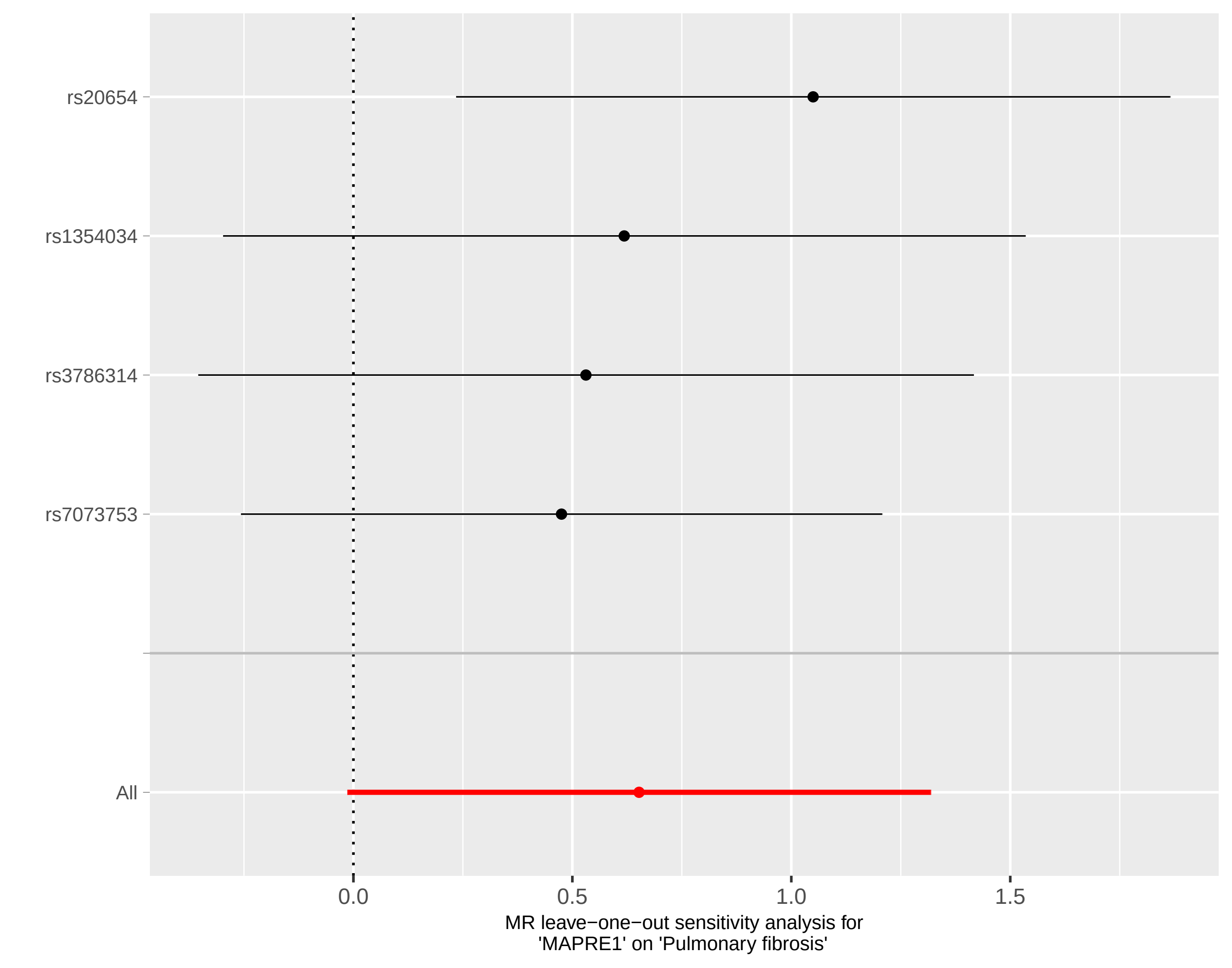

rs20654
rs1354034
rs3786314
rs7073753
All
0.0
0.5
1.0
1.5
MR leave−one−out sensitivity analysis for
'MAPRE1' on 'Pulmonary fibrosis'

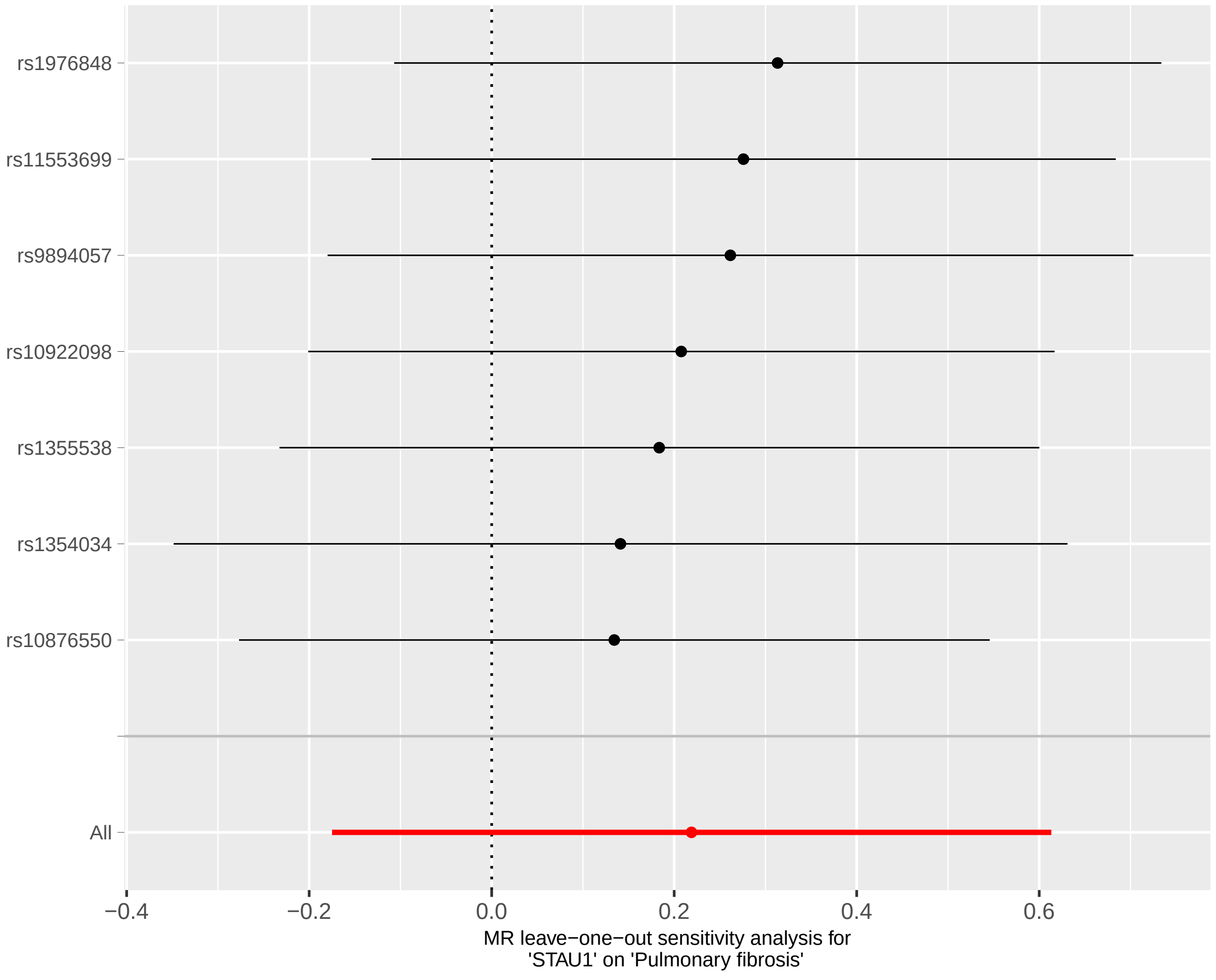
rs1976848
rs11553699
rs9894057
rs10922098
rs1355538
rs1354034
rs10876550
All
−0.4
−0.2
0.0
0.2
0.4
0.6
MR leave−one−out sensitivity analysis for
'STAU1' on 'Pulmonary fibrosis'

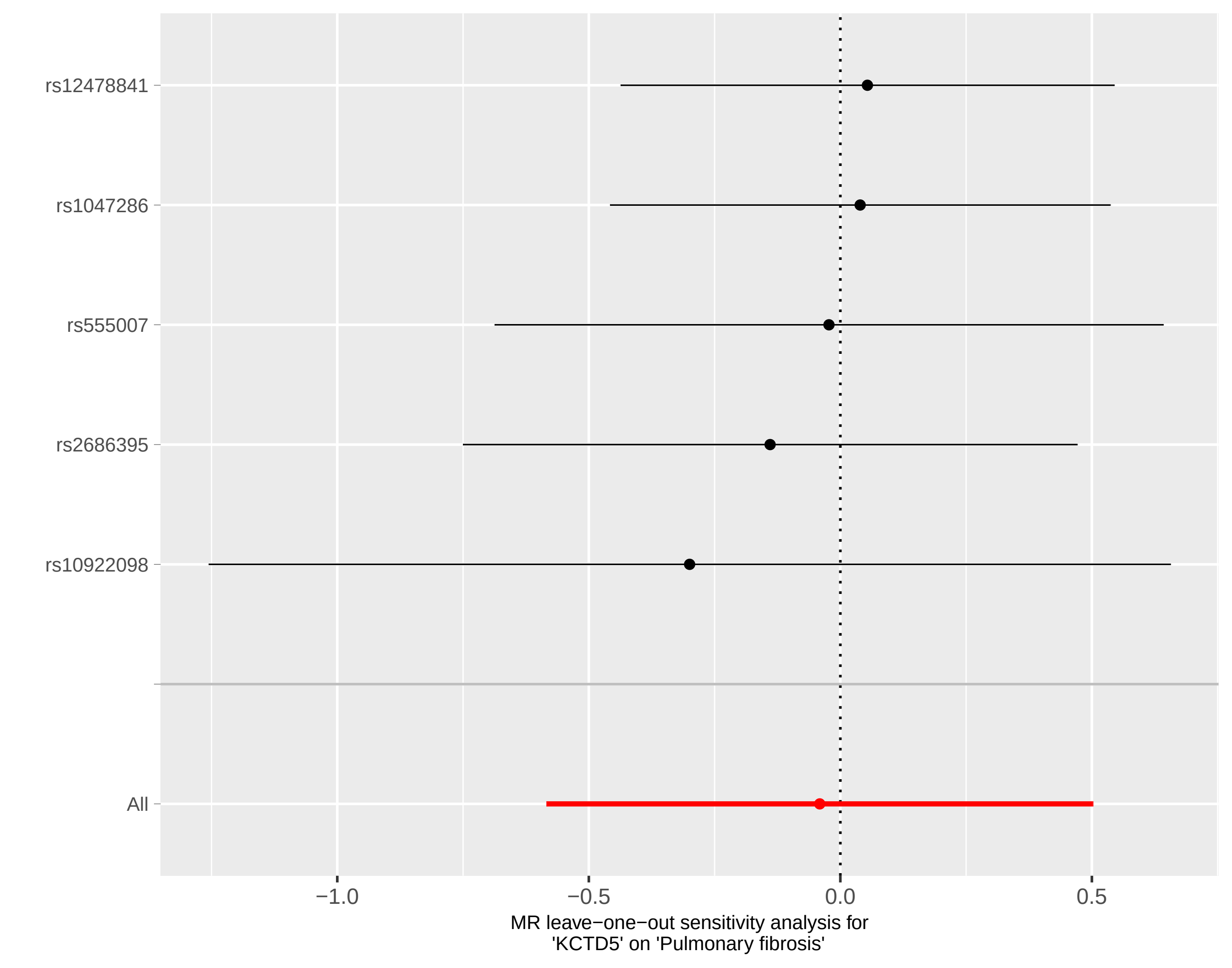
rs12478841
rs1047286
rs555007
rs2686395
rs10922098
All
−1.0
−0.5
0.0
0.5
MR leave−one−out sensitivity analysis for
'KCTD5' on 'Pulmonary fibrosis'

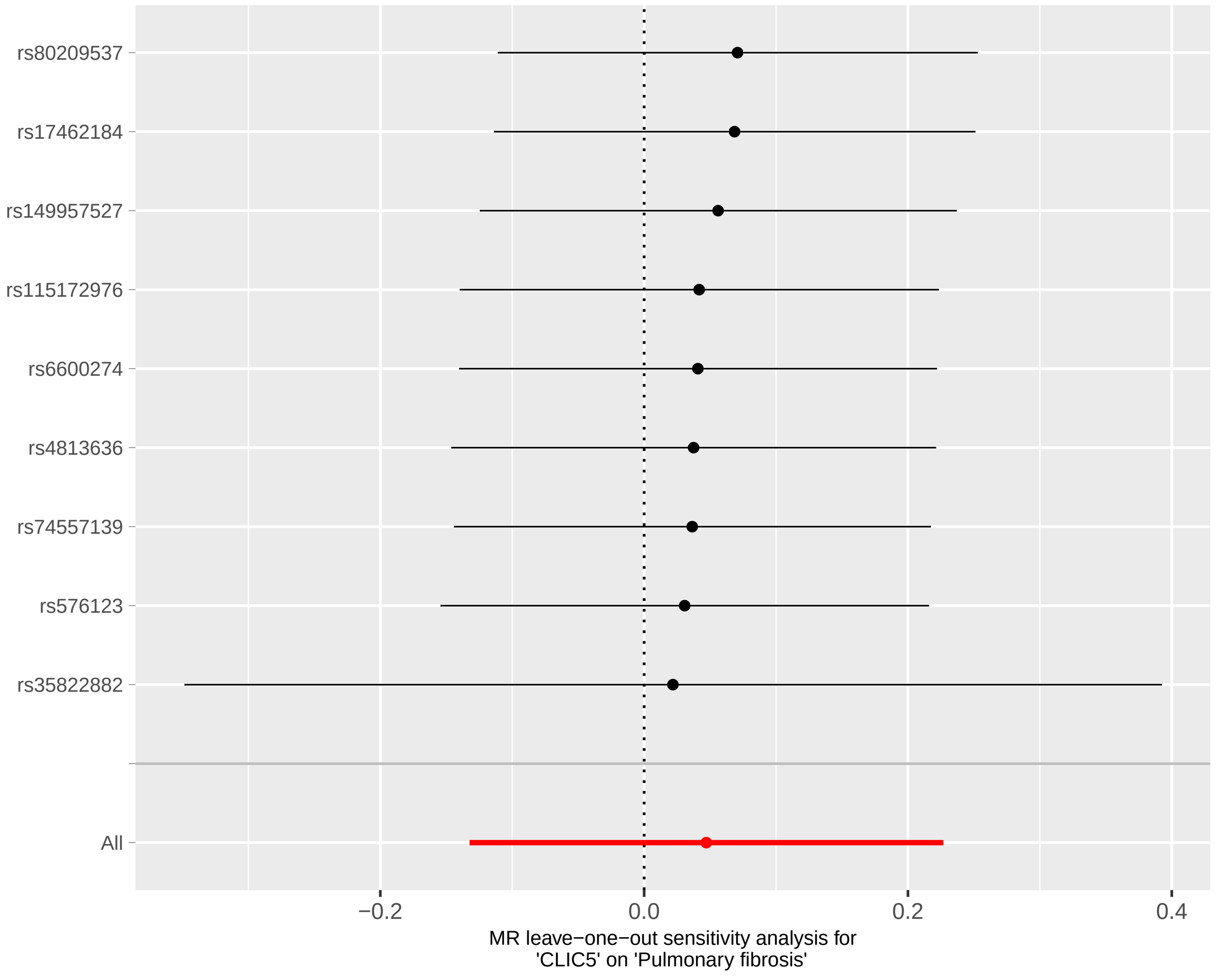

rs80209537
rs17462184
rs149957527
rs115172976
rs6600274
rs4813636
rs74557139
rs576123
rs35822882
All
−0.2
0.0
0.2
0.4
MR leave−one−out sensitivity analysis for
'CLIC5' on 'Pulmonary fibrosis'

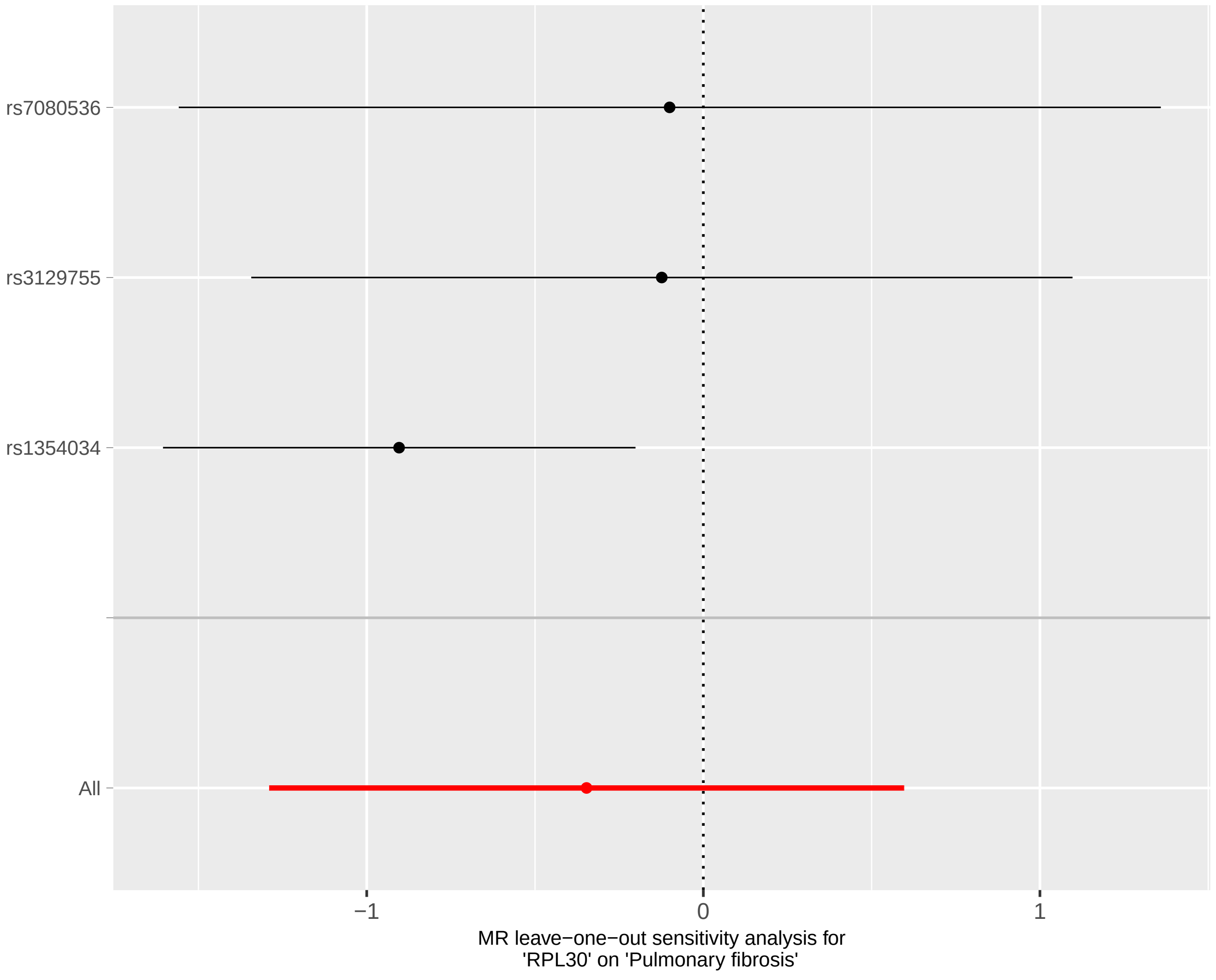

rs7080536
rs3129755
rs1354034
All
−1
0
1
MR leave−one−out sensitivity analysis for
'RPL30' on 'Pulmonary fibrosis'

Insufficient number of SNPs

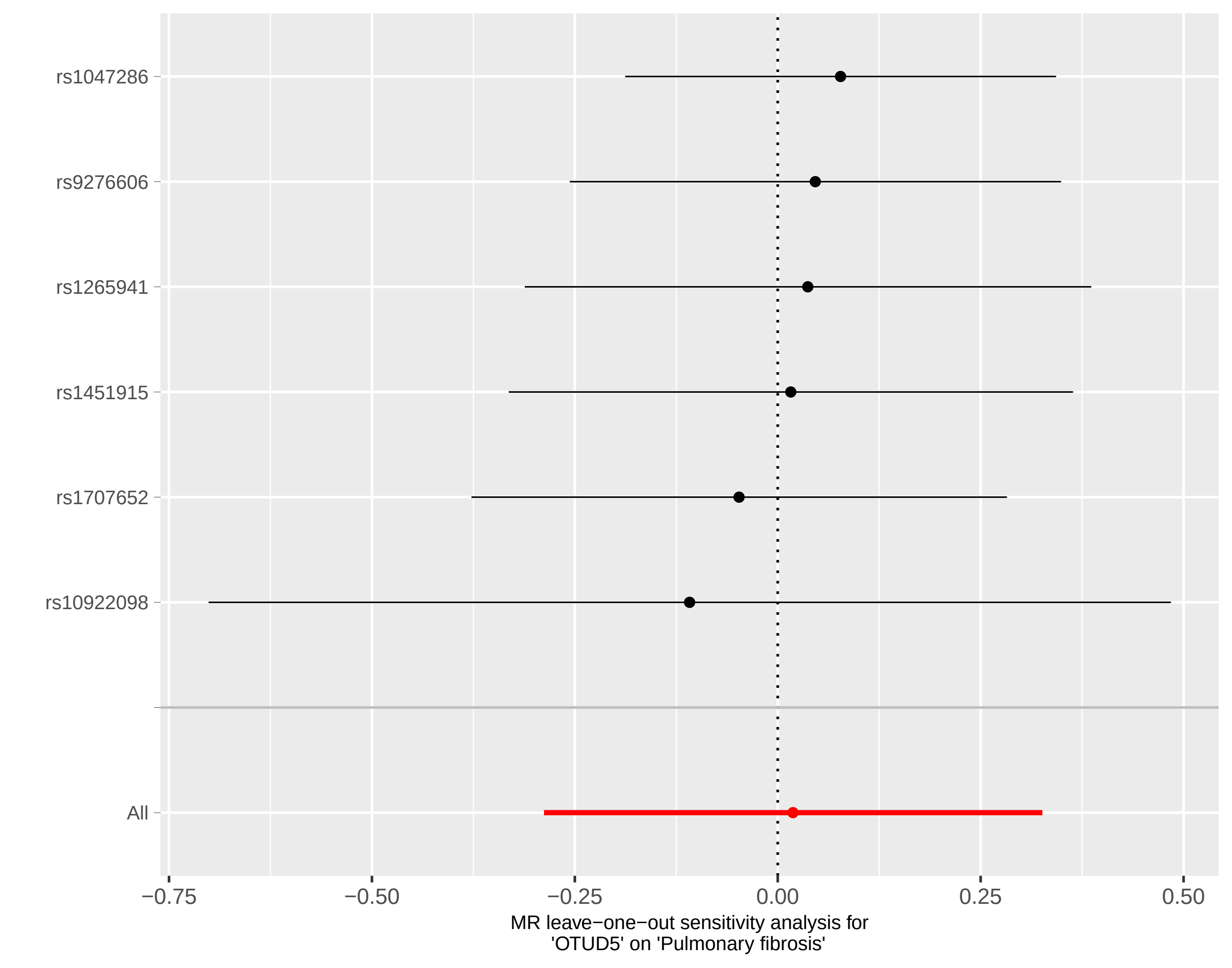
rs1047286
rs9276606
rs1265941
rs1451915
rs1707652
rs10922098
All
−0.75
−0.50
−0.25
0.00
0.25
0.50
MR leave−one−out sensitivity analysis for
'OTUD5' on 'Pulmonary fibrosis'

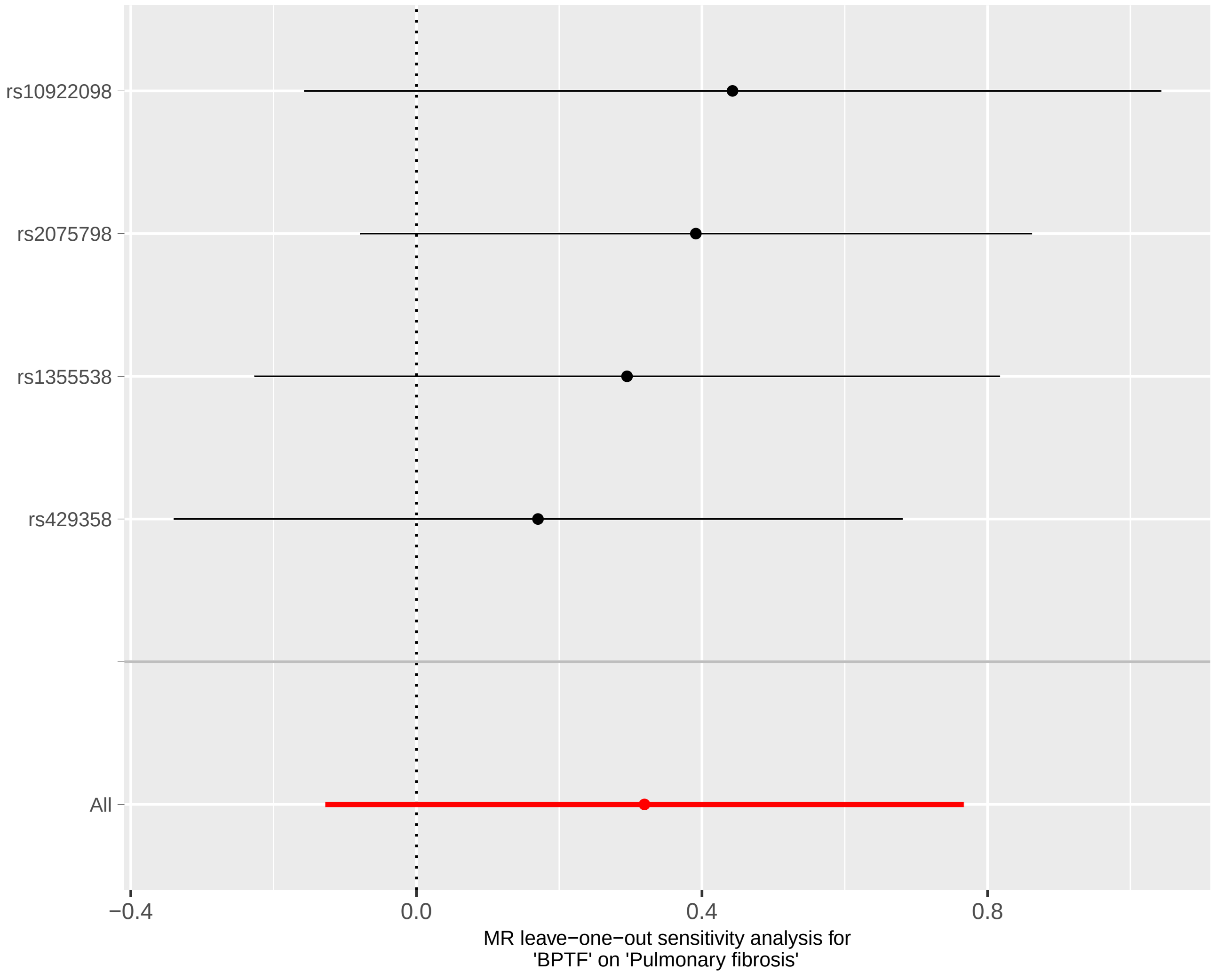
rs10922098
rs2075798
rs1355538
rs429358
All
−0.4
0.0
0.4
0.8
MR leave−one−out sensitivity analysis for
'BPTF' on 'Pulmonary fibrosis'

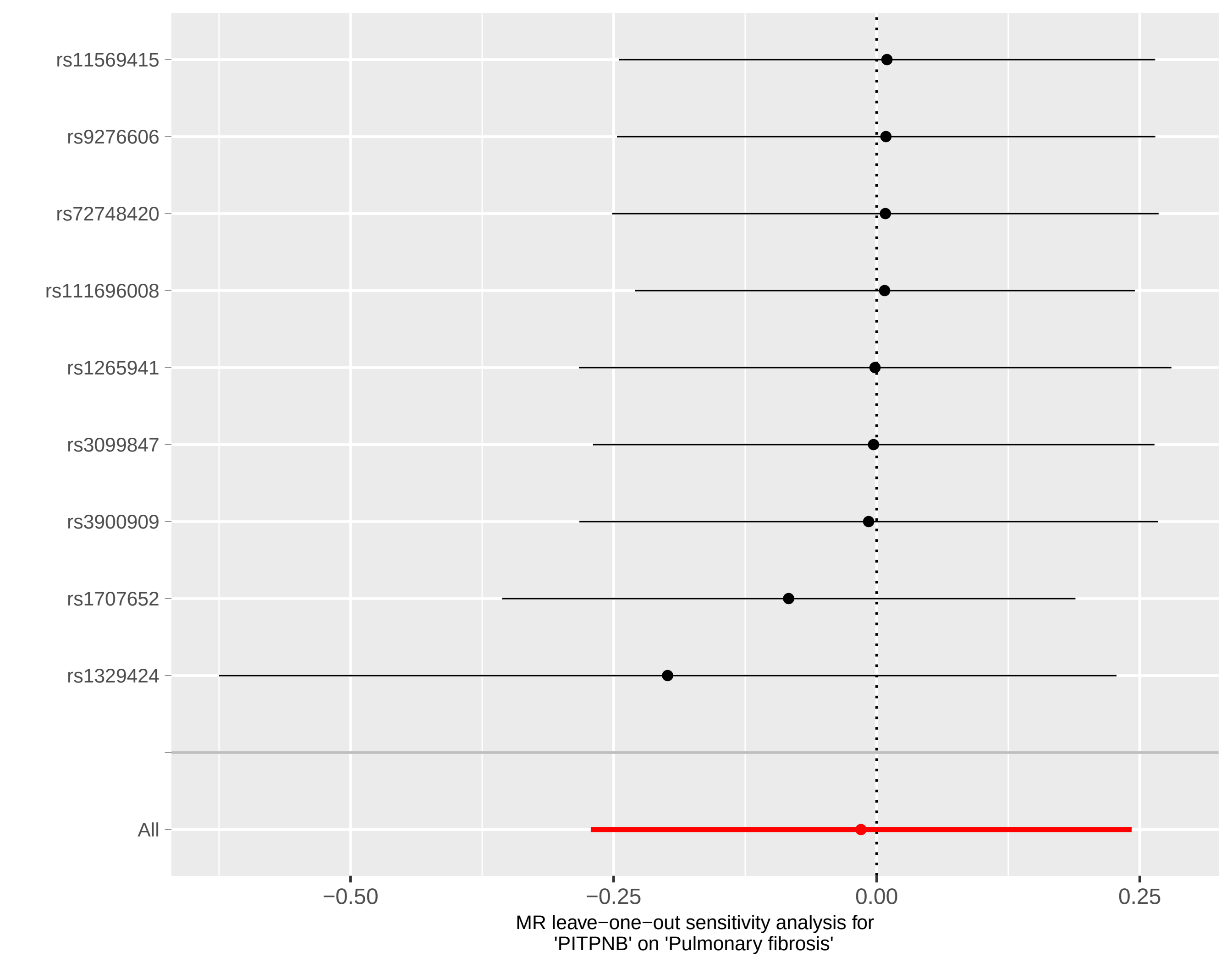
rs11569415
rs9276606
rs72748420
rs111696008
rs1265941
rs3099847
rs3900909
rs1707652
rs1329424
All
−0.50
−0.25
0.00
0.25
MR leave−one−out sensitivity analysis for
'PITPNB' on 'Pulmonary fibrosis'

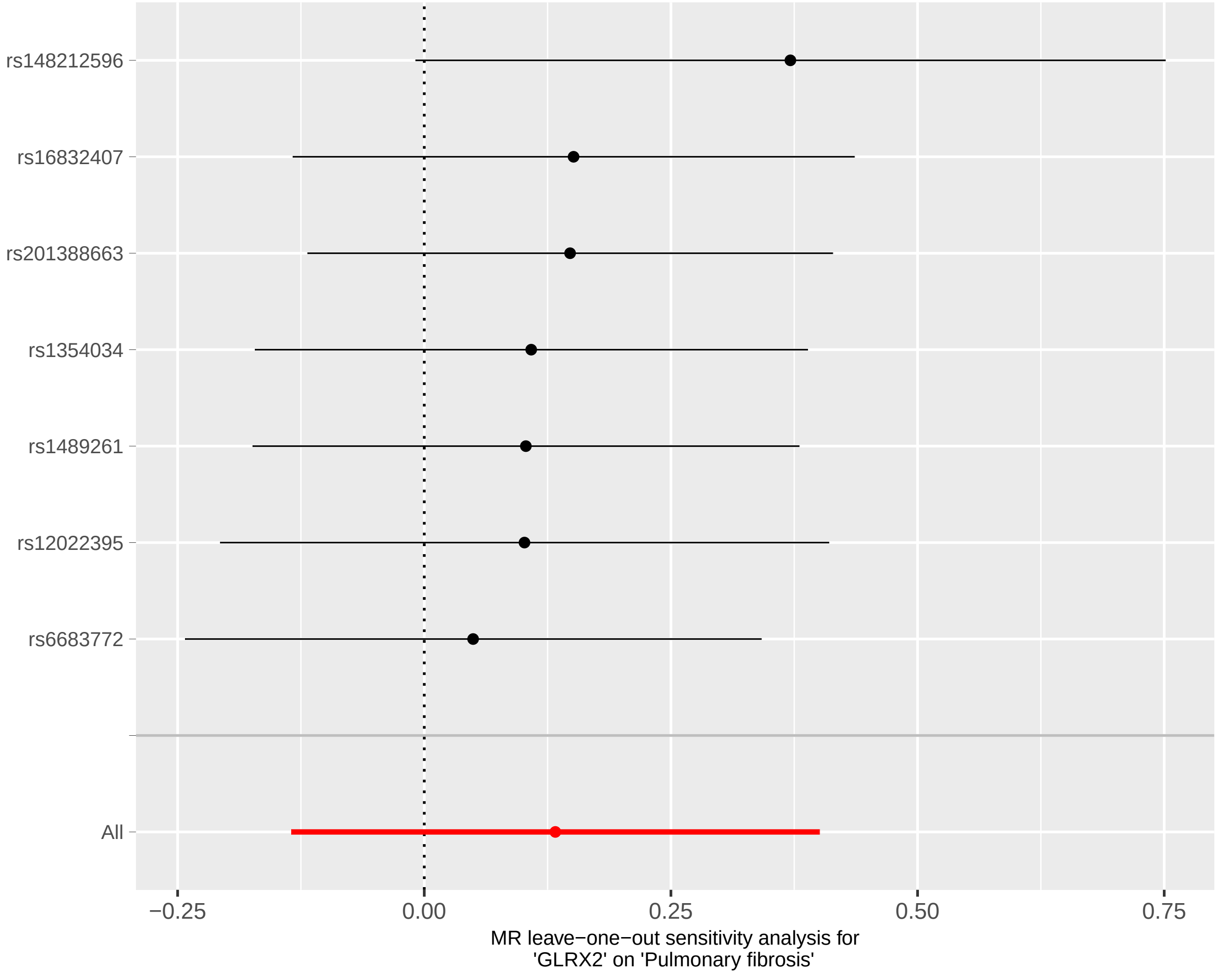

rs148212596
rs16832407
rs201388663
rs1354034
rs1489261
rs12022395
rs6683772
All
−0.25
0.00
0.25
0.50
0.75
MR leave−one−out sensitivity analysis for
'GLRX2' on 'Pulmonary fibrosis'

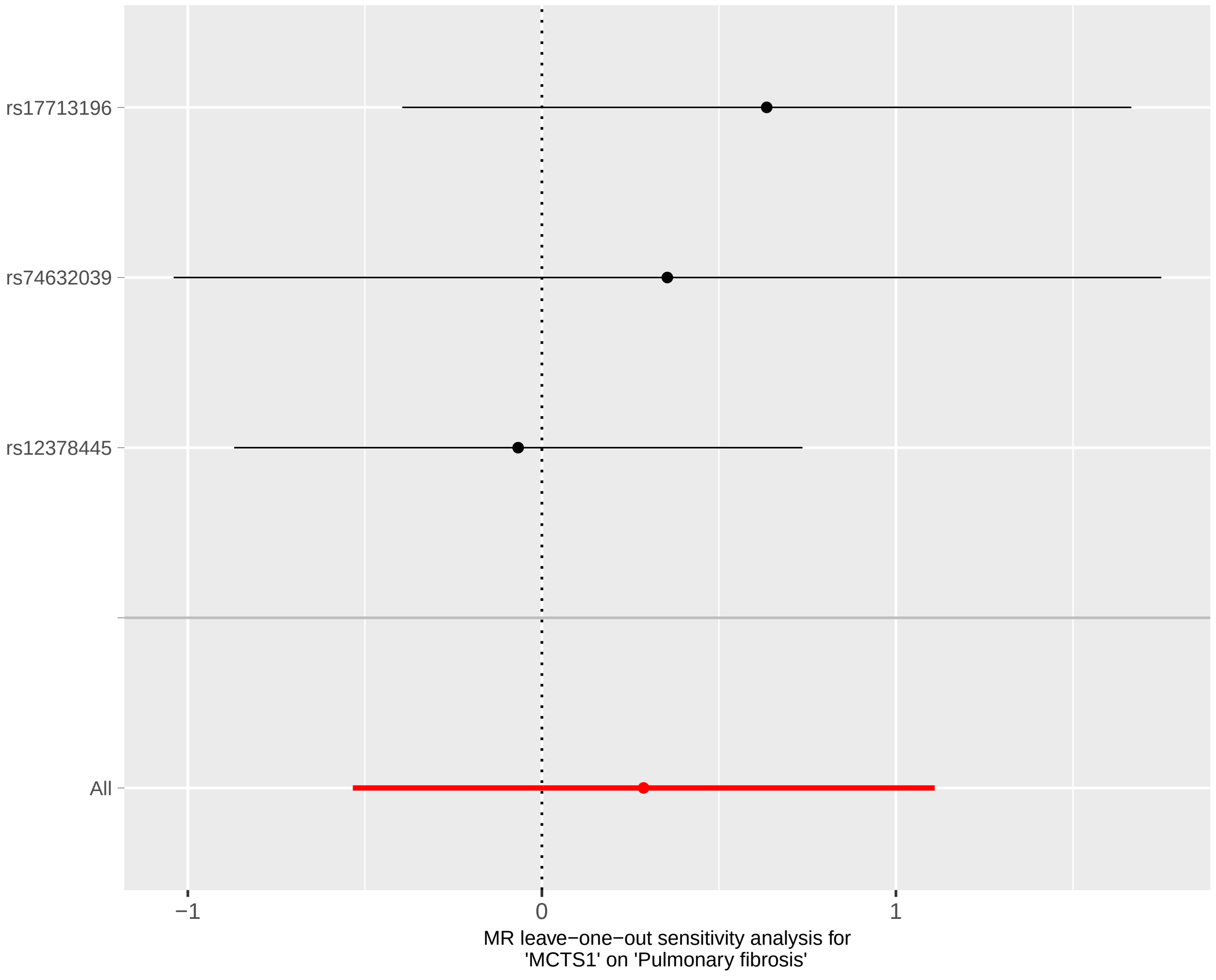
rs17713196
rs74632039
rs12378445
All
−1
0
1
MR leave−one−out sensitivity analysis for
'MCTS1' on 'Pulmonary fibrosis'

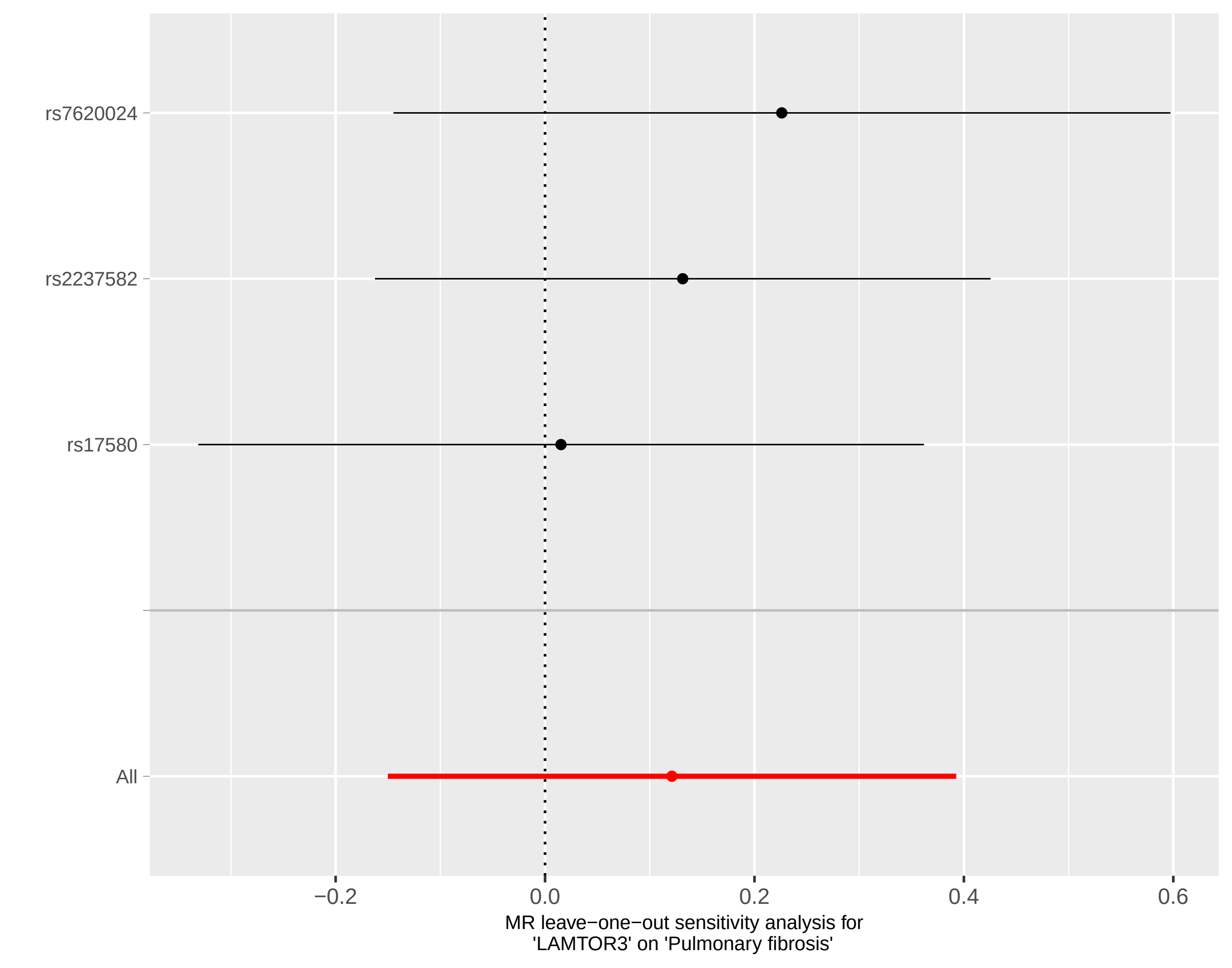
rs7620024
rs2237582
rs17580
All
−0.2
0.0
0.2
0.4
0.6
MR leave−one−out sensitivity analysis for
'LAMTOR3' on 'Pulmonary fibrosis'

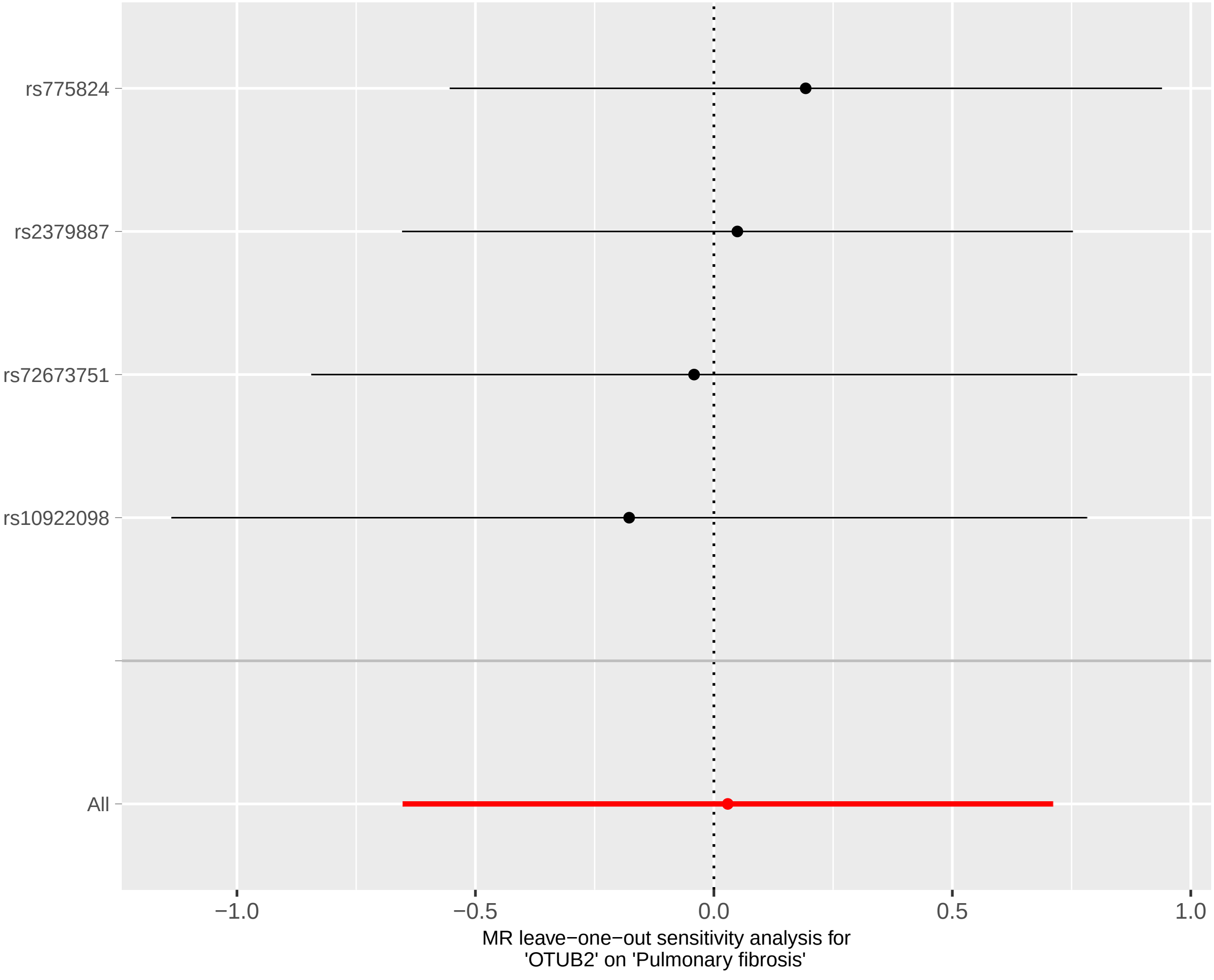
rs775824
rs2379887
rs72673751
rs10922098
All
−1.0
−0.5
0.0
0.5
1.0
MR leave−one−out sensitivity analysis for
'OTUB2' on 'Pulmonary fibrosis'

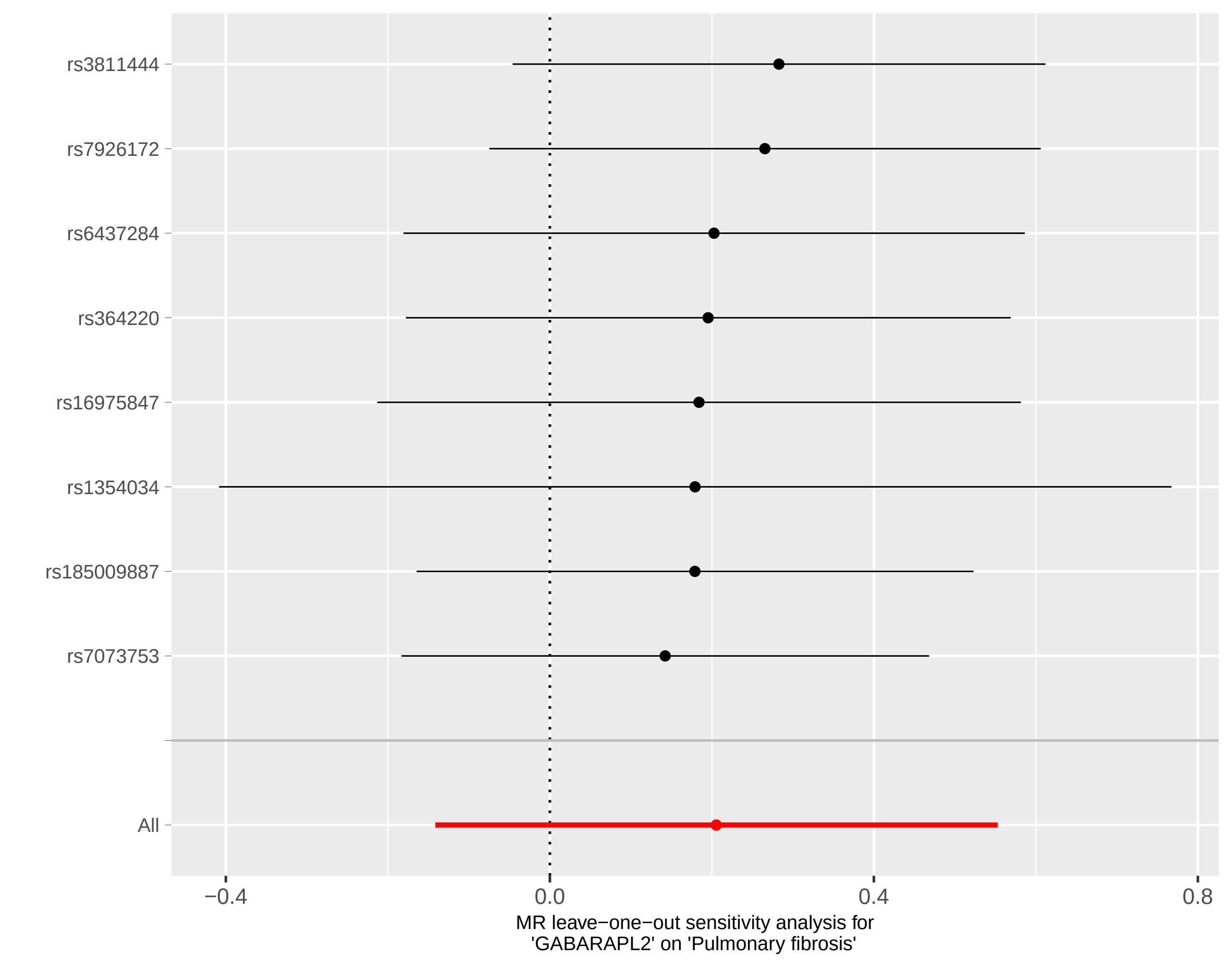
rs3811444
rs7926172
rs6437284
rs364220
rs16975847
rs1354034
rs185009887
rs7073753
All
−0.4
0.0
0.4
0.8
MR leave−one−out sensitivity analysis for
'GABARAPL2' on 'Pulmonary fibrosis'

Insufficient number of SNPs

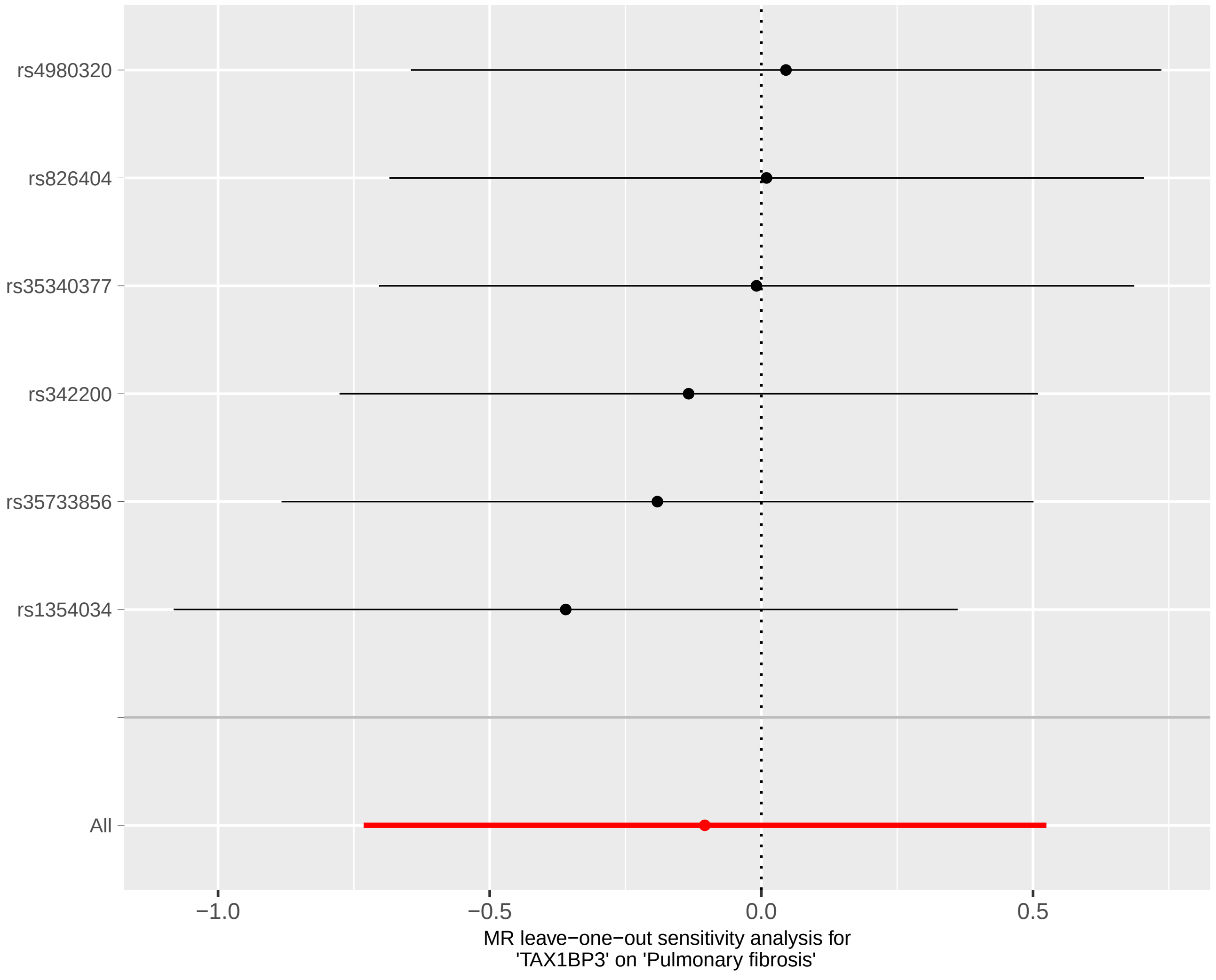
rs4980320
rs826404
rs35340377
rs342200
rs35733856
rs1354034
All
−1.0
−0.5
0.0
0.5
MR leave−one−out sensitivity analysis for
'TAX1BP3' on 'Pulmonary fibrosis'

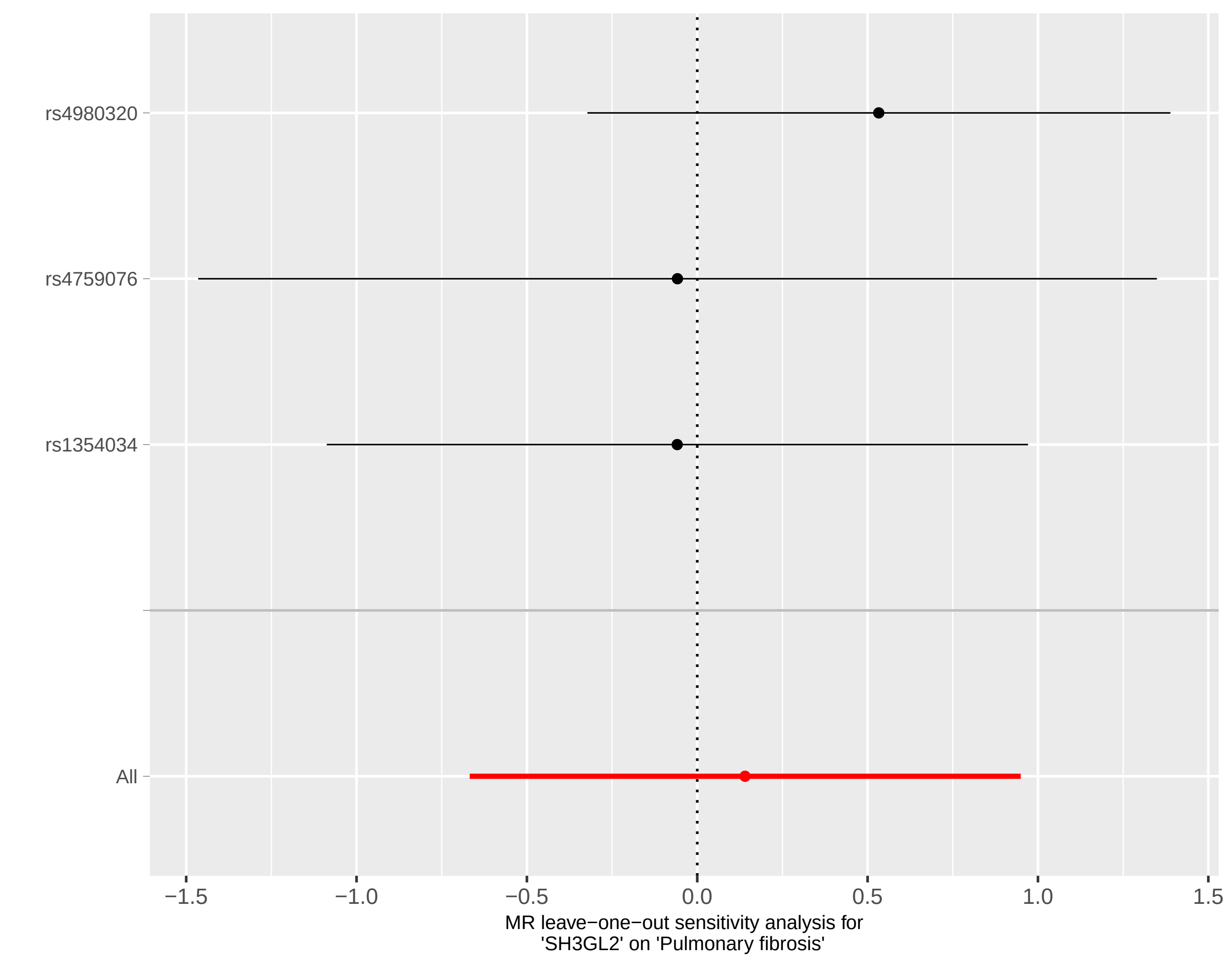
rs4980320
rs4759076
rs1354034
All
−1.5
−1.0
−0.5
0.0
0.5
1.0
1.5
MR leave−one−out sensitivity analysis for
'SH3GL2' on 'Pulmonary fibrosis'

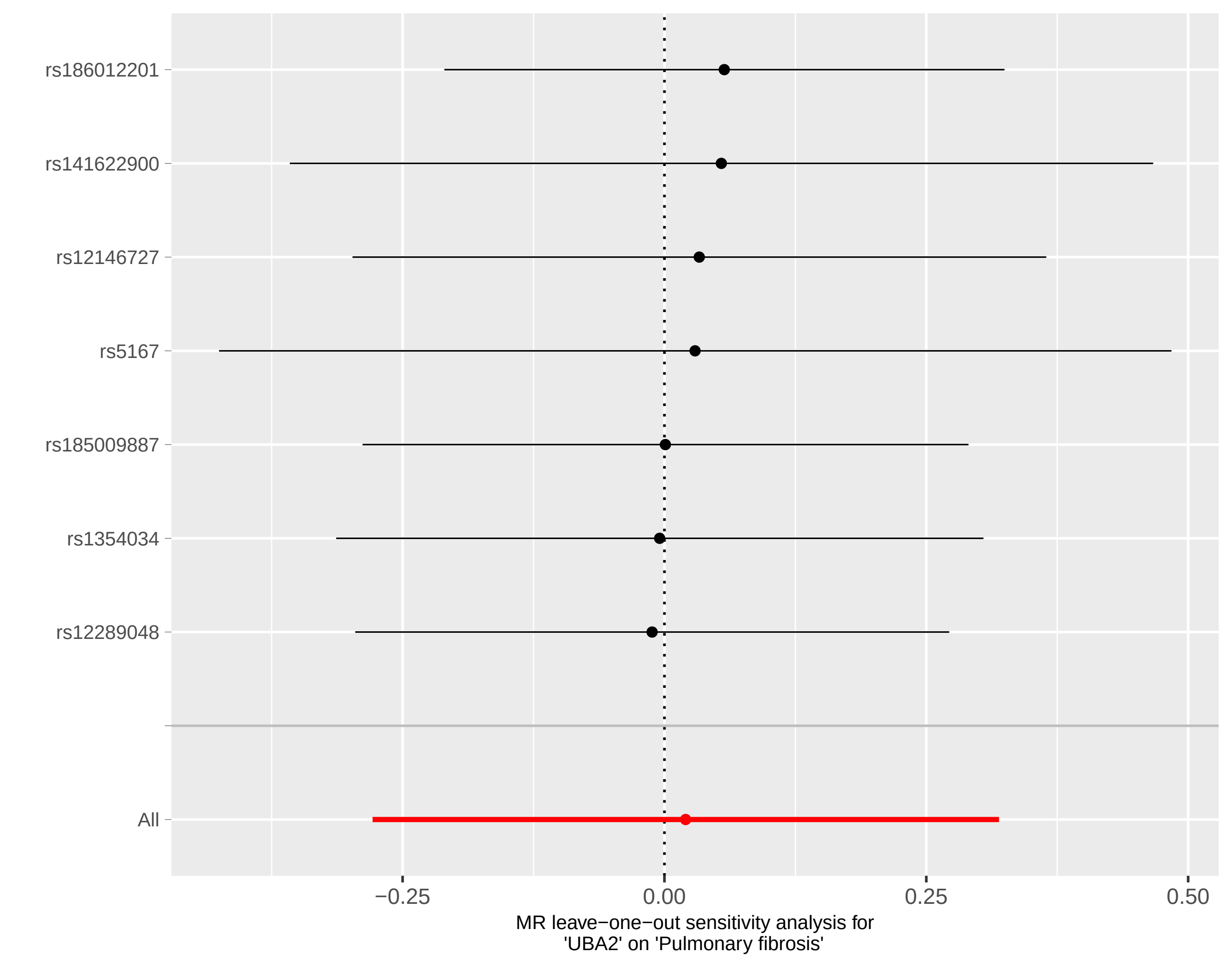
rs186012201
rs141622900
rs12146727
rs5167
rs185009887
rs1354034
rs12289048
All
−0.25
0.00
0.25
0.50
MR leave−one−out sensitivity analysis for
'UBA2' on 'Pulmonary fibrosis'

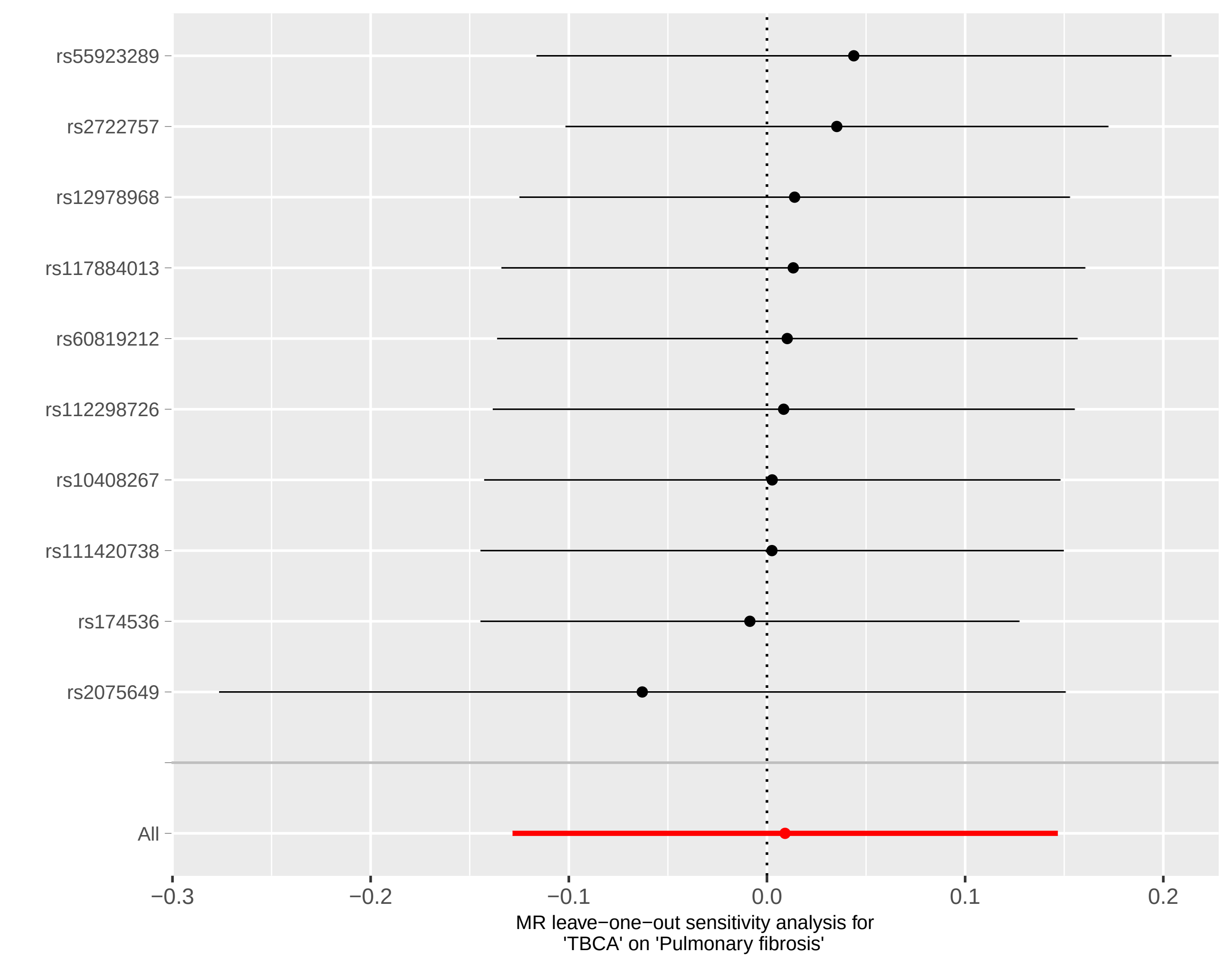

rs55923289
rs2722757
rs12978968
rs117884013
rs60819212
rs112298726
rs10408267
rs111420738
rs174536
rs2075649
All
−0.3
−0.2
−0.1
0.0
0.1
0.2
MR leave−one−out sensitivity analysis for
'TBCA' on 'Pulmonary fibrosis'

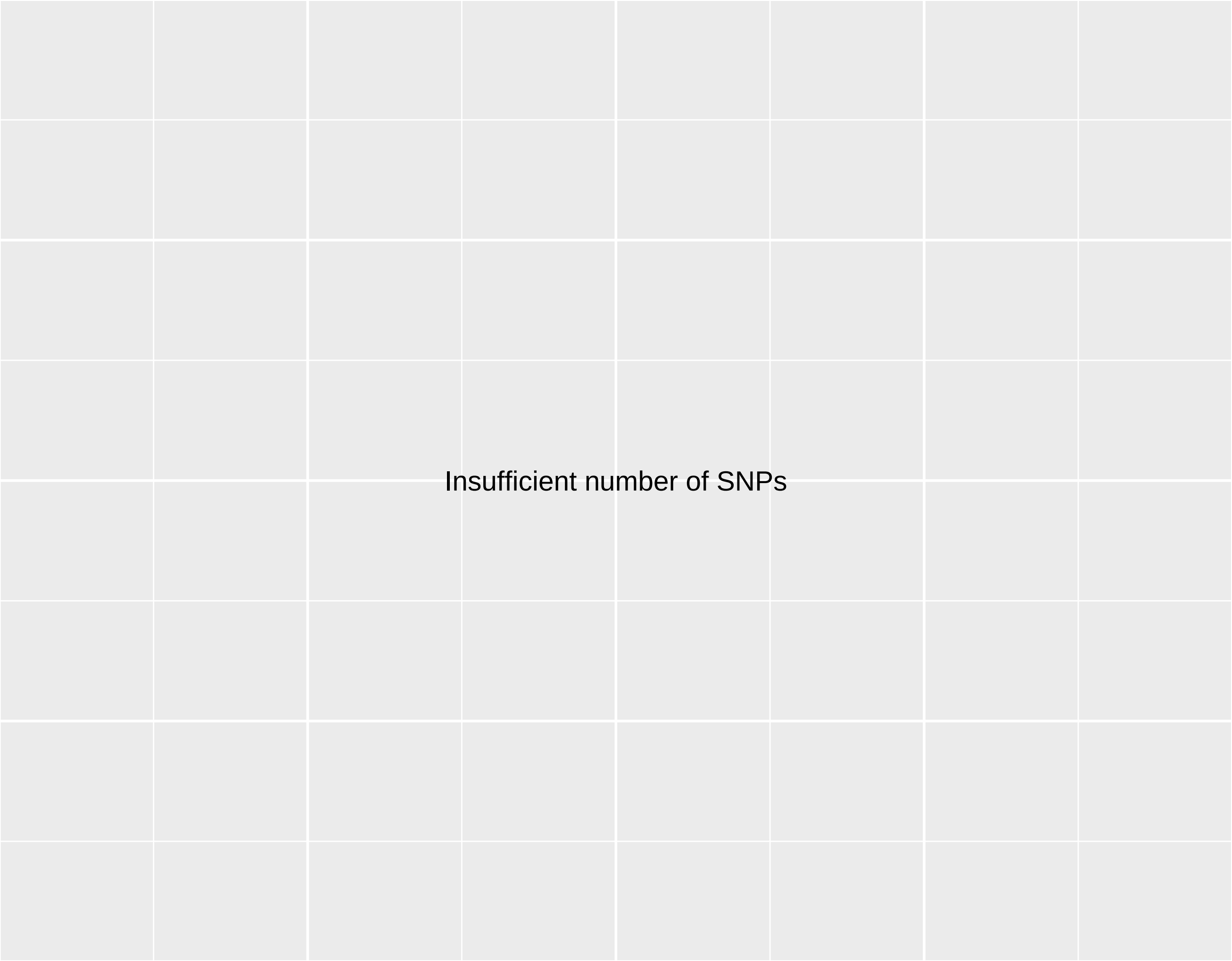
Insufficient number of SNPs

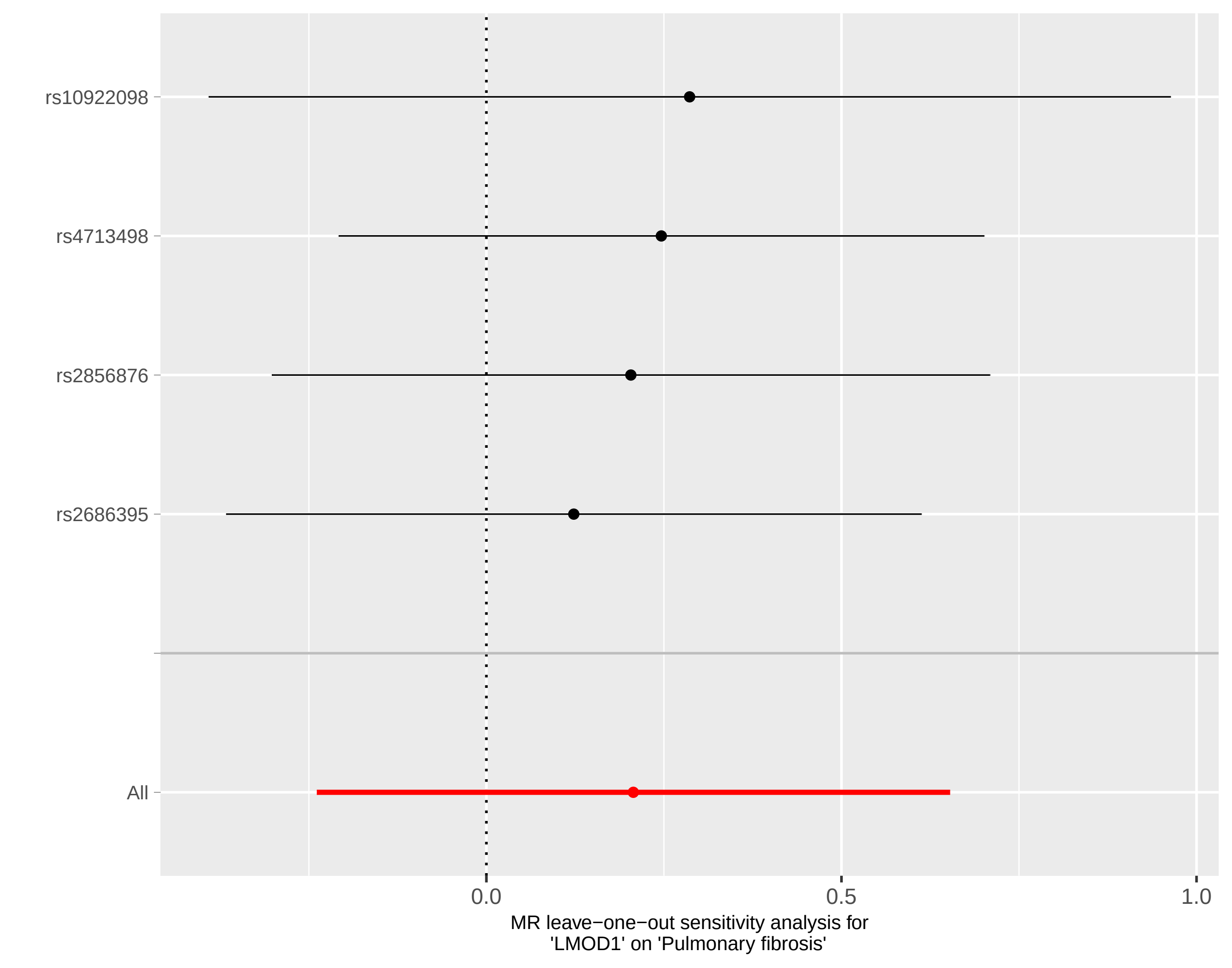
rs10922098
rs4713498
rs2856876
rs2686395
All
0.0
0.5
1.0
MR leave−one−out sensitivity analysis for
'LMOD1' on 'Pulmonary fibrosis'

Insufficient number of SNPs

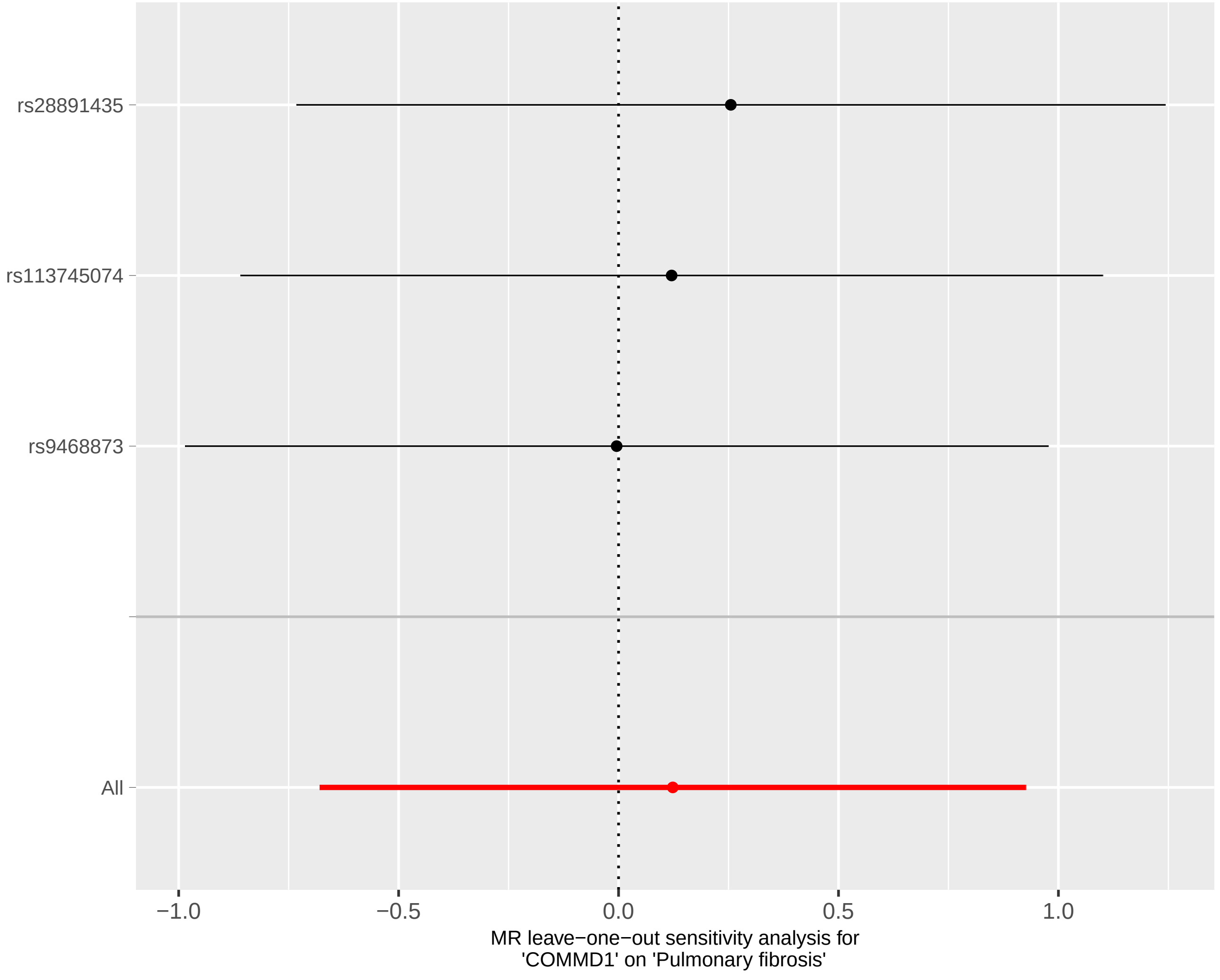
rs28891435
rs113745074
rs9468873
All
−1.0
−0.5
0.0
0.5
1.0
MR leave−one−out sensitivity analysis for
'COMMD1' on 'Pulmonary fibrosis'

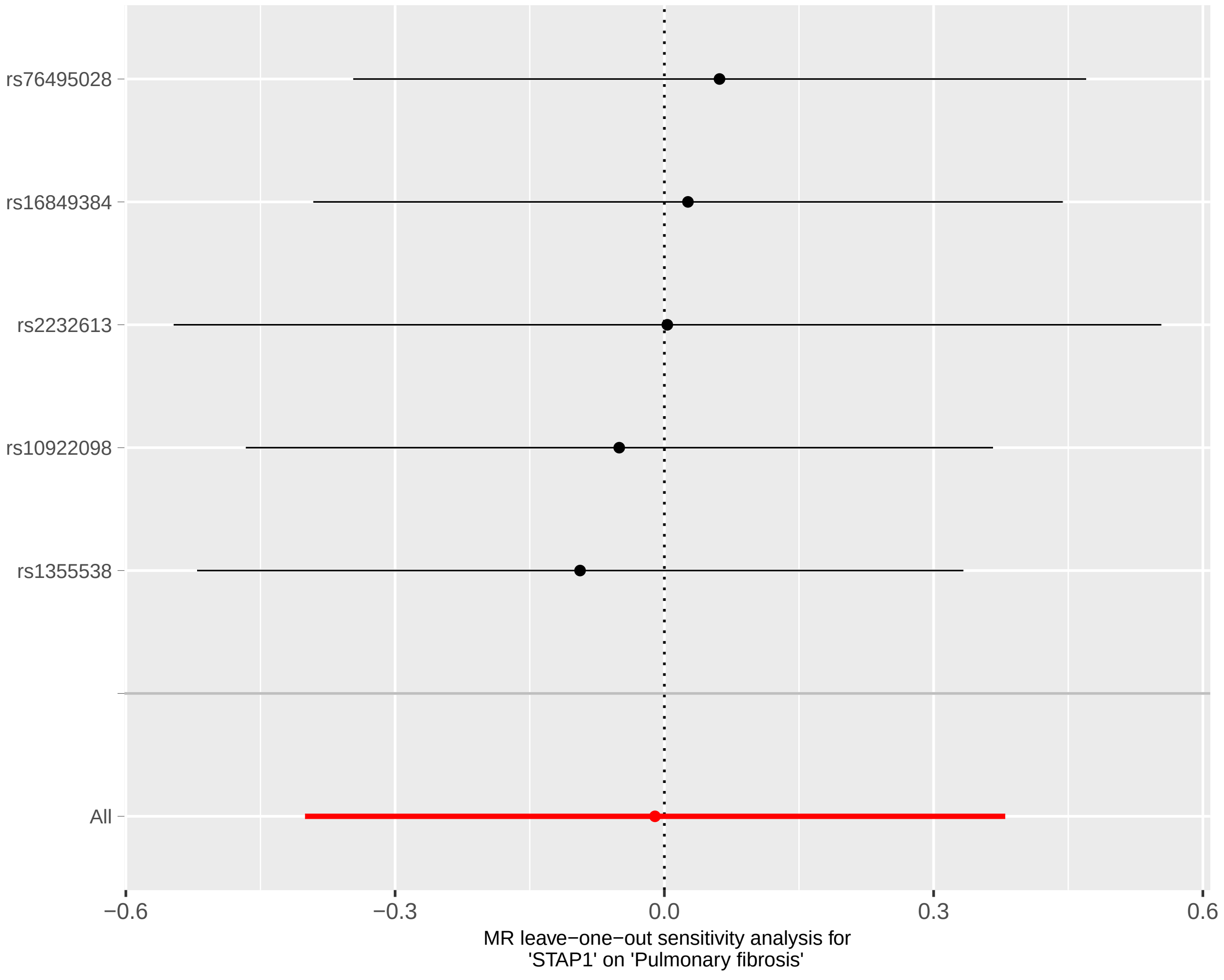
rs76495028
rs16849384
rs2232613
rs10922098
rs1355538
All
−0.6
−0.3
0.0
0.3
0.6
MR leave−one−out sensitivity analysis for
'STAP1' on 'Pulmonary fibrosis'

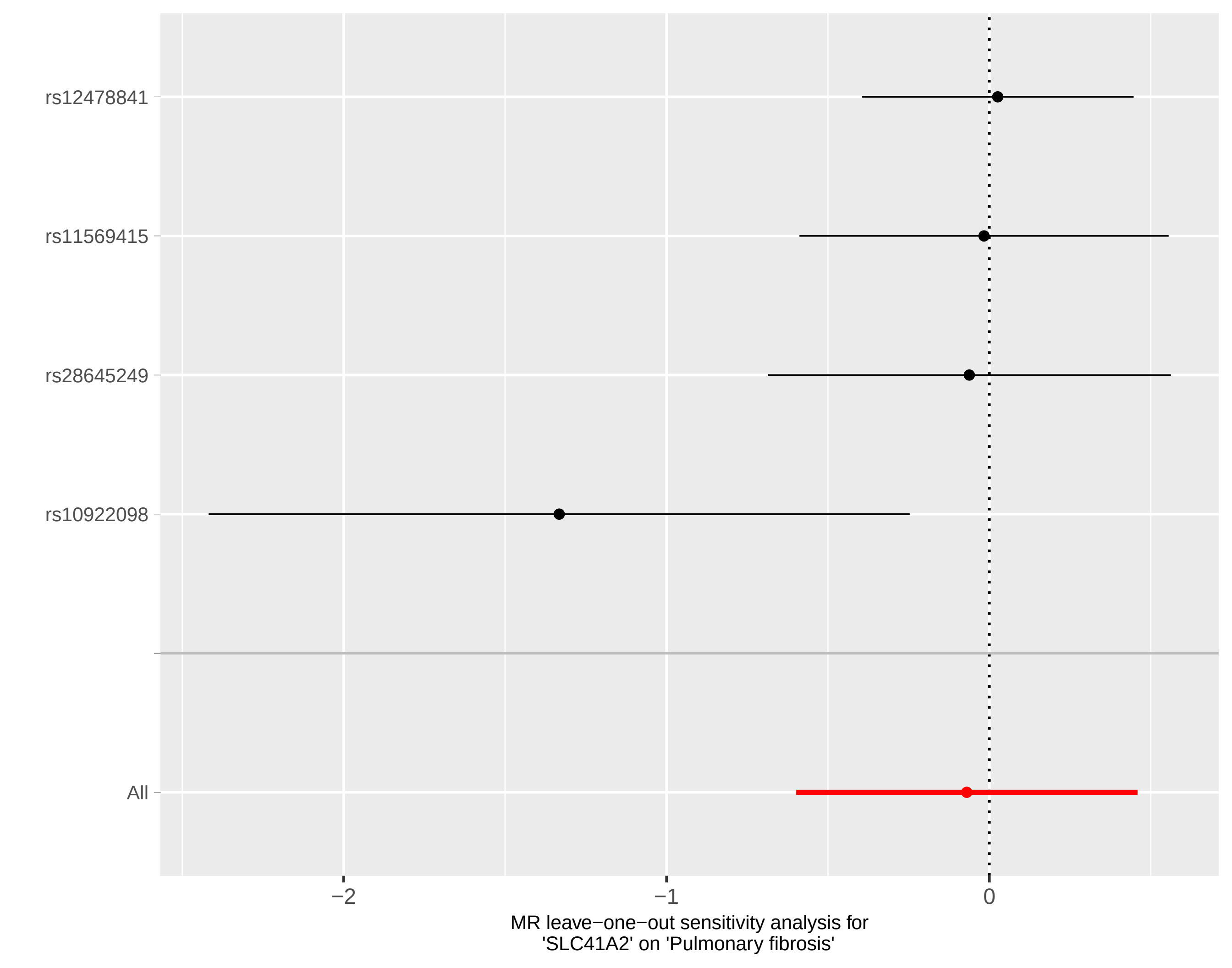
rs12478841
rs11569415
rs28645249
rs10922098
All
−2
−1
0
MR leave−one−out sensitivity analysis for
'SLC41A2' on 'Pulmonary fibrosis'

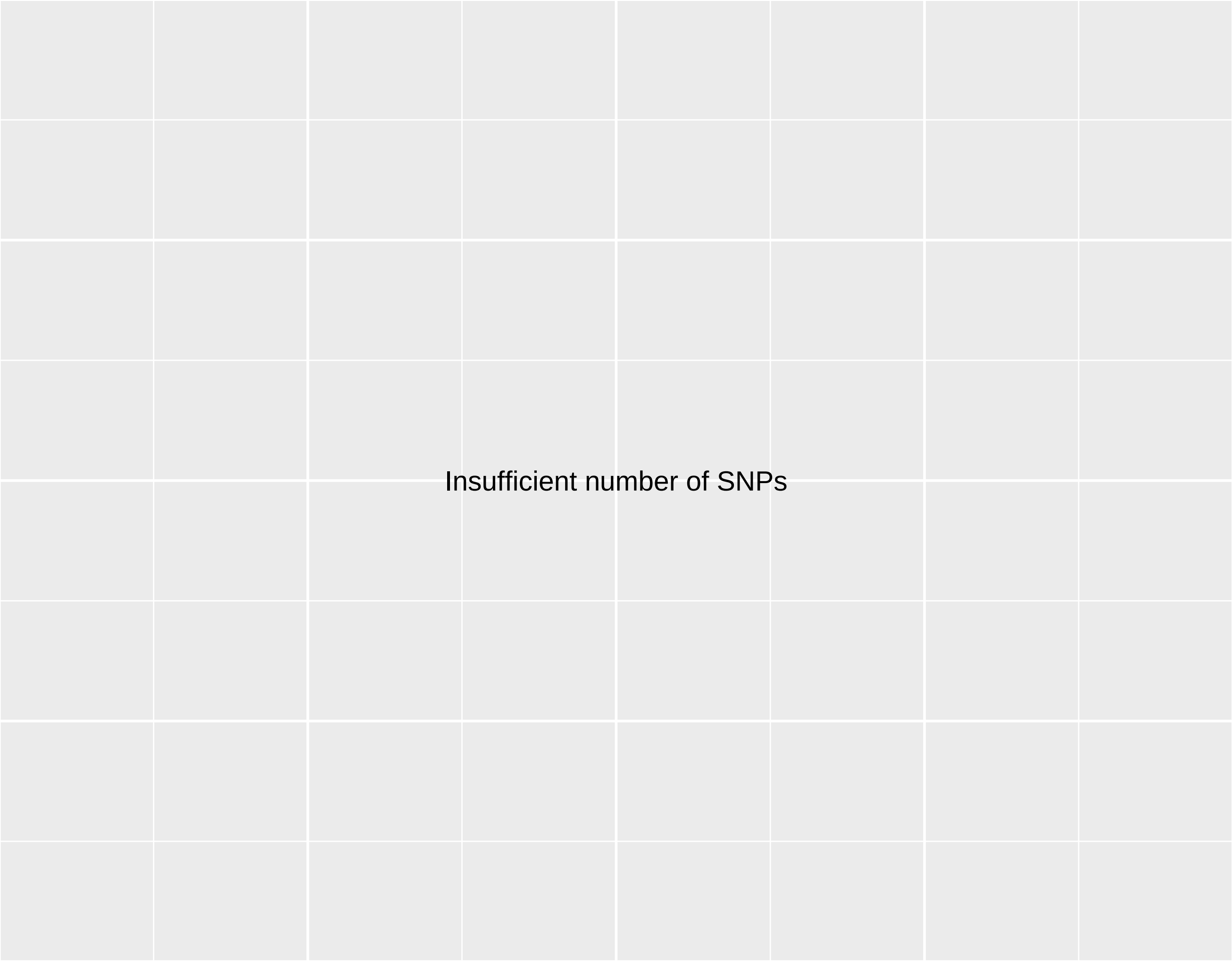
Insufficient number of SNPs

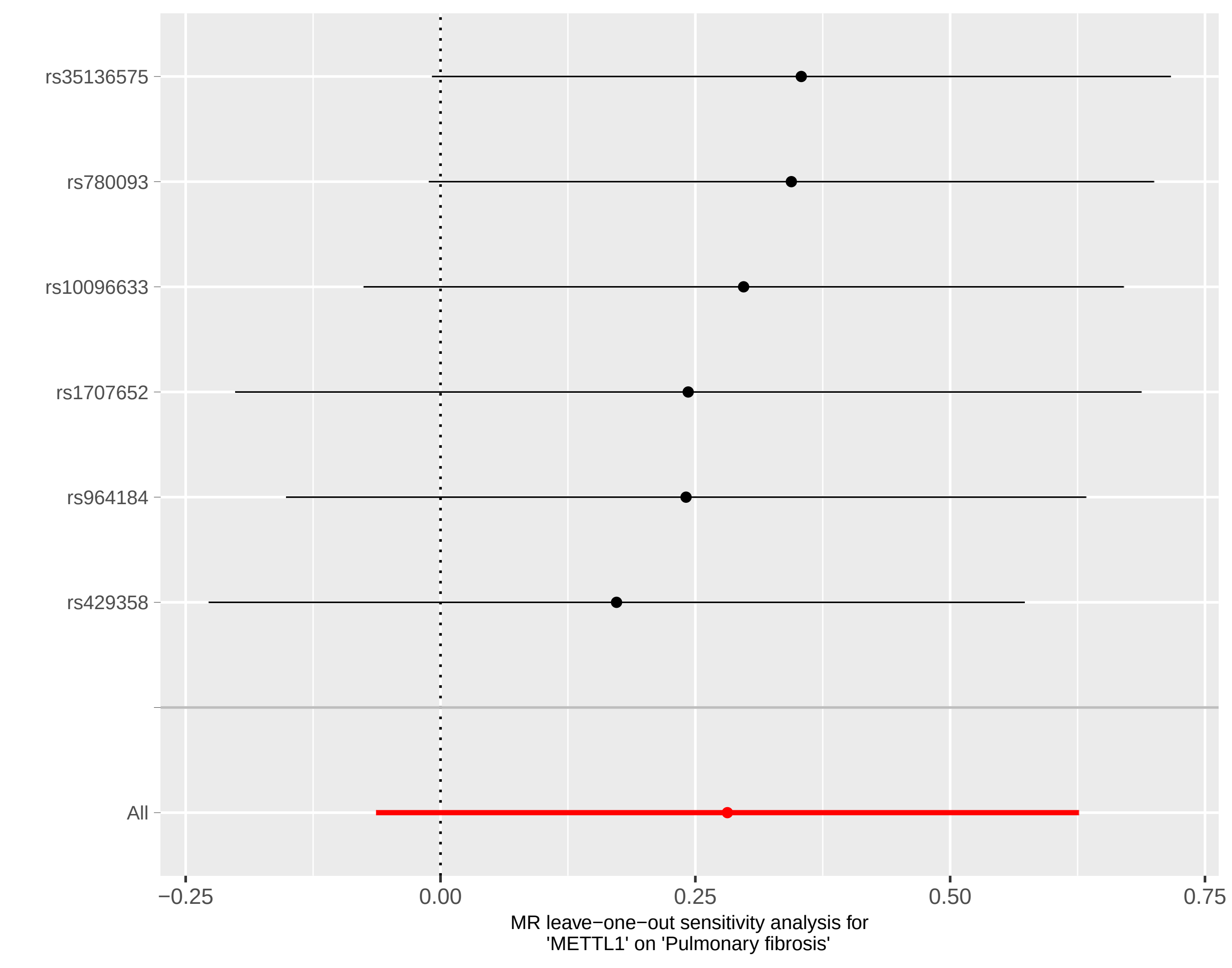
rs35136575
rs780093
rs10096633
rs1707652
rs964184
rs429358
All
−0.25
0.00
0.25
0.50
0.75
MR leave−one−out sensitivity analysis for
'METTL1' on 'Pulmonary fibrosis'

Insufficient number of SNPs

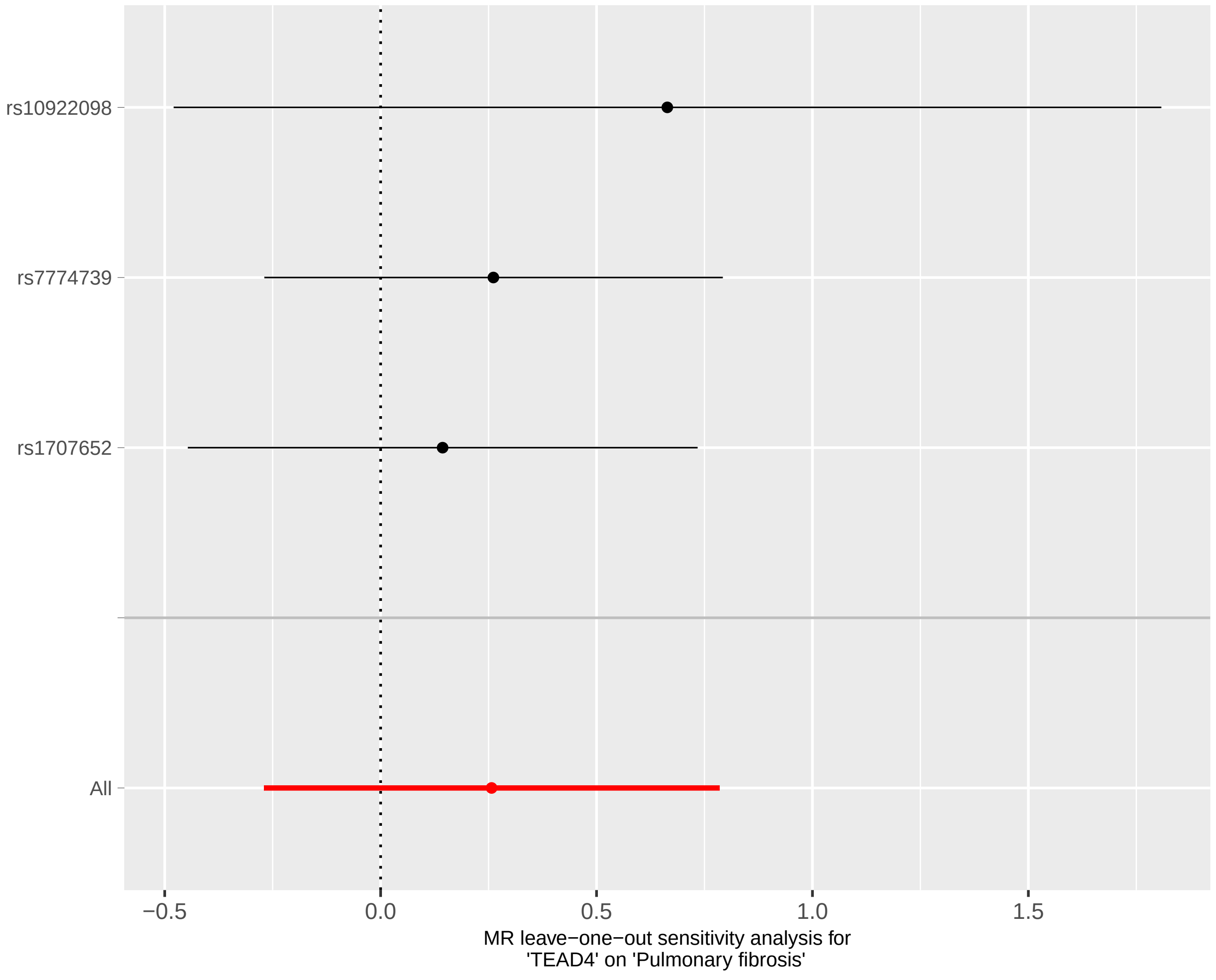

rs10922098
rs7774739
rs1707652
All
−0.5
0.0
0.5
1.0
1.5
MR leave−one−out sensitivity analysis for
'TEAD4' on 'Pulmonary fibrosis'

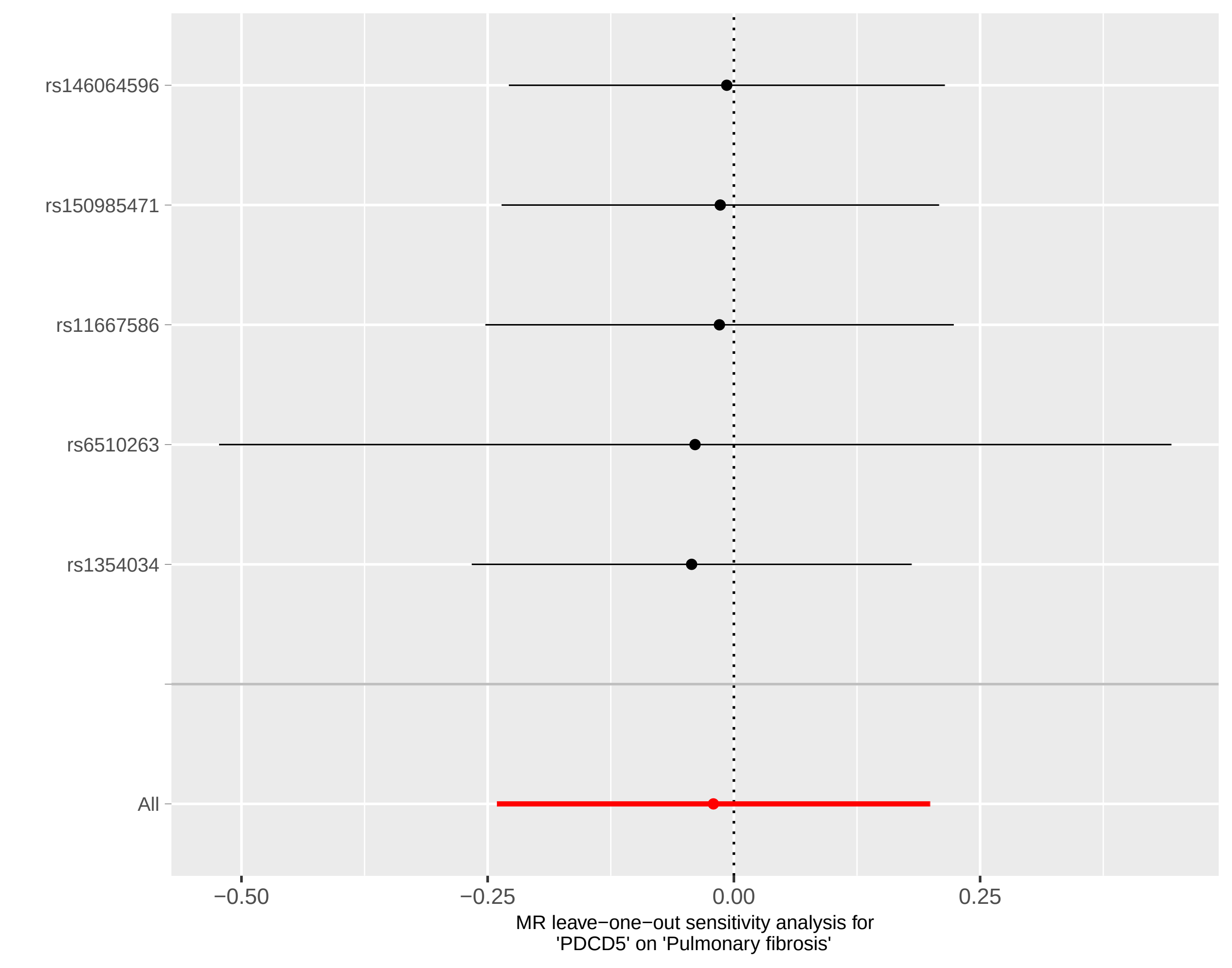

rs146064596
rs150985471
rs11667586
rs6510263
rs1354034
All
−0.50
−0.25
0.00
0.25
MR leave−one−out sensitivity analysis for
'PDCD5' on 'Pulmonary fibrosis'

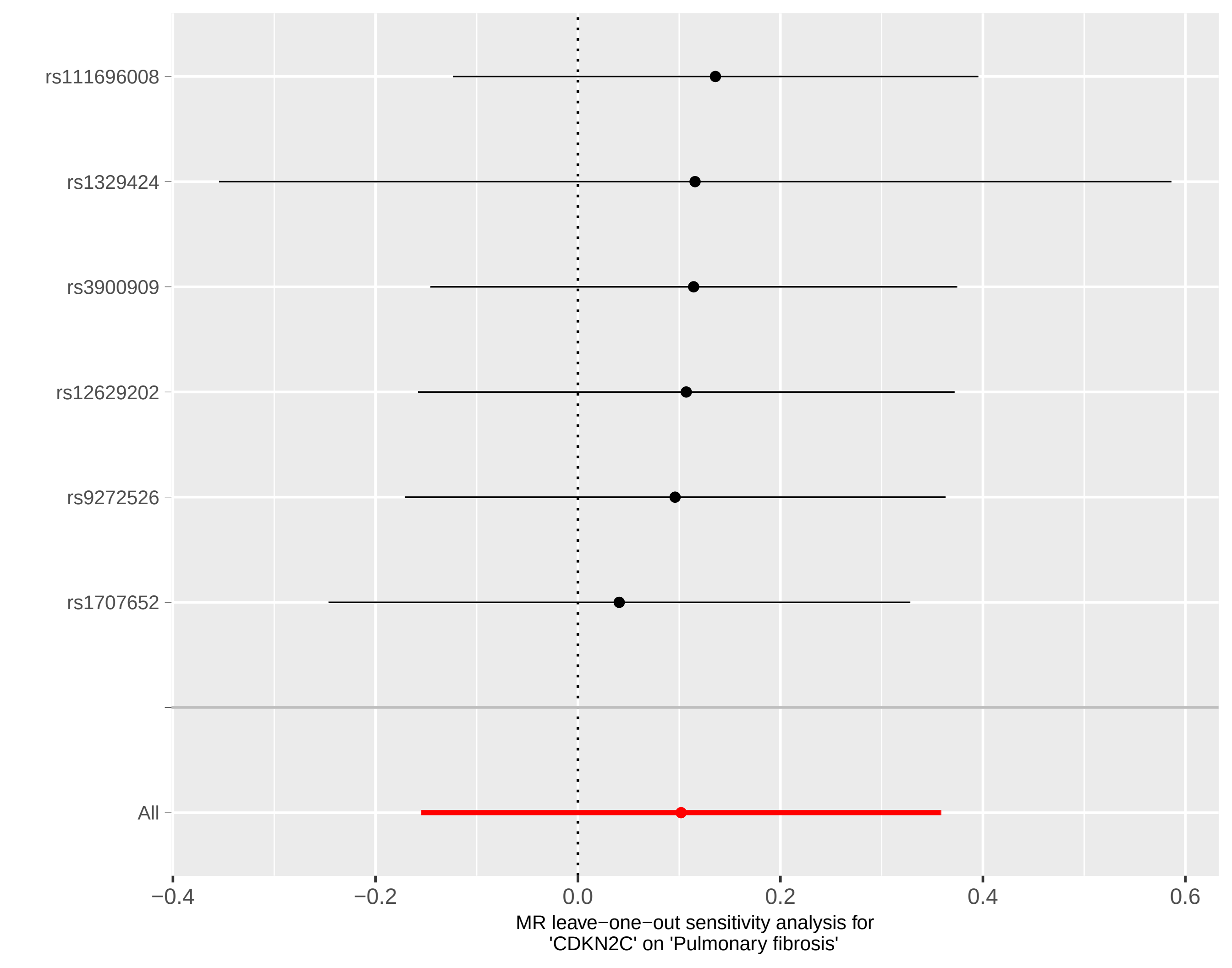
rs111696008
rs1329424
rs3900909
rs12629202
rs9272526
rs1707652
All
−0.4
−0.2
0.0
0.2
0.4
0.6
MR leave−one−out sensitivity analysis for
'CDKN2C' on 'Pulmonary fibrosis'

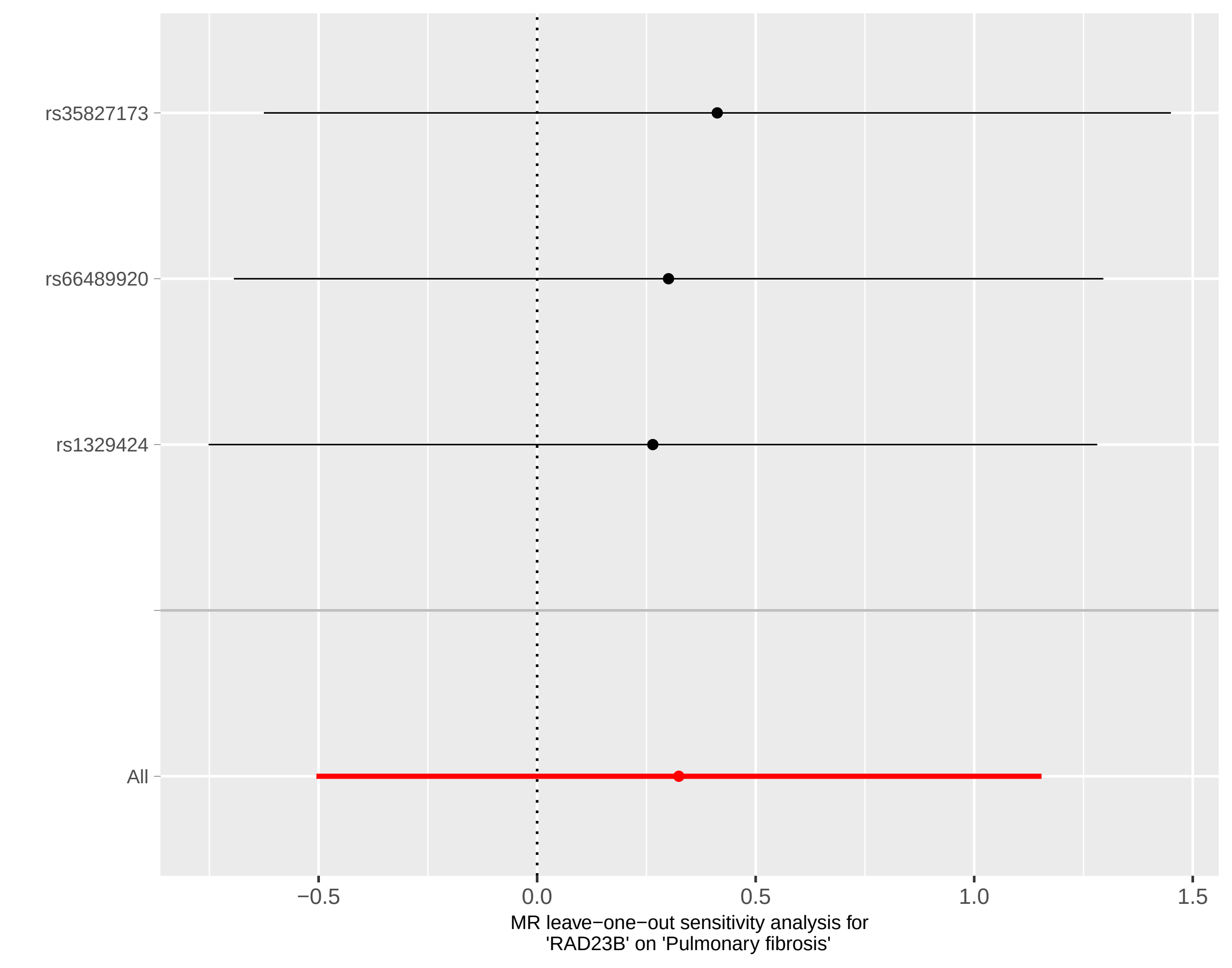

rs35827173
rs66489920
rs1329424
All
−0.5
0.0
0.5
1.0
1.5
MR leave−one−out sensitivity analysis for
'RAD23B' on 'Pulmonary fibrosis'

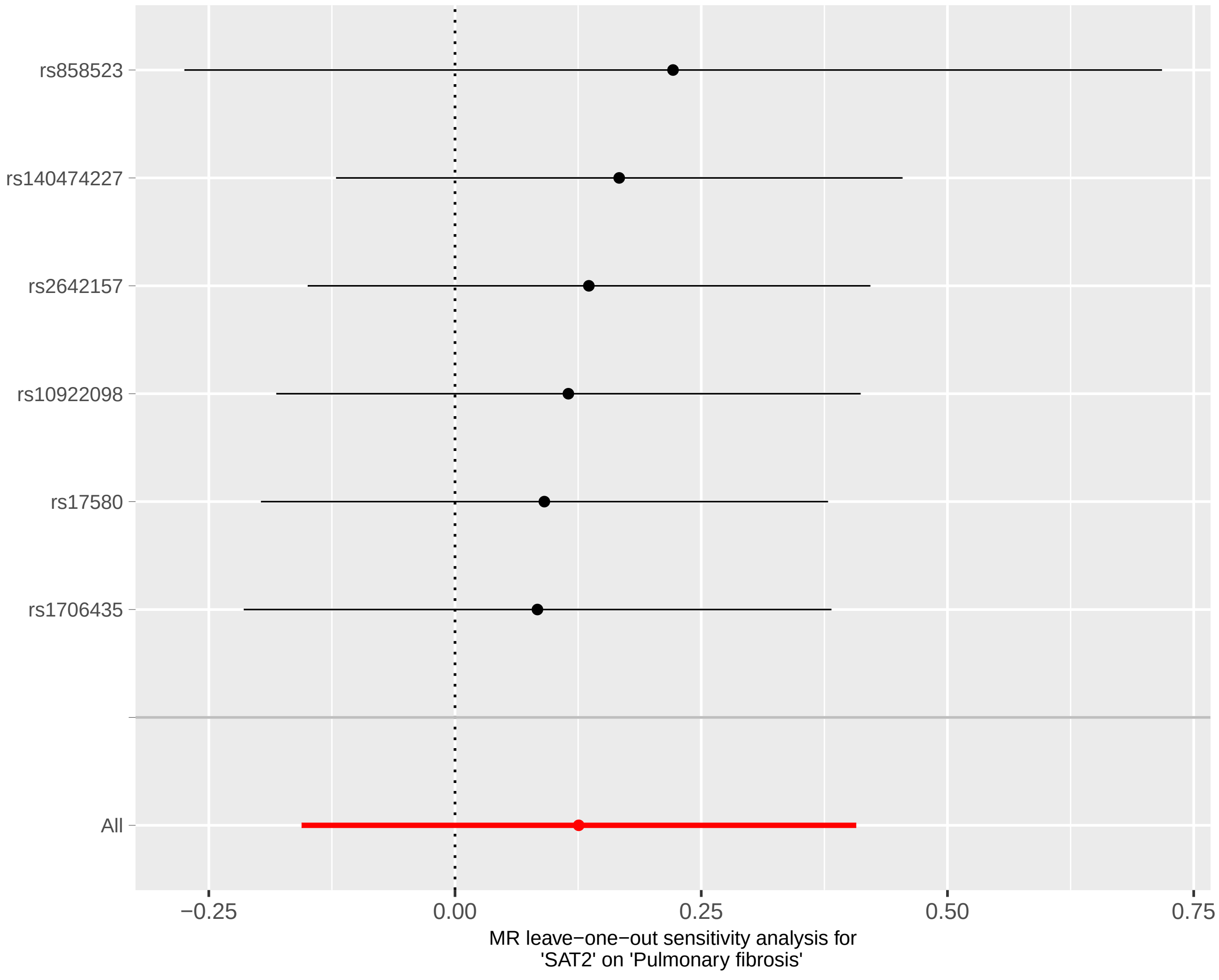

rs858523
rs140474227
rs2642157
rs10922098
rs17580
rs1706435
All
−0.25
0.00
0.25
0.50
0.75
MR leave−one−out sensitivity analysis for
'SAT2' on 'Pulmonary fibrosis'

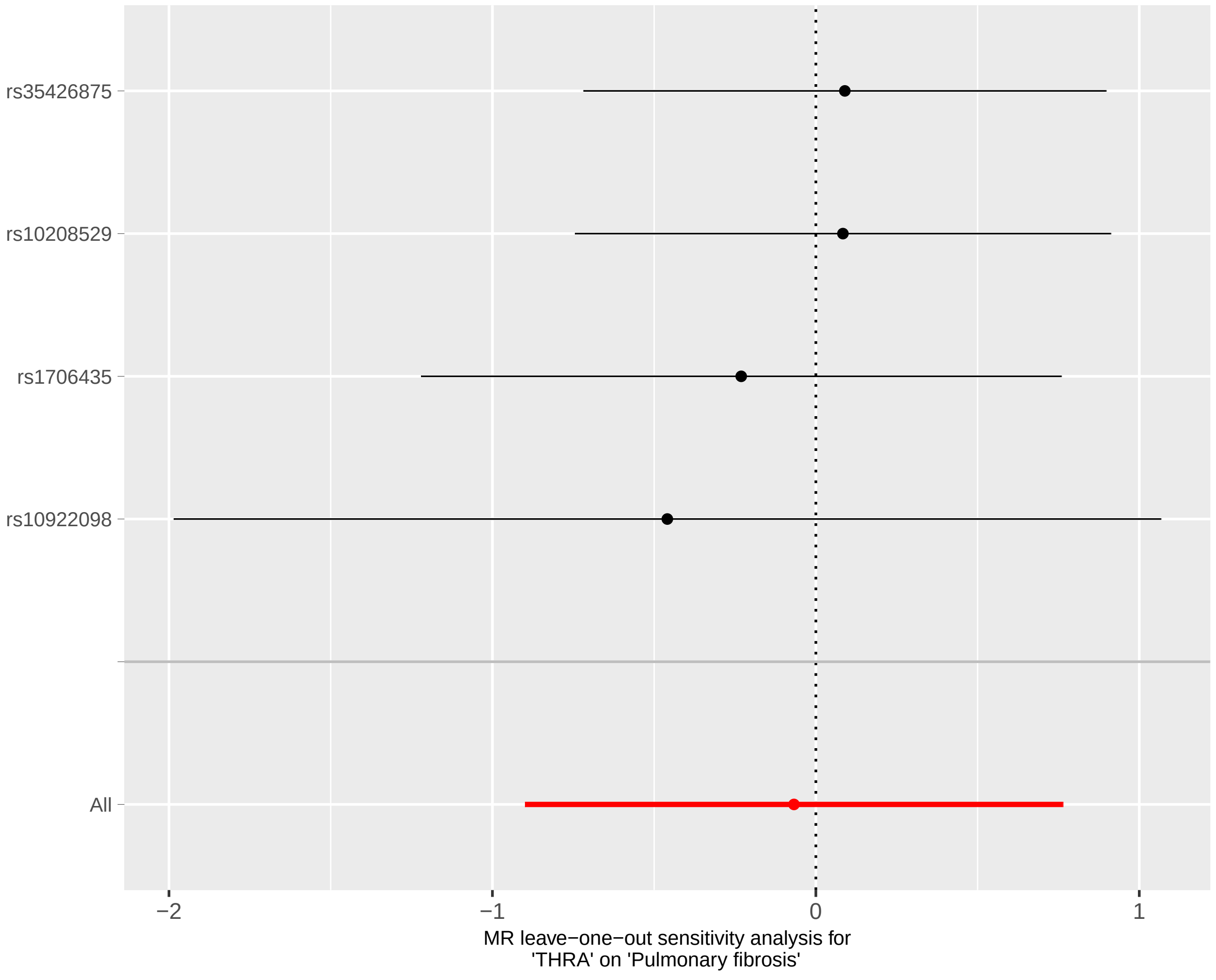

rs35426875
rs10208529
rs1706435
rs10922098
All
−2
−1
0
1
MR leave−one−out sensitivity analysis for
'THRA' on 'Pulmonary fibrosis'

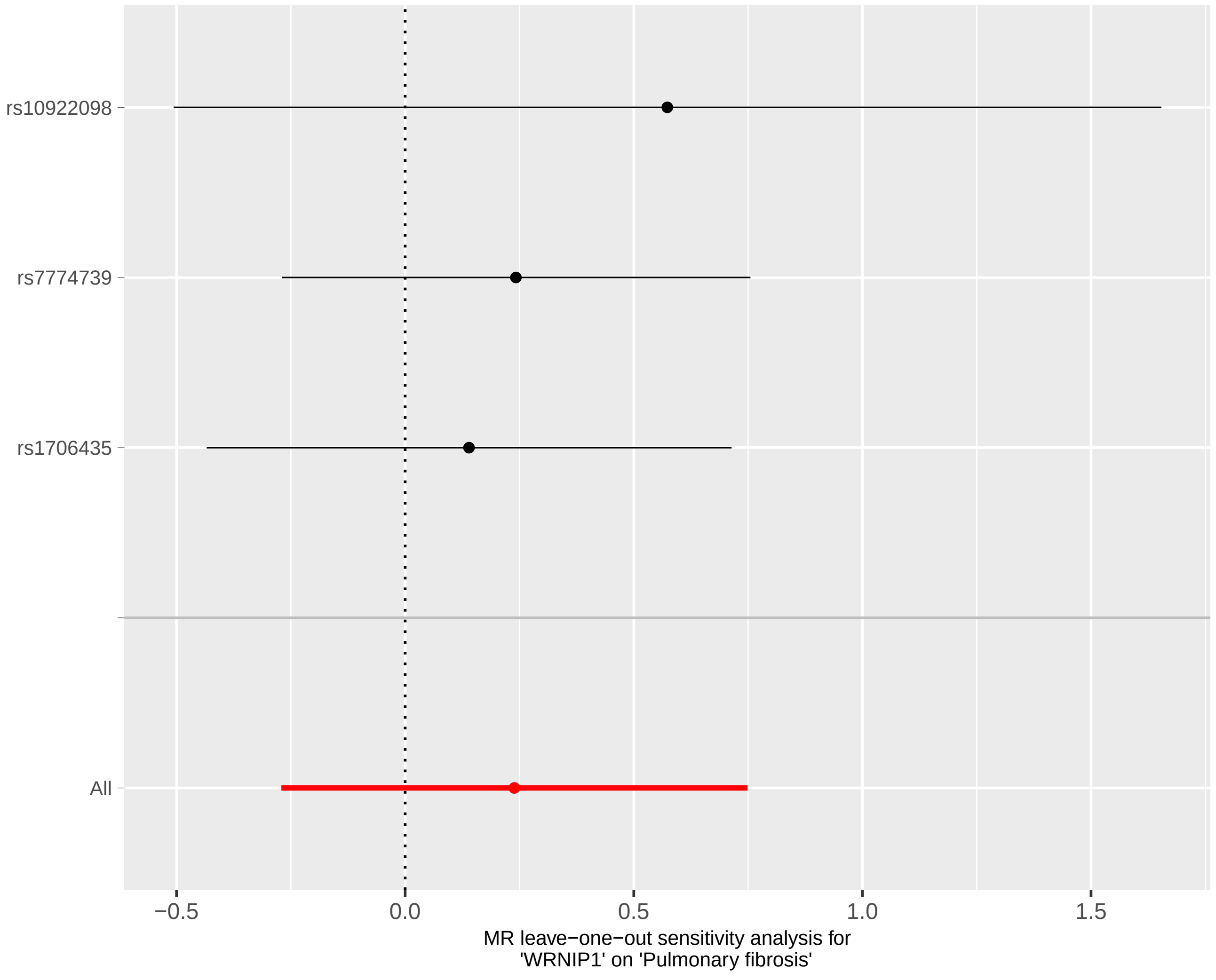
rs10922098
rs7774739
rs1706435
All
−0.5
0.0
0.5
1.0
1.5
MR leave−one−out sensitivity analysis for
'WRNIP1' on 'Pulmonary fibrosis'

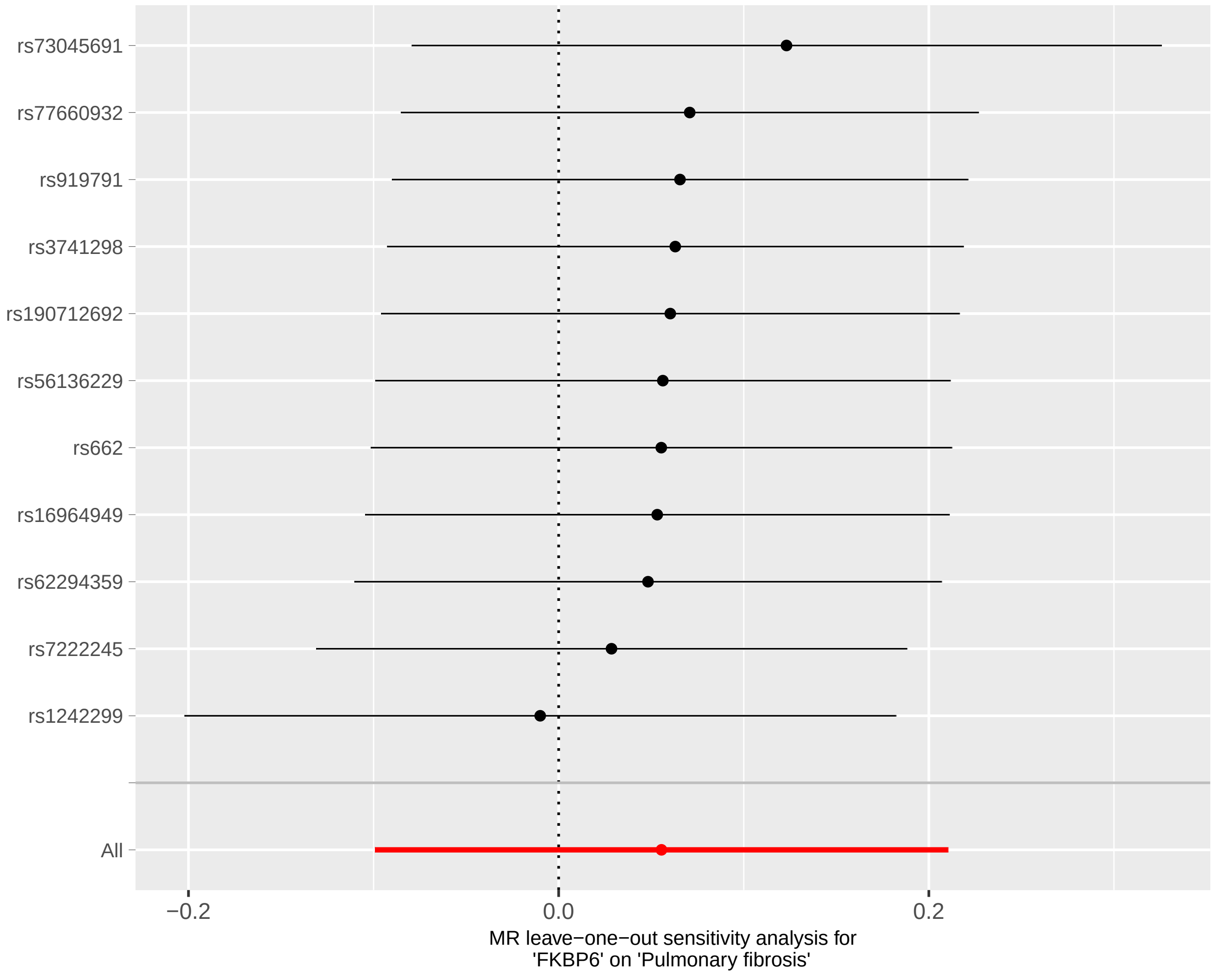

rs73045691
rs77660932
rs919791
rs3741298
rs190712692
rs56136229
rs662
rs16964949
rs62294359
rs7222245
rs1242299
All
−0.2
0.0
0.2
MR leave−one−out sensitivity analysis for
'FKBP6' on 'Pulmonary fibrosis'

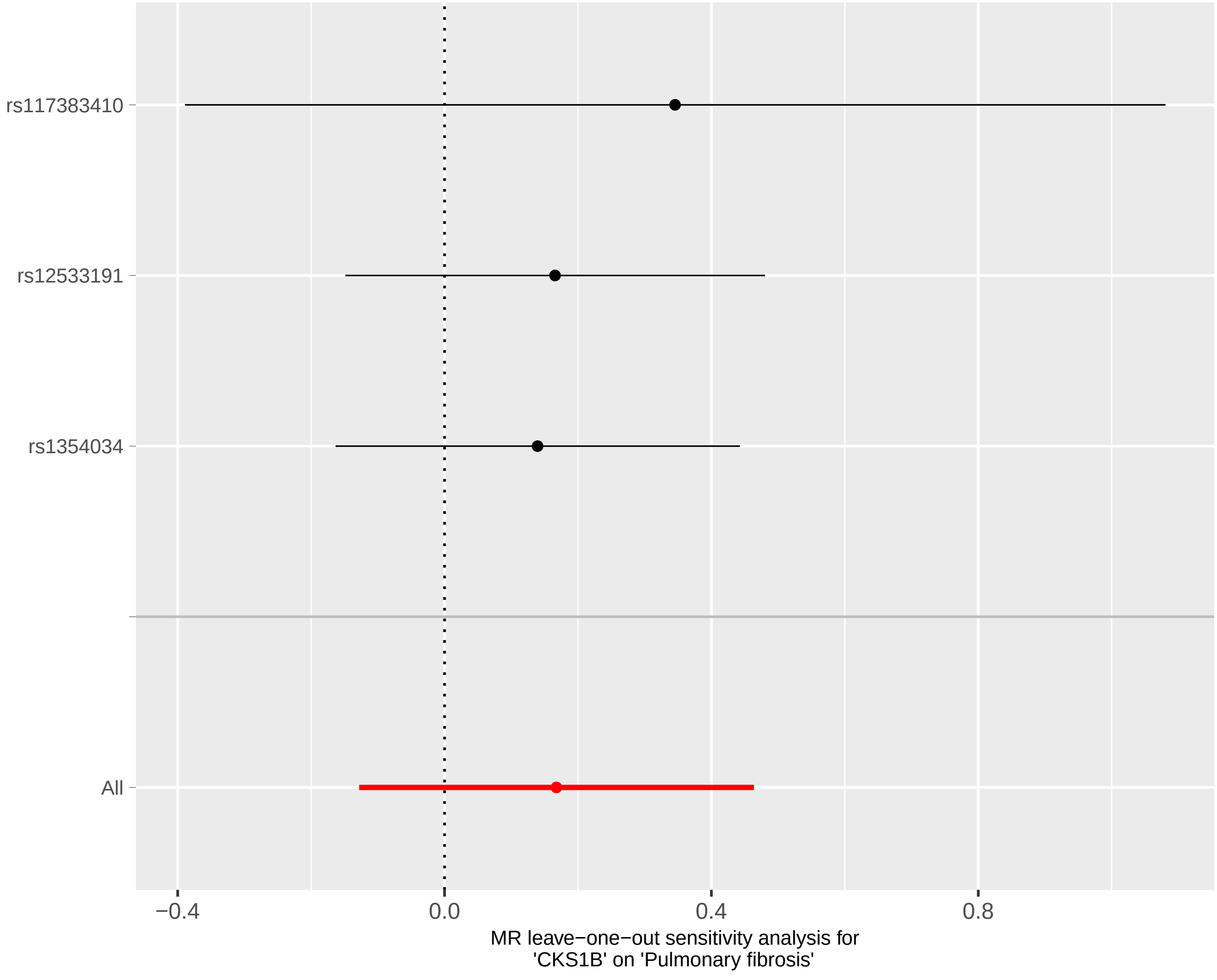

rs117383410
rs12533191
rs1354034
All
−0.4
0.0
0.4
0.8
MR leave−one−out sensitivity analysis for
'CKS1B' on 'Pulmonary fibrosis'

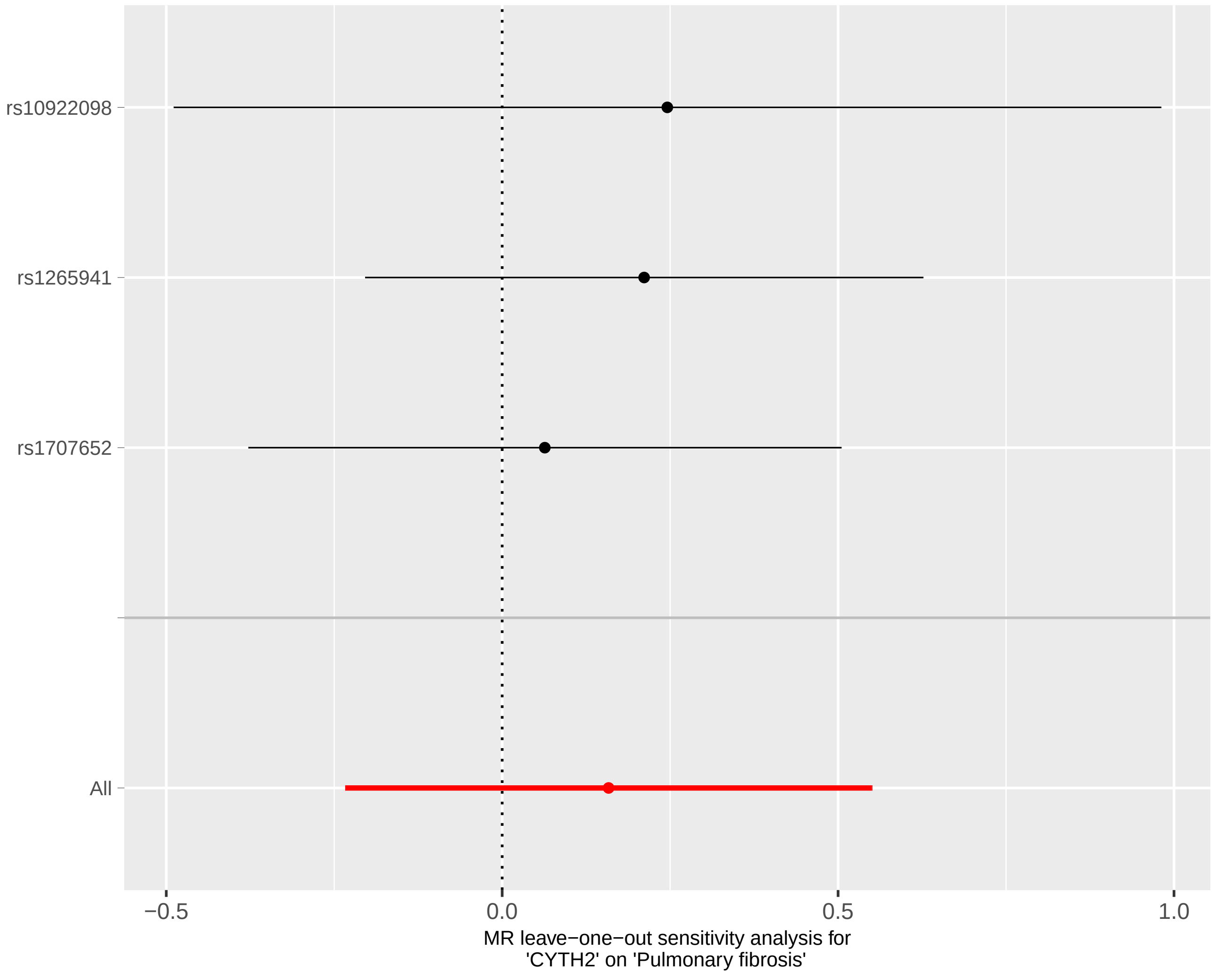

rs10922098
rs1265941
rs1707652
All
−0.5
0.0
0.5
1.0
MR leave−one−out sensitivity analysis for
'CYTH2' on 'Pulmonary fibrosis'

Insufficient number of SNPs

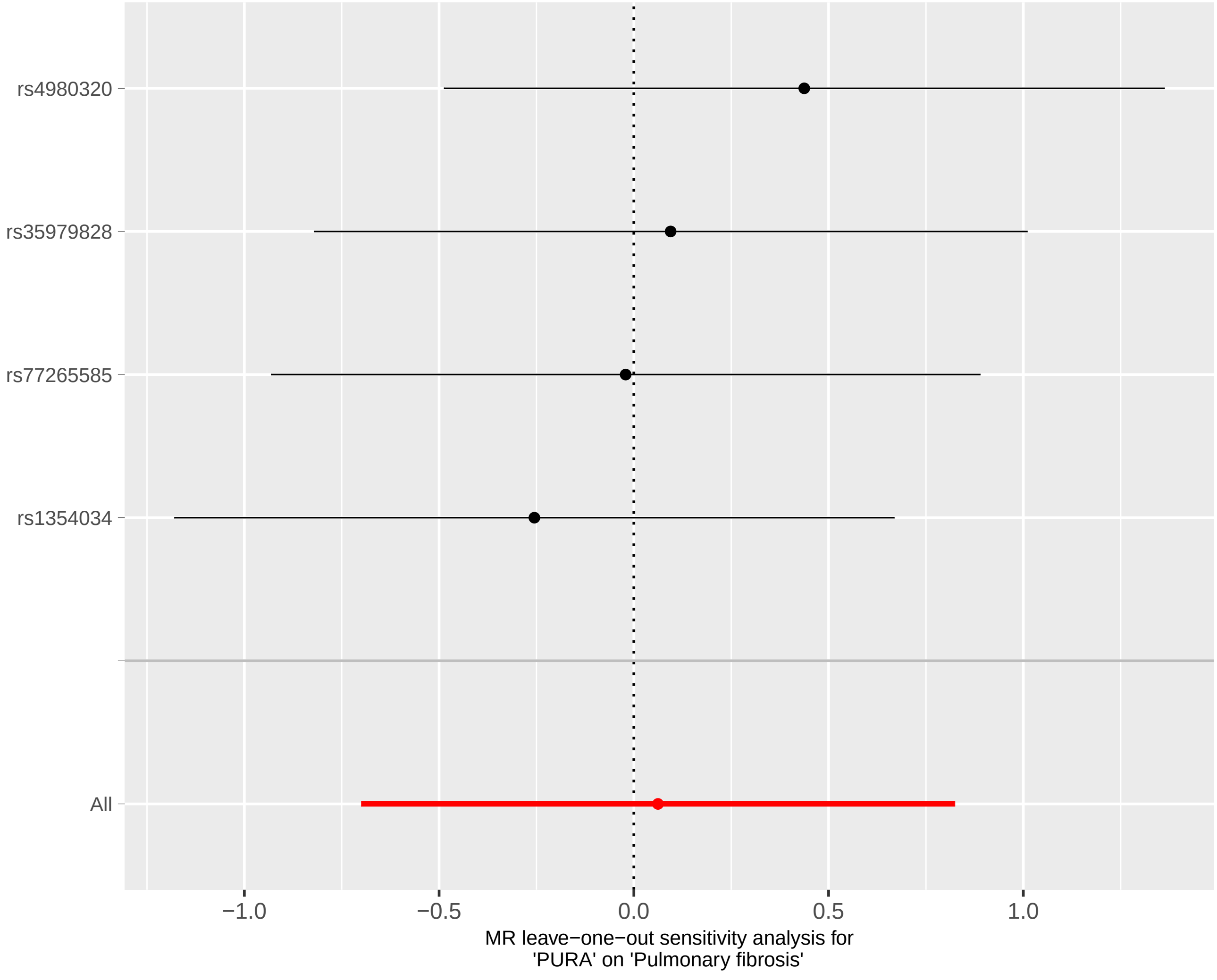

rs4980320
rs35979828
rs77265585
rs1354034
All
−1.0
−0.5
0.0
0.5
1.0
MR leave−one−out sensitivity analysis for
'PURA' on 'Pulmonary fibrosis'

Insufficient number of SNPs

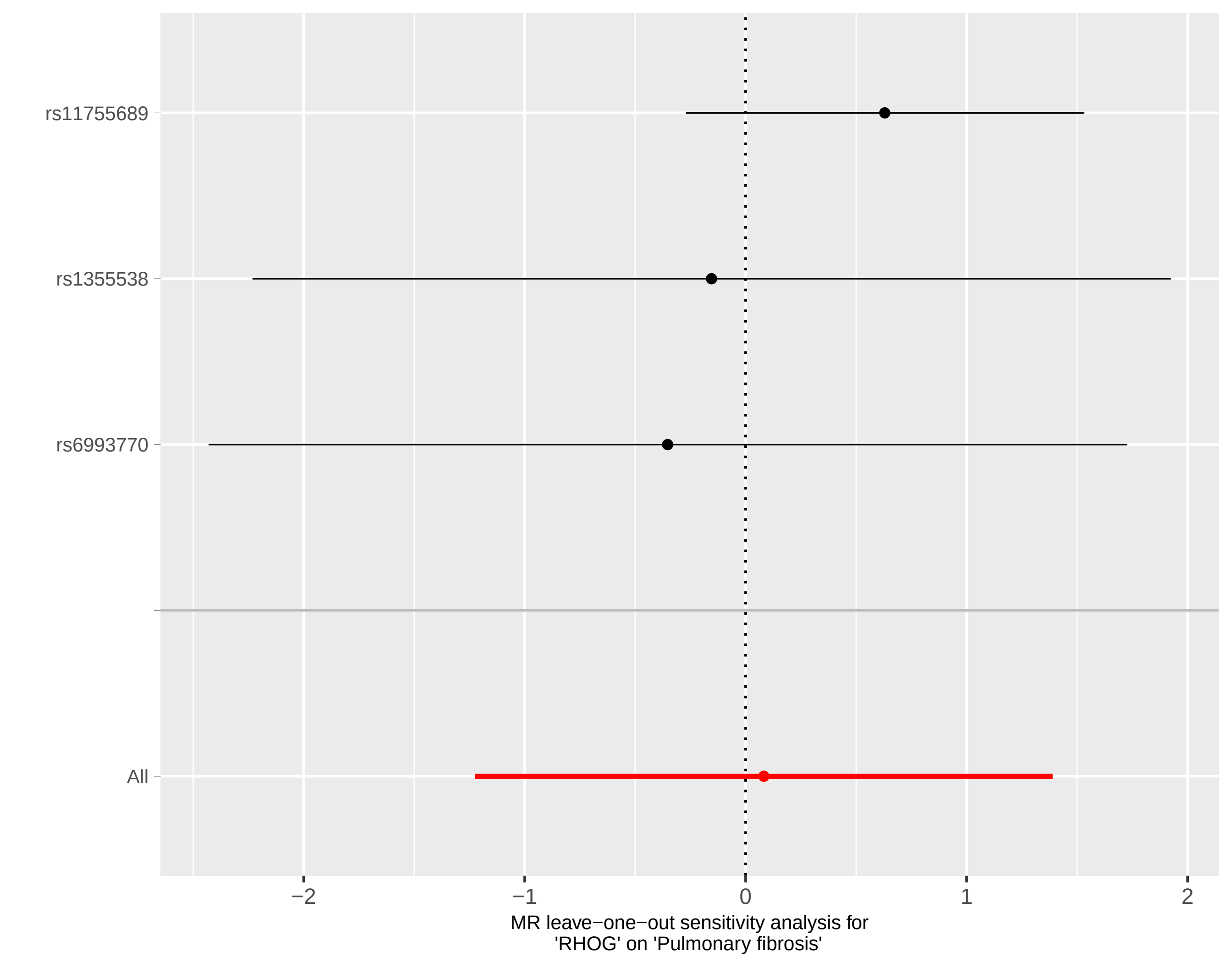

rs11755689
rs1355538
rs6993770
All
−2
−1
0
1
2
MR leave−one−out sensitivity analysis for
'RHOG' on 'Pulmonary fibrosis'

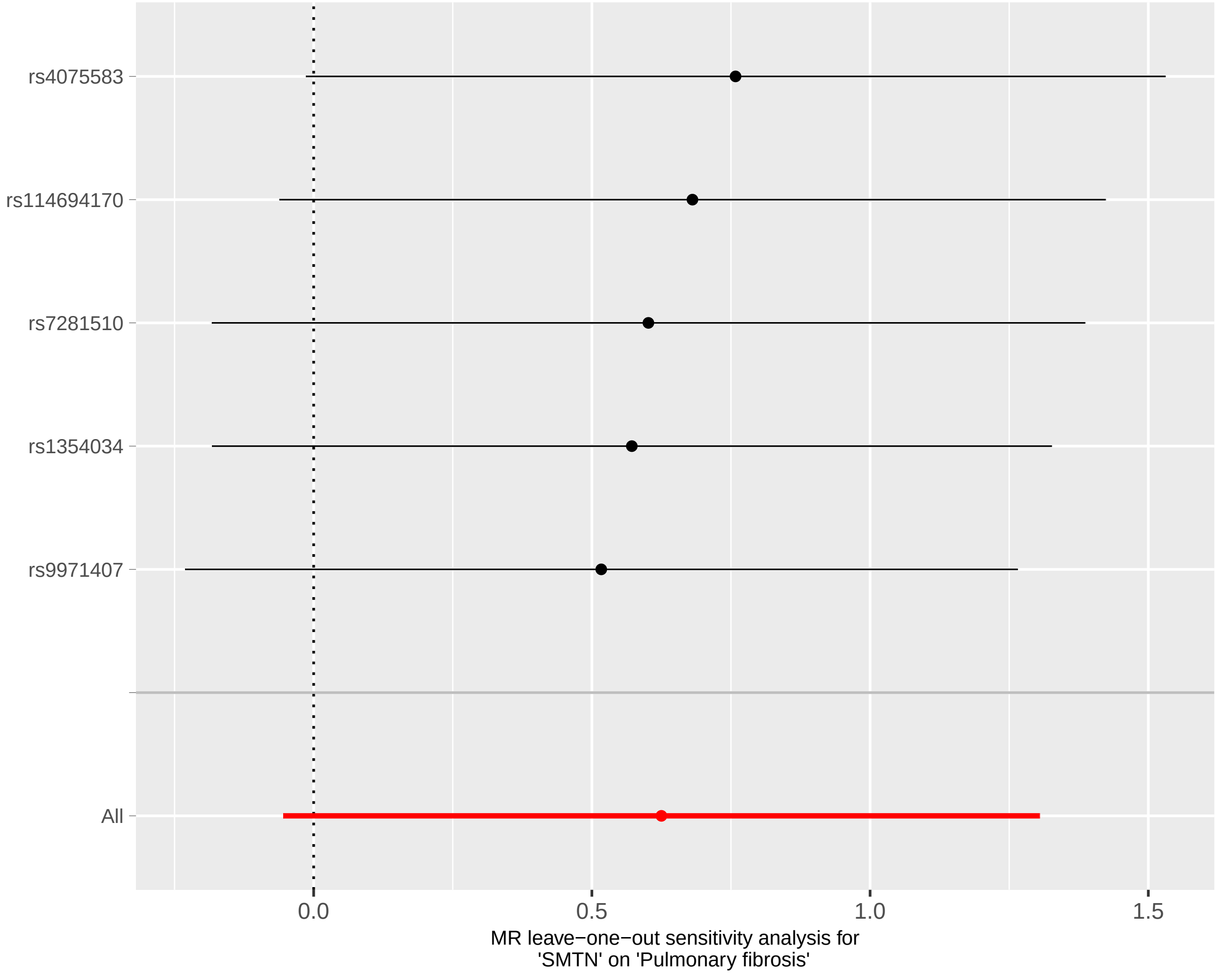

rs4075583
rs114694170
rs7281510
rs1354034
rs9971407
All
0.0
0.5
1.0
1.5
MR leave−one−out sensitivity analysis for
'SMTN' on 'Pulmonary fibrosis'

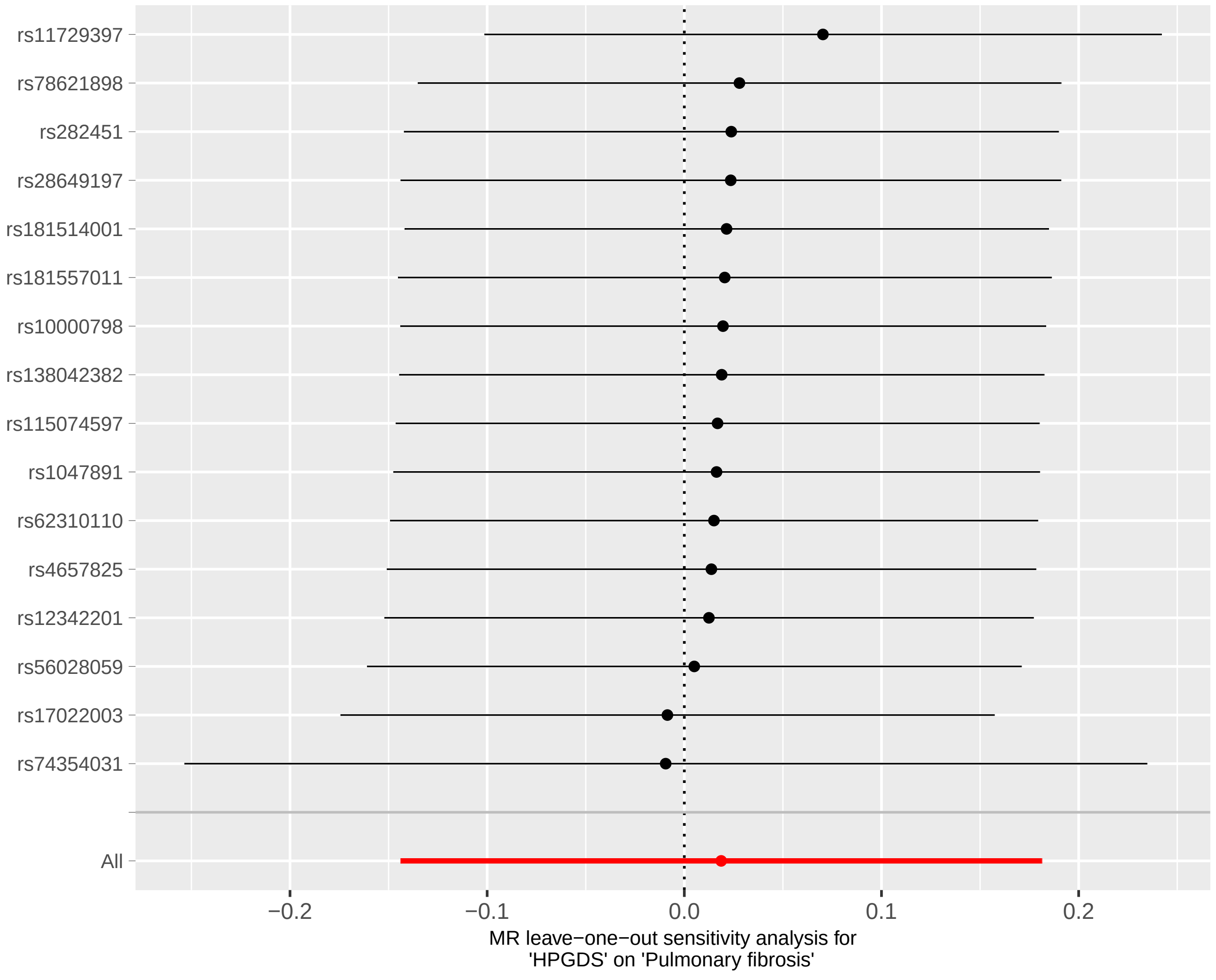
rs11729397
rs78621898
rs282451
rs28649197
rs181514001
rs181557011
rs10000798
rs138042382
rs115074597
rs1047891
rs62310110
rs4657825
rs12342201
rs56028059
rs17022003
rs74354031
All
−0.2
−0.1
0.0
0.1
0.2
MR leave−one−out sensitivity analysis for
'HPGDS' on 'Pulmonary fibrosis'

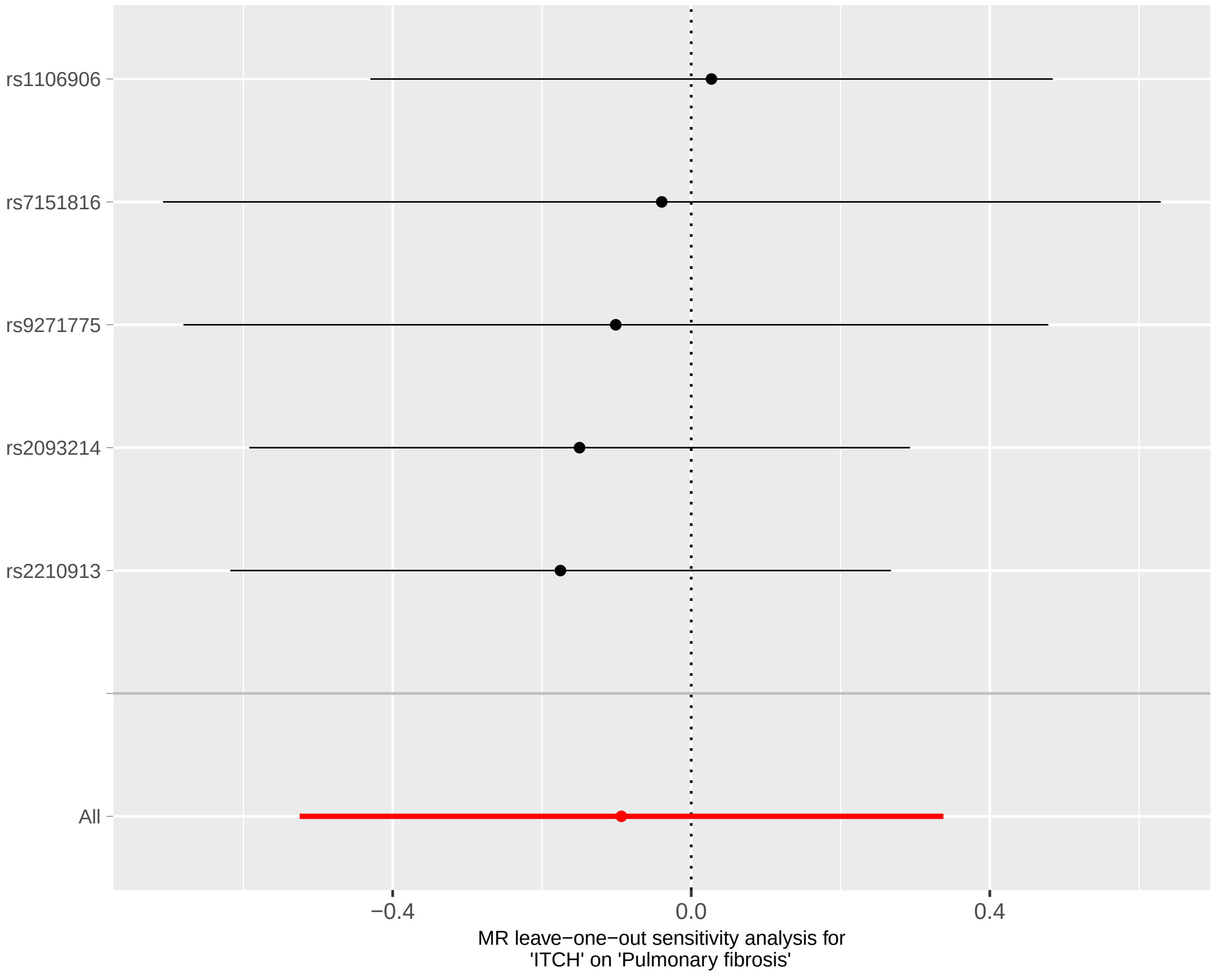
rs1106906
rs7151816
rs9271775
rs2093214
rs2210913
All
−0.4
0.0
0.4
MR leave−one−out sensitivity analysis for
'ITCH' on 'Pulmonary fibrosis'

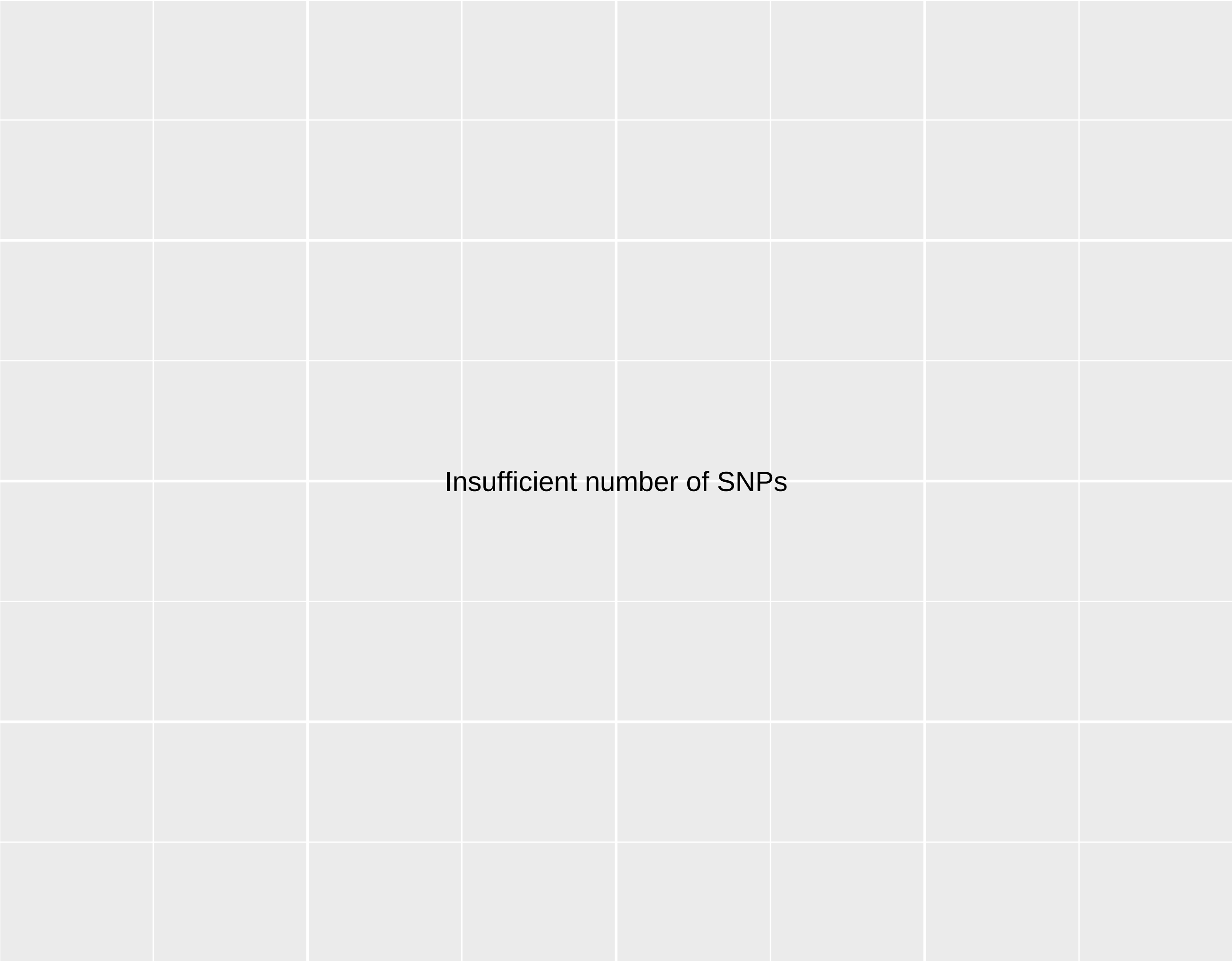
Insufficient number of SNPs

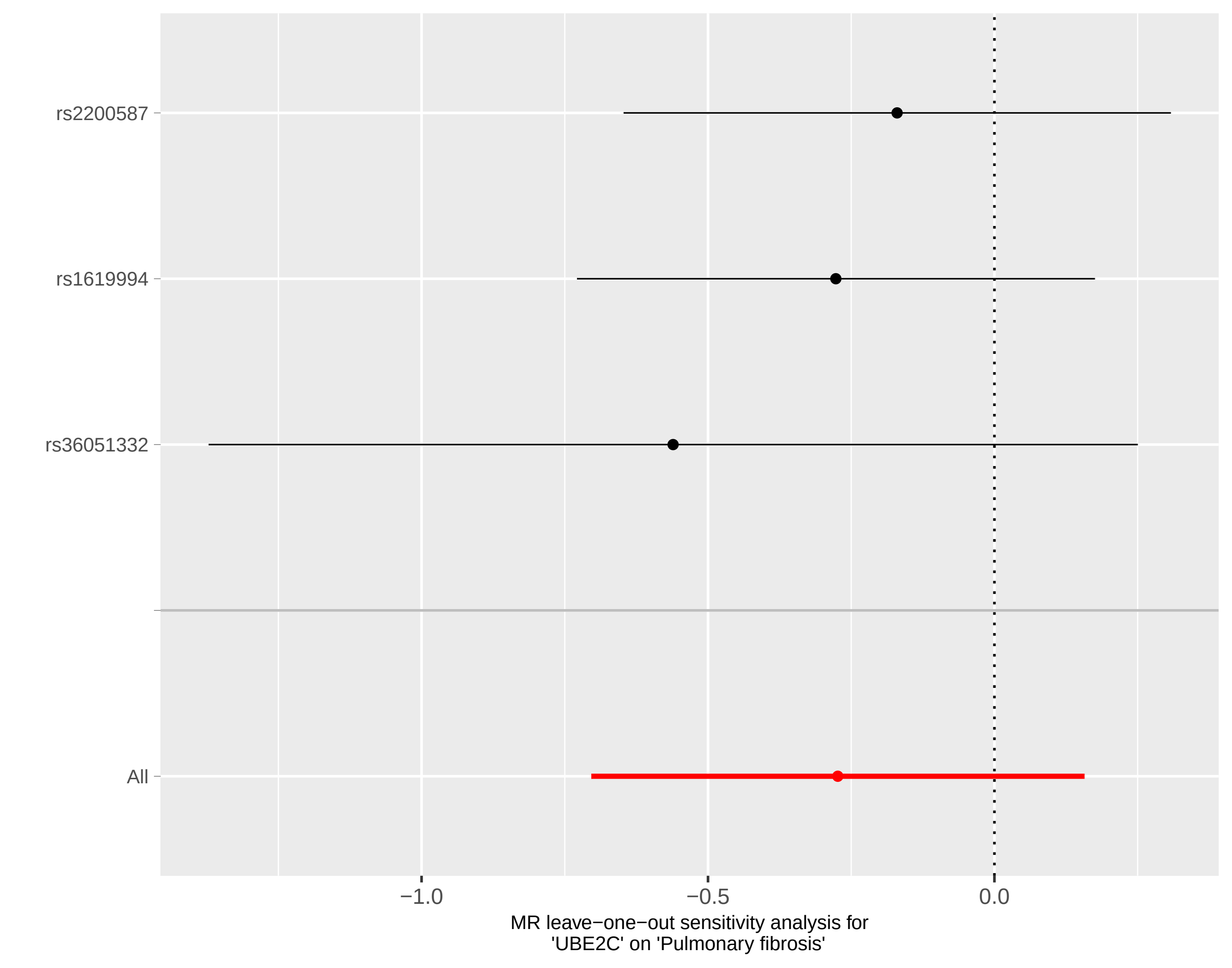
rs2200587
rs1619994
rs36051332
All
−1.0
−0.5
0.0
MR leave−one−out sensitivity analysis for
'UBE2C' on 'Pulmonary fibrosis'

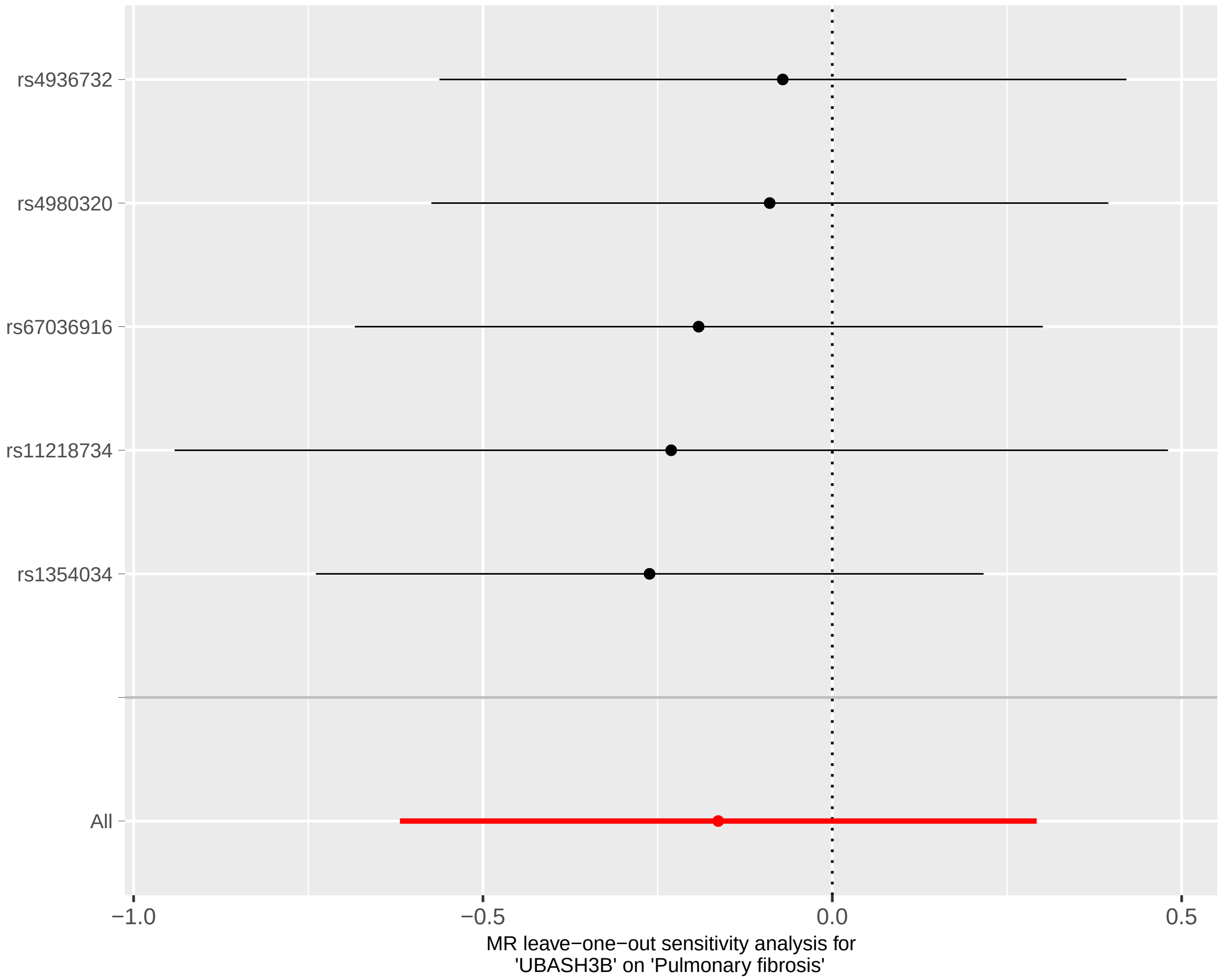
rs4936732
rs4980320
rs67036916
rs11218734
rs1354034
All
−1.0
−0.5
0.0
0.5
MR leave−one−out sensitivity analysis for
'UBASH3B' on 'Pulmonary fibrosis'

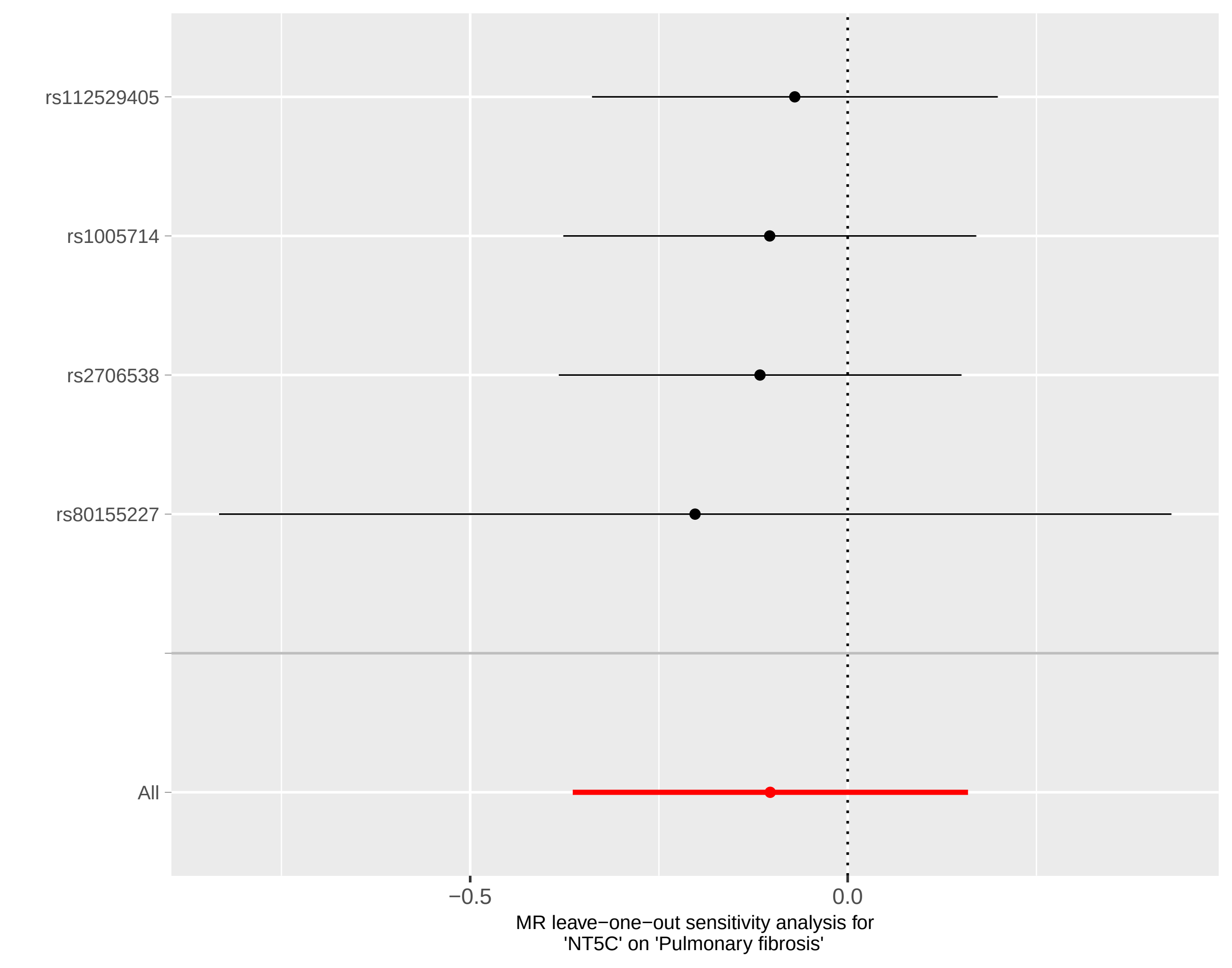
rs112529405
rs1005714
rs2706538
rs80155227
All
−0.5
0.0
MR leave−one−out sensitivity analysis for
'NT5C' on 'Pulmonary fibrosis'

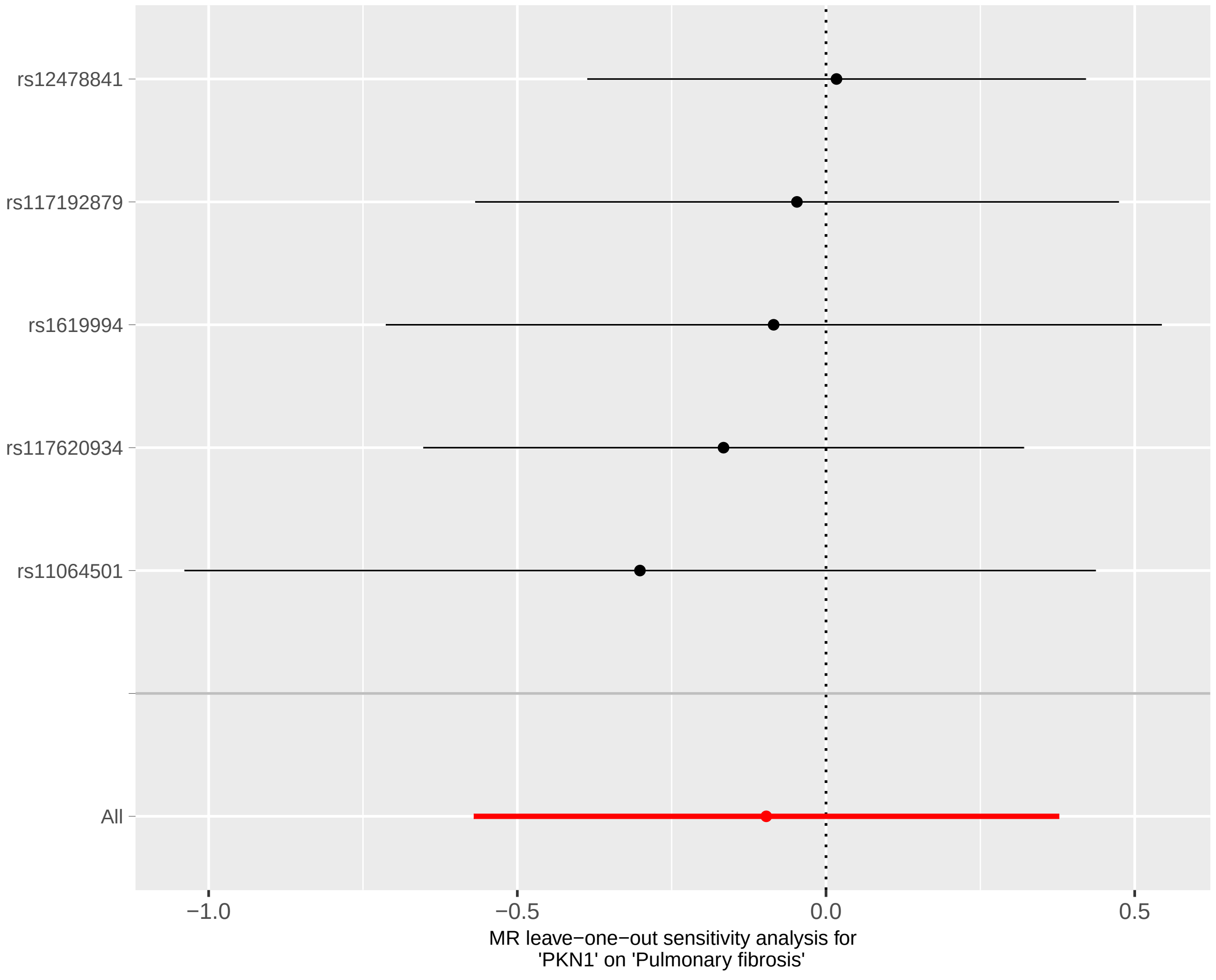

rs12478841
rs117192879
rs1619994
rs117620934
rs11064501
All
−1.0
−0.5
0.0
0.5
MR leave−one−out sensitivity analysis for
'PKN1' on 'Pulmonary fibrosis'

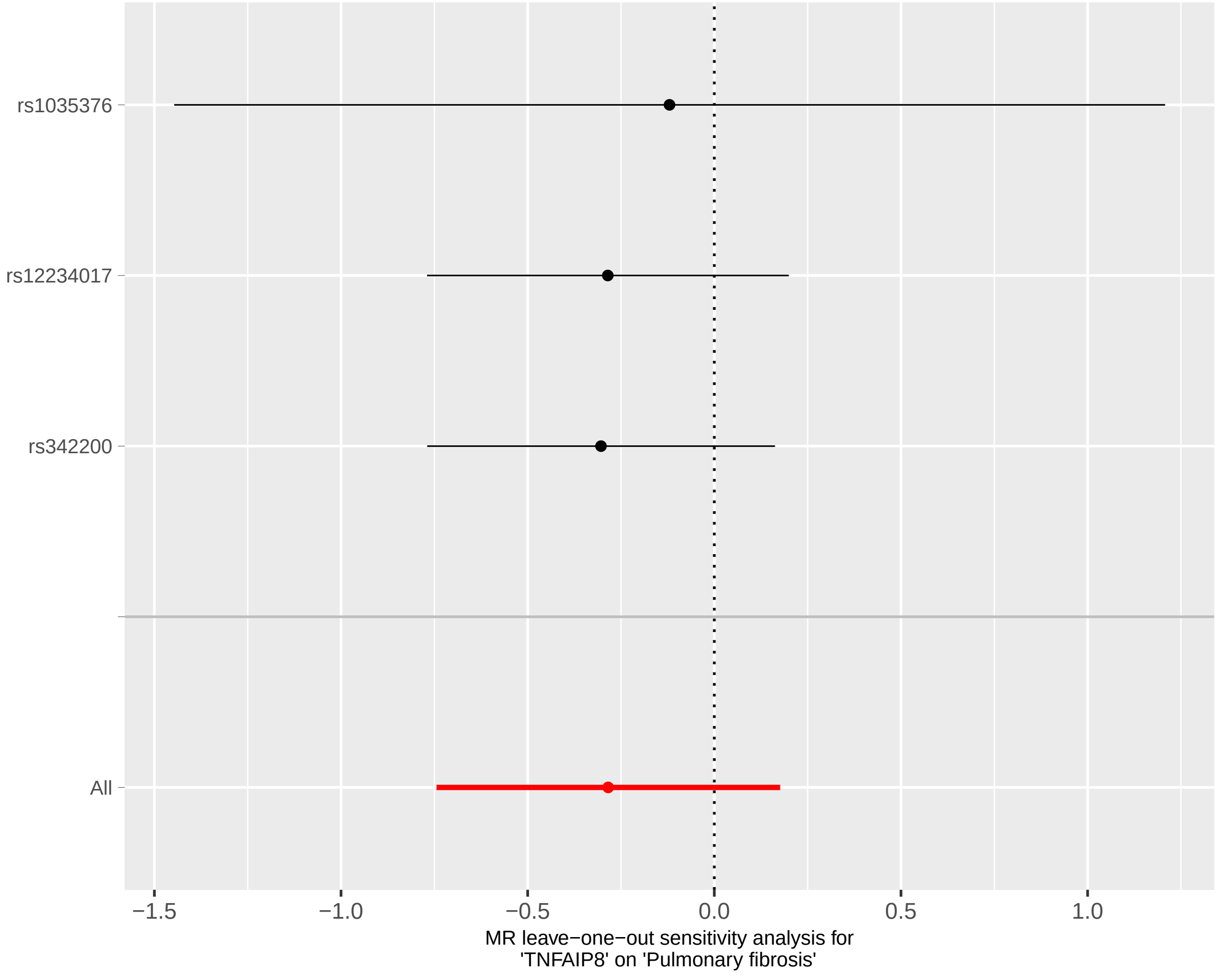
rs1035376
rs12234017
rs342200
All
−1.5
−1.0
−0.5
0.0
0.5
1.0
MR leave−one−out sensitivity analysis for
'TNFAIP8' on 'Pulmonary fibrosis'

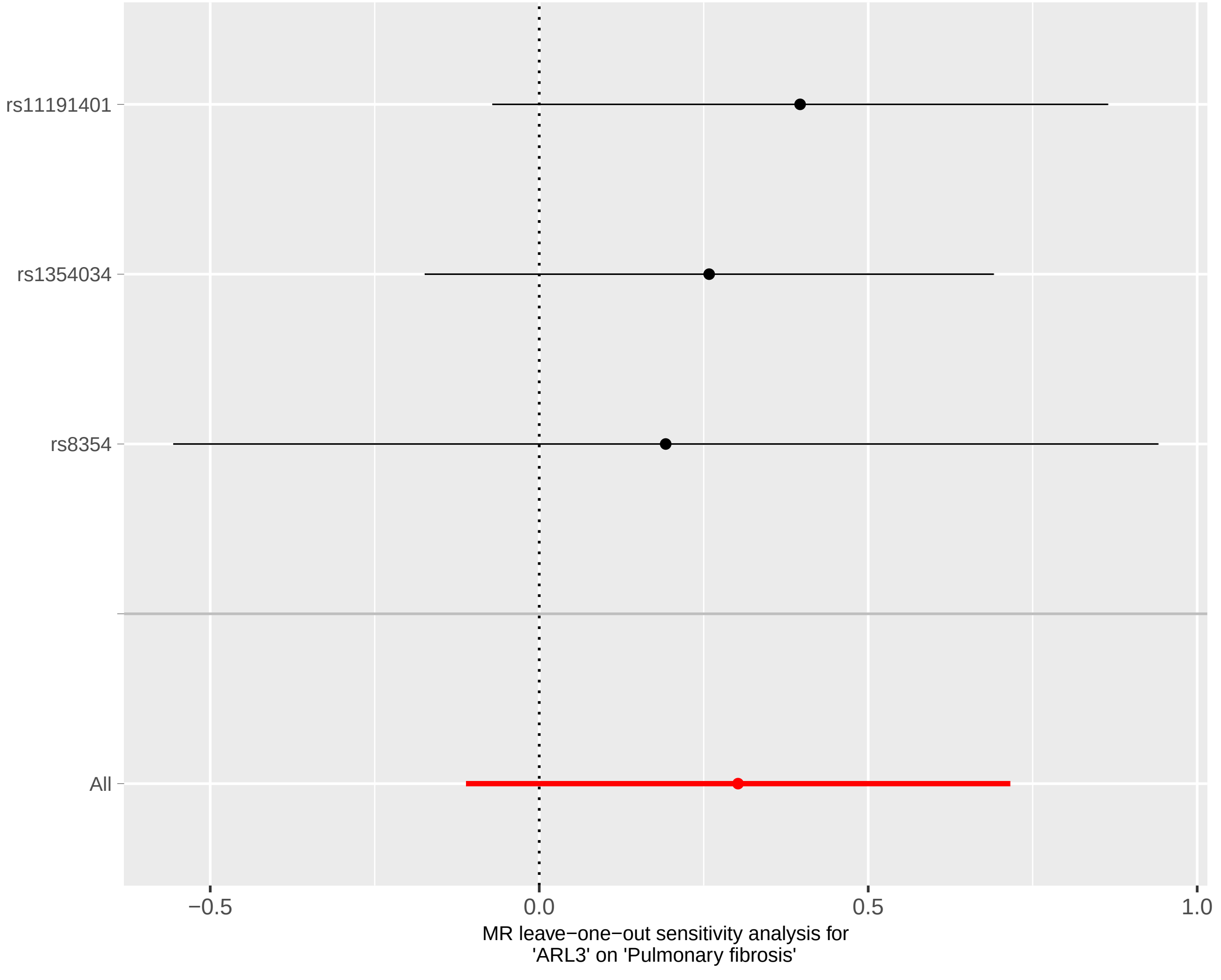
rs11191401
rs1354034
rs8354
All
−0.5
0.0
0.5
1.0
MR leave−one−out sensitivity analysis for
'ARL3' on 'Pulmonary fibrosis'

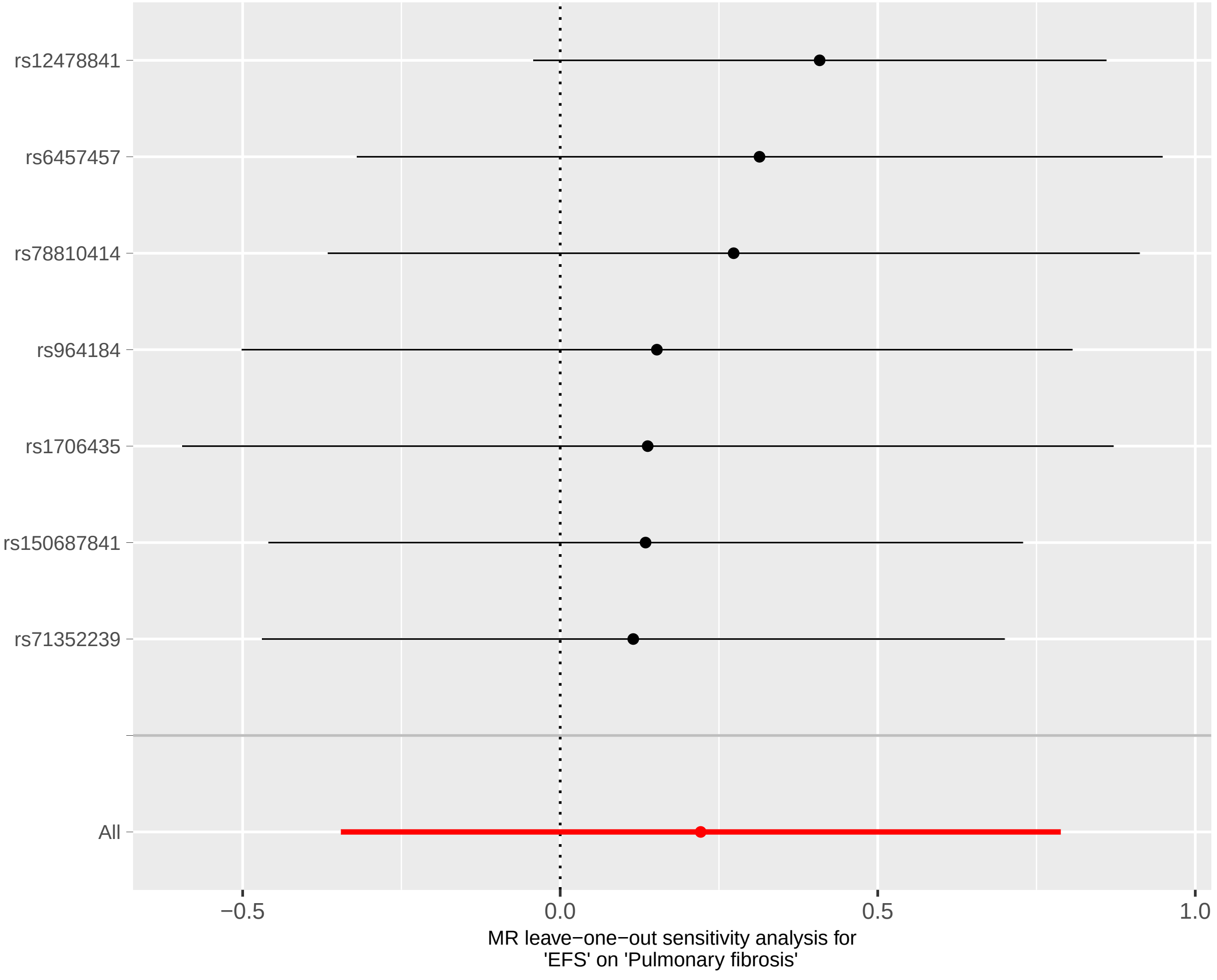
rs12478841
rs6457457
rs78810414
rs964184
rs1706435
rs150687841
rs71352239
All
−0.5
0.0
0.5
1.0
MR leave−one−out sensitivity analysis for
'EFS' on 'Pulmonary fibrosis'

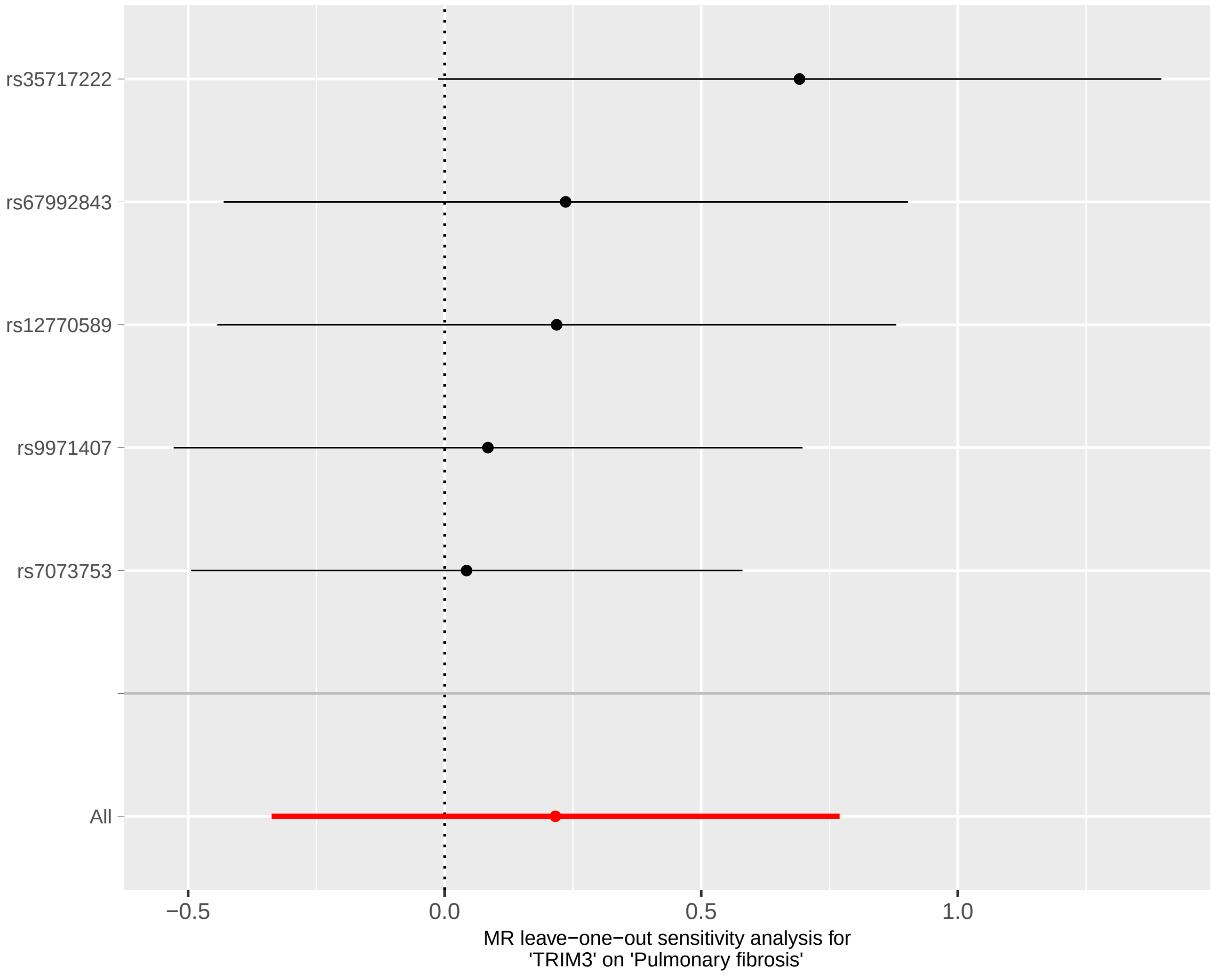
rs35717222
rs67992843
rs12770589
rs9971407
rs7073753
All
−0.5
0.0
0.5
1.0
MR leave−one−out sensitivity analysis for
'TRIM3' on 'Pulmonary fibrosis'

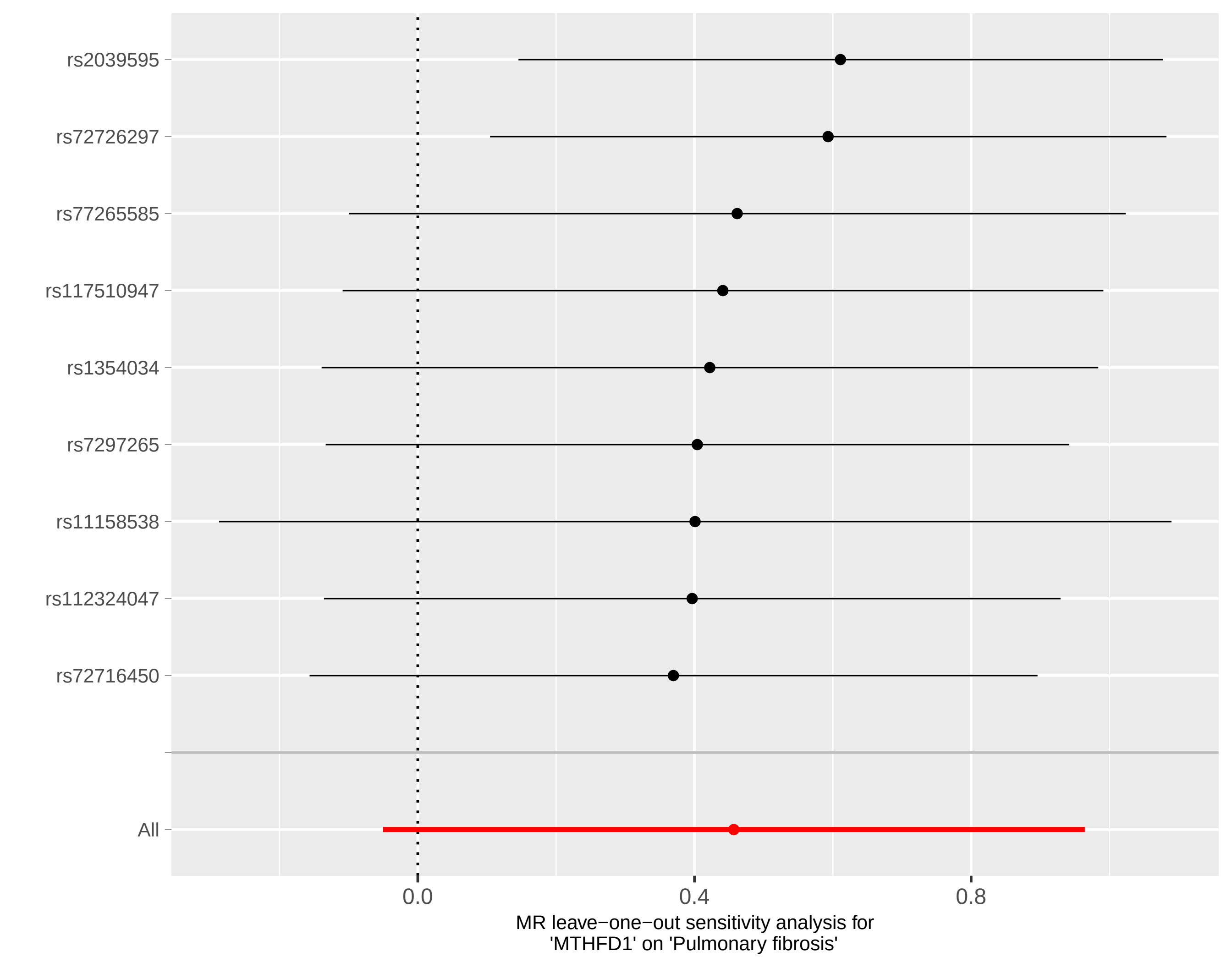

rs2039595
rs72726297
rs77265585
rs117510947
rs1354034
rs7297265
rs11158538
rs112324047
rs72716450
All
0.0
0.4
0.8
MR leave−one−out sensitivity analysis for
'MTHFD1' on 'Pulmonary fibrosis'

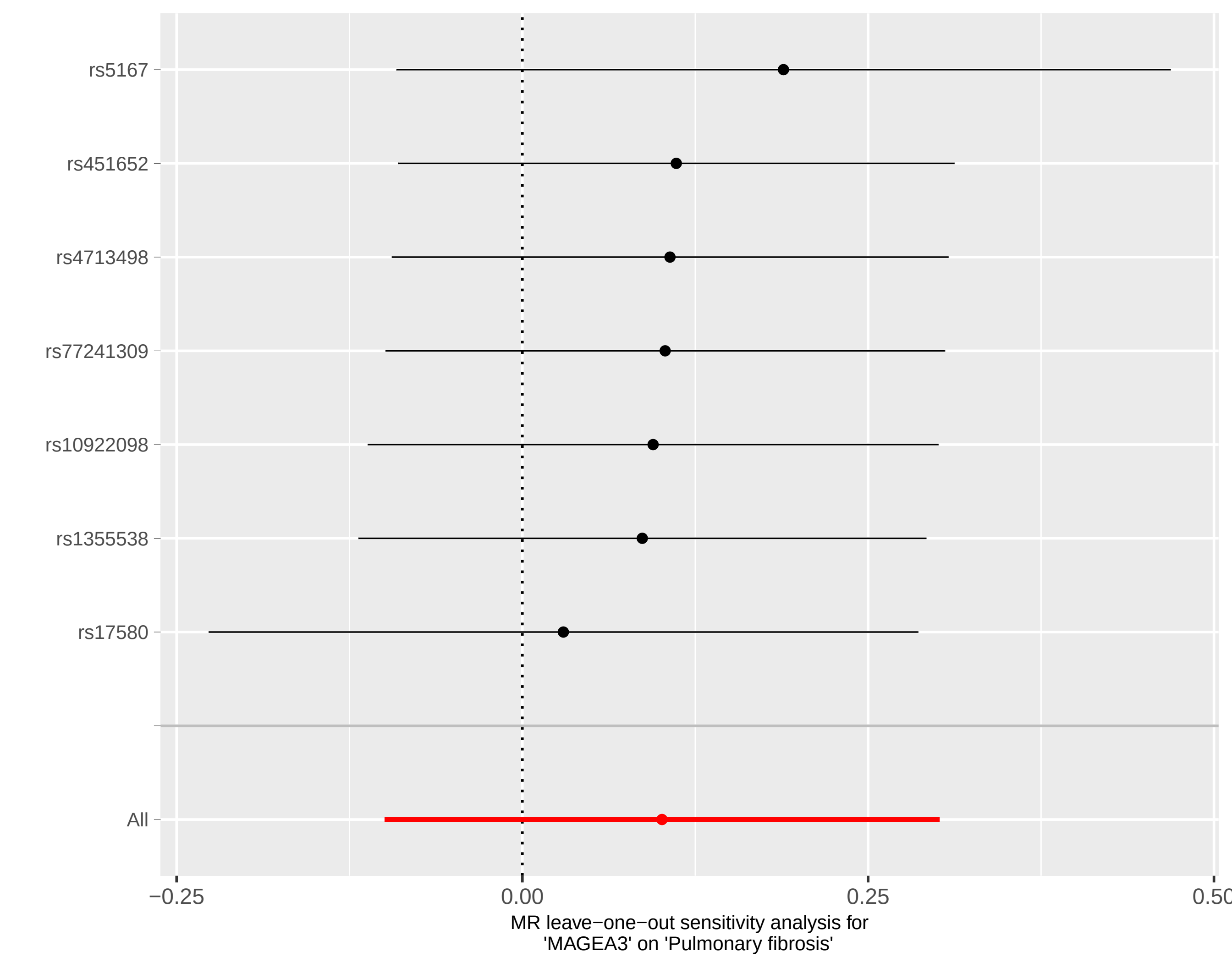
rs5167
rs451652
rs4713498
rs77241309
rs10922098
rs1355538
rs17580
All
−0.25
0.00
0.25
0.50
MR leave−one−out sensitivity analysis for
'MAGEA3' on 'Pulmonary fibrosis'

Insufficient number of SNPs

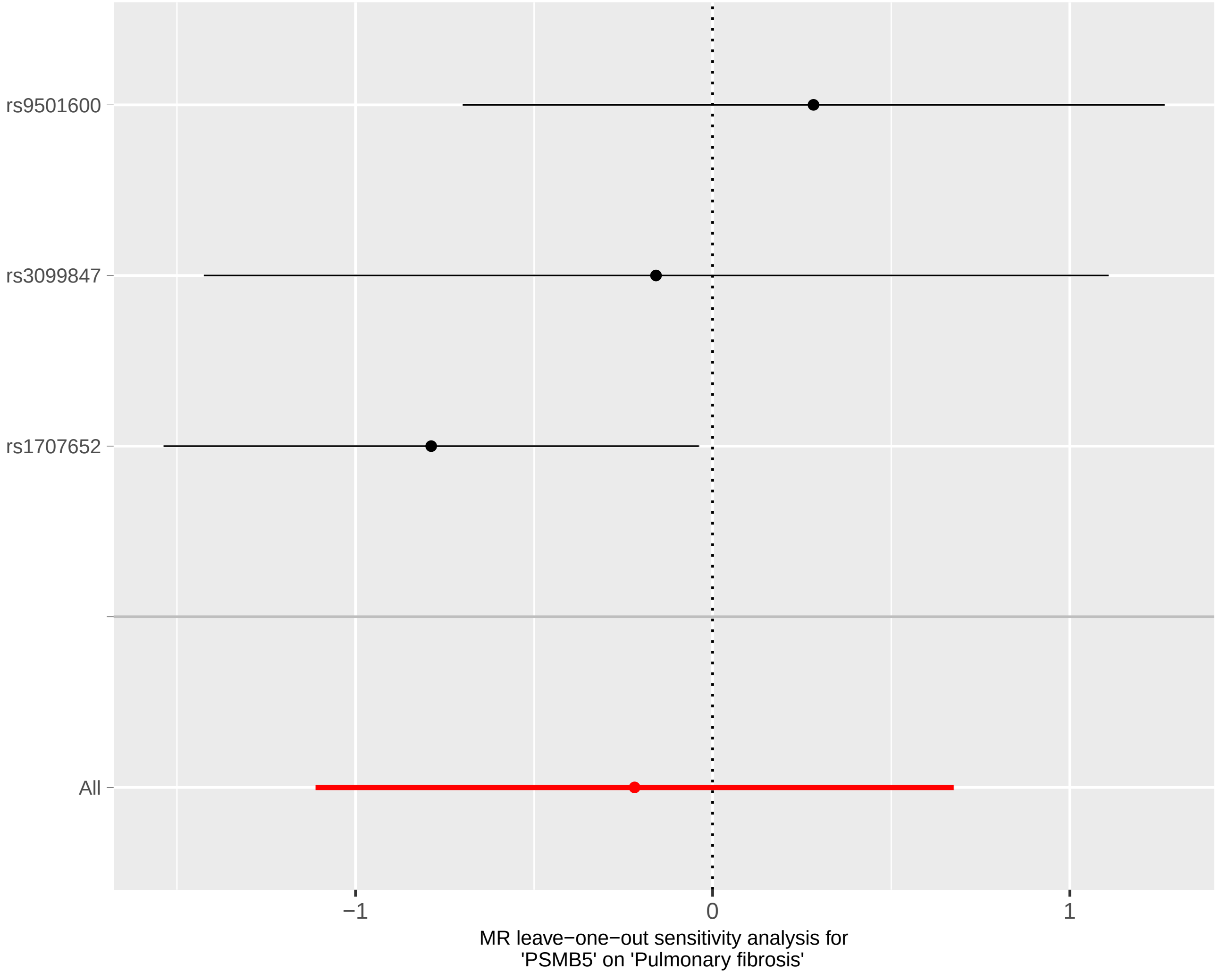
rs9501600
rs3099847
rs1707652
All
−1
0
1
MR leave−one−out sensitivity analysis for
'PSMB5' on 'Pulmonary fibrosis'

Insufficient number of SNPs

Insufficient number of SNPs

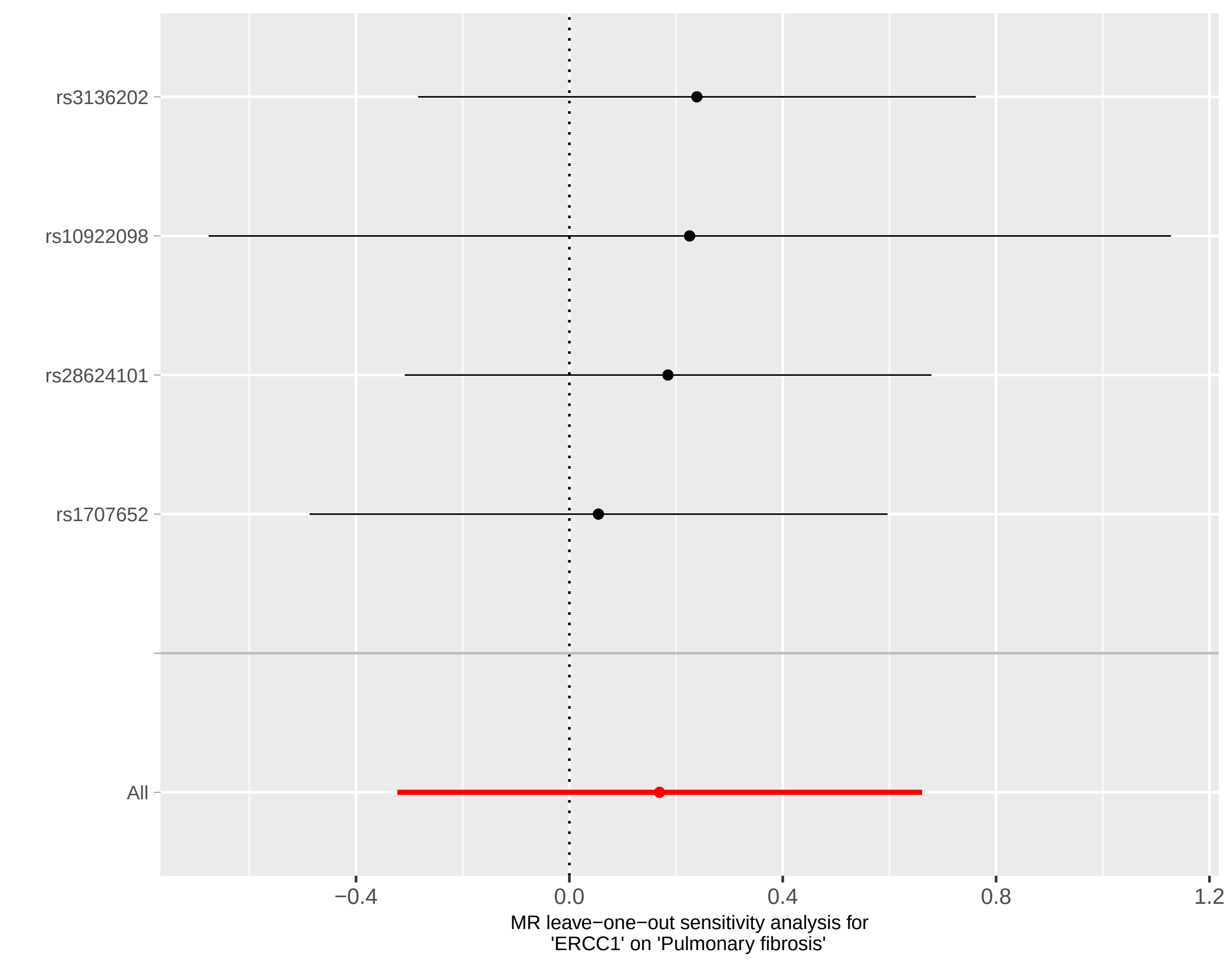
rs3136202
rs10922098
rs28624101
rs1707652
All
−0.4
0.0
0.4
0.8
1.2
MR leave−one−out sensitivity analysis for
'ERCC1' on 'Pulmonary fibrosis'

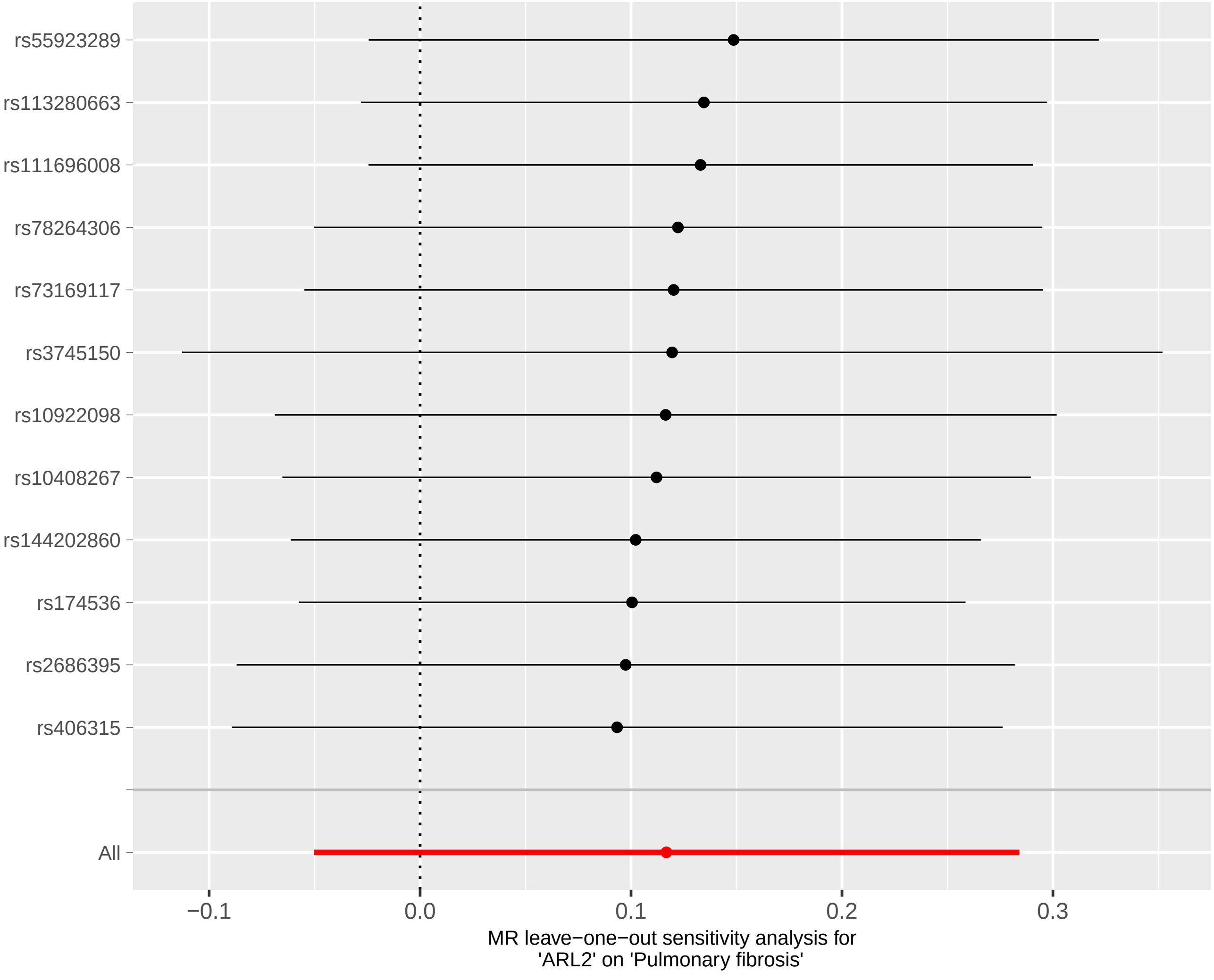

rs55923289
rs113280663
rs111696008
rs78264306
rs73169117
rs3745150
rs10922098
rs10408267
rs144202860
rs174536
rs2686395
rs406315
All
−0.1
0.0
0.1
0.2
0.3
MR leave−one−out sensitivity analysis for
'ARL2' on 'Pulmonary fibrosis'

Insufficient number of SNPs

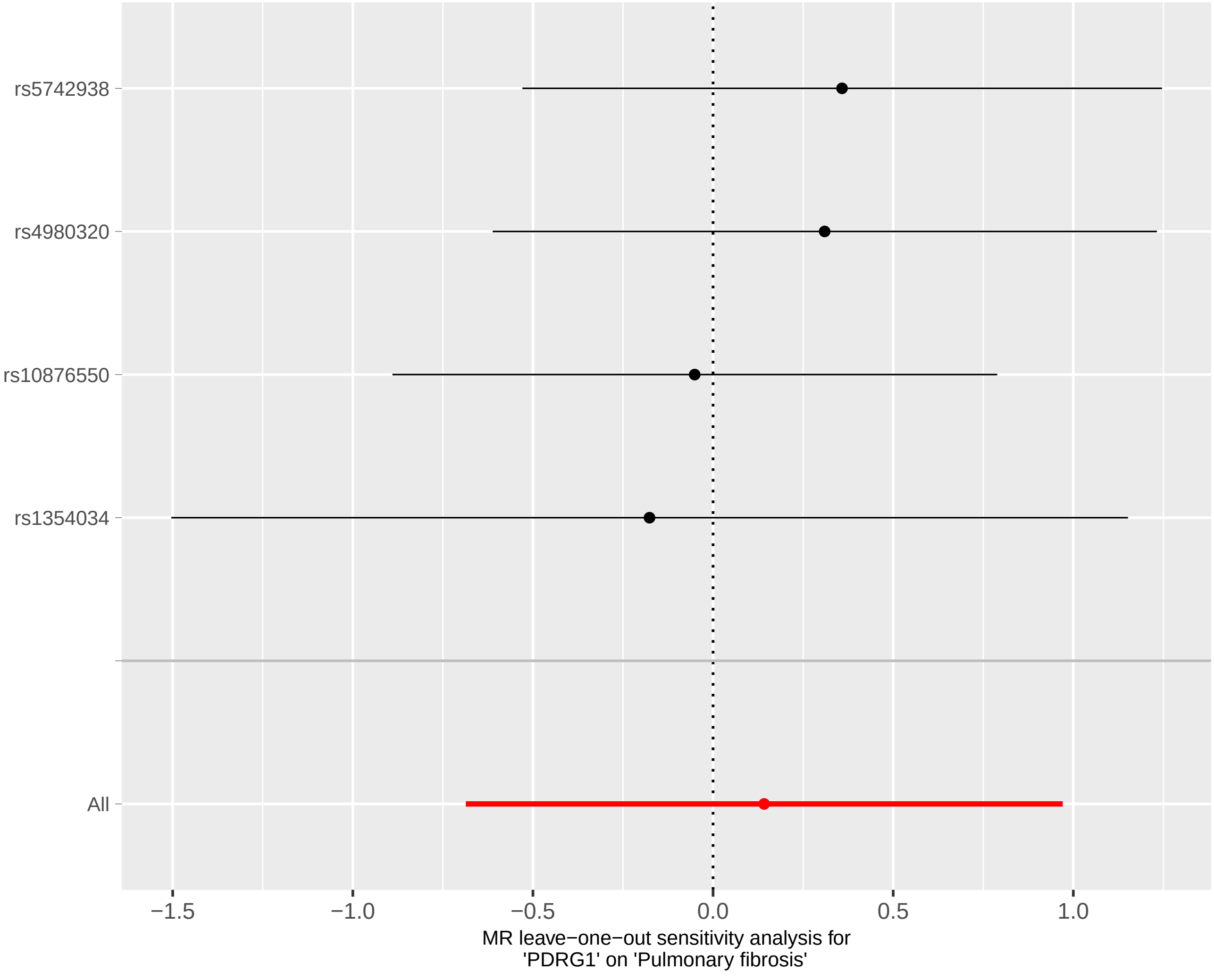

rs5742938
rs4980320
rs10876550
rs1354034
All
−1.5
−1.0
−0.5
0.0
0.5
1.0
MR leave−one−out sensitivity analysis for
'PDRG1' on 'Pulmonary fibrosis'

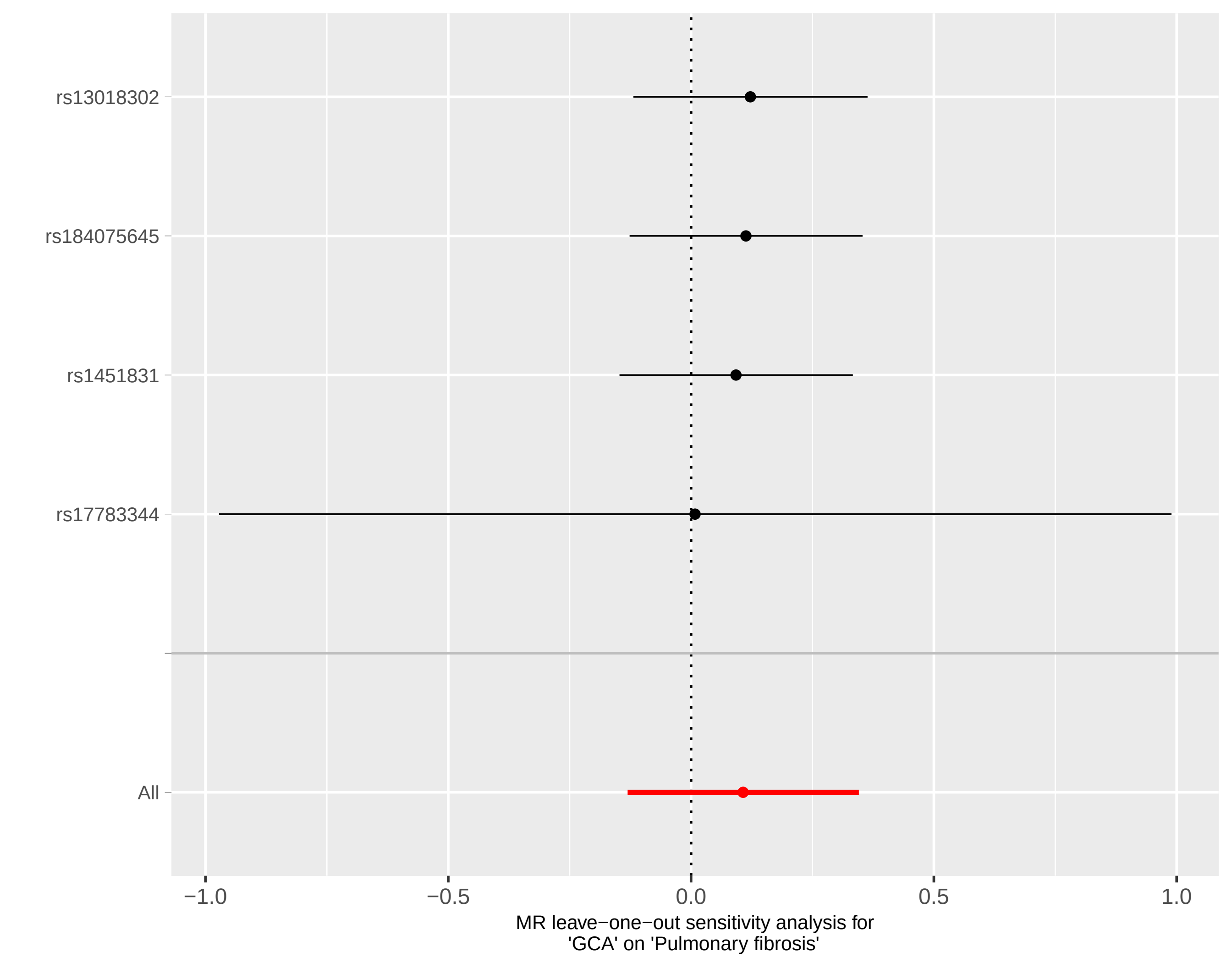
rs13018302
rs184075645
rs1451831
rs17783344
All
−1.0
−0.5
0.0
0.5
1.0
MR leave−one−out sensitivity analysis for
'GCA' on 'Pulmonary fibrosis'

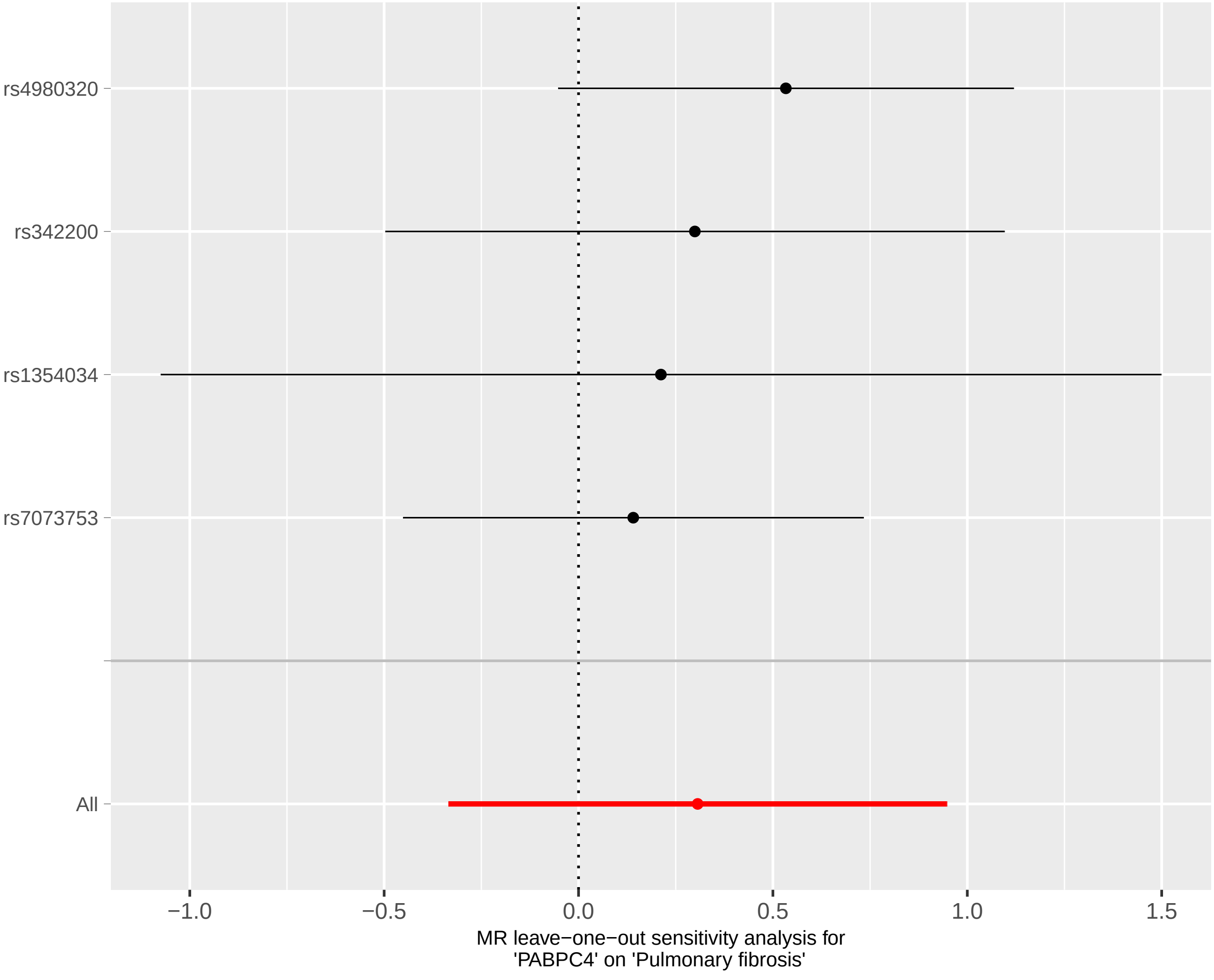
rs4980320
rs342200
rs1354034
rs7073753
All
−1.0
−0.5
0.0
0.5
1.0
1.5
MR leave−one−out sensitivity analysis for
'PABPC4' on 'Pulmonary fibrosis'

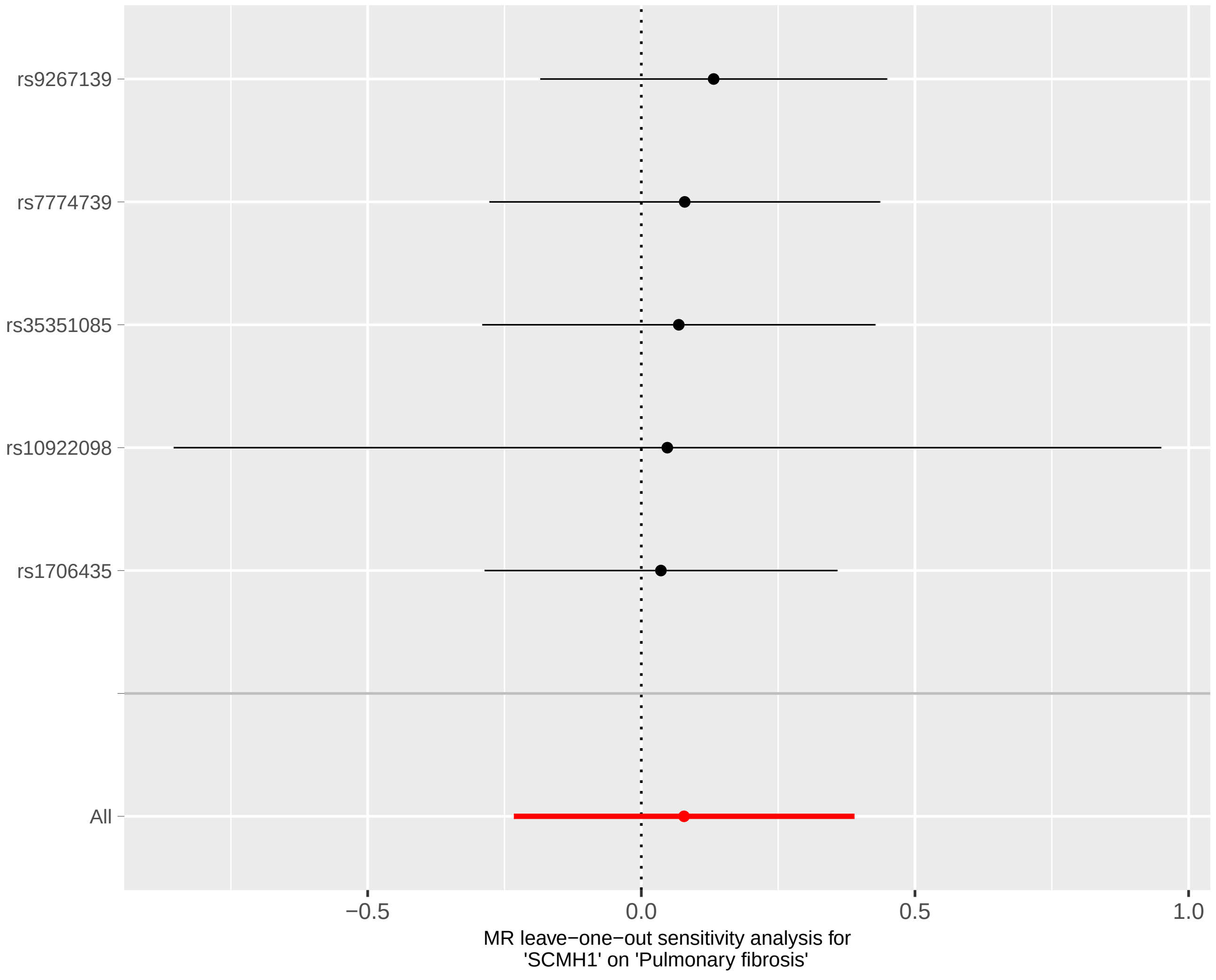

rs9267139
rs7774739
rs35351085
rs10922098
rs1706435
All
−0.5
0.0
0.5
1.0
MR leave−one−out sensitivity analysis for
'SCMH1' on 'Pulmonary fibrosis'

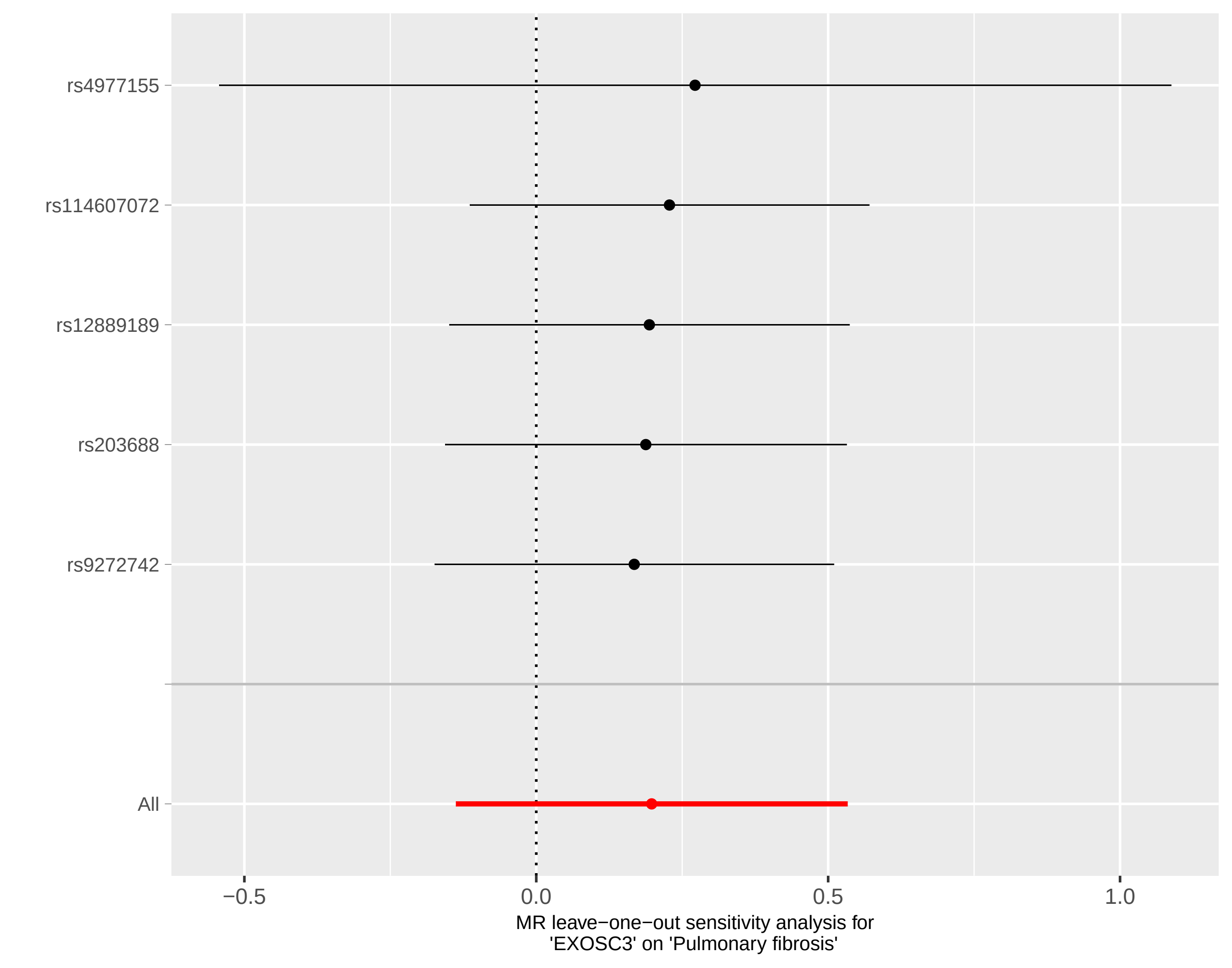
rs4977155
rs114607072
rs12889189
rs203688
rs9272742
All
−0.5
0.0
0.5
1.0
MR leave−one−out sensitivity analysis for
'EXOSC3' on 'Pulmonary fibrosis'

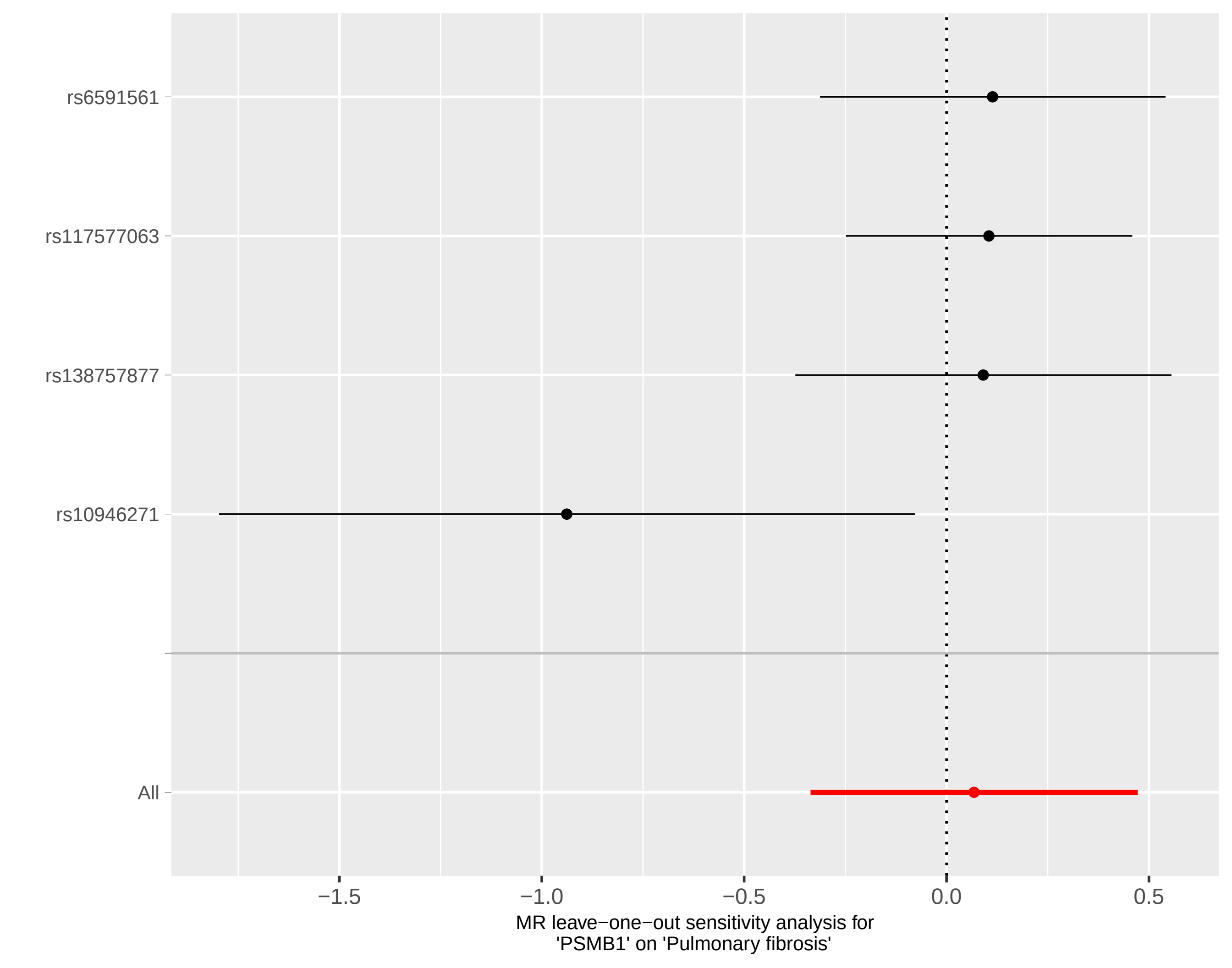

rs6591561
rs117577063
rs138757877
rs10946271
All
−1.5
−1.0
−0.5
0.0
0.5
MR leave−one−out sensitivity analysis for
'PSMB1' on 'Pulmonary fibrosis'

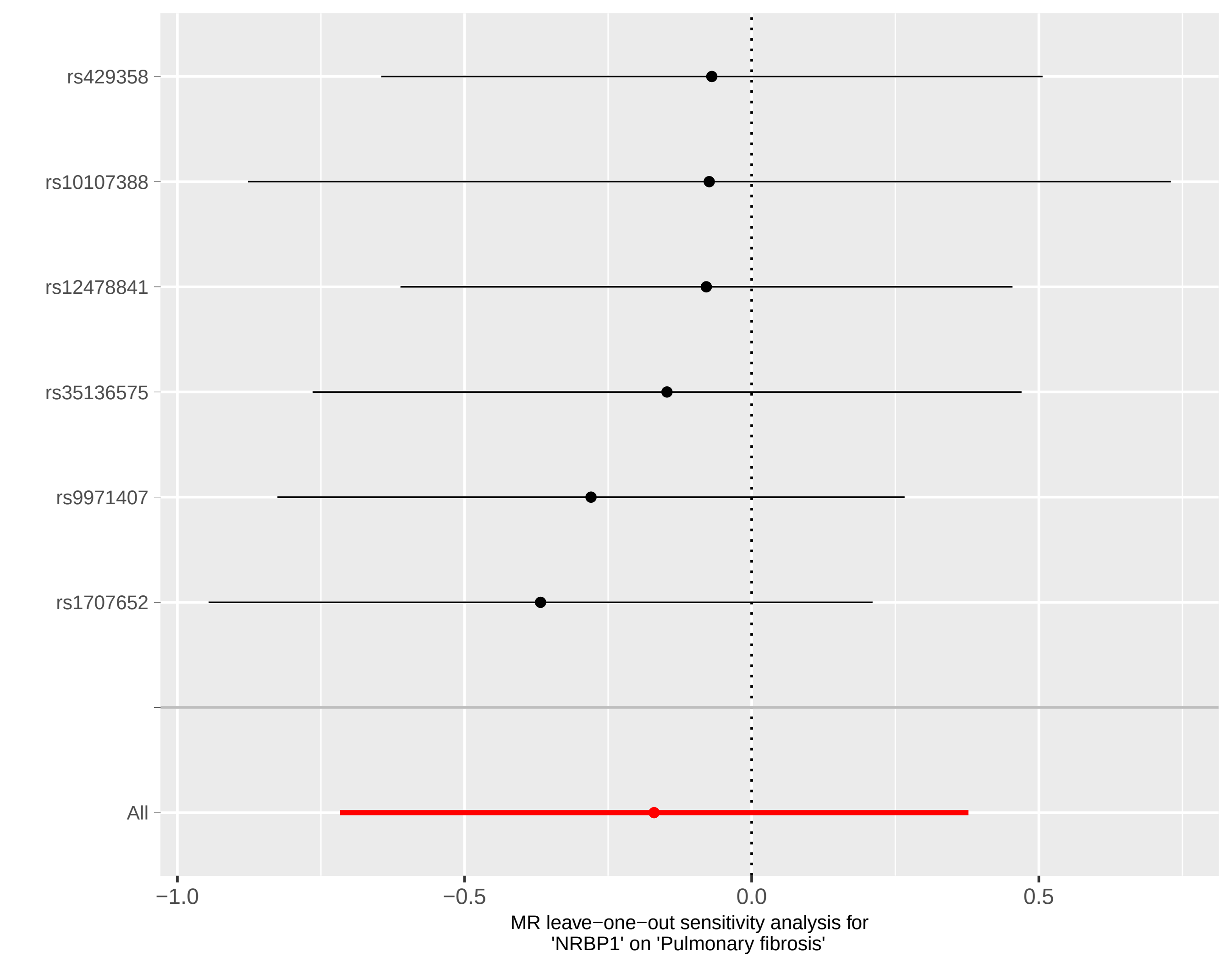
rs429358
rs10107388
rs12478841
rs35136575
rs9971407
rs1707652
All
−1.0
−0.5
0.0
0.5
MR leave−one−out sensitivity analysis for
'NRBP1' on 'Pulmonary fibrosis'

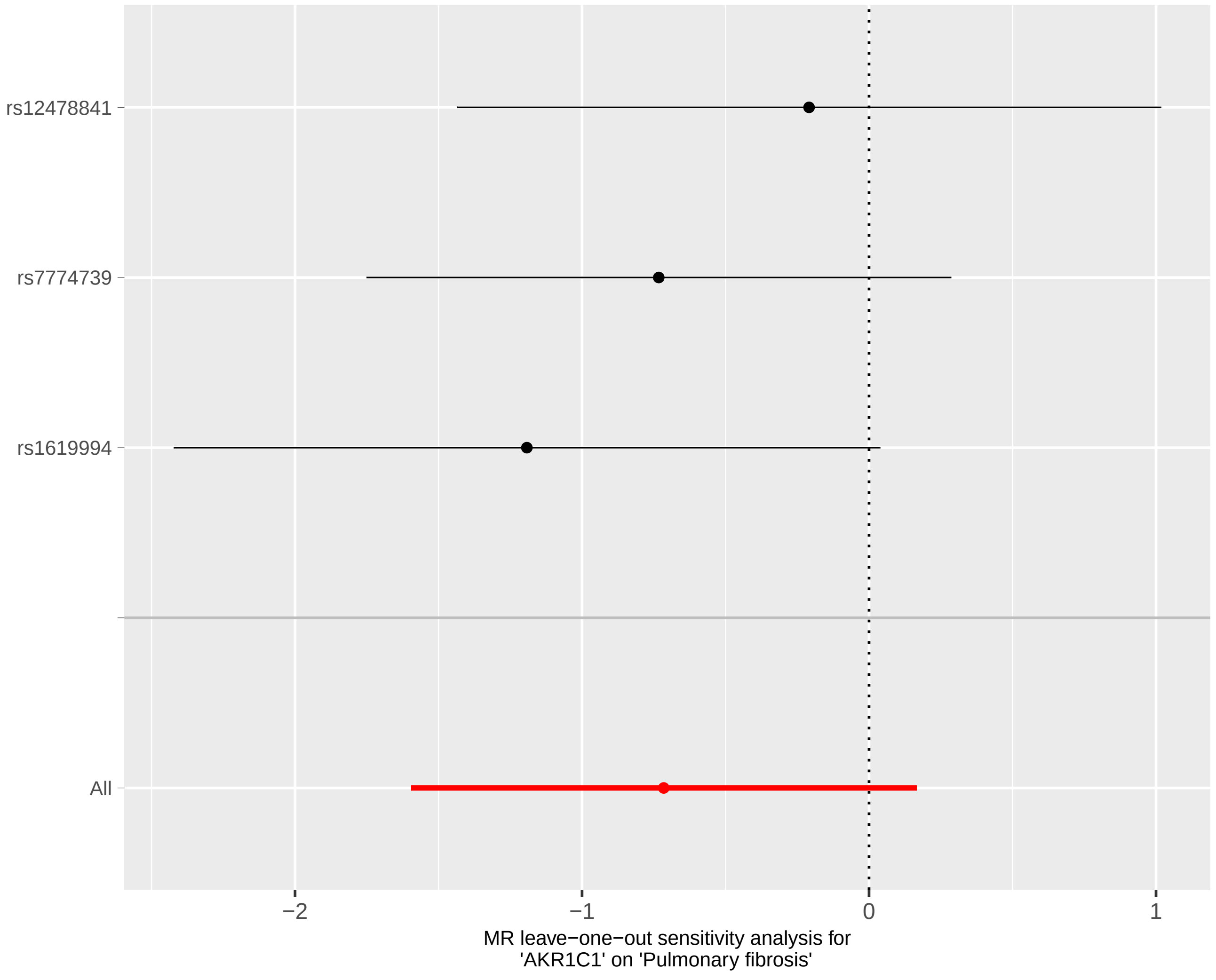
rs12478841
rs7774739
rs1619994
All
−2
−1
0
1
MR leave−one−out sensitivity analysis for
'AKR1C1' on 'Pulmonary fibrosis'

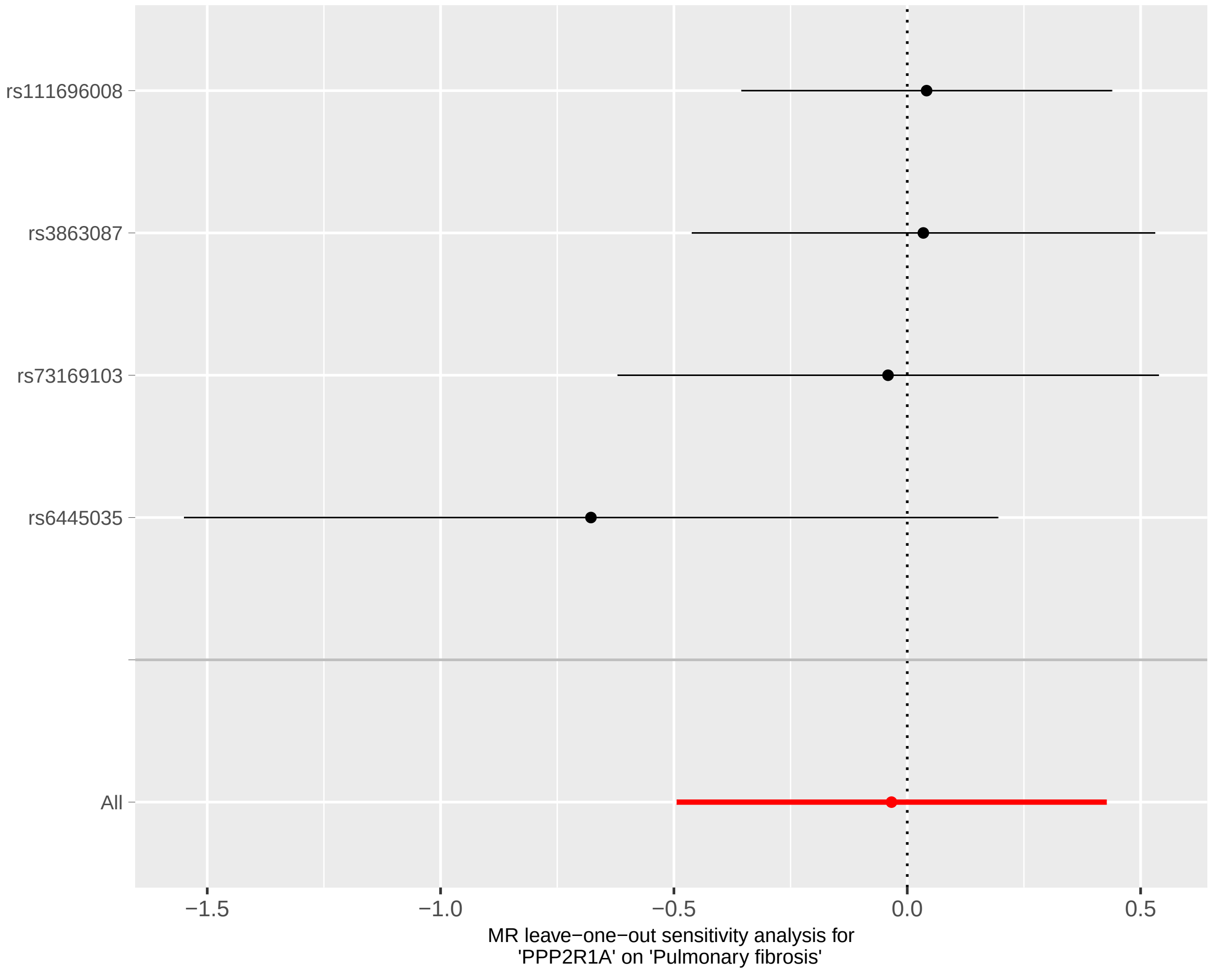

rs111696008
rs3863087
rs73169103
rs6445035
All
−1.5
−1.0
−0.5
0.0
0.5
MR leave−one−out sensitivity analysis for
'PPP2R1A' on 'Pulmonary fibrosis'

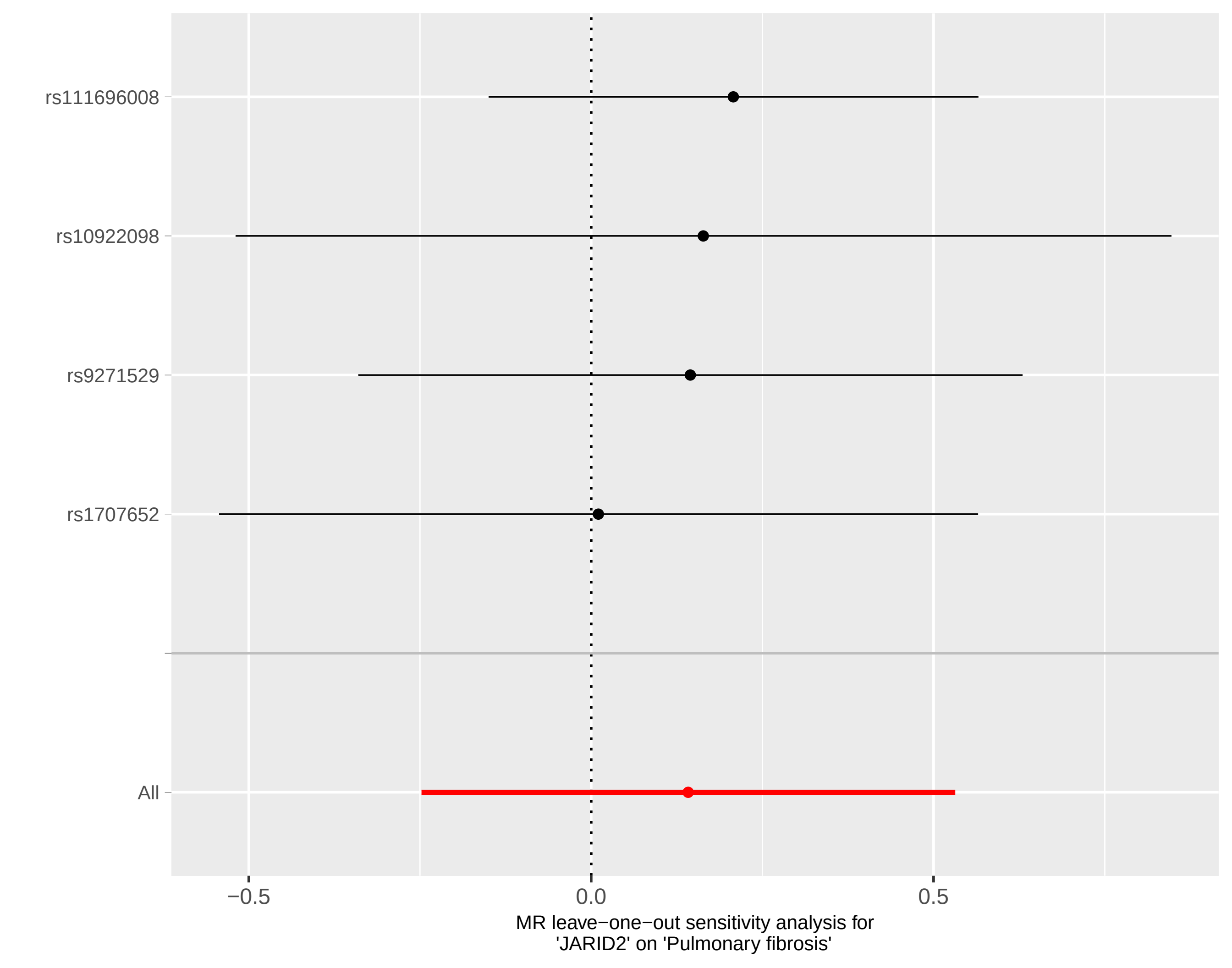
rs111696008
rs10922098
rs9271529
rs1707652
All
−0.5
0.0
0.5
MR leave−one−out sensitivity analysis for
'JARID2' on 'Pulmonary fibrosis'

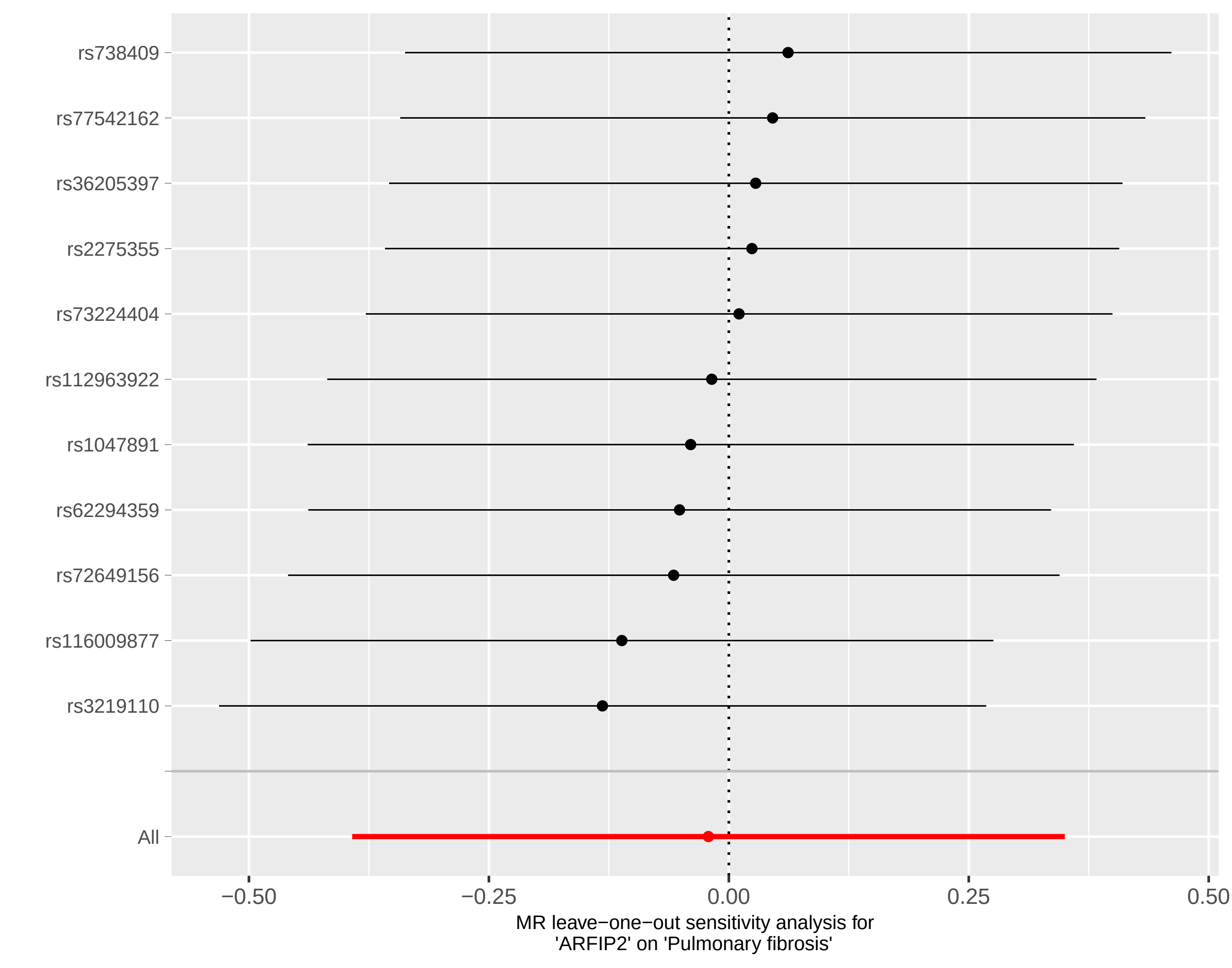

rs738409
rs77542162
rs36205397
rs2275355
rs73224404
rs112963922
rs1047891
rs62294359
rs72649156
rs116009877
rs3219110
All
−0.50
−0.25
0.00
0.25
0.50
MR leave−one−out sensitivity analysis for
'ARFIP2' on 'Pulmonary fibrosis'

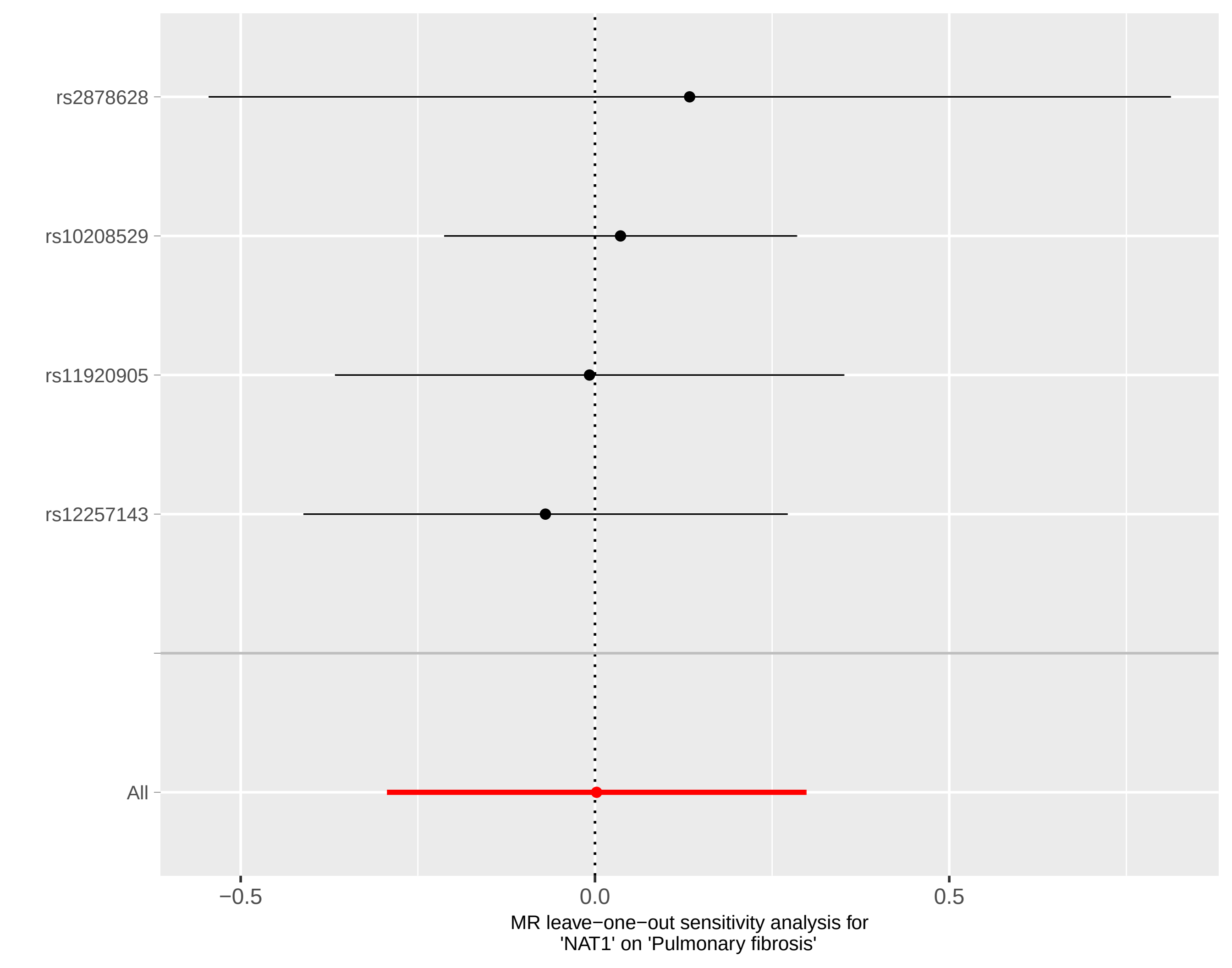
rs2878628
rs10208529
rs11920905
rs12257143
All
−0.5
0.0
0.5
MR leave−one−out sensitivity analysis for
'NAT1' on 'Pulmonary fibrosis'

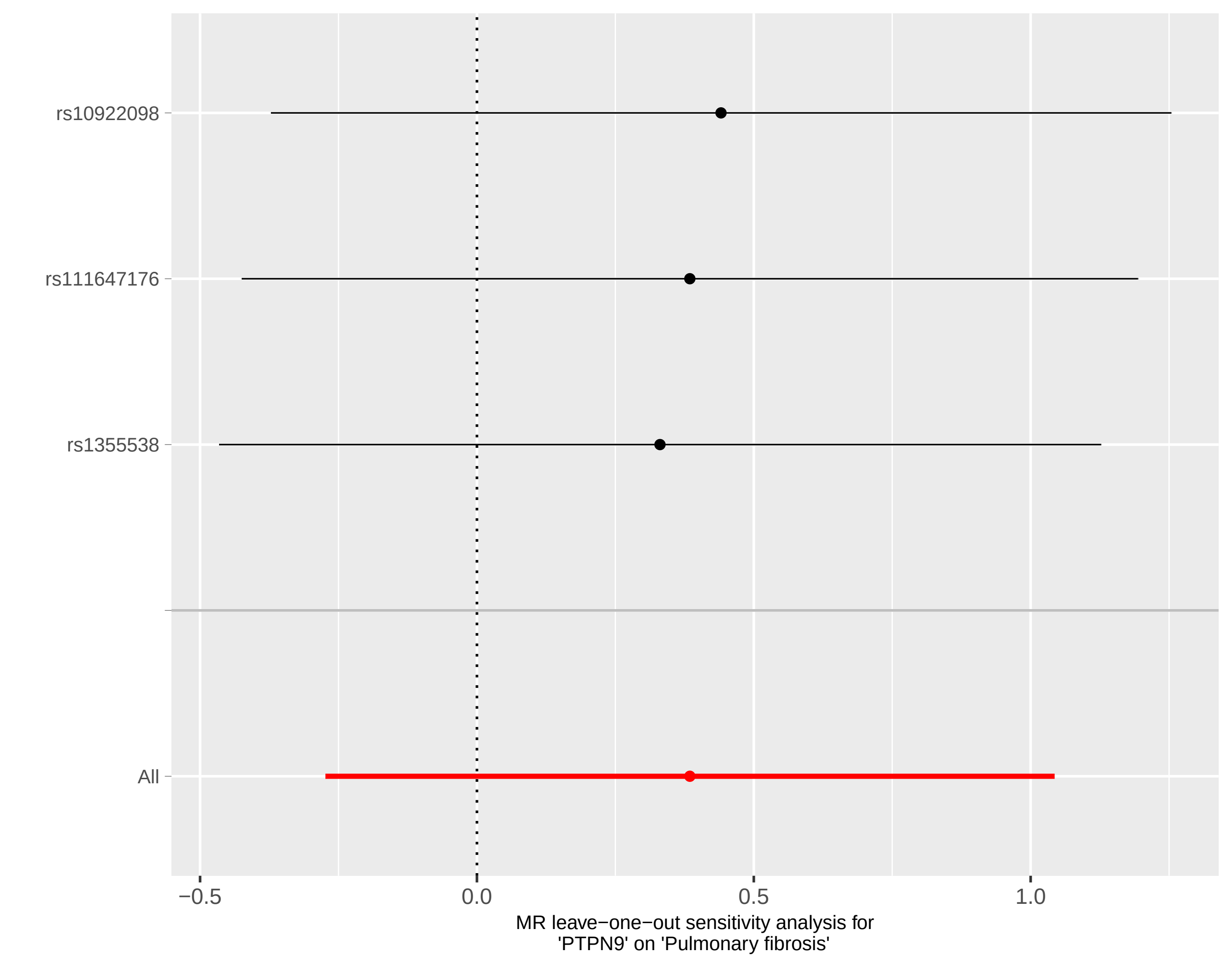
rs10922098
rs111647176
rs1355538
All
−0.5
0.0
0.5
1.0
MR leave−one−out sensitivity analysis for
'PTPN9' on 'Pulmonary fibrosis'

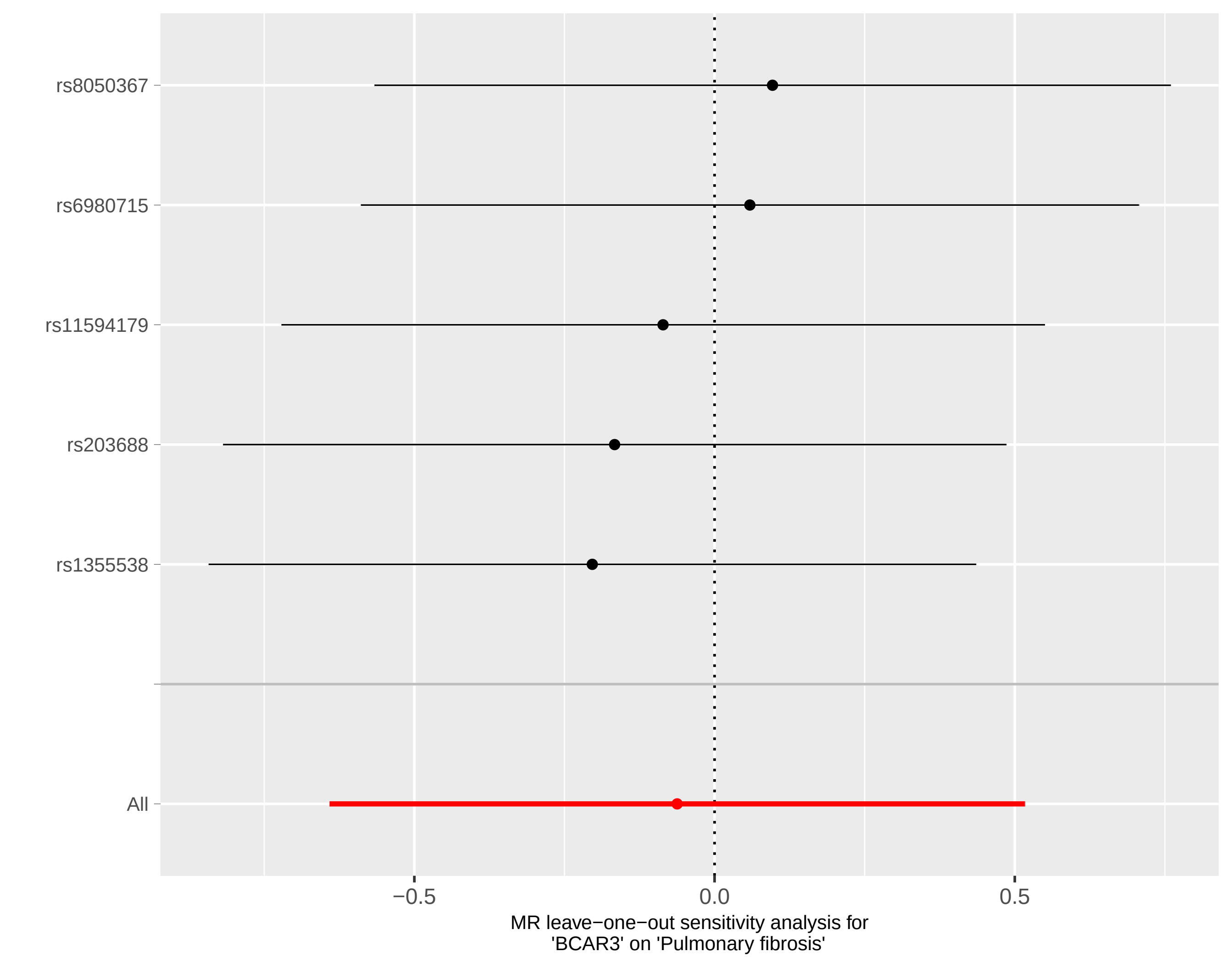
rs8050367
rs6980715
rs11594179
rs203688
rs1355538
All
−0.5
0.0
0.5
MR leave−one−out sensitivity analysis for
'BCAR3' on 'Pulmonary fibrosis'

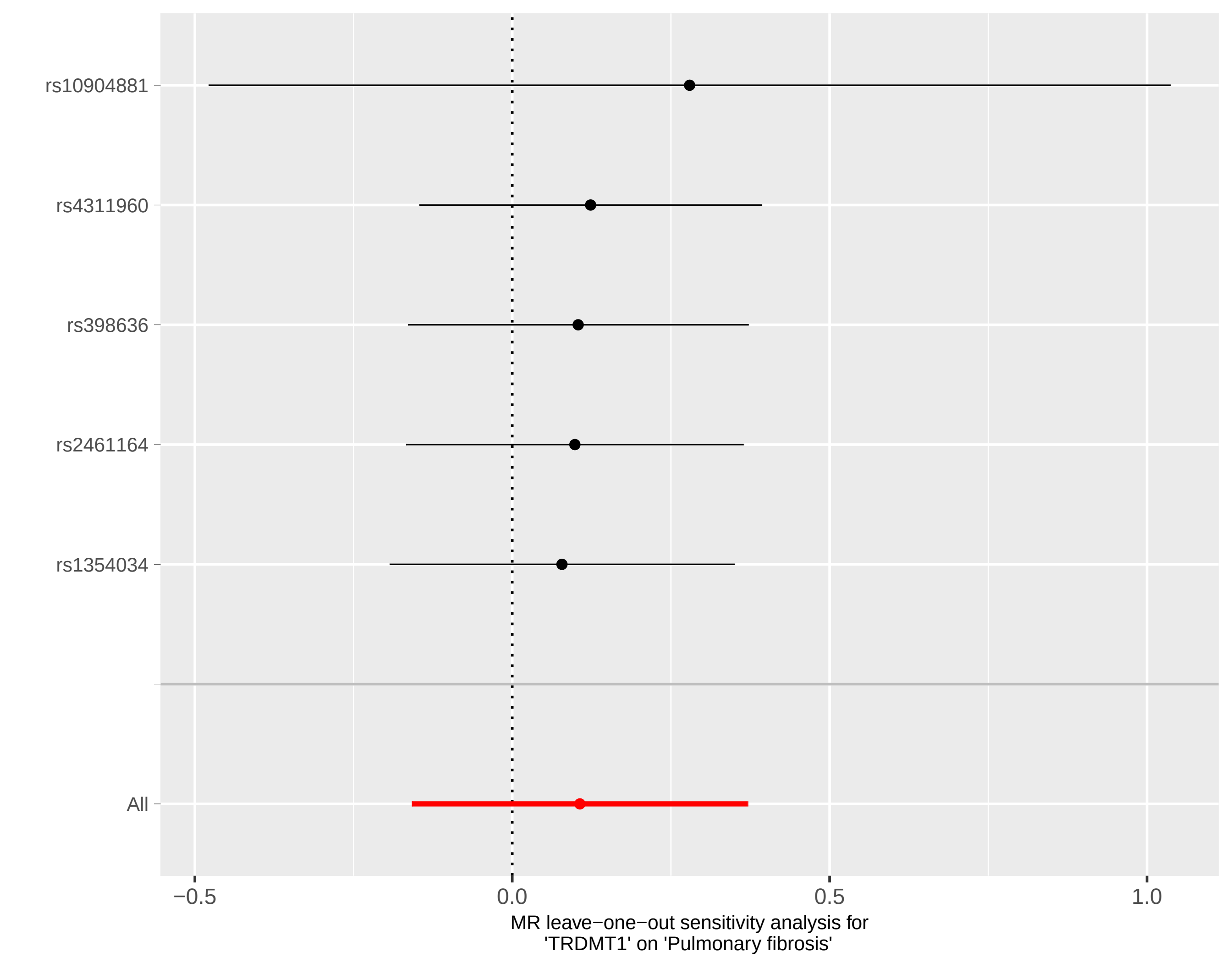
rs10904881
rs4311960
rs398636
rs2461164
rs1354034
All
−0.5
0.0
0.5
1.0
MR leave−one−out sensitivity analysis for
'TRDMT1' on 'Pulmonary fibrosis'

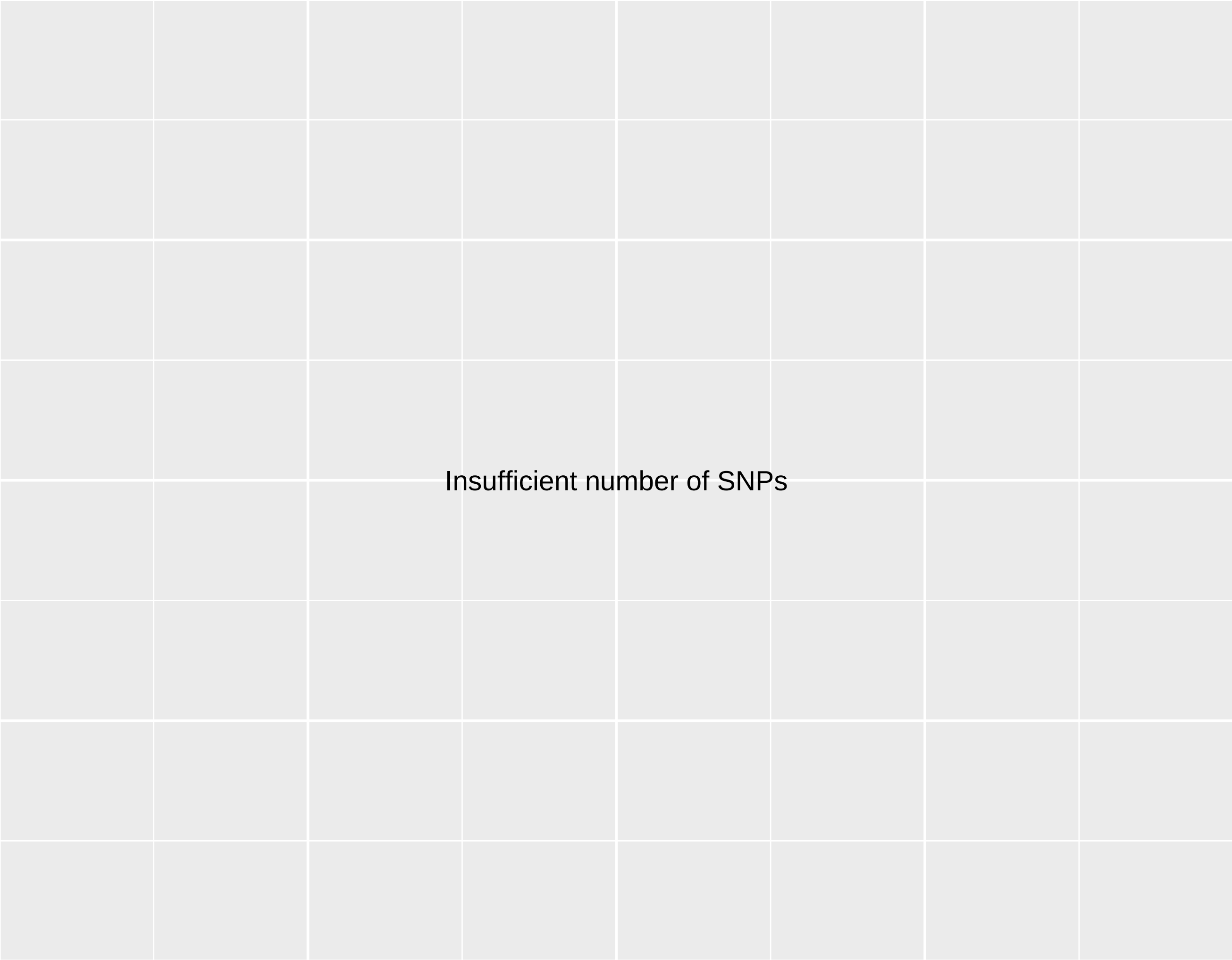
Insufficient number of SNPs

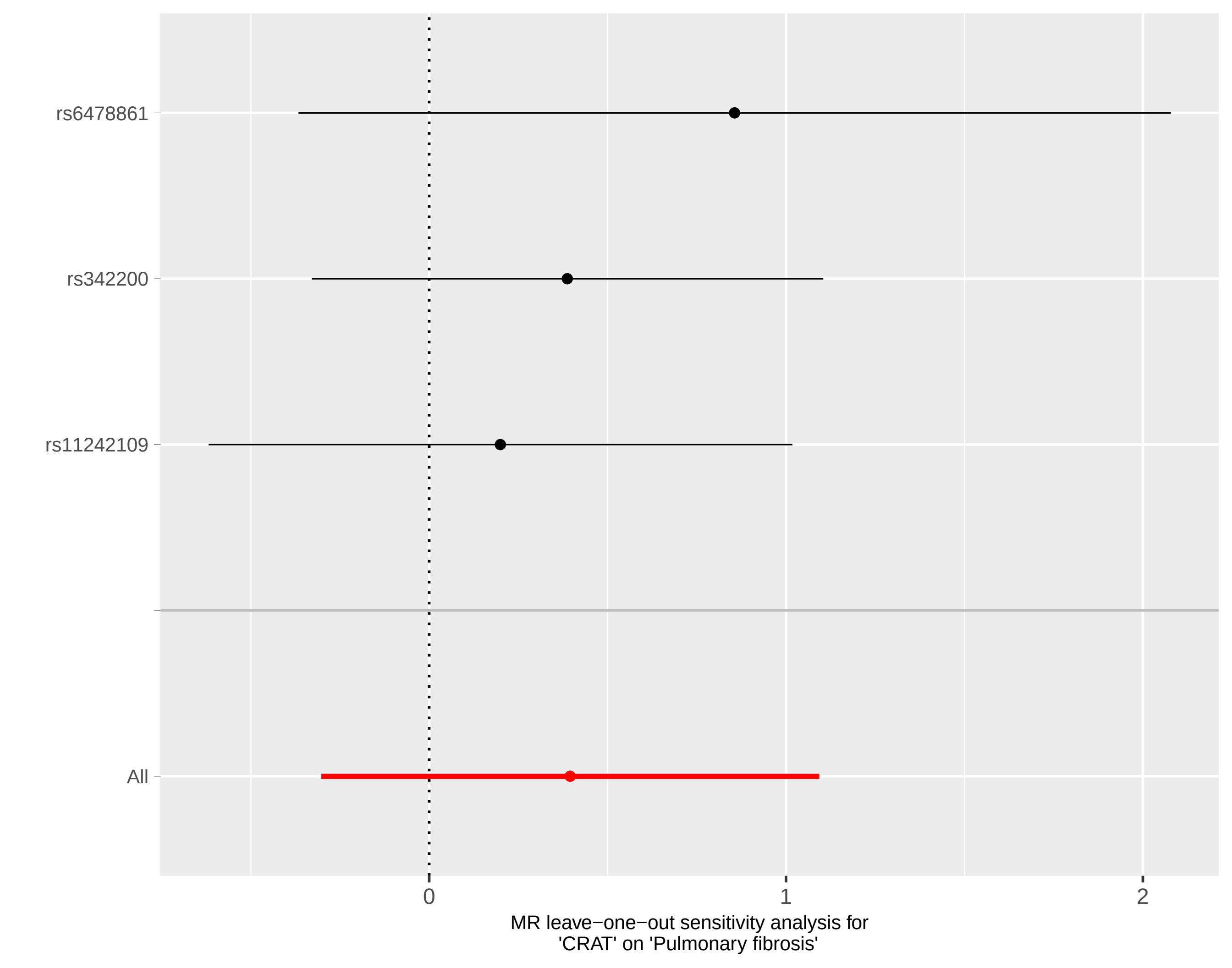
rs6478861
rs342200
rs11242109
All
0
1
2
MR leave−one−out sensitivity analysis for
'CRAT' on 'Pulmonary fibrosis'

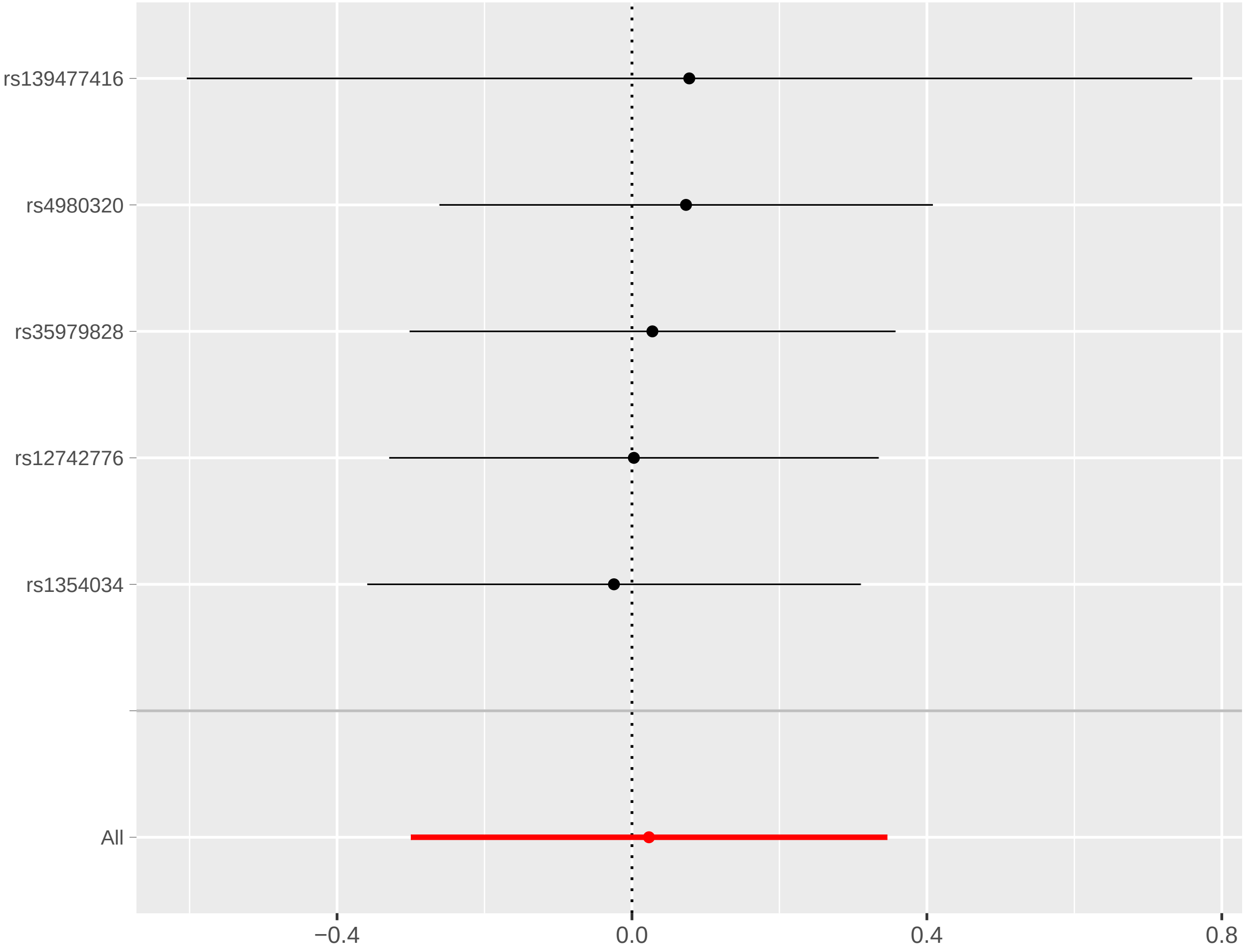

MR leave−one−out sensitivity analysis for
'INPP5B' on 'Pulmonary fibrosis'

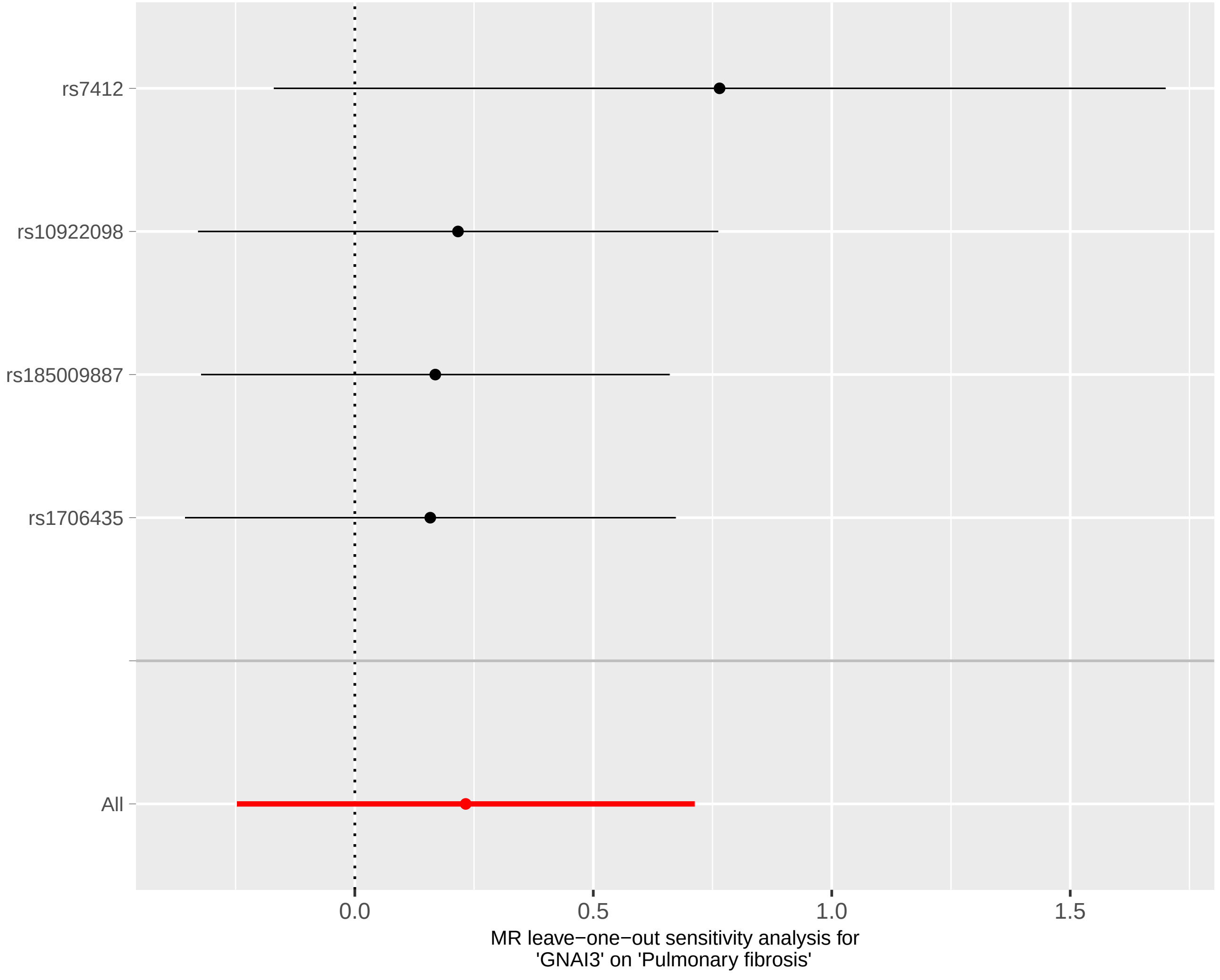
rs7412
rs10922098
rs185009887
rs1706435
All
0.0
0.5
1.0
1.5
MR leave−one−out sensitivity analysis for
'GNAI3' on 'Pulmonary fibrosis'

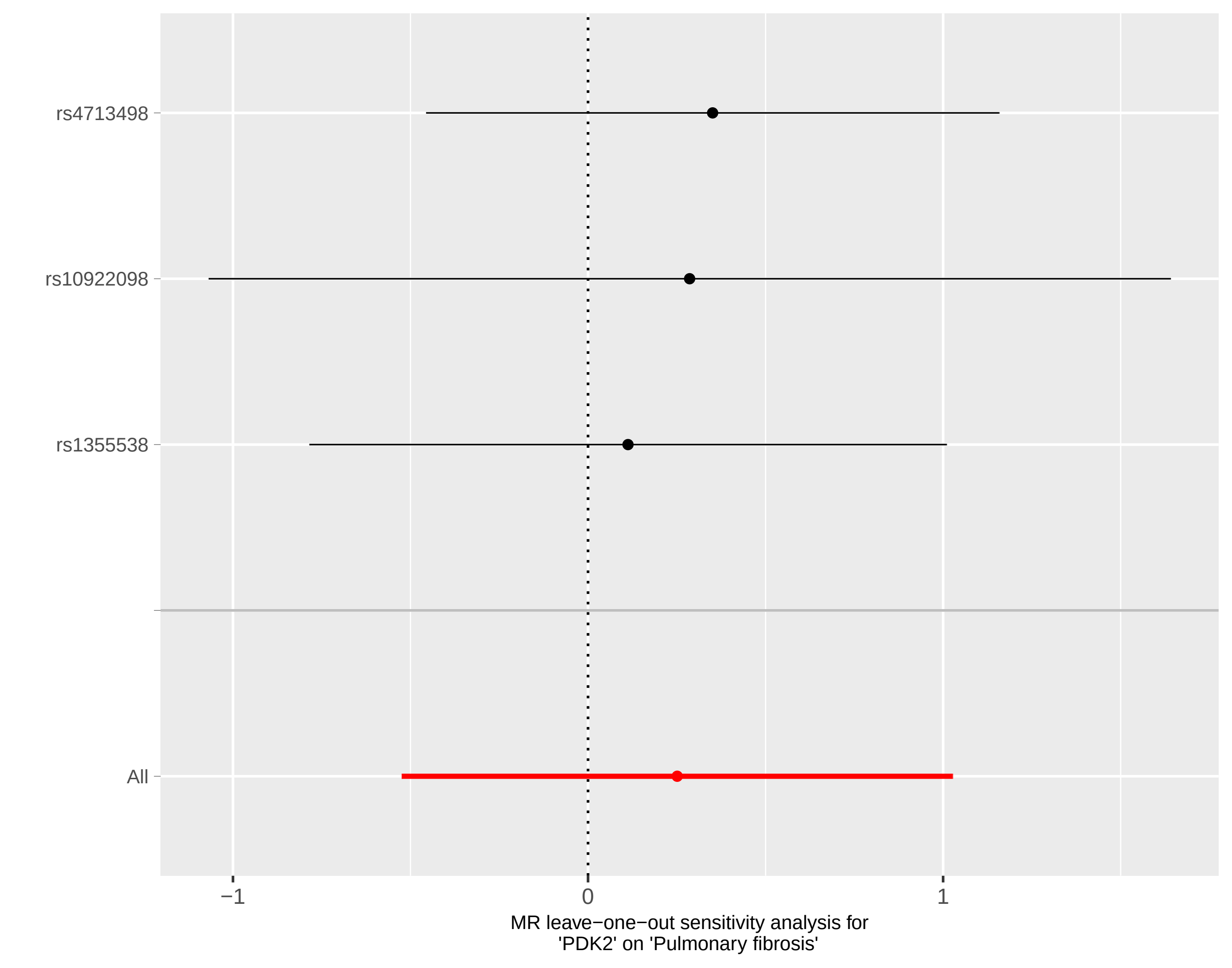

rs4713498
rs10922098
rs1355538
All
−1
0
1
MR leave−one−out sensitivity analysis for
'PDK2' on 'Pulmonary fibrosis'

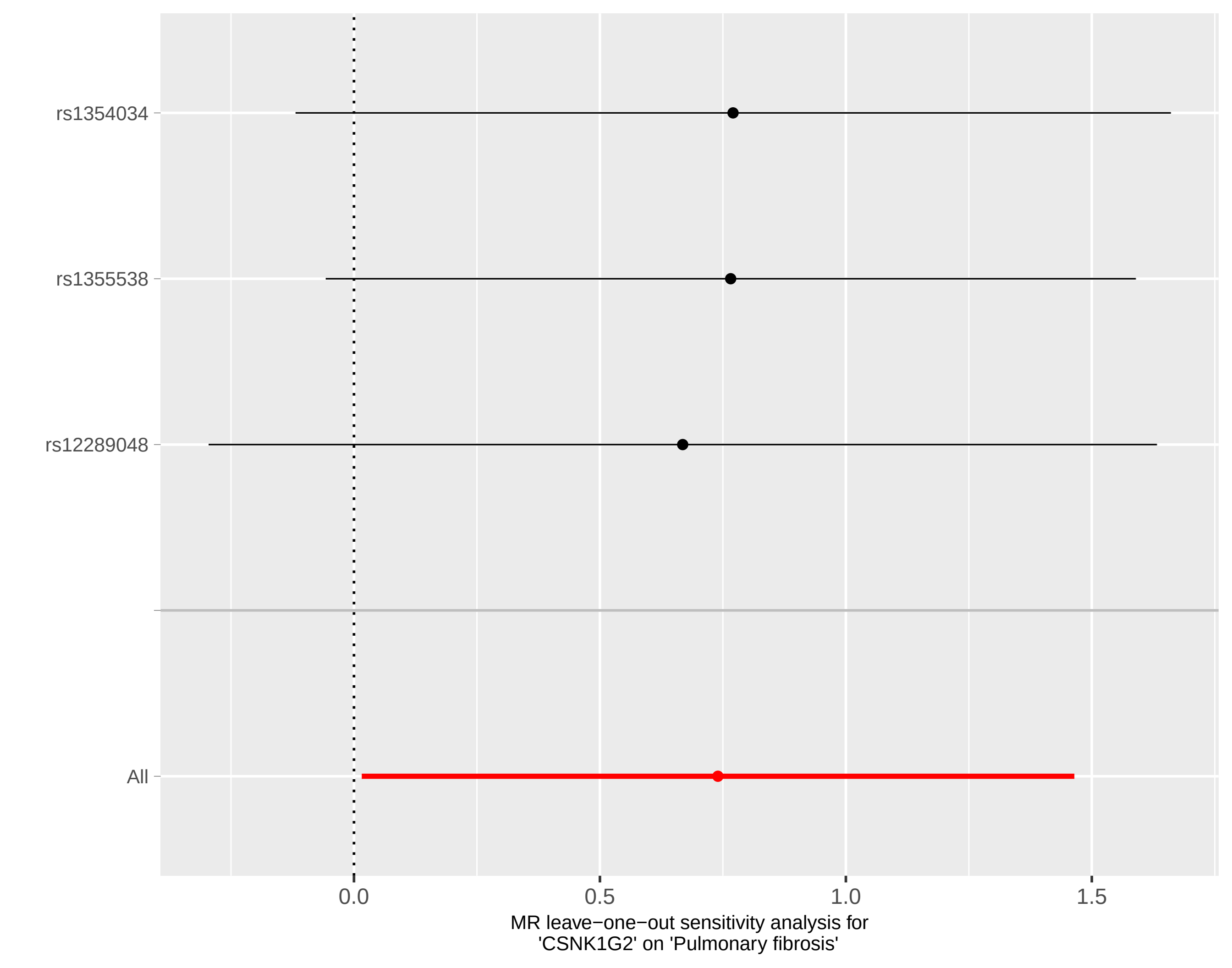

rs1354034
rs1355538
rs12289048
All
0.0
0.5
1.0
1.5
MR leave−one−out sensitivity analysis for
'CSNK1G2' on 'Pulmonary fibrosis'

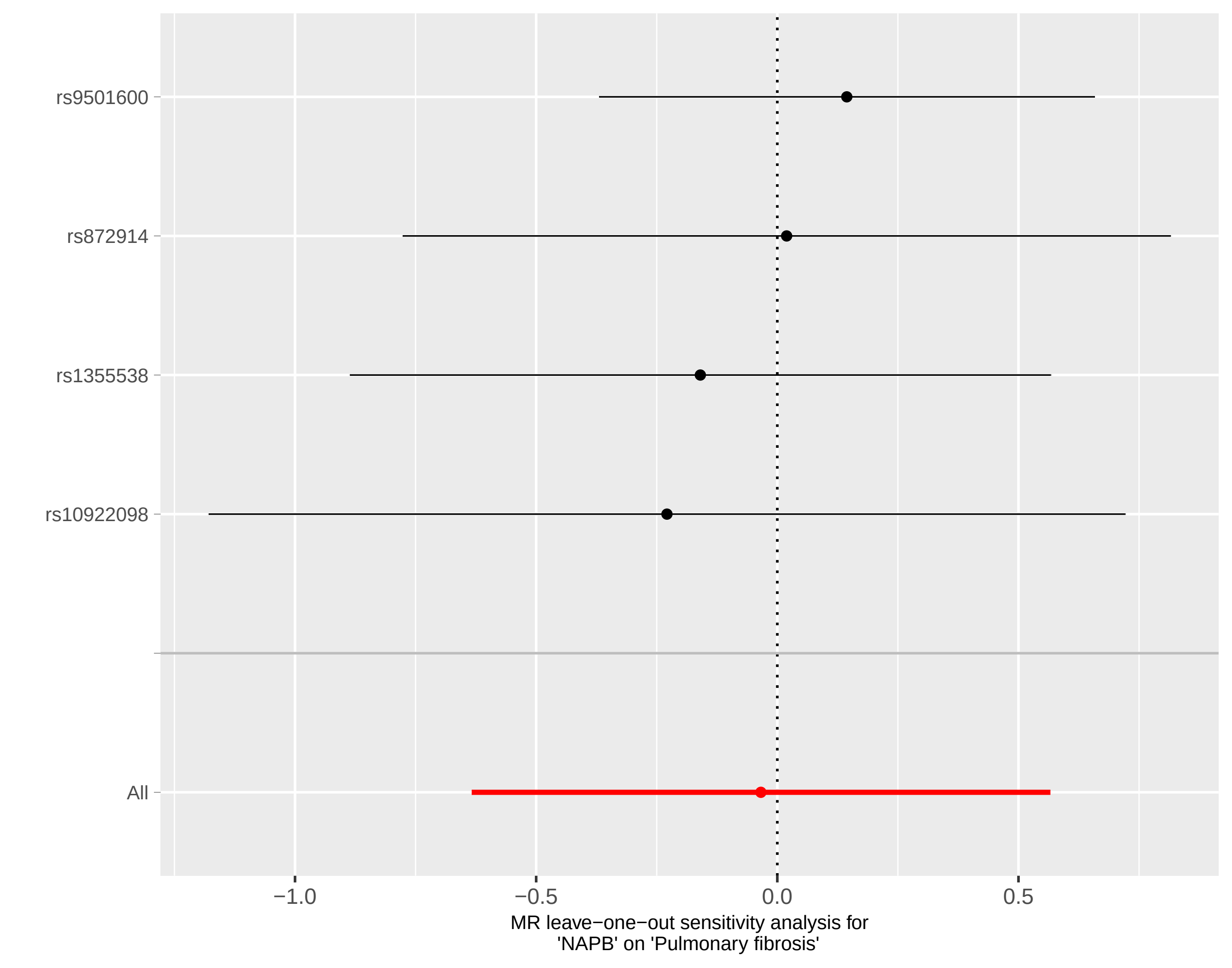
rs9501600
rs872914
rs1355538
rs10922098
All
−1.0
−0.5
0.0
0.5
MR leave−one−out sensitivity analysis for
'NAPB' on 'Pulmonary fibrosis'

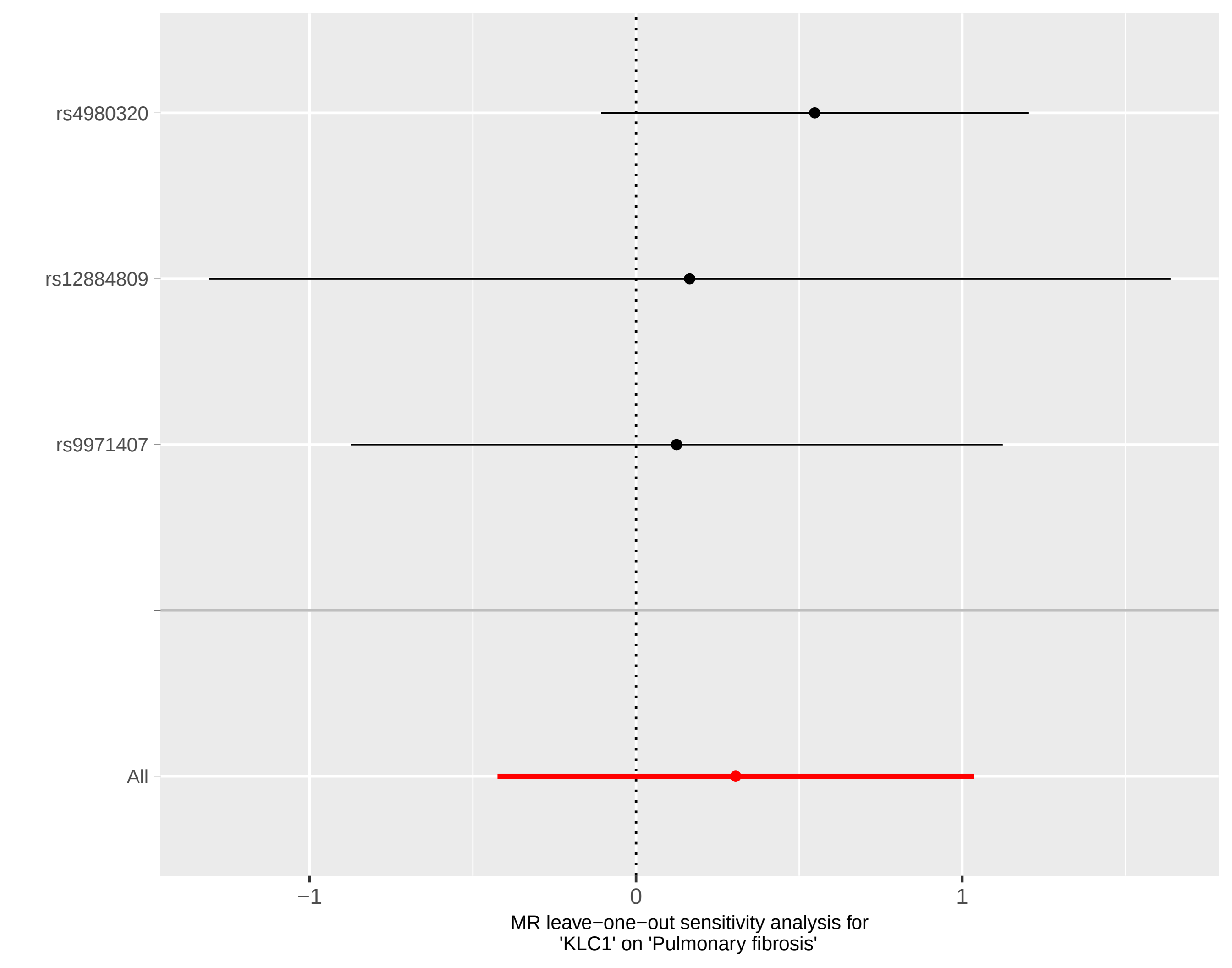
rs4980320
rs12884809
rs9971407
All
−1
0
1
MR leave−one−out sensitivity analysis for
'KLC1' on 'Pulmonary fibrosis'

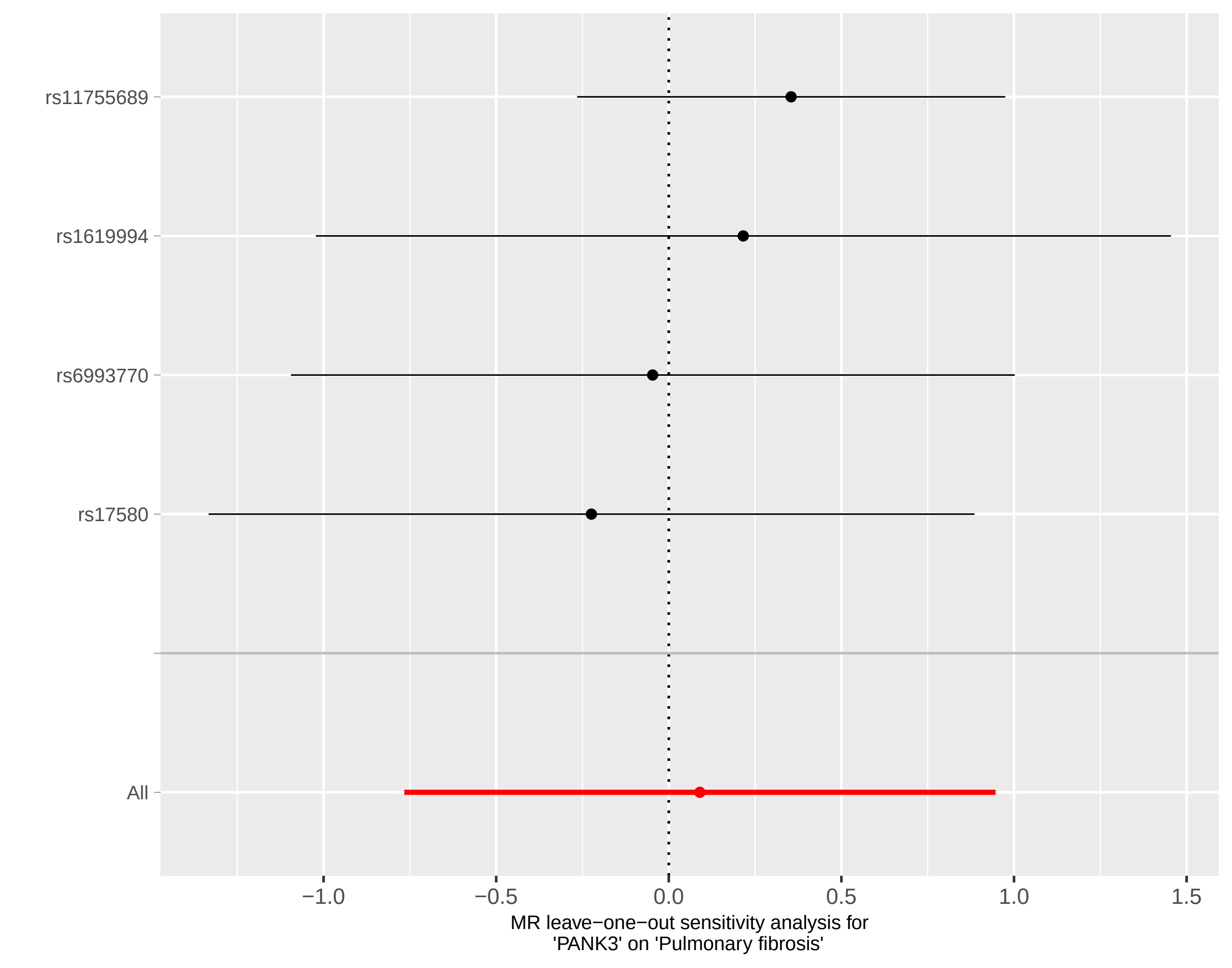
rs11755689
rs1619994
rs6993770
rs17580
All
−1.0
−0.5
0.0
0.5
1.0
1.5
MR leave−one−out sensitivity analysis for
'PANK3' on 'Pulmonary fibrosis'

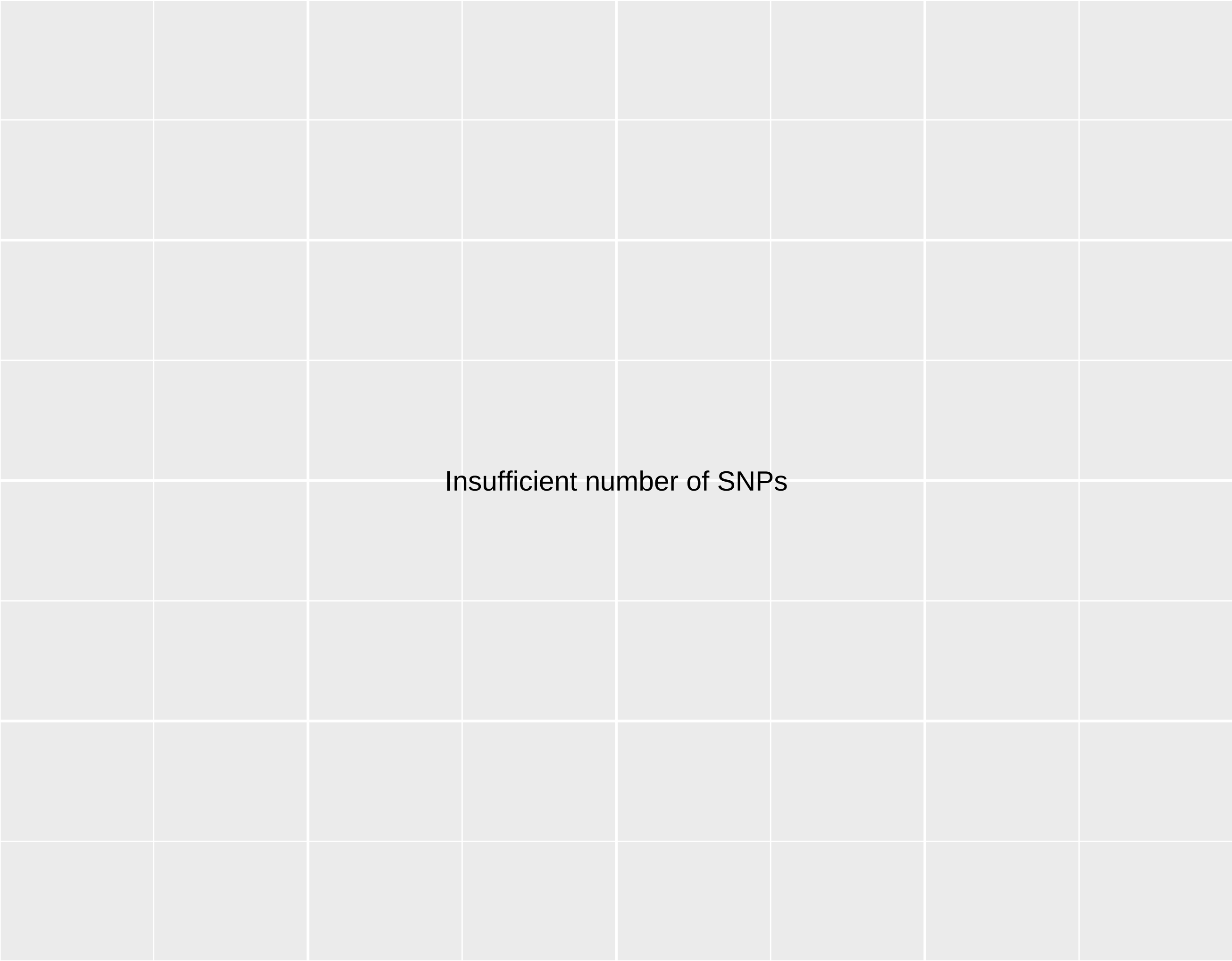
Insufficient number of SNPs

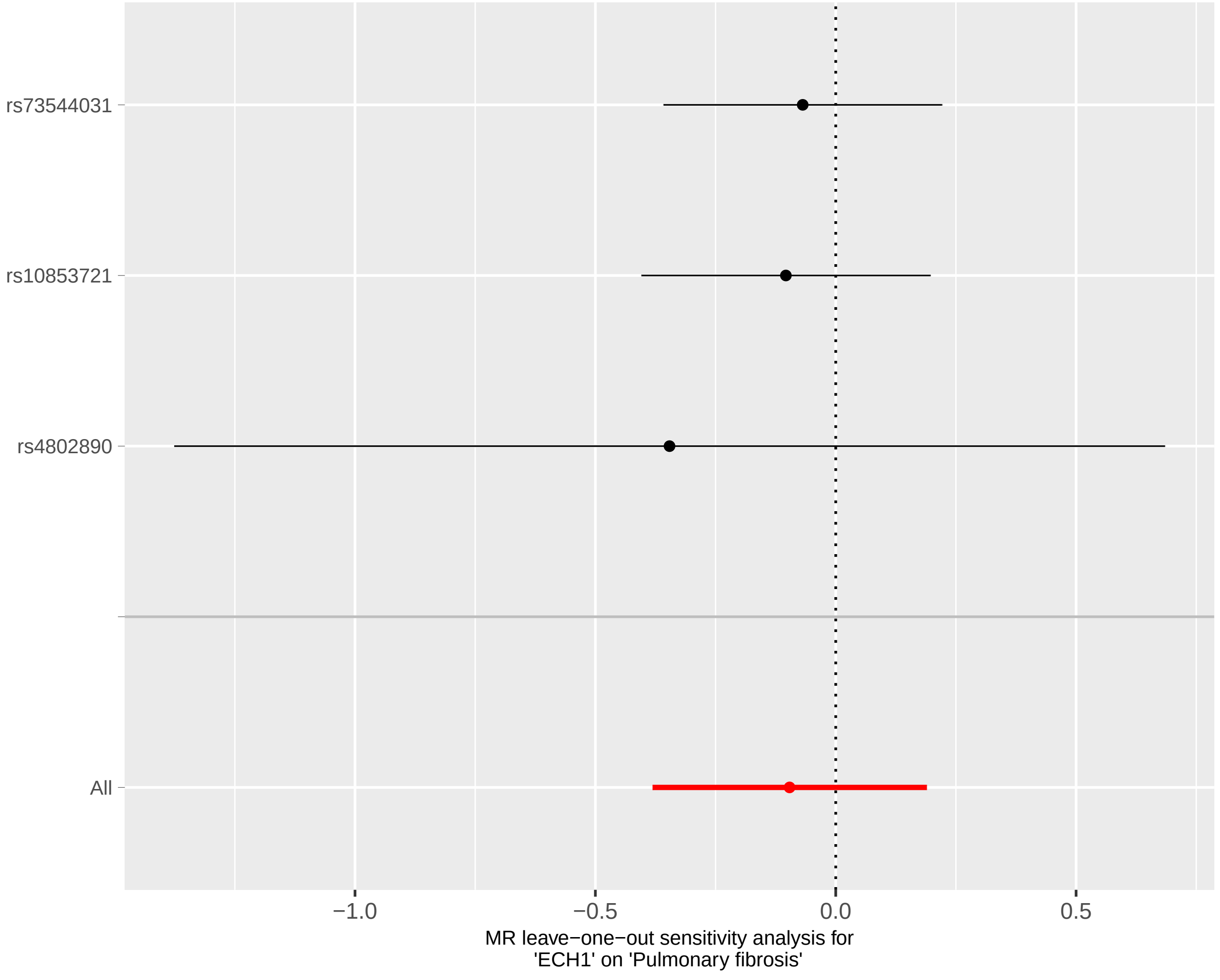

rs73544031
rs10853721
rs4802890
All
−1.0
−0.5
0.0
0.5
MR leave−one−out sensitivity analysis for
'ECH1' on 'Pulmonary fibrosis'

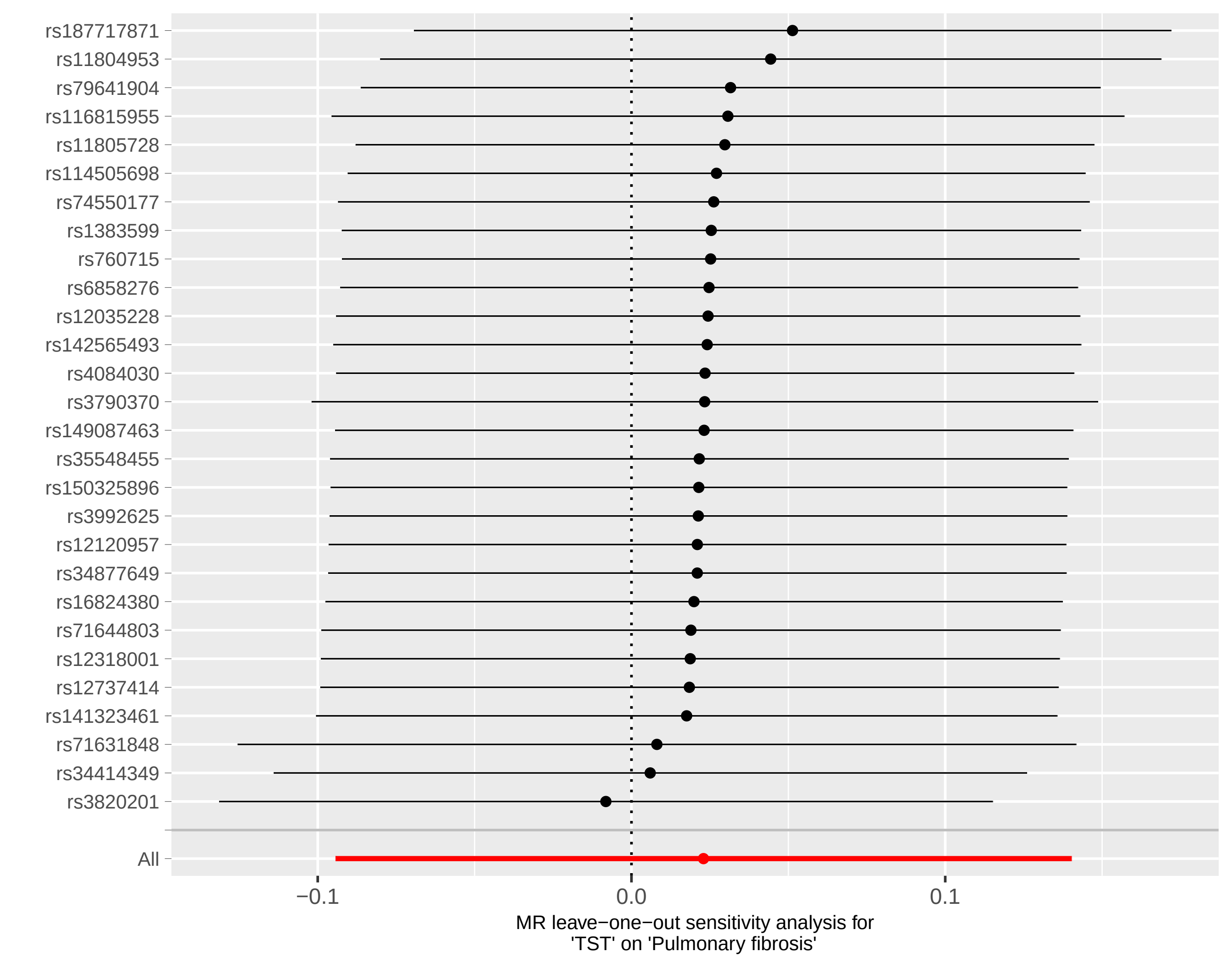
rs187717871
rs11804953
rs79641904
rs116815955
rs11805728
rs114505698
rs74550177
rs1383599
rs760715
rs6858276
rs12035228
rs142565493
rs4084030
rs3790370
rs149087463
rs35548455
rs150325896
rs3992625
rs12120957
rs34877649
rs16824380
rs71644803
rs12318001
rs12737414
rs141323461
rs71631848
rs34414349
rs3820201
All
−0.1
0.0
0.1
MR leave−one−out sensitivity analysis for
'TST' on 'Pulmonary fibrosis'

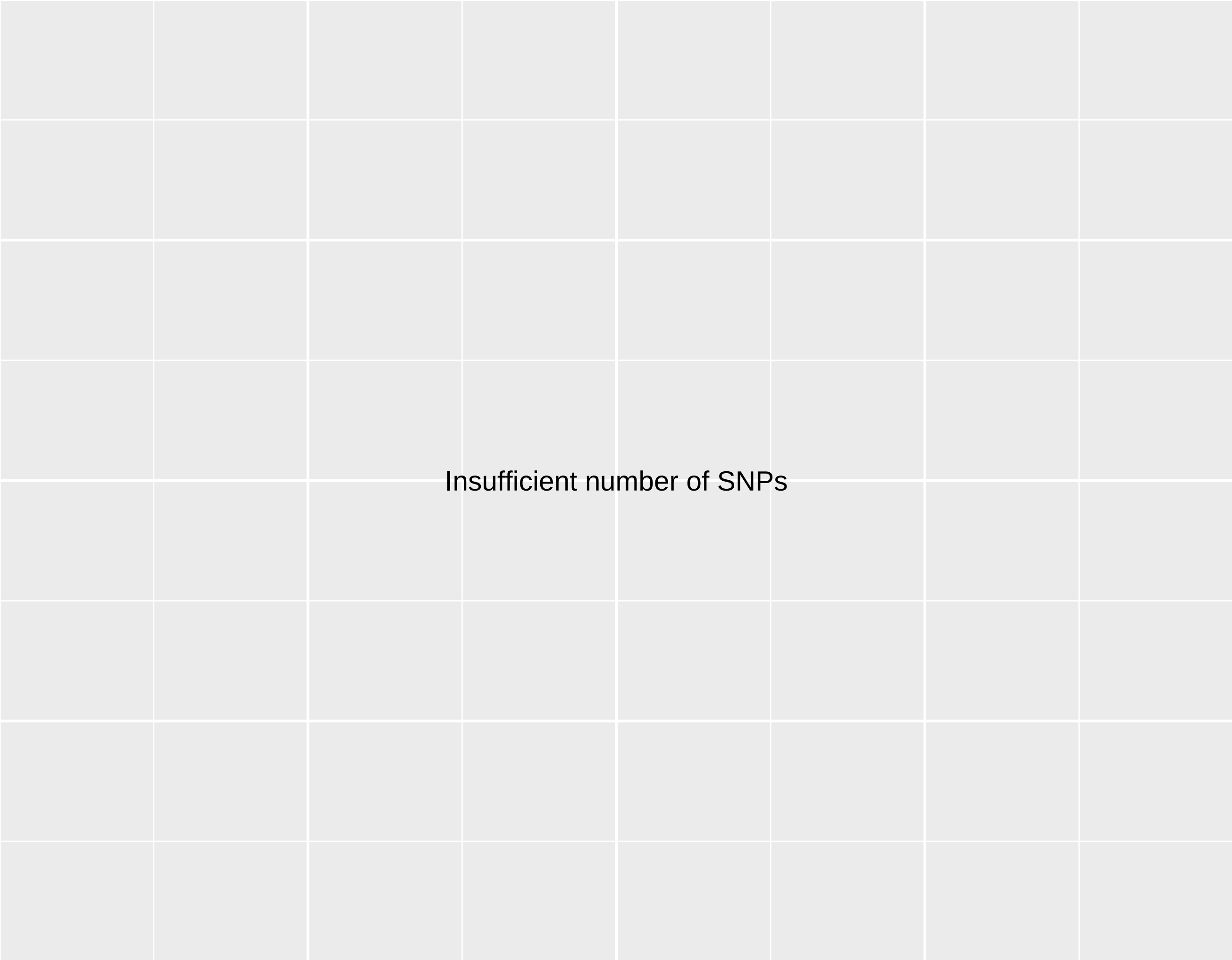
Insufficient number of SNPs

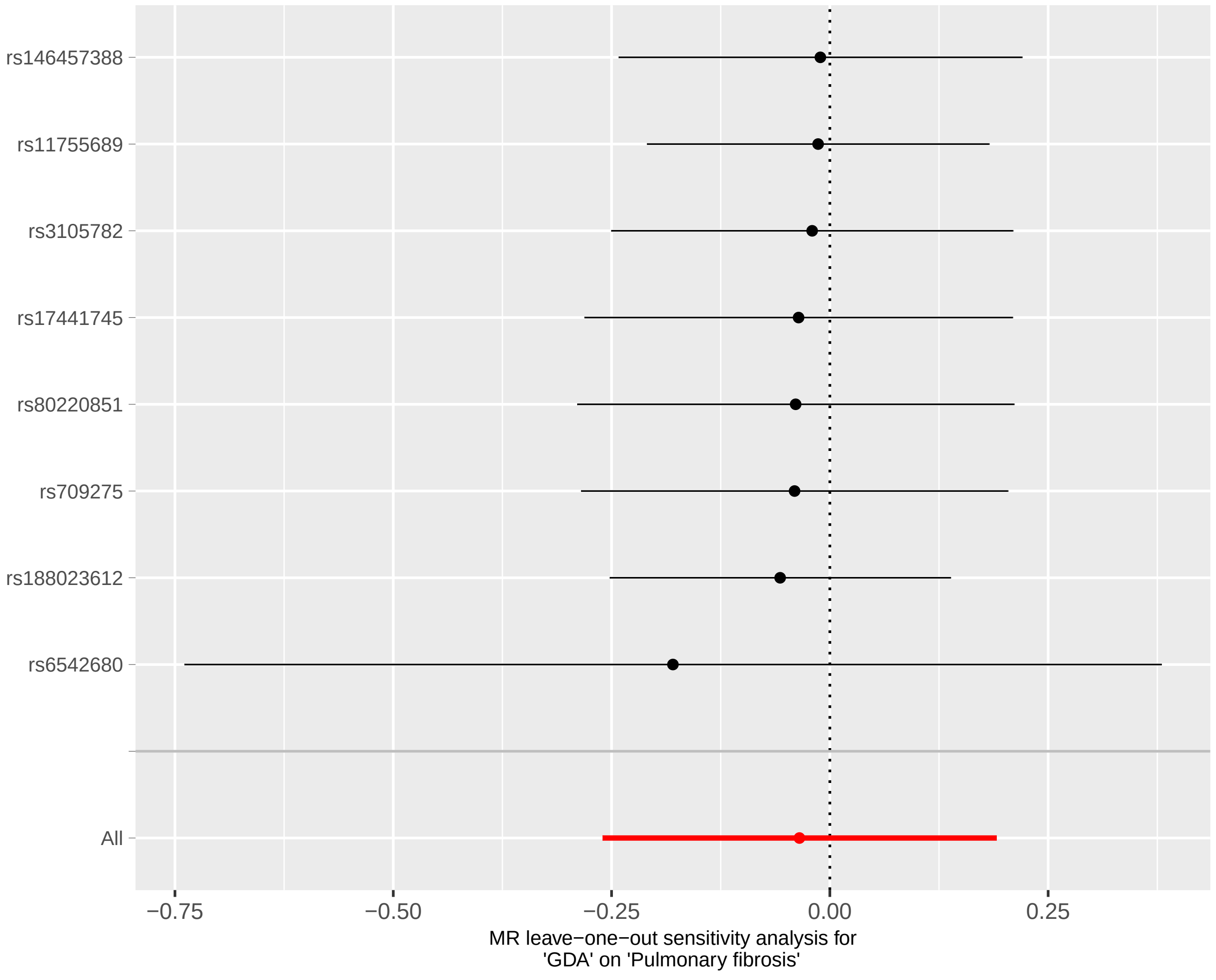
rs146457388
rs11755689
rs3105782
rs17441745
rs80220851
rs709275
rs188023612
rs6542680
All
−0.75
−0.50
−0.25
0.00
0.25
MR leave−one−out sensitivity analysis for
'GDA' on 'Pulmonary fibrosis'

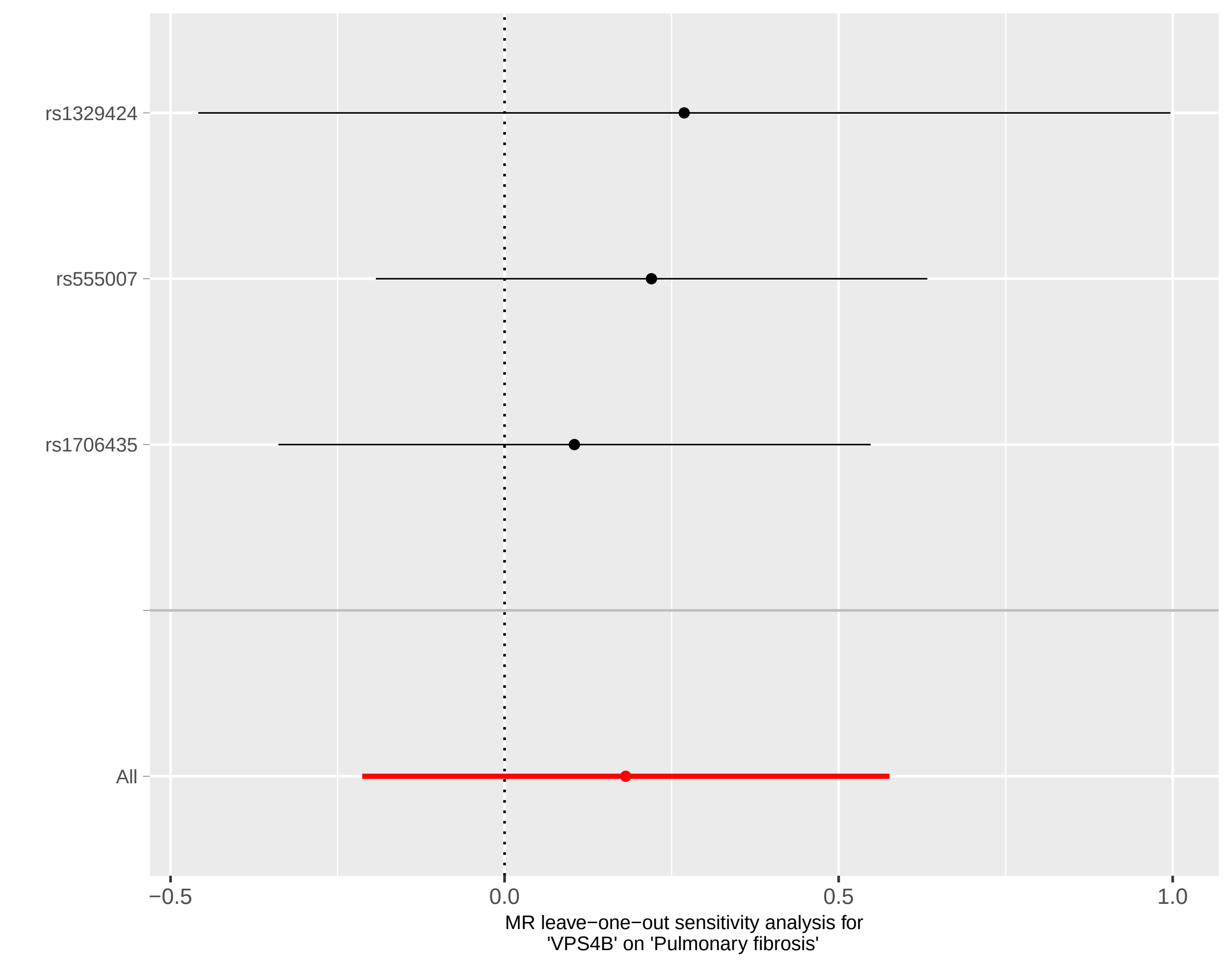
rs1329424
rs555007
rs1706435
All
−0.5
0.0
0.5
1.0
MR leave−one−out sensitivity analysis for
'VPS4B' on 'Pulmonary fibrosis'

Insufficient number of SNPs

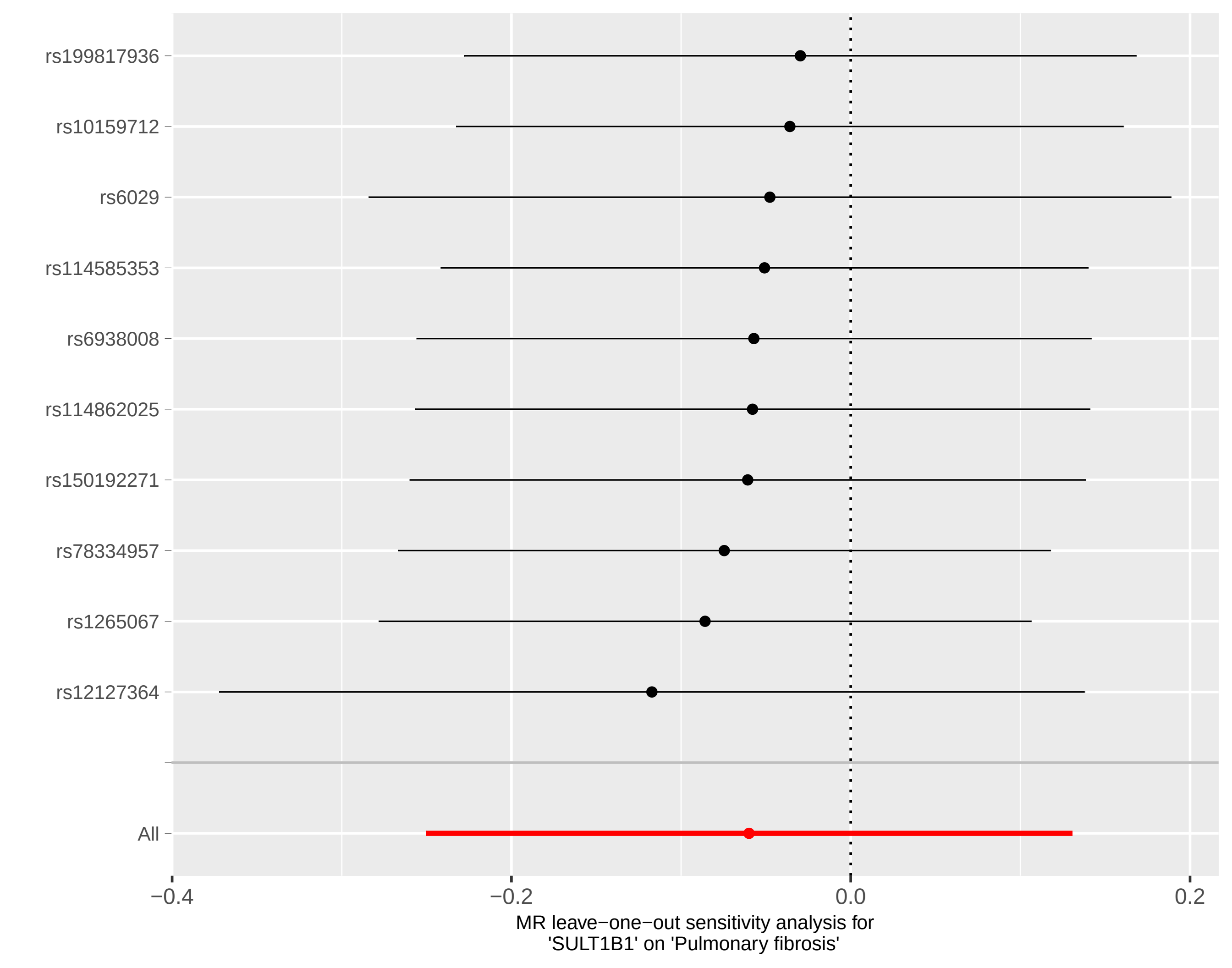

rs199817936
rs10159712
rs6029
rs114585353
rs6938008
rs114862025
rs150192271
rs78334957
rs1265067
rs12127364
All
−0.4
−0.2
0.0
0.2
MR leave−one−out sensitivity analysis for
'SULT1B1' on 'Pulmonary fibrosis'

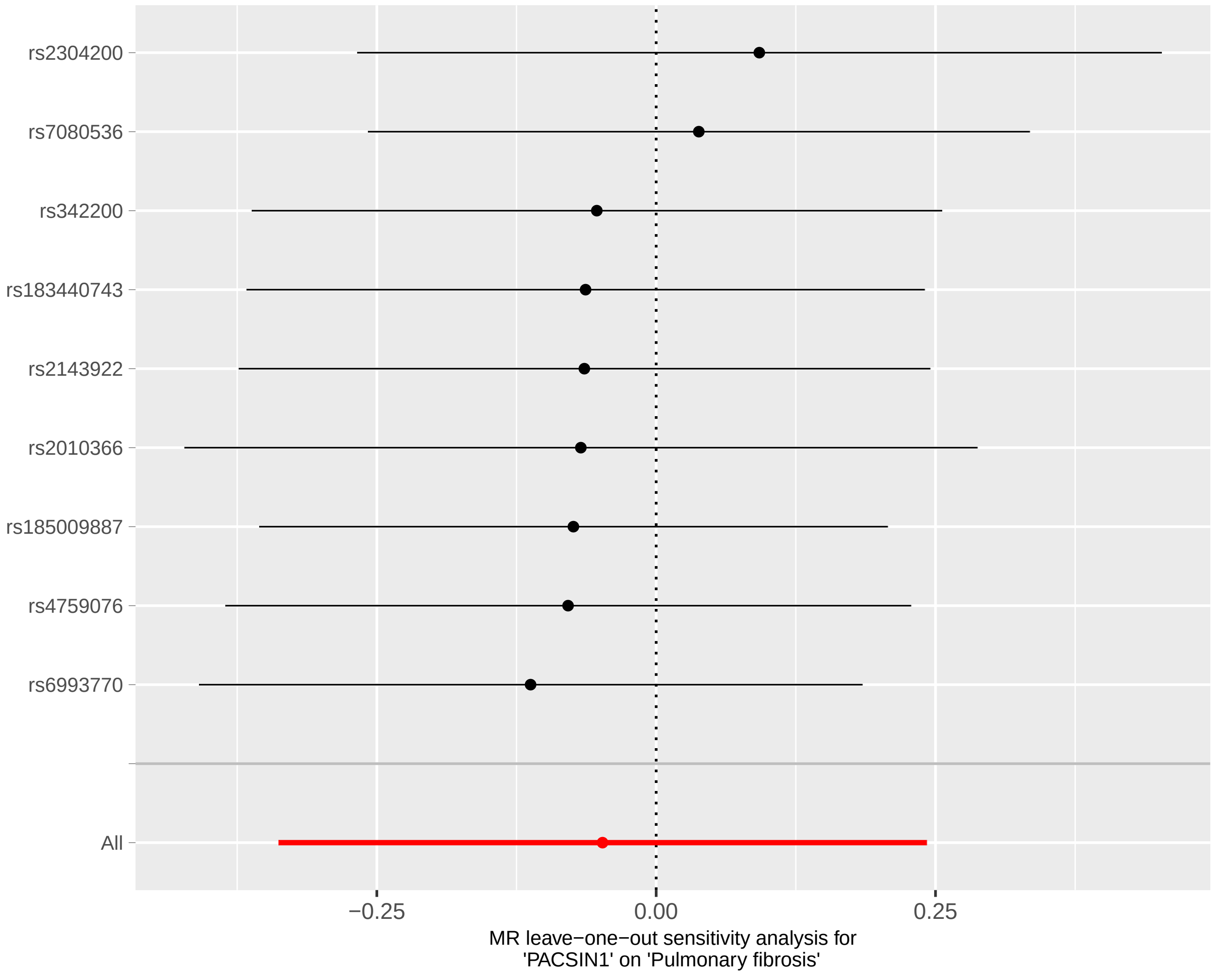

rs2304200
rs7080536
rs342200
rs183440743
rs2143922
rs2010366
rs185009887
rs4759076
rs6993770
All
−0.25
0.00
0.25
MR leave−one−out sensitivity analysis for
'PACSIN1' on 'Pulmonary fibrosis'

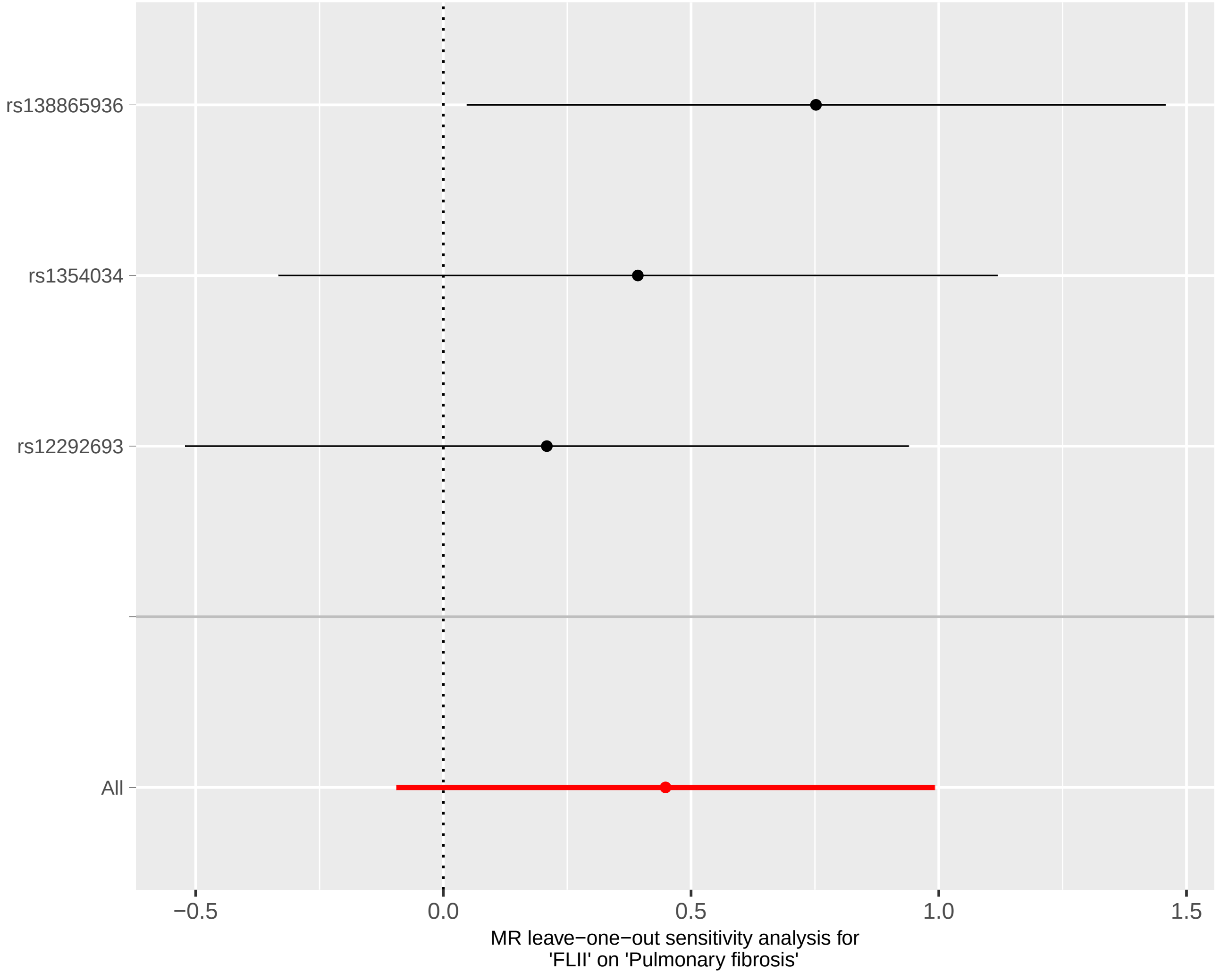

rs138865936
rs1354034
rs12292693
All
−0.5
0.0
0.5
1.0
1.5
MR leave−one−out sensitivity analysis for
'FLII' on 'Pulmonary fibrosis'

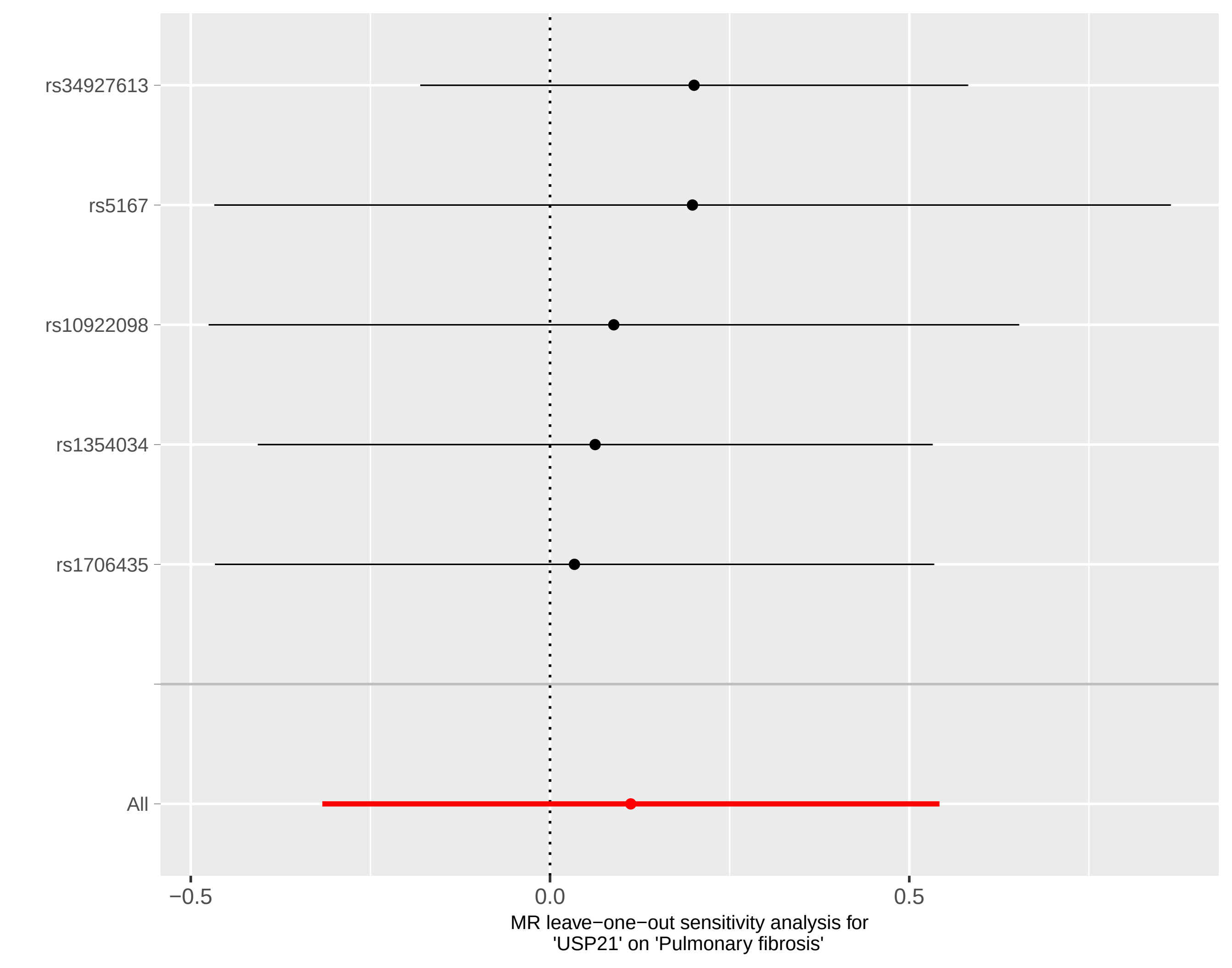
rs34927613
rs5167
rs10922098
rs1354034
rs1706435
All
−0.5
0.0
0.5
MR leave−one−out sensitivity analysis for
'USP21' on 'Pulmonary fibrosis'

Insufficient number of SNPs

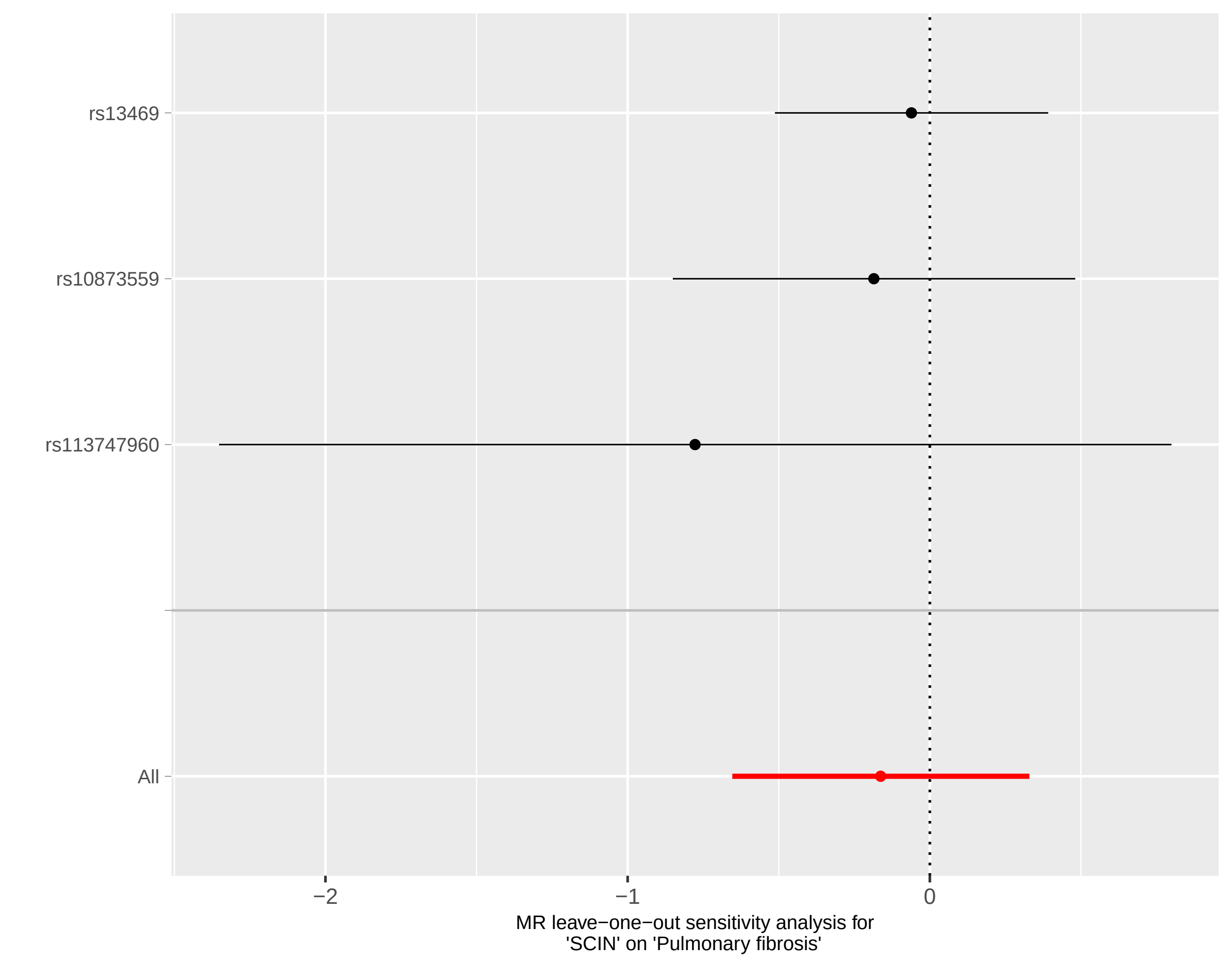
rs13469
rs10873559
rs113747960
All
−2
−1
0
MR leave−one−out sensitivity analysis for
'SCIN' on 'Pulmonary fibrosis'

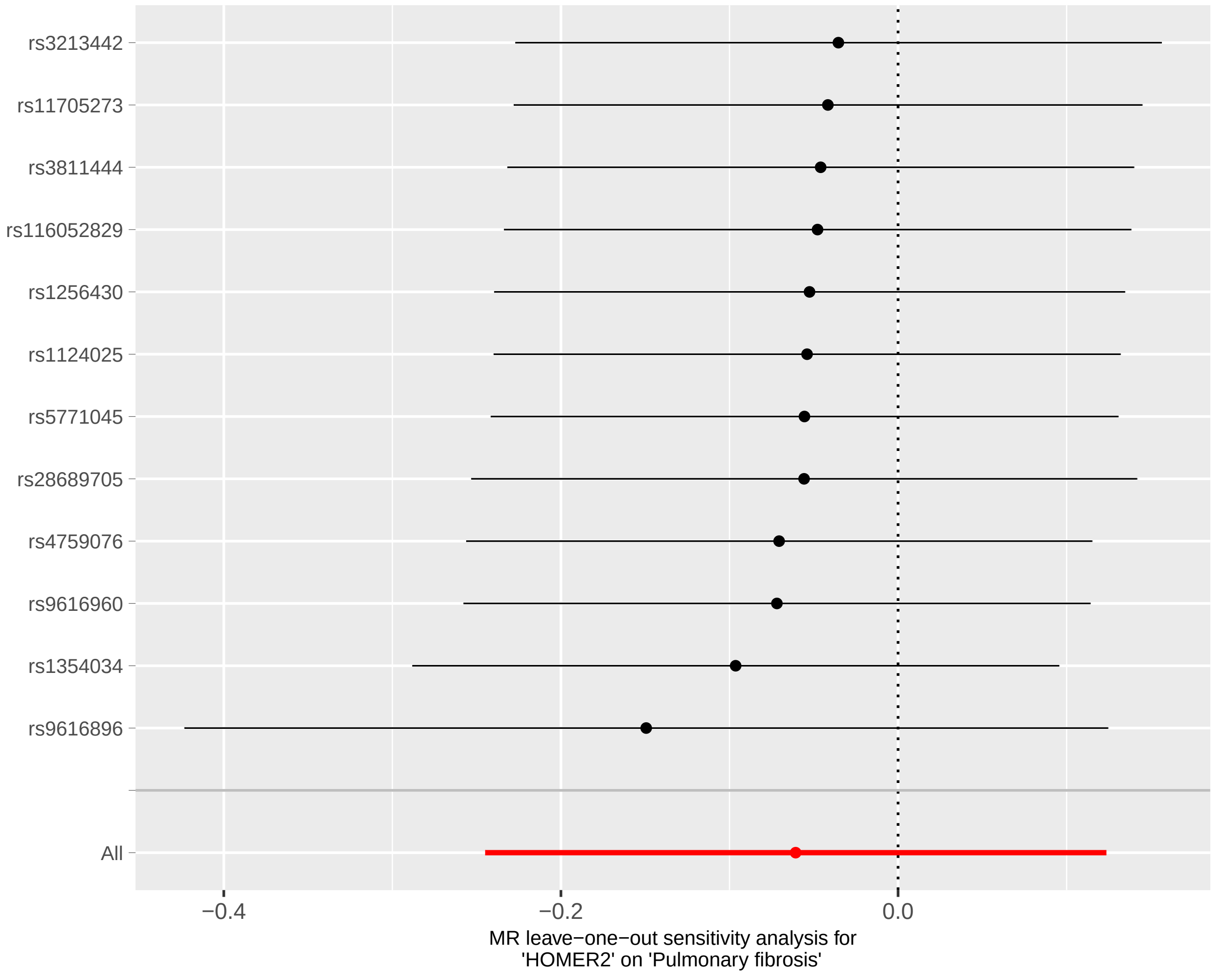

rs3213442
rs11705273
rs3811444
rs116052829
rs1256430
rs1124025
rs5771045
rs28689705
rs4759076
rs9616960
rs1354034
rs9616896
All
−0.4
−0.2
0.0
MR leave−one−out sensitivity analysis for
'HOMER2' on 'Pulmonary fibrosis'

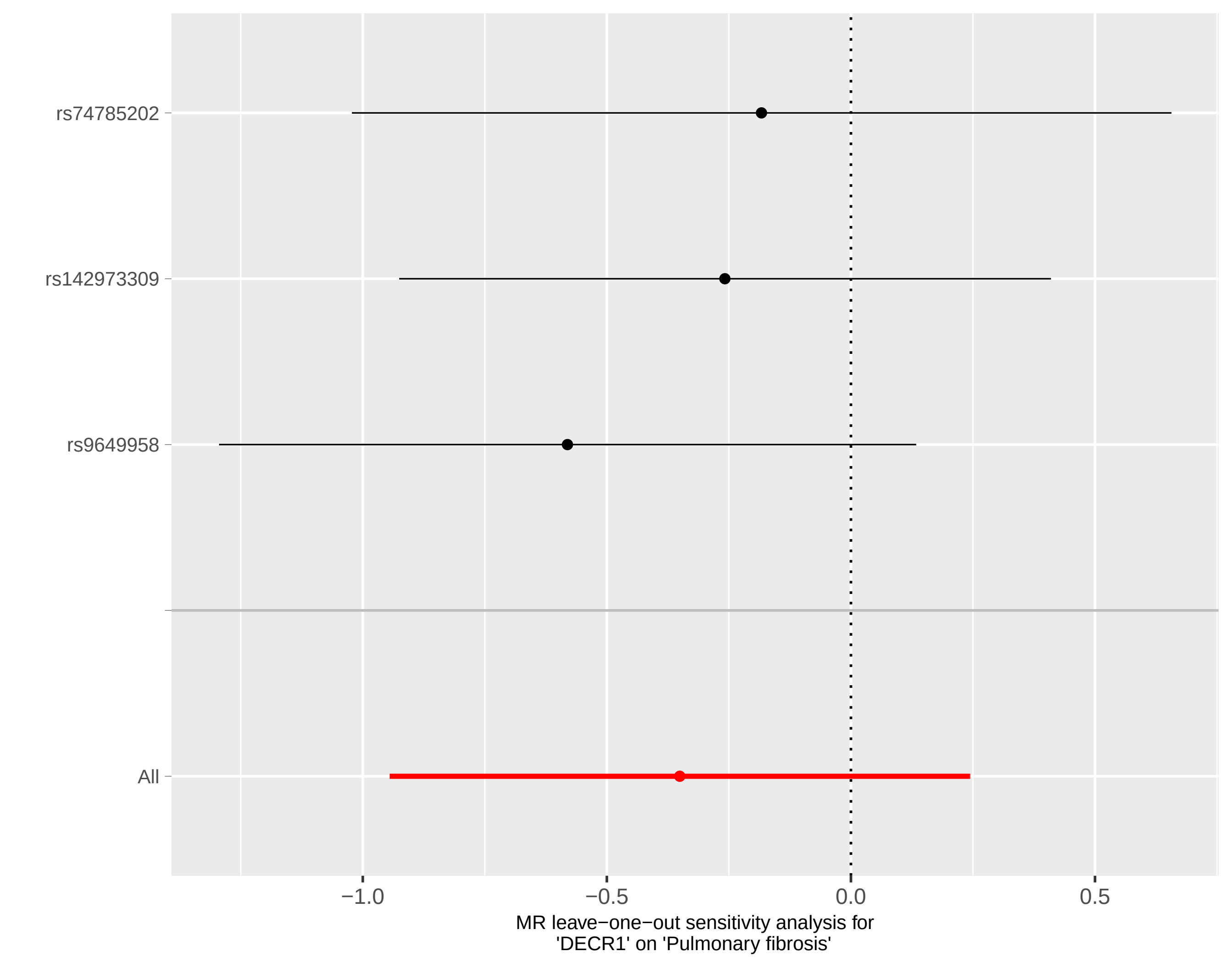

rs74785202
rs142973309
rs9649958
All
−1.0
−0.5
0.0
0.5
MR leave−one−out sensitivity analysis for
'DECR1' on 'Pulmonary fibrosis'

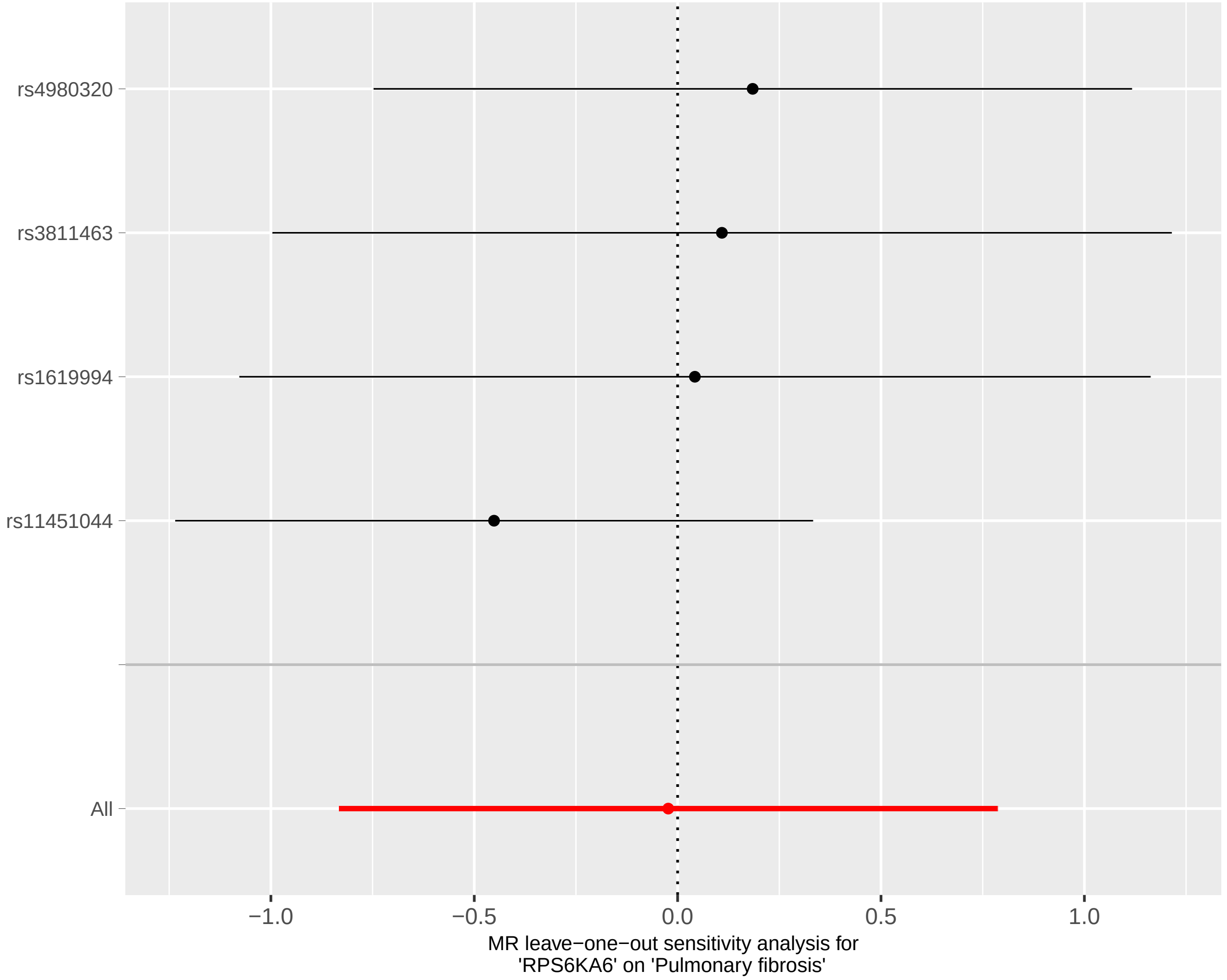
rs4980320
rs3811463
rs1619994
rs11451044
All
−1.0
−0.5
0.0
0.5
1.0
MR leave−one−out sensitivity analysis for
'RPS6KA6' on 'Pulmonary fibrosis'

Insufficient number of SNPs

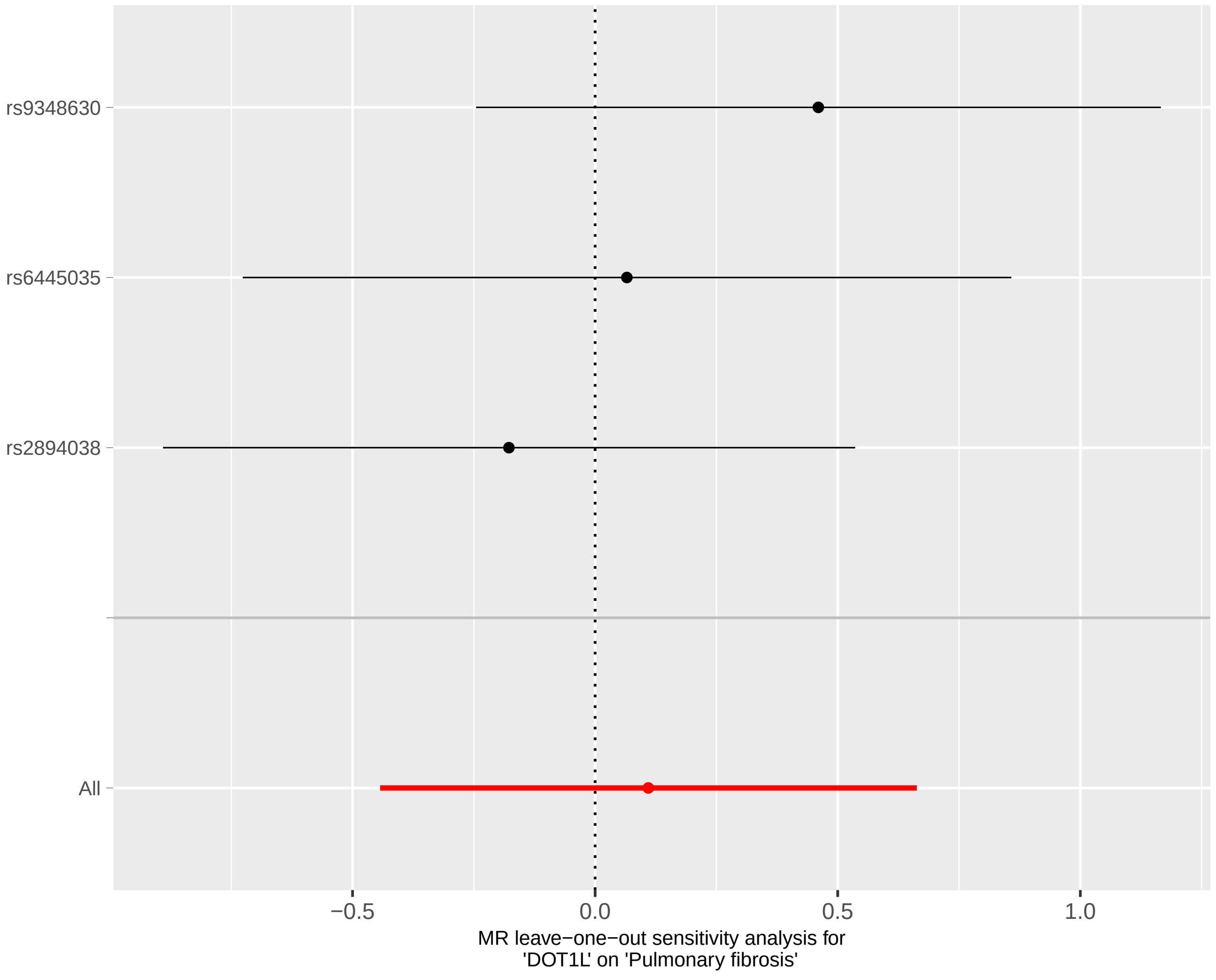
rs9348630
rs6445035
rs2894038
All
−0.5
0.0
0.5
1.0
MR leave−one−out sensitivity analysis for
'DOT1L' on 'Pulmonary fibrosis'

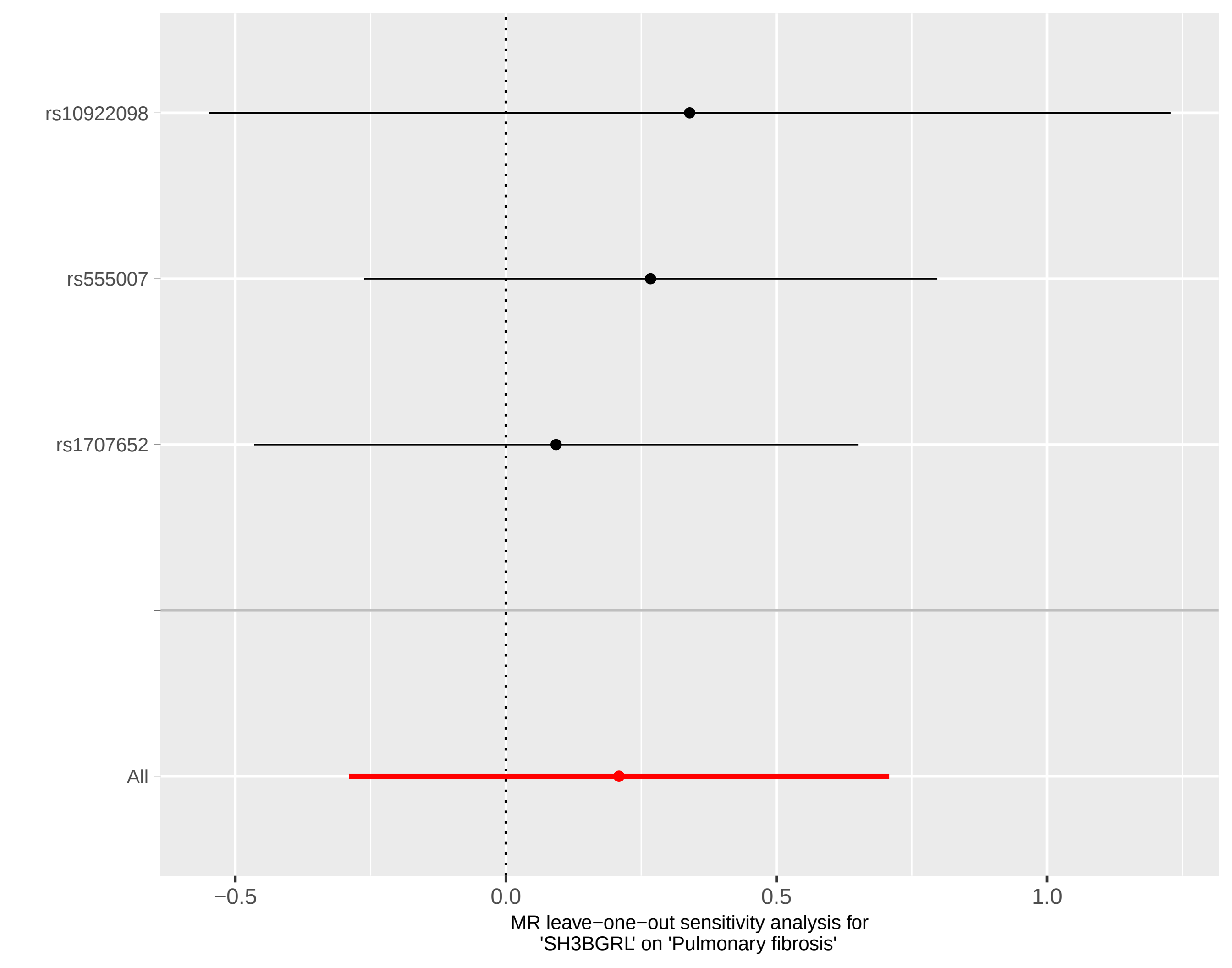

rs10922098
rs555007
rs1707652
All
−0.5
0.0
0.5
1.0
MR leave−one−out sensitivity analysis for
'SH3BGRL' on 'Pulmonary fibrosis'

Insufficient number of SNPs

Insufficient number of SNPs

Insufficient number of SNPs

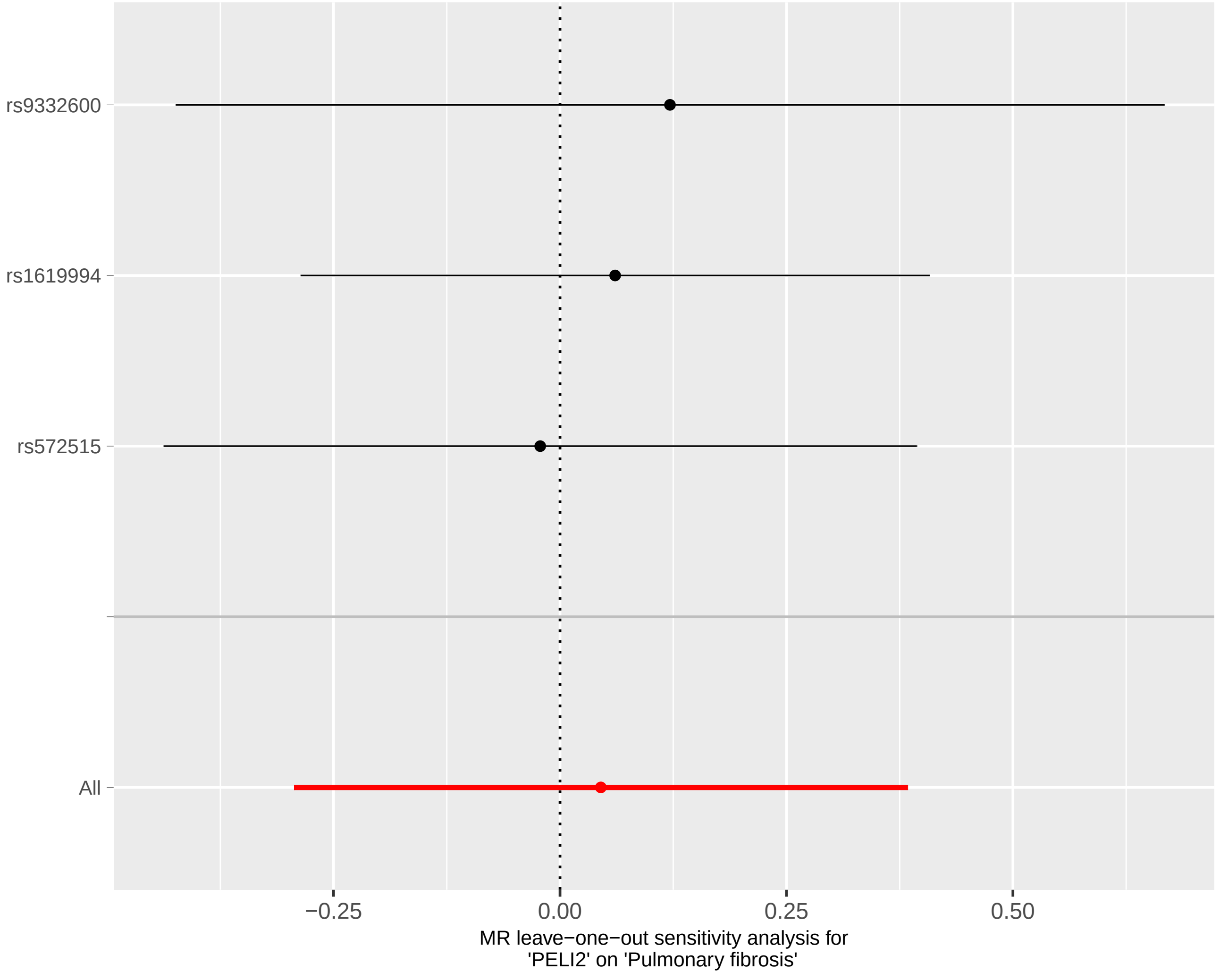

rs9332600
rs1619994
rs572515
All
−0.25
0.00
0.25
0.50
MR leave−one−out sensitivity analysis for
'PELI2' on 'Pulmonary fibrosis'

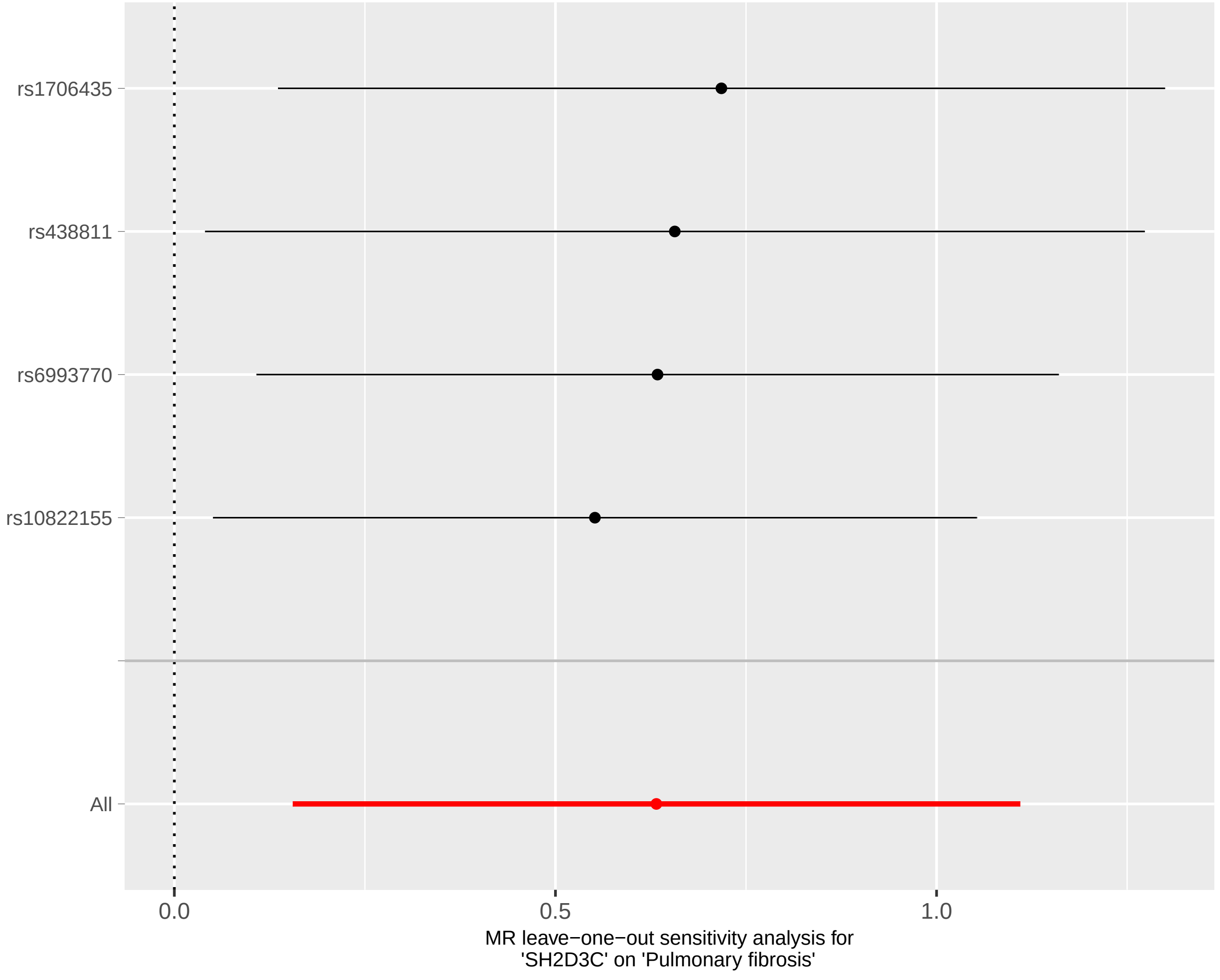
rs1706435
rs438811
rs6993770
rs10822155
All
0.0
0.5
1.0
MR leave−one−out sensitivity analysis for
'SH2D3C' on 'Pulmonary fibrosis'

Insufficient number of SNPs

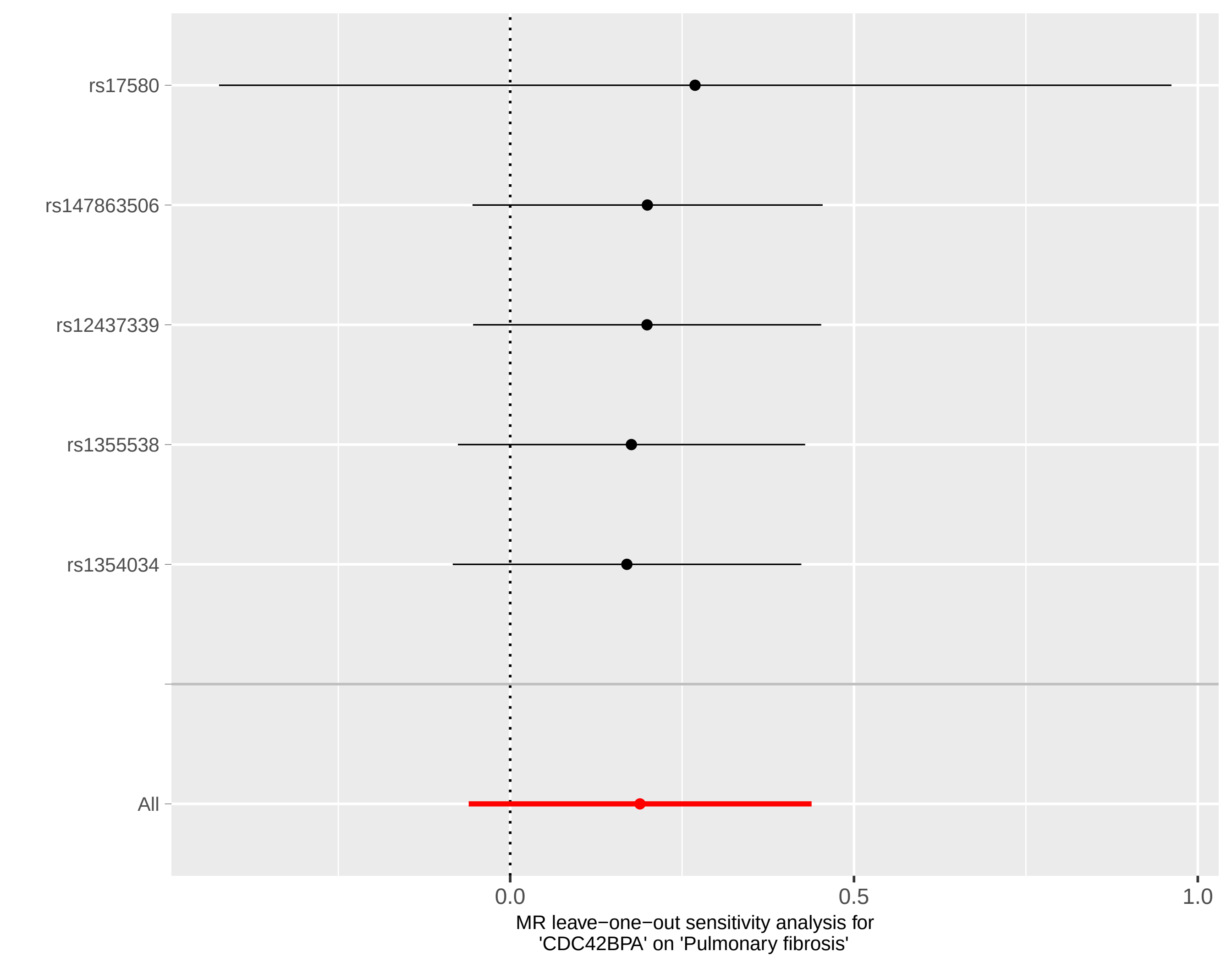
rs17580
rs147863506
rs12437339
rs1355538
rs1354034
All
0.0
0.5
1.0
MR leave−one−out sensitivity analysis for
'CDC42BPA' on 'Pulmonary fibrosis'

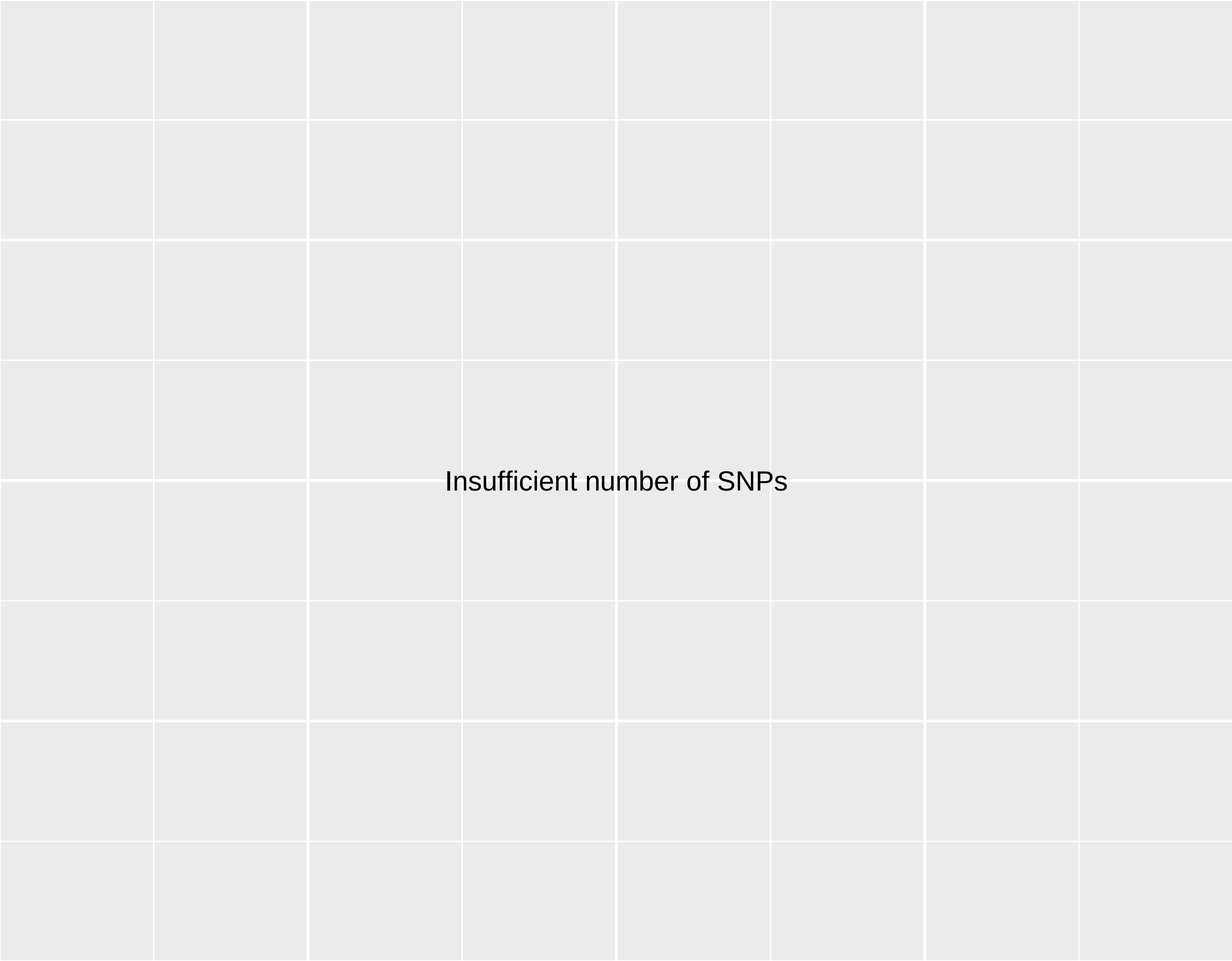
Insufficient number of SNPs

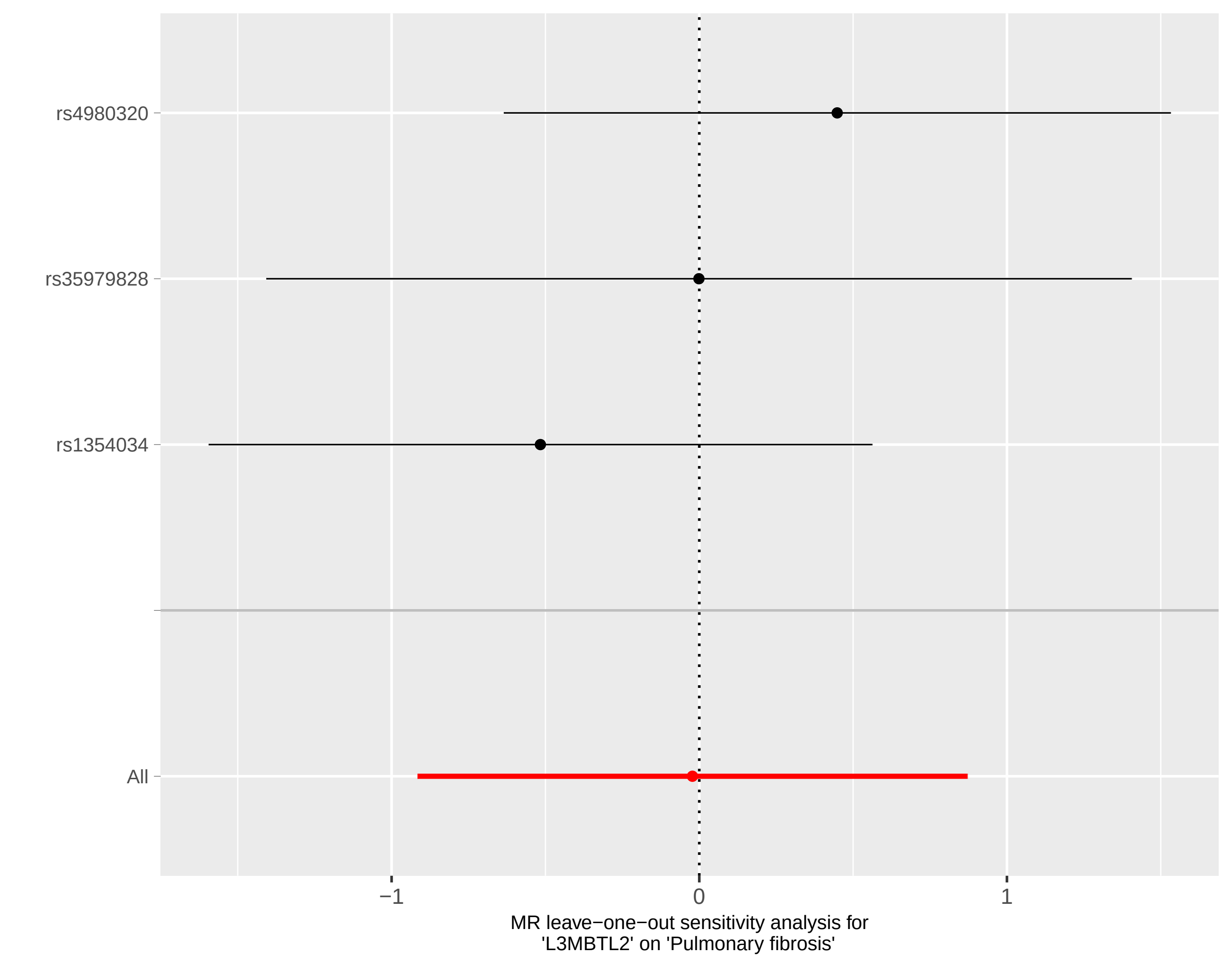

rs4980320
rs35979828
rs1354034
All
−1
0
1
MR leave−one−out sensitivity analysis for
'L3MBTL2' on 'Pulmonary fibrosis'

Insufficient number of SNPs

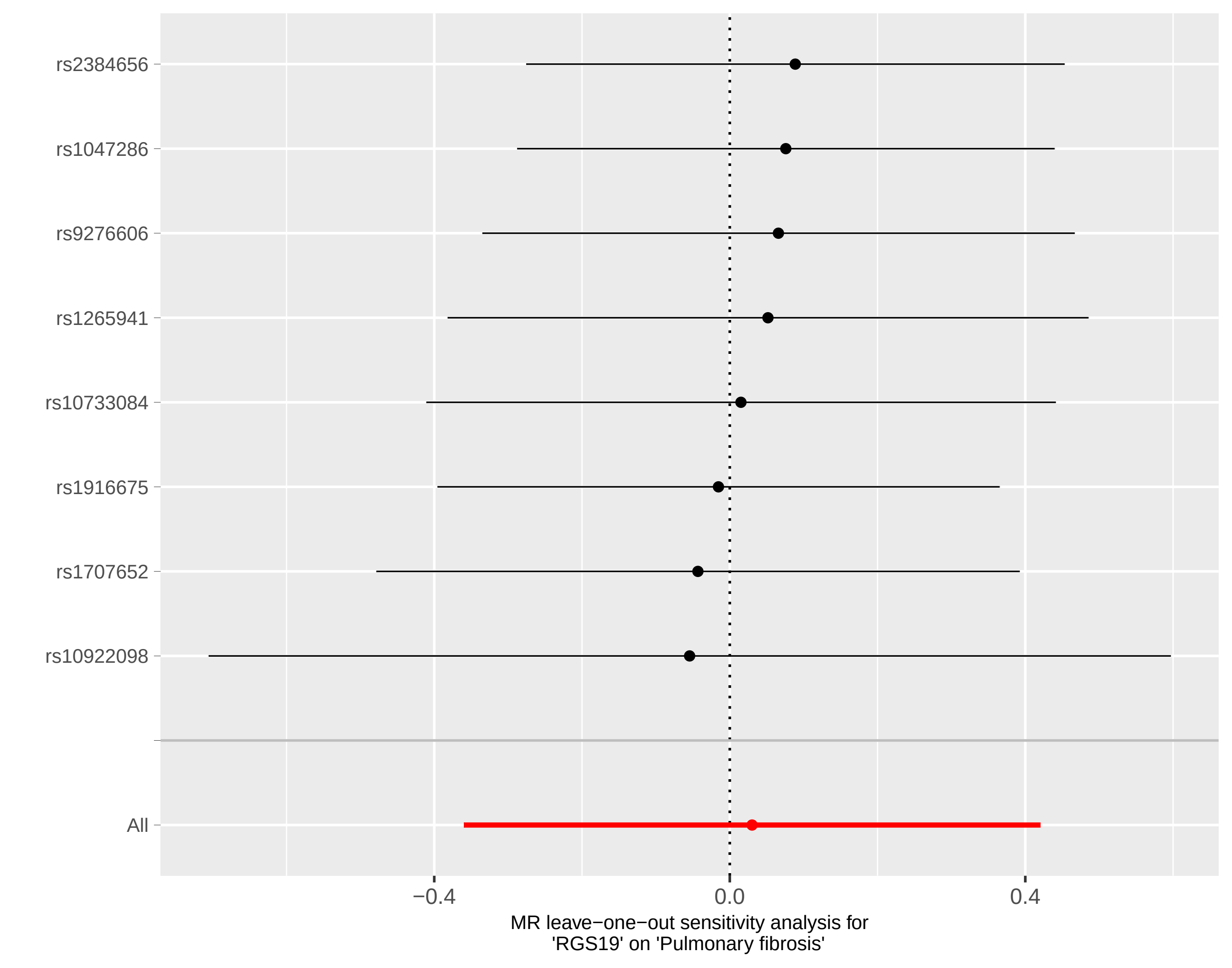
rs2384656
rs1047286
rs9276606
rs1265941
rs10733084
rs1916675
rs1707652
rs10922098
All
−0.4
0.0
0.4
MR leave−one−out sensitivity analysis for
'RGS19' on 'Pulmonary fibrosis'

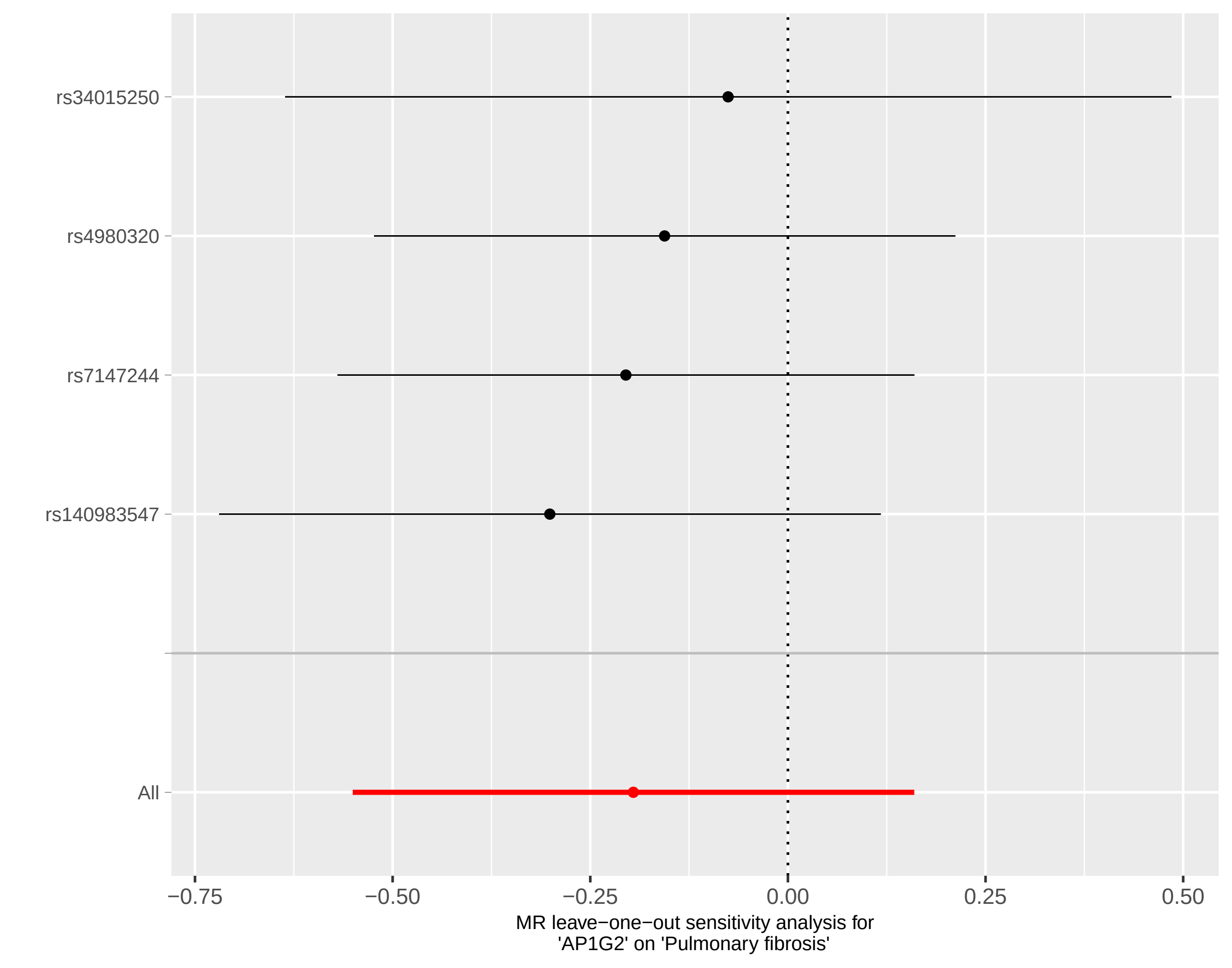

rs34015250
rs4980320
rs7147244
rs140983547
All
−0.75
−0.50
−0.25
0.00
0.25
0.50
MR leave−one−out sensitivity analysis for
'AP1G2' on 'Pulmonary fibrosis'

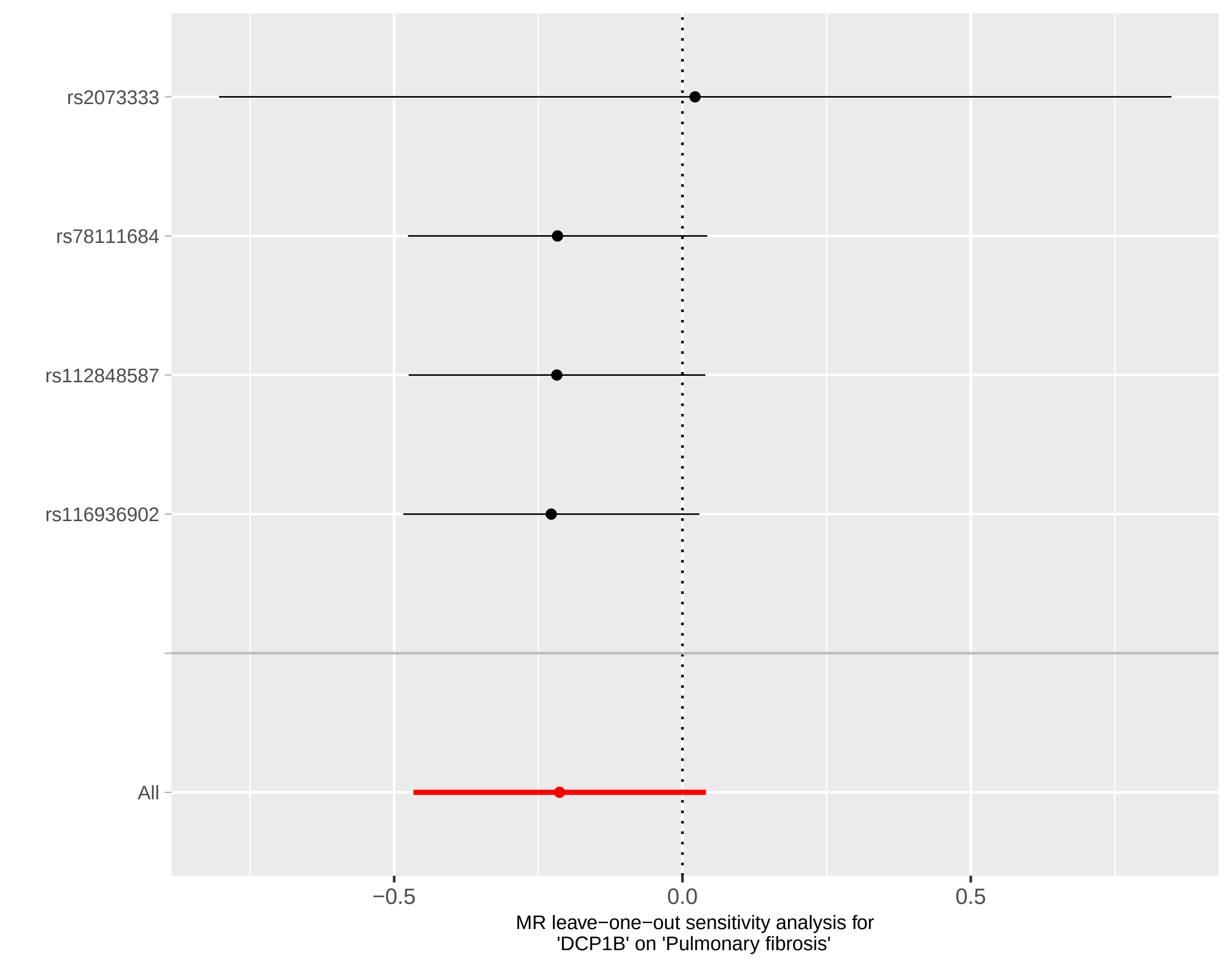
rs2073333
rs78111684
rs112848587
rs116936902
All
−0.5
0.0
0.5
MR leave−one−out sensitivity analysis for
'DCP1B' on 'Pulmonary fibrosis'

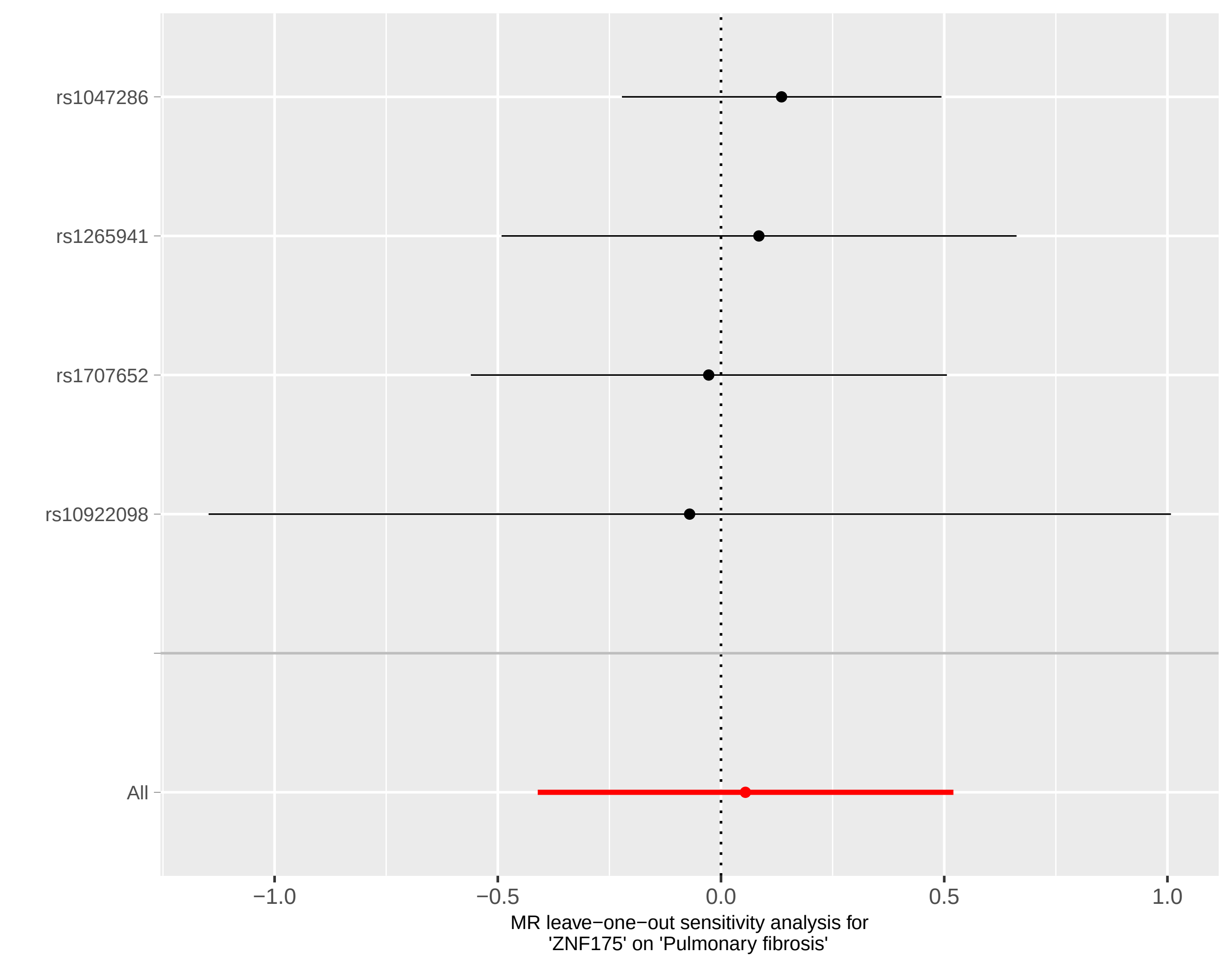

rs1047286
rs1265941
rs1707652
rs10922098
All
−1.0
−0.5
0.0
0.5
1.0
MR leave−one−out sensitivity analysis for
'ZNF175' on 'Pulmonary fibrosis'

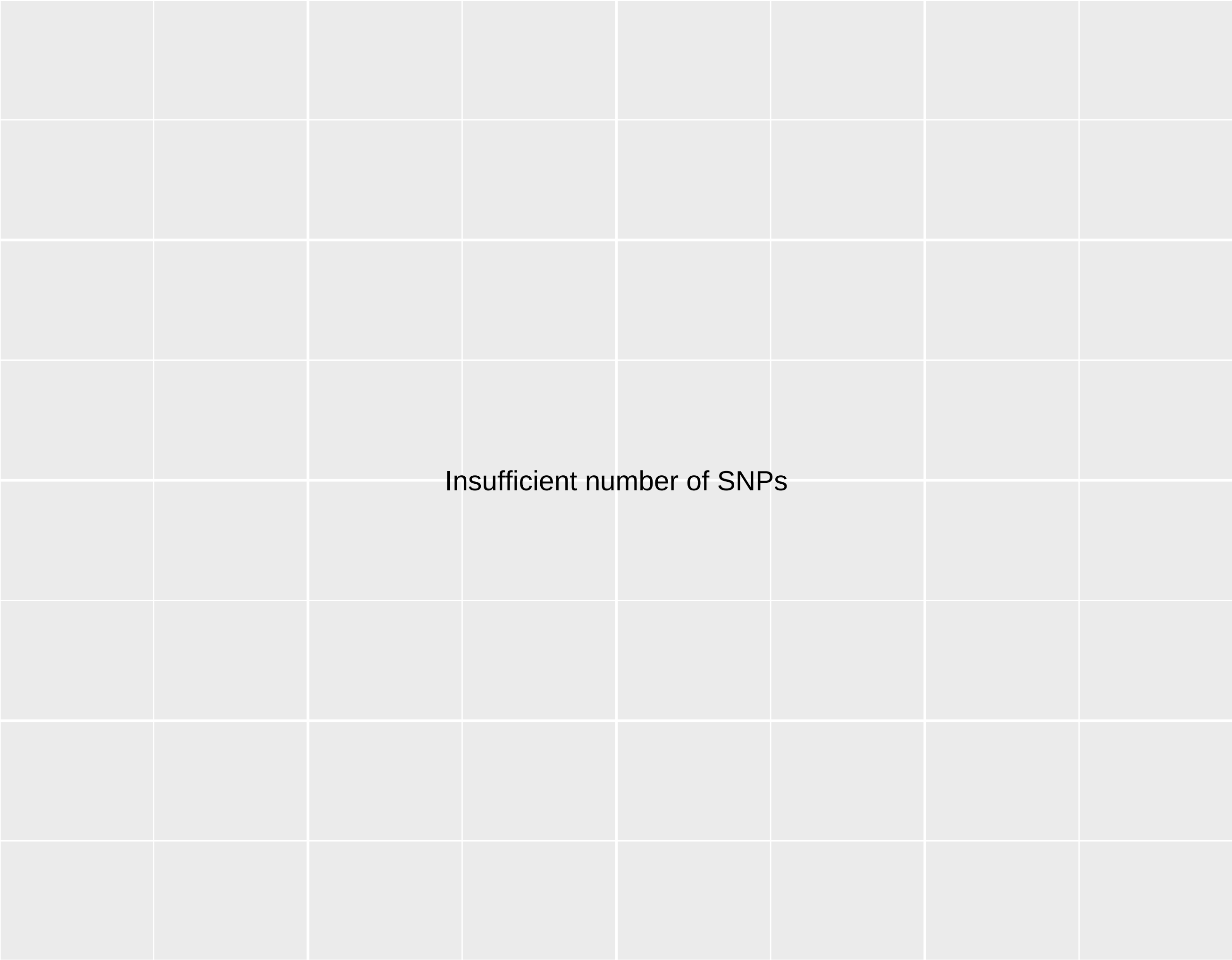
Insufficient number of SNPs

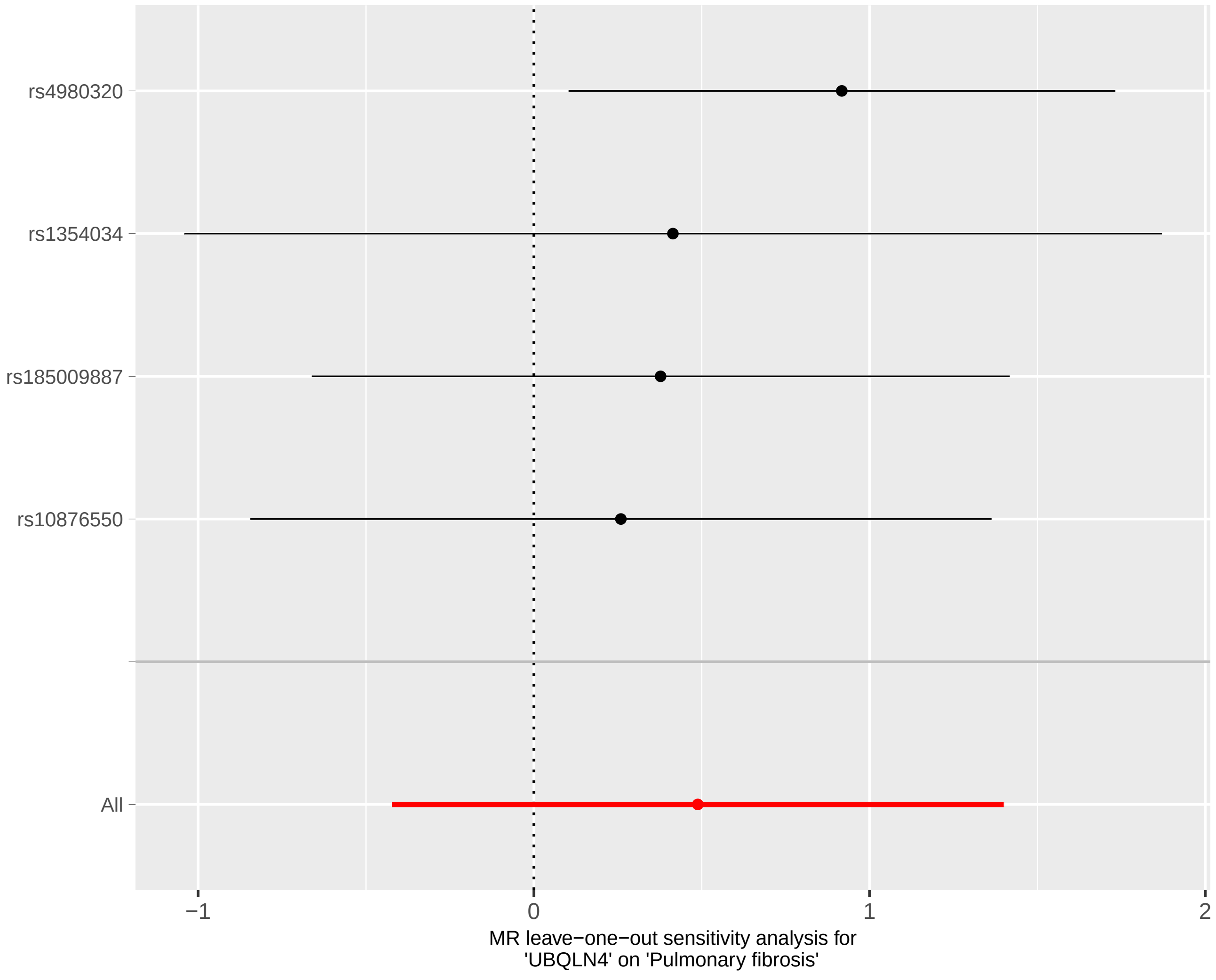
rs4980320
rs1354034
rs185009887
rs10876550
All
−1
0
1
2
MR leave−one−out sensitivity analysis for
'UBQLN4' on 'Pulmonary fibrosis'

Insufficient number of SNPs

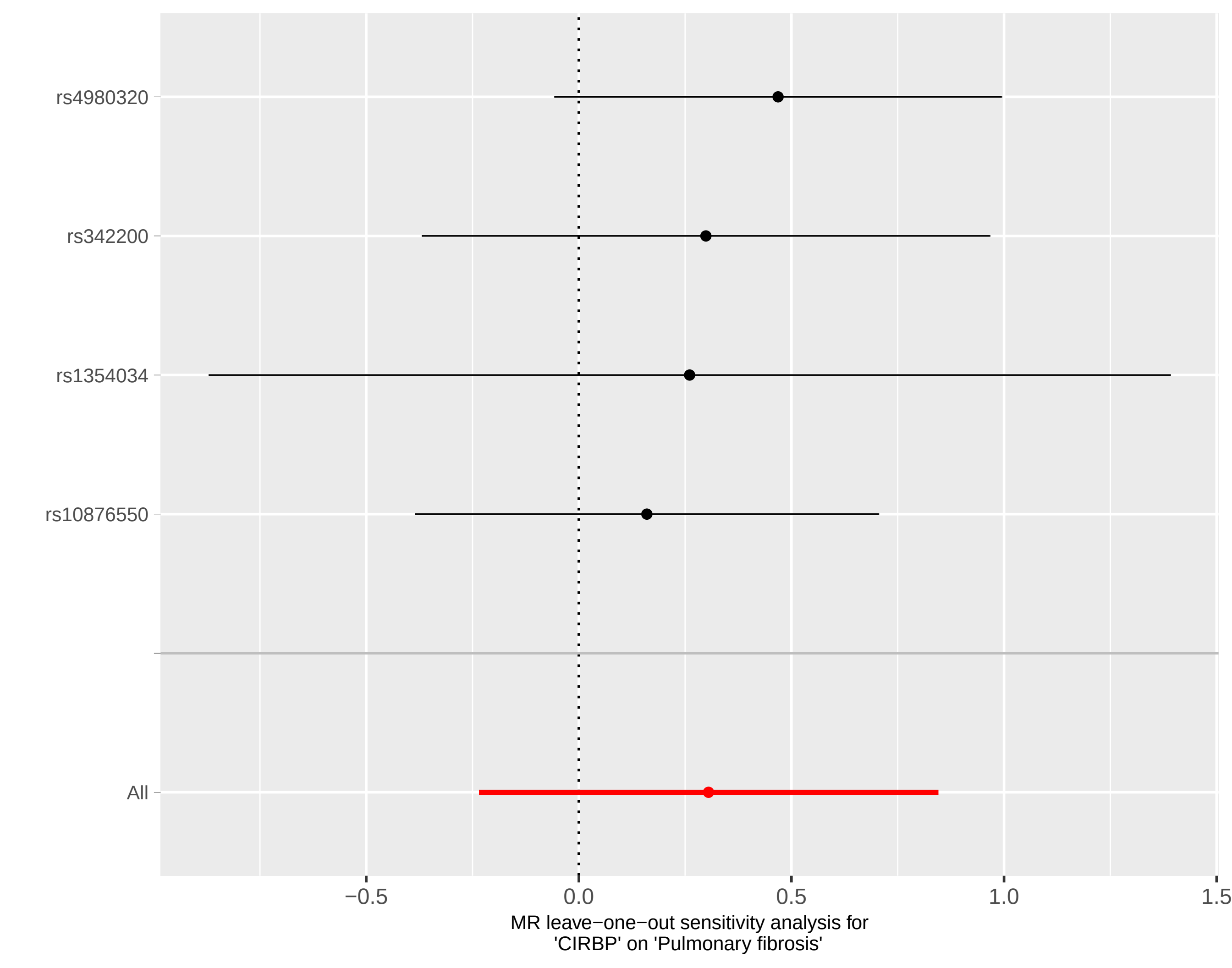
rs4980320
rs342200
rs1354034
rs10876550
All
−0.5
0.0
0.5
1.0
1.5
MR leave−one−out sensitivity analysis for
'CIRBP' on 'Pulmonary fibrosis'

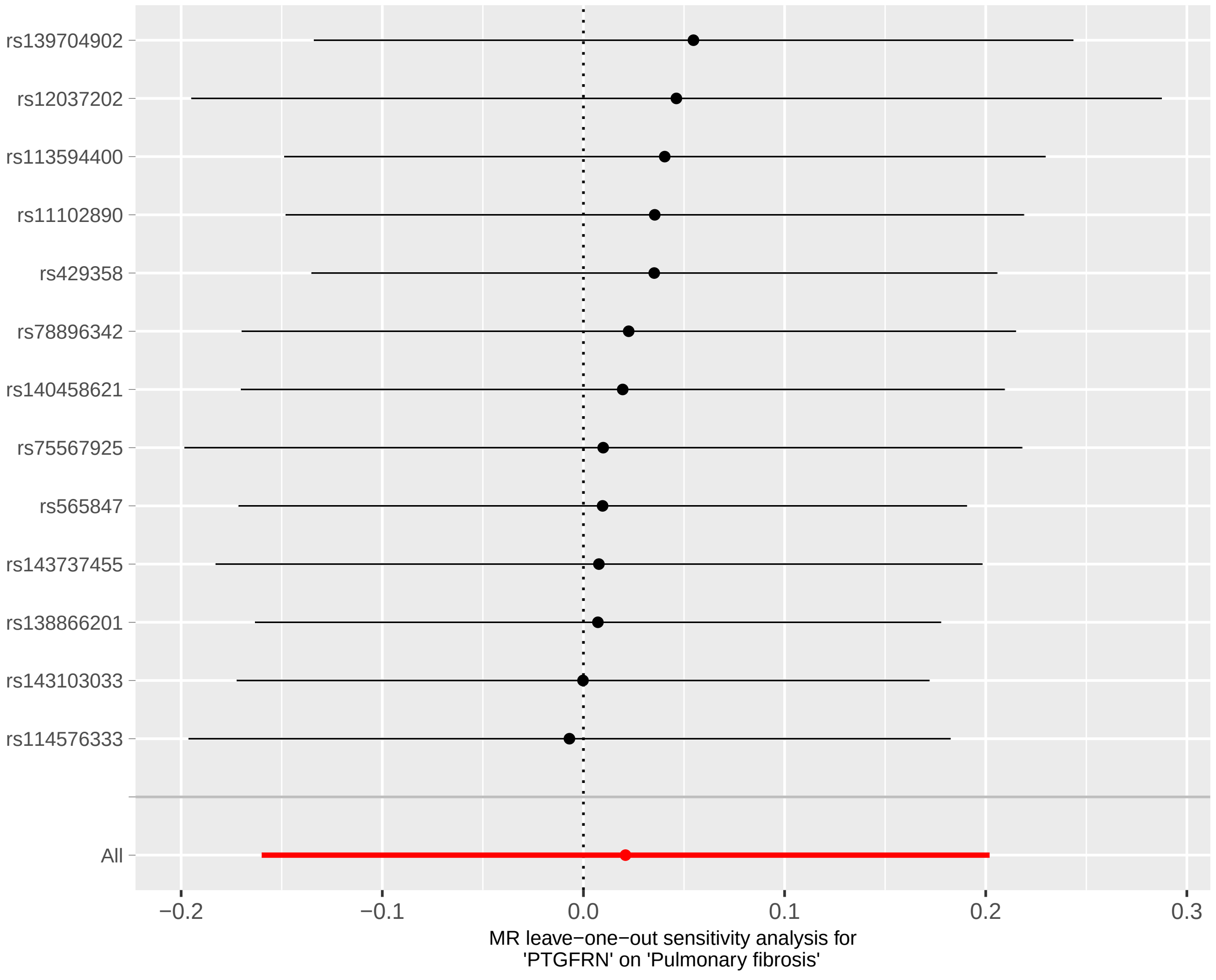
rs139704902
rs12037202
rs113594400
rs11102890
rs429358
rs78896342
rs140458621
rs75567925
rs565847
rs143737455
rs138866201
rs143103033
rs114576333
All
−0.2
−0.1
0.0
0.1
0.2
0.3
MR leave−one−out sensitivity analysis for
'PTGFRN' on 'Pulmonary fibrosis'

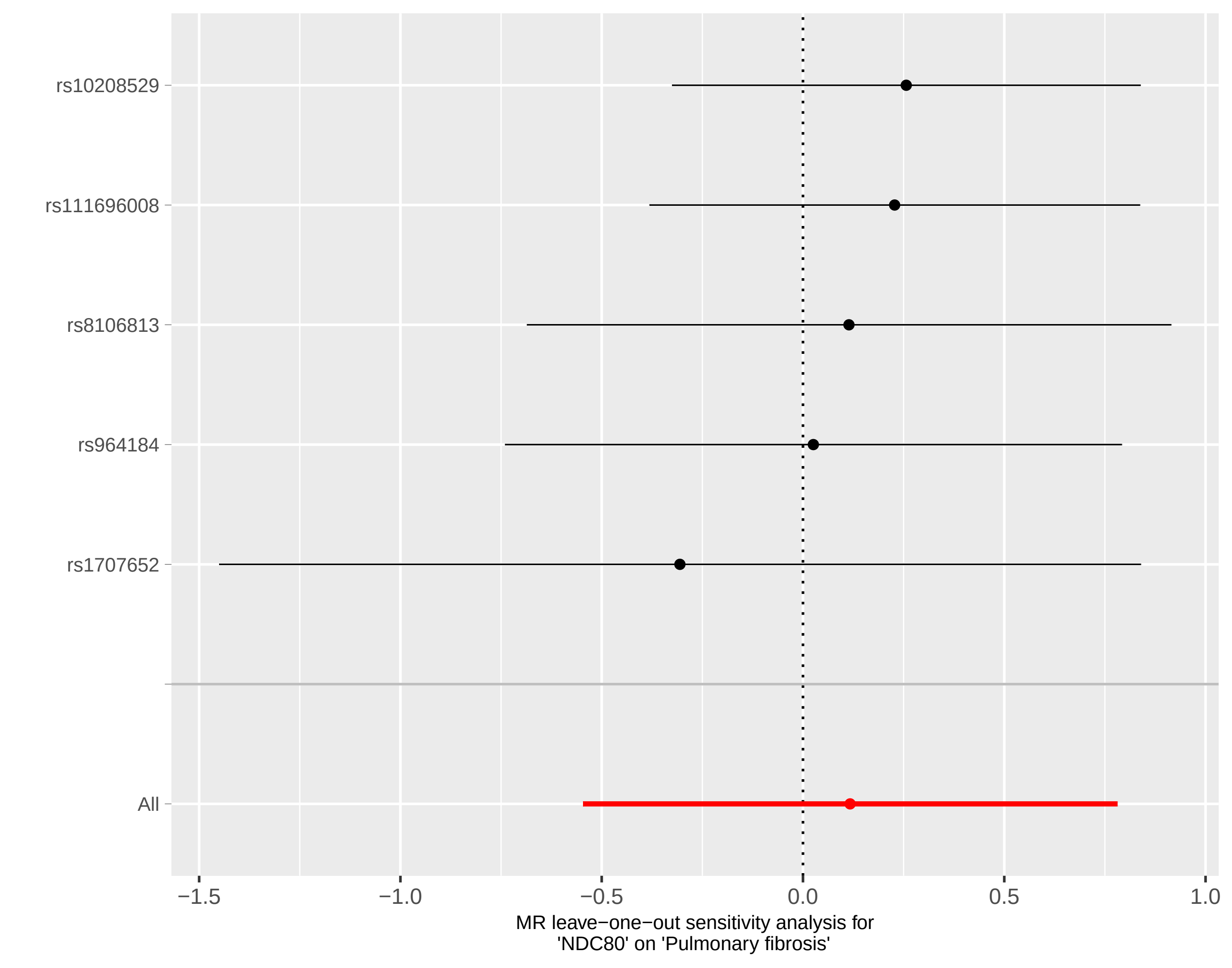

rs10208529
rs111696008
rs8106813
rs964184
rs1707652
All
−1.5
−1.0
−0.5
0.0
0.5
1.0
MR leave−one−out sensitivity analysis for
'NDC80' on 'Pulmonary fibrosis'

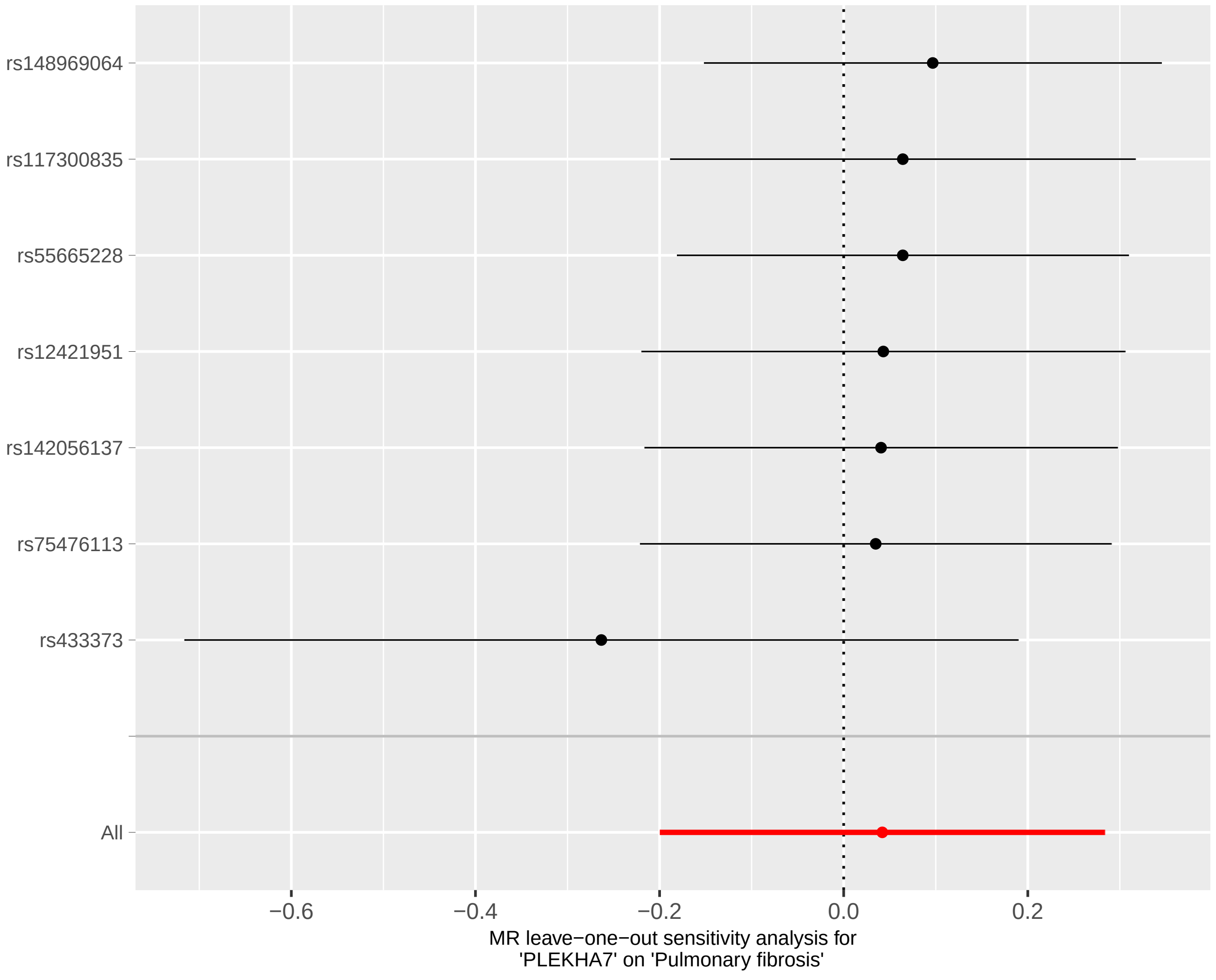
rs148969064
rs117300835
rs55665228
rs12421951
rs142056137
rs75476113
rs433373
All
−0.6
−0.4
−0.2
0.0
0.2
MR leave−one−out sensitivity analysis for
'PLEKHA7' on 'Pulmonary fibrosis'

Insufficient number of SNPs

Insufficient number of SNPs

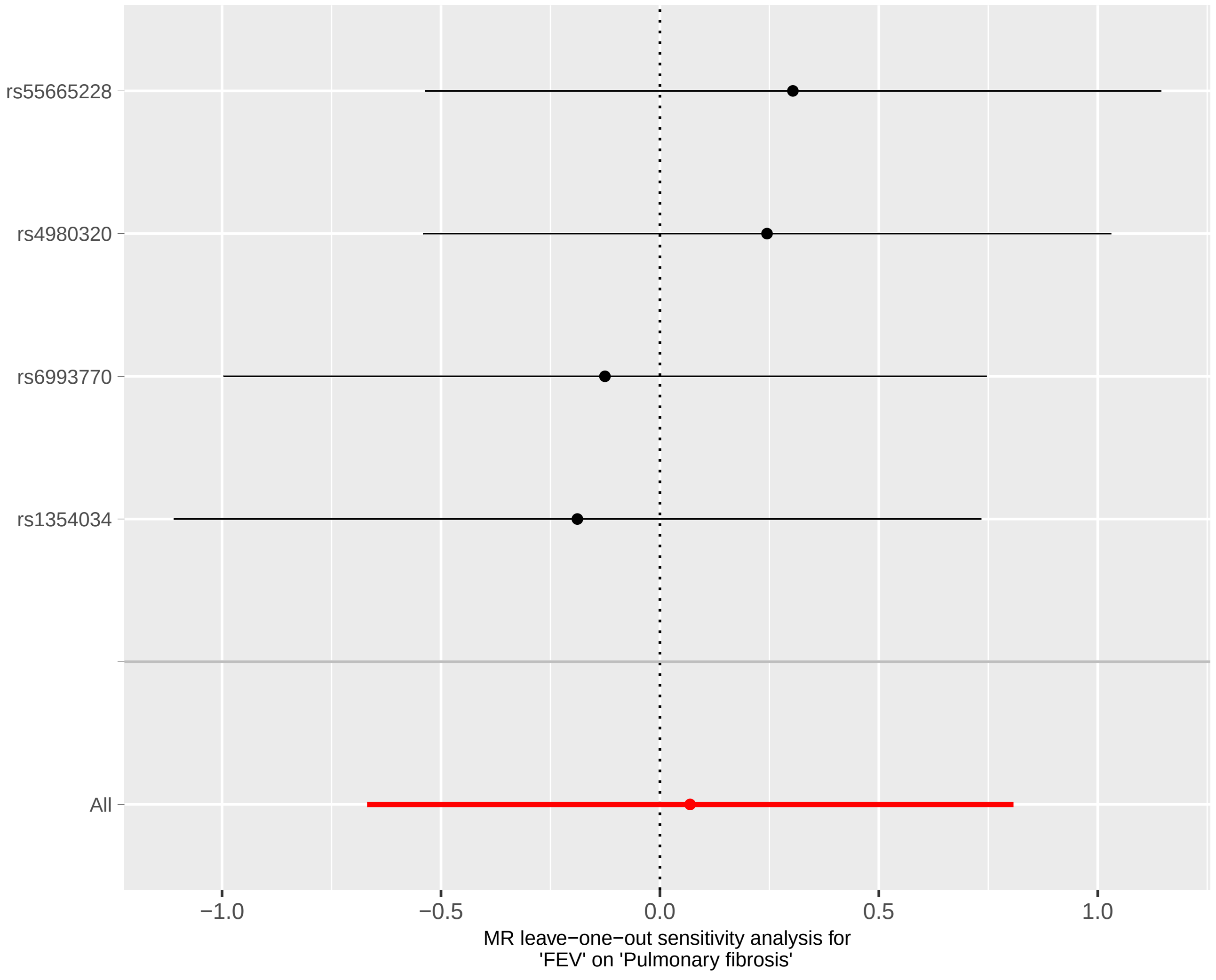

rs55665228
rs4980320
rs6993770
rs1354034
All
−1.0
−0.5
0.0
0.5
1.0
MR leave−one−out sensitivity analysis for
'FEV' on 'Pulmonary fibrosis'

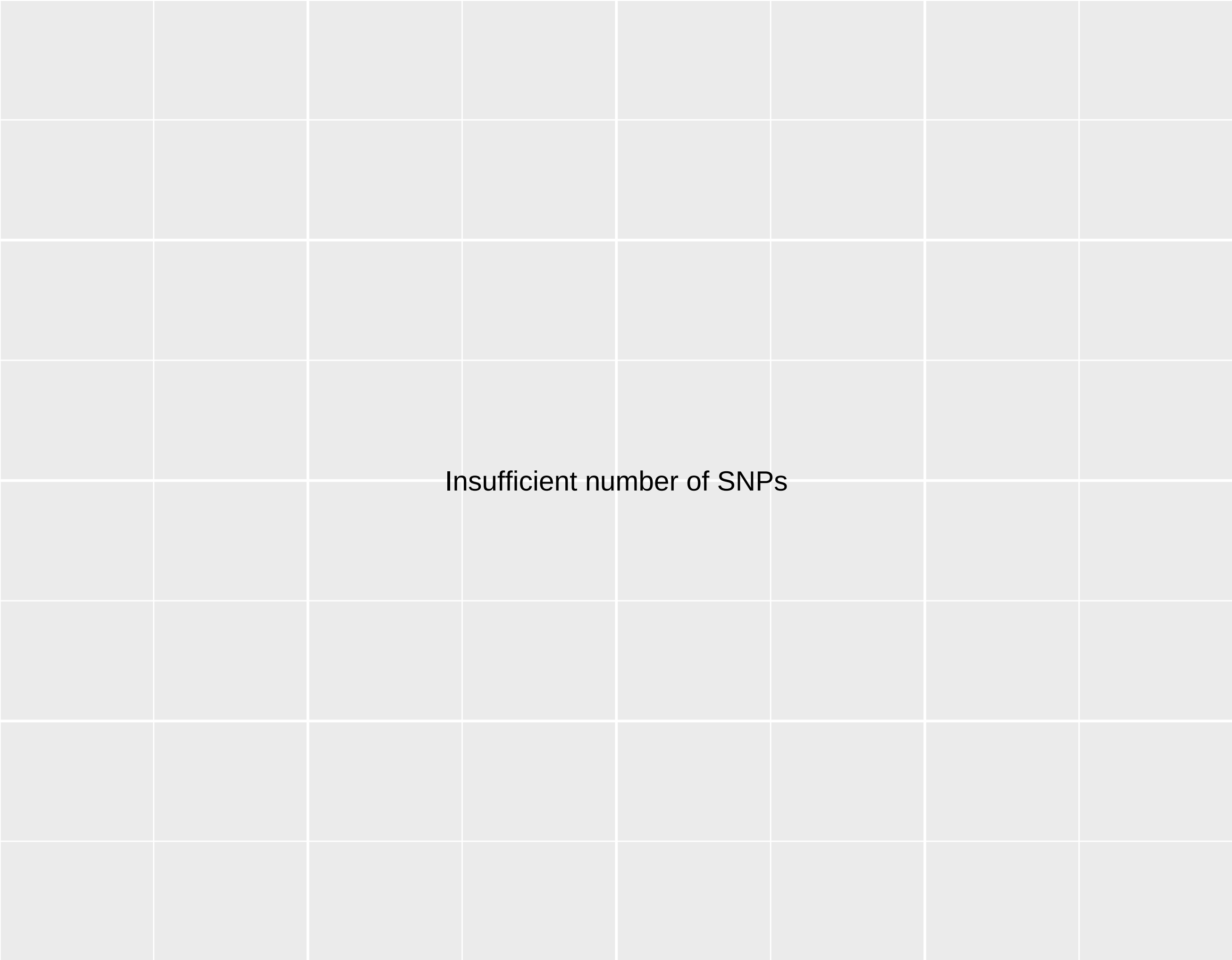
Insufficient number of SNPs

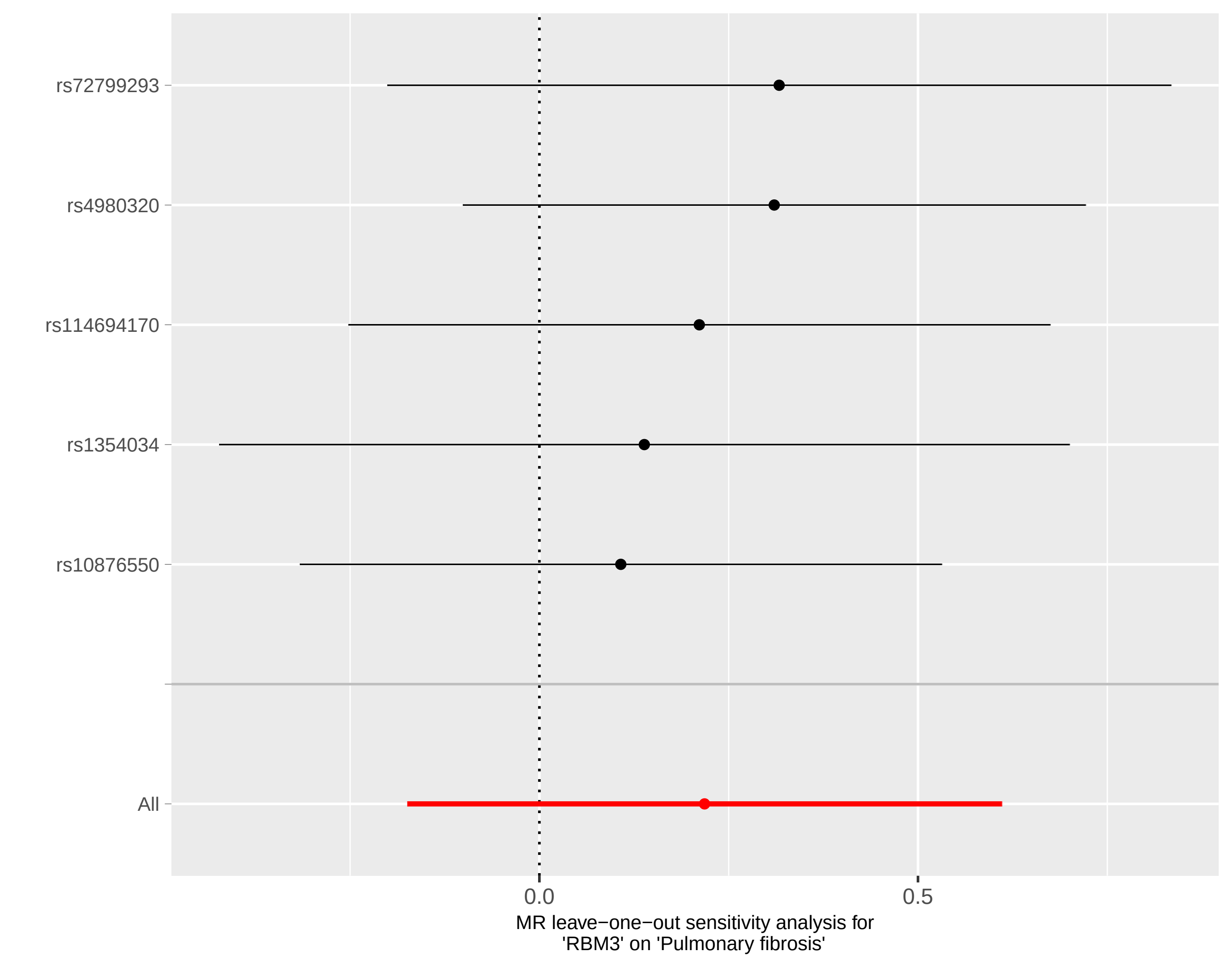
rs72799293
rs4980320
rs114694170
rs1354034
rs10876550
All
0.0
0.5
MR leave−one−out sensitivity analysis for
'RBM3' on 'Pulmonary fibrosis'

Insufficient number of SNPs

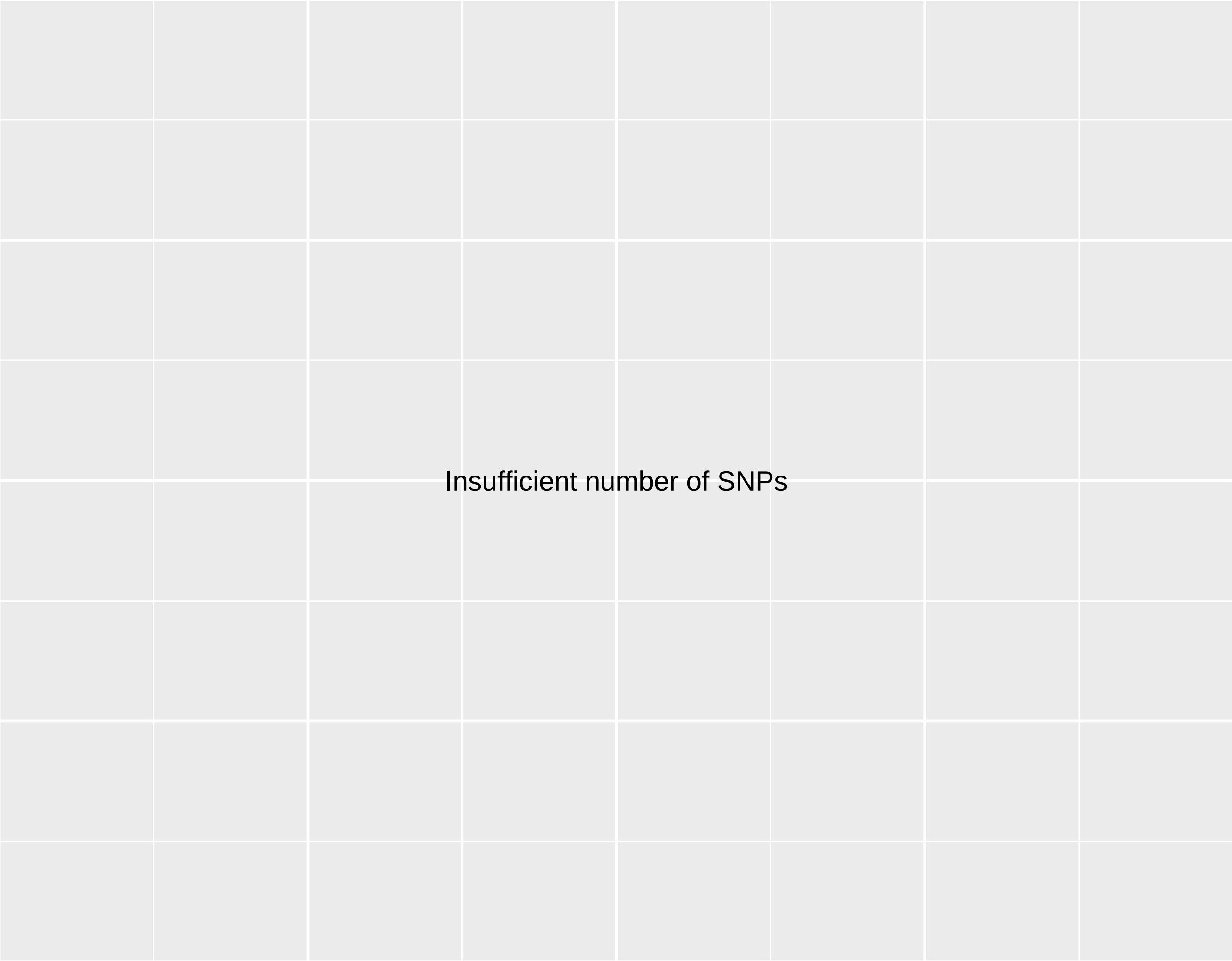
Insufficient number of SNPs

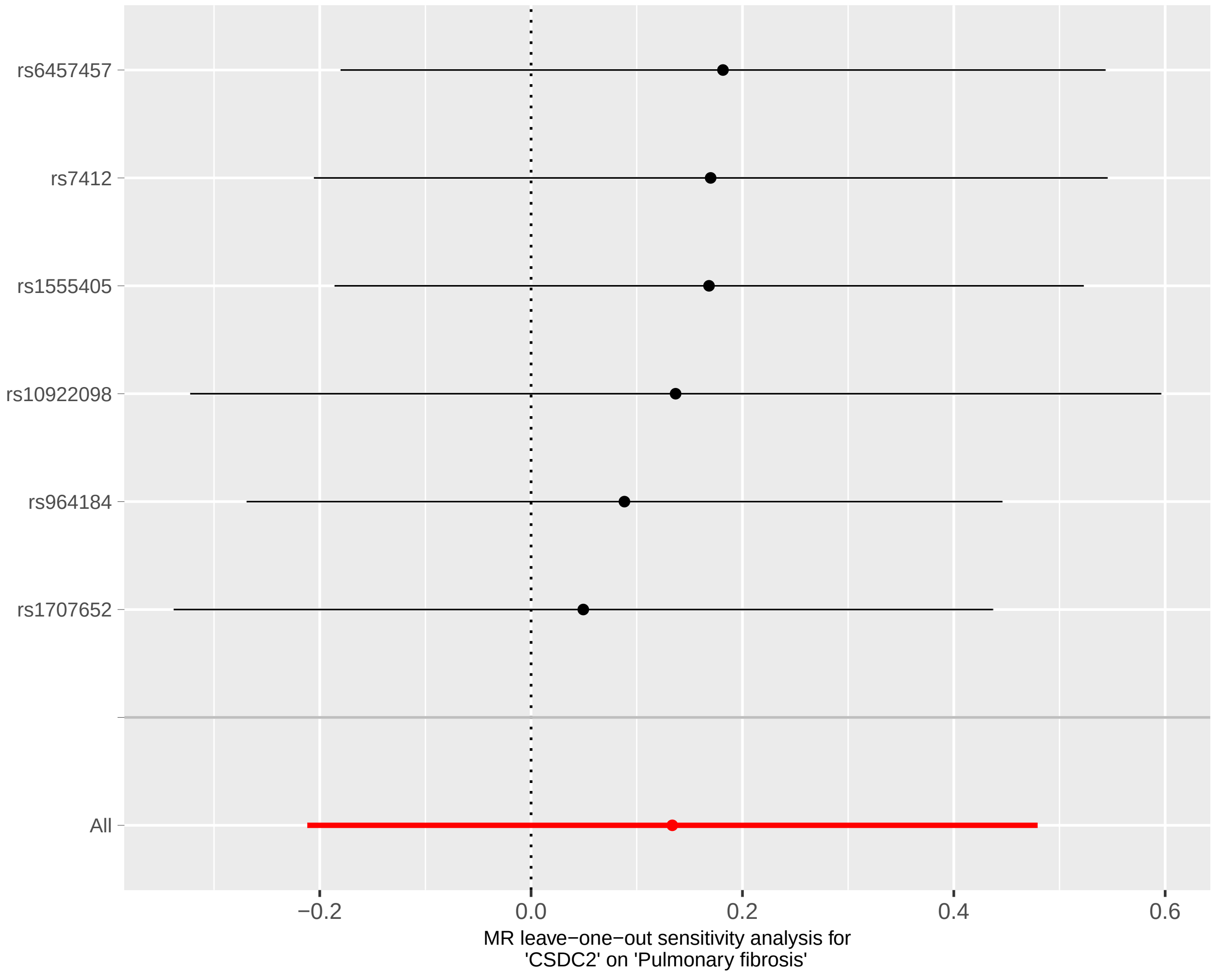
rs6457457
rs7412
rs1555405
rs10922098
rs964184
rs1707652
All
−0.2
0.0
0.2
0.4
0.6
MR leave−one−out sensitivity analysis for
'CSDC2' on 'Pulmonary fibrosis'

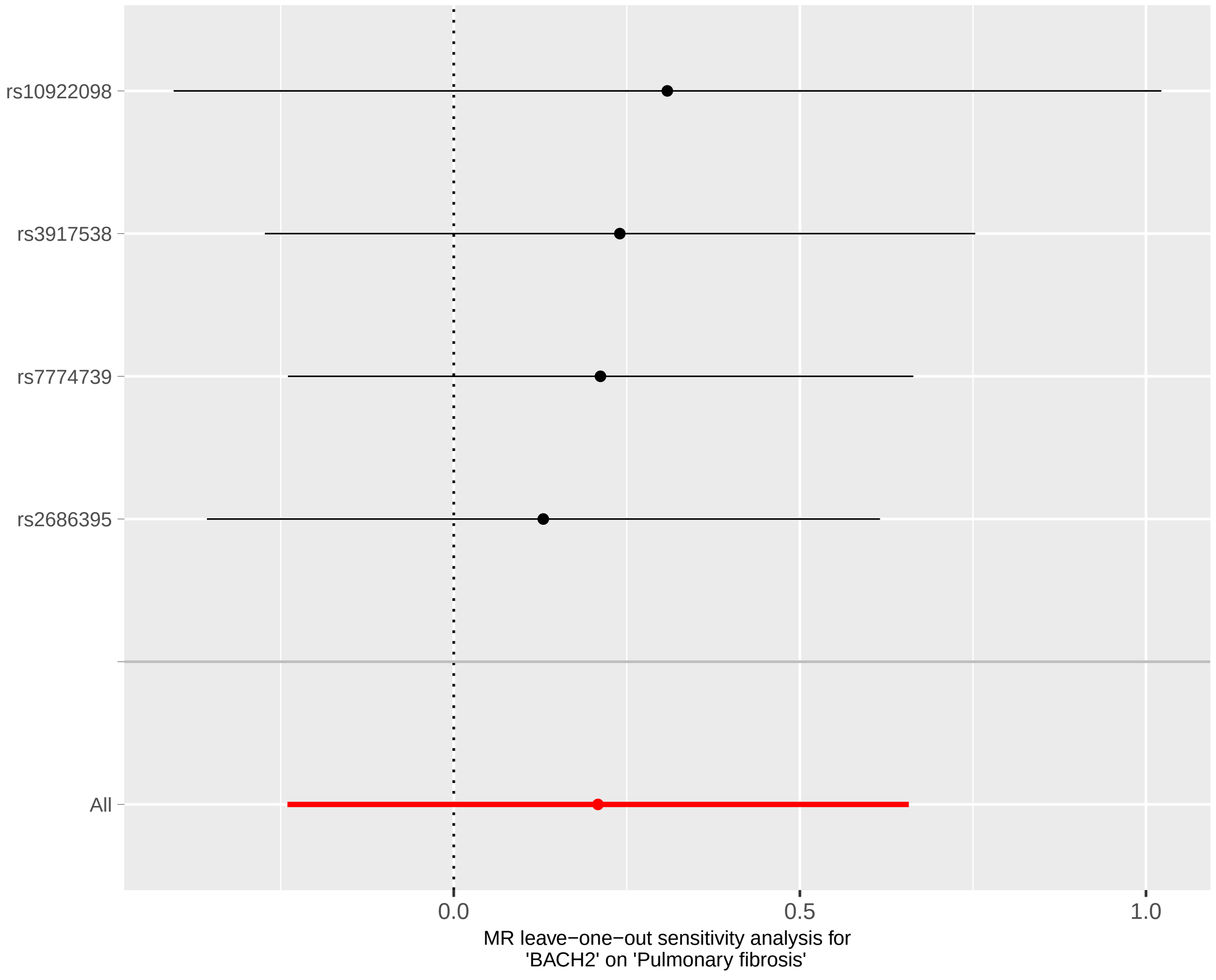
rs10922098
rs3917538
rs7774739
rs2686395
All
0.0
0.5
1.0
MR leave−one−out sensitivity analysis for
'BACH2' on 'Pulmonary fibrosis'

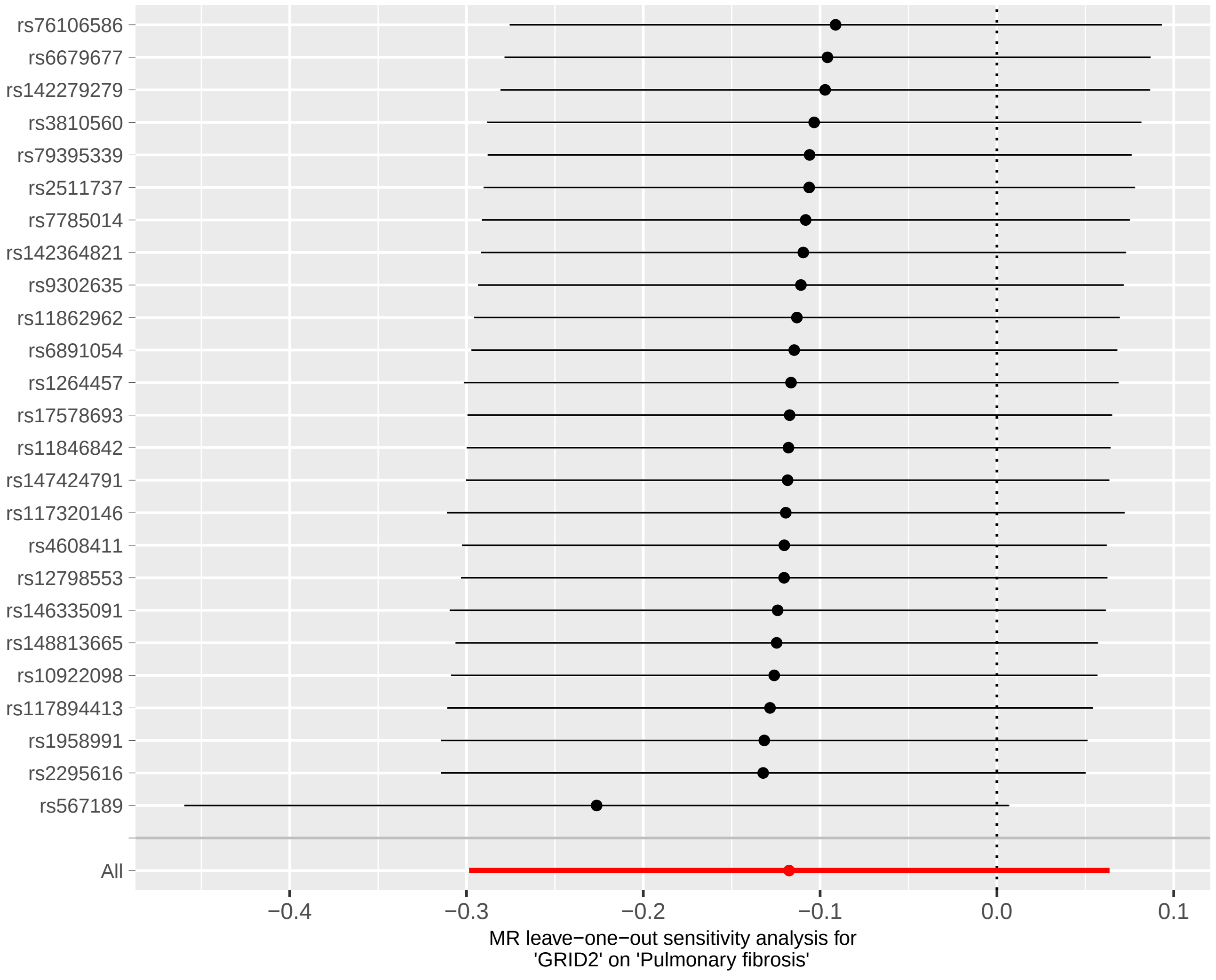
rs76106586
rs6679677
rs142279279
rs3810560
rs79395339
rs2511737
rs7785014
rs142364821
rs9302635
rs11862962
rs6891054
rs1264457
rs17578693
rs11846842
rs147424791
rs117320146
rs4608411
rs12798553
rs146335091
rs148813665
rs10922098
rs117894413
rs1958991
rs2295616
rs567189
All
−0.4
−0.3
−0.2
−0.1
0.0
0.1
MR leave−one−out sensitivity analysis for
'GRID2' on 'Pulmonary fibrosis'

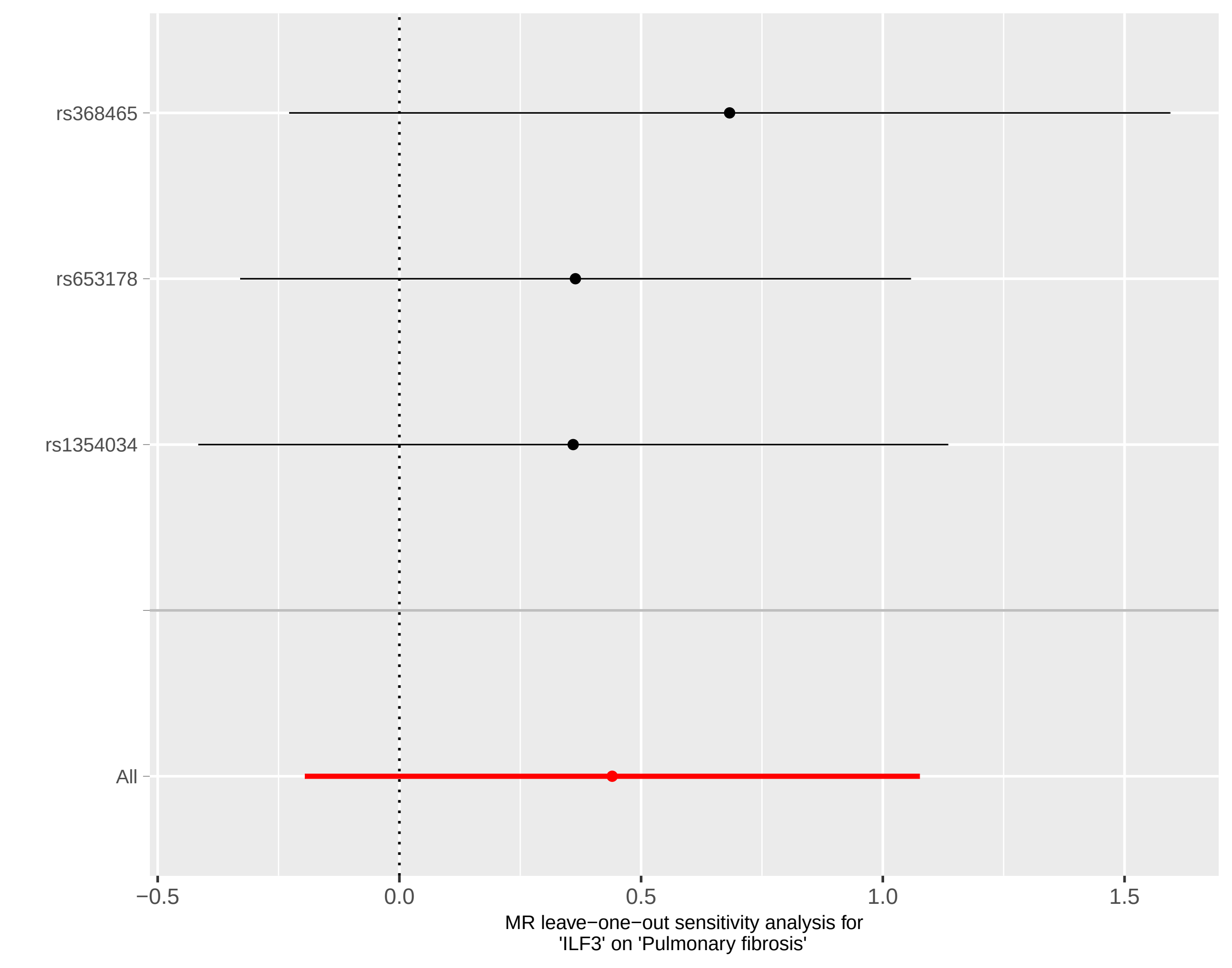
rs368465
rs653178
rs1354034
All
−0.5
0.0
0.5
1.0
1.5
MR leave−one−out sensitivity analysis for
'ILF3' on 'Pulmonary fibrosis'

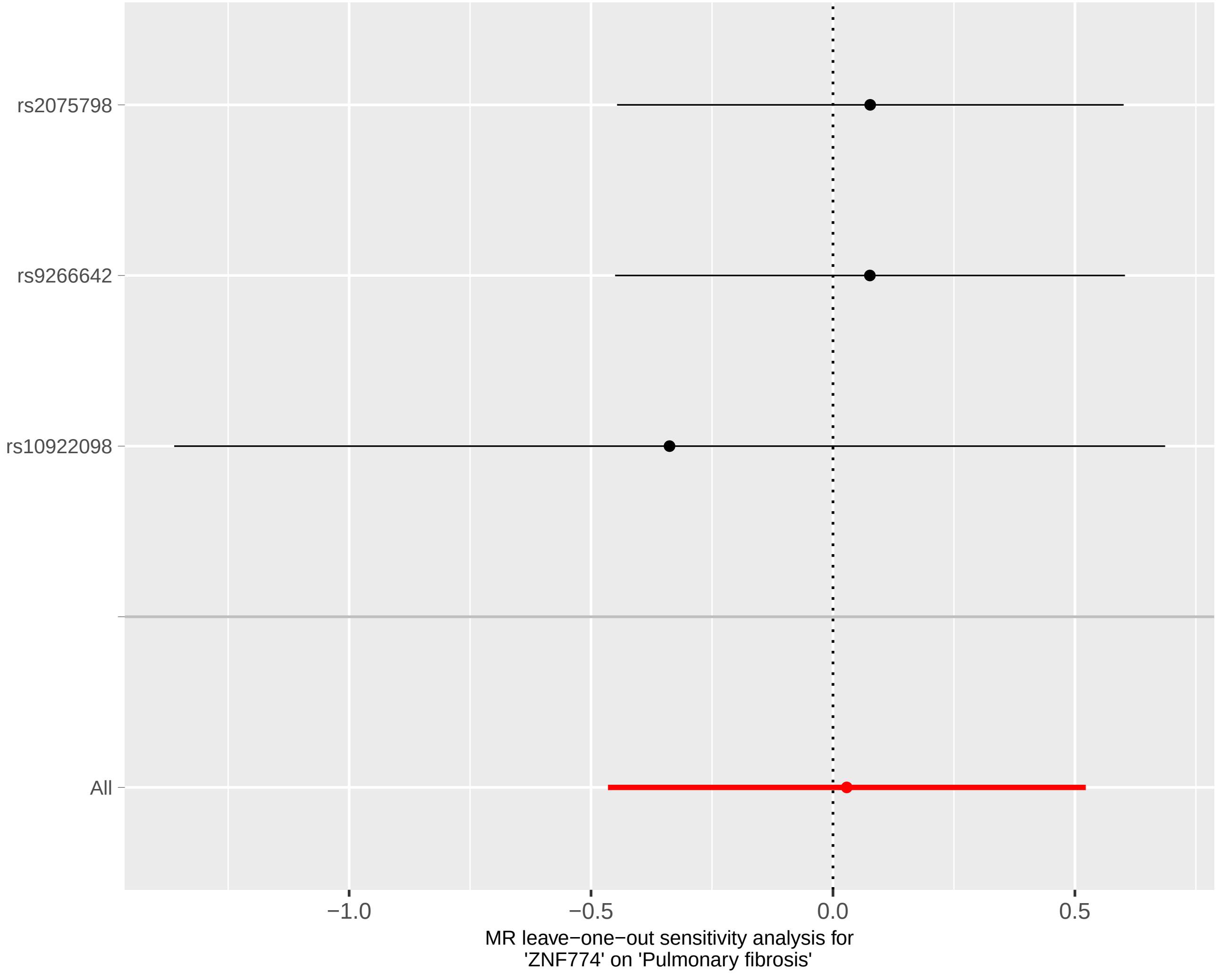
rs2075798
rs9266642
rs10922098
All
−1.0
−0.5
0.0
0.5
MR leave−one−out sensitivity analysis for
'ZNF774' on 'Pulmonary fibrosis'

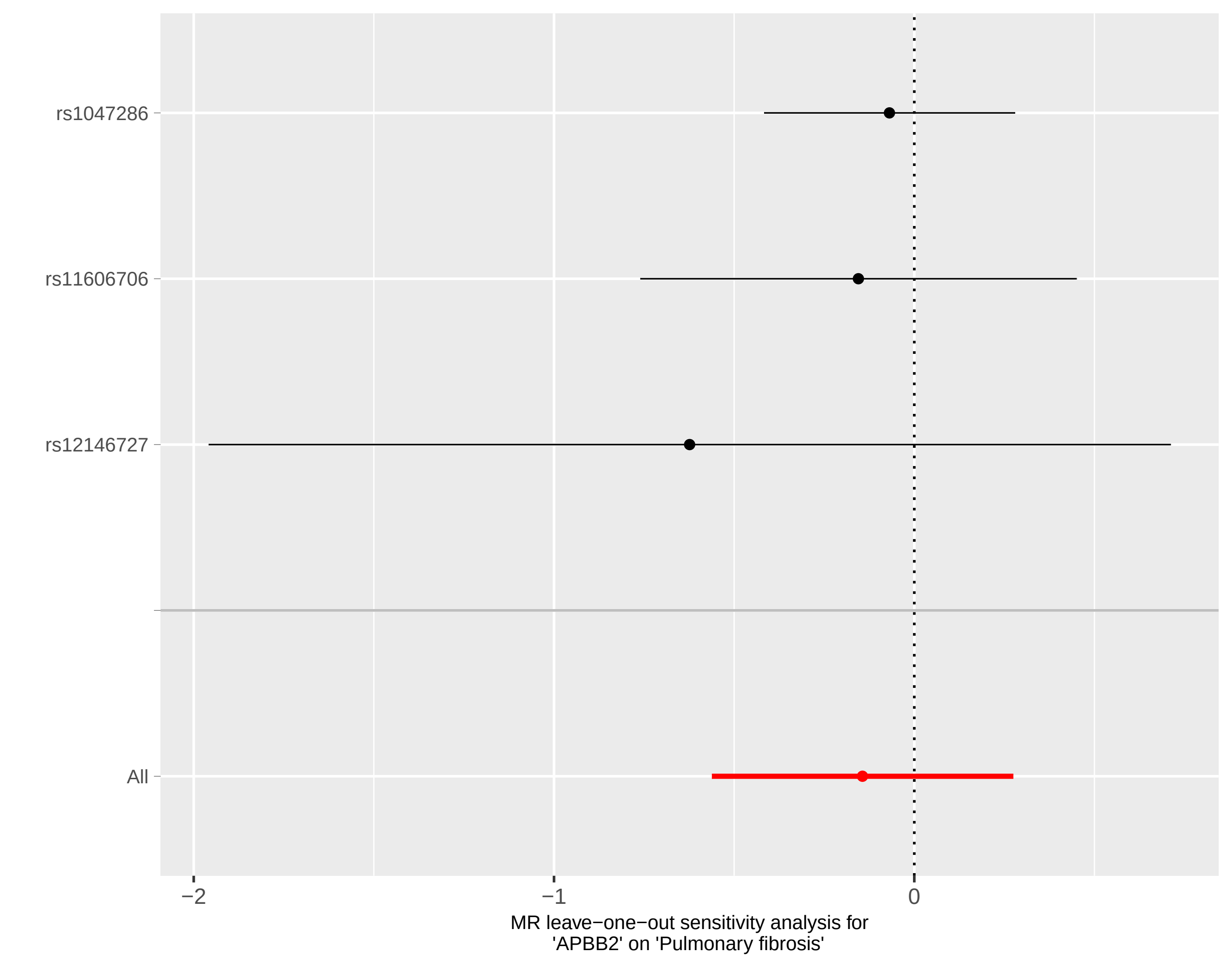
rs1047286
rs11606706
rs12146727
All
−2
−1
0
MR leave−one−out sensitivity analysis for
'APBB2' on 'Pulmonary fibrosis'

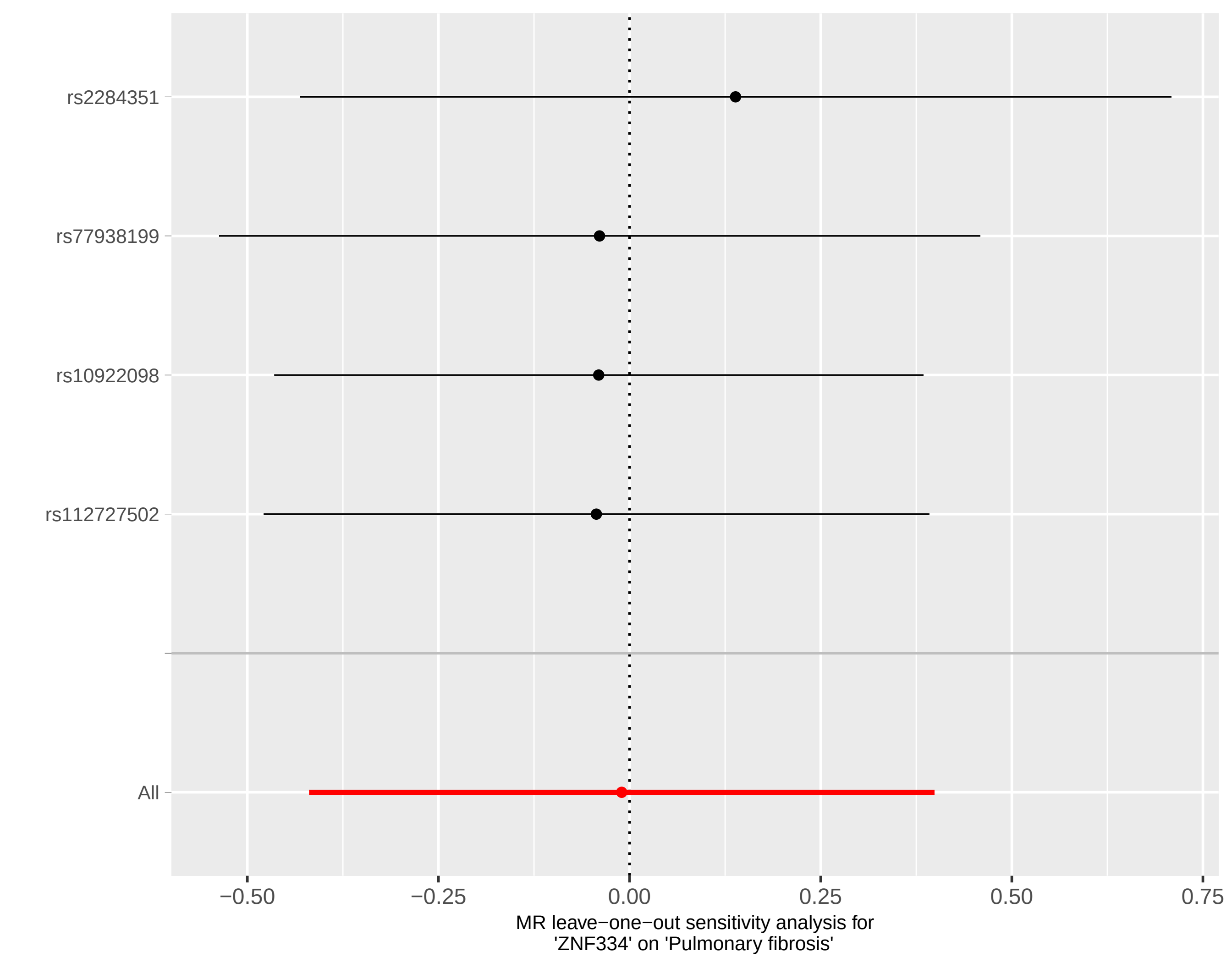
rs2284351
rs77938199
rs10922098
rs112727502
All
−0.50
−0.25
0.00
0.25
0.50
0.75
MR leave−one−out sensitivity analysis for
'ZNF334' on 'Pulmonary fibrosis'

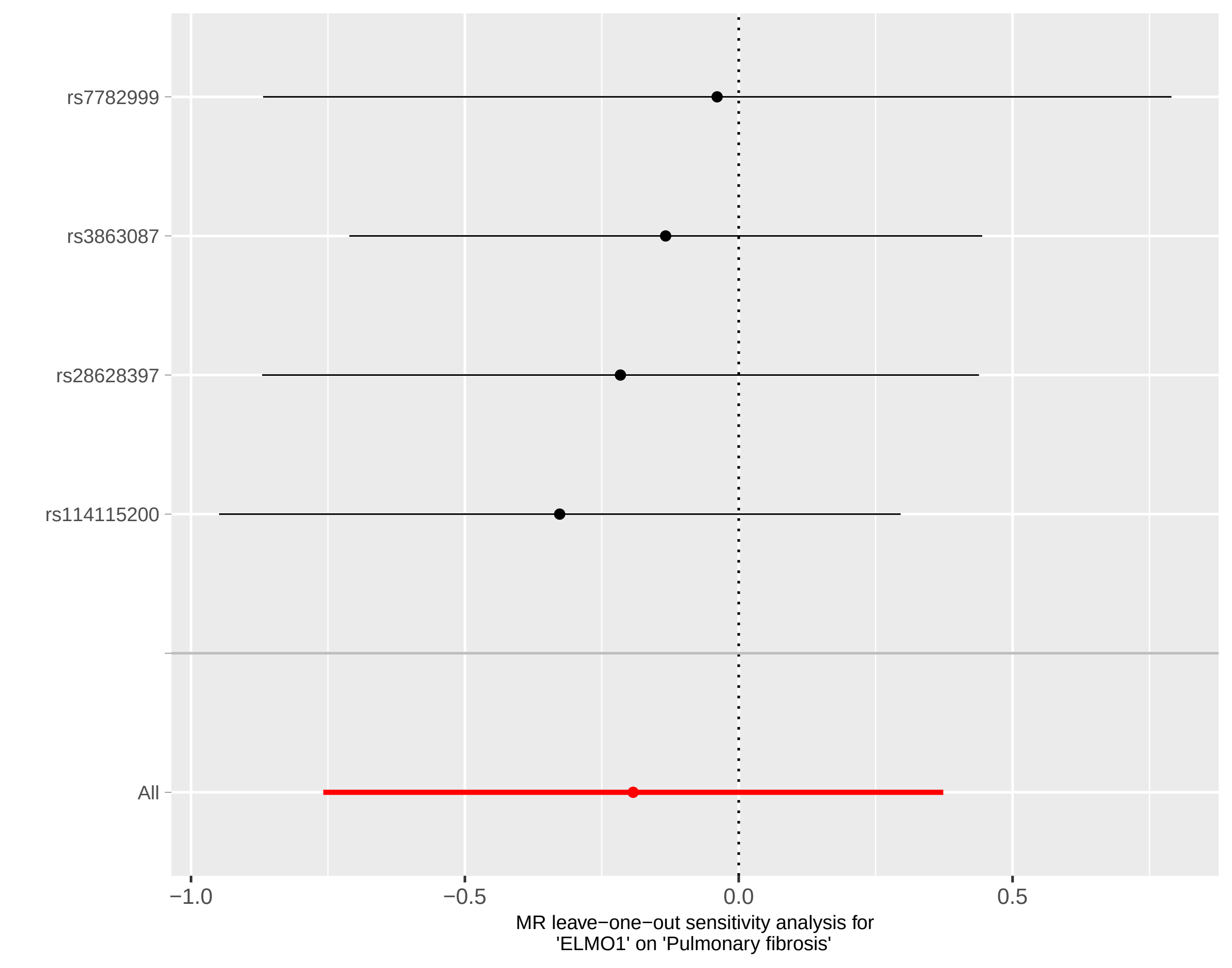
rs7782999
rs3863087
rs28628397
rs114115200
All
−1.0
−0.5
0.0
0.5
MR leave−one−out sensitivity analysis for
'ELMO1' on 'Pulmonary fibrosis'

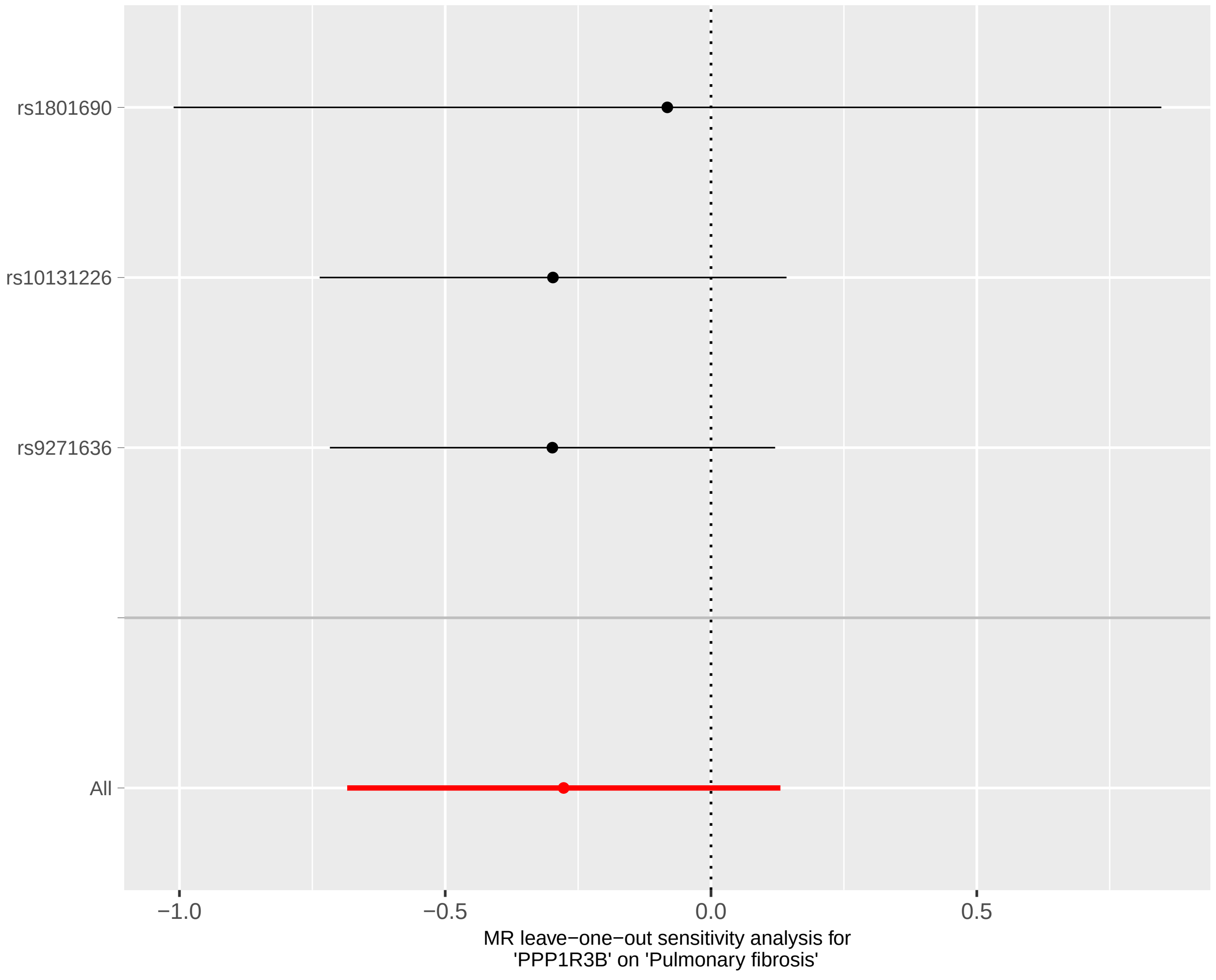
rs1801690
rs10131226
rs9271636
All
−1.0
−0.5
0.0
0.5
MR leave−one−out sensitivity analysis for
'PPP1R3B' on 'Pulmonary fibrosis'

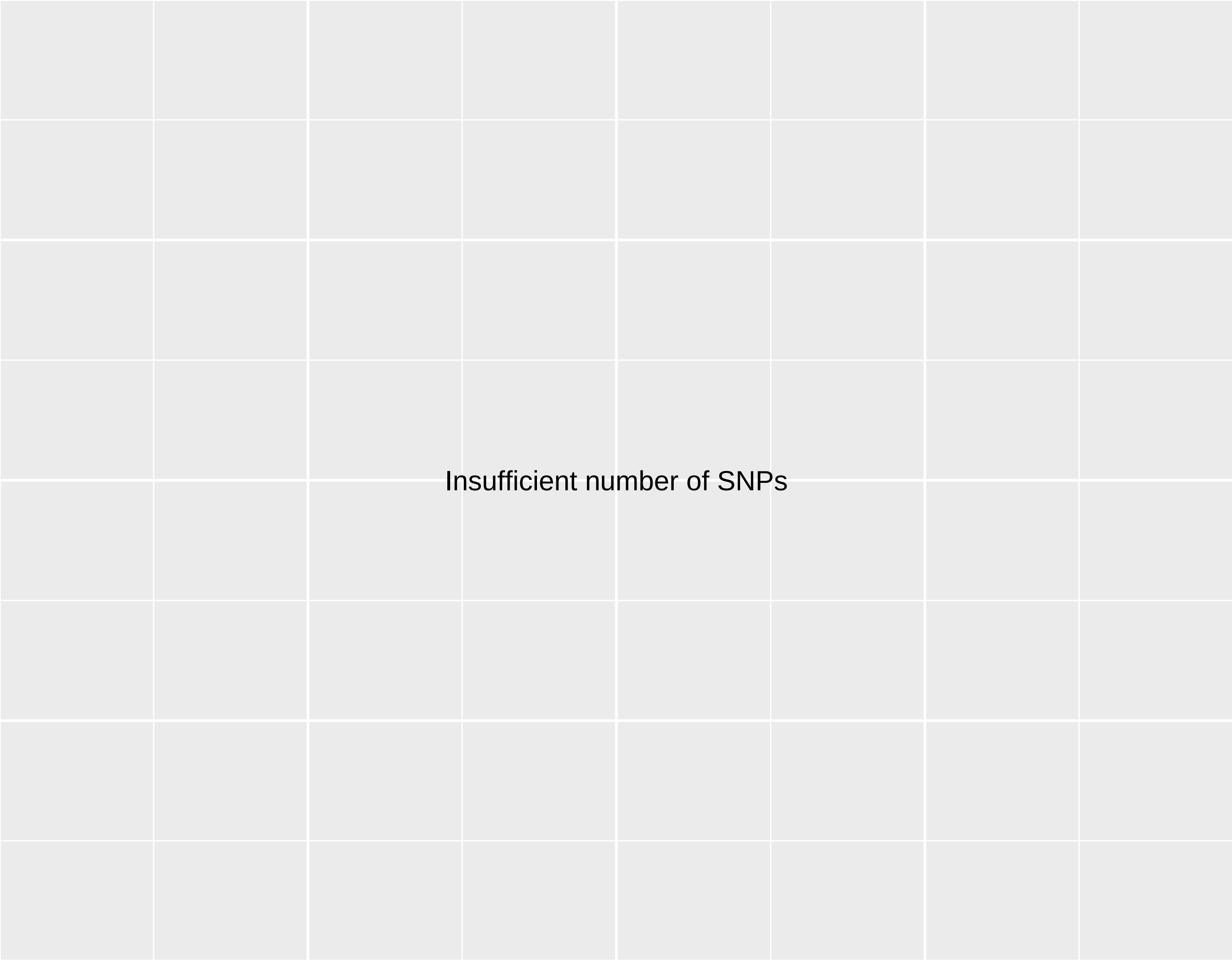
Insufficient number of SNPs

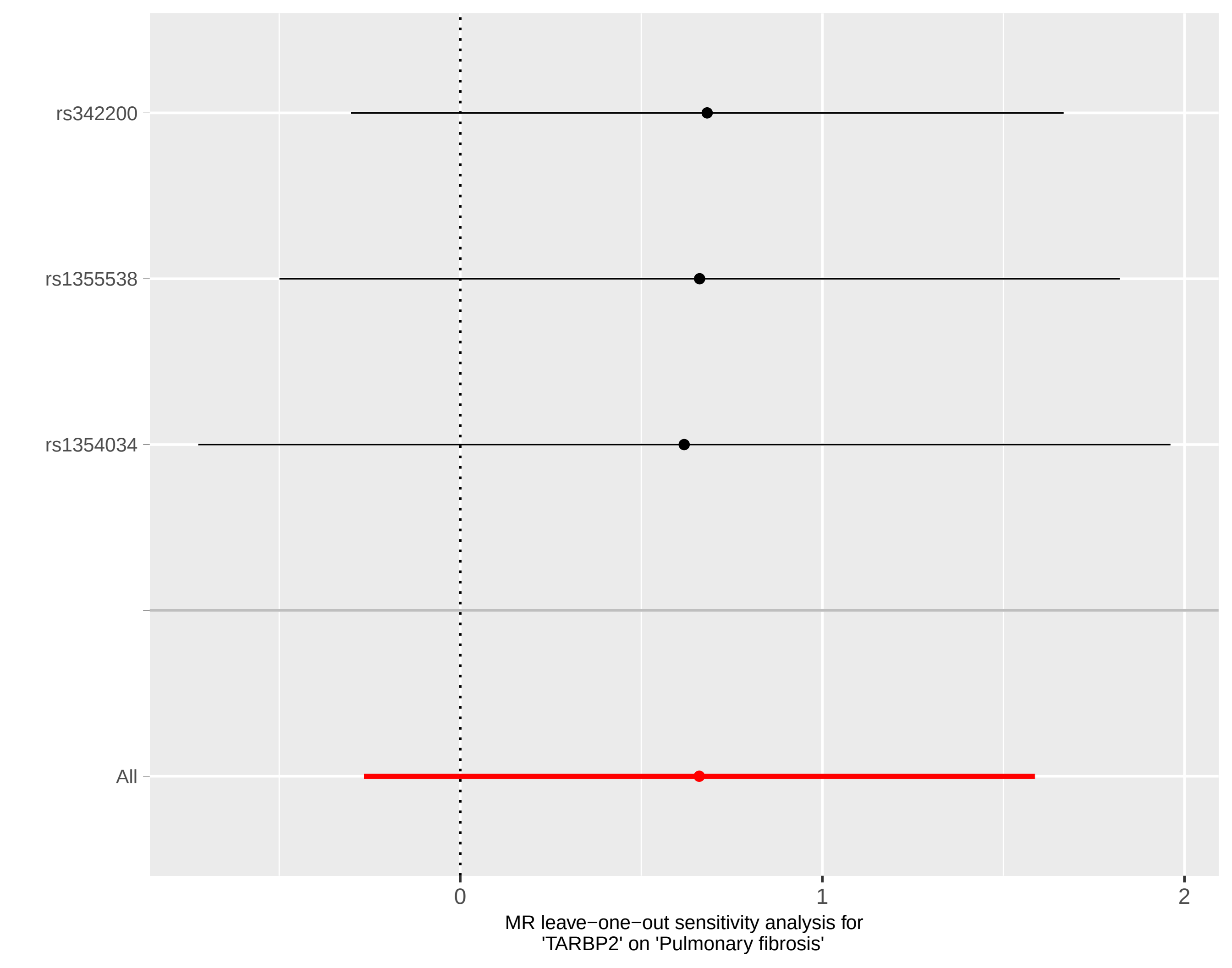
rs342200
rs1355538
rs1354034
All
0
1
2
MR leave−one−out sensitivity analysis for
'TARBP2' on 'Pulmonary fibrosis'

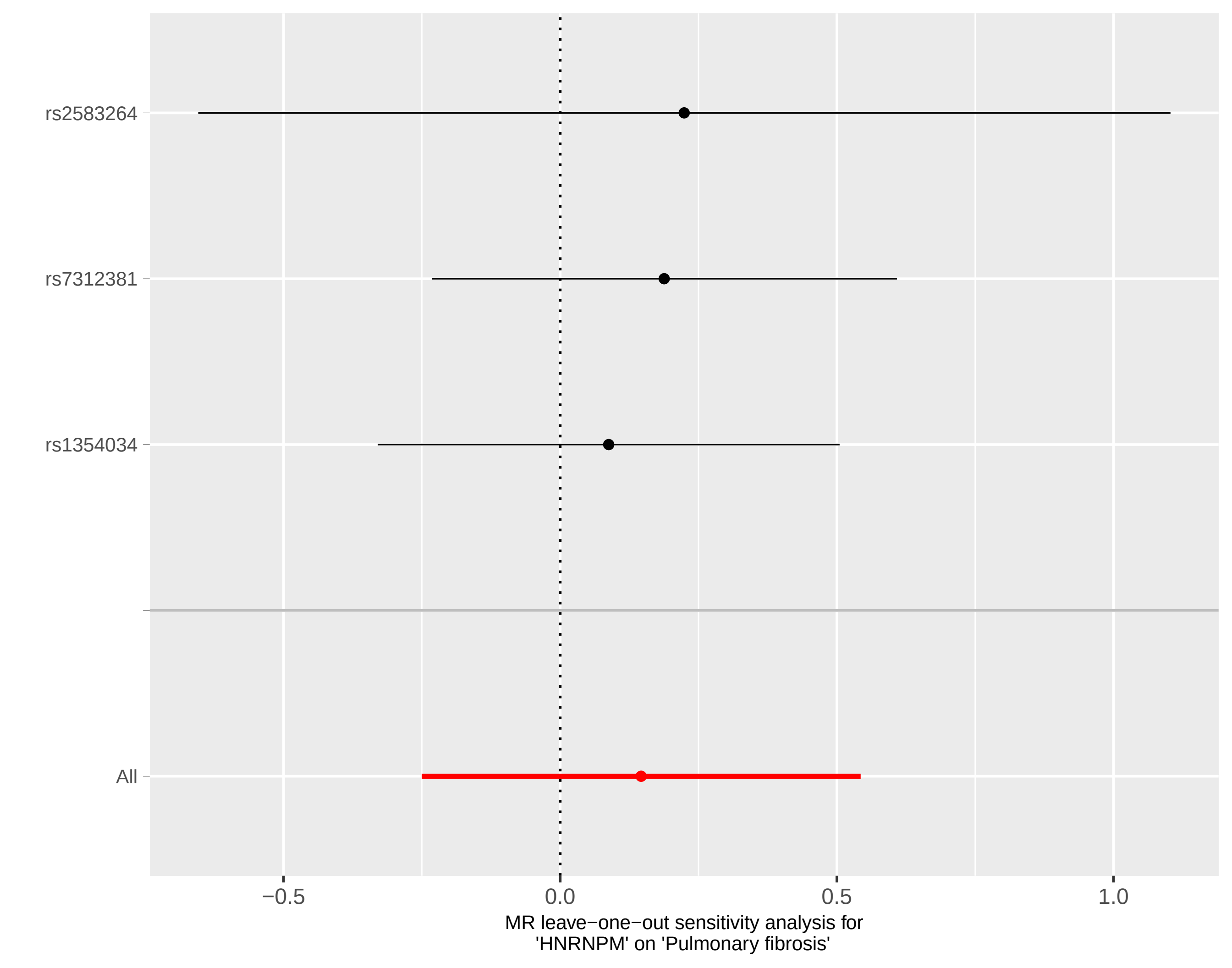

rs2583264
rs7312381
rs1354034
All
−0.5
0.0
0.5
1.0
MR leave−one−out sensitivity analysis for
'HNRNPM' on 'Pulmonary fibrosis'

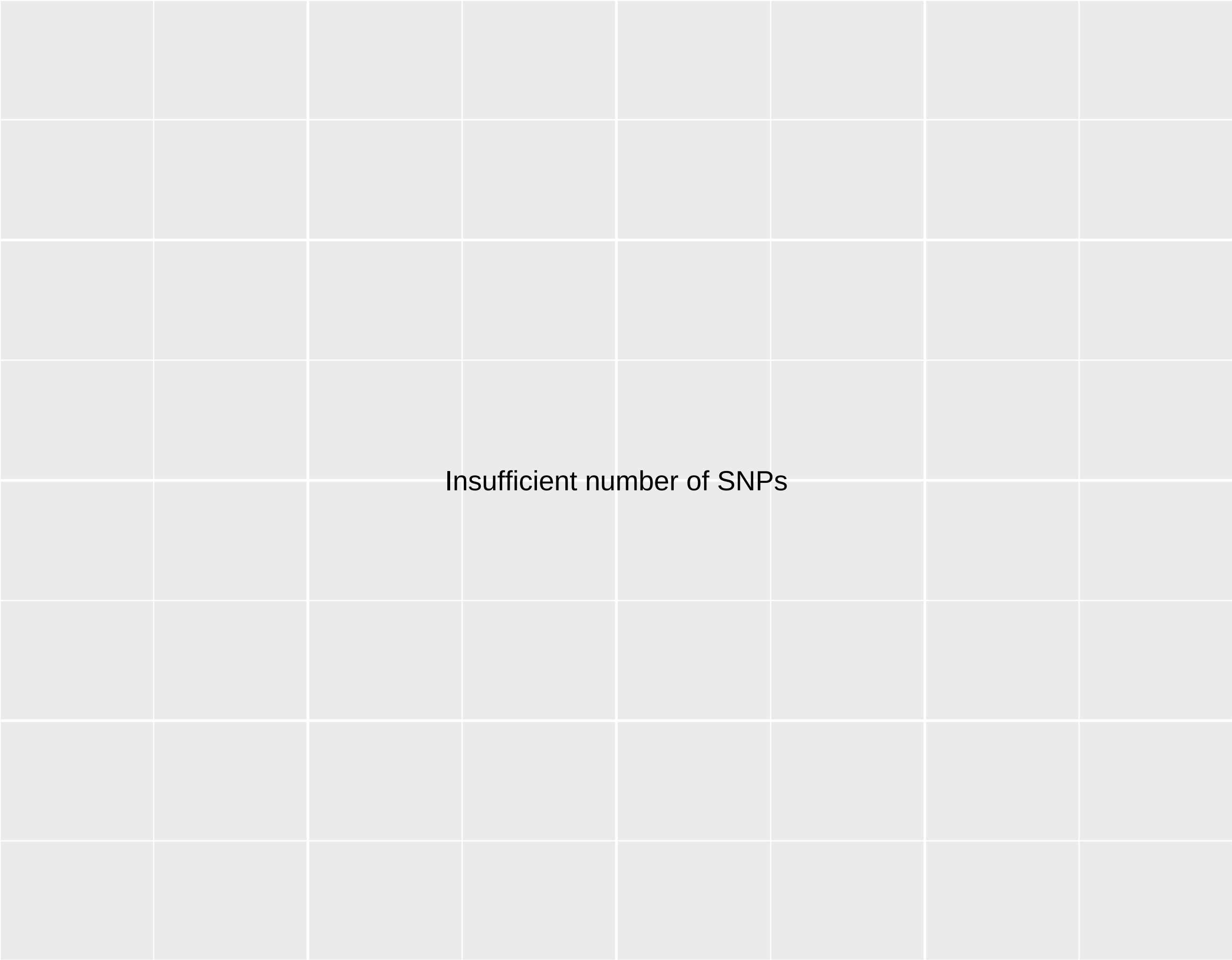
Insufficient number of SNPs

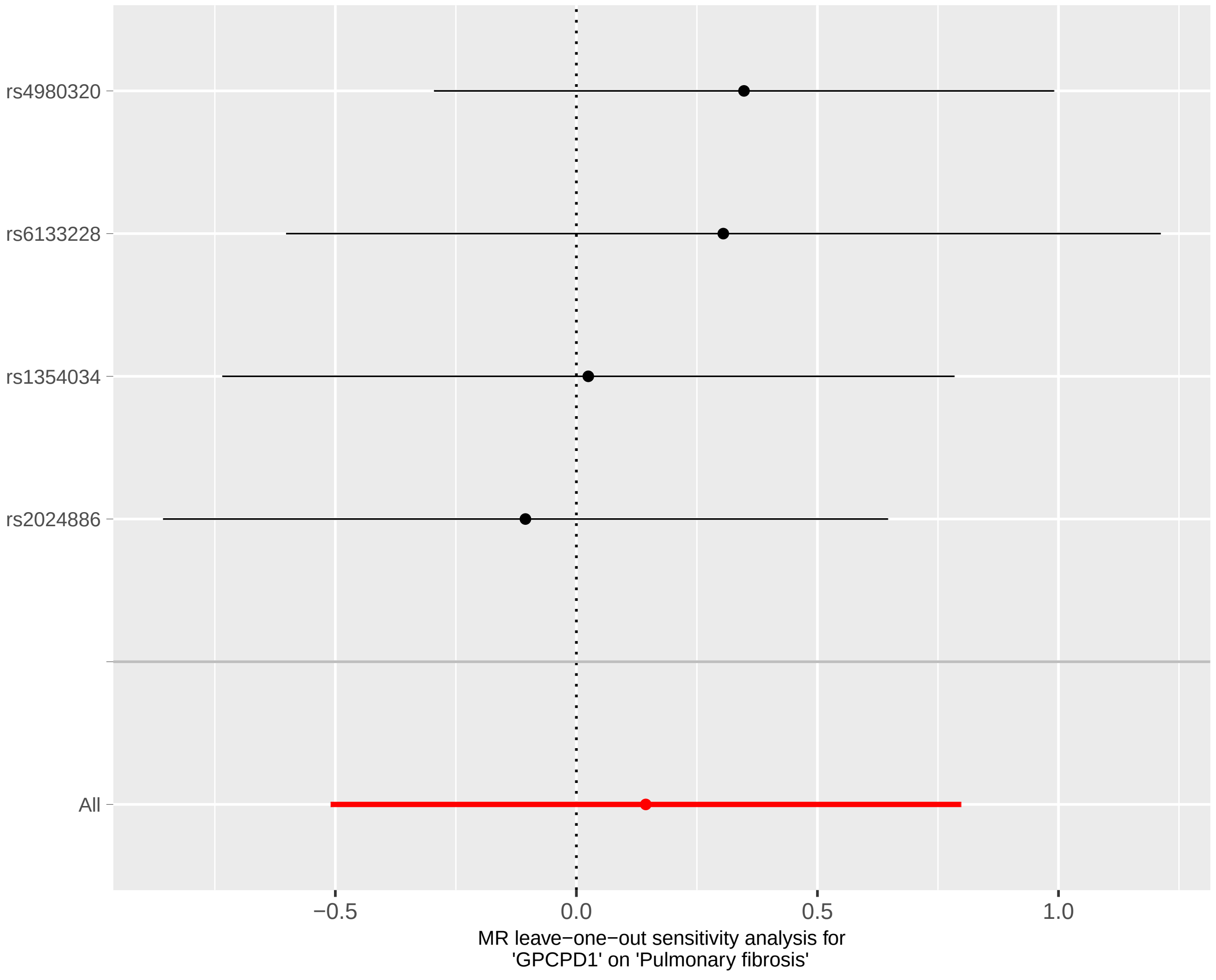
rs4980320
rs6133228
rs1354034
rs2024886
All
−0.5
0.0
0.5
1.0
MR leave−one−out sensitivity analysis for
'GPCPD1' on 'Pulmonary fibrosis'

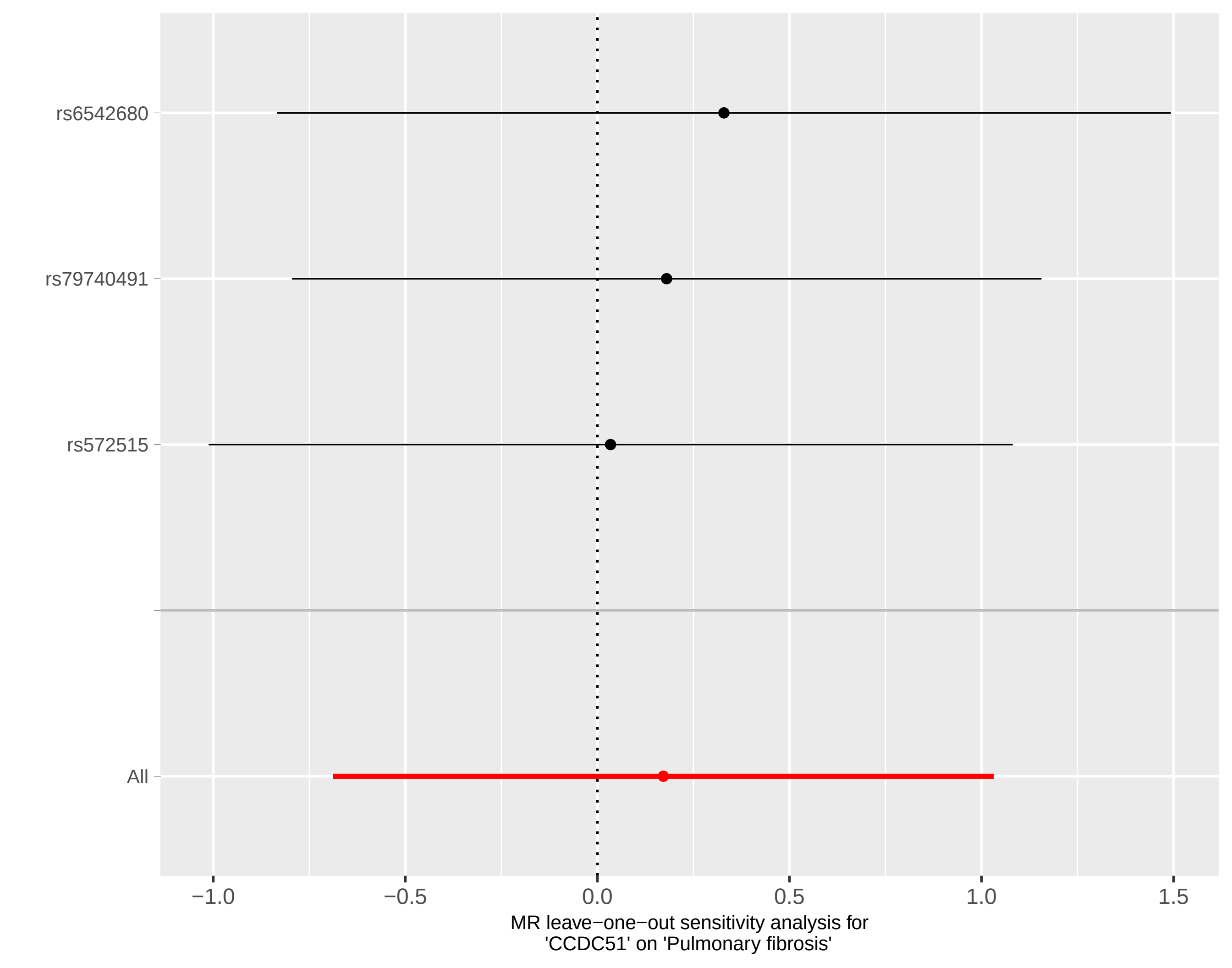
rs6542680
rs79740491
rs572515
All
−1.0
−0.5
0.0
0.5
1.0
1.5
MR leave−one−out sensitivity analysis for
'CCDC51' on 'Pulmonary fibrosis'

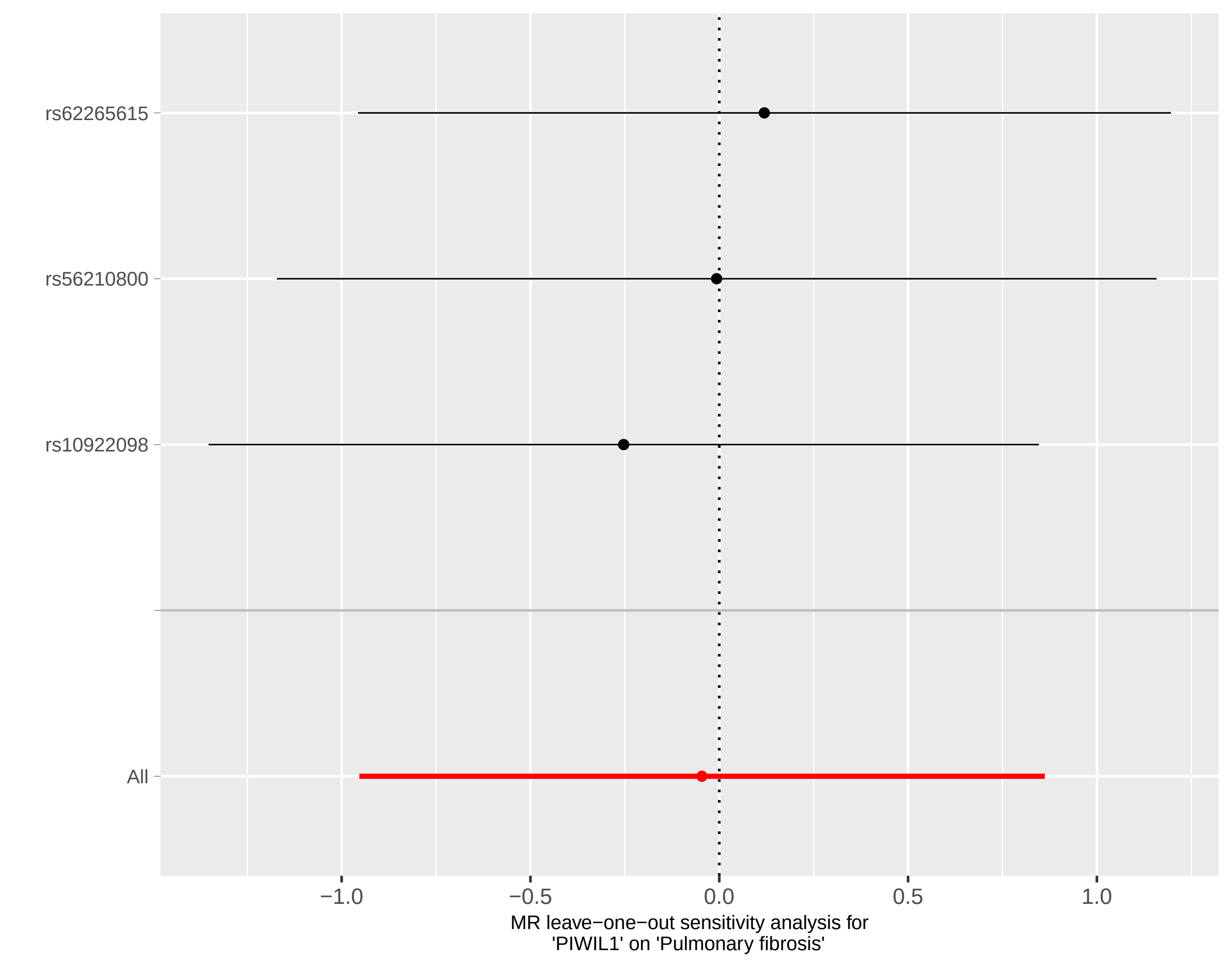
rs62265615
rs56210800
rs10922098
All
−1.0
−0.5
0.0
0.5
1.0
MR leave−one−out sensitivity analysis for
'PIWIL1' on 'Pulmonary fibrosis'

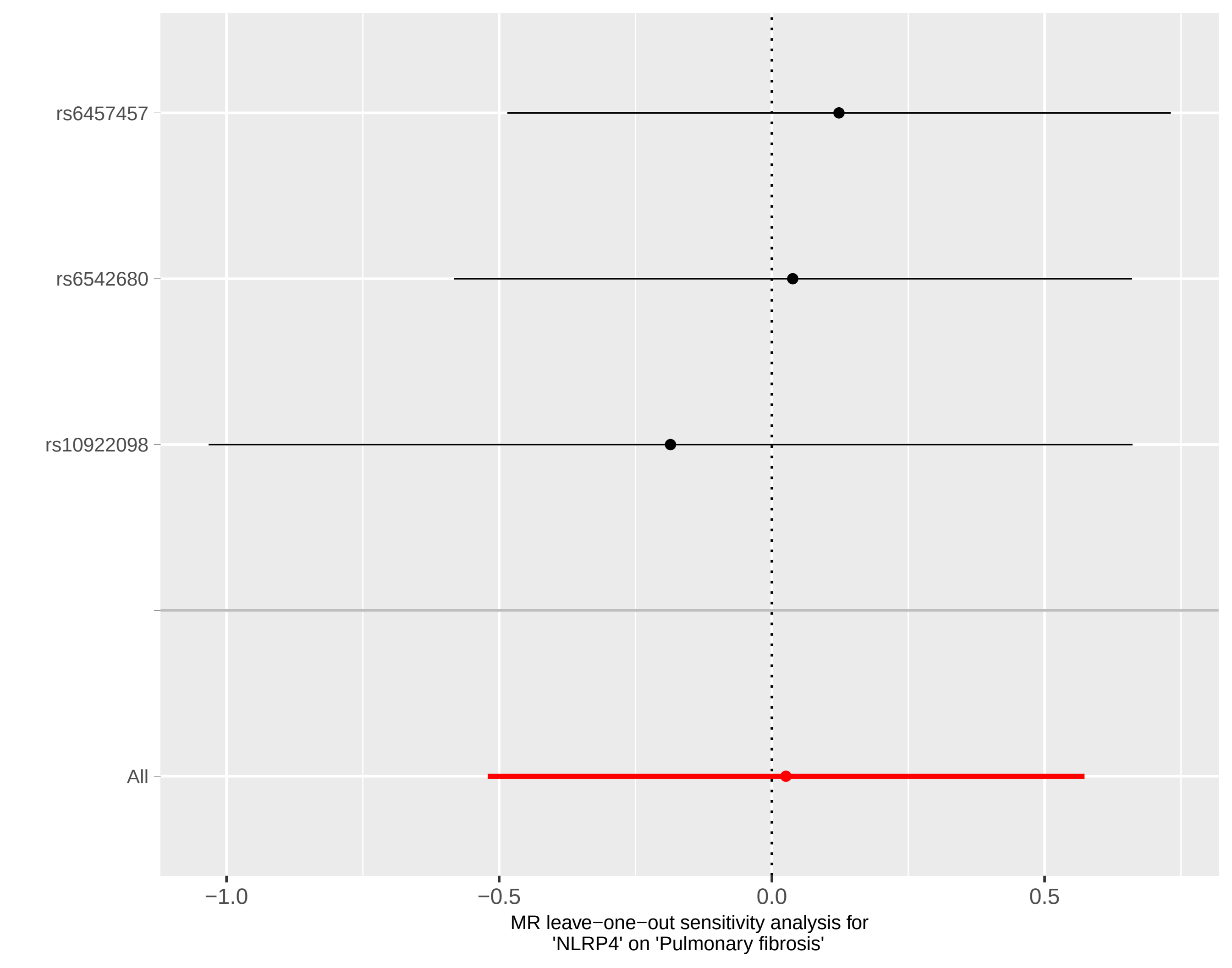
rs6457457
rs6542680
rs10922098
All
−1.0
−0.5
0.0
0.5
MR leave−one−out sensitivity analysis for
'NLRP4' on 'Pulmonary fibrosis'

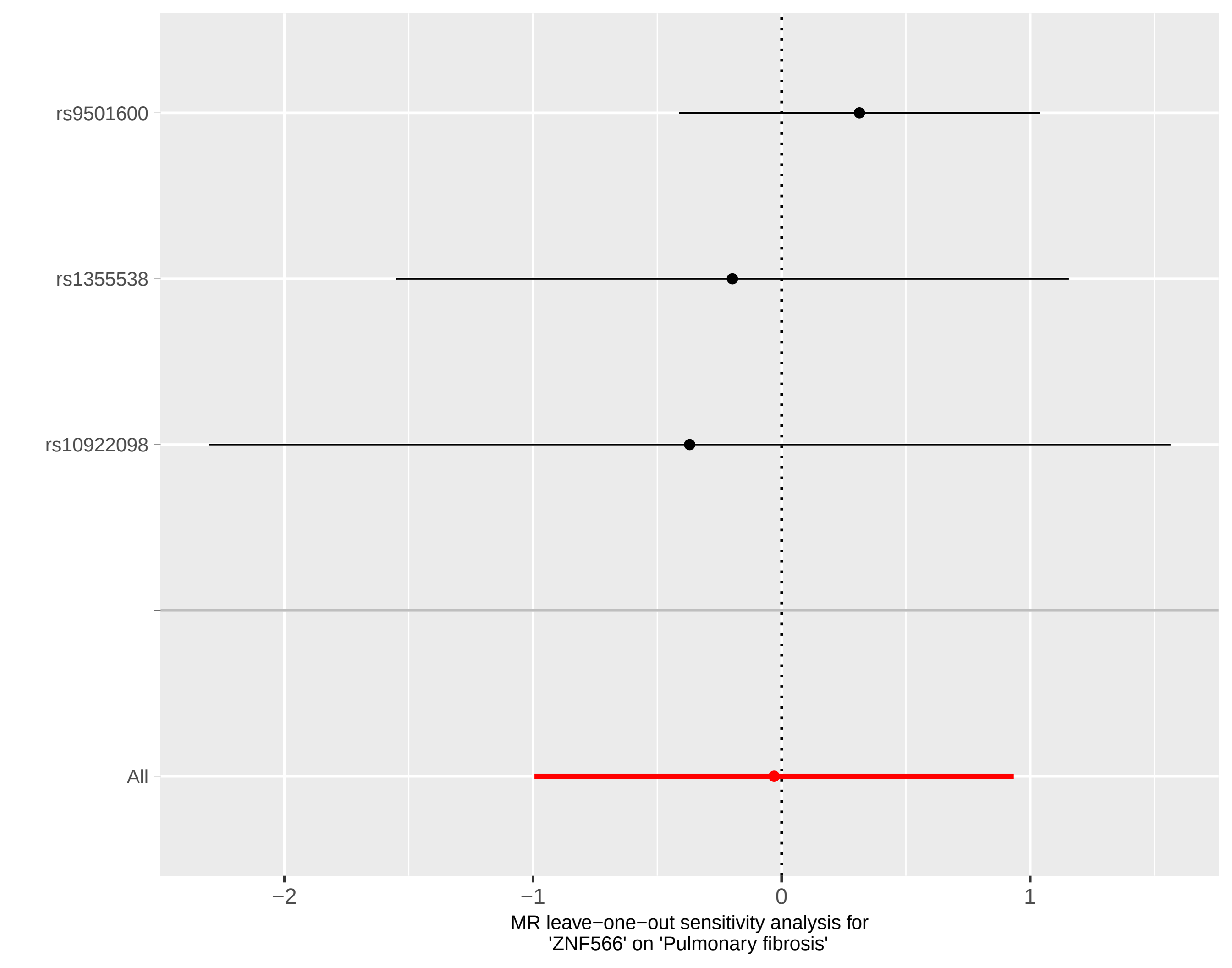
rs9501600
rs1355538
rs10922098
All
−2
−1
0
1
MR leave−one−out sensitivity analysis for
'ZNF566' on 'Pulmonary fibrosis'

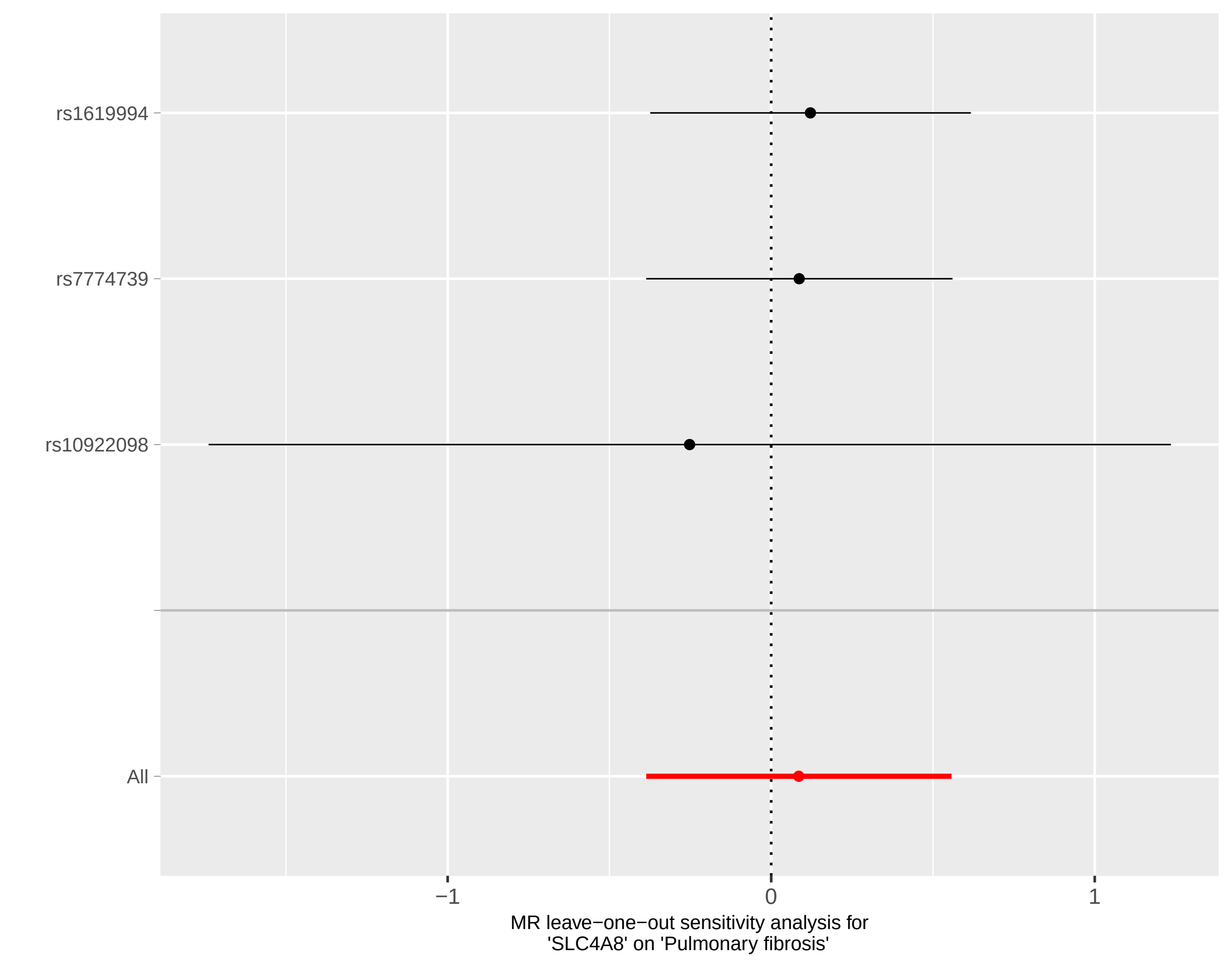

rs1619994
rs7774739
rs10922098
All
−1
0
1
MR leave−one−out sensitivity analysis for
'SLC4A8' on 'Pulmonary fibrosis'

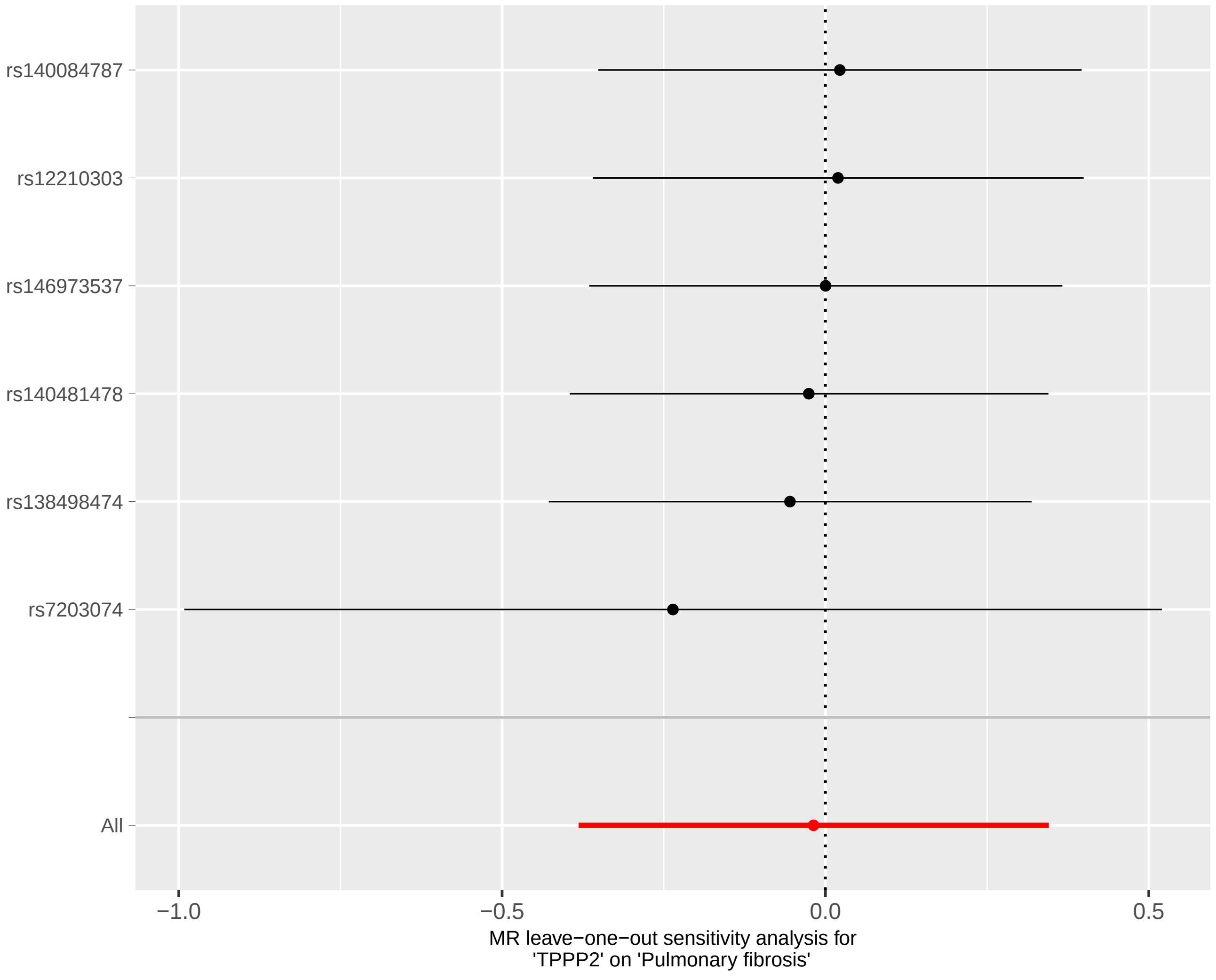

rs140084787
rs12210303
rs146973537
rs140481478
rs138498474
rs7203074
All
−1.0
−0.5
0.0
0.5
MR leave−one−out sensitivity analysis for
'TPPP2' on 'Pulmonary fibrosis'

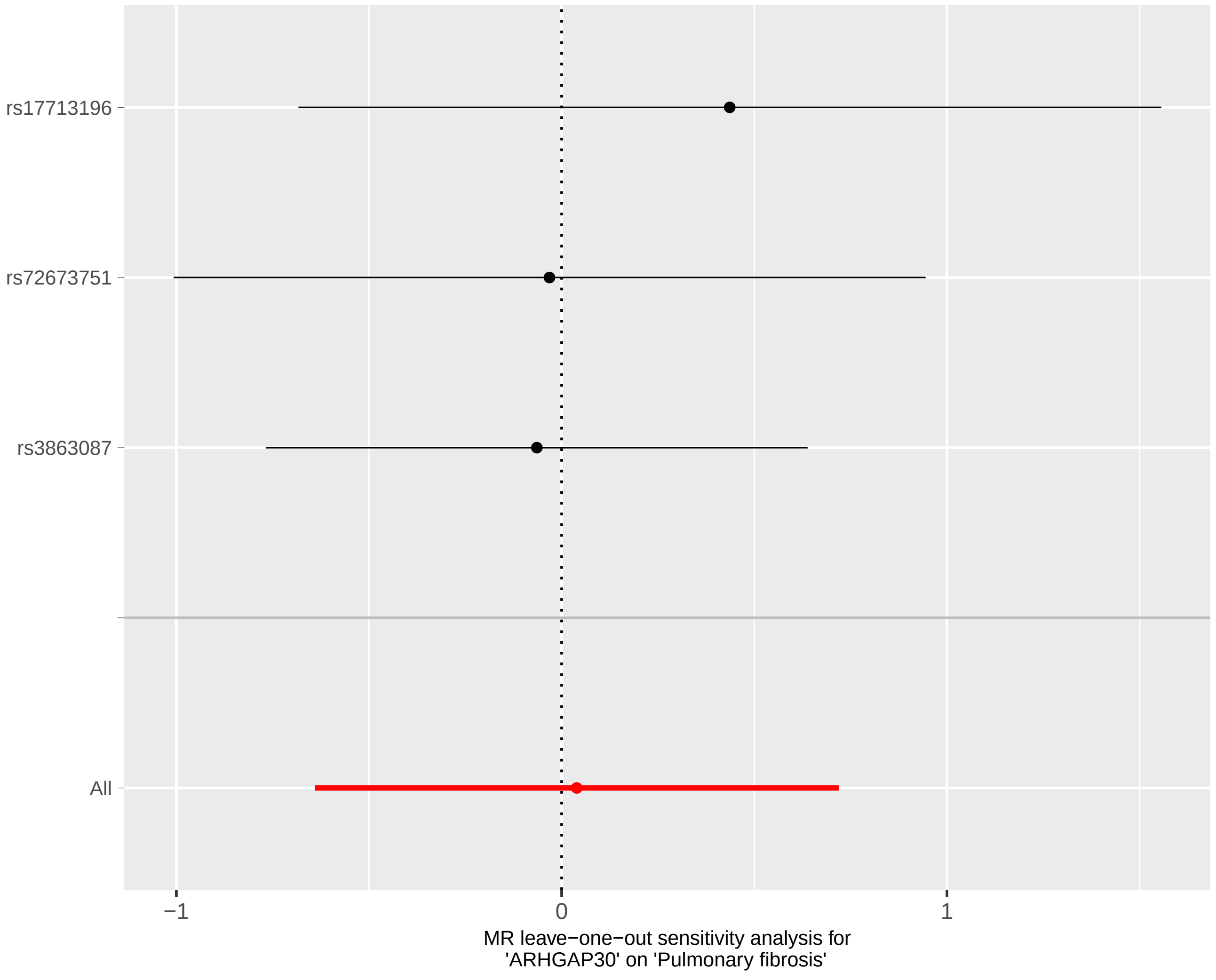

rs17713196
rs72673751
rs3863087
All
−1
0
1
MR leave−one−out sensitivity analysis for
'ARHGAP30' on 'Pulmonary fibrosis'

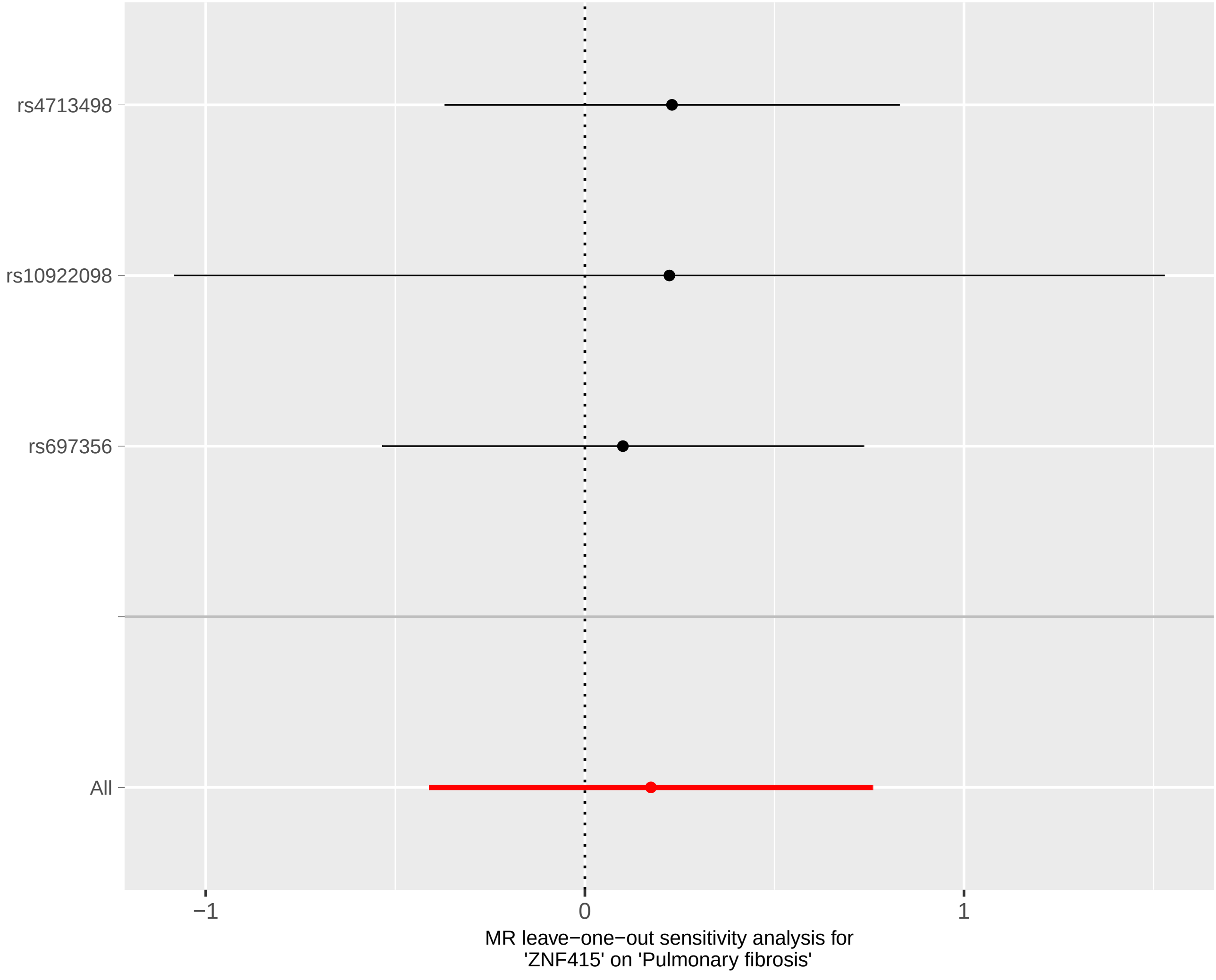

rs4713498
rs10922098
rs697356
All
−1
0
1
MR leave−one−out sensitivity analysis for
'ZNF415' on 'Pulmonary fibrosis'

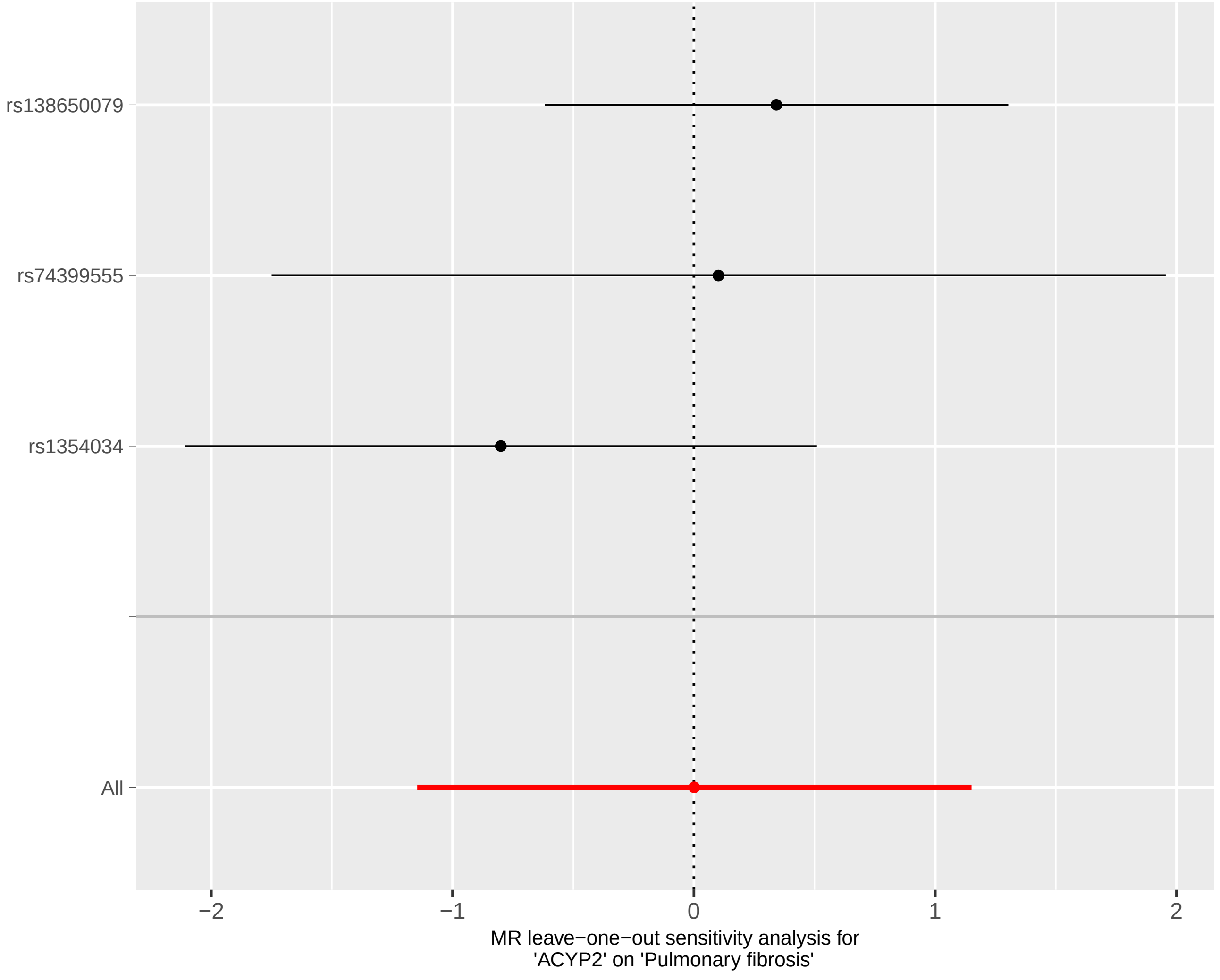
rs138650079
rs74399555
rs1354034
All
−2
−1
0
1
2
MR leave−one−out sensitivity analysis for
'ACYP2' on 'Pulmonary fibrosis'

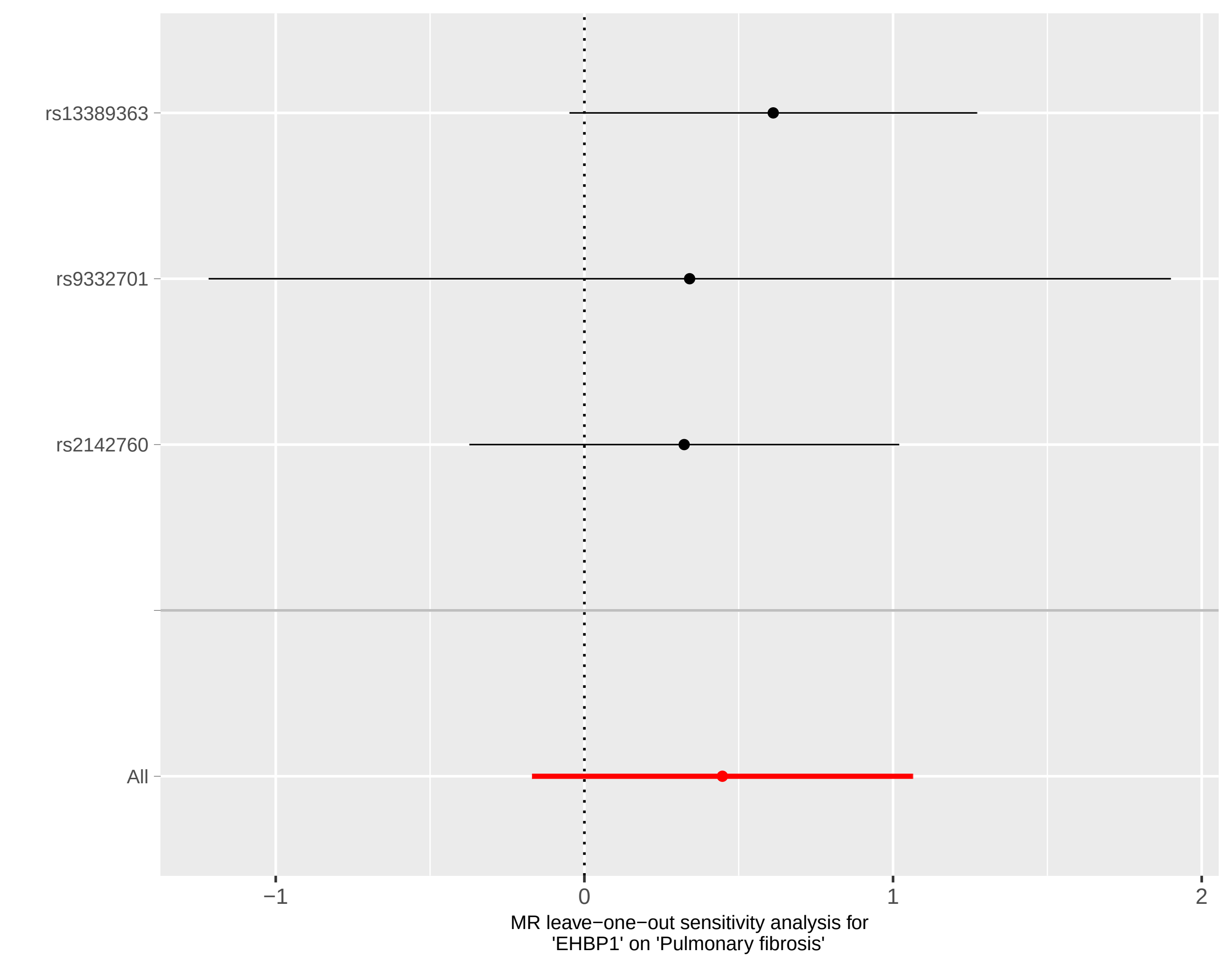

rs13389363
rs9332701
rs2142760
All
−1
0
1
2
MR leave−one−out sensitivity analysis for
'EHBP1' on 'Pulmonary fibrosis'

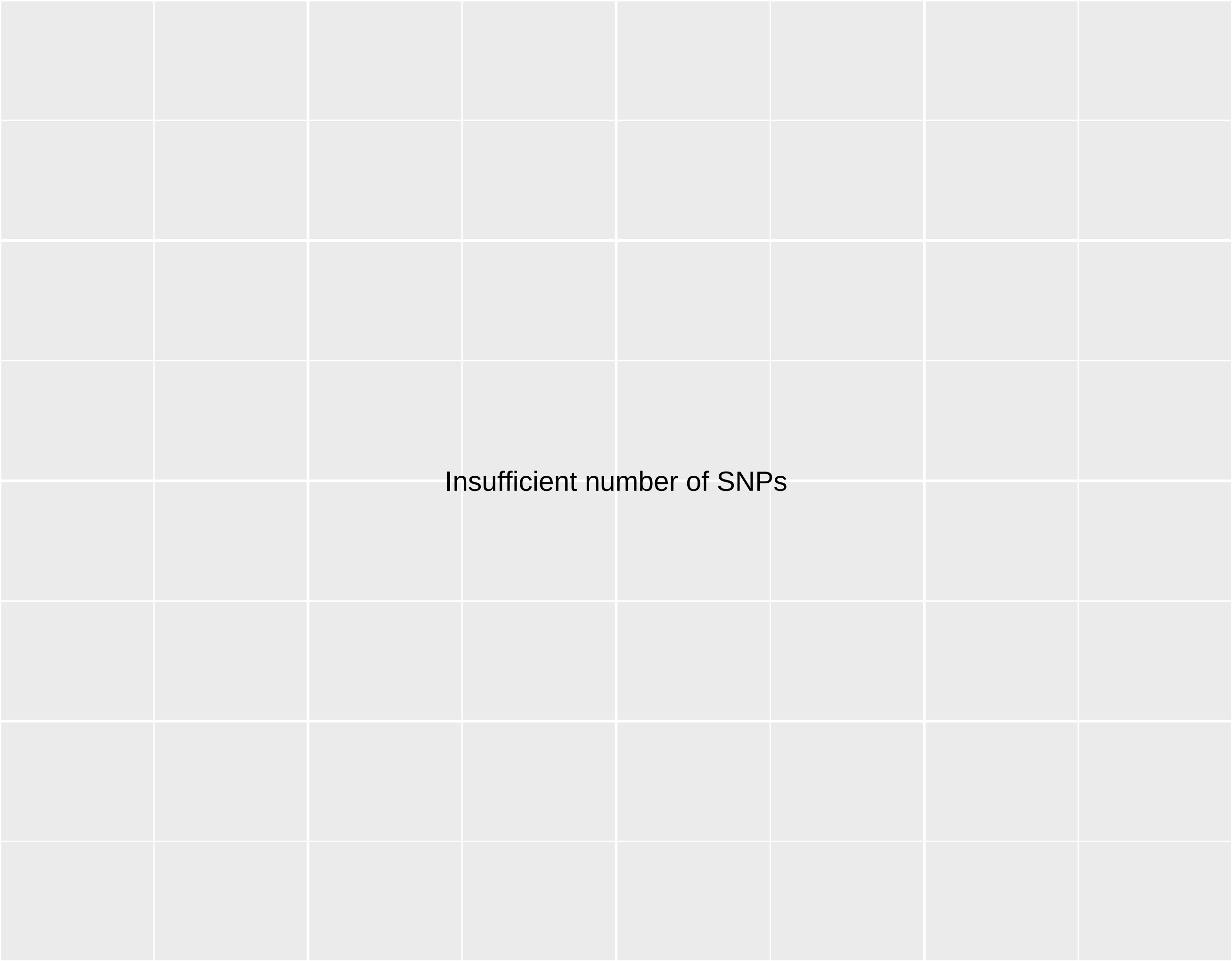
Insufficient number of SNPs

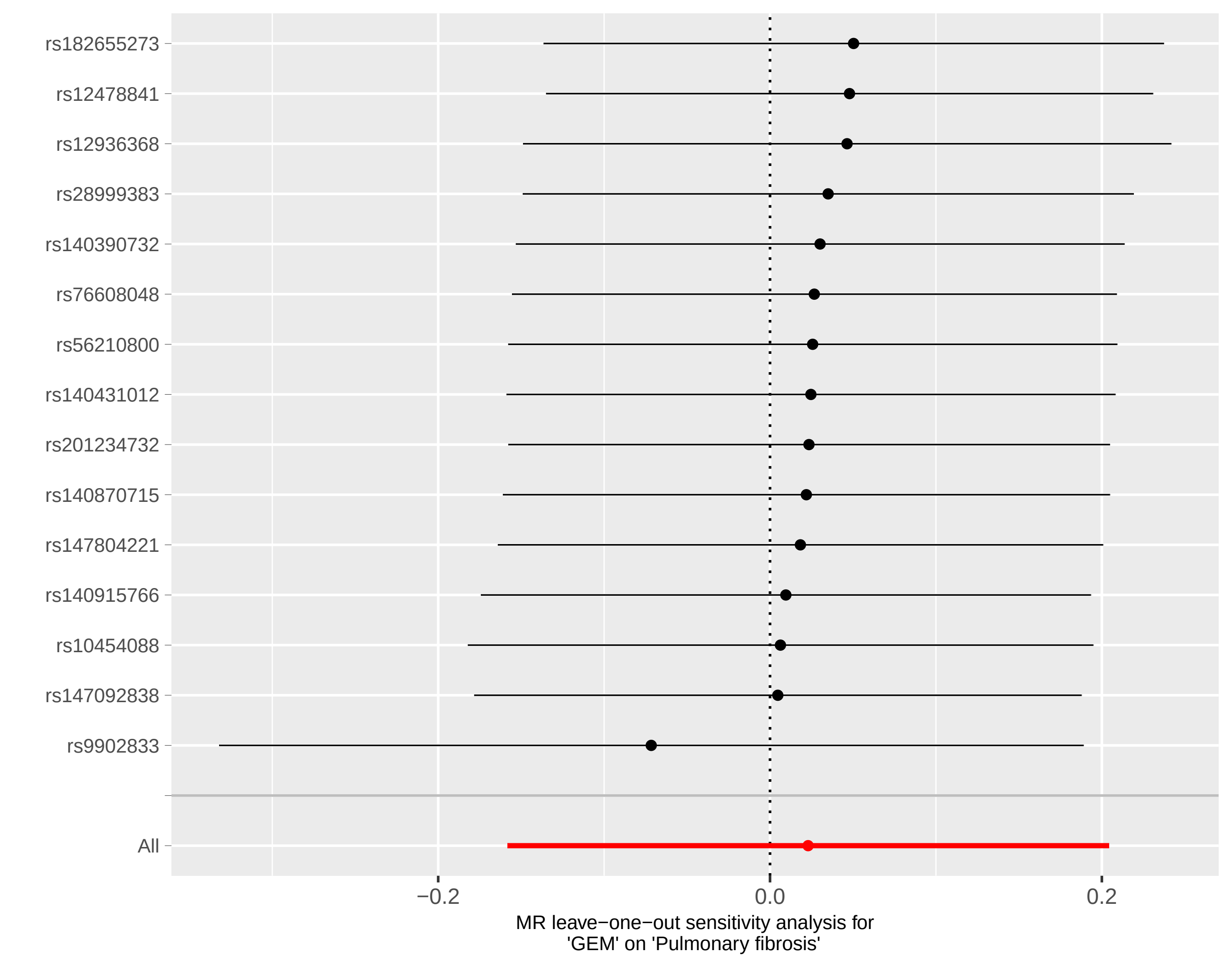
rs182655273
rs12478841
rs12936368
rs28999383
rs140390732
rs76608048
rs56210800
rs140431012
rs201234732
rs140870715
rs147804221
rs140915766
rs10454088
rs147092838
rs9902833
All
−0.2
0.0
0.2
MR leave−one−out sensitivity analysis for
'GEM' on 'Pulmonary fibrosis'

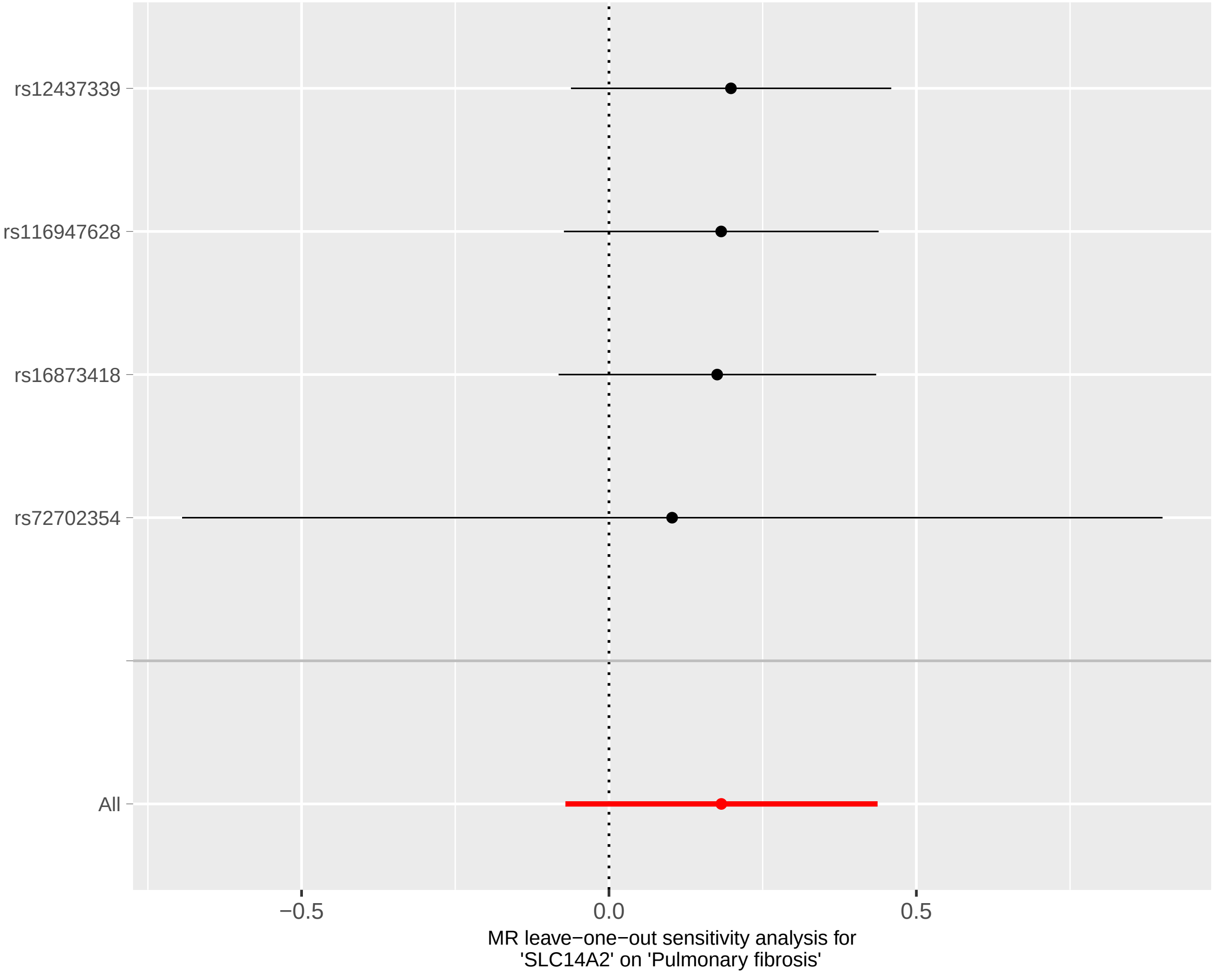

rs12437339
rs116947628
rs16873418
rs72702354
All
−0.5
0.0
0.5
MR leave−one−out sensitivity analysis for
'SLC14A2' on 'Pulmonary fibrosis'

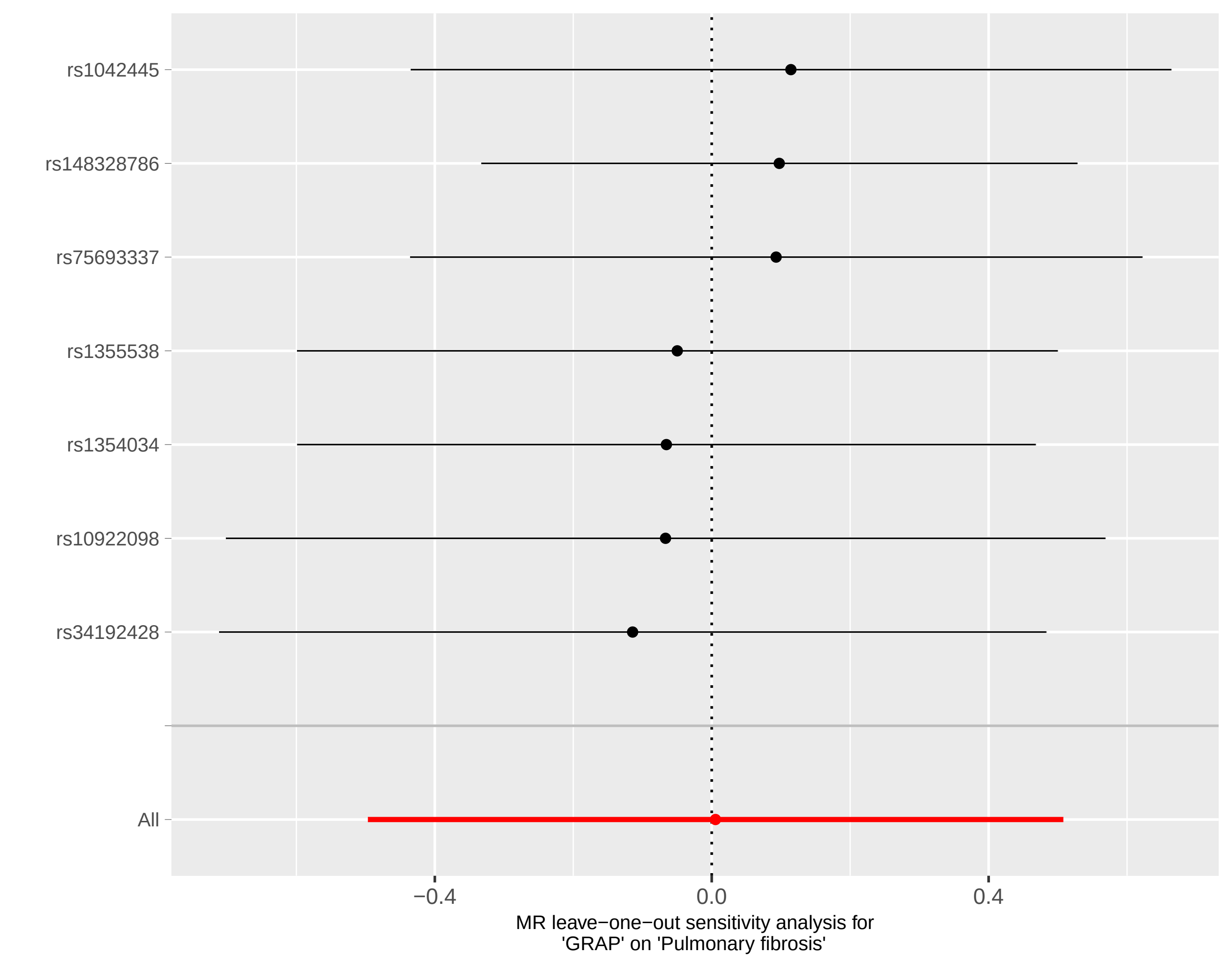

rs1042445
rs148328786
rs75693337
rs1355538
rs1354034
rs10922098
rs34192428
All
−0.4
0.0
0.4
MR leave−one−out sensitivity analysis for
'GRAP' on 'Pulmonary fibrosis'

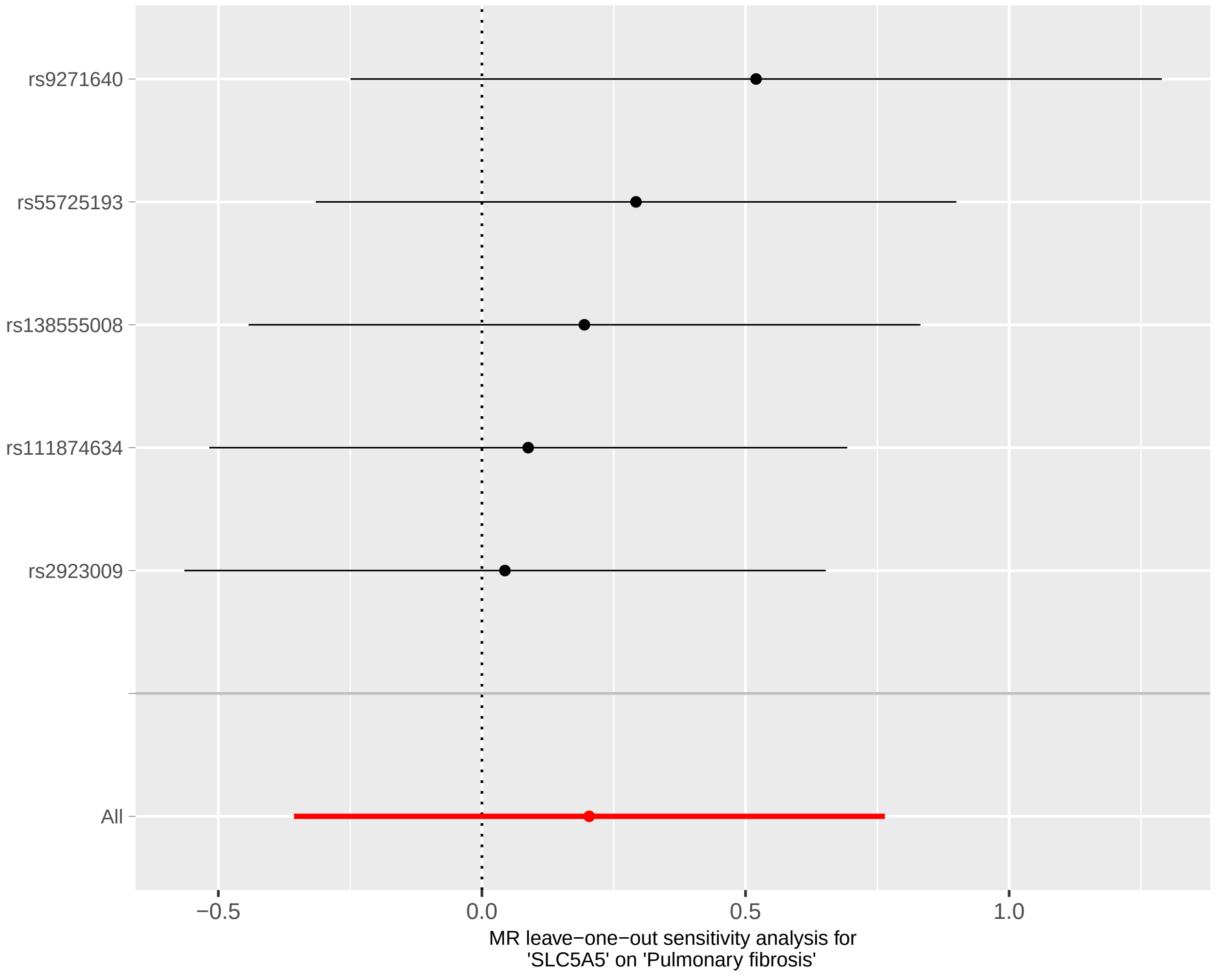
rs9271640
rs55725193
rs138555008
rs111874634
rs2923009
All
−0.5
0.0
0.5
1.0
MR leave−one−out sensitivity analysis for
'SLC5A5' on 'Pulmonary fibrosis'

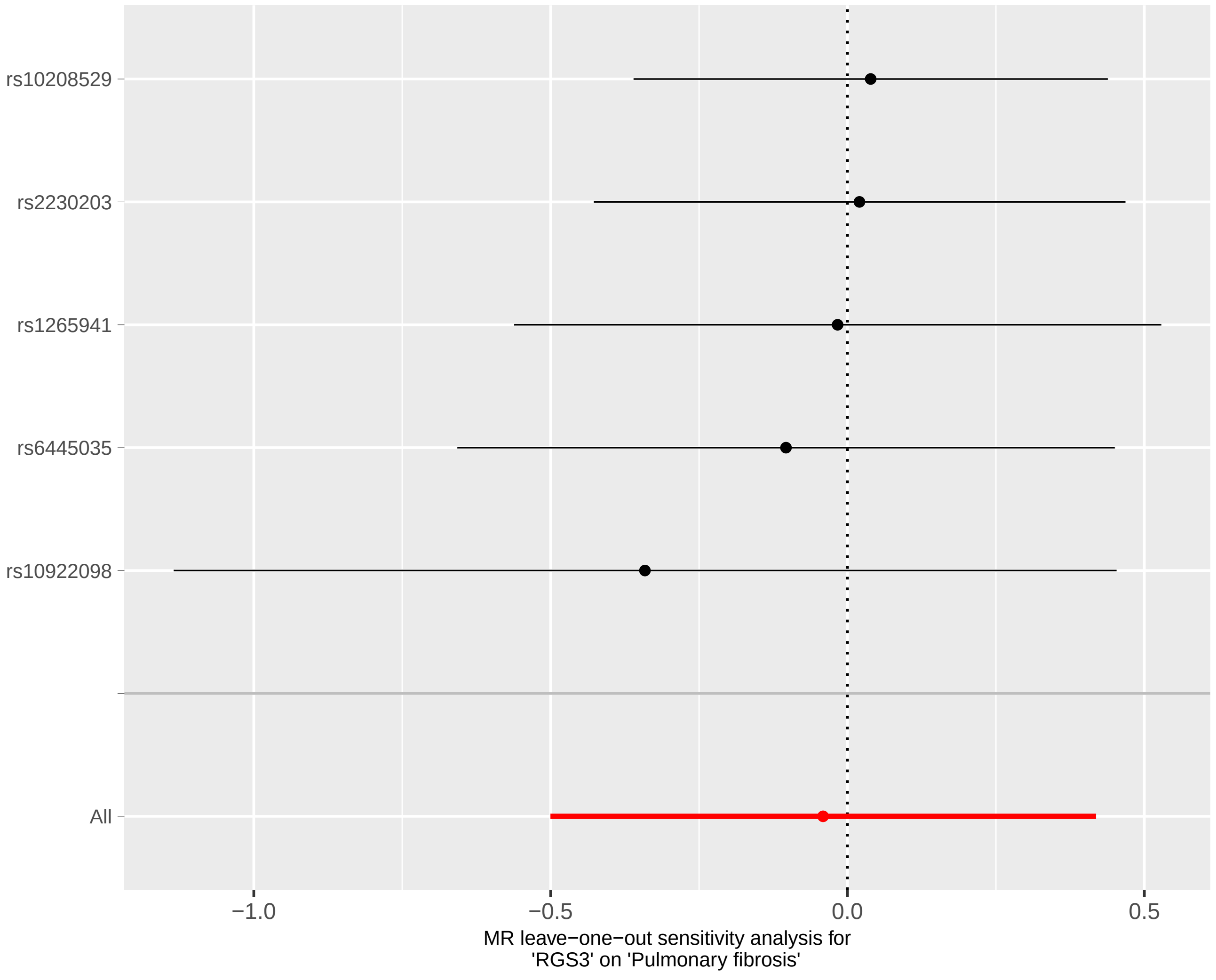

rs10208529
rs2230203
rs1265941
rs6445035
rs10922098
All
−1.0
−0.5
0.0
0.5
MR leave−one−out sensitivity analysis for
'RGS3' on 'Pulmonary fibrosis'

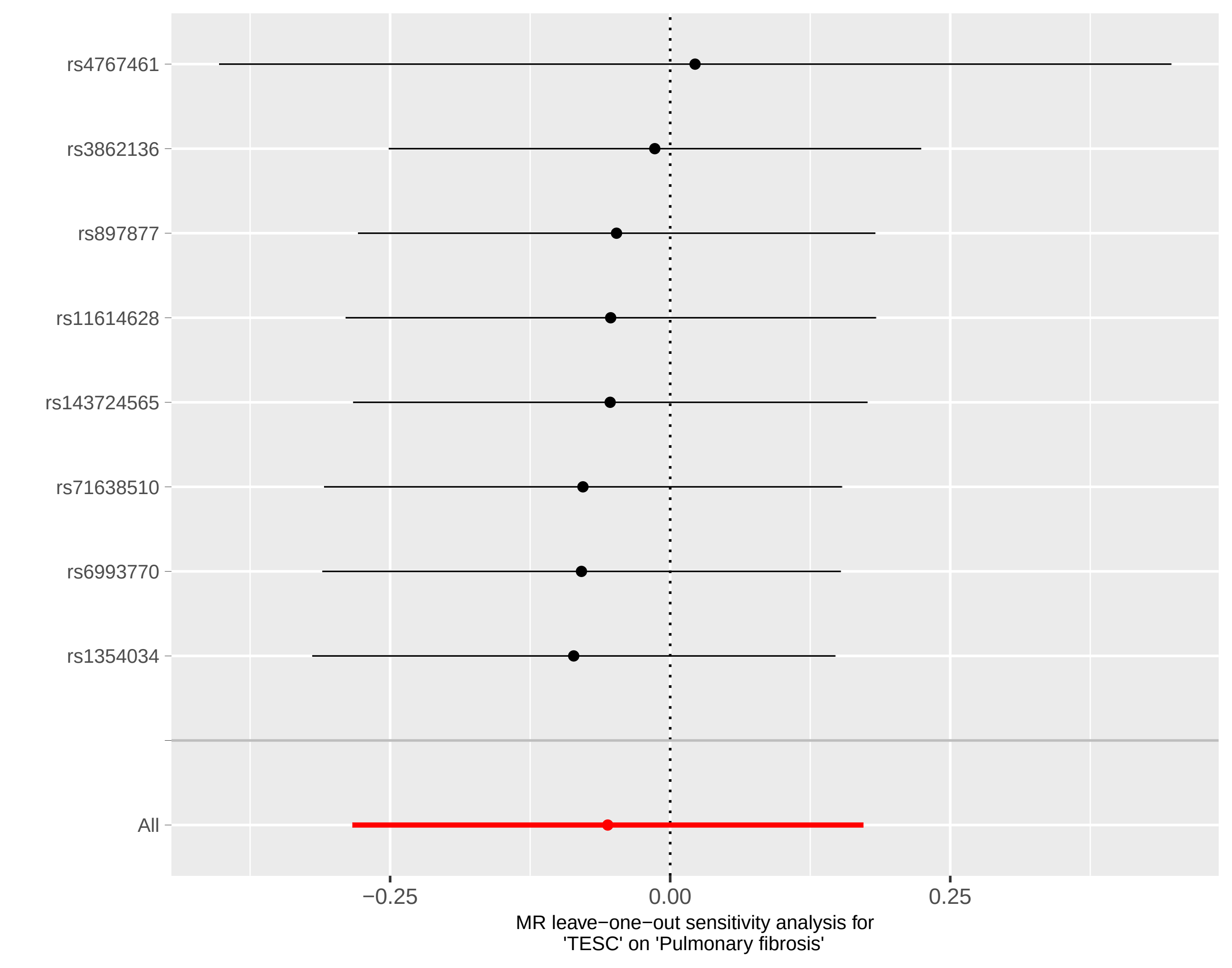

rs4767461
rs3862136
rs897877
rs11614628
rs143724565
rs71638510
rs6993770
rs1354034
All
−0.25
0.00
0.25
MR leave−one−out sensitivity analysis for
'TESC' on 'Pulmonary fibrosis'

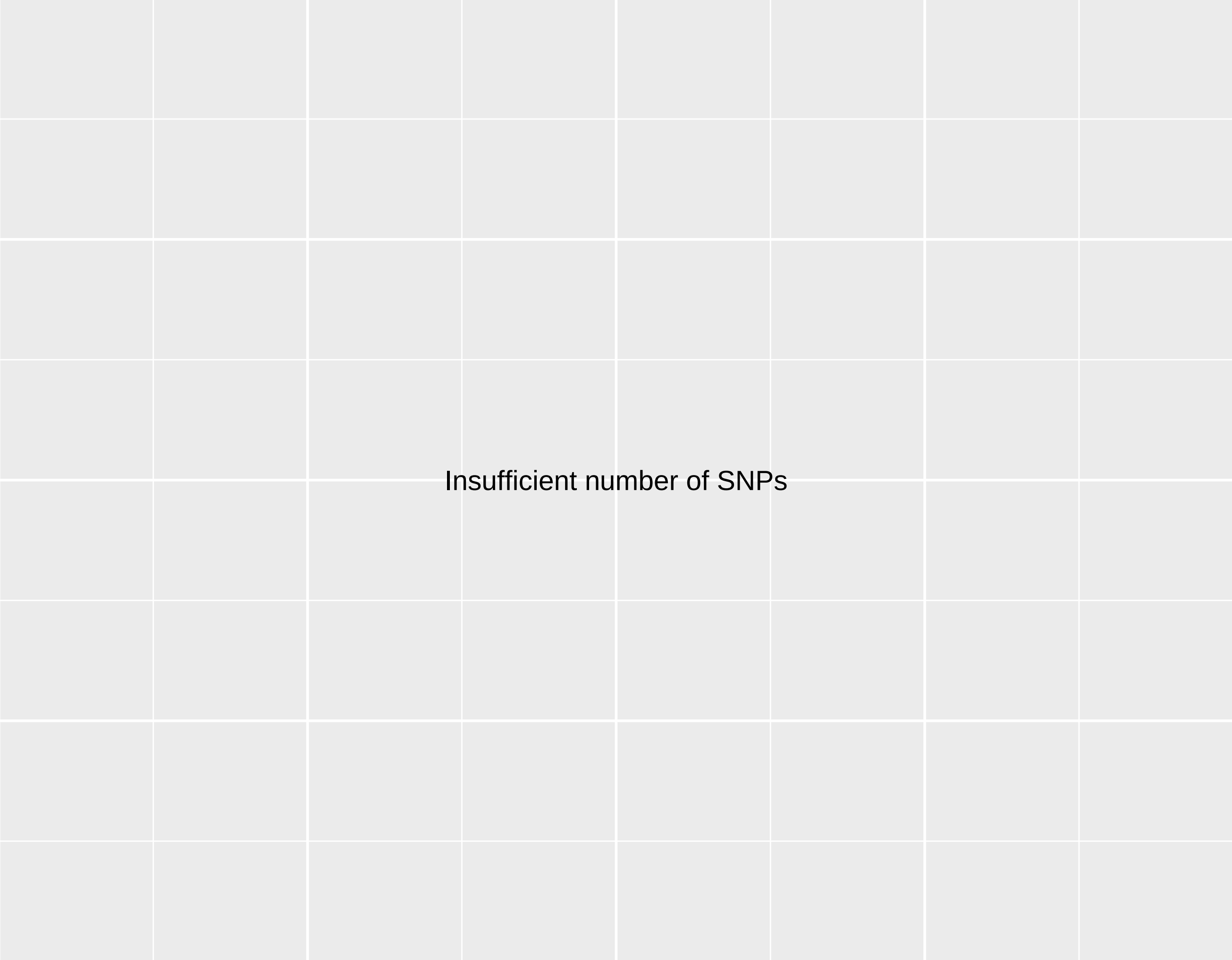
Insufficient number of SNPs

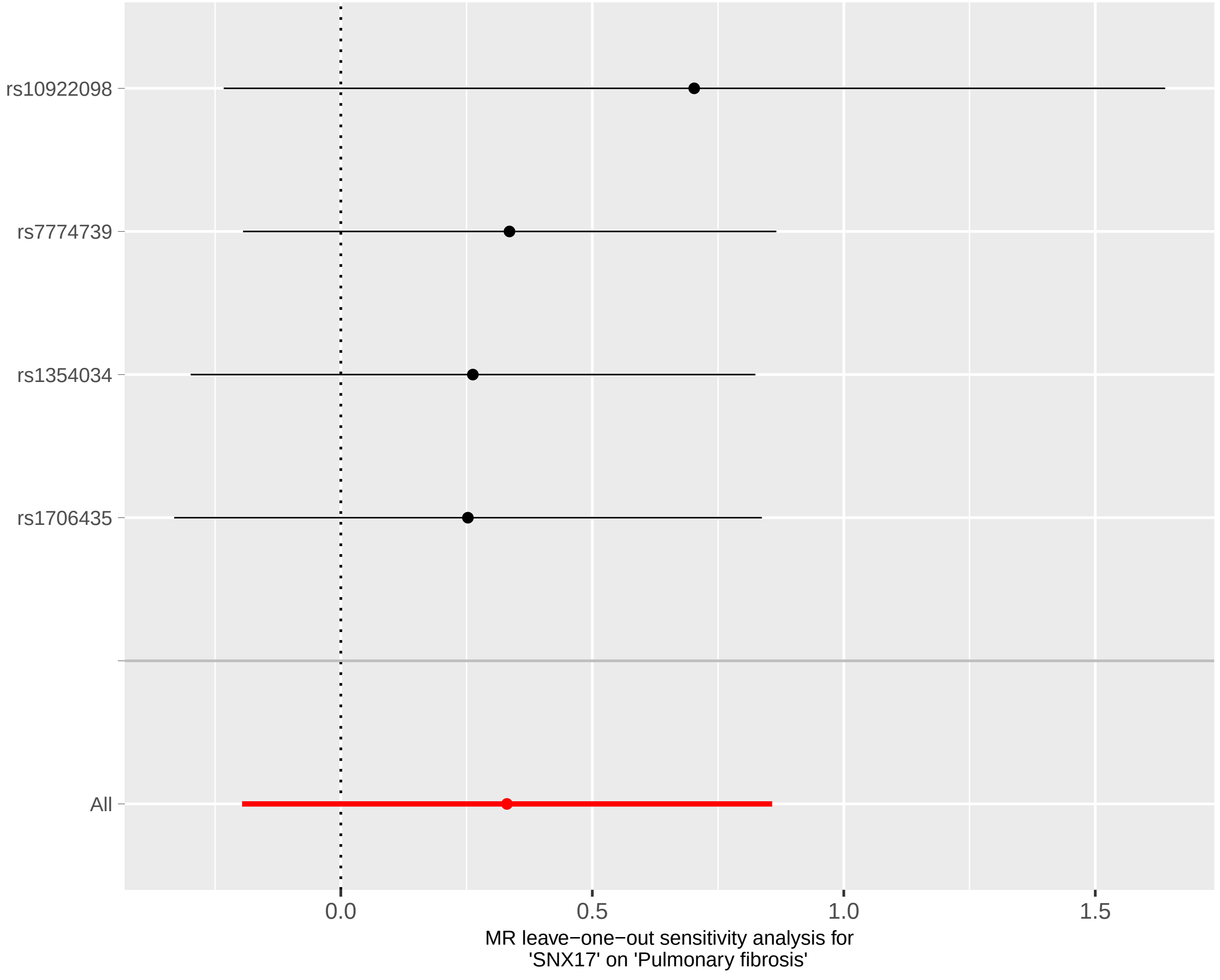
rs10922098
rs7774739
rs1354034
rs1706435
All
0.0
0.5
1.0
1.5
MR leave−one−out sensitivity analysis for
'SNX17' on 'Pulmonary fibrosis'

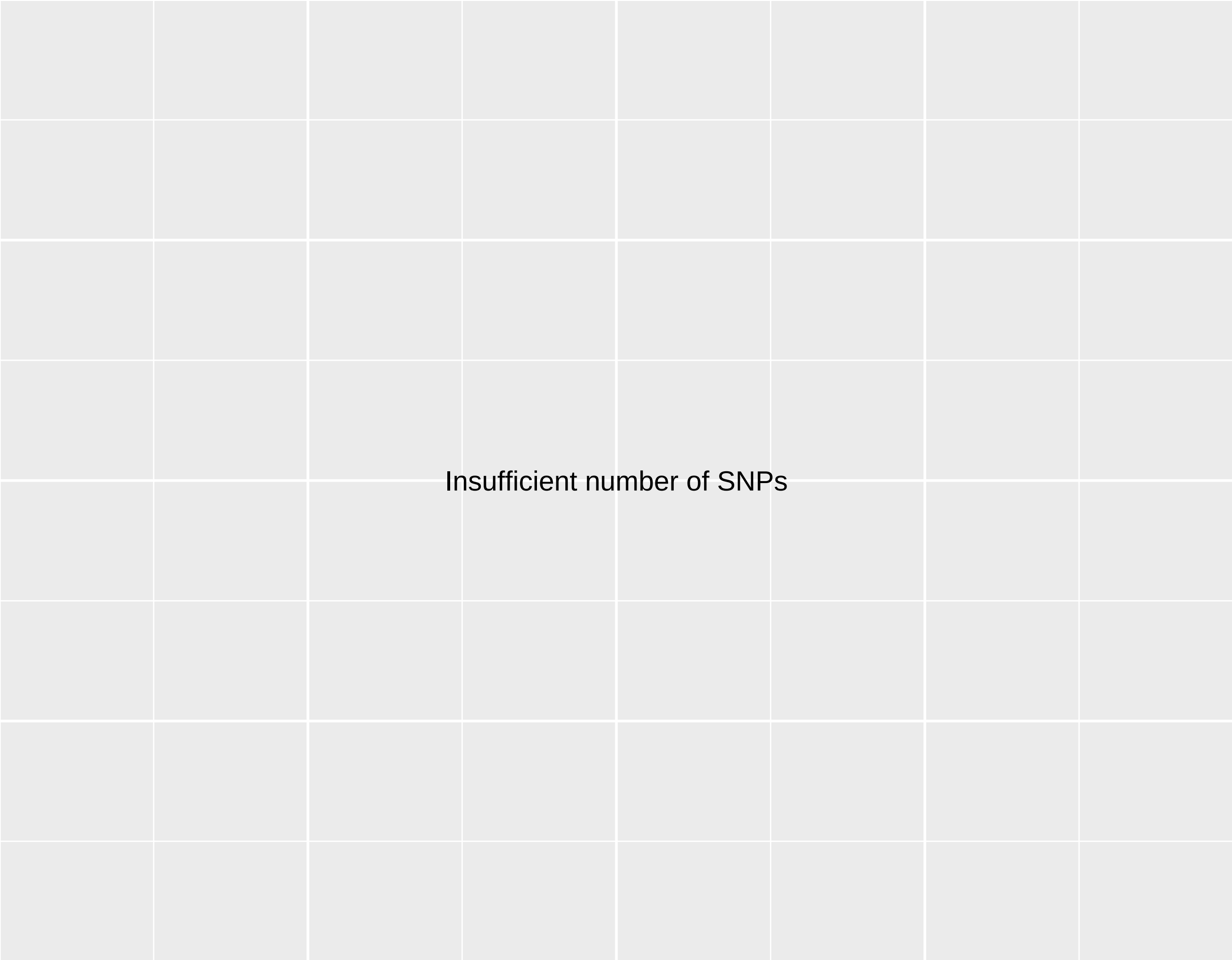
Insufficient number of SNPs

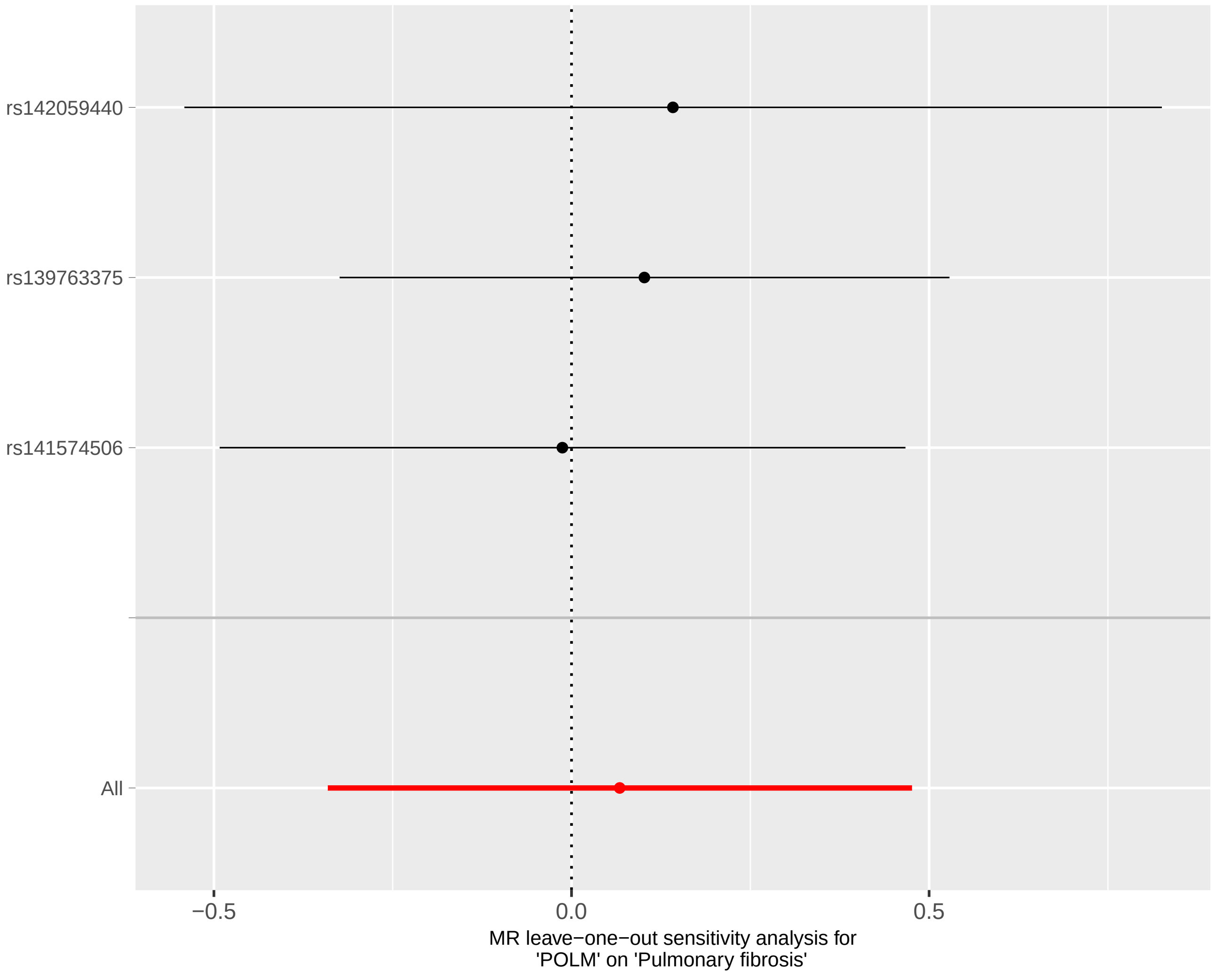

rs142059440
rs139763375
rs141574506
All
−0.5
0.0
0.5
MR leave−one−out sensitivity analysis for
'POLM' on 'Pulmonary fibrosis'

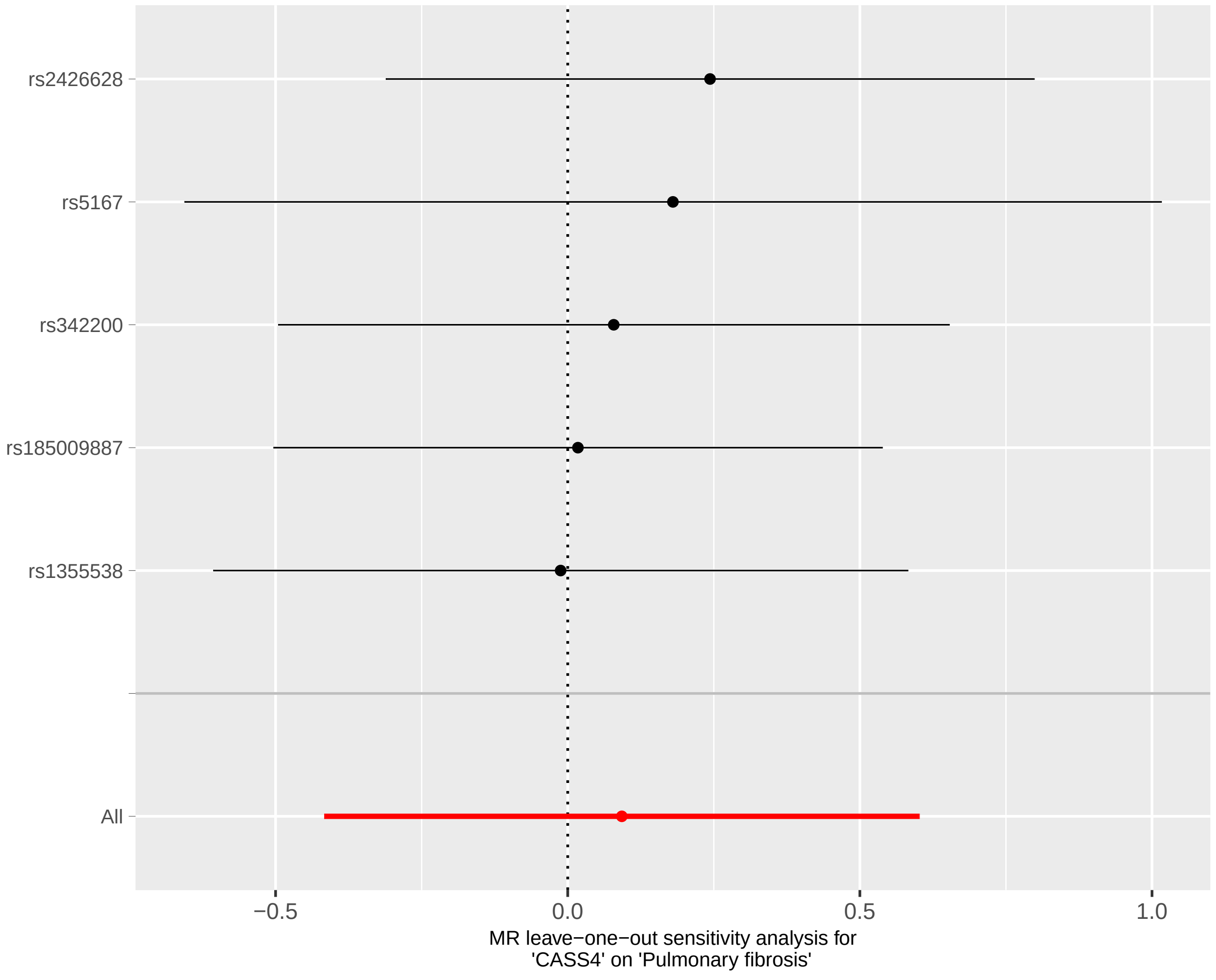
rs2426628
rs5167
rs342200
rs185009887
rs1355538
All
−0.5
0.0
0.5
1.0
MR leave−one−out sensitivity analysis for
'CASS4' on 'Pulmonary fibrosis'

Insufficient number of SNPs

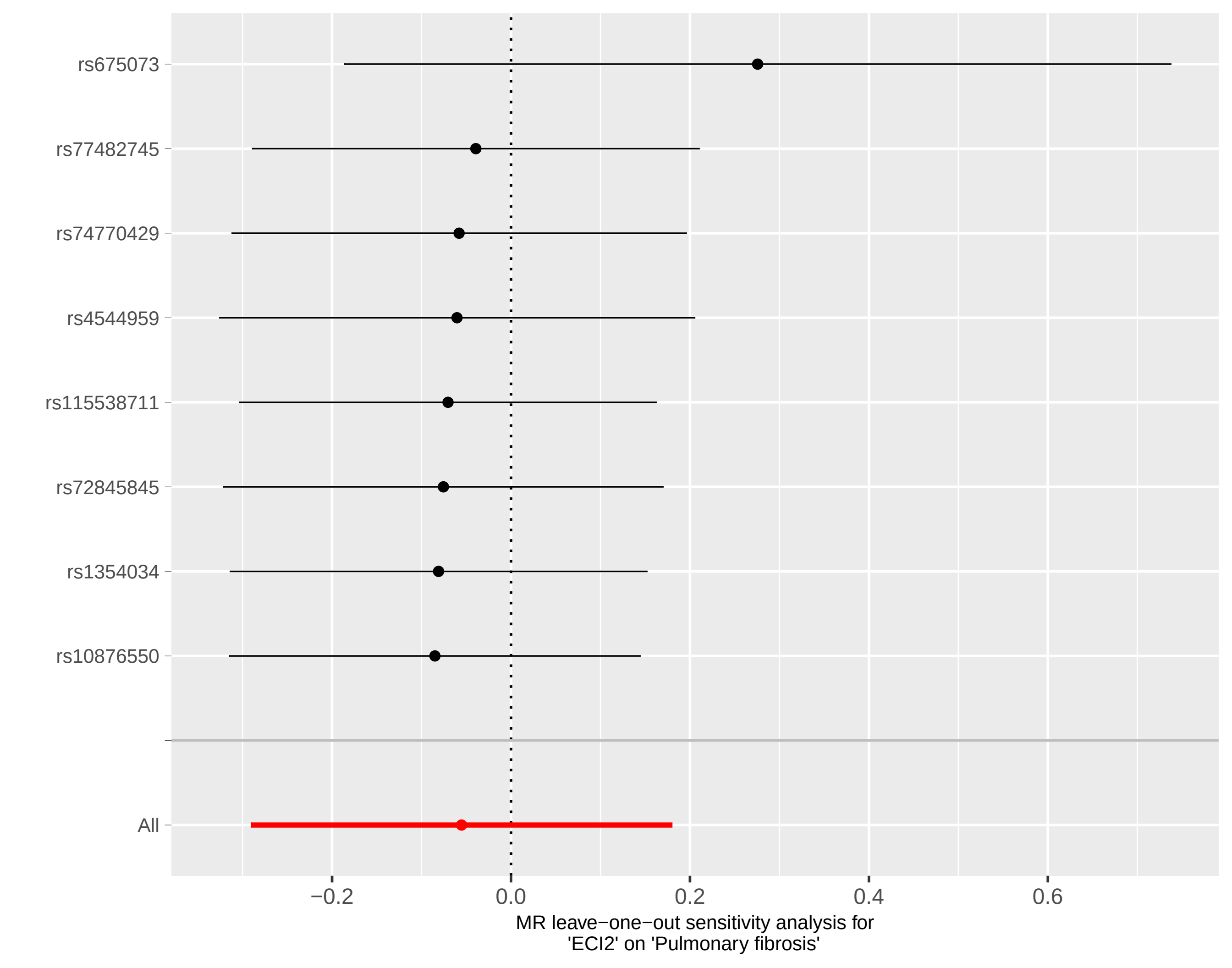
rs675073
rs77482745
rs74770429
rs4544959
rs115538711
rs72845845
rs1354034
rs10876550
All
−0.2
0.0
0.2
0.4
0.6
MR leave−one−out sensitivity analysis for
'ECI2' on 'Pulmonary fibrosis'

Insufficient number of SNPs

Insufficient number of SNPs

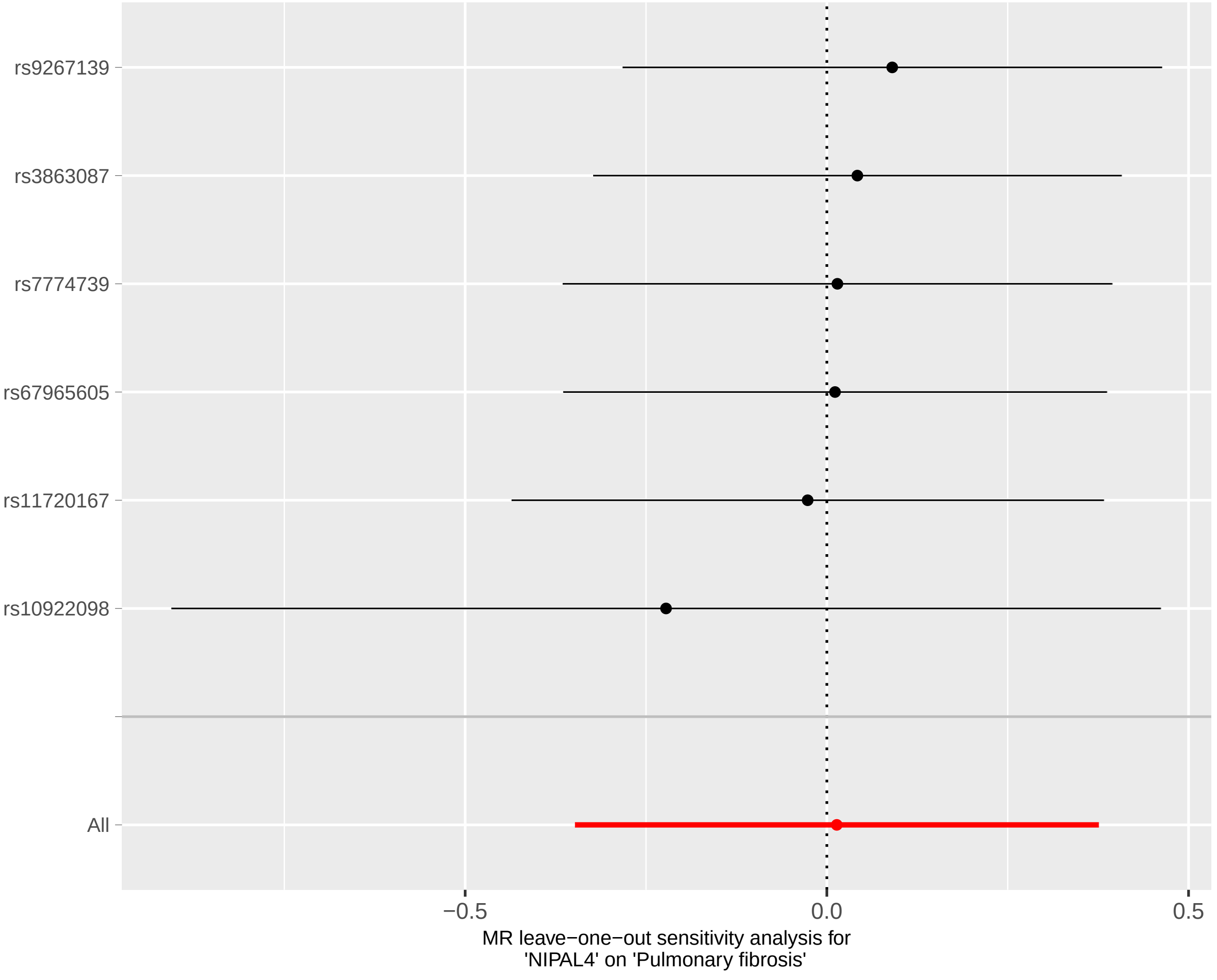
rs9267139
rs3863087
rs7774739
rs67965605
rs11720167
rs10922098
All
−0.5
0.0
0.5
MR leave−one−out sensitivity analysis for
'NIPAL4' on 'Pulmonary fibrosis'

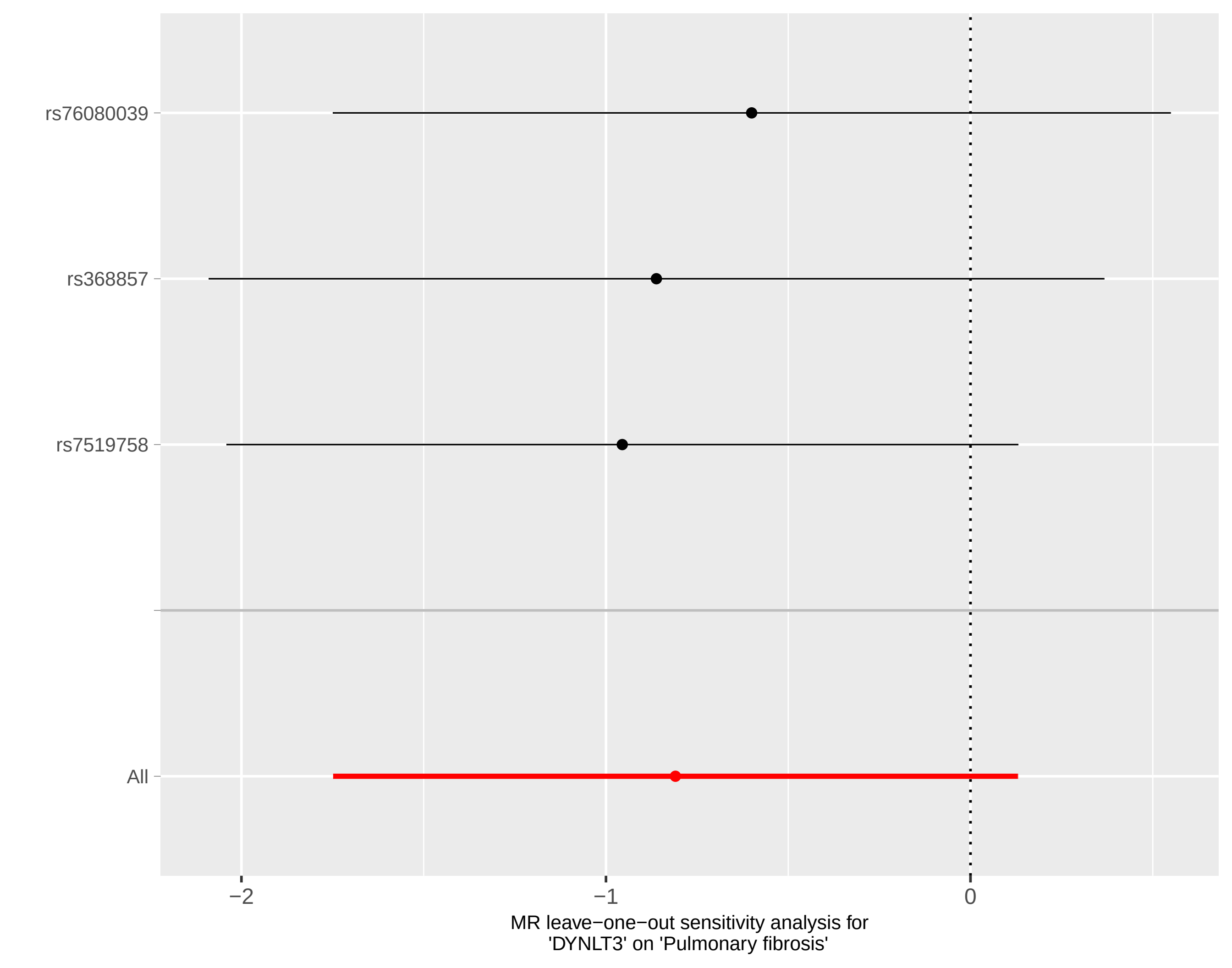
rs76080039
rs368857
rs7519758
All
−2
−1
0
MR leave−one−out sensitivity analysis for
'DYNLT3' on 'Pulmonary fibrosis'

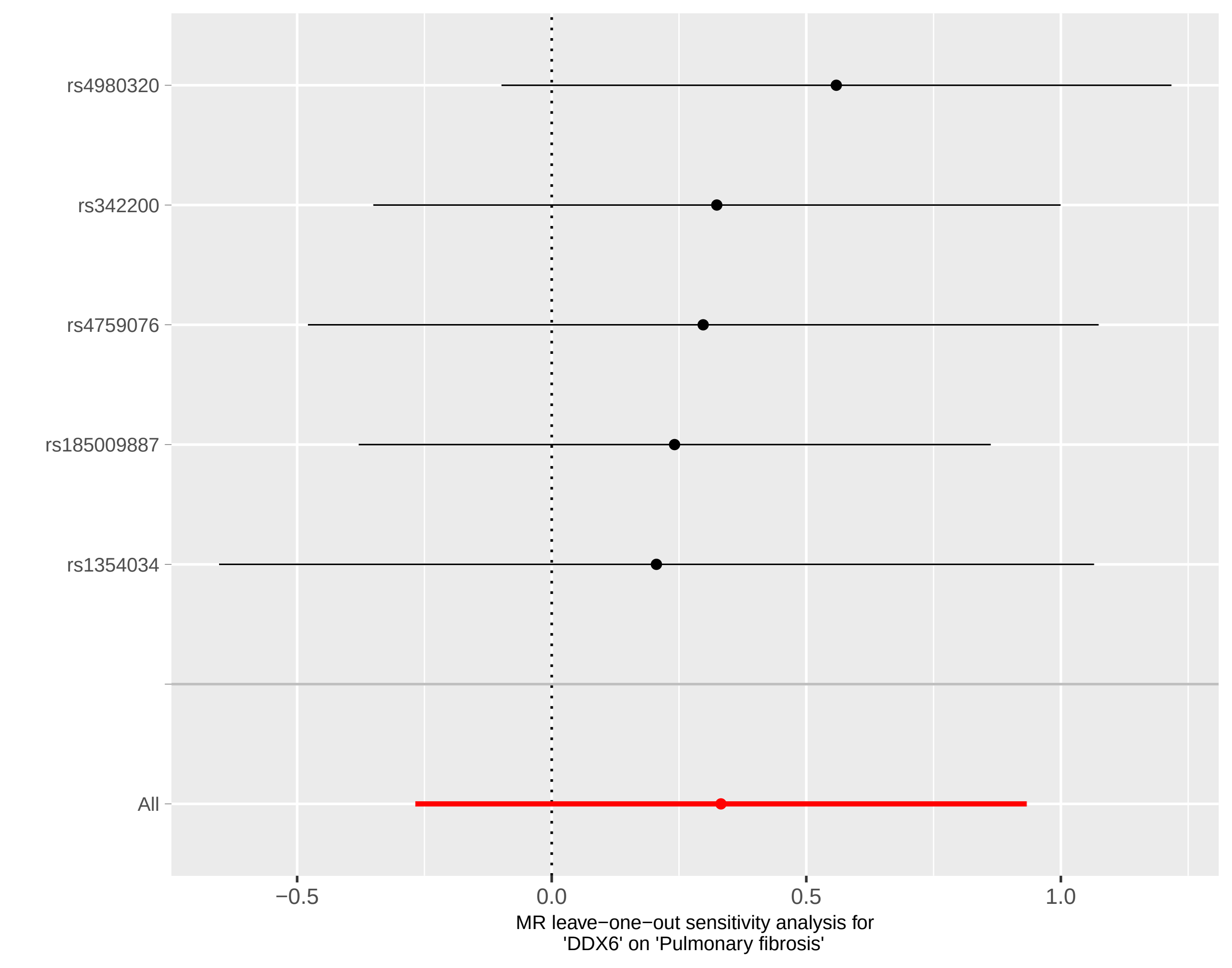
rs4980320
rs342200
rs4759076
rs185009887
rs1354034
All
−0.5
0.0
0.5
1.0
MR leave−one−out sensitivity analysis for
'DDX6' on 'Pulmonary fibrosis'

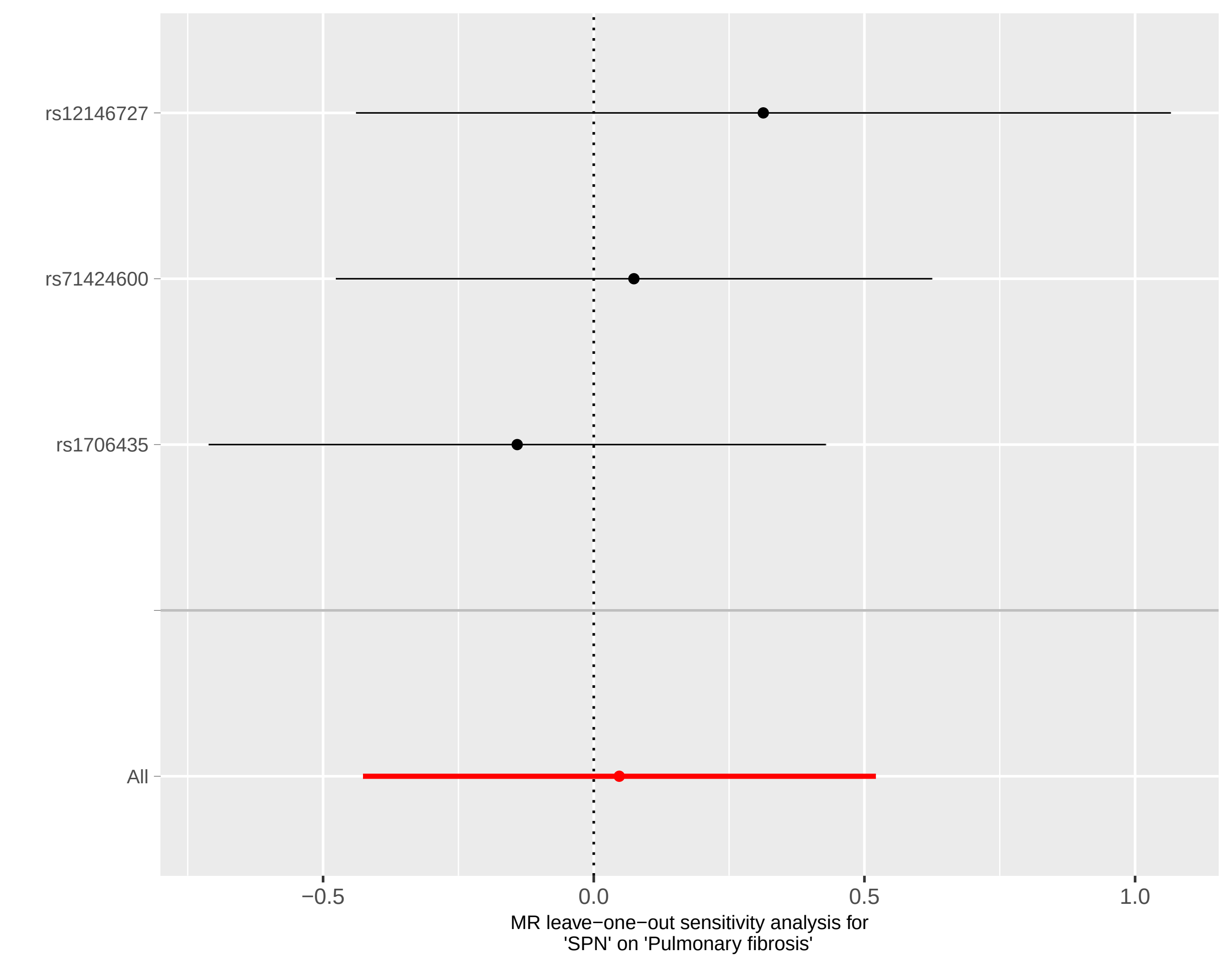
rs12146727
rs71424600
rs1706435
All
−0.5
0.0
0.5
1.0
MR leave−one−out sensitivity analysis for
'SPN' on 'Pulmonary fibrosis'

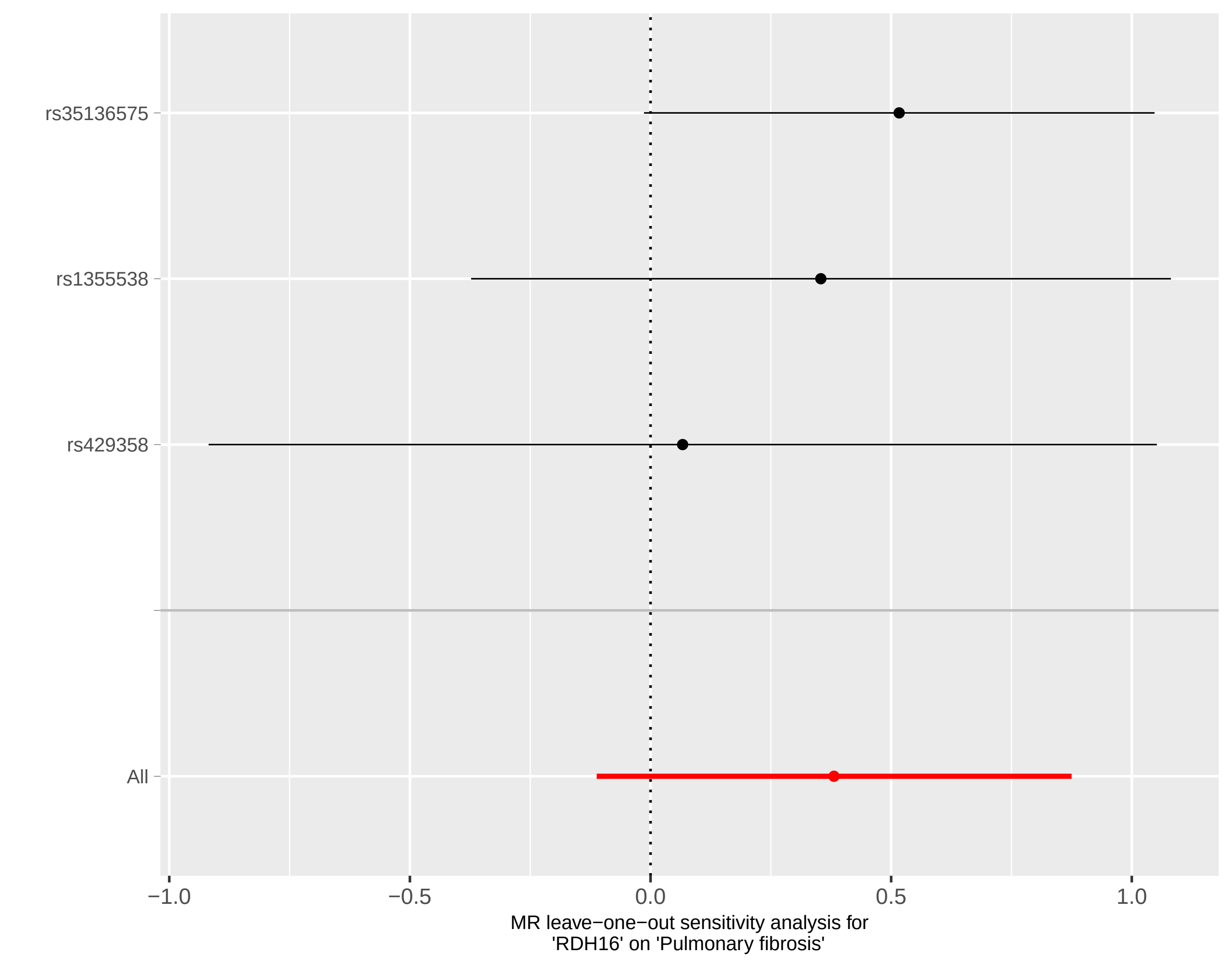
rs35136575
rs1355538
rs429358
All
−1.0
−0.5
0.0
0.5
1.0
MR leave−one−out sensitivity analysis for
'RDH16' on 'Pulmonary fibrosis'

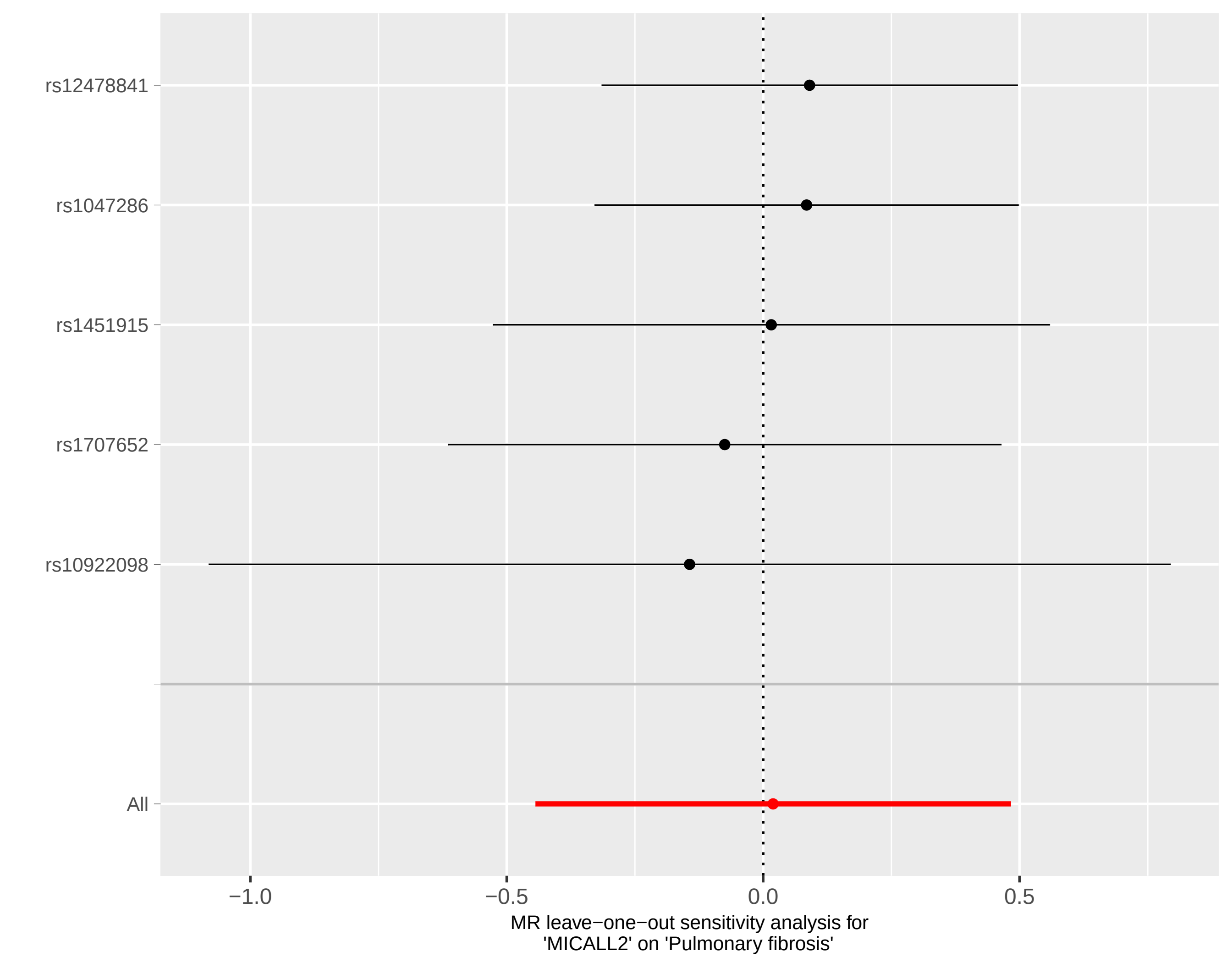
rs12478841
rs1047286
rs1451915
rs1707652
rs10922098
All
−1.0
−0.5
0.0
0.5
MR leave−one−out sensitivity analysis for
'MICALL2' on 'Pulmonary fibrosis'

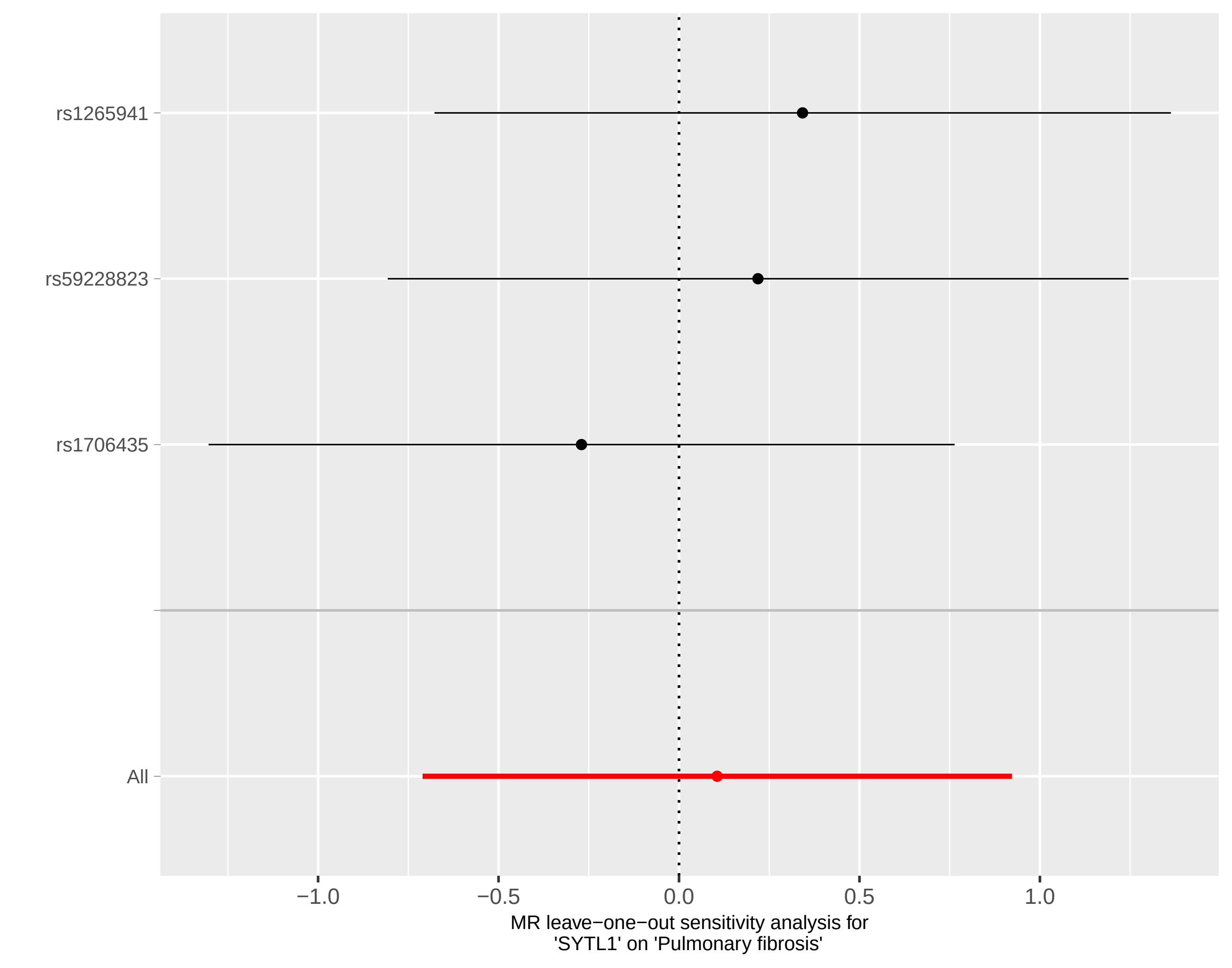
rs1265941
rs59228823
rs1706435
All
−1.0
−0.5
0.0
0.5
1.0
MR leave−one−out sensitivity analysis for
'SYTL1' on 'Pulmonary fibrosis'

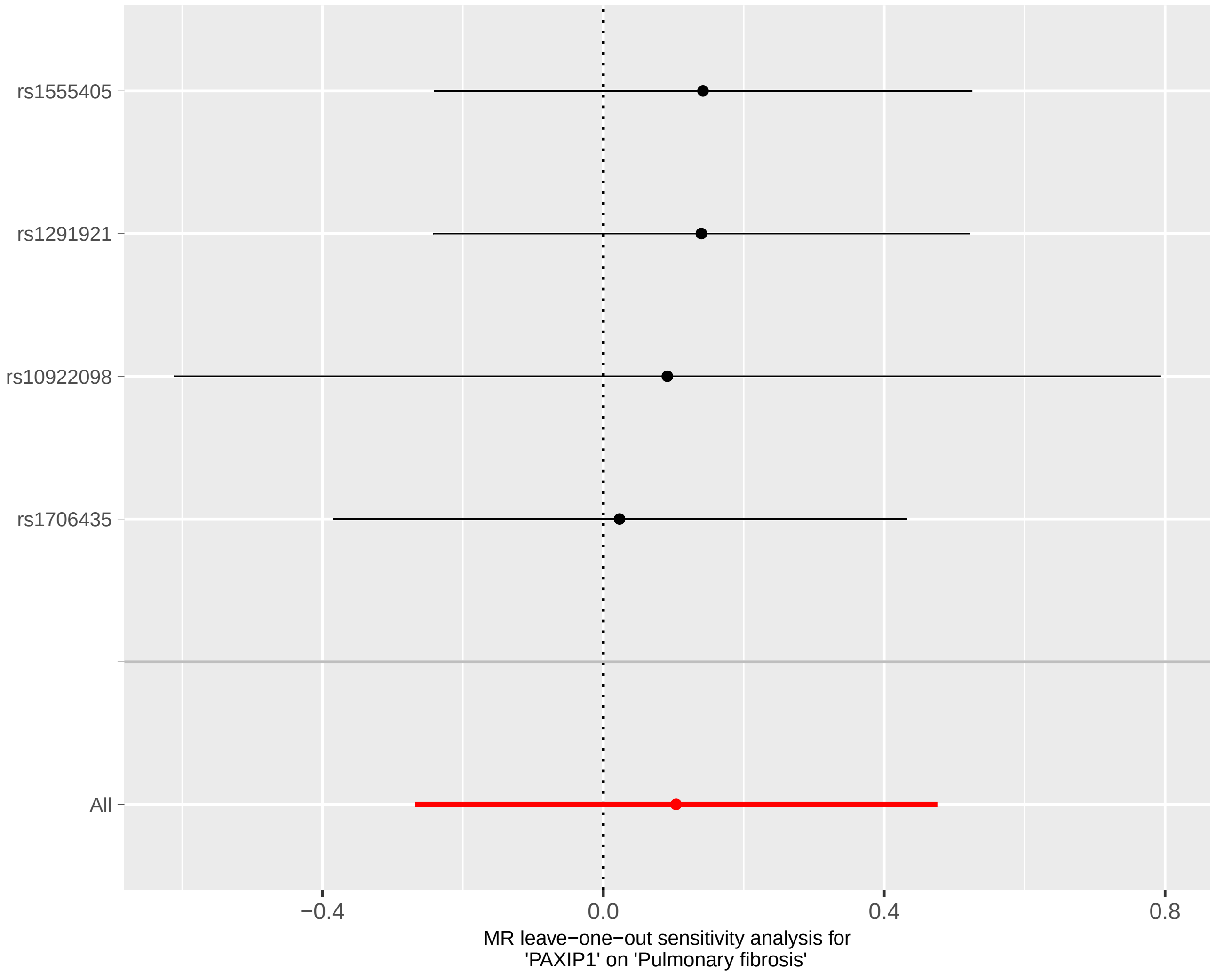
rs1555405
rs1291921
rs10922098
rs1706435
All
−0.4
0.0
0.4
0.8
MR leave−one−out sensitivity analysis for
'PAXIP1' on 'Pulmonary fibrosis'

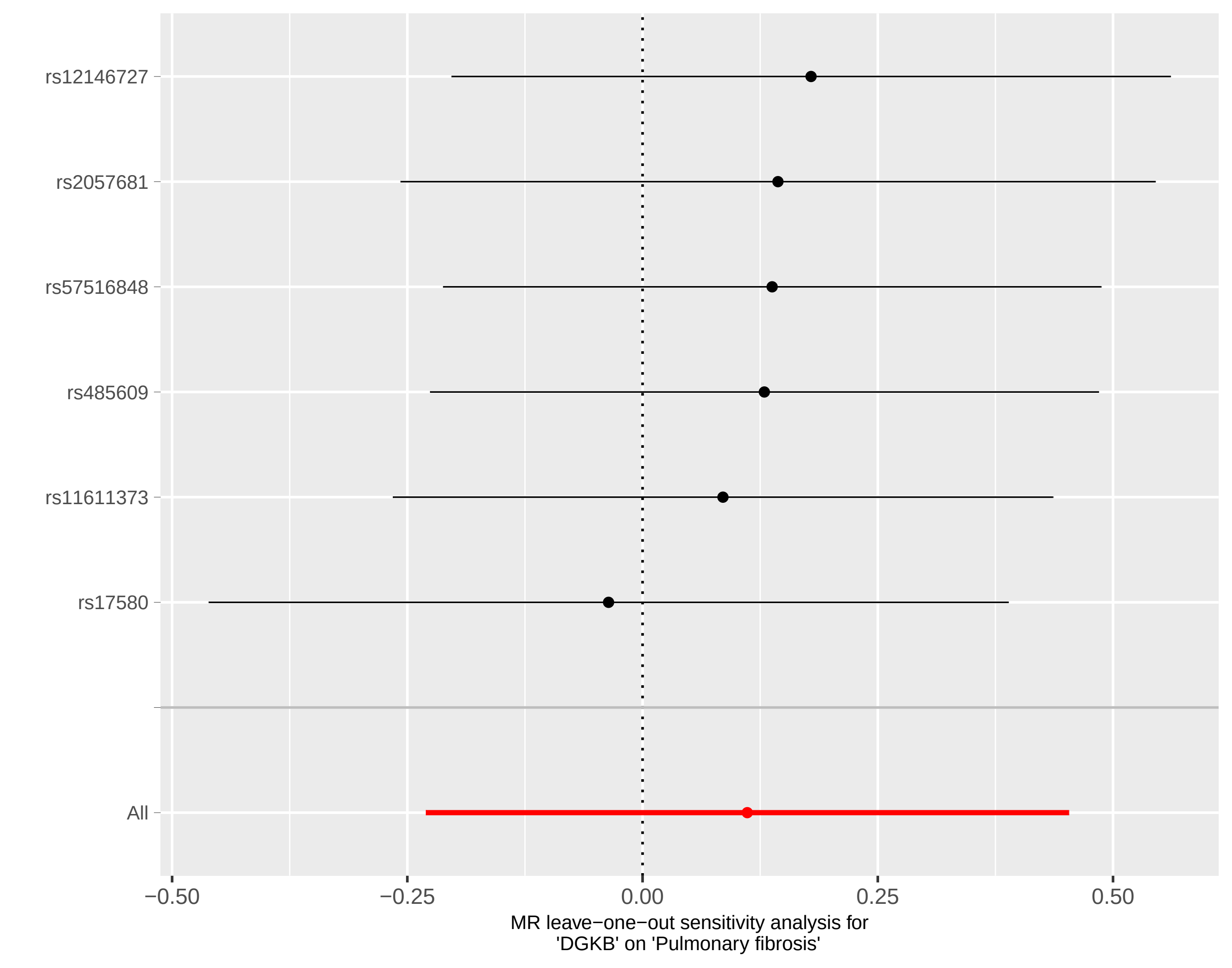
rs12146727
rs2057681
rs57516848
rs485609
rs11611373
rs17580
All
−0.50
−0.25
0.00
0.25
0.50
MR leave−one−out sensitivity analysis for
'DGKB' on 'Pulmonary fibrosis'

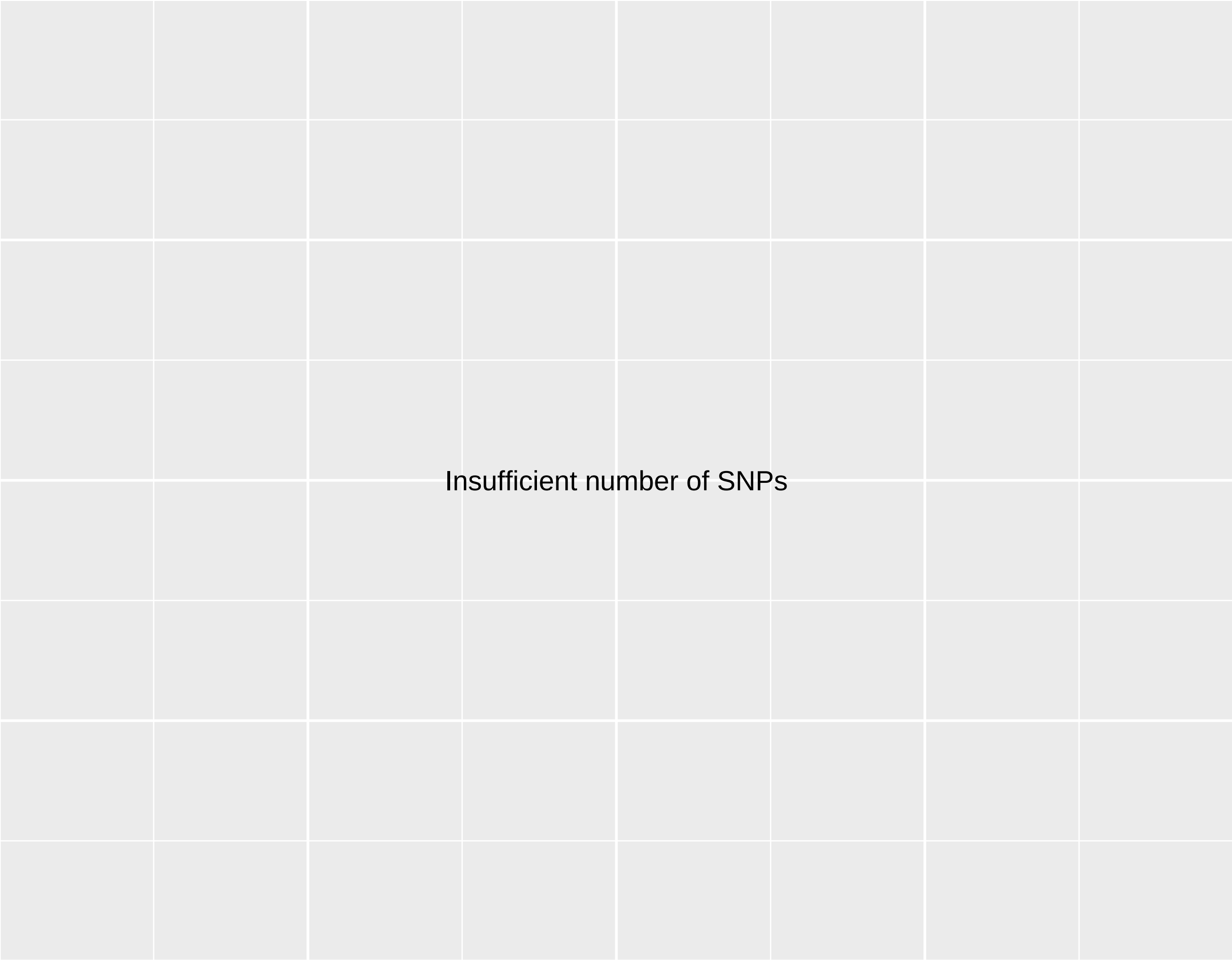
Insufficient number of SNPs

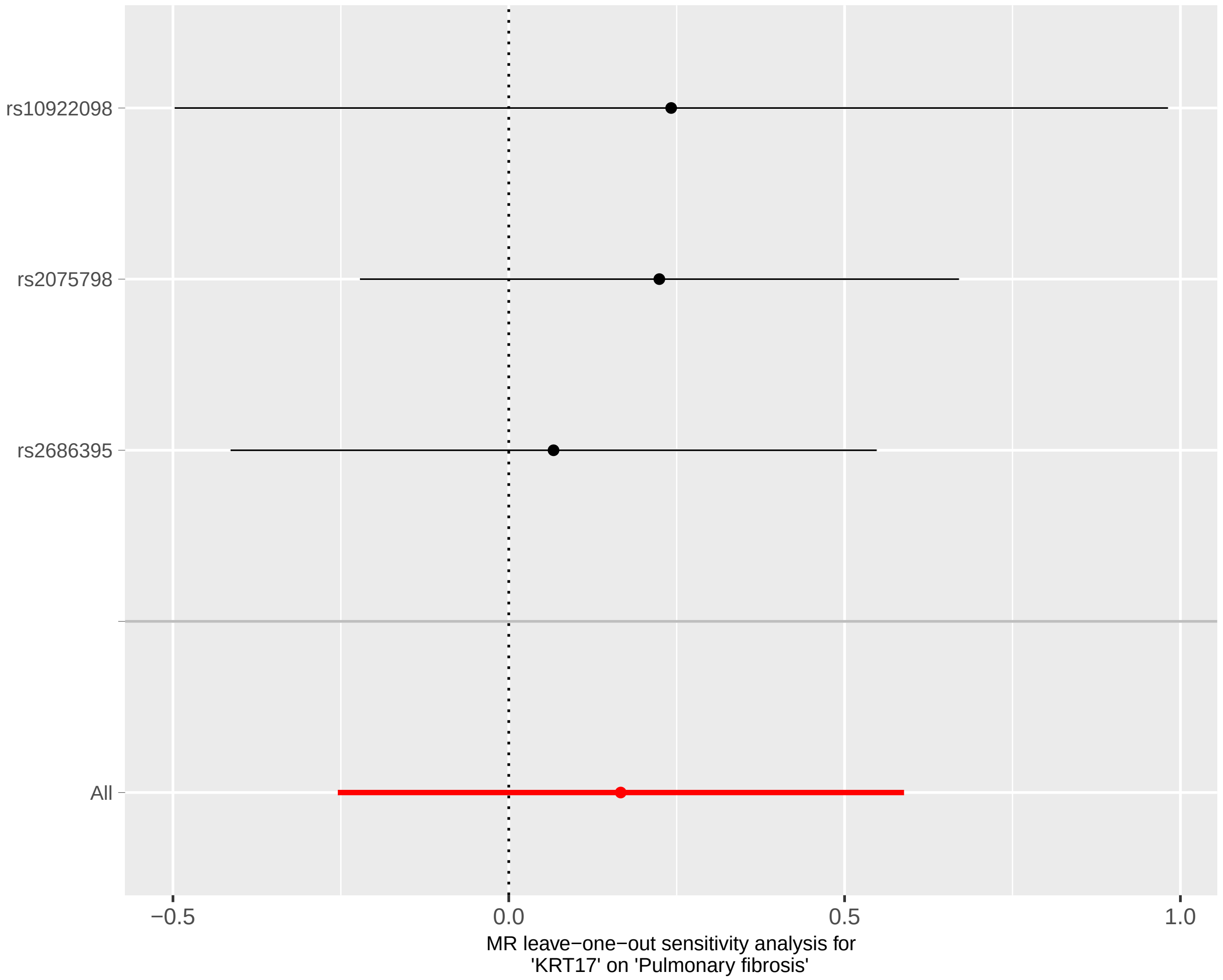
rs10922098
rs2075798
rs2686395
All
−0.5
0.0
0.5
1.0
MR leave−one−out sensitivity analysis for
'KRT17' on 'Pulmonary fibrosis'

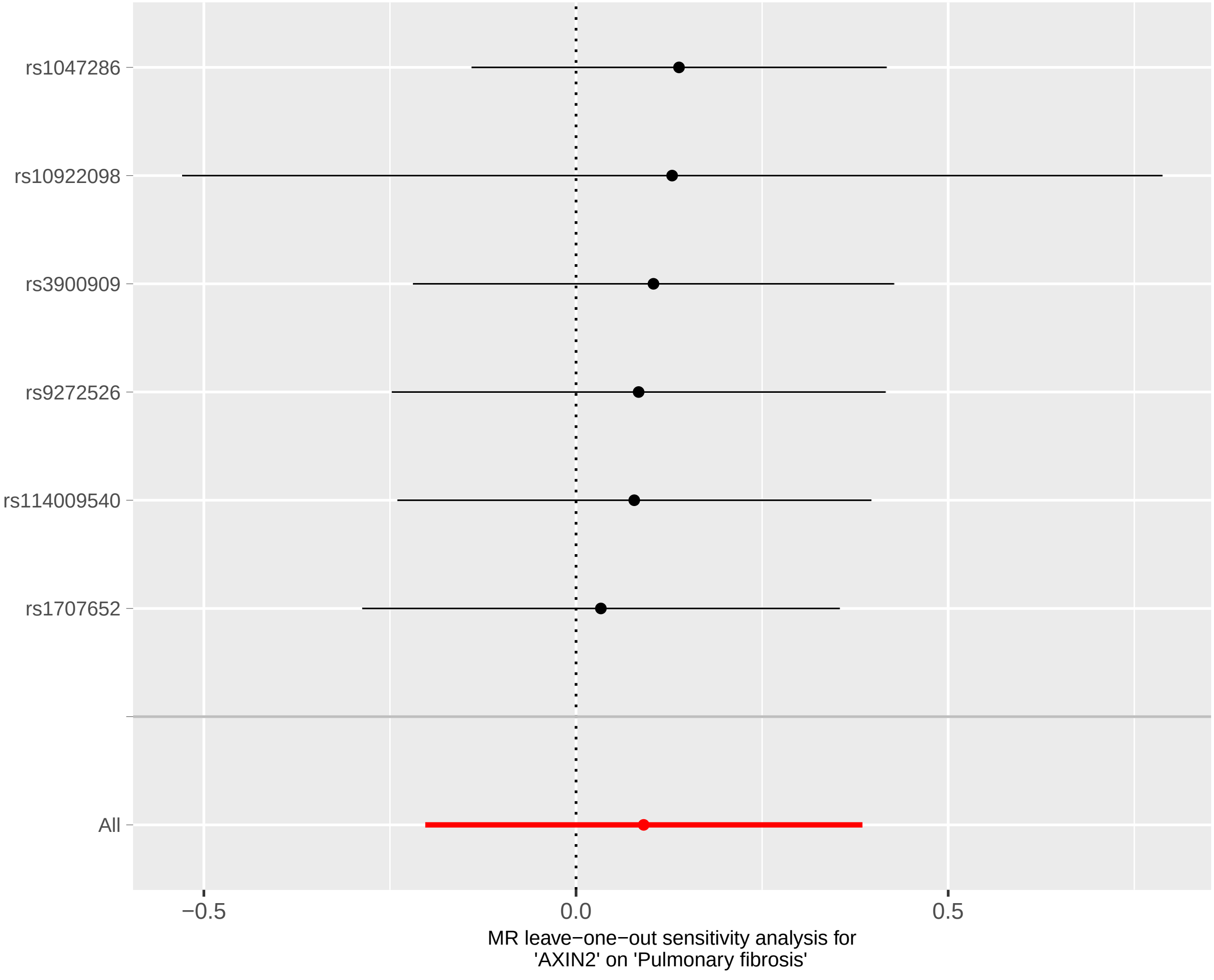

rs1047286
rs10922098
rs3900909
rs9272526
rs114009540
rs1707652
All
−0.5
0.0
0.5
MR leave−one−out sensitivity analysis for
'AXIN2' on 'Pulmonary fibrosis'

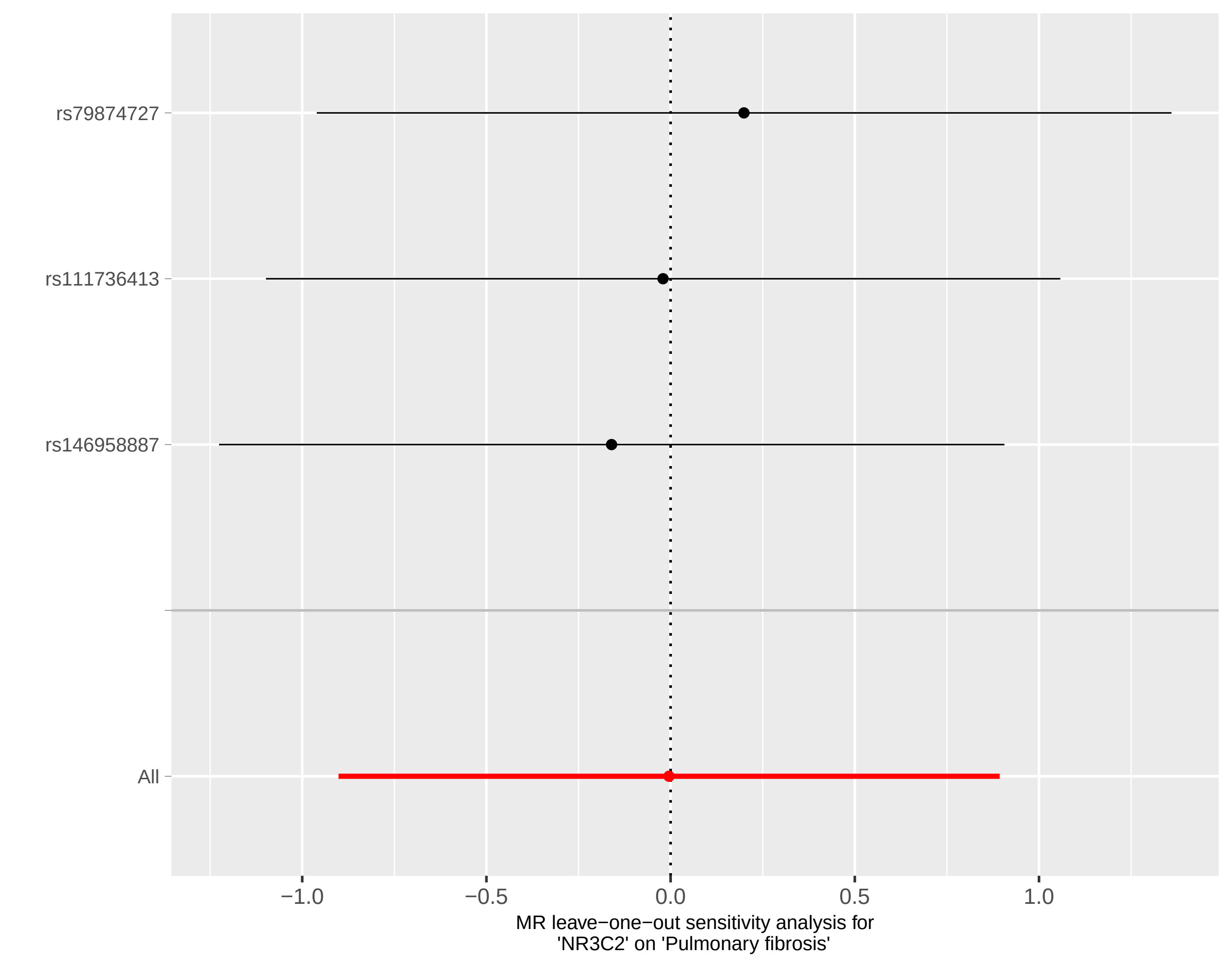

rs79874727
rs111736413
rs146958887
All
−1.0
−0.5
0.0
0.5
1.0
MR leave−one−out sensitivity analysis for
'NR3C2' on 'Pulmonary fibrosis'

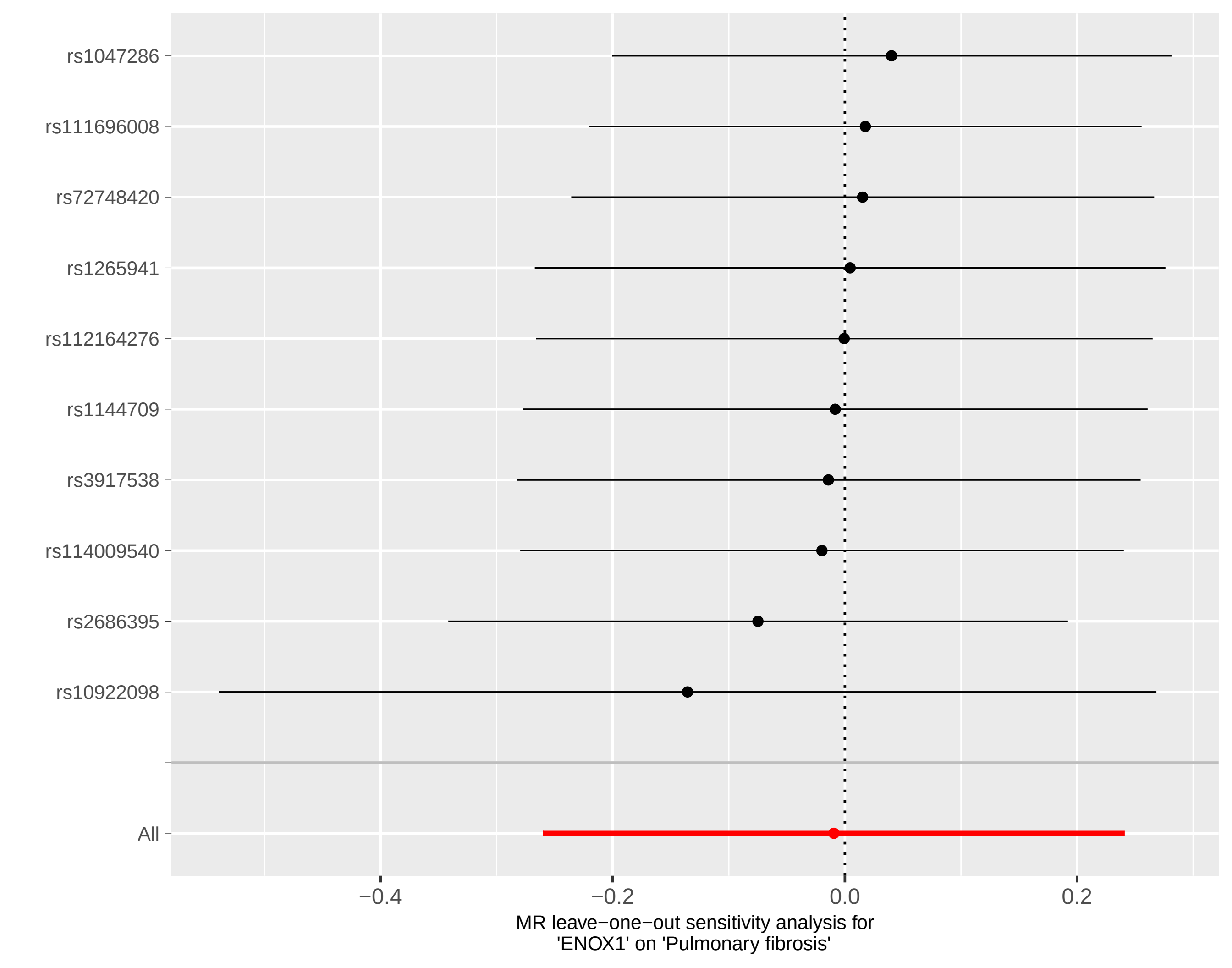
rs1047286
rs111696008
rs72748420
rs1265941
rs112164276
rs1144709
rs3917538
rs114009540
rs2686395
rs10922098
All
−0.4
−0.2
0.0
0.2
MR leave−one−out sensitivity analysis for
'ENOX1' on 'Pulmonary fibrosis'

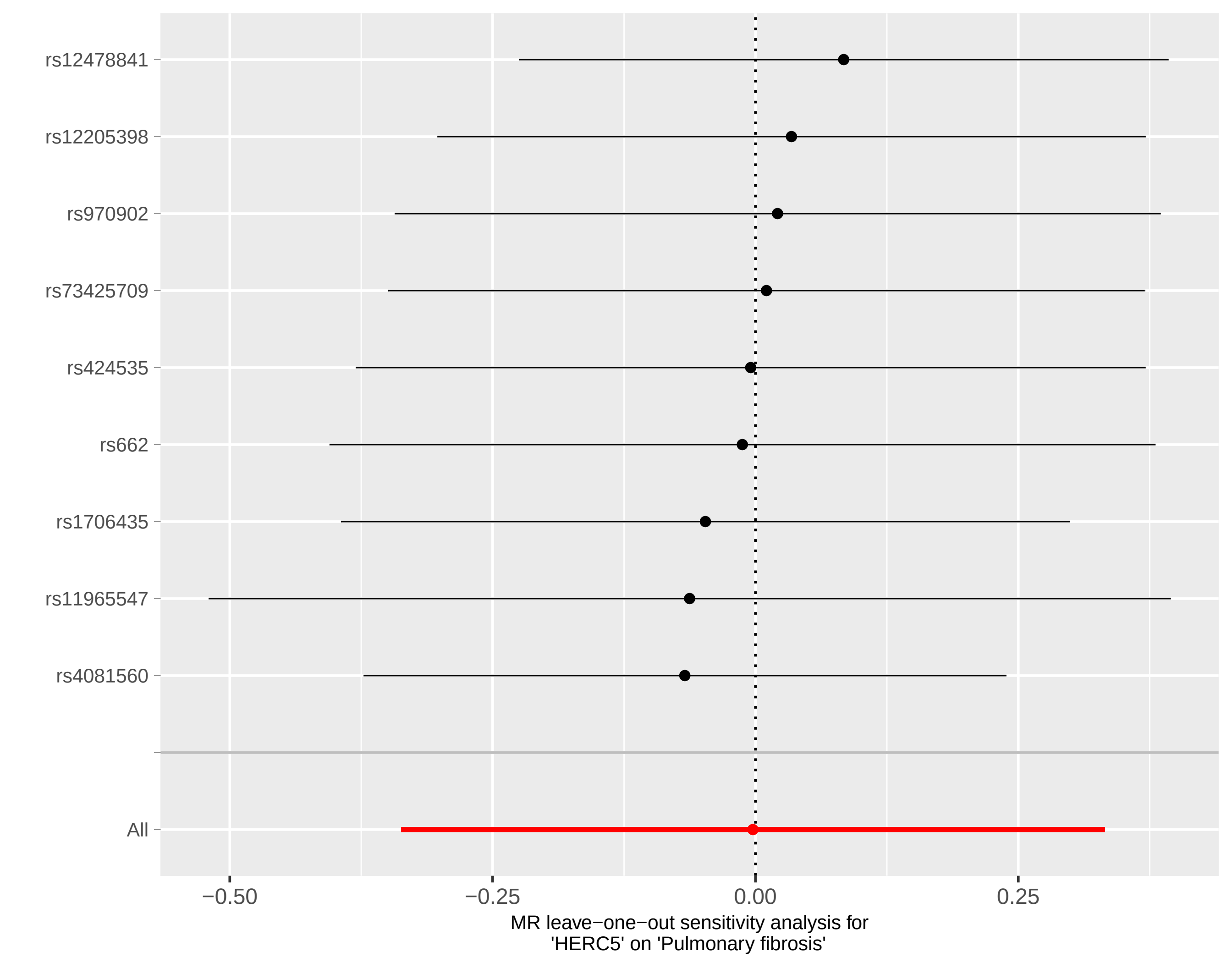

rs12478841
rs12205398
rs970902
rs73425709
rs424535
rs662
rs1706435
rs11965547
rs4081560
All
−0.50
−0.25
0.00
0.25
MR leave−one−out sensitivity analysis for
'HERC5' on 'Pulmonary fibrosis'

Insufficient number of SNPs

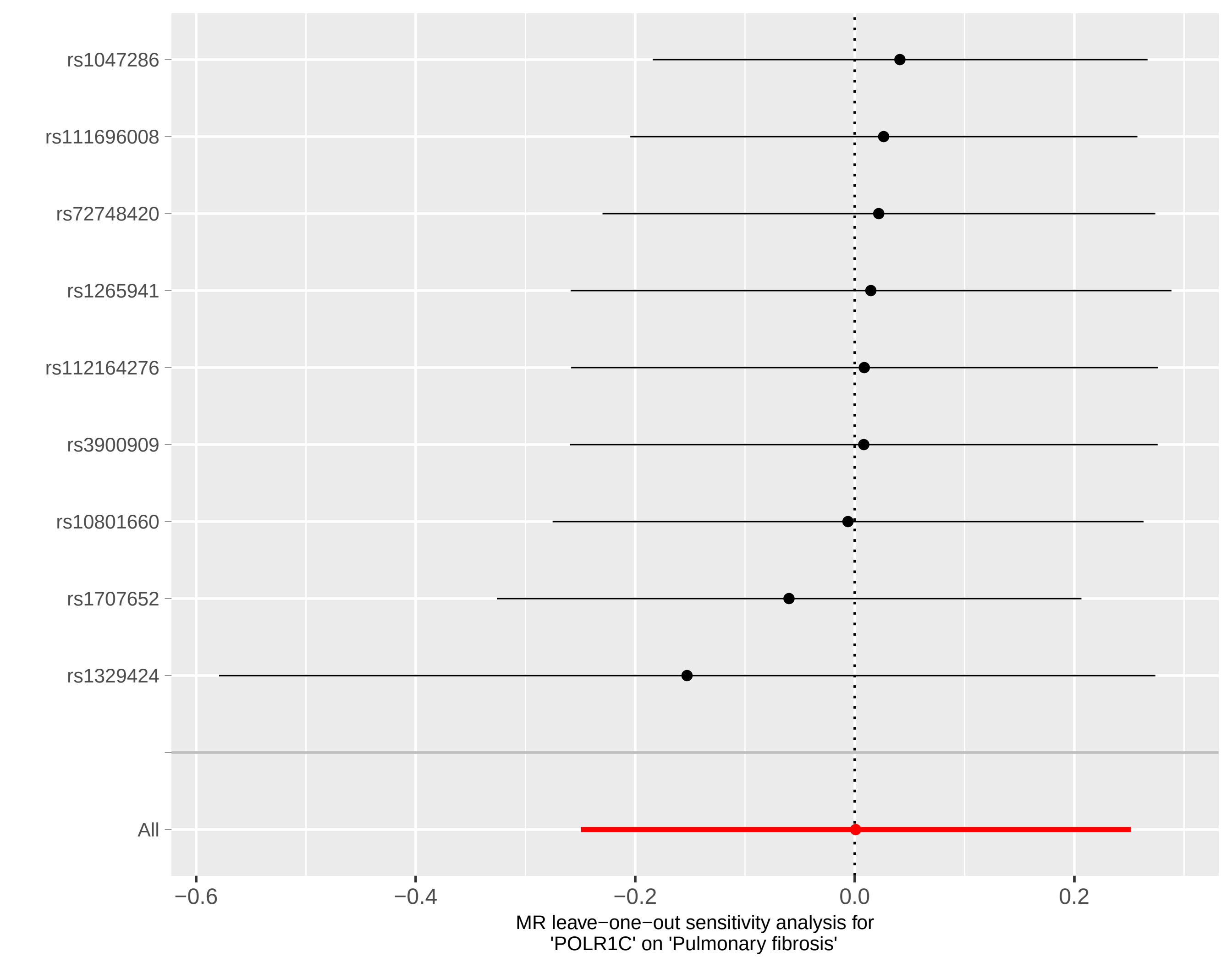

rs1047286
rs111696008
rs72748420
rs1265941
rs112164276
rs3900909
rs10801660
rs1707652
rs1329424
All
−0.6
−0.4
−0.2
0.0
0.2
MR leave−one−out sensitivity analysis for
'POLR1C' on 'Pulmonary fibrosis'

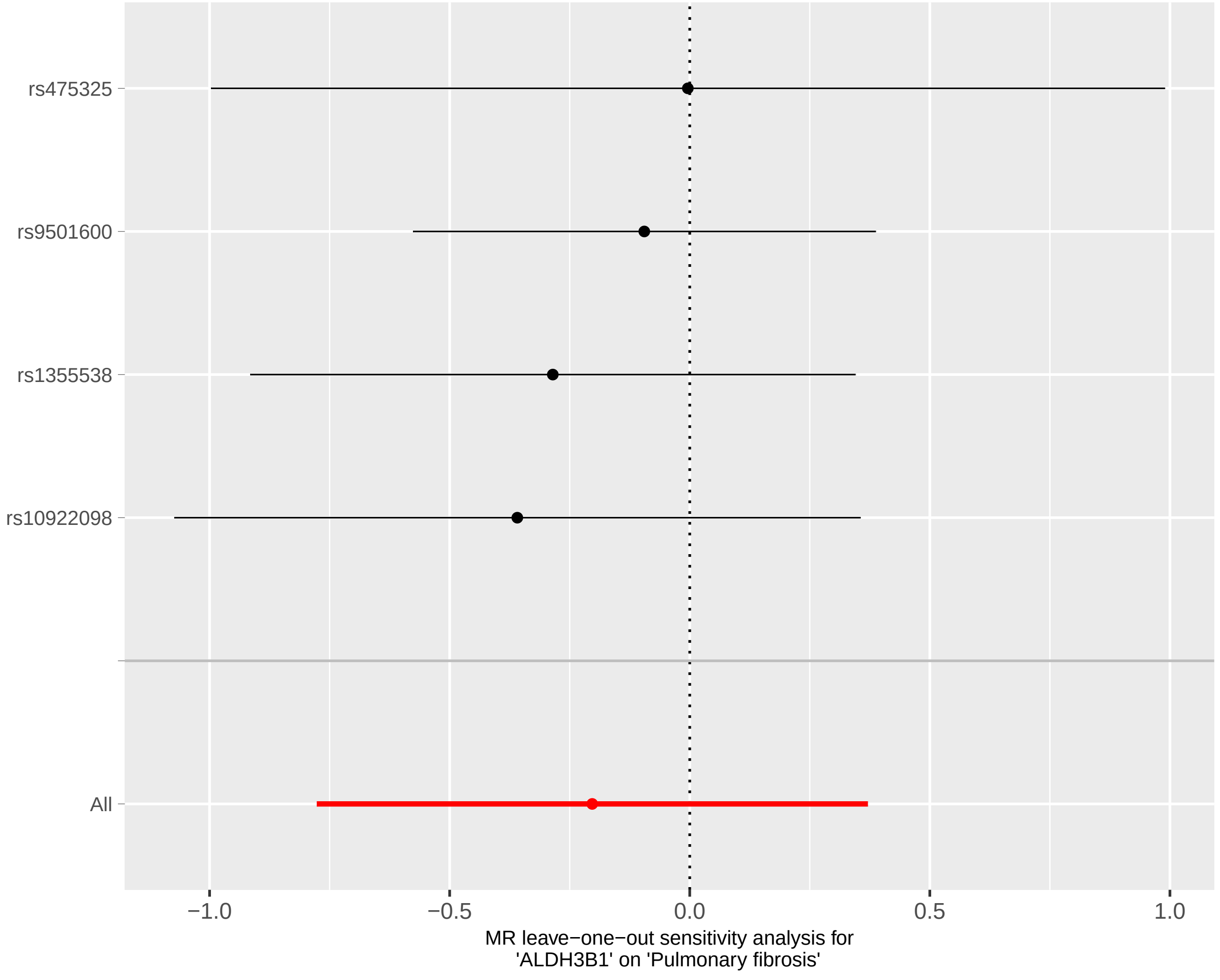

rs475325
rs9501600
rs1355538
rs10922098
All
−1.0
−0.5
0.0
0.5
1.0
MR leave−one−out sensitivity analysis for
'ALDH3B1' on 'Pulmonary fibrosis'

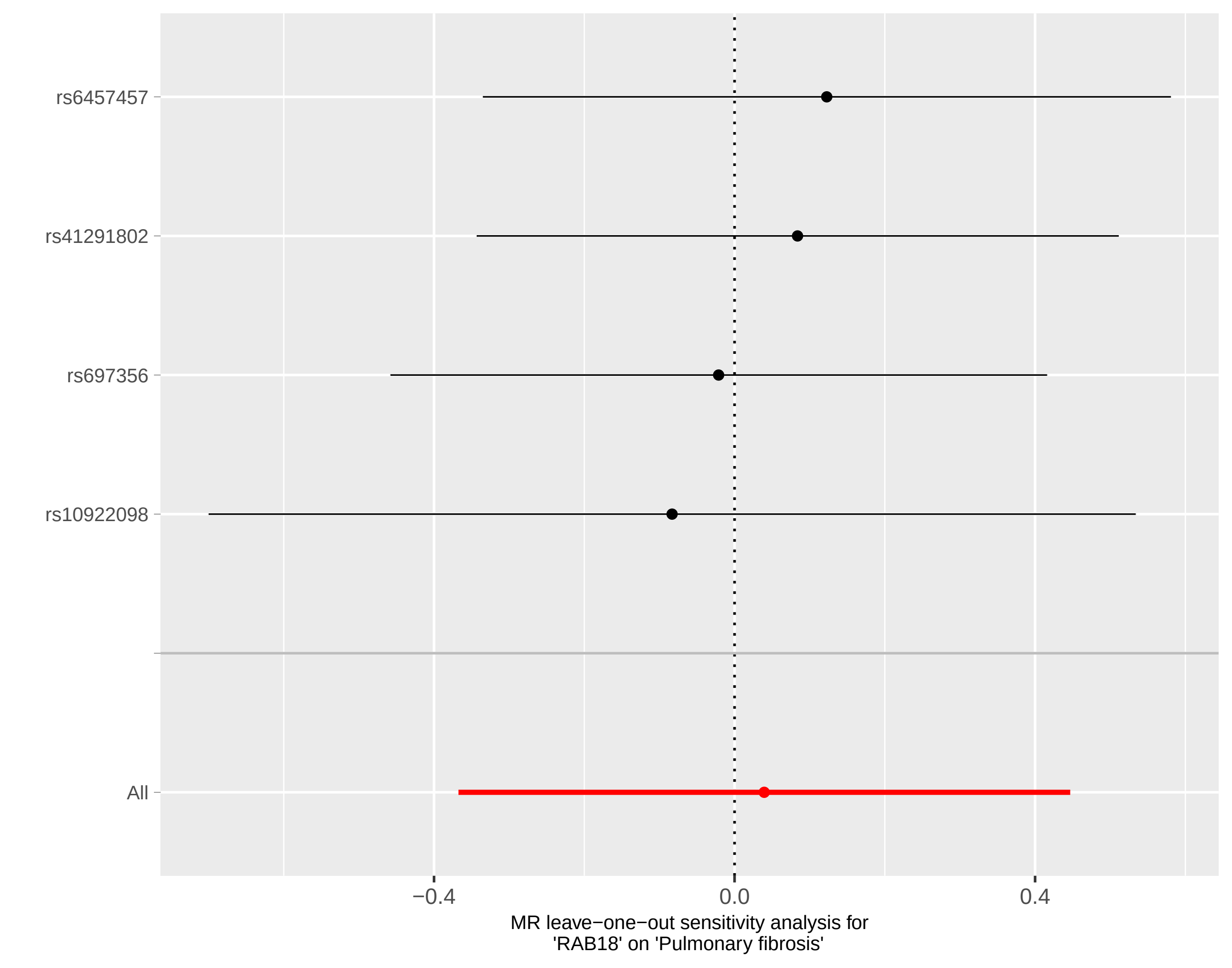
rs6457457
rs41291802
rs697356
rs10922098
All
−0.4
0.0
0.4
MR leave−one−out sensitivity analysis for
'RAB18' on 'Pulmonary fibrosis'

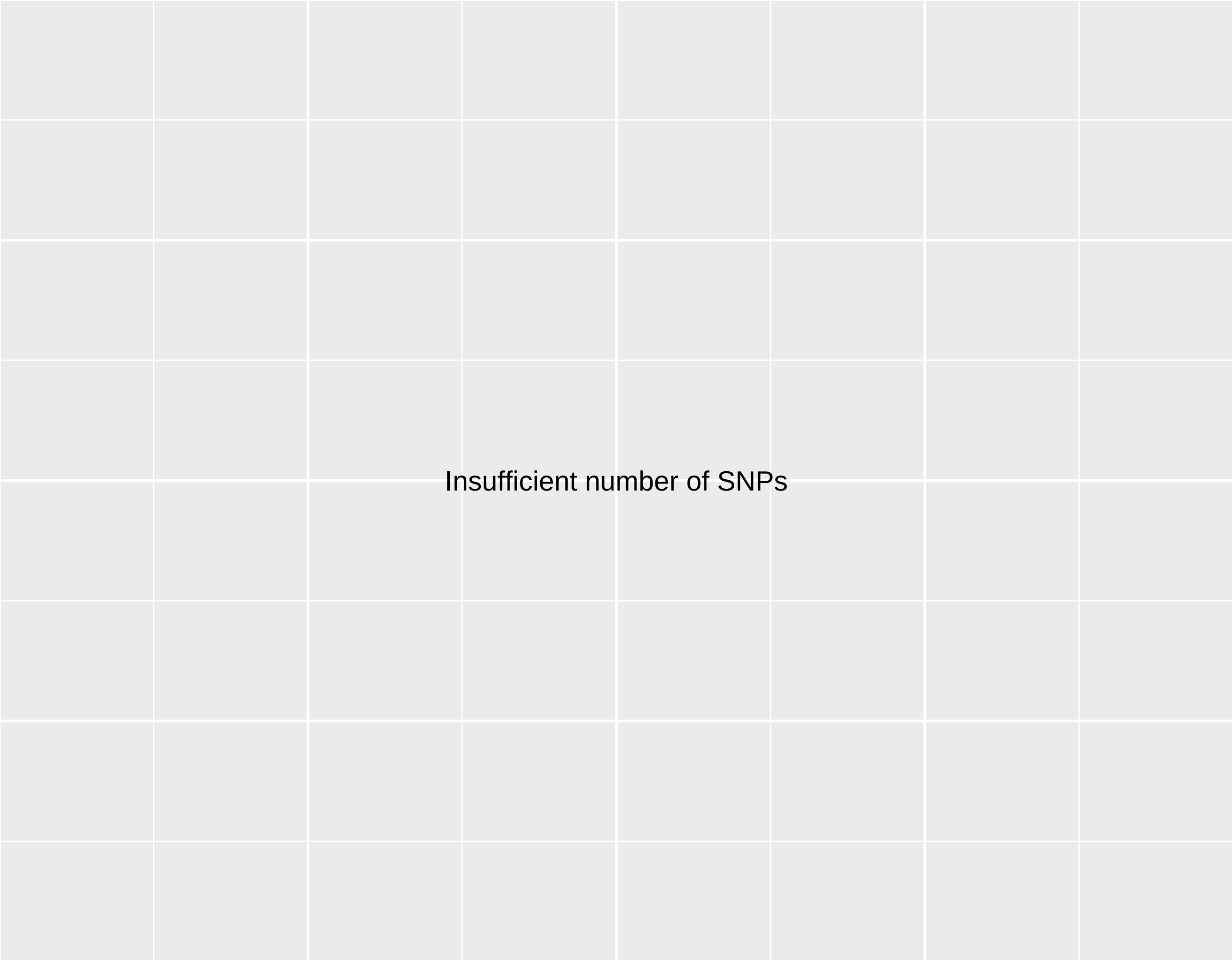
Insufficient number of SNPs

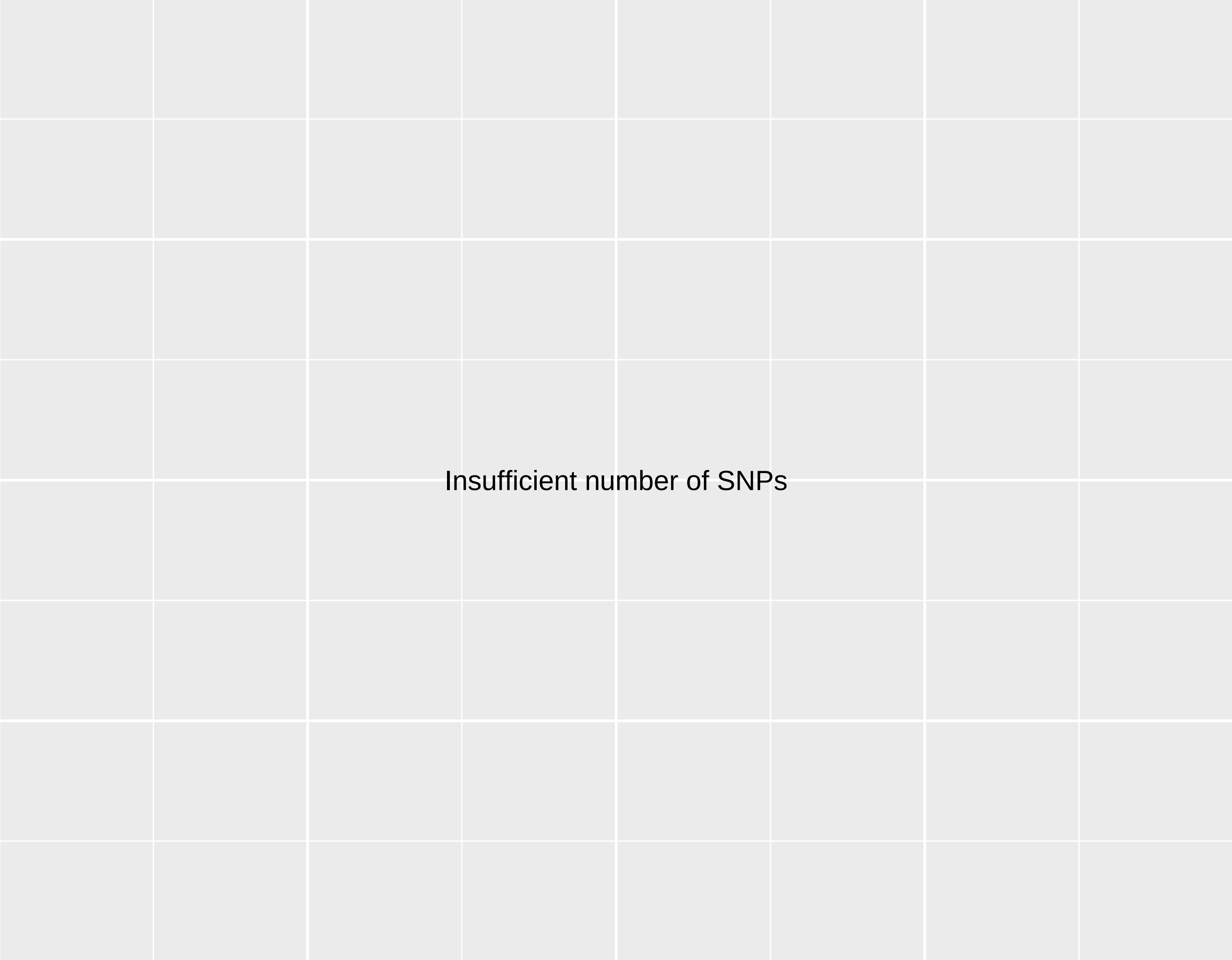
Insufficient number of SNPs

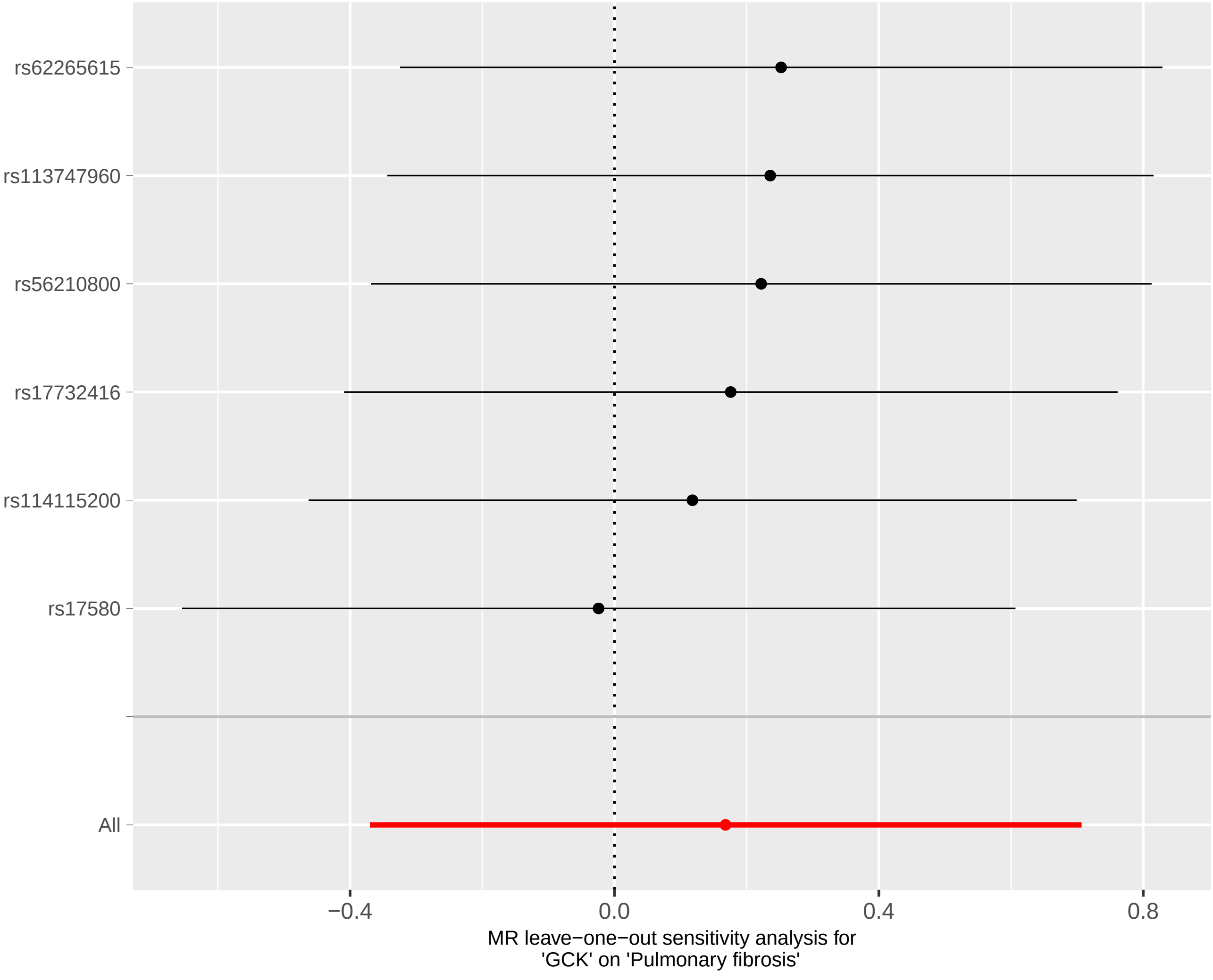

rs62265615
rs113747960
rs56210800
rs17732416
rs114115200
rs17580
All
−0.4
0.0
0.4
0.8
MR leave−one−out sensitivity analysis for
'GCK' on 'Pulmonary fibrosis'

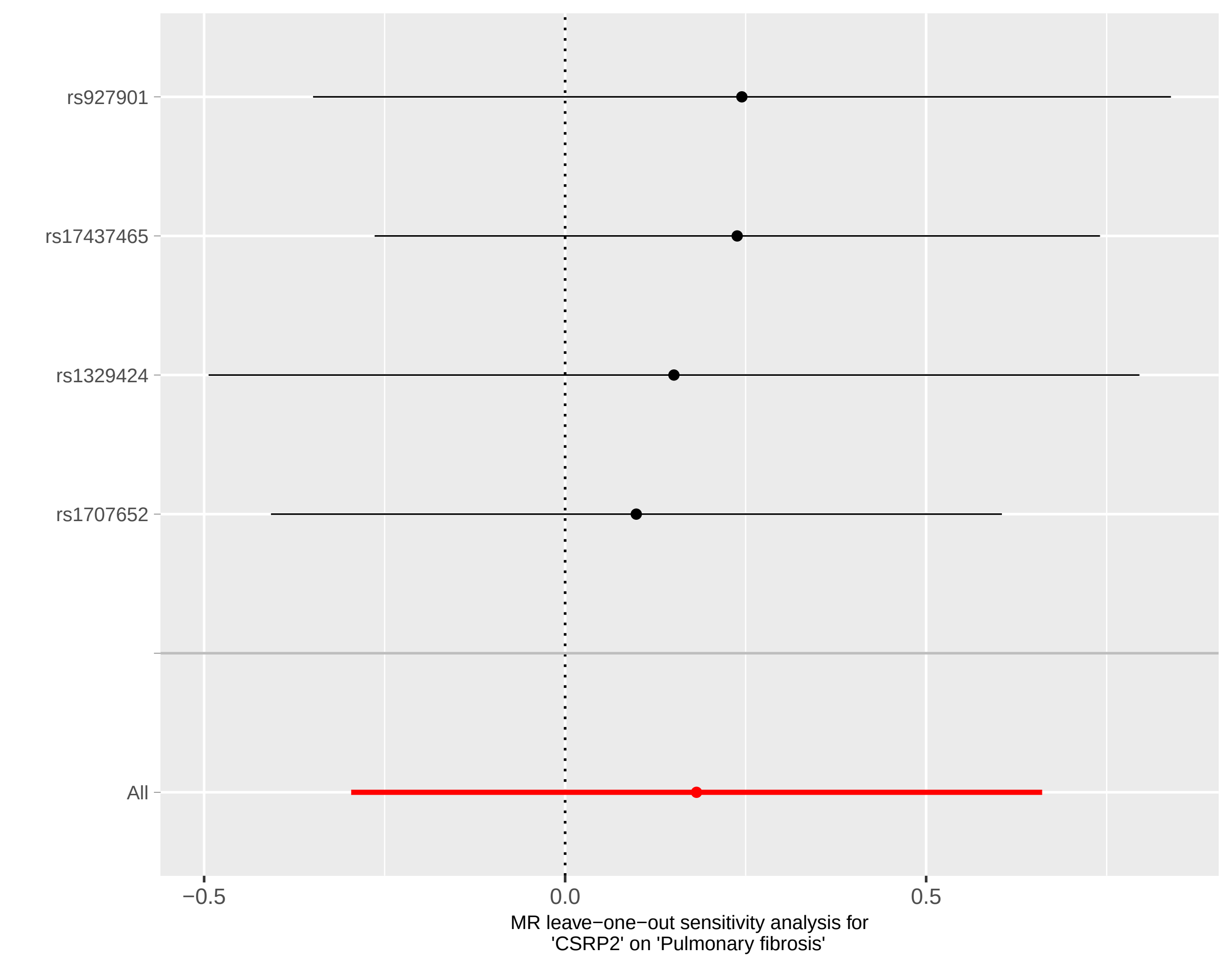
rs927901
rs17437465
rs1329424
rs1707652
All
−0.5
0.0
0.5
MR leave−one−out sensitivity analysis for
'CSRP2' on 'Pulmonary fibrosis'

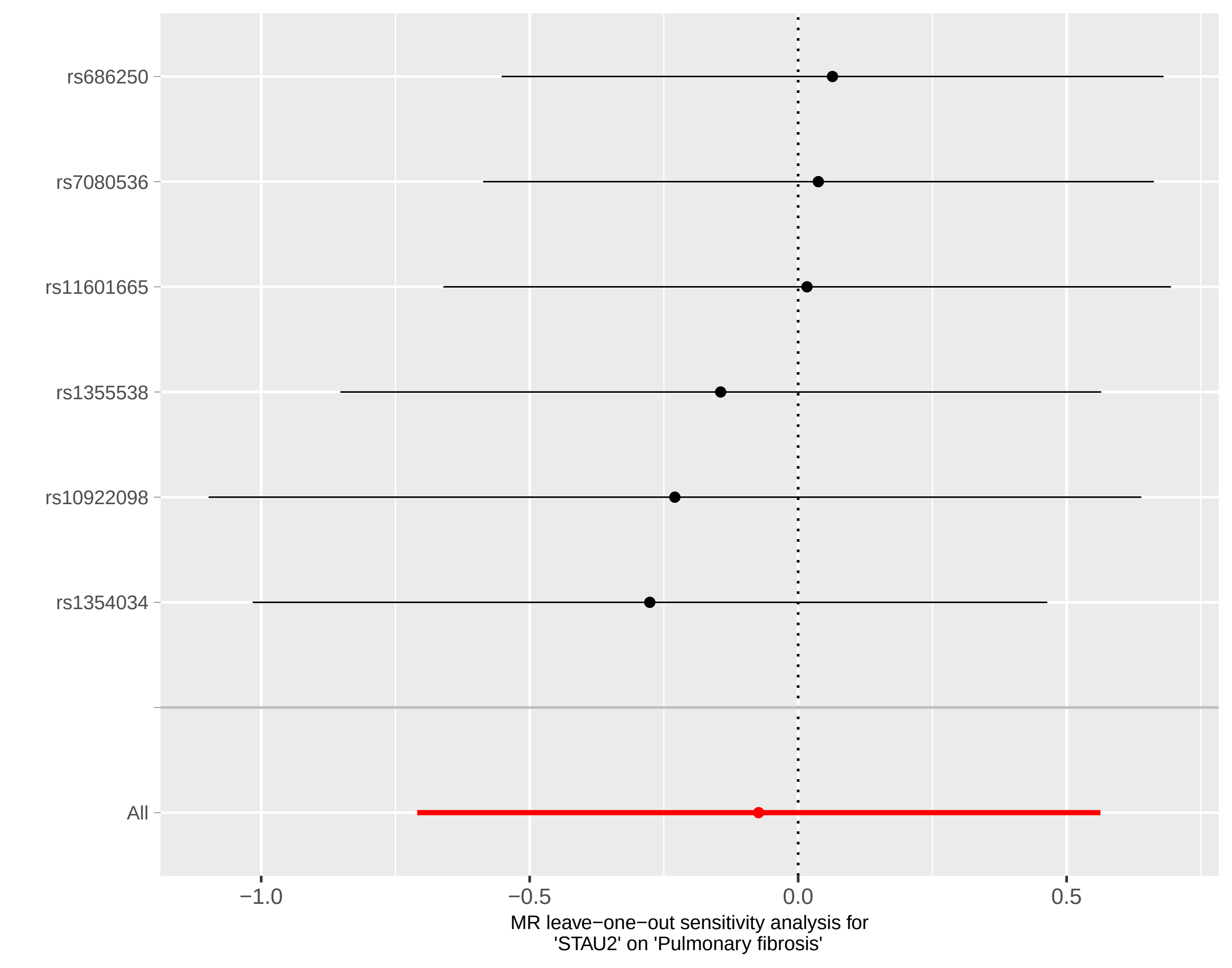
rs686250
rs7080536
rs11601665
rs1355538
rs10922098
rs1354034
All
−1.0
−0.5
0.0
0.5
MR leave−one−out sensitivity analysis for
'STAU2' on 'Pulmonary fibrosis'

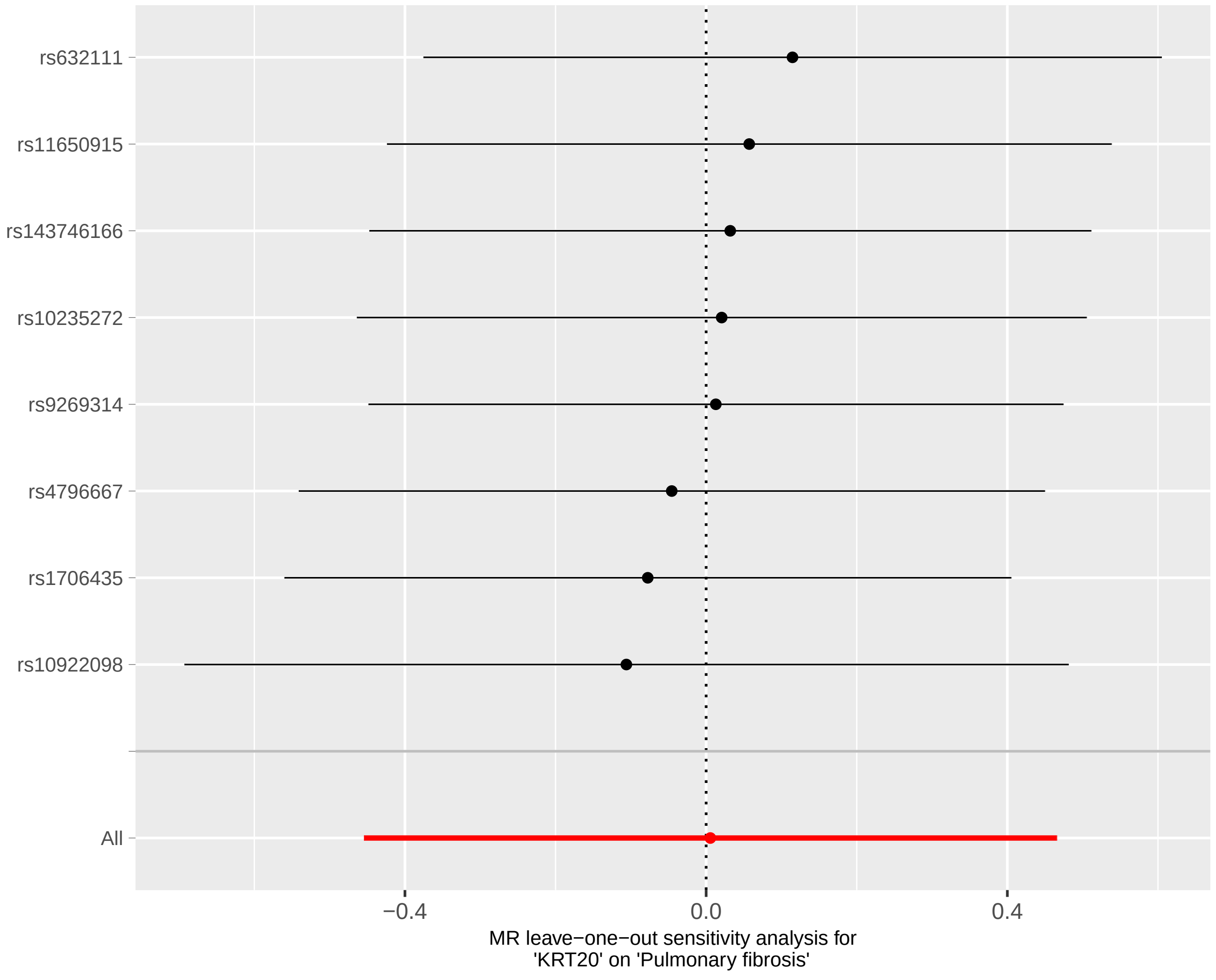
rs632111
rs11650915
rs143746166
rs10235272
rs9269314
rs4796667
rs1706435
rs10922098
All
−0.4
0.0
0.4
MR leave−one−out sensitivity analysis for
'KRT20' on 'Pulmonary fibrosis'

Insufficient number of SNPs

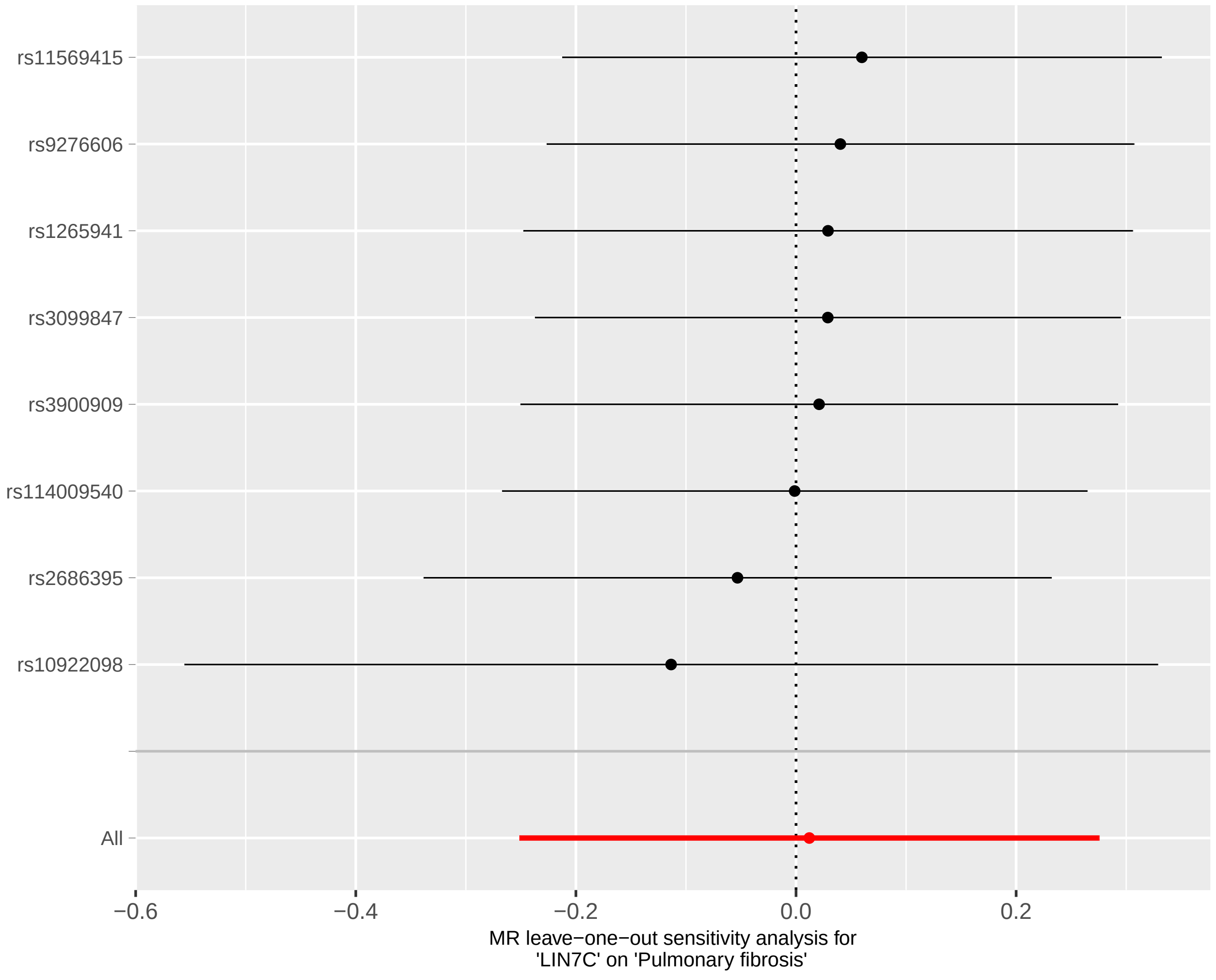
rs11569415
rs9276606
rs1265941
rs3099847
rs3900909
rs114009540
rs2686395
rs10922098
All
−0.6
−0.4
−0.2
0.0
0.2
MR leave−one−out sensitivity analysis for
'LIN7C' on 'Pulmonary fibrosis'

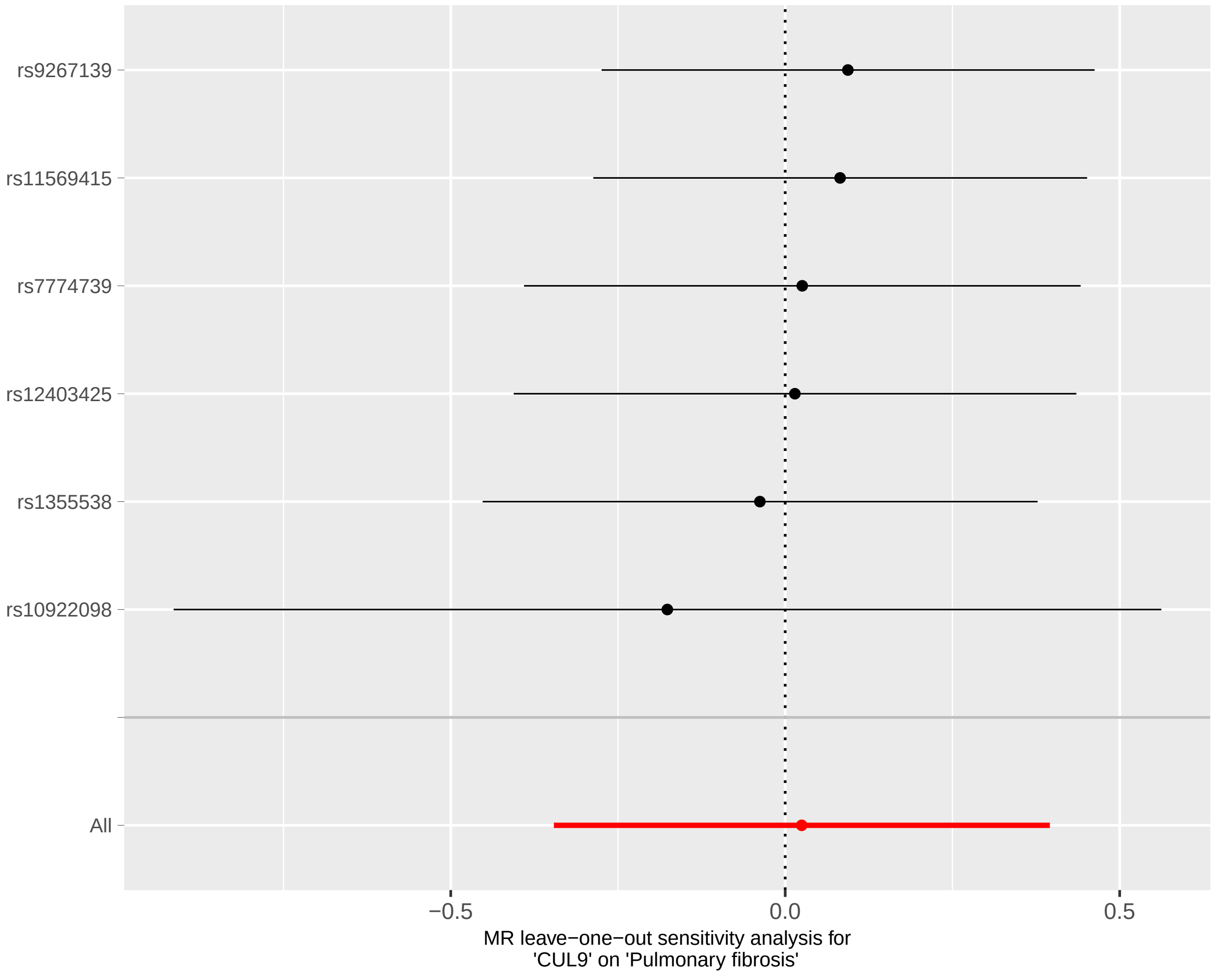
rs9267139
rs11569415
rs7774739
rs12403425
rs1355538
rs10922098
All
−0.5
0.0
0.5
MR leave−one−out sensitivity analysis for
'CUL9' on 'Pulmonary fibrosis'

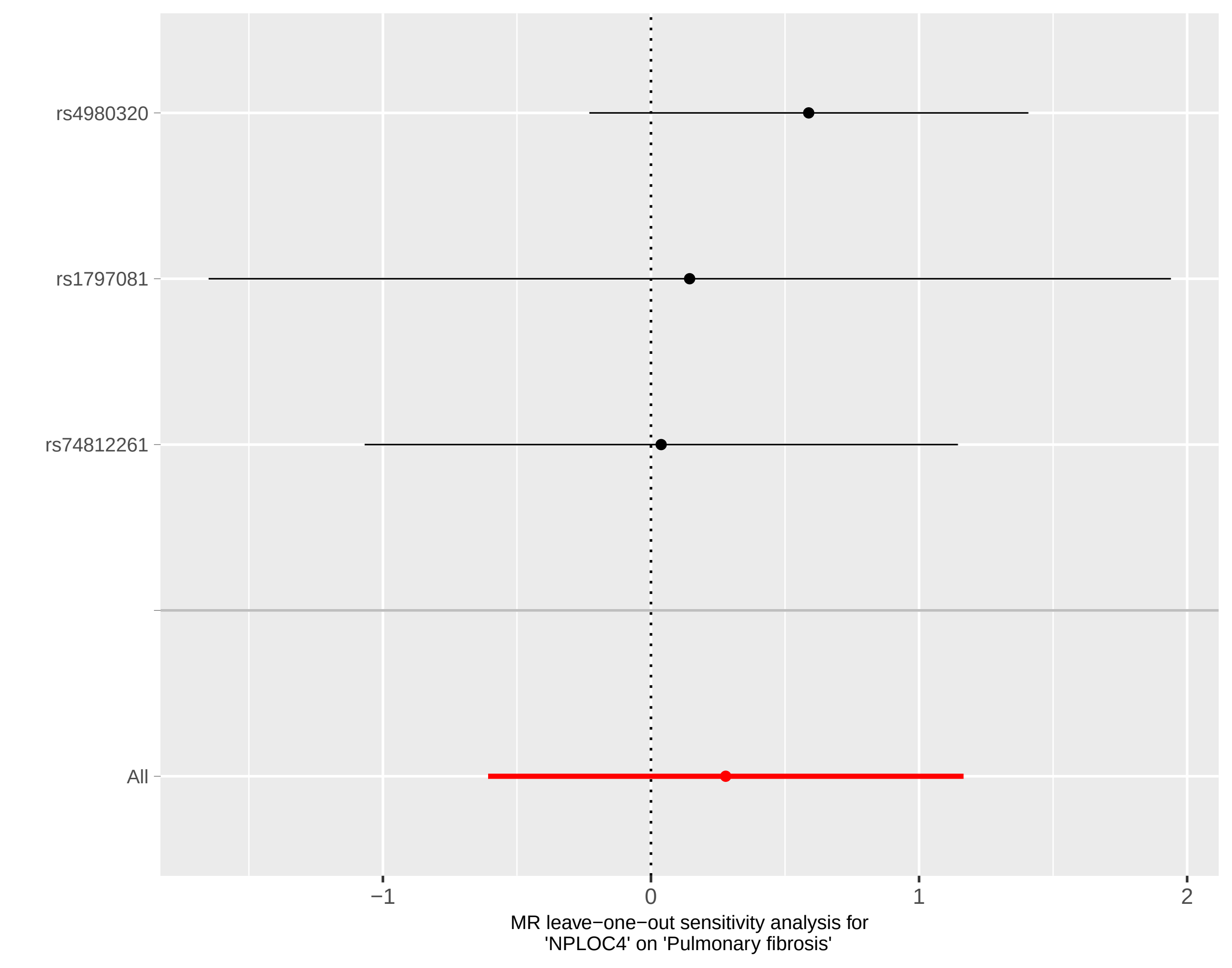
rs4980320
rs1797081
rs74812261
All
−1
0
1
2
MR leave−one−out sensitivity analysis for
'NPLOC4' on 'Pulmonary fibrosis'

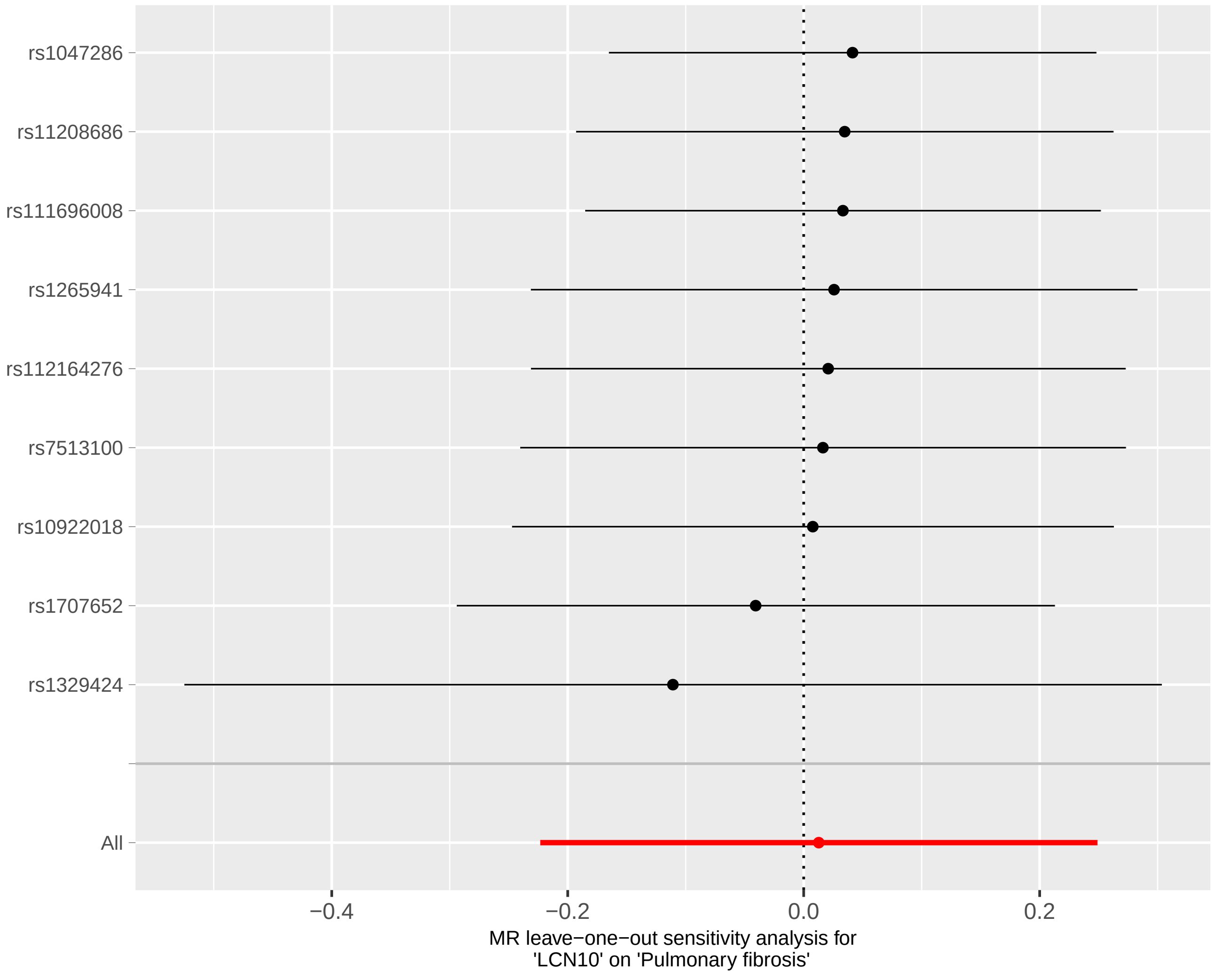
rs1047286
rs11208686
rs111696008
rs1265941
rs112164276
rs7513100
rs10922018
rs1707652
rs1329424
All
−0.4
−0.2
0.0
0.2
MR leave−one−out sensitivity analysis for
'LCN10' on 'Pulmonary fibrosis'

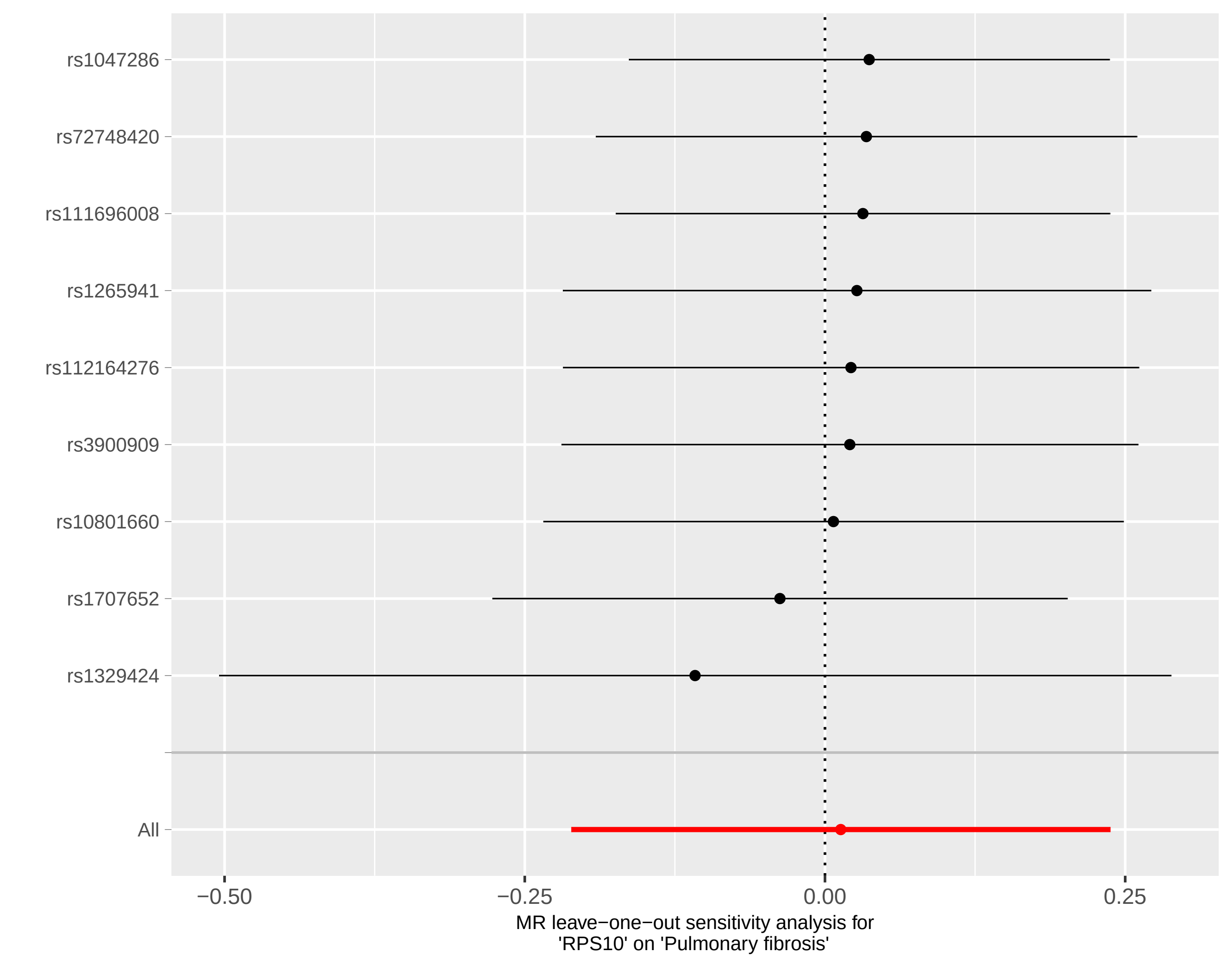
rs1047286
rs72748420
rs111696008
rs1265941
rs112164276
rs3900909
rs10801660
rs1707652
rs1329424
All
−0.50
−0.25
0.00
0.25
MR leave−one−out sensitivity analysis for
'RPS10' on 'Pulmonary fibrosis'

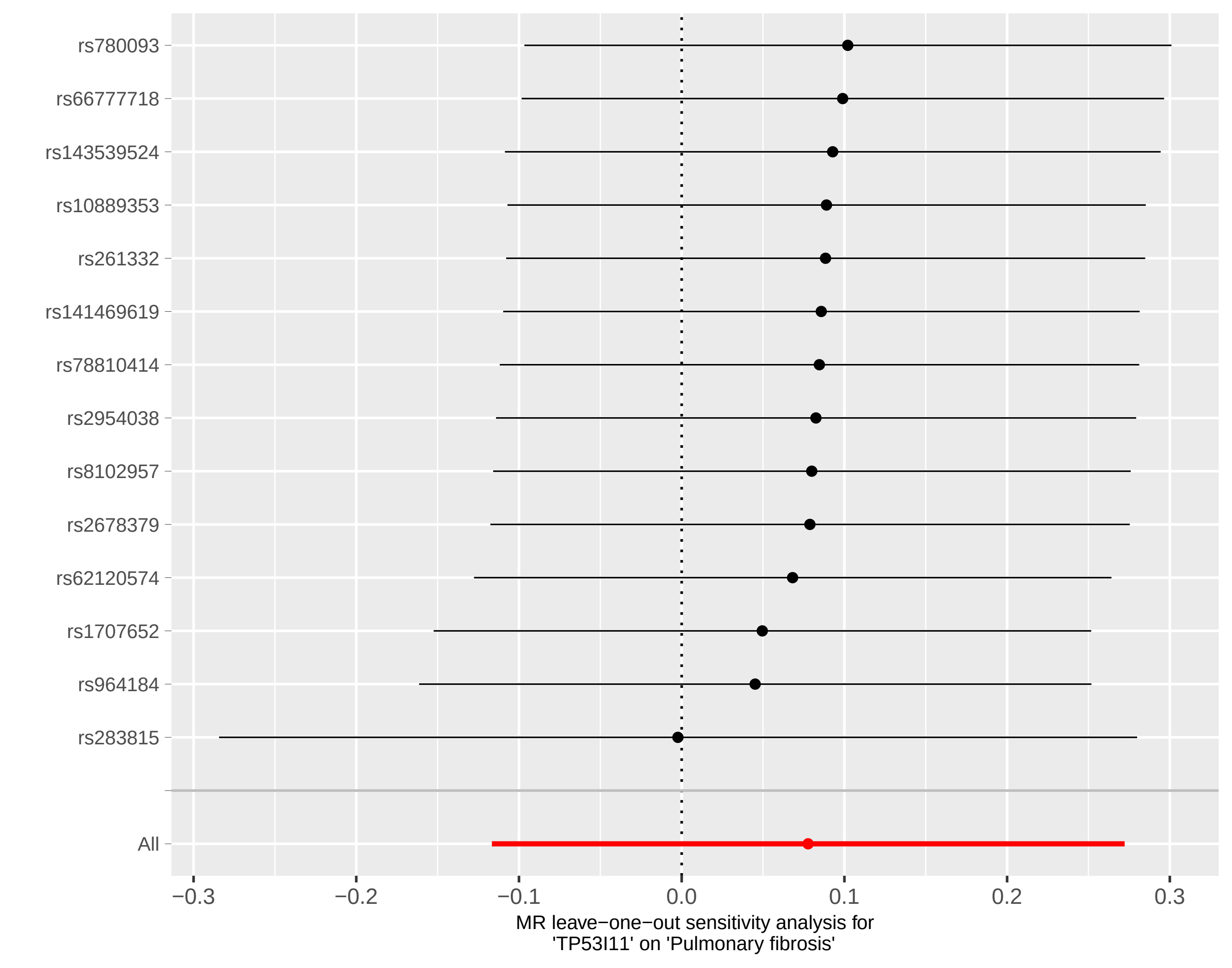
rs780093
rs66777718
rs143539524
rs10889353
rs261332
rs141469619
rs78810414
rs2954038
rs8102957
rs2678379
rs62120574
rs1707652
rs964184
rs283815
All
−0.3
−0.2
−0.1
0.0
0.1
0.2
0.3
MR leave−one−out sensitivity analysis for
'TP53I11' on 'Pulmonary fibrosis'

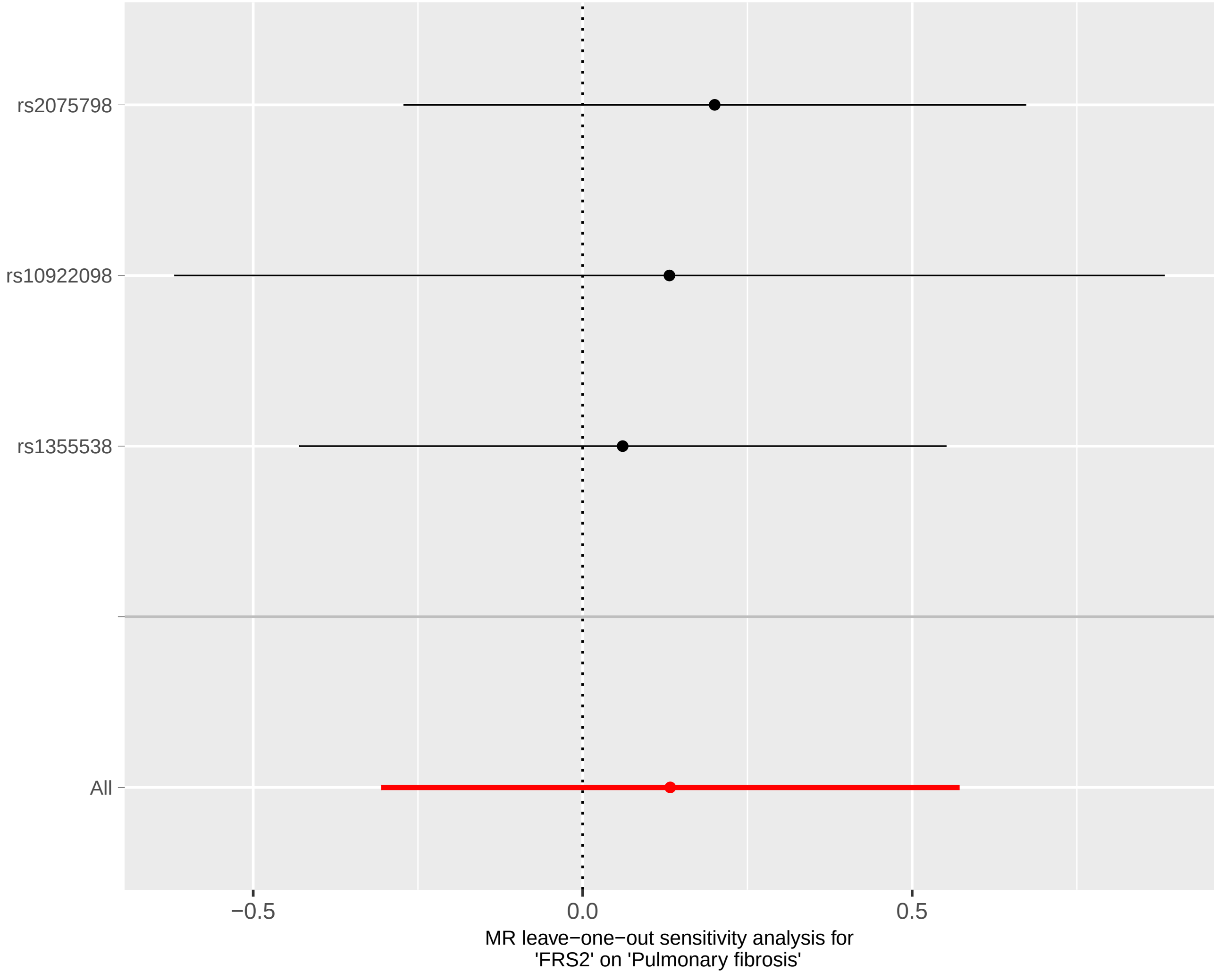
rs2075798
rs10922098
rs1355538
All
−0.5
0.0
0.5
MR leave−one−out sensitivity analysis for
'FRS2' on 'Pulmonary fibrosis'

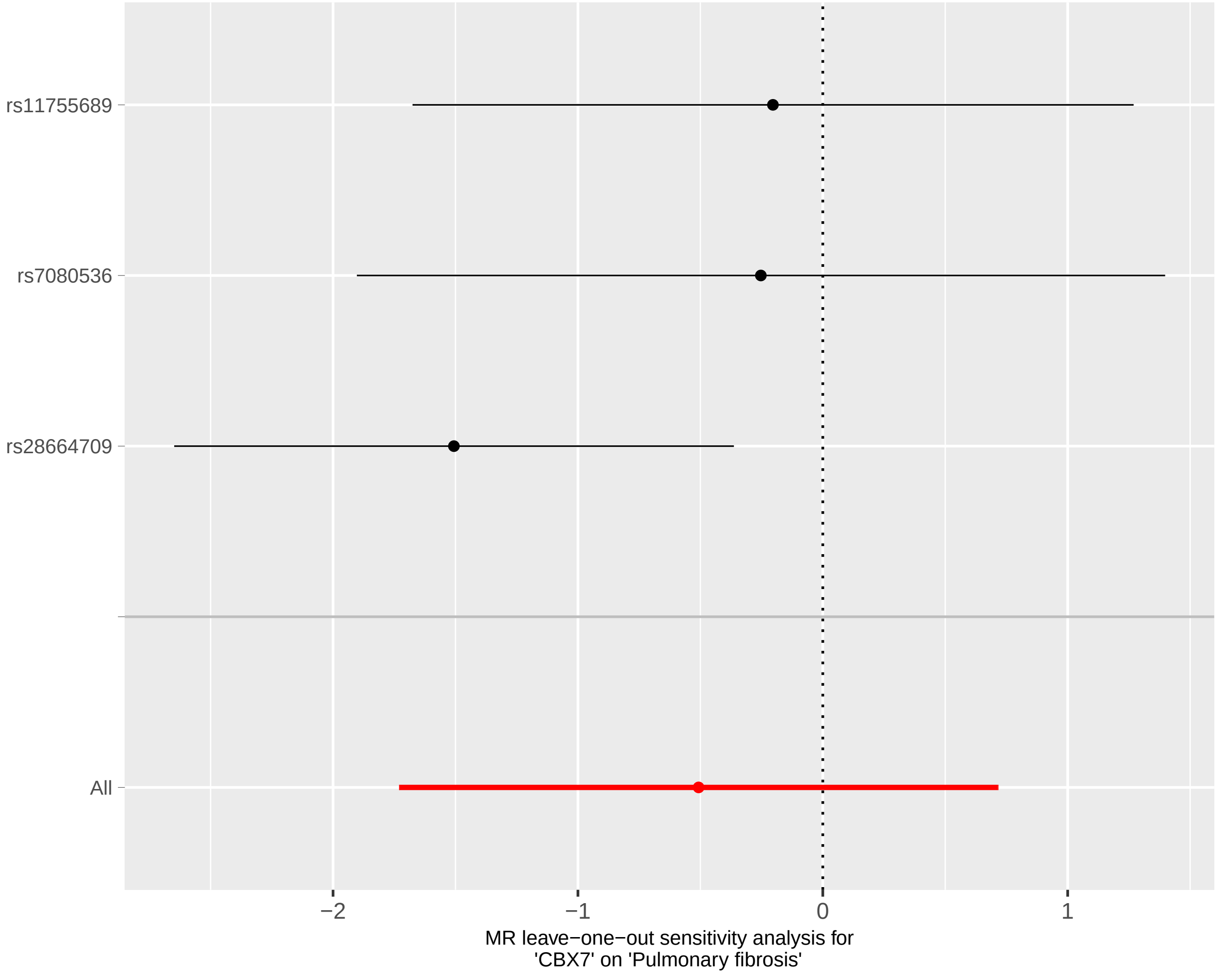
rs11755689
rs7080536
rs28664709
All
−2
−1
0
1
MR leave−one−out sensitivity analysis for
'CBX7' on 'Pulmonary fibrosis'

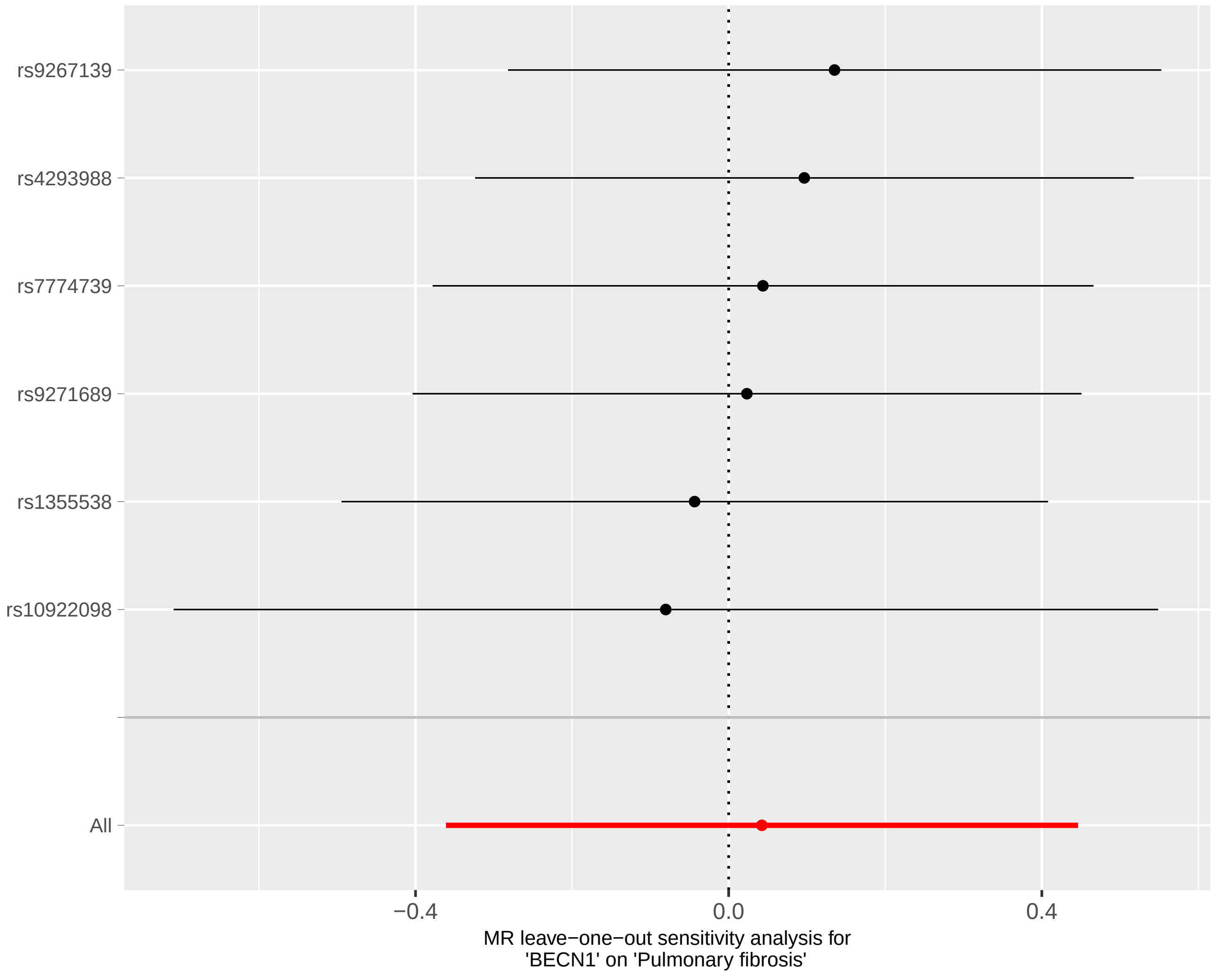
rs9267139
rs4293988
rs7774739
rs9271689
rs1355538
rs10922098
All
−0.4
0.0
0.4
MR leave−one−out sensitivity analysis for
'BECN1' on 'Pulmonary fibrosis'

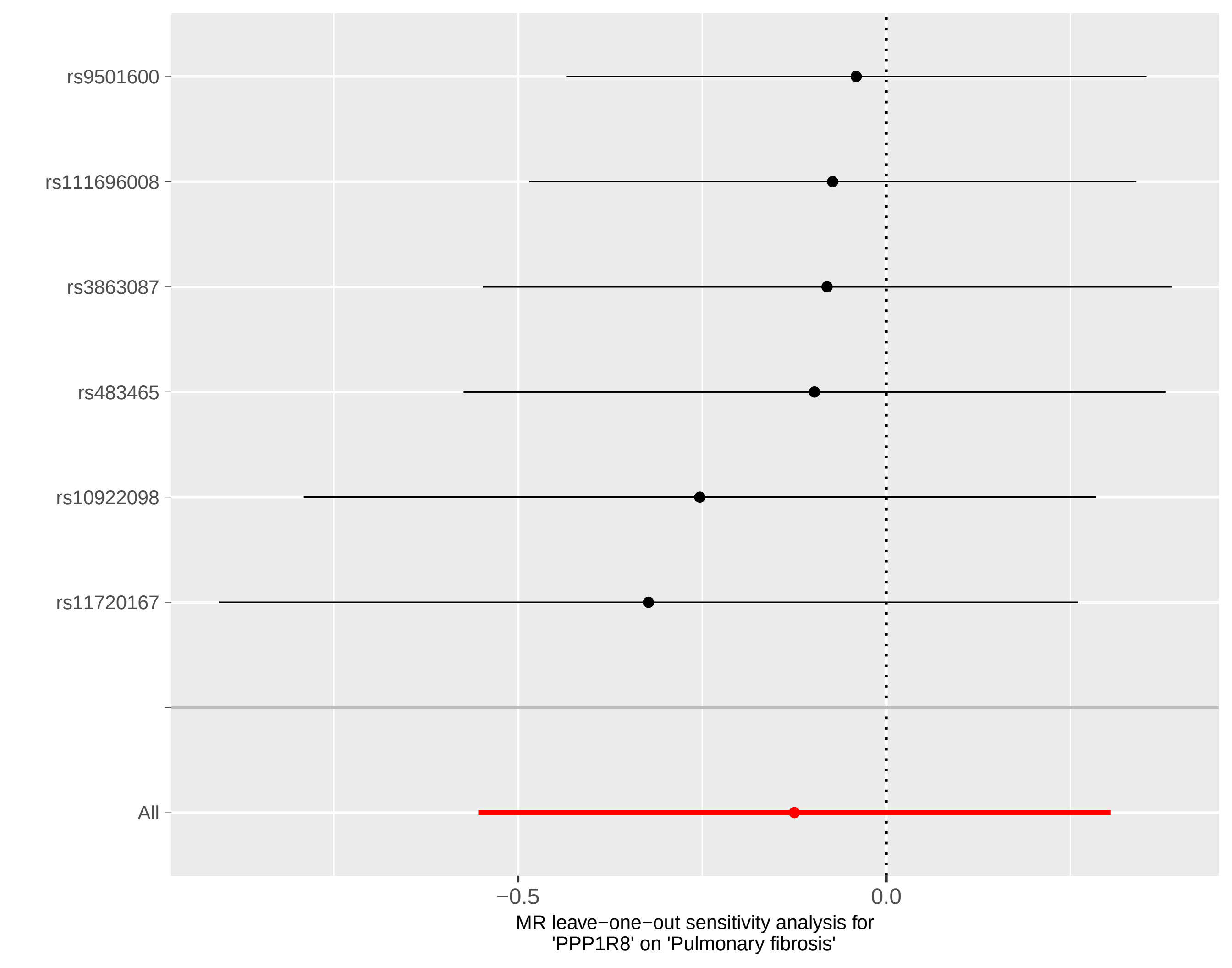
rs9501600
rs111696008
rs3863087
rs483465
rs10922098
rs11720167
All
−0.5
0.0
MR leave−one−out sensitivity analysis for
'PPP1R8' on 'Pulmonary fibrosis'

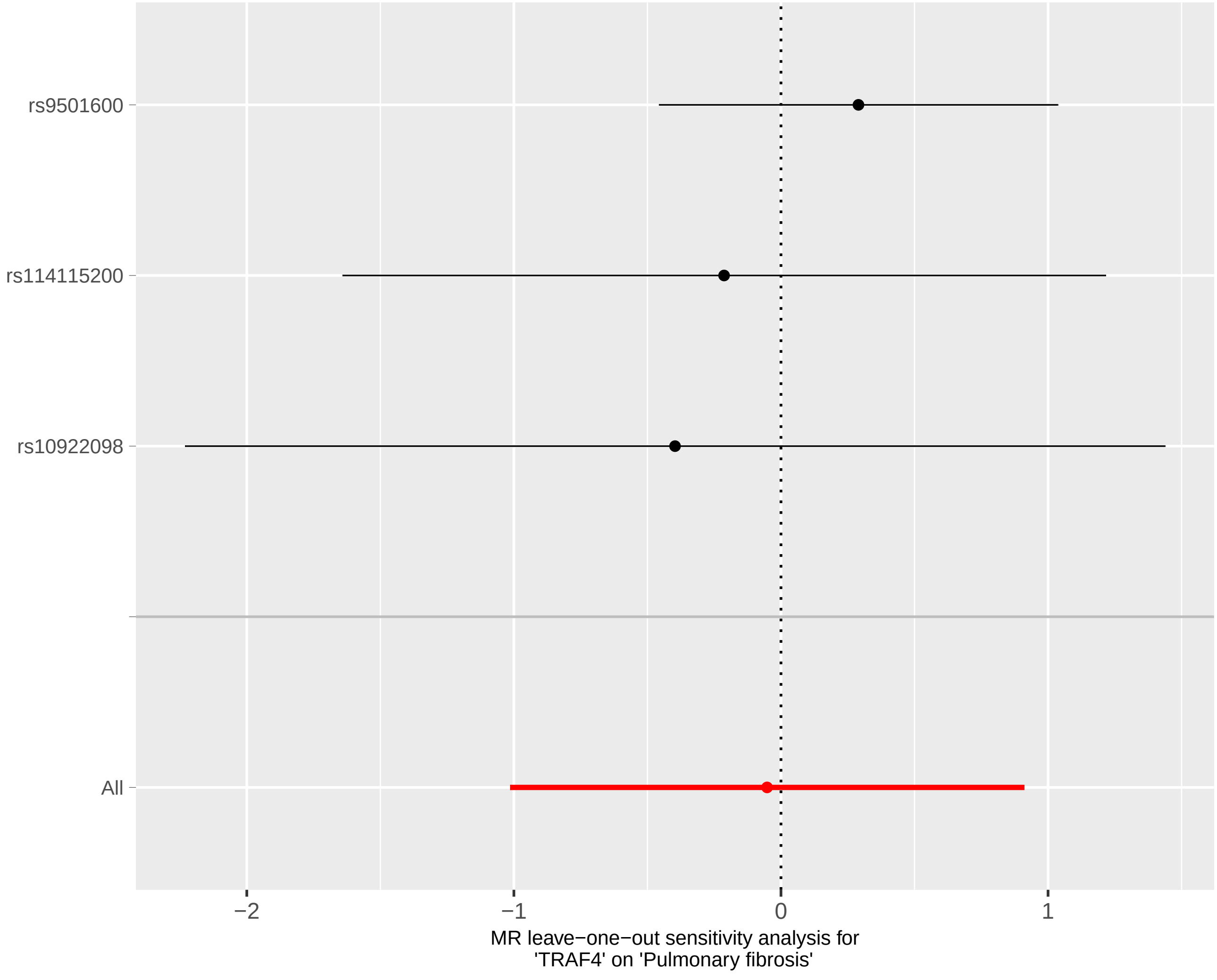

rs9501600
rs114115200
rs10922098
All
−2
−1
0
1
MR leave−one−out sensitivity analysis for
'TRAF4' on 'Pulmonary fibrosis'

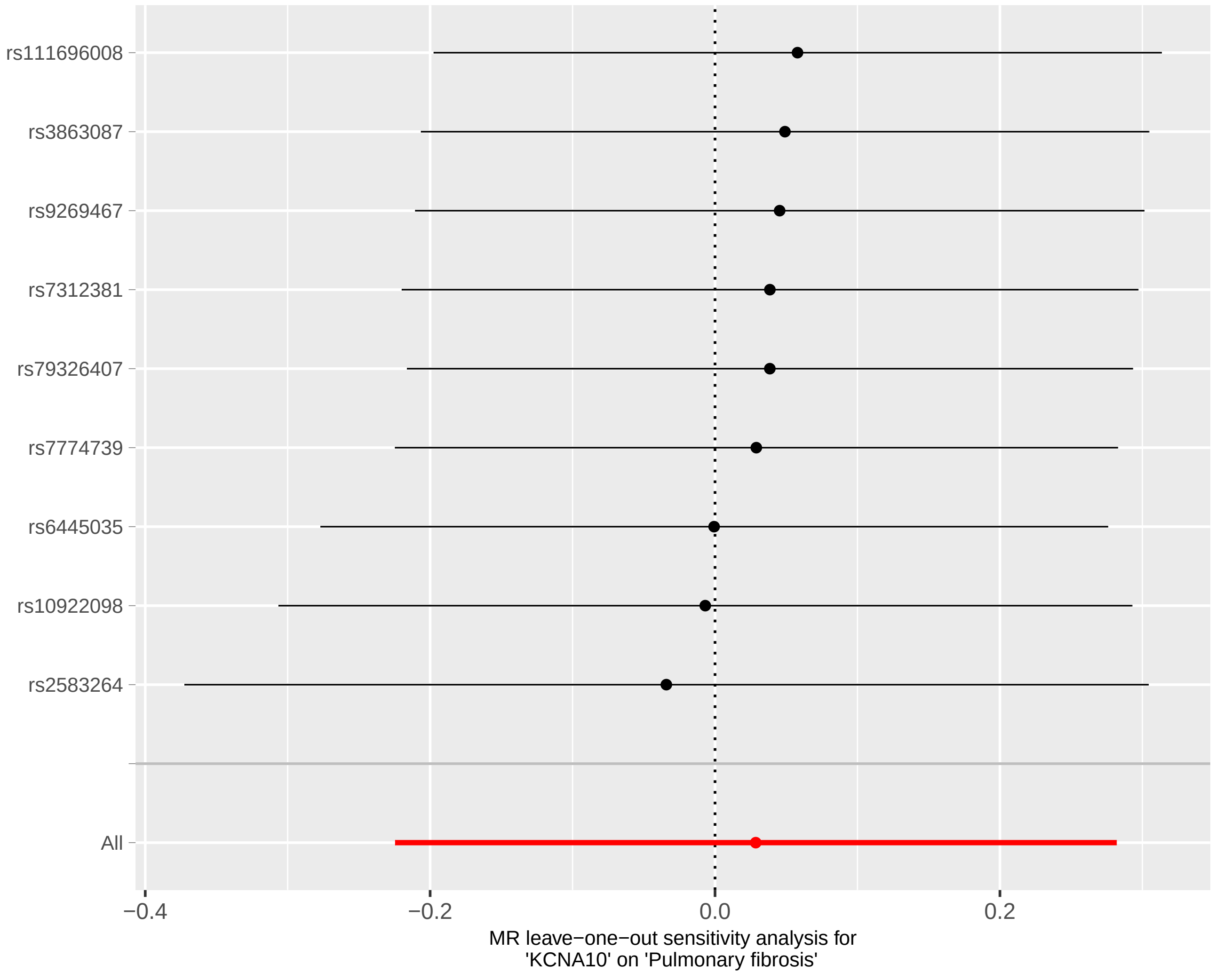

rs111696008
rs3863087
rs9269467
rs7312381
rs79326407
rs7774739
rs6445035
rs10922098
rs2583264
All
−0.4
−0.2
0.0
0.2
MR leave−one−out sensitivity analysis for
'KCNA10' on 'Pulmonary fibrosis'

Insufficient number of SNPs

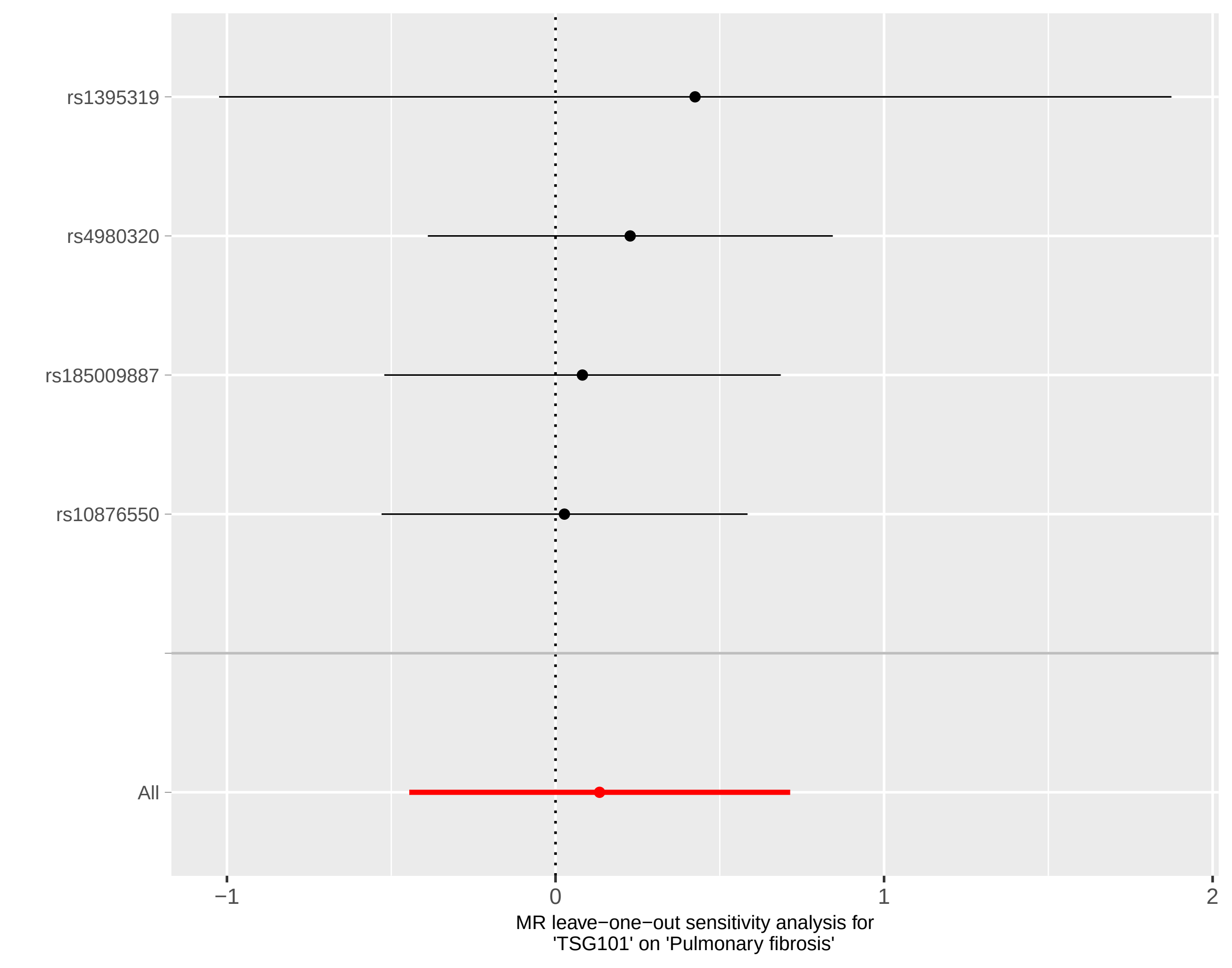
rs1395319
rs4980320
rs185009887
rs10876550
All
−1
0
1
2
MR leave−one−out sensitivity analysis for
'TSG101' on 'Pulmonary fibrosis'

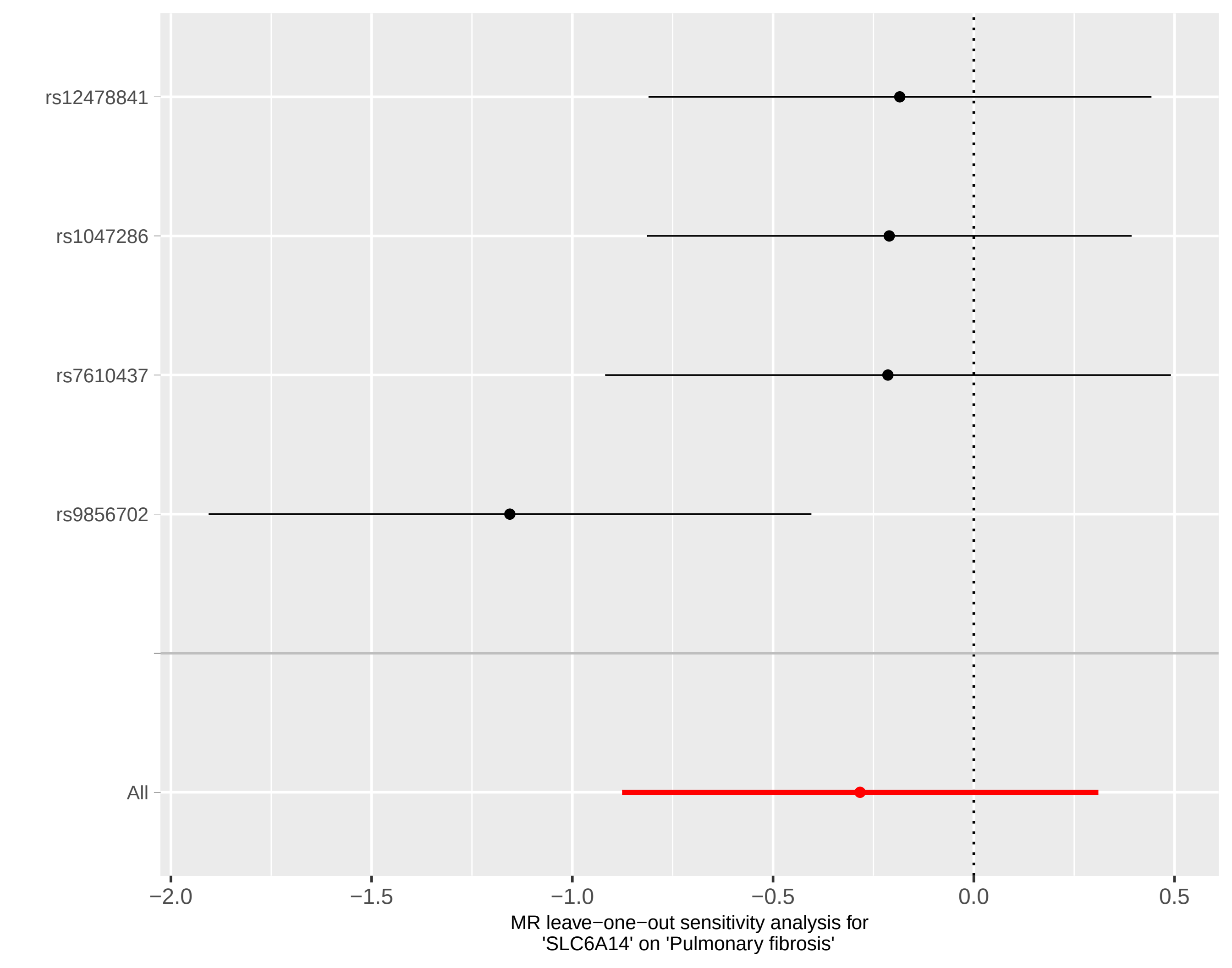
rs12478841
rs1047286
rs7610437
rs9856702
All
−2.0
−1.5
−1.0
−0.5
0.0
0.5
MR leave−one−out sensitivity analysis for
'SLC6A14' on 'Pulmonary fibrosis'

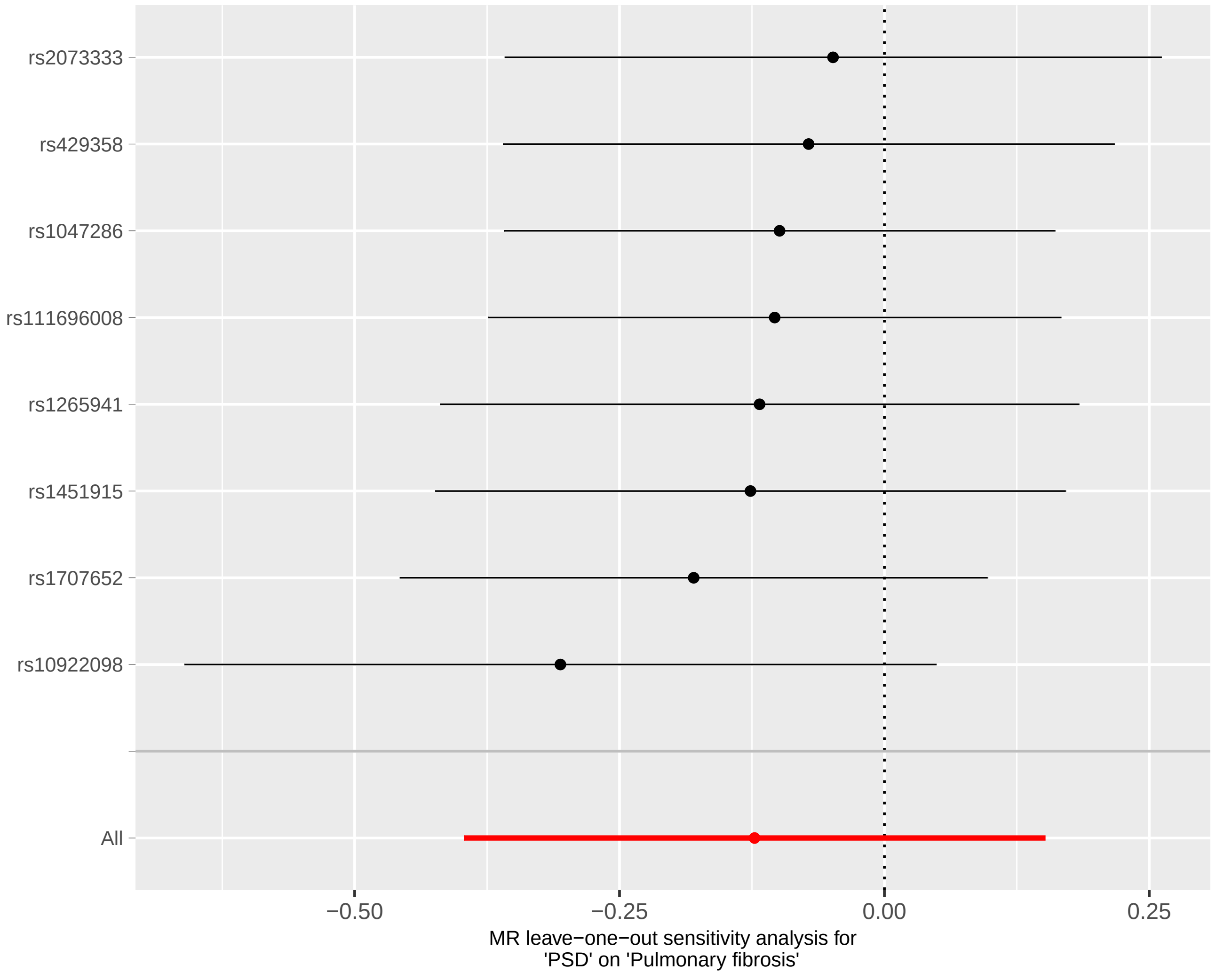

rs2073333
rs429358
rs1047286
rs111696008
rs1265941
rs1451915
rs1707652
rs10922098
All
−0.50
−0.25
0.00
0.25
MR leave−one−out sensitivity analysis for
'PSD' on 'Pulmonary fibrosis'

Insufficient number of SNPs

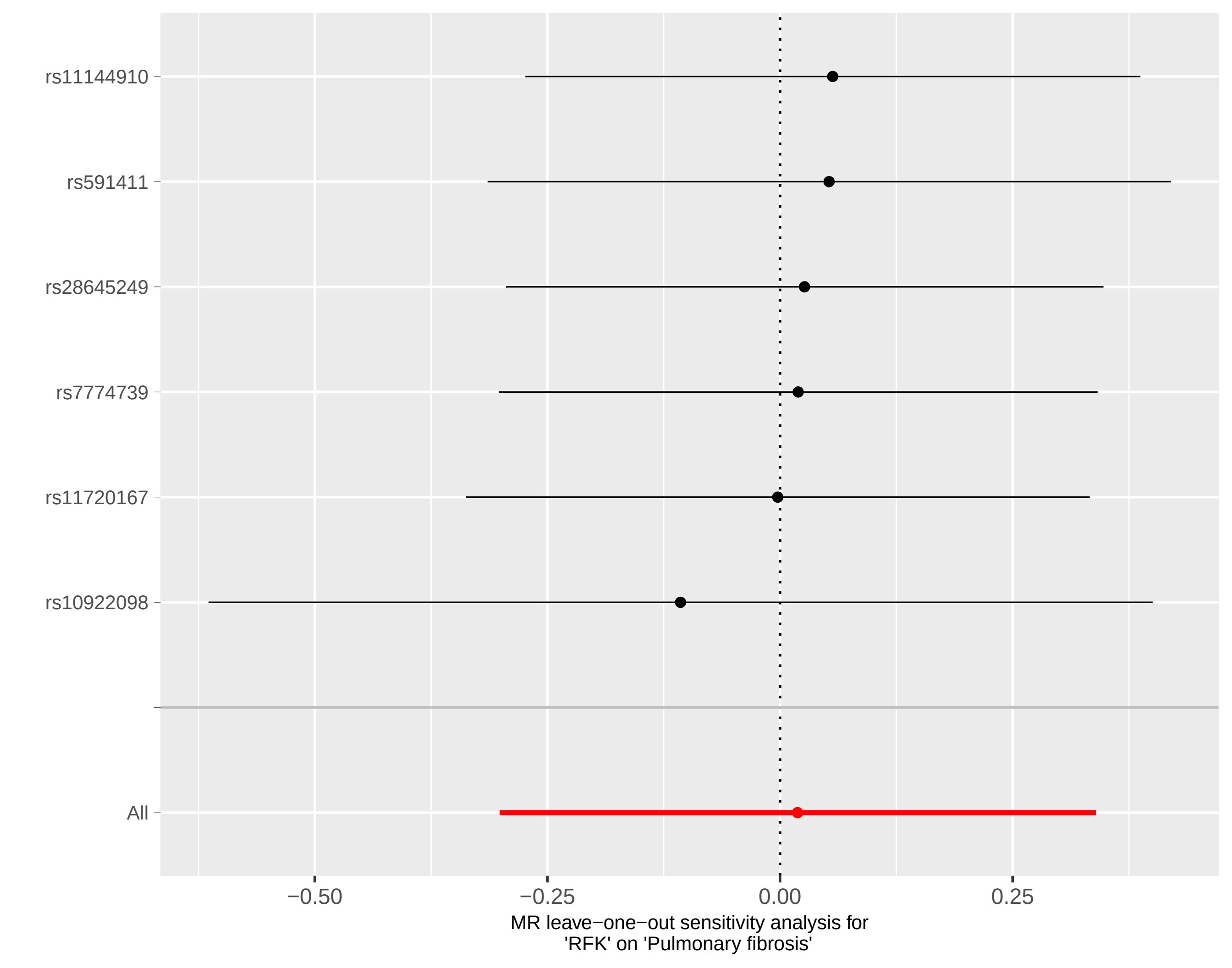
rs11144910
rs591411
rs28645249
rs7774739
rs11720167
rs10922098
All
−0.50
−0.25
0.00
0.25
MR leave−one−out sensitivity analysis for
'RFK' on 'Pulmonary fibrosis'

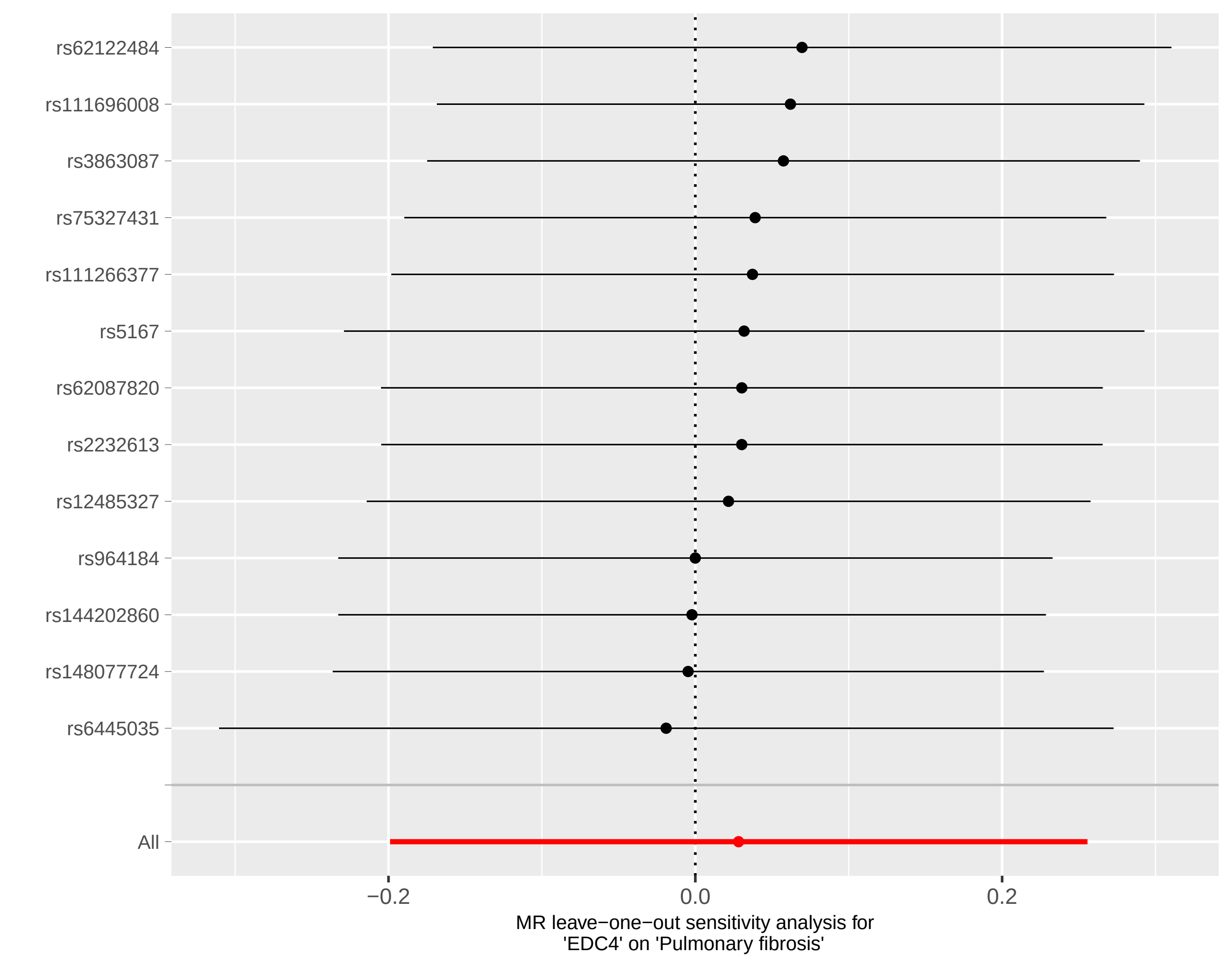
rs62122484
rs111696008
rs3863087
rs75327431
rs111266377
rs5167
rs62087820
rs2232613
rs12485327
rs964184
rs144202860
rs148077724
rs6445035
All
−0.2
0.0
0.2
MR leave−one−out sensitivity analysis for
'EDC4' on 'Pulmonary fibrosis'

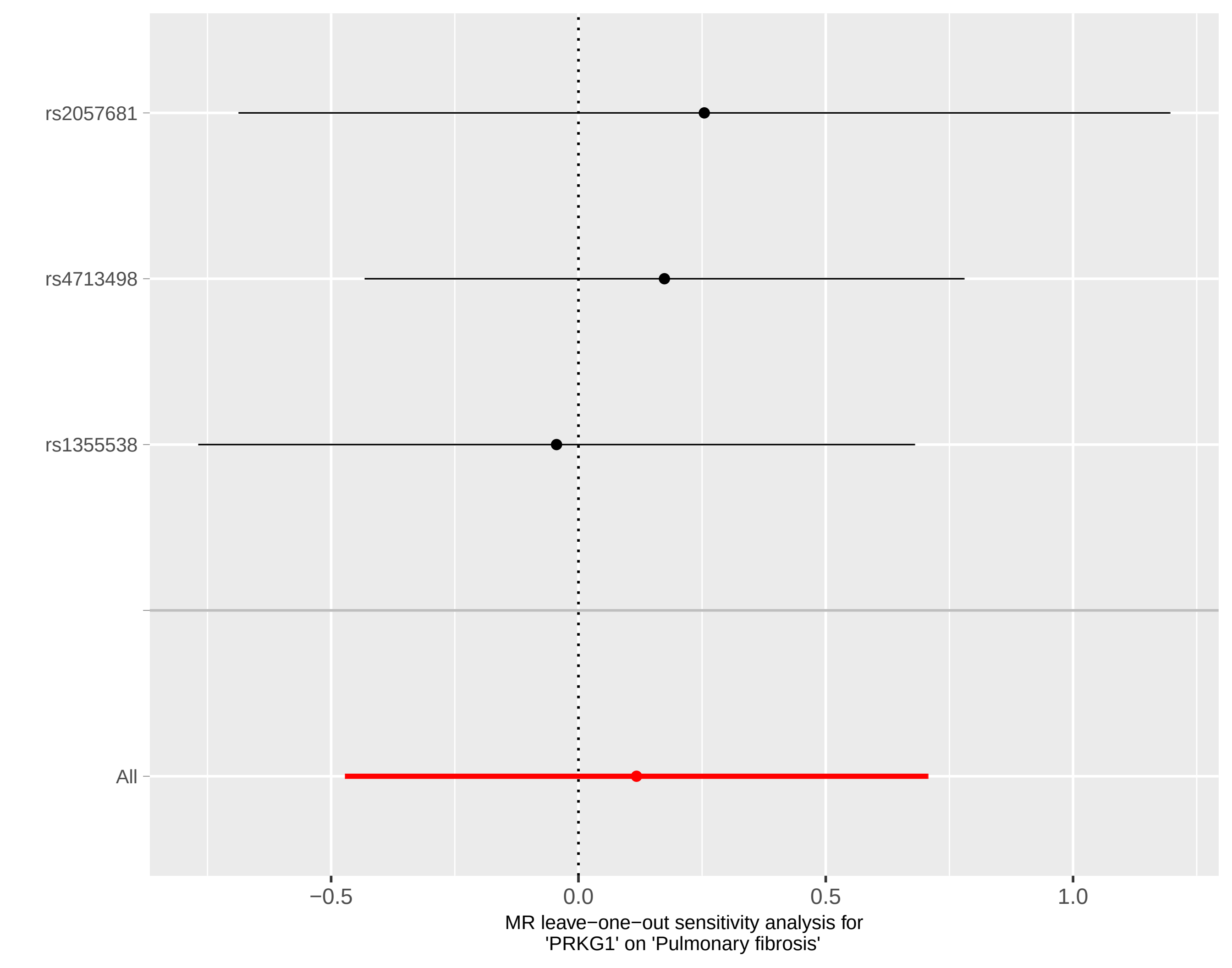
rs2057681
rs4713498
rs1355538
All
−0.5
0.0
0.5
1.0
MR leave−one−out sensitivity analysis for
'PRKG1' on 'Pulmonary fibrosis'

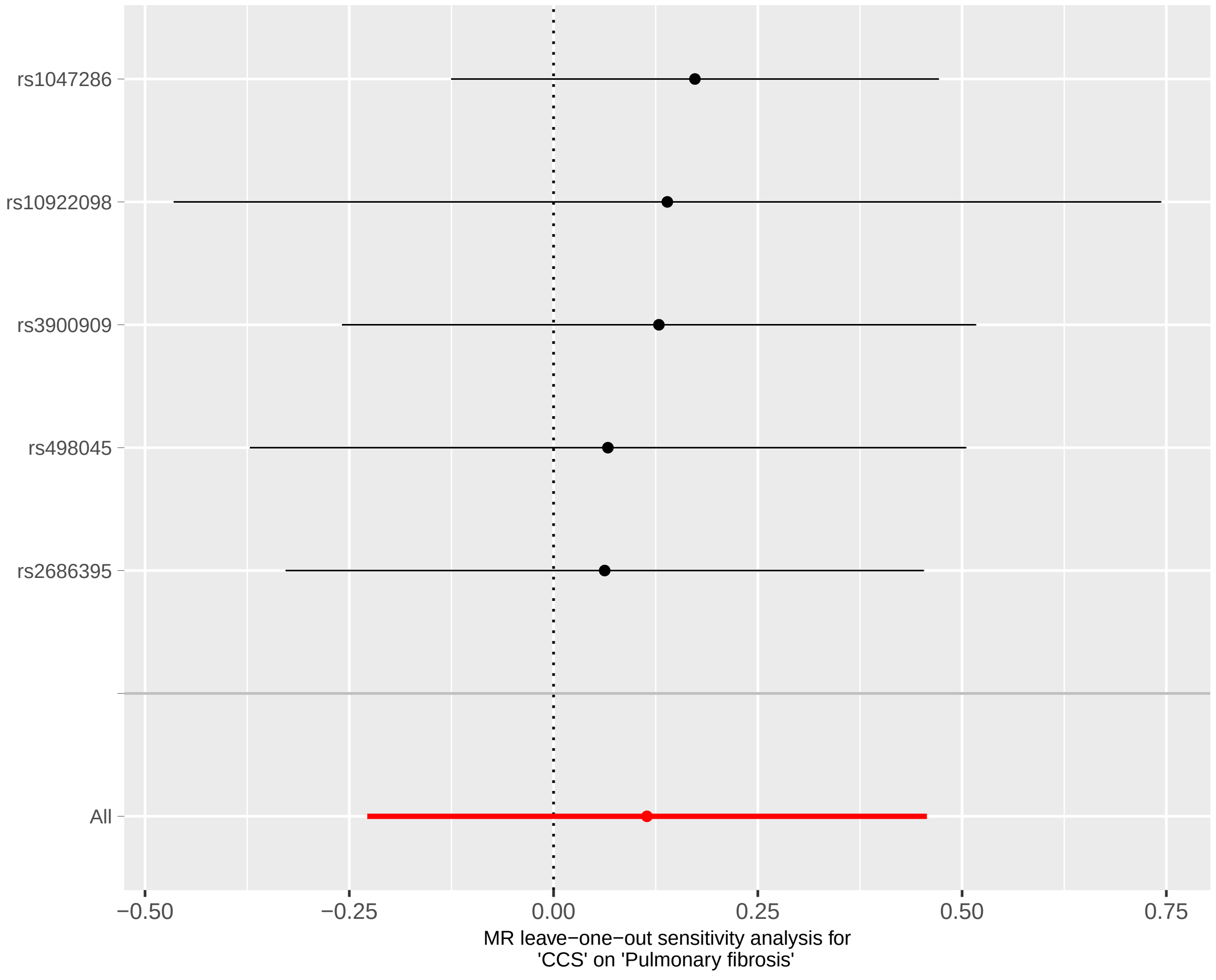
rs1047286
rs10922098
rs3900909
rs498045
rs2686395
All
−0.50
−0.25
0.00
0.25
0.50
0.75
MR leave−one−out sensitivity analysis for
'CCS' on 'Pulmonary fibrosis'

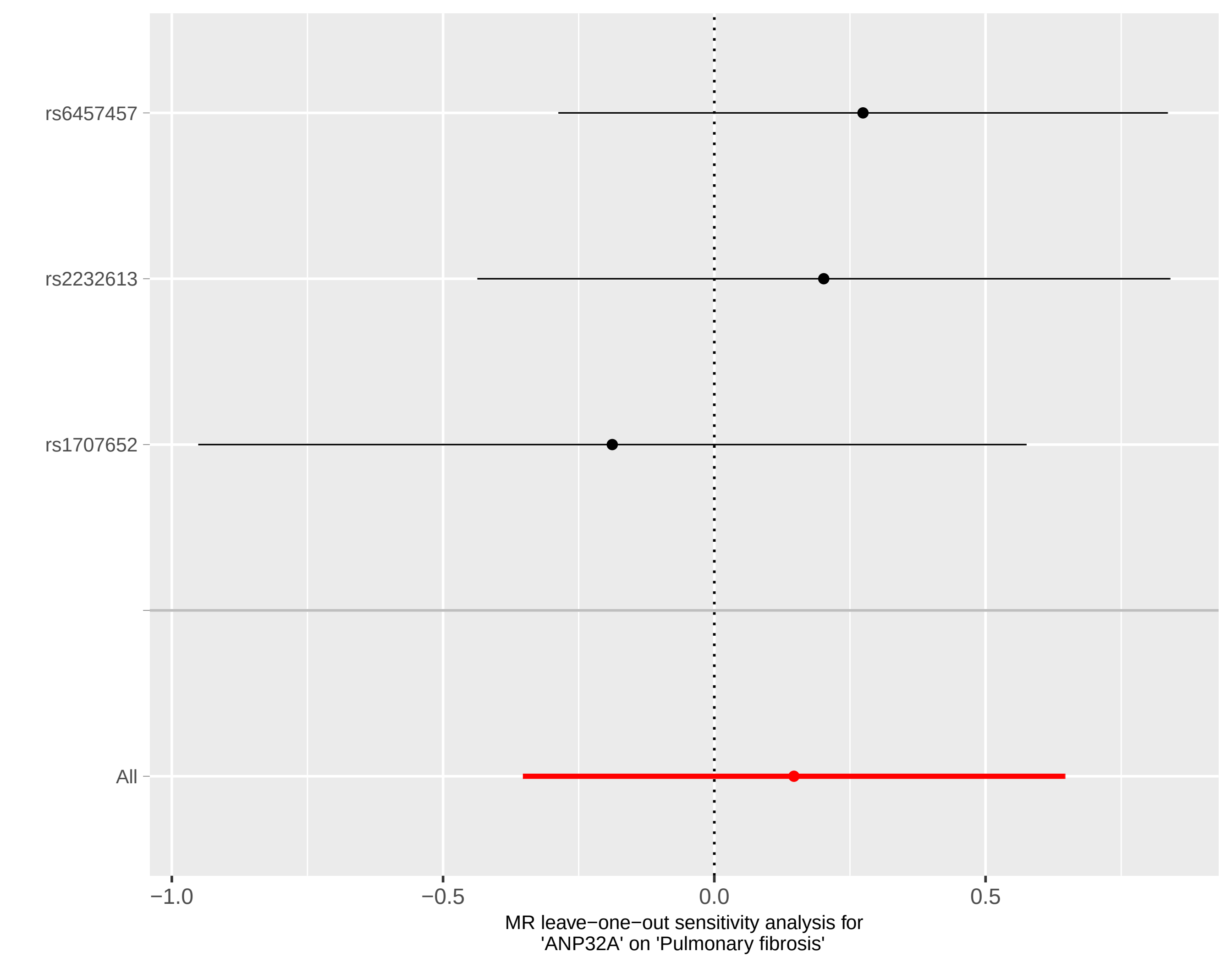

rs6457457
rs2232613
rs1707652
All
−1.0
−0.5
0.0
0.5
MR leave−one−out sensitivity analysis for
'ANP32A' on 'Pulmonary fibrosis'

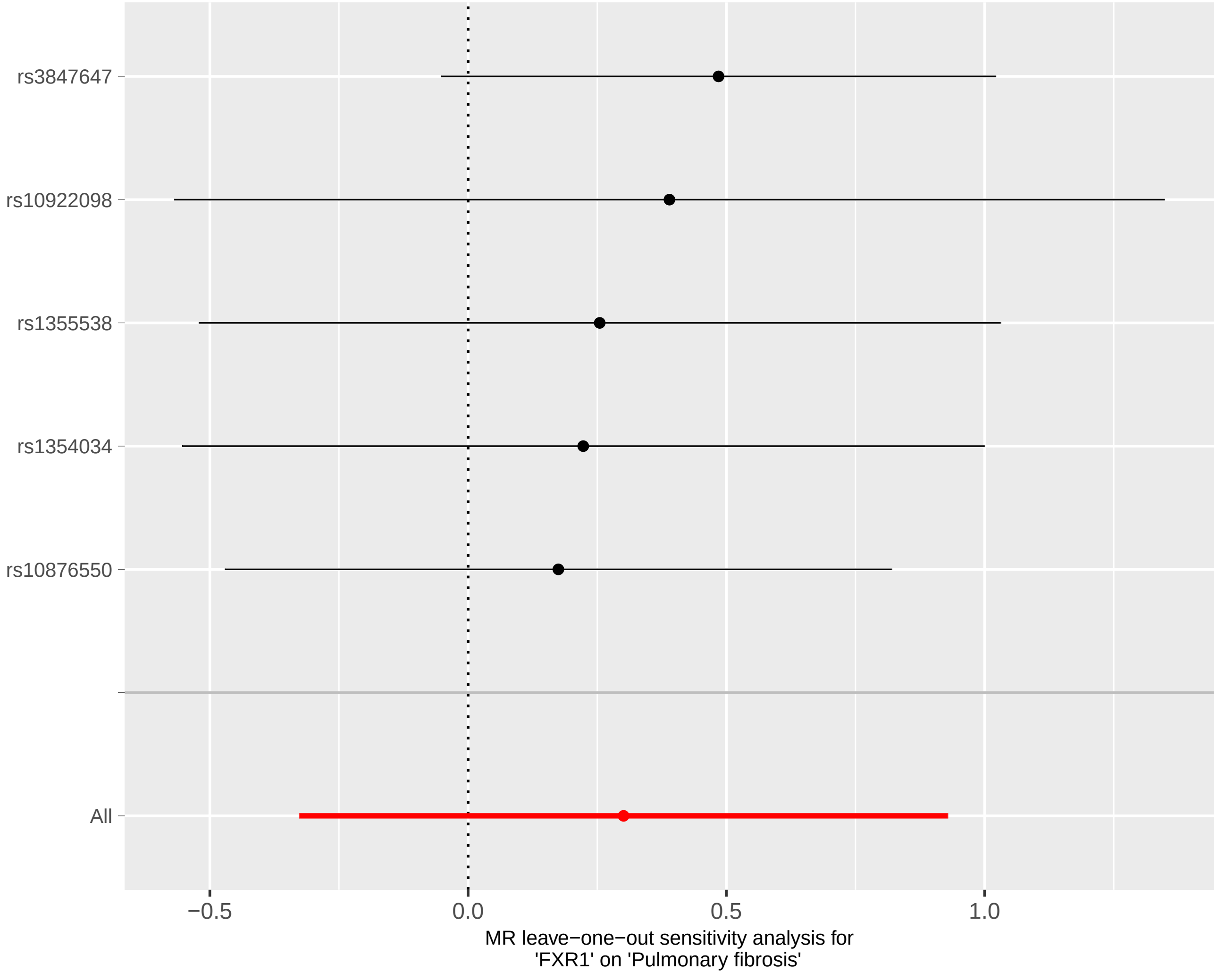
rs3847647
rs10922098
rs1355538
rs1354034
rs10876550
All
−0.5
0.0
0.5
1.0
MR leave−one−out sensitivity analysis for
'FXR1' on 'Pulmonary fibrosis'

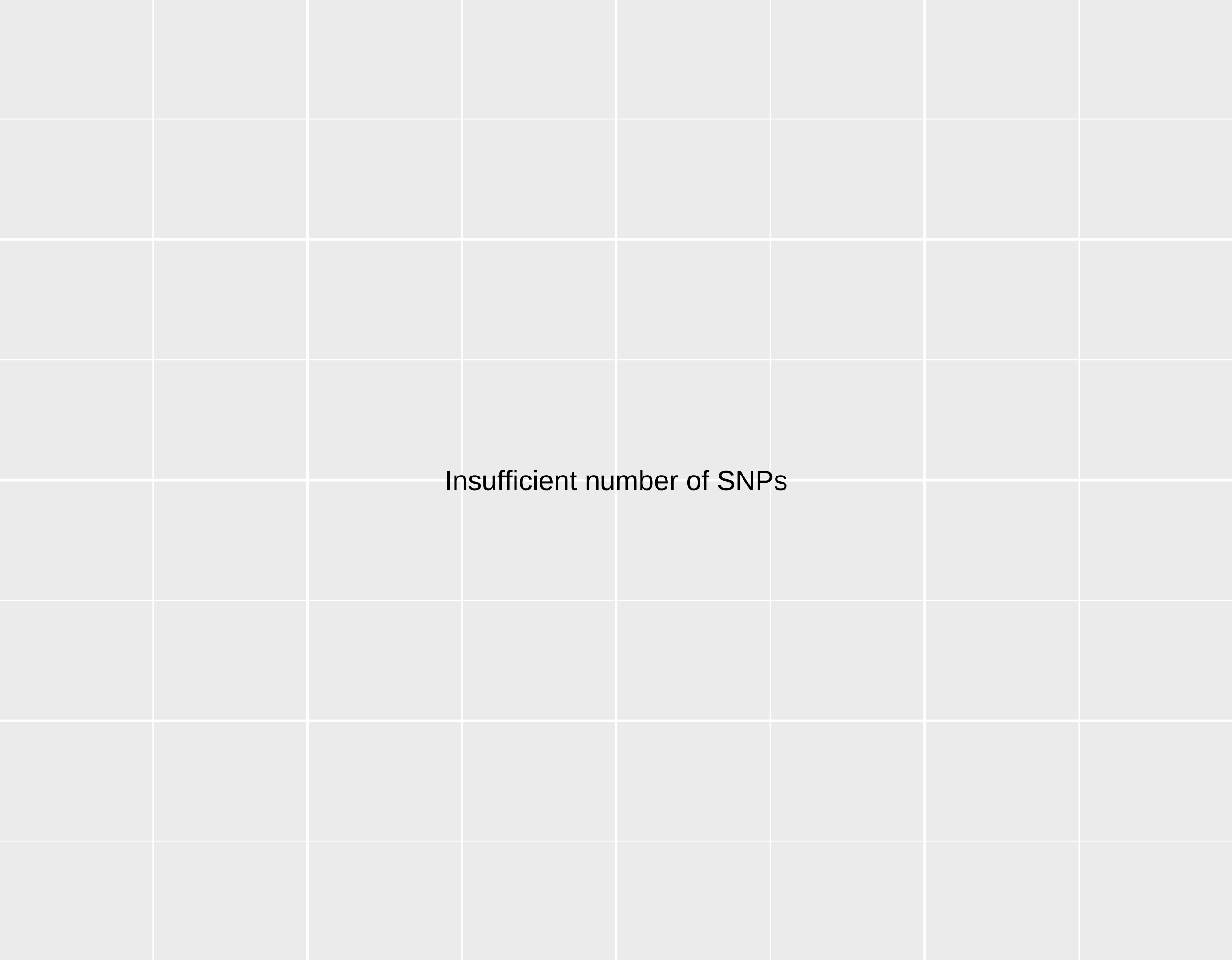
Insufficient number of SNPs

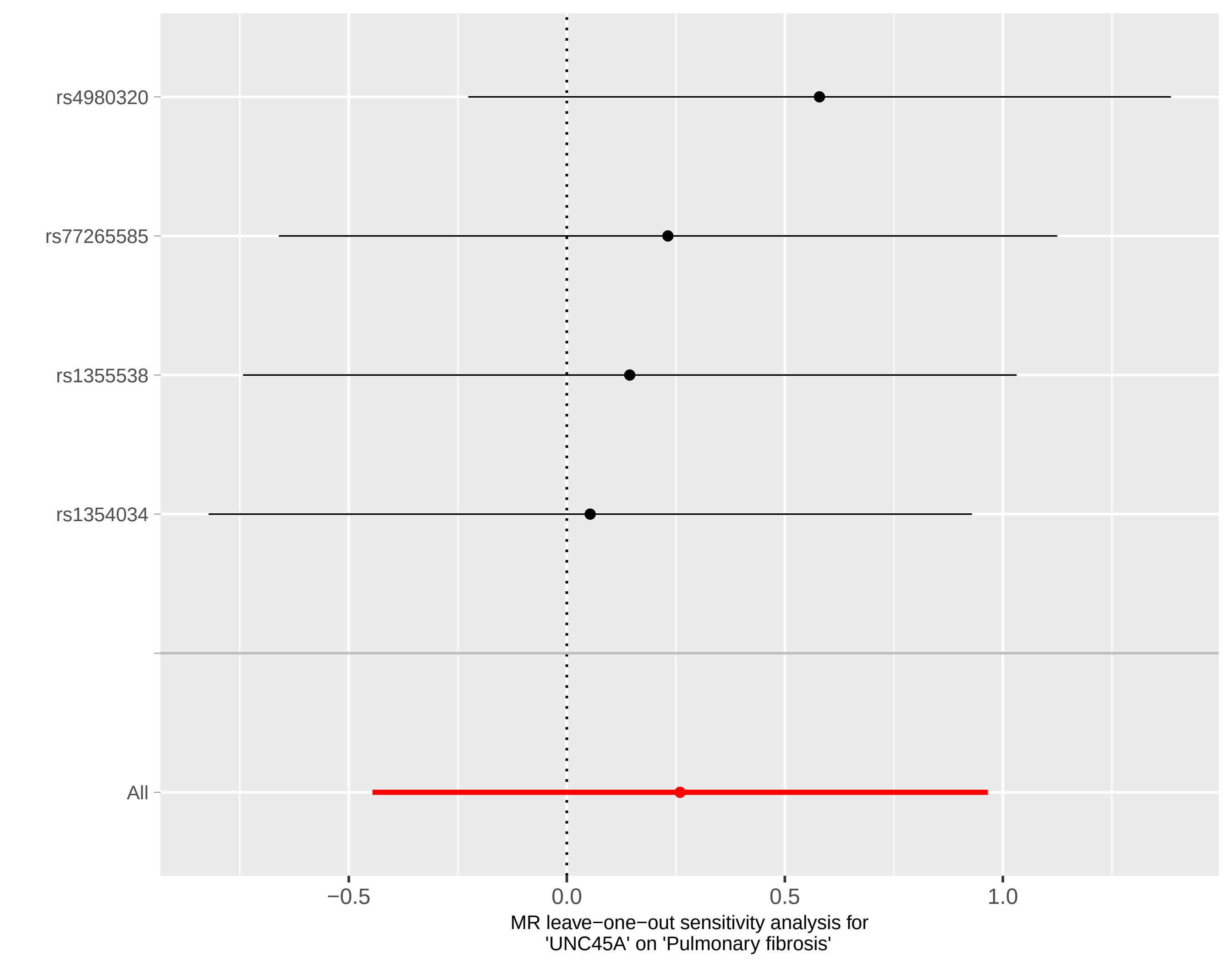
rs4980320
rs77265585
rs1355538
rs1354034
All
−0.5
0.0
0.5
1.0
MR leave−one−out sensitivity analysis for
'UNC45A' on 'Pulmonary fibrosis'

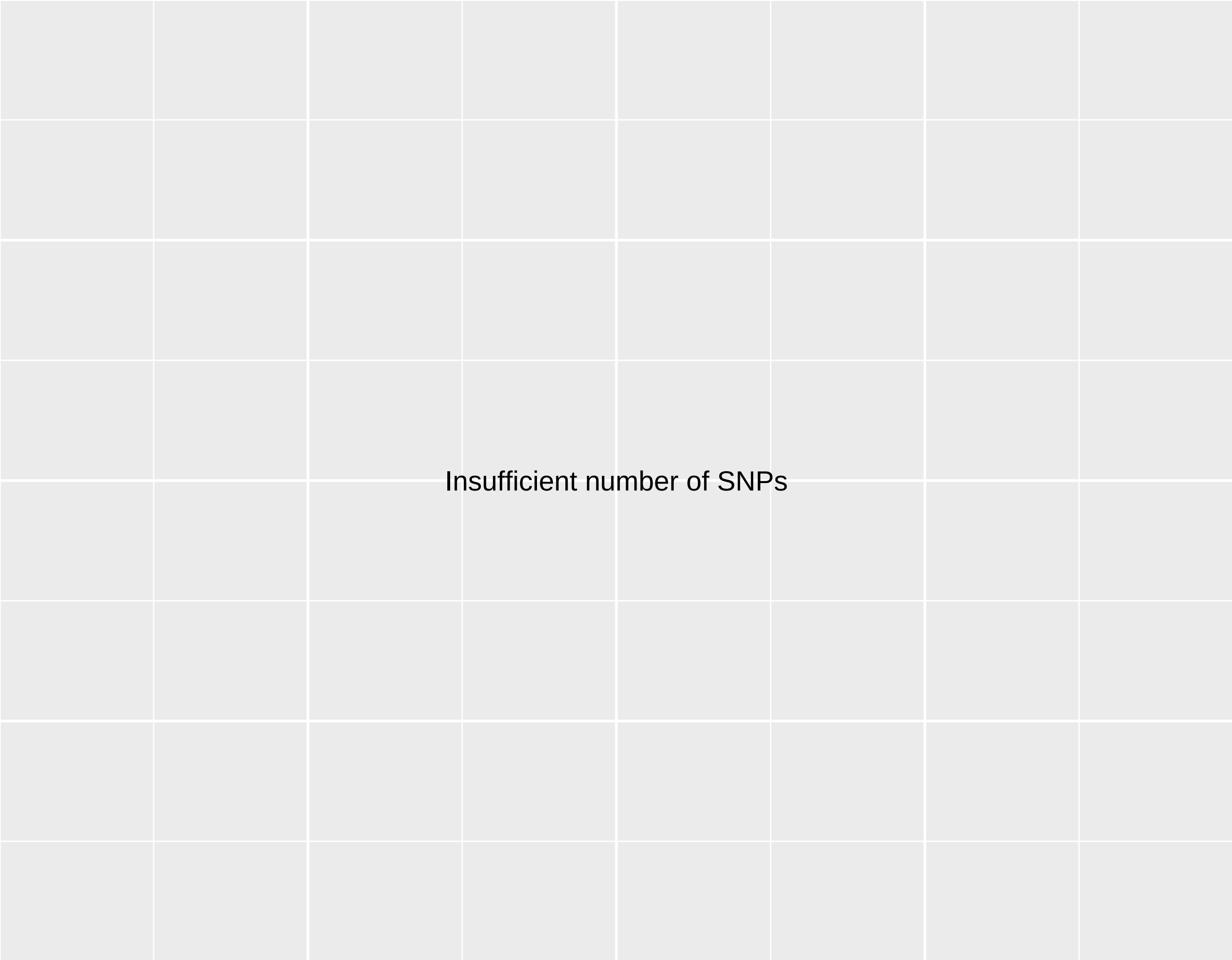
Insufficient number of SNPs

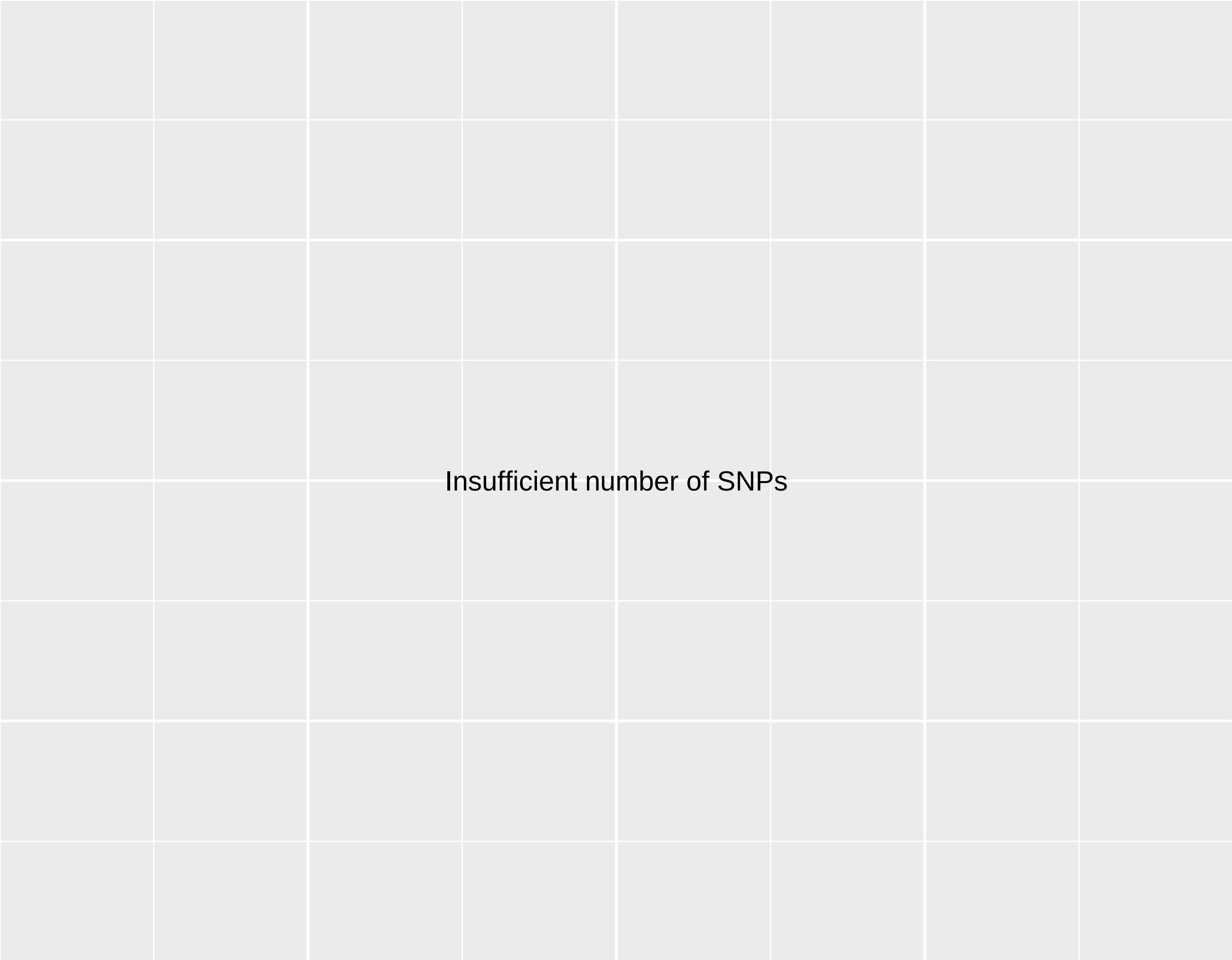
Insufficient number of SNPs

Insufficient number of SNPs

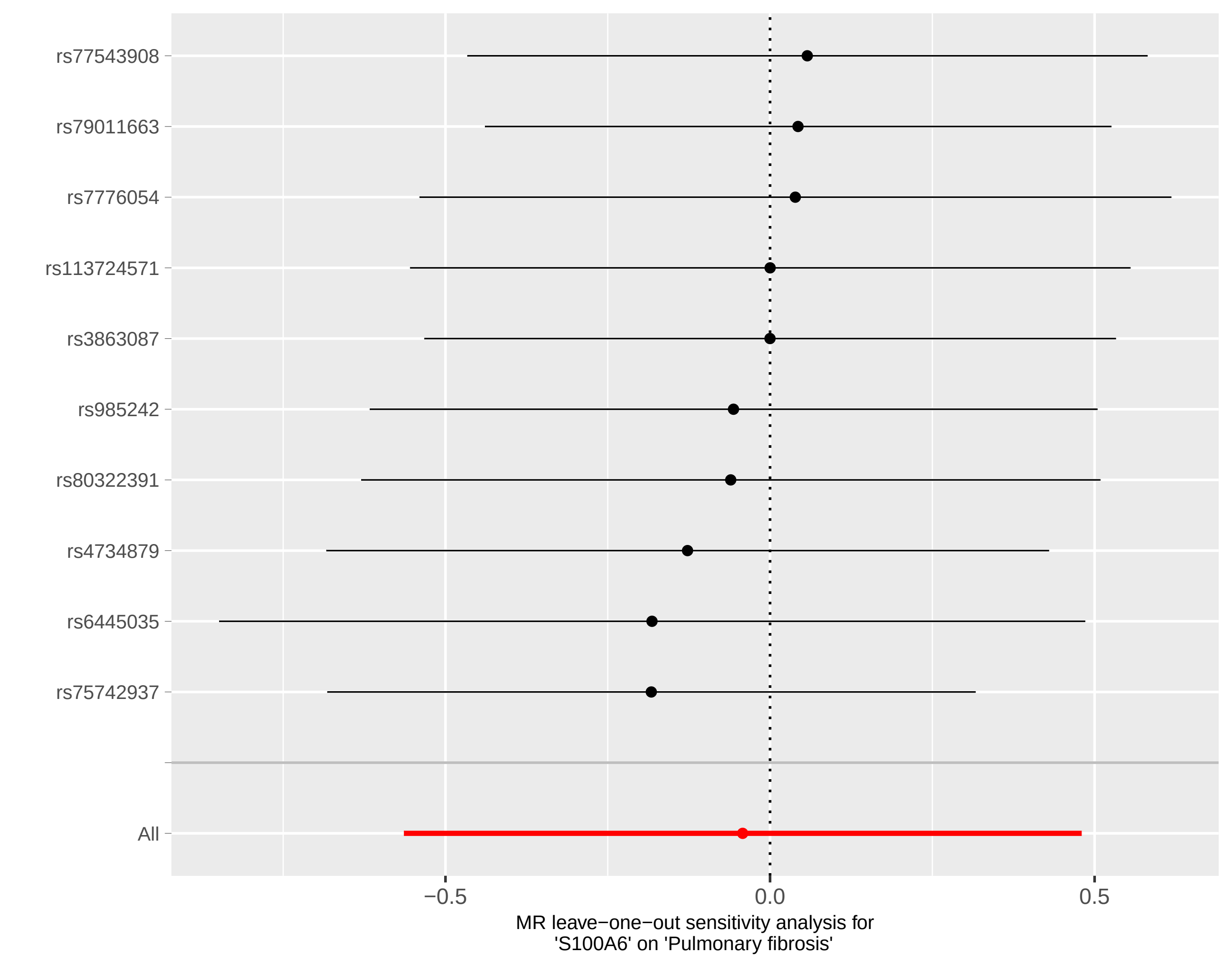
rs77543908
rs79011663
rs7776054
rs113724571
rs3863087
rs985242
rs80322391
rs4734879
rs6445035
rs75742937
All
−0.5
0.0
0.5
MR leave−one−out sensitivity analysis for
'S100A6' on 'Pulmonary fibrosis'

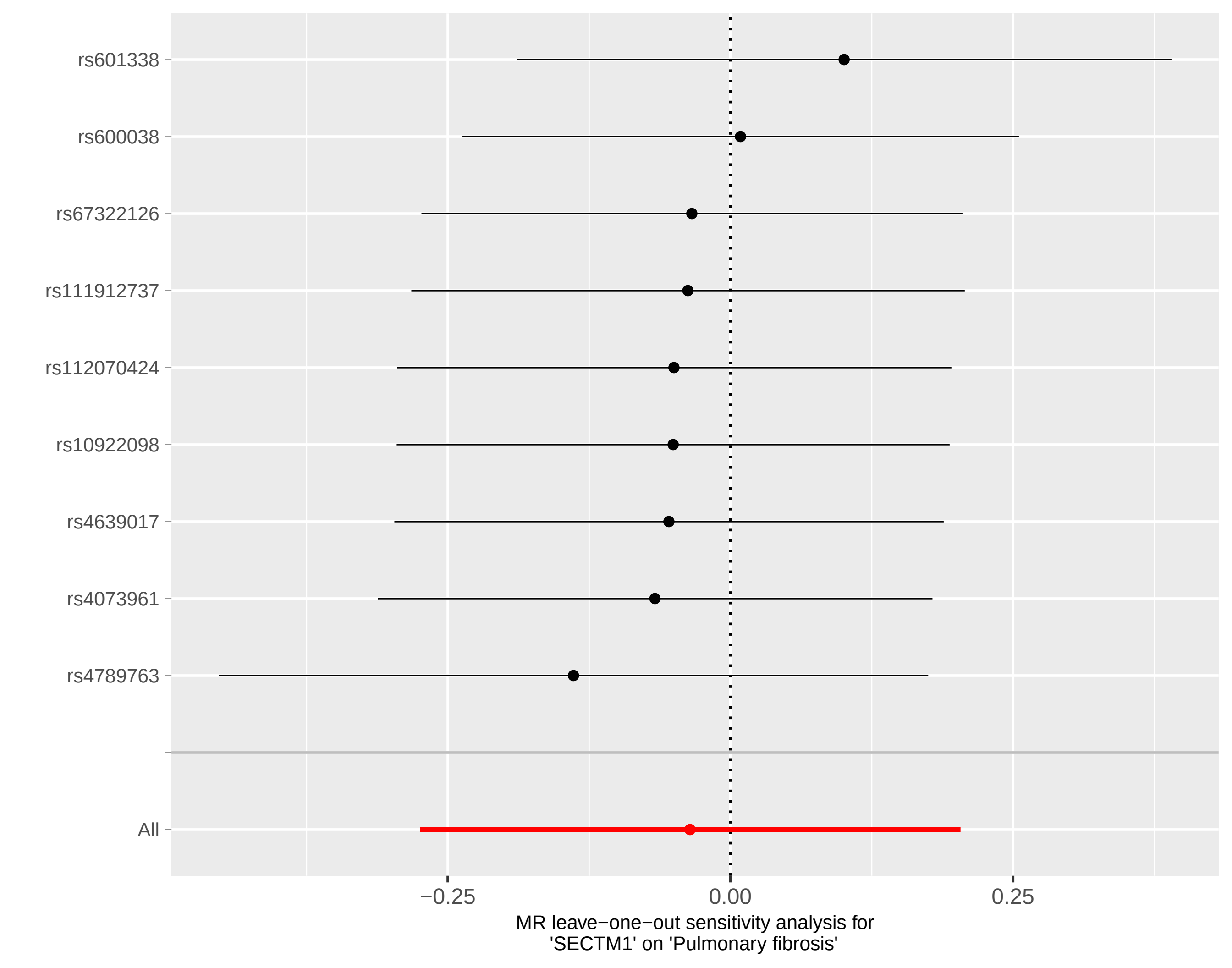

rs601338
rs600038
rs67322126
rs111912737
rs112070424
rs10922098
rs4639017
rs4073961
rs4789763
All
−0.25
0.00
0.25
MR leave−one−out sensitivity analysis for
'SECTM1' on 'Pulmonary fibrosis'

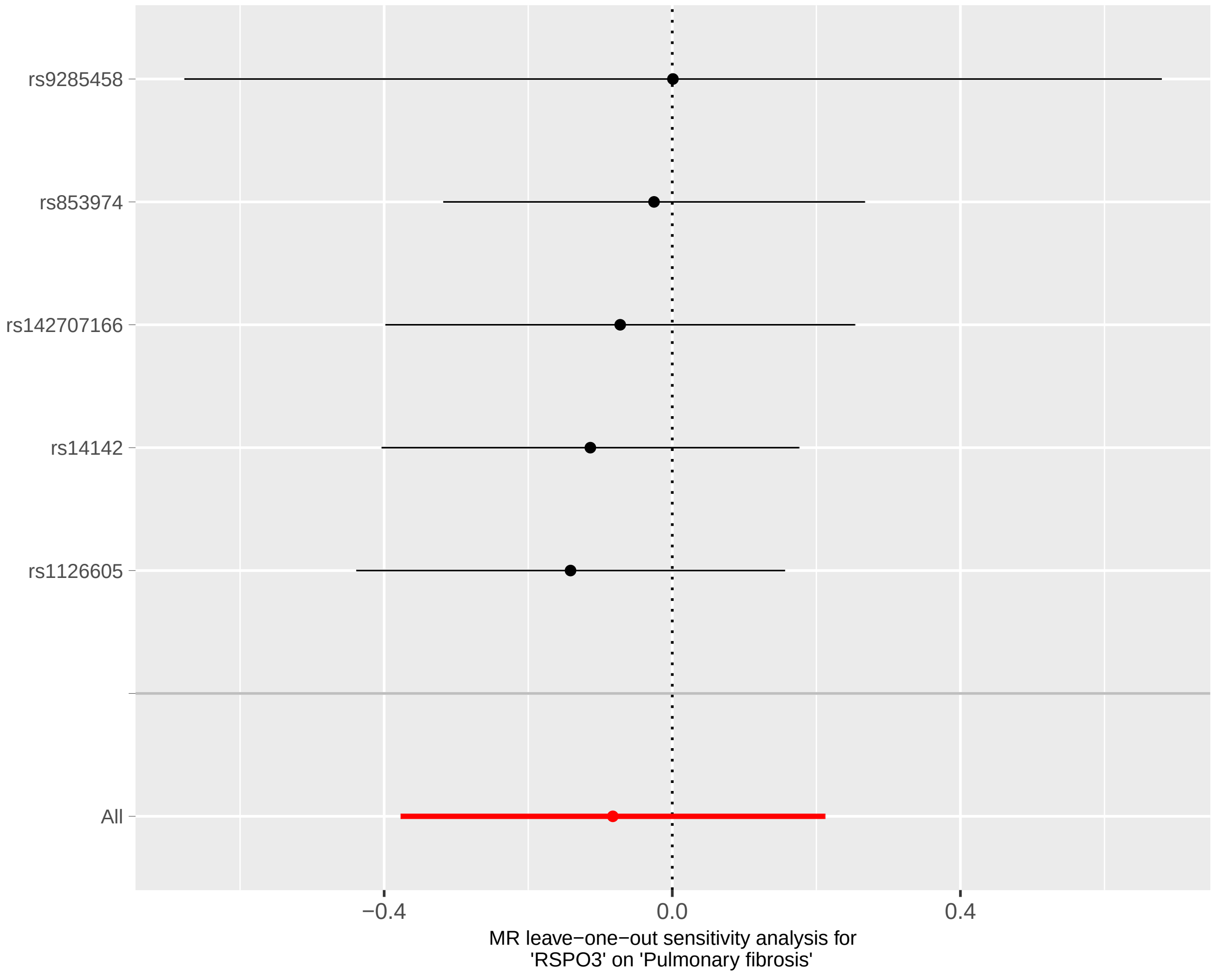

rs9285458
rs853974
rs142707166
rs14142
rs1126605
All
−0.4
0.0
0.4
MR leave−one−out sensitivity analysis for
'RSPO3' on 'Pulmonary fibrosis'

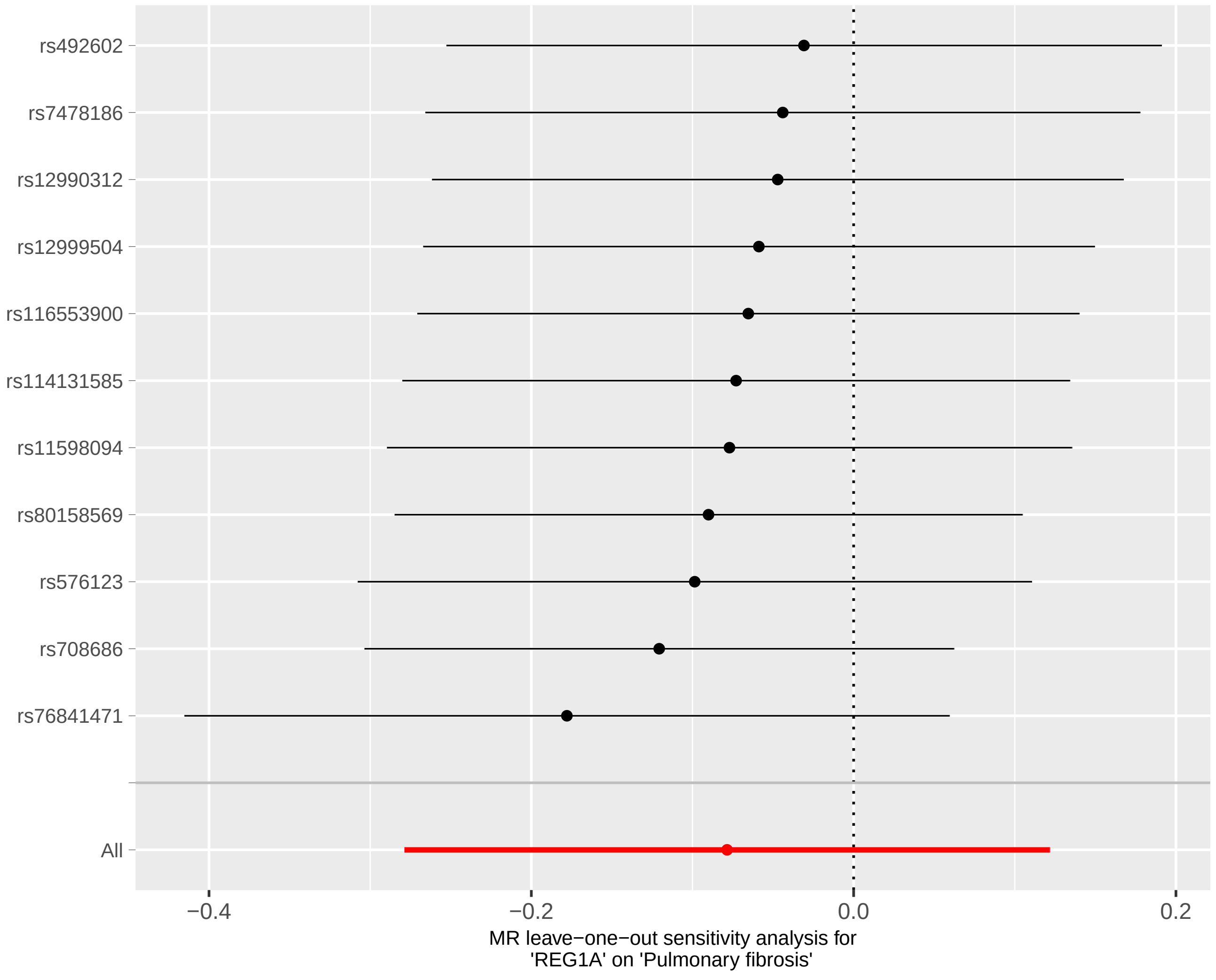

rs492602
rs7478186
rs12990312
rs12999504
rs116553900
rs114131585
rs11598094
rs80158569
rs576123
rs708686
rs76841471
All
−0.4
−0.2
0.0
0.2
MR leave−one−out sensitivity analysis for
'REG1A' on 'Pulmonary fibrosis'

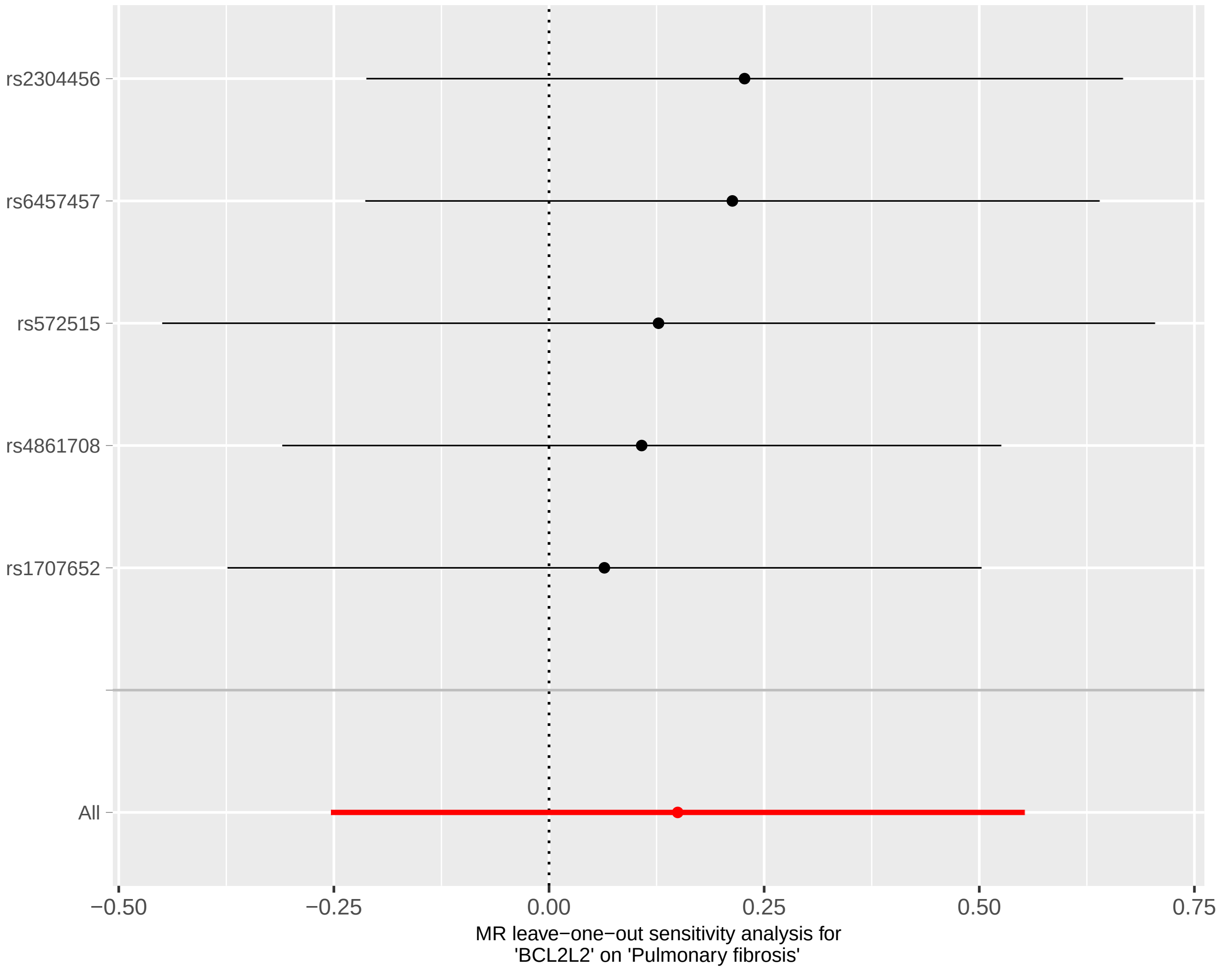

rs2304456
rs6457457
rs572515
rs4861708
rs1707652
All
−0.50
−0.25
0.00
0.25
0.50
0.75
MR leave−one−out sensitivity analysis for
'BCL2L2' on 'Pulmonary fibrosis'

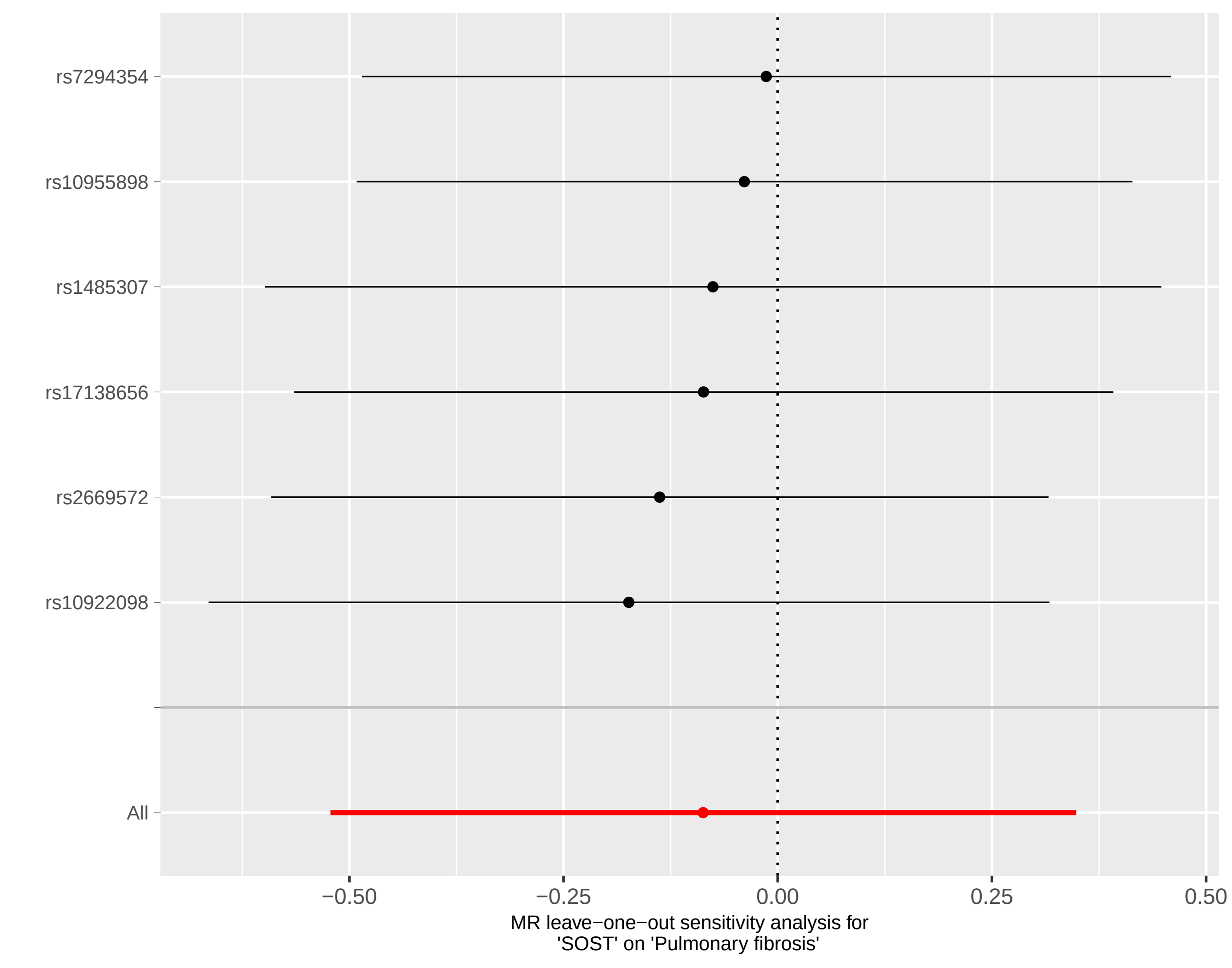
rs7294354
rs10955898
rs1485307
rs17138656
rs2669572
rs10922098
All
−0.50
−0.25
0.00
0.25
0.50
MR leave−one−out sensitivity analysis for
'SOST' on 'Pulmonary fibrosis'

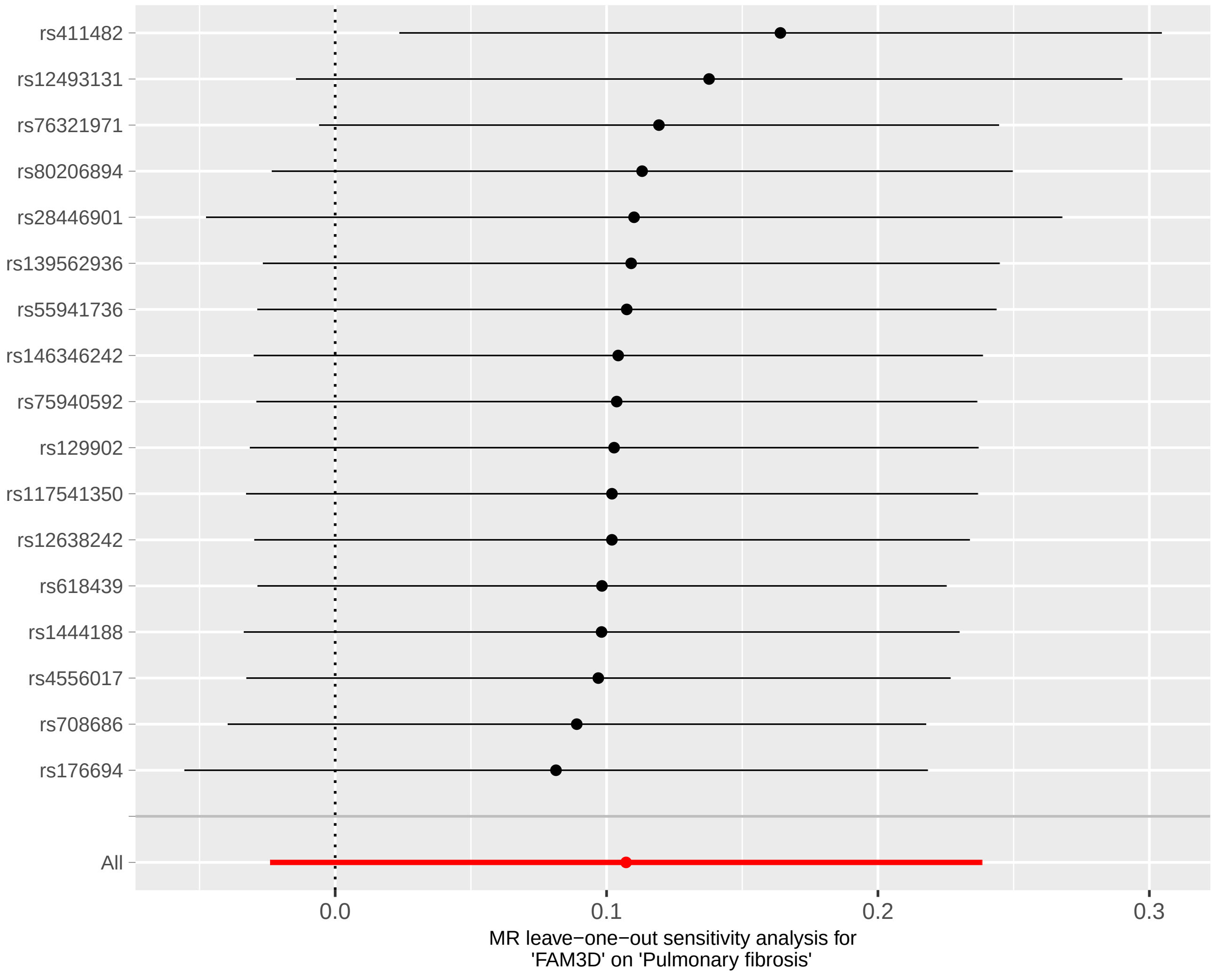

rs411482
rs12493131
rs76321971
rs80206894
rs28446901
rs139562936
rs55941736
rs146346242
rs75940592
rs129902
rs117541350
rs12638242
rs618439
rs1444188
rs4556017
rs708686
rs176694
All
0.0
0.1
0.2
0.3
MR leave−one−out sensitivity analysis for
'FAM3D' on 'Pulmonary fibrosis'

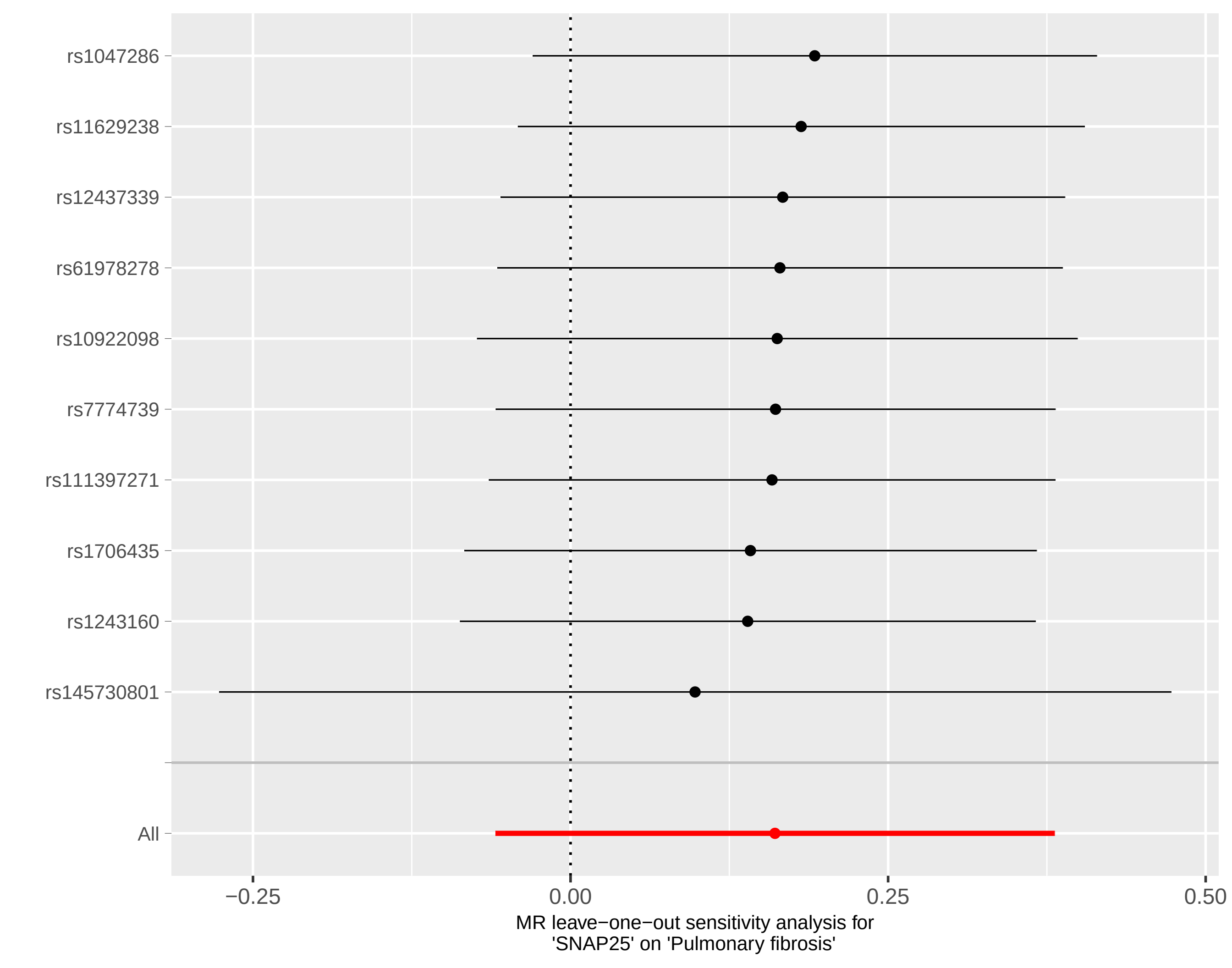

rs1047286
rs11629238
rs12437339
rs61978278
rs10922098
rs7774739
rs111397271
rs1706435
rs1243160
rs145730801
All
−0.25
0.00
0.25
0.50
MR leave−one−out sensitivity analysis for
'SNAP25' on 'Pulmonary fibrosis'

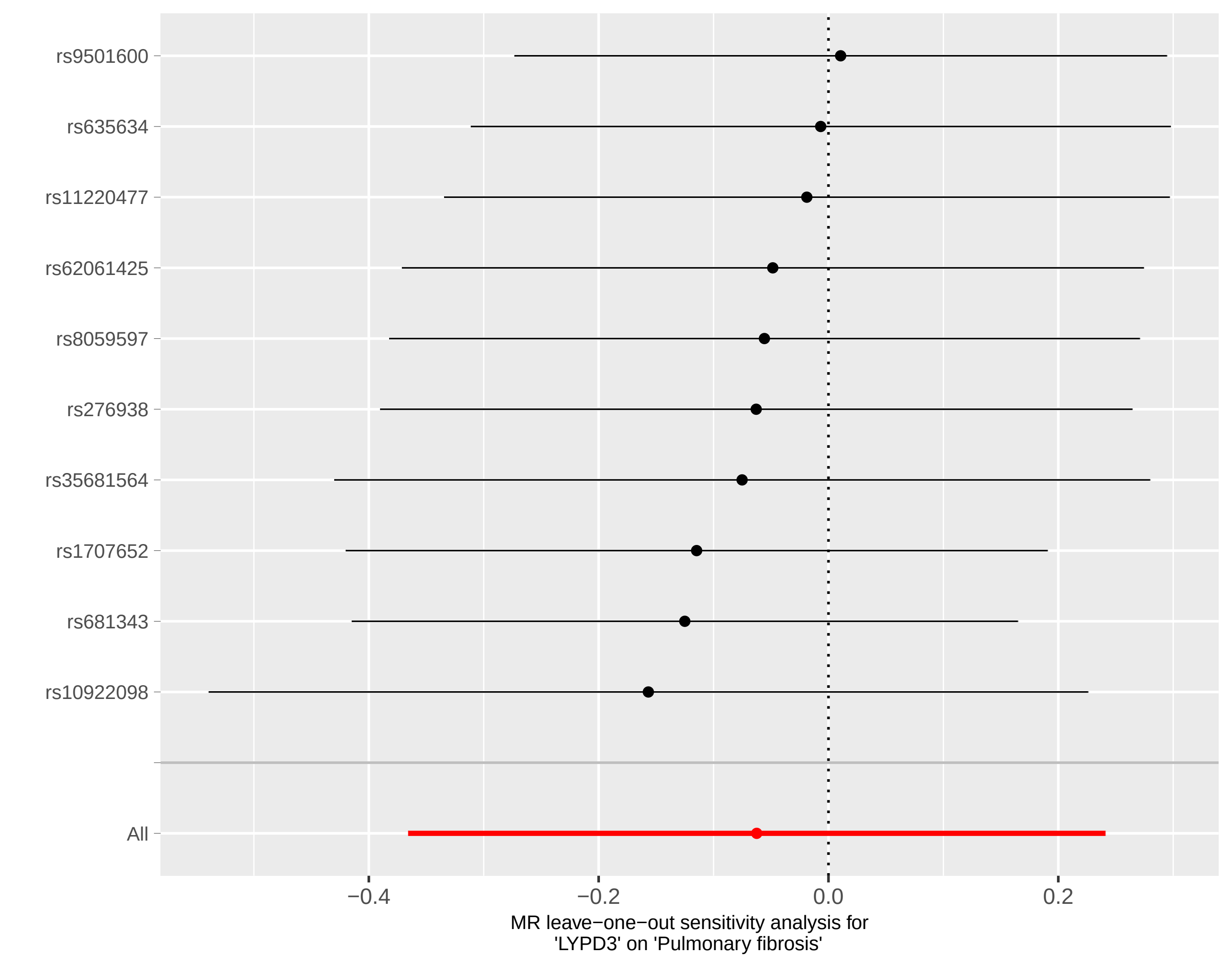
rs9501600
rs635634
rs11220477
rs62061425
rs8059597
rs276938
rs35681564
rs1707652
rs681343
rs10922098
All
−0.4
−0.2
0.0
0.2
MR leave−one−out sensitivity analysis for
'LYPD3' on 'Pulmonary fibrosis'

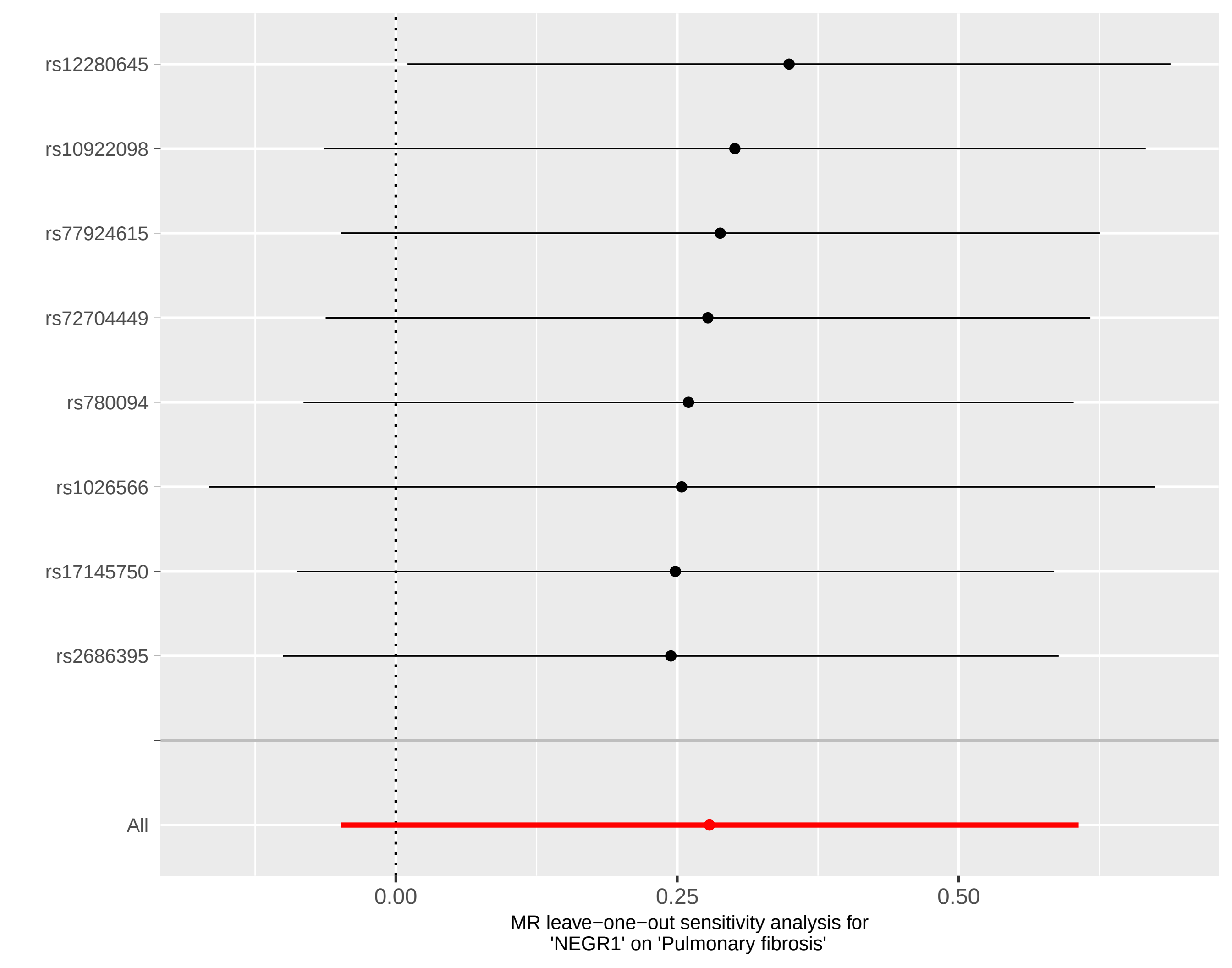

rs12280645
rs10922098
rs77924615
rs72704449
rs780094
rs1026566
rs17145750
rs2686395
All
0.00
0.25
0.50
MR leave−one−out sensitivity analysis for
'NEGR1' on 'Pulmonary fibrosis'

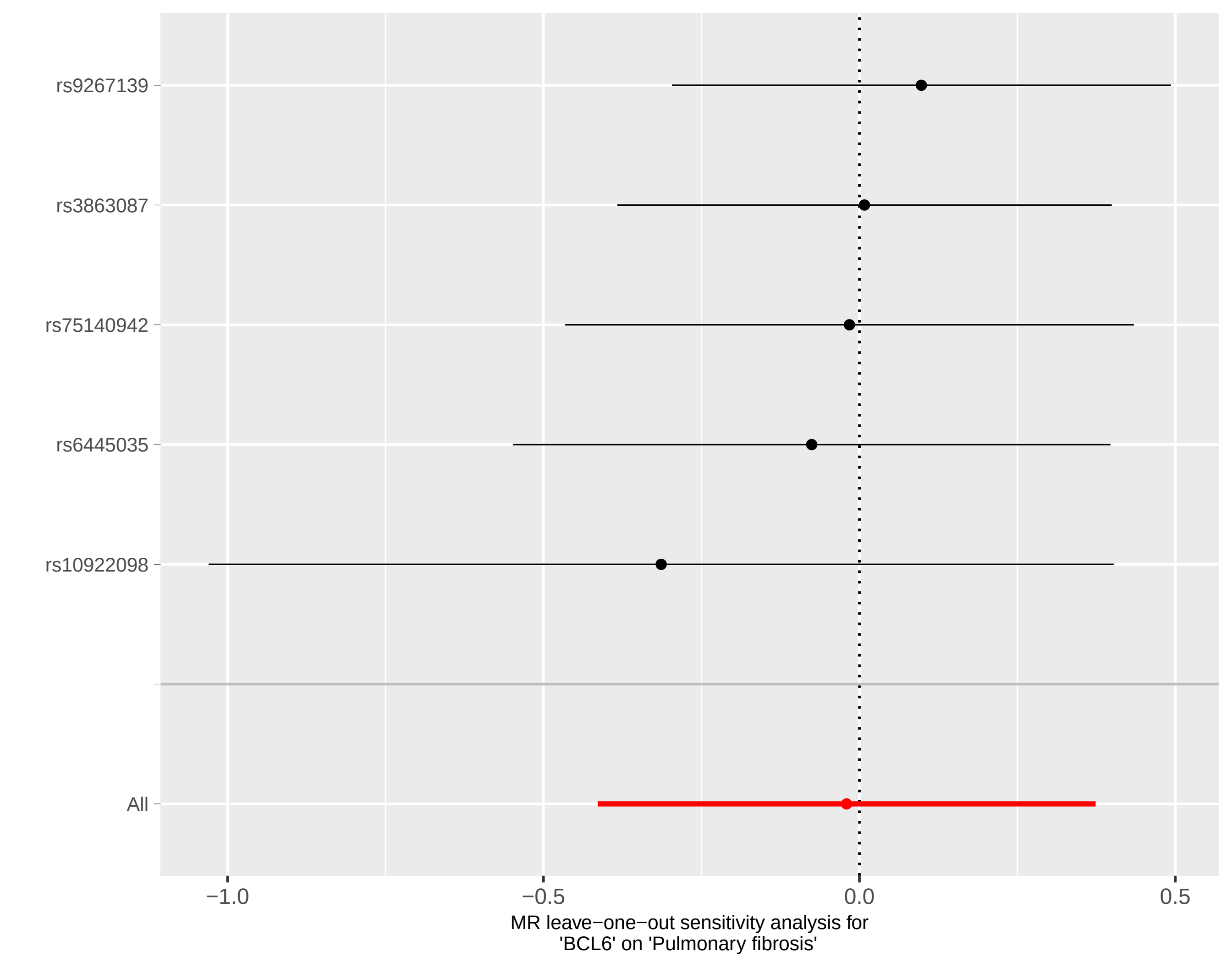

rs9267139
rs3863087
rs75140942
rs6445035
rs10922098
All
−1.0
−0.5
0.0
0.5
MR leave−one−out sensitivity analysis for
'BCL6' on 'Pulmonary fibrosis'

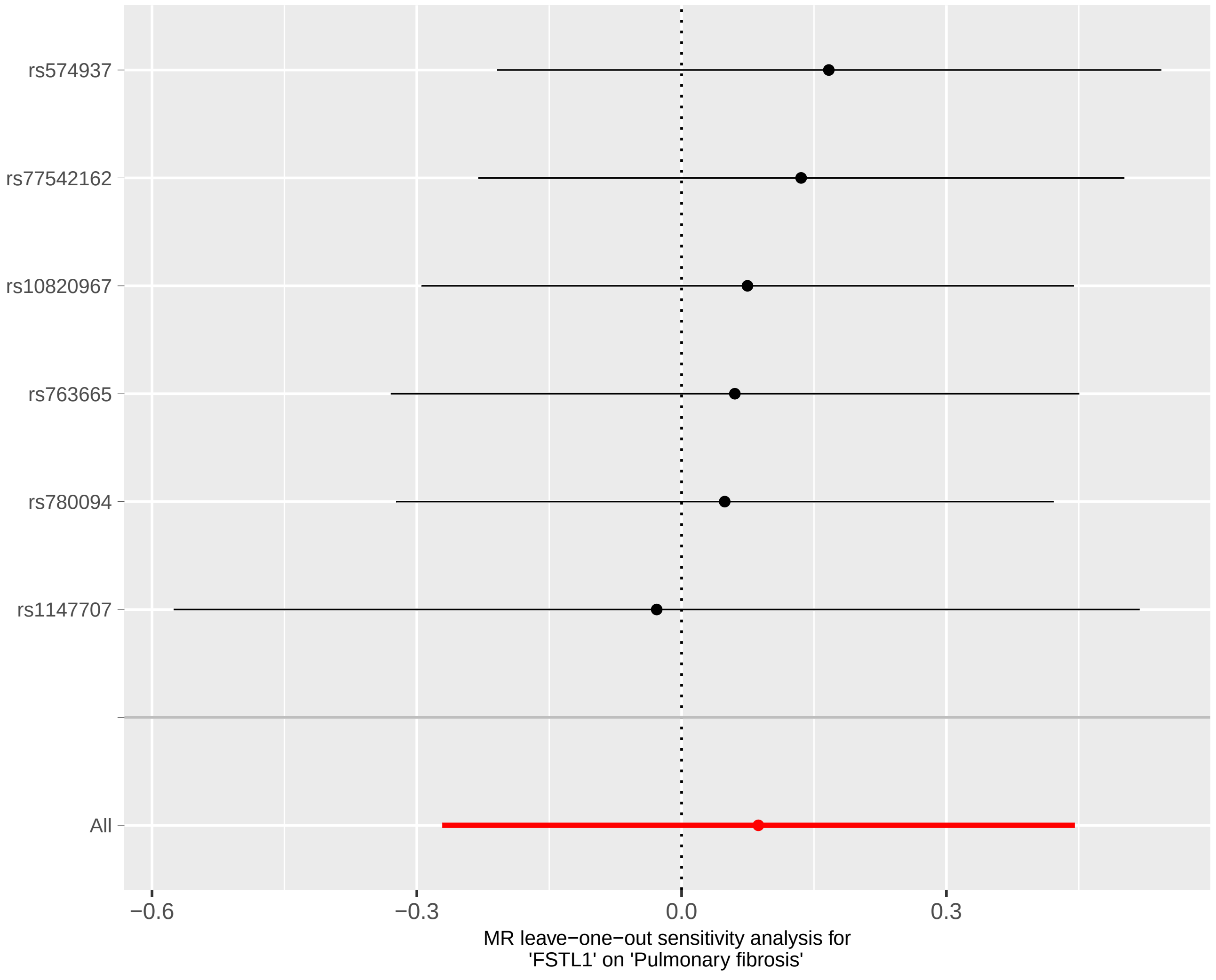
rs574937
rs77542162
rs10820967
rs763665
rs780094
rs1147707
All
−0.6
−0.3
0.0
0.3
MR leave−one−out sensitivity analysis for
'FSTL1' on 'Pulmonary fibrosis'

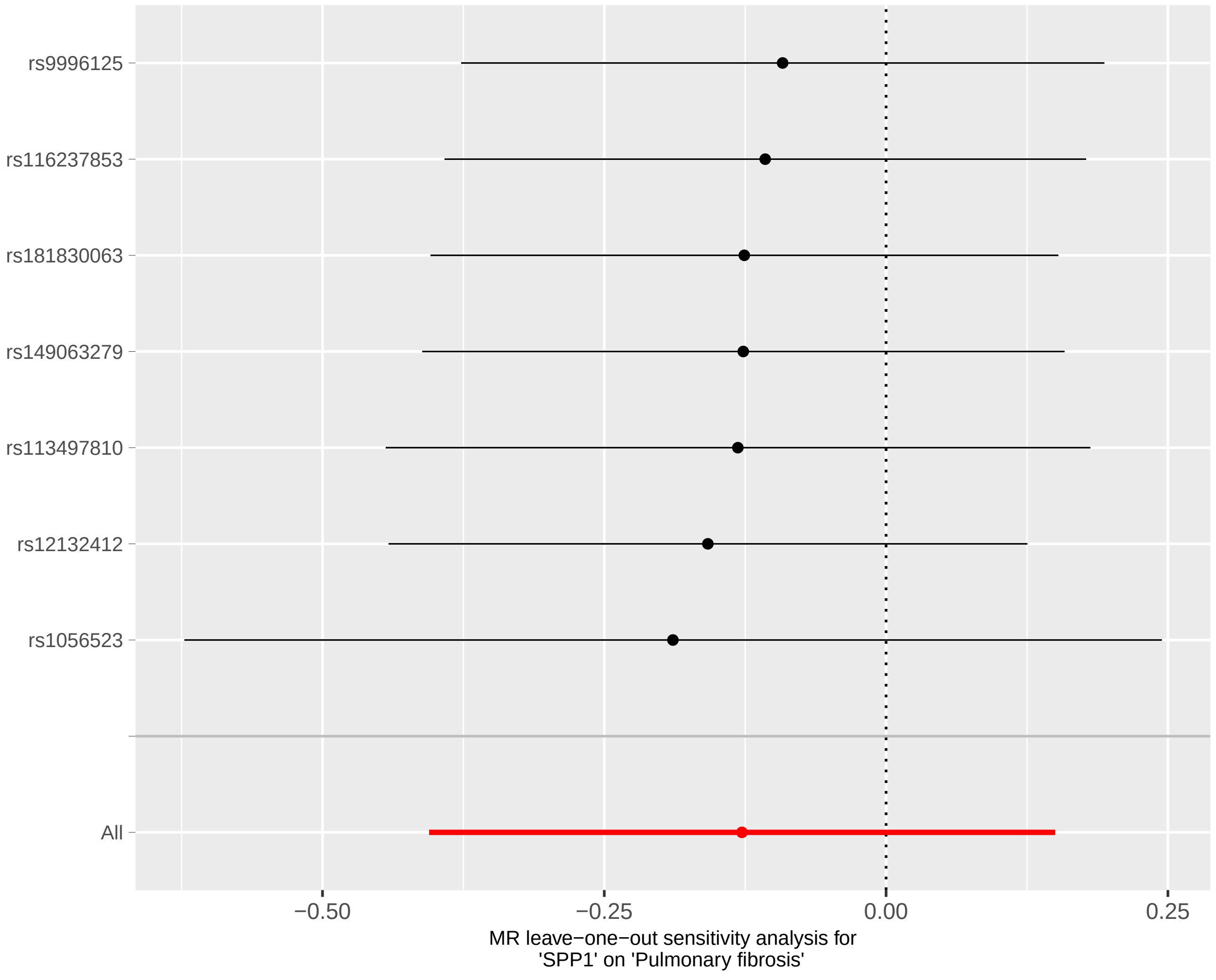

rs9996125
rs116237853
rs181830063
rs149063279
rs113497810
rs12132412
rs1056523
All
−0.50
−0.25
0.00
0.25
MR leave−one−out sensitivity analysis for
'SPP1' on 'Pulmonary fibrosis'

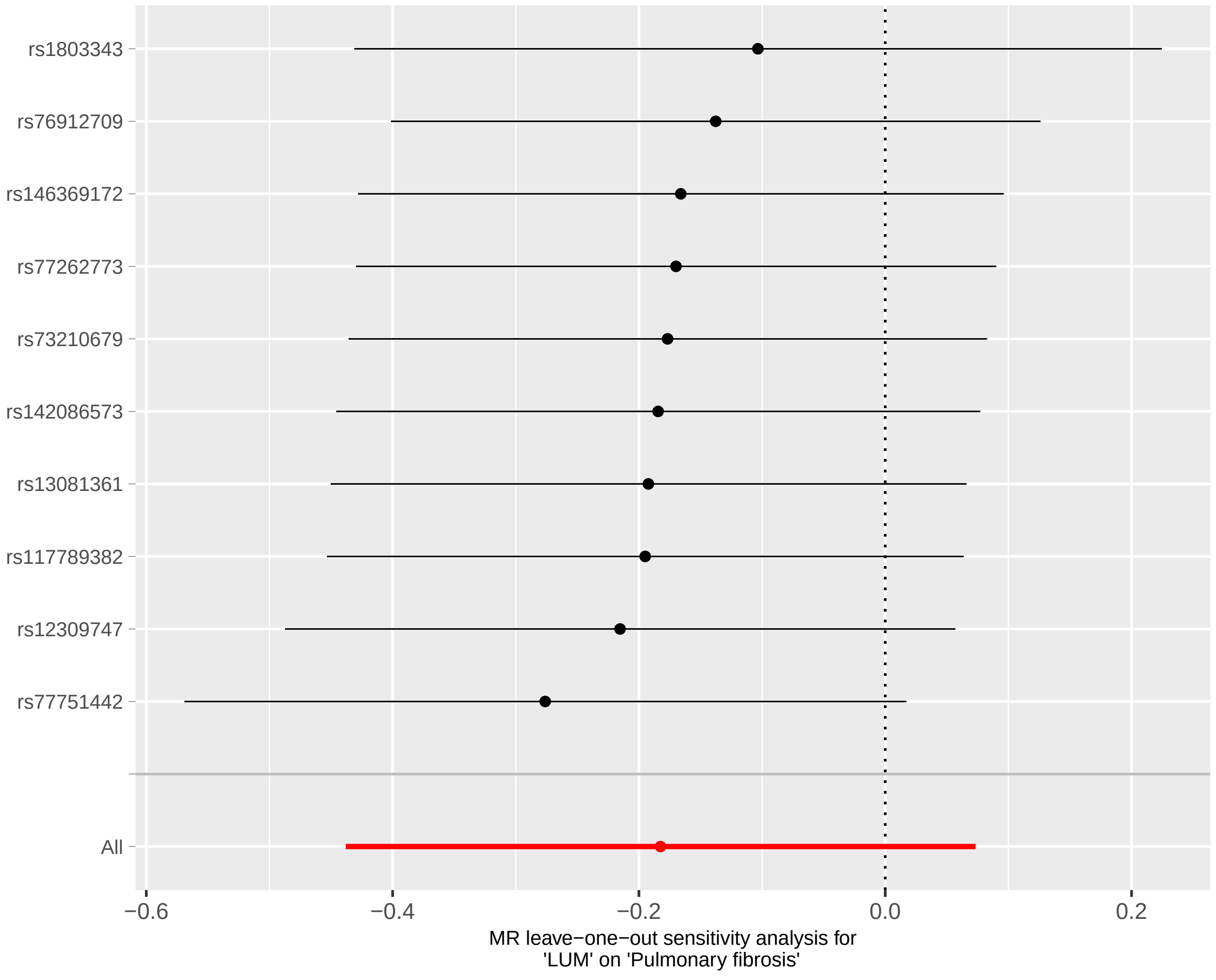
rs1803343
rs76912709
rs146369172
rs77262773
rs73210679
rs142086573
rs13081361
rs117789382
rs12309747
rs77751442
All
−0.6
−0.4
−0.2
0.0
0.2
MR leave−one−out sensitivity analysis for
'LUM' on 'Pulmonary fibrosis'

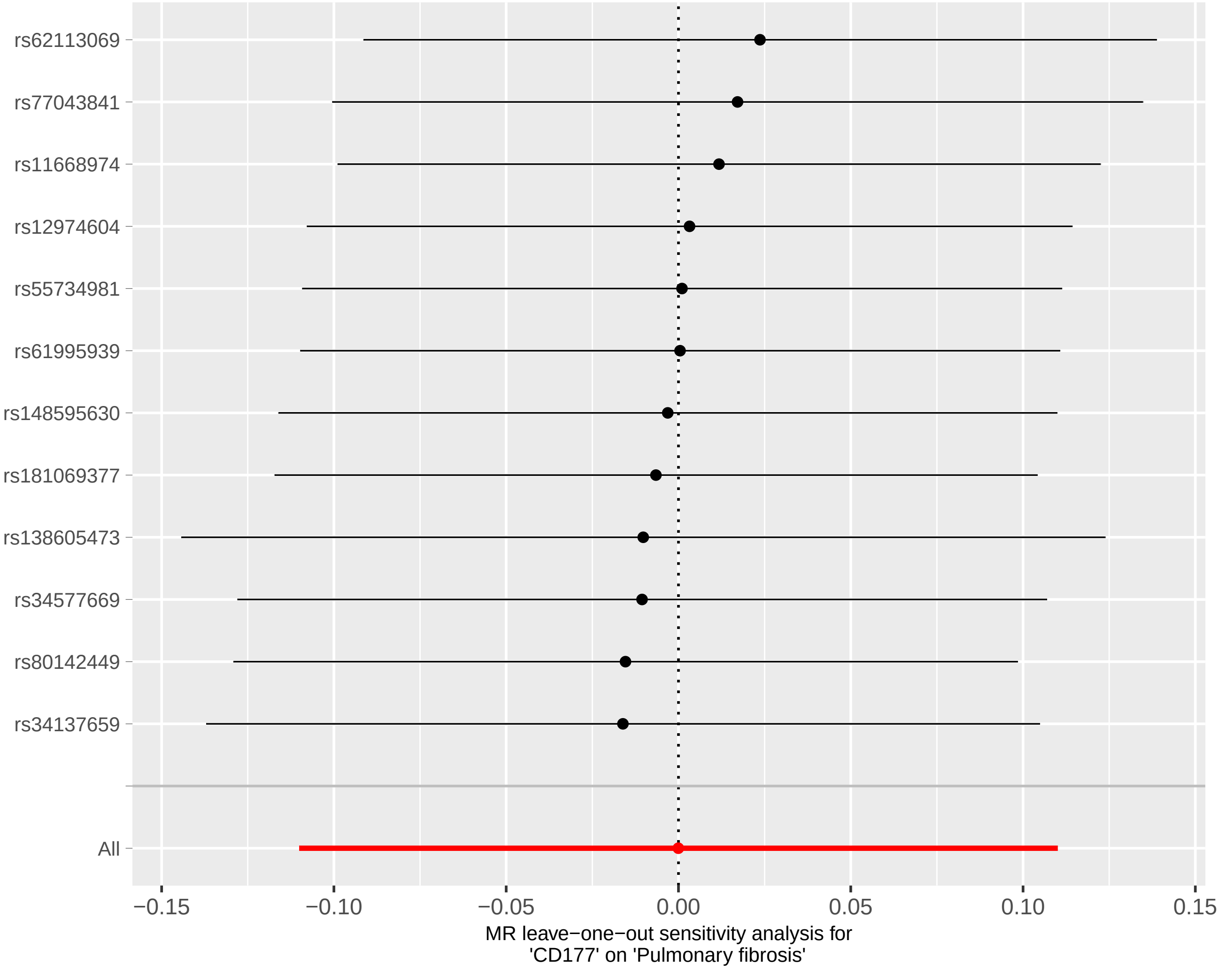

rs62113069
rs77043841
rs11668974
rs12974604
rs55734981
rs61995939
rs148595630
rs181069377
rs138605473
rs34577669
rs80142449
rs34137659
All
−0.15
−0.10
−0.05
0.00
0.05
0.10
0.15
MR leave−one−out sensitivity analysis for
'CD177' on 'Pulmonary fibrosis'

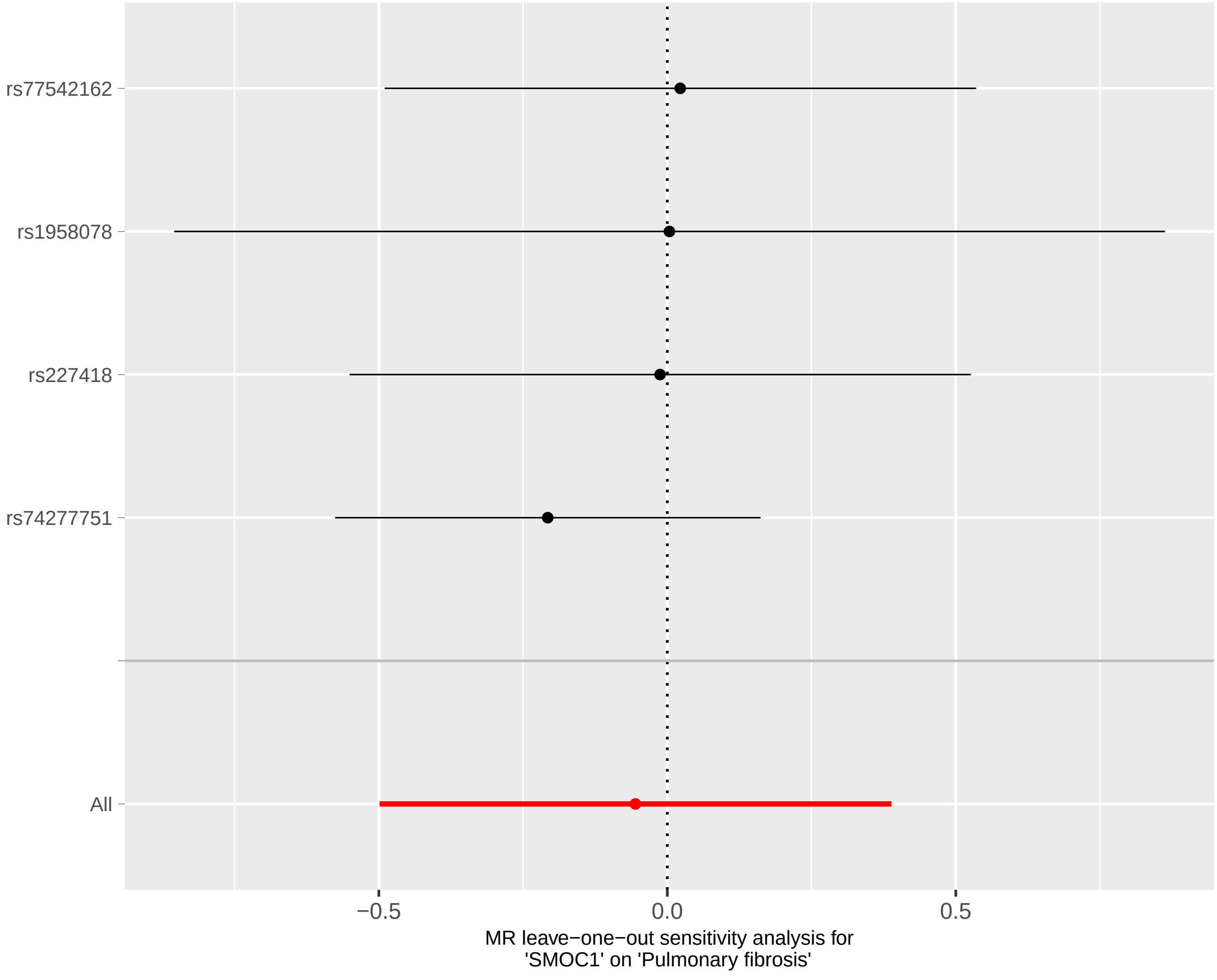
rs77542162
rs1958078
rs227418
rs74277751
All
−0.5
0.0
0.5
MR leave−one−out sensitivity analysis for
'SMOC1' on 'Pulmonary fibrosis'

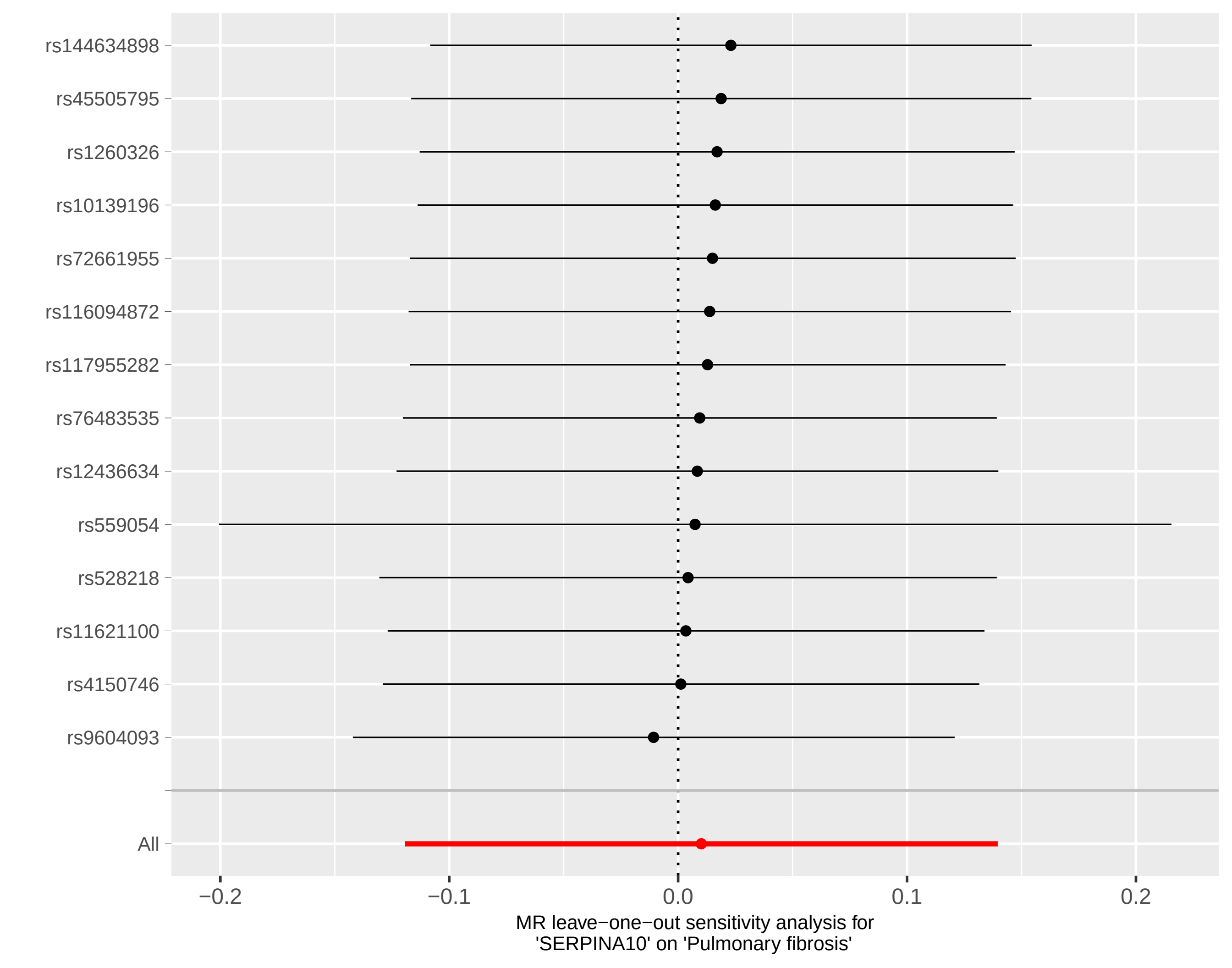

rs144634898
rs45505795
rs1260326
rs10139196
rs72661955
rs116094872
rs117955282
rs76483535
rs12436634
rs559054
rs528218
rs11621100
rs4150746
rs9604093
All
−0.2
−0.1
0.0
0.1
0.2
MR leave−one−out sensitivity analysis for
'SERPINA10' on 'Pulmonary fibrosis'

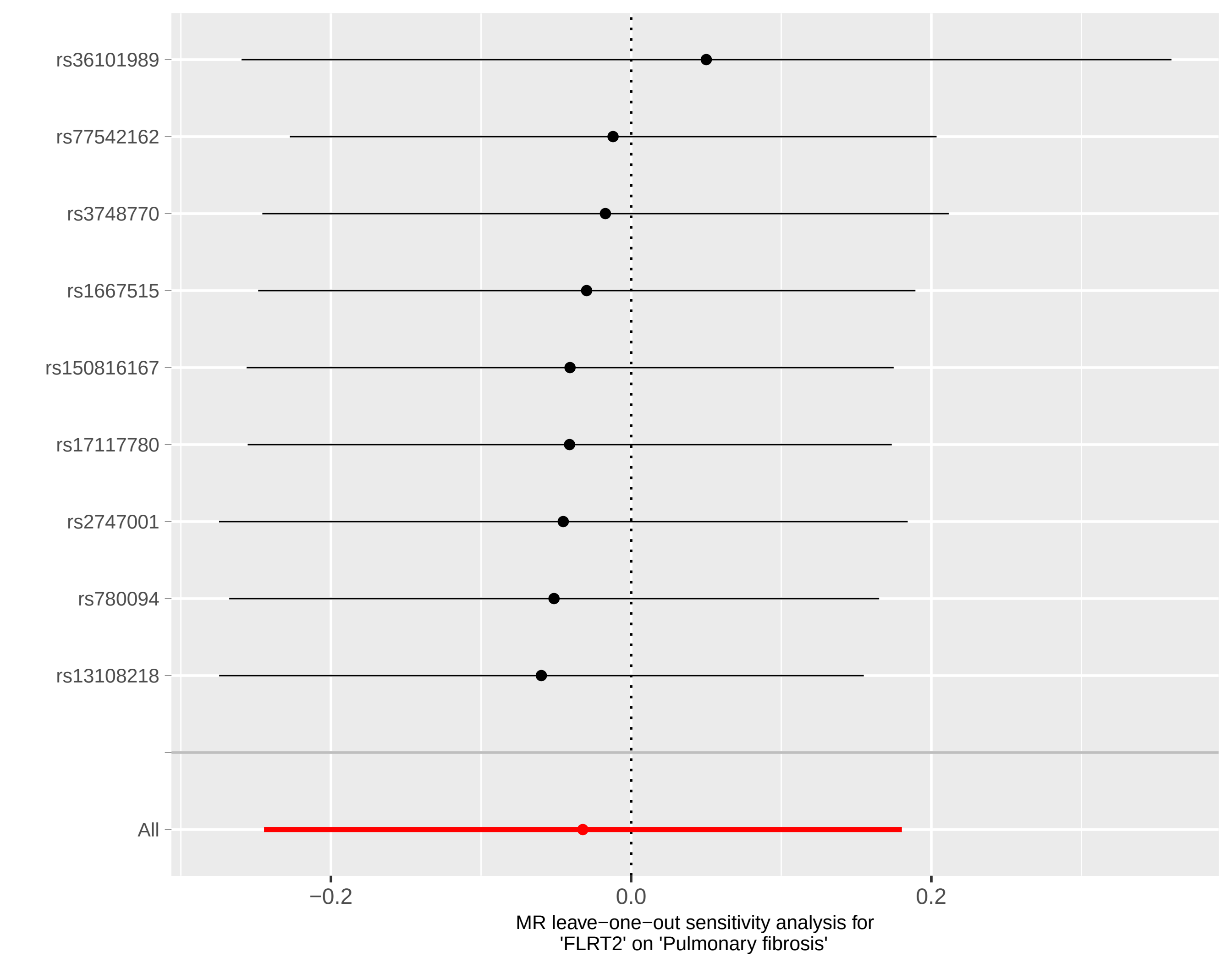

rs36101989
rs77542162
rs3748770
rs1667515
rs150816167
rs17117780
rs2747001
rs780094
rs13108218
All
−0.2
0.0
0.2
MR leave−one−out sensitivity analysis for
'FLRT2' on 'Pulmonary fibrosis'

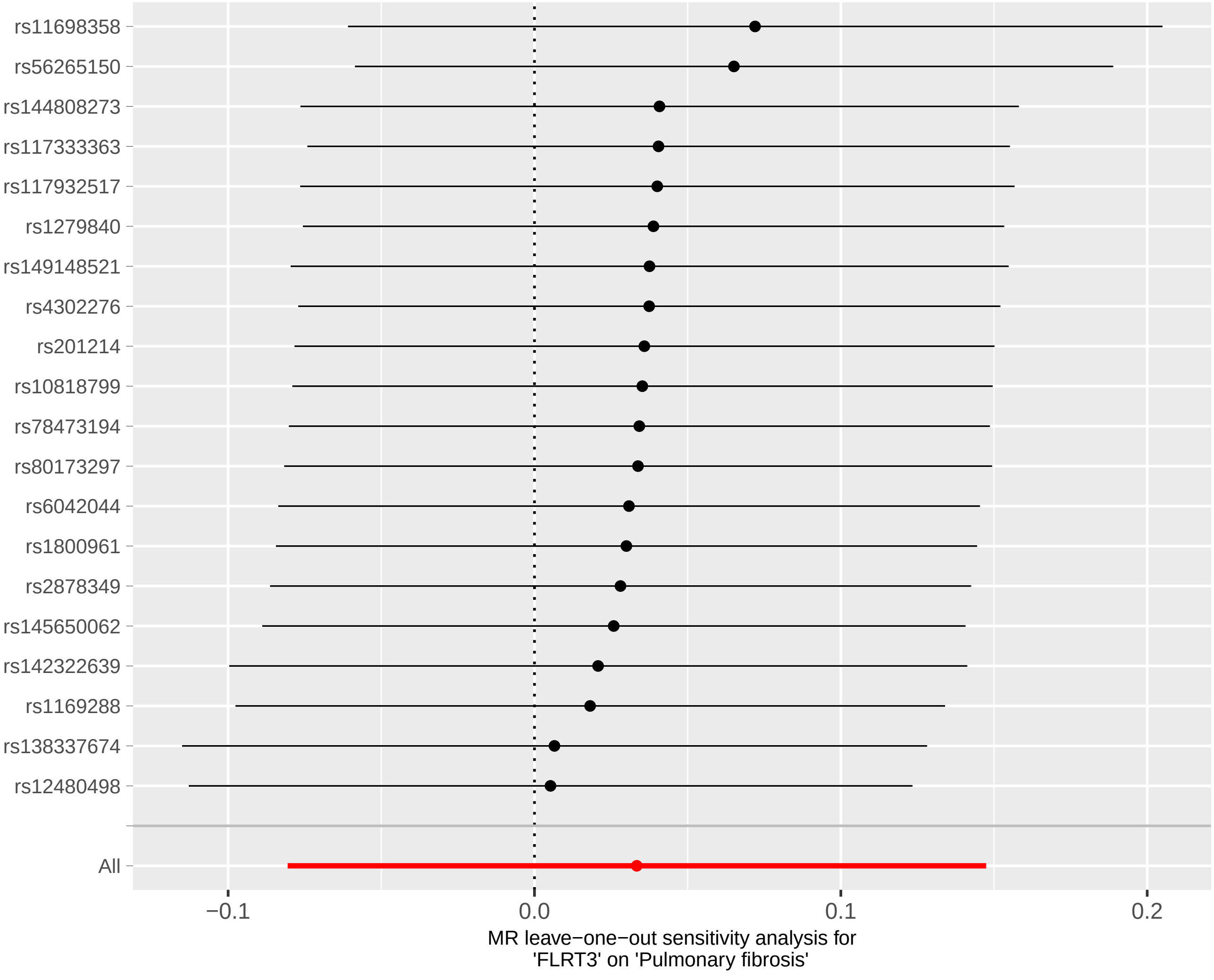
rs11698358
rs56265150
rs144808273
rs117333363
rs117932517
rs1279840
rs149148521
rs4302276
rs201214
rs10818799
rs78473194
rs80173297
rs6042044
rs1800961
rs2878349
rs145650062
rs142322639
rs1169288
rs138337674
rs12480498
All
−0.1
0.0
0.1
0.2
MR leave−one−out sensitivity analysis for
'FLRT3' on 'Pulmonary fibrosis'

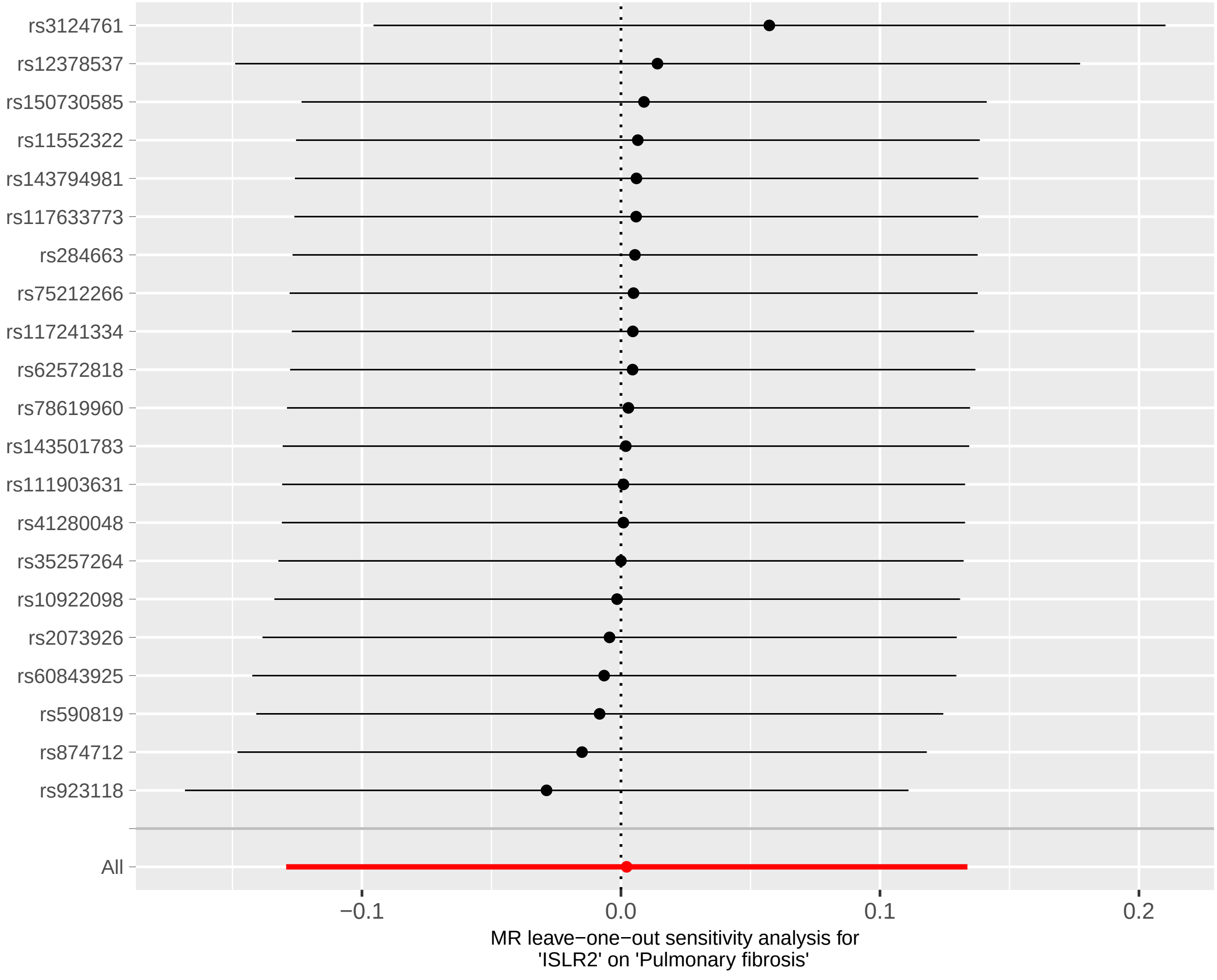
rs3124761
rs12378537
rs150730585
rs11552322
rs143794981
rs117633773
rs284663
rs75212266
rs117241334
rs62572818
rs78619960
rs143501783
rs111903631
rs41280048
rs35257264
rs10922098
rs2073926
rs60843925
rs590819
rs874712
rs923118
All
−0.1
0.0
0.1
0.2
MR leave−one−out sensitivity analysis for
'ISLR2' on 'Pulmonary fibrosis'

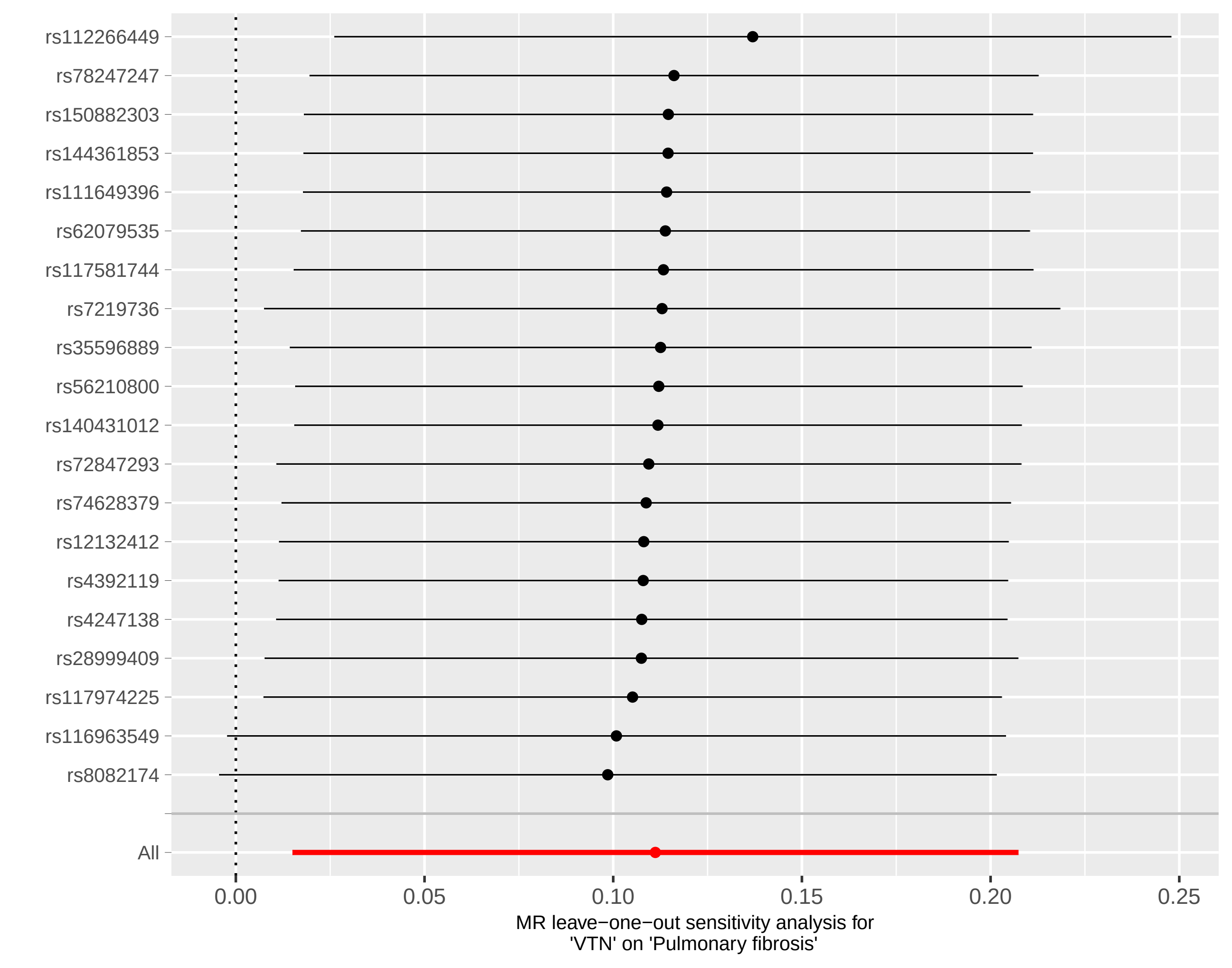

rs112266449
rs78247247
rs150882303
rs144361853
rs111649396
rs62079535
rs117581744
rs7219736
rs35596889
rs56210800
rs140431012
rs72847293
rs74628379
rs12132412
rs4392119
rs4247138
rs28999409
rs117974225
rs116963549
rs8082174
All
0.00
0.05
0.10
0.15
0.20
0.25
MR leave−one−out sensitivity analysis for
'VTN' on 'Pulmonary fibrosis'

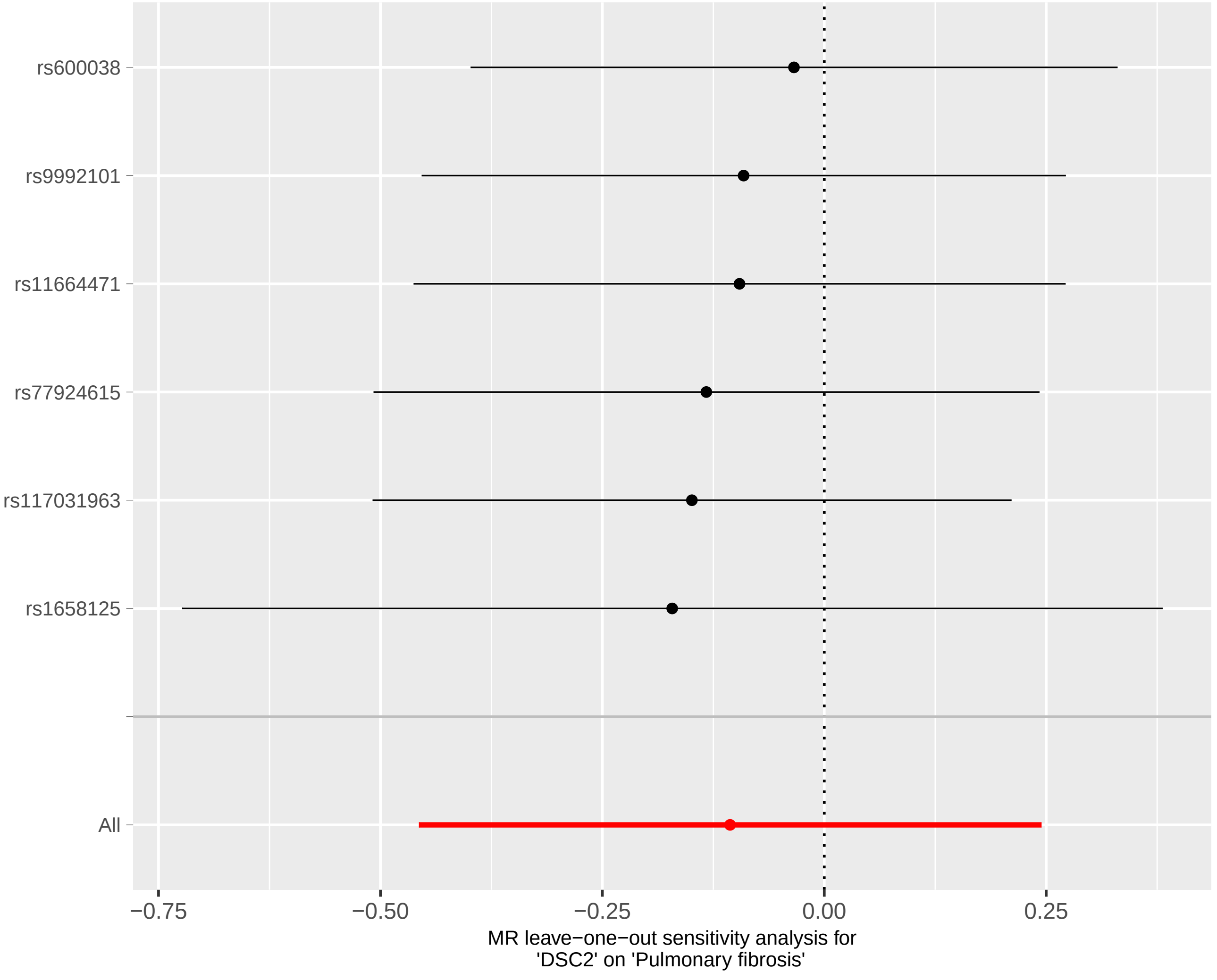
rs600038
rs9992101
rs11664471
rs77924615
rs117031963
rs1658125
All
−0.75
−0.50
−0.25
0.00
0.25
MR leave−one−out sensitivity analysis for
'DSC2' on 'Pulmonary fibrosis'

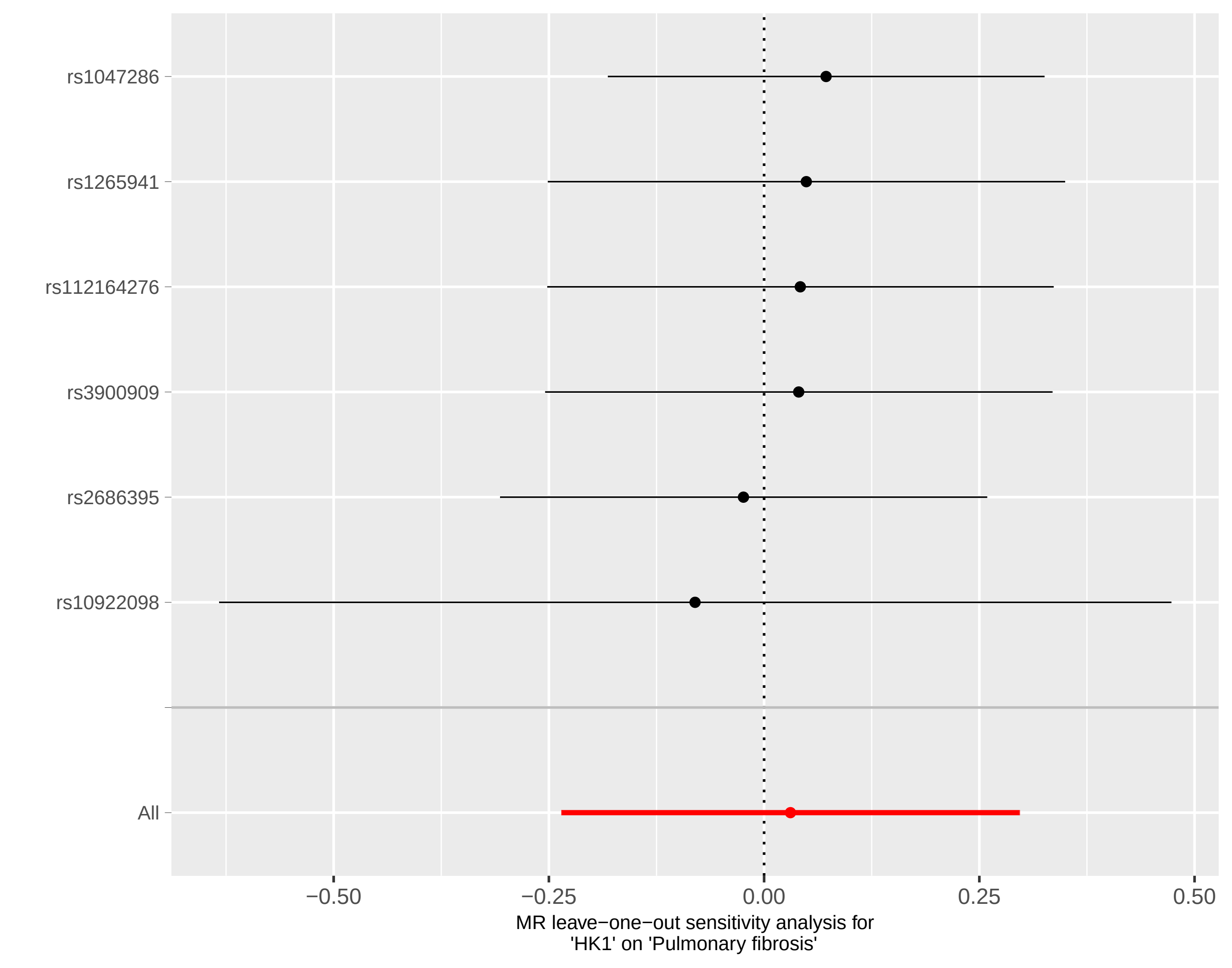
rs1047286
rs1265941
rs112164276
rs3900909
rs2686395
rs10922098
All
−0.50
−0.25
0.00
0.25
0.50
MR leave−one−out sensitivity analysis for
'HK1' on 'Pulmonary fibrosis'

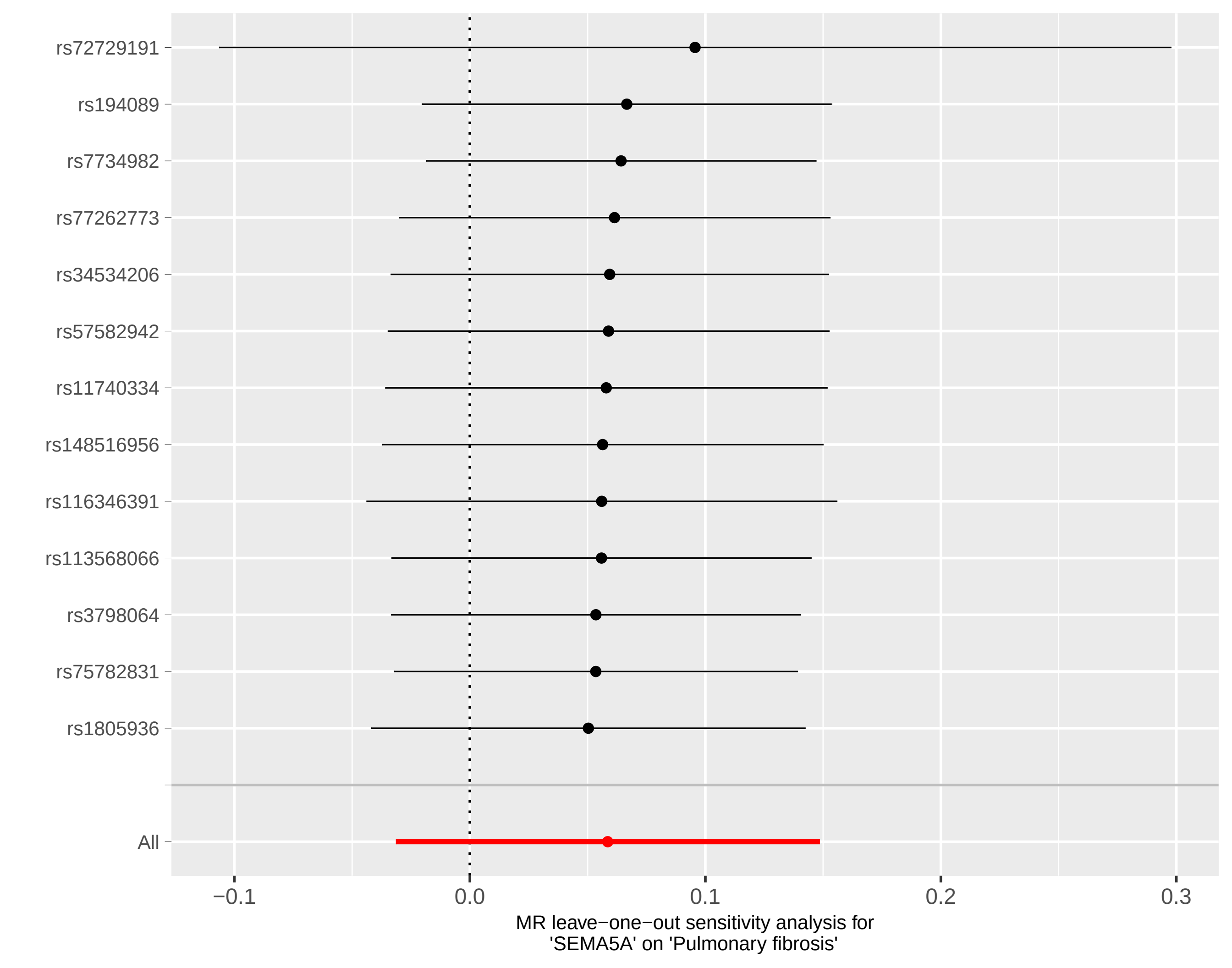

rs72729191
rs194089
rs7734982
rs77262773
rs34534206
rs57582942
rs11740334
rs148516956
rs116346391
rs113568066
rs3798064
rs75782831
rs1805936
All
−0.1
0.0
0.1
0.2
0.3
MR leave−one−out sensitivity analysis for
'SEMA5A' on 'Pulmonary fibrosis'

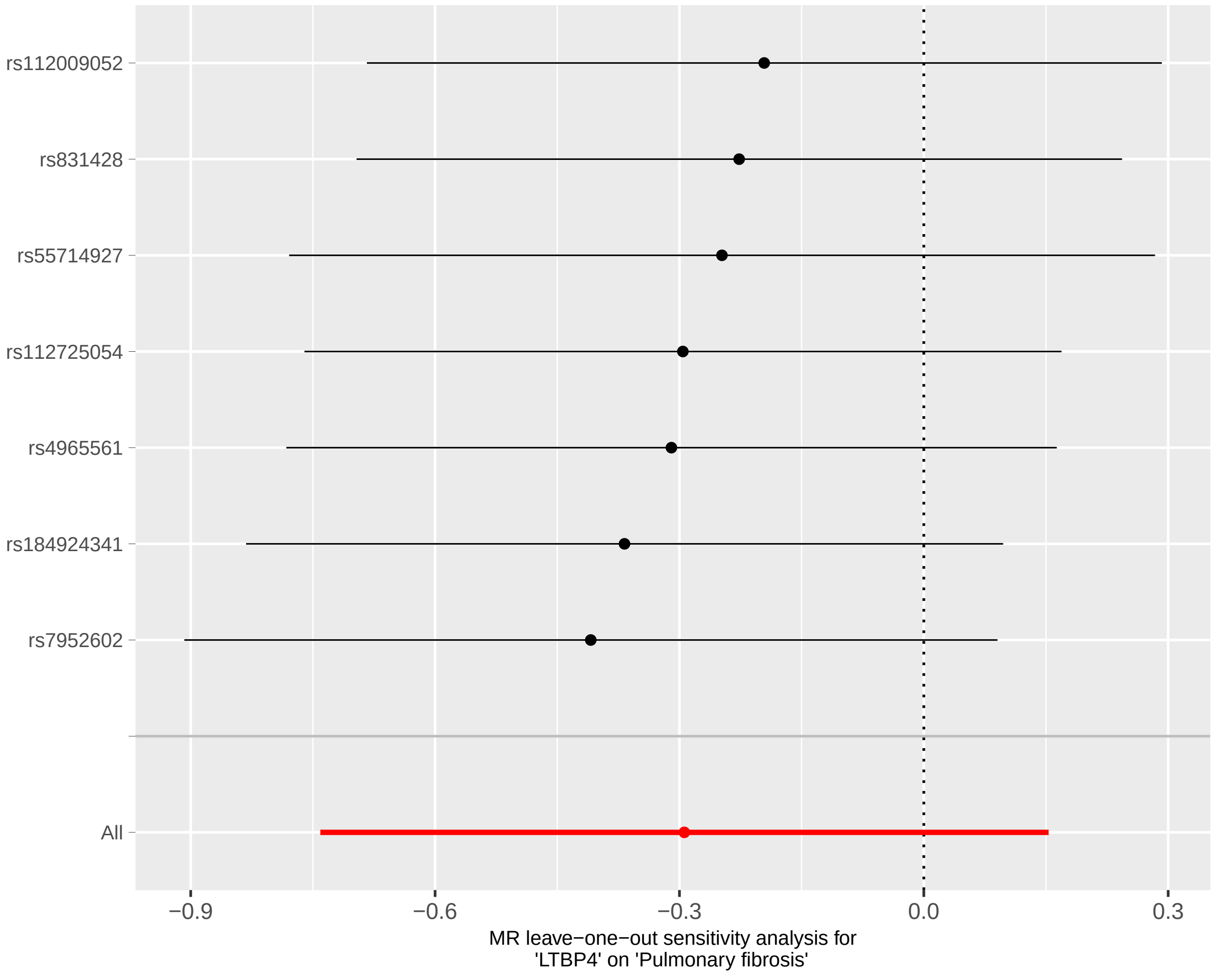
rs112009052
rs831428
rs55714927
rs112725054
rs4965561
rs184924341
rs7952602
All
−0.9
−0.6
−0.3
0.0
0.3
MR leave−one−out sensitivity analysis for
'LTBP4' on 'Pulmonary fibrosis'

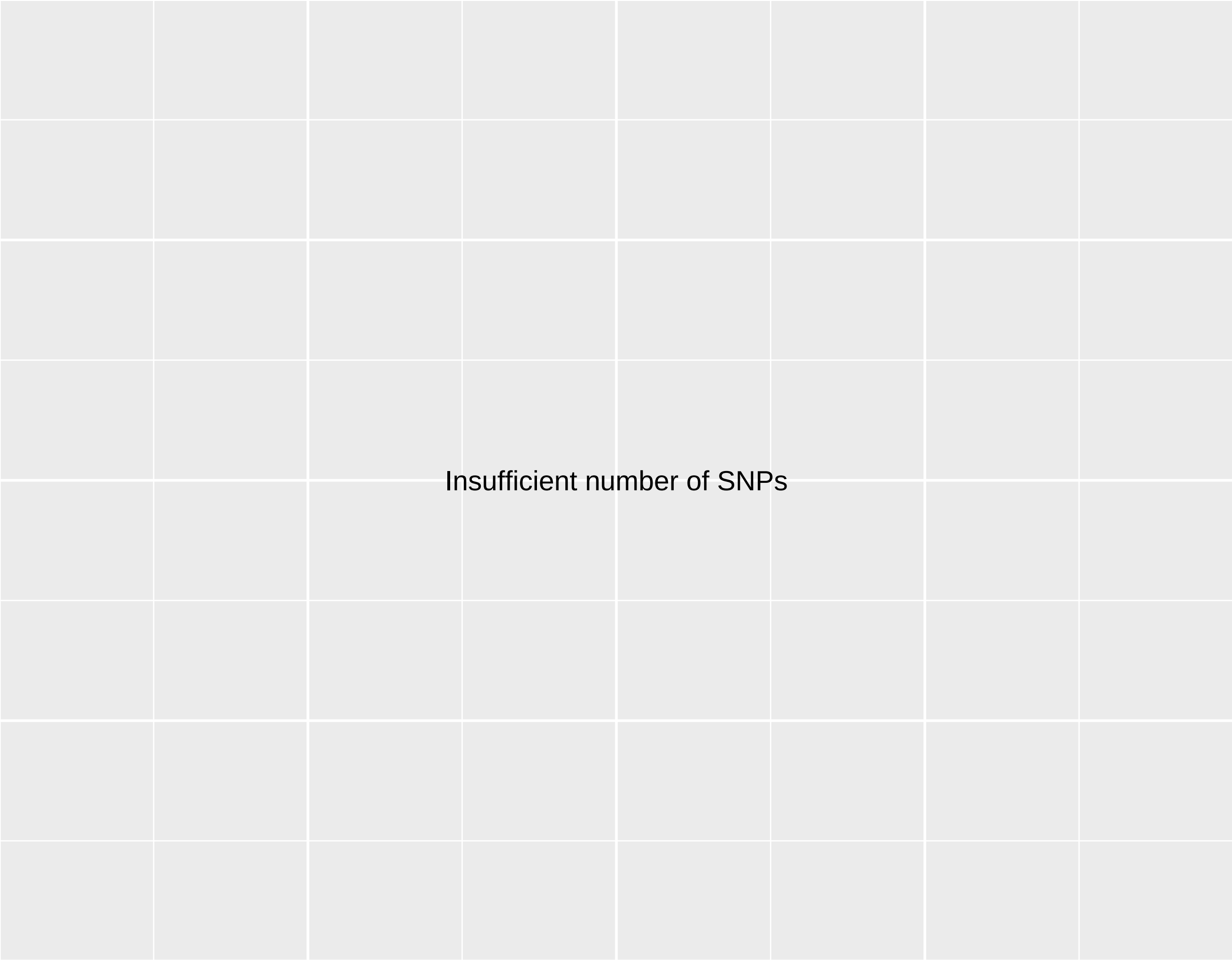
Insufficient number of SNPs

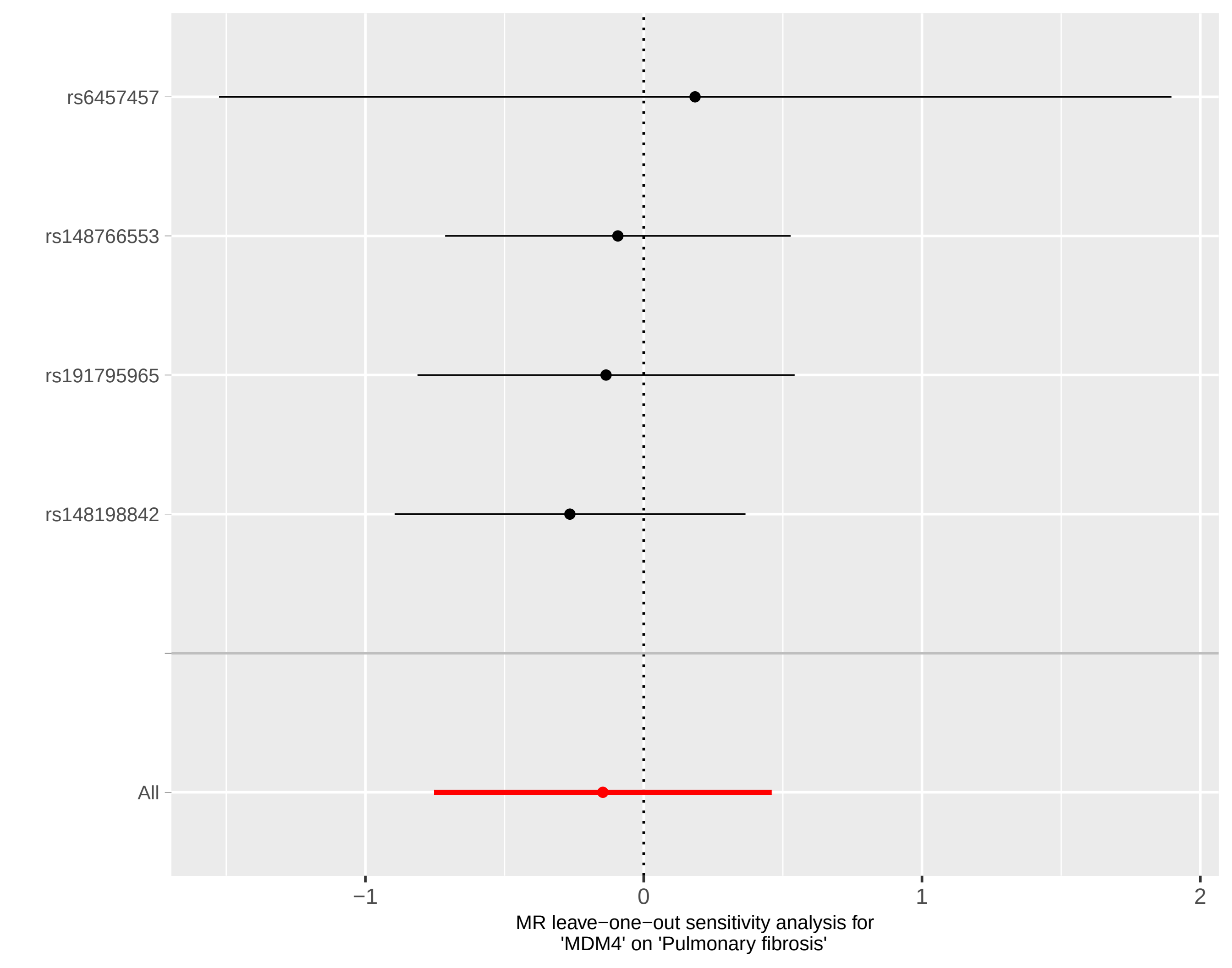

rs6457457
rs148766553
rs191795965
rs148198842
All
−1
0
1
2
MR leave−one−out sensitivity analysis for
'MDM4' on 'Pulmonary fibrosis'

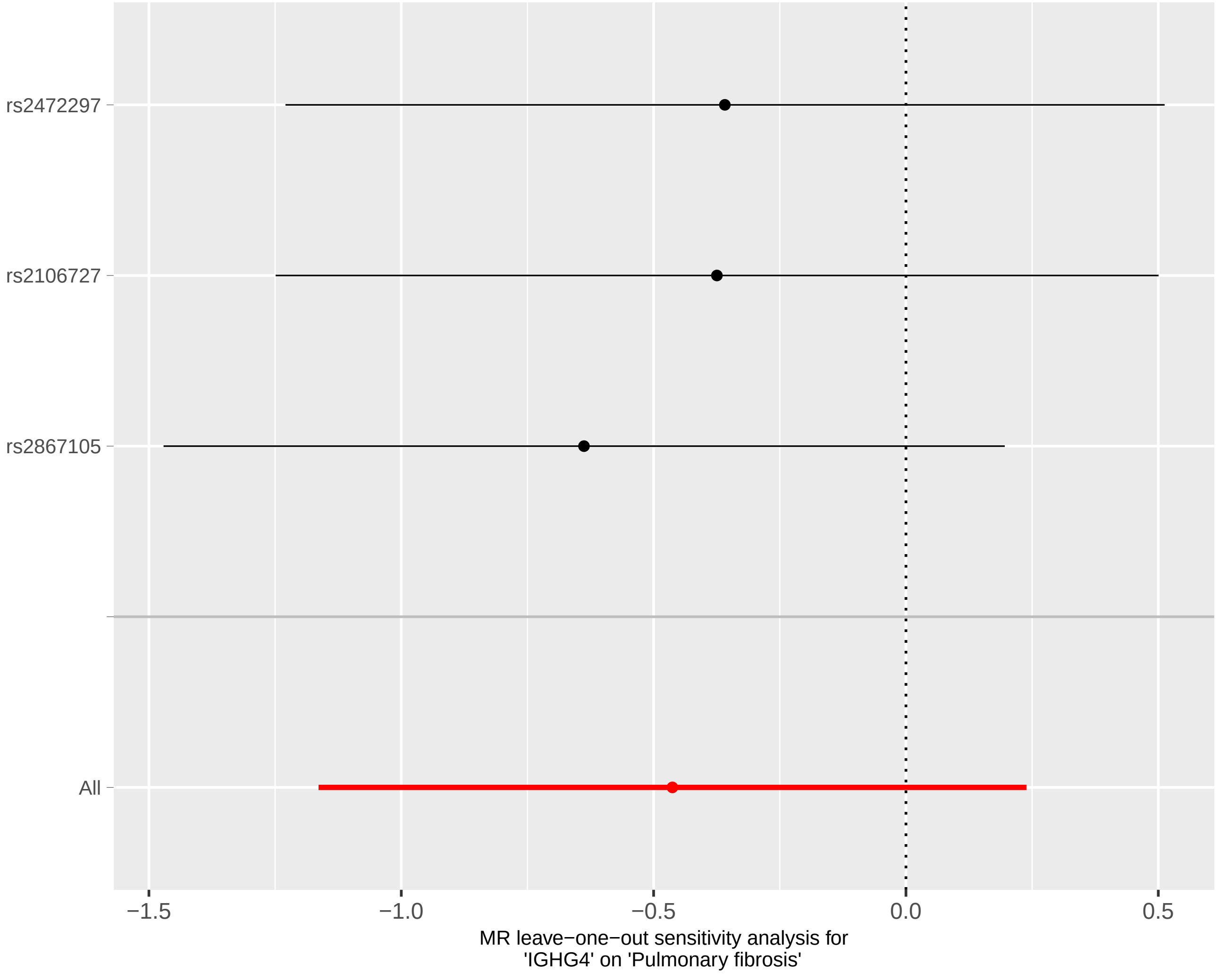
rs2472297
rs2106727
rs2867105
All
−1.5
−1.0
−0.5
0.0
0.5
MR leave−one−out sensitivity analysis for
'IGHG4' on 'Pulmonary fibrosis'

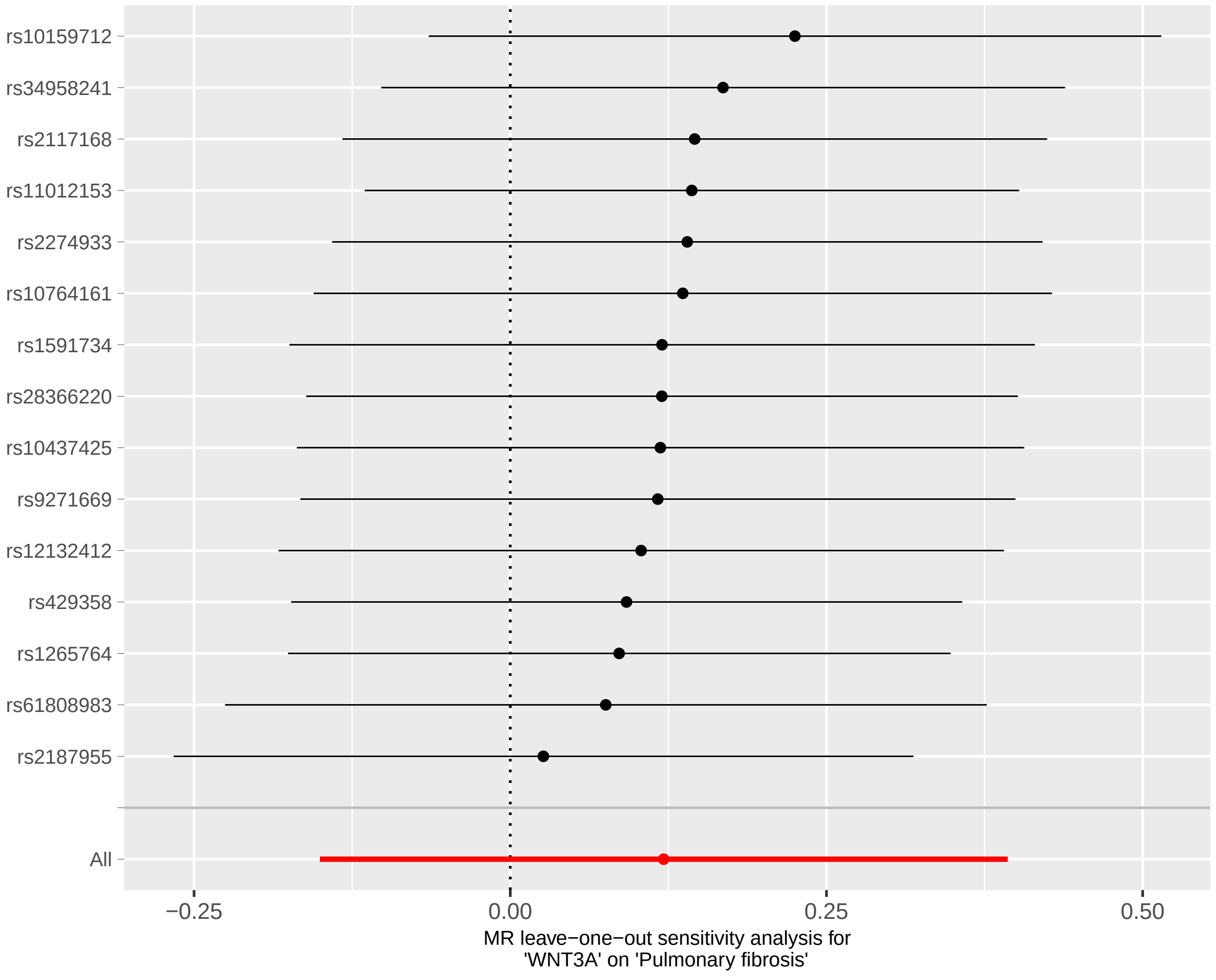
rs10159712
rs34958241
rs2117168
rs11012153
rs2274933
rs10764161
rs1591734
rs28366220
rs10437425
rs9271669
rs12132412
rs429358
rs1265764
rs61808983
rs2187955
All
−0.25
0.00
0.25
0.50
MR leave−one−out sensitivity analysis for
'WNT3A' on 'Pulmonary fibrosis'

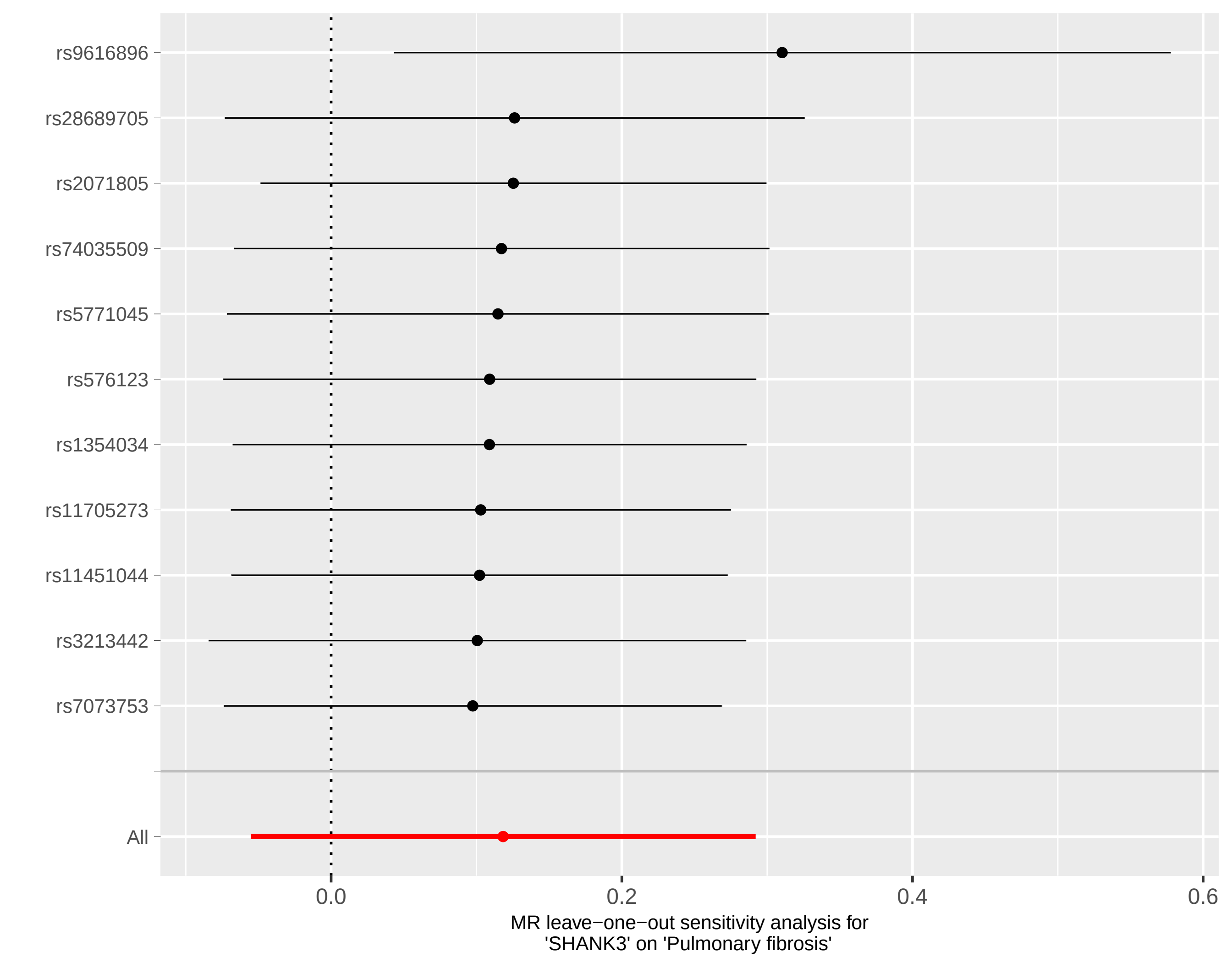

rs9616896
rs28689705
rs2071805
rs74035509
rs5771045
rs576123
rs1354034
rs11705273
rs11451044
rs3213442
rs7073753
All
0.0
0.2
0.4
0.6
MR leave−one−out sensitivity analysis for
'SHANK3' on 'Pulmonary fibrosis'

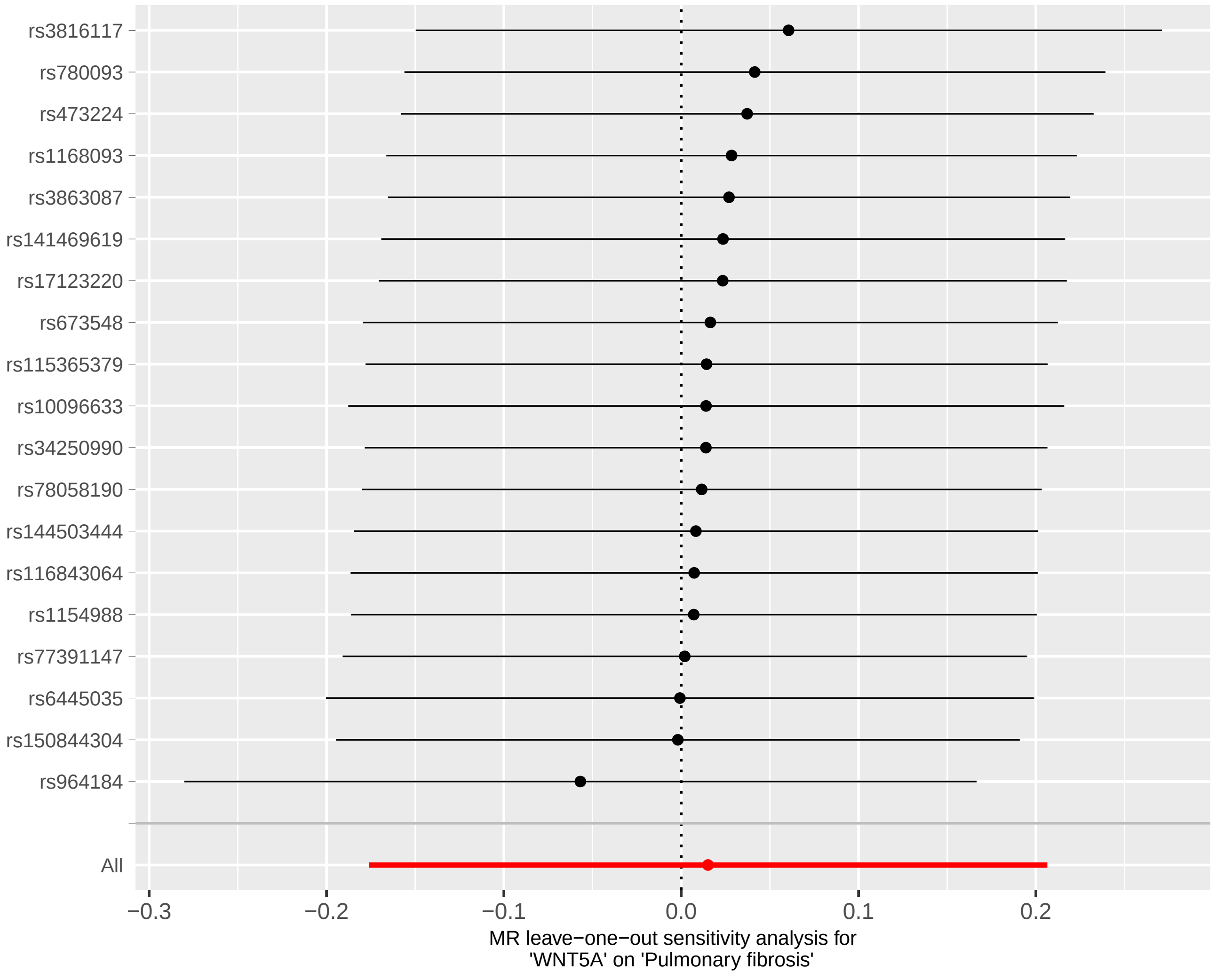
rs3816117
rs780093
rs473224
rs1168093
rs3863087
rs141469619
rs17123220
rs673548
rs115365379
rs10096633
rs34250990
rs78058190
rs144503444
rs116843064
rs1154988
rs77391147
rs6445035
rs150844304
rs964184
All
−0.3
−0.2
−0.1
0.0
0.1
0.2
MR leave−one−out sensitivity analysis for
'WNT5A' on 'Pulmonary fibrosis'

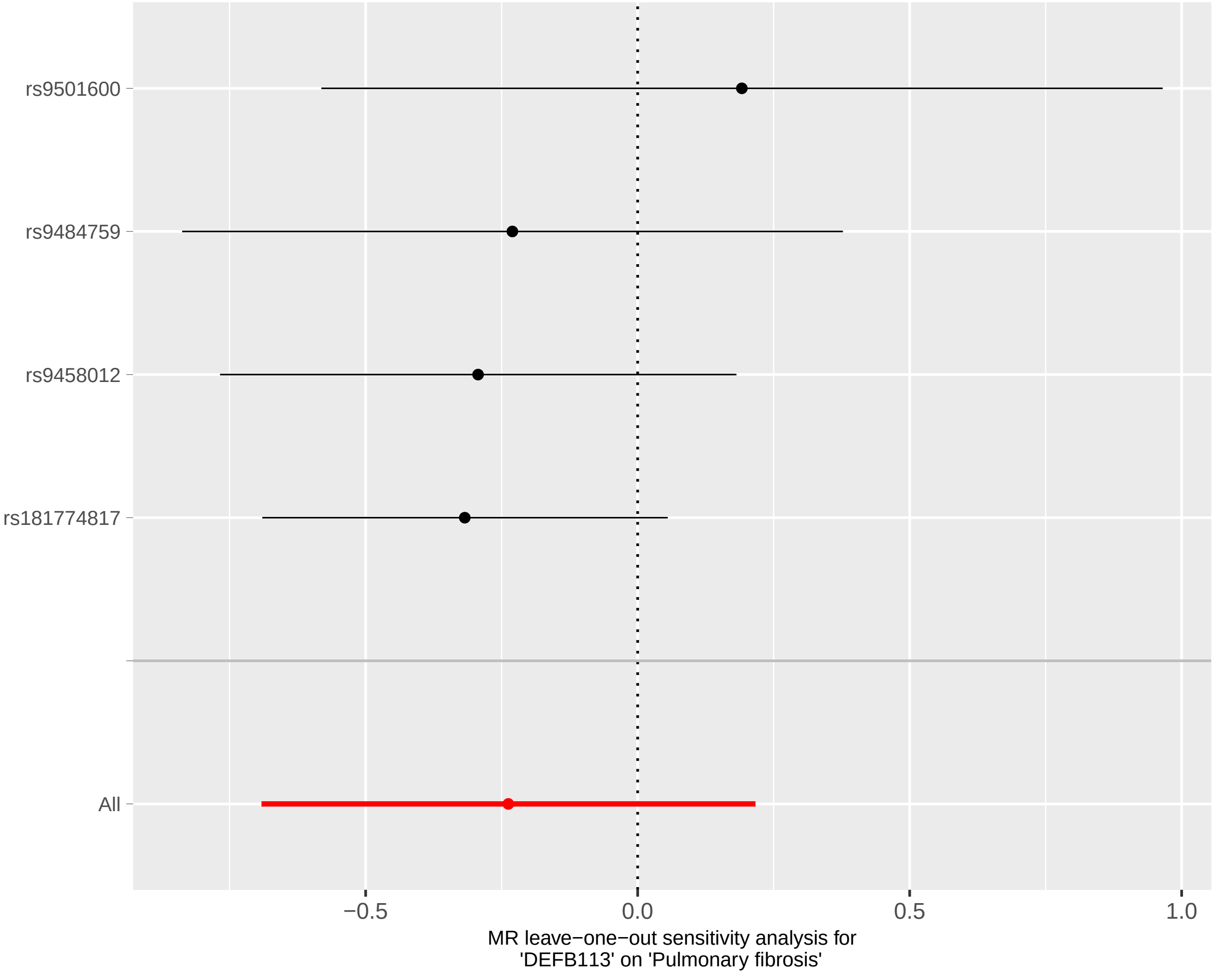
rs9501600
rs9484759
rs9458012
rs181774817
All
−0.5
0.0
0.5
1.0
MR leave−one−out sensitivity analysis for
'DEFB113' on 'Pulmonary fibrosis'

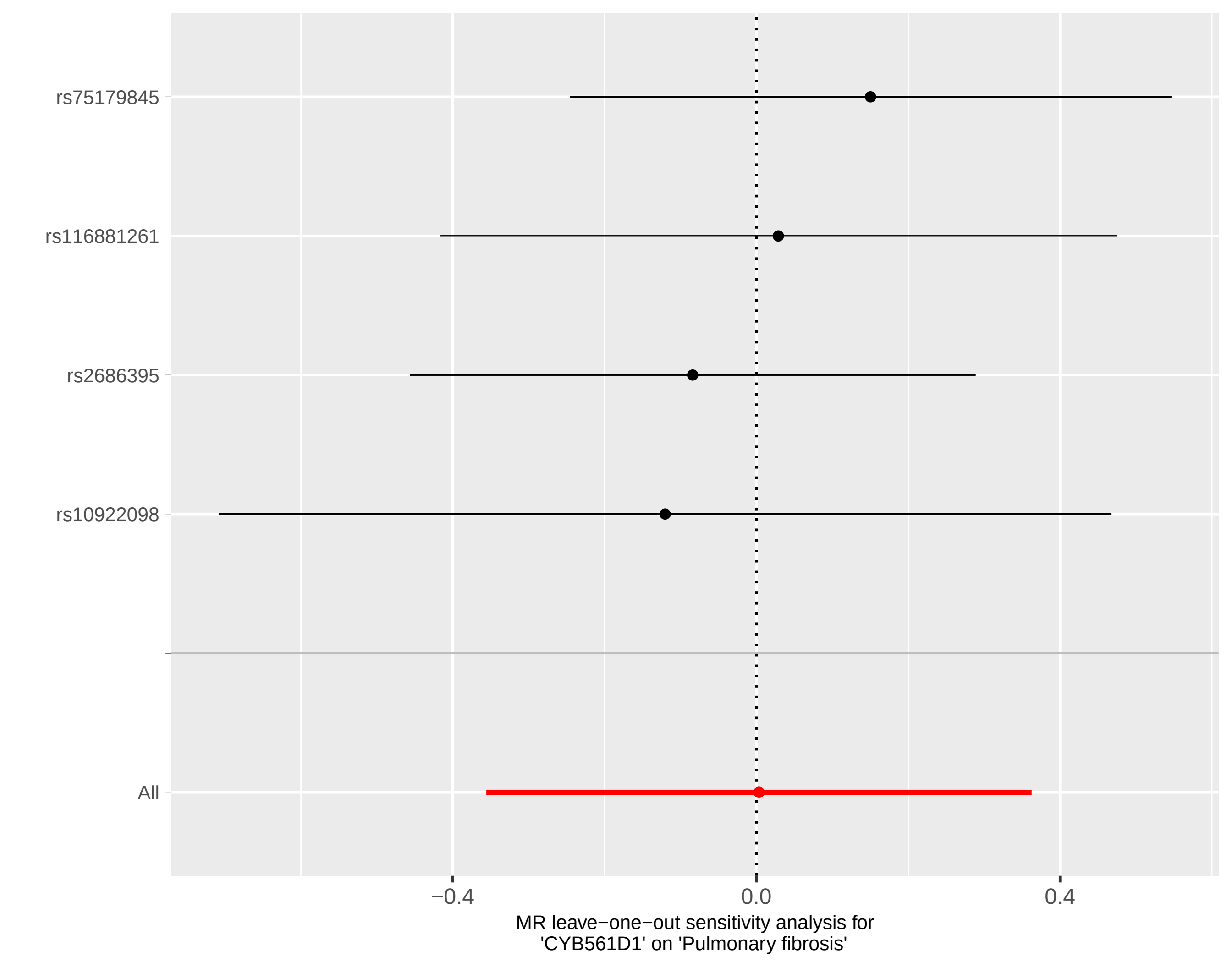
rs75179845
rs116881261
rs2686395
rs10922098
All
−0.4
0.0
0.4
MR leave−one−out sensitivity analysis for
'CYB561D1' on 'Pulmonary fibrosis'

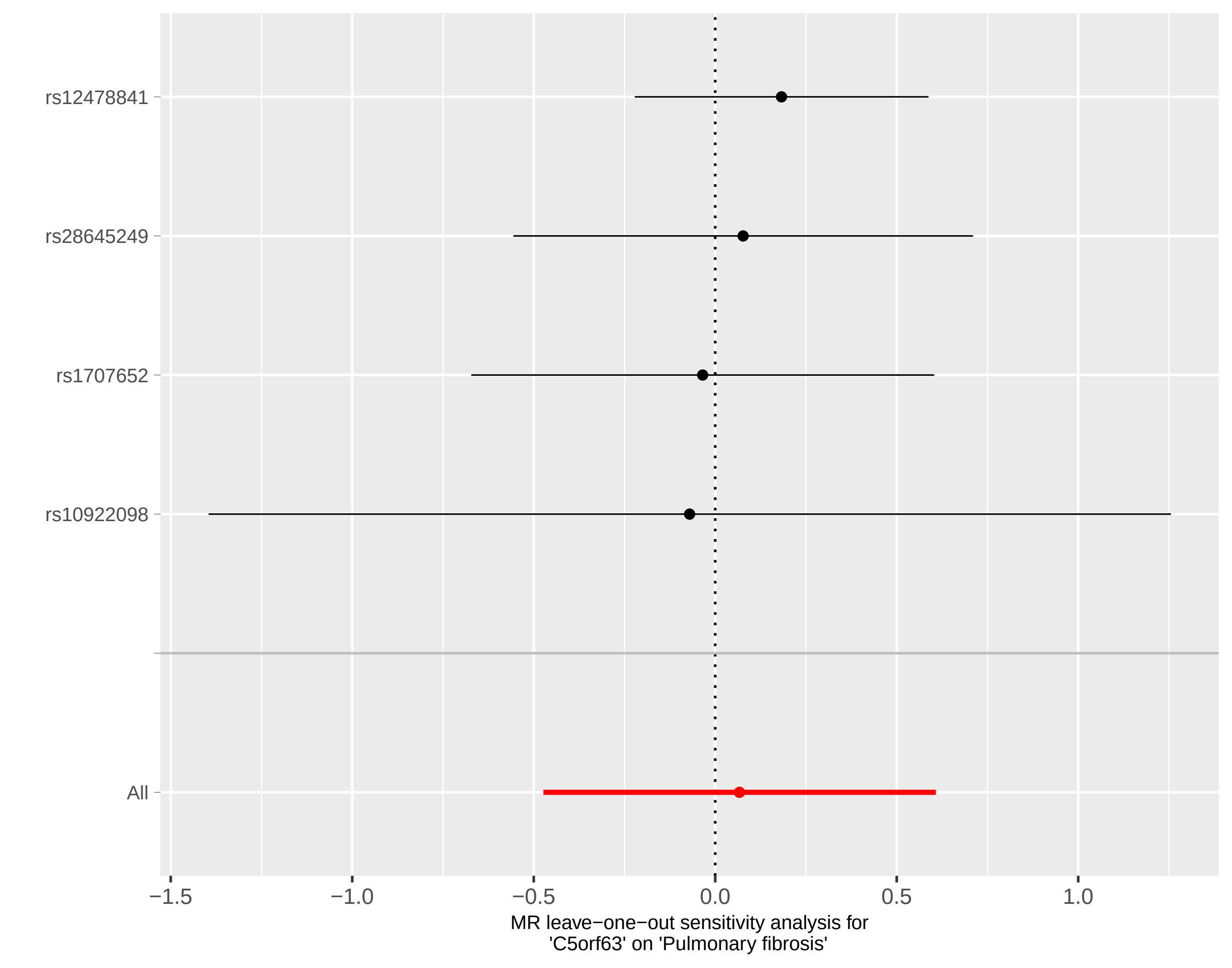

rs12478841
rs28645249
rs1707652
rs10922098
All
−1.5
−1.0
−0.5
0.0
0.5
1.0
MR leave−one−out sensitivity analysis for
'C5orf63' on 'Pulmonary fibrosis'

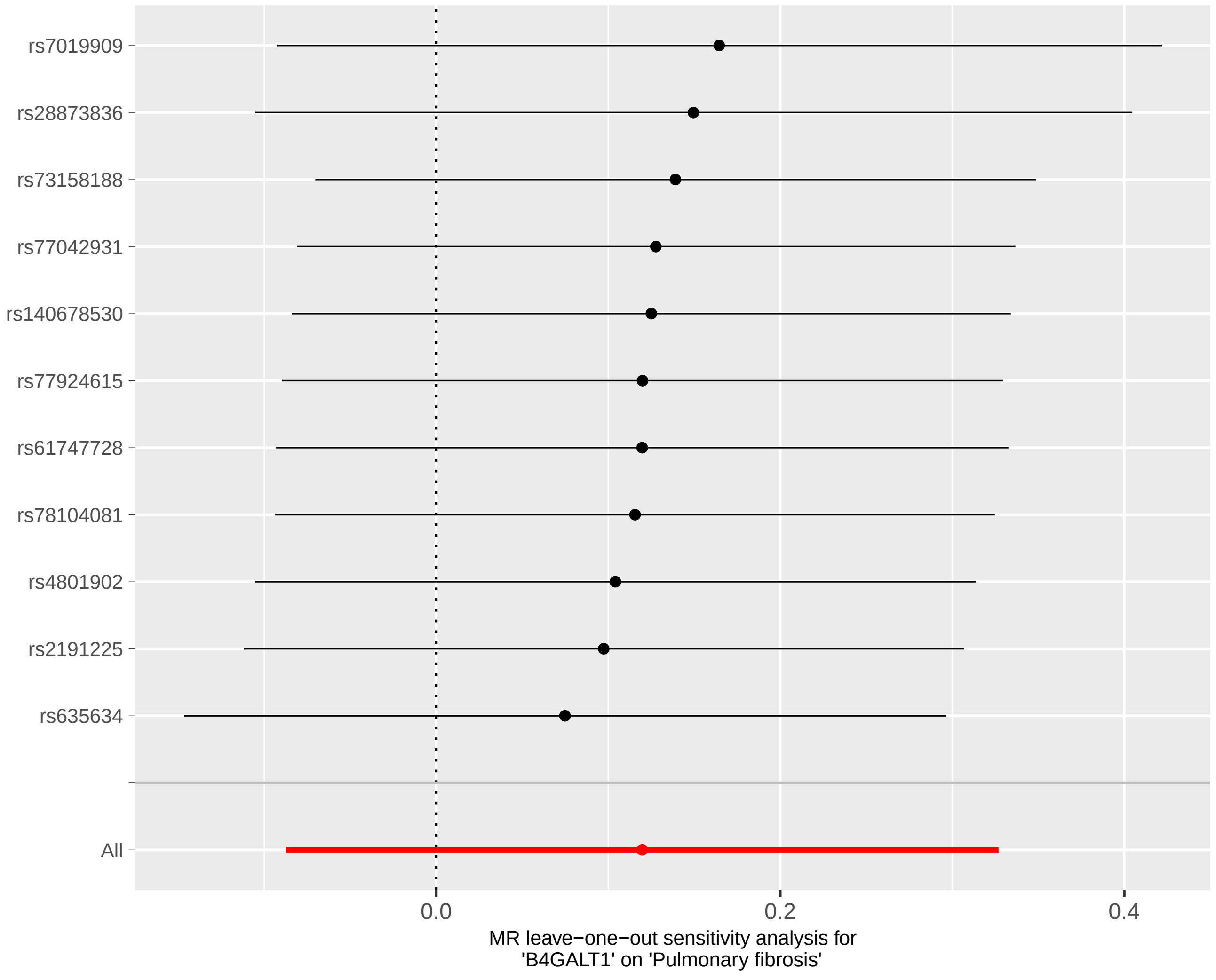
rs7019909
rs28873836
rs73158188
rs77042931
rs140678530
rs77924615
rs61747728
rs78104081
rs4801902
rs2191225
rs635634
All
0.0
0.2
0.4
MR leave−one−out sensitivity analysis for
'B4GALT1' on 'Pulmonary fibrosis'

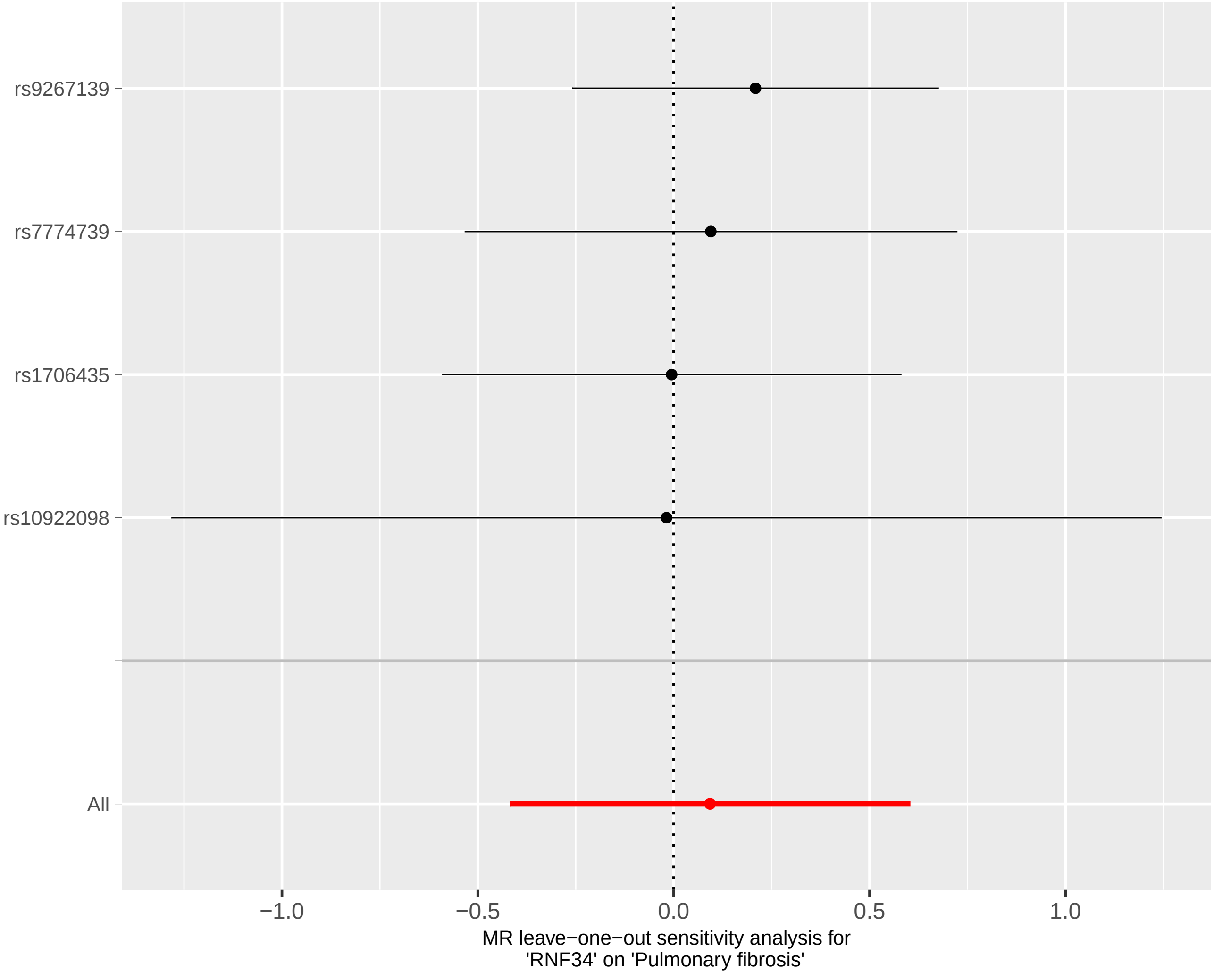
rs9267139
rs7774739
rs1706435
rs10922098
All
−1.0
−0.5
0.0
0.5
1.0
MR leave−one−out sensitivity analysis for
'RNF34' on 'Pulmonary fibrosis'

Insufficient number of SNPs

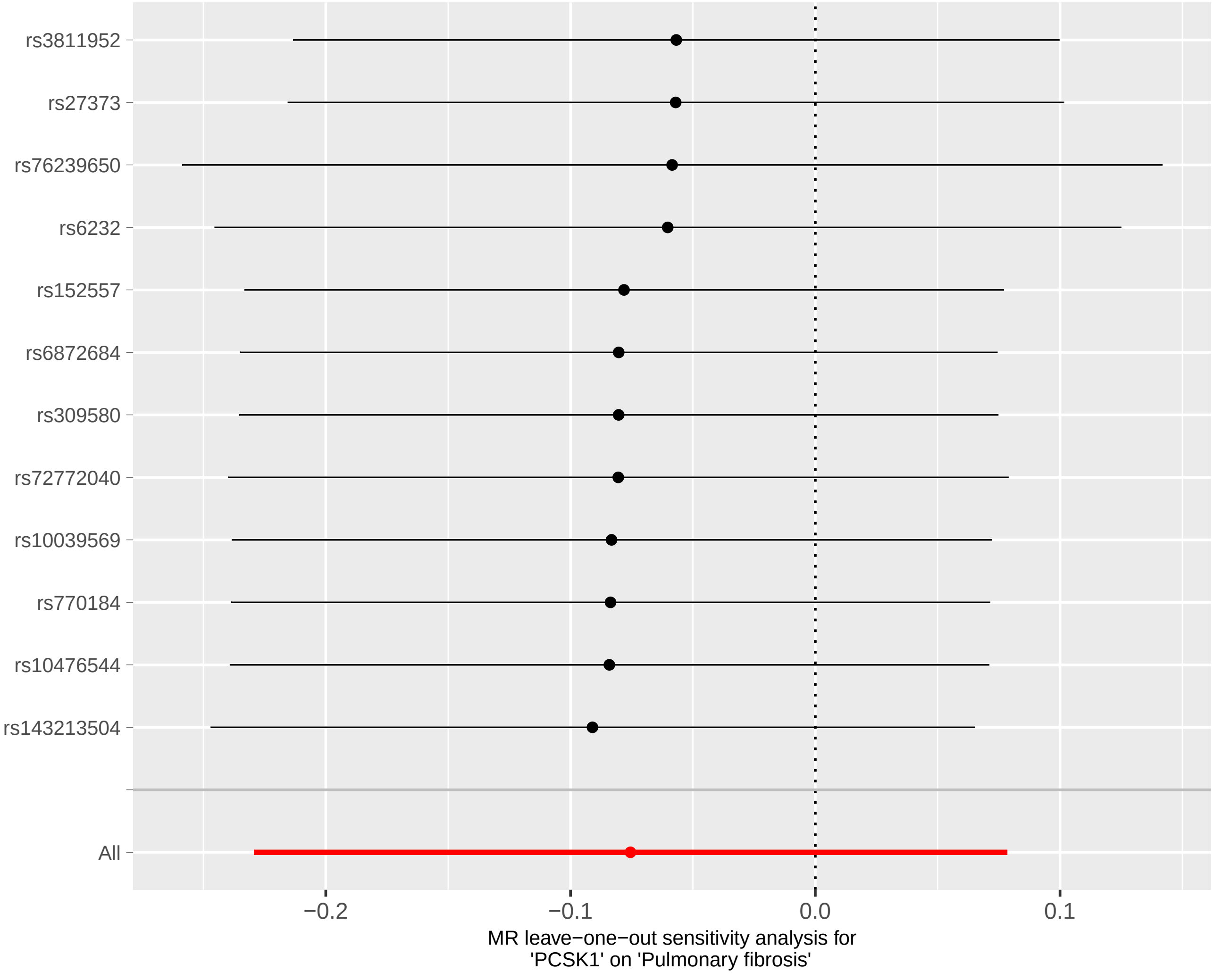

rs3811952
rs27373
rs76239650
rs6232
rs152557
rs6872684
rs309580
rs72772040
rs10039569
rs770184
rs10476544
rs143213504
All
−0.2
−0.1
0.0
0.1
MR leave−one−out sensitivity analysis for
'PCSK1' on 'Pulmonary fibrosis'

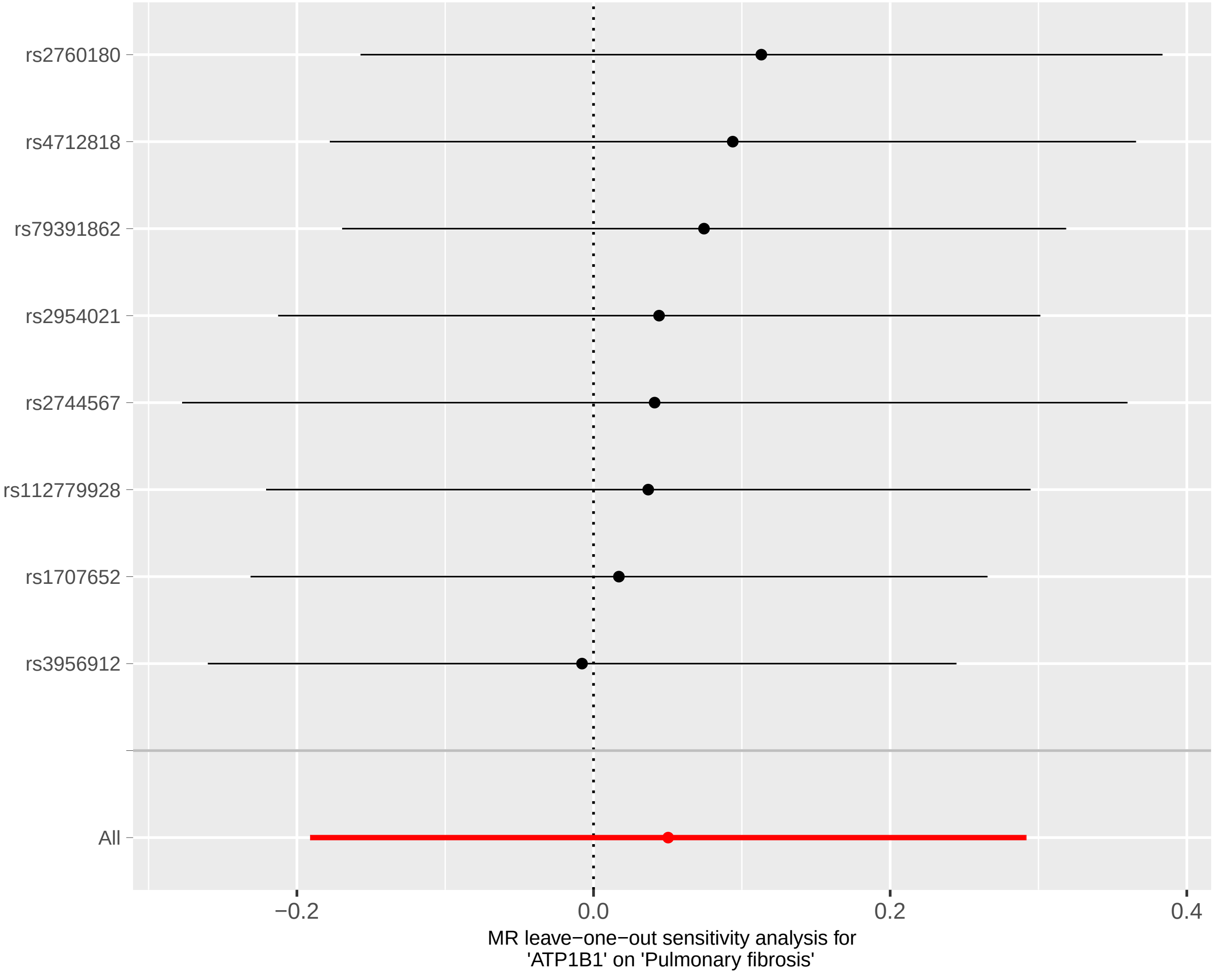
rs2760180
rs4712818
rs79391862
rs2954021
rs2744567
rs112779928
rs1707652
rs3956912
All
−0.2
0.0
0.2
0.4
MR leave−one−out sensitivity analysis for
'ATP1B1' on 'Pulmonary fibrosis'

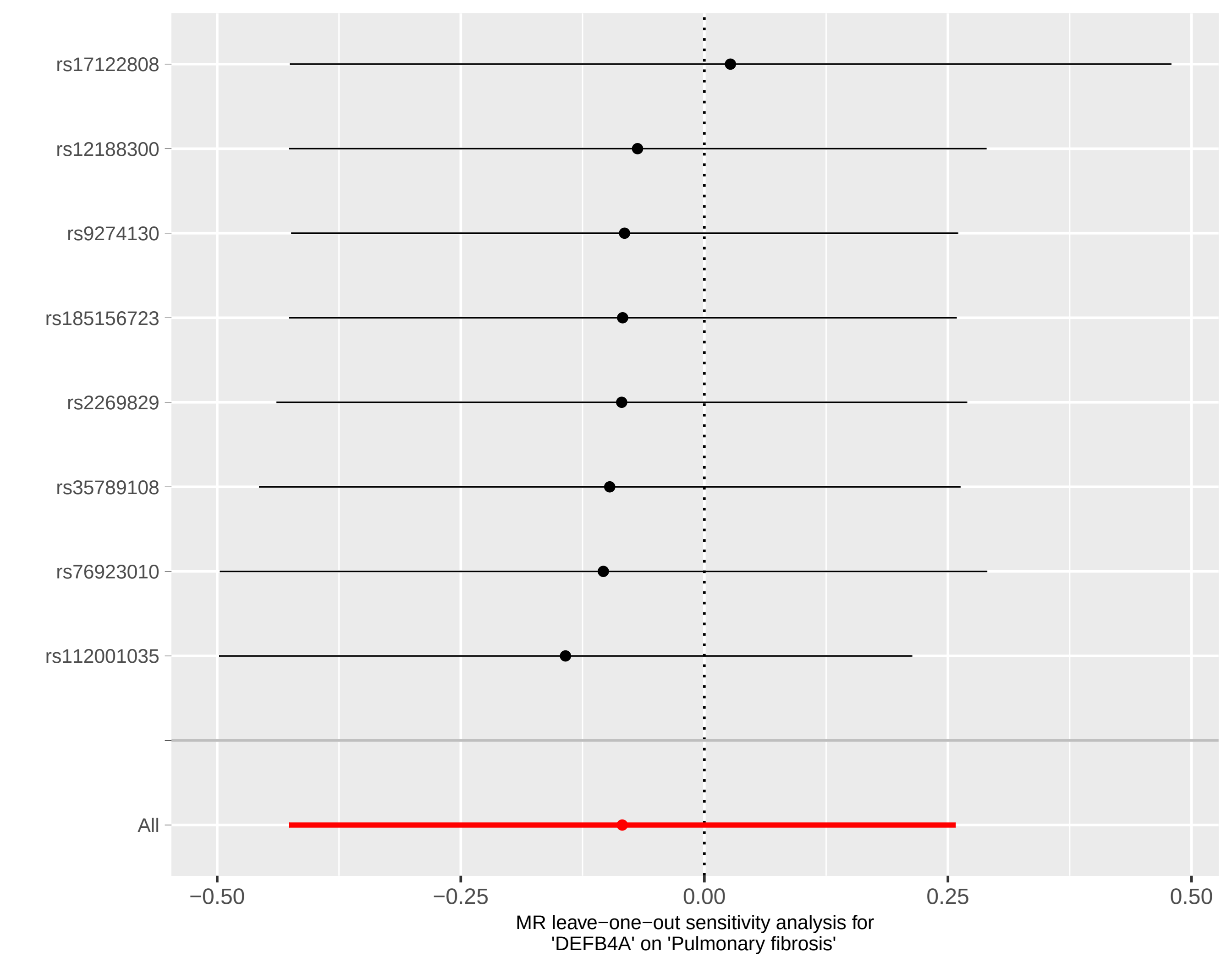
rs17122808
rs12188300
rs9274130
rs185156723
rs2269829
rs35789108
rs76923010
rs112001035
All
−0.50
−0.25
0.00
0.25
0.50
MR leave−one−out sensitivity analysis for
'DEFB4A' on 'Pulmonary fibrosis'

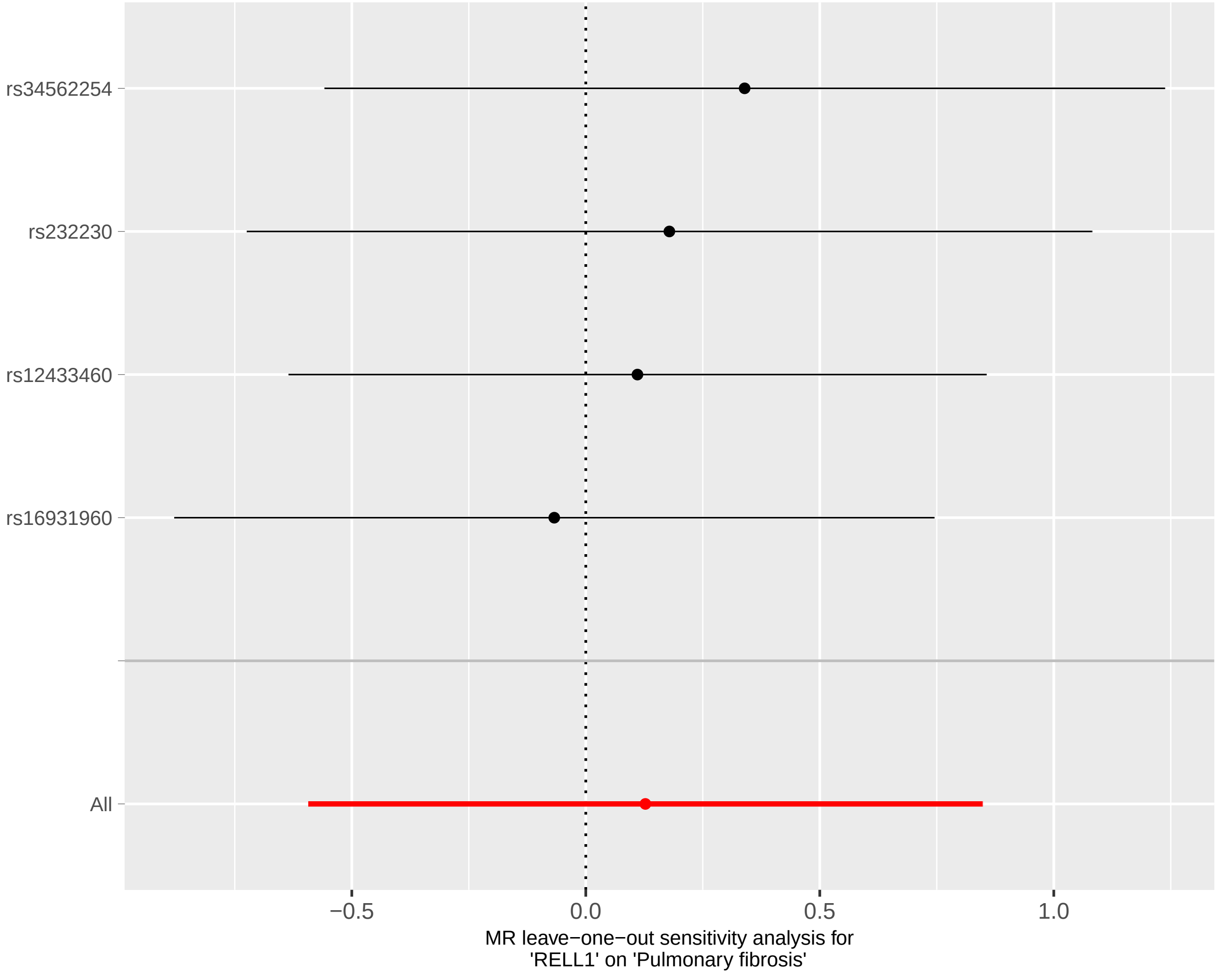

rs34562254
rs232230
rs12433460
rs16931960
All
−0.5
0.0
0.5
1.0
MR leave−one−out sensitivity analysis for
'RELL1' on 'Pulmonary fibrosis'

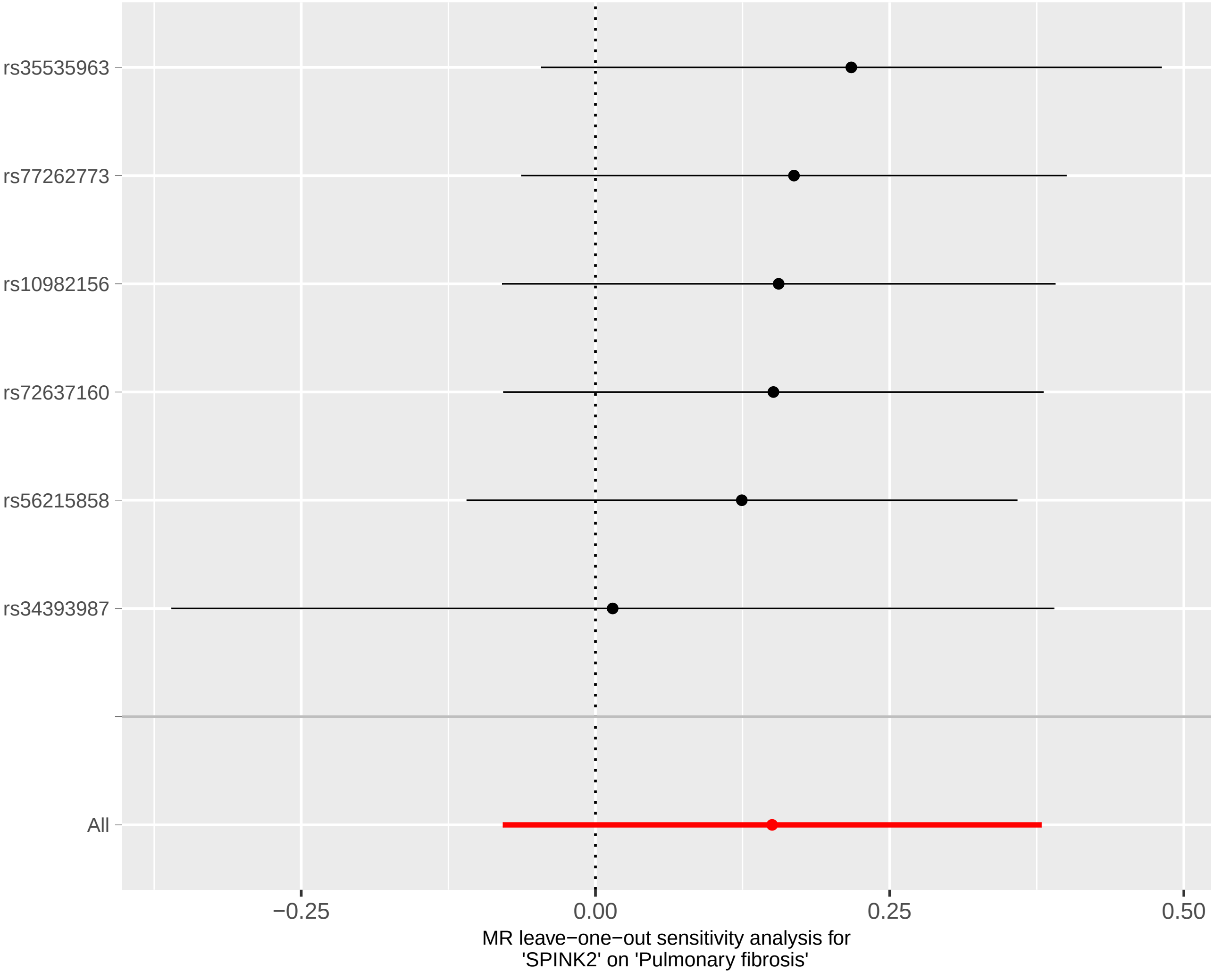
rs35535963
rs77262773
rs10982156
rs72637160
rs56215858
rs34393987
All
−0.25
0.00
0.25
0.50
MR leave−one−out sensitivity analysis for
'SPINK2' on 'Pulmonary fibrosis'

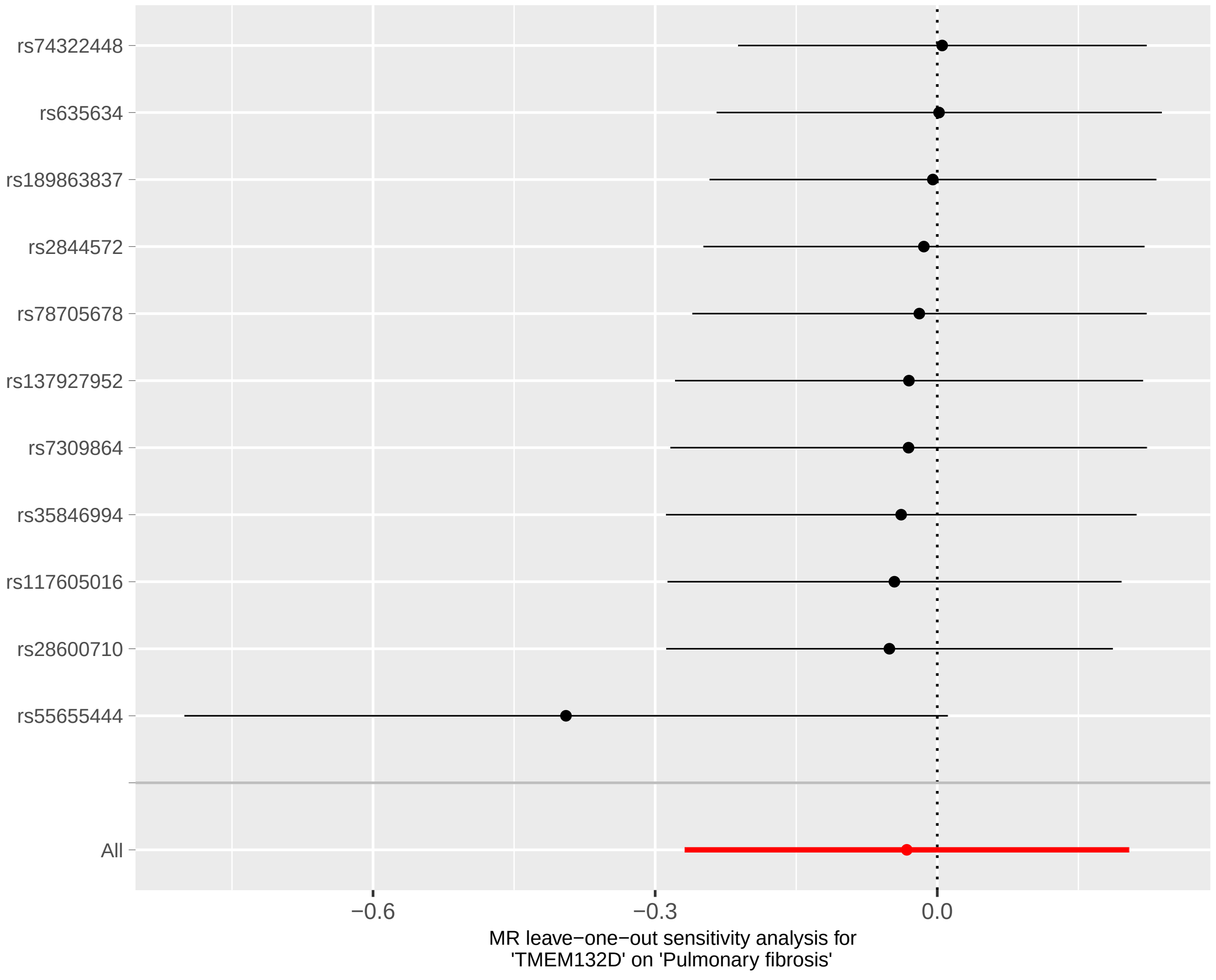
rs74322448
rs635634
rs189863837
rs2844572
rs78705678
rs137927952
rs7309864
rs35846994
rs117605016
rs28600710
rs55655444
All
−0.6
−0.3
0.0
MR leave−one−out sensitivity analysis for
'TMEM132D' on 'Pulmonary fibrosis'

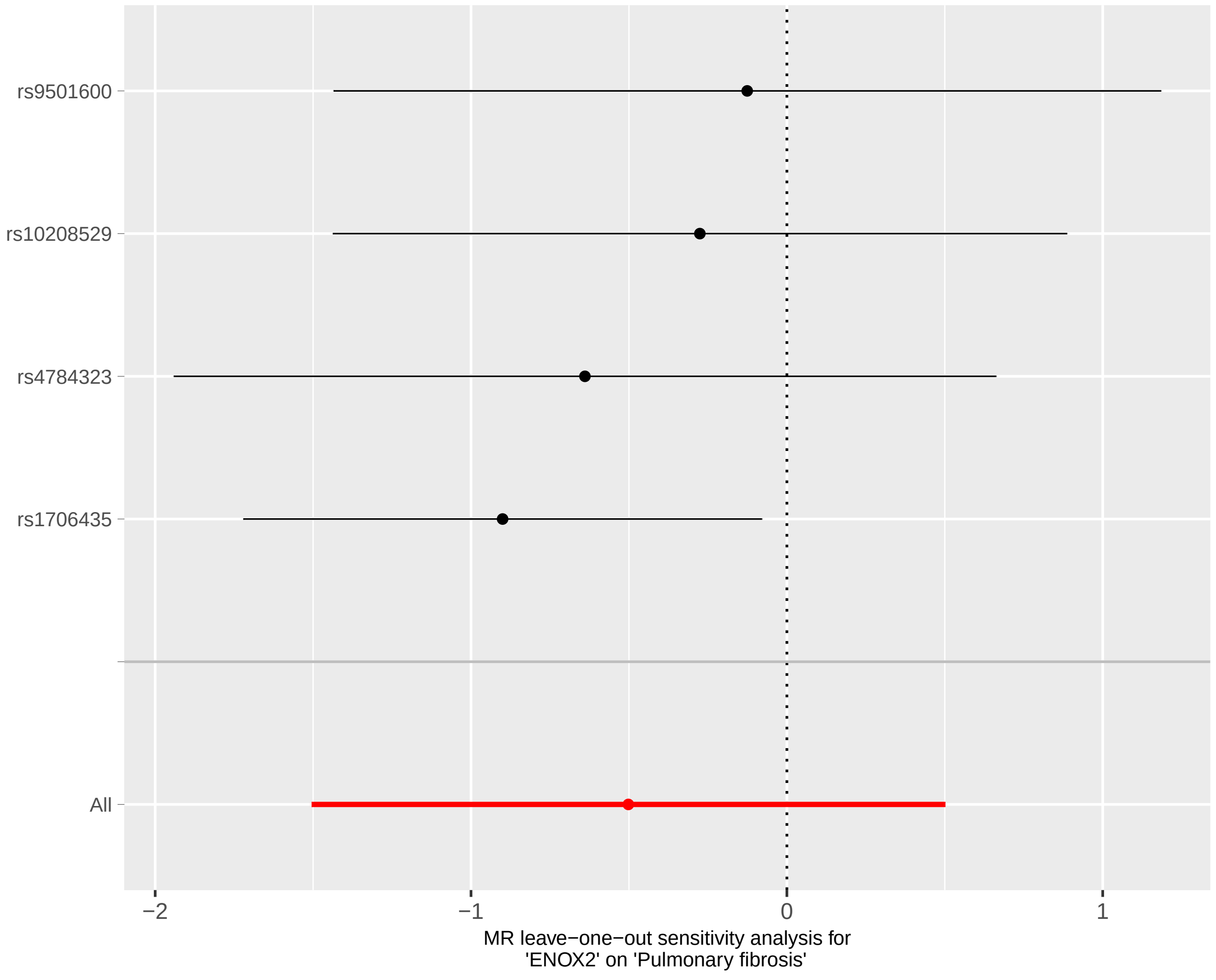
rs9501600
rs10208529
rs4784323
rs1706435
All
−2
−1
0
1
MR leave−one−out sensitivity analysis for
'ENOX2' on 'Pulmonary fibrosis'

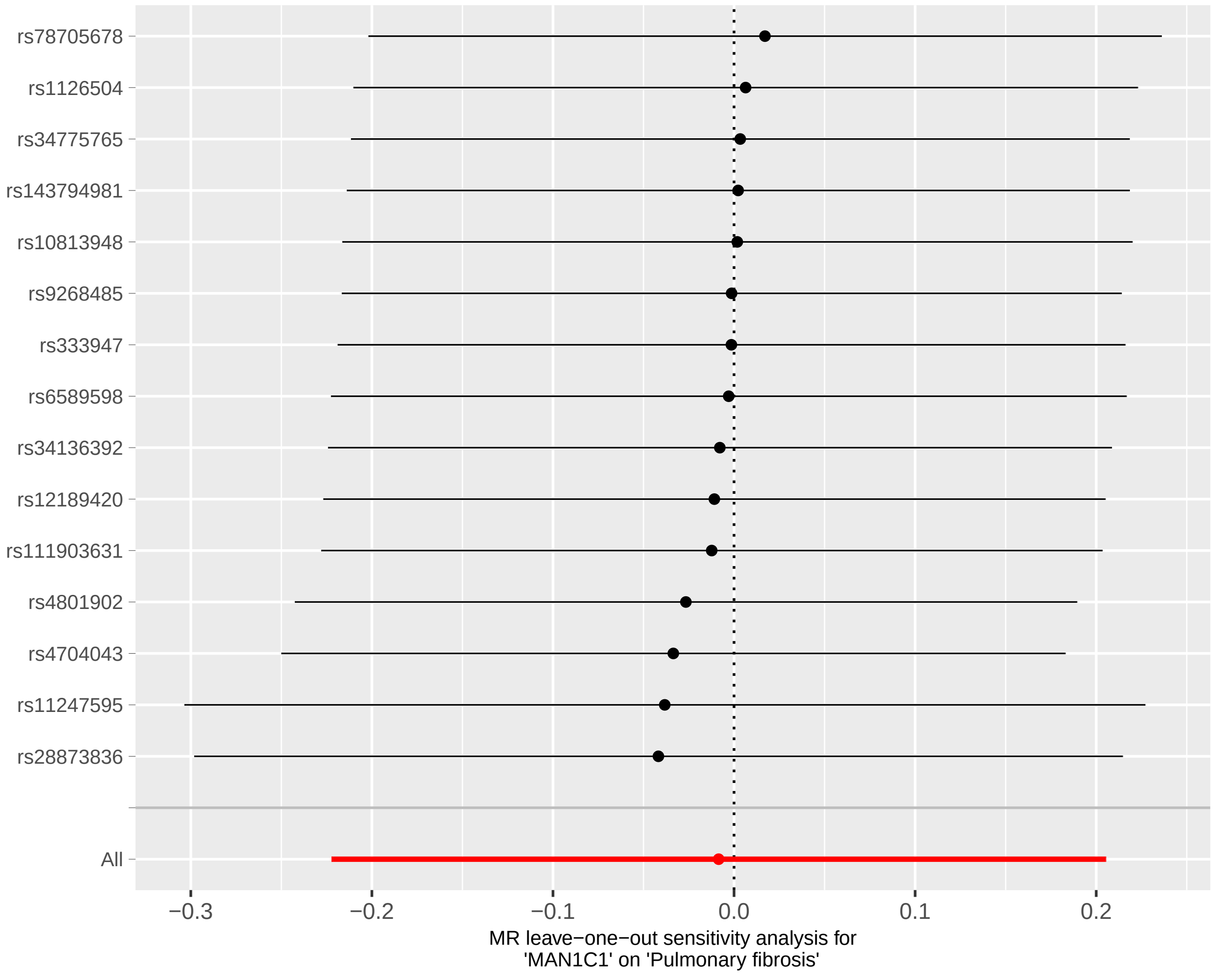
rs78705678
rs1126504
rs34775765
rs143794981
rs10813948
rs9268485
rs333947
rs6589598
rs34136392
rs12189420
rs111903631
rs4801902
rs4704043
rs11247595
rs28873836
All
−0.3
−0.2
−0.1
0.0
0.1
0.2
MR leave−one−out sensitivity analysis for
'MAN1C1' on 'Pulmonary fibrosis'

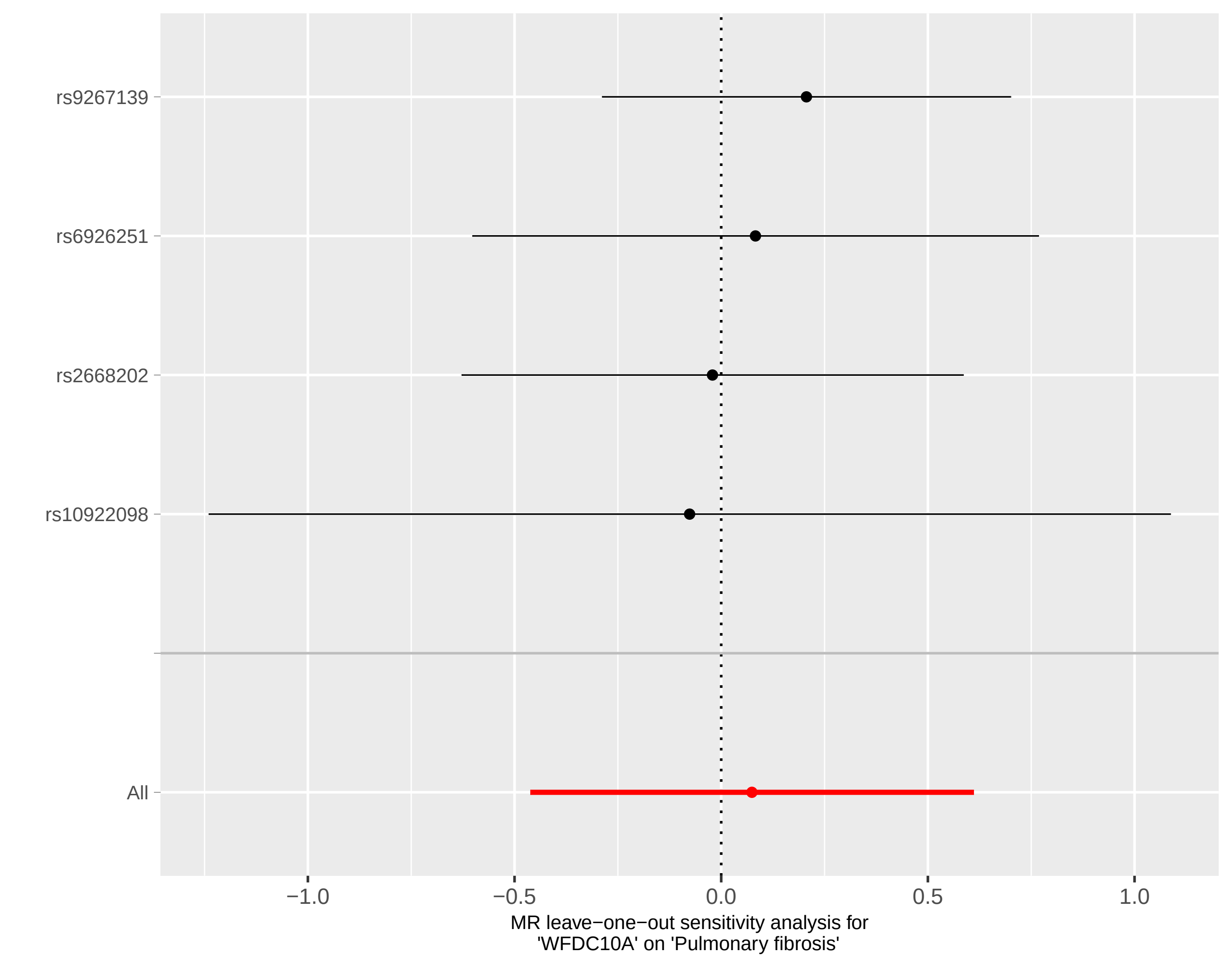

rs9267139
rs6926251
rs2668202
rs10922098
All
−1.0
−0.5
0.0
0.5
1.0
MR leave−one−out sensitivity analysis for
'WFDC10A' on 'Pulmonary fibrosis'

Insufficient number of SNPs

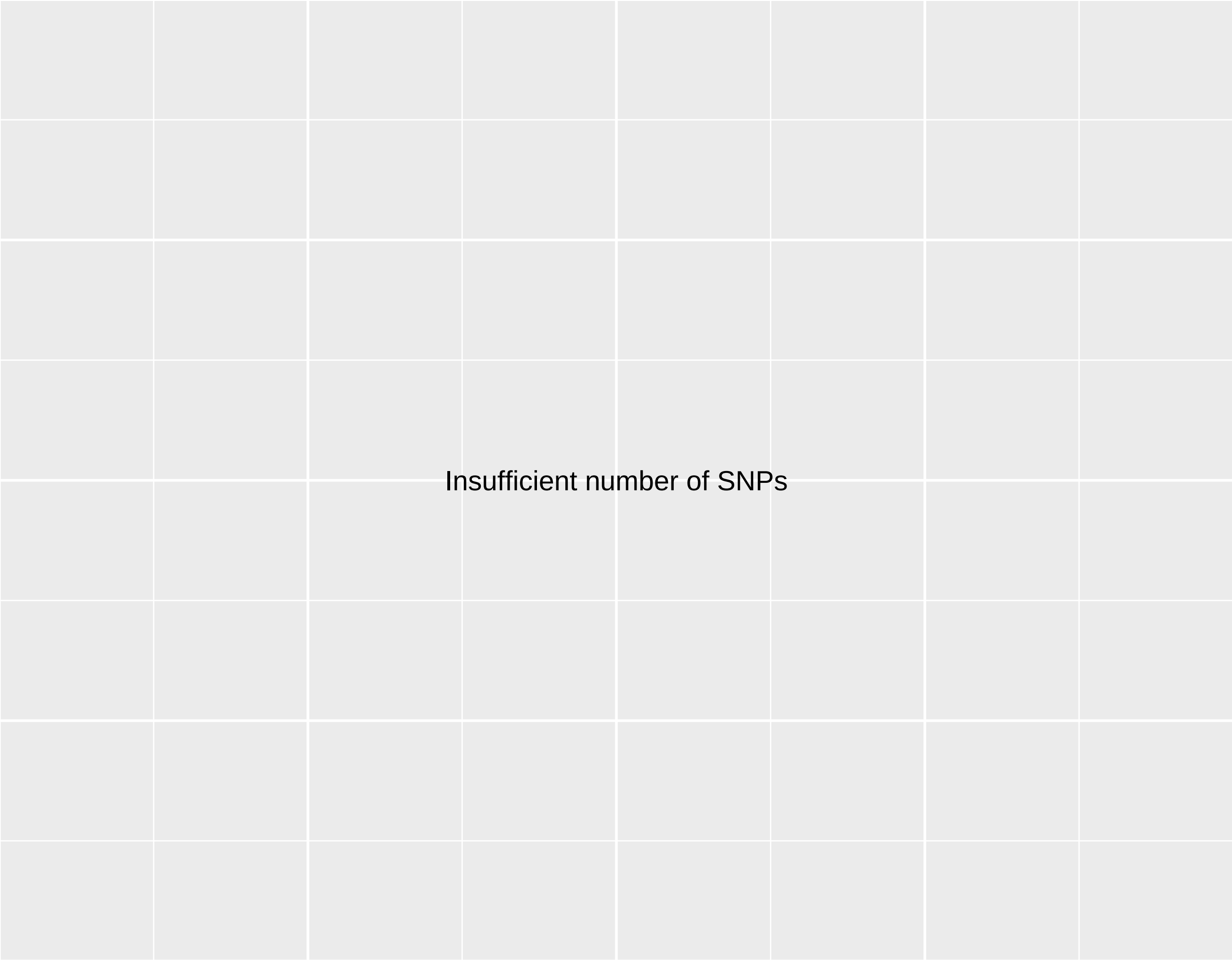
Insufficient number of SNPs

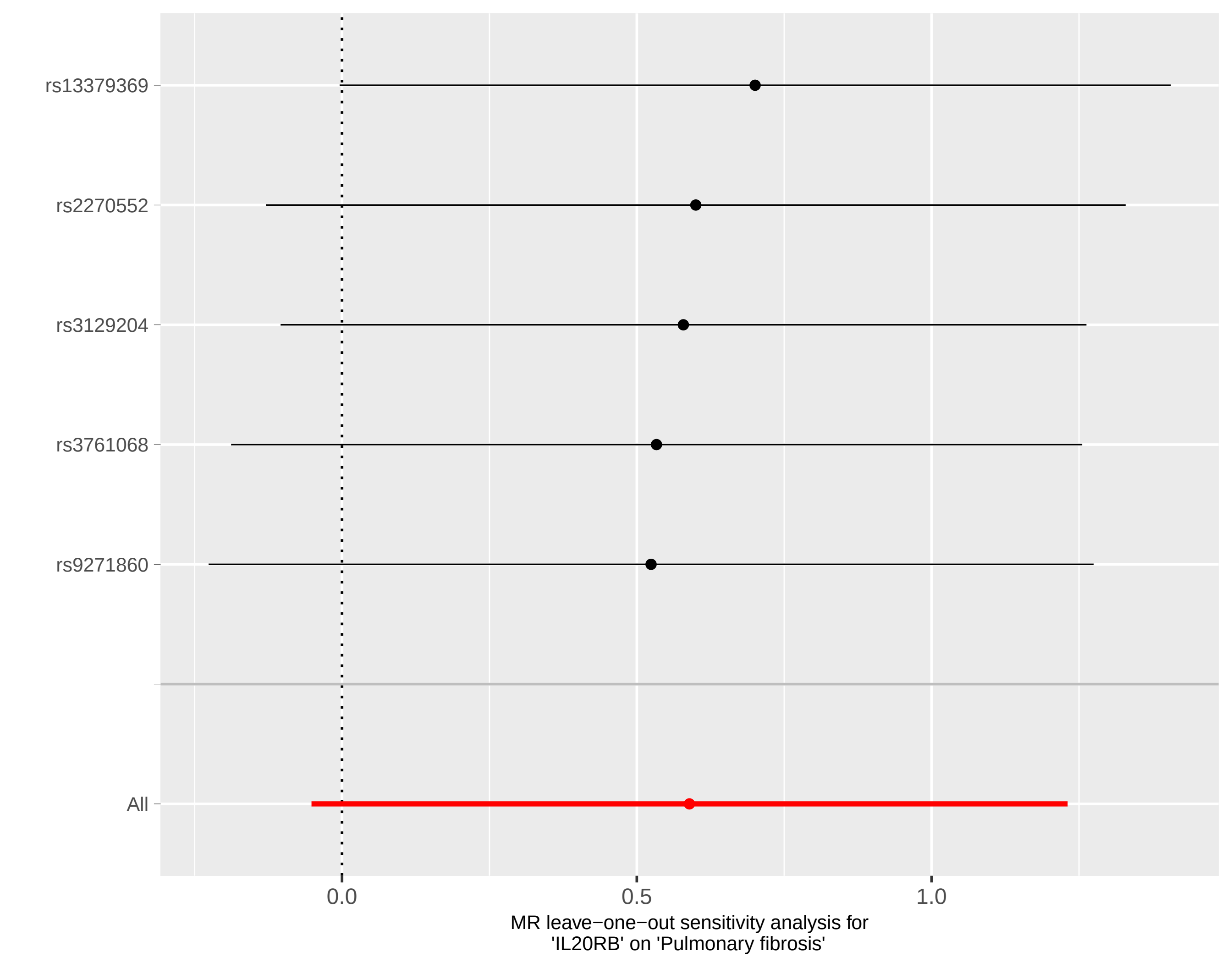
rs13379369
rs2270552
rs3129204
rs3761068
rs9271860
All
0.0
0.5
1.0
MR leave−one−out sensitivity analysis for
'IL20RB' on 'Pulmonary fibrosis'

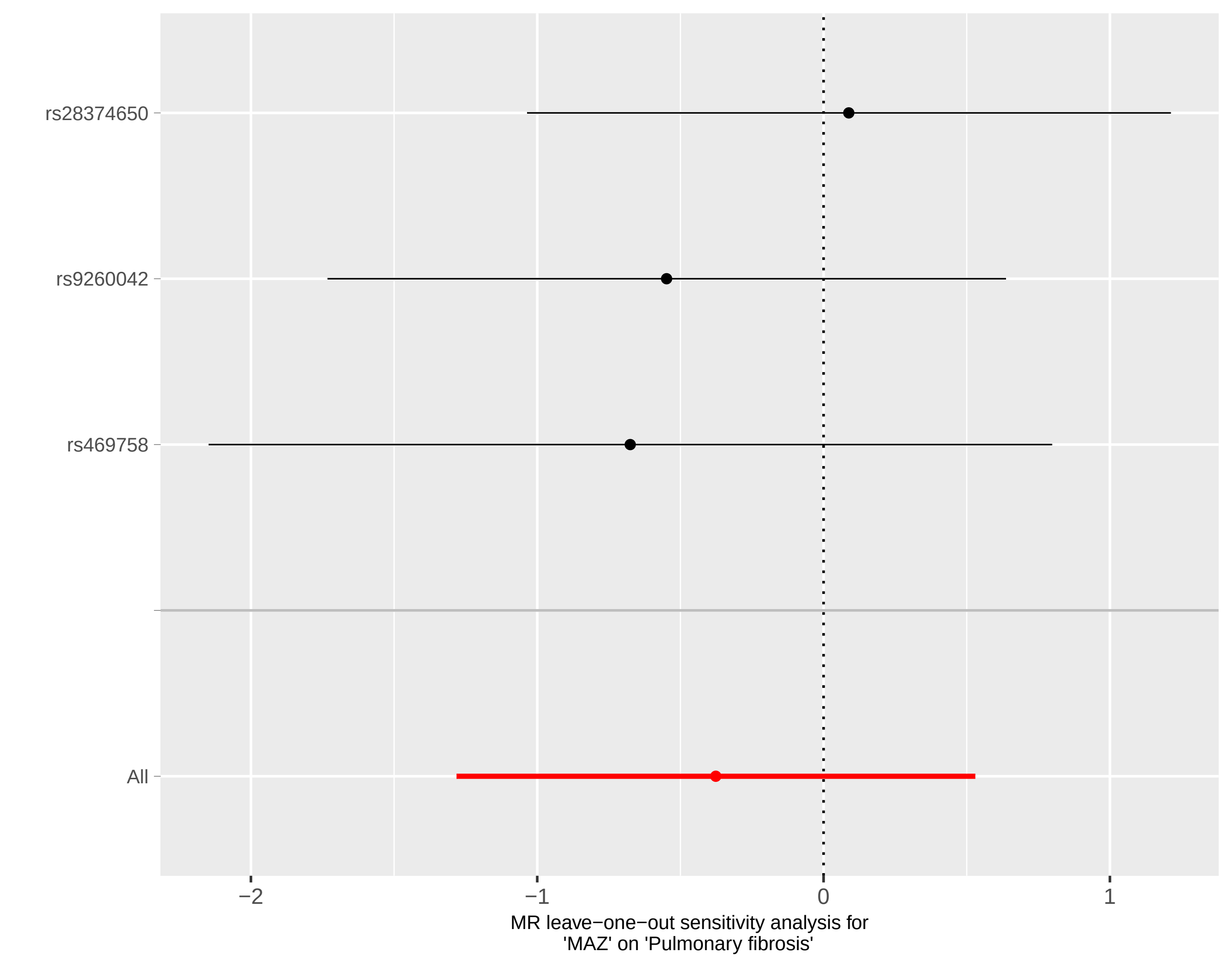

rs28374650
rs9260042
rs469758
All
−2
−1
0
1
MR leave−one−out sensitivity analysis for
'MAZ' on 'Pulmonary fibrosis'

Insufficient number of SNPs

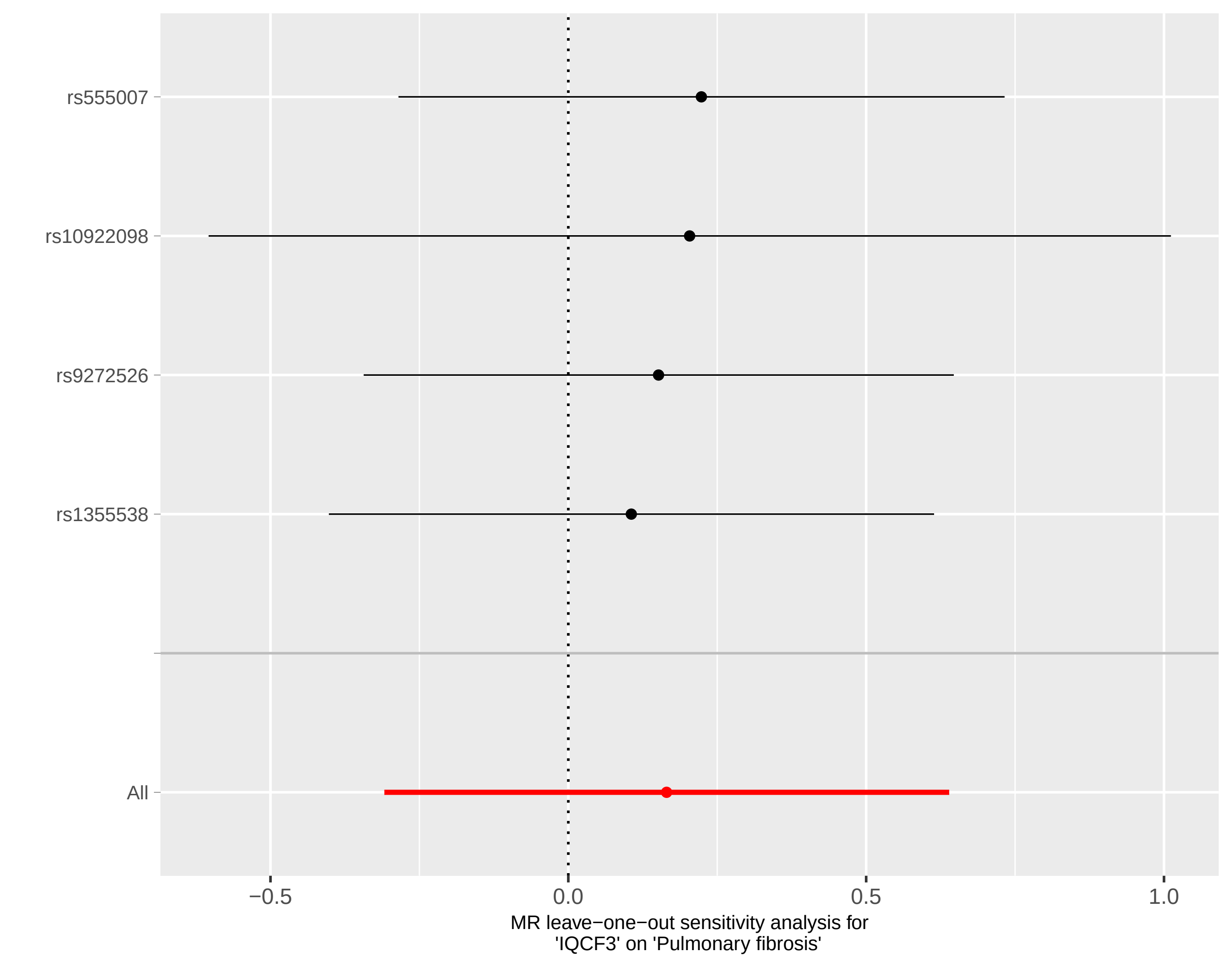
rs555007
rs10922098
rs9272526
rs1355538
All
−0.5
0.0
0.5
1.0
MR leave−one−out sensitivity analysis for
'IQCF3' on 'Pulmonary fibrosis'

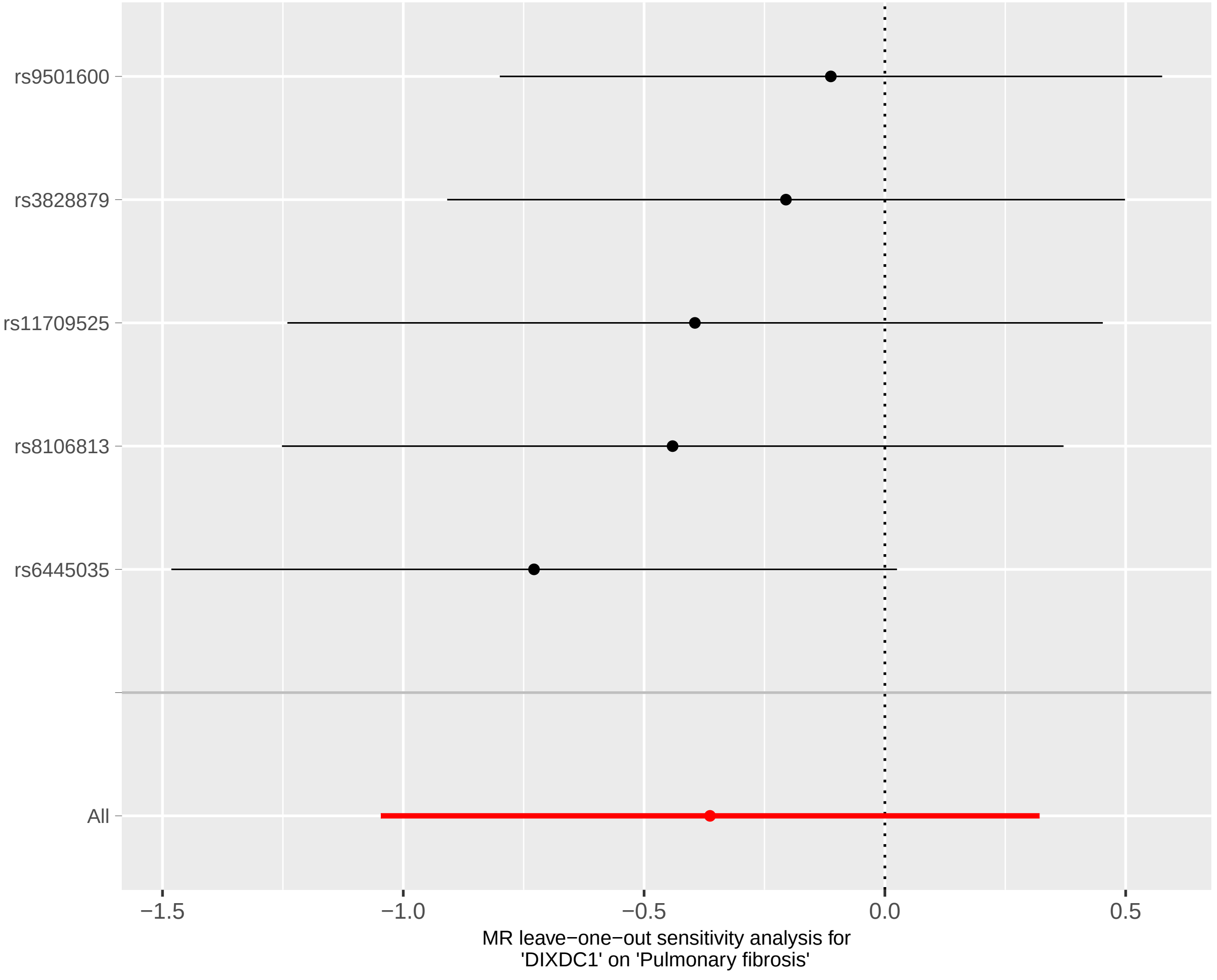

rs9501600
rs3828879
rs11709525
rs8106813
rs6445035
All
−1.5
−1.0
−0.5
0.0
0.5
MR leave−one−out sensitivity analysis for
'DIXDC1' on 'Pulmonary fibrosis'

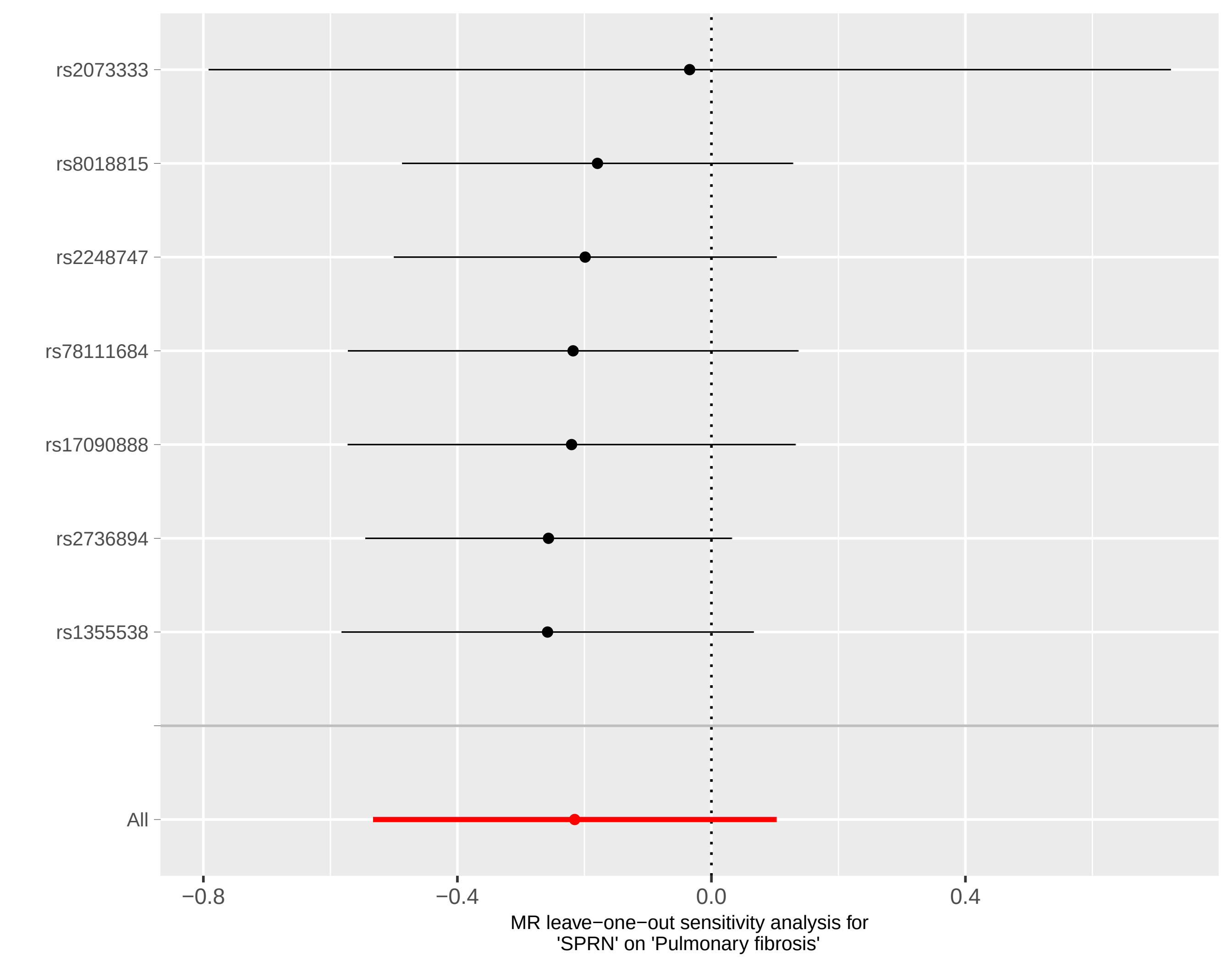

rs2073333
rs8018815
rs2248747
rs78111684
rs17090888
rs2736894
rs1355538
All
−0.8
−0.4
0.0
0.4
MR leave−one−out sensitivity analysis for
'SPRN' on 'Pulmonary fibrosis'

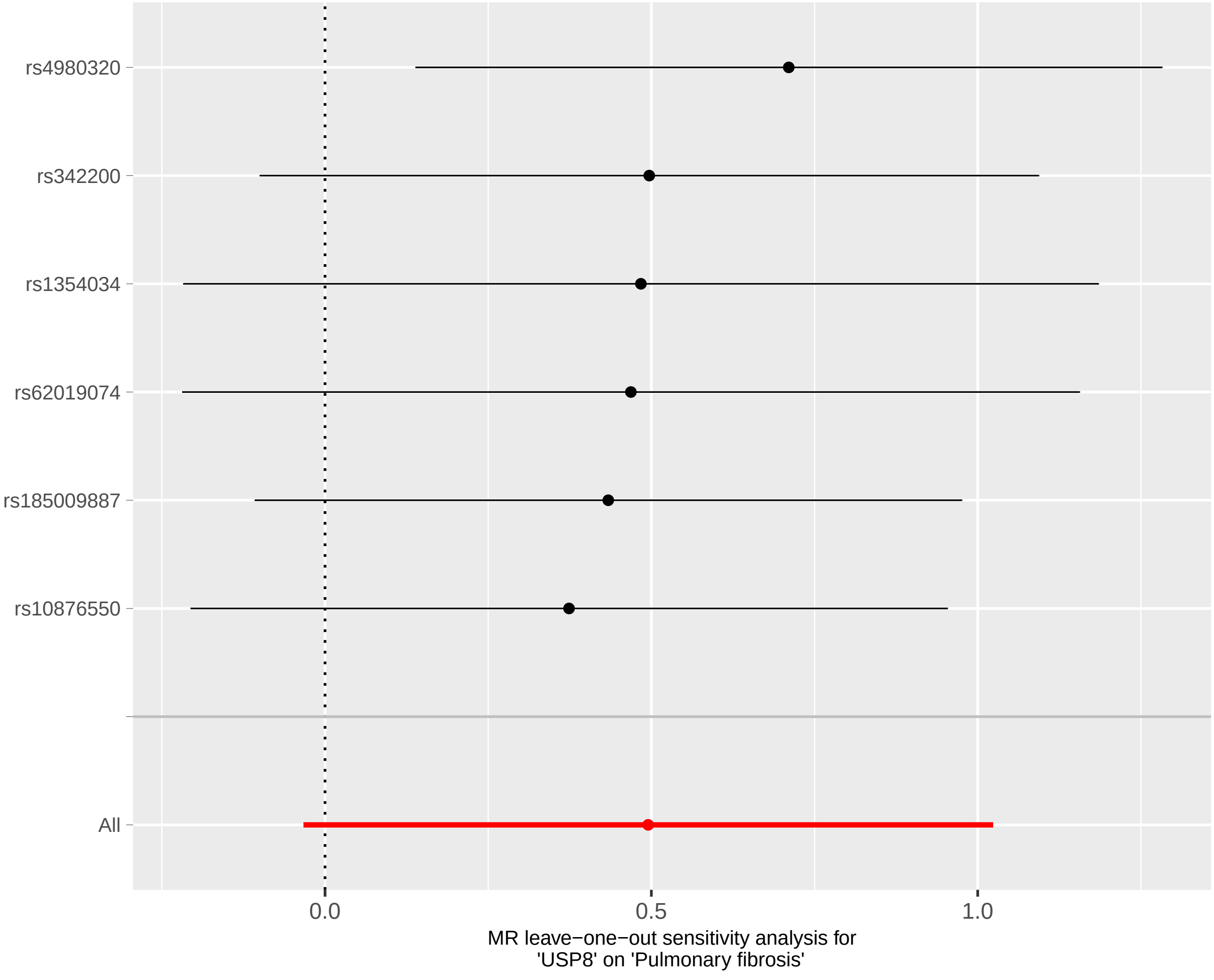
rs4980320
rs342200
rs1354034
rs62019074
rs185009887
rs10876550
All
0.0
0.5
1.0
MR leave−one−out sensitivity analysis for
'USP8' on 'Pulmonary fibrosis'

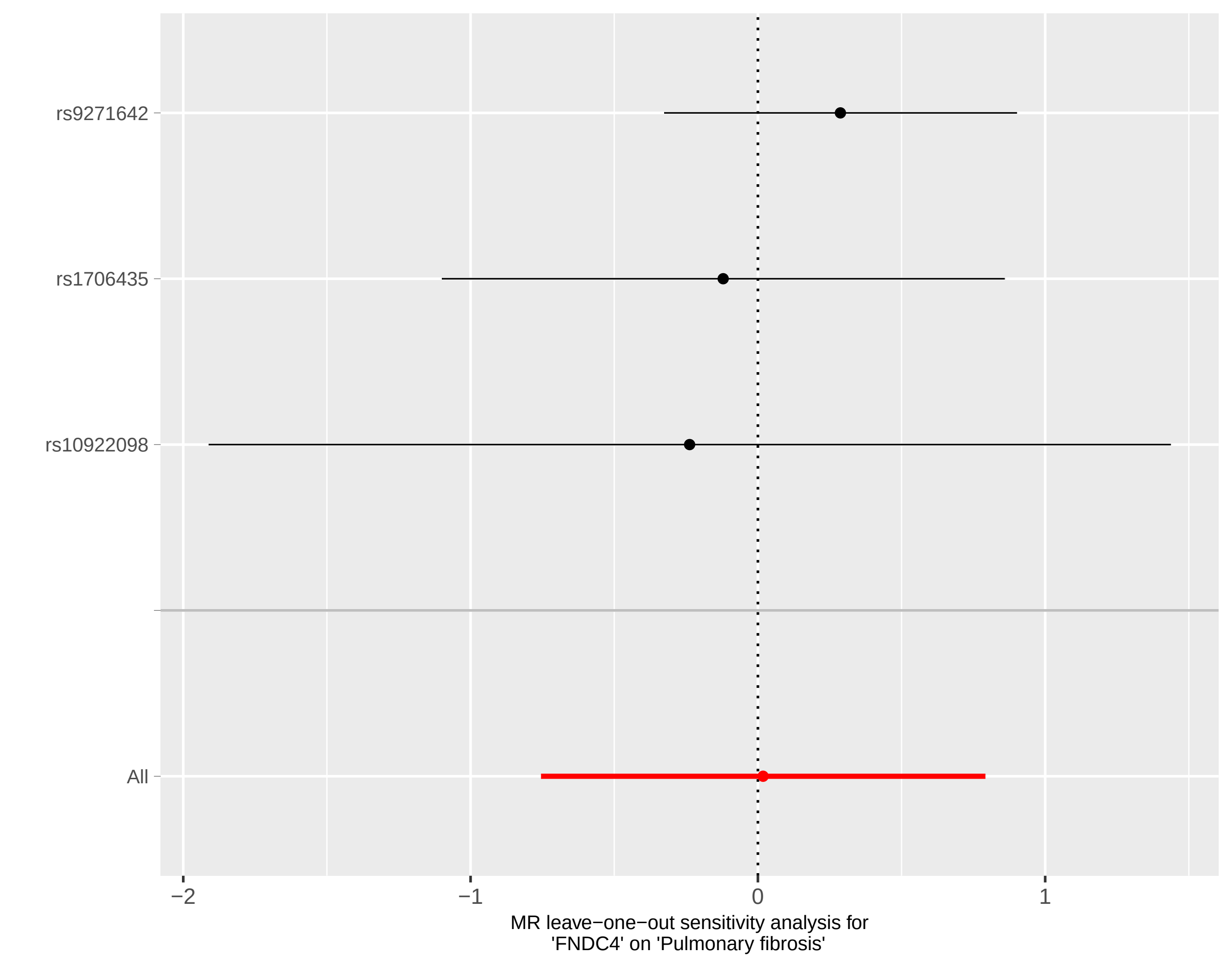
rs9271642
rs1706435
rs10922098
All
−2
−1
0
1
MR leave−one−out sensitivity analysis for
'FNDC4' on 'Pulmonary fibrosis'

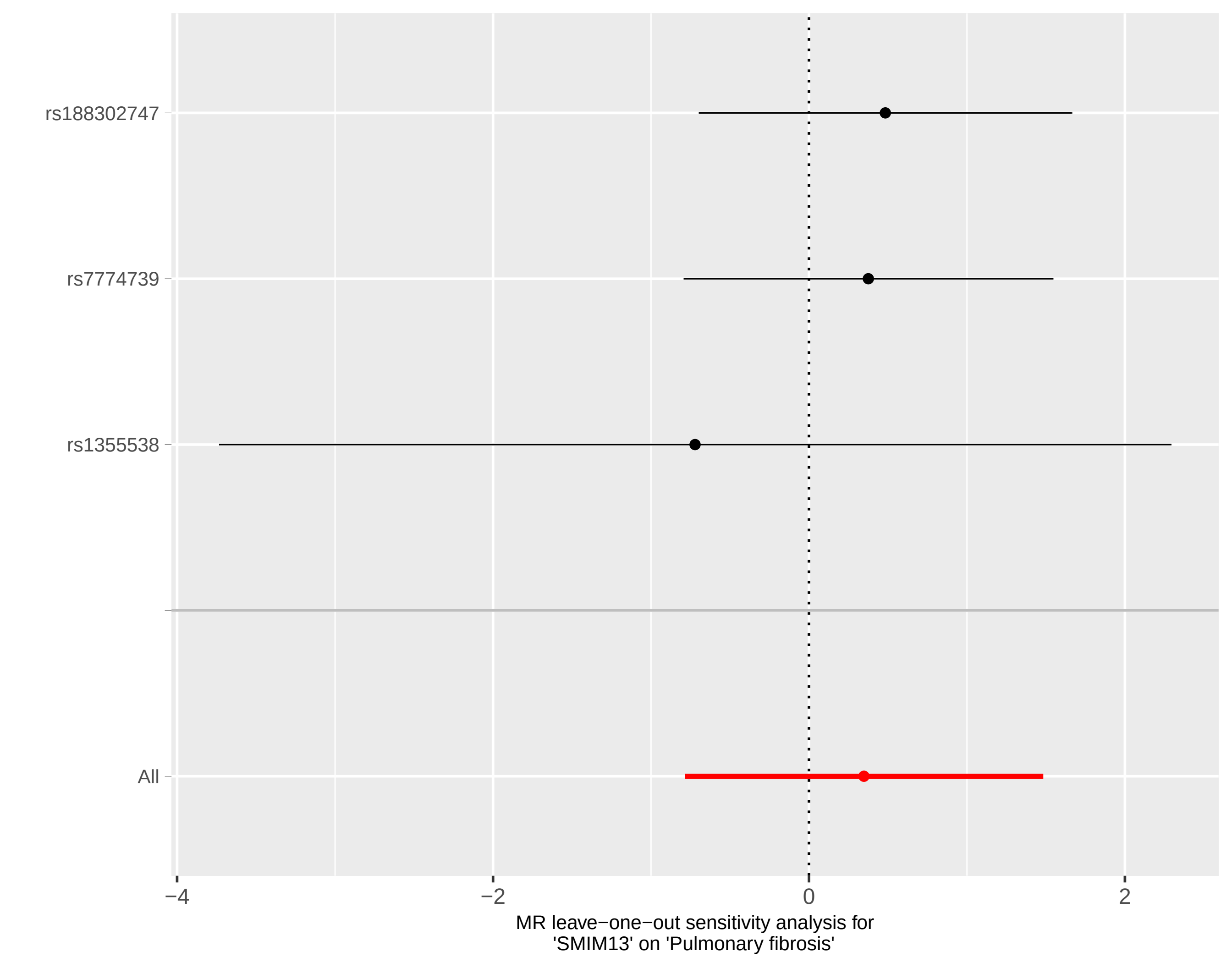
rs188302747
rs7774739
rs1355538
All
−4
−2
0
2
MR leave−one−out sensitivity analysis for
'SMIM13' on 'Pulmonary fibrosis'

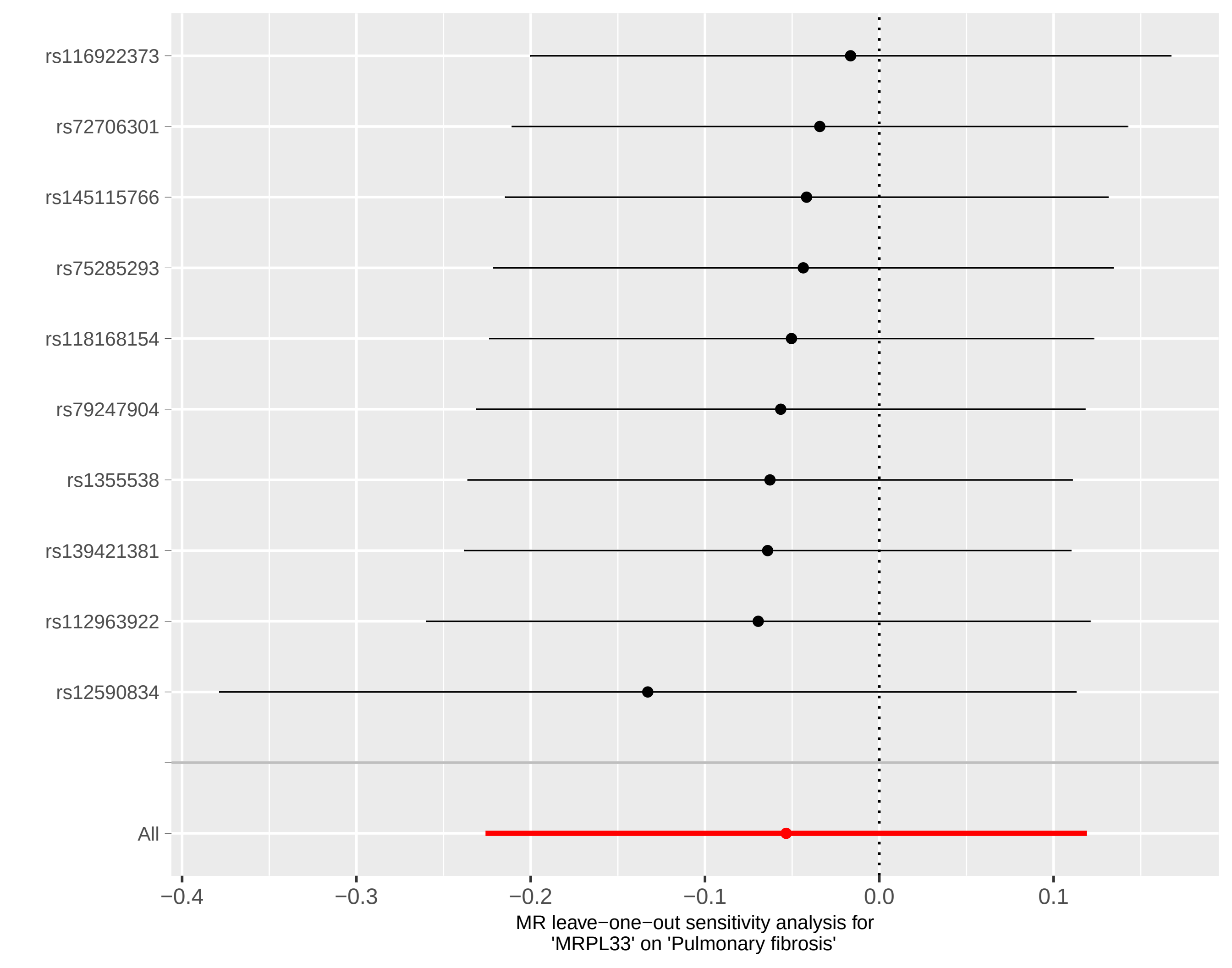
rs116922373
rs72706301
rs145115766
rs75285293
rs118168154
rs79247904
rs1355538
rs139421381
rs112963922
rs12590834
All
−0.4
−0.3
−0.2
−0.1
0.0
0.1
MR leave−one−out sensitivity analysis for
'MRPL33' on 'Pulmonary fibrosis'

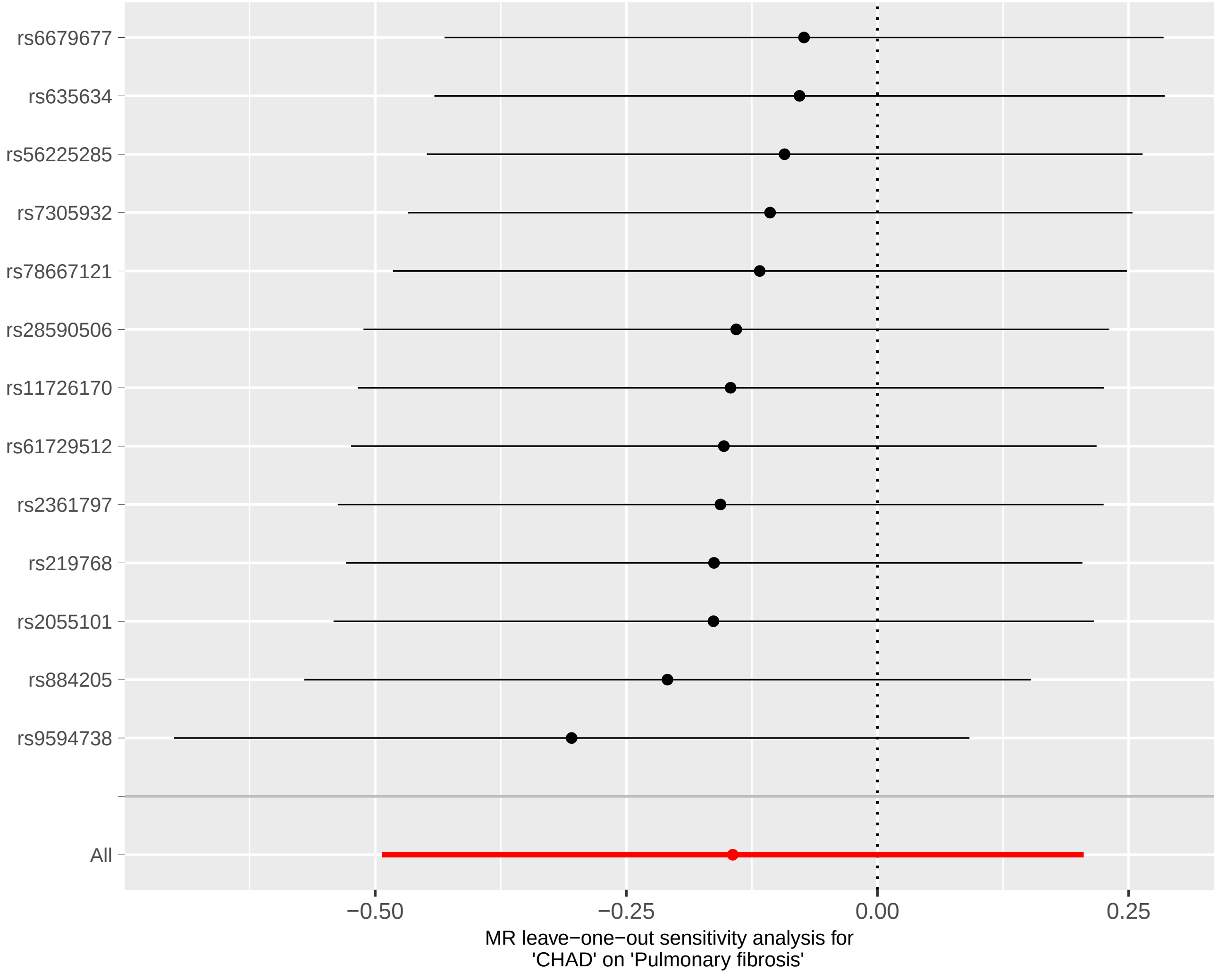

rs6679677
rs635634
rs56225285
rs7305932
rs78667121
rs28590506
rs11726170
rs61729512
rs2361797
rs219768
rs2055101
rs884205
rs9594738
All
−0.50
−0.25
0.00
0.25
MR leave−one−out sensitivity analysis for
'CHAD' on 'Pulmonary fibrosis'

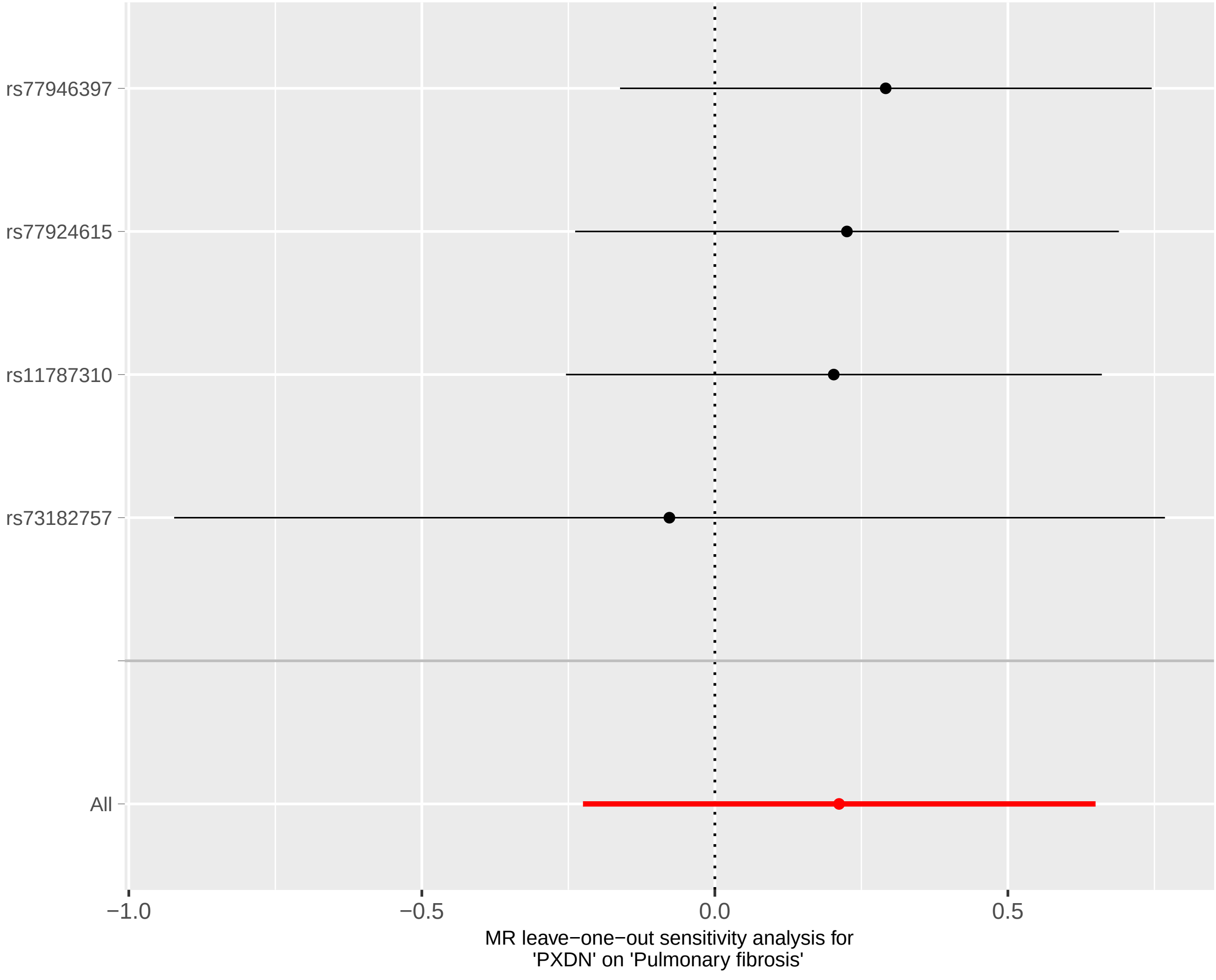
rs77946397
rs77924615
rs11787310
rs73182757
All
−1.0
−0.5
0.0
0.5
MR leave−one−out sensitivity analysis for
'PXDN' on 'Pulmonary fibrosis'

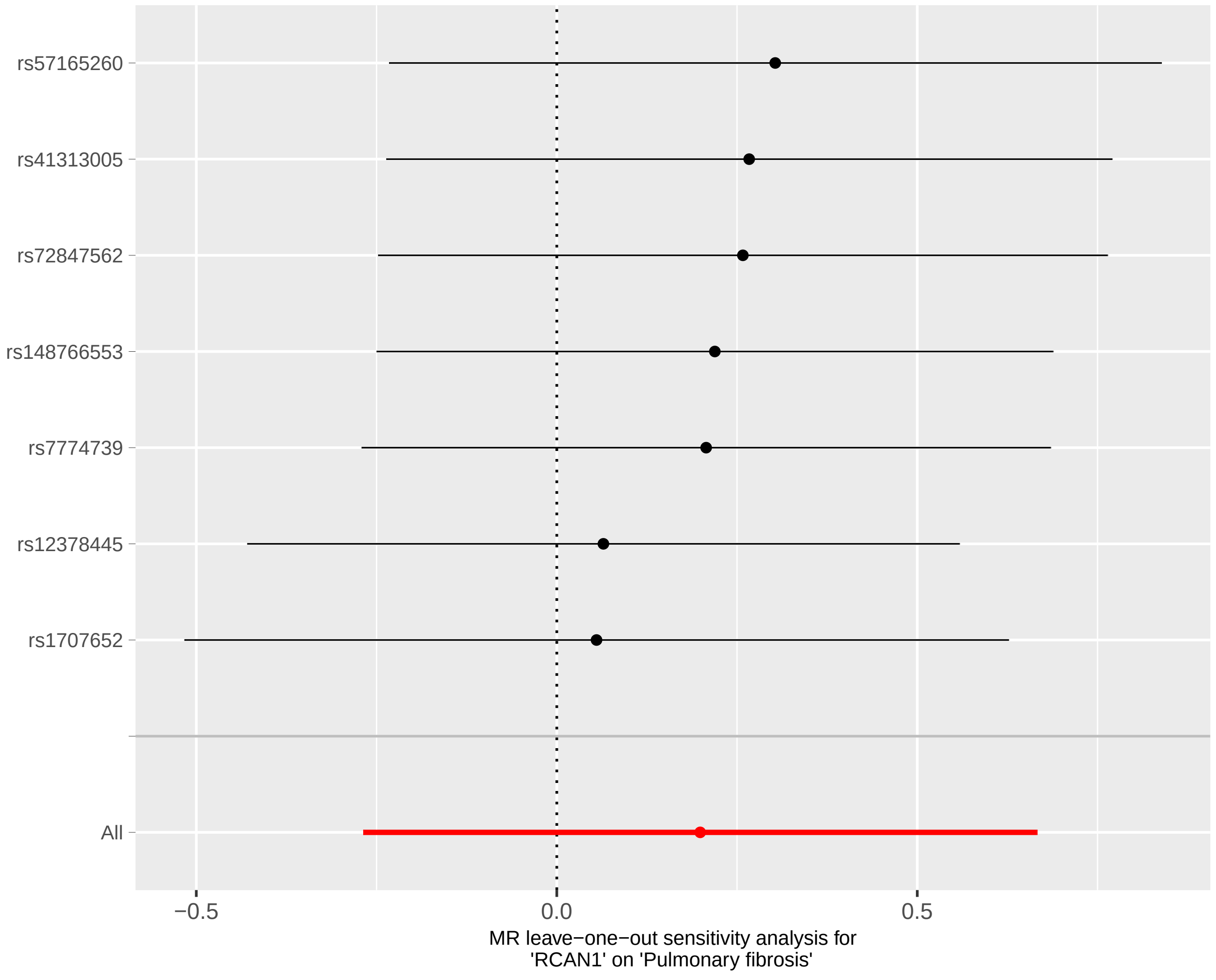

rs57165260
rs41313005
rs72847562
rs148766553
rs7774739
rs12378445
rs1707652
All
−0.5
0.0
0.5
MR leave−one−out sensitivity analysis for
'RCAN1' on 'Pulmonary fibrosis'

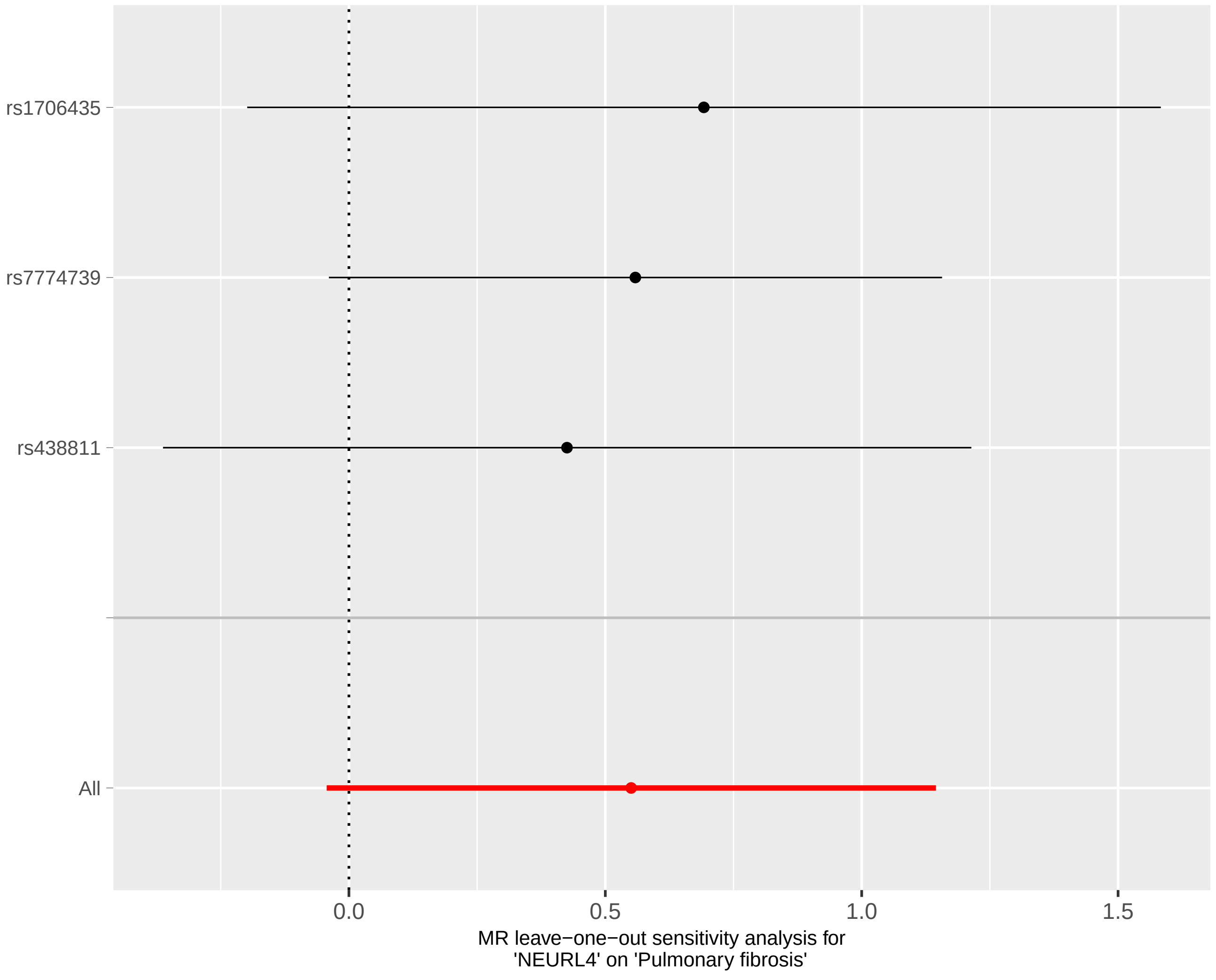

rs1706435
rs7774739
rs438811
All
0.0
0.5
1.0
1.5
MR leave−one−out sensitivity analysis for
'NEURL4' on 'Pulmonary fibrosis'

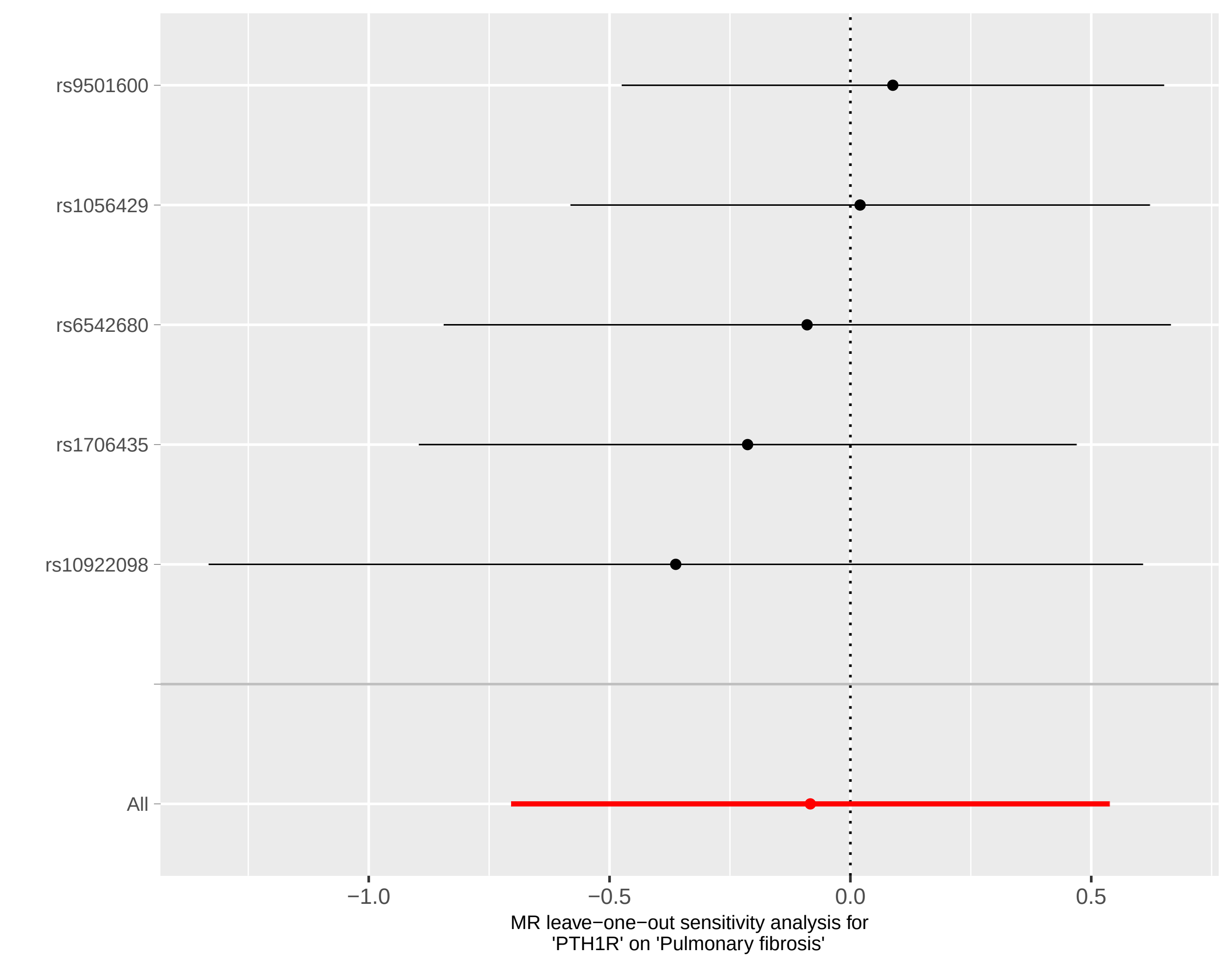
rs9501600
rs1056429
rs6542680
rs1706435
rs10922098
All
−1.0
−0.5
0.0
0.5
MR leave−one−out sensitivity analysis for
'PTH1R' on 'Pulmonary fibrosis'

Insufficient number of SNPs

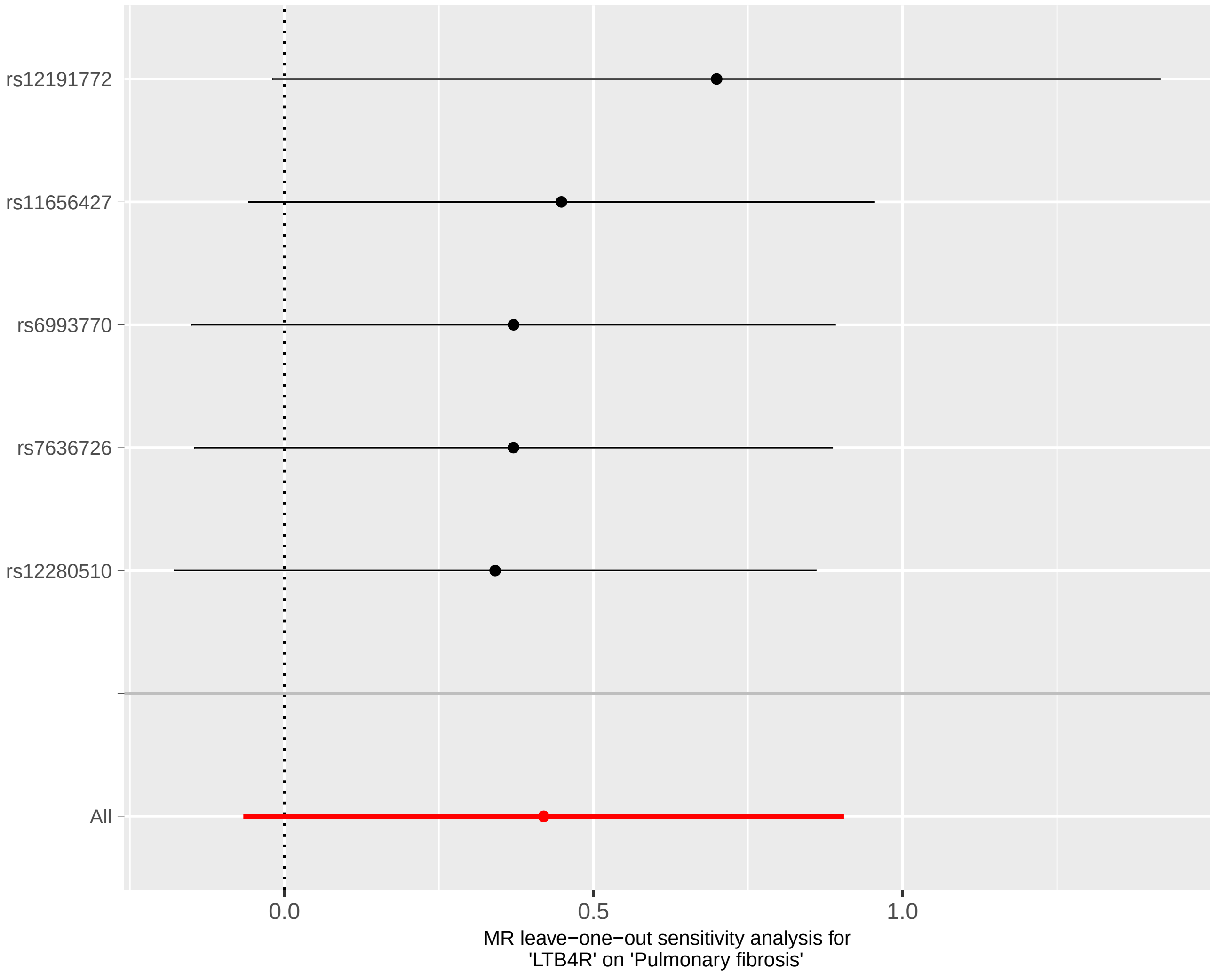

rs12191772
rs11656427
rs6993770
rs7636726
rs12280510
All
0.0
0.5
1.0
MR leave−one−out sensitivity analysis for
'LTB4R' on 'Pulmonary fibrosis'

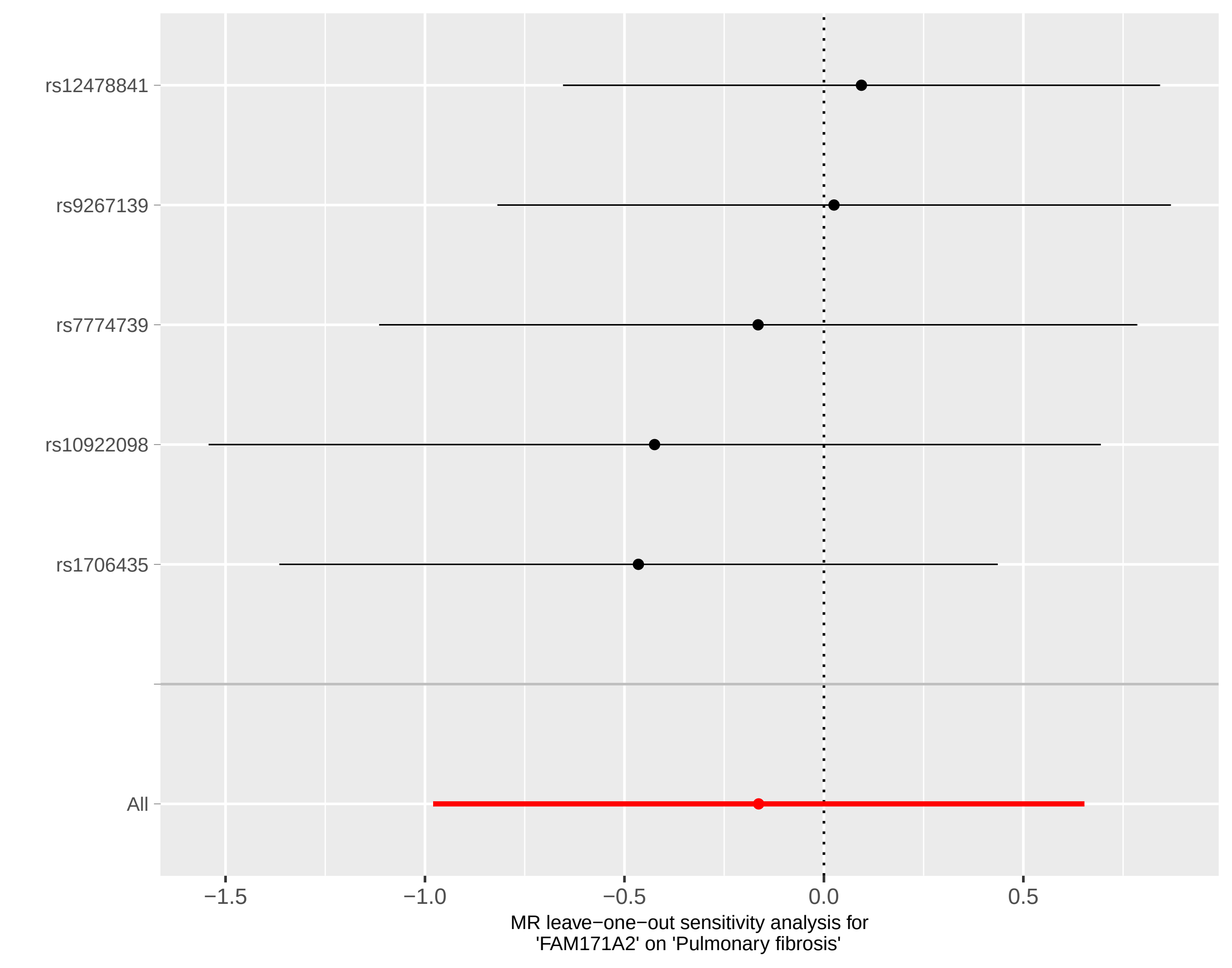

rs12478841
rs9267139
rs7774739
rs10922098
rs1706435
All
−1.5
−1.0
−0.5
0.0
0.5
MR leave−one−out sensitivity analysis for
'FAM171A2' on 'Pulmonary fibrosis'

Insufficient number of SNPs

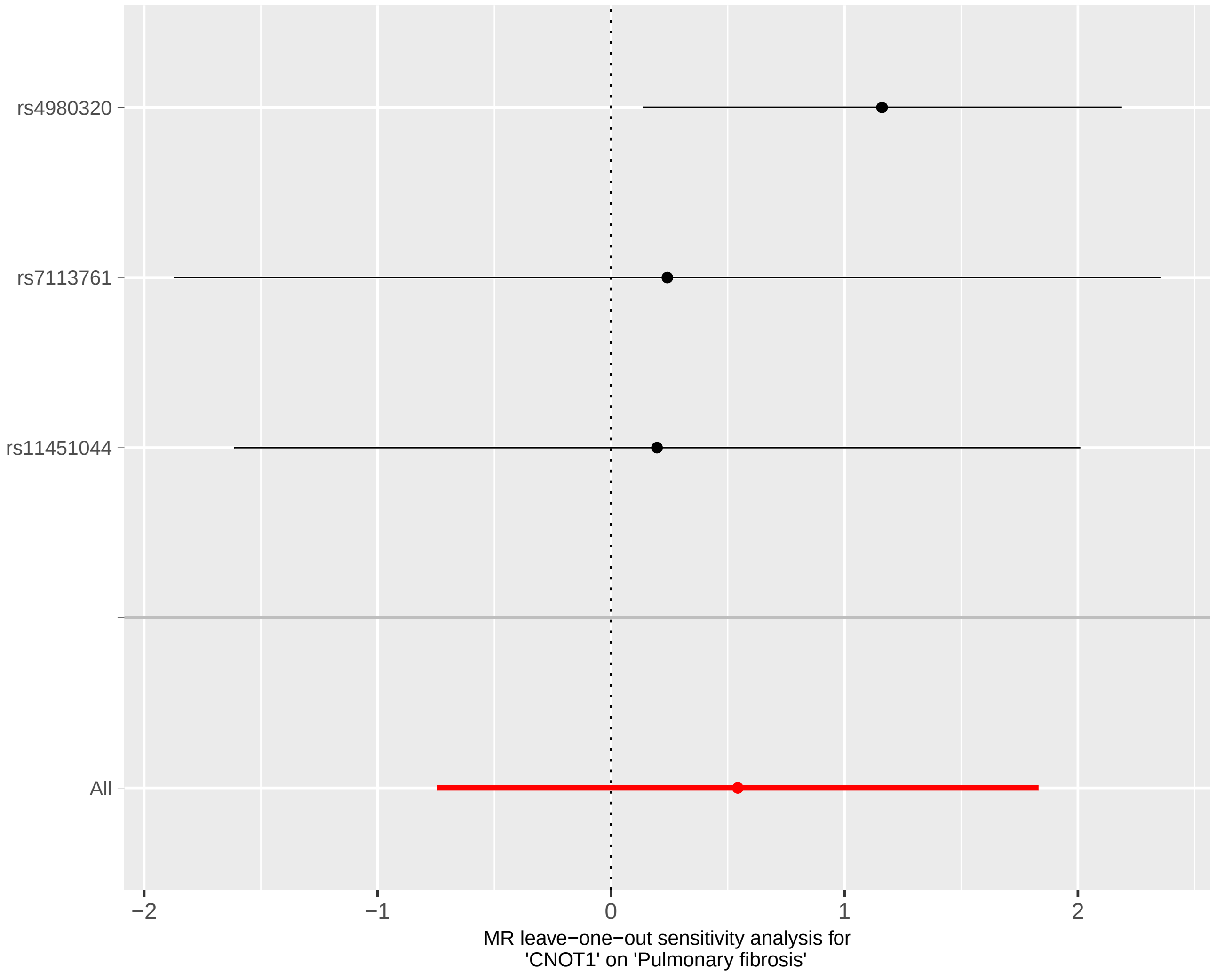
rs4980320
rs7113761
rs11451044
All
−2
−1
0
1
2
MR leave−one−out sensitivity analysis for
'CNOT1' on 'Pulmonary fibrosis'

Insufficient number of SNPs

Insufficient number of SNPs

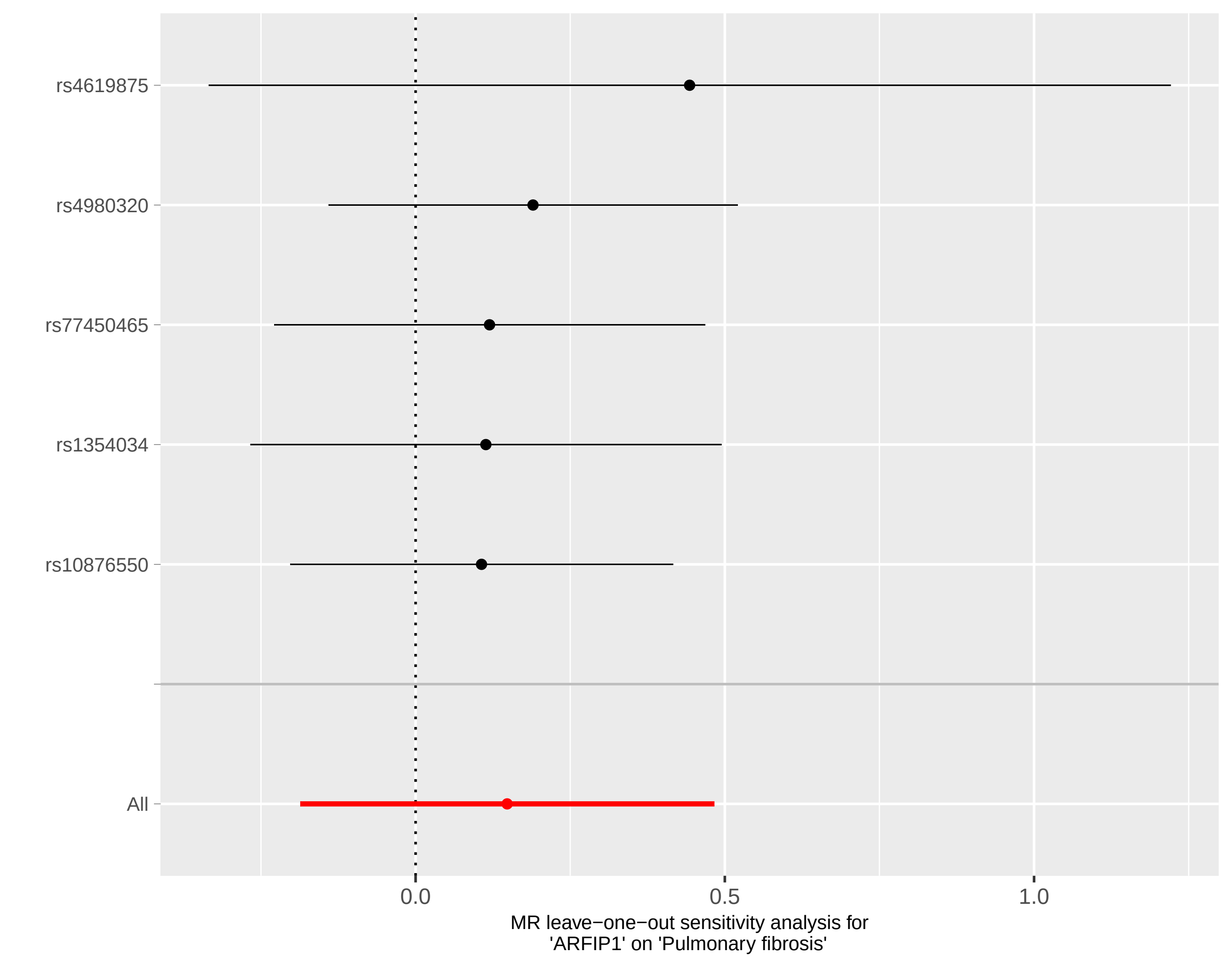

rs4619875
rs4980320
rs77450465
rs1354034
rs10876550
All
0.0
0.5
1.0
MR leave−one−out sensitivity analysis for
'ARFIP1' on 'Pulmonary fibrosis'

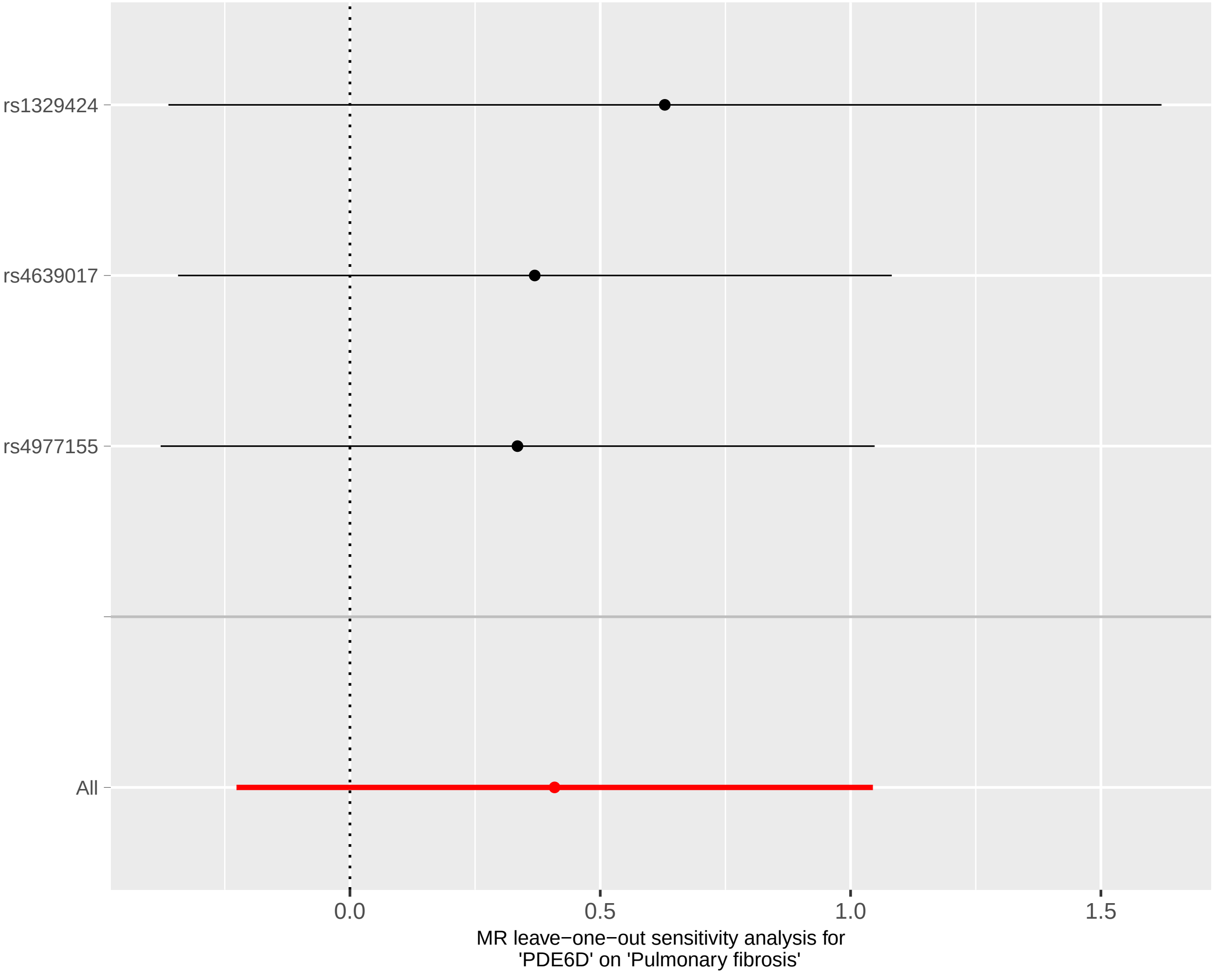
rs1329424
rs4639017
rs4977155
All
0.0
0.5
1.0
1.5
MR leave−one−out sensitivity analysis for
'PDE6D' on 'Pulmonary fibrosis'

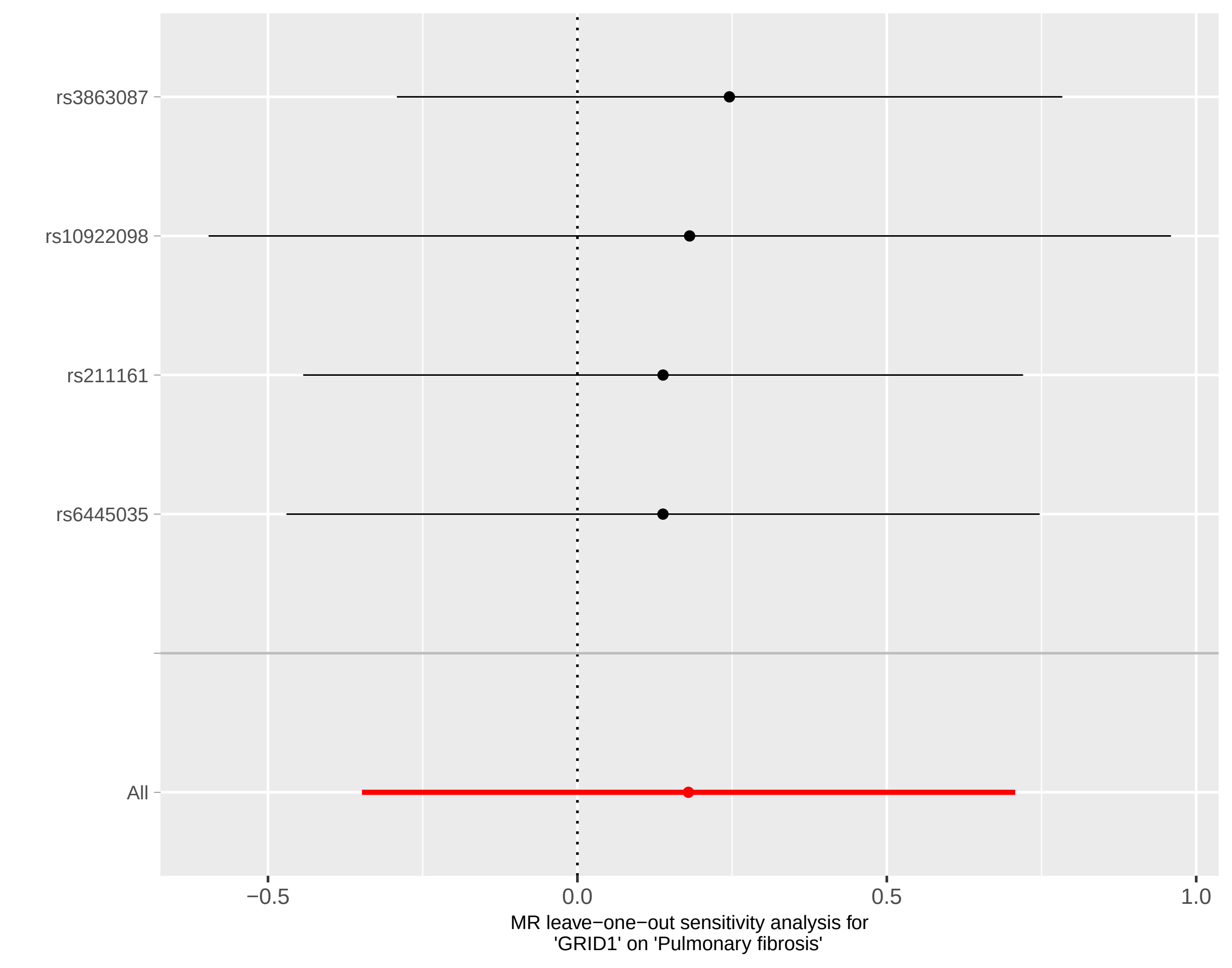

rs3863087
rs10922098
rs211161
rs6445035
All
−0.5
0.0
0.5
1.0
MR leave−one−out sensitivity analysis for
'GRID1' on 'Pulmonary fibrosis'

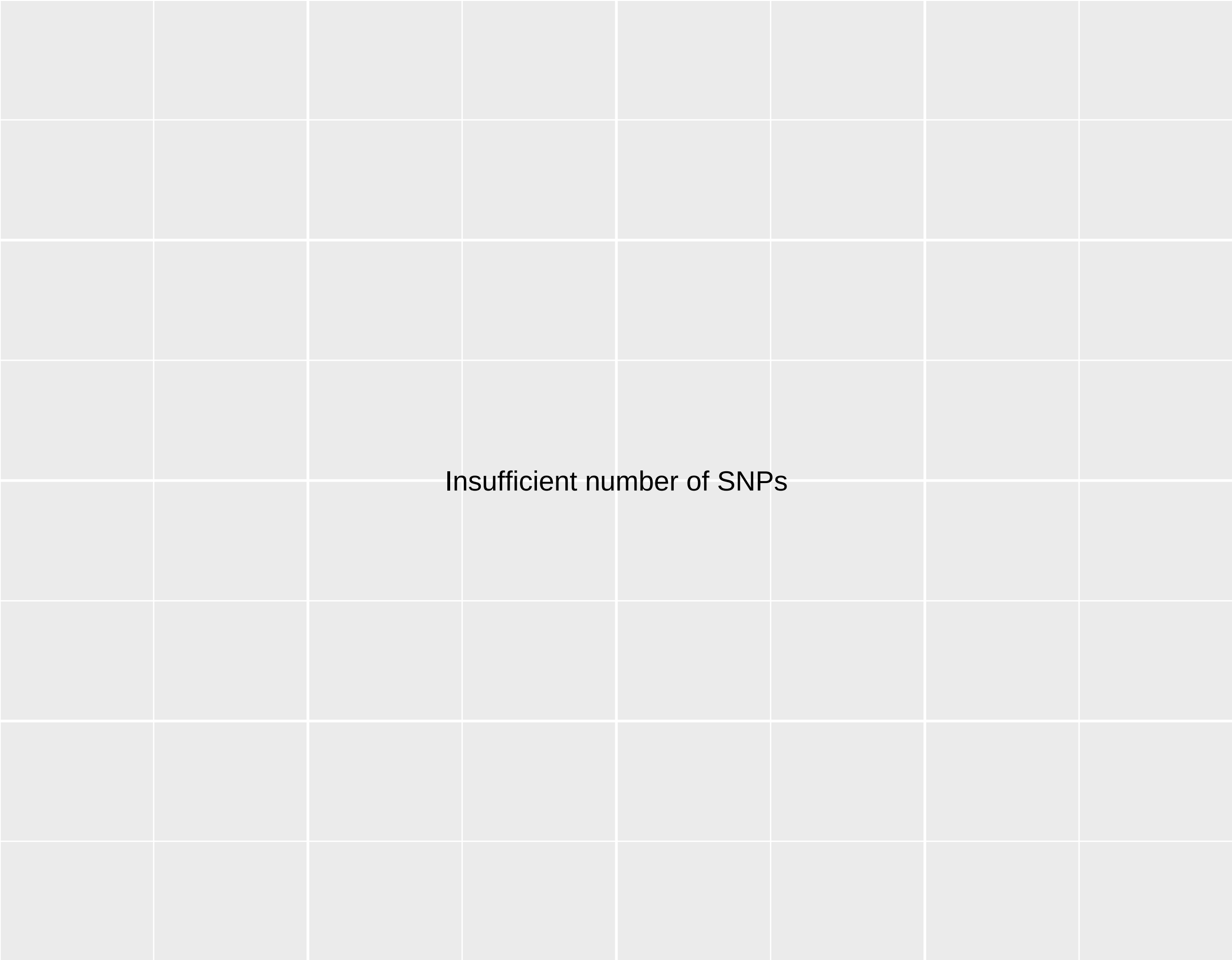
Insufficient number of SNPs

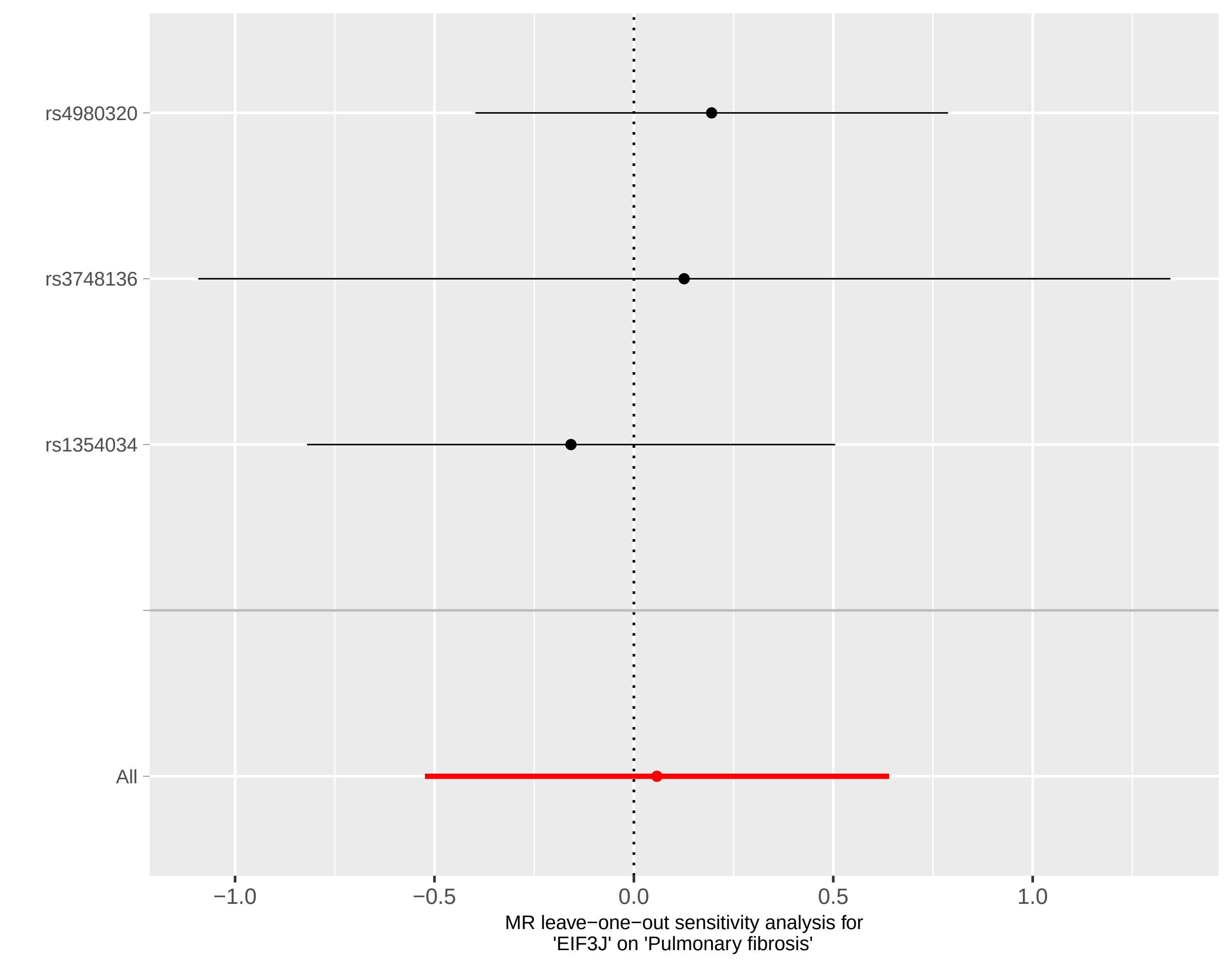
rs4980320
rs3748136
rs1354034
All
−1.0
−0.5
0.0
0.5
1.0
MR leave−one−out sensitivity analysis for
'EIF3J' on 'Pulmonary fibrosis'

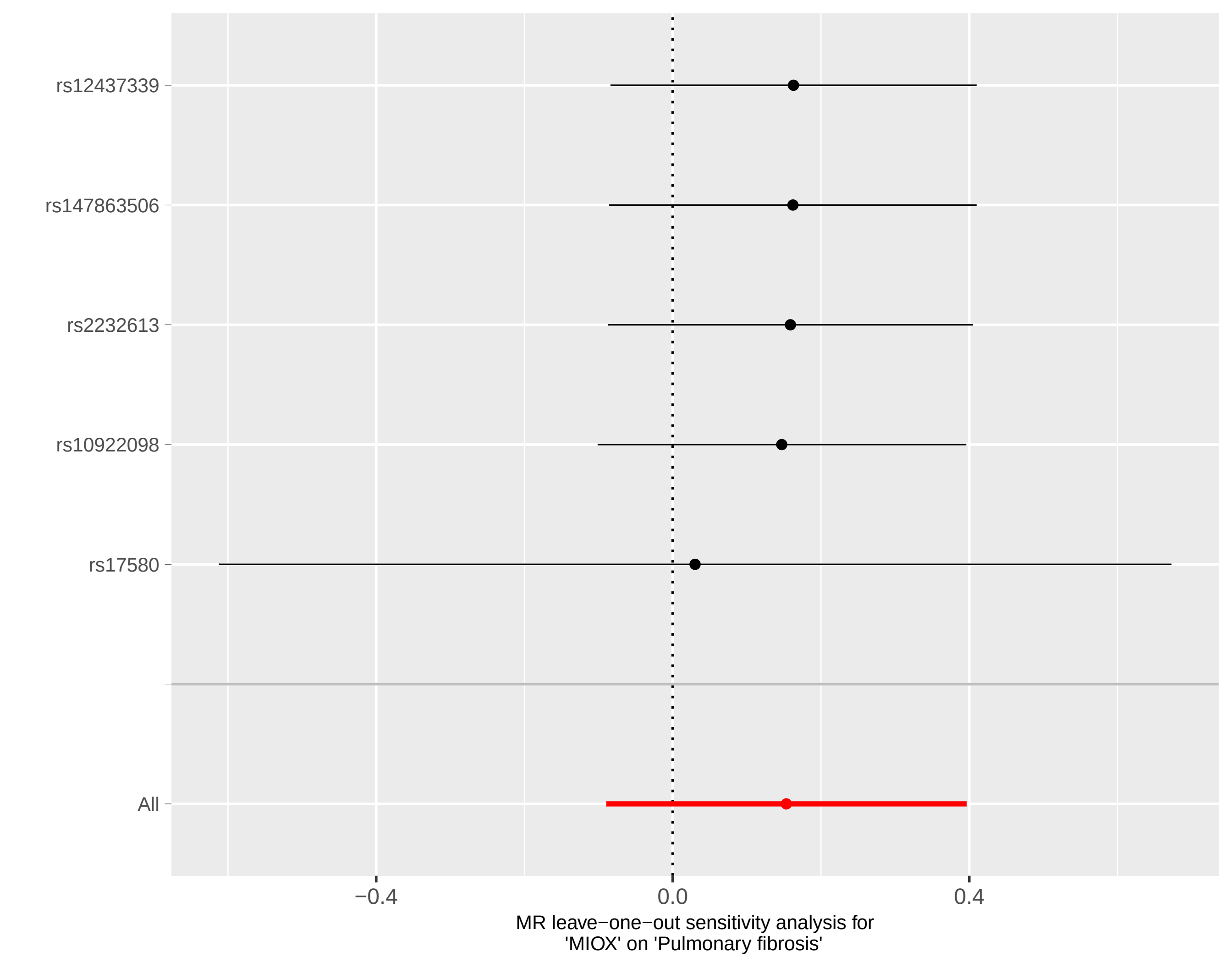
rs12437339
rs147863506
rs2232613
rs10922098
rs17580
All
−0.4
0.0
0.4
MR leave−one−out sensitivity analysis for
'MIOX' on 'Pulmonary fibrosis'

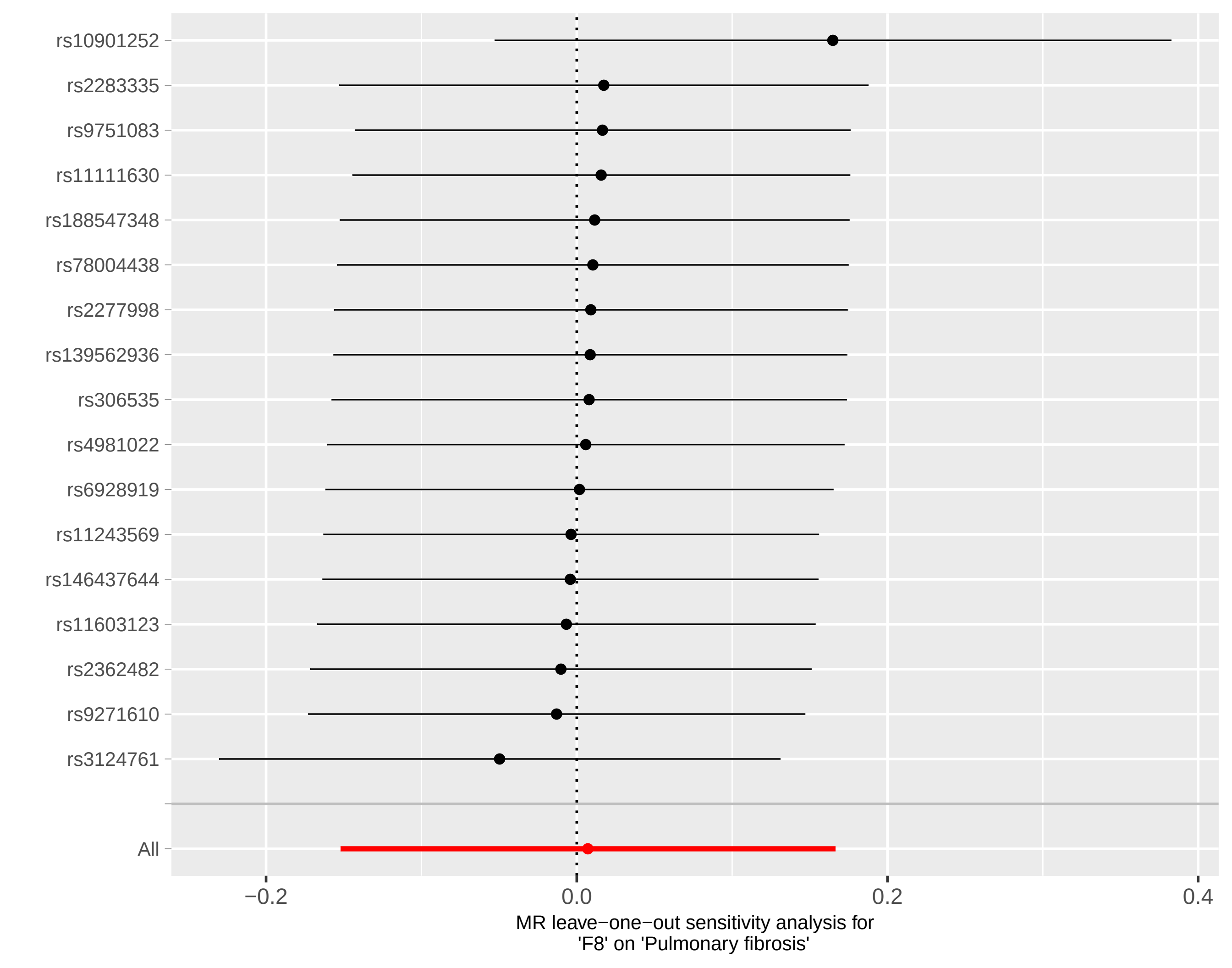
rs10901252
rs2283335
rs9751083
rs11111630
rs188547348
rs78004438
rs2277998
rs139562936
rs306535
rs4981022
rs6928919
rs11243569
rs146437644
rs11603123
rs2362482
rs9271610
rs3124761
All
−0.2
0.0
0.2
0.4
MR leave−one−out sensitivity analysis for
'F8' on 'Pulmonary fibrosis'

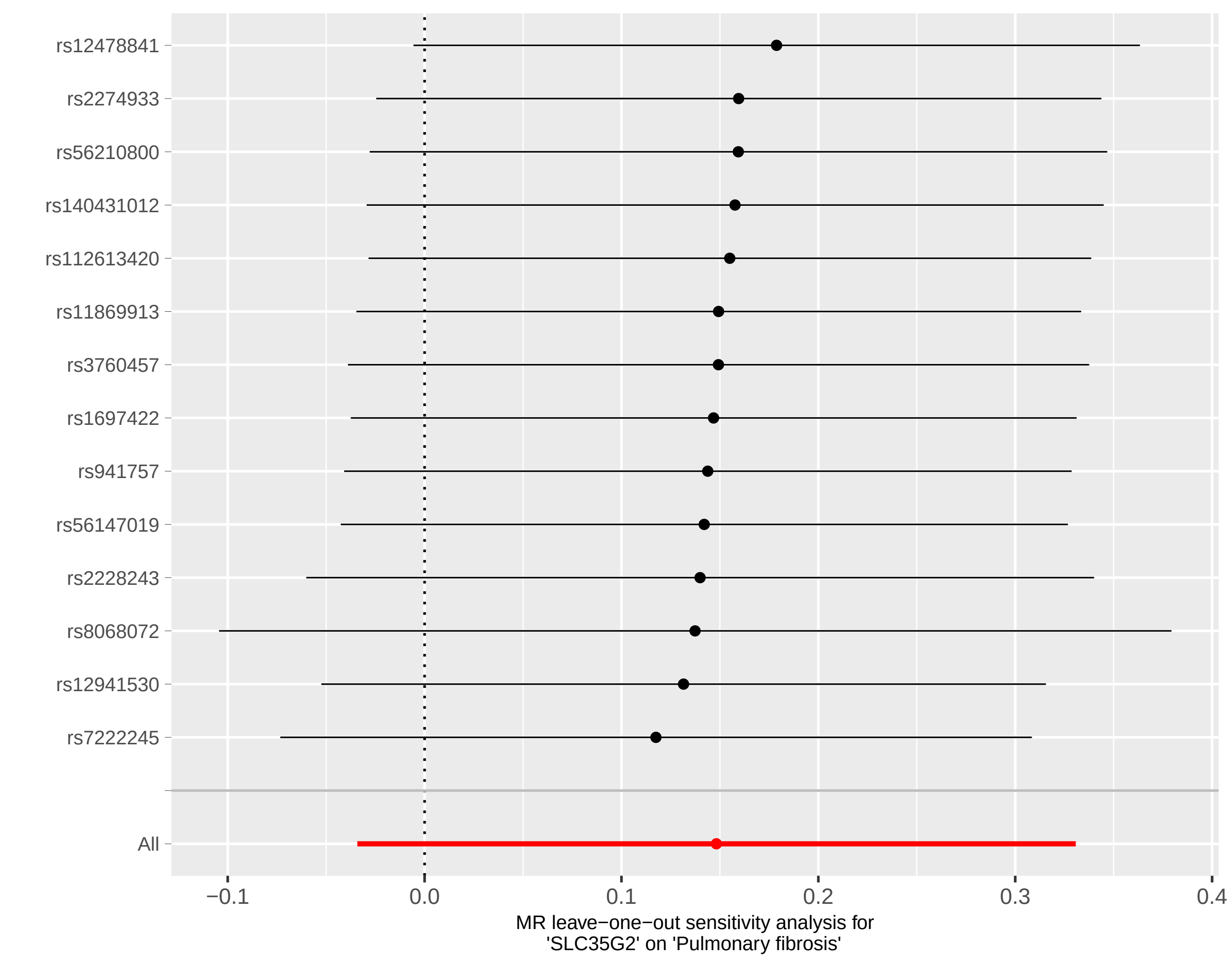
rs12478841
rs2274933
rs56210800
rs140431012
rs112613420
rs11869913
rs3760457
rs1697422
rs941757
rs56147019
rs2228243
rs8068072
rs12941530
rs7222245
All
−0.1
0.0
0.1
0.2
0.3
0.4
MR leave−one−out sensitivity analysis for
'SLC35G2' on 'Pulmonary fibrosis'

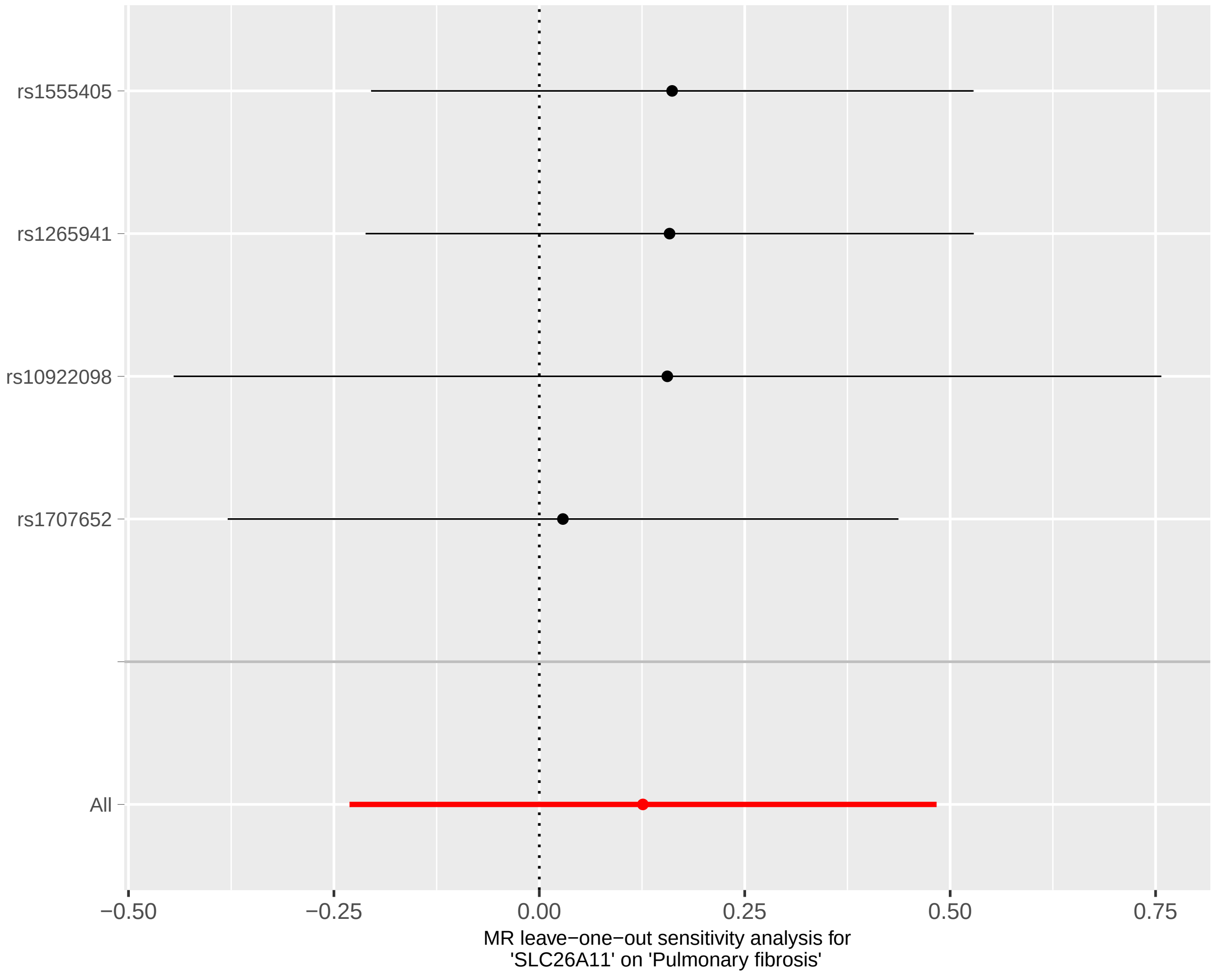
rs1555405
rs1265941
rs10922098
rs1707652
All
−0.50
−0.25
0.00
0.25
0.50
0.75
MR leave−one−out sensitivity analysis for
'SLC26A11' on 'Pulmonary fibrosis'

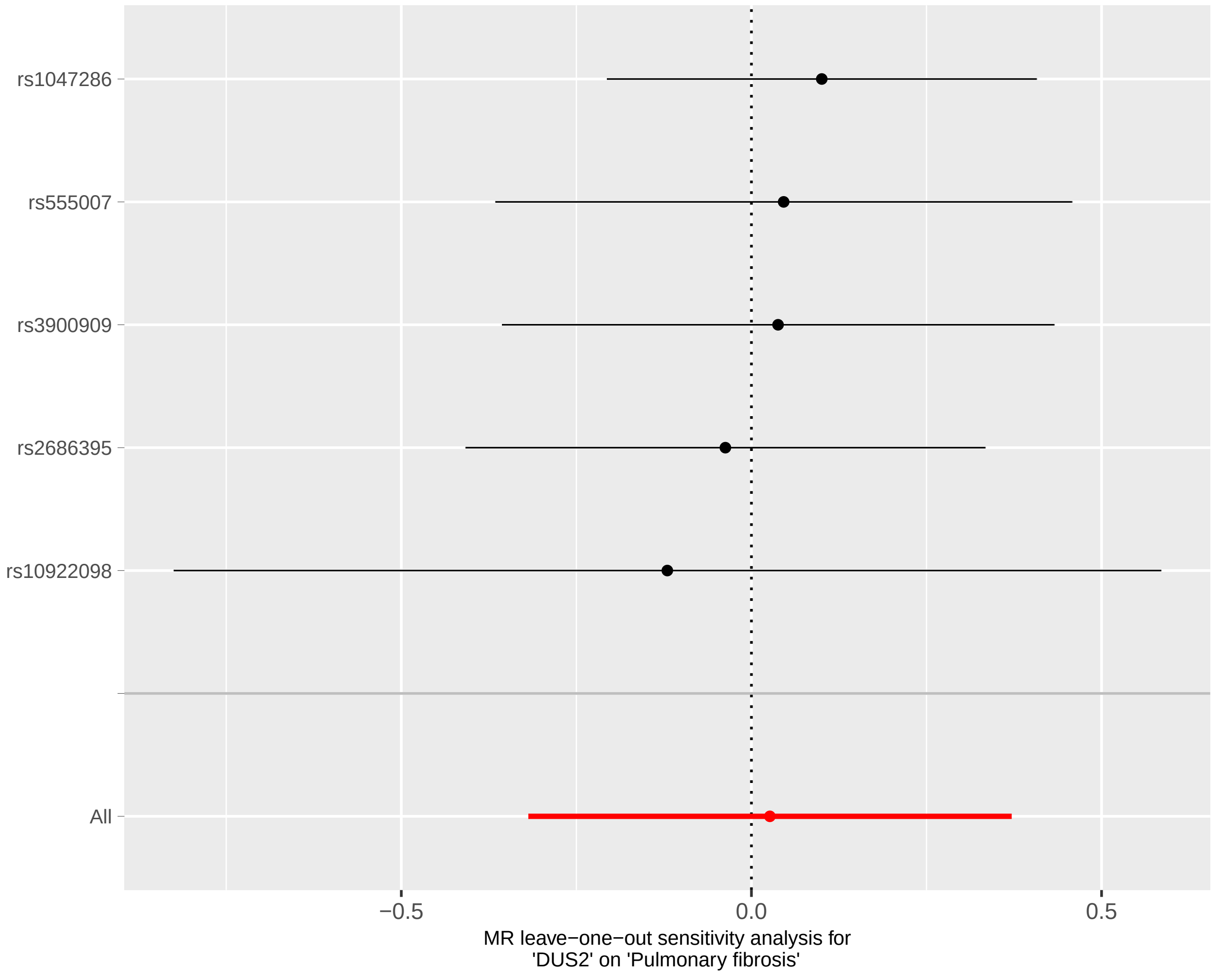
rs1047286
rs555007
rs3900909
rs2686395
rs10922098
All
−0.5
0.0
0.5
MR leave−one−out sensitivity analysis for
'DUS2' on 'Pulmonary fibrosis'

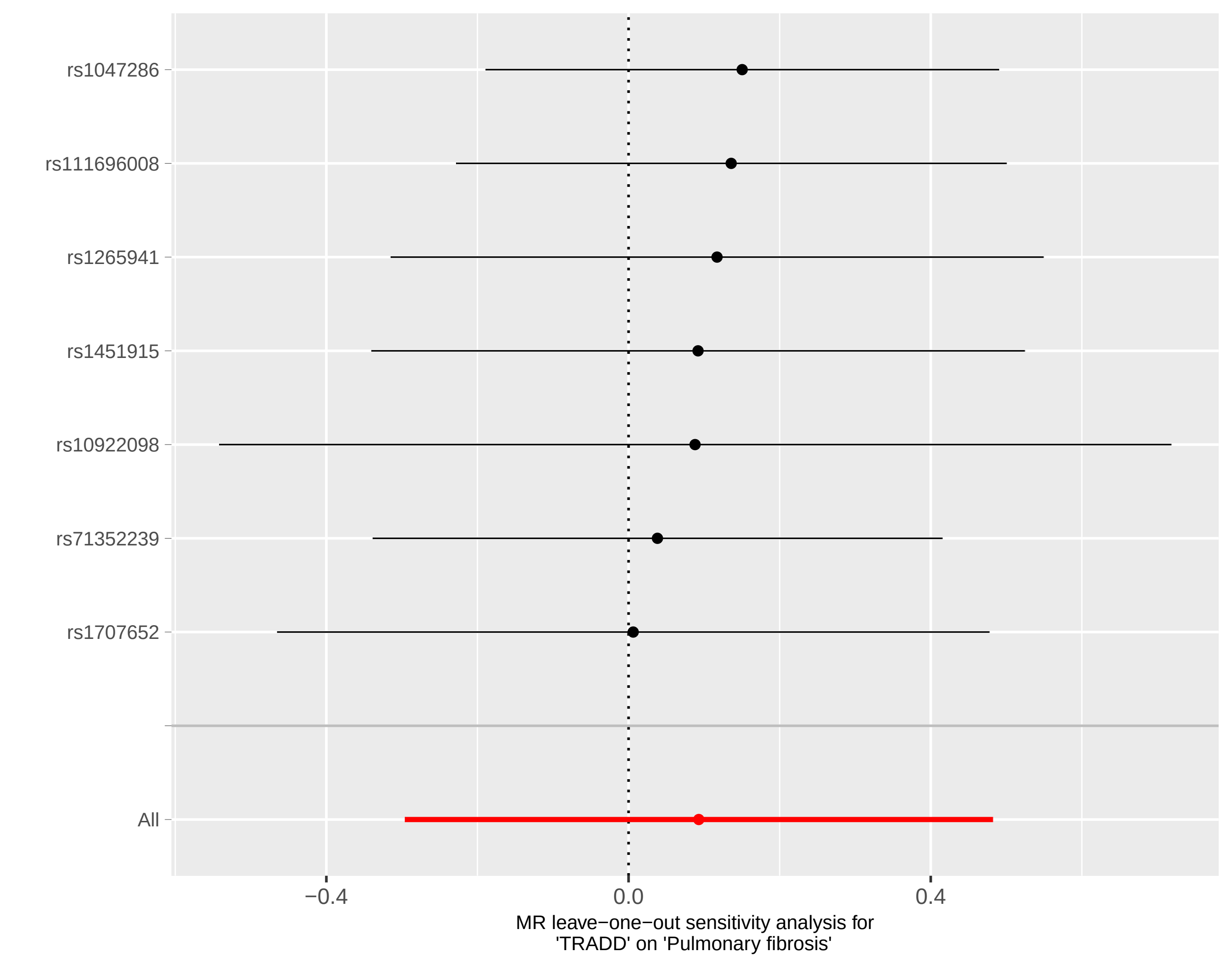
rs1047286
rs111696008
rs1265941
rs1451915
rs10922098
rs71352239
rs1707652
All
−0.4
0.0
0.4
MR leave−one−out sensitivity analysis for
'TRADD' on 'Pulmonary fibrosis'

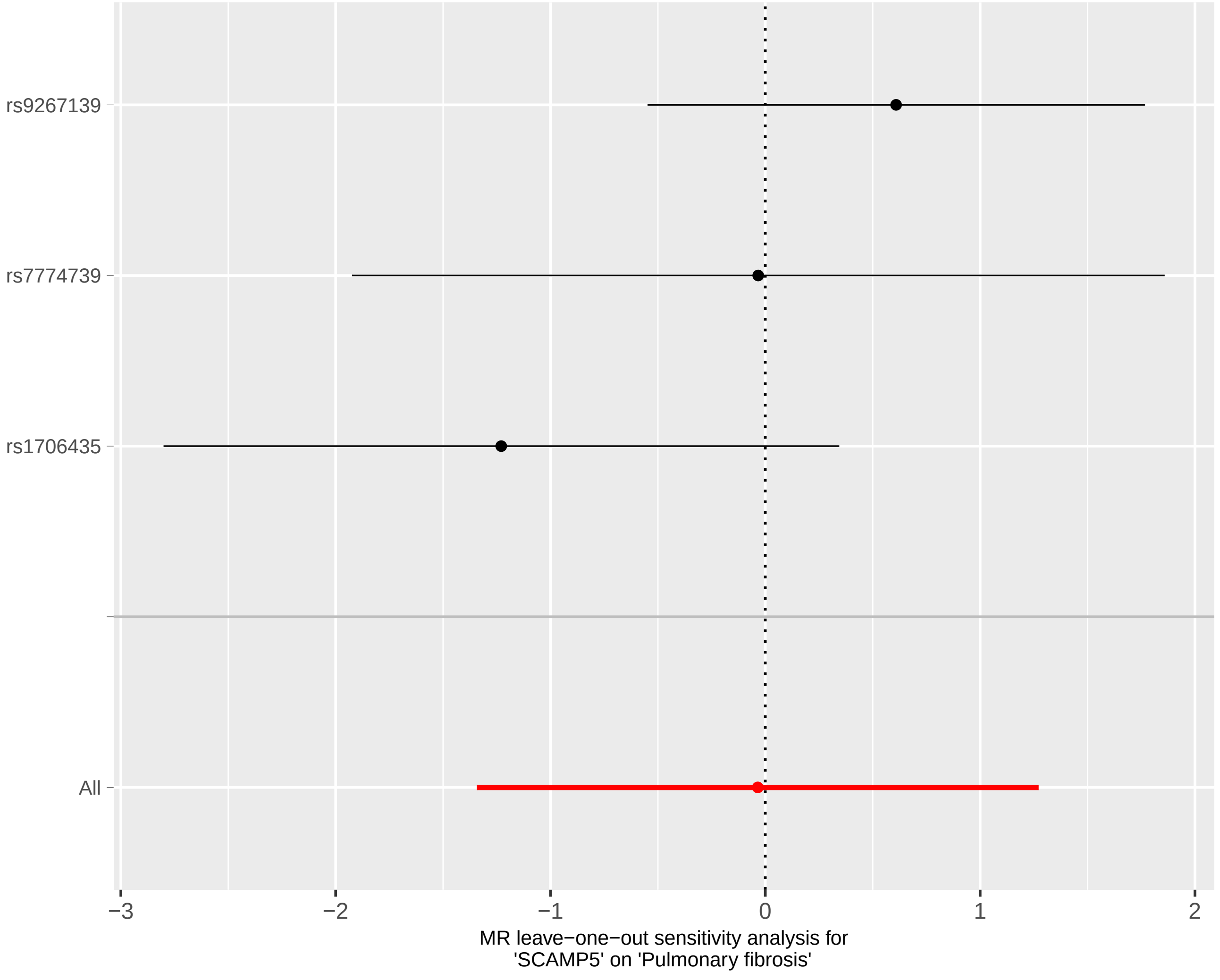
rs9267139
rs7774739
rs1706435
All
−3
−2
−1
0
1
2
MR leave−one−out sensitivity analysis for
'SCAMP5' on 'Pulmonary fibrosis'

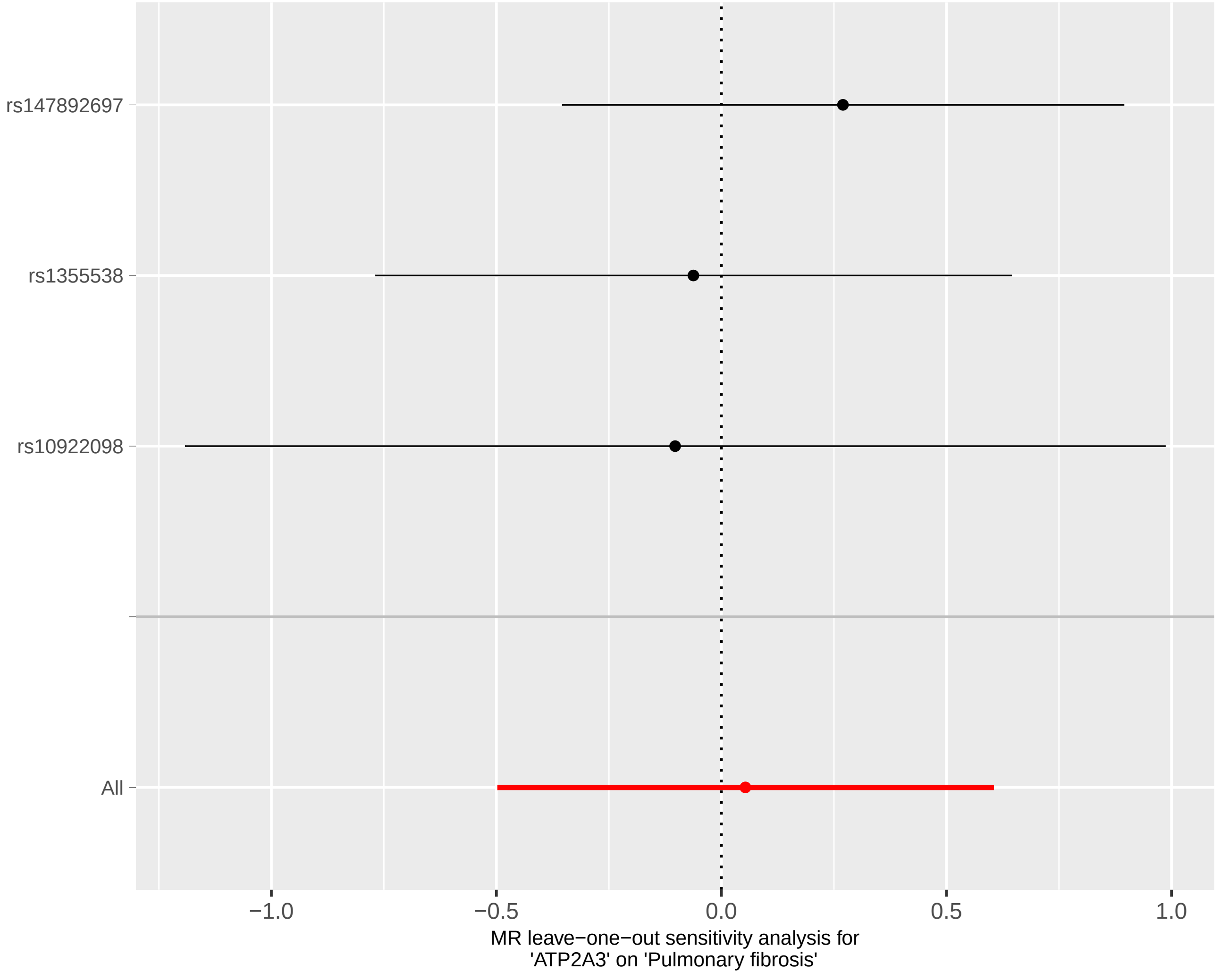
rs147892697
rs1355538
rs10922098
All
−1.0
−0.5
0.0
0.5
1.0
MR leave−one−out sensitivity analysis for
'ATP2A3' on 'Pulmonary fibrosis'

Insufficient number of SNPs

Insufficient number of SNPs

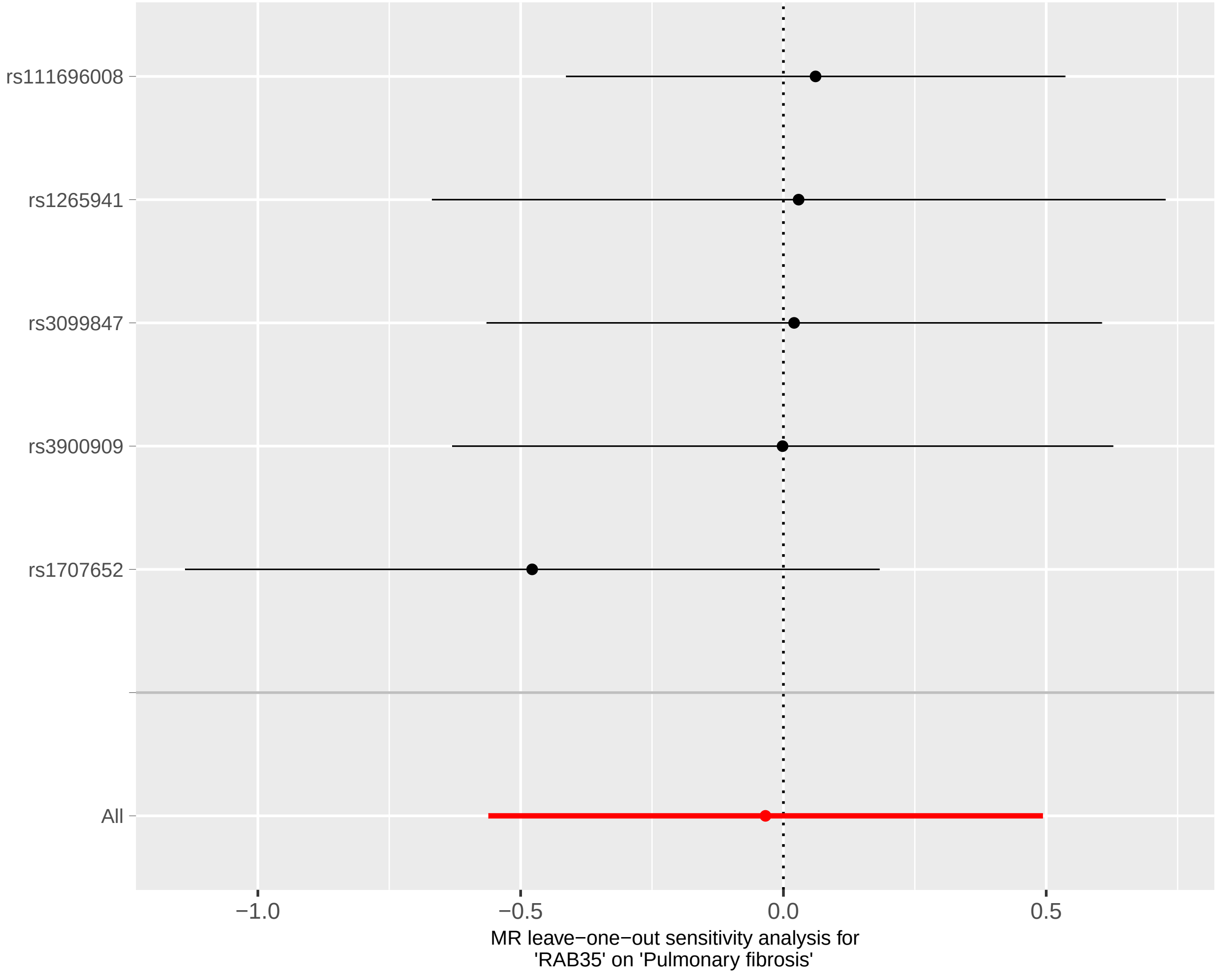
rs111696008
rs1265941
rs3099847
rs3900909
rs1707652
All
−1.0
−0.5
0.0
0.5
MR leave−one−out sensitivity analysis for
'RAB35' on 'Pulmonary fibrosis'

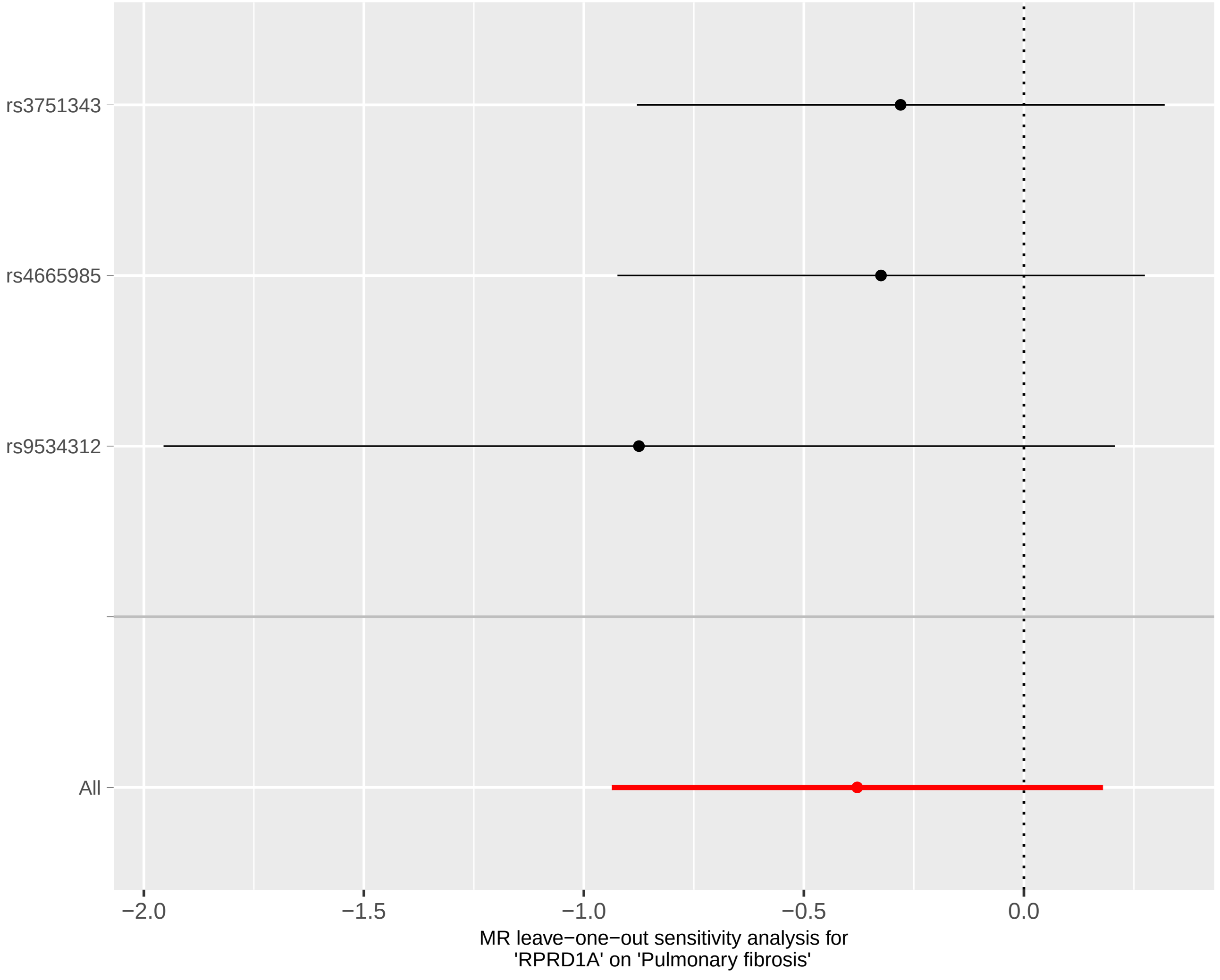

rs3751343
rs4665985
rs9534312
All
−2.0
−1.5
−1.0
−0.5
0.0
MR leave−one−out sensitivity analysis for
'RPRD1A' on 'Pulmonary fibrosis'

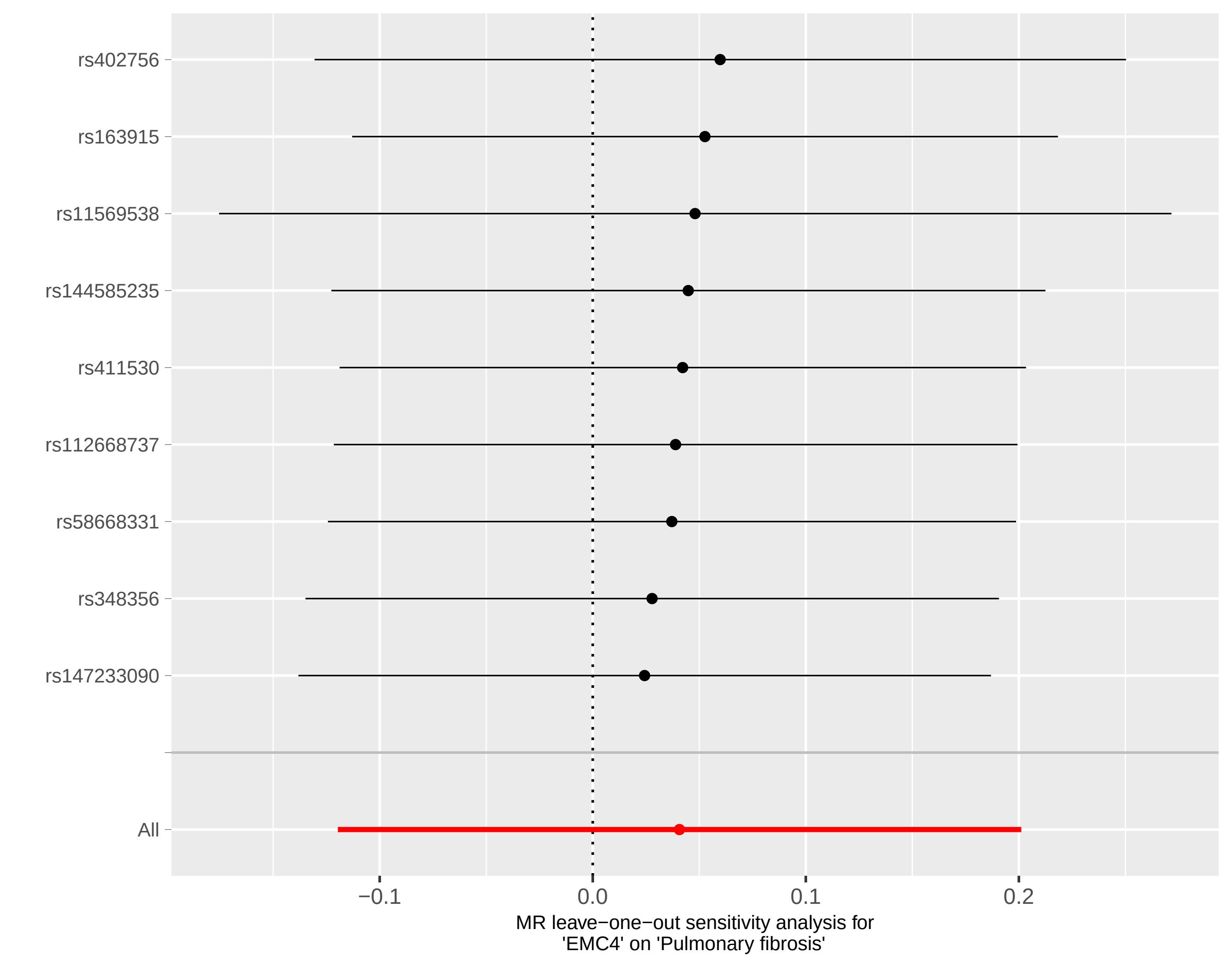
rs402756
rs163915
rs11569538
rs144585235
rs411530
rs112668737
rs58668331
rs348356
rs147233090
All
−0.1
0.0
0.1
0.2
MR leave−one−out sensitivity analysis for
'EMC4' on 'Pulmonary fibrosis'

Insufficient number of SNPs

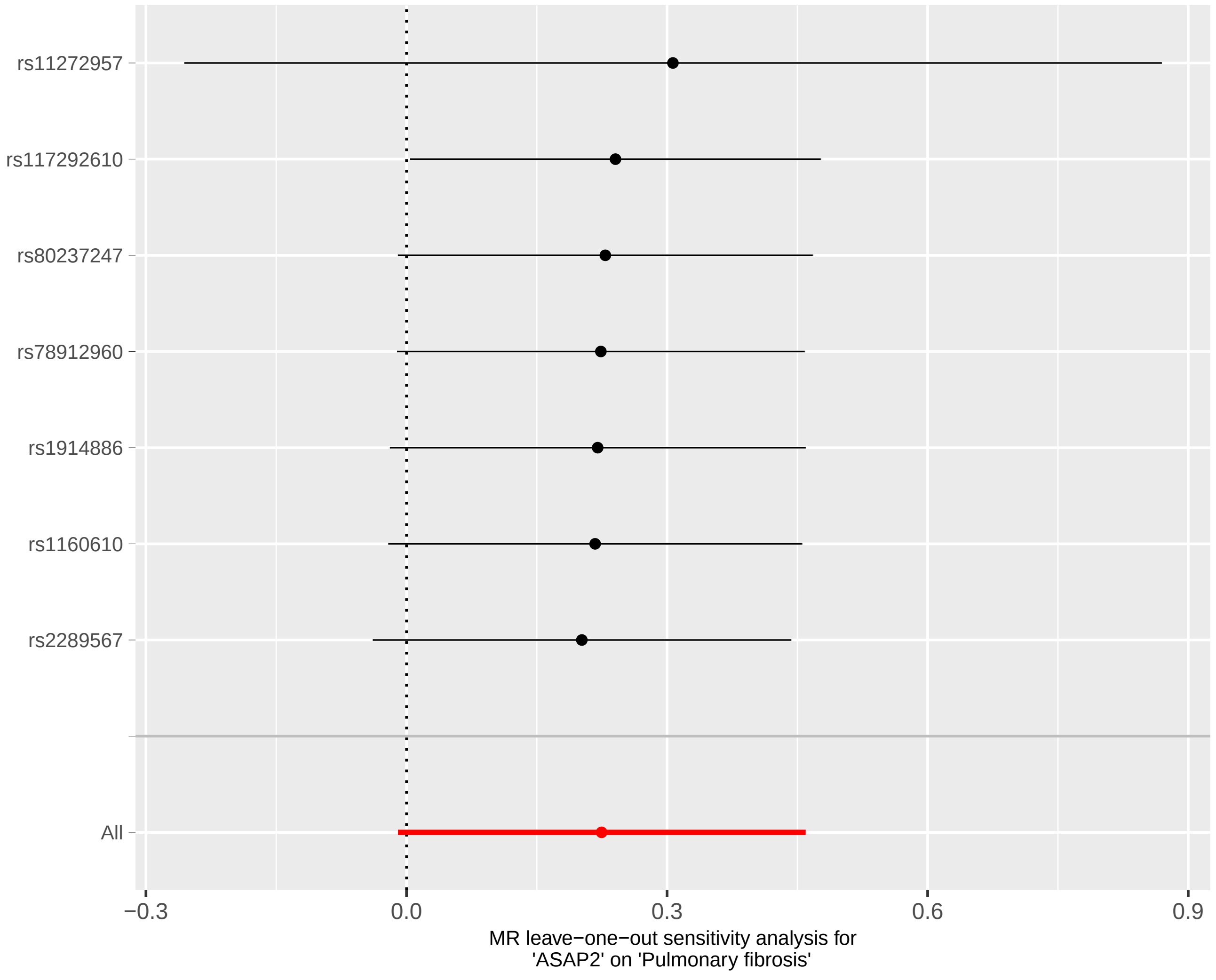
rs11272957
rs117292610
rs80237247
rs78912960
rs1914886
rs1160610
rs2289567
All
−0.3
0.0
0.3
0.6
0.9
MR leave−one−out sensitivity analysis for
'ASAP2' on 'Pulmonary fibrosis'

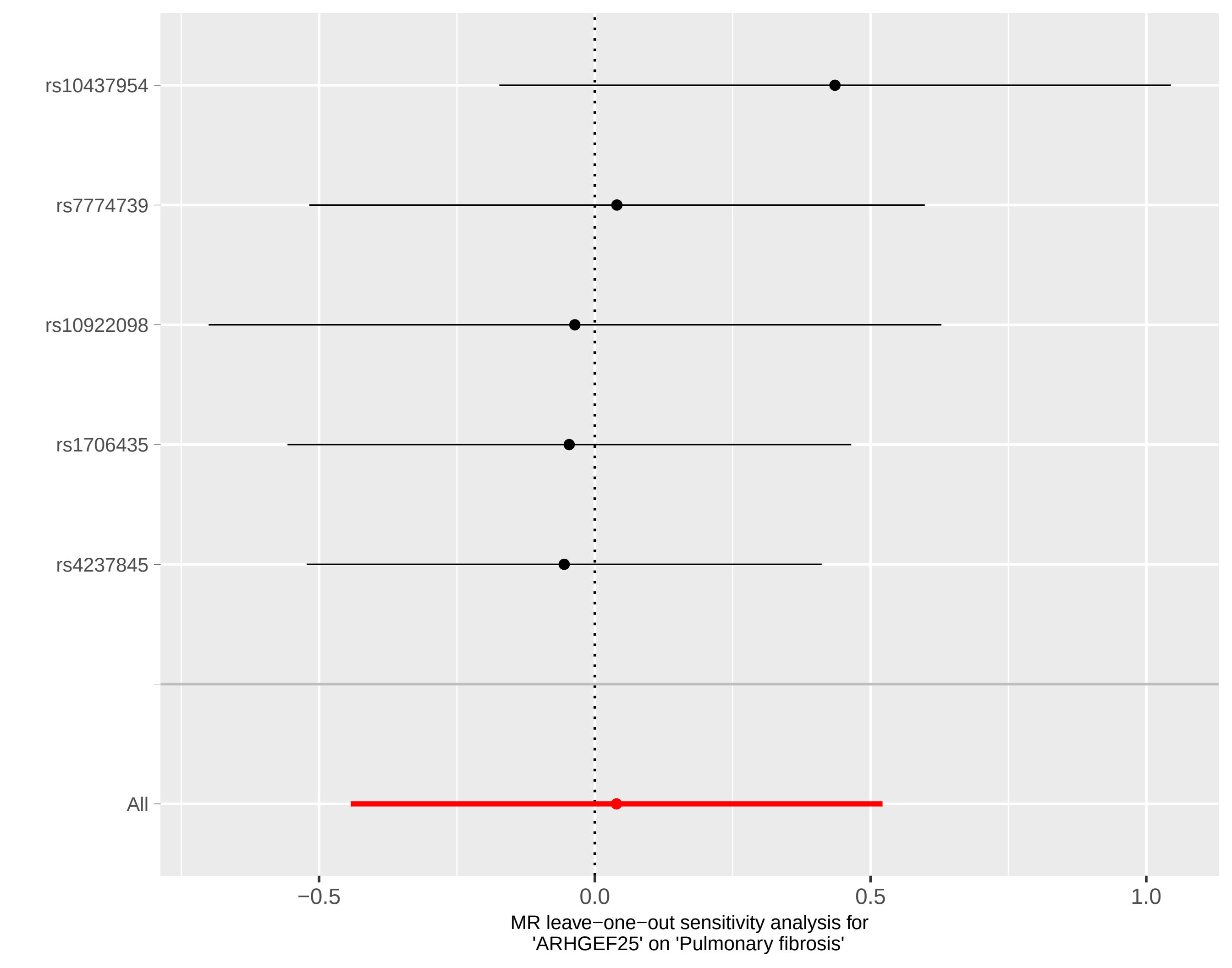
rs10437954
rs7774739
rs10922098
rs1706435
rs4237845
All
−0.5
0.0
0.5
1.0
MR leave−one−out sensitivity analysis for
'ARHGEF25' on 'Pulmonary fibrosis'

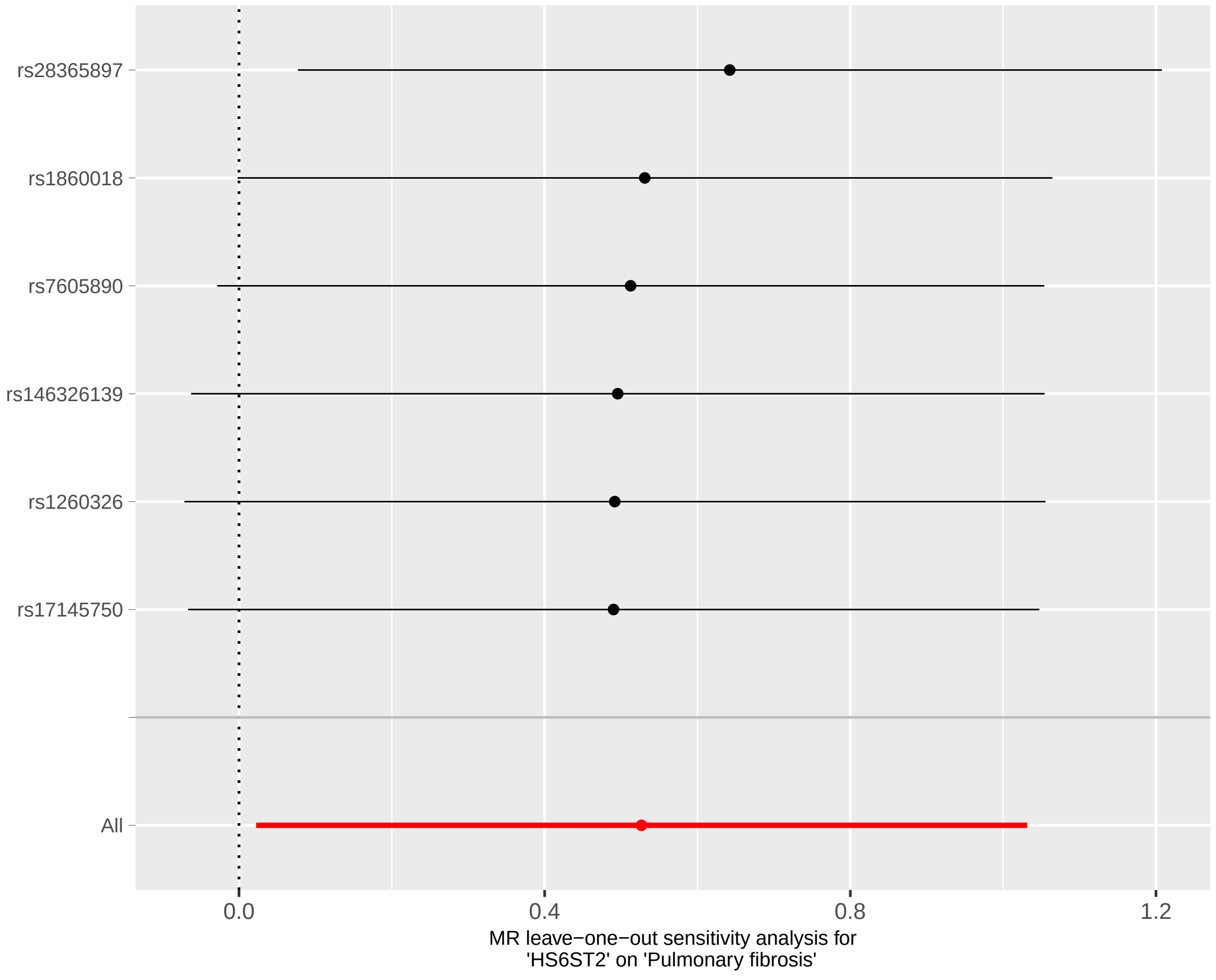
rs28365897
rs1860018
rs7605890
rs146326139
rs1260326
rs17145750
All
0.0
0.4
0.8
1.2
MR leave−one−out sensitivity analysis for
'HS6ST2' on 'Pulmonary fibrosis'

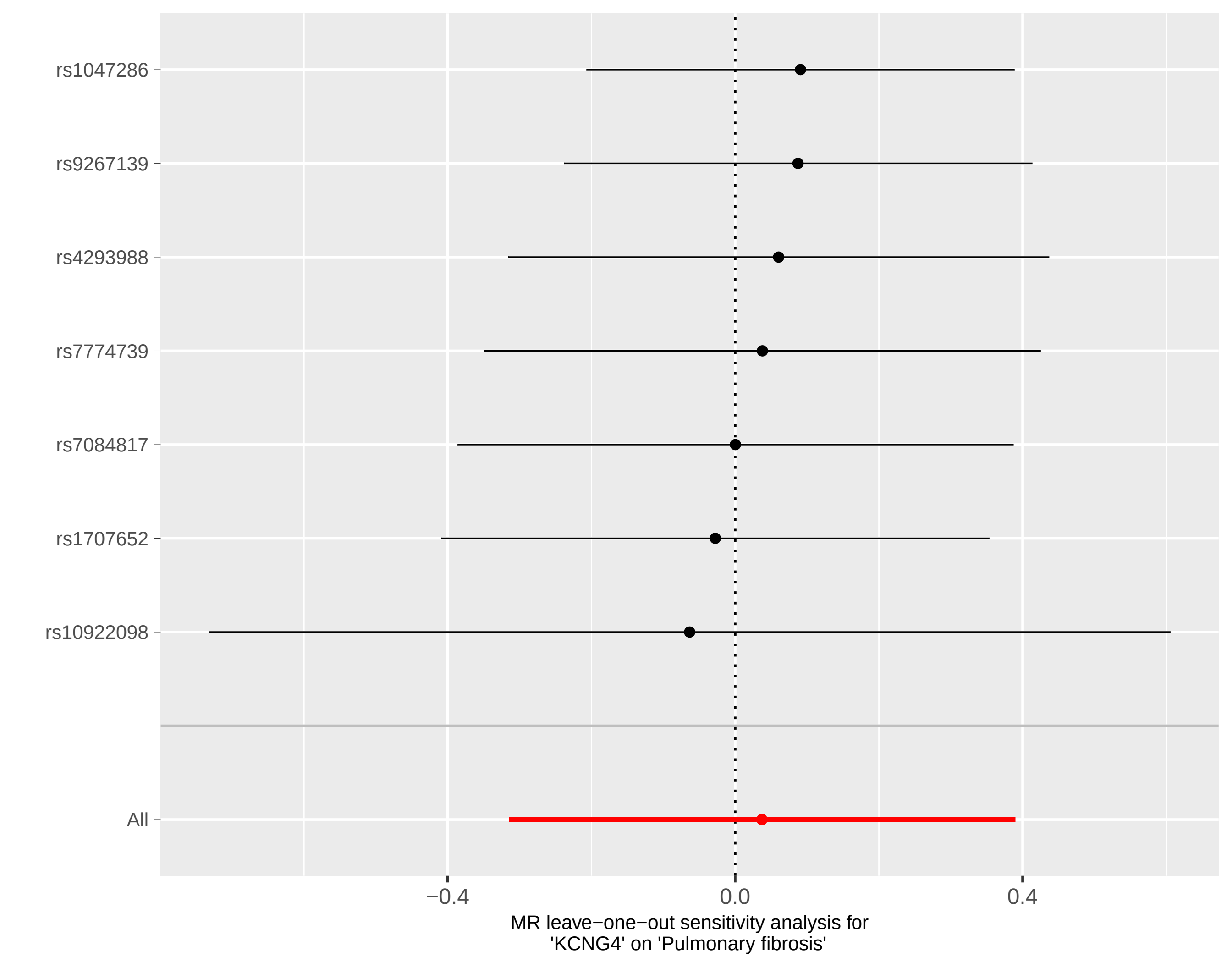
rs1047286
rs9267139
rs4293988
rs7774739
rs7084817
rs1707652
rs10922098
All
−0.4
0.0
0.4
MR leave−one−out sensitivity analysis for
'KCNG4' on 'Pulmonary fibrosis'

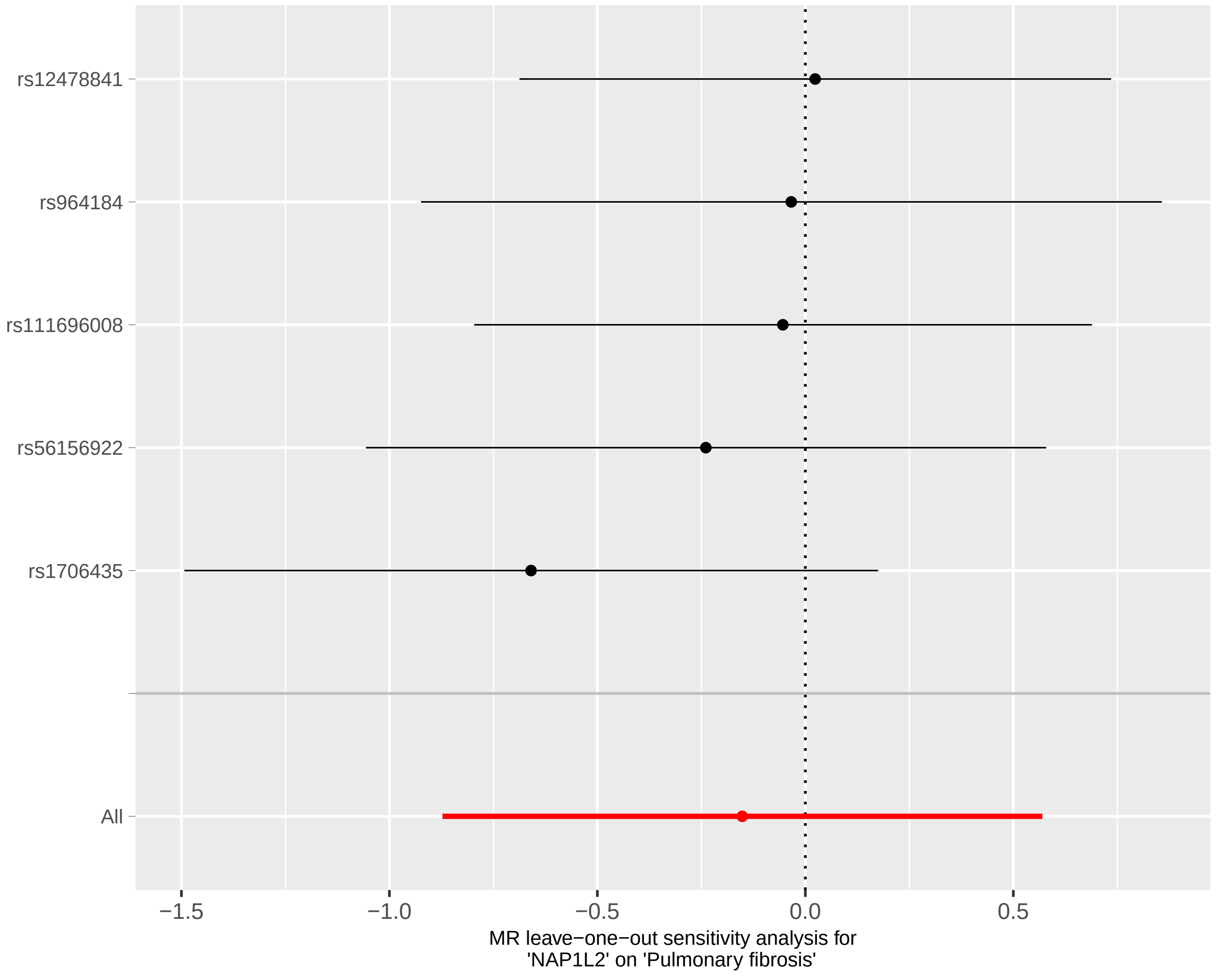
rs12478841
rs964184
rs111696008
rs56156922
rs1706435
All
−1.5
−1.0
−0.5
0.0
0.5
MR leave−one−out sensitivity analysis for
'NAP1L2' on 'Pulmonary fibrosis'

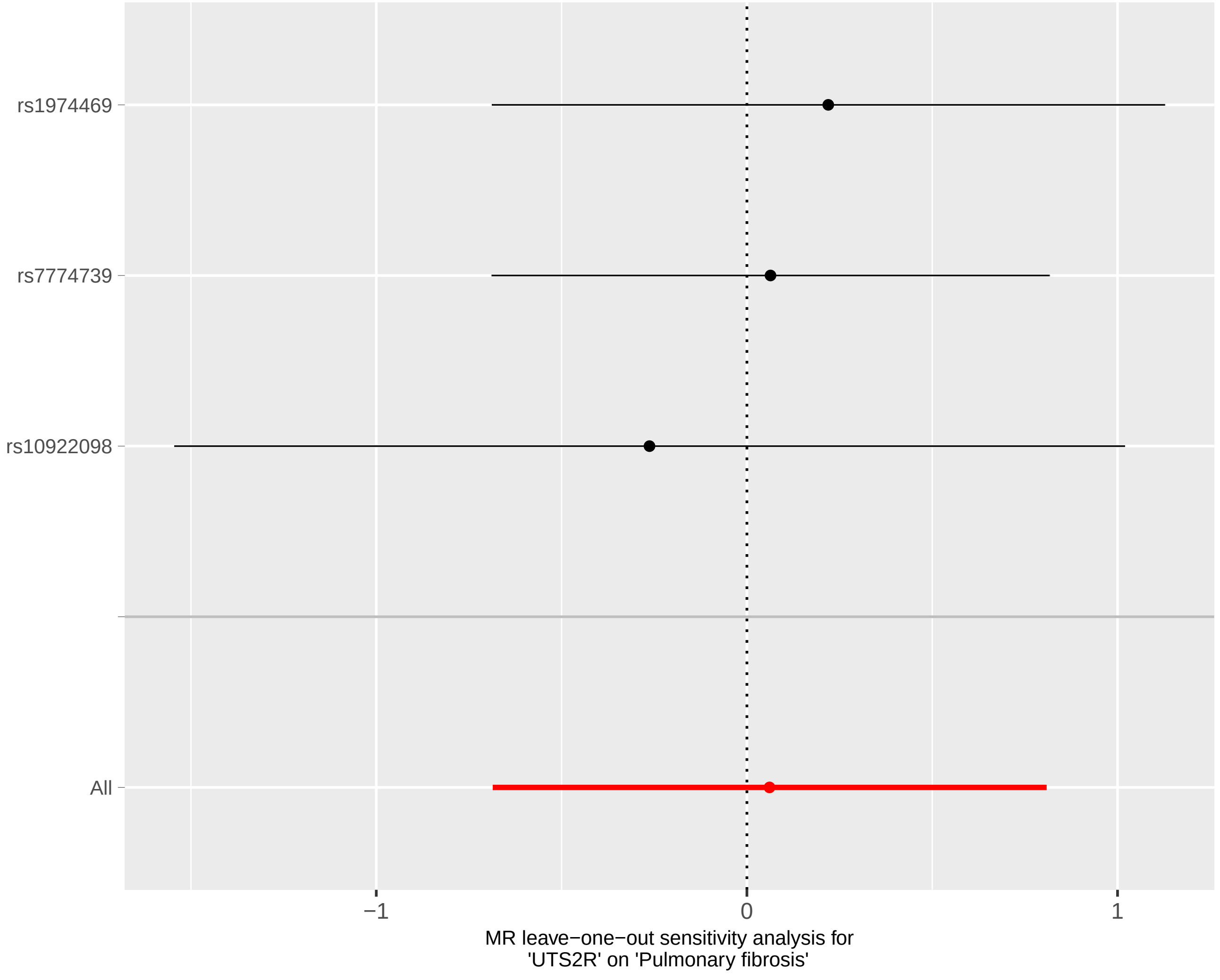

rs1974469
rs7774739
rs10922098
All
−1
0
1
MR leave−one−out sensitivity analysis for
'UTS2R' on 'Pulmonary fibrosis'

Insufficient number of SNPs

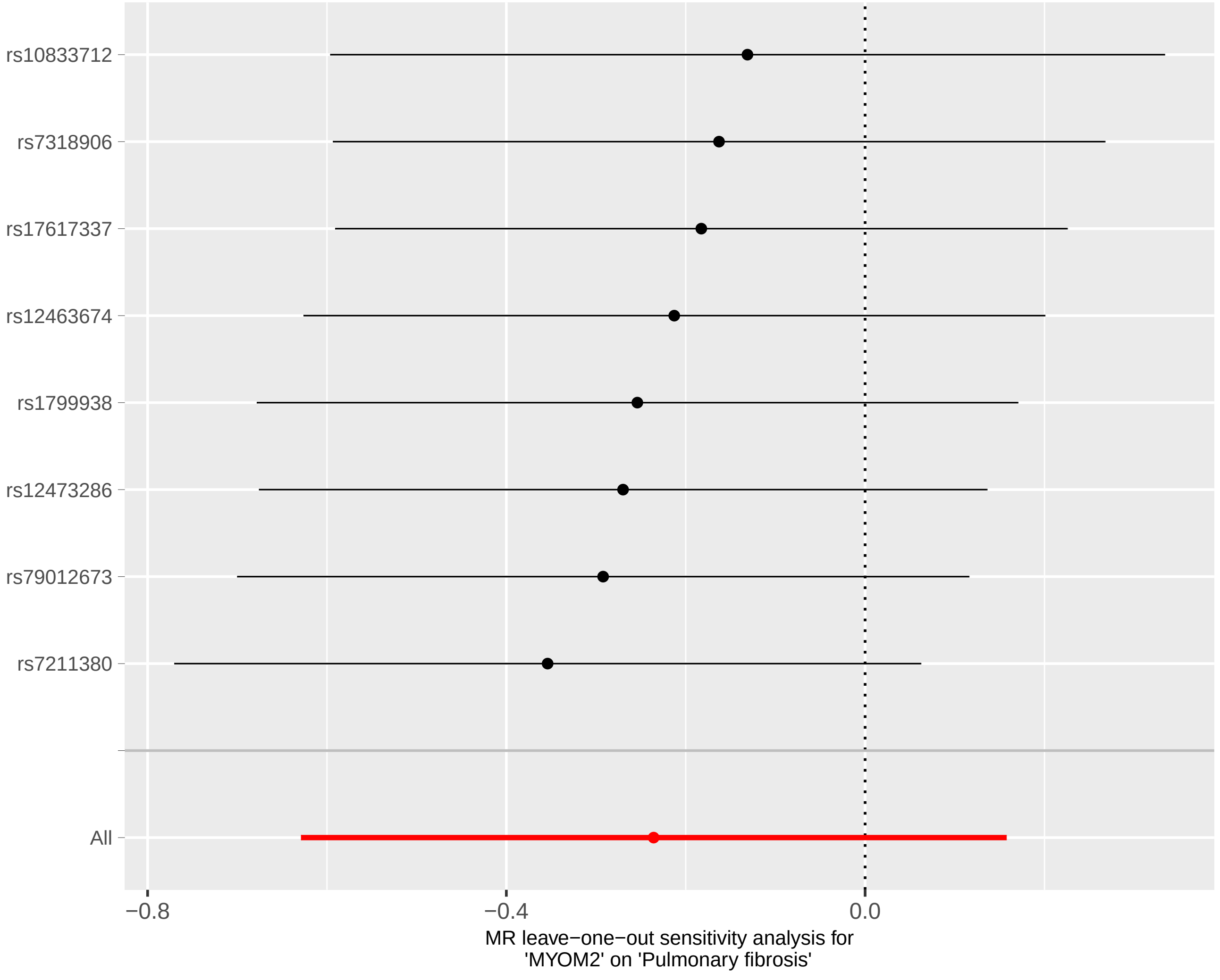

rs10833712
rs7318906
rs17617337
rs12463674
rs1799938
rs12473286
rs79012673
rs7211380
All
−0.8
−0.4
0.0
MR leave−one−out sensitivity analysis for
'MYOM2' on 'Pulmonary fibrosis'

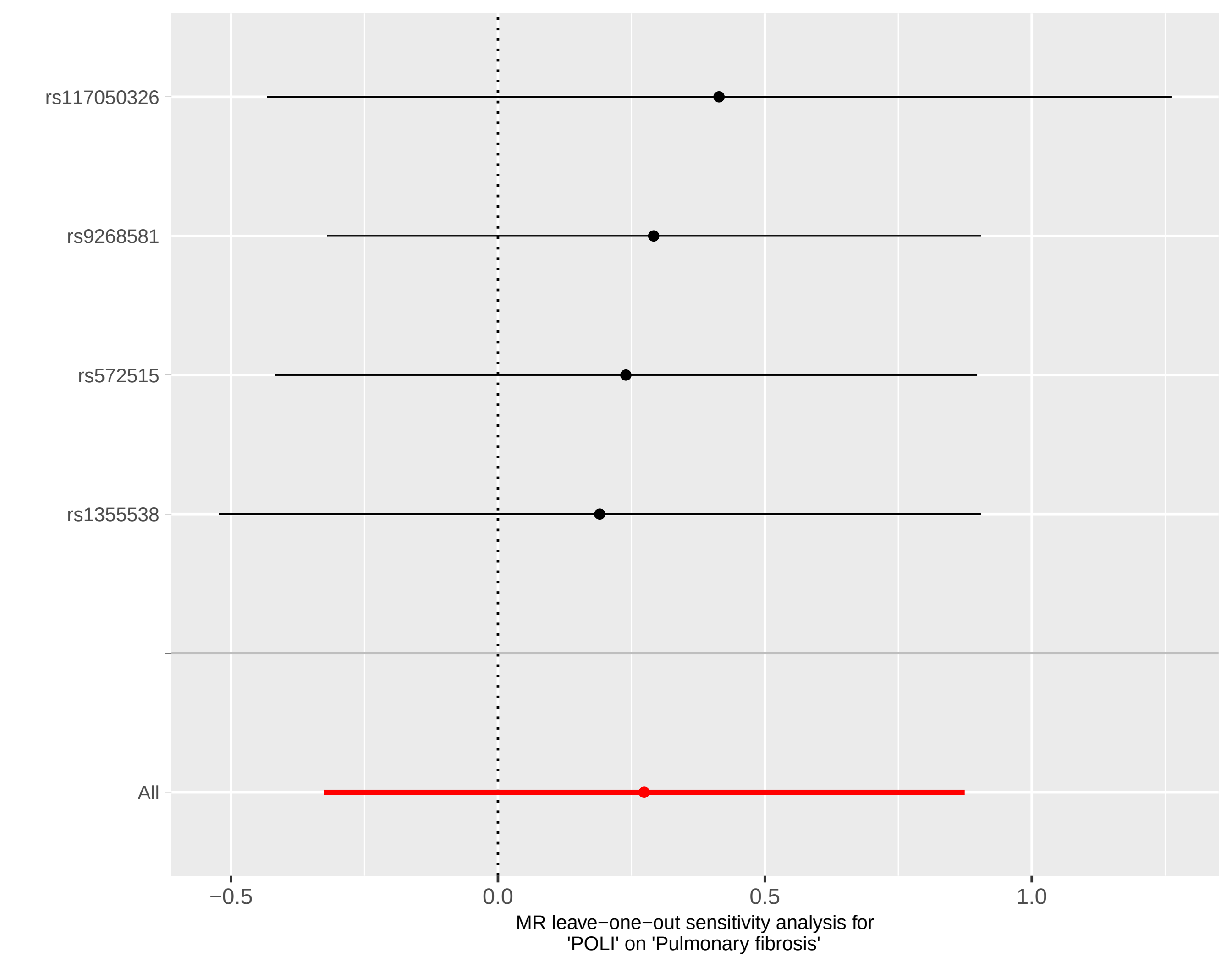

rs117050326
rs9268581
rs572515
rs1355538
All
−0.5
0.0
0.5
1.0
MR leave−one−out sensitivity analysis for
'POLI' on 'Pulmonary fibrosis'

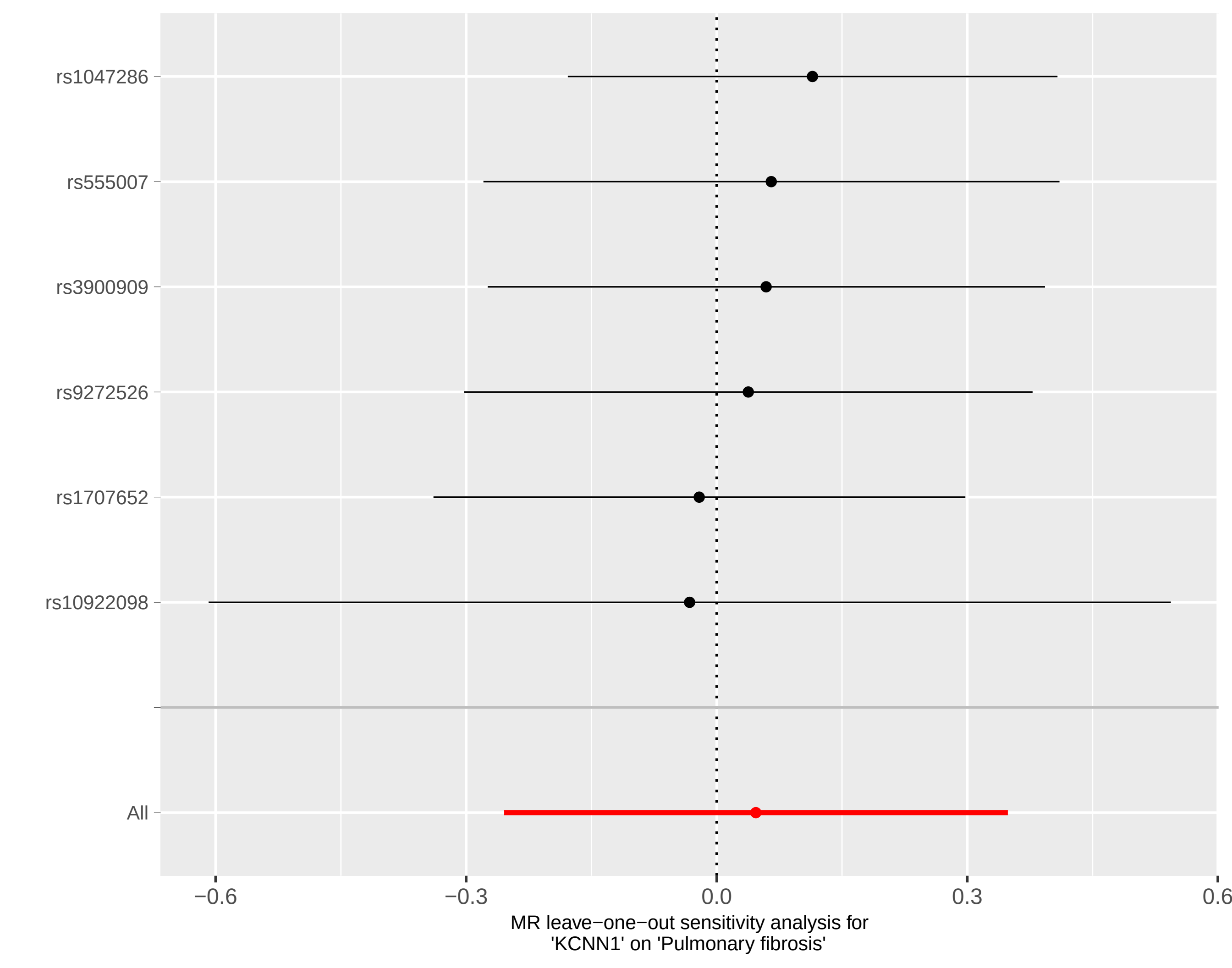
rs1047286
rs555007
rs3900909
rs9272526
rs1707652
rs10922098
All
−0.6
−0.3
0.0
0.3
0.6
MR leave−one−out sensitivity analysis for
'KCNN1' on 'Pulmonary fibrosis'

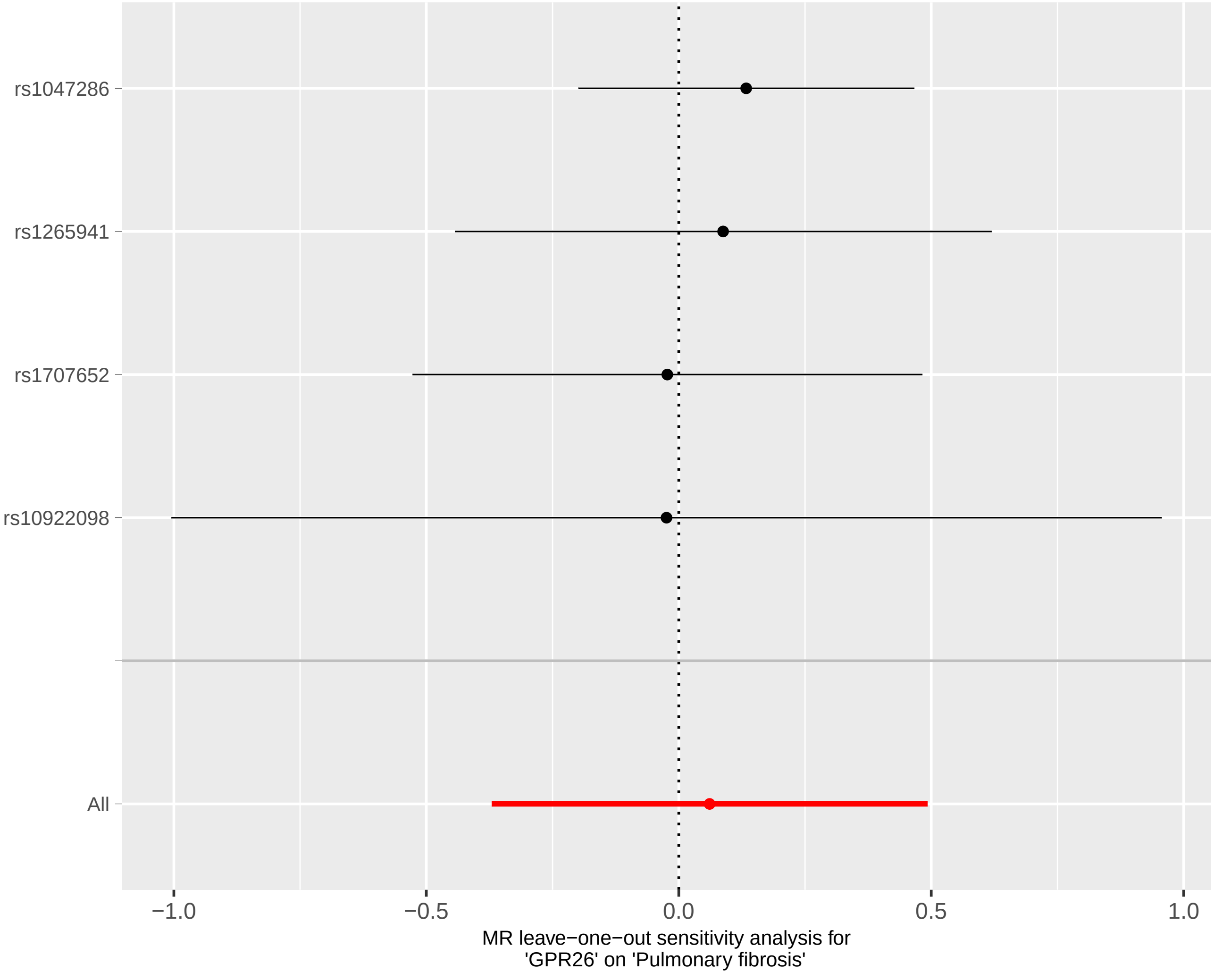
rs1047286
rs1265941
rs1707652
rs10922098
All
−1.0
−0.5
0.0
0.5
1.0
MR leave−one−out sensitivity analysis for
'GPR26' on 'Pulmonary fibrosis'

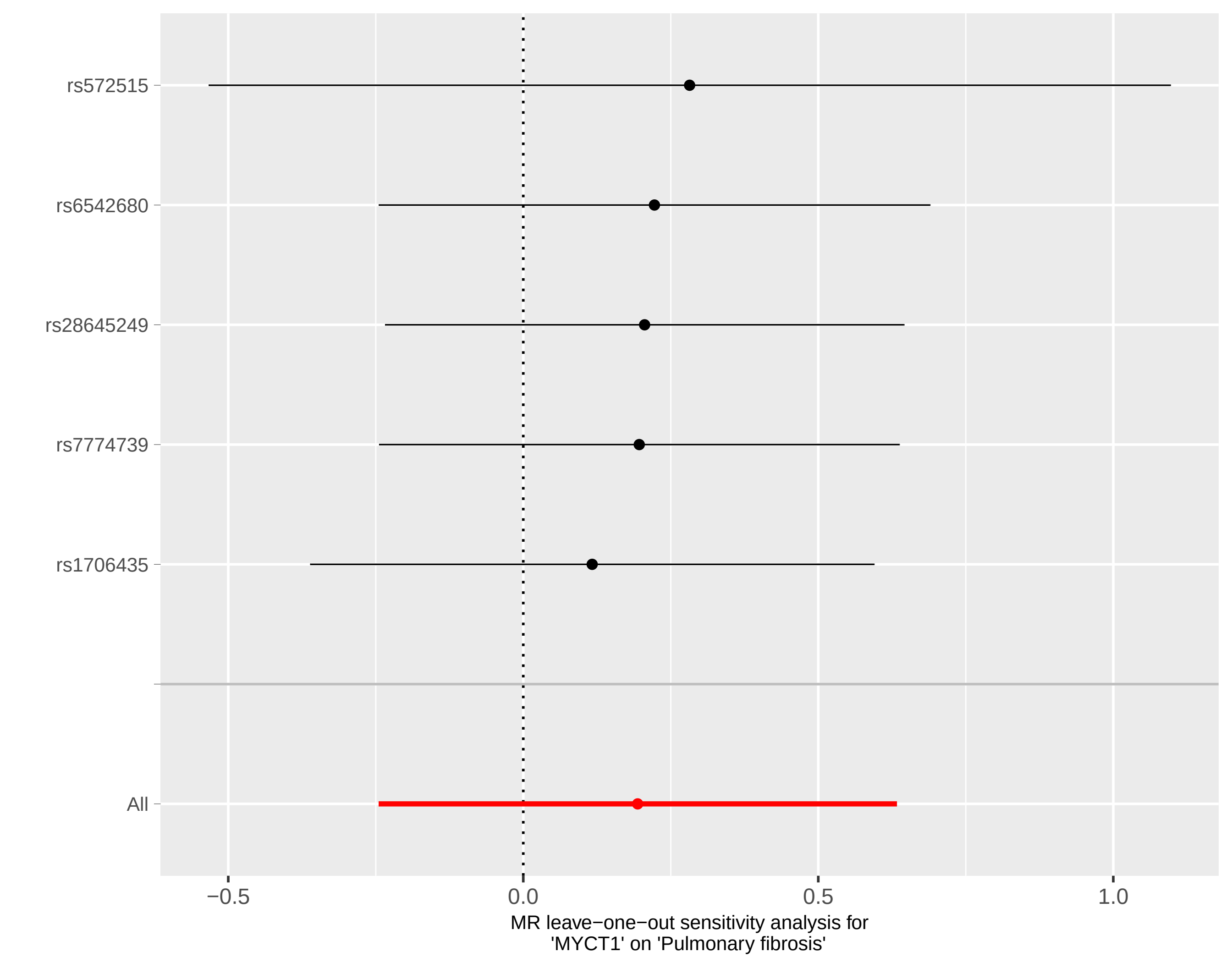

rs572515
rs6542680
rs28645249
rs7774739
rs1706435
All
−0.5
0.0
0.5
1.0
MR leave−one−out sensitivity analysis for
'MYCT1' on 'Pulmonary fibrosis'

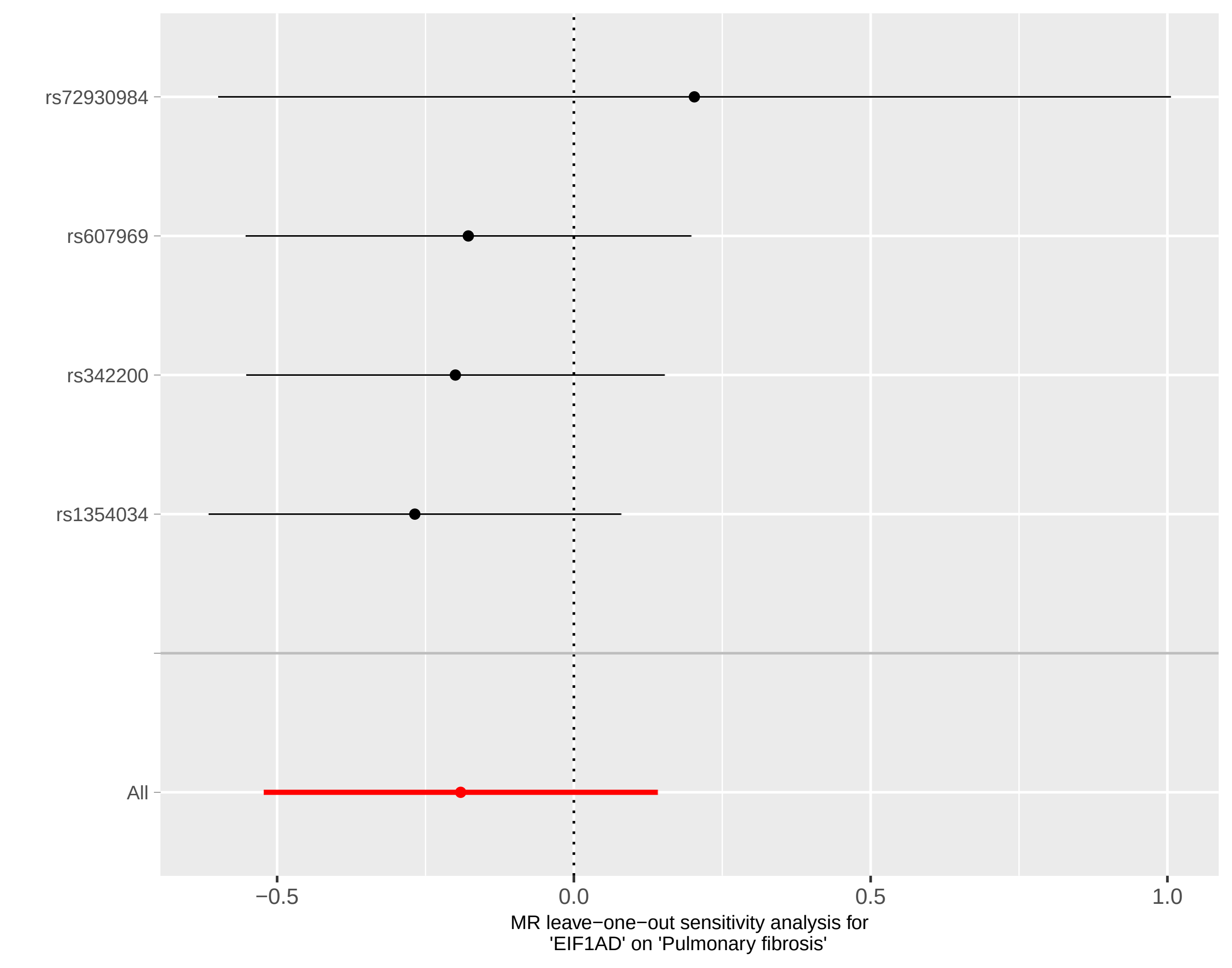

rs72930984
rs607969
rs342200
rs1354034
All
−0.5
0.0
0.5
1.0
MR leave−one−out sensitivity analysis for
'EIF1AD' on 'Pulmonary fibrosis'

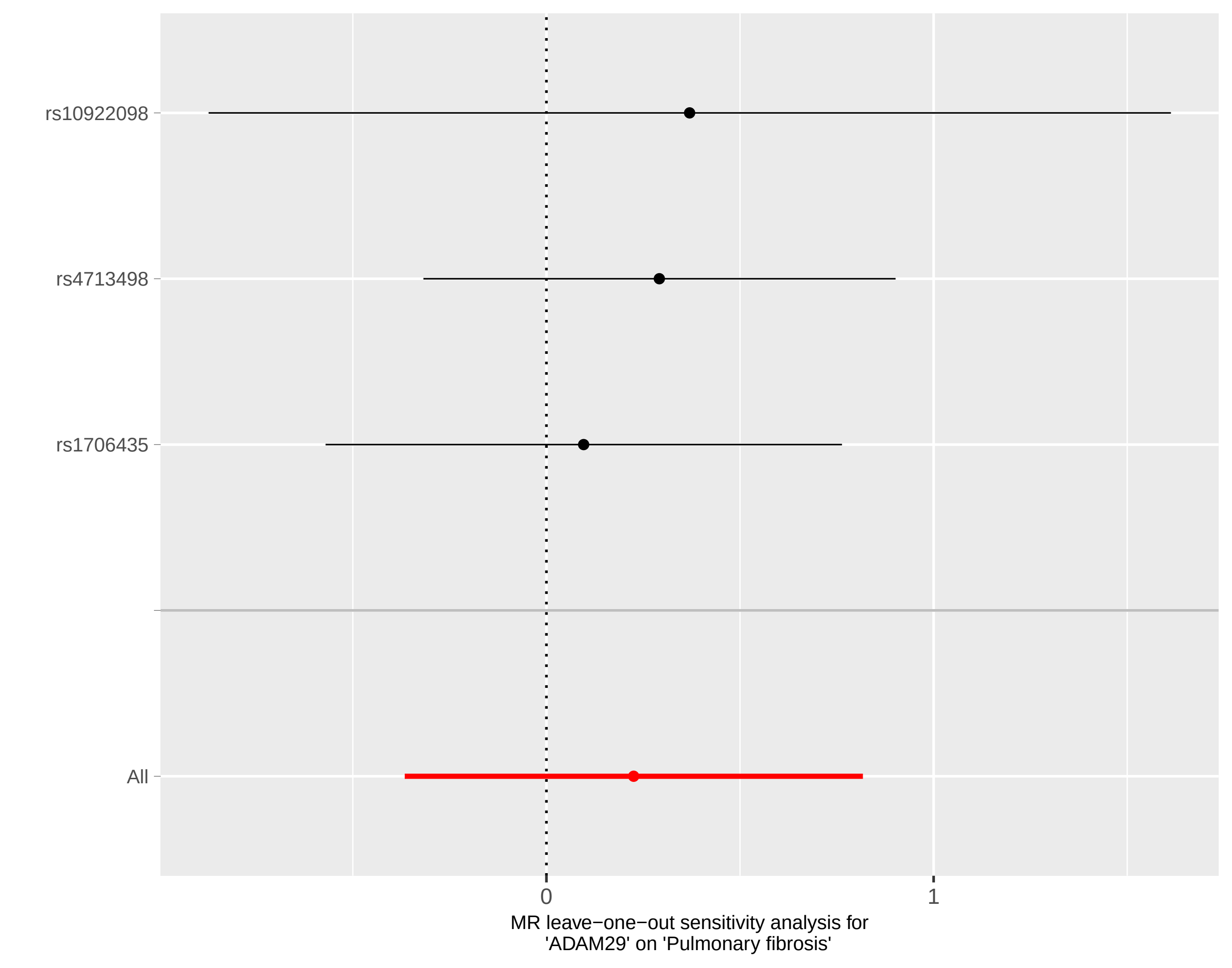
rs10922098
rs4713498
rs1706435
All
0
1
MR leave−one−out sensitivity analysis for
'ADAM29' on 'Pulmonary fibrosis'

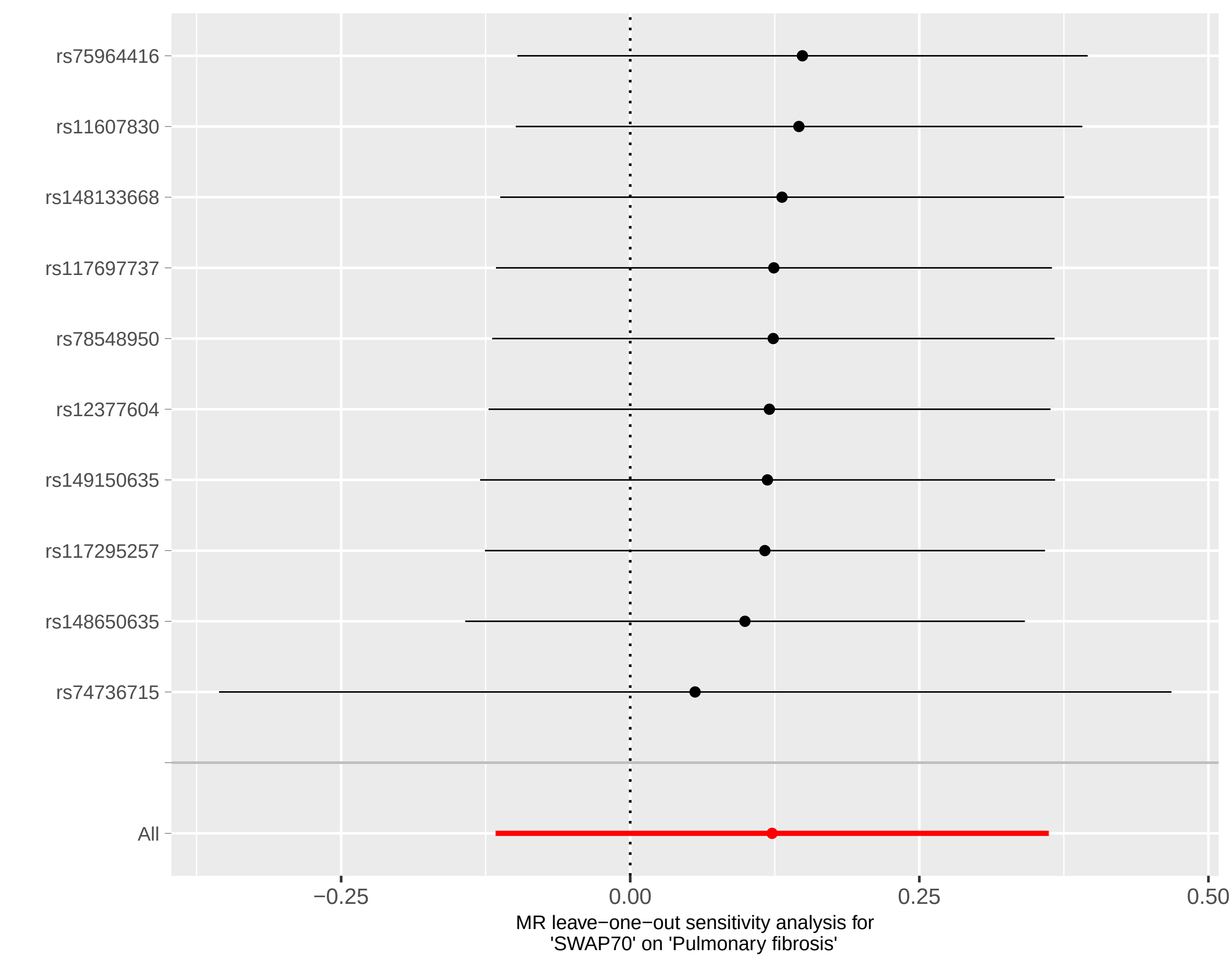
rs75964416
rs11607830
rs148133668
rs117697737
rs78548950
rs12377604
rs149150635
rs117295257
rs148650635
rs74736715
All
−0.25
0.00
0.25
0.50
MR leave−one−out sensitivity analysis for
'SWAP70' on 'Pulmonary fibrosis'

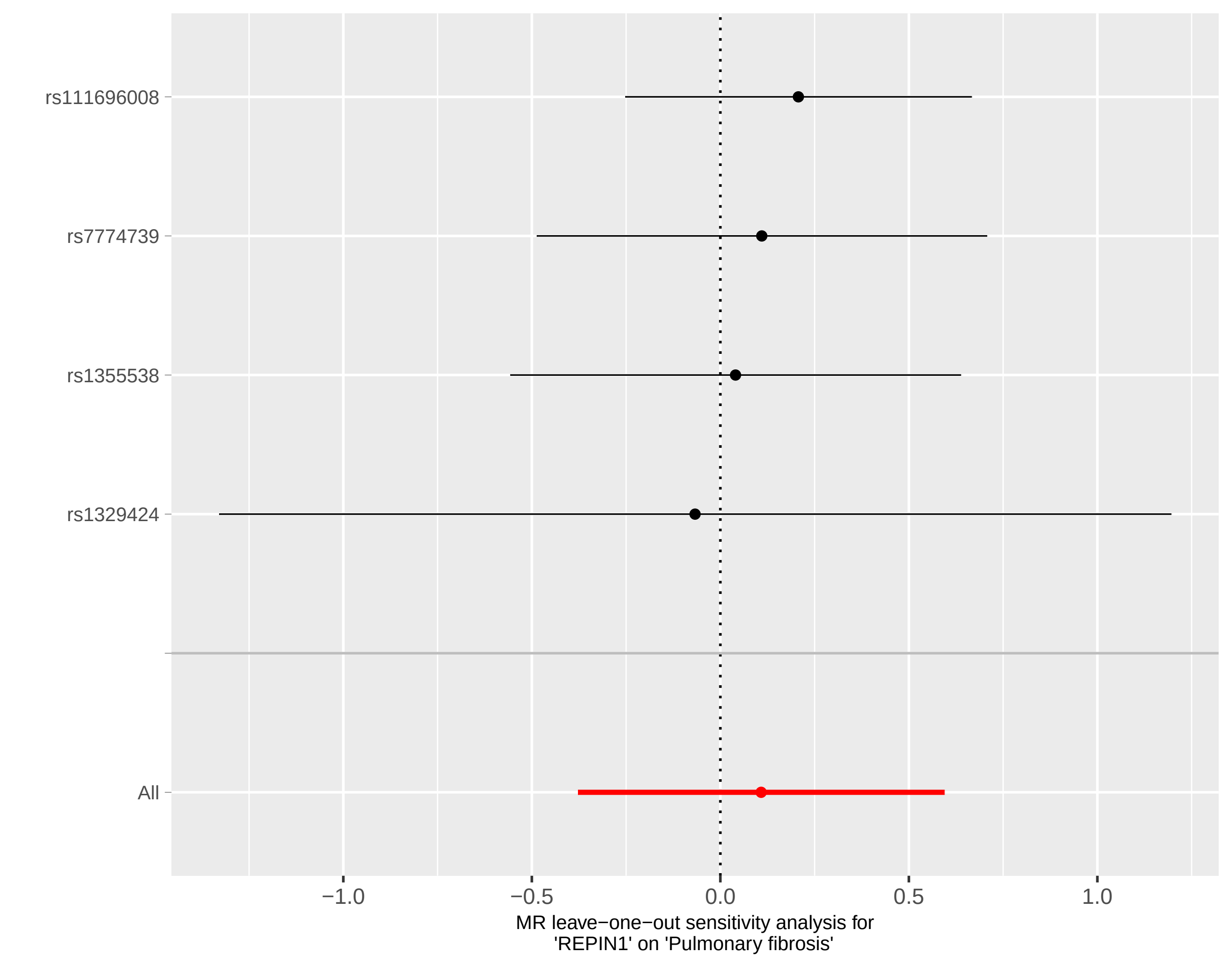
rs111696008
rs7774739
rs1355538
rs1329424
All
−1.0
−0.5
0.0
0.5
1.0
MR leave−one−out sensitivity analysis for
'REPIN1' on 'Pulmonary fibrosis'

Insufficient number of SNPs

Insufficient number of SNPs

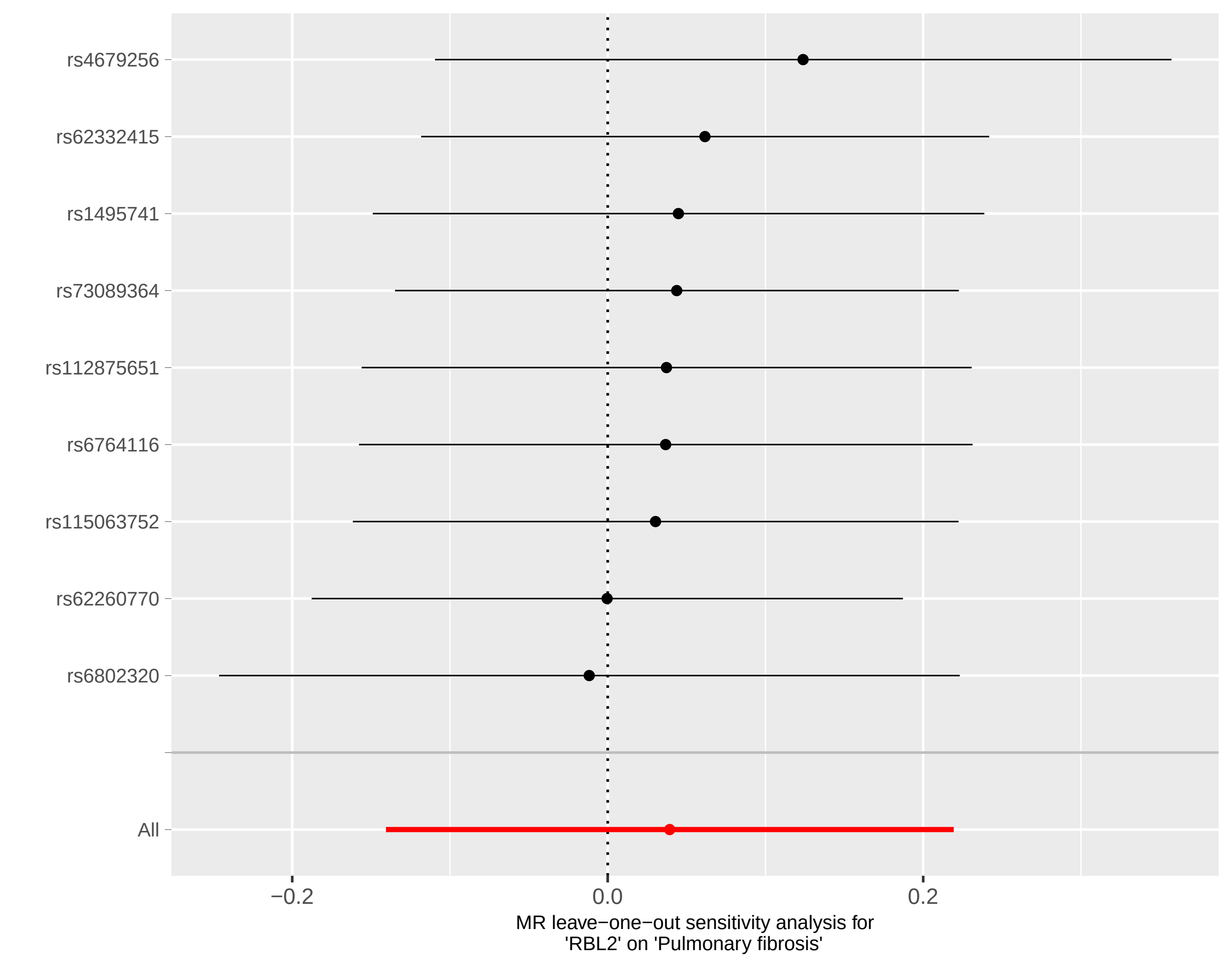

rs4679256
rs62332415
rs1495741
rs73089364
rs112875651
rs6764116
rs115063752
rs62260770
rs6802320
All
−0.2
0.0
0.2
MR leave−one−out sensitivity analysis for
'RBL2' on 'Pulmonary fibrosis'

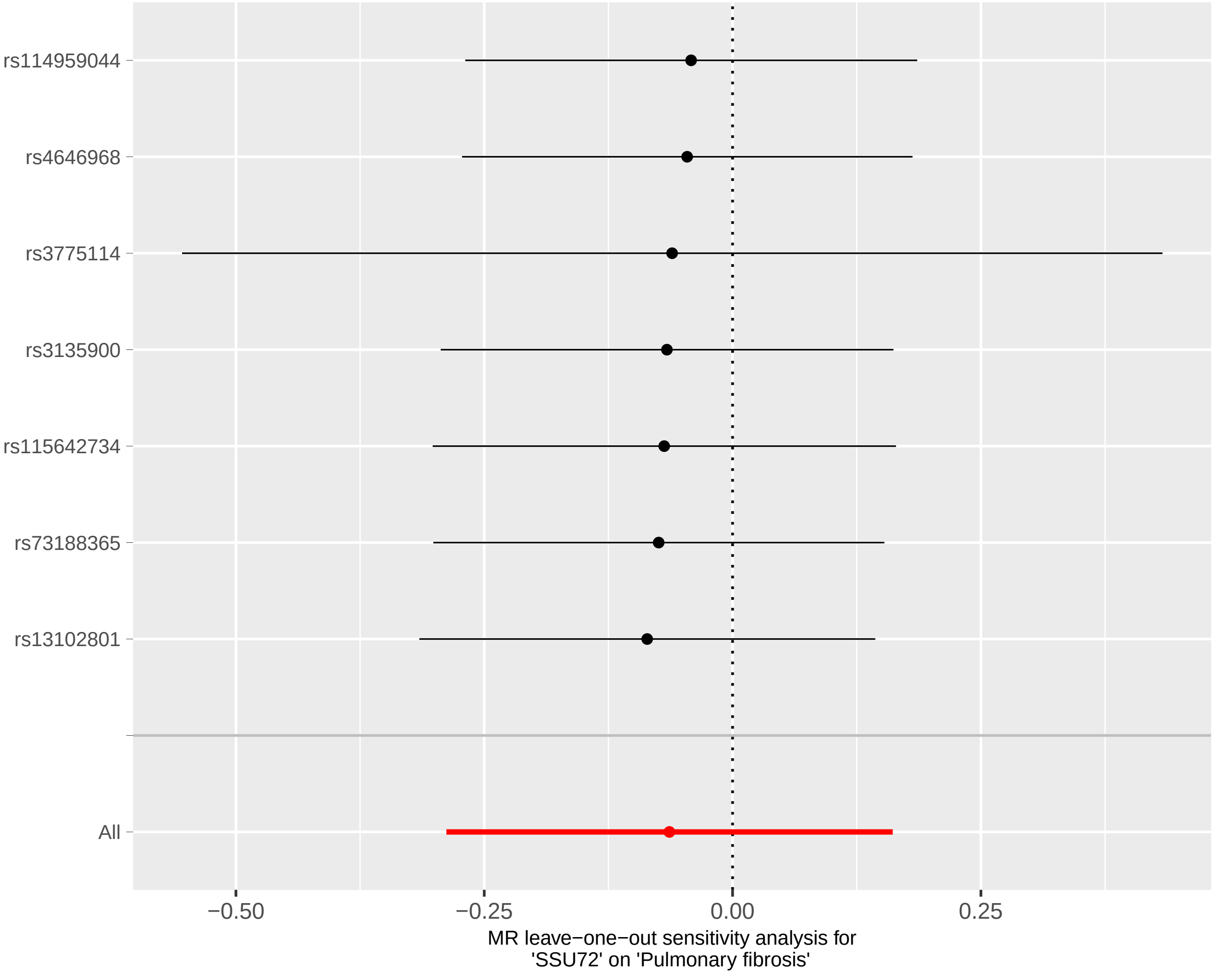

rs114959044
rs4646968
rs3775114
rs3135900
rs115642734
rs73188365
rs13102801
All
−0.50
−0.25
0.00
0.25
MR leave−one−out sensitivity analysis for
'SSU72' on 'Pulmonary fibrosis'

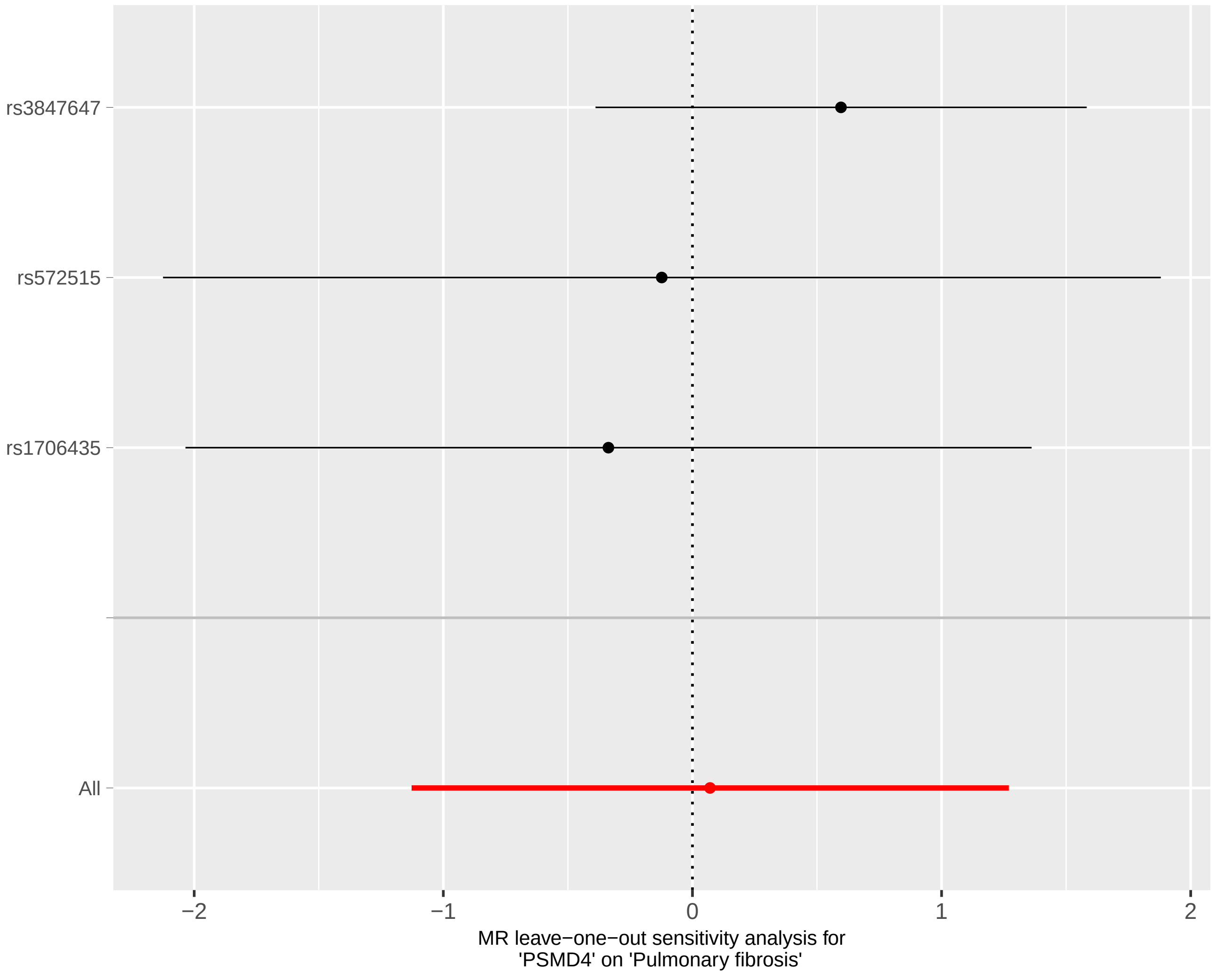

rs3847647
rs572515
rs1706435
All
−2
−1
0
1
2
MR leave−one−out sensitivity analysis for
'PSMD4' on 'Pulmonary fibrosis'

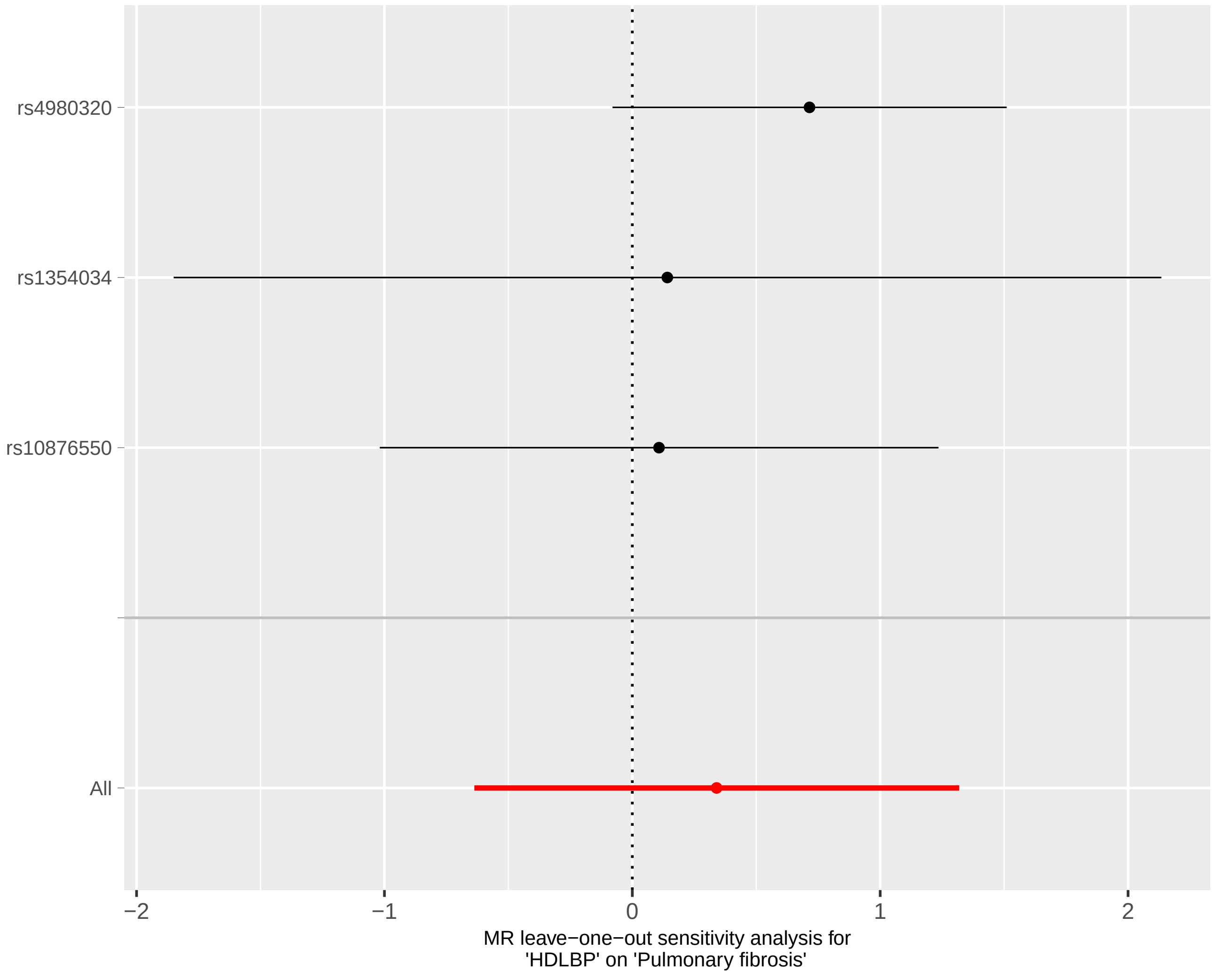

rs4980320
rs1354034
rs10876550
All
−2
−1
0
1
2
MR leave−one−out sensitivity analysis for
'HDLBP' on 'Pulmonary fibrosis'

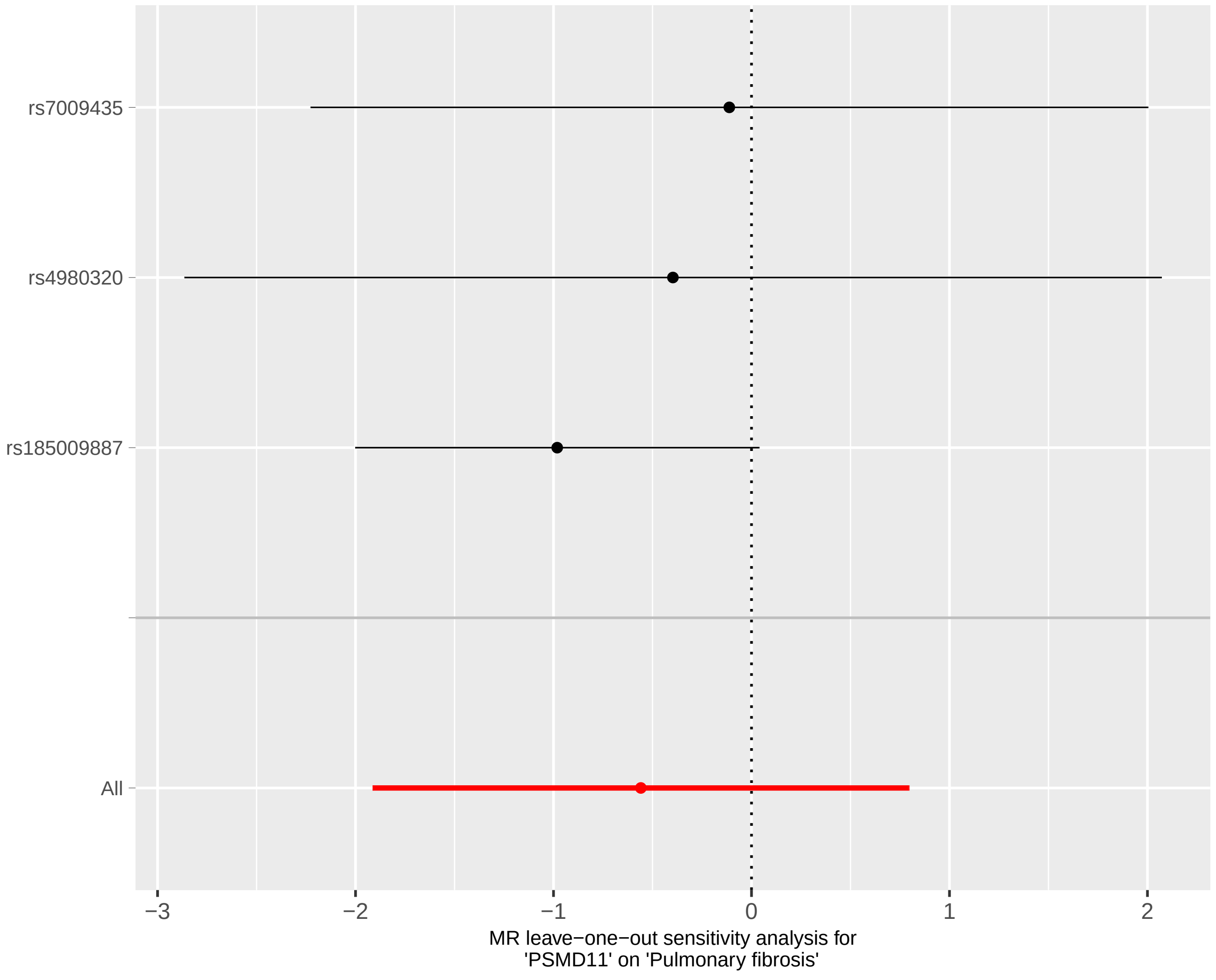
rs7009435
rs4980320
rs185009887
All
−3
−2
−1
0
1
2
MR leave−one−out sensitivity analysis for
'PSMD11' on 'Pulmonary fibrosis'

Insufficient number of SNPs

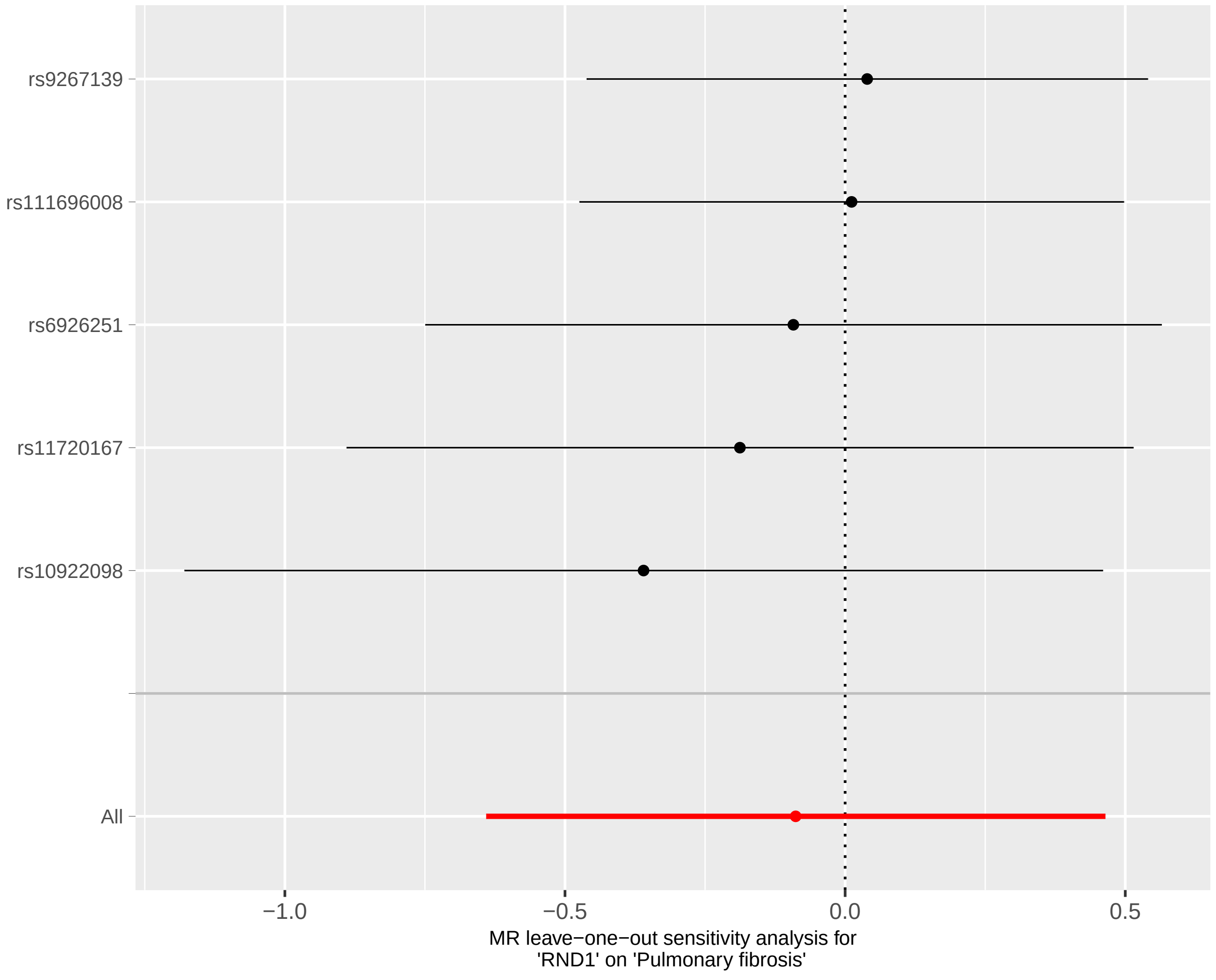

rs9267139
rs111696008
rs6926251
rs11720167
rs10922098
All
−1.0
−0.5
0.0
0.5
MR leave−one−out sensitivity analysis for
'RND1' on 'Pulmonary fibrosis'

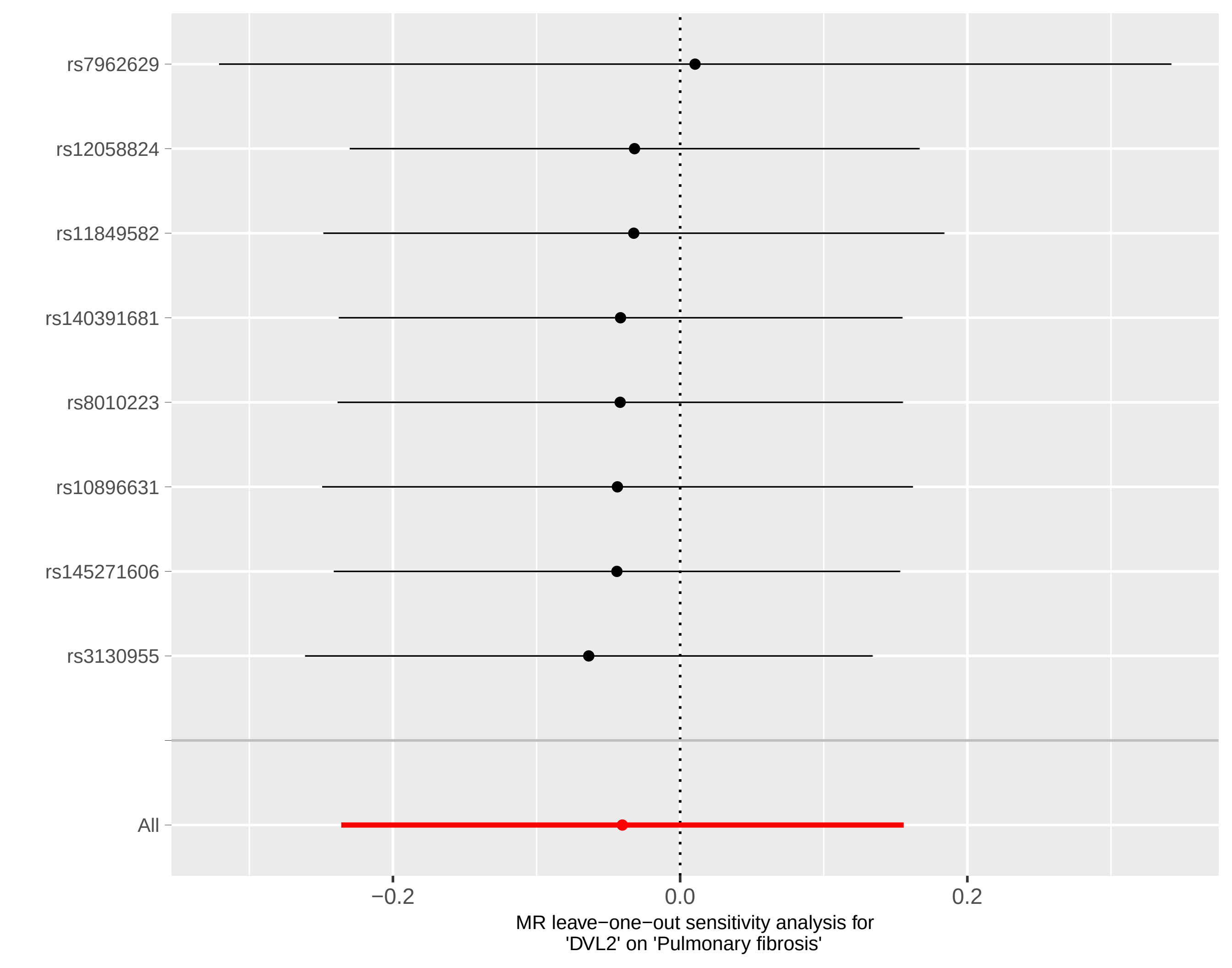
rs7962629
rs12058824
rs11849582
rs140391681
rs8010223
rs10896631
rs145271606
rs3130955
All
−0.2
0.0
0.2
MR leave−one−out sensitivity analysis for
'DVL2' on 'Pulmonary fibrosis'

Insufficient number of SNPs

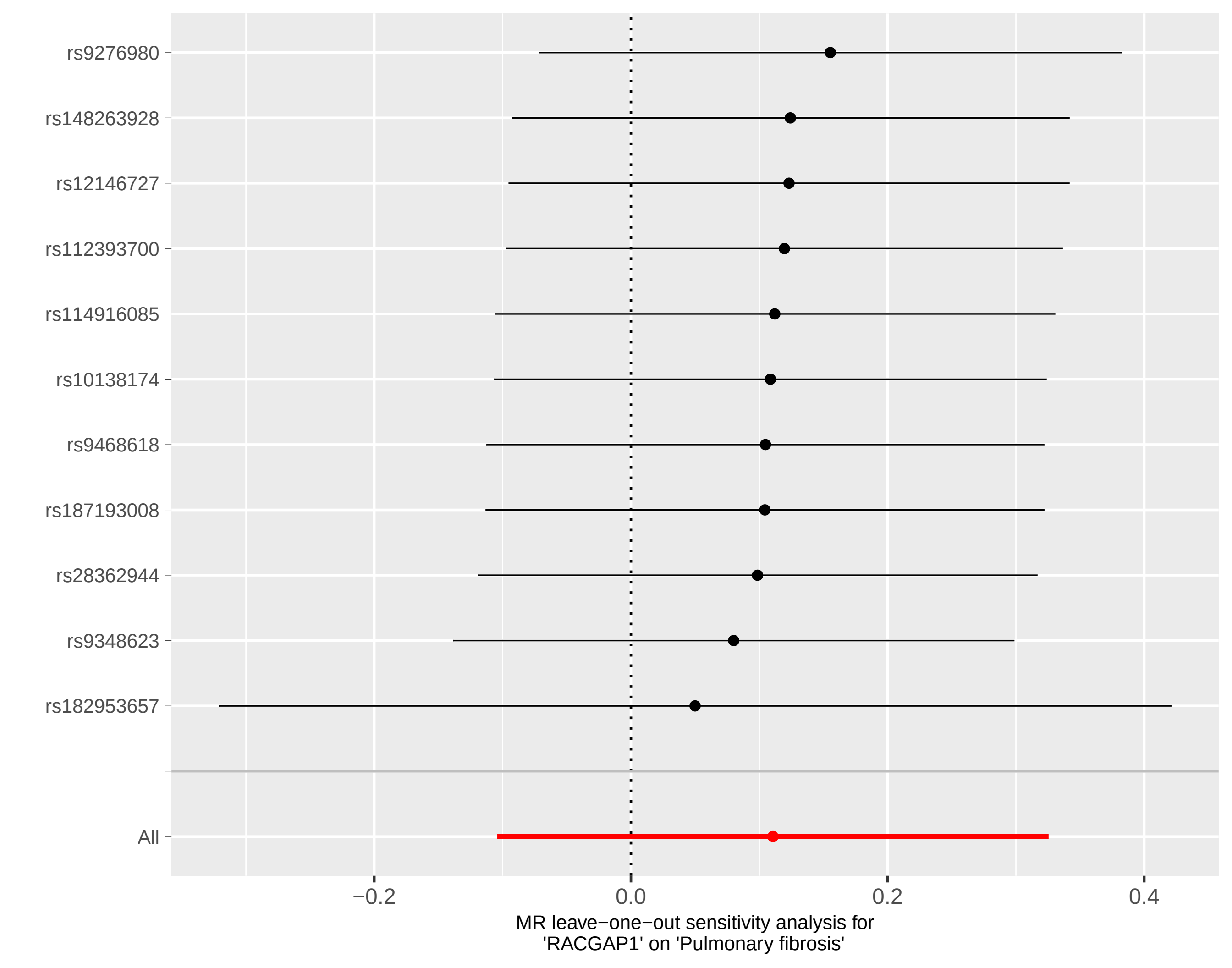
rs9276980
rs148263928
rs12146727
rs112393700
rs114916085
rs10138174
rs9468618
rs187193008
rs28362944
rs9348623
rs182953657
All
−0.2
0.0
0.2
0.4
MR leave−one−out sensitivity analysis for
'RACGAP1' on 'Pulmonary fibrosis'

Insufficient number of SNPs

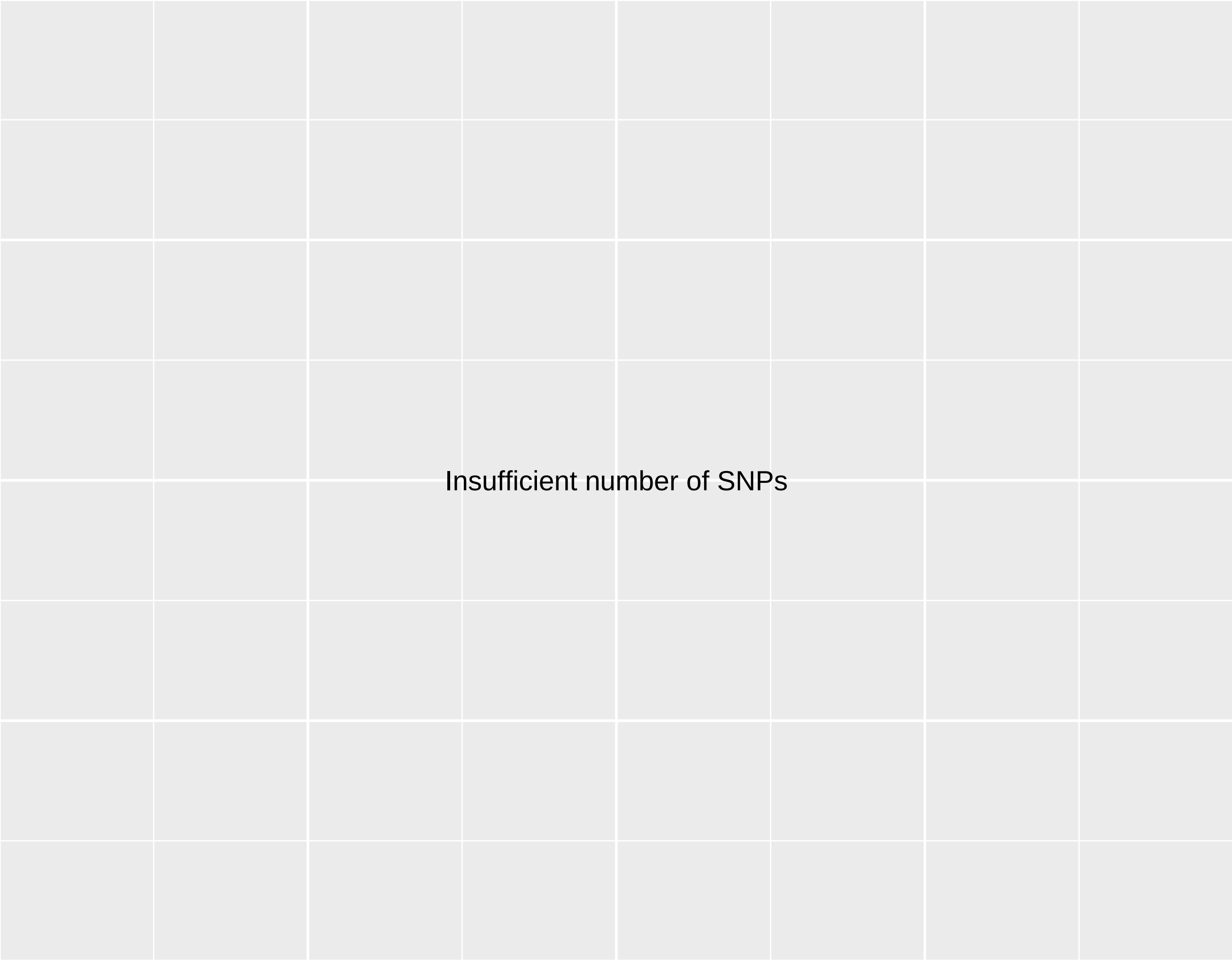
Insufficient number of SNPs

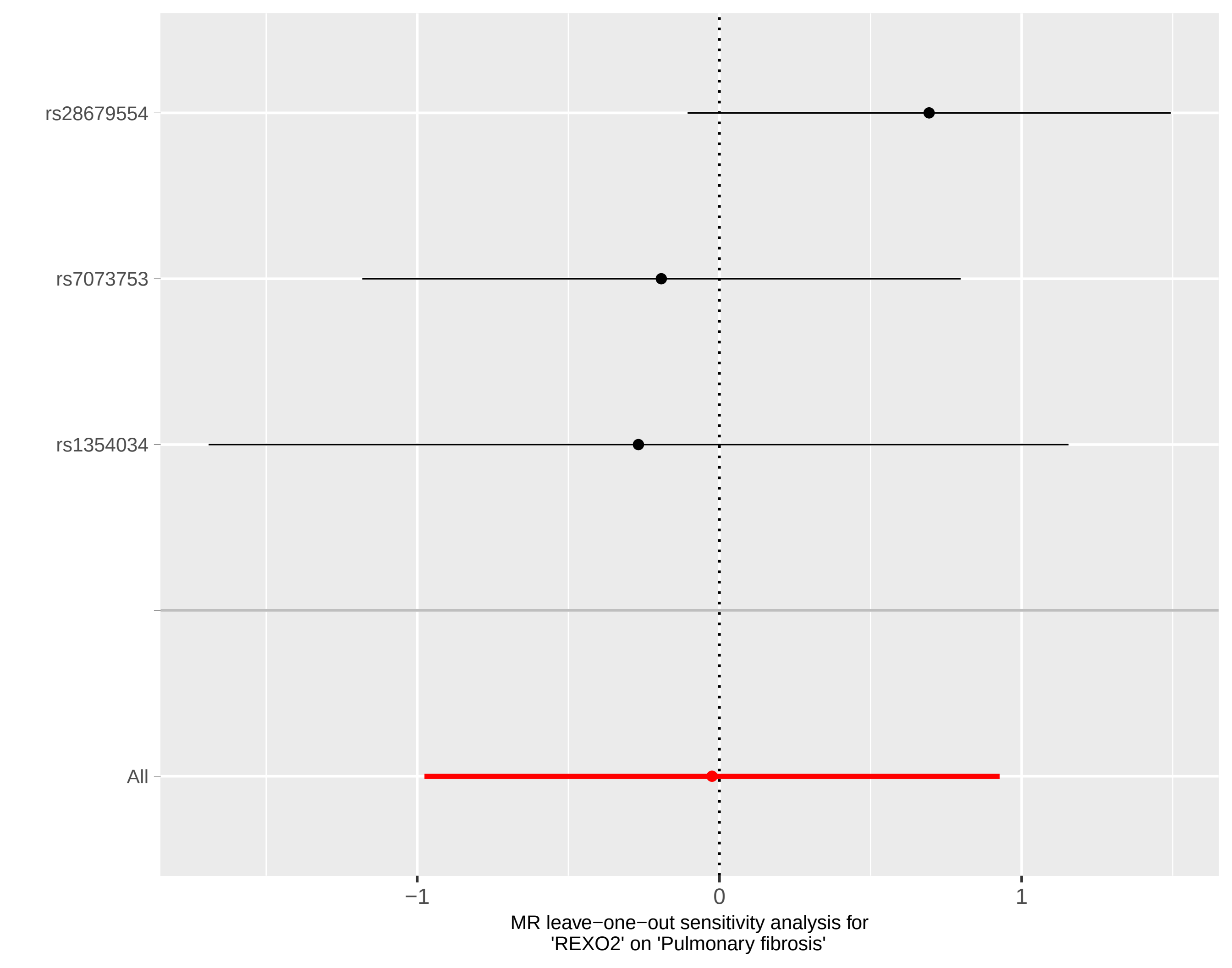
rs28679554
rs7073753
rs1354034
All
−1
0
1
MR leave−one−out sensitivity analysis for
'REXO2' on 'Pulmonary fibrosis'

Insufficient number of SNPs

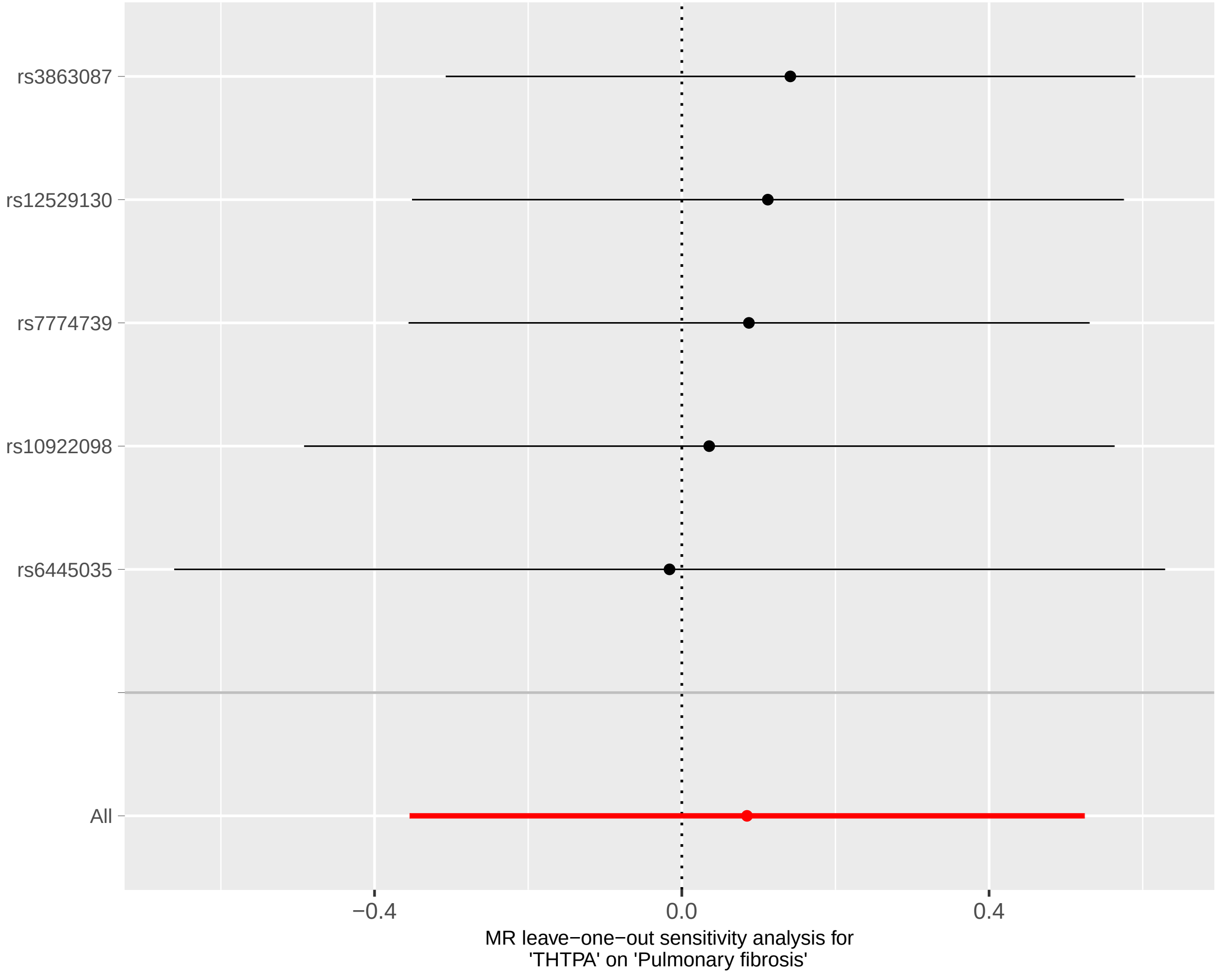

rs3863087
rs12529130
rs7774739
rs10922098
rs6445035
All
−0.4
0.0
0.4
MR leave−one−out sensitivity analysis for
'THTPA' on 'Pulmonary fibrosis'

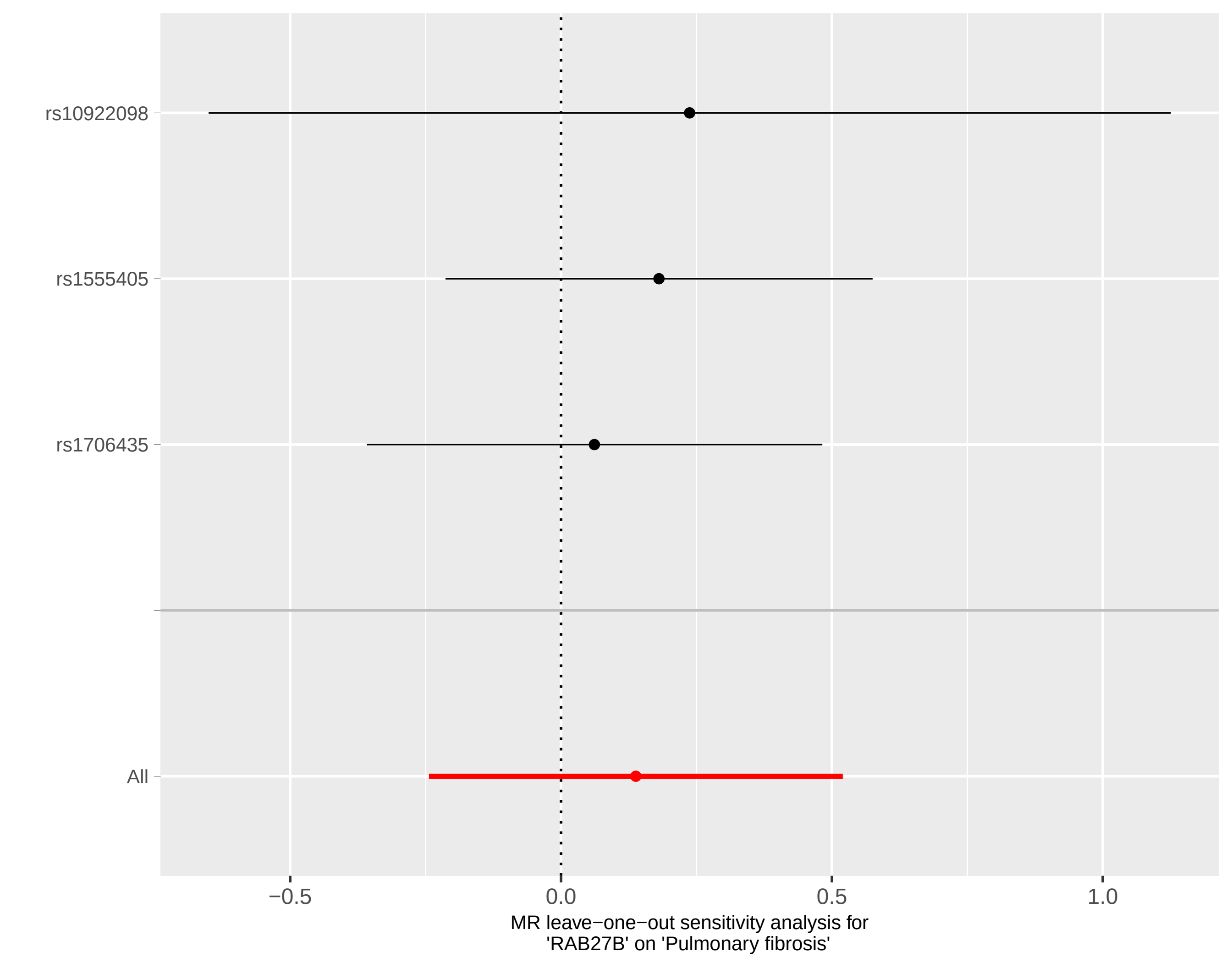
rs10922098
rs1555405
rs1706435
All
−0.5
0.0
0.5
1.0
MR leave−one−out sensitivity analysis for
'RAB27B' on 'Pulmonary fibrosis'

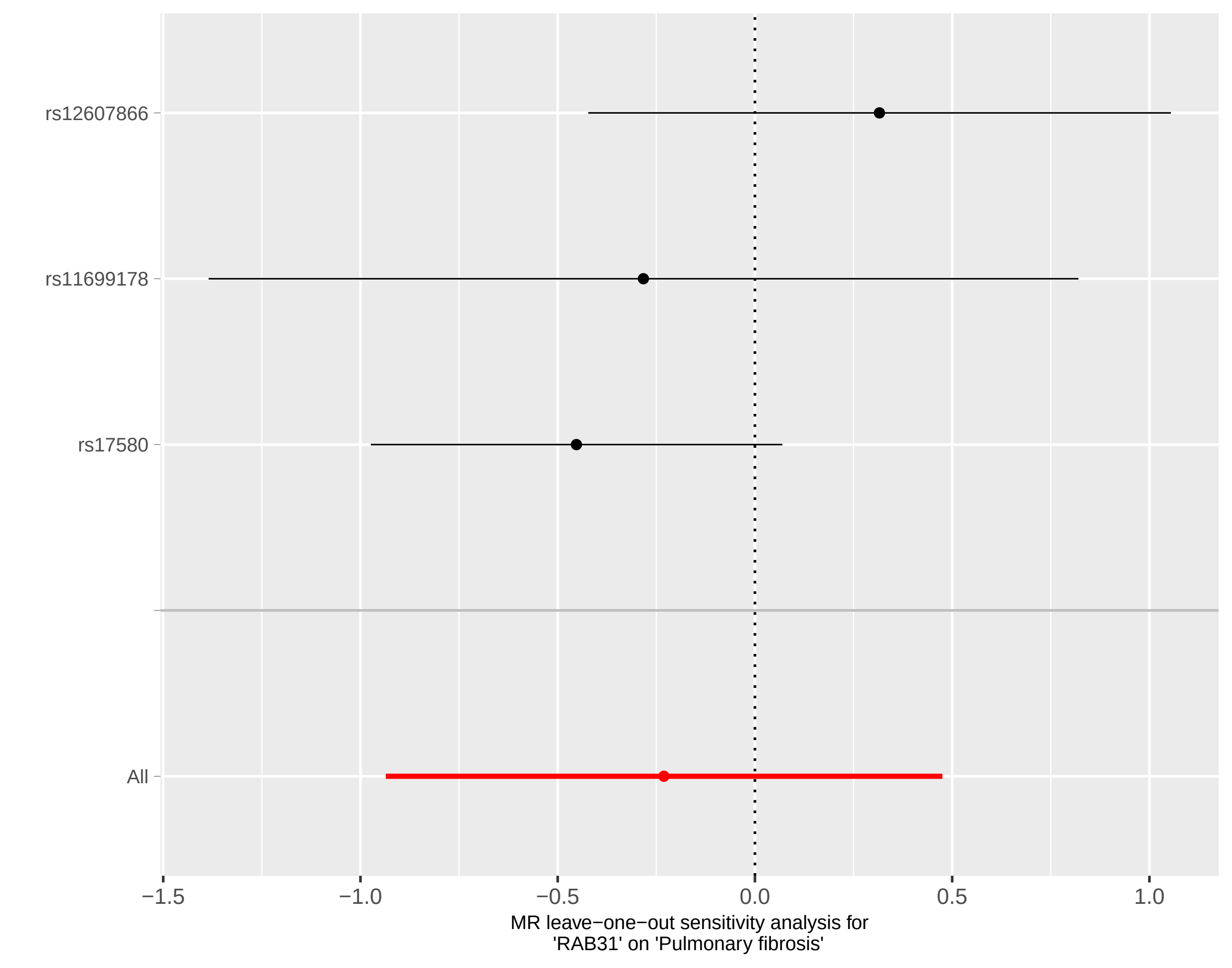
rs12607866
rs11699178
rs17580
All
−1.5
−1.0
−0.5
0.0
0.5
1.0
MR leave−one−out sensitivity analysis for
'RAB31' on 'Pulmonary fibrosis'

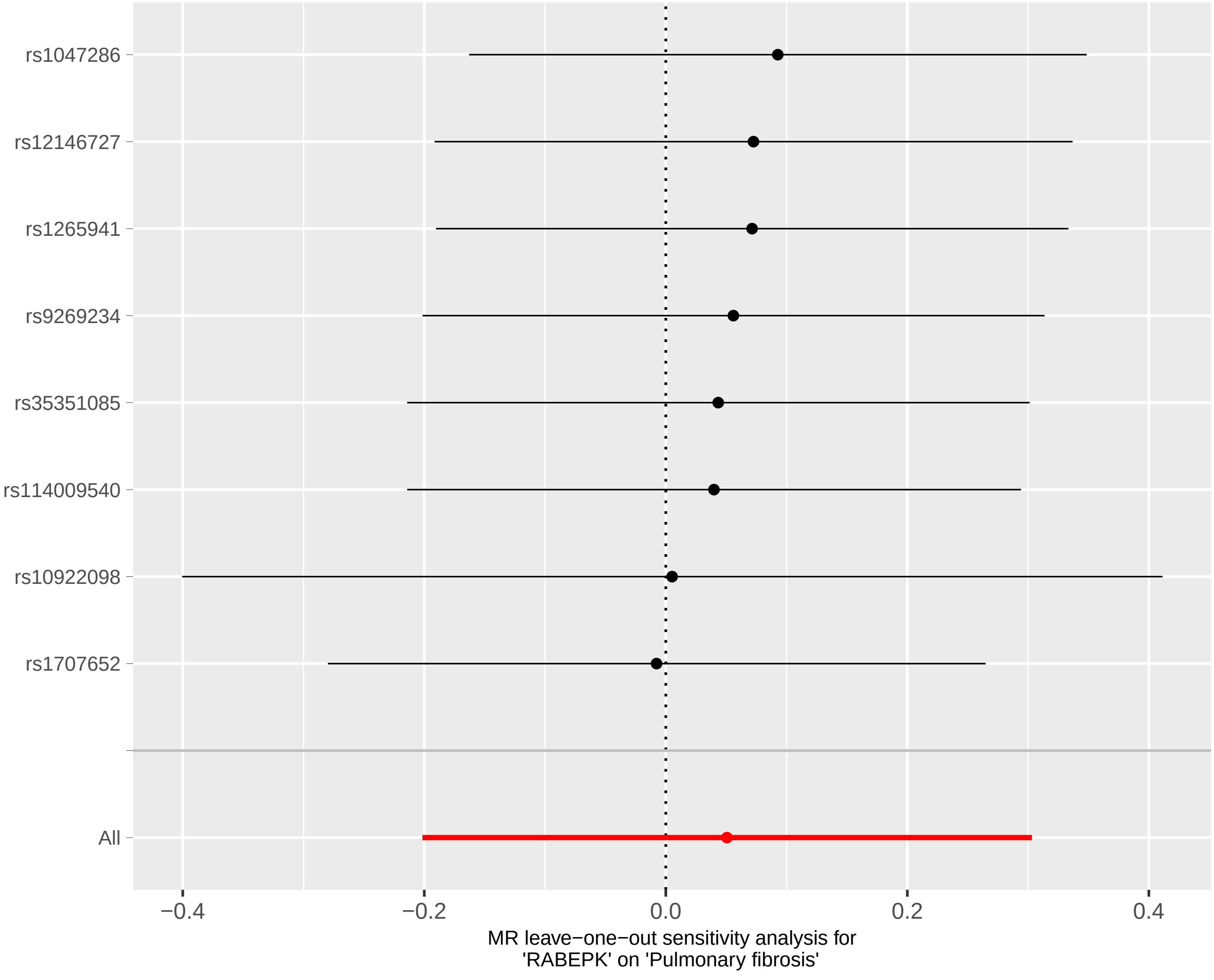
rs1047286
rs12146727
rs1265941
rs9269234
rs35351085
rs114009540
rs10922098
rs1707652
All
−0.4
−0.2
0.0
0.2
0.4
MR leave−one−out sensitivity analysis for
'RABEPK' on 'Pulmonary fibrosis'

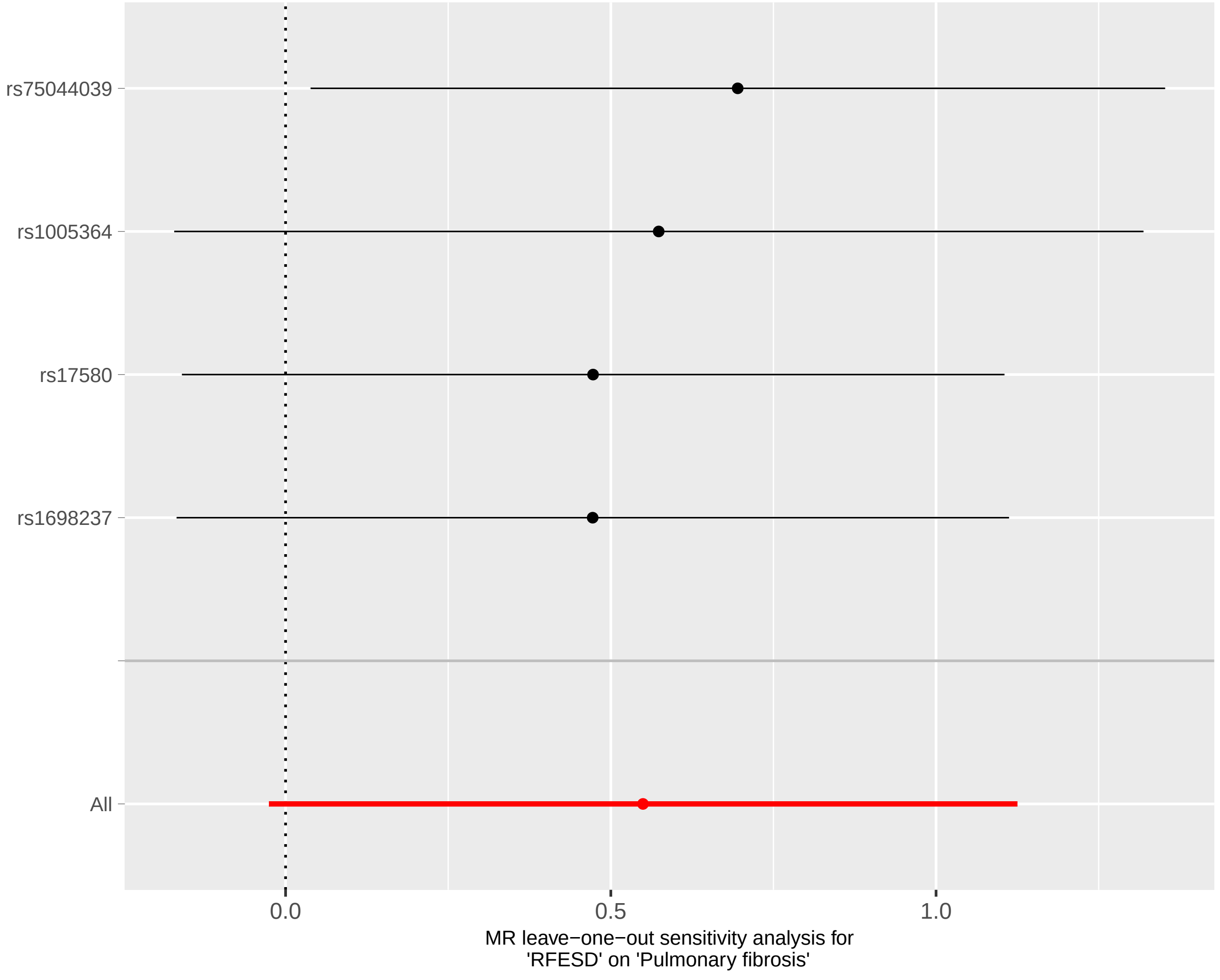
rs75044039
rs1005364
rs17580
rs1698237
All
0.0
0.5
1.0
MR leave−one−out sensitivity analysis for
'RFESD' on 'Pulmonary fibrosis'

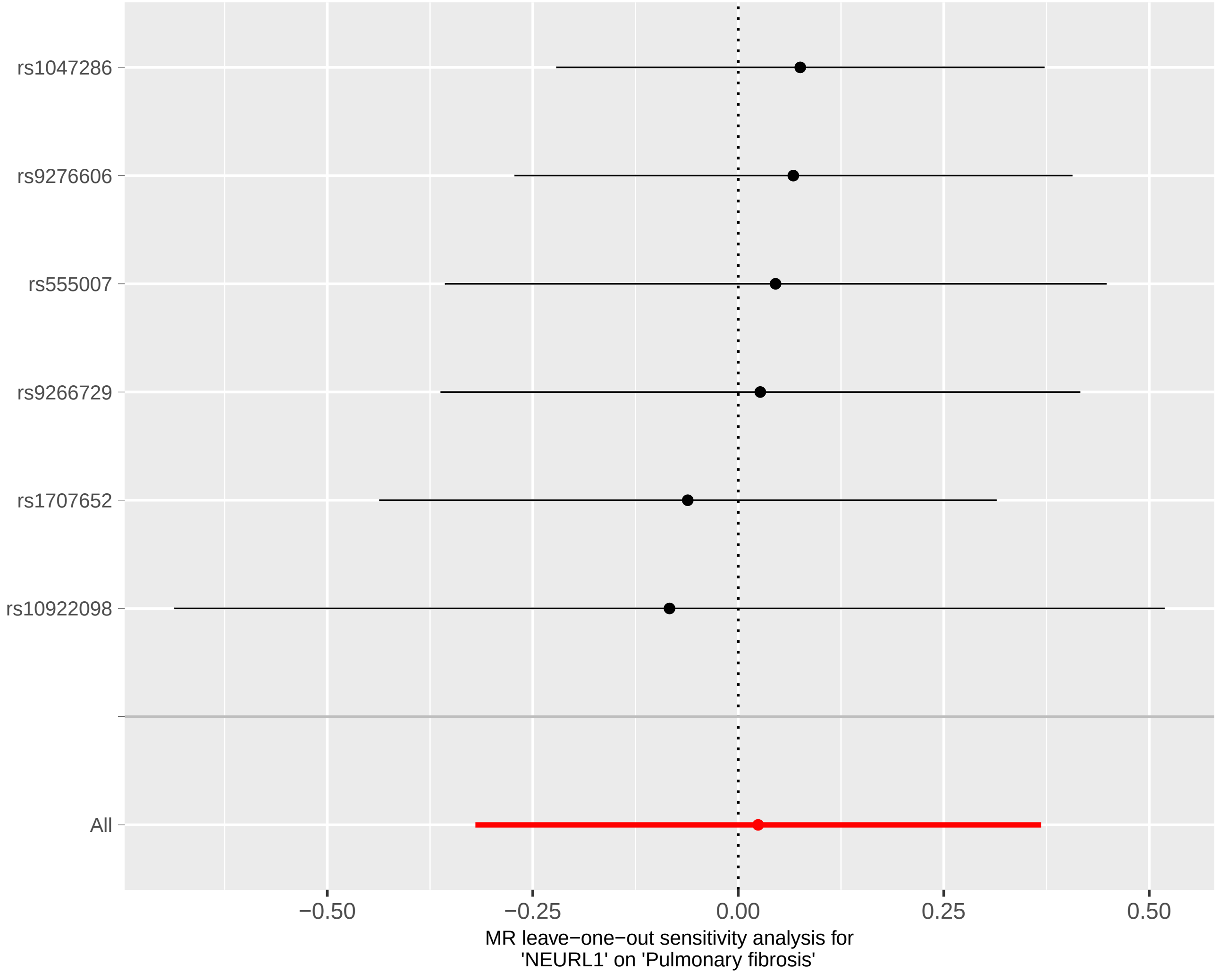
rs1047286
rs9276606
rs555007
rs9266729
rs1707652
rs10922098
All
−0.50
−0.25
0.00
0.25
0.50
MR leave−one−out sensitivity analysis for
'NEURL1' on 'Pulmonary fibrosis'

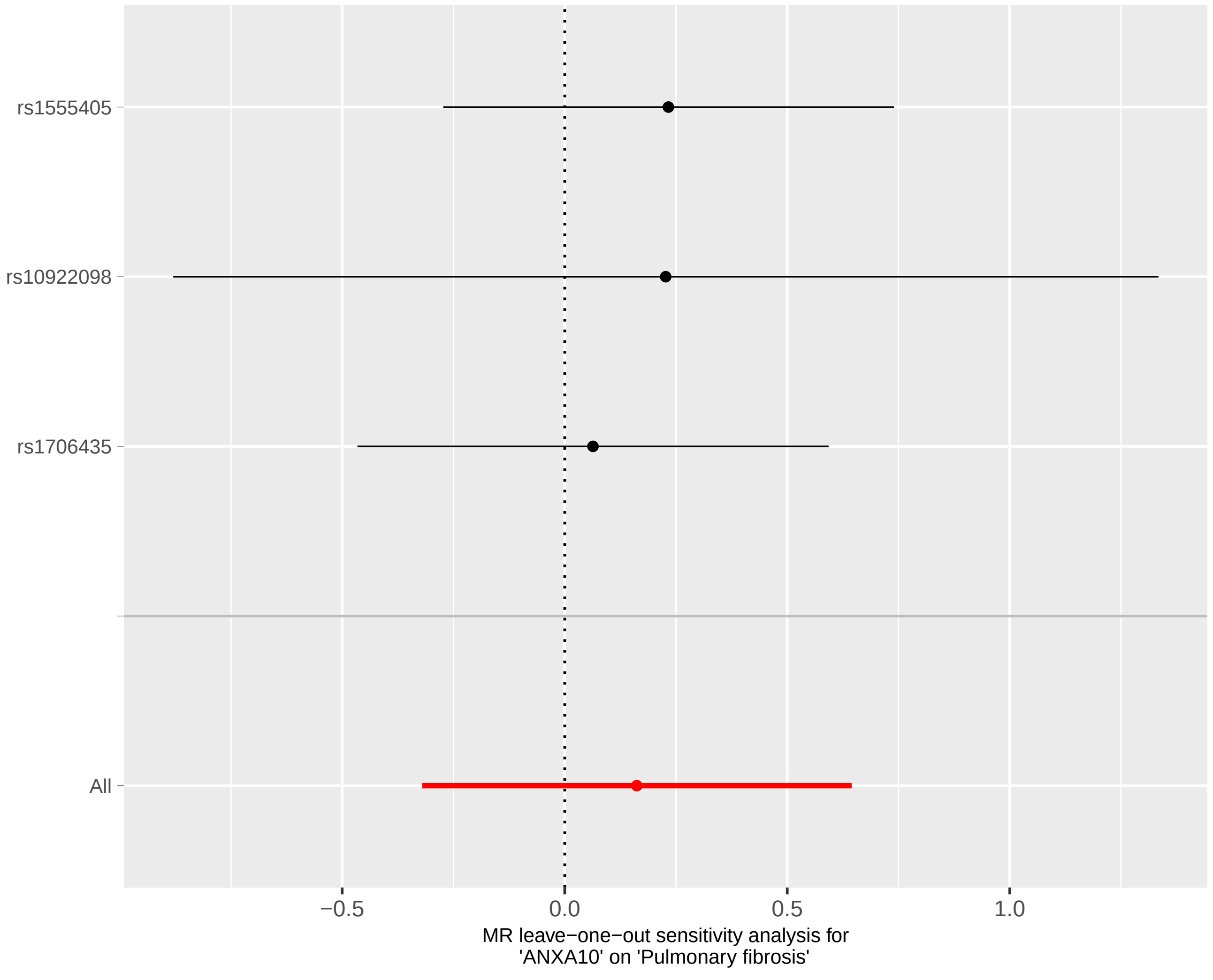
rs1555405
rs10922098
rs1706435
All
−0.5
0.0
0.5
1.0
MR leave−one−out sensitivity analysis for
'ANXA10' on 'Pulmonary fibrosis'

Insufficient number of SNPs

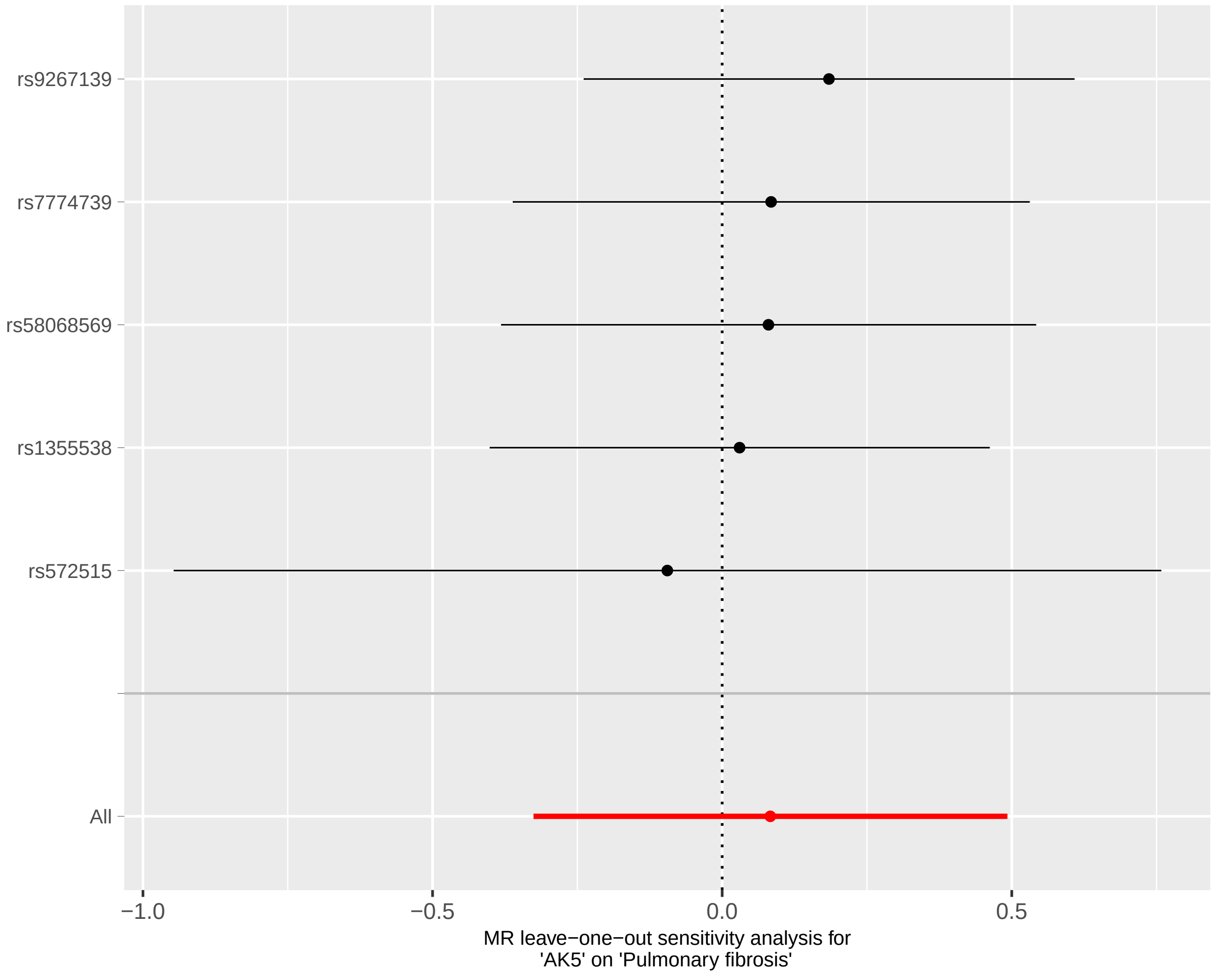

rs9267139
rs7774739
rs58068569
rs1355538
rs572515
All
−1.0
−0.5
0.0
0.5
MR leave−one−out sensitivity analysis for
'AK5' on 'Pulmonary fibrosis'

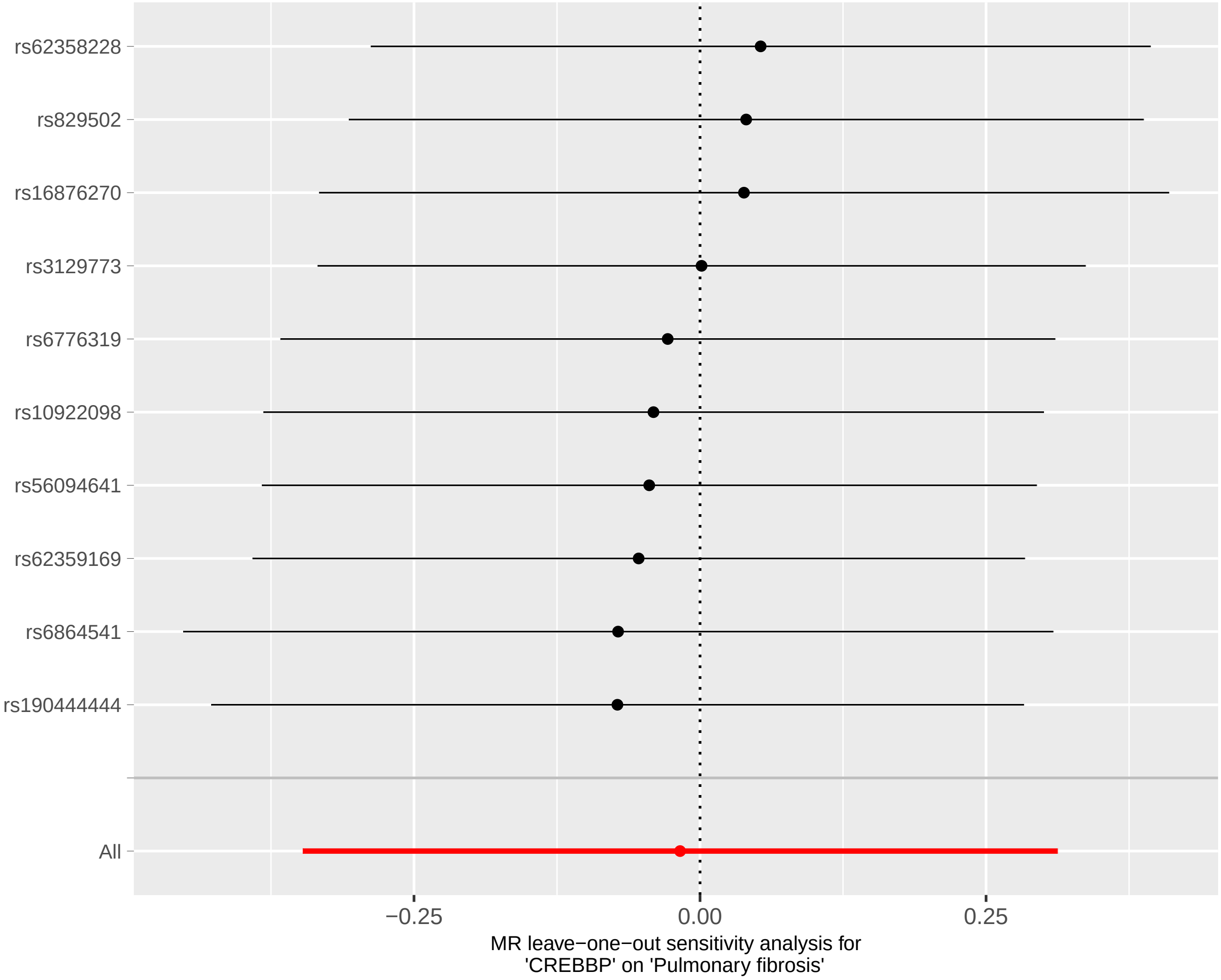
rs62358228
rs829502
rs16876270
rs3129773
rs6776319
rs10922098
rs56094641
rs62359169
rs6864541
rs190444444
All
−0.25
0.00
0.25
MR leave−one−out sensitivity analysis for
'CREBBP' on 'Pulmonary fibrosis'

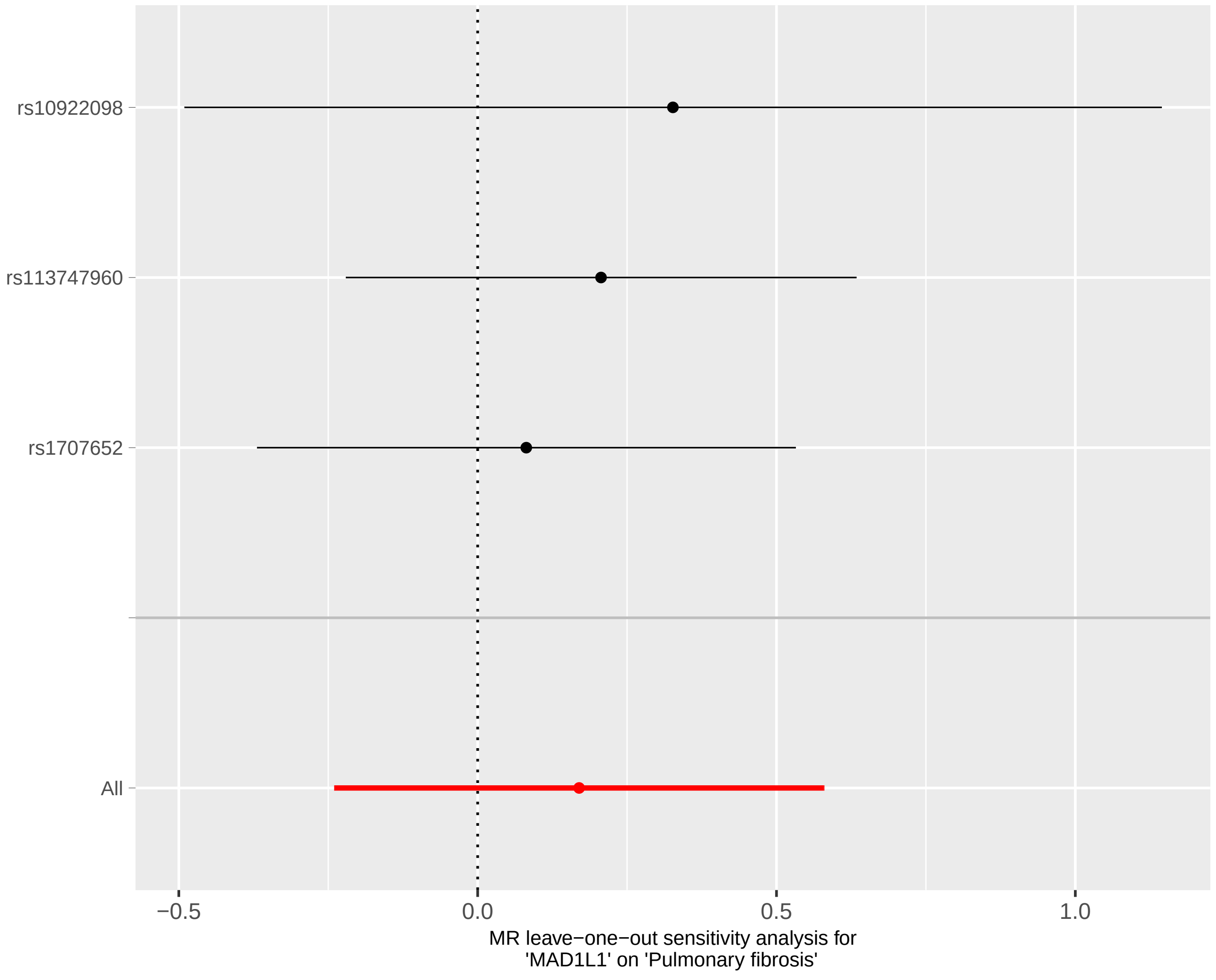

rs10922098
rs113747960
rs1707652
All
−0.5
0.0
0.5
1.0
MR leave−one−out sensitivity analysis for
'MAD1L1' on 'Pulmonary fibrosis'

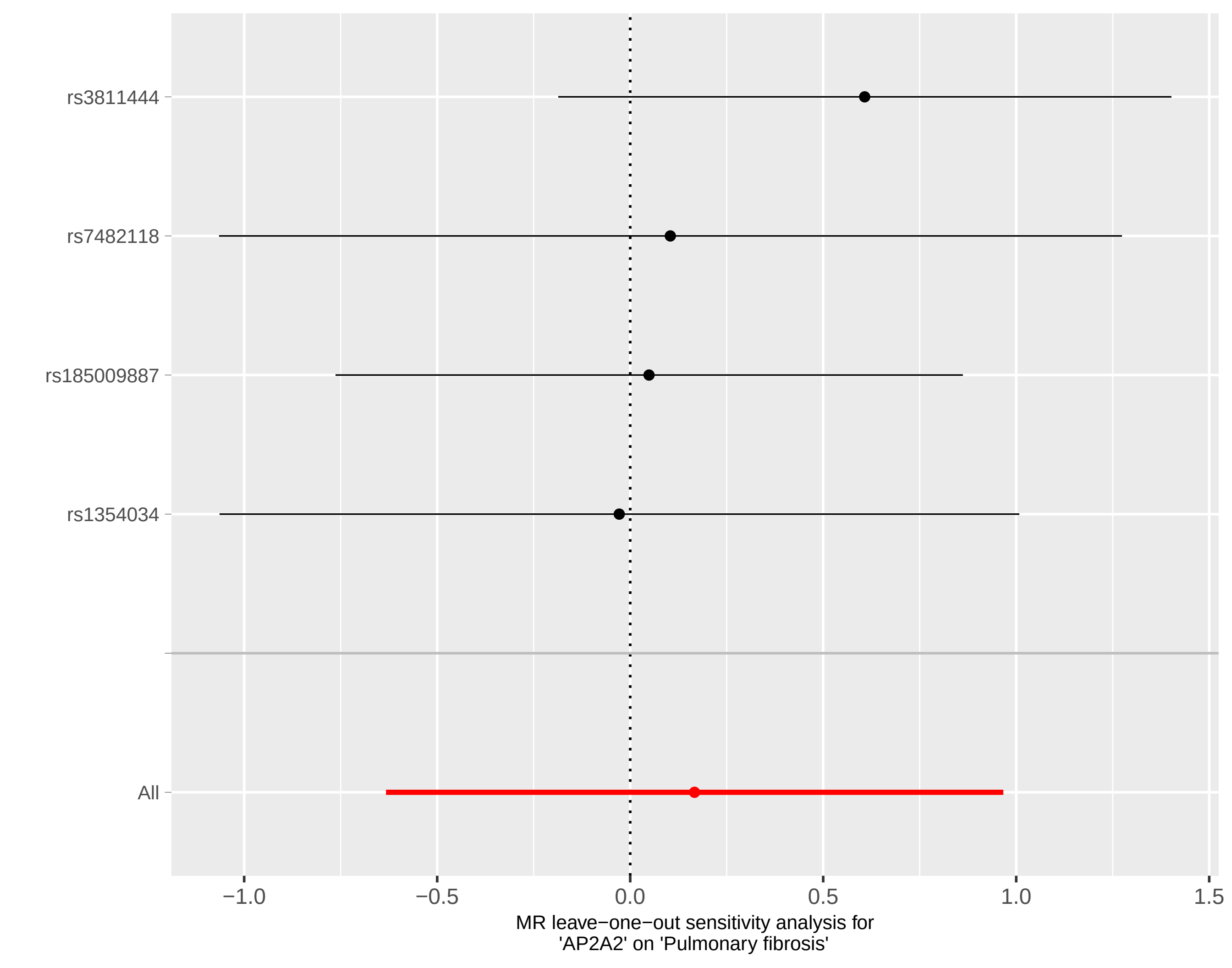

rs3811444
rs7482118
rs185009887
rs1354034
All
−1.0
−0.5
0.0
0.5
1.0
1.5
MR leave−one−out sensitivity analysis for
'AP2A2' on 'Pulmonary fibrosis'

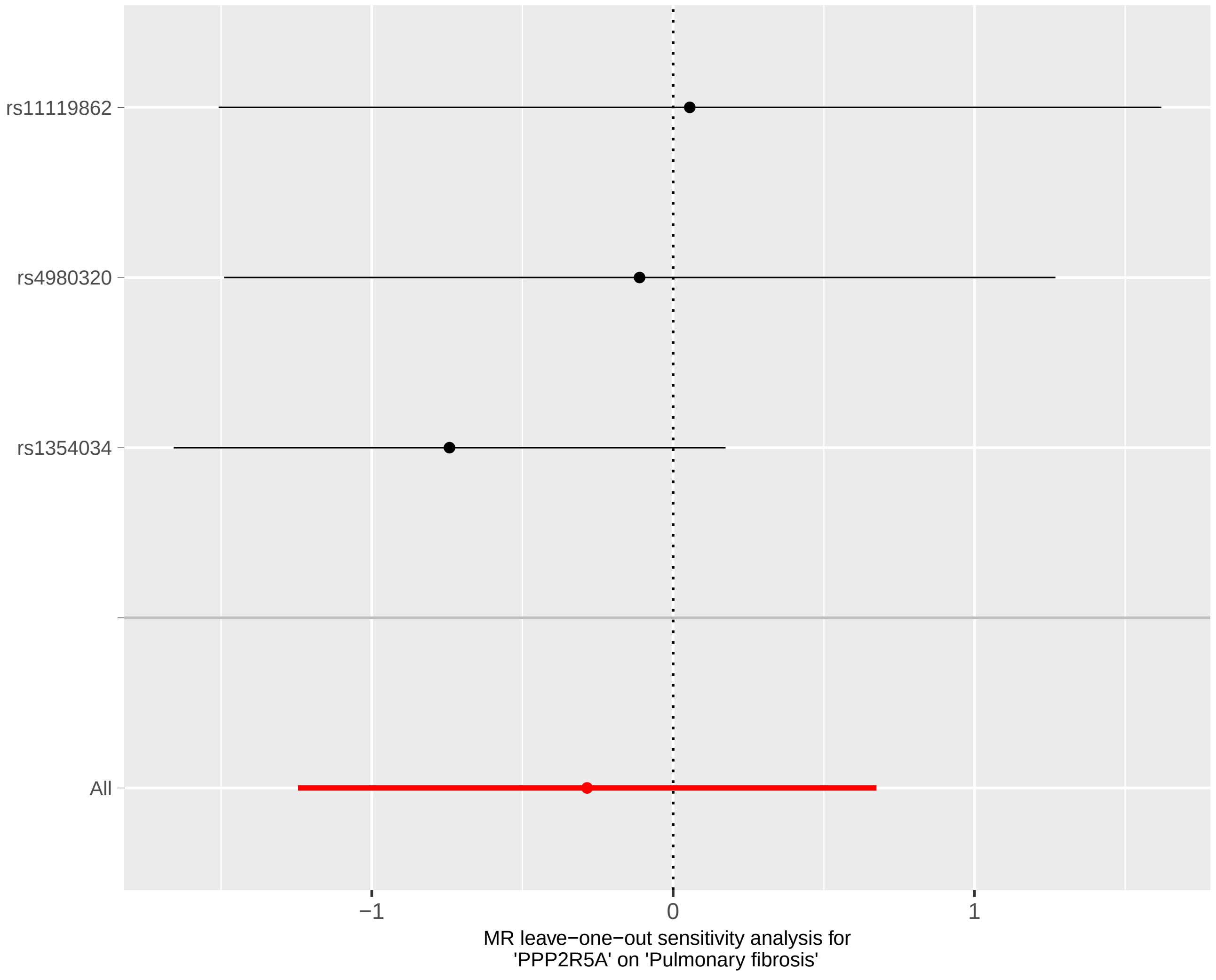

rs11119862
rs4980320
rs1354034
All
−1
0
1
MR leave−one−out sensitivity analysis for
'PPP2R5A' on 'Pulmonary fibrosis'

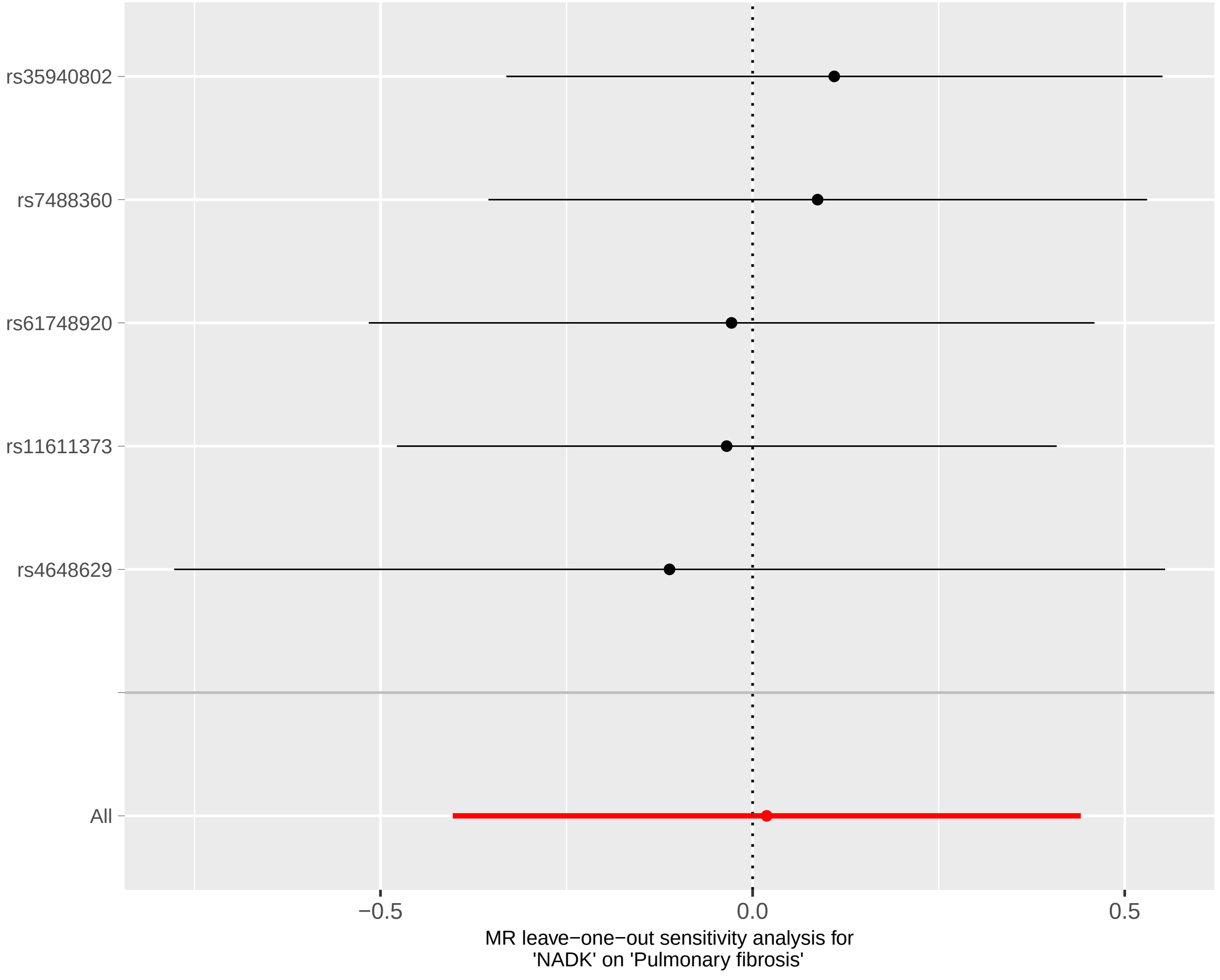
rs35940802
rs7488360
rs61748920
rs11611373
rs4648629
All
−0.5
0.0
0.5
MR leave−one−out sensitivity analysis for
'NADK' on 'Pulmonary fibrosis'

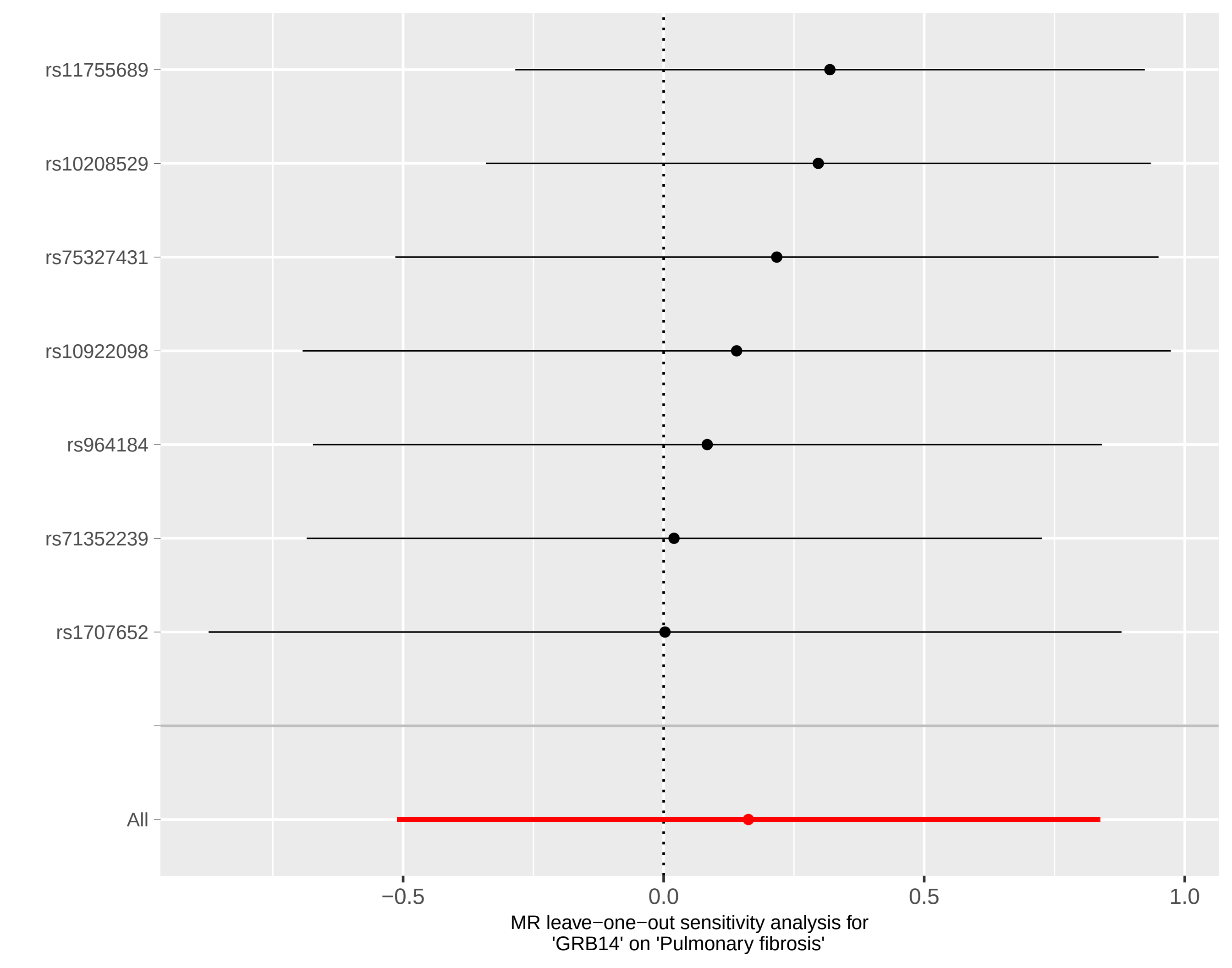
rs11755689
rs10208529
rs75327431
rs10922098
rs964184
rs71352239
rs1707652
All
−0.5
0.0
0.5
1.0
MR leave−one−out sensitivity analysis for
'GRB14' on 'Pulmonary fibrosis'

Insufficient number of SNPs

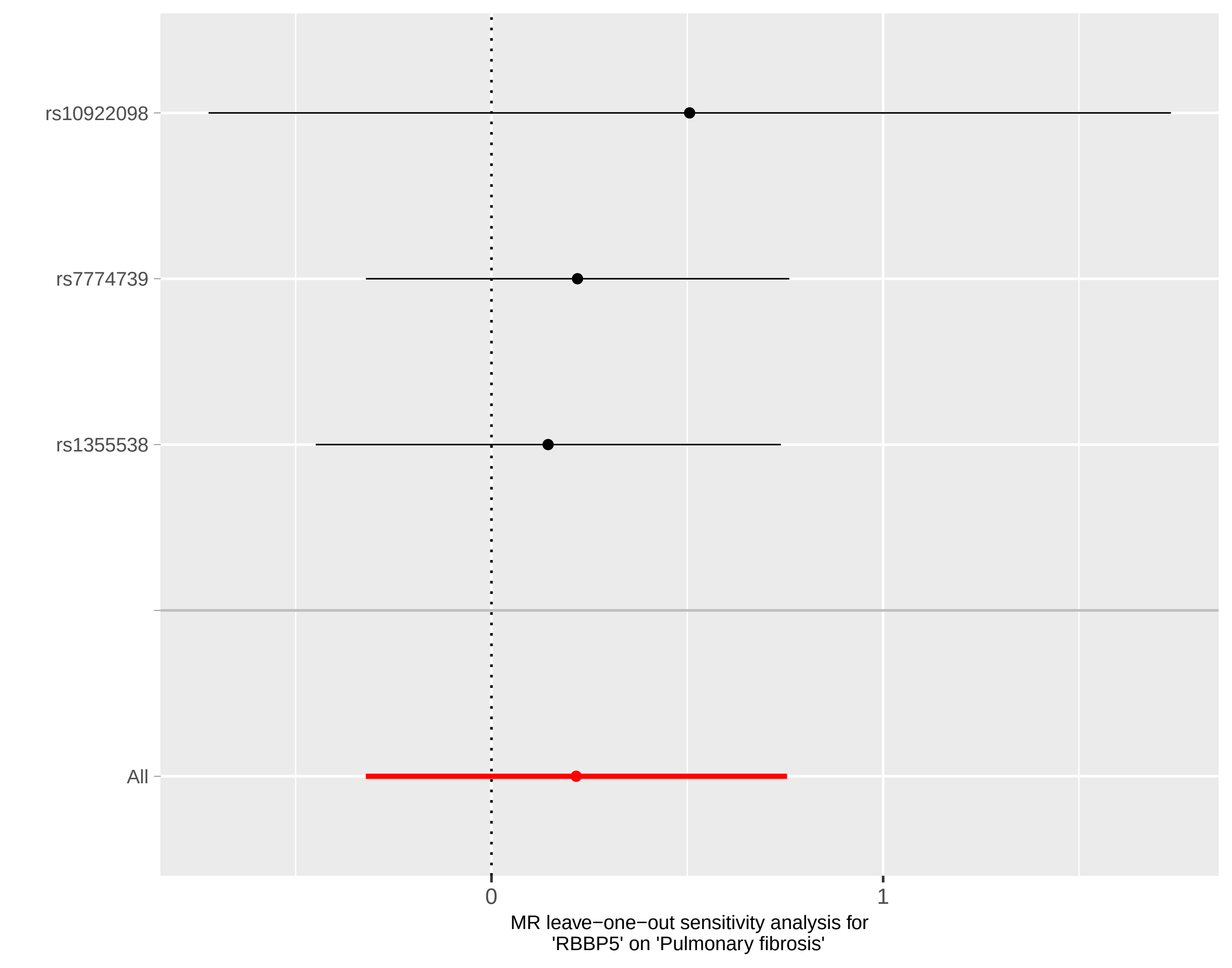

rs10922098
rs7774739
rs1355538
All
0
1
MR leave−one−out sensitivity analysis for
'RBBP5' on 'Pulmonary fibrosis'

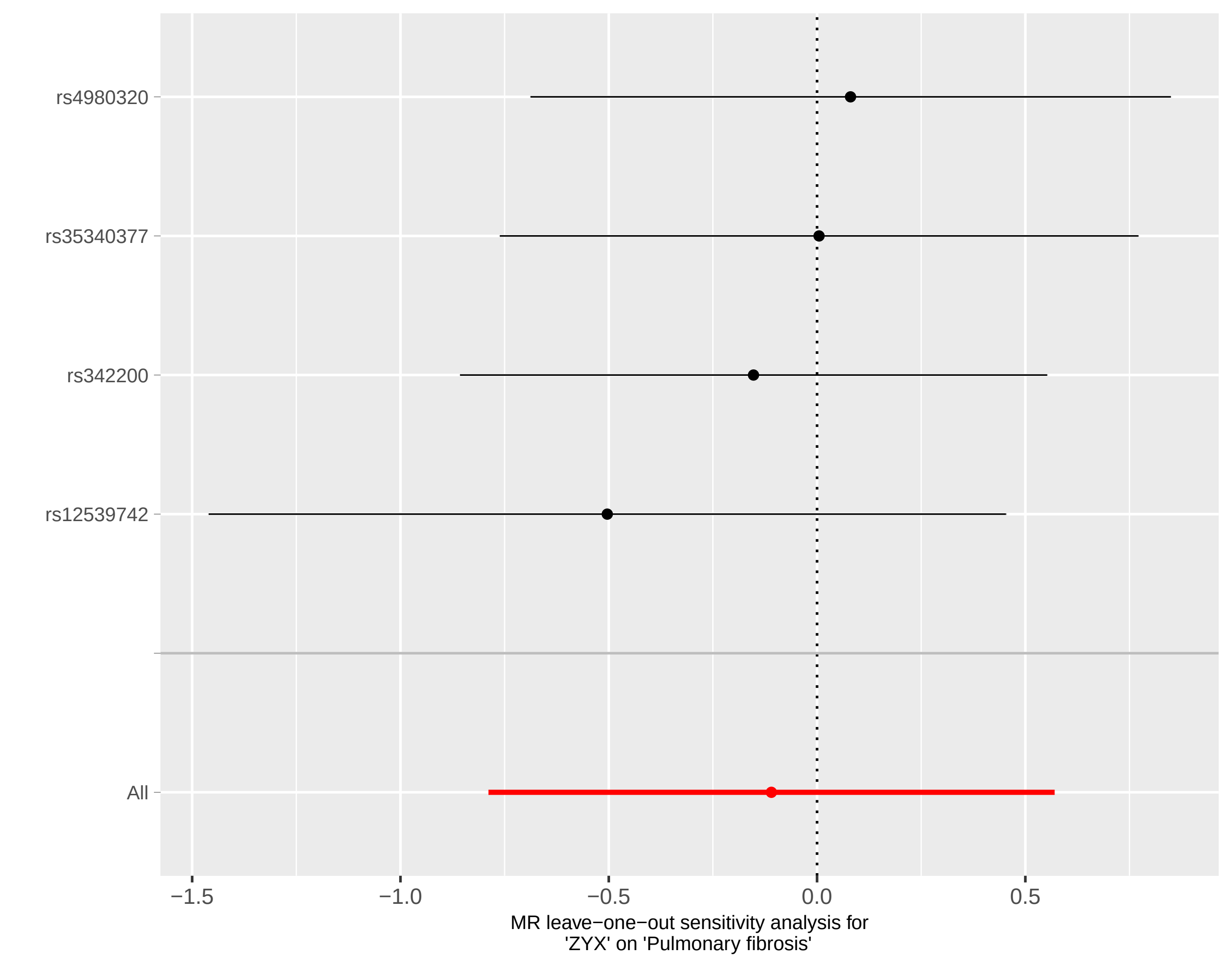
rs4980320
rs35340377
rs342200
rs12539742
All
−1.5
−1.0
−0.5
0.0
0.5
MR leave−one−out sensitivity analysis for
'ZYX' on 'Pulmonary fibrosis'

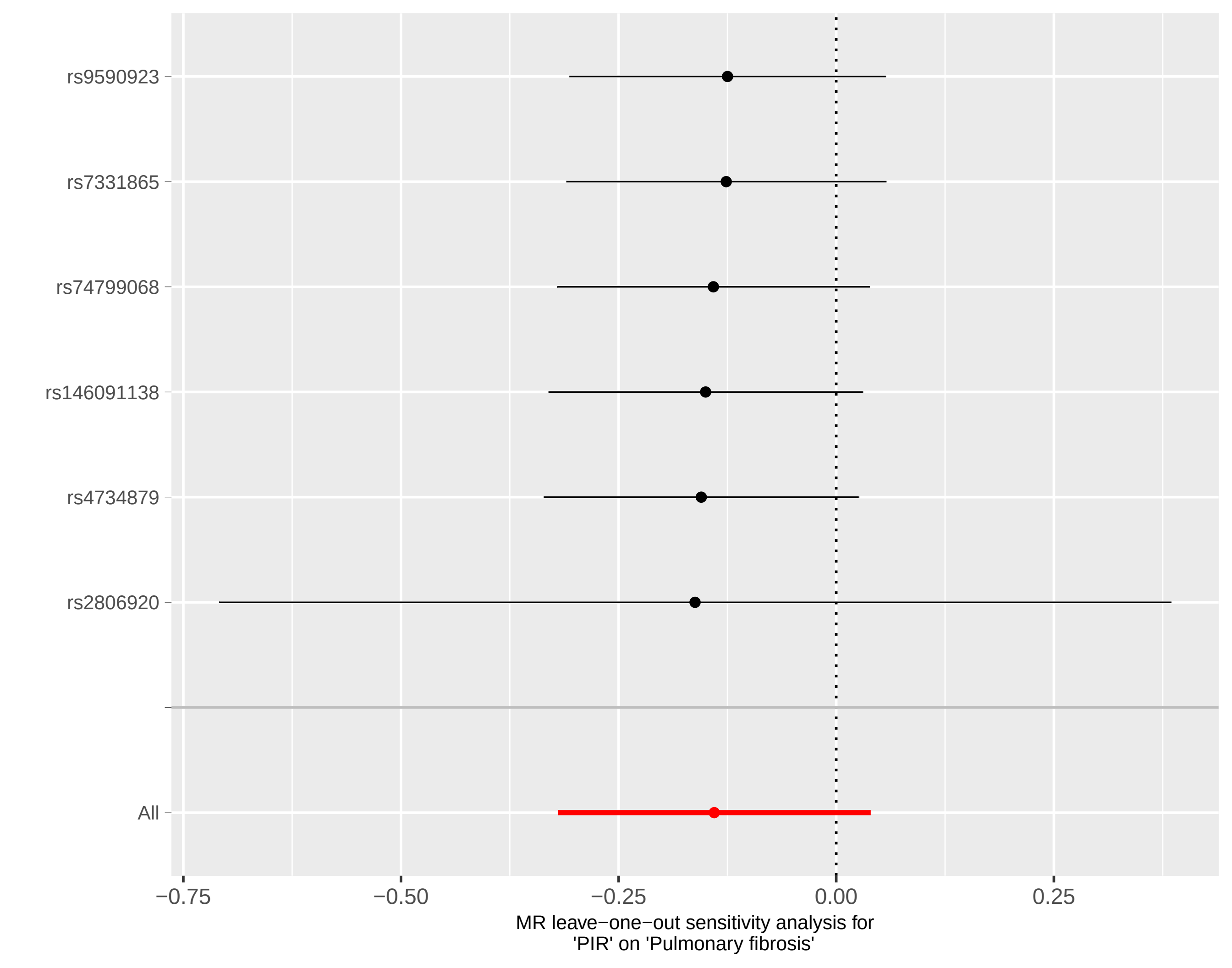

rs9590923
rs7331865
rs74799068
rs146091138
rs4734879
rs2806920
All
−0.75
−0.50
−0.25
0.00
0.25
MR leave−one−out sensitivity analysis for
'PIR' on 'Pulmonary fibrosis'

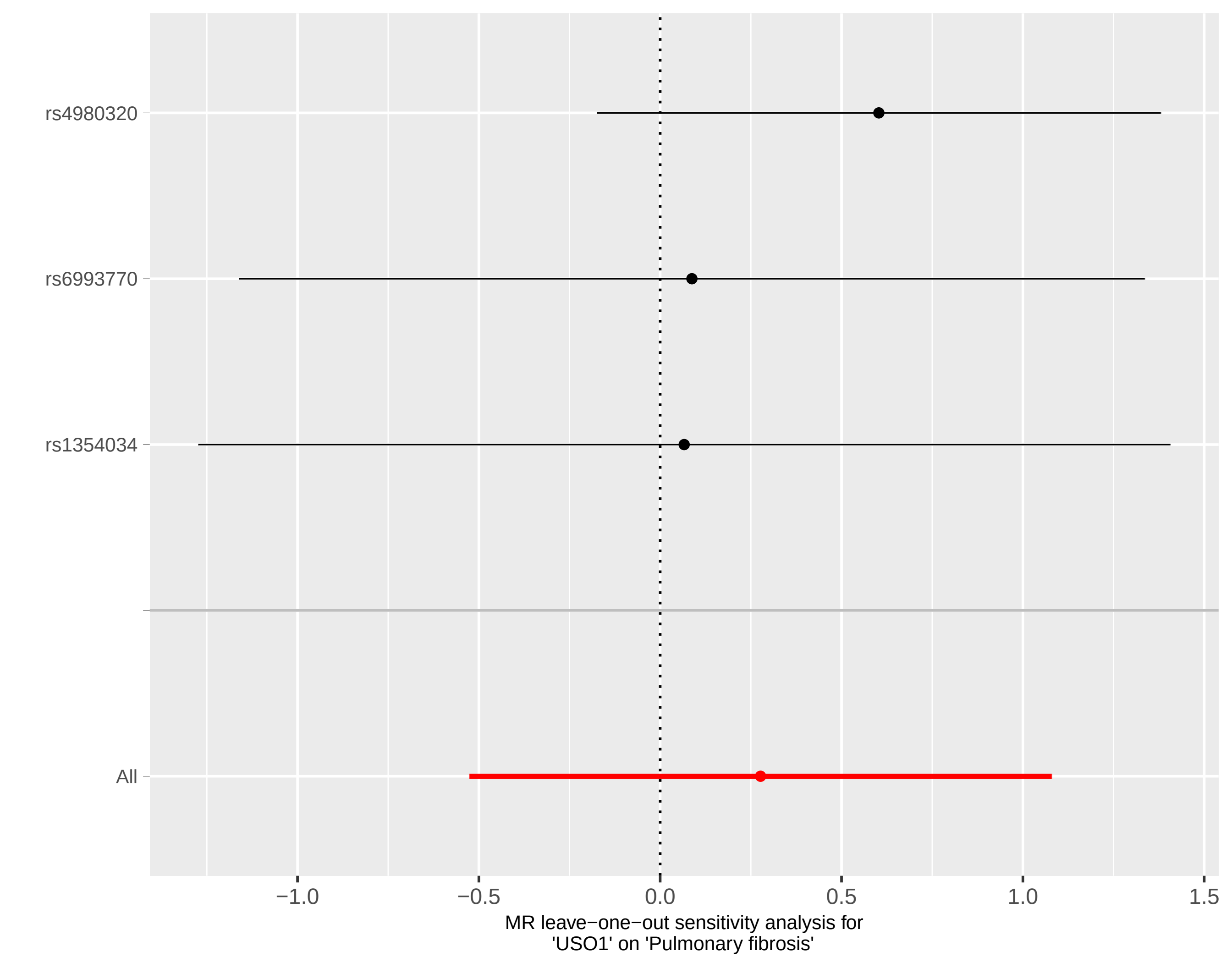
rs4980320
rs6993770
rs1354034
All
−1.0
−0.5
0.0
0.5
1.0
1.5
MR leave−one−out sensitivity analysis for
'USO1' on 'Pulmonary fibrosis'

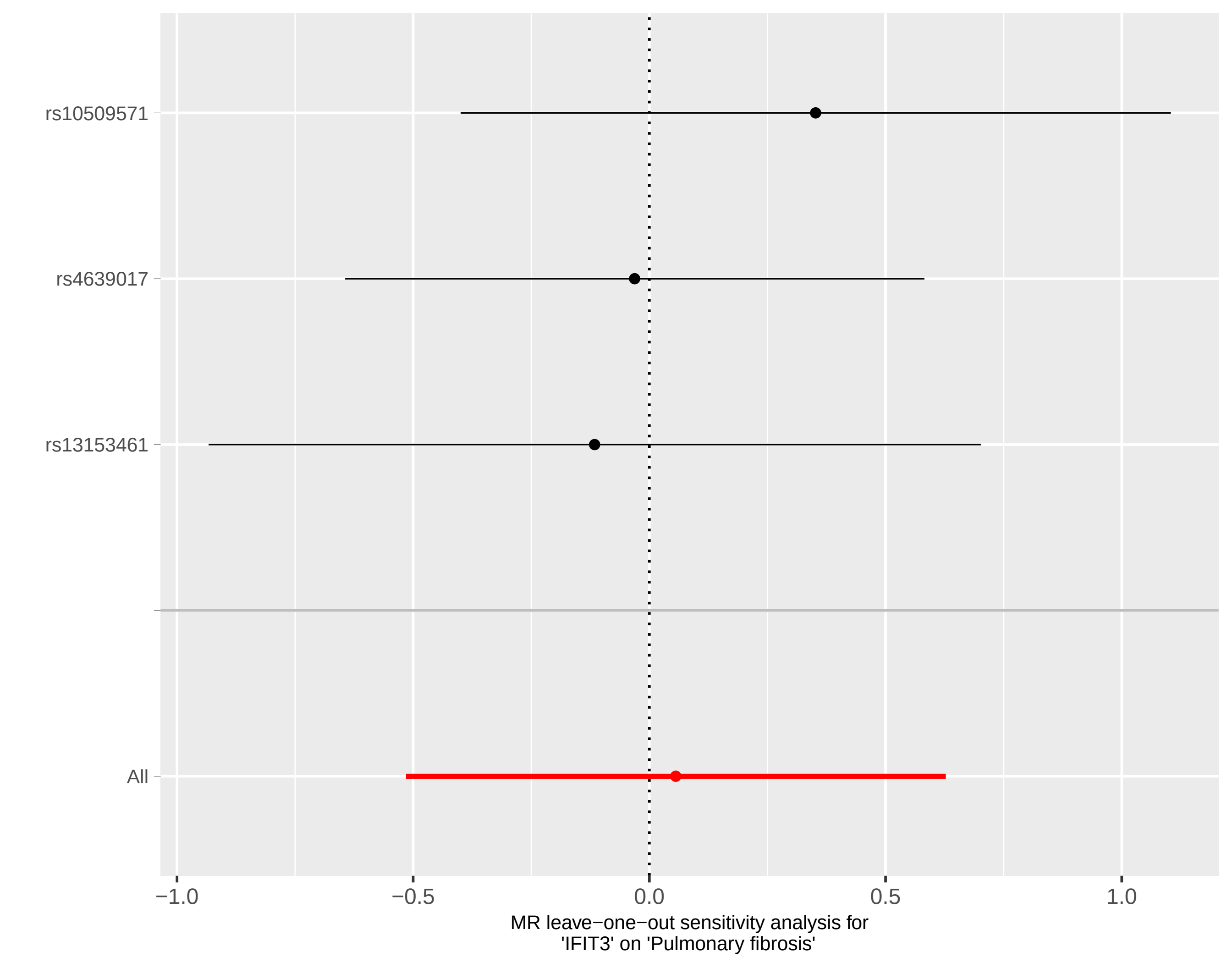
rs10509571
rs4639017
rs13153461
All
−1.0
−0.5
0.0
0.5
1.0
MR leave−one−out sensitivity analysis for
'IFIT3' on 'Pulmonary fibrosis'

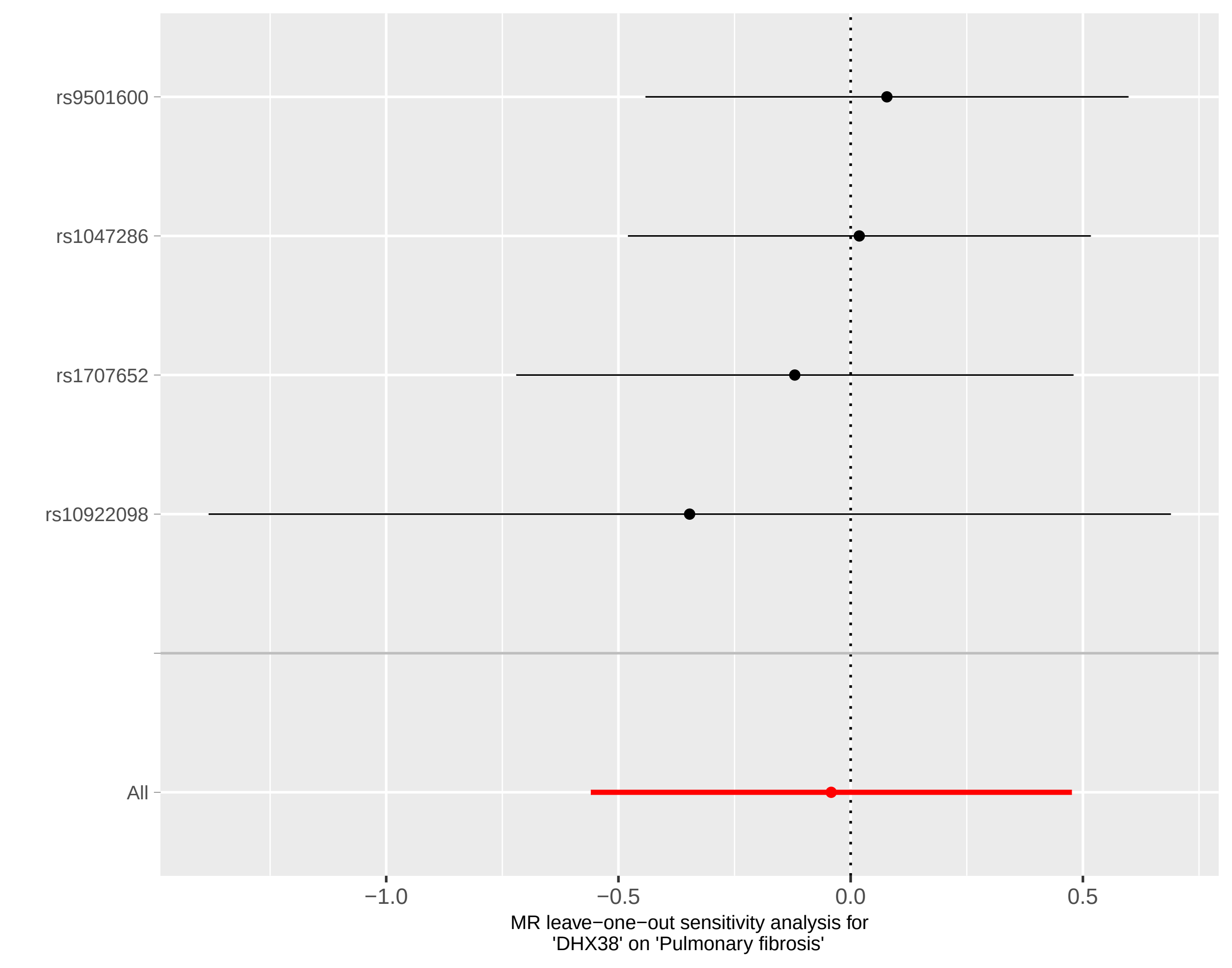
rs9501600
rs1047286
rs1707652
rs10922098
All
−1.0
−0.5
0.0
0.5
MR leave−one−out sensitivity analysis for
'DHX38' on 'Pulmonary fibrosis'

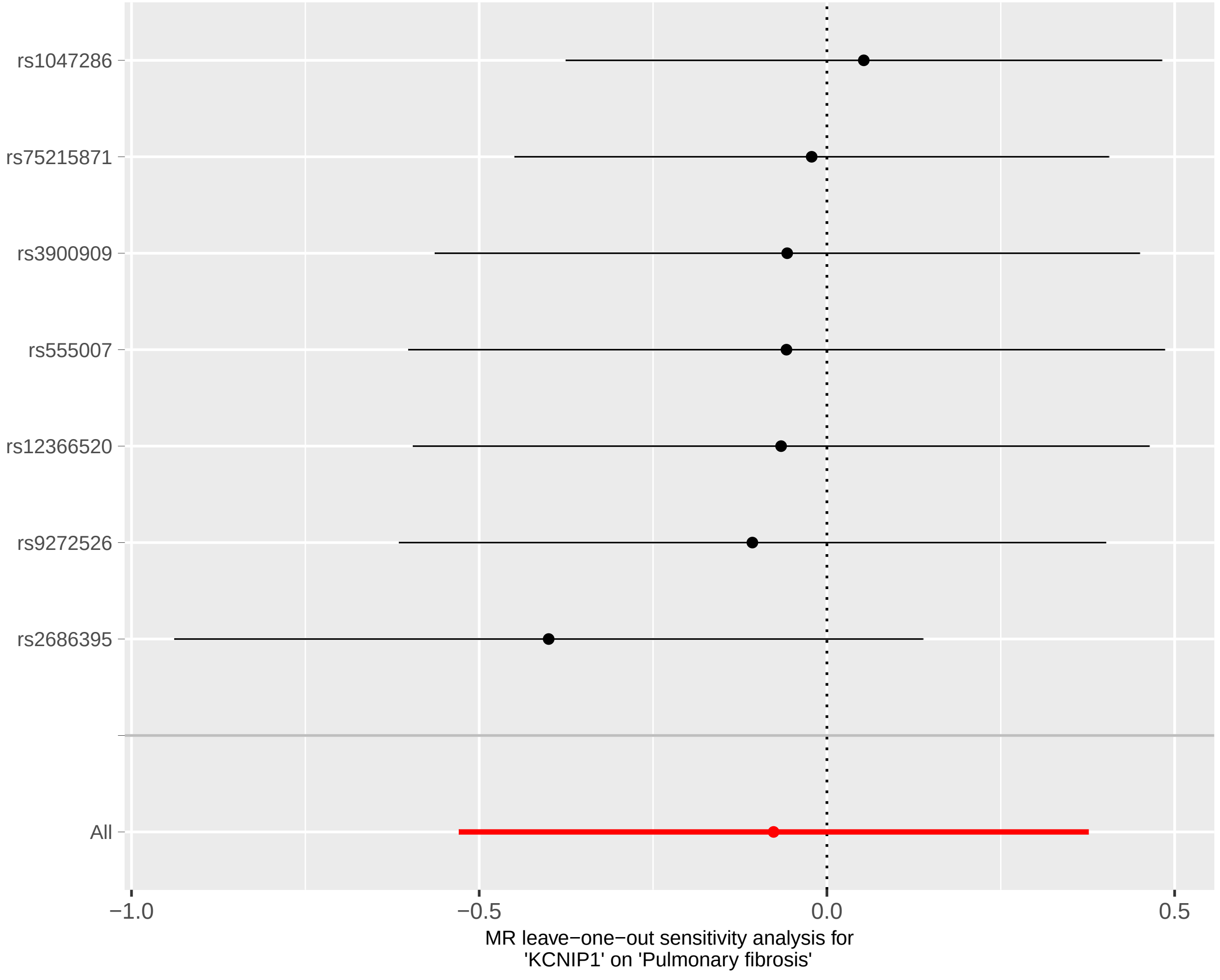

rs1047286
rs75215871
rs3900909
rs555007
rs12366520
rs9272526
rs2686395
All
−1.0
−0.5
0.0
0.5
MR leave−one−out sensitivity analysis for
'KCNIP1' on 'Pulmonary fibrosis'

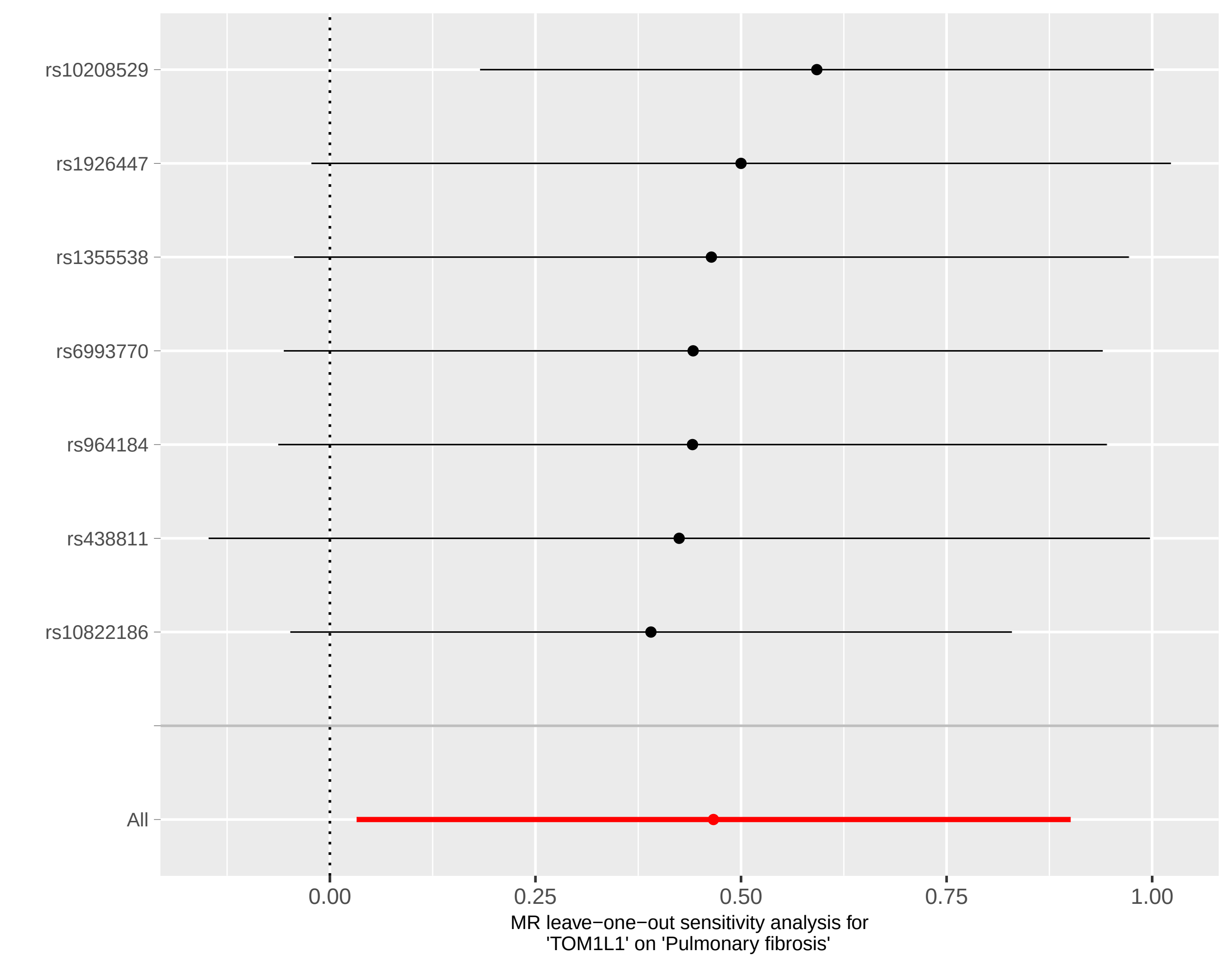
rs10208529
rs1926447
rs1355538
rs6993770
rs964184
rs438811
rs10822186
All
0.00
0.25
0.50
0.75
1.00
MR leave−one−out sensitivity analysis for
'TOM1L1' on 'Pulmonary fibrosis'

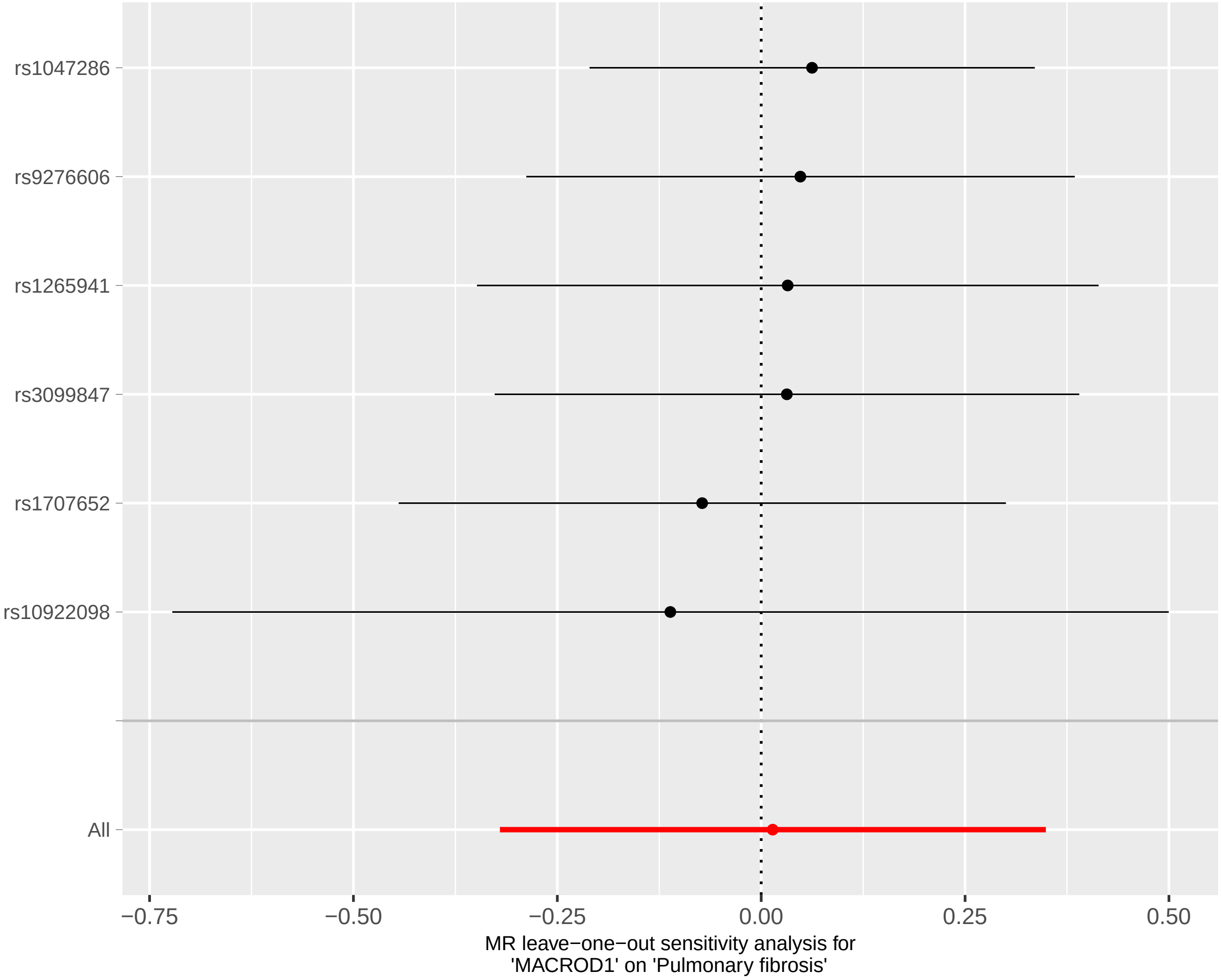

rs1047286
rs9276606
rs1265941
rs3099847
rs1707652
rs10922098
All
−0.75
−0.50
−0.25
0.00
0.25
0.50
MR leave−one−out sensitivity analysis for
'MACROD1' on 'Pulmonary fibrosis'

Insufficient number of SNPs

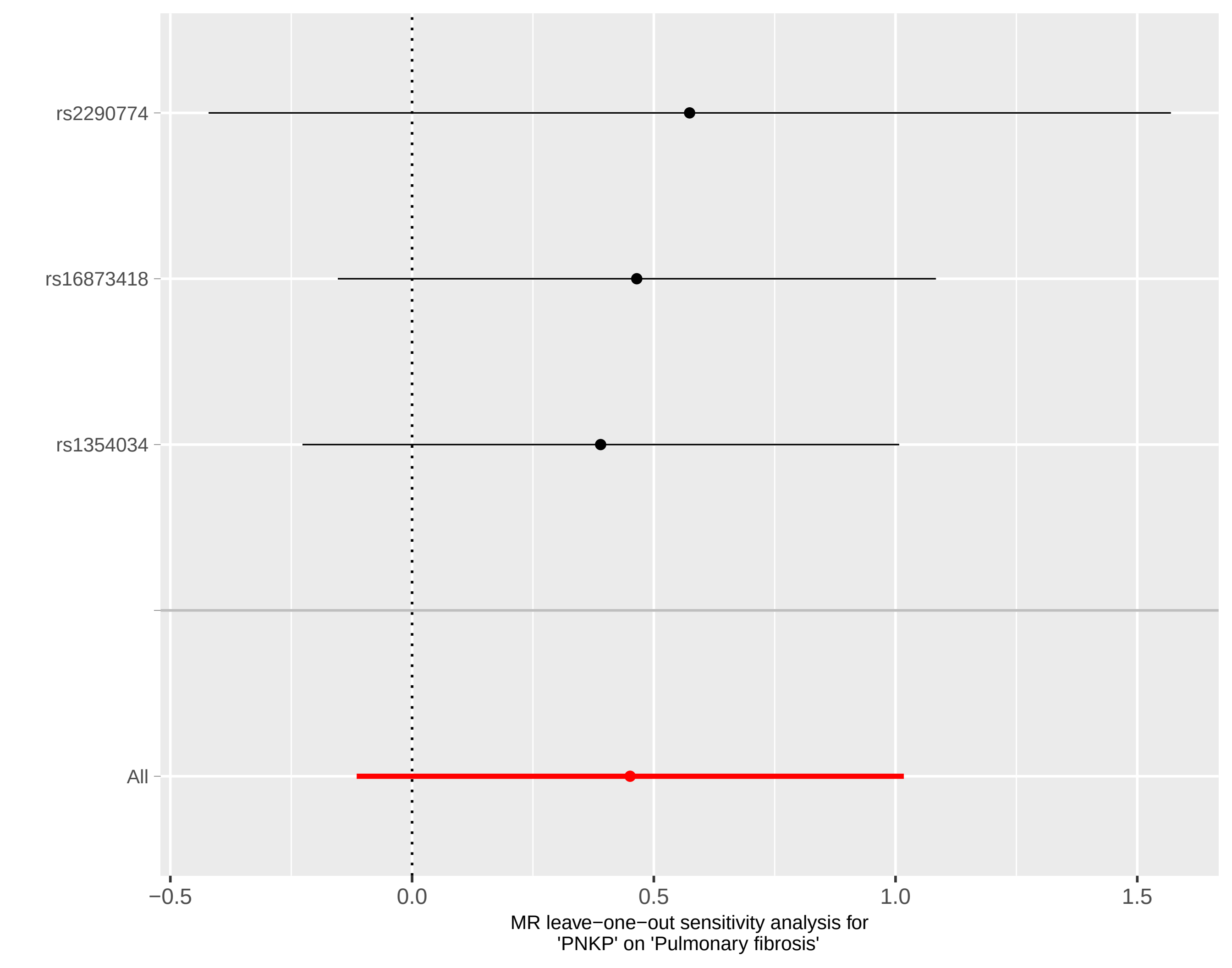
rs2290774
rs16873418
rs1354034
All
−0.5
0.0
0.5
1.0
1.5
MR leave−one−out sensitivity analysis for
'PNKP' on 'Pulmonary fibrosis'

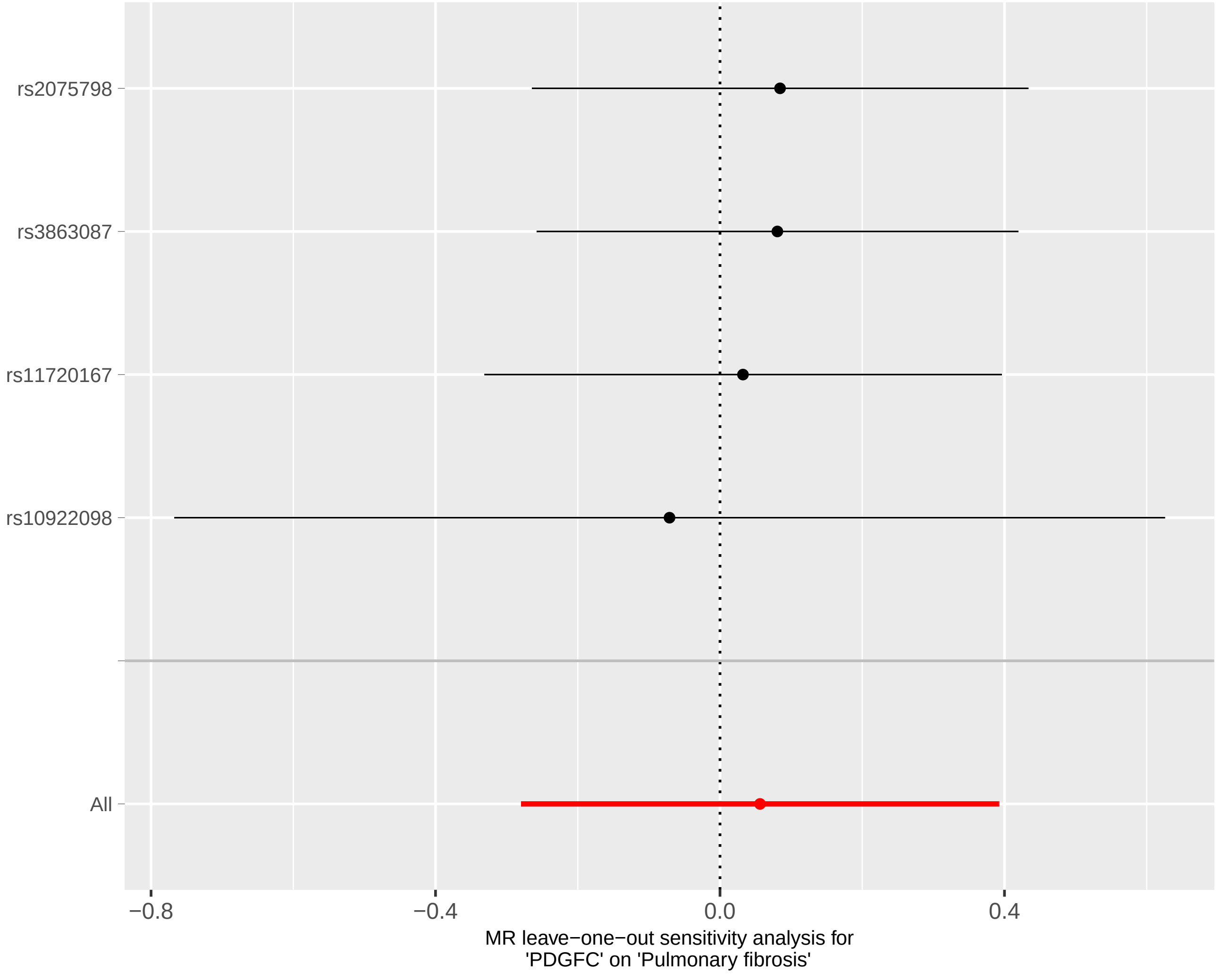

rs2075798
rs3863087
rs11720167
rs10922098
All
−0.8
−0.4
0.0
0.4
MR leave−one−out sensitivity analysis for
'PDGFC' on 'Pulmonary fibrosis'

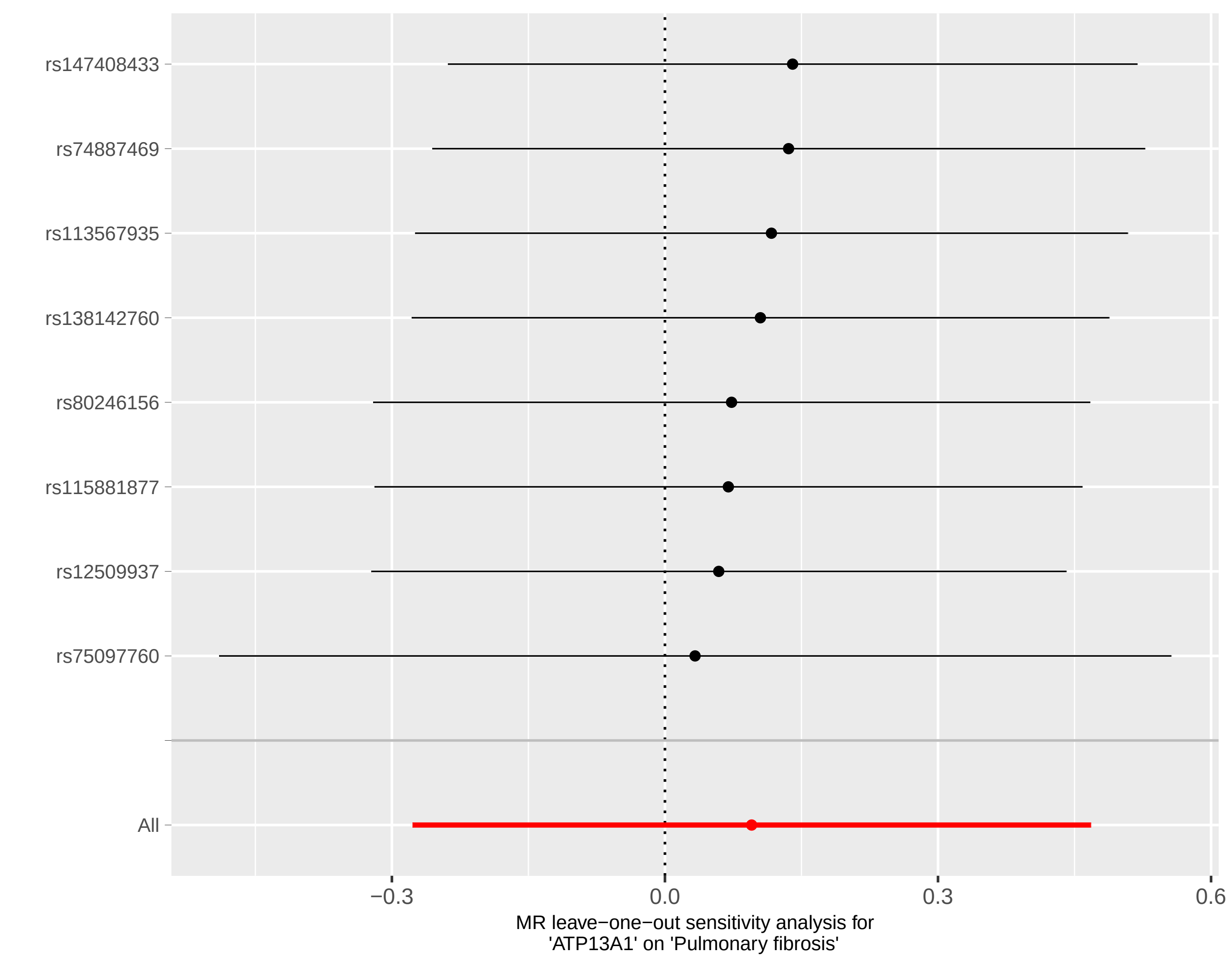
rs147408433
rs74887469
rs113567935
rs138142760
rs80246156
rs115881877
rs12509937
rs75097760
All
−0.3
0.0
0.3
0.6
MR leave−one−out sensitivity analysis for
'ATP13A1' on 'Pulmonary fibrosis'

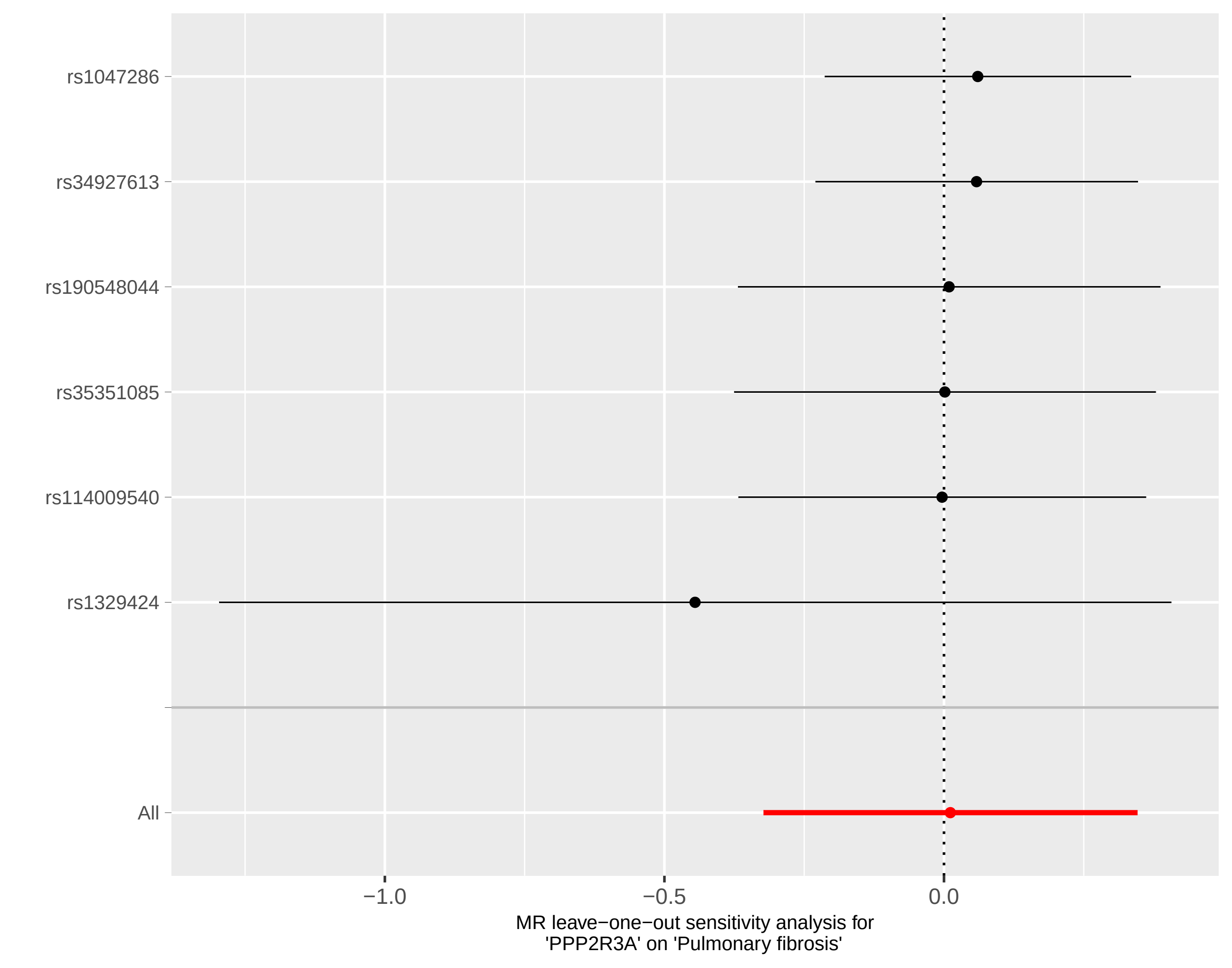

rs1047286
rs34927613
rs190548044
rs35351085
rs114009540
rs1329424
All
−1.0
−0.5
0.0
MR leave−one−out sensitivity analysis for
'PPP2R3A' on 'Pulmonary fibrosis'

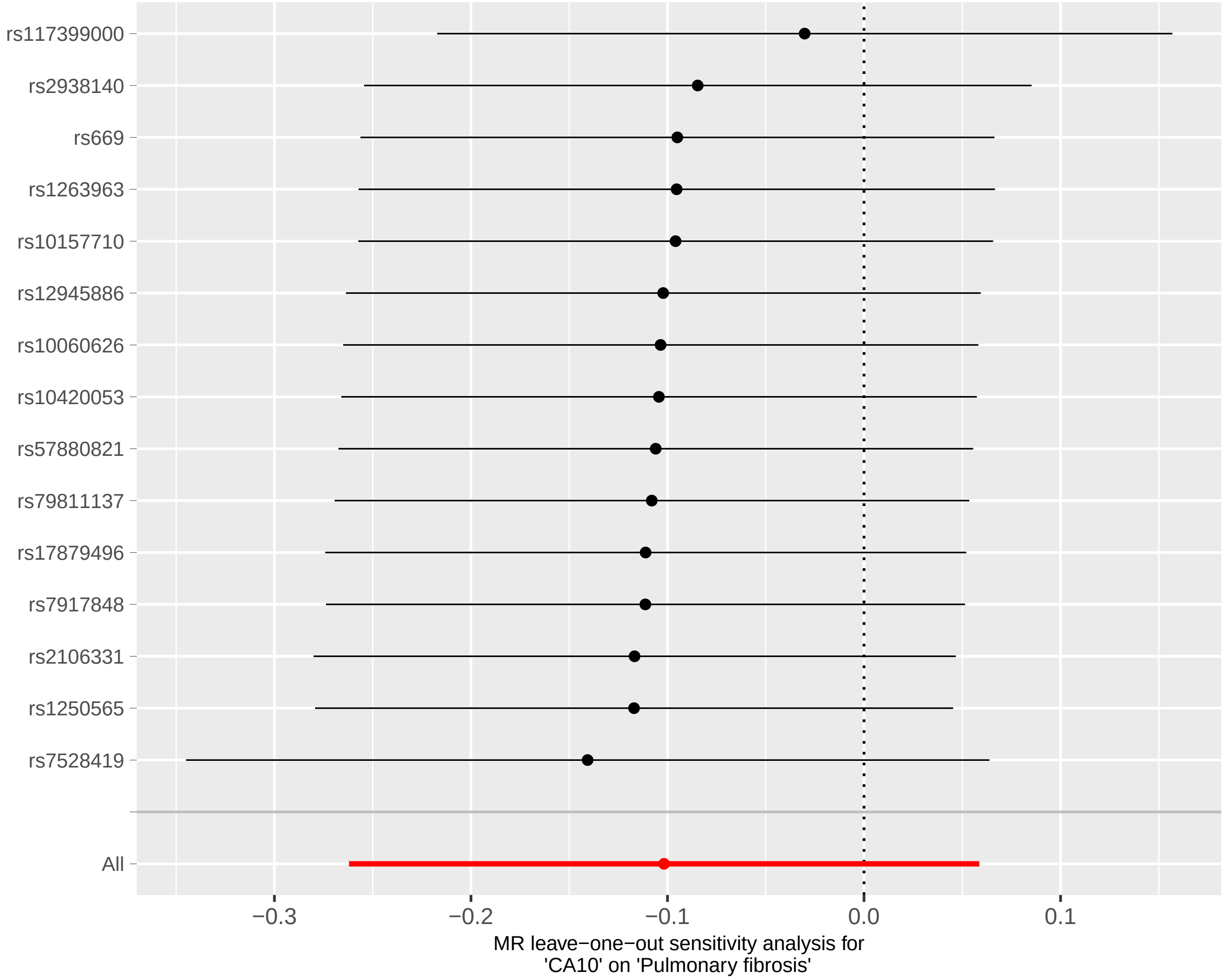
rs117399000
rs2938140
rs669
rs1263963
rs10157710
rs12945886
rs10060626
rs10420053
rs57880821
rs79811137
rs17879496
rs7917848
rs2106331
rs1250565
rs7528419
All
−0.3
−0.2
−0.1
0.0
0.1
MR leave−one−out sensitivity analysis for
'CA10' on 'Pulmonary fibrosis'

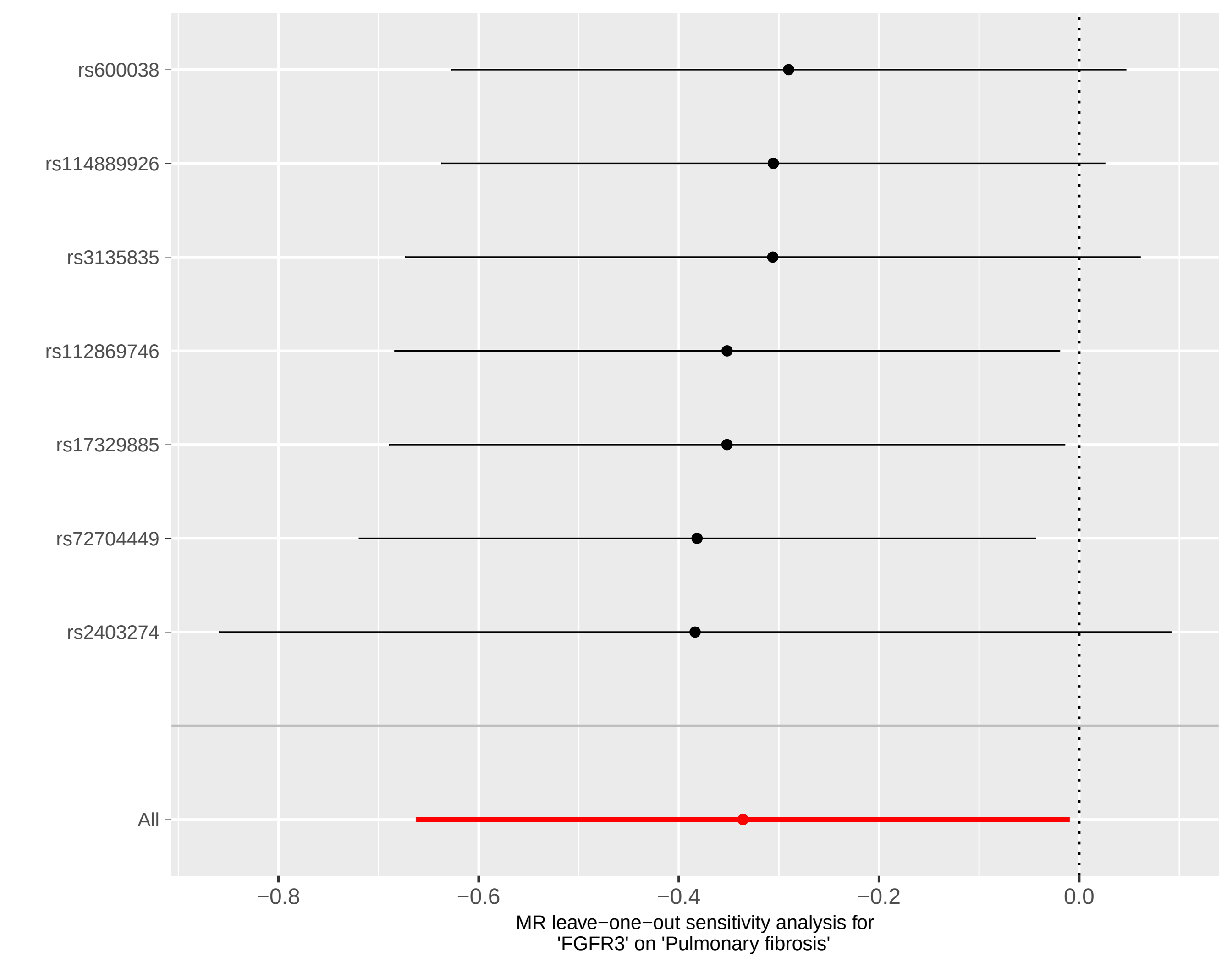
rs600038
rs114889926
rs3135835
rs112869746
rs17329885
rs72704449
rs2403274
All
−0.8
−0.6
−0.4
−0.2
0.0
MR leave−one−out sensitivity analysis for
'FGFR3' on 'Pulmonary fibrosis'

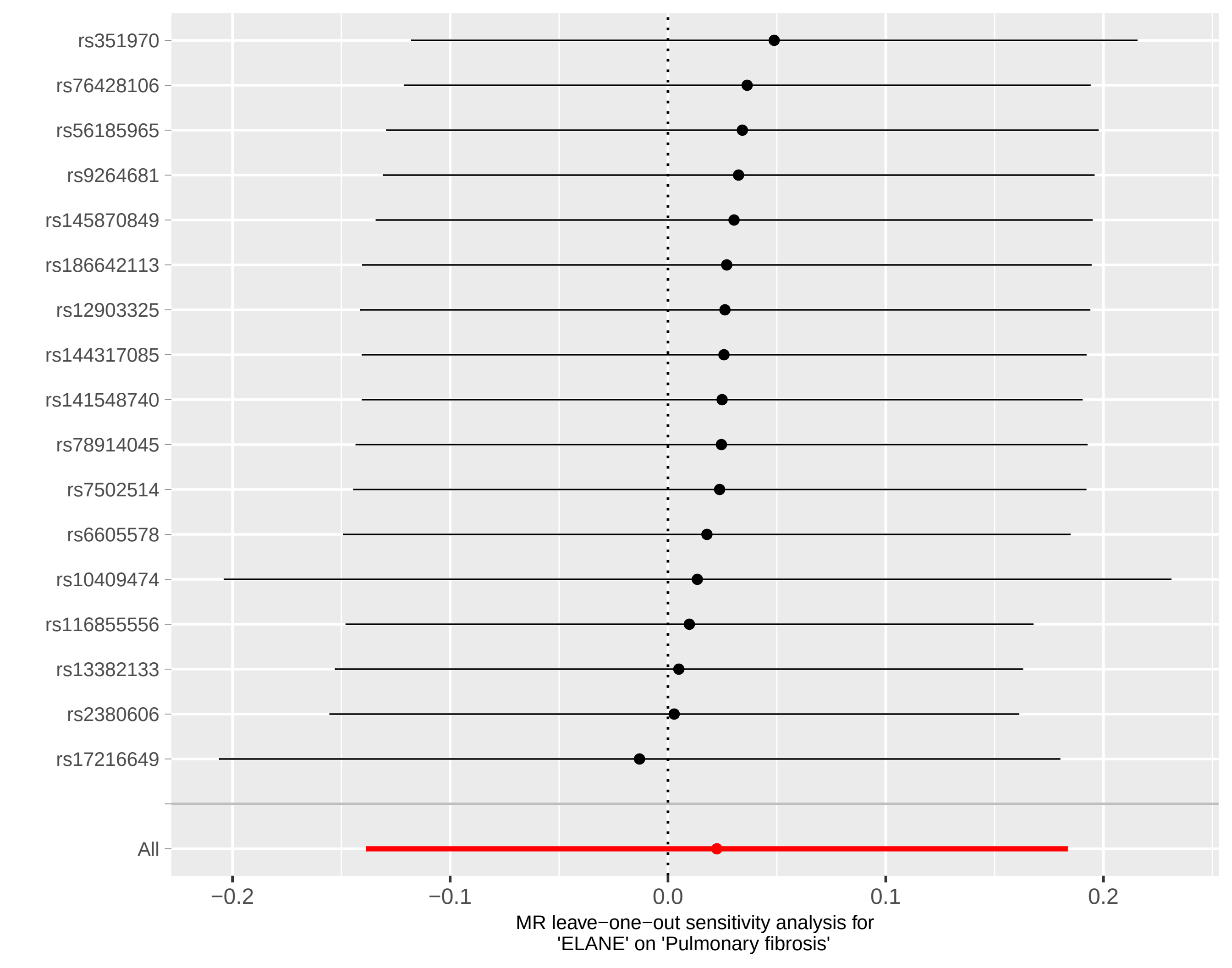
rs351970
rs76428106
rs56185965
rs9264681
rs145870849
rs186642113
rs12903325
rs144317085
rs141548740
rs78914045
rs7502514
rs6605578
rs10409474
rs116855556
rs13382133
rs2380606
rs17216649
All
−0.2
−0.1
0.0
0.1
0.2
MR leave−one−out sensitivity analysis for
'ELANE' on 'Pulmonary fibrosis'

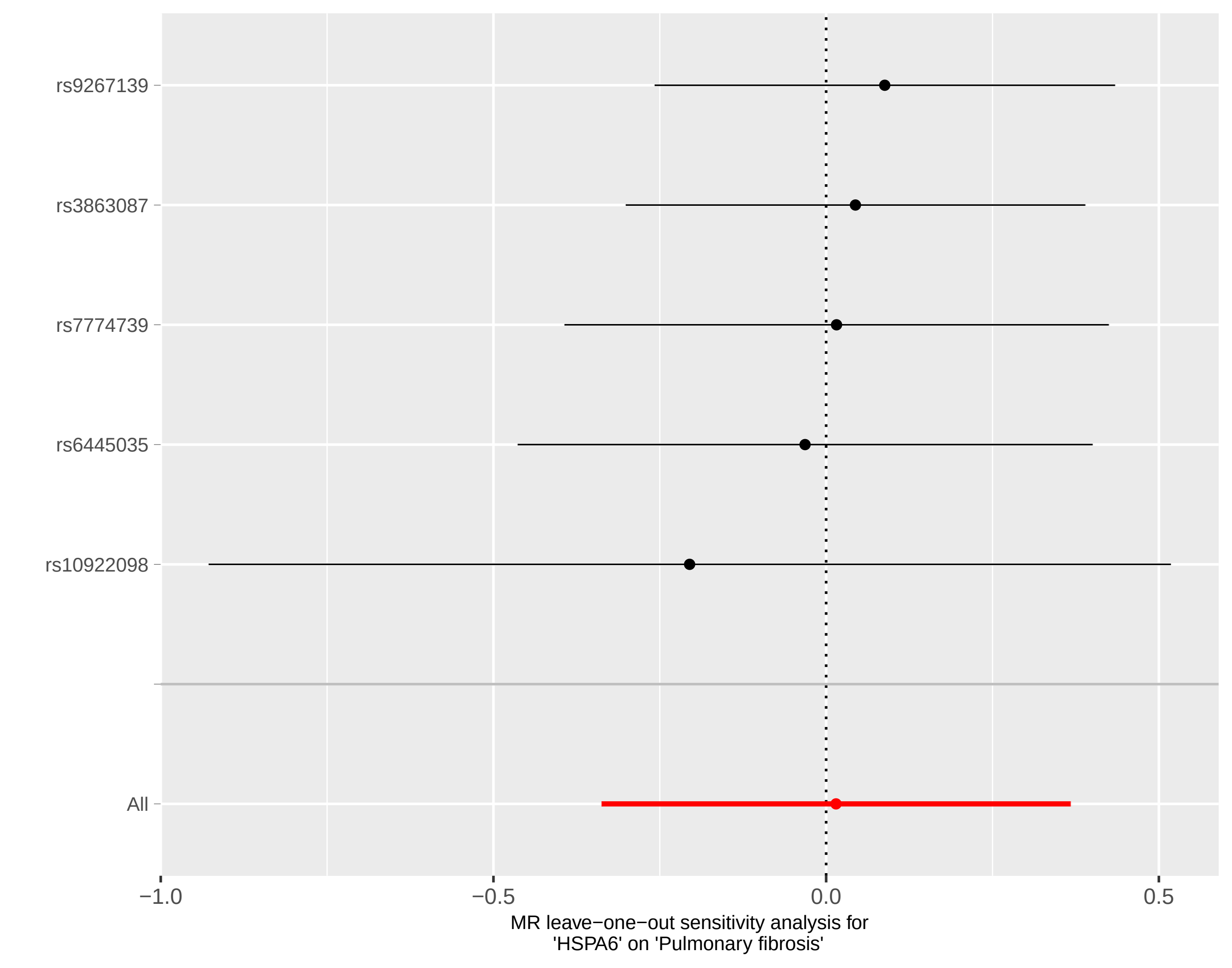
rs9267139
rs3863087
rs7774739
rs6445035
rs10922098
All
−1.0
−0.5
0.0
0.5
MR leave−one−out sensitivity analysis for
'HSPA6' on 'Pulmonary fibrosis'

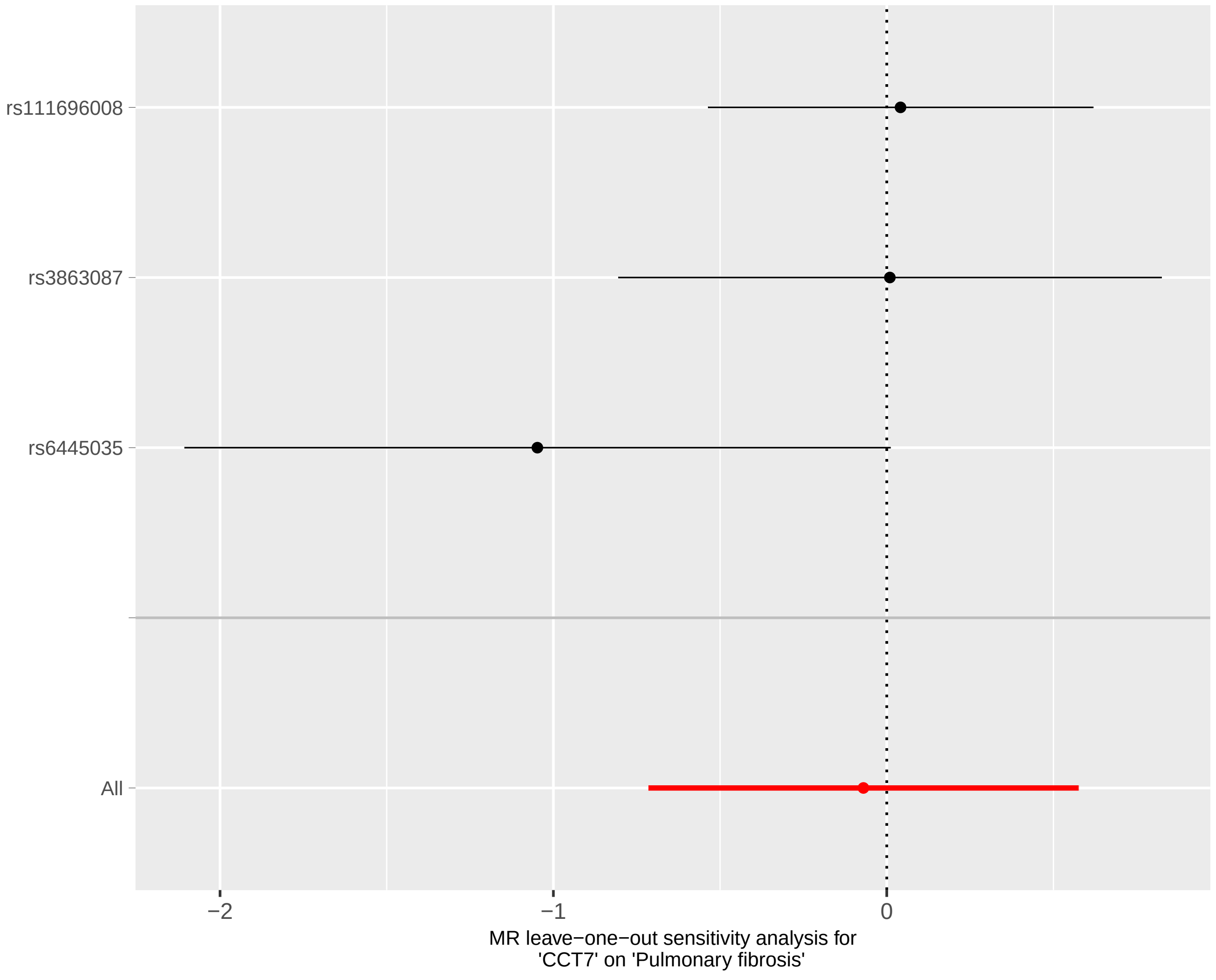
rs111696008
rs3863087
rs6445035
All
−2
−1
0
MR leave−one−out sensitivity analysis for
'CCT7' on 'Pulmonary fibrosis'

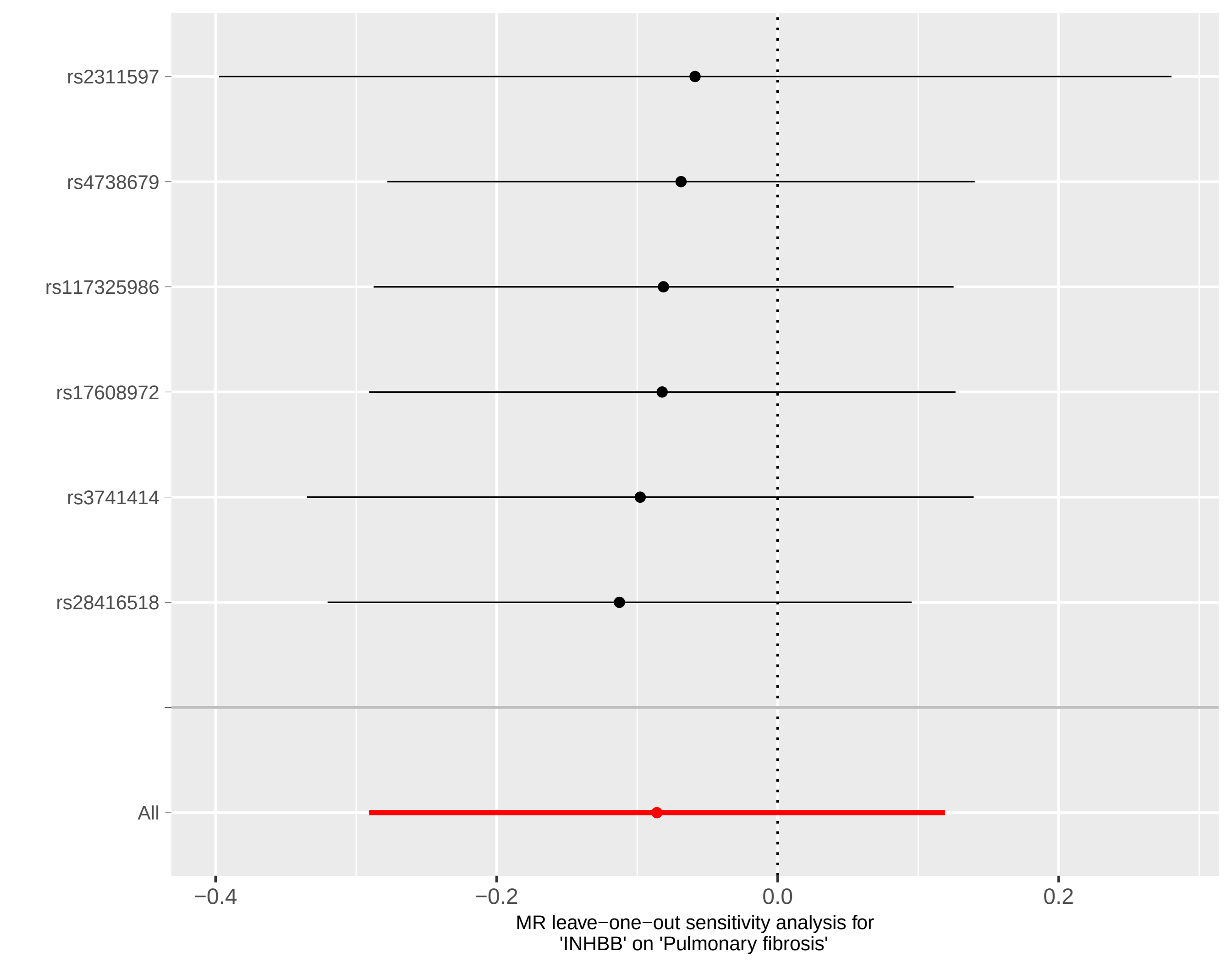
rs2311597
rs4738679
rs117325986
rs17608972
rs3741414
rs28416518
All
−0.4
−0.2
0.0
0.2
MR leave−one−out sensitivity analysis for
'INHBB' on 'Pulmonary fibrosis'

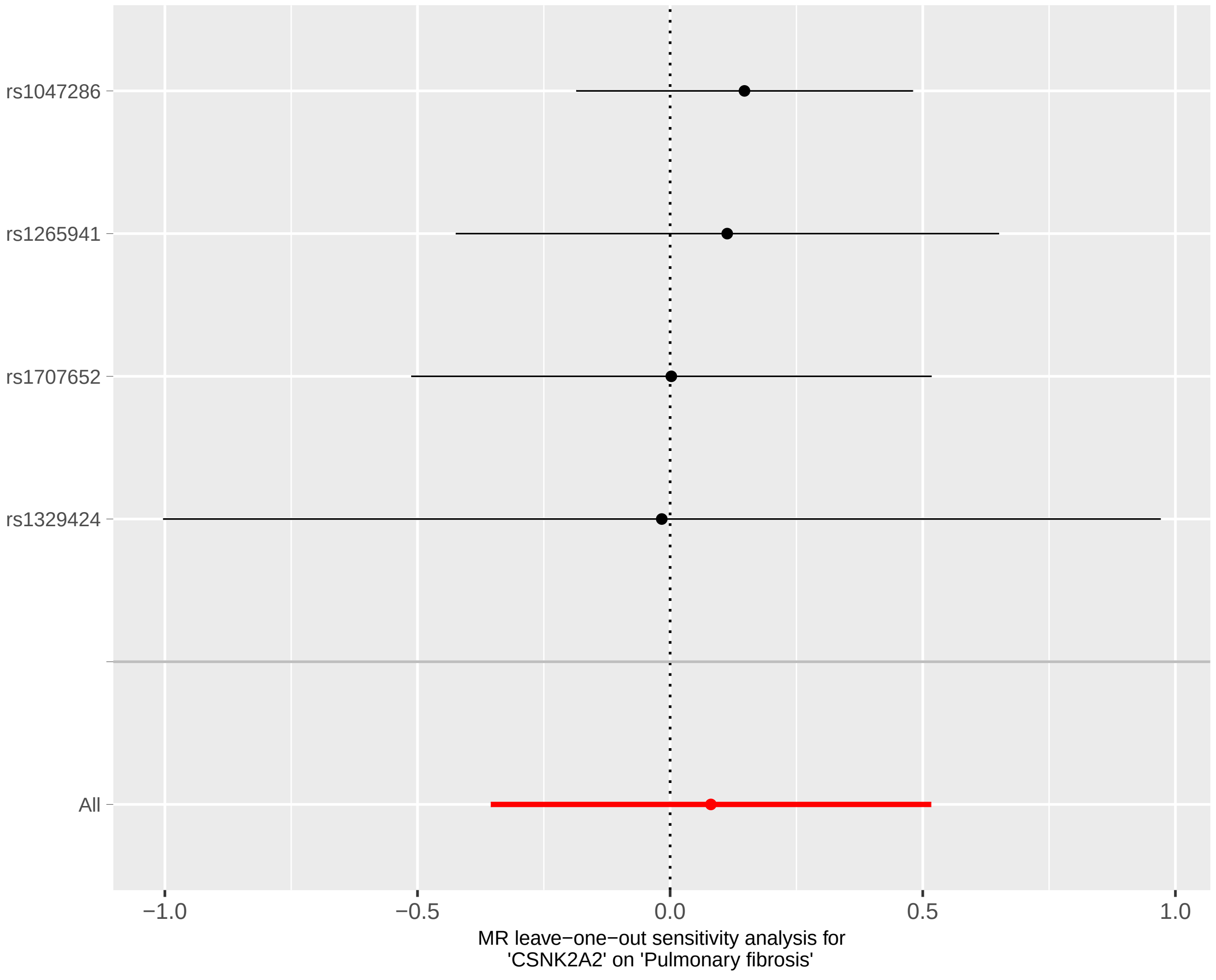
rs1047286
rs1265941
rs1707652
rs1329424
All
−1.0
−0.5
0.0
0.5
1.0
MR leave−one−out sensitivity analysis for
'CSNK2A2' on 'Pulmonary fibrosis'

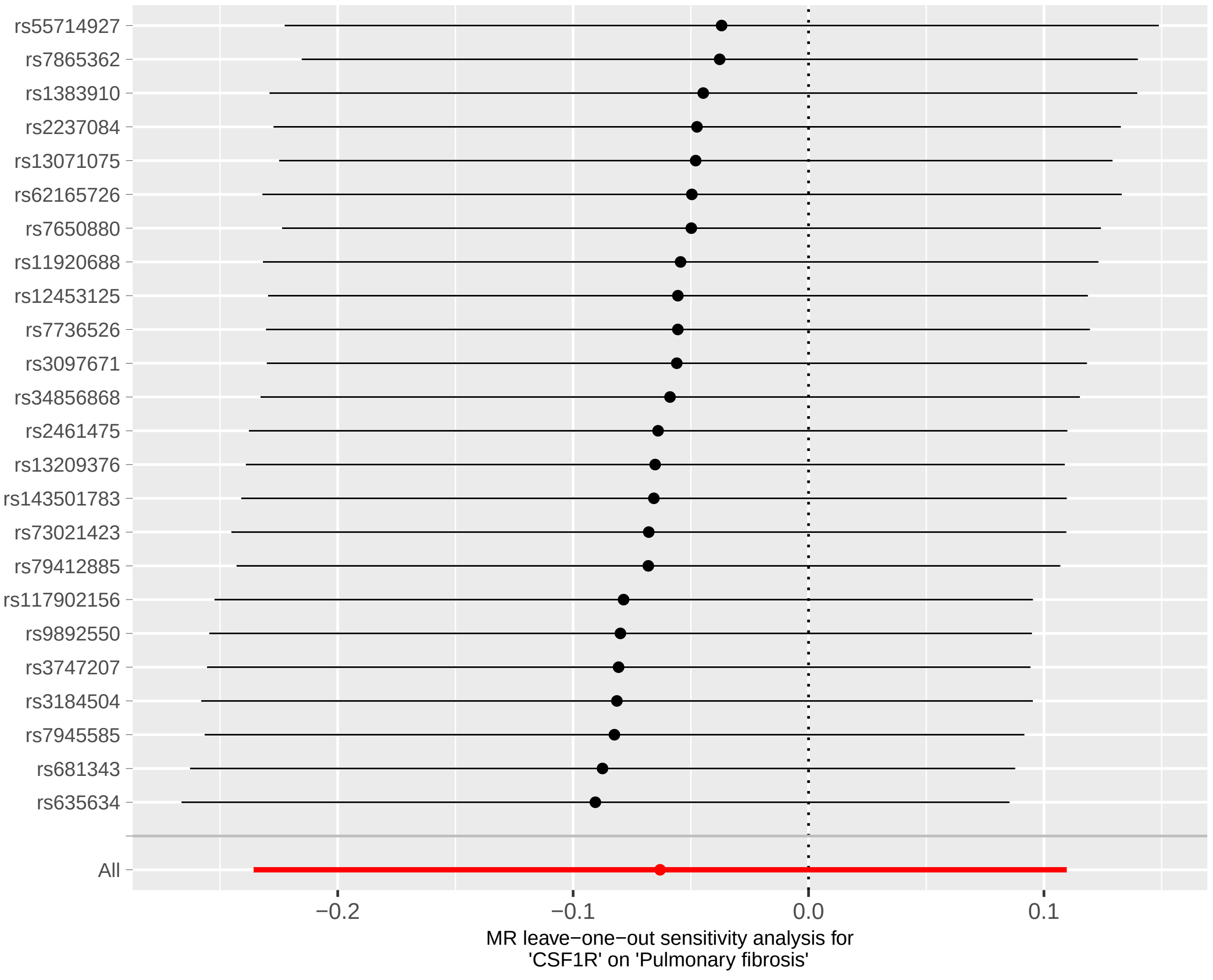

rs55714927
rs7865362
rs1383910
rs2237084
rs13071075
rs62165726
rs7650880
rs11920688
rs12453125
rs7736526
rs3097671
rs34856868
rs2461475
rs13209376
rs143501783
rs73021423
rs79412885
rs117902156
rs9892550
rs3747207
rs3184504
rs7945585
rs681343
rs635634
All
−0.2
−0.1
0.0
0.1
MR leave−one−out sensitivity analysis for
'CSF1R' on 'Pulmonary fibrosis'

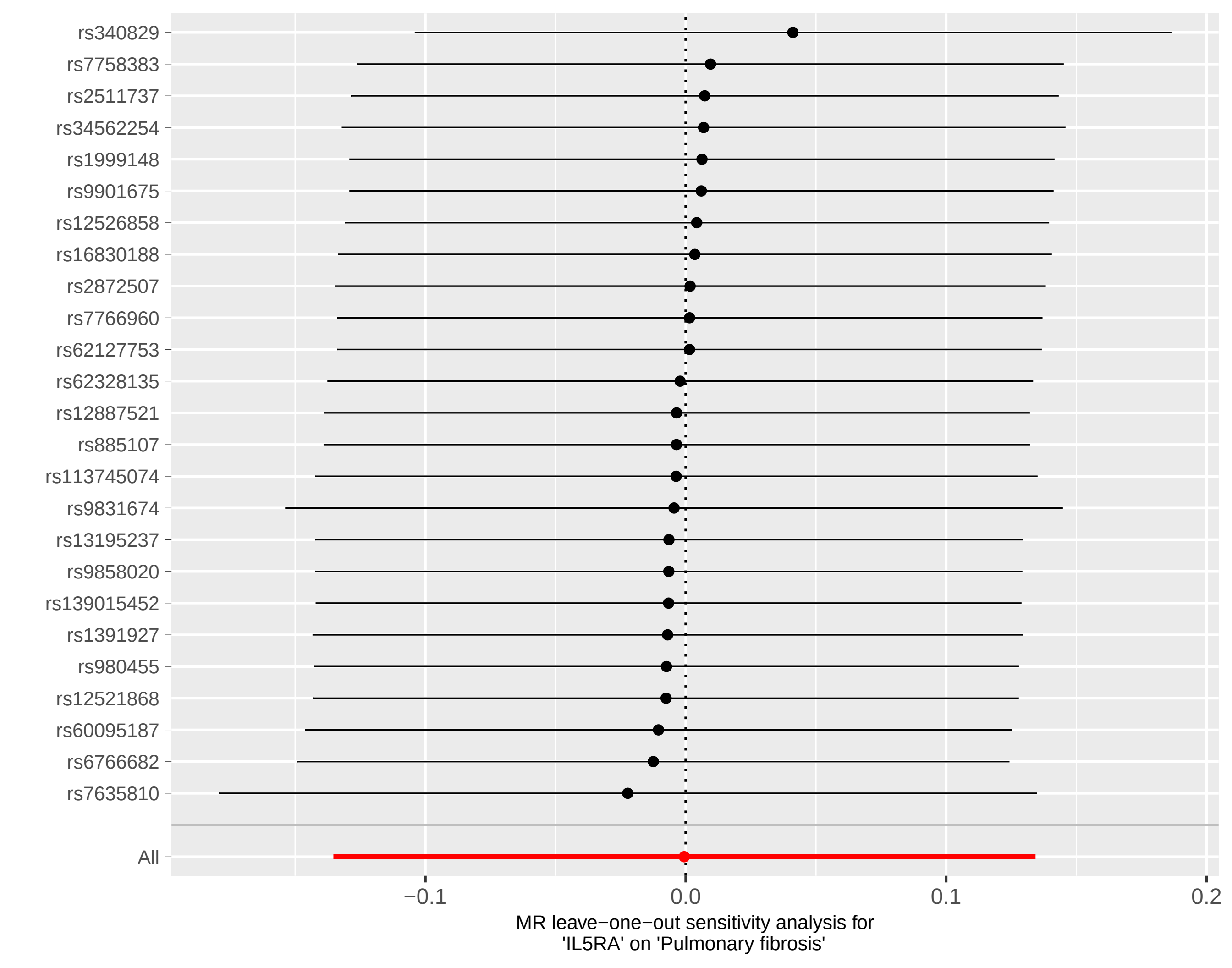
rs340829
rs7758383
rs2511737
rs34562254
rs1999148
rs9901675
rs12526858
rs16830188
rs2872507
rs7766960
rs62127753
rs62328135
rs12887521
rs885107
rs113745074
rs9831674
rs13195237
rs9858020
rs139015452
rs1391927
rs980455
rs12521868
rs60095187
rs6766682
rs7635810
All
−0.1
0.0
0.1
0.2
MR leave−one−out sensitivity analysis for
'IL5RA' on 'Pulmonary fibrosis'

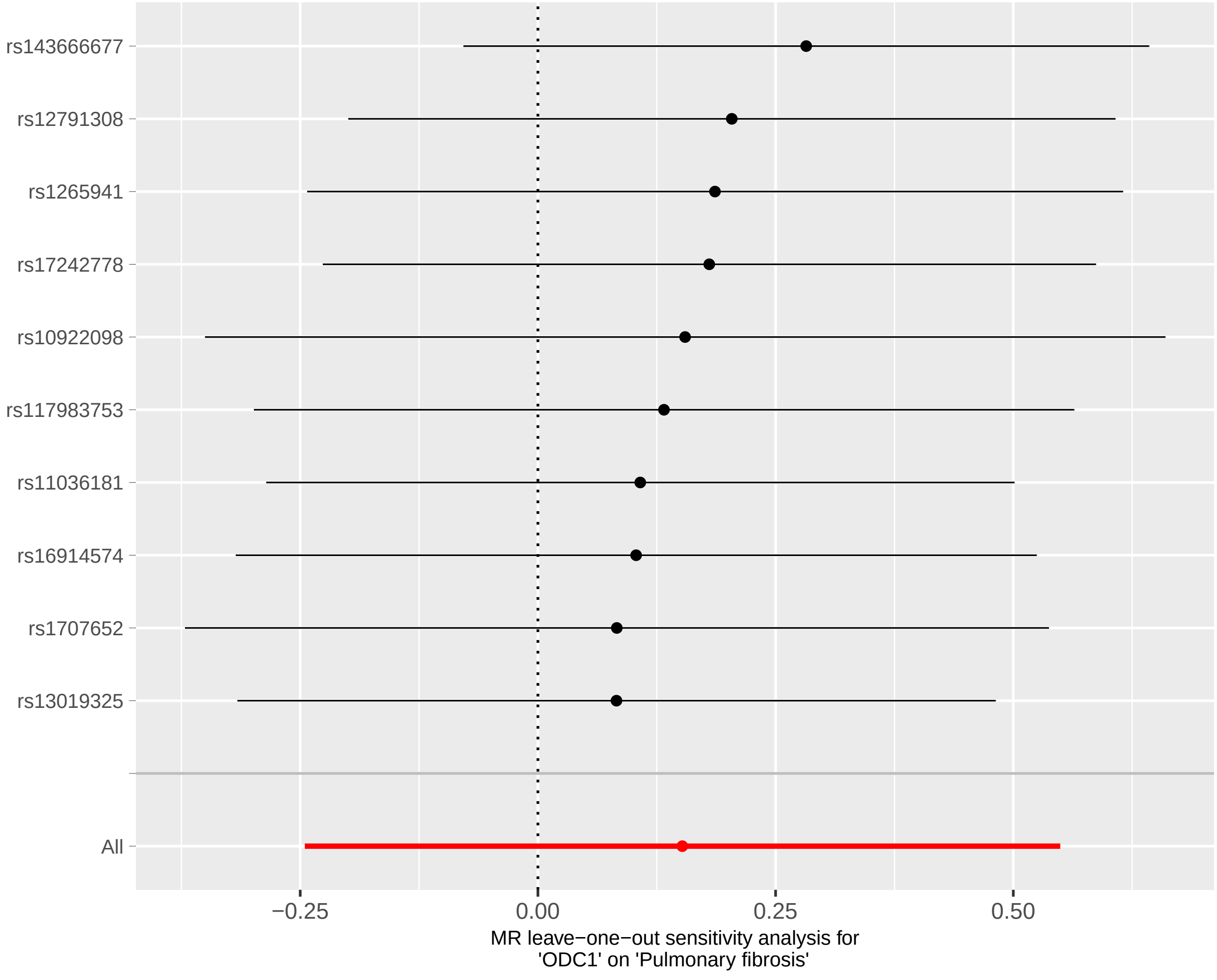

rs143666677
rs12791308
rs1265941
rs17242778
rs10922098
rs117983753
rs11036181
rs16914574
rs1707652
rs13019325
All
−0.25
0.00
0.25
0.50
MR leave−one−out sensitivity analysis for
'ODC1' on 'Pulmonary fibrosis'

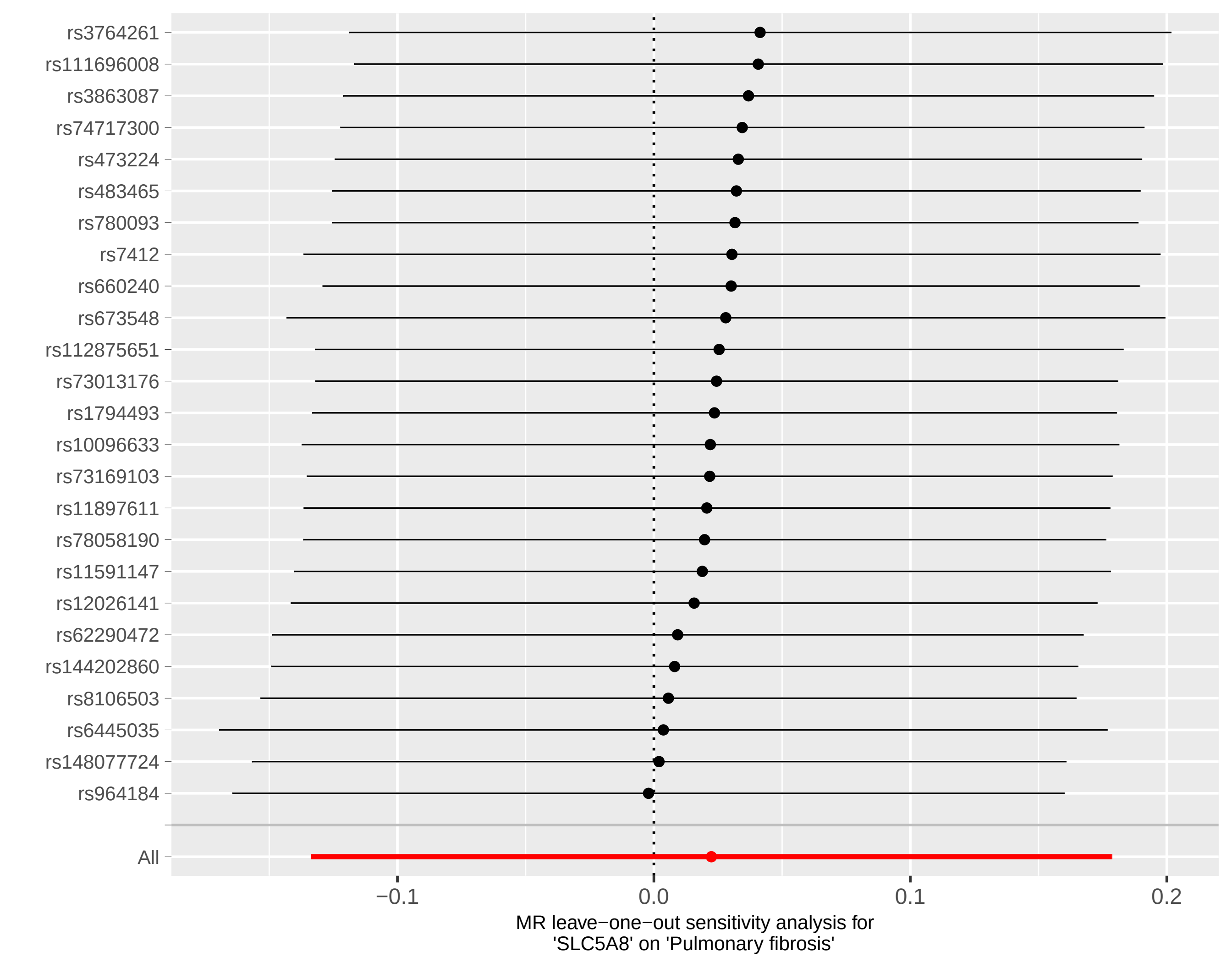
rs3764261
rs111696008
rs3863087
rs74717300
rs473224
rs483465
rs780093
rs7412
rs660240
rs673548
rs112875651
rs73013176
rs1794493
rs10096633
rs73169103
rs11897611
rs78058190
rs11591147
rs12026141
rs62290472
rs144202860
rs8106503
rs6445035
rs148077724
rs964184
All
−0.1
0.0
0.1
0.2
MR leave−one−out sensitivity analysis for
'SLC5A8' on 'Pulmonary fibrosis'

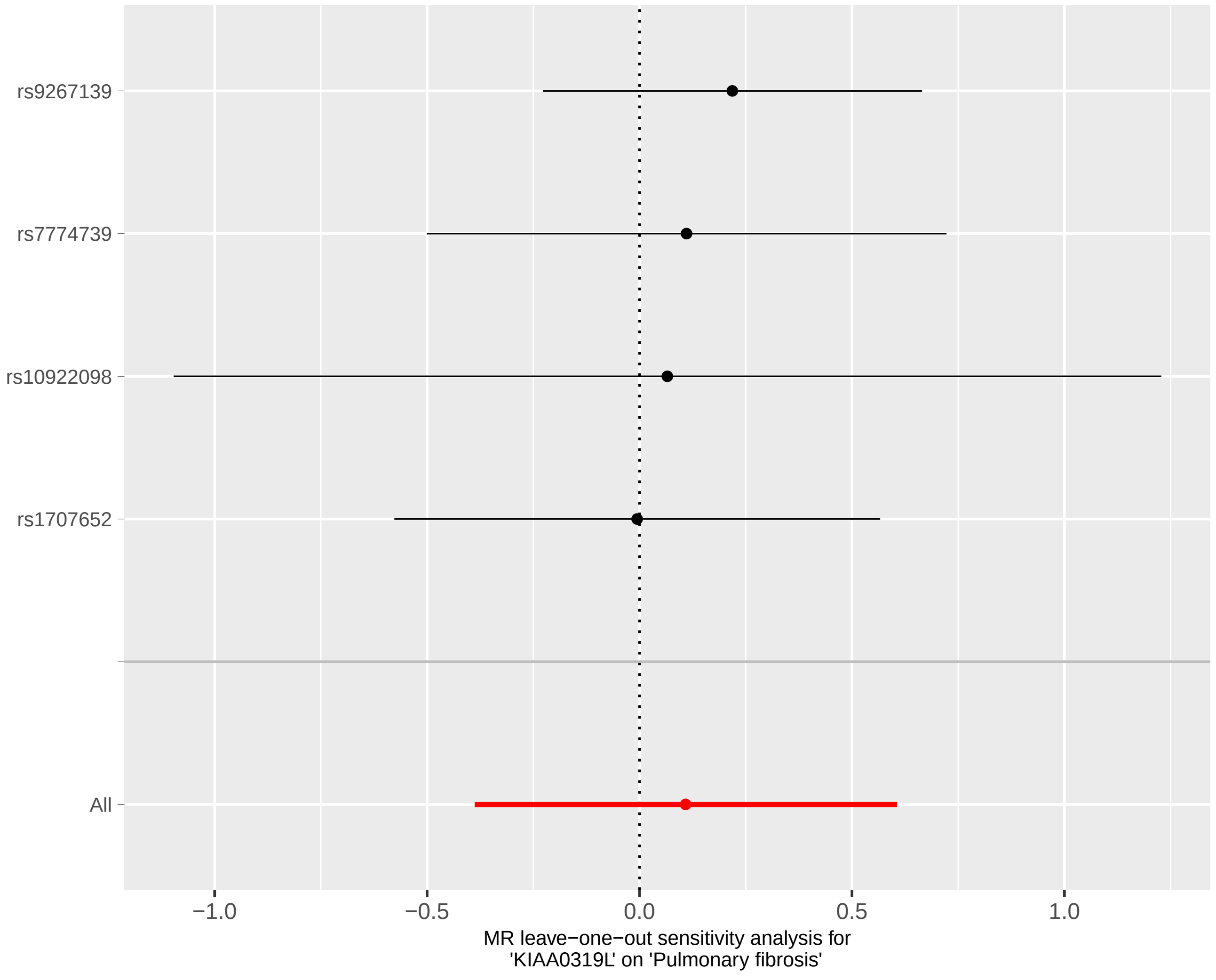

rs9267139
rs7774739
rs10922098
rs1707652
All
−1.0
−0.5
0.0
0.5
1.0
MR leave−one−out sensitivity analysis for
'KIAA0319L' on 'Pulmonary fibrosis'

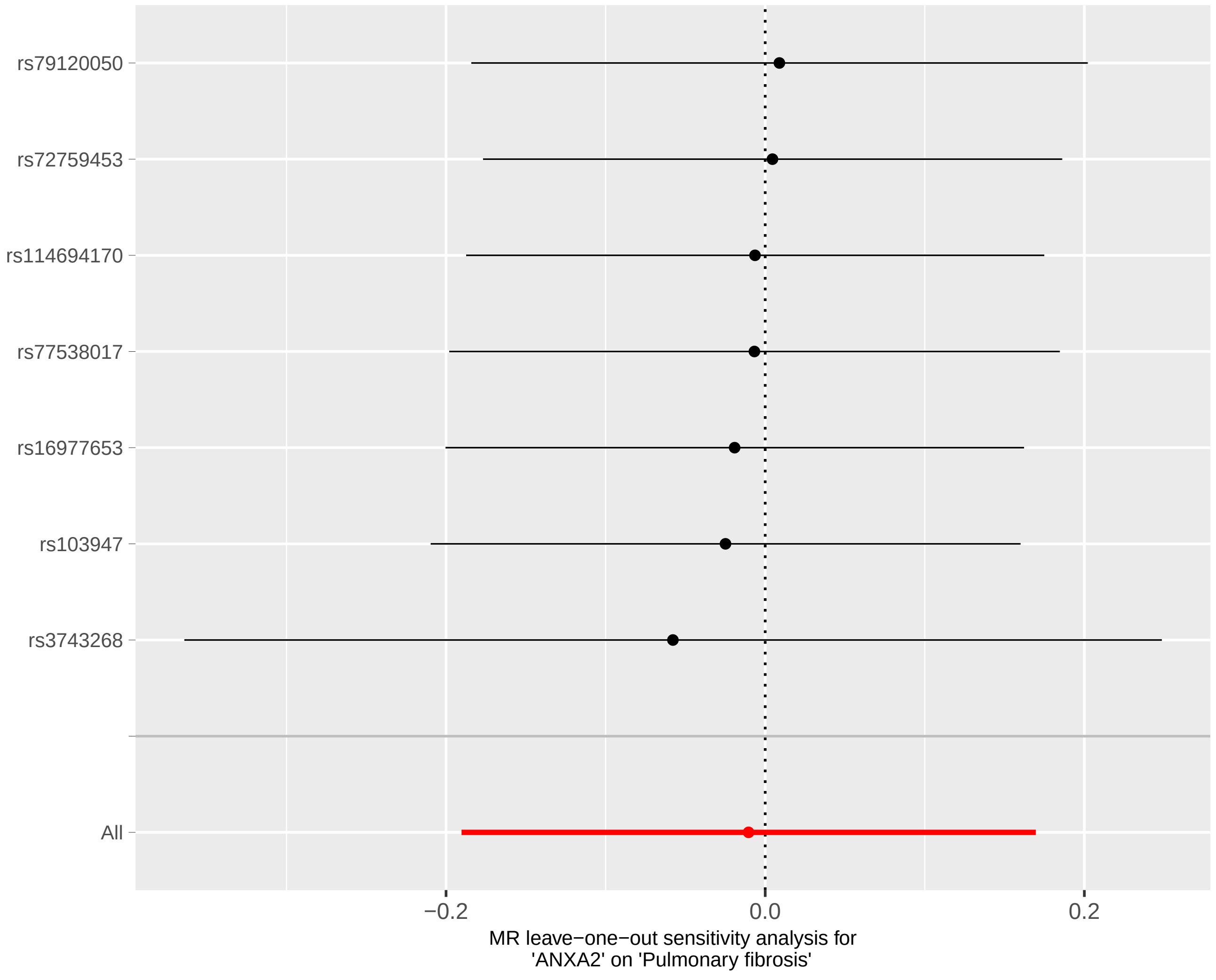

rs79120050
rs72759453
rs114694170
rs77538017
rs16977653
rs103947
rs3743268
All
−0.2
0.0
0.2
MR leave−one−out sensitivity analysis for
'ANXA2' on 'Pulmonary fibrosis'

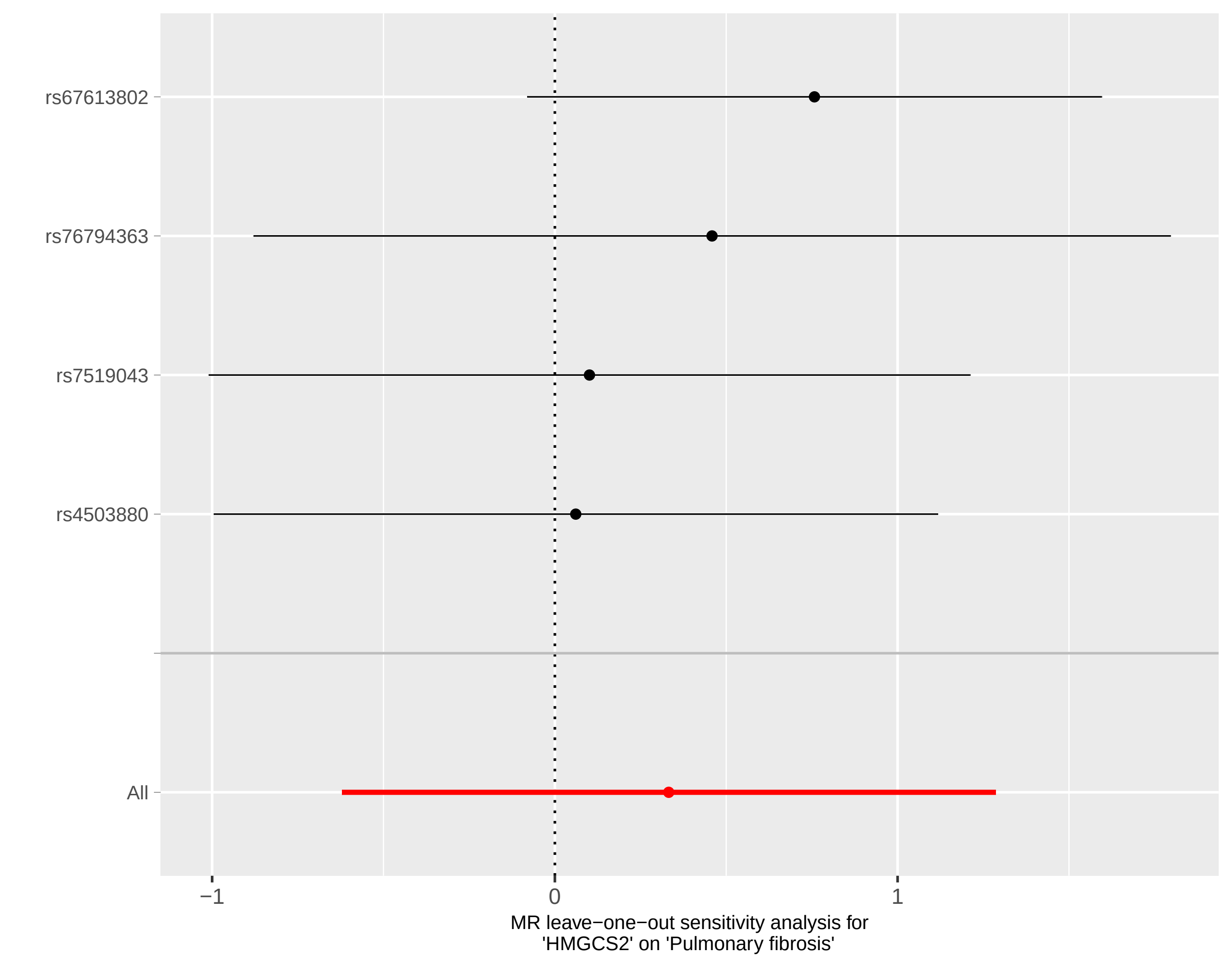
rs67613802
rs76794363
rs7519043
rs4503880
All
−1
0
1
MR leave−one−out sensitivity analysis for
'HMGCS2' on 'Pulmonary fibrosis'

Insufficient number of SNPs

Insufficient number of SNPs

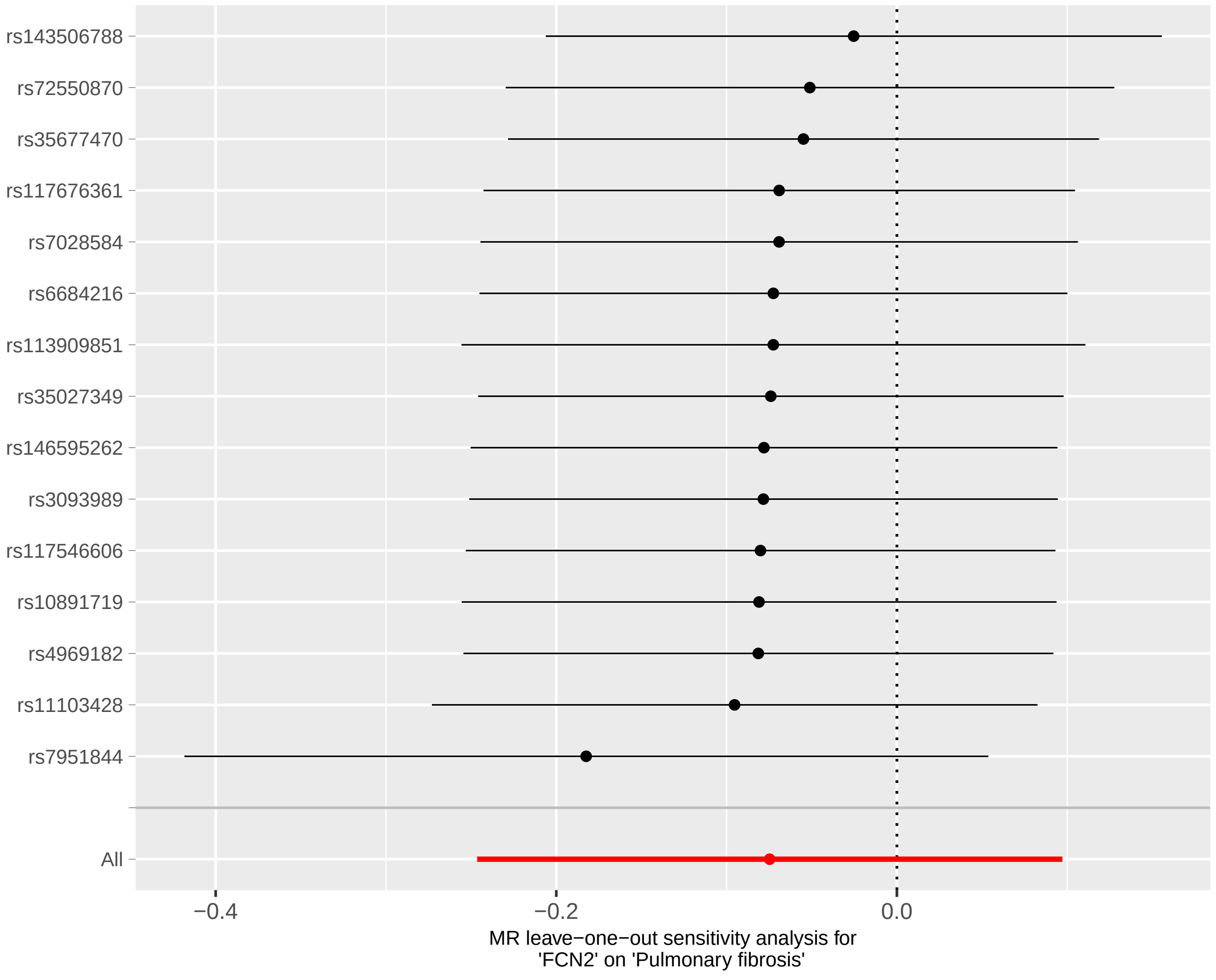
rs143506788
rs72550870
rs35677470
rs117676361
rs7028584
rs6684216
rs113909851
rs35027349
rs146595262
rs3093989
rs117546606
rs10891719
rs4969182
rs11103428
rs7951844
All
−0.4
−0.2
0.0
MR leave−one−out sensitivity analysis for
'FCN2' on 'Pulmonary fibrosis'

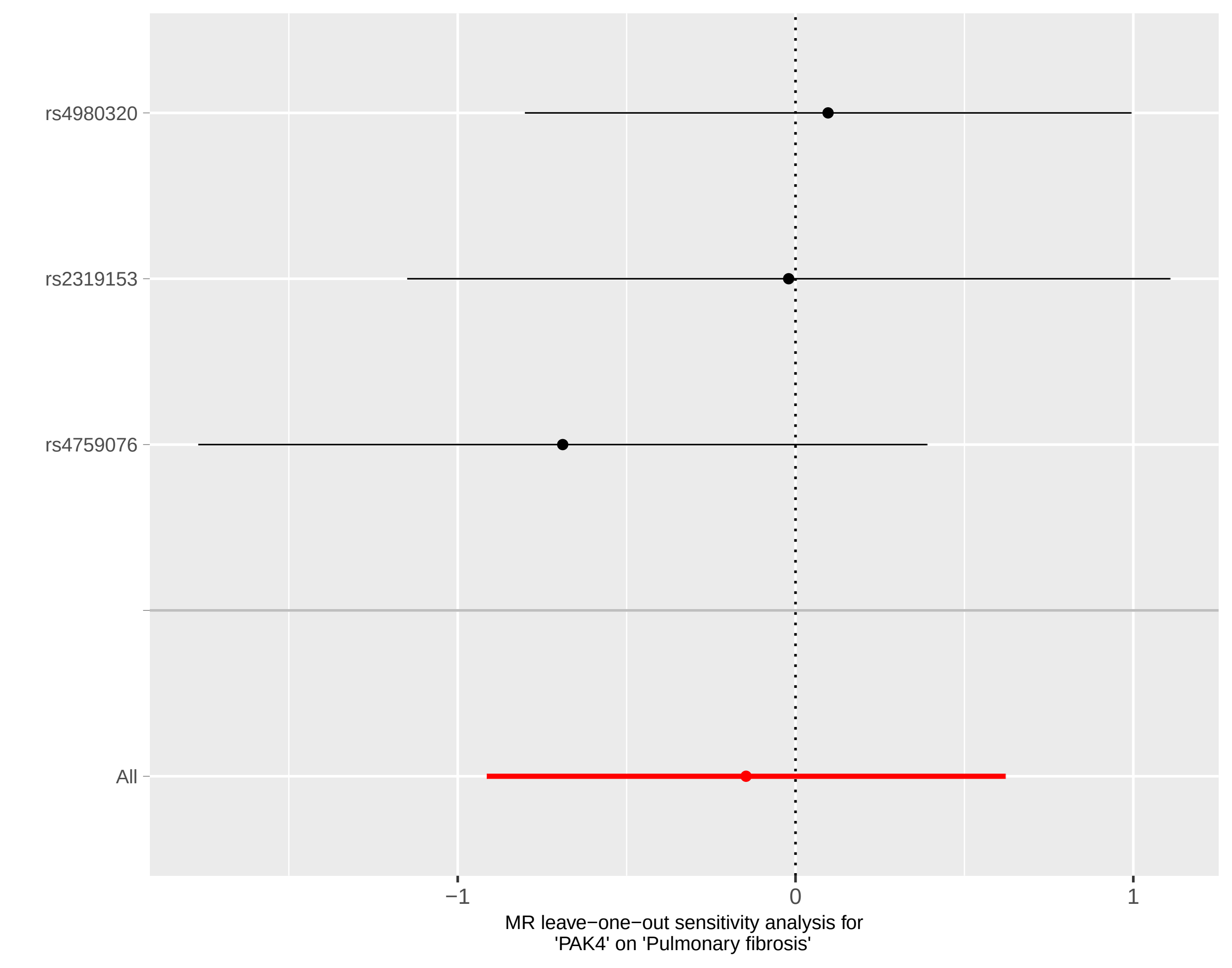
rs4980320
rs2319153
rs4759076
All
−1
0
1
MR leave−one−out sensitivity analysis for
'PAK4' on 'Pulmonary fibrosis'

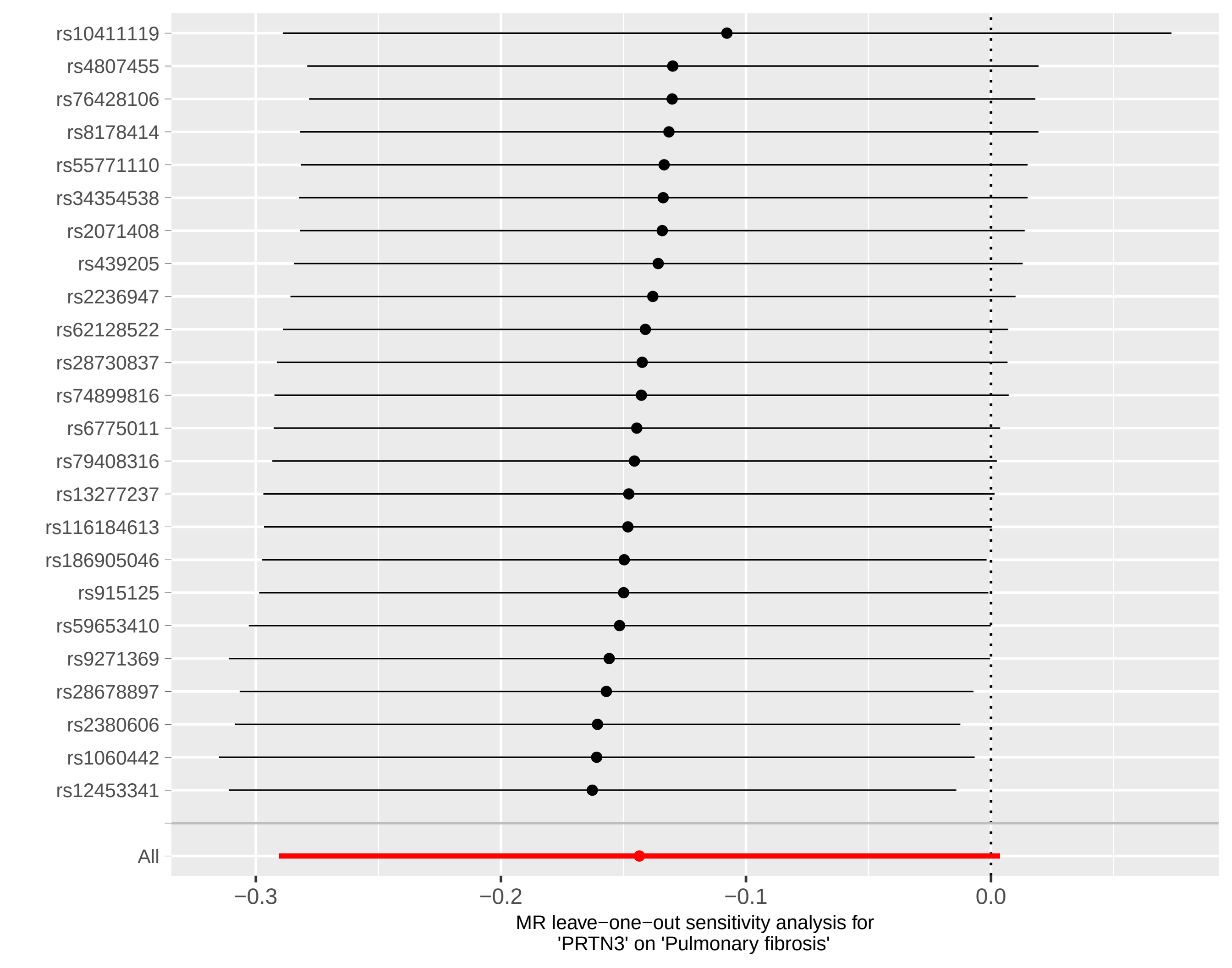
rs10411119
rs4807455
rs76428106
rs8178414
rs55771110
rs34354538
rs2071408
rs439205
rs2236947
rs62128522
rs28730837
rs74899816
rs6775011
rs79408316
rs13277237
rs116184613
rs186905046
rs915125
rs59653410
rs9271369
rs28678897
rs2380606
rs1060442
rs12453341
All
−0.3
−0.2
−0.1
0.0
MR leave−one−out sensitivity analysis for
'PRTN3' on 'Pulmonary fibrosis'

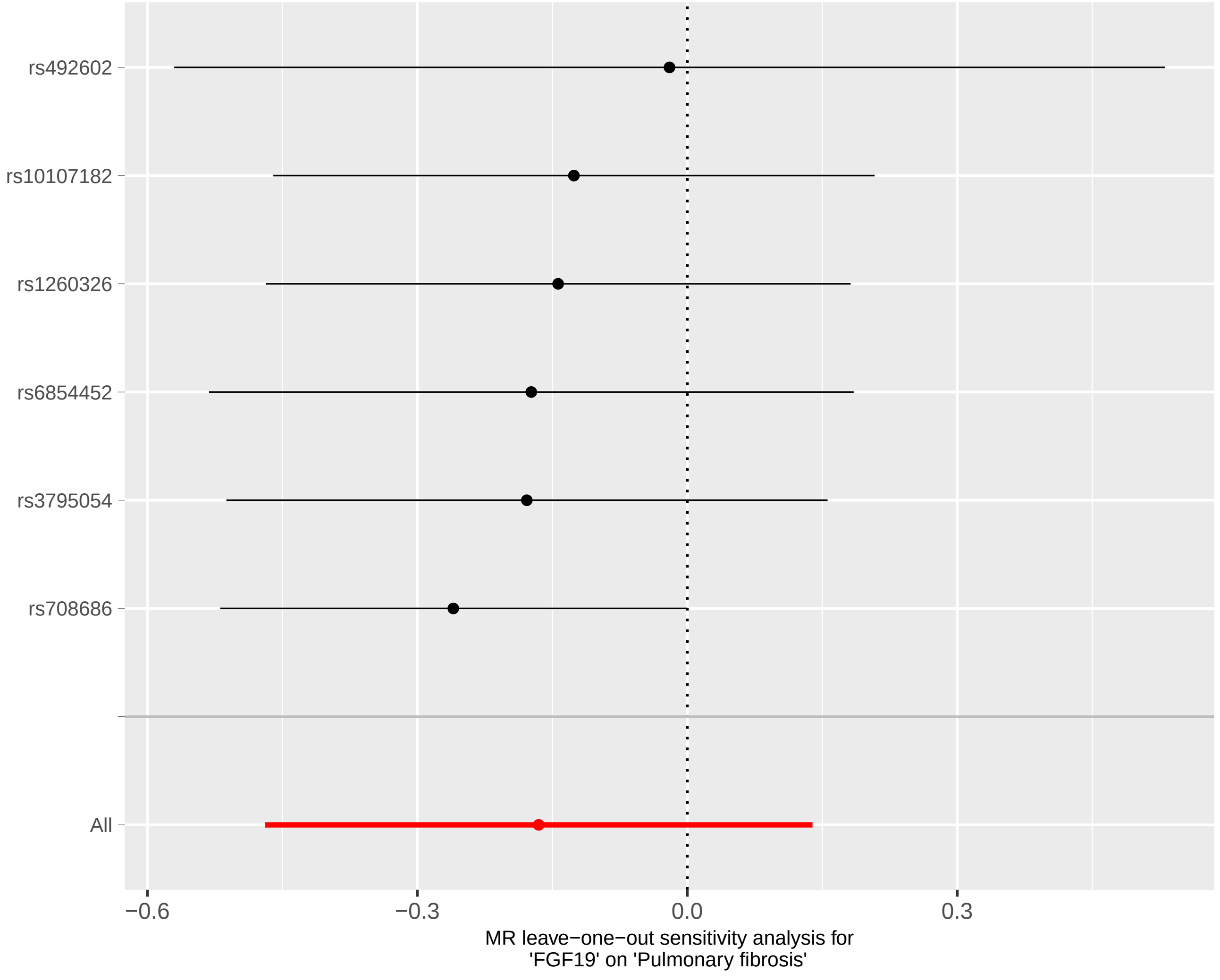
rs492602
rs10107182
rs1260326
rs6854452
rs3795054
rs708686
All
−0.6
−0.3
0.0
0.3
MR leave−one−out sensitivity analysis for
'FGF19' on 'Pulmonary fibrosis'

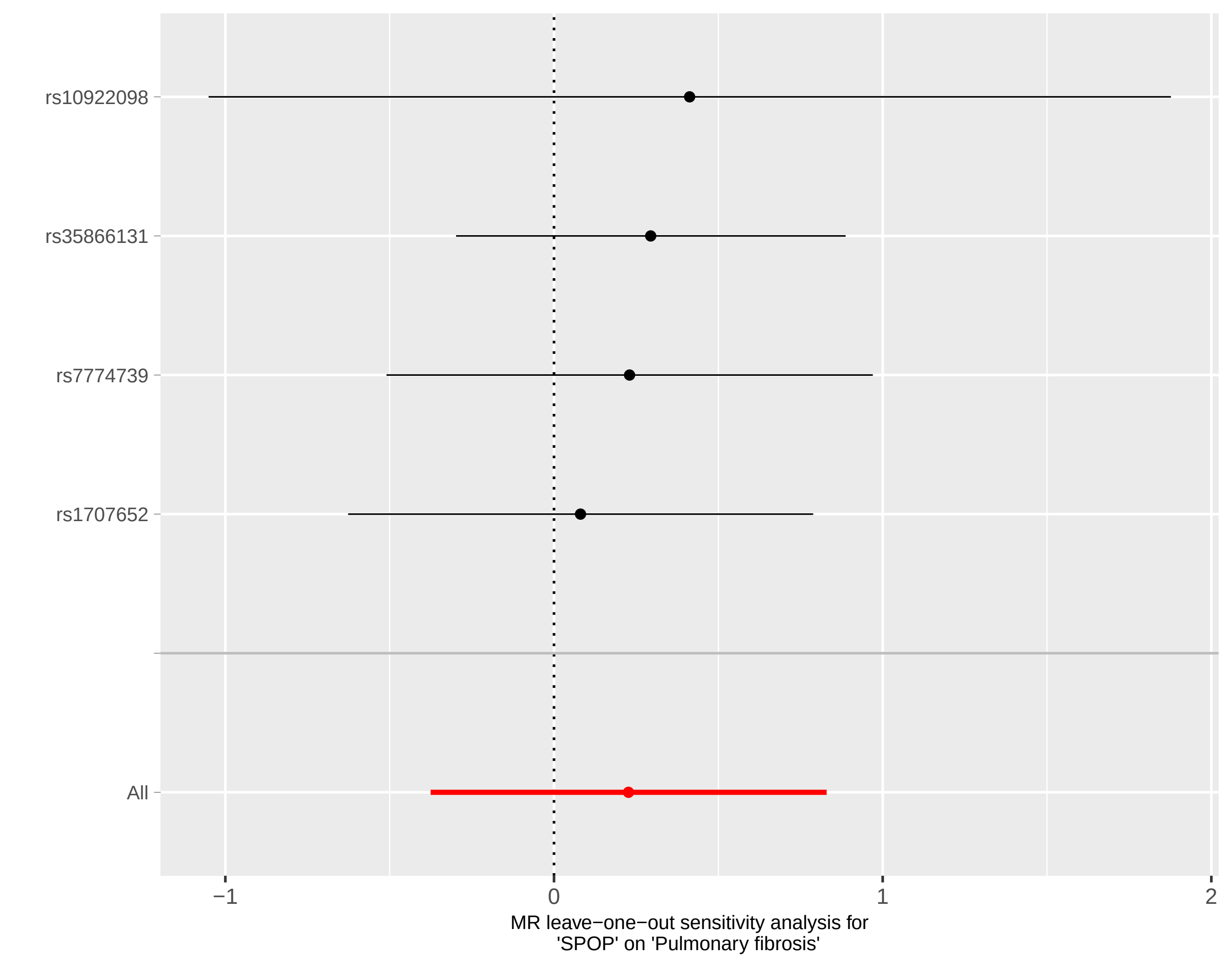
rs10922098
rs35866131
rs7774739
rs1707652
All
−1
0
1
2
MR leave−one−out sensitivity analysis for
'SPOP' on 'Pulmonary fibrosis'

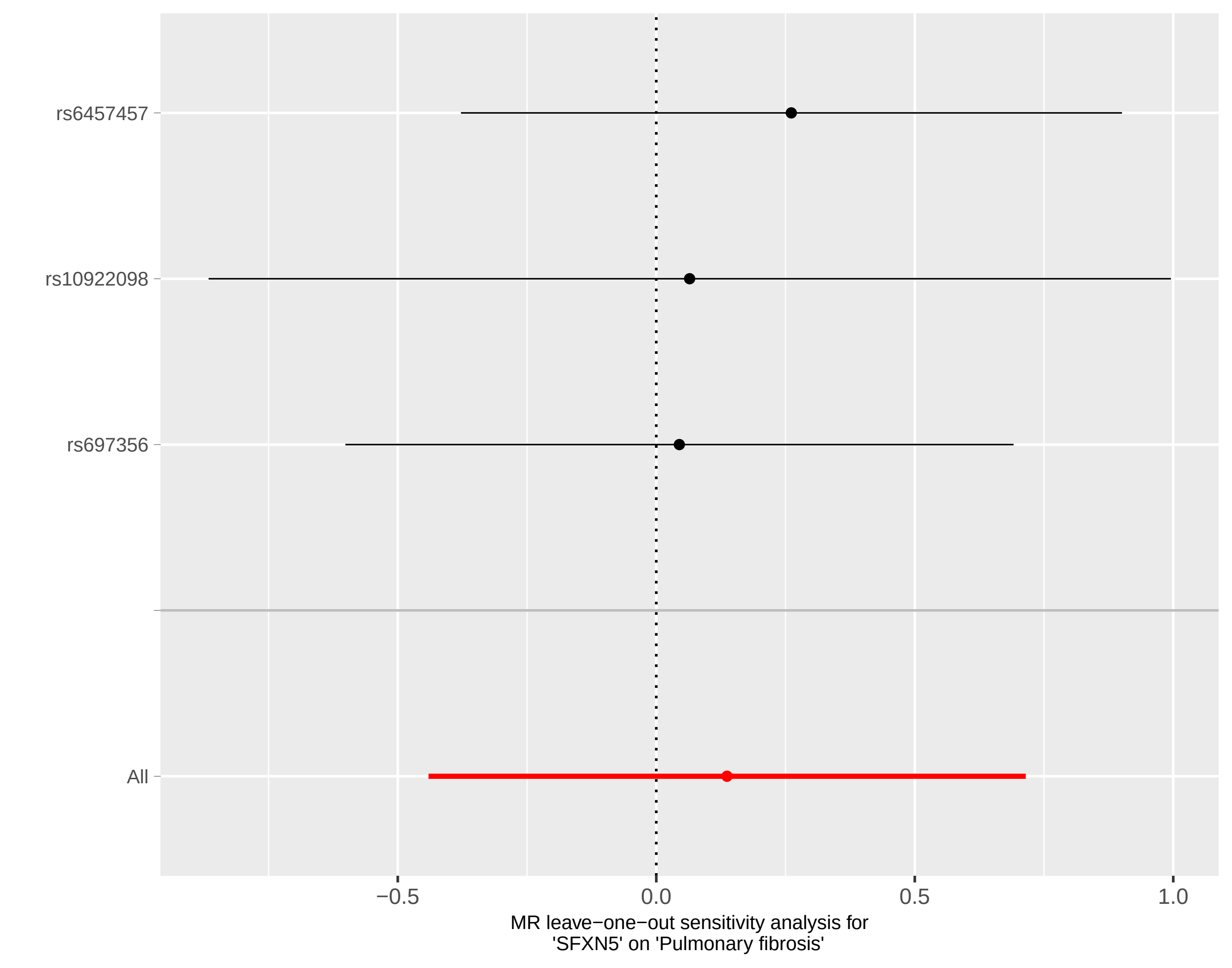
rs6457457
rs10922098
rs697356
All
−0.5
0.0
0.5
1.0
MR leave−one−out sensitivity analysis for
'SFXN5' on 'Pulmonary fibrosis'

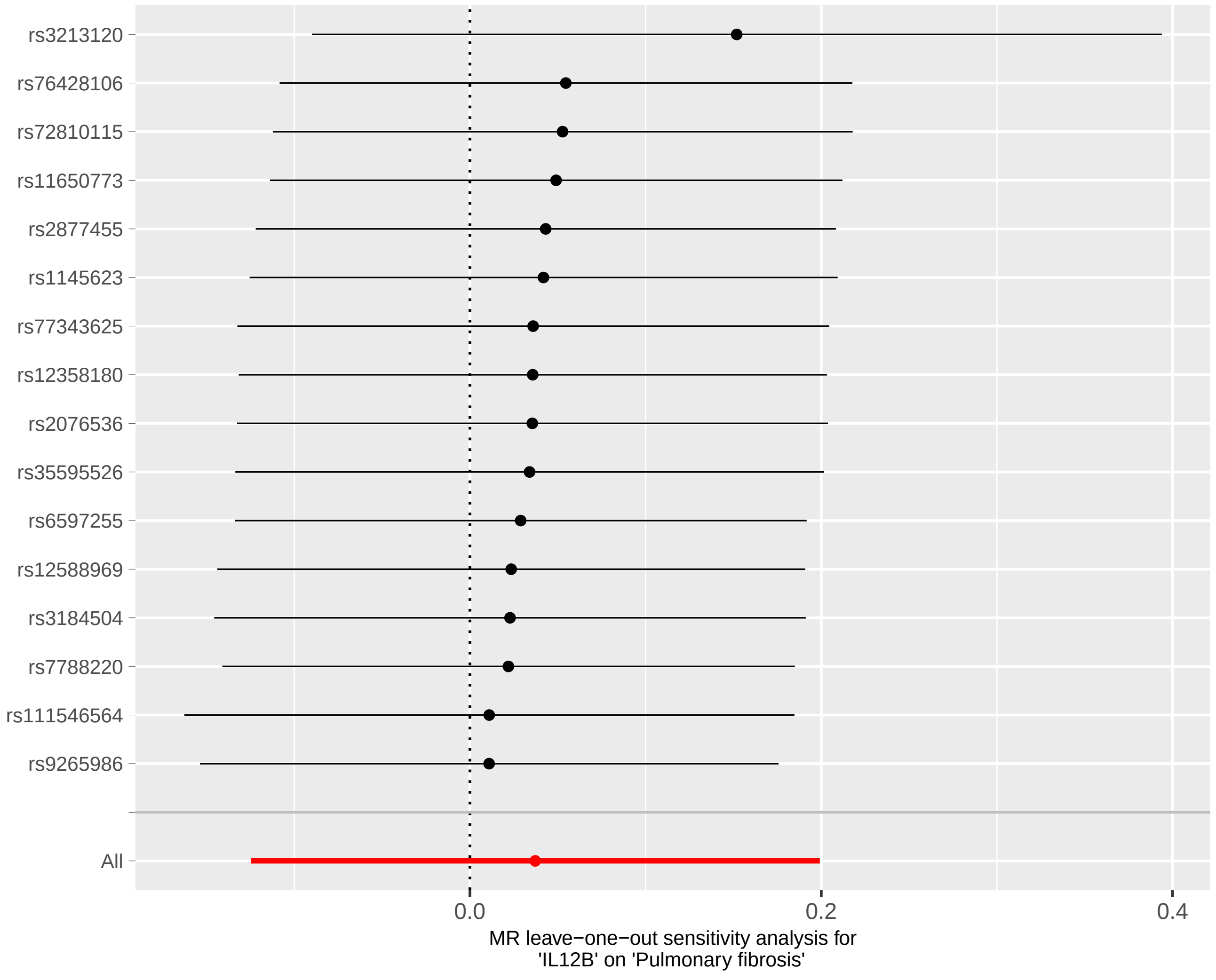

rs3213120
rs76428106
rs72810115
rs11650773
rs2877455
rs1145623
rs77343625
rs12358180
rs2076536
rs35595526
rs6597255
rs12588969
rs3184504
rs7788220
rs111546564
rs9265986
All
0.0
0.2
0.4
MR leave−one−out sensitivity analysis for
'IL12B' on 'Pulmonary fibrosis'

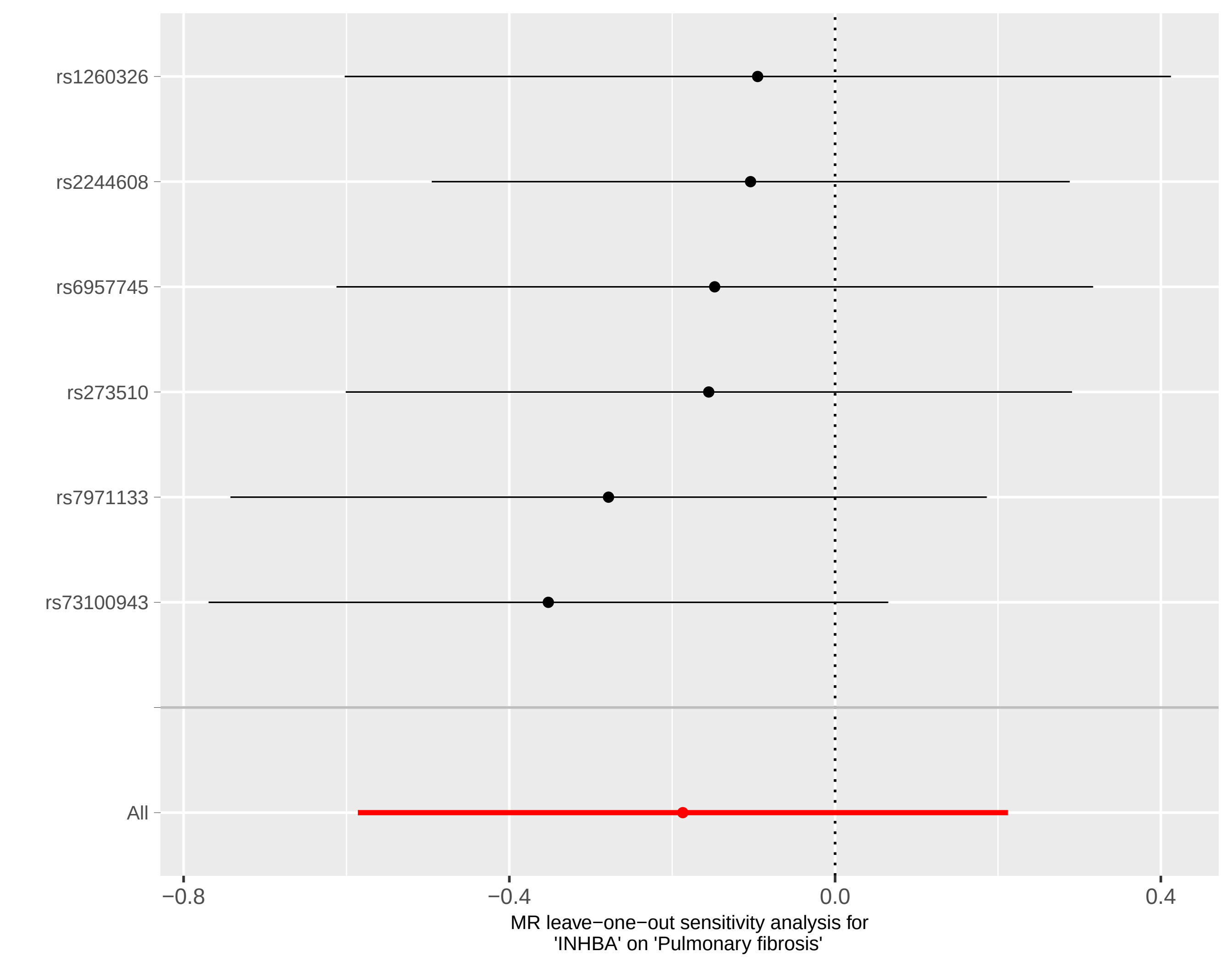
rs1260326
rs2244608
rs6957745
rs273510
rs7971133
rs73100943
All
−0.8
−0.4
0.0
0.4
MR leave−one−out sensitivity analysis for
'INHBA' on 'Pulmonary fibrosis'

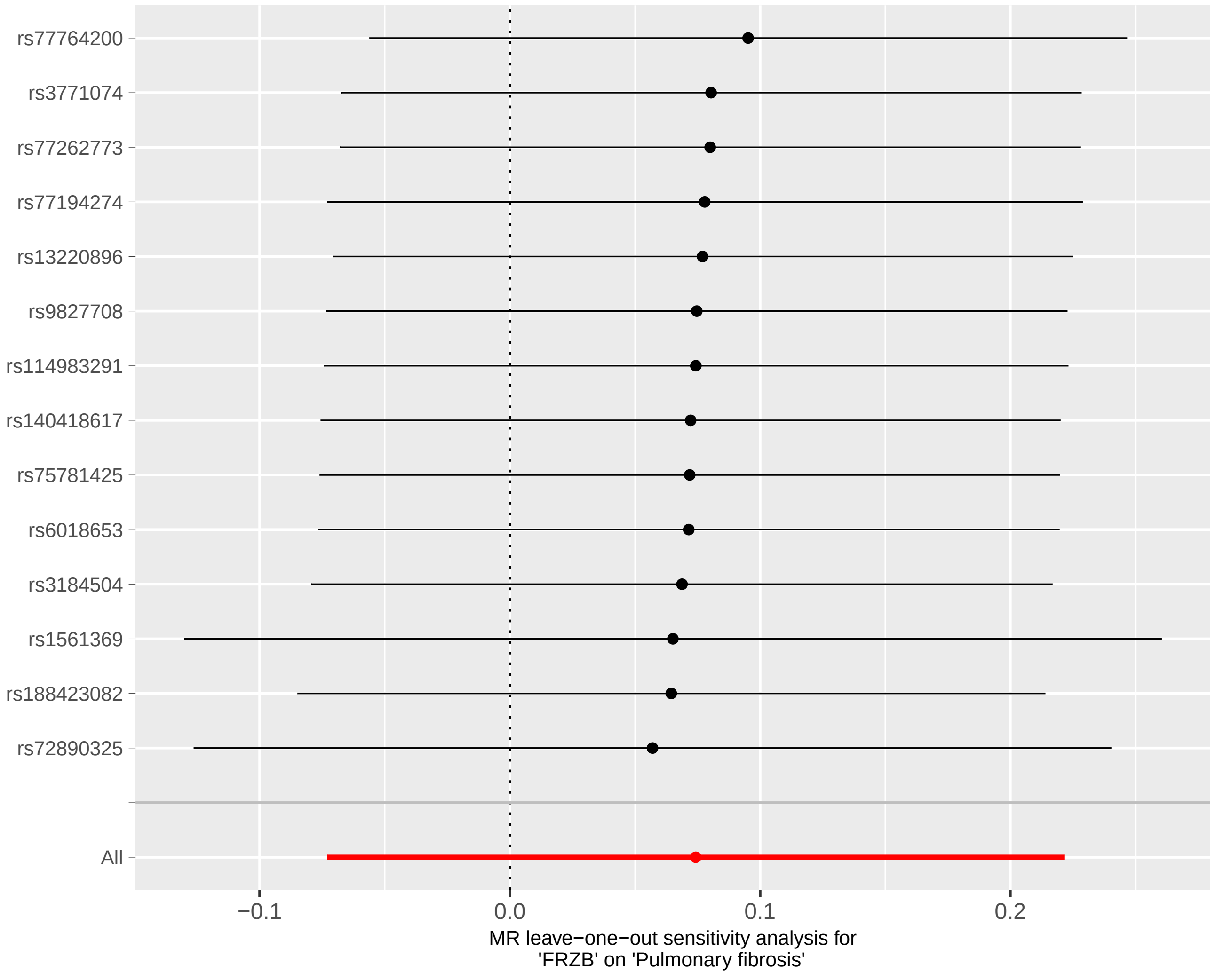
rs77764200
rs3771074
rs77262773
rs77194274
rs13220896
rs9827708
rs114983291
rs140418617
rs75781425
rs6018653
rs3184504
rs1561369
rs188423082
rs72890325
All
−0.1
0.0
0.1
0.2
MR leave−one−out sensitivity analysis for
'FRZB' on 'Pulmonary fibrosis'

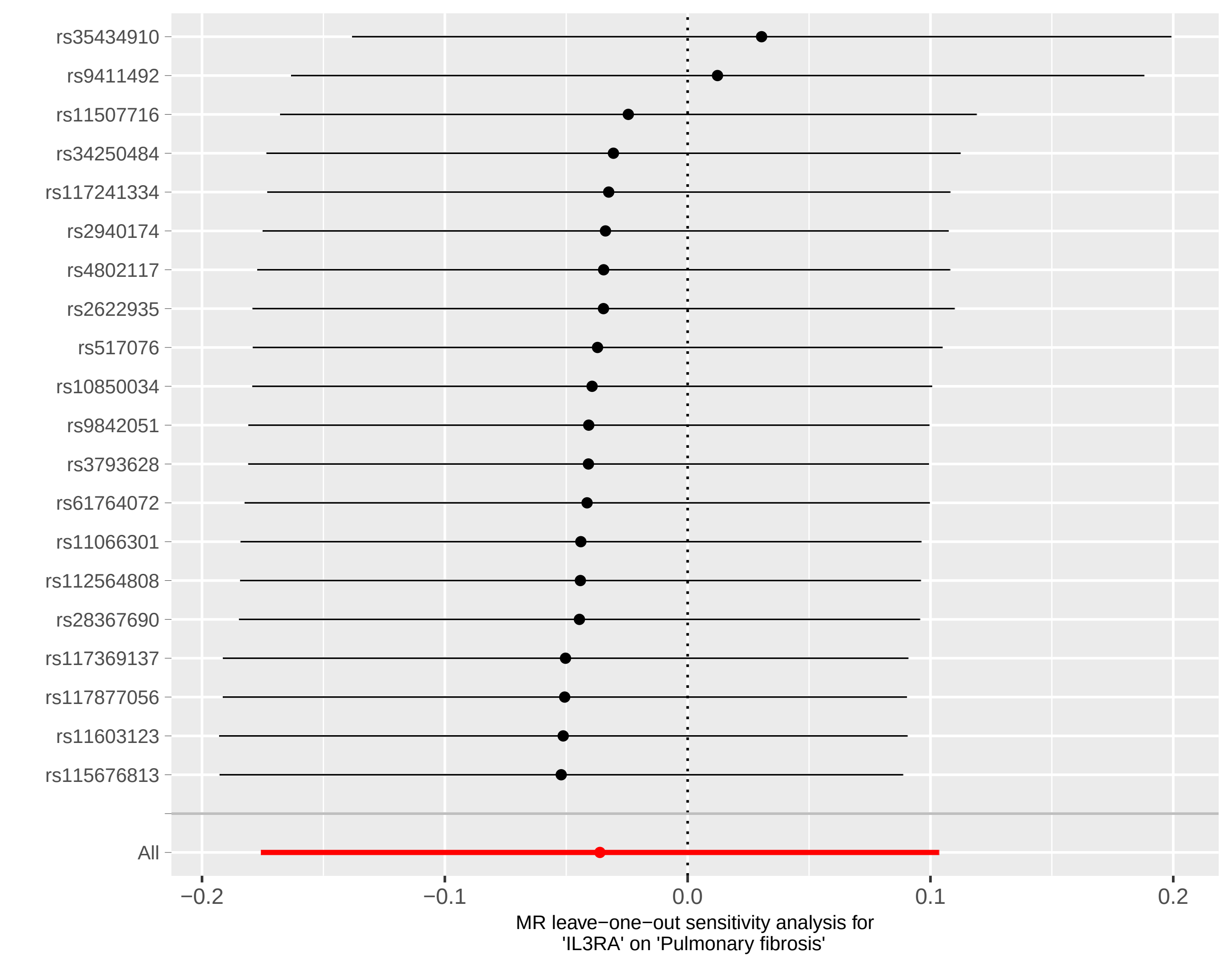

rs35434910
rs9411492
rs11507716
rs34250484
rs117241334
rs2940174
rs4802117
rs2622935
rs517076
rs10850034
rs9842051
rs3793628
rs61764072
rs11066301
rs112564808
rs28367690
rs117369137
rs117877056
rs11603123
rs115676813
All
−0.2
−0.1
0.0
0.1
0.2
MR leave−one−out sensitivity analysis for
'IL3RA' on 'Pulmonary fibrosis'

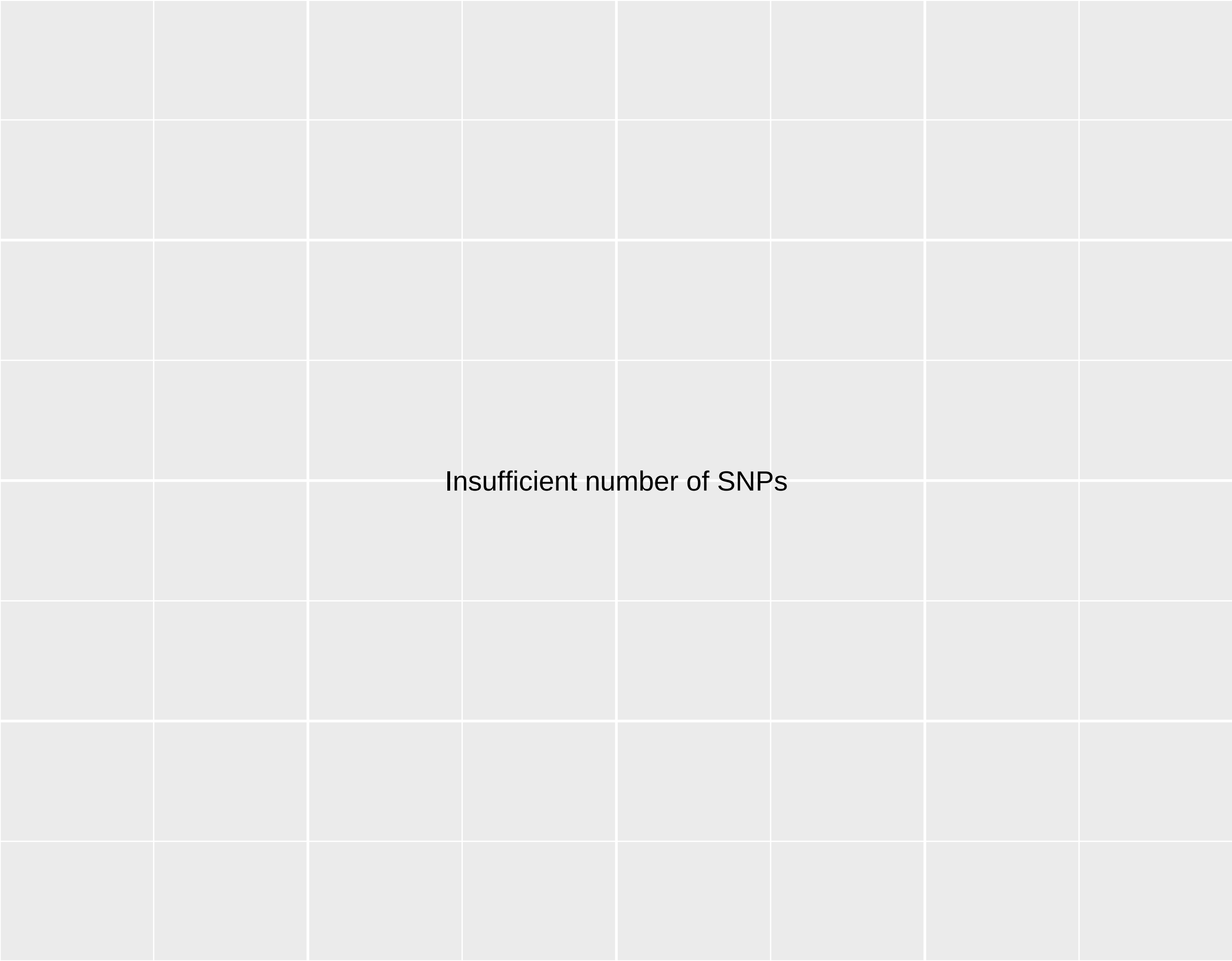
Insufficient number of SNPs

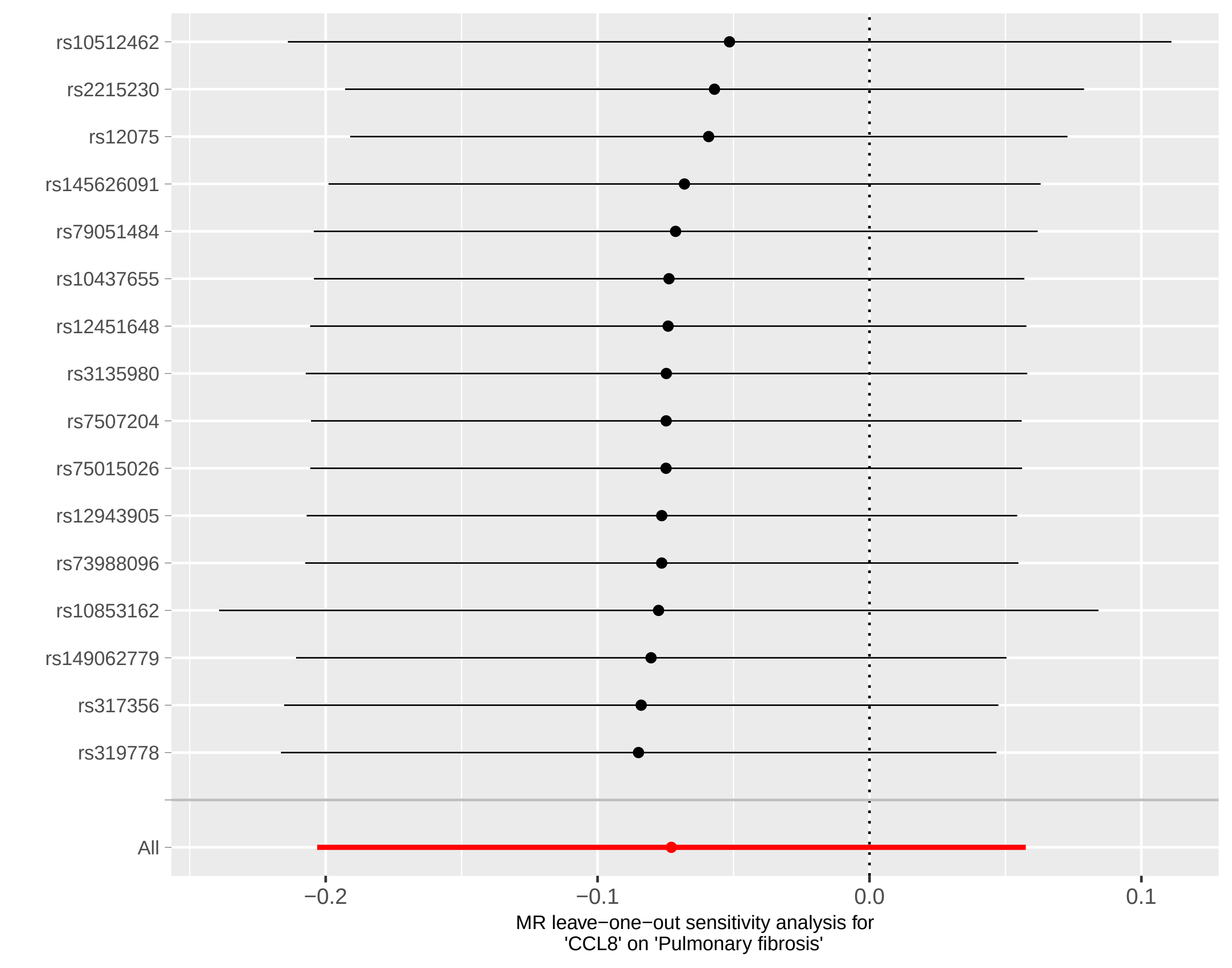
rs10512462
rs2215230
rs12075
rs145626091
rs79051484
rs10437655
rs12451648
rs3135980
rs7507204
rs75015026
rs12943905
rs73988096
rs10853162
rs149062779
rs317356
rs319778
All
−0.2
−0.1
0.0
0.1
MR leave−one−out sensitivity analysis for
'CCL8' on 'Pulmonary fibrosis'

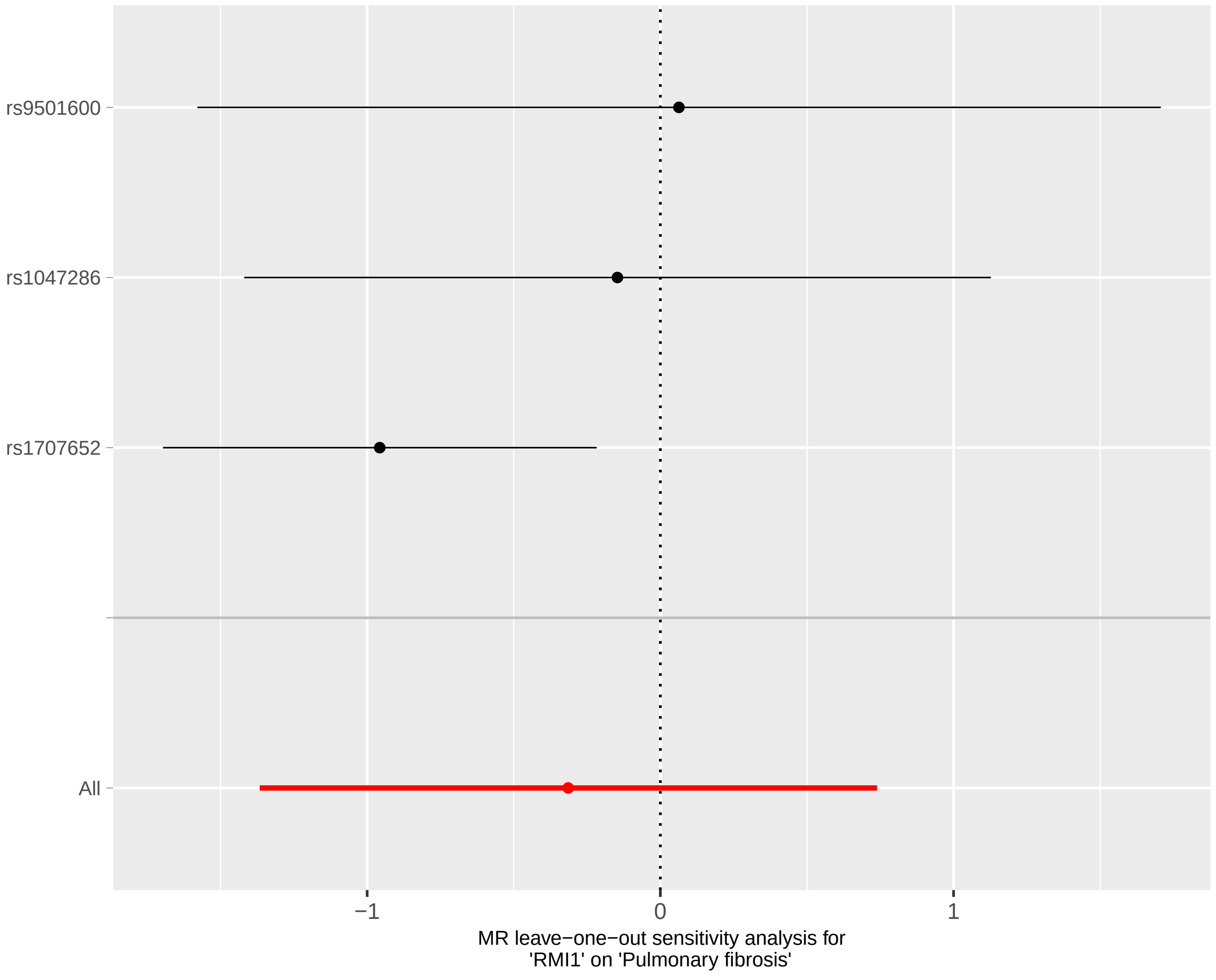

rs9501600
rs1047286
rs1707652
All
−1
0
1
MR leave−one−out sensitivity analysis for
'RMI1' on 'Pulmonary fibrosis'

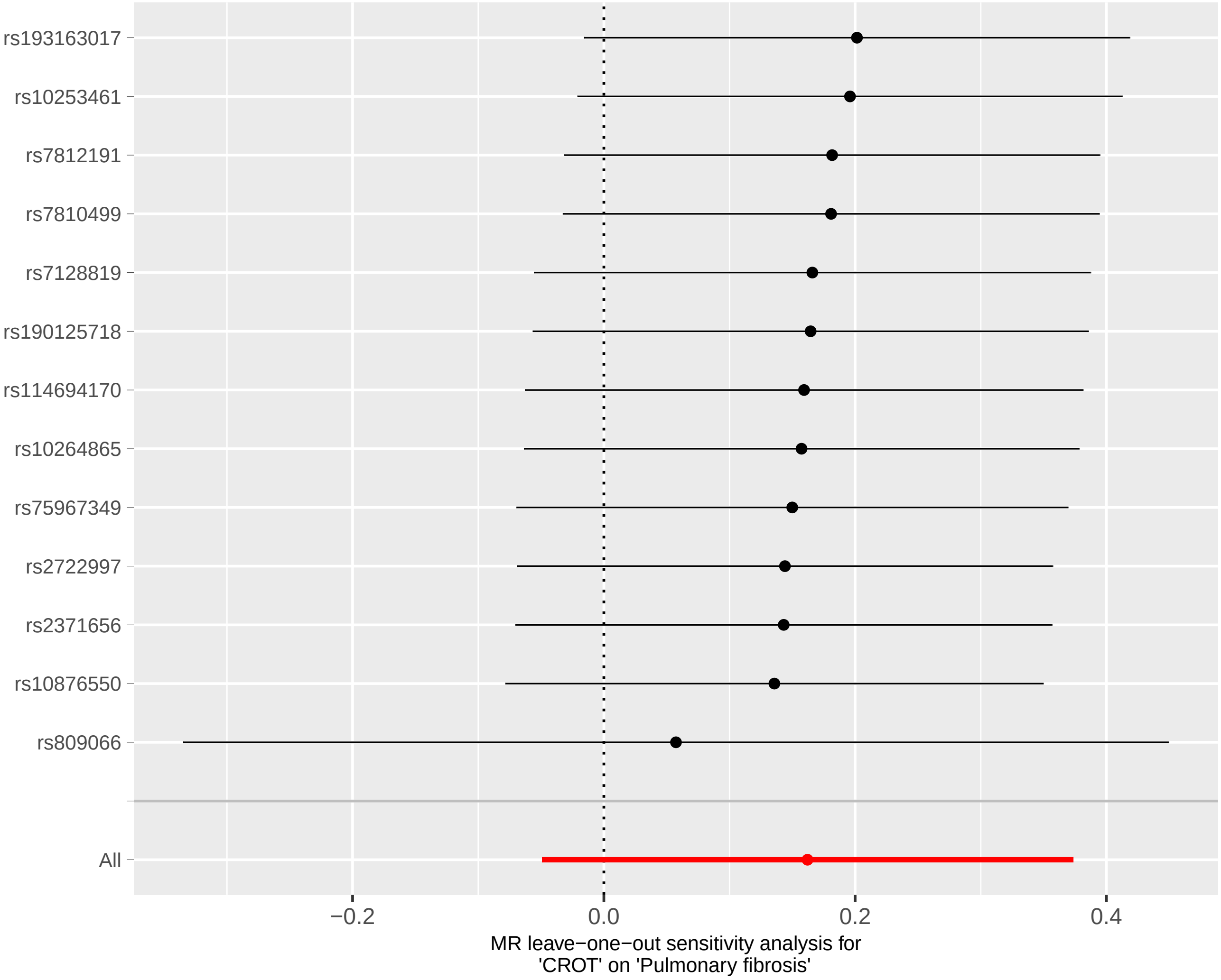

rs193163017
rs10253461
rs7812191
rs7810499
rs7128819
rs190125718
rs114694170
rs10264865
rs75967349
rs2722997
rs2371656
rs10876550
rs809066
All
−0.2
0.0
0.2
0.4
MR leave−one−out sensitivity analysis for
'CROT' on 'Pulmonary fibrosis'

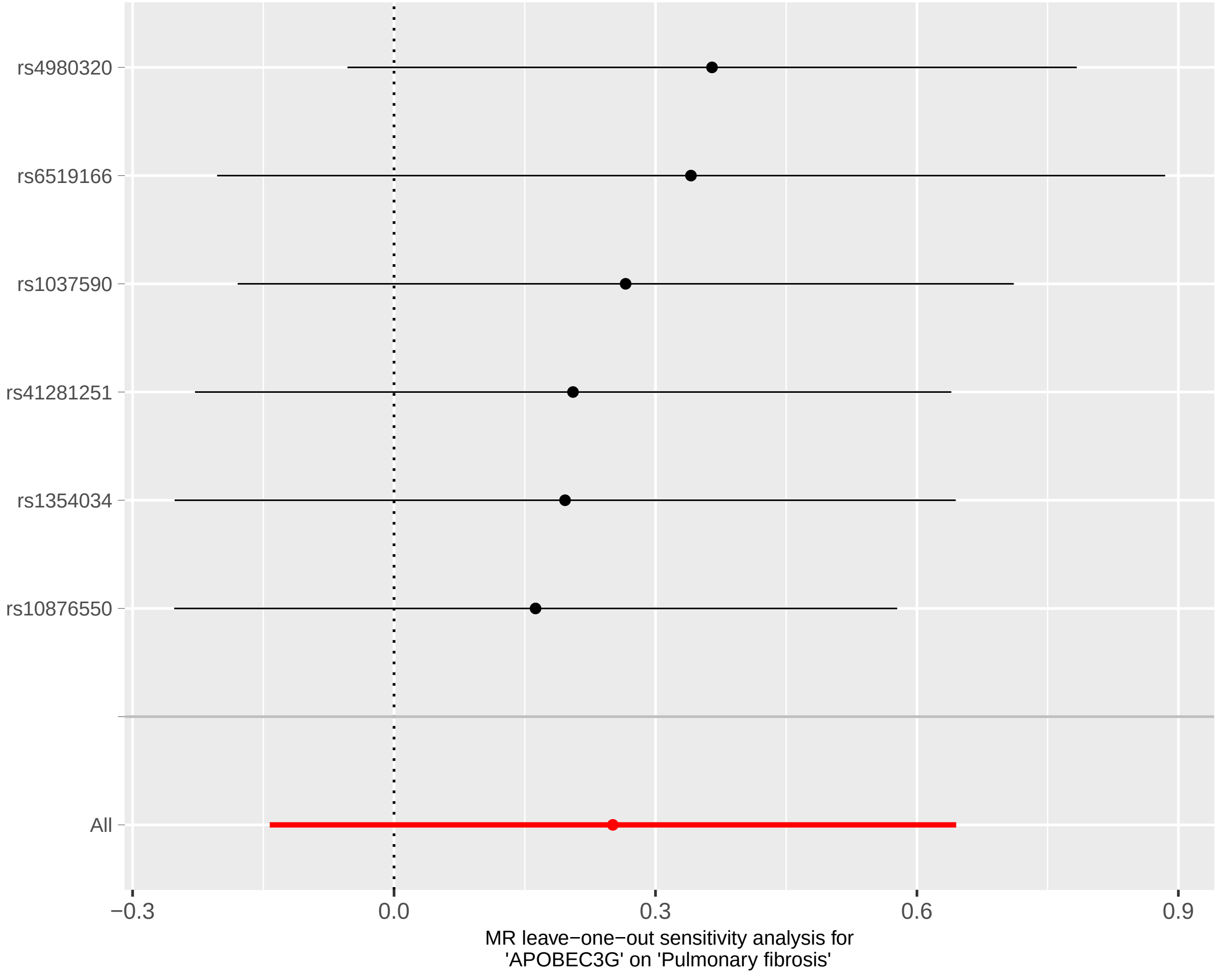

rs4980320
rs6519166
rs1037590
rs41281251
rs1354034
rs10876550
All
−0.3
0.0
0.3
0.6
0.9
MR leave−one−out sensitivity analysis for
'APOBEC3G' on 'Pulmonary fibrosis'

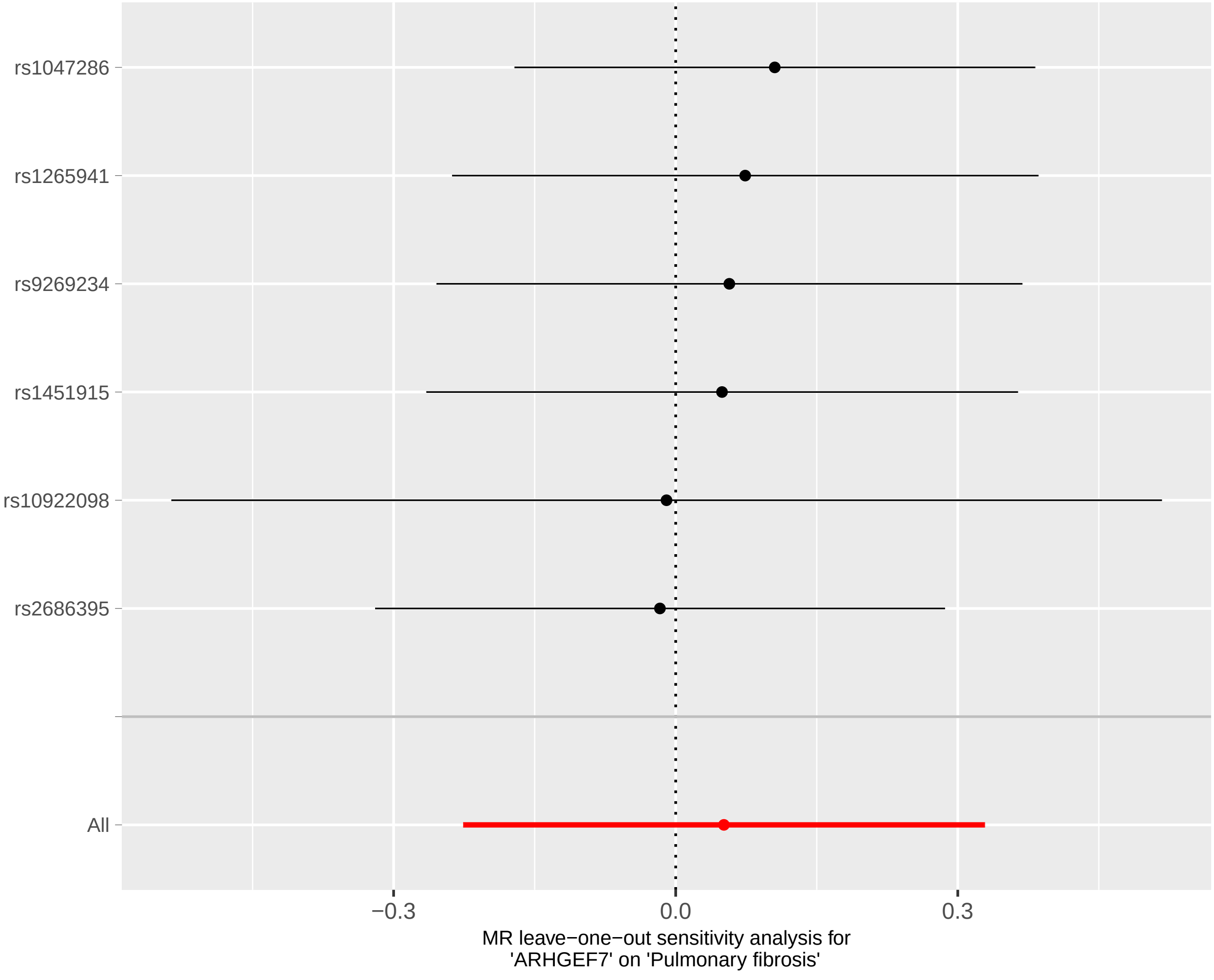

rs1047286
rs1265941
rs9269234
rs1451915
rs10922098
rs2686395
All
−0.3
0.0
0.3
MR leave−one−out sensitivity analysis for
'ARHGEF7' on 'Pulmonary fibrosis'

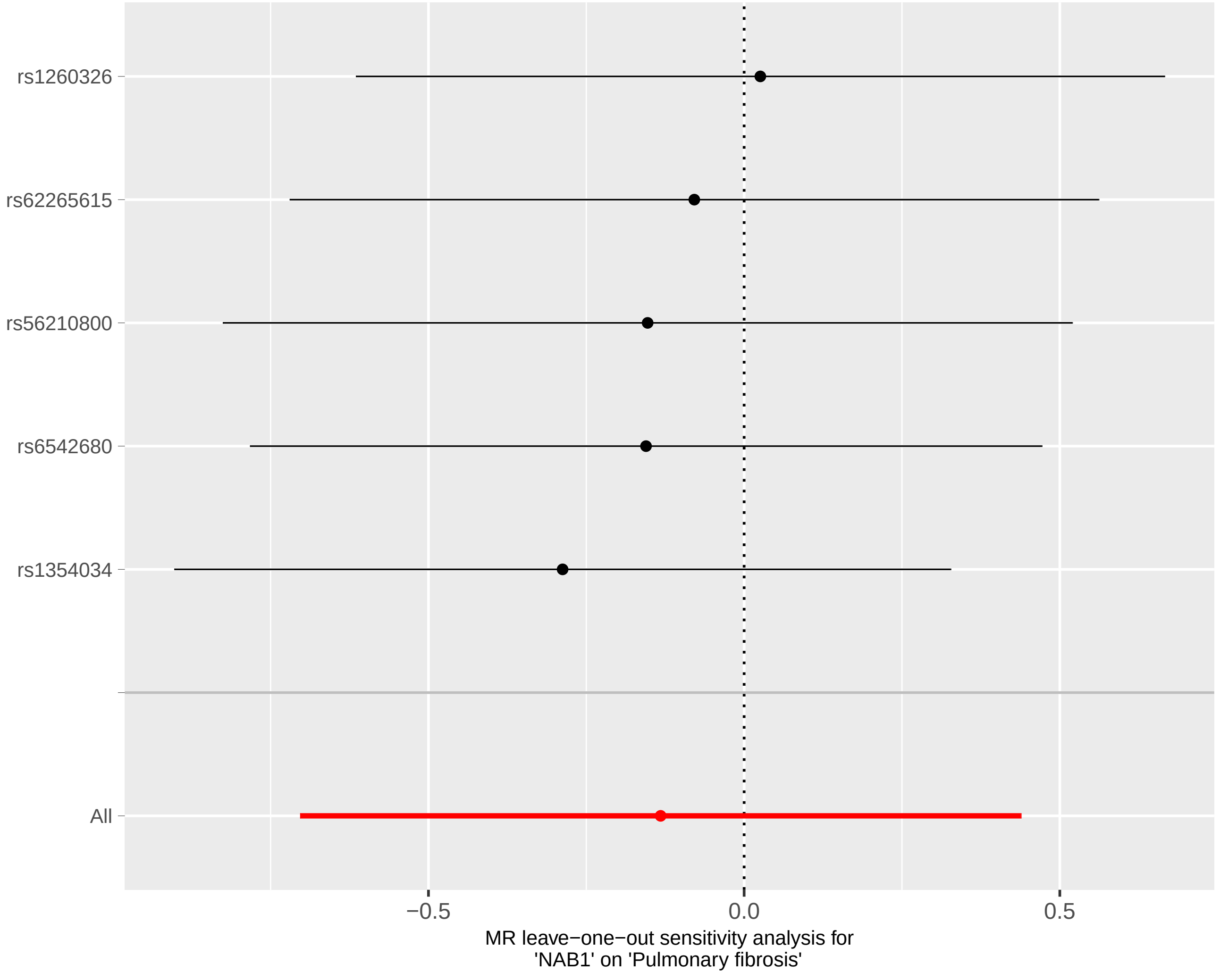
rs1260326
rs62265615
rs56210800
rs6542680
rs1354034
All
−0.5
0.0
0.5
MR leave−one−out sensitivity analysis for
'NAB1' on 'Pulmonary fibrosis'

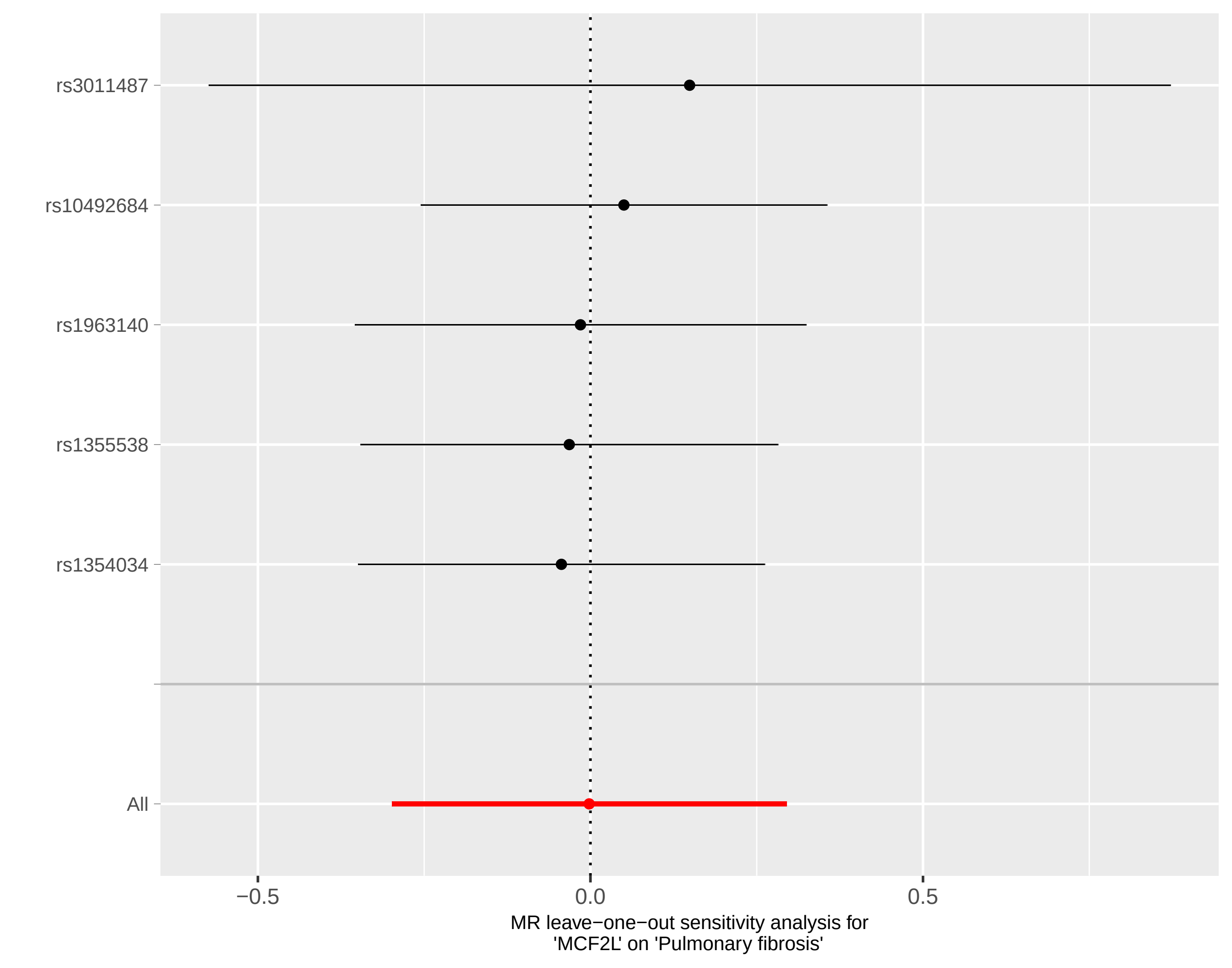

rs3011487
rs10492684
rs1963140
rs1355538
rs1354034
All
−0.5
0.0
0.5
MR leave−one−out sensitivity analysis for
'MCF2L' on 'Pulmonary fibrosis'

Insufficient number of SNPs

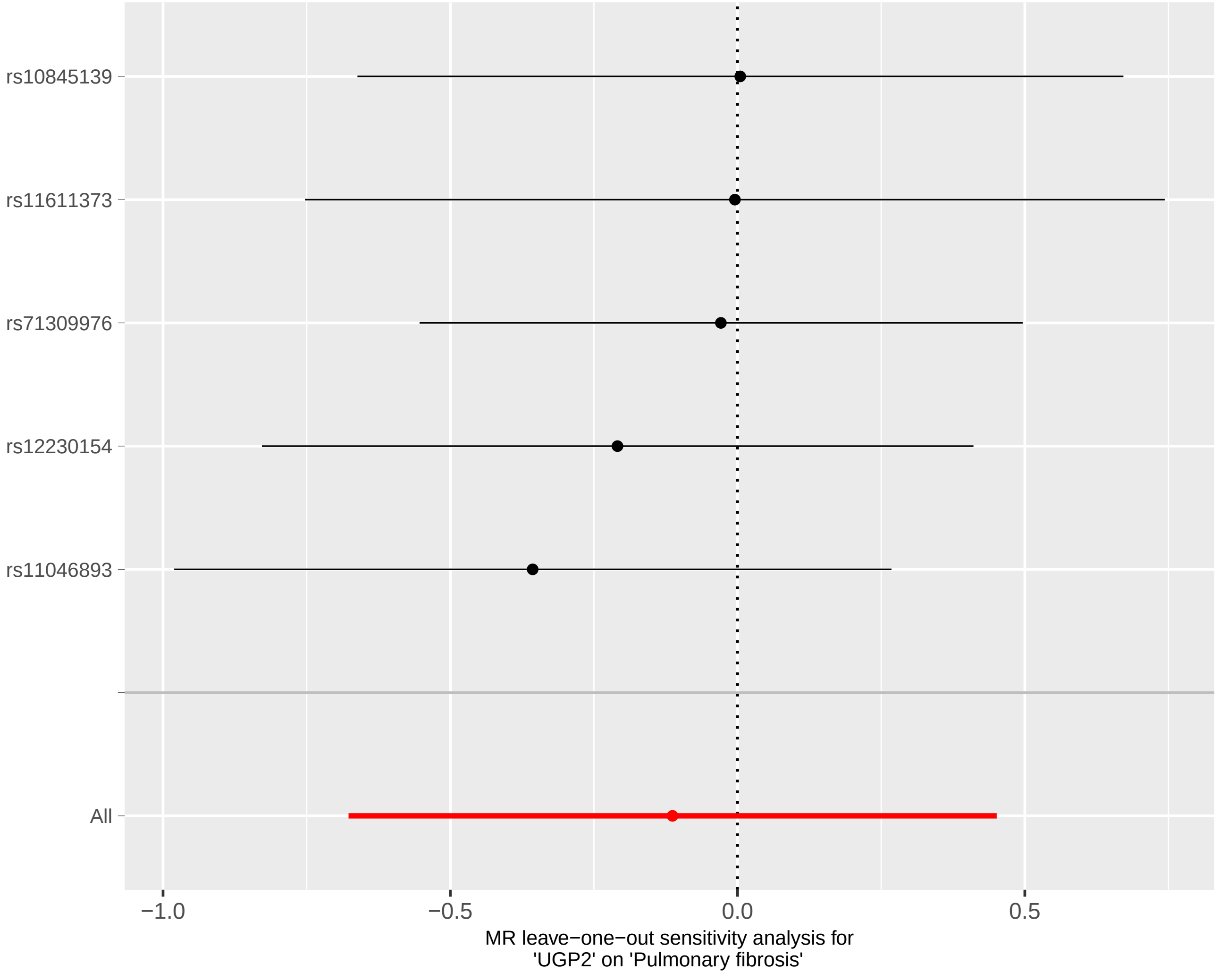

rs10845139
rs11611373
rs71309976
rs12230154
rs11046893
All
−1.0
−0.5
0.0
0.5
MR leave−one−out sensitivity analysis for
'UGP2' on 'Pulmonary fibrosis'

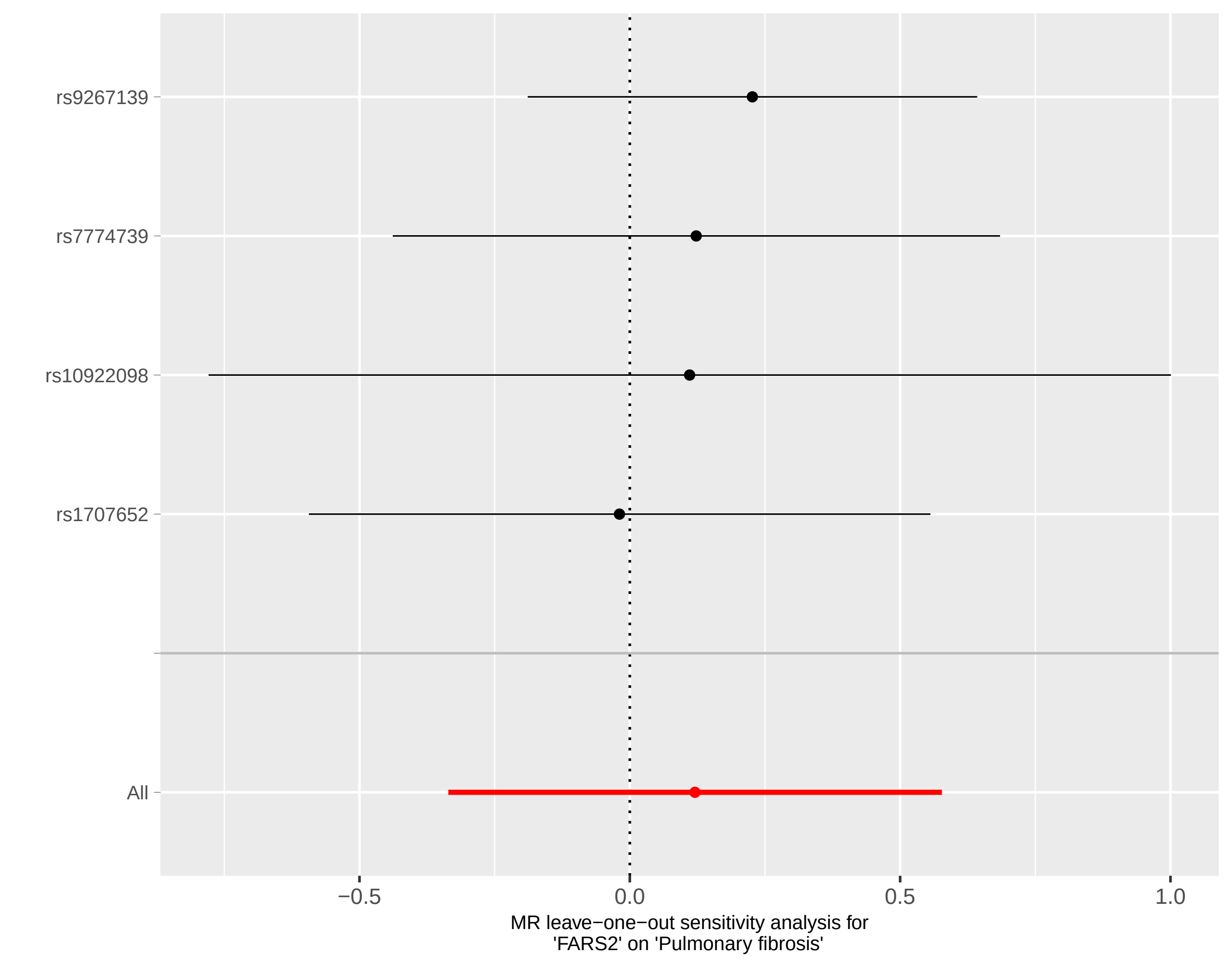
rs9267139
rs7774739
rs10922098
rs1707652
All
−0.5
0.0
0.5
1.0
MR leave−one−out sensitivity analysis for
'FARS2' on 'Pulmonary fibrosis'

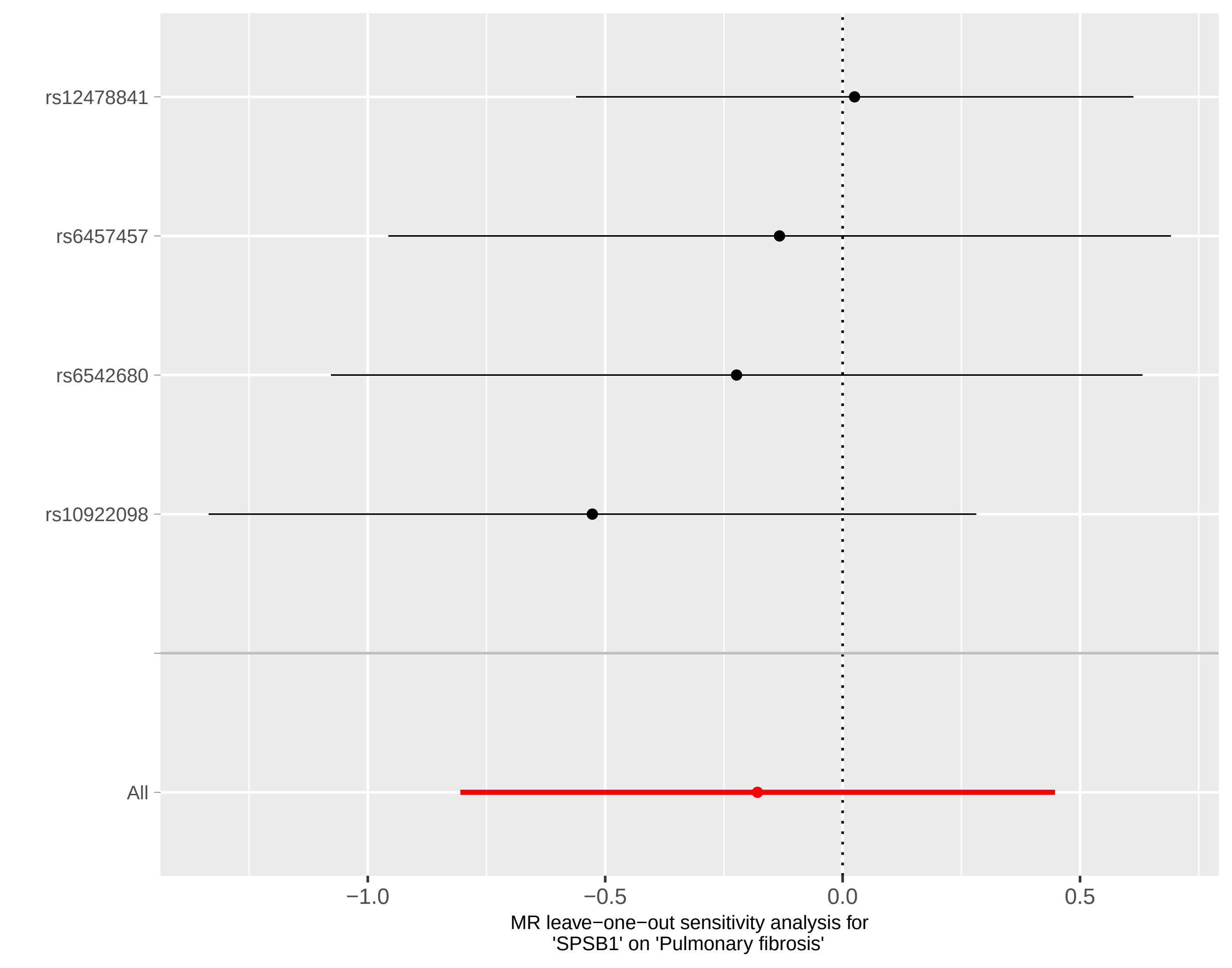
rs12478841
rs6457457
rs6542680
rs10922098
All
−1.0
−0.5
0.0
0.5
MR leave−one−out sensitivity analysis for
'SPSB1' on 'Pulmonary fibrosis'

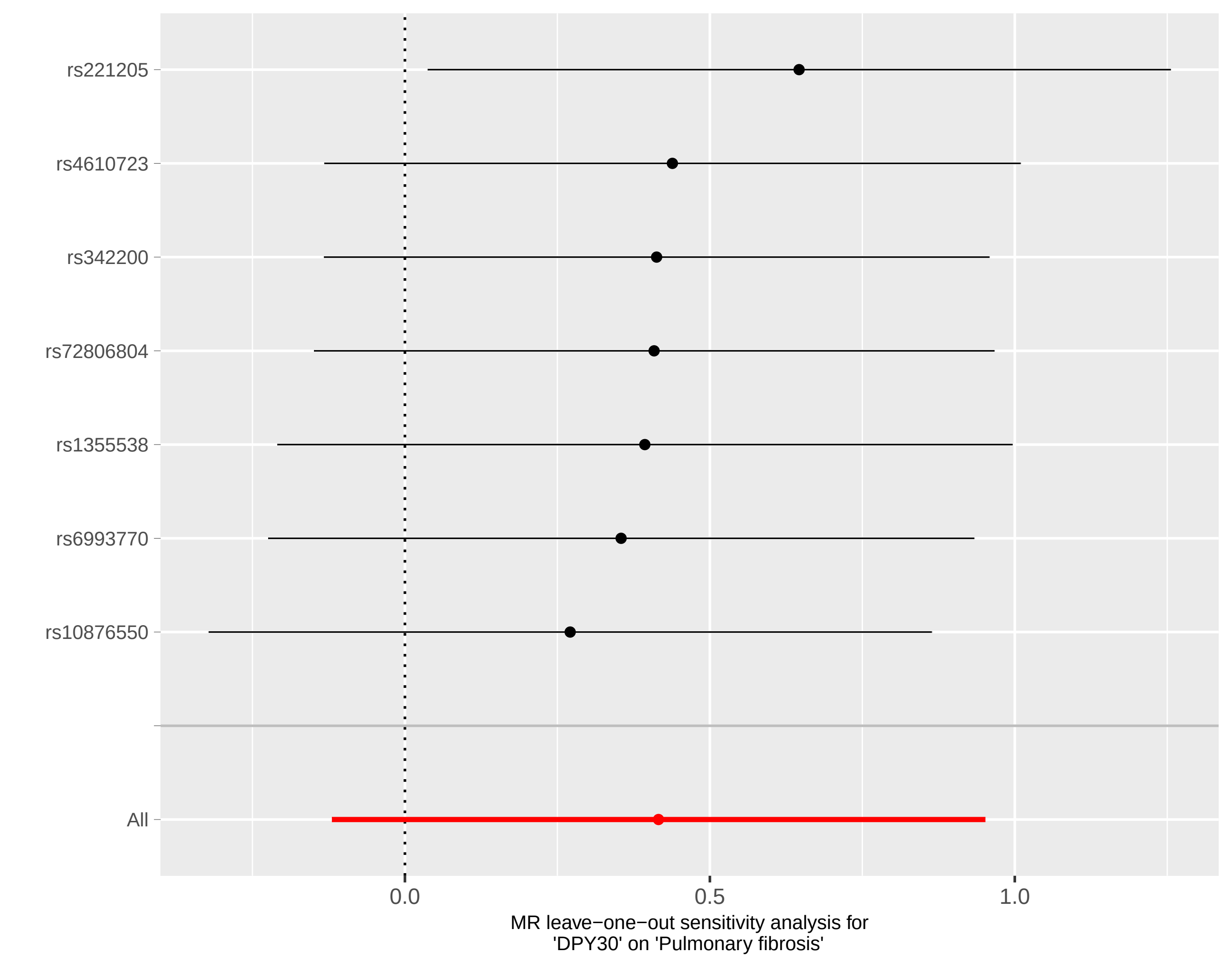
rs221205
rs4610723
rs342200
rs72806804
rs1355538
rs6993770
rs10876550
All
0.0
0.5
1.0
MR leave−one−out sensitivity analysis for
'DPY30' on 'Pulmonary fibrosis'

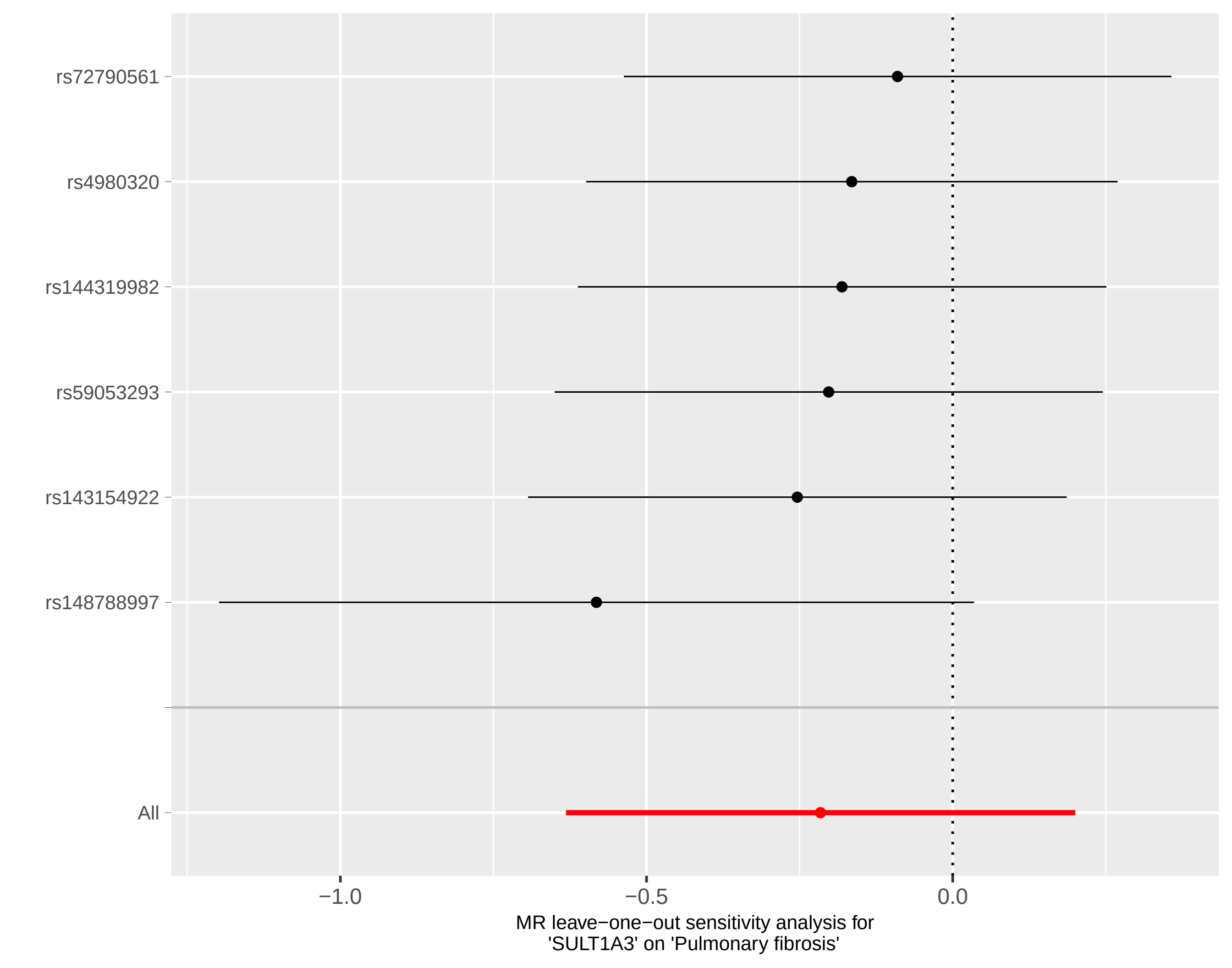
rs72790561
rs4980320
rs144319982
rs59053293
rs143154922
rs148788997
All
−1.0
−0.5
0.0
MR leave−one−out sensitivity analysis for
'SULT1A3' on 'Pulmonary fibrosis'

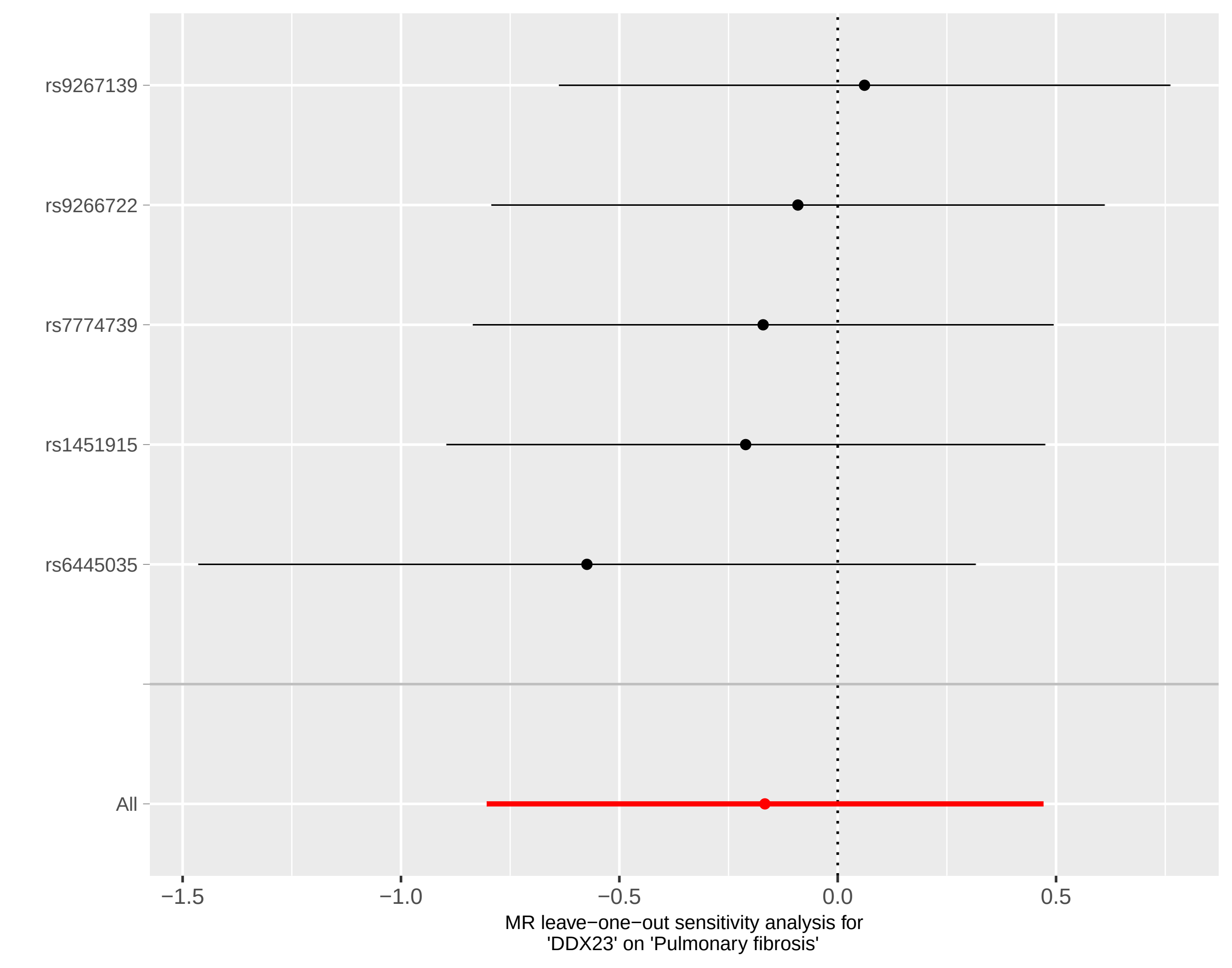

rs9267139
rs9266722
rs7774739
rs1451915
rs6445035
All
−1.5
−1.0
−0.5
0.0
0.5
MR leave−one−out sensitivity analysis for
'DDX23' on 'Pulmonary fibrosis'

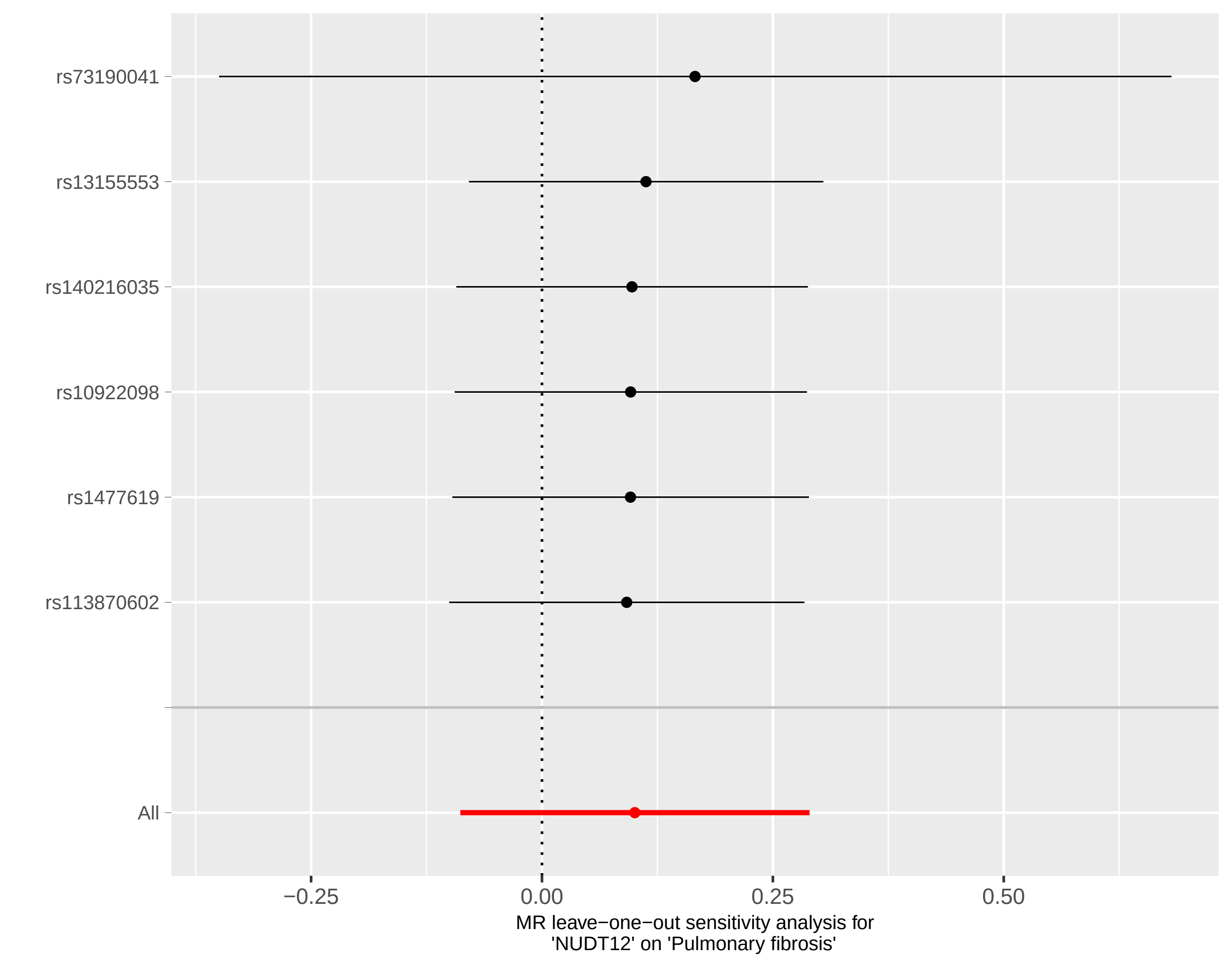

rs73190041
rs13155553
rs140216035
rs10922098
rs1477619
rs113870602
All
−0.25
0.00
0.25
0.50
MR leave−one−out sensitivity analysis for
'NUDT12' on 'Pulmonary fibrosis'

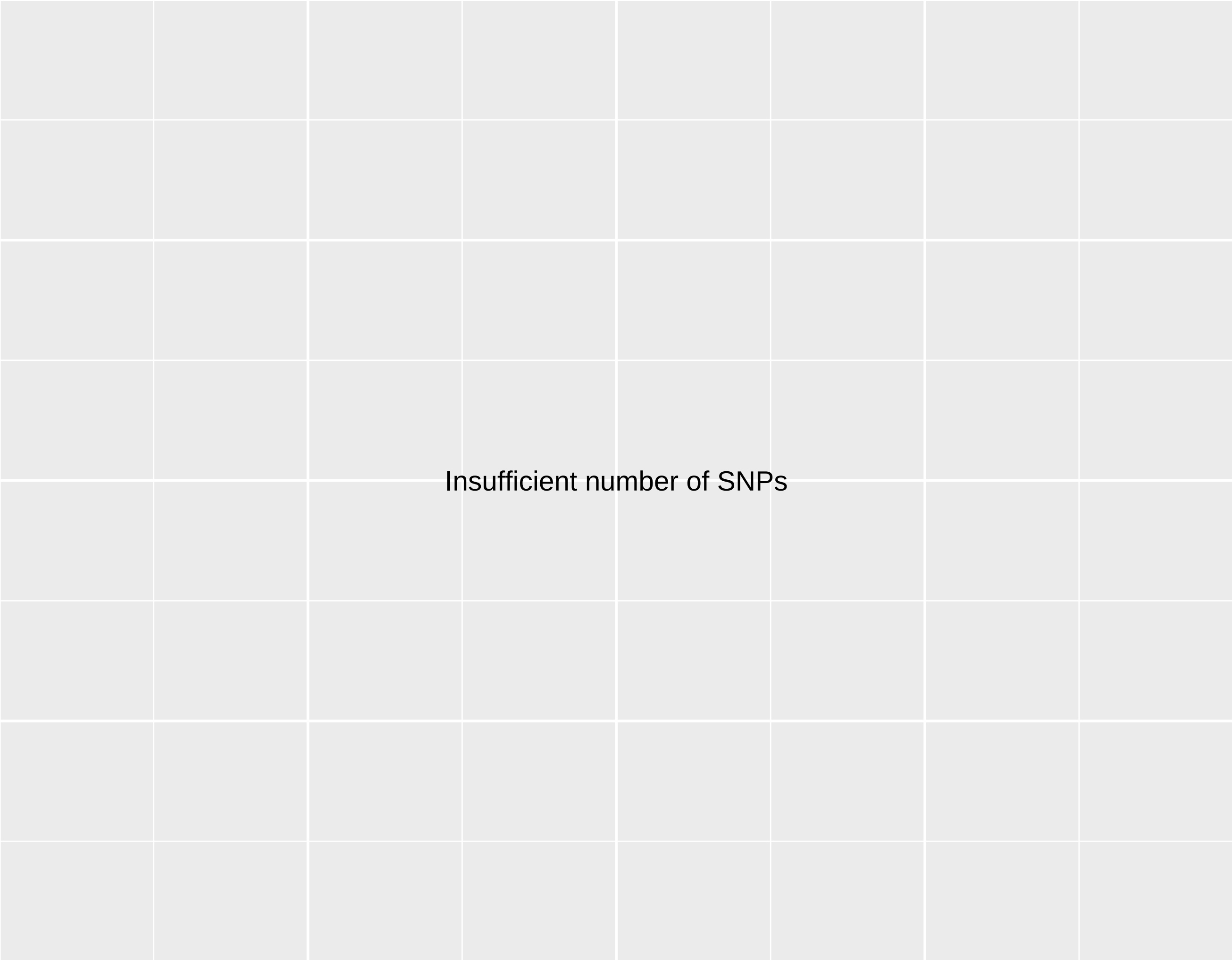
Insufficient number of SNPs

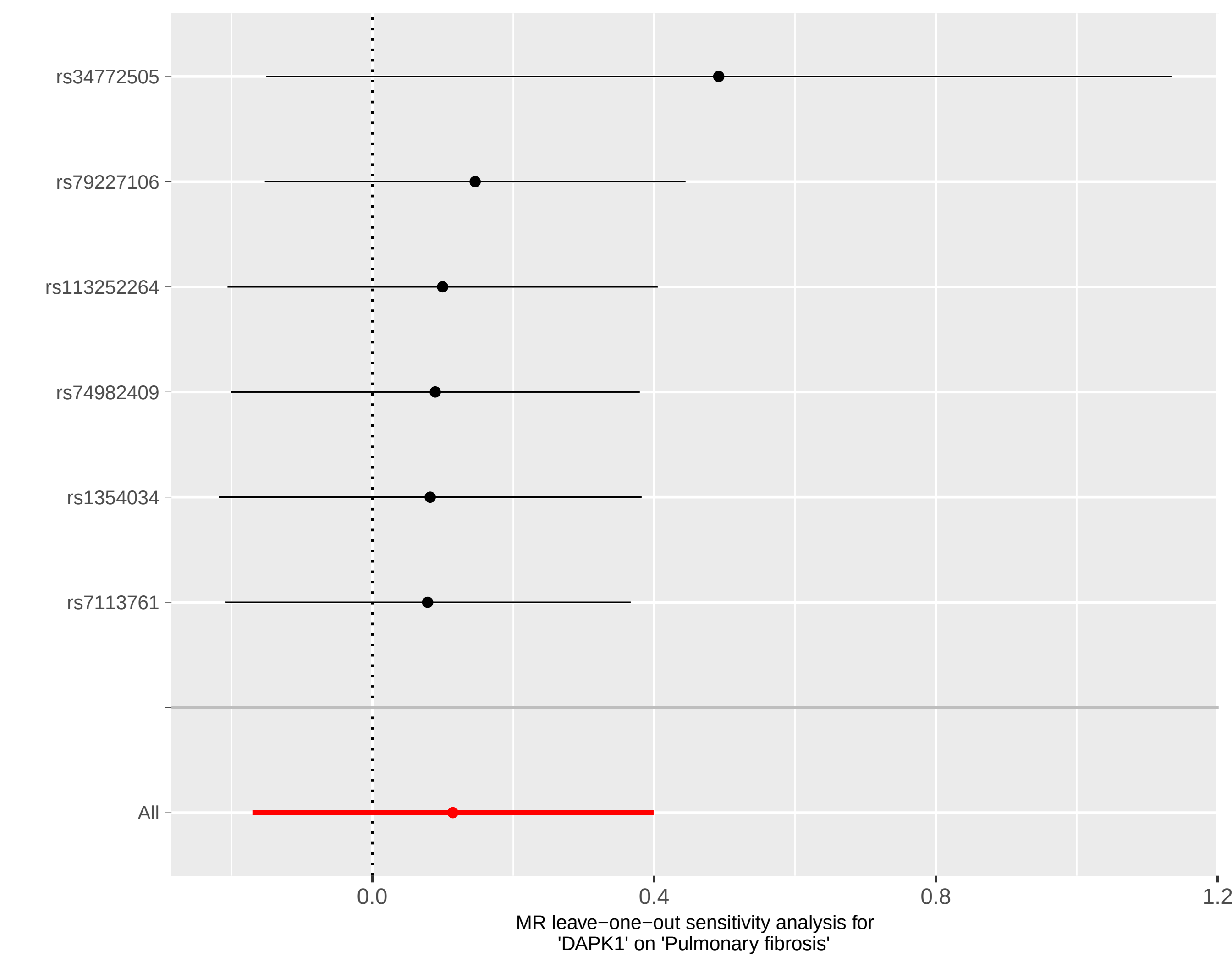
rs34772505
rs79227106
rs113252264
rs74982409
rs1354034
rs7113761
All
0.0
0.4
0.8
1.2
MR leave−one−out sensitivity analysis for
'DAPK1' on 'Pulmonary fibrosis'

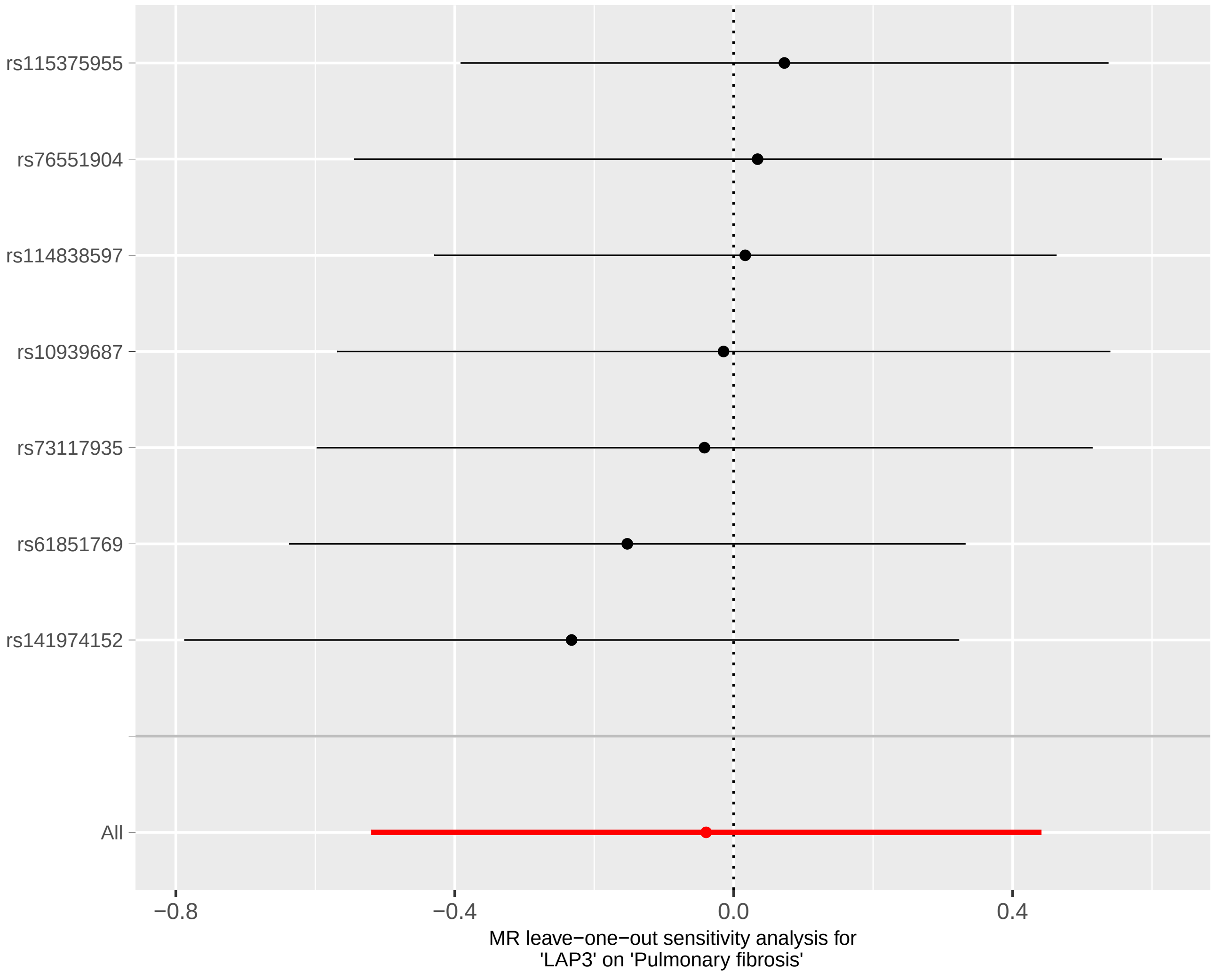
rs115375955
rs76551904
rs114838597
rs10939687
rs73117935
rs61851769
rs141974152
All
−0.8
−0.4
0.0
0.4
MR leave−one−out sensitivity analysis for
'LAP3' on 'Pulmonary fibrosis'

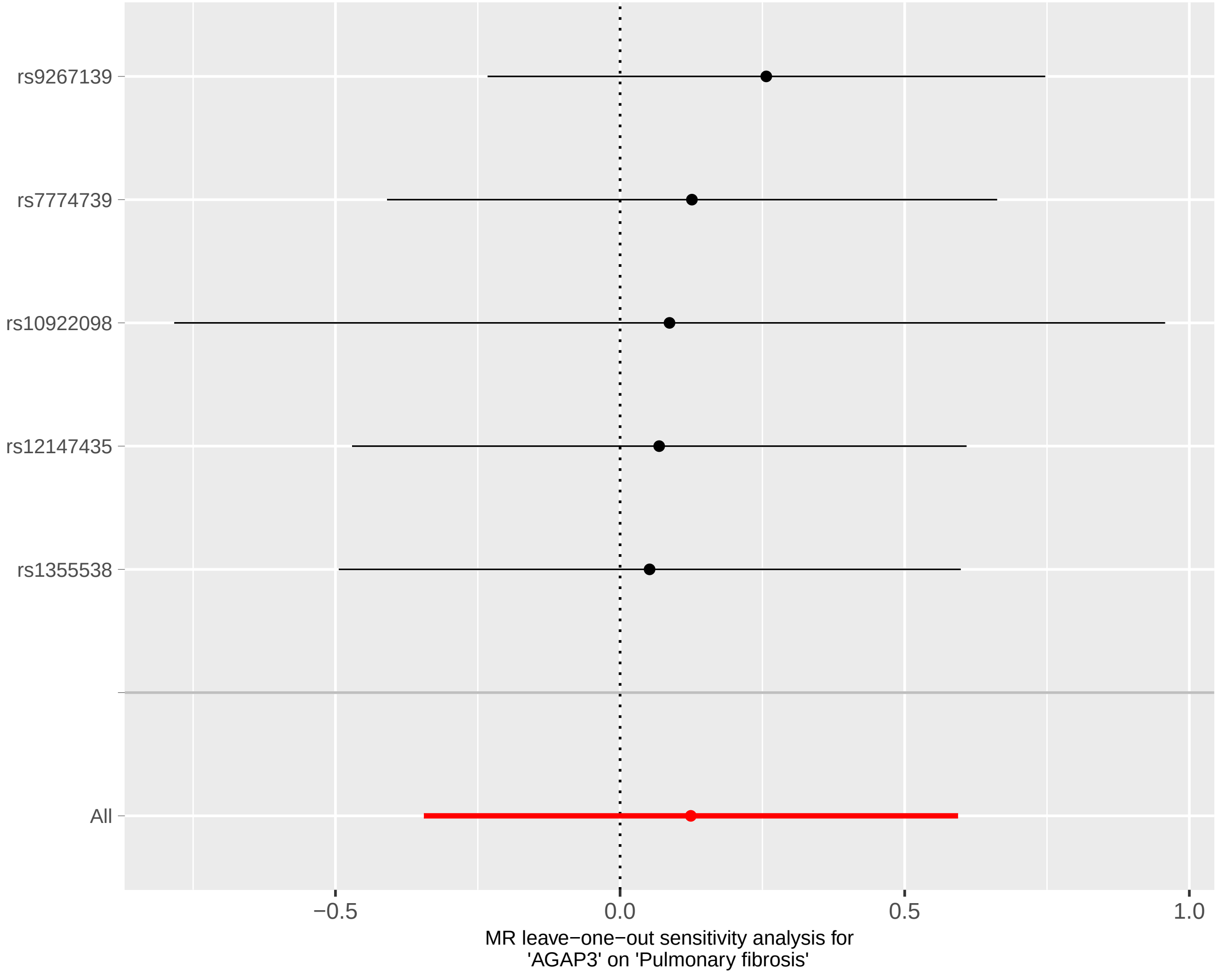

rs9267139
rs7774739
rs10922098
rs12147435
rs1355538
All
−0.5
0.0
0.5
1.0
MR leave−one−out sensitivity analysis for
'AGAP3' on 'Pulmonary fibrosis'

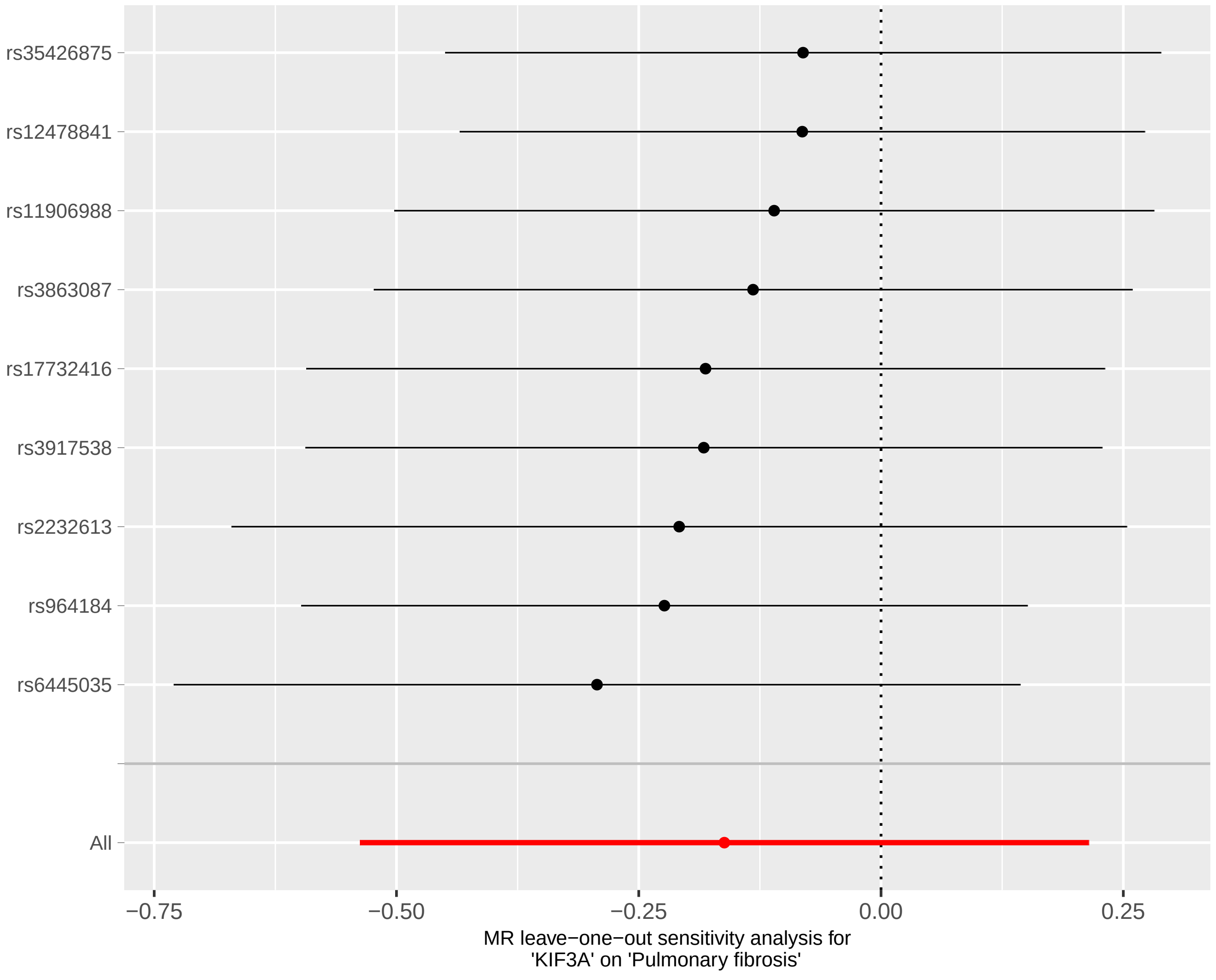
rs35426875
rs12478841
rs11906988
rs3863087
rs17732416
rs3917538
rs2232613
rs964184
rs6445035
All
−0.75
−0.50
−0.25
0.00
0.25
MR leave−one−out sensitivity analysis for
'KIF3A' on 'Pulmonary fibrosis'

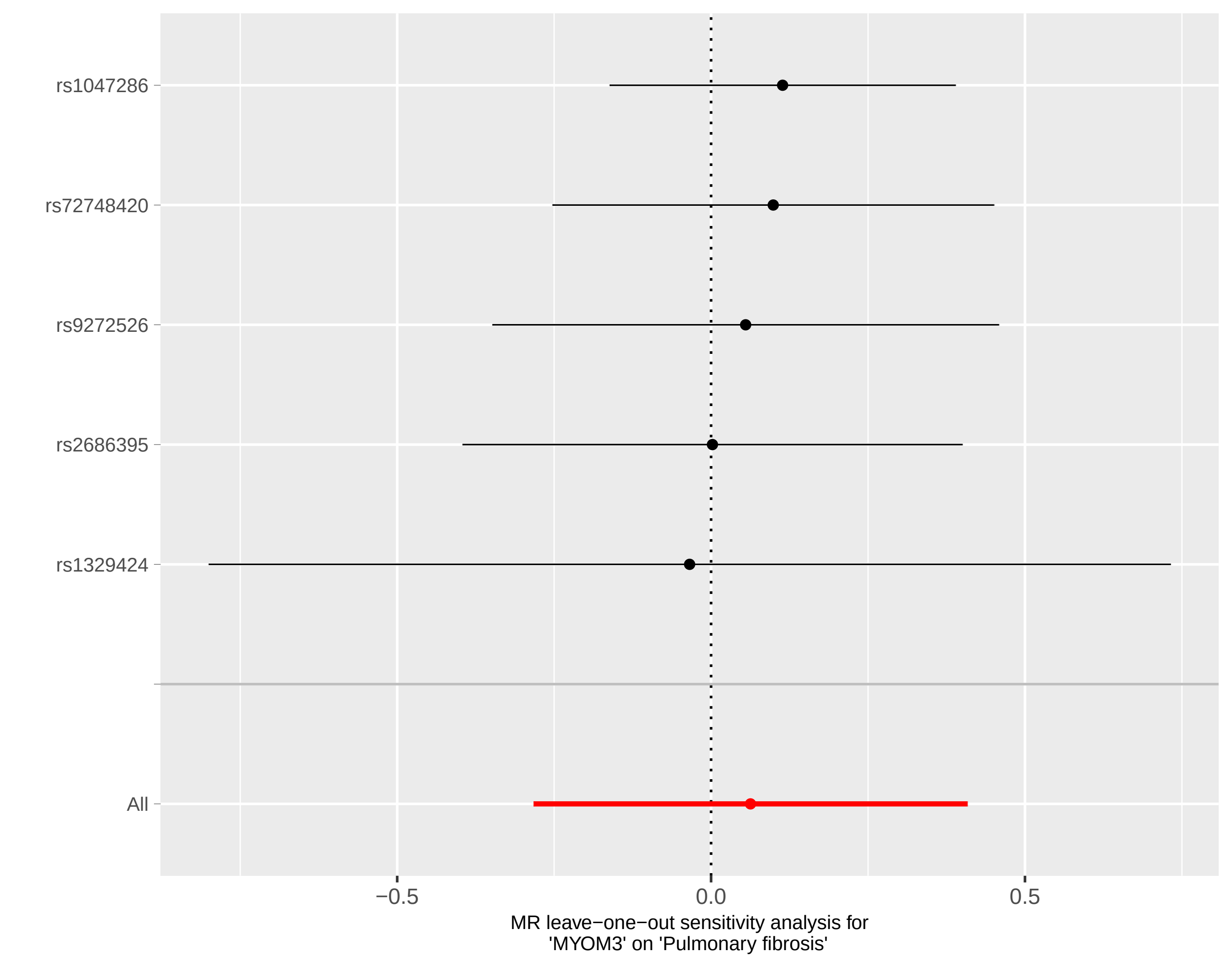

rs1047286
rs72748420
rs9272526
rs2686395
rs1329424
All
−0.5
0.0
0.5
MR leave−one−out sensitivity analysis for
'MYOM3' on 'Pulmonary fibrosis'

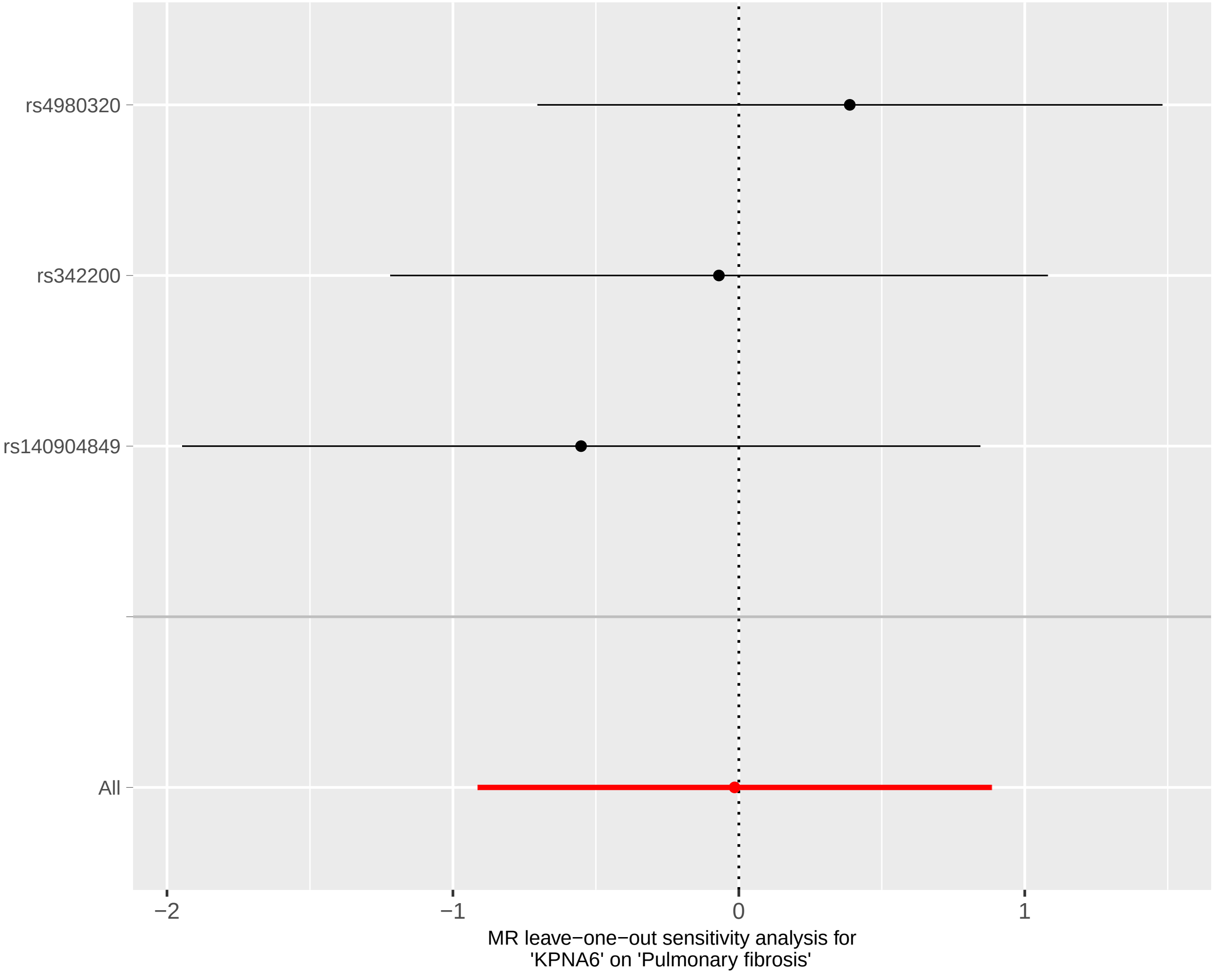

rs4980320
rs342200
rs140904849
All
−2
−1
0
1
MR leave−one−out sensitivity analysis for
'KPNA6' on 'Pulmonary fibrosis'

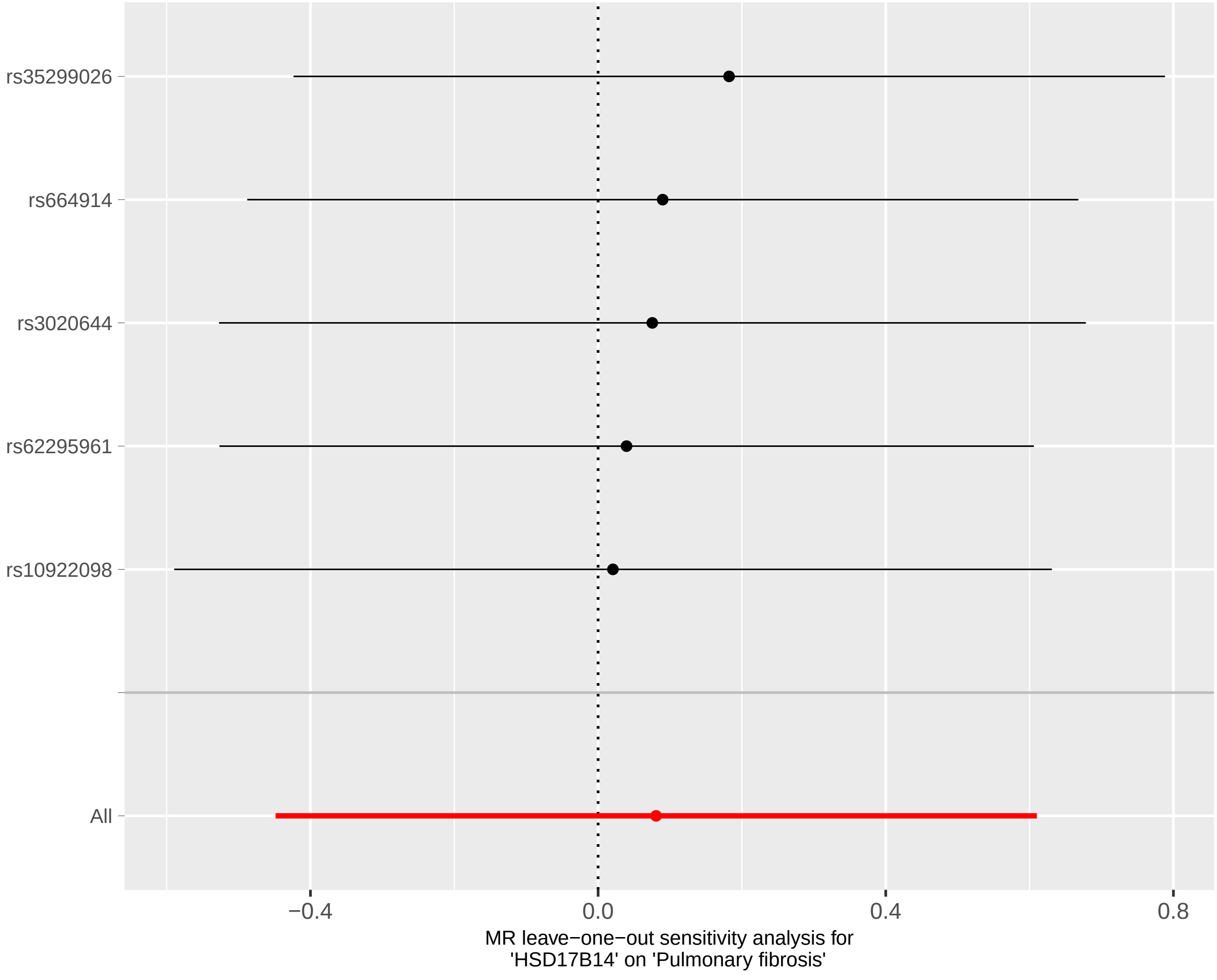
rs35299026
rs664914
rs3020644
rs62295961
rs10922098
All
−0.4
0.0
0.4
0.8
MR leave−one−out sensitivity analysis for
'HSD17B14' on 'Pulmonary fibrosis'

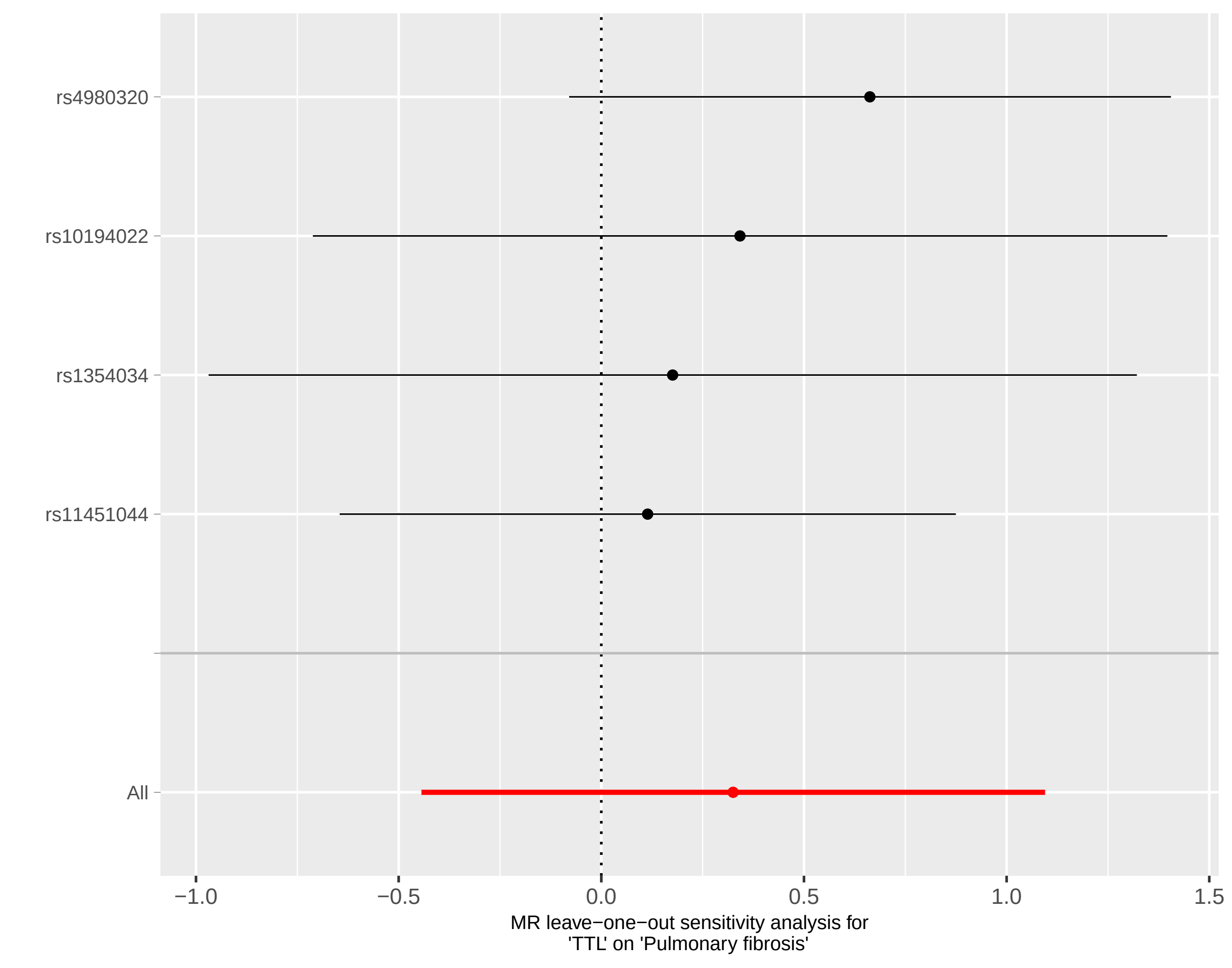
rs4980320
rs10194022
rs1354034
rs11451044
All
−1.0
−0.5
0.0
0.5
1.0
1.5
MR leave−one−out sensitivity analysis for
'TTL' on 'Pulmonary fibrosis'

Insufficient number of SNPs

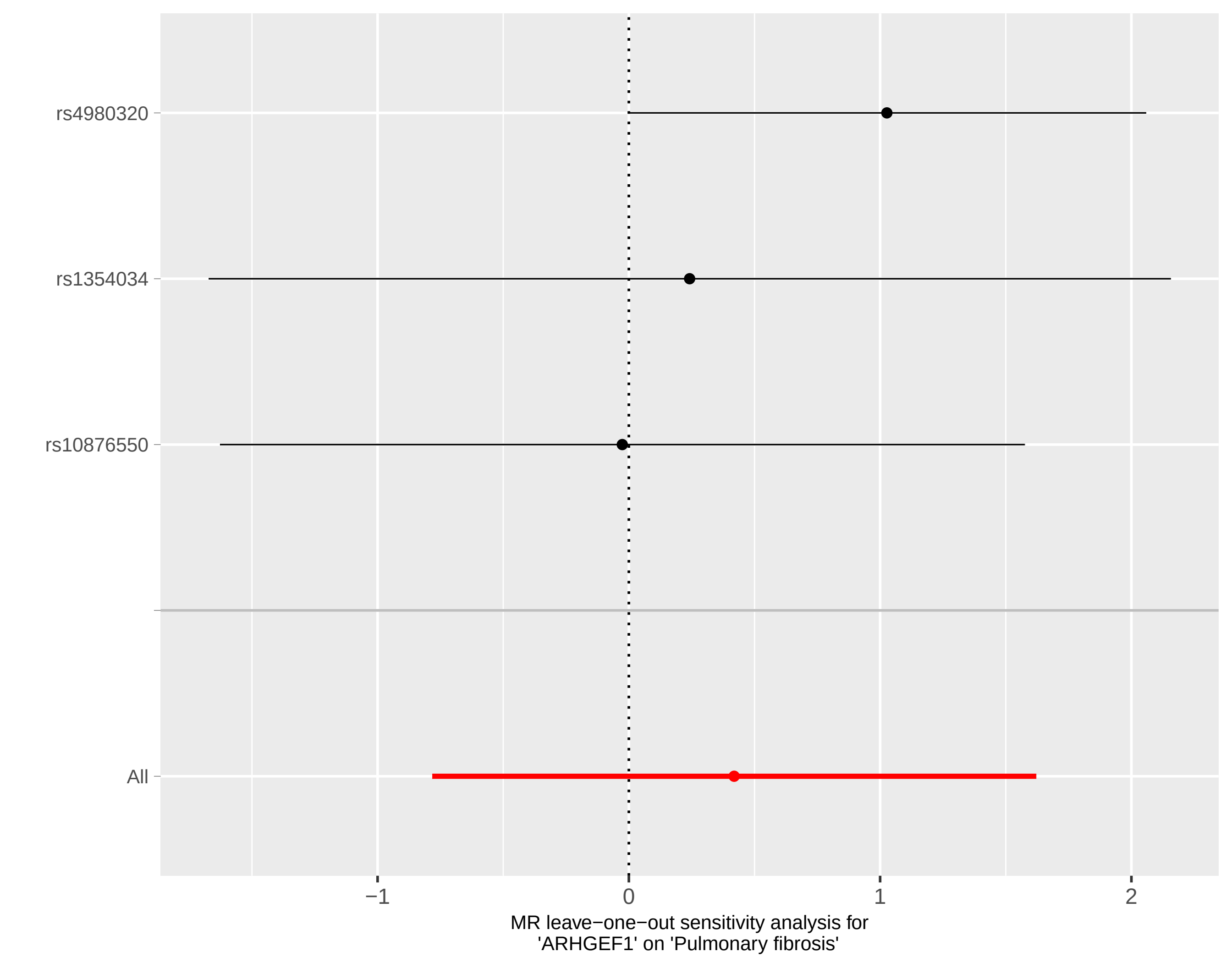

rs4980320
rs1354034
rs10876550
All
−1
0
1
2
MR leave−one−out sensitivity analysis for
'ARHGEF1' on 'Pulmonary fibrosis'

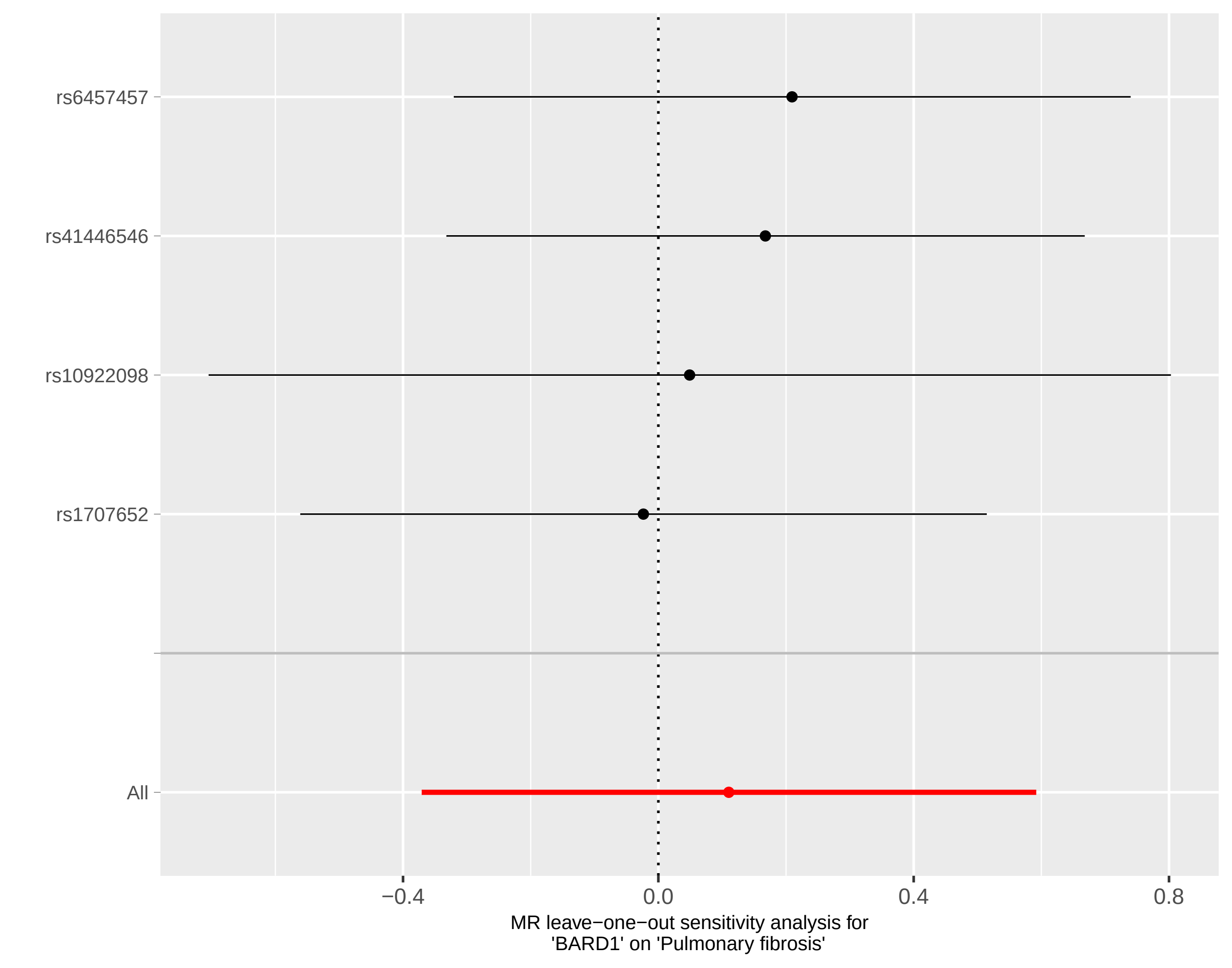
rs6457457
rs41446546
rs10922098
rs1707652
All
−0.4
0.0
0.4
0.8
MR leave−one−out sensitivity analysis for
'BARD1' on 'Pulmonary fibrosis'

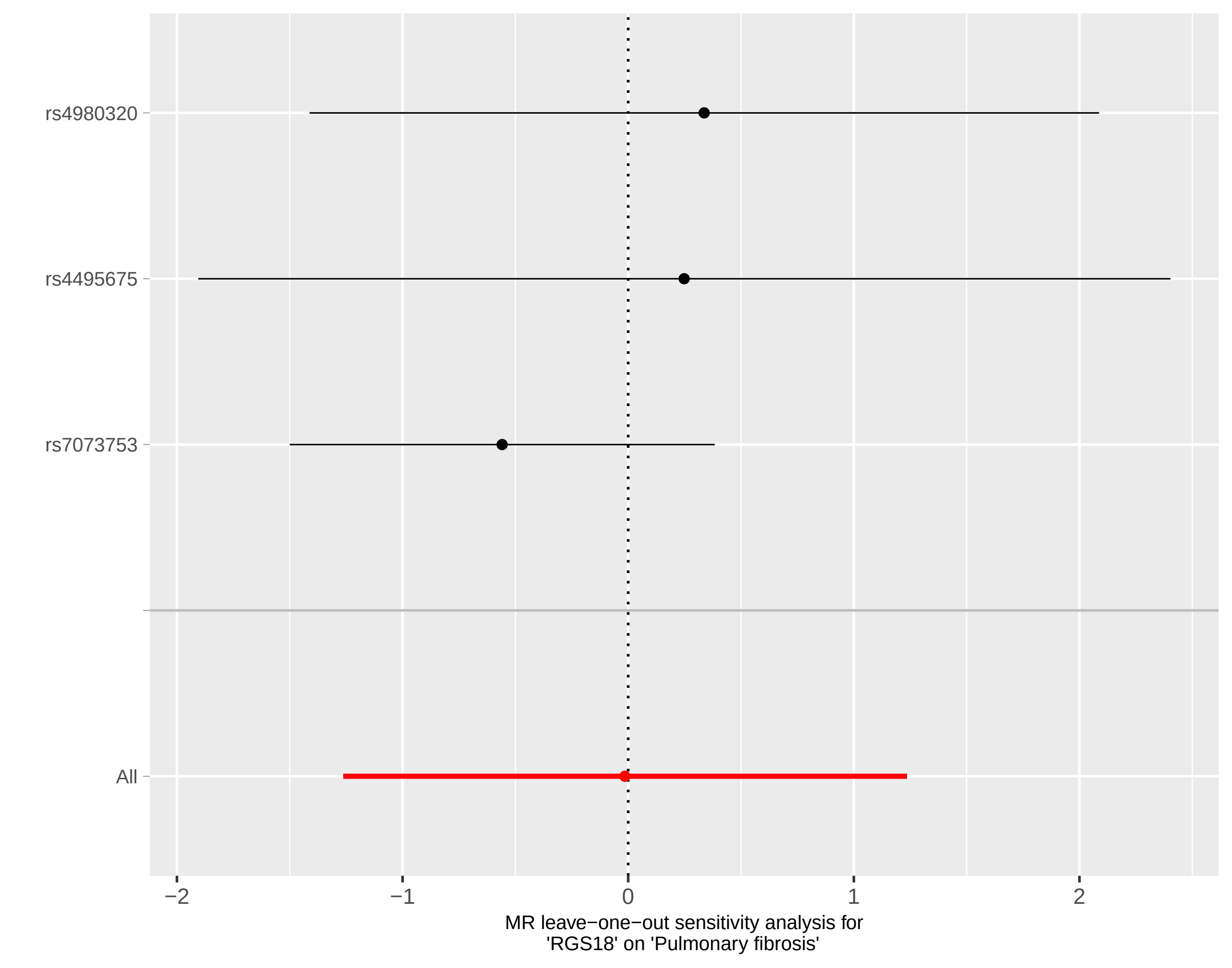
rs4980320
rs4495675
rs7073753
All
−2
−1
0
1
2
MR leave−one−out sensitivity analysis for
'RGS18' on 'Pulmonary fibrosis'

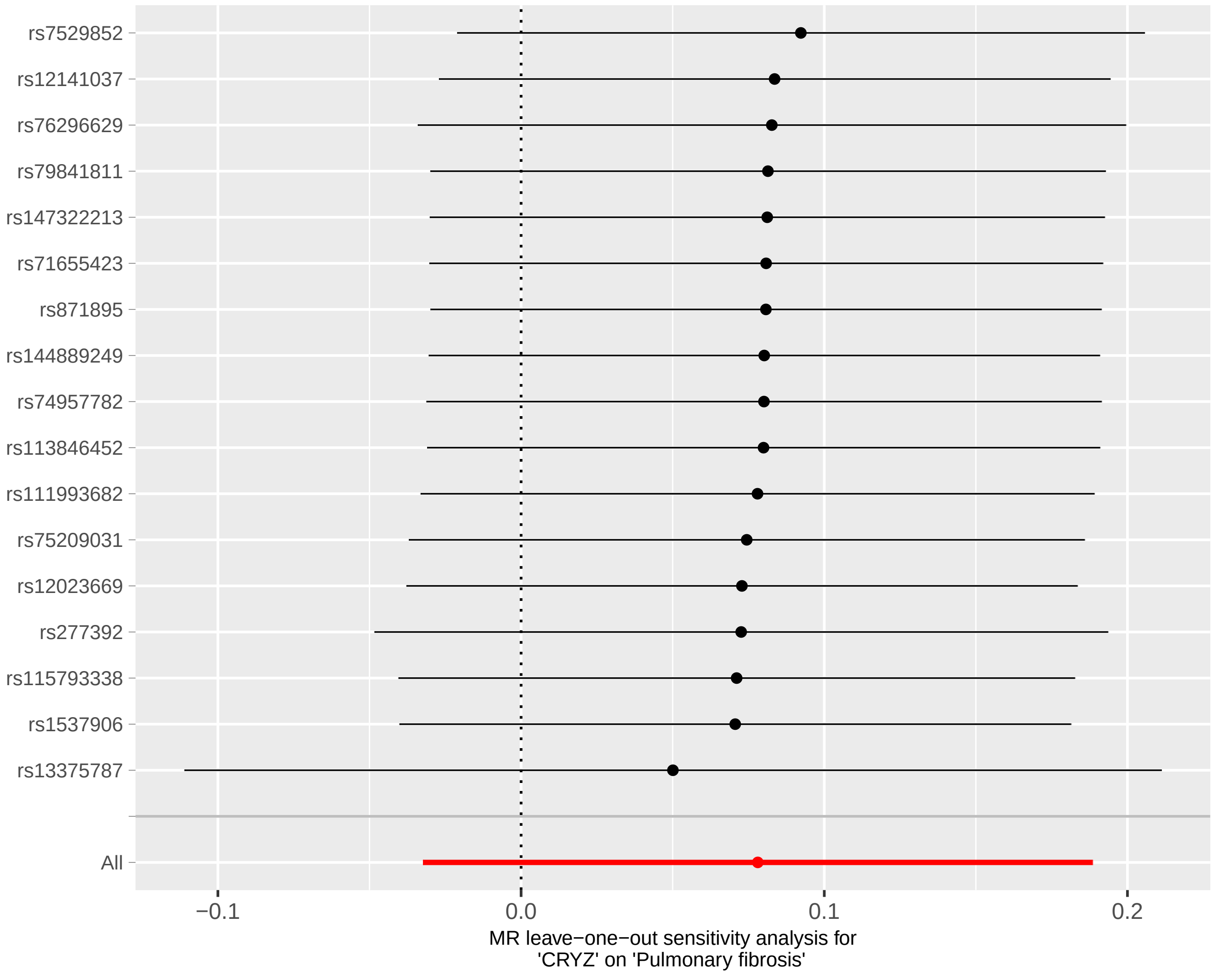

rs7529852
rs12141037
rs76296629
rs79841811
rs147322213
rs71655423
rs871895
rs144889249
rs74957782
rs113846452
rs111993682
rs75209031
rs12023669
rs277392
rs115793338
rs1537906
rs13375787
All
−0.1
0.0
0.1
0.2
MR leave−one−out sensitivity analysis for
'CRYZ' on 'Pulmonary fibrosis'

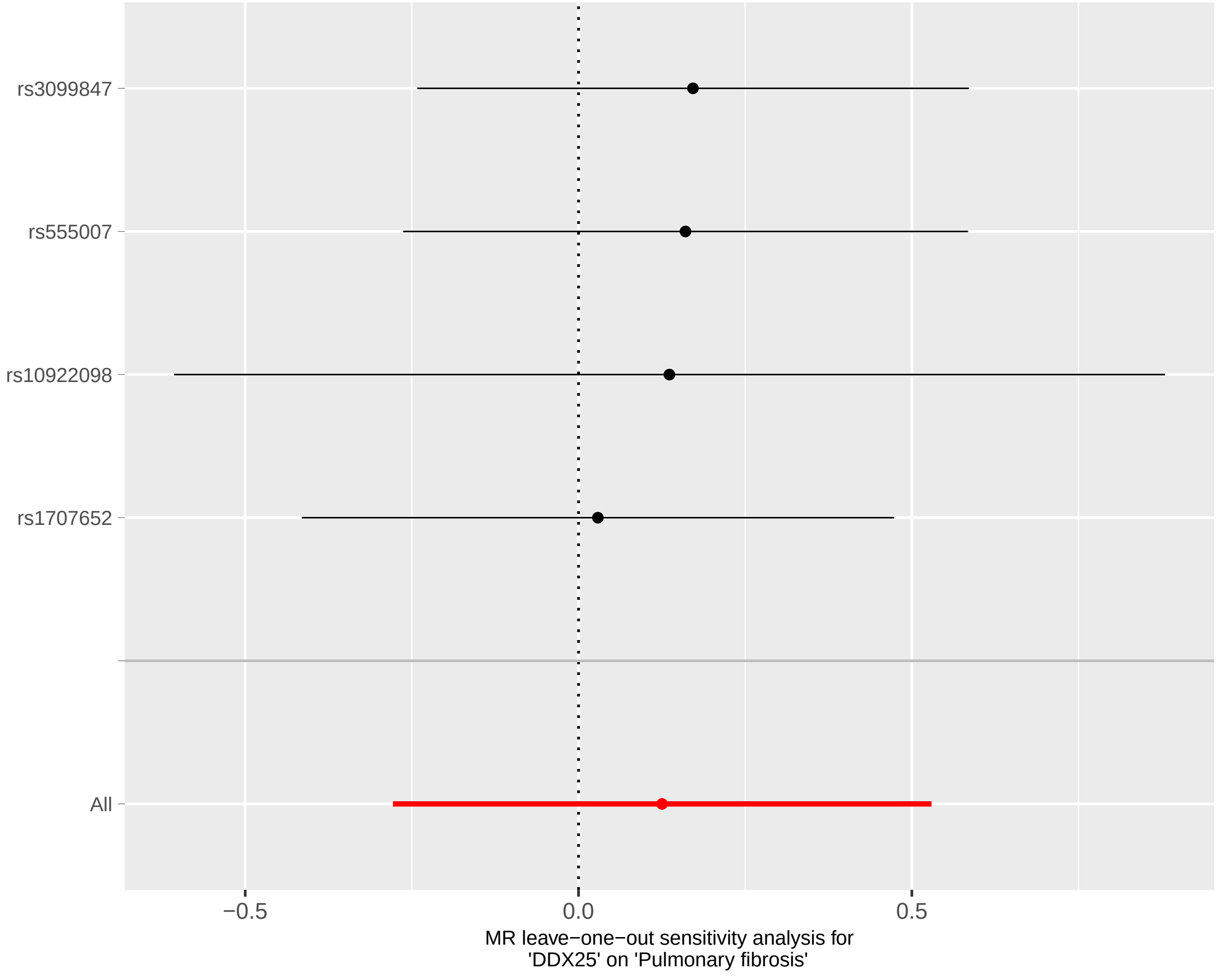
rs3099847
rs555007
rs10922098
rs1707652
All
−0.5
0.0
0.5
MR leave−one−out sensitivity analysis for
'DDX25' on 'Pulmonary fibrosis'

Insufficient number of SNPs

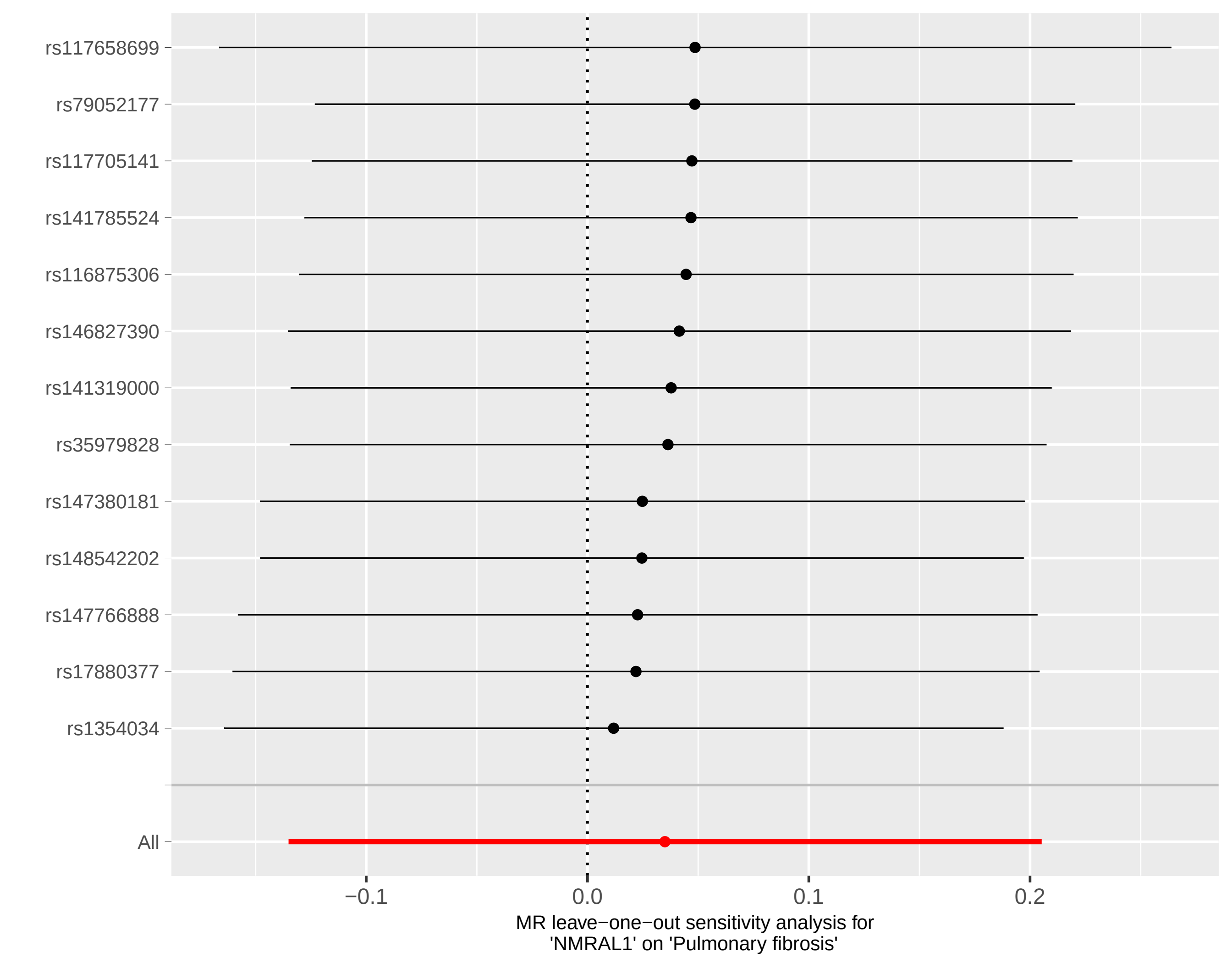
rs117658699
rs79052177
rs117705141
rs141785524
rs116875306
rs146827390
rs141319000
rs35979828
rs147380181
rs148542202
rs147766888
rs17880377
rs1354034
All
−0.1
0.0
0.1
0.2
MR leave−one−out sensitivity analysis for
'NMRAL1' on 'Pulmonary fibrosis'

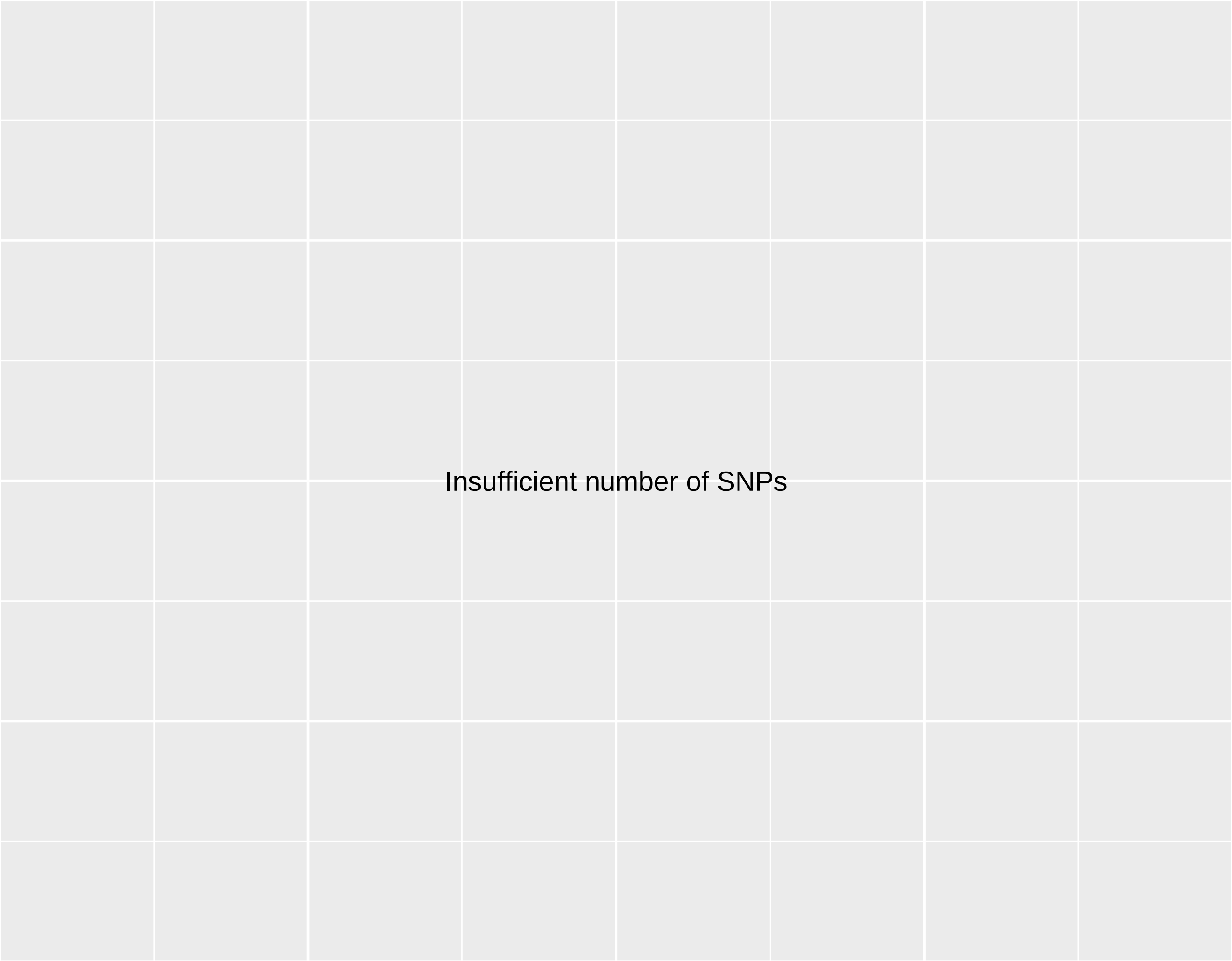
Insufficient number of SNPs

Insufficient number of SNPs

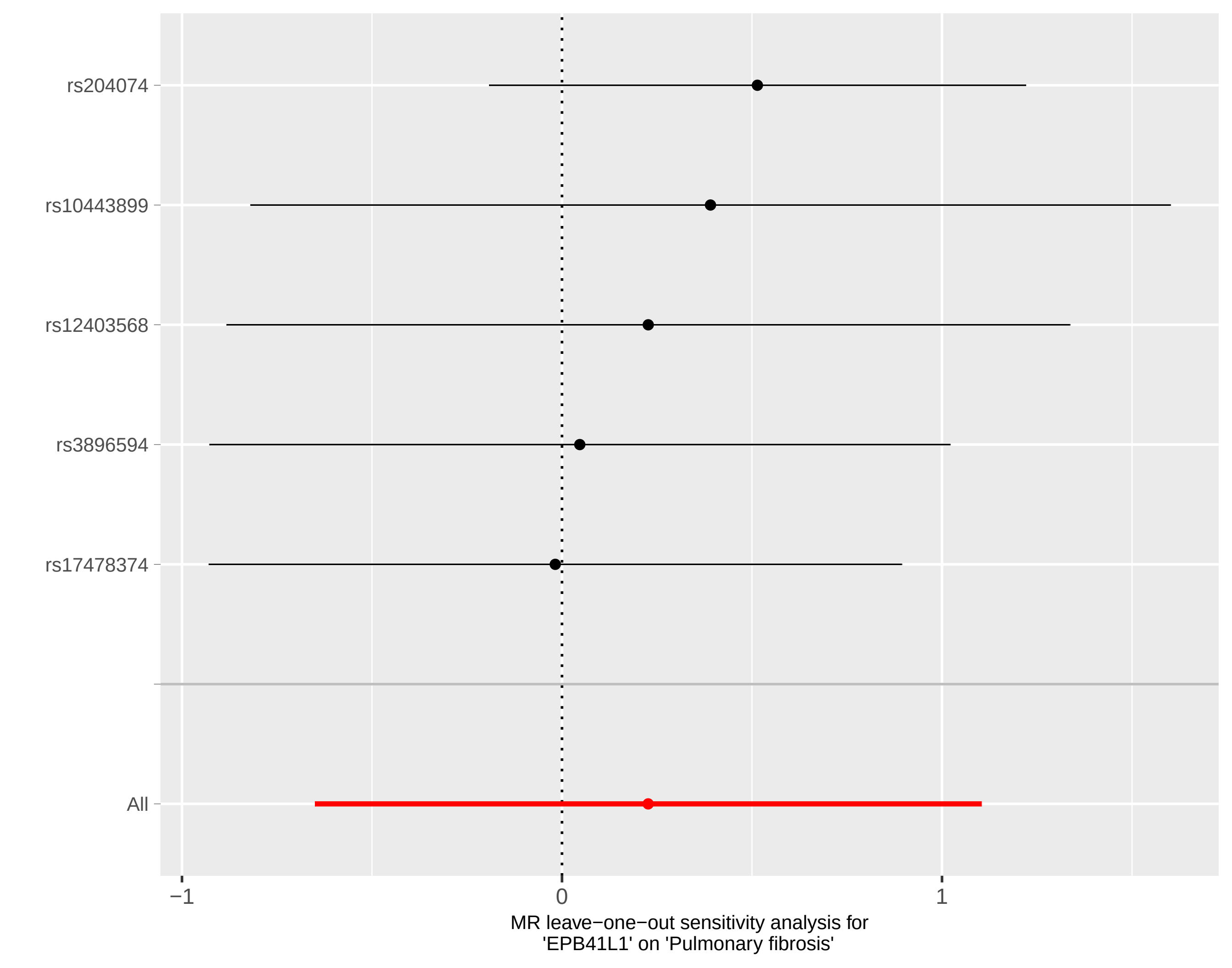
rs204074
rs10443899
rs12403568
rs3896594
rs17478374
All
−1
0
1
MR leave−one−out sensitivity analysis for
'EPB41L1' on 'Pulmonary fibrosis'

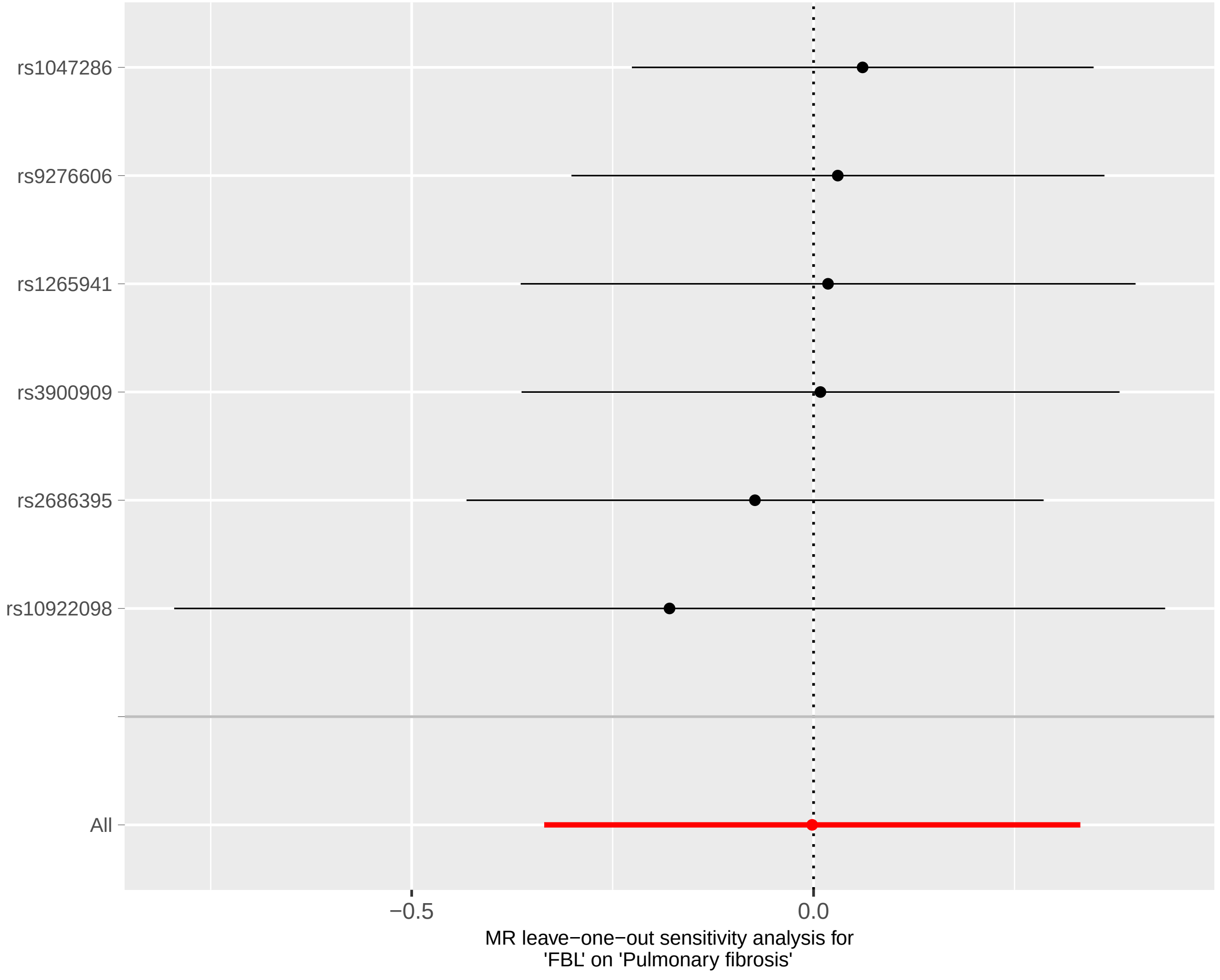

rs1047286
rs9276606
rs1265941
rs3900909
rs2686395
rs10922098
All
−0.5
0.0
MR leave−one−out sensitivity analysis for
'FBL' on 'Pulmonary fibrosis'

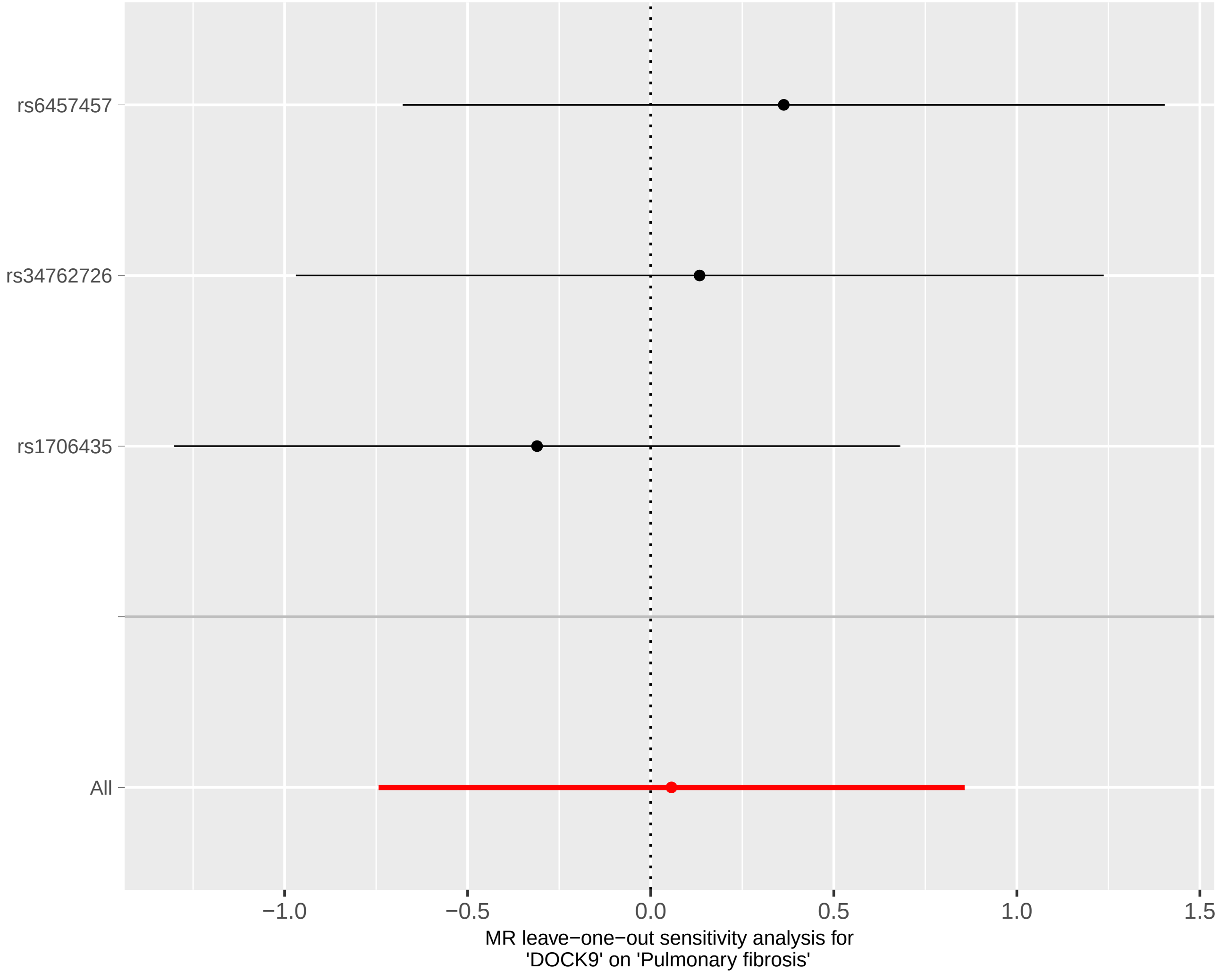
rs6457457
rs34762726
rs1706435
All
−1.0
−0.5
0.0
0.5
1.0
1.5
MR leave−one−out sensitivity analysis for
'DOCK9' on 'Pulmonary fibrosis'

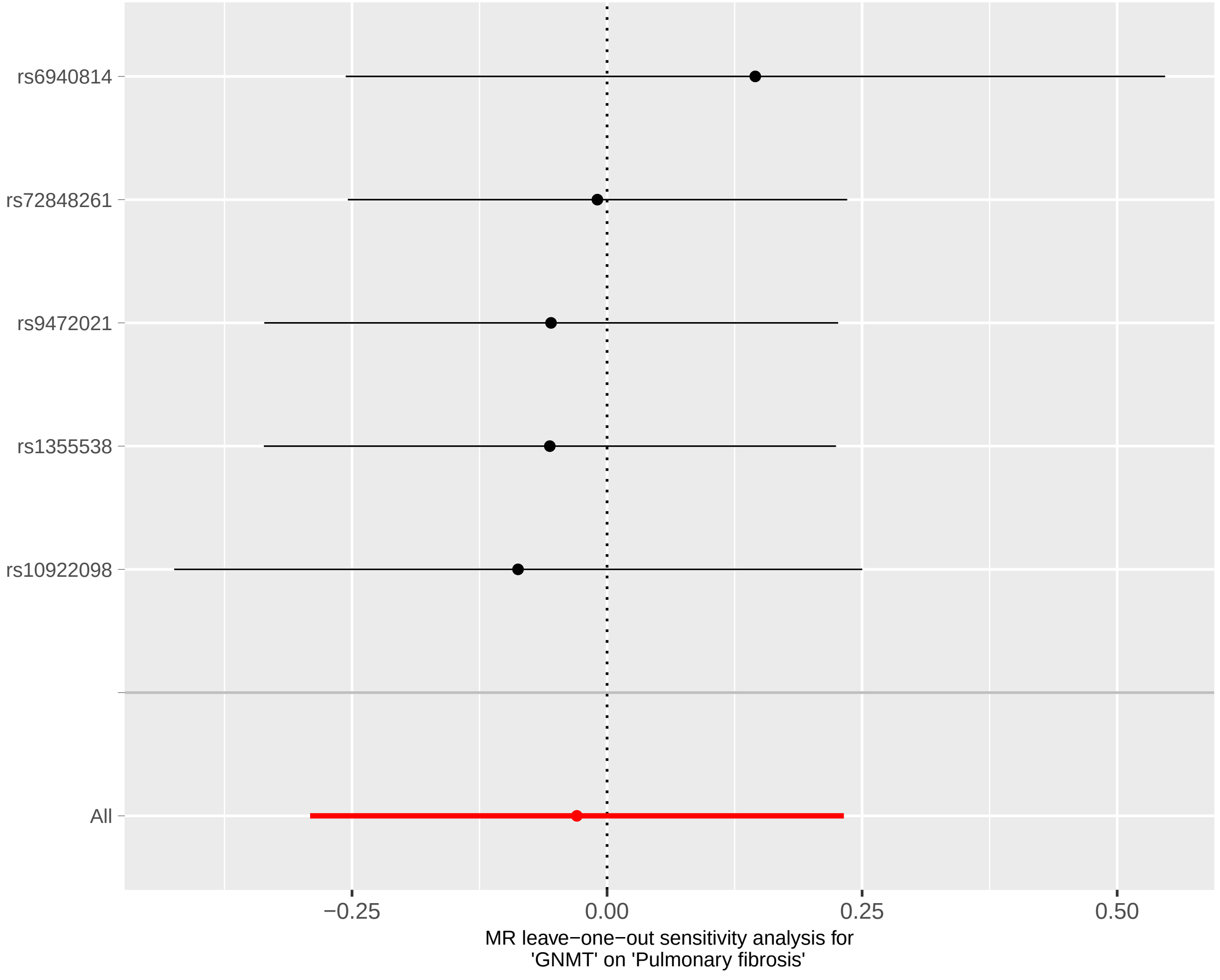
rs6940814
rs72848261
rs9472021
rs1355538
rs10922098
All
−0.25
0.00
0.25
0.50
MR leave−one−out sensitivity analysis for
'GNMT' on 'Pulmonary fibrosis'

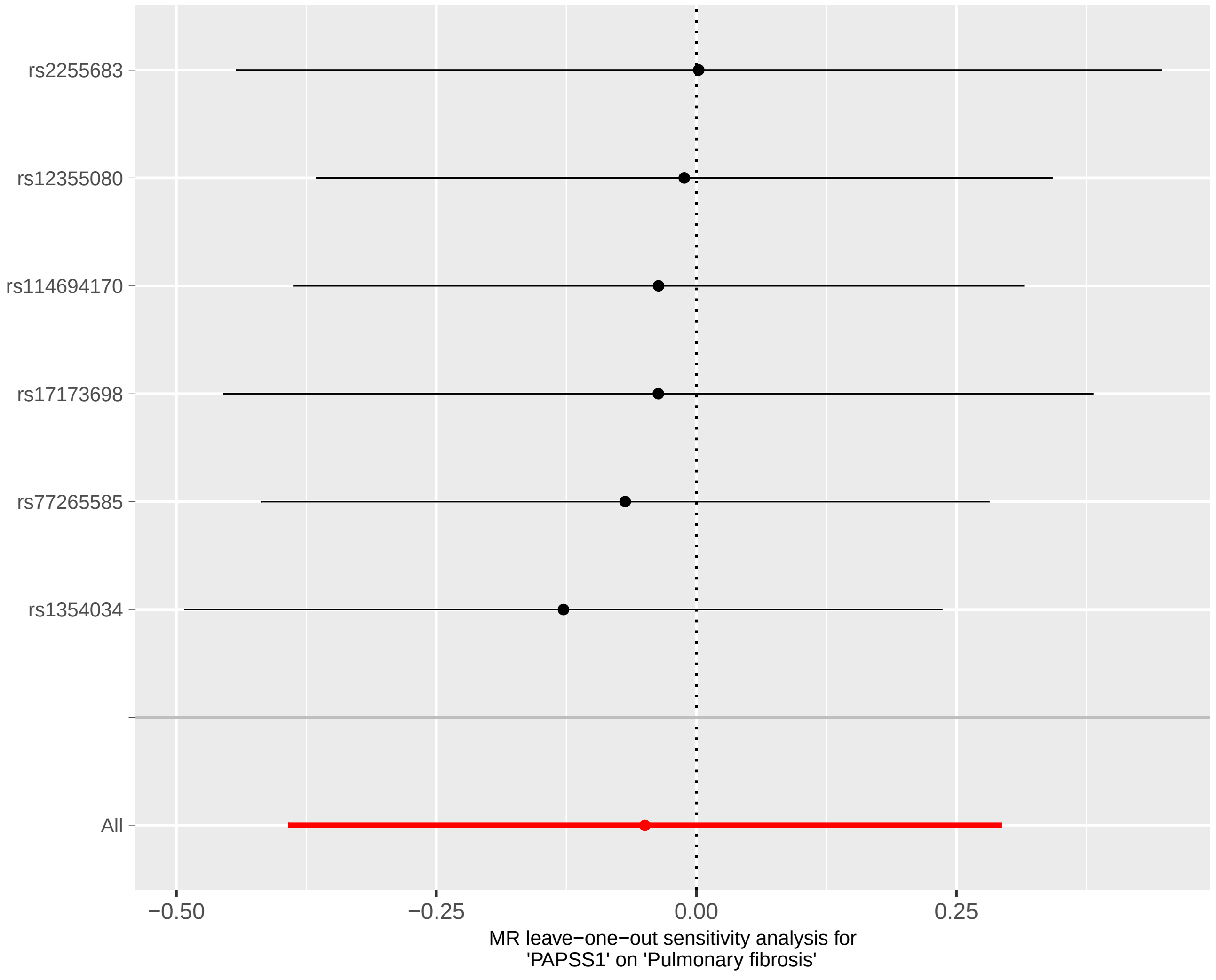
rs2255683
rs12355080
rs114694170
rs17173698
rs77265585
rs1354034
All
−0.50
−0.25
0.00
0.25
MR leave−one−out sensitivity analysis for
'PAPSS1' on 'Pulmonary fibrosis'

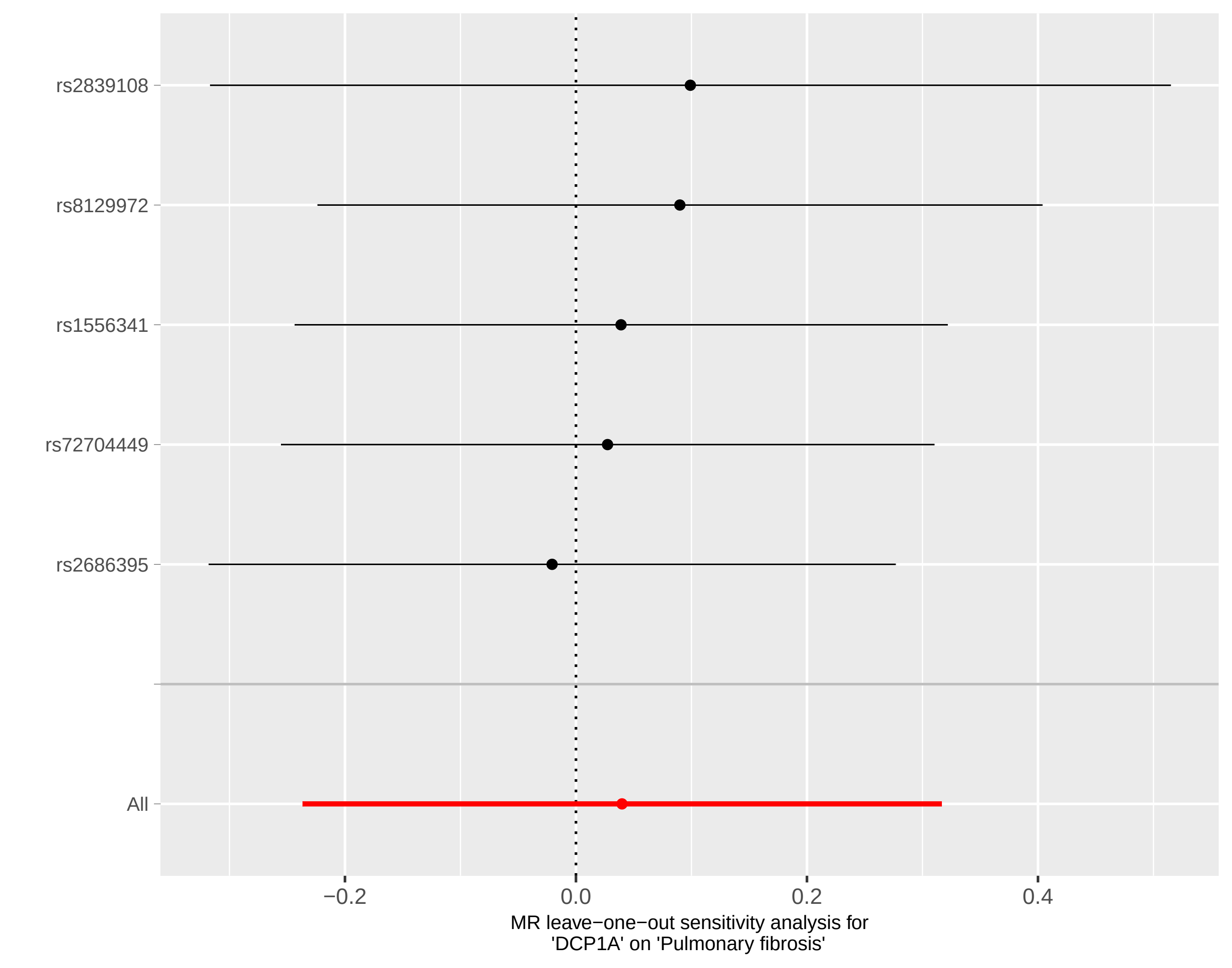
rs2839108
rs8129972
rs1556341
rs72704449
rs2686395
All
−0.2
0.0
0.2
0.4
MR leave−one−out sensitivity analysis for
'DCP1A' on 'Pulmonary fibrosis'

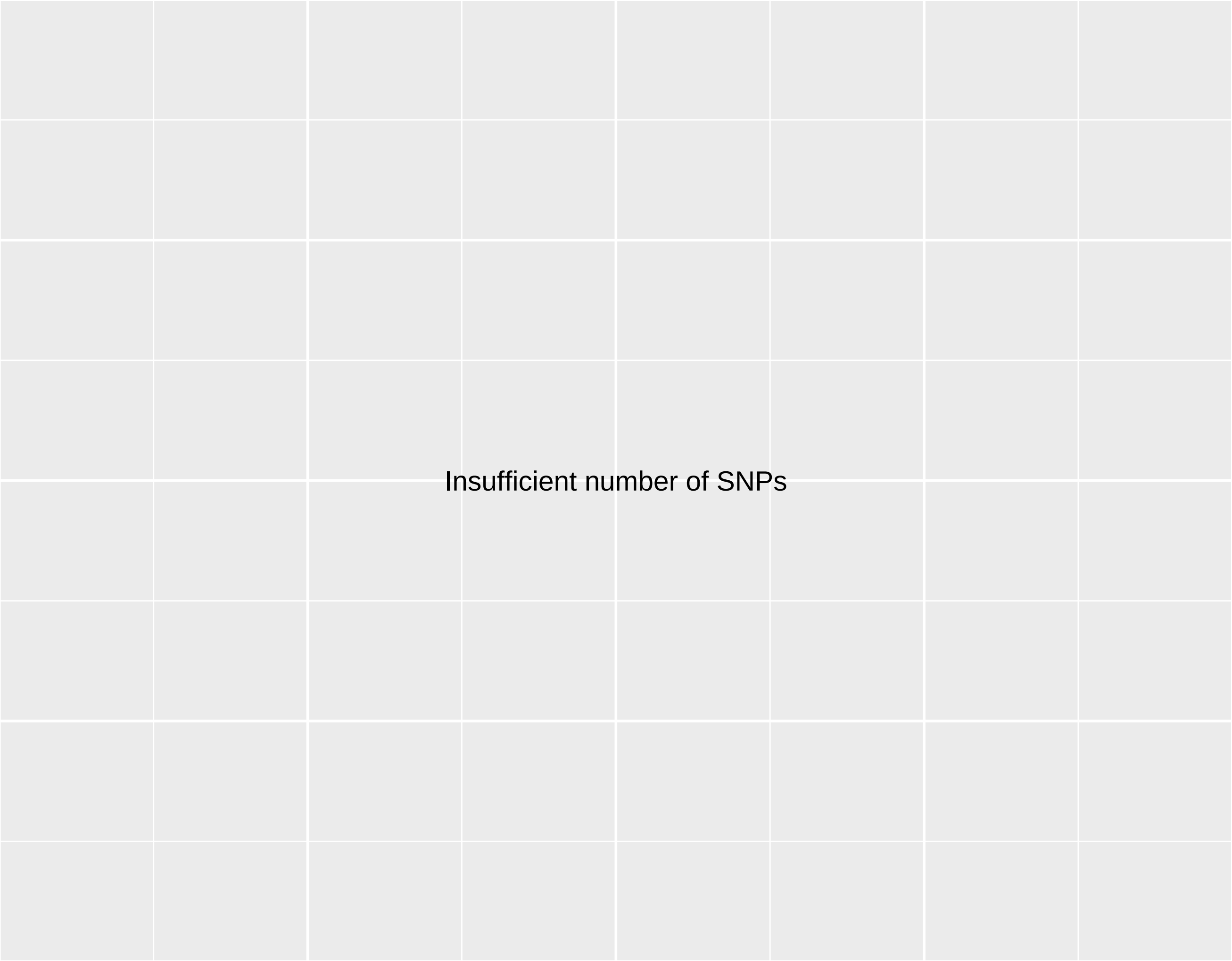
Insufficient number of SNPs

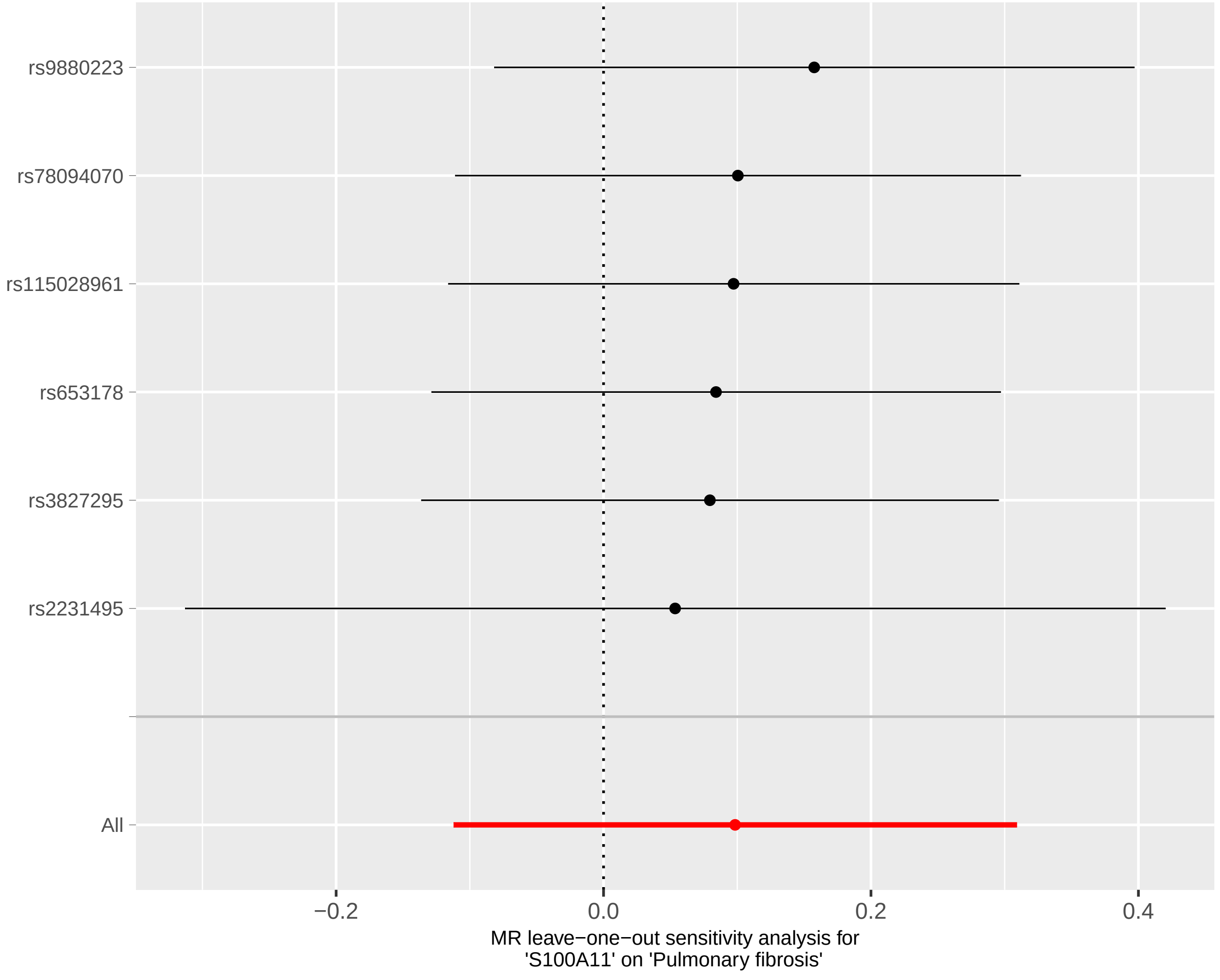
rs9880223
rs78094070
rs115028961
rs653178
rs3827295
rs2231495
All
−0.2
0.0
0.2
0.4
MR leave−one−out sensitivity analysis for
'S100A11' on 'Pulmonary fibrosis'

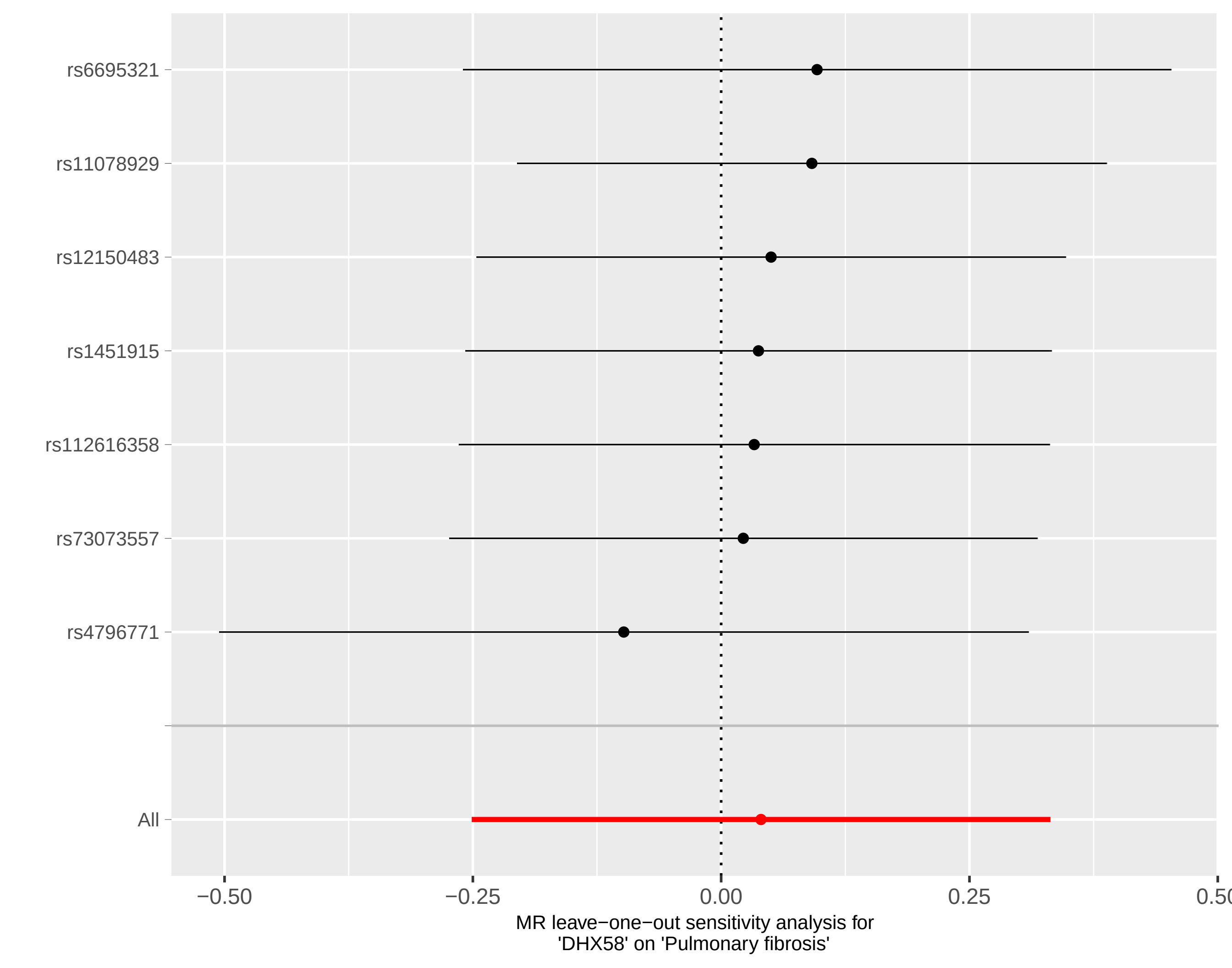
rs6695321
rs11078929
rs12150483
rs1451915
rs112616358
rs73073557
rs4796771
All
−0.50
−0.25
0.00
0.25
0.50
MR leave−one−out sensitivity analysis for
'DHX58' on 'Pulmonary fibrosis'

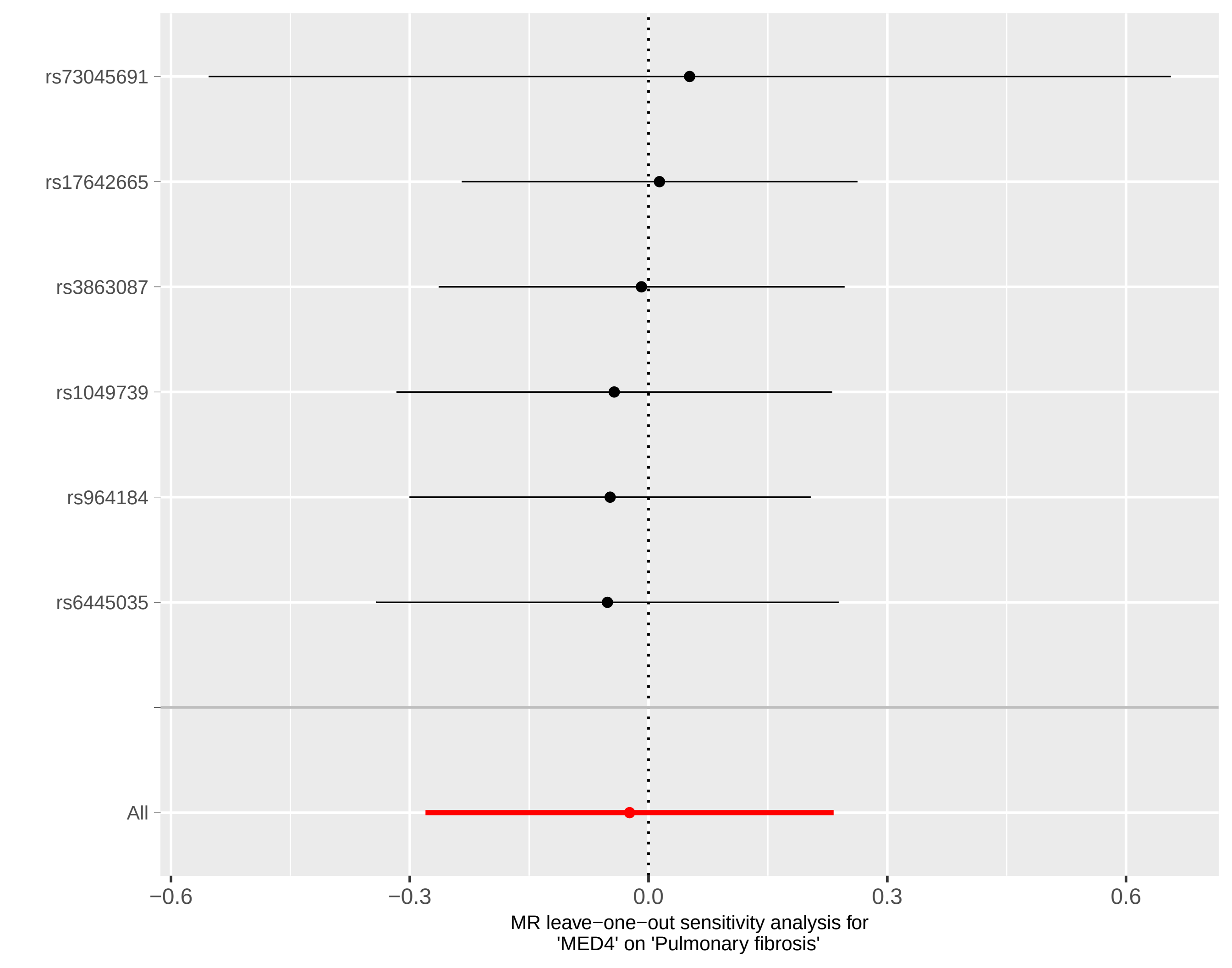
rs73045691
rs17642665
rs3863087
rs1049739
rs964184
rs6445035
All
−0.6
−0.3
0.0
0.3
0.6
MR leave−one−out sensitivity analysis for
'MED4' on 'Pulmonary fibrosis'

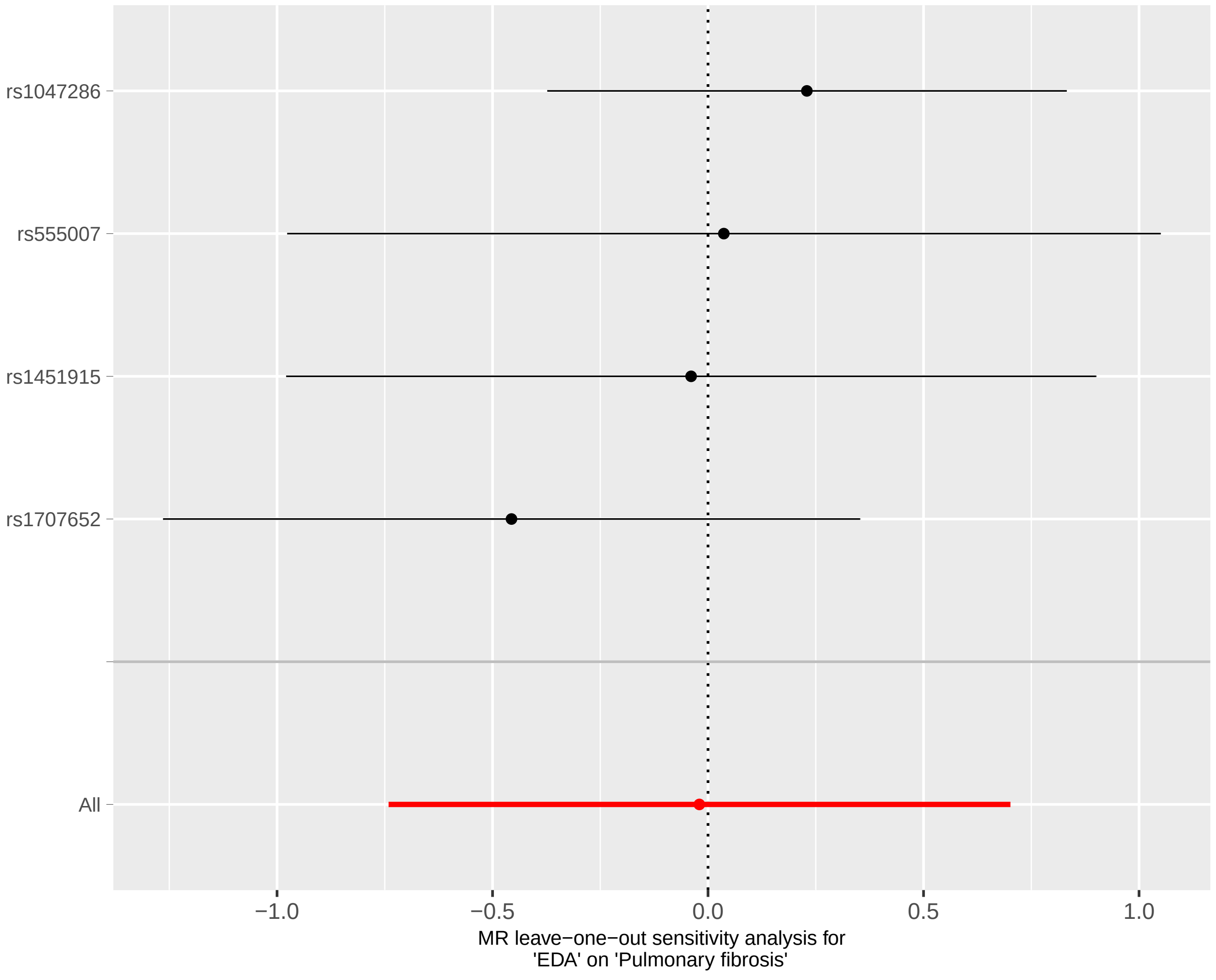

rs1047286
rs555007
rs1451915
rs1707652
All
−1.0
−0.5
0.0
0.5
1.0
MR leave−one−out sensitivity analysis for
'EDA' on 'Pulmonary fibrosis'

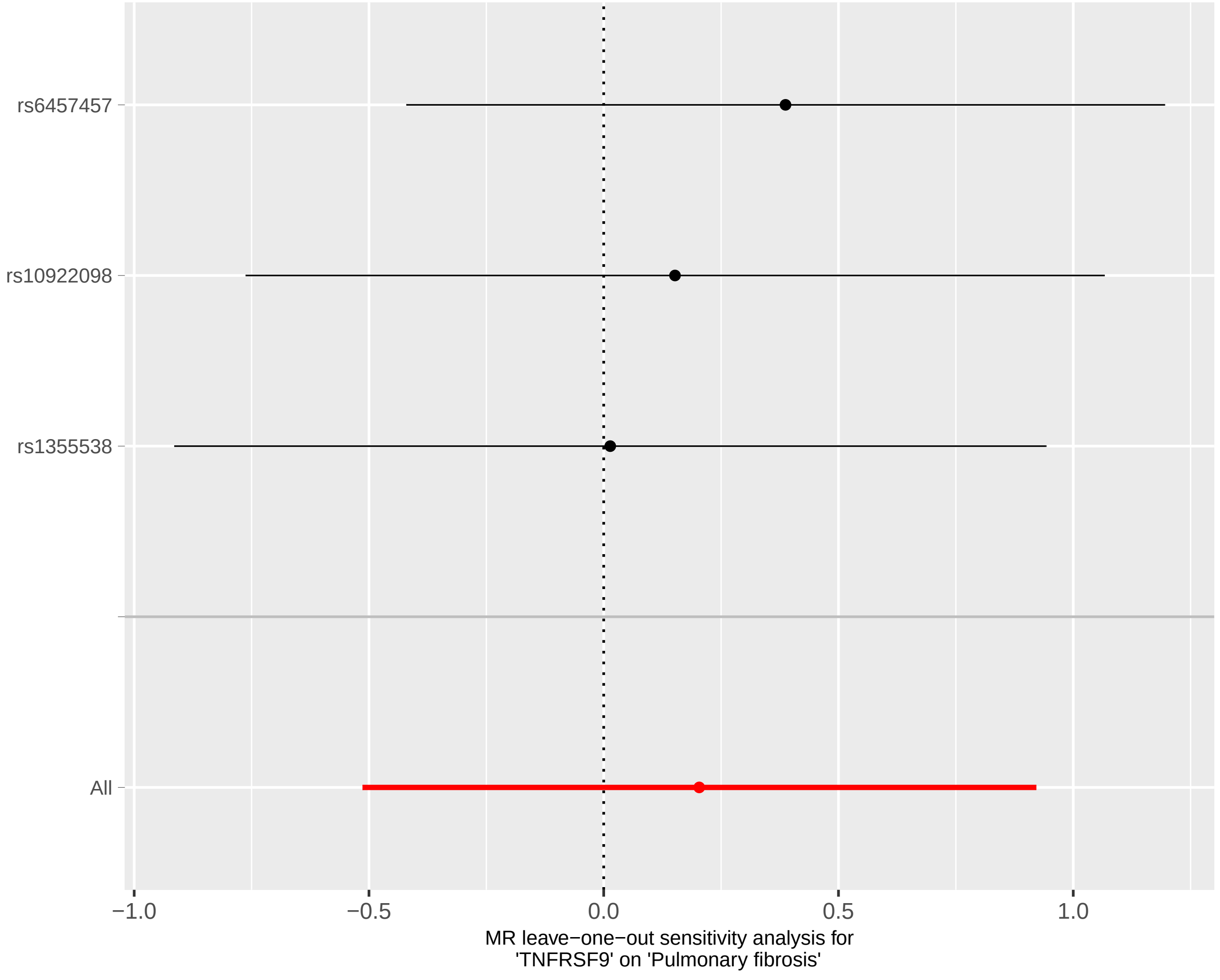
rs6457457
rs10922098
rs1355538
All
−1.0
−0.5
0.0
0.5
1.0
MR leave−one−out sensitivity analysis for
'TNFRSF9' on 'Pulmonary fibrosis'

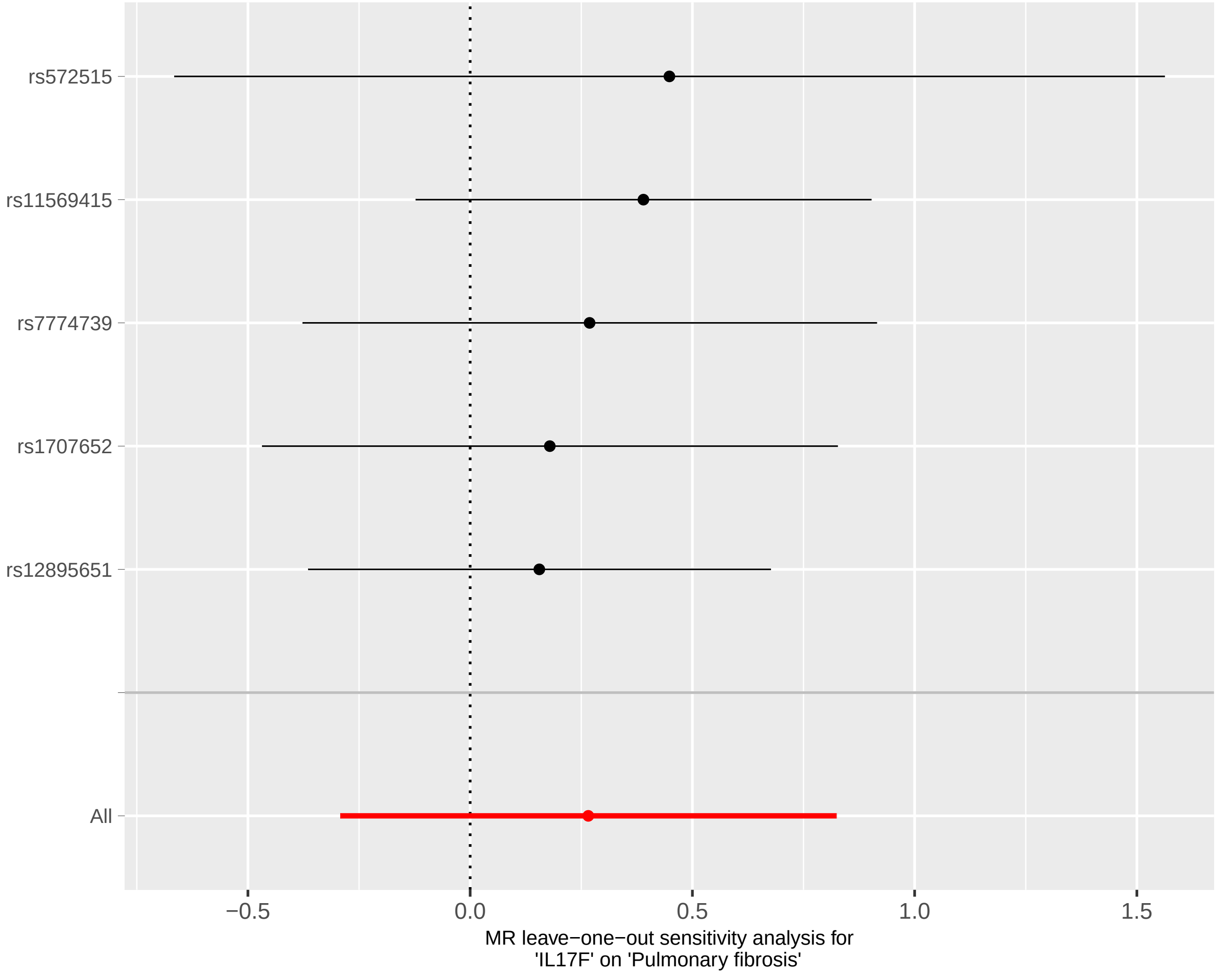
rs572515
rs11569415
rs7774739
rs1707652
rs12895651
All
−0.5
0.0
0.5
1.0
1.5
MR leave−one−out sensitivity analysis for
'IL17F' on 'Pulmonary fibrosis'

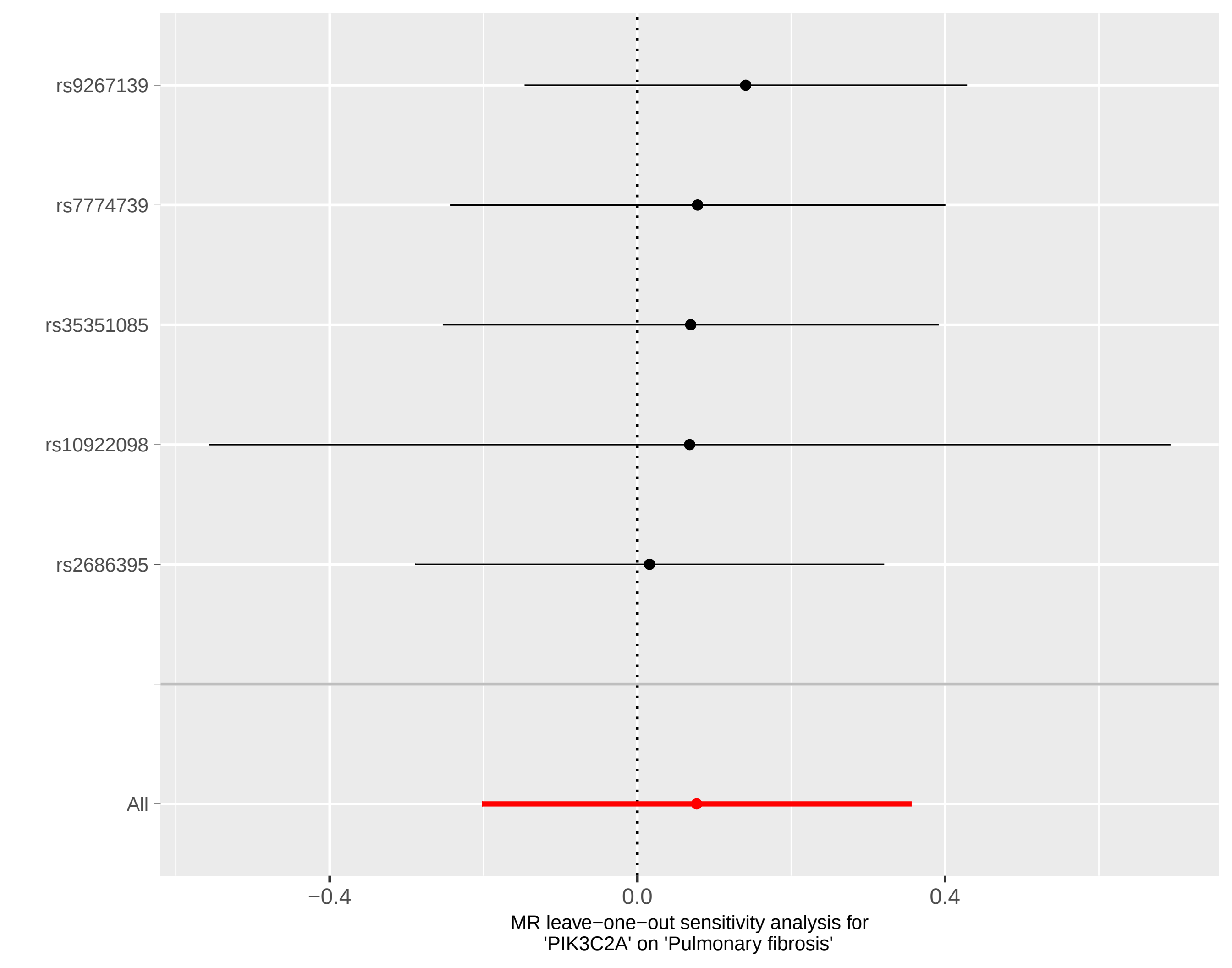
rs9267139
rs7774739
rs35351085
rs10922098
rs2686395
All
−0.4
0.0
0.4
MR leave−one−out sensitivity analysis for
'PIK3C2A' on 'Pulmonary fibrosis'

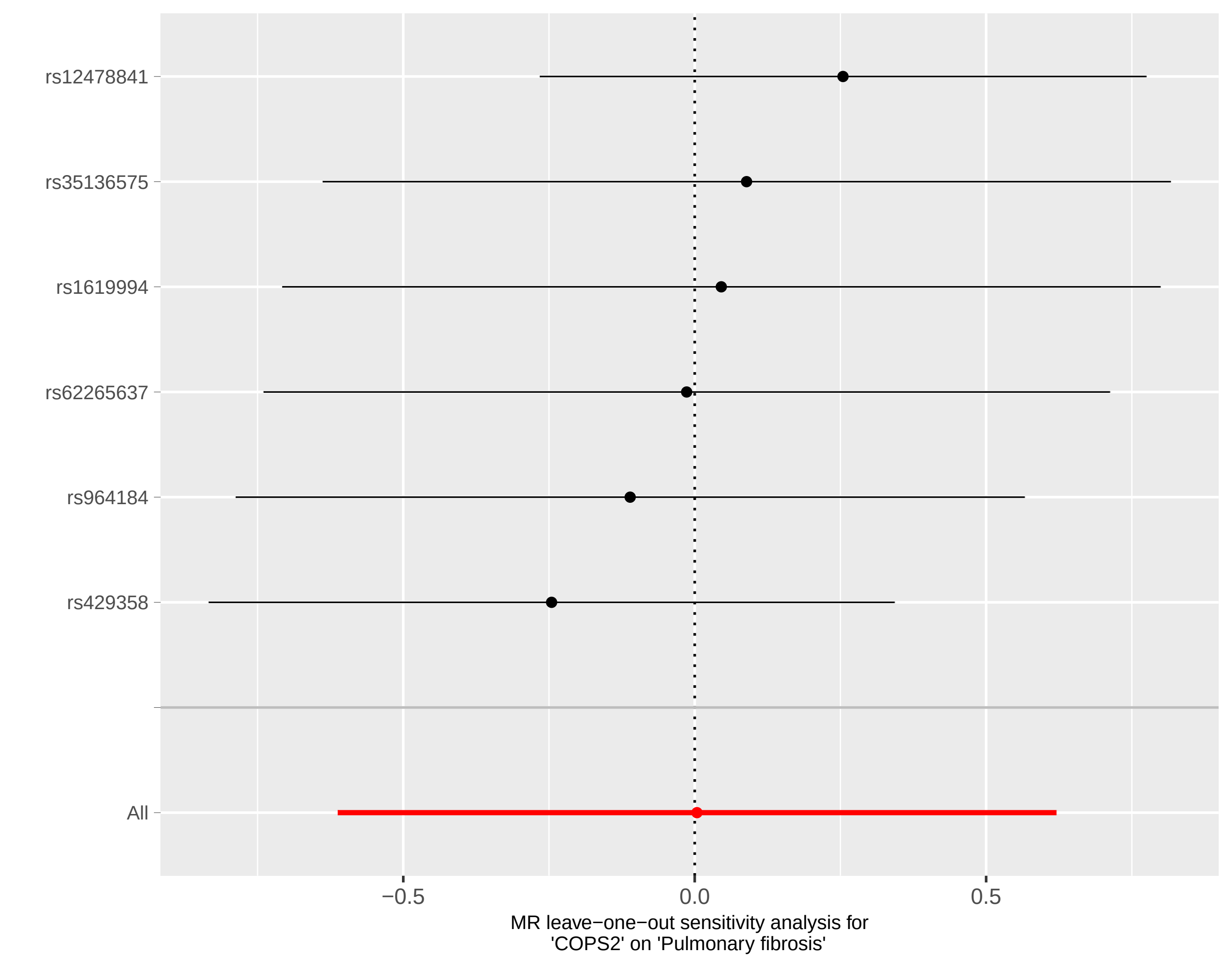
rs12478841
rs35136575
rs1619994
rs62265637
rs964184
rs429358
All
−0.5
0.0
0.5
MR leave−one−out sensitivity analysis for
'COPS2' on 'Pulmonary fibrosis'

Insufficient number of SNPs

Insufficient number of SNPs

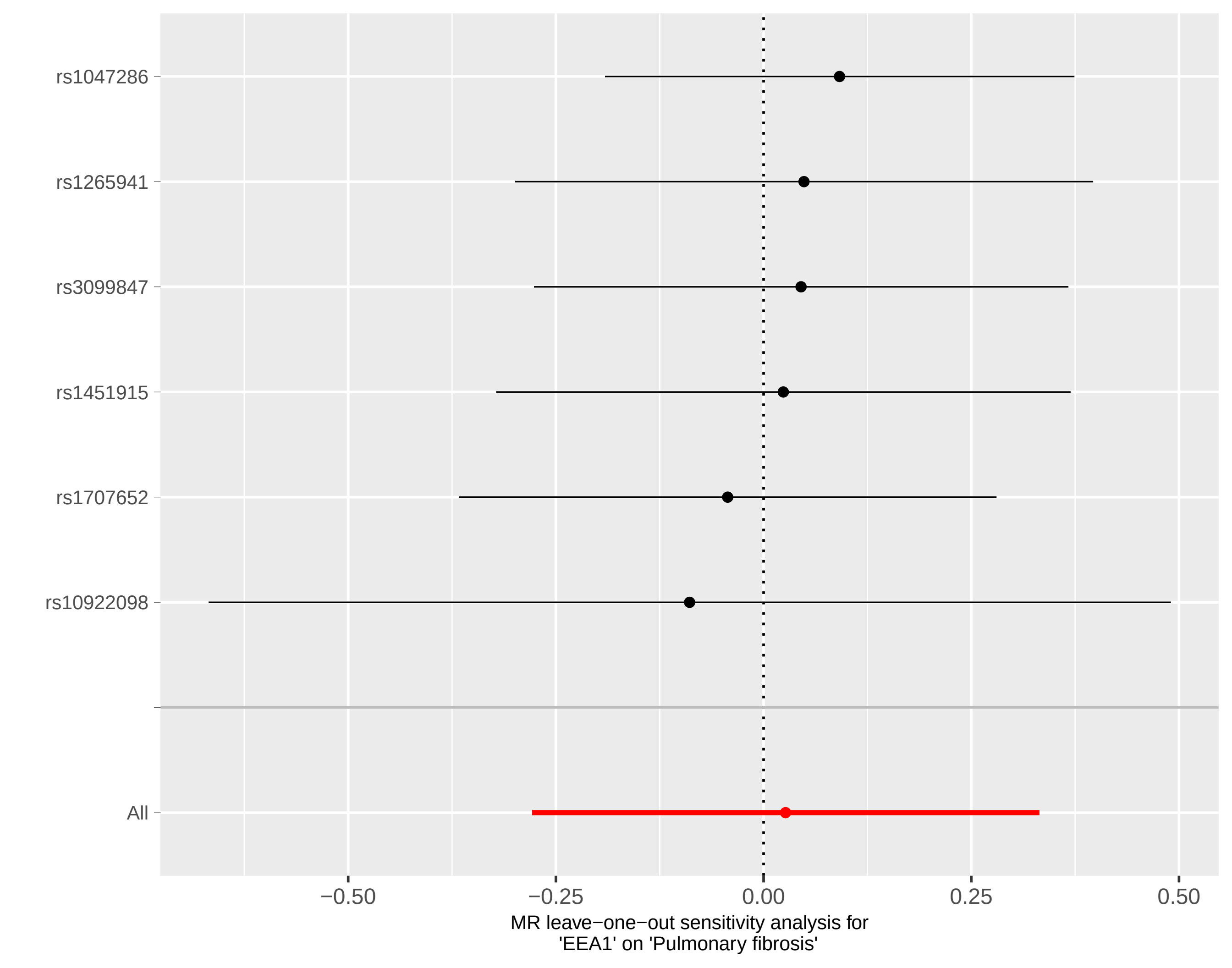

rs1047286
rs1265941
rs3099847
rs1451915
rs1707652
rs10922098
All
−0.50
−0.25
0.00
0.25
0.50
MR leave−one−out sensitivity analysis for
'EEA1' on 'Pulmonary fibrosis'

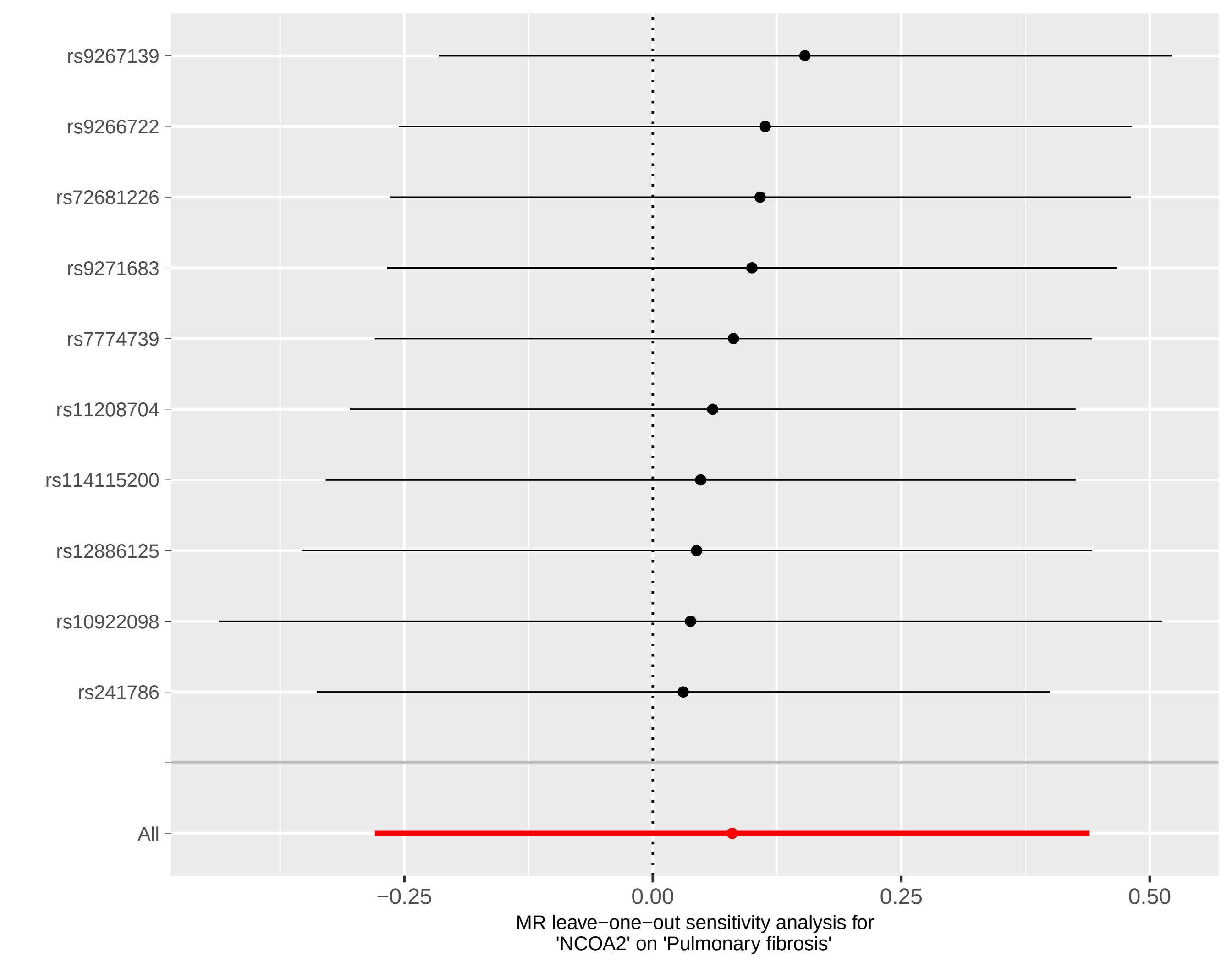
rs9267139
rs9266722
rs72681226
rs9271683
rs7774739
rs11208704
rs114115200
rs12886125
rs10922098
rs241786
All
−0.25
0.00
0.25
0.50
MR leave−one−out sensitivity analysis for
'NCOA2' on 'Pulmonary fibrosis'

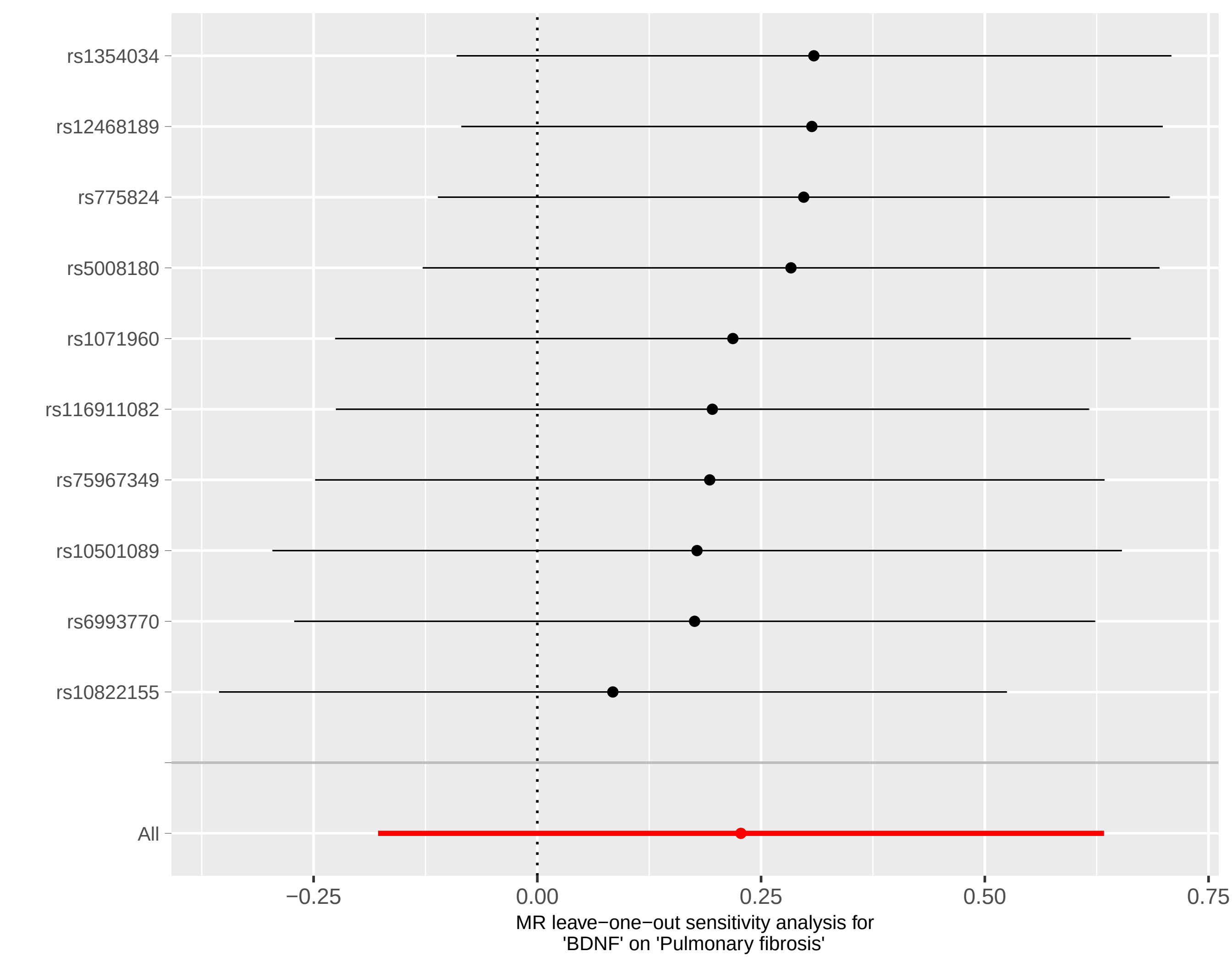
rs1354034
rs12468189
rs775824
rs5008180
rs1071960
rs116911082
rs75967349
rs10501089
rs6993770
rs10822155
All
−0.25
0.00
0.25
0.50
0.75
MR leave−one−out sensitivity analysis for
'BDNF' on 'Pulmonary fibrosis'

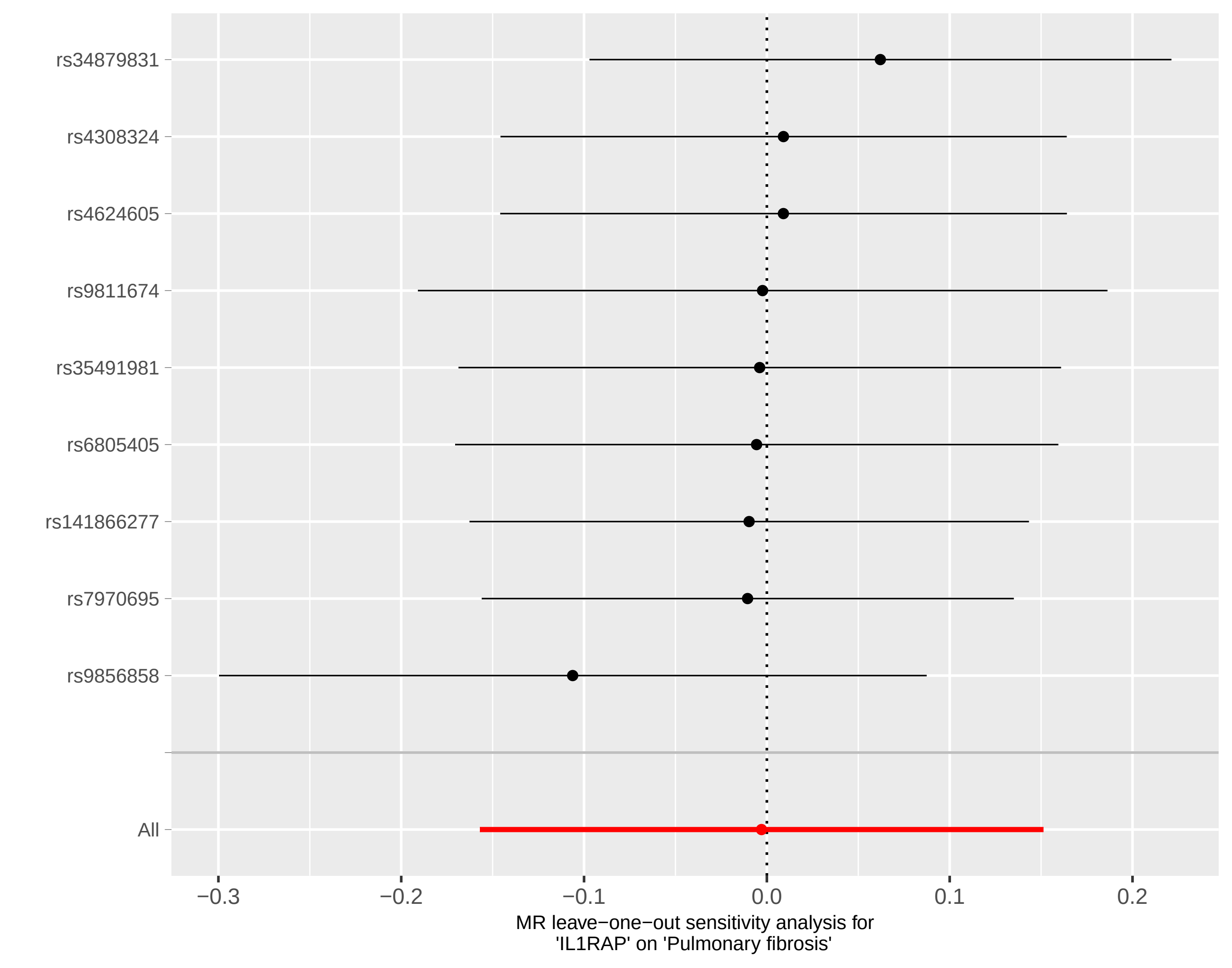
rs34879831
rs4308324
rs4624605
rs9811674
rs35491981
rs6805405
rs141866277
rs7970695
rs9856858
All
−0.3
−0.2
−0.1
0.0
0.1
0.2
MR leave−one−out sensitivity analysis for
'IL1RAP' on 'Pulmonary fibrosis'

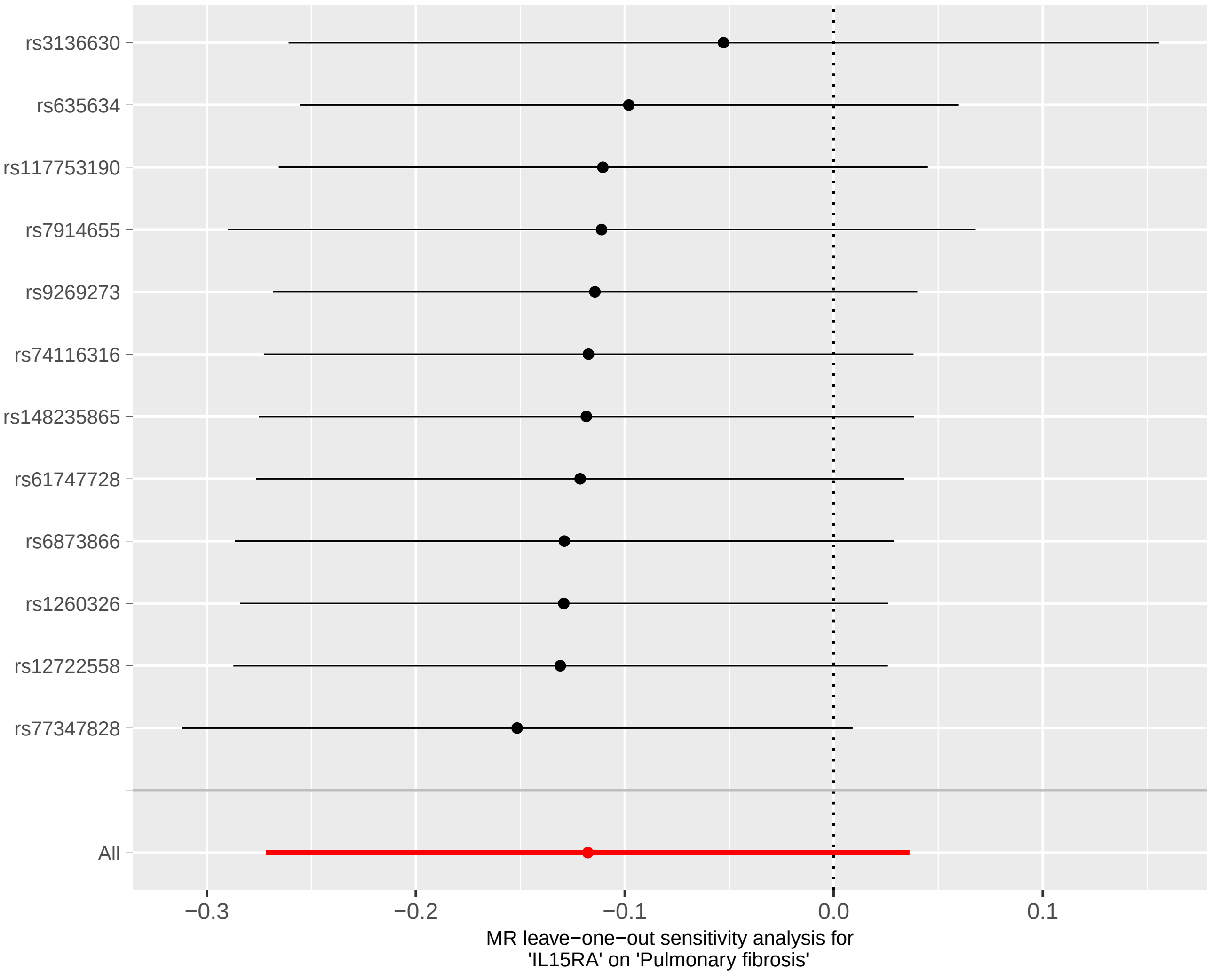
rs3136630
rs635634
rs117753190
rs7914655
rs9269273
rs74116316
rs148235865
rs61747728
rs6873866
rs1260326
rs12722558
rs77347828
All
−0.3
−0.2
−0.1
0.0
0.1
MR leave−one−out sensitivity analysis for
'IL15RA' on 'Pulmonary fibrosis'

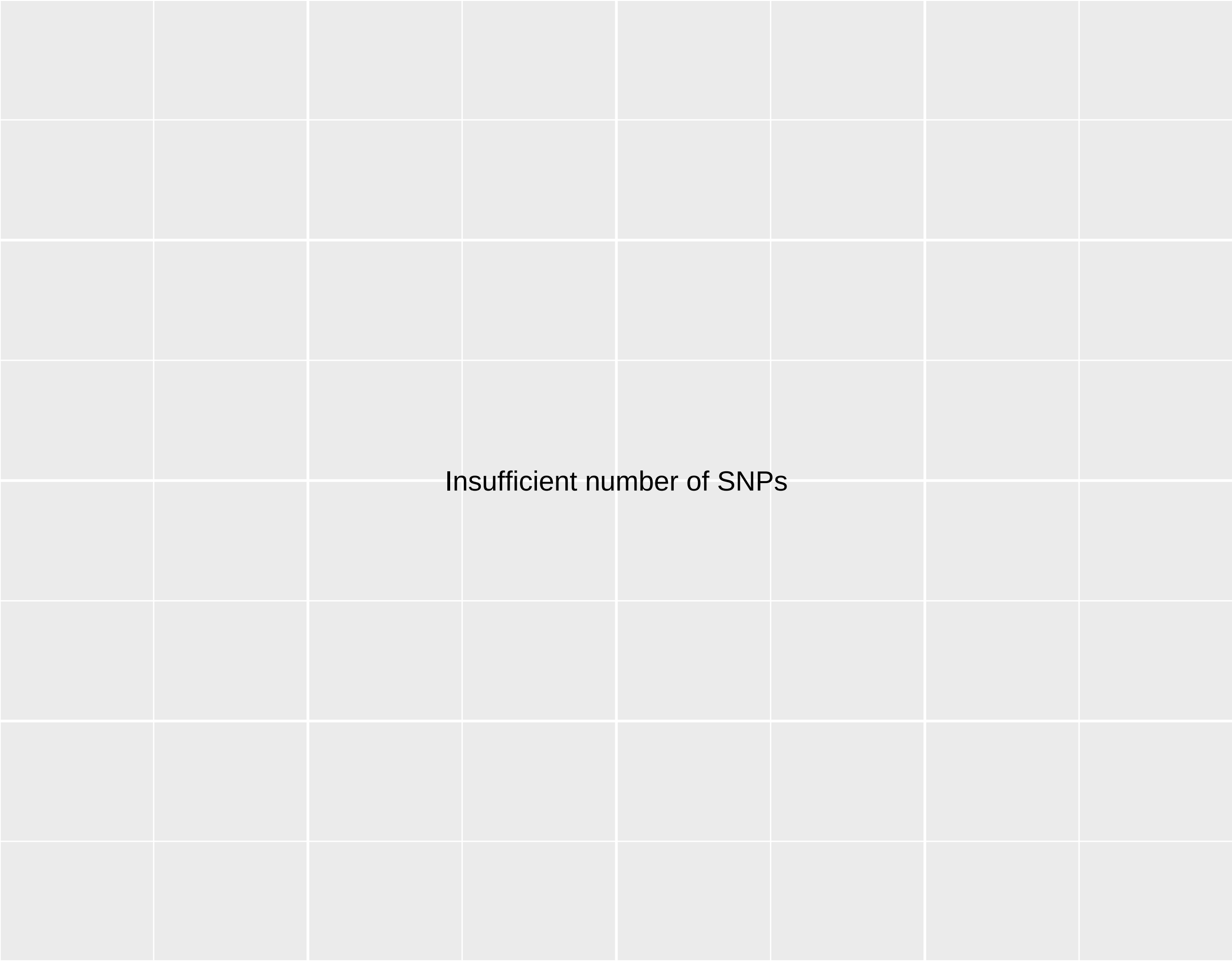
Insufficient number of SNPs

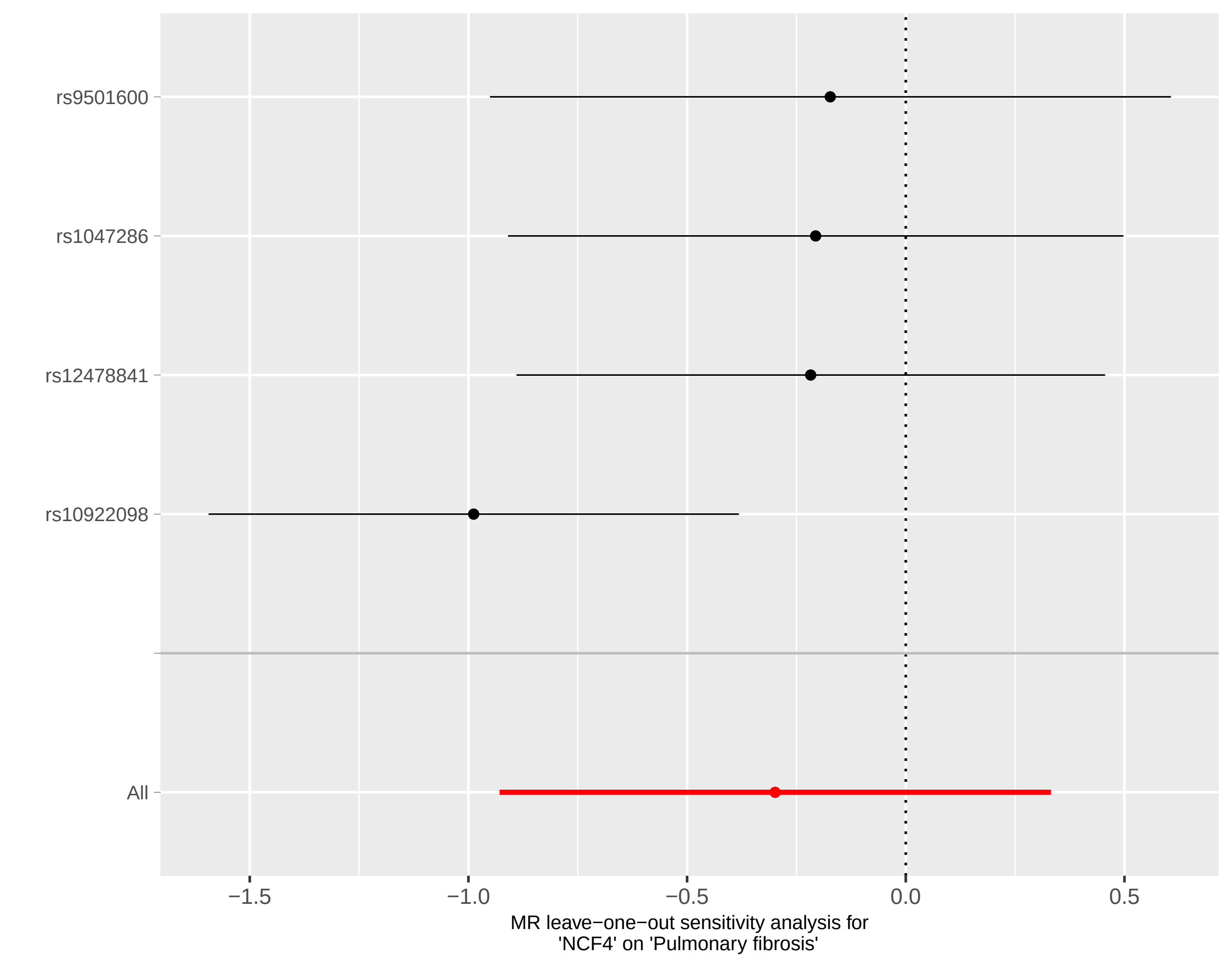
rs9501600
rs1047286
rs12478841
rs10922098
All
−1.5
−1.0
−0.5
0.0
0.5
MR leave−one−out sensitivity analysis for
'NCF4' on 'Pulmonary fibrosis'

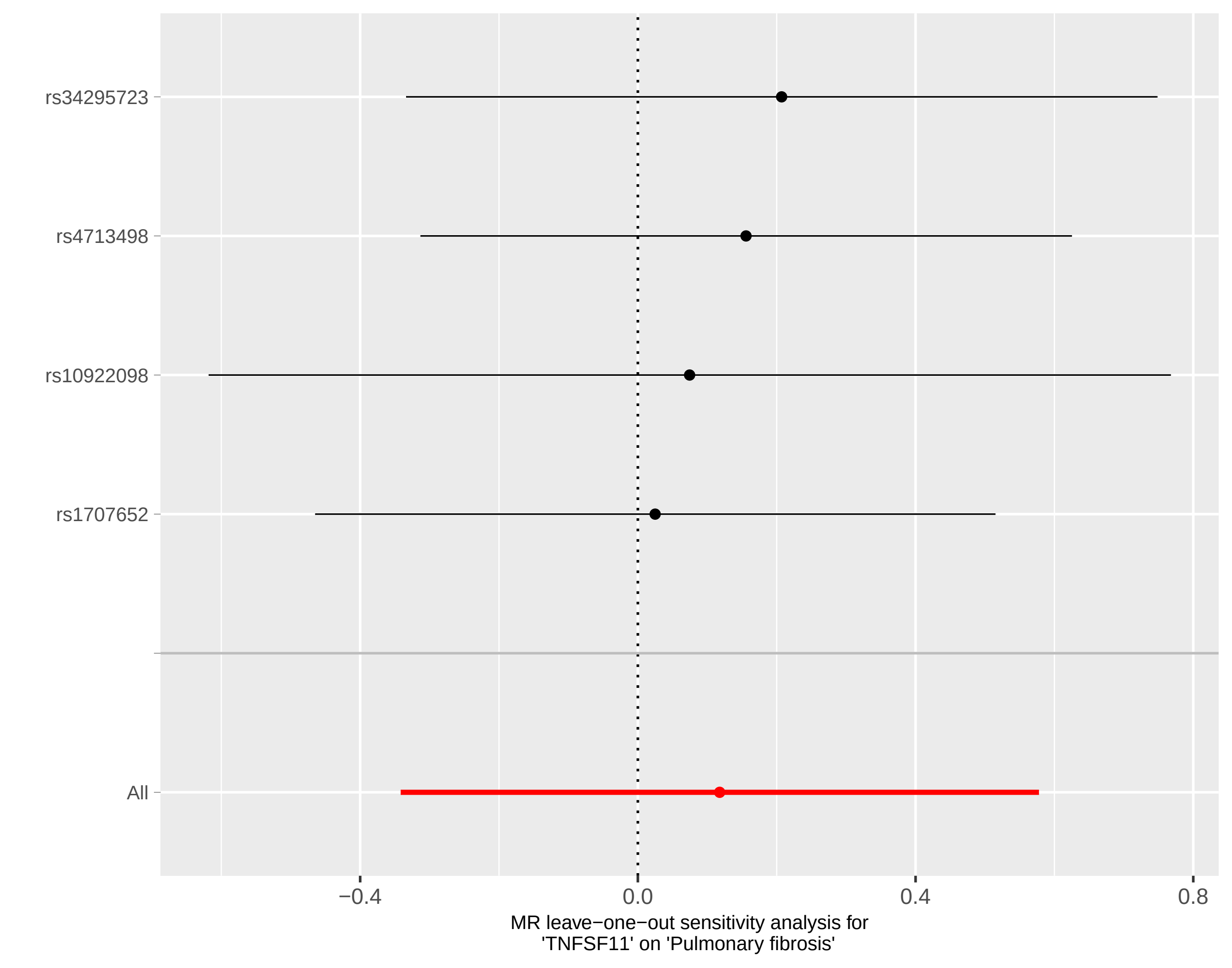
rs34295723
rs4713498
rs10922098
rs1707652
All
−0.4
0.0
0.4
0.8
MR leave−one−out sensitivity analysis for
'TNFSF11' on 'Pulmonary fibrosis'

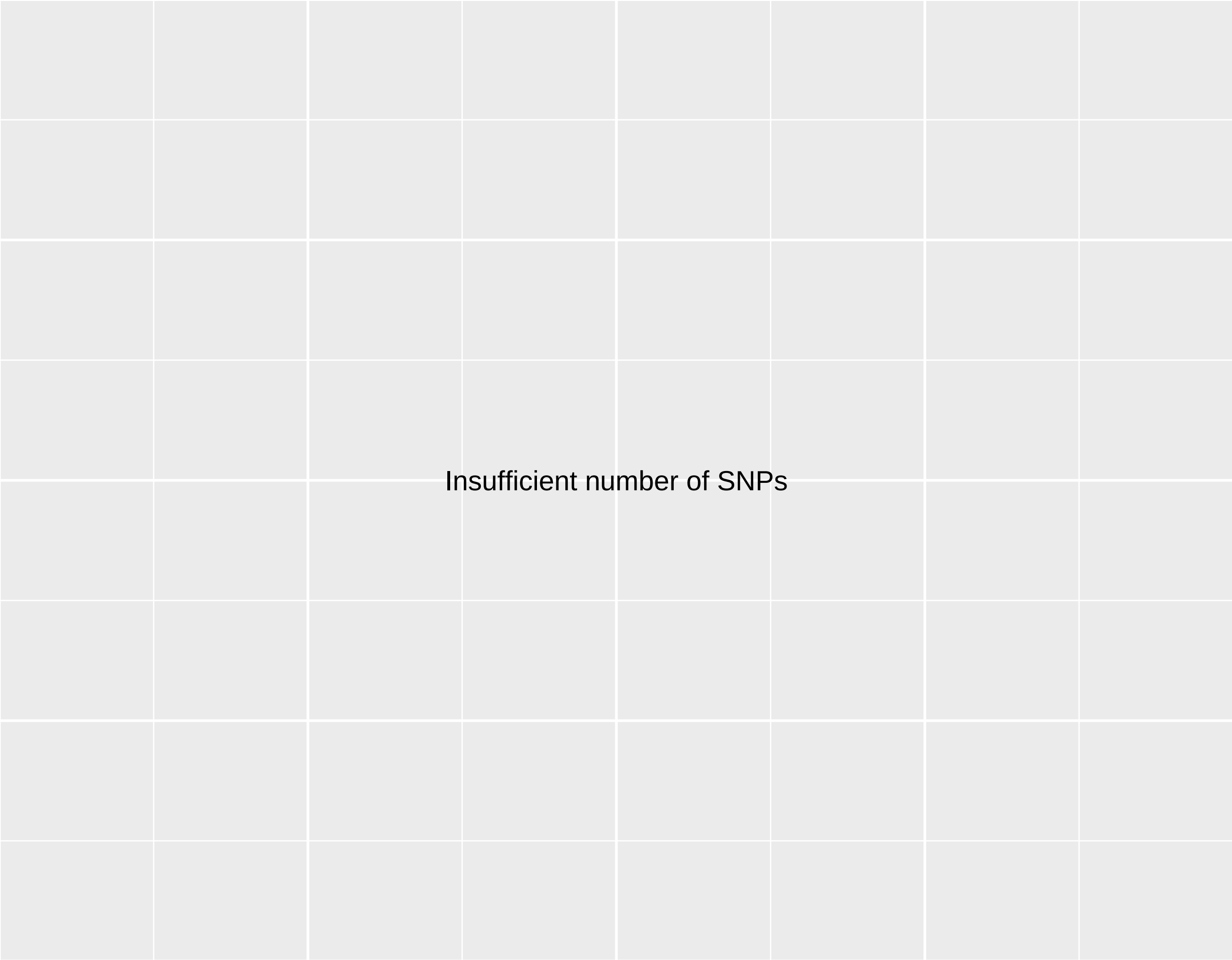
Insufficient number of SNPs

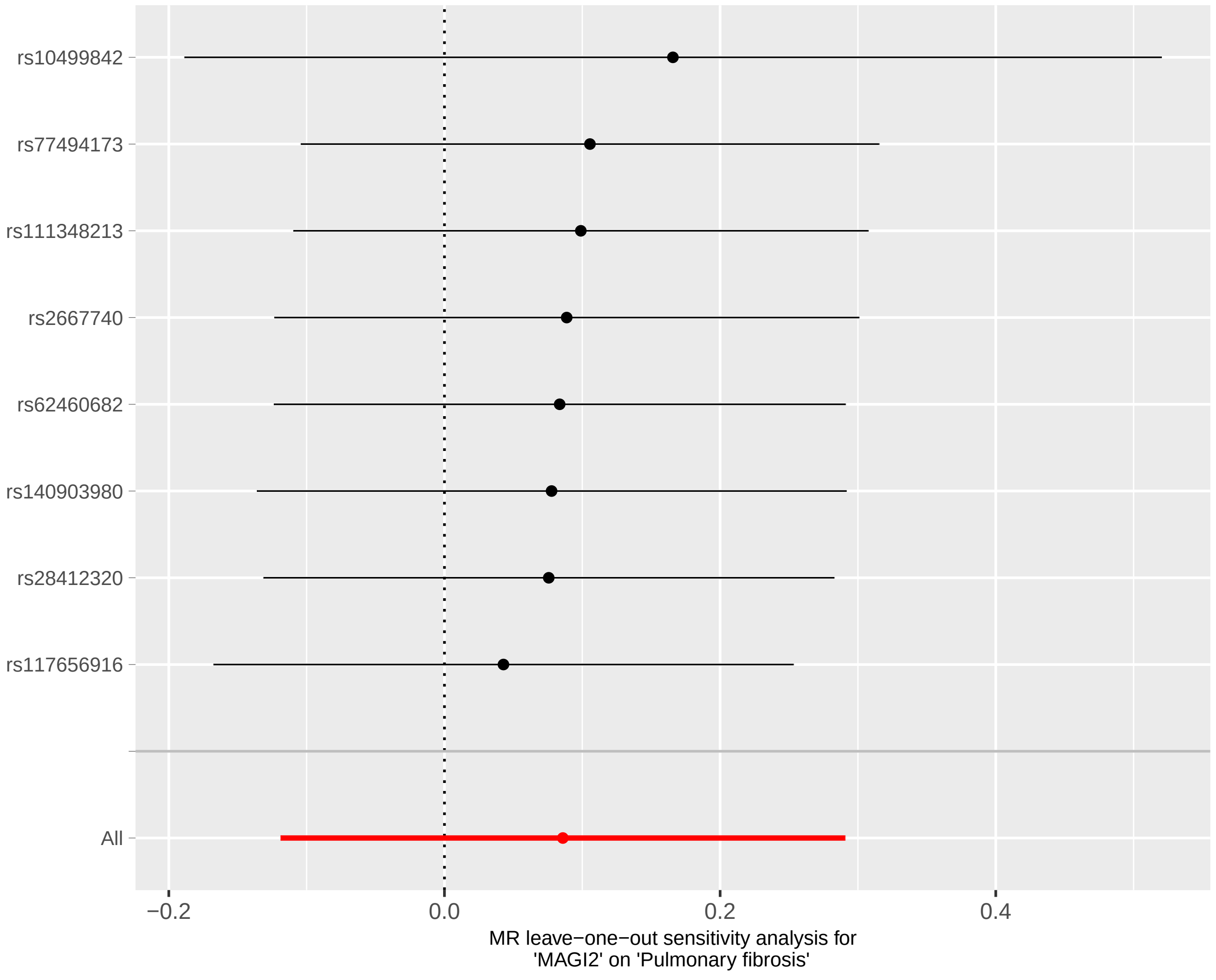

rs10499842
rs77494173
rs111348213
rs2667740
rs62460682
rs140903980
rs28412320
rs117656916
All
−0.2
0.0
0.2
0.4
MR leave−one−out sensitivity analysis for
'MAGI2' on 'Pulmonary fibrosis'

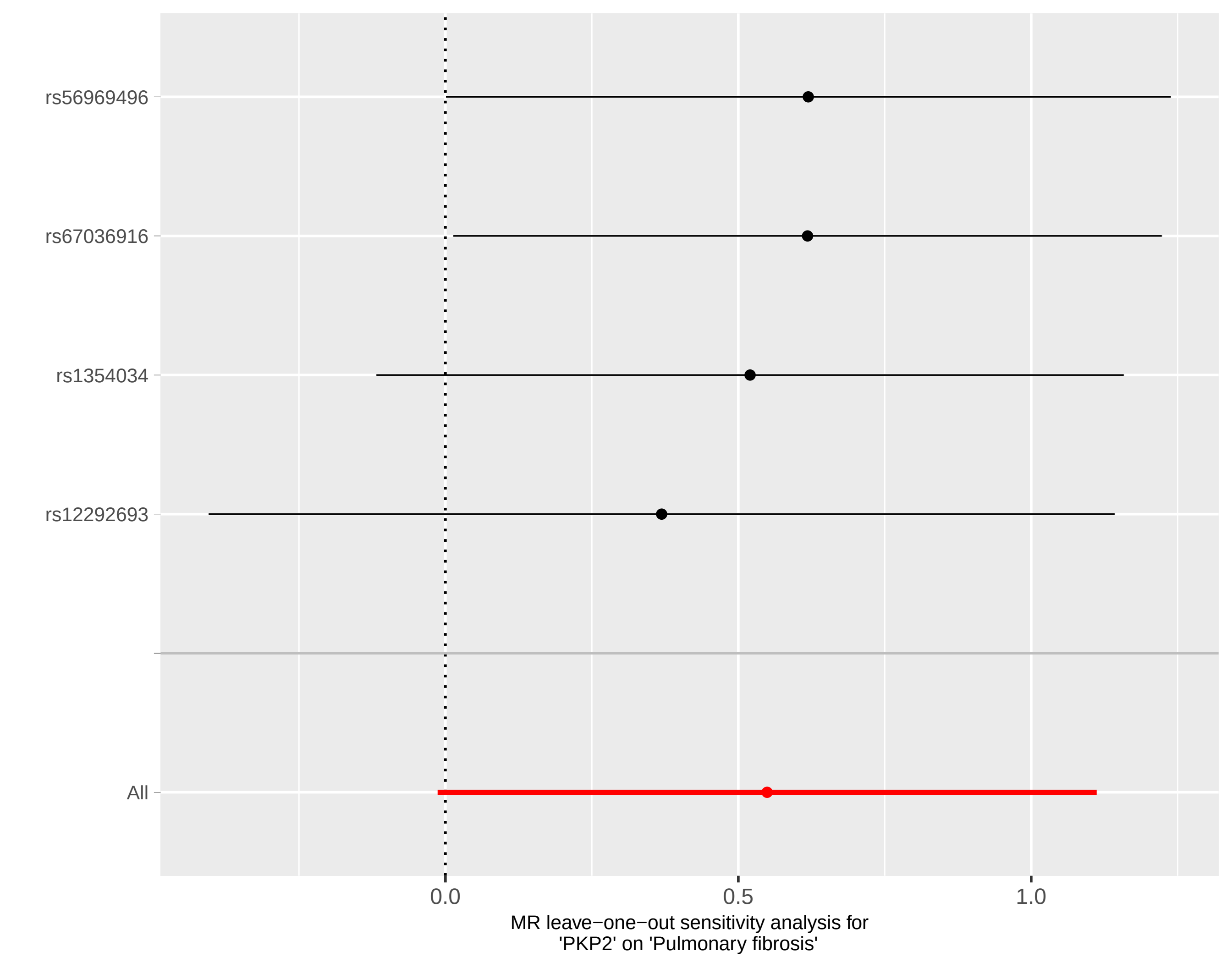
rs56969496
rs67036916
rs1354034
rs12292693
All
0.0
0.5
1.0
MR leave−one−out sensitivity analysis for
'PKP2' on 'Pulmonary fibrosis'

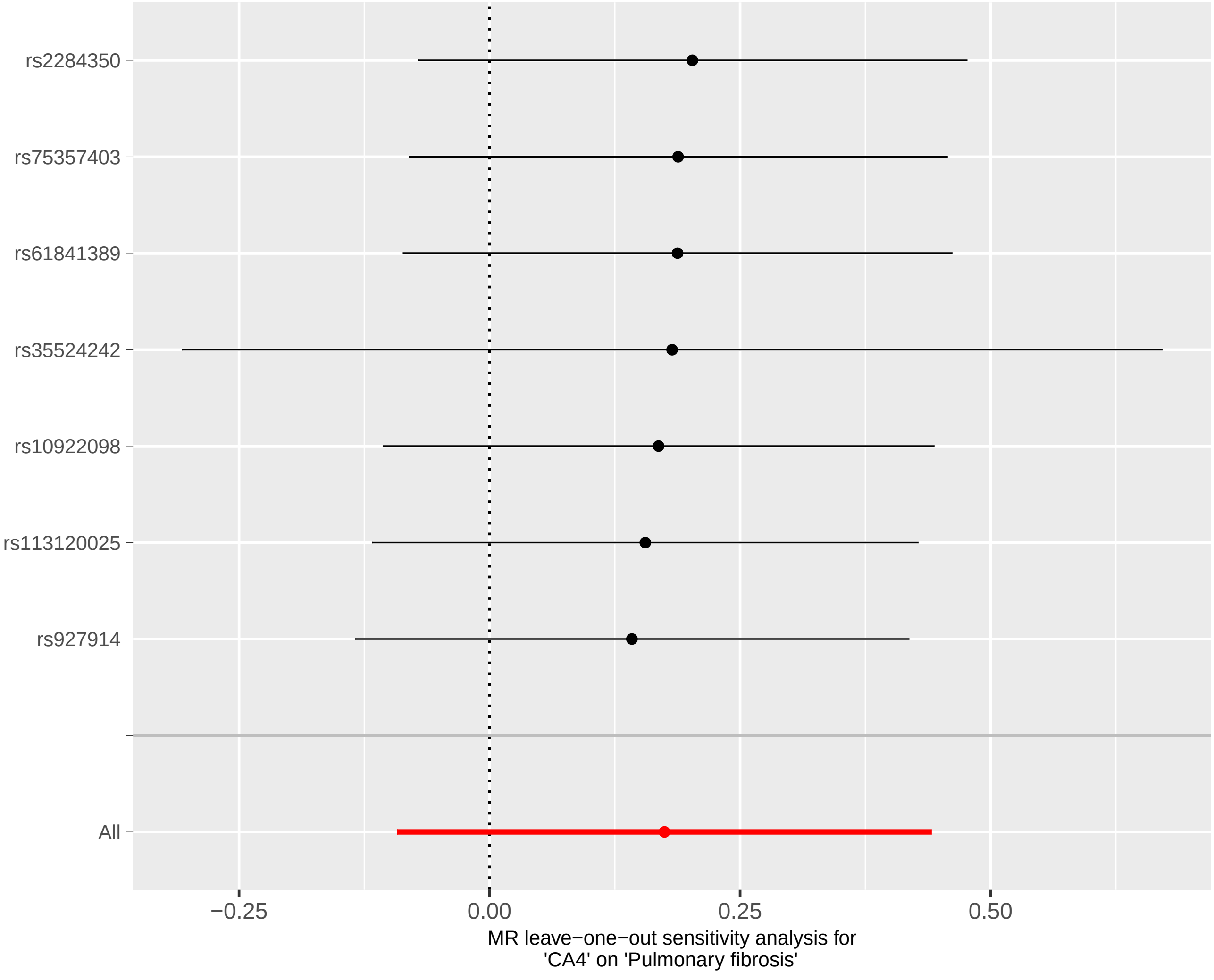
rs2284350
rs75357403
rs61841389
rs35524242
rs10922098
rs113120025
rs927914
All
−0.25
0.00
0.25
0.50
MR leave−one−out sensitivity analysis for
'CA4' on 'Pulmonary fibrosis'

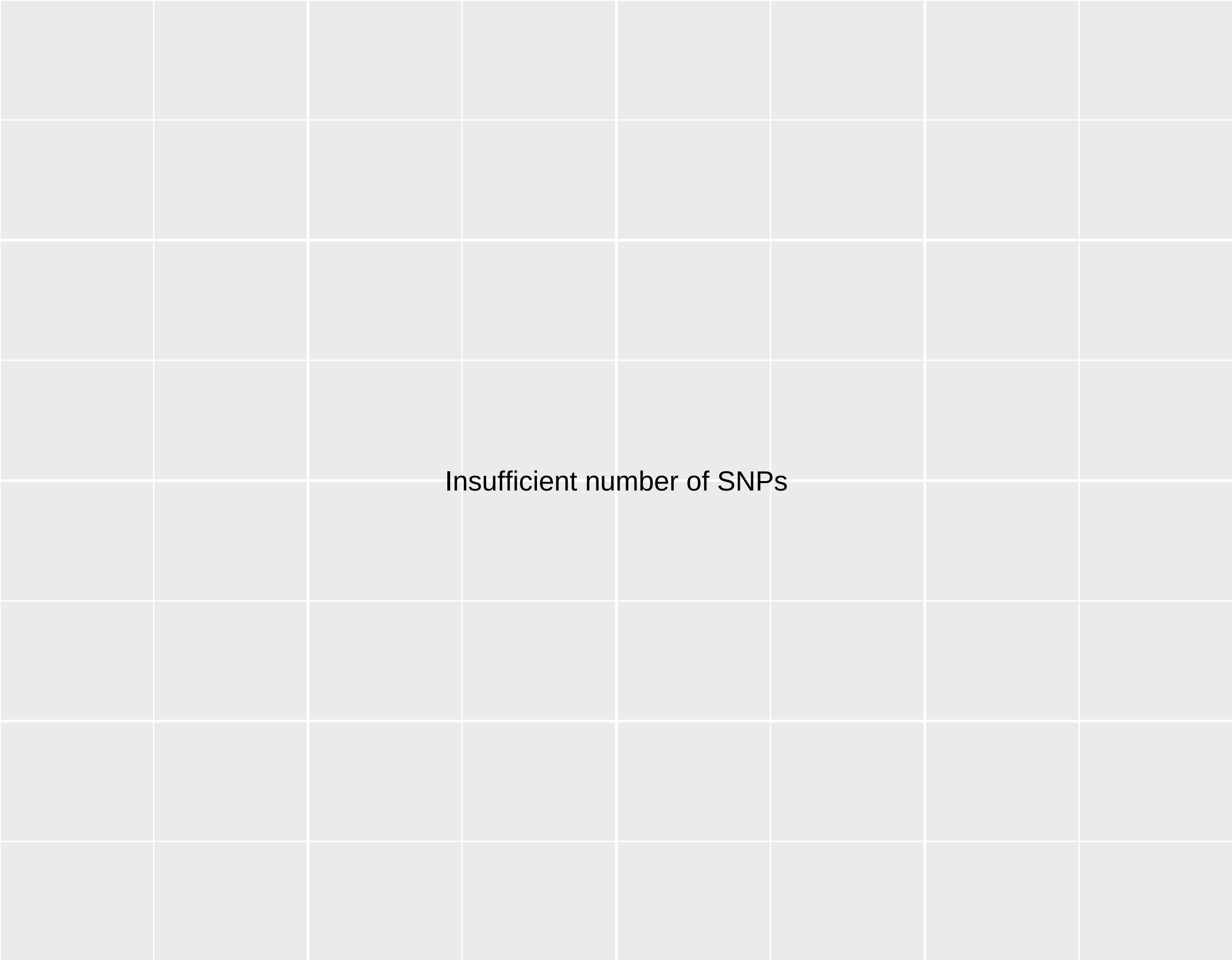
Insufficient number of SNPs

Insufficient number of SNPs

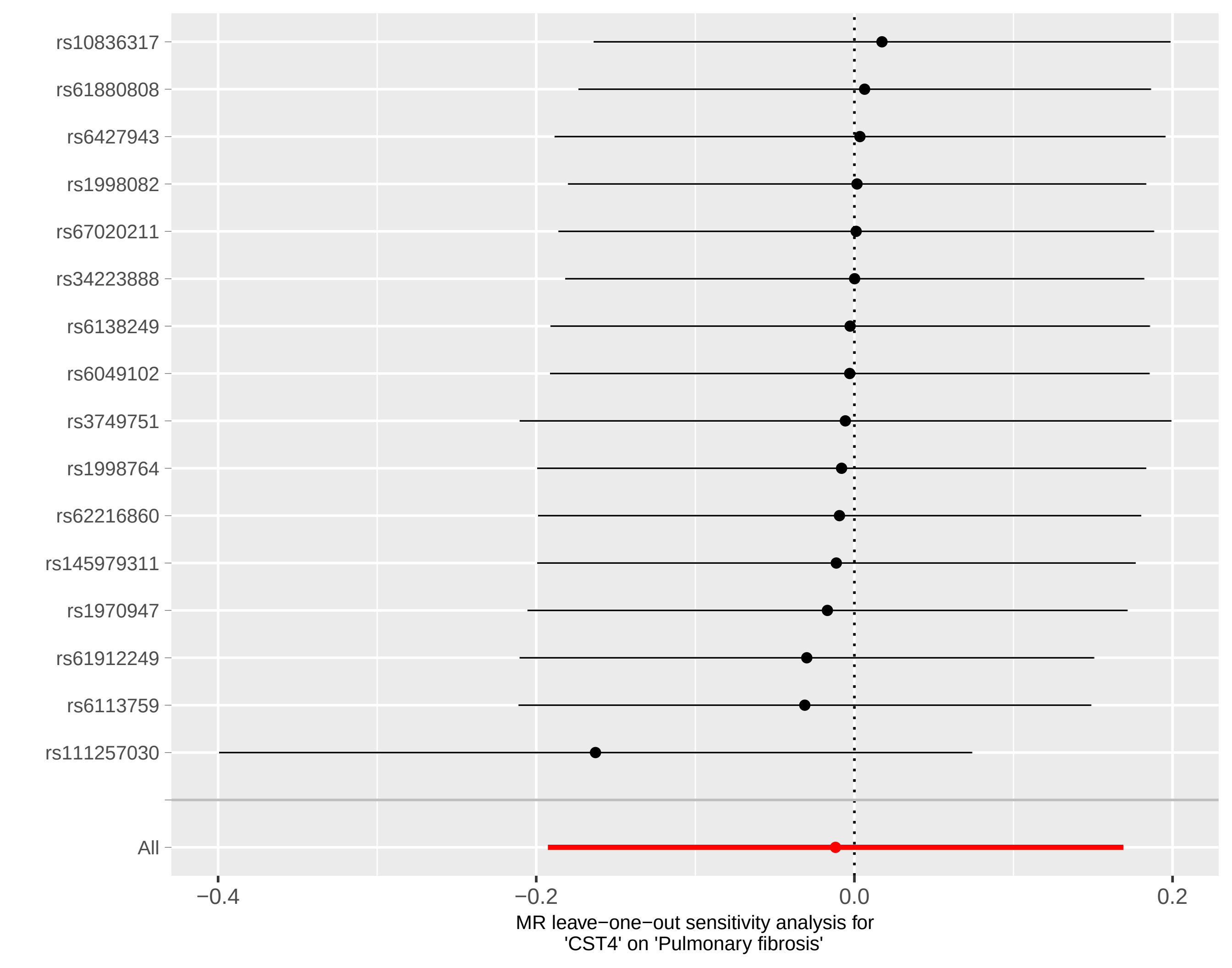

rs10836317
rs61880808
rs6427943
rs1998082
rs67020211
rs34223888
rs6138249
rs6049102
rs3749751
rs1998764
rs62216860
rs145979311
rs1970947
rs61912249
rs6113759
rs111257030
All
−0.4
−0.2
0.0
0.2
MR leave−one−out sensitivity analysis for
'CST4' on 'Pulmonary fibrosis'

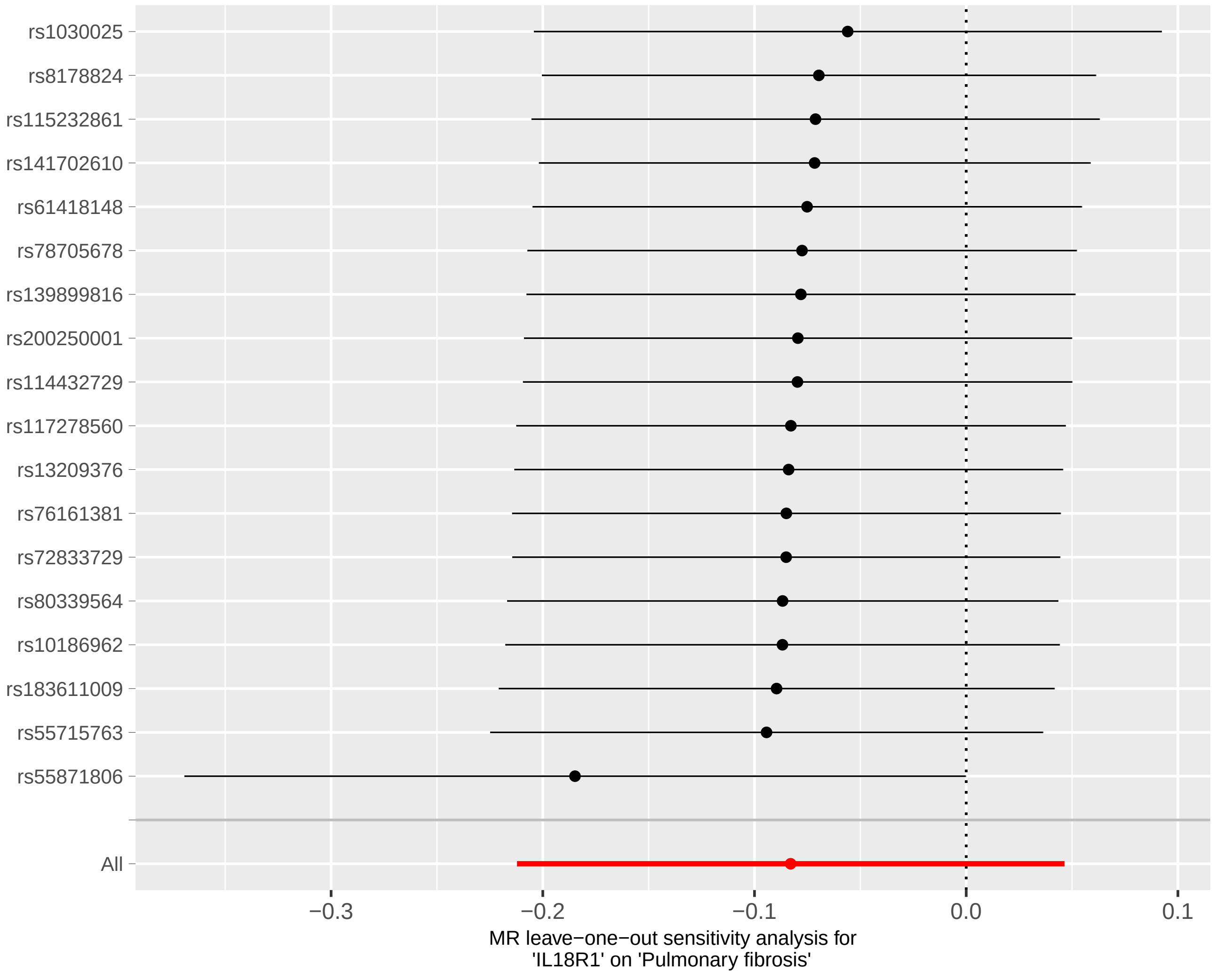

rs1030025
rs8178824
rs115232861
rs141702610
rs61418148
rs78705678
rs139899816
rs200250001
rs114432729
rs117278560
rs13209376
rs76161381
rs72833729
rs80339564
rs10186962
rs183611009
rs55715763
rs55871806
All
−0.3
−0.2
−0.1
0.0
0.1
MR leave−one−out sensitivity analysis for
'IL18R1' on 'Pulmonary fibrosis'

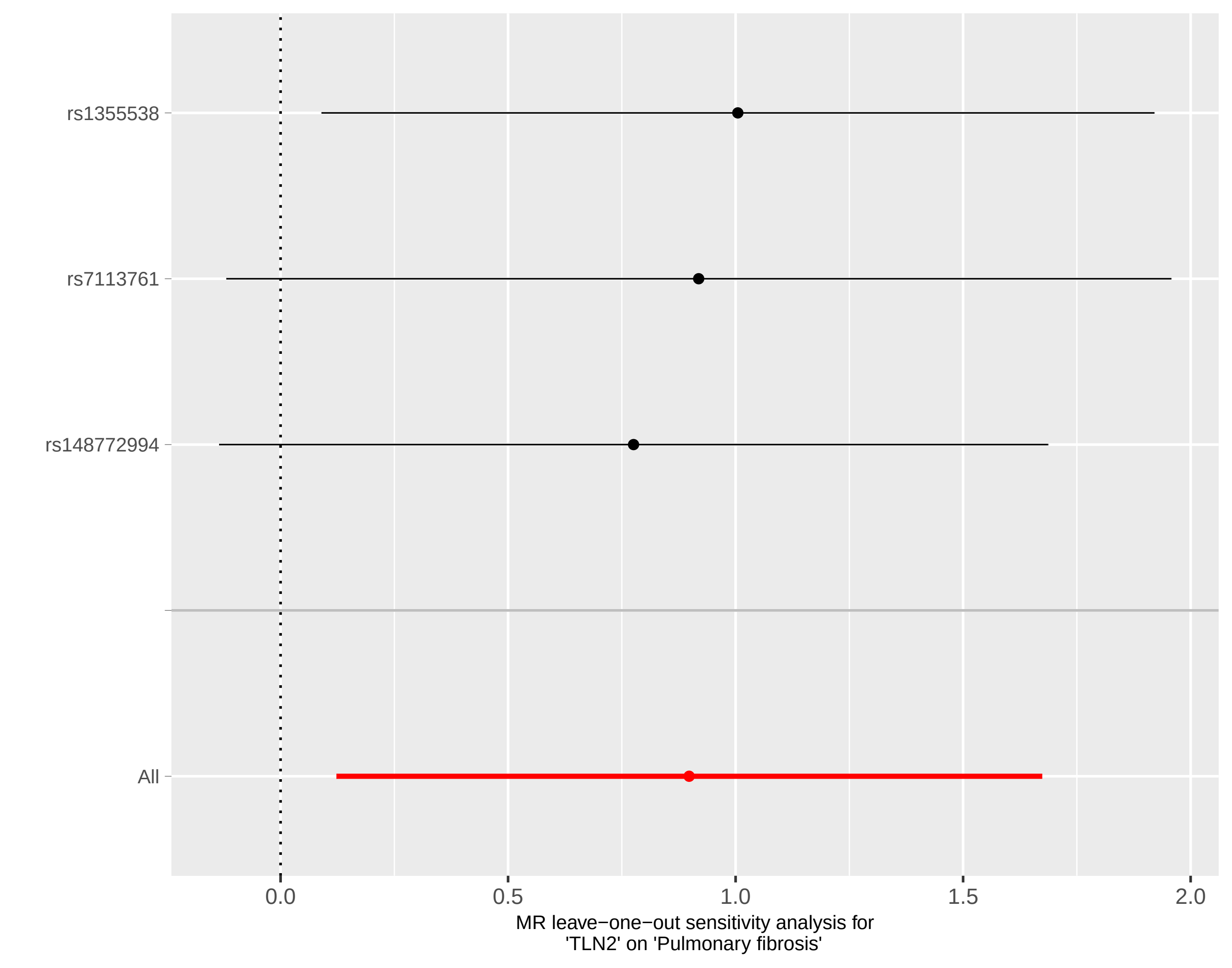

rs1355538
rs7113761
rs148772994
All
0.0
0.5
1.0
1.5
2.0
MR leave−one−out sensitivity analysis for
'TLN2' on 'Pulmonary fibrosis'

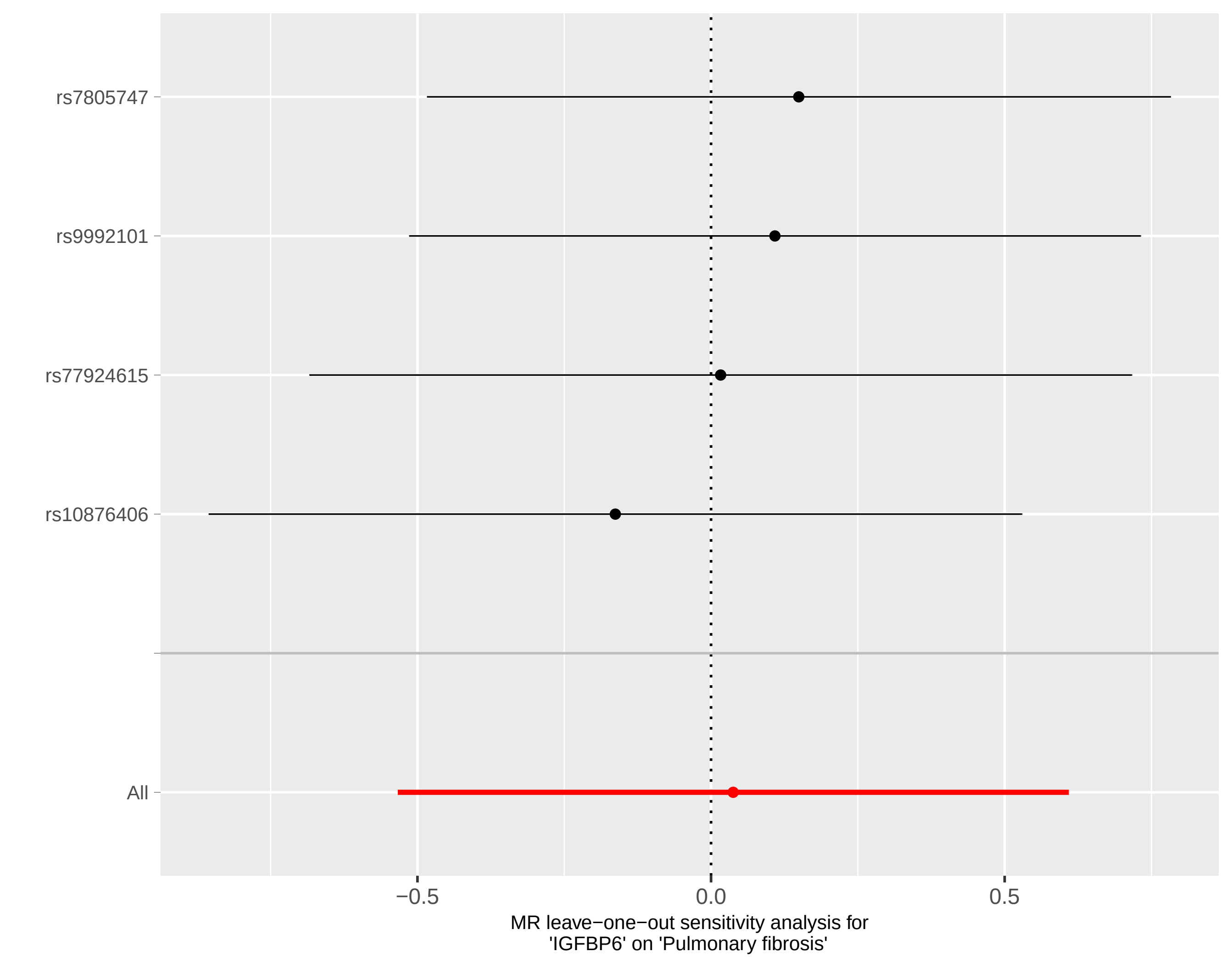
rs7805747
rs9992101
rs77924615
rs10876406
All
−0.5
0.0
0.5
MR leave−one−out sensitivity analysis for
'IGFBP6' on 'Pulmonary fibrosis'

Insufficient number of SNPs

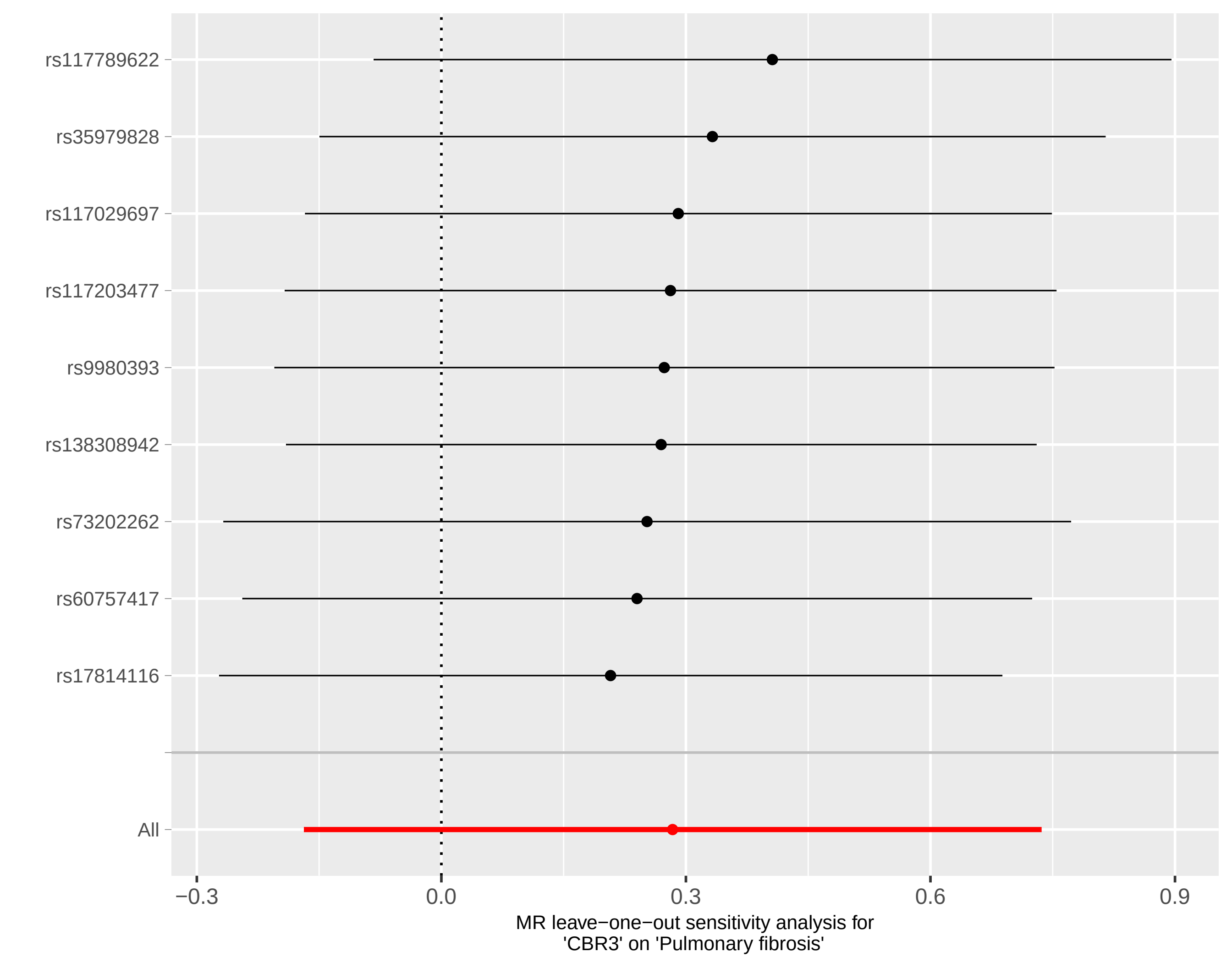
rs117789622
rs35979828
rs117029697
rs117203477
rs9980393
rs138308942
rs73202262
rs60757417
rs17814116
All
−0.3
0.0
0.3
0.6
0.9
MR leave−one−out sensitivity analysis for
'CBR3' on 'Pulmonary fibrosis'

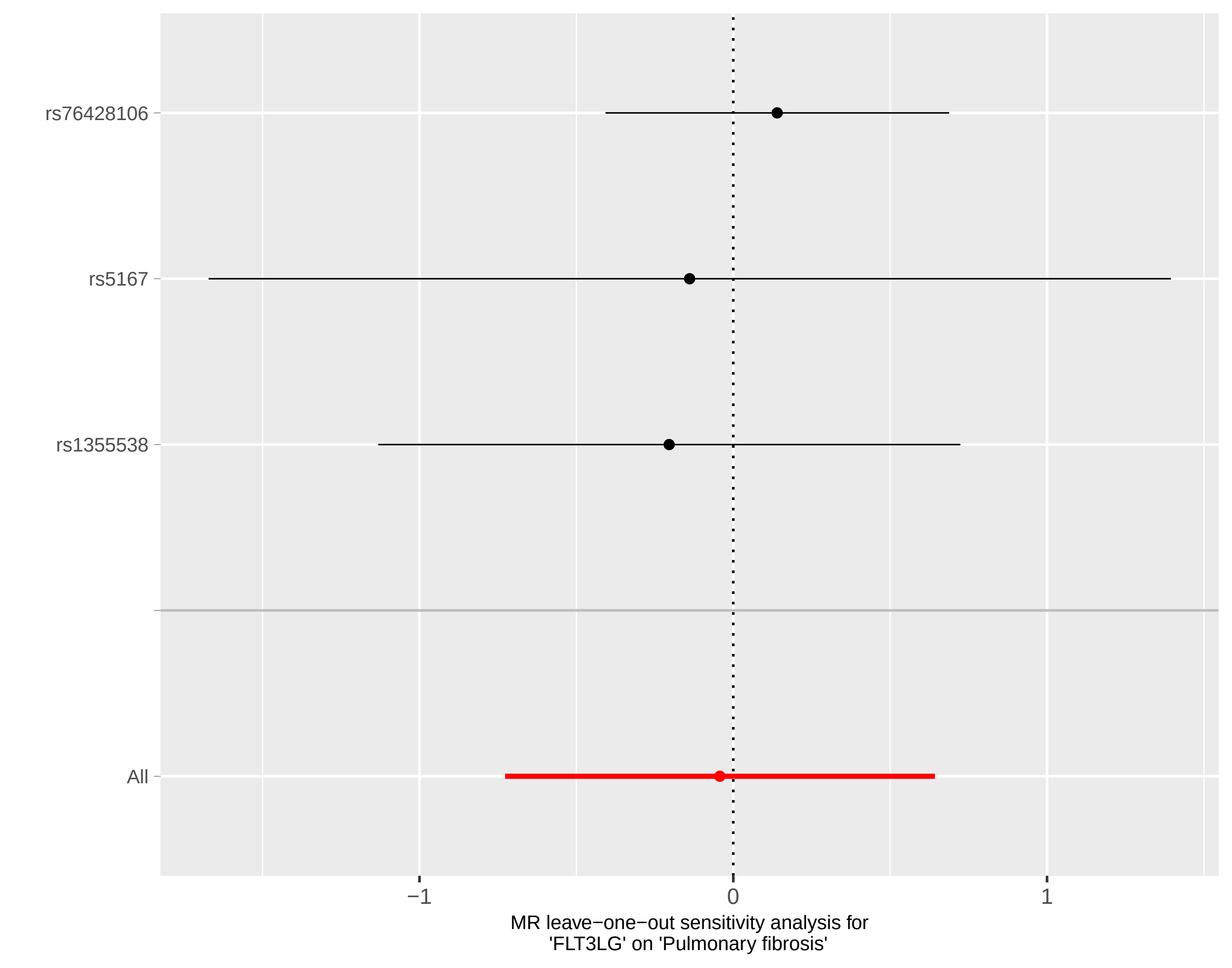
rs76428106
rs5167
rs1355538
All
−1
0
1
MR leave−one−out sensitivity analysis for
'FLT3LG' on 'Pulmonary fibrosis'

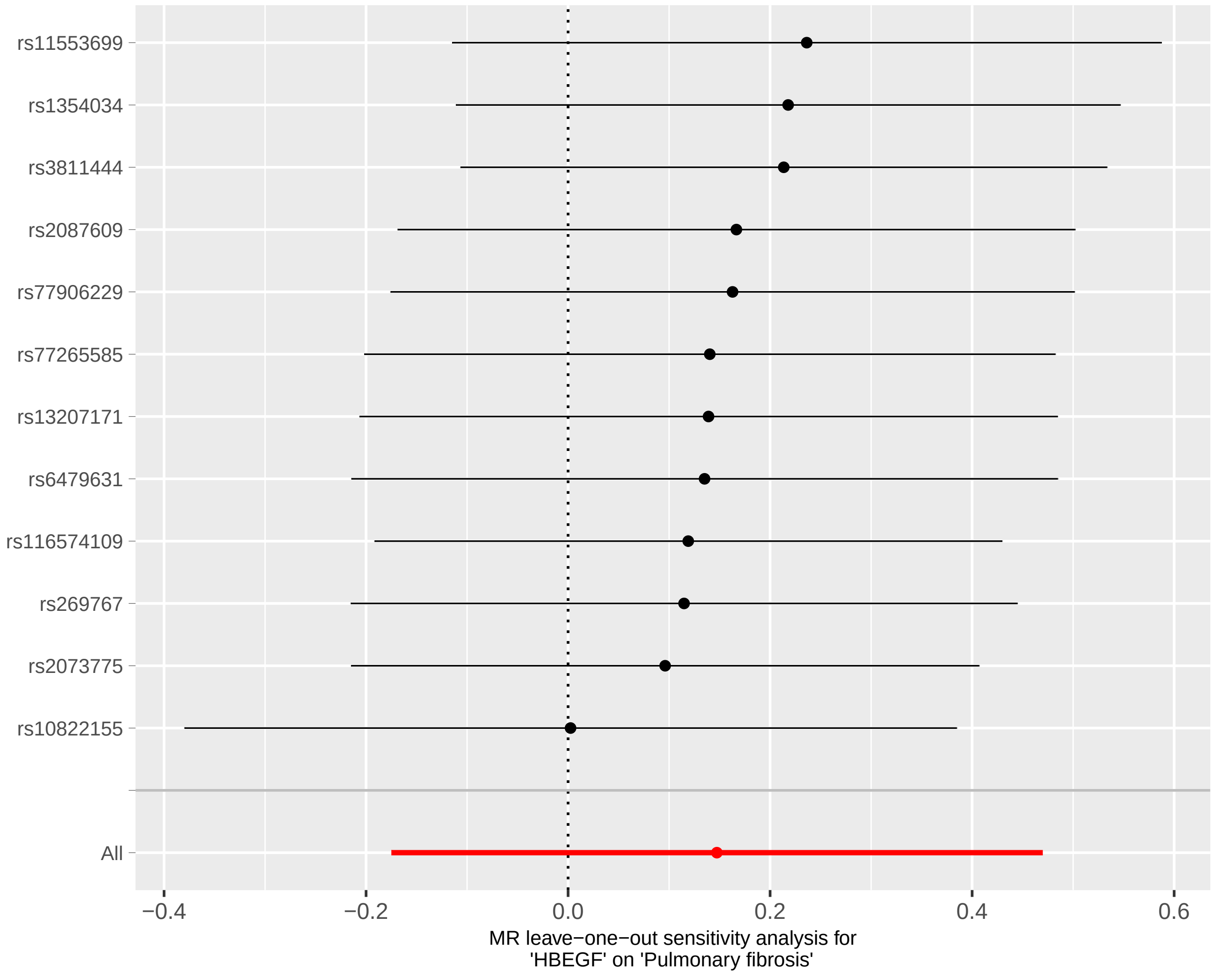
rs11553699
rs1354034
rs3811444
rs2087609
rs77906229
rs77265585
rs13207171
rs6479631
rs116574109
rs269767
rs2073775
rs10822155
All
−0.4
−0.2
0.0
0.2
0.4
0.6
MR leave−one−out sensitivity analysis for
'HBEGF' on 'Pulmonary fibrosis'

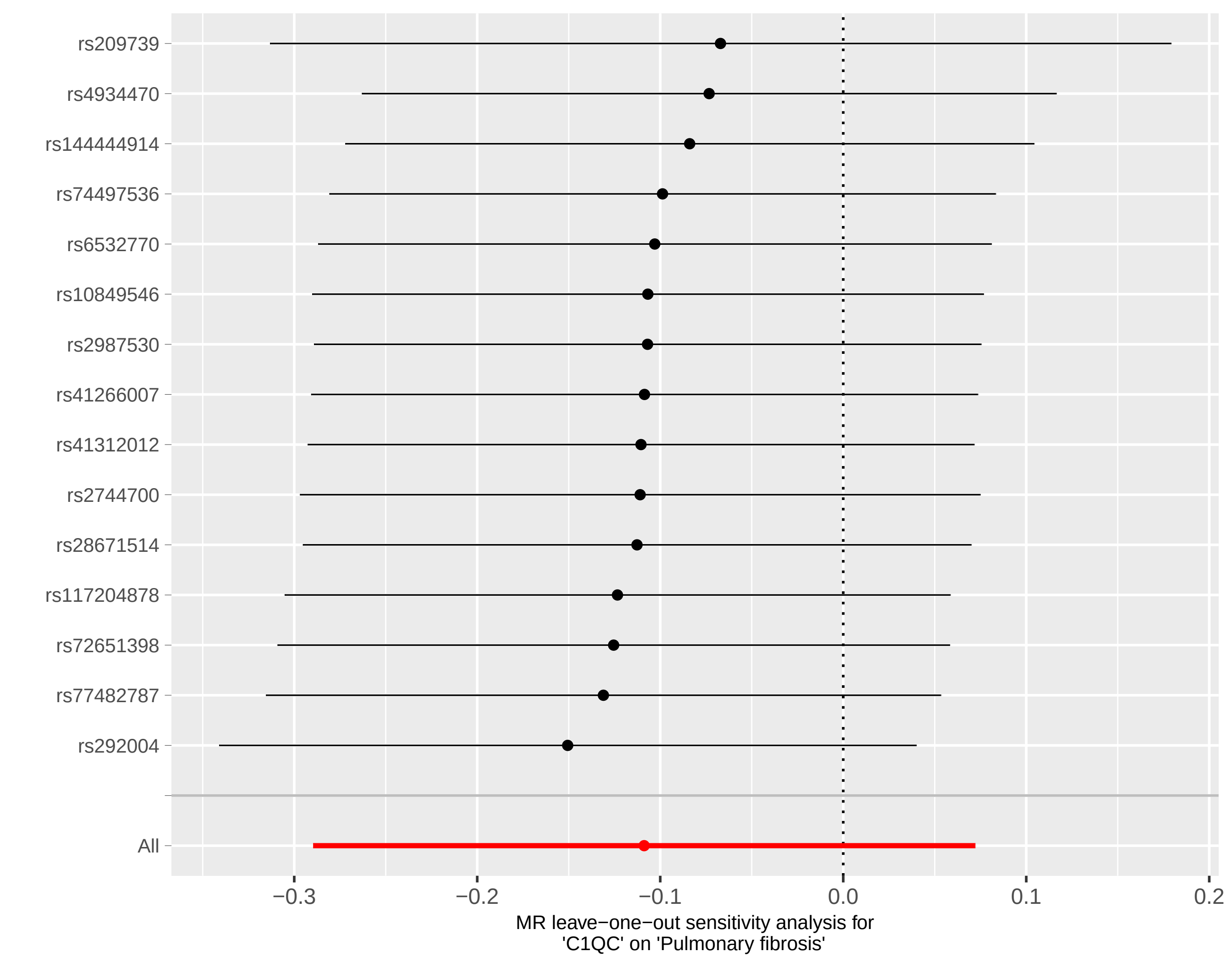
rs209739
rs4934470
rs144444914
rs74497536
rs6532770
rs10849546
rs2987530
rs41266007
rs41312012
rs2744700
rs28671514
rs117204878
rs72651398
rs77482787
rs292004
All
−0.3
−0.2
−0.1
0.0
0.1
0.2
MR leave−one−out sensitivity analysis for
'C1QC' on 'Pulmonary fibrosis'

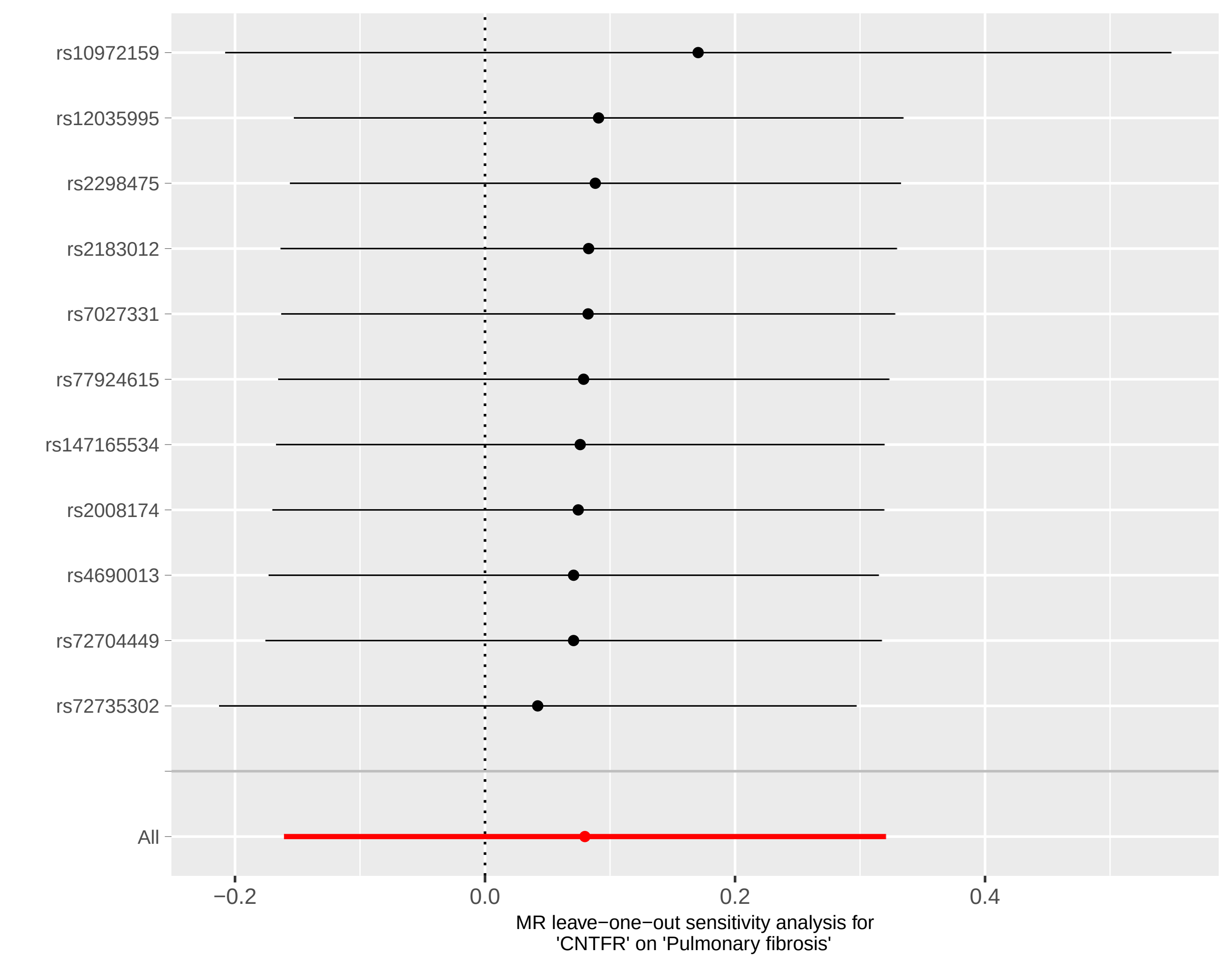
rs10972159
rs12035995
rs2298475
rs2183012
rs7027331
rs77924615
rs147165534
rs2008174
rs4690013
rs72704449
rs72735302
All
−0.2
0.0
0.2
0.4
MR leave−one−out sensitivity analysis for
'CNTFR' on 'Pulmonary fibrosis'

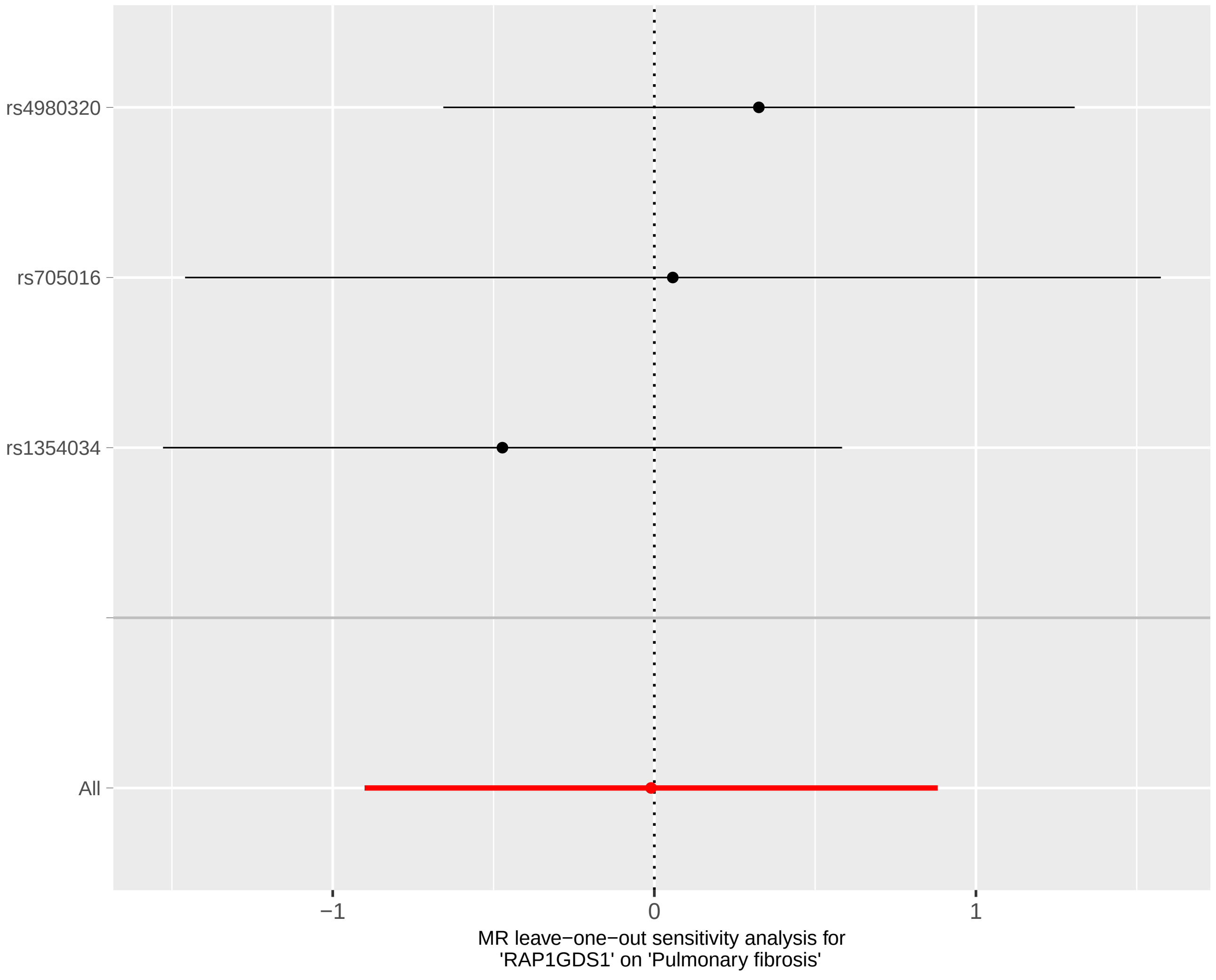
rs4980320
rs705016
rs1354034
All
−1
0
1
MR leave−one−out sensitivity analysis for
'RAP1GDS1' on 'Pulmonary fibrosis'

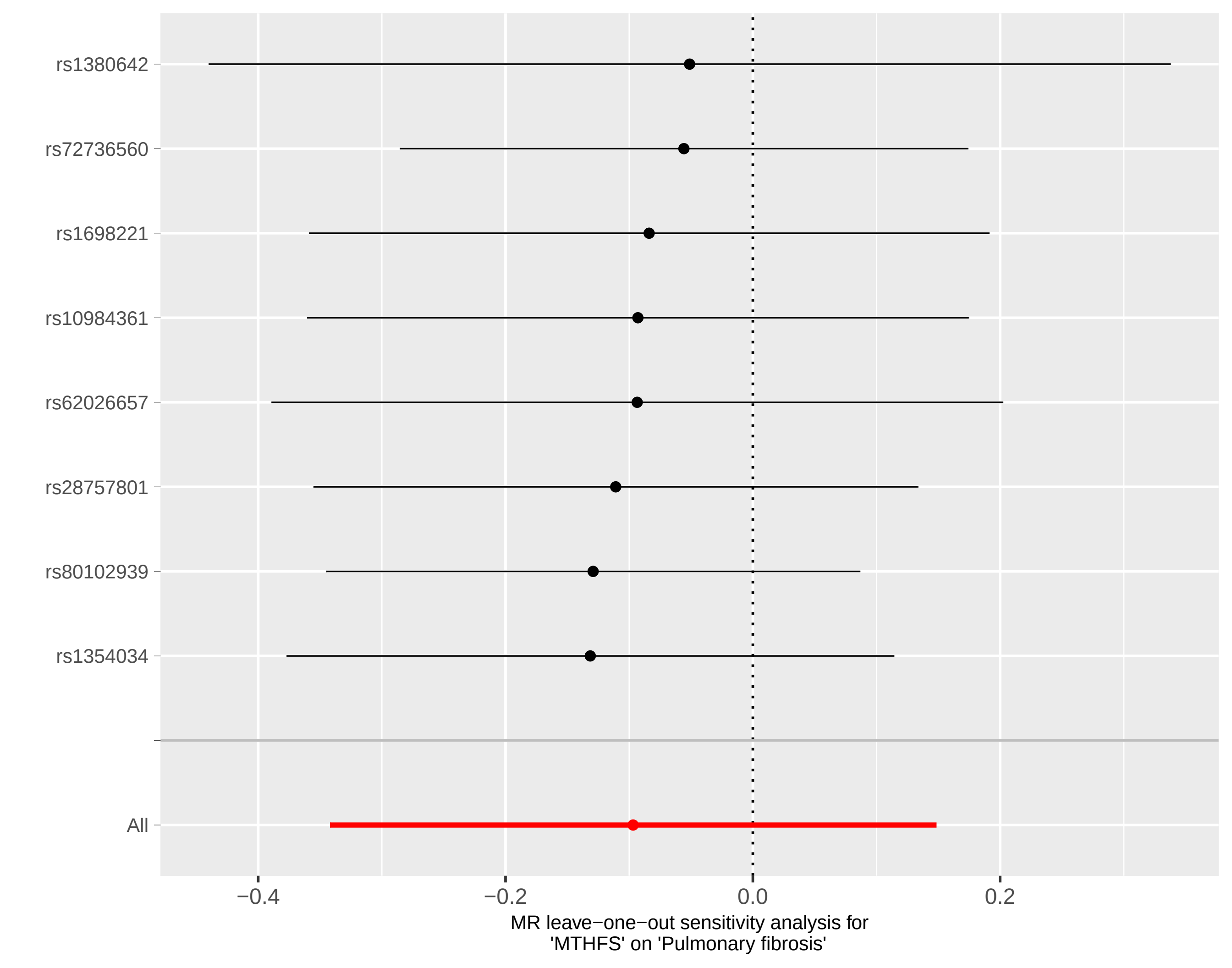
rs1380642
rs72736560
rs1698221
rs10984361
rs62026657
rs28757801
rs80102939
rs1354034
All
−0.4
−0.2
0.0
0.2
MR leave−one−out sensitivity analysis for
'MTHFS' on 'Pulmonary fibrosis'

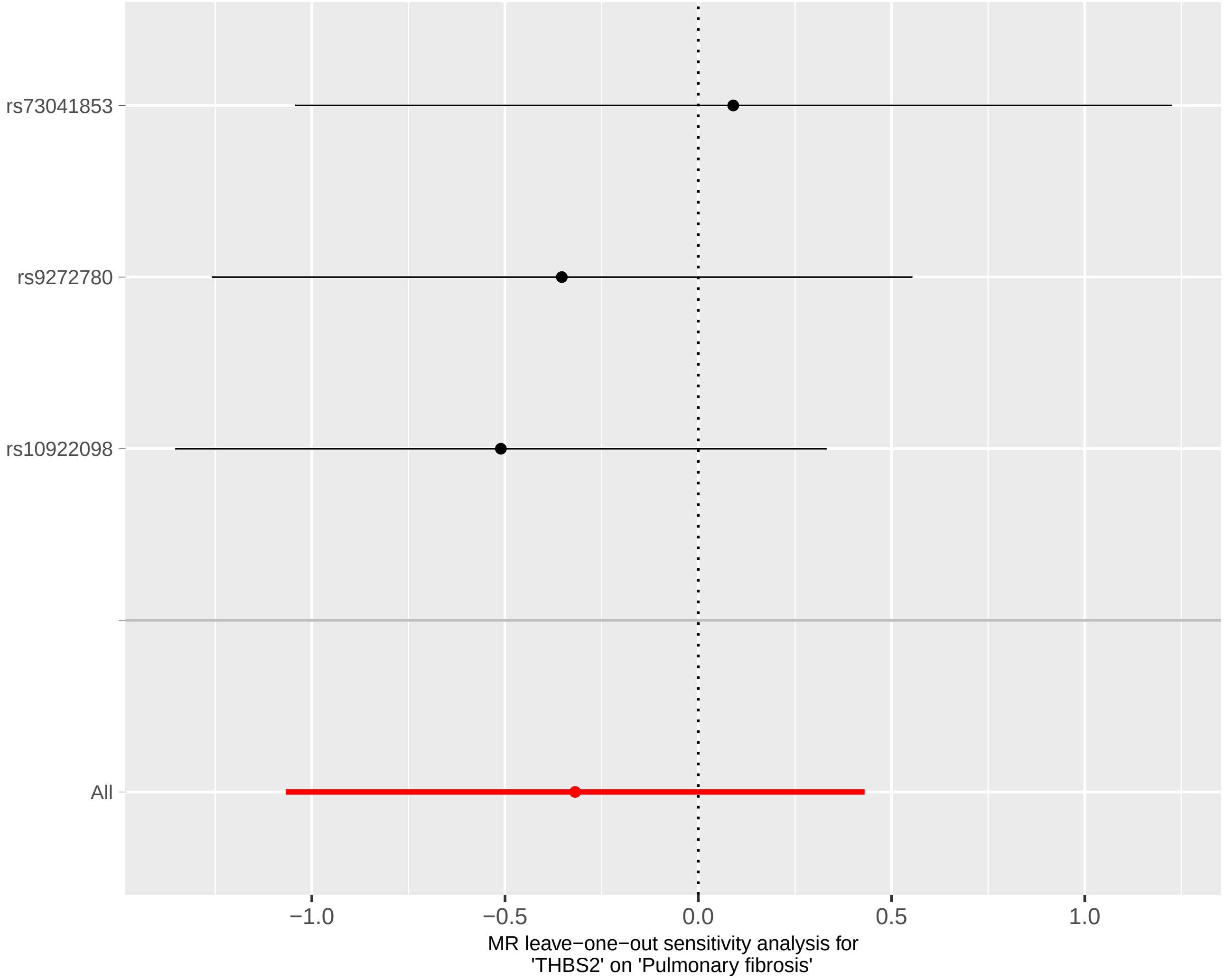
rs73041853
rs9272780
rs10922098
All
−1.0
−0.5
0.0
0.5
1.0
MR leave−one−out sensitivity analysis for
'THBS2' on 'Pulmonary fibrosis'

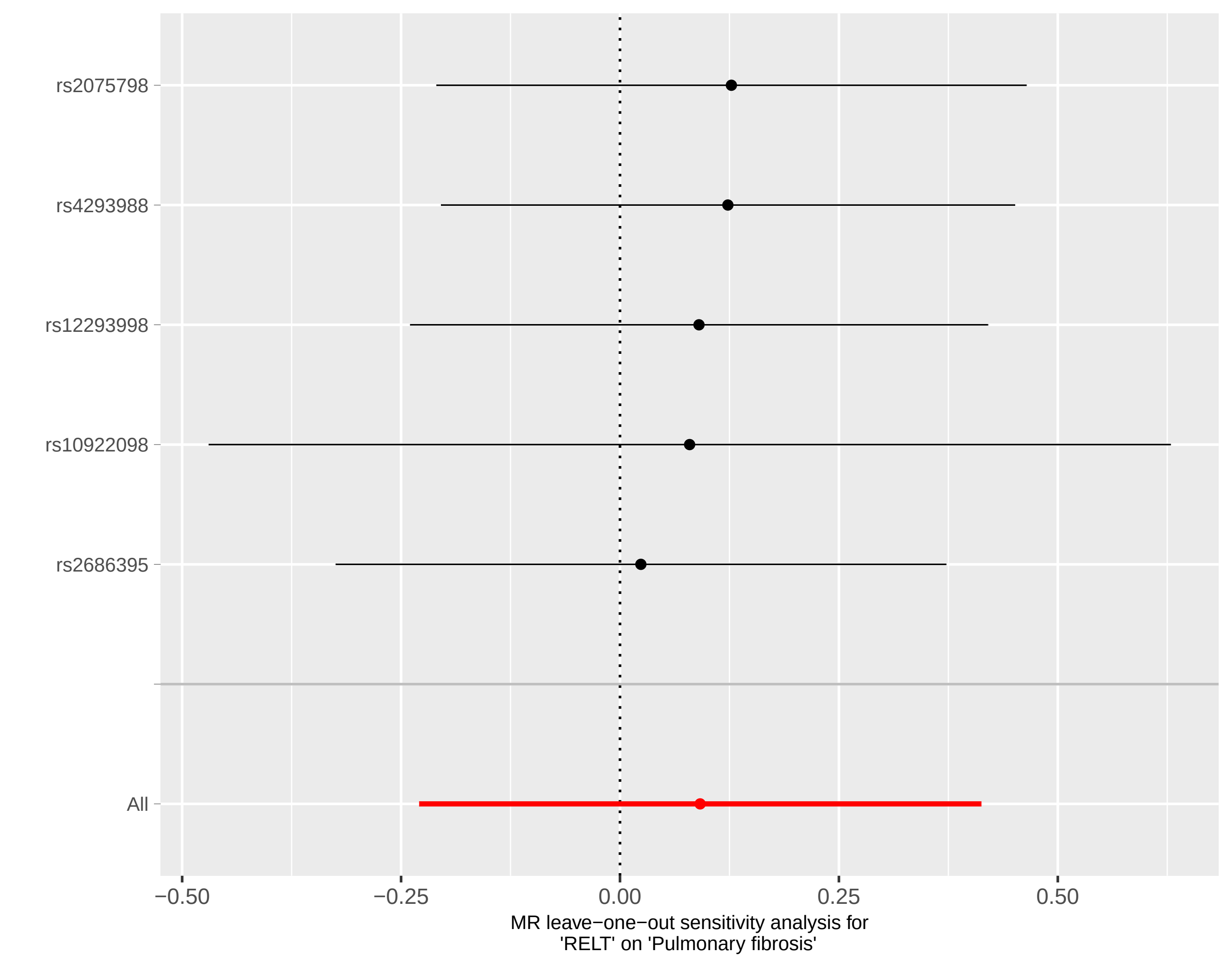
rs2075798
rs4293988
rs12293998
rs10922098
rs2686395
All
−0.50
−0.25
0.00
0.25
0.50
MR leave−one−out sensitivity analysis for
'RELT' on 'Pulmonary fibrosis'

Insufficient number of SNPs

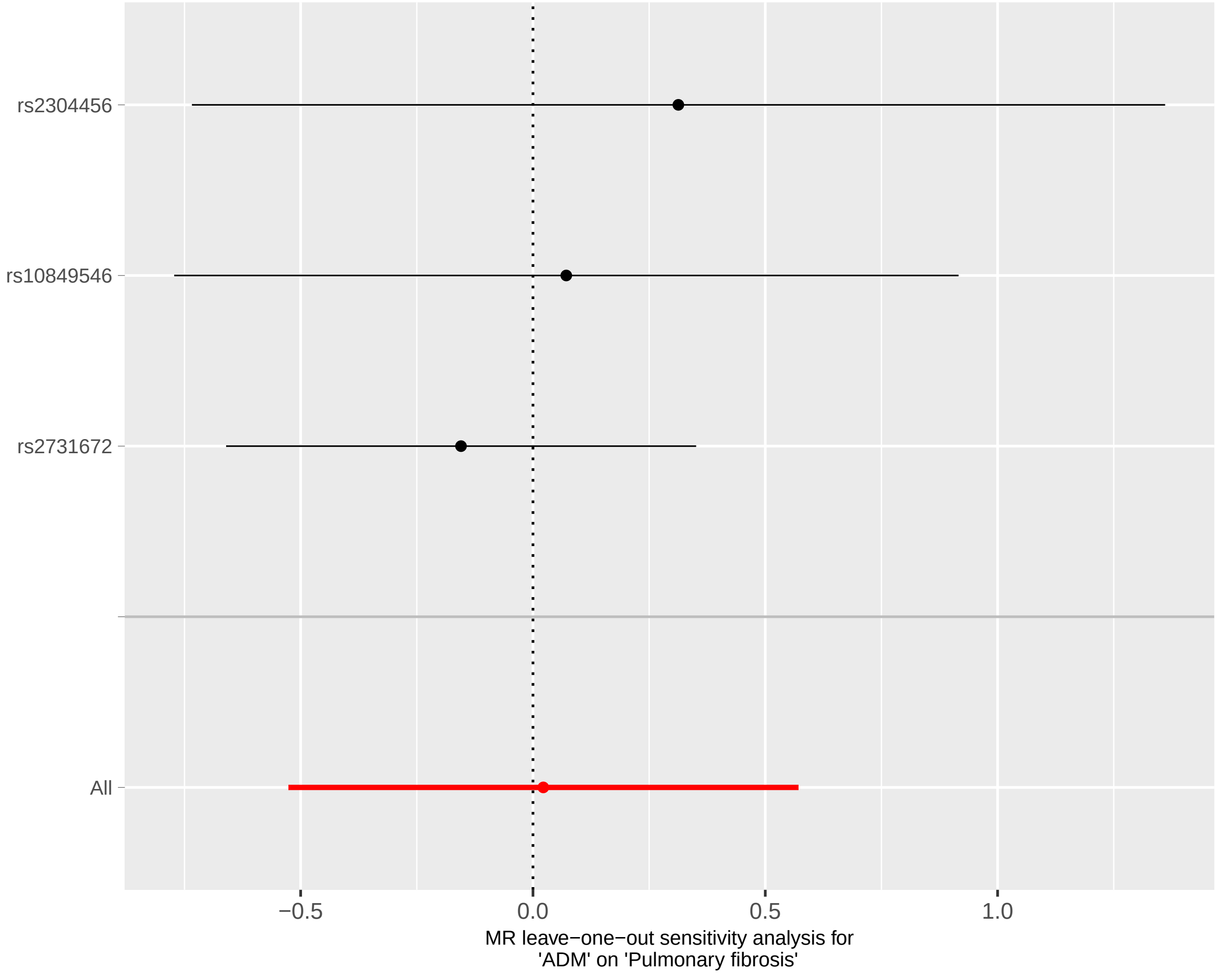
rs2304456
rs10849546
rs2731672
All
−0.5
0.0
0.5
1.0
MR leave−one−out sensitivity analysis for
'ADM' on 'Pulmonary fibrosis'

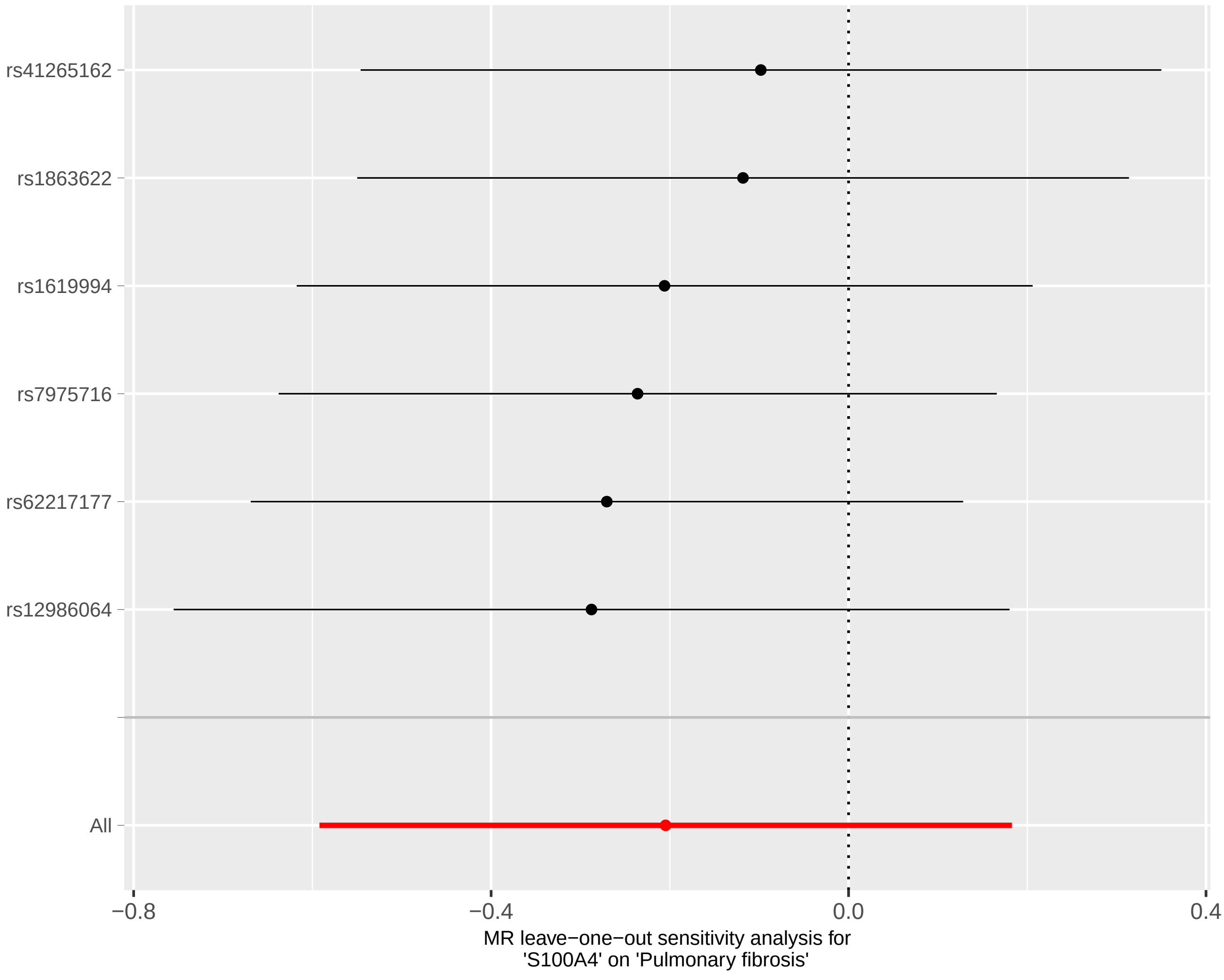
rs41265162
rs1863622
rs1619994
rs7975716
rs62217177
rs12986064
All
−0.8
−0.4
0.0
0.4
MR leave−one−out sensitivity analysis for
'S100A4' on 'Pulmonary fibrosis'

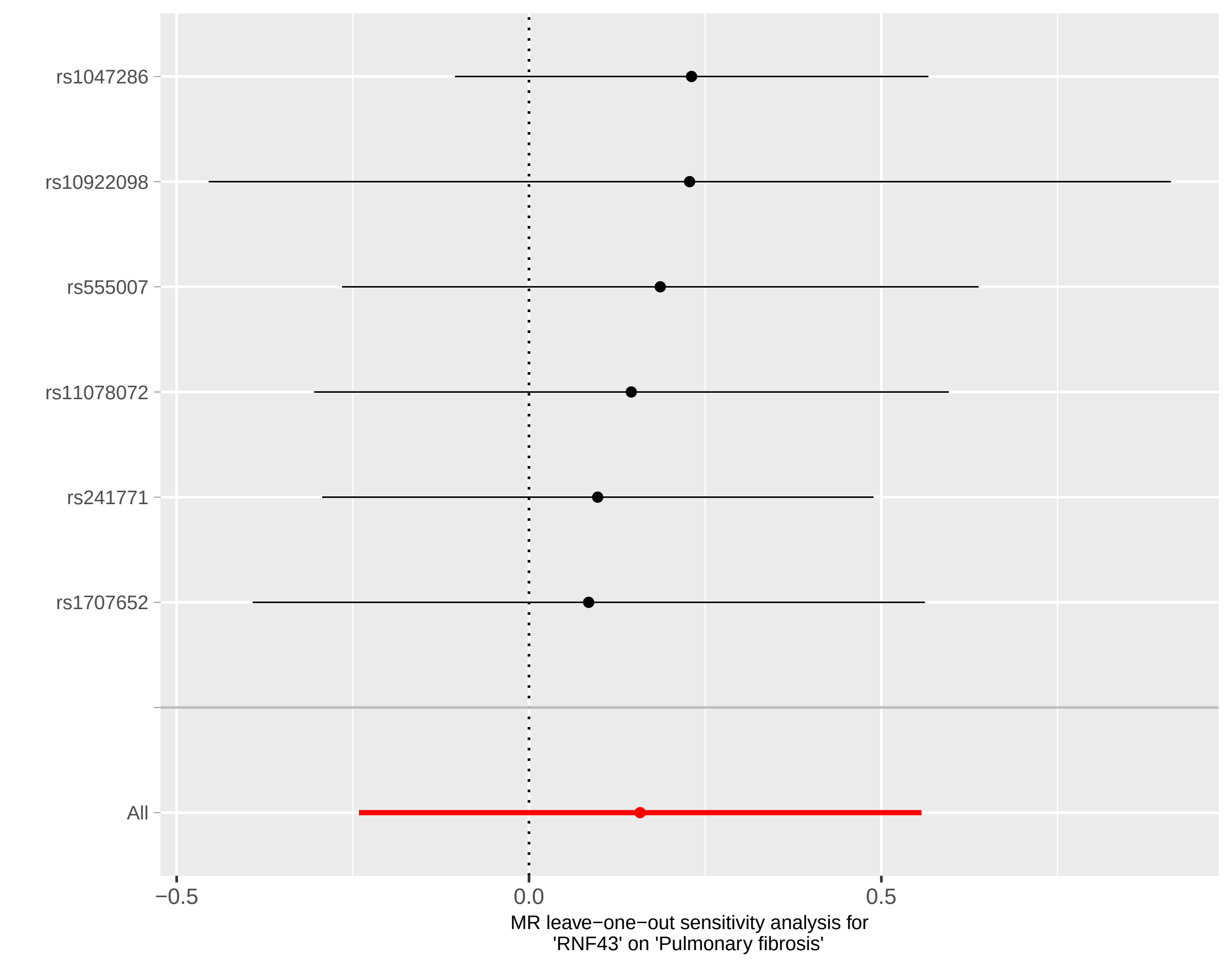
rs1047286
rs10922098
rs555007
rs11078072
rs241771
rs1707652
All
−0.5
0.0
0.5
MR leave−one−out sensitivity analysis for
'RNF43' on 'Pulmonary fibrosis'

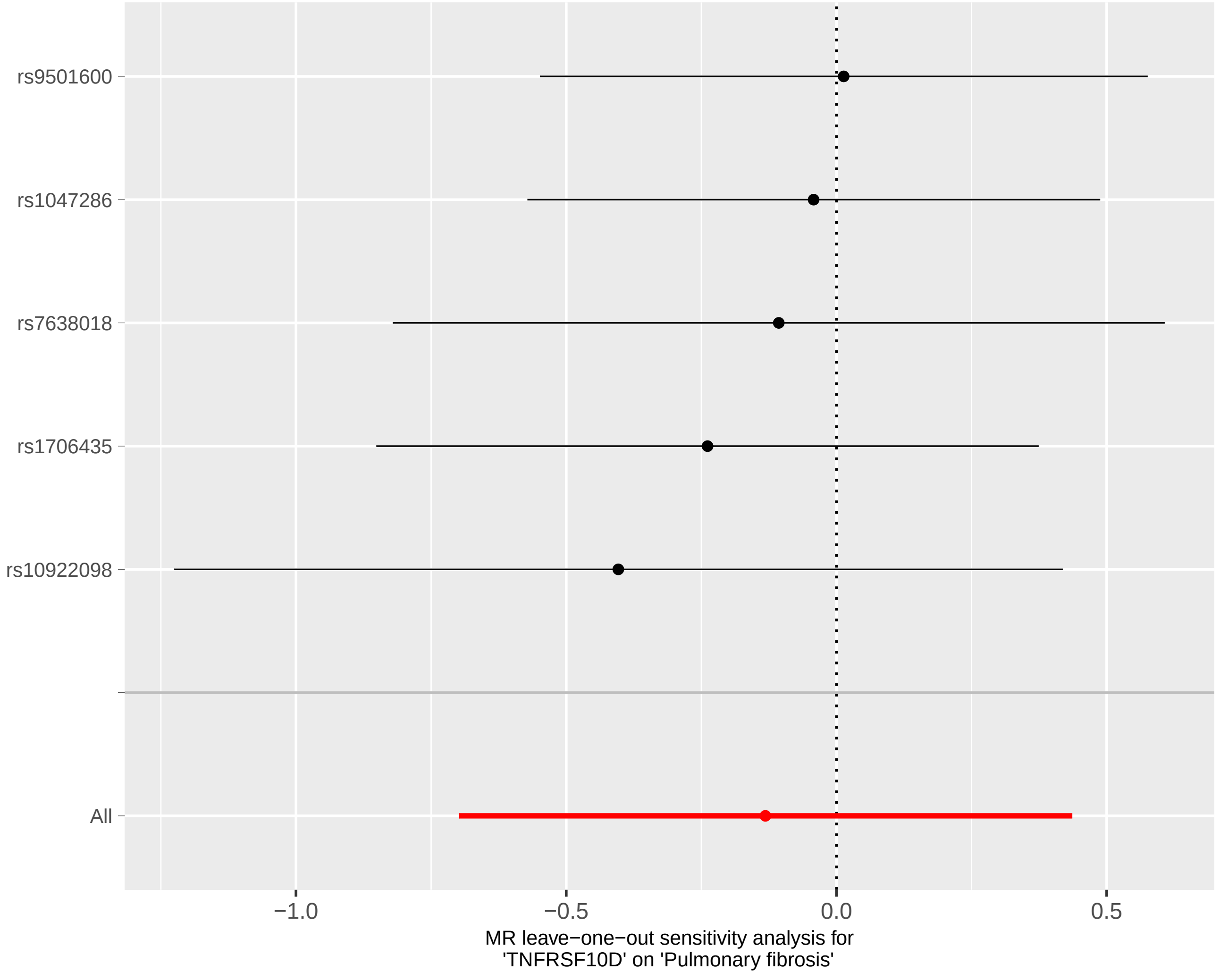

rs9501600
rs1047286
rs7638018
rs1706435
rs10922098
All
−1.0
−0.5
0.0
0.5
MR leave−one−out sensitivity analysis for
'TNFRSF10D' on 'Pulmonary fibrosis'

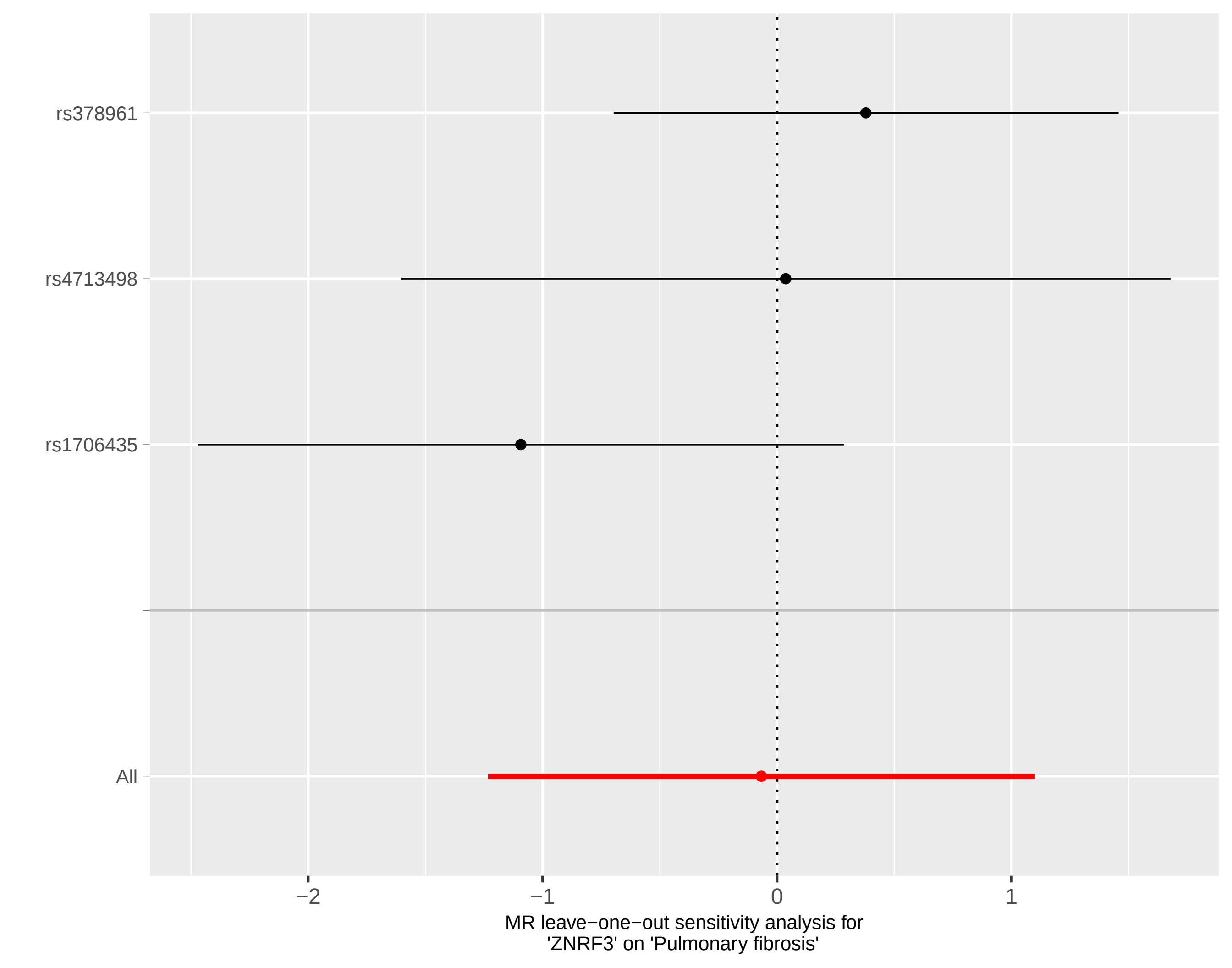
rs378961
rs4713498
rs1706435
All
−2
−1
0
1
MR leave−one−out sensitivity analysis for
'ZNRF3' on 'Pulmonary fibrosis'

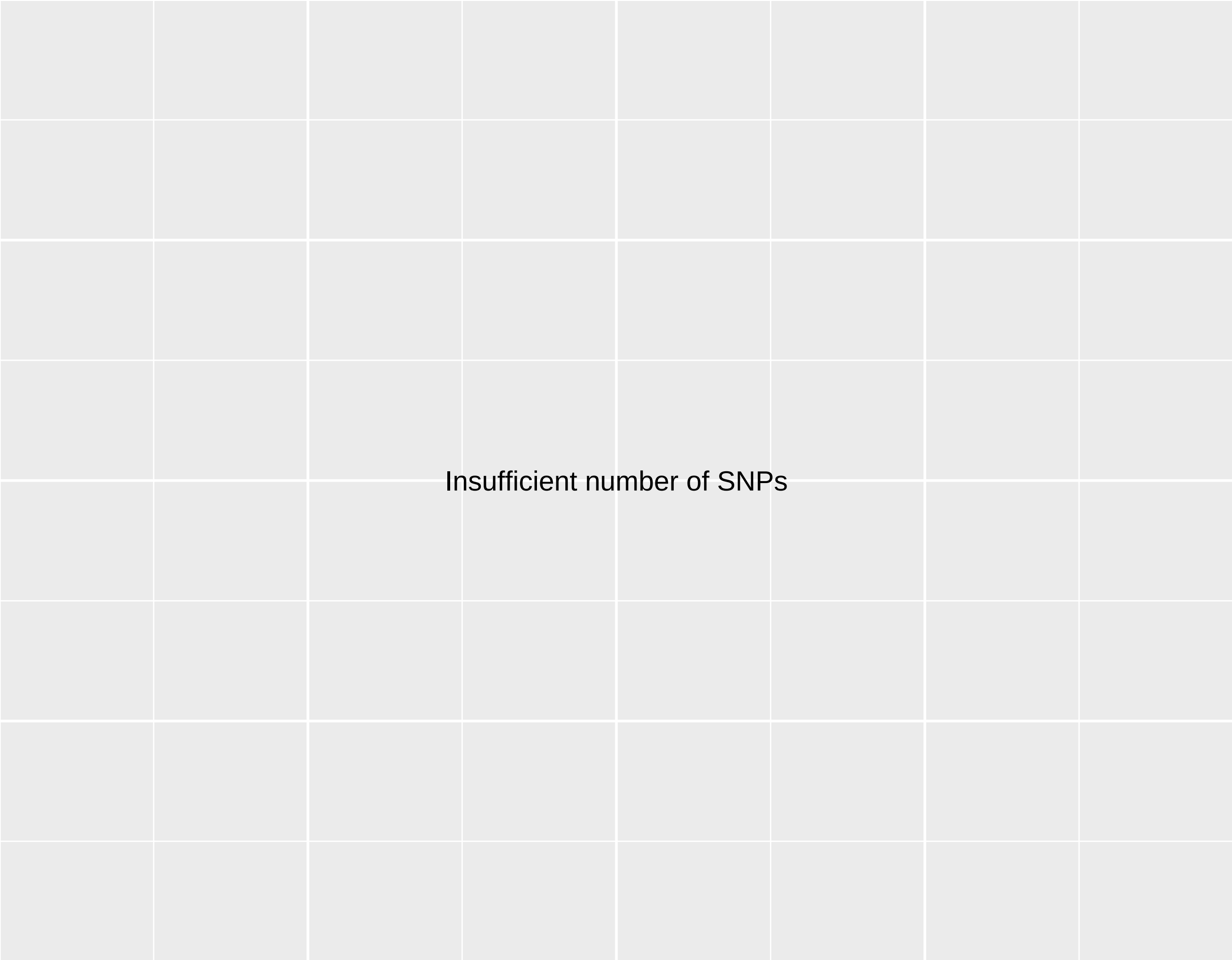
Insufficient number of SNPs

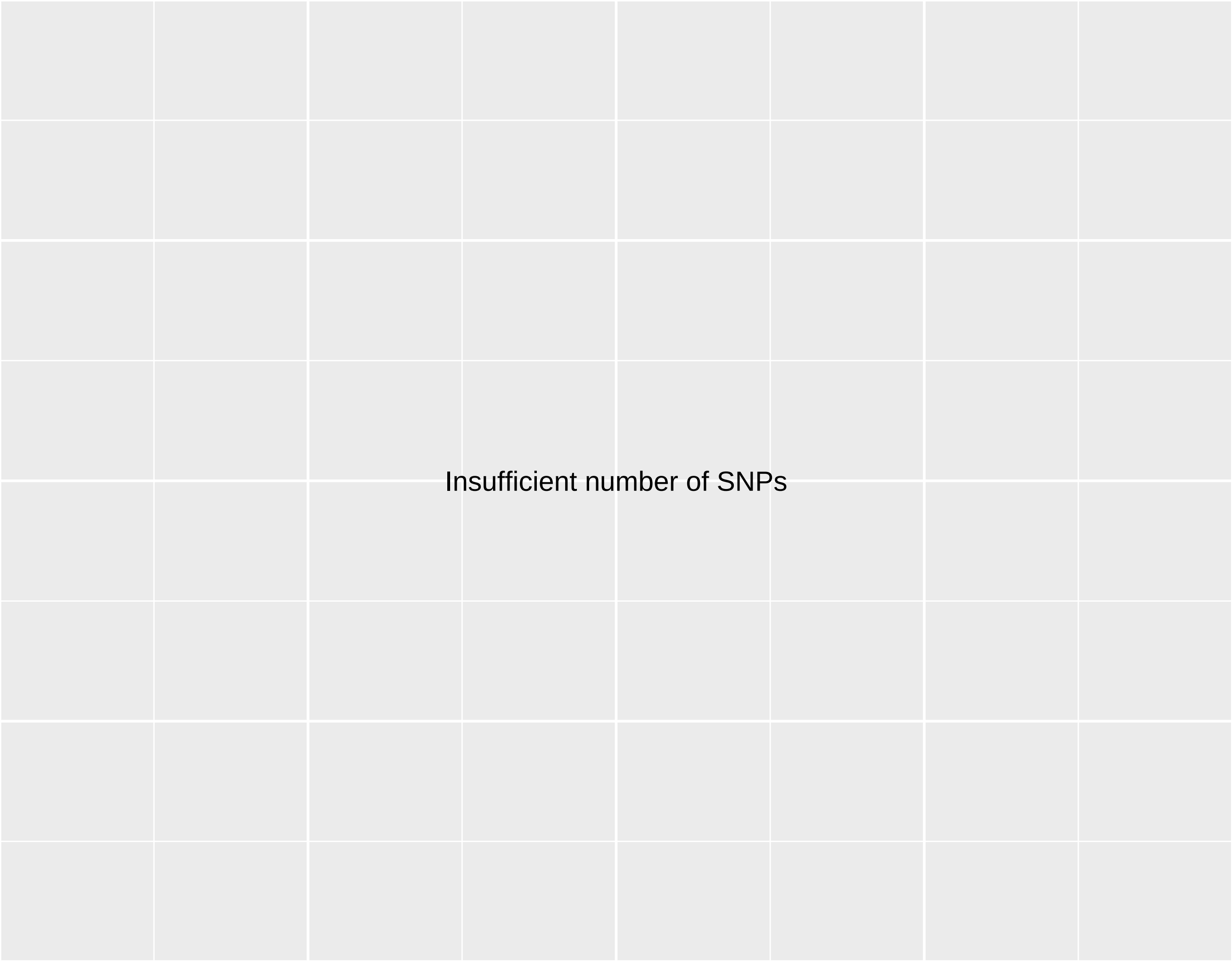
Insufficient number of SNPs

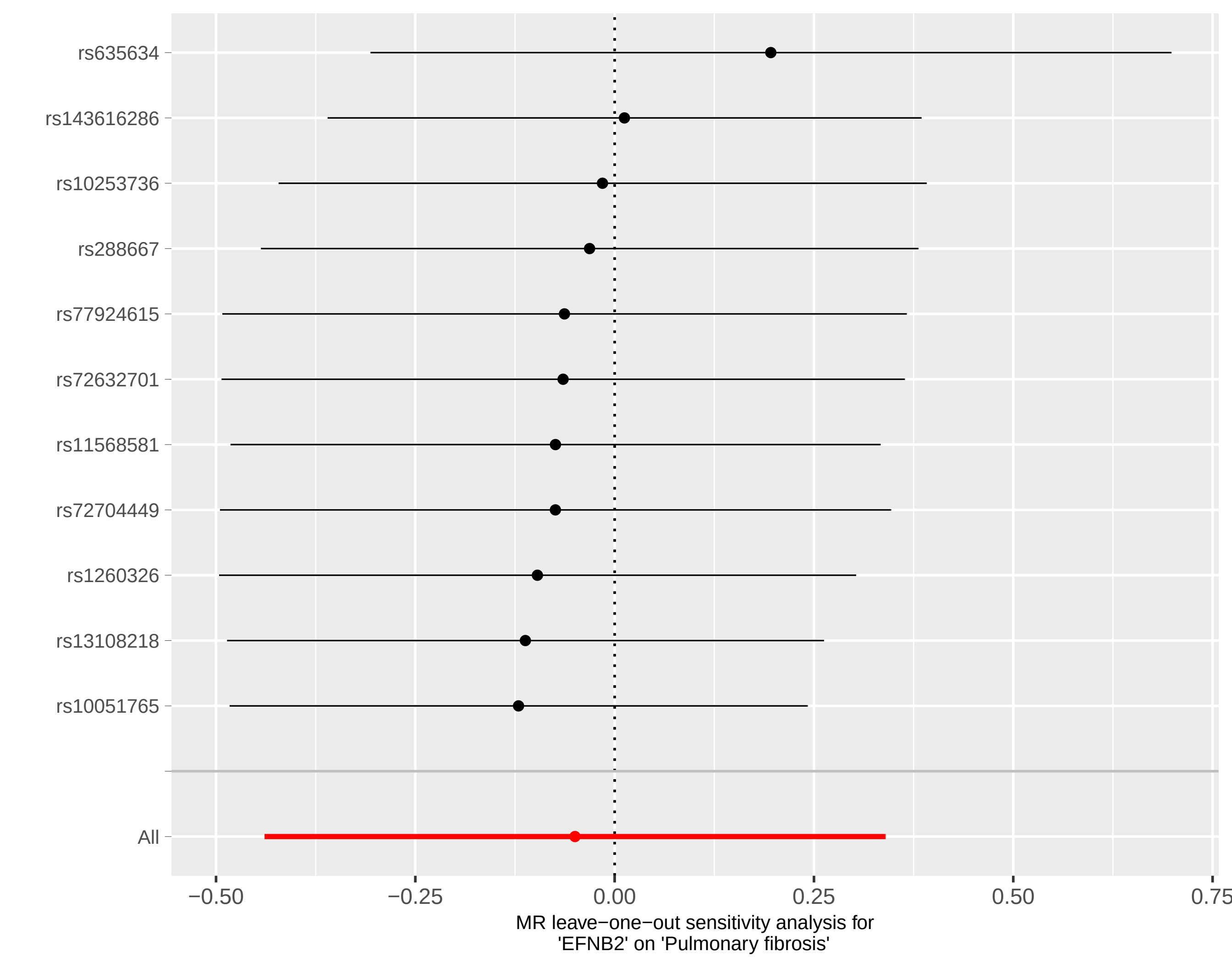

rs635634
rs143616286
rs10253736
rs288667
rs77924615
rs72632701
rs11568581
rs72704449
rs1260326
rs13108218
rs10051765
All
−0.50
−0.25
0.00
0.25
0.50
0.75
MR leave−one−out sensitivity analysis for
'EFNB2' on 'Pulmonary fibrosis'

Insufficient number of SNPs

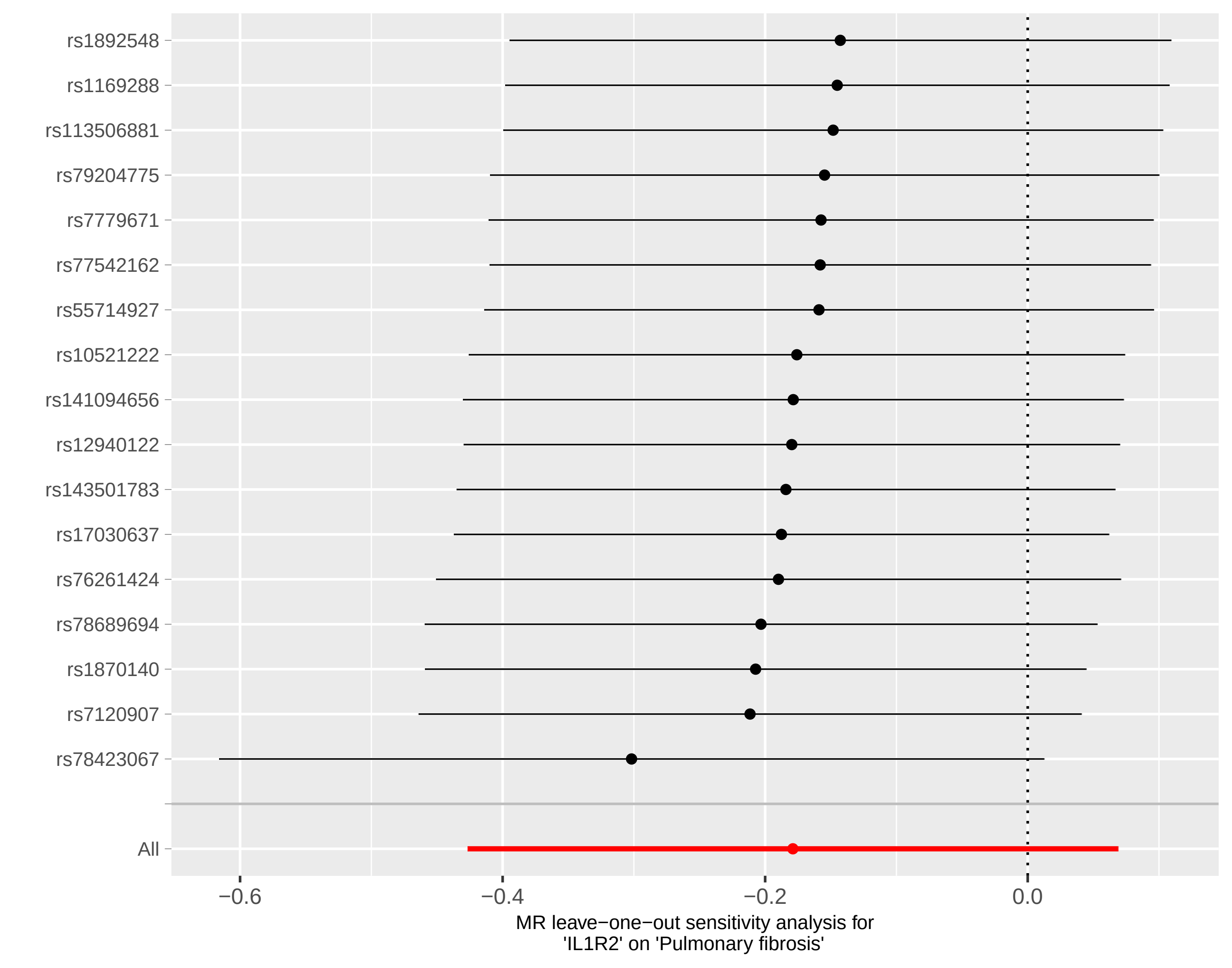
rs1892548
rs1169288
rs113506881
rs79204775
rs7779671
rs77542162
rs55714927
rs10521222
rs141094656
rs12940122
rs143501783
rs17030637
rs76261424
rs78689694
rs1870140
rs7120907
rs78423067
All
−0.6
−0.4
−0.2
0.0
MR leave−one−out sensitivity analysis for
'IL1R2' on 'Pulmonary fibrosis'

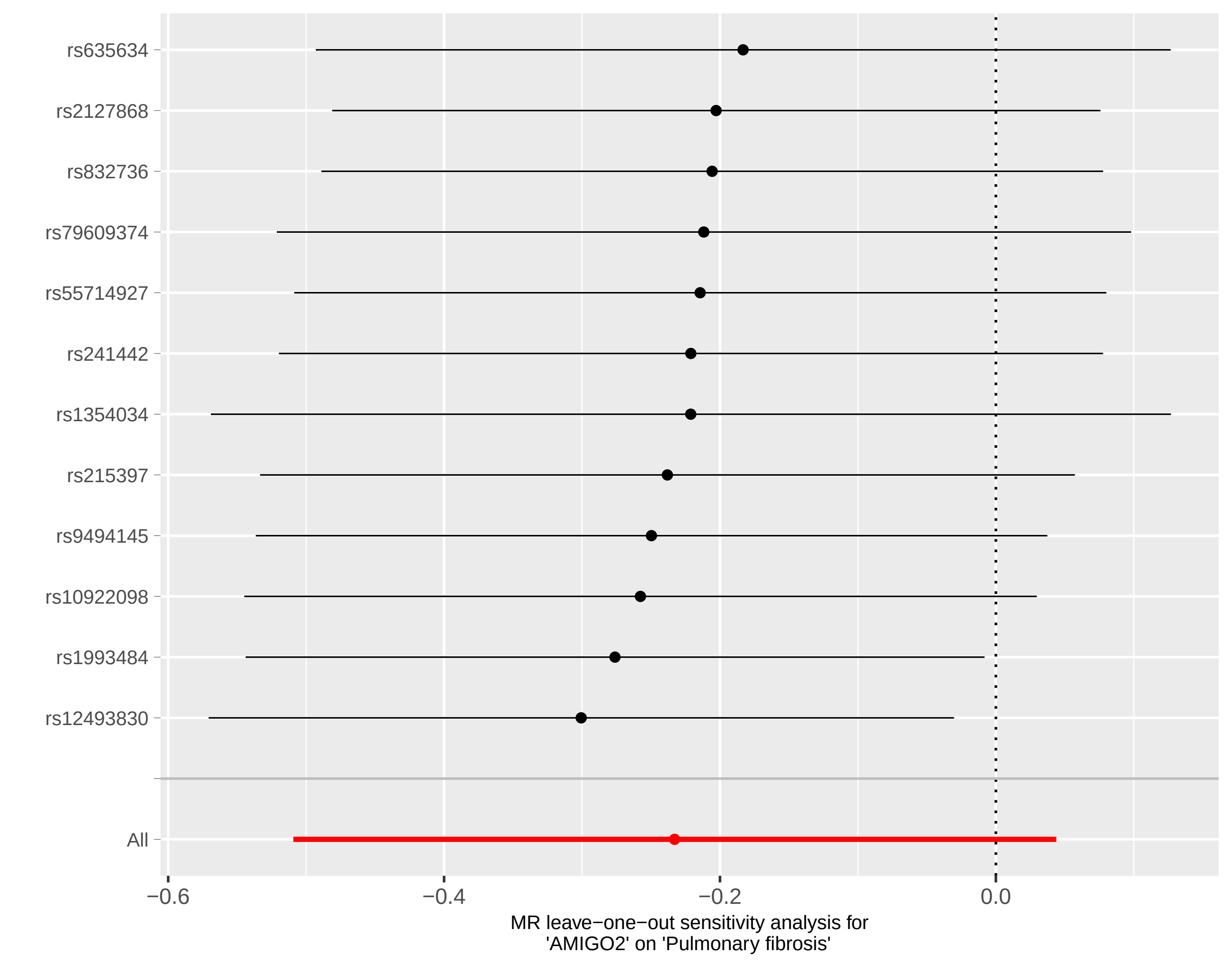
rs635634
rs2127868
rs832736
rs79609374
rs55714927
rs241442
rs1354034
rs215397
rs9494145
rs10922098
rs1993484
rs12493830
All
−0.6
−0.4
−0.2
0.0
MR leave−one−out sensitivity analysis for
'AMIGO2' on 'Pulmonary fibrosis'

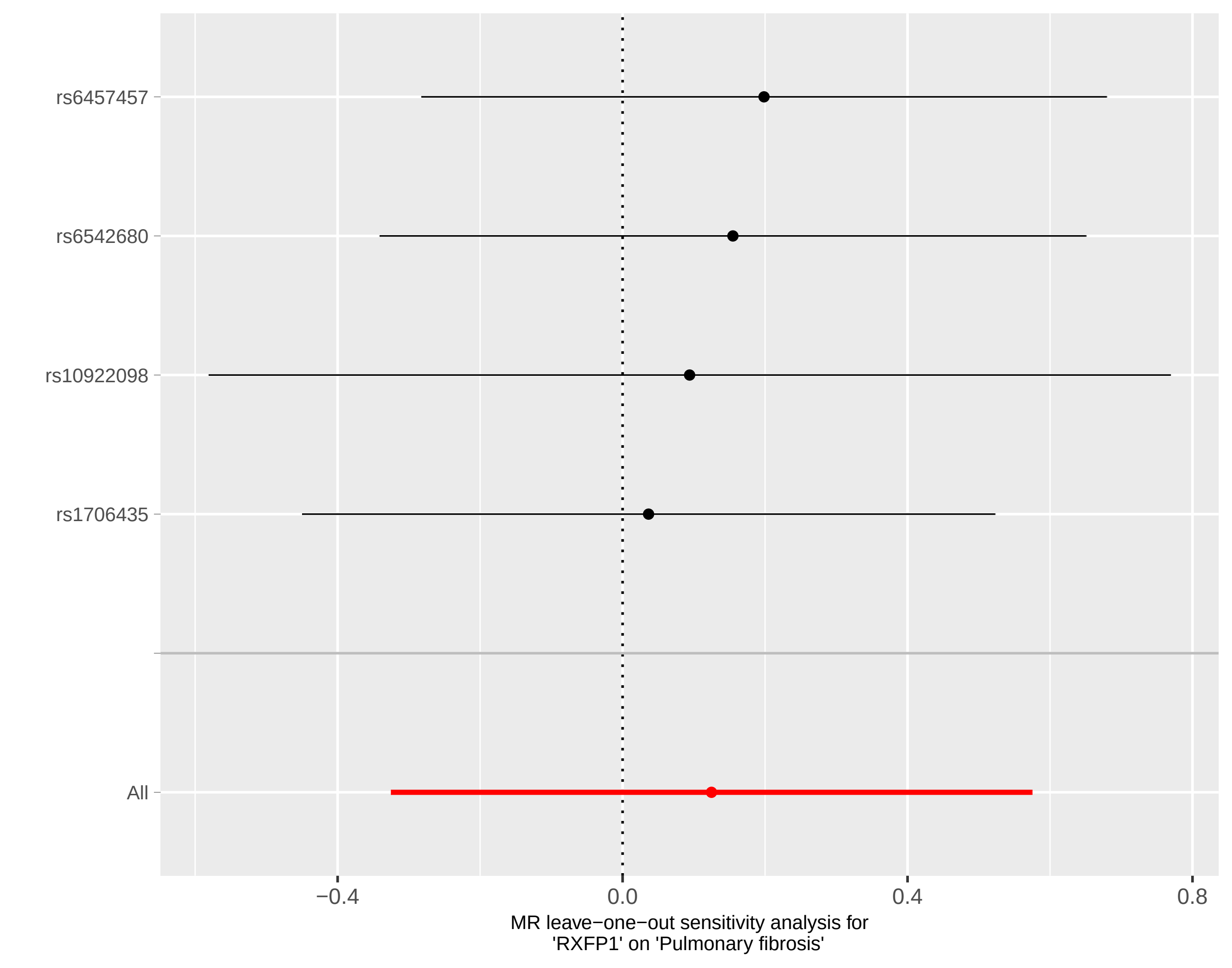
rs6457457
rs6542680
rs10922098
rs1706435
All
−0.4
0.0
0.4
0.8
MR leave−one−out sensitivity analysis for
'RXFP1' on 'Pulmonary fibrosis'

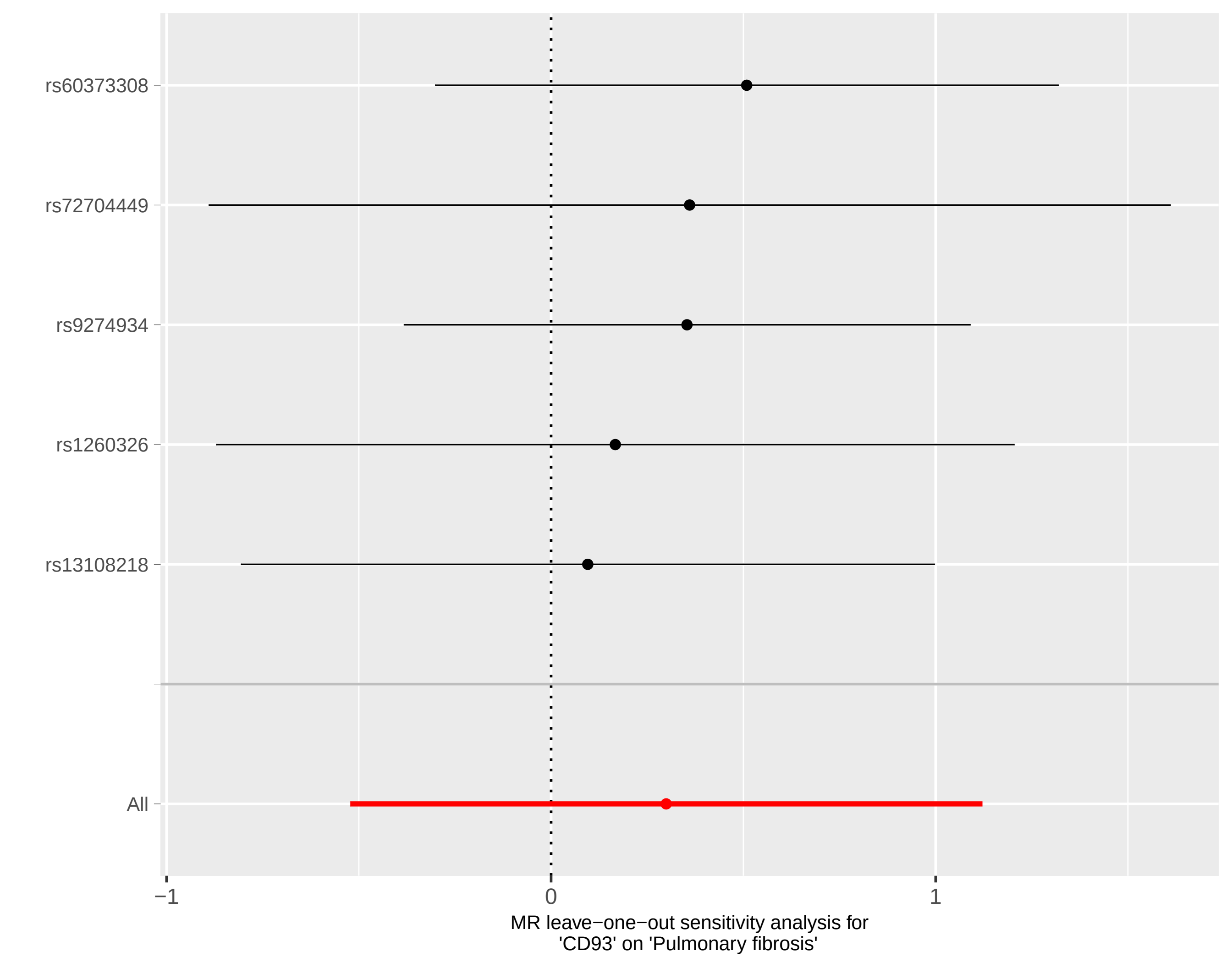
rs60373308
rs72704449
rs9274934
rs1260326
rs13108218
All
−1
0
1
MR leave−one−out sensitivity analysis for
'CD93' on 'Pulmonary fibrosis'

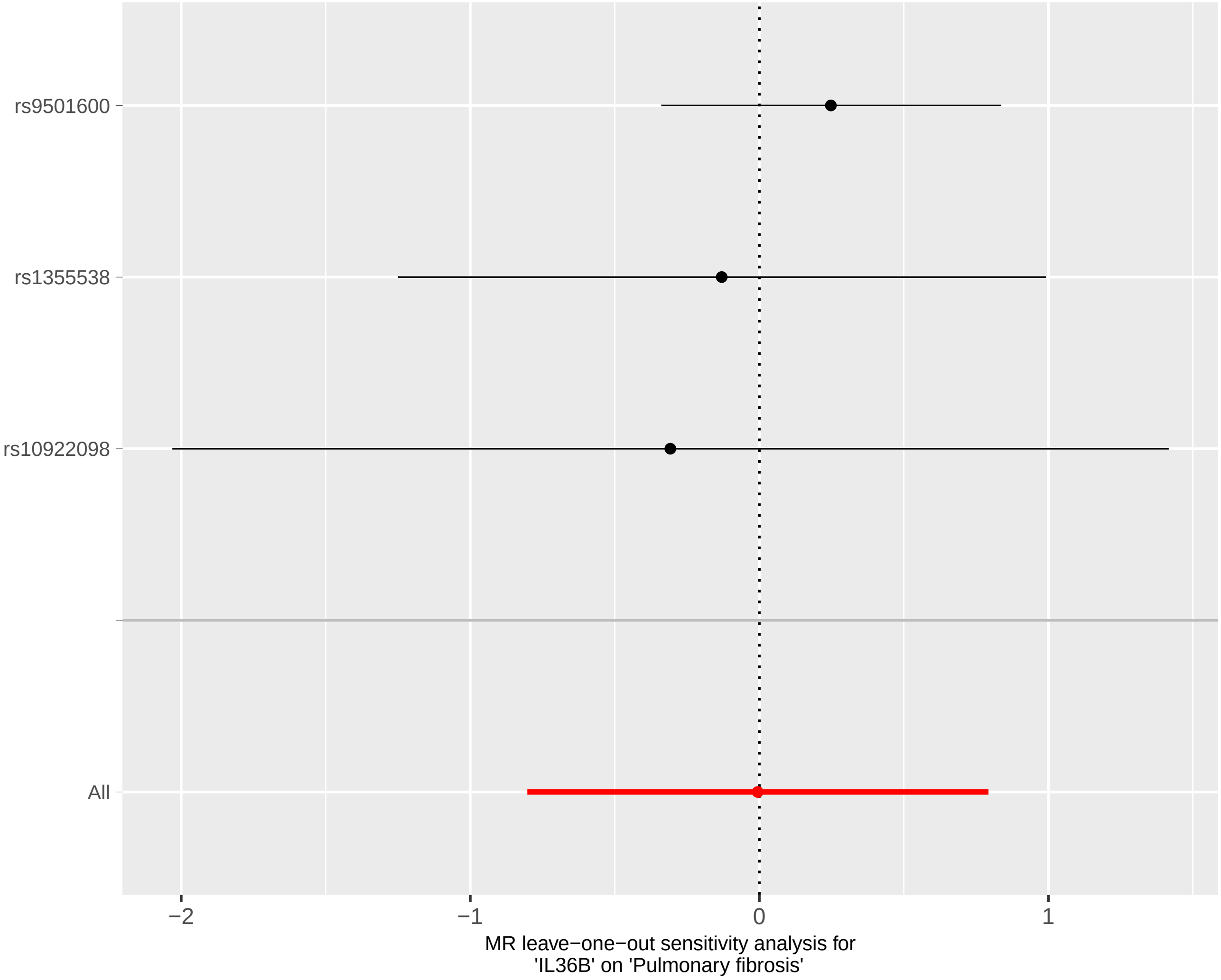
rs9501600
rs1355538
rs10922098
All
−2
−1
0
1
MR leave−one−out sensitivity analysis for
'IL36B' on 'Pulmonary fibrosis'

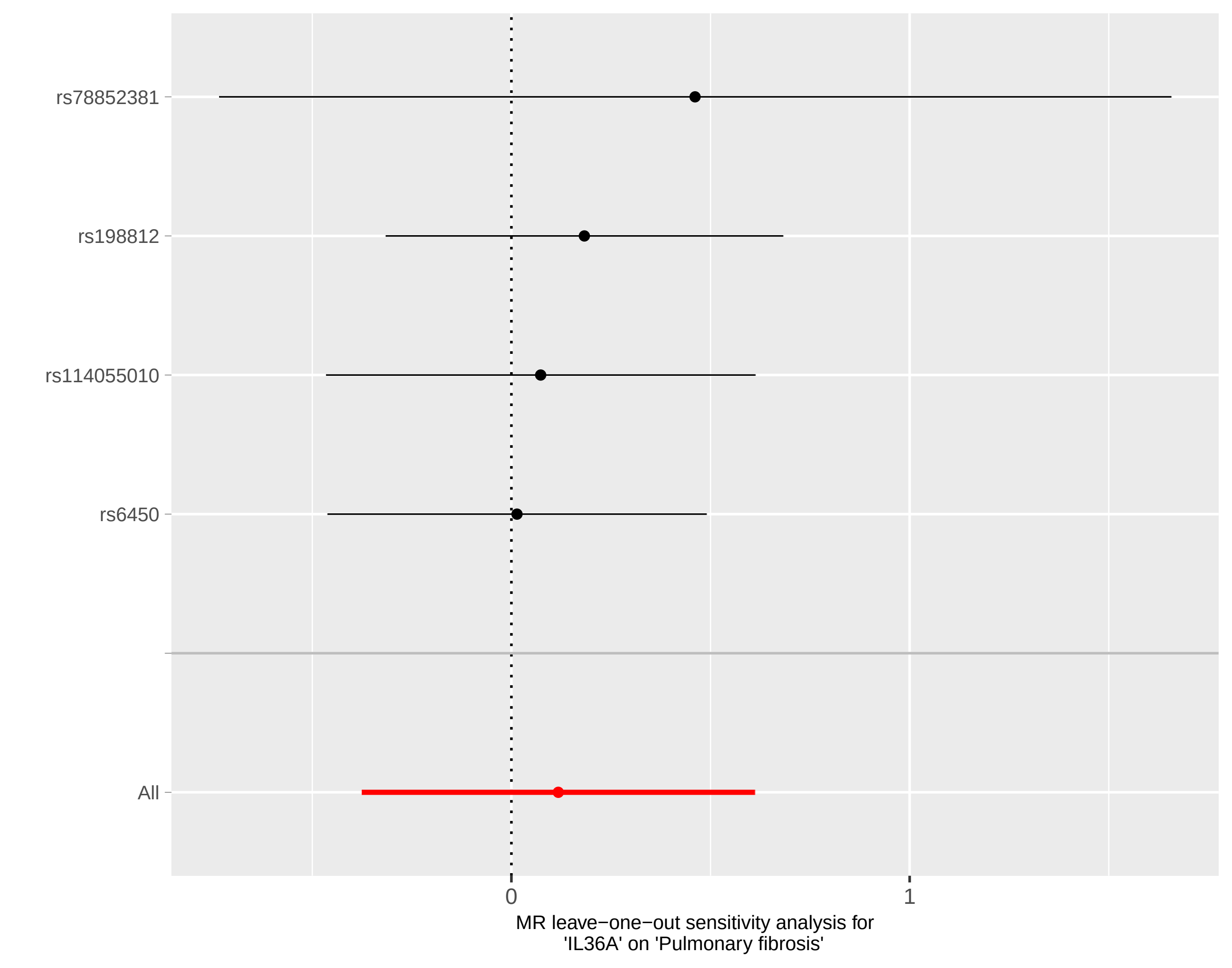
rs78852381
rs198812
rs114055010
rs6450
All
0
1
MR leave−one−out sensitivity analysis for
'IL36A' on 'Pulmonary fibrosis'

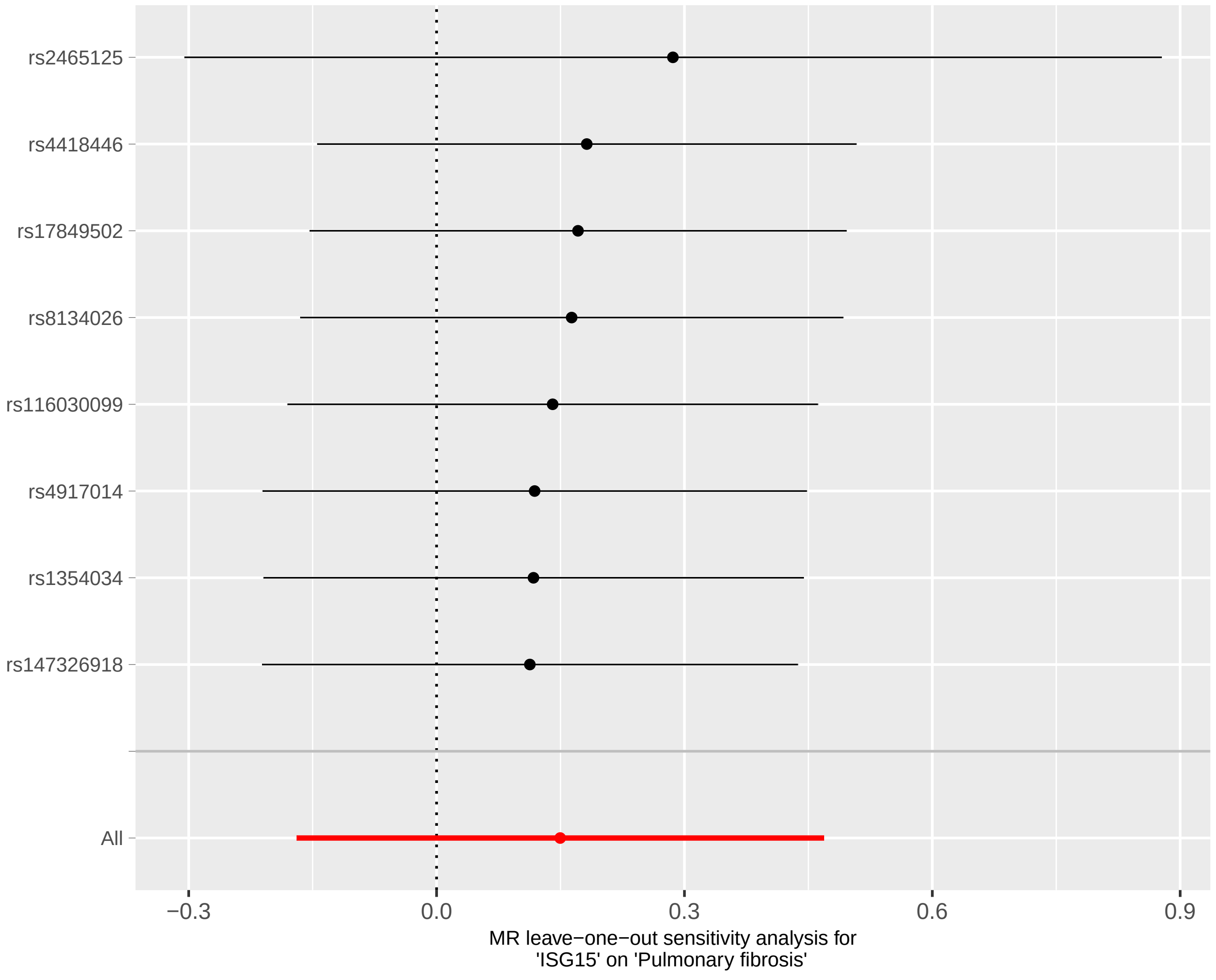
rs2465125
rs4418446
rs17849502
rs8134026
rs116030099
rs4917014
rs1354034
rs147326918
All
−0.3
0.0
0.3
0.6
0.9
MR leave−one−out sensitivity analysis for
'ISG15' on 'Pulmonary fibrosis'

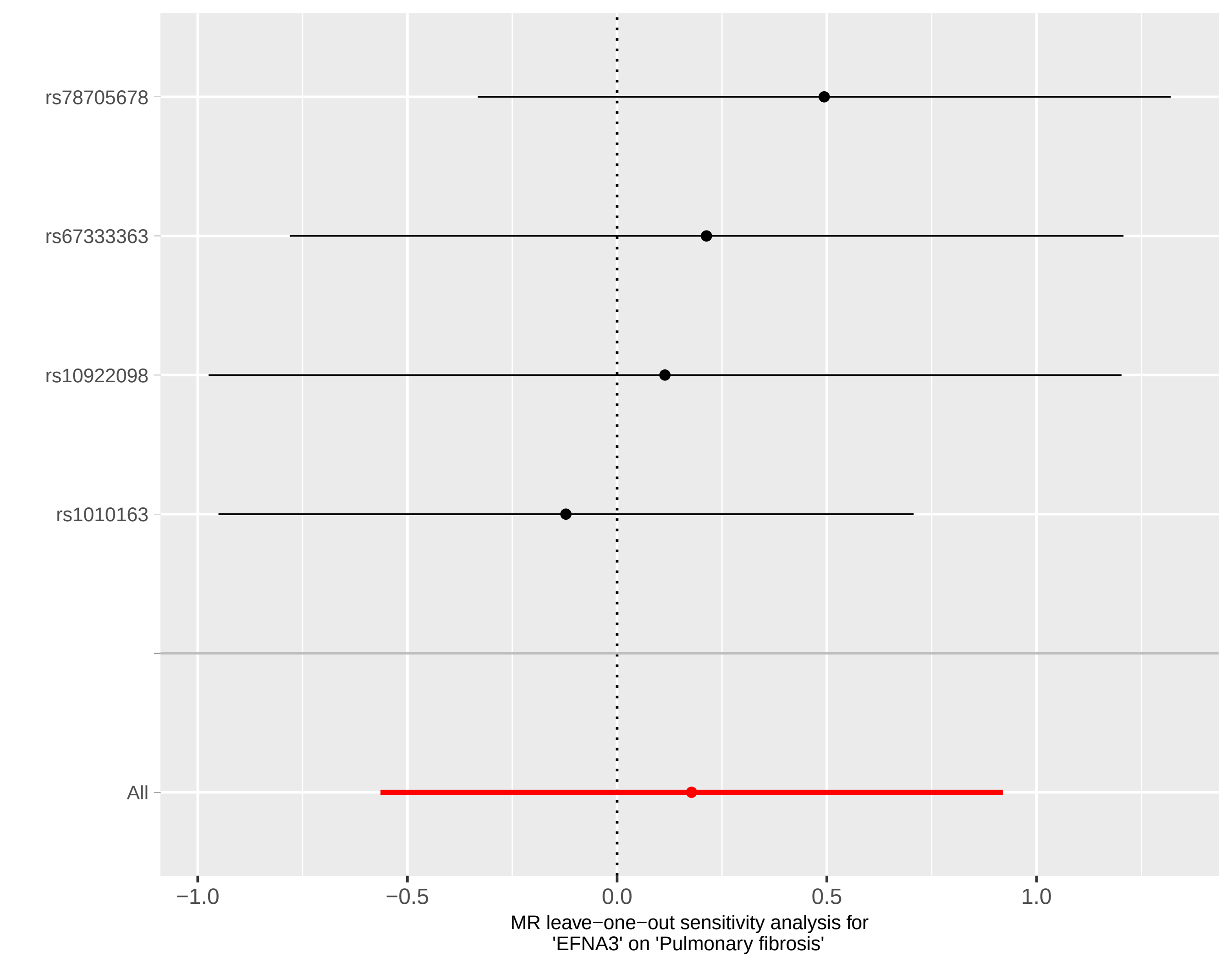
rs78705678
rs67333363
rs10922098
rs1010163
All
−1.0
−0.5
0.0
0.5
1.0
MR leave−one−out sensitivity analysis for
'EFNA3' on 'Pulmonary fibrosis'

Insufficient number of SNPs

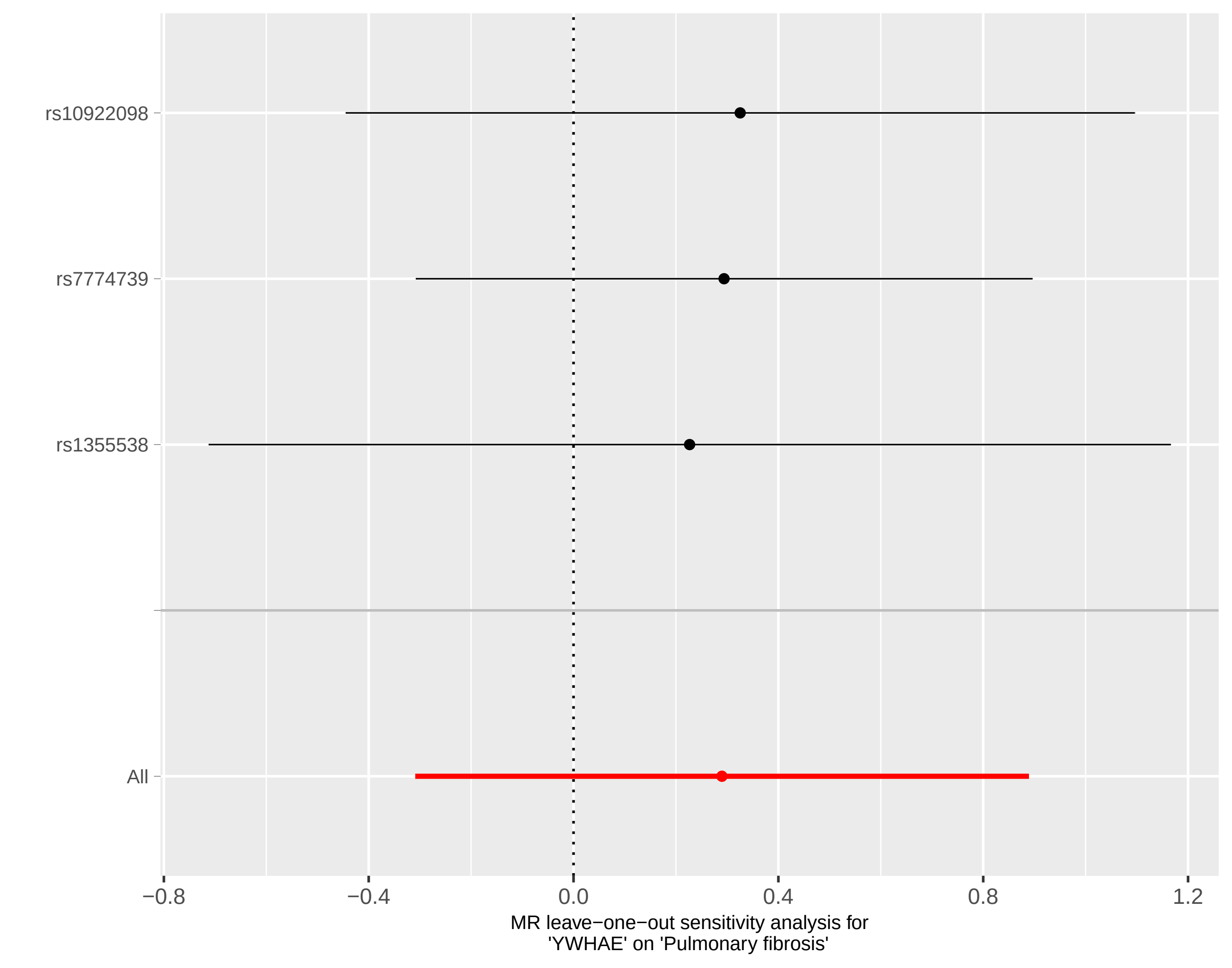
rs10922098
rs7774739
rs1355538
All
−0.8
−0.4
0.0
0.4
0.8
1.2
MR leave−one−out sensitivity analysis for
'YWHAE' on 'Pulmonary fibrosis'

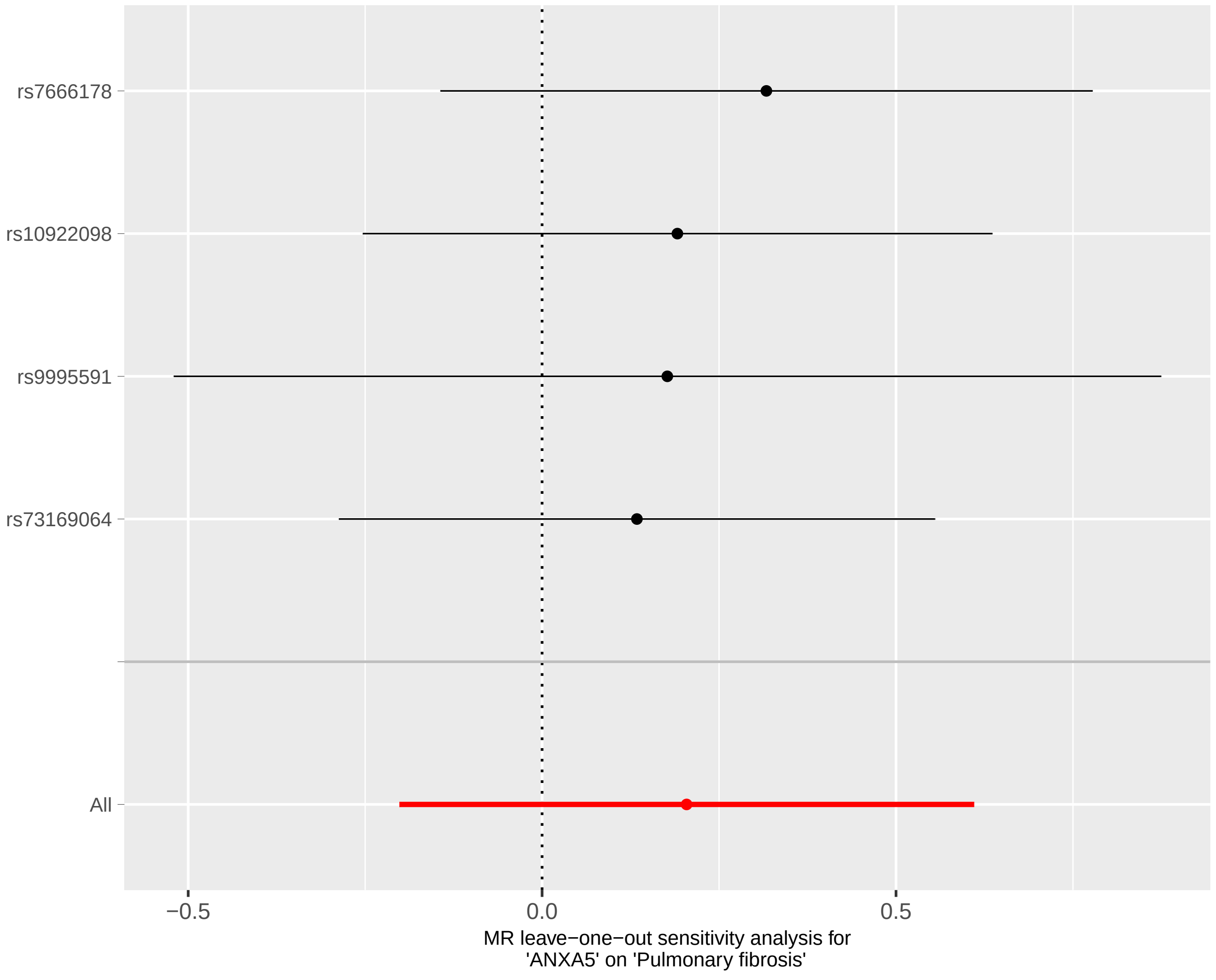

rs7666178
rs10922098
rs9995591
rs73169064
All
−0.5
0.0
0.5
MR leave−one−out sensitivity analysis for
'ANXA5' on 'Pulmonary fibrosis'

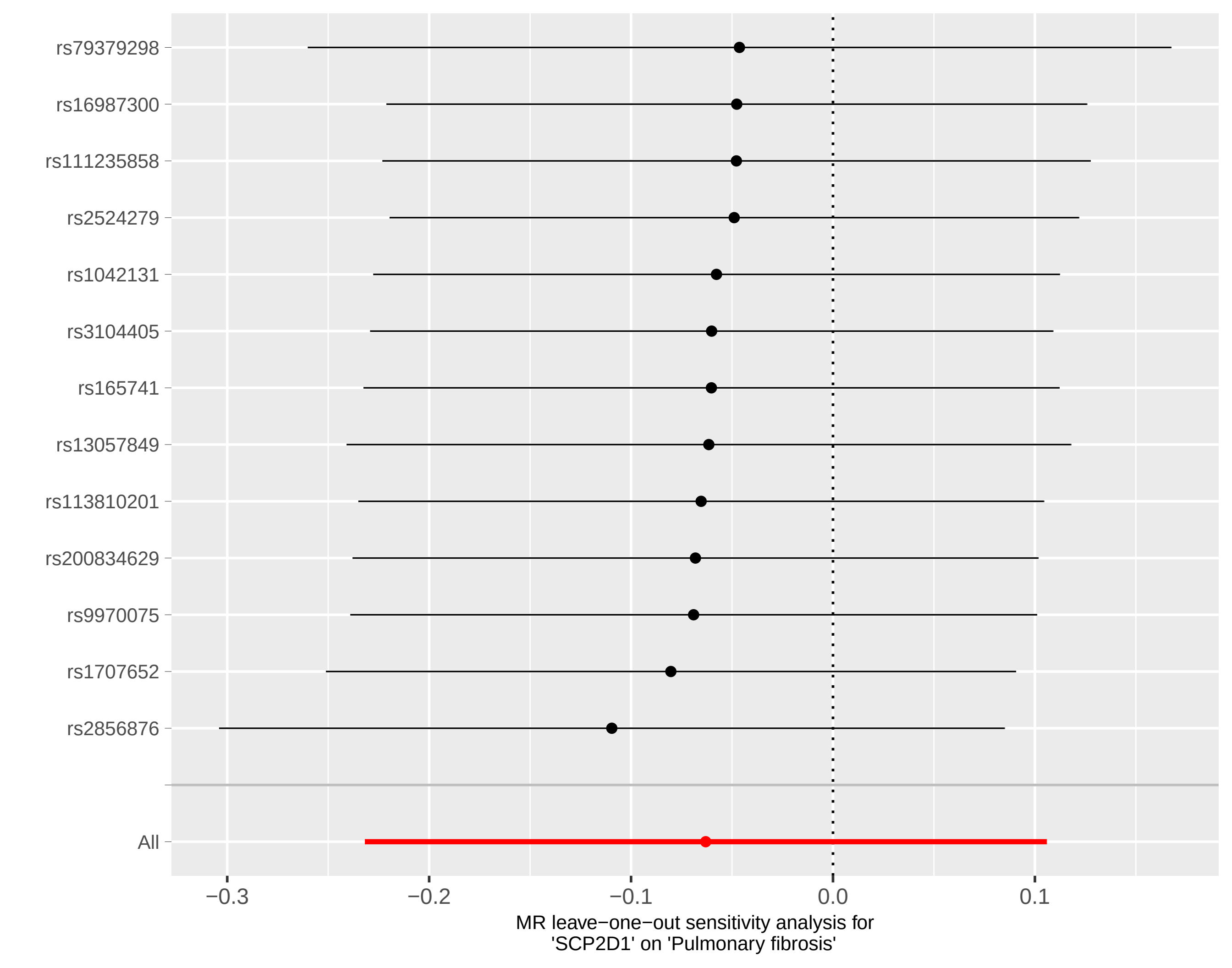
rs79379298
rs16987300
rs111235858
rs2524279
rs1042131
rs3104405
rs165741
rs13057849
rs113810201
rs200834629
rs9970075
rs1707652
rs2856876
All
−0.3
−0.2
−0.1
0.0
0.1
MR leave−one−out sensitivity analysis for
'SCP2D1' on 'Pulmonary fibrosis'

Insufficient number of SNPs

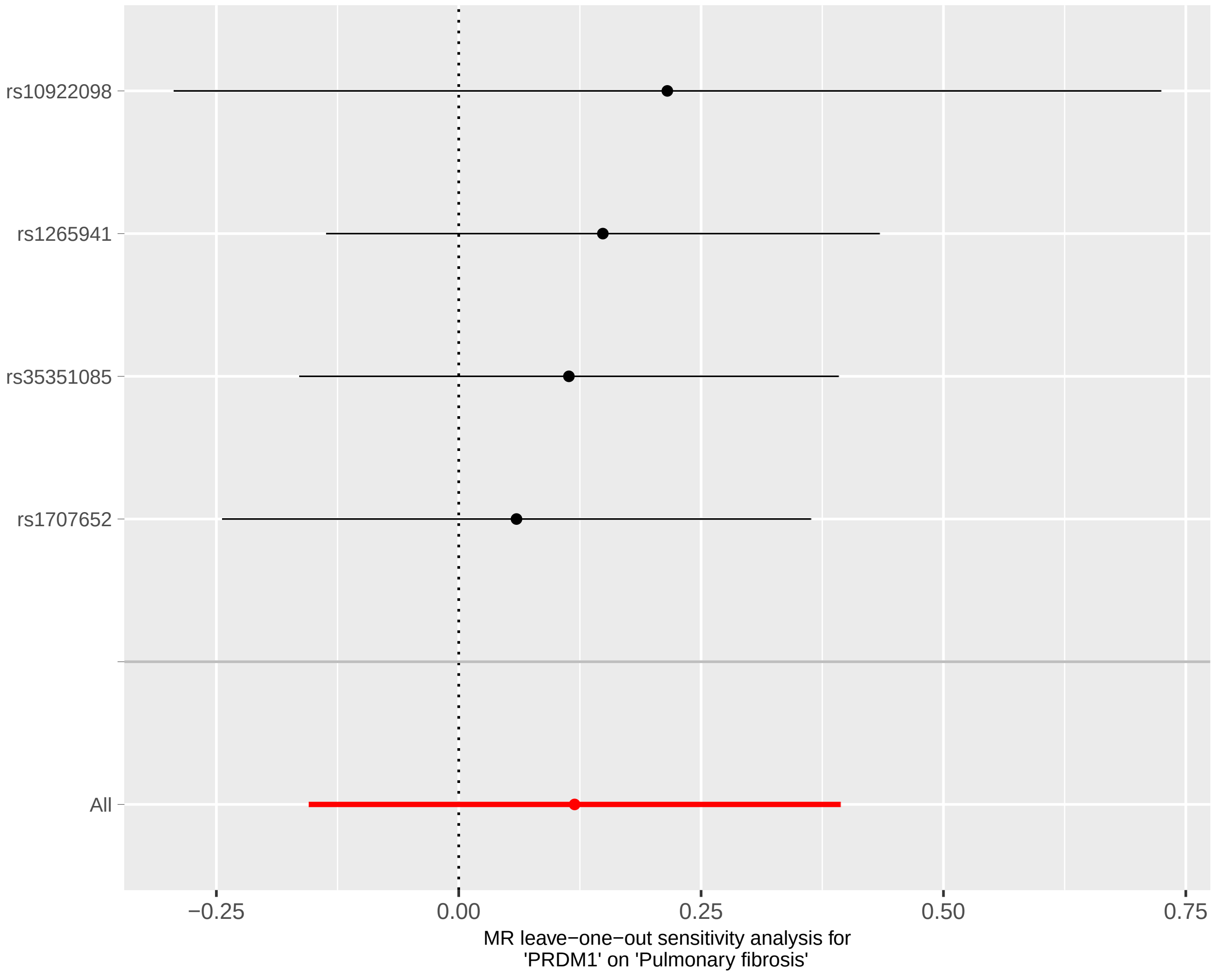
rs10922098
rs1265941
rs35351085
rs1707652
All
−0.25
0.00
0.25
0.50
0.75
MR leave−one−out sensitivity analysis for
'PRDM1' on 'Pulmonary fibrosis'

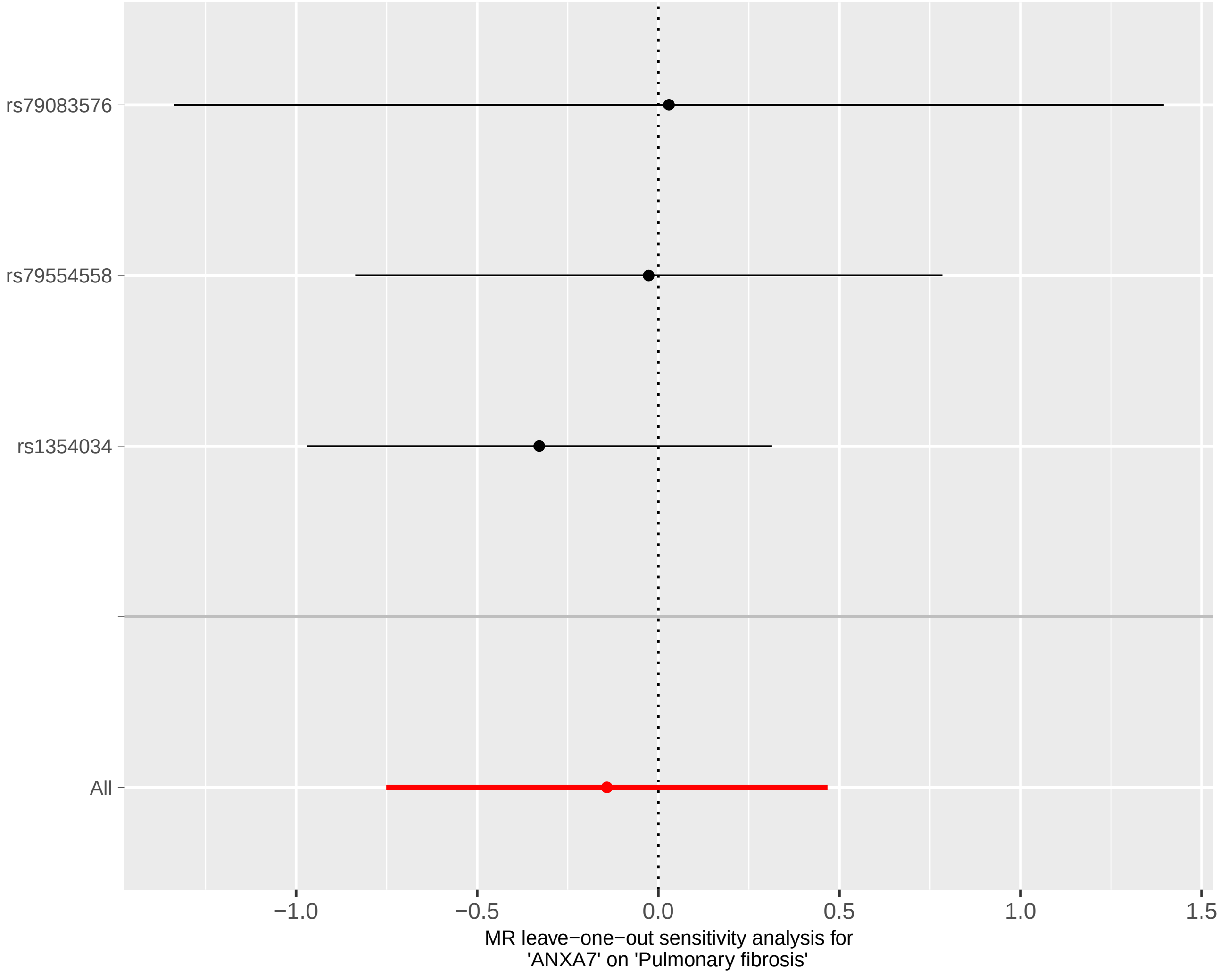
rs79083576
rs79554558
rs1354034
All
−1.0
−0.5
0.0
0.5
1.0
1.5
MR leave−one−out sensitivity analysis for
'ANXA7' on 'Pulmonary fibrosis'

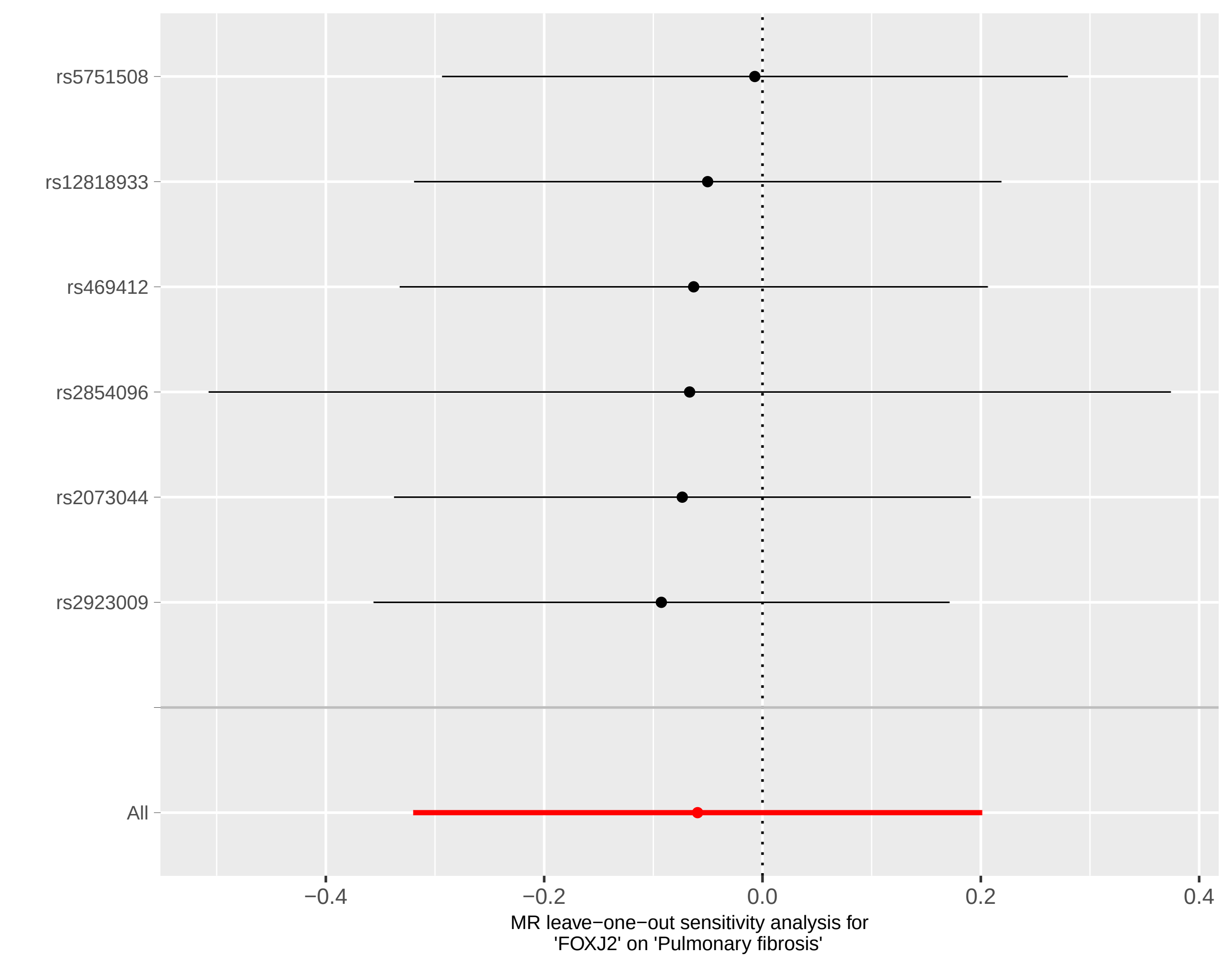
rs5751508
rs12818933
rs469412
rs2854096
rs2073044
rs2923009
All
−0.4
−0.2
0.0
0.2
0.4
MR leave−one−out sensitivity analysis for
'FOXJ2' on 'Pulmonary fibrosis'

Insufficient number of SNPs

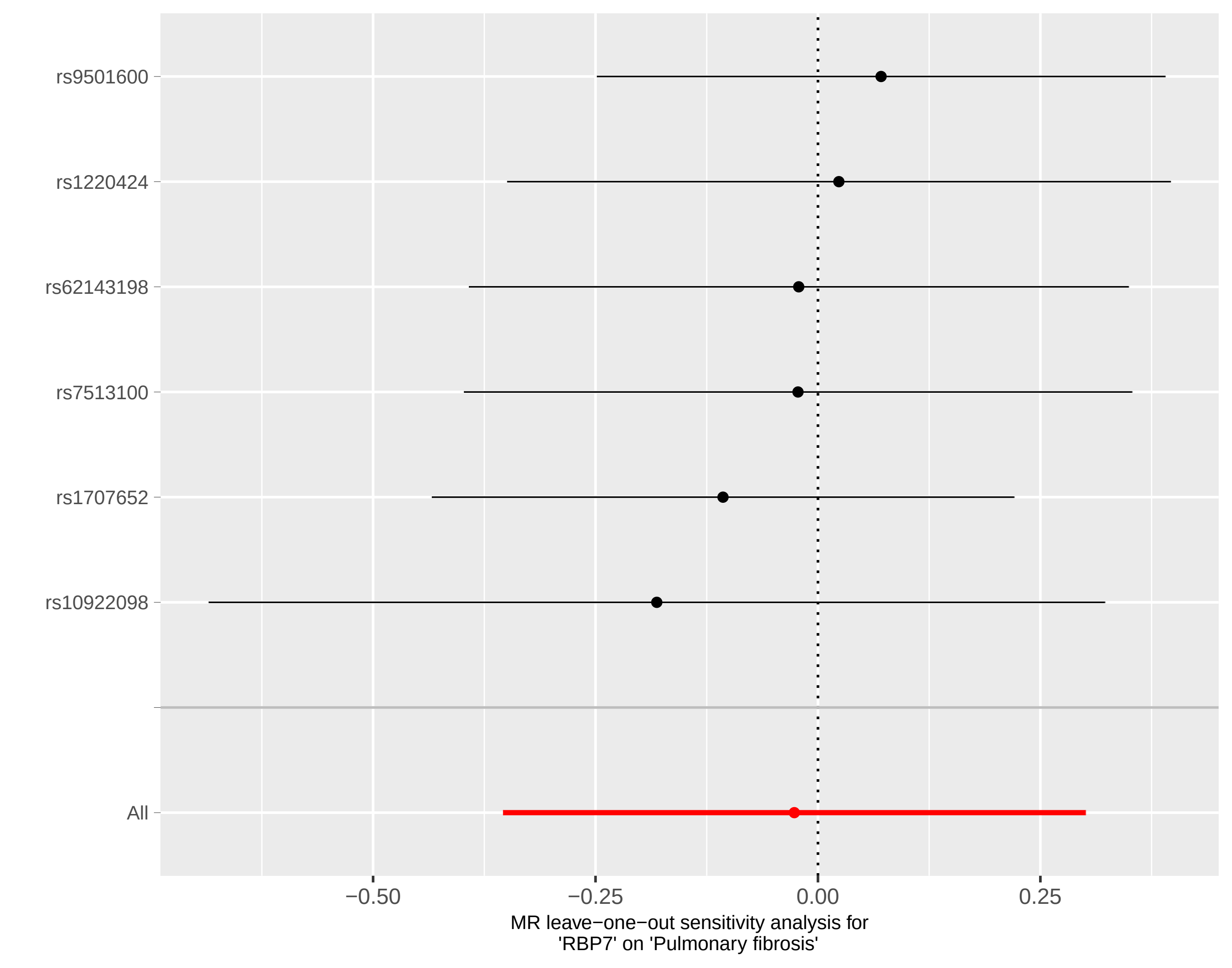
rs9501600
rs1220424
rs62143198
rs7513100
rs1707652
rs10922098
All
−0.50
−0.25
0.00
0.25
MR leave−one−out sensitivity analysis for
'RBP7' on 'Pulmonary fibrosis'

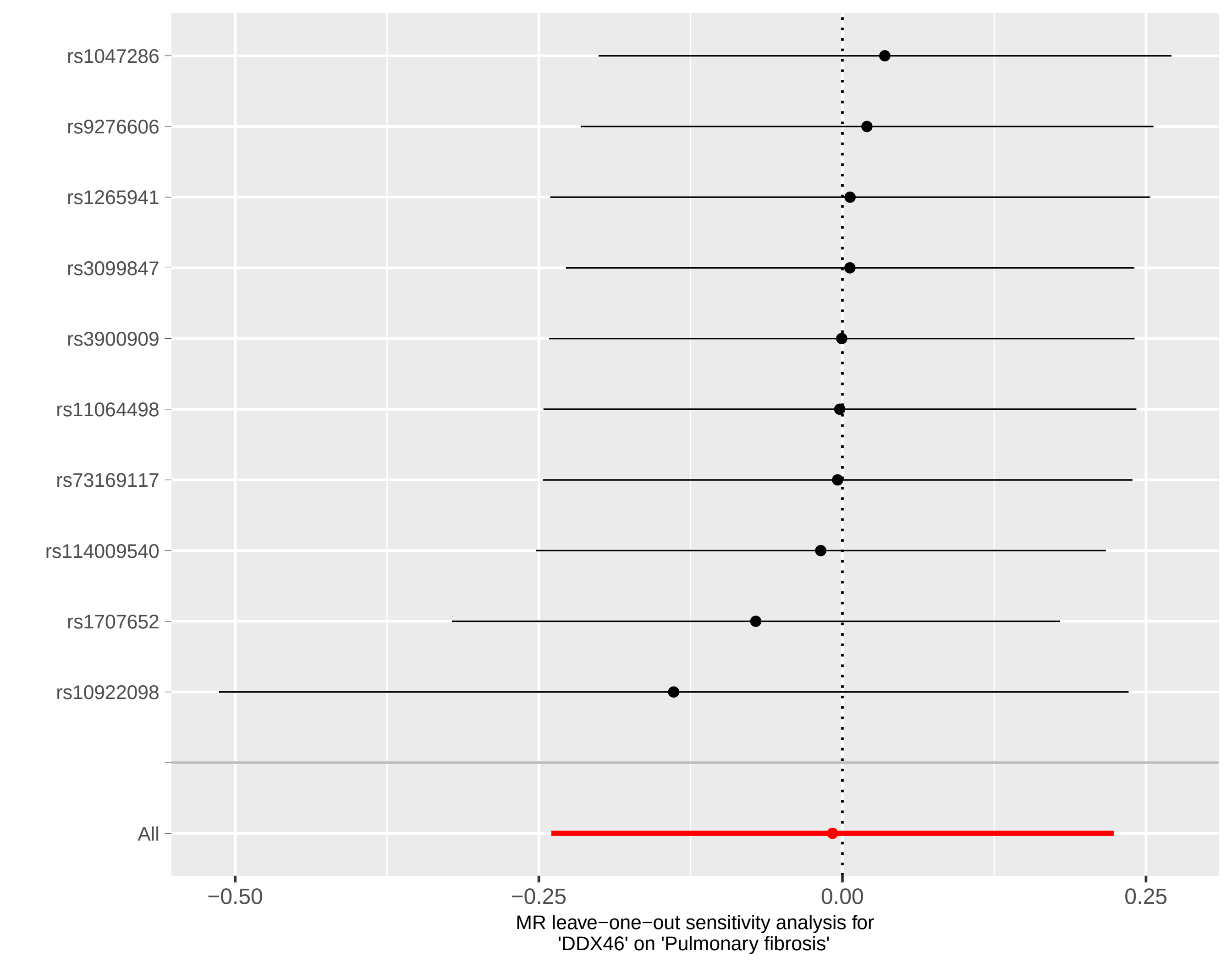
rs1047286
rs9276606
rs1265941
rs3099847
rs3900909
rs11064498
rs73169117
rs114009540
rs1707652
rs10922098
All
−0.50
−0.25
0.00
0.25
MR leave−one−out sensitivity analysis for
'DDX46' on 'Pulmonary fibrosis'

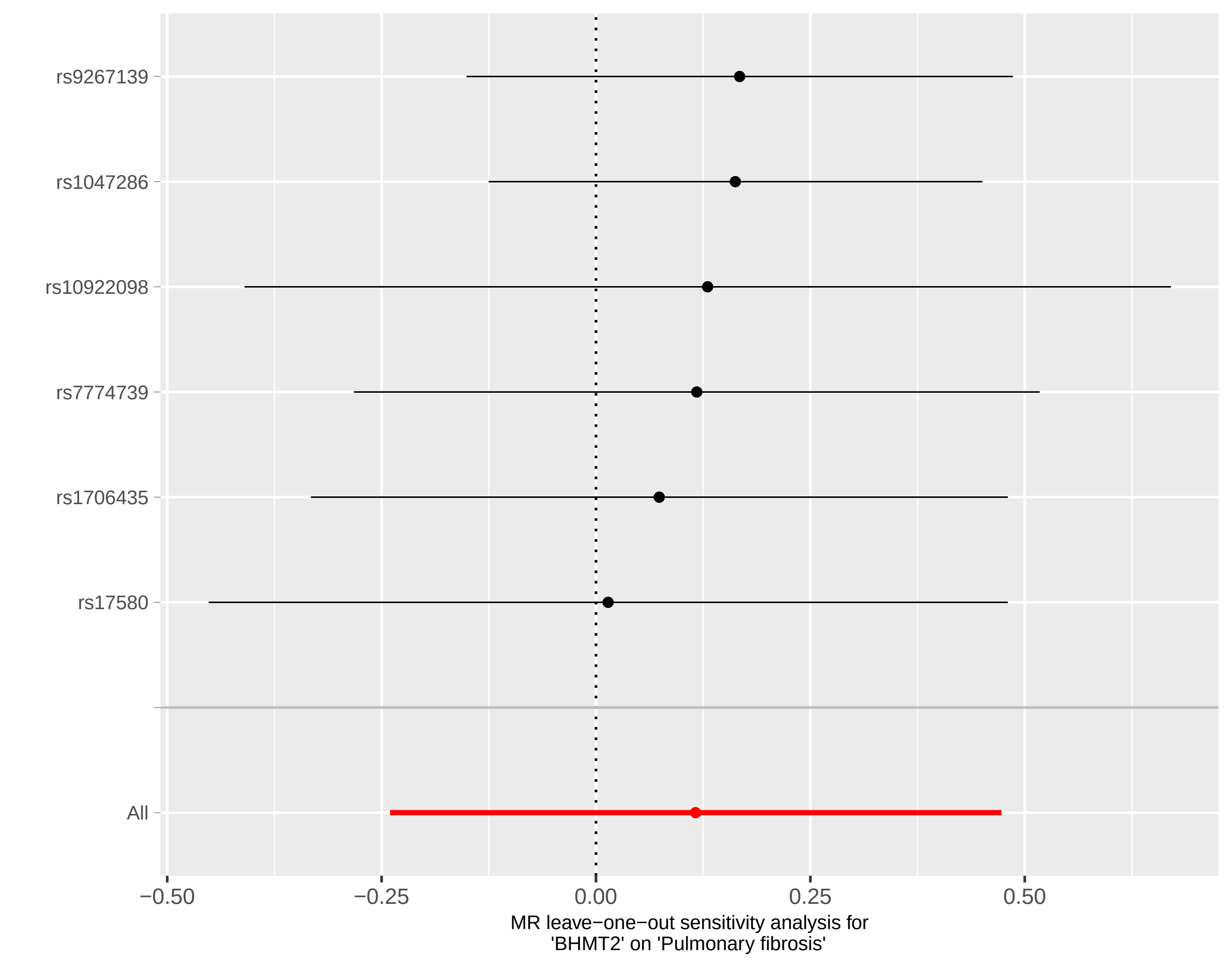
rs9267139
rs1047286
rs10922098
rs7774739
rs1706435
rs17580
All
−0.50
−0.25
0.00
0.25
0.50
MR leave−one−out sensitivity analysis for
'BHMT2' on 'Pulmonary fibrosis'

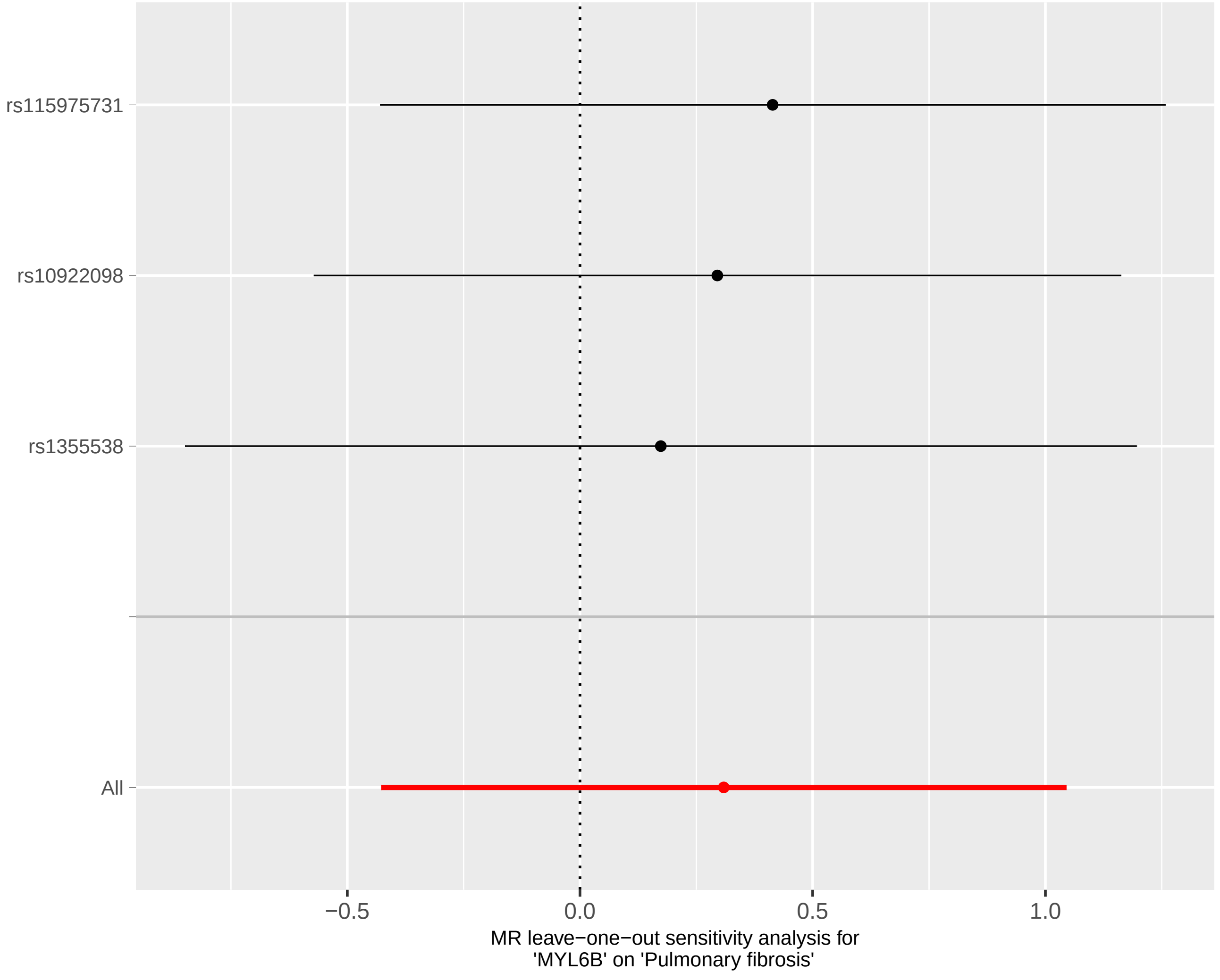
rs115975731
rs10922098
rs1355538
All
−0.5
0.0
0.5
1.0
MR leave−one−out sensitivity analysis for
'MYL6B' on 'Pulmonary fibrosis'

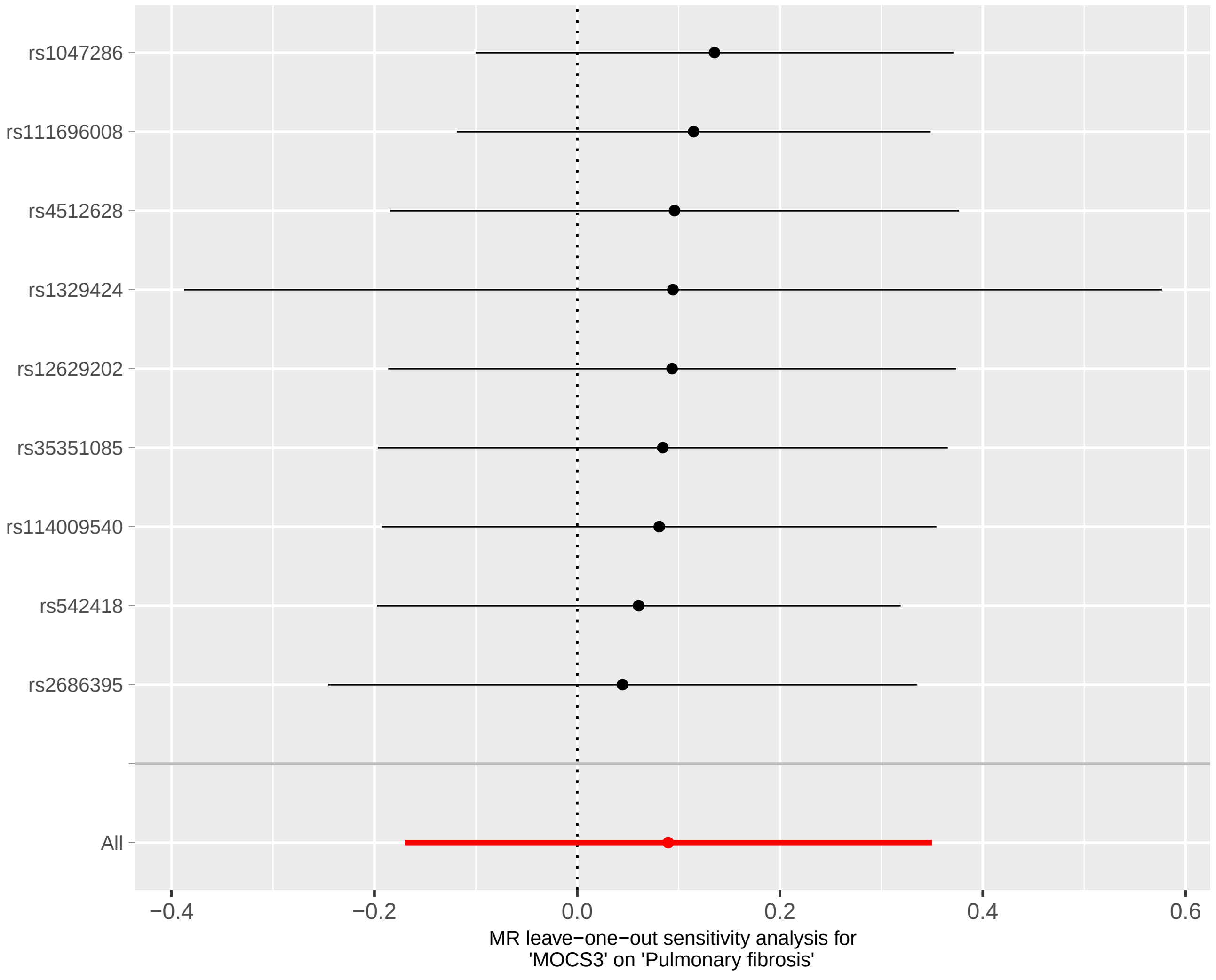
rs1047286
rs111696008
rs4512628
rs1329424
rs12629202
rs35351085
rs114009540
rs542418
rs2686395
All
−0.4
−0.2
0.0
0.2
0.4
0.6
MR leave−one−out sensitivity analysis for
'MOCS3' on 'Pulmonary fibrosis'

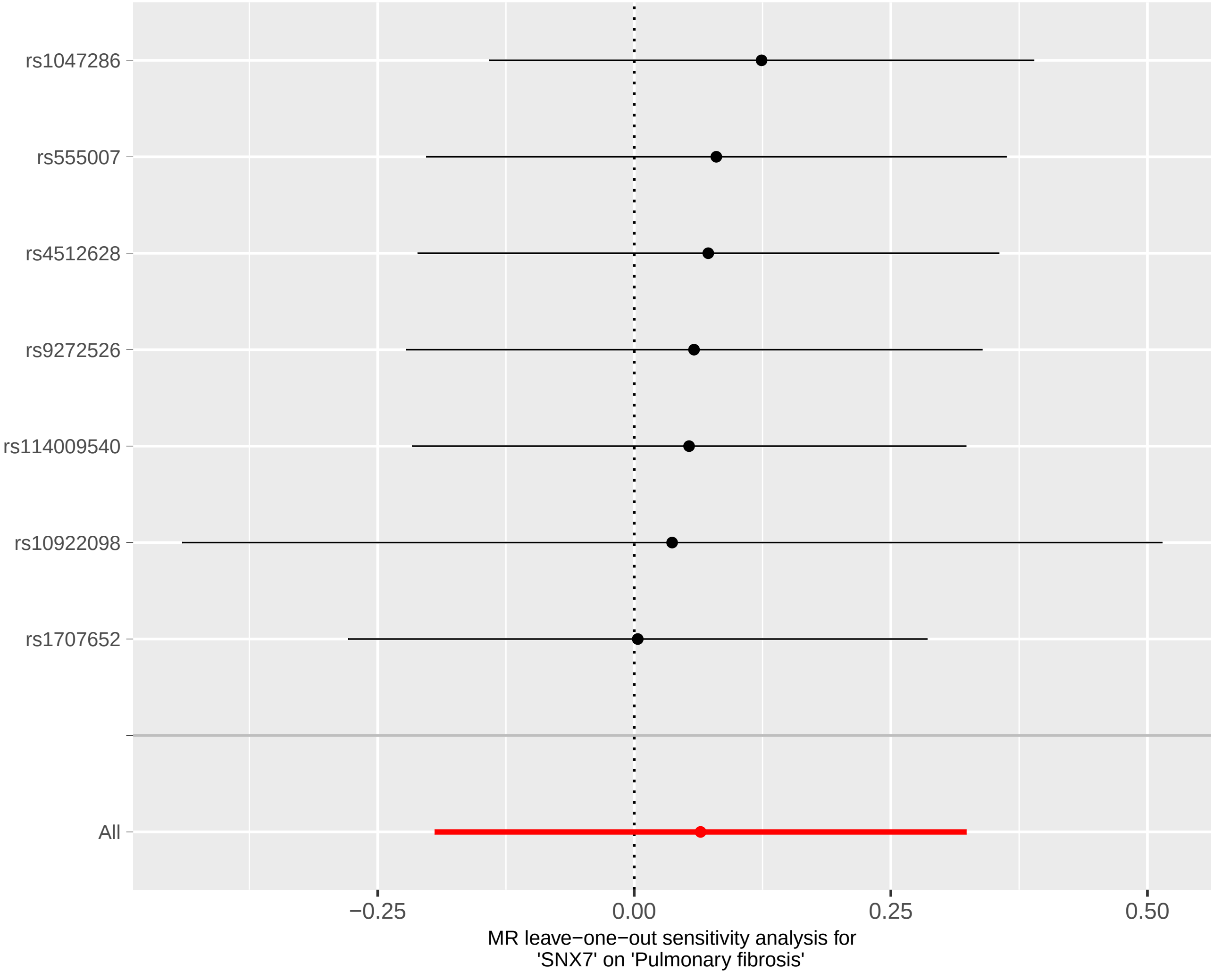
rs1047286
rs555007
rs4512628
rs9272526
rs114009540
rs10922098
rs1707652
All
−0.25
0.00
0.25
0.50
MR leave−one−out sensitivity analysis for
'SNX7' on 'Pulmonary fibrosis'

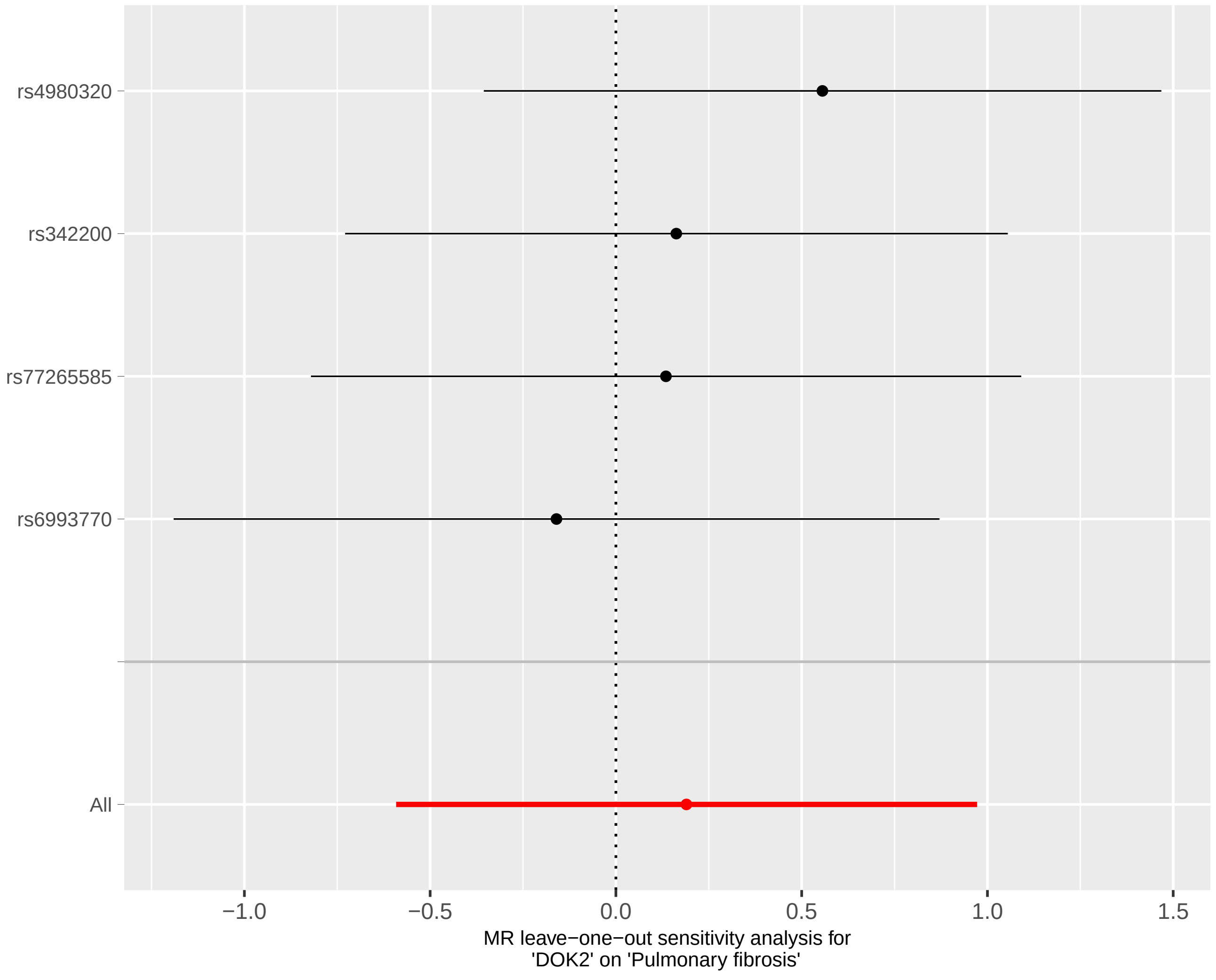
rs4980320
rs342200
rs77265585
rs6993770
All
−1.0
−0.5
0.0
0.5
1.0
1.5
MR leave−one−out sensitivity analysis for
'DOK2' on 'Pulmonary fibrosis'

Insufficient number of SNPs

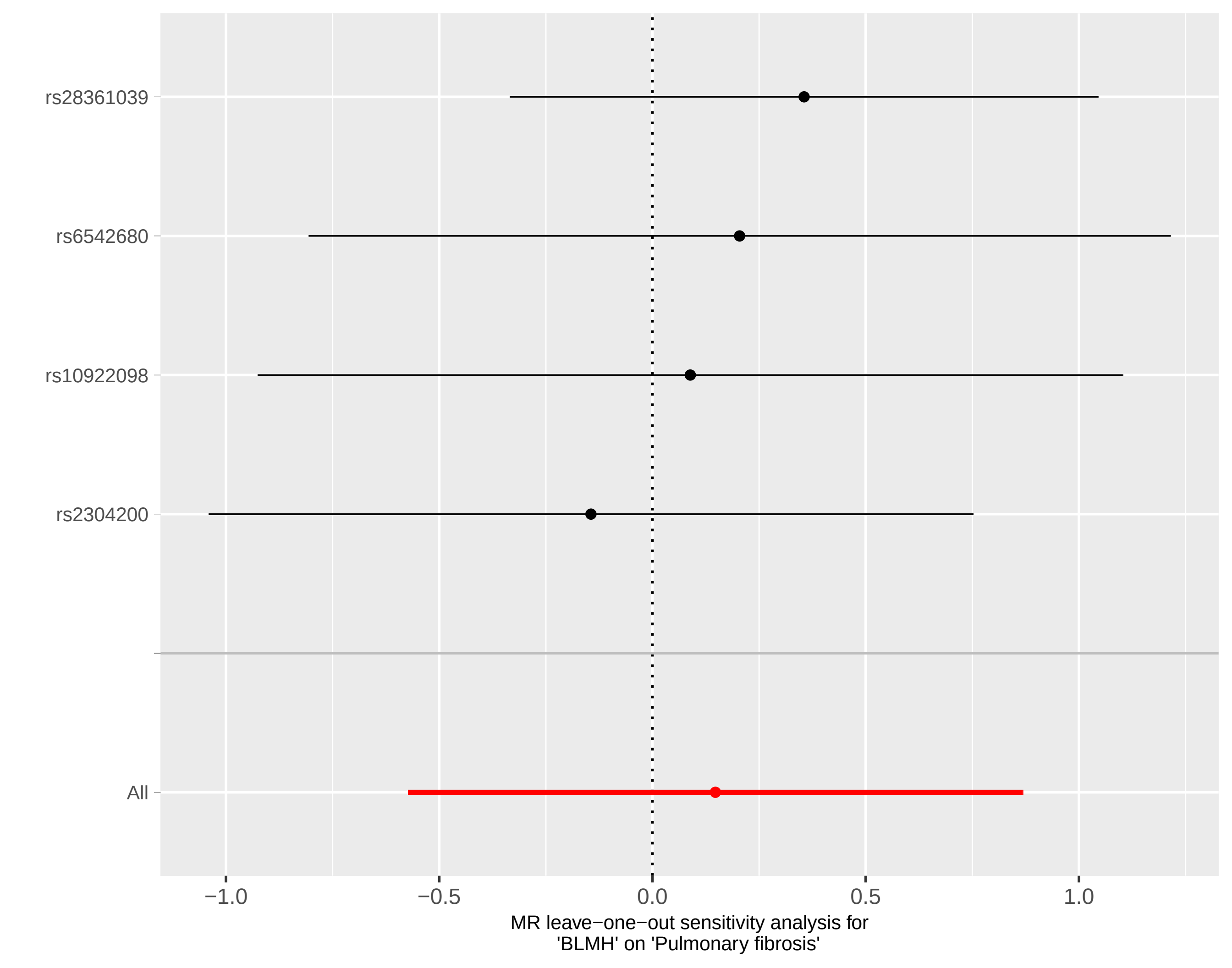
rs28361039
rs6542680
rs10922098
rs2304200
All
−1.0
−0.5
0.0
0.5
1.0
MR leave−one−out sensitivity analysis for
'BLMH' on 'Pulmonary fibrosis'

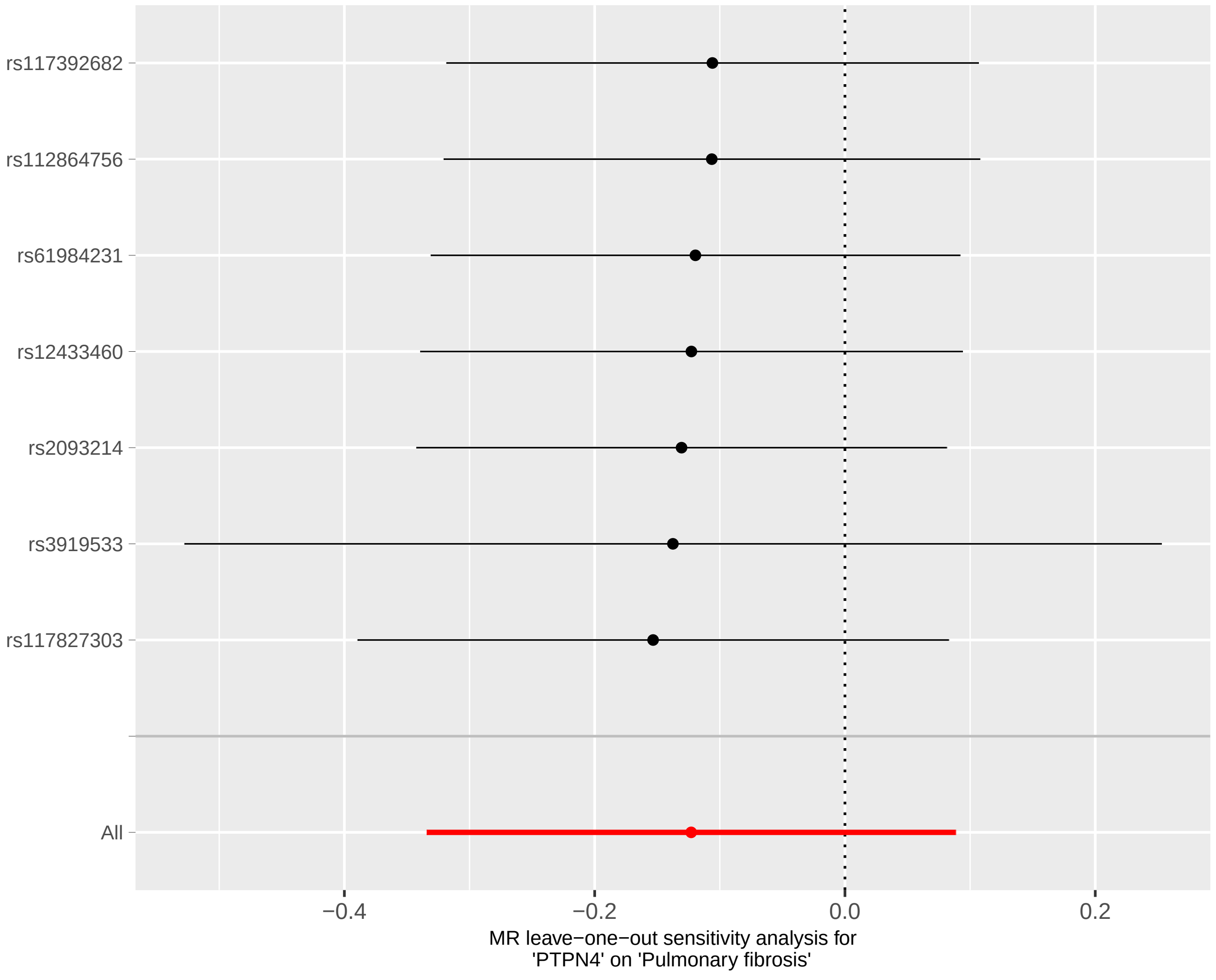
rs117392682
rs112864756
rs61984231
rs12433460
rs2093214
rs3919533
rs117827303
All
−0.4
−0.2
0.0
0.2
MR leave−one−out sensitivity analysis for
'PTPN4' on 'Pulmonary fibrosis'

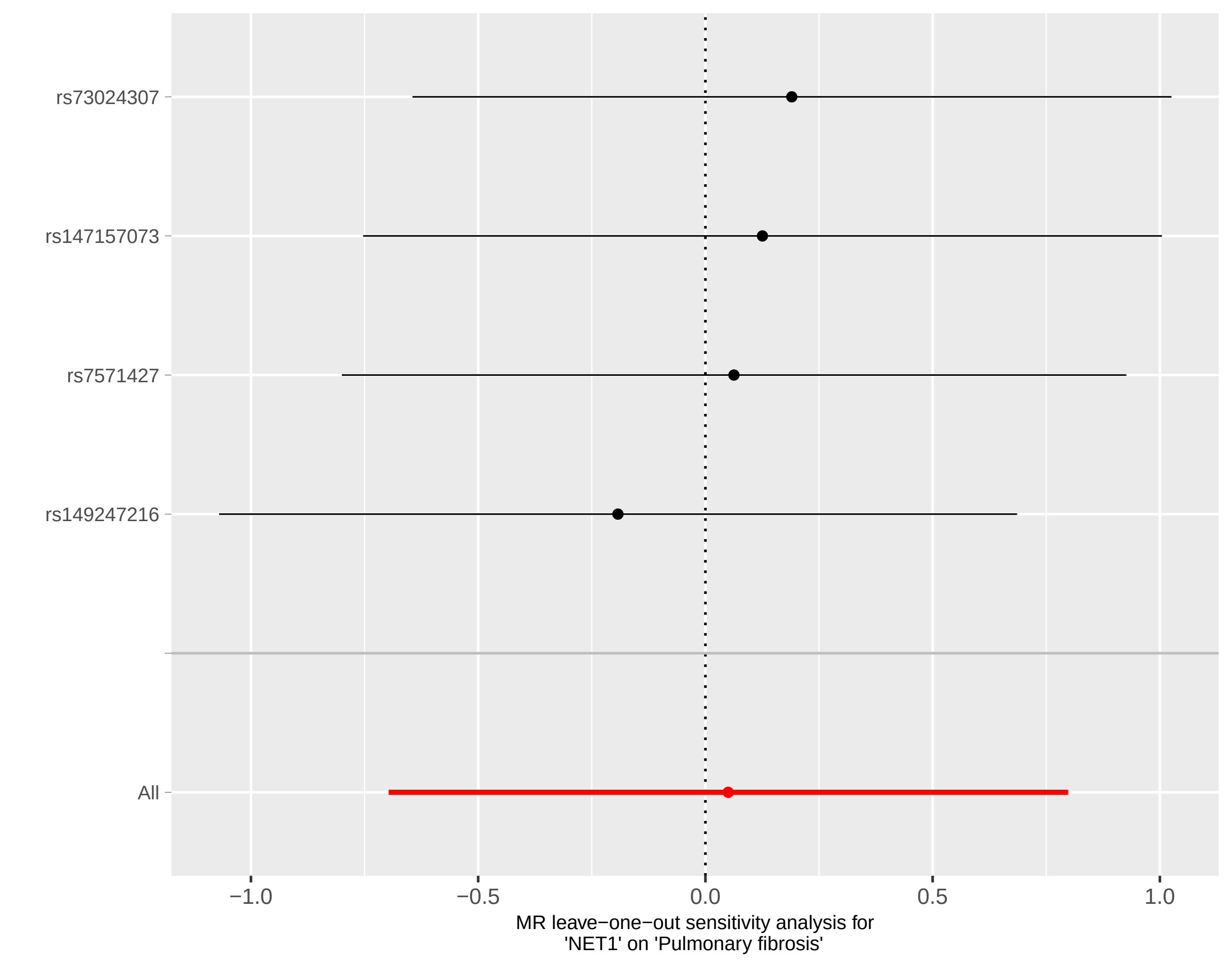
rs73024307
rs147157073
rs7571427
rs149247216
All
−1.0
−0.5
0.0
0.5
1.0
MR leave−one−out sensitivity analysis for
'NET1' on 'Pulmonary fibrosis'

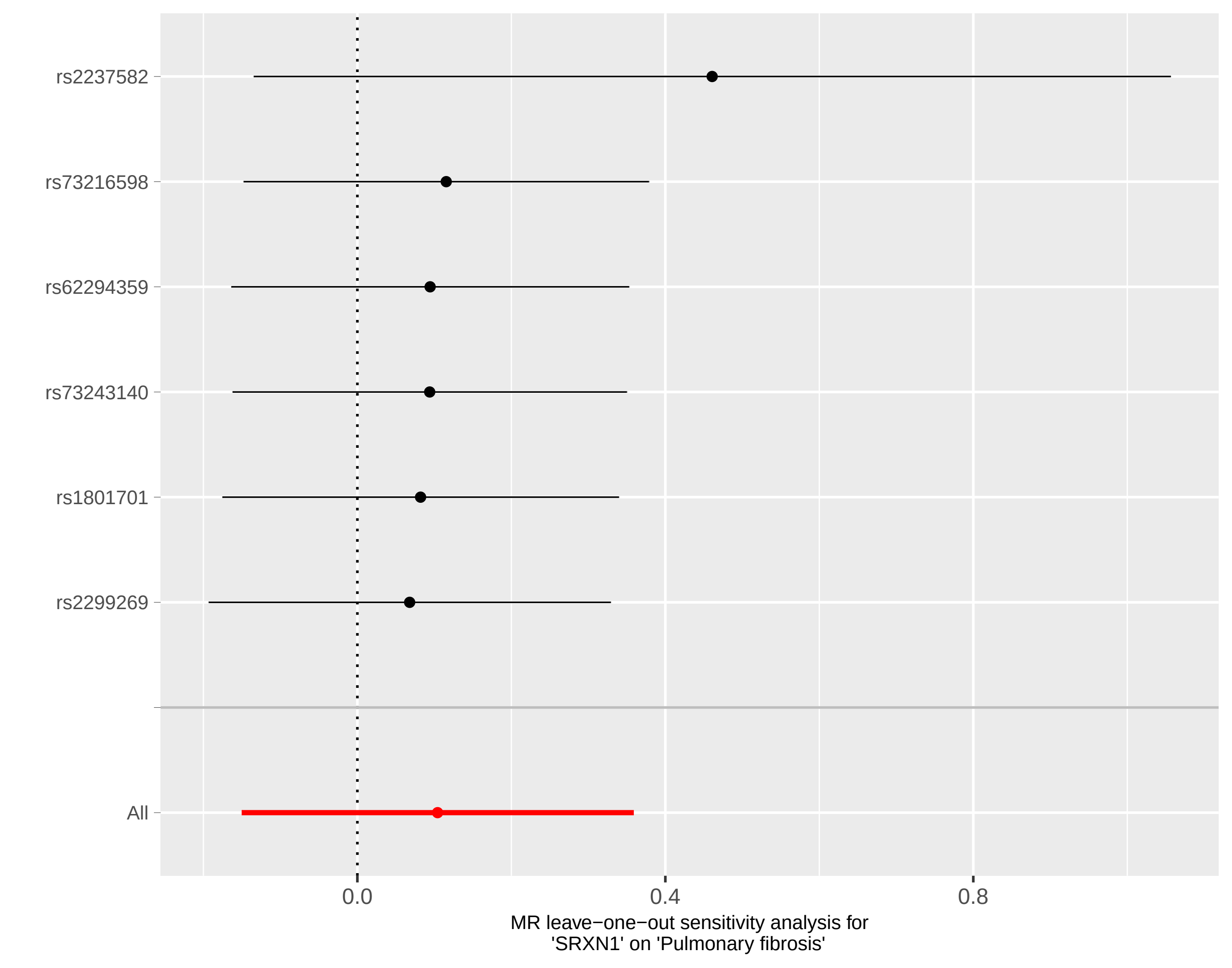
rs2237582
rs73216598
rs62294359
rs73243140
rs1801701
rs2299269
All
0.0
0.4
0.8
MR leave−one−out sensitivity analysis for
'SRXN1' on 'Pulmonary fibrosis'

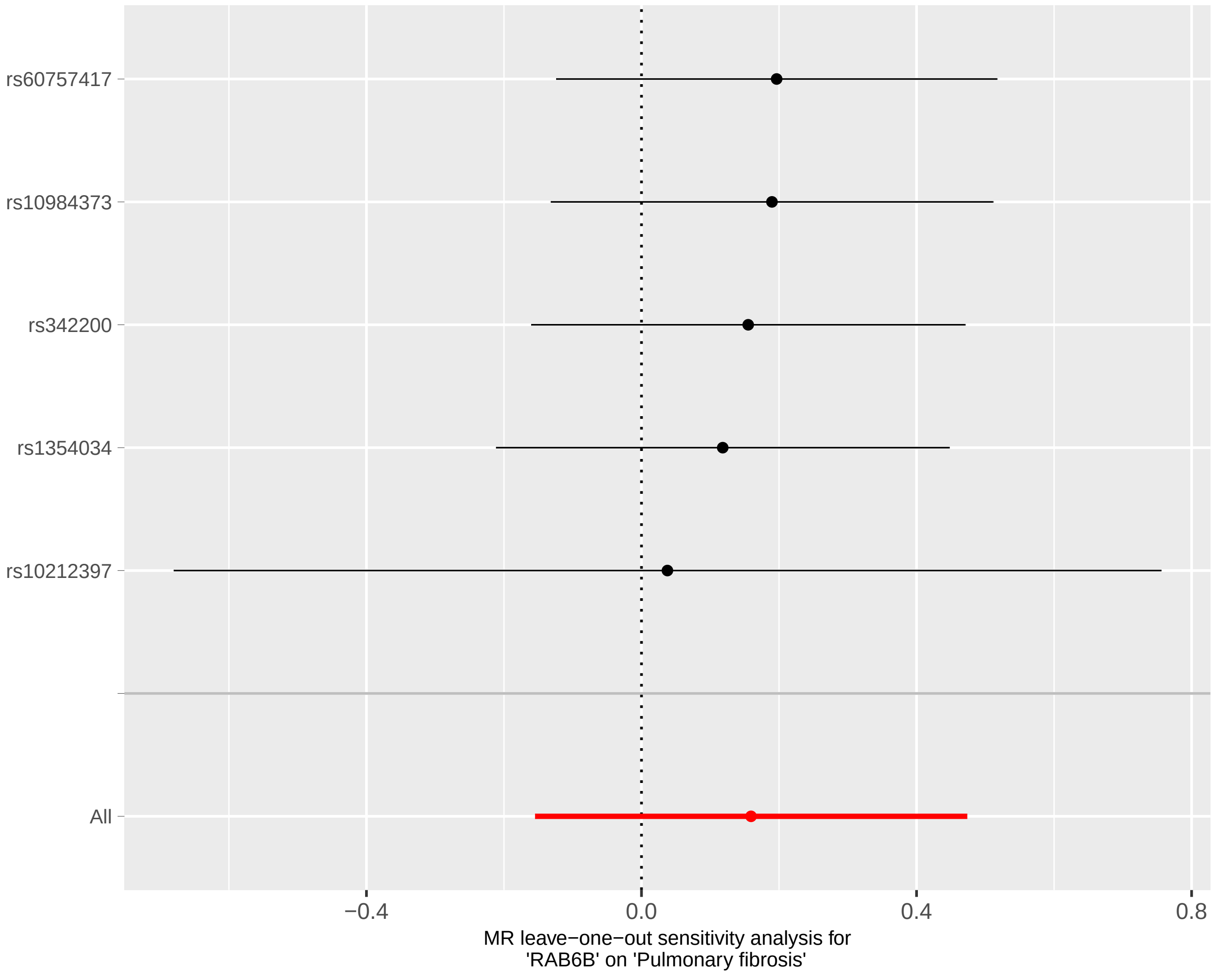
rs60757417
rs10984373
rs342200
rs1354034
rs10212397
All
−0.4
0.0
0.4
0.8
MR leave−one−out sensitivity analysis for
'RAB6B' on 'Pulmonary fibrosis'

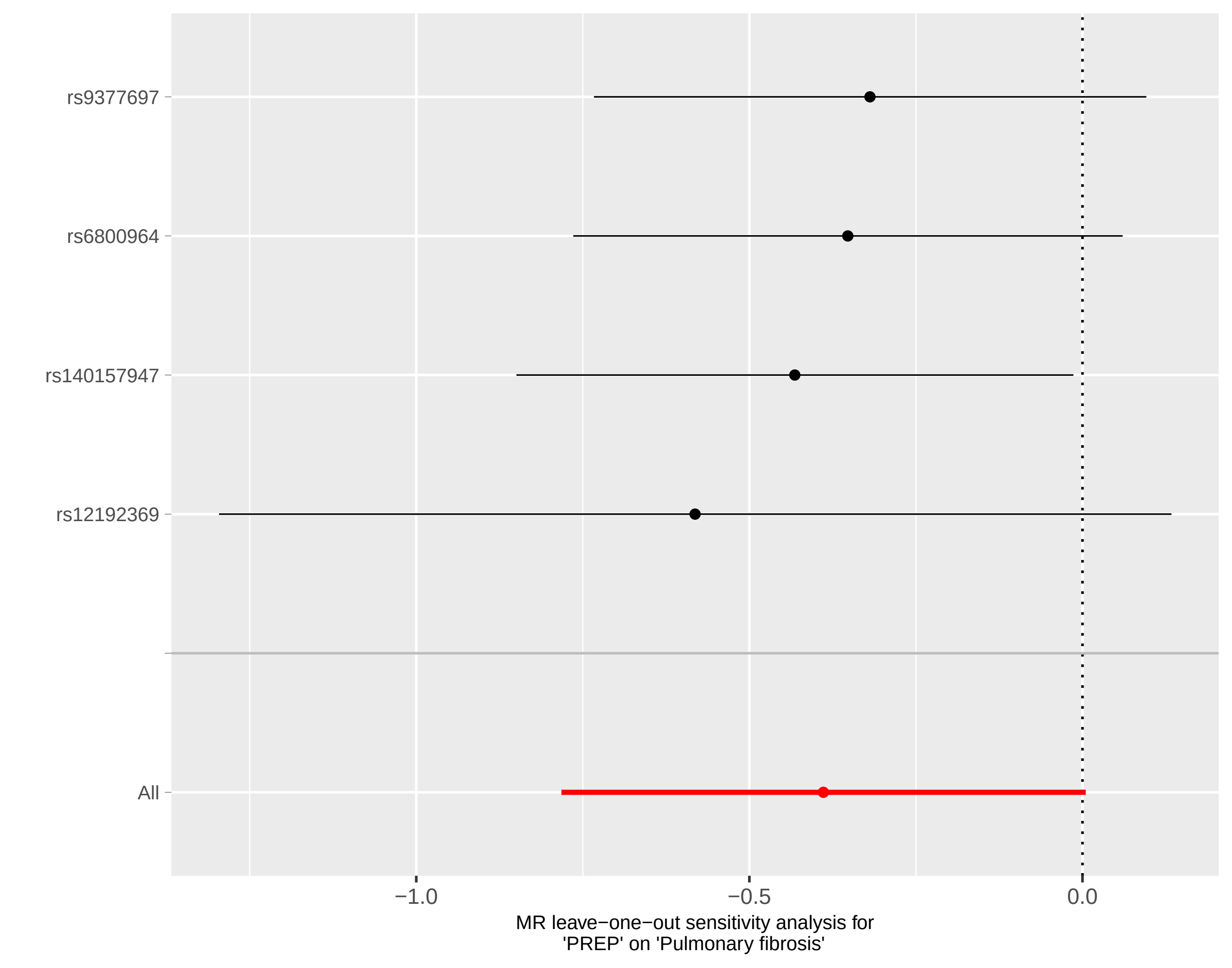
rs9377697
rs6800964
rs140157947
rs12192369
All
−1.0
−0.5
0.0
MR leave−one−out sensitivity analysis for
'PREP' on 'Pulmonary fibrosis'

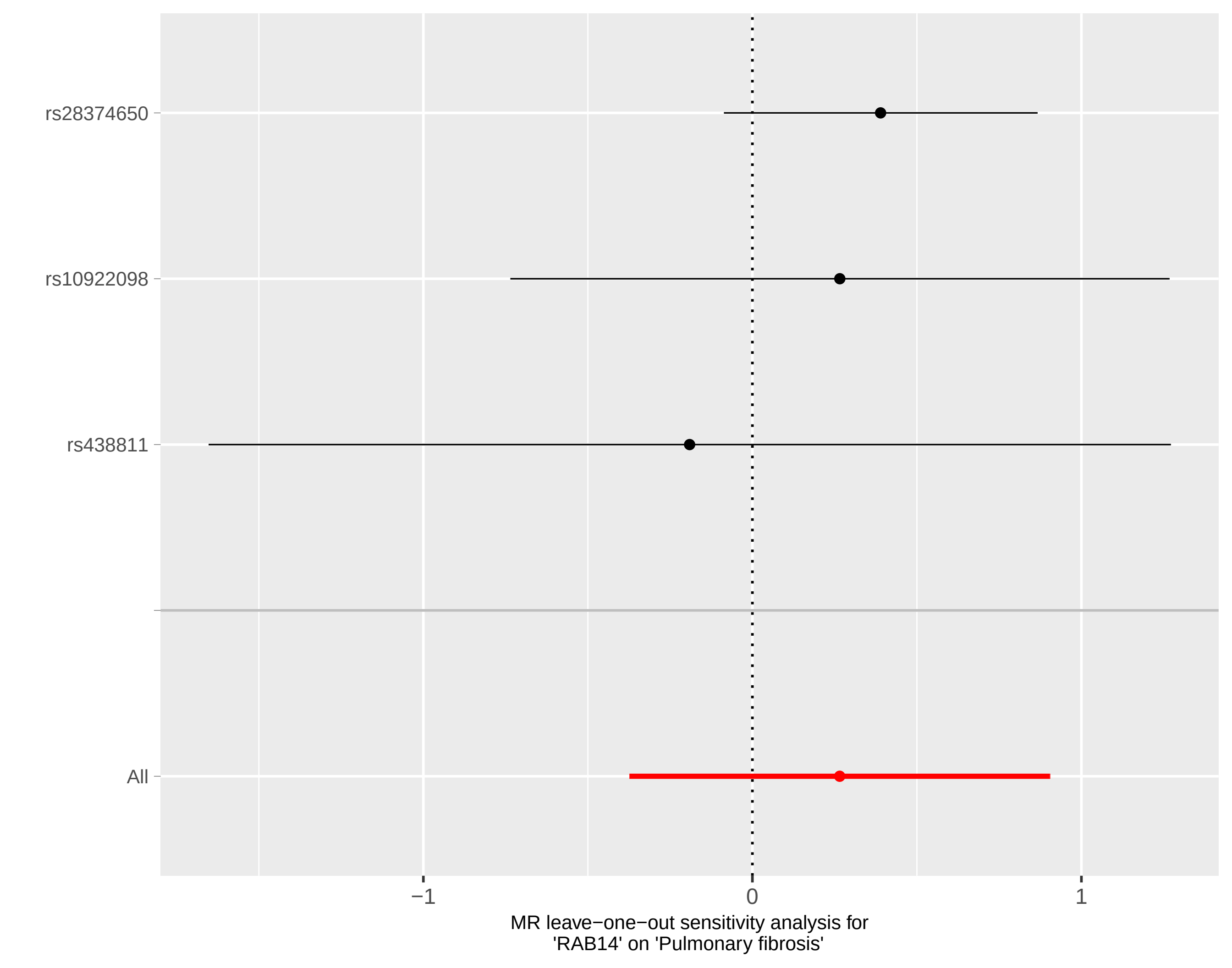
rs28374650
rs10922098
rs438811
All
−1
0
1
MR leave−one−out sensitivity analysis for
'RAB14' on 'Pulmonary fibrosis'

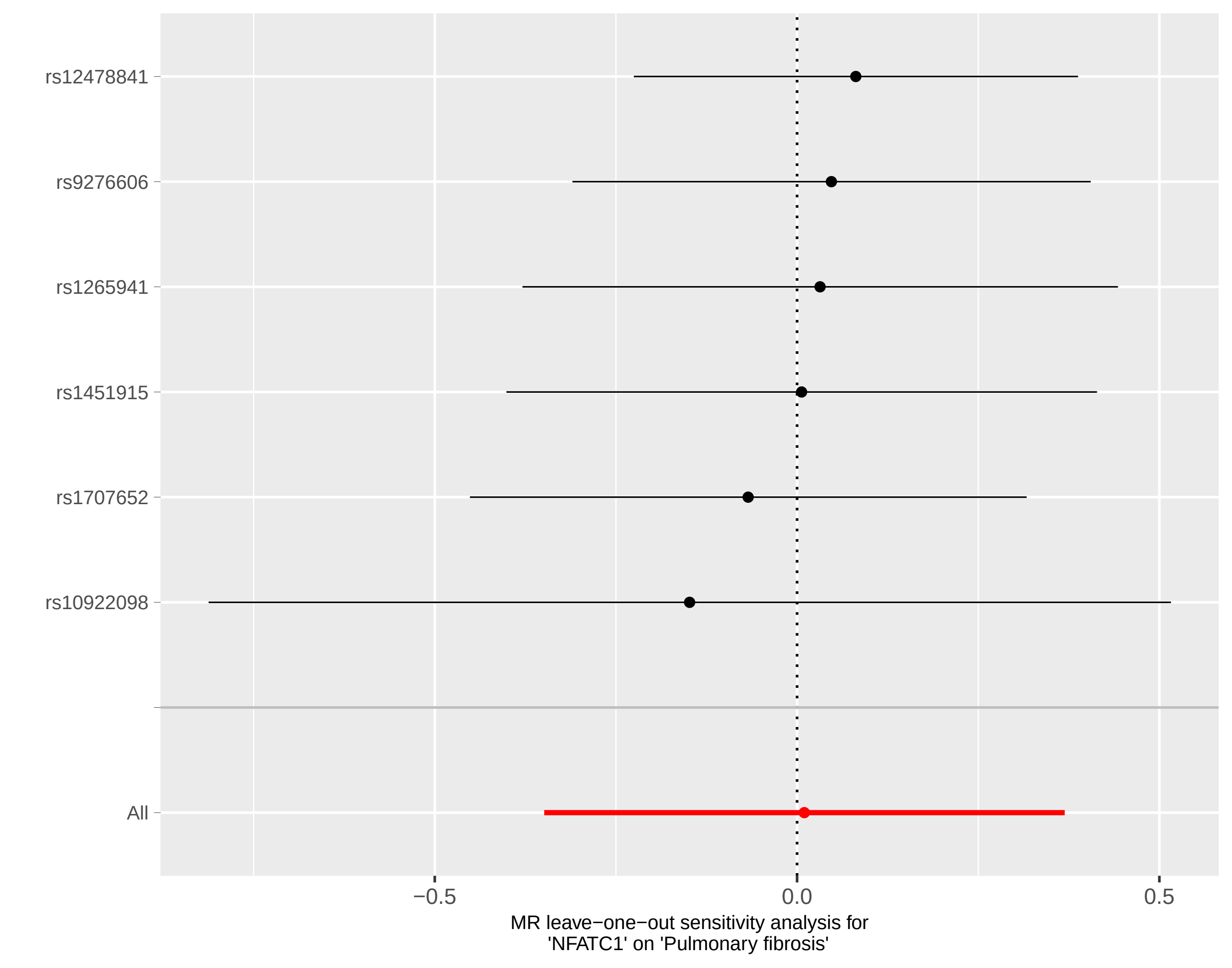

rs12478841
rs9276606
rs1265941
rs1451915
rs1707652
rs10922098
All
−0.5
0.0
0.5
MR leave−one−out sensitivity analysis for
'NFATC1' on 'Pulmonary fibrosis'

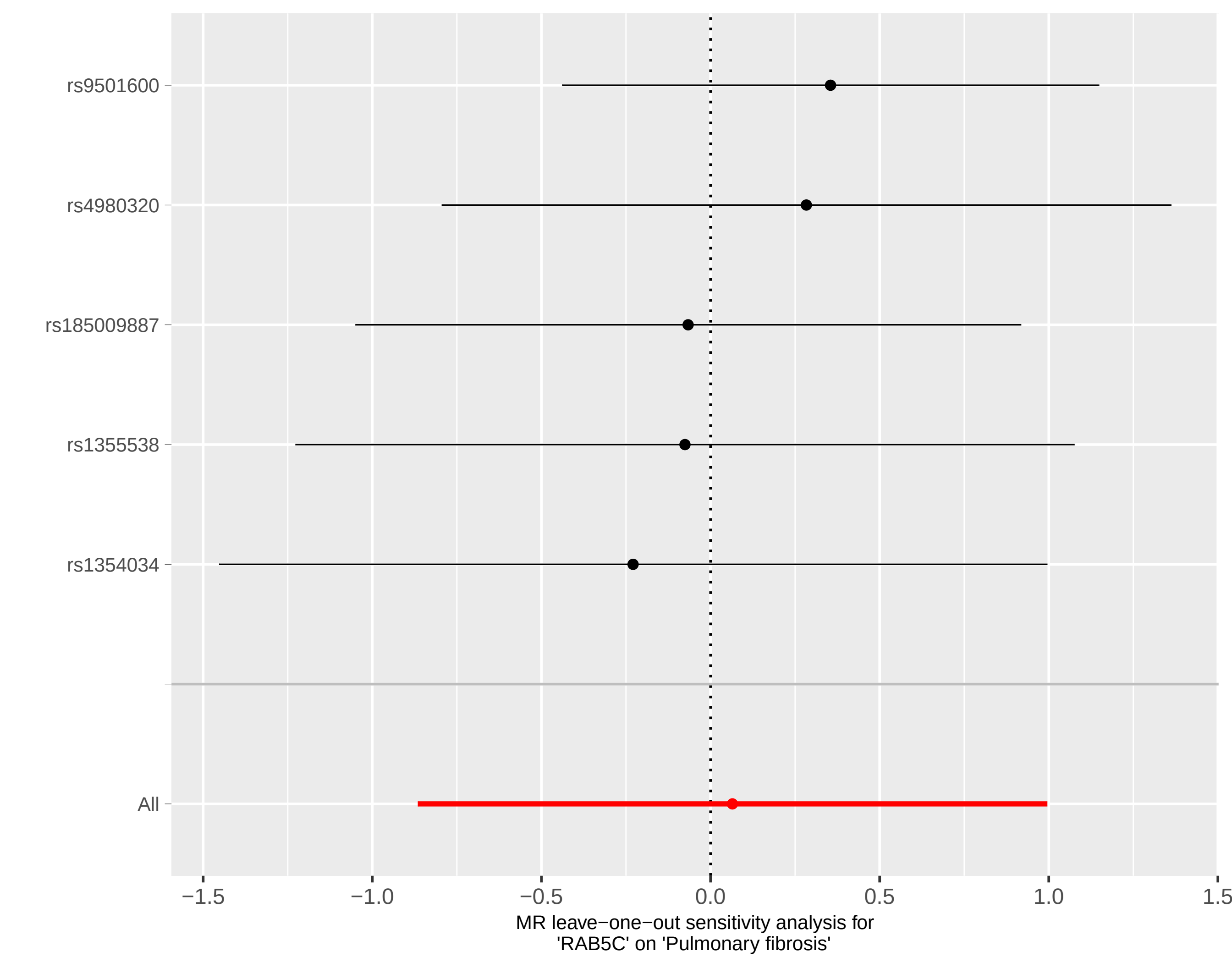
rs9501600
rs4980320
rs185009887
rs1355538
rs1354034
All
−1.5
−1.0
−0.5
0.0
0.5
1.0
1.5
MR leave−one−out sensitivity analysis for
'RAB5C' on 'Pulmonary fibrosis'

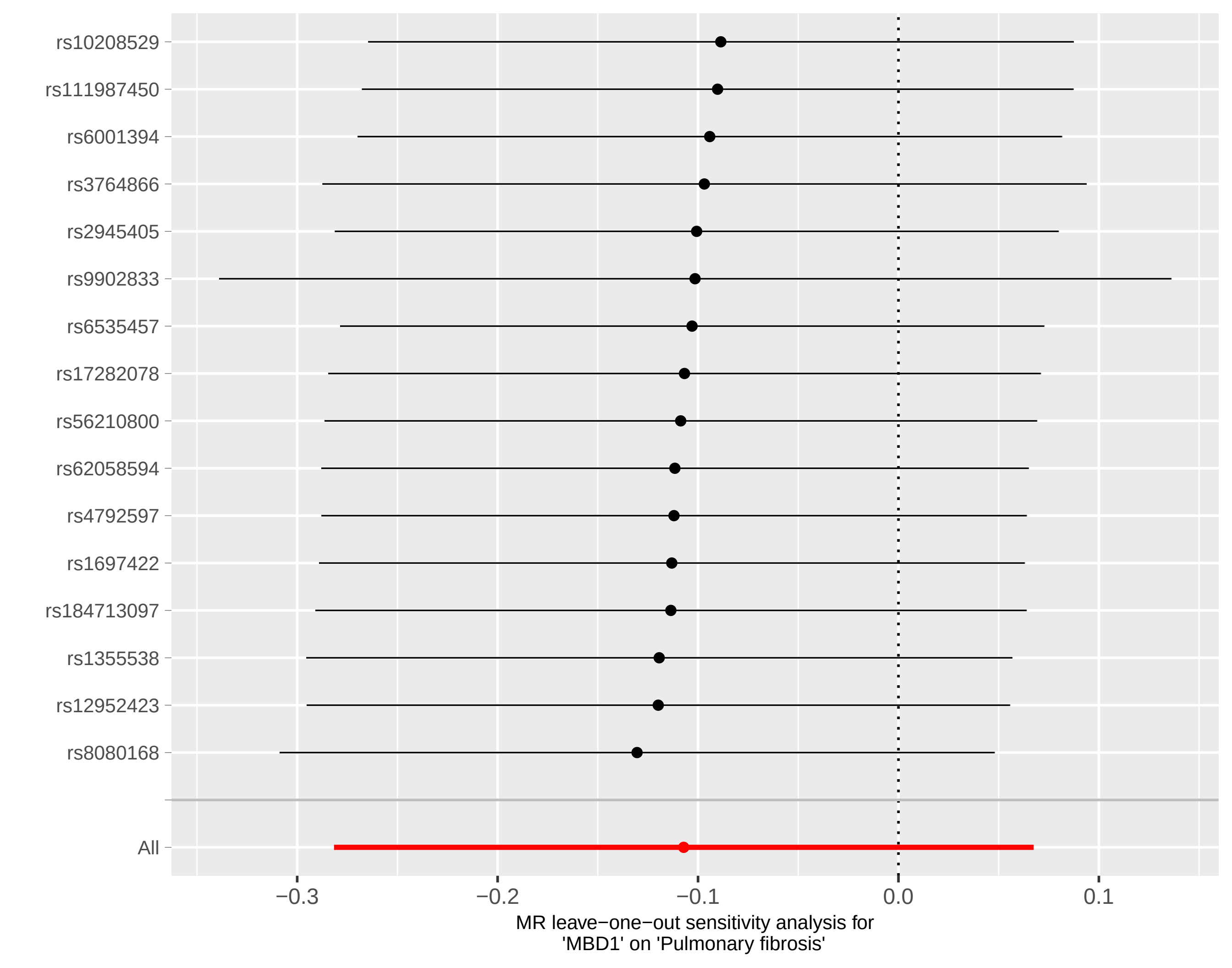
rs10208529
rs111987450
rs6001394
rs3764866
rs2945405
rs9902833
rs6535457
rs17282078
rs56210800
rs62058594
rs4792597
rs1697422
rs184713097
rs1355538
rs12952423
rs8080168
All
−0.3
−0.2
−0.1
0.0
0.1
MR leave−one−out sensitivity analysis for
'MBD1' on 'Pulmonary fibrosis'

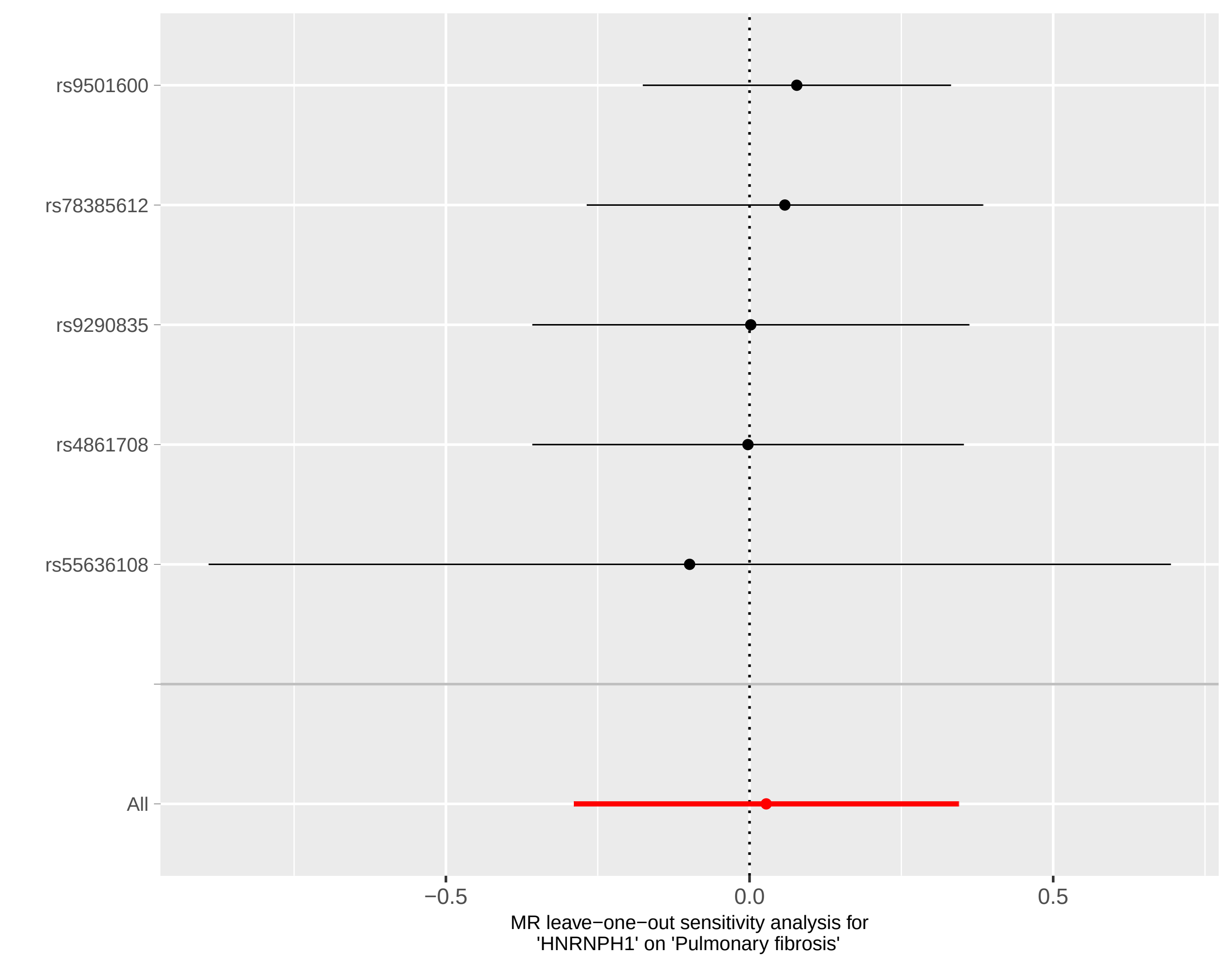
rs9501600
rs78385612
rs9290835
rs4861708
rs55636108
All
−0.5
0.0
0.5
MR leave−one−out sensitivity analysis for
'HNRNPH1' on 'Pulmonary fibrosis'

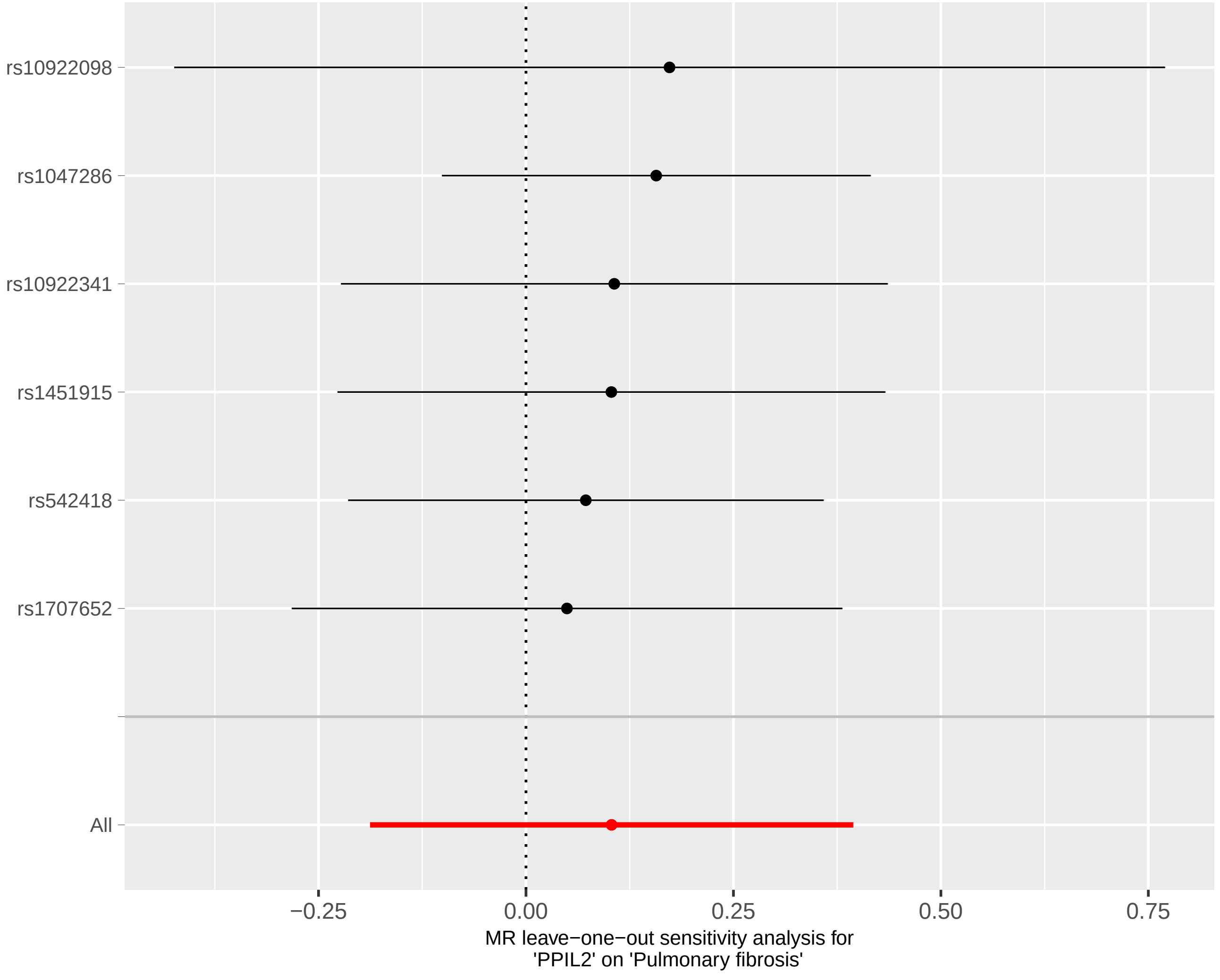
rs10922098
rs1047286
rs10922341
rs1451915
rs542418
rs1707652
All
−0.25
0.00
0.25
0.50
0.75
MR leave−one−out sensitivity analysis for
'PPIL2' on 'Pulmonary fibrosis'

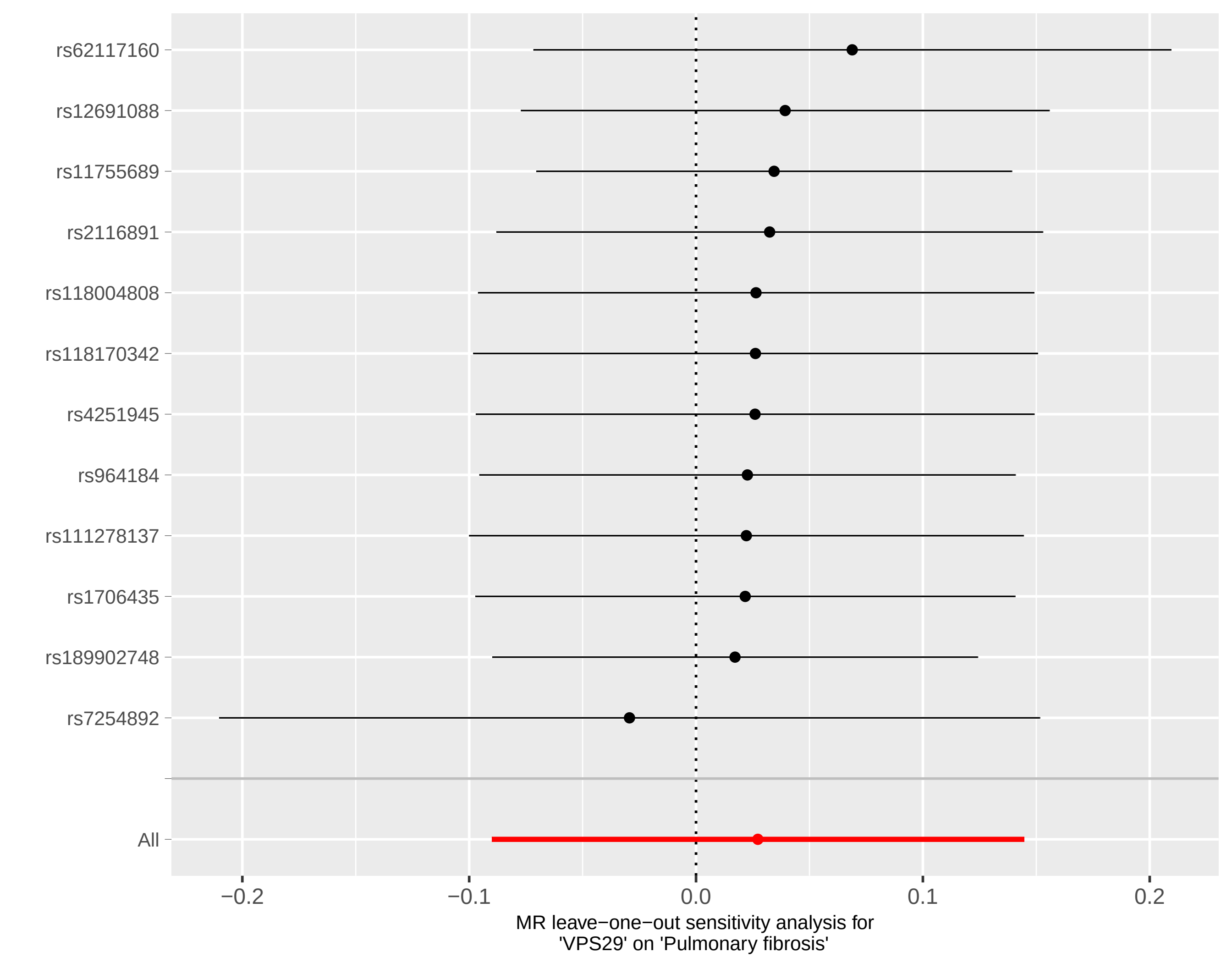
rs62117160
rs12691088
rs11755689
rs2116891
rs118004808
rs118170342
rs4251945
rs964184
rs111278137
rs1706435
rs189902748
rs7254892
All
−0.2
−0.1
0.0
0.1
0.2
MR leave−one−out sensitivity analysis for
'VPS29' on 'Pulmonary fibrosis'

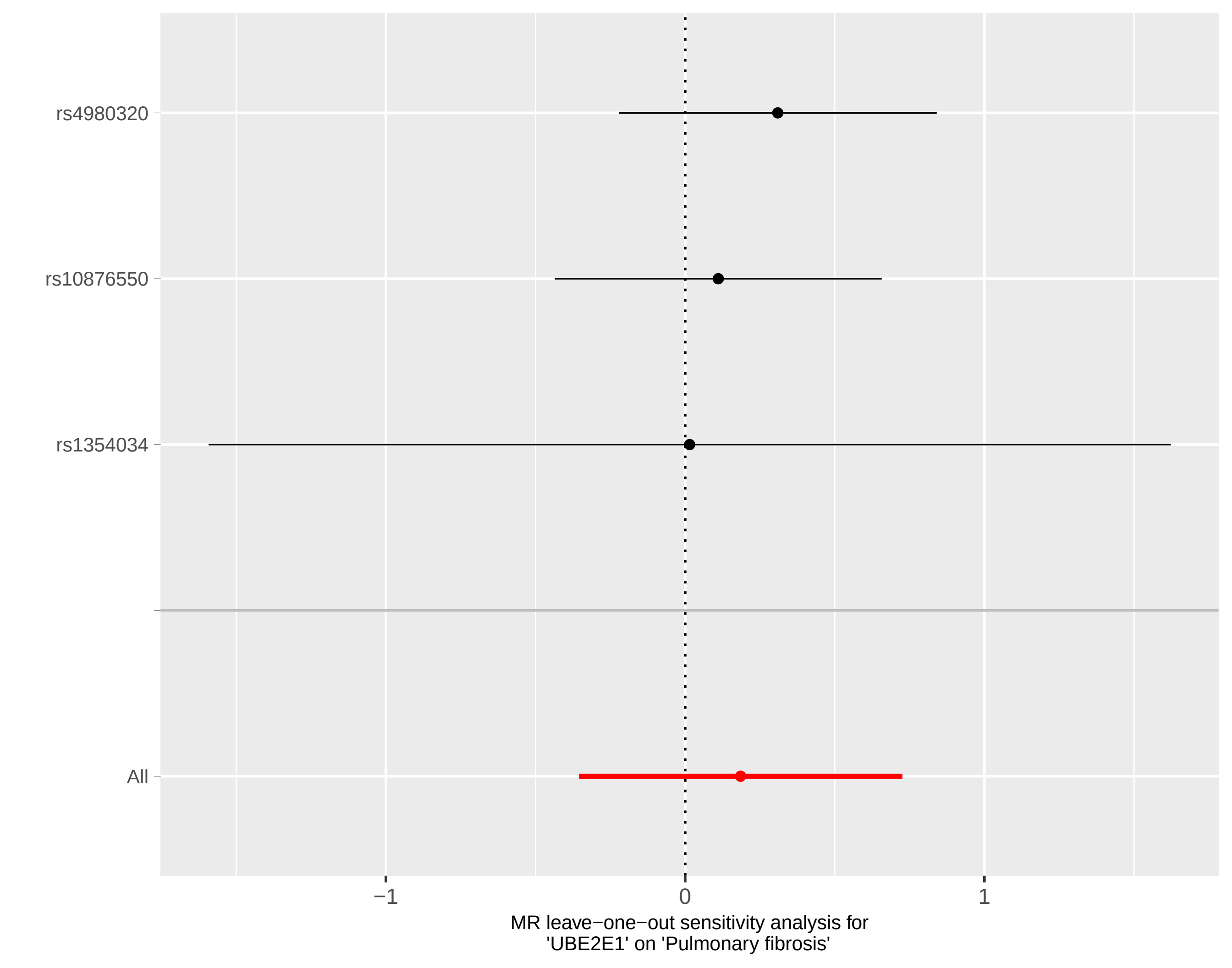
rs4980320
rs10876550
rs1354034
All
−1
0
1
MR leave−one−out sensitivity analysis for
'UBE2E1' on 'Pulmonary fibrosis'

Insufficient number of SNPs

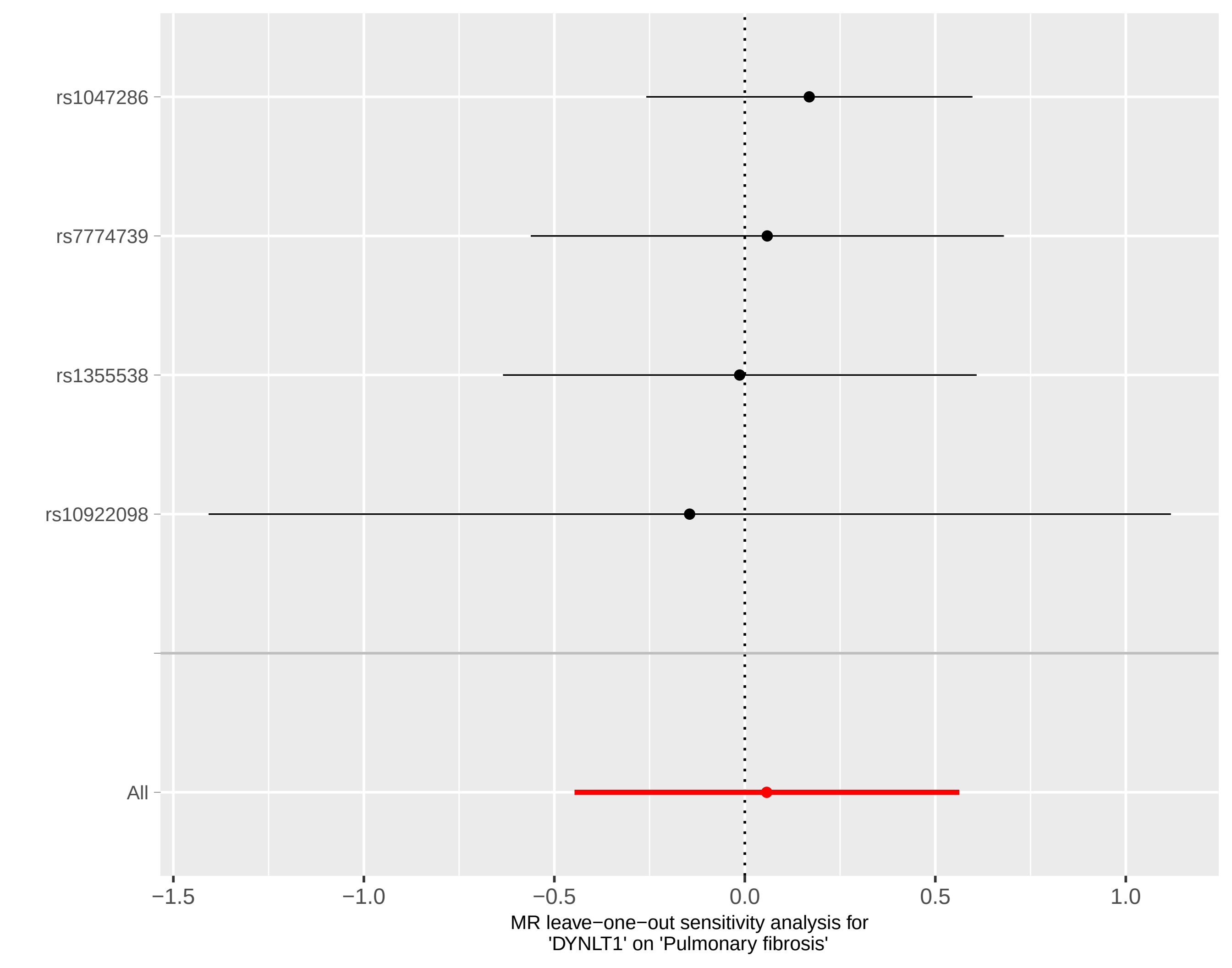
rs1047286
rs7774739
rs1355538
rs10922098
All
−1.5
−1.0
−0.5
0.0
0.5
1.0
MR leave−one−out sensitivity analysis for
'DYNLT1' on 'Pulmonary fibrosis'

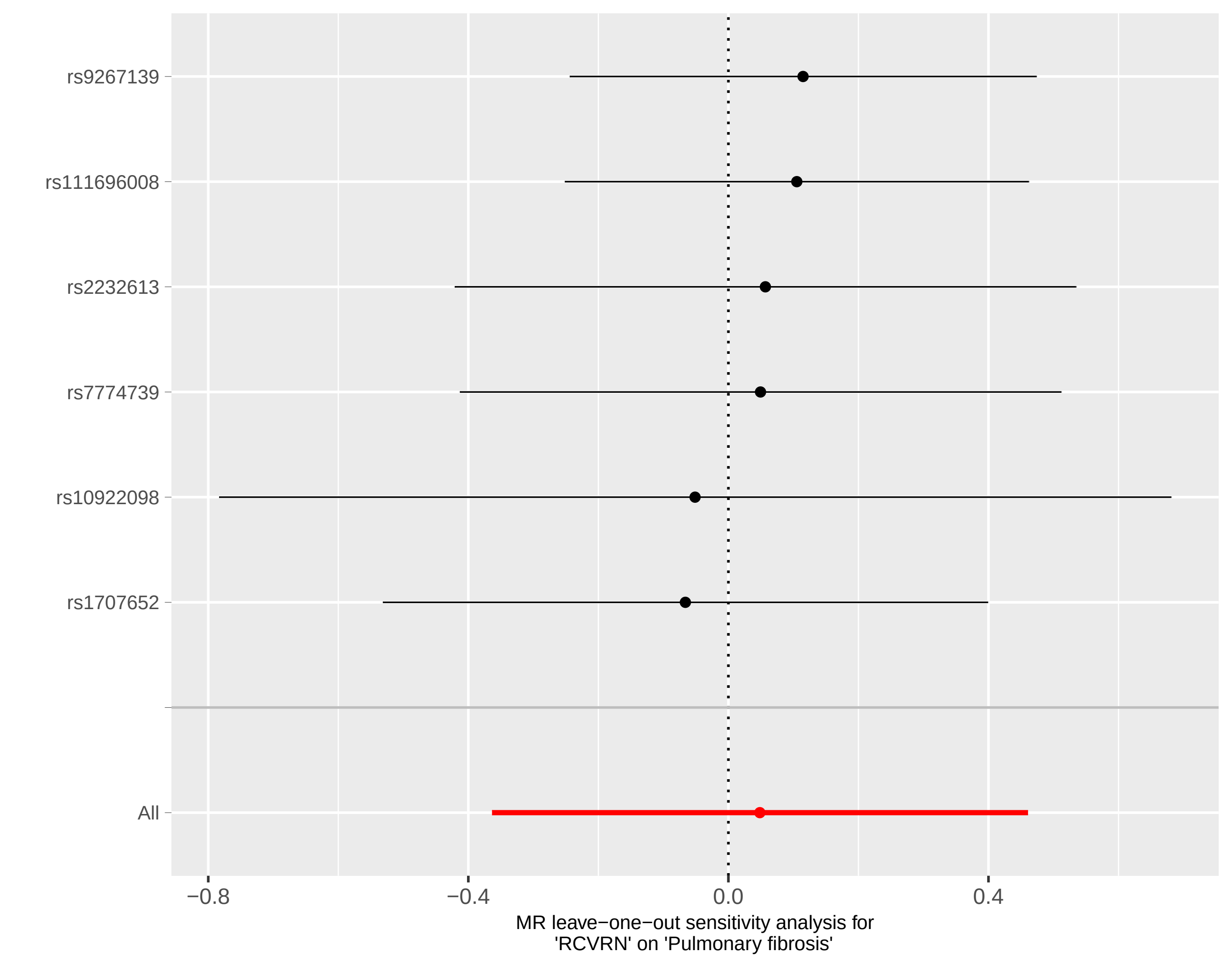
rs9267139
rs111696008
rs2232613
rs7774739
rs10922098
rs1707652
All
−0.8
−0.4
0.0
0.4
MR leave−one−out sensitivity analysis for
'RCVRN' on 'Pulmonary fibrosis'

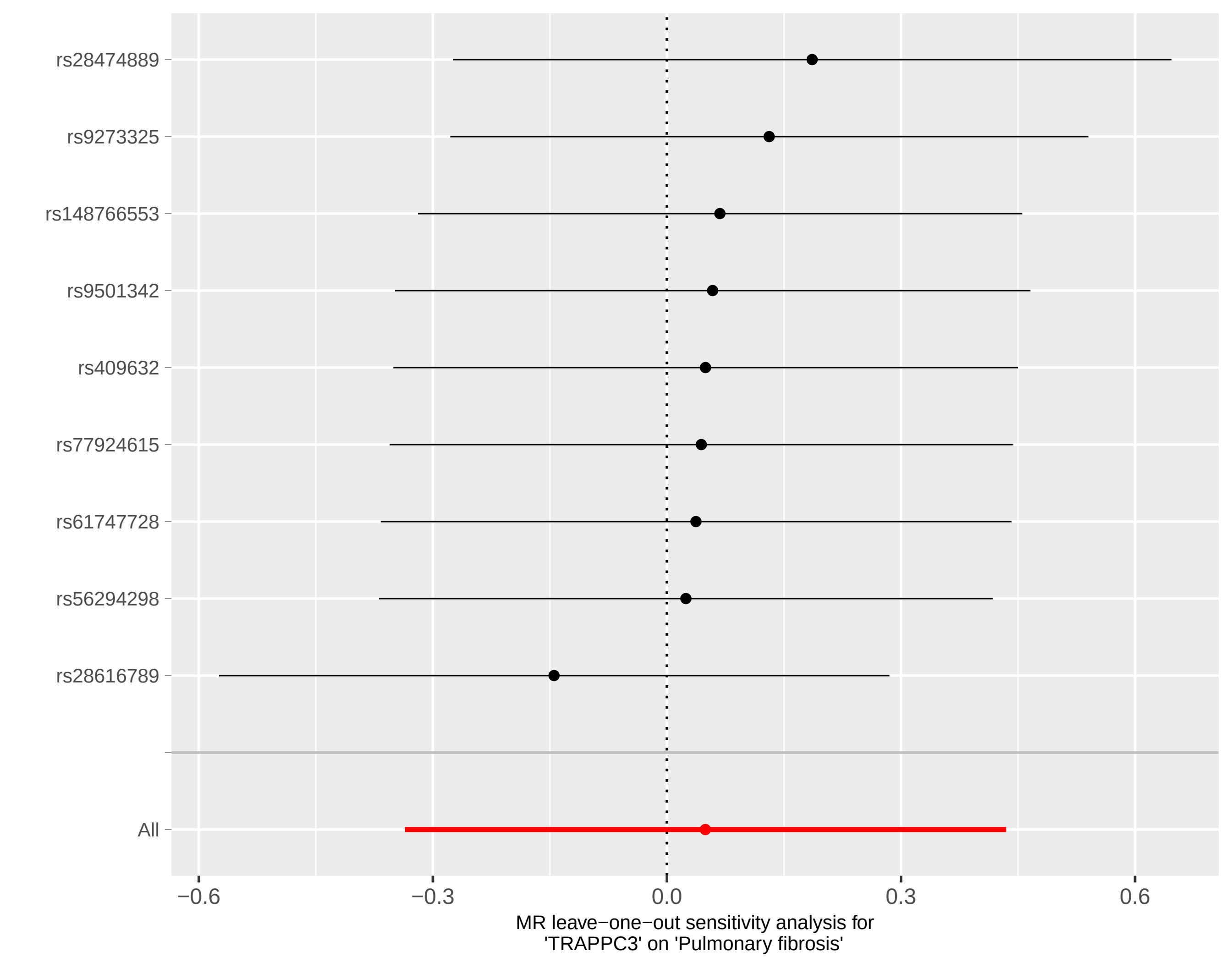
rs28474889
rs9273325
rs148766553
rs9501342
rs409632
rs77924615
rs61747728
rs56294298
rs28616789
All
−0.6
−0.3
0.0
0.3
0.6
MR leave−one−out sensitivity analysis for
'TRAPPC3' on 'Pulmonary fibrosis'

Insufficient number of SNPs

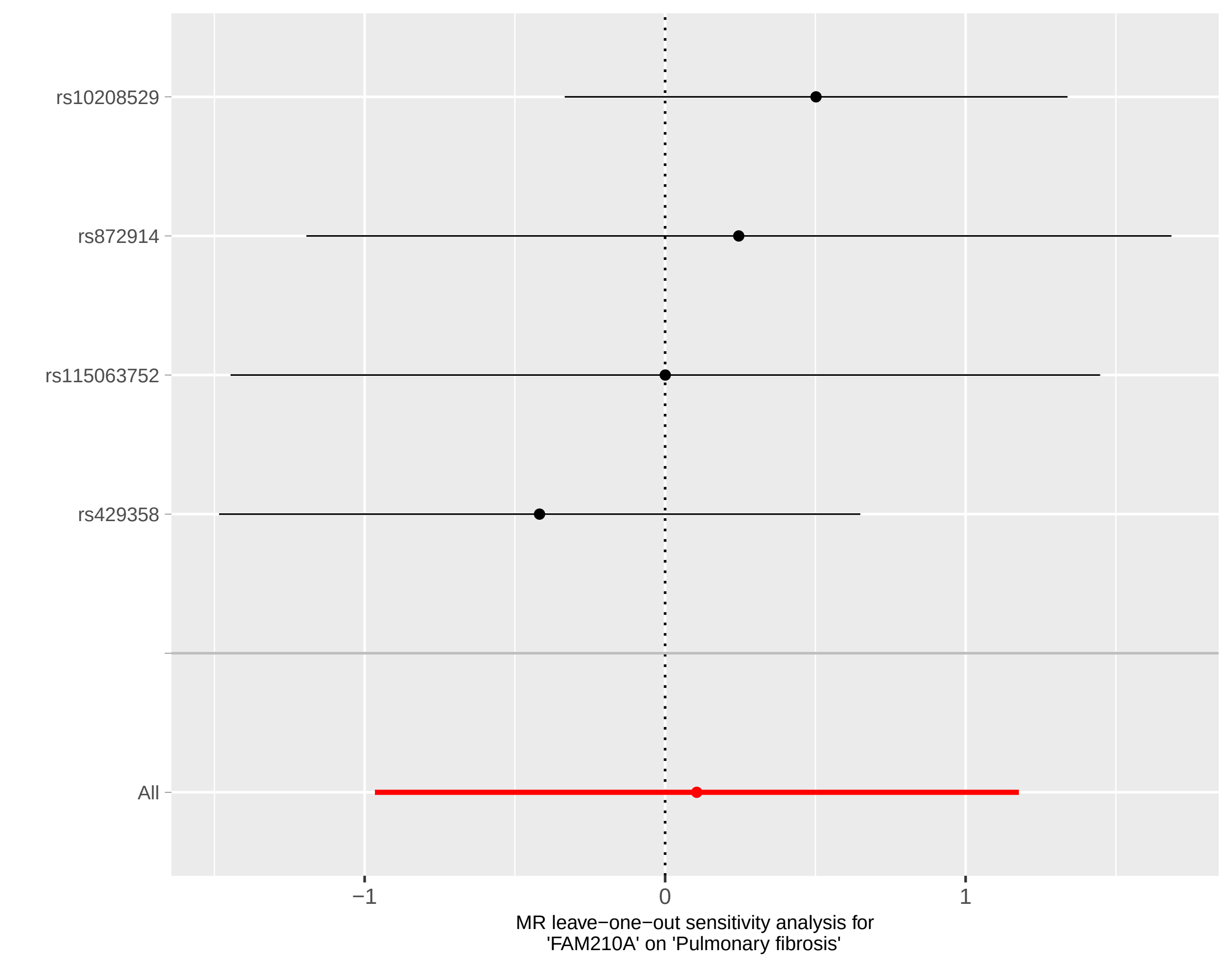
rs10208529
rs872914
rs115063752
rs429358
All
−1
0
1
MR leave−one−out sensitivity analysis for
'FAM210A' on 'Pulmonary fibrosis'

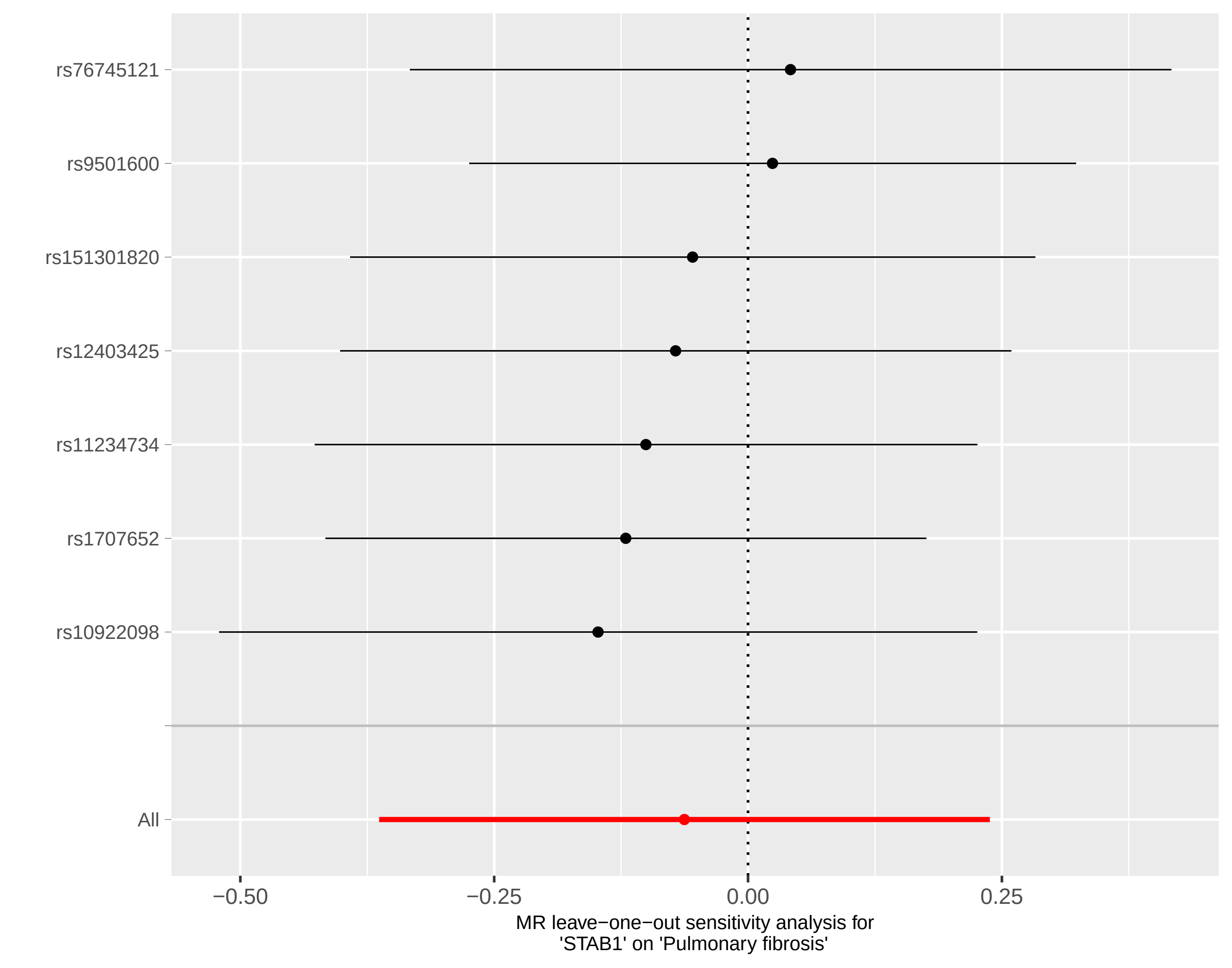
rs76745121
rs9501600
rs151301820
rs12403425
rs11234734
rs1707652
rs10922098
All
−0.50
−0.25
0.00
0.25
MR leave−one−out sensitivity analysis for
'STAB1' on 'Pulmonary fibrosis'

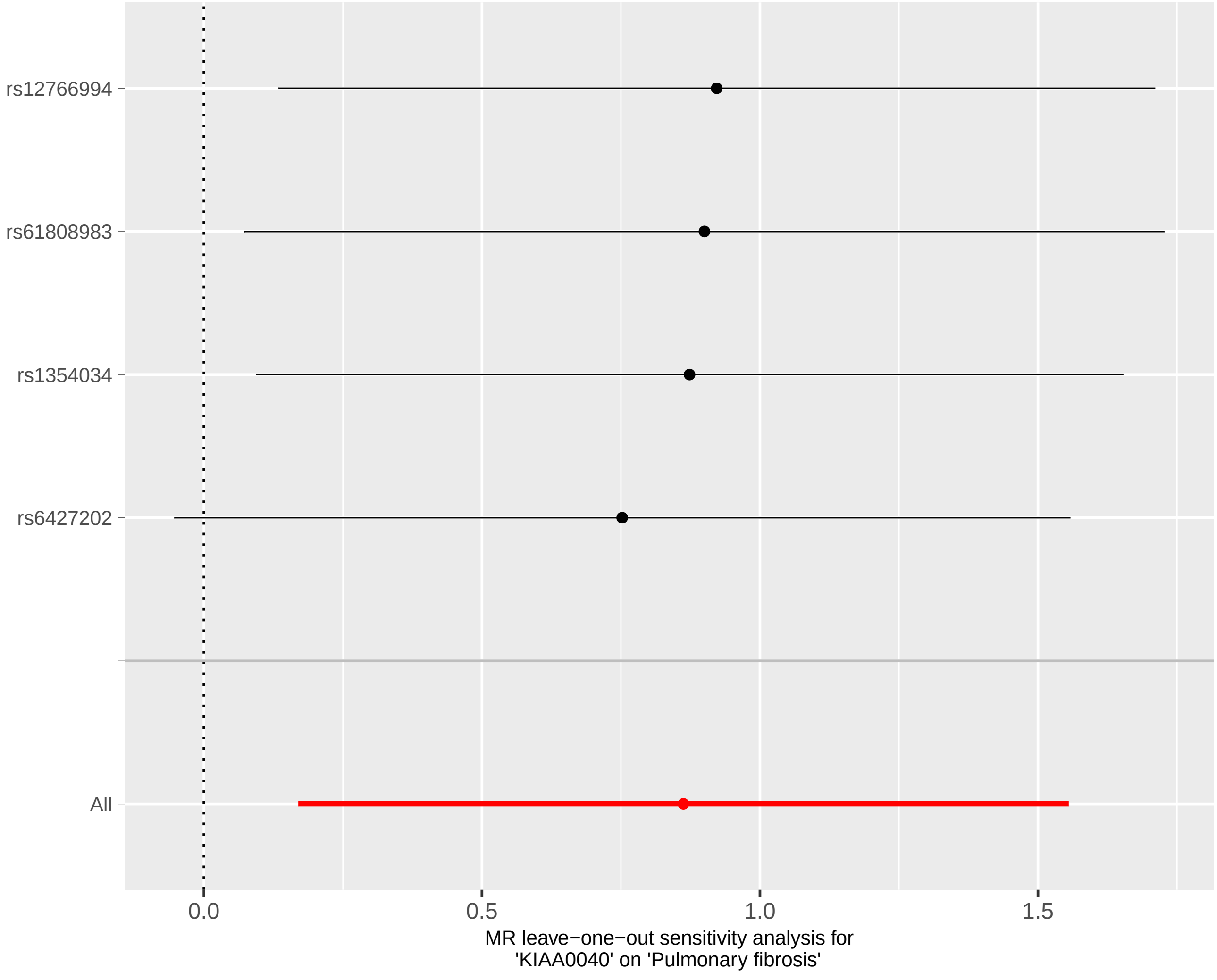

rs12766994
rs61808983
rs1354034
rs6427202
All
0.0
0.5
1.0
1.5
MR leave−one−out sensitivity analysis for
'KIAA0040' on 'Pulmonary fibrosis'

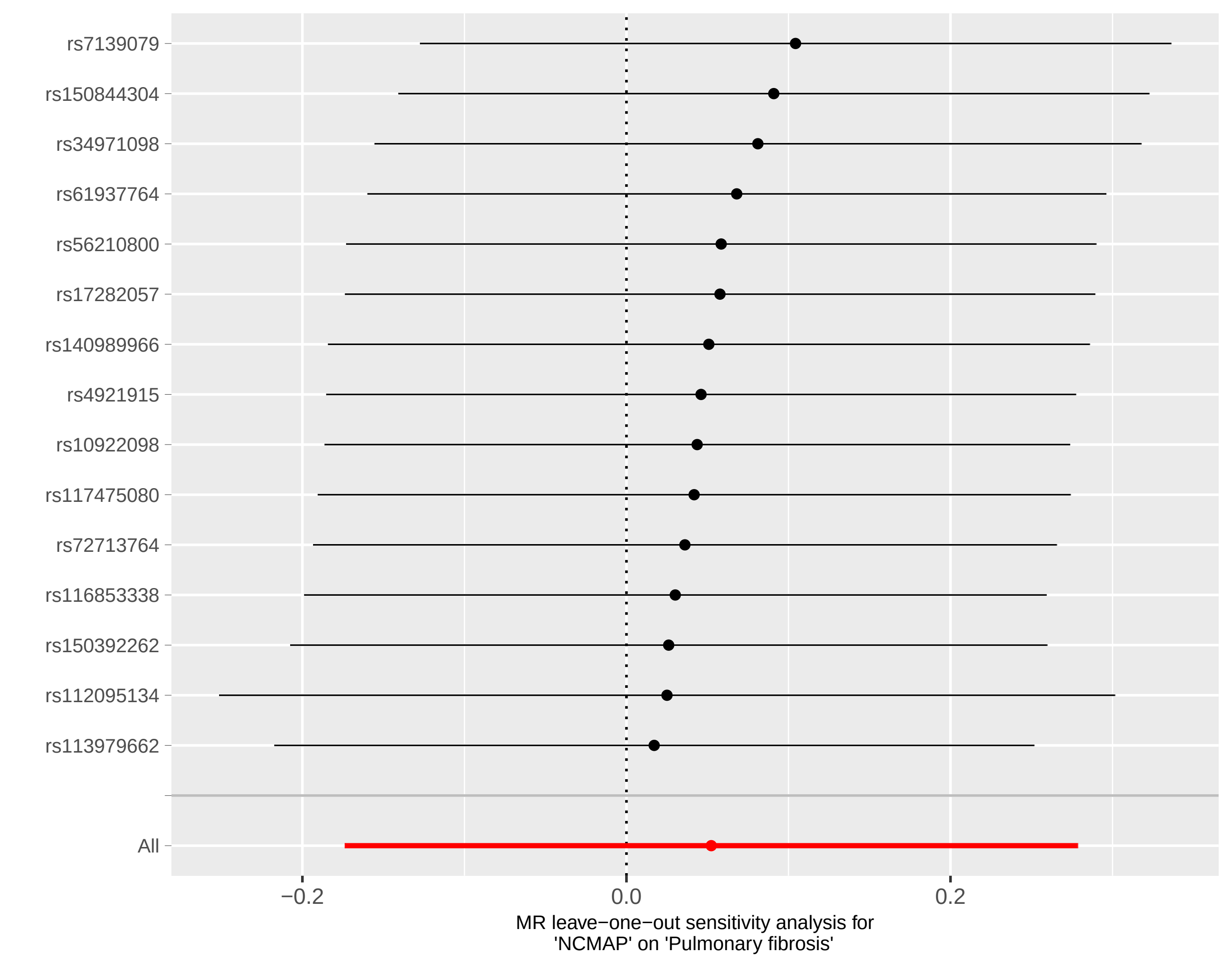
rs7139079
rs150844304
rs34971098
rs61937764
rs56210800
rs17282057
rs140989966
rs4921915
rs10922098
rs117475080
rs72713764
rs116853338
rs150392262
rs112095134
rs113979662
All
−0.2
0.0
0.2
MR leave−one−out sensitivity analysis for
'NCMAP' on 'Pulmonary fibrosis'

Insufficient number of SNPs

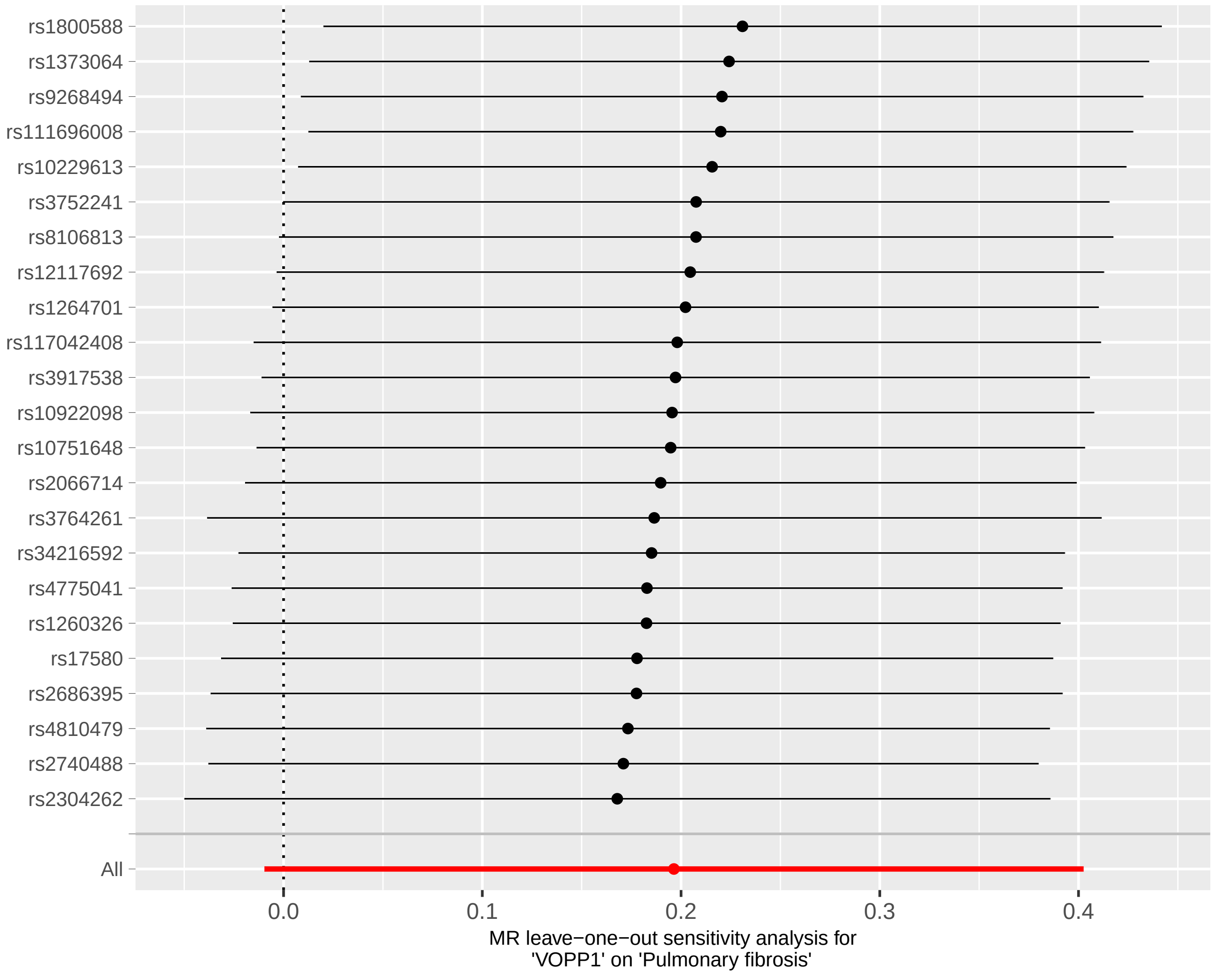
rs1800588
rs1373064
rs9268494
rs111696008
rs10229613
rs3752241
rs8106813
rs12117692
rs1264701
rs117042408
rs3917538
rs10922098
rs10751648
rs2066714
rs3764261
rs34216592
rs4775041
rs1260326
rs17580
rs2686395
rs4810479
rs2740488
rs2304262
All
0.0
0.1
0.2
0.3
0.4
MR leave−one−out sensitivity analysis for
'VOPP1' on 'Pulmonary fibrosis'

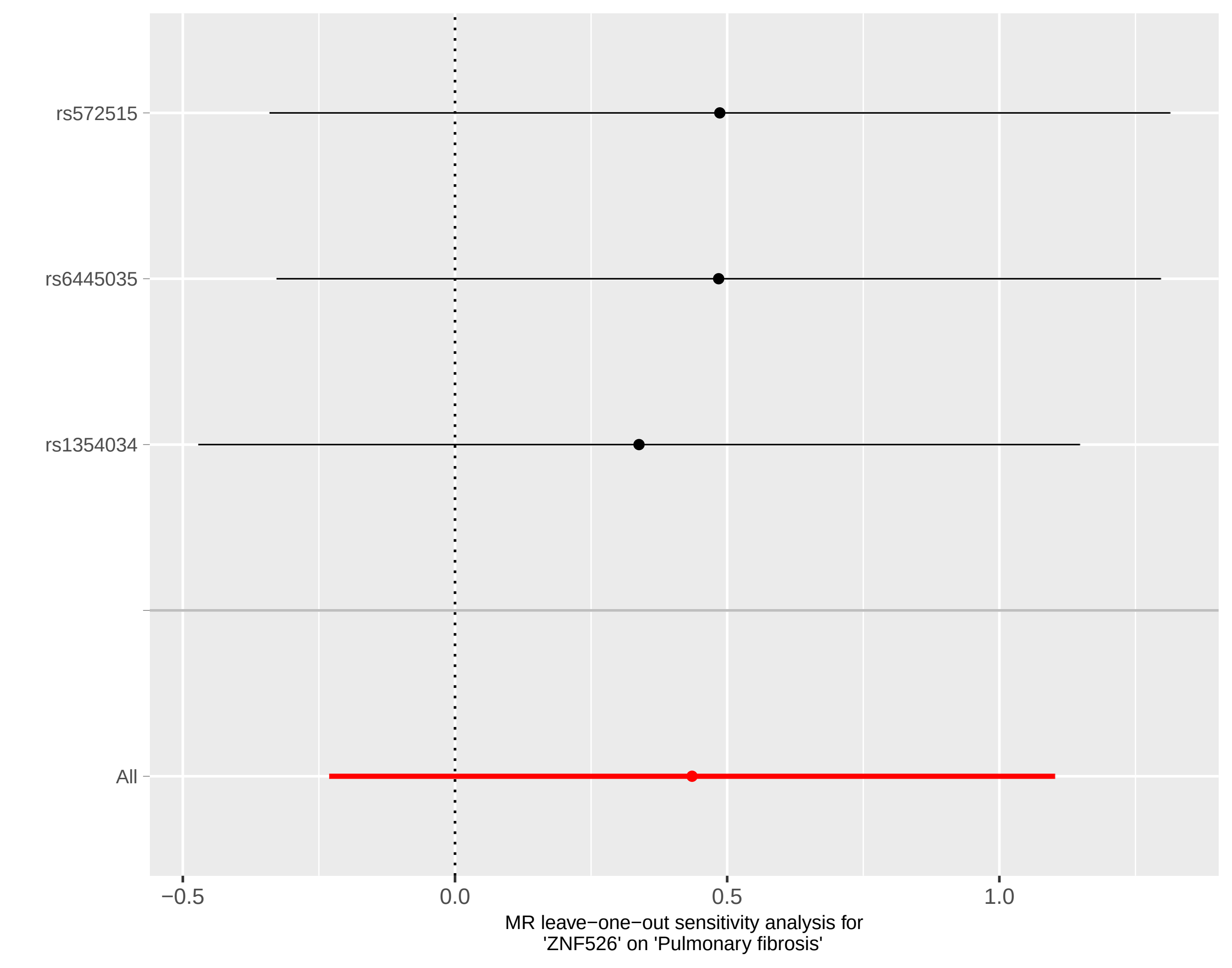
rs572515
rs6445035
rs1354034
All
−0.5
0.0
0.5
1.0
MR leave−one−out sensitivity analysis for
'ZNF526' on 'Pulmonary fibrosis'

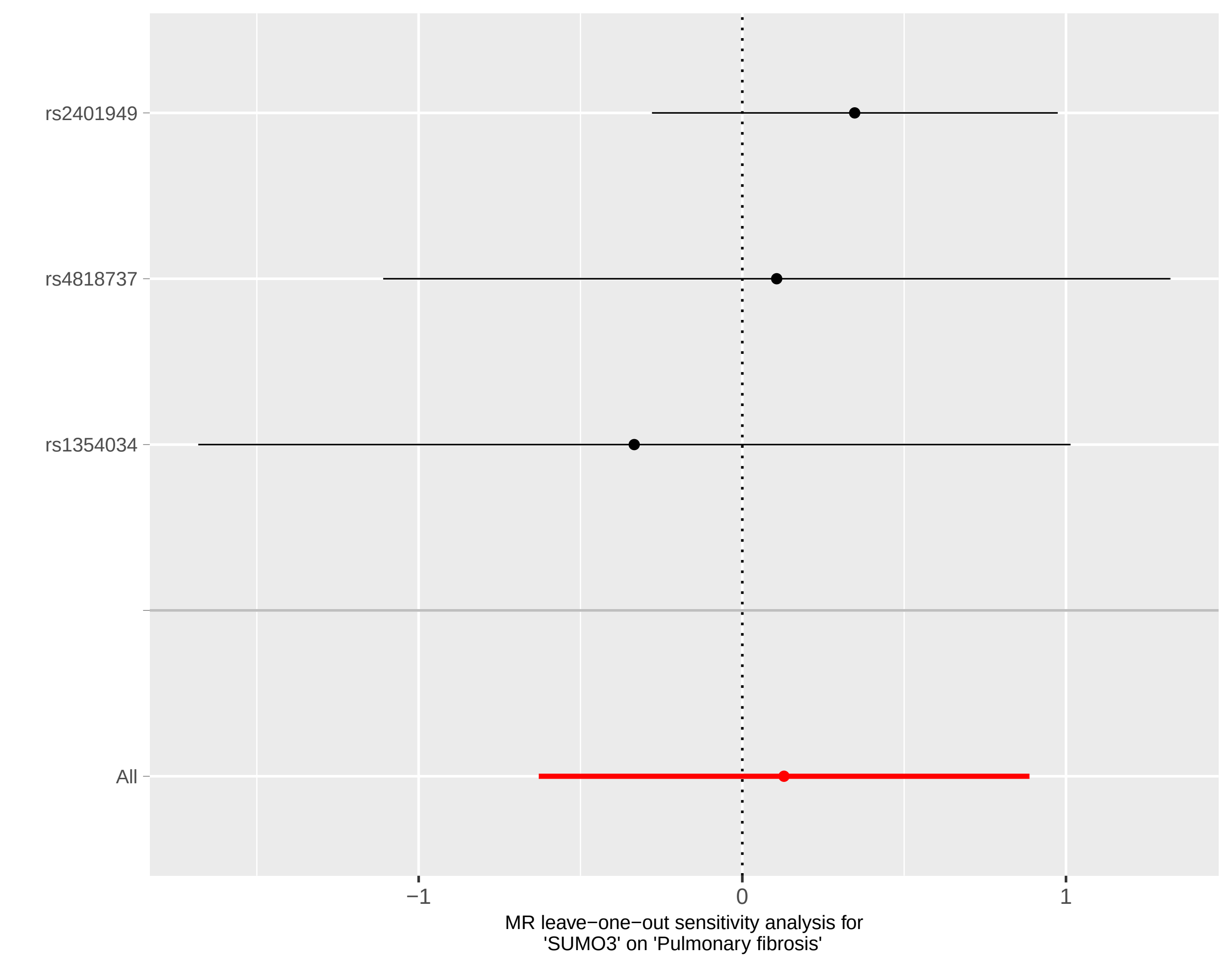
rs2401949
rs4818737
rs1354034
All
−1
0
1
MR leave−one−out sensitivity analysis for
'SUMO3' on 'Pulmonary fibrosis'

Insufficient number of SNPs

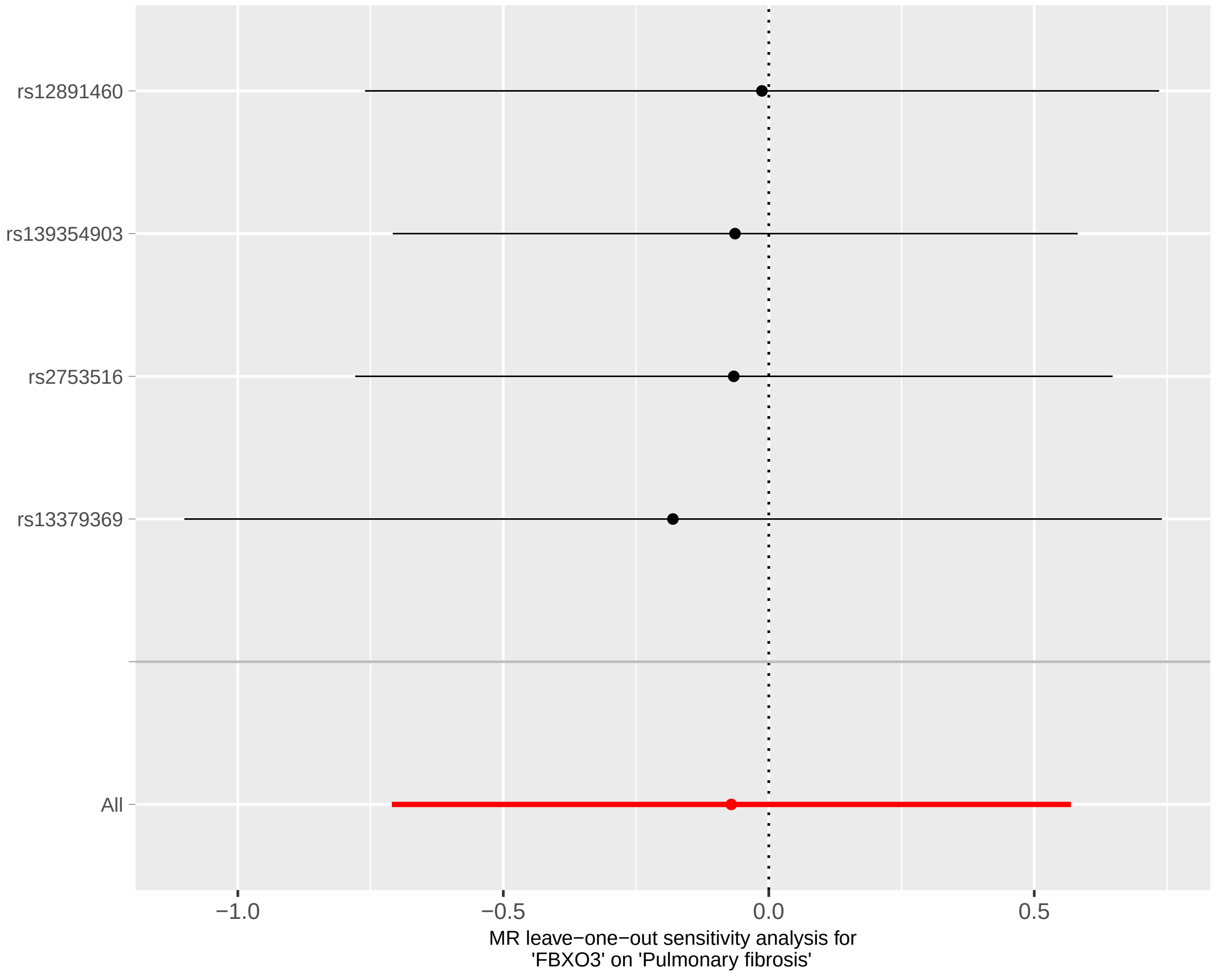
rs12891460
rs139354903
rs2753516
rs13379369
All
−1.0
−0.5
0.0
0.5
MR leave−one−out sensitivity analysis for
'FBXO3' on 'Pulmonary fibrosis'

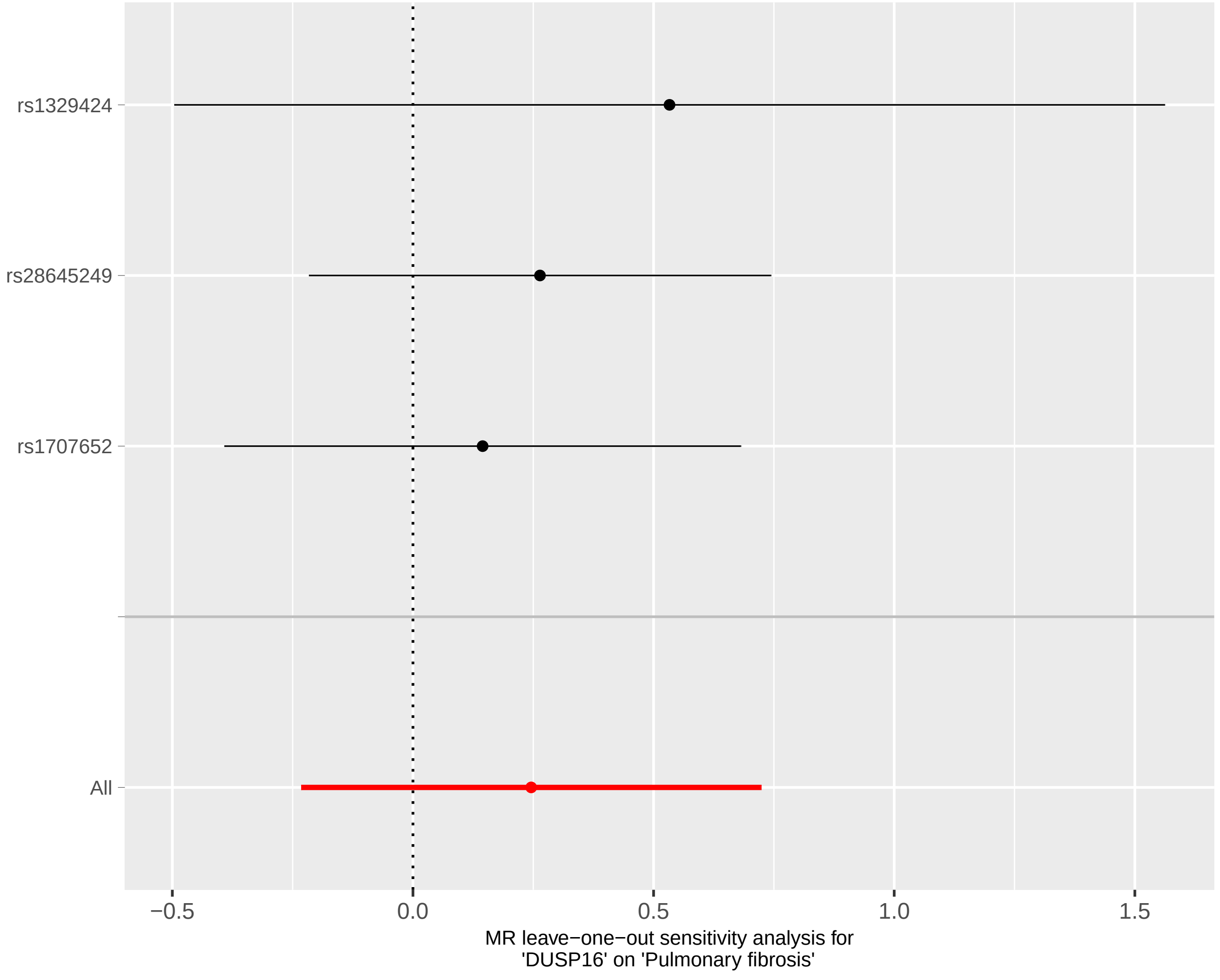
rs1329424
rs28645249
rs1707652
All
−0.5
0.0
0.5
1.0
1.5
MR leave−one−out sensitivity analysis for
'DUSP16' on 'Pulmonary fibrosis'

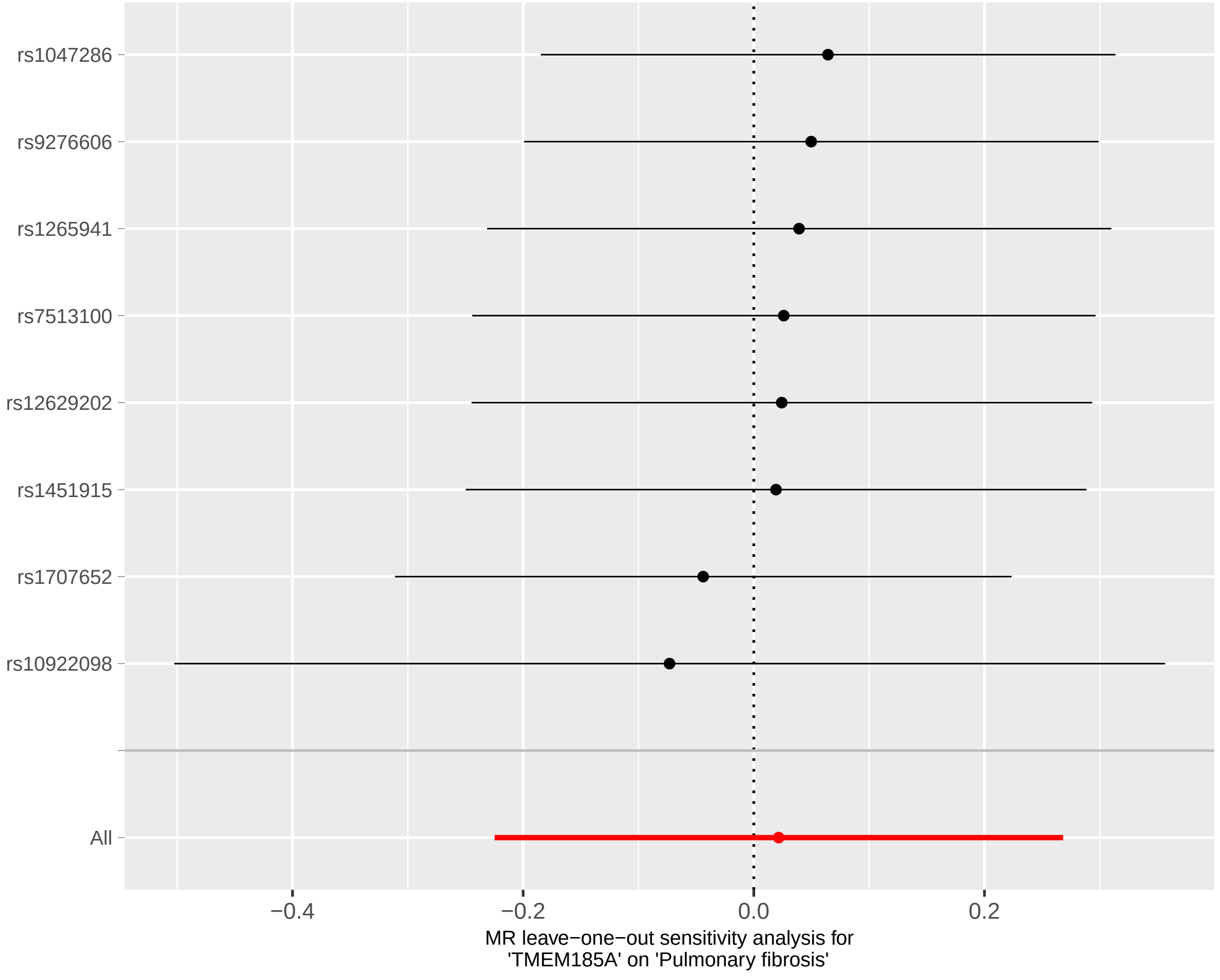

rs1047286
rs9276606
rs1265941
rs7513100
rs12629202
rs1451915
rs1707652
rs10922098
All
−0.4
−0.2
0.0
0.2
MR leave−one−out sensitivity analysis for
'TMEM185A' on 'Pulmonary fibrosis'

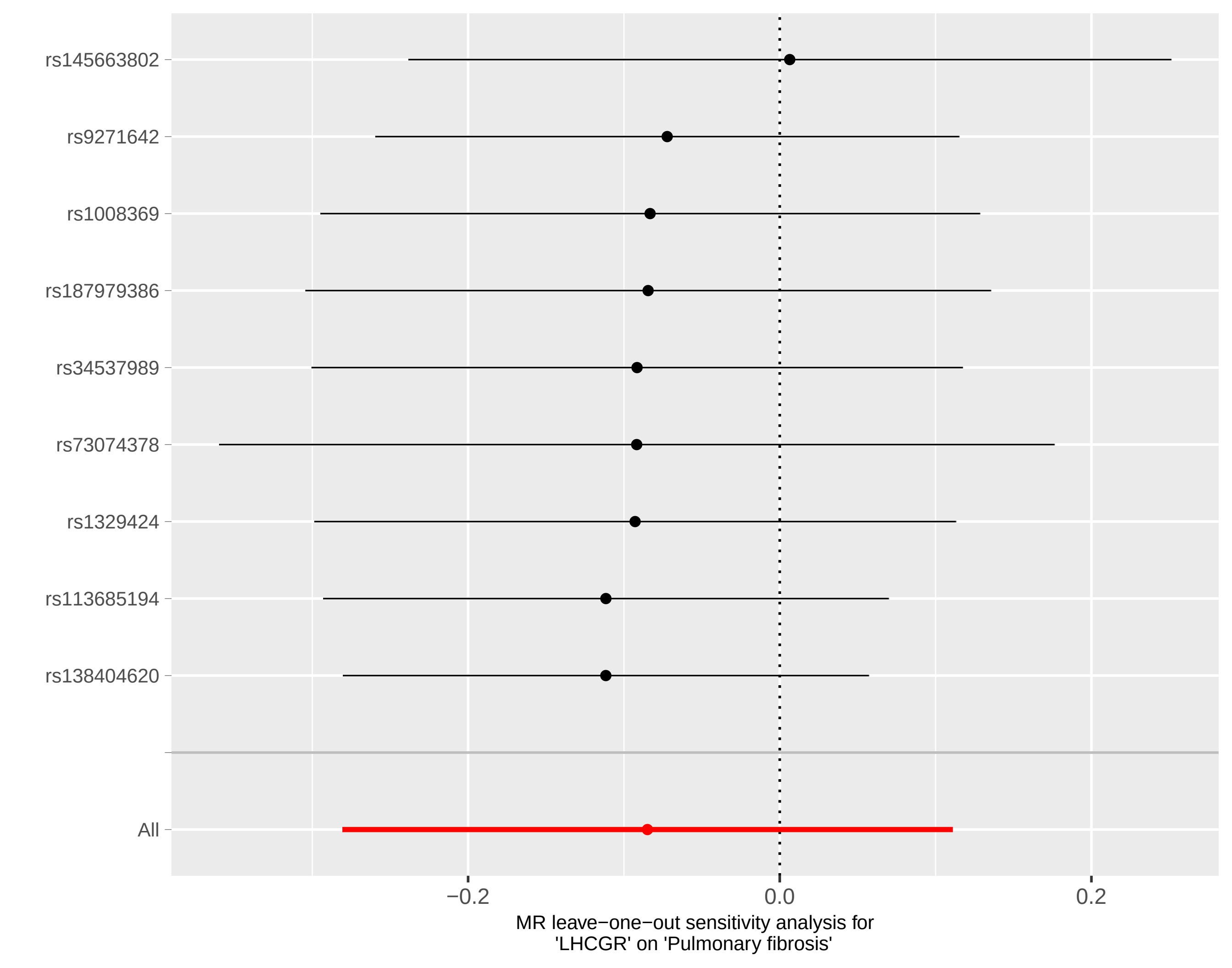
rs145663802
rs9271642
rs1008369
rs187979386
rs34537989
rs73074378
rs1329424
rs113685194
rs138404620
All
−0.2
0.0
0.2
MR leave−one−out sensitivity analysis for
'LHCGR' on 'Pulmonary fibrosis'

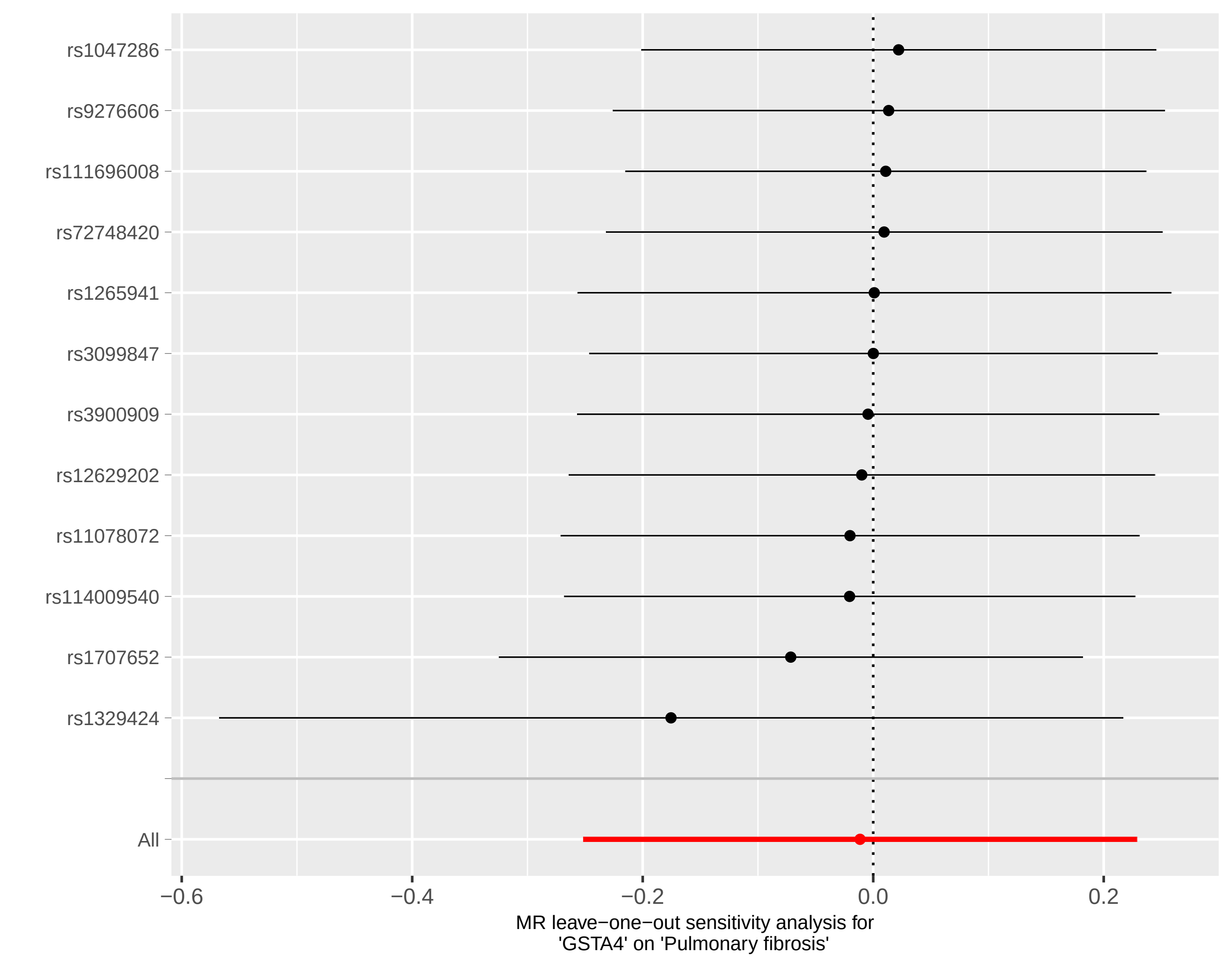
rs1047286
rs9276606
rs111696008
rs72748420
rs1265941
rs3099847
rs3900909
rs12629202
rs11078072
rs114009540
rs1707652
rs1329424
All
−0.6
−0.4
−0.2
0.0
0.2
MR leave−one−out sensitivity analysis for
'GSTA4' on 'Pulmonary fibrosis'

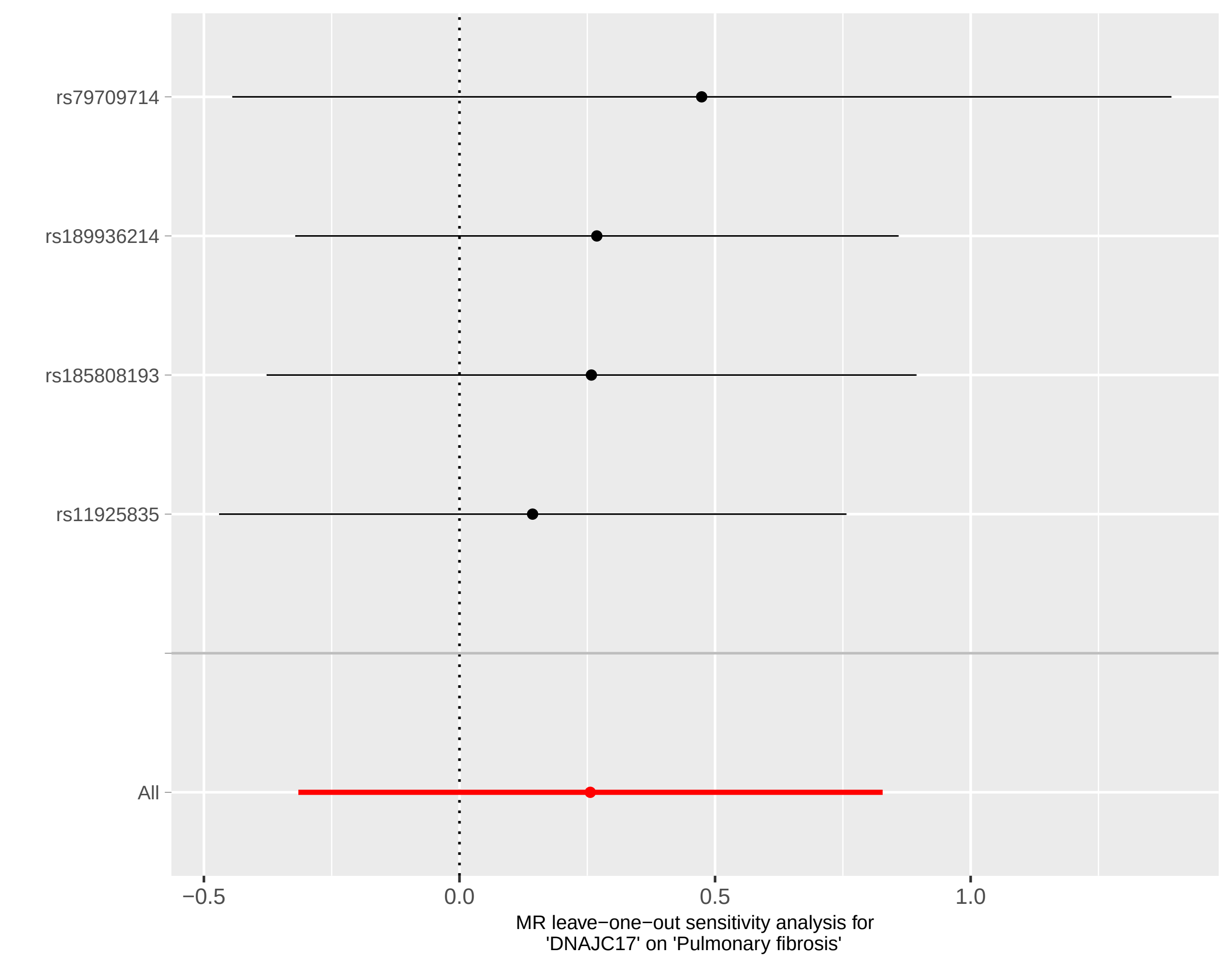
rs79709714
rs189936214
rs185808193
rs11925835
All
−0.5
0.0
0.5
1.0
MR leave−one−out sensitivity analysis for
'DNAJC17' on 'Pulmonary fibrosis'

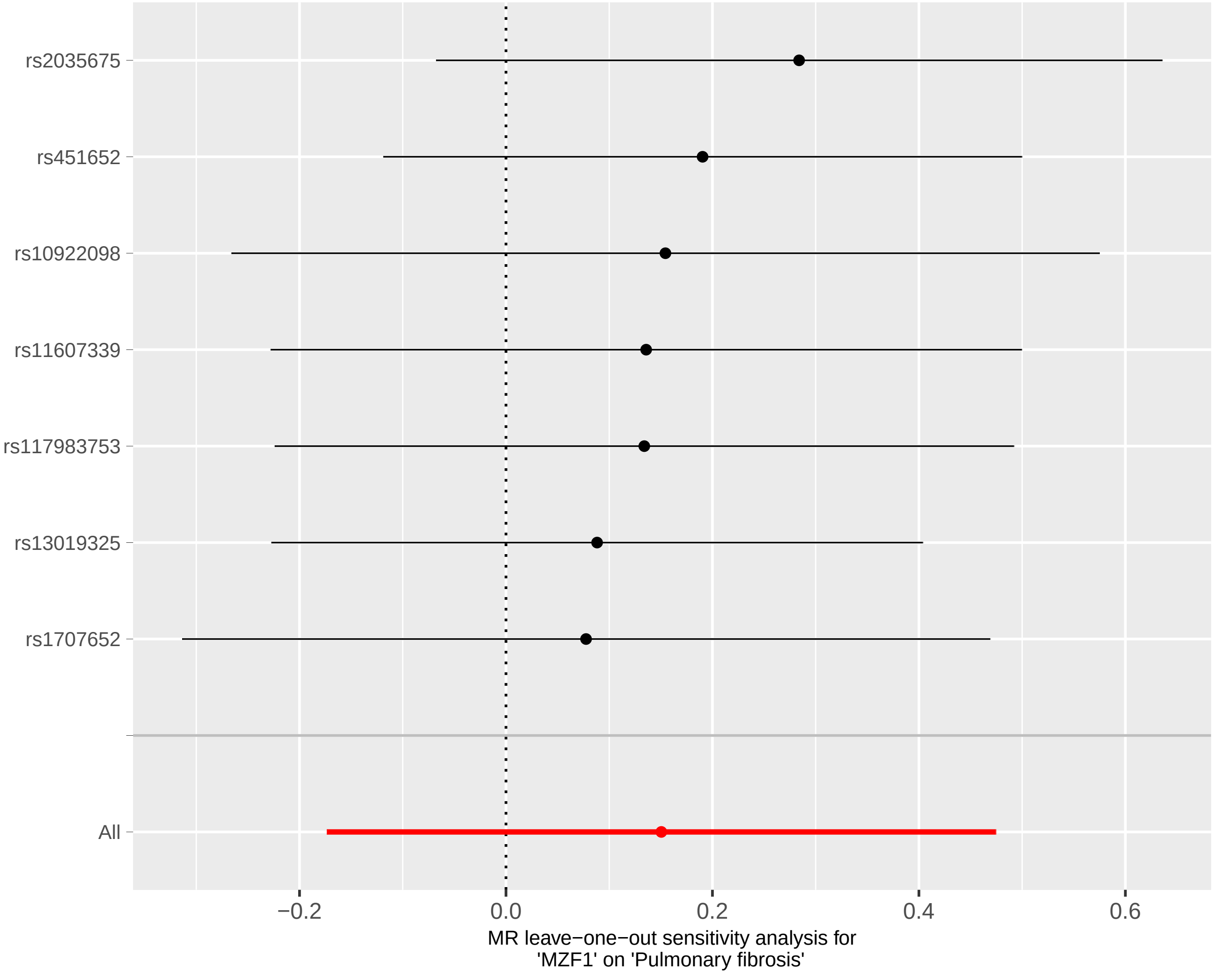
rs2035675
rs451652
rs10922098
rs11607339
rs117983753
rs13019325
rs1707652
All
−0.2
0.0
0.2
0.4
0.6
MR leave−one−out sensitivity analysis for
'MZF1' on 'Pulmonary fibrosis'

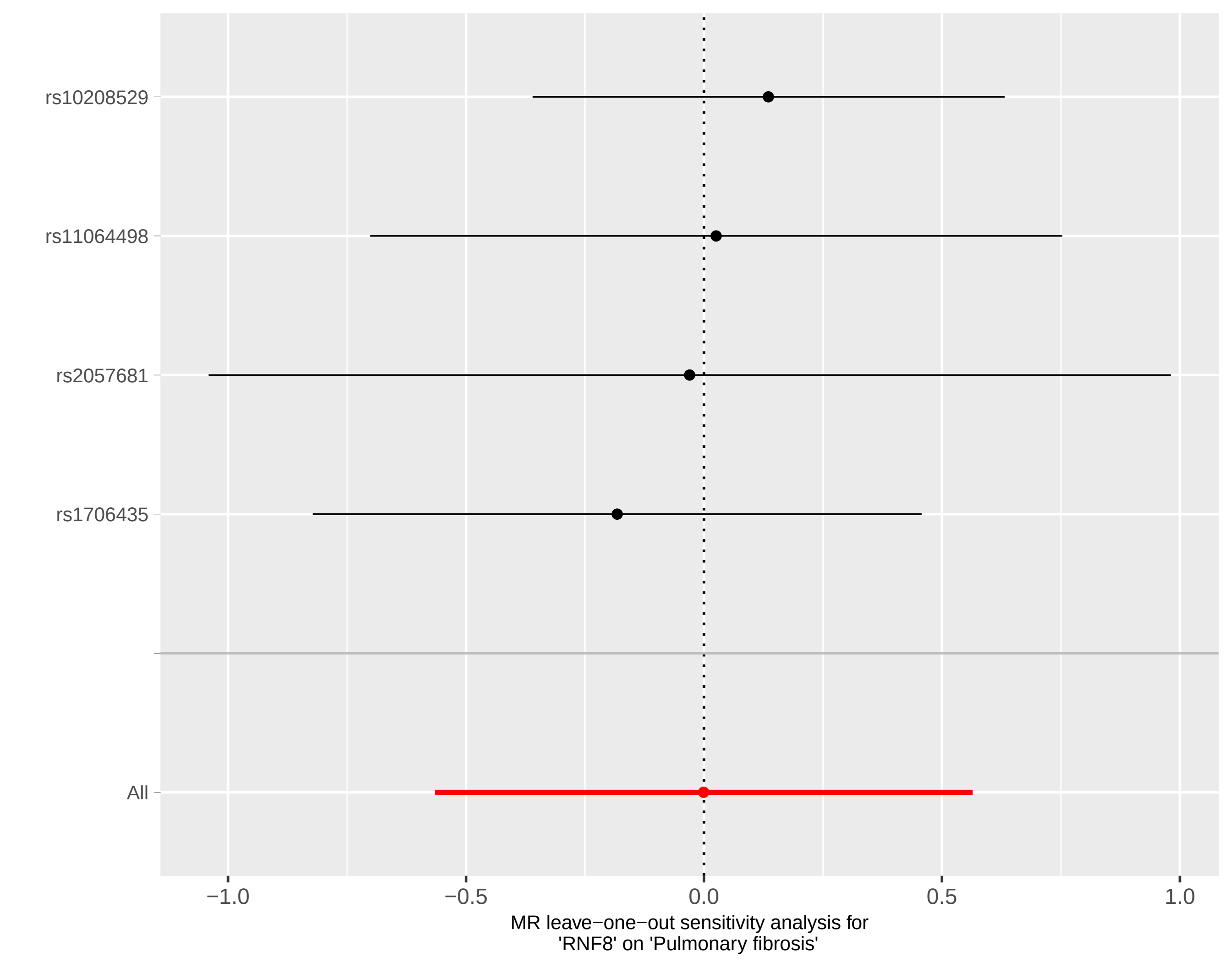

rs10208529
rs11064498
rs2057681
rs1706435
All
−1.0
−0.5
0.0
0.5
1.0
MR leave−one−out sensitivity analysis for
'RNF8' on 'Pulmonary fibrosis'

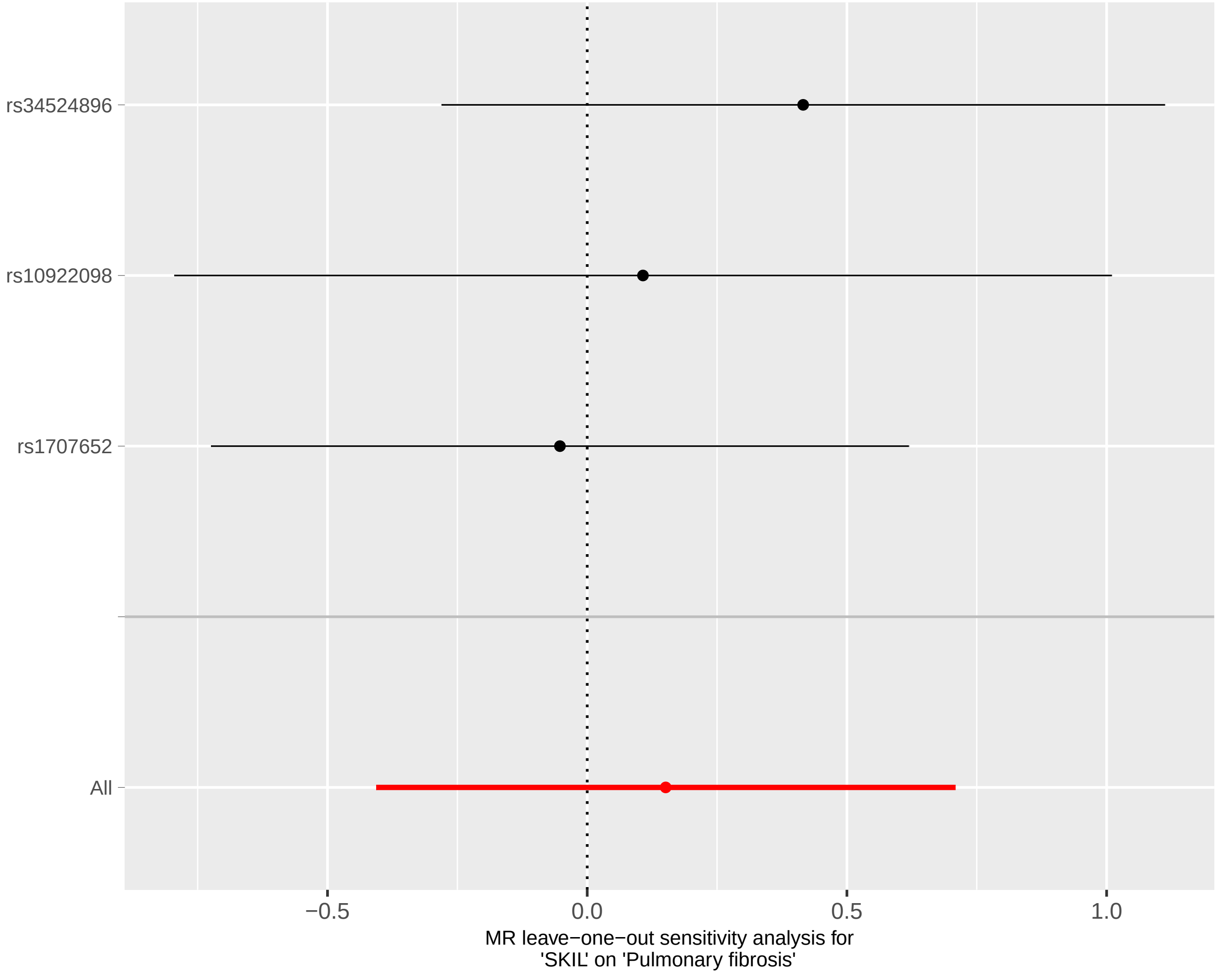

rs34524896
rs10922098
rs1707652
All
−0.5
0.0
0.5
1.0
MR leave−one−out sensitivity analysis for
'SKIL' on 'Pulmonary fibrosis'

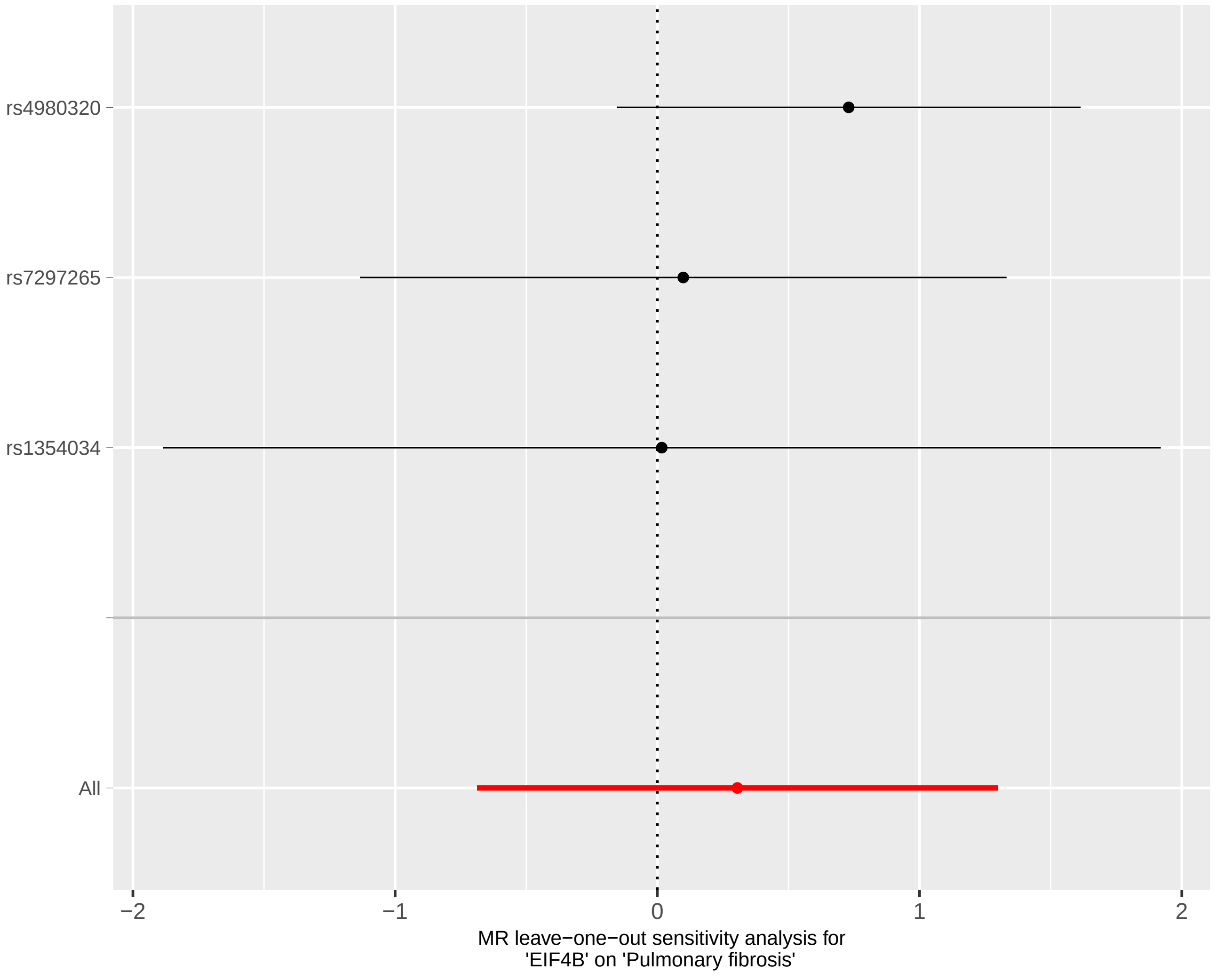
rs4980320
rs7297265
rs1354034
All
−2
−1
0
1
2
MR leave−one−out sensitivity analysis for
'EIF4B' on 'Pulmonary fibrosis'

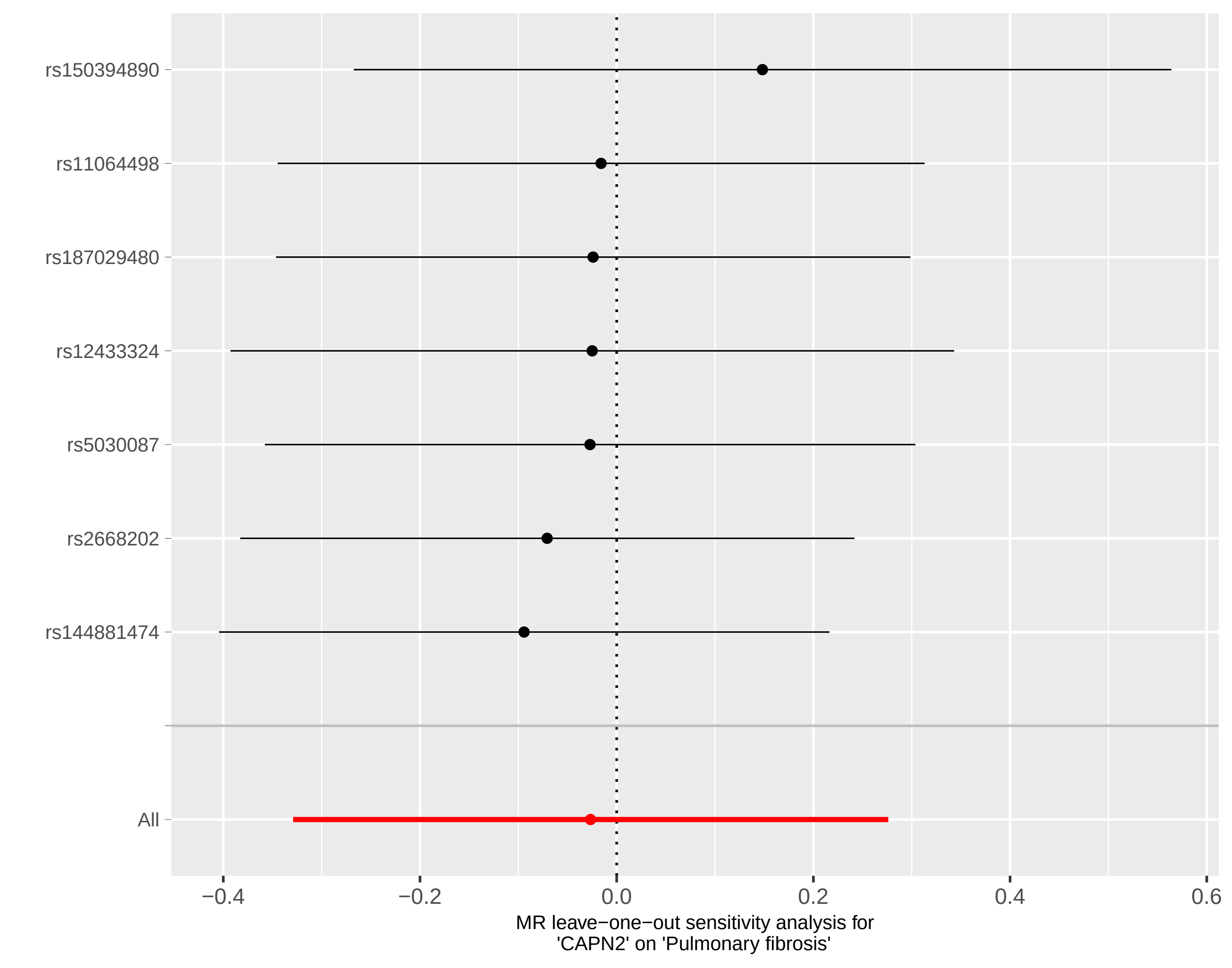

rs150394890
rs11064498
rs187029480
rs12433324
rs5030087
rs2668202
rs144881474
All
−0.4
−0.2
0.0
0.2
0.4
0.6
MR leave−one−out sensitivity analysis for
'CAPN2' on 'Pulmonary fibrosis'

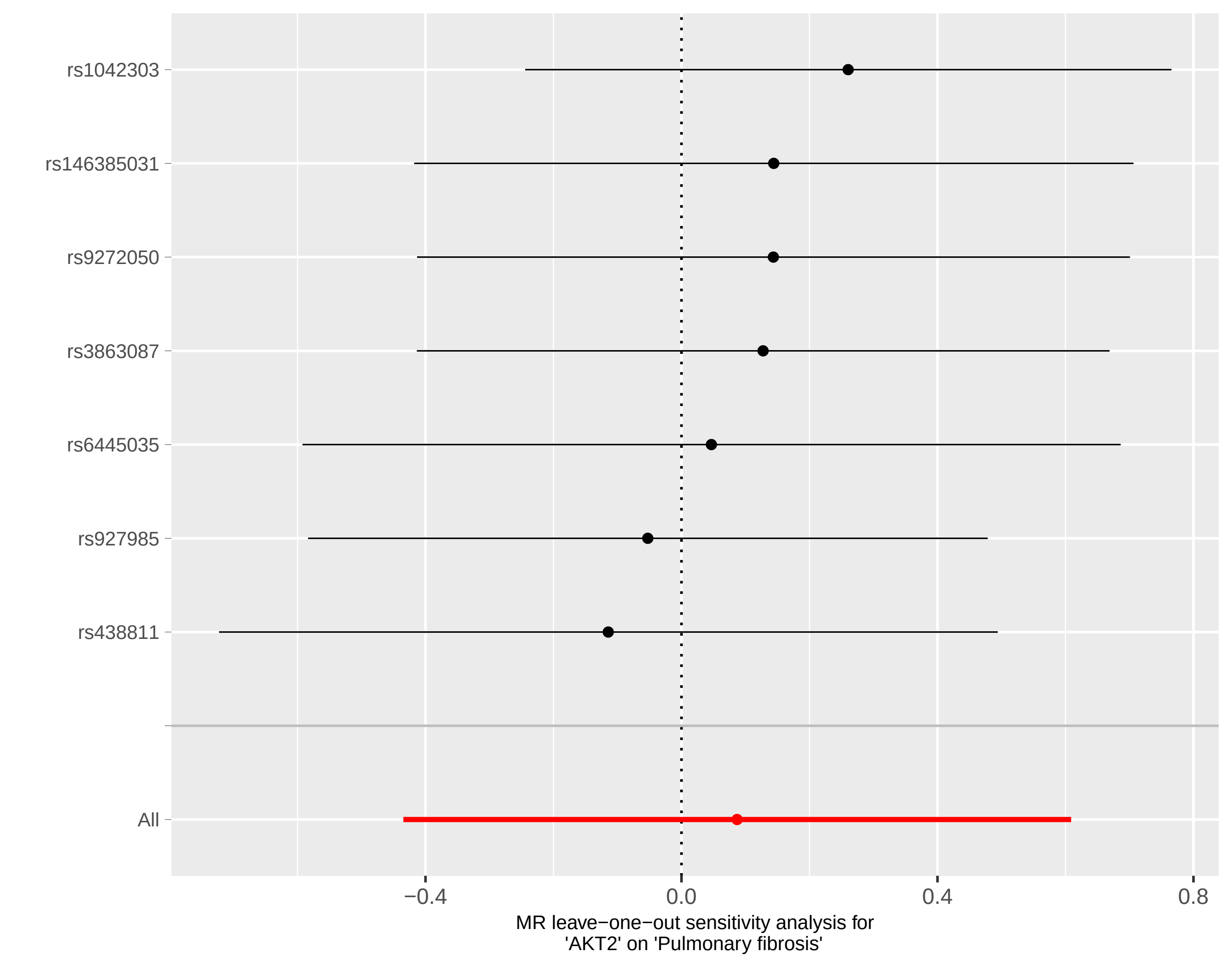
rs1042303
rs146385031
rs9272050
rs3863087
rs6445035
rs927985
rs438811
All
−0.4
0.0
0.4
0.8
MR leave−one−out sensitivity analysis for
'AKT2' on 'Pulmonary fibrosis'

Insufficient number of SNPs

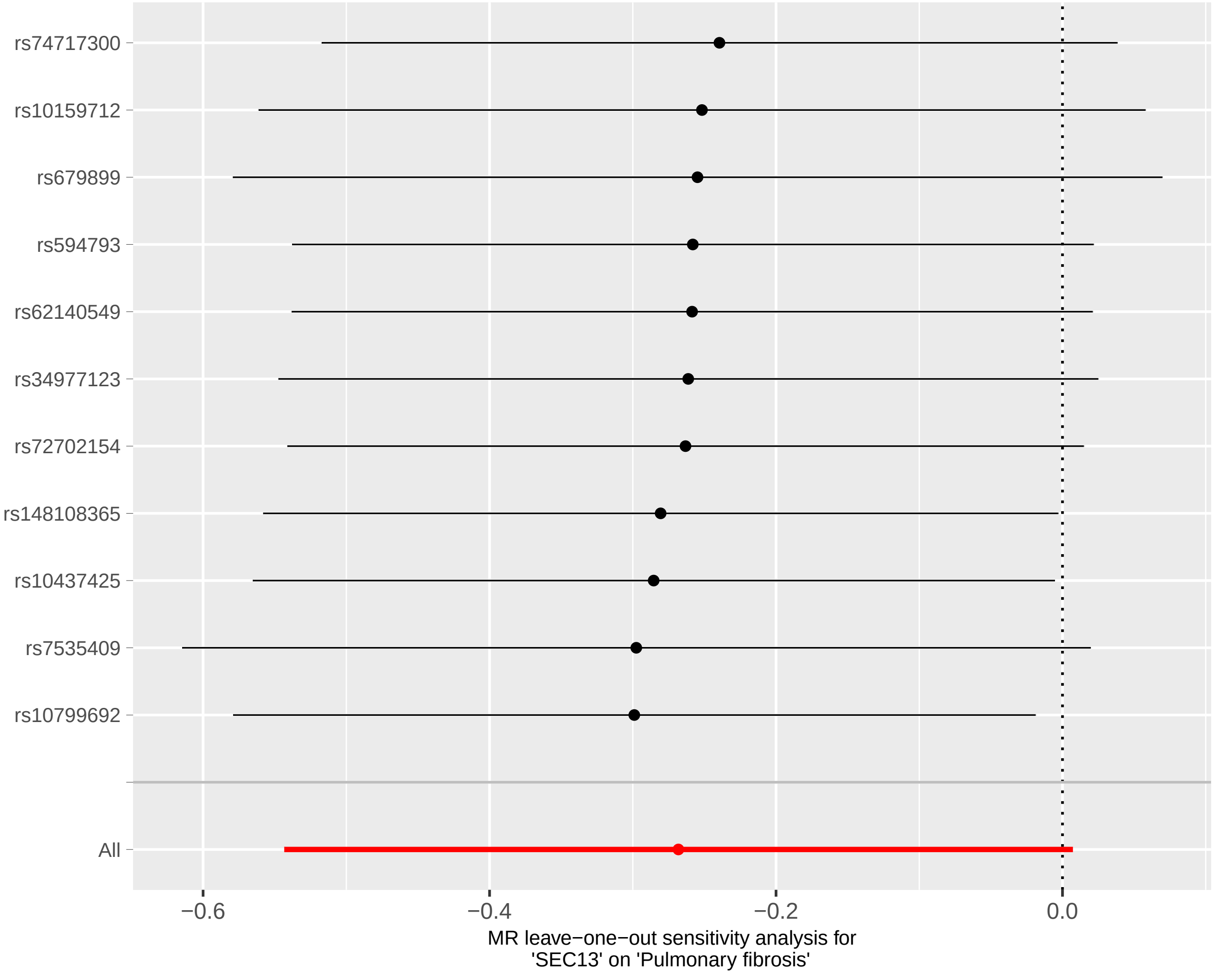

rs74717300
rs10159712
rs679899
rs594793
rs62140549
rs34977123
rs72702154
rs148108365
rs10437425
rs7535409
rs10799692
All
−0.6
−0.4
−0.2
0.0
MR leave−one−out sensitivity analysis for
'SEC13' on 'Pulmonary fibrosis'

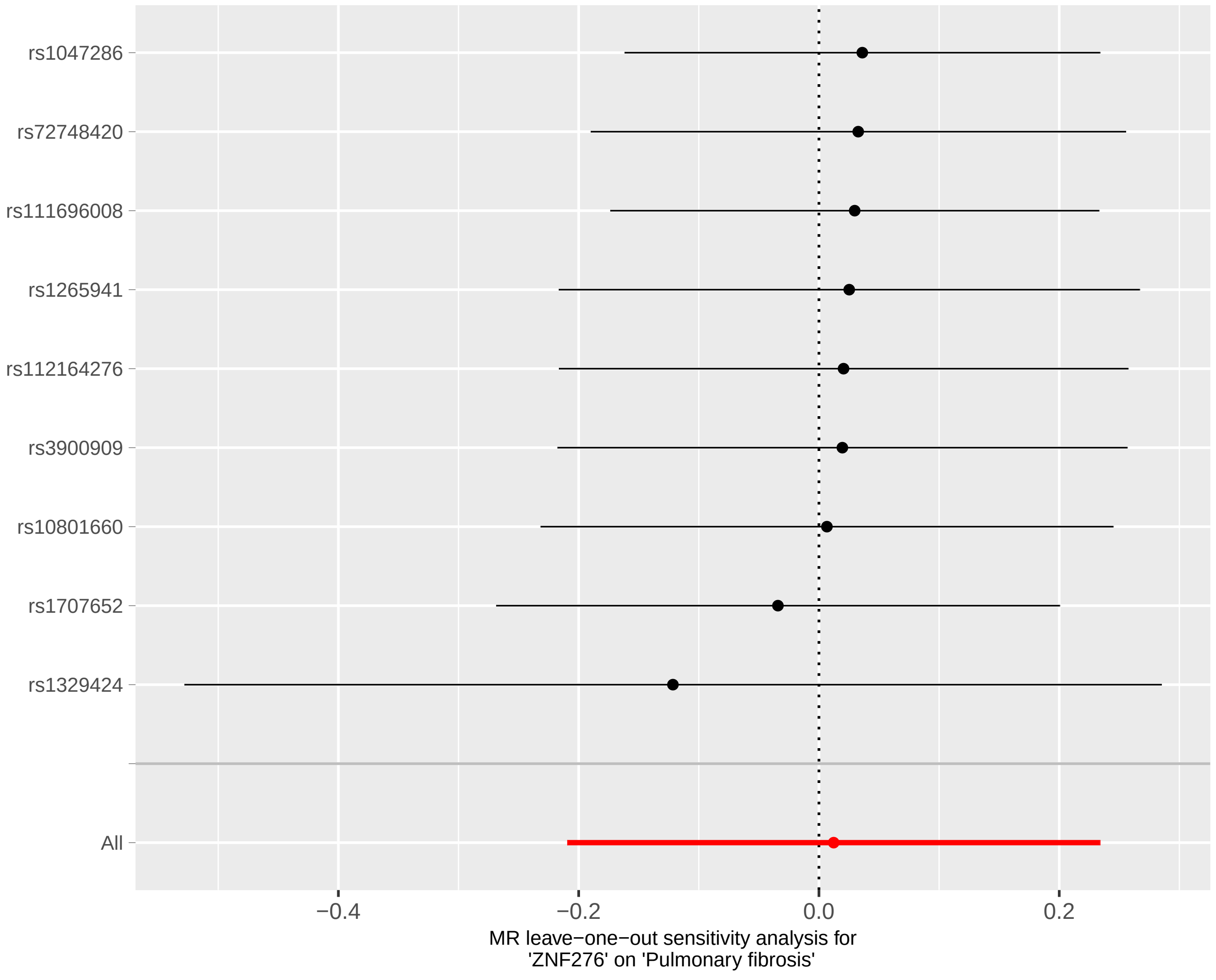
rs1047286
rs72748420
rs111696008
rs1265941
rs112164276
rs3900909
rs10801660
rs1707652
rs1329424
All
−0.4
−0.2
0.0
0.2
MR leave−one−out sensitivity analysis for
'ZNF276' on 'Pulmonary fibrosis'

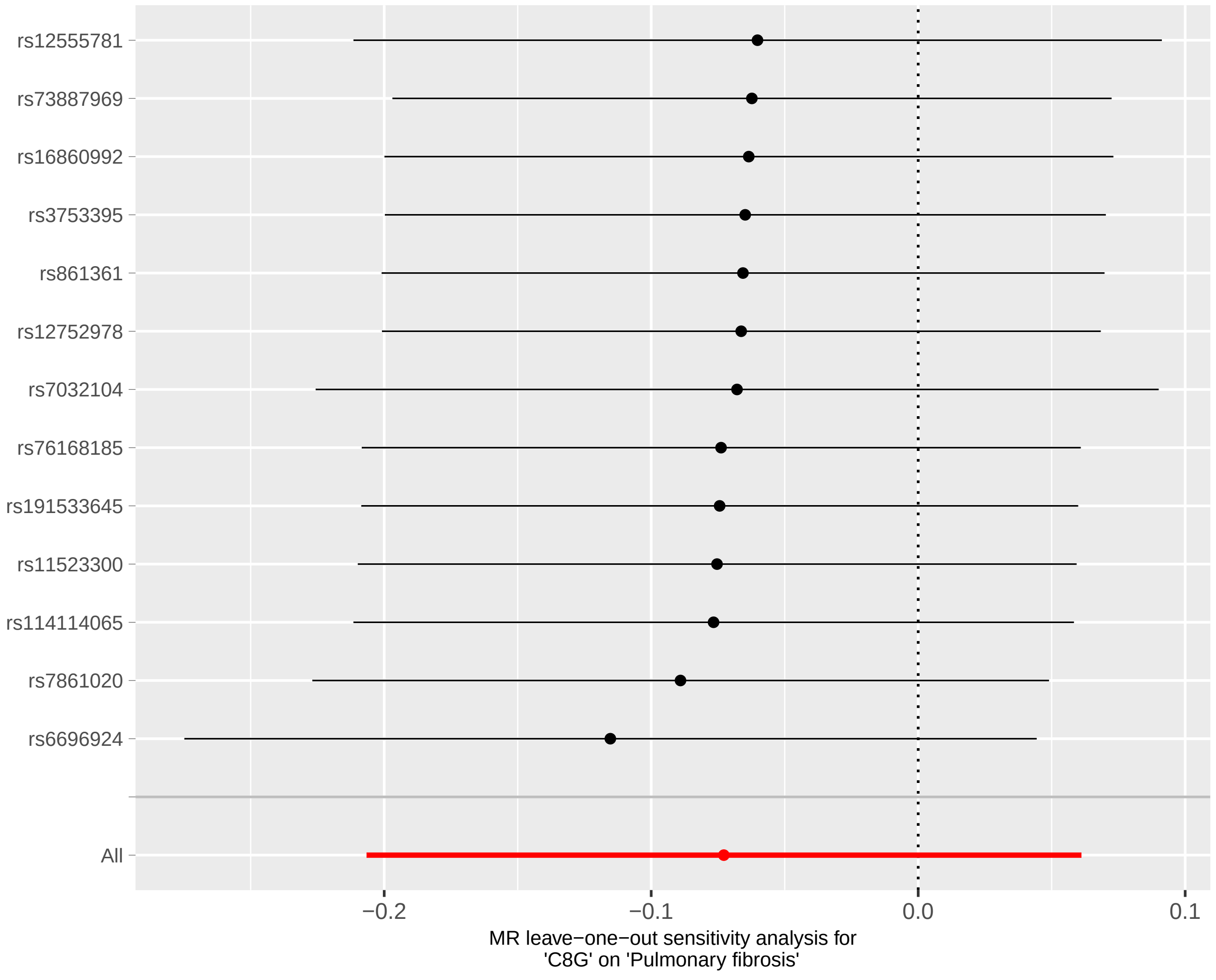
rs12555781
rs73887969
rs16860992
rs3753395
rs861361
rs12752978
rs7032104
rs76168185
rs191533645
rs11523300
rs114114065
rs7861020
rs6696924
All
−0.2
−0.1
0.0
0.1
MR leave−one−out sensitivity analysis for
'C8G' on 'Pulmonary fibrosis'

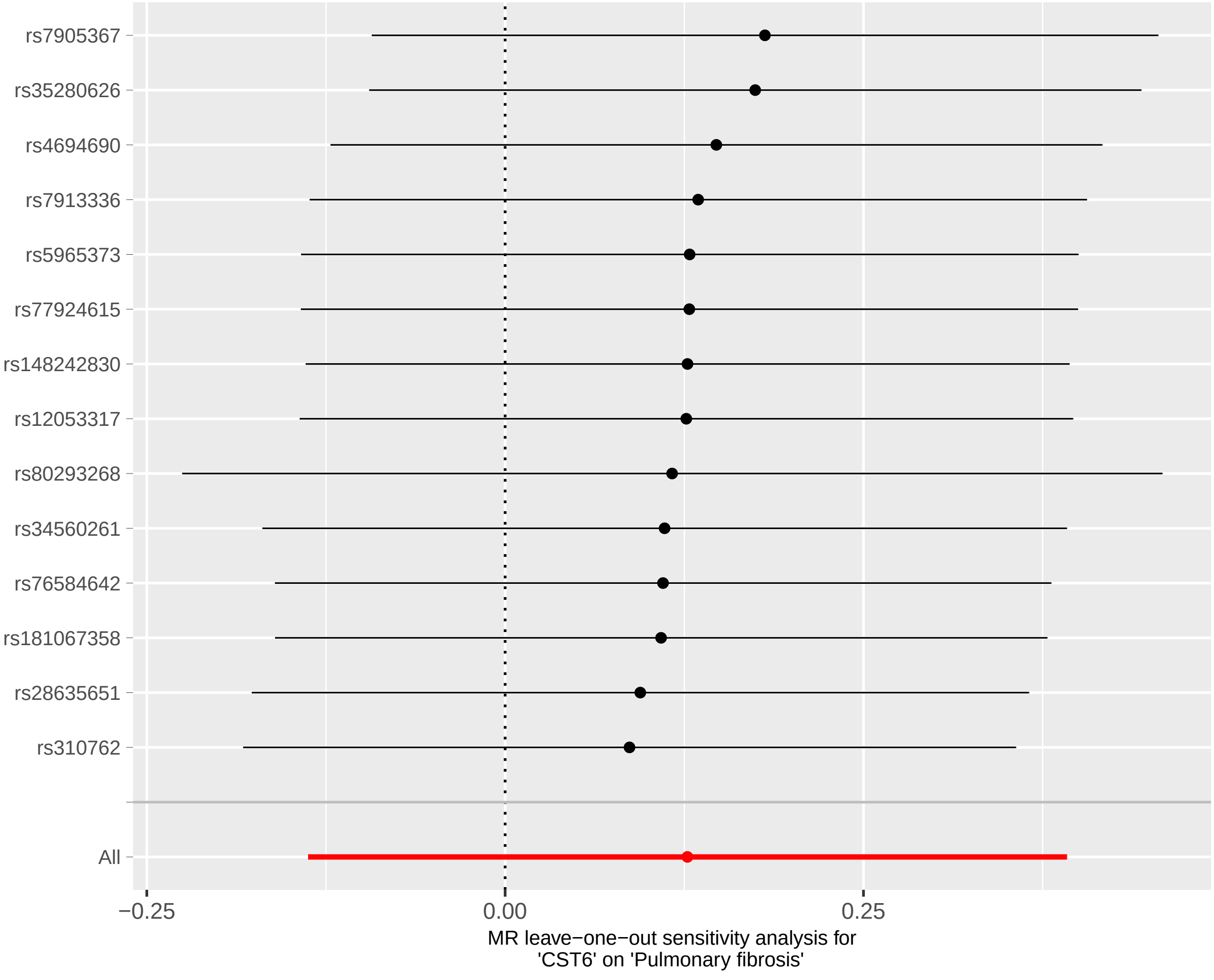
rs7905367
rs35280626
rs4694690
rs7913336
rs5965373
rs77924615
rs148242830
rs12053317
rs80293268
rs34560261
rs76584642
rs181067358
rs28635651
rs310762
All
−0.25
0.00
0.25
MR leave−one−out sensitivity analysis for
'CST6' on 'Pulmonary fibrosis'

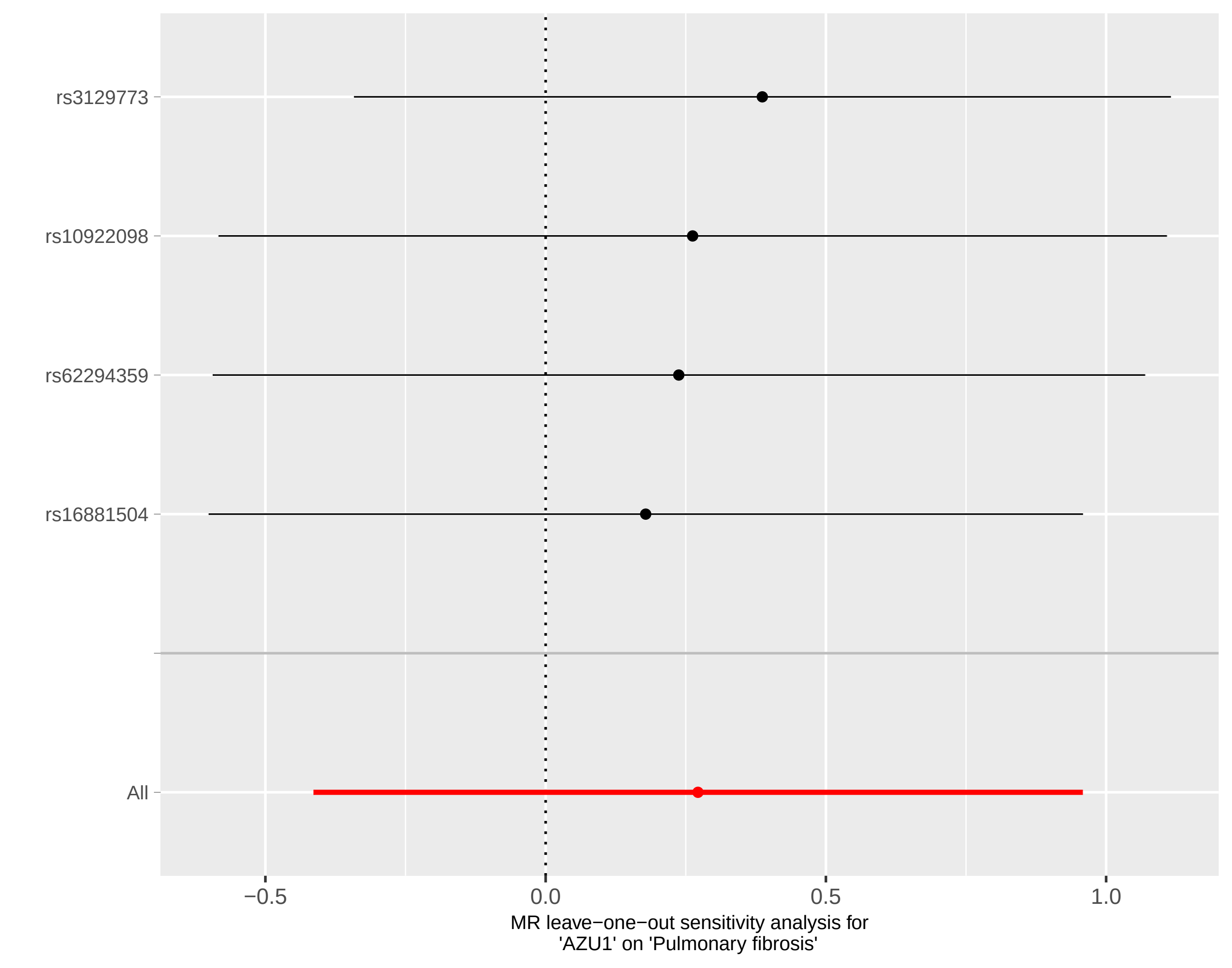
rs3129773
rs10922098
rs62294359
rs16881504
All
−0.5
0.0
0.5
1.0
MR leave−one−out sensitivity analysis for
'AZU1' on 'Pulmonary fibrosis'

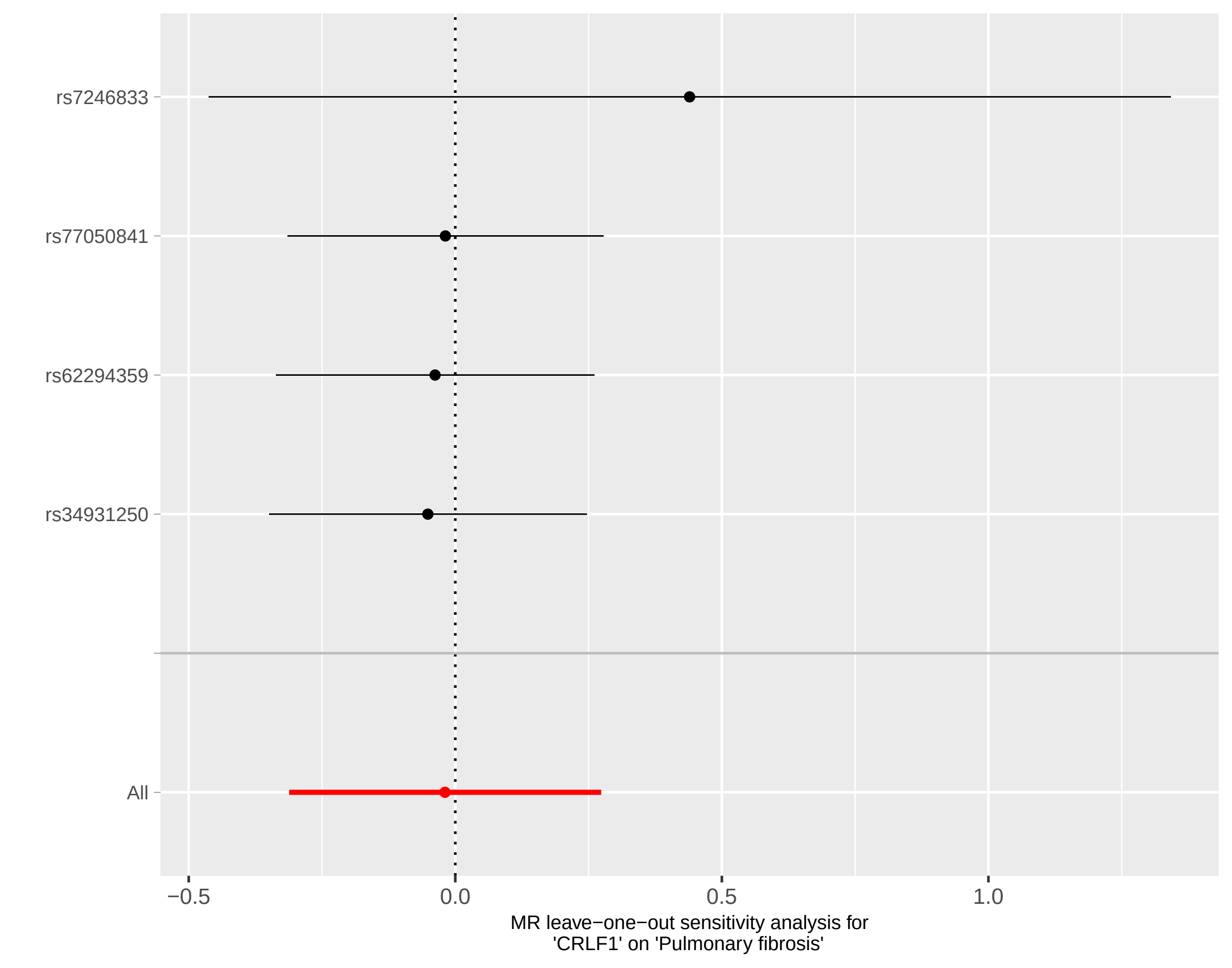
rs7246833
rs77050841
rs62294359
rs34931250
All
−0.5
0.0
0.5
1.0
MR leave−one−out sensitivity analysis for
'CRLF1' on 'Pulmonary fibrosis'

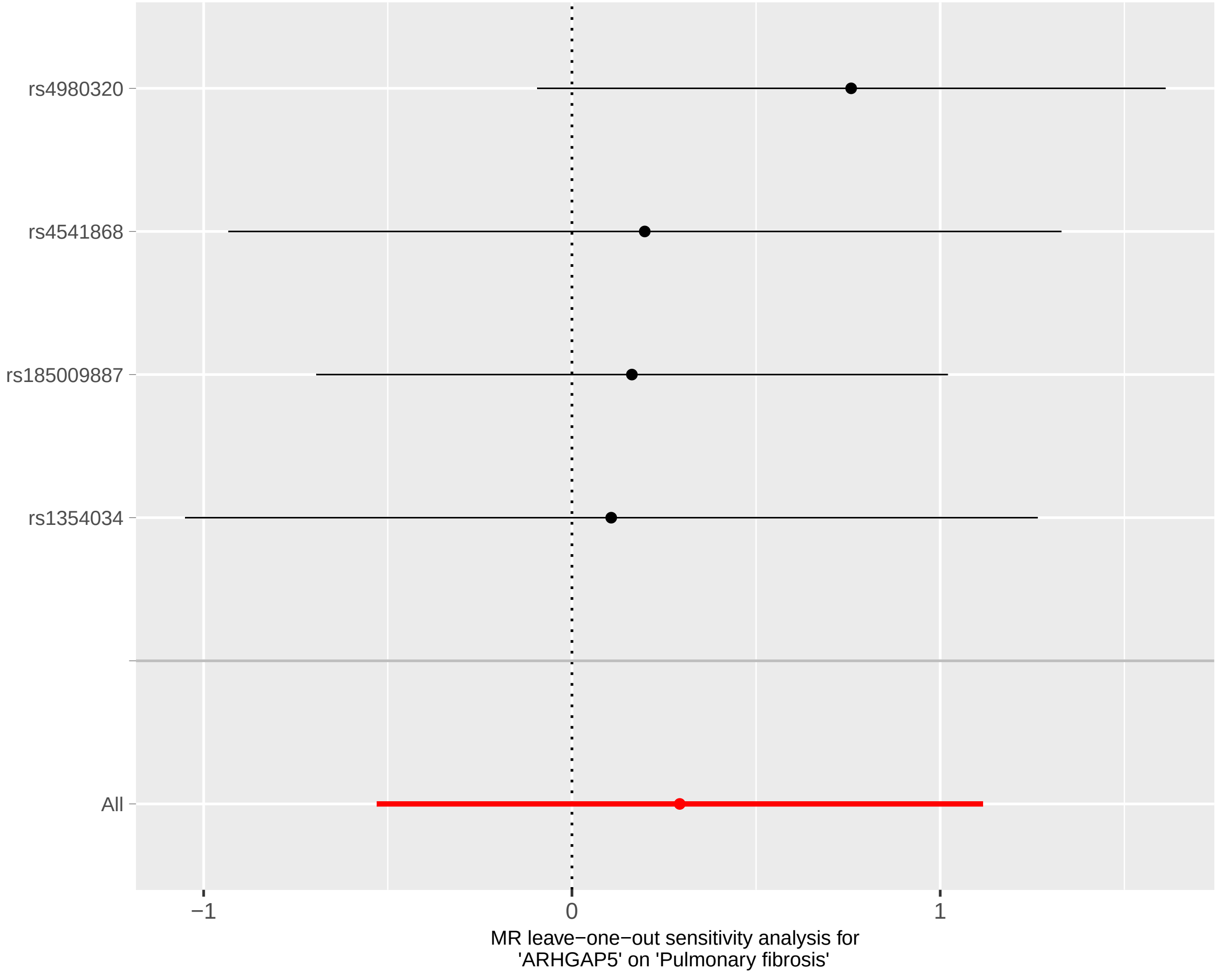

rs4980320
rs4541868
rs185009887
rs1354034
All
−1
0
1
MR leave−one−out sensitivity analysis for
'ARHGAP5' on 'Pulmonary fibrosis'

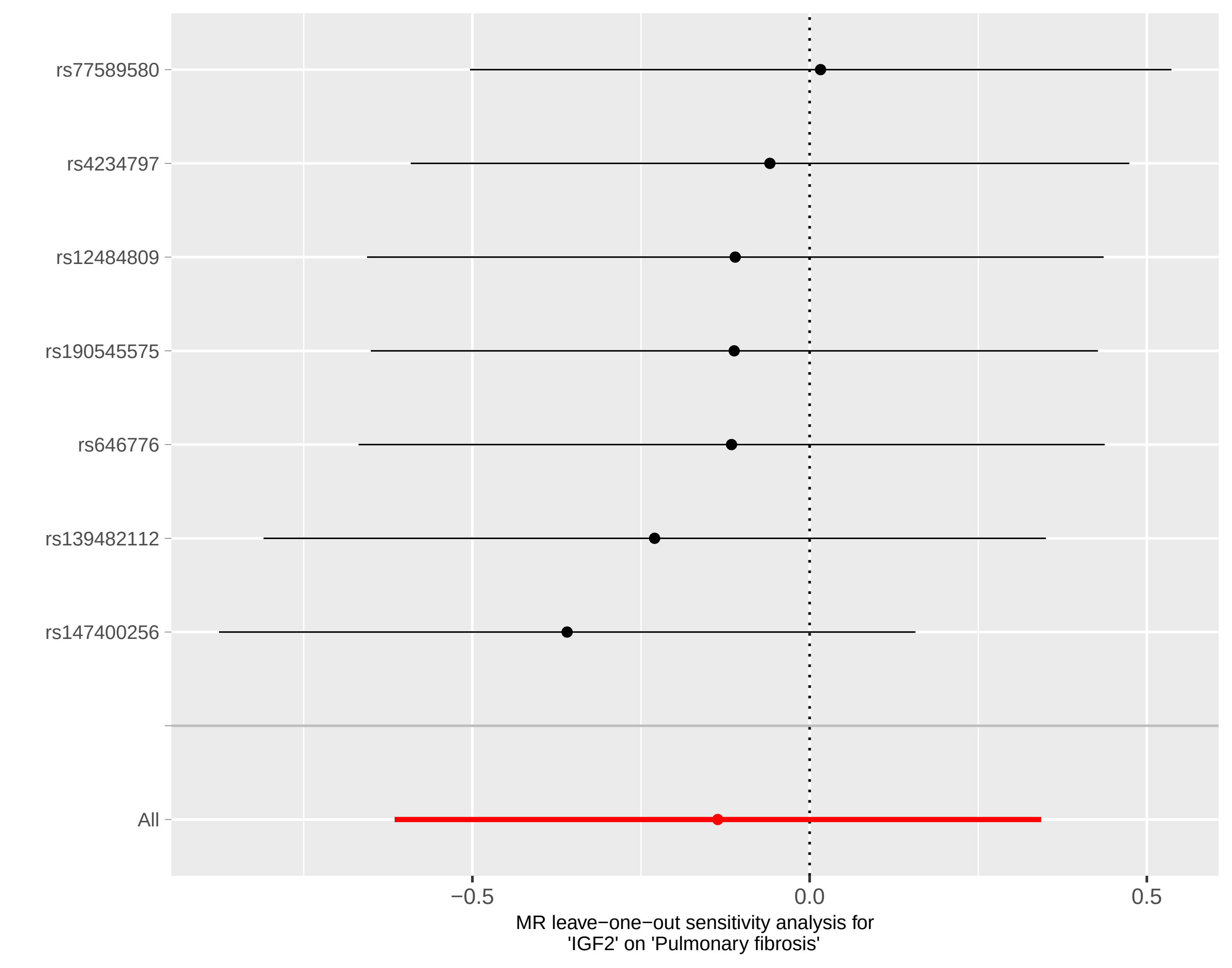
rs77589580
rs4234797
rs12484809
rs190545575
rs646776
rs139482112
rs147400256
All
−0.5
0.0
0.5
MR leave−one−out sensitivity analysis for
'IGF2' on 'Pulmonary fibrosis'

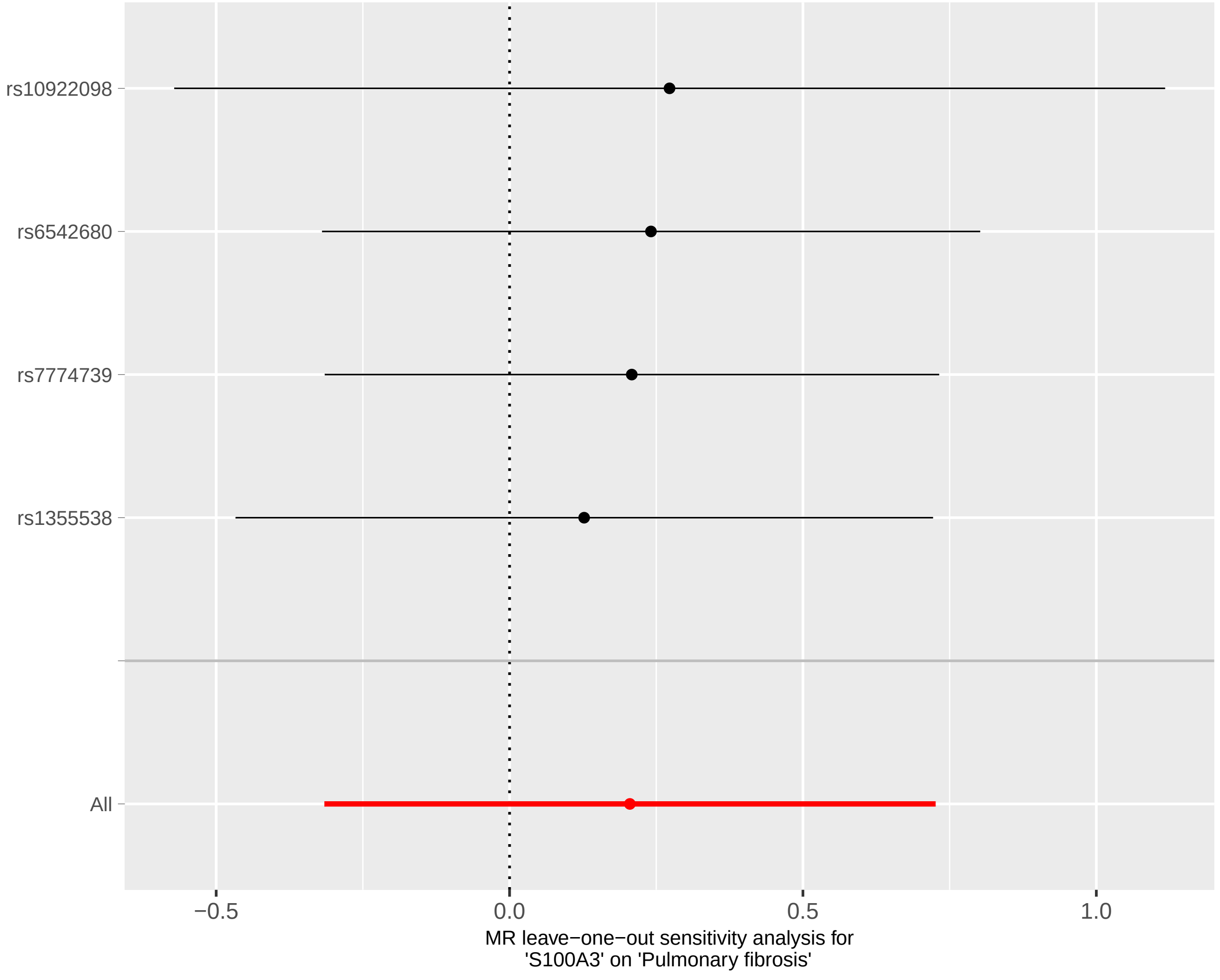
rs10922098
rs6542680
rs7774739
rs1355538
All
−0.5
0.0
0.5
1.0
MR leave−one−out sensitivity analysis for
'S100A3' on 'Pulmonary fibrosis'

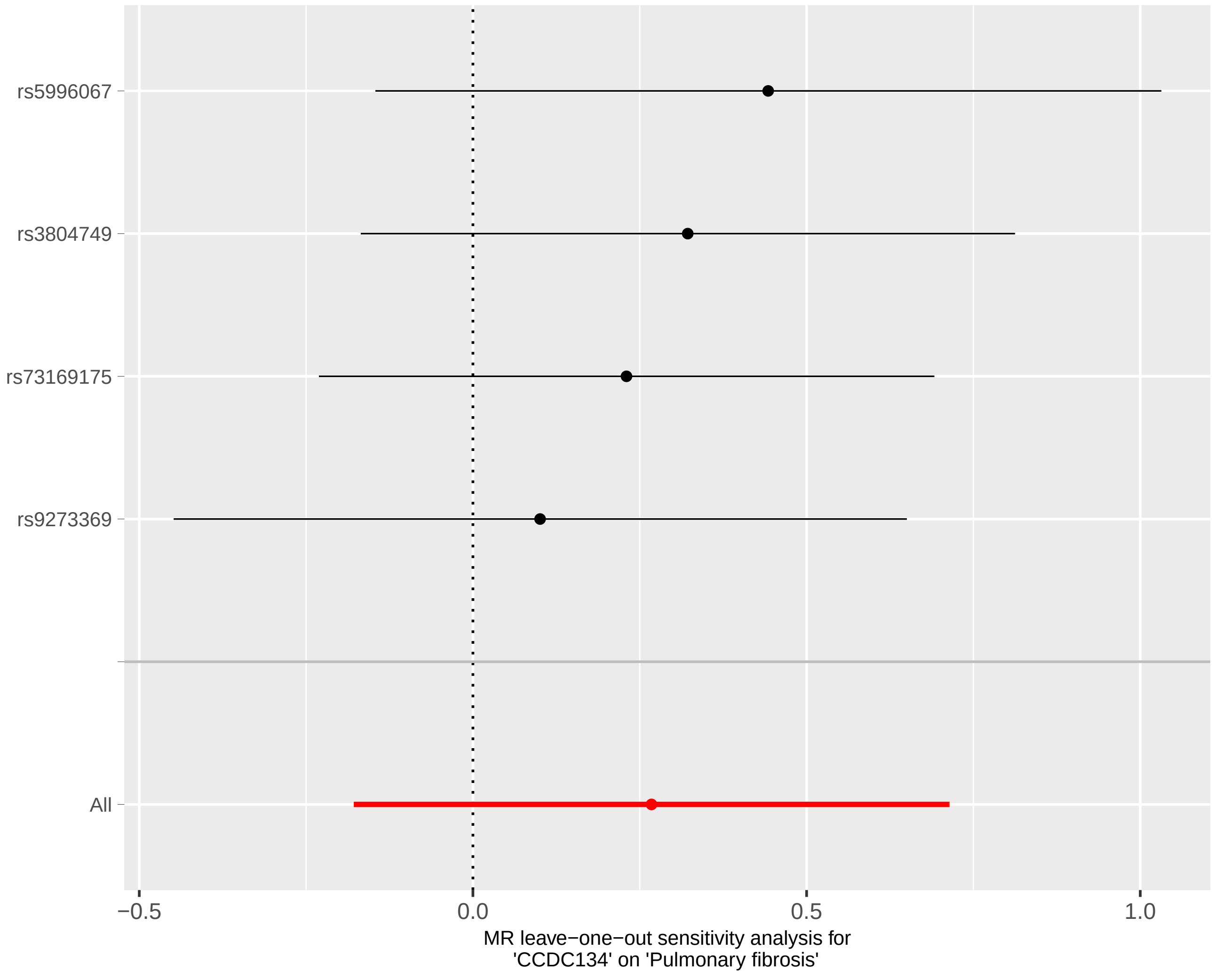
rs5996067
rs3804749
rs73169175
rs9273369
All
−0.5
0.0
0.5
1.0
MR leave−one−out sensitivity analysis for
'CCDC134' on 'Pulmonary fibrosis'

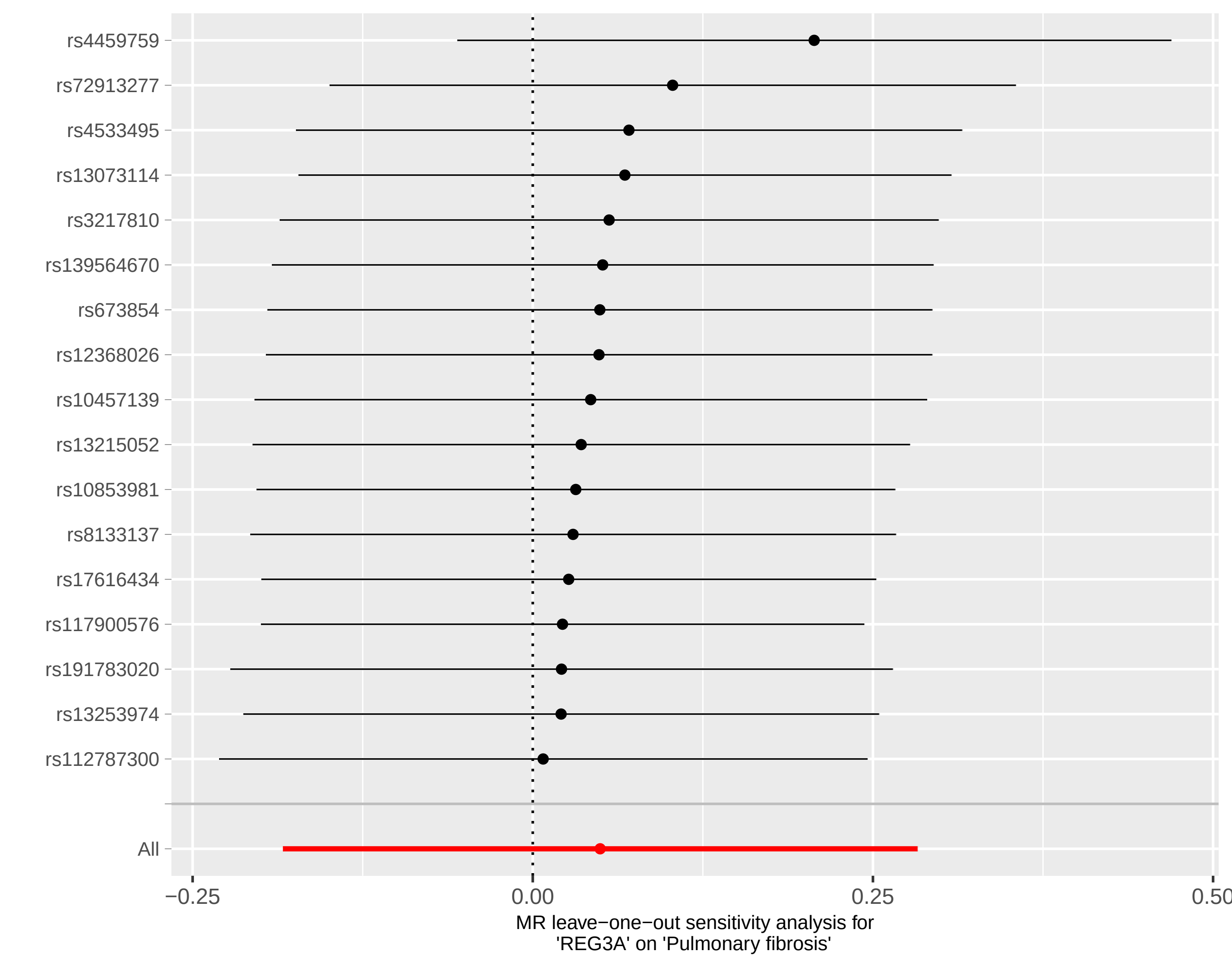
rs4459759
rs72913277
rs4533495
rs13073114
rs3217810
rs139564670
rs673854
rs12368026
rs10457139
rs13215052
rs10853981
rs8133137
rs17616434
rs117900576
rs191783020
rs13253974
rs112787300
All
−0.25
0.00
0.25
0.50
MR leave−one−out sensitivity analysis for
'REG3A' on 'Pulmonary fibrosis'

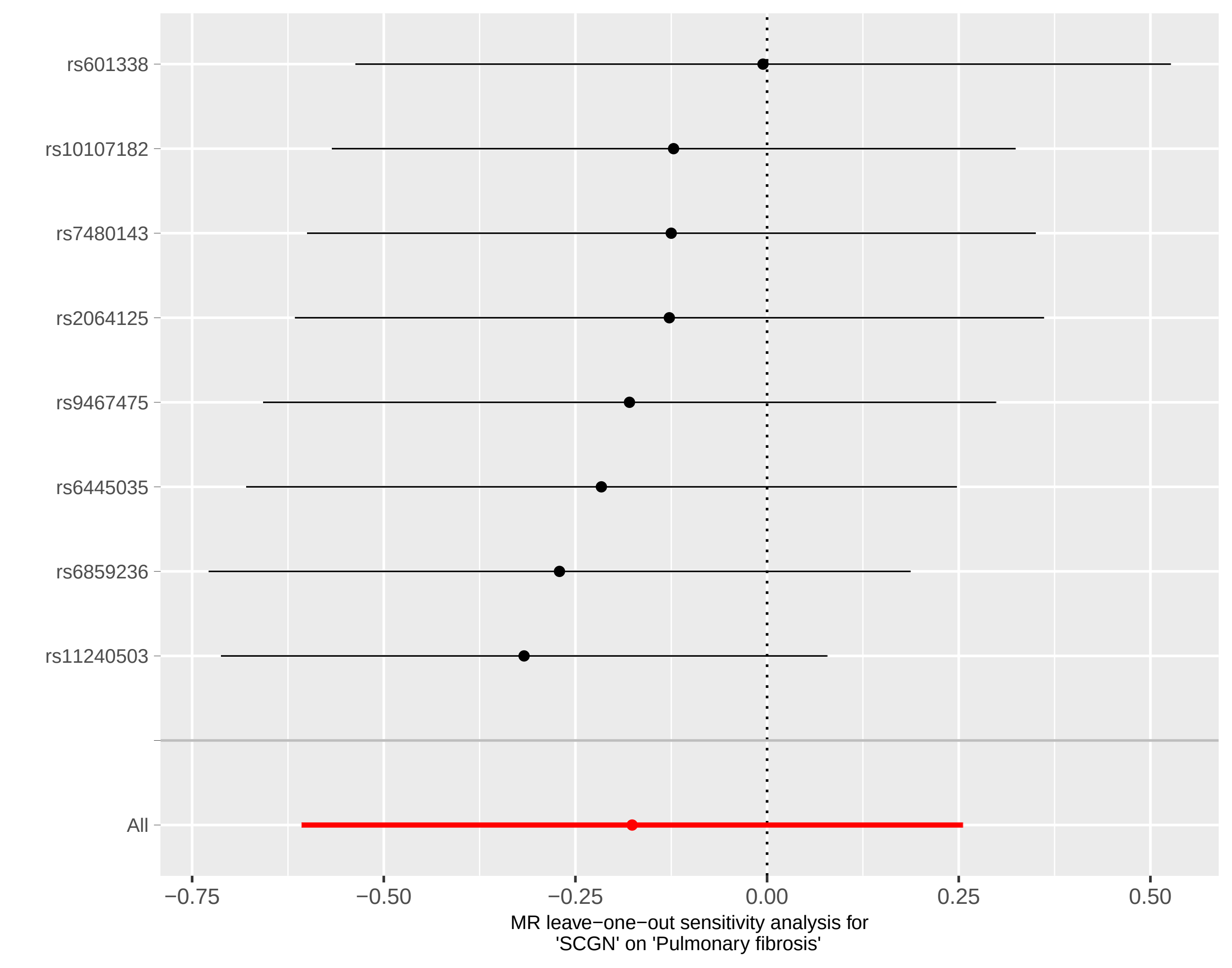
rs601338
rs10107182
rs7480143
rs2064125
rs9467475
rs6445035
rs6859236
rs11240503
All
−0.75
−0.50
−0.25
0.00
0.25
0.50
MR leave−one−out sensitivity analysis for
'SCGN' on 'Pulmonary fibrosis'

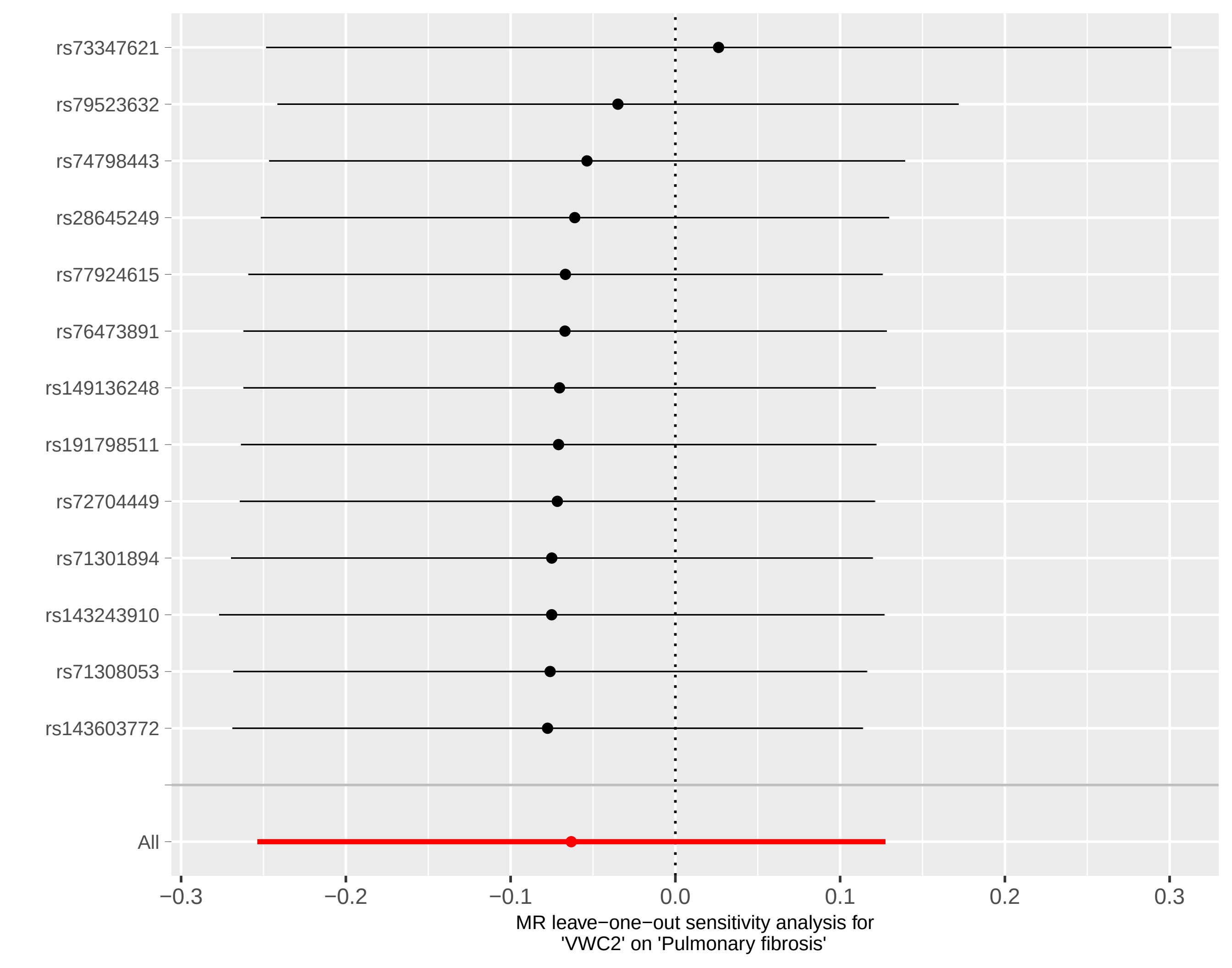

rs73347621
rs79523632
rs74798443
rs28645249
rs77924615
rs76473891
rs149136248
rs191798511
rs72704449
rs71301894
rs143243910
rs71308053
rs143603772
All
−0.3
−0.2
−0.1
0.0
0.1
0.2
0.3
MR leave−one−out sensitivity analysis for
'VWC2' on 'Pulmonary fibrosis'

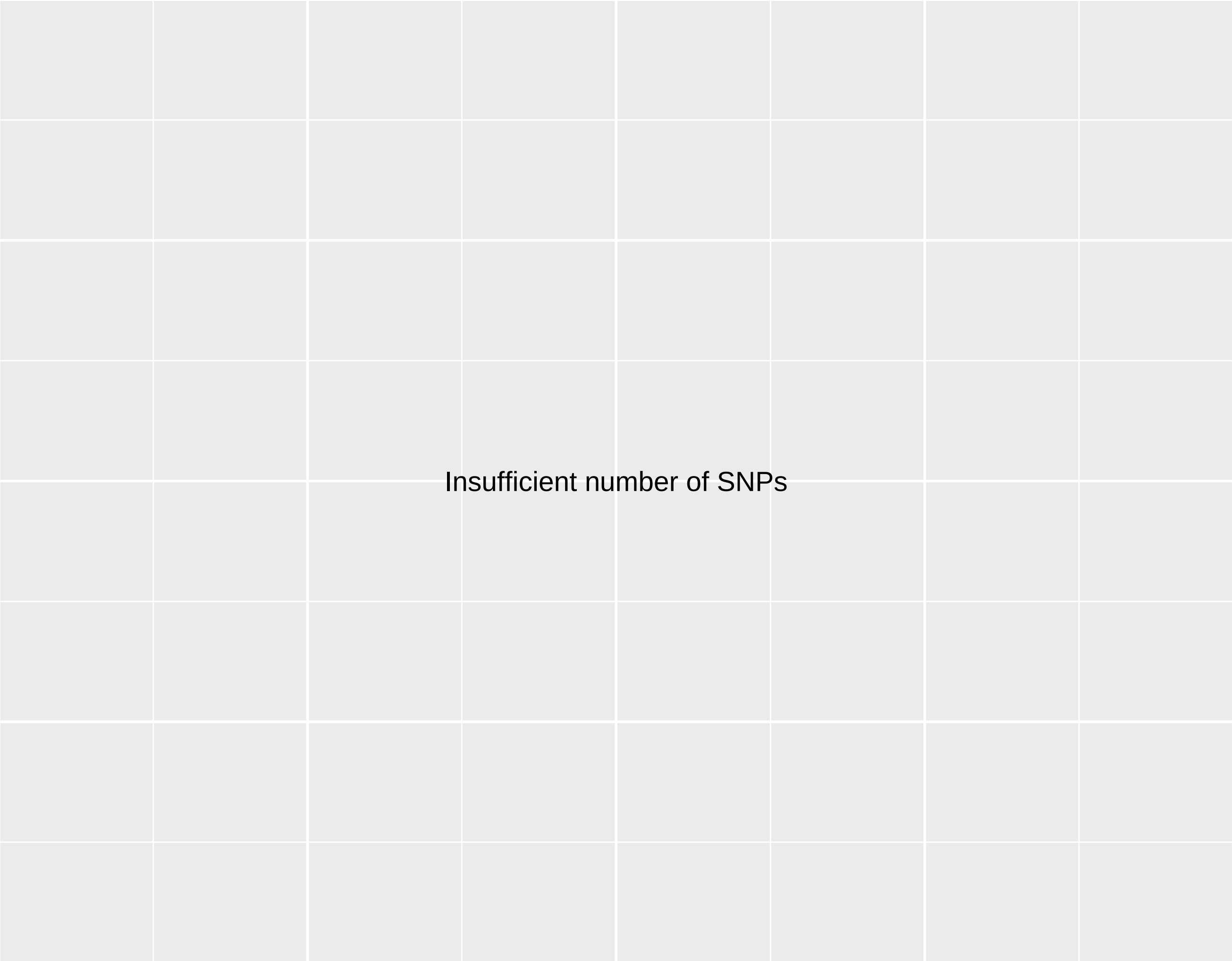
Insufficient number of SNPs

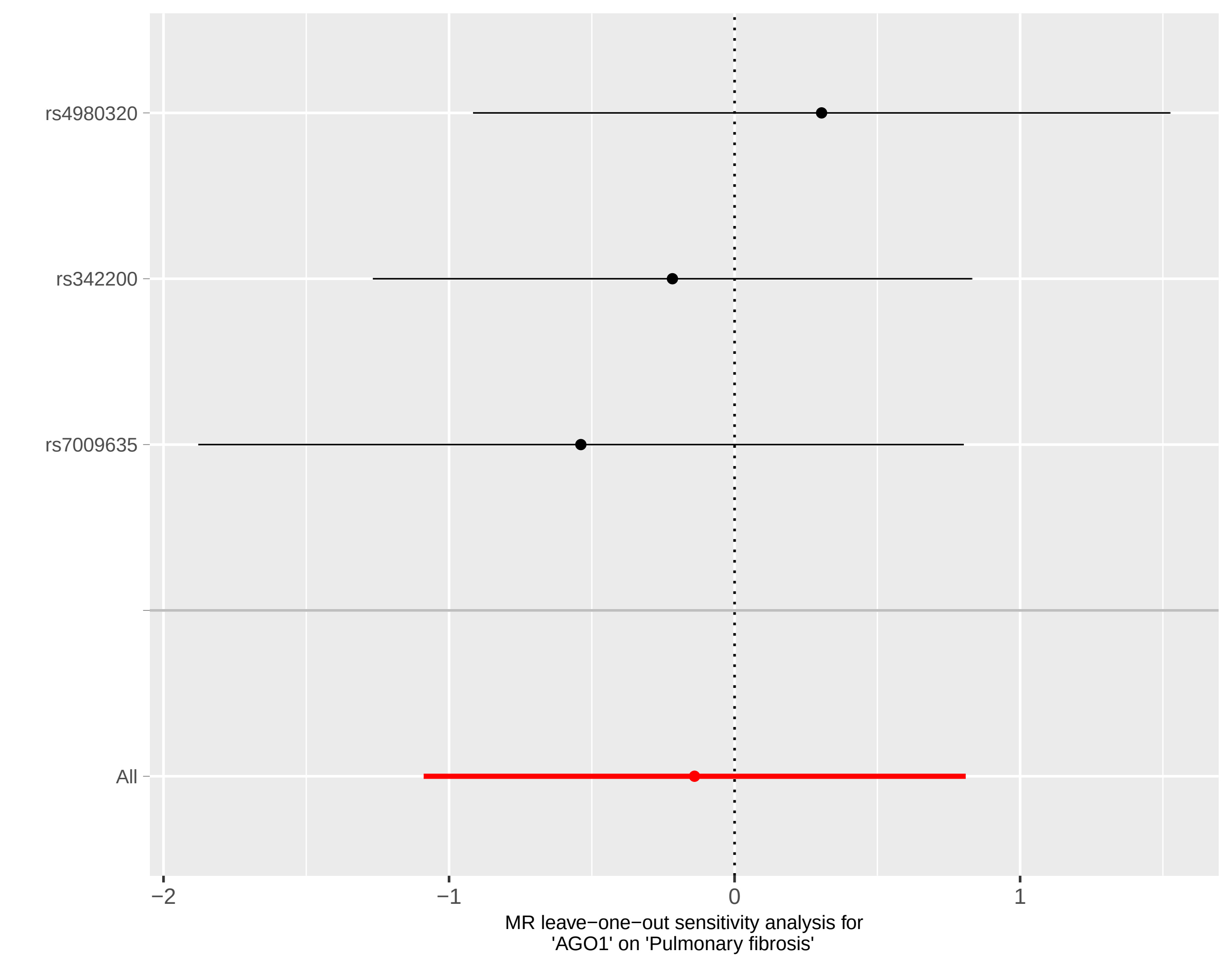
rs4980320
rs342200
rs7009635
All
−2
−1
0
1
MR leave−one−out sensitivity analysis for
'AGO1' on 'Pulmonary fibrosis'

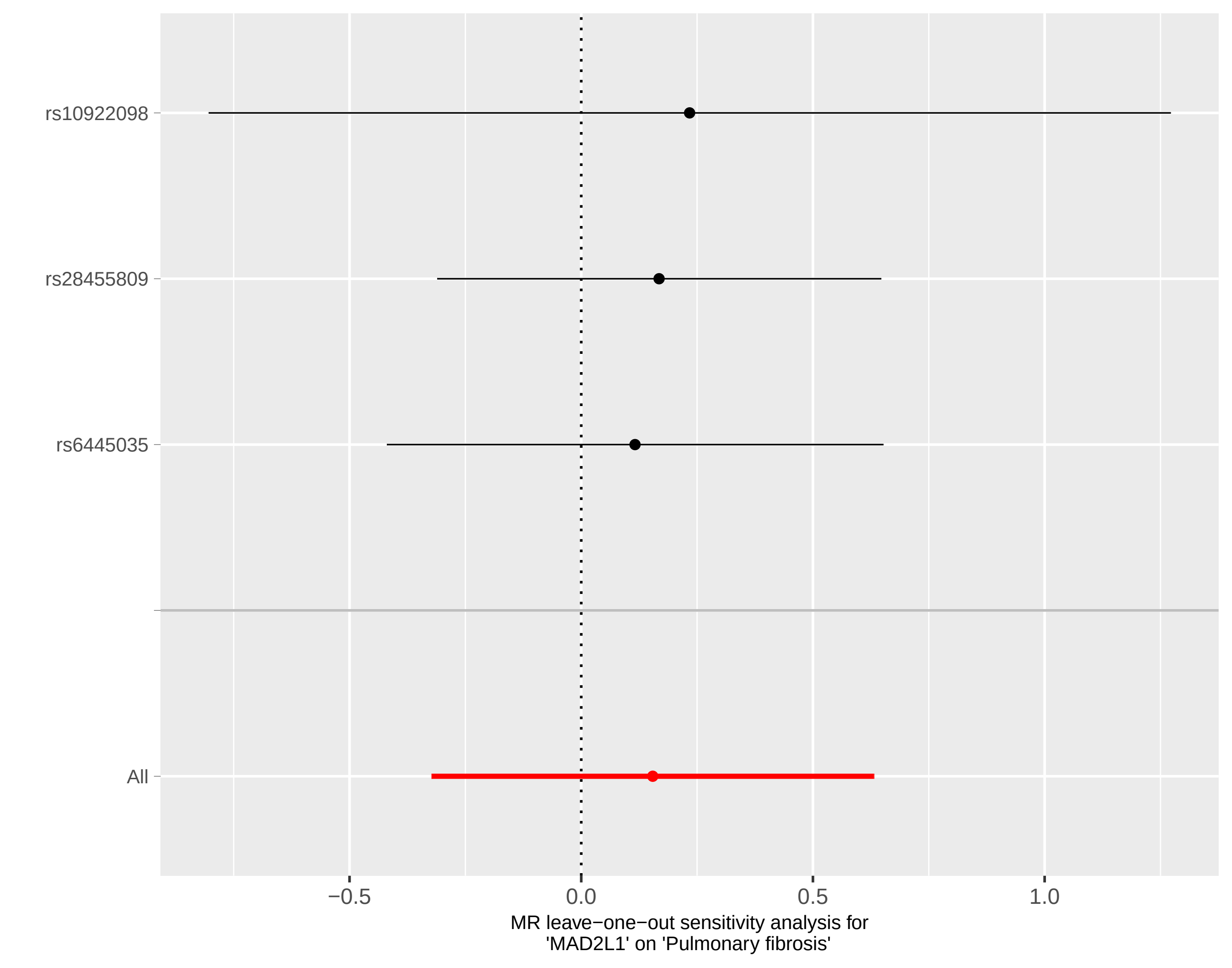
rs10922098
rs28455809
rs6445035
All
−0.5
0.0
0.5
1.0
MR leave−one−out sensitivity analysis for
'MAD2L1' on 'Pulmonary fibrosis'

Insufficient number of SNPs

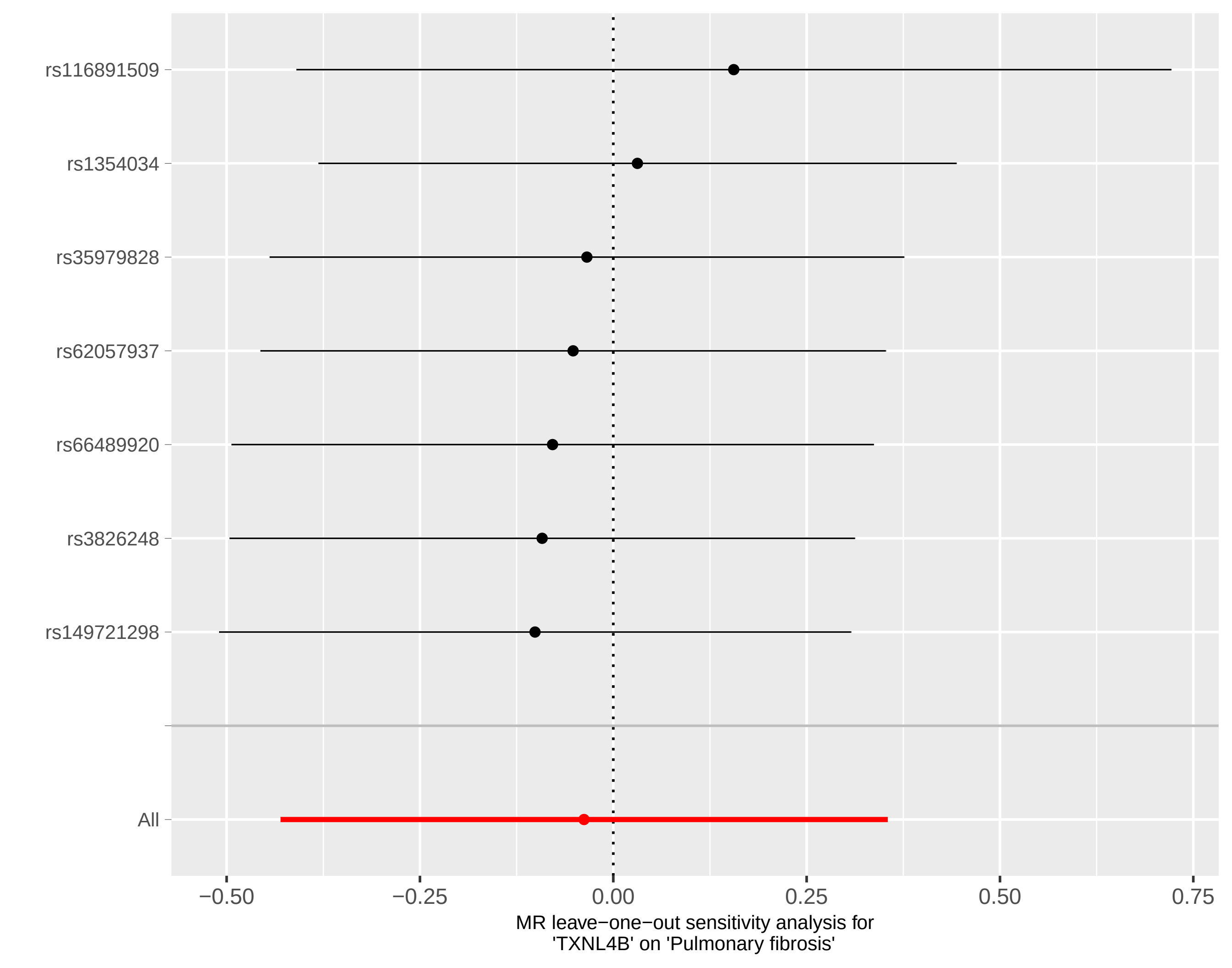
rs116891509
rs1354034
rs35979828
rs62057937
rs66489920
rs3826248
rs149721298
All
−0.50
−0.25
0.00
0.25
0.50
0.75
MR leave−one−out sensitivity analysis for
'TXNL4B' on 'Pulmonary fibrosis'

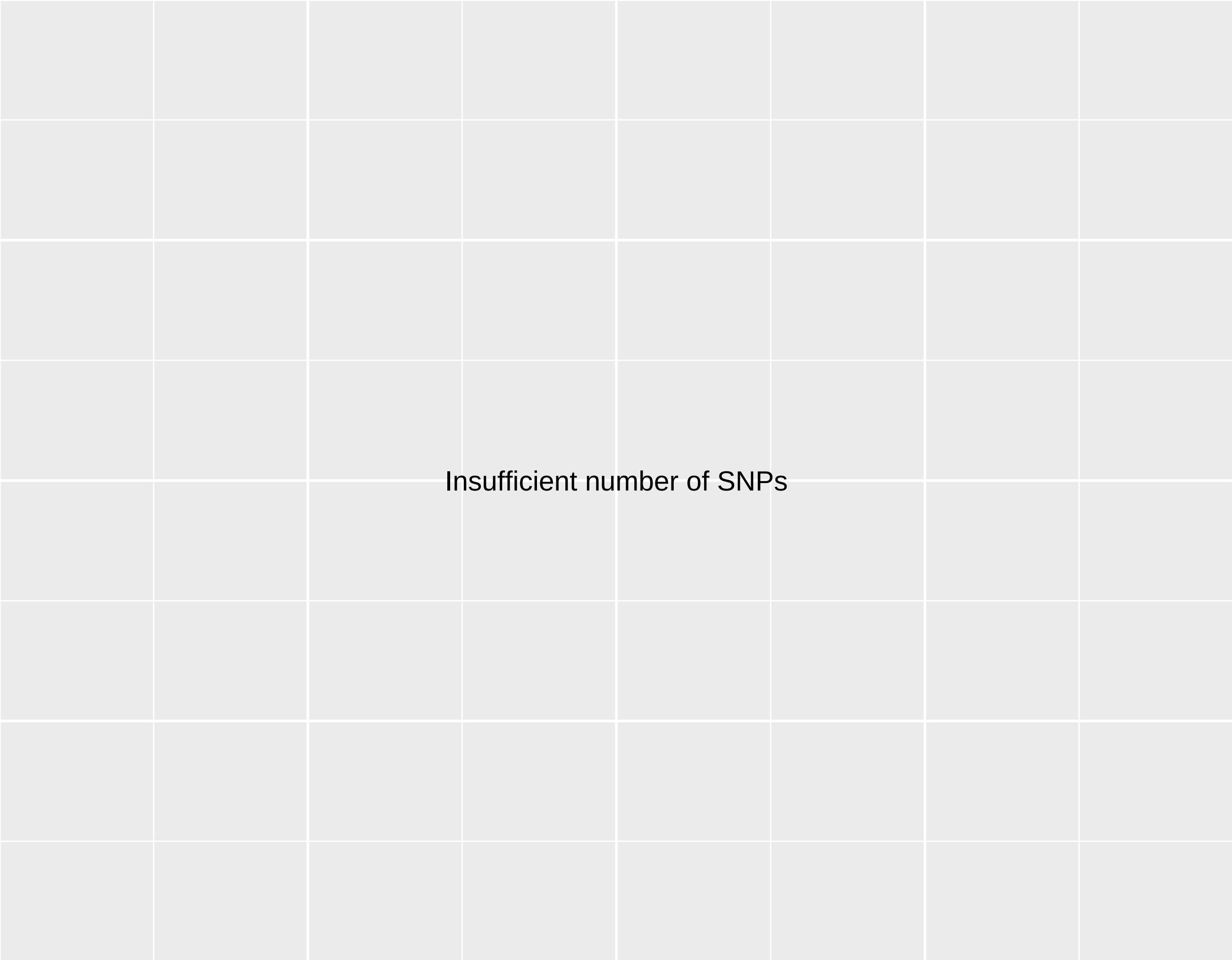
Insufficient number of SNPs

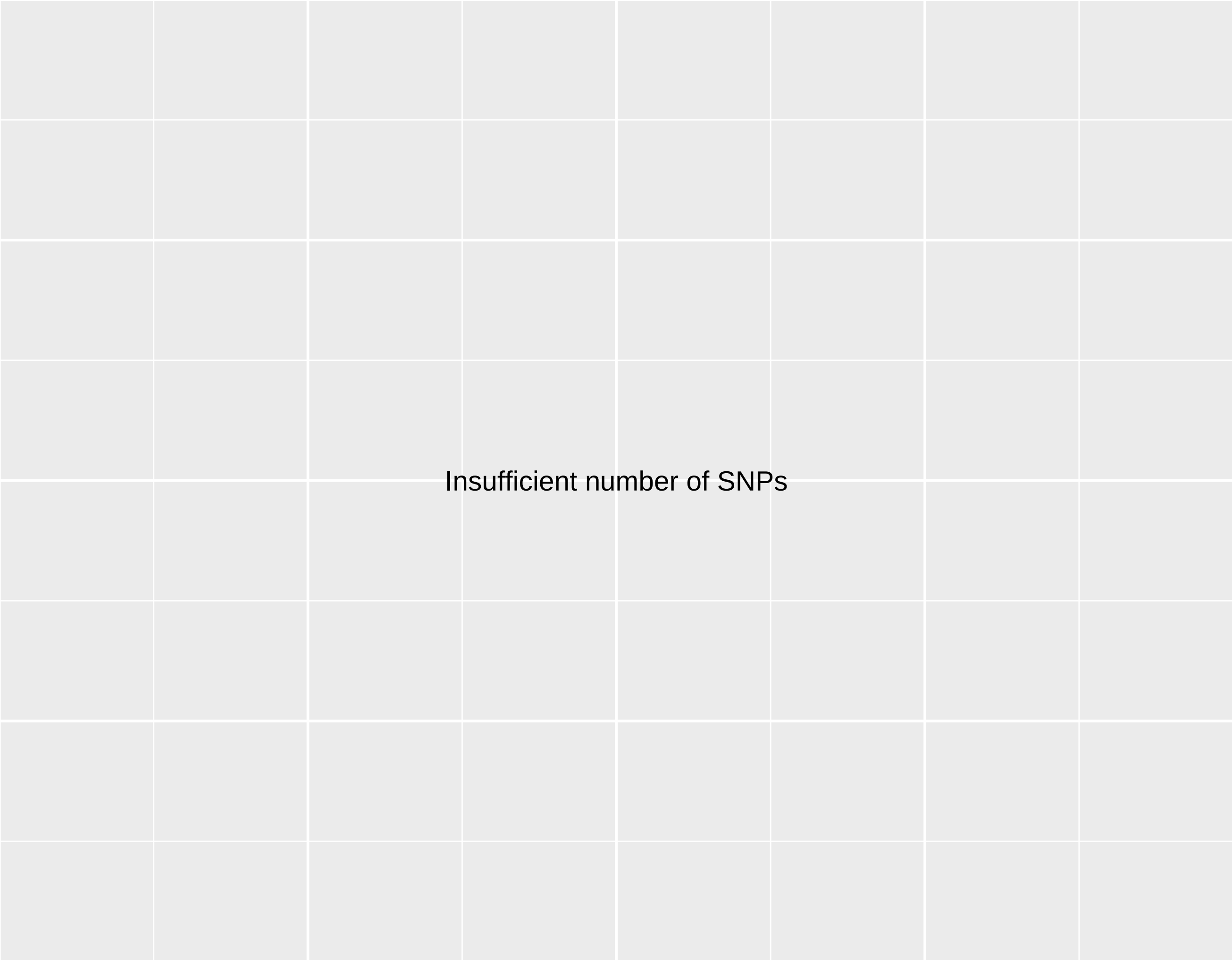
Insufficient number of SNPs

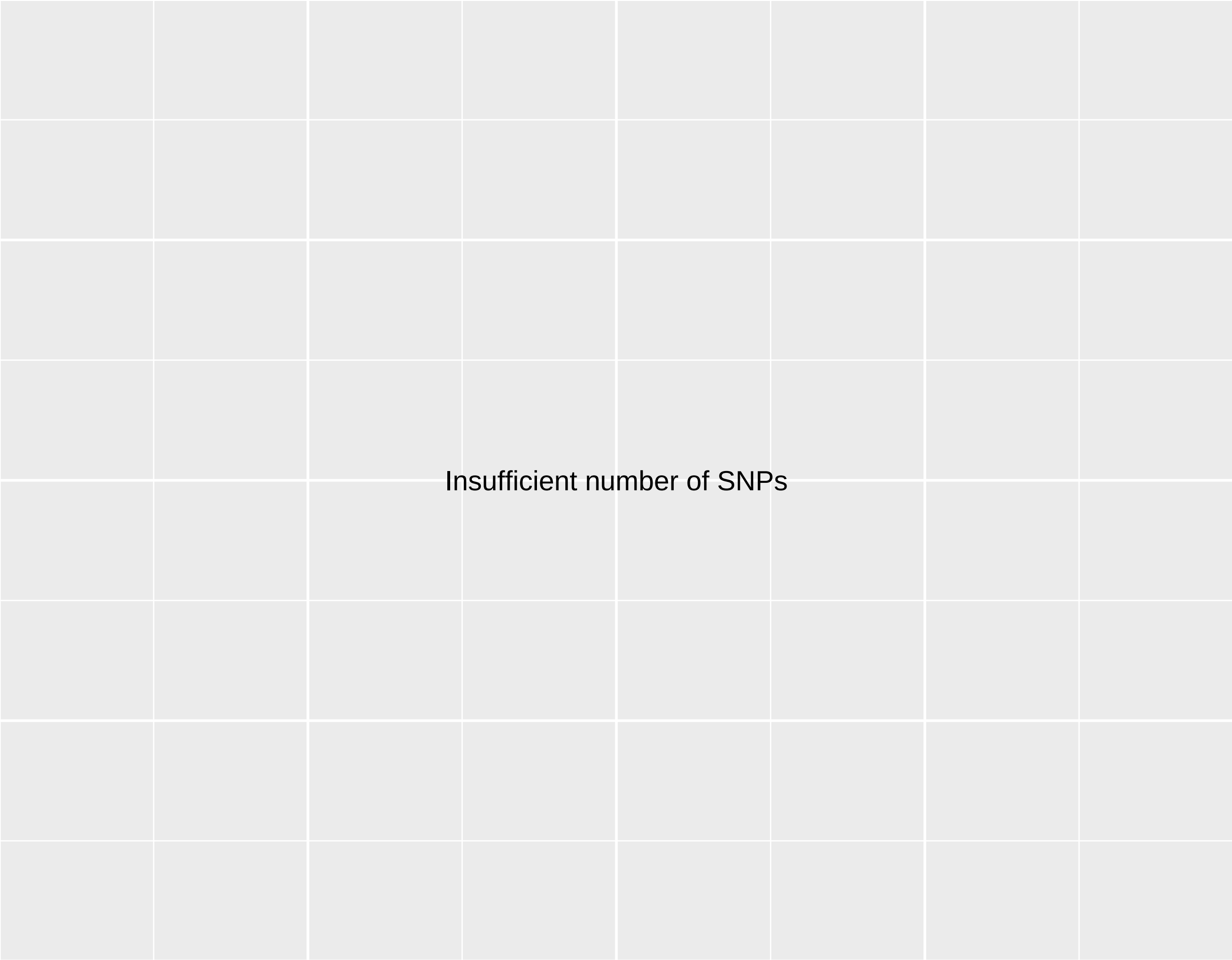
Insufficient number of SNPs

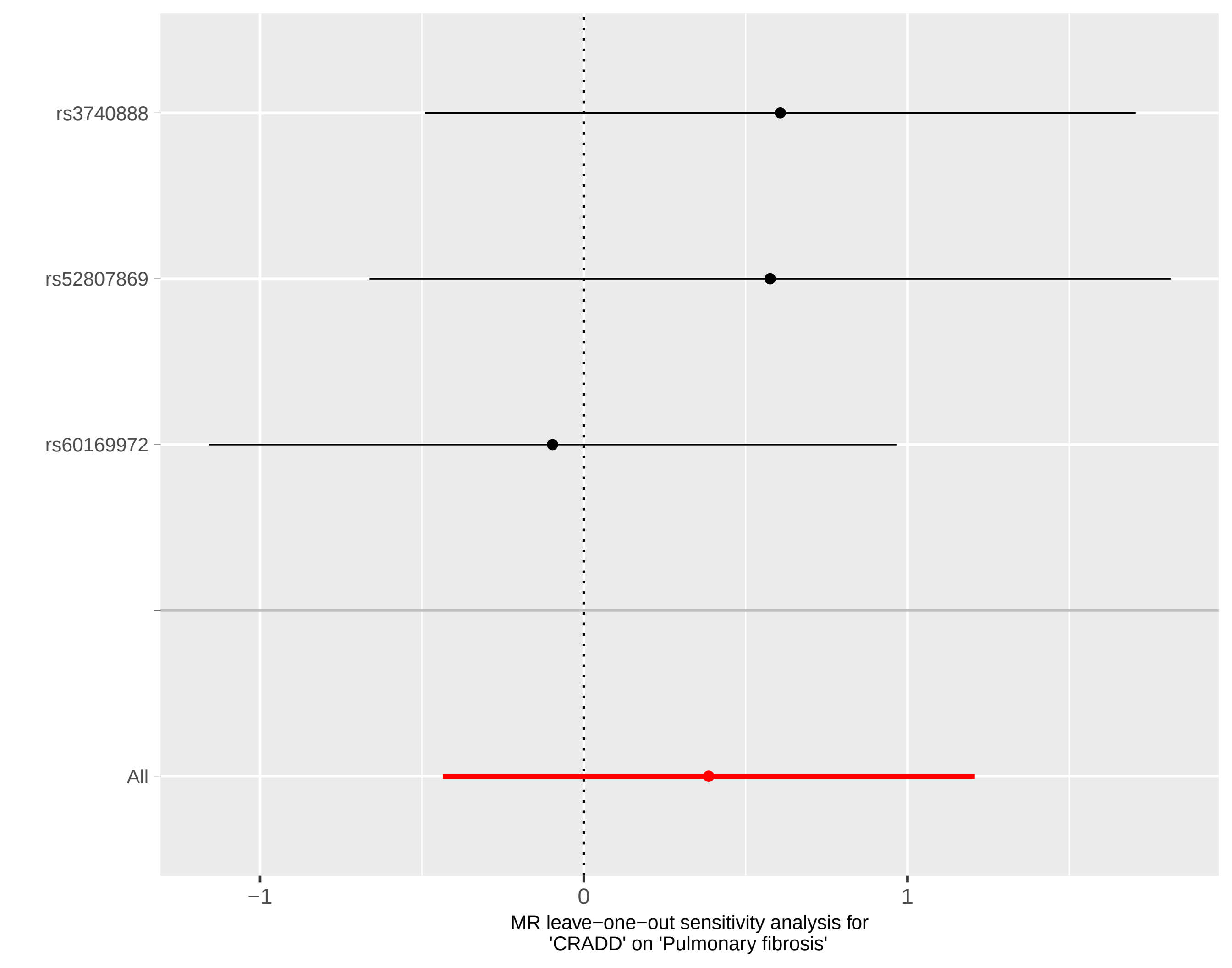
rs3740888
rs52807869
rs60169972
All
−1
0
1
MR leave−one−out sensitivity analysis for
'CRADD' on 'Pulmonary fibrosis'

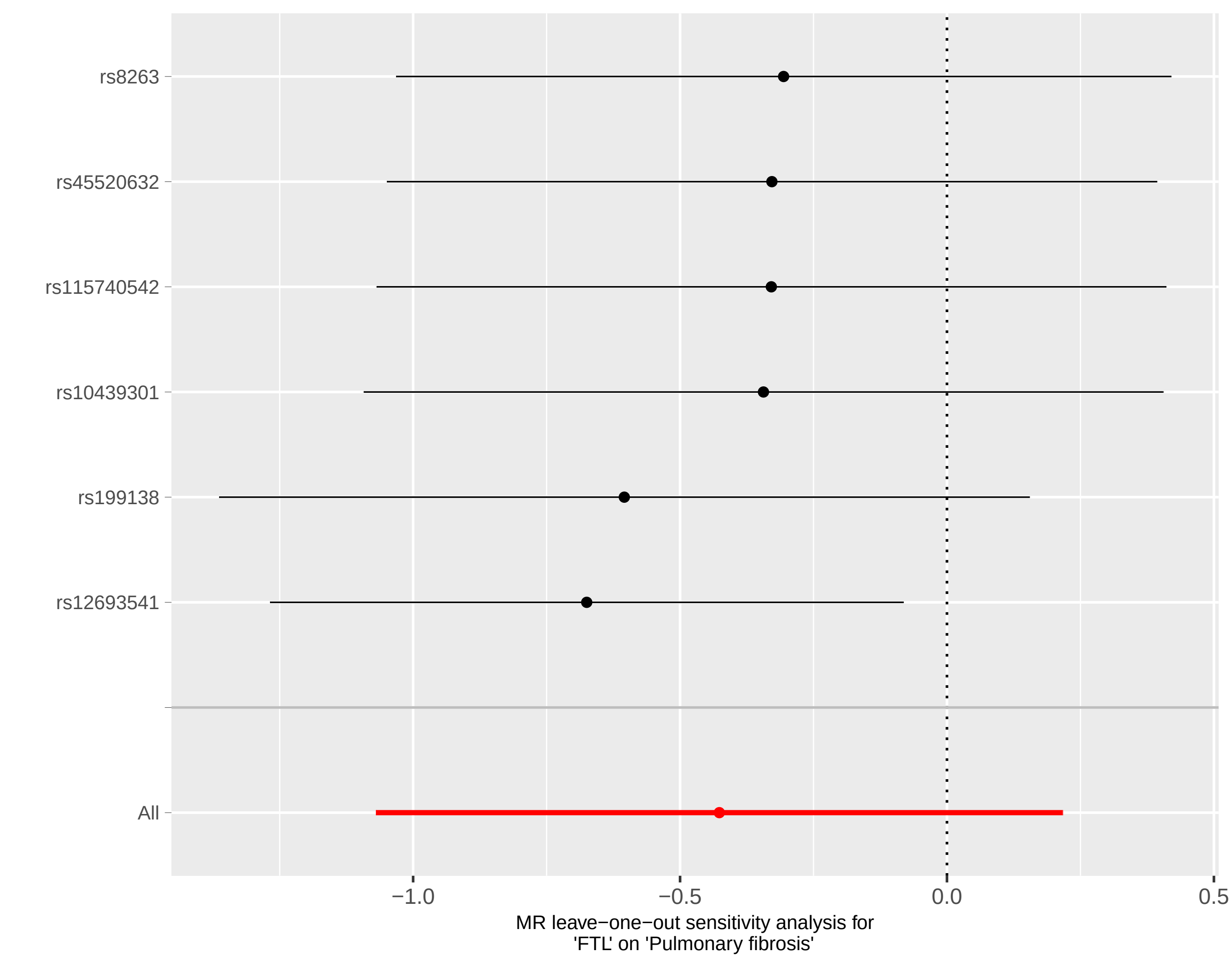
rs8263
rs45520632
rs115740542
rs10439301
rs199138
rs12693541
All
−1.0
−0.5
0.0
0.5
MR leave−one−out sensitivity analysis for
'FTL' on 'Pulmonary fibrosis'

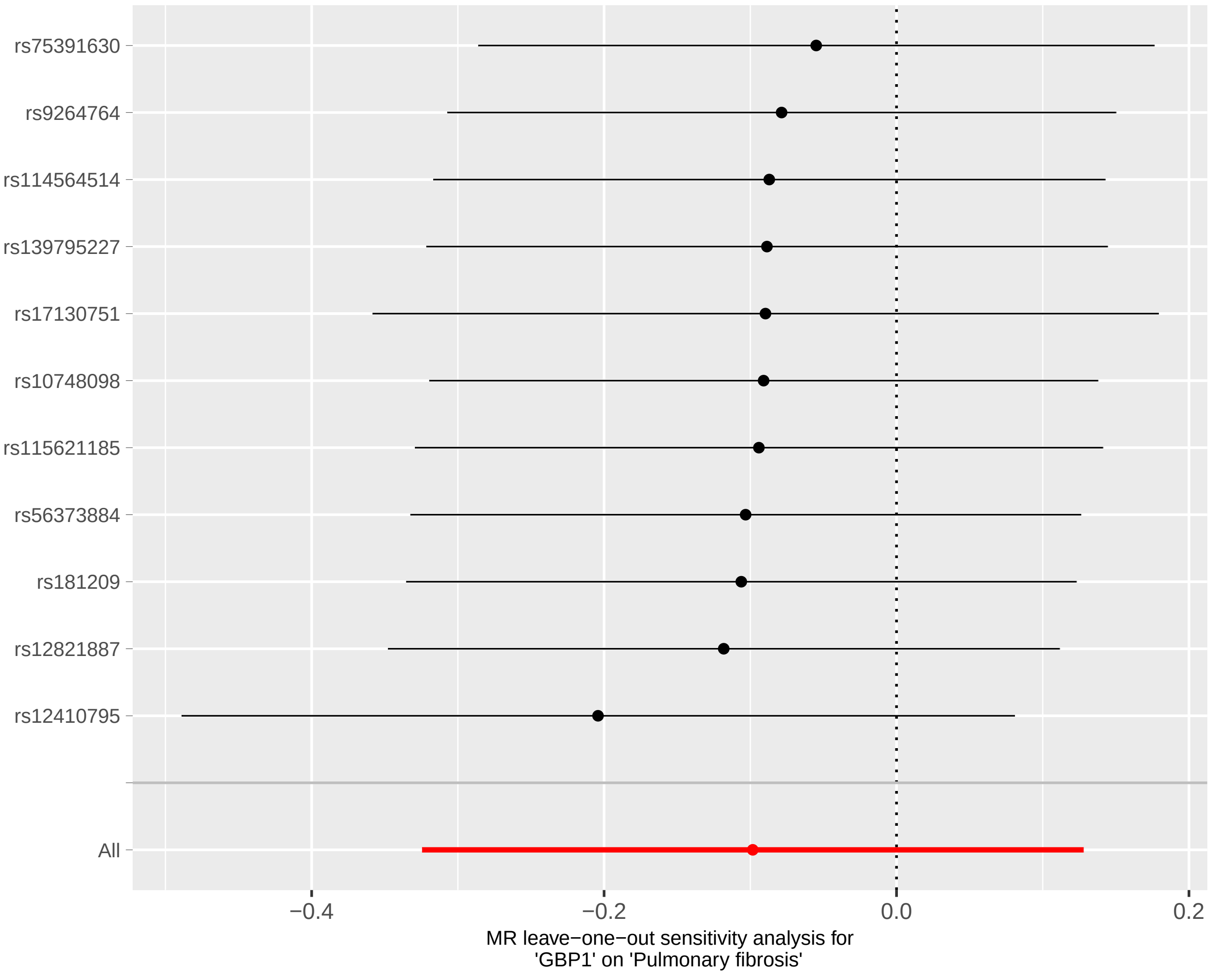
rs75391630
rs9264764
rs114564514
rs139795227
rs17130751
rs10748098
rs115621185
rs56373884
rs181209
rs12821887
rs12410795
All
−0.4
−0.2
0.0
0.2
MR leave−one−out sensitivity analysis for
'GBP1' on 'Pulmonary fibrosis'

Insufficient number of SNPs

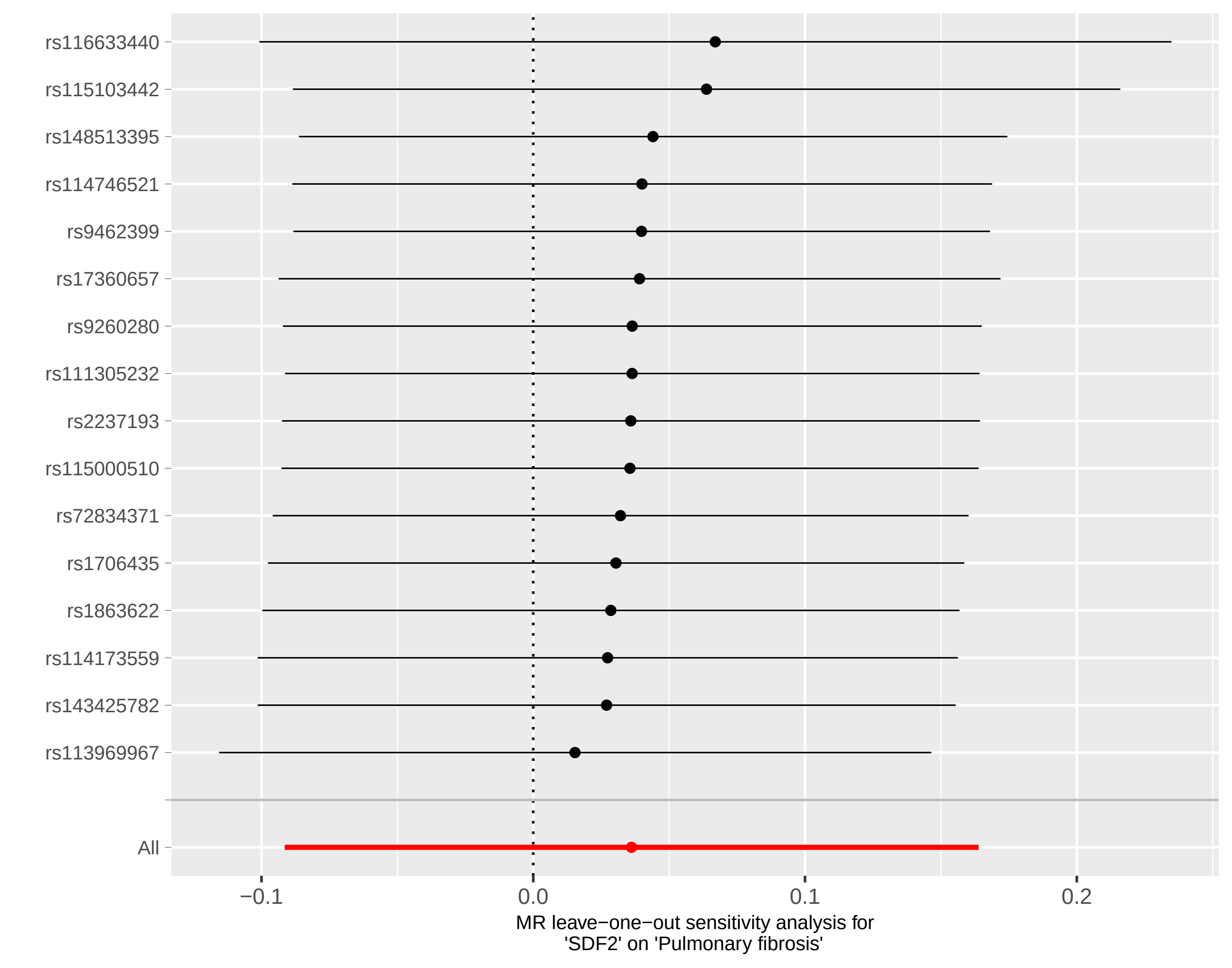
rs116633440
rs115103442
rs148513395
rs114746521
rs9462399
rs17360657
rs9260280
rs111305232
rs2237193
rs115000510
rs72834371
rs1706435
rs1863622
rs114173559
rs143425782
rs113969967
All
−0.1
0.0
0.1
0.2
MR leave−one−out sensitivity analysis for
'SDF2' on 'Pulmonary fibrosis'

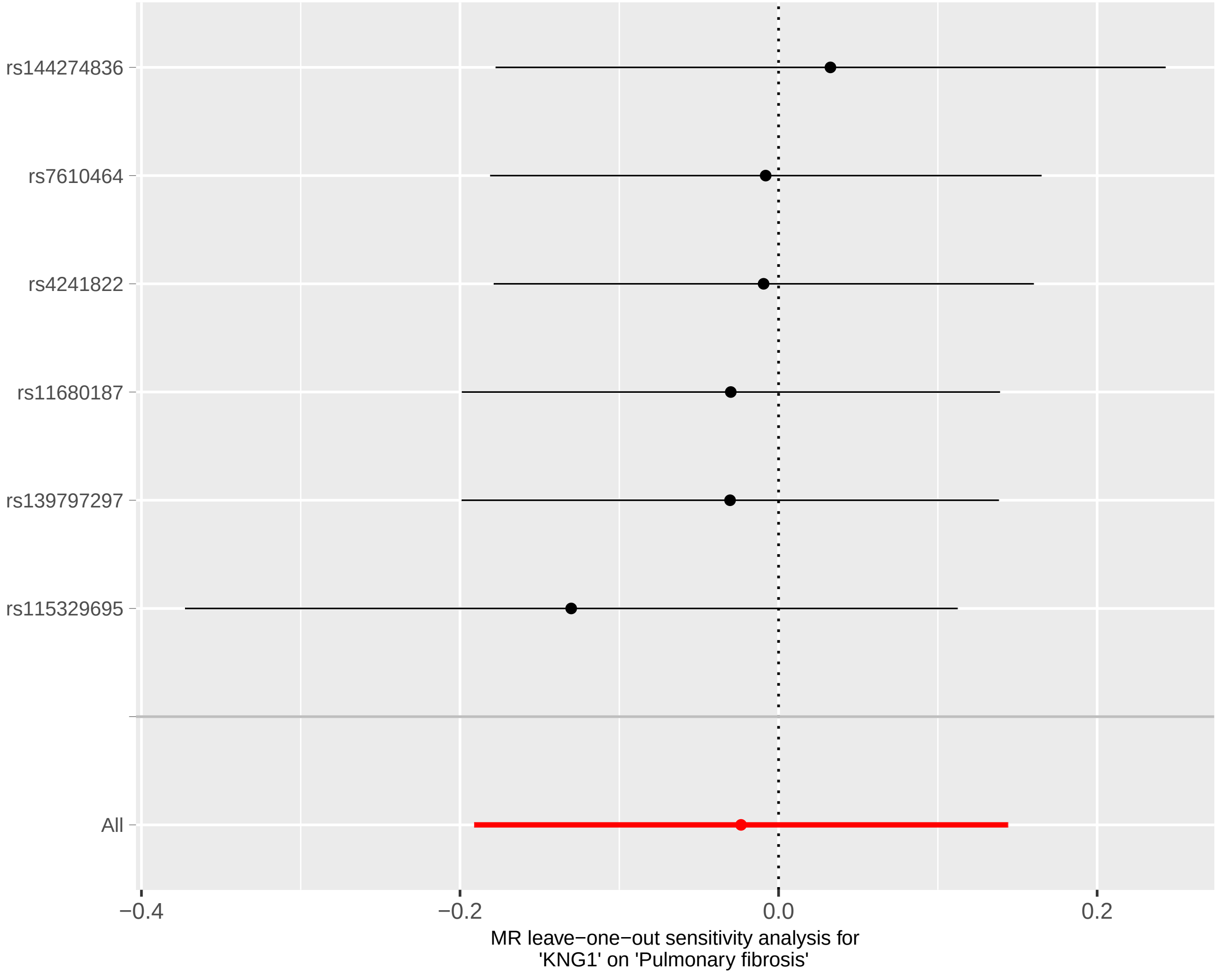

rs144274836
rs7610464
rs4241822
rs11680187
rs139797297
rs115329695
All
−0.4
−0.2
0.0
0.2
MR leave−one−out sensitivity analysis for
'KNG1' on 'Pulmonary fibrosis'

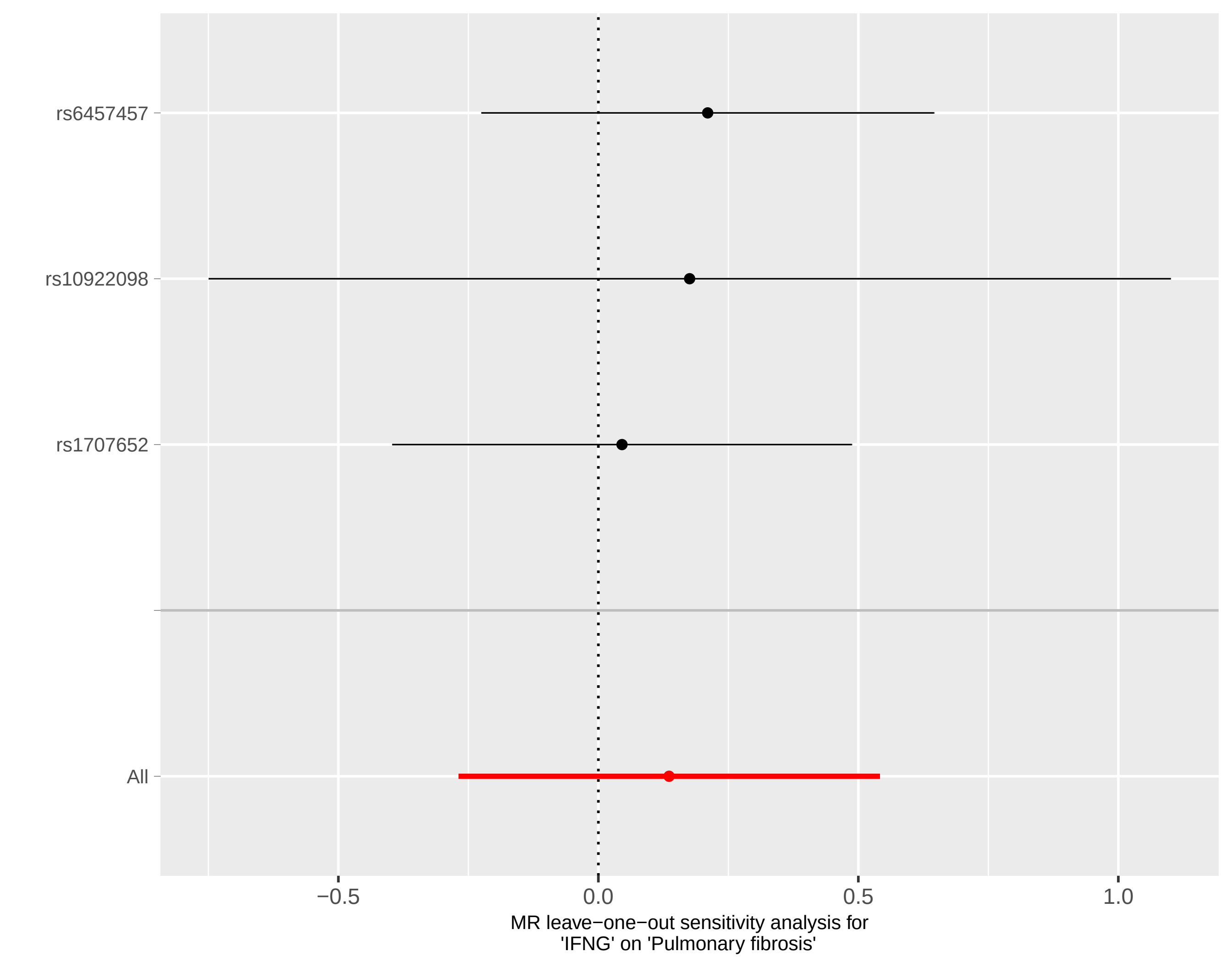
rs6457457
rs10922098
rs1707652
All
−0.5
0.0
0.5
1.0
MR leave−one−out sensitivity analysis for
'IFNG' on 'Pulmonary fibrosis'

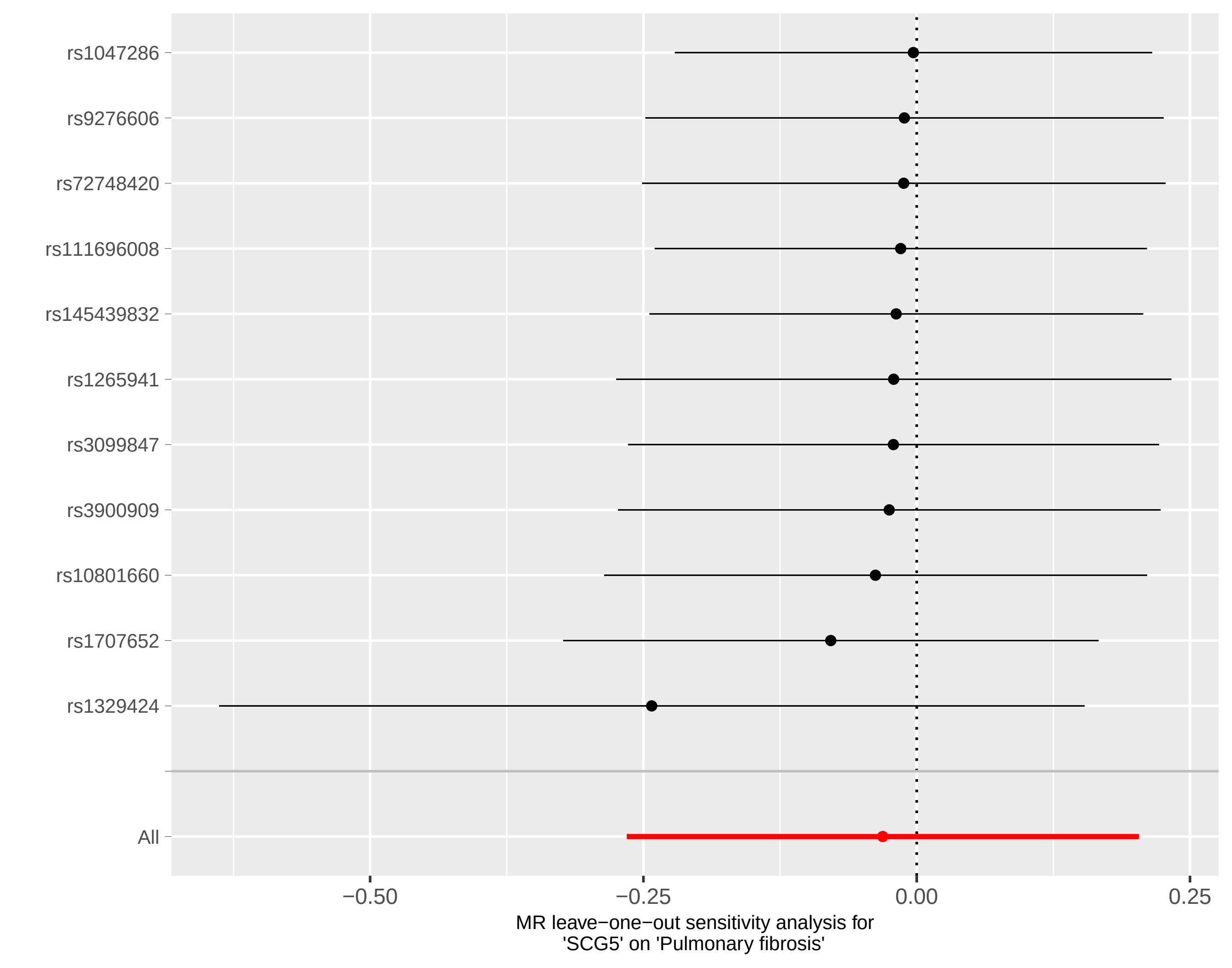

rs1047286
rs9276606
rs72748420
rs111696008
rs145439832
rs1265941
rs3099847
rs3900909
rs10801660
rs1707652
rs1329424
All
−0.50
−0.25
0.00
0.25
MR leave−one−out sensitivity analysis for
'SCG5' on 'Pulmonary fibrosis'

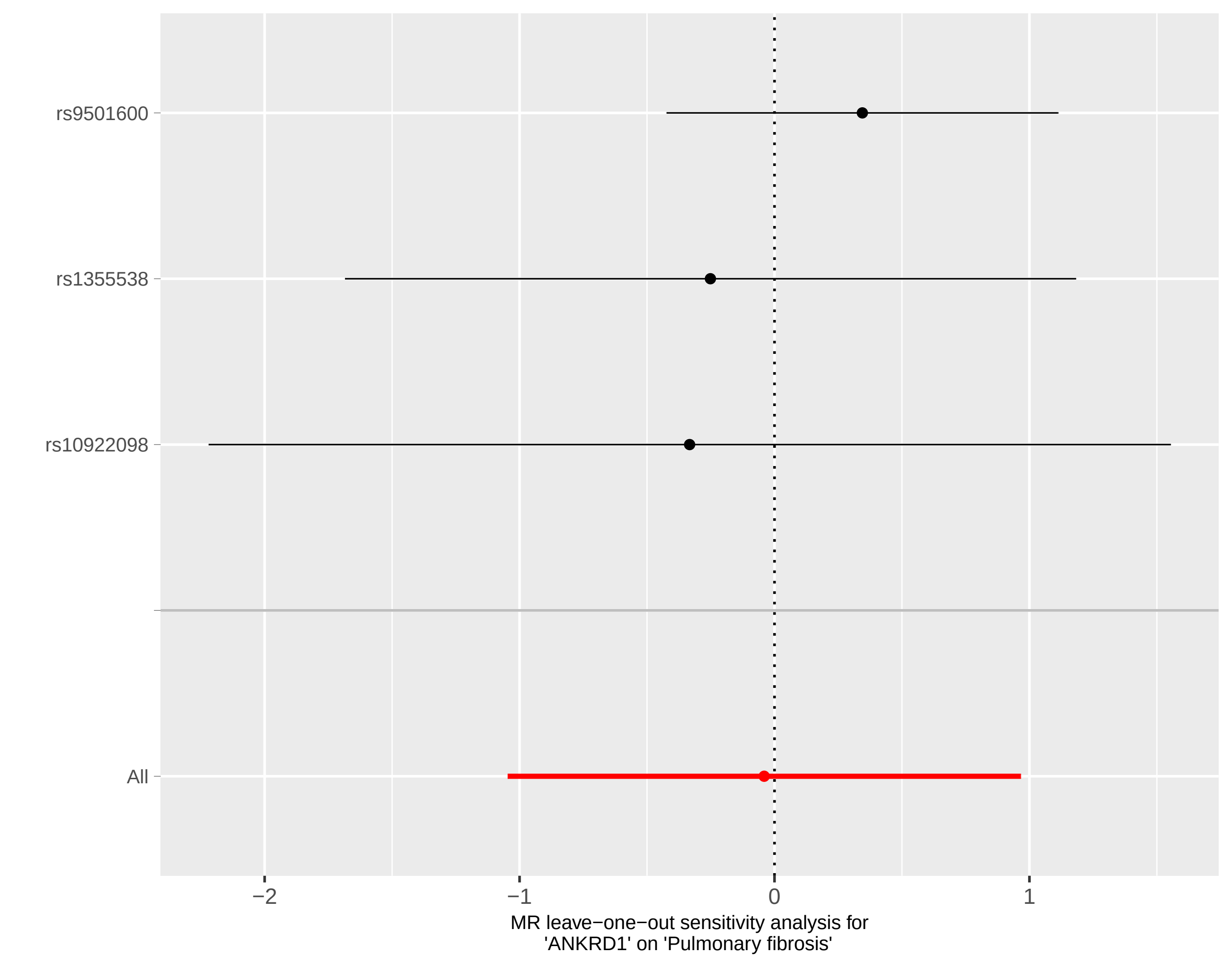
rs9501600
rs1355538
rs10922098
All
−2
−1
0
1
MR leave−one−out sensitivity analysis for
'ANKRD1' on 'Pulmonary fibrosis'

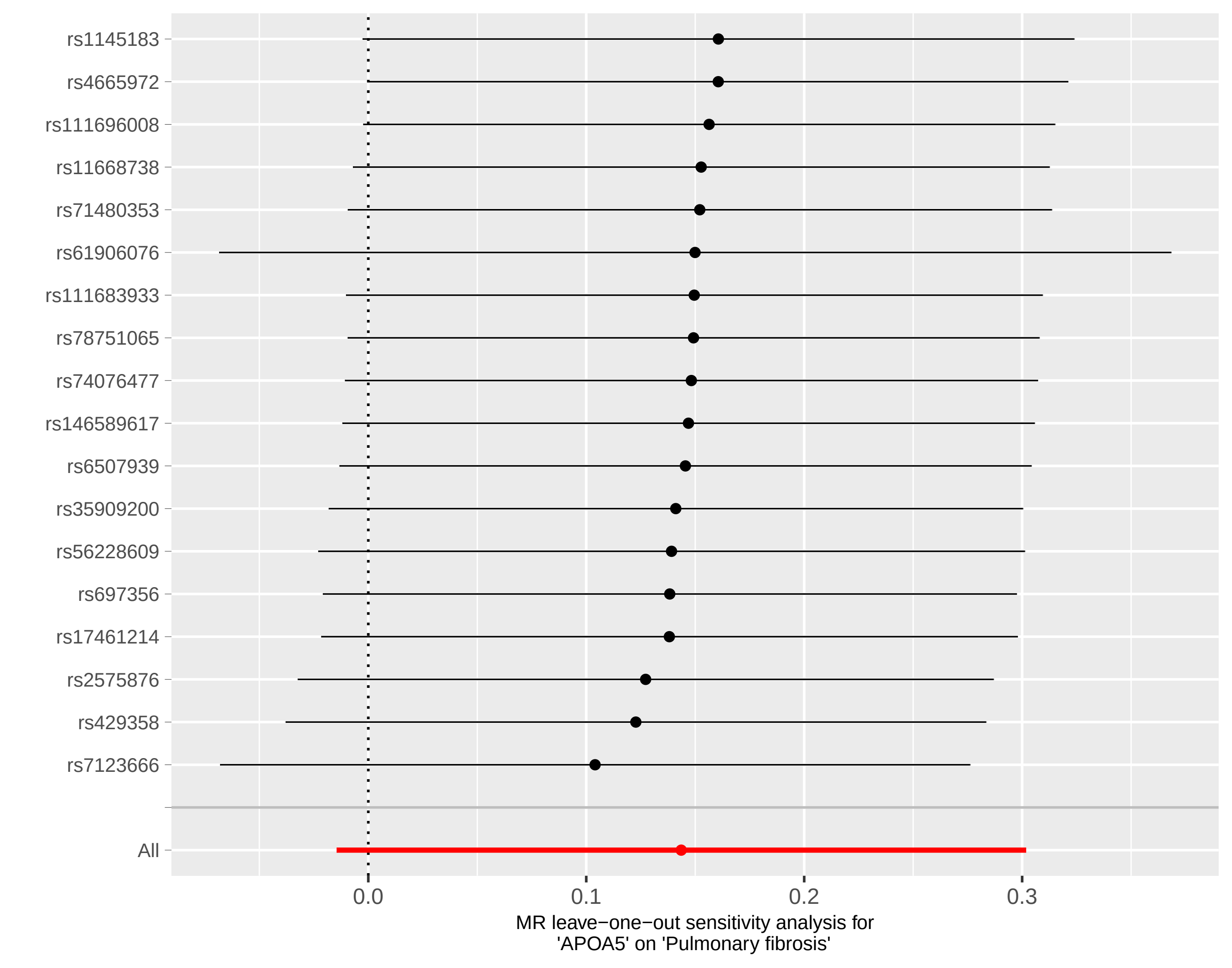
rs1145183
rs4665972
rs111696008
rs11668738
rs71480353
rs61906076
rs111683933
rs78751065
rs74076477
rs146589617
rs6507939
rs35909200
rs56228609
rs697356
rs17461214
rs2575876
rs429358
rs7123666
All
0.0
0.1
0.2
0.3
MR leave−one−out sensitivity analysis for
'APOA5' on 'Pulmonary fibrosis'

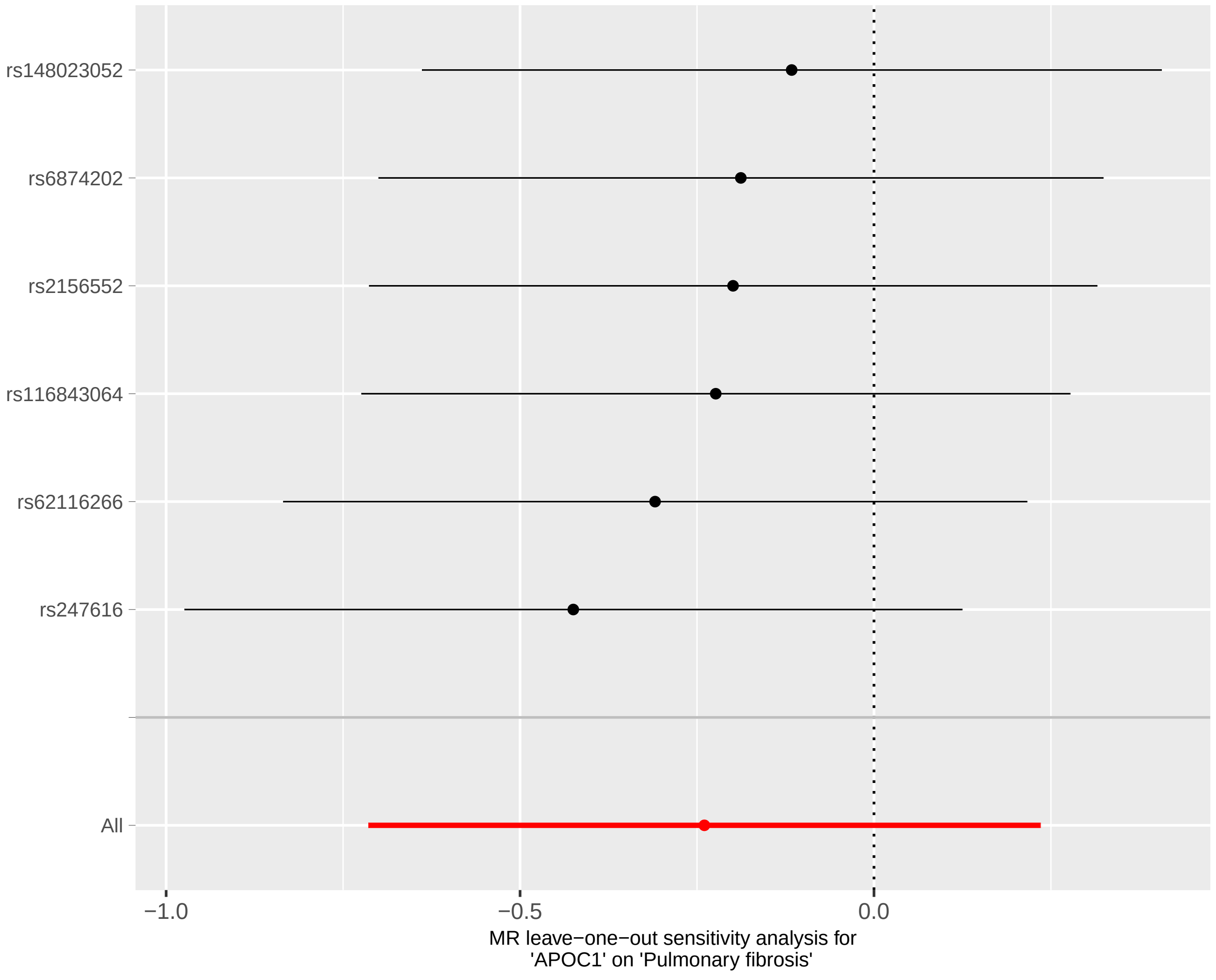
rs148023052
rs6874202
rs2156552
rs116843064
rs62116266
rs247616
All
−1.0
−0.5
0.0
MR leave−one−out sensitivity analysis for
'APOC1' on 'Pulmonary fibrosis'

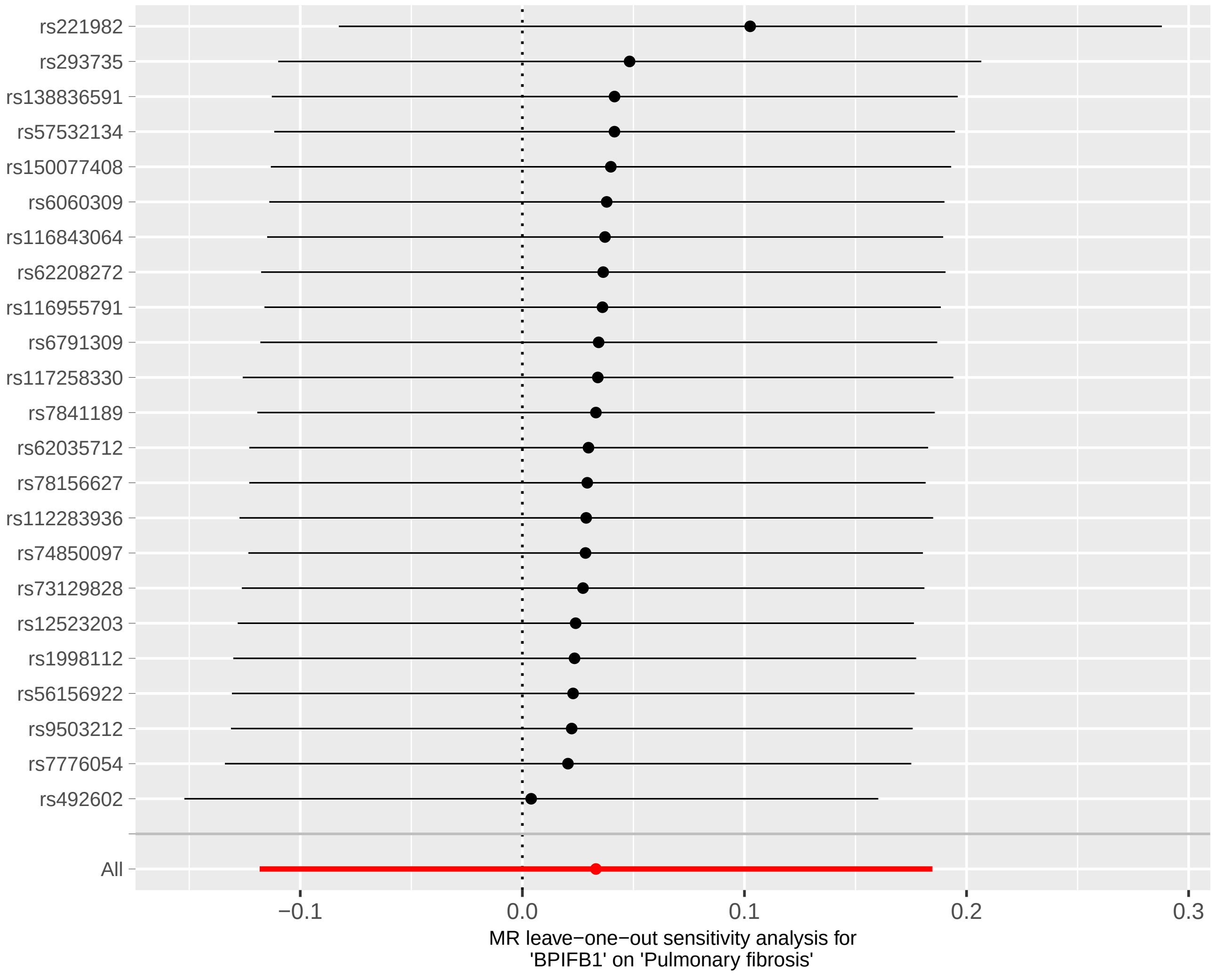

rs221982
rs293735
rs138836591
rs57532134
rs150077408
rs6060309
rs116843064
rs62208272
rs116955791
rs6791309
rs117258330
rs7841189
rs62035712
rs78156627
rs112283936
rs74850097
rs73129828
rs12523203
rs1998112
rs56156922
rs9503212
rs7776054
rs492602
All
−0.1
0.0
0.1
0.2
0.3
MR leave−one−out sensitivity analysis for
'BPIFB1' on 'Pulmonary fibrosis'

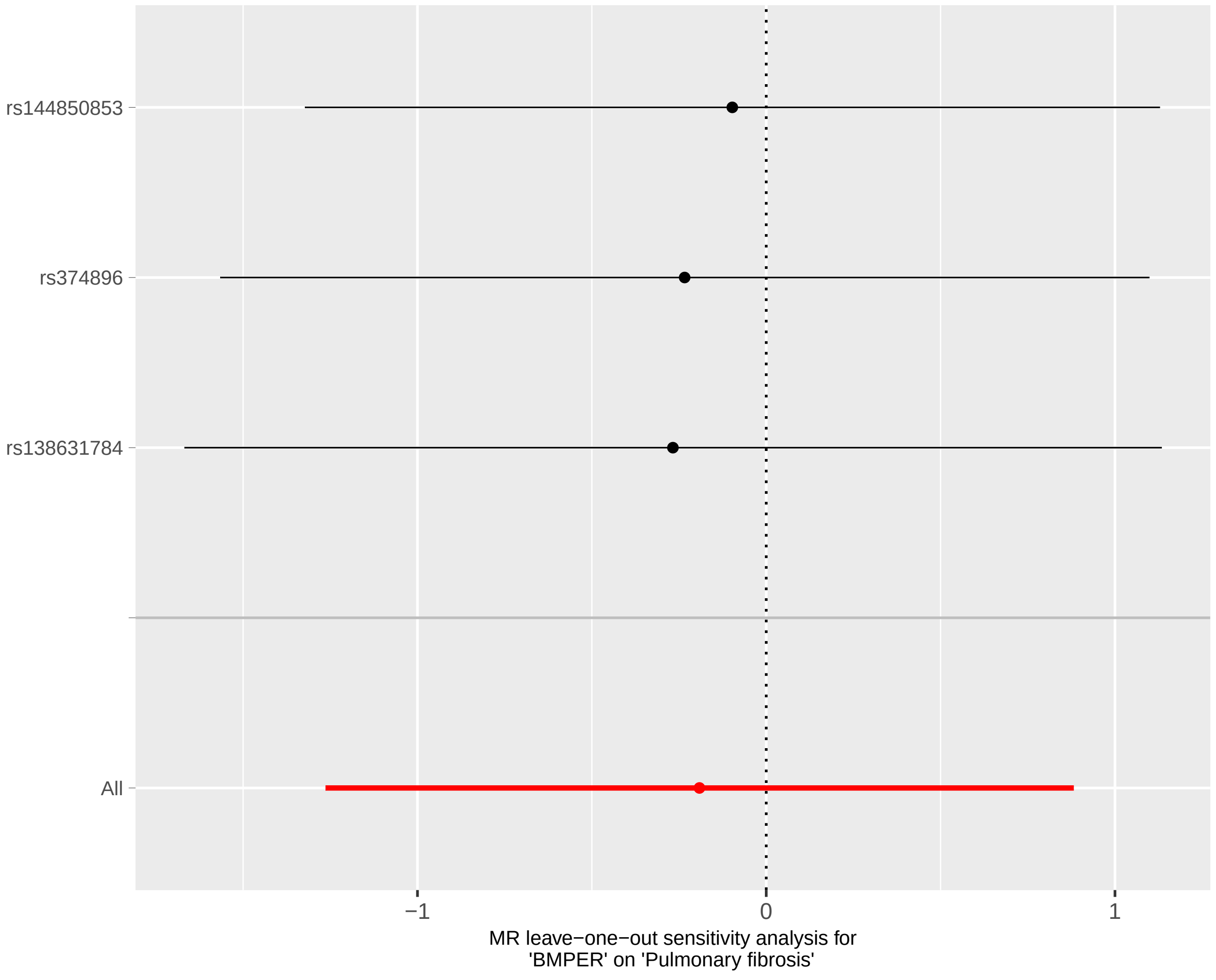

rs144850853
rs374896
rs138631784
All
−1
0
1
MR leave−one−out sensitivity analysis for
'BMPER' on 'Pulmonary fibrosis'

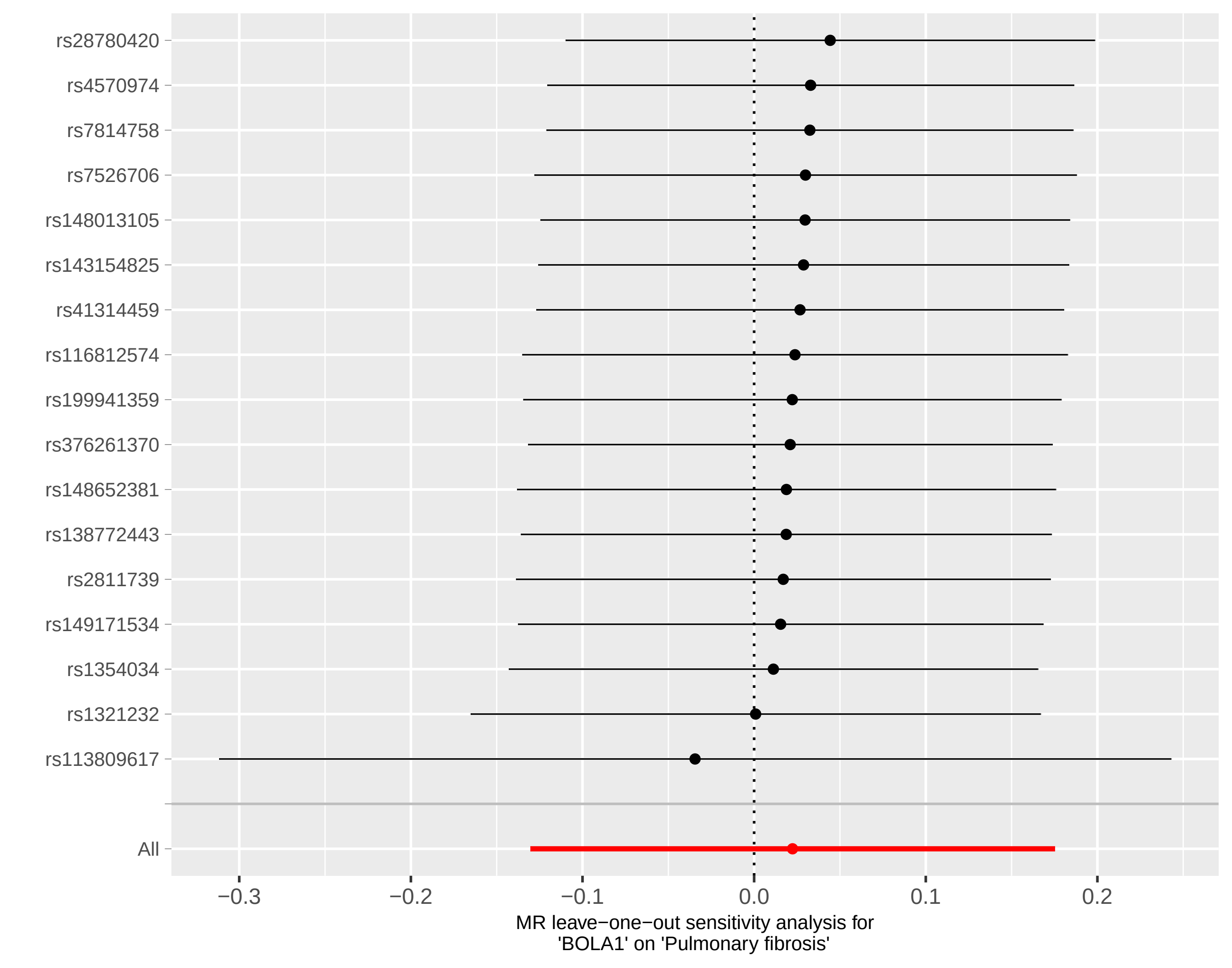
rs28780420
rs4570974
rs7814758
rs7526706
rs148013105
rs143154825
rs41314459
rs116812574
rs199941359
rs376261370
rs148652381
rs138772443
rs2811739
rs149171534
rs1354034
rs1321232
rs113809617
All
−0.3
−0.2
−0.1
0.0
0.1
0.2
MR leave−one−out sensitivity analysis for
'BOLA1' on 'Pulmonary fibrosis'

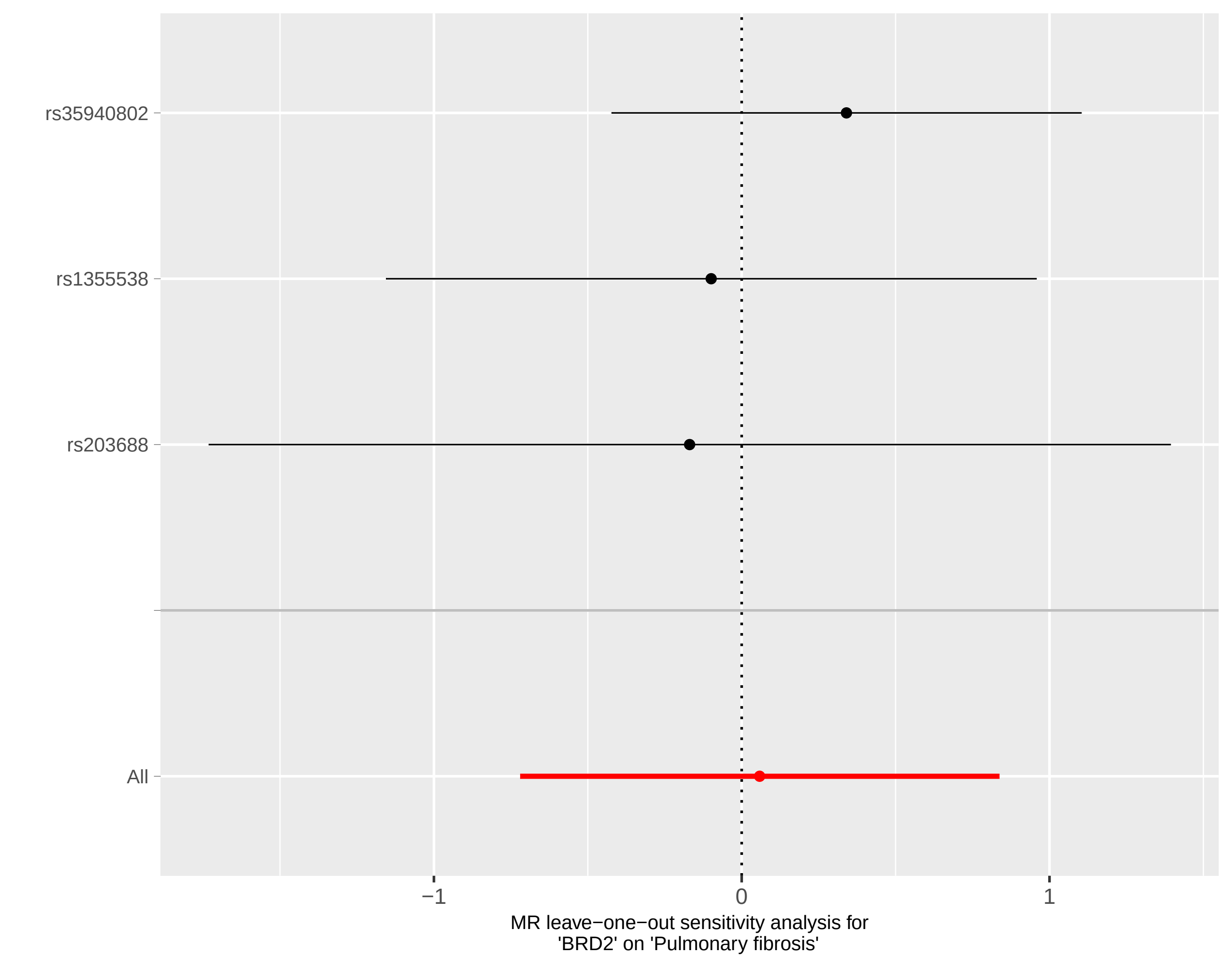
rs35940802
rs1355538
rs203688
All
−1
0
1
MR leave−one−out sensitivity analysis for
'BRD2' on 'Pulmonary fibrosis'

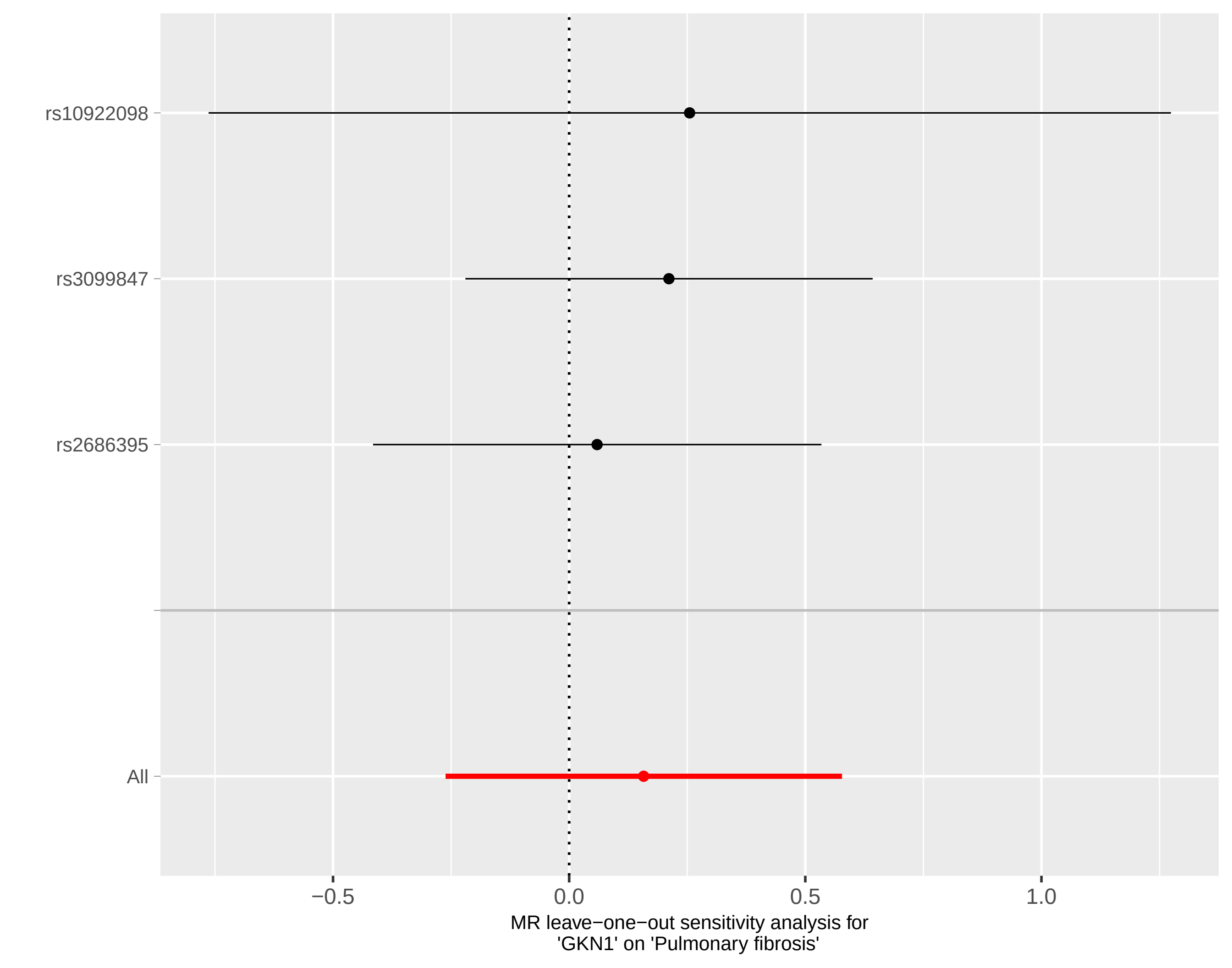

rs10922098
rs3099847
rs2686395
All
−0.5
0.0
0.5
1.0
MR leave−one−out sensitivity analysis for
'GKN1' on 'Pulmonary fibrosis'

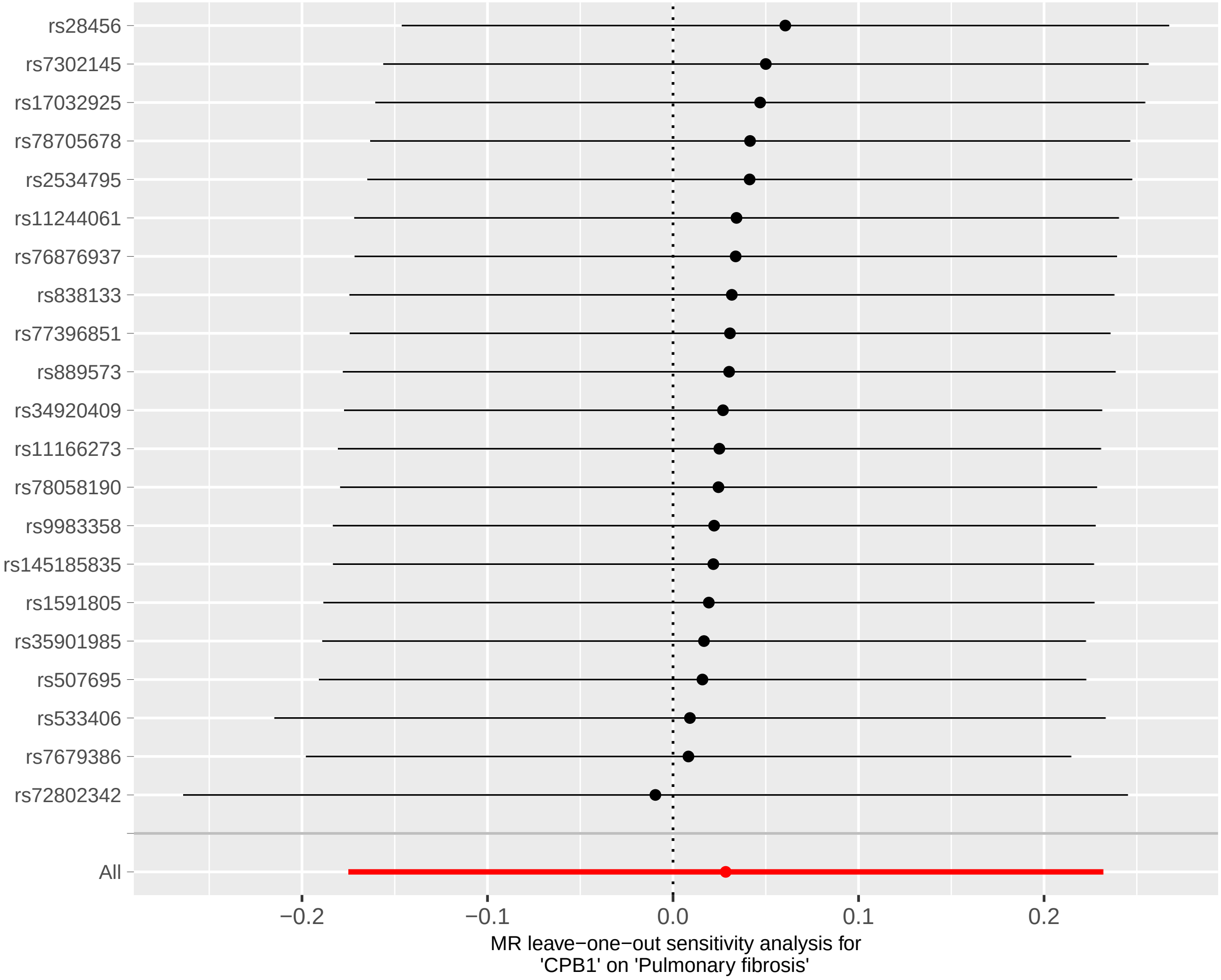
rs28456
rs7302145
rs17032925
rs78705678
rs2534795
rs11244061
rs76876937
rs838133
rs77396851
rs889573
rs34920409
rs11166273
rs78058190
rs9983358
rs145185835
rs1591805
rs35901985
rs507695
rs533406
rs7679386
rs72802342
All
−0.2
−0.1
0.0
0.1
0.2
MR leave−one−out sensitivity analysis for
'CPB1' on 'Pulmonary fibrosis'

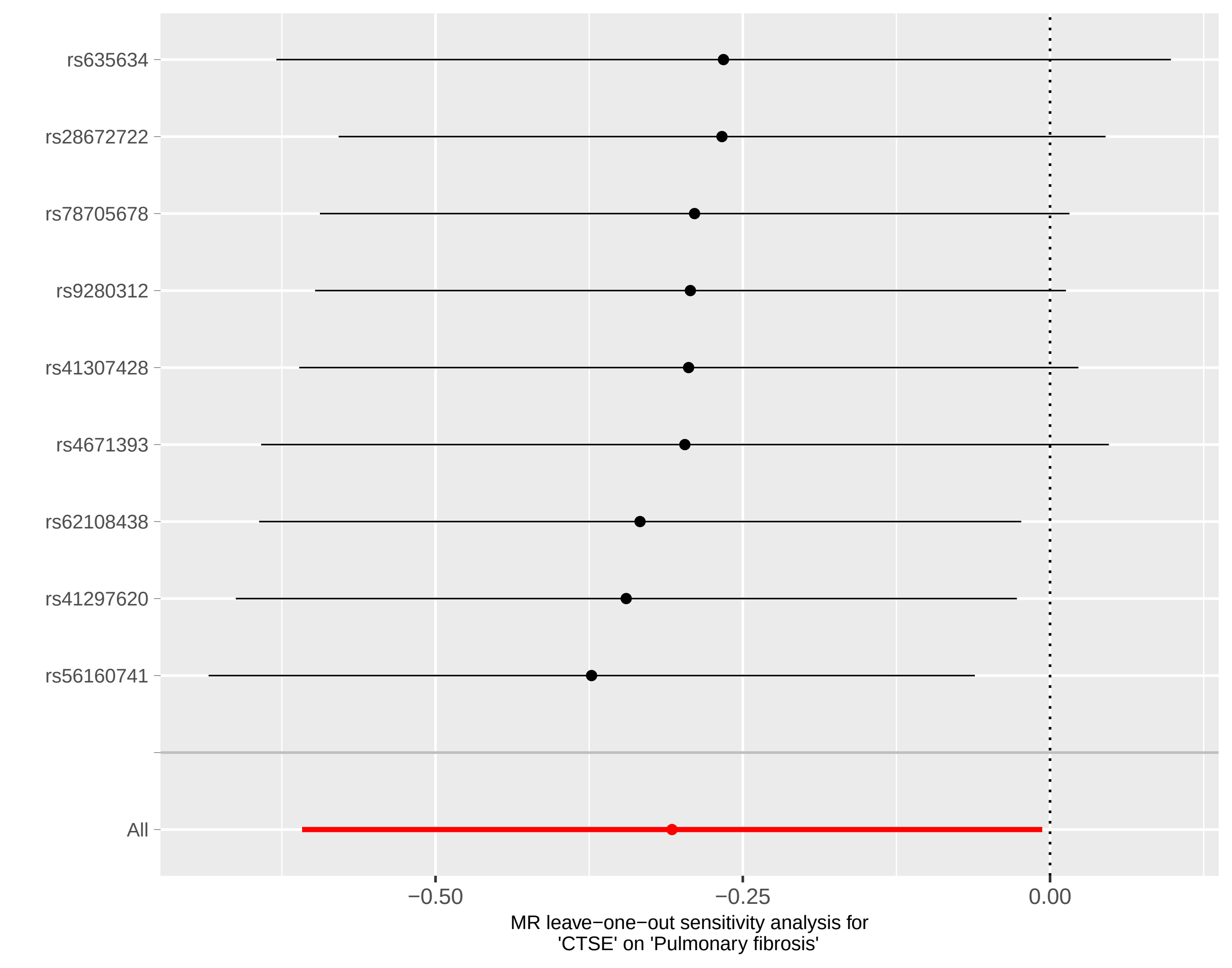
rs635634
rs28672722
rs78705678
rs9280312
rs41307428
rs4671393
rs62108438
rs41297620
rs56160741
All
−0.50
−0.25
0.00
MR leave−one−out sensitivity analysis for
'CTSE' on 'Pulmonary fibrosis'

Insufficient number of SNPs

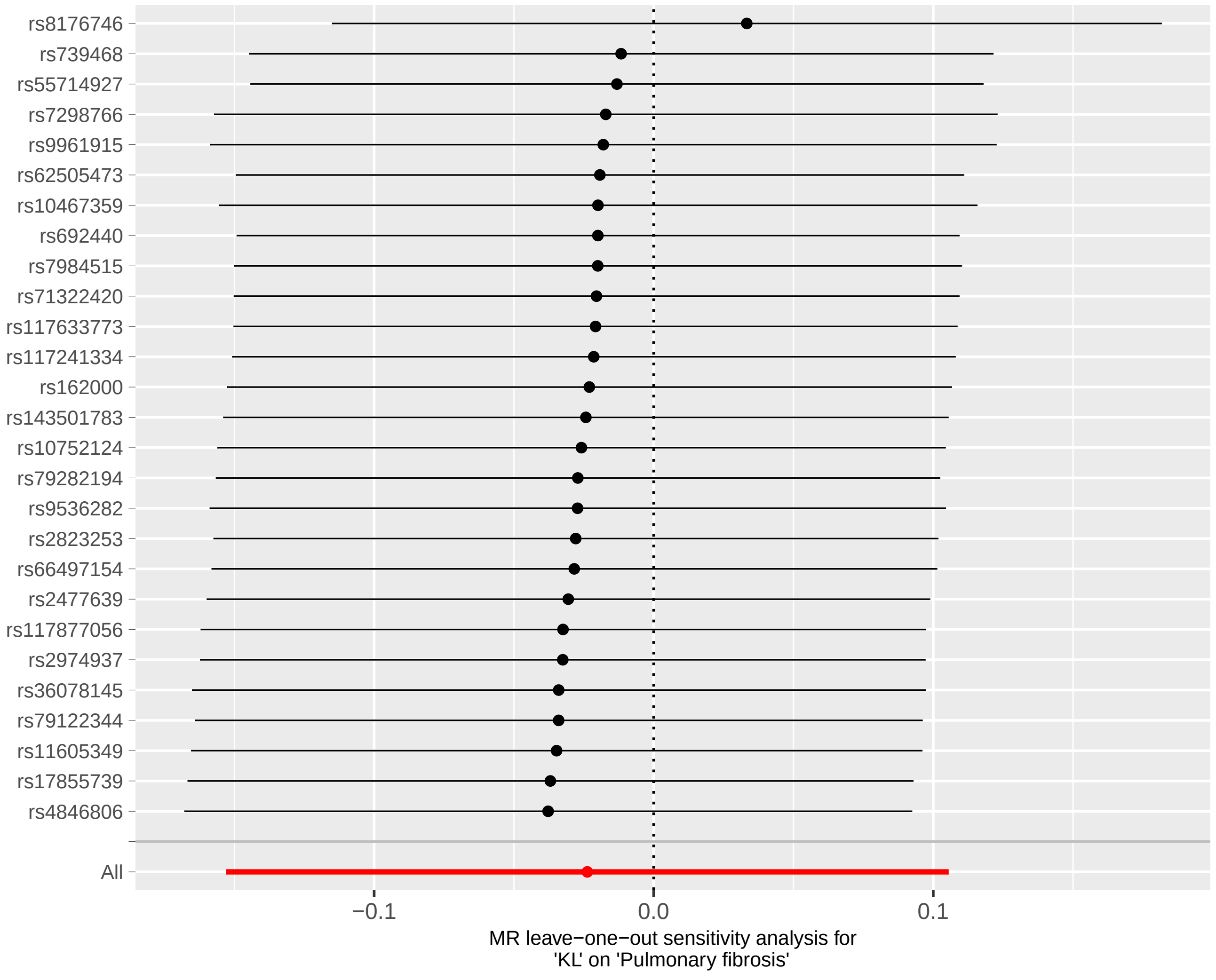
rs8176746
rs739468
rs55714927
rs7298766
rs9961915
rs62505473
rs10467359
rs692440
rs7984515
rs71322420
rs117633773
rs117241334
rs162000
rs143501783
rs10752124
rs79282194
rs9536282
rs2823253
rs66497154
rs2477639
rs117877056
rs2974937
rs36078145
rs79122344
rs11605349
rs17855739
rs4846806
All
−0.1
0.0
0.1
MR leave−one−out sensitivity analysis for
'KL' on 'Pulmonary fibrosis'

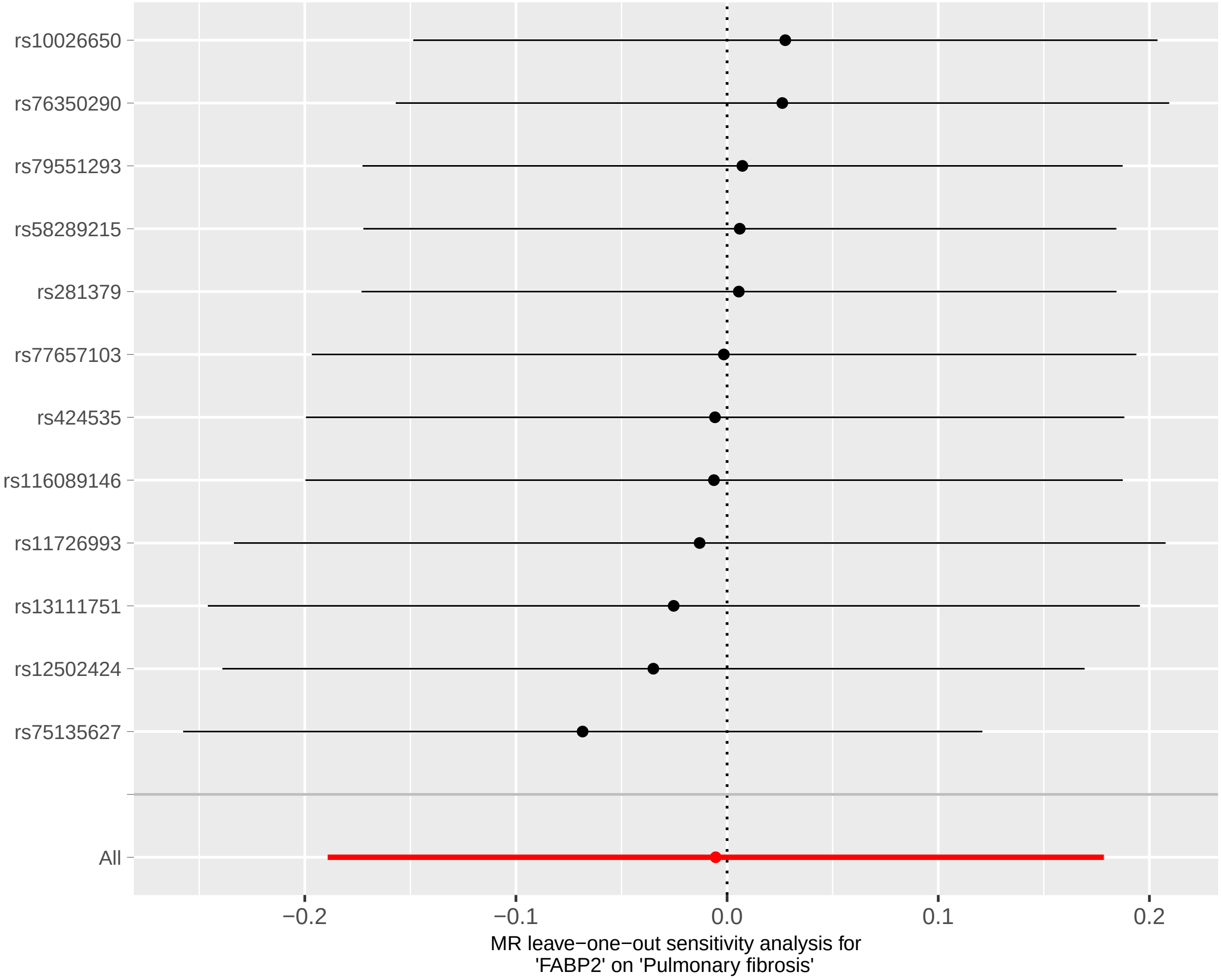

rs10026650
rs76350290
rs79551293
rs58289215
rs281379
rs77657103
rs424535
rs116089146
rs11726993
rs13111751
rs12502424
rs75135627
All
−0.2
−0.1
0.0
0.1
0.2
MR leave−one−out sensitivity analysis for
'FABP2' on 'Pulmonary fibrosis'

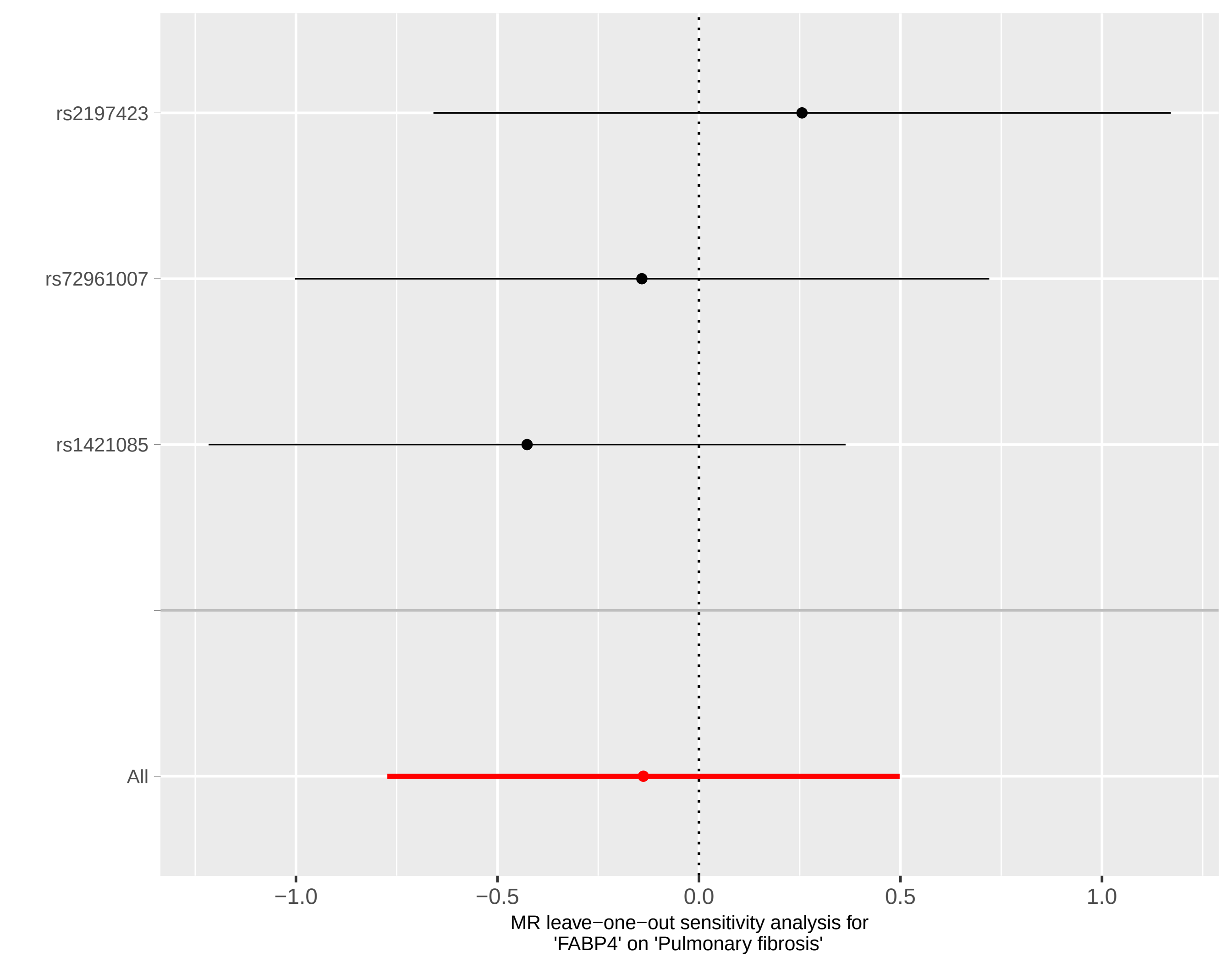
rs2197423
rs72961007
rs1421085
All
−1.0
−0.5
0.0
0.5
1.0
MR leave−one−out sensitivity analysis for
'FABP4' on 'Pulmonary fibrosis'

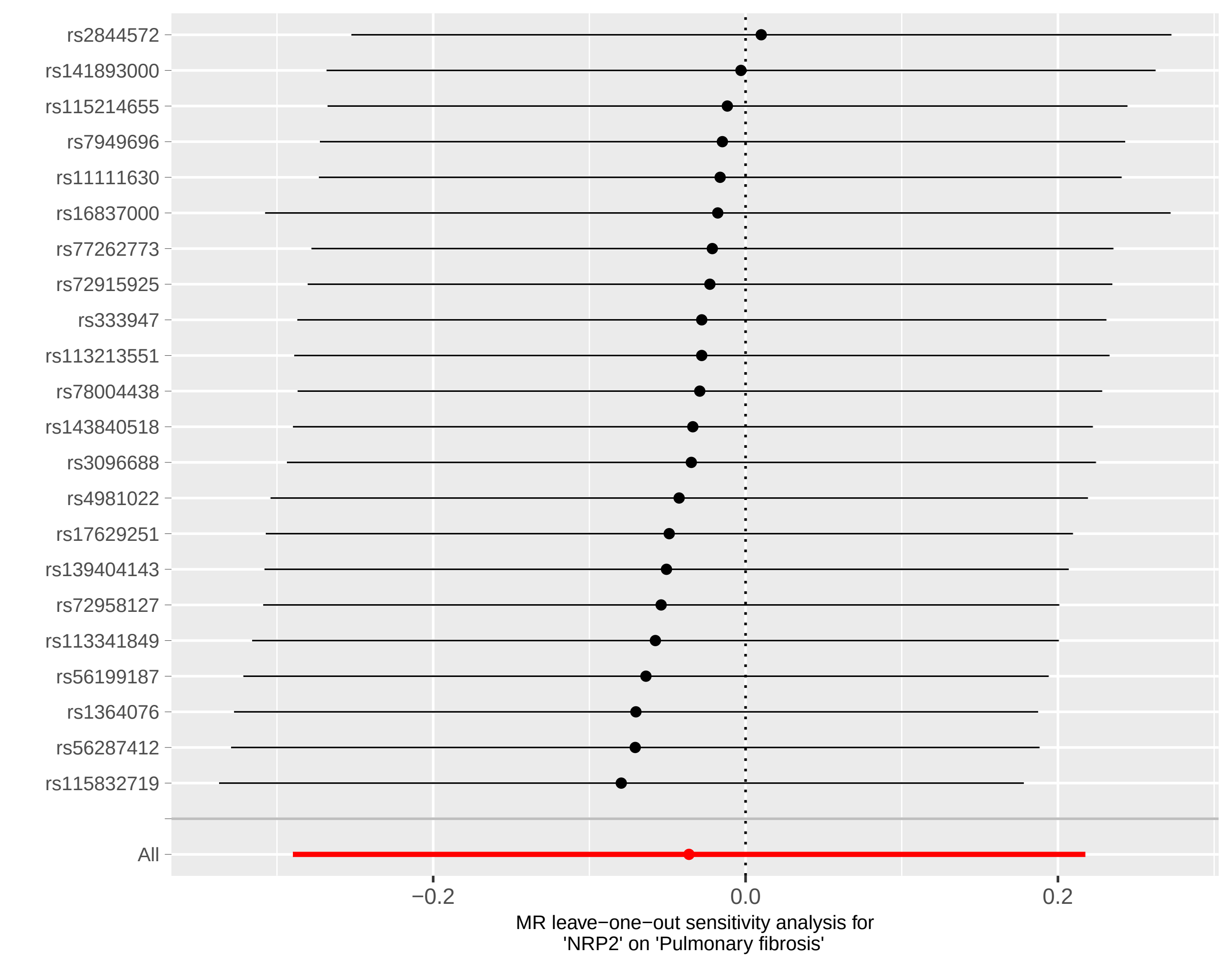
rs2844572
rs141893000
rs115214655
rs7949696
rs11111630
rs16837000
rs77262773
rs72915925
rs333947
rs113213551
rs78004438
rs143840518
rs3096688
rs4981022
rs17629251
rs139404143
rs72958127
rs113341849
rs56199187
rs1364076
rs56287412
rs115832719
All
−0.2
0.0
0.2
MR leave−one−out sensitivity analysis for
'NRP2' on 'Pulmonary fibrosis'

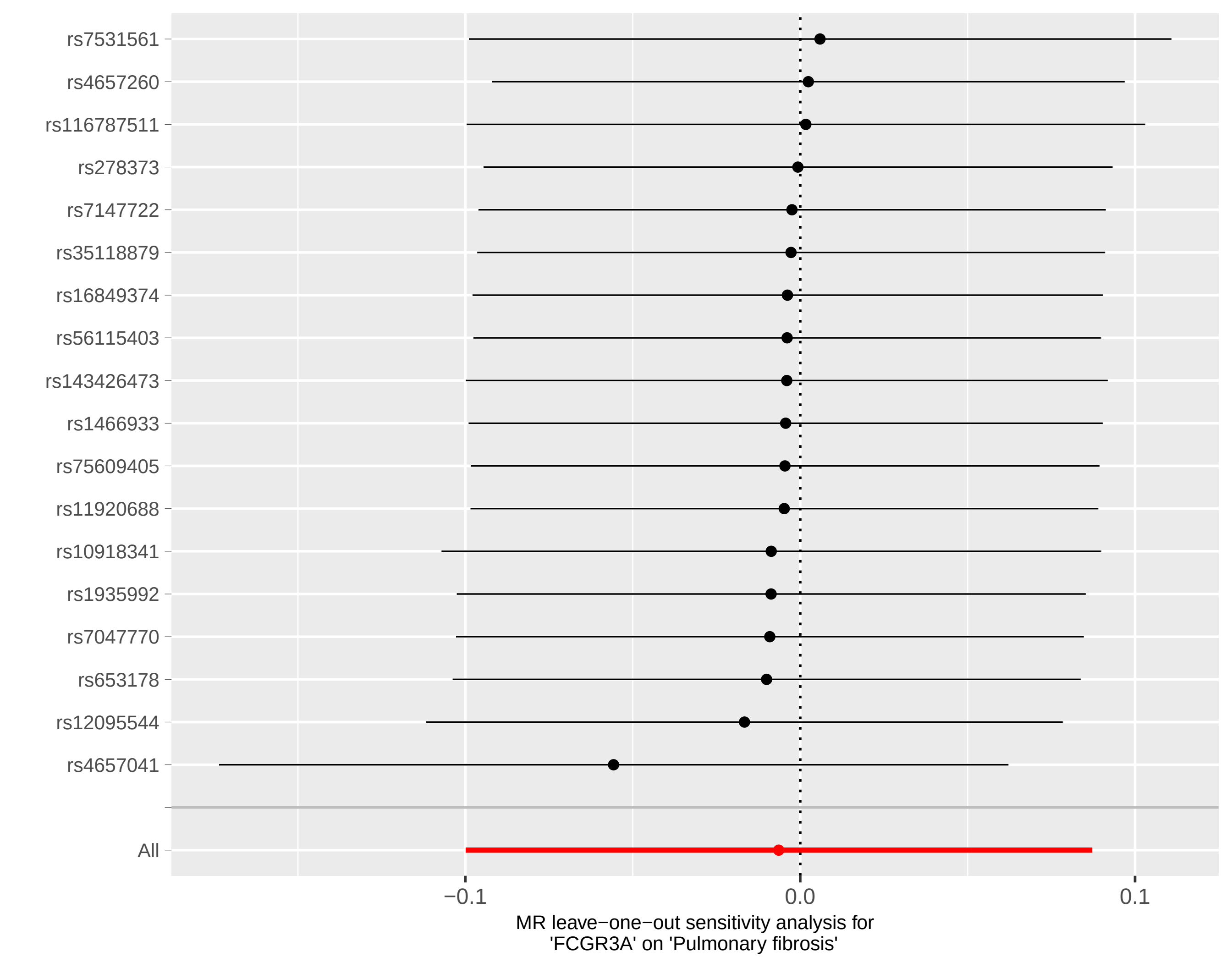
rs7531561
rs4657260
rs116787511
rs278373
rs7147722
rs35118879
rs16849374
rs56115403
rs143426473
rs1466933
rs75609405
rs11920688
rs10918341
rs1935992
rs7047770
rs653178
rs12095544
rs4657041
All
−0.1
0.0
0.1
MR leave−one−out sensitivity analysis for
'FCGR3A' on 'Pulmonary fibrosis'

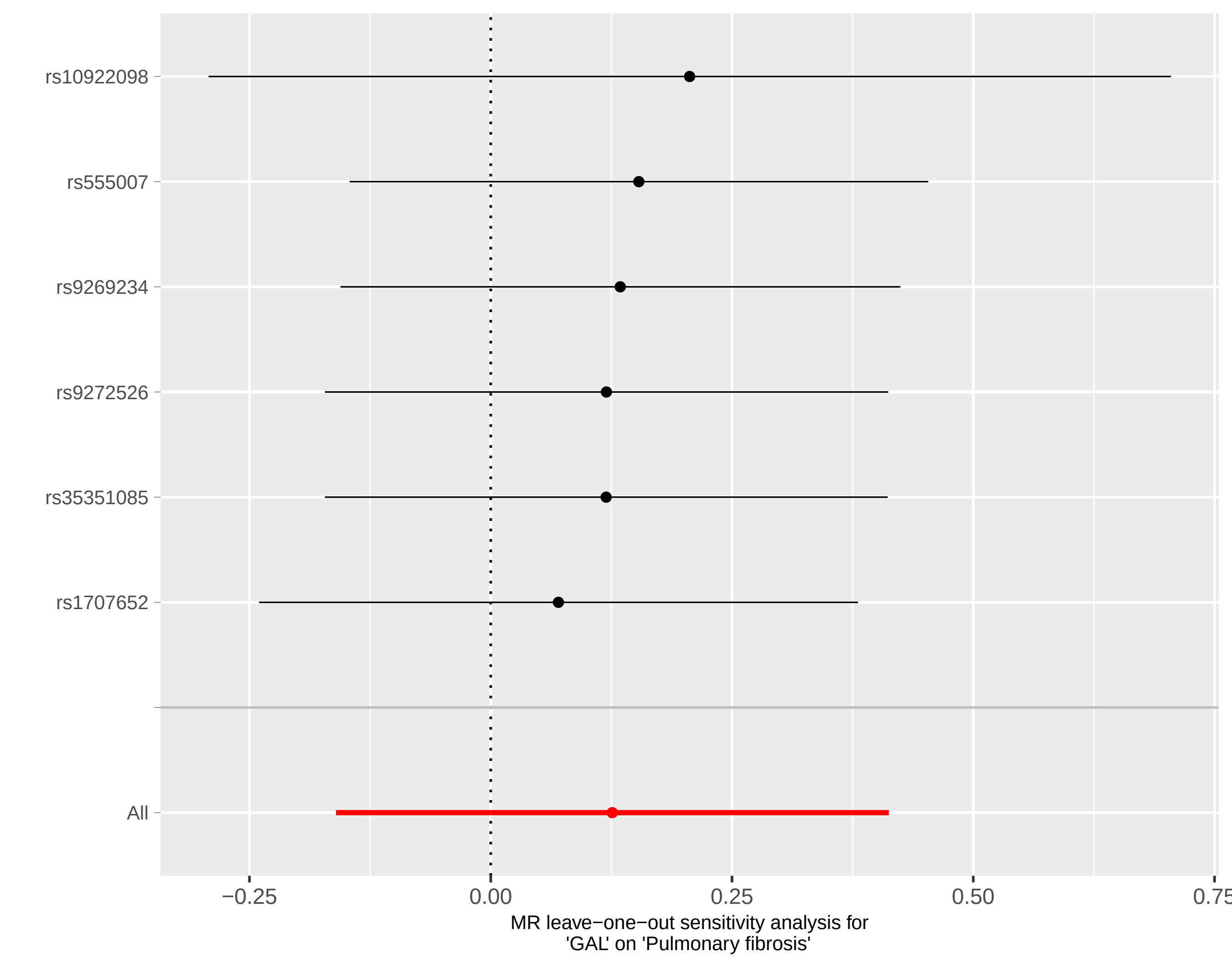
rs10922098
rs555007
rs9269234
rs9272526
rs35351085
rs1707652
All
−0.25
0.00
0.25
0.50
0.75
MR leave−one−out sensitivity analysis for
'GAL' on 'Pulmonary fibrosis'

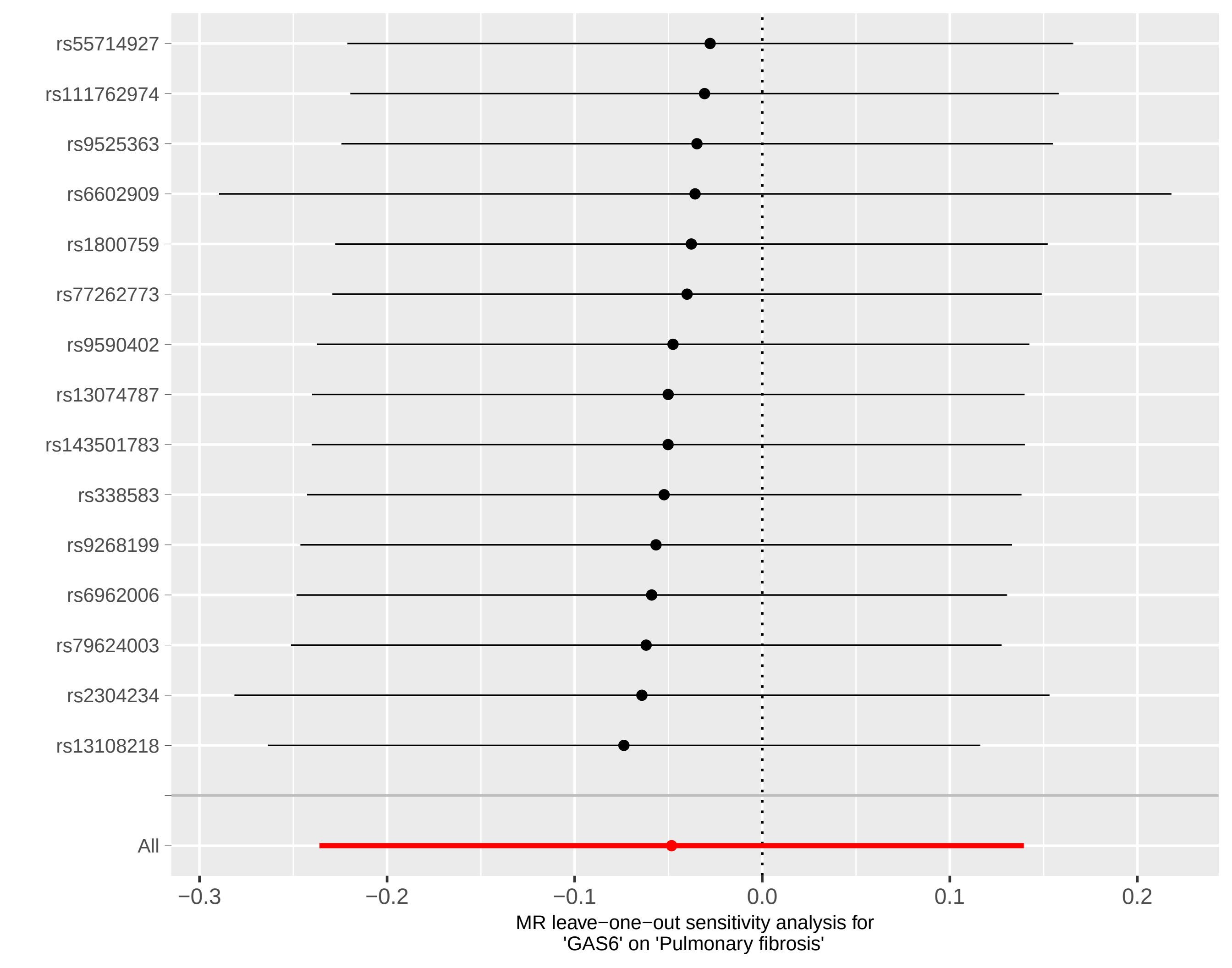
rs55714927
rs111762974
rs9525363
rs6602909
rs1800759
rs77262773
rs9590402
rs13074787
rs143501783
rs338583
rs9268199
rs6962006
rs79624003
rs2304234
rs13108218
All
−0.3
−0.2
−0.1
0.0
0.1
0.2
MR leave−one−out sensitivity analysis for
'GAS6' on 'Pulmonary fibrosis'

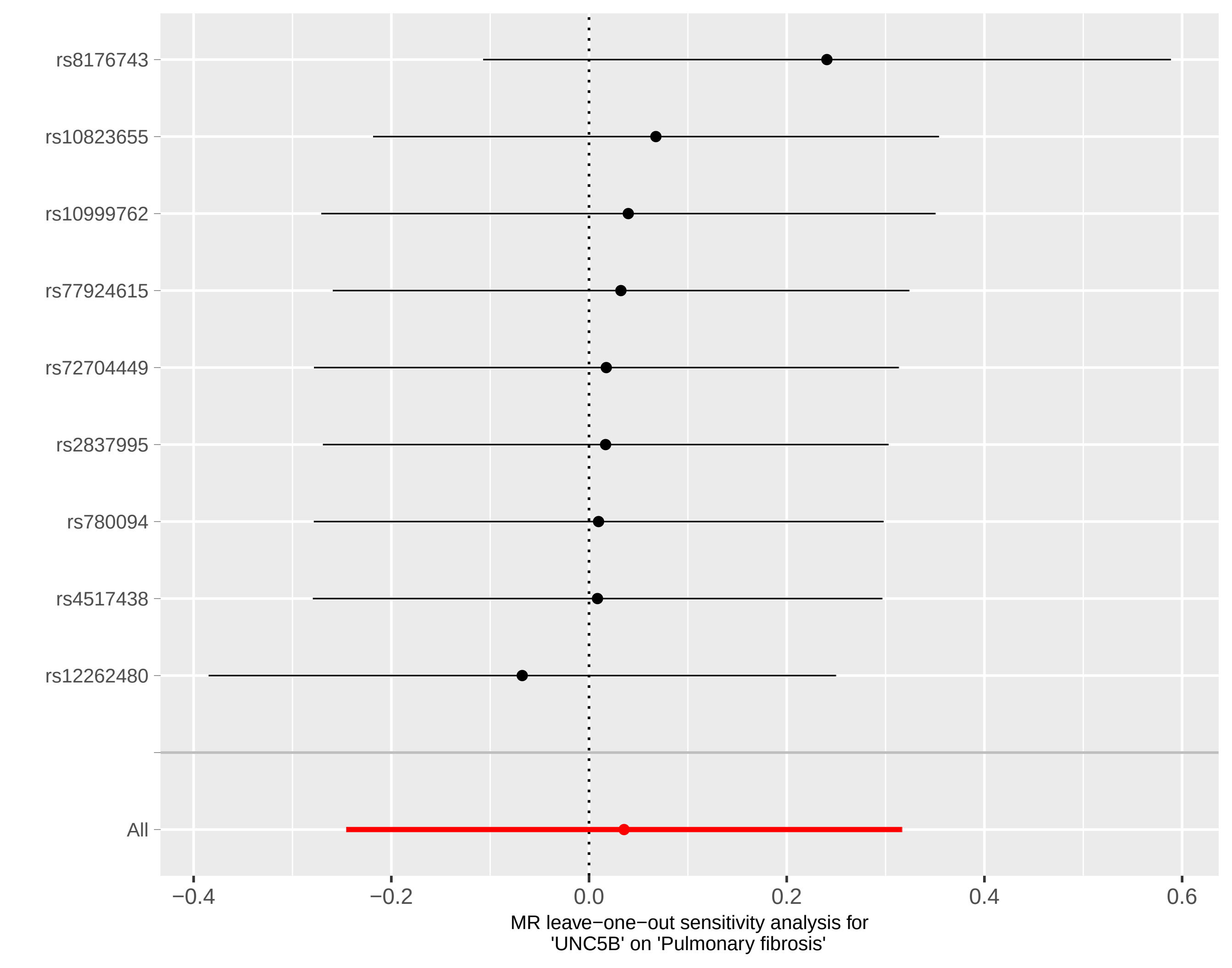
rs8176743
rs10823655
rs10999762
rs77924615
rs72704449
rs2837995
rs780094
rs4517438
rs12262480
All
−0.4
−0.2
0.0
0.2
0.4
0.6
MR leave−one−out sensitivity analysis for
'UNC5B' on 'Pulmonary fibrosis'

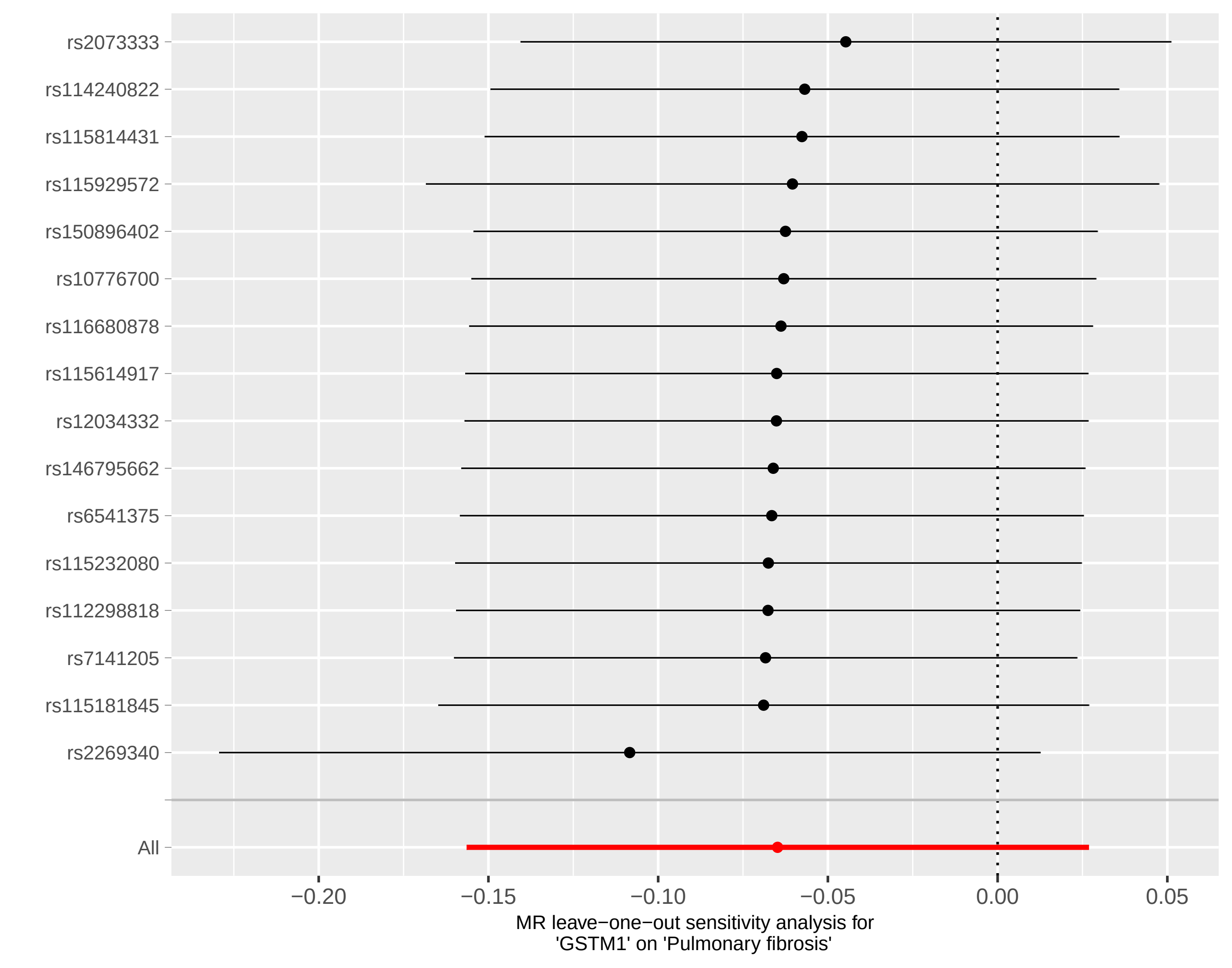
rs2073333
rs114240822
rs115814431
rs115929572
rs150896402
rs10776700
rs116680878
rs115614917
rs12034332
rs146795662
rs6541375
rs115232080
rs112298818
rs7141205
rs115181845
rs2269340
All
−0.20
−0.15
−0.10
−0.05
0.00
0.05
MR leave−one−out sensitivity analysis for
'GSTM1' on 'Pulmonary fibrosis'

Insufficient number of SNPs

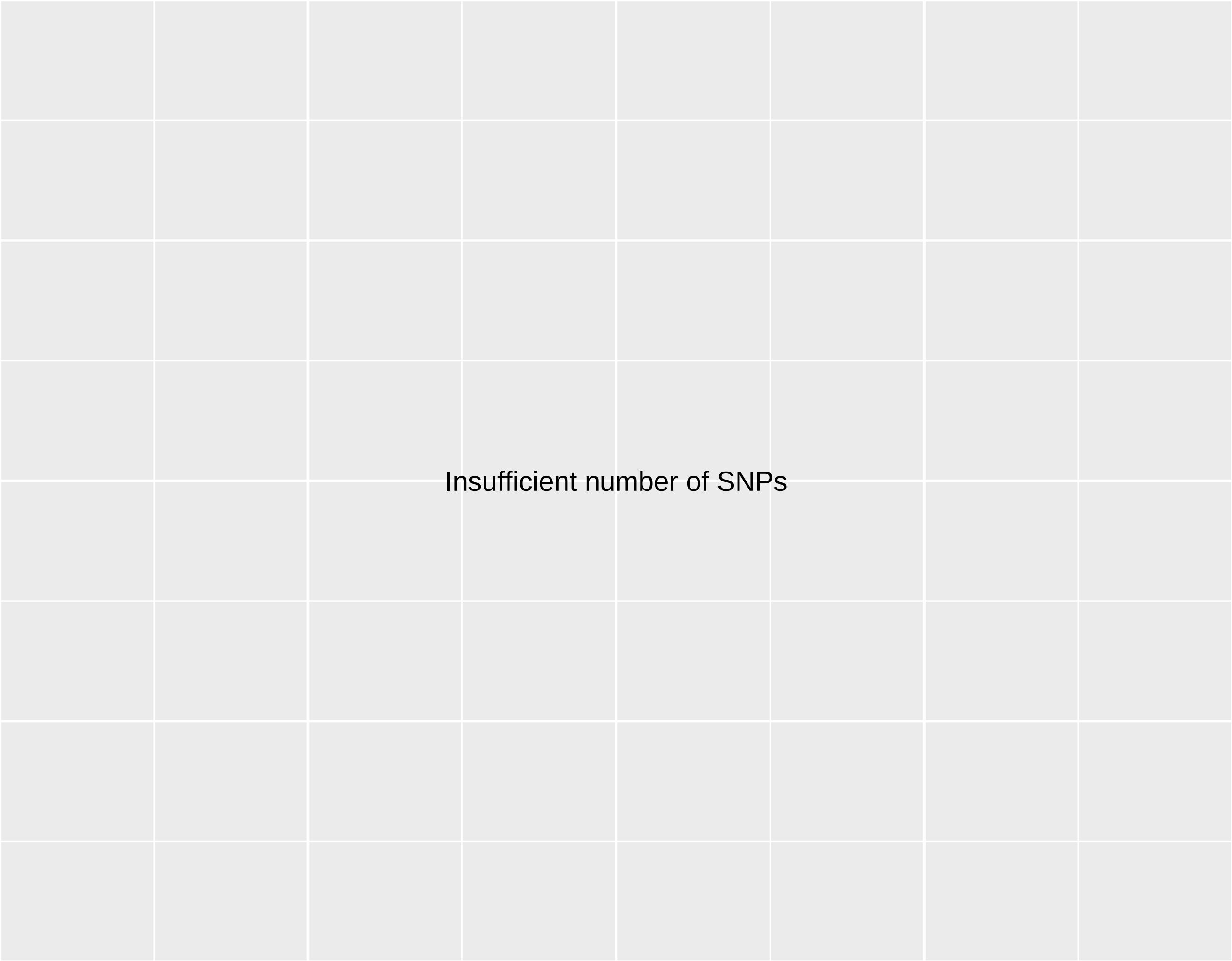
Insufficient number of SNPs

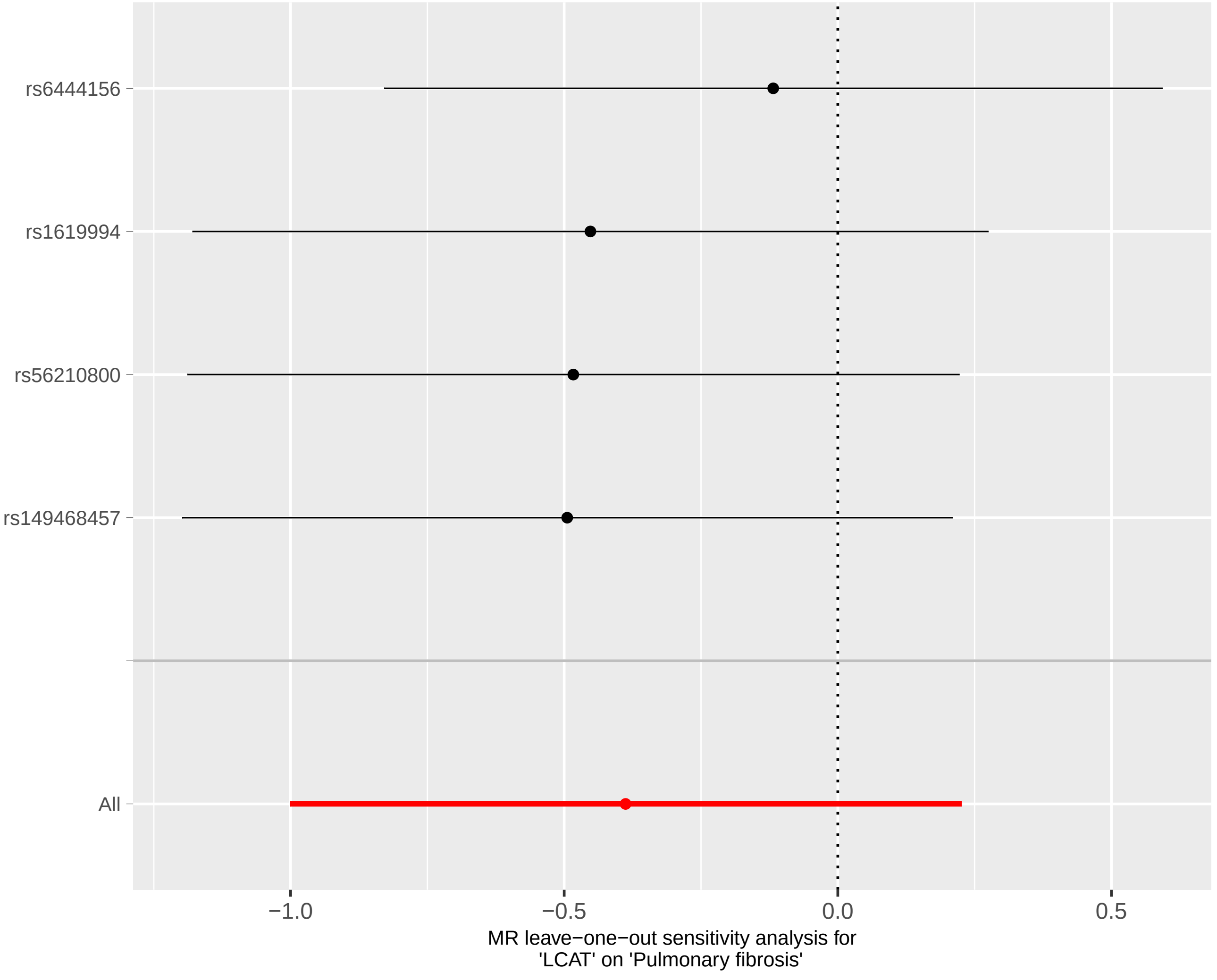

rs6444156
rs1619994
rs56210800
rs149468457
All
−1.0
−0.5
0.0
0.5
MR leave−one−out sensitivity analysis for
'LCAT' on 'Pulmonary fibrosis'

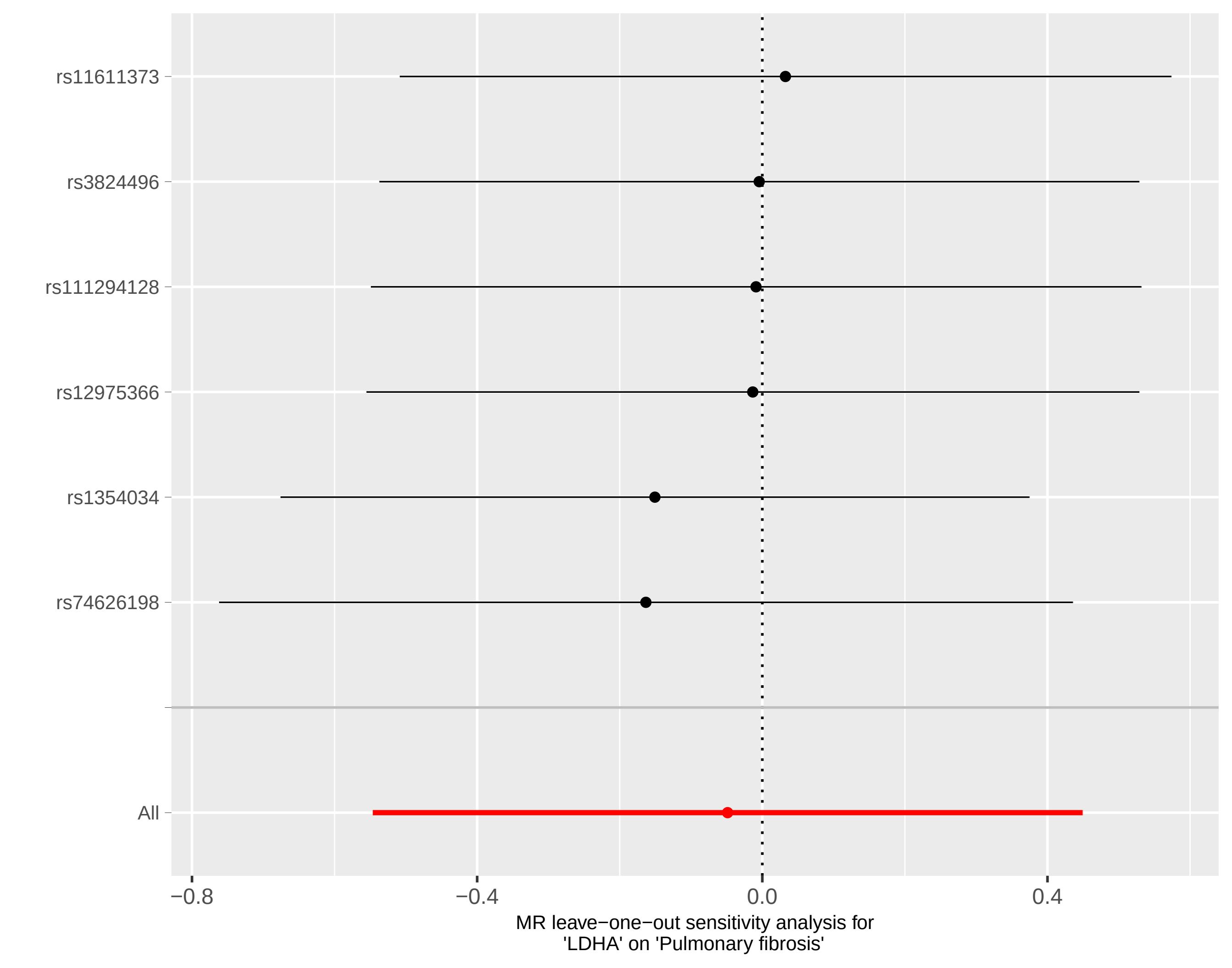
rs11611373
rs3824496
rs111294128
rs12975366
rs1354034
rs74626198
All
−0.8
−0.4
0.0
0.4
MR leave−one−out sensitivity analysis for
'LDHA' on 'Pulmonary fibrosis'

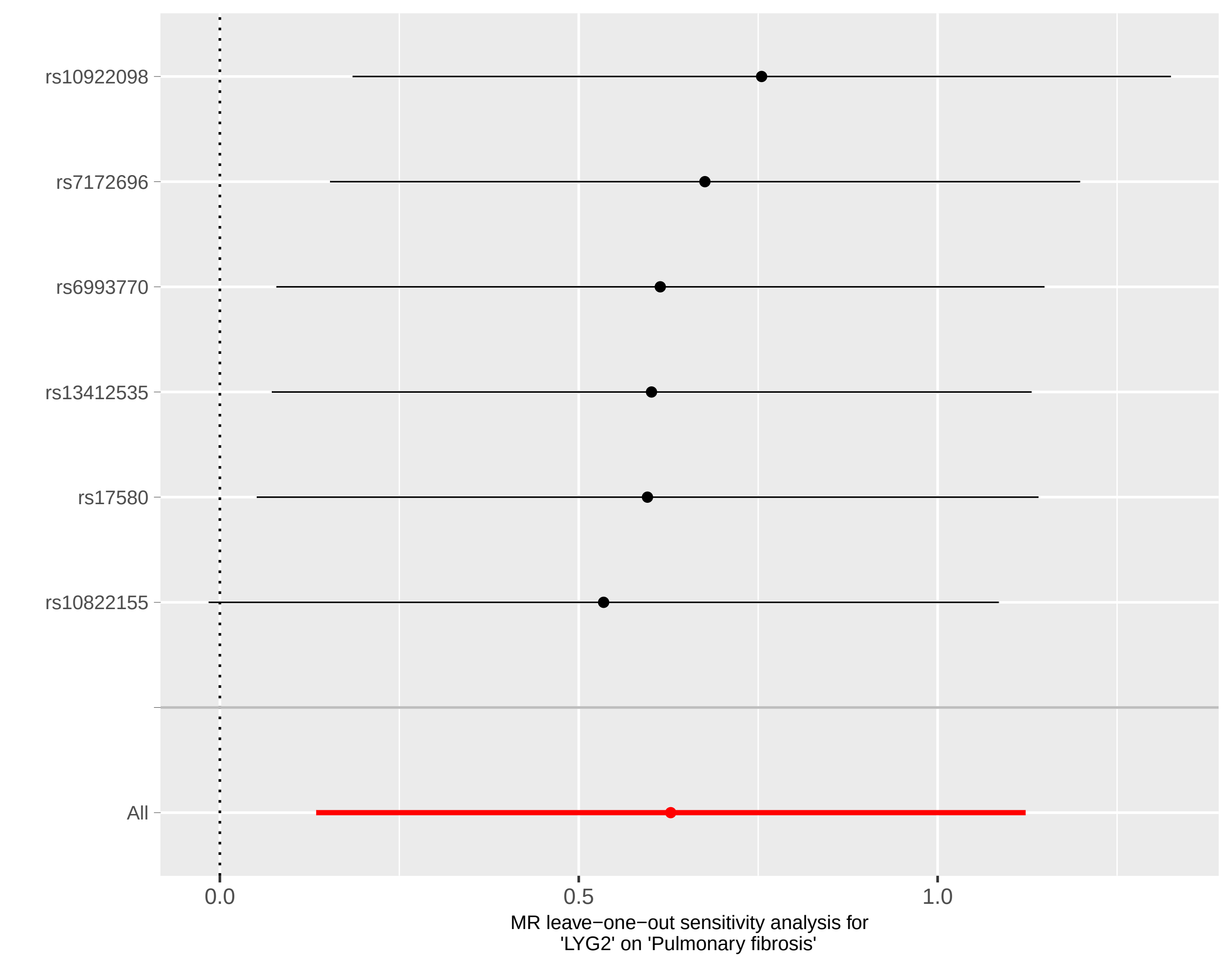
rs10922098
rs7172696
rs6993770
rs13412535
rs17580
rs10822155
All
0.0
0.5
1.0
MR leave−one−out sensitivity analysis for
'LYG2' on 'Pulmonary fibrosis'

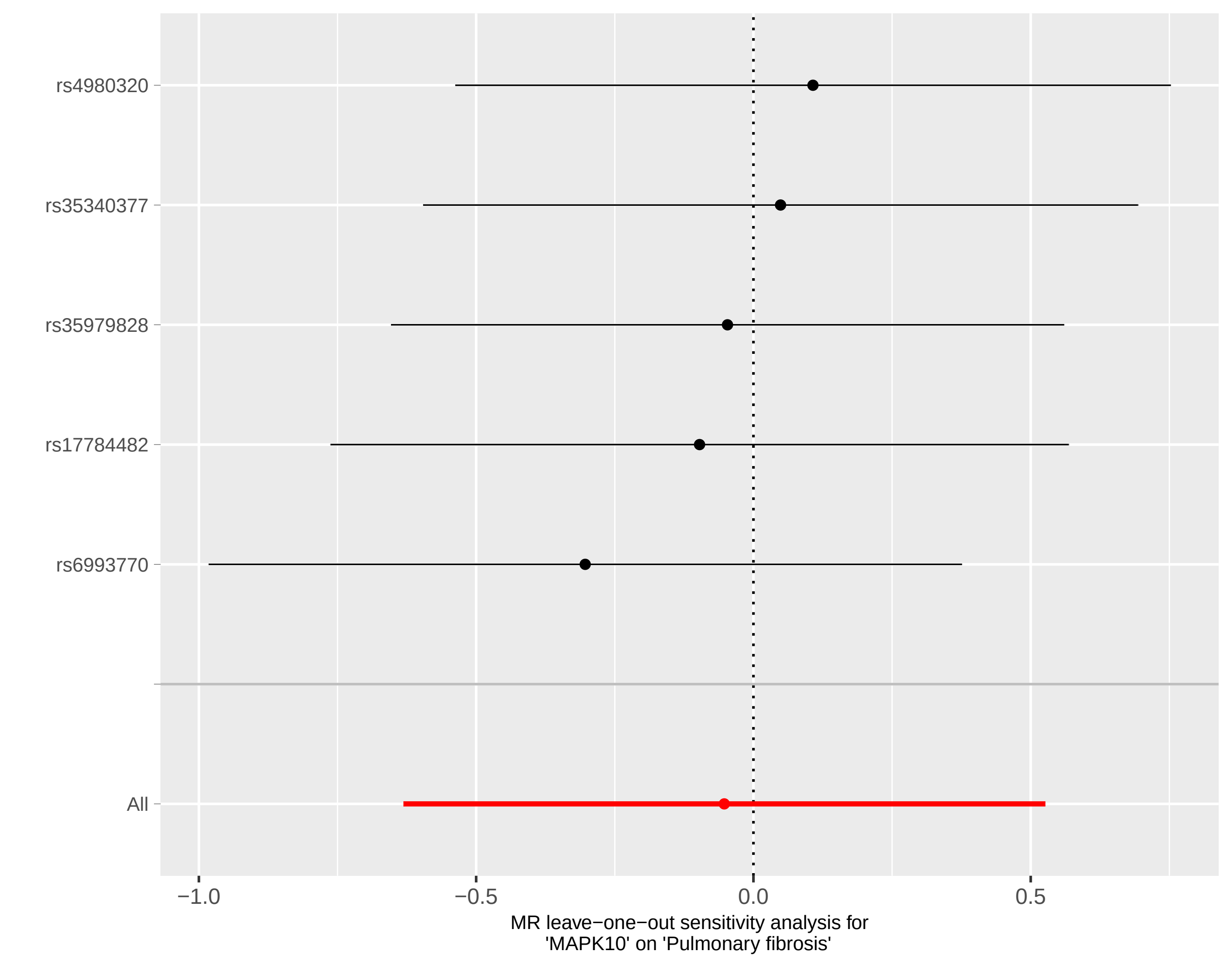
rs4980320
rs35340377
rs35979828
rs17784482
rs6993770
All
−1.0
−0.5
0.0
0.5
MR leave−one−out sensitivity analysis for
'MAPK10' on 'Pulmonary fibrosis'

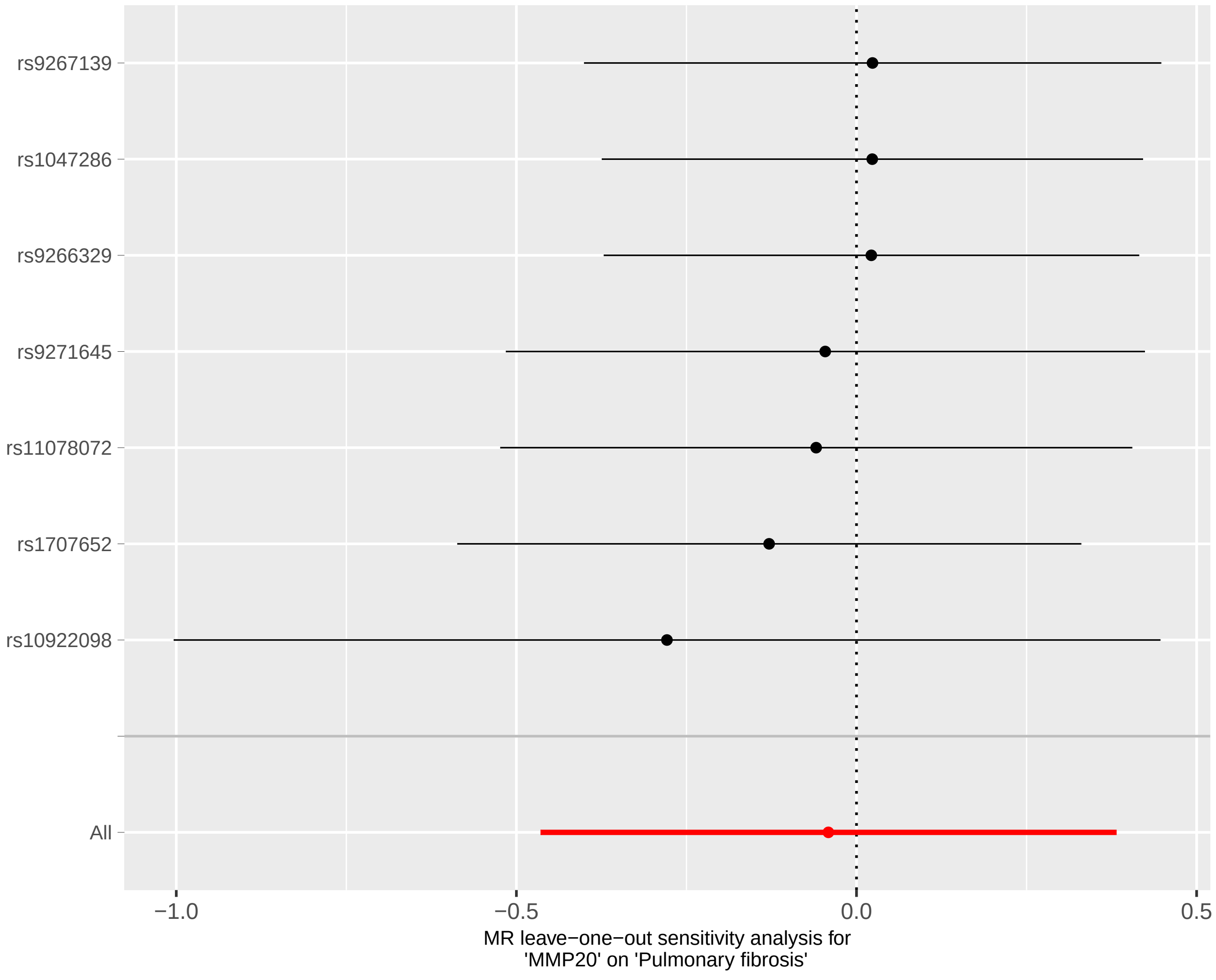
rs9267139
rs1047286
rs9266329
rs9271645
rs11078072
rs1707652
rs10922098
All
−1.0
−0.5
0.0
0.5
MR leave−one−out sensitivity analysis for
'MMP20' on 'Pulmonary fibrosis'

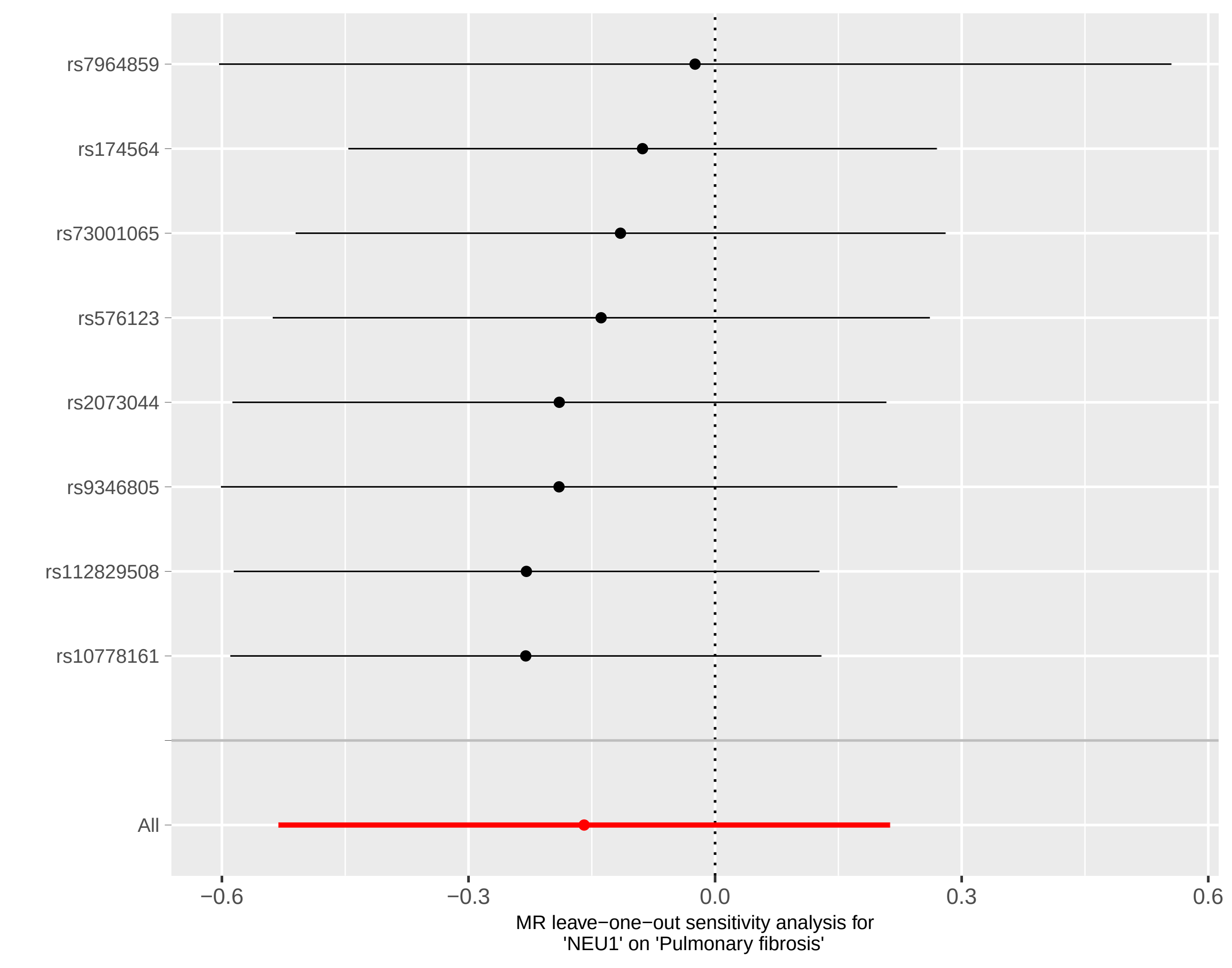
rs7964859
rs174564
rs73001065
rs576123
rs2073044
rs9346805
rs112829508
rs10778161
All
−0.6
−0.3
0.0
0.3
0.6
MR leave−one−out sensitivity analysis for
'NEU1' on 'Pulmonary fibrosis'

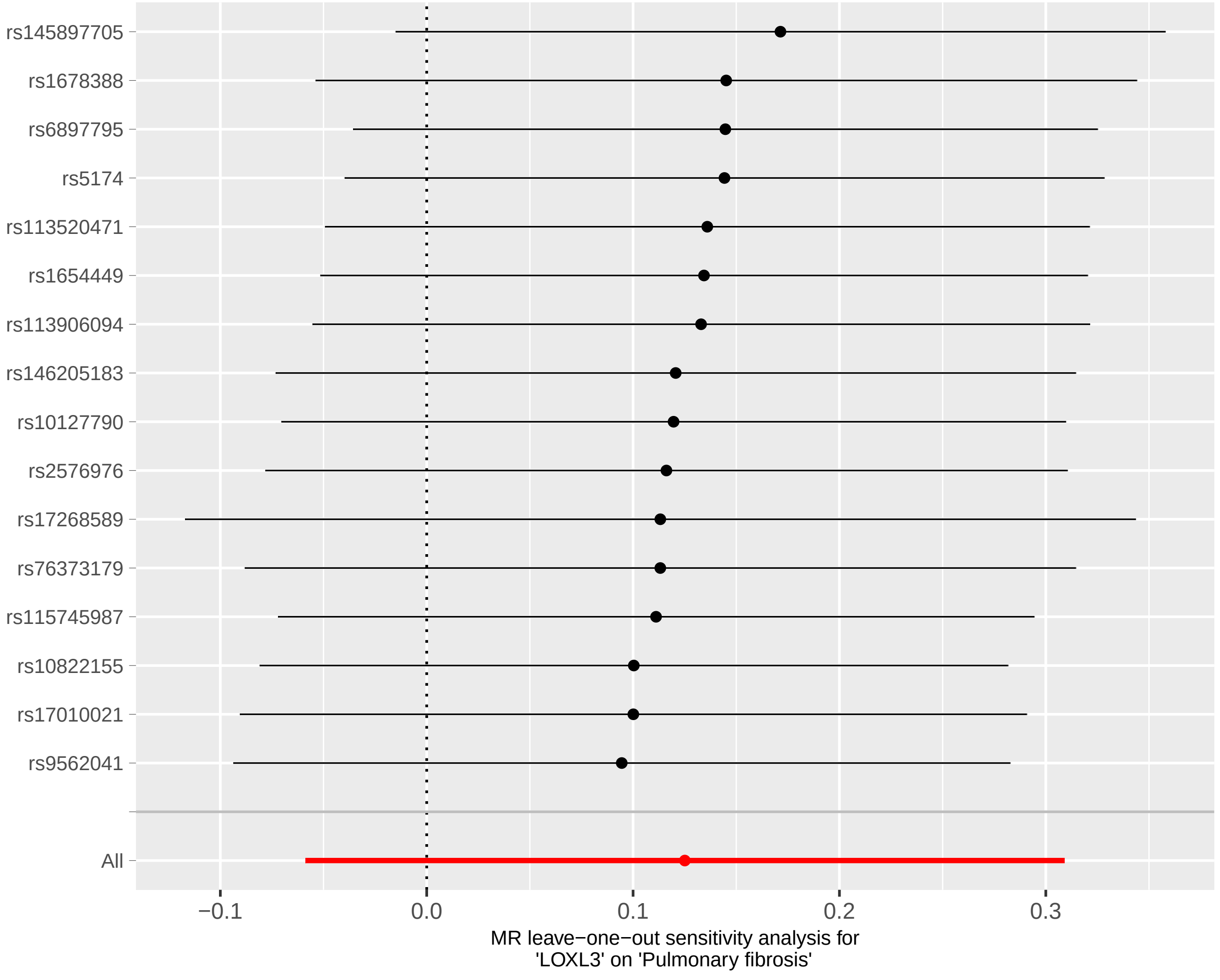
rs145897705
rs1678388
rs6897795
rs5174
rs113520471
rs1654449
rs113906094
rs146205183
rs10127790
rs2576976
rs17268589
rs76373179
rs115745987
rs10822155
rs17010021
rs9562041
All
−0.1
0.0
0.1
0.2
0.3
MR leave−one−out sensitivity analysis for
'LOXL3' on 'Pulmonary fibrosis'

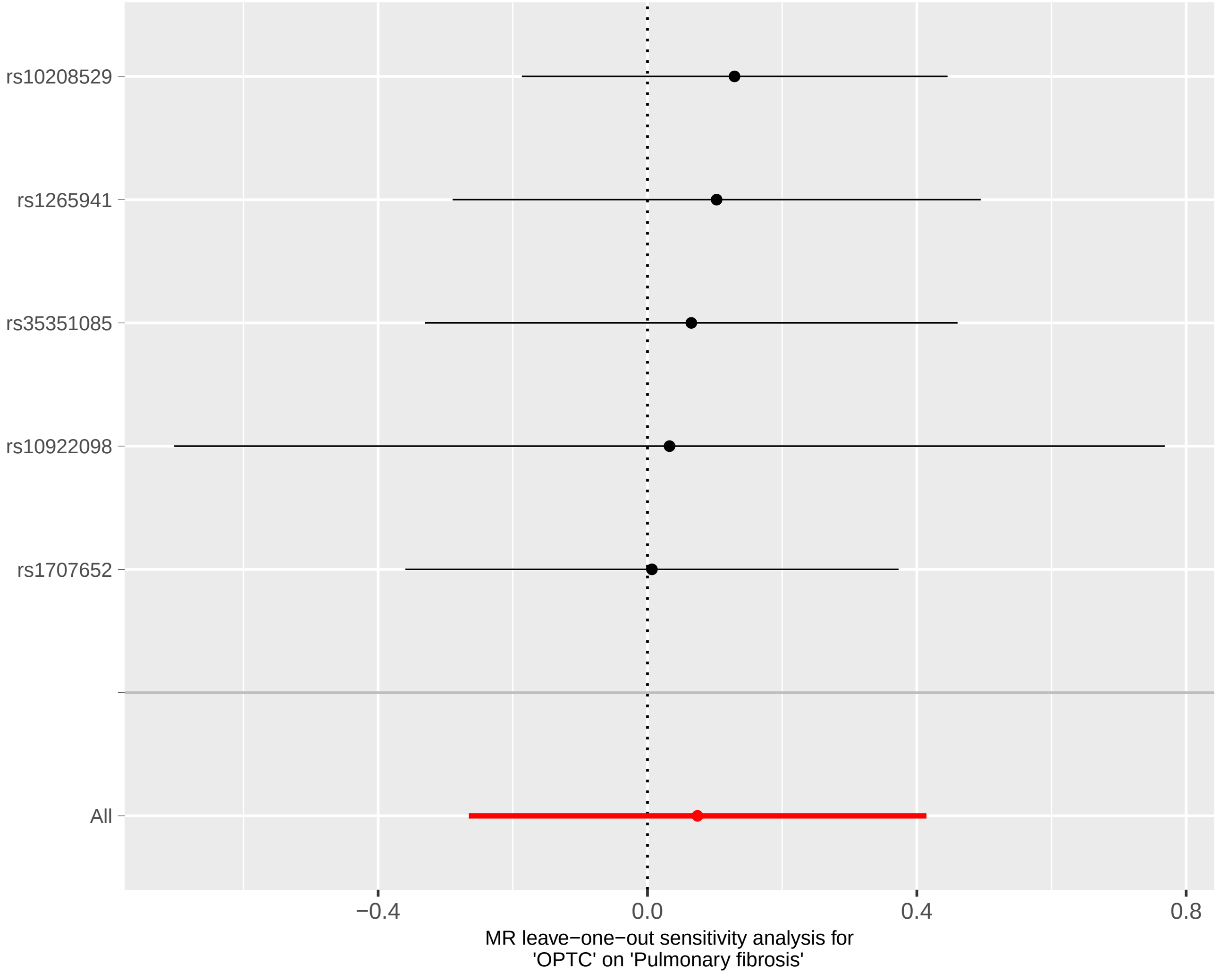
rs10208529
rs1265941
rs35351085
rs10922098
rs1707652
All
−0.4
0.0
0.4
0.8
MR leave−one−out sensitivity analysis for
'OPTC' on 'Pulmonary fibrosis'

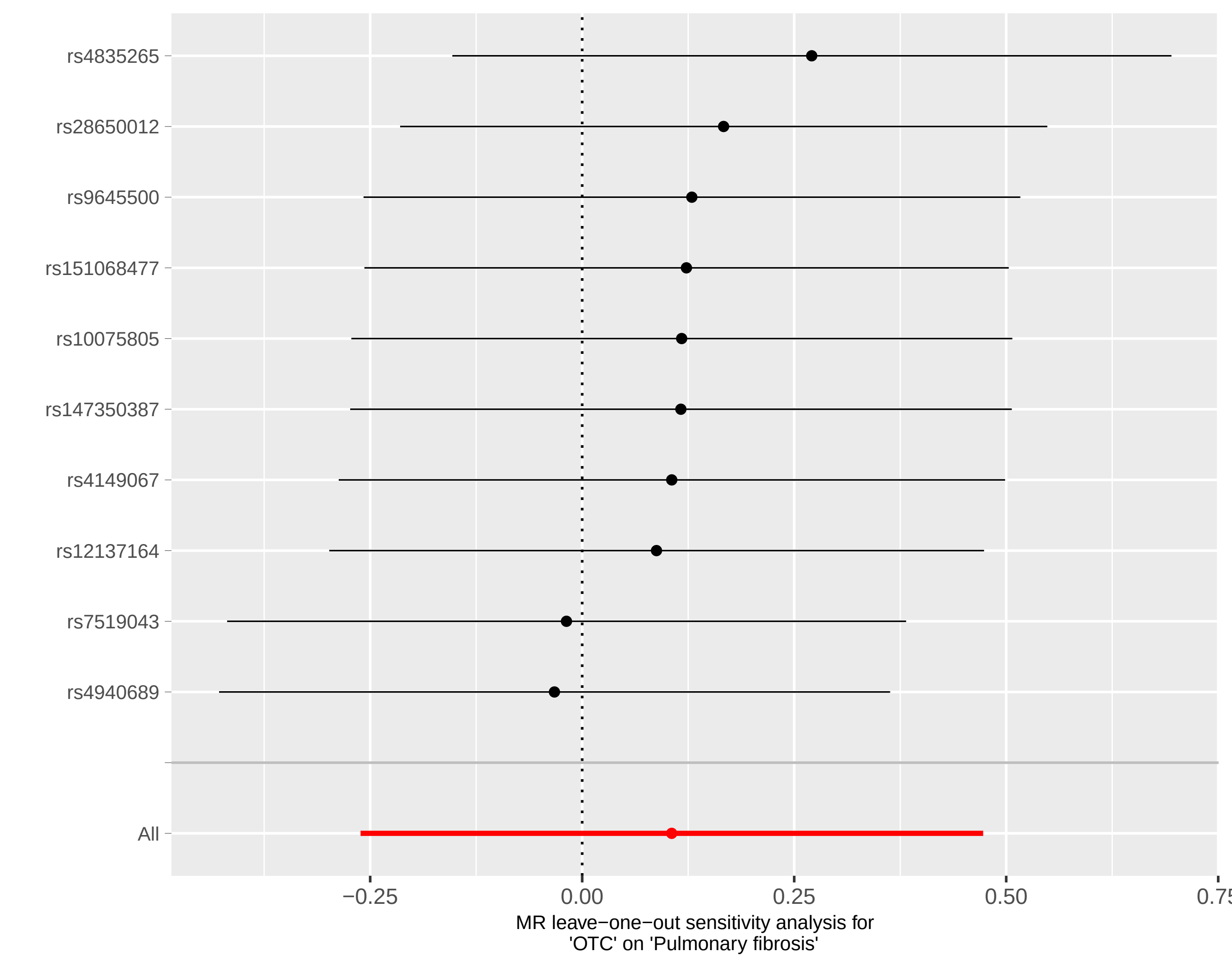
rs4835265
rs28650012
rs9645500
rs151068477
rs10075805
rs147350387
rs4149067
rs12137164
rs7519043
rs4940689
All
−0.25
0.00
0.25
0.50
MR leave−one−out sensitivity analysis for
'OTC' on 'Pulmonary fibrosis'

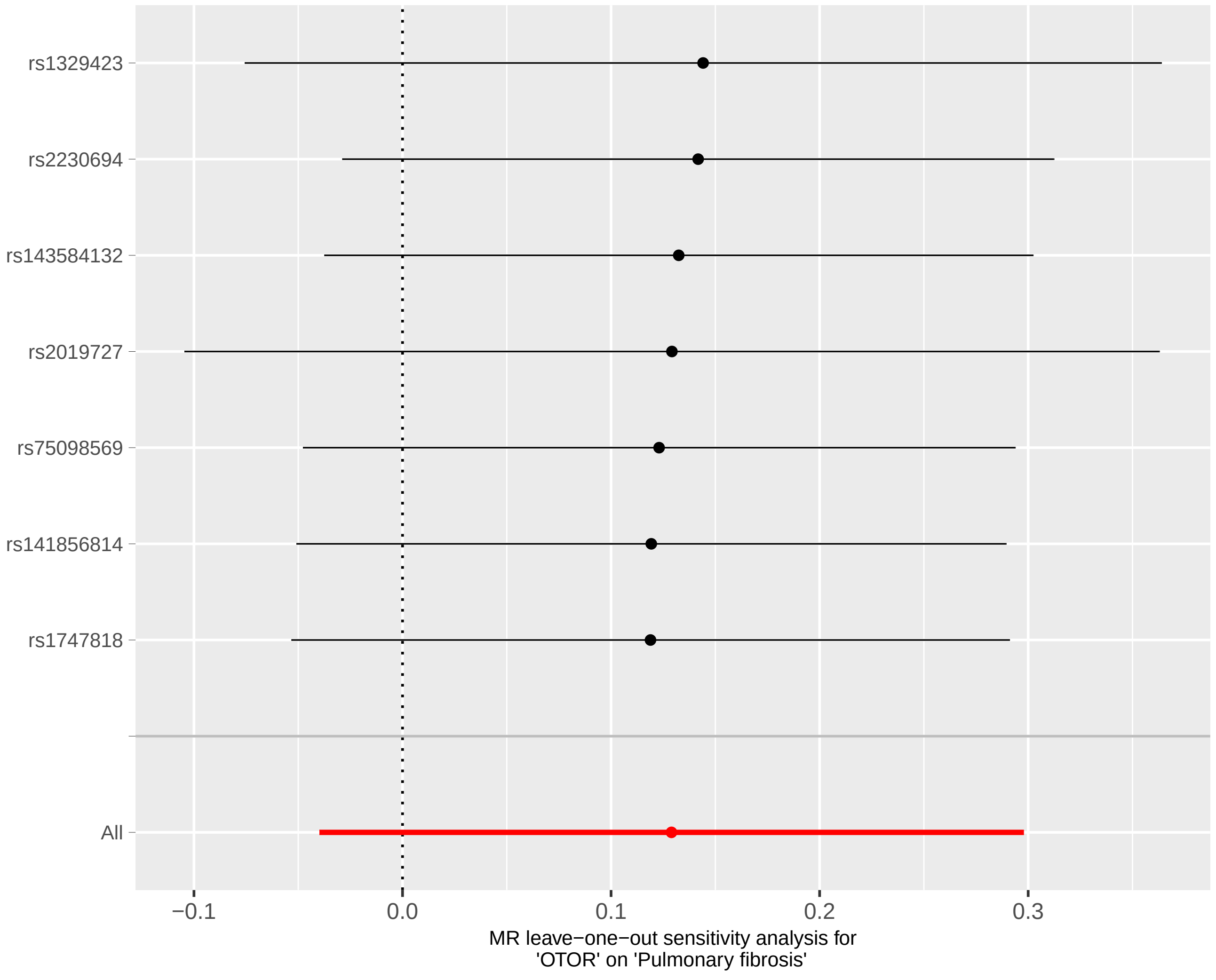

rs1329423
rs2230694
rs143584132
rs2019727
rs75098569
rs141856814
rs1747818
All
−0.1
0.0
0.1
0.2
0.3
MR leave−one−out sensitivity analysis for
'OTOR' on 'Pulmonary fibrosis'

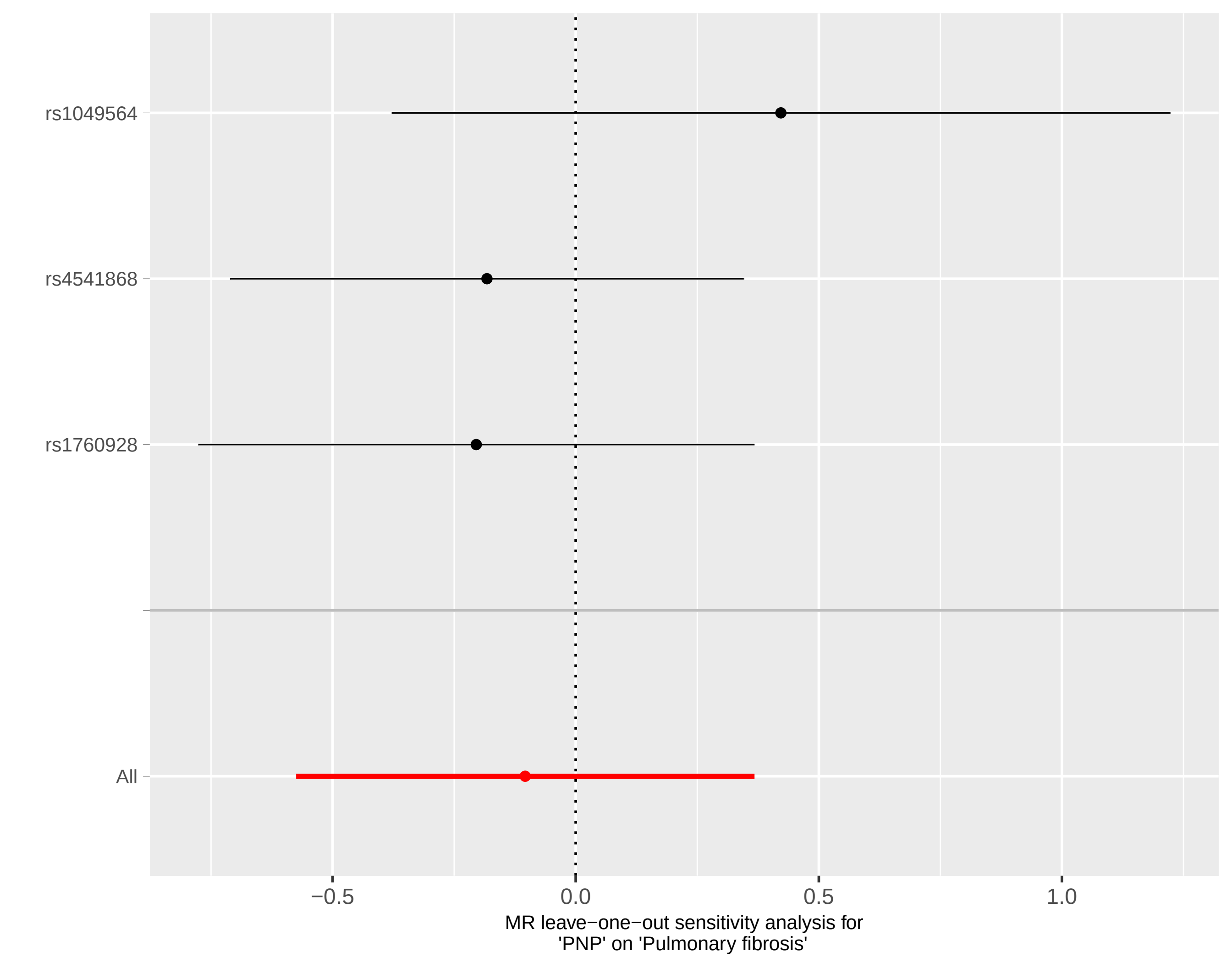
rs1049564
rs4541868
rs1760928
All
−0.5
0.0
0.5
1.0
MR leave−one−out sensitivity analysis for
'PNP' on 'Pulmonary fibrosis'

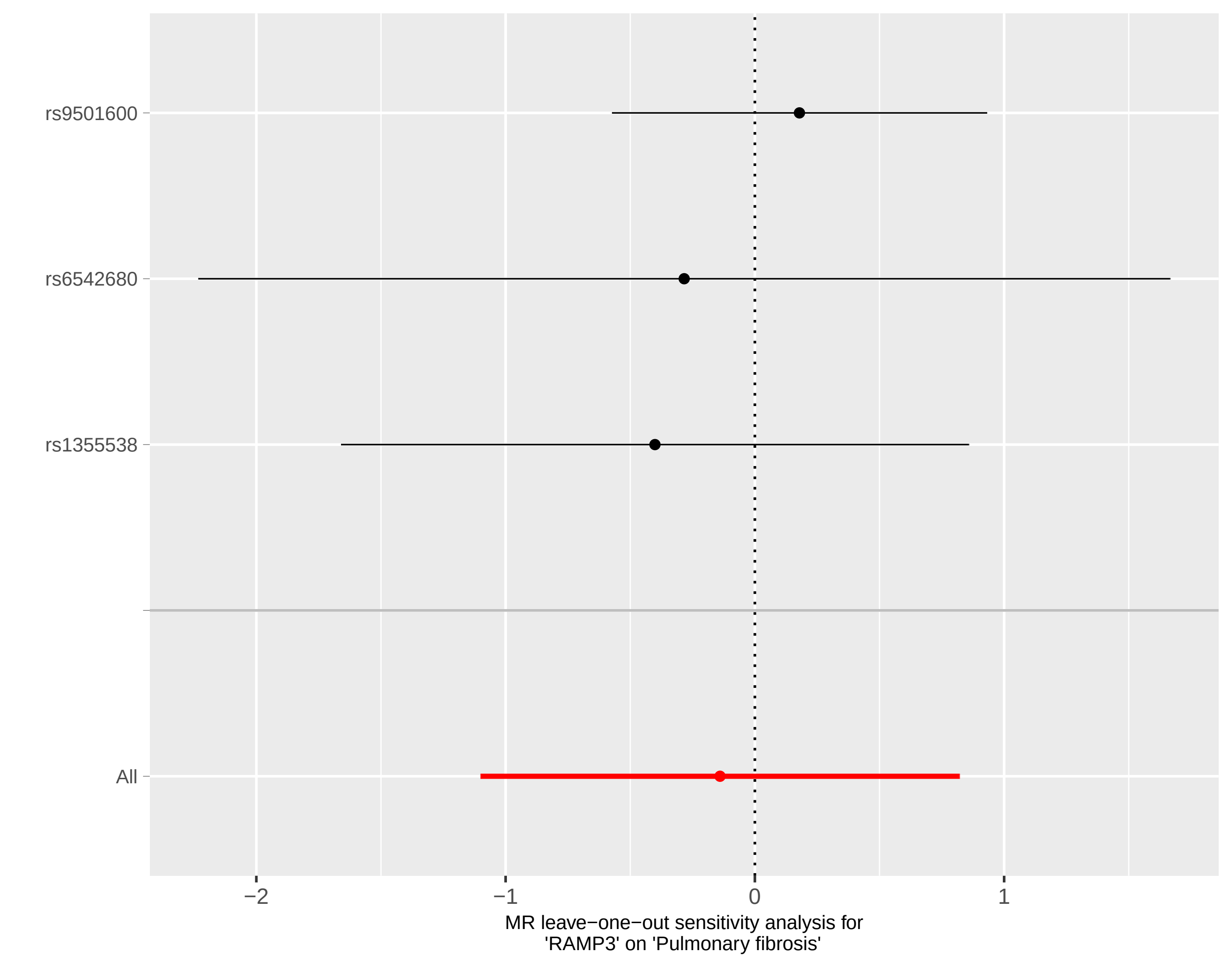
rs9501600
rs6542680
rs1355538
All
−2
−1
0
1
MR leave−one−out sensitivity analysis for
'RAMP3' on 'Pulmonary fibrosis'

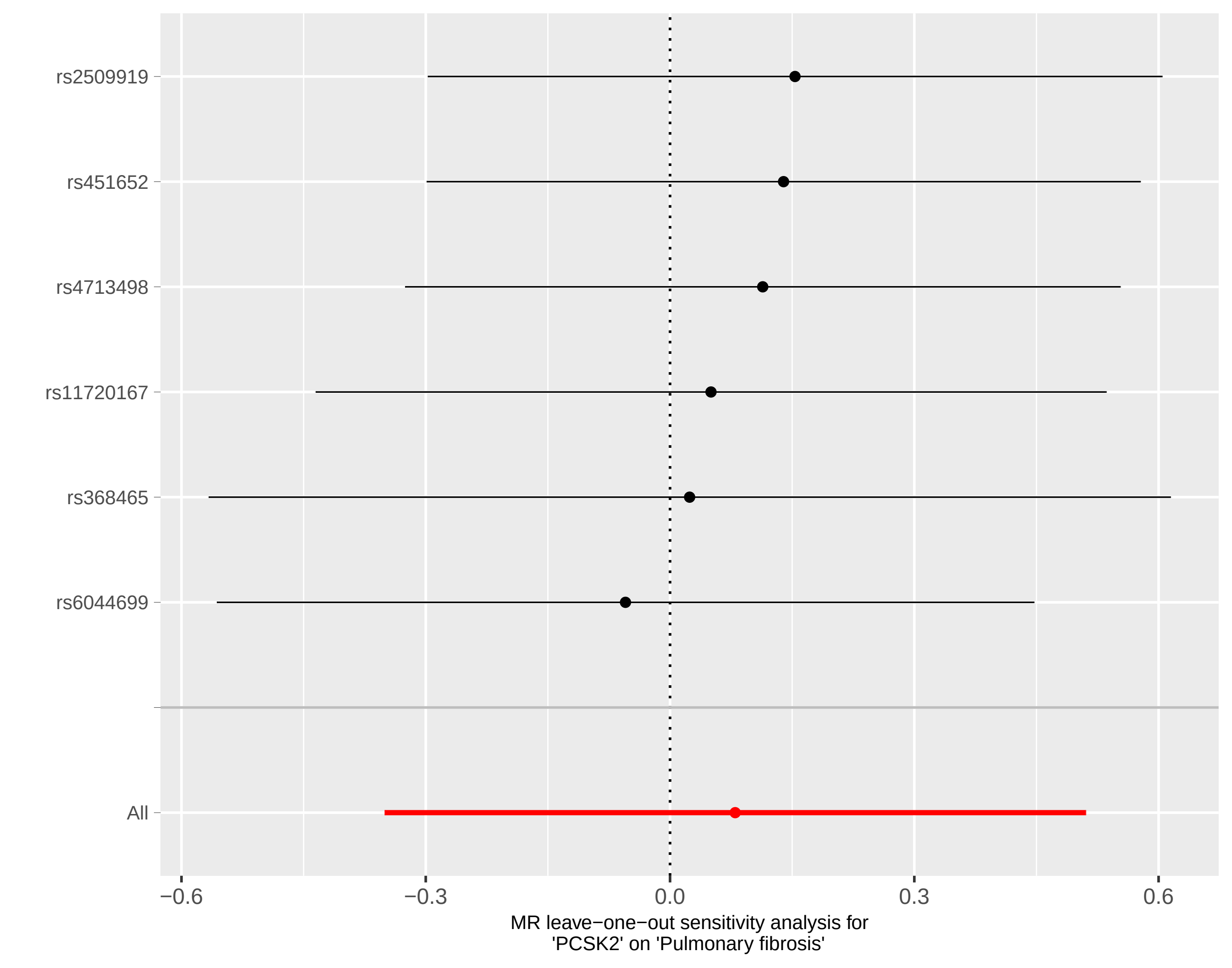

rs2509919
rs451652
rs4713498
rs11720167
rs368465
rs6044699
All
−0.6
−0.3
0.0
0.3
0.6
MR leave−one−out sensitivity analysis for
'PCSK2' on 'Pulmonary fibrosis'

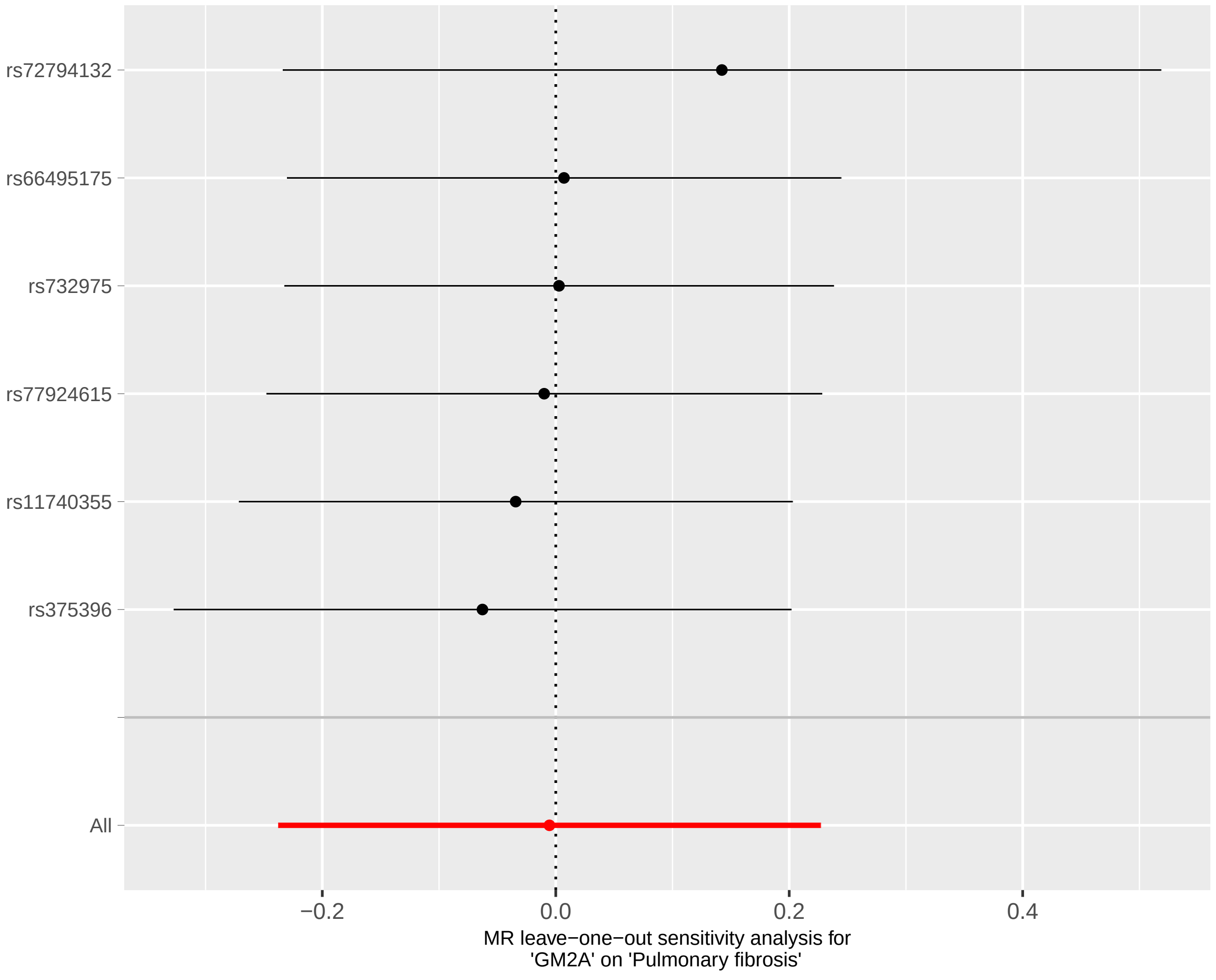
rs72794132
rs66495175
rs732975
rs77924615
rs11740355
rs375396
All
−0.2
0.0
0.2
0.4
MR leave−one−out sensitivity analysis for
'GM2A' on 'Pulmonary fibrosis'

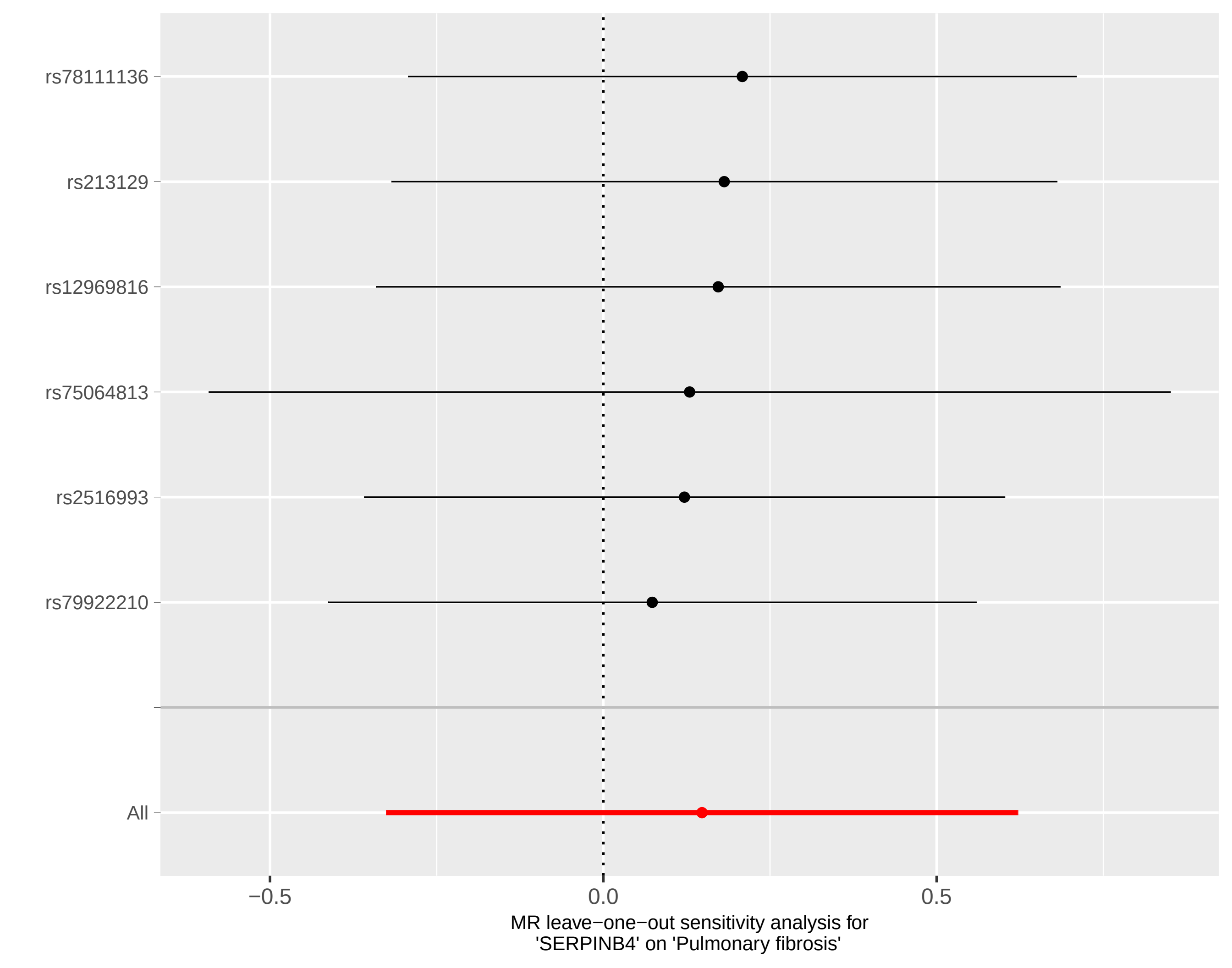
rs78111136
rs213129
rs12969816
rs75064813
rs2516993
rs79922210
All
−0.5
0.0
0.5
MR leave−one−out sensitivity analysis for
'SERPINB4' on 'Pulmonary fibrosis'

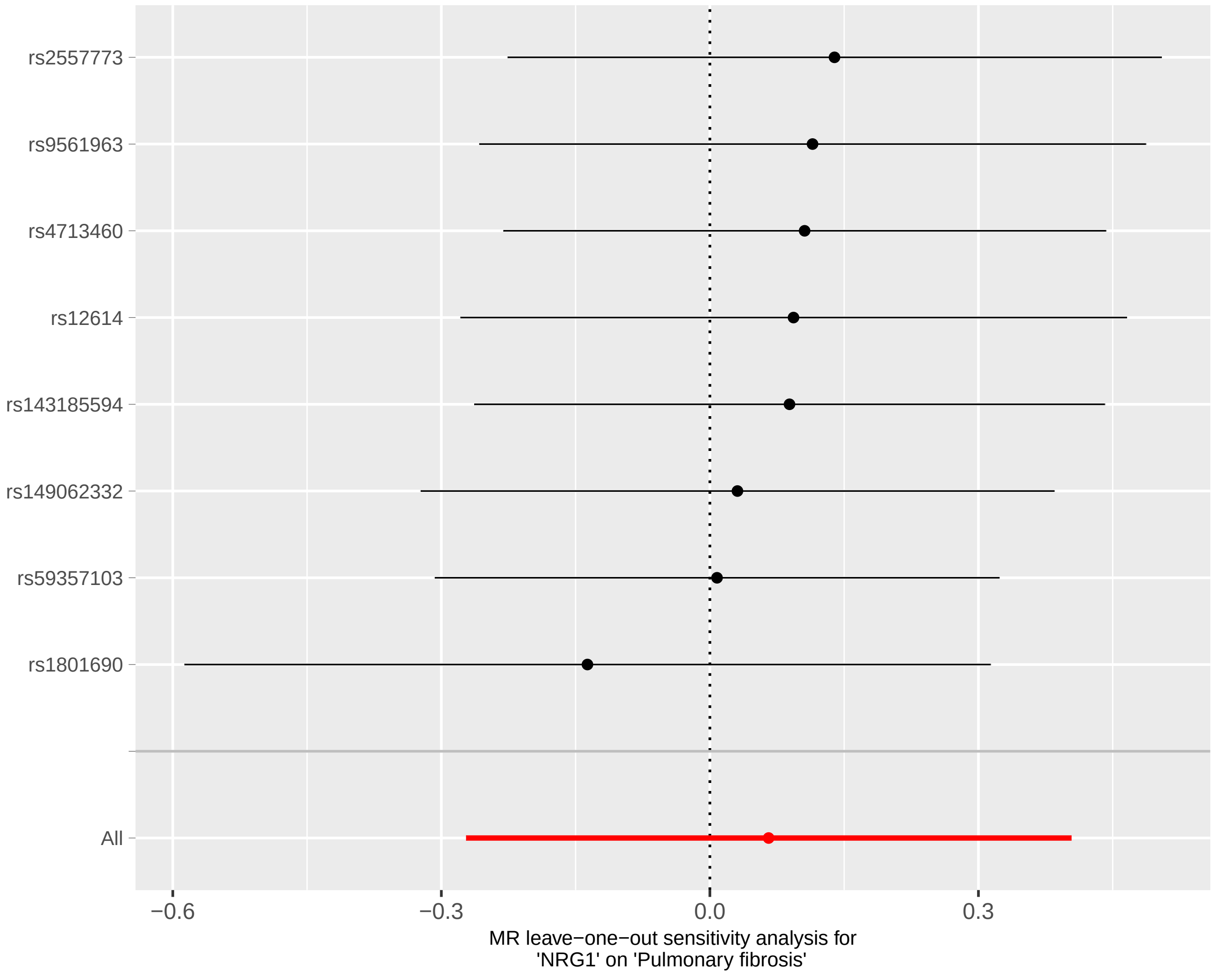
rs2557773
rs9561963
rs4713460
rs12614
rs143185594
rs149062332
rs59357103
rs1801690
All
−0.6
−0.3
0.0
0.3
MR leave−one−out sensitivity analysis for
'NRG1' on 'Pulmonary fibrosis'

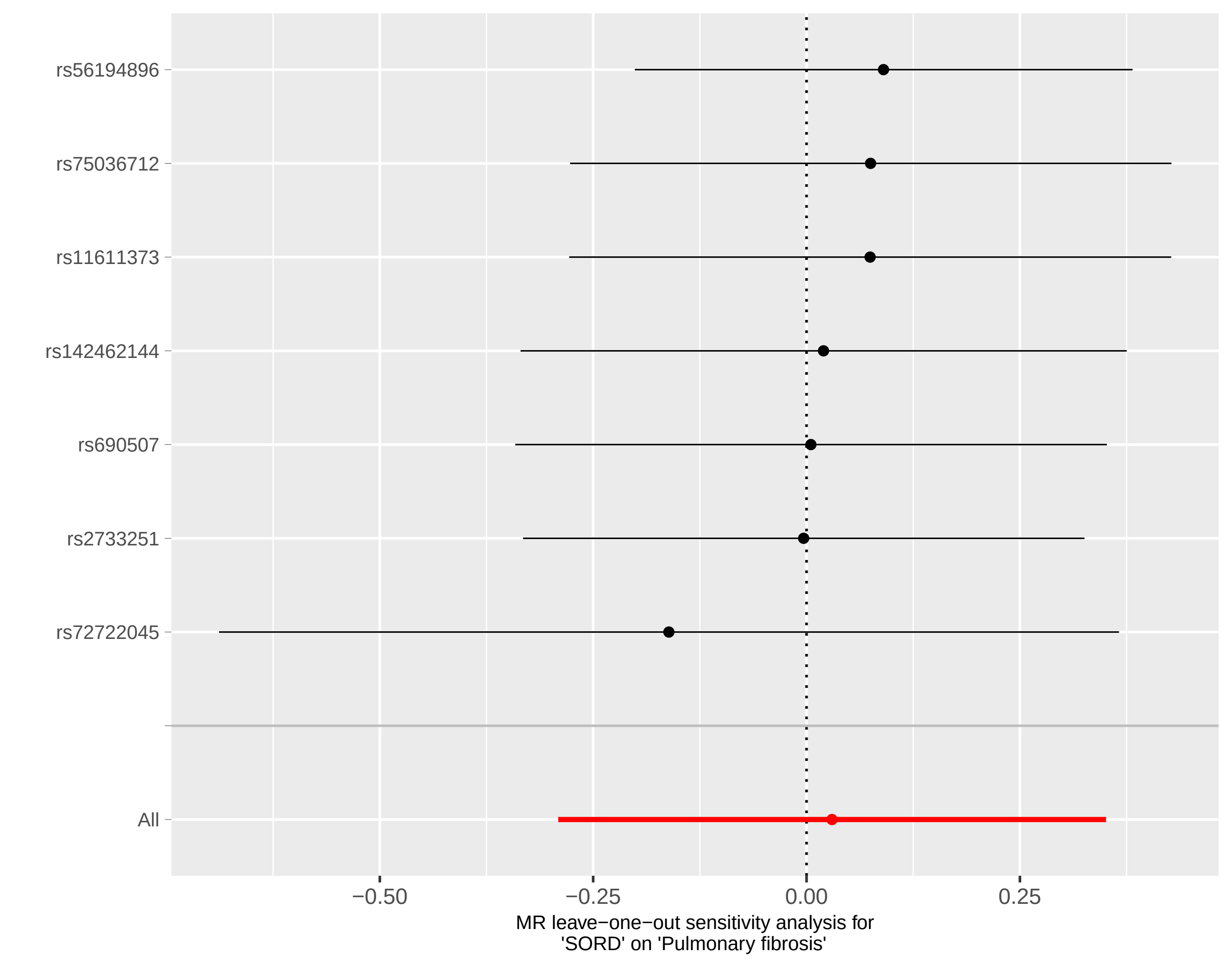
rs56194896
rs75036712
rs11611373
rs142462144
rs690507
rs2733251
rs72722045
All
−0.50
−0.25
0.00
0.25
MR leave−one−out sensitivity analysis for
'SORD' on 'Pulmonary fibrosis'

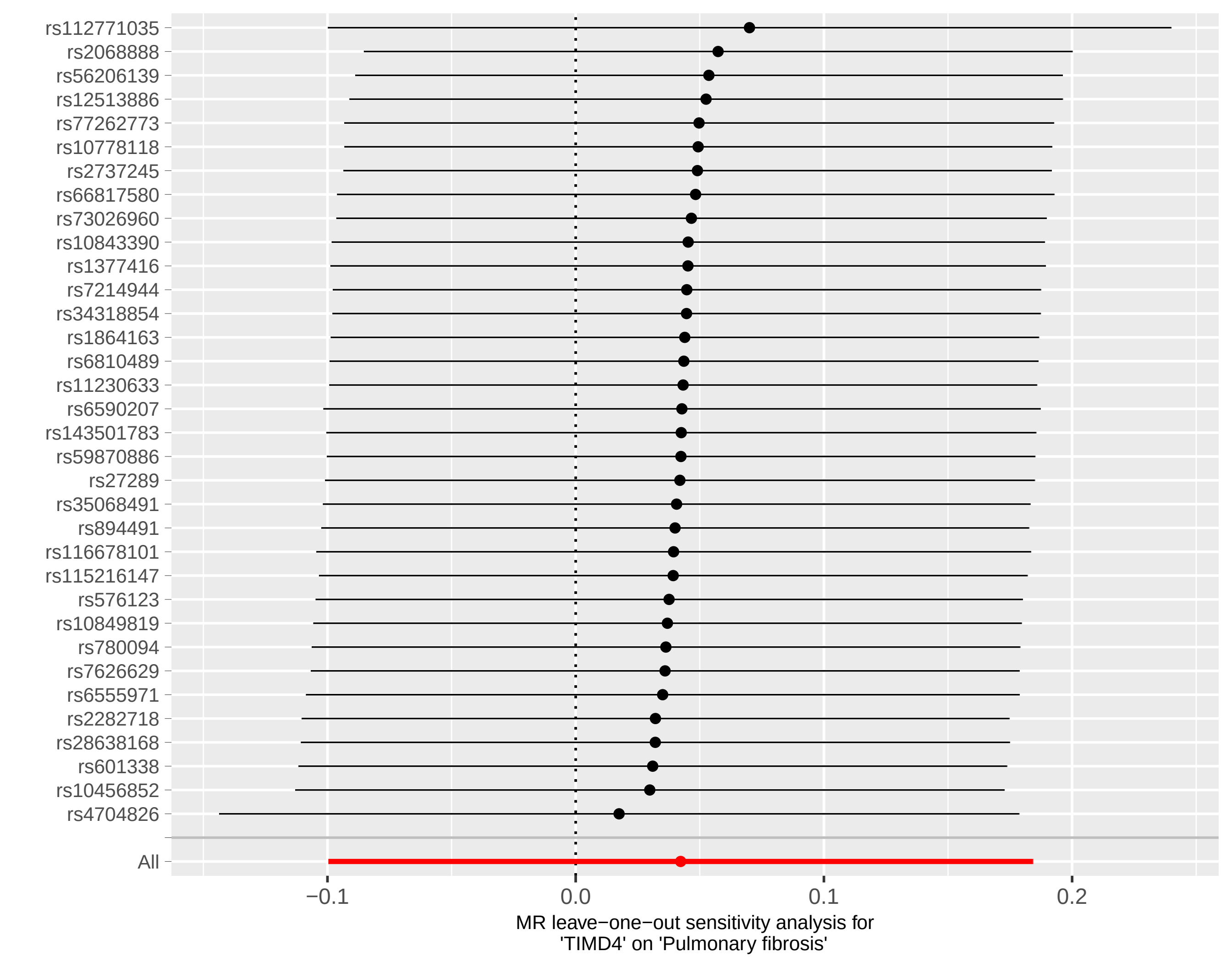
rs112771035
rs2068888
rs56206139
rs12513886
rs77262773
rs10778118
rs2737245
rs66817580
rs73026960
rs10843390
rs1377416
rs7214944
rs34318854
rs1864163
rs6810489
rs11230633
rs6590207
rs143501783
rs59870886
rs27289
rs35068491
rs894491
rs116678101
rs115216147
rs576123
rs10849819
rs780094
rs7626629
rs6555971
rs2282718
rs28638168
rs601338
rs10456852
rs4704826
All
−0.1
0.0
0.1
0.2
MR leave−one−out sensitivity analysis for
'TIMD4' on 'Pulmonary fibrosis'

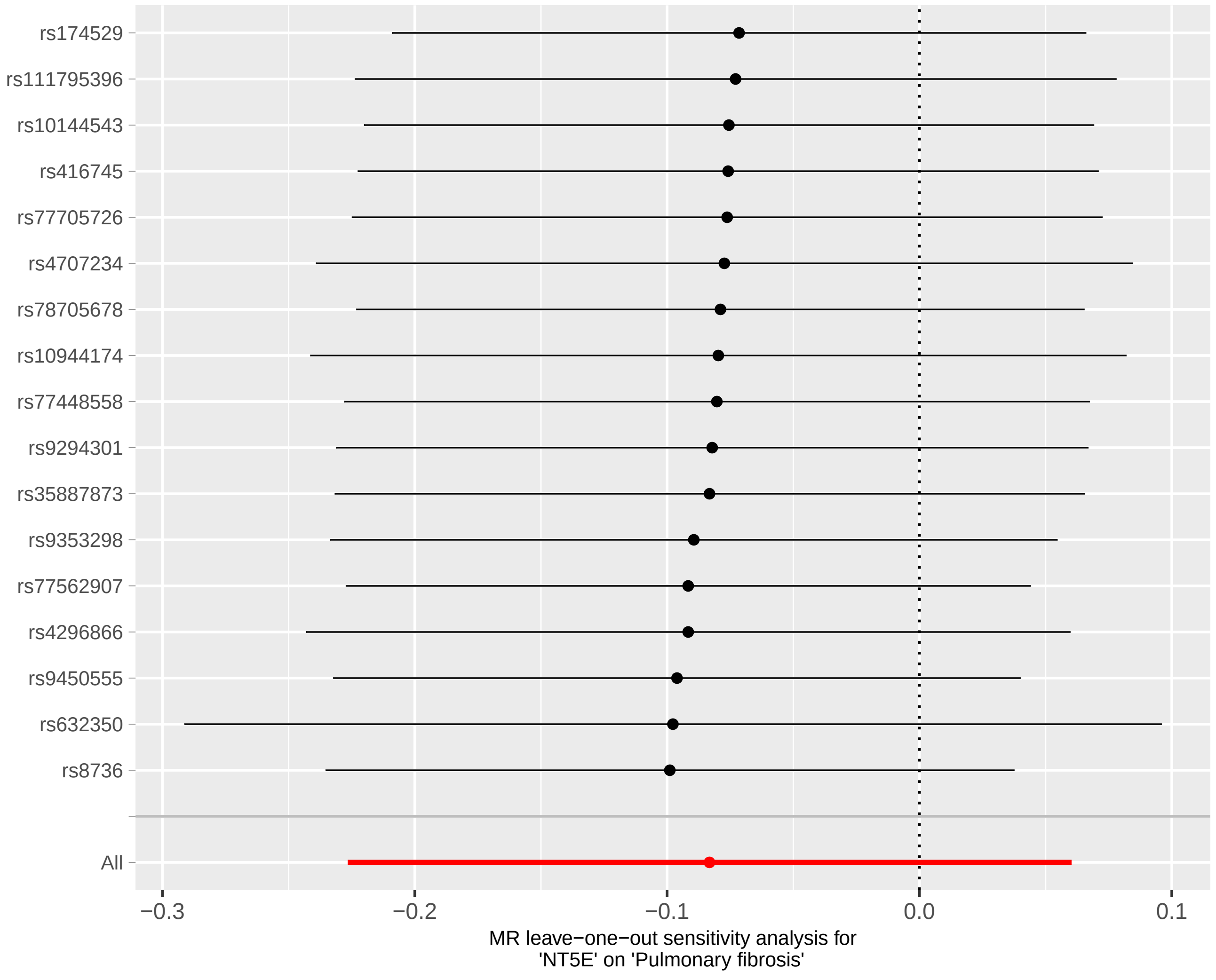

rs174529
rs111795396
rs10144543
rs416745
rs77705726
rs4707234
rs78705678
rs10944174
rs77448558
rs9294301
rs35887873
rs9353298
rs77562907
rs4296866
rs9450555
rs632350
rs8736
All
−0.3
−0.2
−0.1
0.0
0.1
MR leave−one−out sensitivity analysis for
'NT5E' on 'Pulmonary fibrosis'

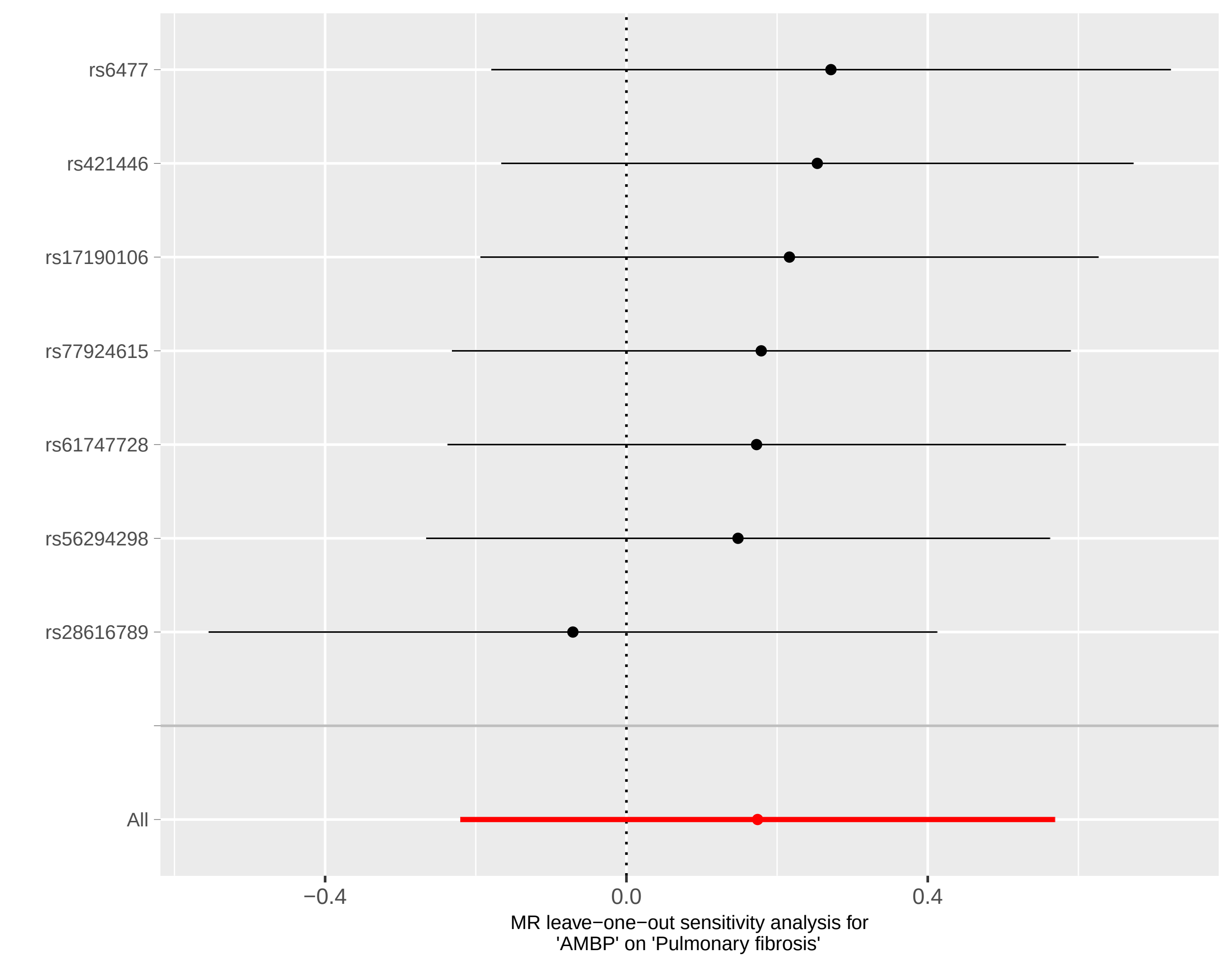
rs6477
rs421446
rs17190106
rs77924615
rs61747728
rs56294298
rs28616789
All
−0.4
0.0
0.4
MR leave−one−out sensitivity analysis for
'AMBP' on 'Pulmonary fibrosis'

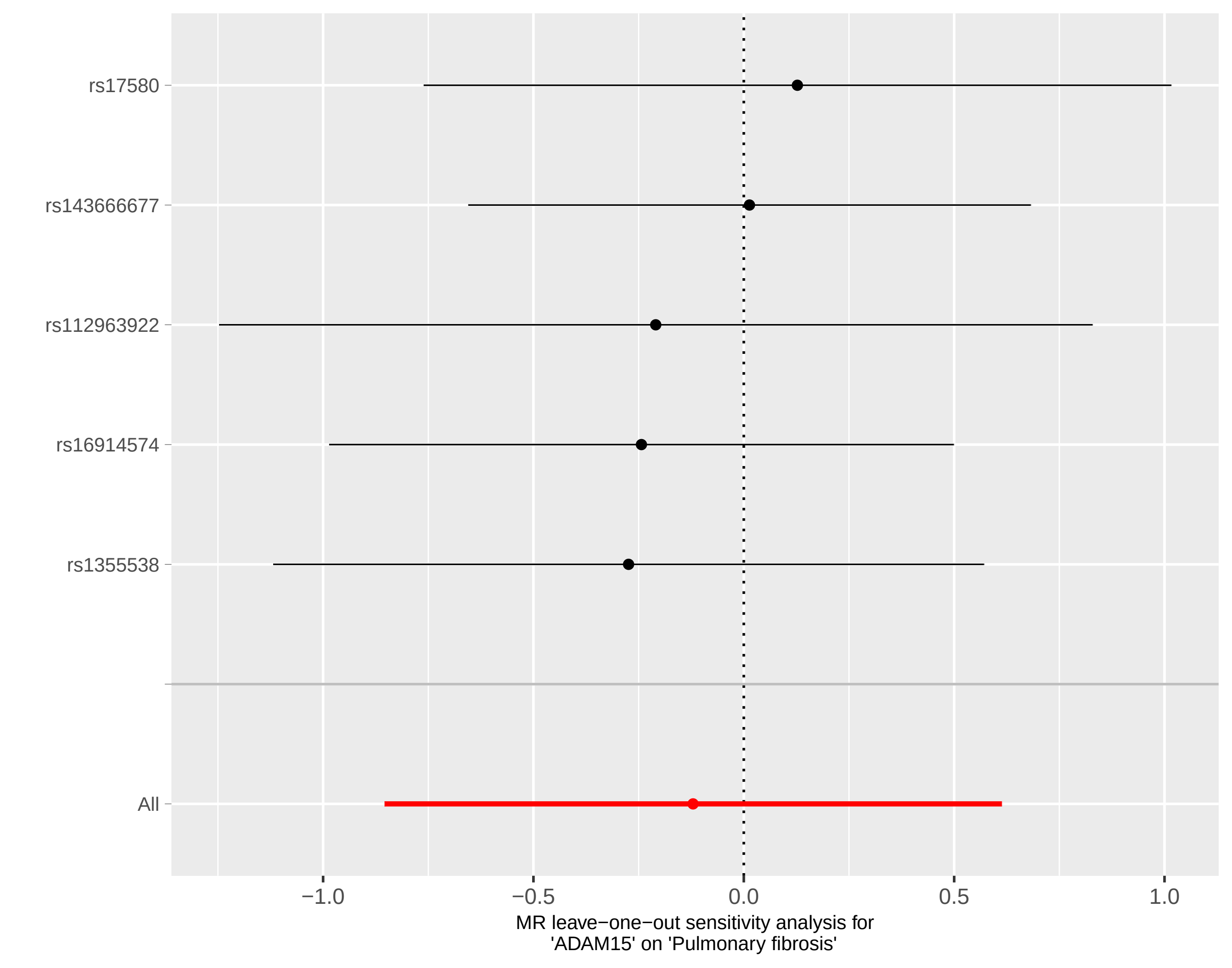
rs17580
rs143666677
rs112963922
rs16914574
rs1355538
All
−1.0
−0.5
0.0
0.5
1.0
MR leave−one−out sensitivity analysis for
'ADAM15' on 'Pulmonary fibrosis'

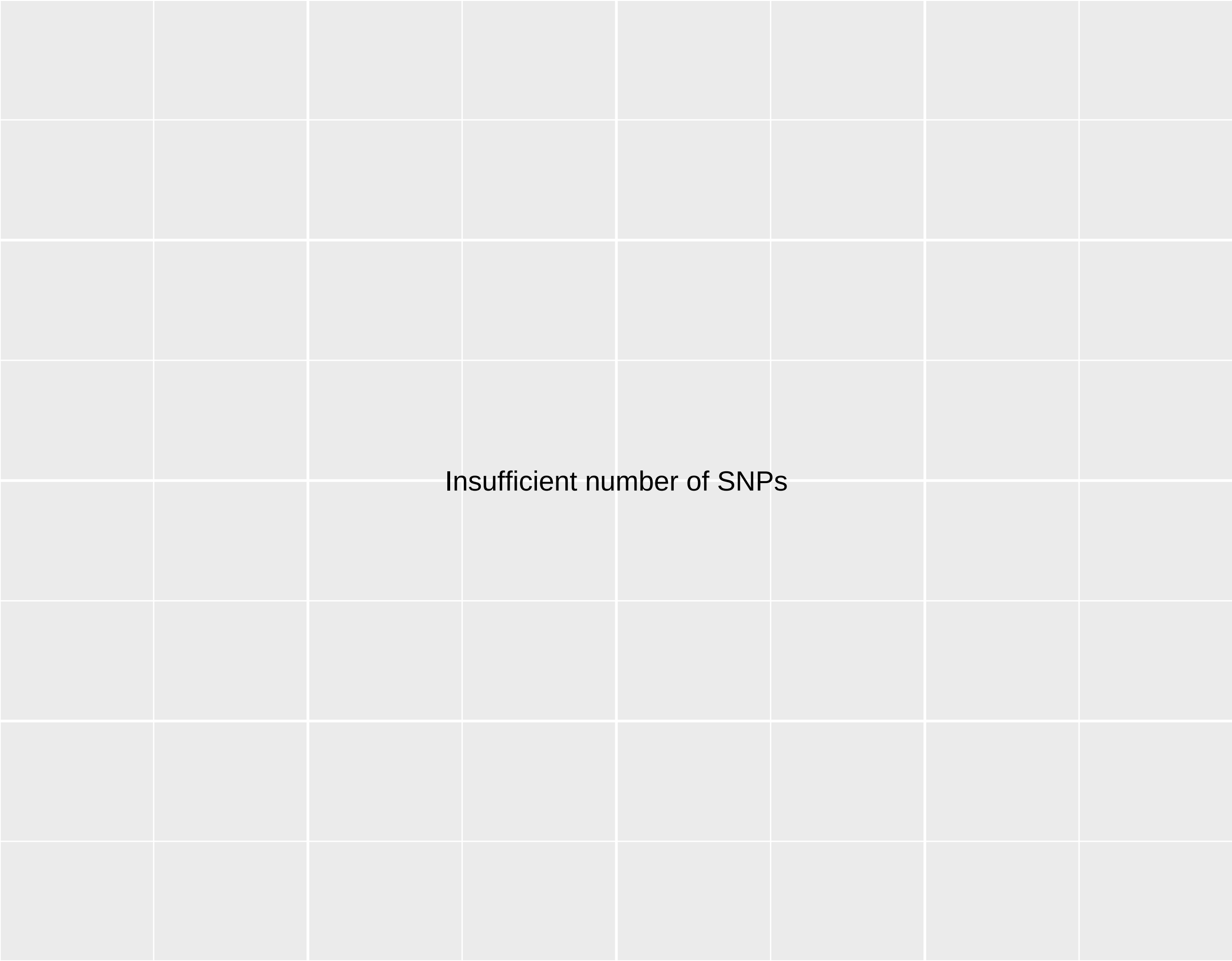
Insufficient number of SNPs

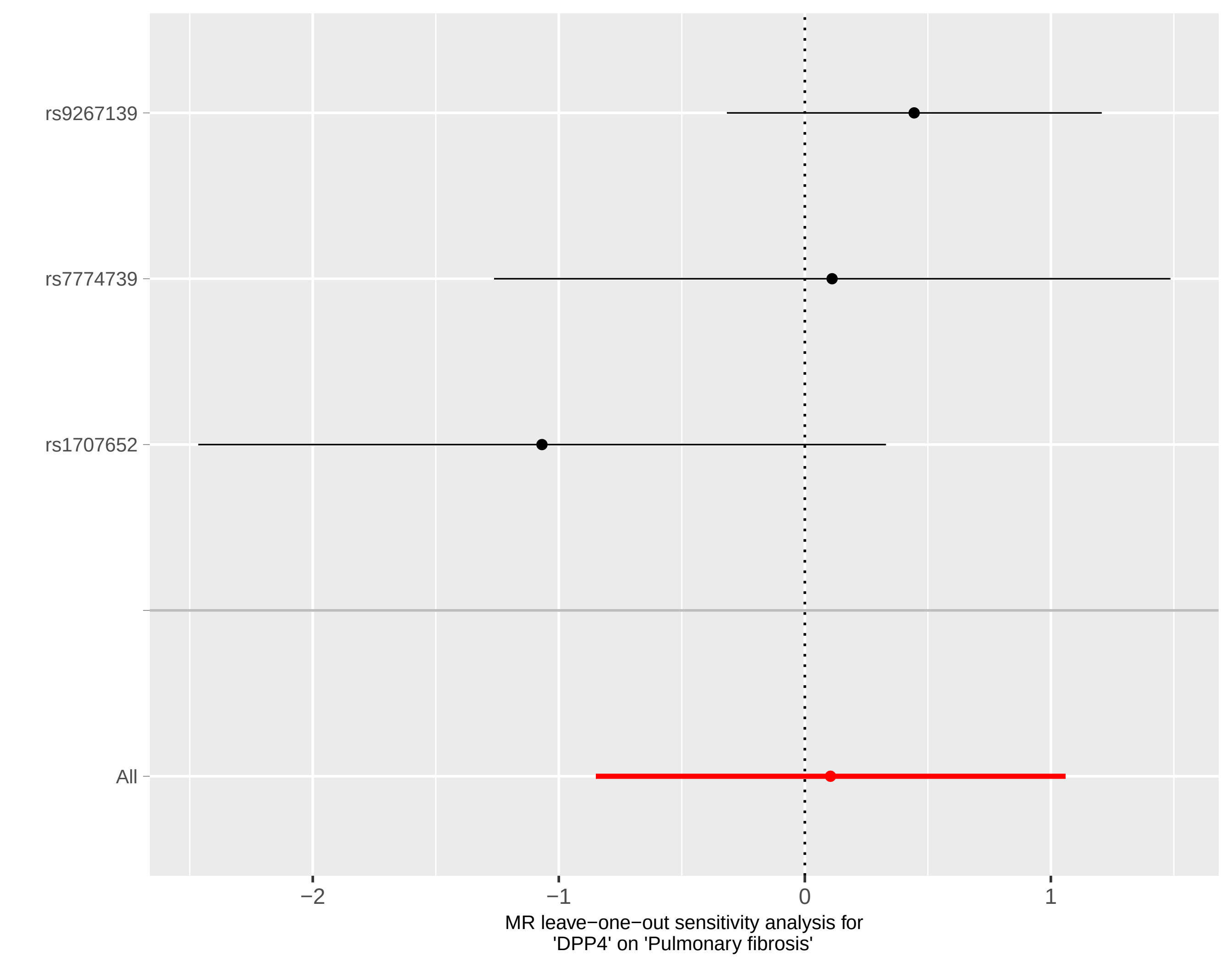
rs9267139
rs7774739
rs1707652
All
−2
−1
0
1
MR leave−one−out sensitivity analysis for
'DPP4' on 'Pulmonary fibrosis'

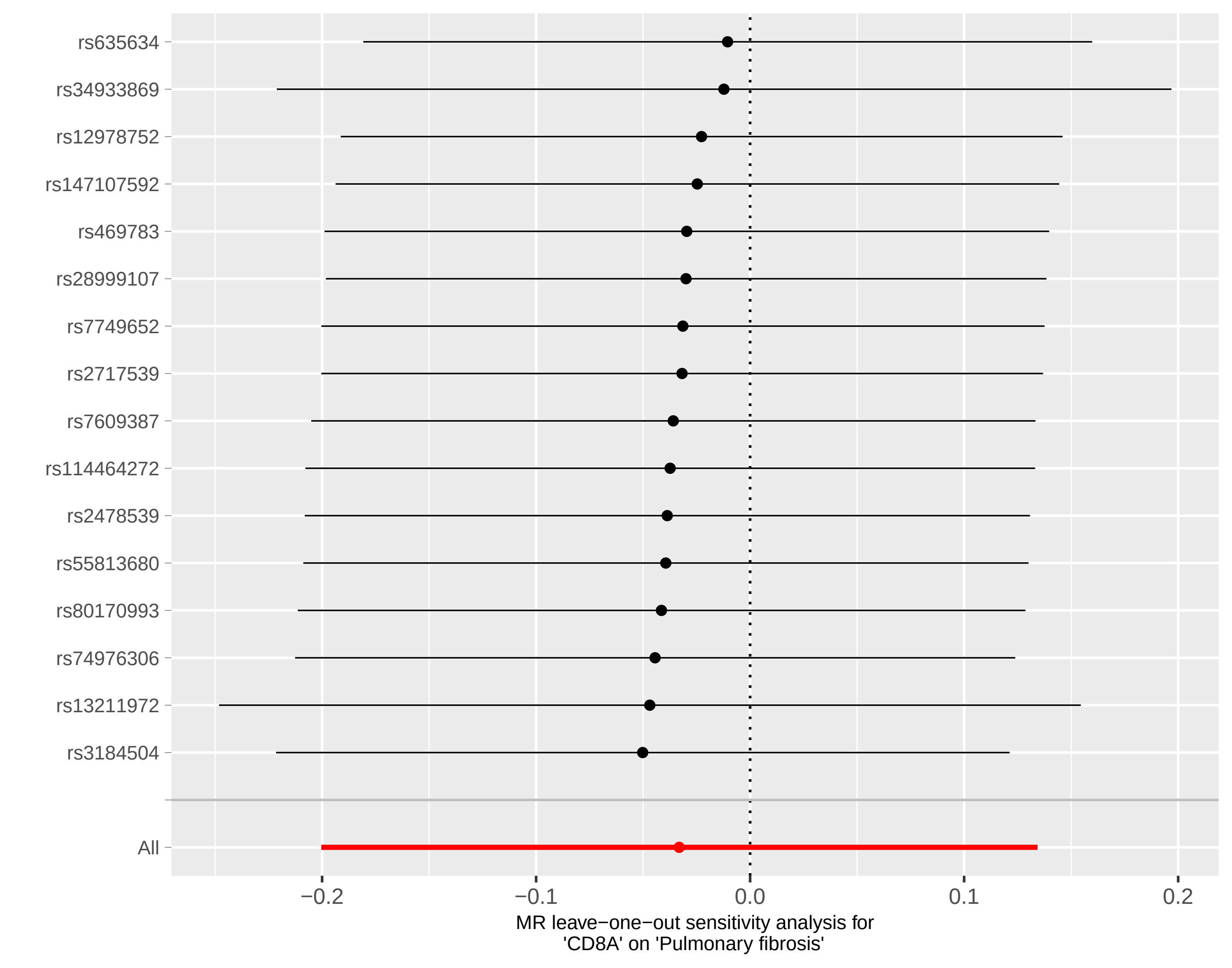

rs635634
rs34933869
rs12978752
rs147107592
rs469783
rs28999107
rs7749652
rs2717539
rs7609387
rs114464272
rs2478539
rs55813680
rs80170993
rs74976306
rs13211972
rs3184504
All
−0.2
−0.1
0.0
0.1
0.2
MR leave−one−out sensitivity analysis for
'CD8A' on 'Pulmonary fibrosis'

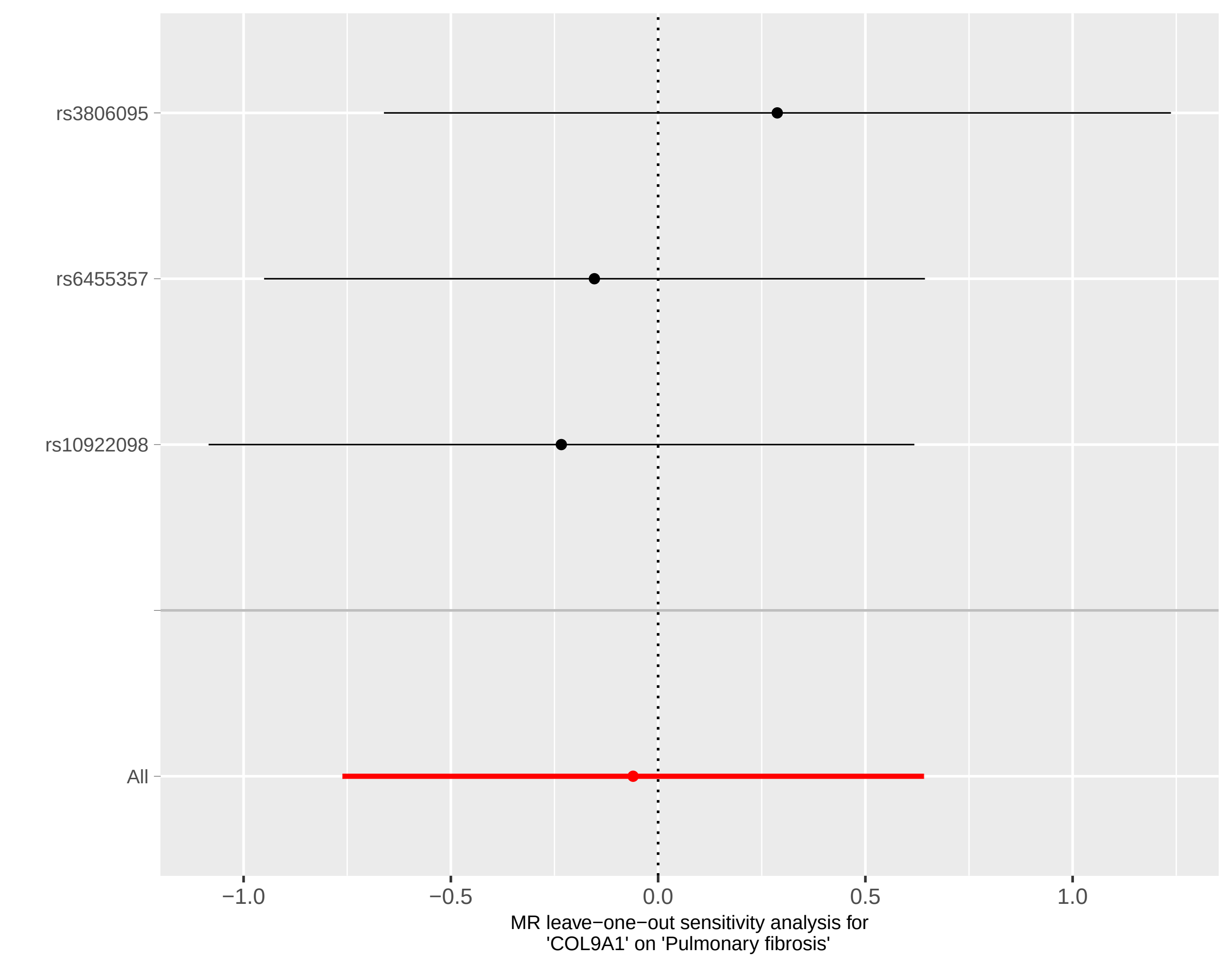
rs3806095
rs6455357
rs10922098
All
−1.0
−0.5
0.0
0.5
1.0
MR leave−one−out sensitivity analysis for
'COL9A1' on 'Pulmonary fibrosis'

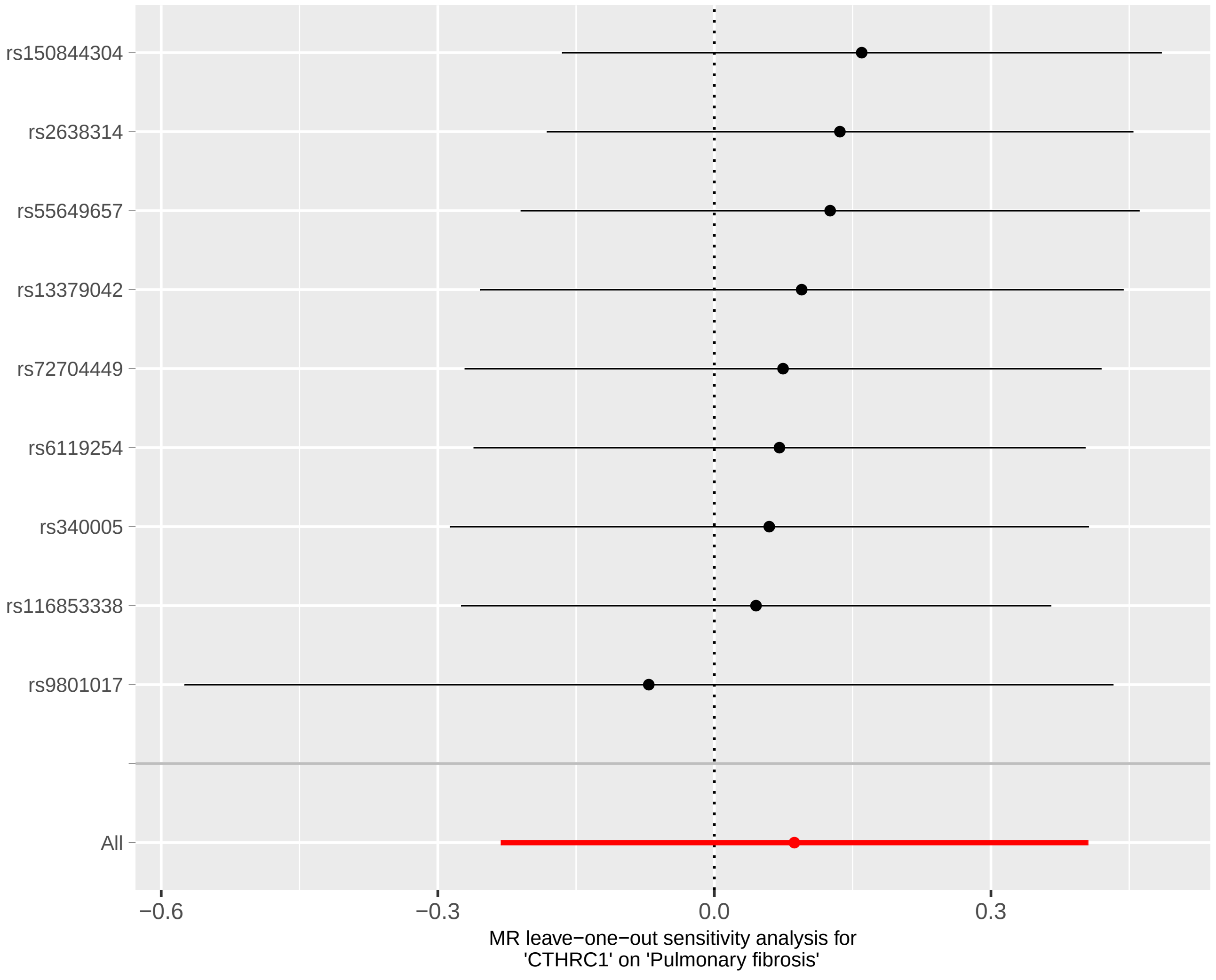
rs150844304
rs2638314
rs55649657
rs13379042
rs72704449
rs6119254
rs340005
rs116853338
rs9801017
All
−0.6
−0.3
0.0
0.3
MR leave−one−out sensitivity analysis for
'CTHRC1' on 'Pulmonary fibrosis'

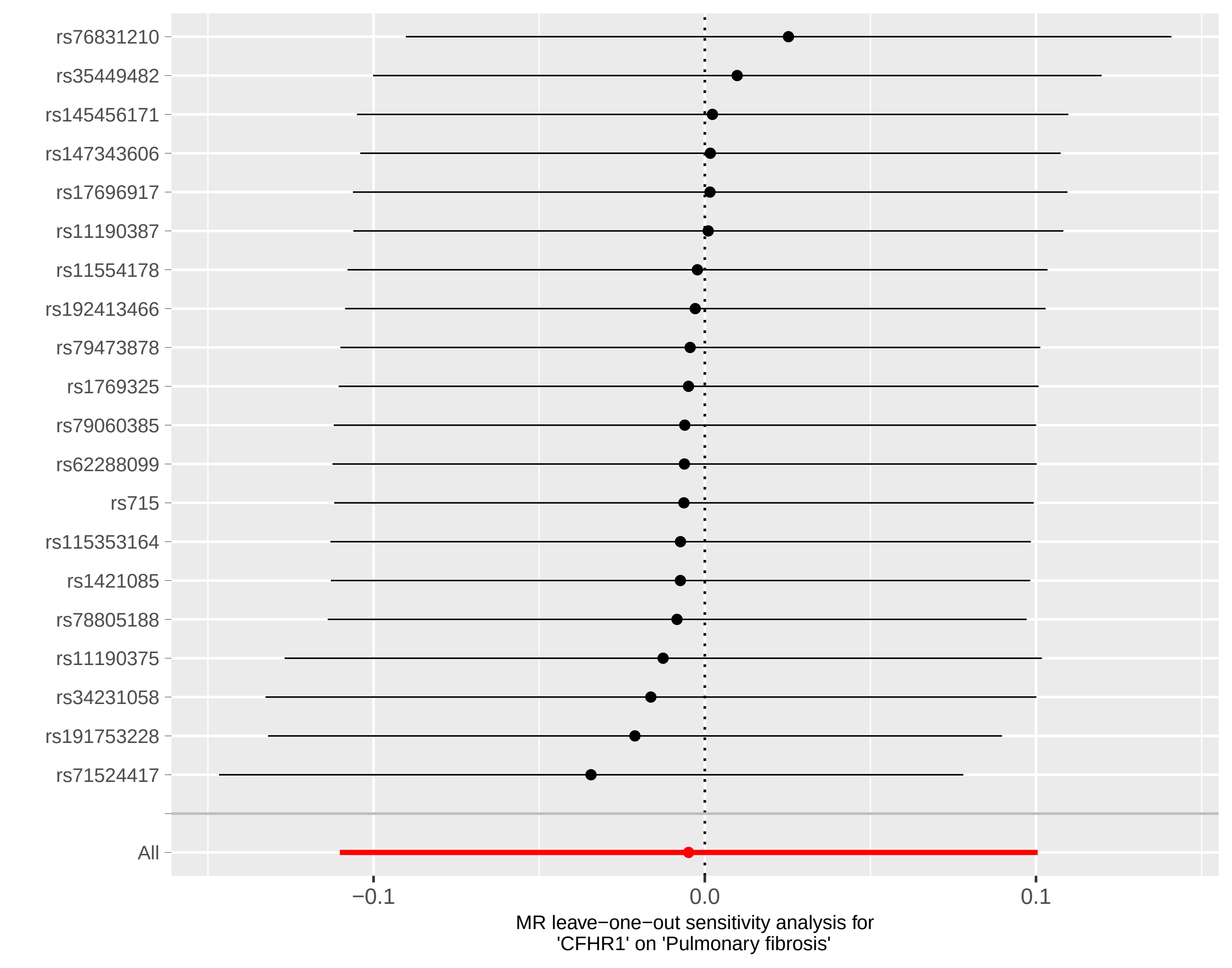

rs76831210
rs35449482
rs145456171
rs147343606
rs17696917
rs11190387
rs11554178
rs192413466
rs79473878
rs1769325
rs79060385
rs62288099
rs715
rs115353164
rs1421085
rs78805188
rs11190375
rs34231058
rs191753228
rs71524417
All
−0.1
0.0
0.1
MR leave−one−out sensitivity analysis for
'CFHR1' on 'Pulmonary fibrosis'

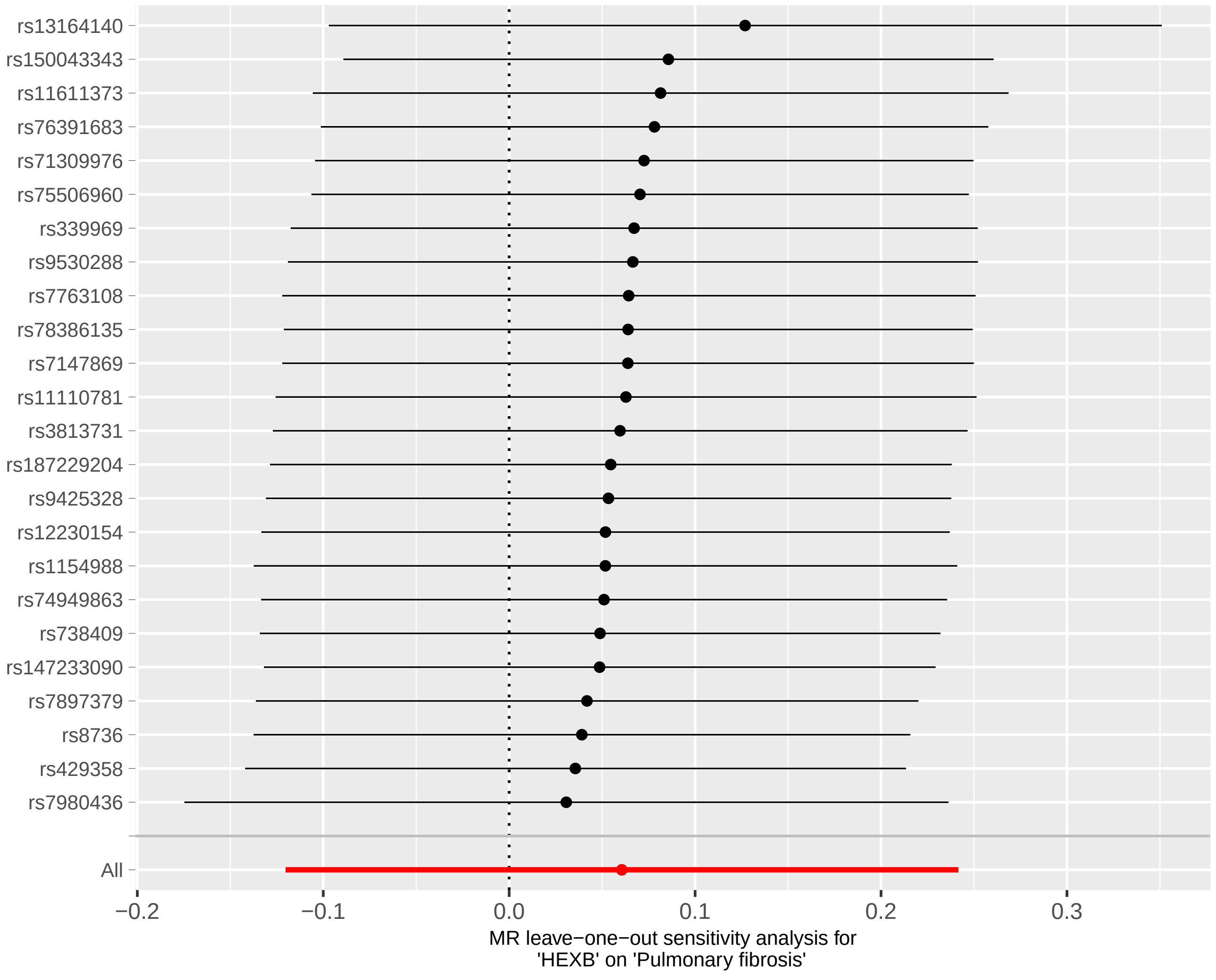

rs13164140
rs150043343
rs11611373
rs76391683
rs71309976
rs75506960
rs339969
rs9530288
rs7763108
rs78386135
rs7147869
rs11110781
rs3813731
rs187229204
rs9425328
rs12230154
rs1154988
rs74949863
rs738409
rs147233090
rs7897379
rs8736
rs429358
rs7980436
All
−0.2
−0.1
0.0
0.1
0.2
0.3
MR leave−one−out sensitivity analysis for
'HEXB' on 'Pulmonary fibrosis'

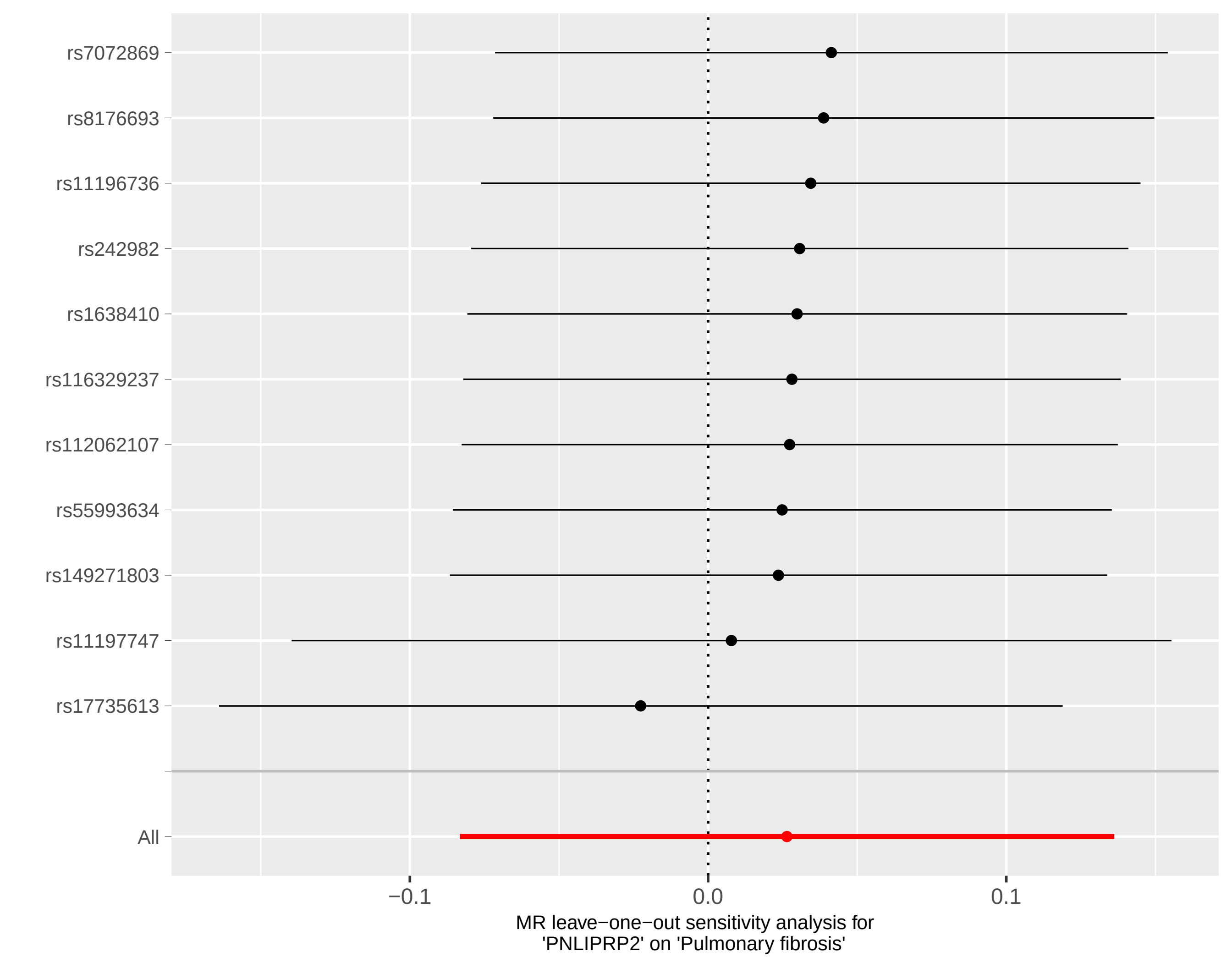
rs7072869
rs8176693
rs11196736
rs242982
rs1638410
rs116329237
rs112062107
rs55993634
rs149271803
rs11197747
rs17735613
All
−0.1
0.0
0.1
MR leave−one−out sensitivity analysis for
'PNLIPRP2' on 'Pulmonary fibrosis'

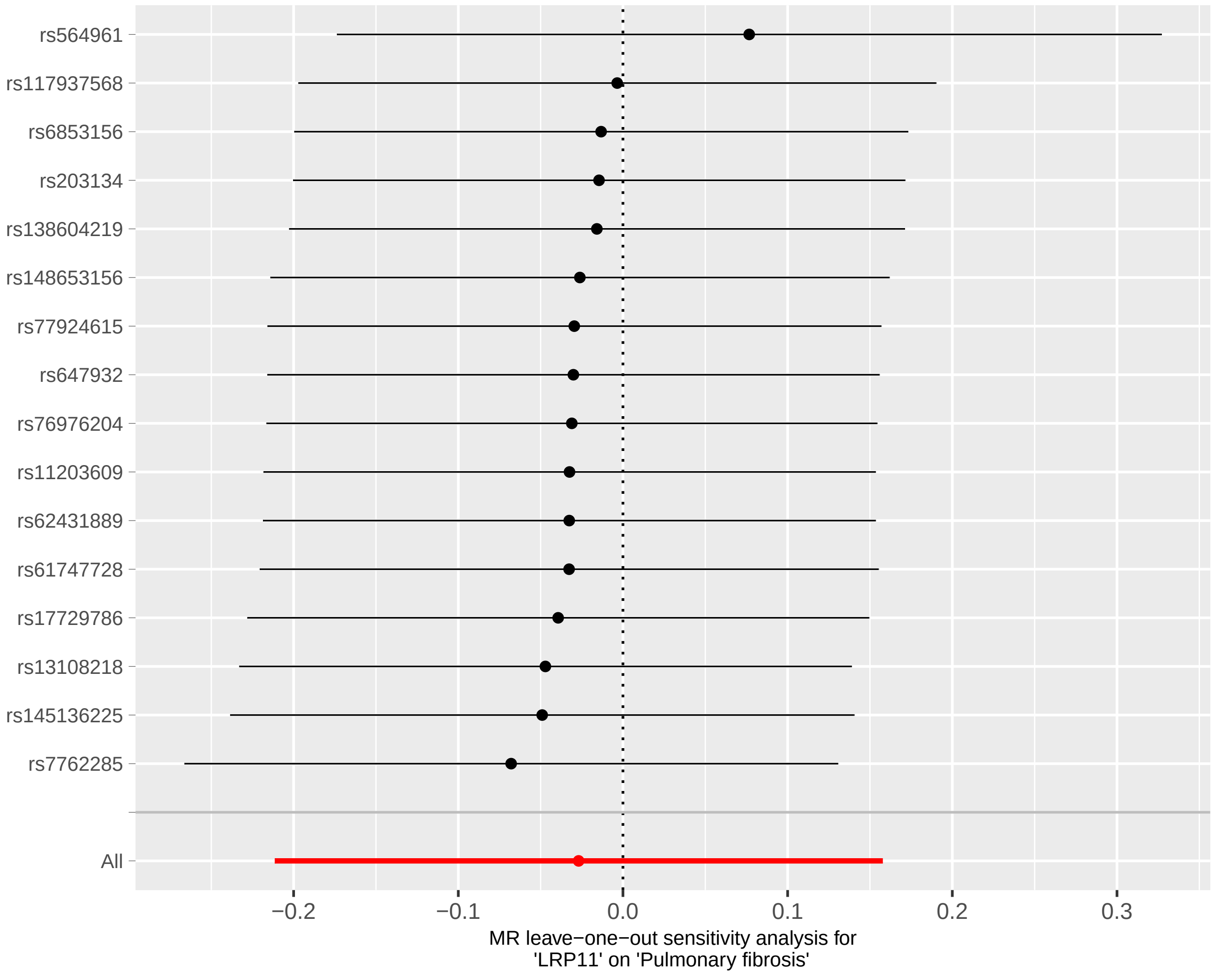
rs564961
rs117937568
rs6853156
rs203134
rs138604219
rs148653156
rs77924615
rs647932
rs76976204
rs11203609
rs62431889
rs61747728
rs17729786
rs13108218
rs145136225
rs7762285
All
−0.2
−0.1
0.0
0.1
0.2
0.3
MR leave−one−out sensitivity analysis for
'LRP11' on 'Pulmonary fibrosis'

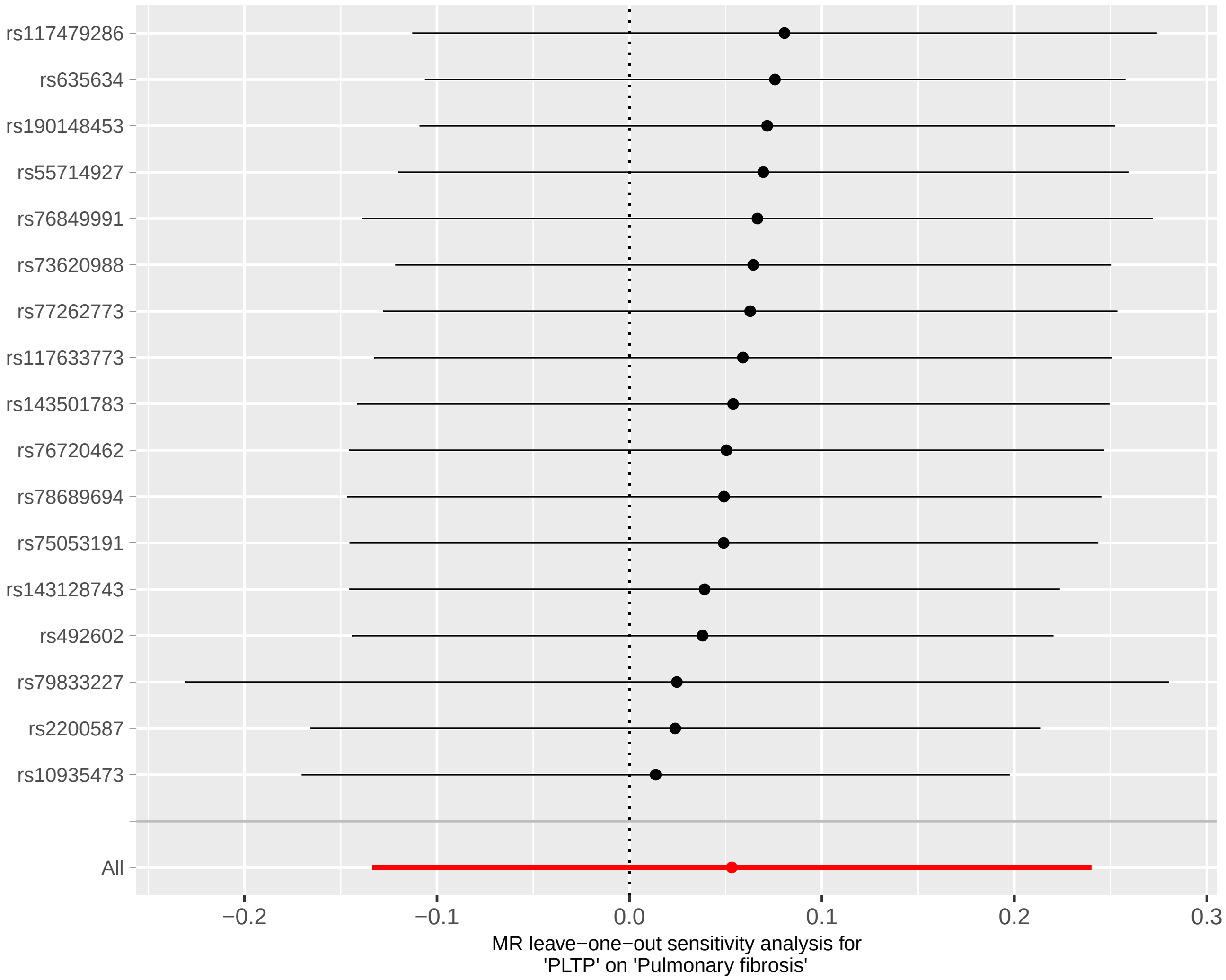
rs117479286
rs635634
rs190148453
rs55714927
rs76849991
rs73620988
rs77262773
rs117633773
rs143501783
rs76720462
rs78689694
rs75053191
rs143128743
rs492602
rs79833227
rs2200587
rs10935473
All
−0.2
−0.1
0.0
0.1
0.2
0.3
MR leave−one−out sensitivity analysis for
'PLTP' on 'Pulmonary fibrosis'

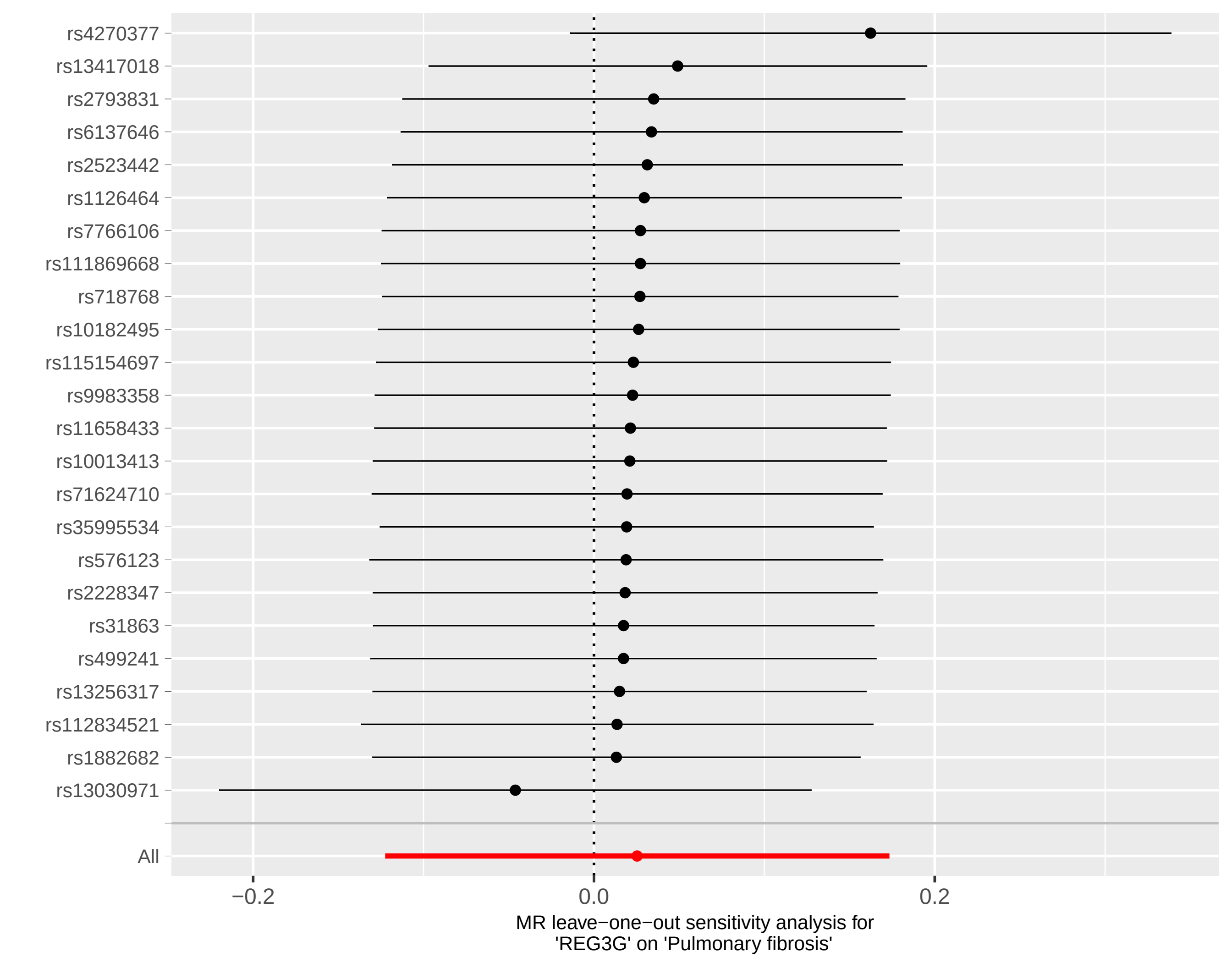
rs4270377
rs13417018
rs2793831
rs6137646
rs2523442
rs1126464
rs7766106
rs111869668
rs718768
rs10182495
rs115154697
rs9983358
rs11658433
rs10013413
rs71624710
rs35995534
rs576123
rs2228347
rs31863
rs499241
rs13256317
rs112834521
rs1882682
rs13030971
All
−0.2
0.0
0.2
MR leave−one−out sensitivity analysis for
'REG3G' on 'Pulmonary fibrosis'

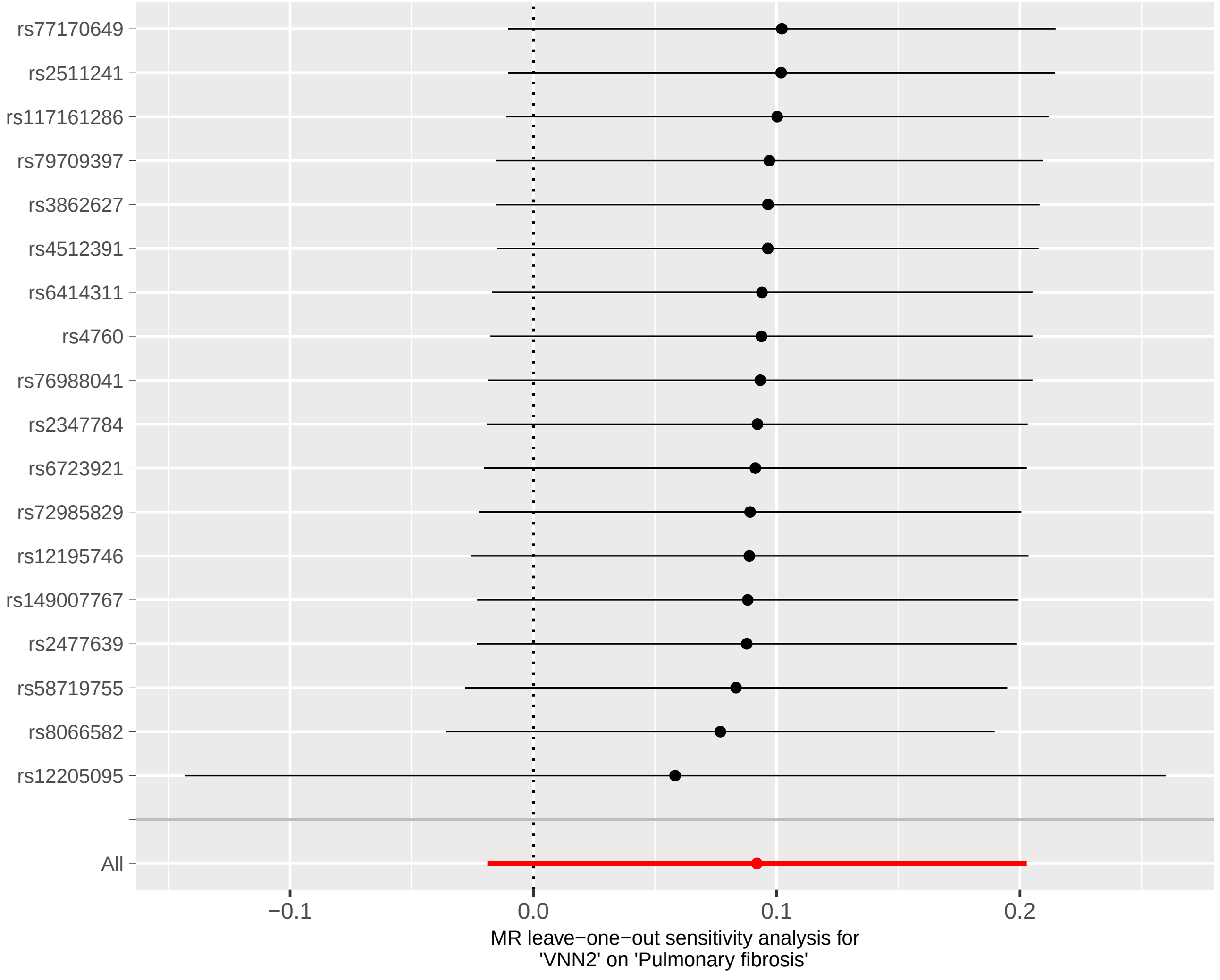
rs77170649
rs2511241
rs117161286
rs79709397
rs3862627
rs4512391
rs6414311
rs4760
rs76988041
rs2347784
rs6723921
rs72985829
rs12195746
rs149007767
rs2477639
rs58719755
rs8066582
rs12205095
All
−0.1
0.0
0.1
0.2
MR leave−one−out sensitivity analysis for
'VNN2' on 'Pulmonary fibrosis'

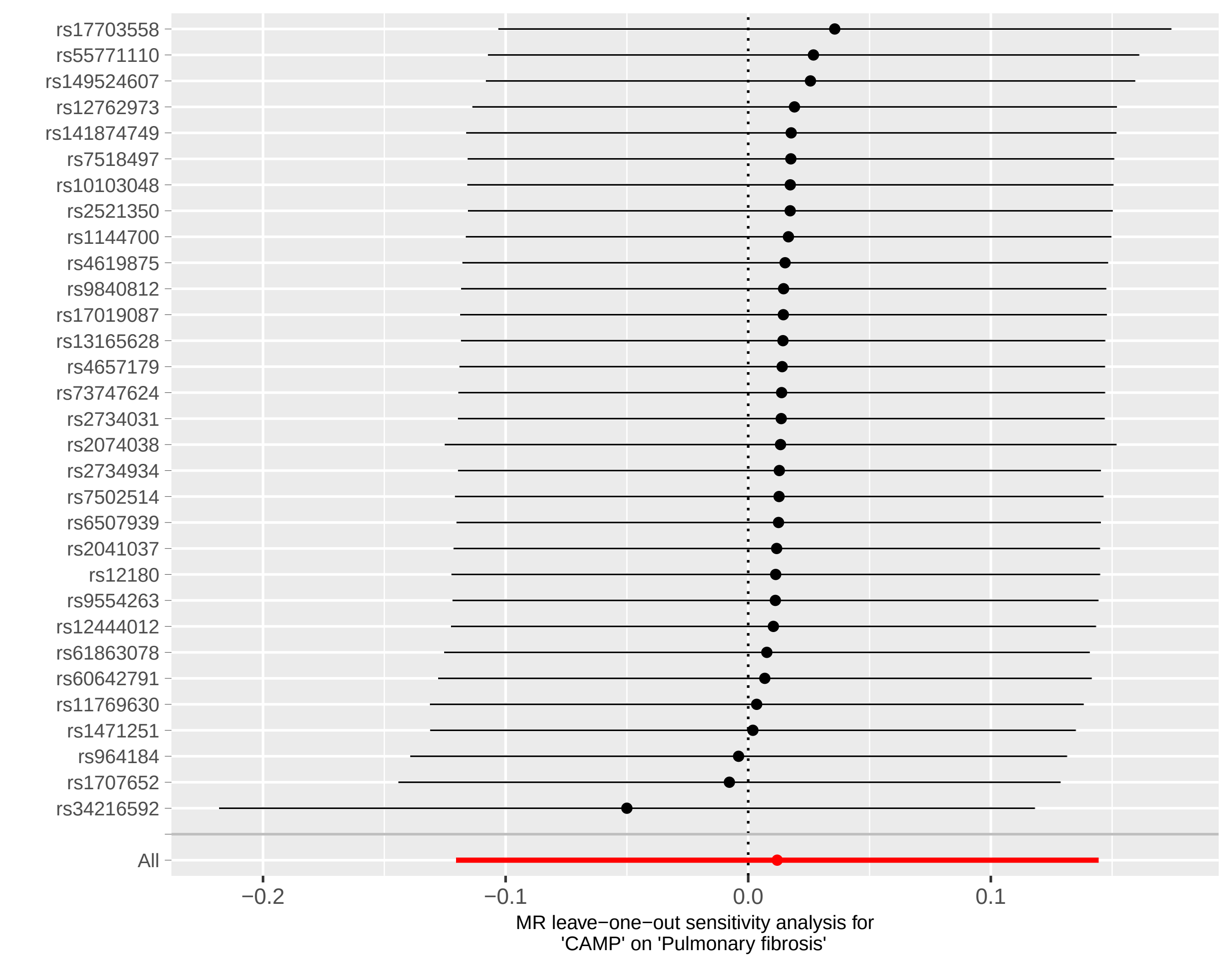
rs17703558
rs55771110
rs149524607
rs12762973
rs141874749
rs7518497
rs10103048
rs2521350
rs1144700
rs4619875
rs9840812
rs17019087
rs13165628
rs4657179
rs73747624
rs2734031
rs2074038
rs2734934
rs7502514
rs6507939
rs2041037
rs12180
rs9554263
rs12444012
rs61863078
rs60642791
rs11769630
rs1471251
rs964184
rs1707652
rs34216592
All
−0.2
−0.1
0.0
0.1
MR leave−one−out sensitivity analysis for
'CAMP' on 'Pulmonary fibrosis'

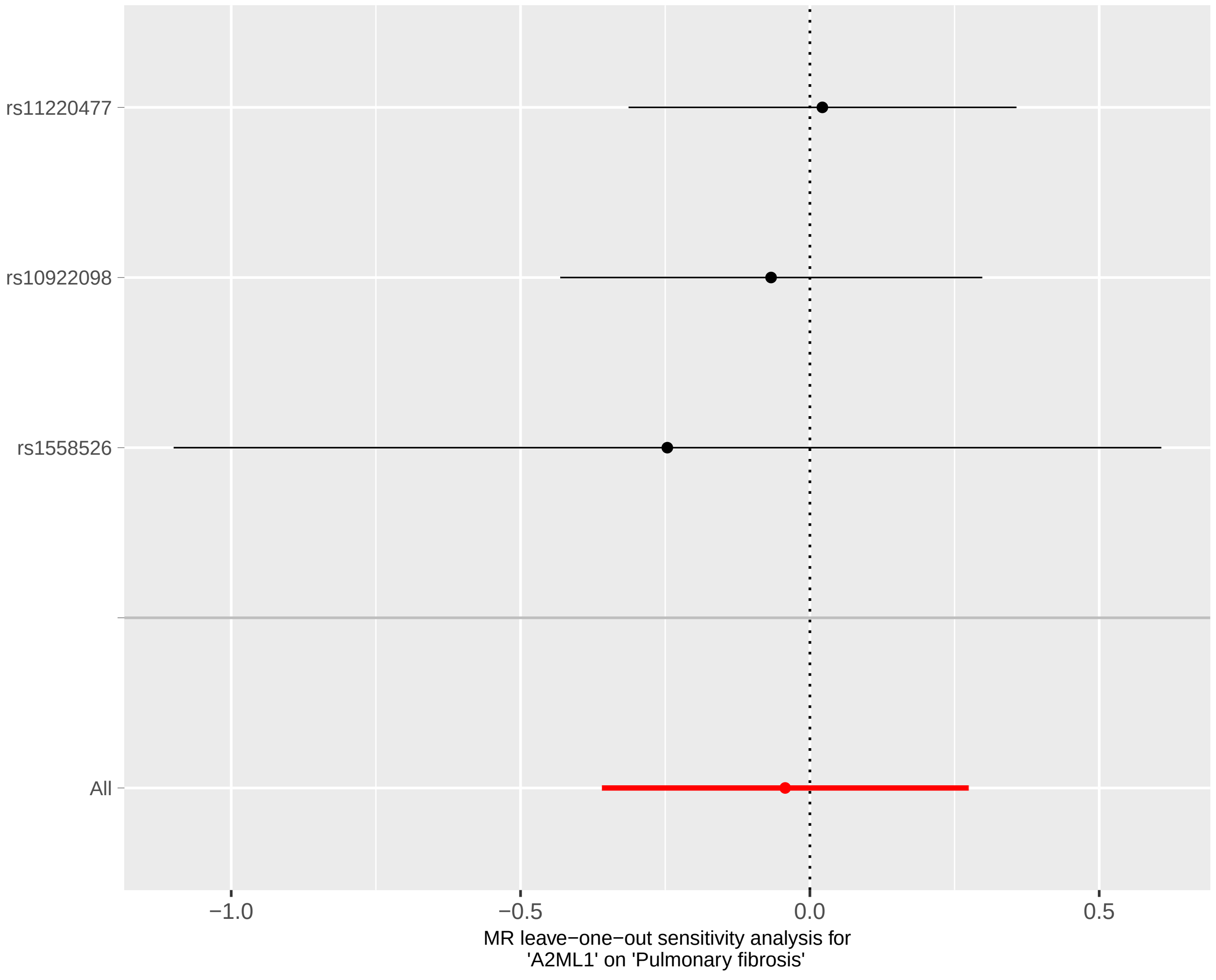
rs11220477
rs10922098
rs1558526
All
−1.0
−0.5
0.0
0.5
MR leave−one−out sensitivity analysis for
'A2ML1' on 'Pulmonary fibrosis'

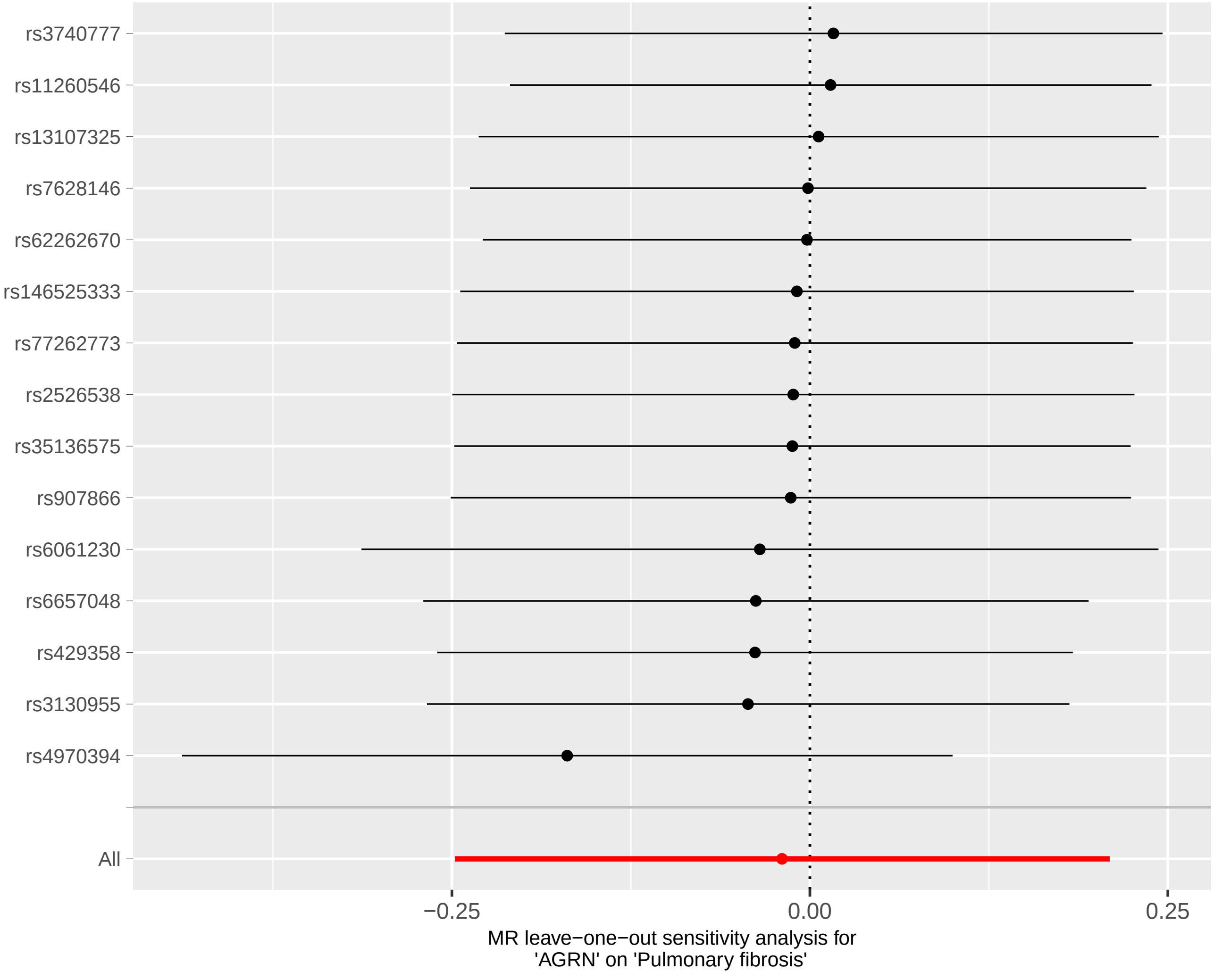
rs3740777
rs11260546
rs13107325
rs7628146
rs62262670
rs146525333
rs77262773
rs2526538
rs35136575
rs907866
rs6061230
rs6657048
rs429358
rs3130955
rs4970394
All
−0.25
0.00
0.25
MR leave−one−out sensitivity analysis for
'AGRN' on 'Pulmonary fibrosis'

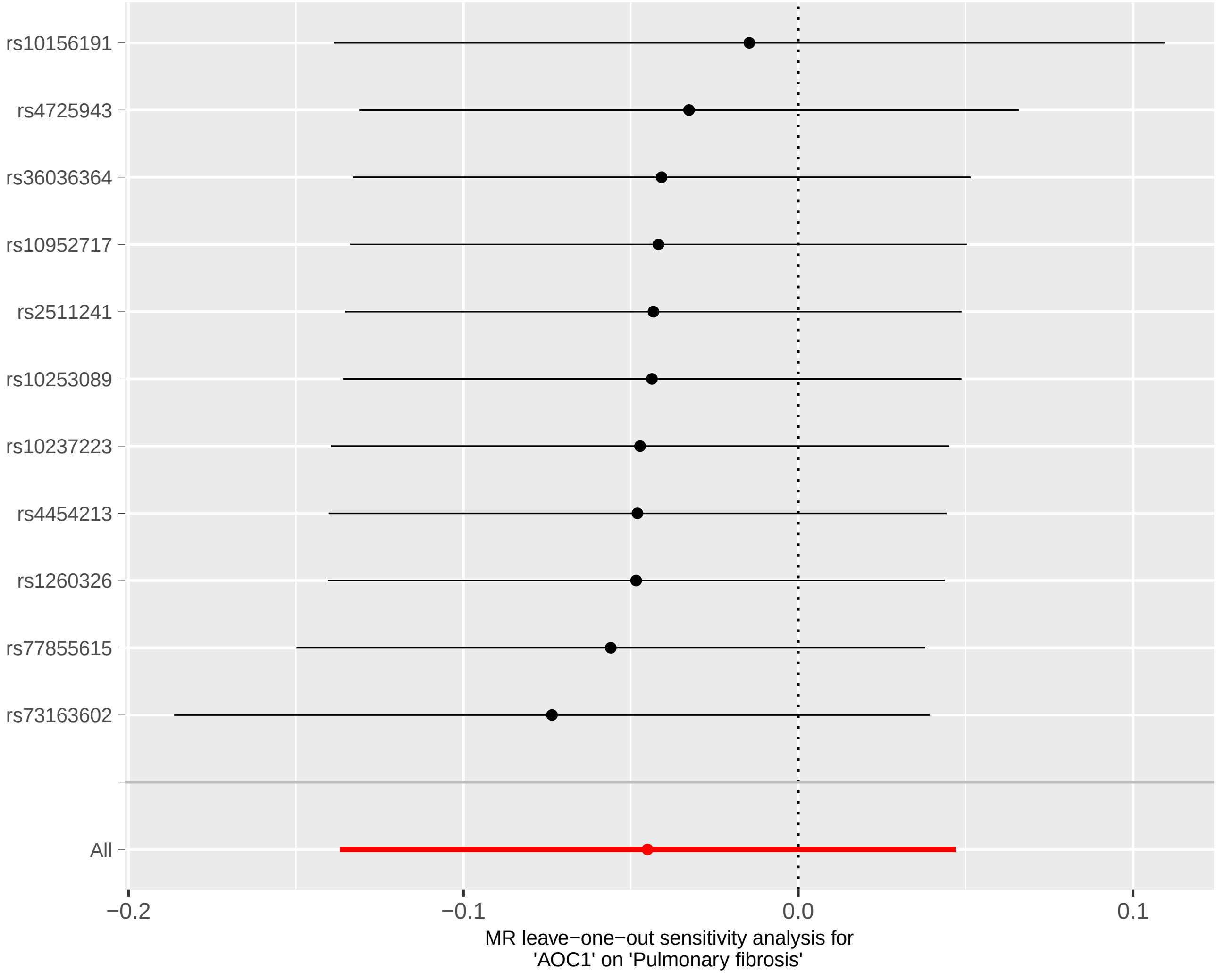
rs10156191
rs4725943
rs36036364
rs10952717
rs2511241
rs10253089
rs10237223
rs4454213
rs1260326
rs77855615
rs73163602
All
−0.2
−0.1
0.0
0.1
MR leave−one−out sensitivity analysis for
'AOC1' on 'Pulmonary fibrosis'

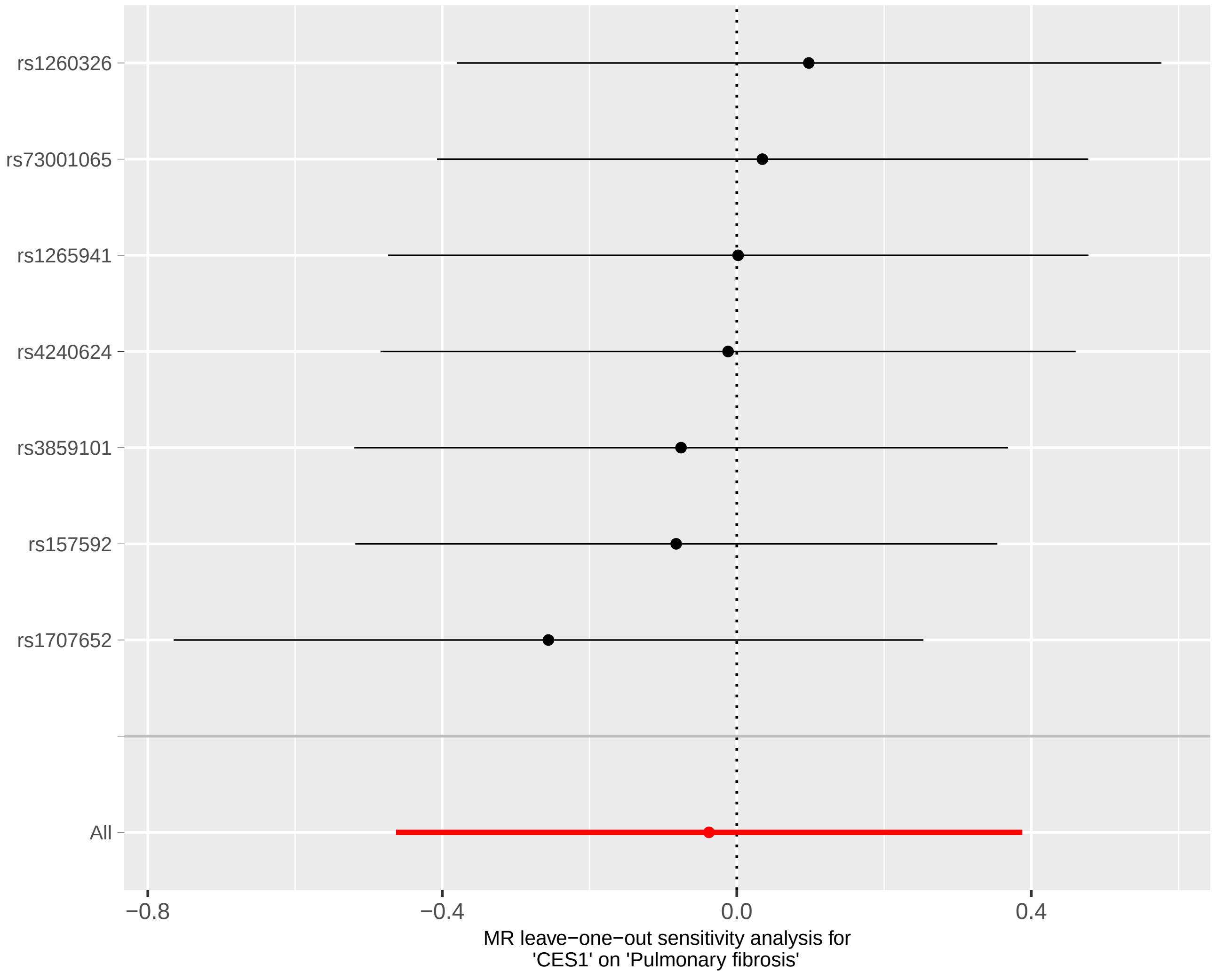

rs1260326
rs73001065
rs1265941
rs4240624
rs3859101
rs157592
rs1707652
All
−0.8
−0.4
0.0
0.4
MR leave−one−out sensitivity analysis for
'CES1' on 'Pulmonary fibrosis'

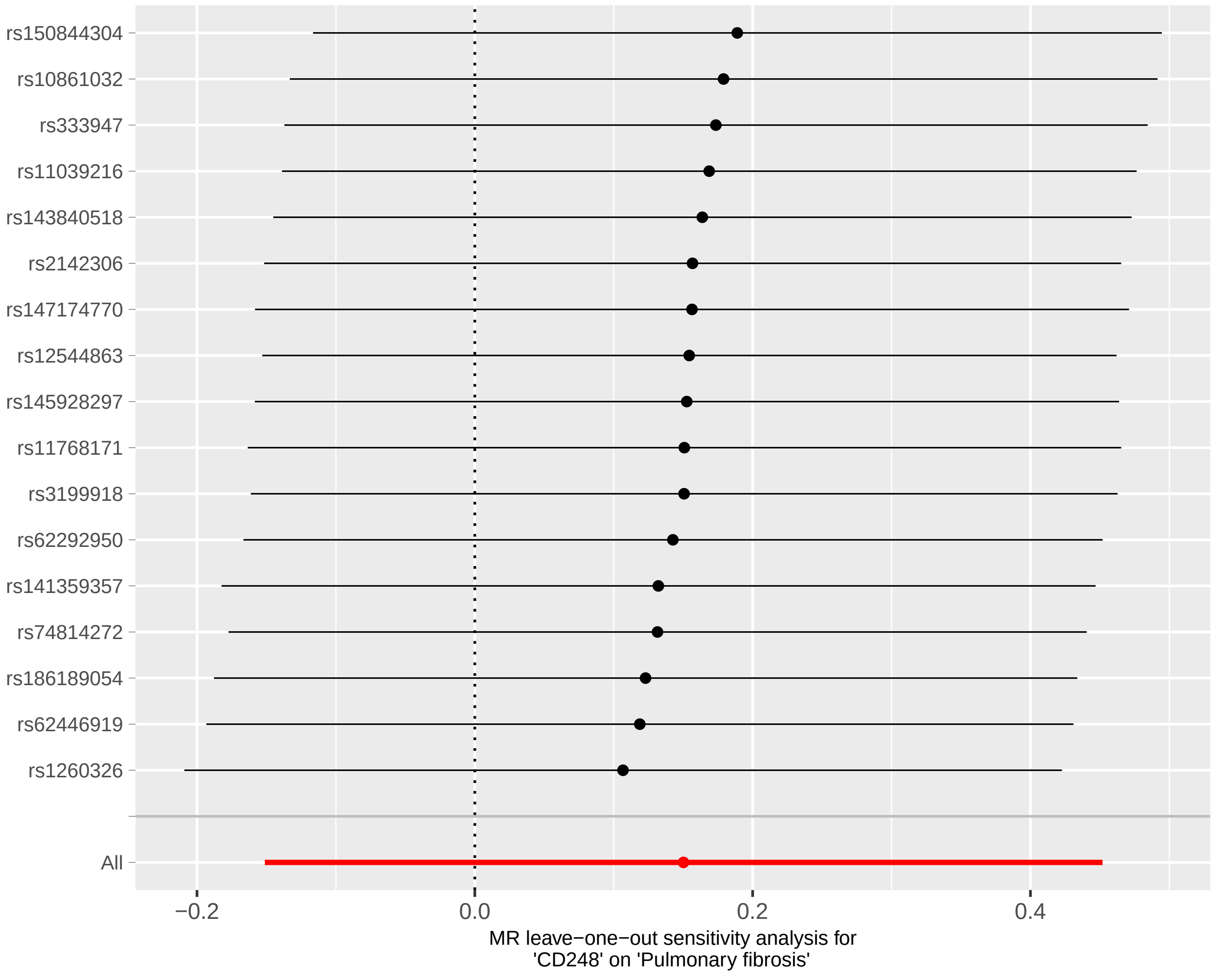

rs150844304
rs10861032
rs333947
rs11039216
rs143840518
rs2142306
rs147174770
rs12544863
rs145928297
rs11768171
rs3199918
rs62292950
rs141359357
rs74814272
rs186189054
rs62446919
rs1260326
All
−0.2
0.0
0.2
0.4
MR leave−one−out sensitivity analysis for
'CD248' on 'Pulmonary fibrosis'

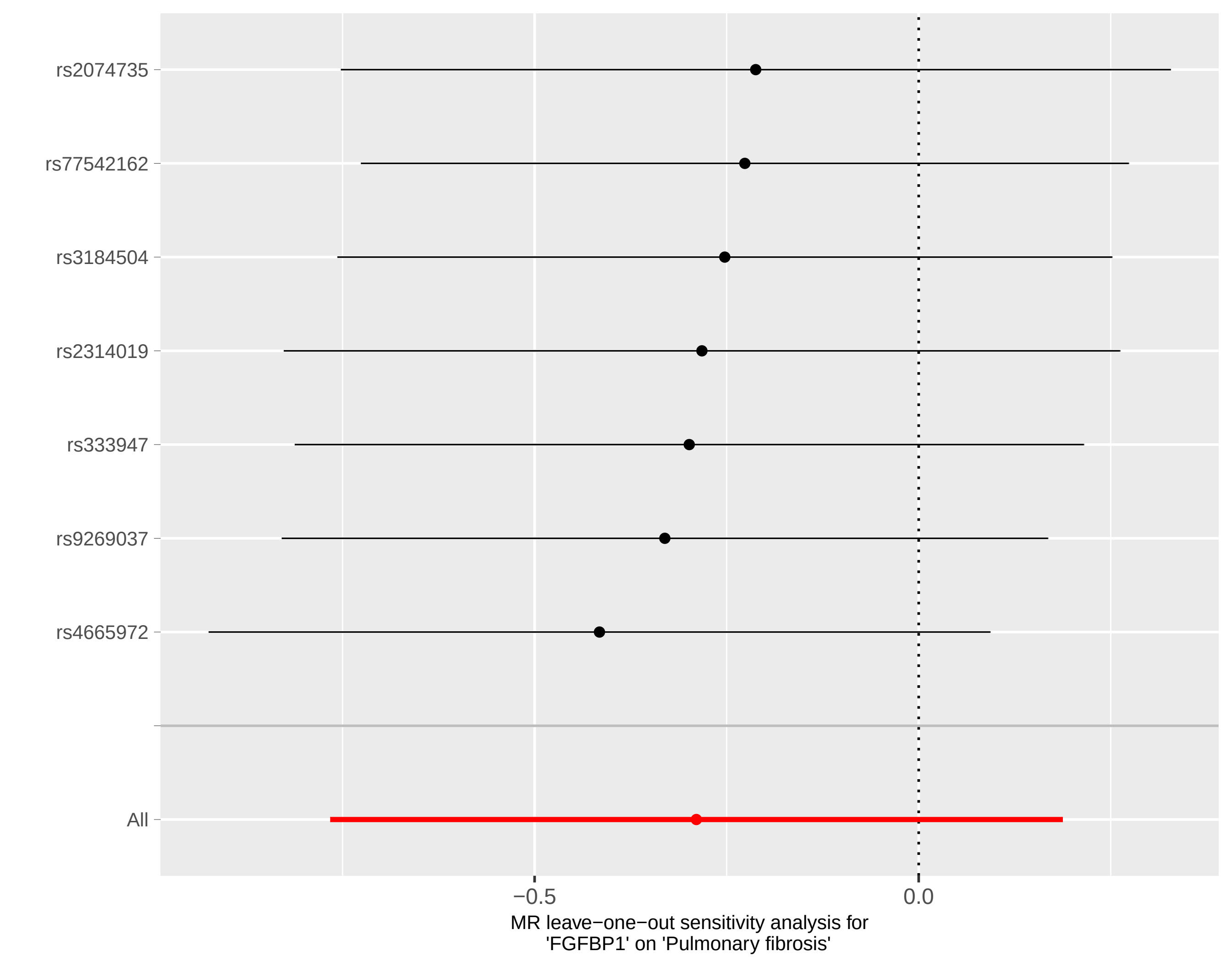
rs2074735
rs77542162
rs3184504
rs2314019
rs333947
rs9269037
rs4665972
All
−0.5
0.0
MR leave−one−out sensitivity analysis for
'FGFBP1' on 'Pulmonary fibrosis'

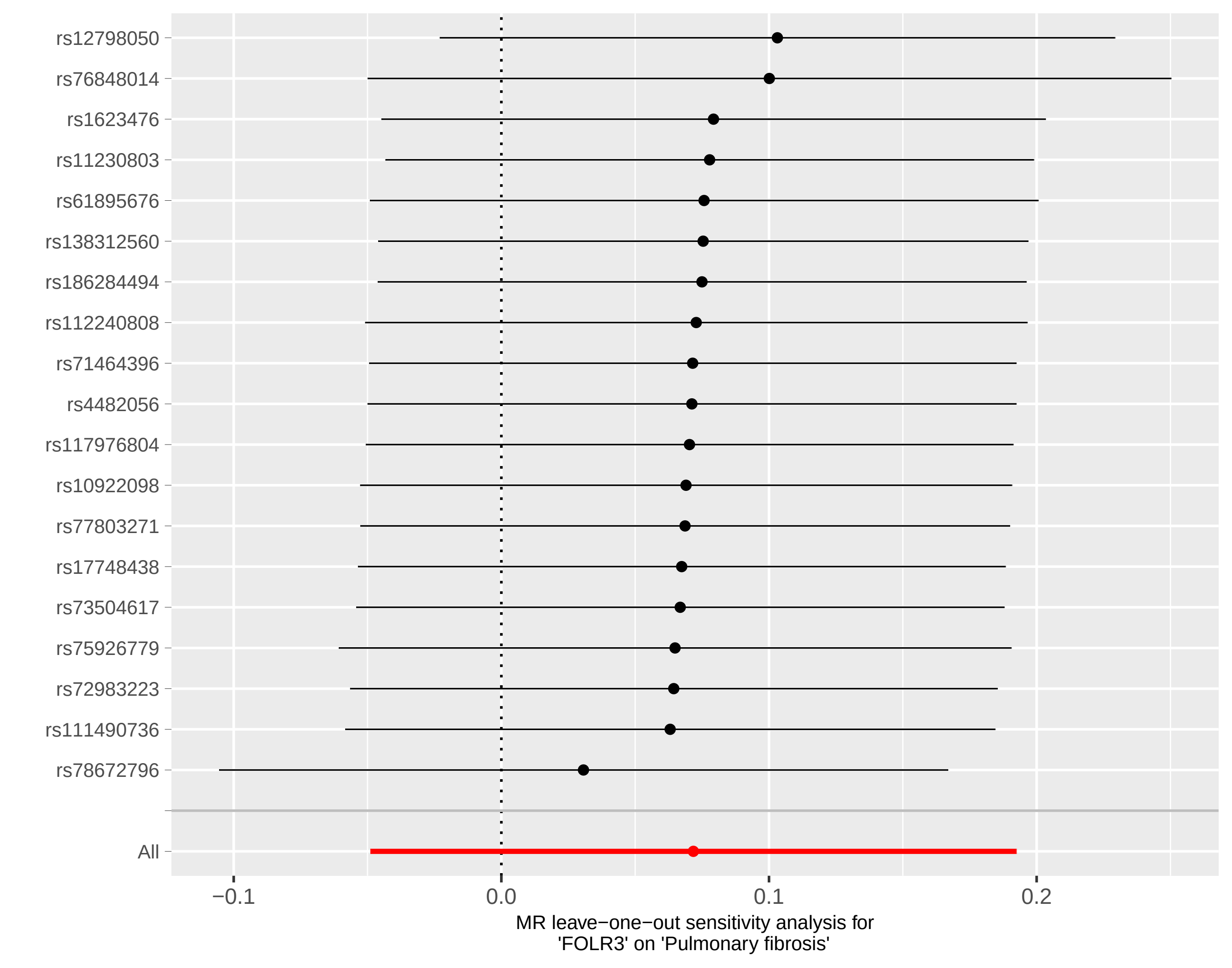
rs12798050
rs76848014
rs1623476
rs11230803
rs61895676
rs138312560
rs186284494
rs112240808
rs71464396
rs4482056
rs117976804
rs10922098
rs77803271
rs17748438
rs73504617
rs75926779
rs72983223
rs111490736
rs78672796
All
−0.1
0.0
0.1
0.2
MR leave−one−out sensitivity analysis for
'FOLR3' on 'Pulmonary fibrosis'

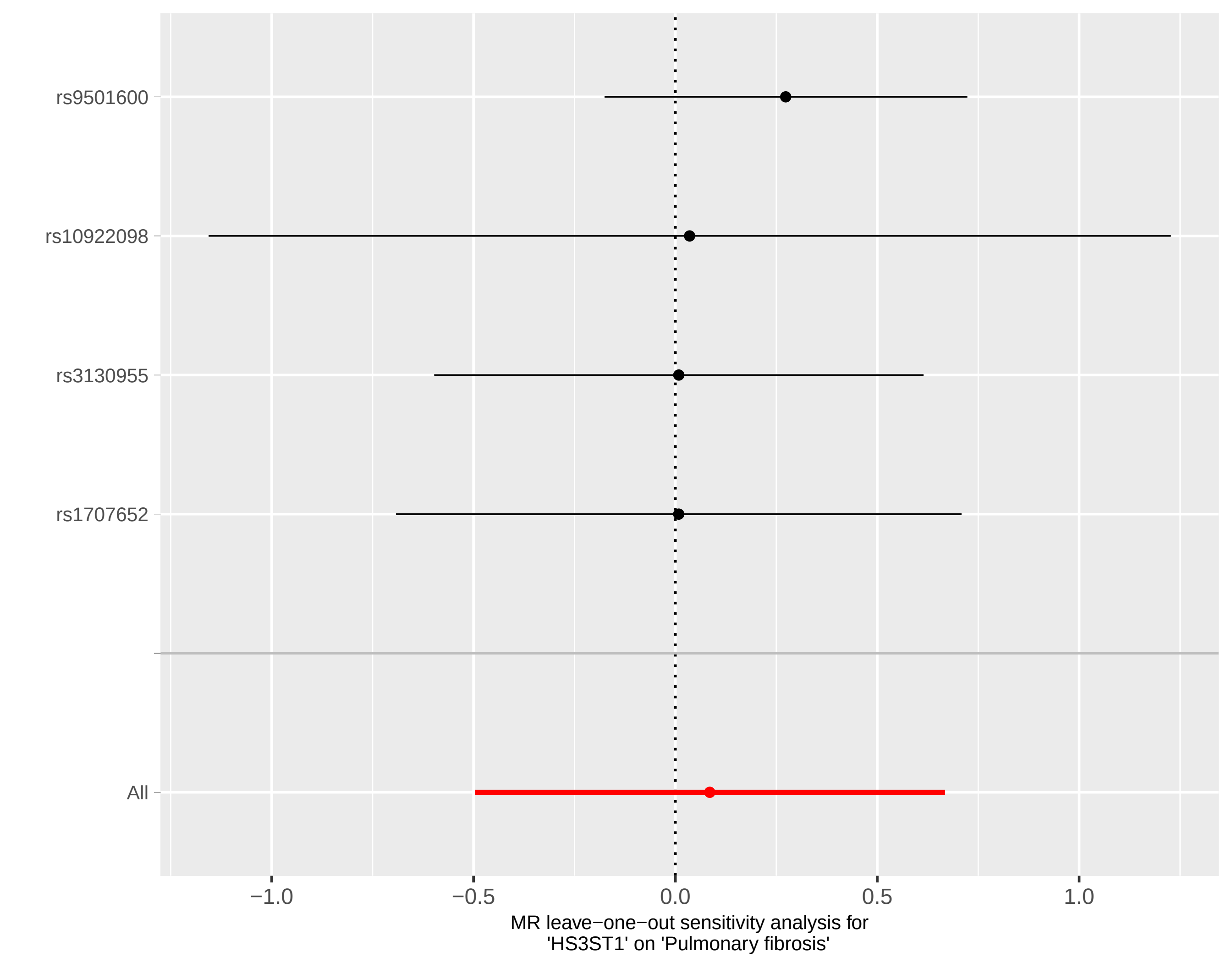
rs9501600
rs10922098
rs3130955
rs1707652
All
−1.0
−0.5
0.0
0.5
1.0
MR leave−one−out sensitivity analysis for
'HS3ST1' on 'Pulmonary fibrosis'

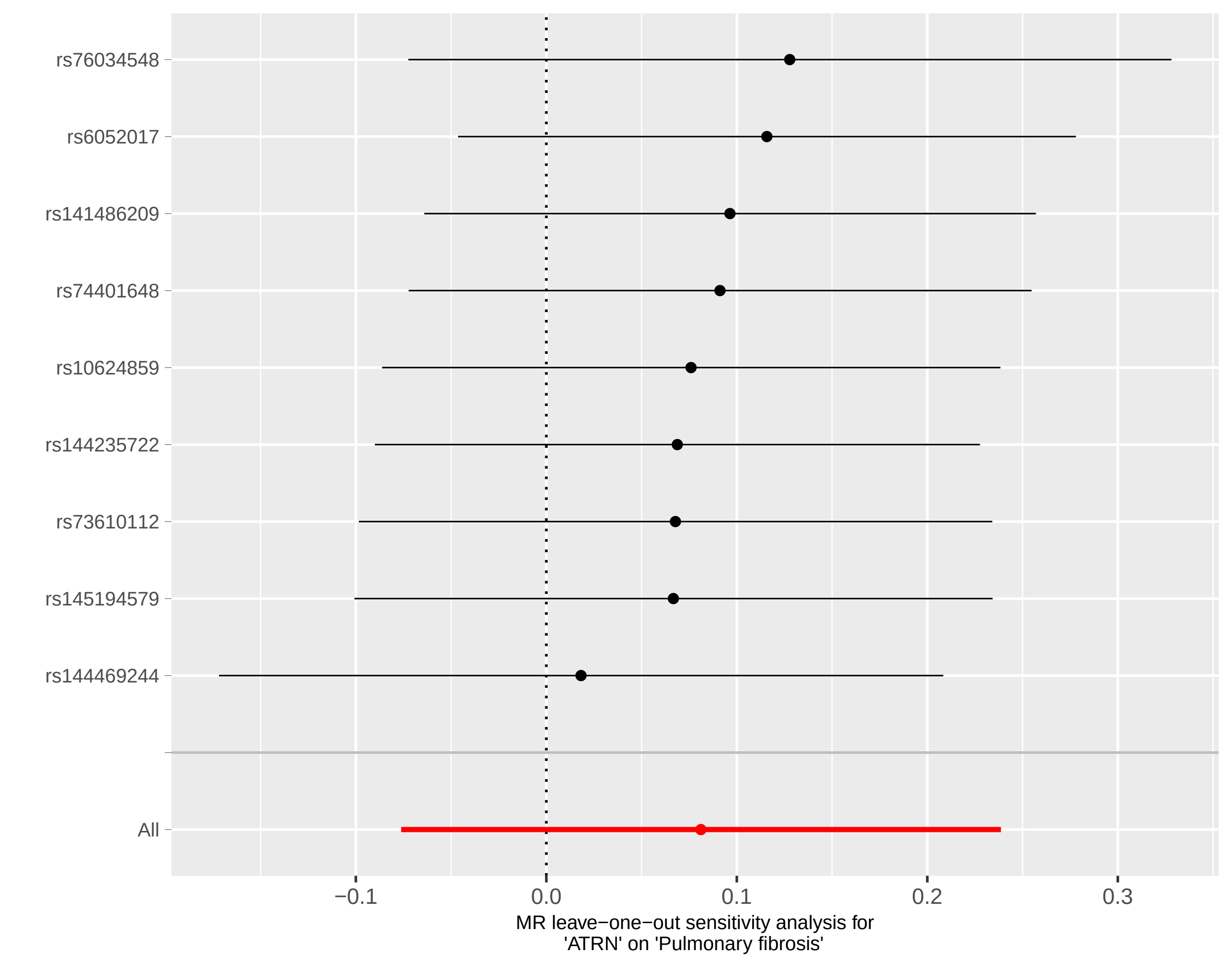
rs76034548
rs6052017
rs141486209
rs74401648
rs10624859
rs144235722
rs73610112
rs145194579
rs144469244
All
−0.1
0.0
0.1
0.2
0.3
MR leave−one−out sensitivity analysis for
'ATRN' on 'Pulmonary fibrosis'

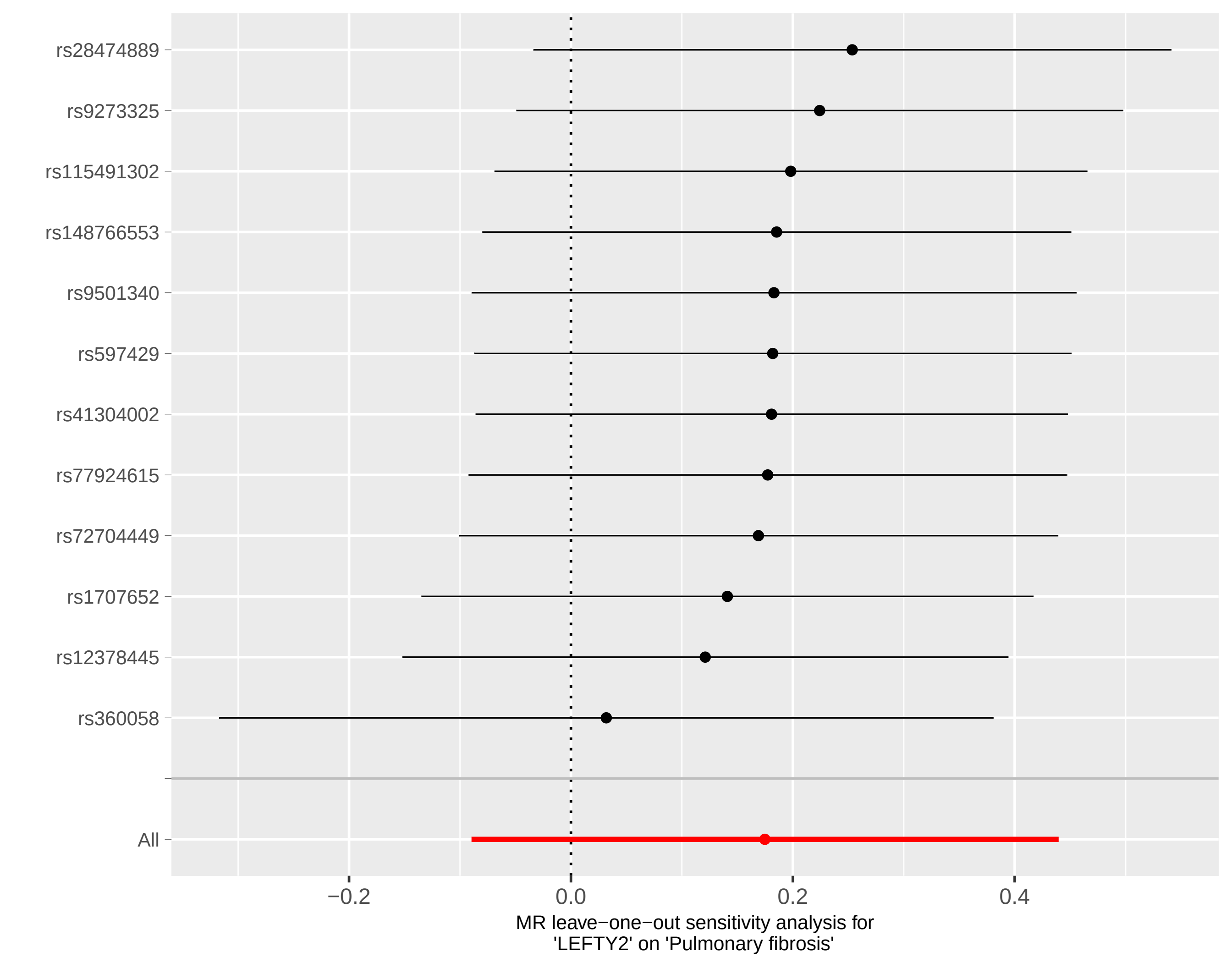
rs28474889
rs9273325
rs115491302
rs148766553
rs9501340
rs597429
rs41304002
rs77924615
rs72704449
rs1707652
rs12378445
rs360058
All
−0.2
0.0
0.2
0.4
MR leave−one−out sensitivity analysis for
'LEFTY2' on 'Pulmonary fibrosis'

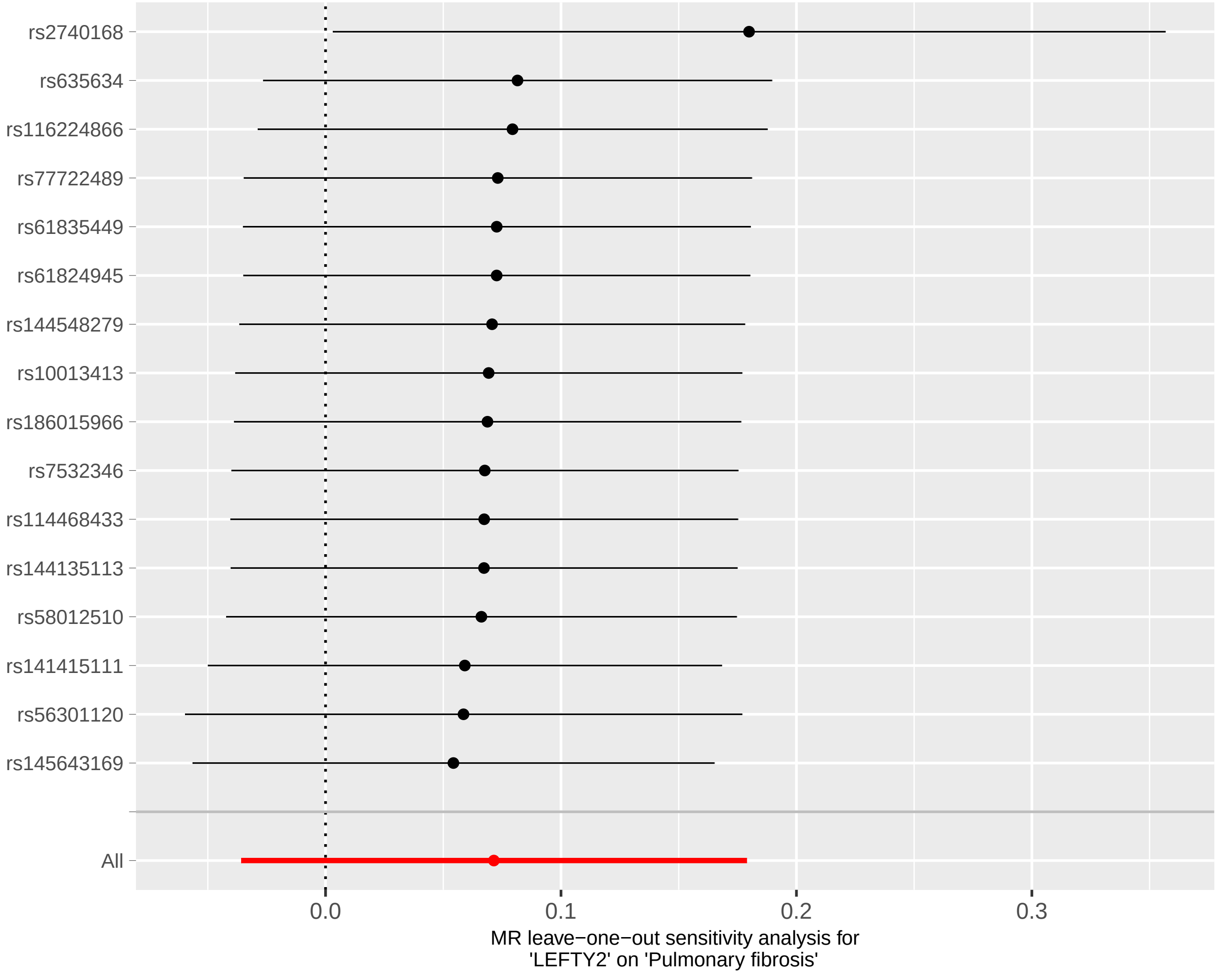

rs2740168
rs635634
rs116224866
rs77722489
rs61835449
rs61824945
rs144548279
rs10013413
rs186015966
rs7532346
rs114468433
rs144135113
rs58012510
rs141415111
rs56301120
rs145643169
All
0.0
0.1
0.2
0.3
MR leave−one−out sensitivity analysis for
'LEFTY2' on 'Pulmonary fibrosis'

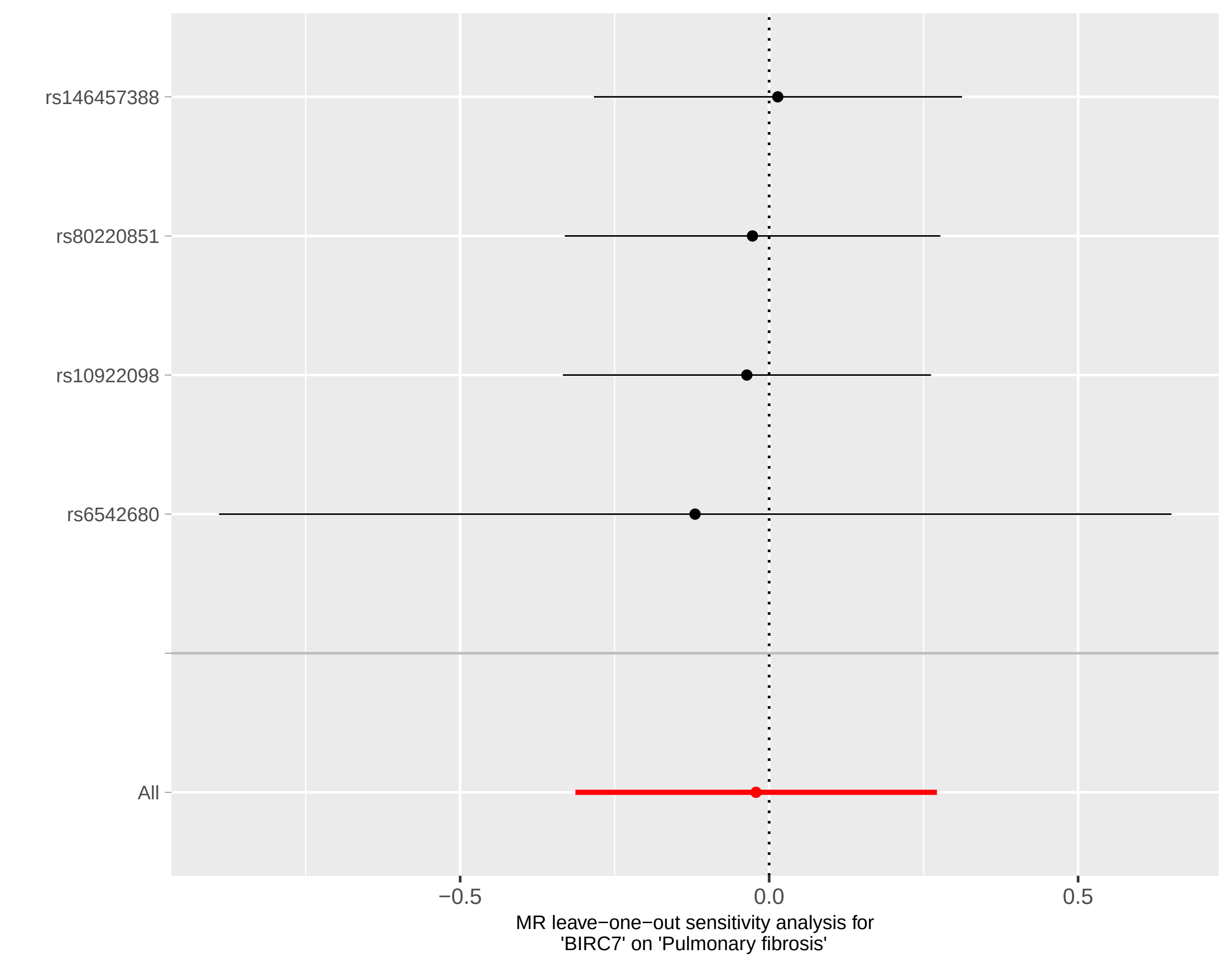
rs146457388
rs80220851
rs10922098
rs6542680
All
−0.5
0.0
0.5
MR leave−one−out sensitivity analysis for
'BIRC7' on 'Pulmonary fibrosis'

Insufficient number of SNPs

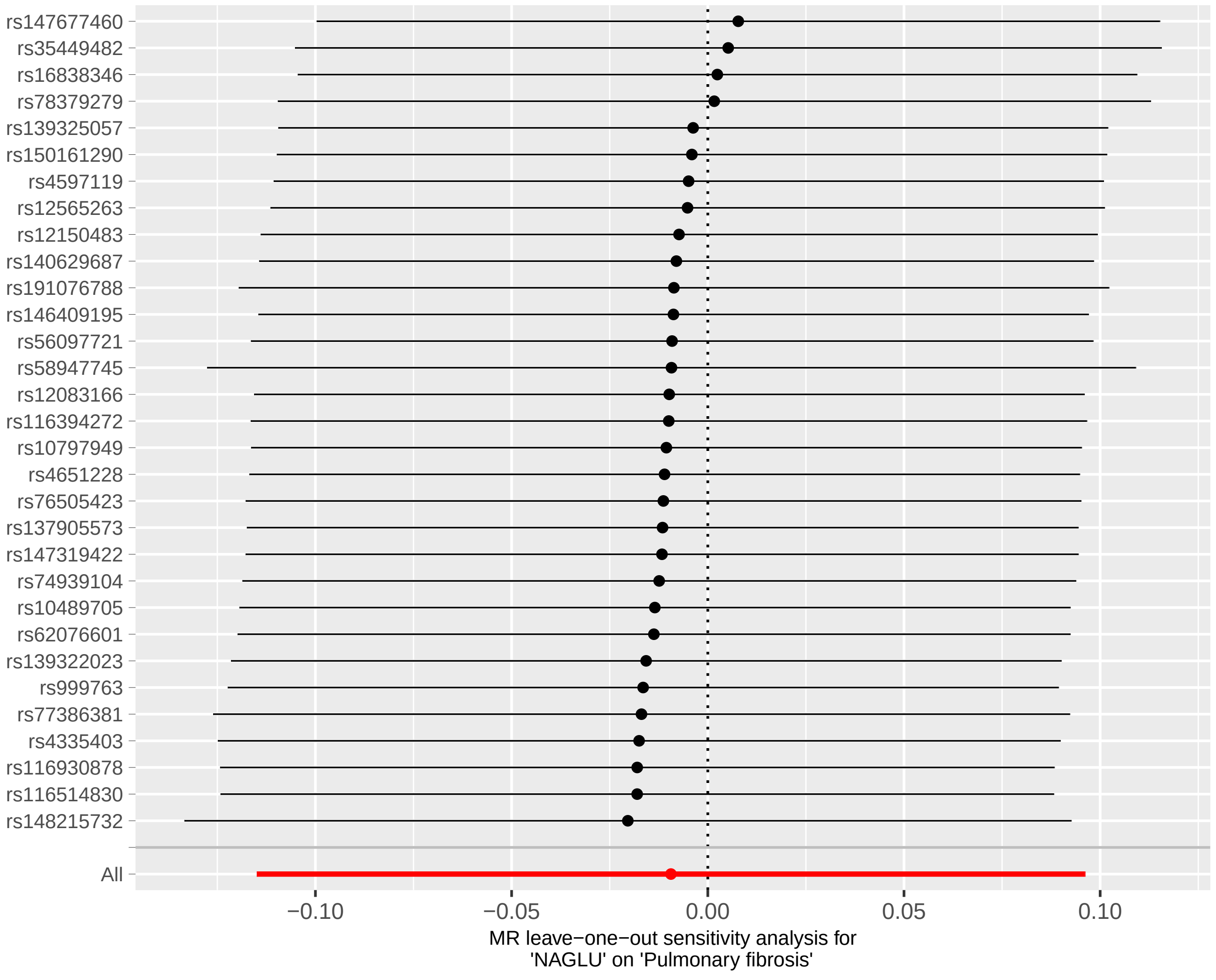
rs147677460
rs35449482
rs16838346
rs78379279
rs139325057
rs150161290
rs4597119
rs12565263
rs12150483
rs140629687
rs191076788
rs146409195
rs56097721
rs58947745
rs12083166
rs116394272
rs10797949
rs4651228
rs76505423
rs137905573
rs147319422
rs74939104
rs10489705
rs62076601
rs139322023
rs999763
rs77386381
rs4335403
rs116930878
rs116514830
rs148215732
All
−0.10
−0.05
0.00
0.05
0.10
MR leave−one−out sensitivity analysis for
'NAGLU' on 'Pulmonary fibrosis'

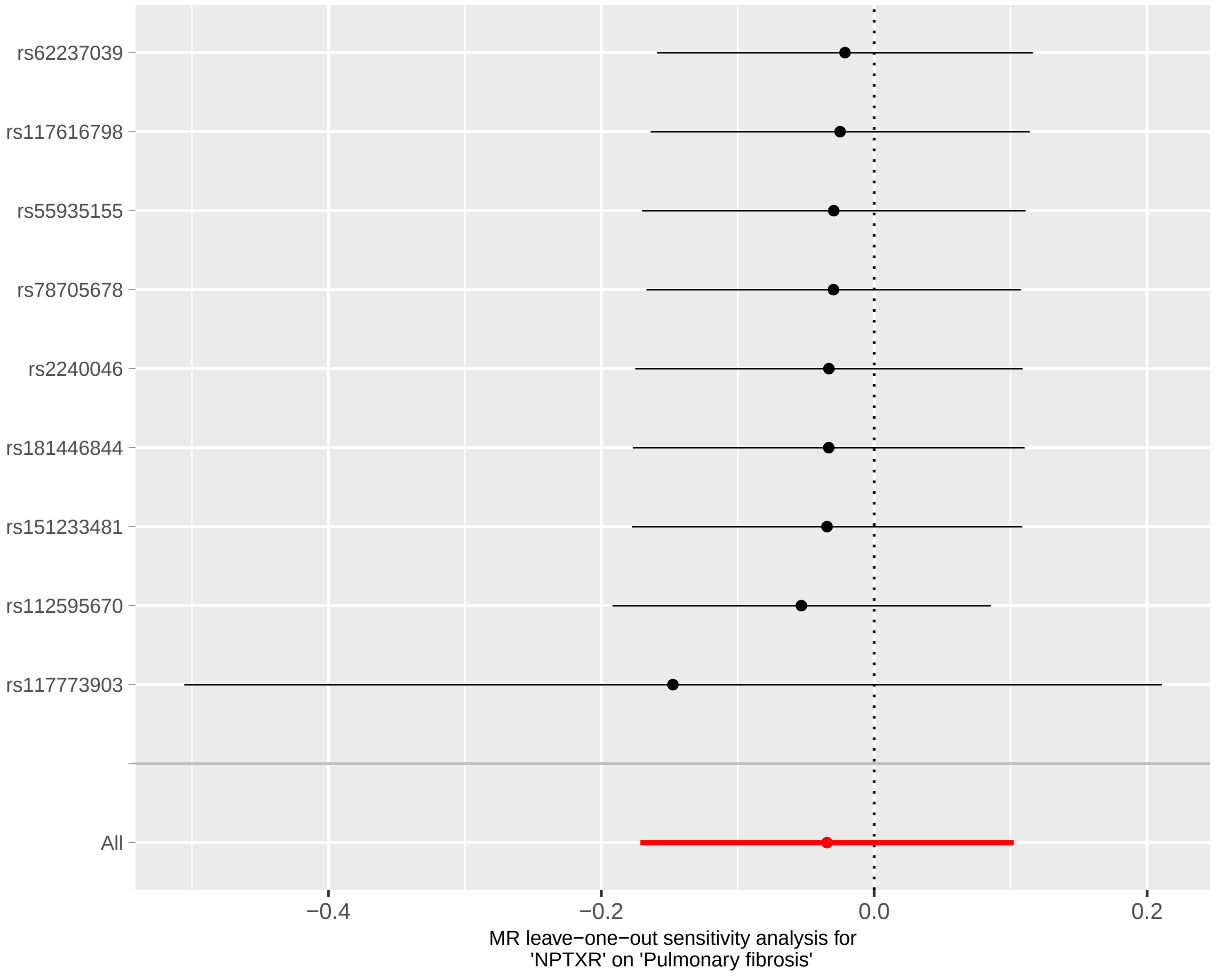
rs62237039
rs117616798
rs55935155
rs78705678
rs2240046
rs181446844
rs151233481
rs112595670
rs117773903
All
−0.4
−0.2
0.0
0.2
MR leave−one−out sensitivity analysis for
'NPTXR' on 'Pulmonary fibrosis'

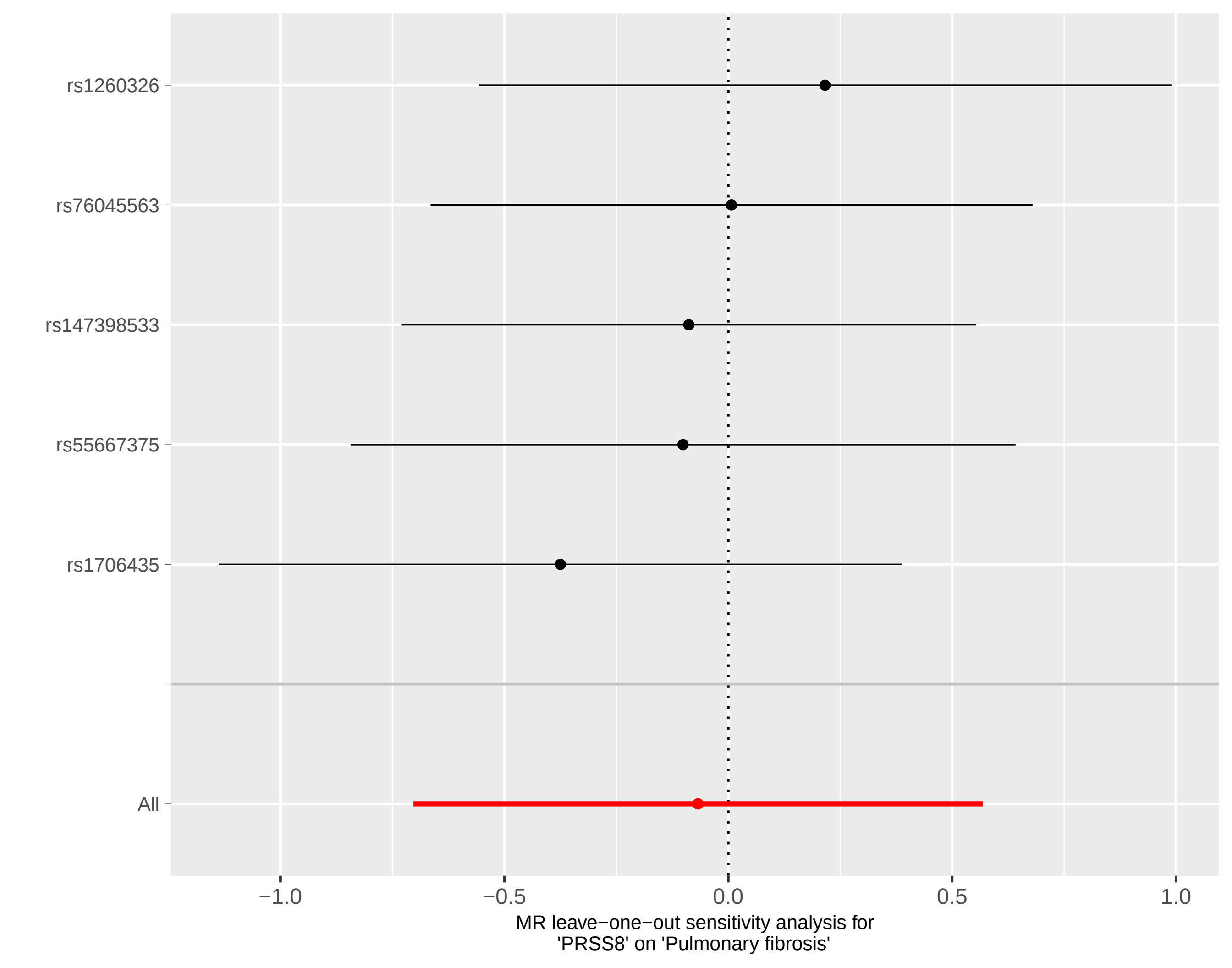

rs1260326
rs76045563
rs147398533
rs55667375
rs1706435
All
−1.0
−0.5
0.0
0.5
1.0
MR leave−one−out sensitivity analysis for
'PRSS8' on 'Pulmonary fibrosis'

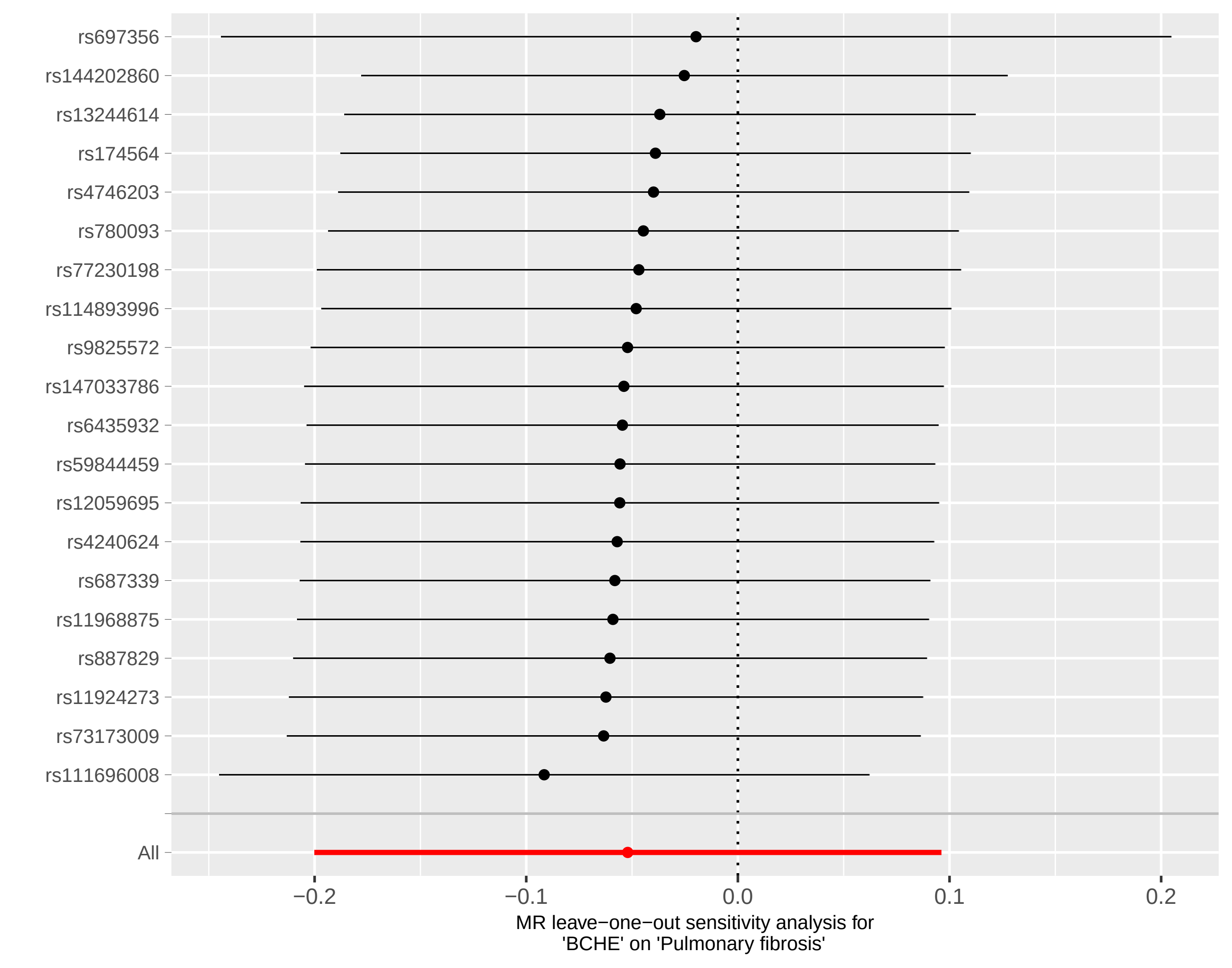
rs697356
rs144202860
rs13244614
rs174564
rs4746203
rs780093
rs77230198
rs114893996
rs9825572
rs147033786
rs6435932
rs59844459
rs12059695
rs4240624
rs687339
rs11968875
rs887829
rs11924273
rs73173009
rs111696008
All
−0.2
−0.1
0.0
0.1
0.2
MR leave−one−out sensitivity analysis for
'BCHE' on 'Pulmonary fibrosis'

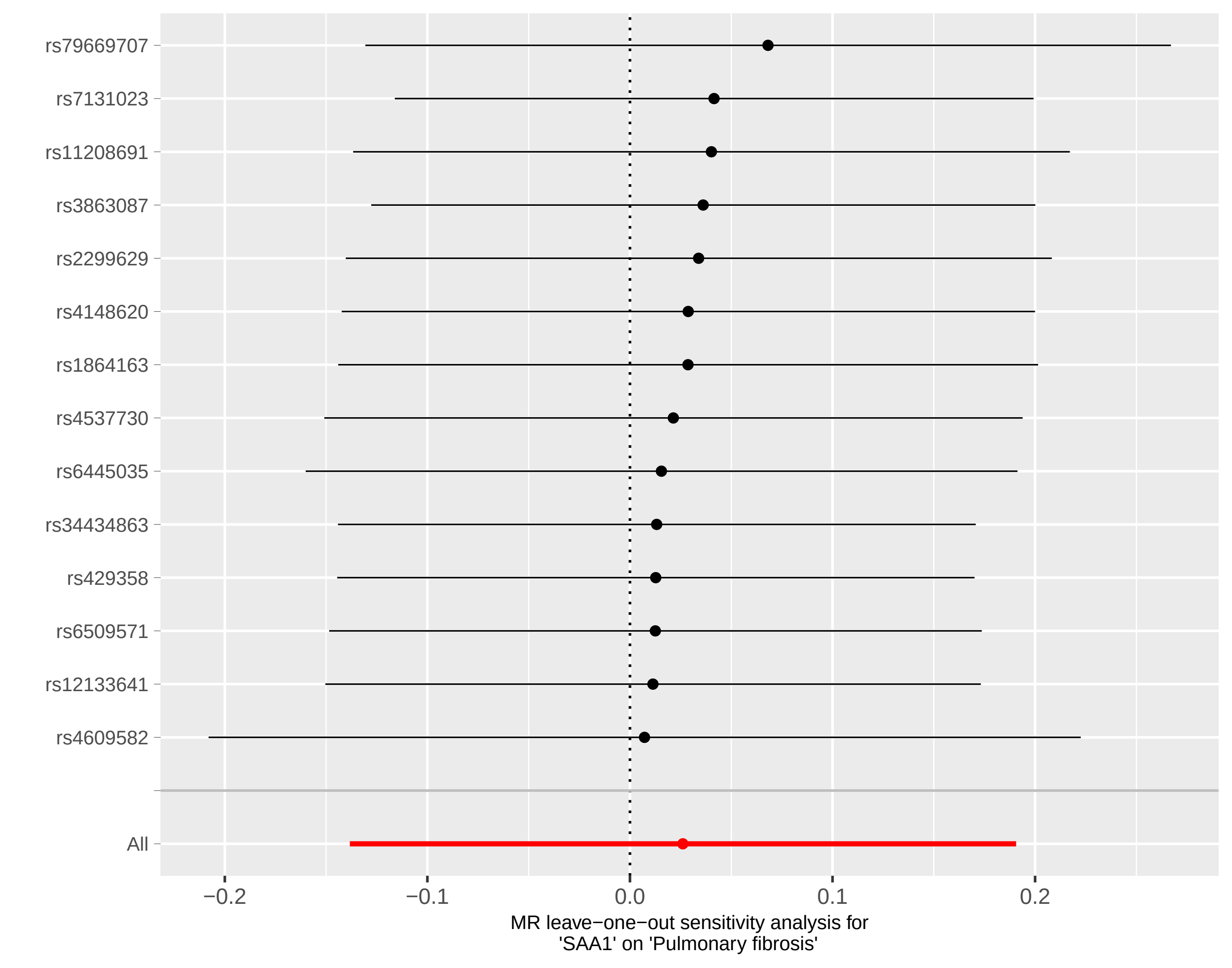
rs79669707
rs7131023
rs11208691
rs3863087
rs2299629
rs4148620
rs1864163
rs4537730
rs6445035
rs34434863
rs429358
rs6509571
rs12133641
rs4609582
All
−0.2
−0.1
0.0
0.1
0.2
MR leave−one−out sensitivity analysis for
'SAA1' on 'Pulmonary fibrosis'

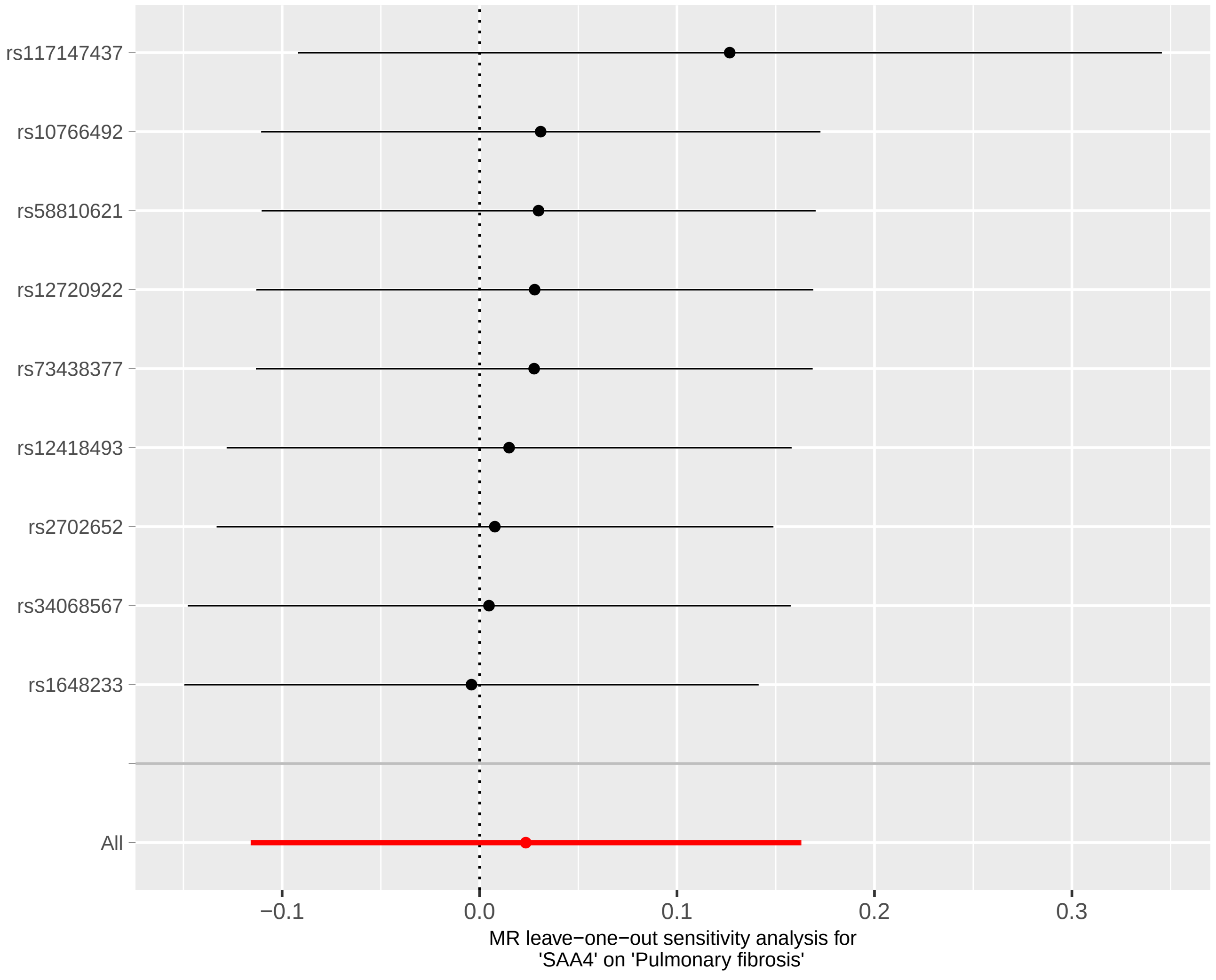
rs117147437
rs10766492
rs58810621
rs12720922
rs73438377
rs12418493
rs2702652
rs34068567
rs1648233
All
−0.1
0.0
0.1
0.2
0.3
MR leave−one−out sensitivity analysis for
'SAA4' on 'Pulmonary fibrosis'

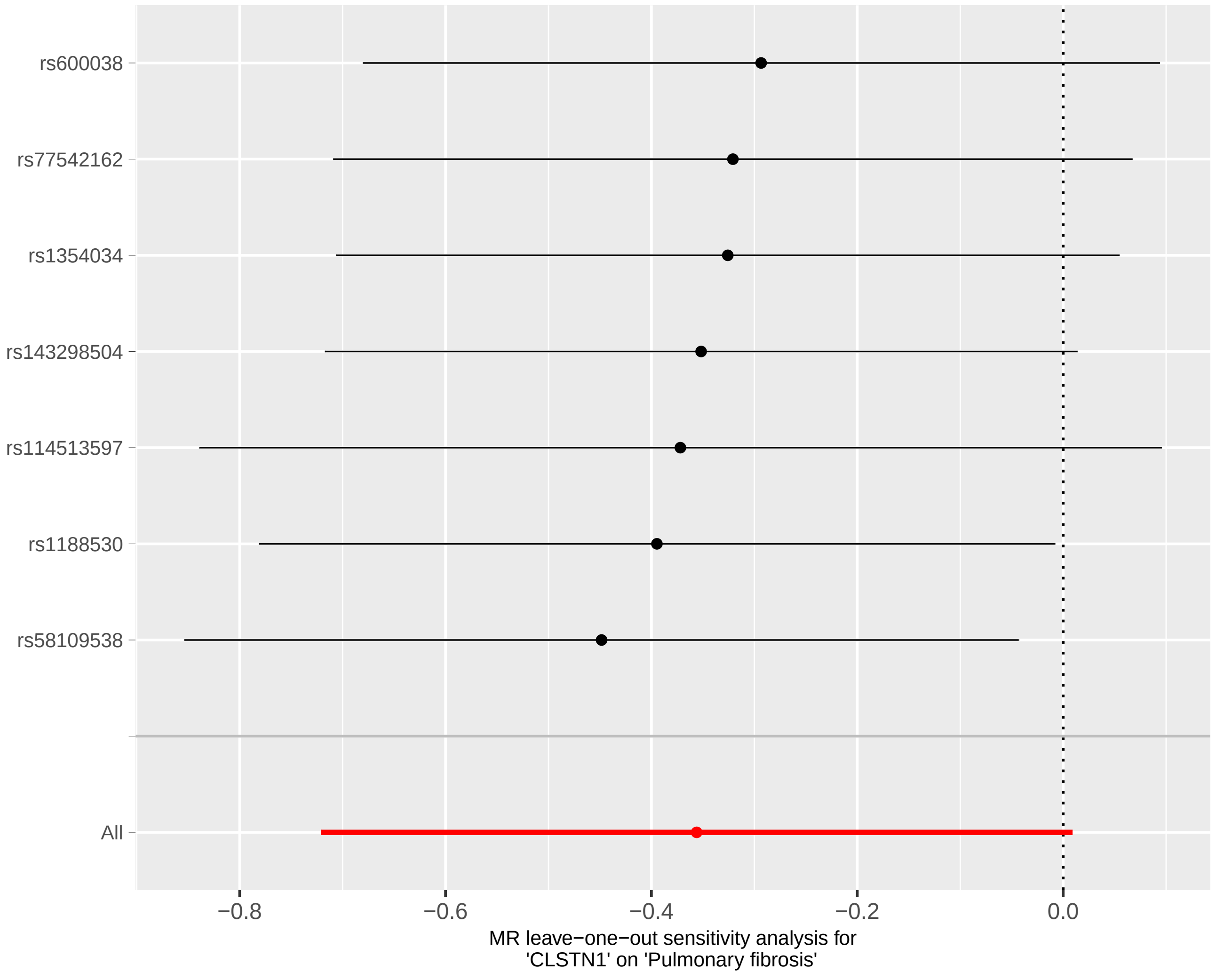
rs600038
rs77542162
rs1354034
rs143298504
rs114513597
rs1188530
rs58109538
All
−0.8
−0.6
−0.4
−0.2
0.0
MR leave−one−out sensitivity analysis for
'CLSTN1' on 'Pulmonary fibrosis'

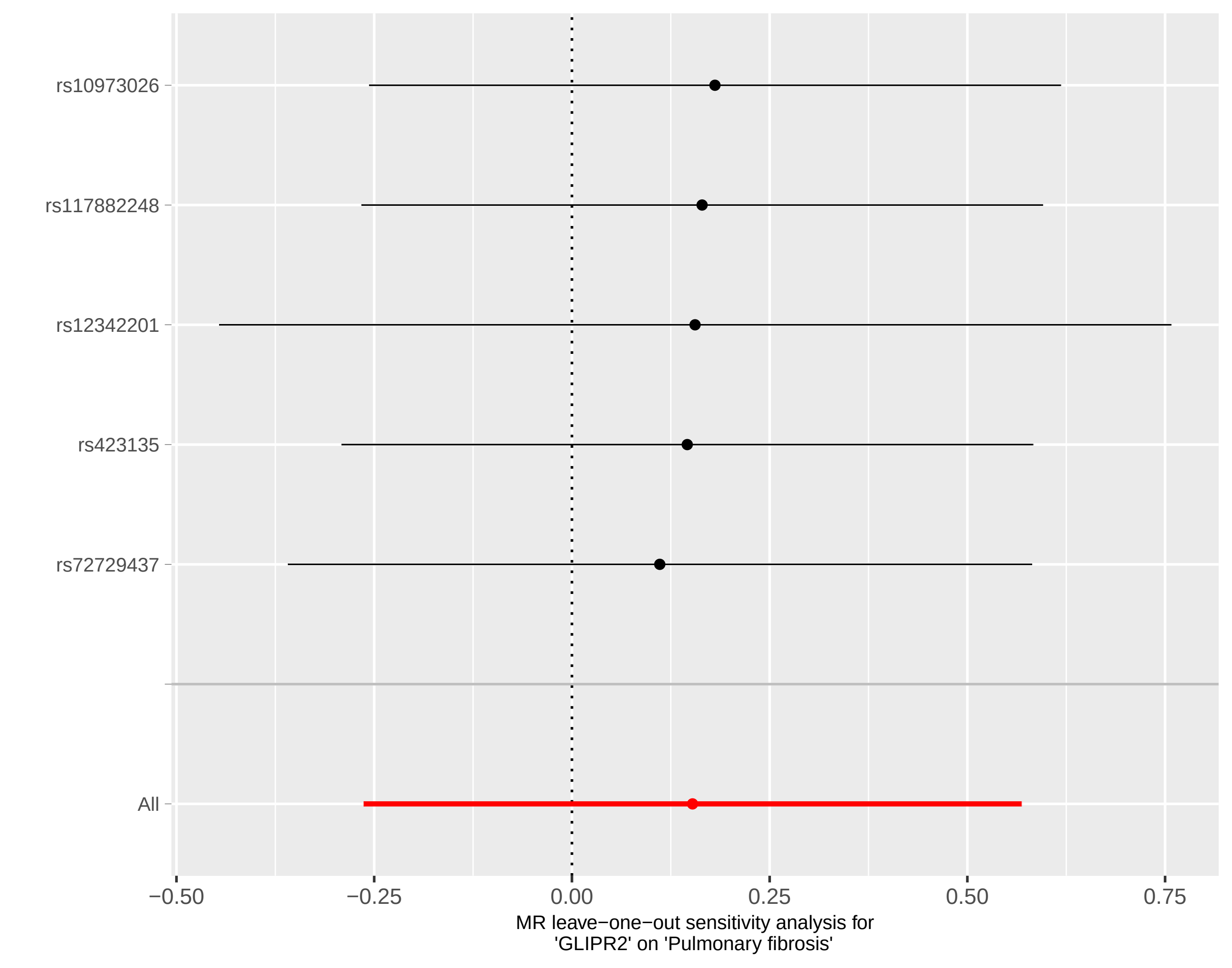
rs10973026
rs117882248
rs12342201
rs423135
rs72729437
All
−0.50
−0.25
0.00
0.25
0.50
0.75
MR leave−one−out sensitivity analysis for
'GLIPR2' on 'Pulmonary fibrosis'

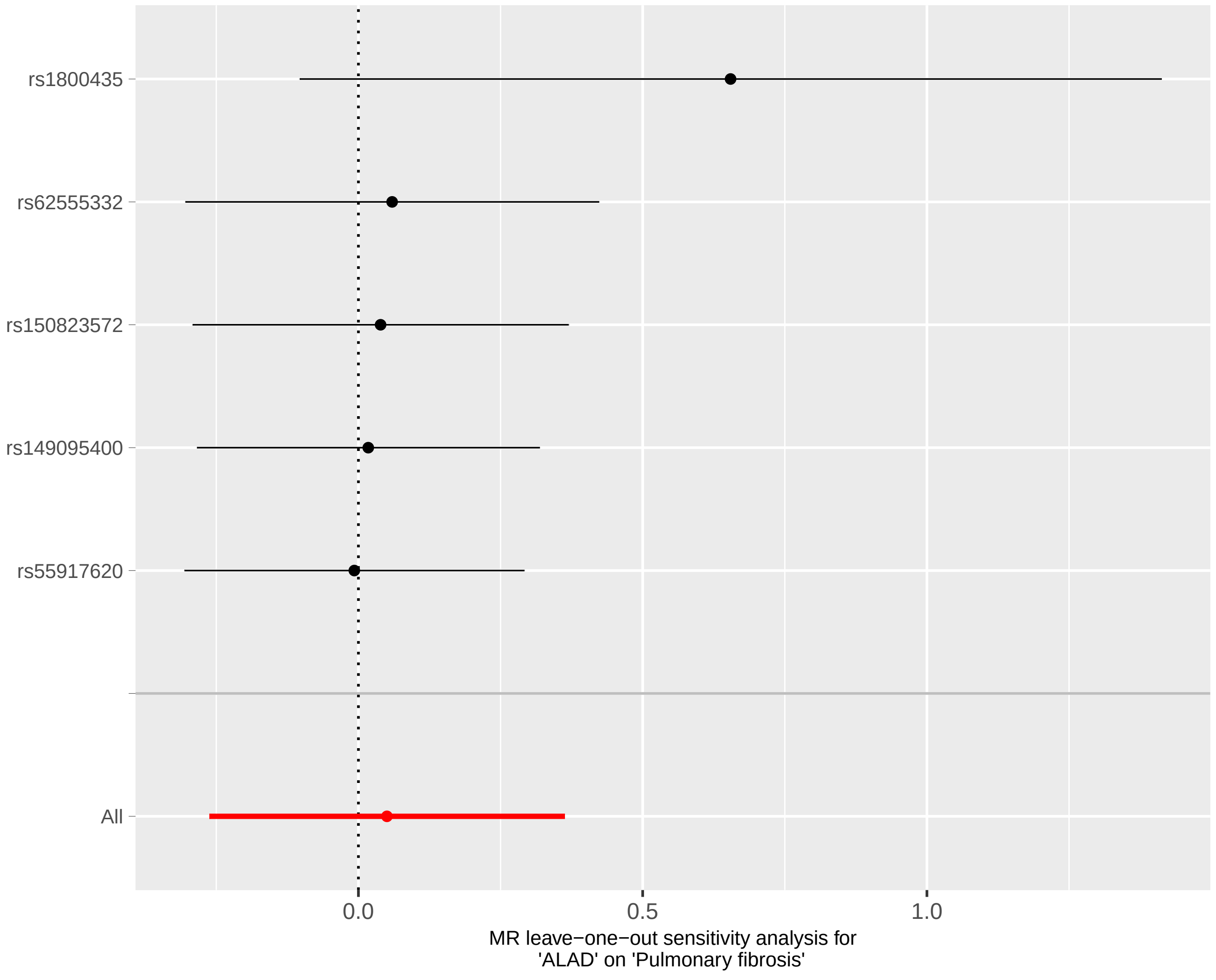
rs1800435
rs62555332
rs150823572
rs149095400
rs55917620
All
0.0
0.5
1.0
MR leave−one−out sensitivity analysis for
'ALAD' on 'Pulmonary fibrosis'

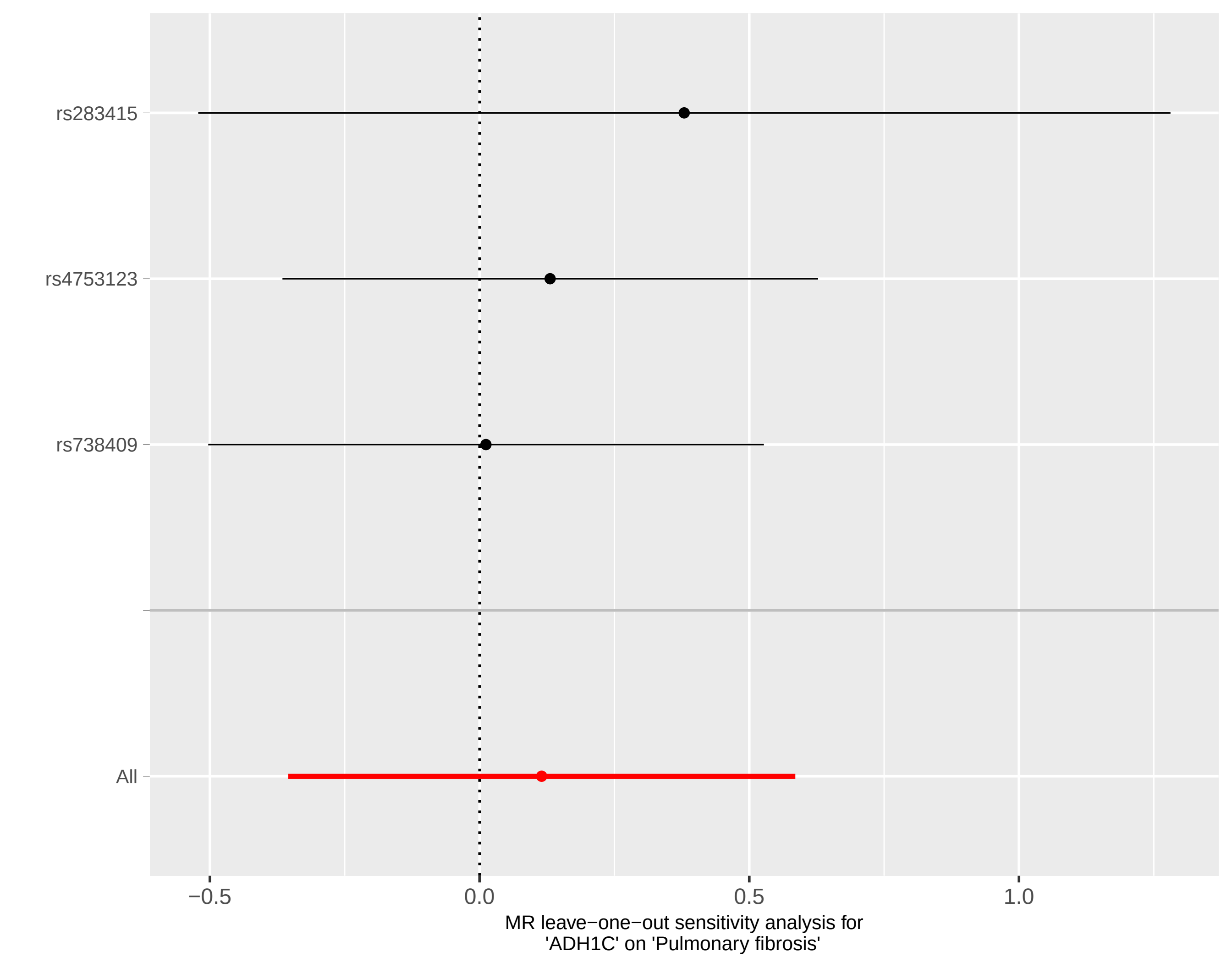
rs283415
rs4753123
rs738409
All
−0.5
0.0
0.5
1.0
MR leave−one−out sensitivity analysis for
'ADH1C' on 'Pulmonary fibrosis'

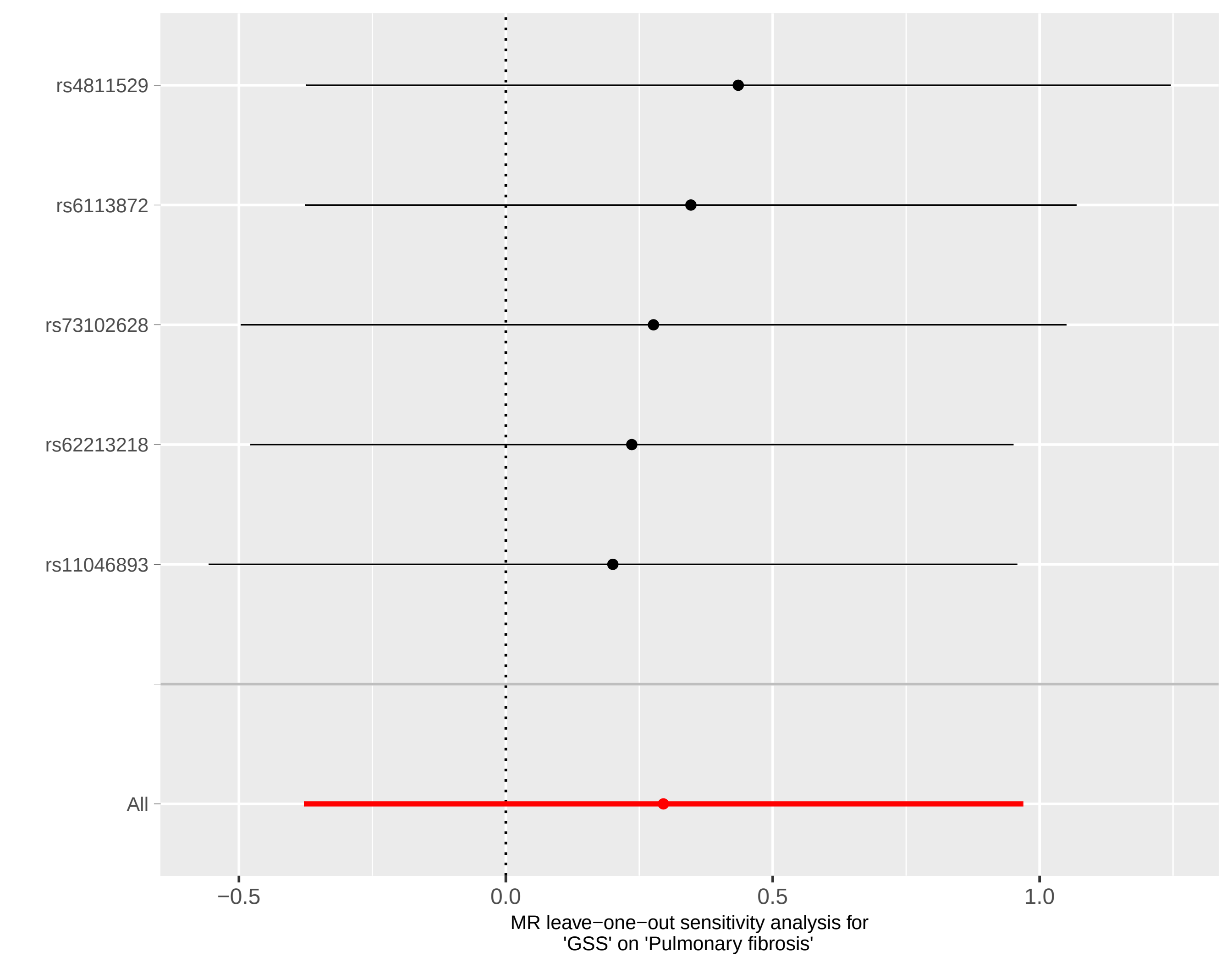

rs4811529
rs6113872
rs73102628
rs62213218
rs11046893
All
−0.5
0.0
0.5
1.0
MR leave−one−out sensitivity analysis for
'GSS' on 'Pulmonary fibrosis'

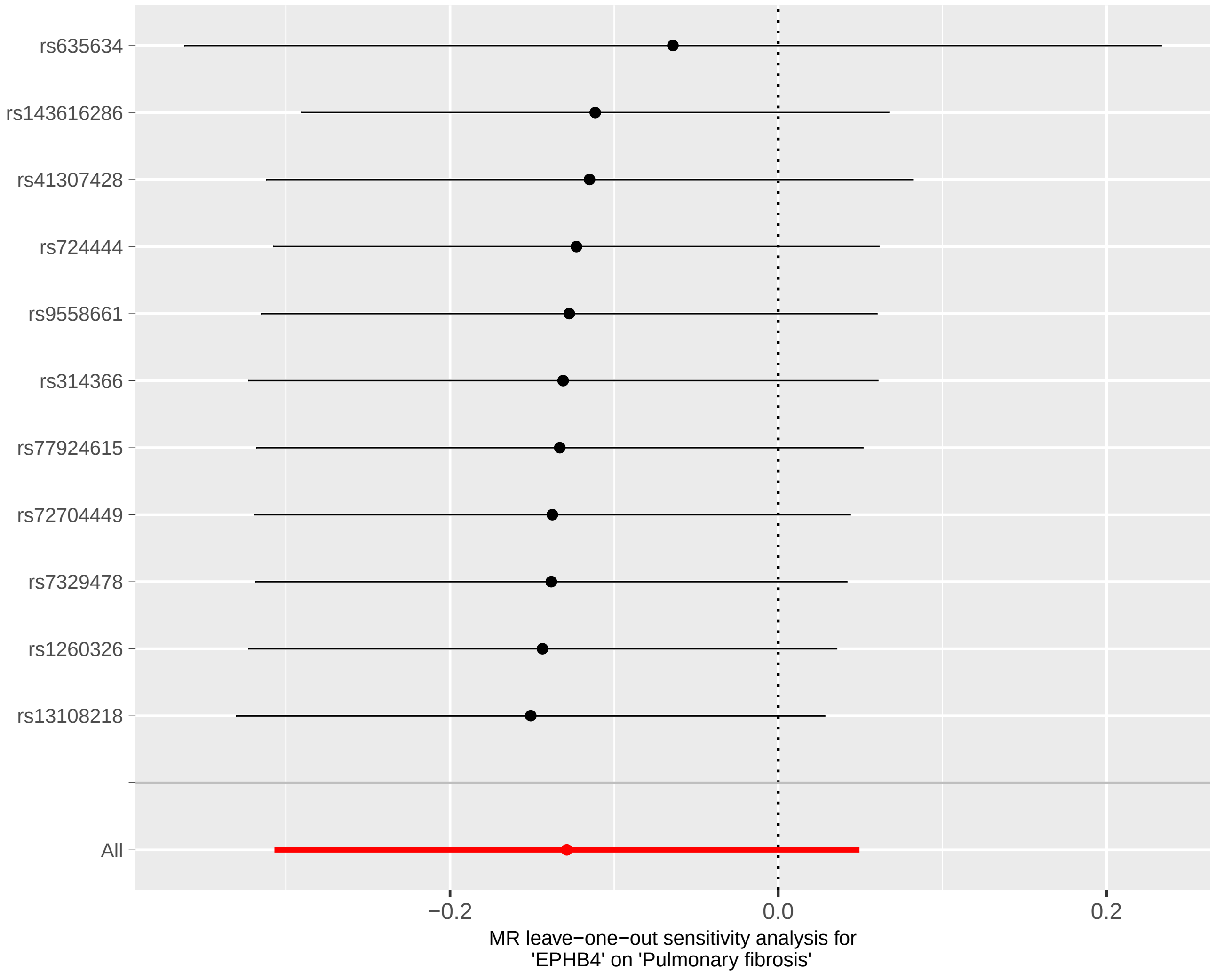
rs635634
rs143616286
rs41307428
rs724444
rs9558661
rs314366
rs77924615
rs72704449
rs7329478
rs1260326
rs13108218
All
−0.2
0.0
0.2
MR leave−one−out sensitivity analysis for
'EPHB4' on 'Pulmonary fibrosis'

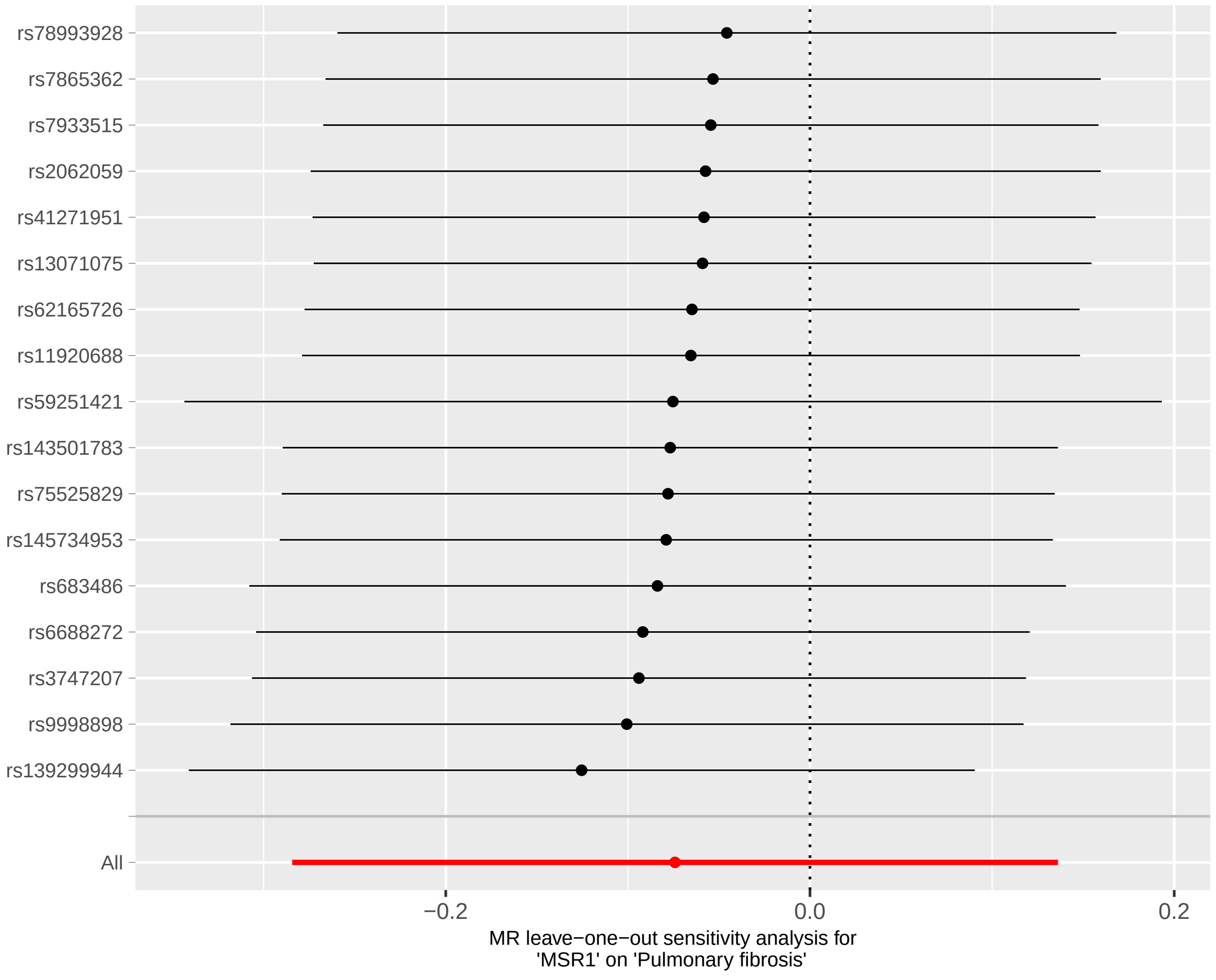
rs78993928
rs7865362
rs7933515
rs2062059
rs41271951
rs13071075
rs62165726
rs11920688
rs59251421
rs143501783
rs75525829
rs145734953
rs683486
rs6688272
rs3747207
rs9998898
rs139299944
All
−0.2
0.0
0.2
MR leave−one−out sensitivity analysis for
'MSR1' on 'Pulmonary fibrosis'

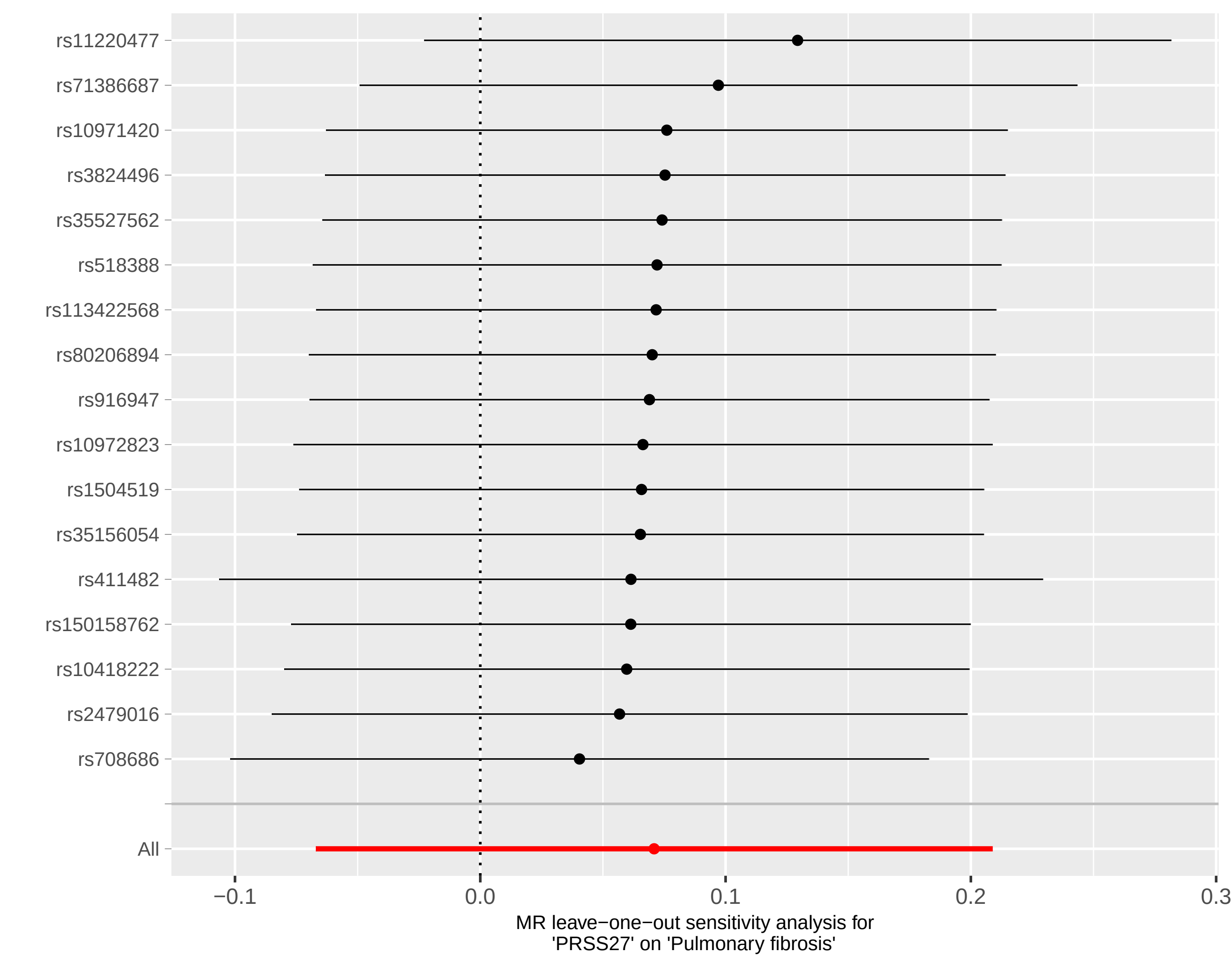
rs11220477
rs71386687
rs10971420
rs3824496
rs35527562
rs518388
rs113422568
rs80206894
rs916947
rs10972823
rs1504519
rs35156054
rs411482
rs150158762
rs10418222
rs2479016
rs708686
All
−0.1
0.0
0.1
0.2
0.3
MR leave−one−out sensitivity analysis for
'PRSS27' on 'Pulmonary fibrosis'

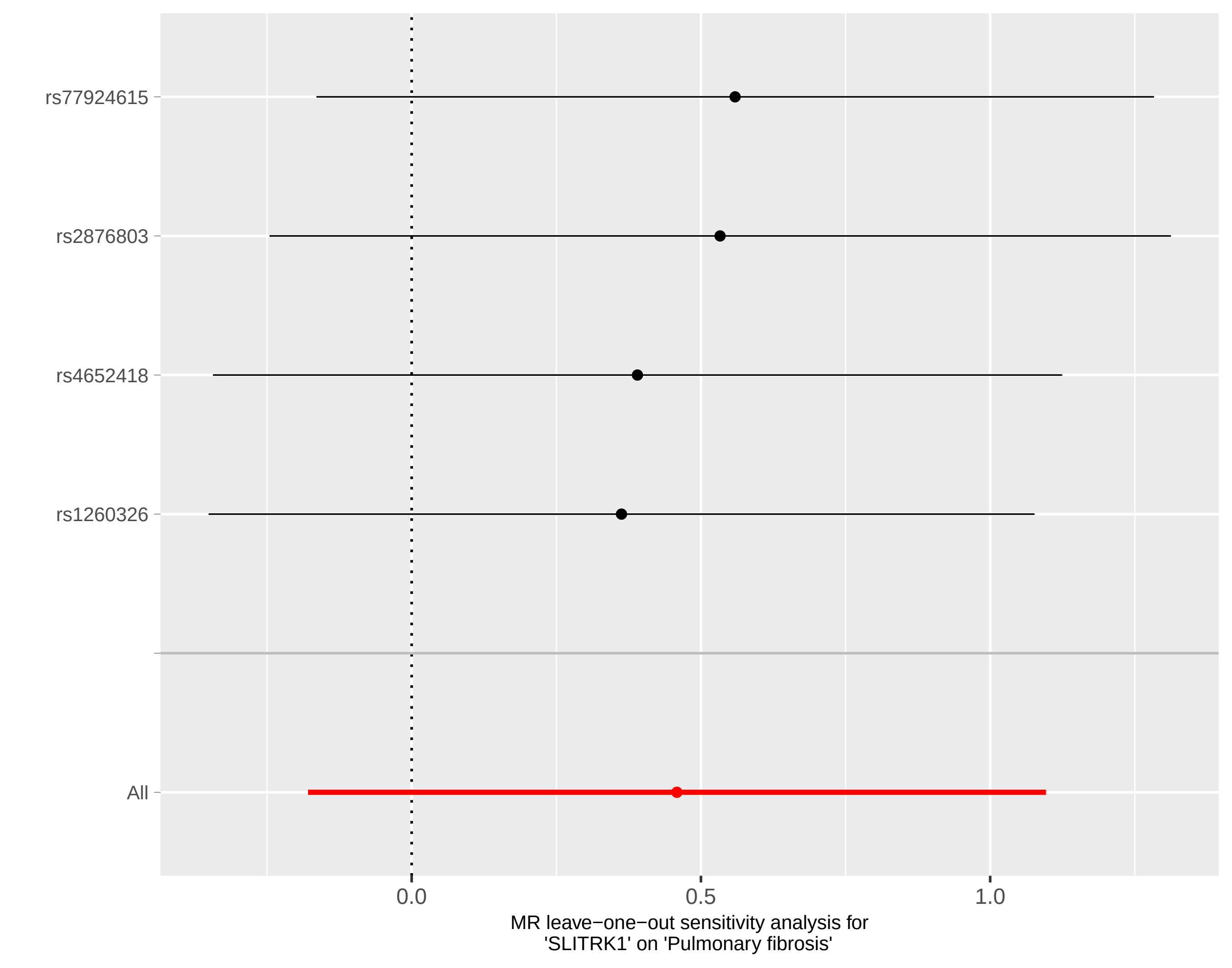

rs77924615
rs2876803
rs4652418
rs1260326
All
0.0
0.5
1.0
MR leave−one−out sensitivity analysis for
'SLITRK1' on 'Pulmonary fibrosis'

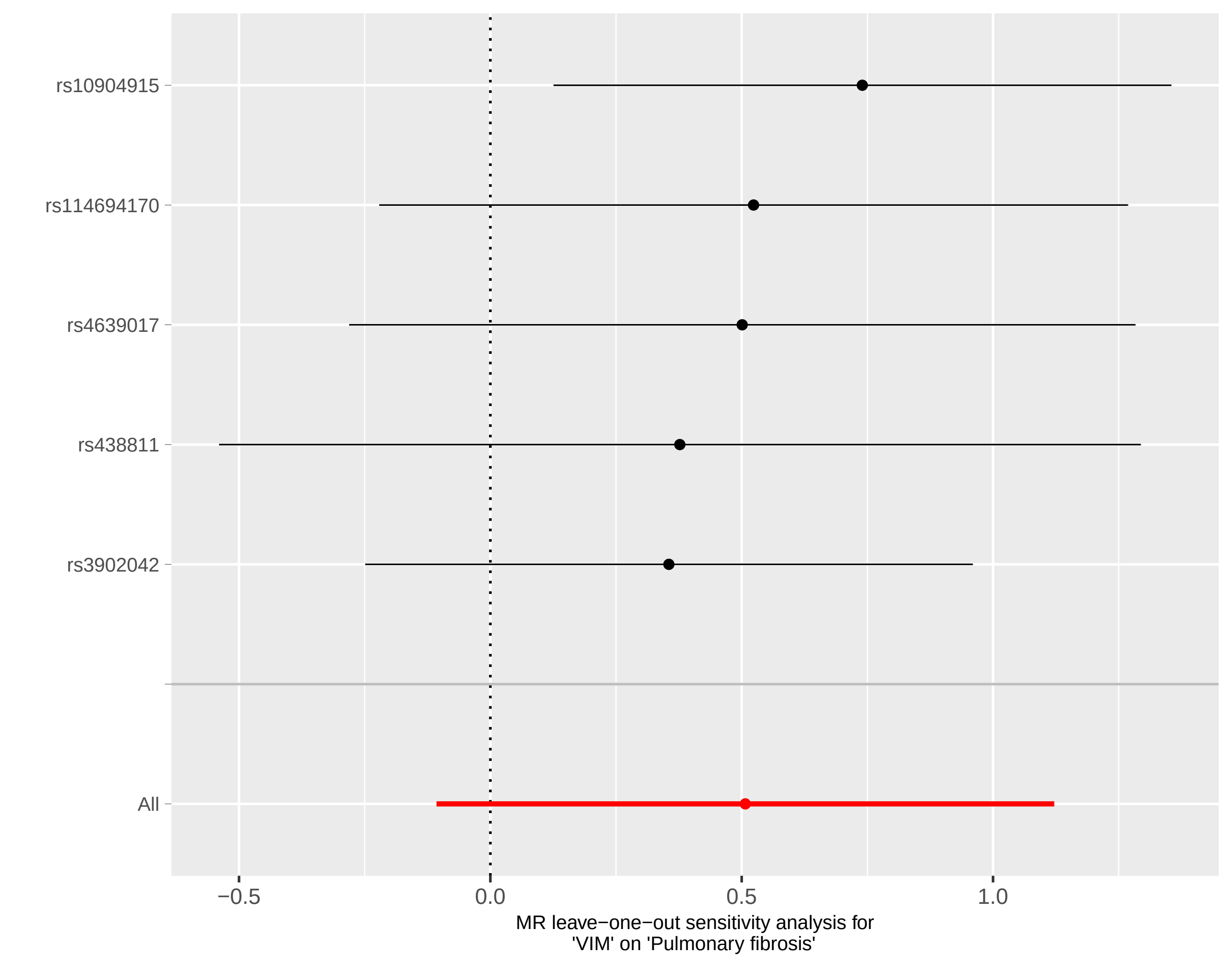
rs10904915
rs114694170
rs4639017
rs438811
rs3902042
All
−0.5
0.0
0.5
1.0
MR leave−one−out sensitivity analysis for
'VIM' on 'Pulmonary fibrosis'

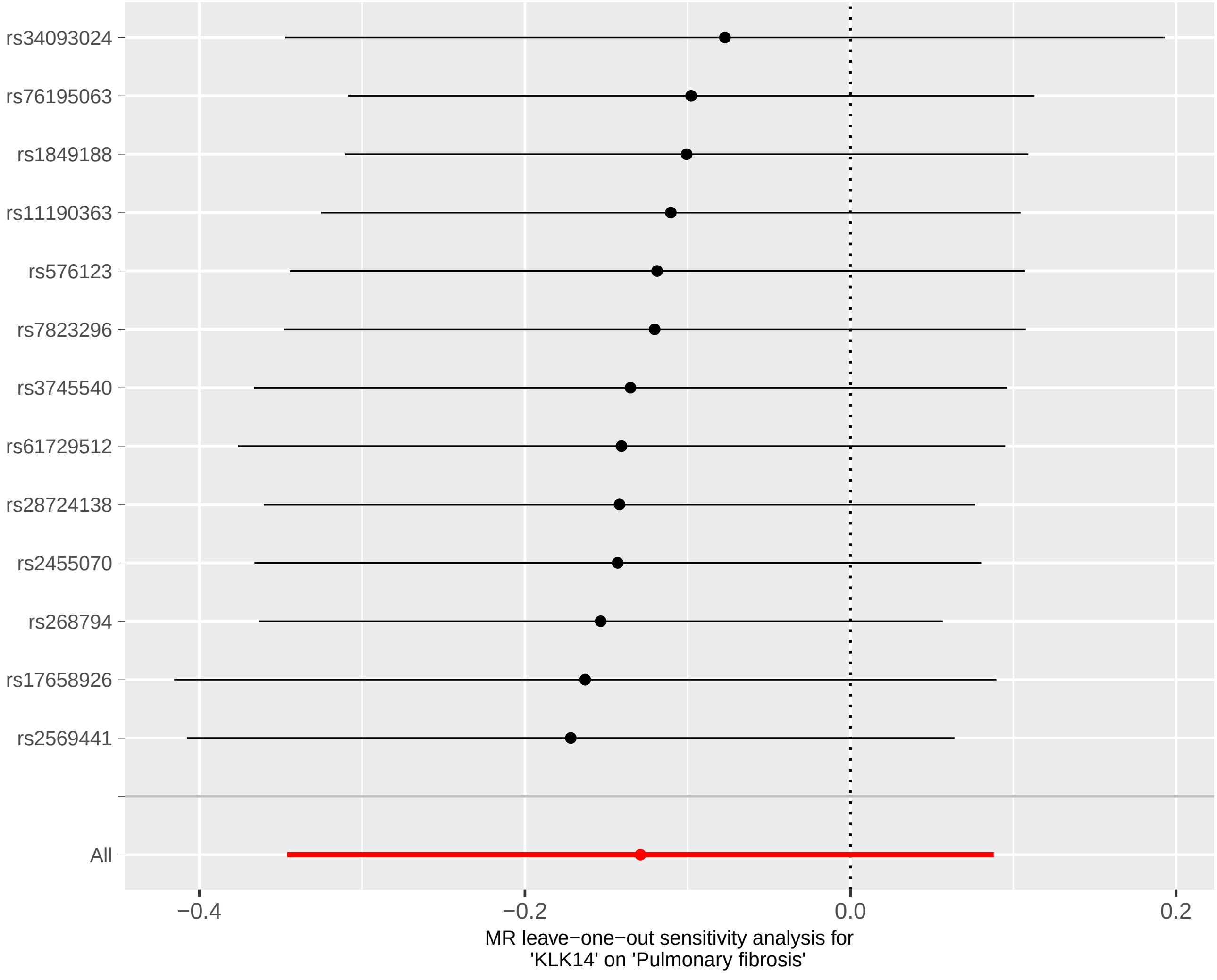
rs34093024
rs76195063
rs1849188
rs11190363
rs576123
rs7823296
rs3745540
rs61729512
rs28724138
rs2455070
rs268794
rs17658926
rs2569441
All
−0.4
−0.2
0.0
0.2
MR leave−one−out sensitivity analysis for
'KLK14' on 'Pulmonary fibrosis'

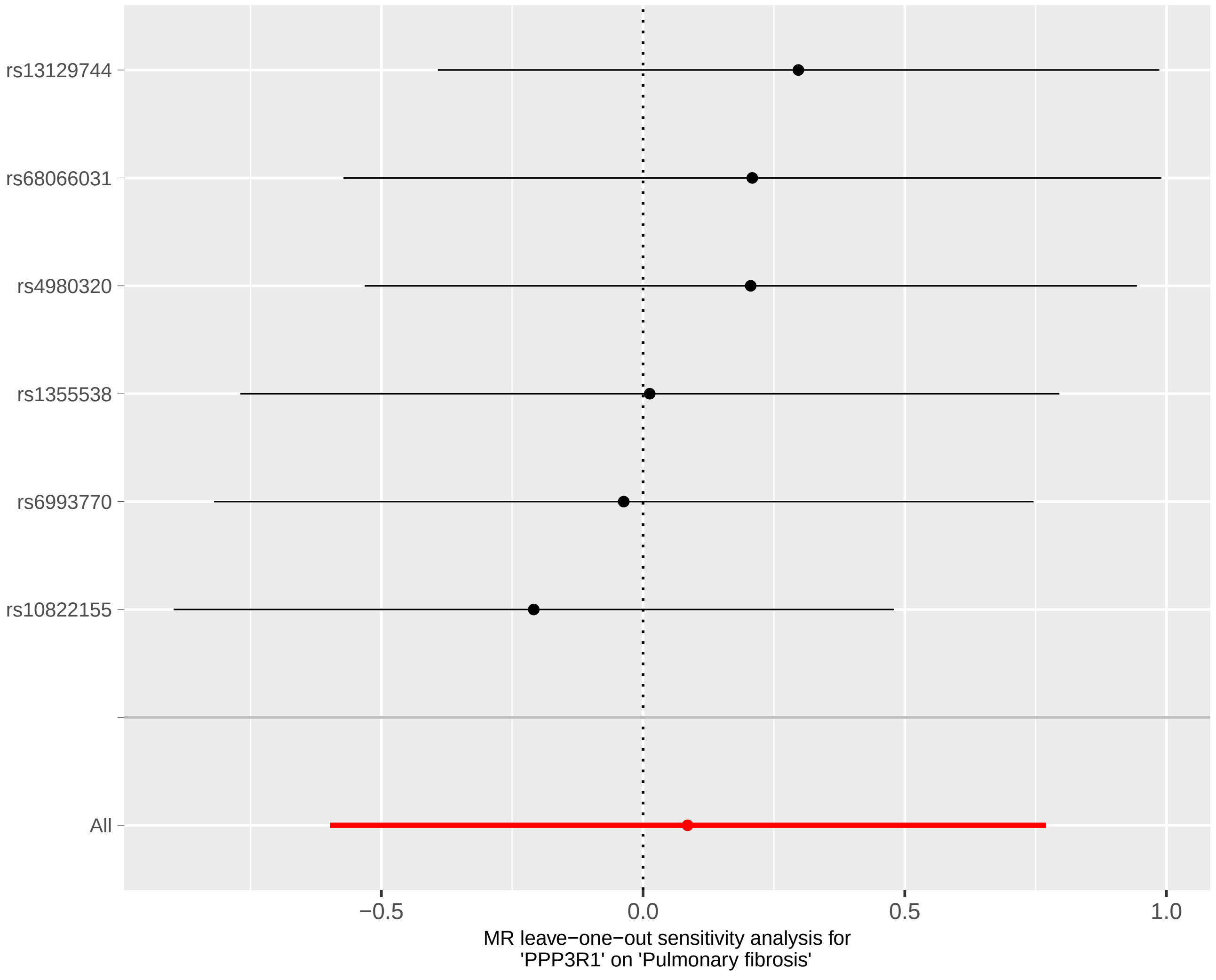
rs13129744
rs68066031
rs4980320
rs1355538
rs6993770
rs10822155
All
−0.5
0.0
0.5
1.0
MR leave−one−out sensitivity analysis for
'PPP3R1' on 'Pulmonary fibrosis'

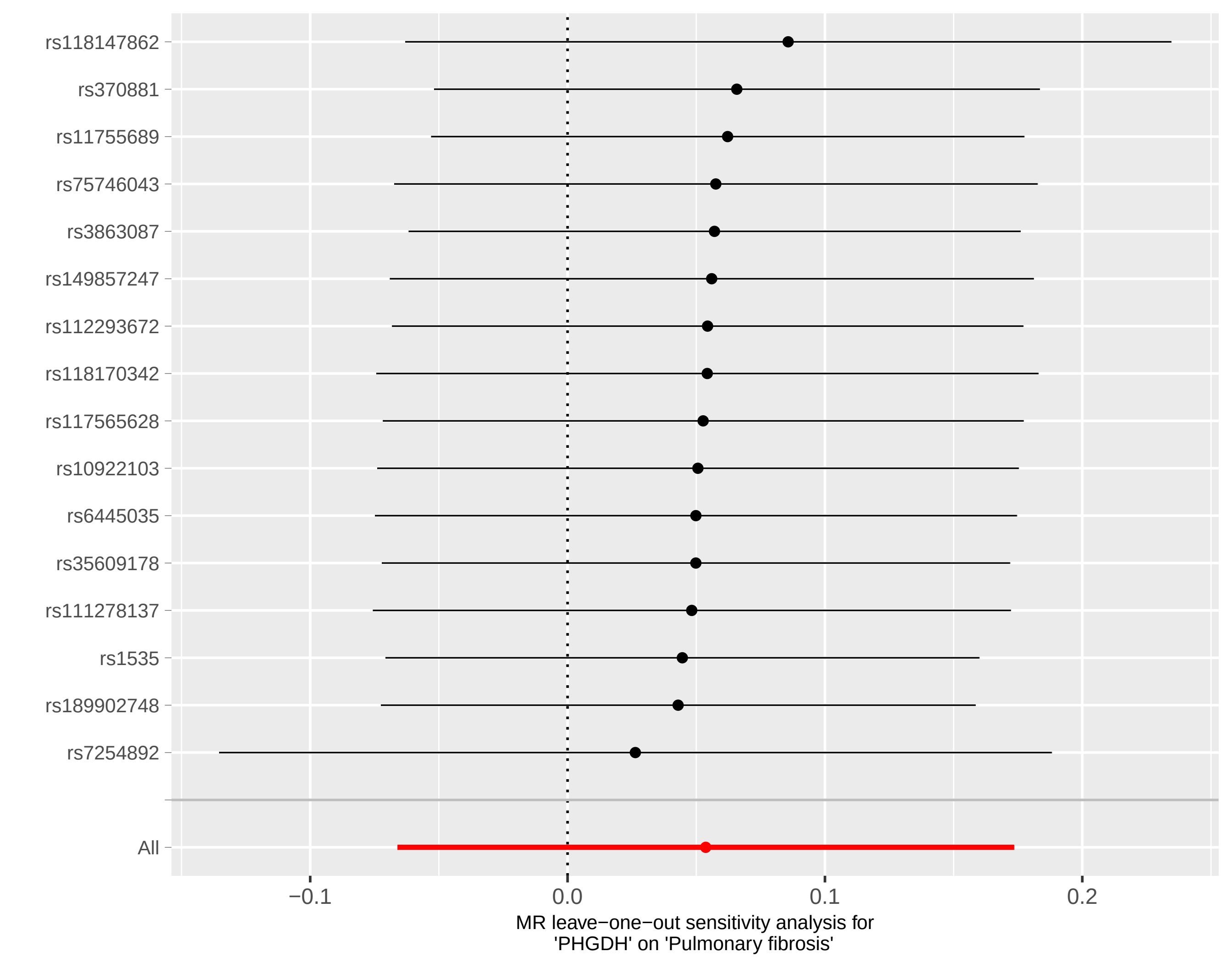
rs118147862
rs370881
rs11755689
rs75746043
rs3863087
rs149857247
rs112293672
rs118170342
rs117565628
rs10922103
rs6445035
rs35609178
rs111278137
rs1535
rs189902748
rs7254892
All
−0.1
0.0
0.1
0.2
MR leave−one−out sensitivity analysis for
'PHGDH' on 'Pulmonary fibrosis'

Insufficient number of SNPs

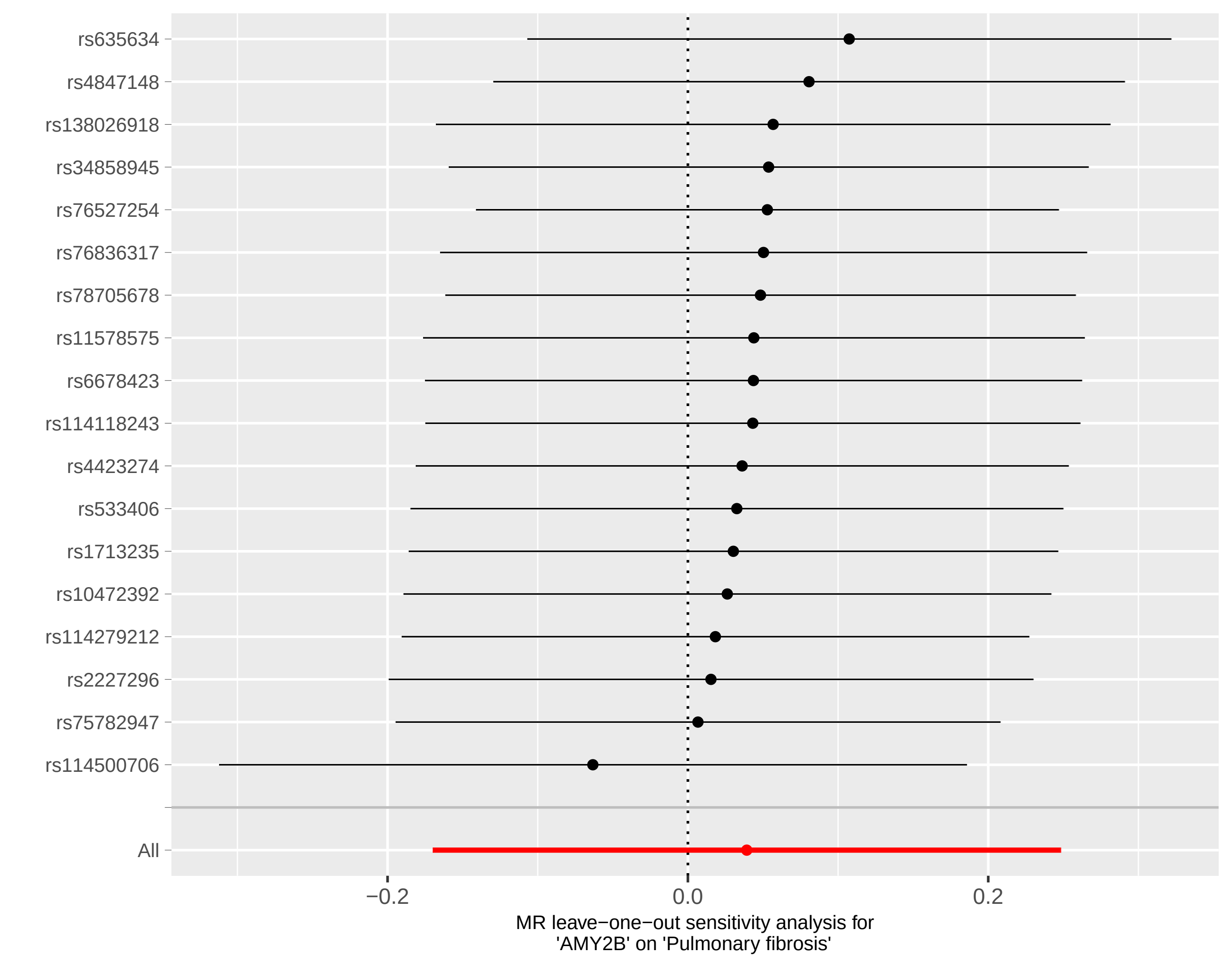
rs635634
rs4847148
rs138026918
rs34858945
rs76527254
rs76836317
rs78705678
rs11578575
rs6678423
rs114118243
rs4423274
rs533406
rs1713235
rs10472392
rs114279212
rs2227296
rs75782947
rs114500706
All
−0.2
0.0
0.2
MR leave−one−out sensitivity analysis for
'AMY2B' on 'Pulmonary fibrosis'

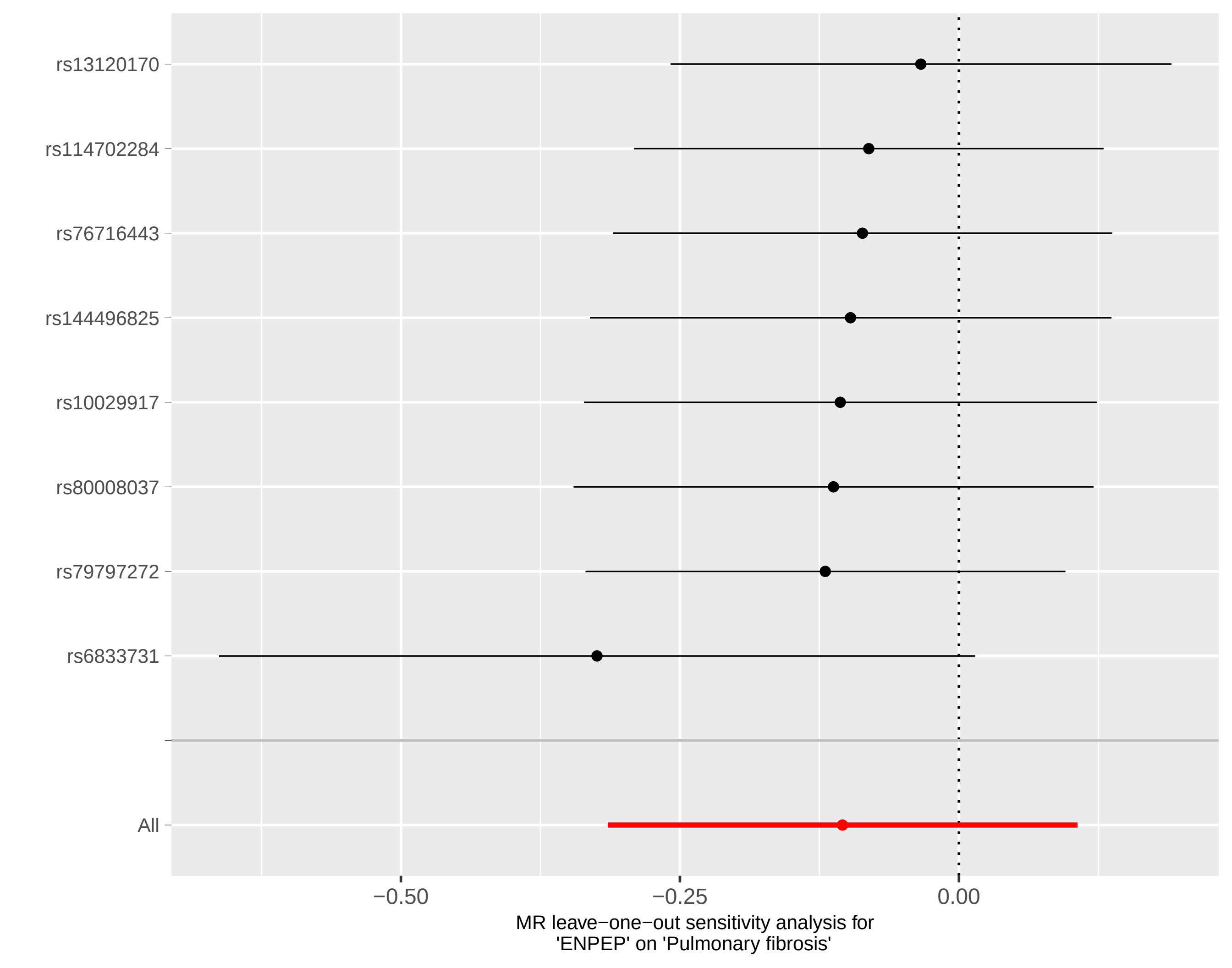
rs13120170
rs114702284
rs76716443
rs144496825
rs10029917
rs80008037
rs79797272
rs6833731
All
−0.50
−0.25
0.00
MR leave−one−out sensitivity analysis for
'ENPEP' on 'Pulmonary fibrosis'

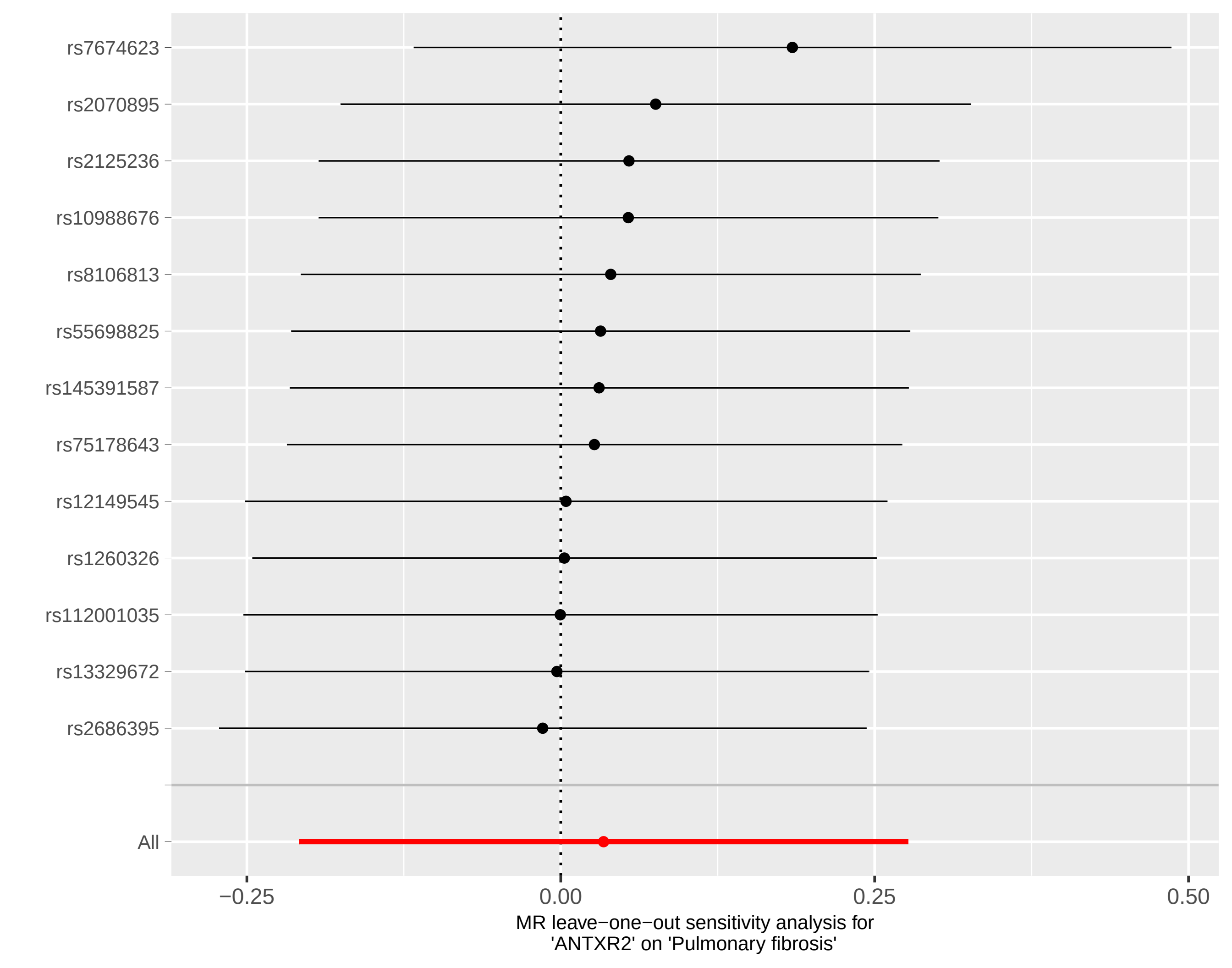

rs7674623
rs2070895
rs2125236
rs10988676
rs8106813
rs55698825
rs145391587
rs75178643
rs12149545
rs1260326
rs112001035
rs13329672
rs2686395
All
−0.25
0.00
0.25
0.50
MR leave−one−out sensitivity analysis for
'ANTXR2' on 'Pulmonary fibrosis'

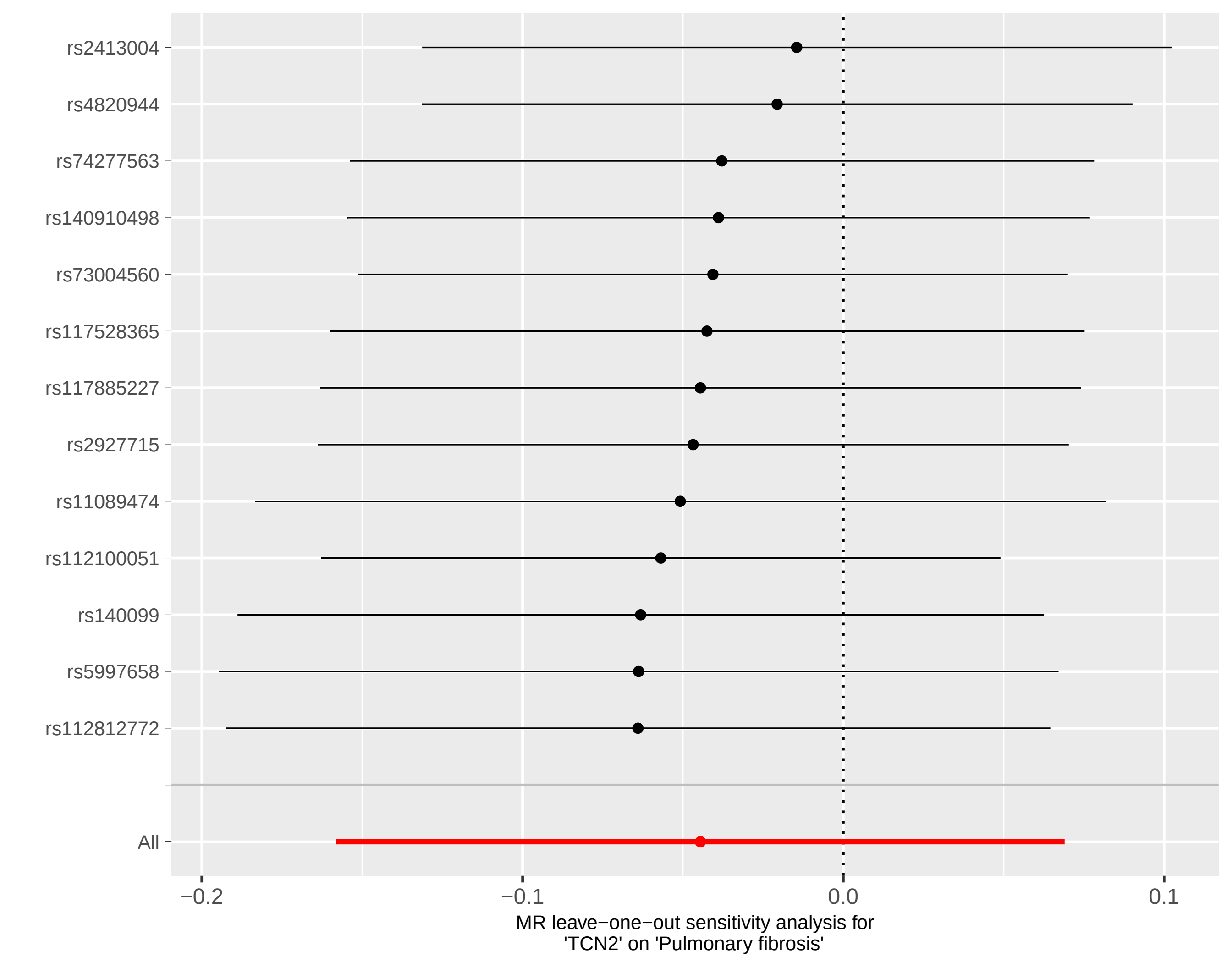
rs2413004
rs4820944
rs74277563
rs140910498
rs73004560
rs117528365
rs117885227
rs2927715
rs11089474
rs112100051
rs140099
rs5997658
rs112812772
All
−0.2
−0.1
0.0
0.1
MR leave−one−out sensitivity analysis for
'TCN2' on 'Pulmonary fibrosis'

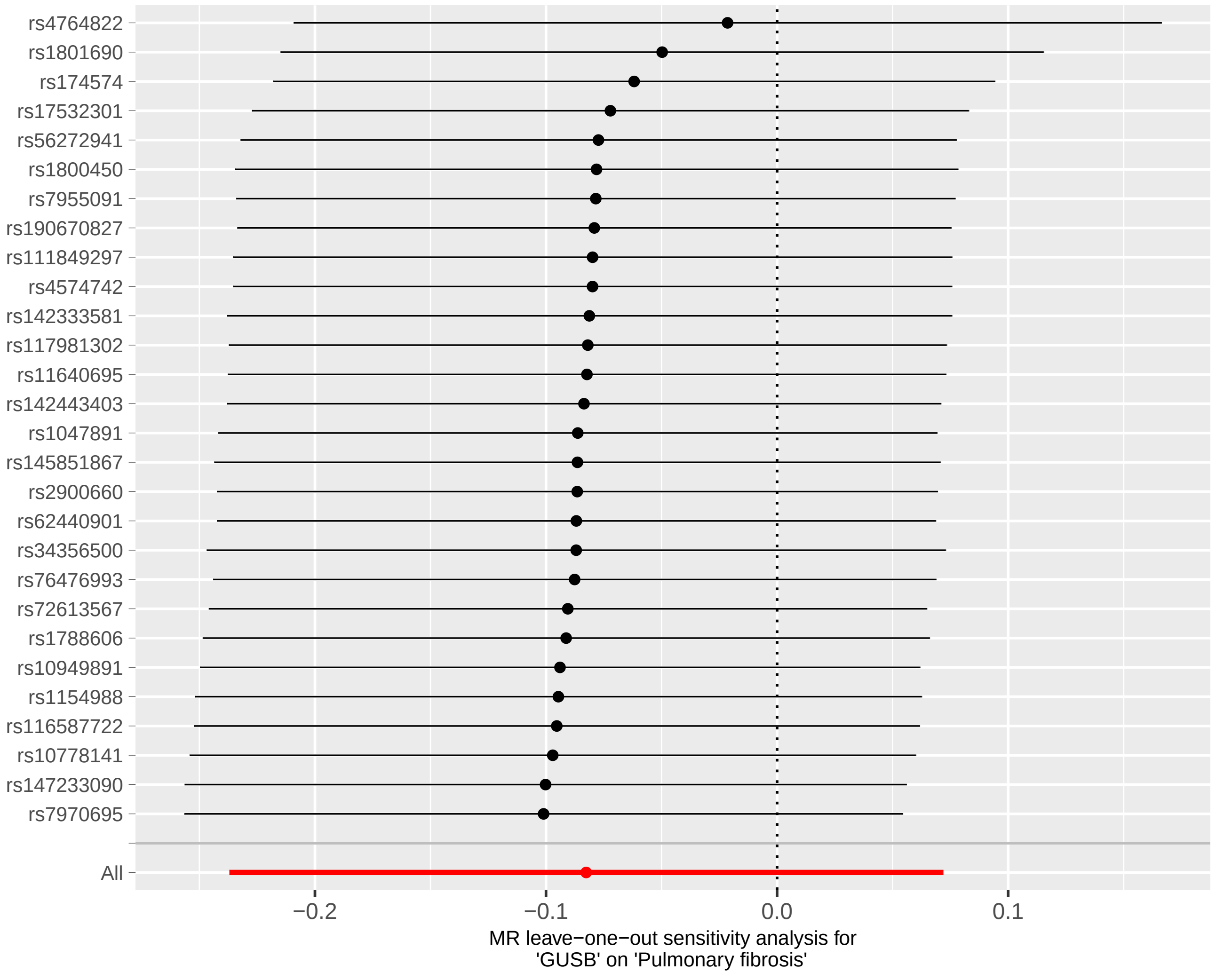
rs4764822
rs1801690
rs174574
rs17532301
rs56272941
rs1800450
rs7955091
rs190670827
rs111849297
rs4574742
rs142333581
rs117981302
rs11640695
rs142443403
rs1047891
rs145851867
rs2900660
rs62440901
rs34356500
rs76476993
rs72613567
rs1788606
rs10949891
rs1154988
rs116587722
rs10778141
rs147233090
rs7970695
All
−0.2
−0.1
0.0
0.1
MR leave−one−out sensitivity analysis for
'GUSB' on 'Pulmonary fibrosis'

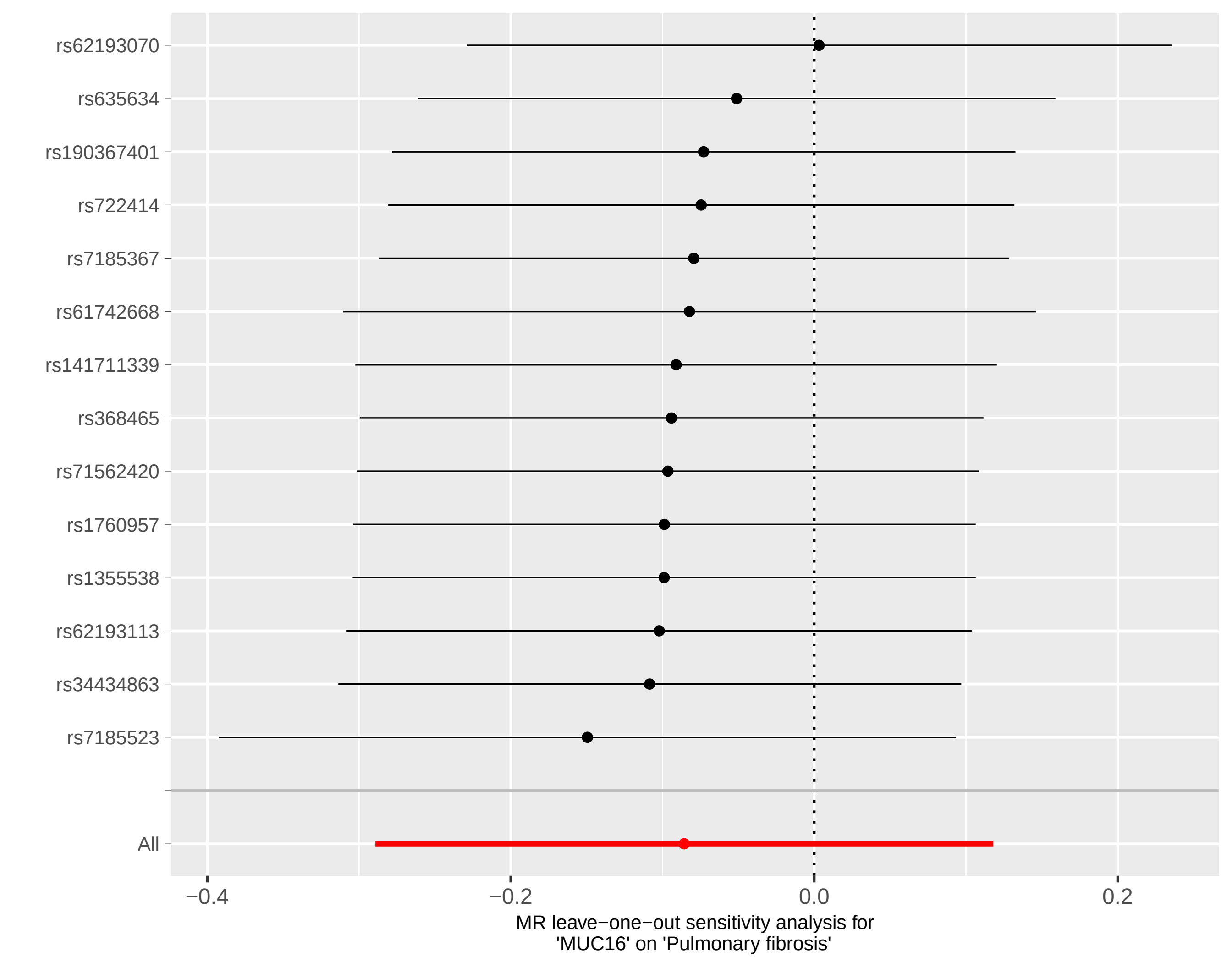
rs62193070
rs635634
rs190367401
rs722414
rs7185367
rs61742668
rs141711339
rs368465
rs71562420
rs1760957
rs1355538
rs62193113
rs34434863
rs7185523
All
−0.4
−0.2
0.0
0.2
MR leave−one−out sensitivity analysis for
'MUC16' on 'Pulmonary fibrosis'

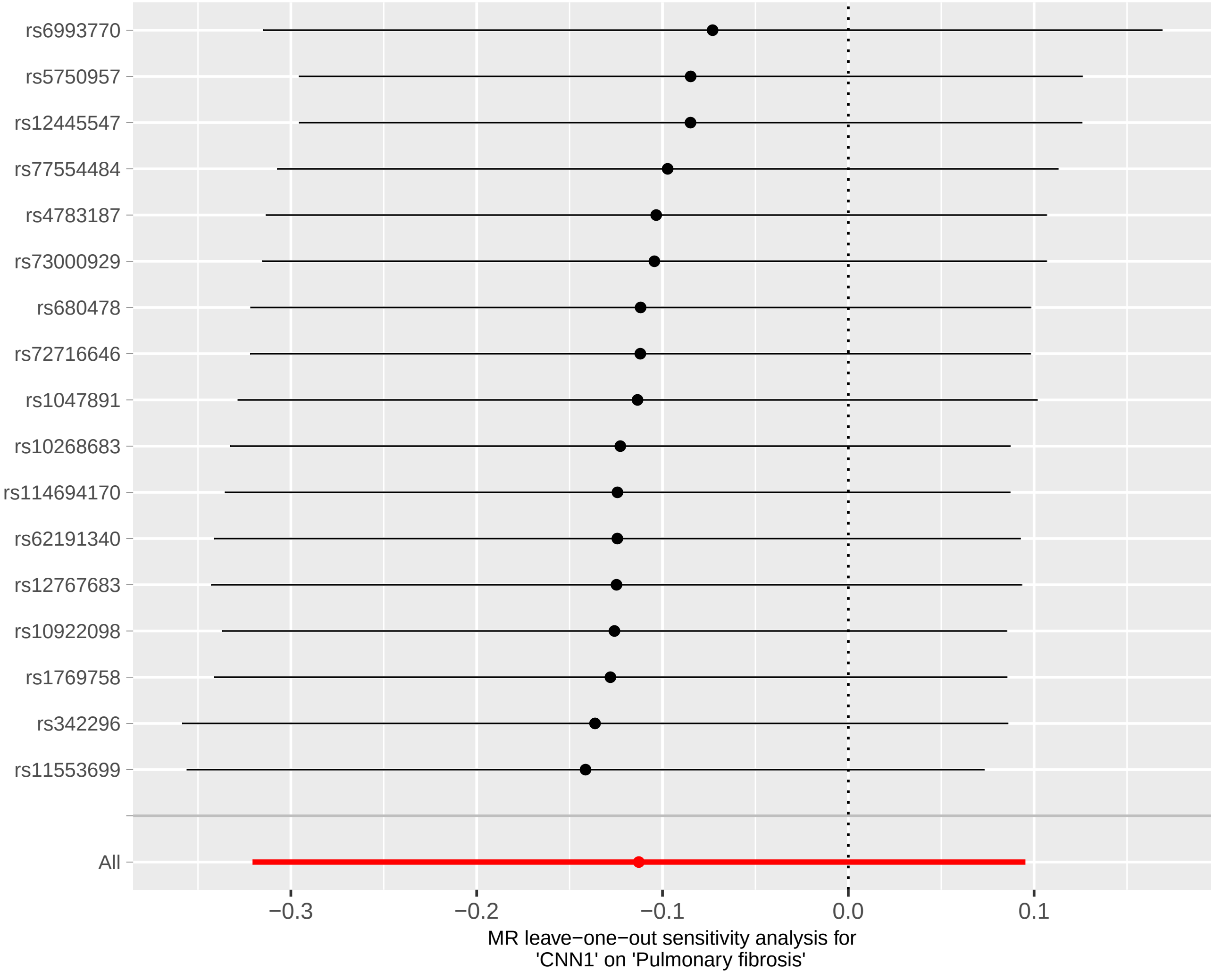
rs6993770
rs5750957
rs12445547
rs77554484
rs4783187
rs73000929
rs680478
rs72716646
rs1047891
rs10268683
rs114694170
rs62191340
rs12767683
rs10922098
rs1769758
rs342296
rs11553699
All
−0.3
−0.2
−0.1
0.0
0.1
MR leave−one−out sensitivity analysis for
'CNN1' on 'Pulmonary fibrosis'

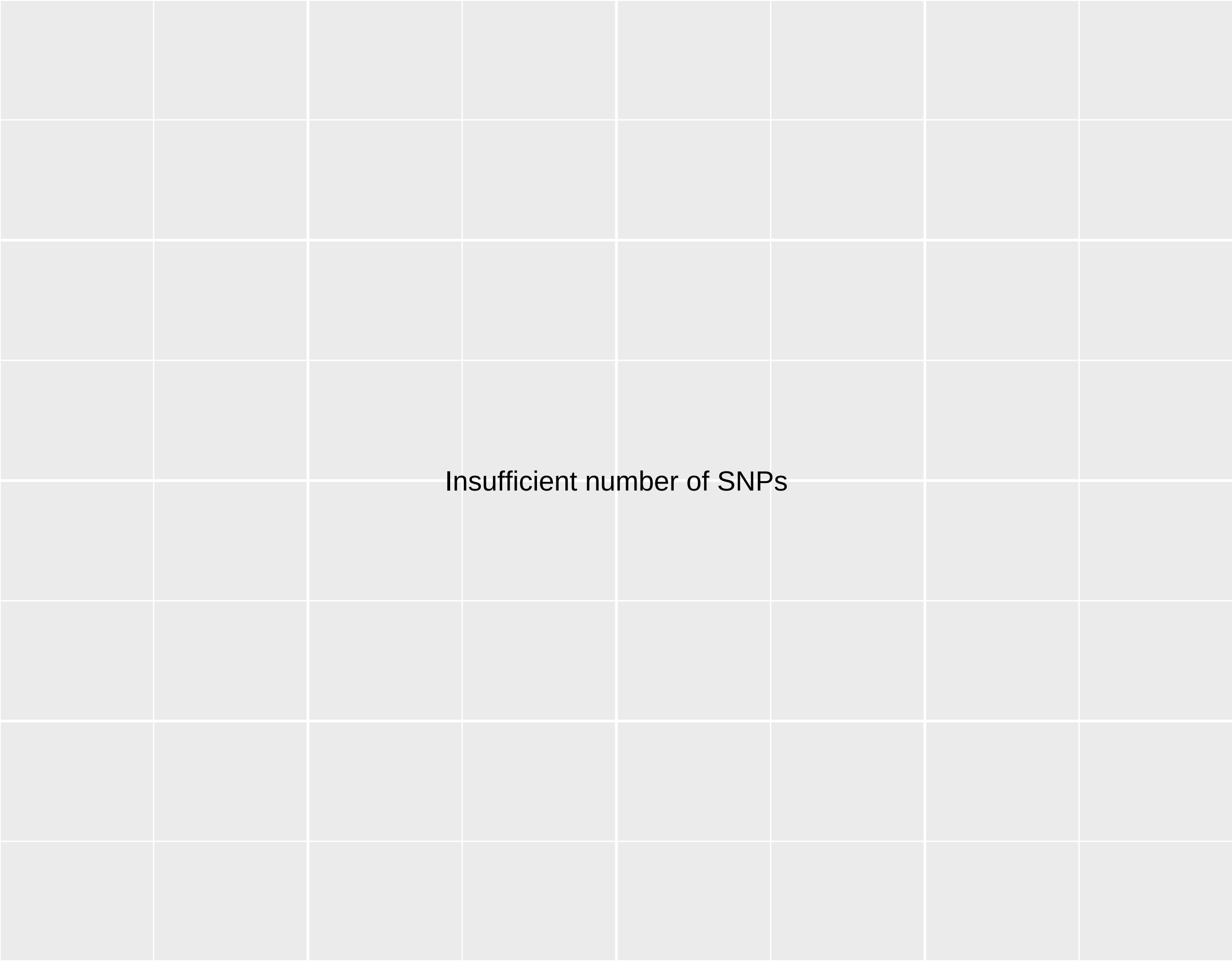
Insufficient number of SNPs

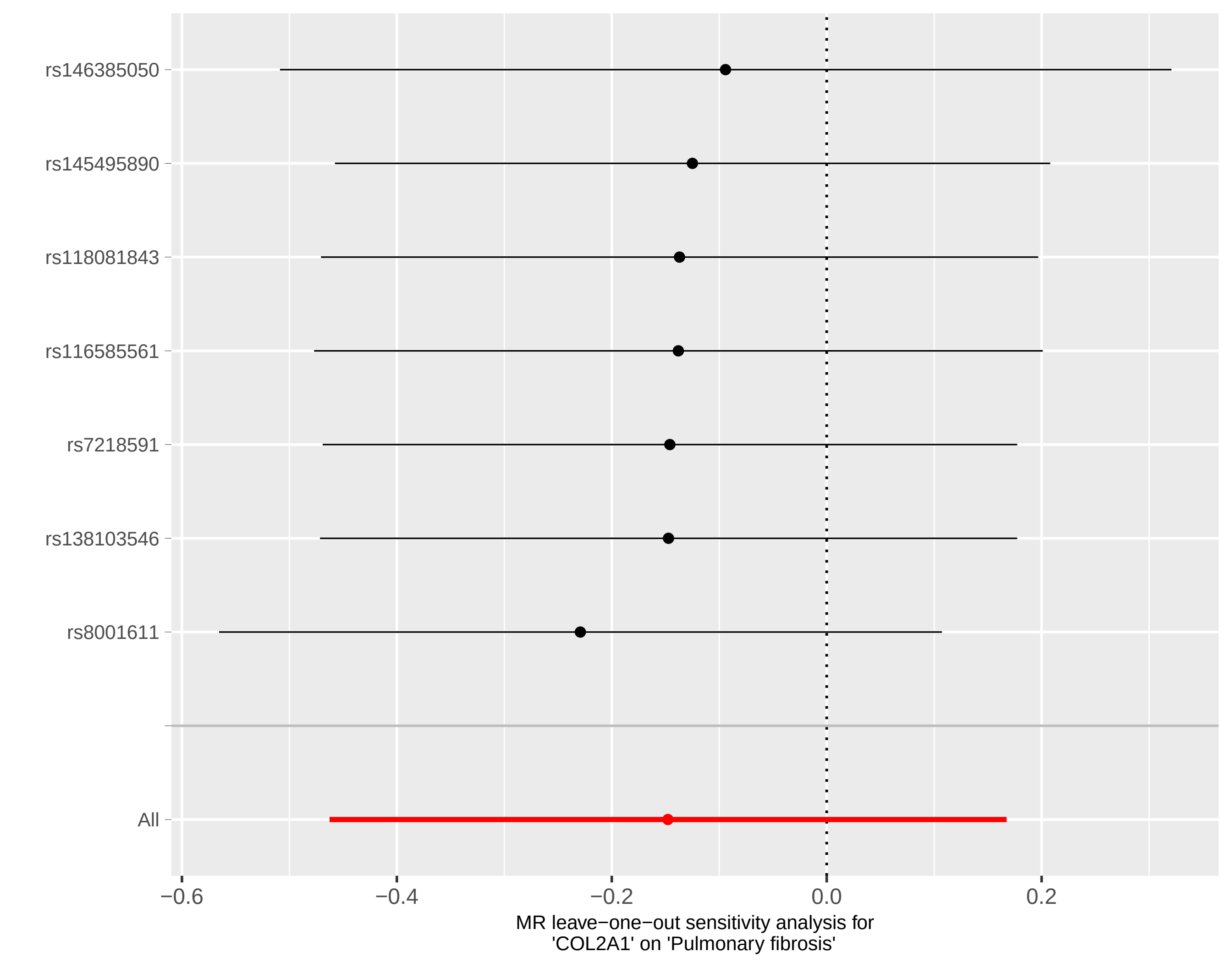
rs146385050
rs145495890
rs118081843
rs116585561
rs7218591
rs138103546
rs8001611
All
−0.6
−0.4
−0.2
0.0
0.2
MR leave−one−out sensitivity analysis for
'COL2A1' on 'Pulmonary fibrosis'

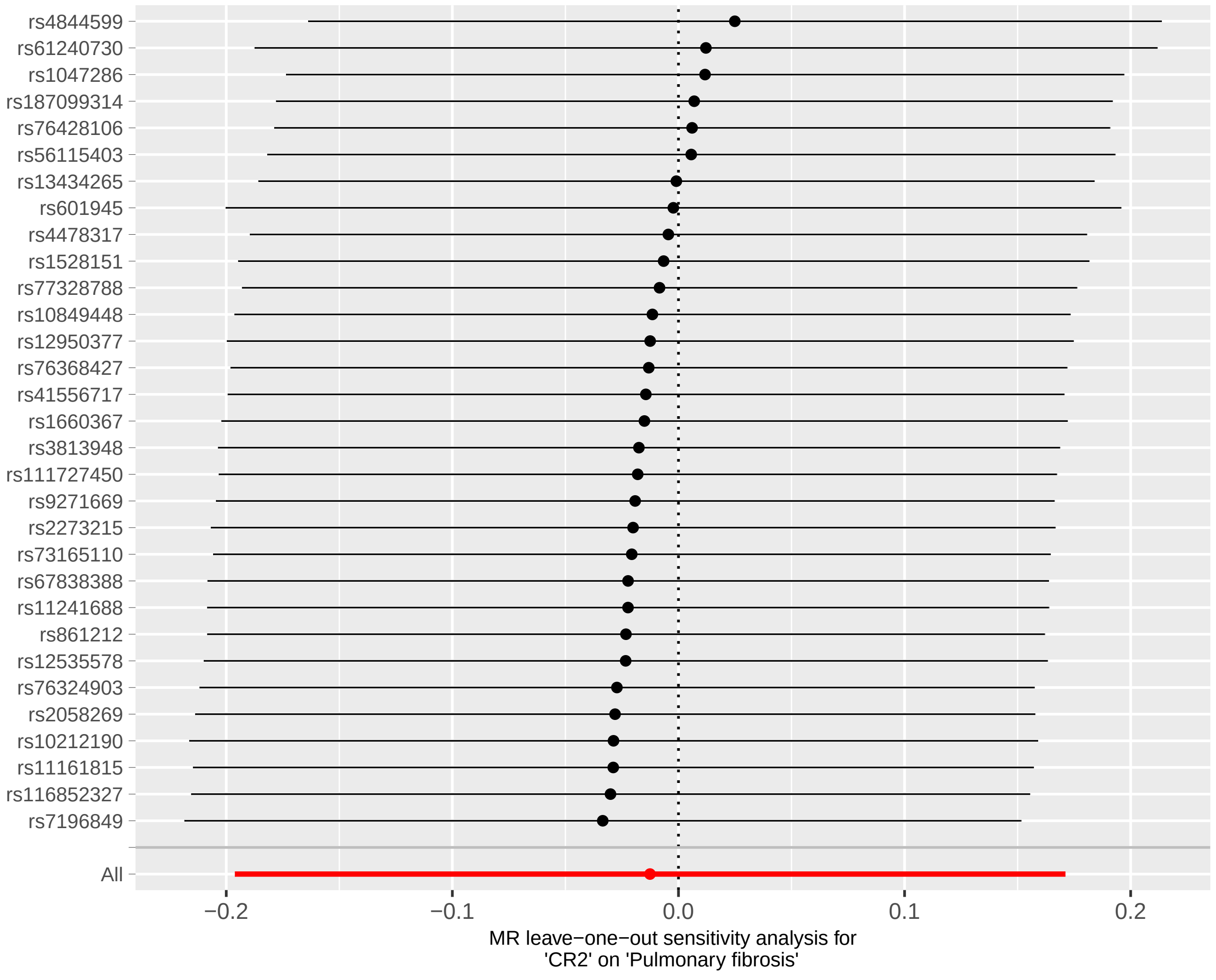
rs4844599
rs61240730
rs1047286
rs187099314
rs76428106
rs56115403
rs13434265
rs601945
rs4478317
rs1528151
rs77328788
rs10849448
rs12950377
rs76368427
rs41556717
rs1660367
rs3813948
rs111727450
rs9271669
rs2273215
rs73165110
rs67838388
rs11241688
rs861212
rs12535578
rs76324903
rs2058269
rs10212190
rs11161815
rs116852327
rs7196849
All
−0.2
−0.1
0.0
0.1
0.2
MR leave−one−out sensitivity analysis for
'CR2' on 'Pulmonary fibrosis'

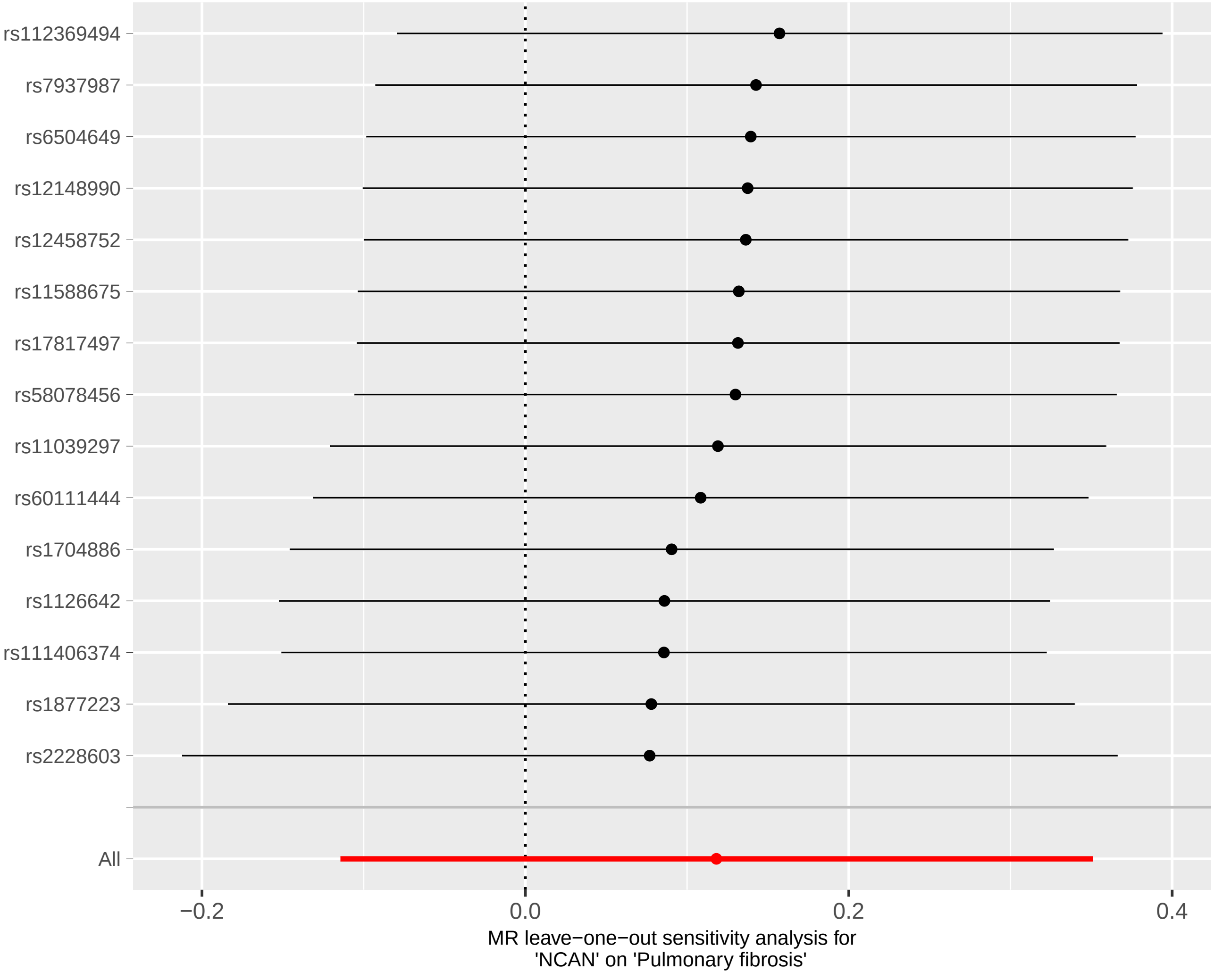

rs112369494
rs7937987
rs6504649
rs12148990
rs12458752
rs11588675
rs17817497
rs58078456
rs11039297
rs60111444
rs1704886
rs1126642
rs111406374
rs1877223
rs2228603
All
−0.2
0.0
0.2
0.4
MR leave−one−out sensitivity analysis for
'NCAN' on 'Pulmonary fibrosis'

Insufficient number of SNPs

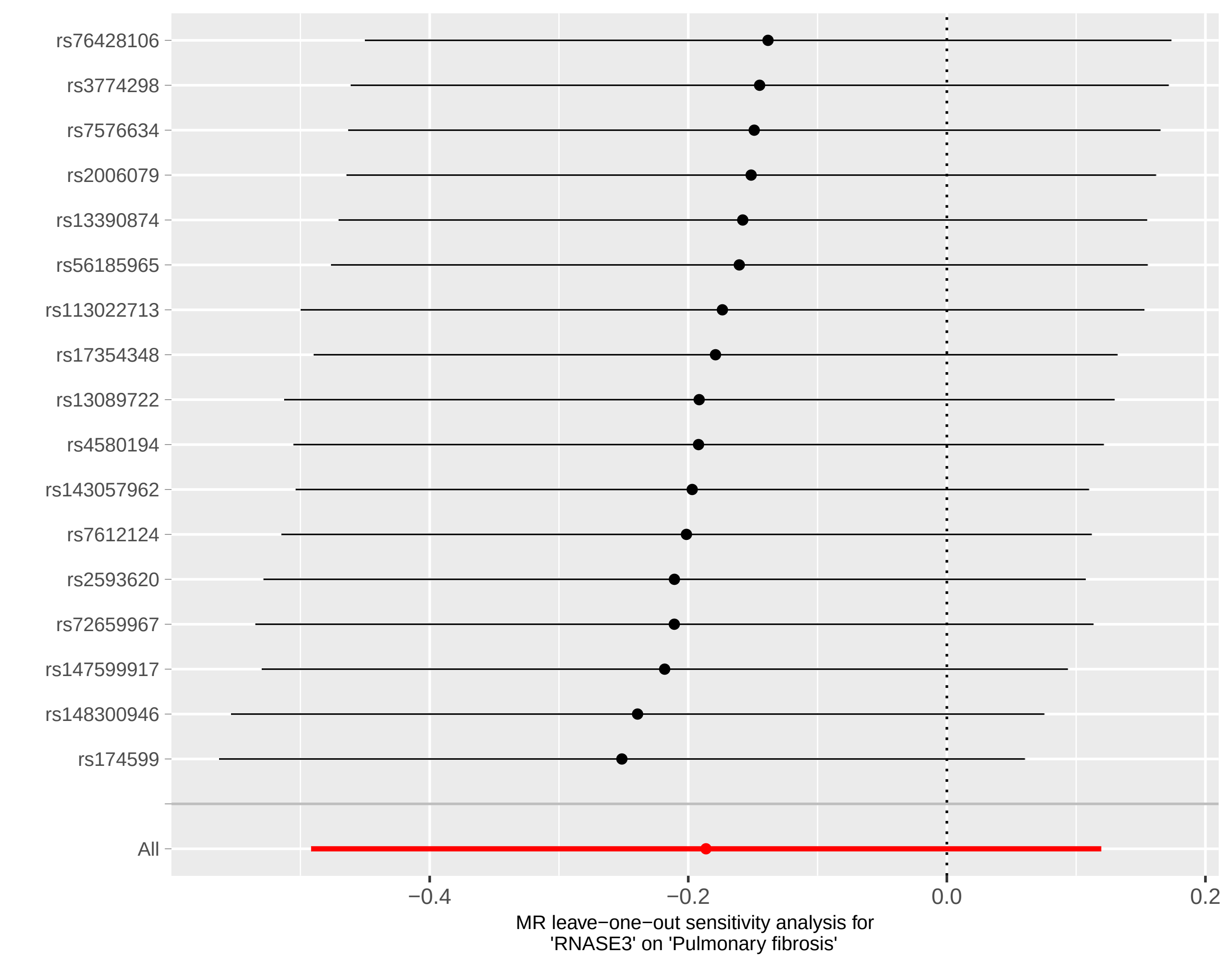

rs76428106
rs3774298
rs7576634
rs2006079
rs13390874
rs56185965
rs113022713
rs17354348
rs13089722
rs4580194
rs143057962
rs7612124
rs2593620
rs72659967
rs147599917
rs148300946
rs174599
All
−0.4
−0.2
0.0
0.2
MR leave−one−out sensitivity analysis for
'RNASE3' on 'Pulmonary fibrosis'

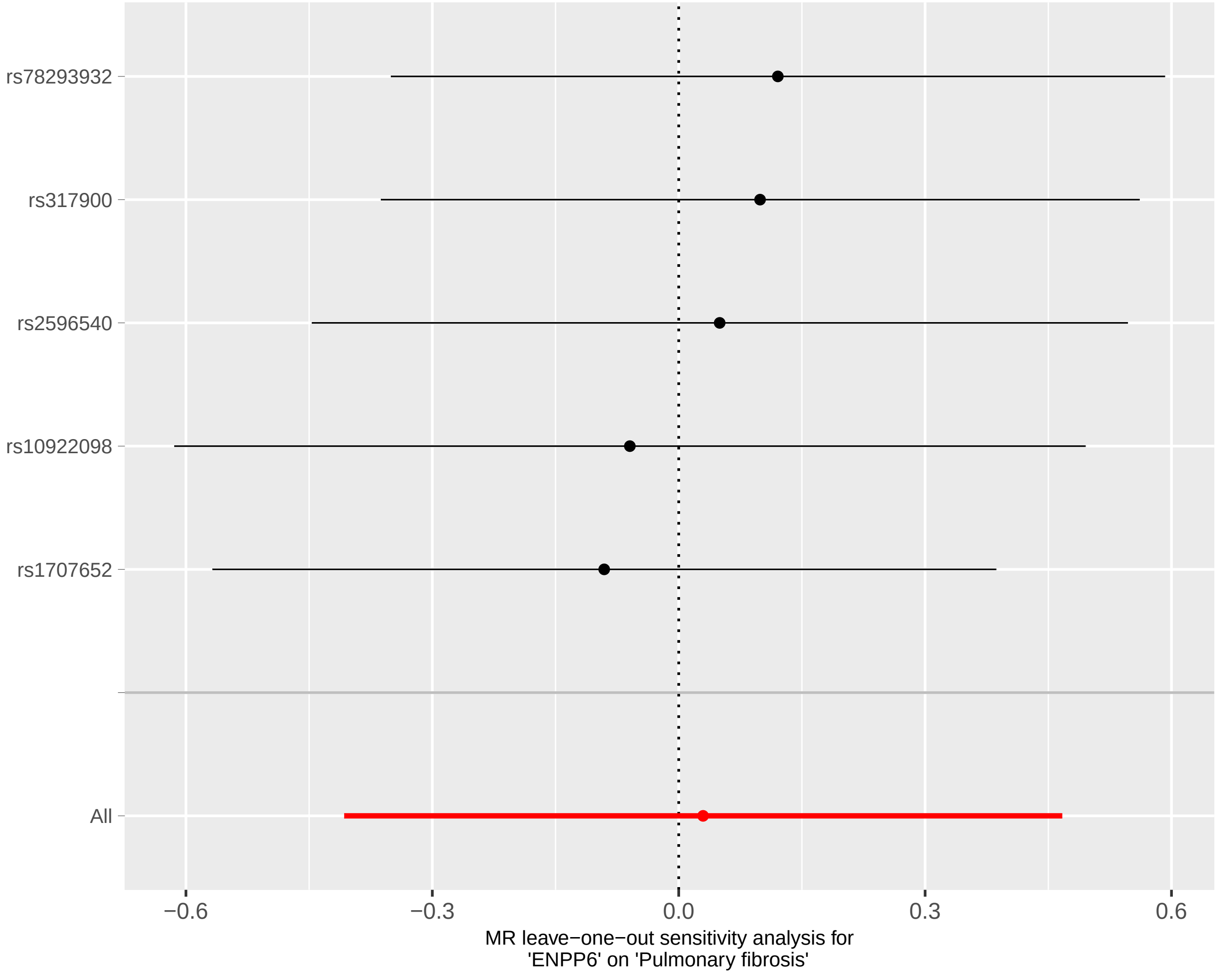
rs78293932
rs317900
rs2596540
rs10922098
rs1707652
All
−0.6
−0.3
0.0
0.3
0.6
MR leave−one−out sensitivity analysis for
'ENPP6' on 'Pulmonary fibrosis'

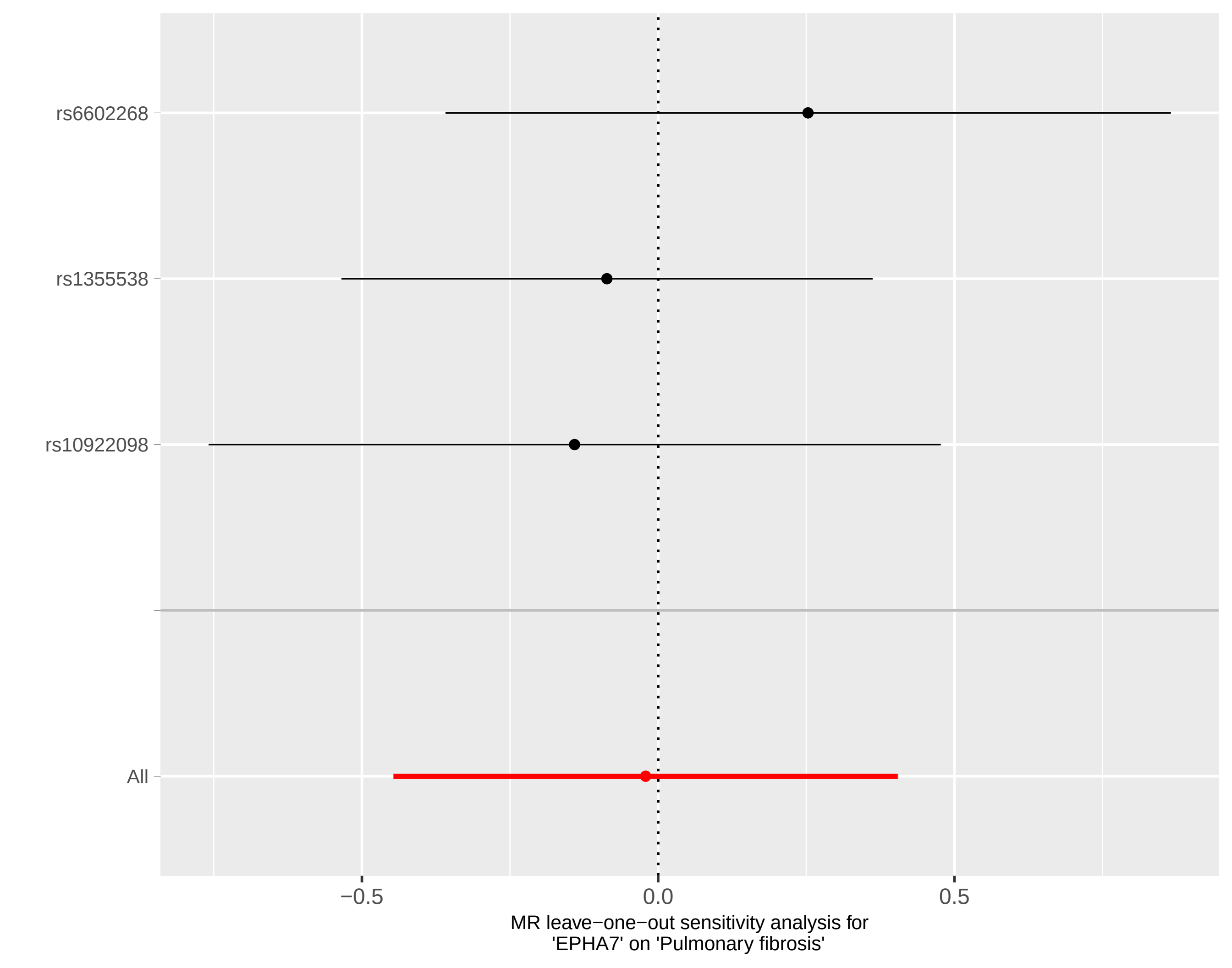
rs6602268
rs1355538
rs10922098
All
−0.5
0.0
0.5
MR leave−one−out sensitivity analysis for
'EPHA7' on 'Pulmonary fibrosis'

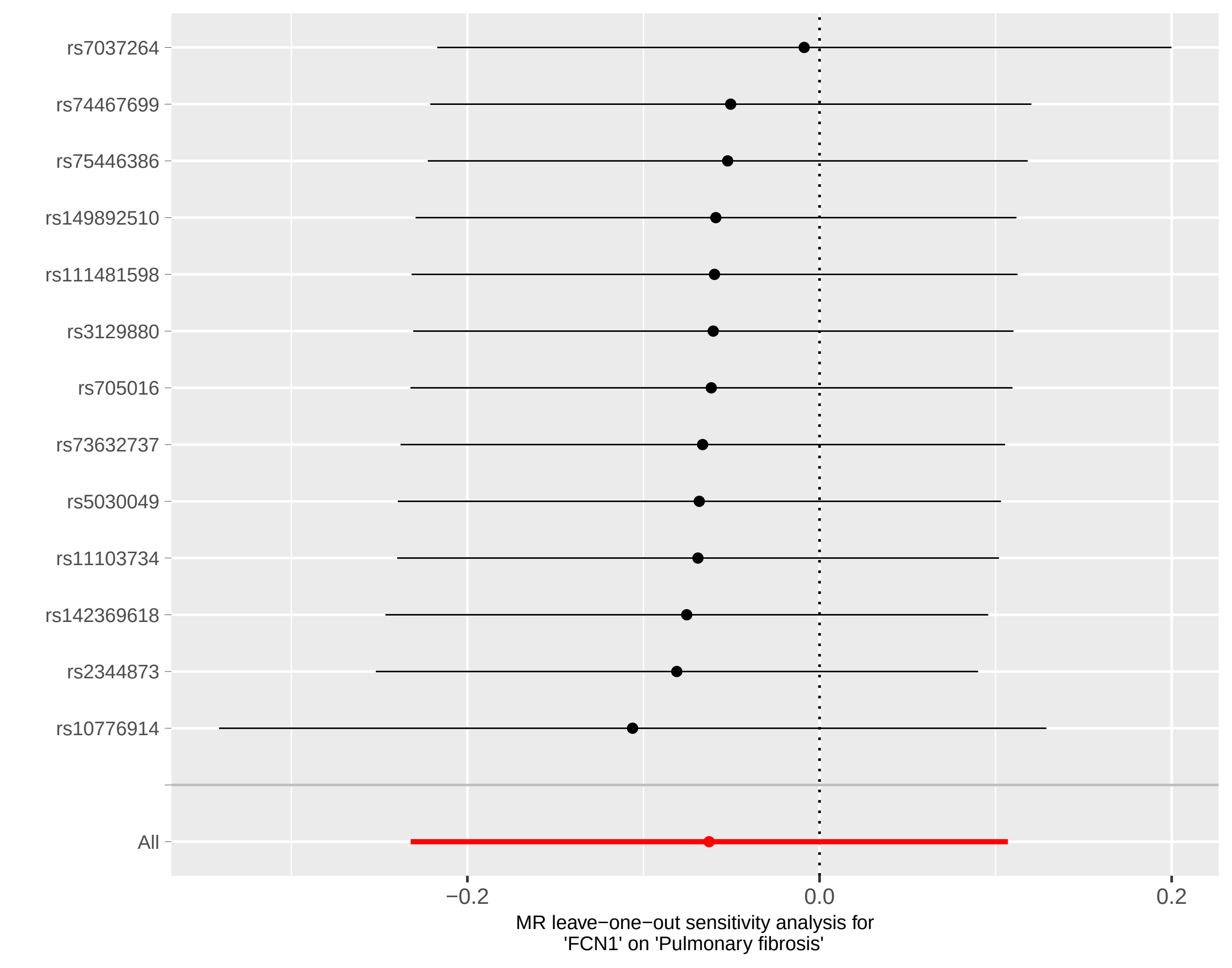
rs7037264
rs74467699
rs75446386
rs149892510
rs111481598
rs3129880
rs705016
rs73632737
rs5030049
rs11103734
rs142369618
rs2344873
rs10776914
All
−0.2
0.0
0.2
MR leave−one−out sensitivity analysis for
'FCN1' on 'Pulmonary fibrosis'

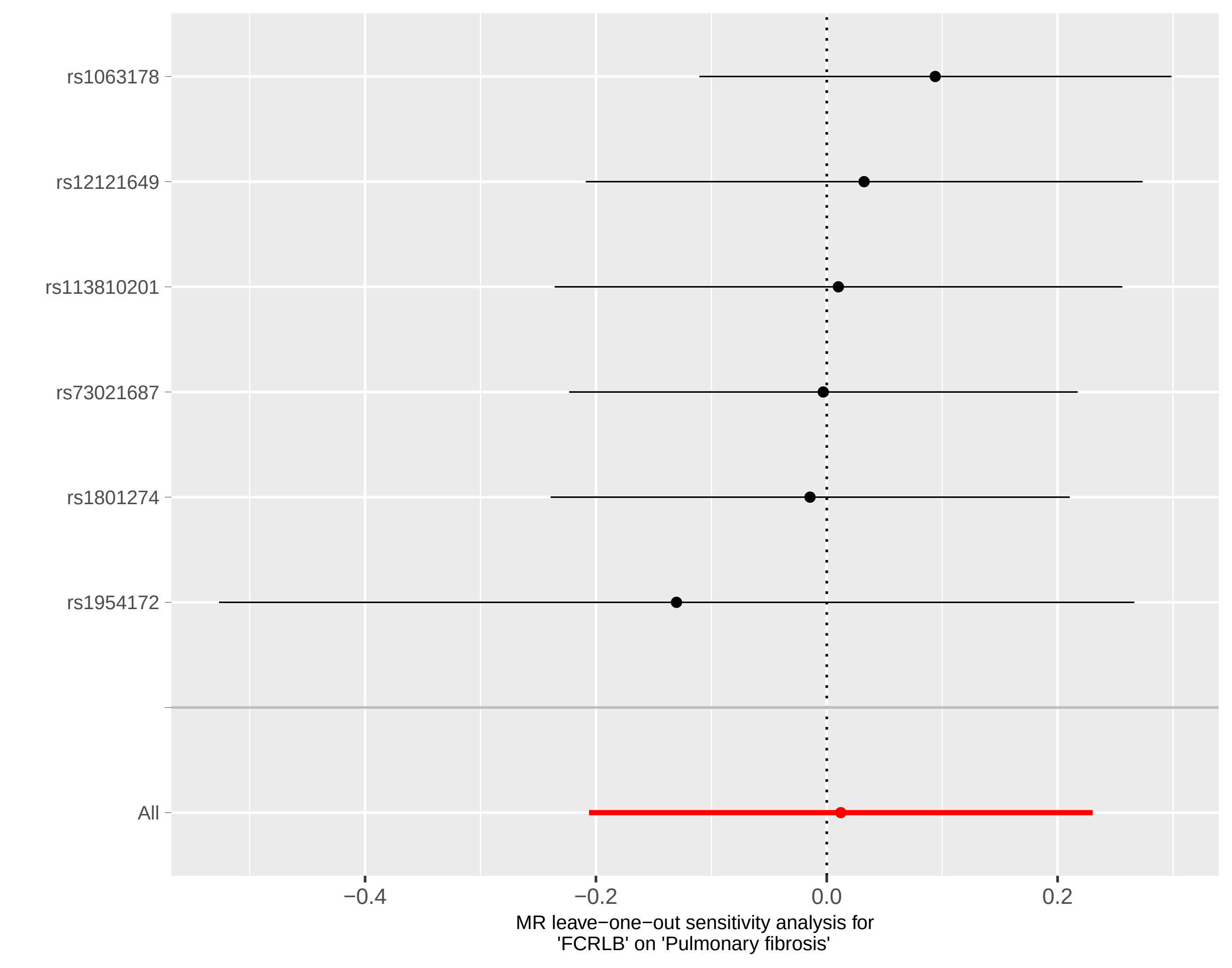
rs1063178
rs12121649
rs113810201
rs73021687
rs1801274
rs1954172
All
−0.4
−0.2
0.0
0.2
MR leave−one−out sensitivity analysis for
'FCRLB' on 'Pulmonary fibrosis'

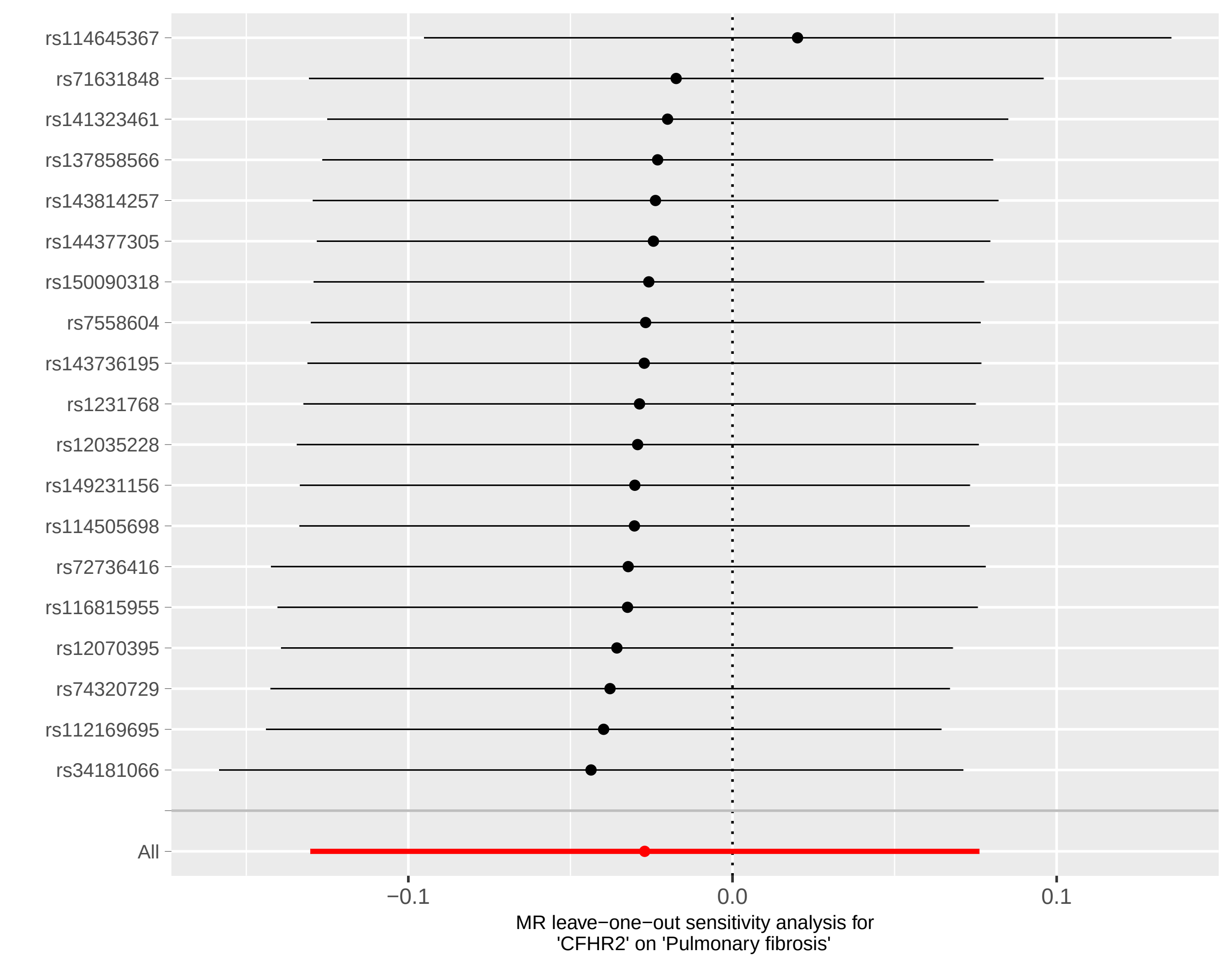
rs114645367
rs71631848
rs141323461
rs137858566
rs143814257
rs144377305
rs150090318
rs7558604
rs143736195
rs1231768
rs12035228
rs149231156
rs114505698
rs72736416
rs116815955
rs12070395
rs74320729
rs112169695
rs34181066
All
−0.1
0.0
0.1
MR leave−one−out sensitivity analysis for
'CFHR2' on 'Pulmonary fibrosis'

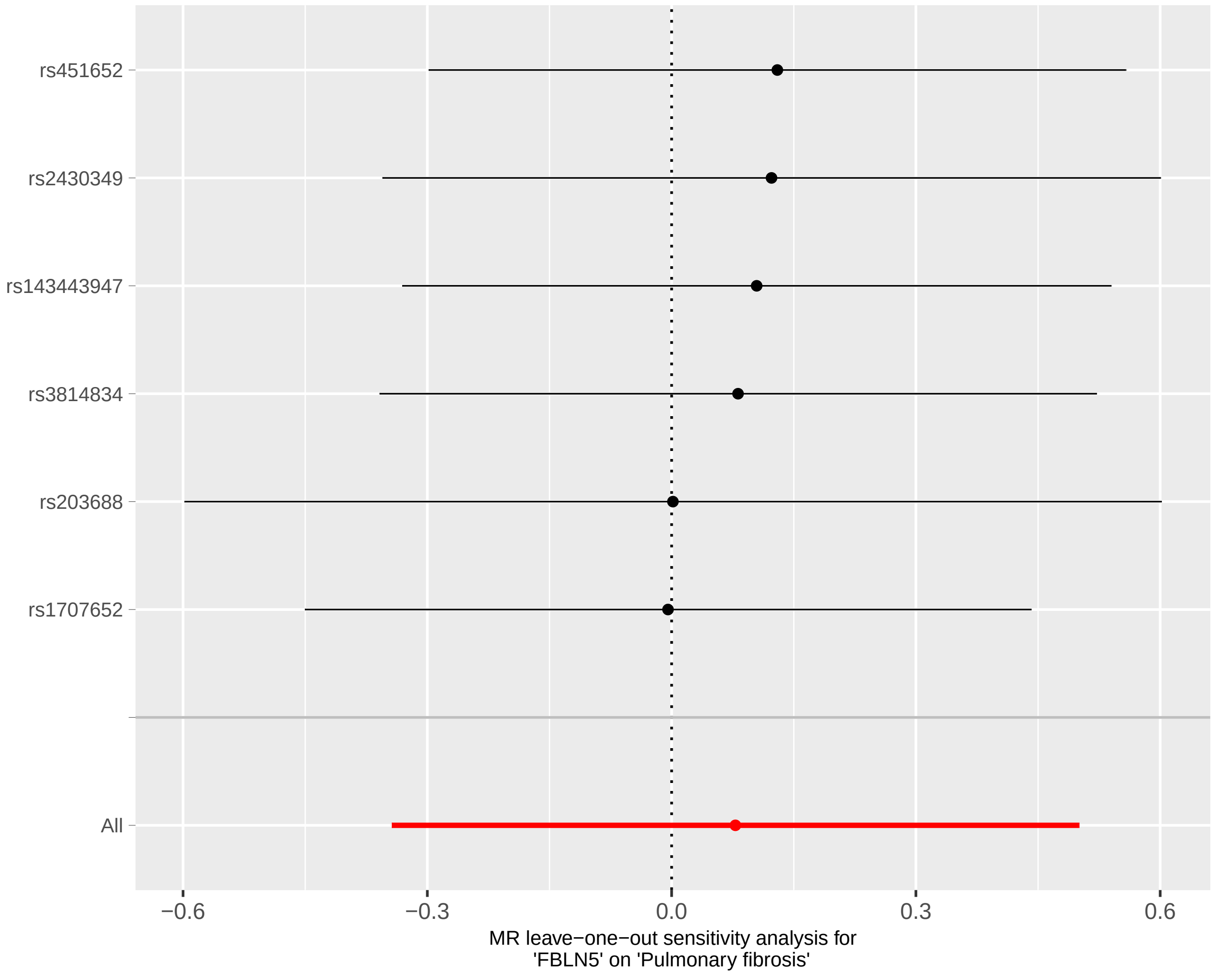
rs451652
rs2430349
rs143443947
rs3814834
rs203688
rs1707652
All
−0.6
−0.3
0.0
0.3
0.6
MR leave−one−out sensitivity analysis for
'FBLN5' on 'Pulmonary fibrosis'

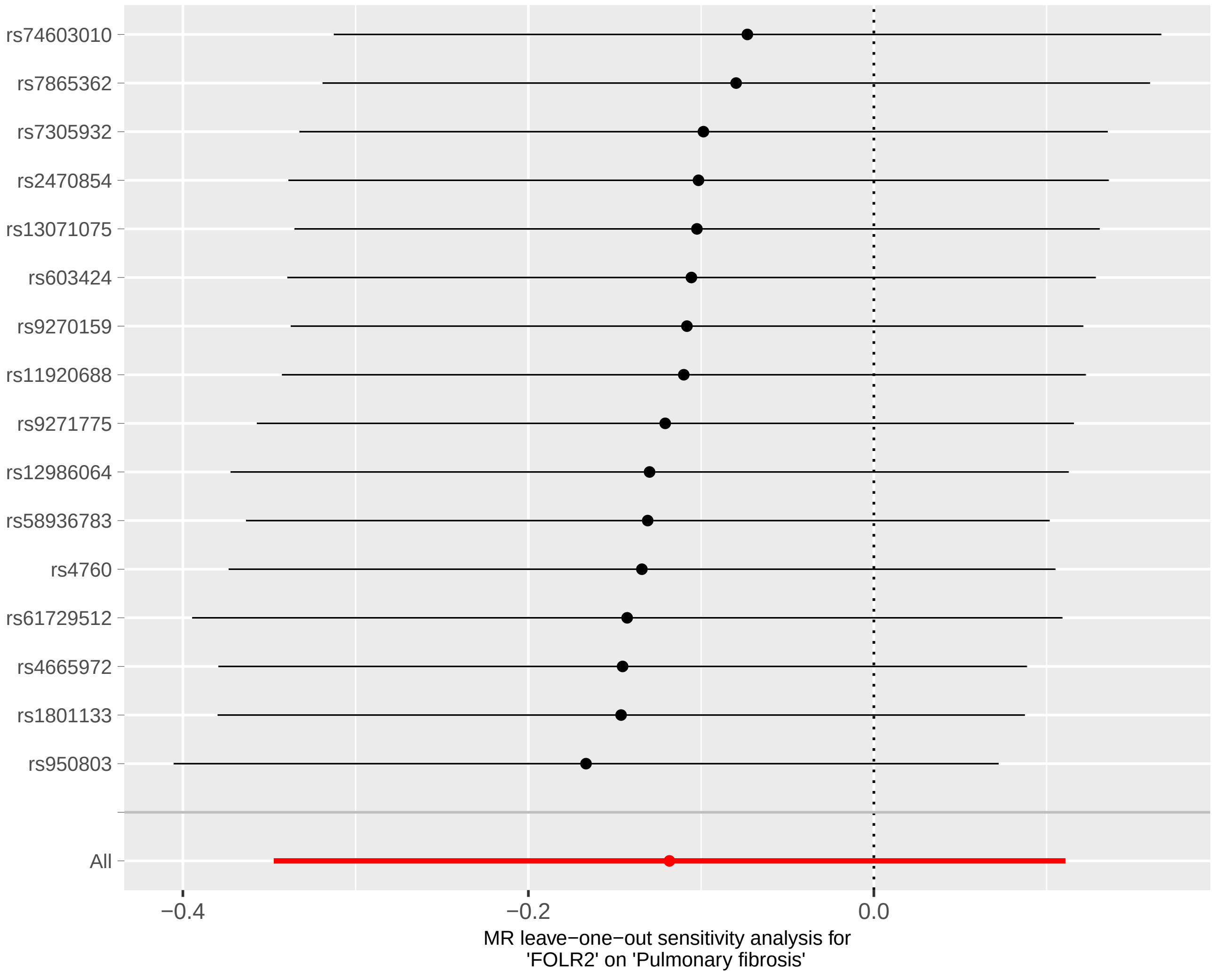
rs74603010
rs7865362
rs7305932
rs2470854
rs13071075
rs603424
rs9270159
rs11920688
rs9271775
rs12986064
rs58936783
rs4760
rs61729512
rs4665972
rs1801133
rs950803
All
−0.4
−0.2
0.0
MR leave−one−out sensitivity analysis for
'FOLR2' on 'Pulmonary fibrosis'

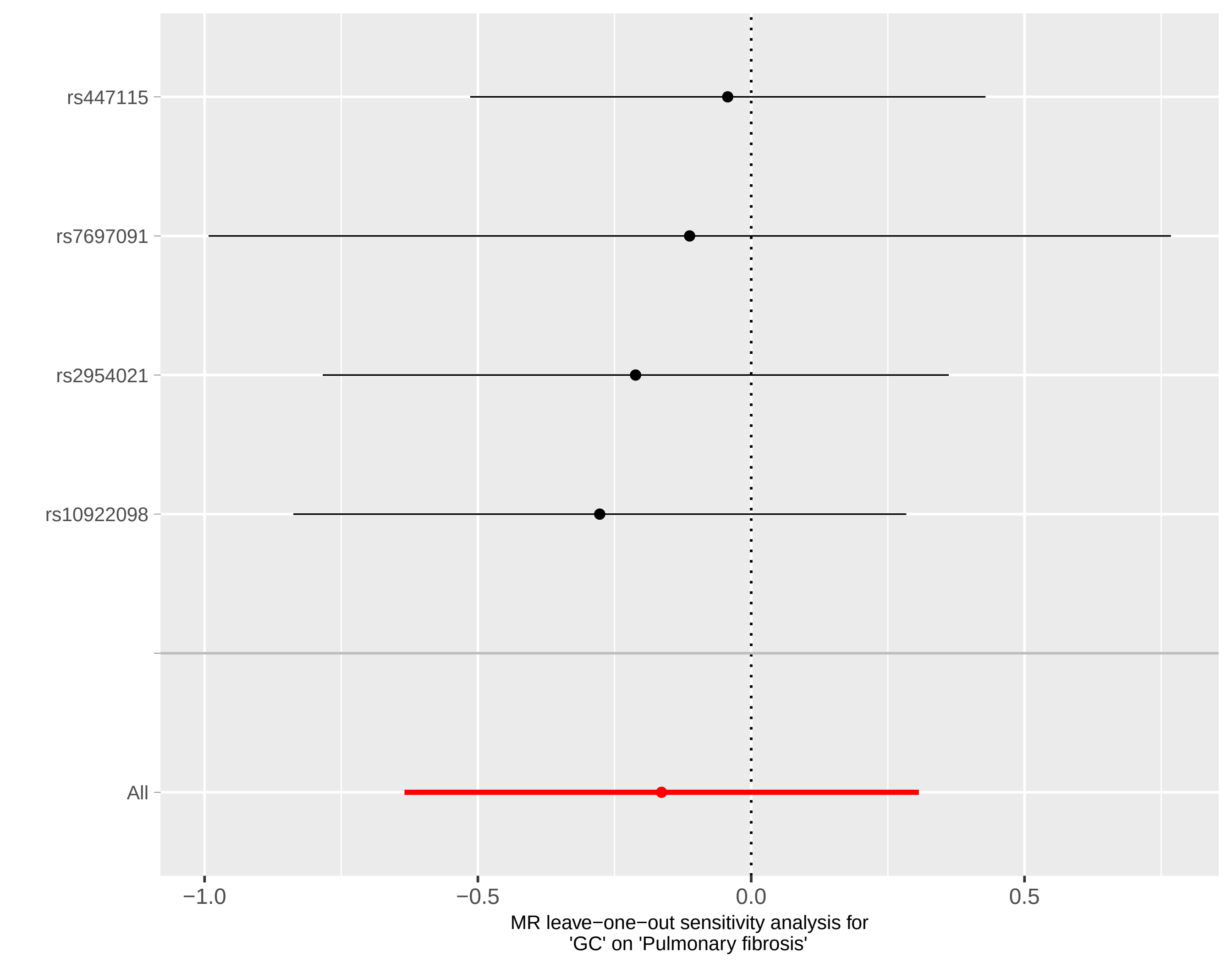
rs447115
rs7697091
rs2954021
rs10922098
All
−1.0
−0.5
0.0
0.5
MR leave−one−out sensitivity analysis for
'GC' on 'Pulmonary fibrosis'

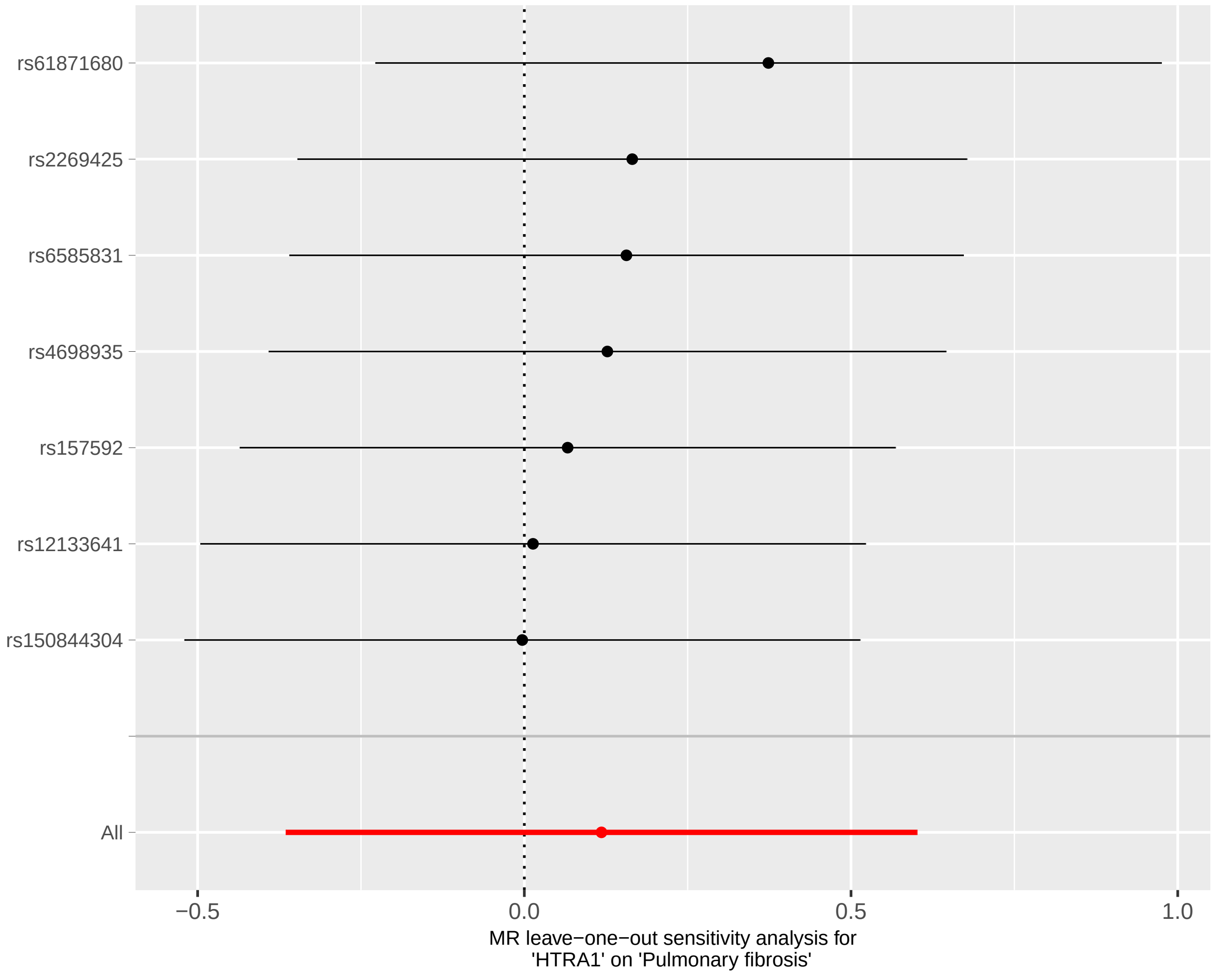
rs61871680
rs2269425
rs6585831
rs4698935
rs157592
rs12133641
rs150844304
All
−0.5
0.0
0.5
1.0
MR leave−one−out sensitivity analysis for
'HTRA1' on 'Pulmonary fibrosis'

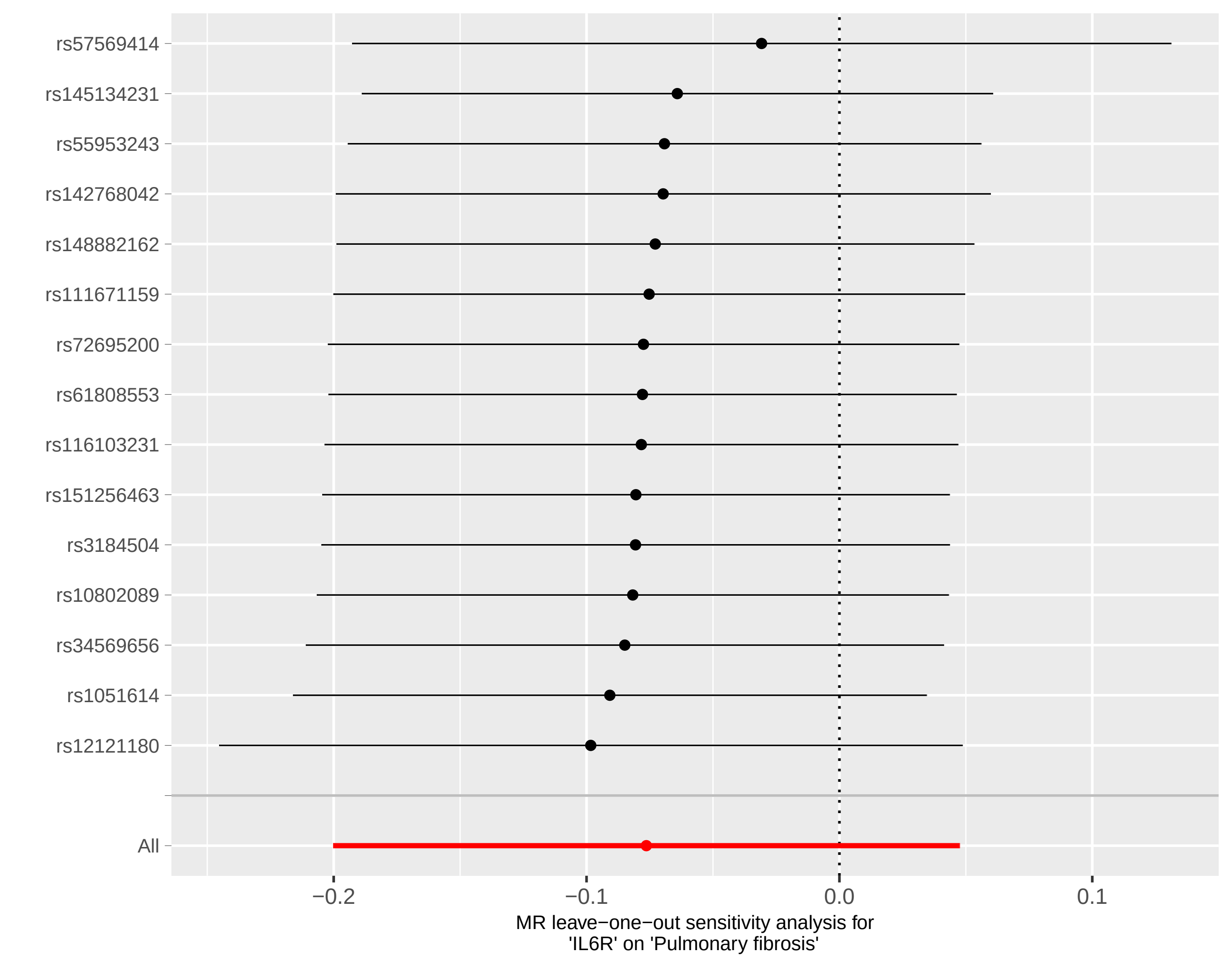
rs57569414
rs145134231
rs55953243
rs142768042
rs148882162
rs111671159
rs72695200
rs61808553
rs116103231
rs151256463
rs3184504
rs10802089
rs34569656
rs1051614
rs12121180
All
−0.2
−0.1
0.0
0.1
MR leave−one−out sensitivity analysis for
'IL6R' on 'Pulmonary fibrosis'

Insufficient number of SNPs

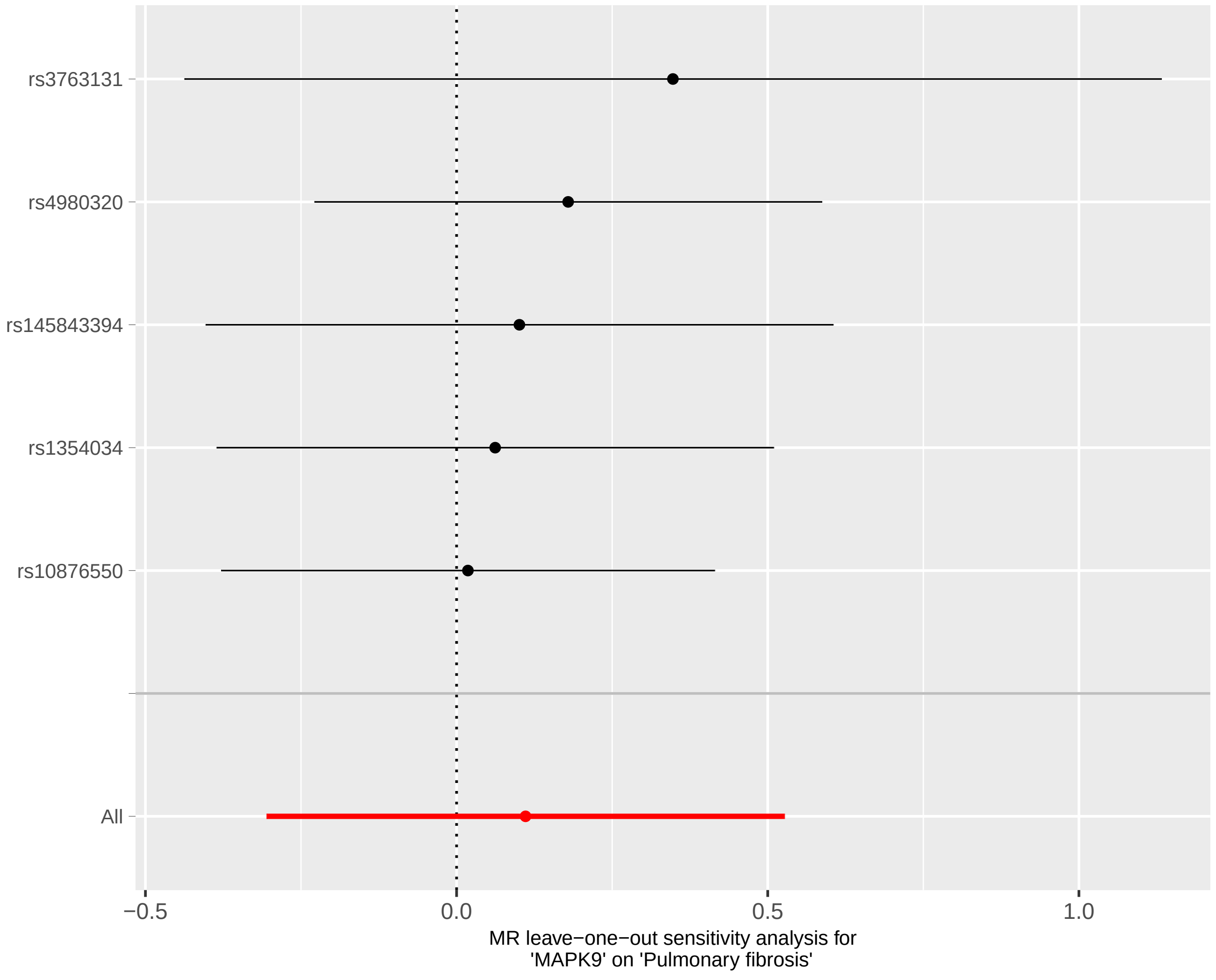
rs3763131
rs4980320
rs145843394
rs1354034
rs10876550
All
−0.5
0.0
0.5
1.0
MR leave−one−out sensitivity analysis for
'MAPK9' on 'Pulmonary fibrosis'

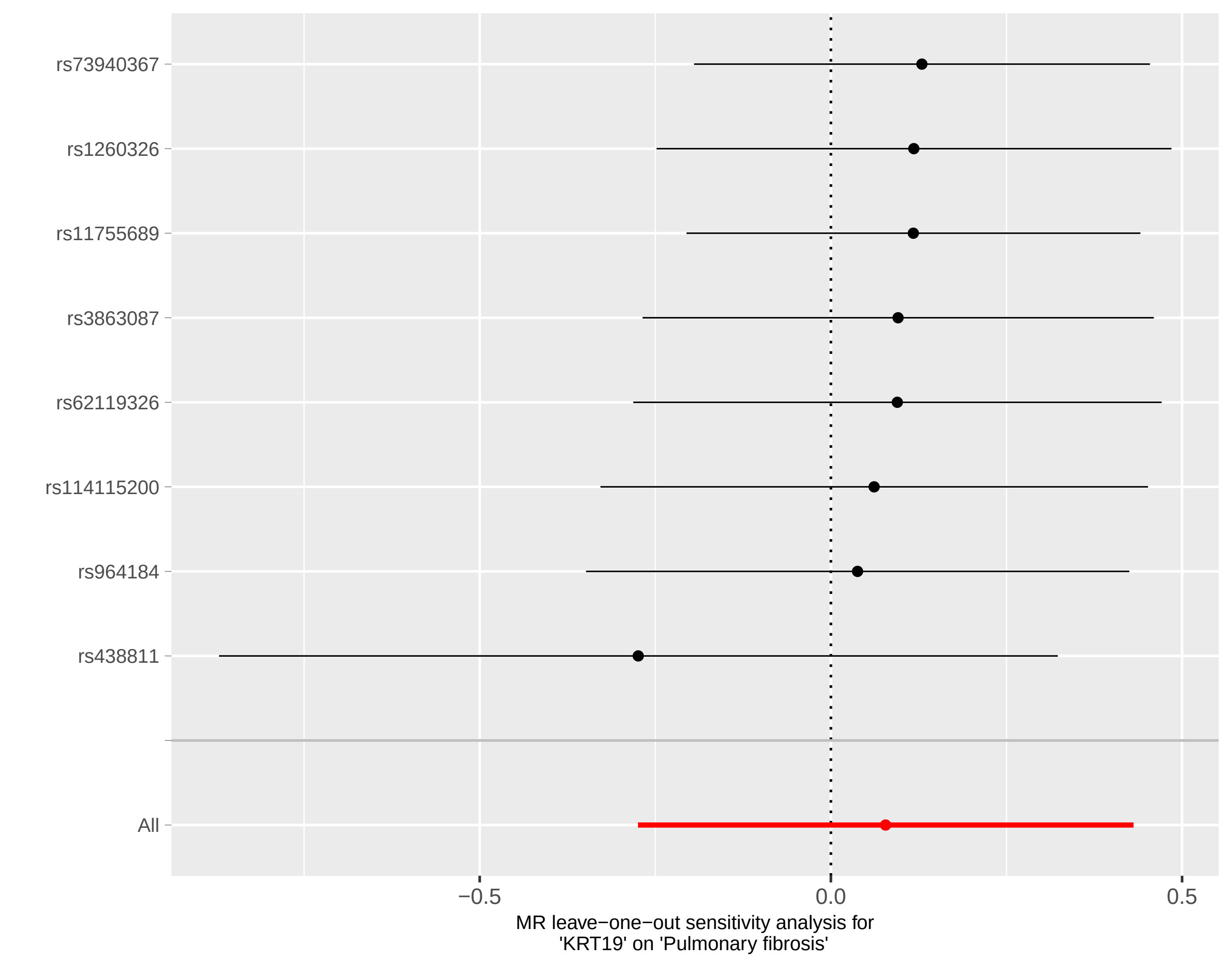
rs73940367
rs1260326
rs11755689
rs3863087
rs62119326
rs114115200
rs964184
rs438811
All
−0.5
0.0
0.5
MR leave−one−out sensitivity analysis for
'KRT19' on 'Pulmonary fibrosis'

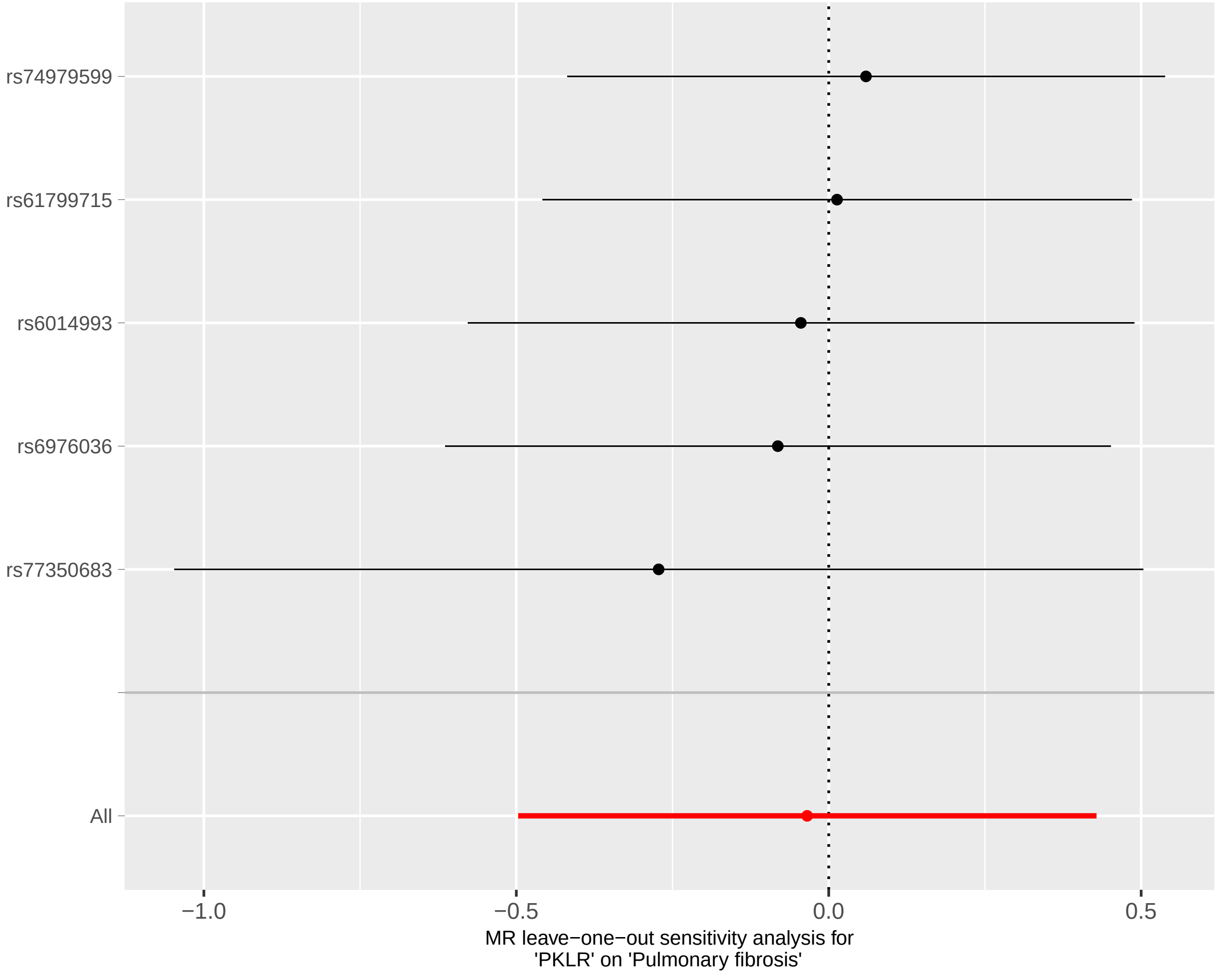
rs74979599
rs61799715
rs6014993
rs6976036
rs77350683
All
−1.0
−0.5
0.0
0.5
MR leave−one−out sensitivity analysis for
'PKLR' on 'Pulmonary fibrosis'

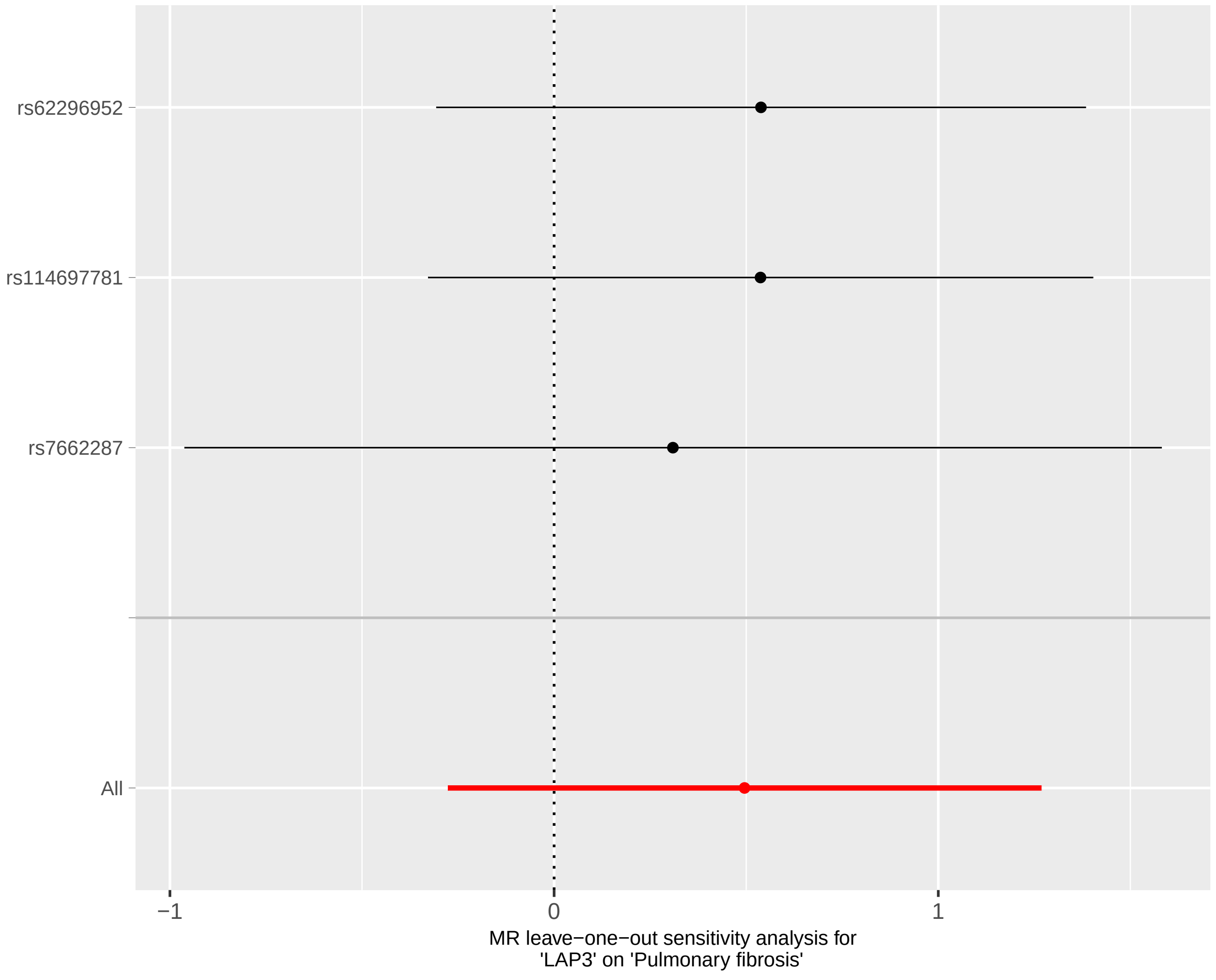
rs62296952
rs114697781
rs7662287
All
−1
0
1
MR leave−one−out sensitivity analysis for
'LAP3' on 'Pulmonary fibrosis'

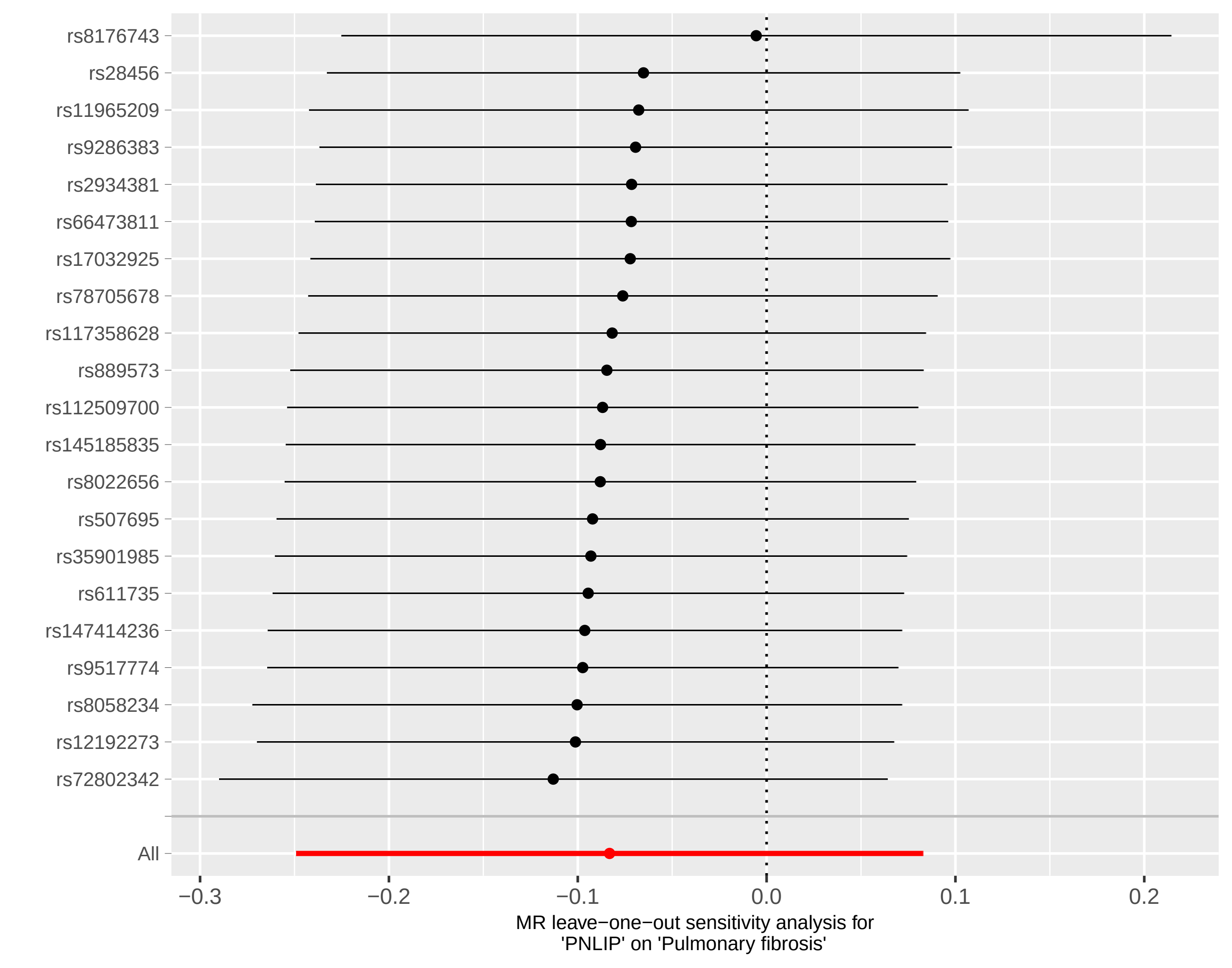
rs8176743
rs28456
rs11965209
rs9286383
rs2934381
rs66473811
rs17032925
rs78705678
rs117358628
rs889573
rs112509700
rs145185835
rs8022656
rs507695
rs35901985
rs611735
rs147414236
rs9517774
rs8058234
rs12192273
rs72802342
All
−0.3
−0.2
−0.1
0.0
0.1
0.2
MR leave−one−out sensitivity analysis for
'PNLIP' on 'Pulmonary fibrosis'

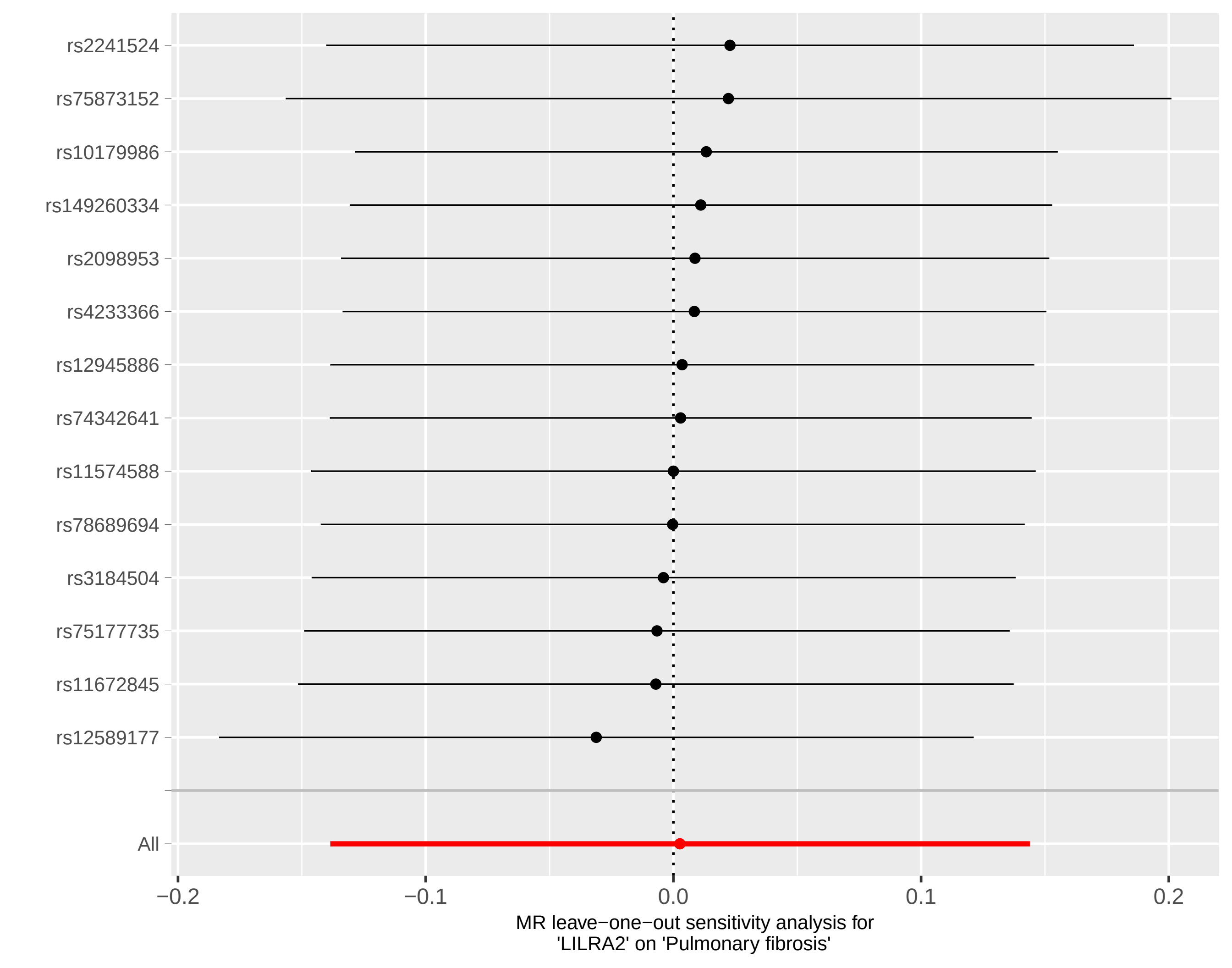
rs2241524
rs75873152
rs10179986
rs149260334
rs2098953
rs4233366
rs12945886
rs74342641
rs11574588
rs78689694
rs3184504
rs75177735
rs11672845
rs12589177
All
−0.2
−0.1
0.0
0.1
0.2
MR leave−one−out sensitivity analysis for
'LILRA2' on 'Pulmonary fibrosis'

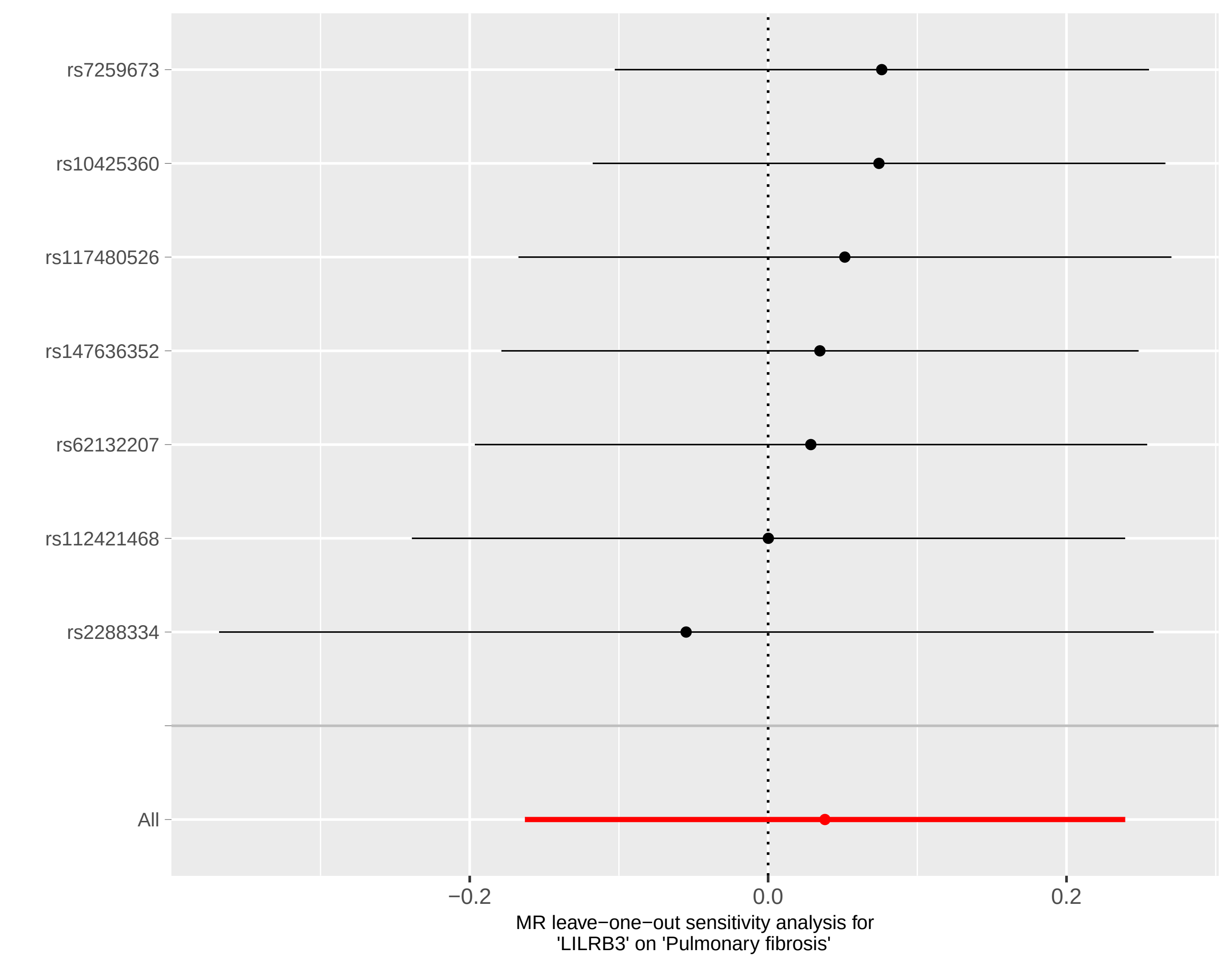
rs7259673
rs10425360
rs117480526
rs147636352
rs62132207
rs112421468
rs2288334
All
−0.2
0.0
0.2
MR leave−one−out sensitivity analysis for
'LILRB3' on 'Pulmonary fibrosis'

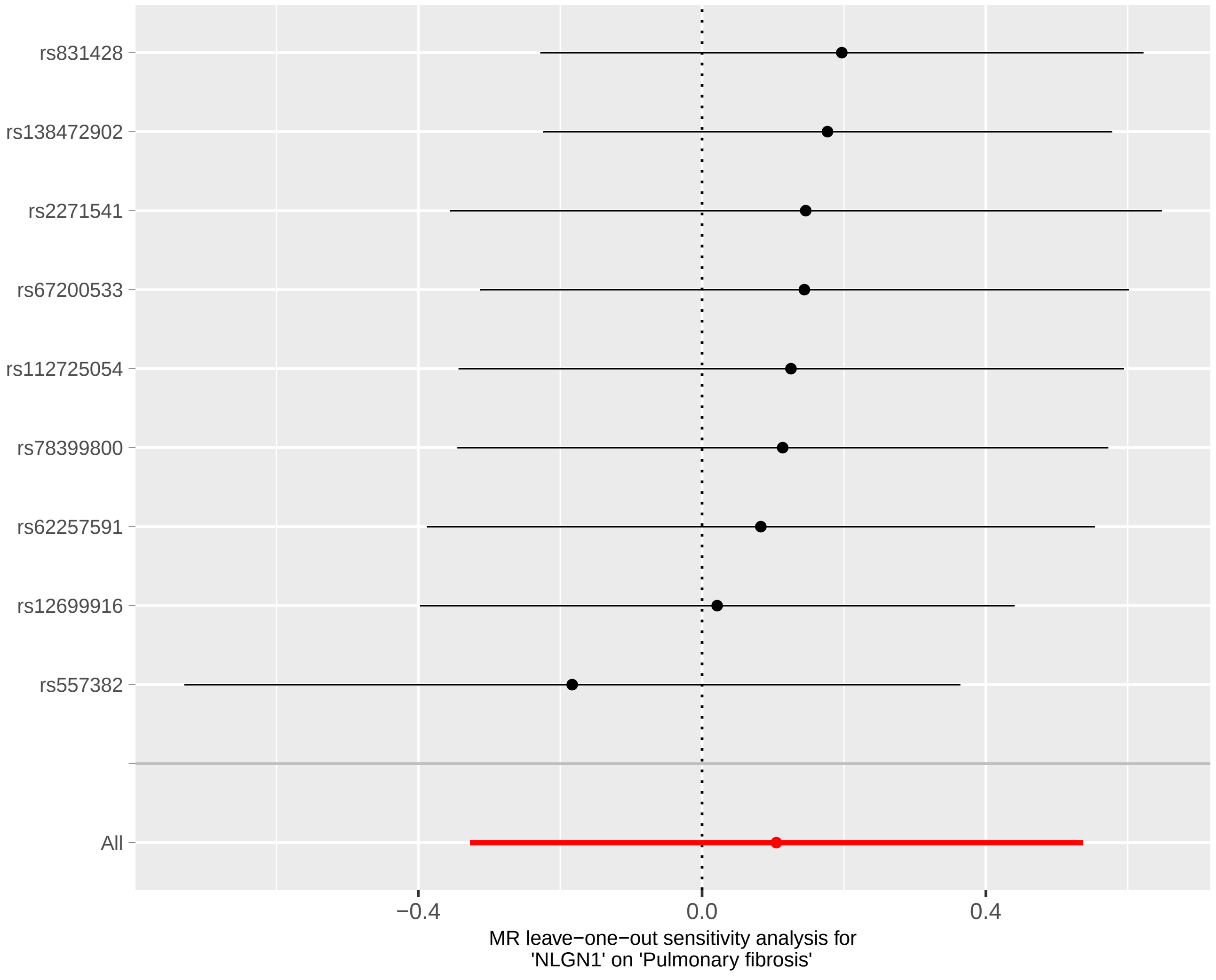
rs831428
rs138472902
rs2271541
rs67200533
rs112725054
rs78399800
rs62257591
rs12699916
rs557382
All
−0.4
0.0
0.4
MR leave−one−out sensitivity analysis for
'NLGN1' on 'Pulmonary fibrosis'

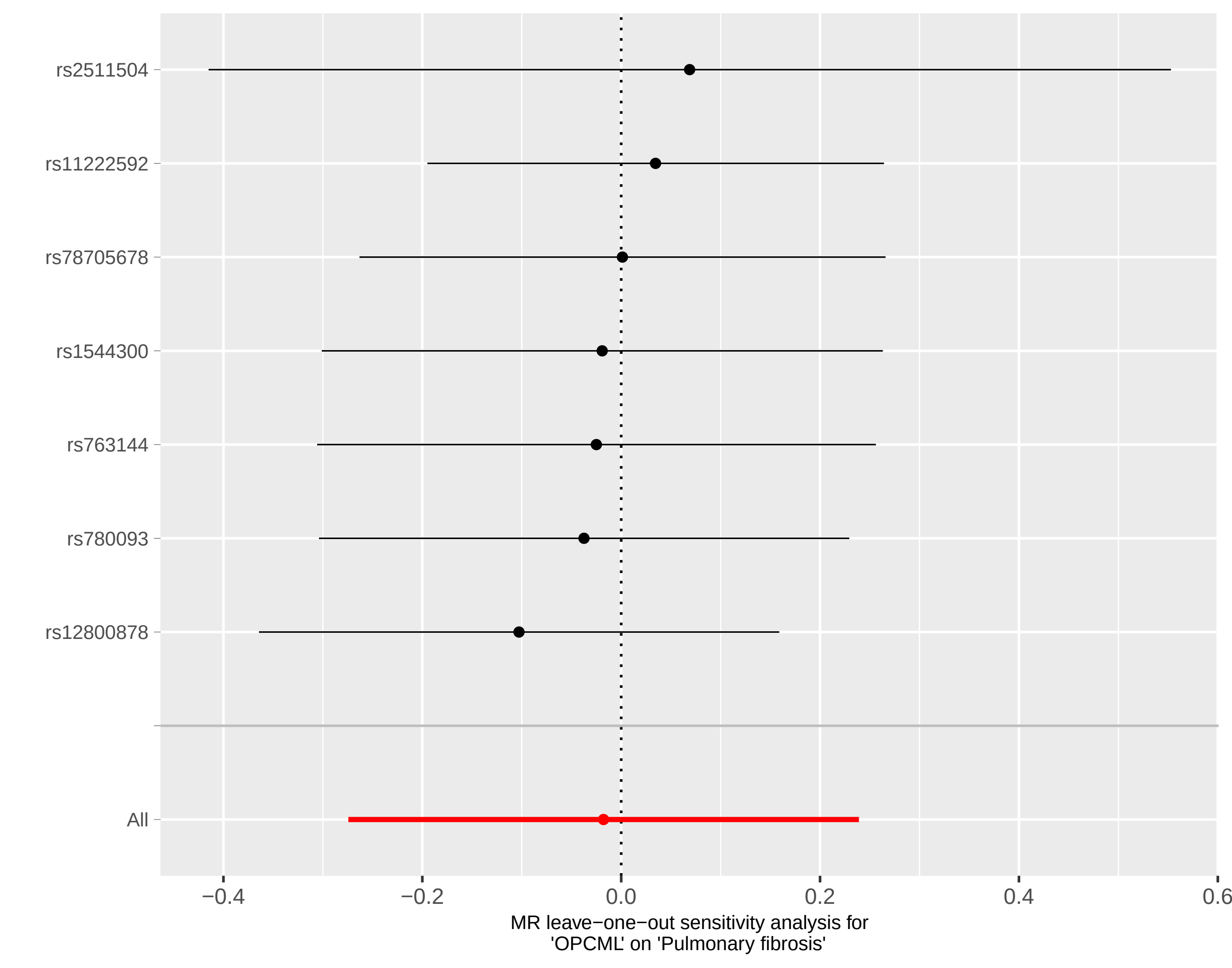
rs2511504
rs11222592
rs78705678
rs1544300
rs763144
rs780093
rs12800878
All
−0.4
−0.2
0.0
0.2
0.4
0.6
MR leave−one−out sensitivity analysis for
'OPCML' on 'Pulmonary fibrosis'

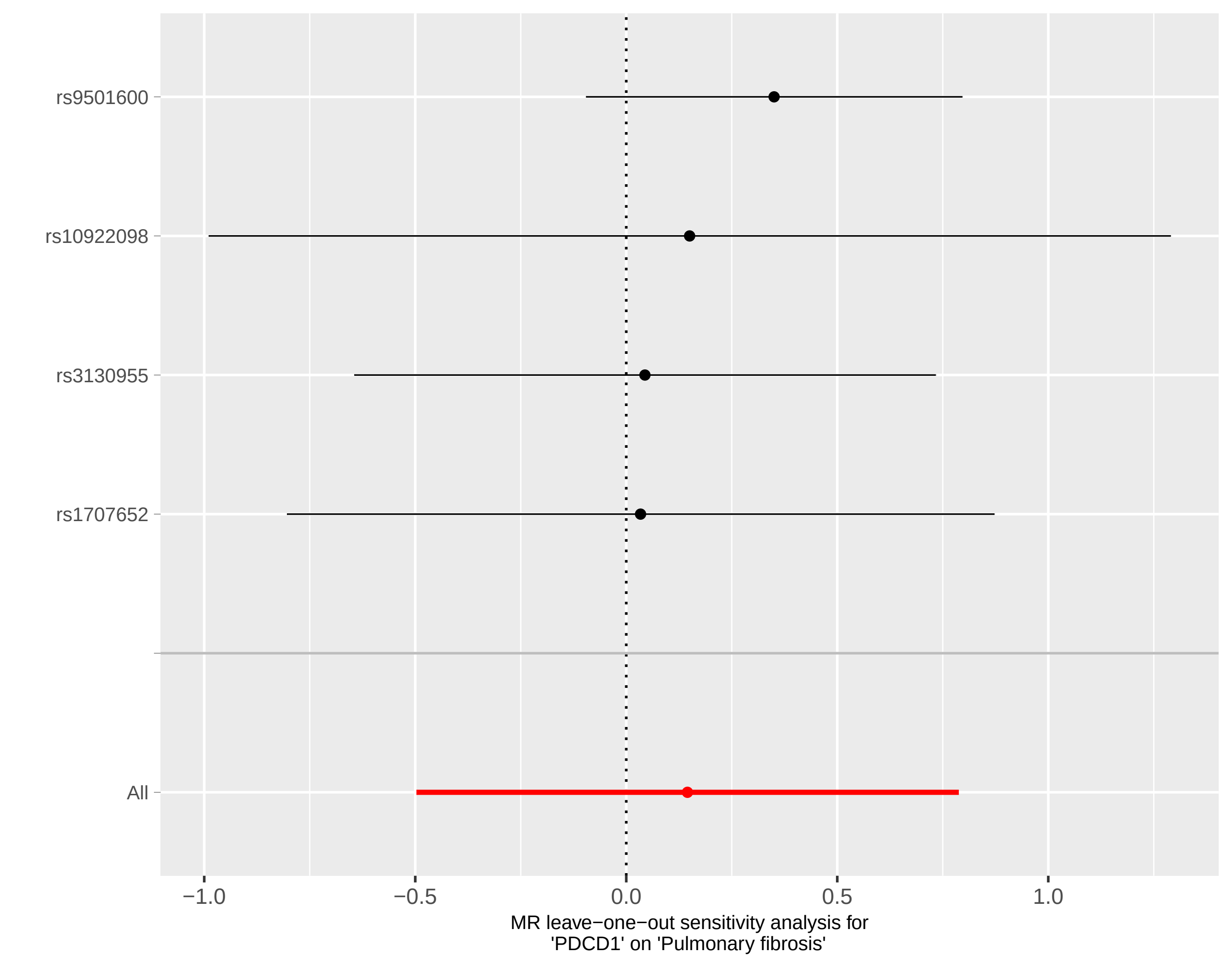
rs9501600
rs10922098
rs3130955
rs1707652
All
−1.0
−0.5
0.0
0.5
1.0
MR leave−one−out sensitivity analysis for
'PDCD1' on 'Pulmonary fibrosis'

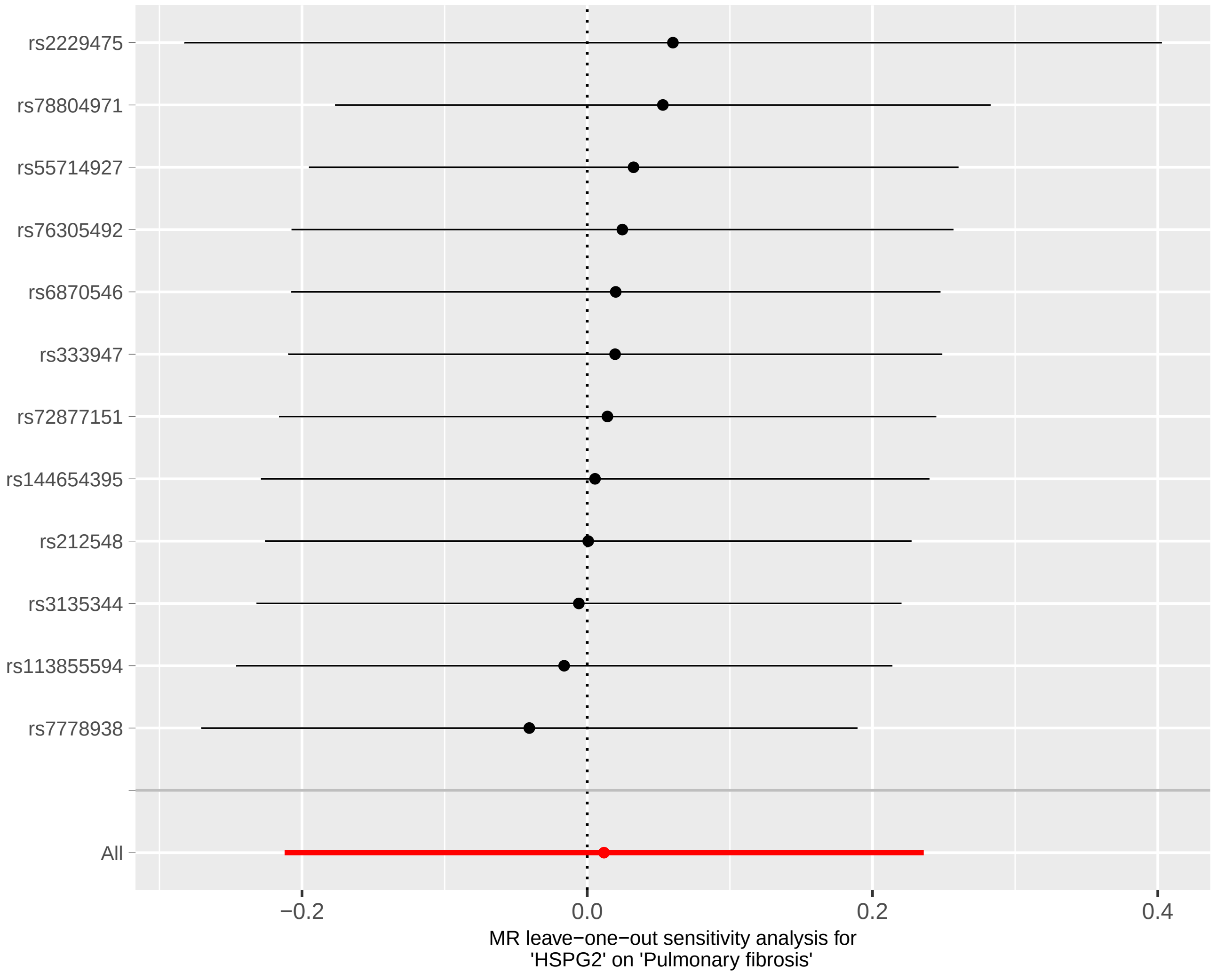

rs2229475
rs78804971
rs55714927
rs76305492
rs6870546
rs333947
rs72877151
rs144654395
rs212548
rs3135344
rs113855594
rs7778938
All
−0.2
0.0
0.2
0.4
MR leave−one−out sensitivity analysis for
'HSPG2' on 'Pulmonary fibrosis'

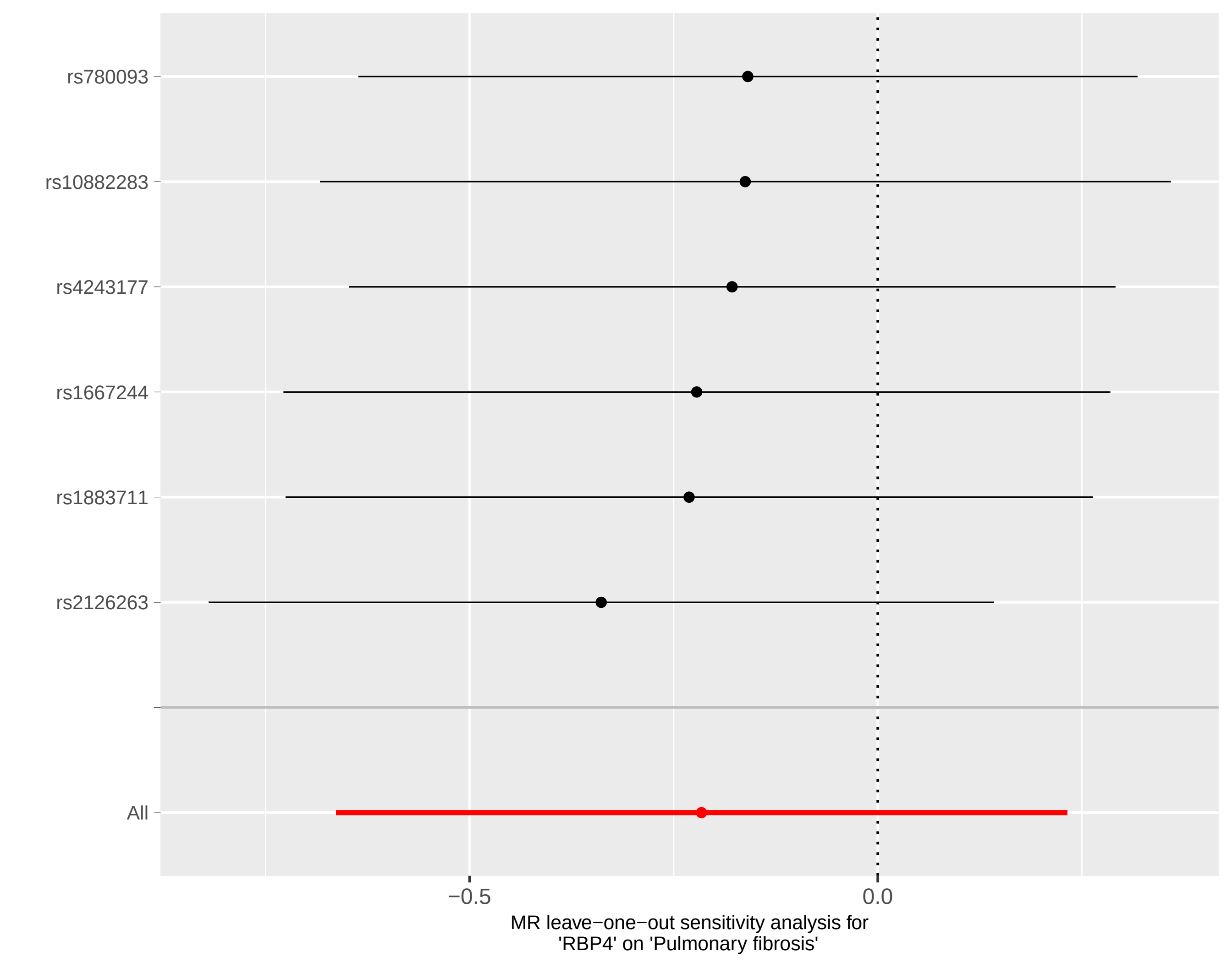
rs780093
rs10882283
rs4243177
rs1667244
rs1883711
rs2126263
All
−0.5
0.0
MR leave−one−out sensitivity analysis for
'RBP4' on 'Pulmonary fibrosis'

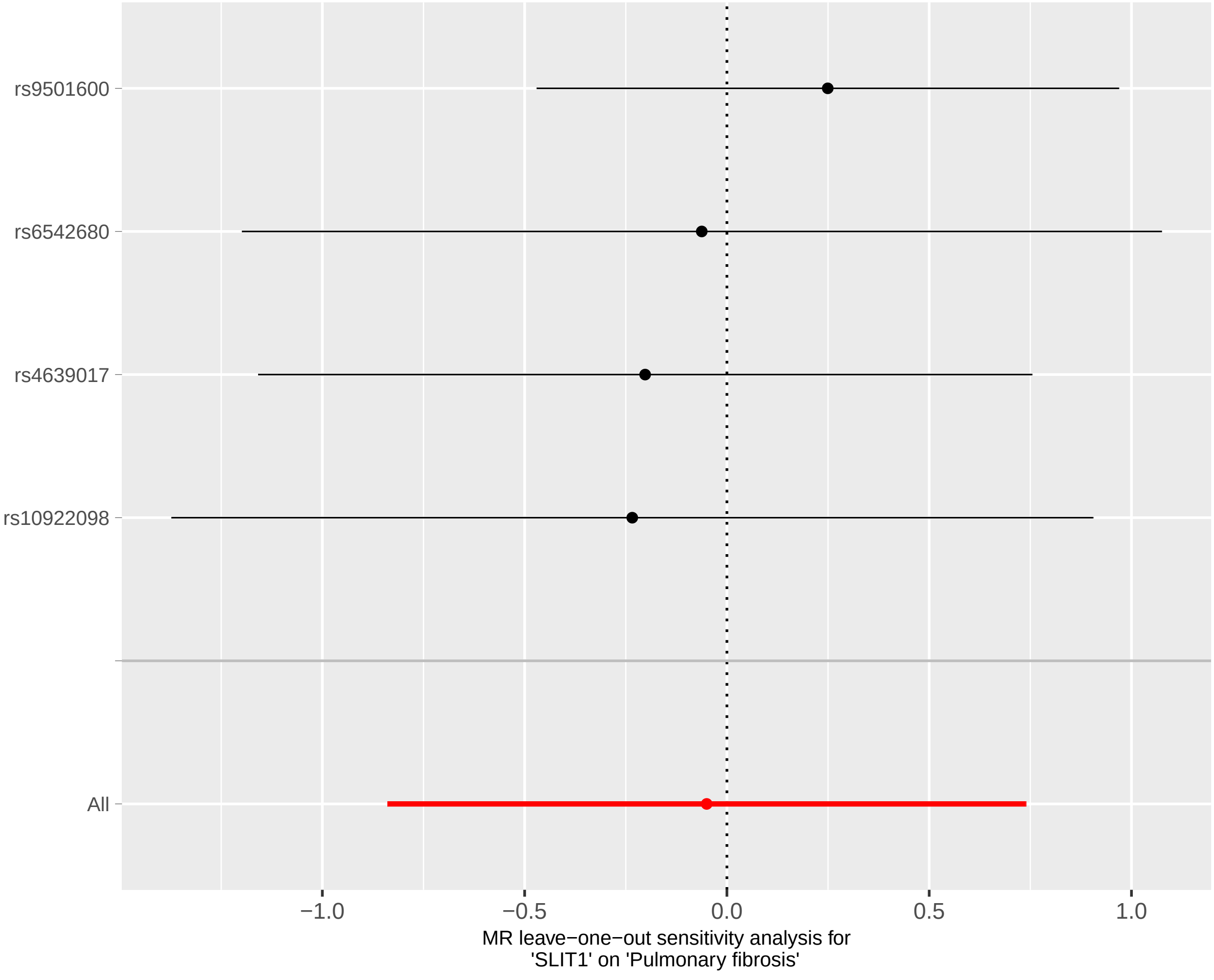
rs9501600
rs6542680
rs4639017
rs10922098
All
−1.0
−0.5
0.0
0.5
1.0
MR leave−one−out sensitivity analysis for
'SLIT1' on 'Pulmonary fibrosis'

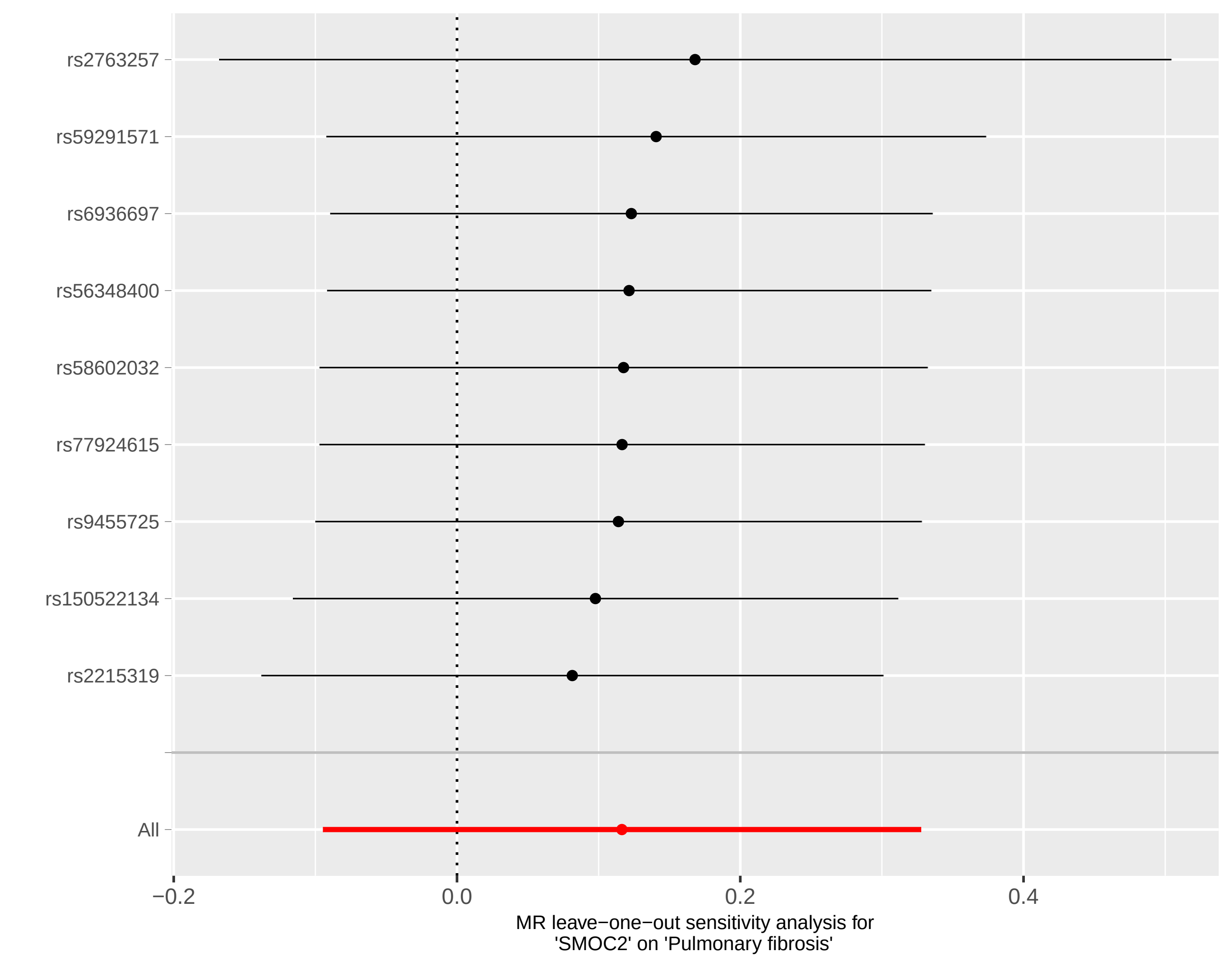
rs2763257
rs59291571
rs6936697
rs56348400
rs58602032
rs77924615
rs9455725
rs150522134
rs2215319
All
−0.2
0.0
0.2
0.4
MR leave−one−out sensitivity analysis for
'SMOC2' on 'Pulmonary fibrosis'

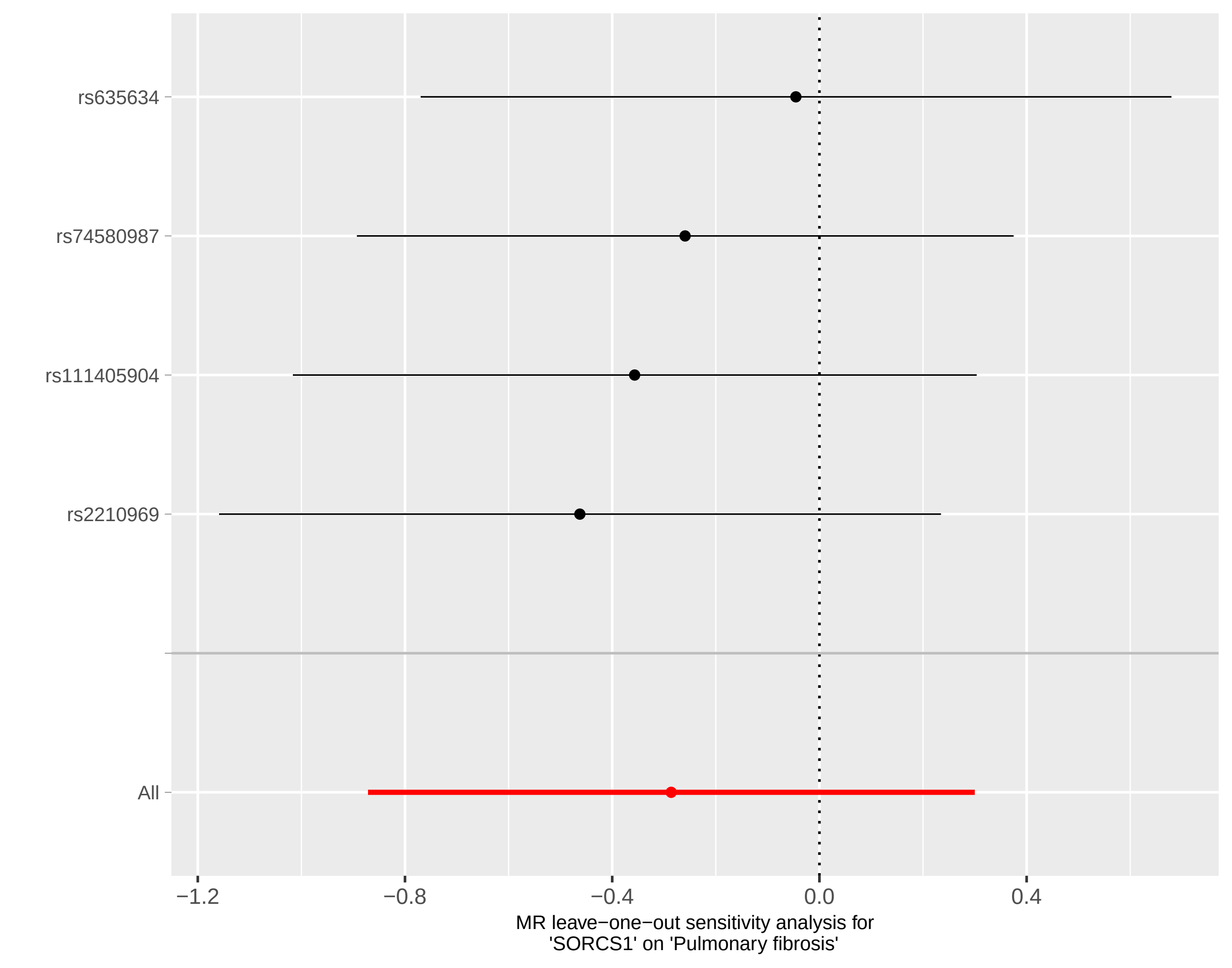

rs635634
rs74580987
rs111405904
rs2210969
All
−1.2
−0.8
−0.4
0.0
0.4
MR leave−one−out sensitivity analysis for
'SORCS1' on 'Pulmonary fibrosis'

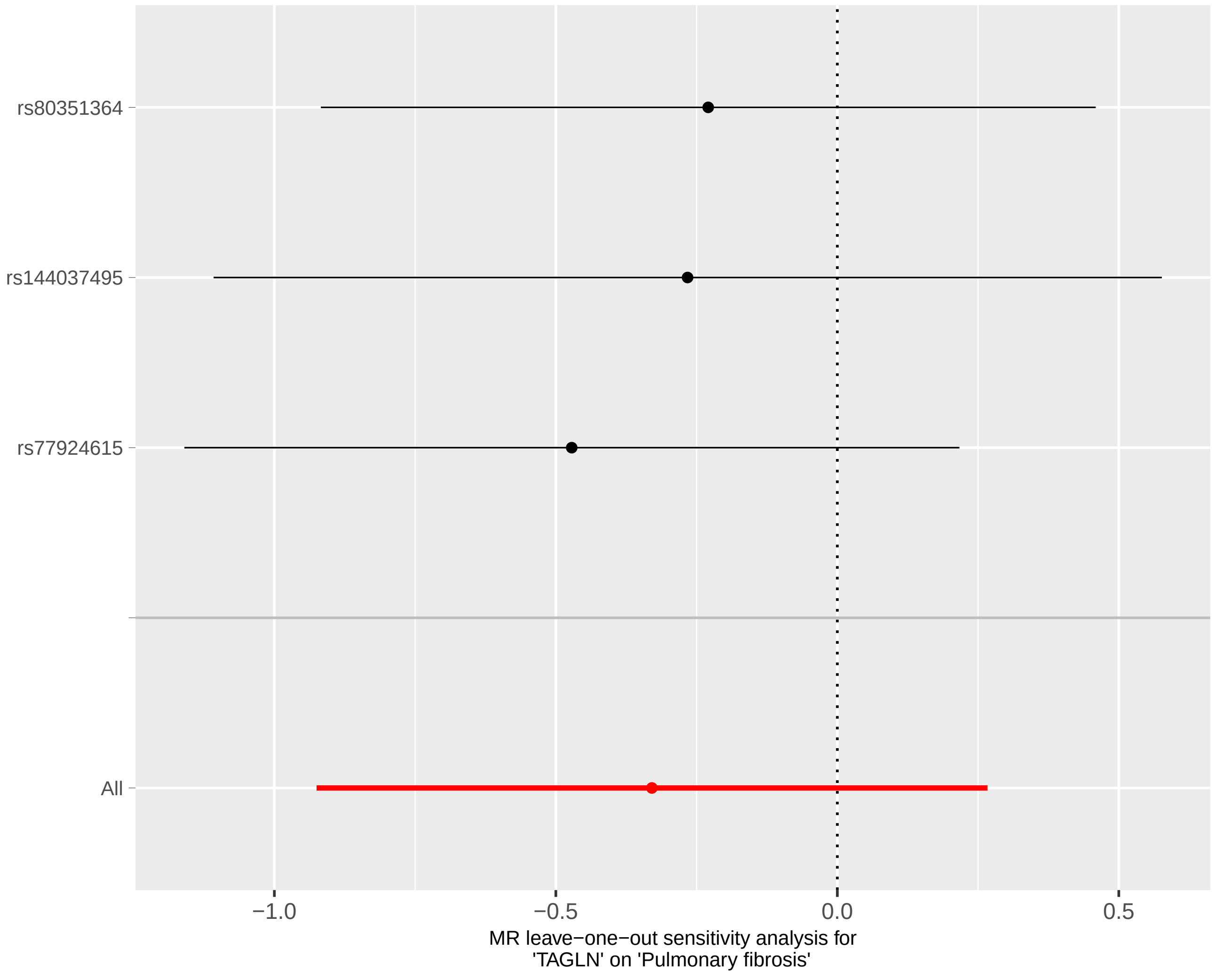

rs80351364
rs144037495
rs77924615
All
−1.0
−0.5
0.0
0.5
MR leave−one−out sensitivity analysis for
'TAGLN' on 'Pulmonary fibrosis'

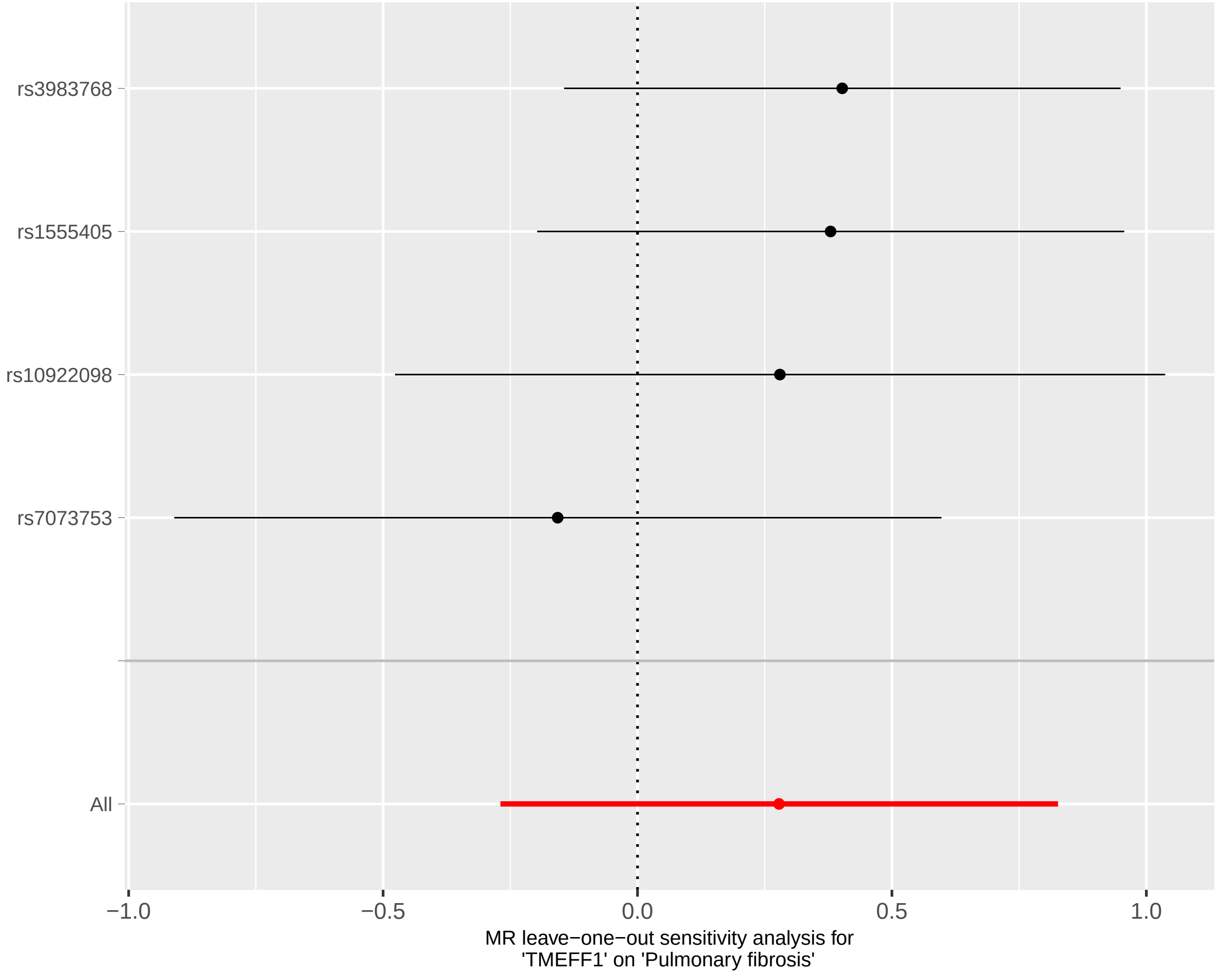
rs3983768
rs1555405
rs10922098
rs7073753
All
−1.0
−0.5
0.0
0.5
1.0
MR leave−one−out sensitivity analysis for
'TMEFF1' on 'Pulmonary fibrosis'

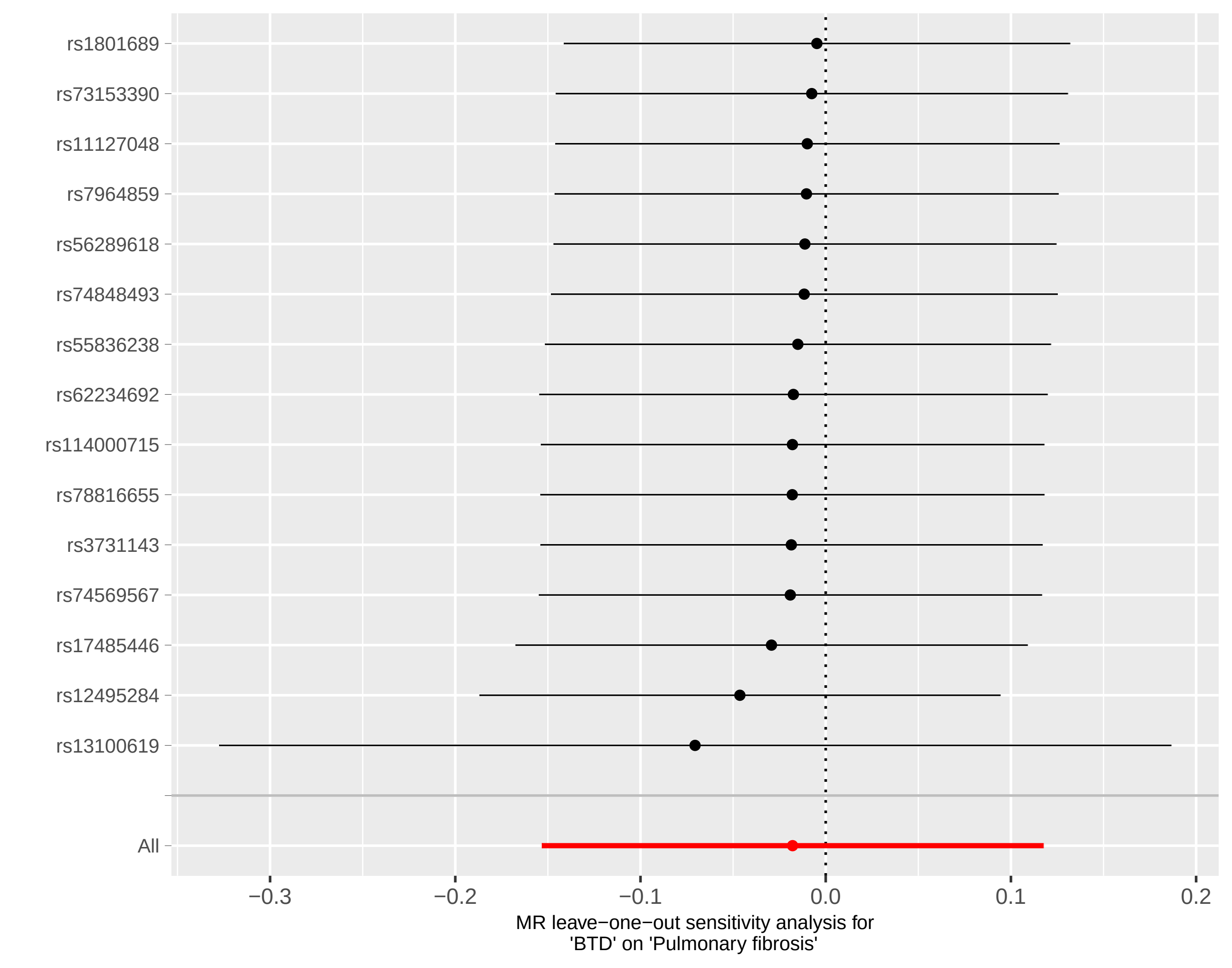
rs1801689
rs73153390
rs11127048
rs7964859
rs56289618
rs74848493
rs55836238
rs62234692
rs114000715
rs78816655
rs3731143
rs74569567
rs17485446
rs12495284
rs13100619
All
−0.3
−0.2
−0.1
0.0
0.1
0.2
MR leave−one−out sensitivity analysis for
'BTD' on 'Pulmonary fibrosis'

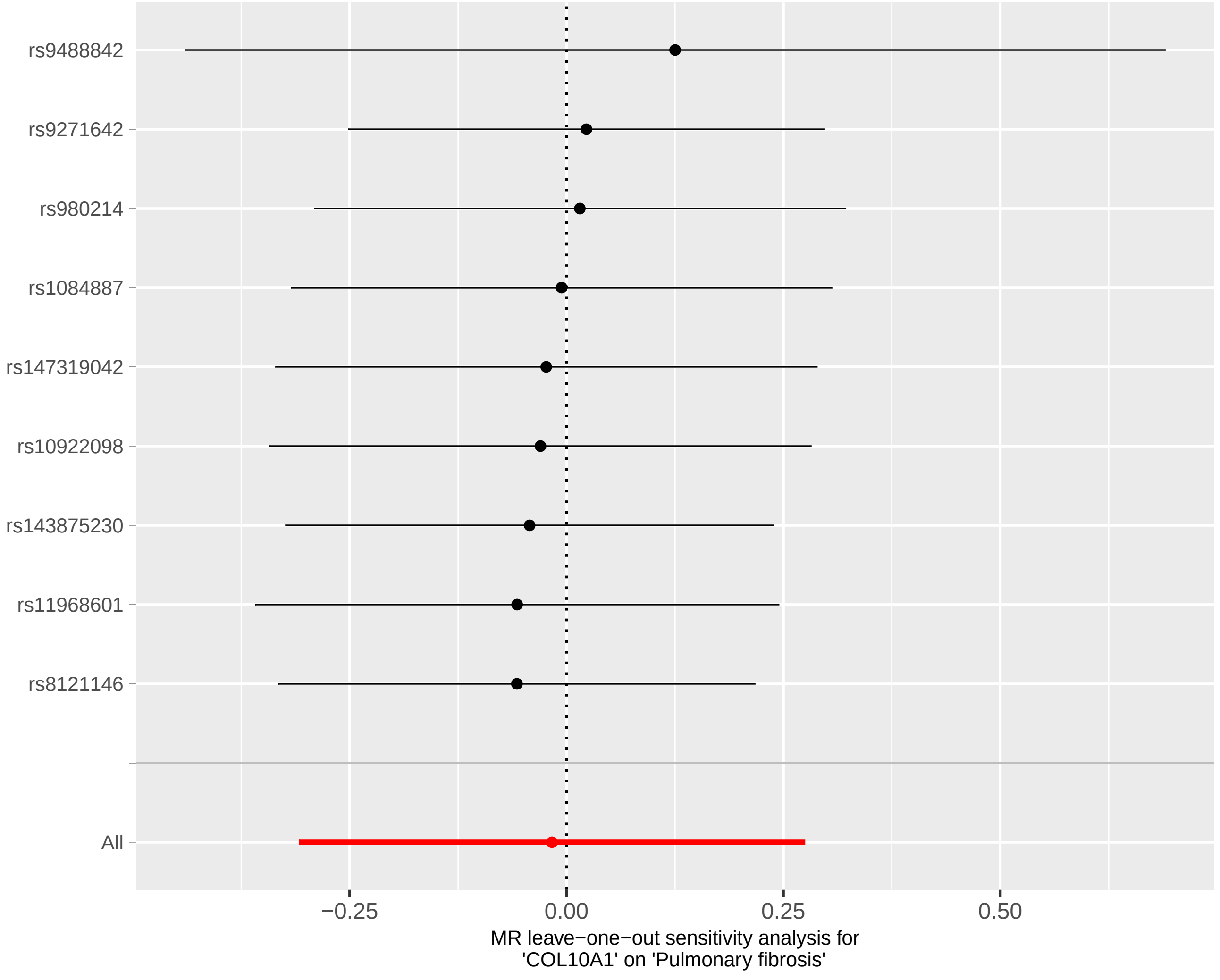
rs9488842
rs9271642
rs980214
rs1084887
rs147319042
rs10922098
rs143875230
rs11968601
rs8121146
All
−0.25
0.00
0.25
0.50
MR leave−one−out sensitivity analysis for
'COL10A1' on 'Pulmonary fibrosis'

Insufficient number of SNPs

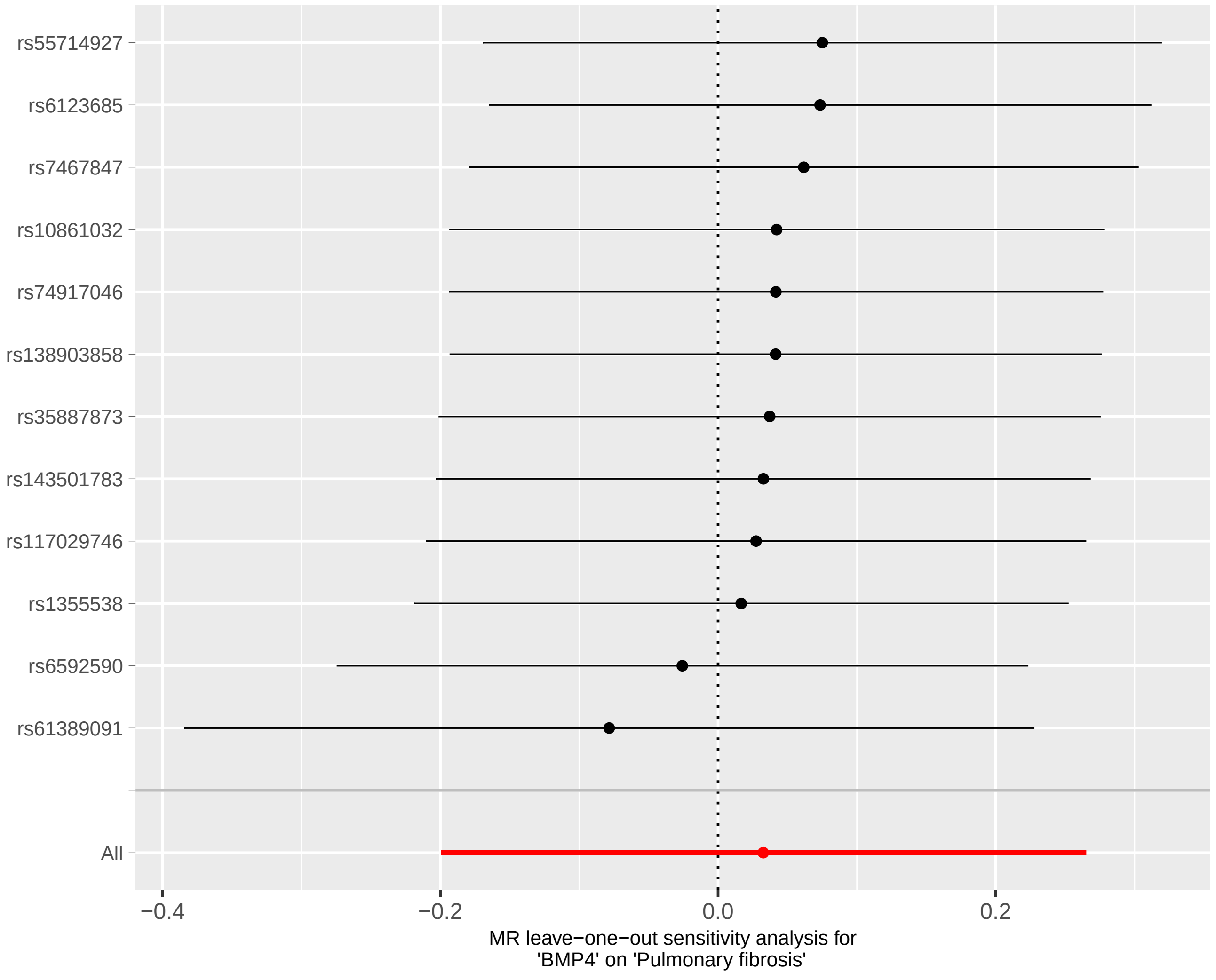
rs55714927
rs6123685
rs7467847
rs10861032
rs74917046
rs138903858
rs35887873
rs143501783
rs117029746
rs1355538
rs6592590
rs61389091
All
−0.4
−0.2
0.0
0.2
MR leave−one−out sensitivity analysis for
'BMP4' on 'Pulmonary fibrosis'

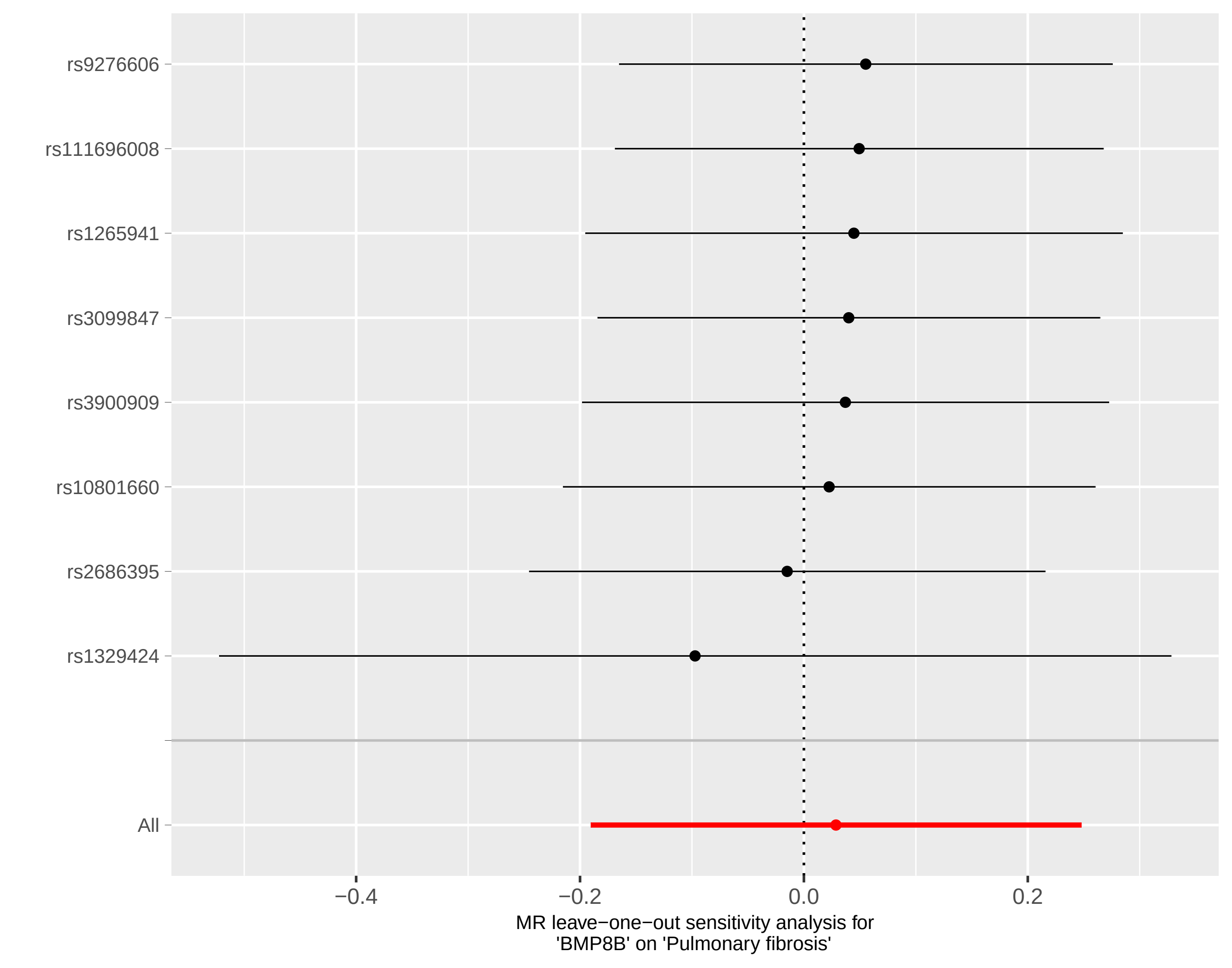

rs9276606
rs111696008
rs1265941
rs3099847
rs3900909
rs10801660
rs2686395
rs1329424
All
−0.4
−0.2
0.0
0.2
MR leave−one−out sensitivity analysis for
'BMP8B' on 'Pulmonary fibrosis'

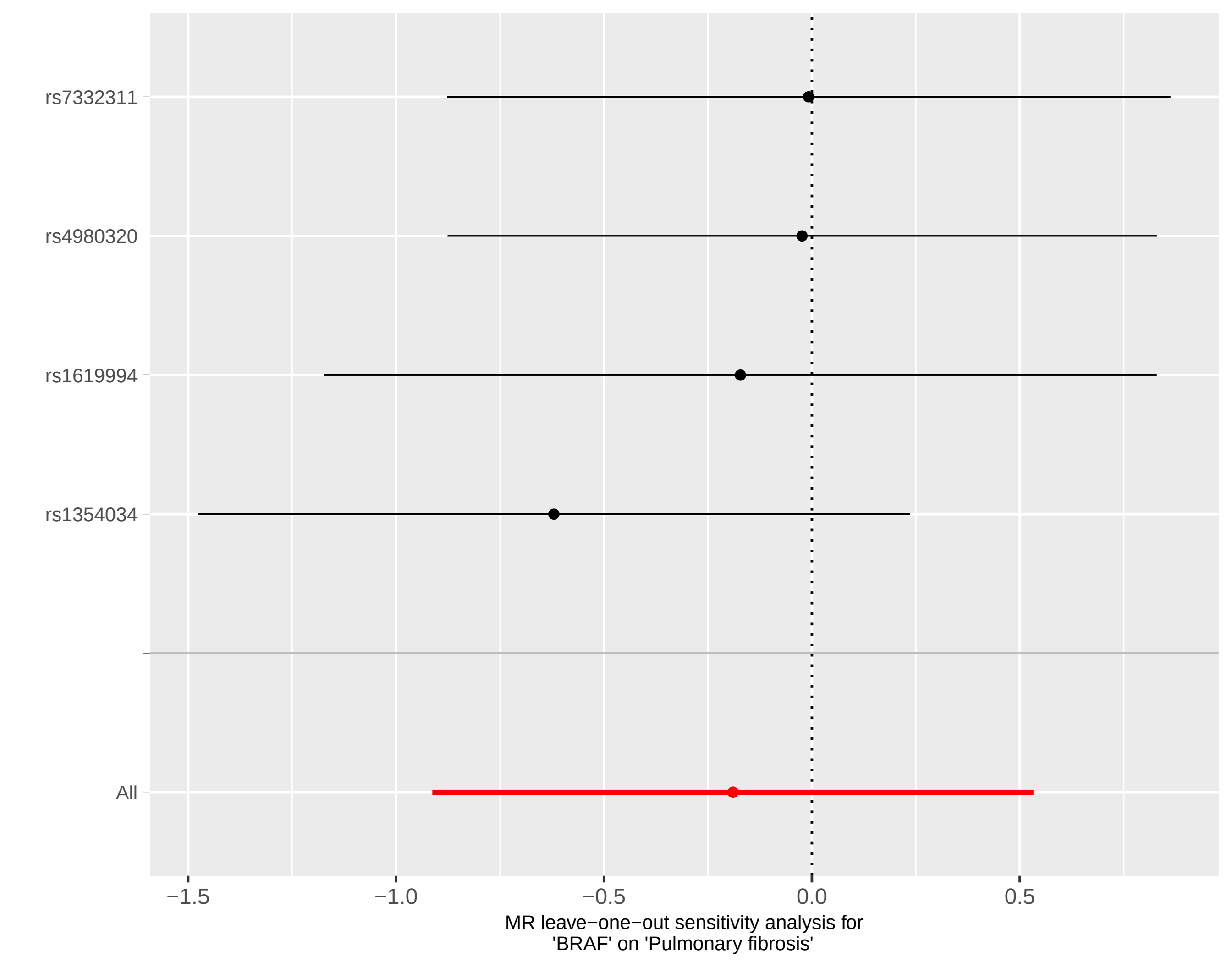
rs7332311
rs4980320
rs1619994
rs1354034
All
−1.5
−1.0
−0.5
0.0
0.5
MR leave−one−out sensitivity analysis for
'BRAF' on 'Pulmonary fibrosis'

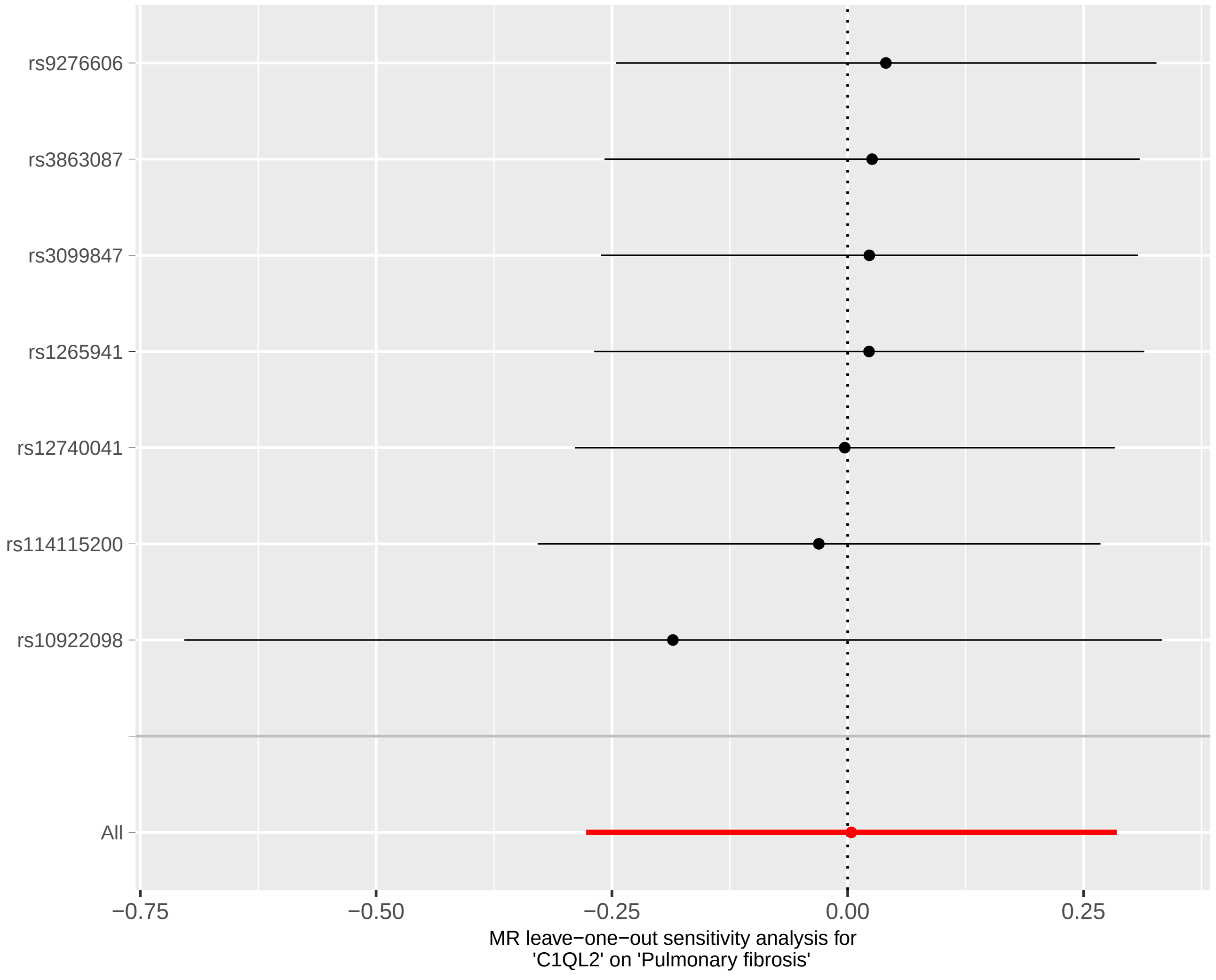
rs9276606
rs3863087
rs3099847
rs1265941
rs12740041
rs114115200
rs10922098
All
−0.75
−0.50
−0.25
0.00
0.25
MR leave−one−out sensitivity analysis for
'C1QL2' on 'Pulmonary fibrosis'

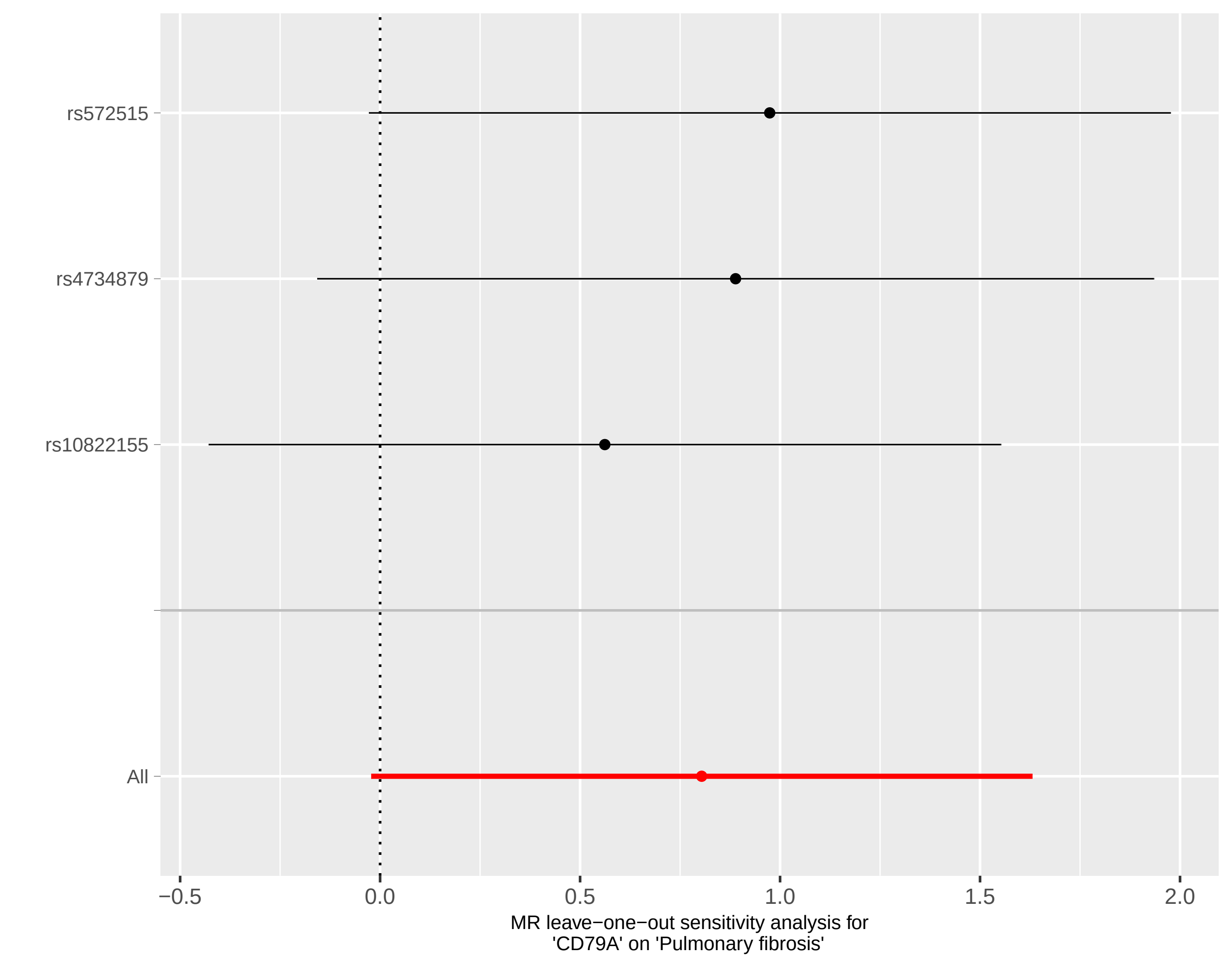
rs572515
rs4734879
rs10822155
All
−0.5
0.0
0.5
1.0
1.5
2.0
MR leave−one−out sensitivity analysis for
'CD79A' on 'Pulmonary fibrosis'

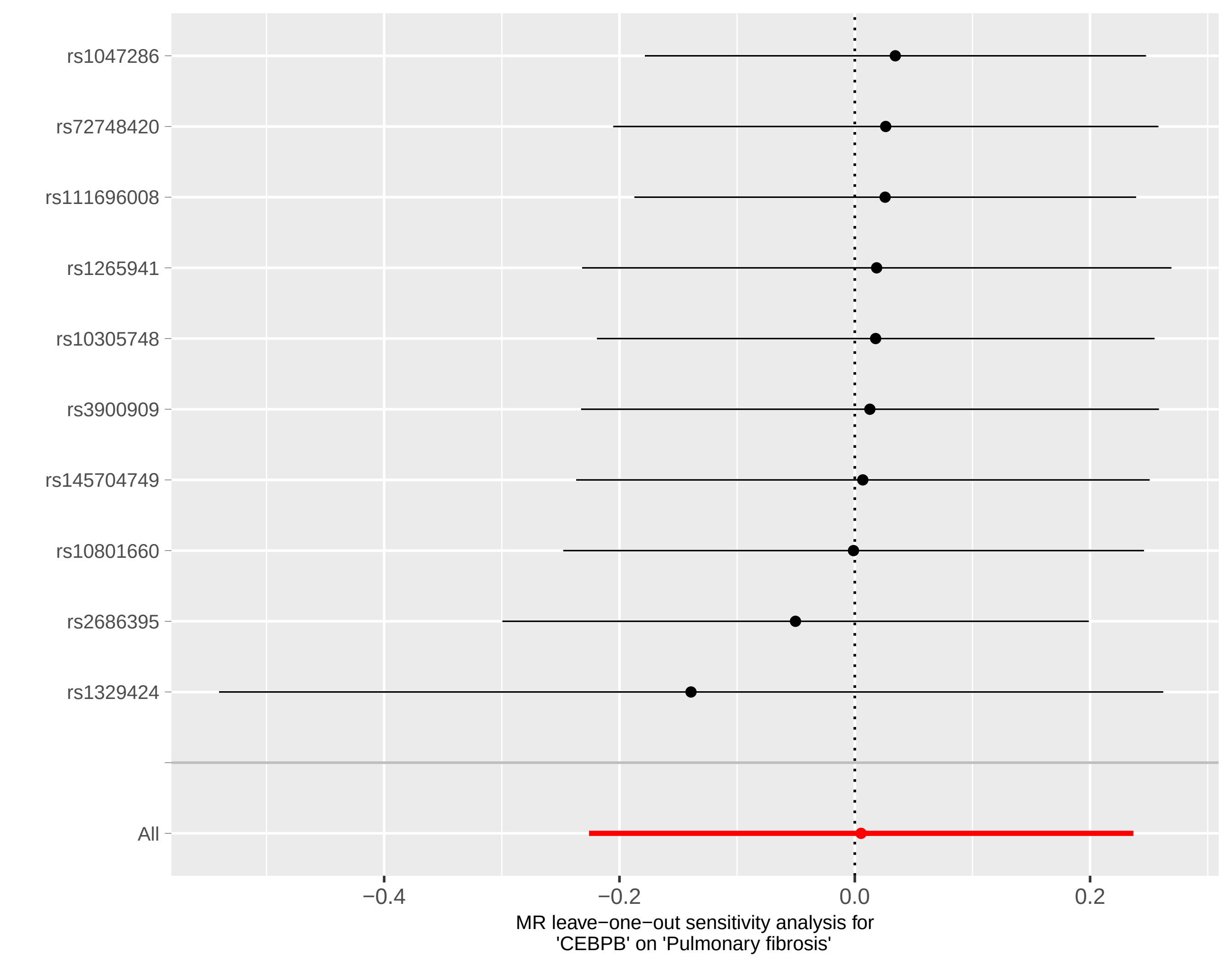

rs1047286
rs72748420
rs111696008
rs1265941
rs10305748
rs3900909
rs145704749
rs10801660
rs2686395
rs1329424
All
−0.4
−0.2
0.0
0.2
MR leave−one−out sensitivity analysis for
'CEBPB' on 'Pulmonary fibrosis'

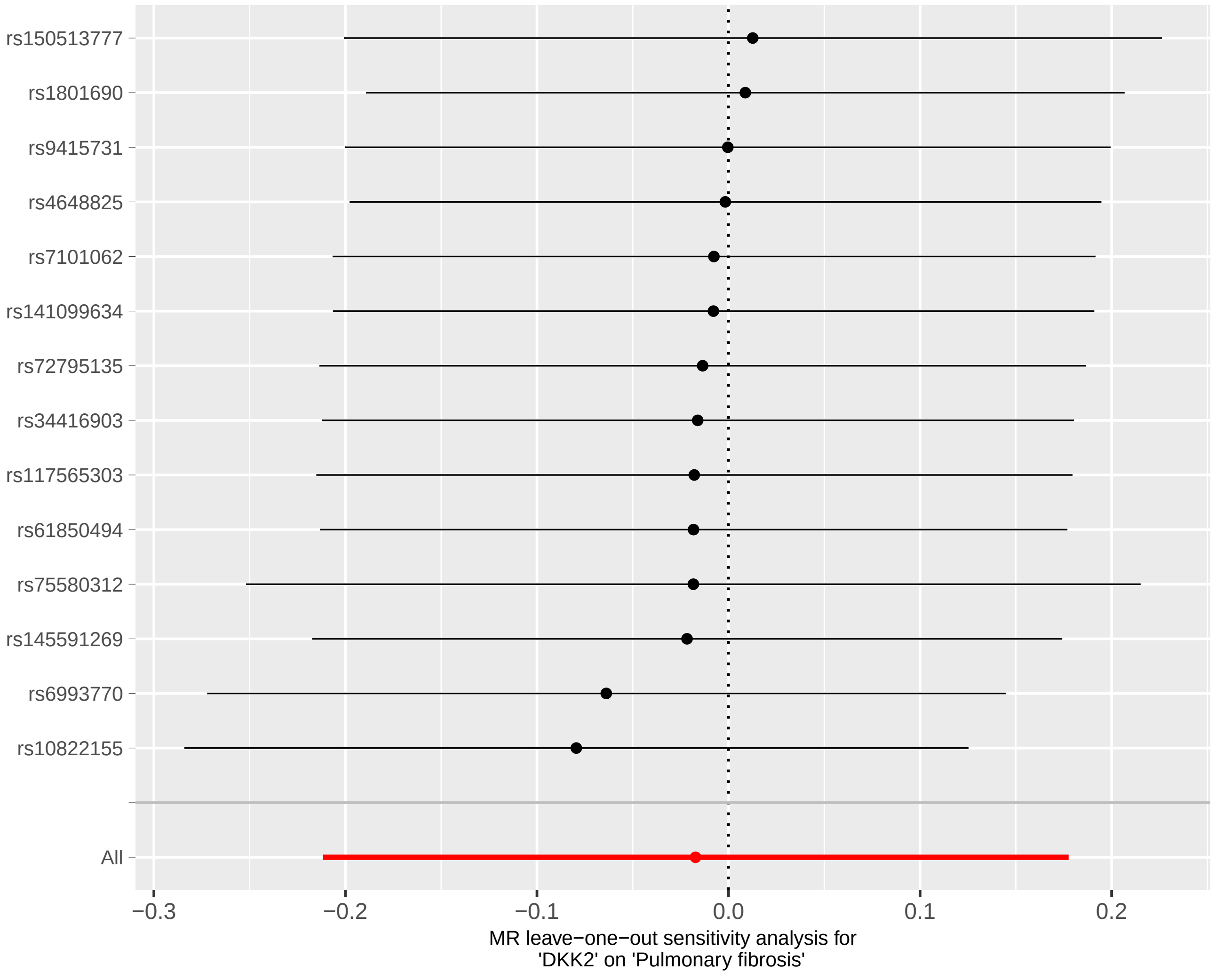
rs150513777
rs1801690
rs9415731
rs4648825
rs7101062
rs141099634
rs72795135
rs34416903
rs117565303
rs61850494
rs75580312
rs145591269
rs6993770
rs10822155
All
−0.3
−0.2
−0.1
0.0
0.1
0.2
MR leave−one−out sensitivity analysis for
'DKK2' on 'Pulmonary fibrosis'

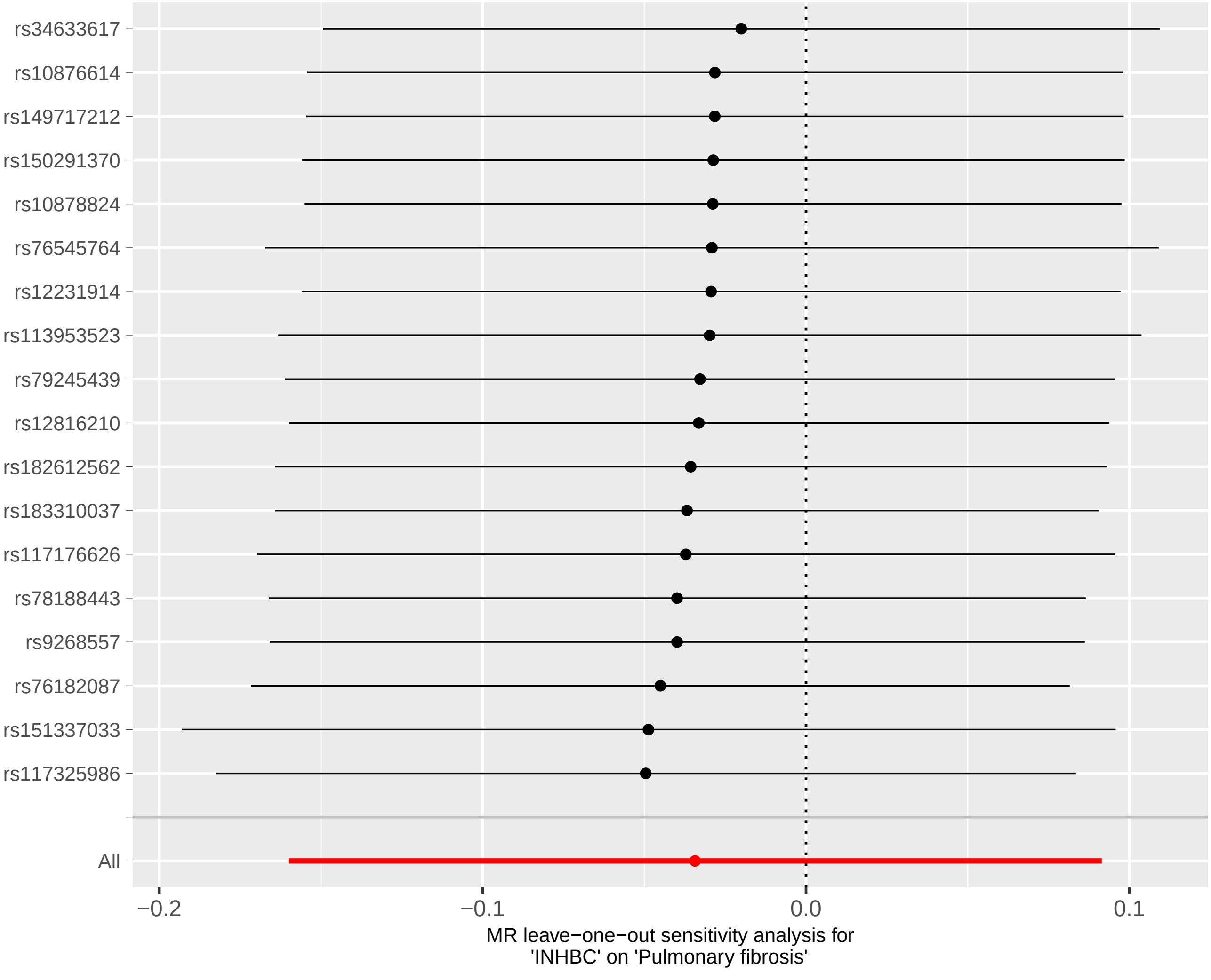
rs34633617
rs10876614
rs149717212
rs150291370
rs10878824
rs76545764
rs12231914
rs113953523
rs79245439
rs12816210
rs182612562
rs183310037
rs117176626
rs78188443
rs9268557
rs76182087
rs151337033
rs117325986
All
−0.2
−0.1
0.0
0.1
MR leave−one−out sensitivity analysis for
'INHBC' on 'Pulmonary fibrosis'

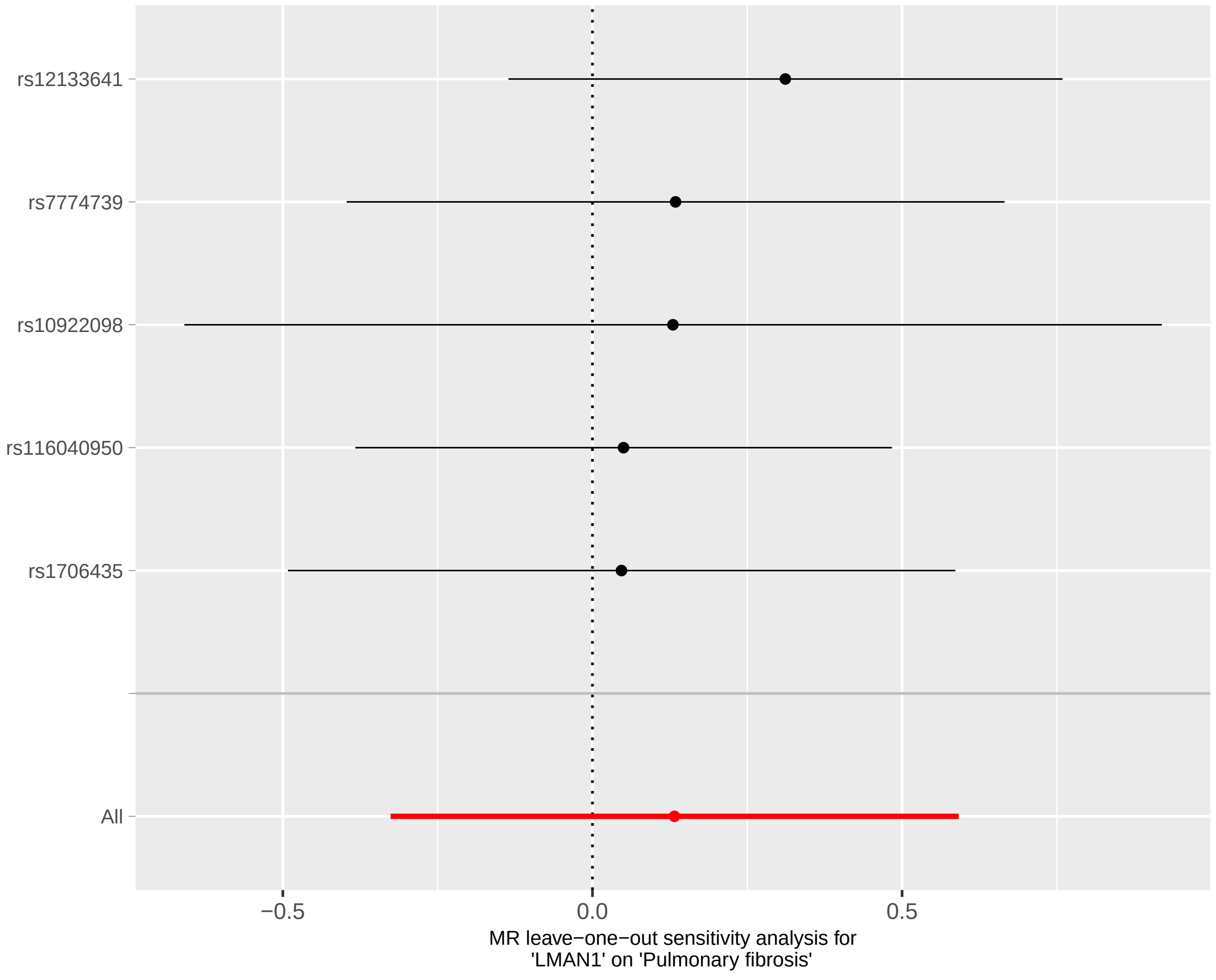

rs12133641
rs7774739
rs10922098
rs116040950
rs1706435
All
−0.5
0.0
0.5
MR leave−one−out sensitivity analysis for
'LMAN1' on 'Pulmonary fibrosis'

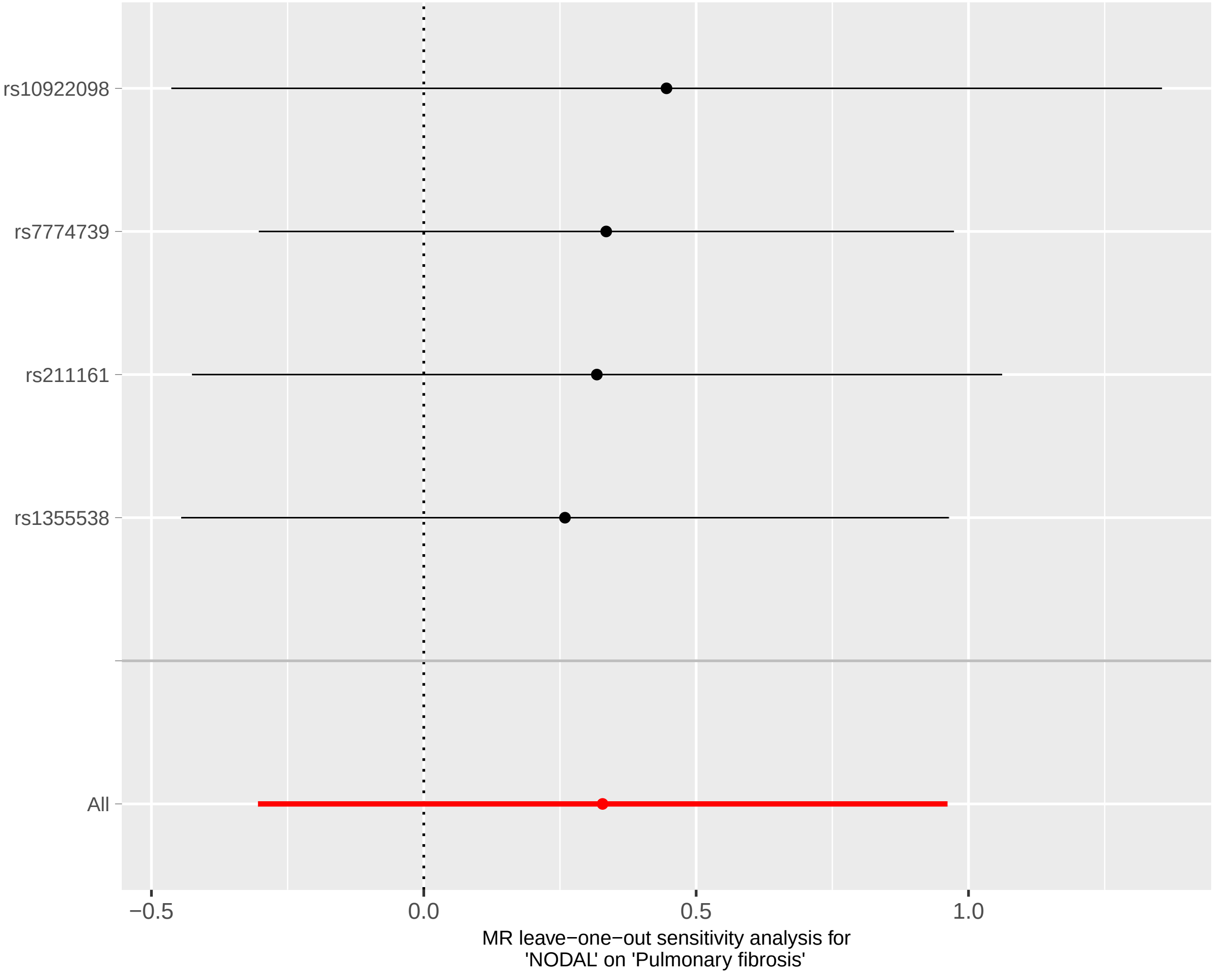
rs10922098
rs7774739
rs211161
rs1355538
All
−0.5
0.0
0.5
1.0
MR leave−one−out sensitivity analysis for
'NODAL' on 'Pulmonary fibrosis'

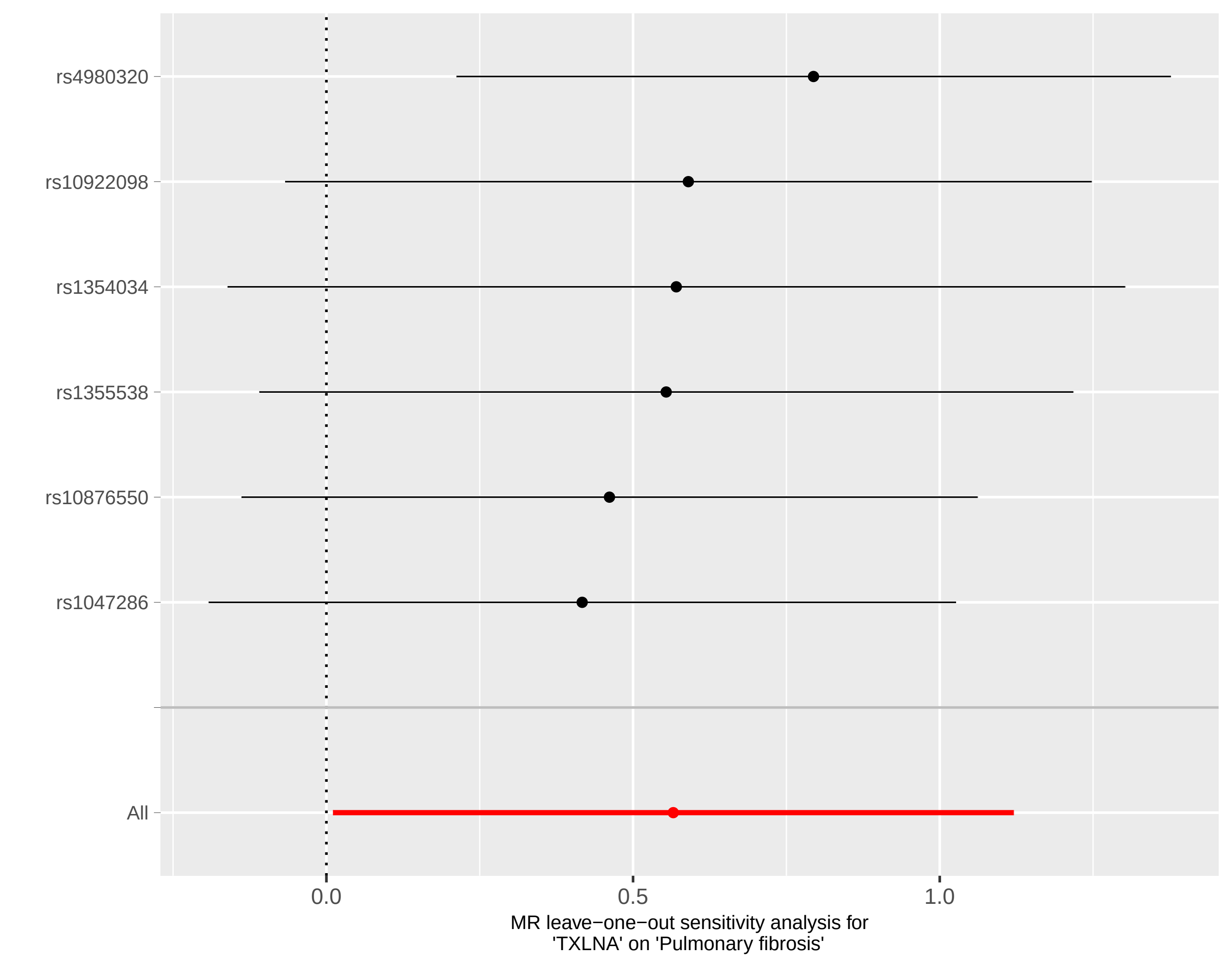

rs4980320
rs10922098
rs1354034
rs1355538
rs10876550
rs1047286
All
0.0
0.5
1.0
MR leave−one−out sensitivity analysis for
'TXLNA' on 'Pulmonary fibrosis'

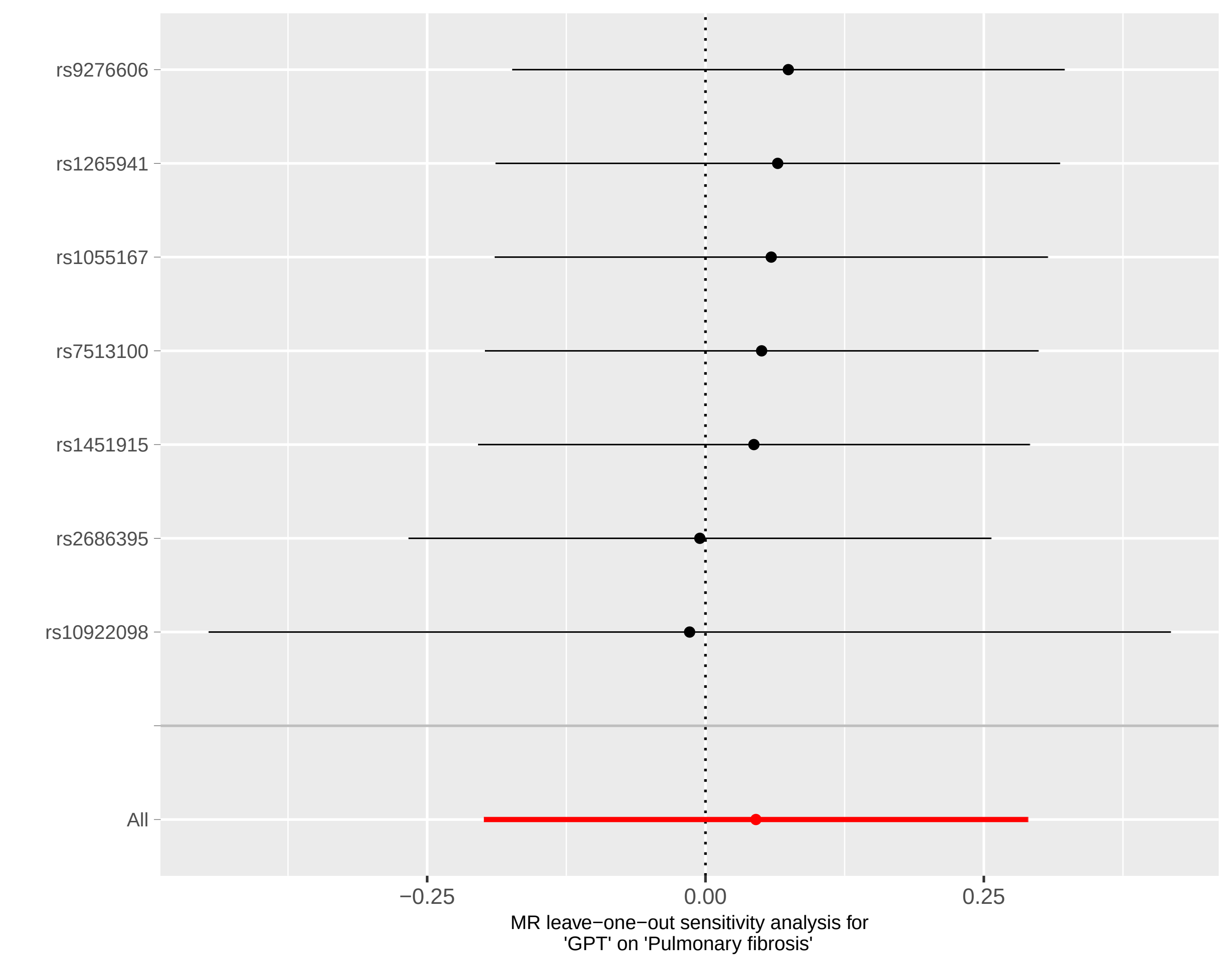
rs9276606
rs1265941
rs1055167
rs7513100
rs1451915
rs2686395
rs10922098
All
−0.25
0.00
0.25
MR leave−one−out sensitivity analysis for
'GPT' on 'Pulmonary fibrosis'

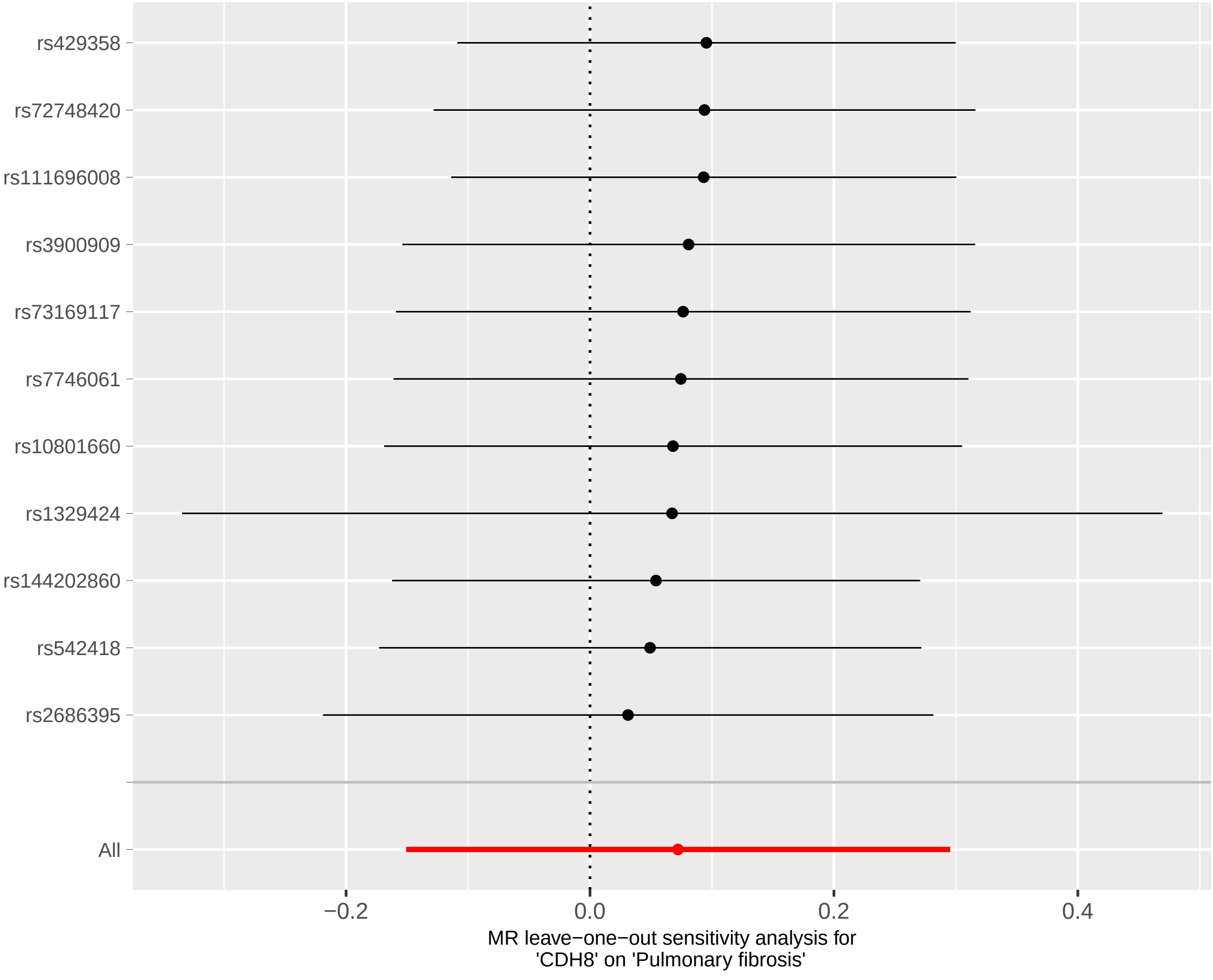

rs429358
rs72748420
rs111696008
rs3900909
rs73169117
rs7746061
rs10801660
rs1329424
rs144202860
rs542418
rs2686395
All
−0.2
0.0
0.2
0.4
MR leave−one−out sensitivity analysis for
'CDH8' on 'Pulmonary fibrosis'

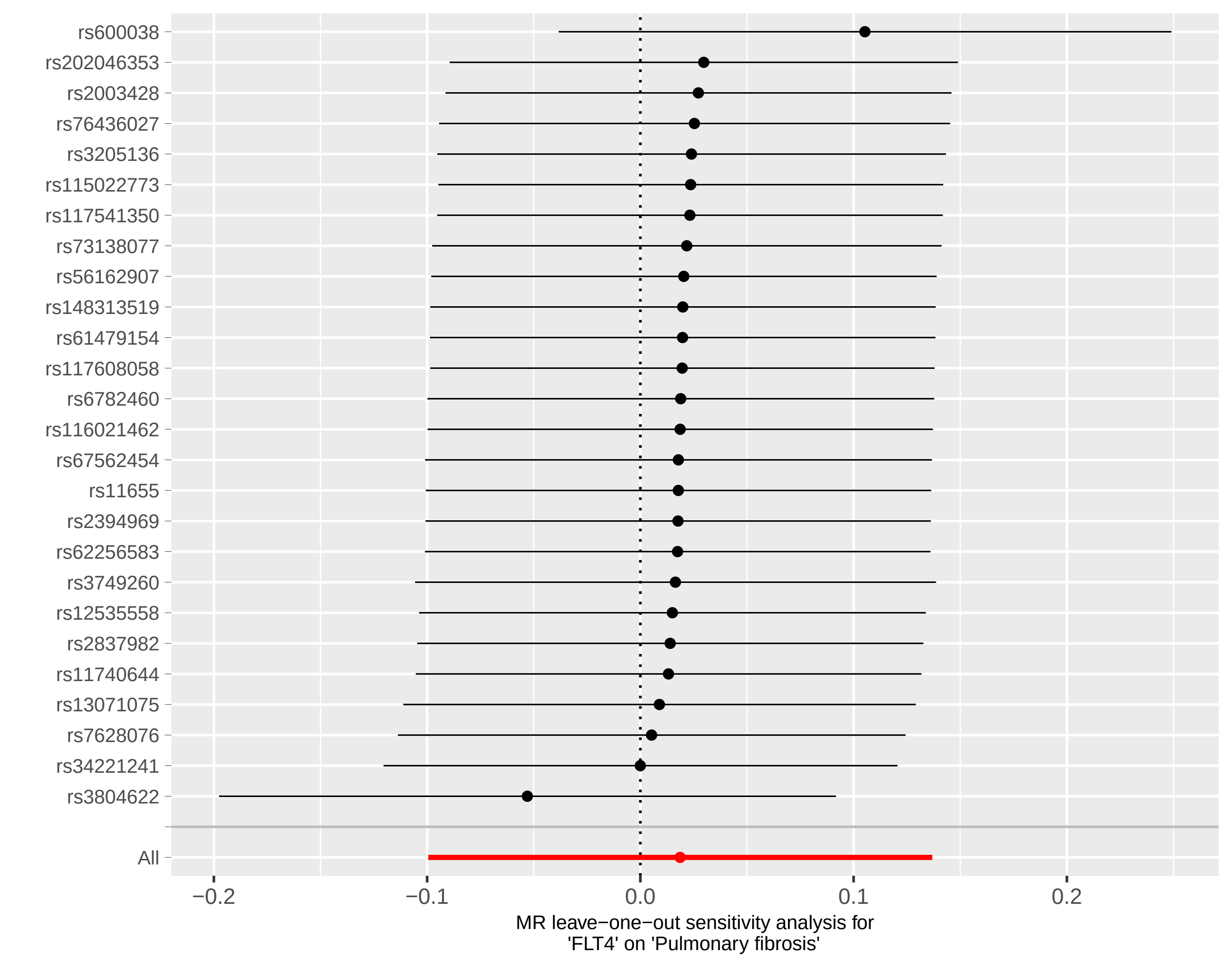
rs600038
rs202046353
rs2003428
rs76436027
rs3205136
rs115022773
rs117541350
rs73138077
rs56162907
rs148313519
rs61479154
rs117608058
rs6782460
rs116021462
rs67562454
rs11655
rs2394969
rs62256583
rs3749260
rs12535558
rs2837982
rs11740644
rs13071075
rs7628076
rs34221241
rs3804622
All
−0.2
−0.1
0.0
0.1
0.2
MR leave−one−out sensitivity analysis for
'FLT4' on 'Pulmonary fibrosis'

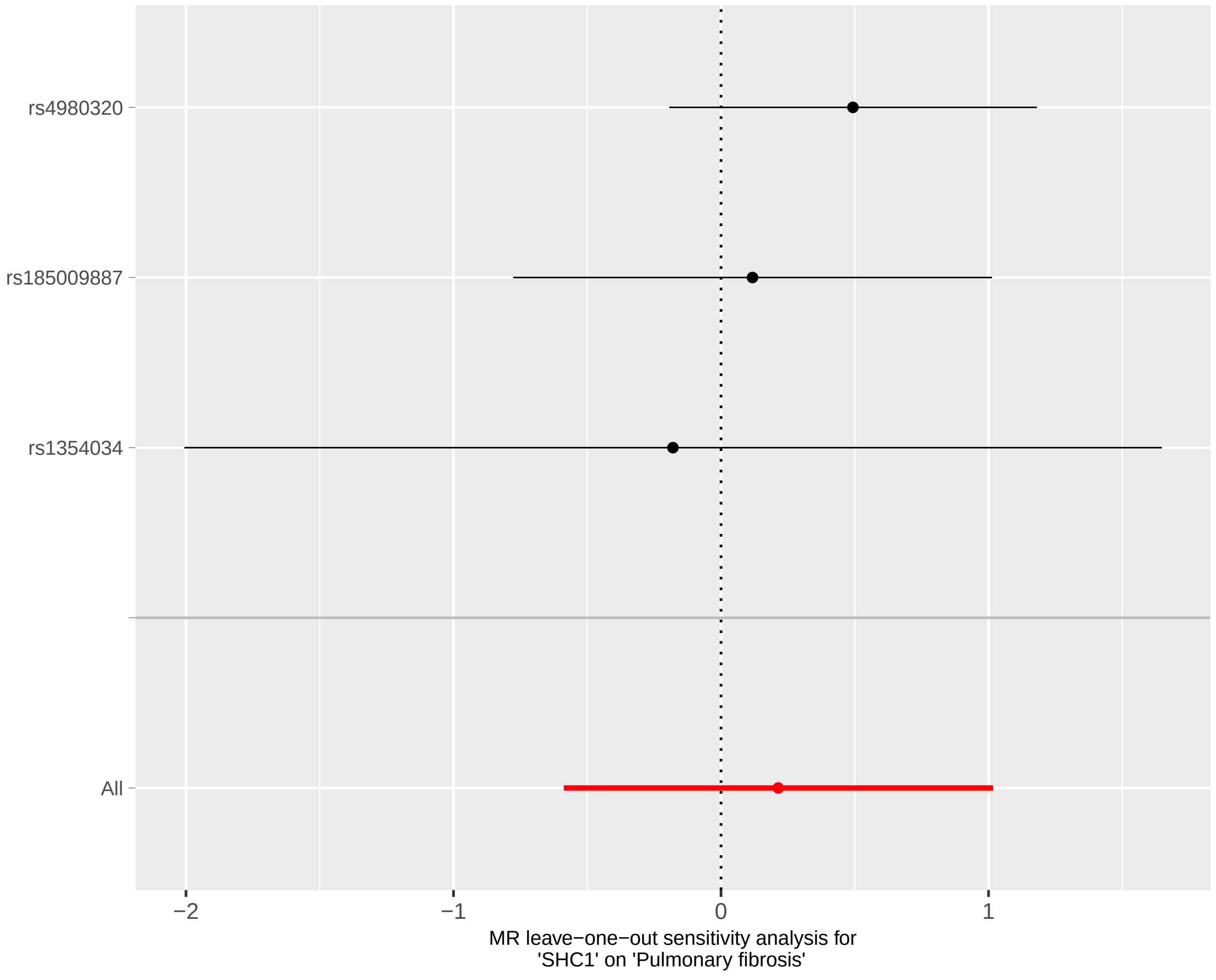
rs4980320
rs185009887
rs1354034
All
−2
−1
0
1
MR leave−one−out sensitivity analysis for
'SHC1' on 'Pulmonary fibrosis'

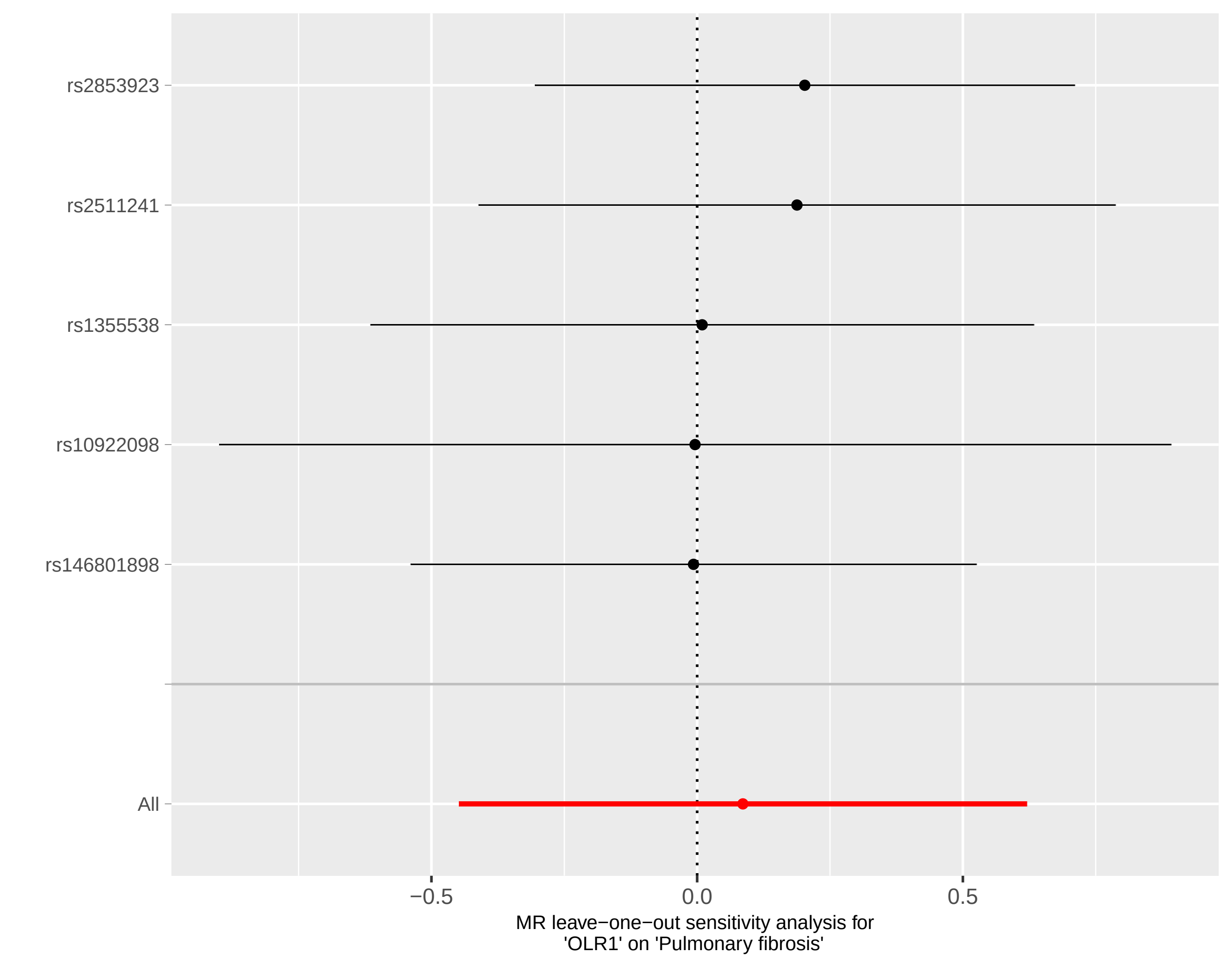
rs2853923
rs2511241
rs1355538
rs10922098
rs146801898
All
−0.5
0.0
0.5
MR leave−one−out sensitivity analysis for
'OLR1' on 'Pulmonary fibrosis'

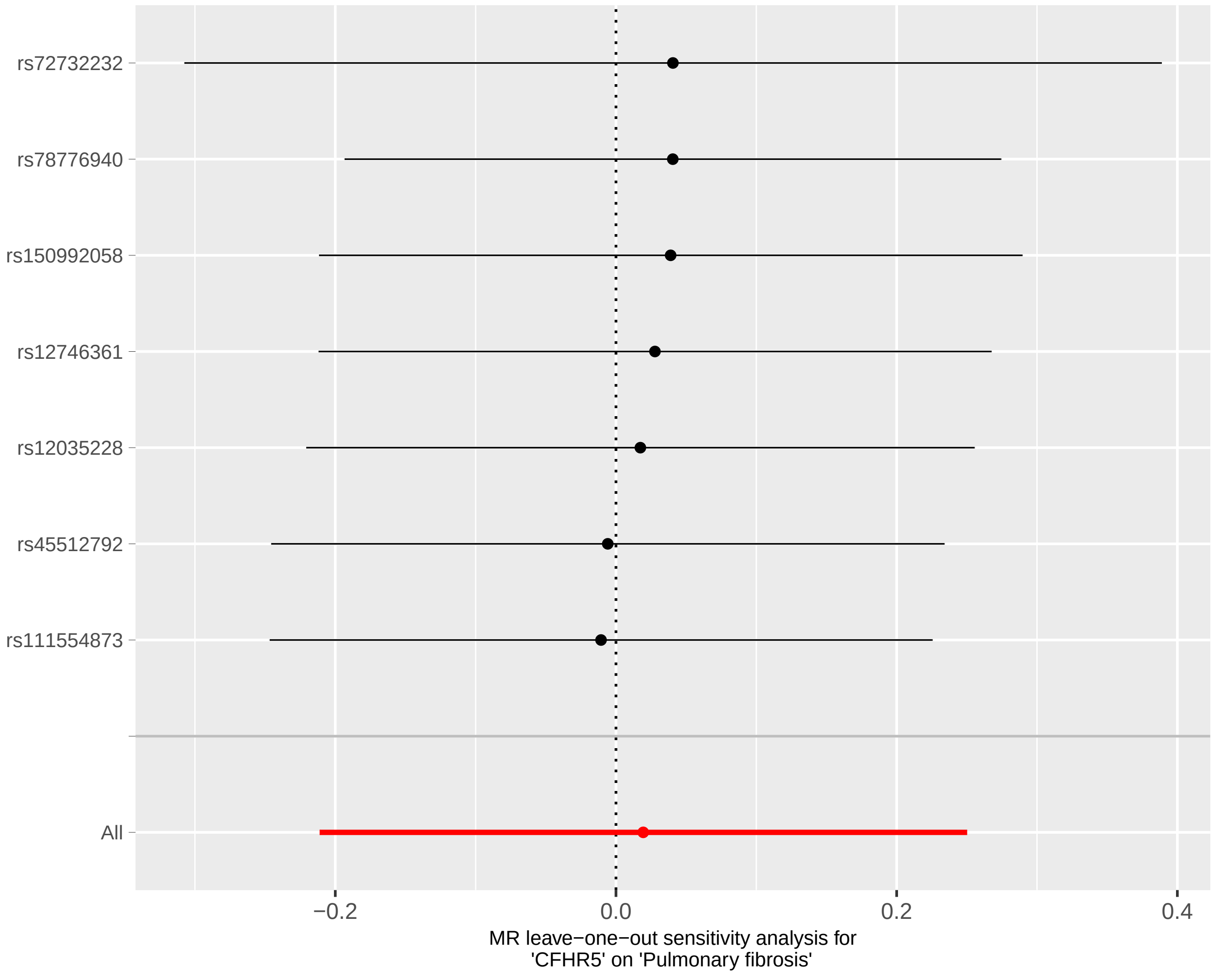
rs72732232
rs78776940
rs150992058
rs12746361
rs12035228
rs45512792
rs111554873
All
−0.2
0.0
0.2
0.4
MR leave−one−out sensitivity analysis for
'CFHR5' on 'Pulmonary fibrosis'

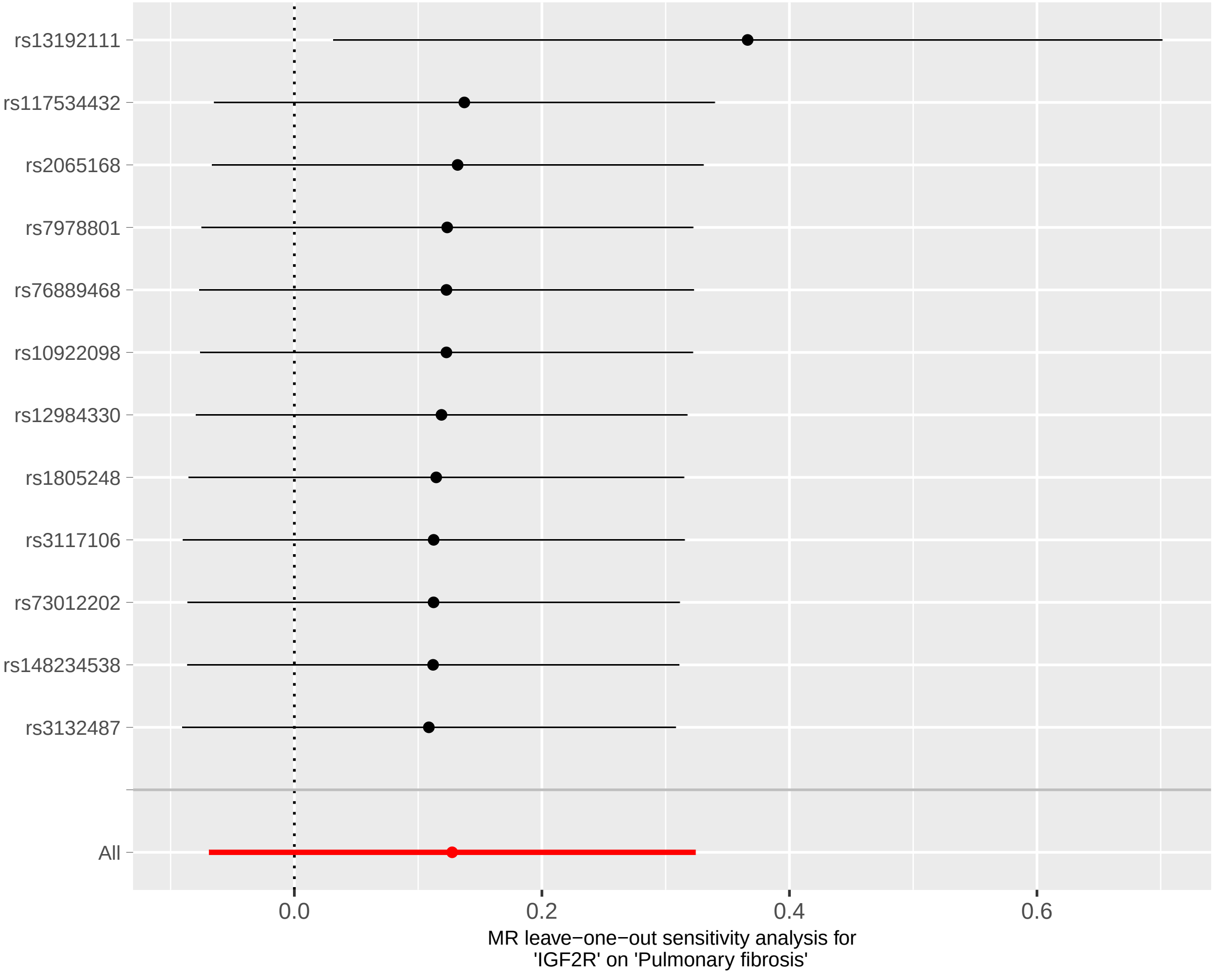
rs13192111
rs117534432
rs2065168
rs7978801
rs76889468
rs10922098
rs12984330
rs1805248
rs3117106
rs73012202
rs148234538
rs3132487
All
0.0
0.2
0.4
0.6
MR leave−one−out sensitivity analysis for
'IGF2R' on 'Pulmonary fibrosis'

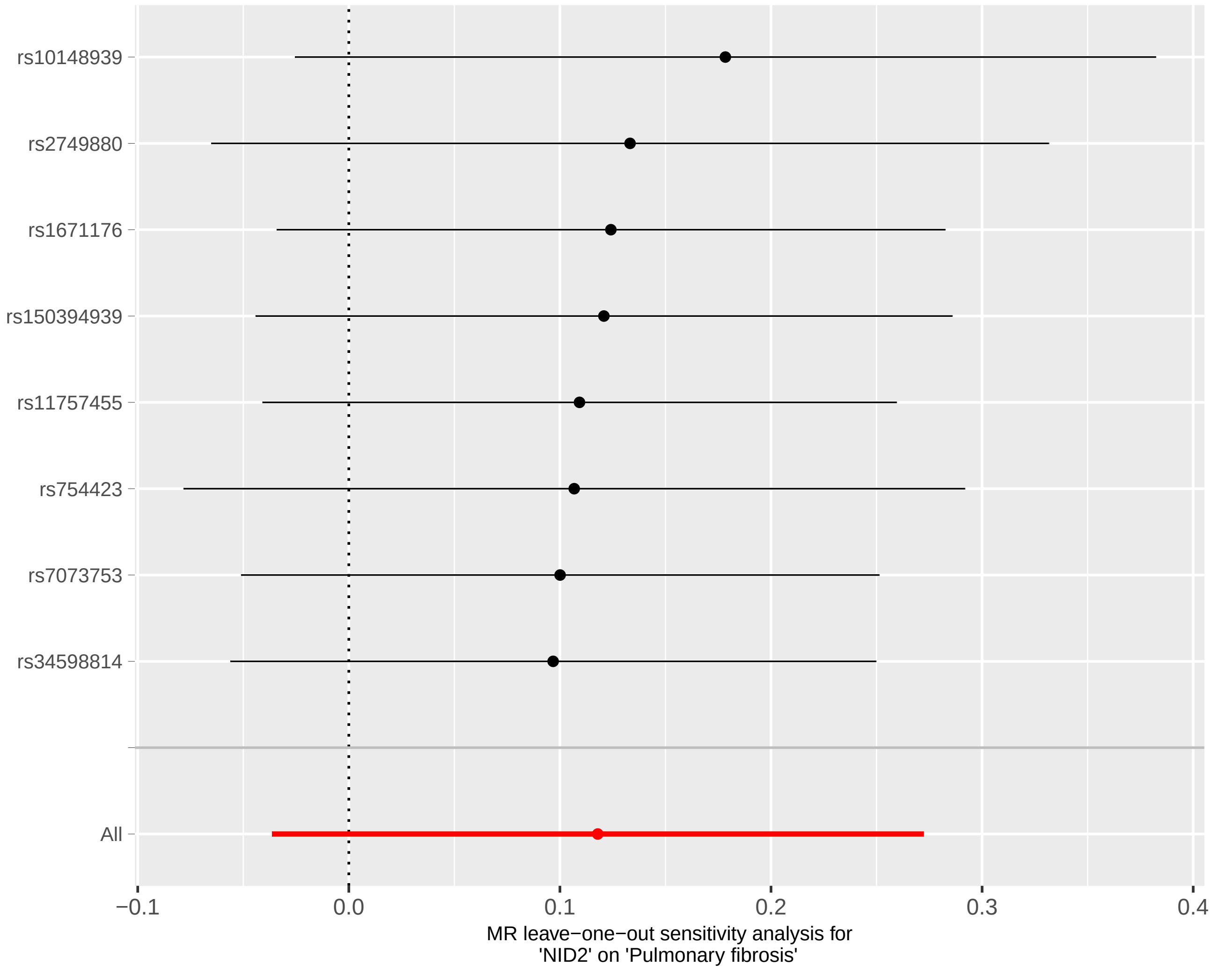
rs10148939
rs2749880
rs1671176
rs150394939
rs11757455
rs754423
rs7073753
rs34598814
All
−0.1
0.0
0.1
0.2
0.3
0.4
MR leave−one−out sensitivity analysis for
'NID2' on 'Pulmonary fibrosis'

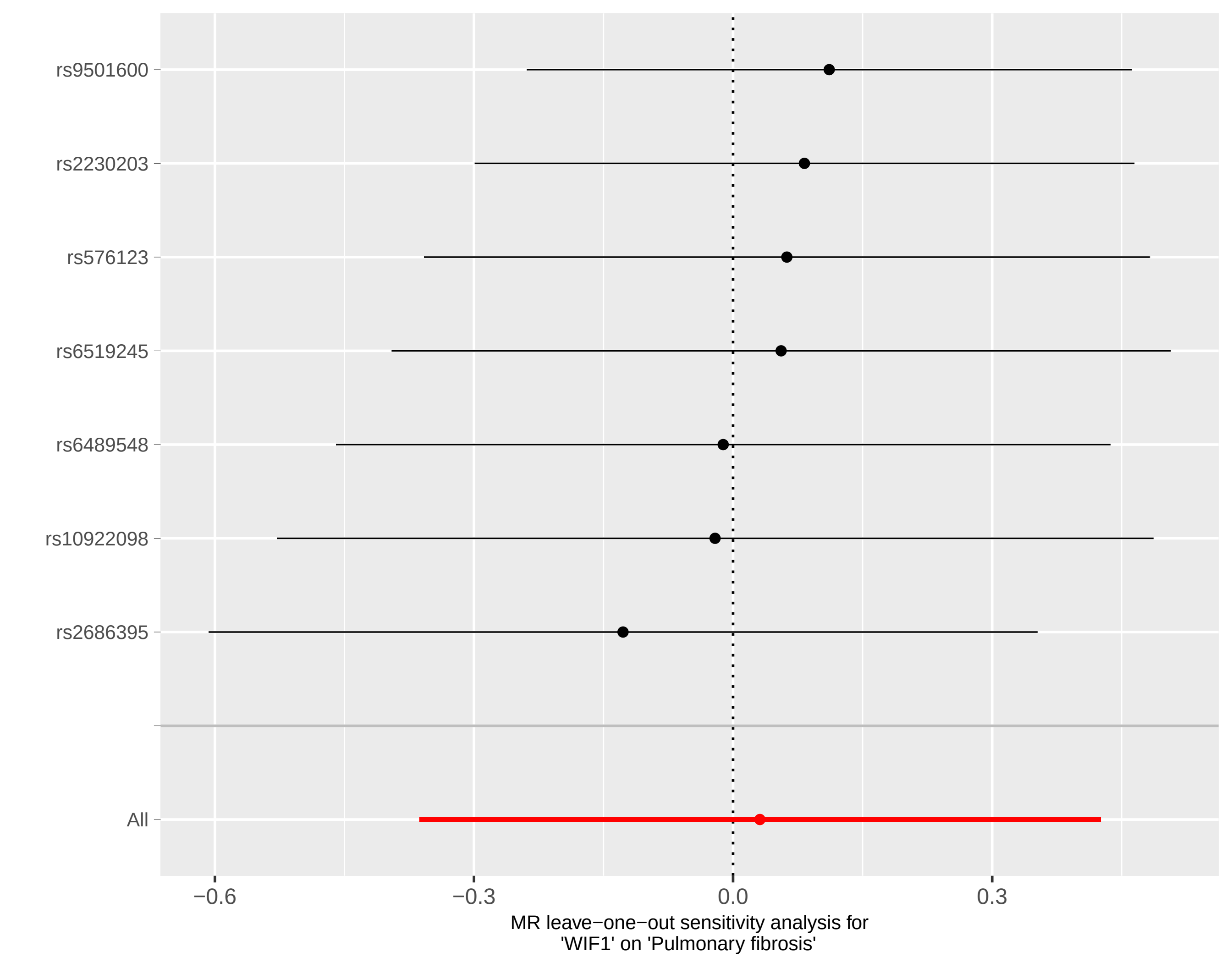
rs9501600
rs2230203
rs576123
rs6519245
rs6489548
rs10922098
rs2686395
All
−0.6
−0.3
0.0
0.3
MR leave−one−out sensitivity analysis for
'WIF1' on 'Pulmonary fibrosis'

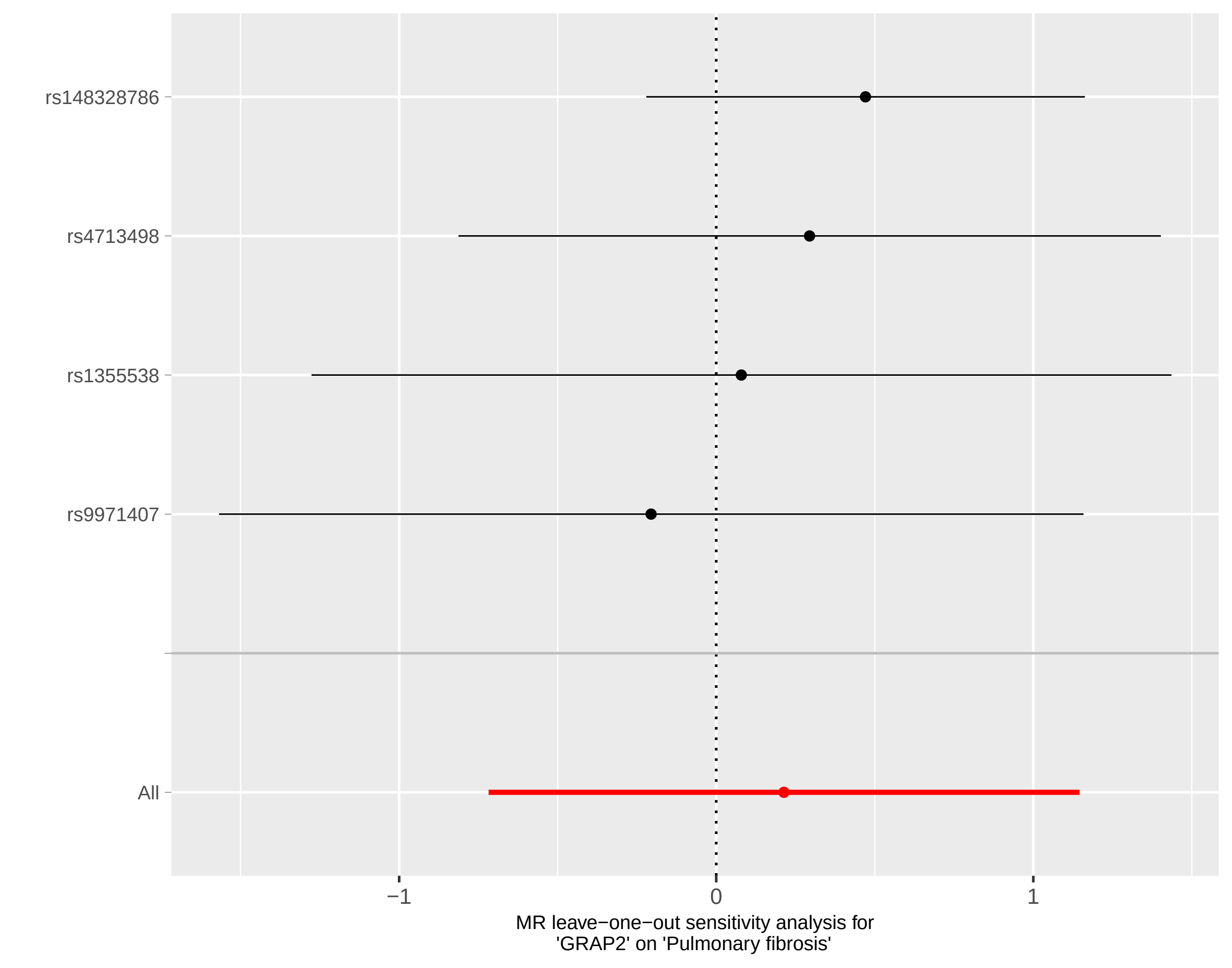

rs148328786
rs4713498
rs1355538
rs9971407
All
−1
0
1
MR leave−one−out sensitivity analysis for
'GRAP2' on 'Pulmonary fibrosis'

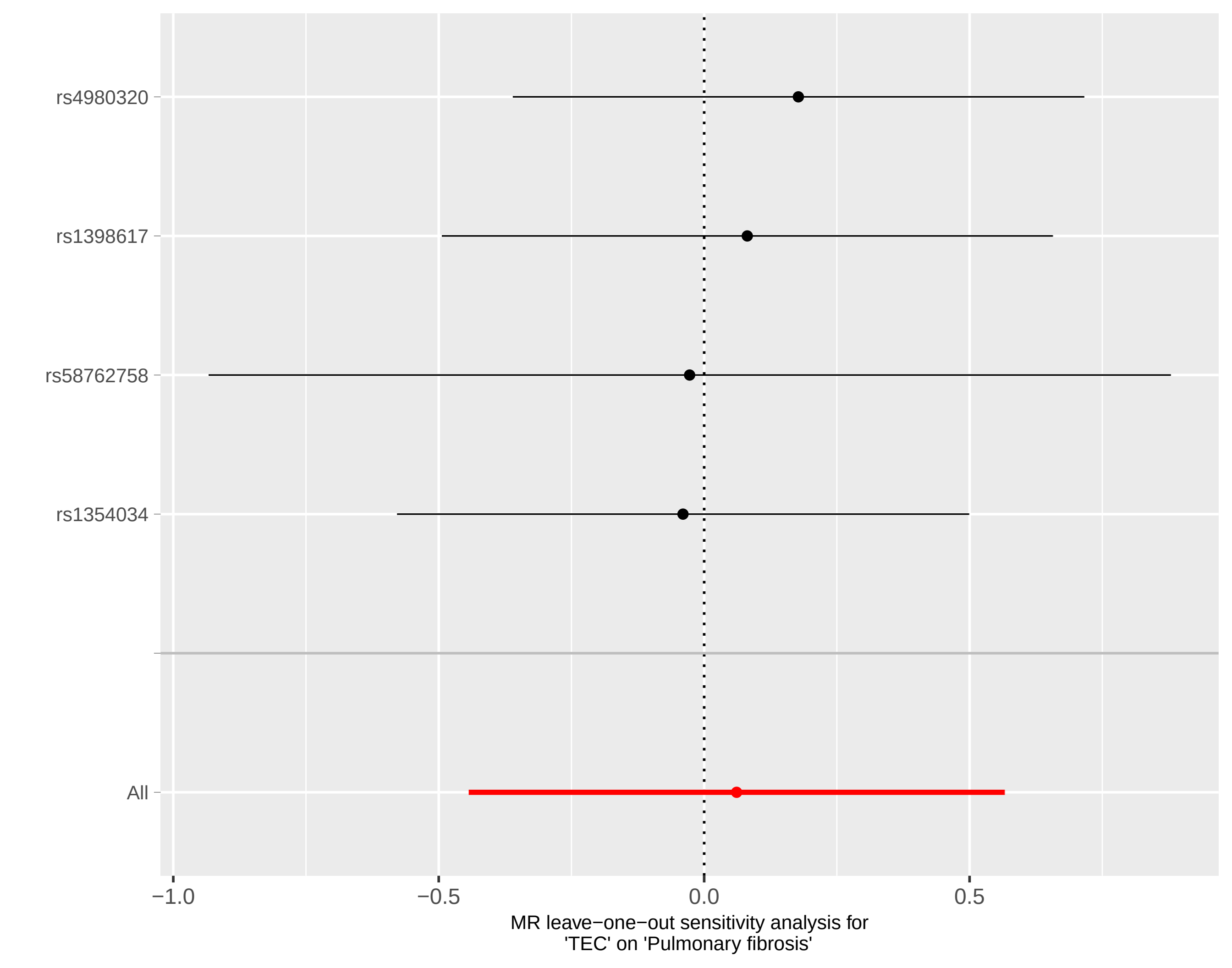
rs4980320
rs1398617
rs58762758
rs1354034
All
−1.0
−0.5
0.0
0.5
MR leave−one−out sensitivity analysis for
'TEC' on 'Pulmonary fibrosis'

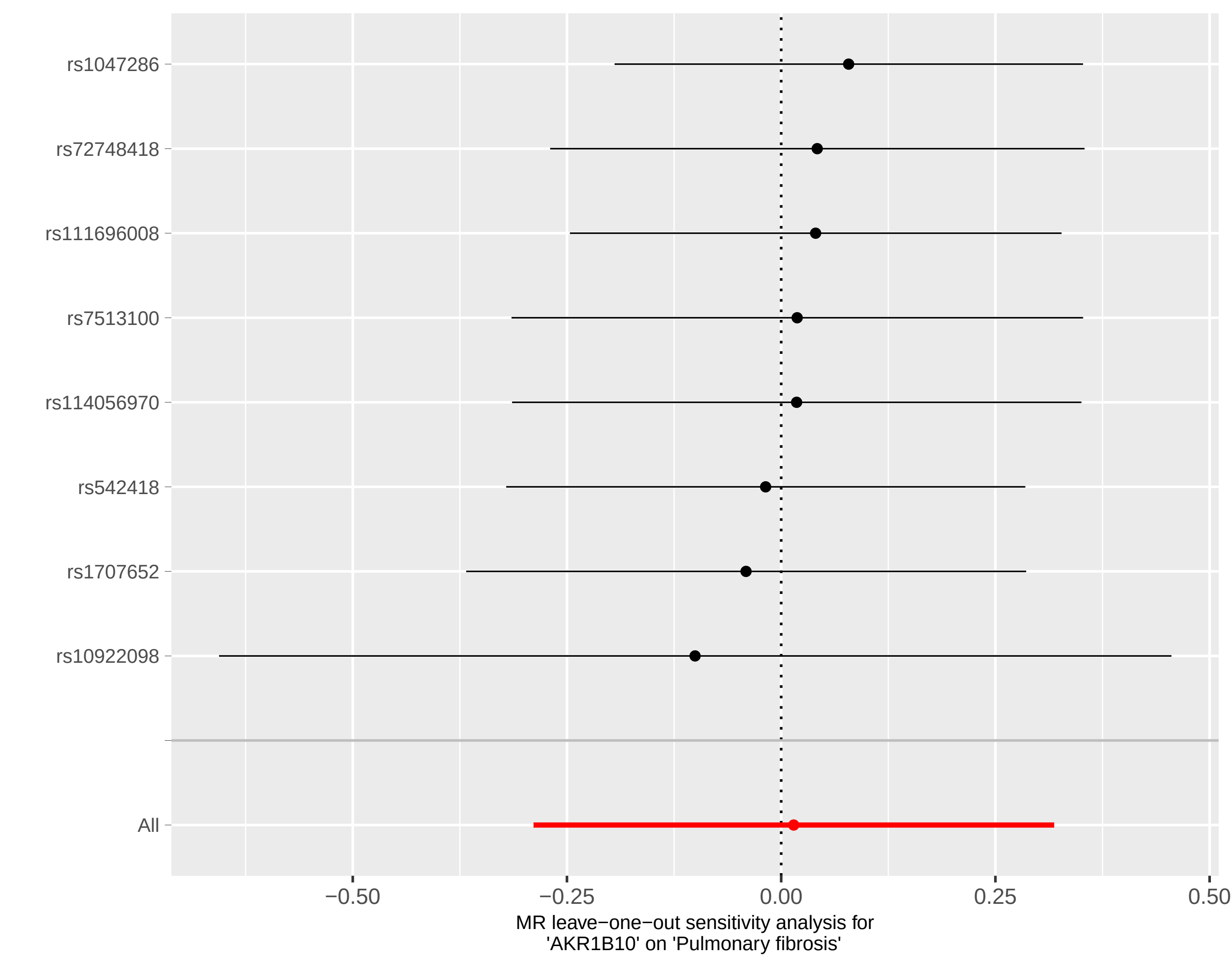
rs1047286
rs72748418
rs111696008
rs7513100
rs114056970
rs542418
rs1707652
rs10922098
All
−0.50
−0.25
0.00
0.25
0.50
MR leave−one−out sensitivity analysis for
'AKR1B10' on 'Pulmonary fibrosis'

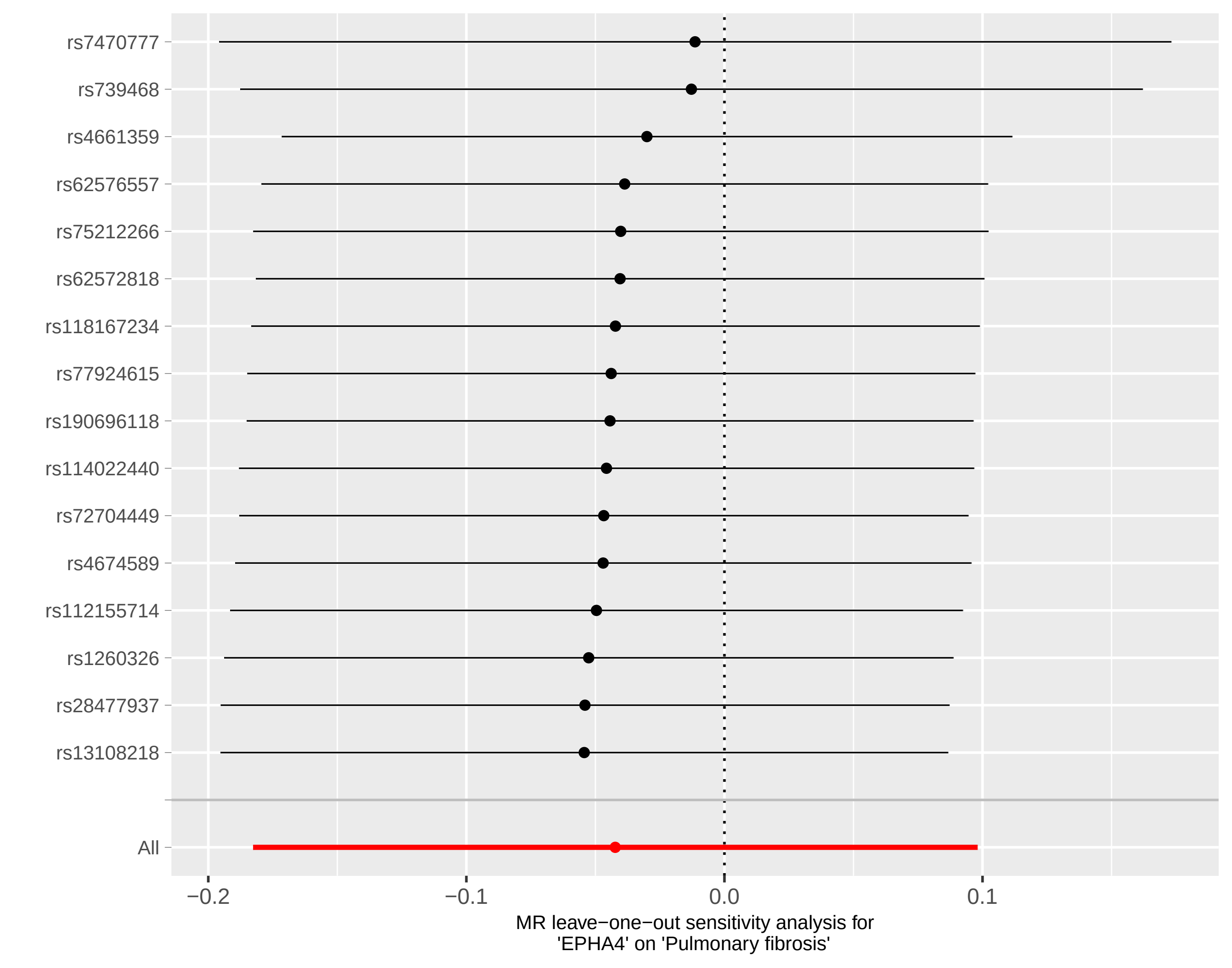
rs7470777
rs739468
rs4661359
rs62576557
rs75212266
rs62572818
rs118167234
rs77924615
rs190696118
rs114022440
rs72704449
rs4674589
rs112155714
rs1260326
rs28477937
rs13108218
All
−0.2
−0.1
0.0
0.1
MR leave−one−out sensitivity analysis for
'EPHA4' on 'Pulmonary fibrosis'

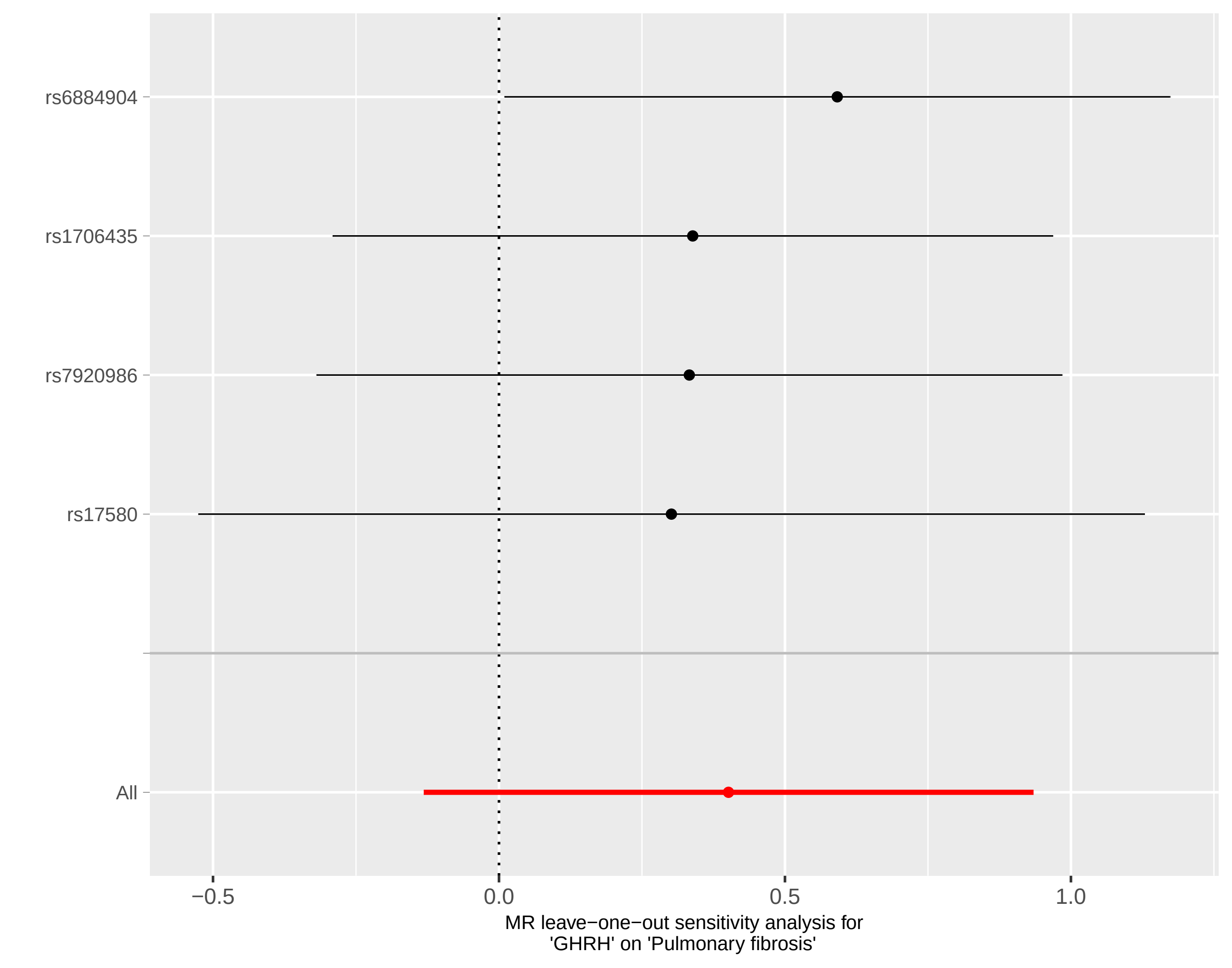
rs6884904
rs1706435
rs7920986
rs17580
All
−0.5
0.0
0.5
1.0
MR leave−one−out sensitivity analysis for
'GHRH' on 'Pulmonary fibrosis'

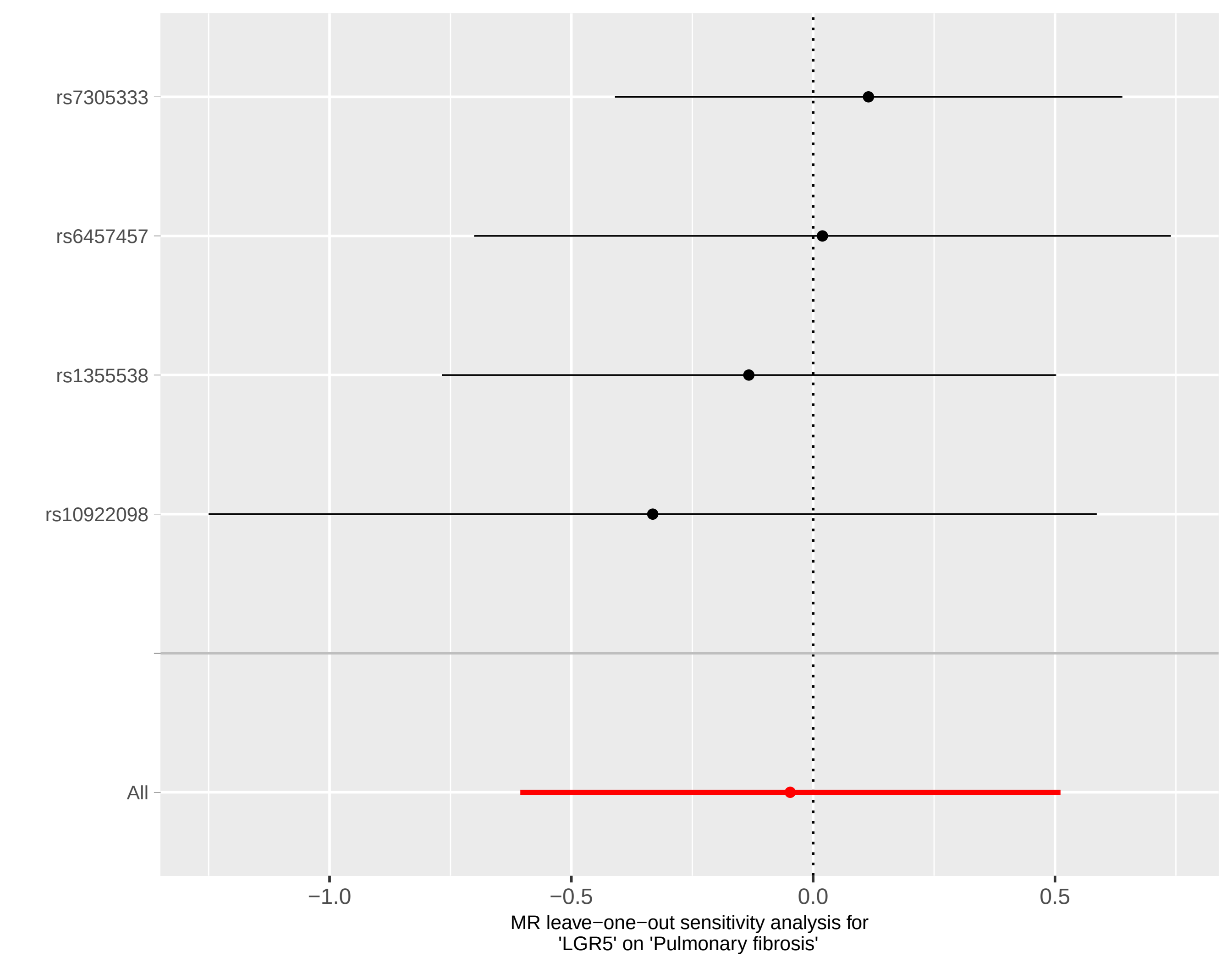
rs7305333
rs6457457
rs1355538
rs10922098
All
−1.0
−0.5
0.0
0.5
MR leave−one−out sensitivity analysis for
'LGR5' on 'Pulmonary fibrosis'

Insufficient number of SNPs

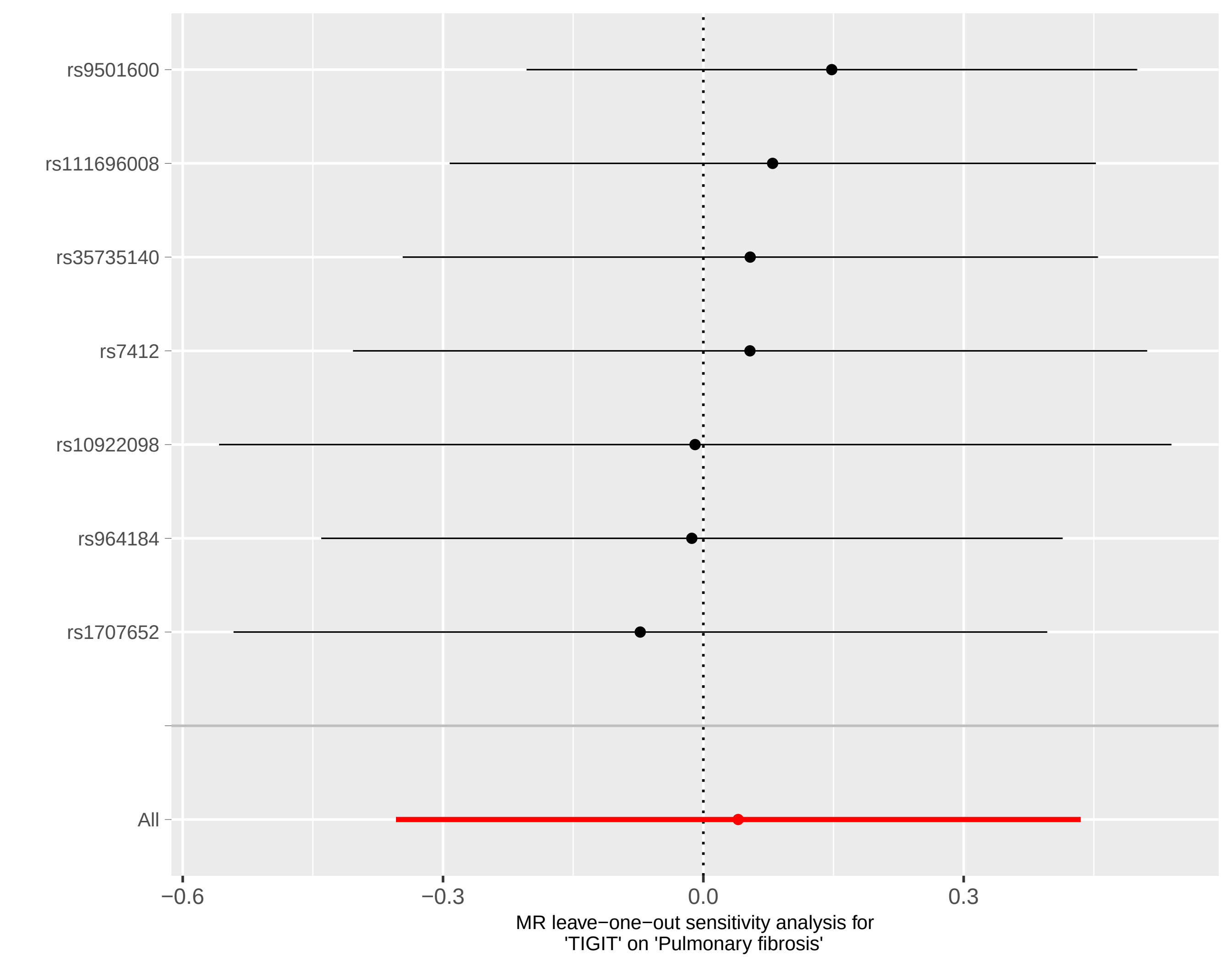
rs9501600
rs111696008
rs35735140
rs7412
rs10922098
rs964184
rs1707652
All
−0.6
−0.3
0.0
0.3
MR leave−one−out sensitivity analysis for
'TIGIT' on 'Pulmonary fibrosis'

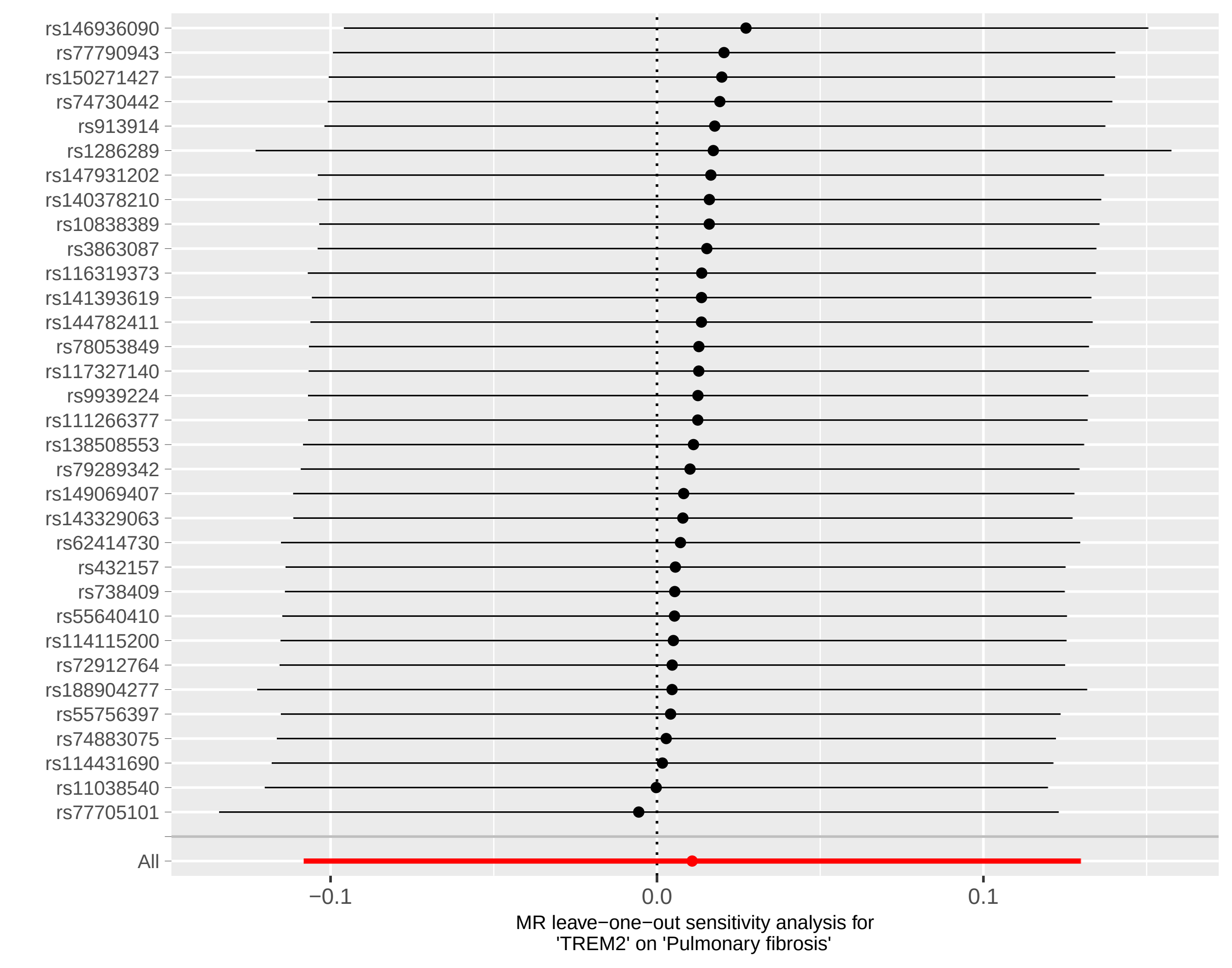

rs146936090
rs77790943
rs150271427
rs74730442
rs913914
rs1286289
rs147931202
rs140378210
rs10838389
rs3863087
rs116319373
rs141393619
rs144782411
rs78053849
rs117327140
rs9939224
rs111266377
rs138508553
rs79289342
rs149069407
rs143329063
rs62414730
rs432157
rs738409
rs55640410
rs114115200
rs72912764
rs188904277
rs55756397
rs74883075
rs114431690
rs11038540
rs77705101
All
−0.1
0.0
0.1
MR leave−one−out sensitivity analysis for
'TREM2' on 'Pulmonary fibrosis'

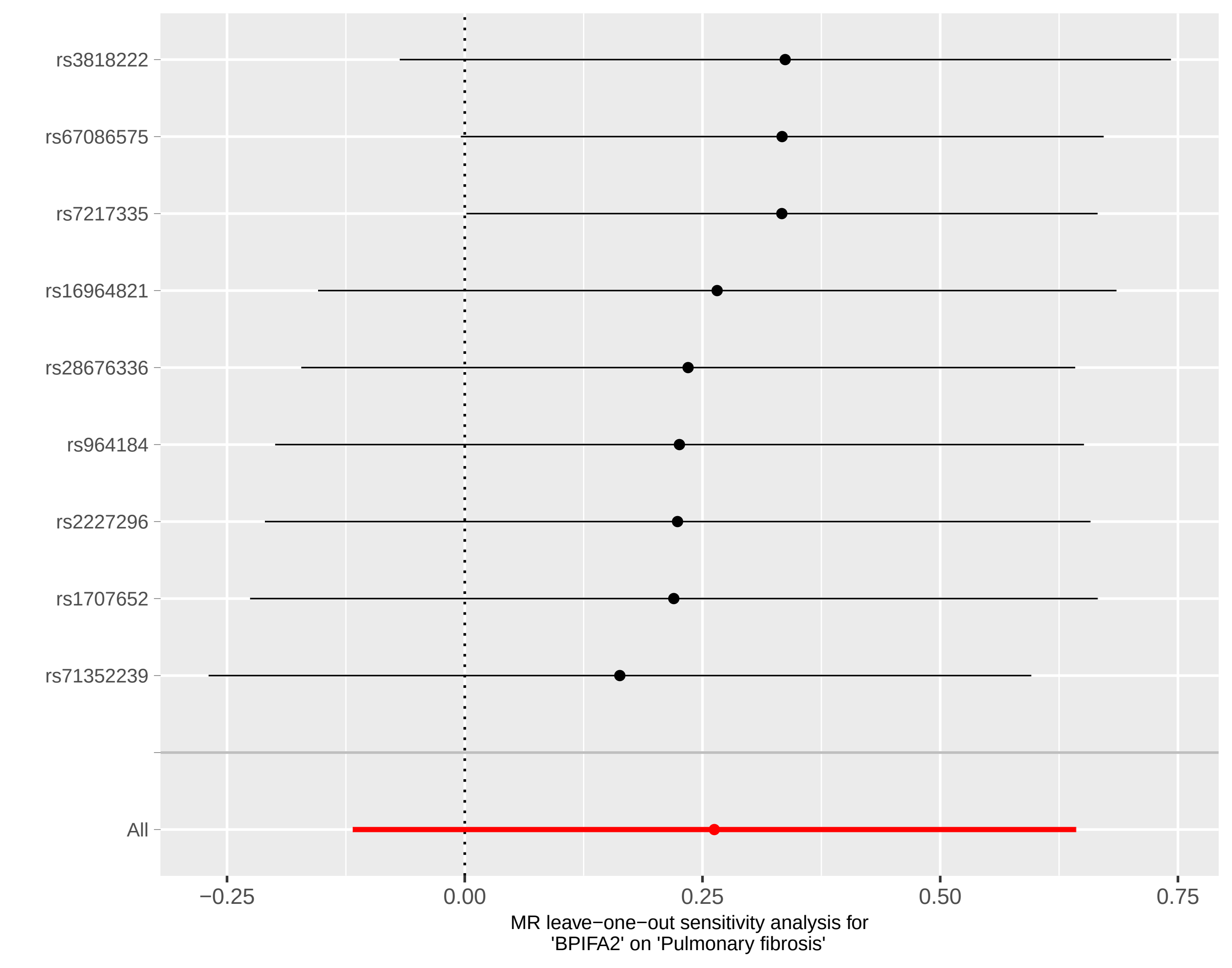
rs3818222
rs67086575
rs7217335
rs16964821
rs28676336
rs964184
rs2227296
rs1707652
rs71352239
All
−0.25
0.00
0.25
0.50
0.75
MR leave−one−out sensitivity analysis for
'BPIFA2' on 'Pulmonary fibrosis'

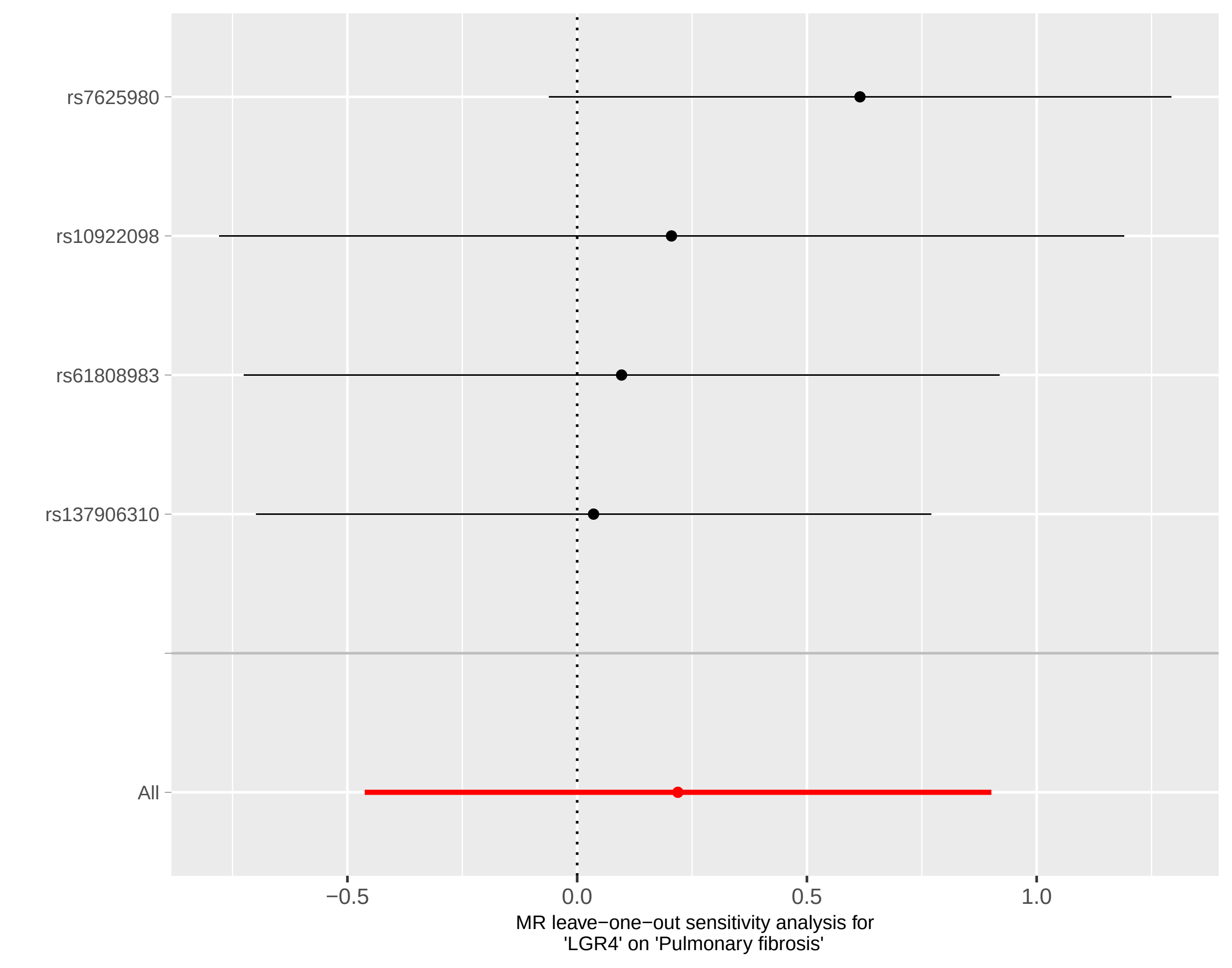
rs7625980
rs10922098
rs61808983
rs137906310
All
−0.5
0.0
0.5
1.0
MR leave−one−out sensitivity analysis for
'LGR4' on 'Pulmonary fibrosis'

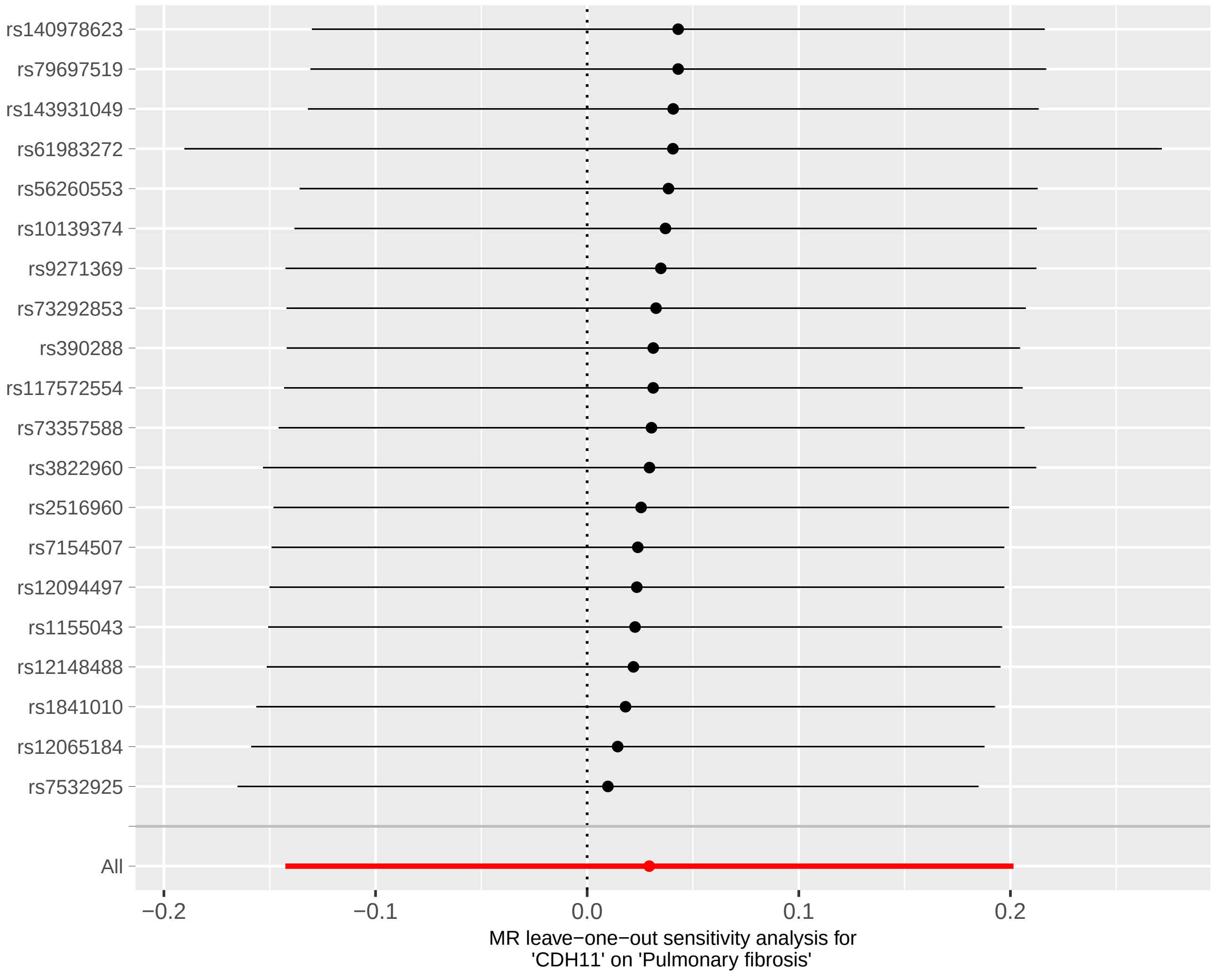

rs140978623
rs79697519
rs143931049
rs61983272
rs56260553
rs10139374
rs9271369
rs73292853
rs390288
rs117572554
rs73357588
rs3822960
rs2516960
rs7154507
rs12094497
rs1155043
rs12148488
rs1841010
rs12065184
rs7532925
All
−0.2
−0.1
0.0
0.1
0.2
MR leave−one−out sensitivity analysis for
'CDH11' on 'Pulmonary fibrosis'

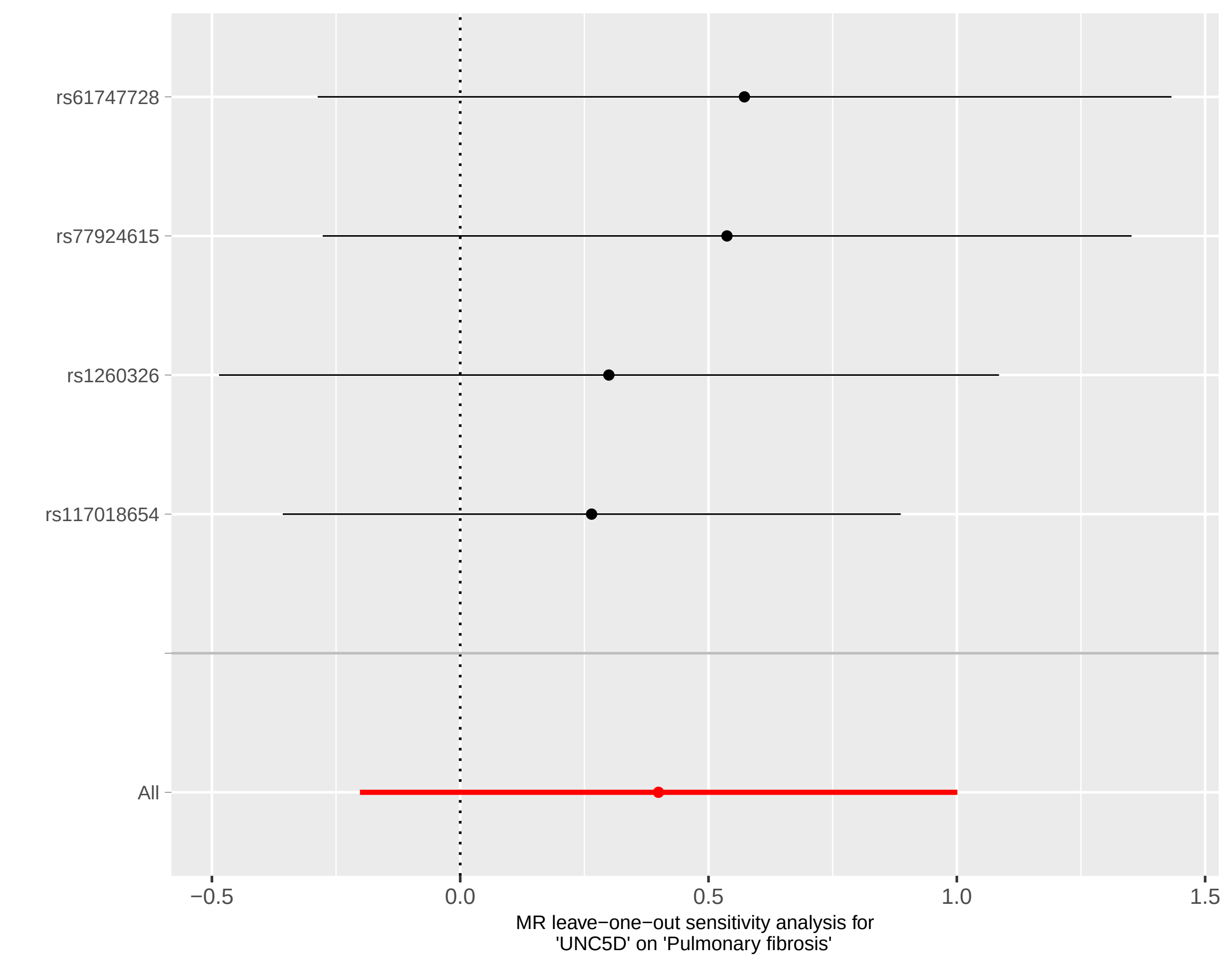

rs61747728
rs77924615
rs1260326
rs117018654
All
−0.5
0.0
0.5
1.0
1.5
MR leave−one−out sensitivity analysis for
'UNC5D' on 'Pulmonary fibrosis'

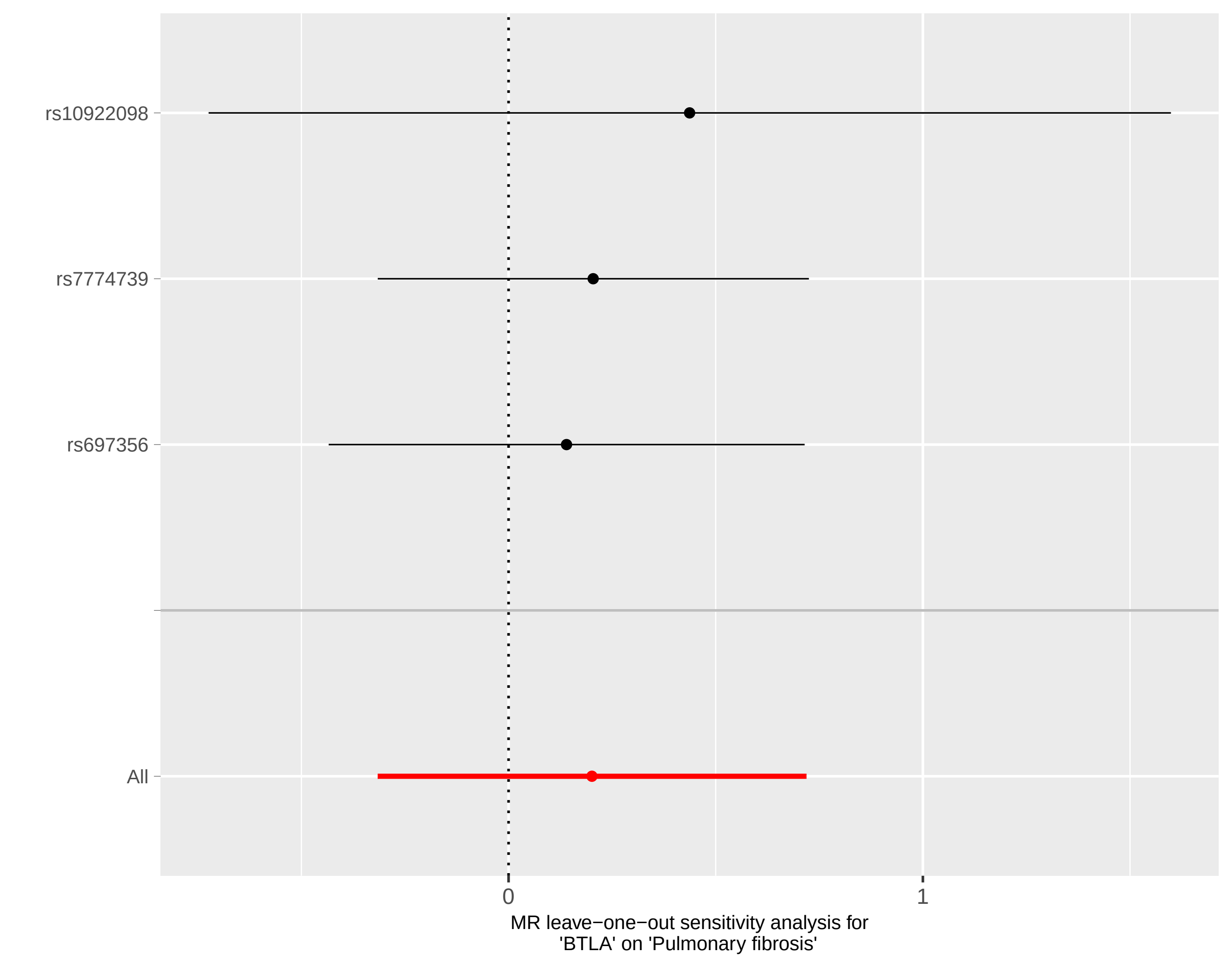
rs10922098
rs7774739
rs697356
All
0
1
MR leave−one−out sensitivity analysis for
'BTLA' on 'Pulmonary fibrosis'

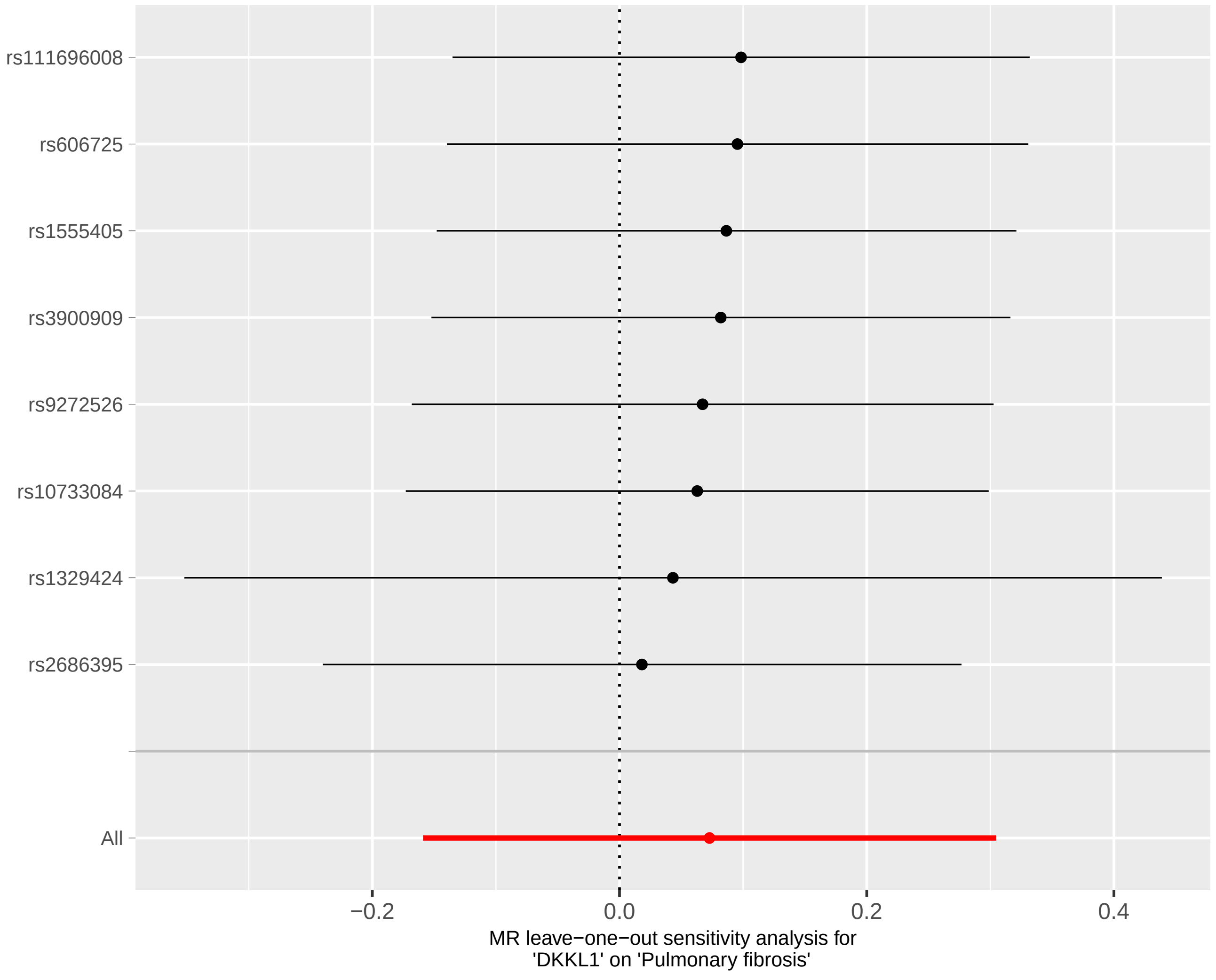

rs111696008
rs606725
rs1555405
rs3900909
rs9272526
rs10733084
rs1329424
rs2686395
All
−0.2
0.0
0.2
0.4
MR leave−one−out sensitivity analysis for
'DKKL1' on 'Pulmonary fibrosis'

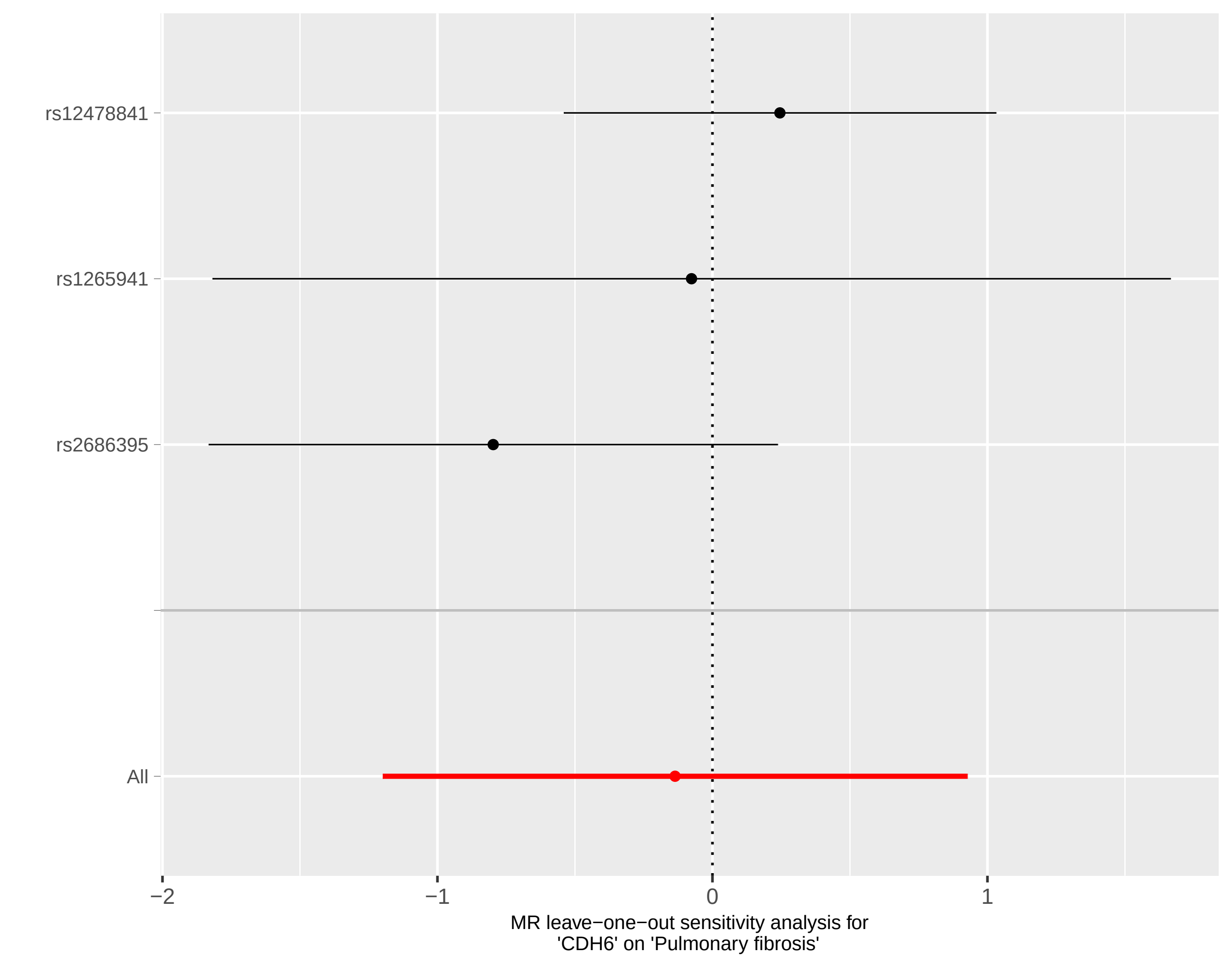
rs12478841
rs1265941
rs2686395
All
−2
−1
0
1
MR leave−one−out sensitivity analysis for
'CDH6' on 'Pulmonary fibrosis'

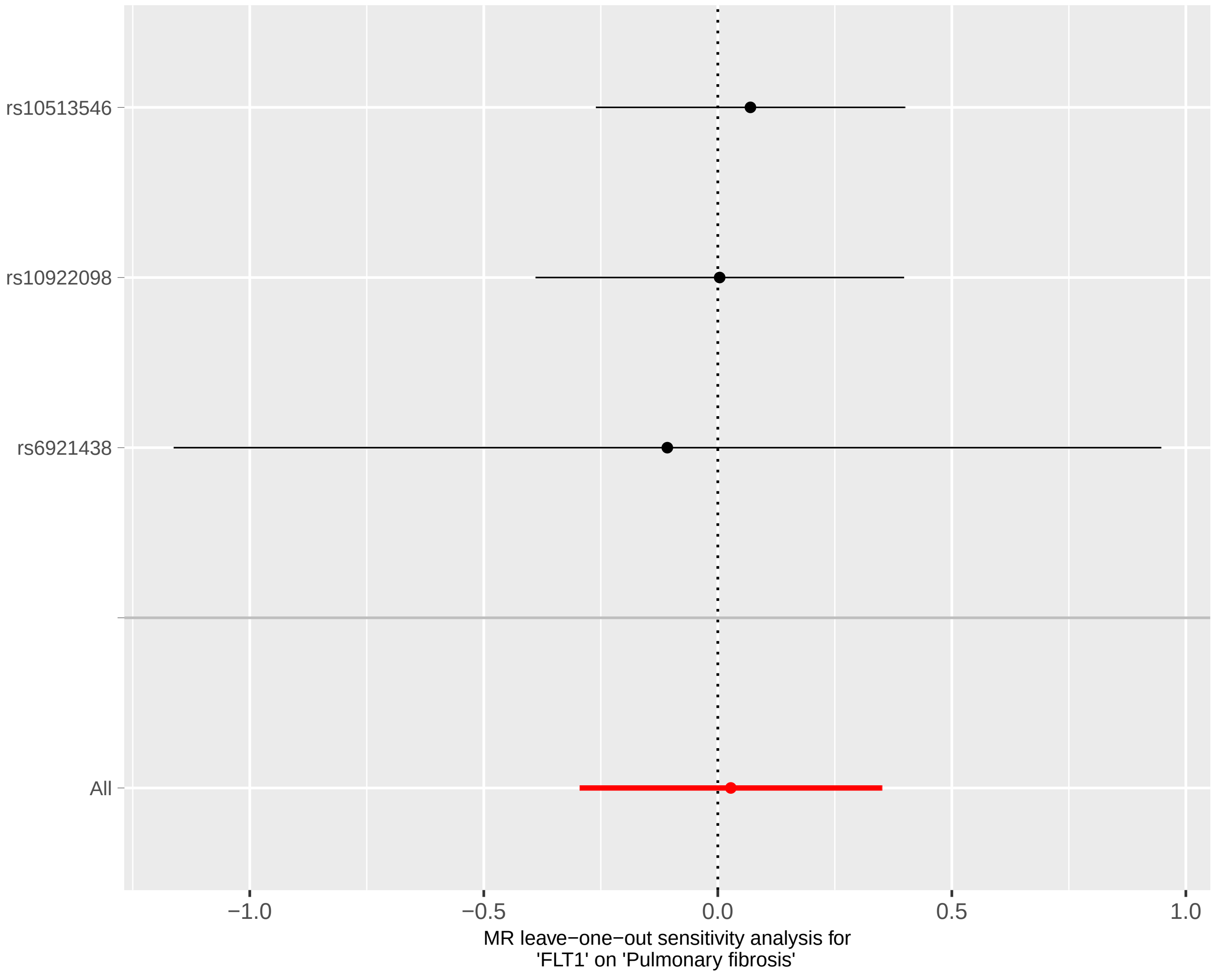
rs10513546
rs10922098
rs6921438
All
−1.0
−0.5
0.0
0.5
1.0
MR leave−one−out sensitivity analysis for
'FLT1' on 'Pulmonary fibrosis'

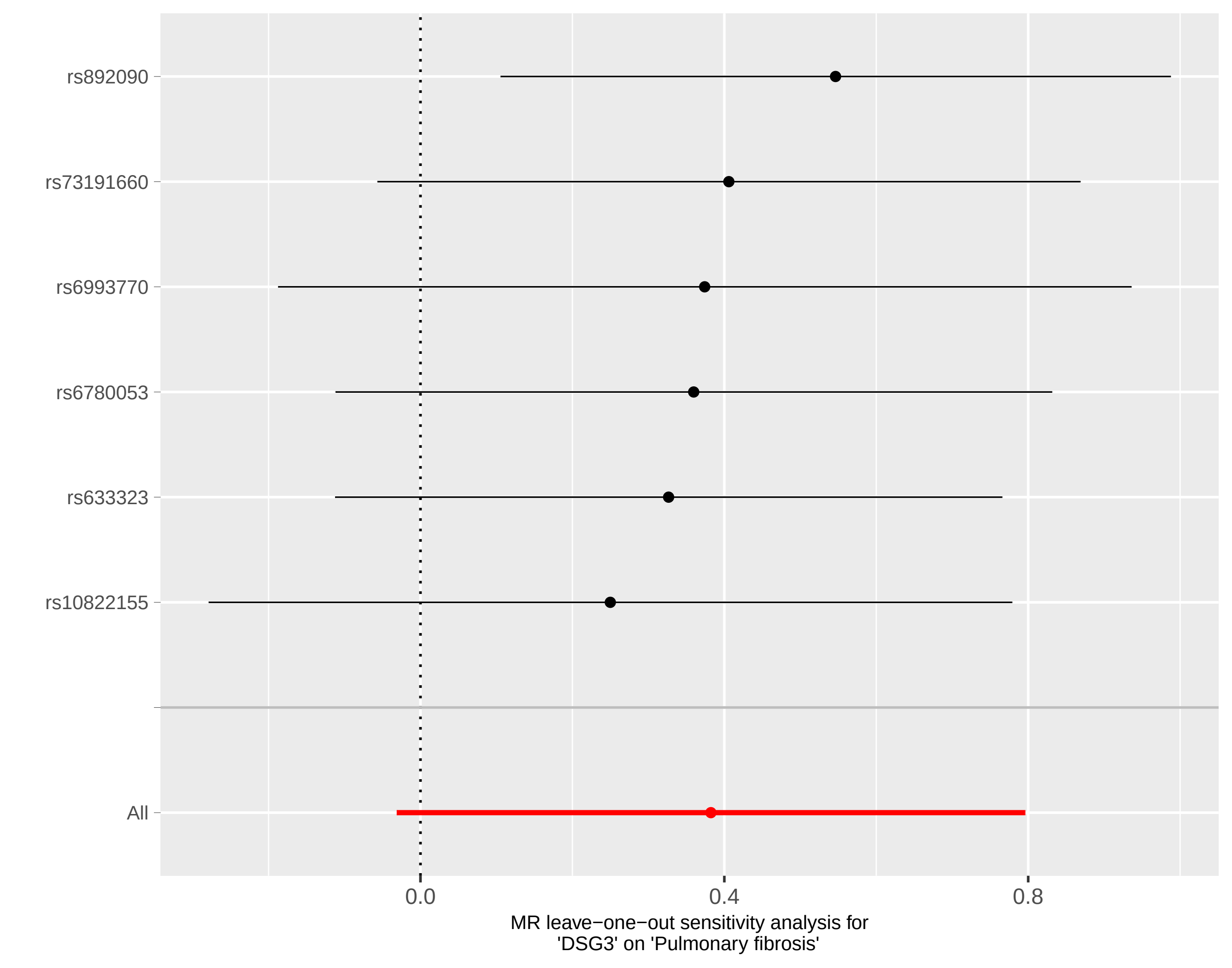
rs892090
rs73191660
rs6993770
rs6780053
rs633323
rs10822155
All
0.0
0.4
0.8
MR leave−one−out sensitivity analysis for
'DSG3' on 'Pulmonary fibrosis'

Insufficient number of SNPs

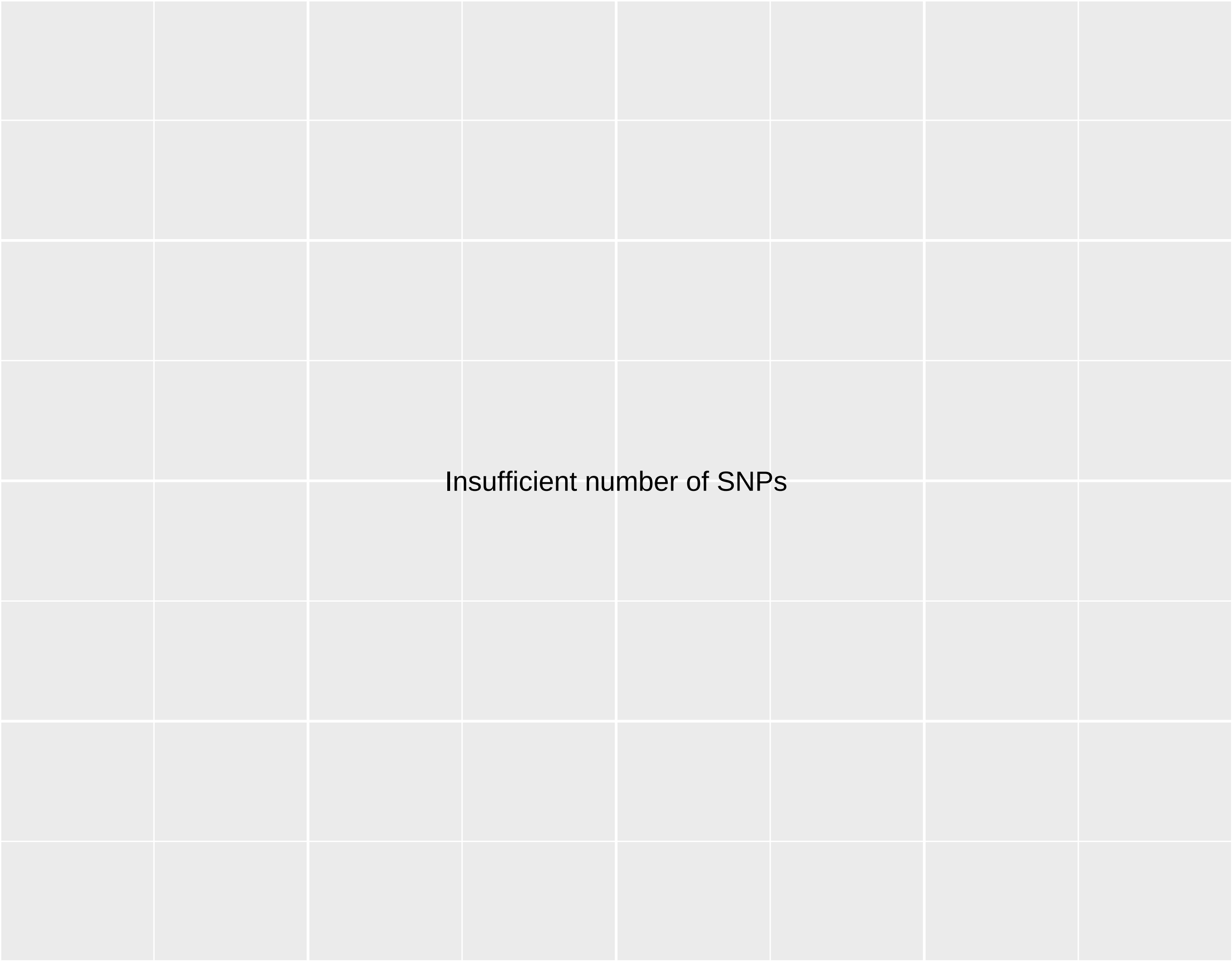
Insufficient number of SNPs

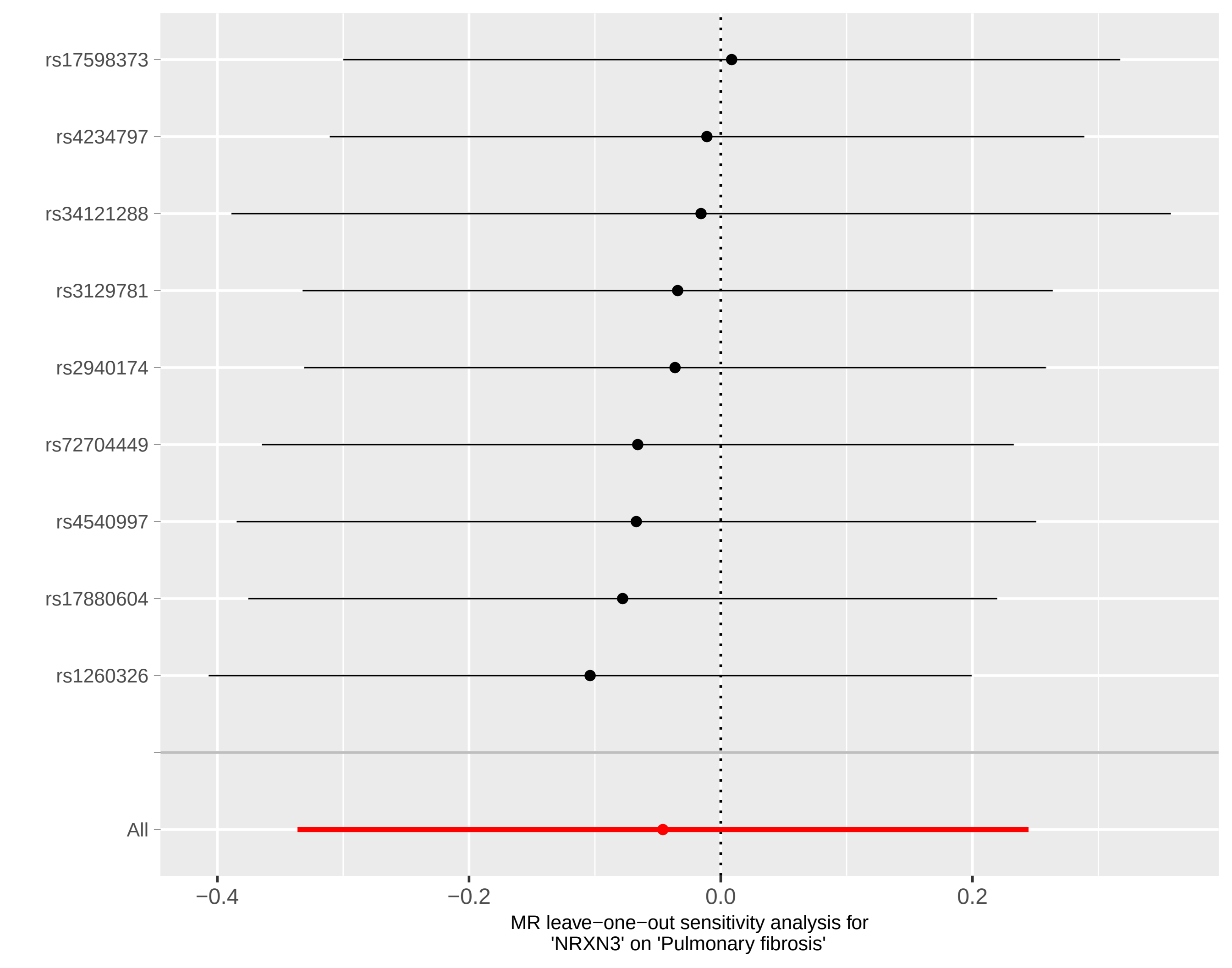
rs17598373
rs4234797
rs34121288
rs3129781
rs2940174
rs72704449
rs4540997
rs17880604
rs1260326
All
−0.4
−0.2
0.0
0.2
MR leave−one−out sensitivity analysis for
'NRXN3' on 'Pulmonary fibrosis'

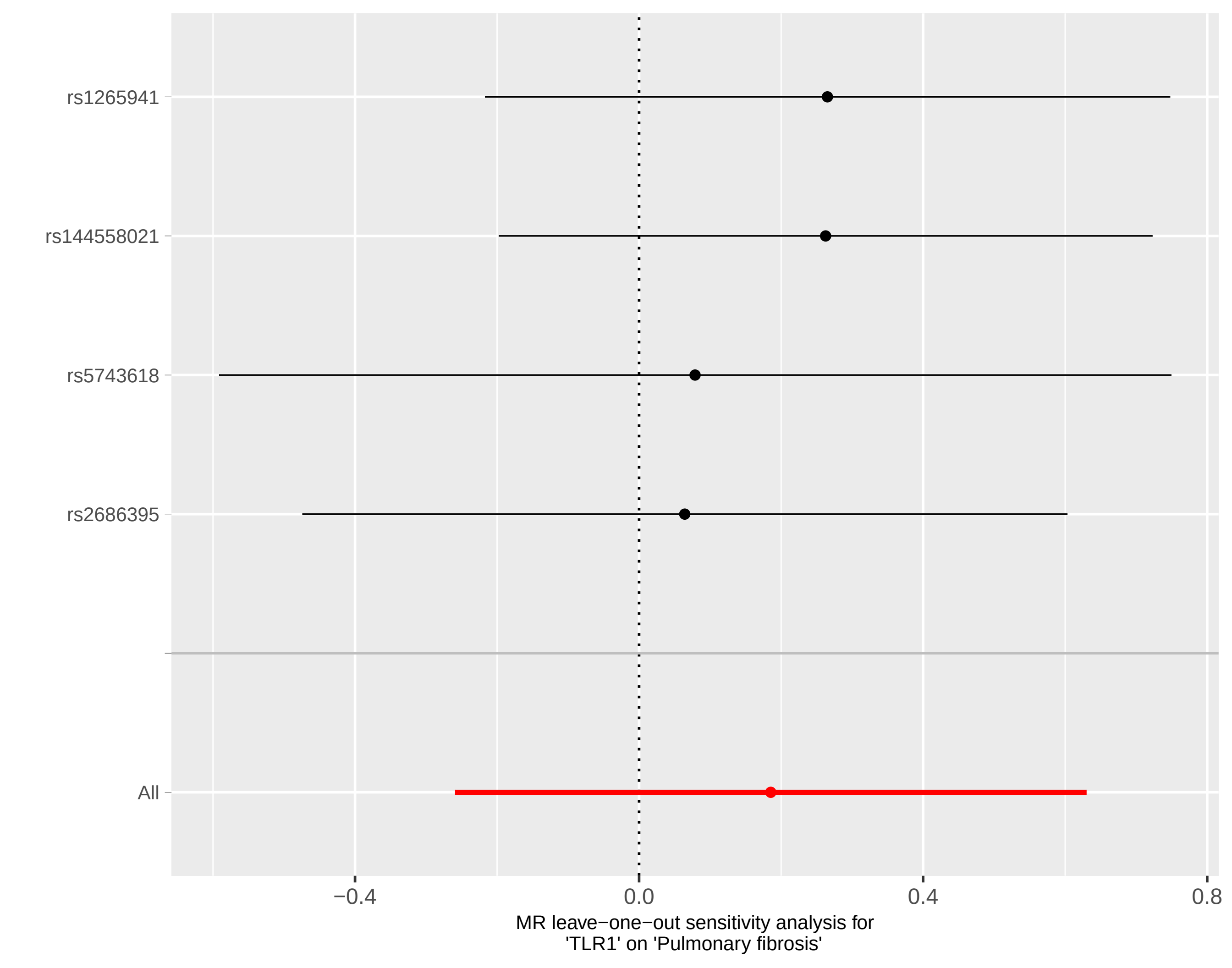

rs1265941
rs144558021
rs5743618
rs2686395
All
−0.4
0.0
0.4
0.8
MR leave−one−out sensitivity analysis for
'TLR1' on 'Pulmonary fibrosis'

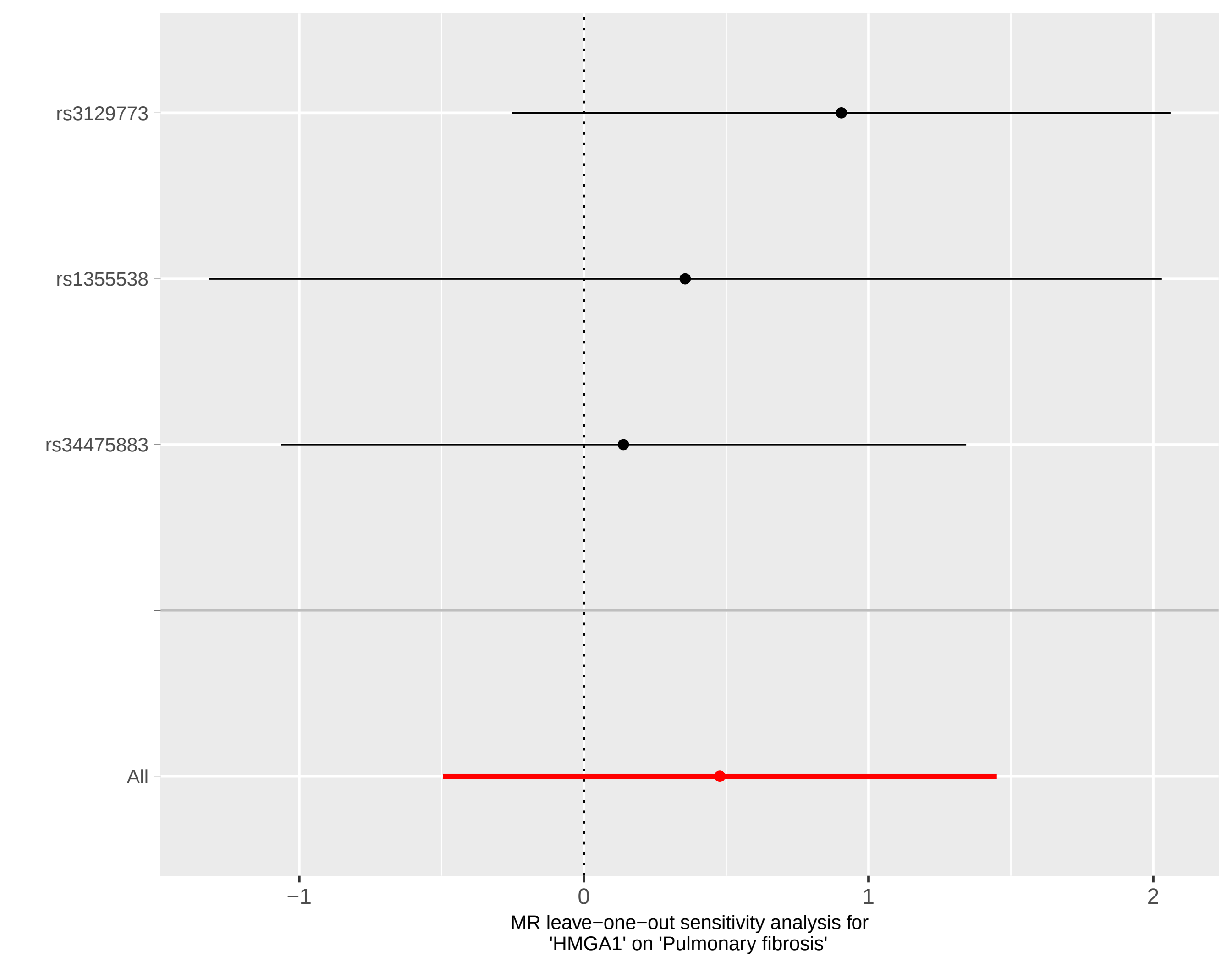
rs3129773
rs1355538
rs34475883
All
−1
0
1
2
MR leave−one−out sensitivity analysis for
'HMGA1' on 'Pulmonary fibrosis'

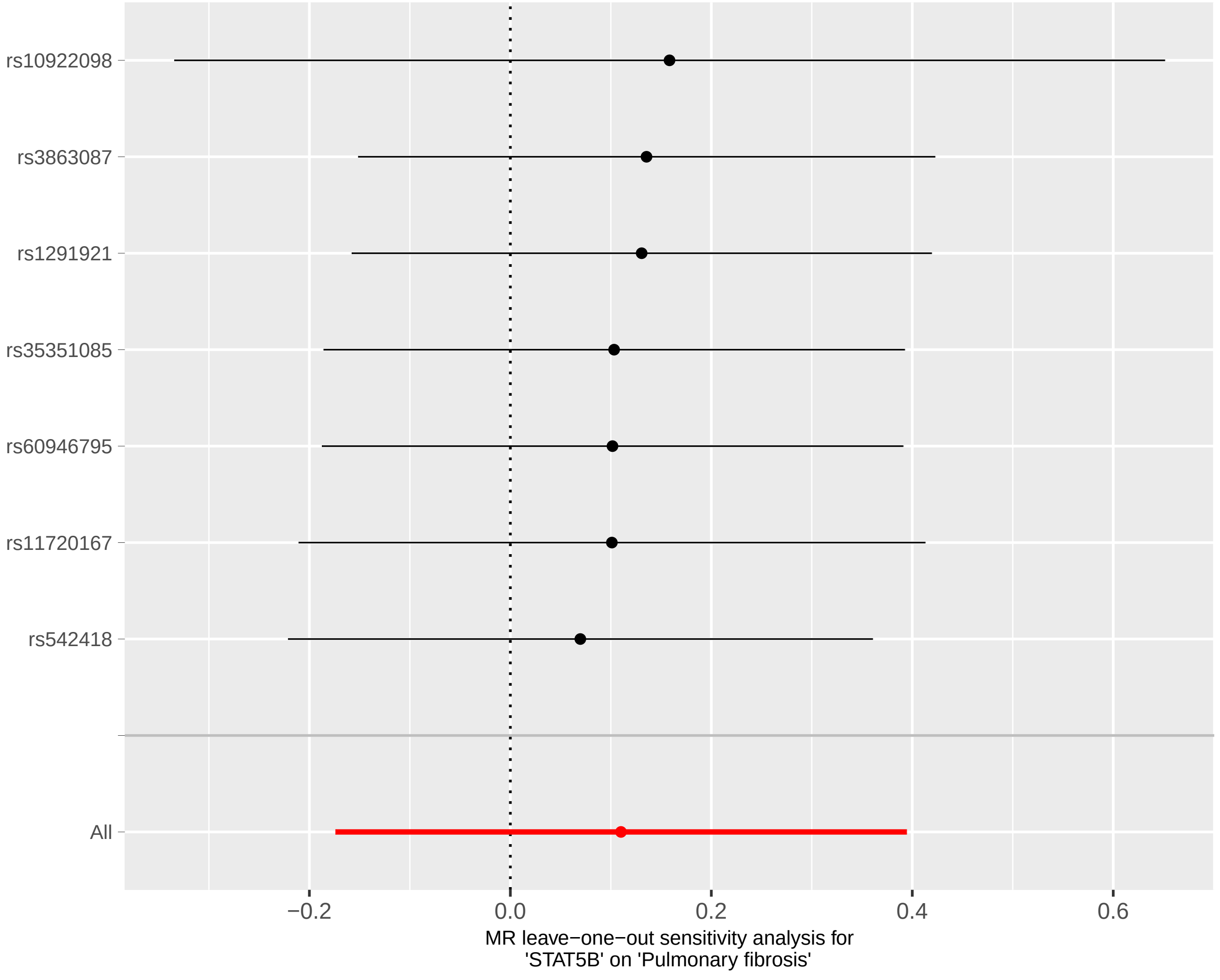
rs10922098
rs3863087
rs1291921
rs35351085
rs60946795
rs11720167
rs542418
All
−0.2
0.0
0.2
0.4
0.6
MR leave−one−out sensitivity analysis for
'STAT5B' on 'Pulmonary fibrosis'

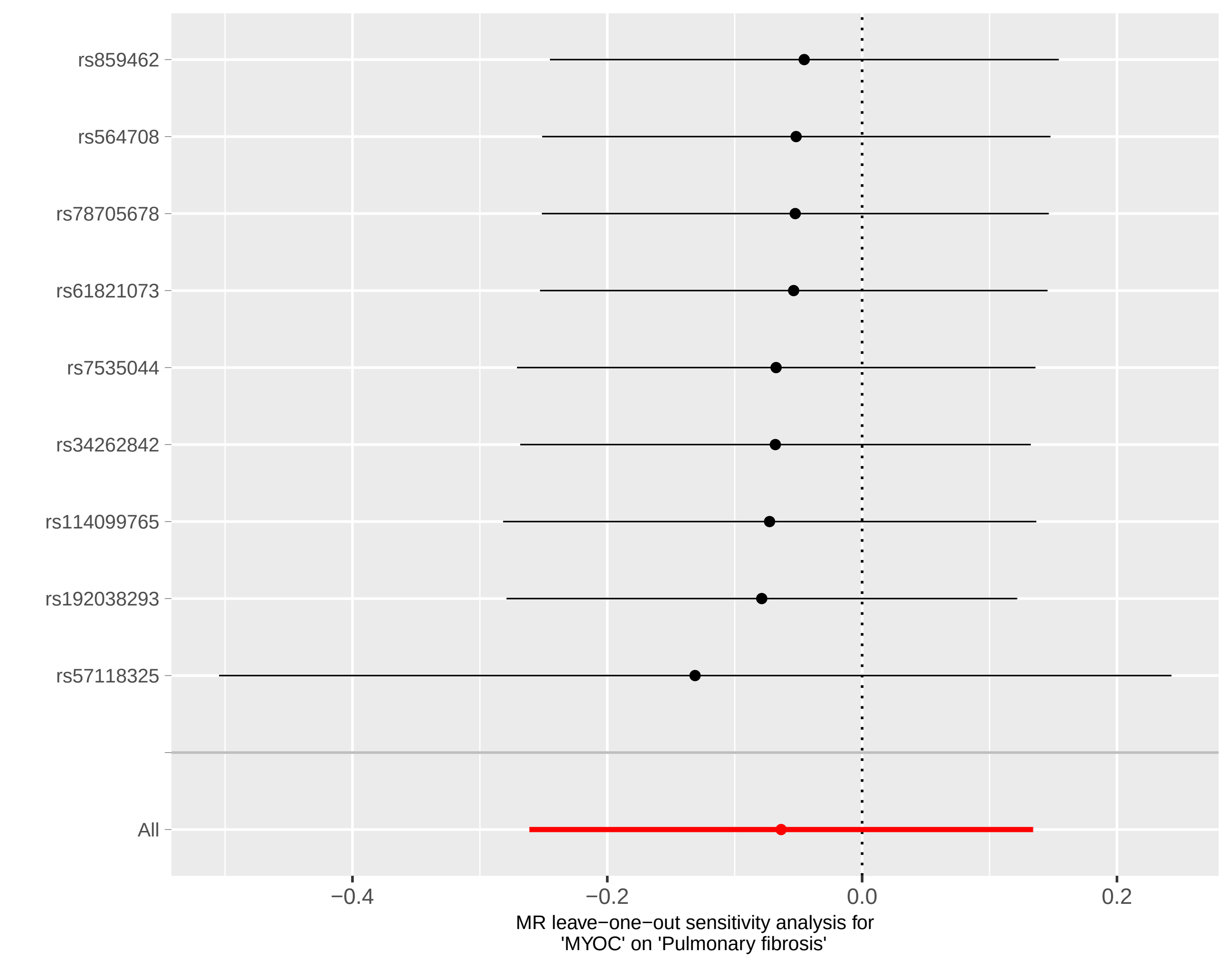
rs859462
rs564708
rs78705678
rs61821073
rs7535044
rs34262842
rs114099765
rs192038293
rs57118325
All
−0.4
−0.2
0.0
0.2
MR leave−one−out sensitivity analysis for
'MYOC' on 'Pulmonary fibrosis'

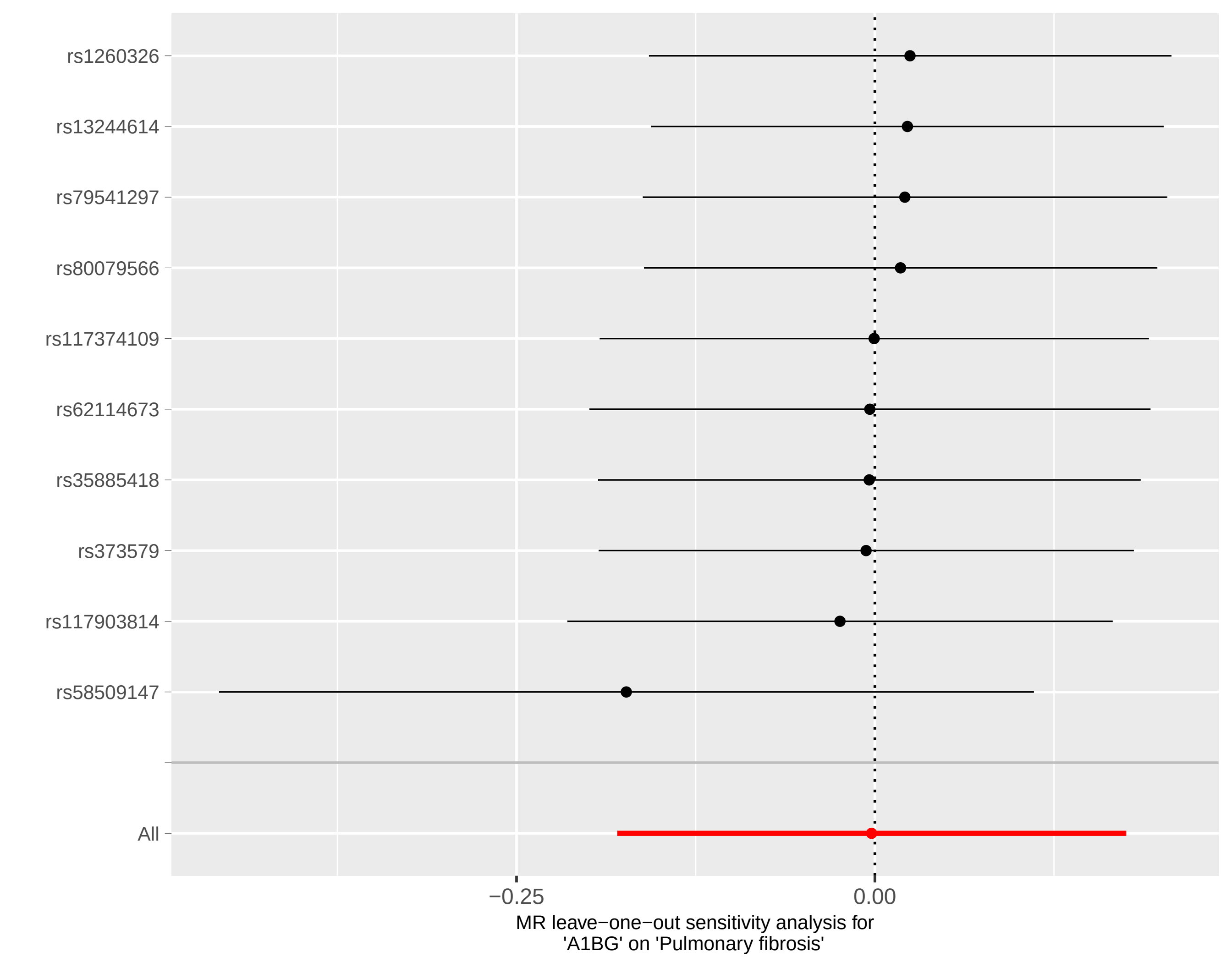
rs1260326
rs13244614
rs79541297
rs80079566
rs117374109
rs62114673
rs35885418
rs373579
rs117903814
rs58509147
All
−0.25
0.00
MR leave−one−out sensitivity analysis for
'A1BG' on 'Pulmonary fibrosis'

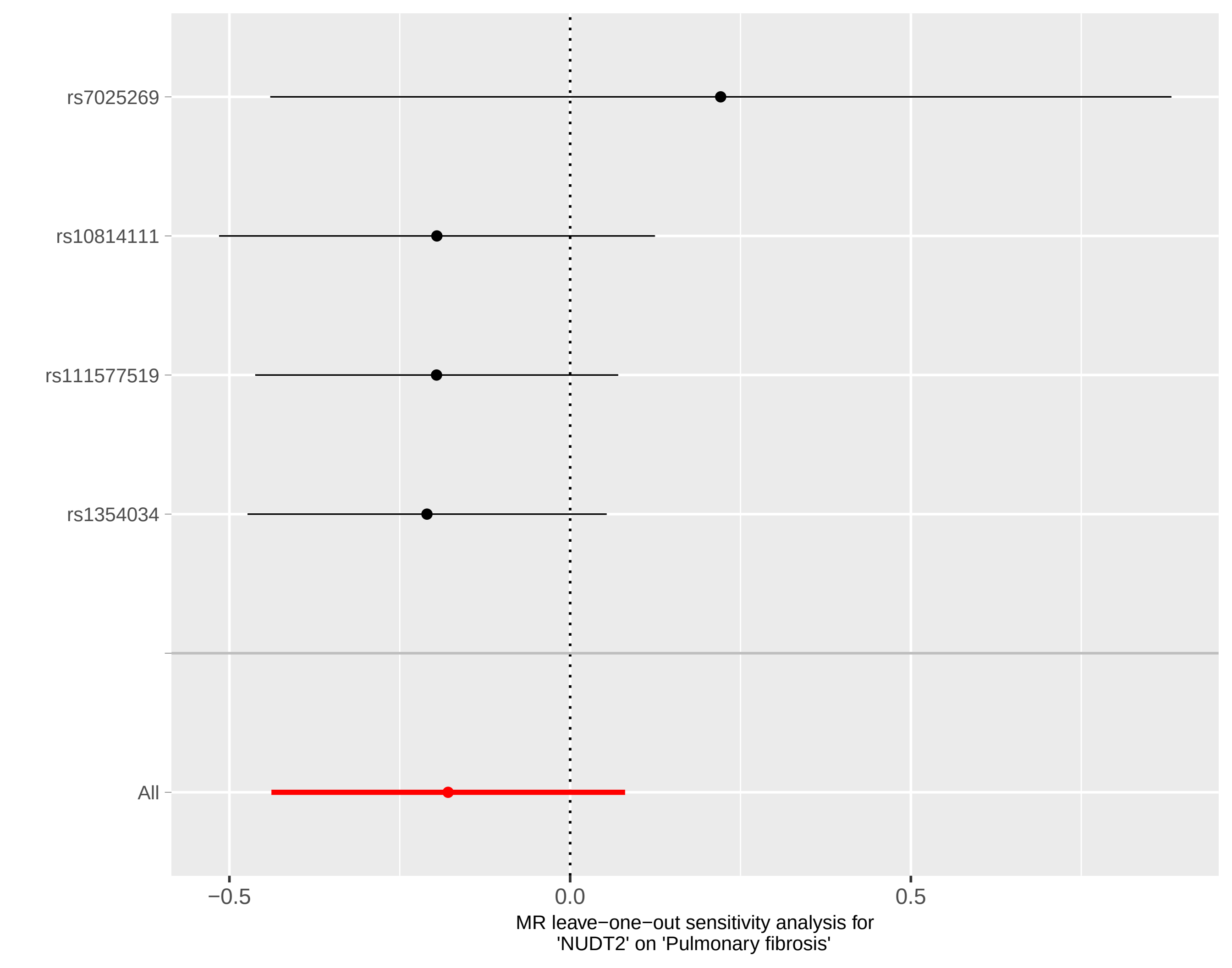
rs7025269
rs10814111
rs111577519
rs1354034
All
−0.5
0.0
0.5
MR leave−one−out sensitivity analysis for
'NUDT2' on 'Pulmonary fibrosis'

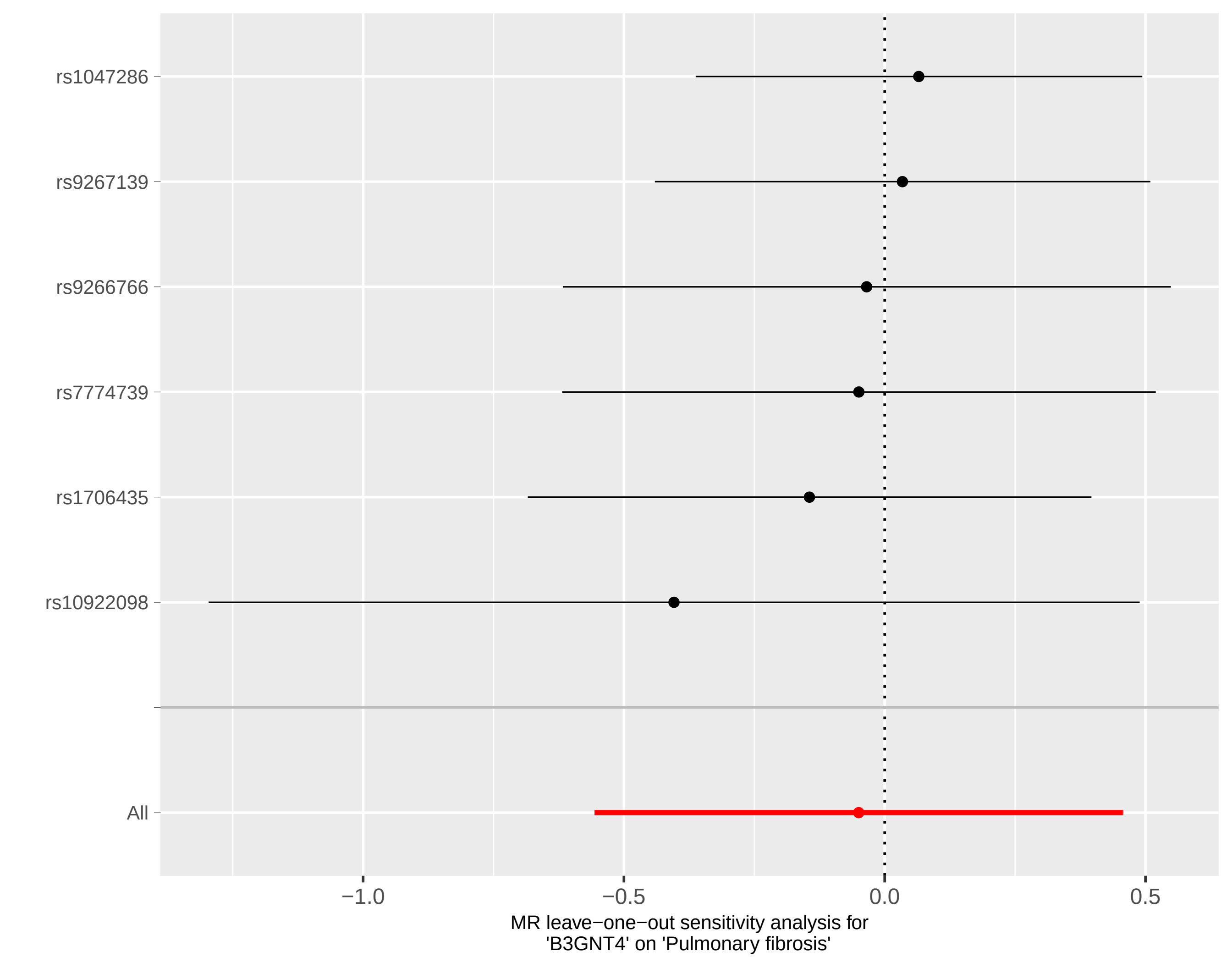

rs1047286
rs9267139
rs9266766
rs7774739
rs1706435
rs10922098
All
−1.0
−0.5
0.0
0.5
MR leave−one−out sensitivity analysis for
'B3GNT4' on 'Pulmonary fibrosis'

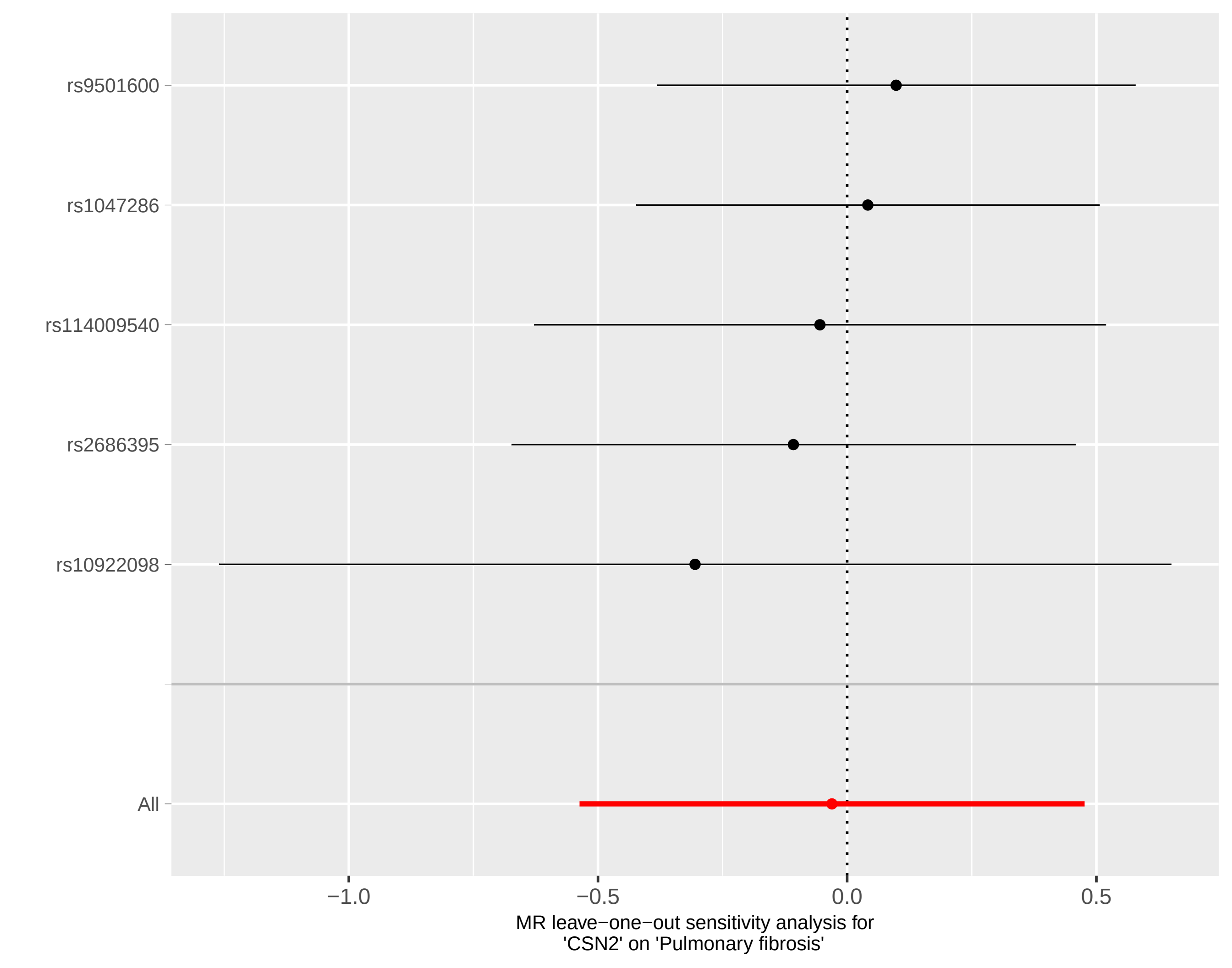

rs9501600
rs1047286
rs114009540
rs2686395
rs10922098
All
−1.0
−0.5
0.0
0.5
MR leave−one−out sensitivity analysis for
'CSN2' on 'Pulmonary fibrosis'

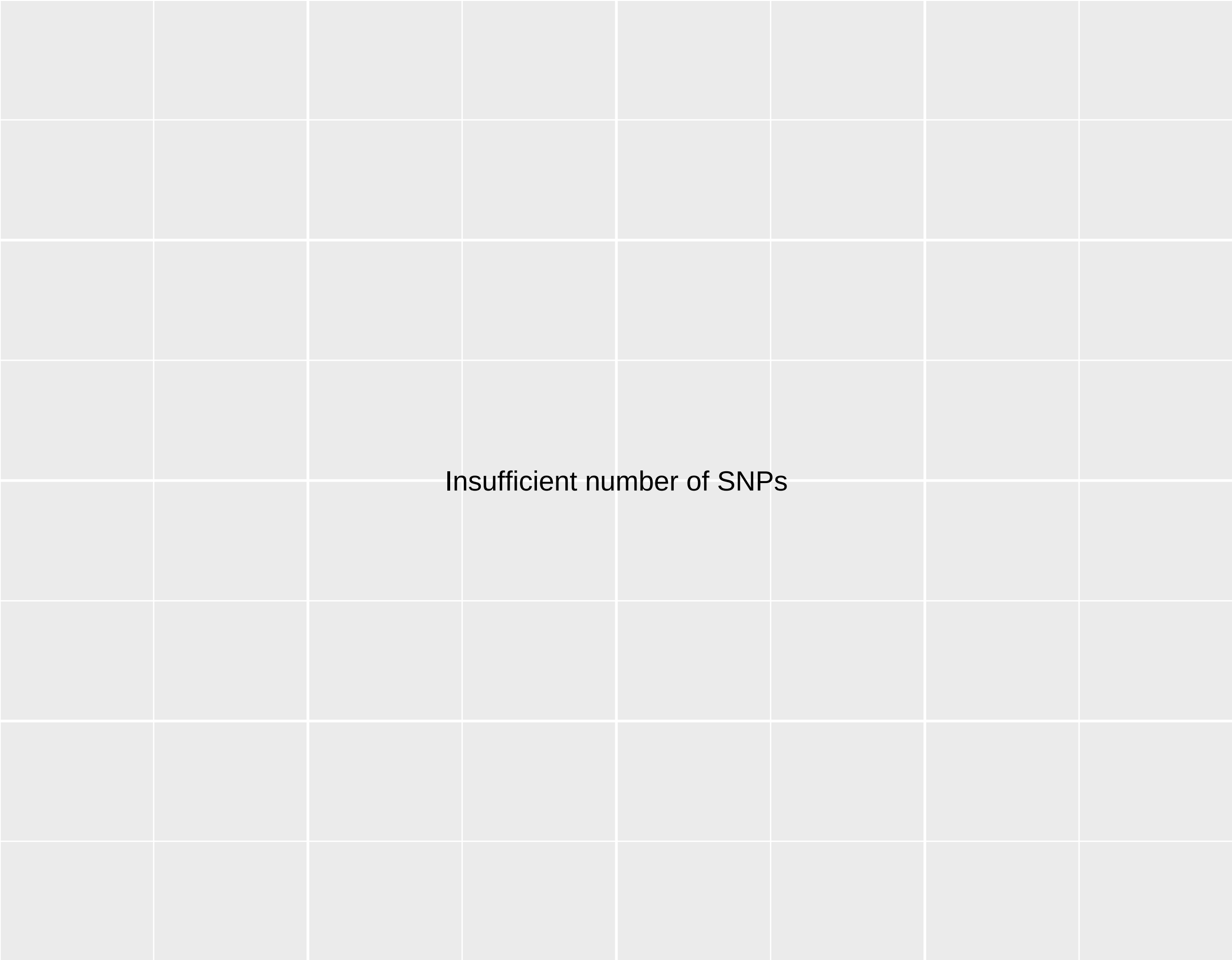
Insufficient number of SNPs

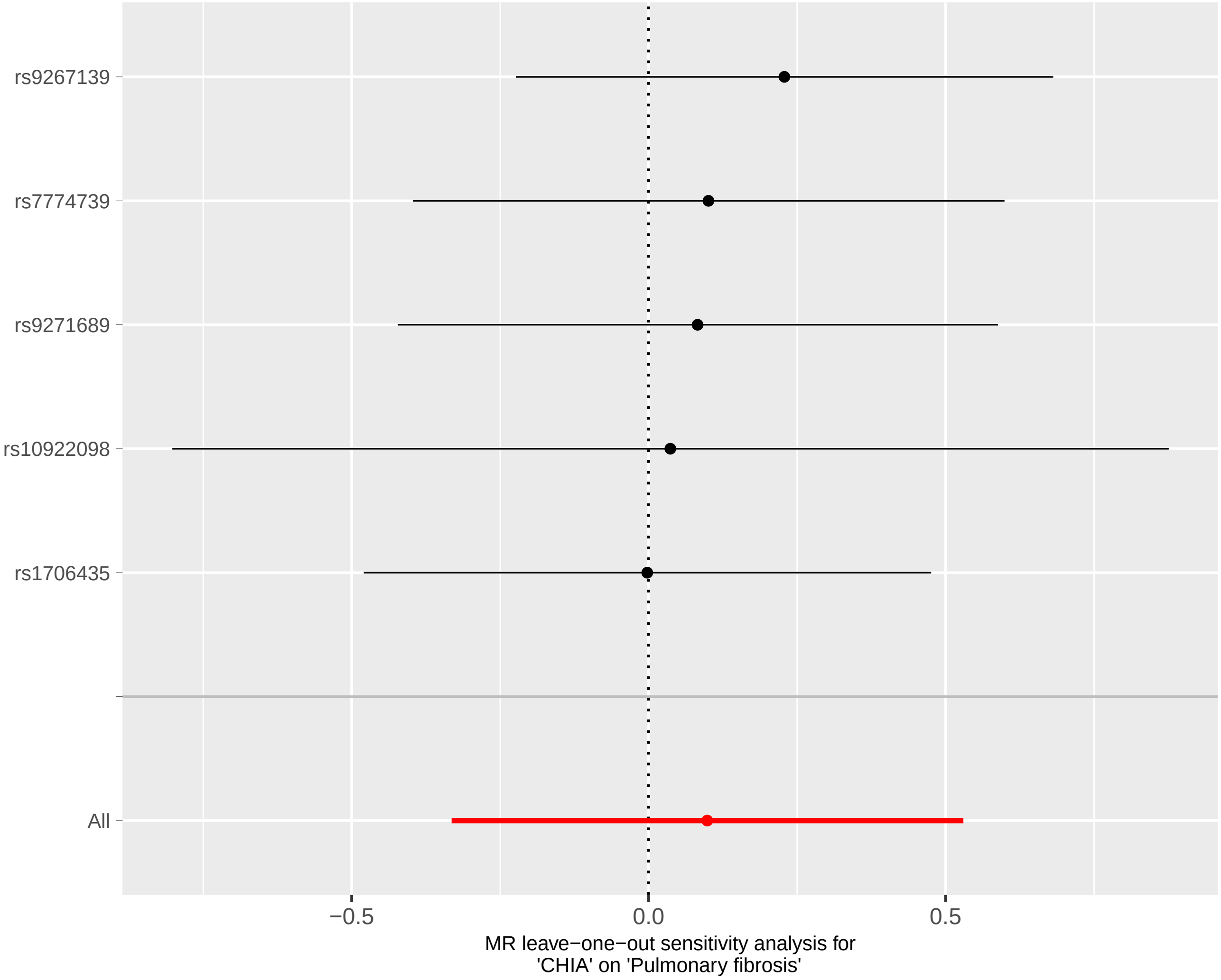

rs9267139
rs7774739
rs9271689
rs10922098
rs1706435
All
−0.5
0.0
0.5
MR leave−one−out sensitivity analysis for
'CHIA' on 'Pulmonary fibrosis'

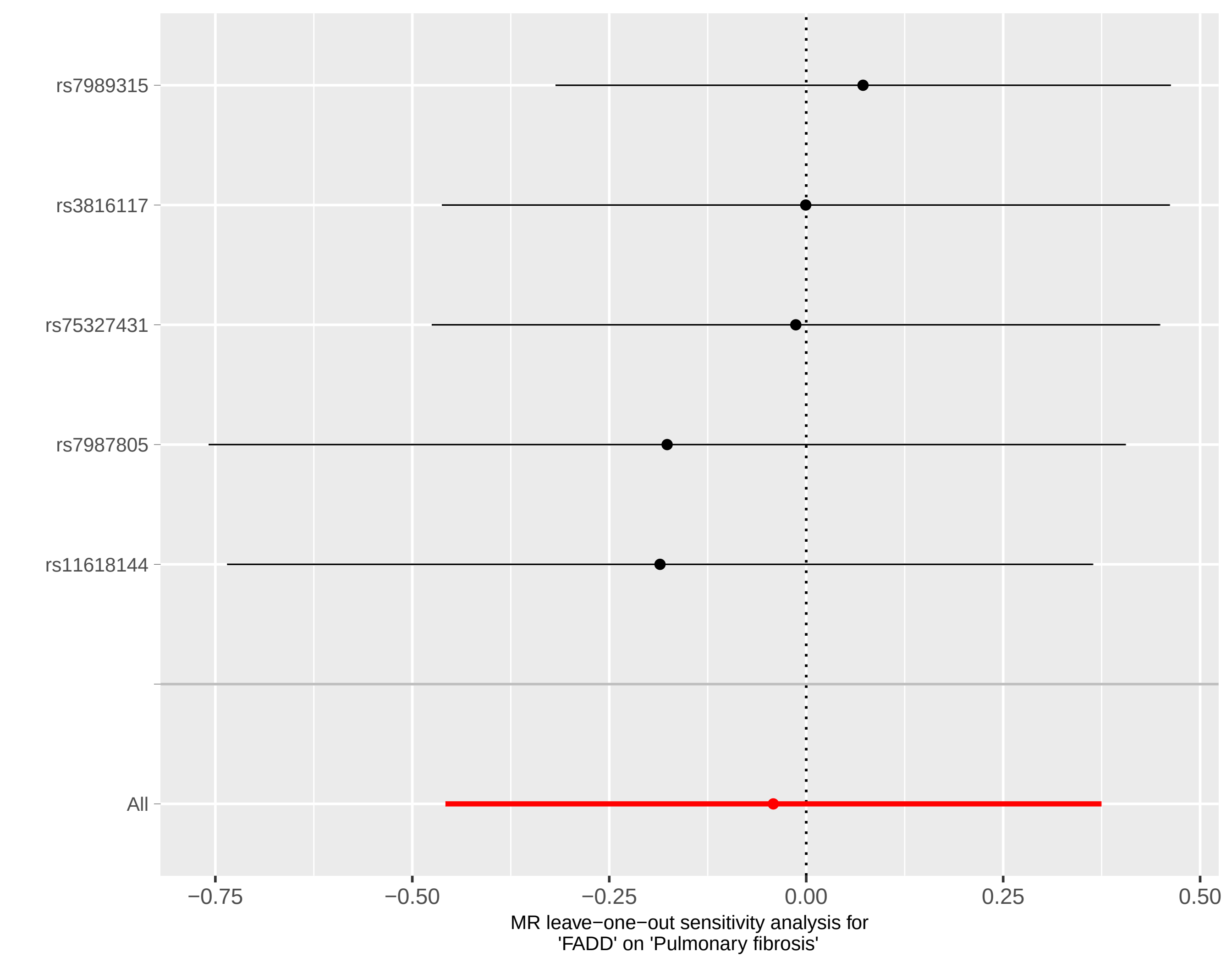
rs7989315
rs3816117
rs75327431
rs7987805
rs11618144
All
−0.75
−0.50
−0.25
0.00
0.25
0.50
MR leave−one−out sensitivity analysis for
'FADD' on 'Pulmonary fibrosis'

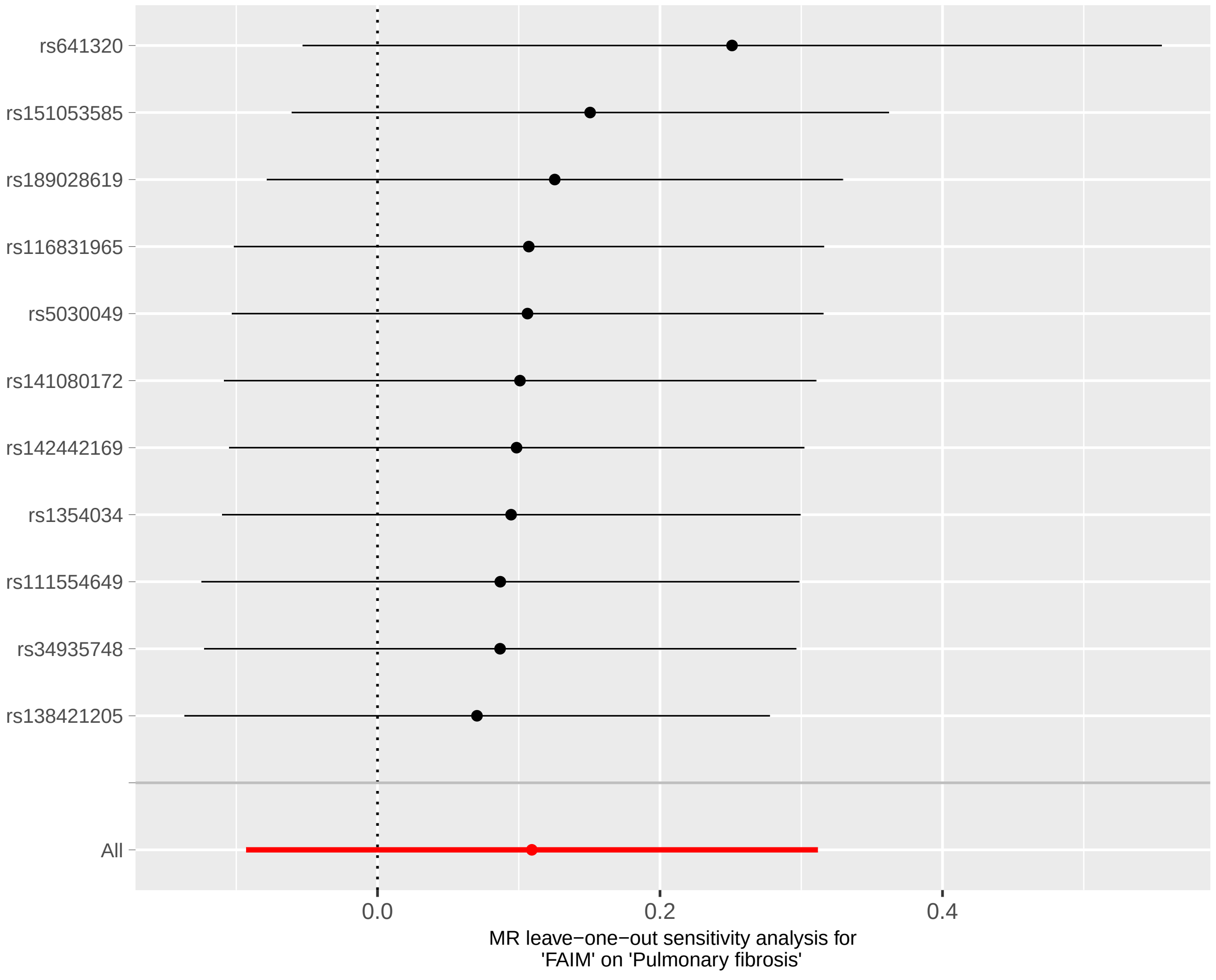
rs641320
rs151053585
rs189028619
rs116831965
rs5030049
rs141080172
rs142442169
rs1354034
rs111554649
rs34935748
rs138421205
All
0.0
0.2
0.4
MR leave−one−out sensitivity analysis for
'FAIM' on 'Pulmonary fibrosis'

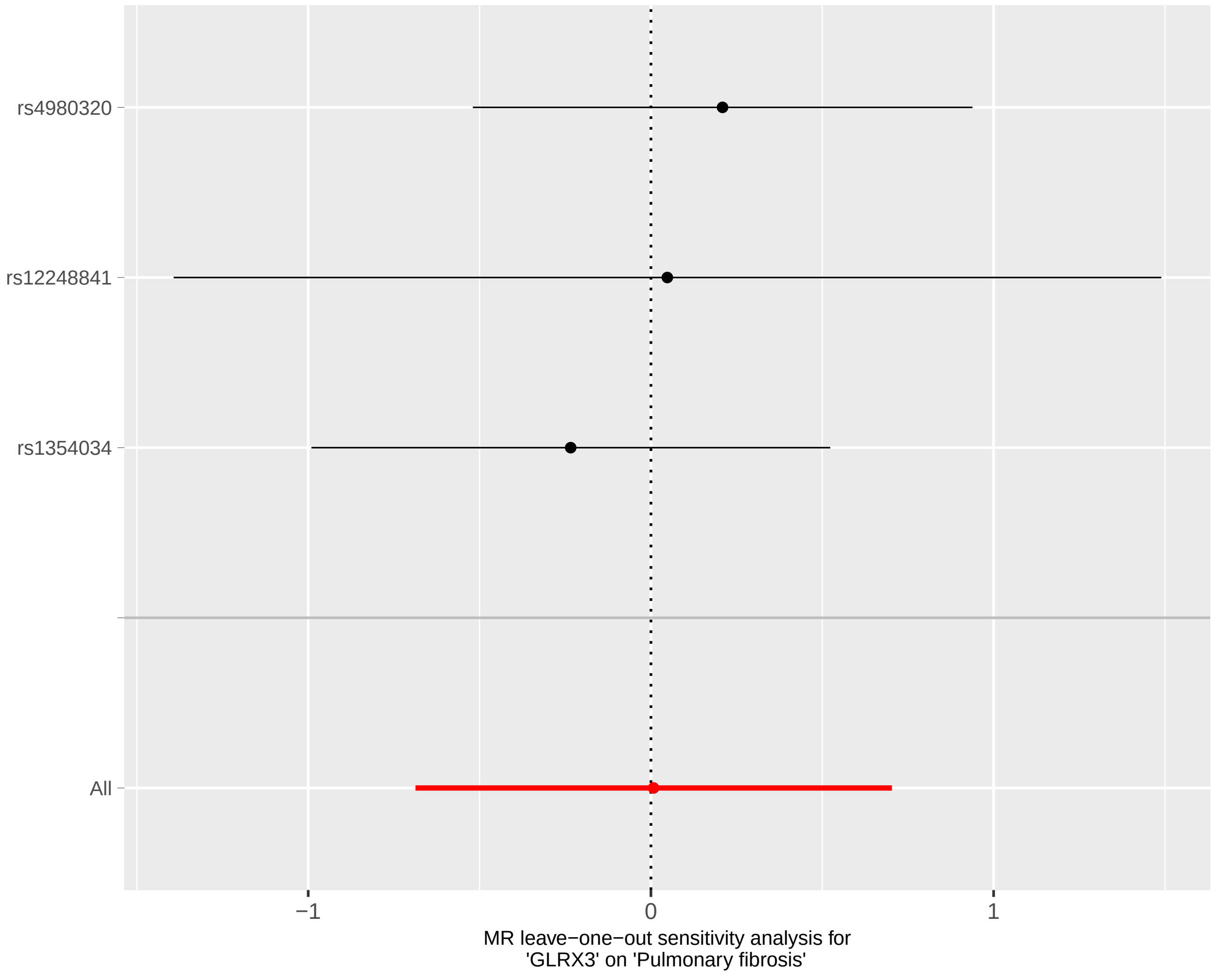
rs4980320
rs12248841
rs1354034
All
−1
0
1
MR leave−one−out sensitivity analysis for
'GLRX3' on 'Pulmonary fibrosis'

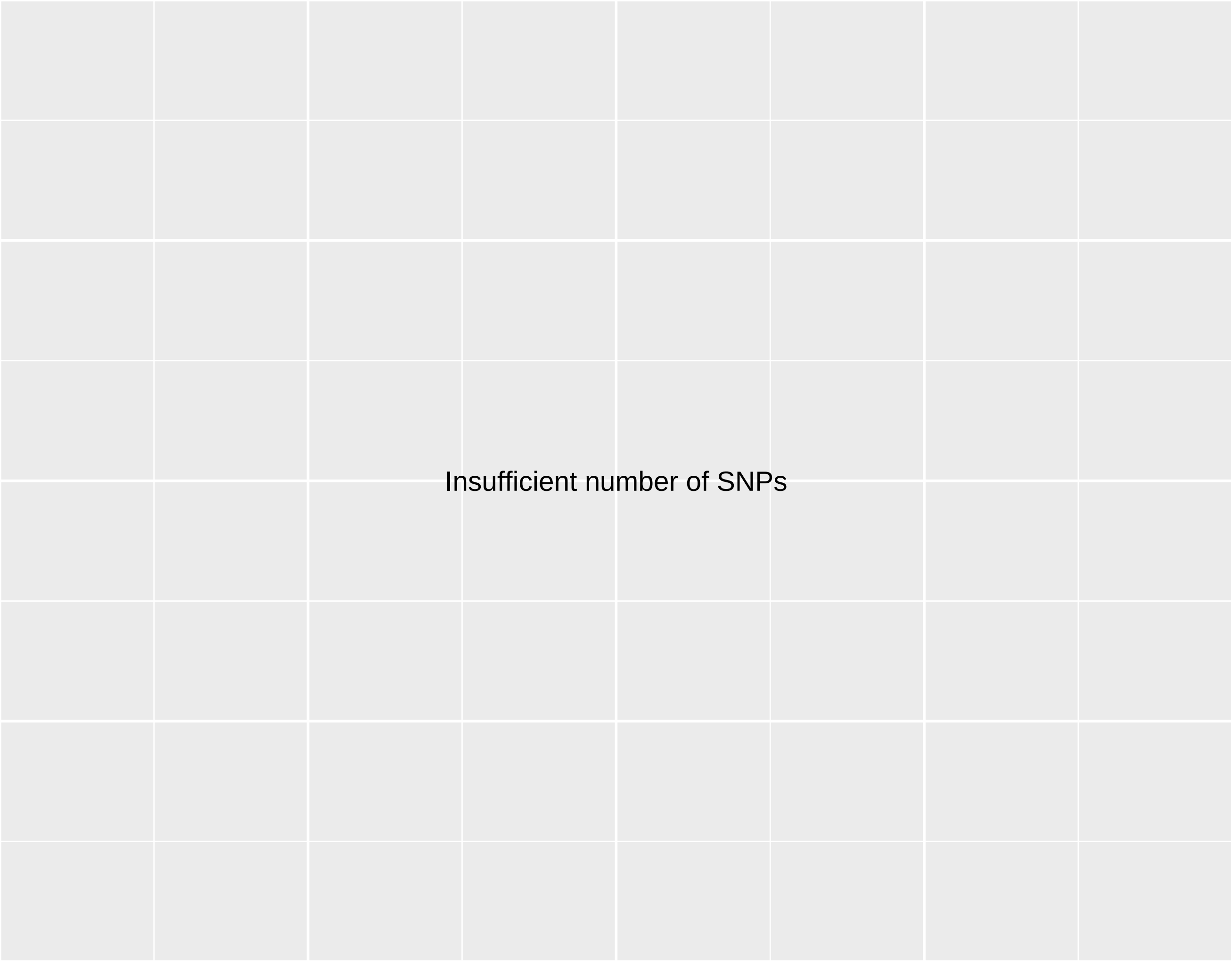
Insufficient number of SNPs

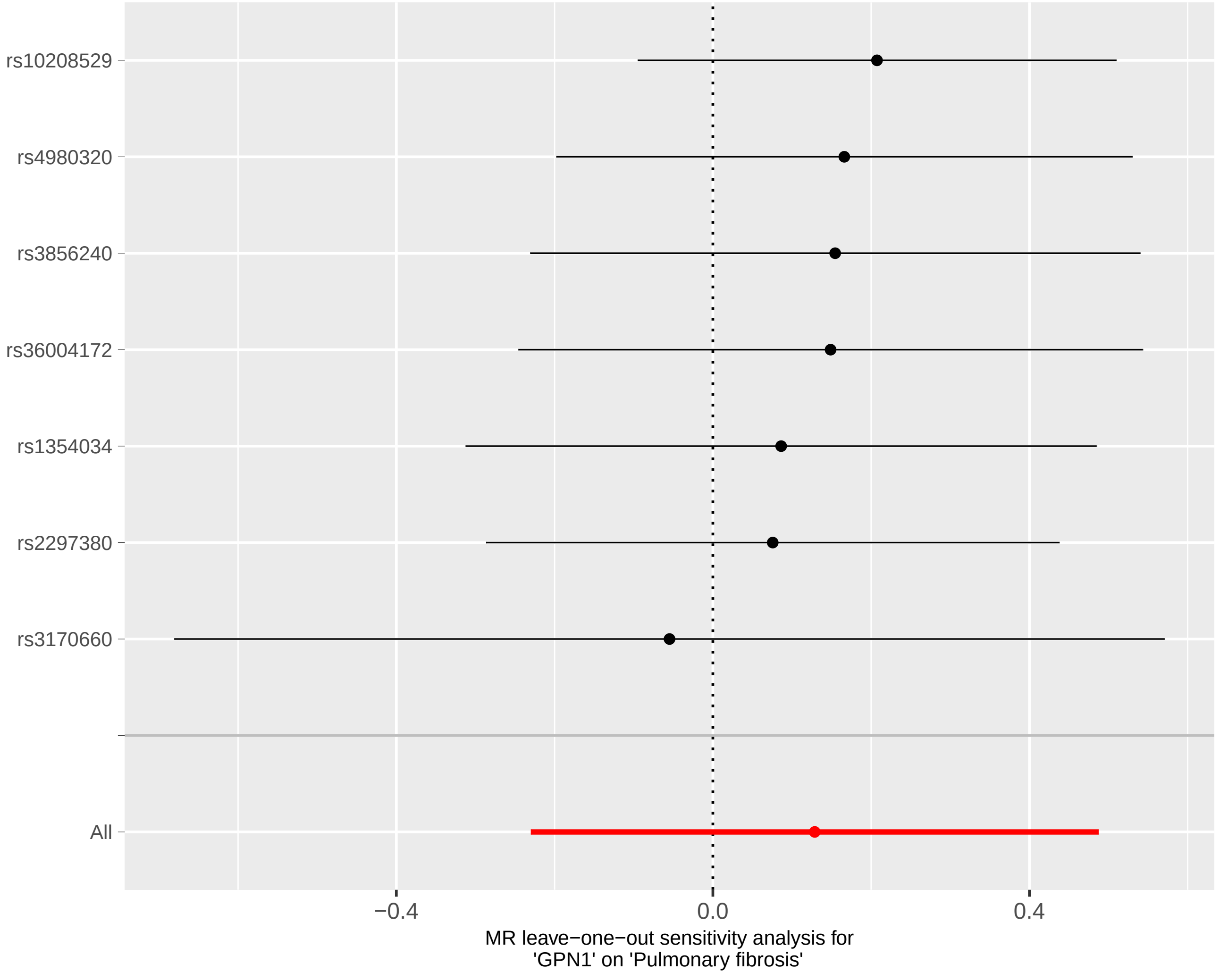
rs10208529
rs4980320
rs3856240
rs36004172
rs1354034
rs2297380
rs3170660
All
−0.4
0.0
0.4
MR leave−one−out sensitivity analysis for
'GPN1' on 'Pulmonary fibrosis'

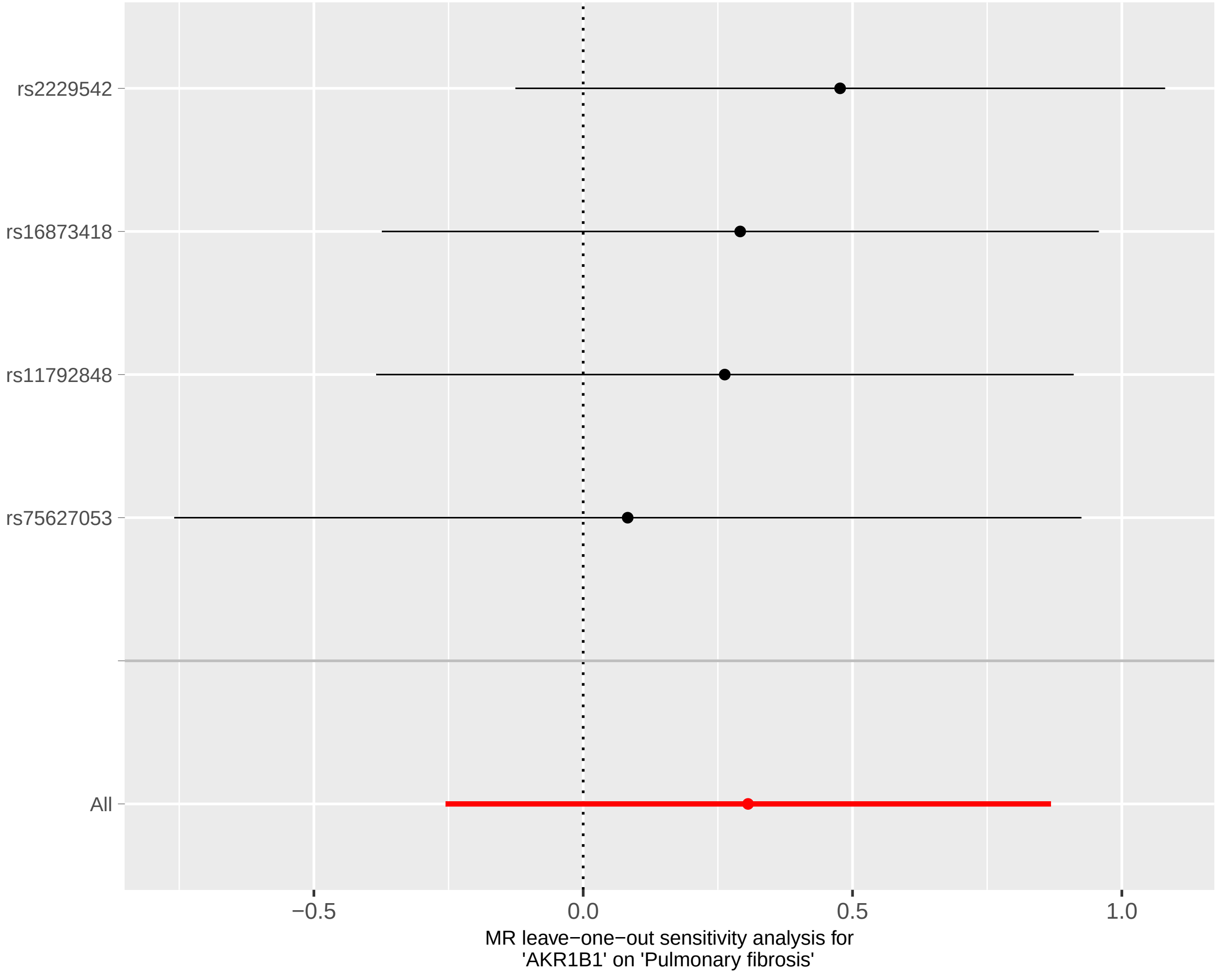
rs2229542
rs16873418
rs11792848
rs75627053
All
−0.5
0.0
0.5
1.0
MR leave−one−out sensitivity analysis for
'AKR1B1' on 'Pulmonary fibrosis'

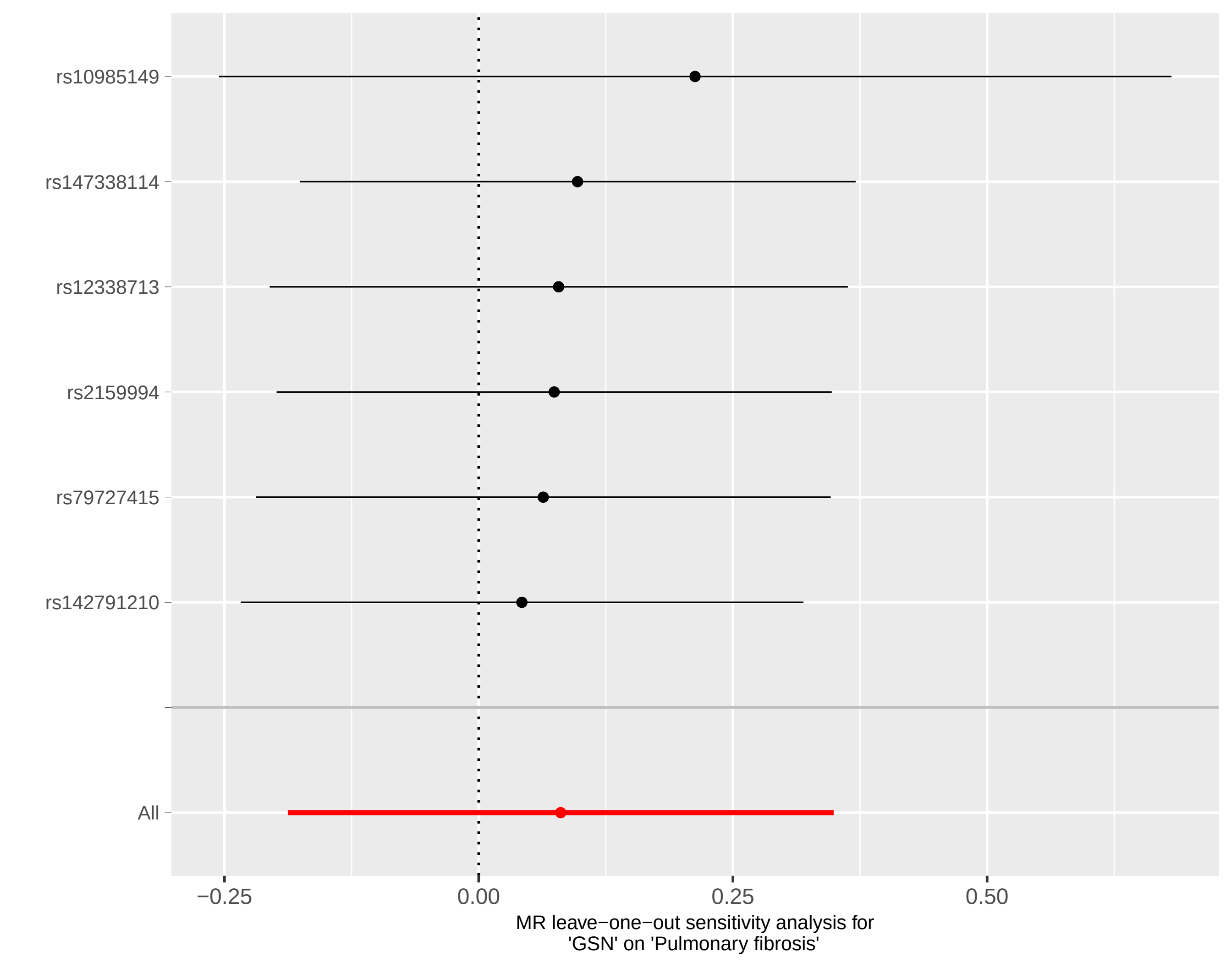

rs10985149
rs147338114
rs12338713
rs2159994
rs79727415
rs142791210
All
−0.25
0.00
0.25
0.50
MR leave−one−out sensitivity analysis for
'GSN' on 'Pulmonary fibrosis'

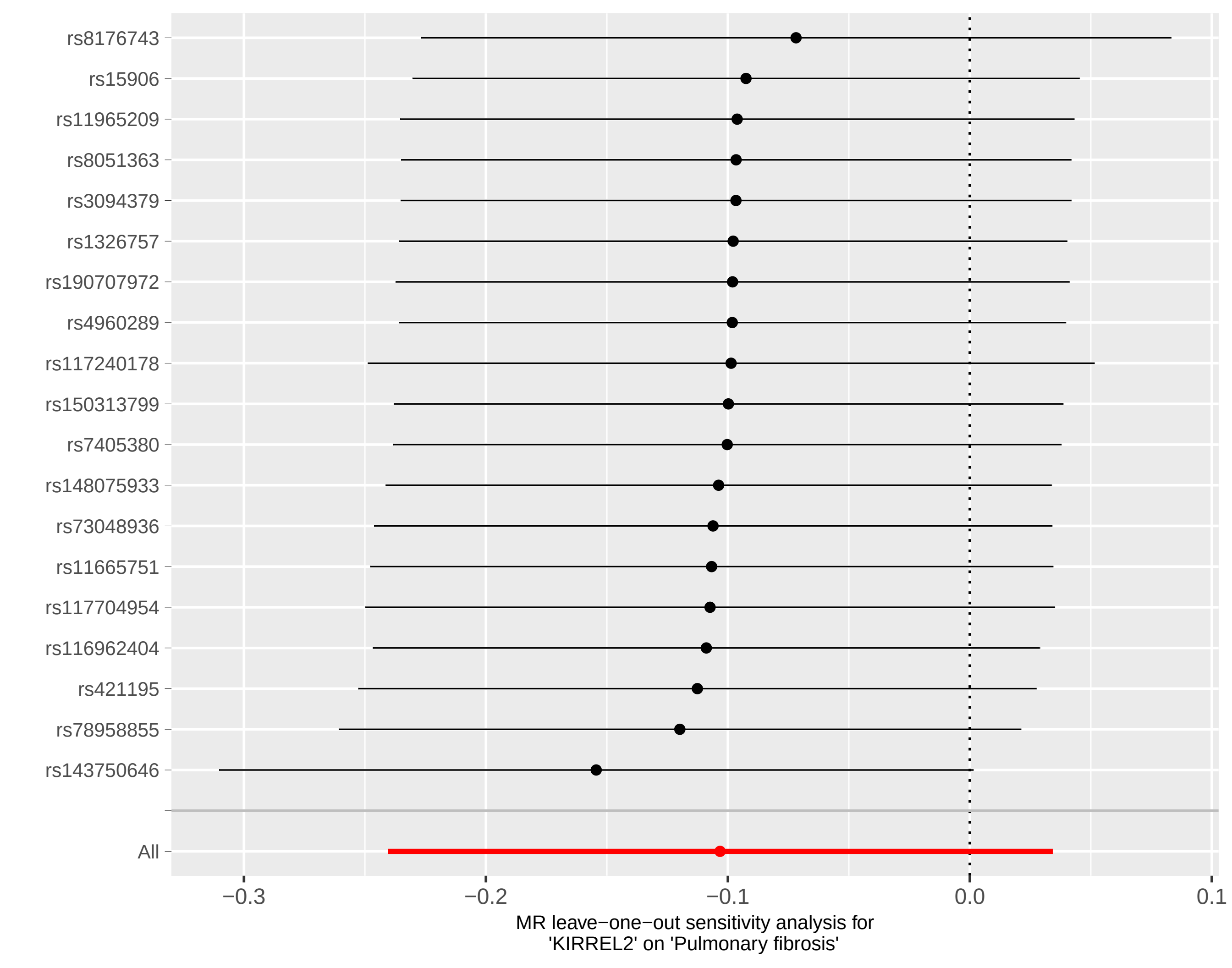

rs8176743
rs15906
rs11965209
rs8051363
rs3094379
rs1326757
rs190707972
rs4960289
rs117240178
rs150313799
rs7405380
rs148075933
rs73048936
rs11665751
rs117704954
rs116962404
rs421195
rs78958855
rs143750646
All
−0.3
−0.2
−0.1
0.0
0.1
MR leave−one−out sensitivity analysis for
'KIRREL2' on 'Pulmonary fibrosis'

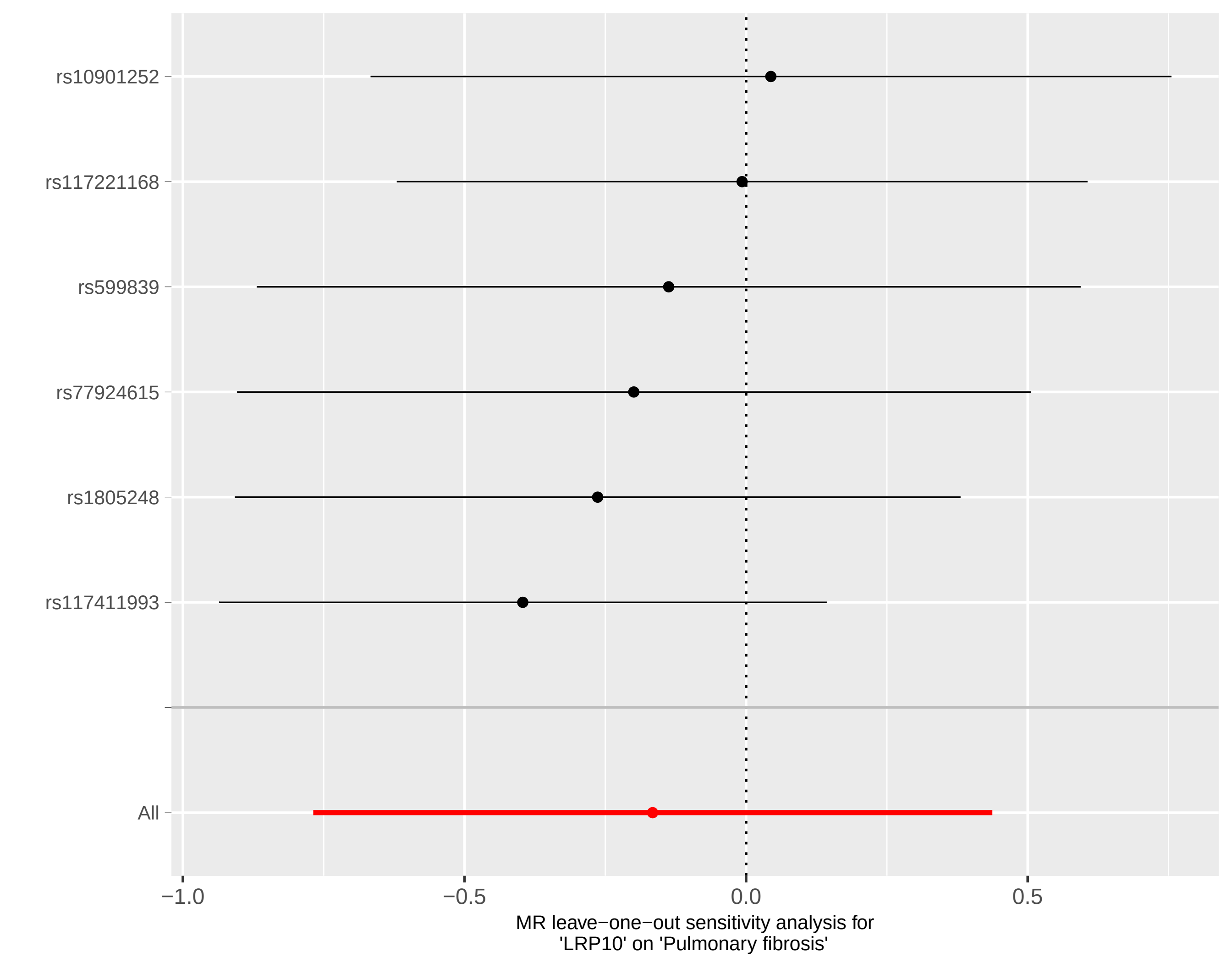

rs10901252
rs117221168
rs599839
rs77924615
rs1805248
rs117411993
All
−1.0
−0.5
0.0
0.5
MR leave−one−out sensitivity analysis for
'LRP10' on 'Pulmonary fibrosis'

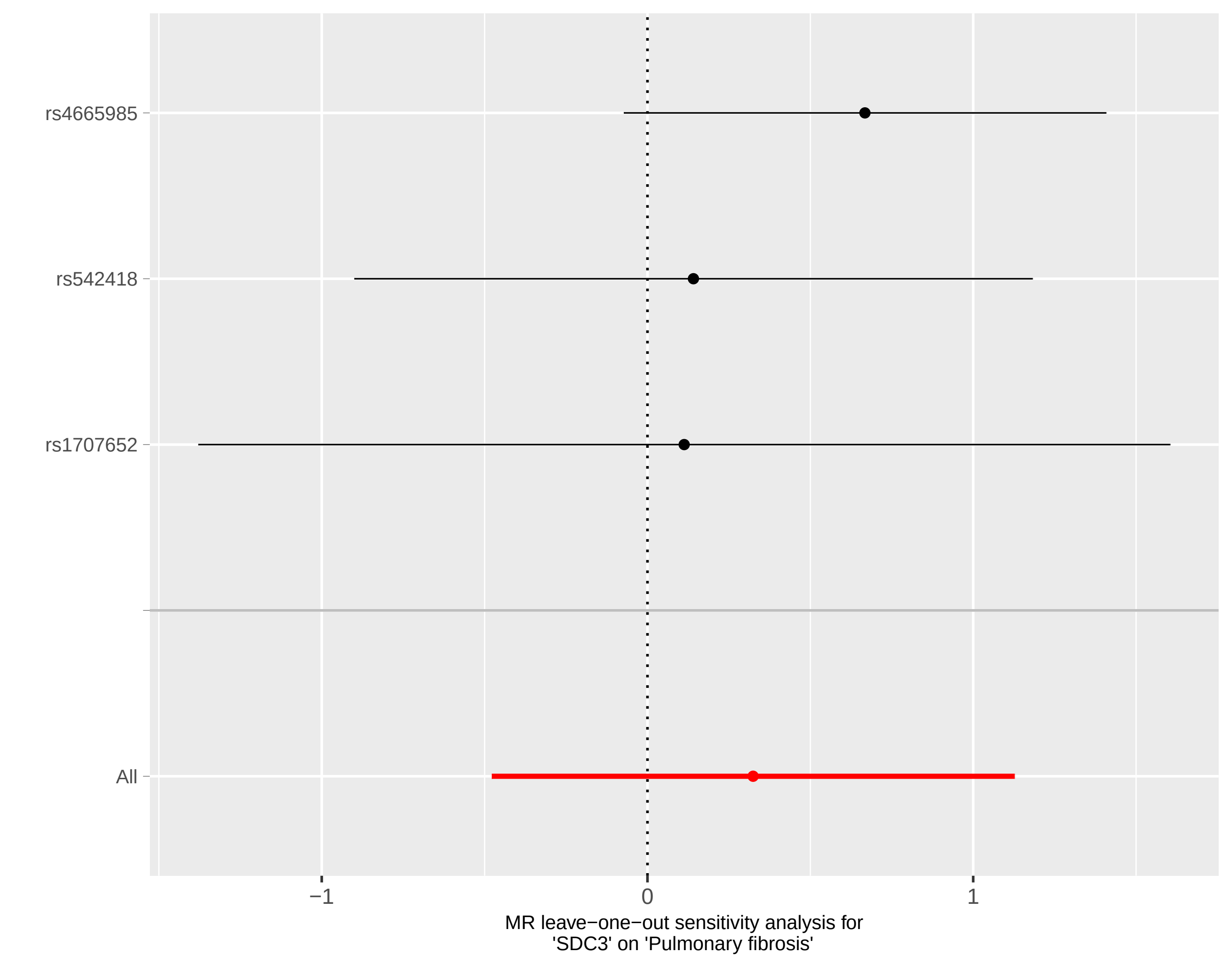
rs4665985
rs542418
rs1707652
All
−1
0
1
MR leave−one−out sensitivity analysis for
'SDC3' on 'Pulmonary fibrosis'

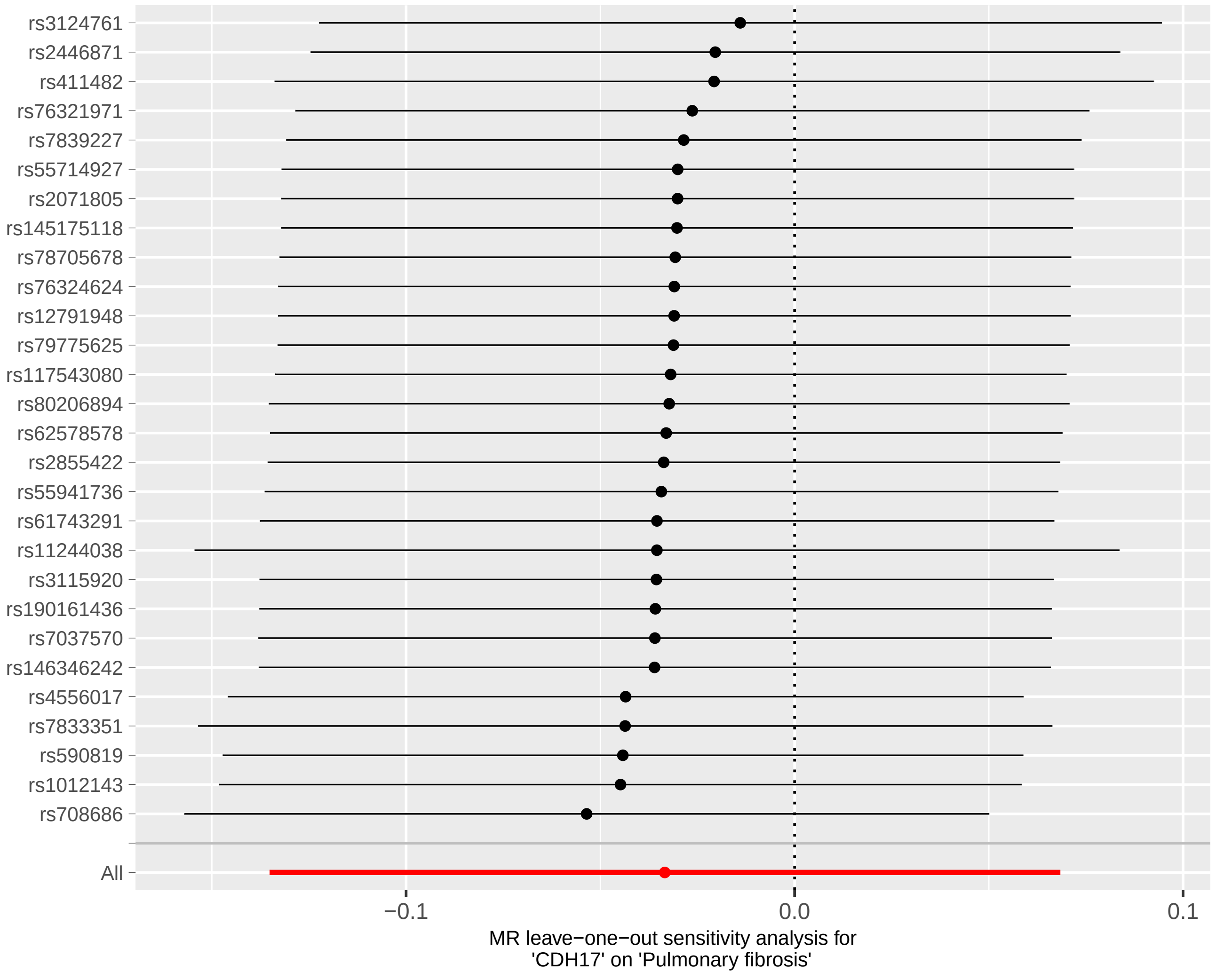
rs3124761
rs2446871
rs411482
rs76321971
rs7839227
rs55714927
rs2071805
rs145175118
rs78705678
rs76324624
rs12791948
rs79775625
rs117543080
rs80206894
rs62578578
rs2855422
rs55941736
rs61743291
rs11244038
rs3115920
rs190161436
rs7037570
rs146346242
rs4556017
rs7833351
rs590819
rs1012143
rs708686
All
−0.1
0.0
0.1
MR leave−one−out sensitivity analysis for
'CDH17' on 'Pulmonary fibrosis'

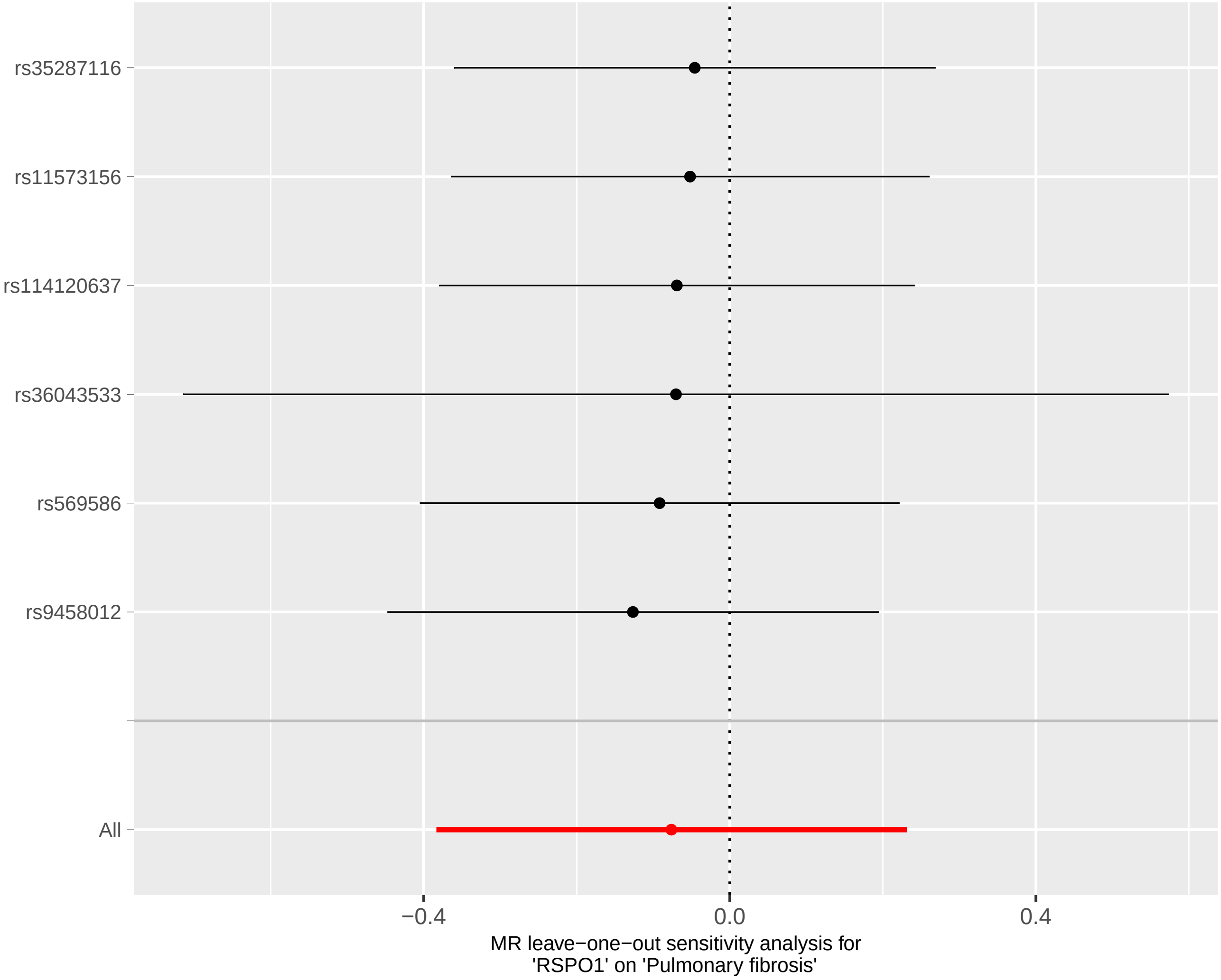
rs35287116
rs11573156
rs114120637
rs36043533
rs569586
rs9458012
All
−0.4
0.0
0.4
MR leave−one−out sensitivity analysis for
'RSPO1' on 'Pulmonary fibrosis'

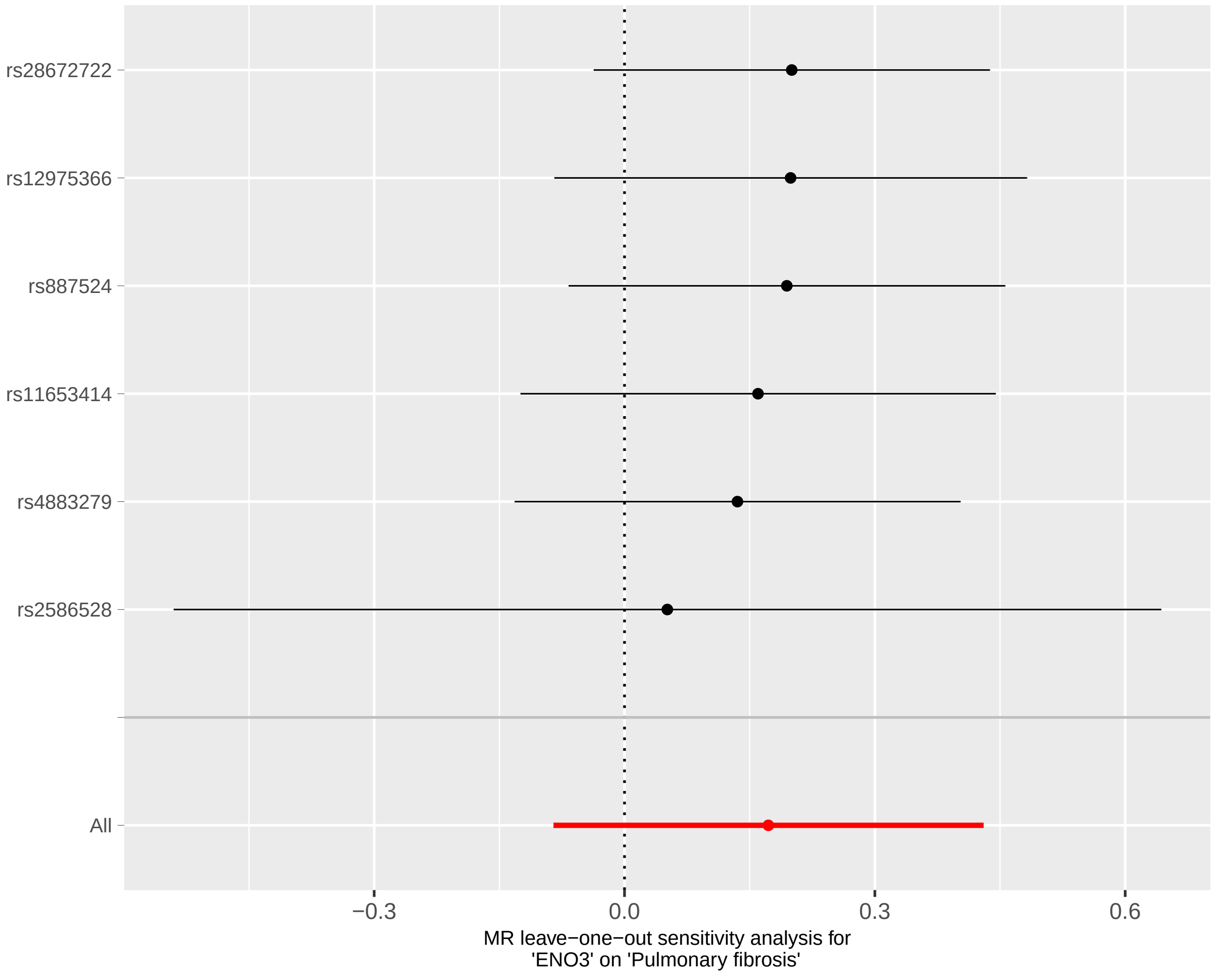
rs28672722
rs12975366
rs887524
rs11653414
rs4883279
rs2586528
All
−0.3
0.0
0.3
0.6
MR leave−one−out sensitivity analysis for
'ENO3' on 'Pulmonary fibrosis'

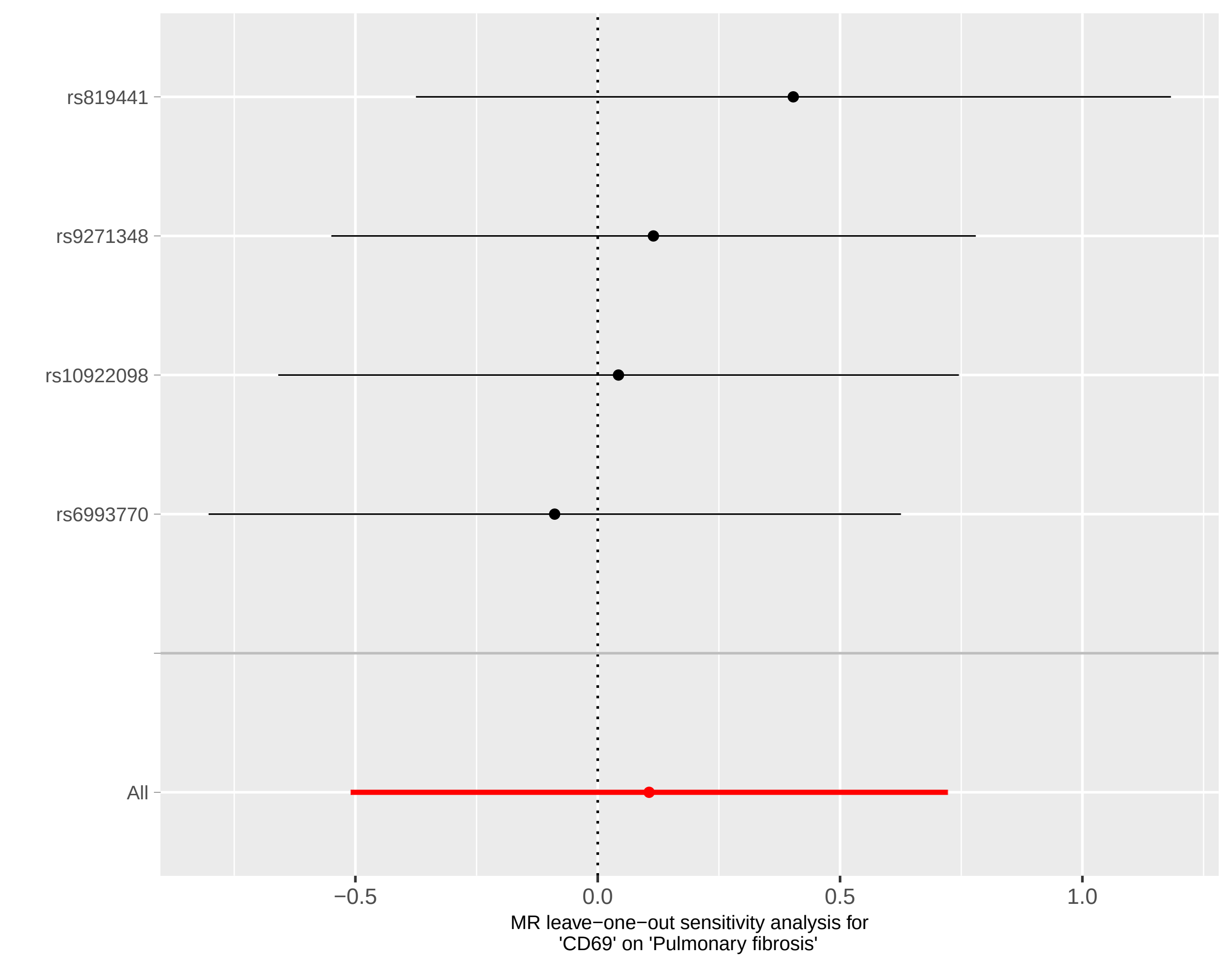

rs819441
rs9271348
rs10922098
rs6993770
All
−0.5
0.0
0.5
1.0
MR leave−one−out sensitivity analysis for
'CD69' on 'Pulmonary fibrosis'

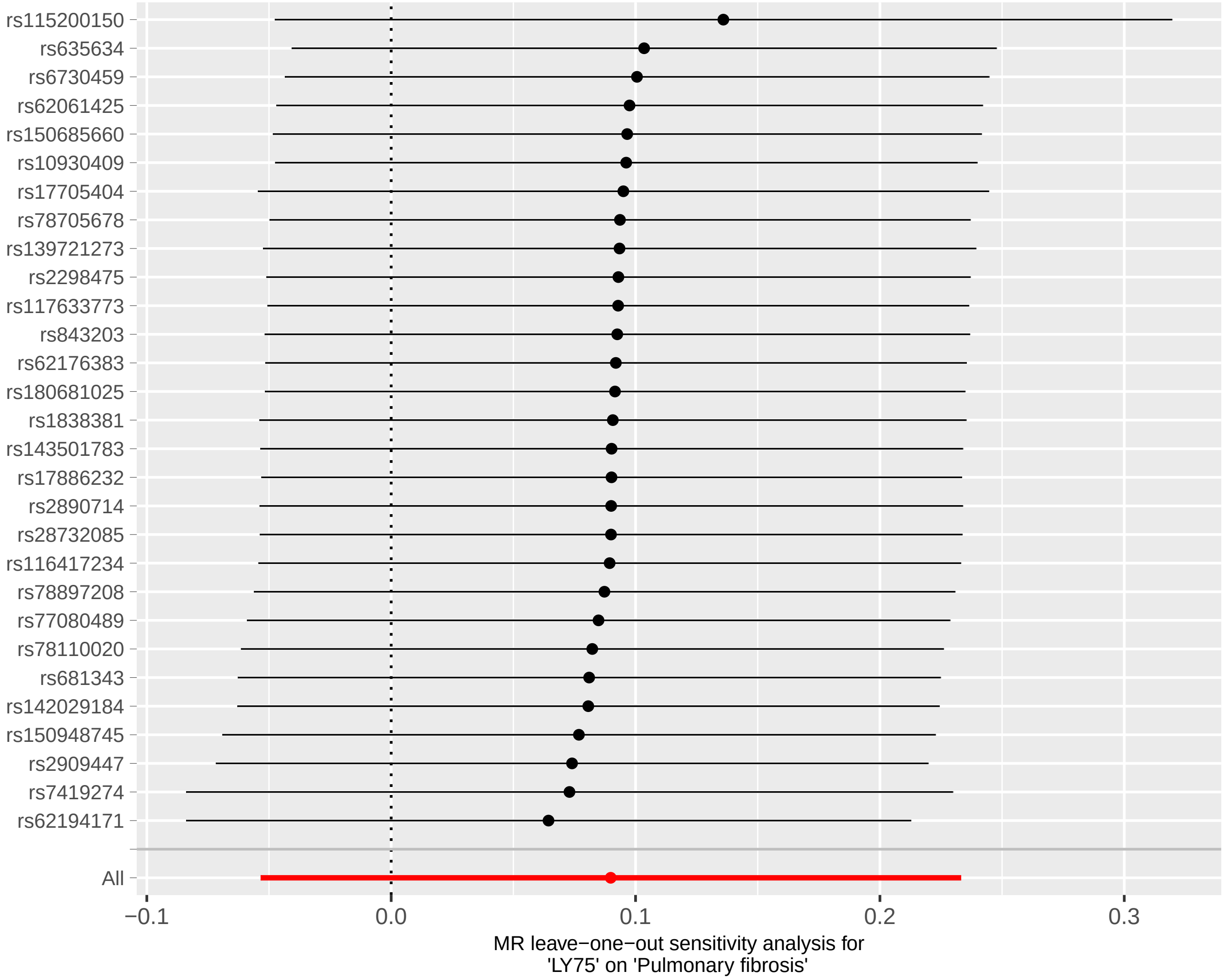
rs115200150
rs635634
rs6730459
rs62061425
rs150685660
rs10930409
rs17705404
rs78705678
rs139721273
rs2298475
rs117633773
rs843203
rs62176383
rs180681025
rs1838381
rs143501783
rs17886232
rs2890714
rs28732085
rs116417234
rs78897208
rs77080489
rs78110020
rs681343
rs142029184
rs150948745
rs2909447
rs7419274
rs62194171
All
−0.1
0.0
0.1
0.2
0.3
MR leave−one−out sensitivity analysis for
'LY75' on 'Pulmonary fibrosis'

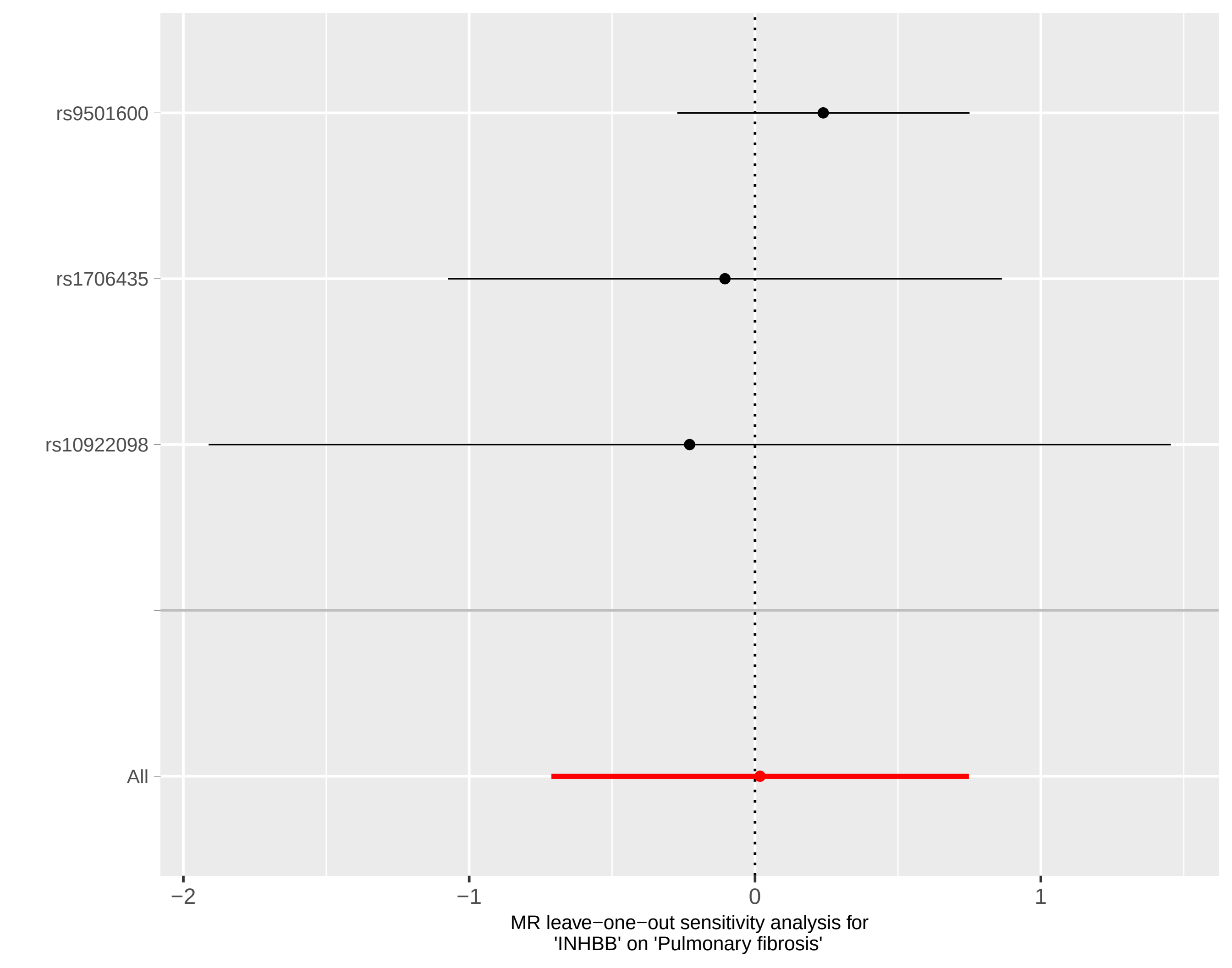
rs9501600
rs1706435
rs10922098
All
−2
−1
0
1
MR leave−one−out sensitivity analysis for
'INHBB' on 'Pulmonary fibrosis'

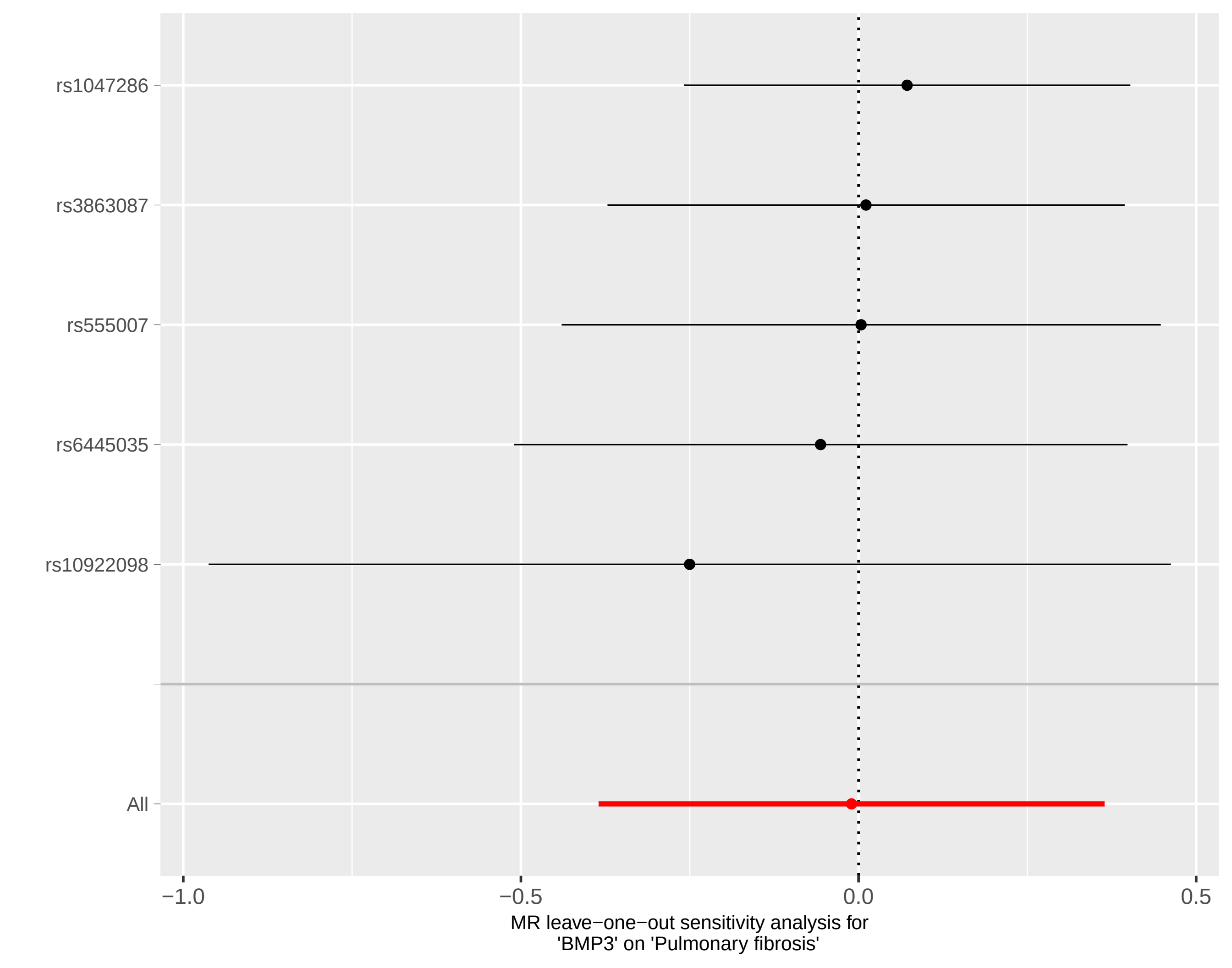
rs1047286
rs3863087
rs555007
rs6445035
rs10922098
All
−1.0
−0.5
0.0
0.5
MR leave−one−out sensitivity analysis for
'BMP3' on 'Pulmonary fibrosis'

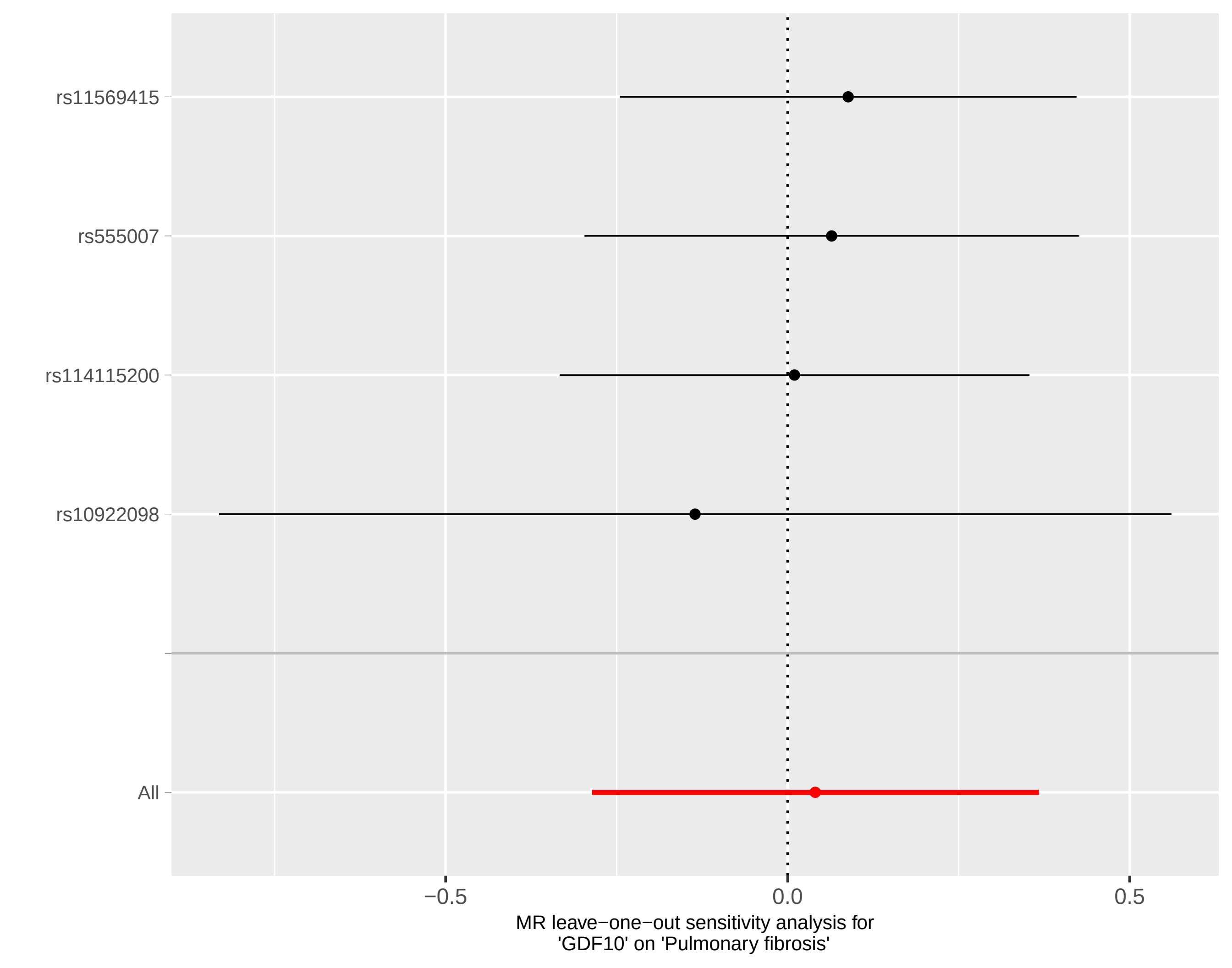
rs11569415
rs555007
rs114115200
rs10922098
All
−0.5
0.0
0.5
MR leave−one−out sensitivity analysis for
'GDF10' on 'Pulmonary fibrosis'

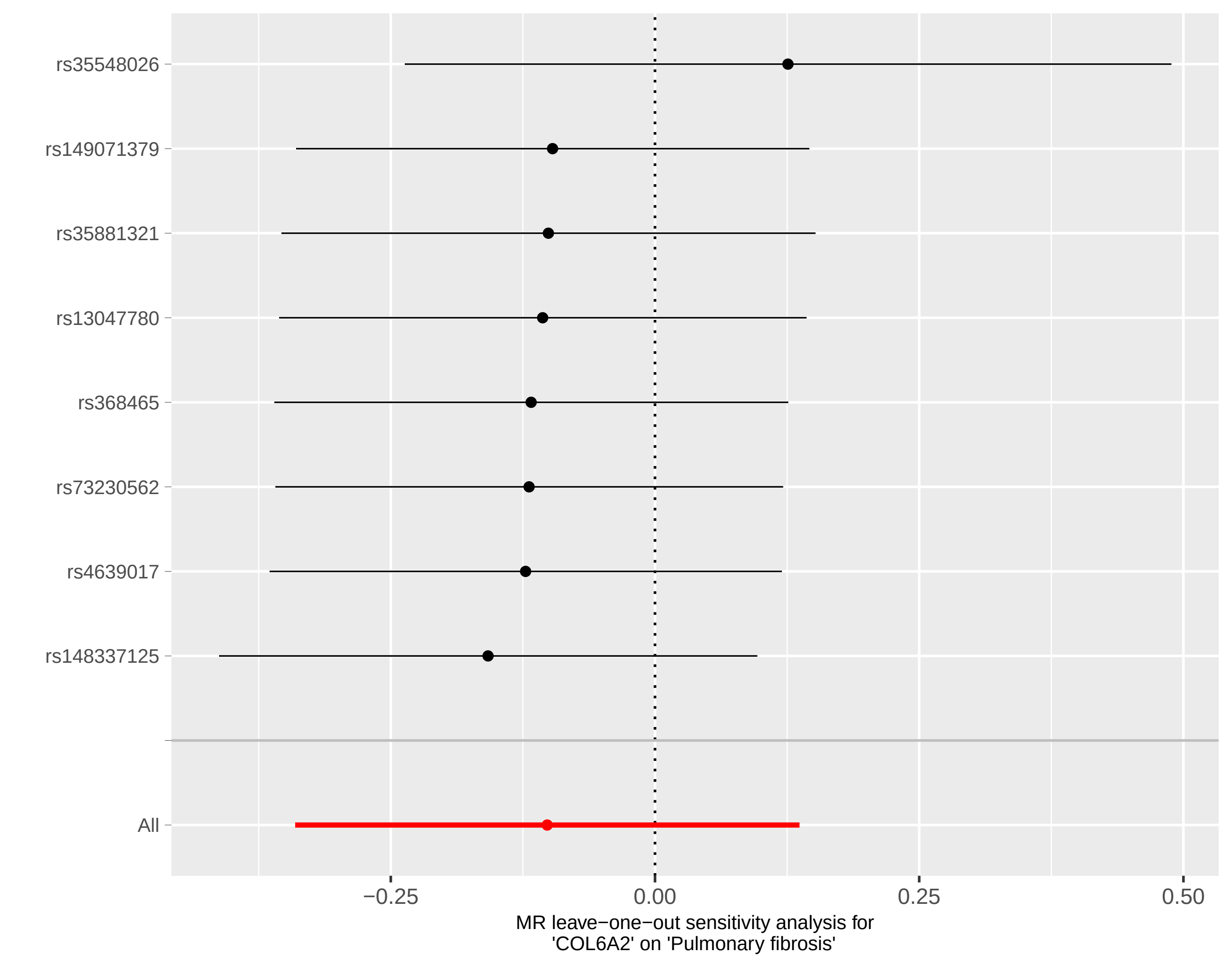

rs35548026
rs149071379
rs35881321
rs13047780
rs368465
rs73230562
rs4639017
rs148337125
All
−0.25
0.00
0.25
0.50
MR leave−one−out sensitivity analysis for
'COL6A2' on 'Pulmonary fibrosis'

Insufficient number of SNPs

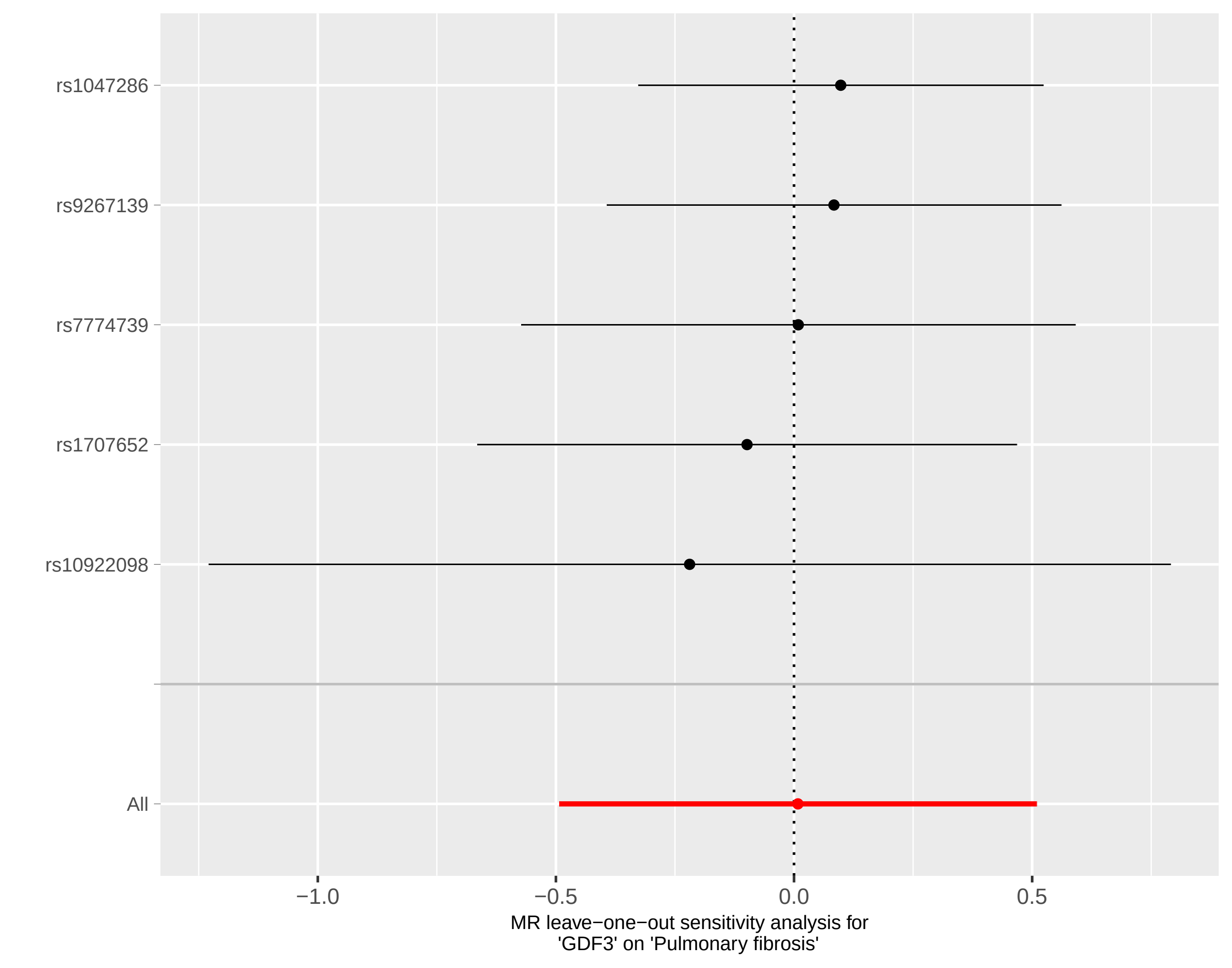
rs1047286
rs9267139
rs7774739
rs1707652
rs10922098
All
−1.0
−0.5
0.0
0.5
MR leave−one−out sensitivity analysis for
'GDF3' on 'Pulmonary fibrosis'

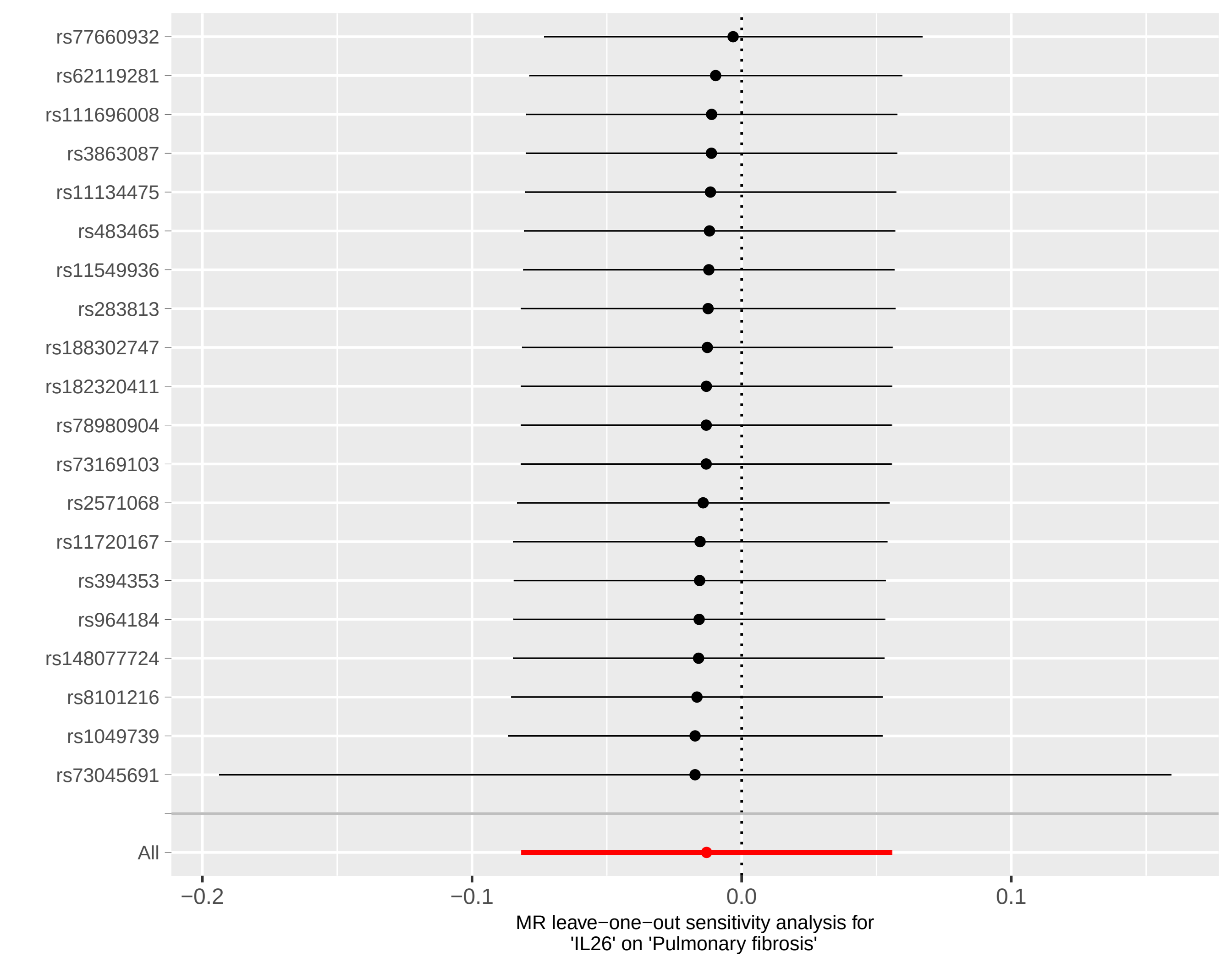
rs77660932
rs62119281
rs111696008
rs3863087
rs11134475
rs483465
rs11549936
rs283813
rs188302747
rs182320411
rs78980904
rs73169103
rs2571068
rs11720167
rs394353
rs964184
rs148077724
rs8101216
rs1049739
rs73045691
All
−0.2
−0.1
0.0
0.1
MR leave−one−out sensitivity analysis for
'IL26' on 'Pulmonary fibrosis'

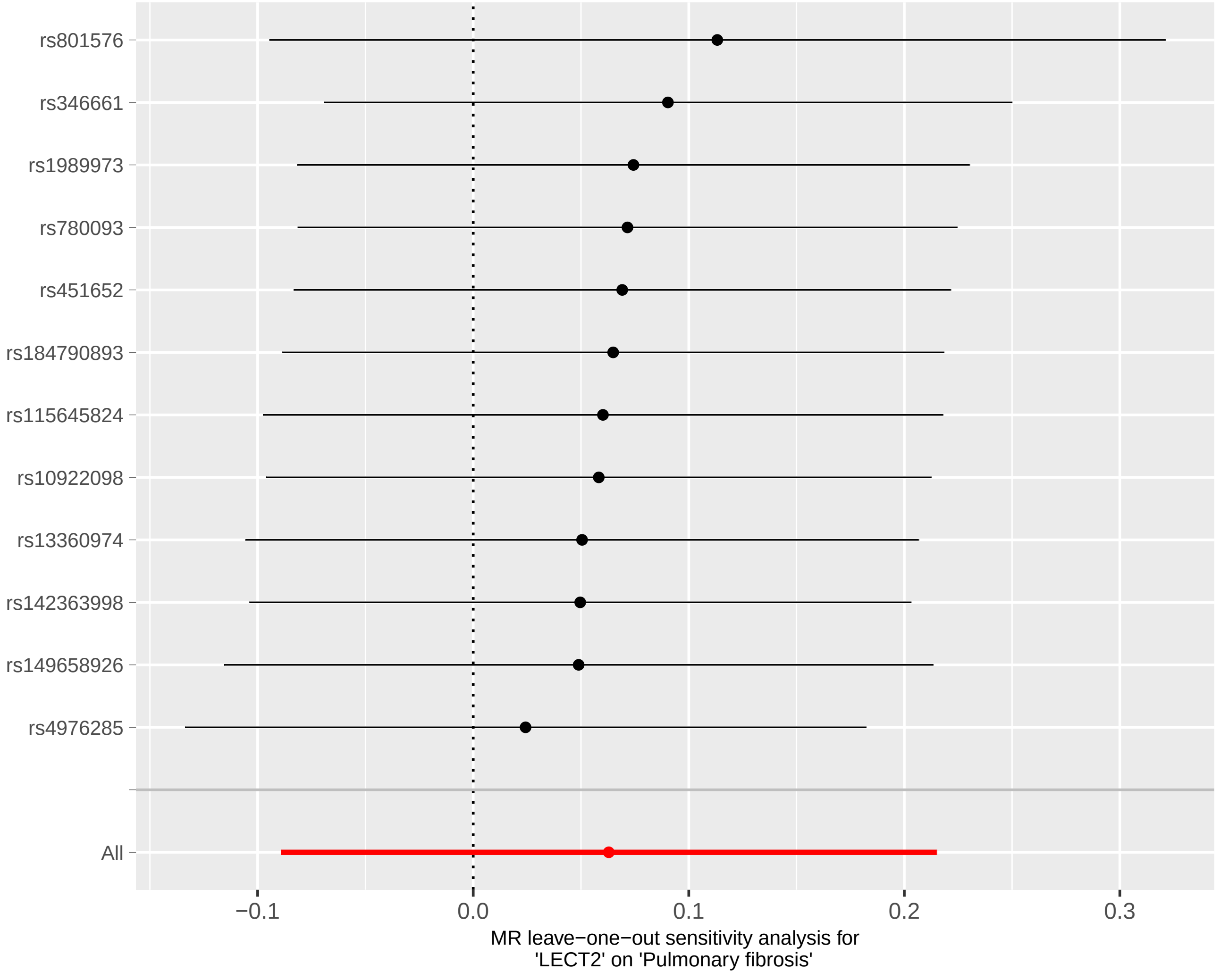
rs801576
rs346661
rs1989973
rs780093
rs451652
rs184790893
rs115645824
rs10922098
rs13360974
rs142363998
rs149658926
rs4976285
All
−0.1
0.0
0.1
0.2
0.3
MR leave−one−out sensitivity analysis for
'LECT2' on 'Pulmonary fibrosis'

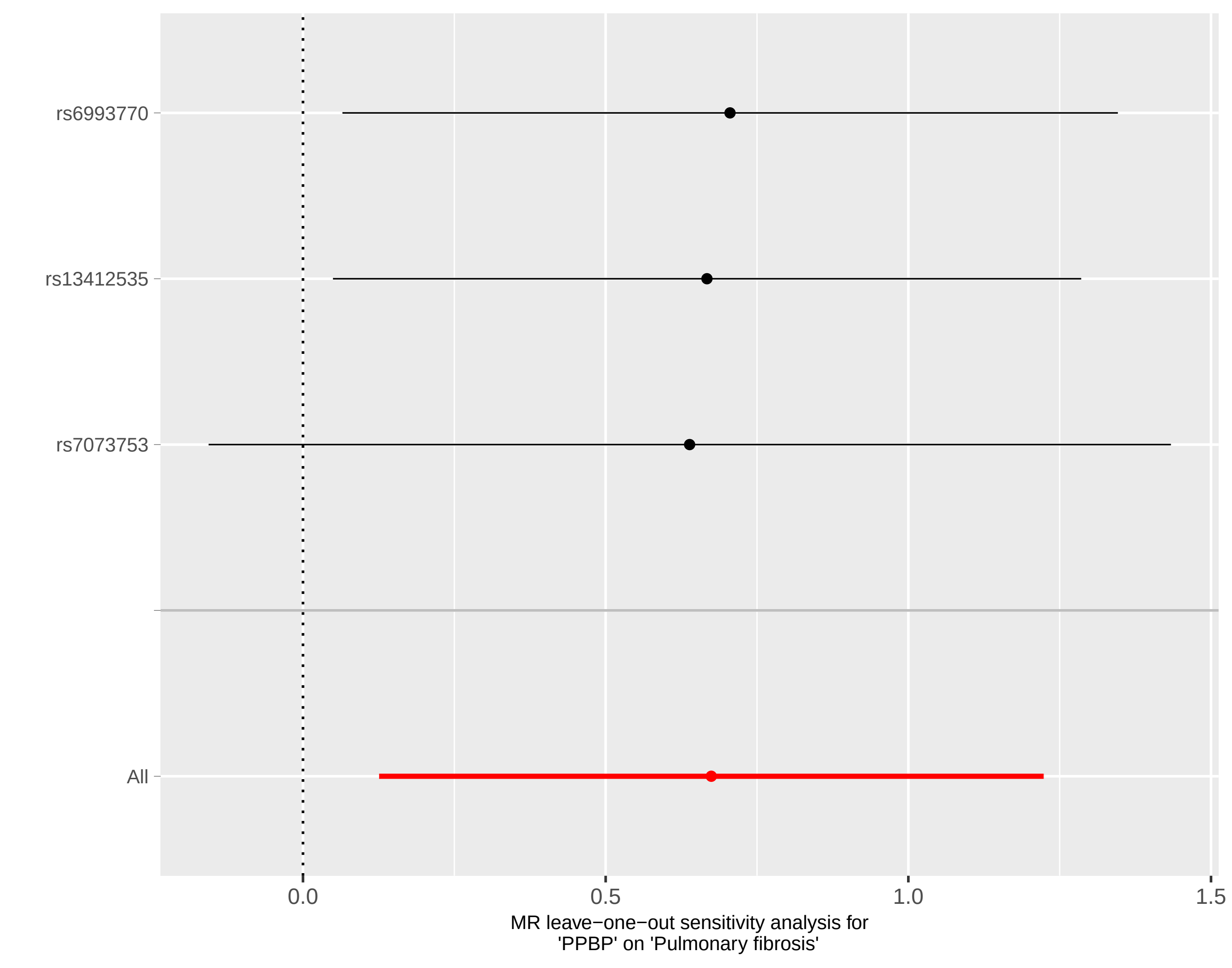
rs6993770
rs13412535
rs7073753
All
0.0
0.5
1.0
1.5
MR leave−one−out sensitivity analysis for
'PPBP' on 'Pulmonary fibrosis'

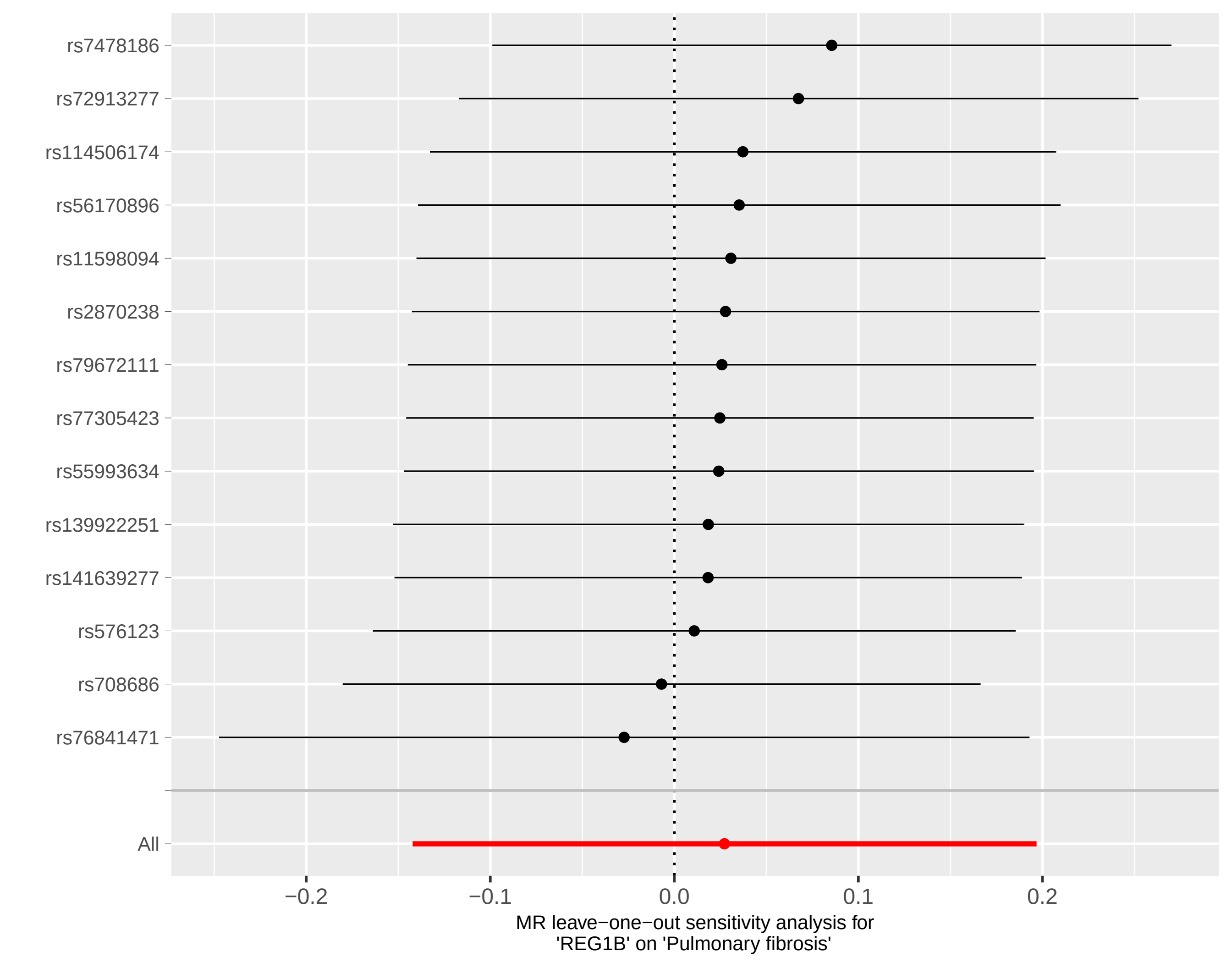
rs7478186
rs72913277
rs114506174
rs56170896
rs11598094
rs2870238
rs79672111
rs77305423
rs55993634
rs139922251
rs141639277
rs576123
rs708686
rs76841471
All
−0.2
−0.1
0.0
0.1
0.2
MR leave−one−out sensitivity analysis for
'REG1B' on 'Pulmonary fibrosis'

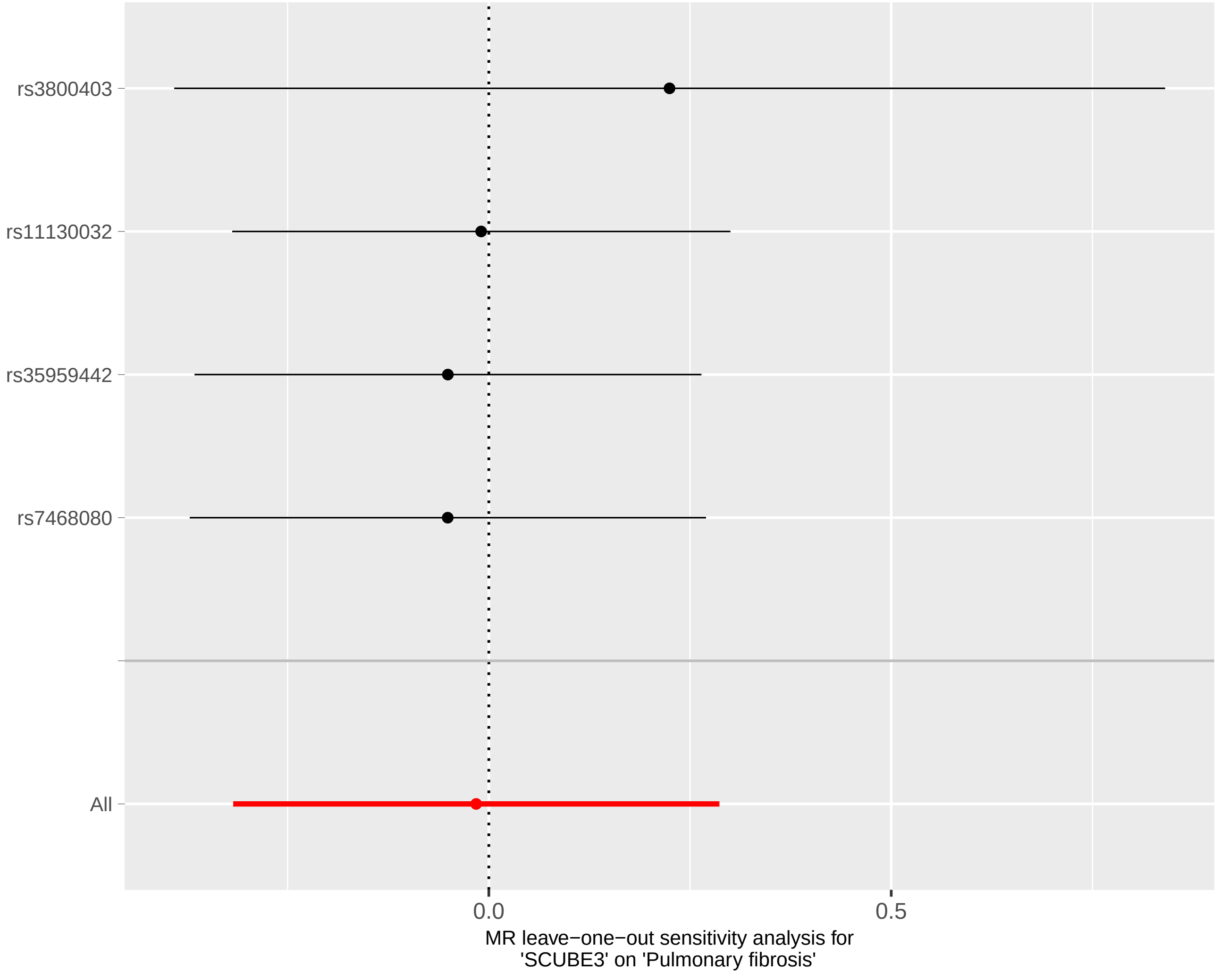
rs3800403
rs11130032
rs35959442
rs7468080
All
0.0
0.5
MR leave−one−out sensitivity analysis for
'SCUBE3' on 'Pulmonary fibrosis'

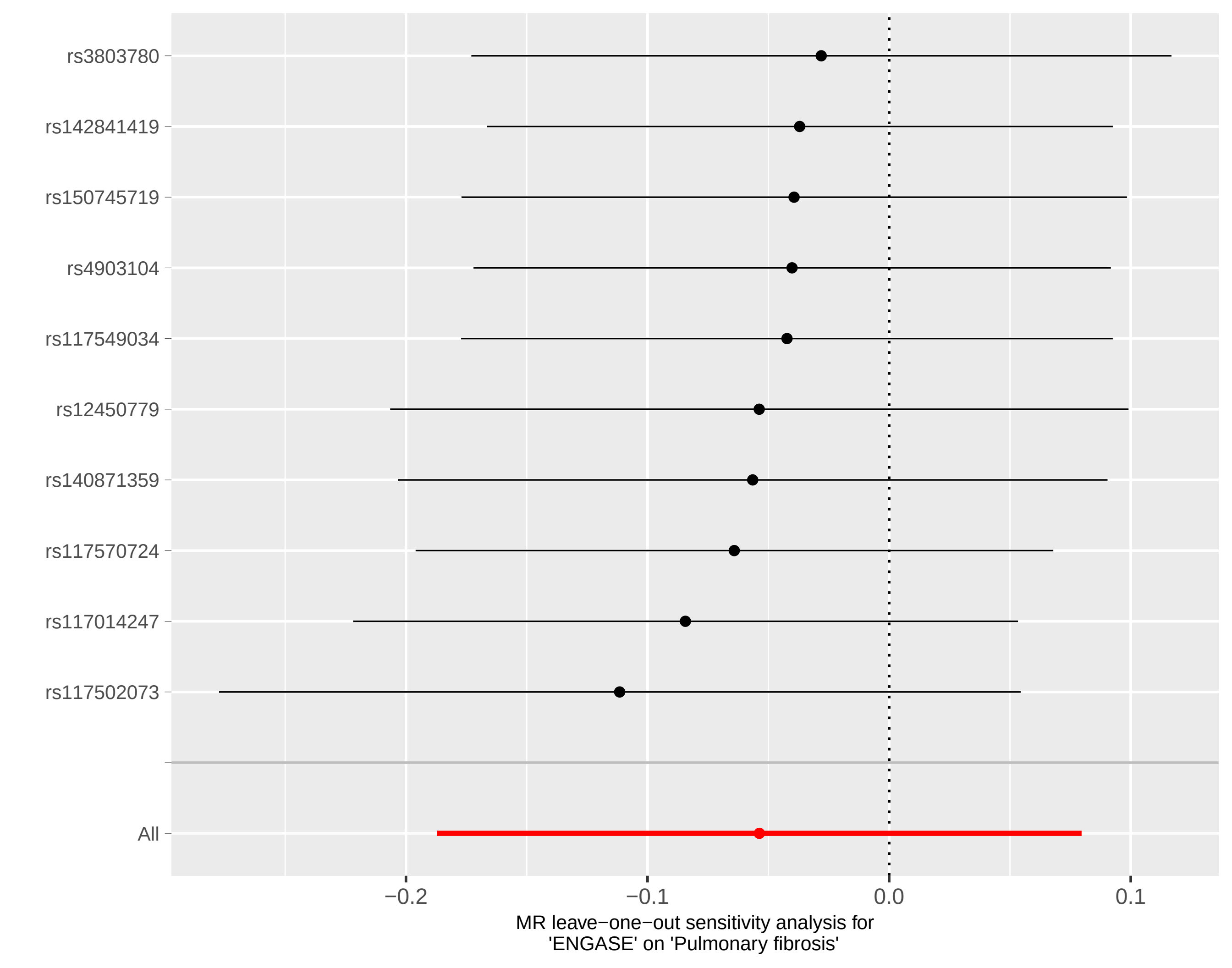
rs3803780
rs142841419
rs150745719
rs4903104
rs117549034
rs12450779
rs140871359
rs117570724
rs117014247
rs117502073
All
−0.2
−0.1
0.0
0.1
MR leave−one−out sensitivity analysis for
'ENGASE' on 'Pulmonary fibrosis'

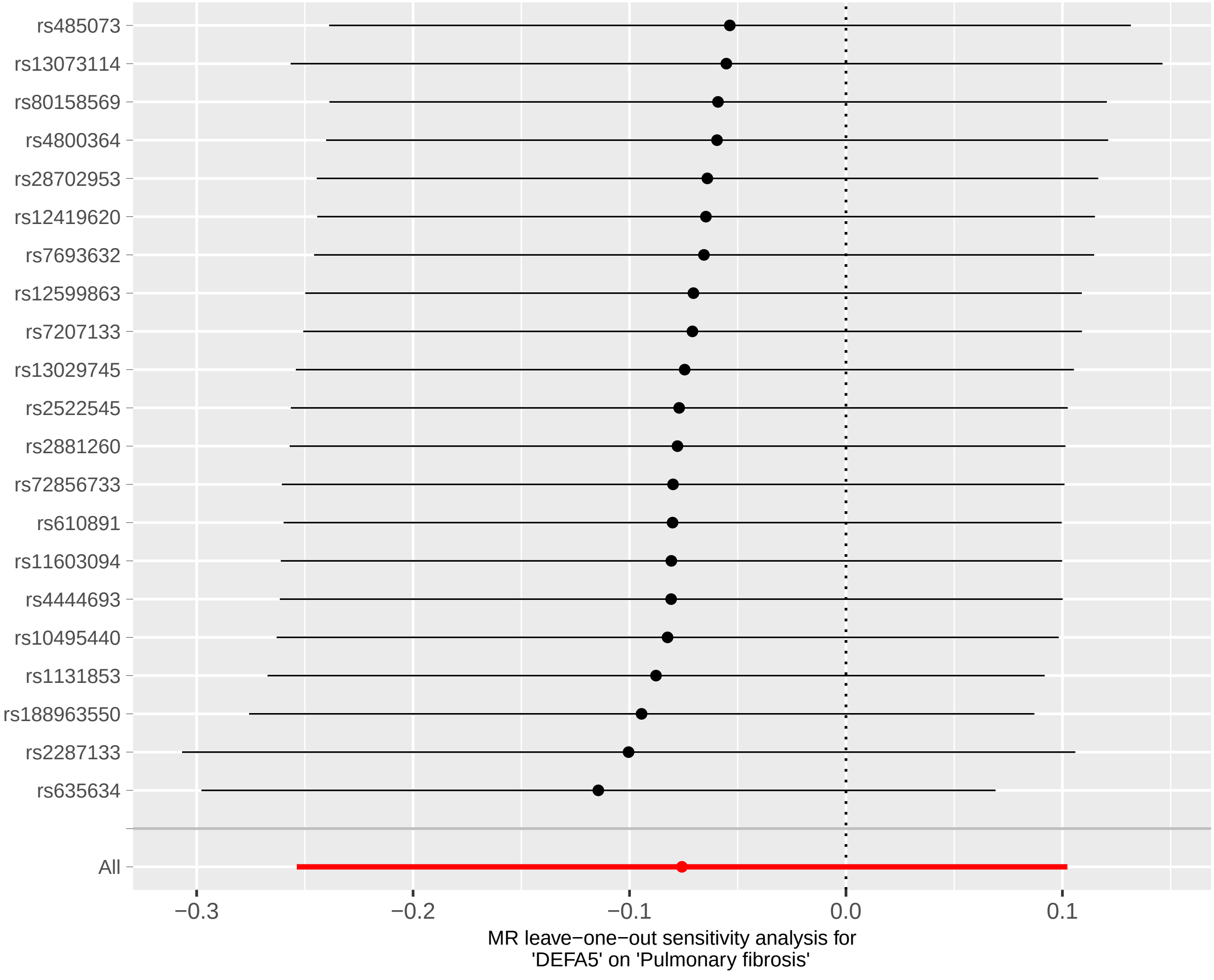
rs485073
rs13073114
rs80158569
rs4800364
rs28702953
rs12419620
rs7693632
rs12599863
rs7207133
rs13029745
rs2522545
rs2881260
rs72856733
rs610891
rs11603094
rs4444693
rs10495440
rs1131853
rs188963550
rs2287133
rs635634
All
−0.3
−0.2
−0.1
0.0
0.1
MR leave−one−out sensitivity analysis for
'DEFA5' on 'Pulmonary fibrosis'

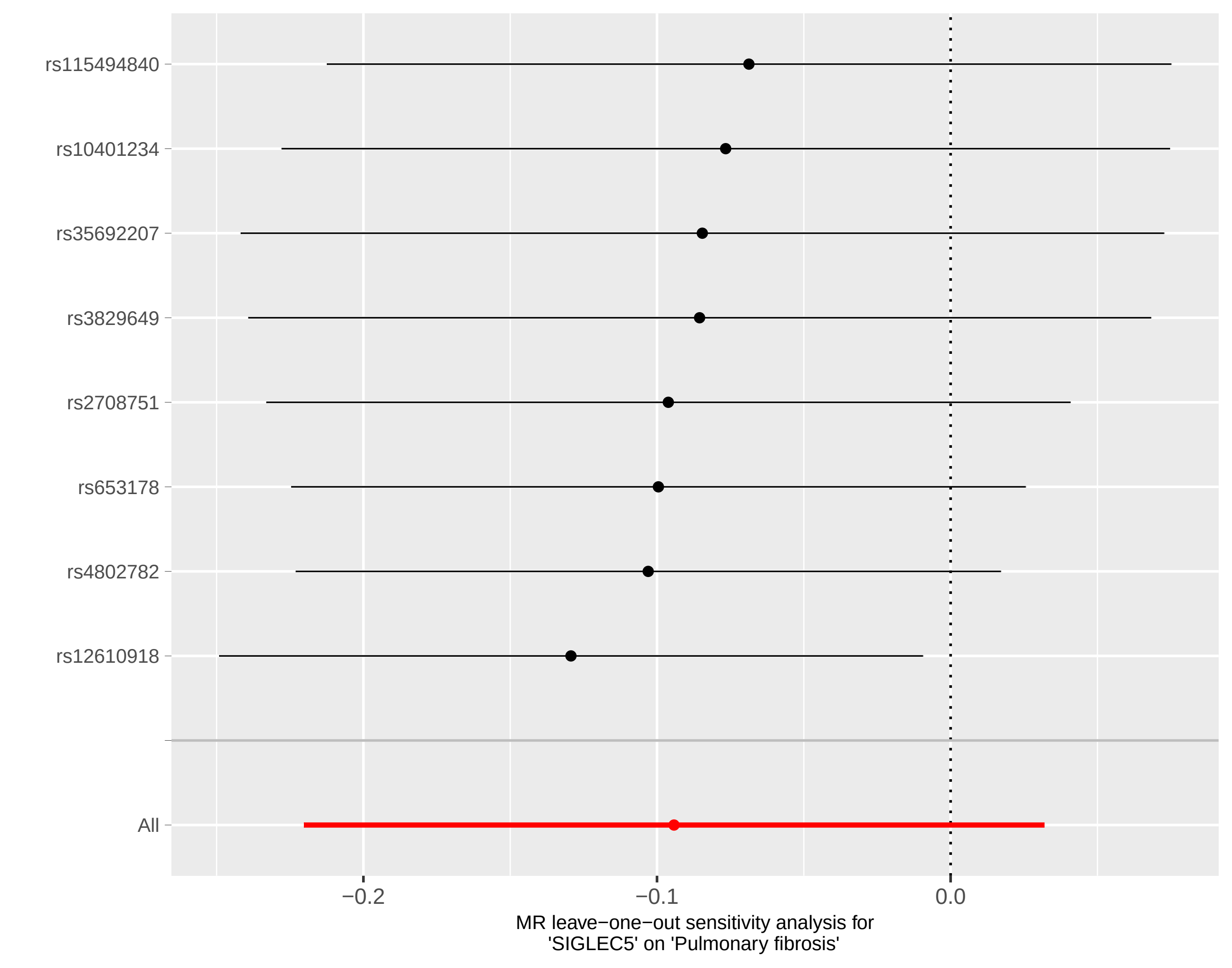
rs115494840
rs10401234
rs35692207
rs3829649
rs2708751
rs653178
rs4802782
rs12610918
All
−0.2
−0.1
0.0
MR leave−one−out sensitivity analysis for
'SIGLEC5' on 'Pulmonary fibrosis'

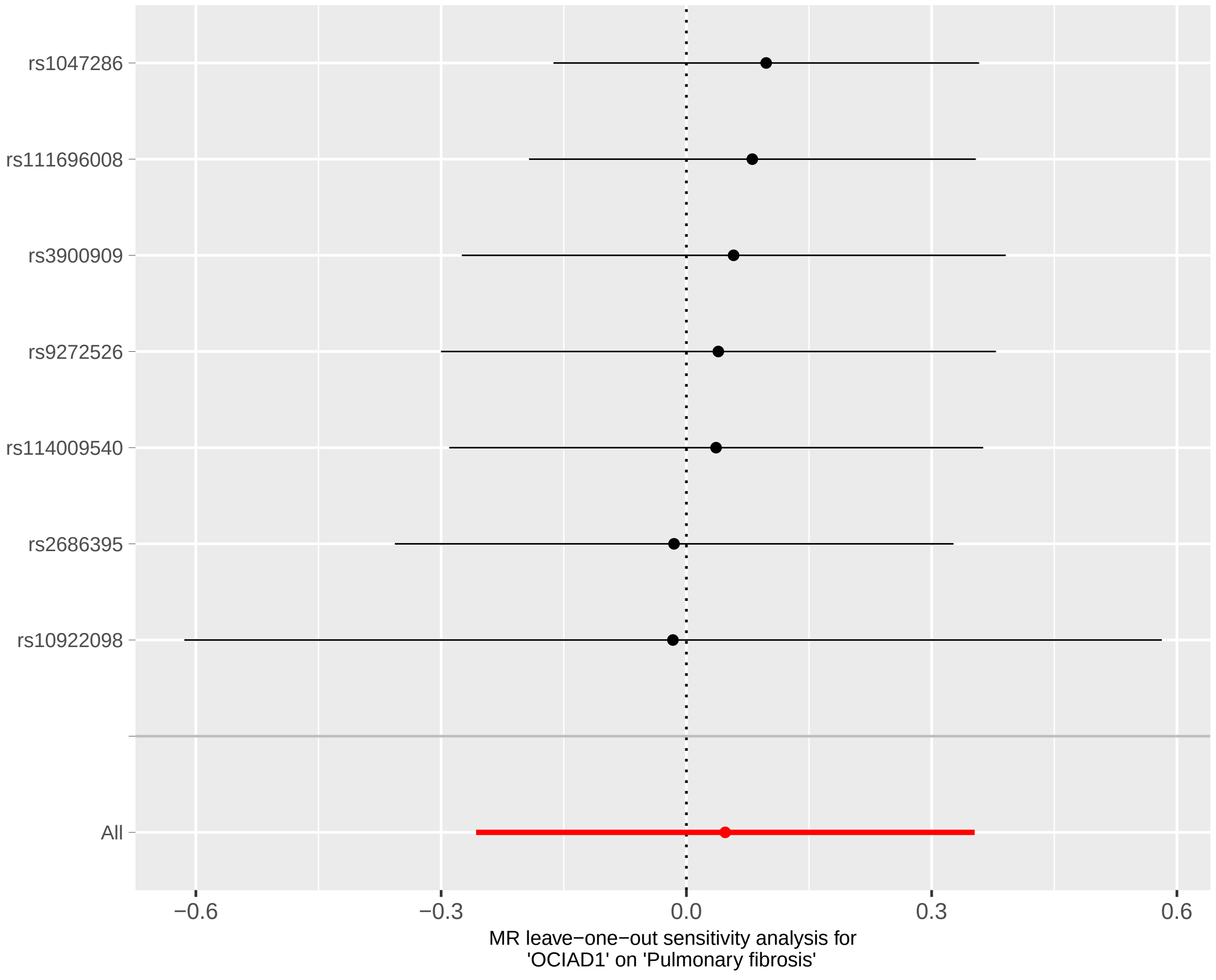

rs1047286
rs111696008
rs3900909
rs9272526
rs114009540
rs2686395
rs10922098
All
−0.6
−0.3
0.0
0.3
0.6
MR leave−one−out sensitivity analysis for
'OCIAD1' on 'Pulmonary fibrosis'

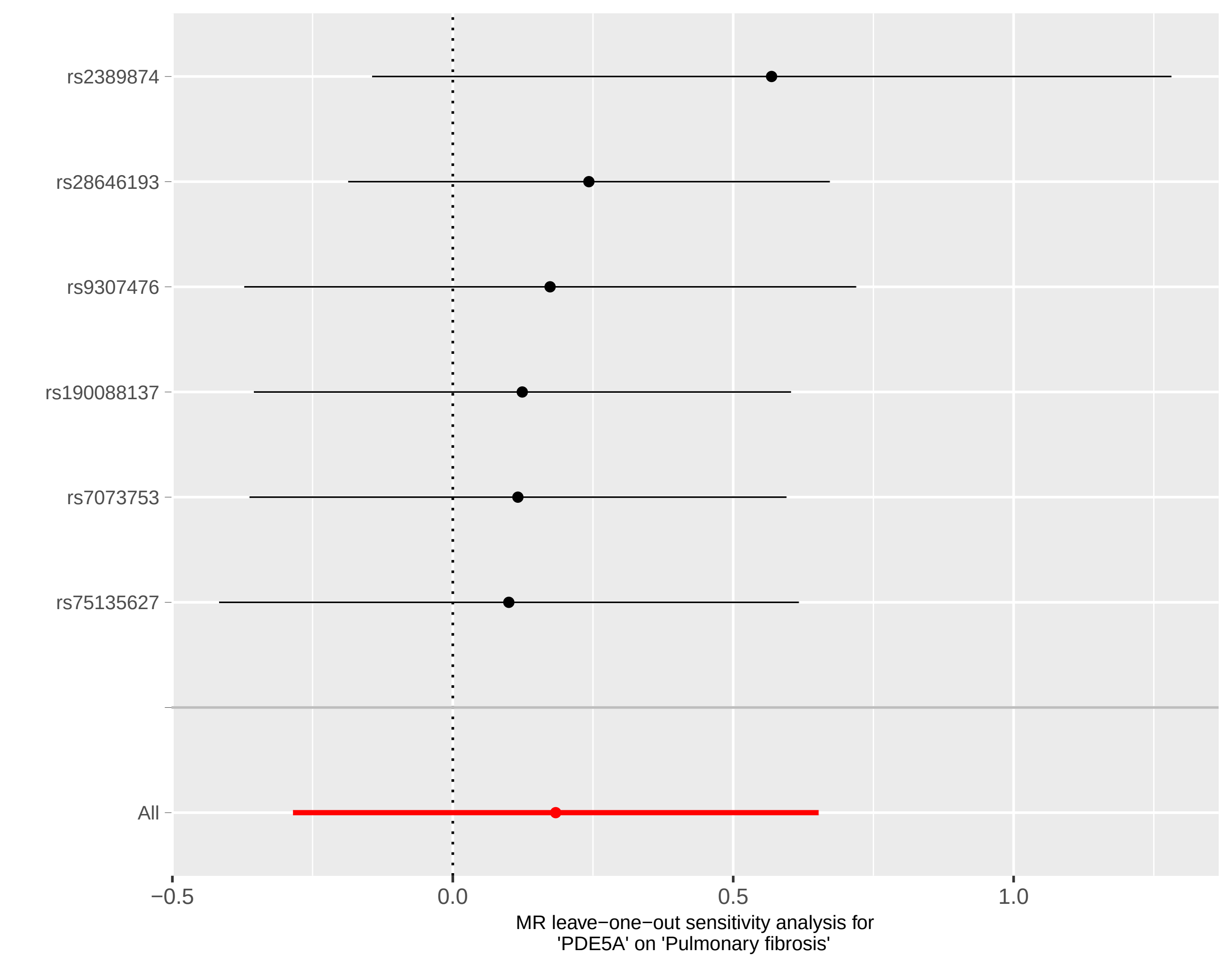
rs2389874
rs28646193
rs9307476
rs190088137
rs7073753
rs75135627
All
−0.5
0.0
0.5
1.0
MR leave−one−out sensitivity analysis for
'PDE5A' on 'Pulmonary fibrosis'

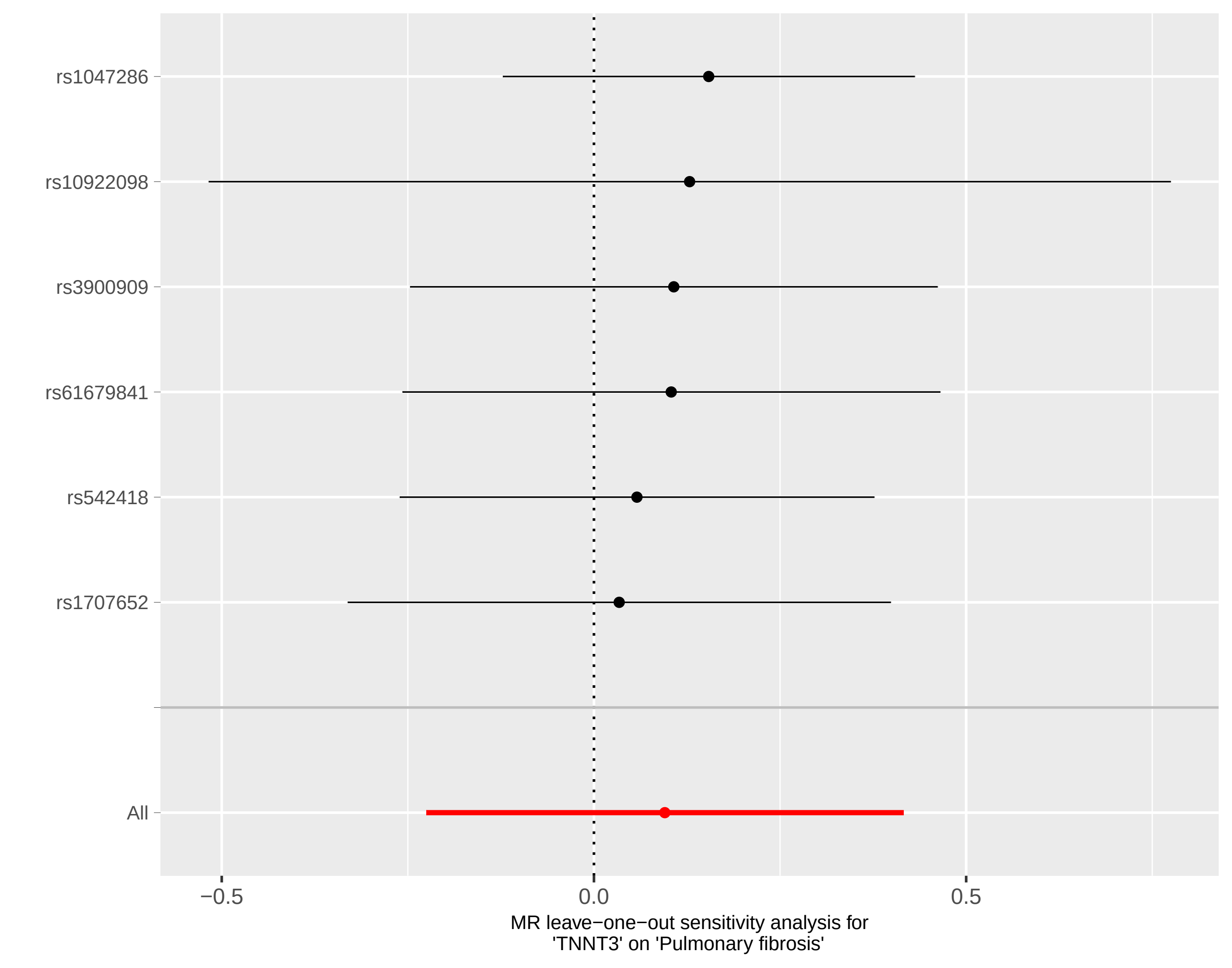
rs1047286
rs10922098
rs3900909
rs61679841
rs542418
rs1707652
All
−0.5
0.0
0.5
MR leave−one−out sensitivity analysis for
'TNNT3' on 'Pulmonary fibrosis'

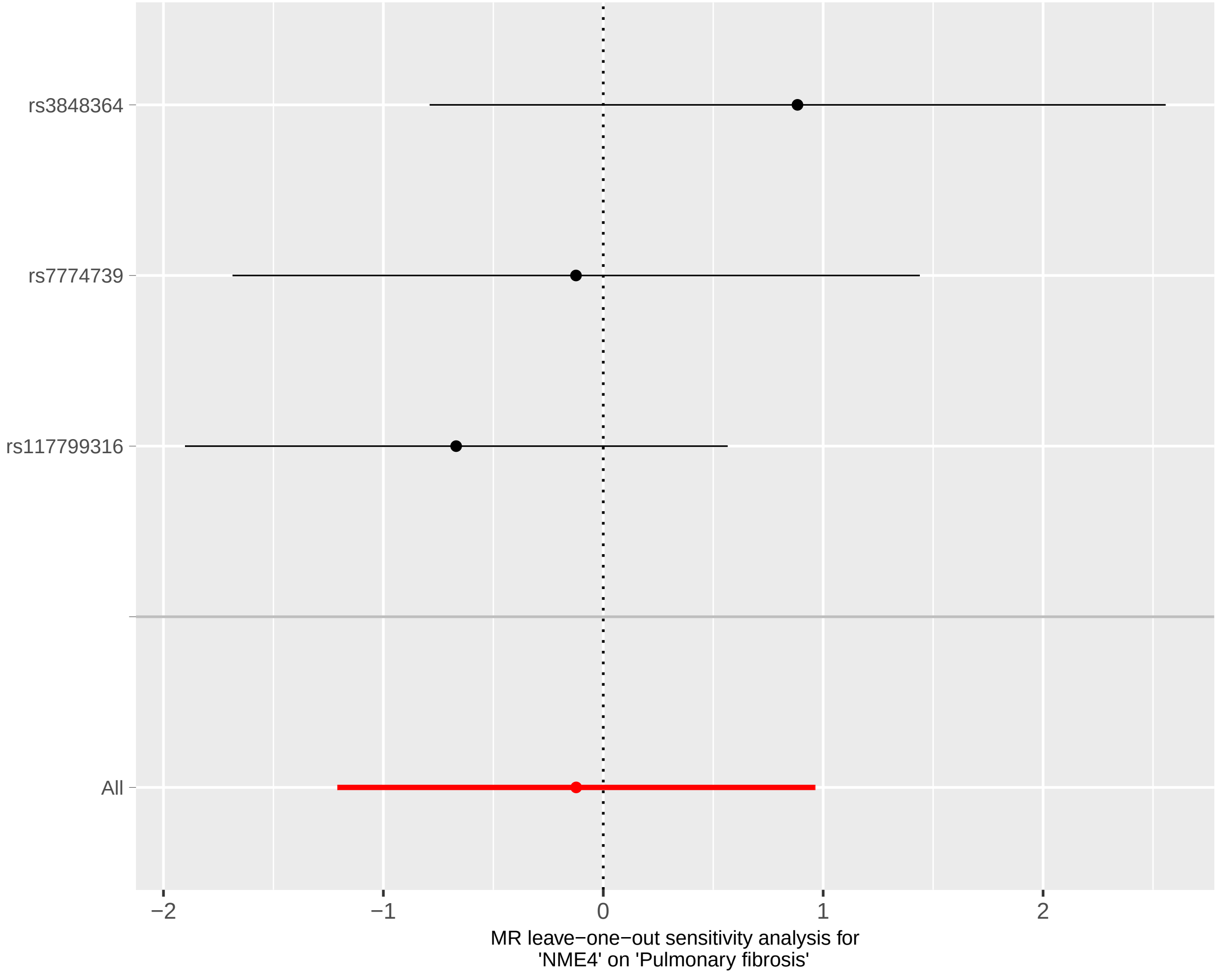
rs3848364
rs7774739
rs117799316
All
−2
−1
0
1
2
MR leave−one−out sensitivity analysis for
'NME4' on 'Pulmonary fibrosis'

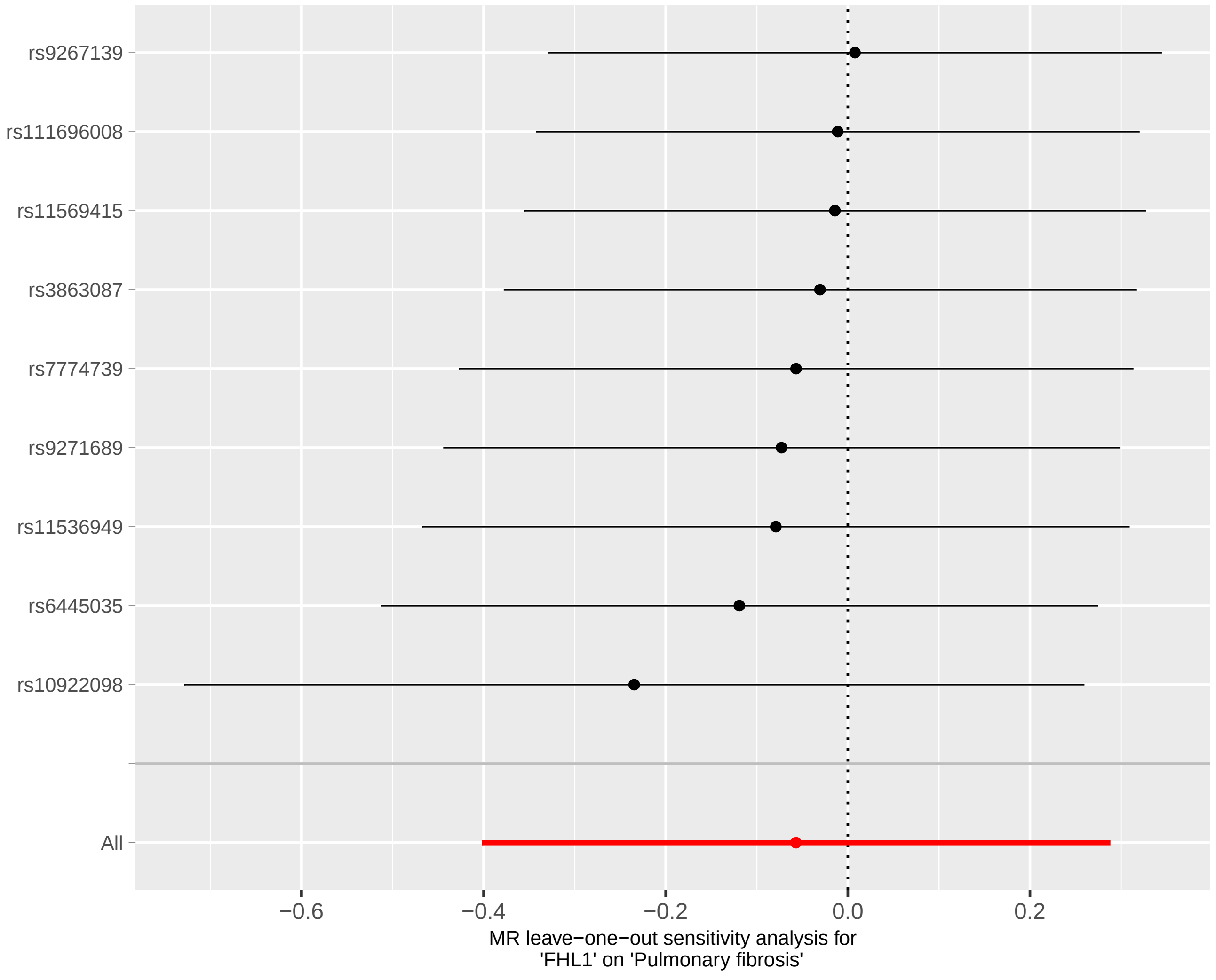

rs9267139
rs111696008
rs11569415
rs3863087
rs7774739
rs9271689
rs11536949
rs6445035
rs10922098
All
−0.6
−0.4
−0.2
0.0
0.2
MR leave−one−out sensitivity analysis for
'FHL1' on 'Pulmonary fibrosis'

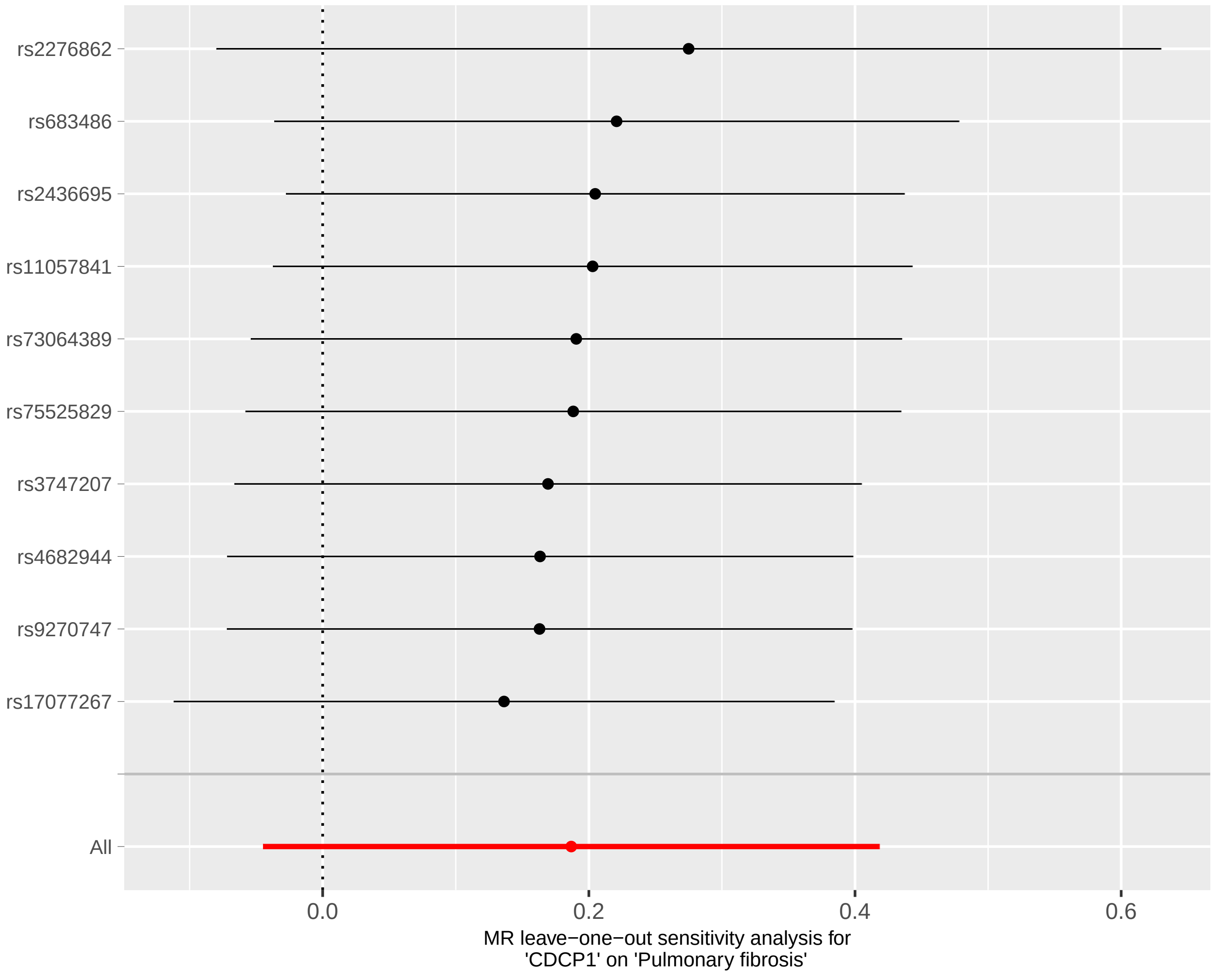

rs2276862
rs683486
rs2436695
rs11057841
rs73064389
rs75525829
rs3747207
rs4682944
rs9270747
rs17077267
All
0.0
0.2
0.4
0.6
MR leave−one−out sensitivity analysis for
'CDCP1' on 'Pulmonary fibrosis'

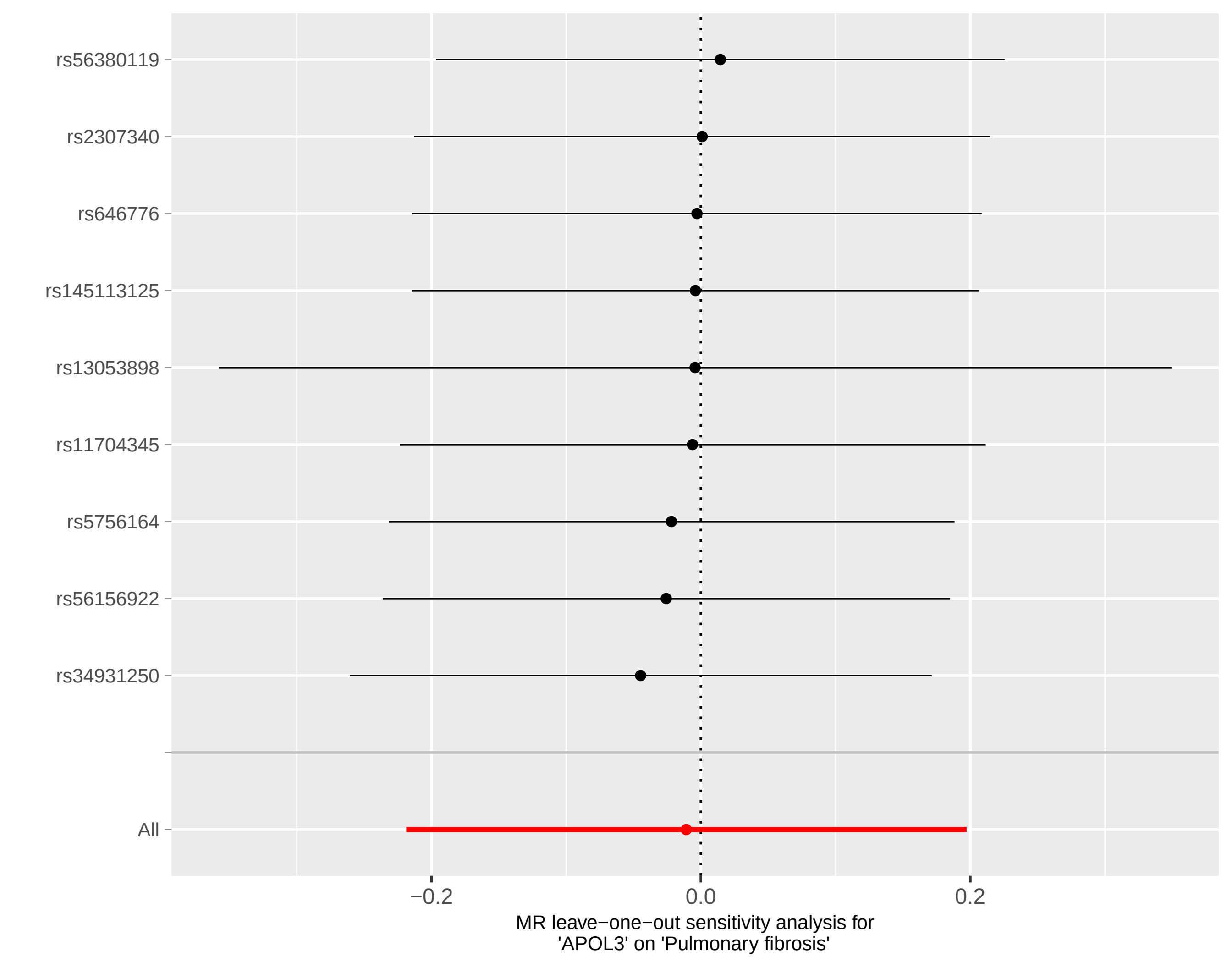
rs56380119
rs2307340
rs646776
rs145113125
rs13053898
rs11704345
rs5756164
rs56156922
rs34931250
All
−0.2
0.0
0.2
MR leave−one−out sensitivity analysis for
'APOL3' on 'Pulmonary fibrosis'

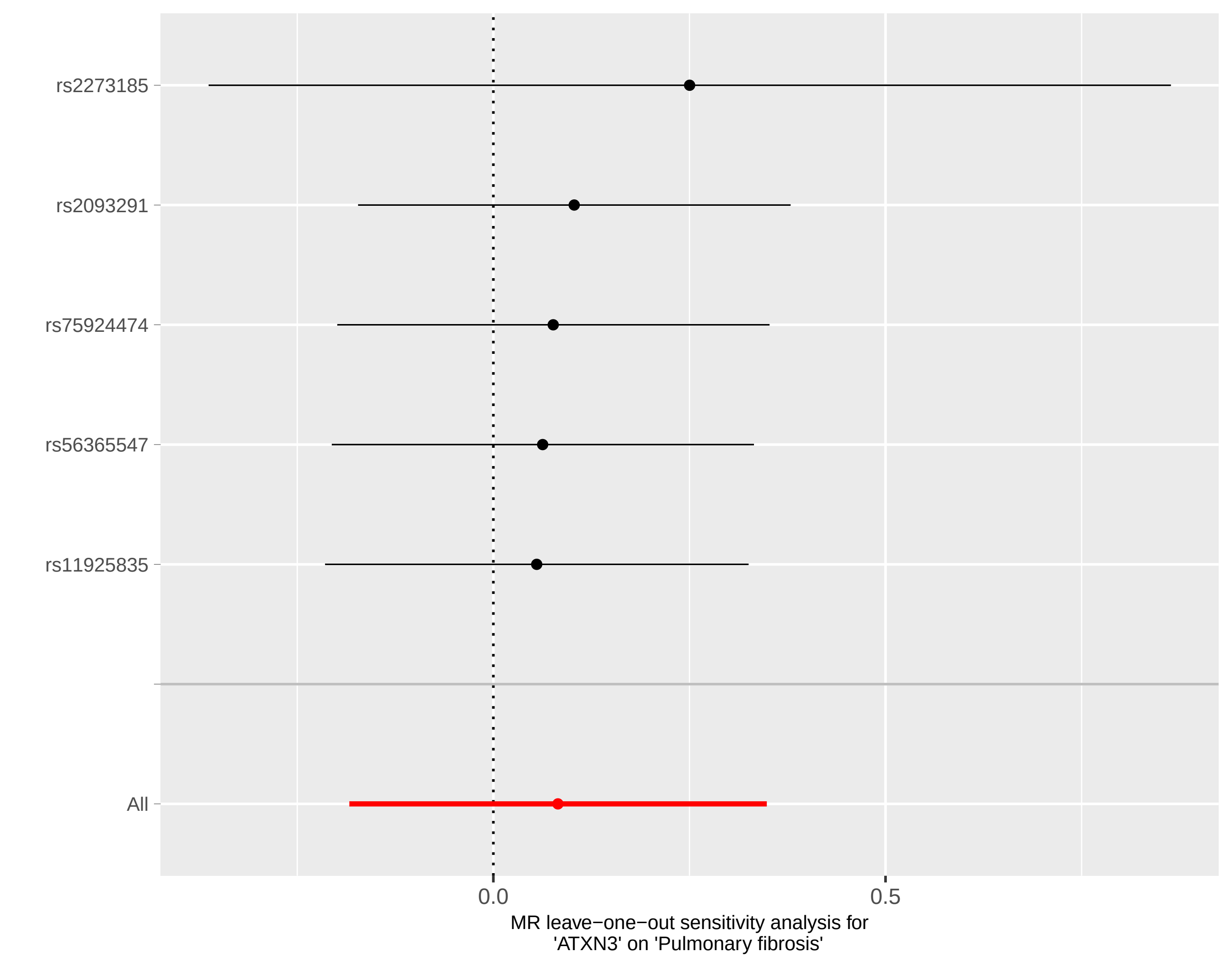
rs2273185
rs2093291
rs75924474
rs56365547
rs11925835
All
0.0
0.5
MR leave−one−out sensitivity analysis for
'ATXN3' on 'Pulmonary fibrosis'

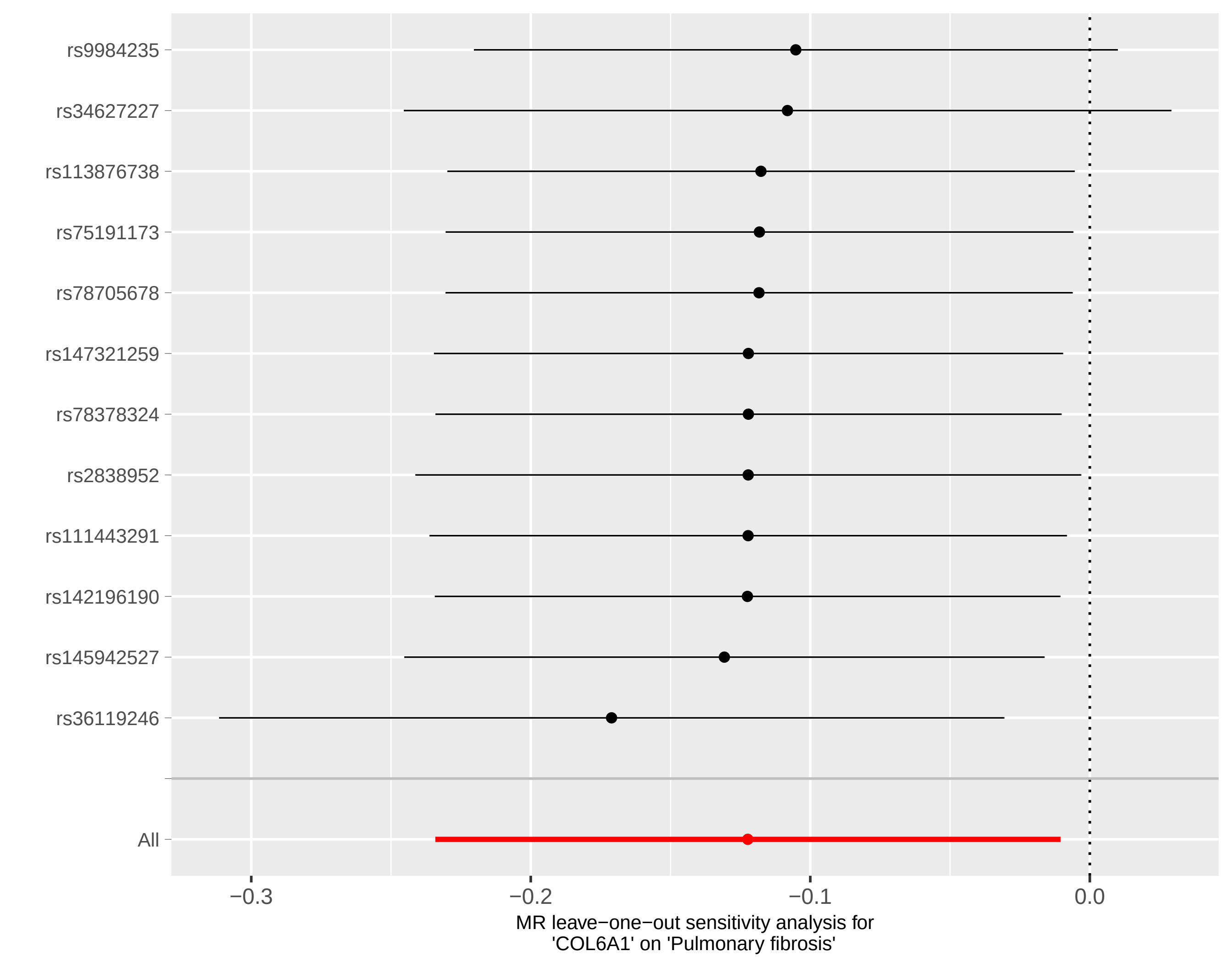
rs9984235
rs34627227
rs113876738
rs75191173
rs78705678
rs147321259
rs78378324
rs2838952
rs111443291
rs142196190
rs145942527
rs36119246
All
−0.3
−0.2
−0.1
0.0
MR leave−one−out sensitivity analysis for
'COL6A1' on 'Pulmonary fibrosis'

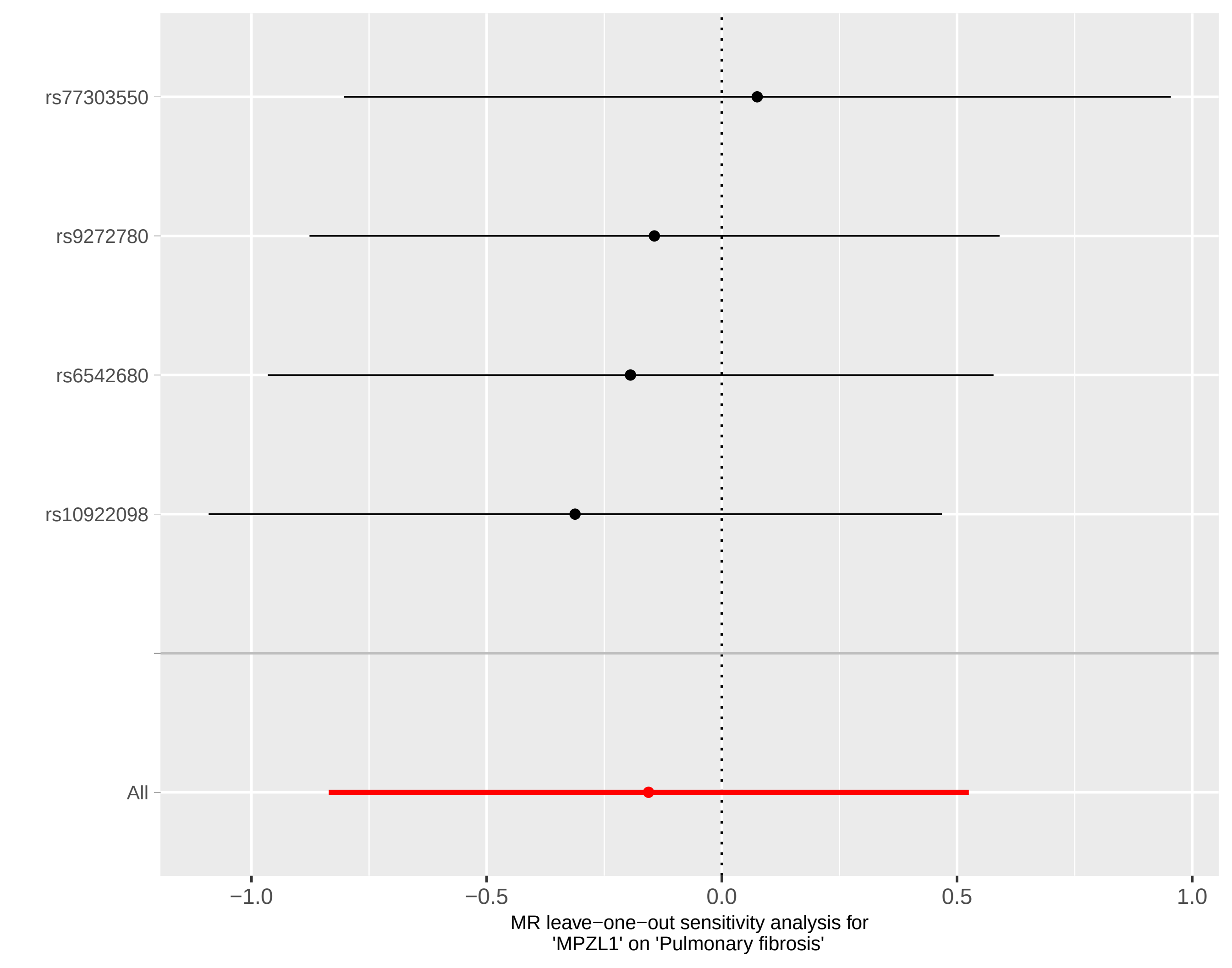
rs77303550
rs9272780
rs6542680
rs10922098
All
−1.0
−0.5
0.0
0.5
1.0
MR leave−one−out sensitivity analysis for
'MPZL1' on 'Pulmonary fibrosis'

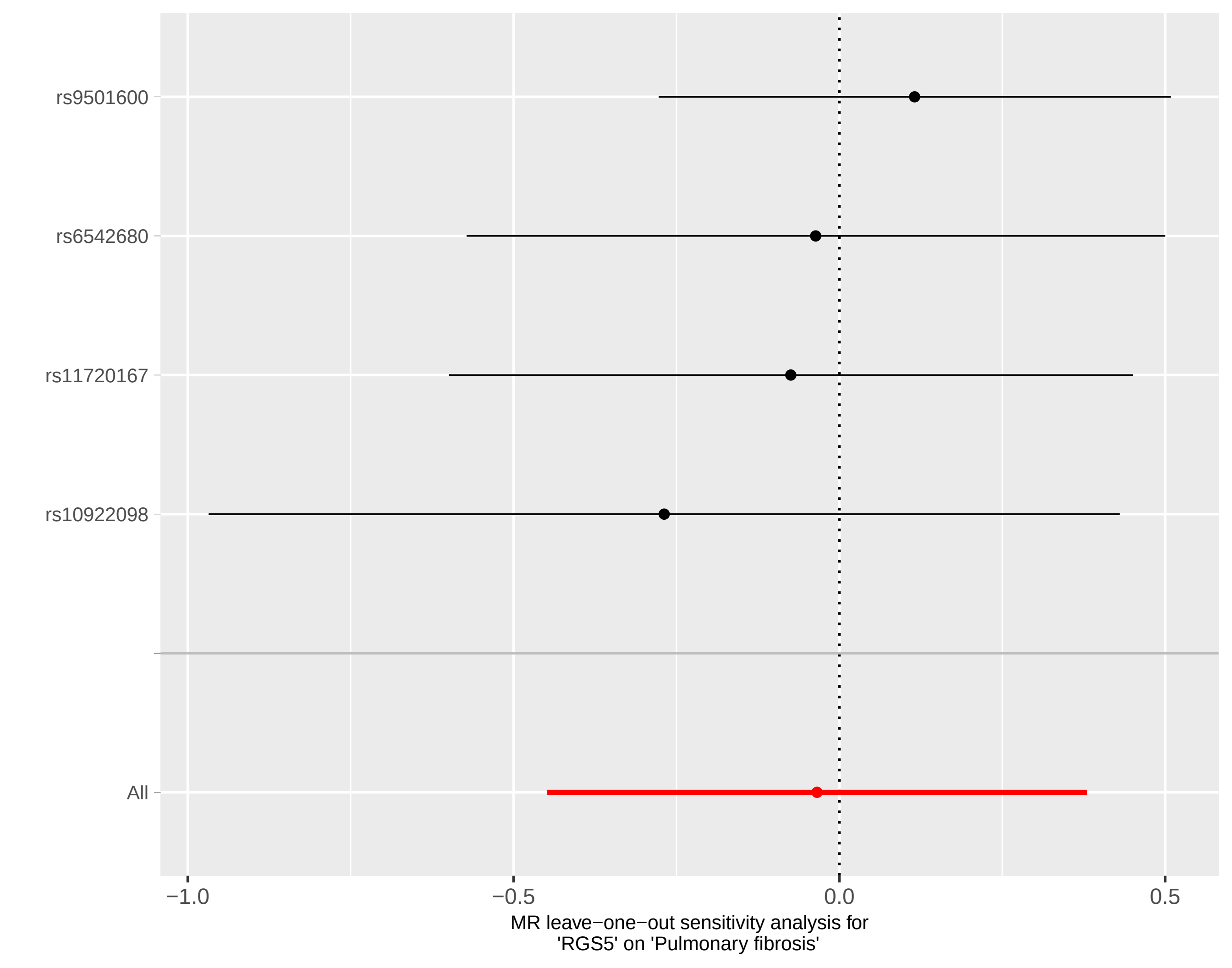
rs9501600
rs6542680
rs11720167
rs10922098
All
−1.0
−0.5
0.0
0.5
MR leave−one−out sensitivity analysis for
'RGS5' on 'Pulmonary fibrosis'

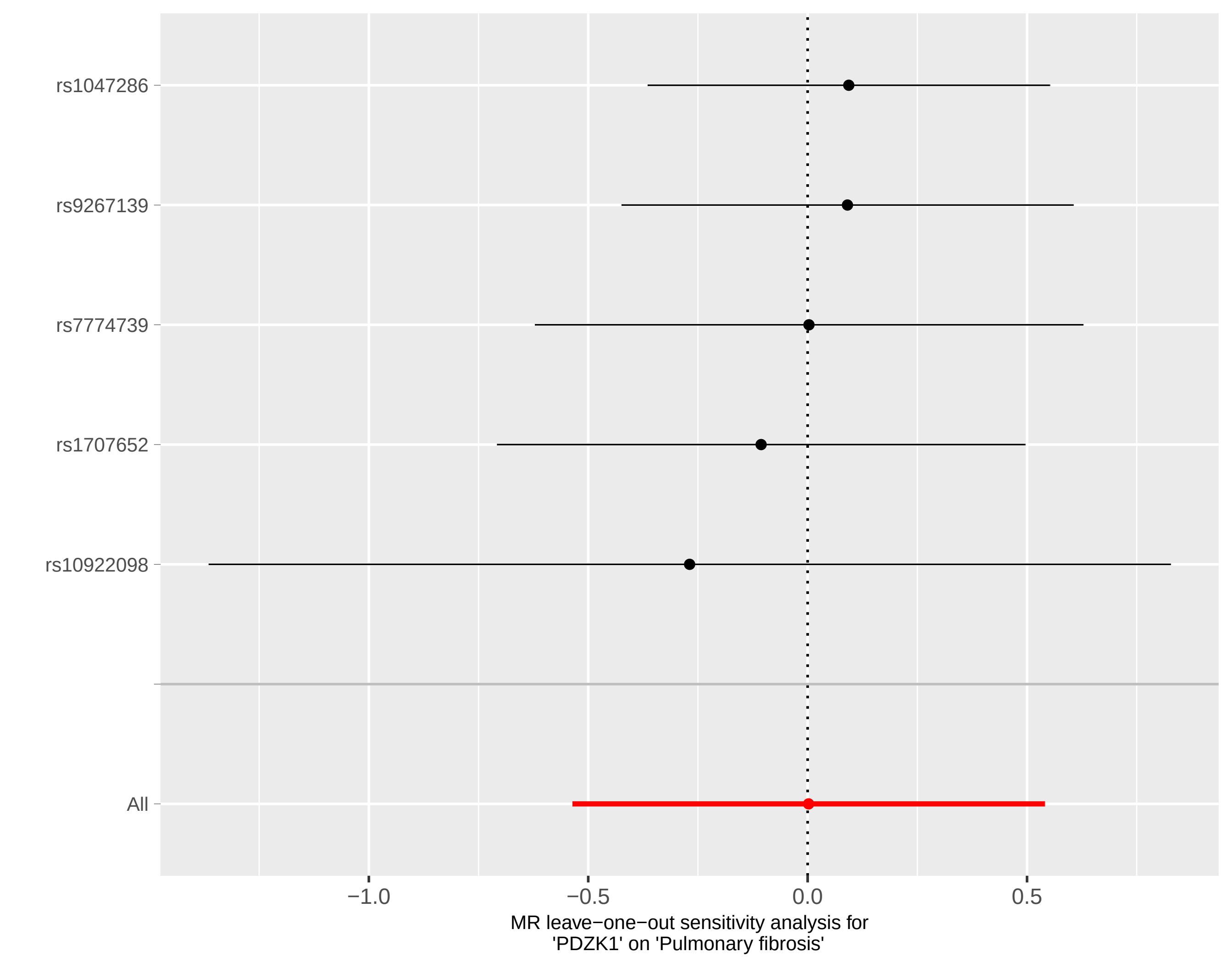
rs1047286
rs9267139
rs7774739
rs1707652
rs10922098
All
−1.0
−0.5
0.0
0.5
MR leave−one−out sensitivity analysis for
'PDZK1' on 'Pulmonary fibrosis'

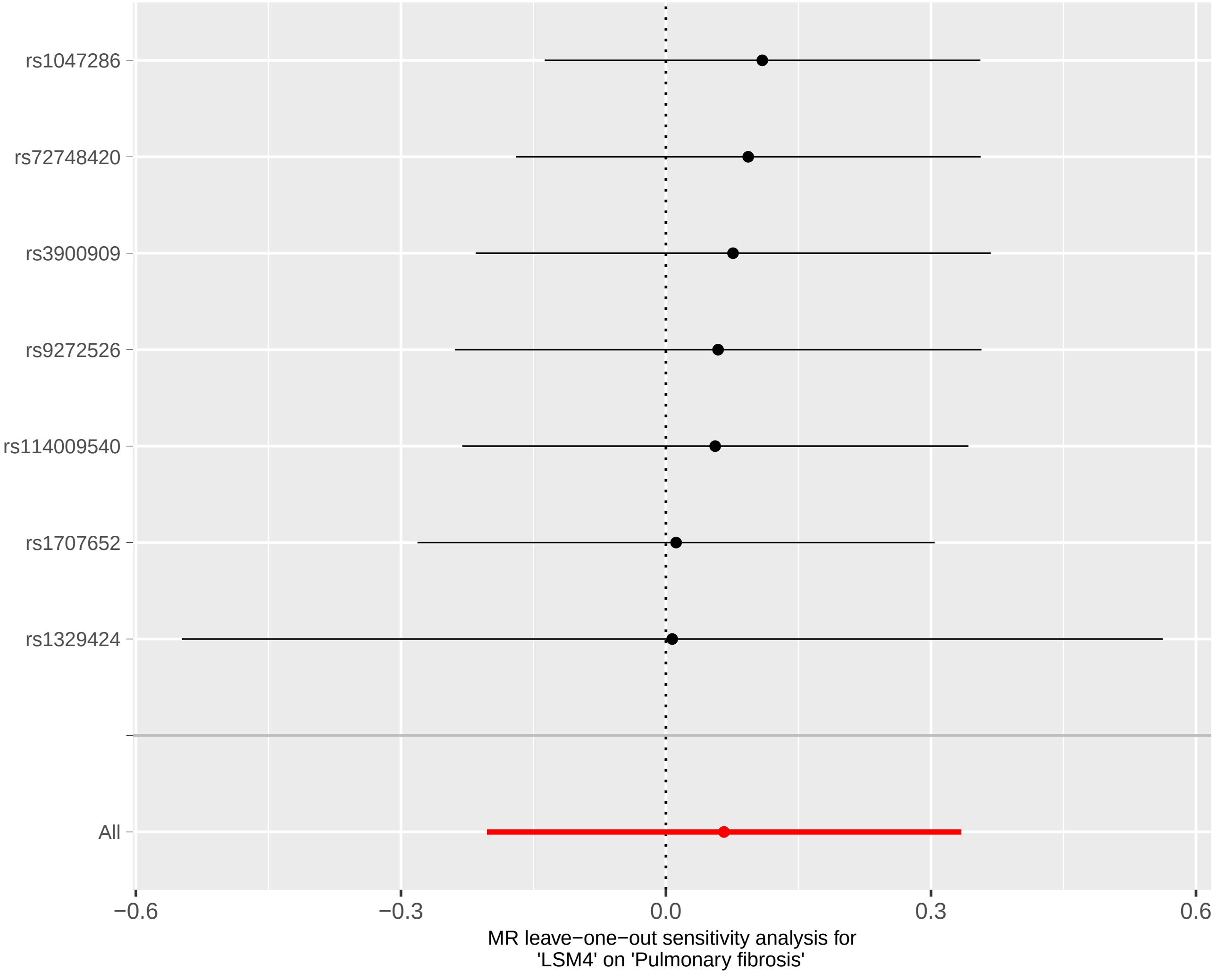
rs1047286
rs72748420
rs3900909
rs9272526
rs114009540
rs1707652
rs1329424
All
−0.6
−0.3
0.0
0.3
0.6
MR leave−one−out sensitivity analysis for
'LSM4' on 'Pulmonary fibrosis'

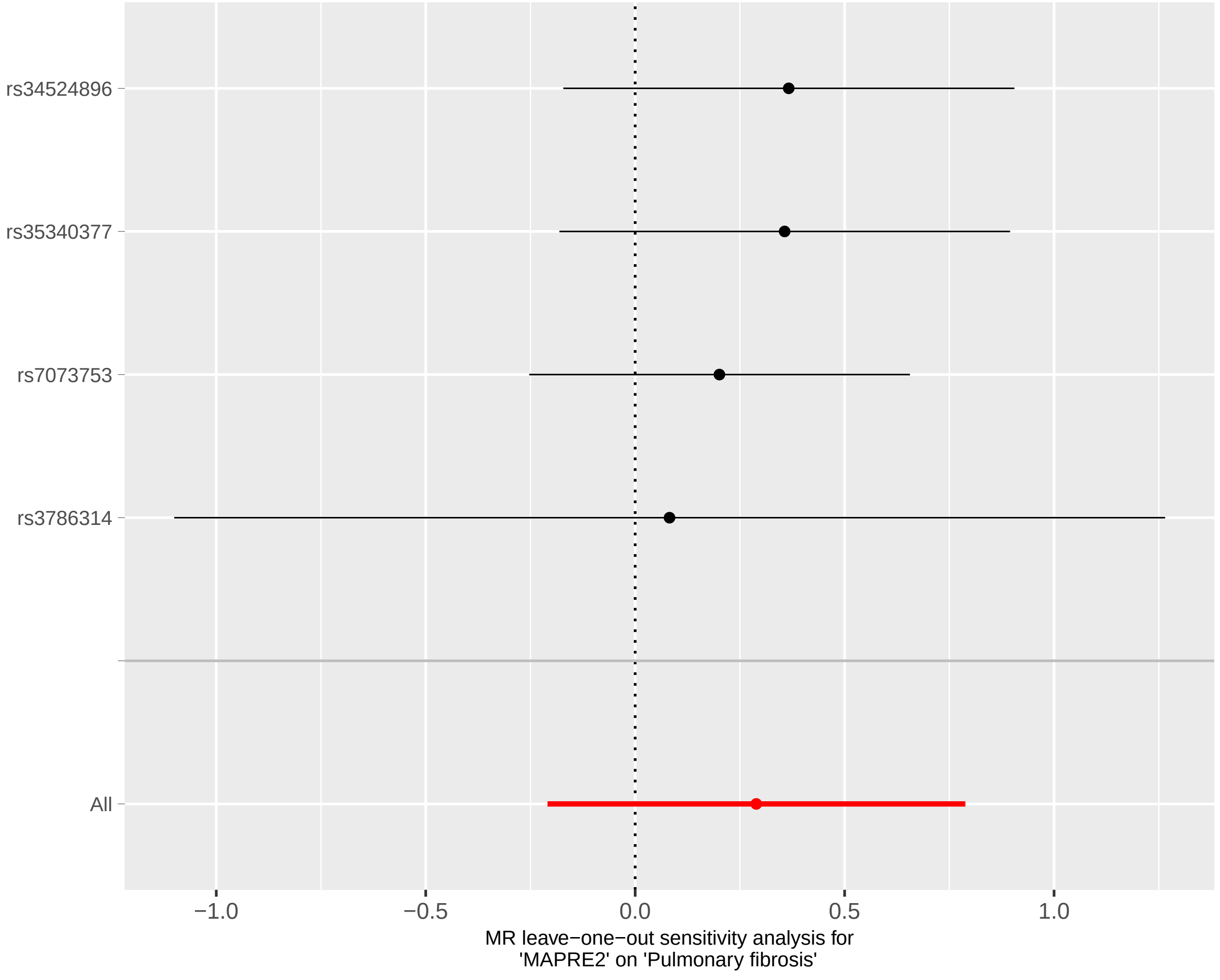
rs34524896
rs35340377
rs7073753
rs3786314
All
−1.0
−0.5
0.0
0.5
1.0
MR leave−one−out sensitivity analysis for
'MAPRE2' on 'Pulmonary fibrosis'

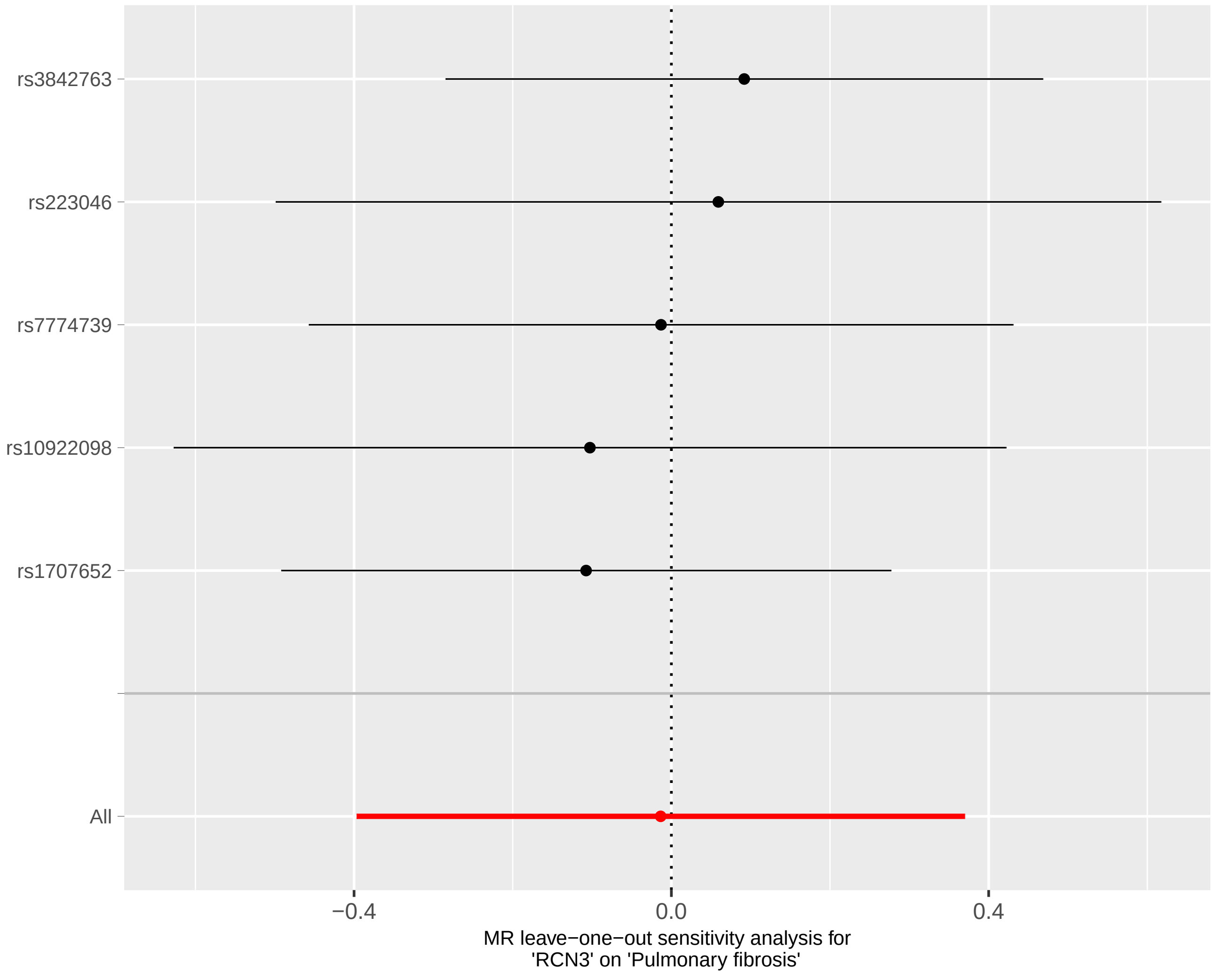
rs3842763
rs223046
rs7774739
rs10922098
rs1707652
All
−0.4
0.0
0.4
MR leave−one−out sensitivity analysis for
'RCN3' on 'Pulmonary fibrosis'

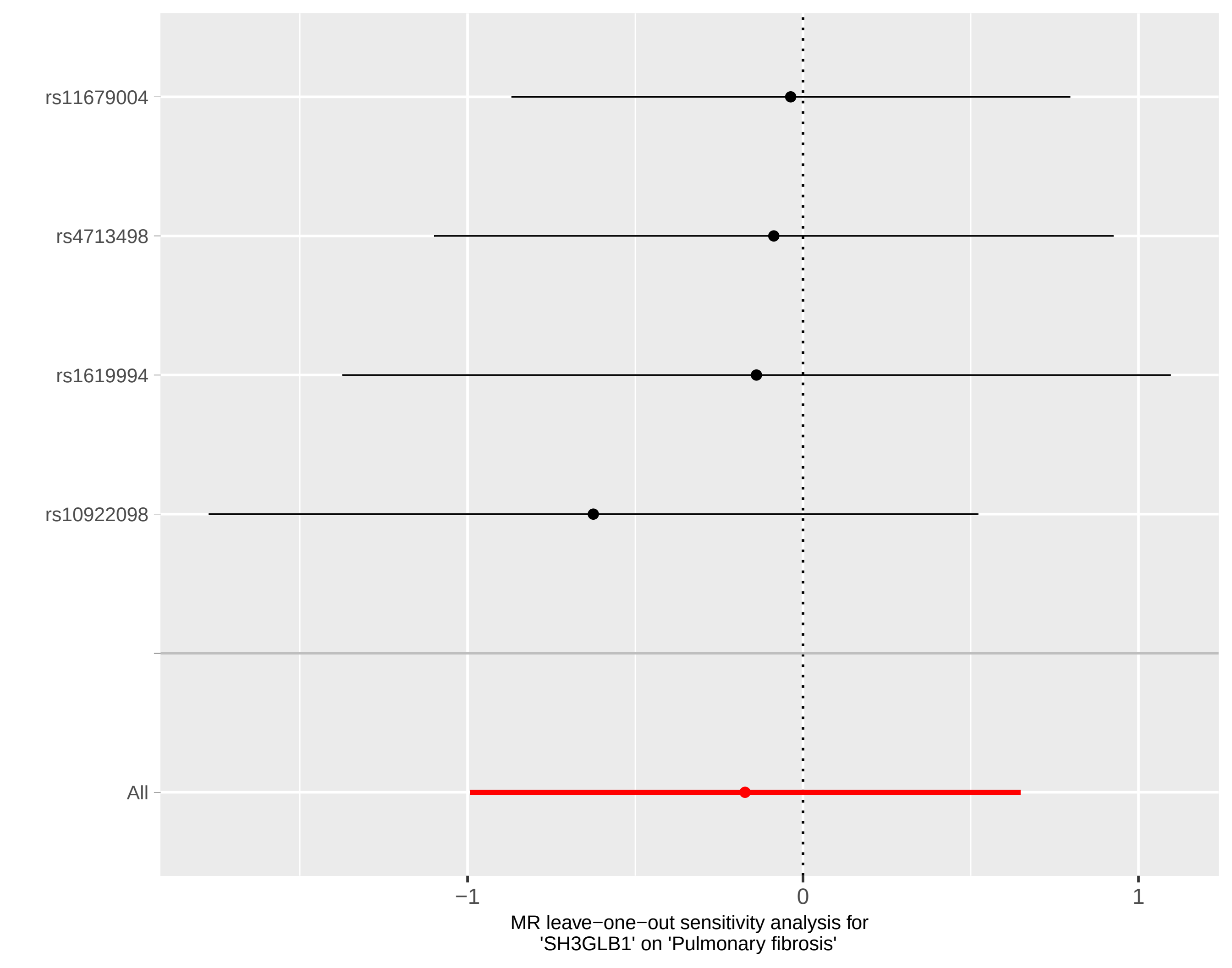
rs11679004
rs4713498
rs1619994
rs10922098
All
−1
0
1
MR leave−one−out sensitivity analysis for
'SH3GLB1' on 'Pulmonary fibrosis'

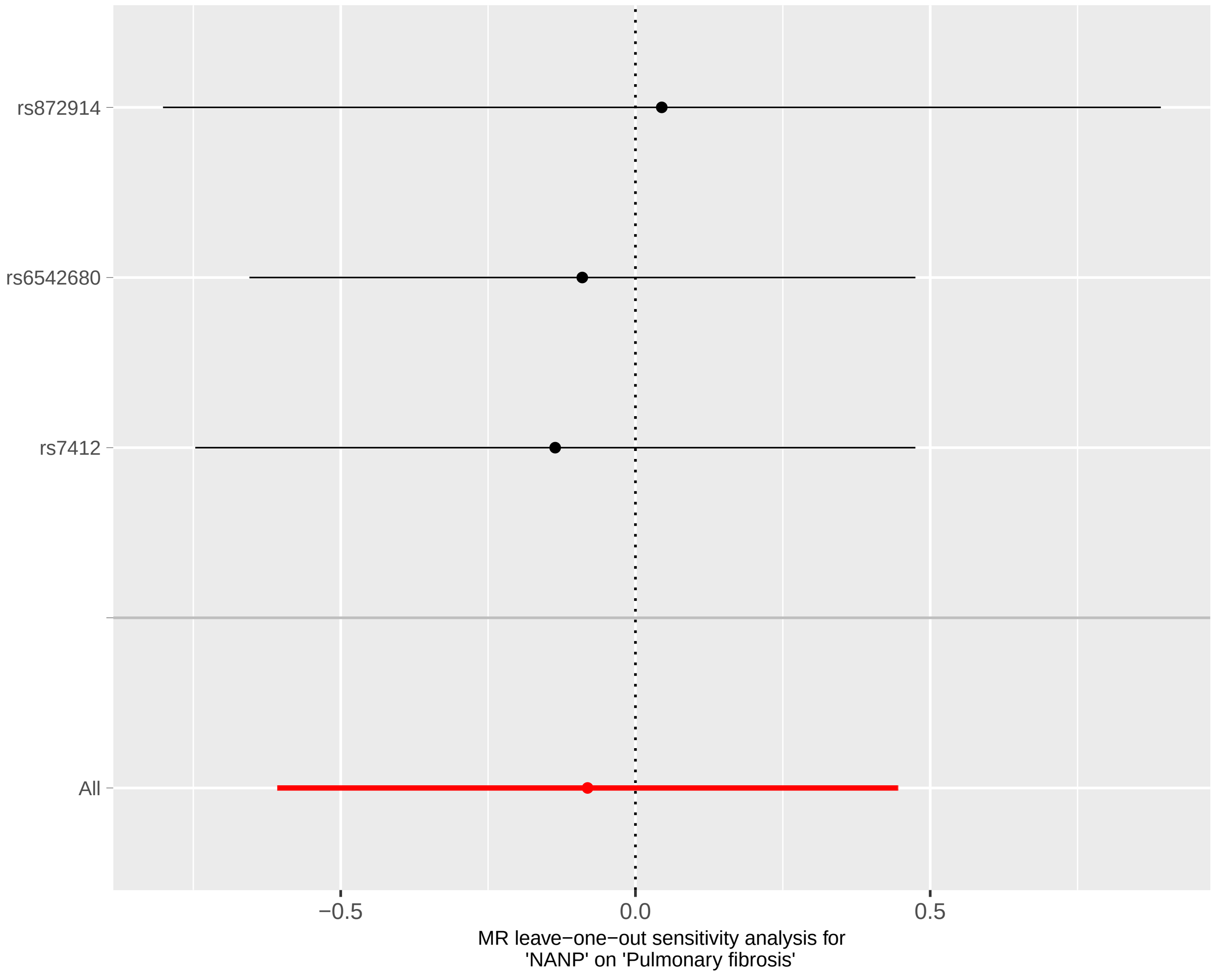

rs872914
rs6542680
rs7412
All
−0.5
0.0
0.5
MR leave−one−out sensitivity analysis for
'NANP' on 'Pulmonary fibrosis'

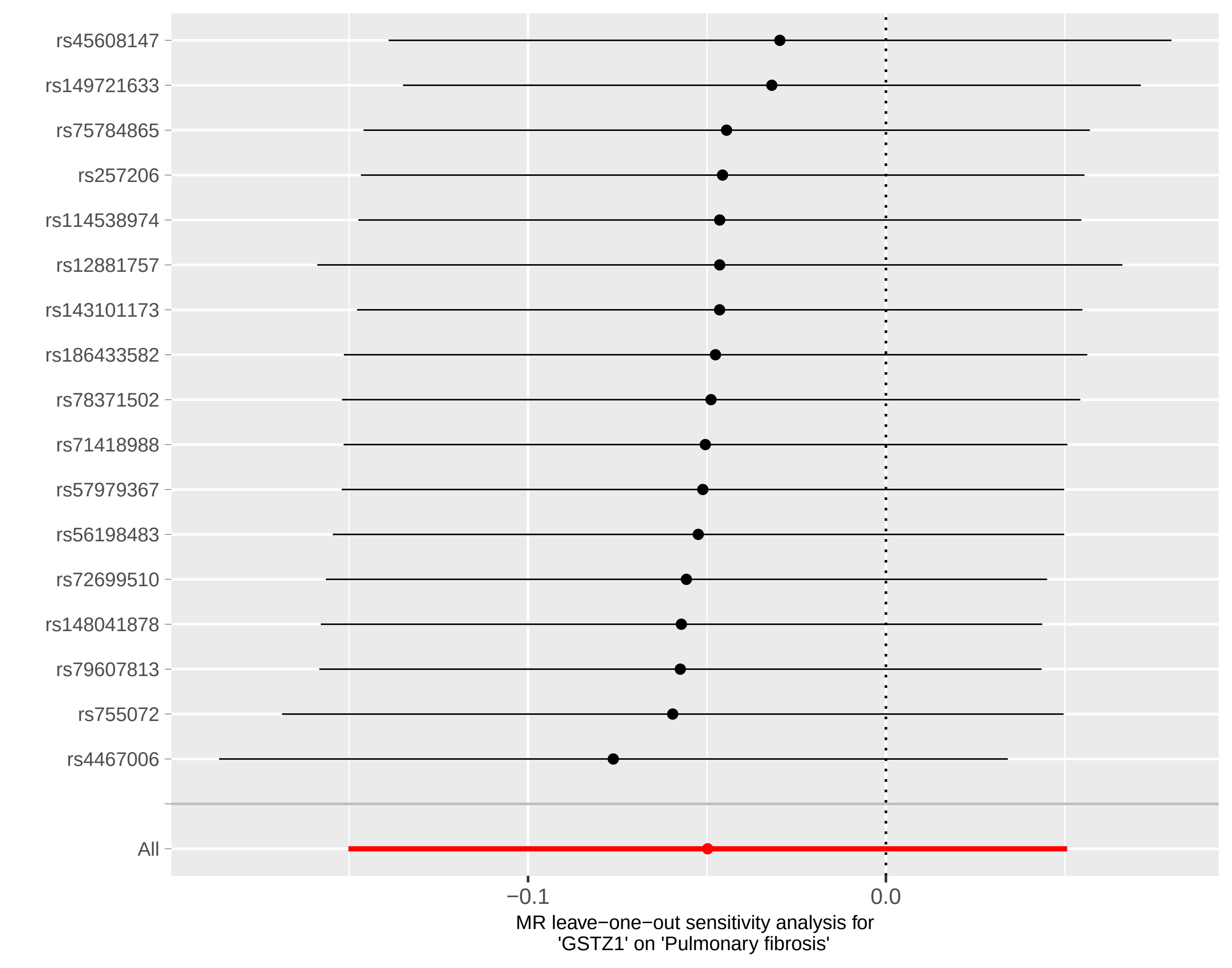
rs45608147
rs149721633
rs75784865
rs257206
rs114538974
rs12881757
rs143101173
rs186433582
rs78371502
rs71418988
rs57979367
rs56198483
rs72699510
rs148041878
rs79607813
rs755072
rs4467006
All
−0.1
0.0
MR leave−one−out sensitivity analysis for
'GSTZ1' on 'Pulmonary fibrosis'

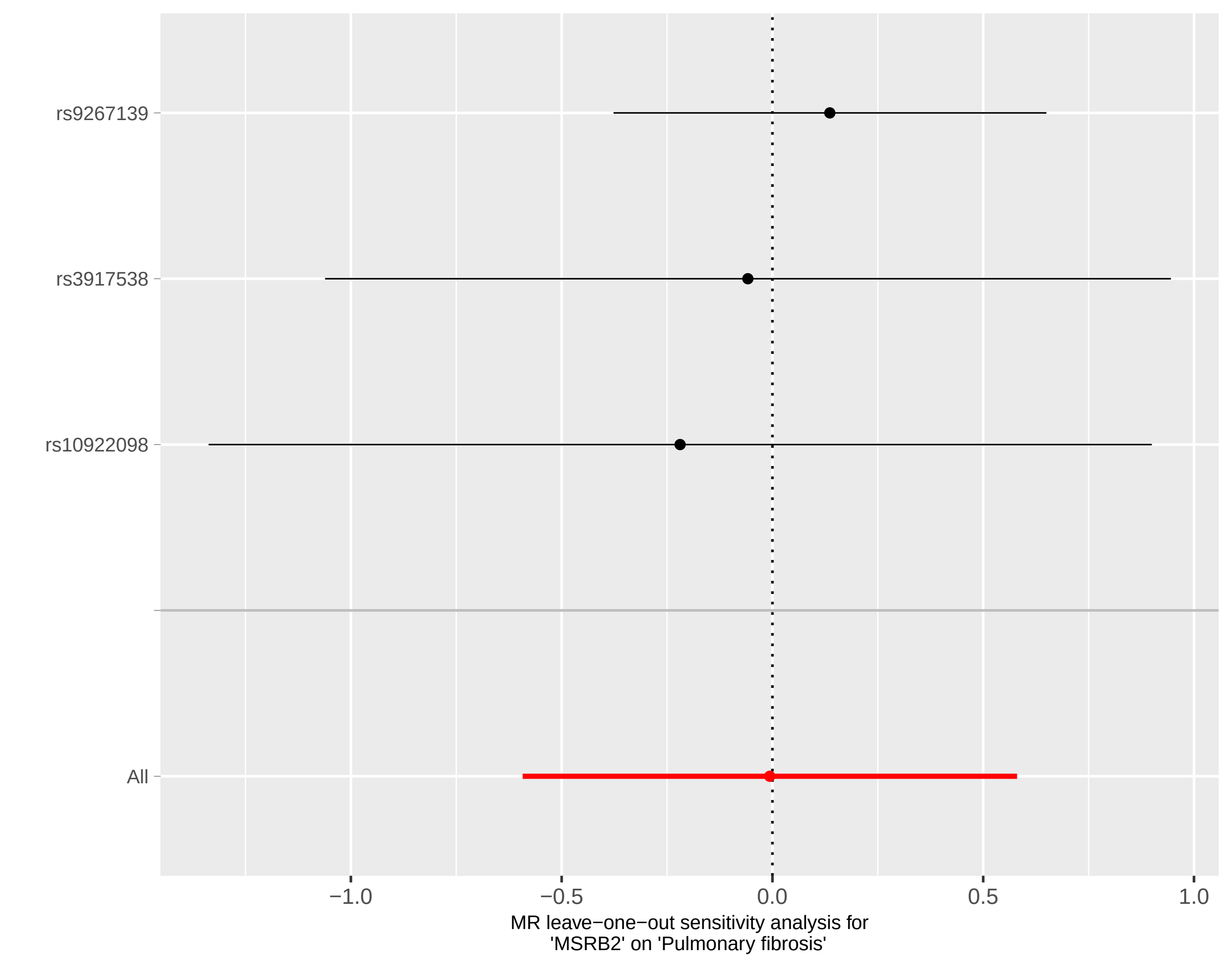
rs9267139
rs3917538
rs10922098
All
−1.0
−0.5
0.0
0.5
1.0
MR leave−one−out sensitivity analysis for
'MSRB2' on 'Pulmonary fibrosis'

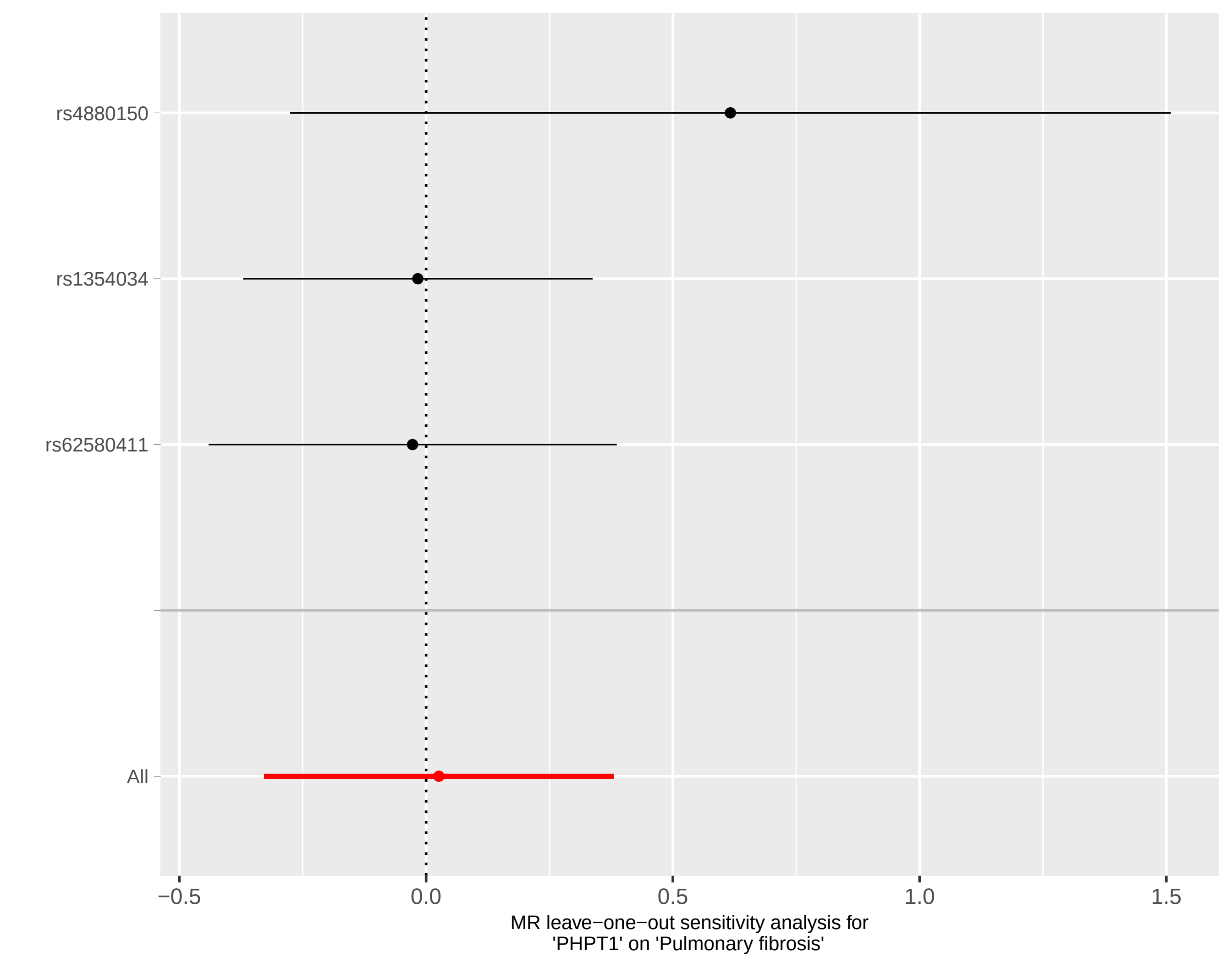
rs4880150
rs1354034
rs62580411
All
−0.5
0.0
0.5
1.0
1.5
MR leave−one−out sensitivity analysis for
'PHPT1' on 'Pulmonary fibrosis'

Insufficient number of SNPs

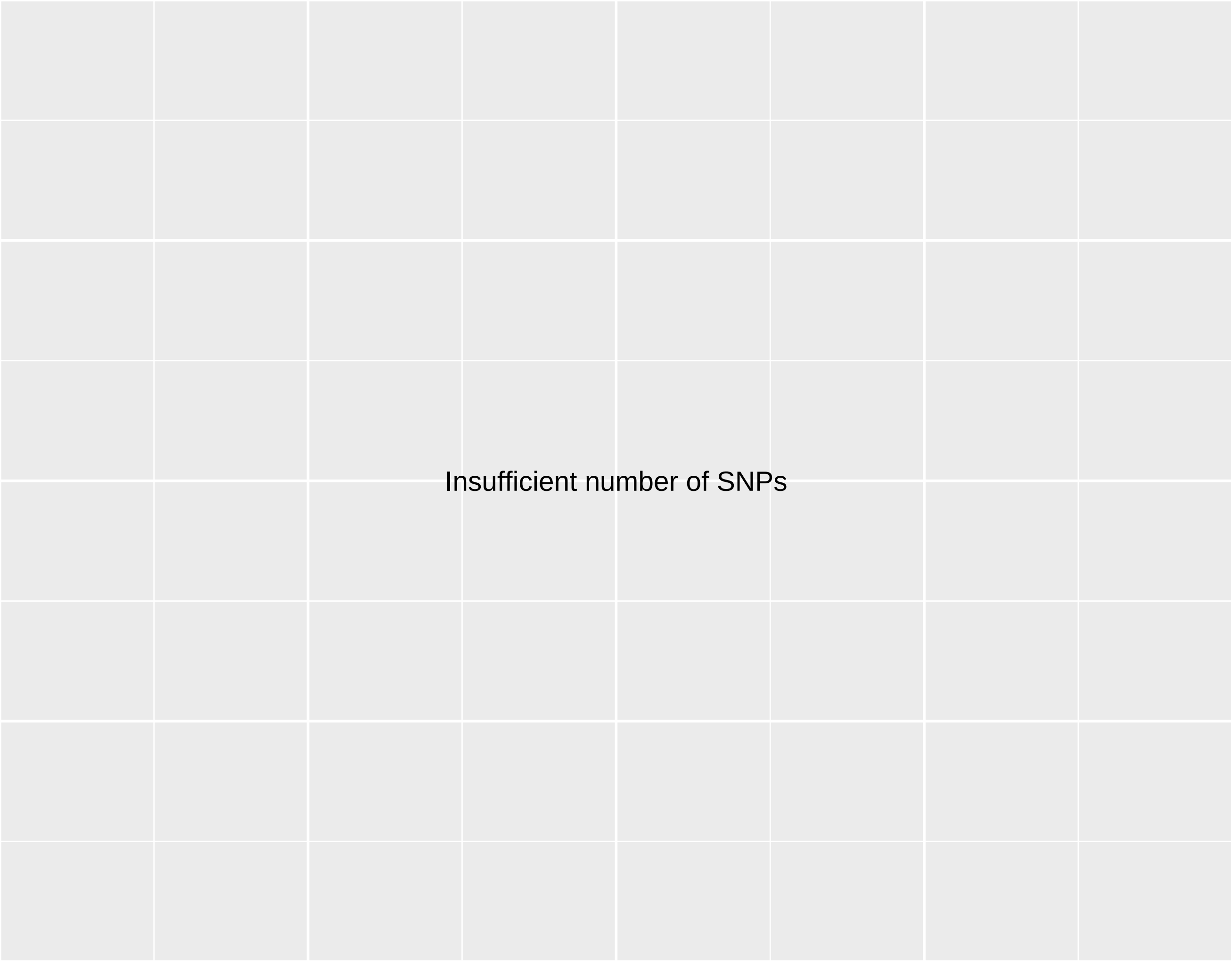
Insufficient number of SNPs

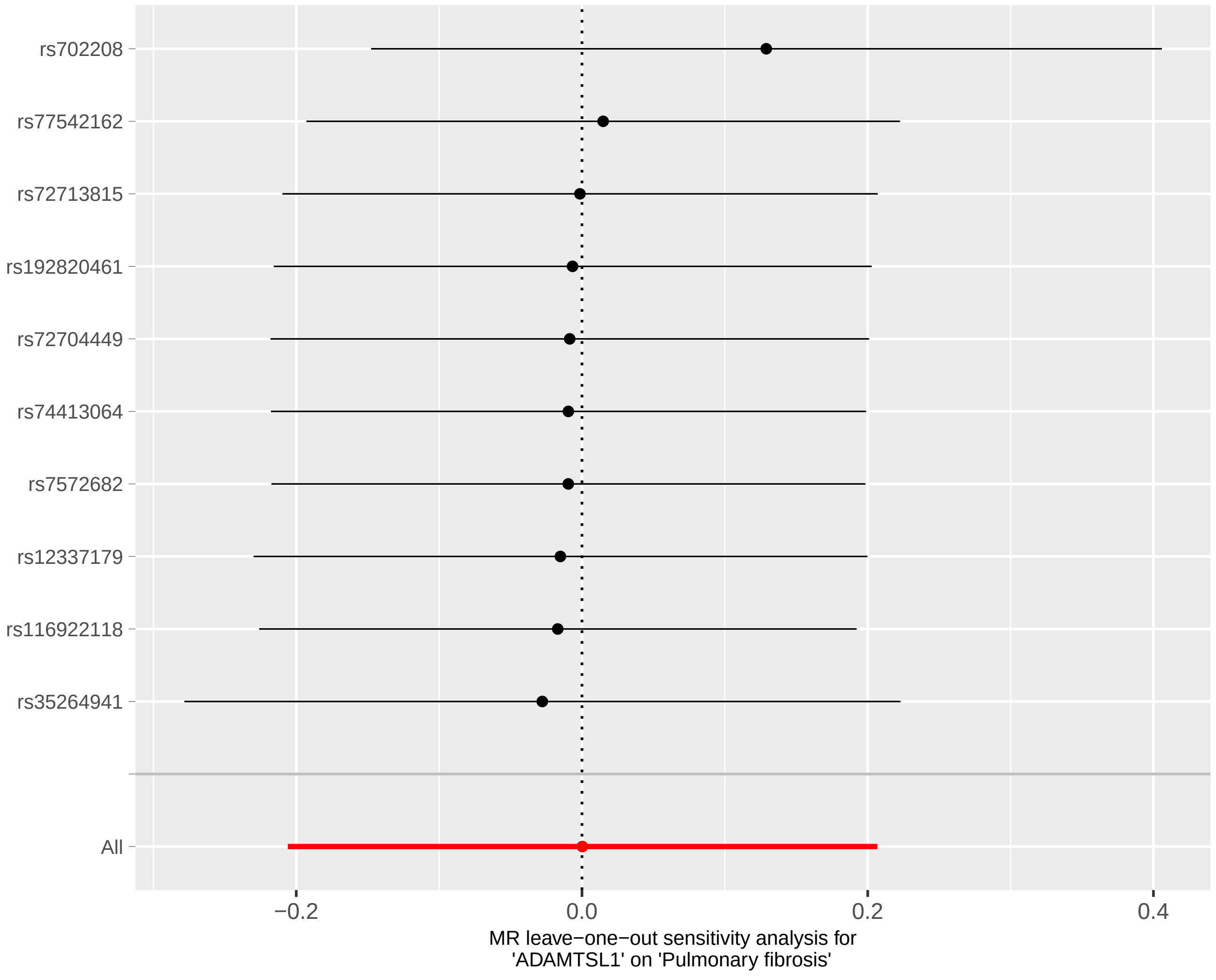
rs702208
rs77542162
rs72713815
rs192820461
rs72704449
rs74413064
rs7572682
rs12337179
rs116922118
rs35264941
All
−0.2
0.0
0.2
0.4
MR leave−one−out sensitivity analysis for
'ADAMTSL1' on 'Pulmonary fibrosis'

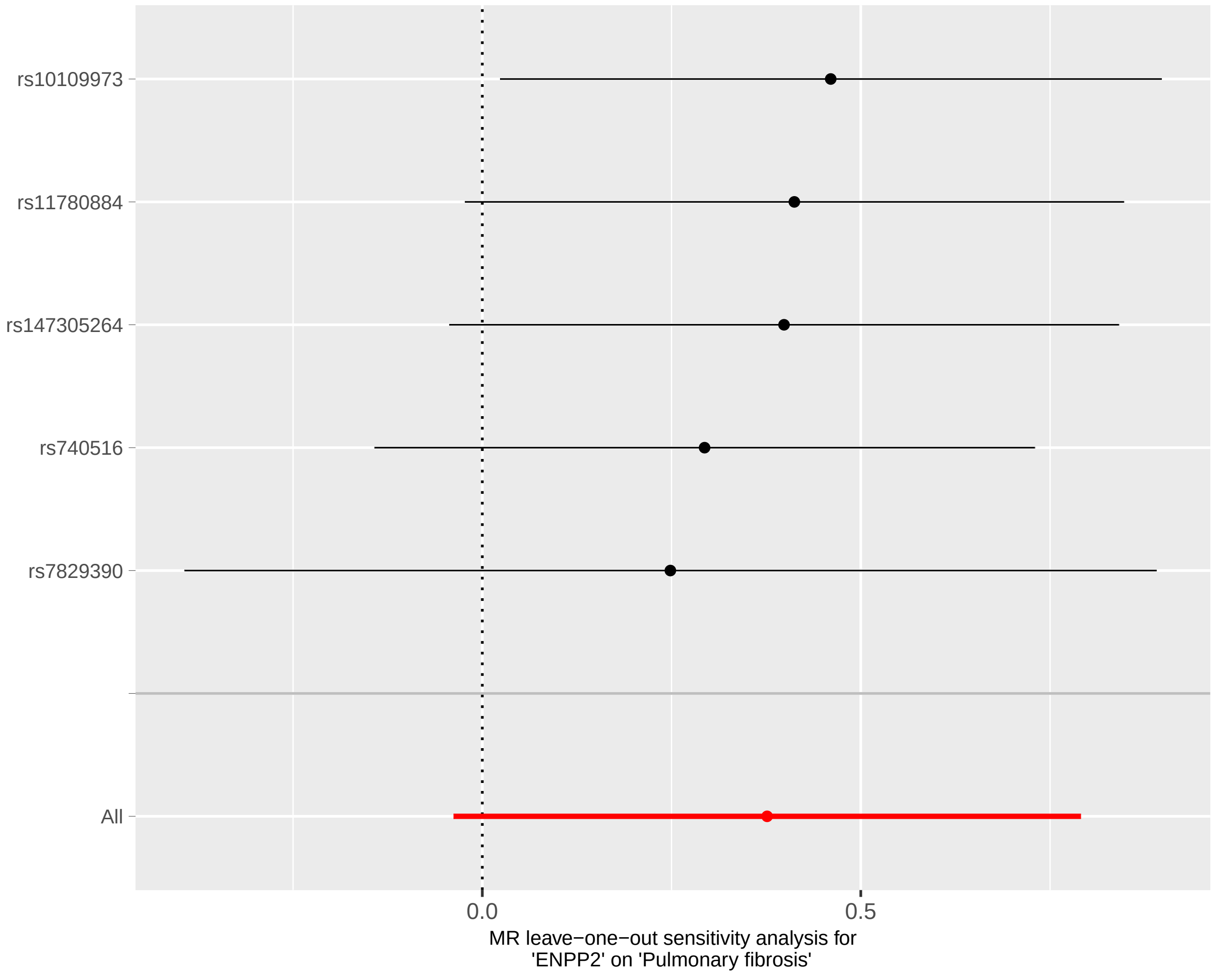

rs10109973
rs11780884
rs147305264
rs740516
rs7829390
All
0.0
0.5
MR leave−one−out sensitivity analysis for
'ENPP2' on 'Pulmonary fibrosis'

Insufficient number of SNPs

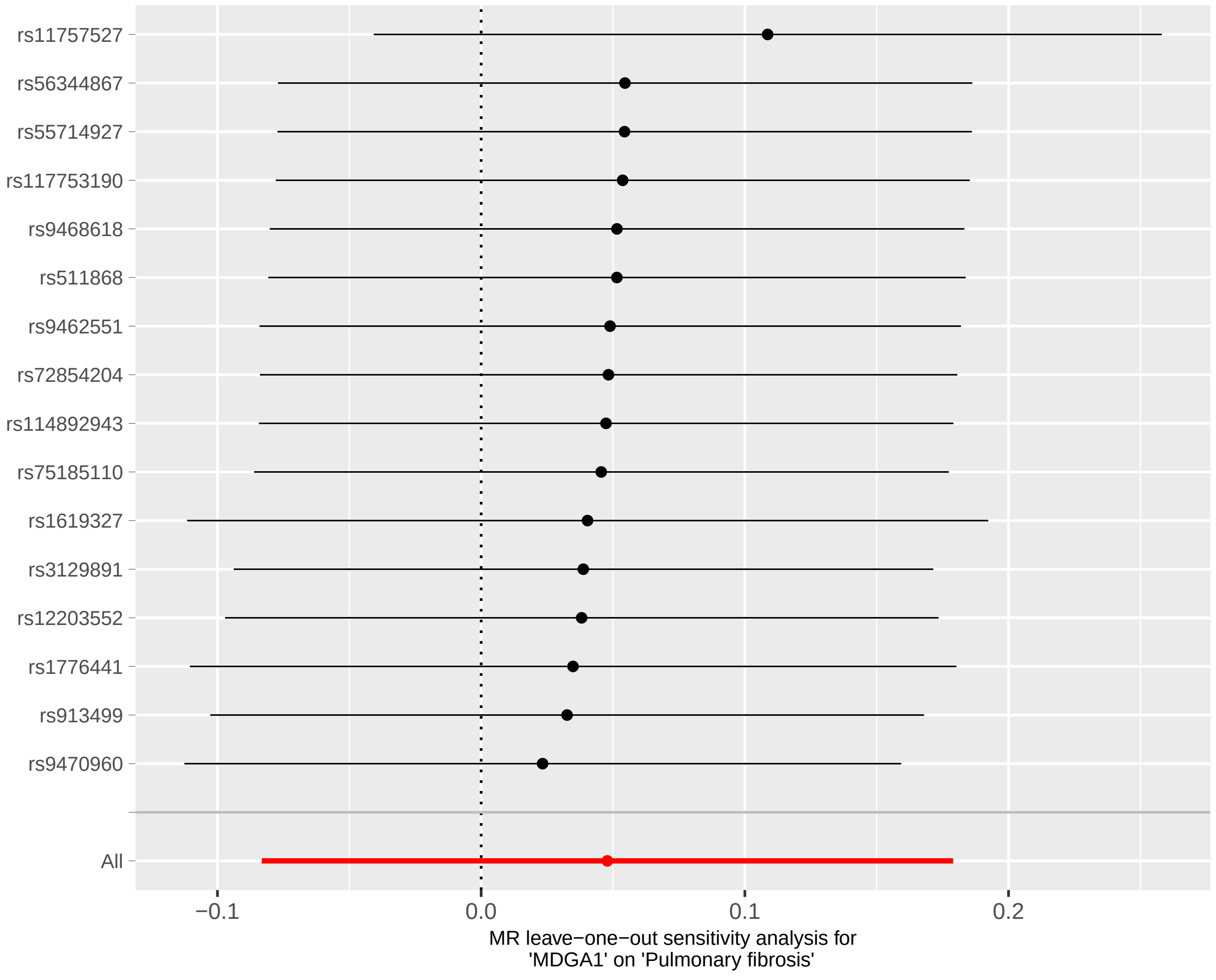

rs11757527
rs56344867
rs55714927
rs117753190
rs9468618
rs511868
rs9462551
rs72854204
rs114892943
rs75185110
rs1619327
rs3129891
rs12203552
rs1776441
rs913499
rs9470960
All
−0.1
0.0
0.1
0.2
MR leave−one−out sensitivity analysis for
'MDGA1' on 'Pulmonary fibrosis'

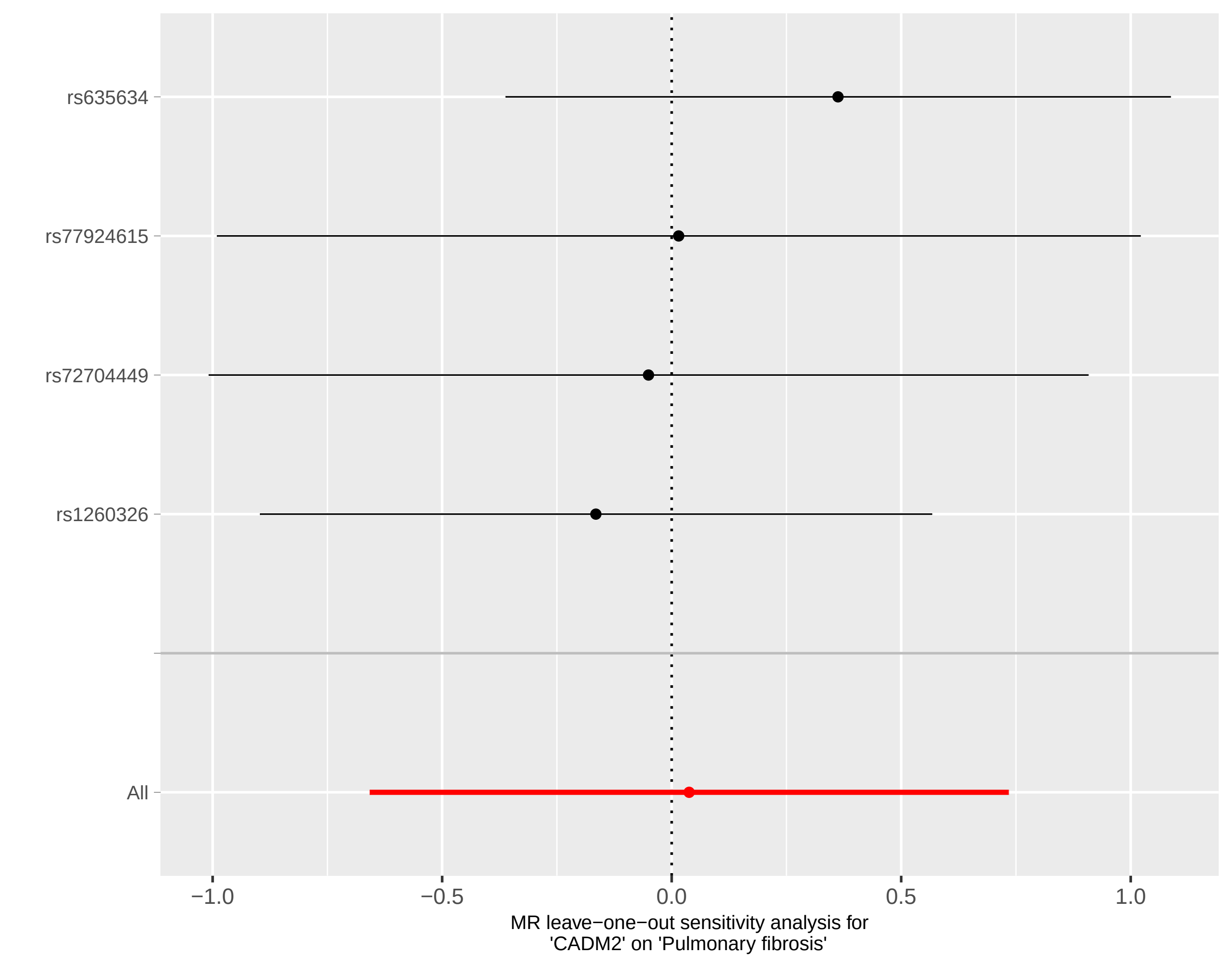
rs635634
rs77924615
rs72704449
rs1260326
All
−1.0
−0.5
0.0
0.5
1.0
MR leave−one−out sensitivity analysis for
'CADM2' on 'Pulmonary fibrosis'

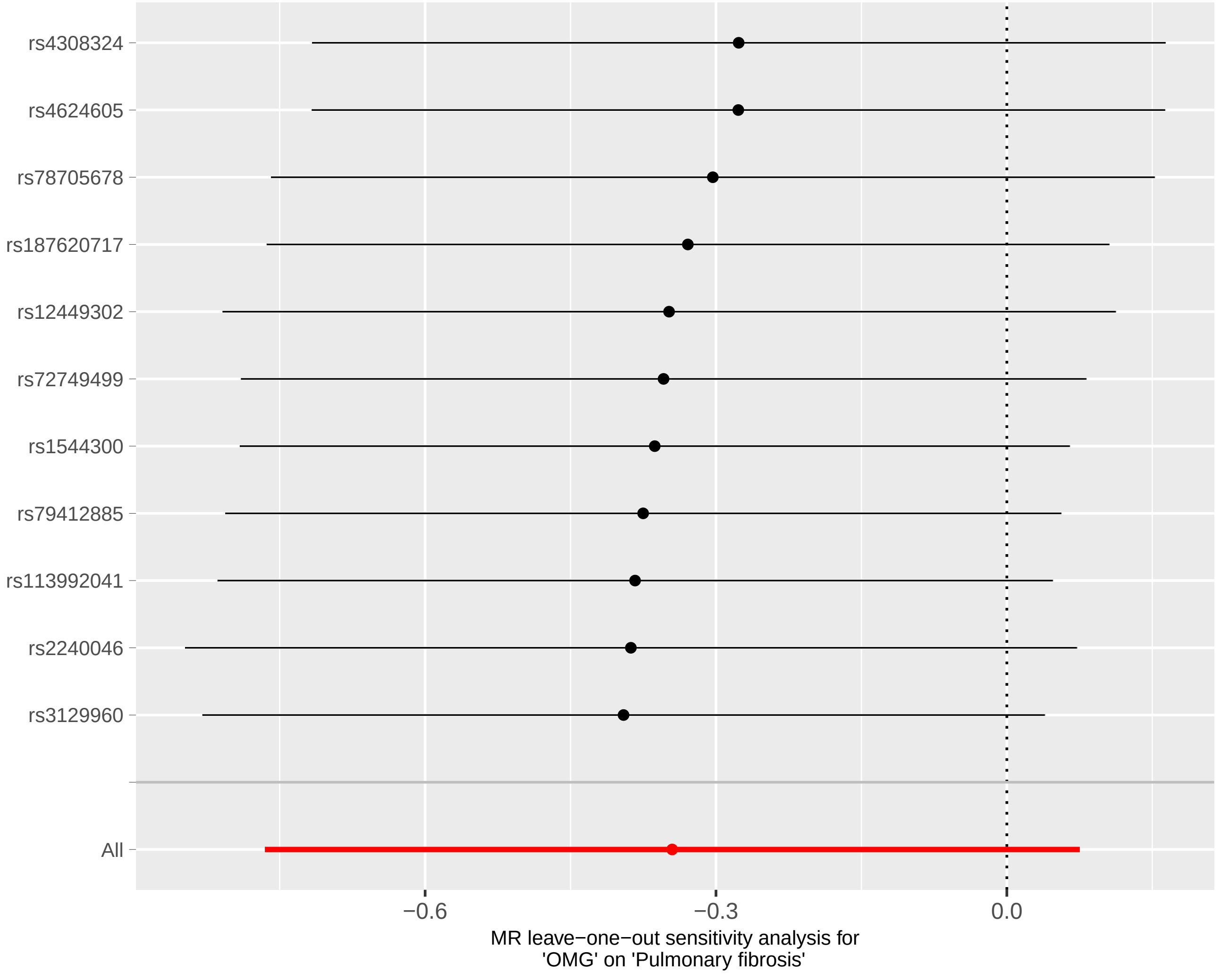
rs4308324
rs4624605
rs78705678
rs187620717
rs12449302
rs72749499
rs1544300
rs79412885
rs113992041
rs2240046
rs3129960
All
−0.6
−0.3
0.0
MR leave−one−out sensitivity analysis for
'OMG' on 'Pulmonary fibrosis'

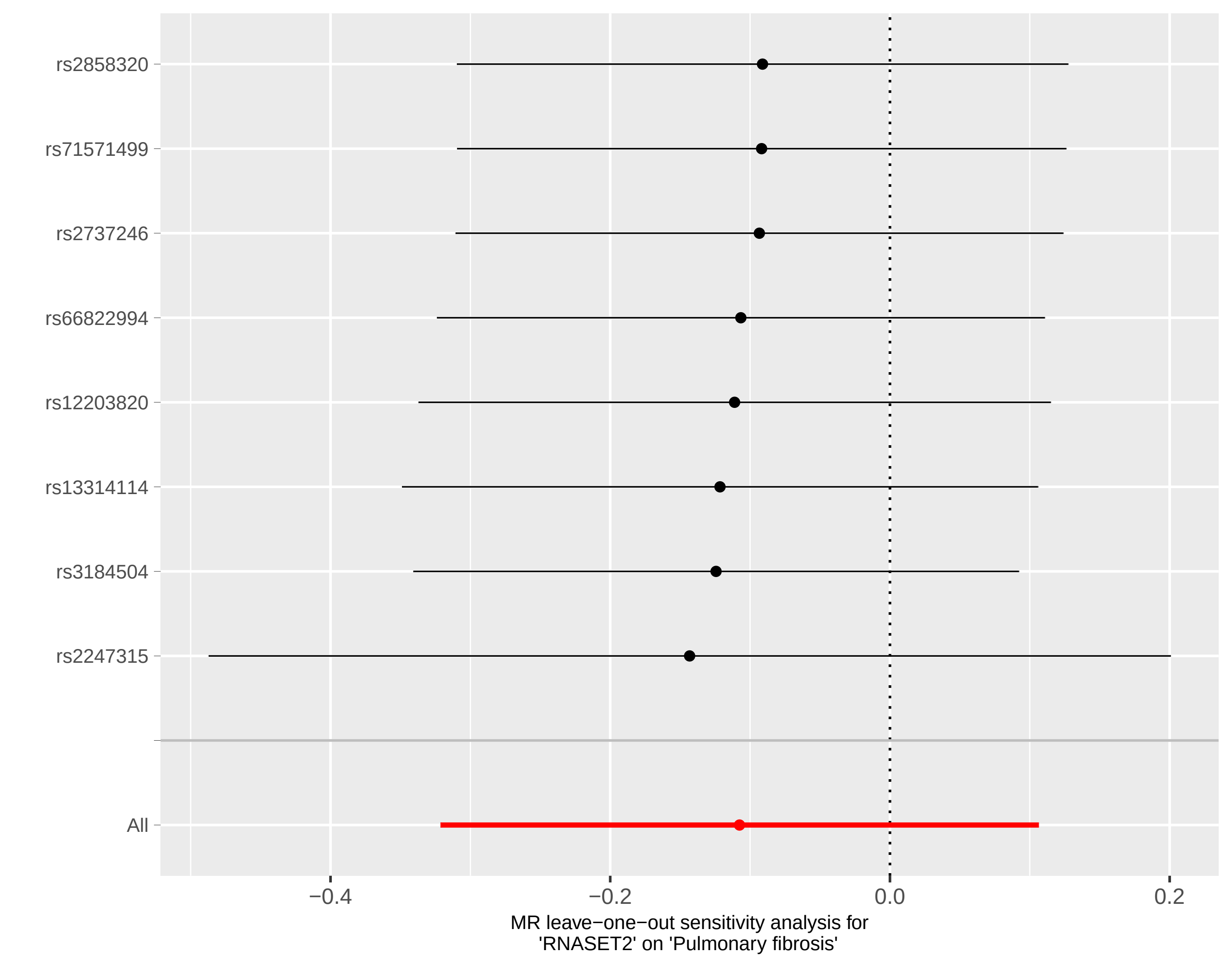

rs2858320
rs71571499
rs2737246
rs66822994
rs12203820
rs13314114
rs3184504
rs2247315
All
−0.4
−0.2
0.0
0.2
MR leave−one−out sensitivity analysis for
'RNASET2' on 'Pulmonary fibrosis'

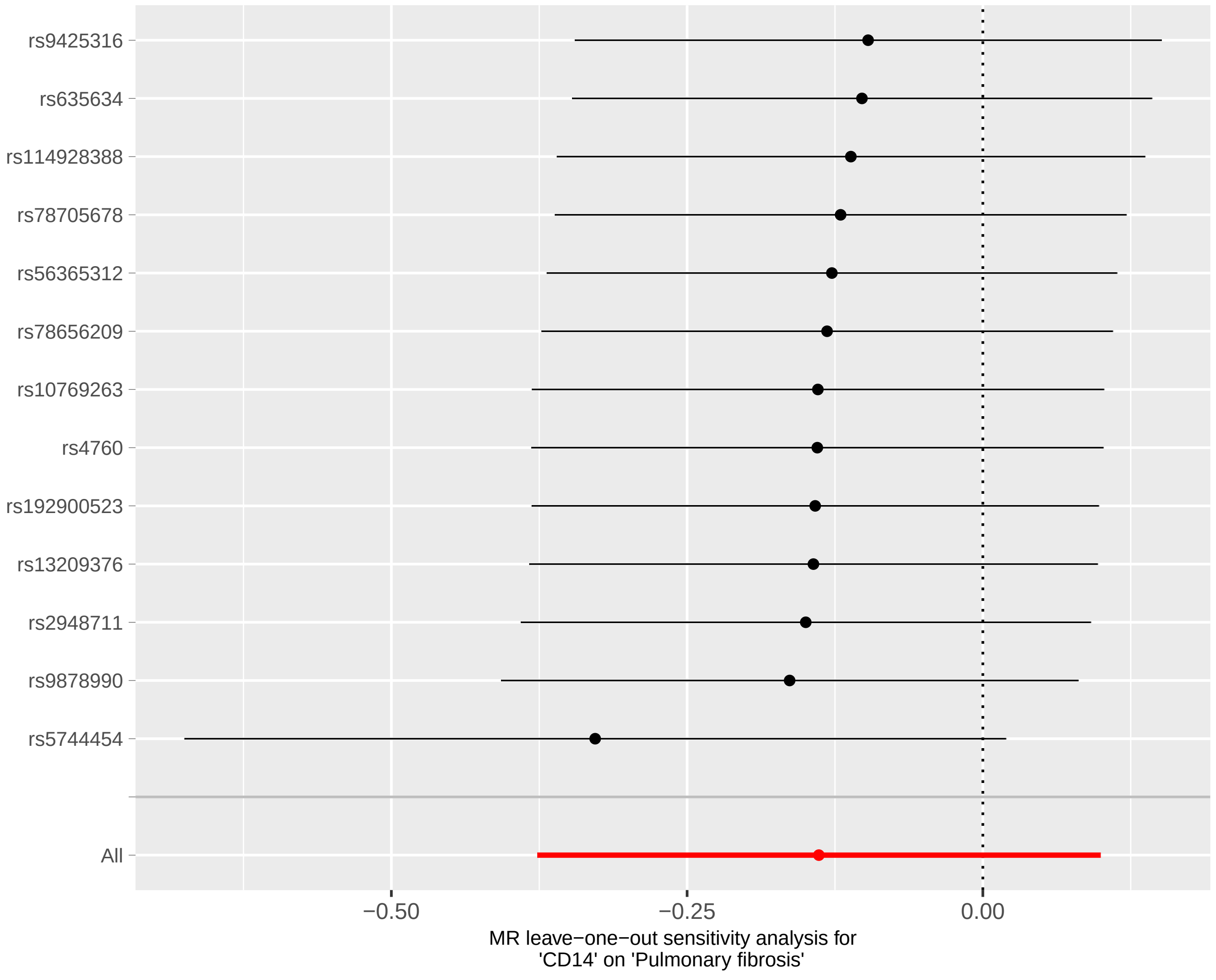
rs9425316
rs635634
rs114928388
rs78705678
rs56365312
rs78656209
rs10769263
rs4760
rs192900523
rs13209376
rs2948711
rs9878990
rs5744454
All
−0.50
−0.25
0.00
MR leave−one−out sensitivity analysis for
'CD14' on 'Pulmonary fibrosis'

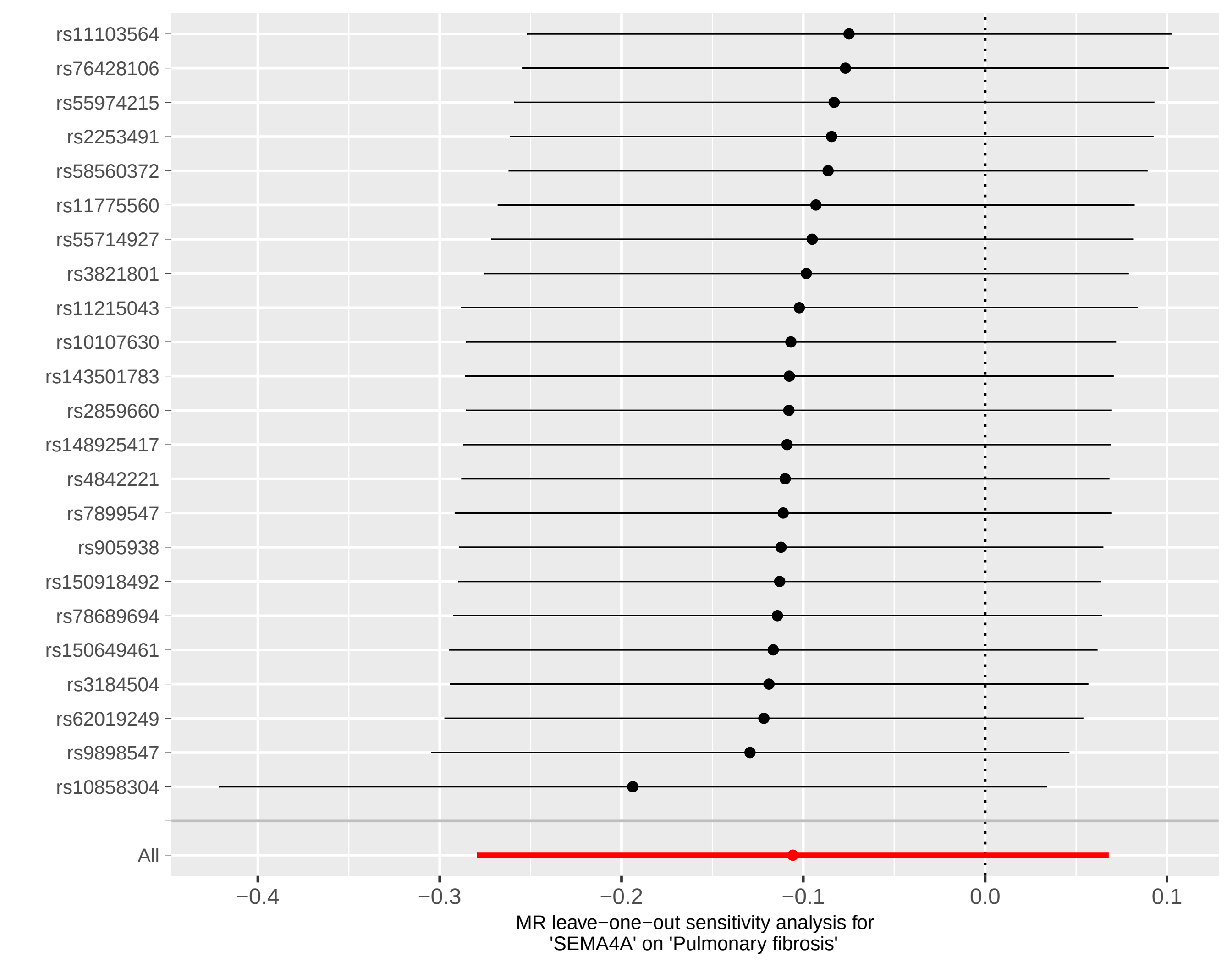
rs11103564
rs76428106
rs55974215
rs2253491
rs58560372
rs11775560
rs55714927
rs3821801
rs11215043
rs10107630
rs143501783
rs2859660
rs148925417
rs4842221
rs7899547
rs905938
rs150918492
rs78689694
rs150649461
rs3184504
rs62019249
rs9898547
rs10858304
All
−0.4
−0.3
−0.2
−0.1
0.0
0.1
MR leave−one−out sensitivity analysis for
'SEMA4A' on 'Pulmonary fibrosis'

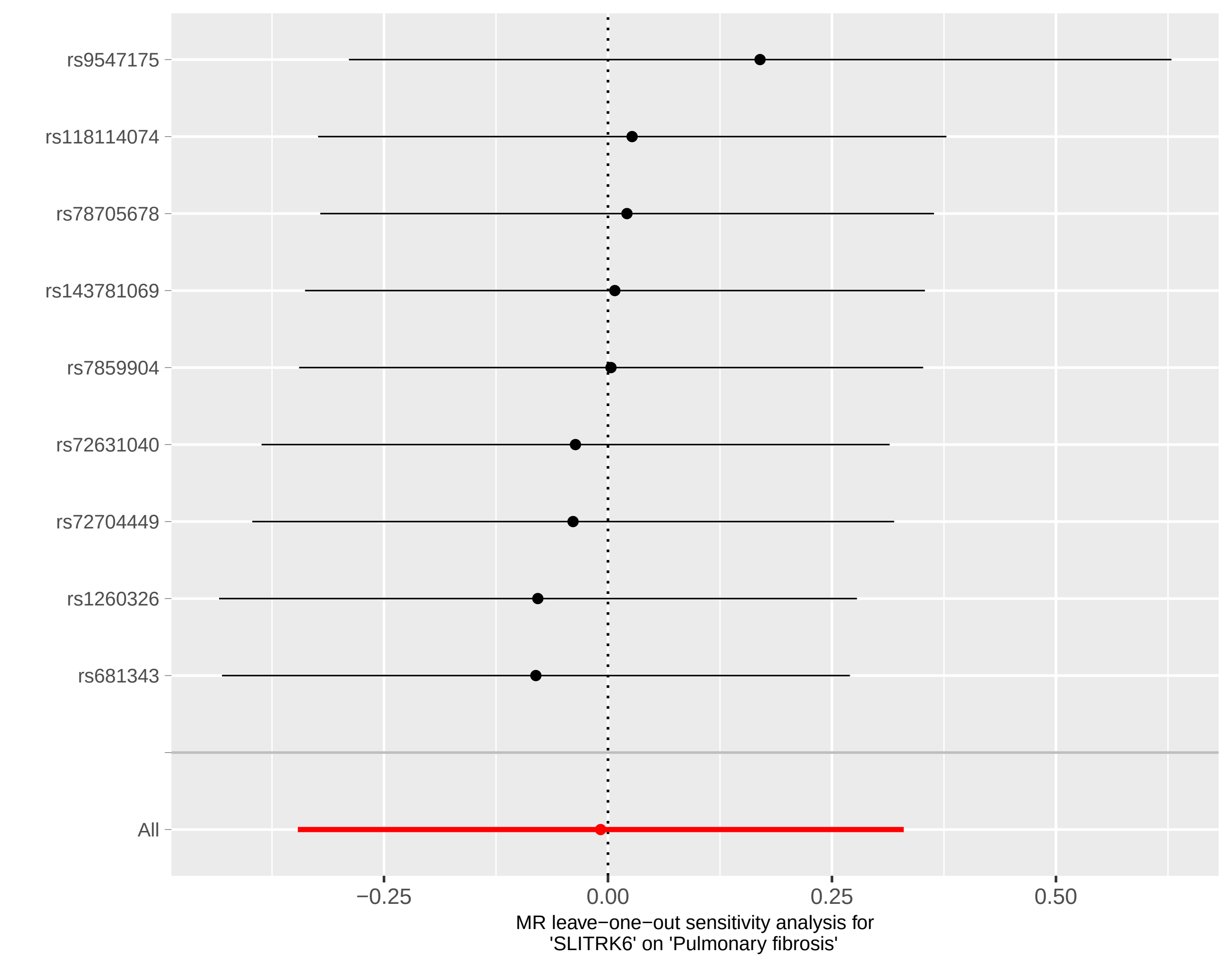
rs9547175
rs118114074
rs78705678
rs143781069
rs7859904
rs72631040
rs72704449
rs1260326
rs681343
All
−0.25
0.00
0.25
0.50
MR leave−one−out sensitivity analysis for
'SLITRK6' on 'Pulmonary fibrosis'

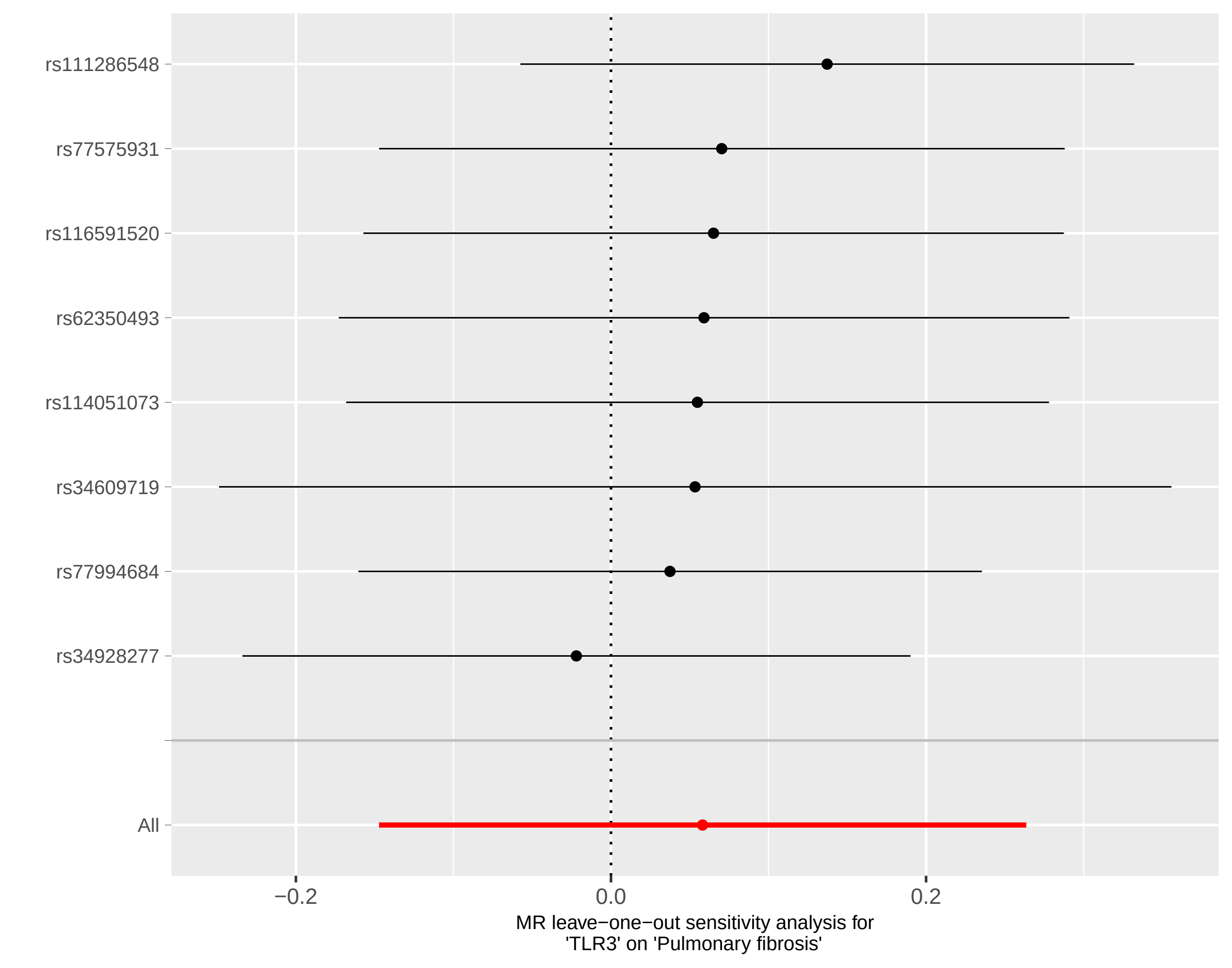
rs111286548
rs77575931
rs116591520
rs62350493
rs114051073
rs34609719
rs77994684
rs34928277
All
−0.2
0.0
0.2
MR leave−one−out sensitivity analysis for
'TLR3' on 'Pulmonary fibrosis'

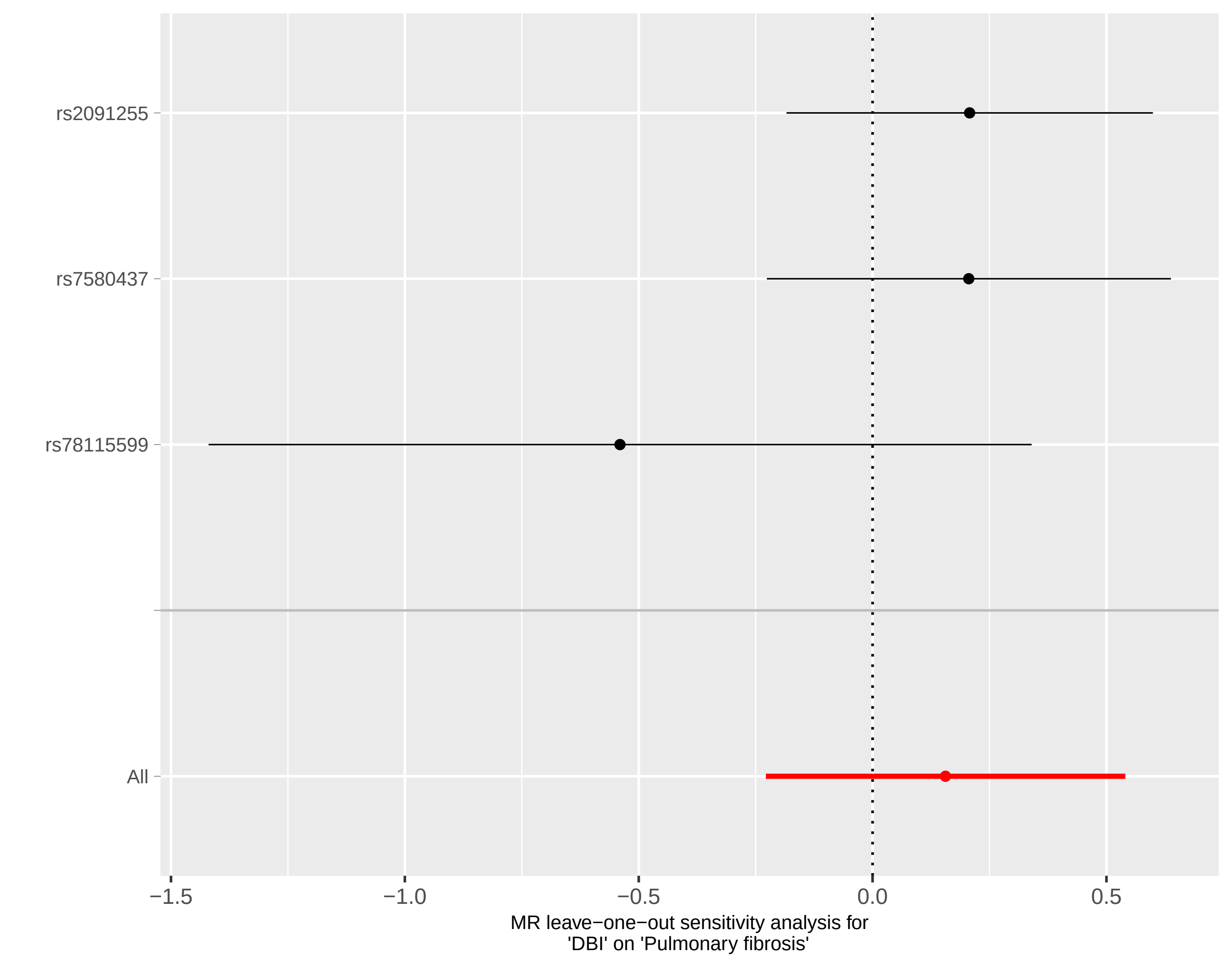
rs2091255
rs7580437
rs78115599
All
−1.5
−1.0
−0.5
0.0
0.5
MR leave−one−out sensitivity analysis for
'DBI' on 'Pulmonary fibrosis'

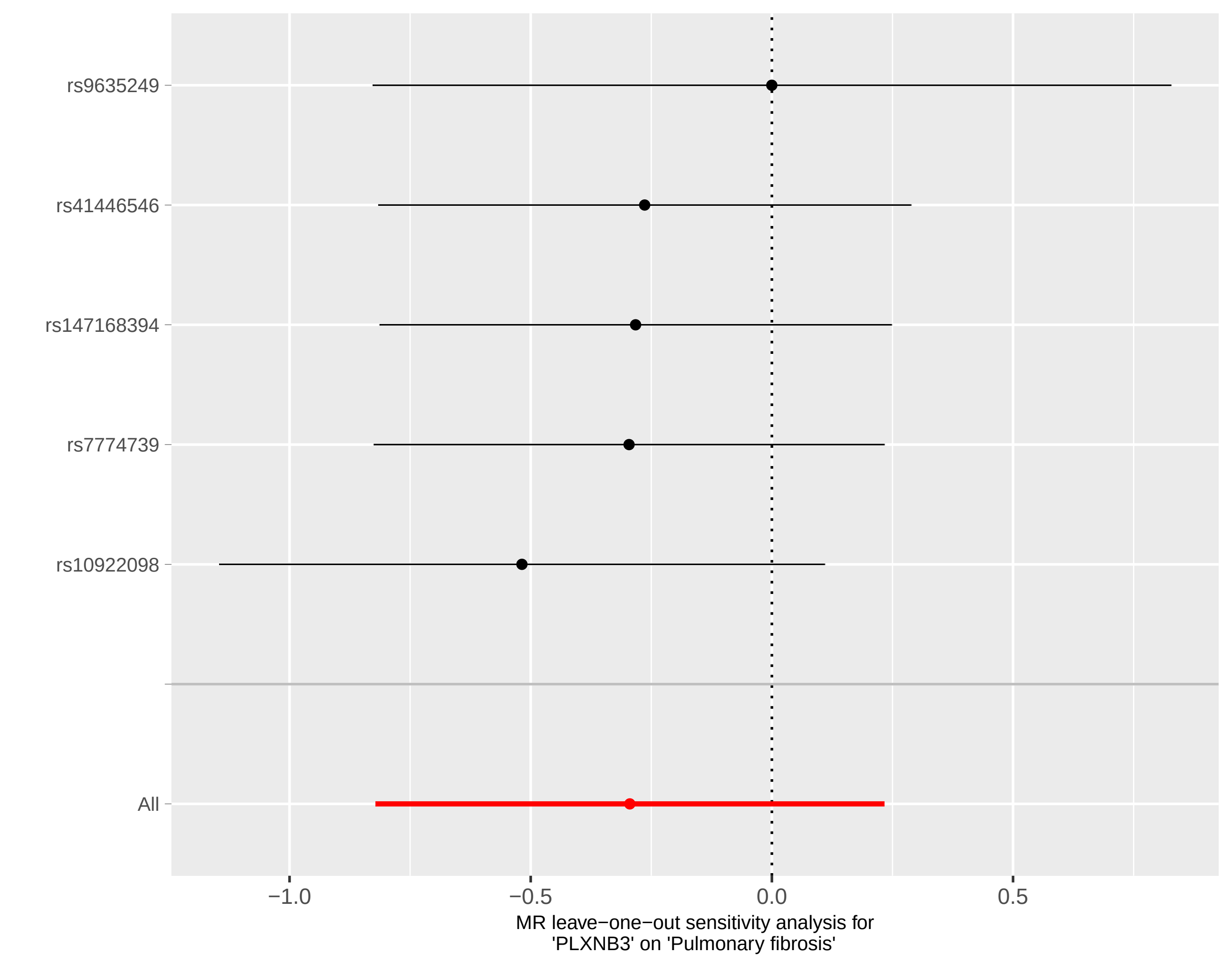
rs9635249
rs41446546
rs147168394
rs7774739
rs10922098
All
−1.0
−0.5
0.0
0.5
MR leave−one−out sensitivity analysis for
'PLXNB3' on 'Pulmonary fibrosis'

Insufficient number of SNPs

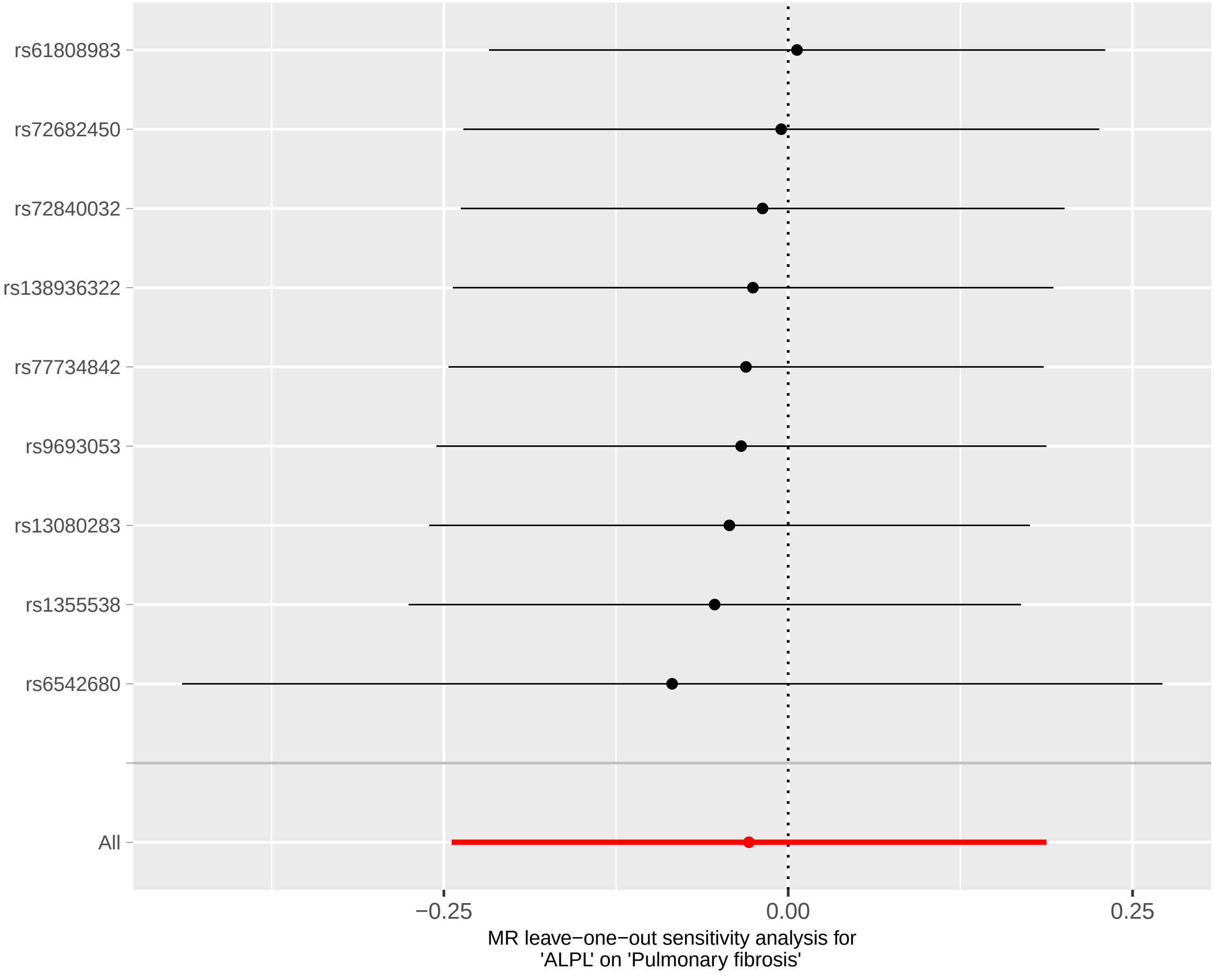
rs61808983
rs72682450
rs72840032
rs138936322
rs77734842
rs9693053
rs13080283
rs1355538
rs6542680
All
−0.25
0.00
0.25
MR leave−one−out sensitivity analysis for
'ALPL' on 'Pulmonary fibrosis'

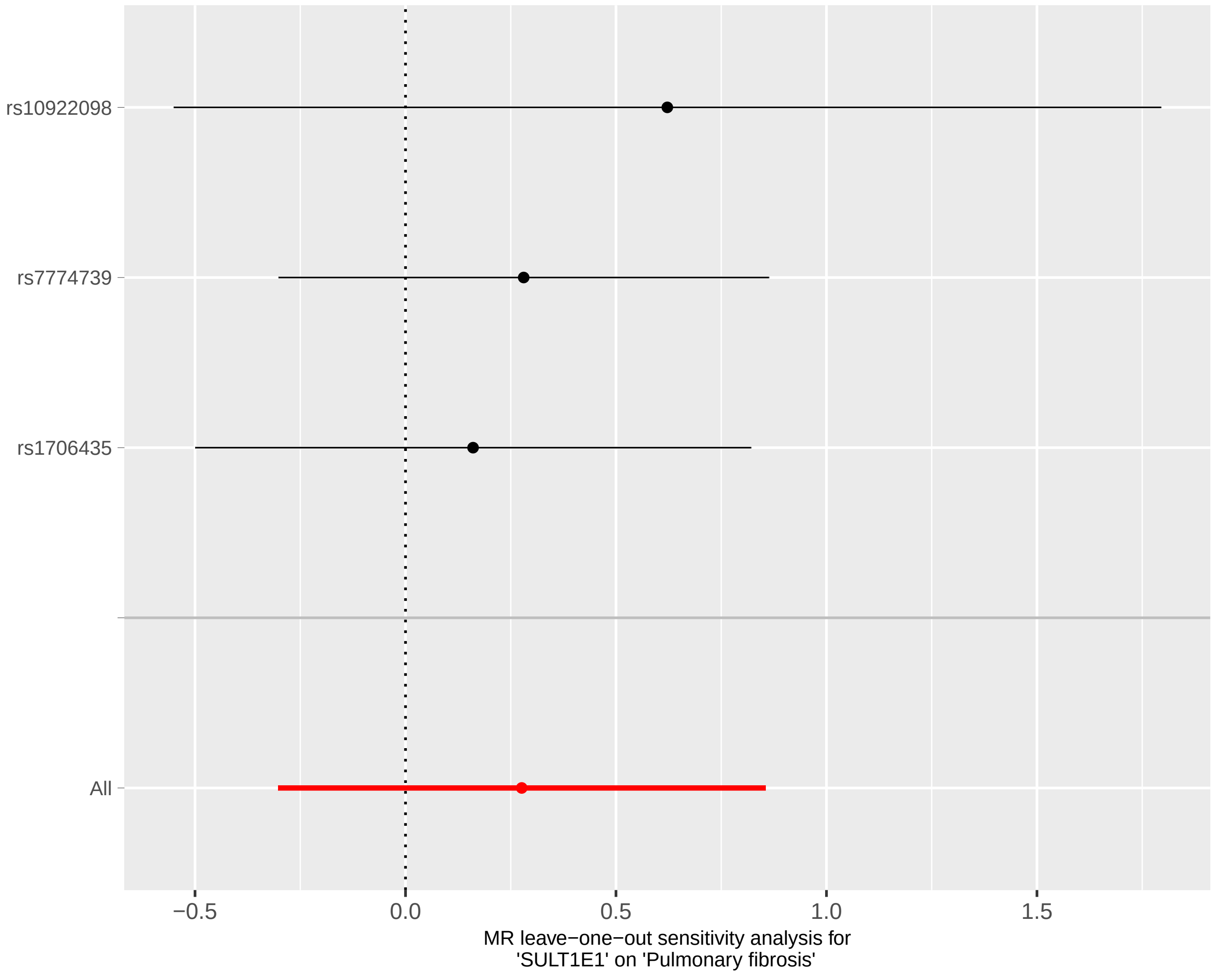

rs10922098
rs7774739
rs1706435
All
−0.5
0.0
0.5
1.0
1.5
MR leave−one−out sensitivity analysis for
'SULT1E1' on 'Pulmonary fibrosis'

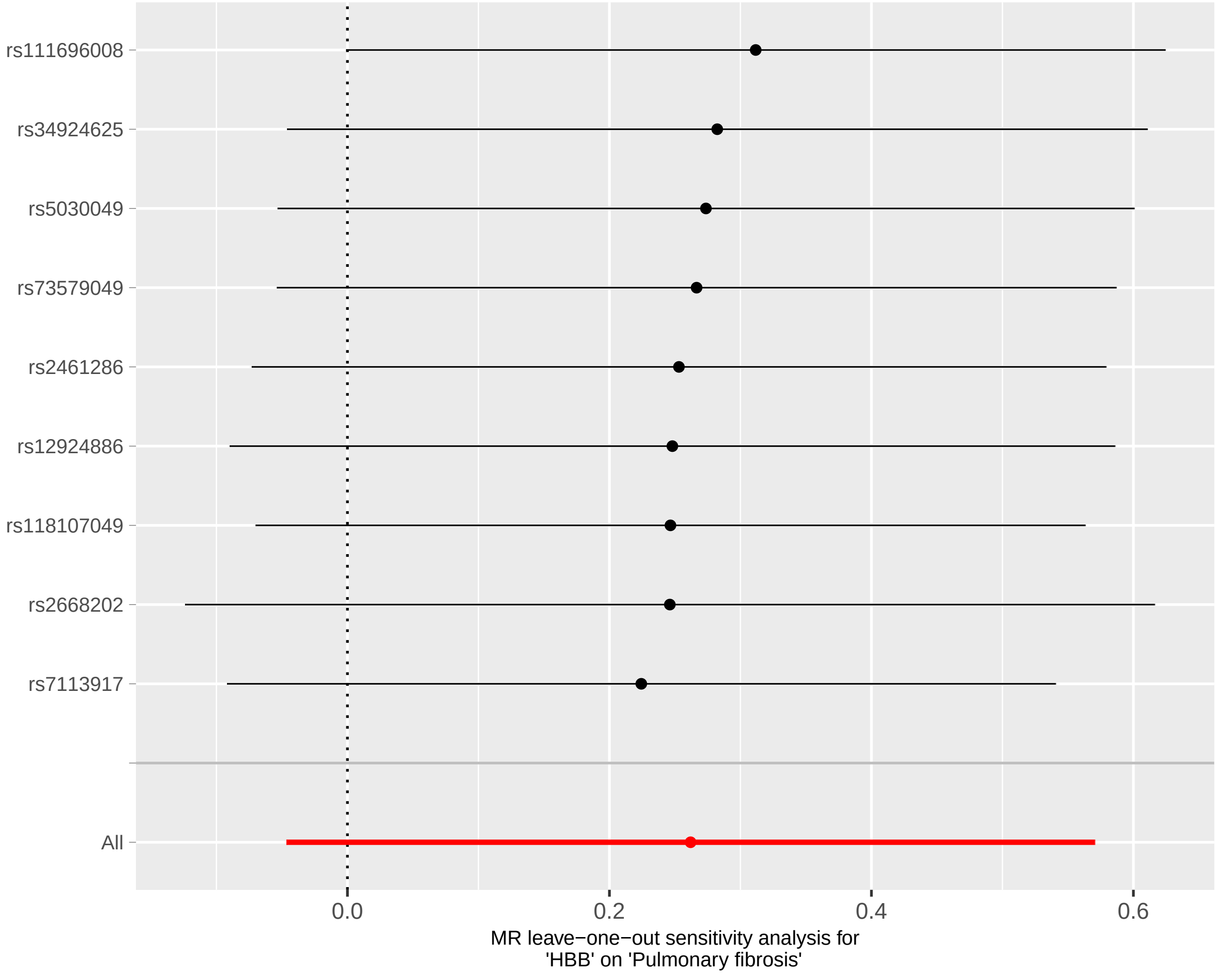
rs111696008
rs34924625
rs5030049
rs73579049
rs2461286
rs12924886
rs118107049
rs2668202
rs7113917
All
0.0
0.2
0.4
0.6
MR leave−one−out sensitivity analysis for
'HBB' on 'Pulmonary fibrosis'

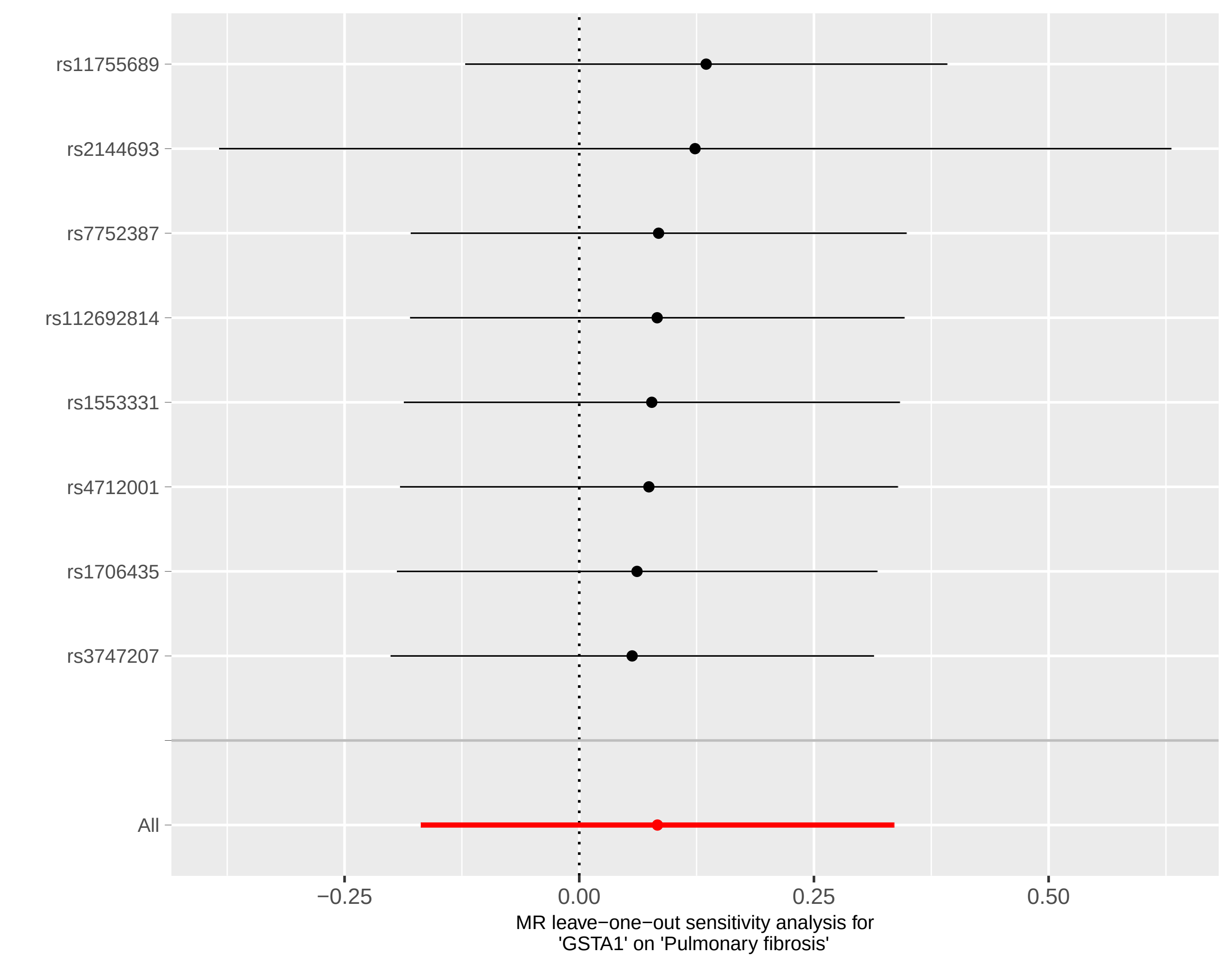
rs11755689
rs2144693
rs7752387
rs112692814
rs1553331
rs4712001
rs1706435
rs3747207
All
−0.25
0.00
0.25
0.50
MR leave−one−out sensitivity analysis for
'GSTA1' on 'Pulmonary fibrosis'

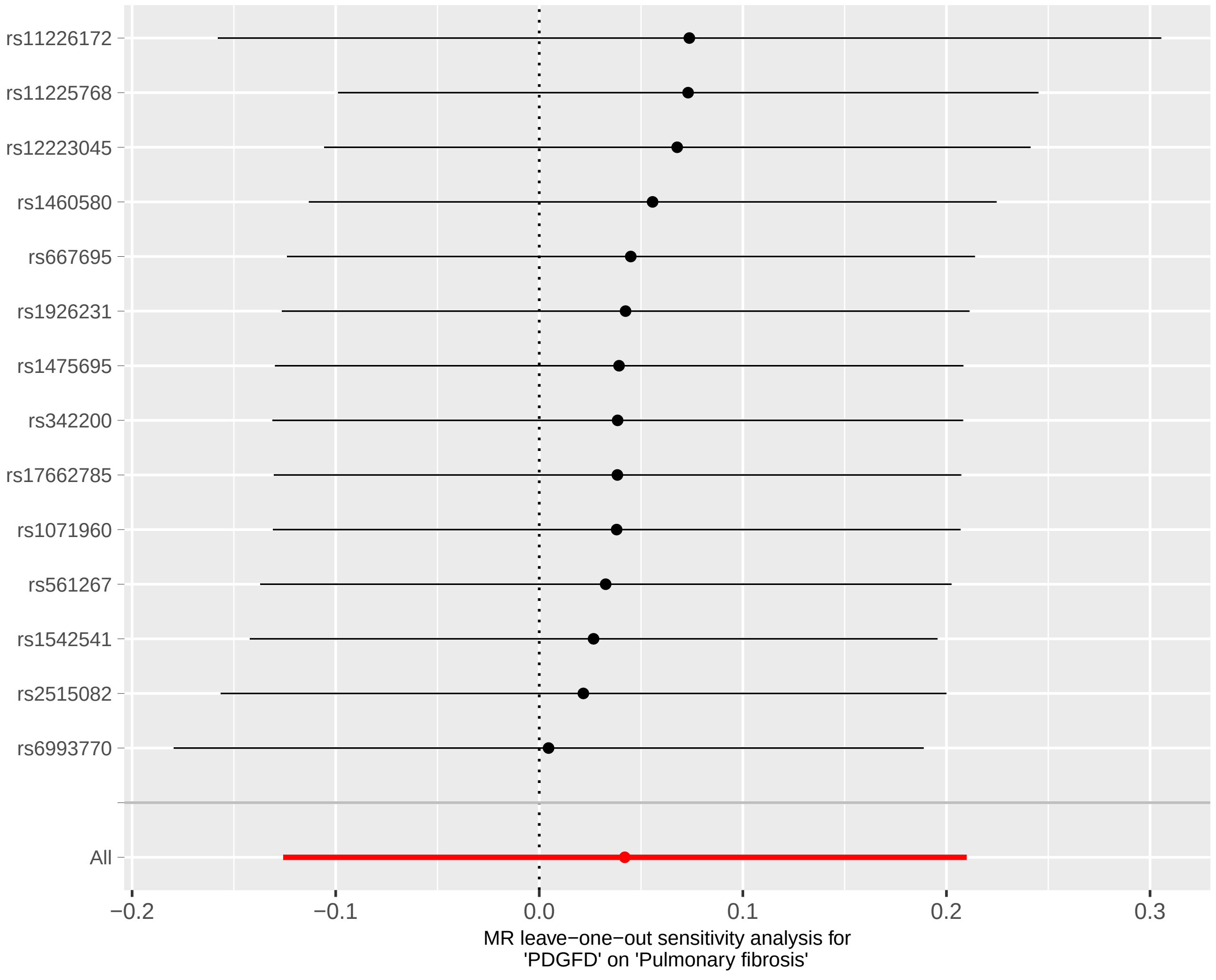
rs11226172
rs11225768
rs12223045
rs1460580
rs667695
rs1926231
rs1475695
rs342200
rs17662785
rs1071960
rs561267
rs1542541
rs2515082
rs6993770
All
−0.2
−0.1
0.0
0.1
0.2
0.3
MR leave−one−out sensitivity analysis for
'PDGFD' on 'Pulmonary fibrosis'

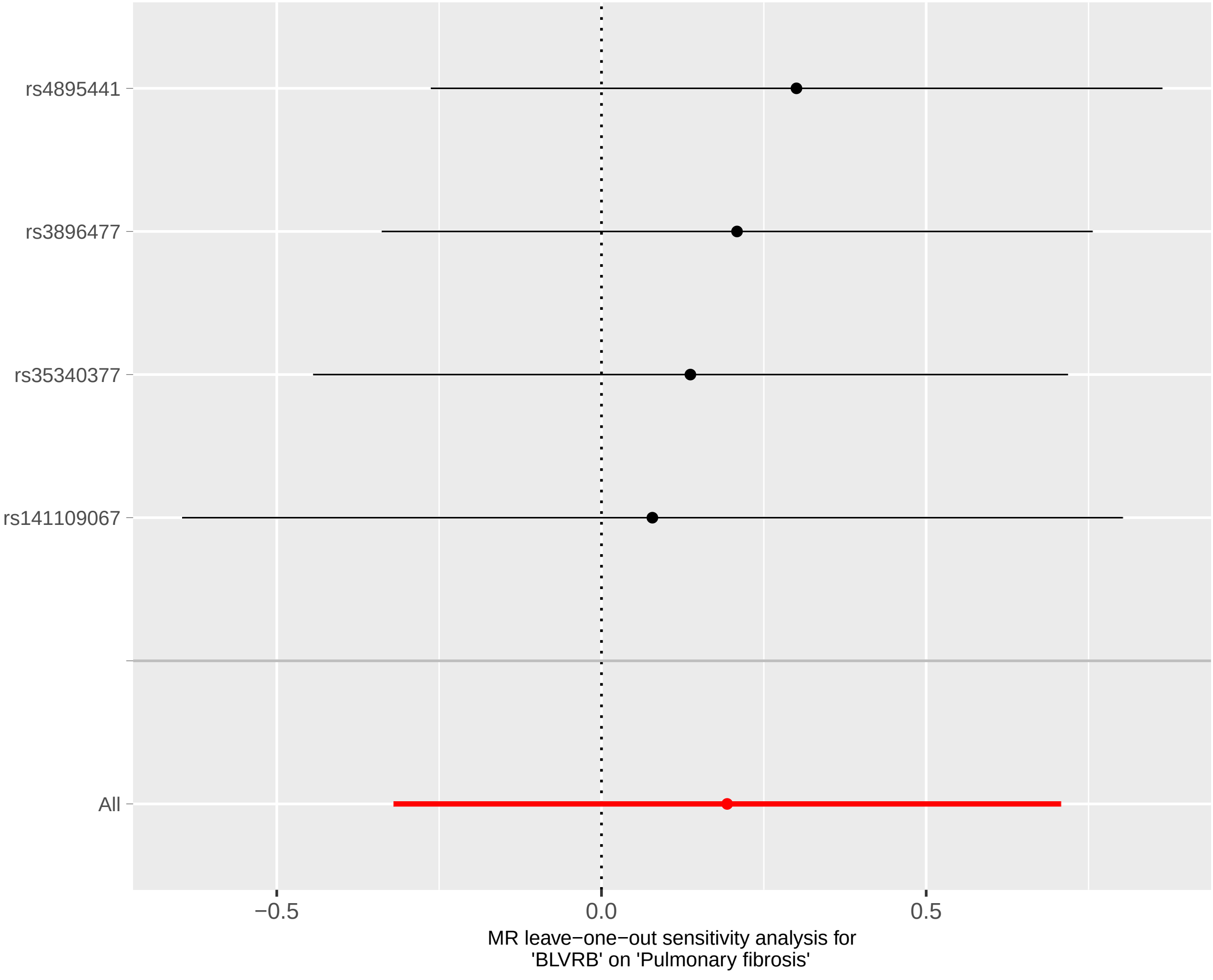
rs4895441
rs3896477
rs35340377
rs141109067
All
−0.5
0.0
0.5
MR leave−one−out sensitivity analysis for
'BLVRB' on 'Pulmonary fibrosis'

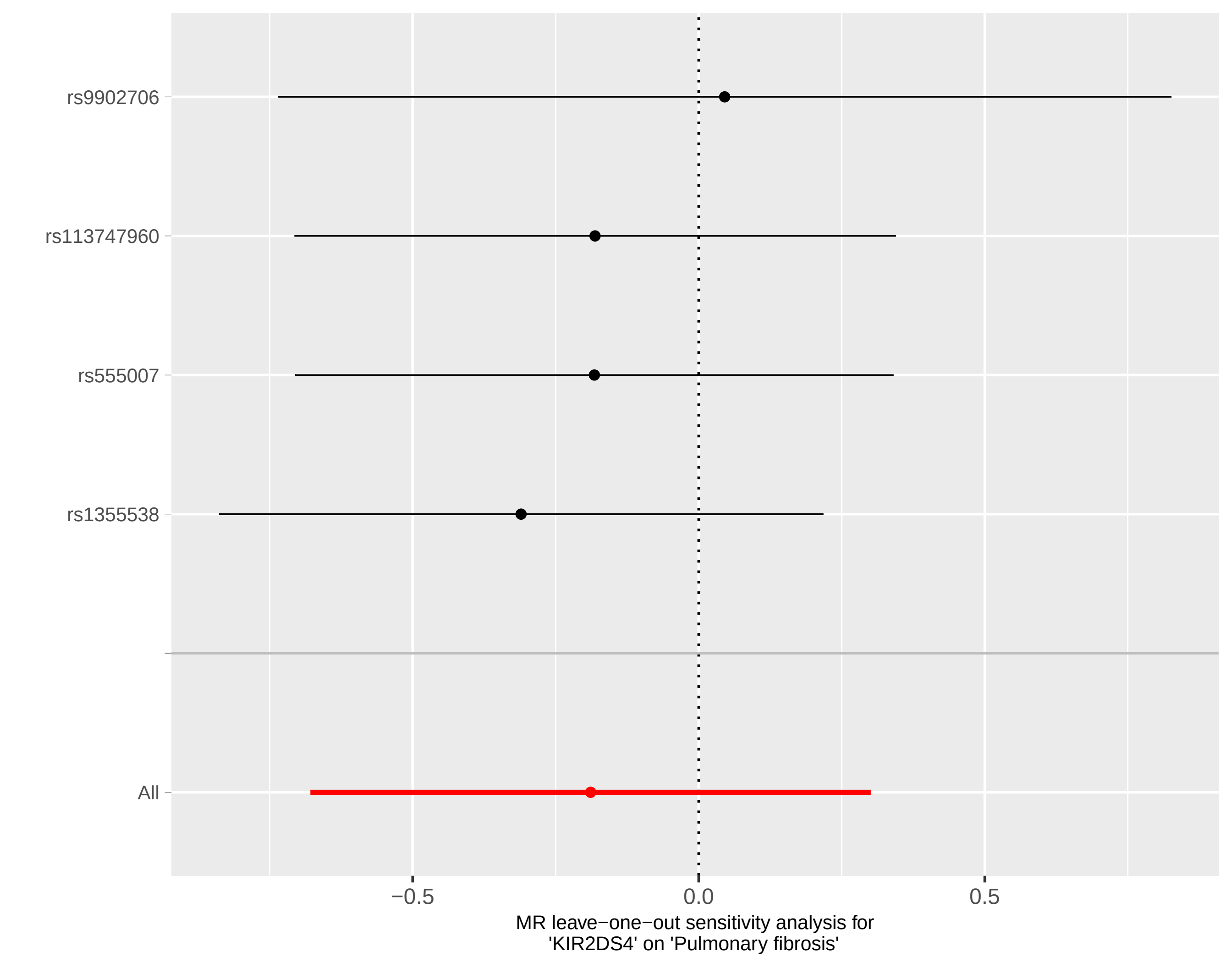
rs9902706
rs113747960
rs555007
rs1355538
All
−0.5
0.0
0.5
MR leave−one−out sensitivity analysis for
'KIR2DS4' on 'Pulmonary fibrosis'

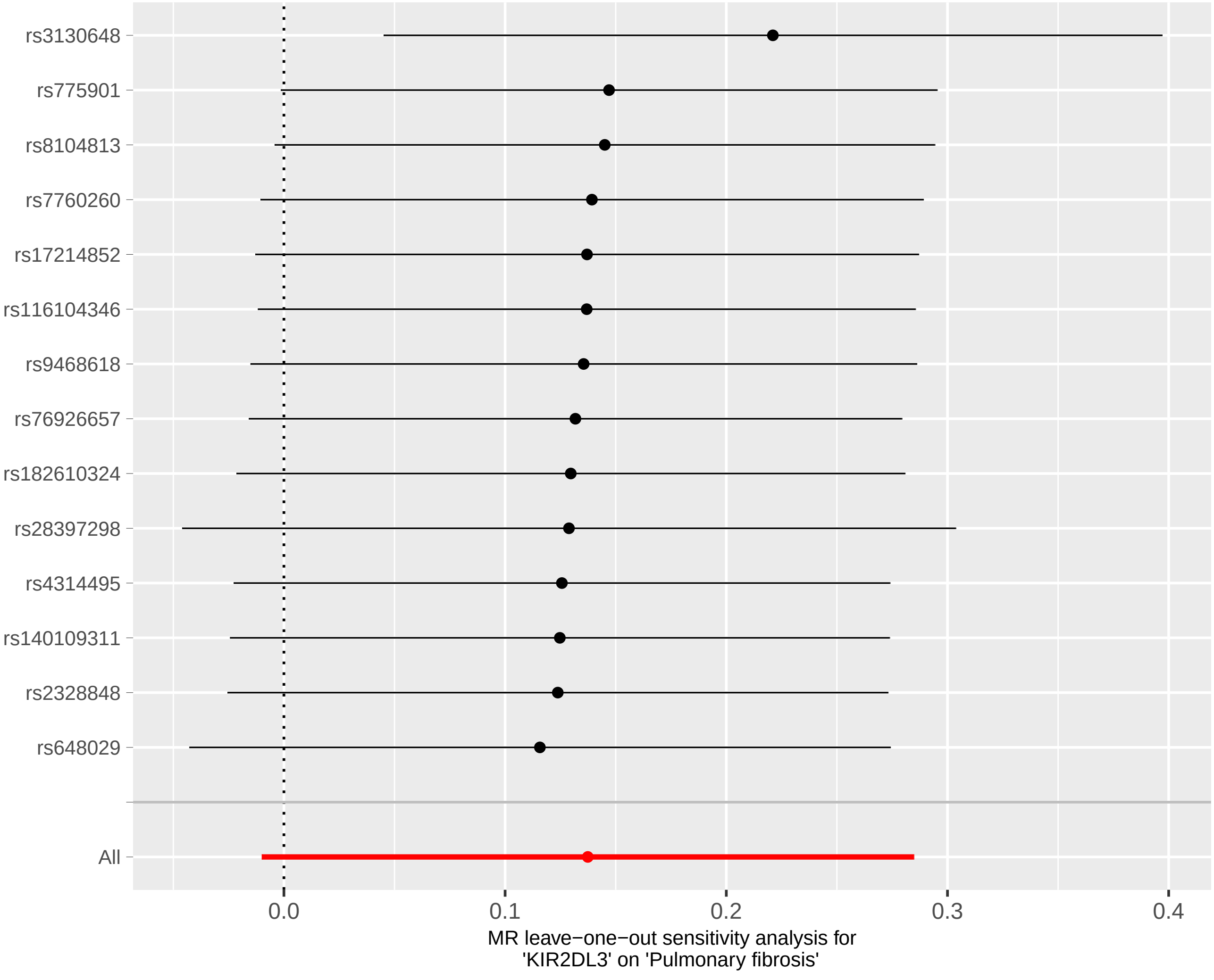
rs3130648
rs775901
rs8104813
rs7760260
rs17214852
rs116104346
rs9468618
rs76926657
rs182610324
rs28397298
rs4314495
rs140109311
rs2328848
rs648029
All
0.0
0.1
0.2
0.3
0.4
MR leave−one−out sensitivity analysis for
'KIR2DL3' on 'Pulmonary fibrosis'

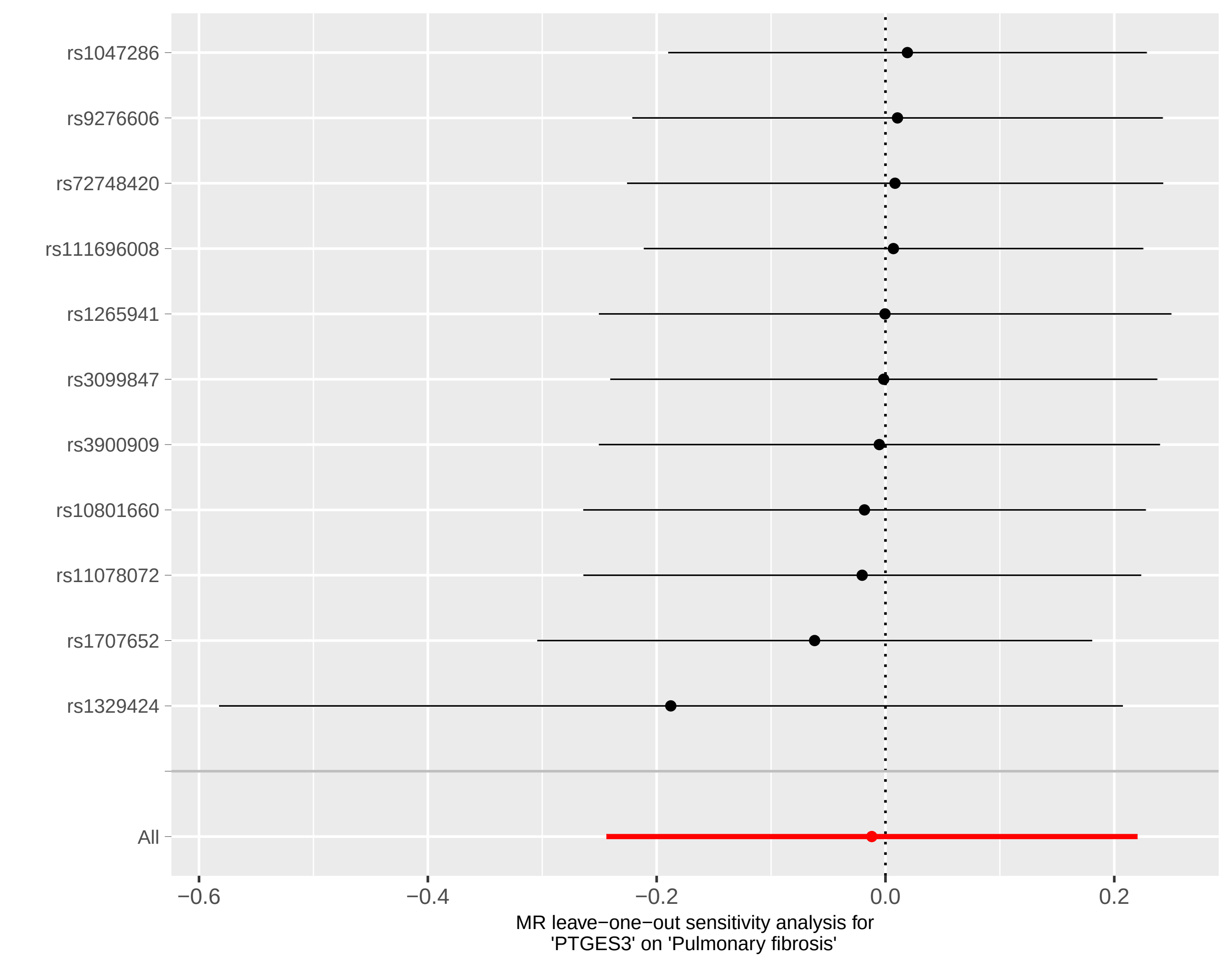

rs1047286
rs9276606
rs72748420
rs111696008
rs1265941
rs3099847
rs3900909
rs10801660
rs11078072
rs1707652
rs1329424
All
−0.6
−0.4
−0.2
0.0
0.2
MR leave−one−out sensitivity analysis for
'PTGES3' on 'Pulmonary fibrosis'

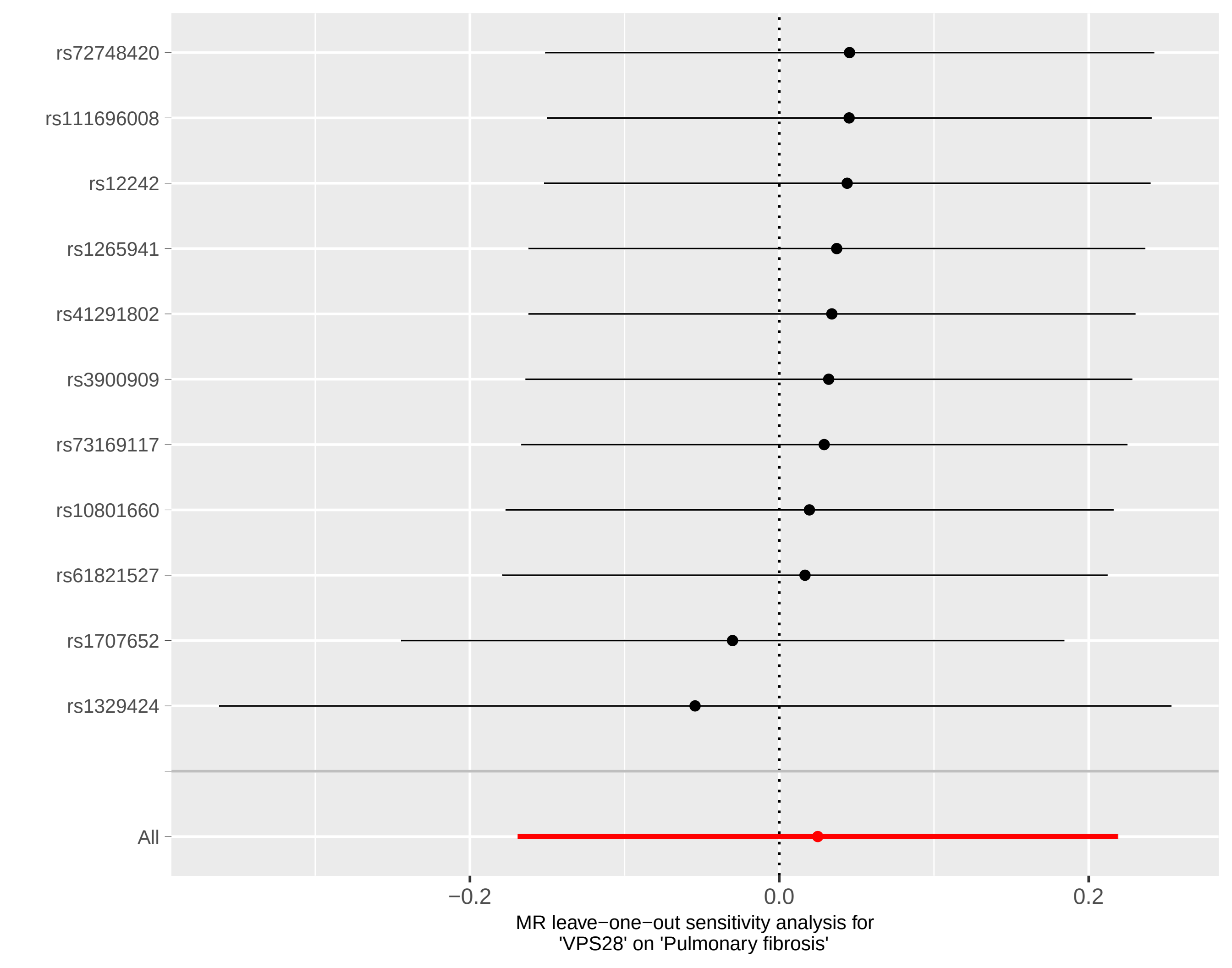

rs72748420
rs111696008
rs12242
rs1265941
rs41291802
rs3900909
rs73169117
rs10801660
rs61821527
rs1707652
rs1329424
All
−0.2
0.0
0.2
MR leave−one−out sensitivity analysis for
'VPS28' on 'Pulmonary fibrosis'

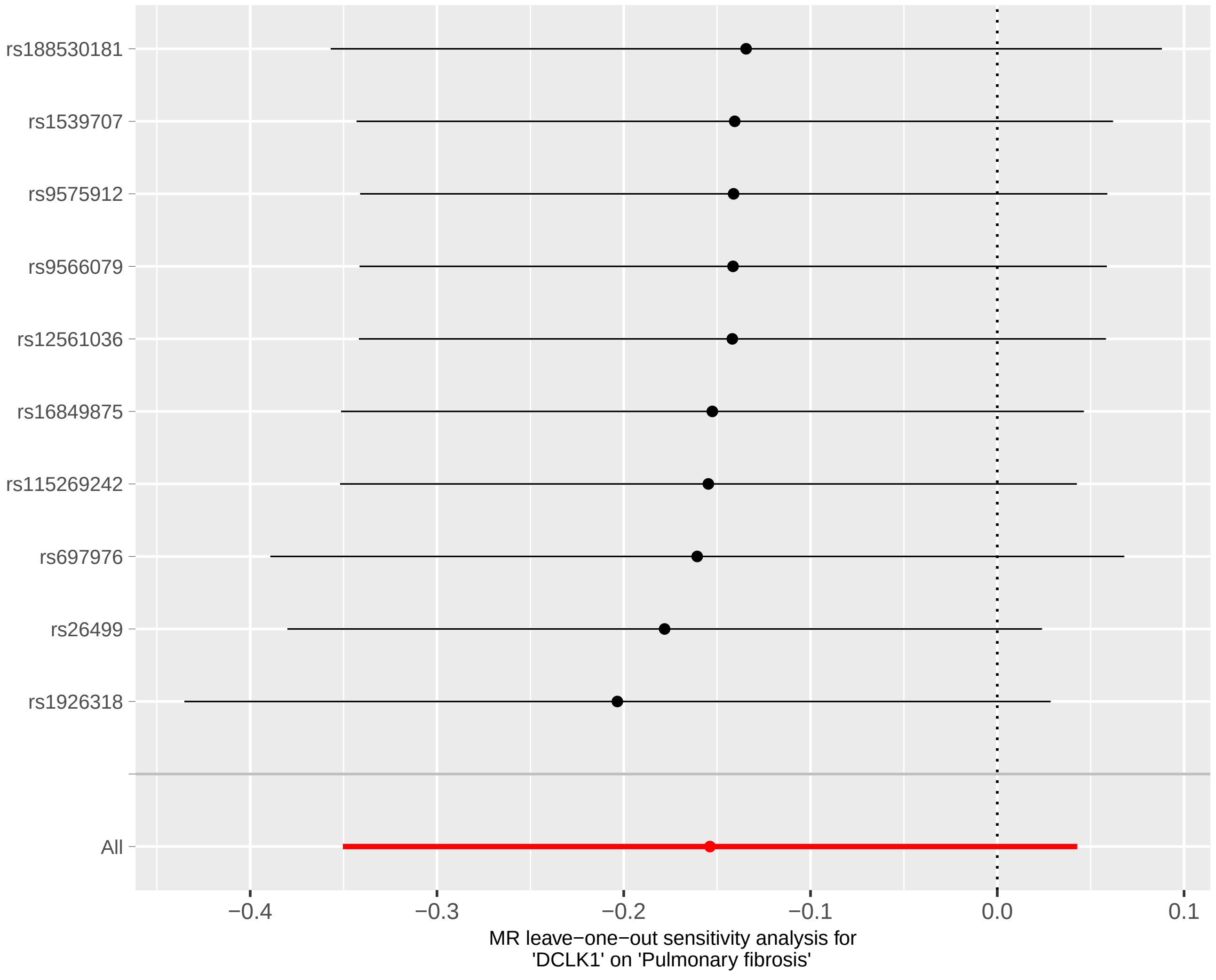
rs188530181
rs1539707
rs9575912
rs9566079
rs12561036
rs16849875
rs115269242
rs697976
rs26499
rs1926318
All
−0.4
−0.3
−0.2
−0.1
0.0
0.1
MR leave−one−out sensitivity analysis for
'DCLK1' on 'Pulmonary fibrosis'

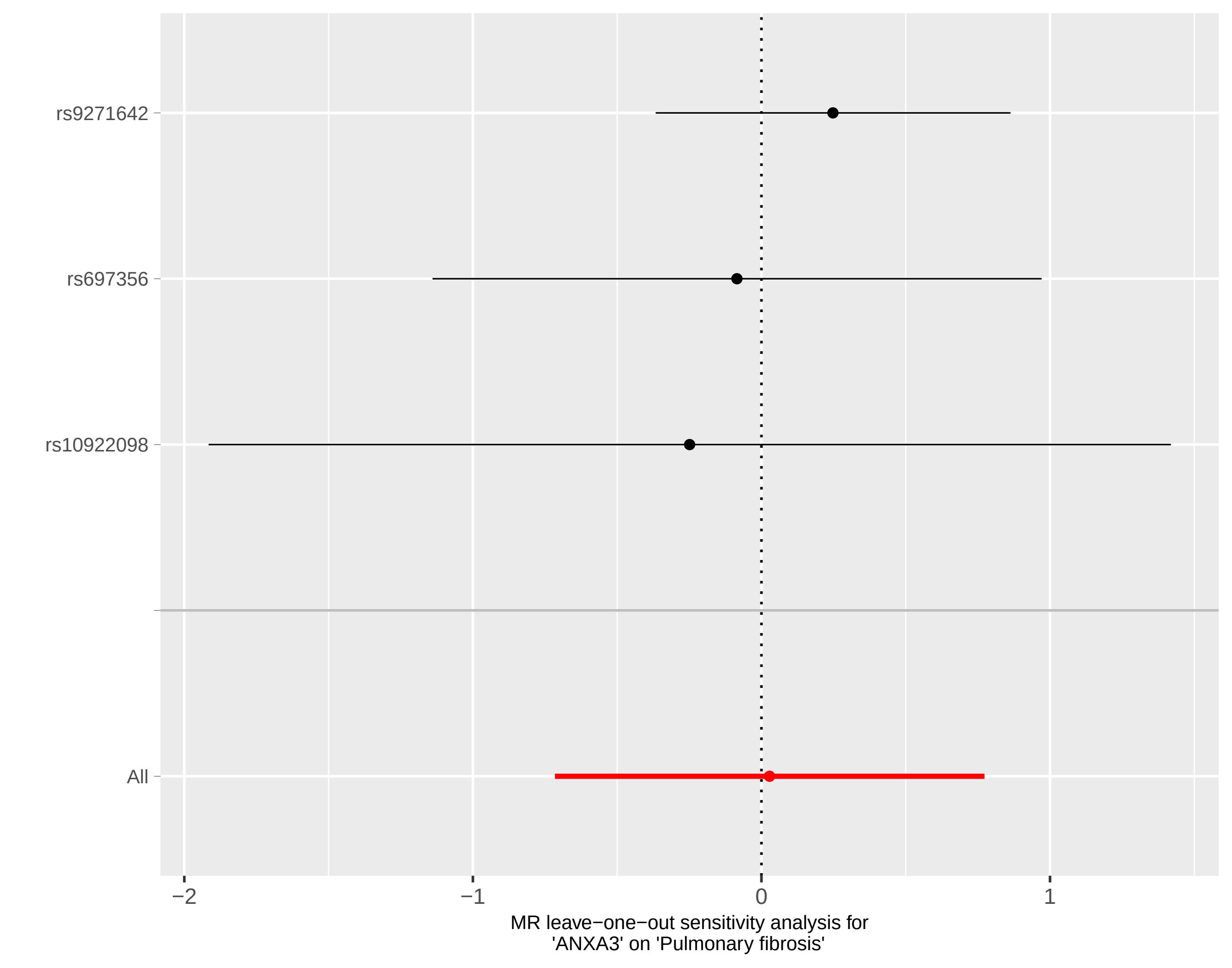

rs9271642
rs697356
rs10922098
All
−2
−1
0
1
MR leave−one−out sensitivity analysis for
'ANXA3' on 'Pulmonary fibrosis'

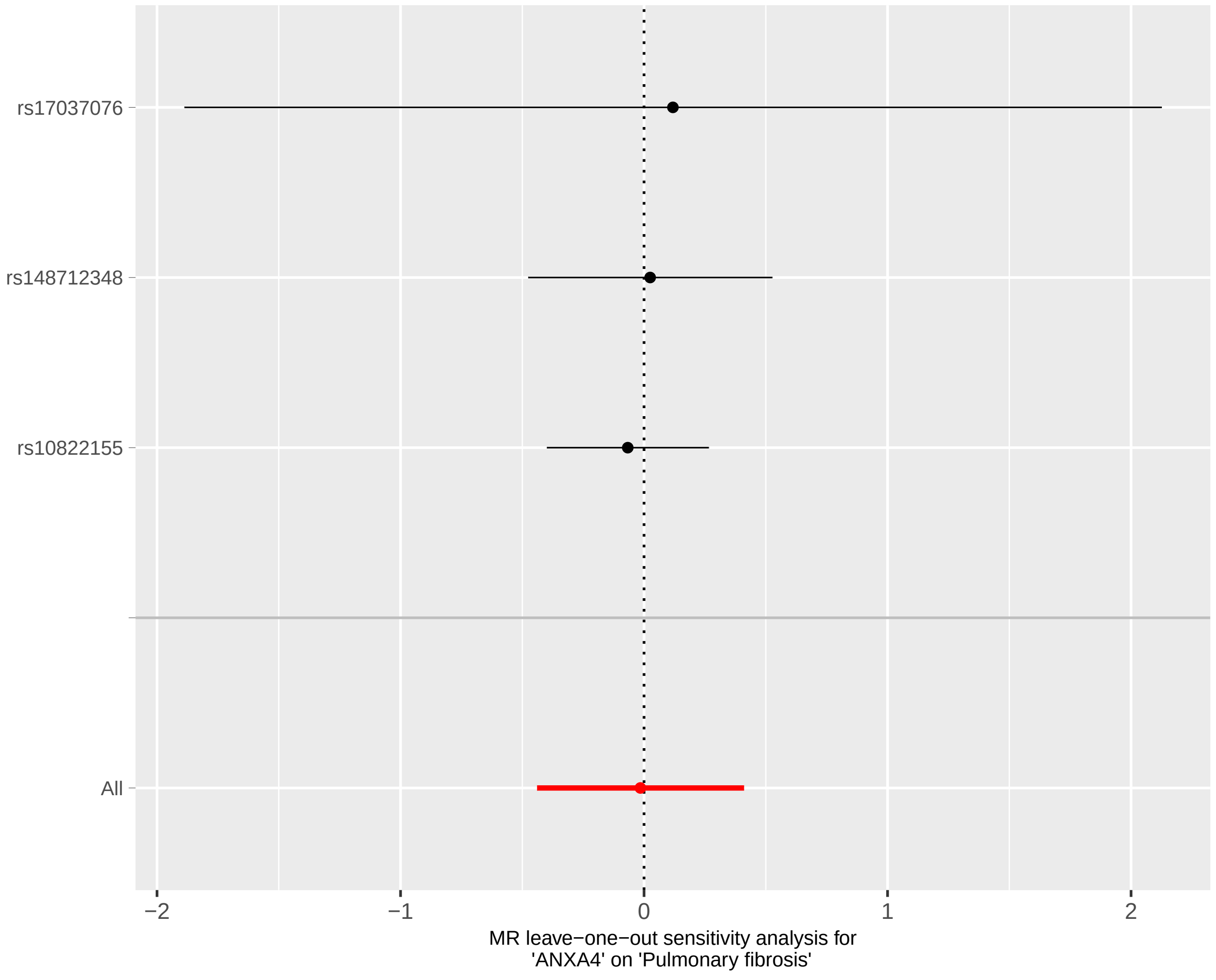
rs17037076
rs148712348
rs10822155
All
−2
−1
0
1
2
MR leave−one−out sensitivity analysis for
'ANXA4' on 'Pulmonary fibrosis'

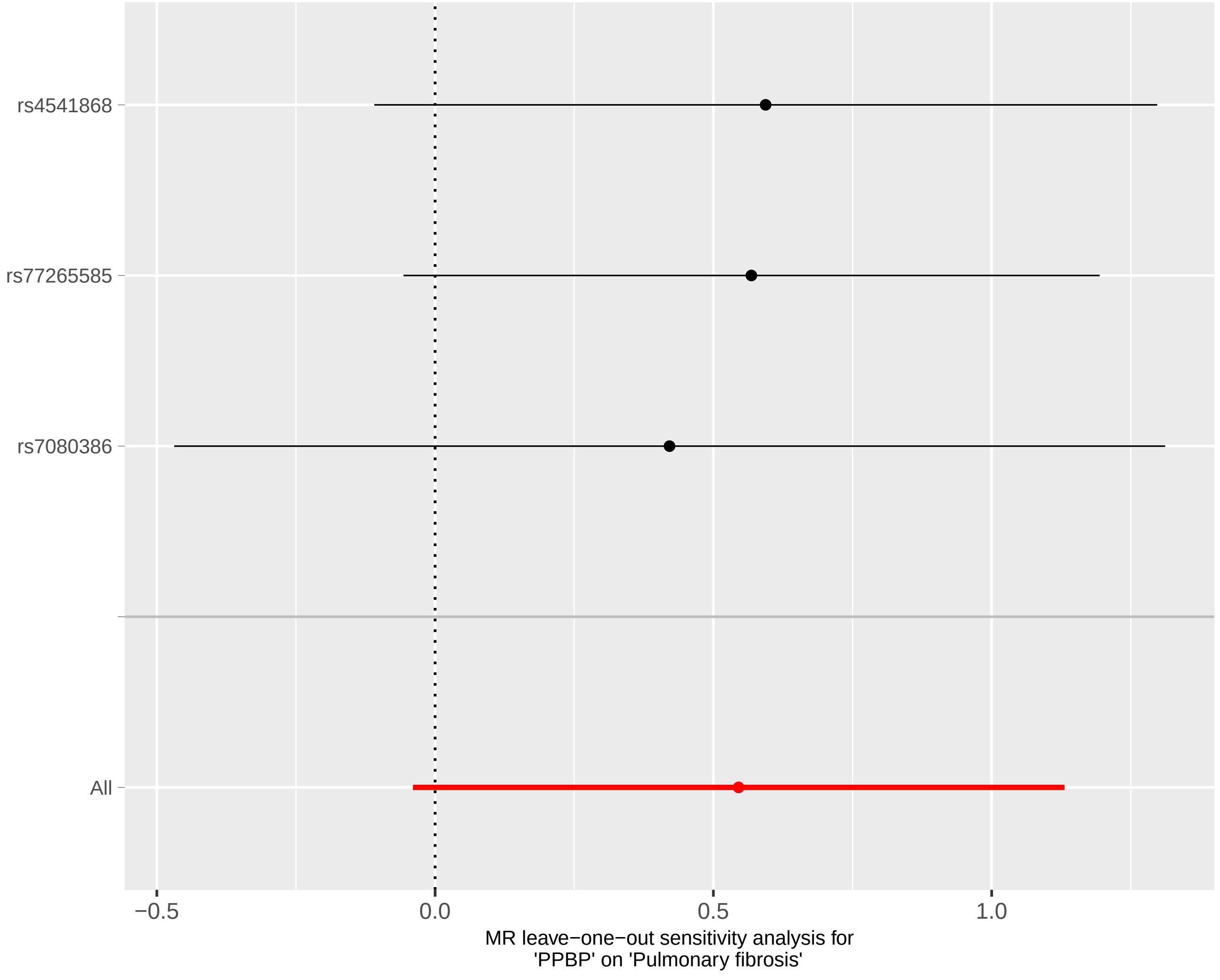
rs4541868
rs77265585
rs7080386
All
−0.5
0.0
0.5
1.0
MR leave−one−out sensitivity analysis for
'PPBP' on 'Pulmonary fibrosis'

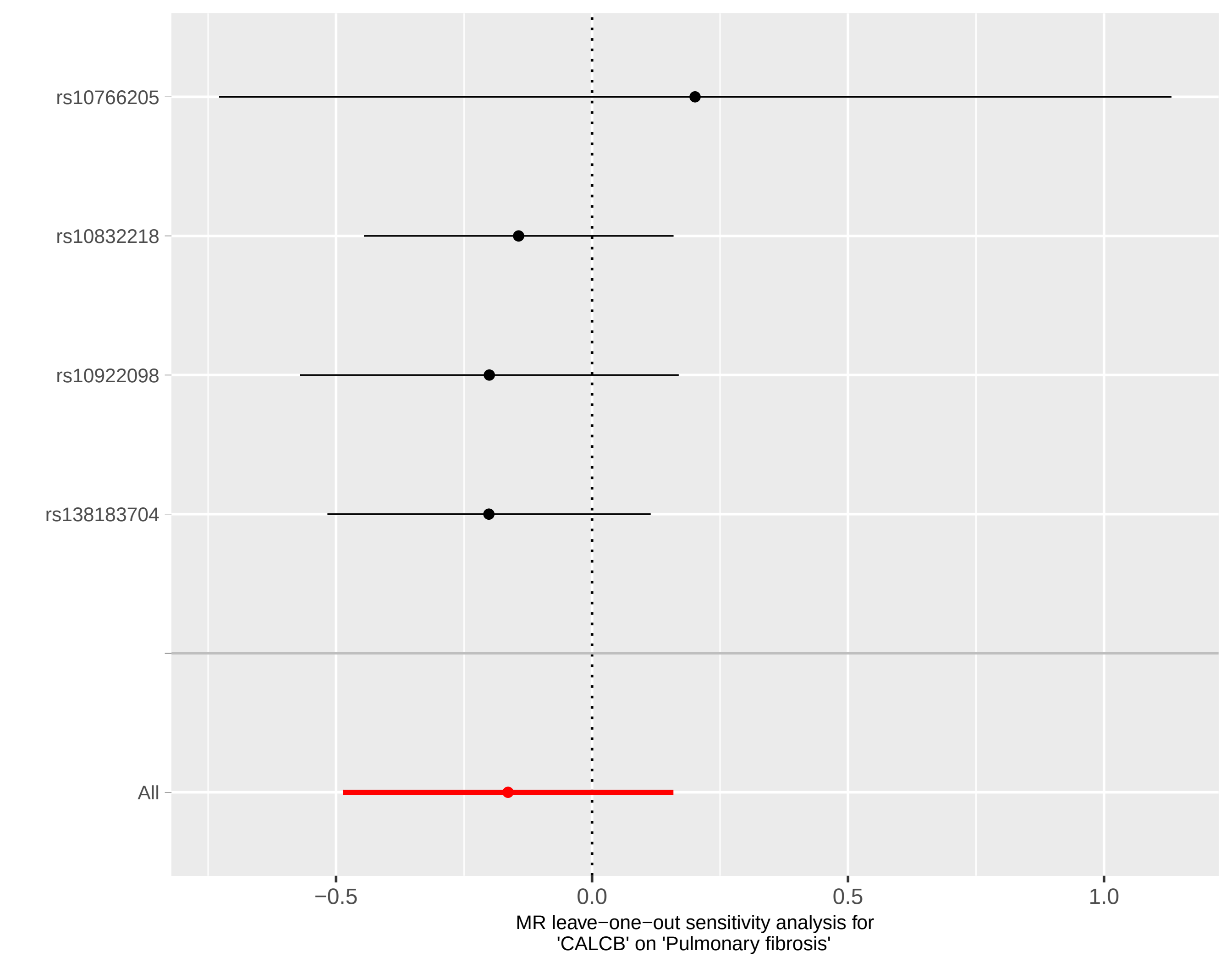

rs10766205
rs10832218
rs10922098
rs138183704
All
−0.5
0.0
0.5
1.0
MR leave−one−out sensitivity analysis for
'CALCB' on 'Pulmonary fibrosis'

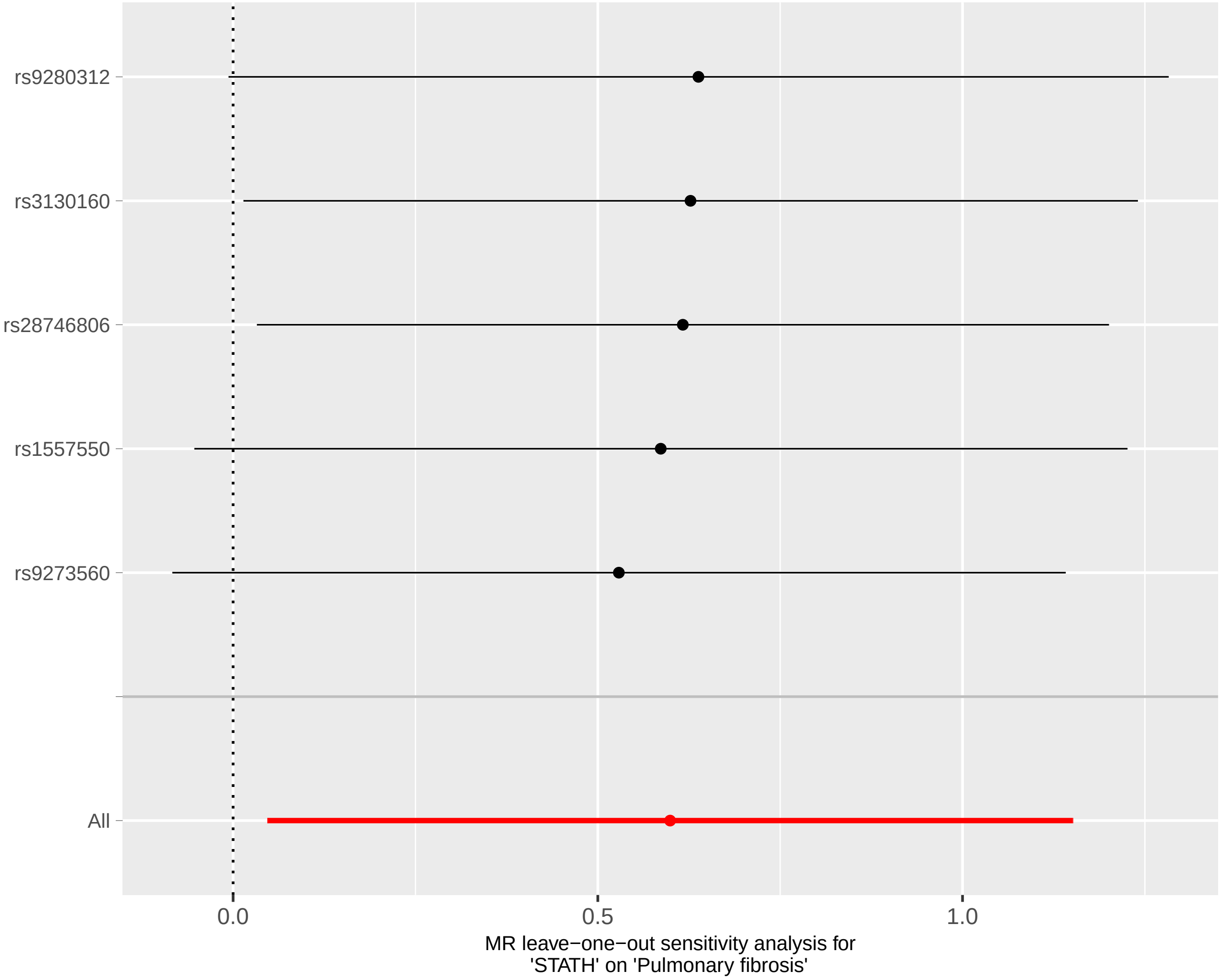
rs9280312
rs3130160
rs28746806
rs1557550
rs9273560
All
0.0
0.5
1.0
MR leave−one−out sensitivity analysis for
'STATH' on 'Pulmonary fibrosis'

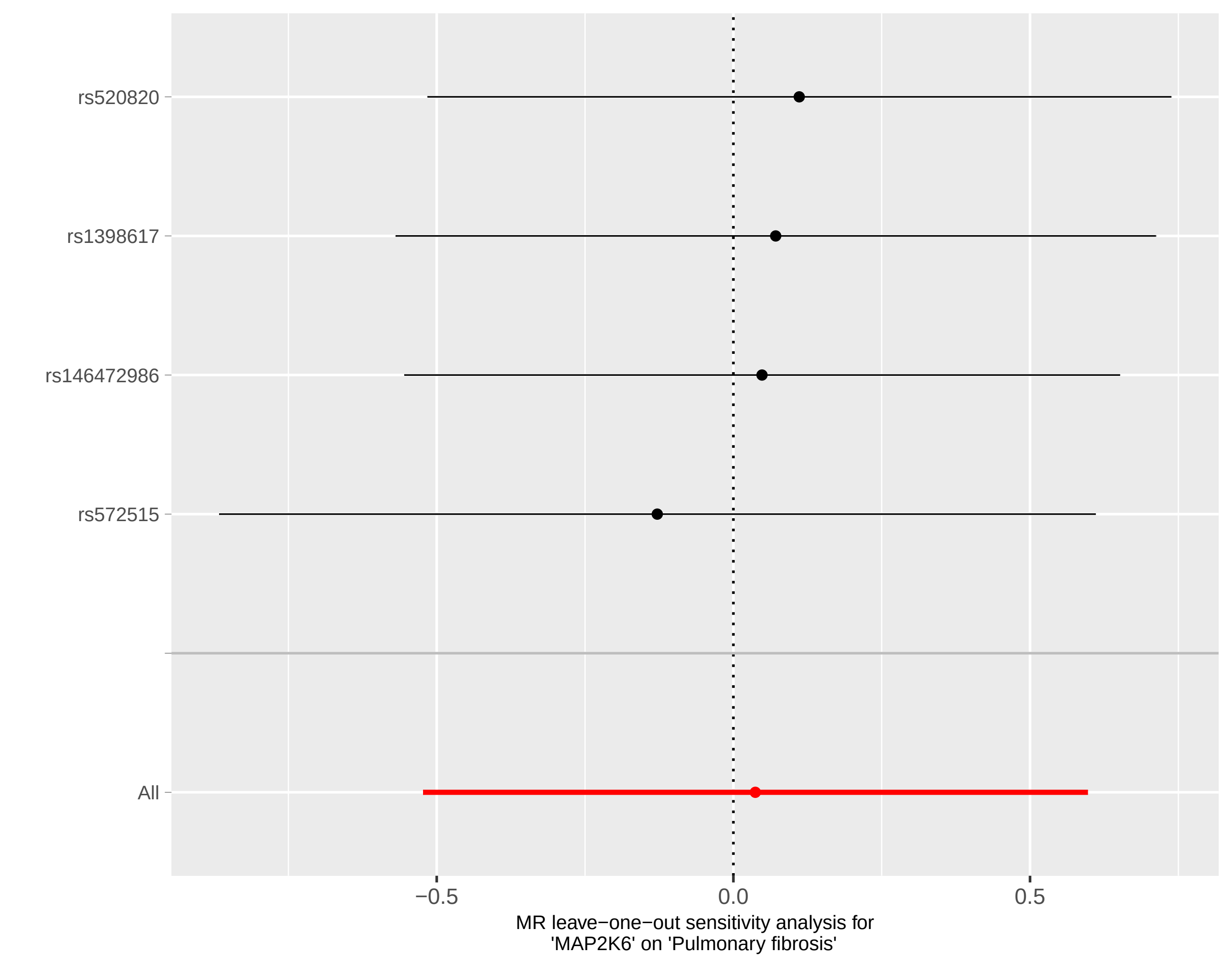
rs520820
rs1398617
rs146472986
rs572515
All
−0.5
0.0
0.5
MR leave−one−out sensitivity analysis for
'MAP2K6' on 'Pulmonary fibrosis'

Insufficient number of SNPs

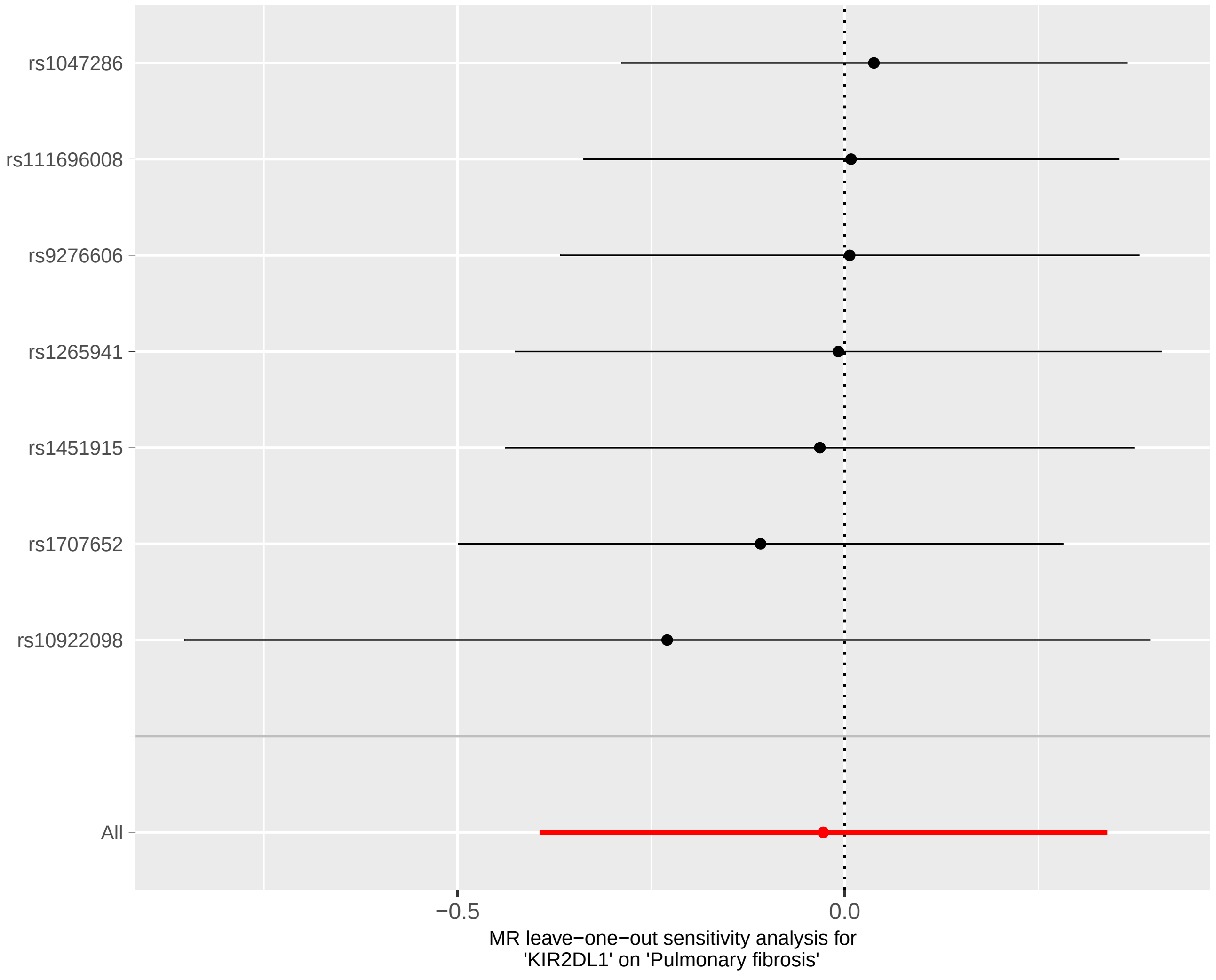
rs1047286
rs111696008
rs9276606
rs1265941
rs1451915
rs1707652
rs10922098
All
−0.5
0.0
MR leave−one−out sensitivity analysis for
'KIR2DL1' on 'Pulmonary fibrosis'

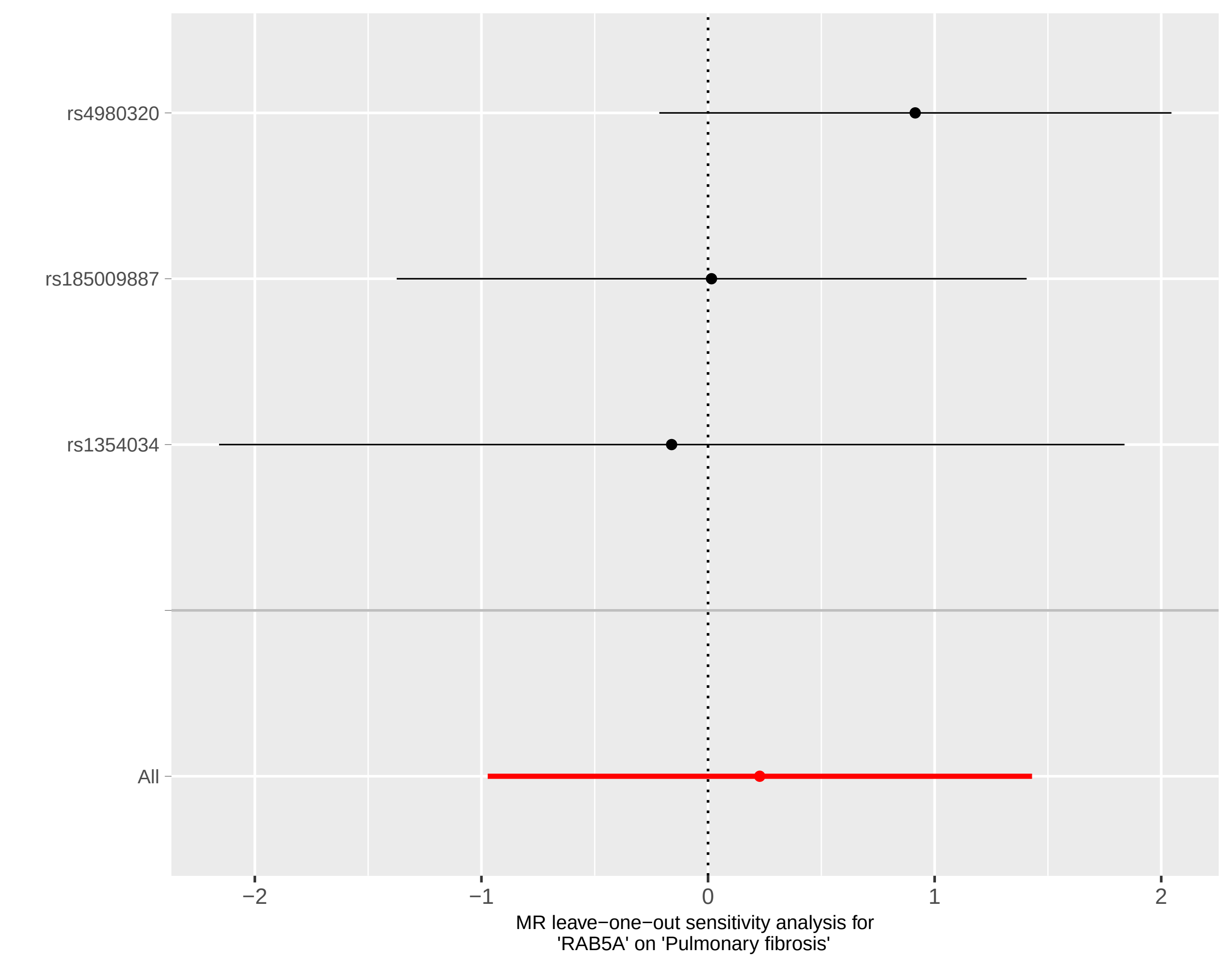
rs4980320
rs185009887
rs1354034
All
−2
−1
0
1
2
MR leave−one−out sensitivity analysis for
'RAB5A' on 'Pulmonary fibrosis'

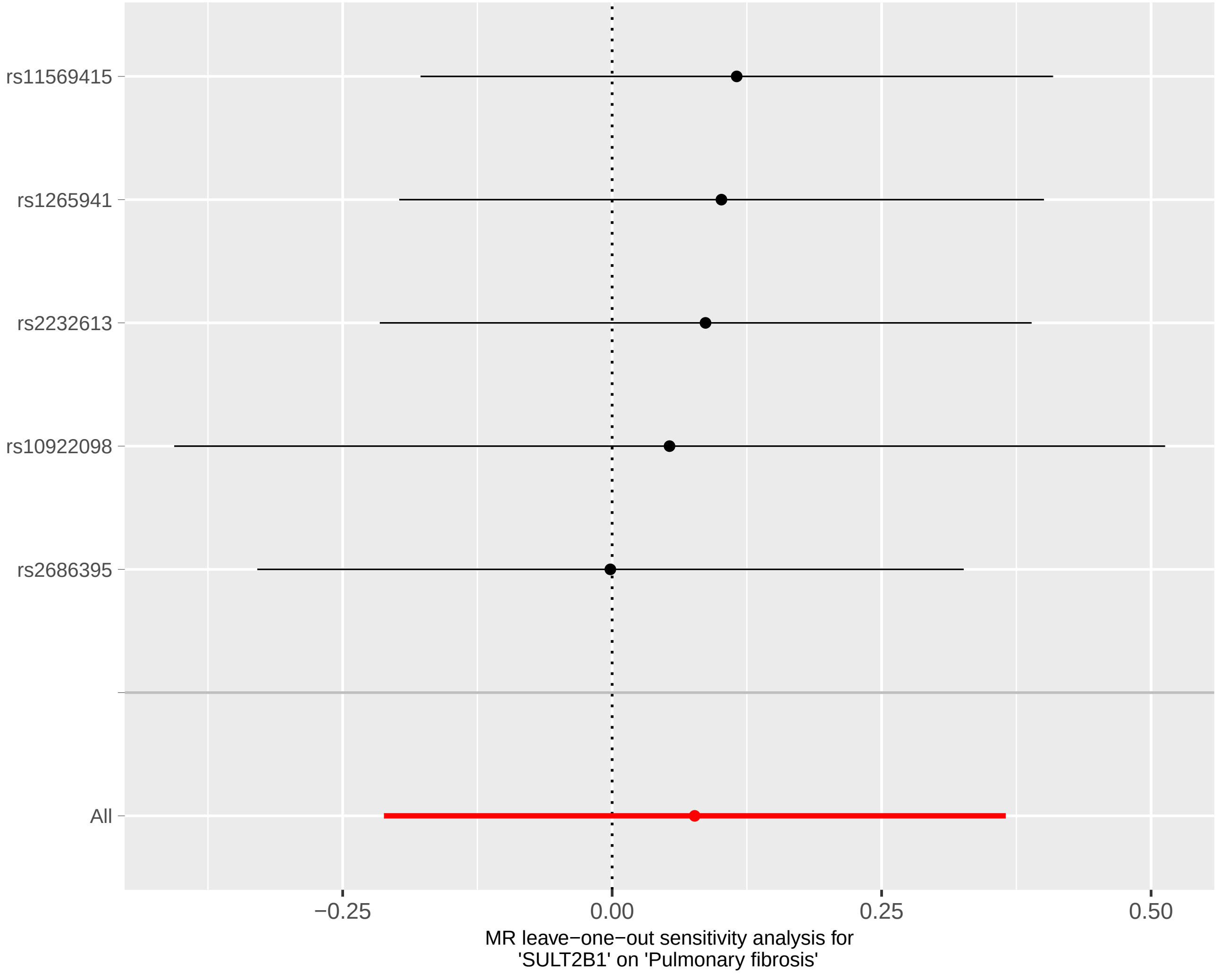
rs11569415
rs1265941
rs2232613
rs10922098
rs2686395
All
−0.25
0.00
0.25
0.50
MR leave−one−out sensitivity analysis for
'SULT2B1' on 'Pulmonary fibrosis'

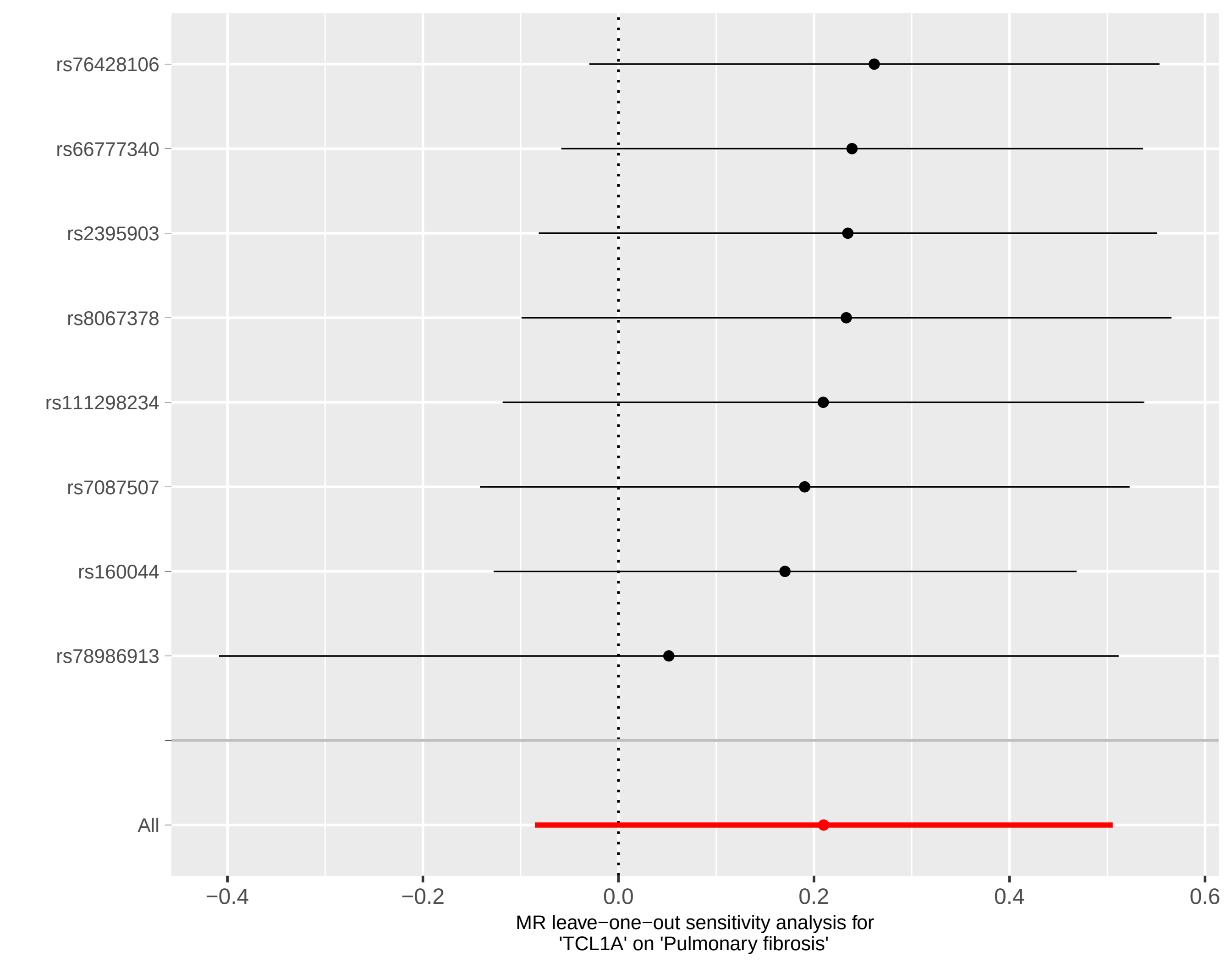
rs76428106
rs66777340
rs2395903
rs8067378
rs111298234
rs7087507
rs160044
rs78986913
All
−0.4
−0.2
0.0
0.2
0.4
0.6
MR leave−one−out sensitivity analysis for
'TCL1A' on 'Pulmonary fibrosis'

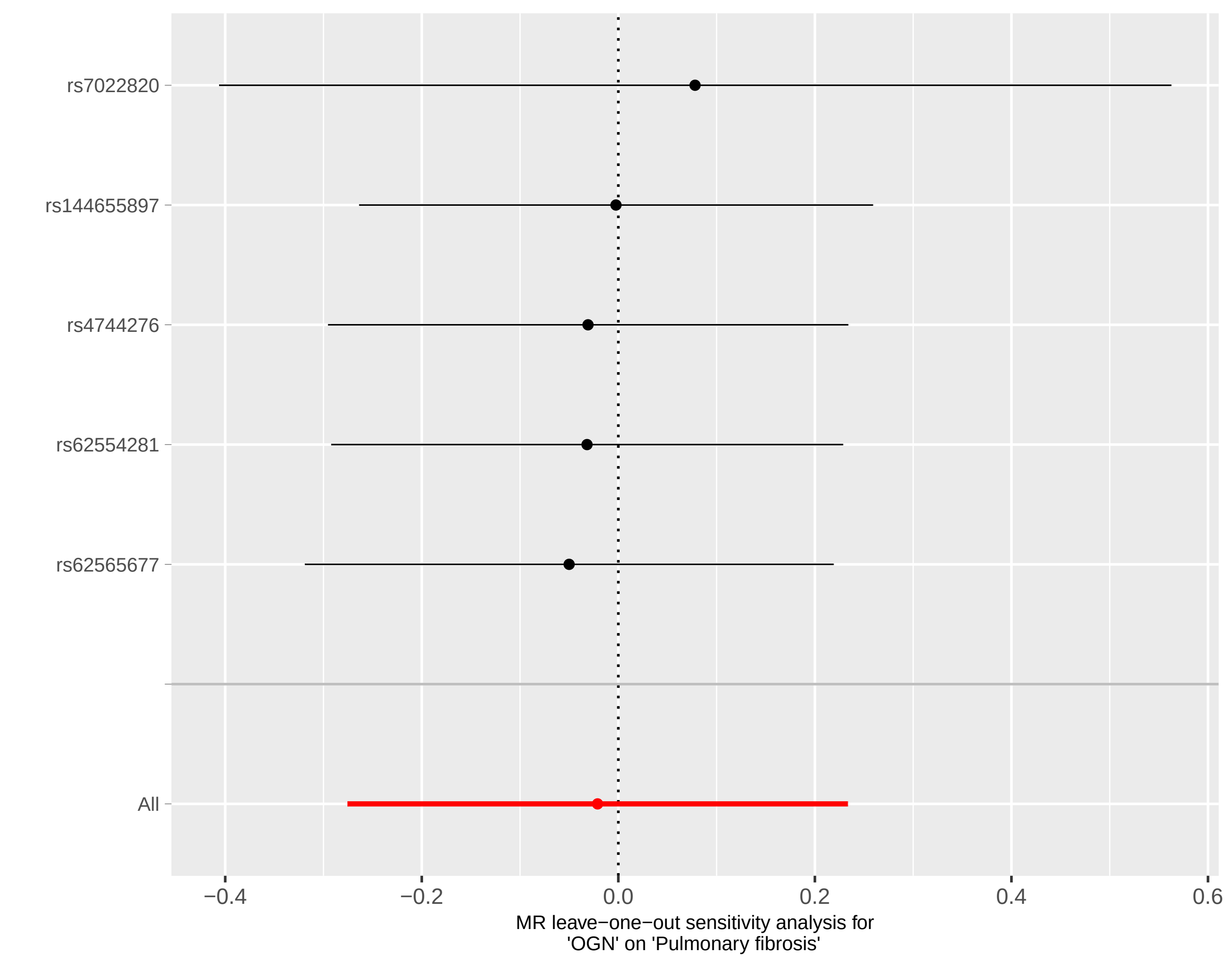
rs7022820
rs144655897
rs4744276
rs62554281
rs62565677
All
−0.4
−0.2
0.0
0.2
0.4
0.6
MR leave−one−out sensitivity analysis for
'OGN' on 'Pulmonary fibrosis'

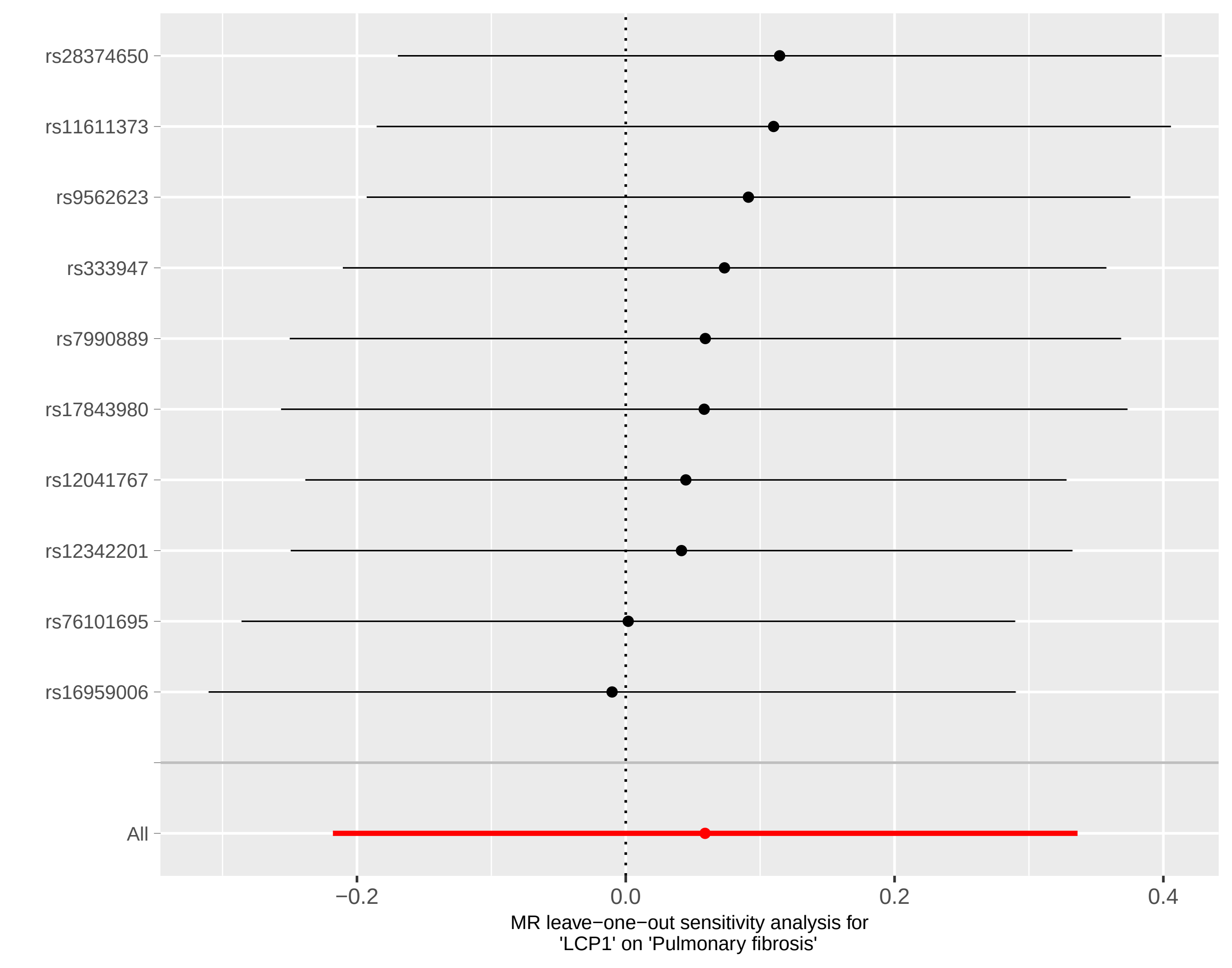
rs28374650
rs11611373
rs9562623
rs333947
rs7990889
rs17843980
rs12041767
rs12342201
rs76101695
rs16959006
All
−0.2
0.0
0.2
0.4
MR leave−one−out sensitivity analysis for
'LCP1' on 'Pulmonary fibrosis'

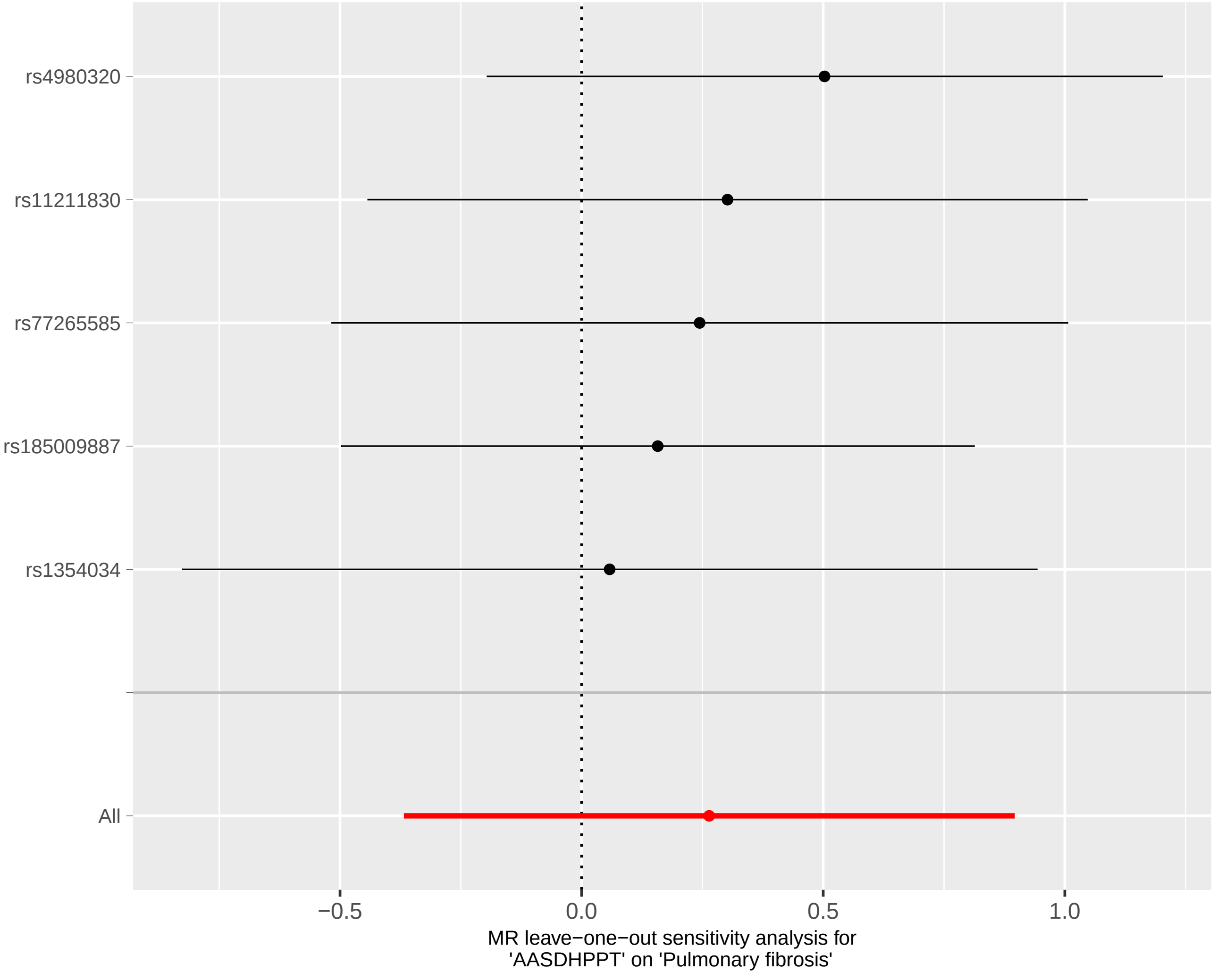
rs4980320
rs11211830
rs77265585
rs185009887
rs1354034
All
−0.5
0.0
0.5
1.0
MR leave−one−out sensitivity analysis for
'AASDHPPT' on 'Pulmonary fibrosis'

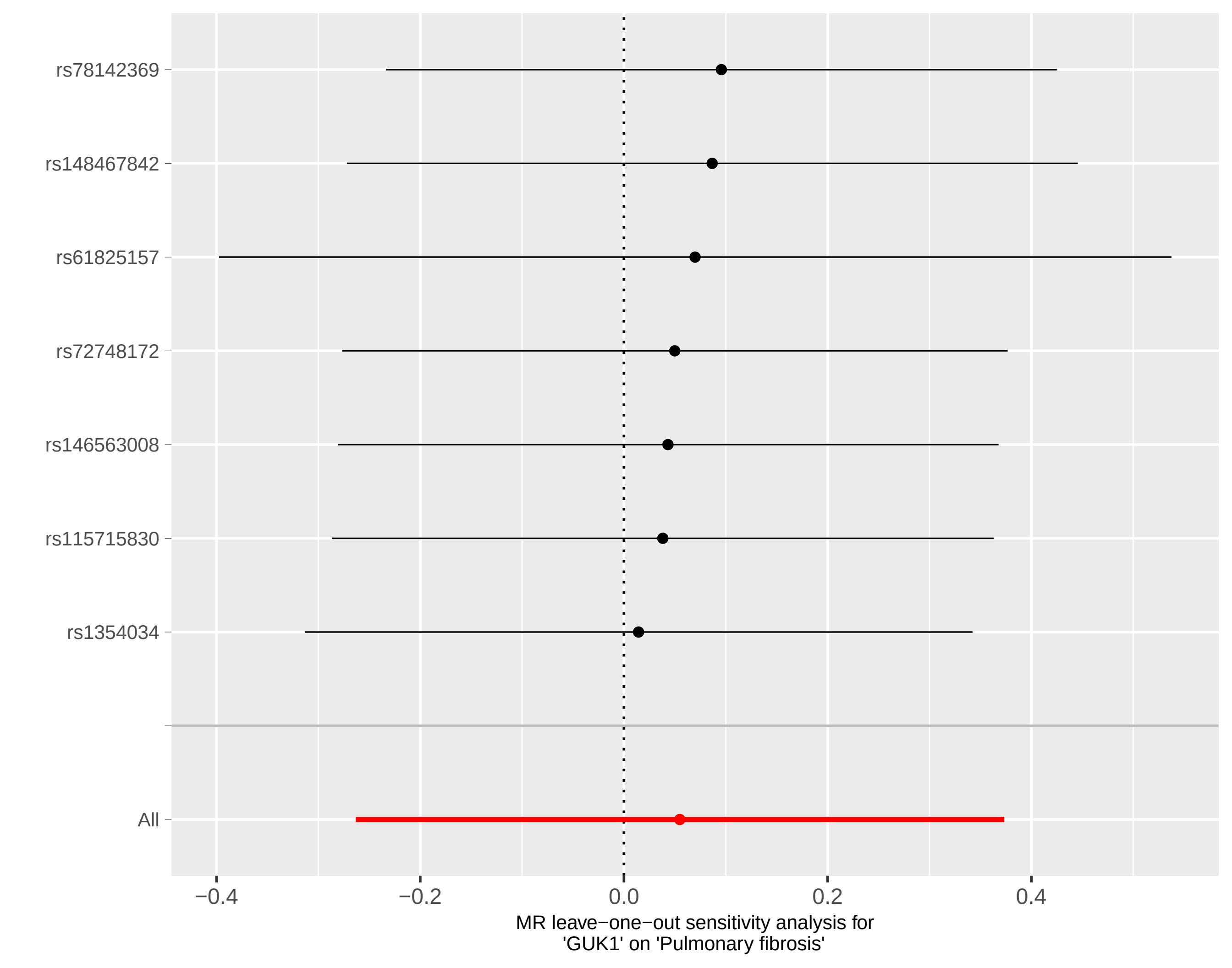
rs78142369
rs148467842
rs61825157
rs72748172
rs146563008
rs115715830
rs1354034
All
−0.4
−0.2
0.0
0.2
0.4
MR leave−one−out sensitivity analysis for
'GUK1' on 'Pulmonary fibrosis'

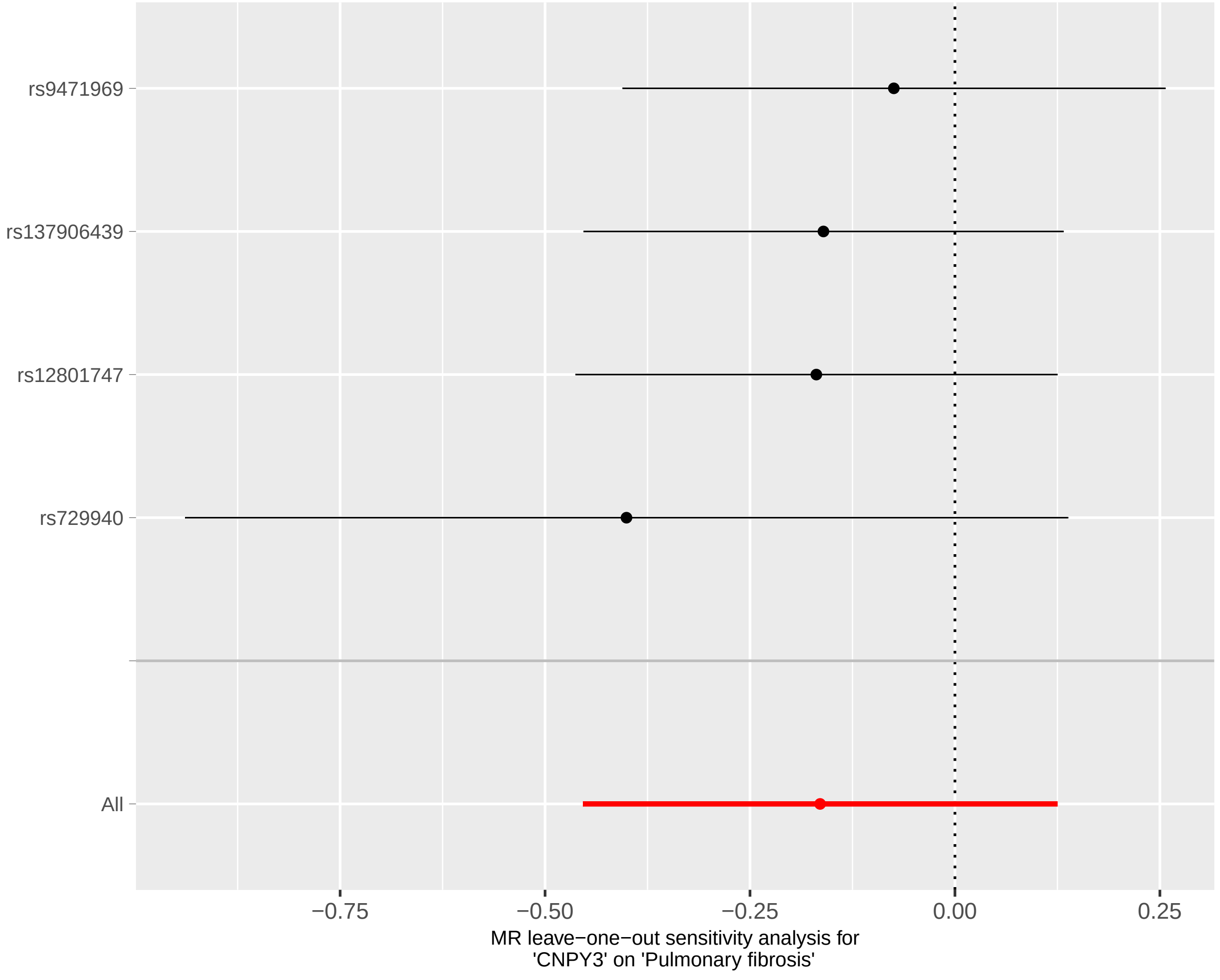
rs9471969
rs137906439
rs12801747
rs729940
All
−0.75
−0.50
−0.25
0.00
0.25
MR leave−one−out sensitivity analysis for
'CNPY3' on 'Pulmonary fibrosis'

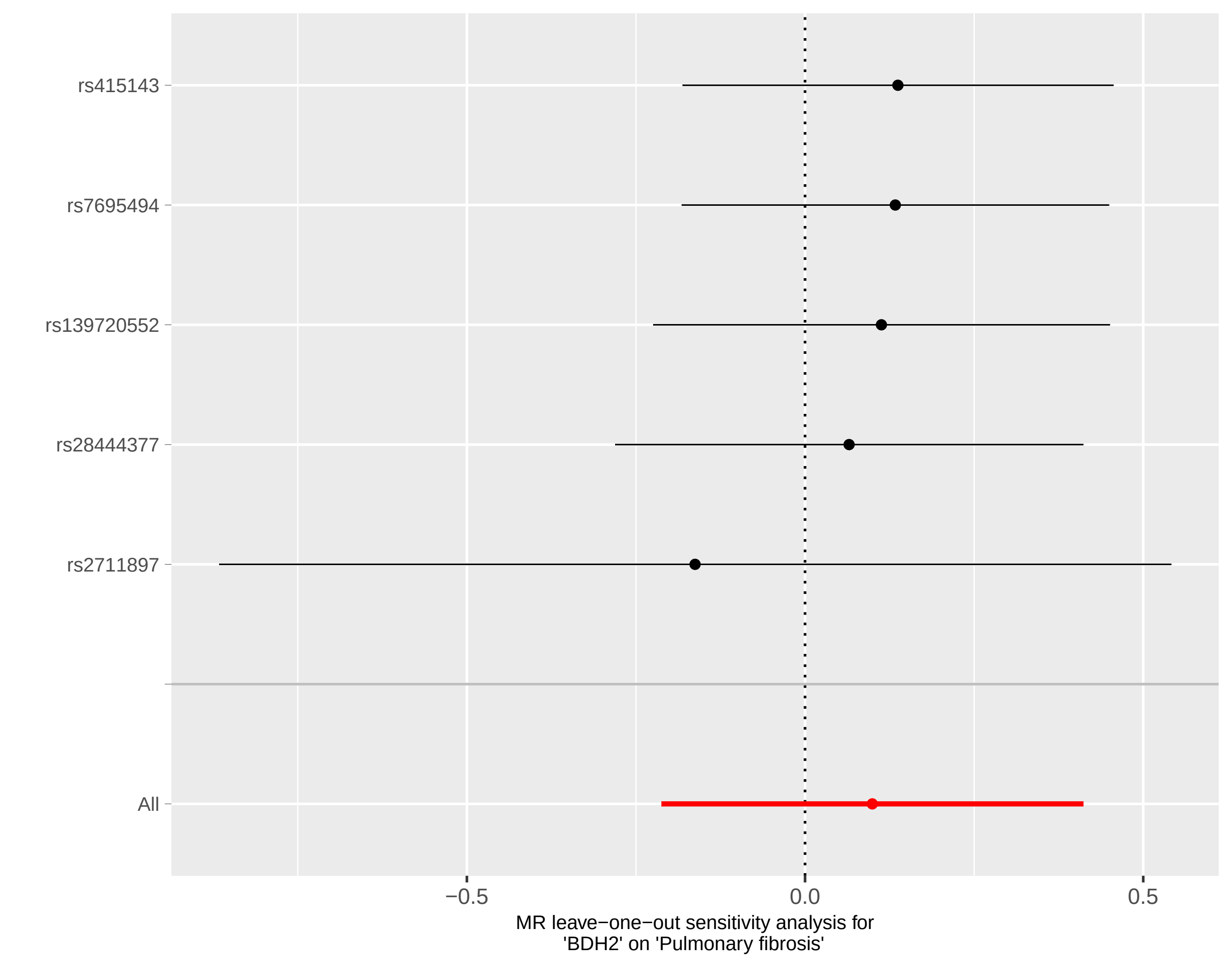
rs415143
rs7695494
rs139720552
rs28444377
rs2711897
All
−0.5
0.0
0.5
MR leave−one−out sensitivity analysis for
'BDH2' on 'Pulmonary fibrosis'

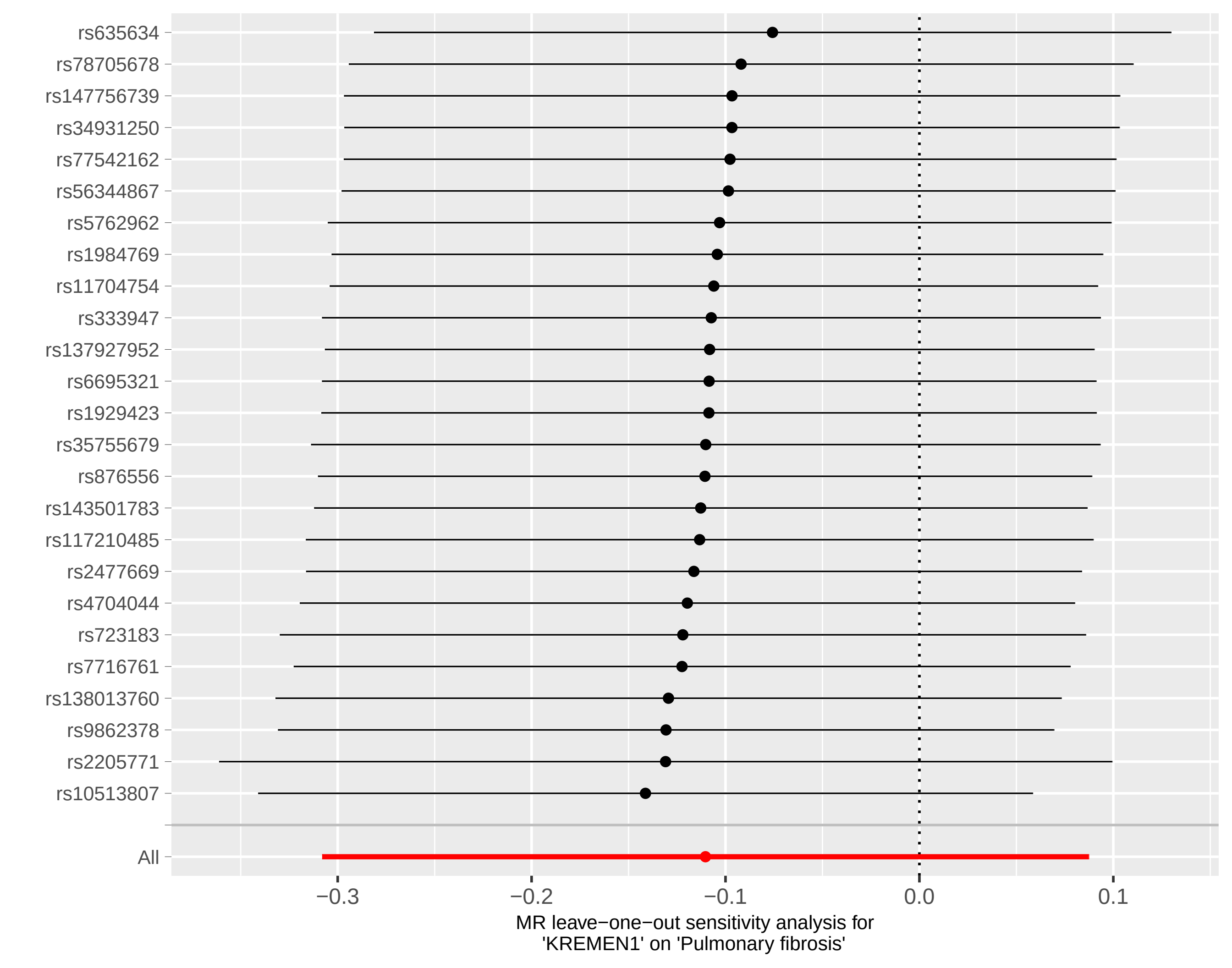
rs635634
rs78705678
rs147756739
rs34931250
rs77542162
rs56344867
rs5762962
rs1984769
rs11704754
rs333947
rs137927952
rs6695321
rs1929423
rs35755679
rs876556
rs143501783
rs117210485
rs2477669
rs4704044
rs723183
rs7716761
rs138013760
rs9862378
rs2205771
rs10513807
All
−0.3
−0.2
−0.1
0.0
0.1
MR leave−one−out sensitivity analysis for
'KREMEN1' on 'Pulmonary fibrosis'

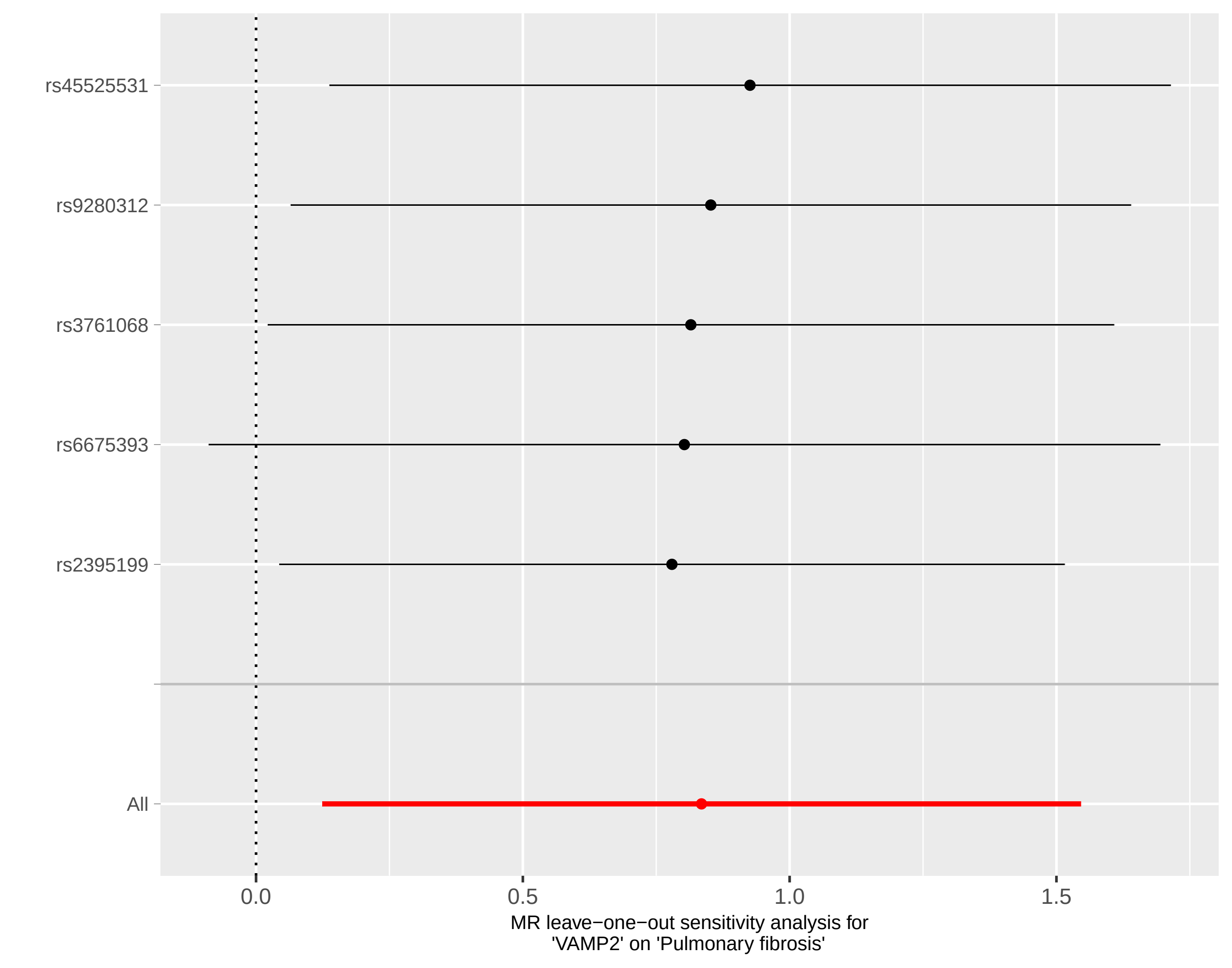
rs45525531
rs9280312
rs3761068
rs6675393
rs2395199
All
0.0
0.5
1.0
1.5
MR leave−one−out sensitivity analysis for
'VAMP2' on 'Pulmonary fibrosis'

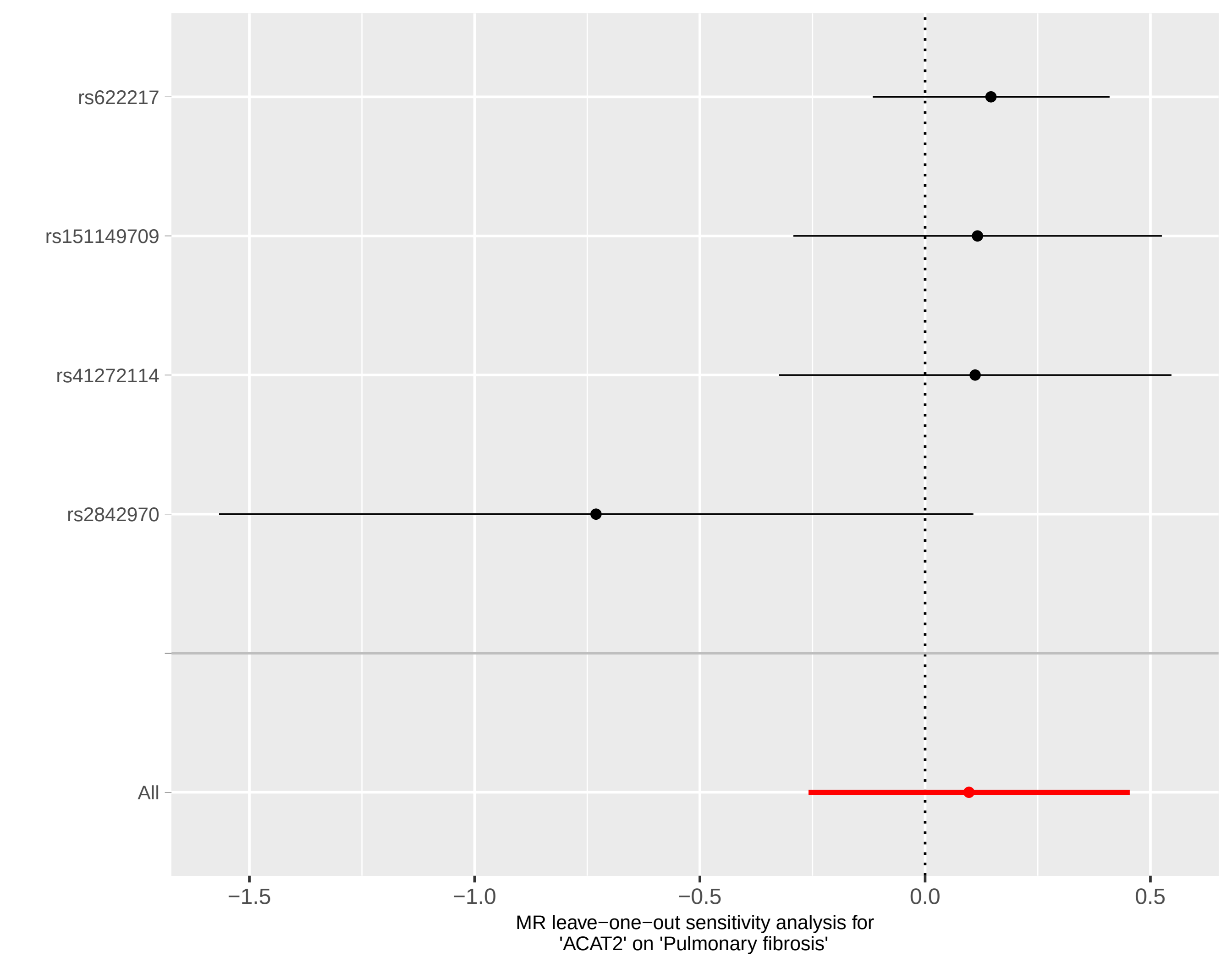
rs622217
rs151149709
rs41272114
rs2842970
All
−1.5
−1.0
−0.5
0.0
0.5
MR leave−one−out sensitivity analysis for
'ACAT2' on 'Pulmonary fibrosis'

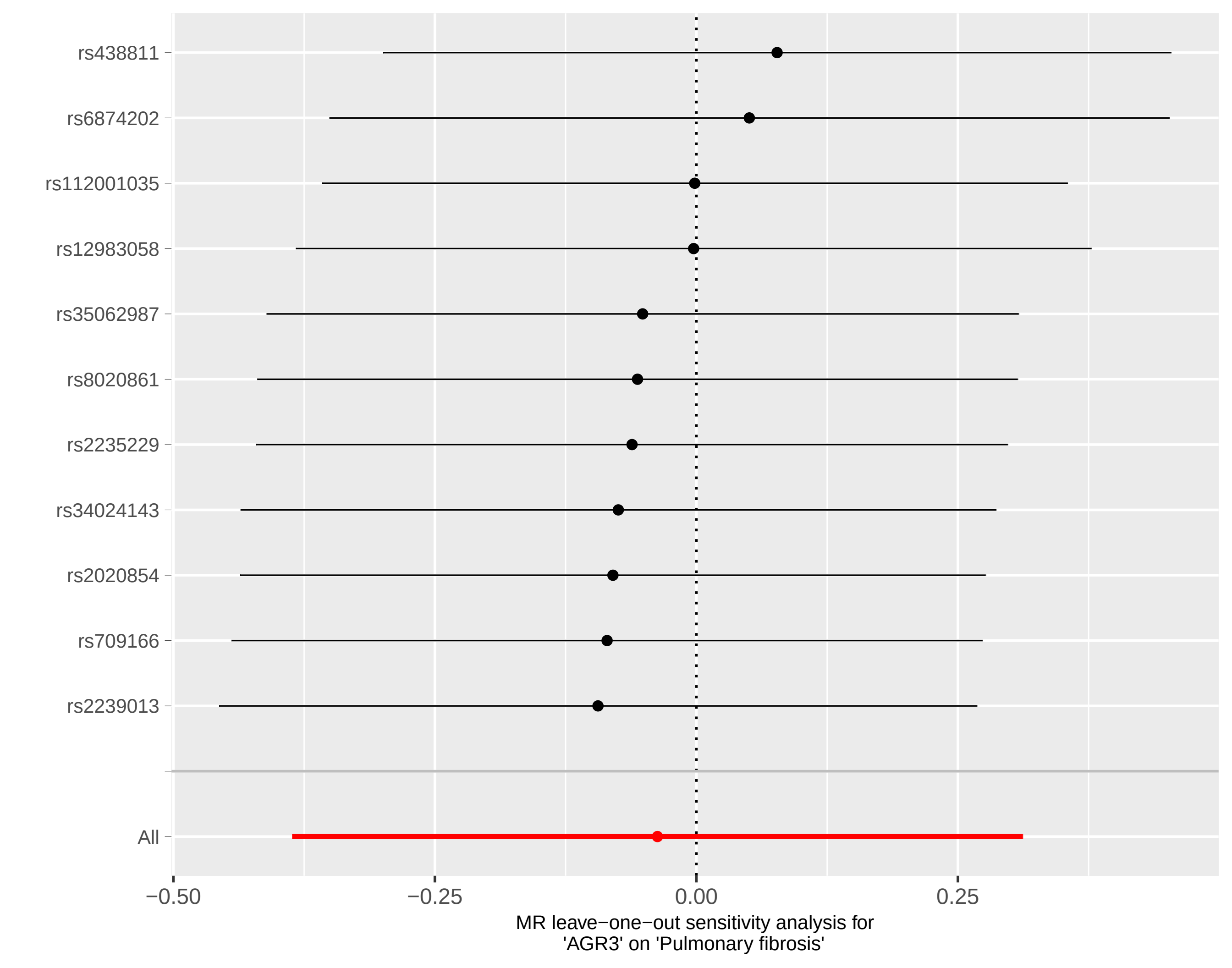
rs438811
rs6874202
rs112001035
rs12983058
rs35062987
rs8020861
rs2235229
rs34024143
rs2020854
rs709166
rs2239013
All
−0.50
−0.25
0.00
0.25
MR leave−one−out sensitivity analysis for
'AGR3' on 'Pulmonary fibrosis'

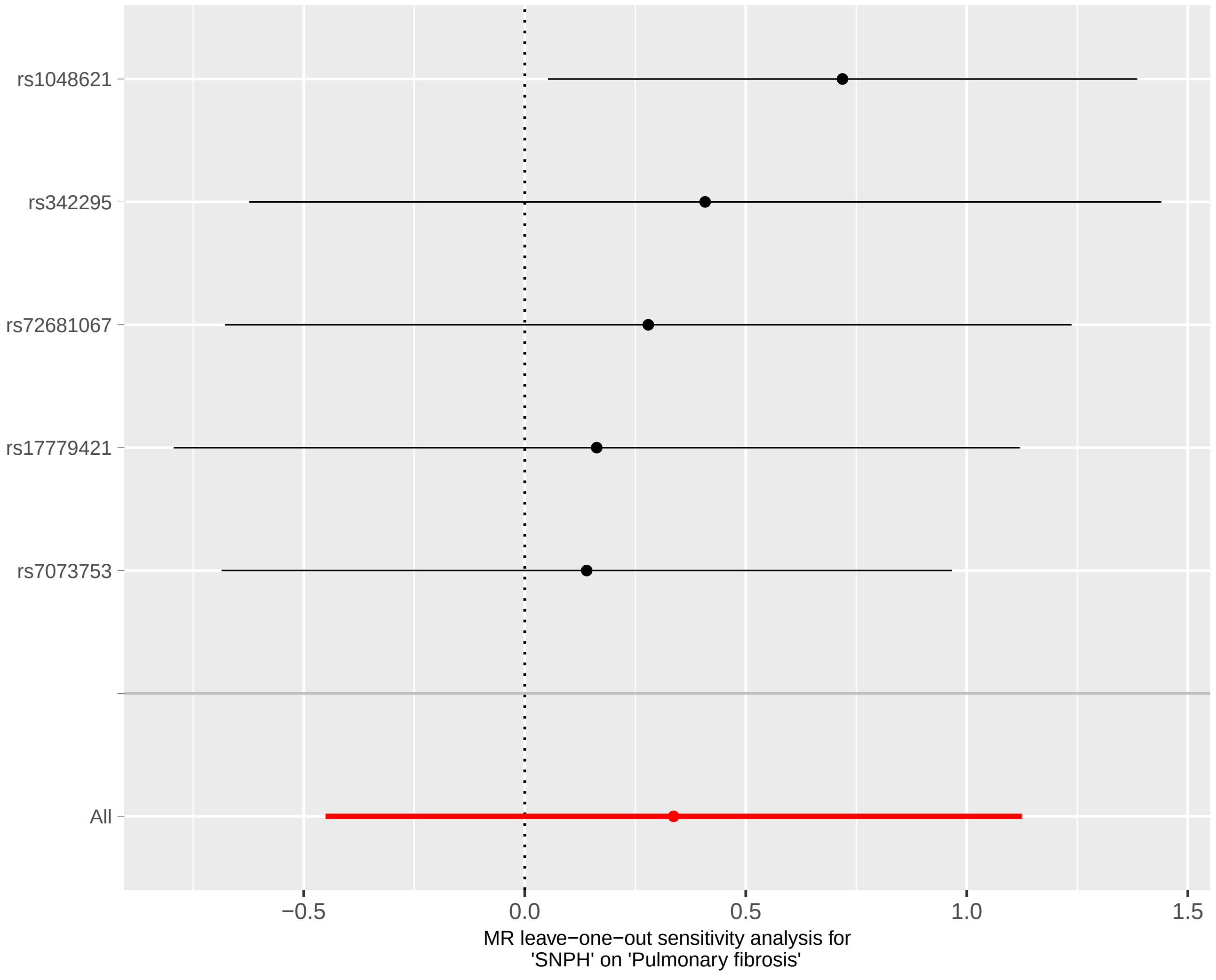

rs1048621
rs342295
rs72681067
rs17779421
rs7073753
All
−0.5
0.0
0.5
1.0
1.5
MR leave−one−out sensitivity analysis for
'SNPH' on 'Pulmonary fibrosis'

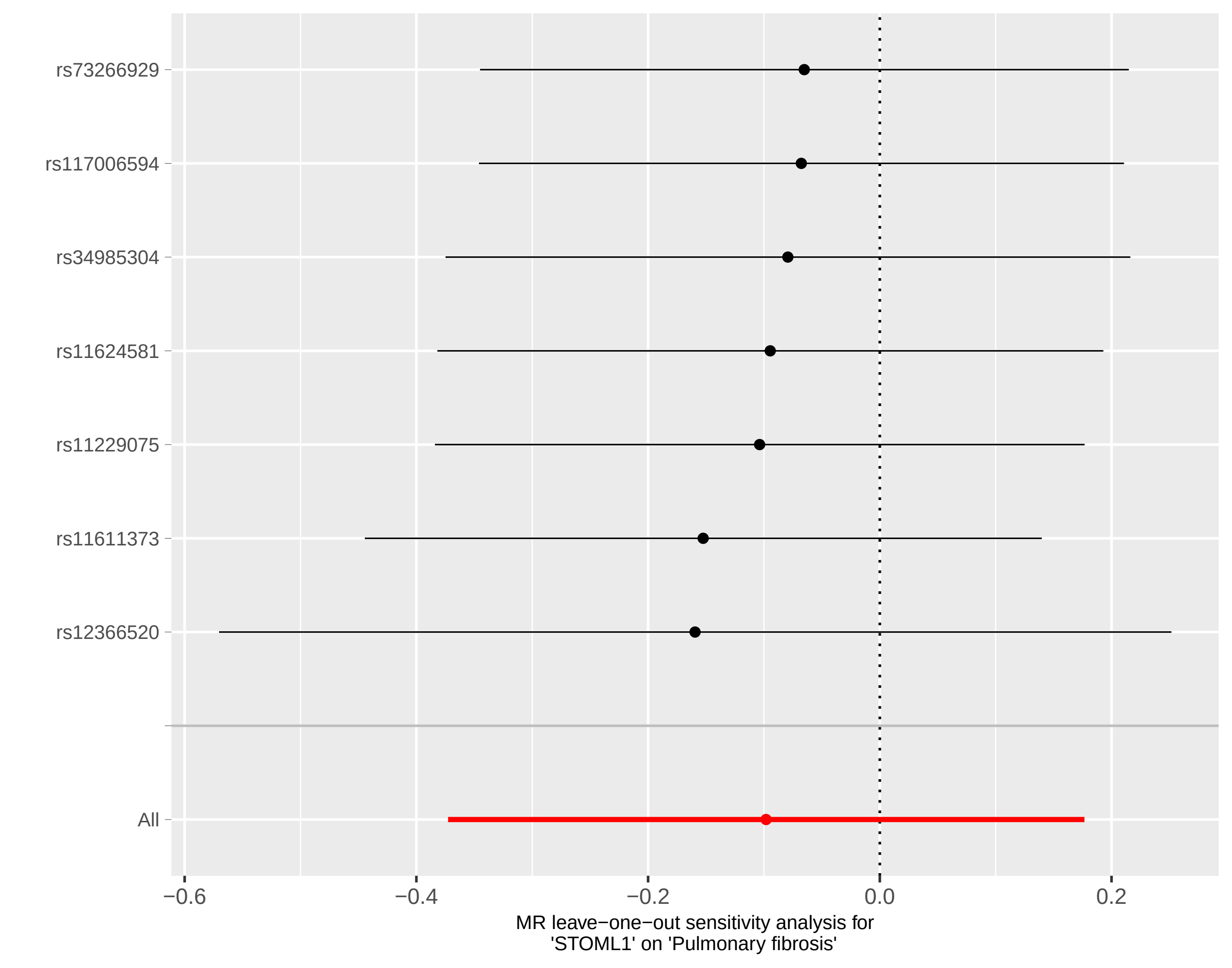
rs73266929
rs117006594
rs34985304
rs11624581
rs11229075
rs11611373
rs12366520
All
−0.6
−0.4
−0.2
0.0
0.2
MR leave−one−out sensitivity analysis for
'STOML1' on 'Pulmonary fibrosis'

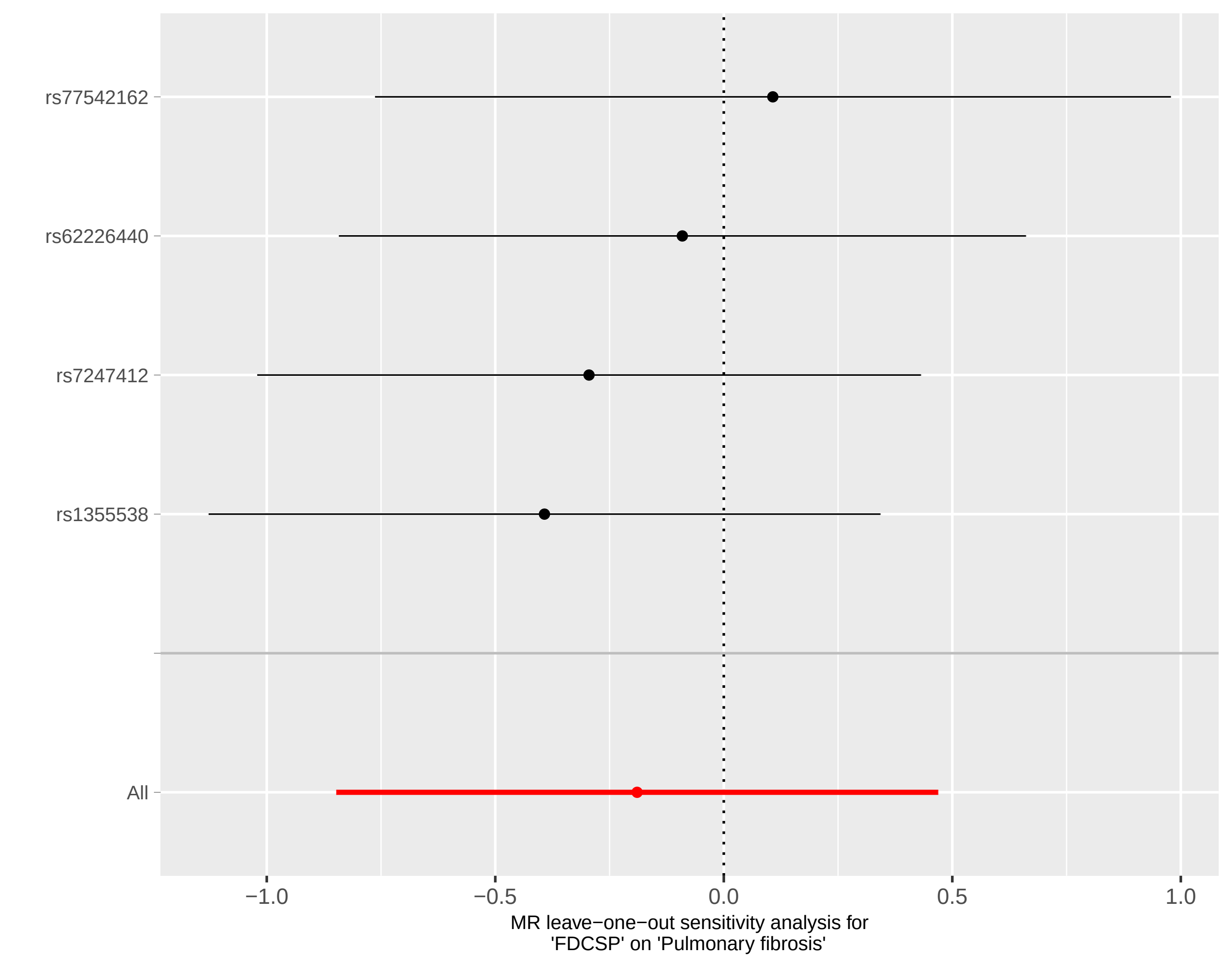

rs77542162
rs62226440
rs7247412
rs1355538
All
−1.0
−0.5
0.0
0.5
1.0
MR leave−one−out sensitivity analysis for
'FDCSP' on 'Pulmonary fibrosis'

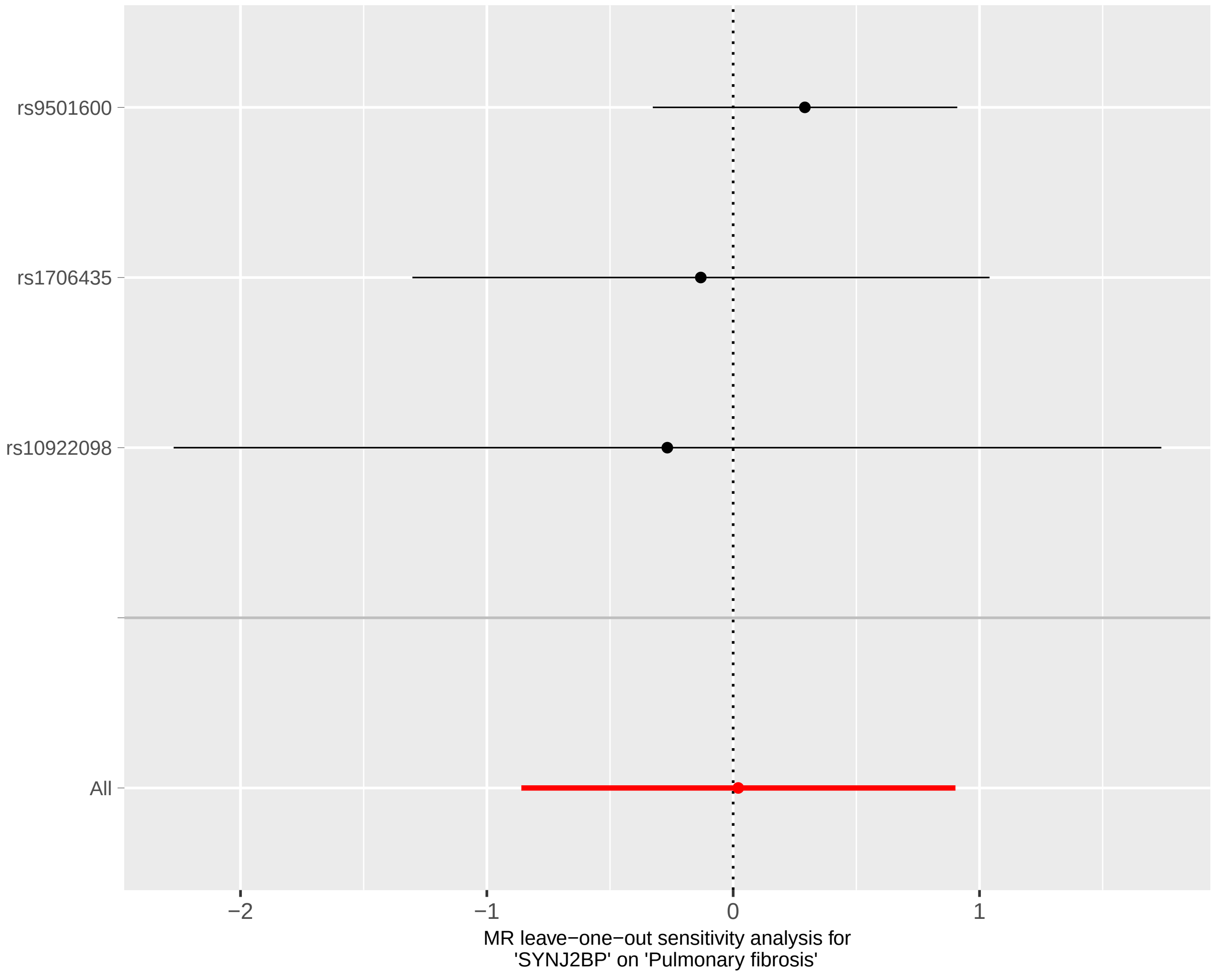
rs9501600
rs1706435
rs10922098
All
−2
−1
0
1
MR leave−one−out sensitivity analysis for
'SYNJ2BP' on 'Pulmonary fibrosis'

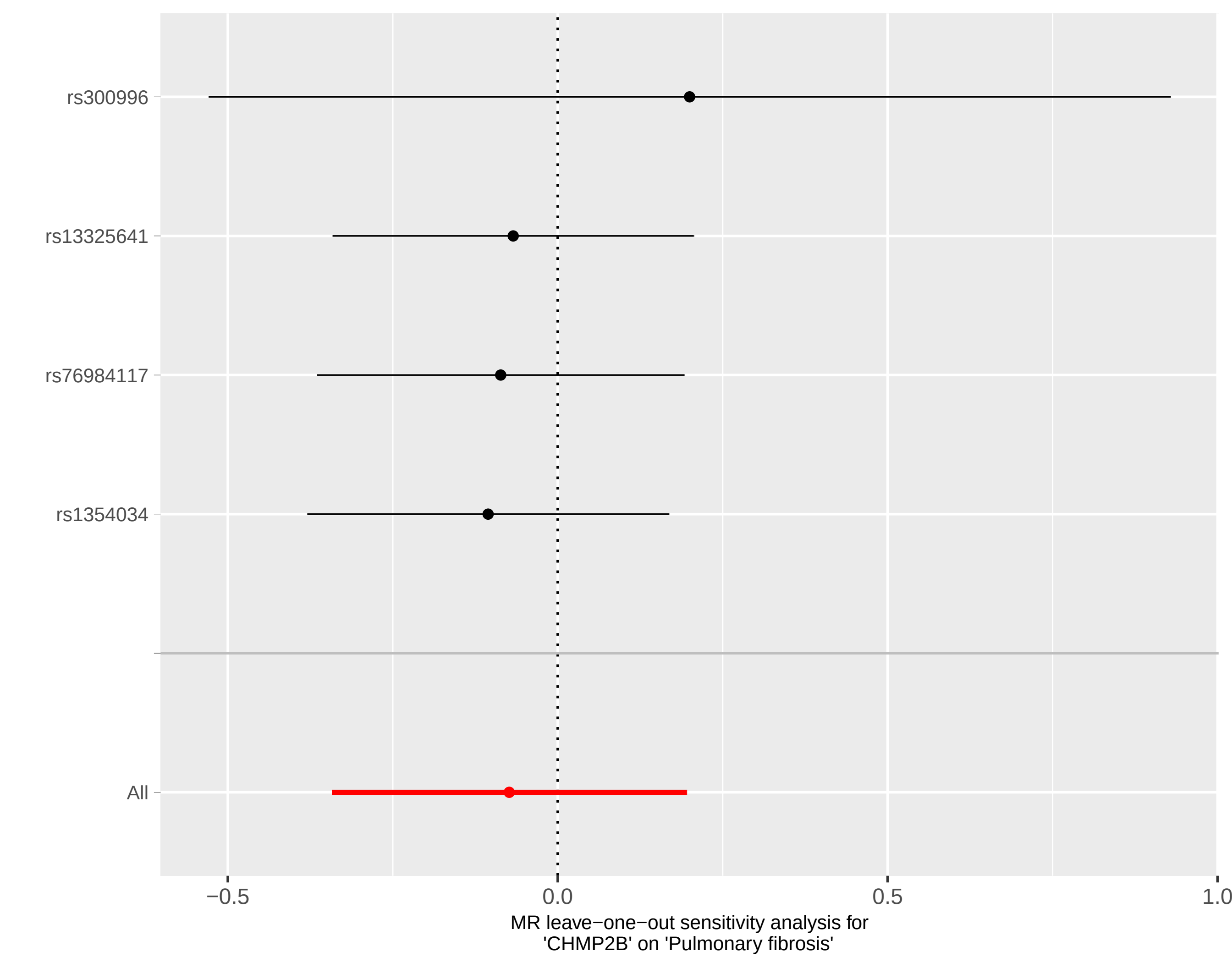

rs300996
rs13325641
rs76984117
rs1354034
All
−0.5
0.0
0.5
1.0
MR leave−one−out sensitivity analysis for
'CHMP2B' on 'Pulmonary fibrosis'

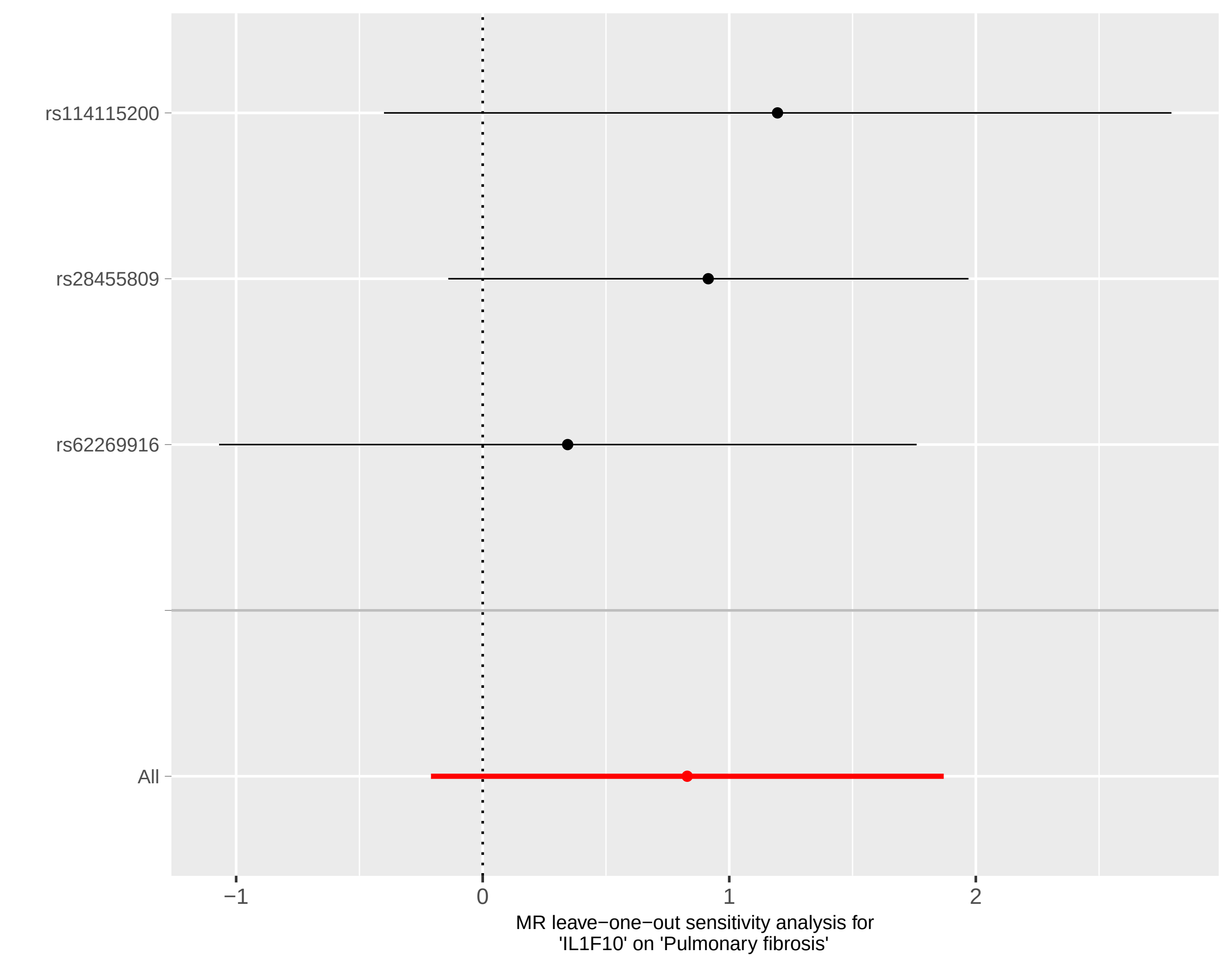

rs114115200
rs28455809
rs62269916
All
−1
0
1
2
MR leave−one−out sensitivity analysis for
'IL1F10' on 'Pulmonary fibrosis'

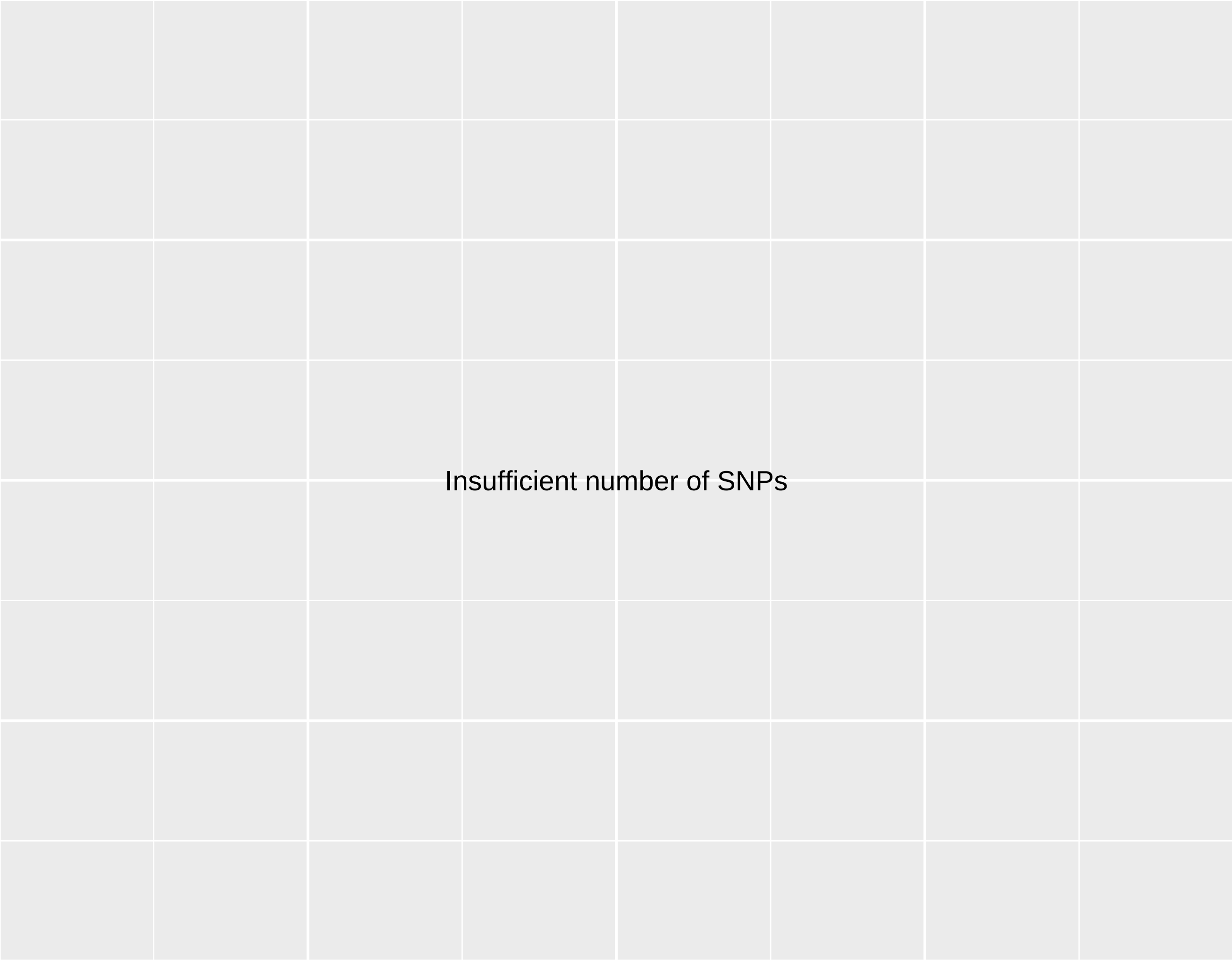
Insufficient number of SNPs

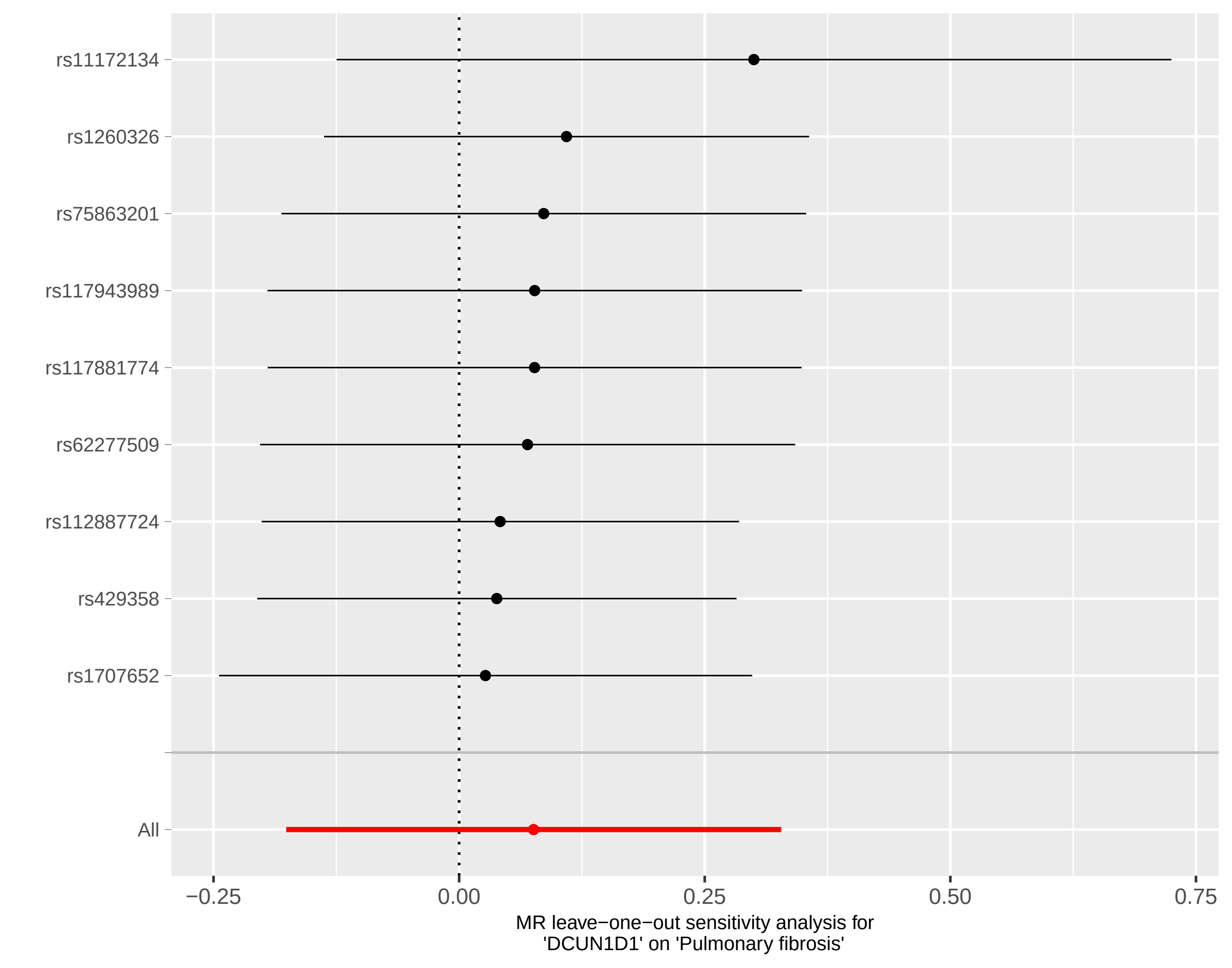
rs11172134
rs1260326
rs75863201
rs117943989
rs117881774
rs62277509
rs112887724
rs429358
rs1707652
All
−0.25
0.00
0.25
0.50
0.75
MR leave−one−out sensitivity analysis for
'DCUN1D1' on 'Pulmonary fibrosis'

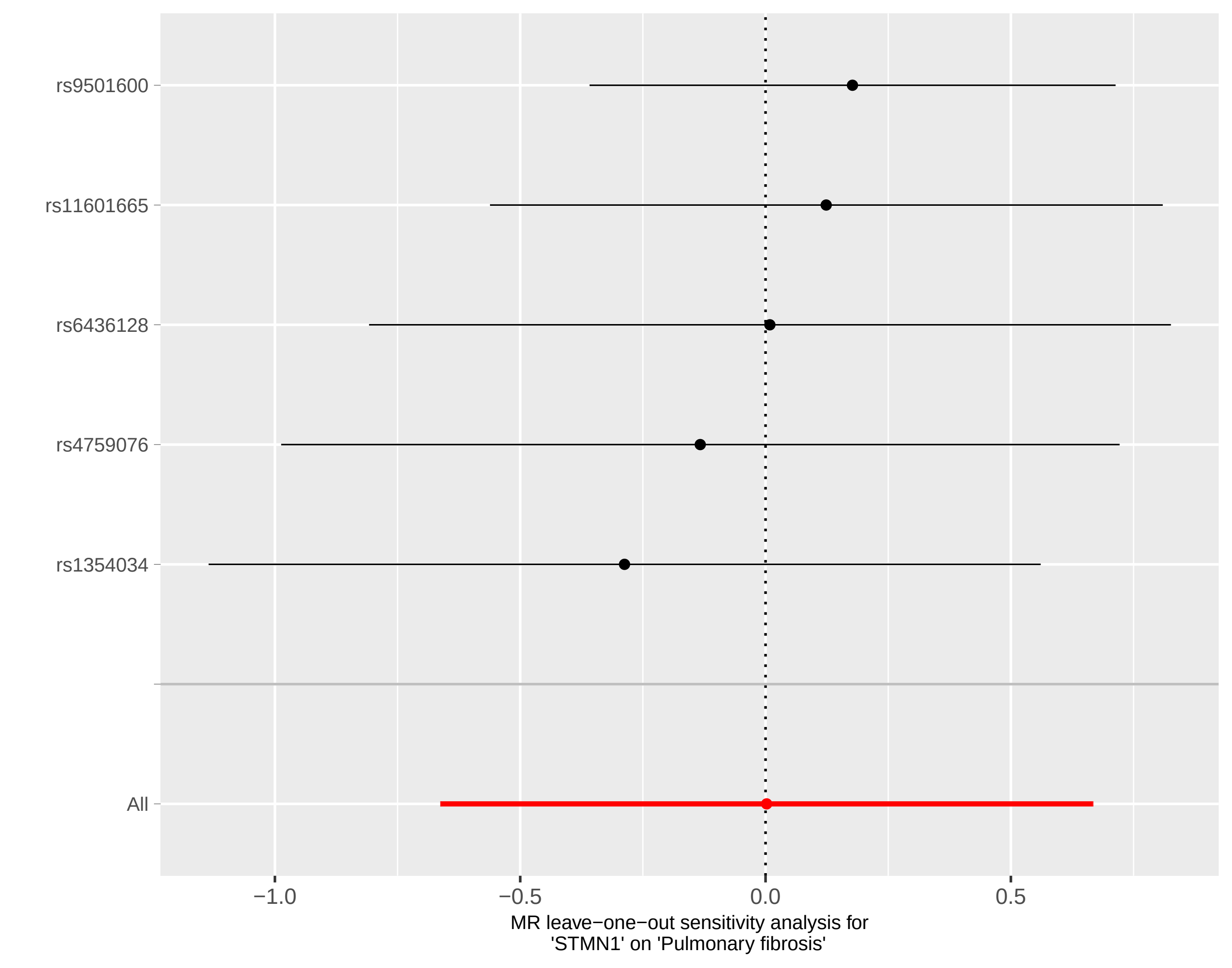
rs9501600
rs11601665
rs6436128
rs4759076
rs1354034
All
−1.0
−0.5
0.0
0.5
MR leave−one−out sensitivity analysis for
'STMN1' on 'Pulmonary fibrosis'

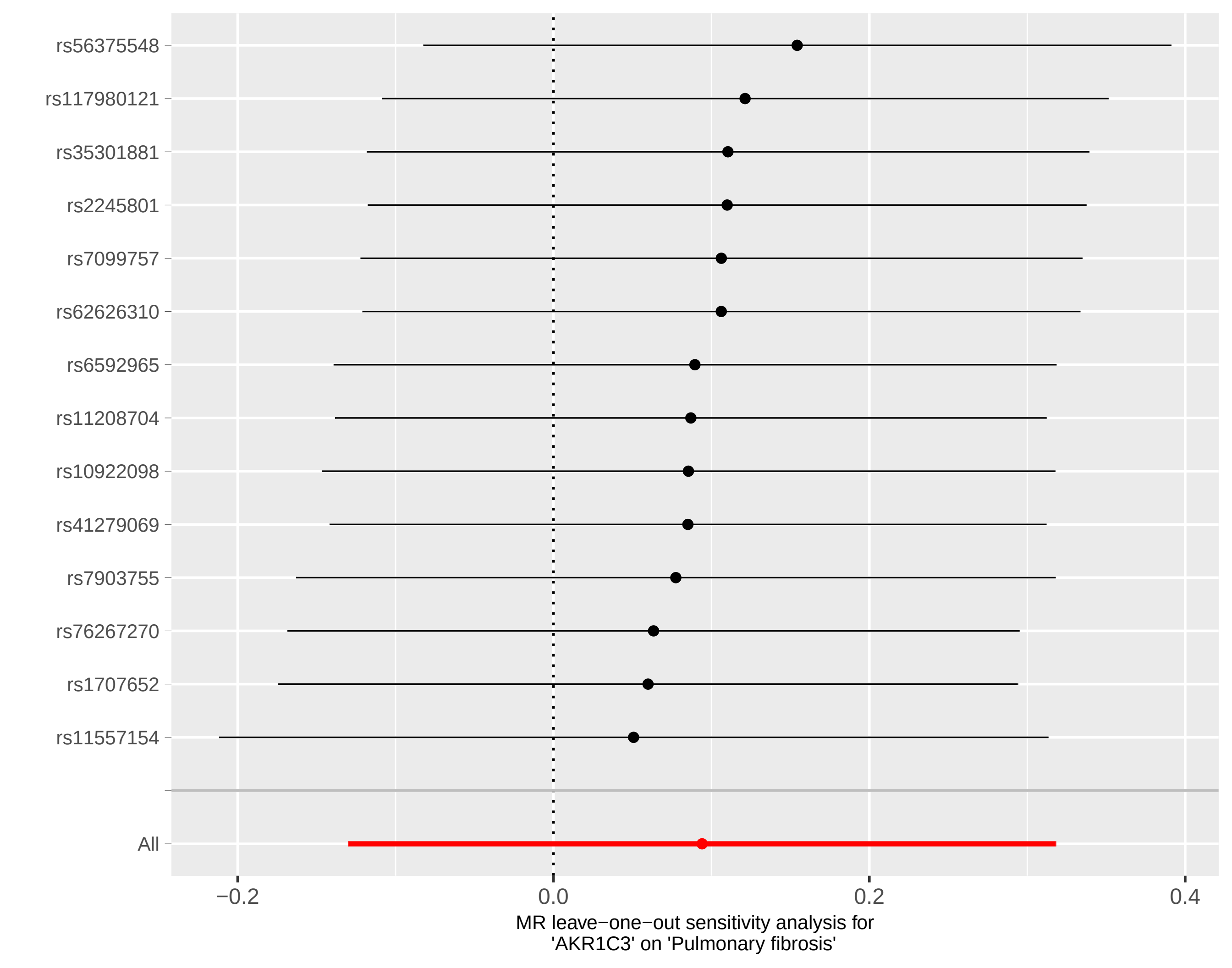
rs56375548
rs117980121
rs35301881
rs2245801
rs7099757
rs62626310
rs6592965
rs11208704
rs10922098
rs41279069
rs7903755
rs76267270
rs1707652
rs11557154
All
−0.2
0.0
0.2
0.4
MR leave−one−out sensitivity analysis for
'AKR1C3' on 'Pulmonary fibrosis'

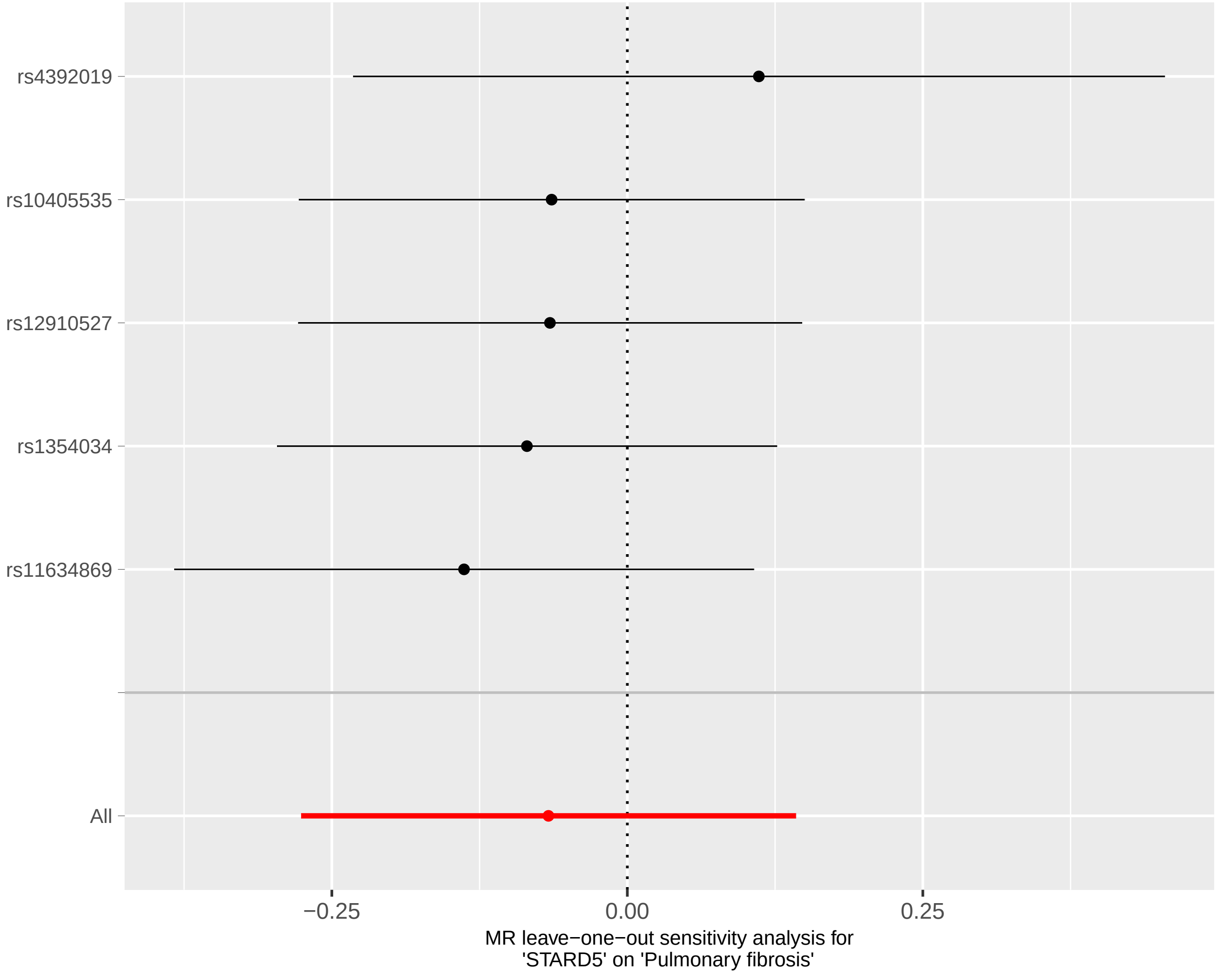
rs4392019
rs10405535
rs12910527
rs1354034
rs11634869
All
−0.25
0.00
0.25
MR leave−one−out sensitivity analysis for
'STARD5' on 'Pulmonary fibrosis'

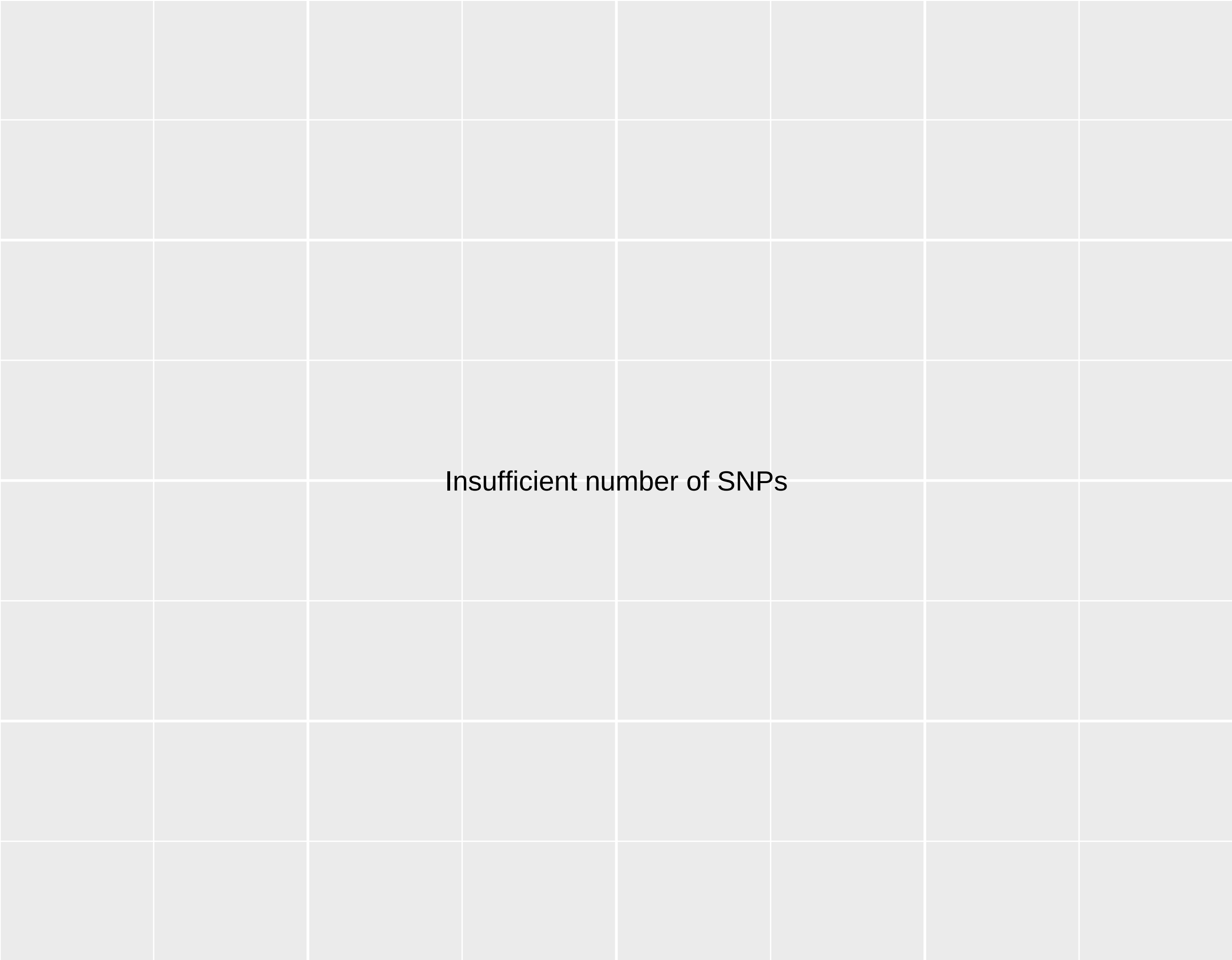
Insufficient number of SNPs

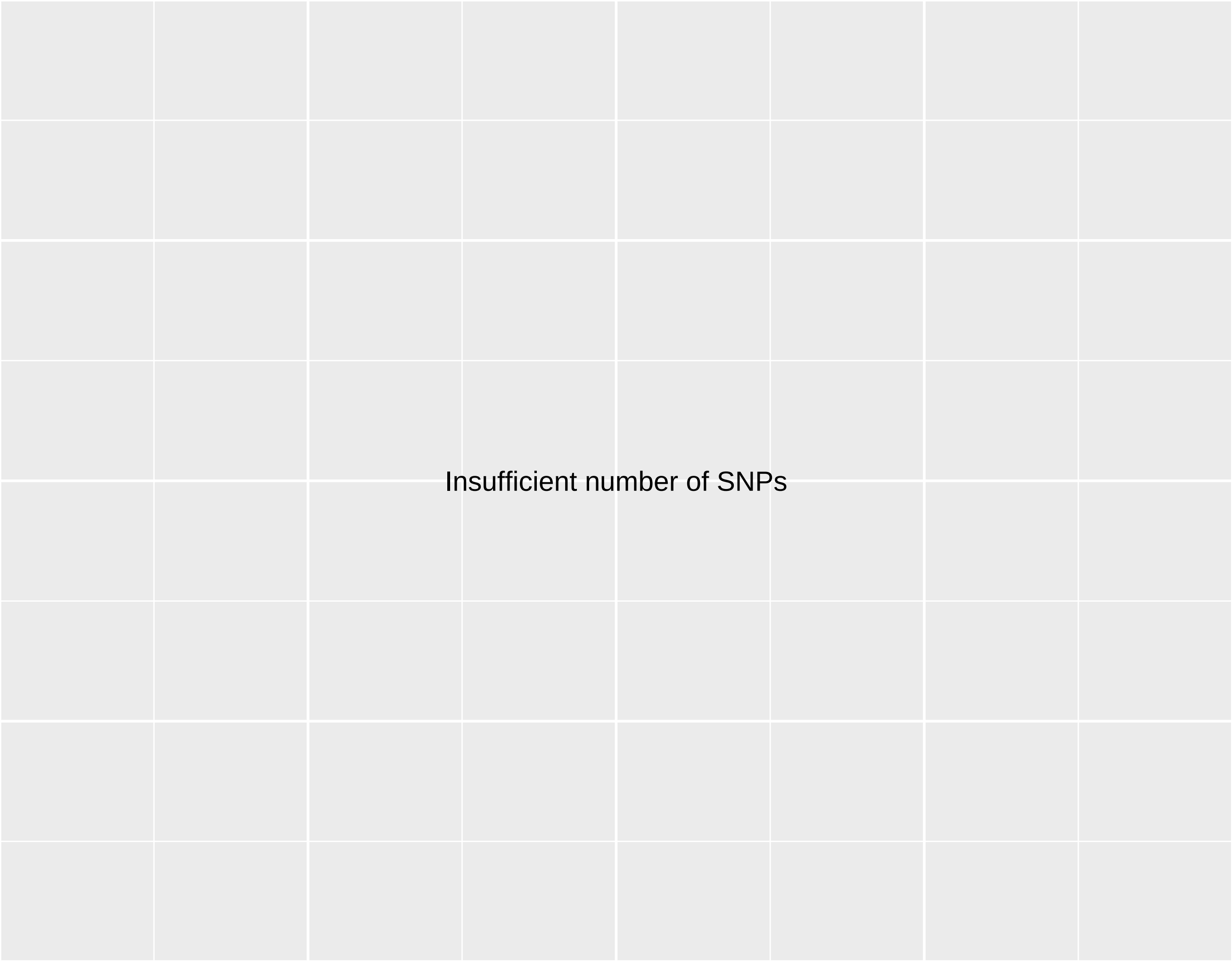
Insufficient number of SNPs

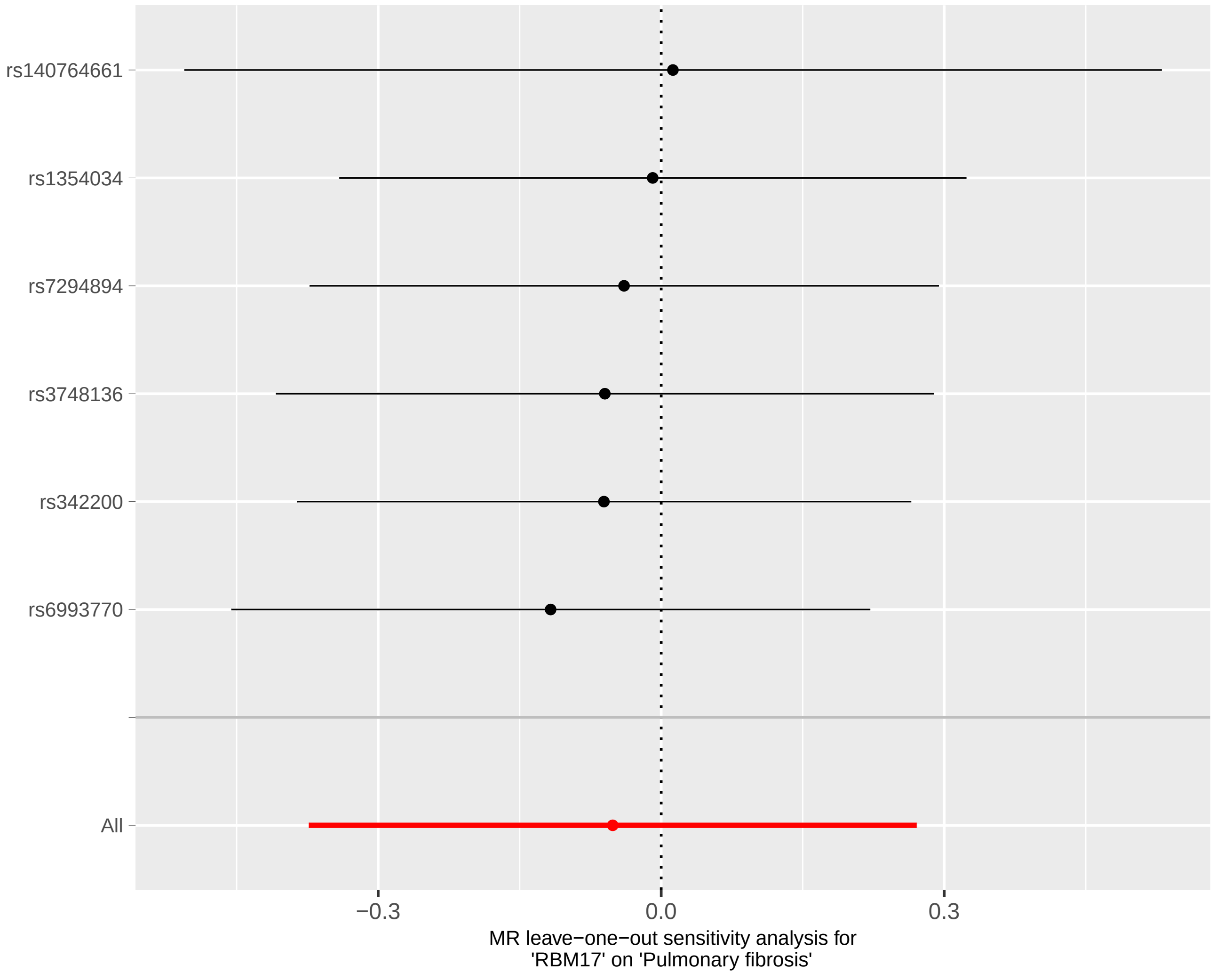

rs140764661
rs1354034
rs7294894
rs3748136
rs342200
rs6993770
All
−0.3
0.0
0.3
MR leave−one−out sensitivity analysis for
'RBM17' on 'Pulmonary fibrosis'

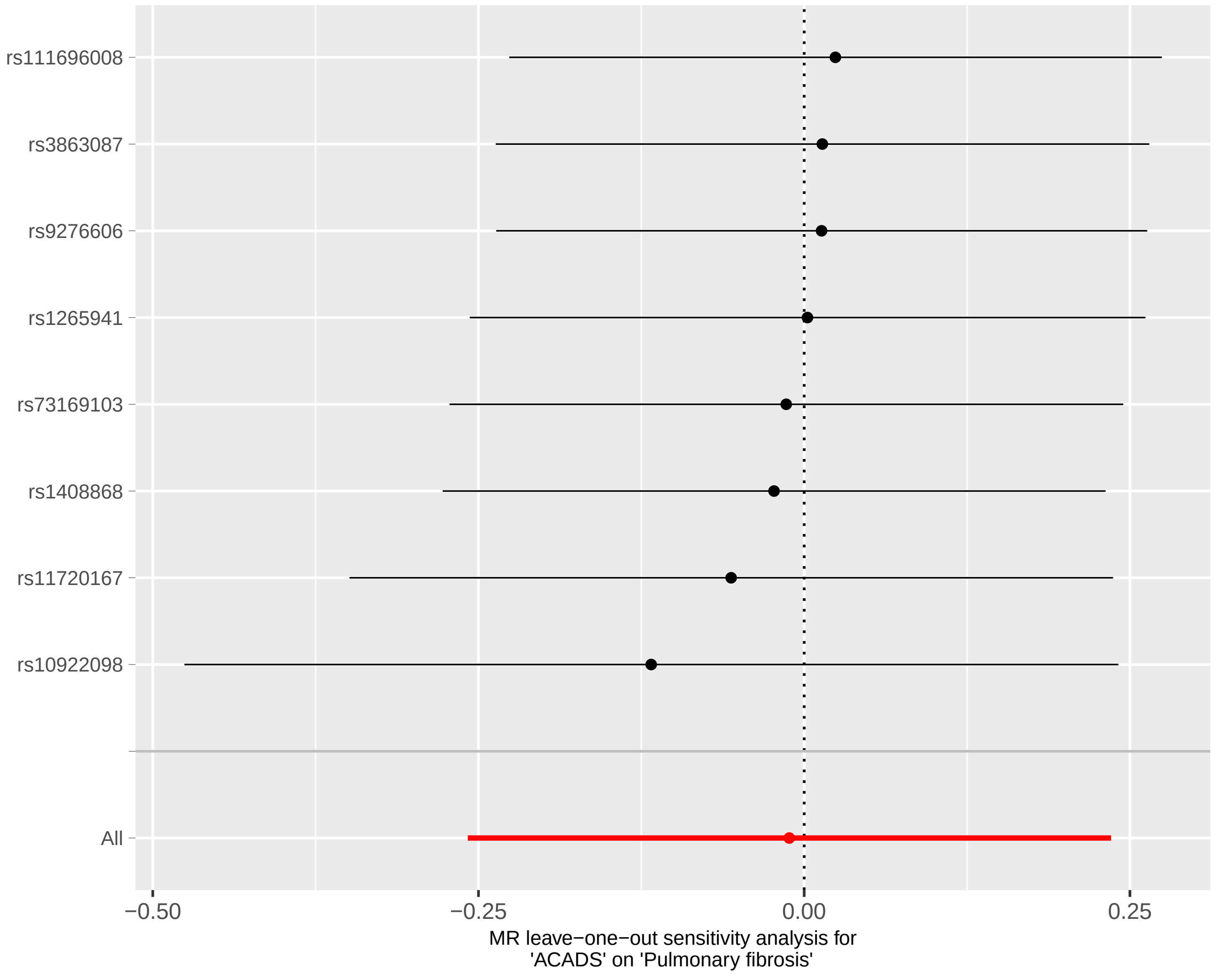
rs111696008
rs3863087
rs9276606
rs1265941
rs73169103
rs1408868
rs11720167
rs10922098
All
−0.50
−0.25
0.00
0.25
MR leave−one−out sensitivity analysis for
'ACADS' on 'Pulmonary fibrosis'

Insufficient number of SNPs

Insufficient number of SNPs

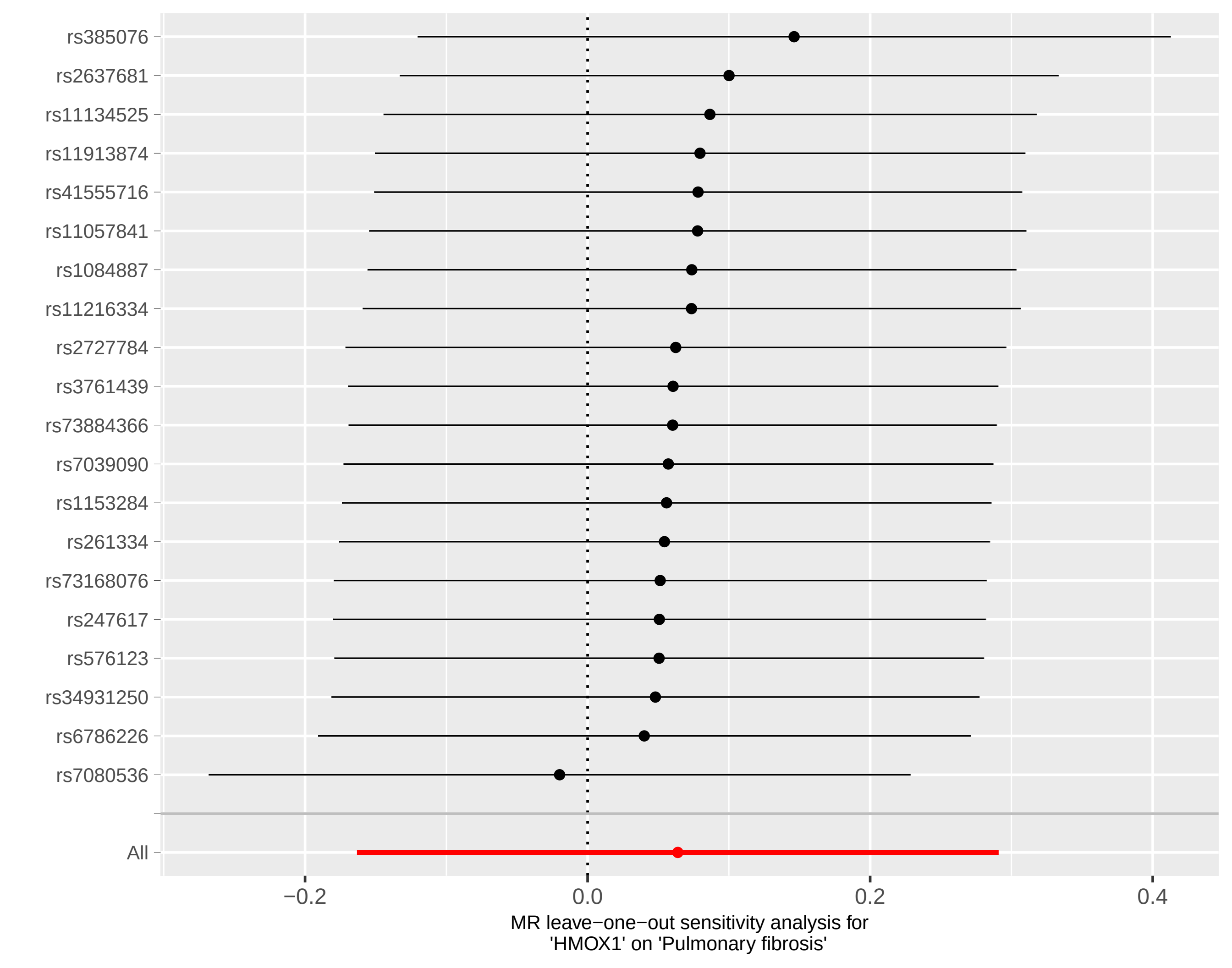
rs385076
rs2637681
rs11134525
rs11913874
rs41555716
rs11057841
rs1084887
rs11216334
rs2727784
rs3761439
rs73884366
rs7039090
rs1153284
rs261334
rs73168076
rs247617
rs576123
rs34931250
rs6786226
rs7080536
All
−0.2
0.0
0.2
0.4
MR leave−one−out sensitivity analysis for
'HMOX1' on 'Pulmonary fibrosis'

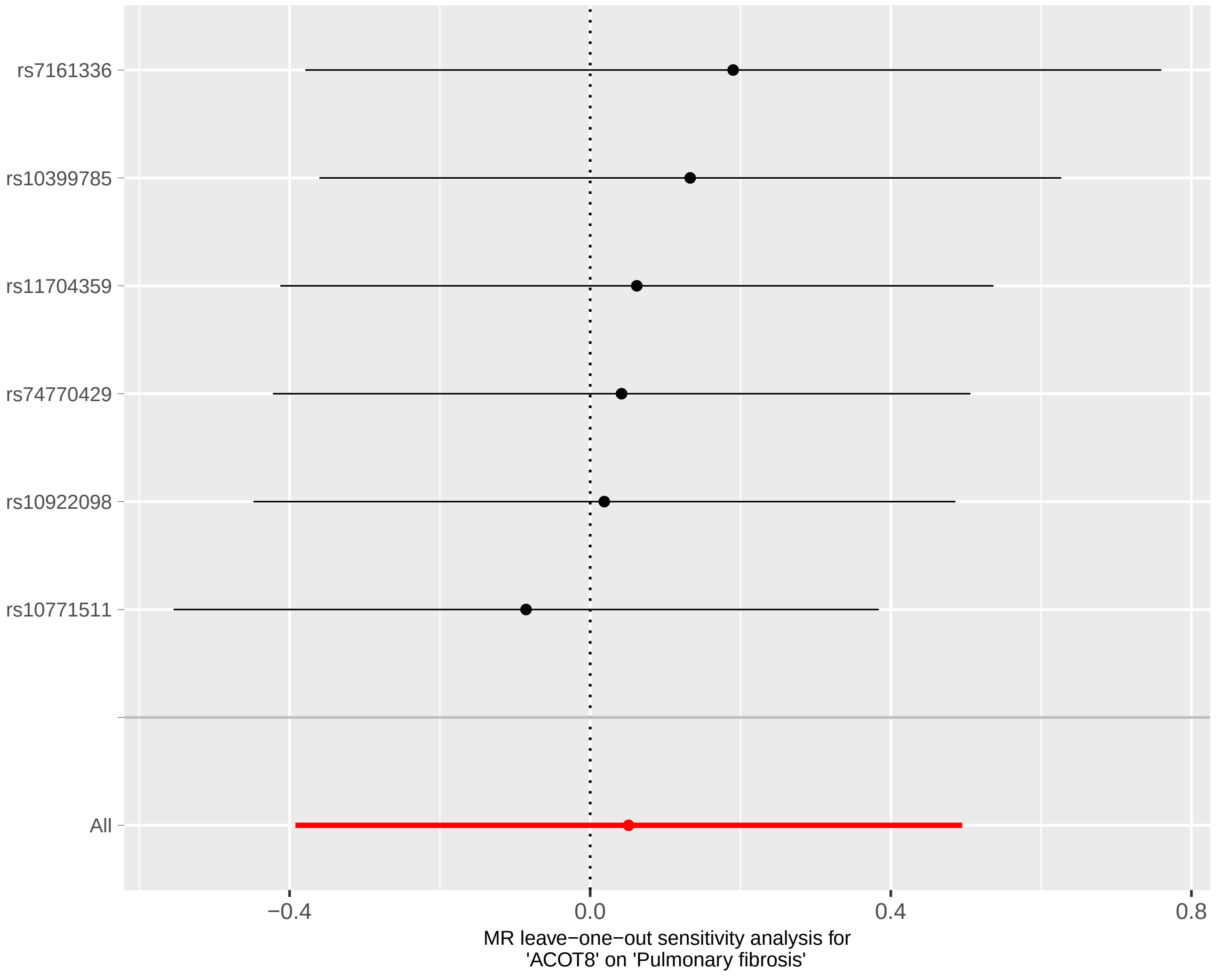
rs7161336
rs10399785
rs11704359
rs74770429
rs10922098
rs10771511
All
−0.4
0.0
0.4
0.8
MR leave−one−out sensitivity analysis for
'ACOT8' on 'Pulmonary fibrosis'

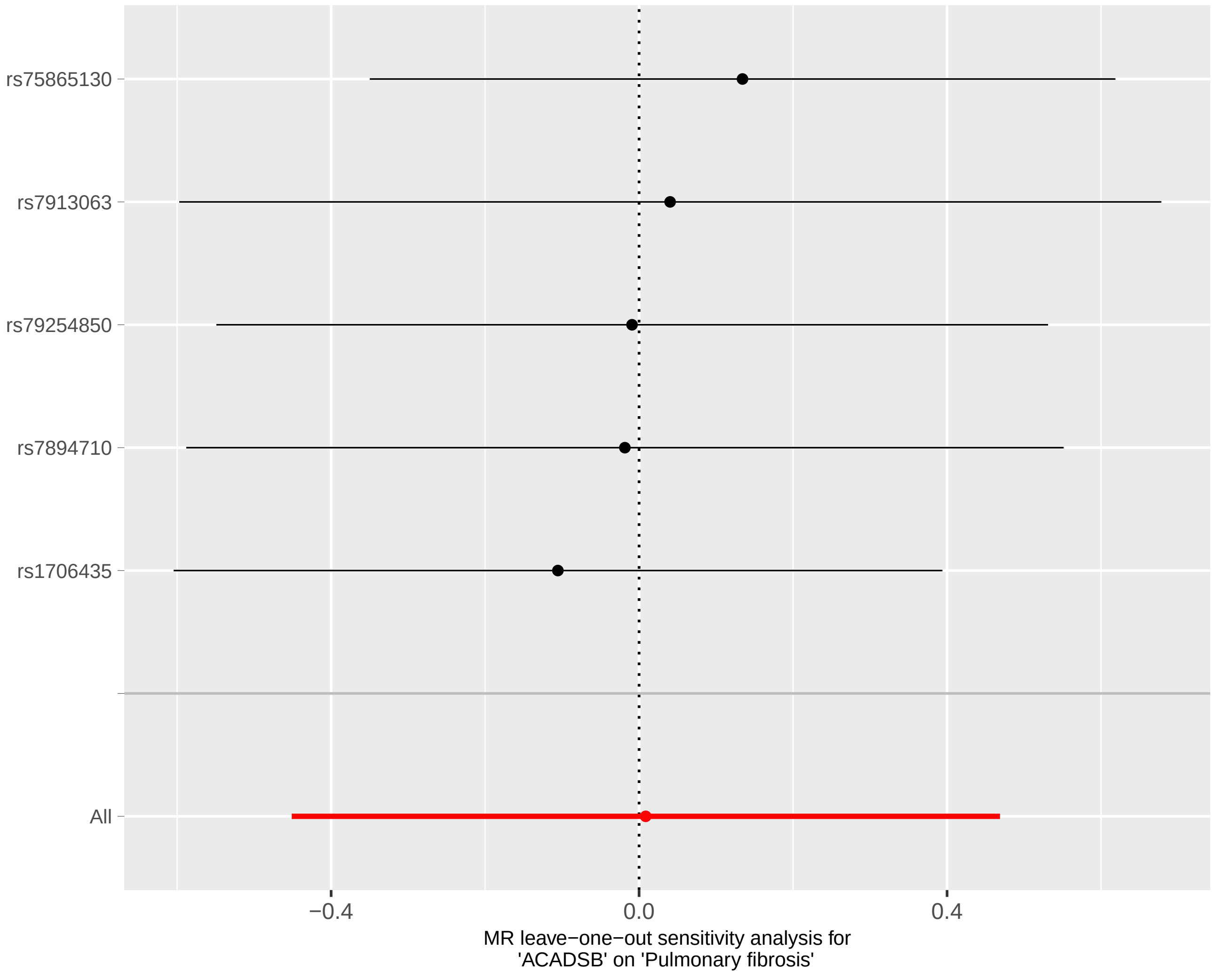
rs75865130
rs7913063
rs79254850
rs7894710
rs1706435
All
−0.4
0.0
0.4
MR leave−one−out sensitivity analysis for
'ACADSB' on 'Pulmonary fibrosis'

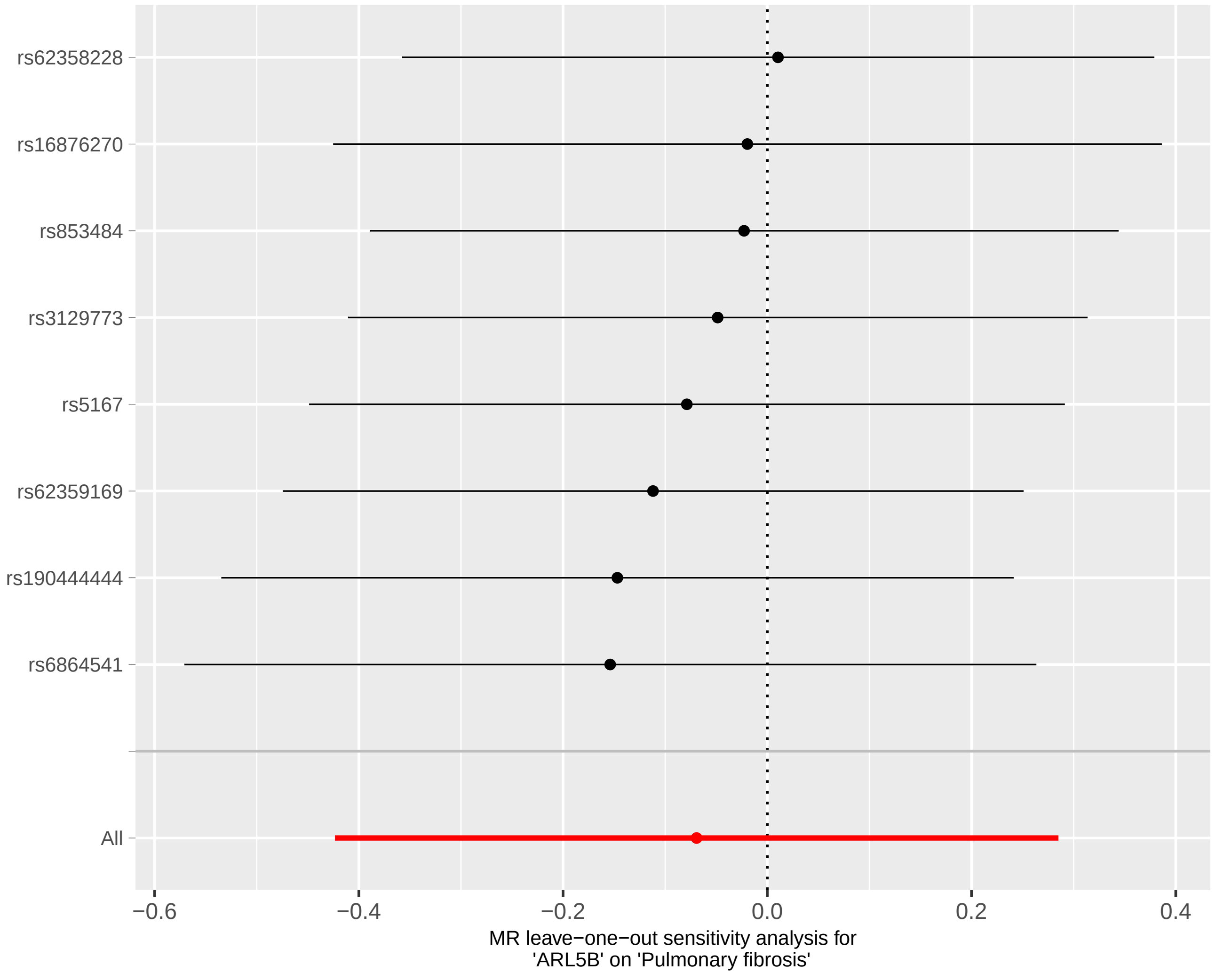
rs62358228
rs16876270
rs853484
rs3129773
rs5167
rs62359169
rs190444444
rs6864541
All
−0.6
−0.4
−0.2
0.0
0.2
0.4
MR leave−one−out sensitivity analysis for
'ARL5B' on 'Pulmonary fibrosis'

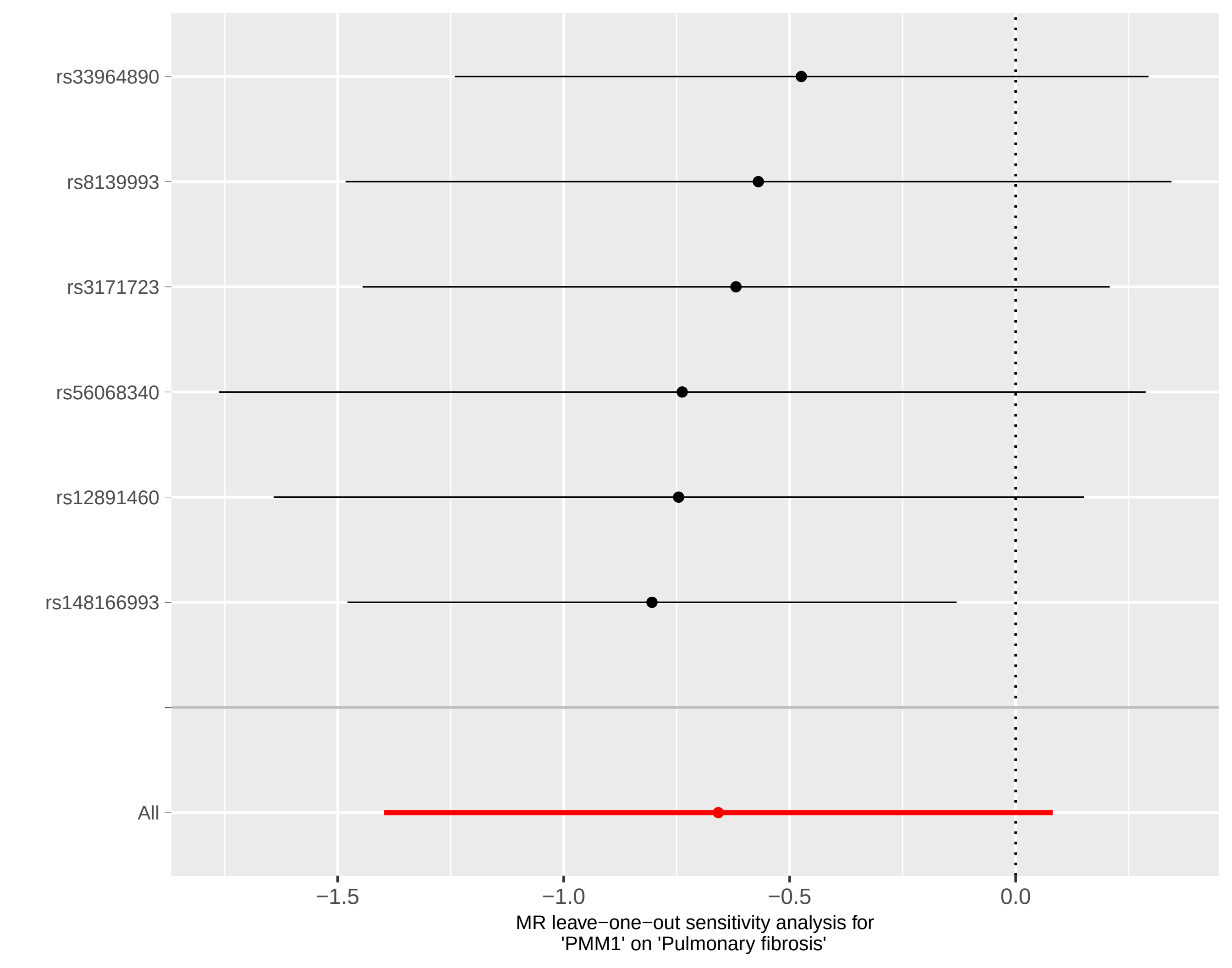
rs33964890
rs8139993
rs3171723
rs56068340
rs12891460
rs148166993
All
−1.5
−1.0
−0.5
0.0
MR leave−one−out sensitivity analysis for
'PMM1' on 'Pulmonary fibrosis'

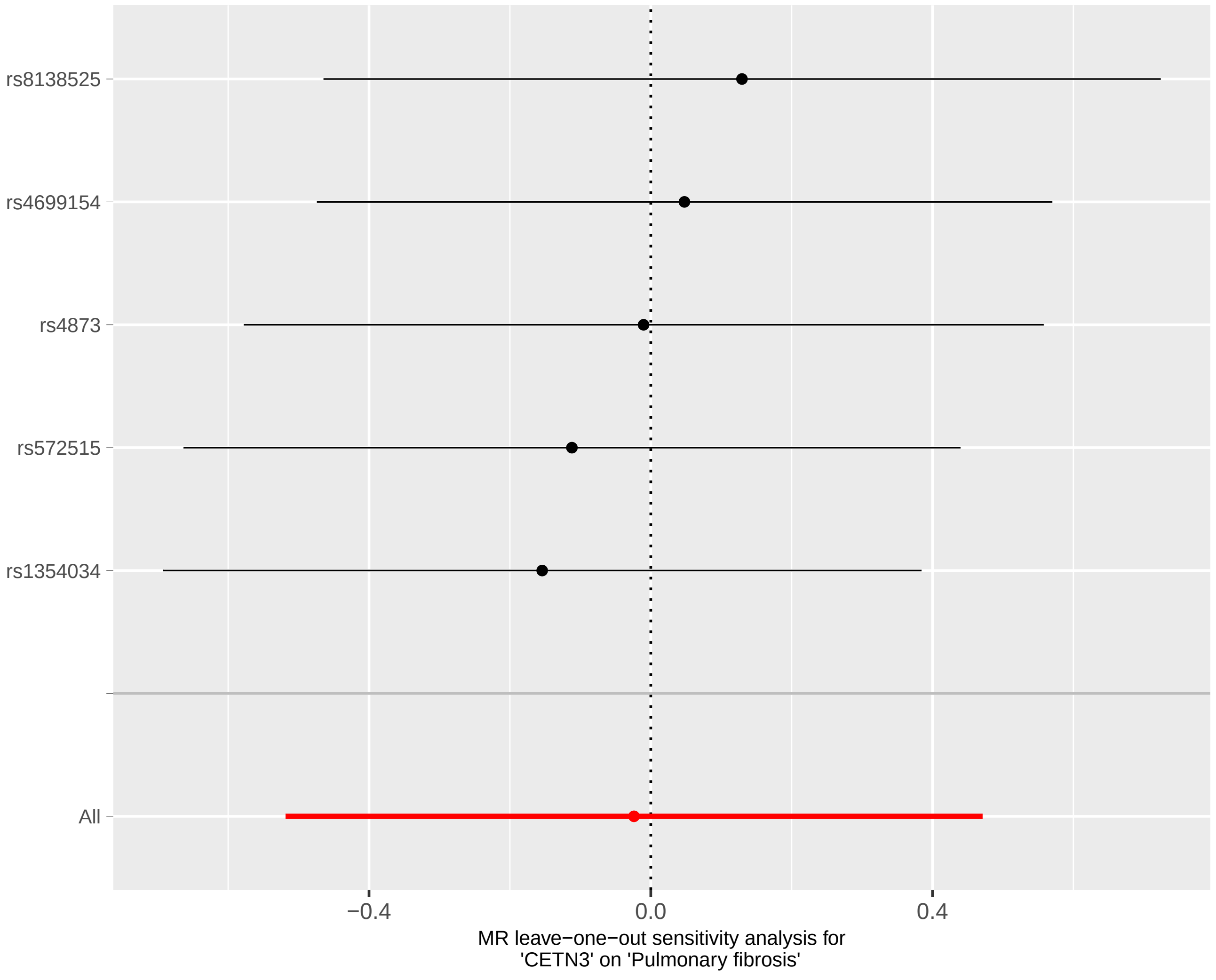

rs8138525
rs4699154
rs4873
rs572515
rs1354034
All
−0.4
0.0
0.4
MR leave−one−out sensitivity analysis for
'CETN3' on 'Pulmonary fibrosis'

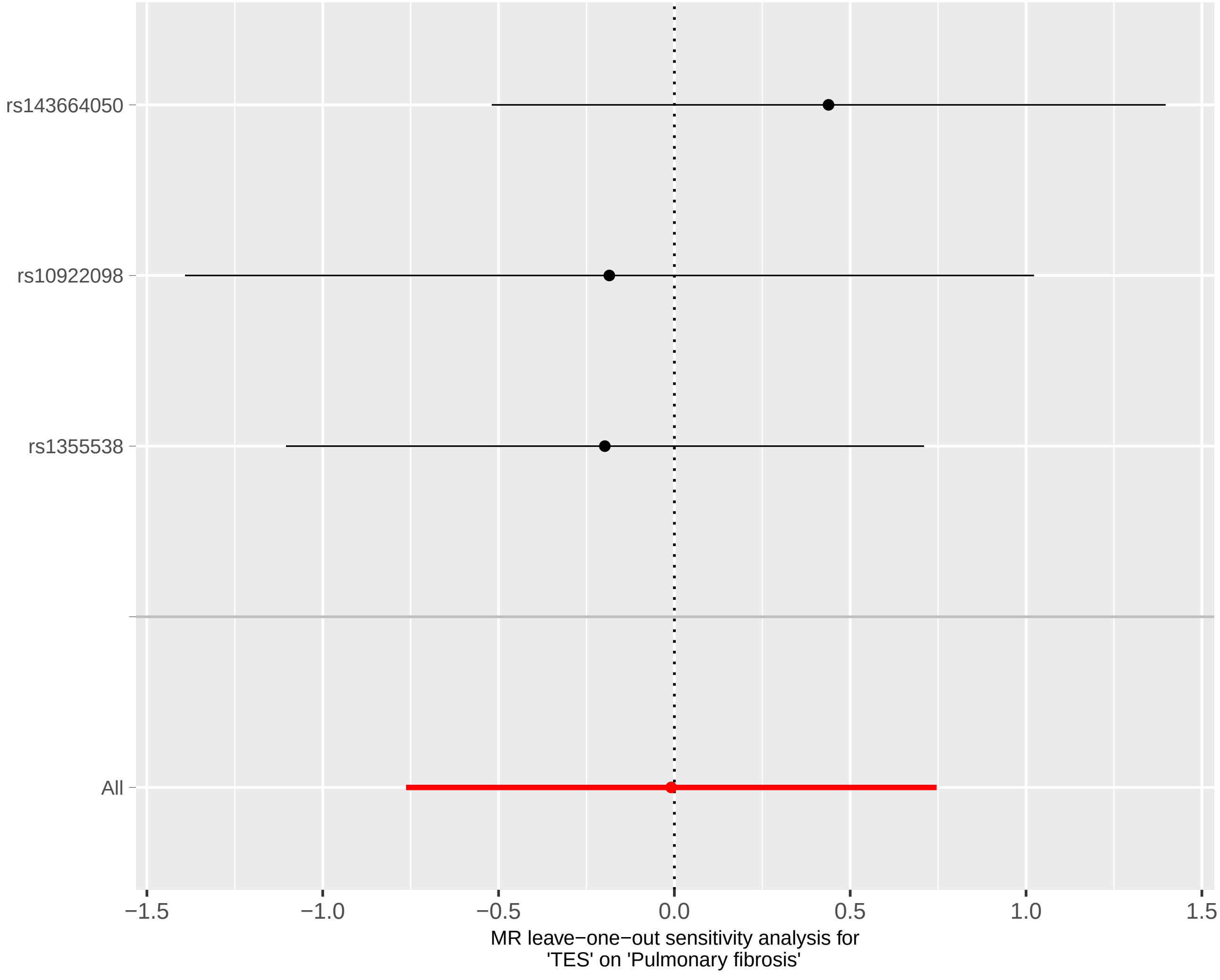

rs143664050
rs10922098
rs1355538
All
−1.5
−1.0
−0.5
0.0
0.5
1.0
1.5
MR leave−one−out sensitivity analysis for
'TES' on 'Pulmonary fibrosis'

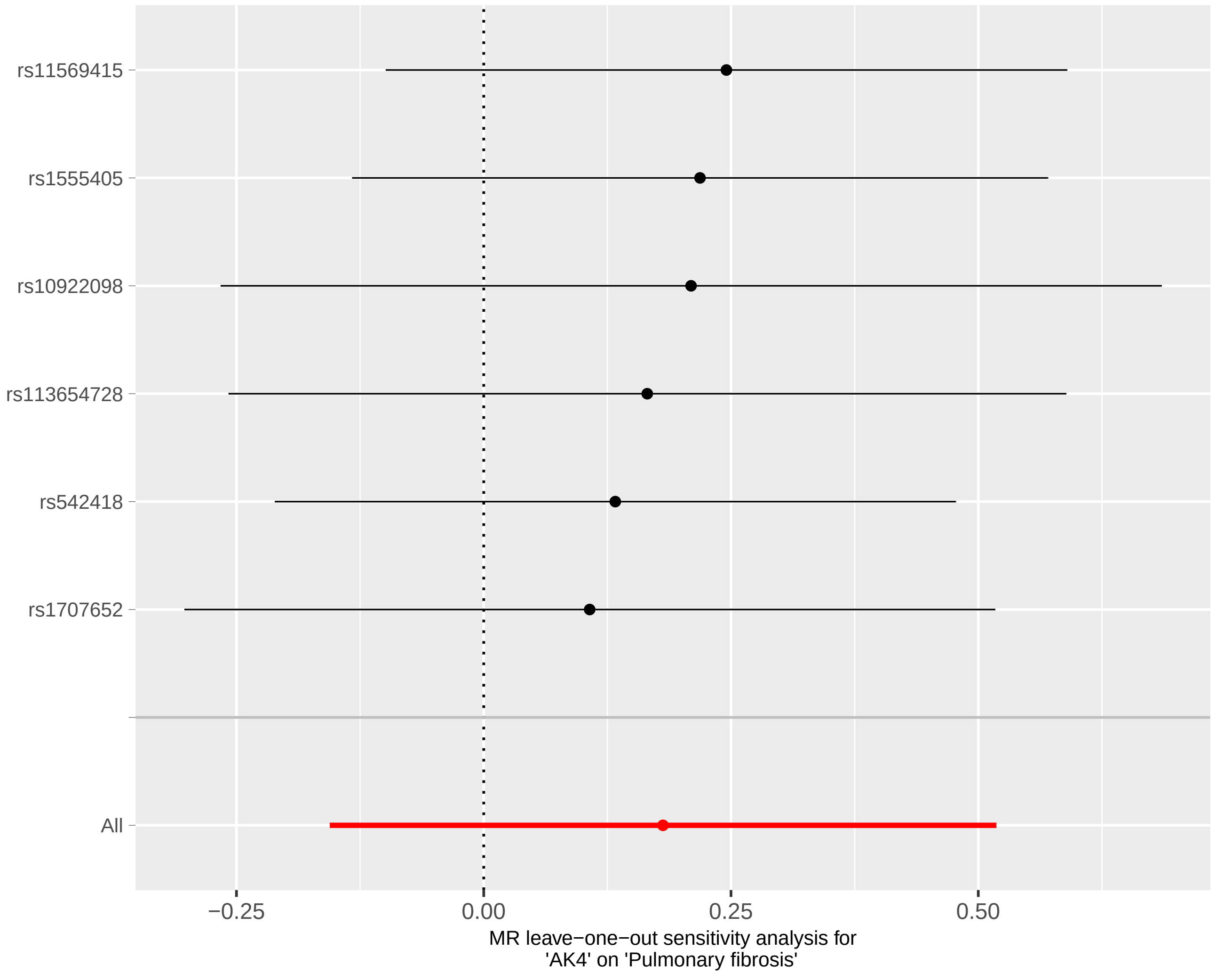
rs11569415
rs1555405
rs10922098
rs113654728
rs542418
rs1707652
All
−0.25
0.00
0.25
0.50
MR leave−one−out sensitivity analysis for
'AK4' on 'Pulmonary fibrosis'

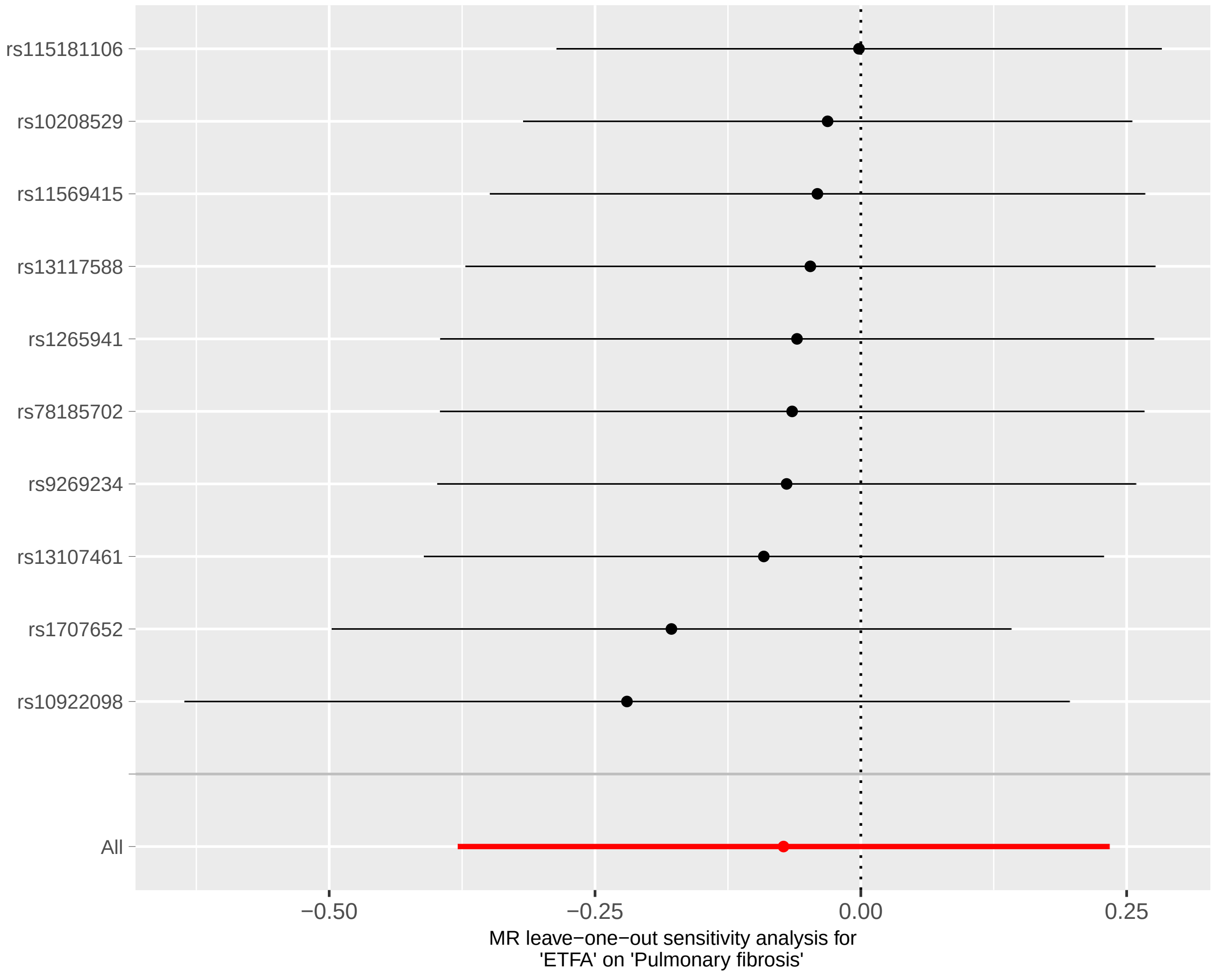
rs115181106
rs10208529
rs11569415
rs13117588
rs1265941
rs78185702
rs9269234
rs13107461
rs1707652
rs10922098
All
−0.50
−0.25
0.00
0.25
MR leave−one−out sensitivity analysis for
'ETFA' on 'Pulmonary fibrosis'

Insufficient number of SNPs

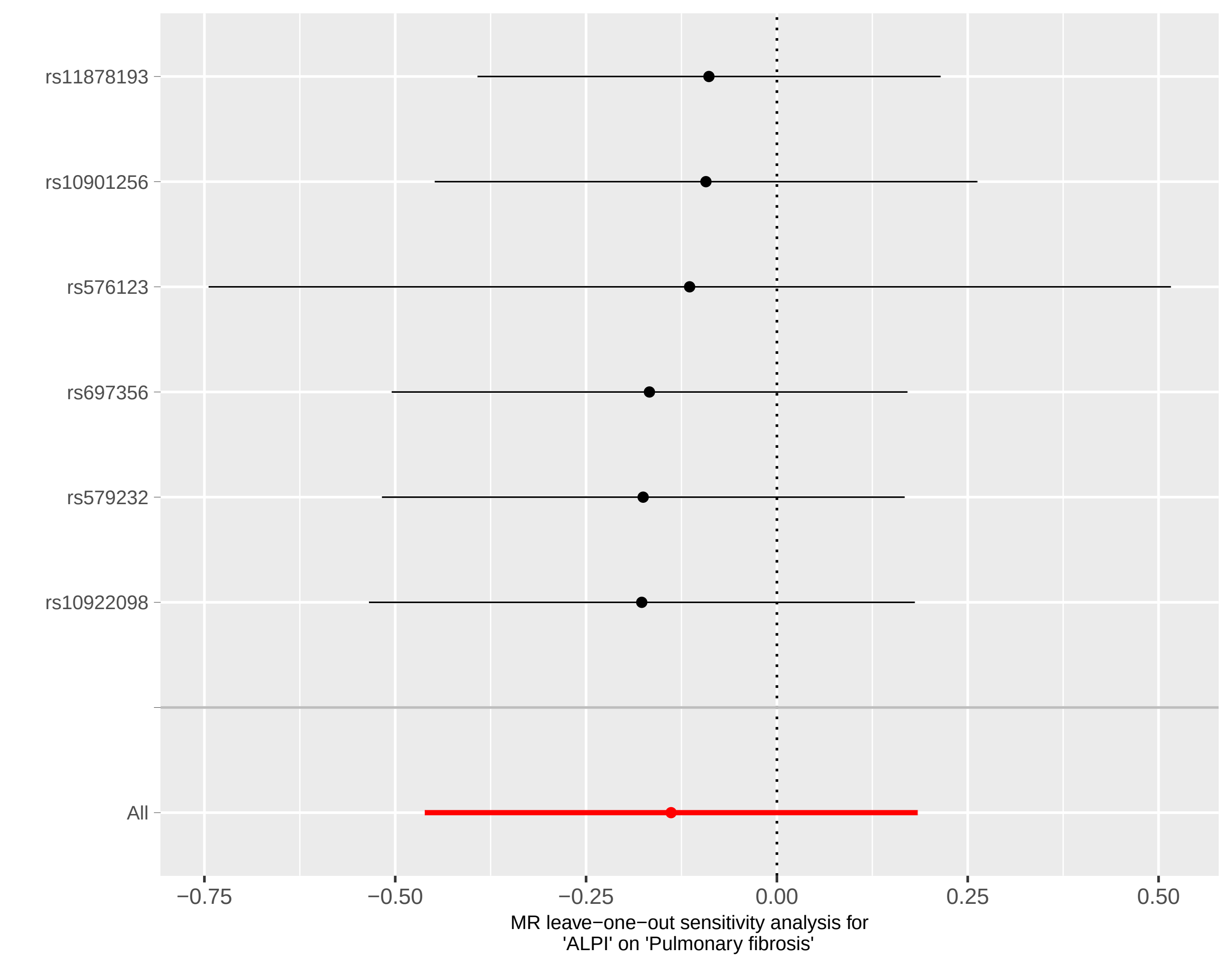
rs11878193
rs10901256
rs576123
rs697356
rs579232
rs10922098
All
−0.75
−0.50
−0.25
0.00
0.25
0.50
MR leave−one−out sensitivity analysis for
'ALPI' on 'Pulmonary fibrosis'

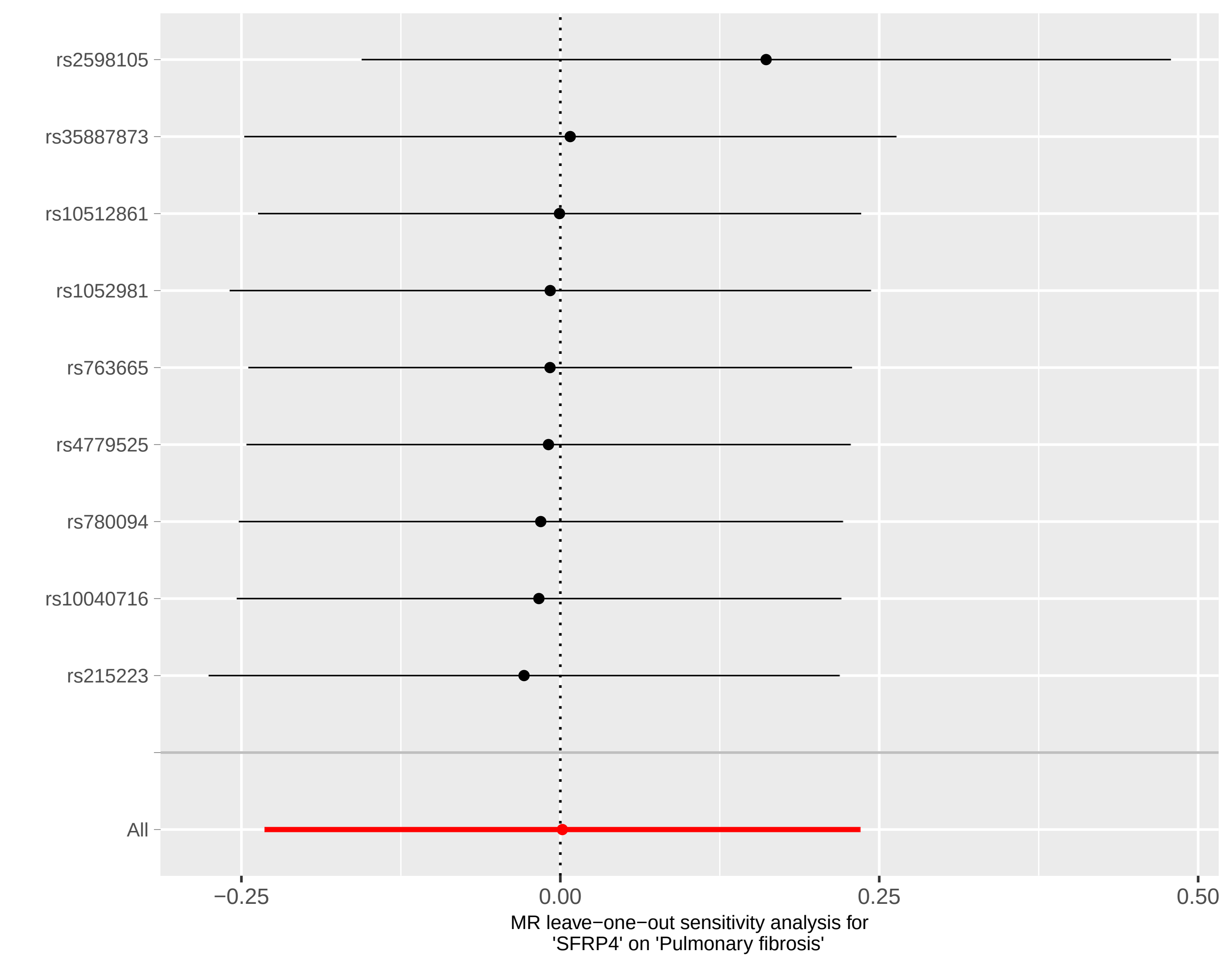
rs2598105
rs35887873
rs10512861
rs1052981
rs763665
rs4779525
rs780094
rs10040716
rs215223
All
−0.25
0.00
0.25
0.50
MR leave−one−out sensitivity analysis for
'SFRP4' on 'Pulmonary fibrosis'

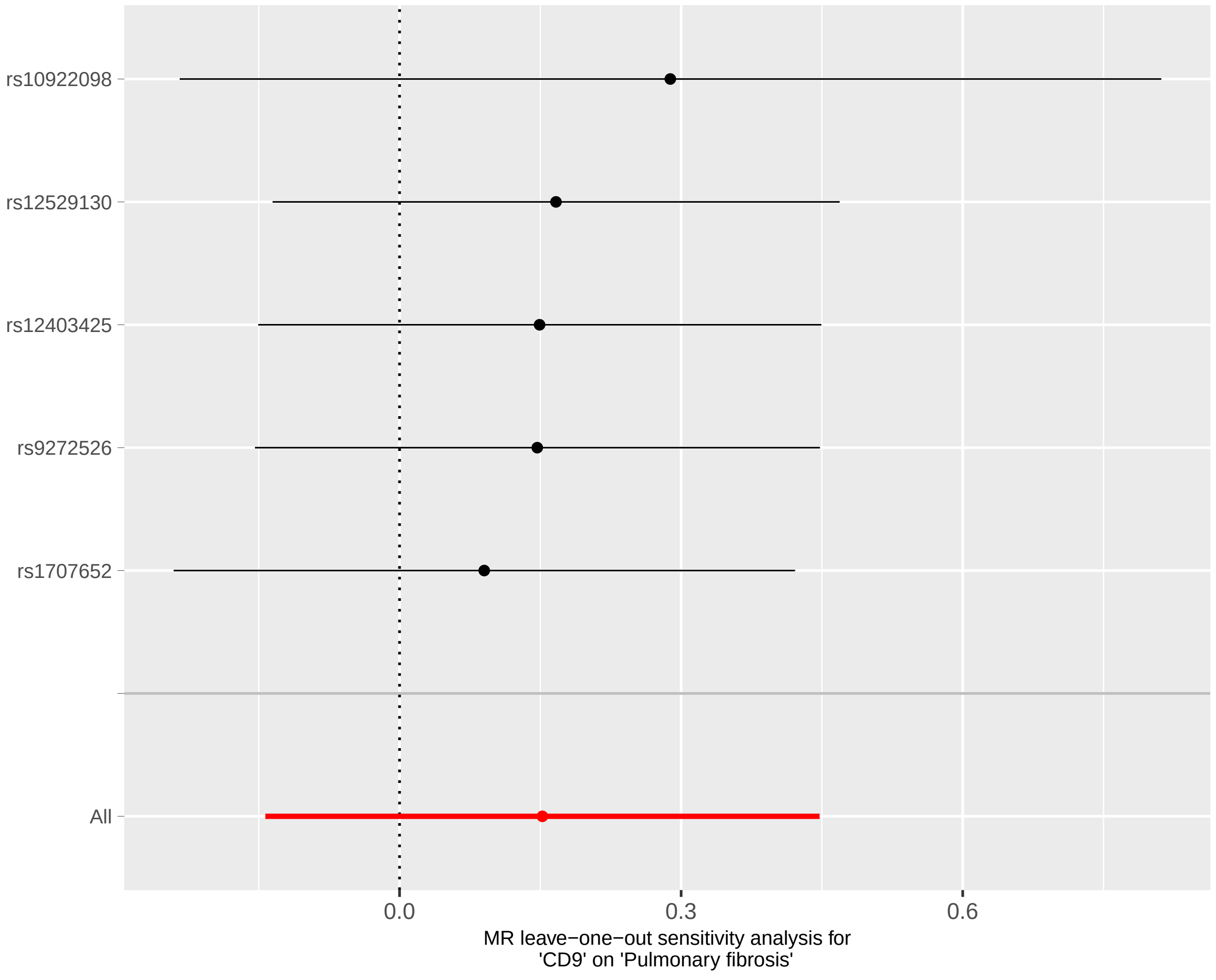

rs10922098
rs12529130
rs12403425
rs9272526
rs1707652
All
0.0
0.3
0.6
MR leave−one−out sensitivity analysis for
'CD9' on 'Pulmonary fibrosis'

Insufficient number of SNPs

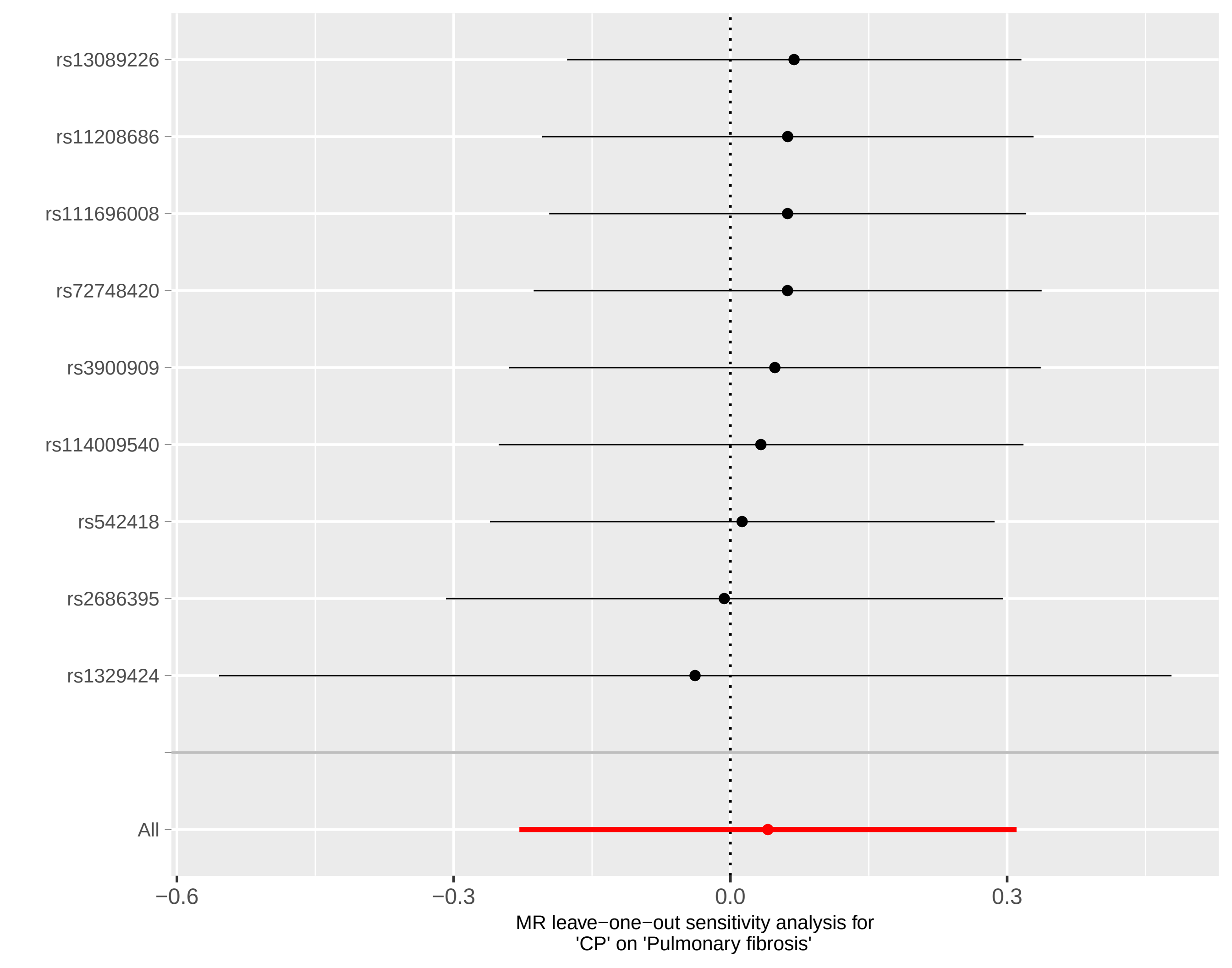
rs13089226
rs11208686
rs111696008
rs72748420
rs3900909
rs114009540
rs542418
rs2686395
rs1329424
All
−0.6
−0.3
0.0
0.3
MR leave−one−out sensitivity analysis for
'CP' on 'Pulmonary fibrosis'

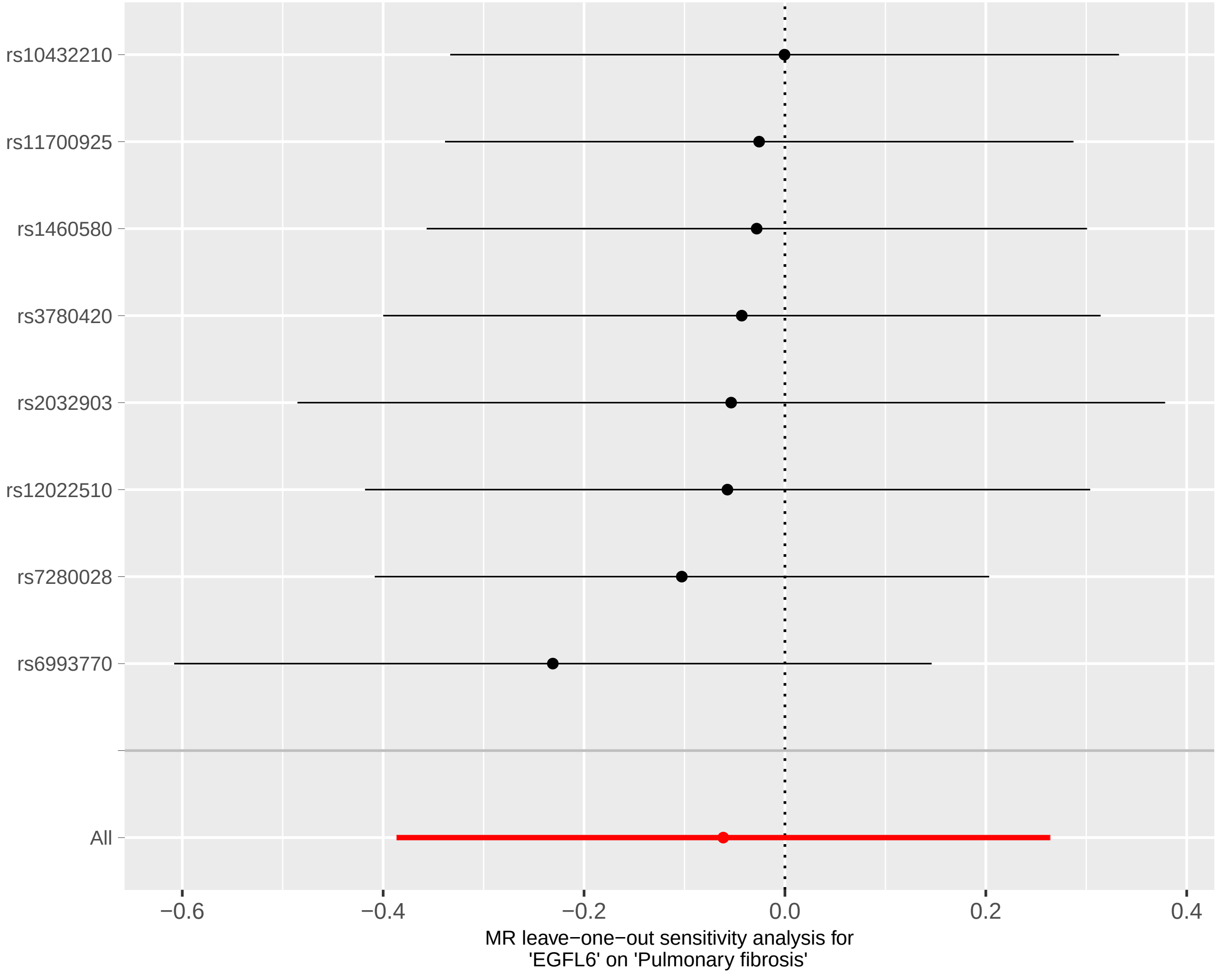
rs10432210
rs11700925
rs1460580
rs3780420
rs2032903
rs12022510
rs7280028
rs6993770
All
−0.6
−0.4
−0.2
0.0
0.2
0.4
MR leave−one−out sensitivity analysis for
'EGFL6' on 'Pulmonary fibrosis'

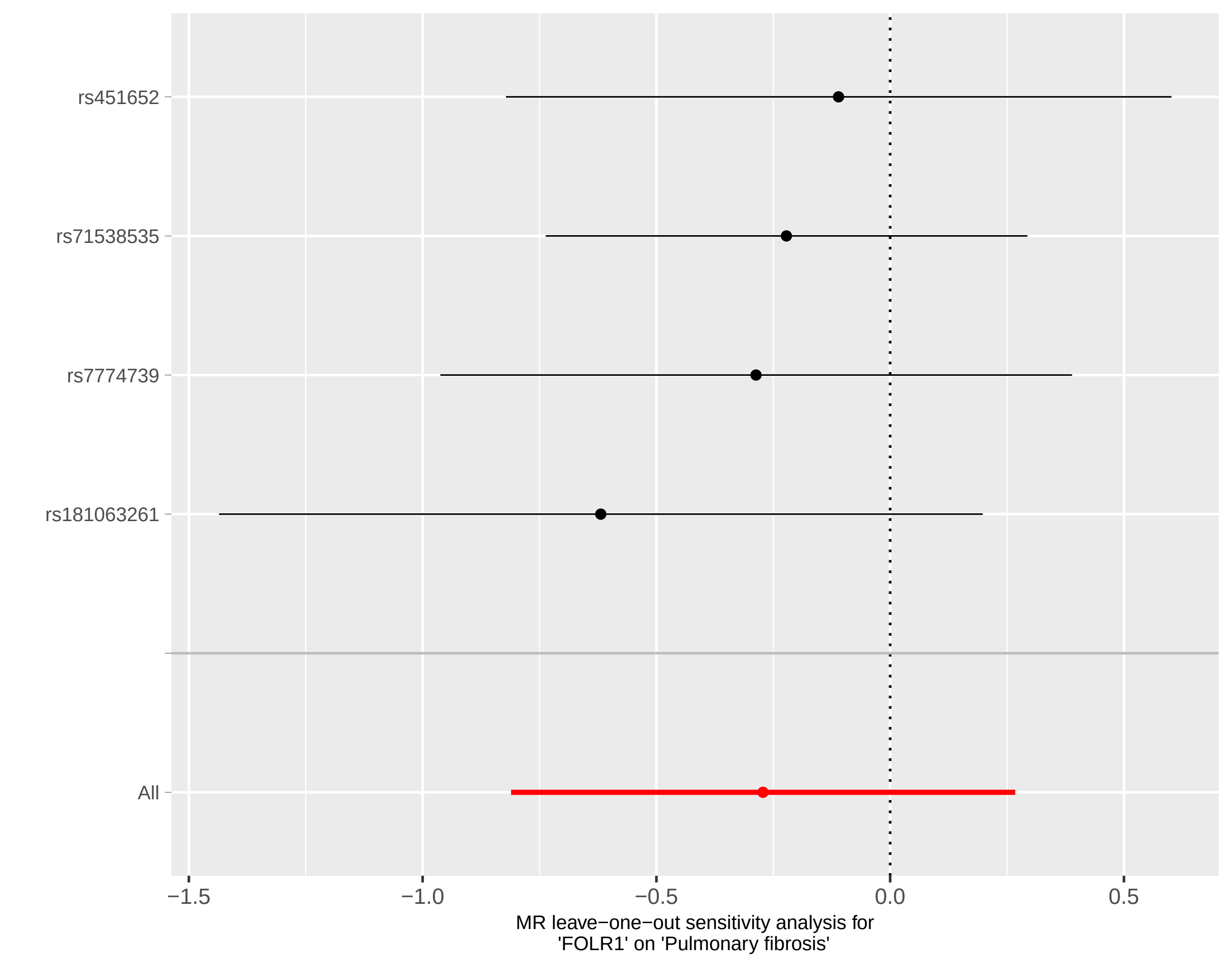
rs451652
rs71538535
rs7774739
rs181063261
All
−1.5
−1.0
−0.5
0.0
0.5
MR leave−one−out sensitivity analysis for
'FOLR1' on 'Pulmonary fibrosis'

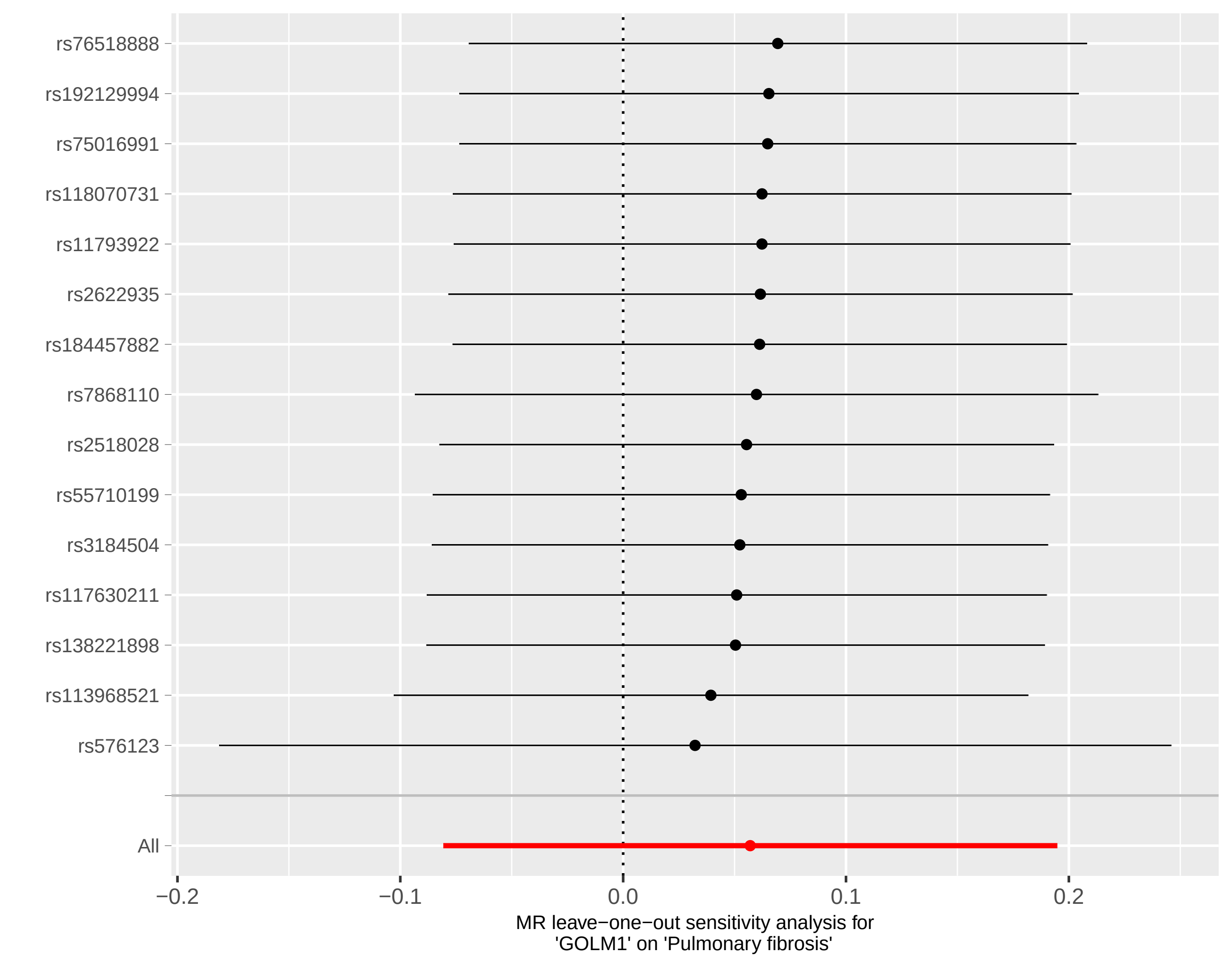
rs76518888
rs192129994
rs75016991
rs118070731
rs11793922
rs2622935
rs184457882
rs7868110
rs2518028
rs55710199
rs3184504
rs117630211
rs138221898
rs113968521
rs576123
All
−0.2
−0.1
0.0
0.1
0.2
MR leave−one−out sensitivity analysis for
'GOLM1' on 'Pulmonary fibrosis'

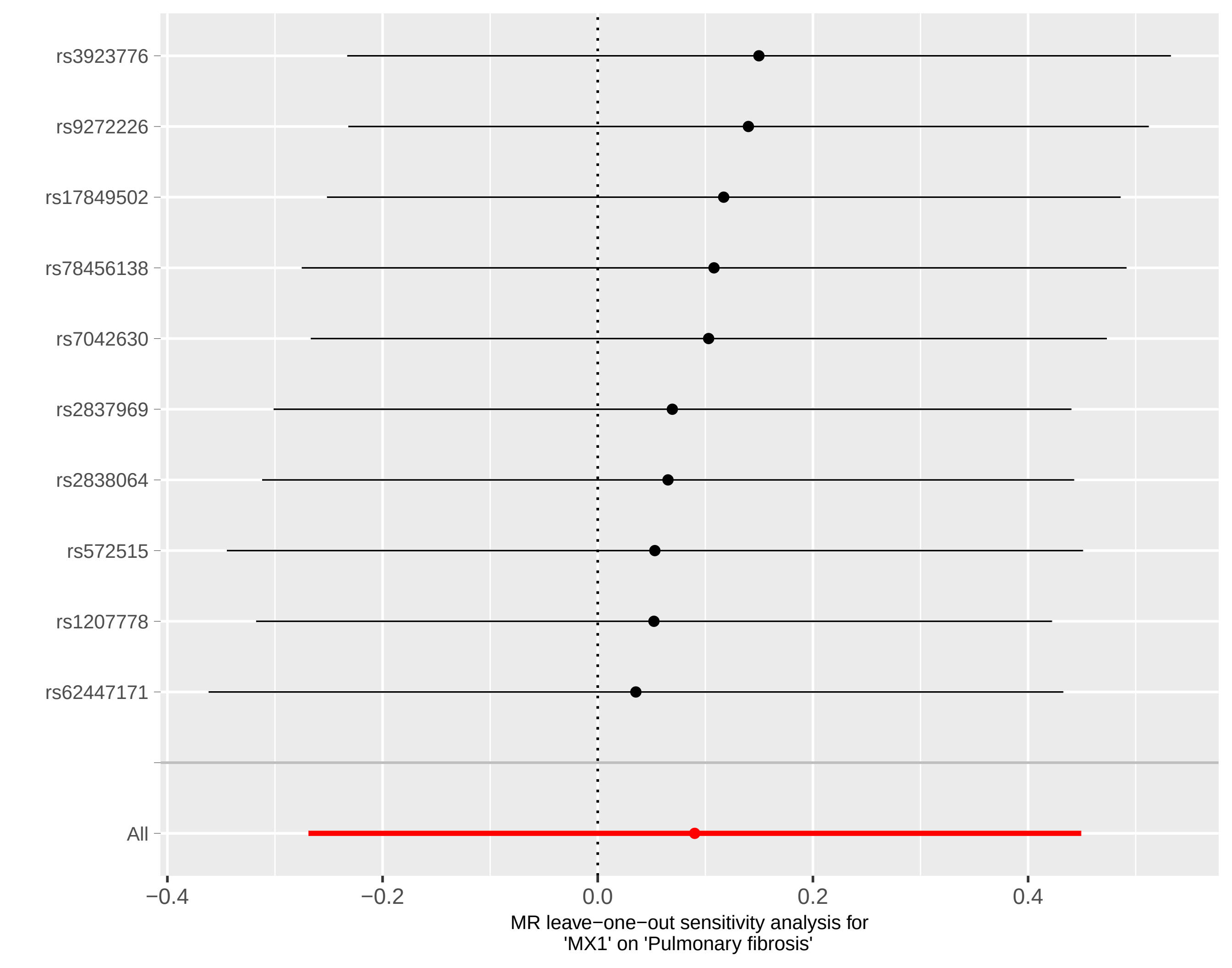
rs3923776
rs9272226
rs17849502
rs78456138
rs7042630
rs2837969
rs2838064
rs572515
rs1207778
rs62447171
All
−0.4
−0.2
0.0
0.2
0.4
MR leave−one−out sensitivity analysis for
'MX1' on 'Pulmonary fibrosis'

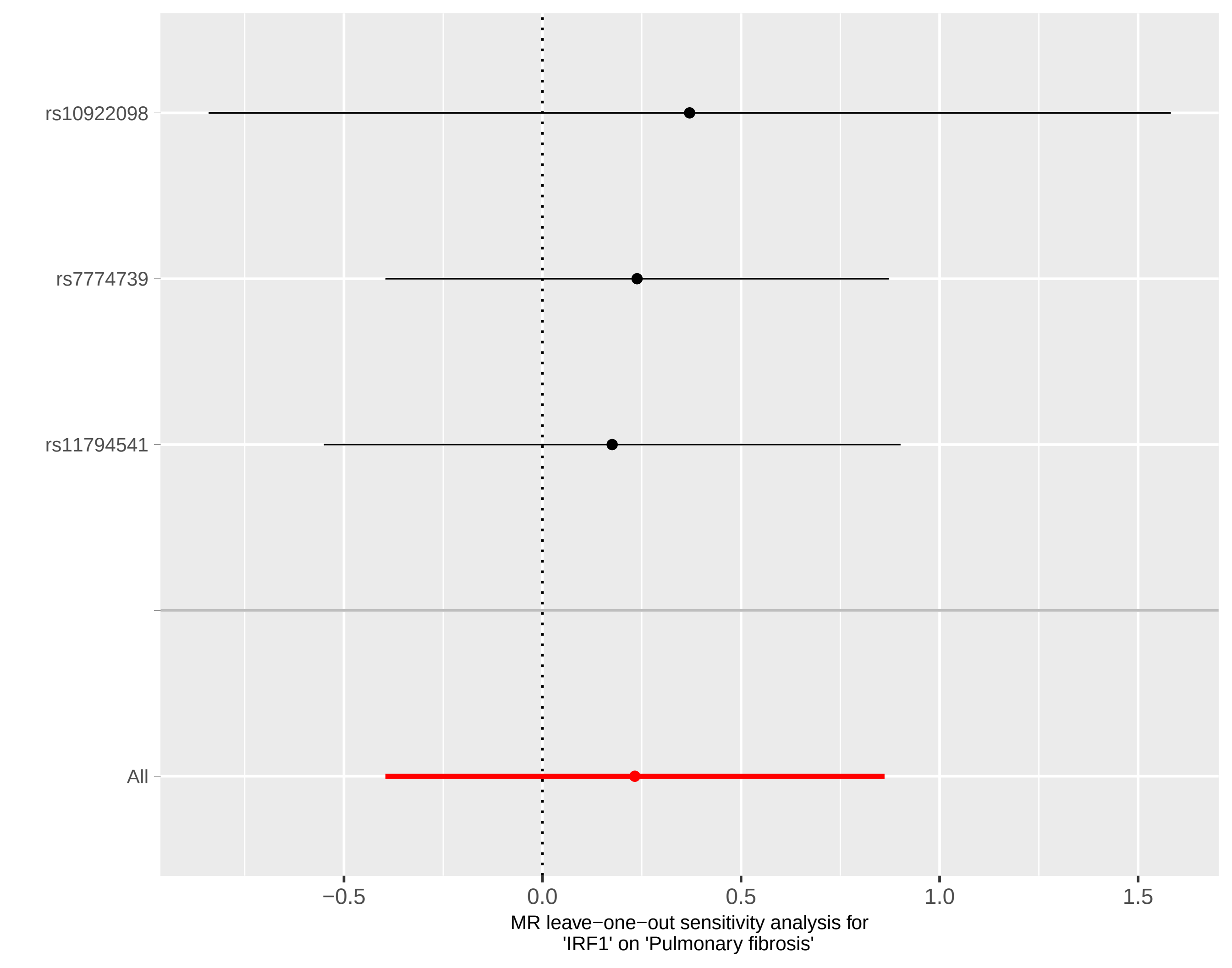
rs10922098
rs7774739
rs11794541
All
−0.5
0.0
0.5
1.0
1.5
MR leave−one−out sensitivity analysis for
'IRF1' on 'Pulmonary fibrosis'

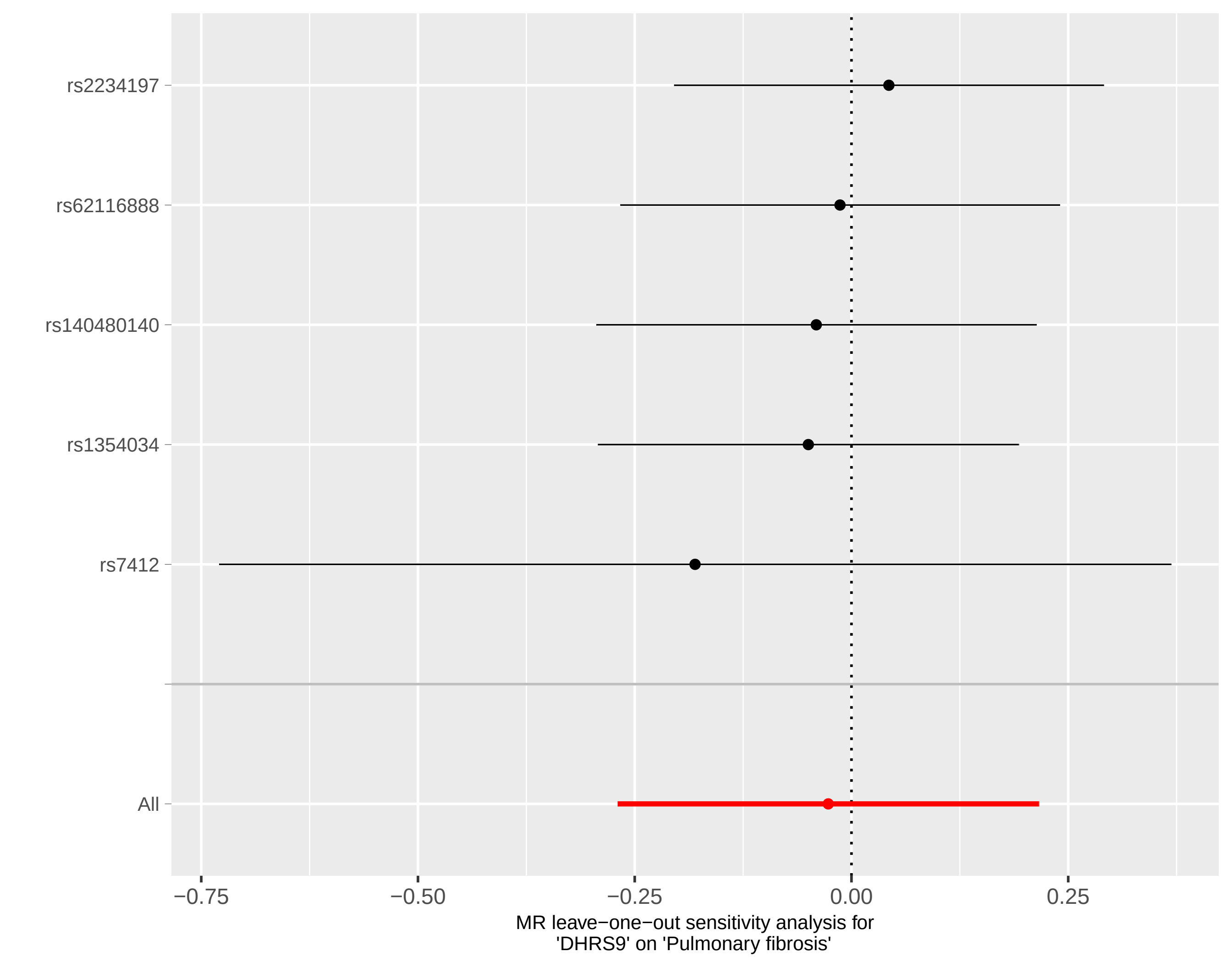
rs2234197
rs62116888
rs140480140
rs1354034
rs7412
All
−0.75
−0.50
−0.25
0.00
0.25
MR leave−one−out sensitivity analysis for
'DHRS9' on 'Pulmonary fibrosis'

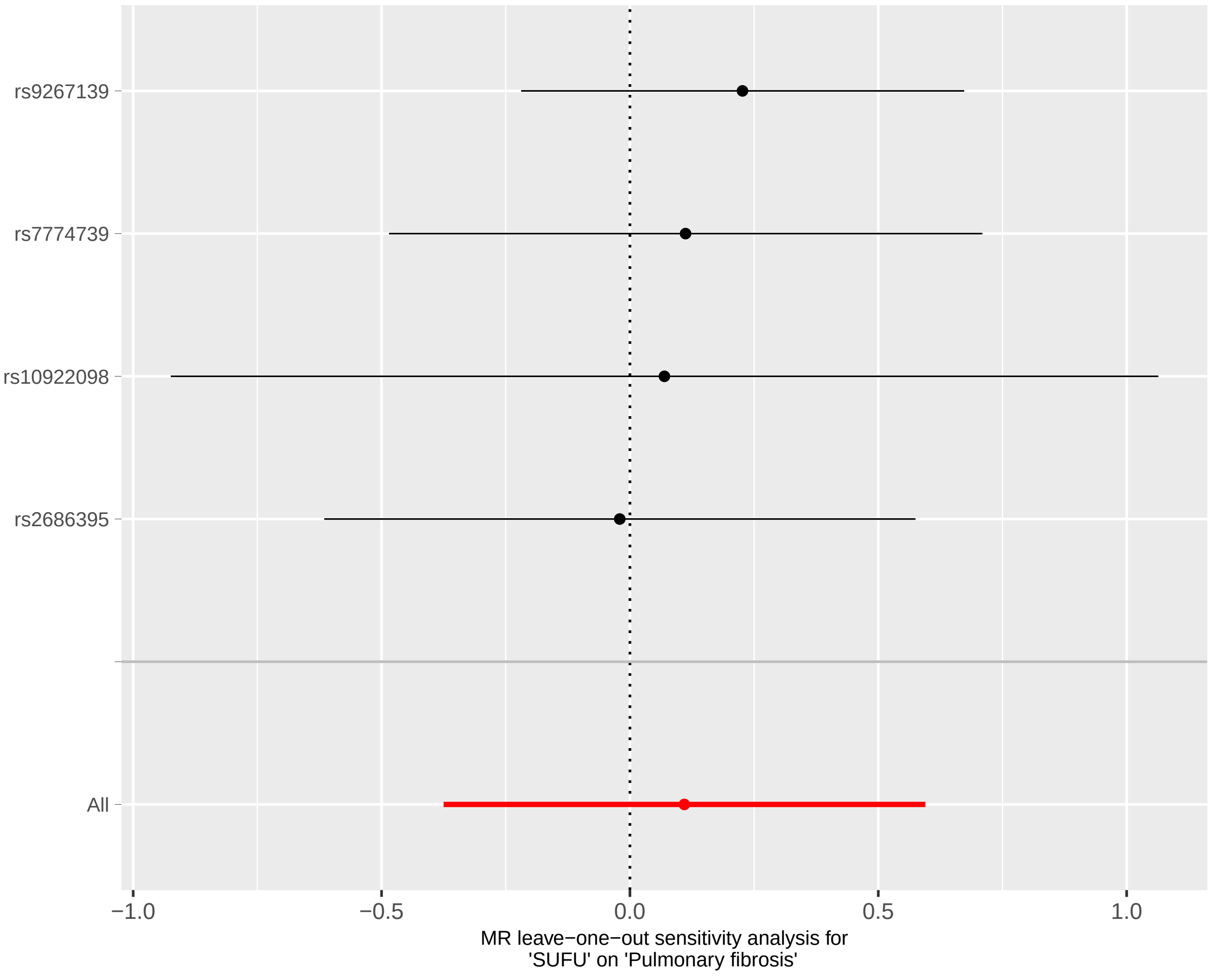

rs9267139
rs7774739
rs10922098
rs2686395
All
−1.0
−0.5
0.0
0.5
1.0
MR leave−one−out sensitivity analysis for
'SUFU' on 'Pulmonary fibrosis'

Insufficient number of SNPs

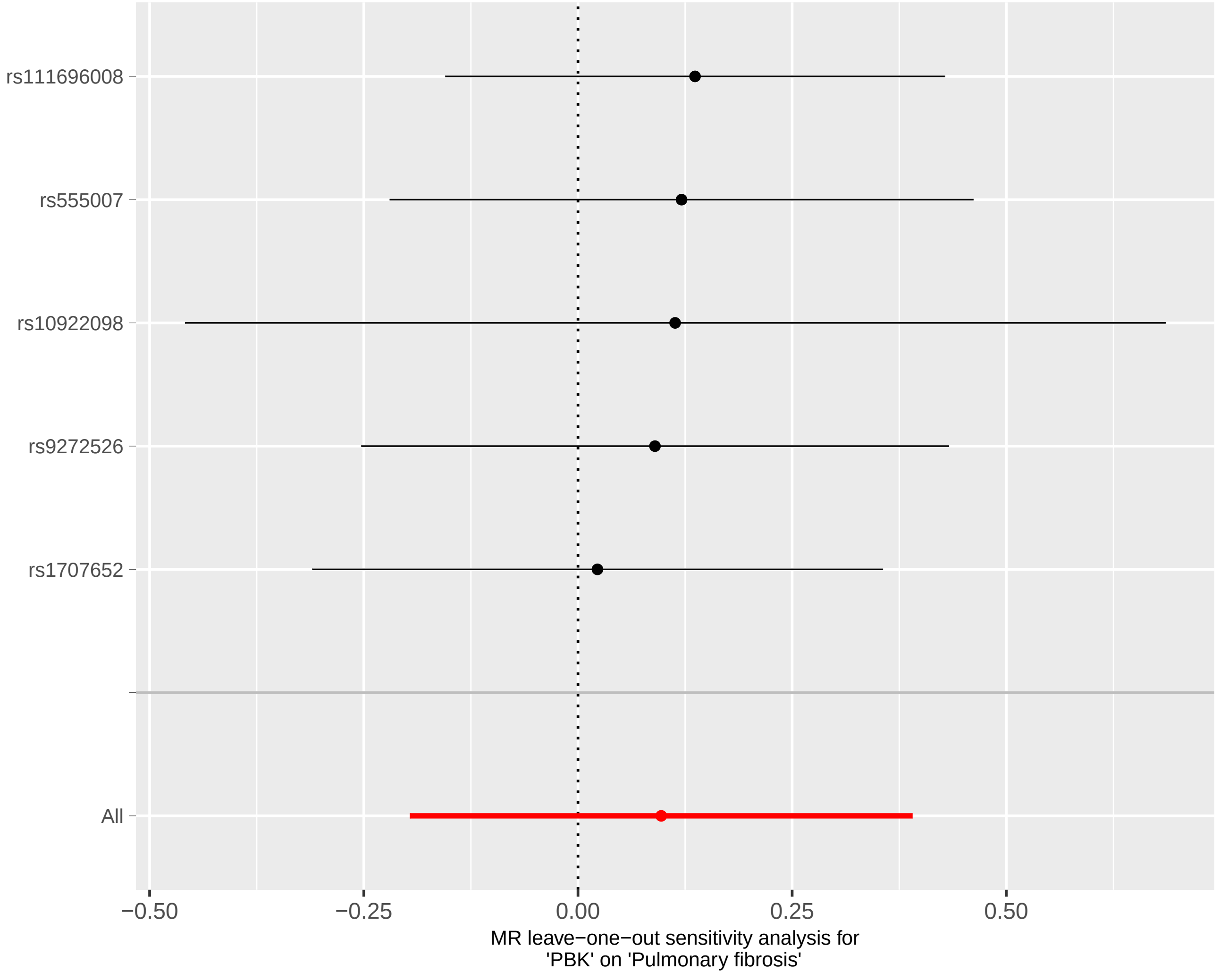
rs111696008
rs555007
rs10922098
rs9272526
rs1707652
All
−0.50
−0.25
0.00
0.25
0.50
MR leave−one−out sensitivity analysis for
'PBK' on 'Pulmonary fibrosis'

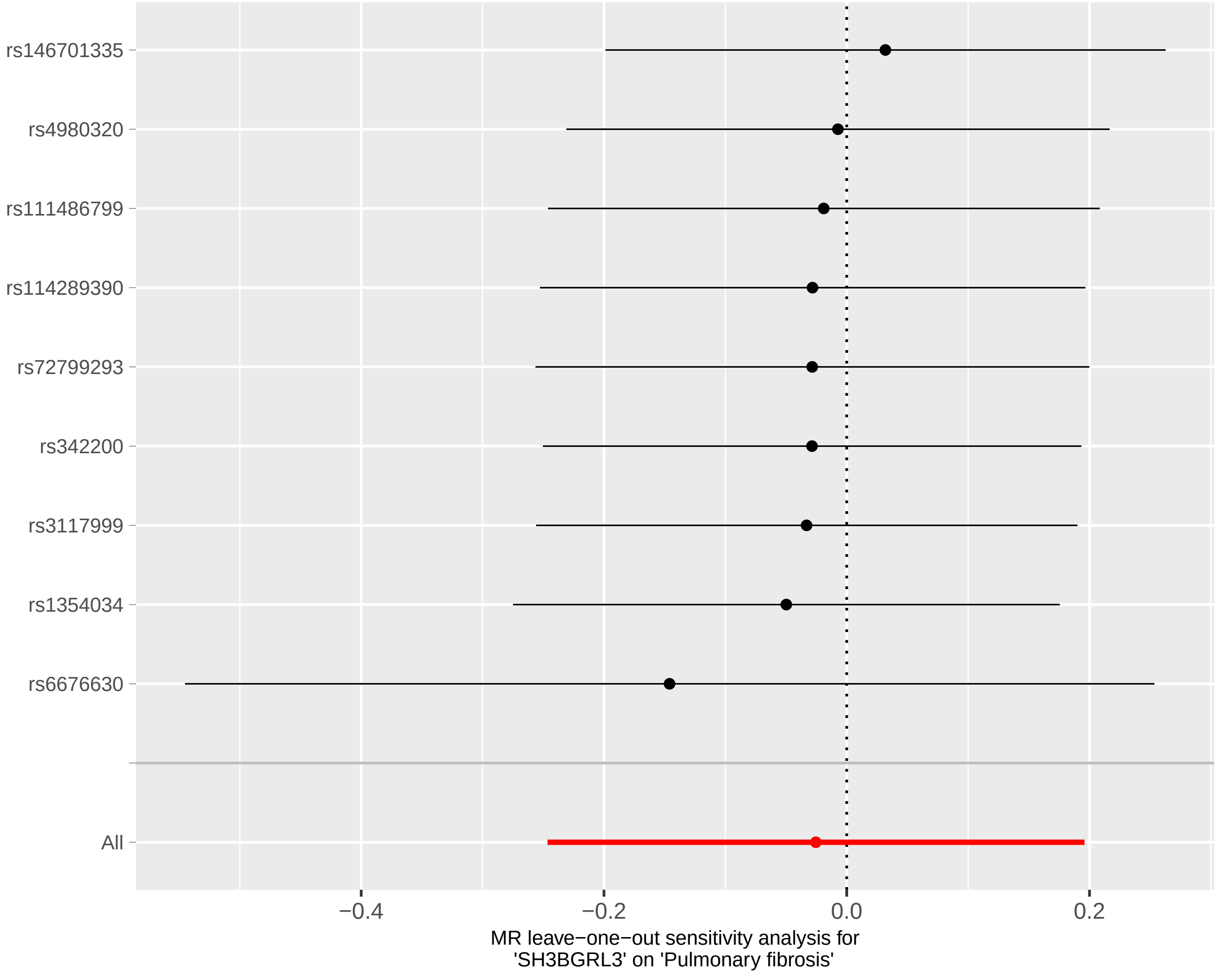
rs146701335
rs4980320
rs111486799
rs114289390
rs72799293
rs342200
rs3117999
rs1354034
rs6676630
All
−0.4
−0.2
0.0
0.2
MR leave−one−out sensitivity analysis for
'SH3BGRL3' on 'Pulmonary fibrosis'

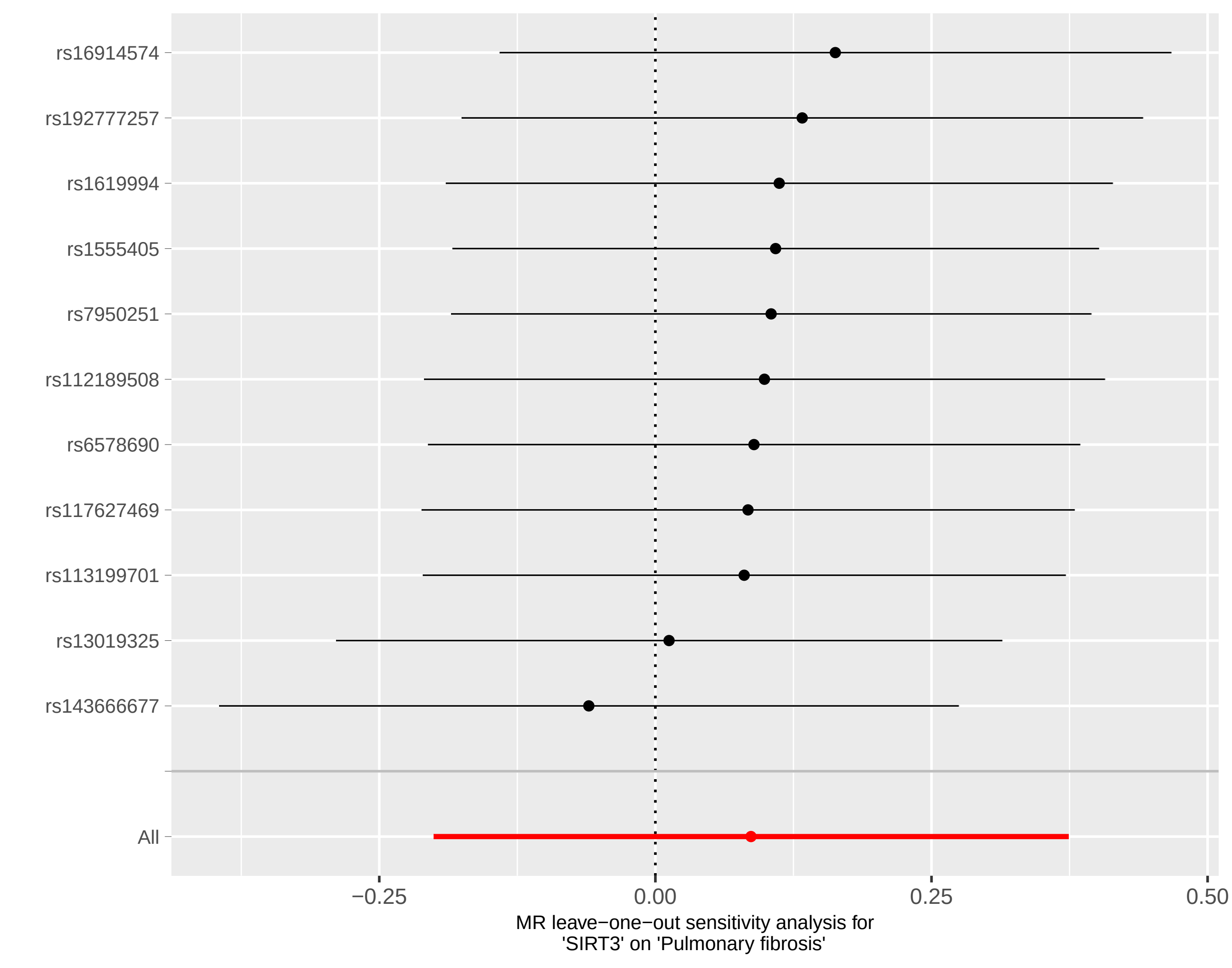
rs16914574
rs192777257
rs1619994
rs1555405
rs7950251
rs112189508
rs6578690
rs117627469
rs113199701
rs13019325
rs143666677
All
−0.25
0.00
0.25
0.50
MR leave−one−out sensitivity analysis for
'SIRT3' on 'Pulmonary fibrosis'

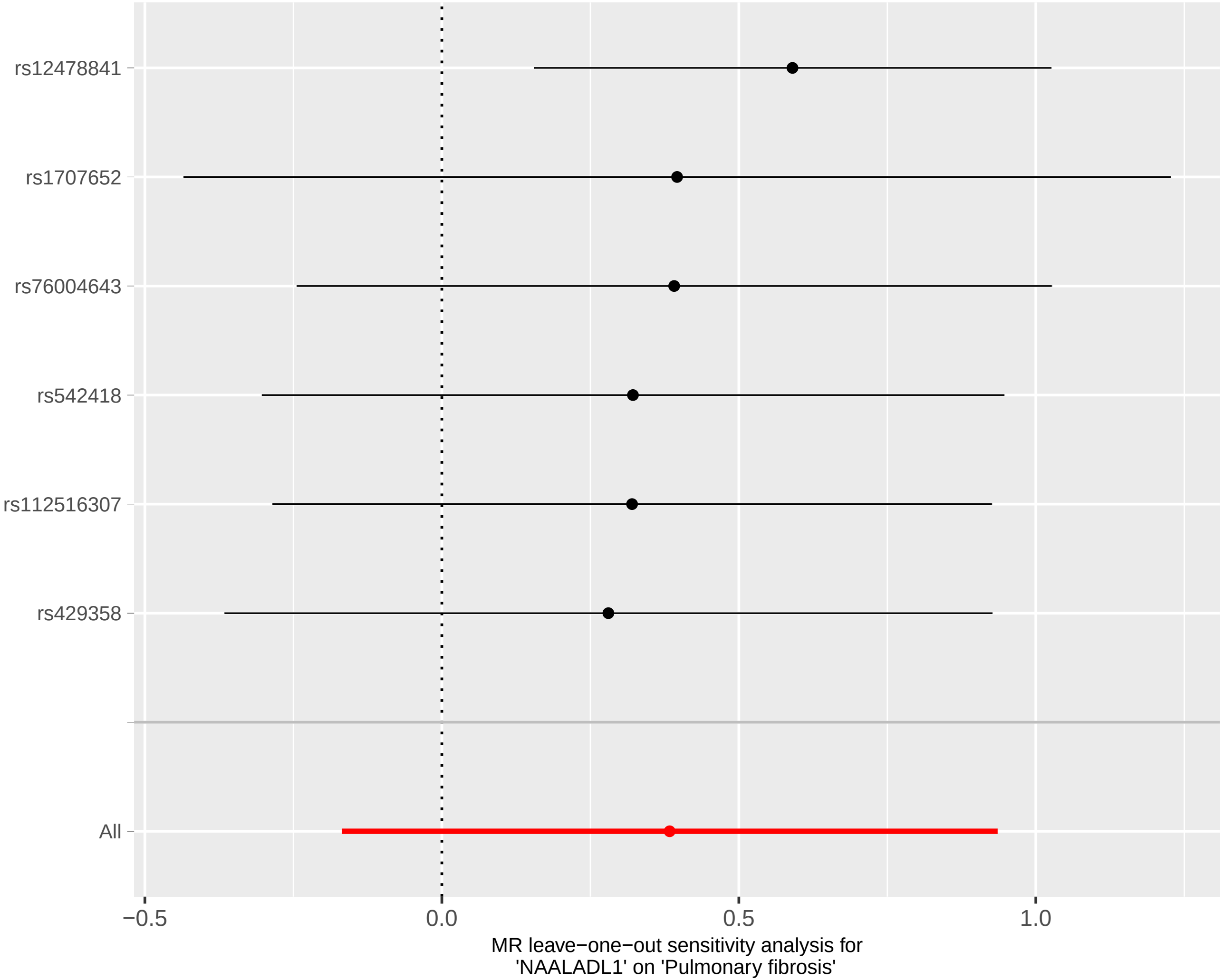
rs12478841
rs1707652
rs76004643
rs542418
rs112516307
rs429358
All
−0.5
0.0
0.5
1.0
MR leave−one−out sensitivity analysis for
'NAALADL1' on 'Pulmonary fibrosis'

Insufficient number of SNPs

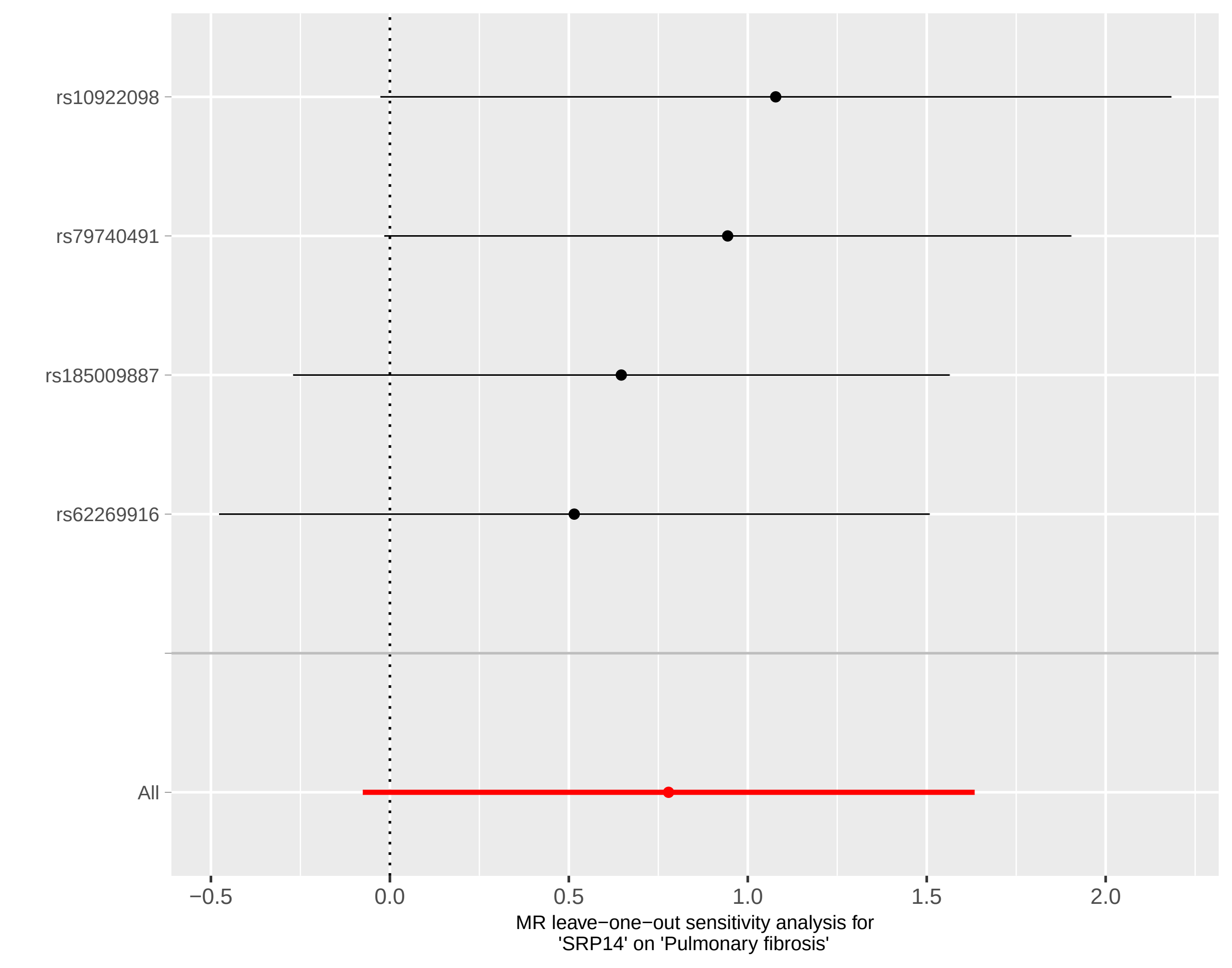
rs10922098
rs79740491
rs185009887
rs62269916
All
−0.5
0.0
0.5
1.0
1.5
2.0
MR leave−one−out sensitivity analysis for
'SRP14' on 'Pulmonary fibrosis'

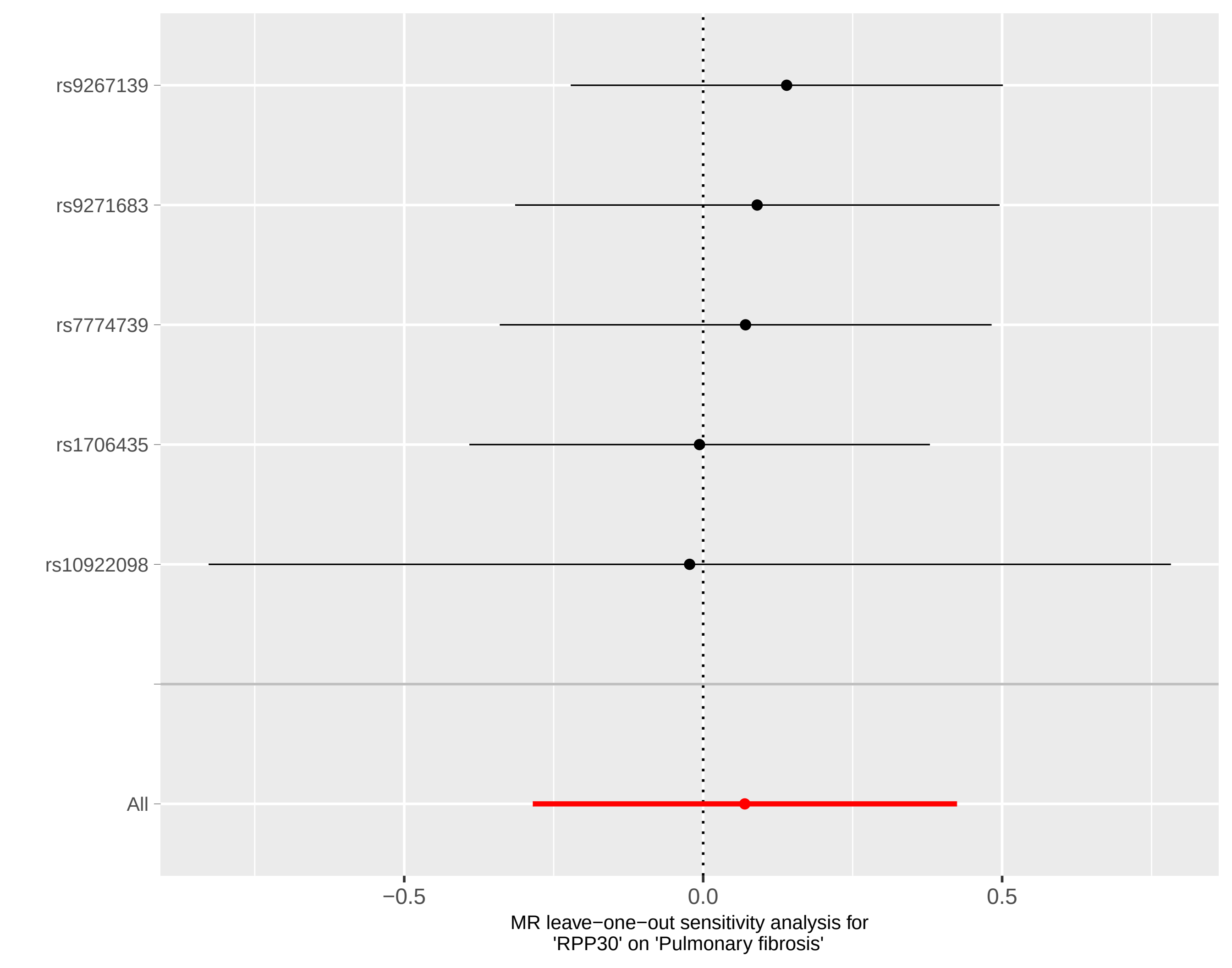

rs9267139
rs9271683
rs7774739
rs1706435
rs10922098
All
−0.5
0.0
0.5
MR leave−one−out sensitivity analysis for
'RPP30' on 'Pulmonary fibrosis'

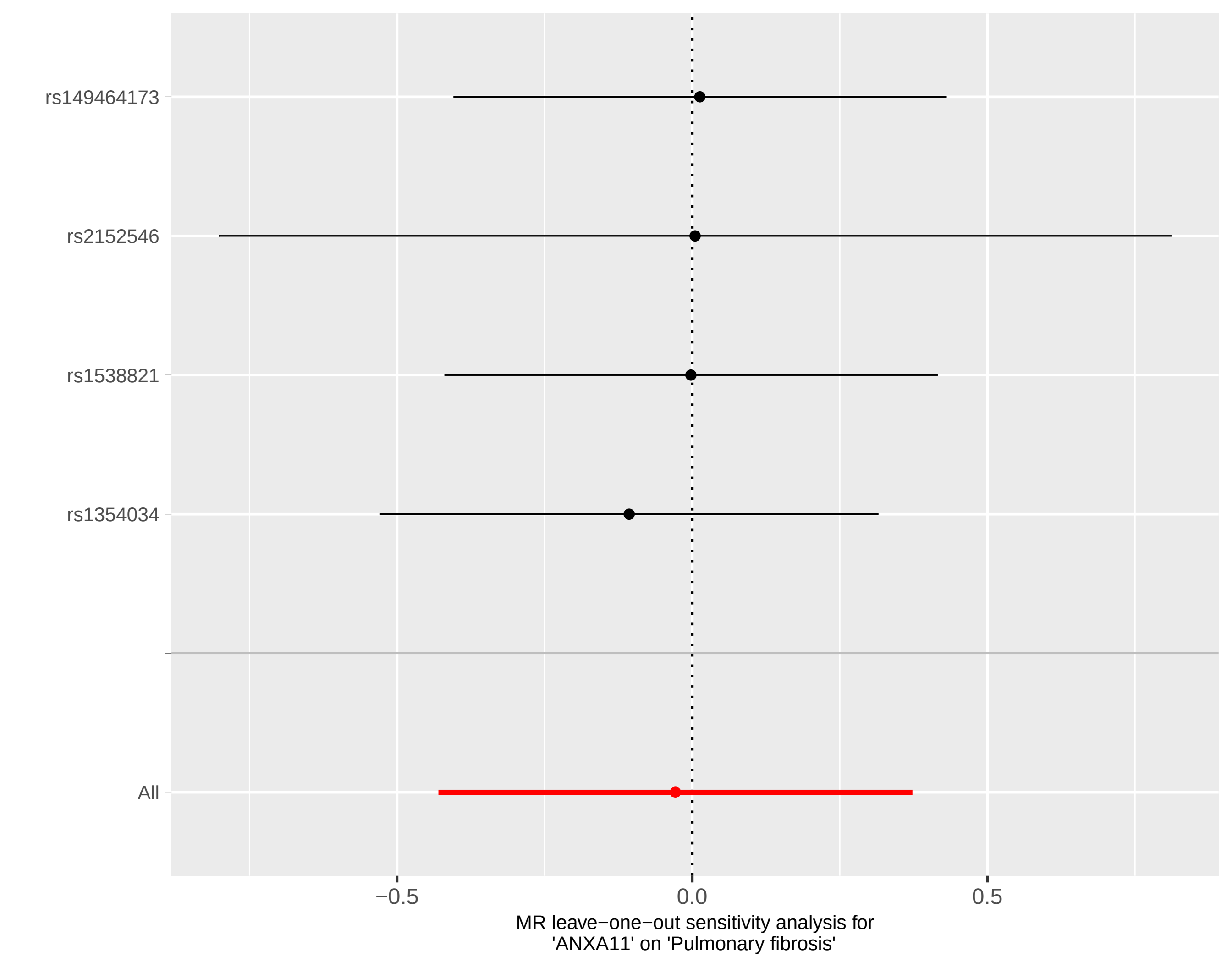
rs149464173
rs2152546
rs1538821
rs1354034
All
−0.5
0.0
0.5
MR leave−one−out sensitivity analysis for
'ANXA11' on 'Pulmonary fibrosis'

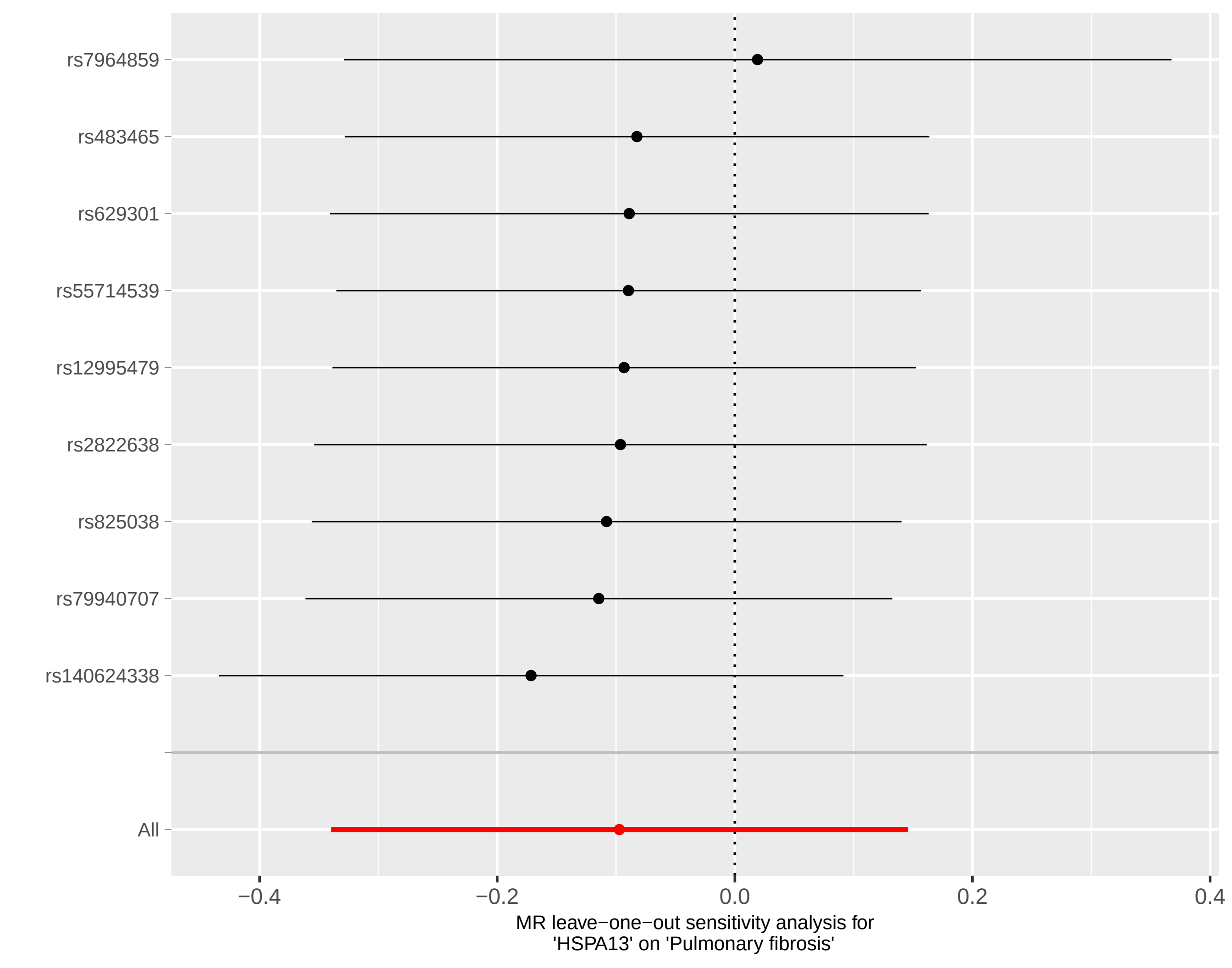

rs7964859
rs483465
rs629301
rs55714539
rs12995479
rs2822638
rs825038
rs79940707
rs140624338
All
−0.4
−0.2
0.0
0.2
0.4
MR leave−one−out sensitivity analysis for
'HSPA13' on 'Pulmonary fibrosis'

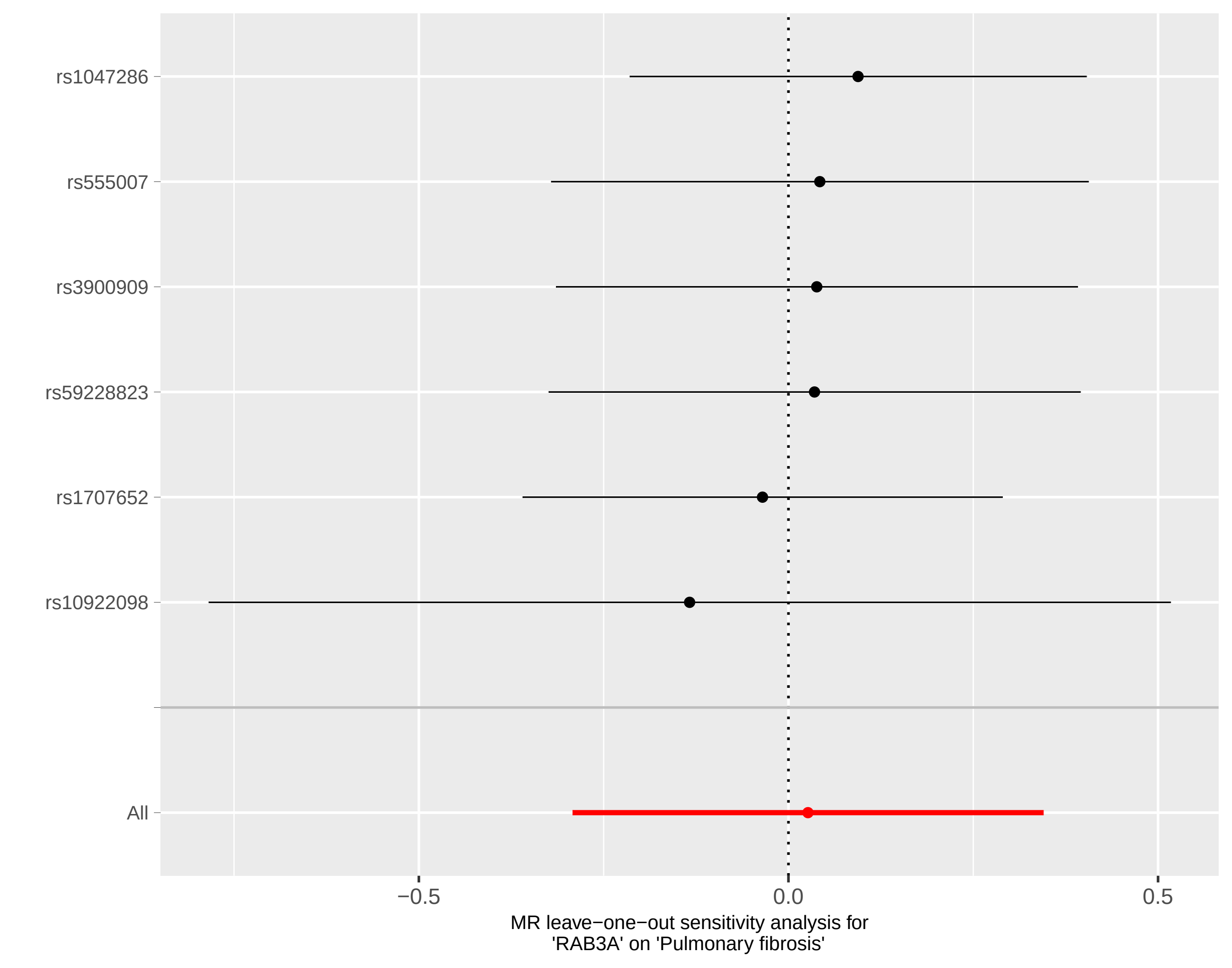
rs1047286
rs555007
rs3900909
rs59228823
rs1707652
rs10922098
All
−0.5
0.0
0.5
MR leave−one−out sensitivity analysis for
'RAB3A' on 'Pulmonary fibrosis'

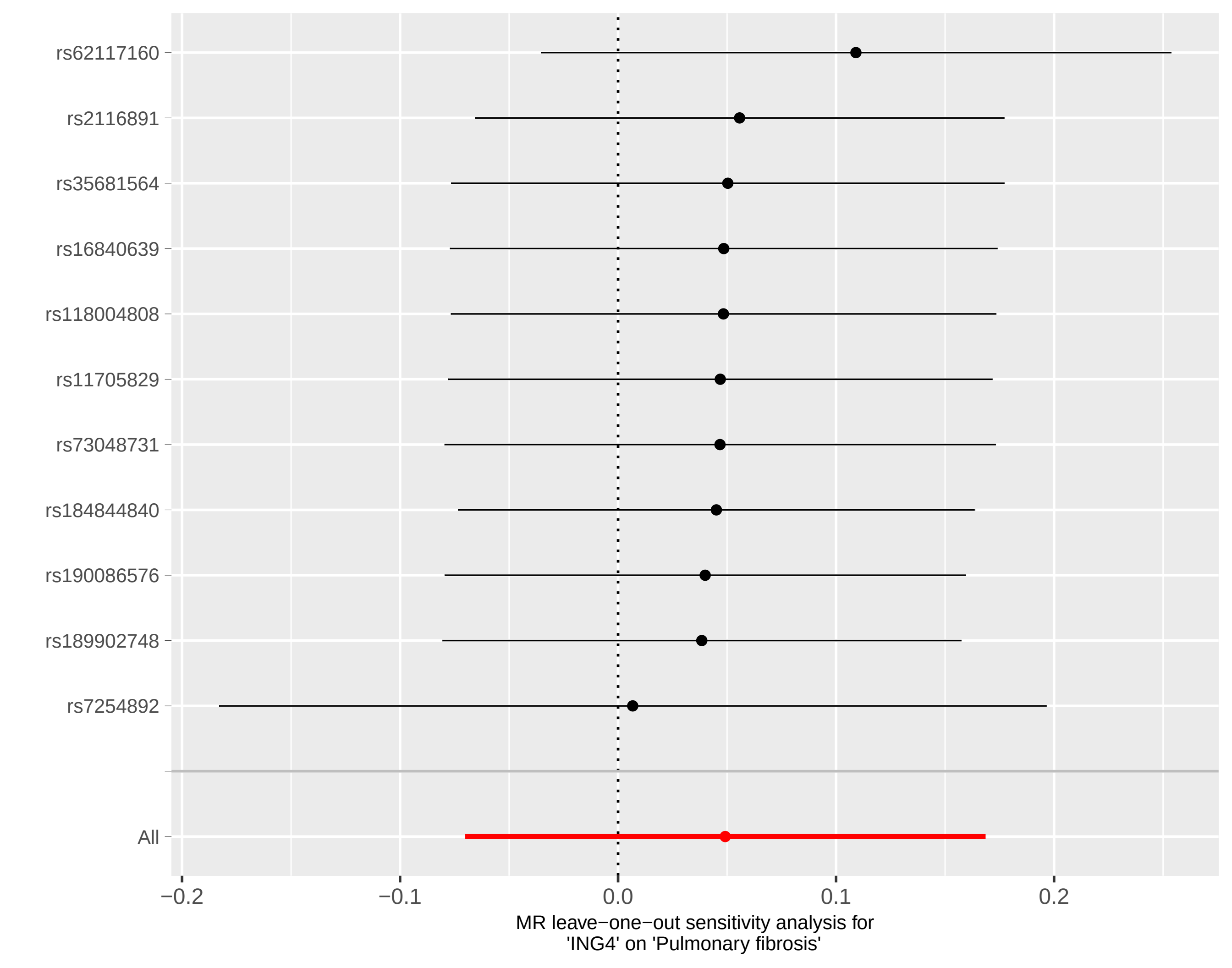
rs62117160
rs2116891
rs35681564
rs16840639
rs118004808
rs11705829
rs73048731
rs184844840
rs190086576
rs189902748
rs7254892
All
−0.2
−0.1
0.0
0.1
0.2
MR leave−one−out sensitivity analysis for
'ING4' on 'Pulmonary fibrosis'

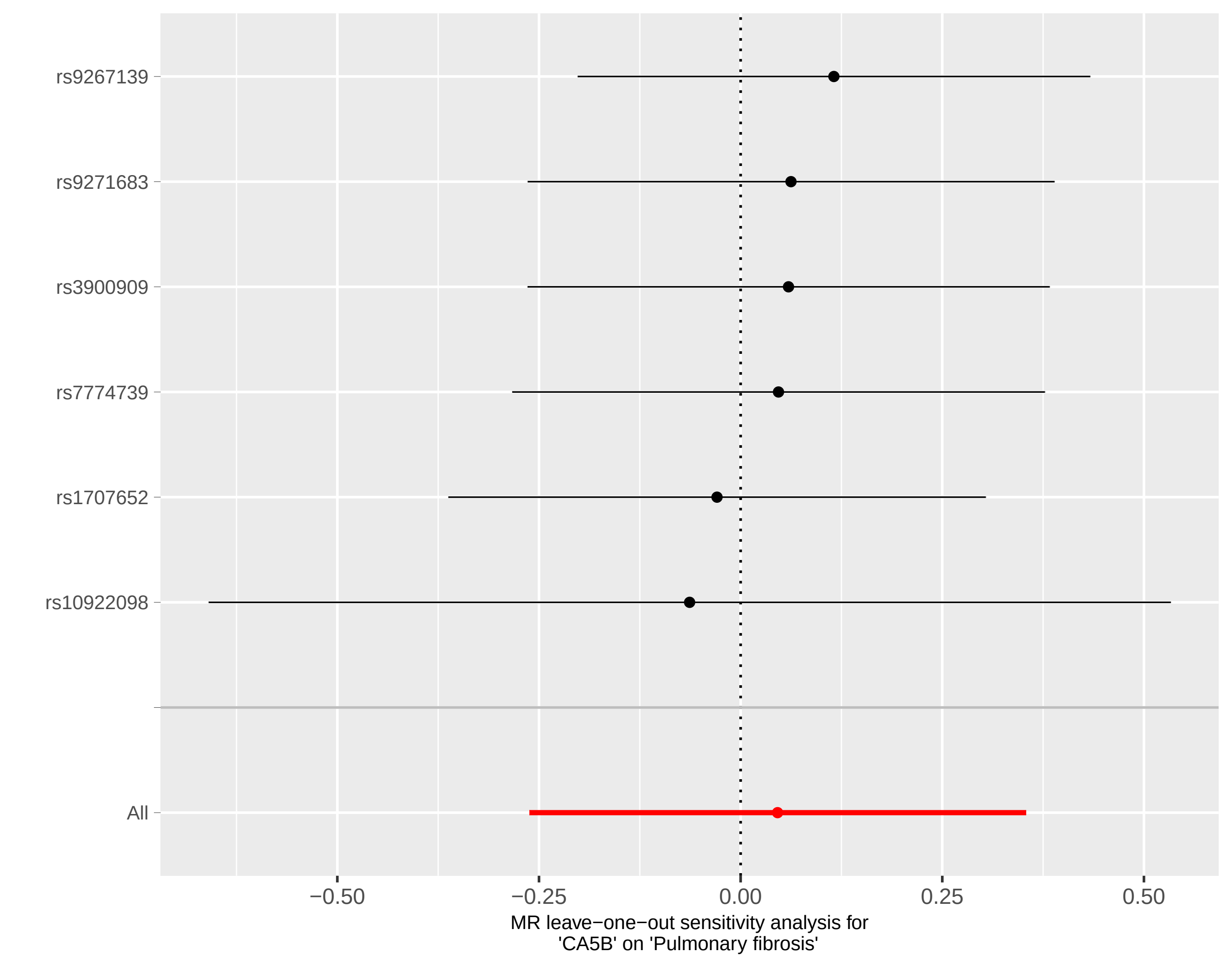
rs9267139
rs9271683
rs3900909
rs7774739
rs1707652
rs10922098
All
−0.50
−0.25
0.00
0.25
0.50
MR leave−one−out sensitivity analysis for
'CA5B' on 'Pulmonary fibrosis'

Insufficient number of SNPs

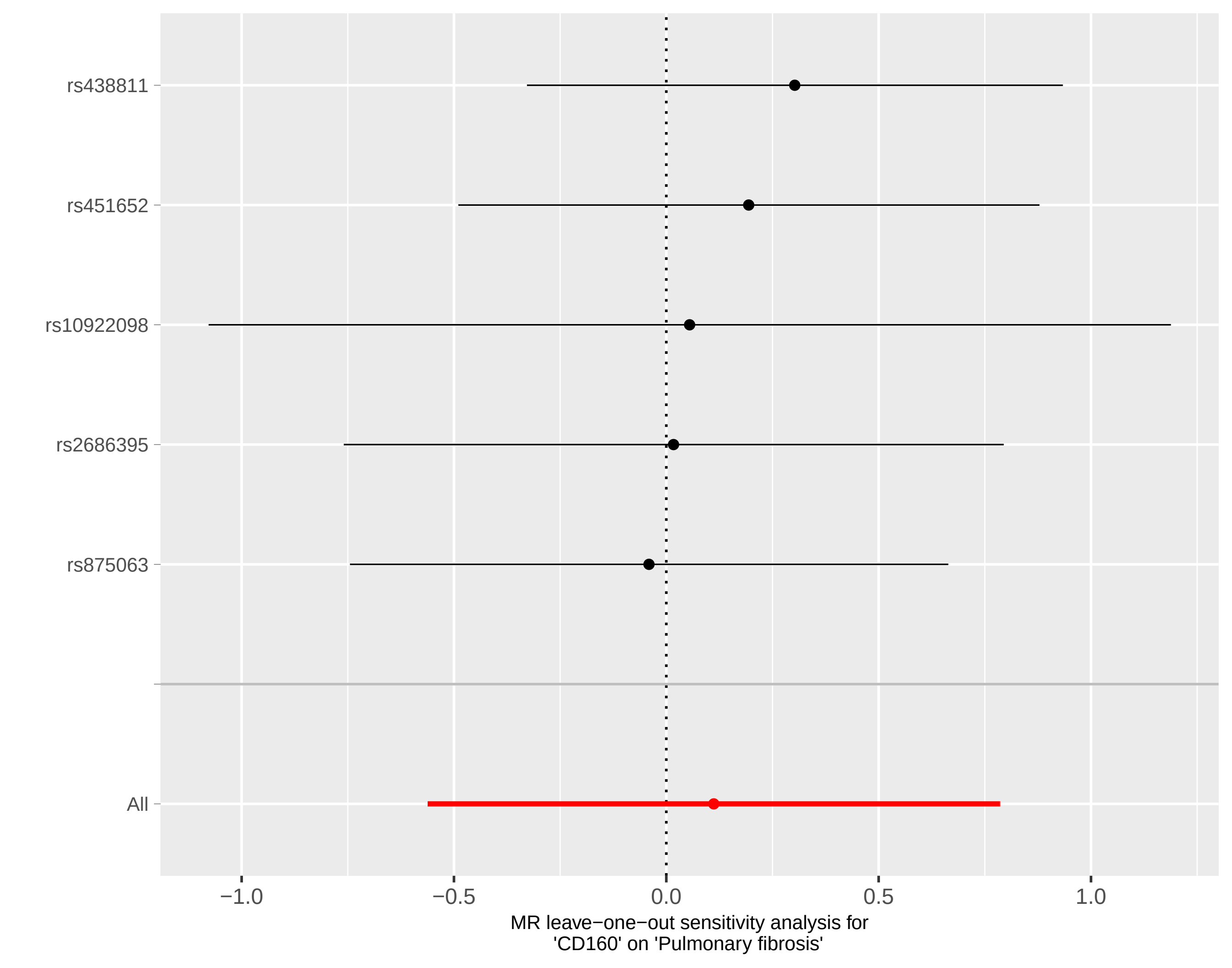
rs438811
rs451652
rs10922098
rs2686395
rs875063
All
−1.0
−0.5
0.0
0.5
1.0
MR leave−one−out sensitivity analysis for
'CD160' on 'Pulmonary fibrosis'

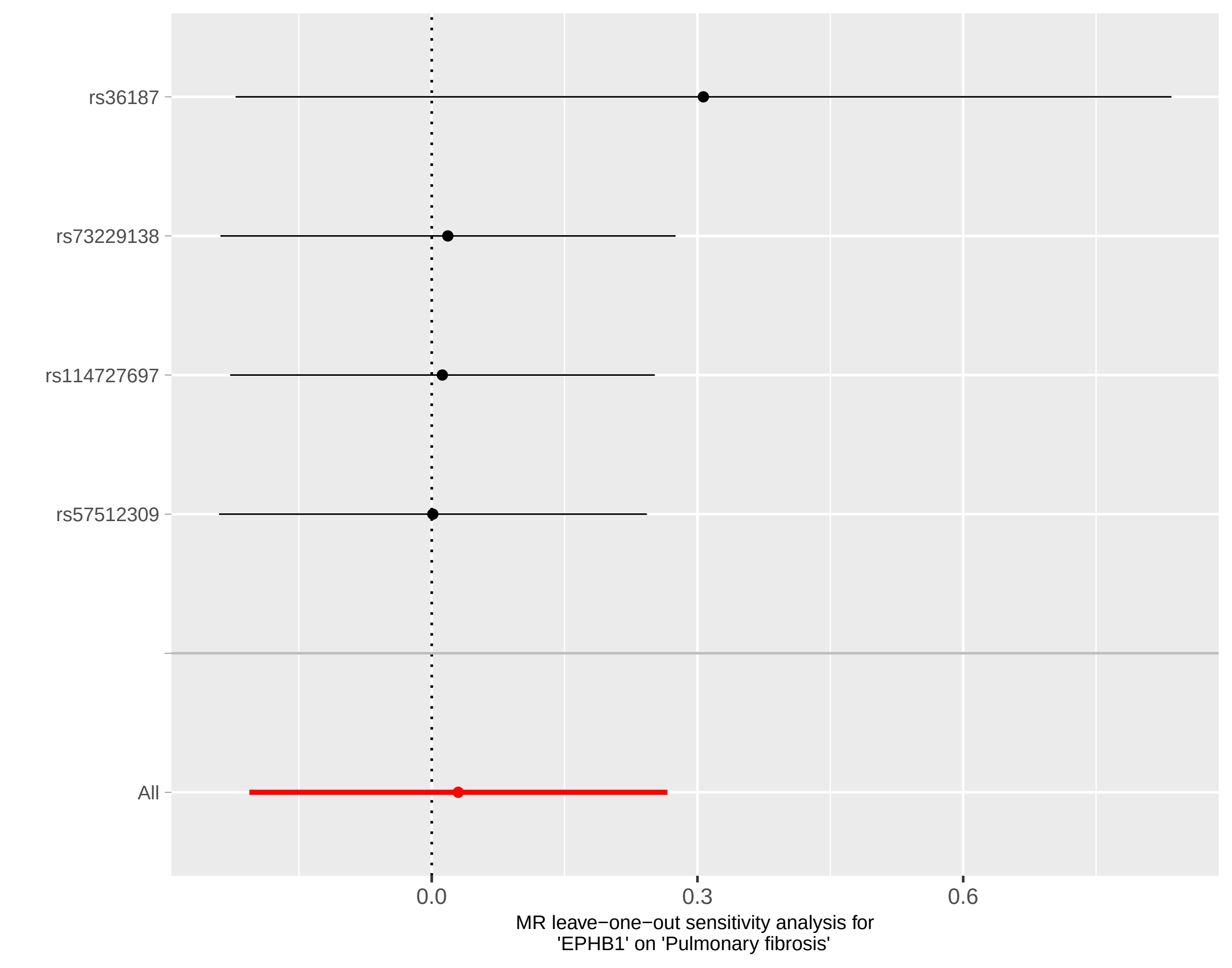
rs36187
rs73229138
rs114727697
rs57512309
All
0.0
0.3
0.6
MR leave−one−out sensitivity analysis for
'EPHB1' on 'Pulmonary fibrosis'

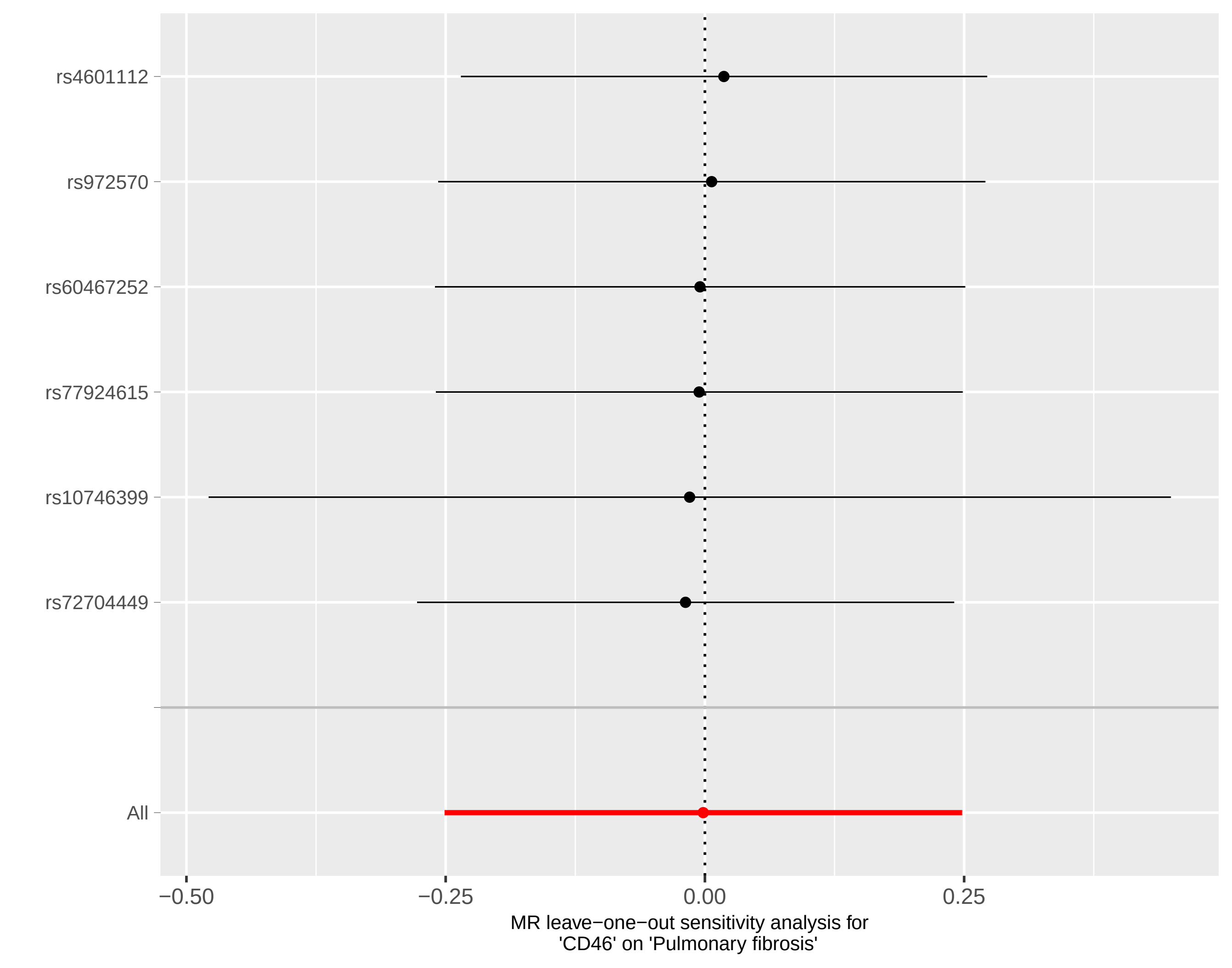

rs4601112
rs972570
rs60467252
rs77924615
rs10746399
rs72704449
All
−0.50
−0.25
0.00
0.25
MR leave−one−out sensitivity analysis for
'CD46' on 'Pulmonary fibrosis'

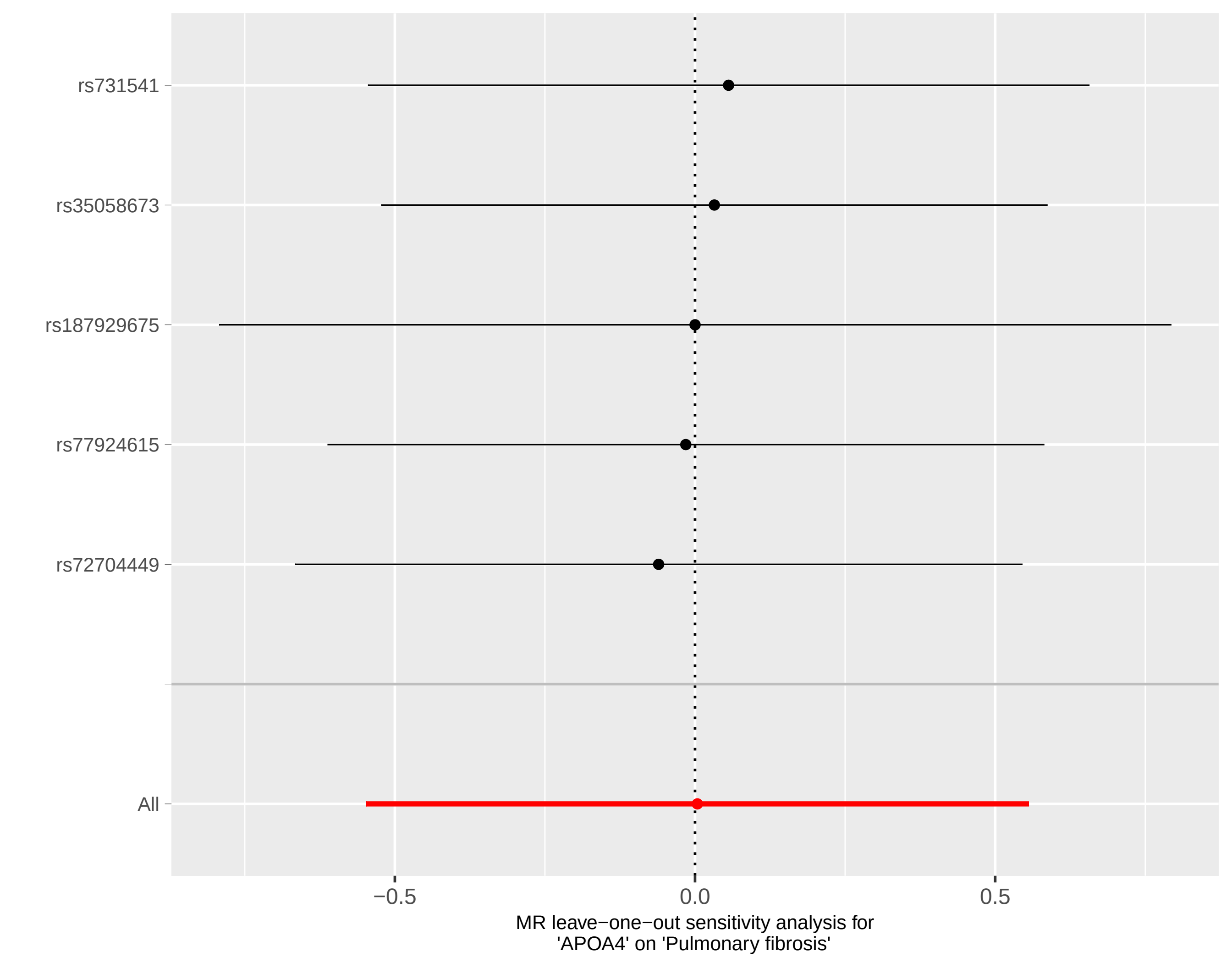
rs731541
rs35058673
rs187929675
rs77924615
rs72704449
All
−0.5
0.0
0.5
MR leave−one−out sensitivity analysis for
'APOA4' on 'Pulmonary fibrosis'

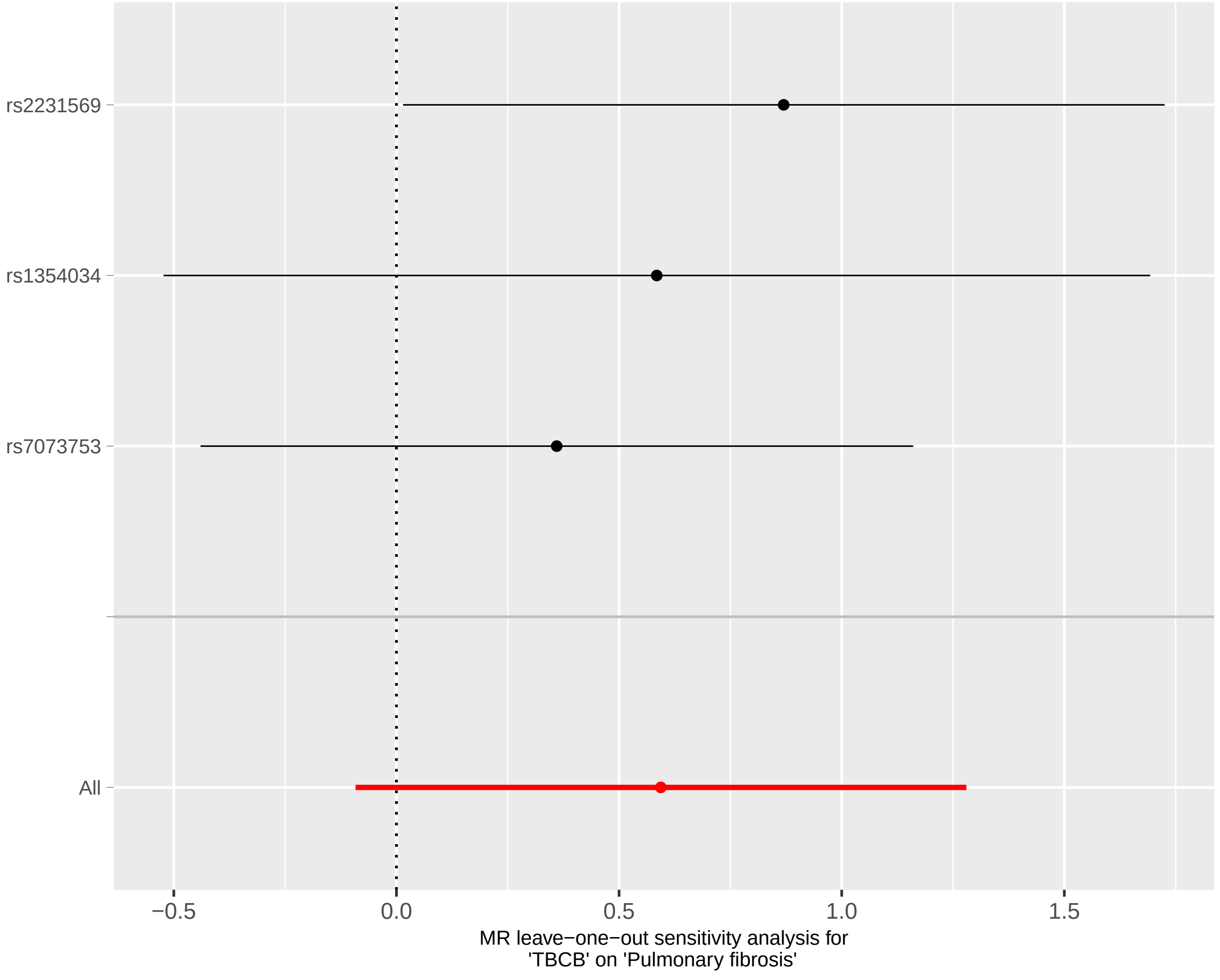

rs2231569
rs1354034
rs7073753
All
−0.5
0.0
0.5
1.0
1.5
MR leave−one−out sensitivity analysis for
'TBCB' on 'Pulmonary fibrosis'

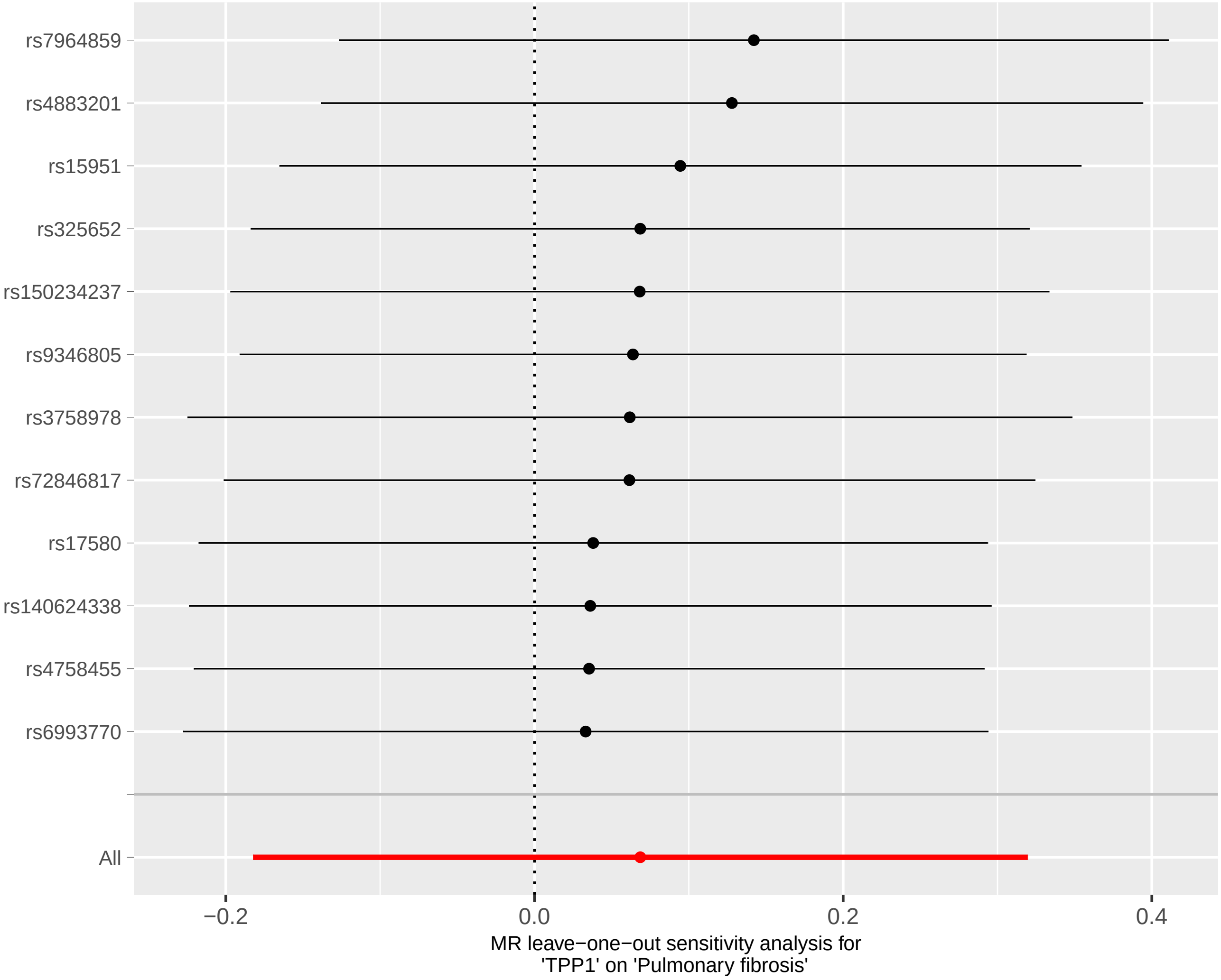
rs7964859
rs4883201
rs15951
rs325652
rs150234237
rs9346805
rs3758978
rs72846817
rs17580
rs140624338
rs4758455
rs6993770
All
−0.2
0.0
0.2
0.4
MR leave−one−out sensitivity analysis for
'TPP1' on 'Pulmonary fibrosis'

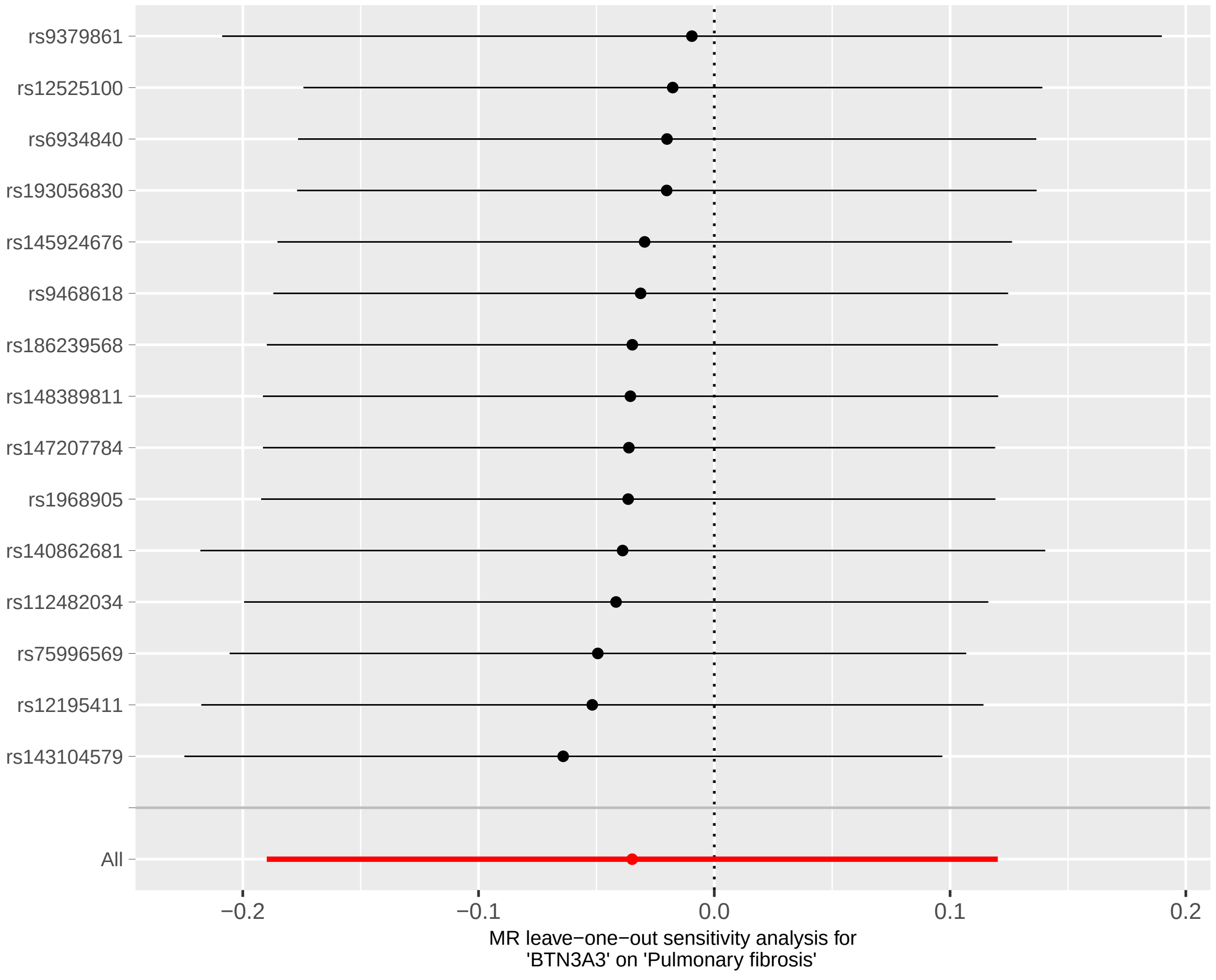
rs9379861
rs12525100
rs6934840
rs193056830
rs145924676
rs9468618
rs186239568
rs148389811
rs147207784
rs1968905
rs140862681
rs112482034
rs75996569
rs12195411
rs143104579
All
−0.2
−0.1
0.0
0.1
0.2
MR leave−one−out sensitivity analysis for
'BTN3A3' on 'Pulmonary fibrosis'

Insufficient number of SNPs

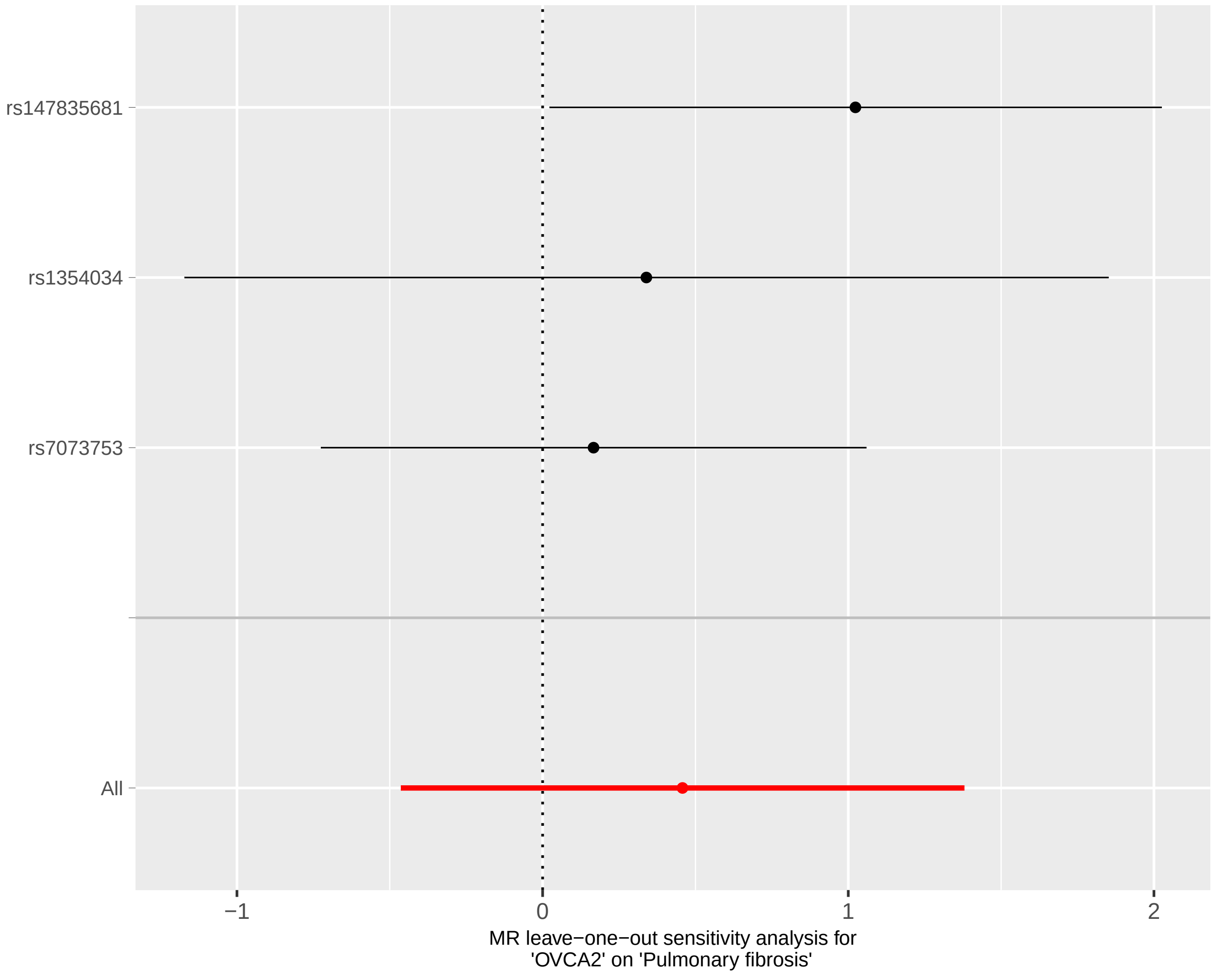
rs147835681
rs1354034
rs7073753
All
−1
0
1
2
MR leave−one−out sensitivity analysis for
'OVCA2' on 'Pulmonary fibrosis'

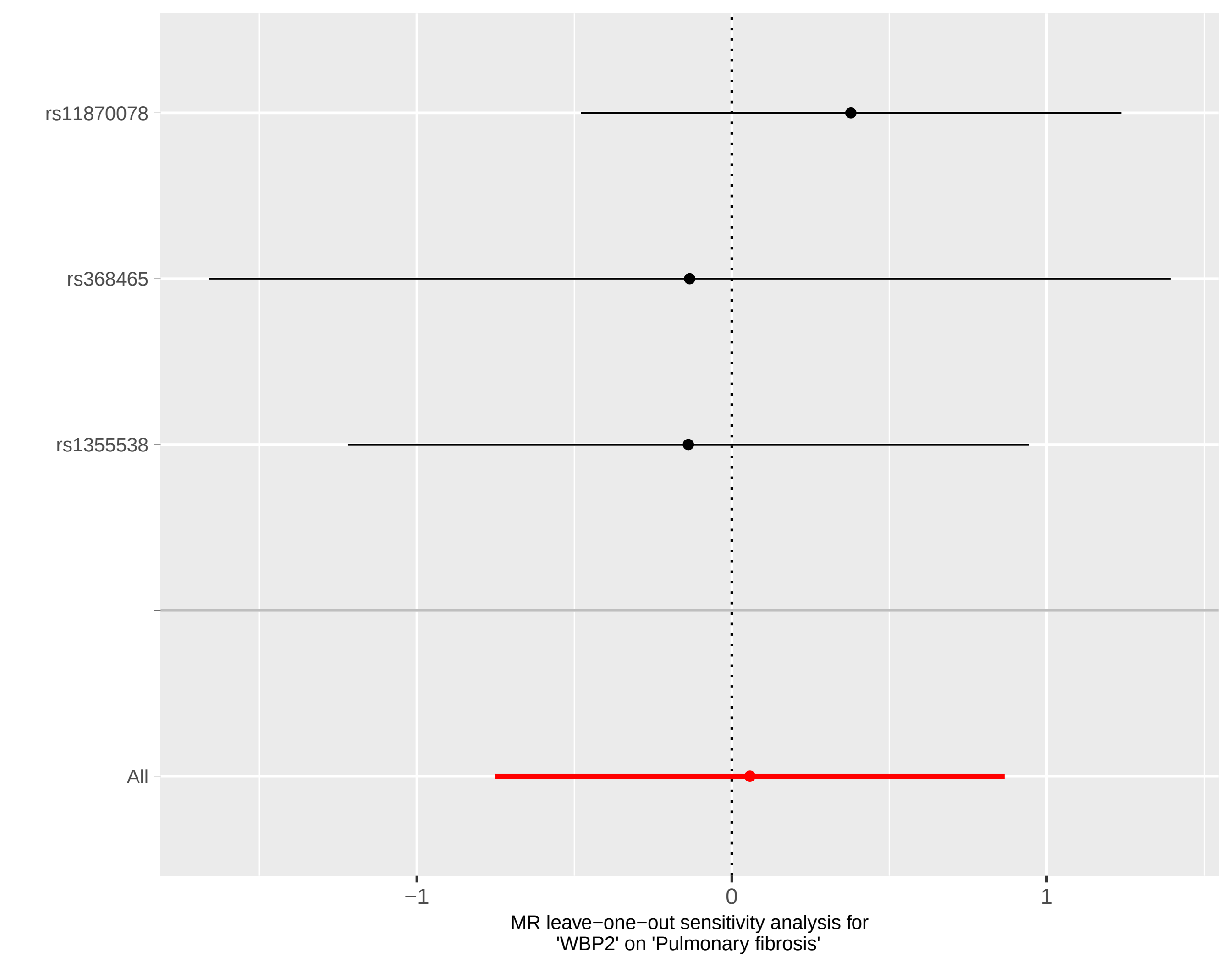
rs11870078
rs368465
rs1355538
All
−1
0
1
MR leave−one−out sensitivity analysis for
'WBP2' on 'Pulmonary fibrosis'

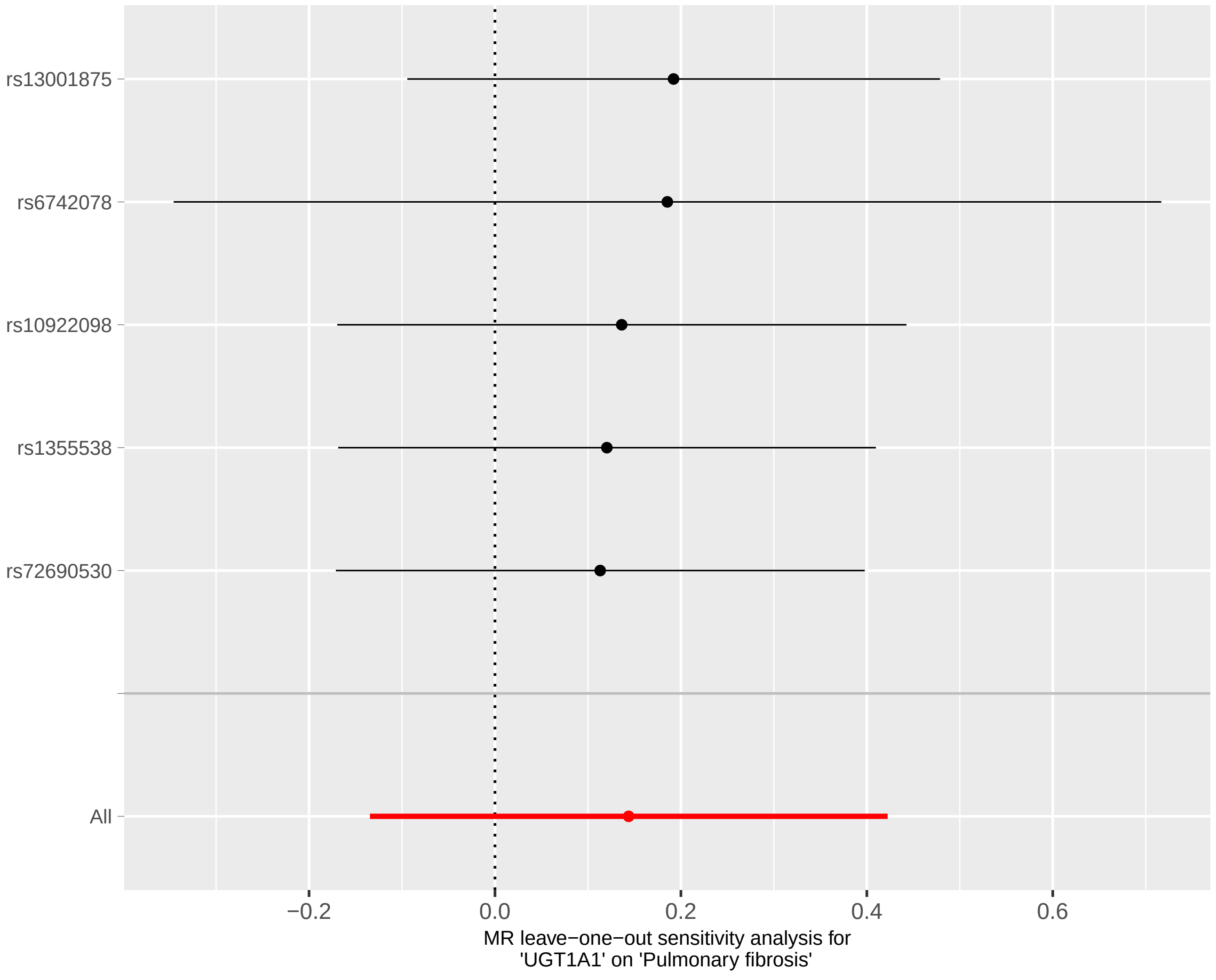
rs13001875
rs6742078
rs10922098
rs1355538
rs72690530
All
−0.2
0.0
0.2
0.4
0.6
MR leave−one−out sensitivity analysis for
'UGT1A1' on 'Pulmonary fibrosis'

Insufficient number of SNPs

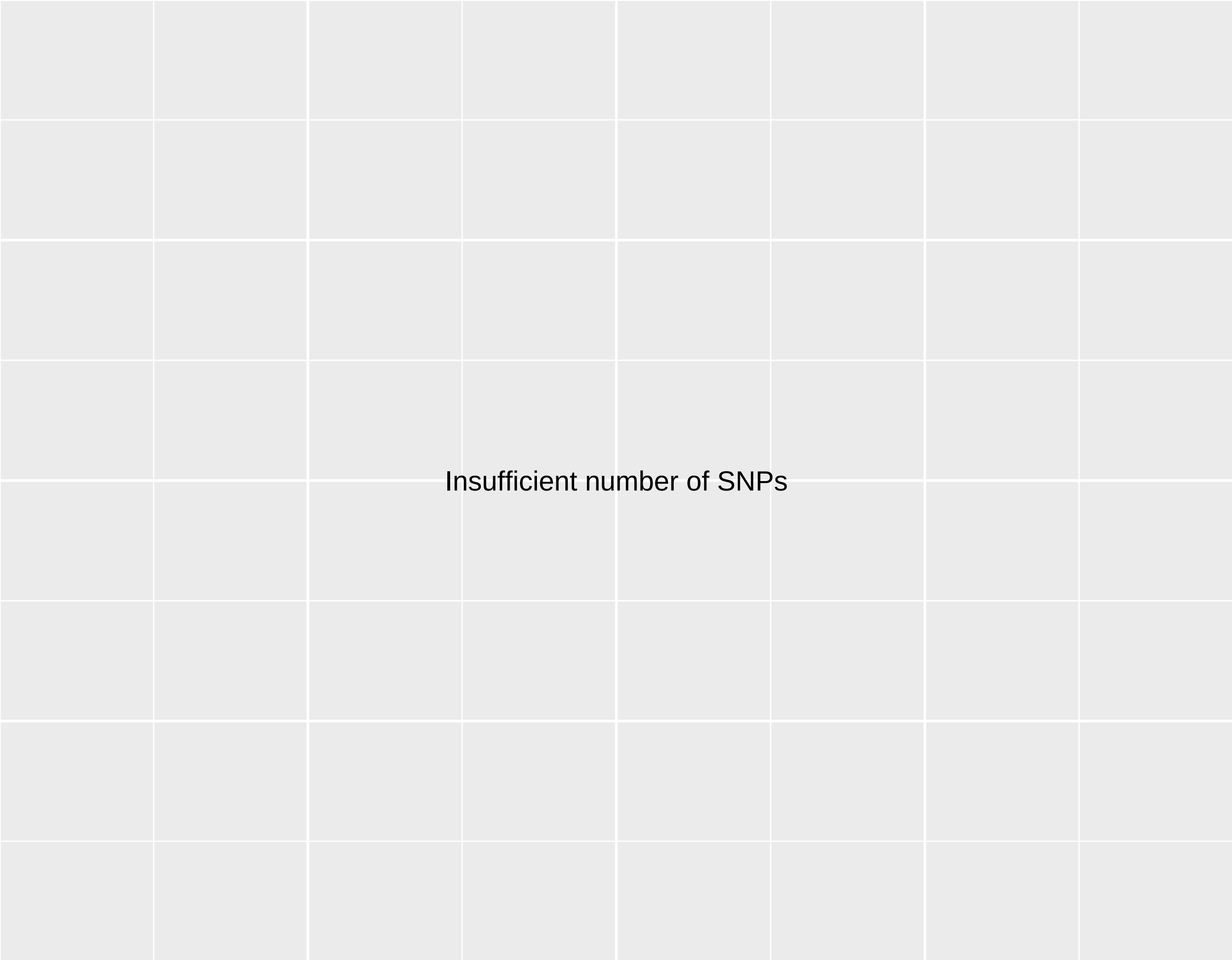
Insufficient number of SNPs

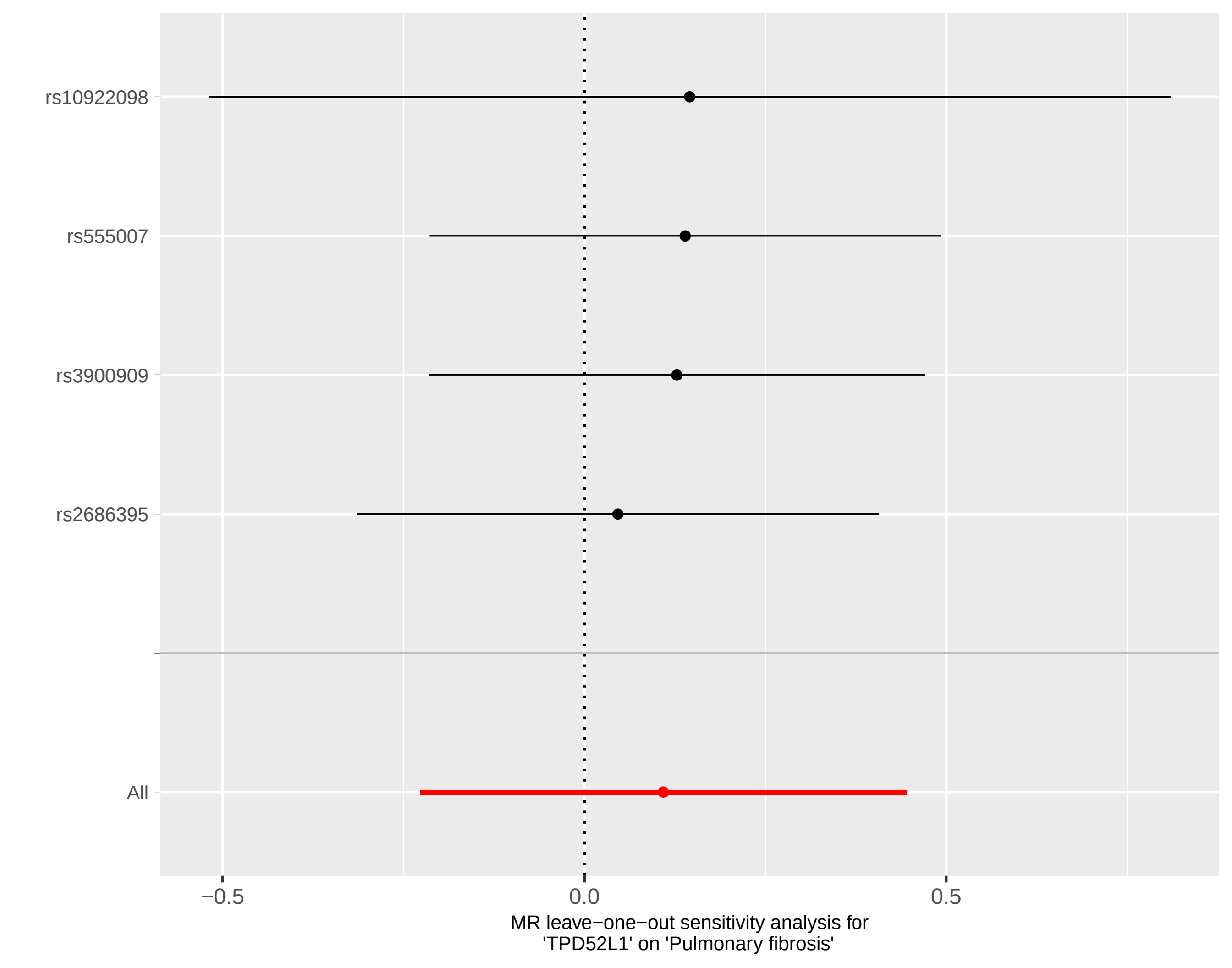
rs10922098
rs555007
rs3900909
rs2686395
All
−0.5
0.0
0.5
MR leave−one−out sensitivity analysis for
'TPD52L1' on 'Pulmonary fibrosis'

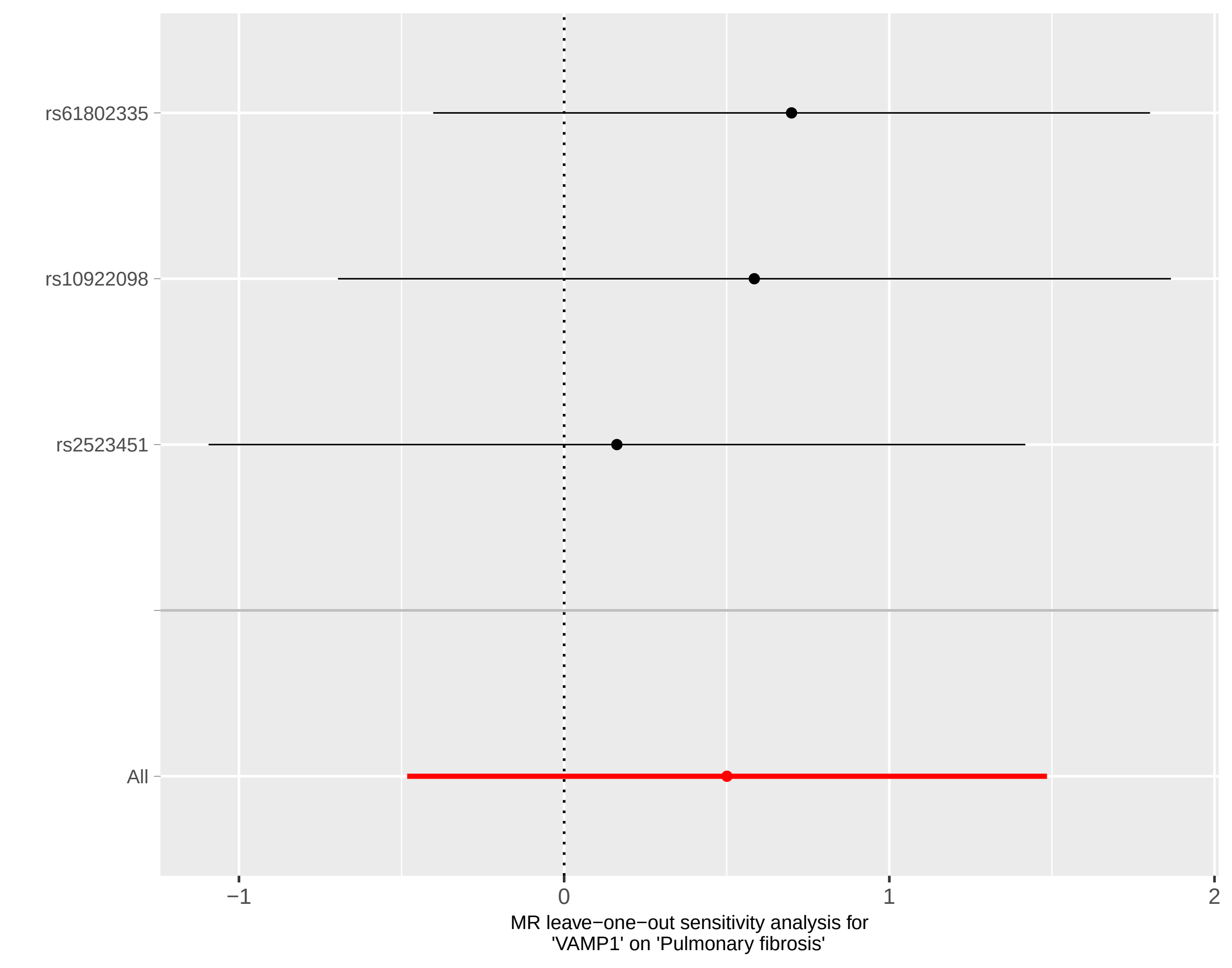
rs61802335
rs10922098
rs2523451
All
−1
0
1
2
MR leave−one−out sensitivity analysis for
'VAMP1' on 'Pulmonary fibrosis'

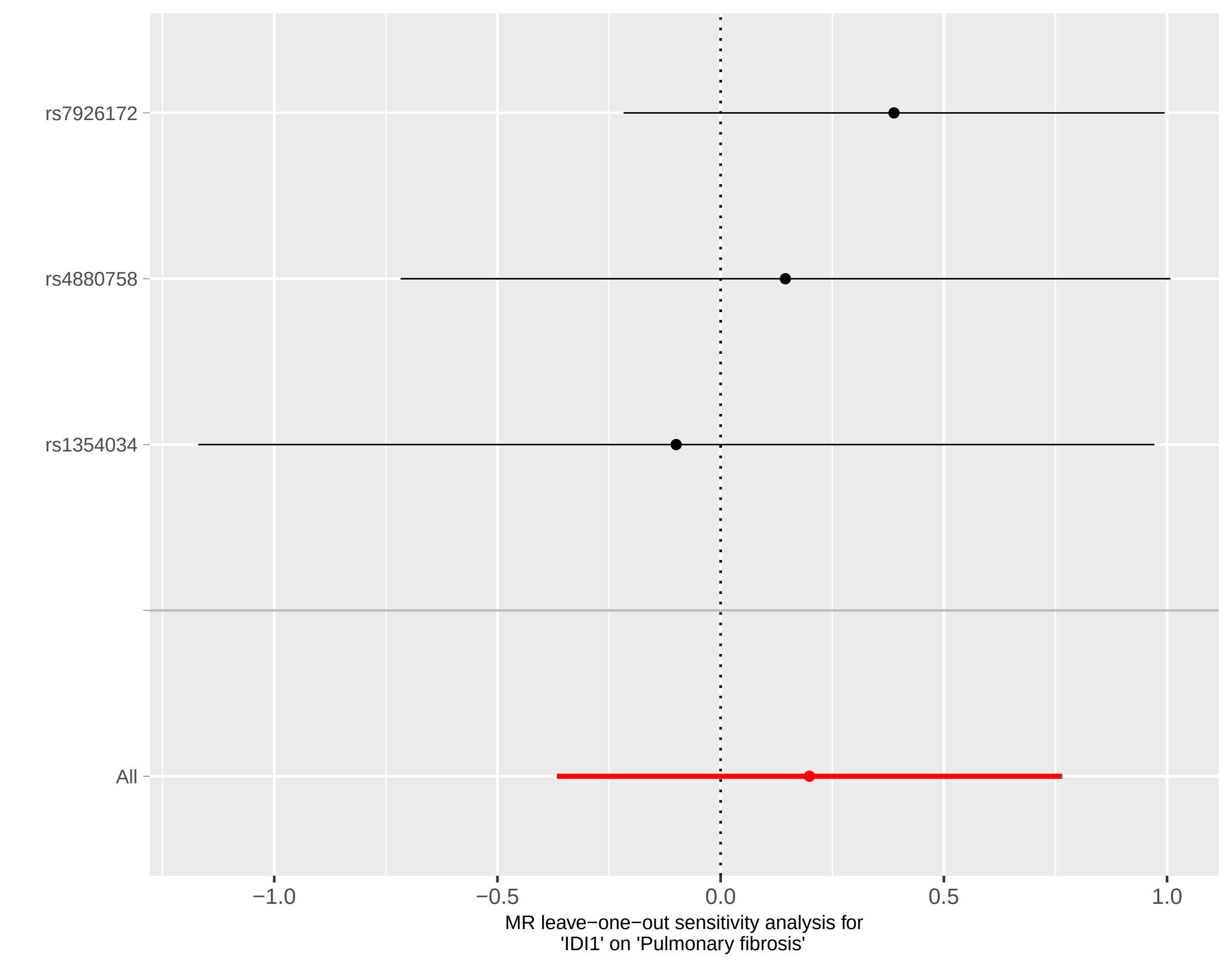
rs7926172
rs4880758
rs1354034
All
−1.0
−0.5
0.0
0.5
1.0
MR leave−one−out sensitivity analysis for
'IDI1' on 'Pulmonary fibrosis'

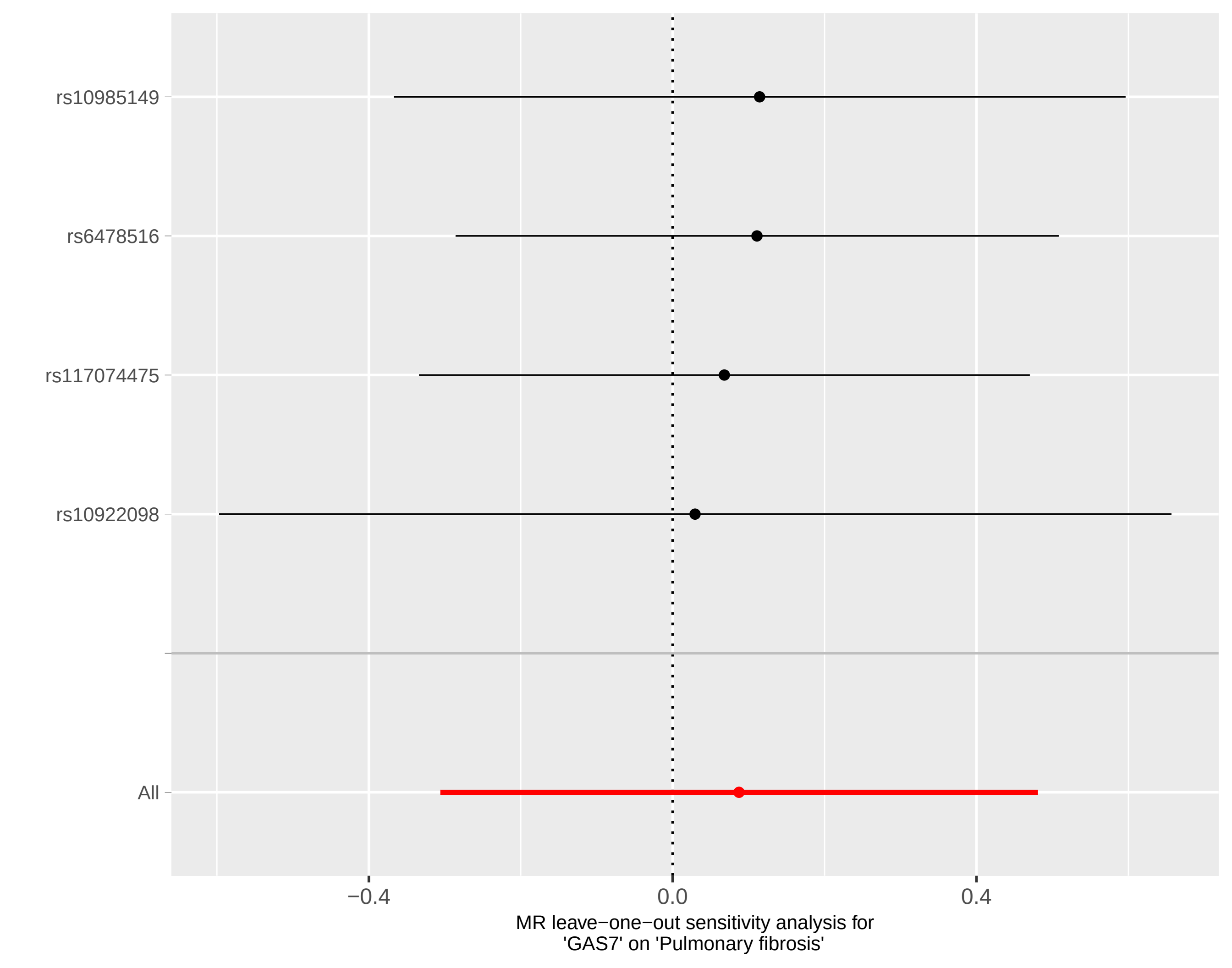
rs10985149
rs6478516
rs117074475
rs10922098
All
−0.4
0.0
0.4
MR leave−one−out sensitivity analysis for
'GAS7' on 'Pulmonary fibrosis'

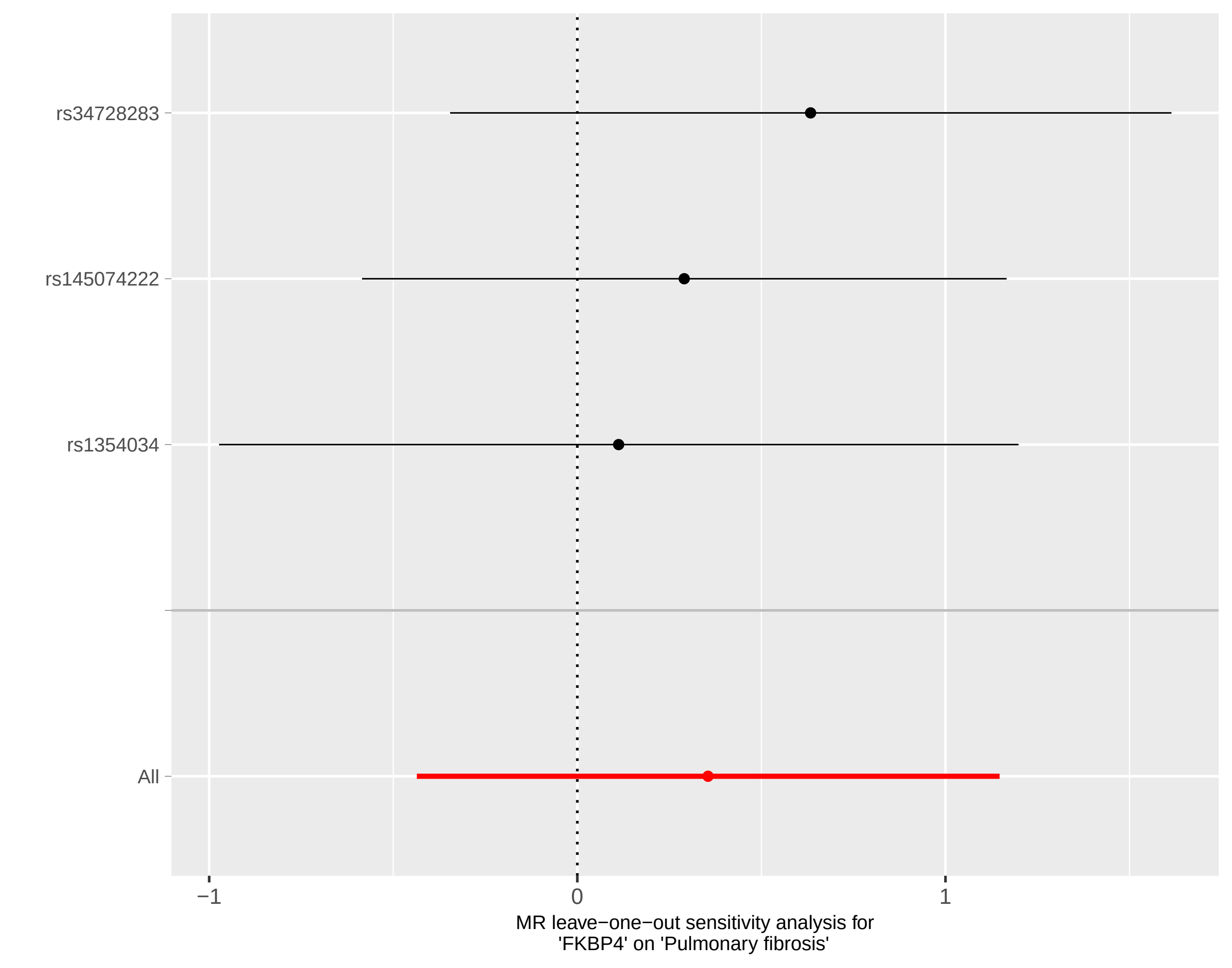
rs34728283
rs145074222
rs1354034
All
−1
0
1
MR leave−one−out sensitivity analysis for
'FKBP4' on 'Pulmonary fibrosis'

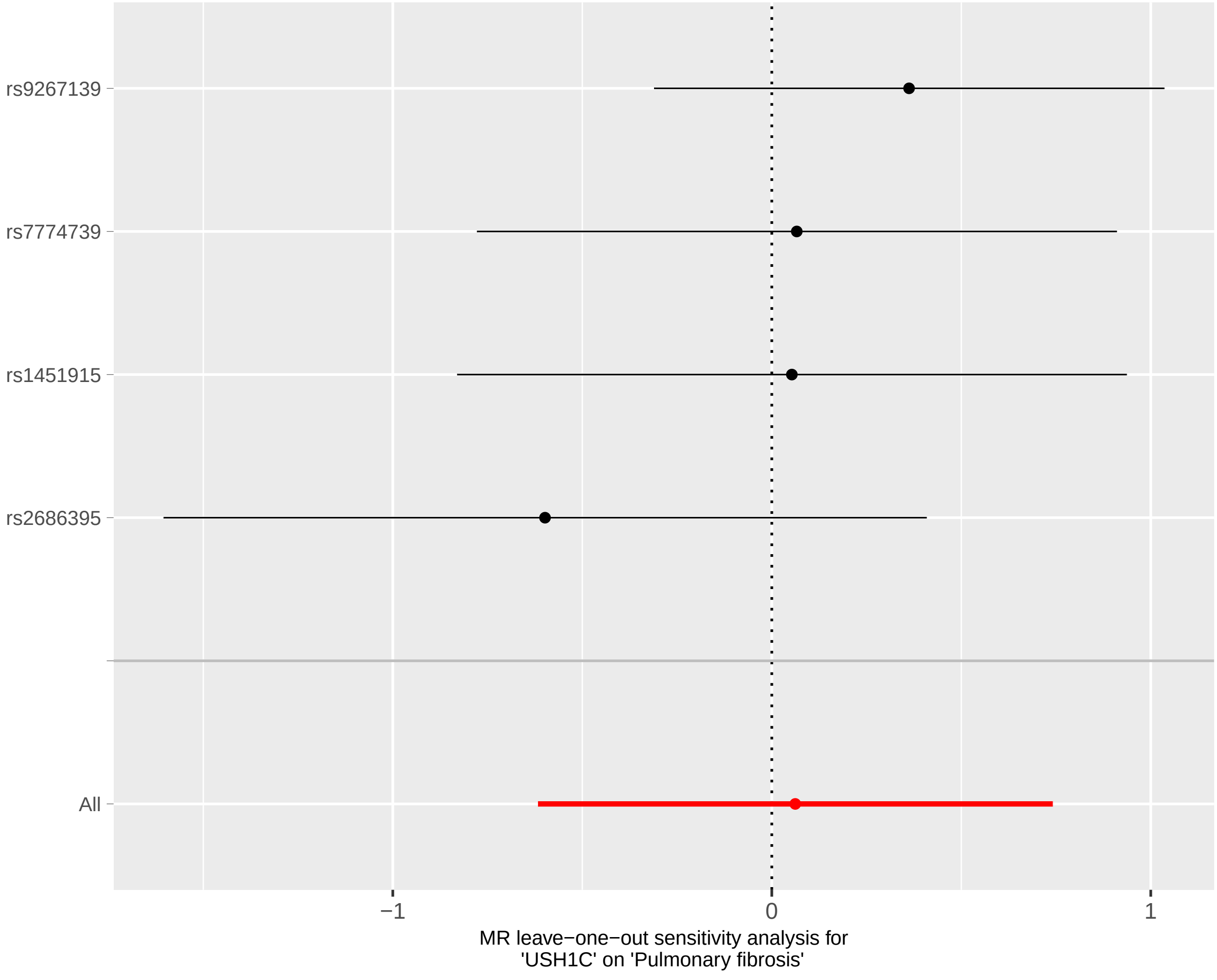
rs9267139
rs7774739
rs1451915
rs2686395
All
−1
0
1
MR leave−one−out sensitivity analysis for
'USH1C' on 'Pulmonary fibrosis'

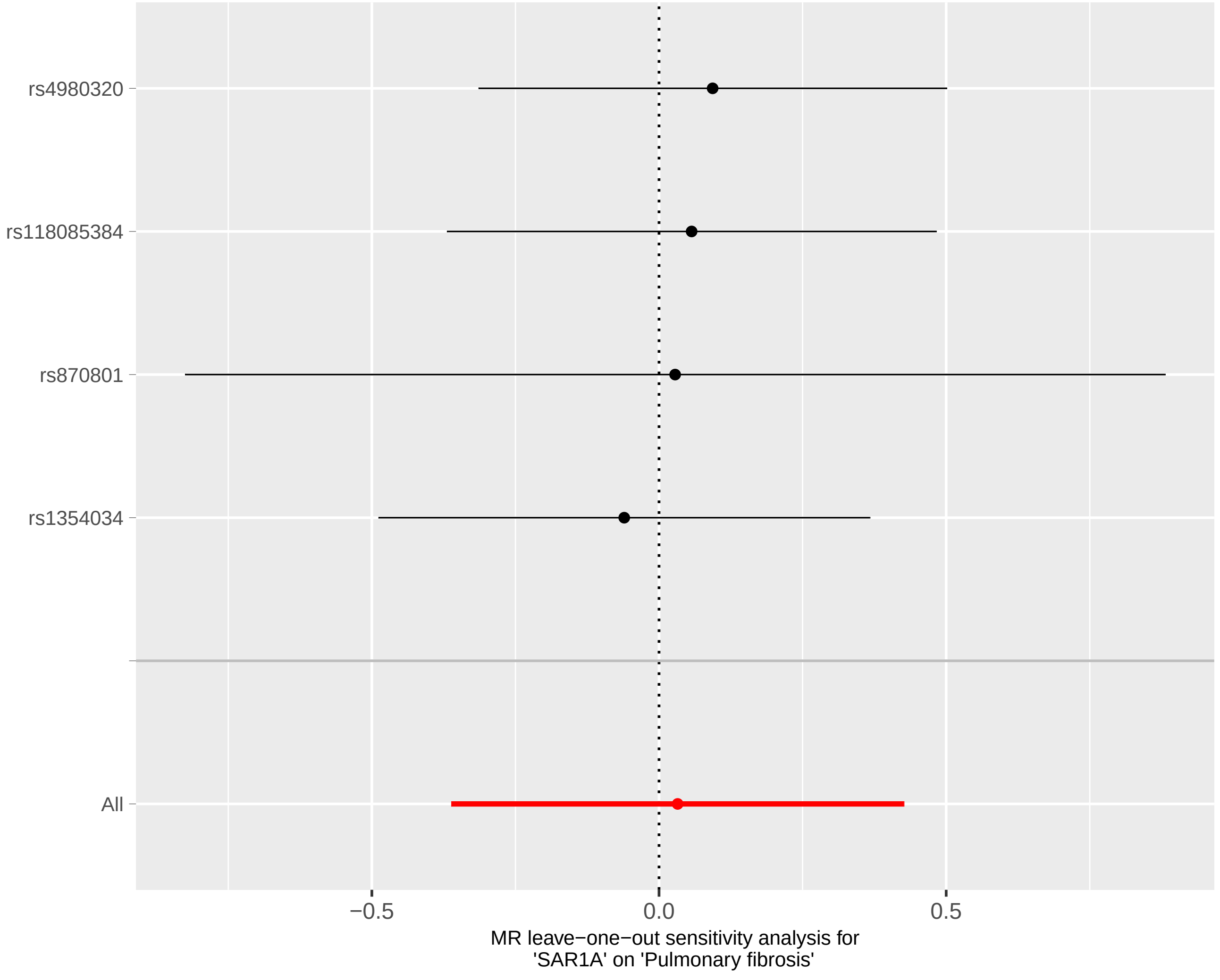
rs4980320
rs118085384
rs870801
rs1354034
All
−0.5
0.0
0.5
MR leave−one−out sensitivity analysis for
'SAR1A' on 'Pulmonary fibrosis'

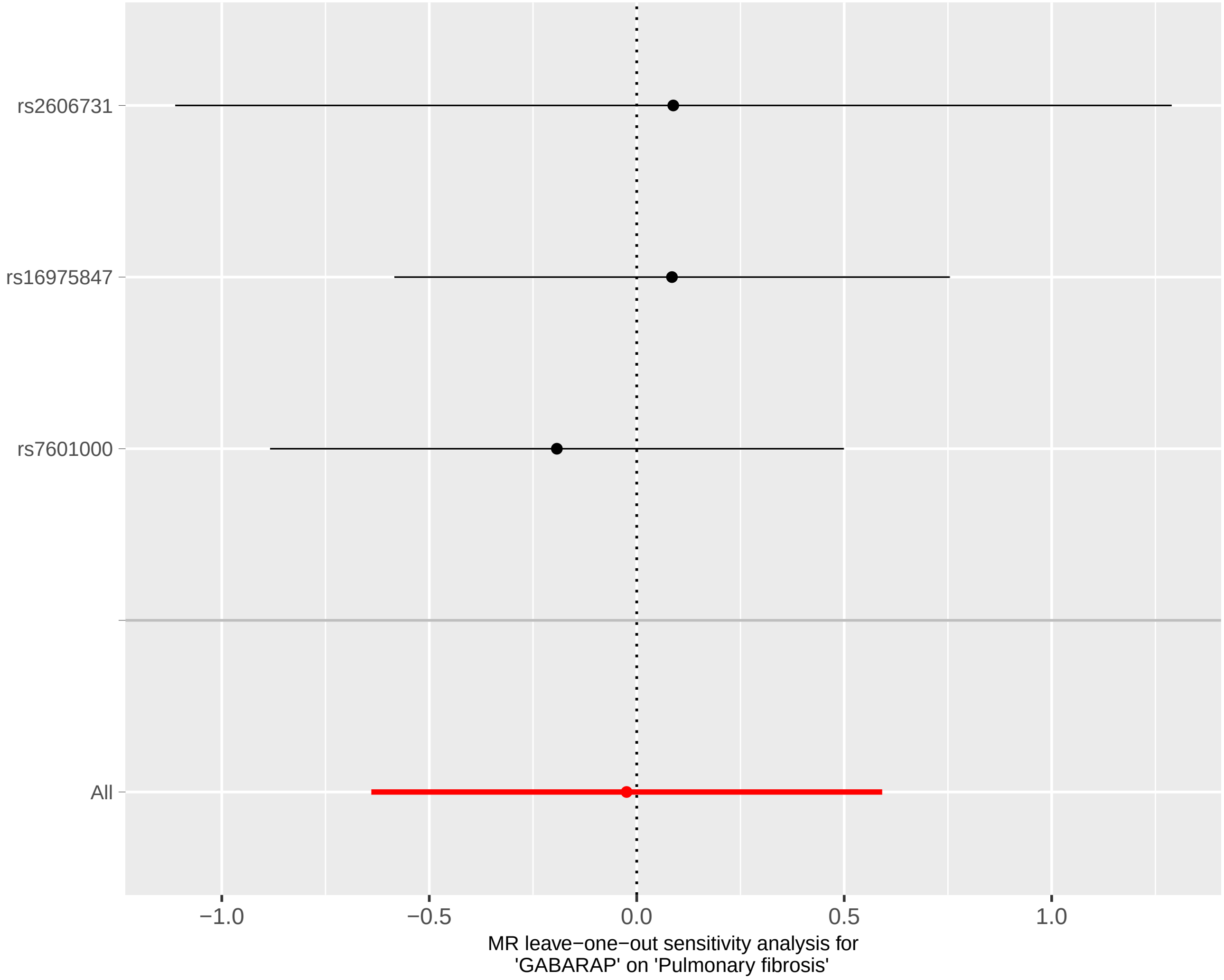
rs2606731
rs16975847
rs7601000
All
−1.0
−0.5
0.0
0.5
1.0
MR leave−one−out sensitivity analysis for
'GABARAP' on 'Pulmonary fibrosis'

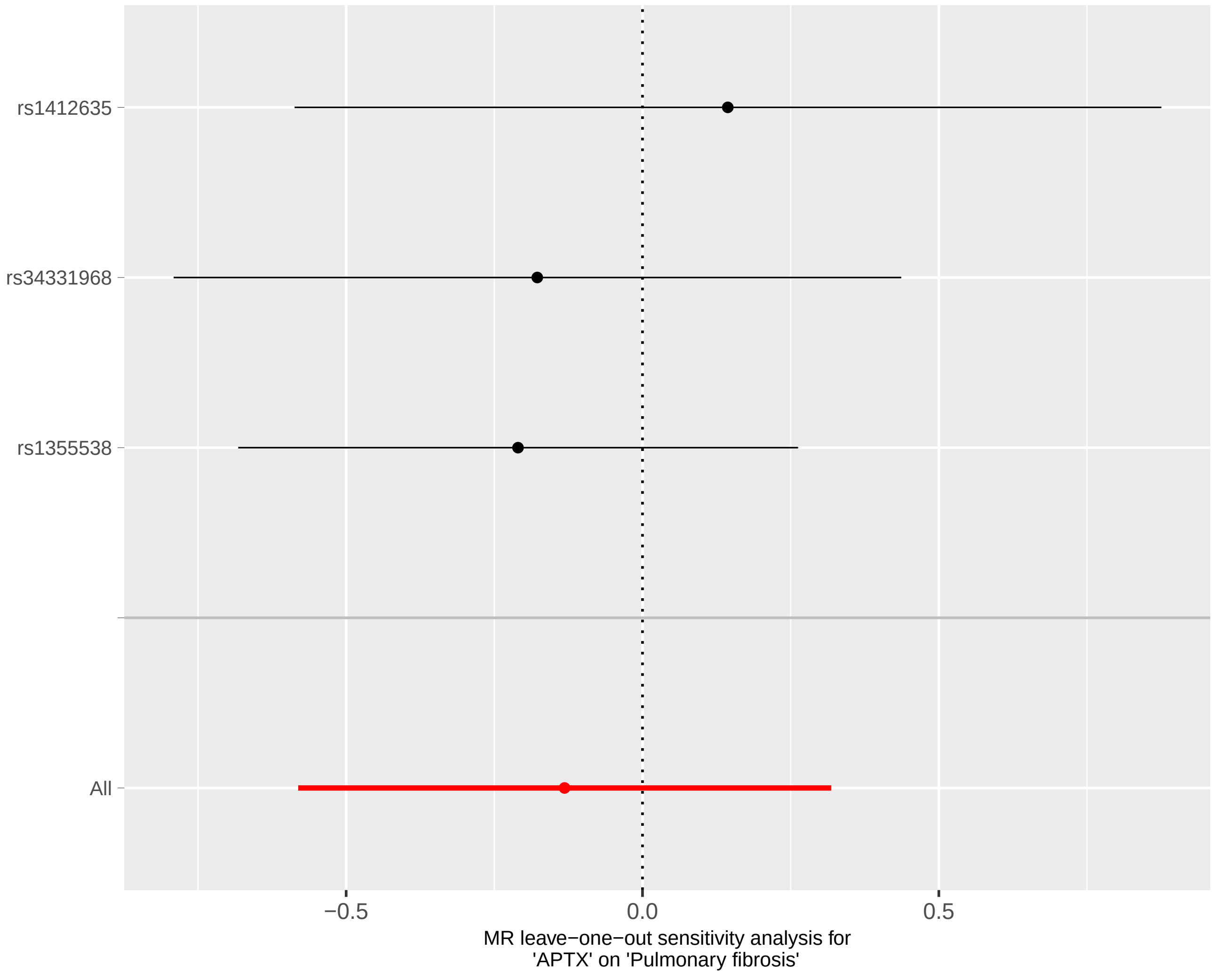
rs1412635
rs34331968
rs1355538
All
−0.5
0.0
0.5
MR leave−one−out sensitivity analysis for
'APTX' on 'Pulmonary fibrosis'

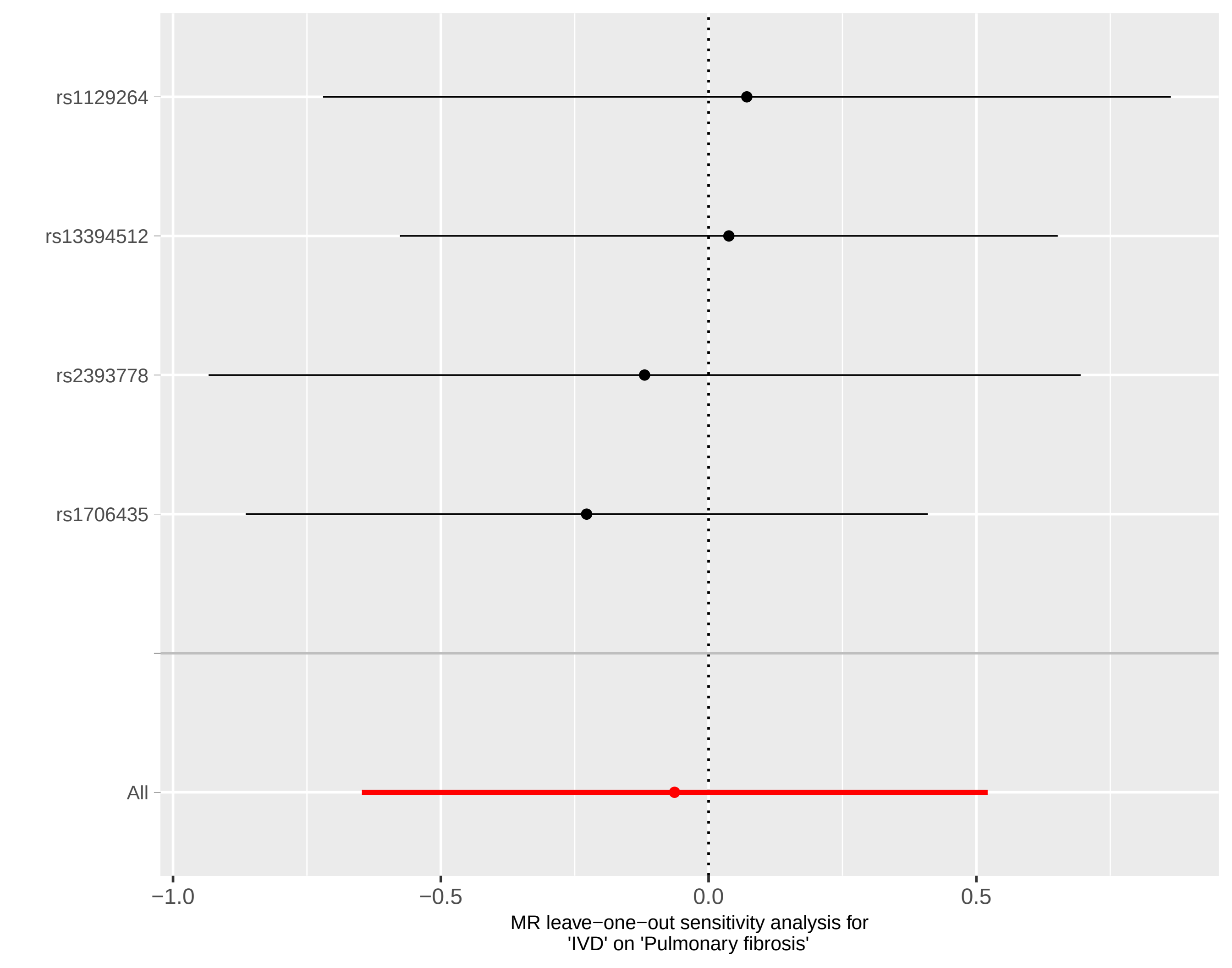
rs1129264
rs13394512
rs2393778
rs1706435
All
−1.0
−0.5
0.0
0.5
MR leave−one−out sensitivity analysis for
'IVD' on 'Pulmonary fibrosis'

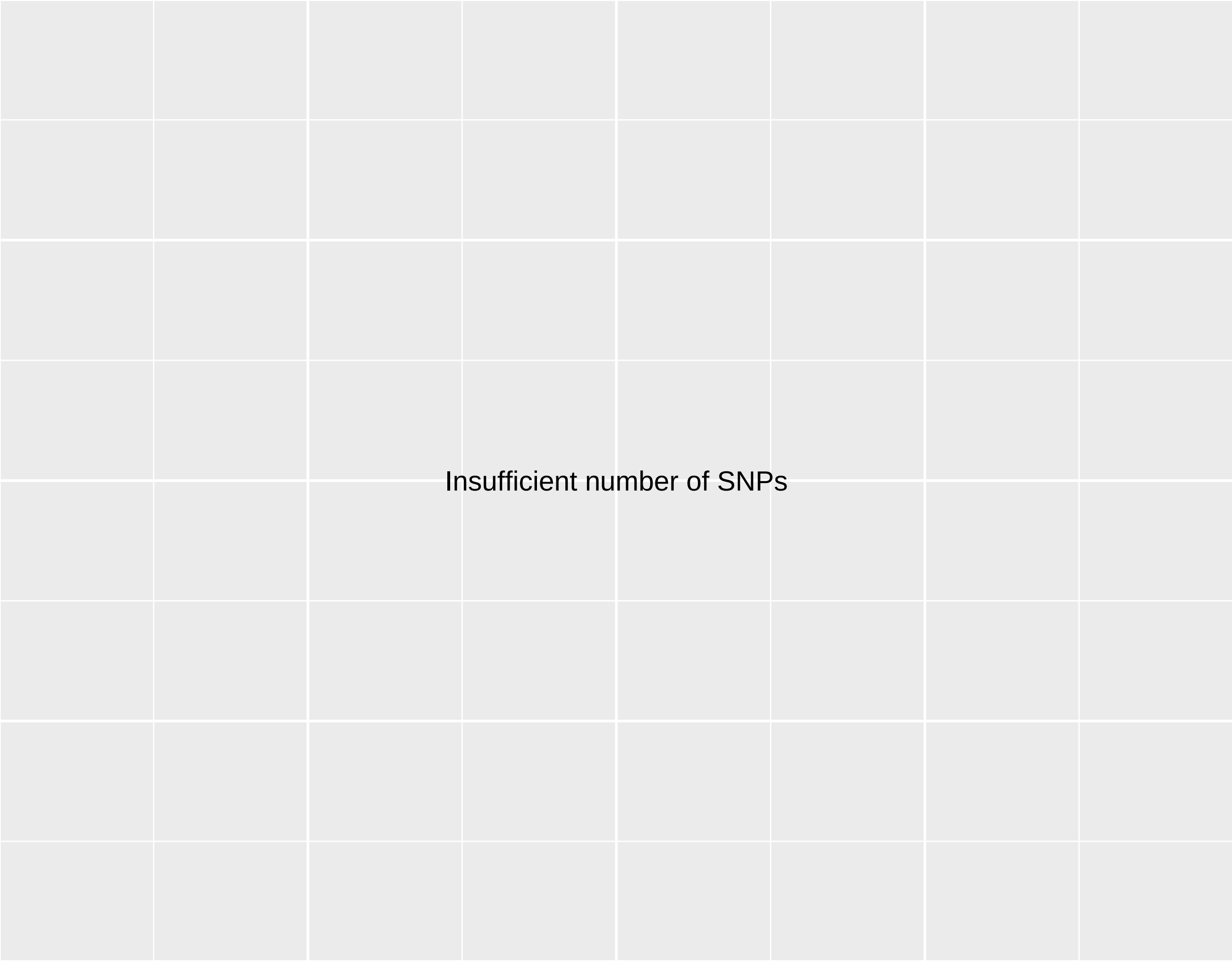
Insufficient number of SNPs

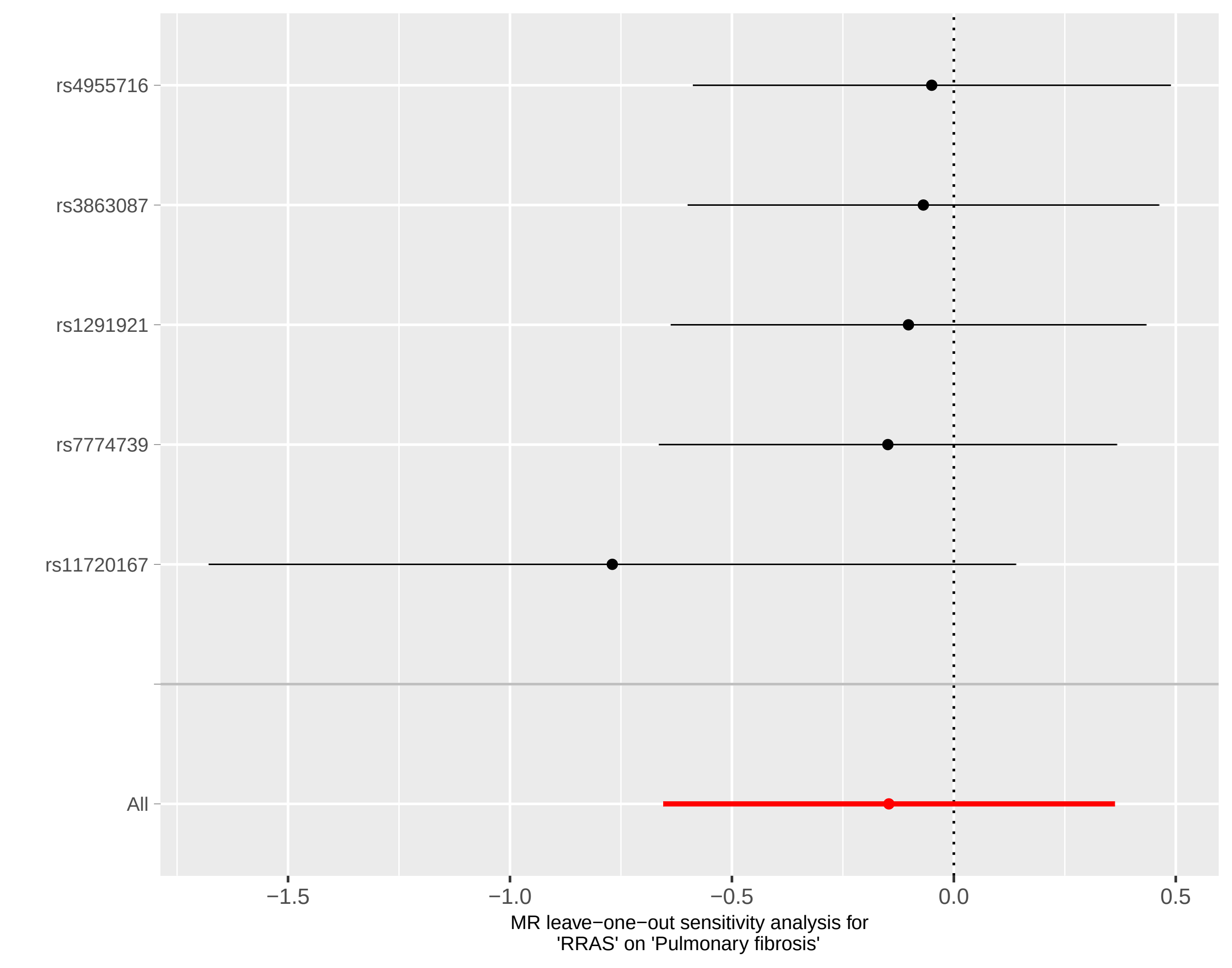
rs4955716
rs3863087
rs1291921
rs7774739
rs11720167
All
−1.5
−1.0
−0.5
0.0
0.5
MR leave−one−out sensitivity analysis for
'RRAS' on 'Pulmonary fibrosis'

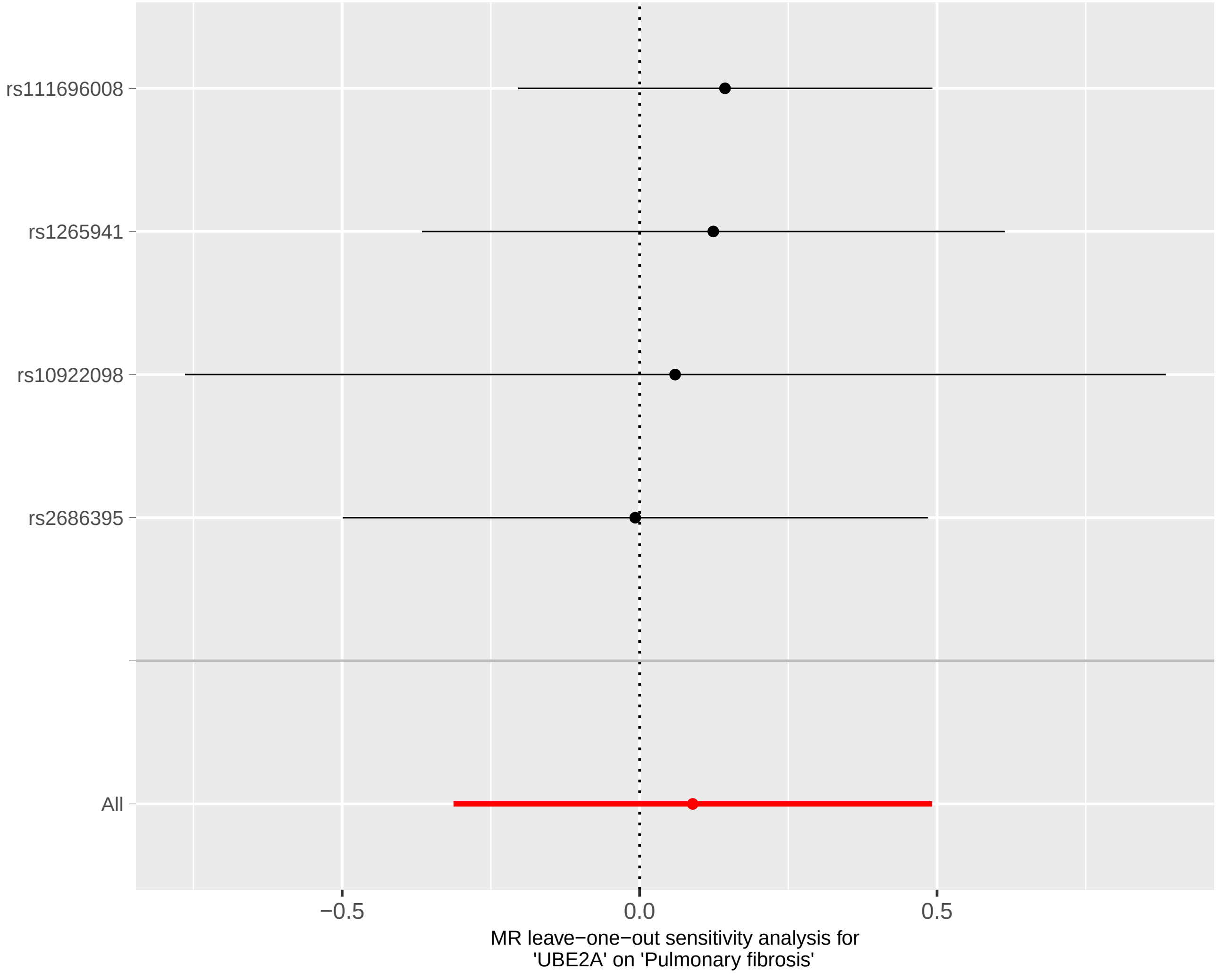

rs111696008
rs1265941
rs10922098
rs2686395
All
−0.5
0.0
0.5
MR leave−one−out sensitivity analysis for
'UBE2A' on 'Pulmonary fibrosis'

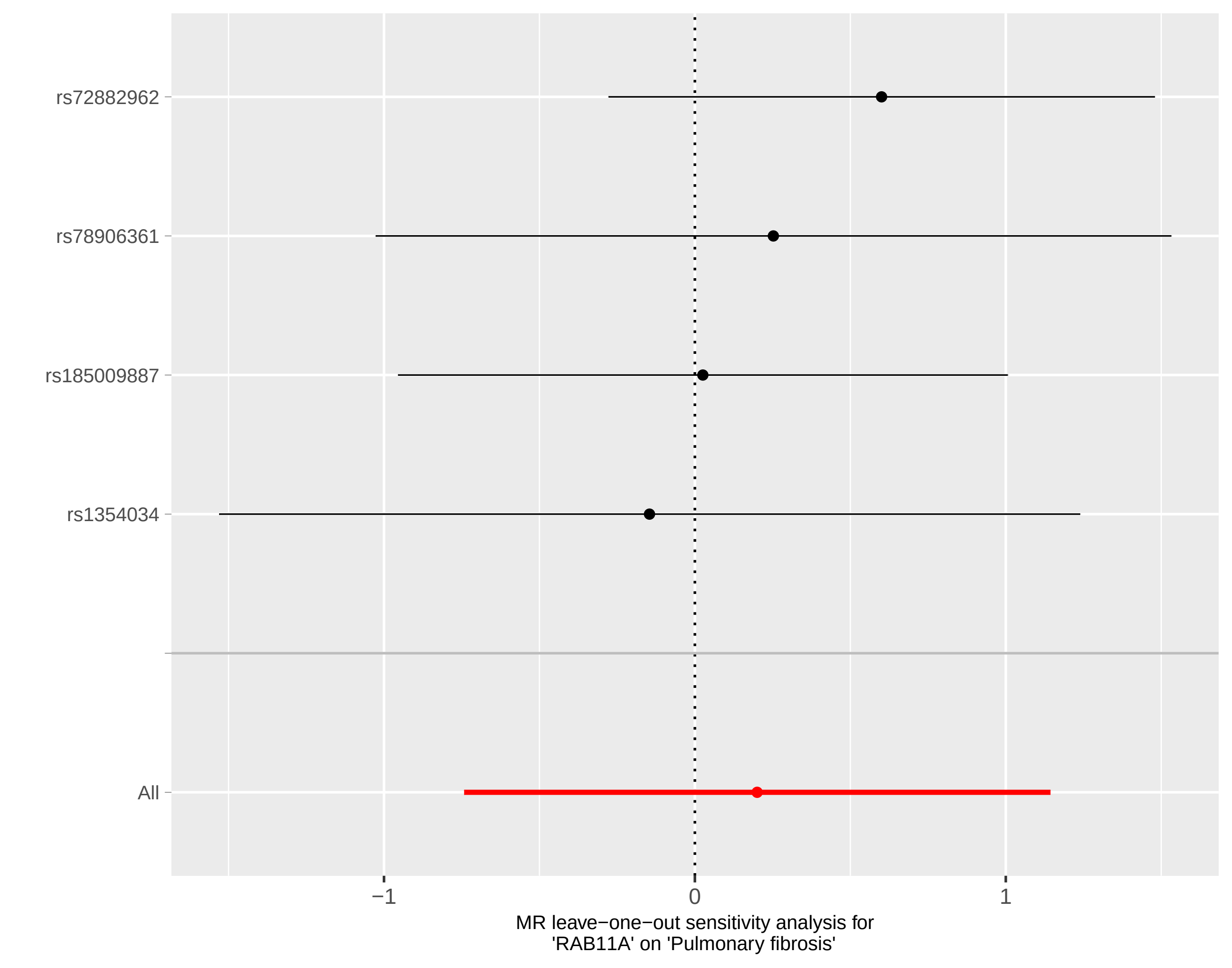

rs72882962
rs78906361
rs185009887
rs1354034
All
−1
0
1
MR leave−one−out sensitivity analysis for
'RAB11A' on 'Pulmonary fibrosis'

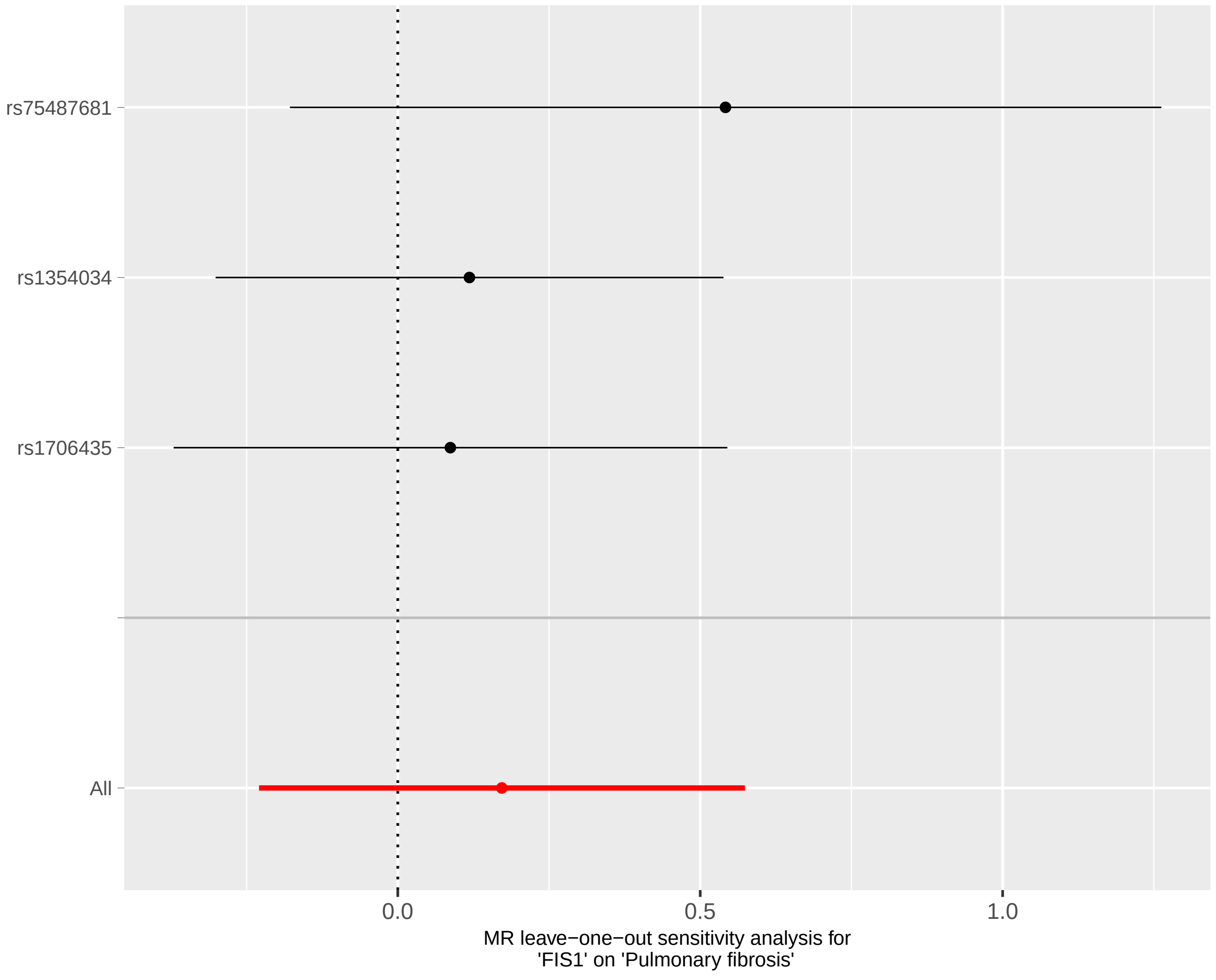
rs75487681
rs1354034
rs1706435
All
0.0
0.5
1.0
MR leave−one−out sensitivity analysis for
'FIS1' on 'Pulmonary fibrosis'

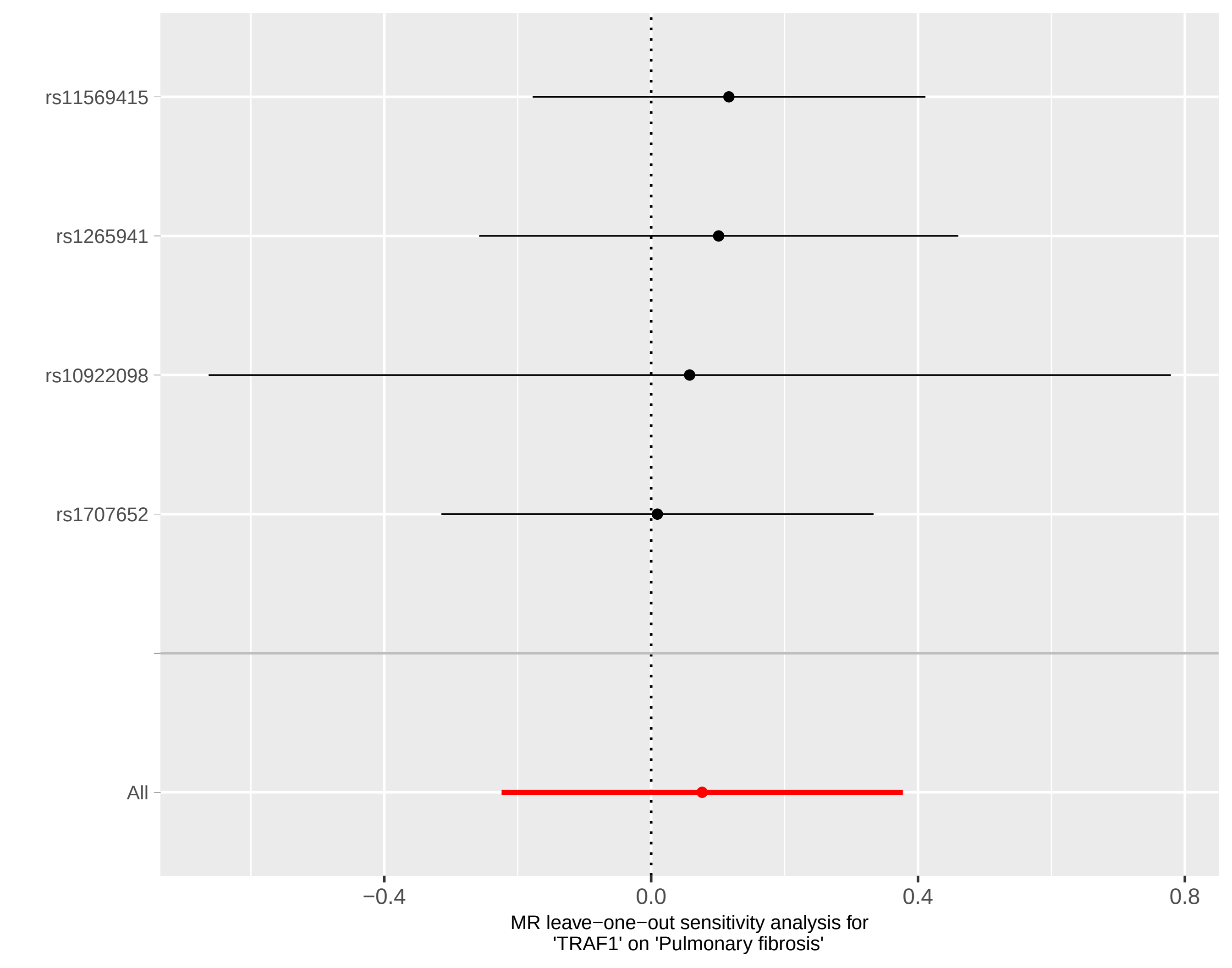

rs11569415
rs1265941
rs10922098
rs1707652
All
−0.4
0.0
0.4
0.8
MR leave−one−out sensitivity analysis for
'TRAF1' on 'Pulmonary fibrosis'

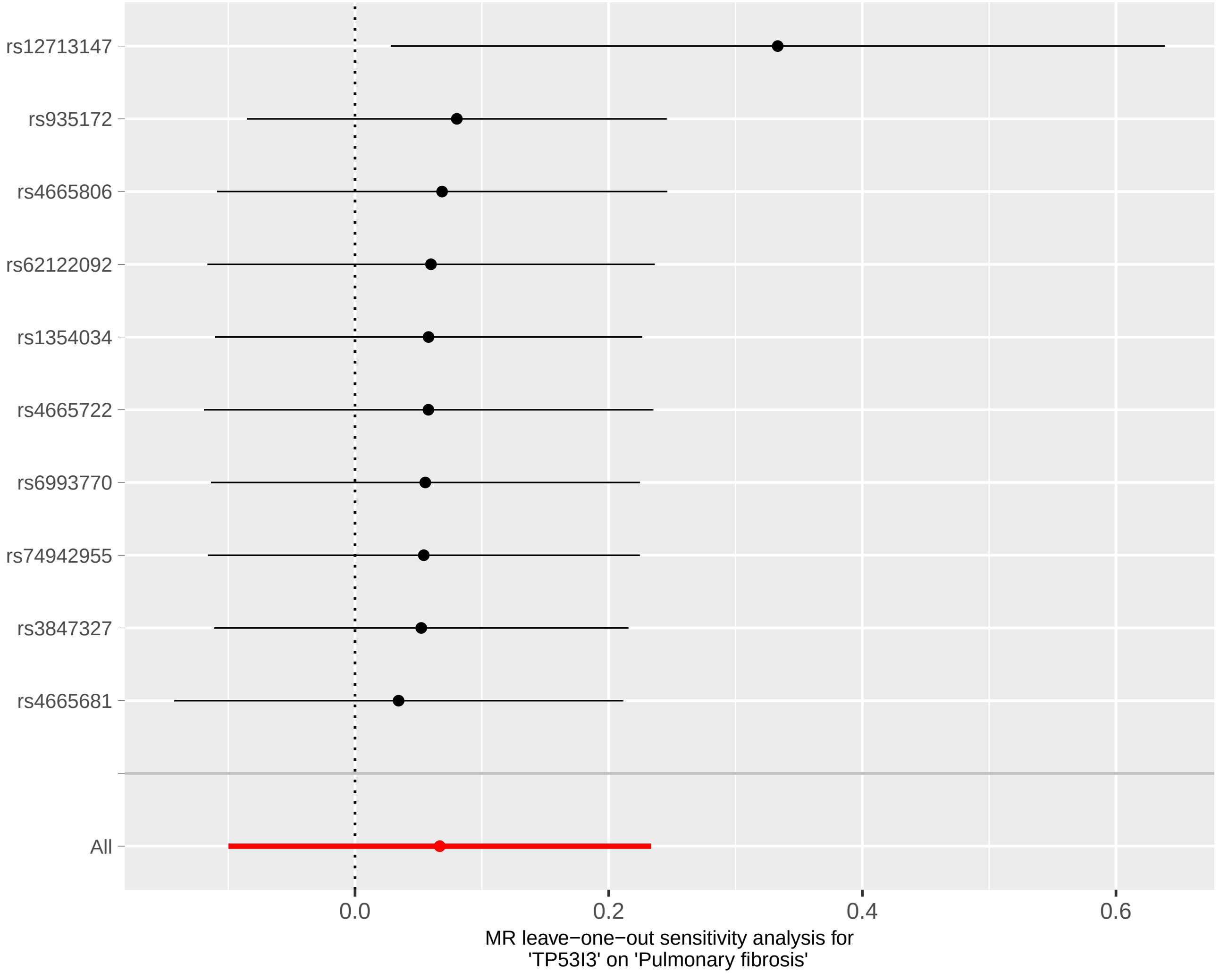

rs12713147
rs935172
rs4665806
rs62122092
rs1354034
rs4665722
rs6993770
rs74942955
rs3847327
rs4665681
All
0.0
0.2
0.4
0.6
MR leave−one−out sensitivity analysis for
'TP53I3' on 'Pulmonary fibrosis'

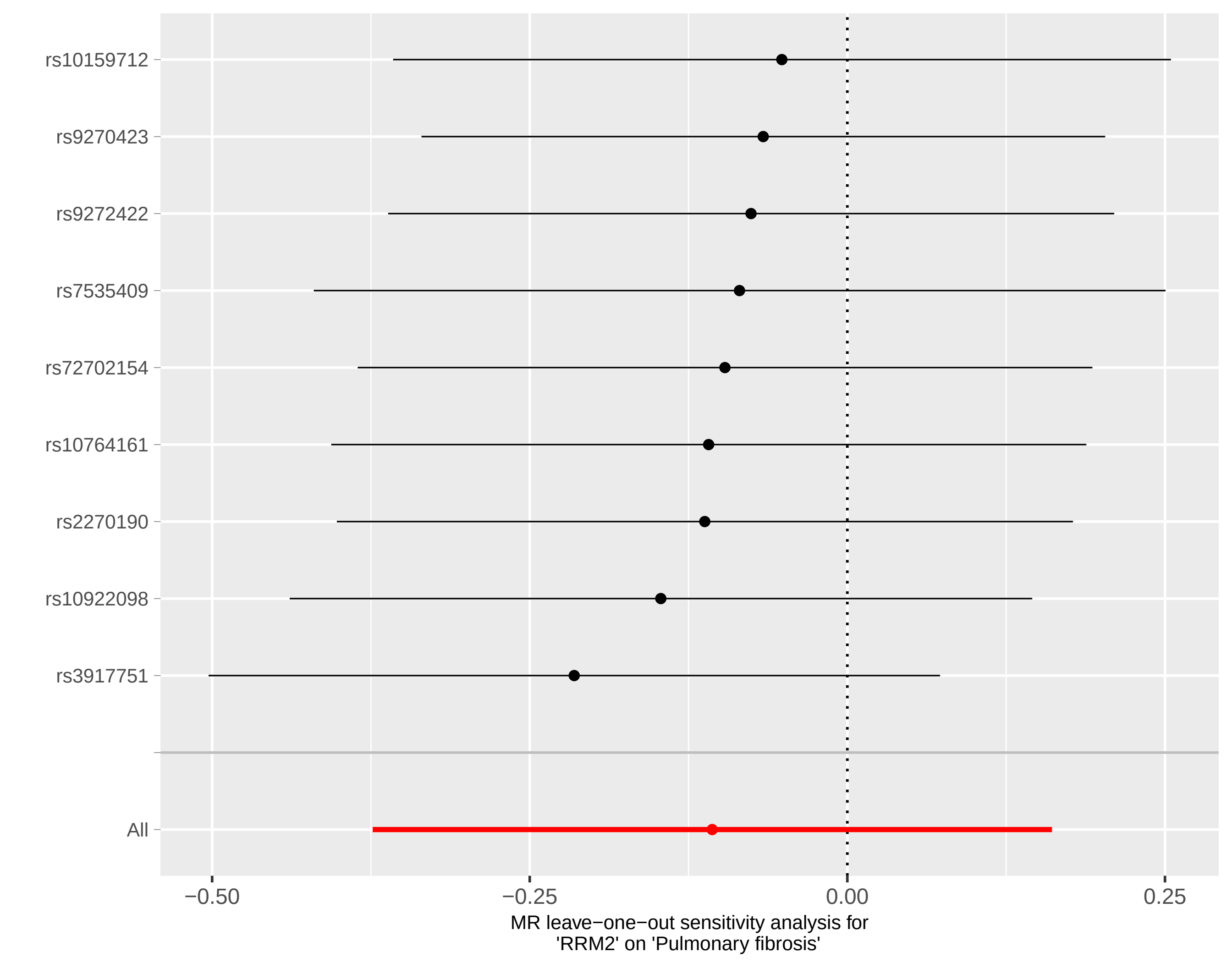
rs10159712
rs9270423
rs9272422
rs7535409
rs72702154
rs10764161
rs2270190
rs10922098
rs3917751
All
−0.50
−0.25
0.00
0.25
MR leave−one−out sensitivity analysis for
'RRM2' on 'Pulmonary fibrosis'

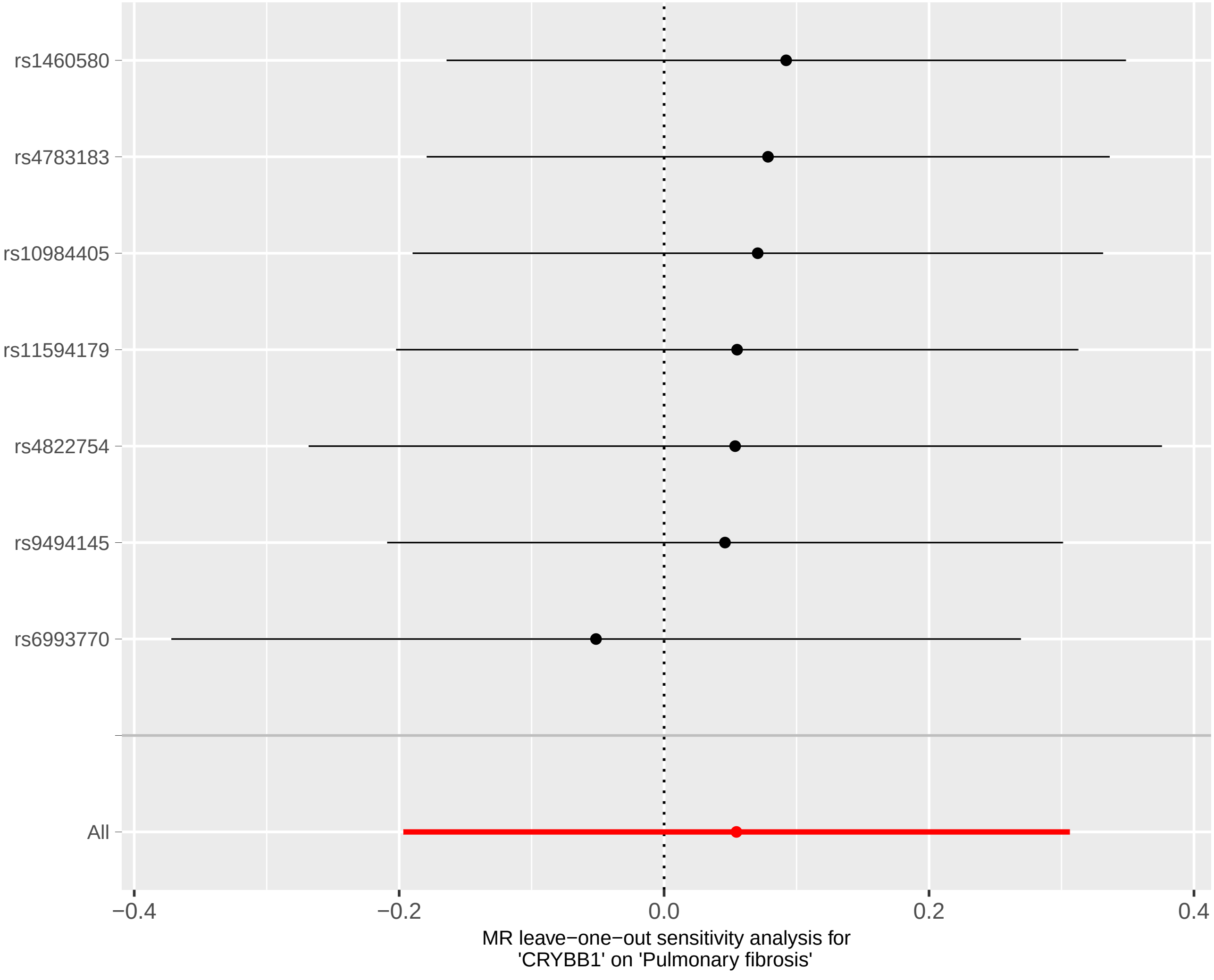
rs1460580
rs4783183
rs10984405
rs11594179
rs4822754
rs9494145
rs6993770
All
−0.4
−0.2
0.0
0.2
0.4
MR leave−one−out sensitivity analysis for
'CRYBB1' on 'Pulmonary fibrosis'

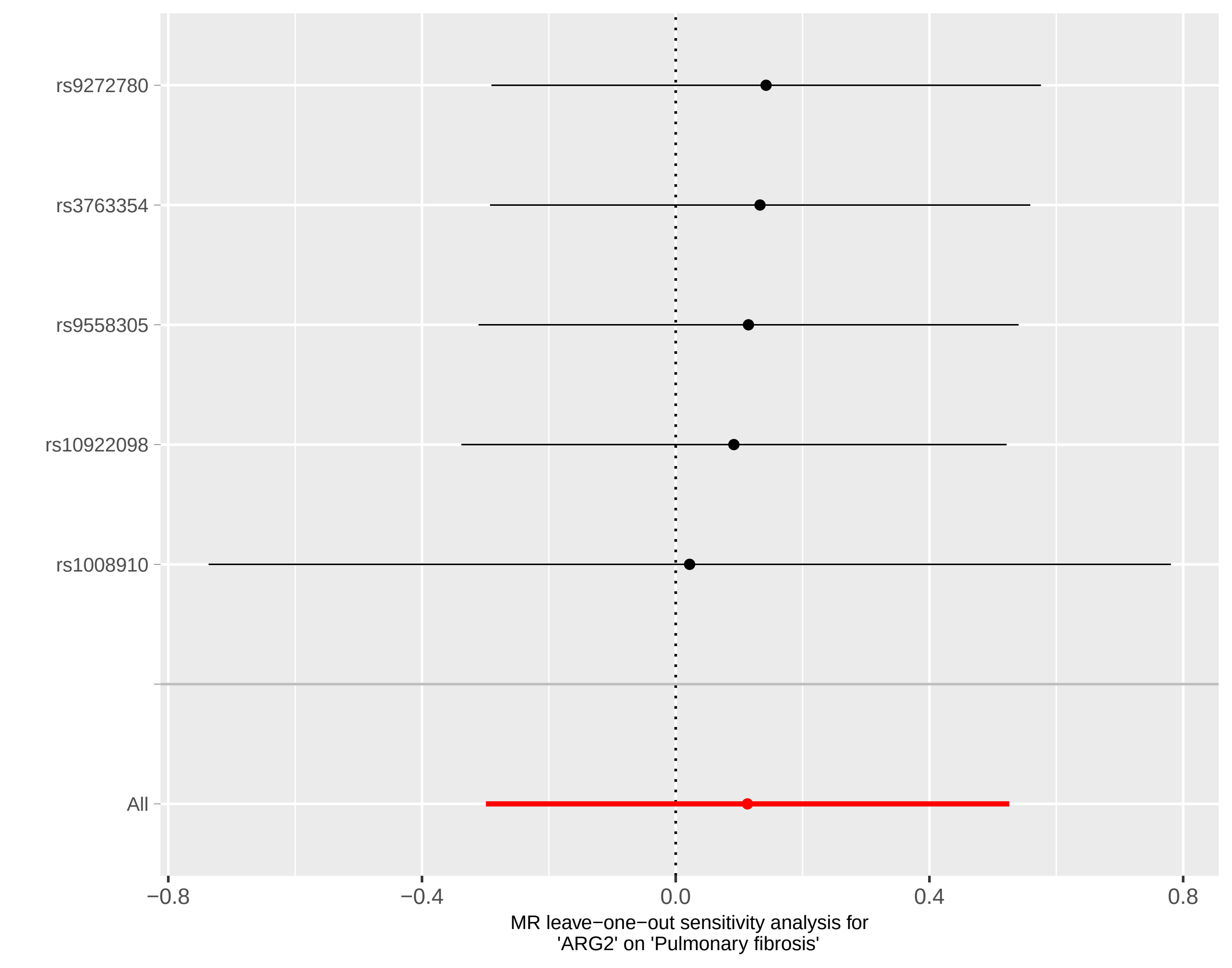

rs9272780
rs3763354
rs9558305
rs10922098
rs1008910
All
−0.8
−0.4
0.0
0.4
0.8
MR leave−one−out sensitivity analysis for
'ARG2' on 'Pulmonary fibrosis'

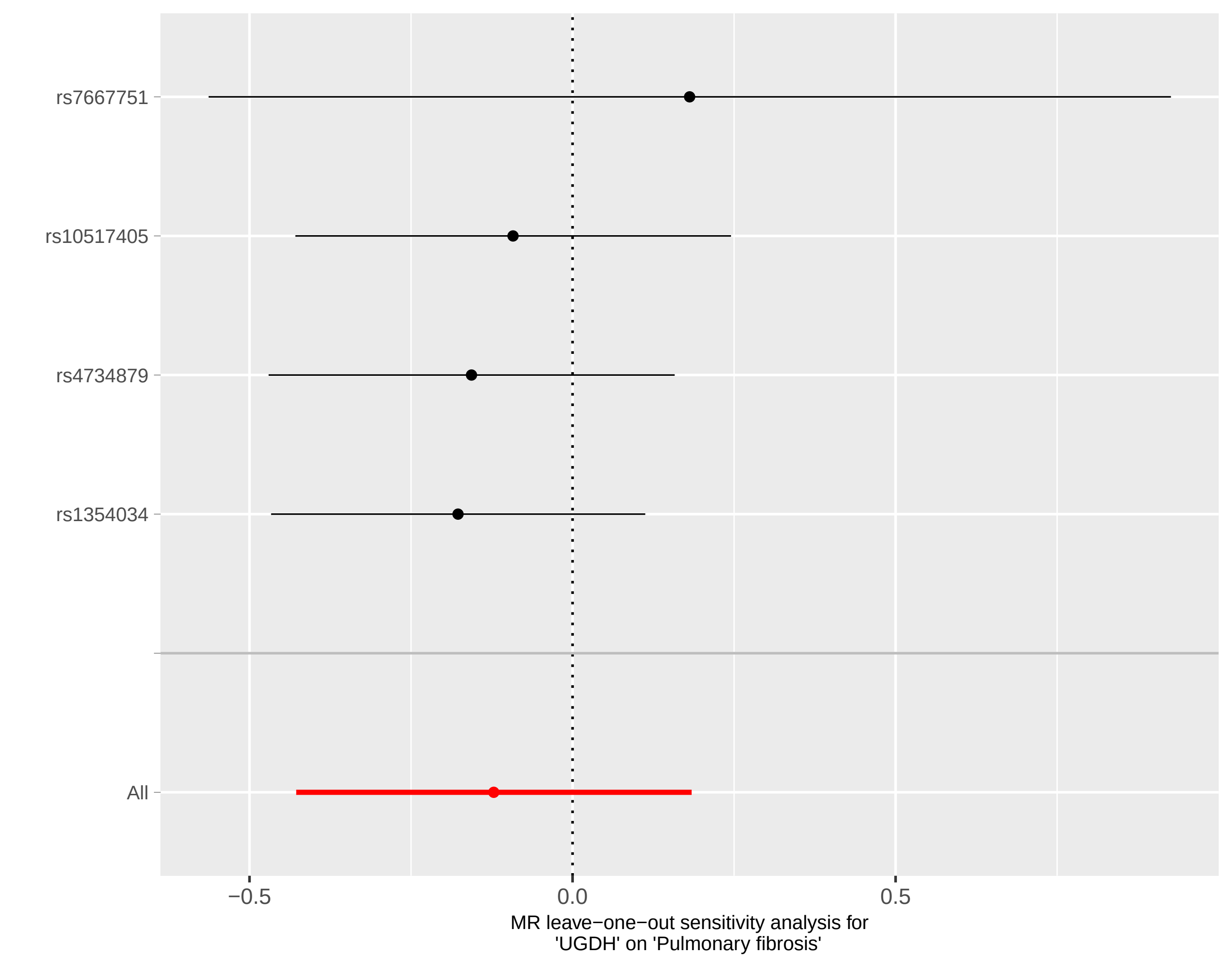
rs7667751
rs10517405
rs4734879
rs1354034
All
−0.5
0.0
0.5
MR leave−one−out sensitivity analysis for
'UGDH' on 'Pulmonary fibrosis'

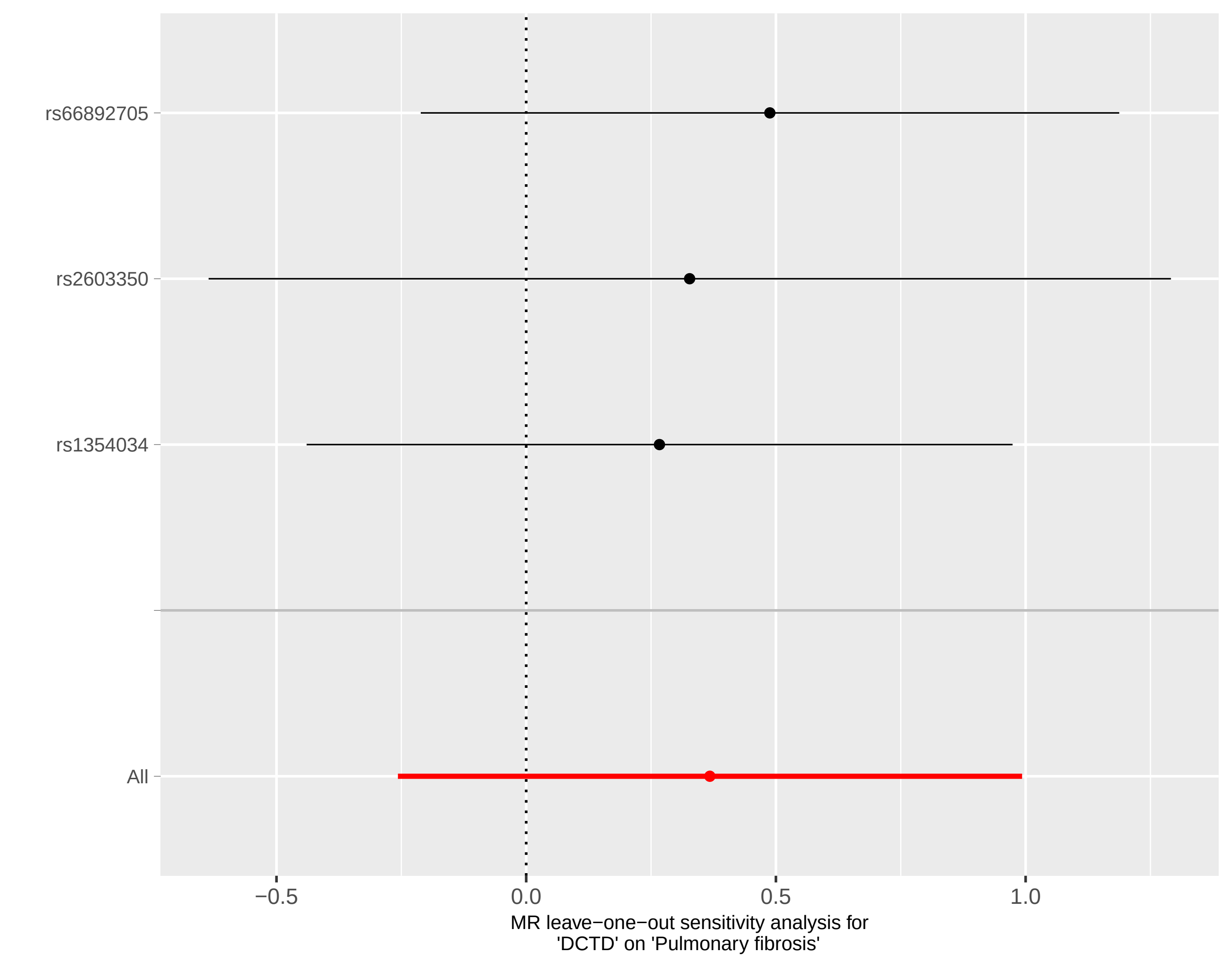
rs66892705
rs2603350
rs1354034
All
−0.5
0.0
0.5
1.0
MR leave−one−out sensitivity analysis for
'DCTD' on 'Pulmonary fibrosis'

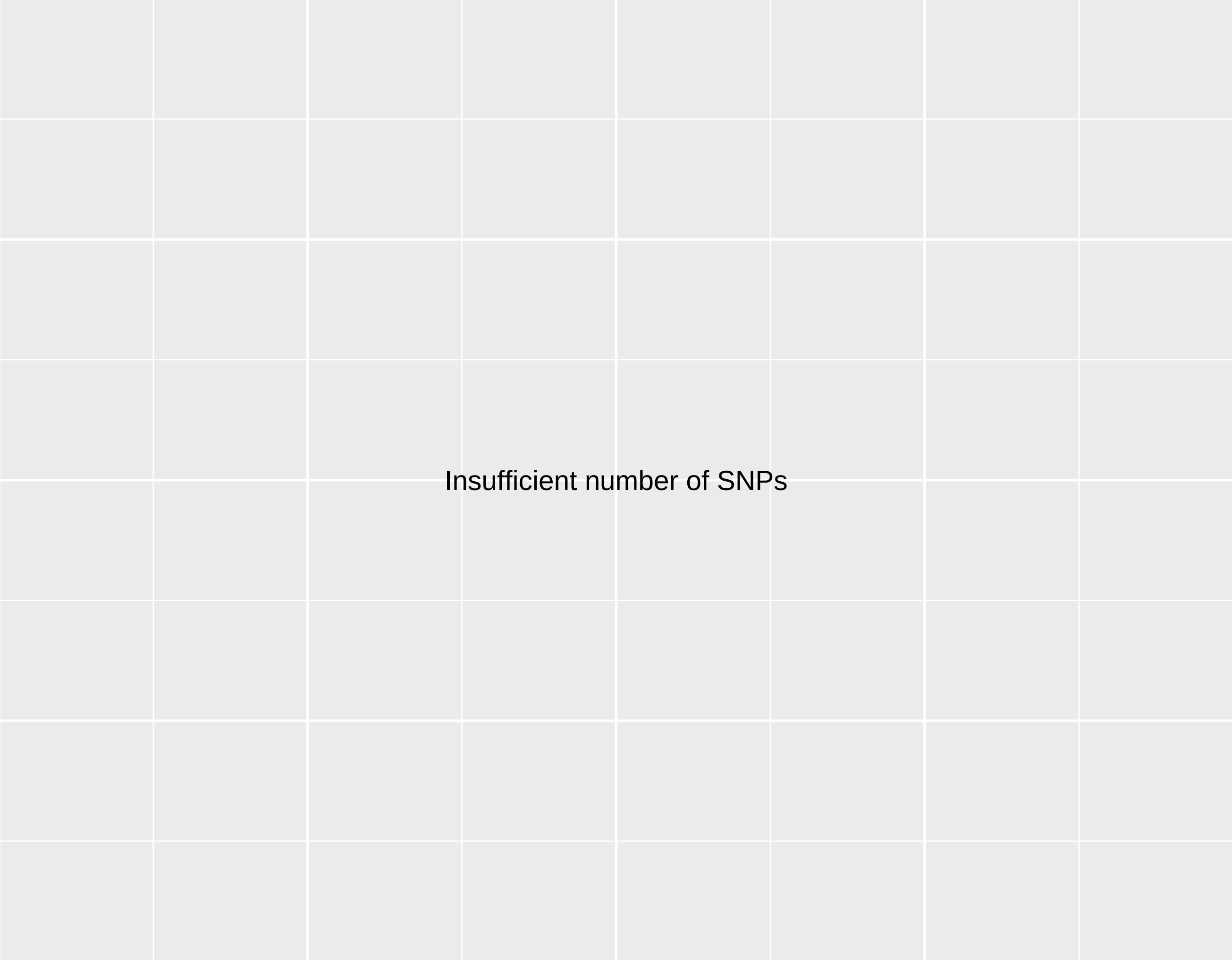
Insufficient number of SNPs

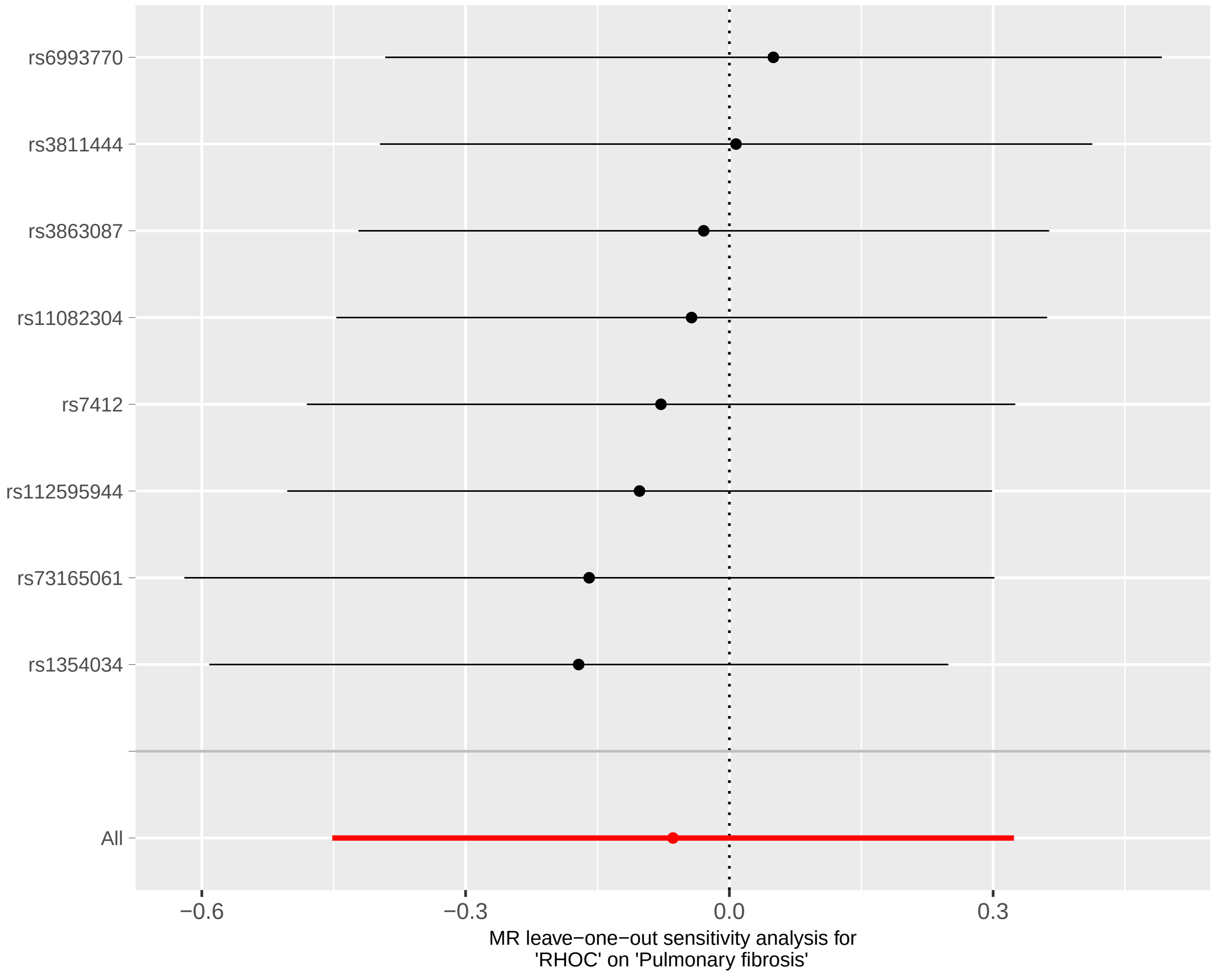

rs6993770
rs3811444
rs3863087
rs11082304
rs7412
rs112595944
rs73165061
rs1354034
All
−0.6
−0.3
0.0
0.3
MR leave−one−out sensitivity analysis for
'RHOC' on 'Pulmonary fibrosis'

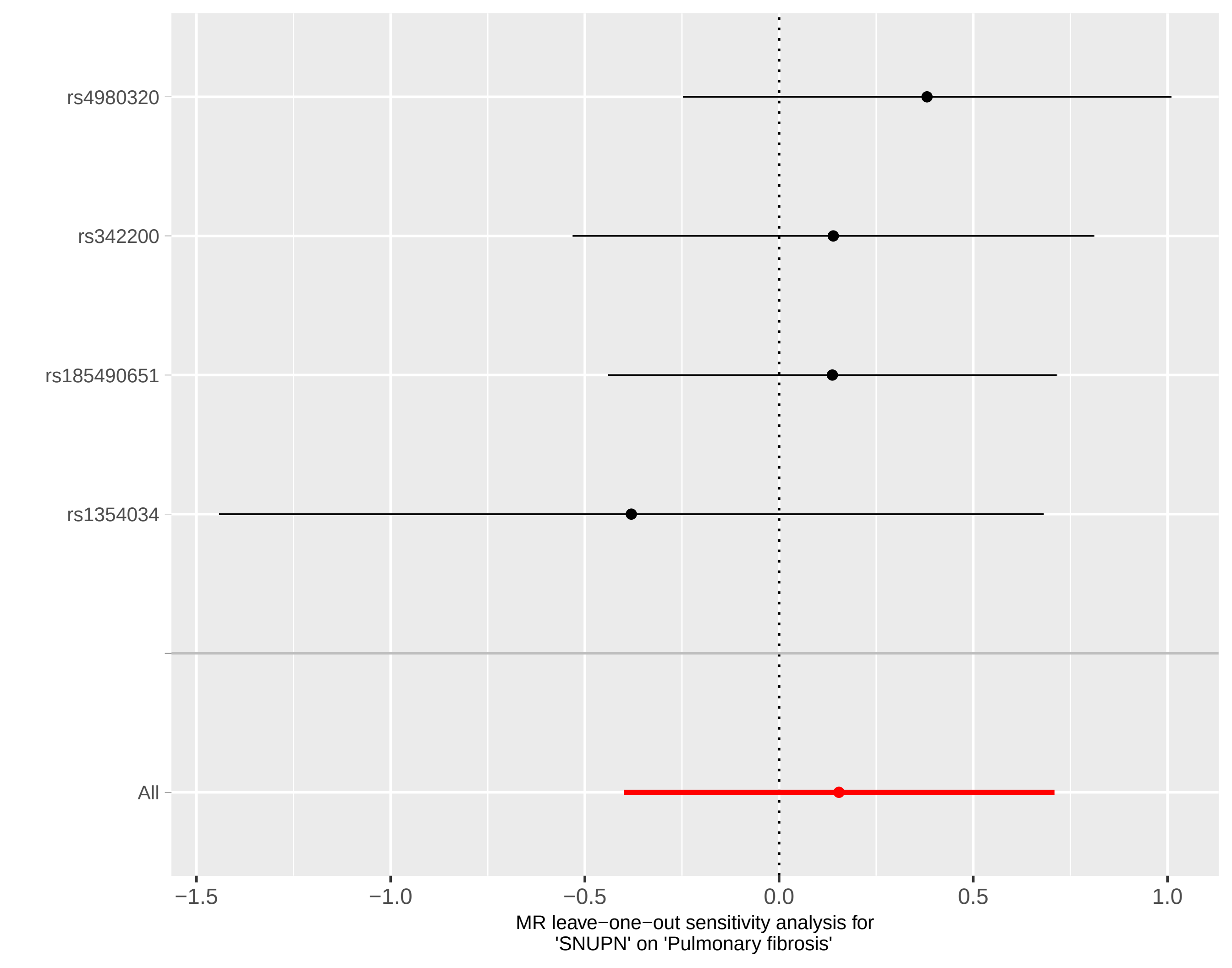
rs4980320
rs342200
rs185490651
rs1354034
All
−1.5
−1.0
−0.5
0.0
0.5
1.0
MR leave−one−out sensitivity analysis for
'SNUPN' on 'Pulmonary fibrosis'

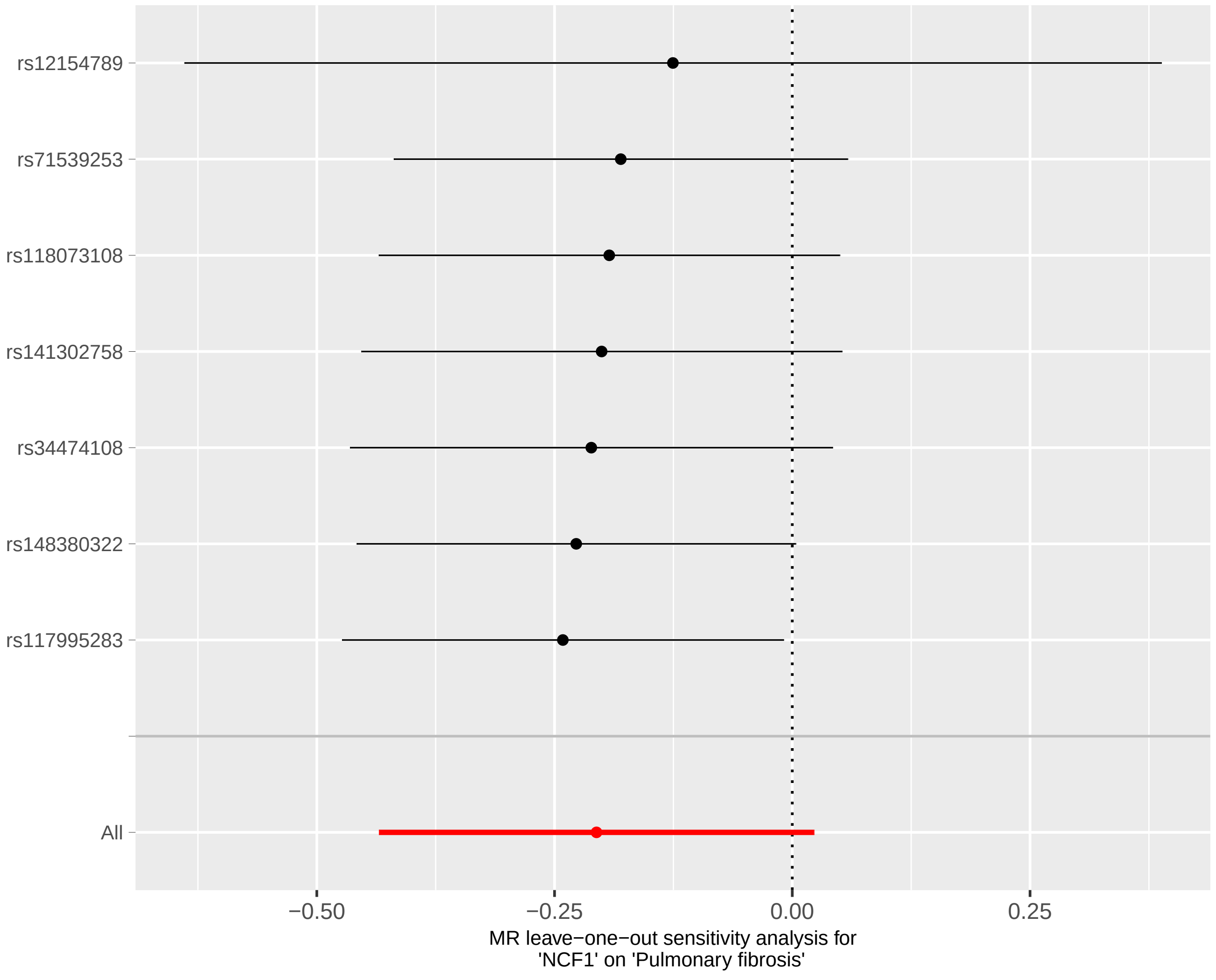

rs12154789
rs71539253
rs118073108
rs141302758
rs34474108
rs148380322
rs117995283
All
−0.50
−0.25
0.00
0.25
MR leave−one−out sensitivity analysis for
'NCF1' on 'Pulmonary fibrosis'

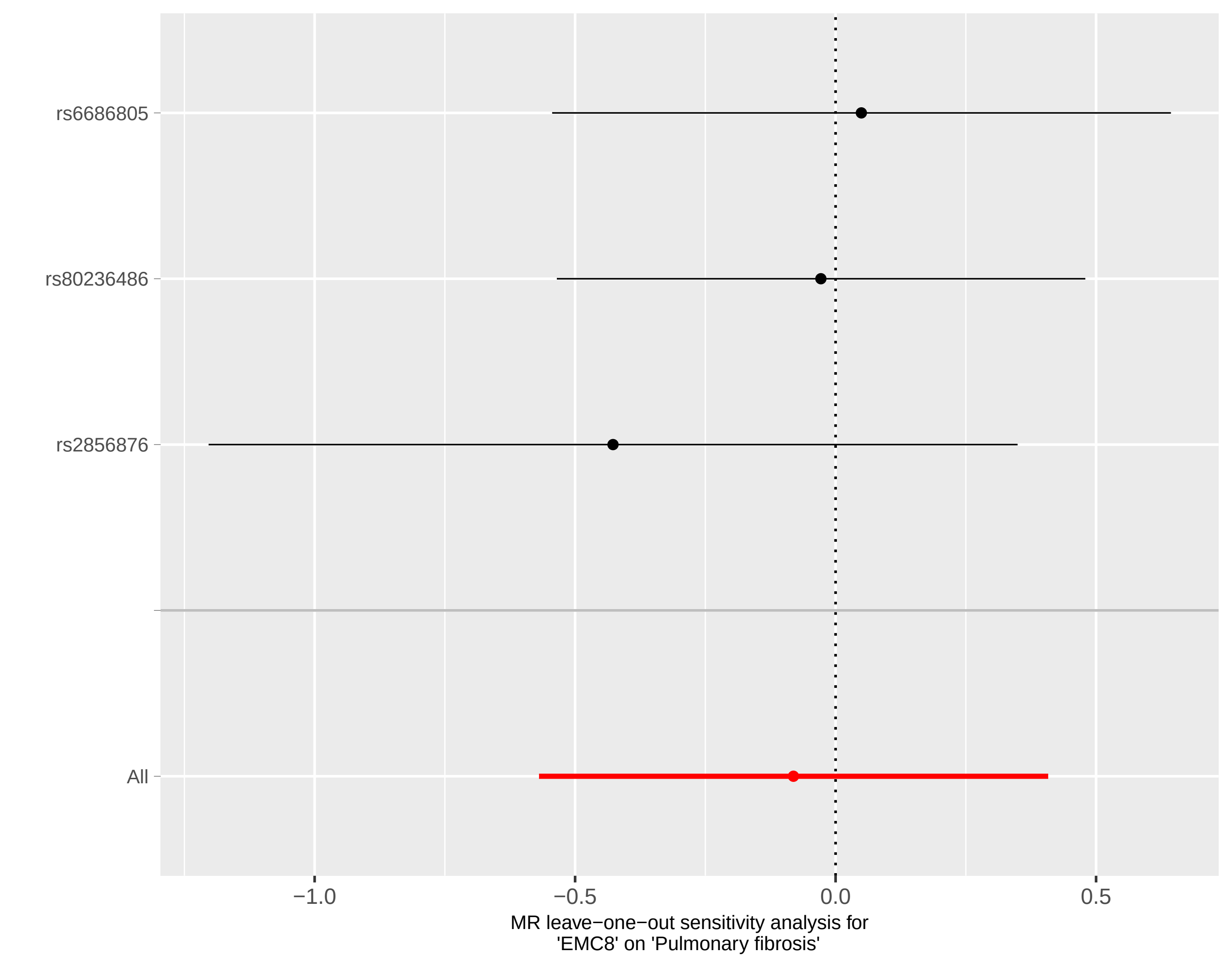
rs6686805
rs80236486
rs2856876
All
−1.0
−0.5
0.0
0.5
MR leave−one−out sensitivity analysis for
'EMC8' on 'Pulmonary fibrosis'

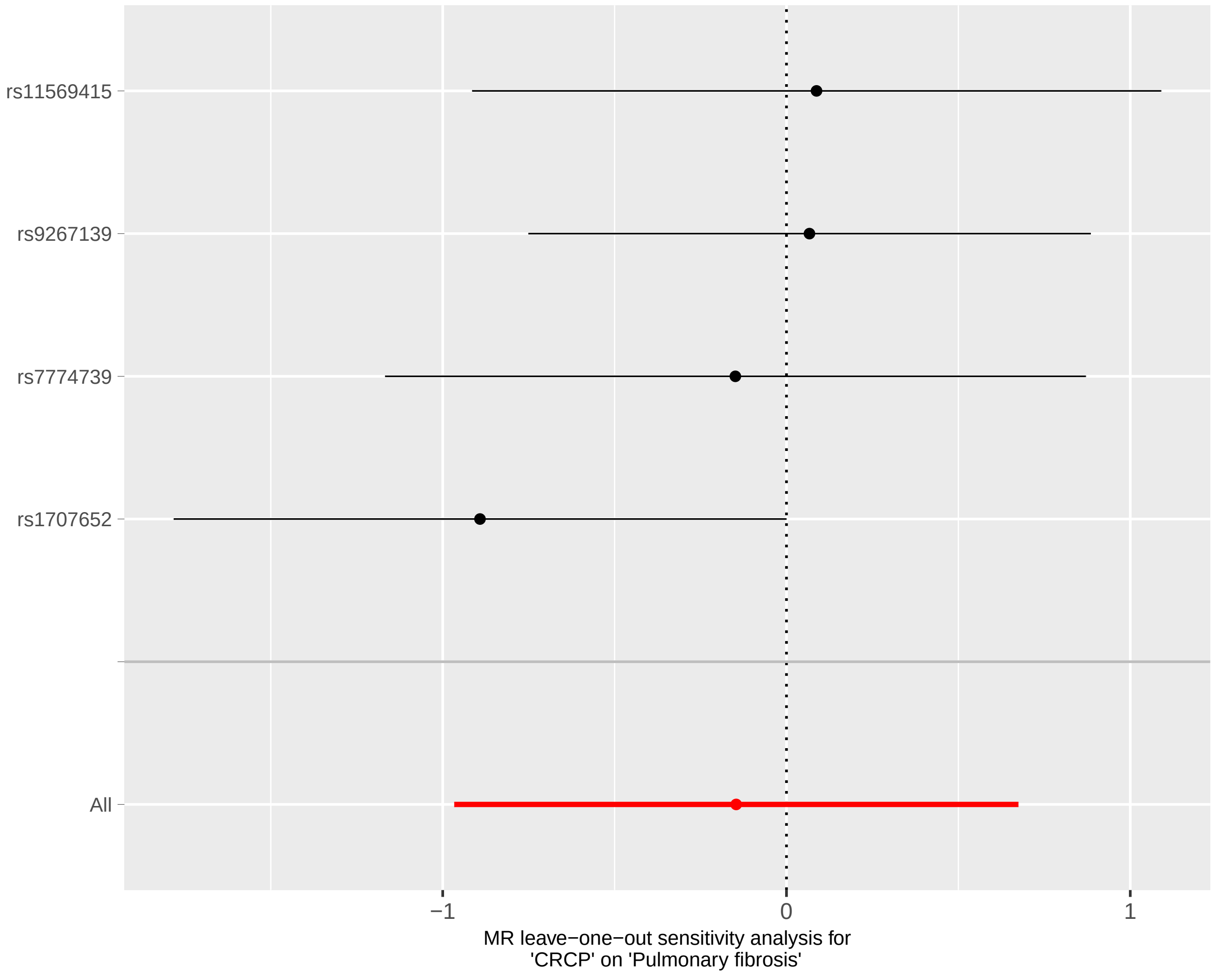
rs11569415
rs9267139
rs7774739
rs1707652
All
−1
0
1
MR leave−one−out sensitivity analysis for
'CRCP' on 'Pulmonary fibrosis'

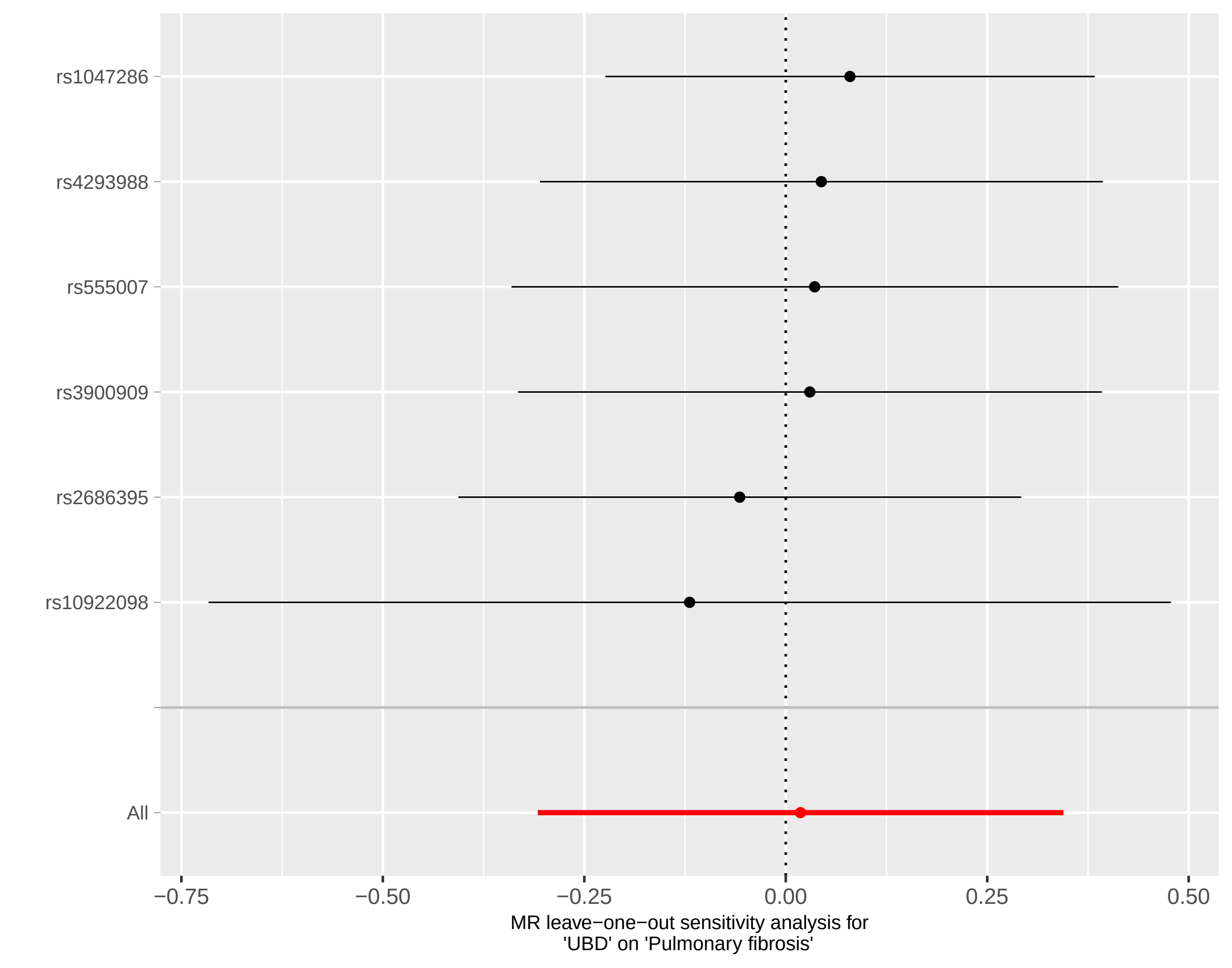
rs1047286
rs4293988
rs555007
rs3900909
rs2686395
rs10922098
All
−0.75
−0.50
−0.25
0.00
0.25
0.50
MR leave−one−out sensitivity analysis for
'UBD' on 'Pulmonary fibrosis'

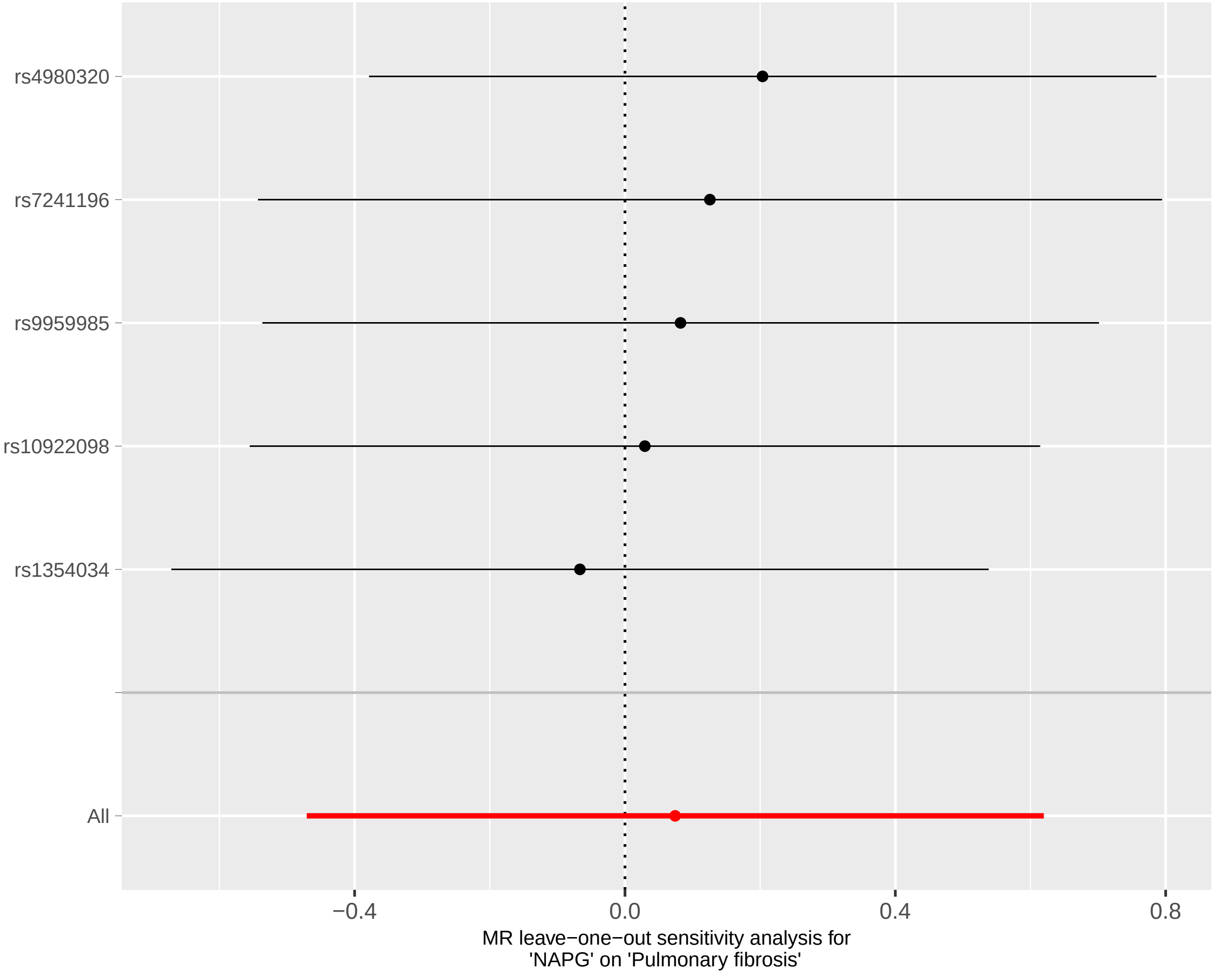

rs4980320
rs7241196
rs9959985
rs10922098
rs1354034
All
−0.4
0.0
0.4
0.8
MR leave−one−out sensitivity analysis for
'NAPG' on 'Pulmonary fibrosis'

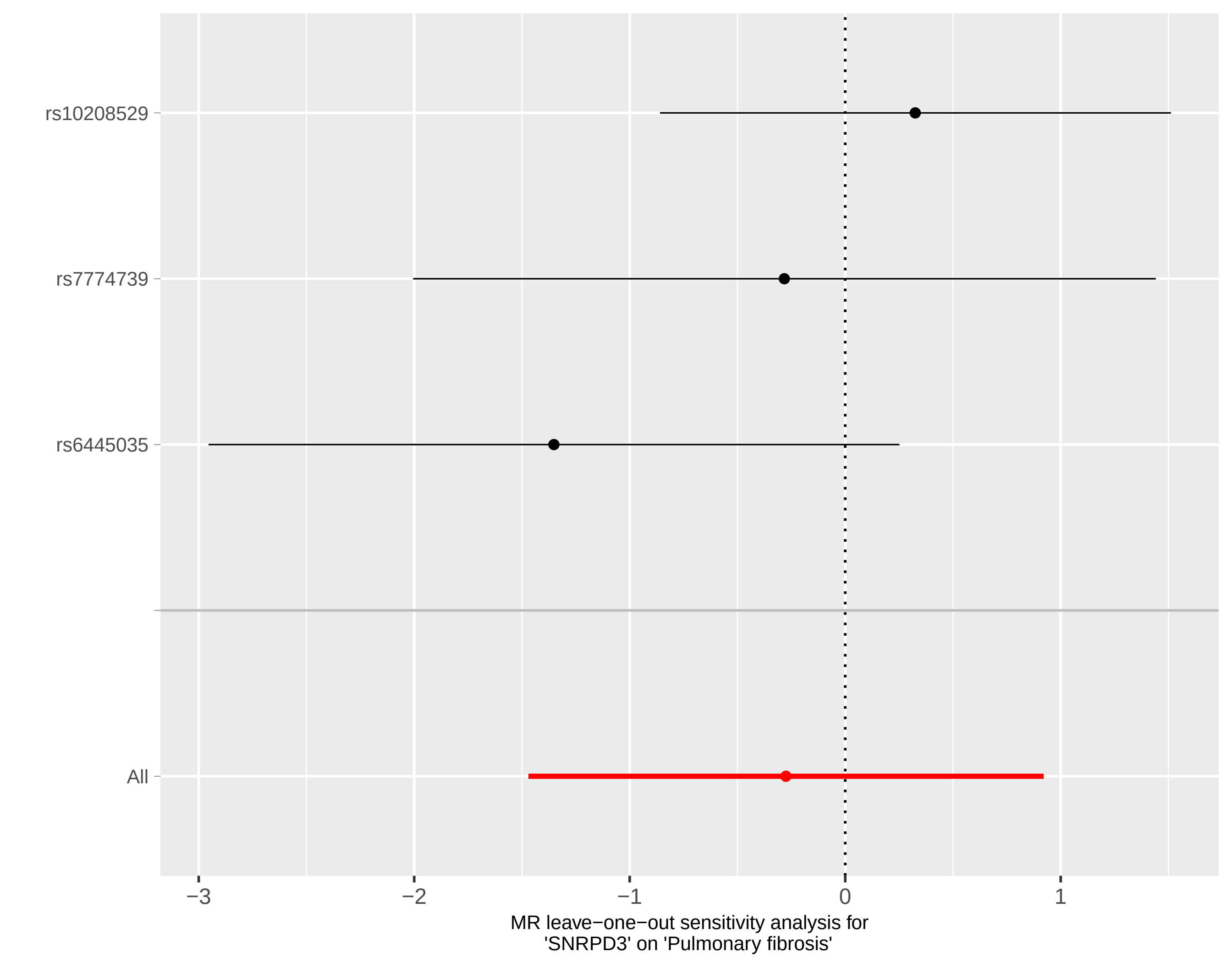
rs10208529
rs7774739
rs6445035
All
−3
−2
−1
0
1
MR leave−one−out sensitivity analysis for
'SNRPD3' on 'Pulmonary fibrosis'

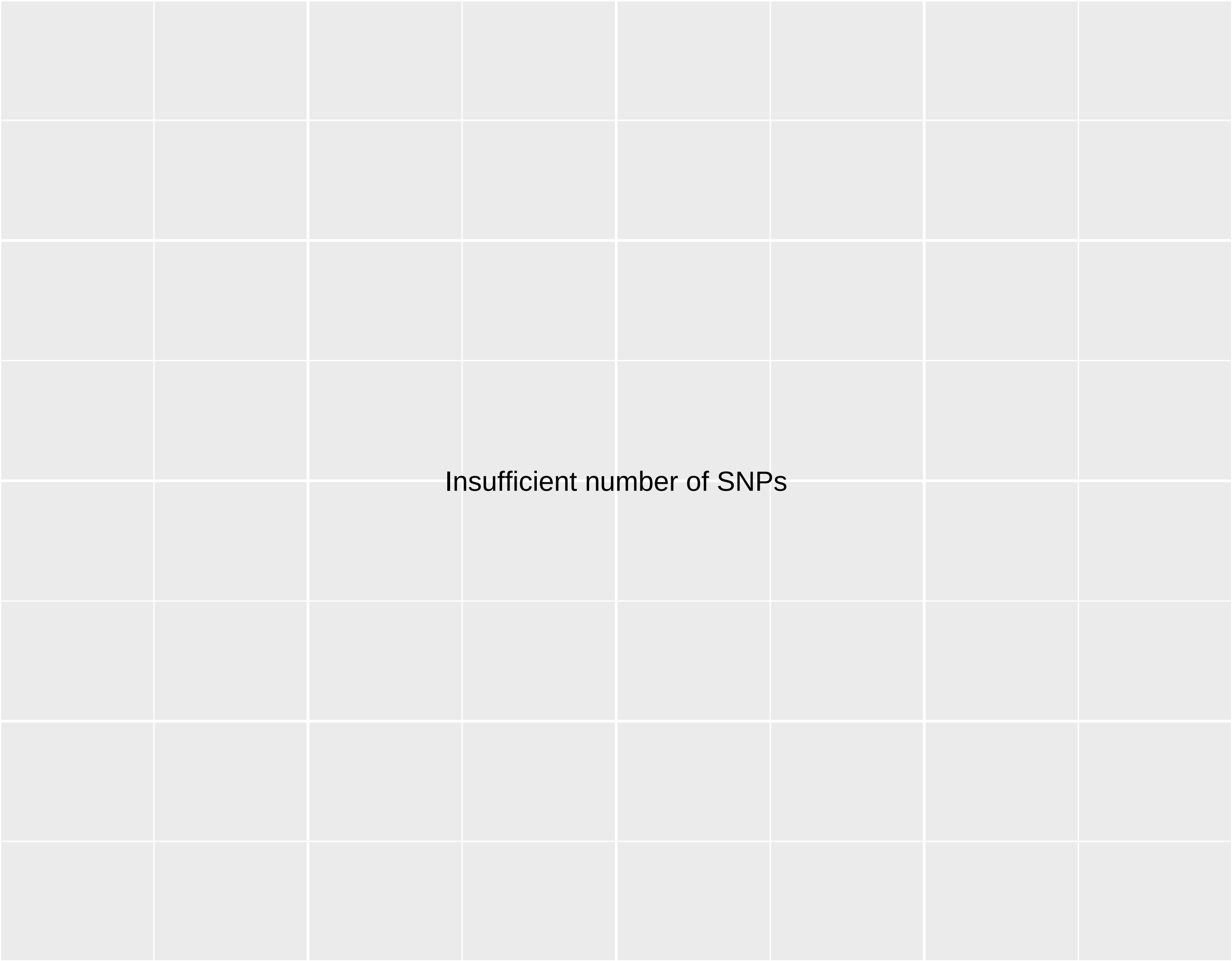
Insufficient number of SNPs

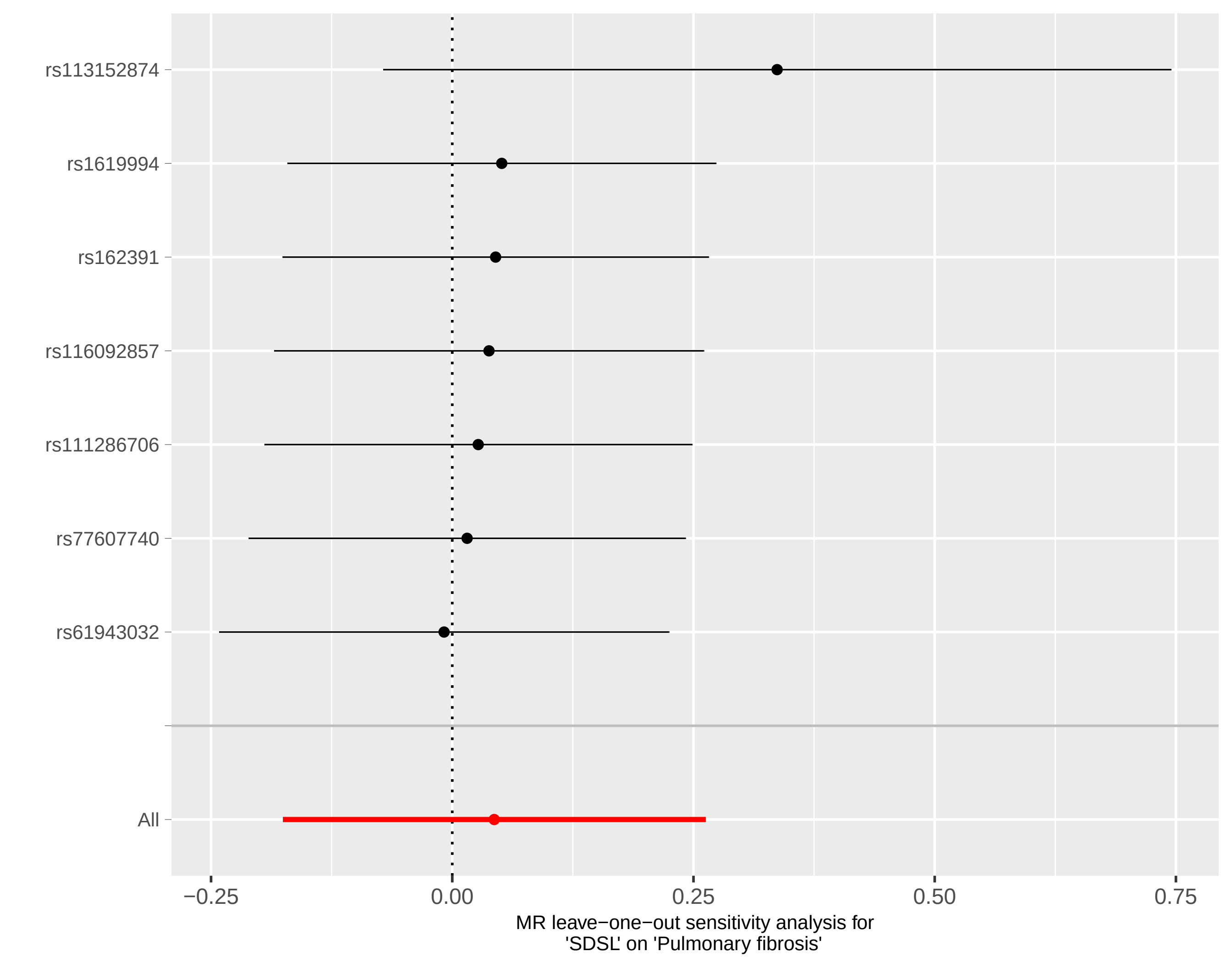
rs113152874
rs1619994
rs162391
rs116092857
rs111286706
rs77607740
rs61943032
All
−0.25
0.00
0.25
0.50
0.75
MR leave−one−out sensitivity analysis for
'SDSL' on 'Pulmonary fibrosis'

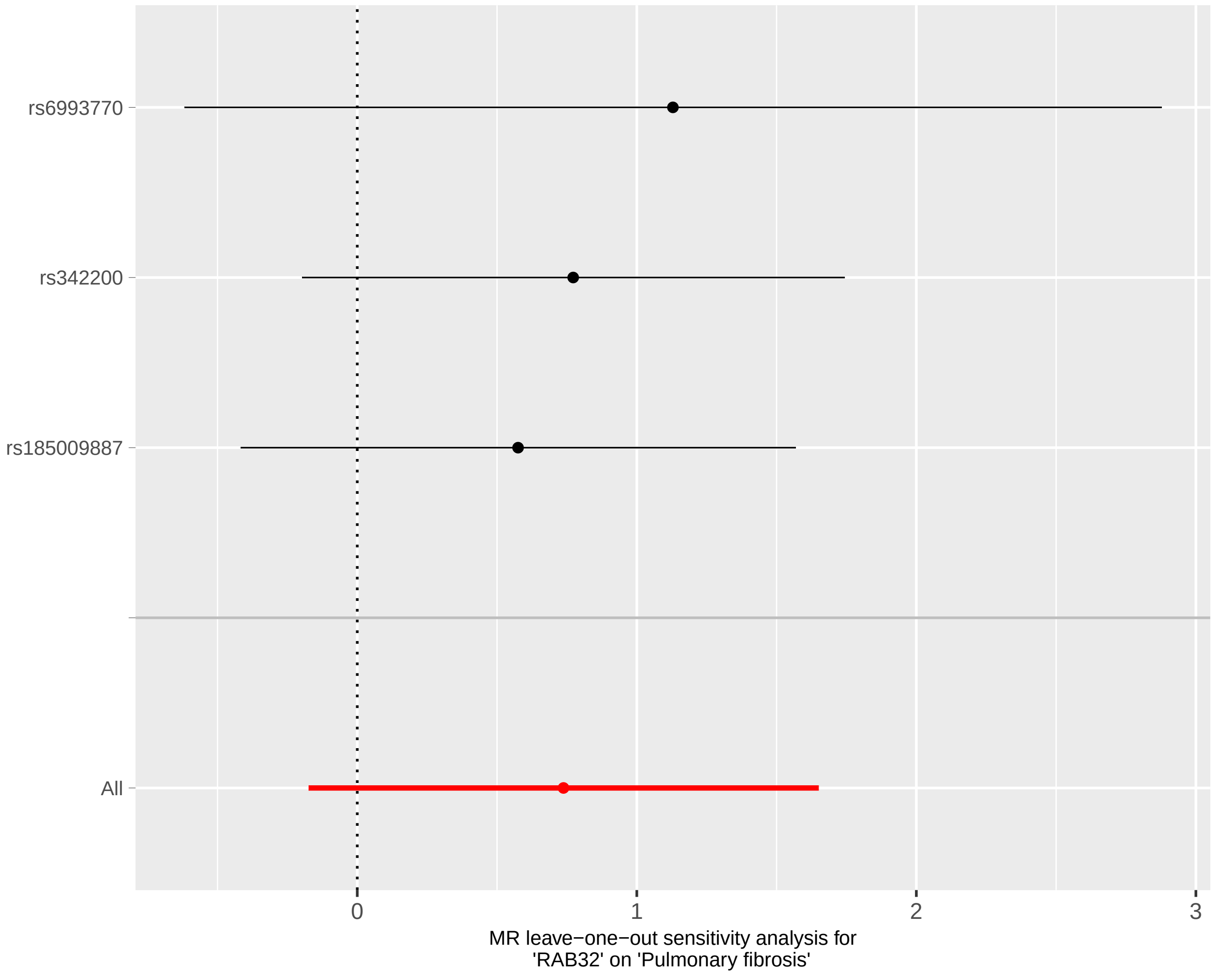
rs6993770
rs342200
rs185009887
All
0
1
2
3
MR leave−one−out sensitivity analysis for
'RAB32' on 'Pulmonary fibrosis'

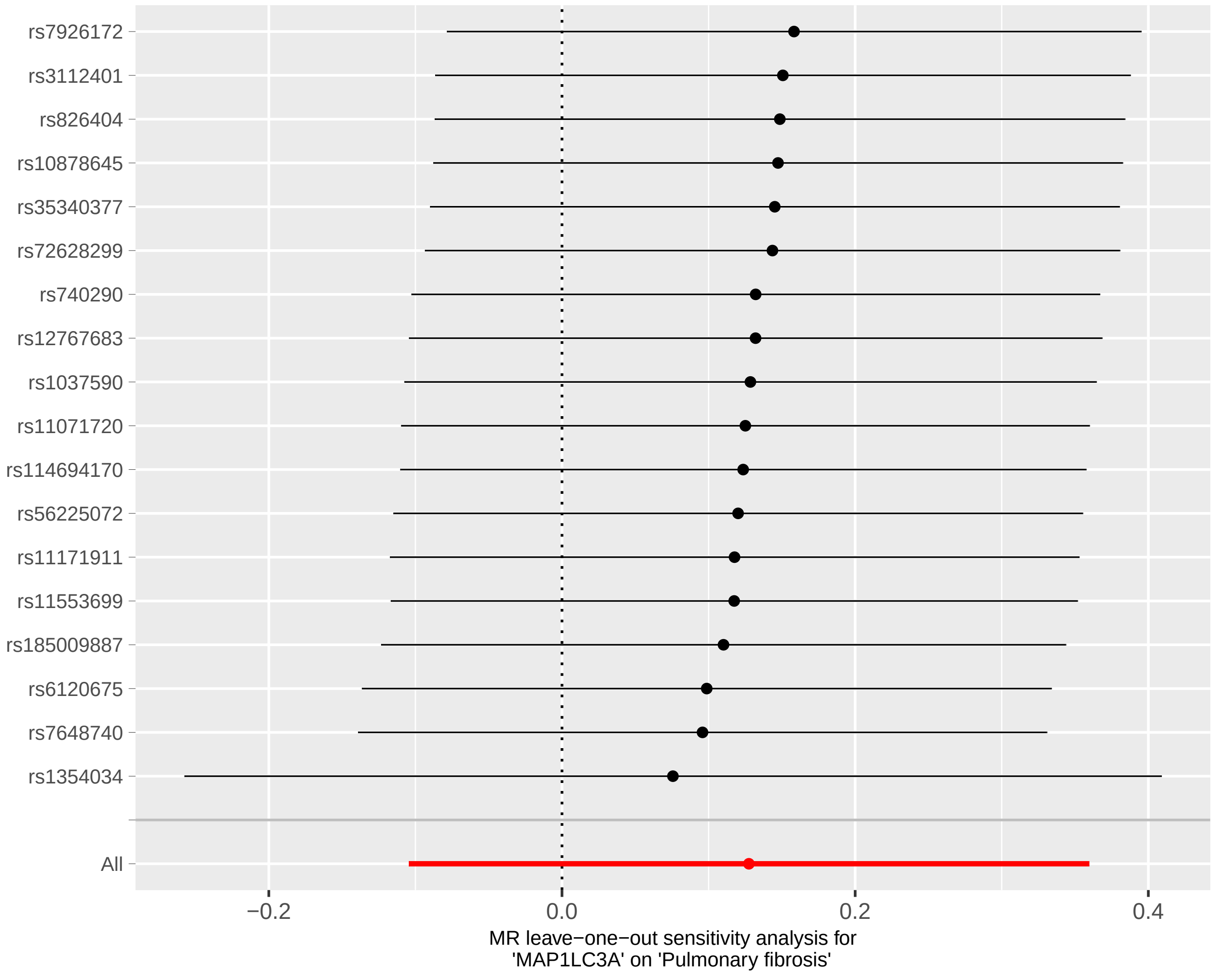
rs7926172
rs3112401
rs826404
rs10878645
rs35340377
rs72628299
rs740290
rs12767683
rs1037590
rs11071720
rs114694170
rs56225072
rs11171911
rs11553699
rs185009887
rs6120675
rs7648740
rs1354034
All
−0.2
0.0
0.2
0.4
MR leave−one−out sensitivity analysis for
'MAP1LC3A' on 'Pulmonary fibrosis'

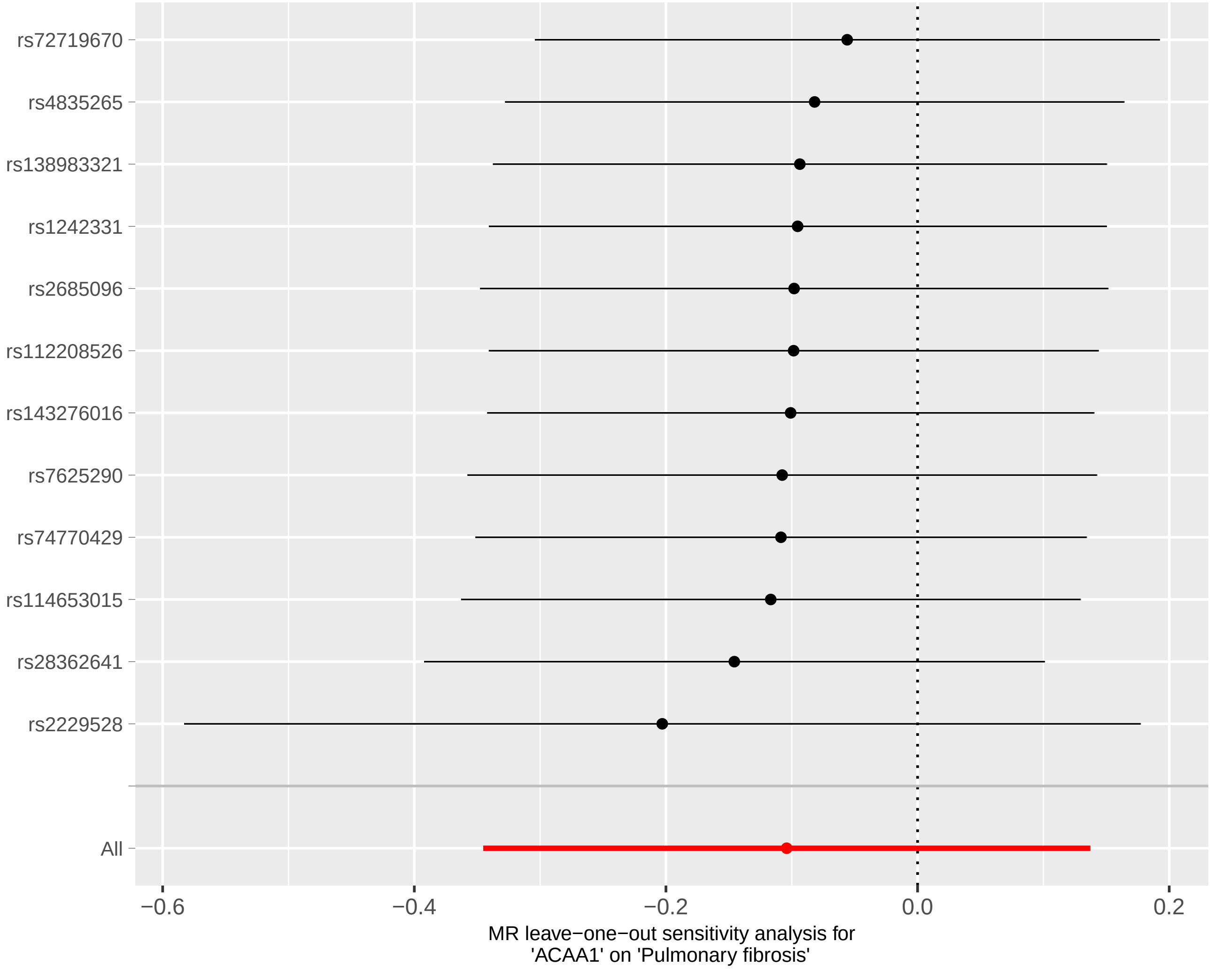

rs72719670
rs4835265
rs138983321
rs1242331
rs2685096
rs112208526
rs143276016
rs7625290
rs74770429
rs114653015
rs28362641
rs2229528
All
−0.6
−0.4
−0.2
0.0
0.2
MR leave−one−out sensitivity analysis for
'ACAA1' on 'Pulmonary fibrosis'

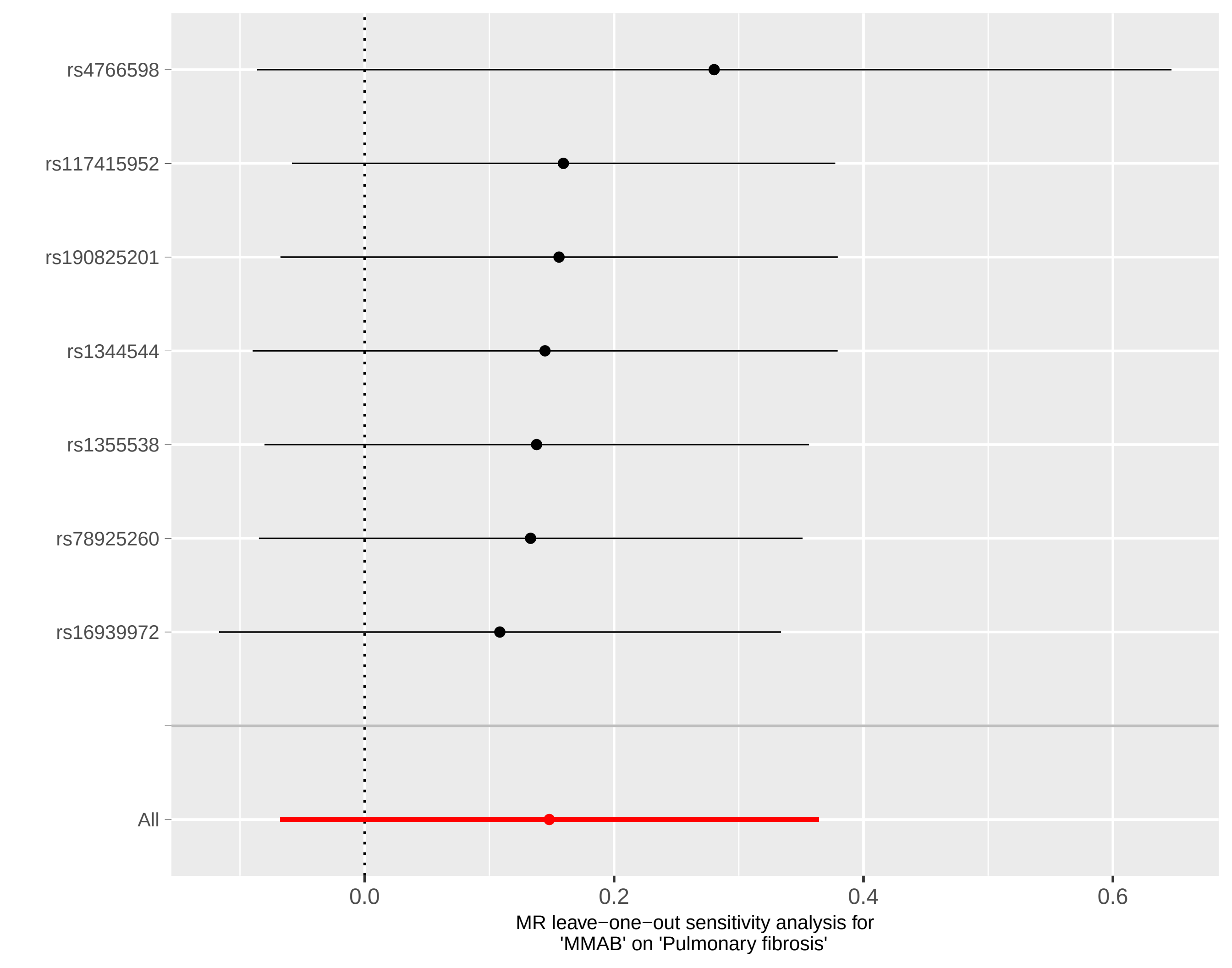
rs4766598
rs117415952
rs190825201
rs1344544
rs1355538
rs78925260
rs16939972
All
0.0
0.2
0.4
0.6
MR leave−one−out sensitivity analysis for
'MMAB' on 'Pulmonary fibrosis'

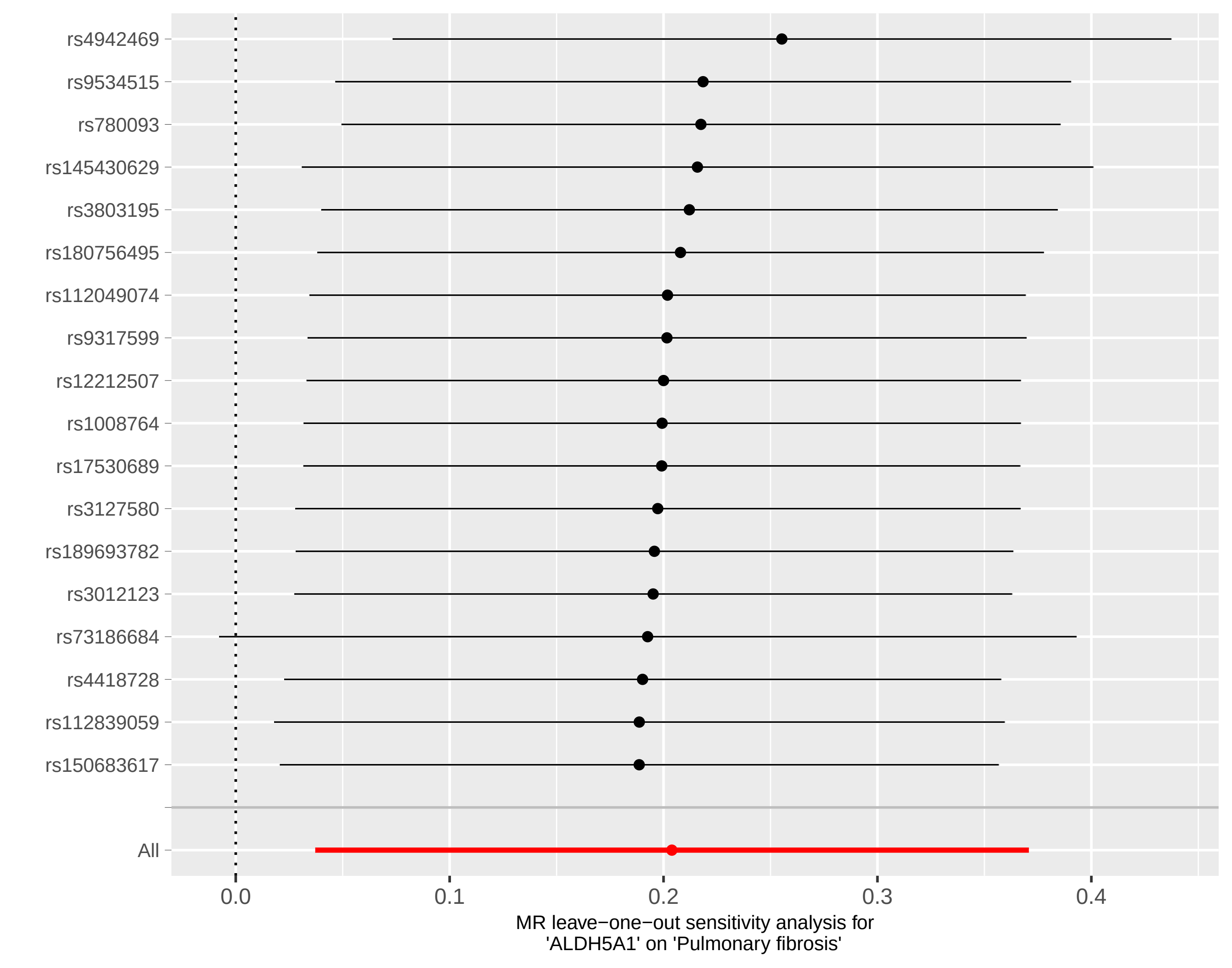
rs4942469
rs9534515
rs780093
rs145430629
rs3803195
rs180756495
rs112049074
rs9317599
rs12212507
rs1008764
rs17530689
rs3127580
rs189693782
rs3012123
rs73186684
rs4418728
rs112839059
rs150683617
All
0.0
0.1
0.2
0.3
0.4
MR leave−one−out sensitivity analysis for
'ALDH5A1' on 'Pulmonary fibrosis'

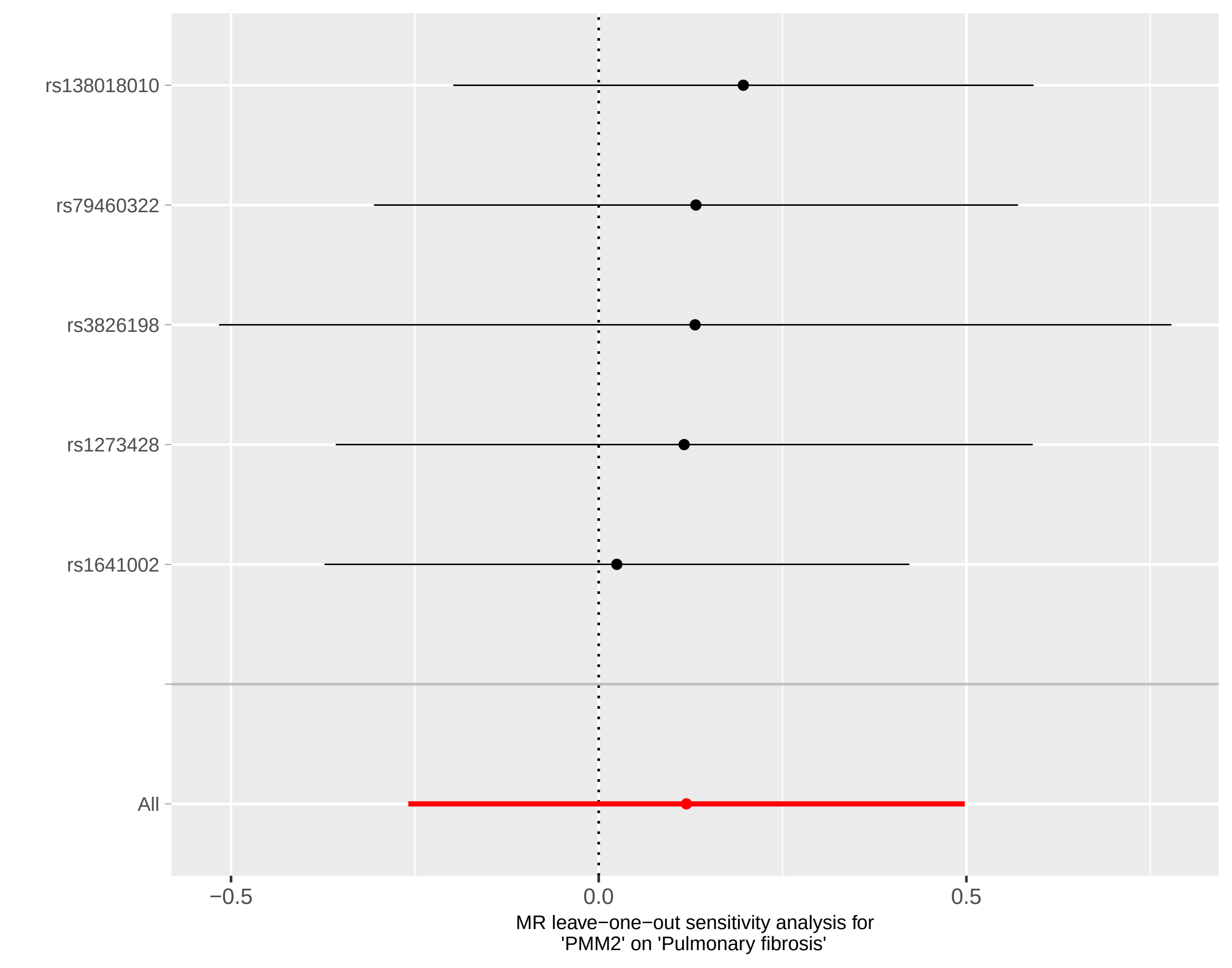
rs138018010
rs79460322
rs3826198
rs1273428
rs1641002
All
−0.5
0.0
0.5
MR leave−one−out sensitivity analysis for
'PMM2' on 'Pulmonary fibrosis'

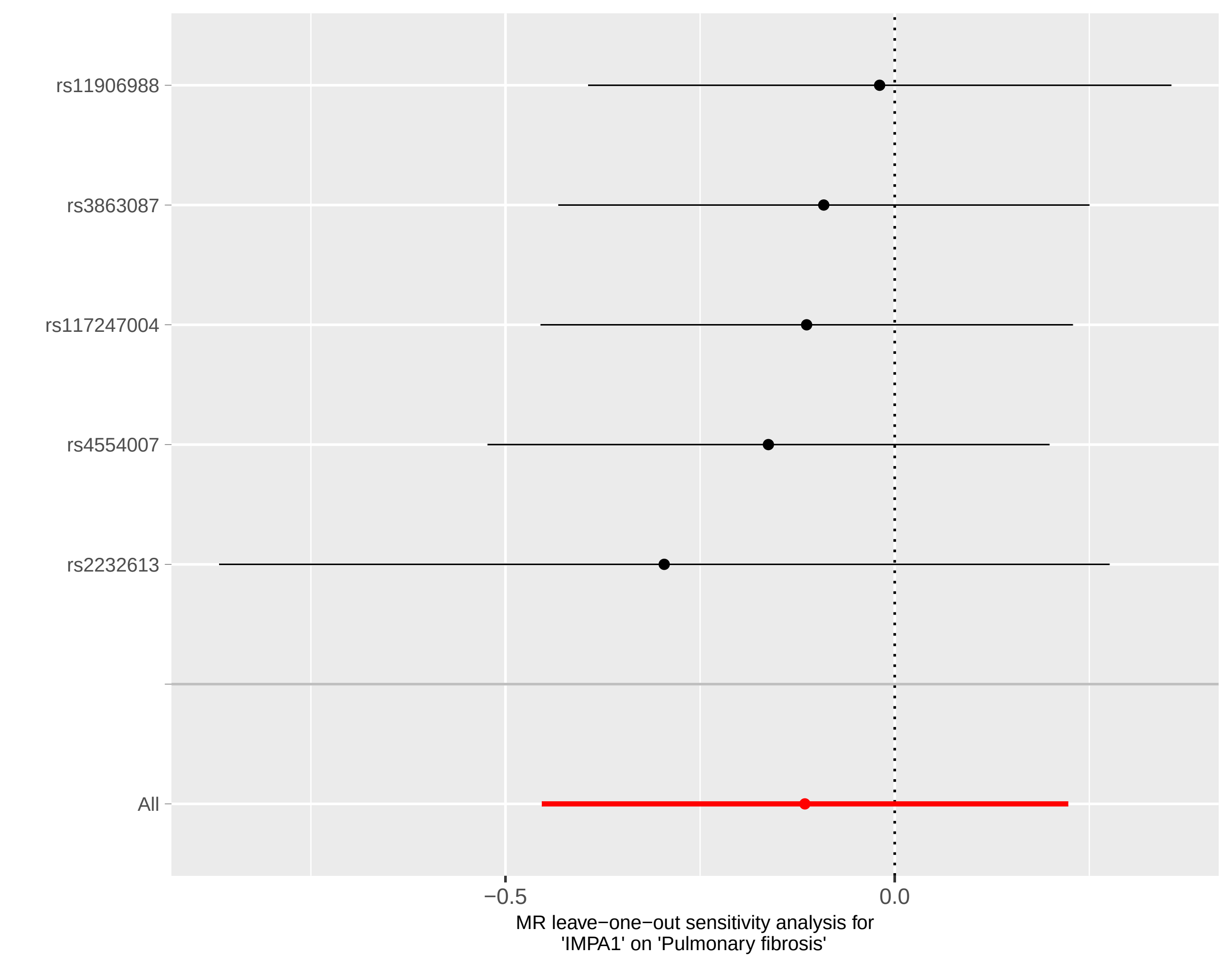
rs11906988
rs3863087
rs117247004
rs4554007
rs2232613
All
−0.5
0.0
MR leave−one−out sensitivity analysis for
'IMPA1' on 'Pulmonary fibrosis'

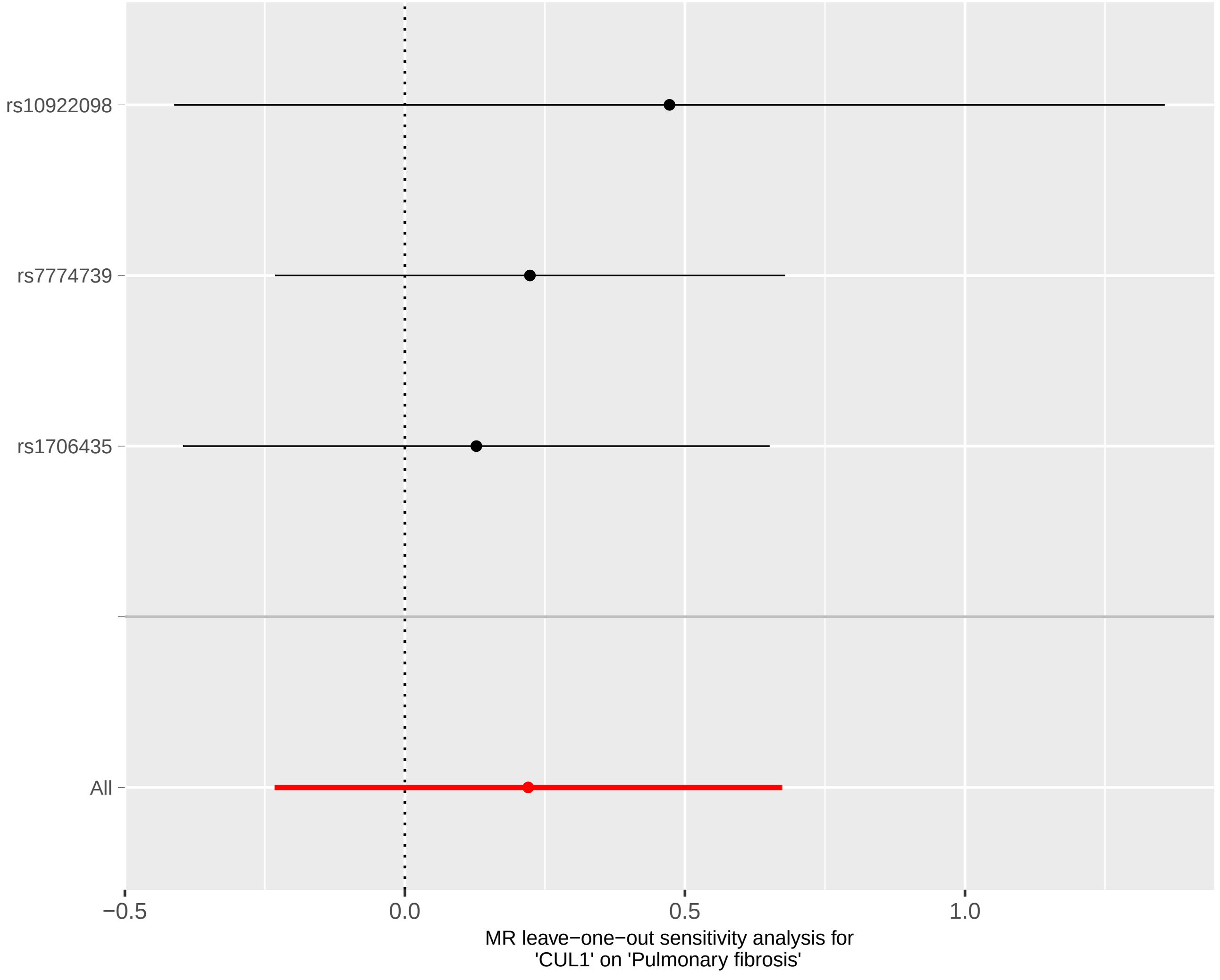
rs10922098
rs7774739
rs1706435
All
−0.5
0.0
0.5
1.0
MR leave−one−out sensitivity analysis for
'CUL1' on 'Pulmonary fibrosis'

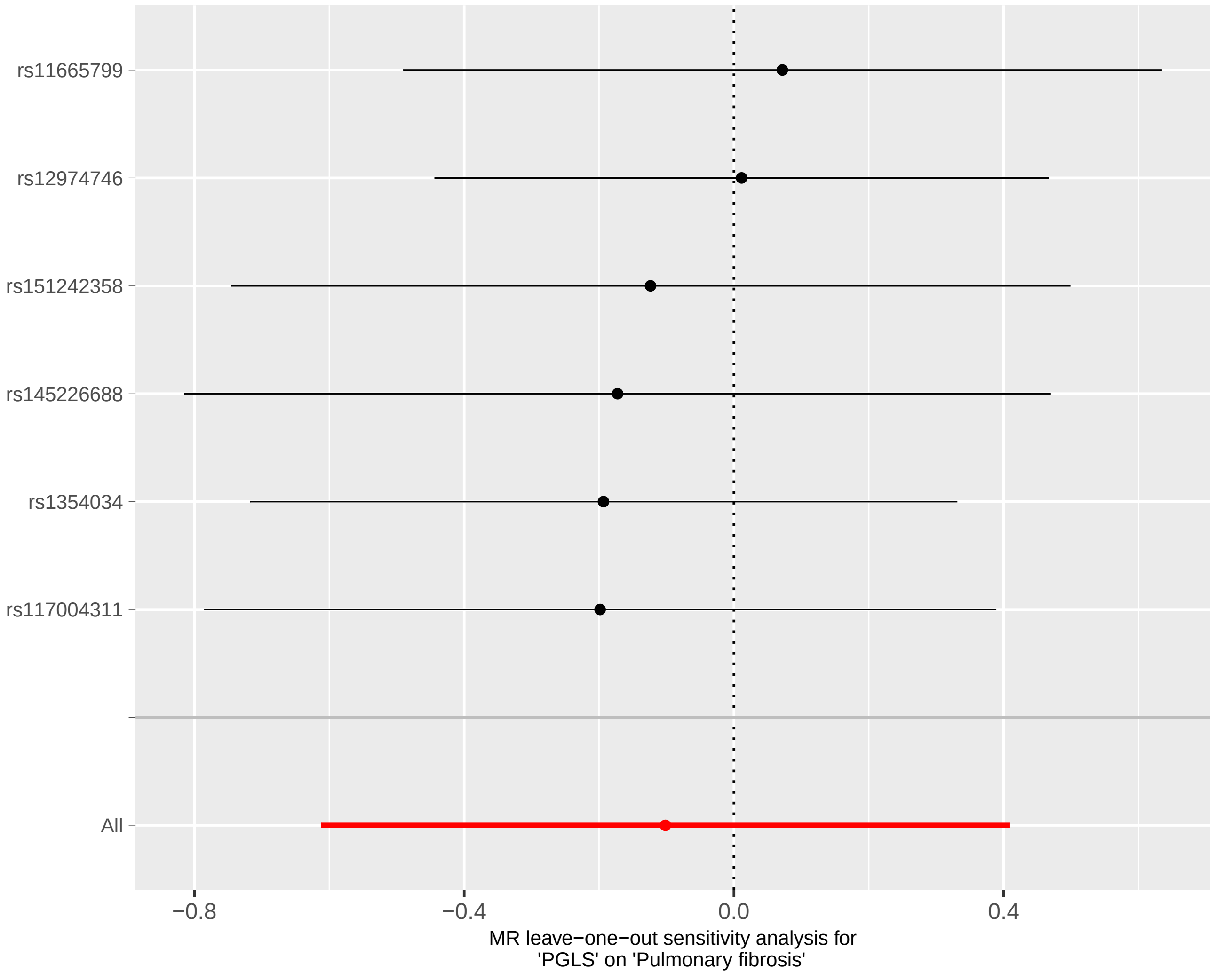
rs11665799
rs12974746
rs151242358
rs145226688
rs1354034
rs117004311
All
−0.8
−0.4
0.0
0.4
MR leave−one−out sensitivity analysis for
'PGLS' on 'Pulmonary fibrosis'

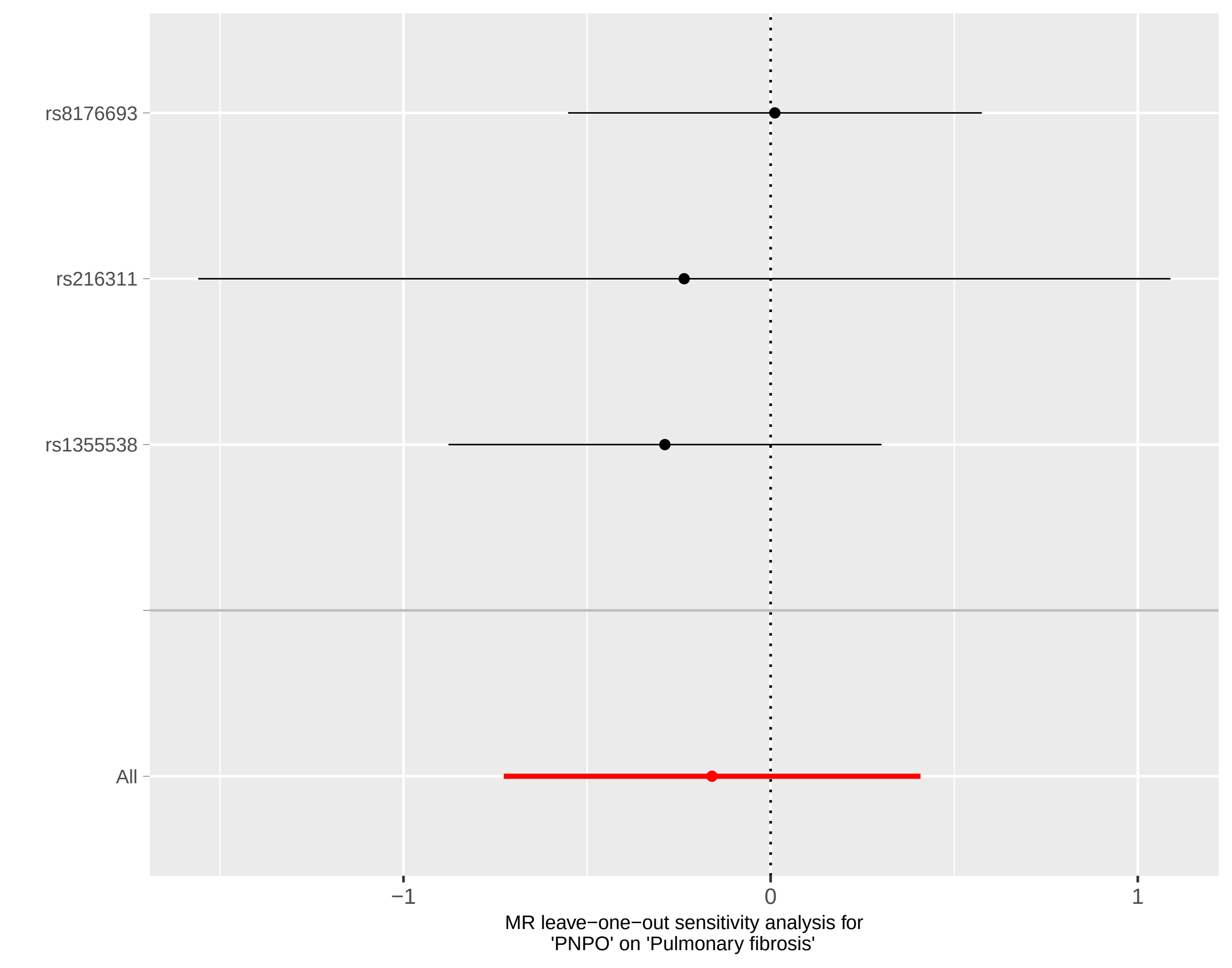
rs8176693
rs216311
rs1355538
All
−1
0
1
MR leave−one−out sensitivity analysis for
'PNPO' on 'Pulmonary fibrosis'

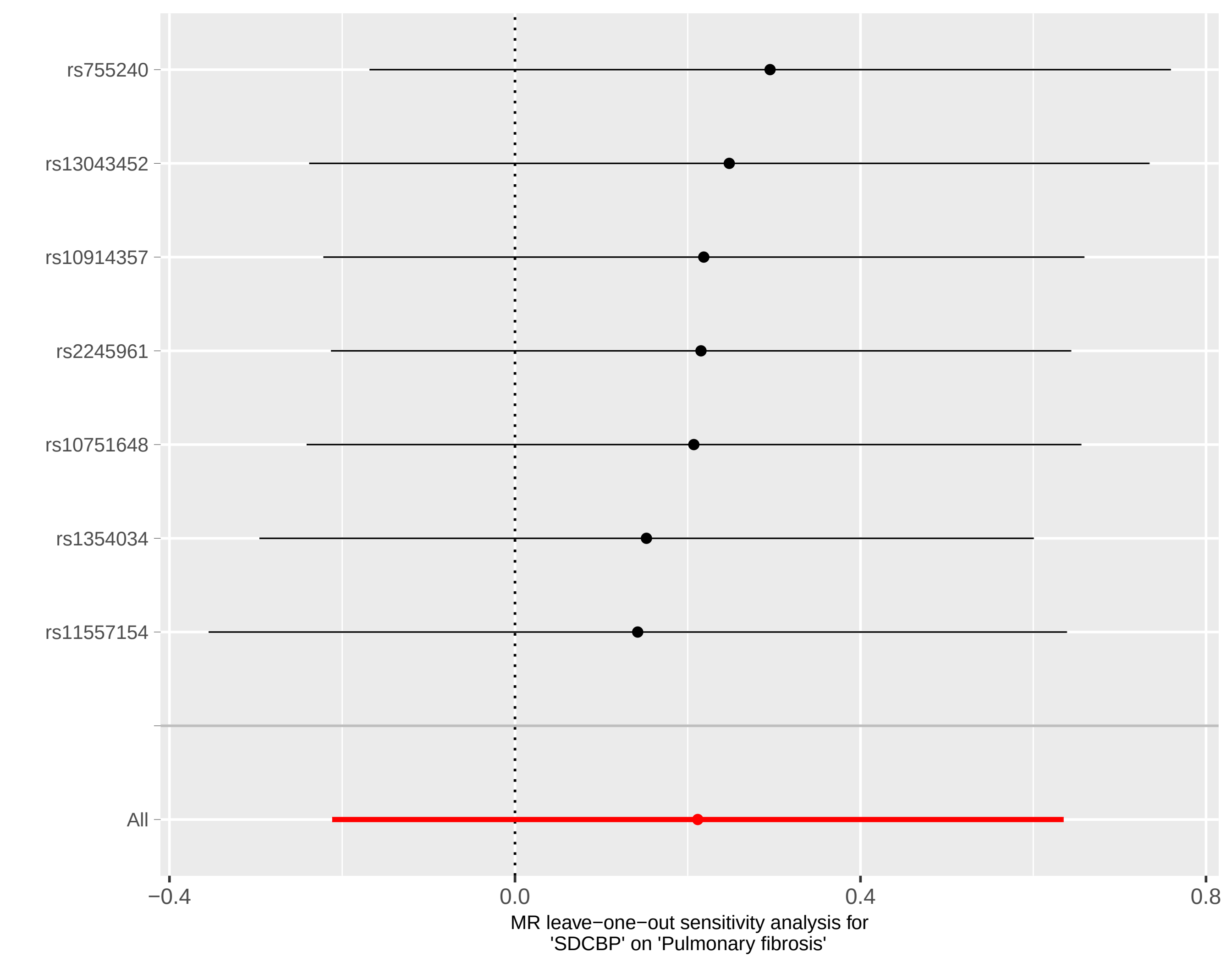
rs755240
rs13043452
rs10914357
rs2245961
rs10751648
rs1354034
rs11557154
All
−0.4
0.0
0.4
0.8
MR leave−one−out sensitivity analysis for
'SDCBP' on 'Pulmonary fibrosis'

Insufficient number of SNPs

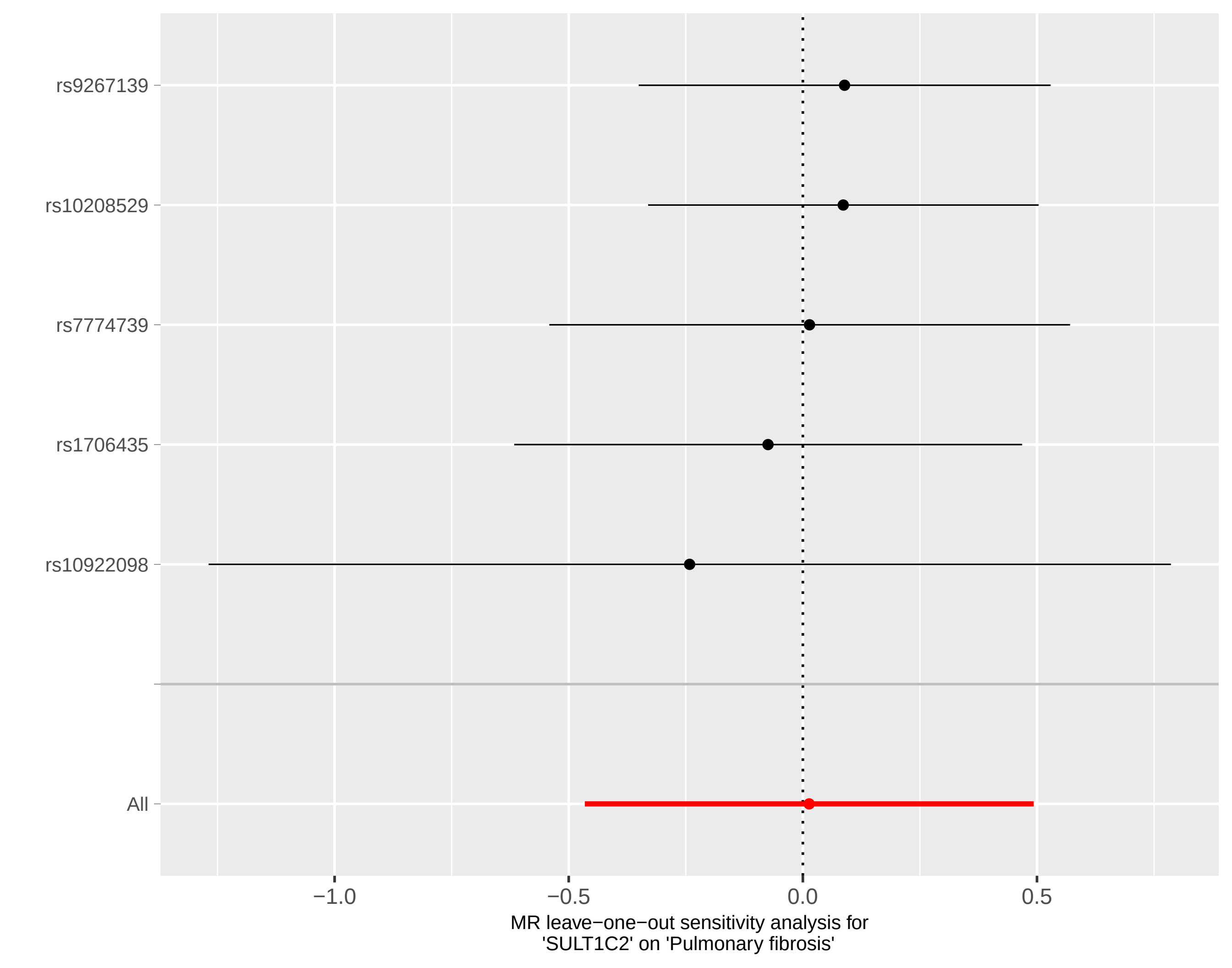
rs9267139
rs10208529
rs7774739
rs1706435
rs10922098
All
−1.0
−0.5
0.0
0.5
MR leave−one−out sensitivity analysis for
'SULT1C2' on 'Pulmonary fibrosis'

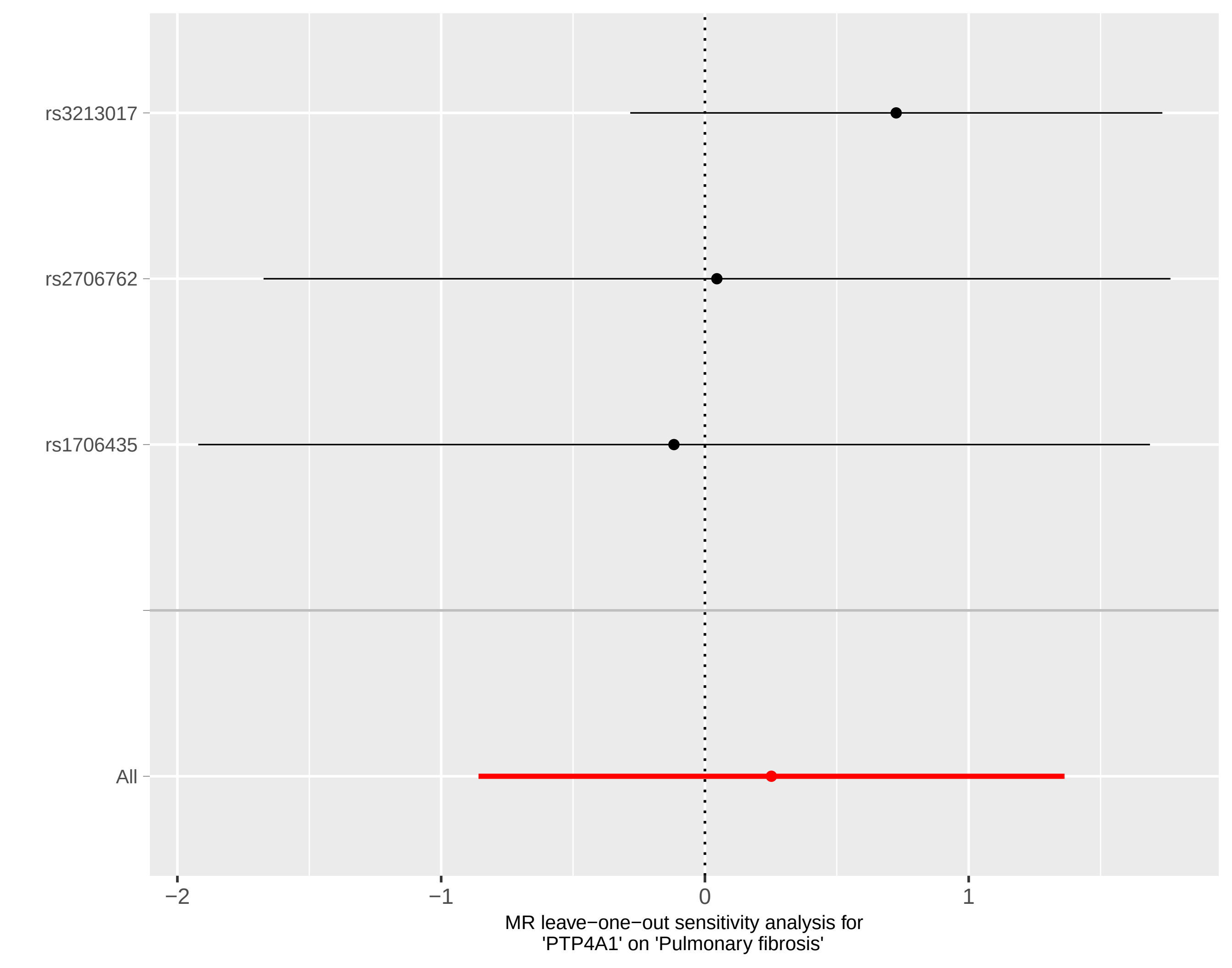

rs3213017
rs2706762
rs1706435
All
−2
−1
0
1
MR leave−one−out sensitivity analysis for
'PTP4A1' on 'Pulmonary fibrosis'

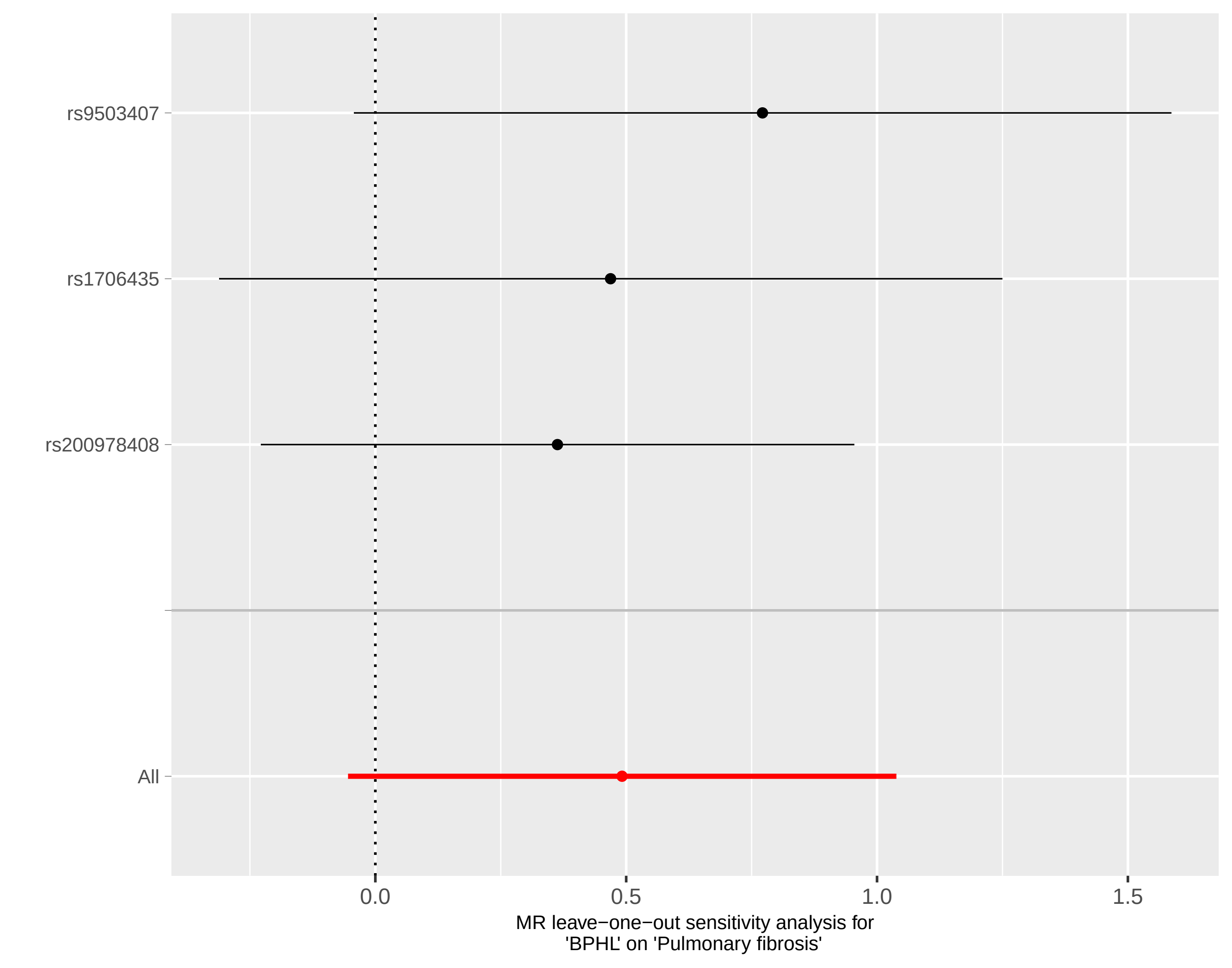
rs9503407
rs1706435
rs200978408
All
0.0
0.5
1.0
1.5
MR leave−one−out sensitivity analysis for
'BPHL' on 'Pulmonary fibrosis'

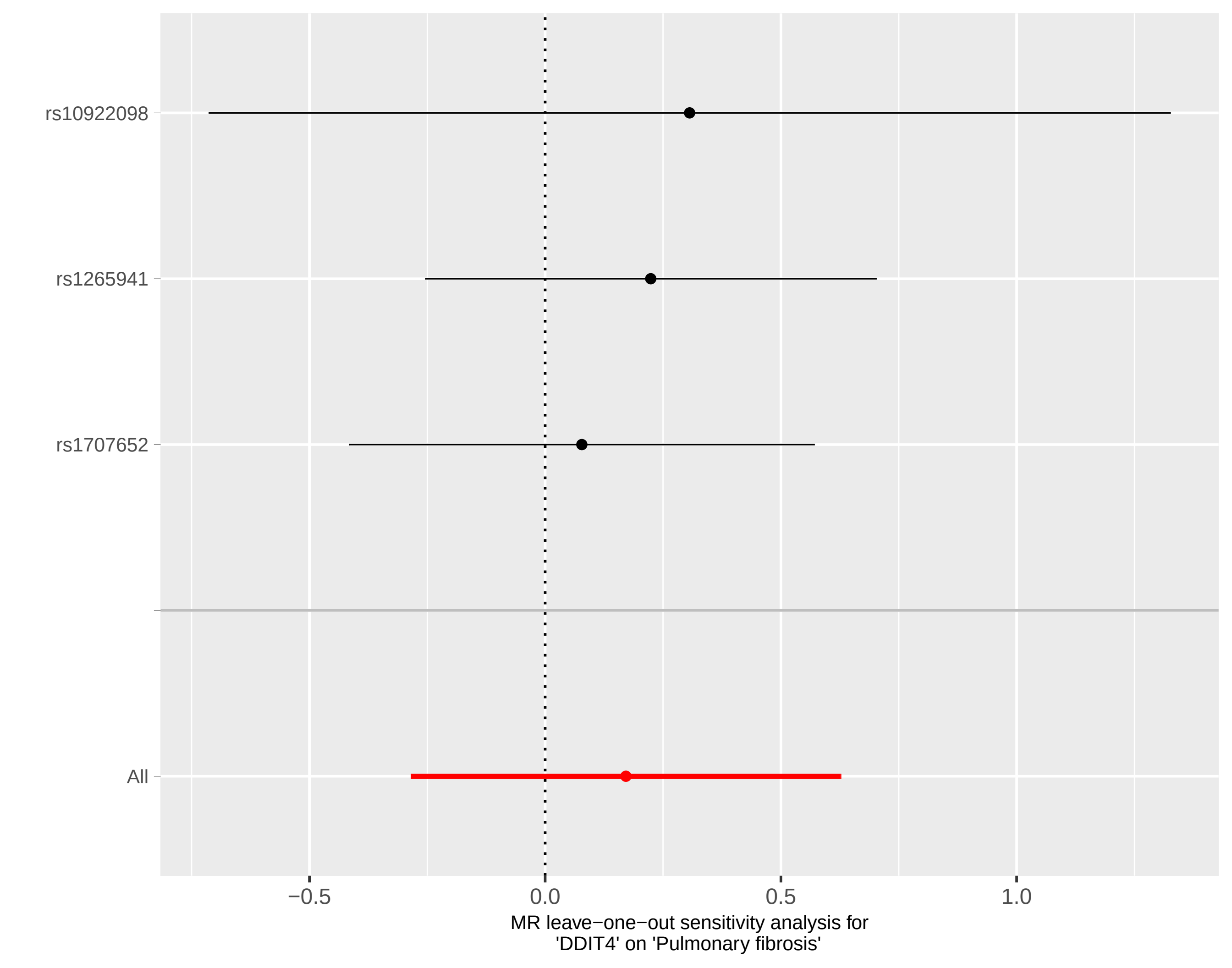
rs10922098
rs1265941
rs1707652
All
−0.5
0.0
0.5
1.0
MR leave−one−out sensitivity analysis for
'DDIT4' on 'Pulmonary fibrosis'

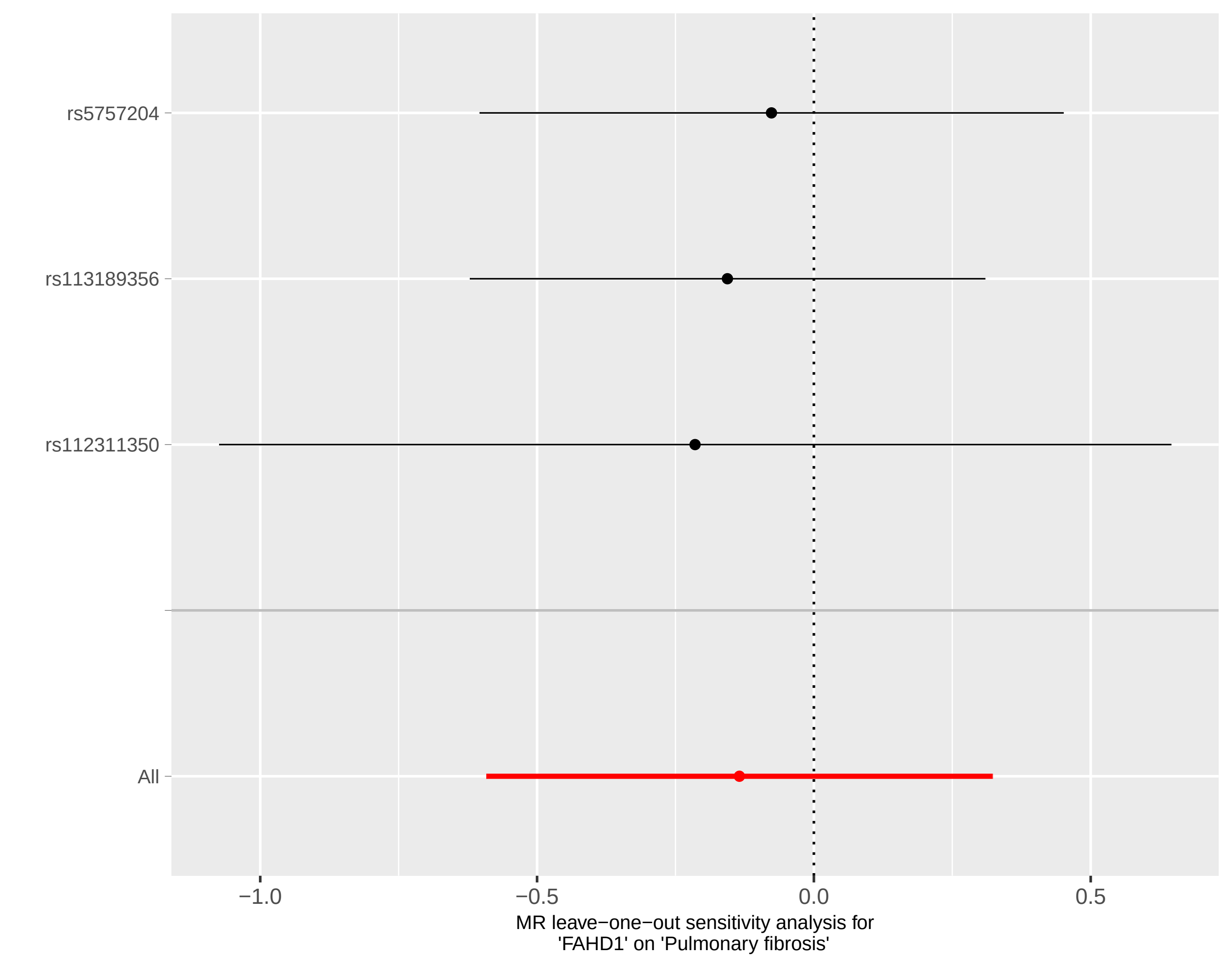
rs5757204
rs113189356
rs112311350
All
−1.0
−0.5
0.0
0.5
MR leave−one−out sensitivity analysis for
'FAHD1' on 'Pulmonary fibrosis'

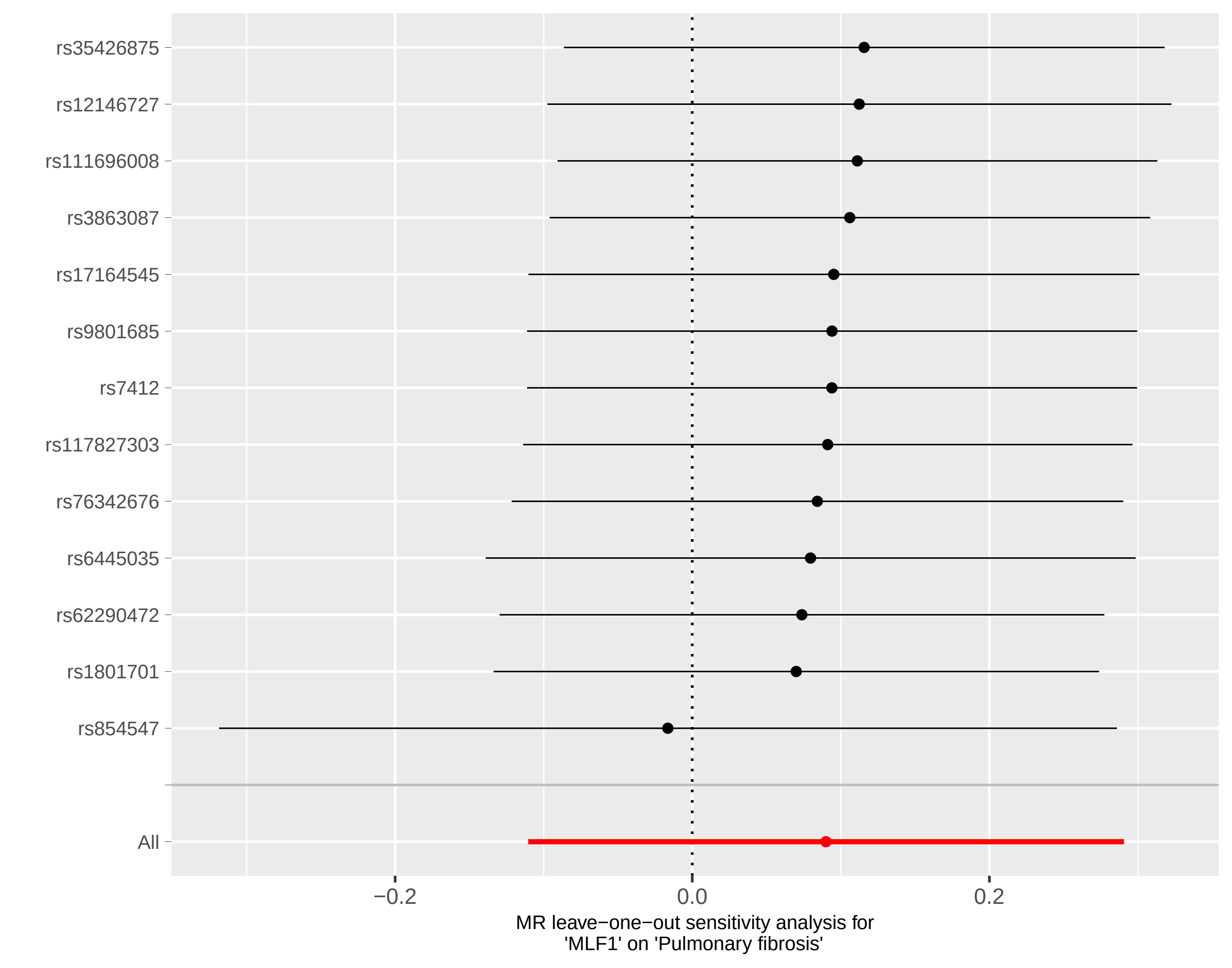
rs35426875
rs12146727
rs111696008
rs3863087
rs17164545
rs9801685
rs7412
rs117827303
rs76342676
rs6445035
rs62290472
rs1801701
rs854547
All
−0.2
0.0
0.2
MR leave−one−out sensitivity analysis for
'MLF1' on 'Pulmonary fibrosis'

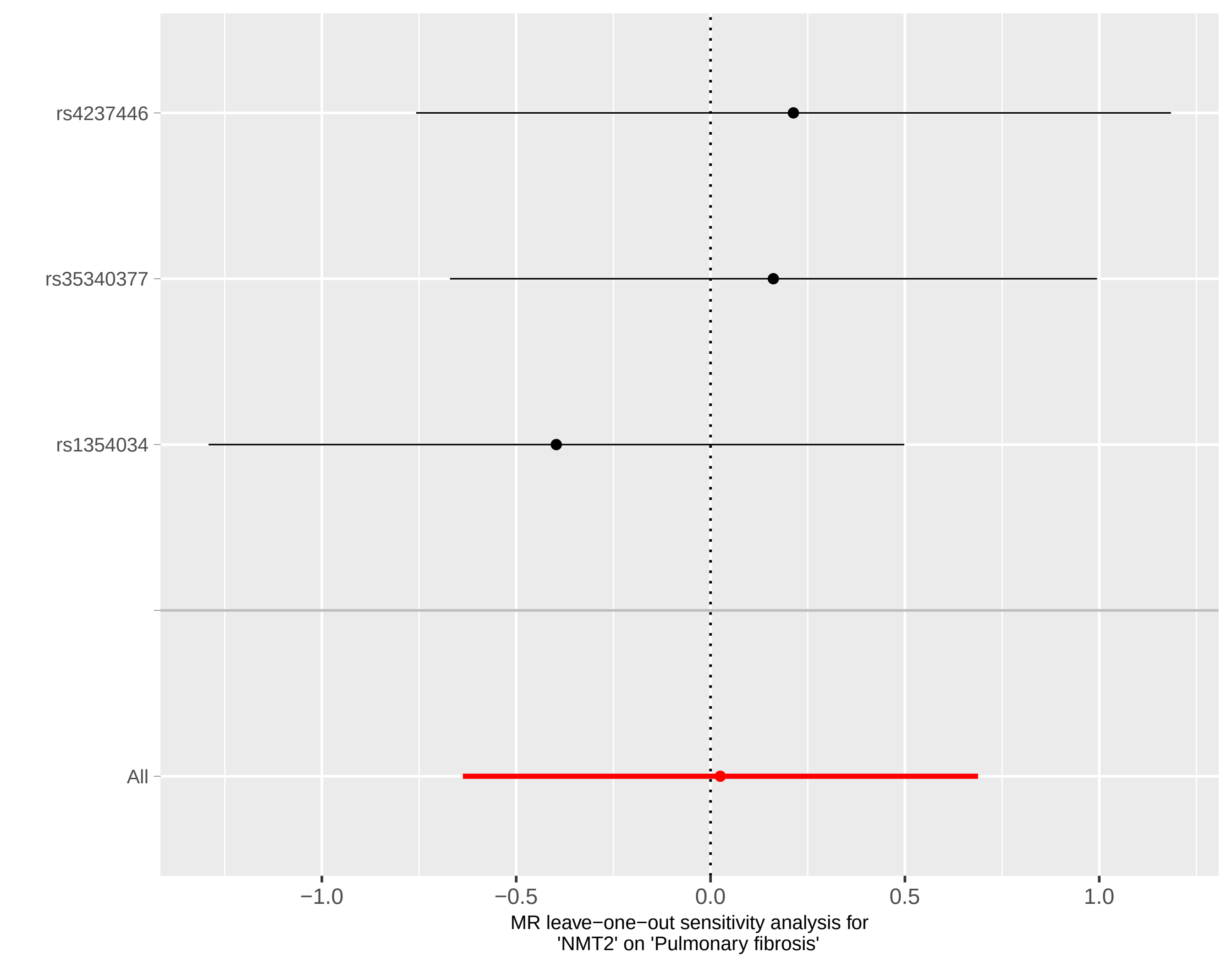
rs4237446
rs35340377
rs1354034
All
−1.0
−0.5
0.0
0.5
1.0
MR leave−one−out sensitivity analysis for
'NMT2' on 'Pulmonary fibrosis'

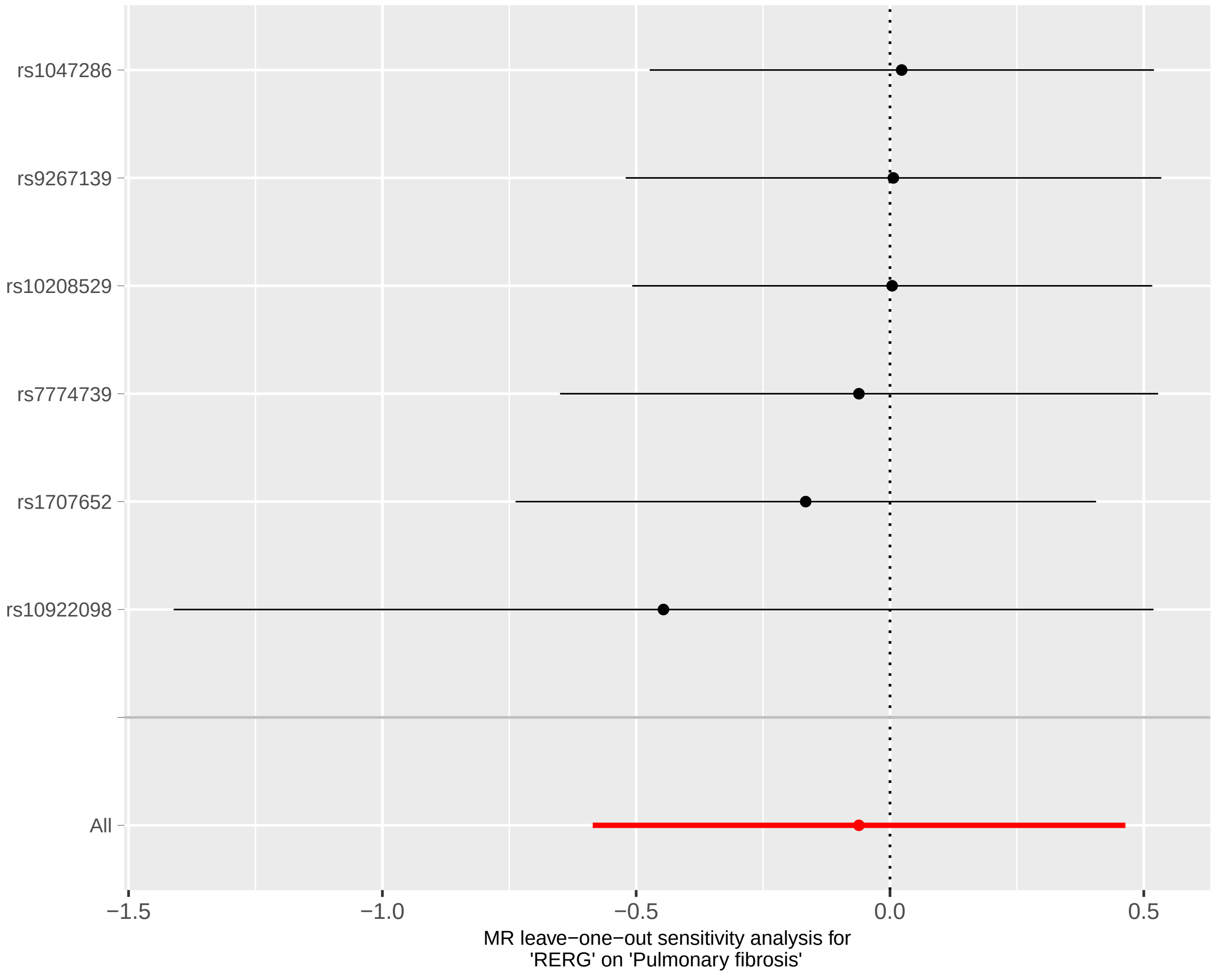

rs1047286
rs9267139
rs10208529
rs7774739
rs1707652
rs10922098
All
−1.5
−1.0
−0.5
0.0
0.5
MR leave−one−out sensitivity analysis for
'RERG' on 'Pulmonary fibrosis'

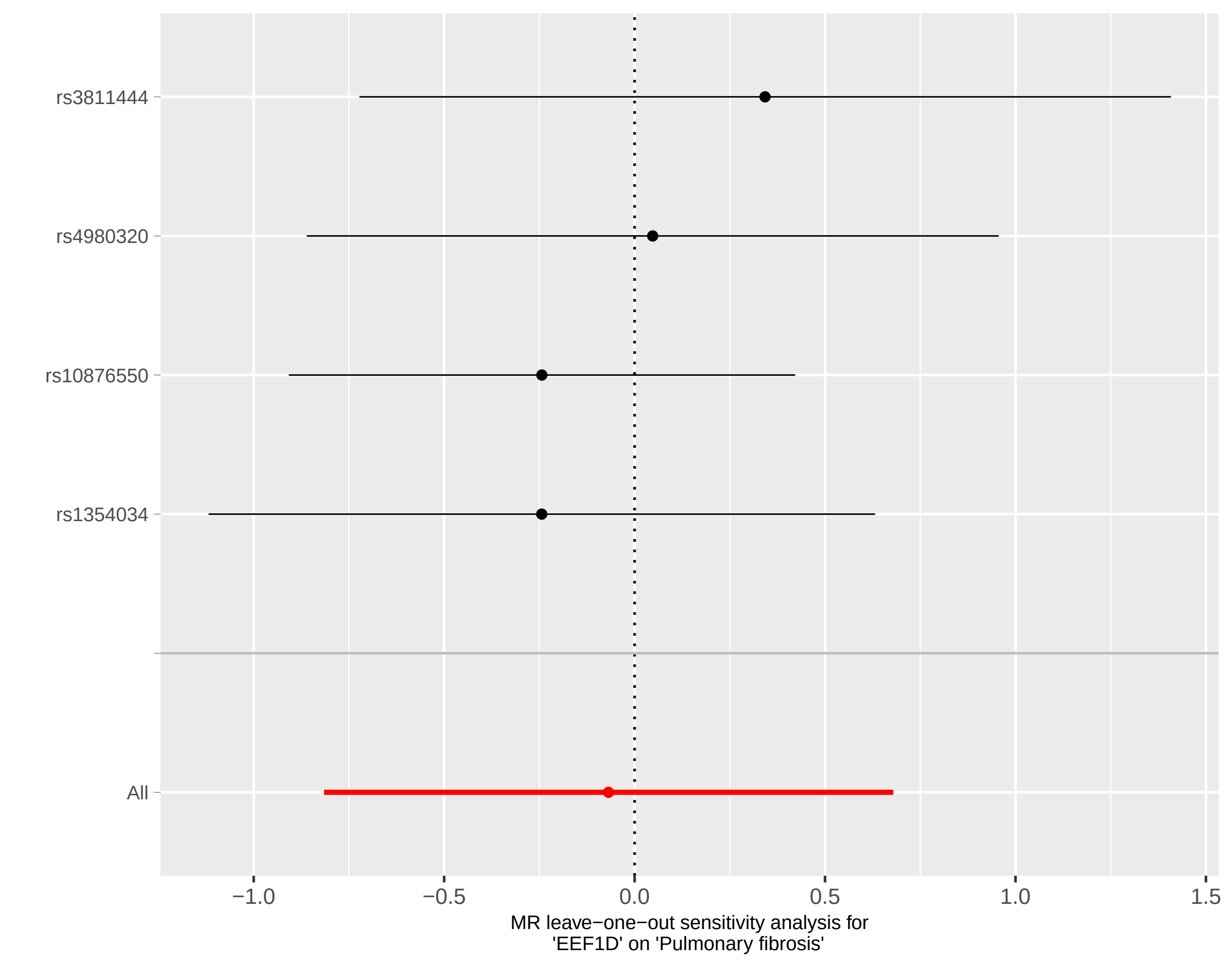

rs3811444
rs4980320
rs10876550
rs1354034
All
−1.0
−0.5
0.0
0.5
1.0
1.5
MR leave−one−out sensitivity analysis for
'EEF1D' on 'Pulmonary fibrosis'

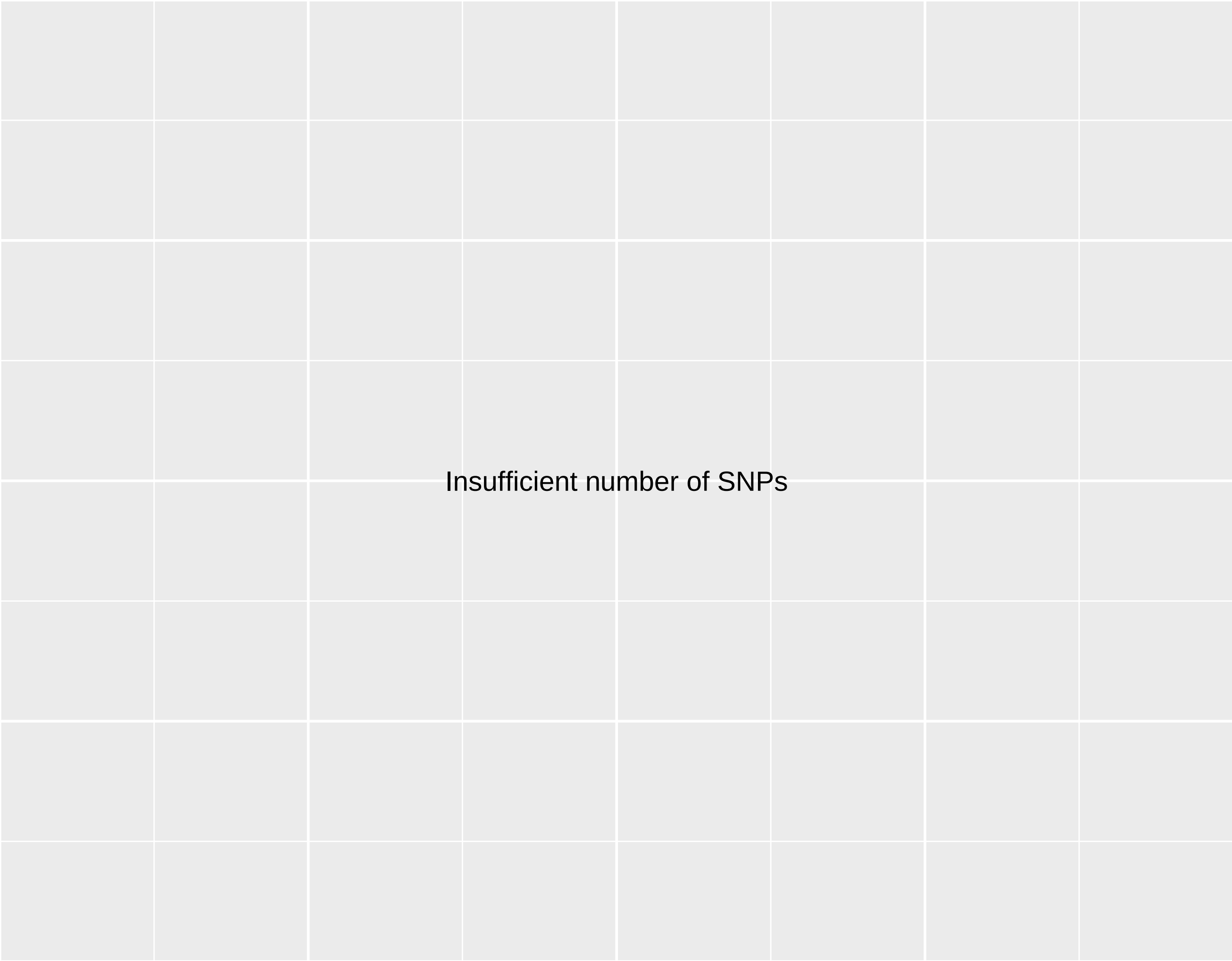
Insufficient number of SNPs

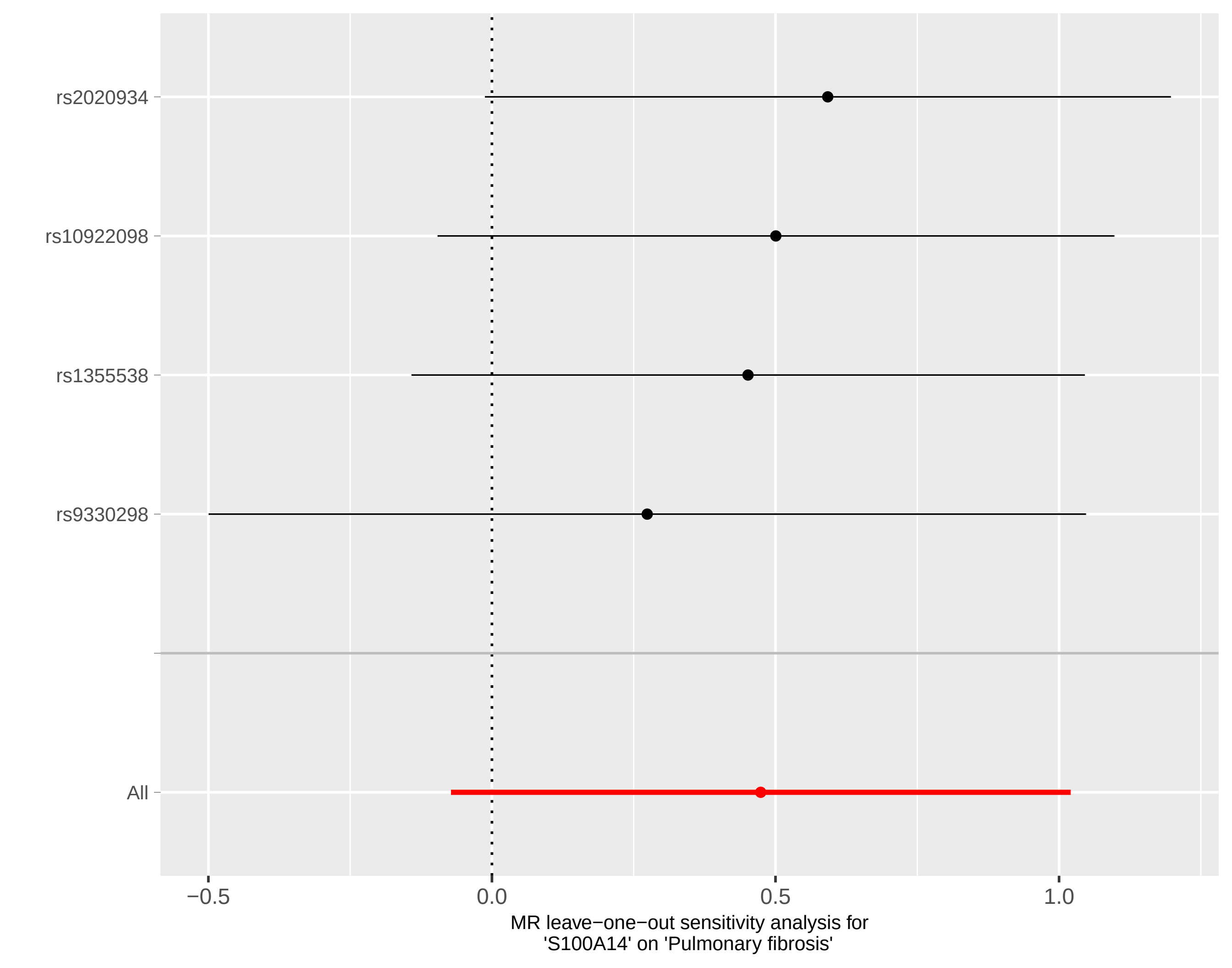

rs2020934
rs10922098
rs1355538
rs9330298
All
−0.5
0.0
0.5
1.0
MR leave−one−out sensitivity analysis for
'S100A14' on 'Pulmonary fibrosis'

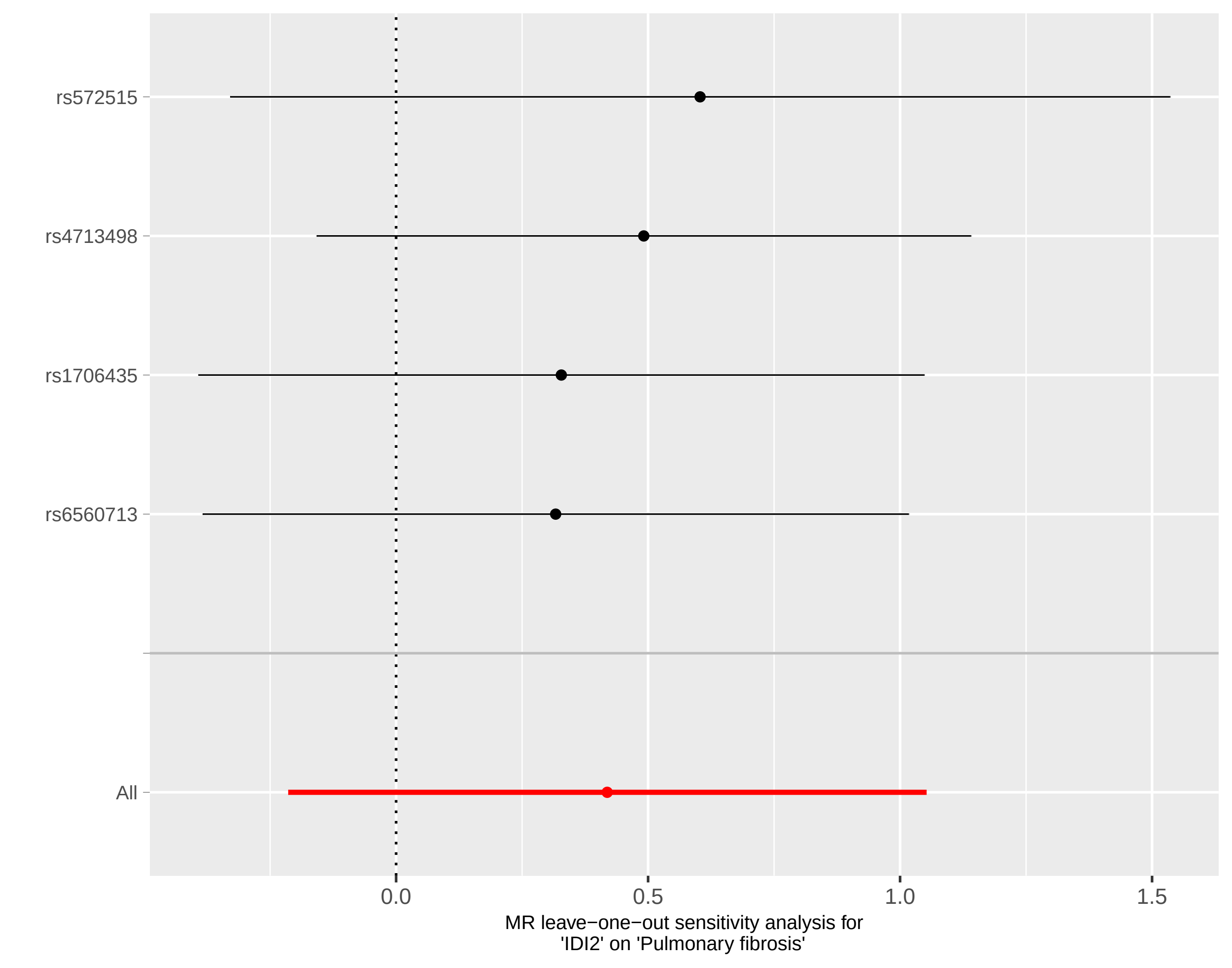

rs572515
rs4713498
rs1706435
rs6560713
All
0.0
0.5
1.0
1.5
MR leave−one−out sensitivity analysis for
'IDI2' on 'Pulmonary fibrosis'

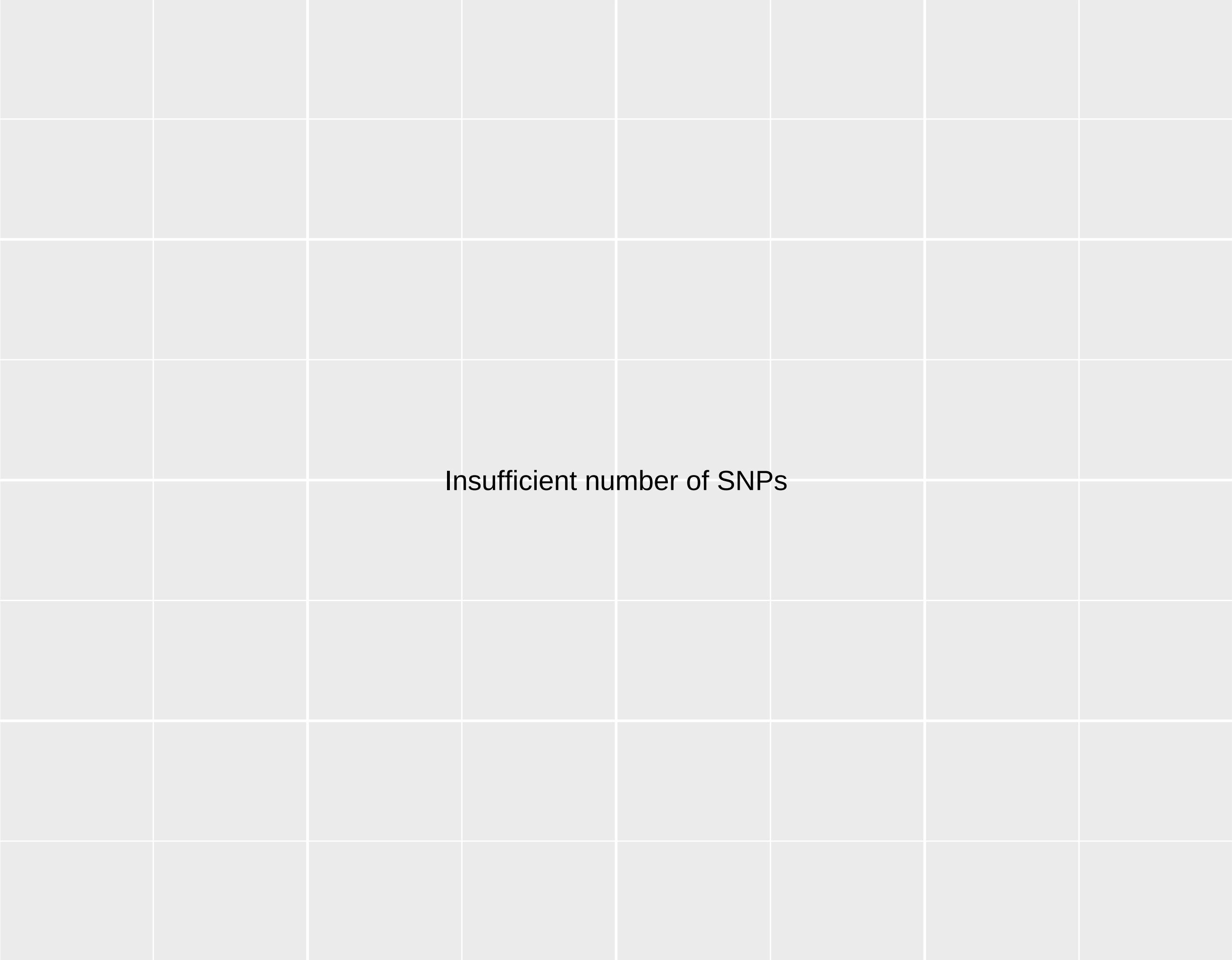
Insufficient number of SNPs

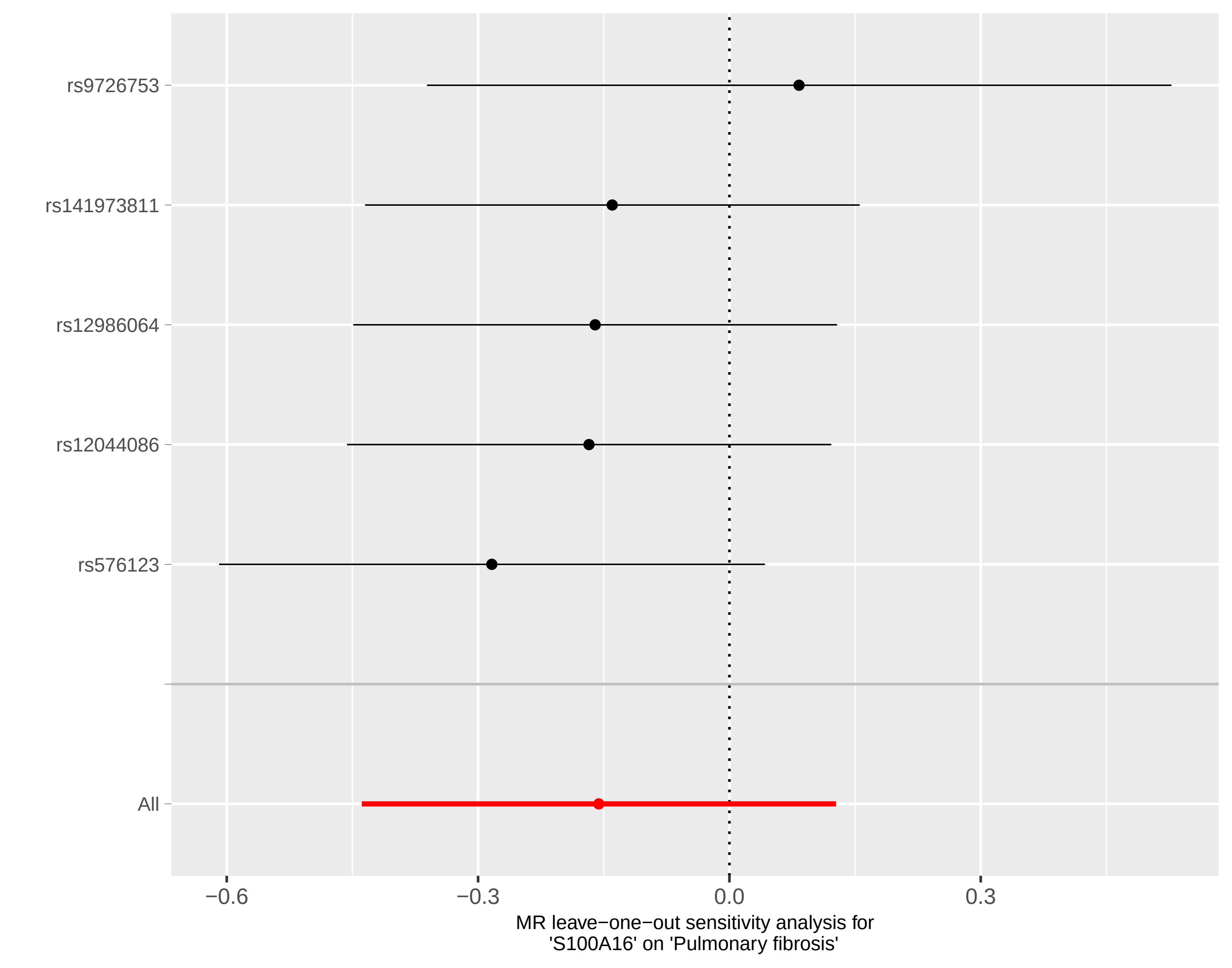

rs9726753
rs141973811
rs12986064
rs12044086
rs576123
All
−0.6
−0.3
0.0
0.3
MR leave−one−out sensitivity analysis for
'S100A16' on 'Pulmonary fibrosis'

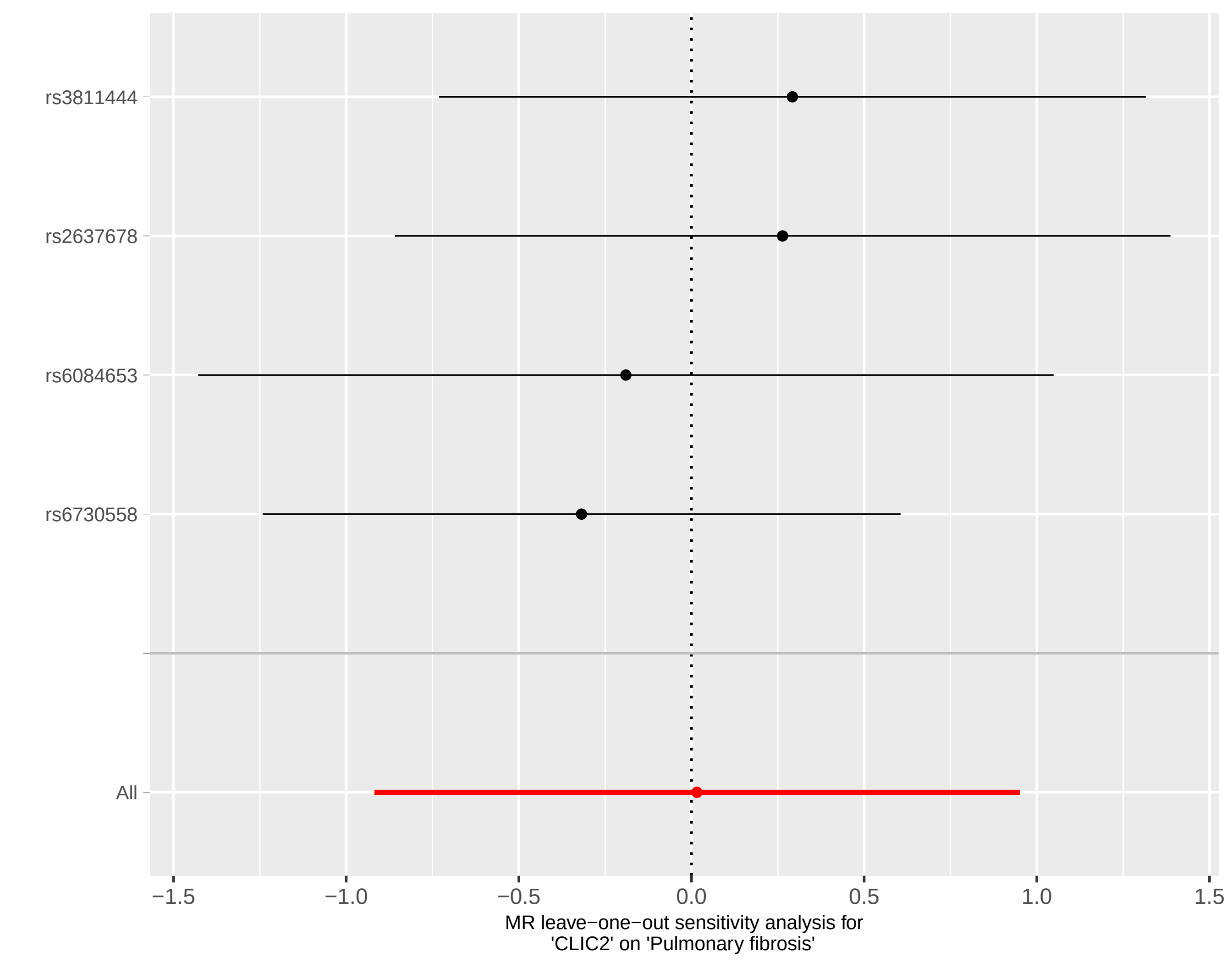
rs3811444
rs2637678
rs6084653
rs6730558
All
−1.5
−1.0
−0.5
0.0
0.5
1.0
1.5
MR leave−one−out sensitivity analysis for
'CLIC2' on 'Pulmonary fibrosis'

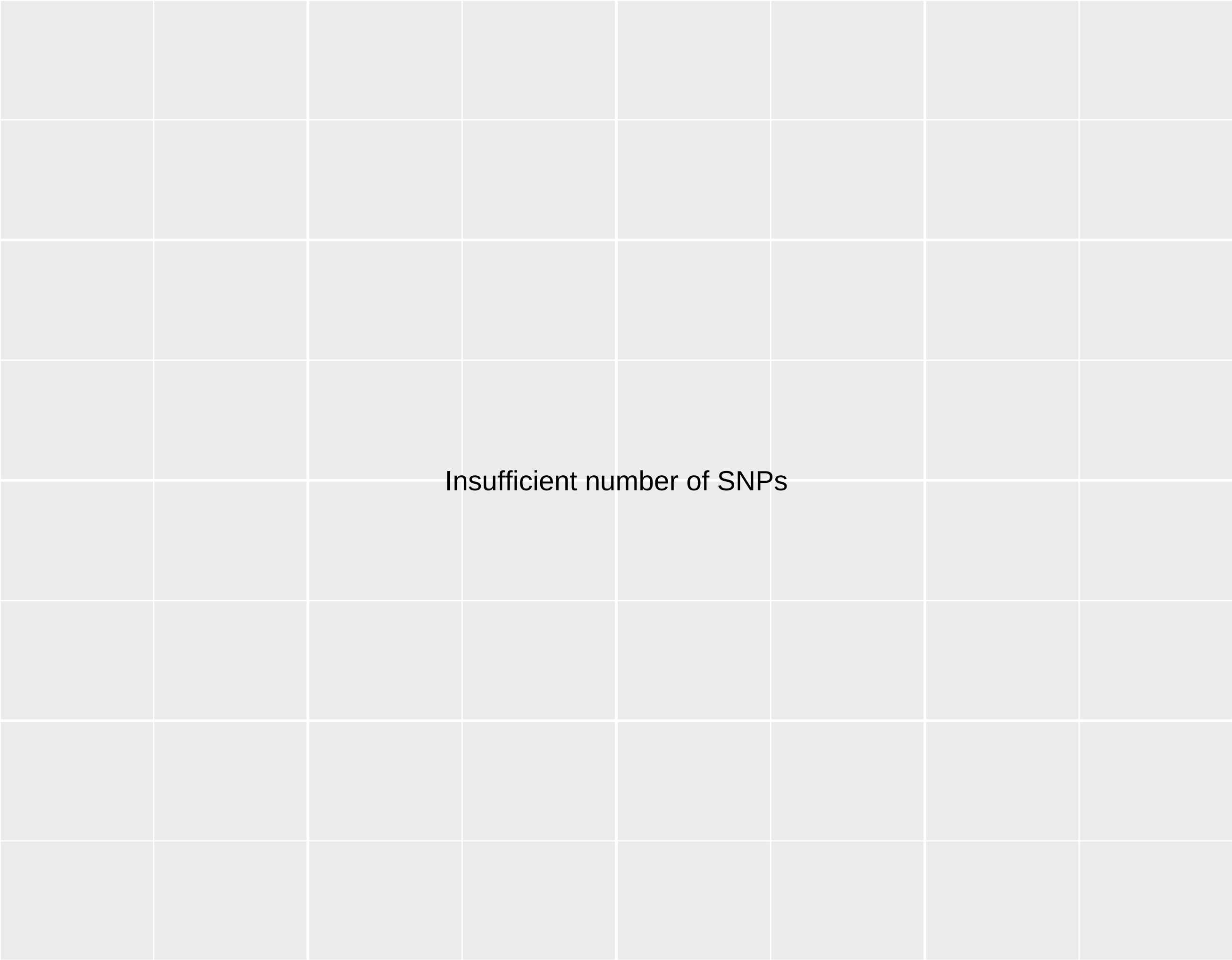
Insufficient number of SNPs

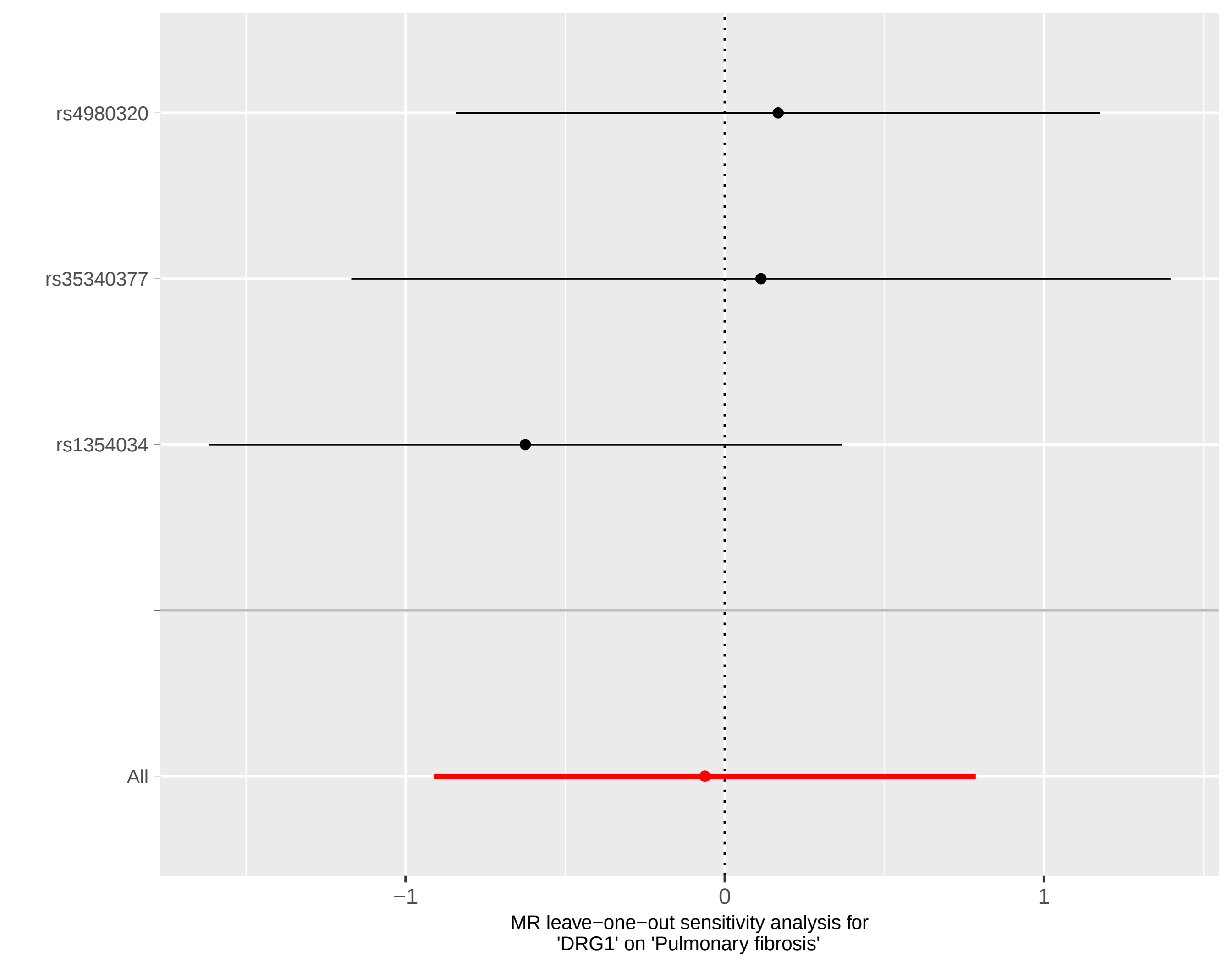
rs4980320
rs35340377
rs1354034
All
−1
0
1
MR leave−one−out sensitivity analysis for
'DRG1' on 'Pulmonary fibrosis'

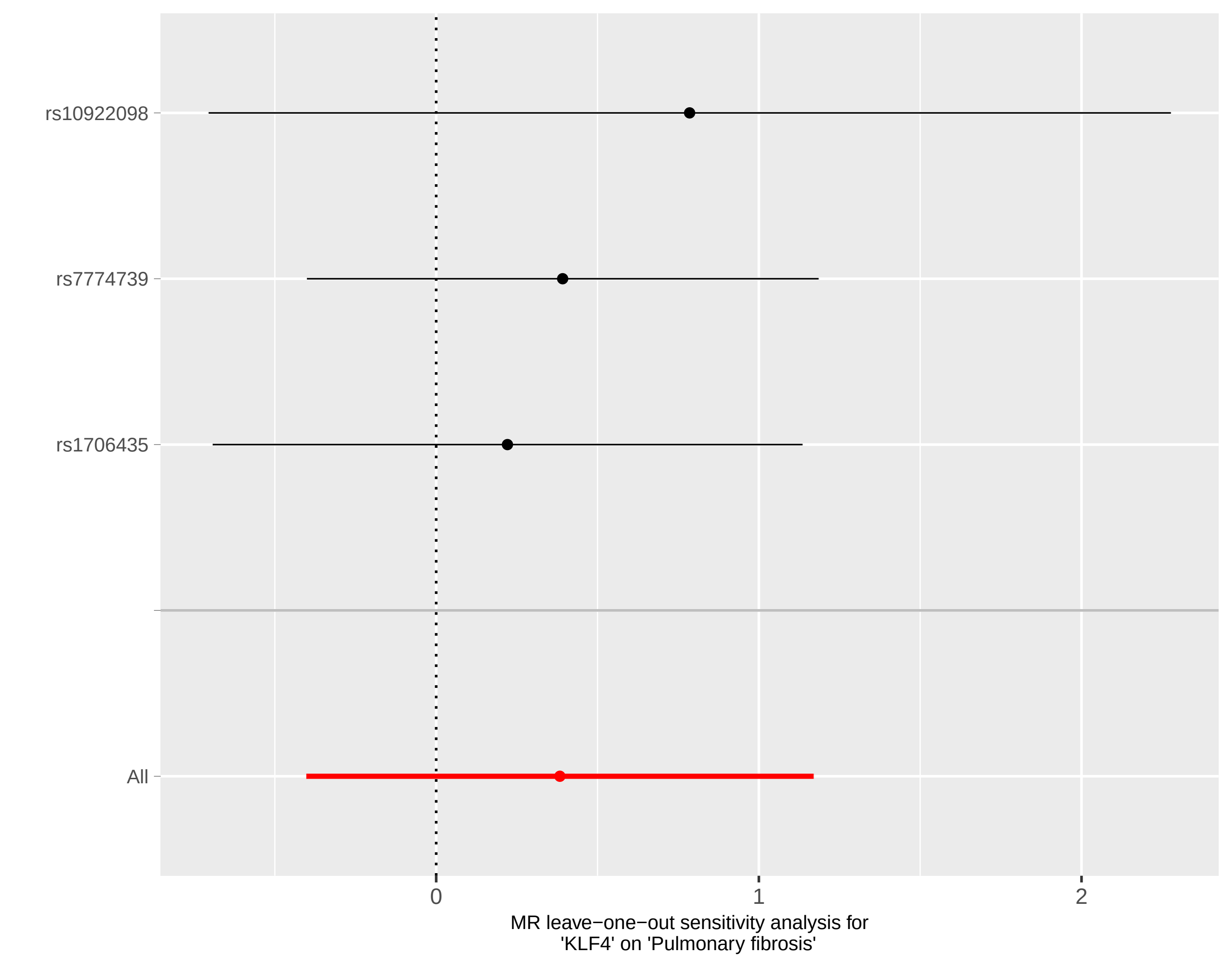
rs10922098
rs7774739
rs1706435
All
0
1
2
MR leave−one−out sensitivity analysis for
'KLF4' on 'Pulmonary fibrosis'

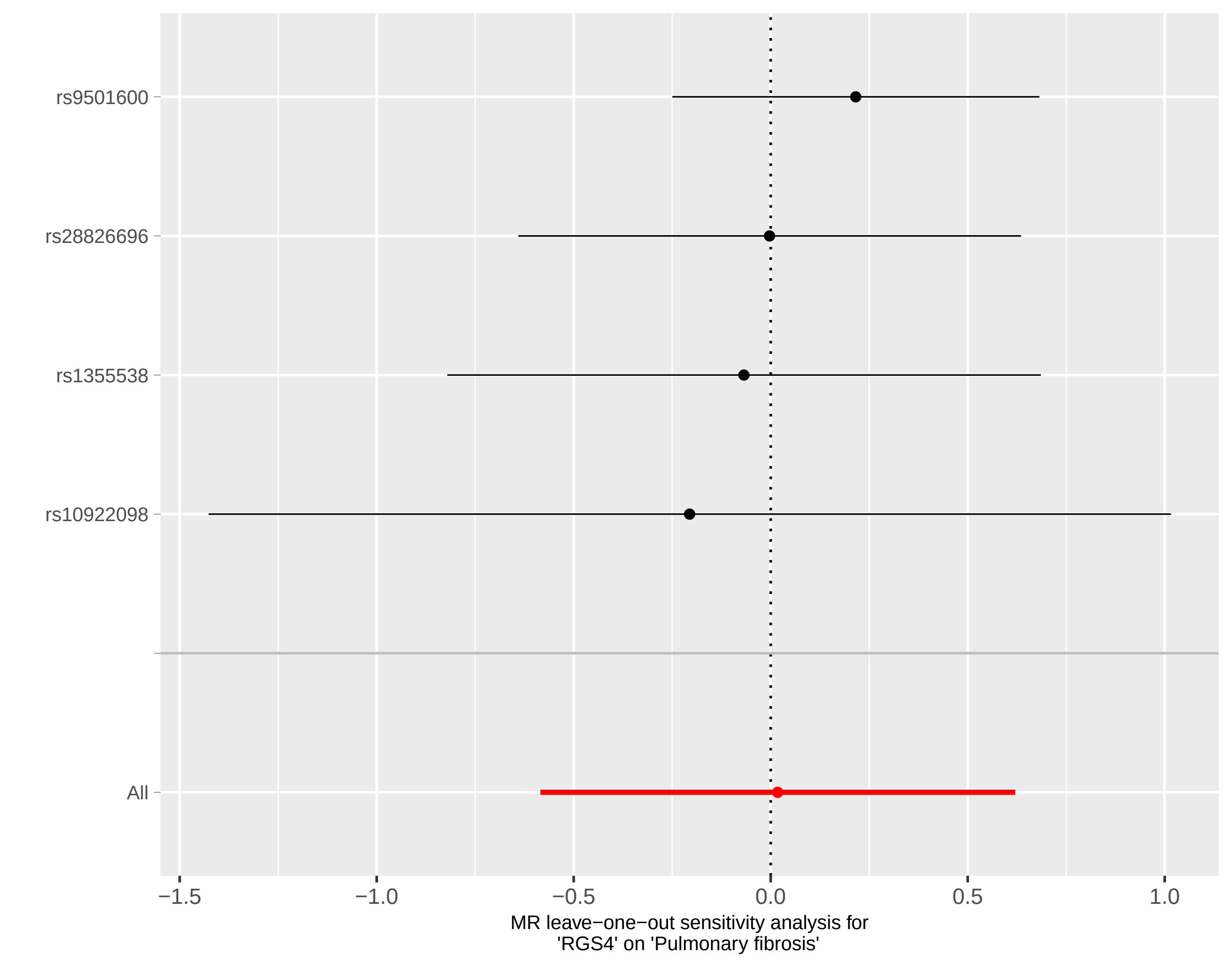
rs9501600
rs28826696
rs1355538
rs10922098
All
−1.5
−1.0
−0.5
0.0
0.5
1.0
MR leave−one−out sensitivity analysis for
'RGS4' on 'Pulmonary fibrosis'

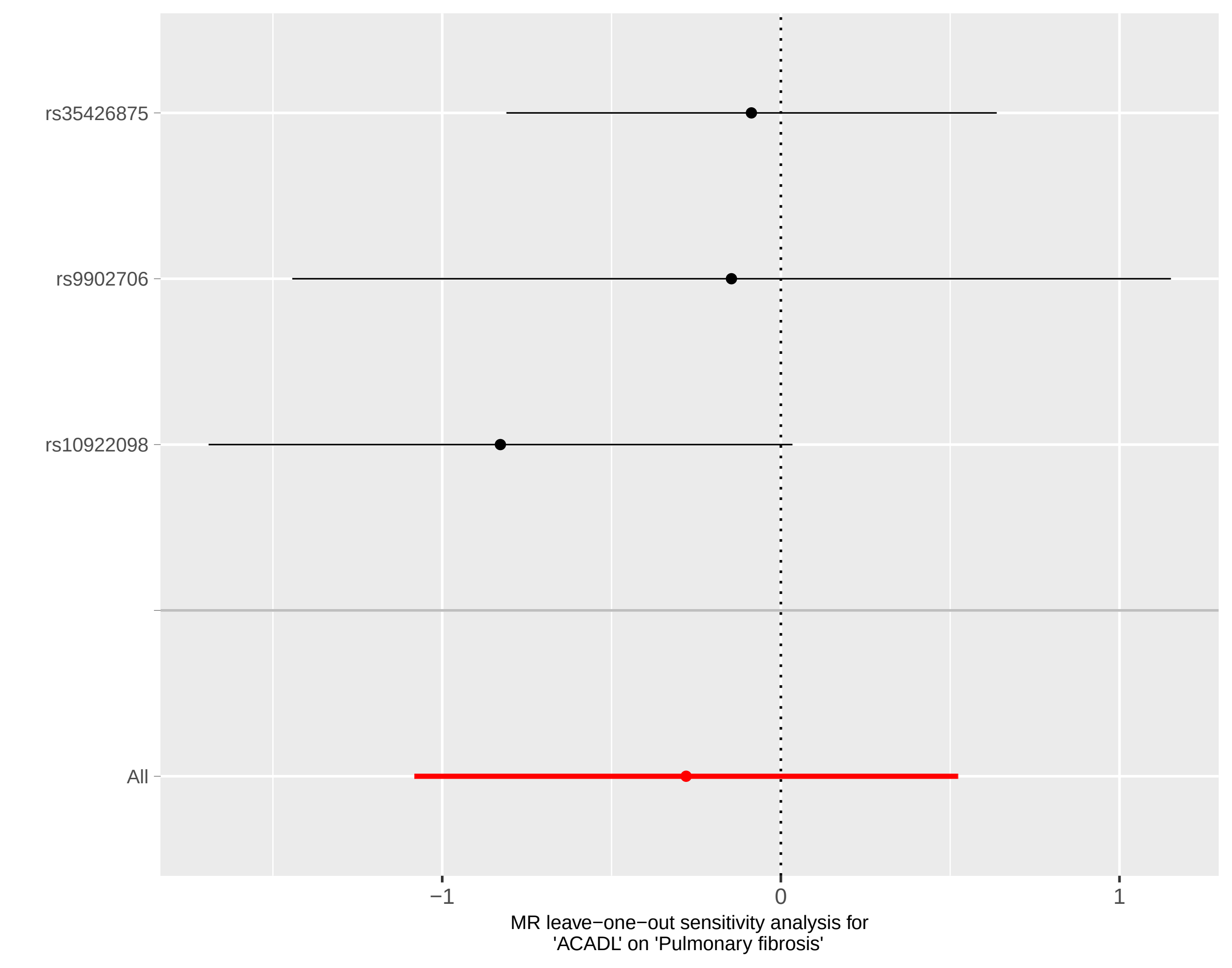

rs35426875
rs9902706
rs10922098
All
−1
0
1
MR leave−one−out sensitivity analysis for
'ACADL' on 'Pulmonary fibrosis'

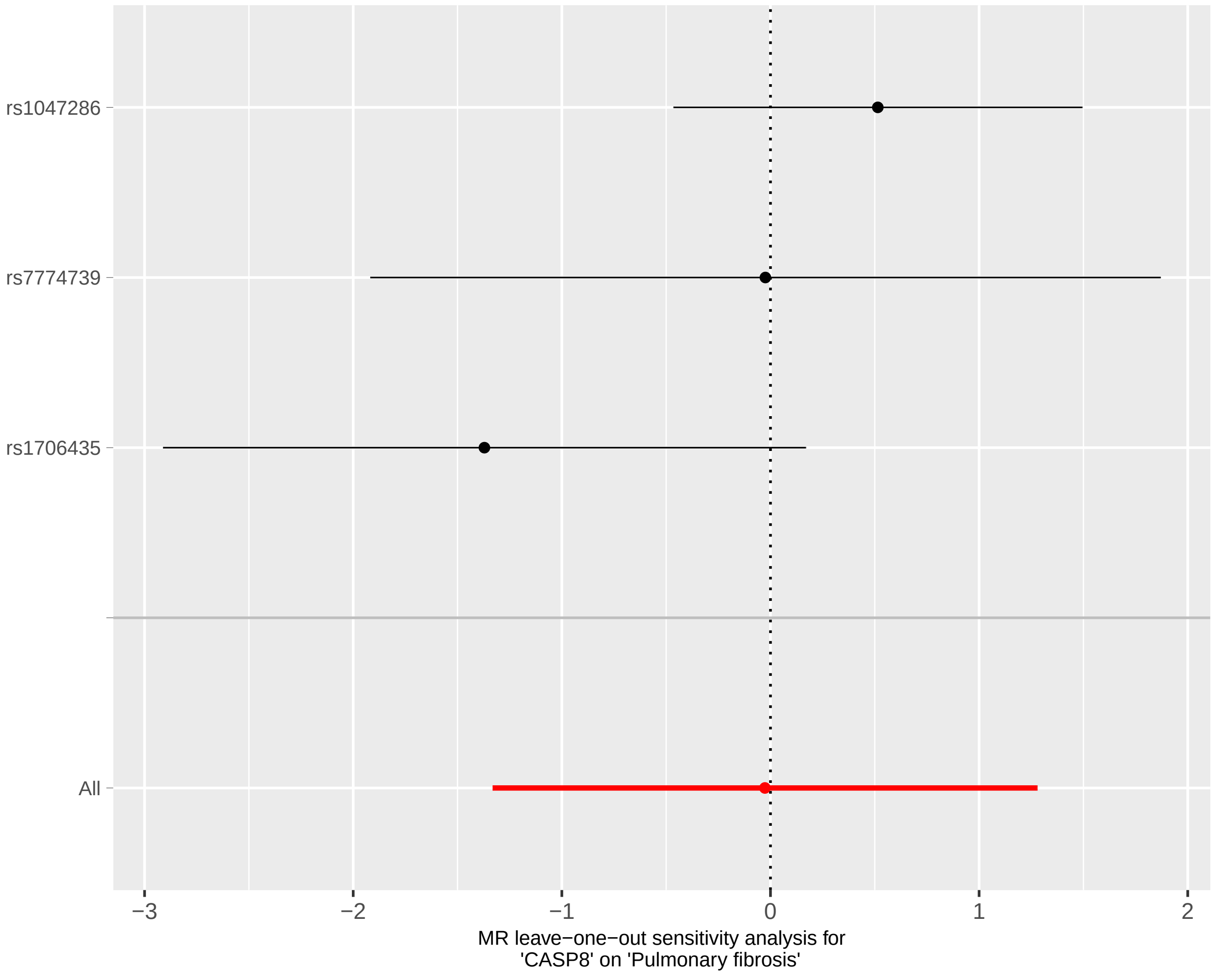

rs1047286
rs7774739
rs1706435
All
−3
−2
−1
0
1
2
MR leave−one−out sensitivity analysis for
'CASP8' on 'Pulmonary fibrosis'

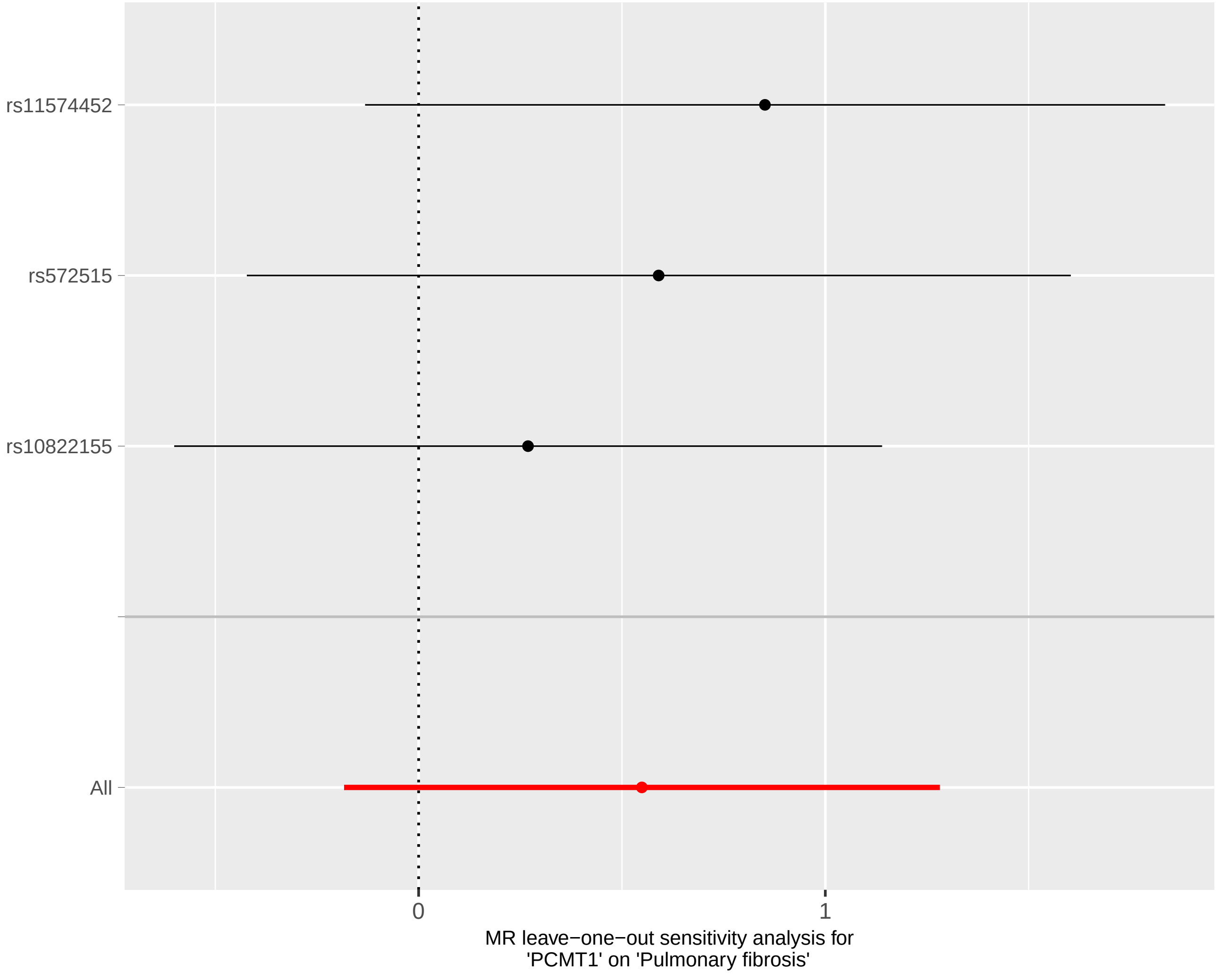
rs11574452
rs572515
rs10822155
All
0
1
MR leave−one−out sensitivity analysis for
'PCMT1' on 'Pulmonary fibrosis'

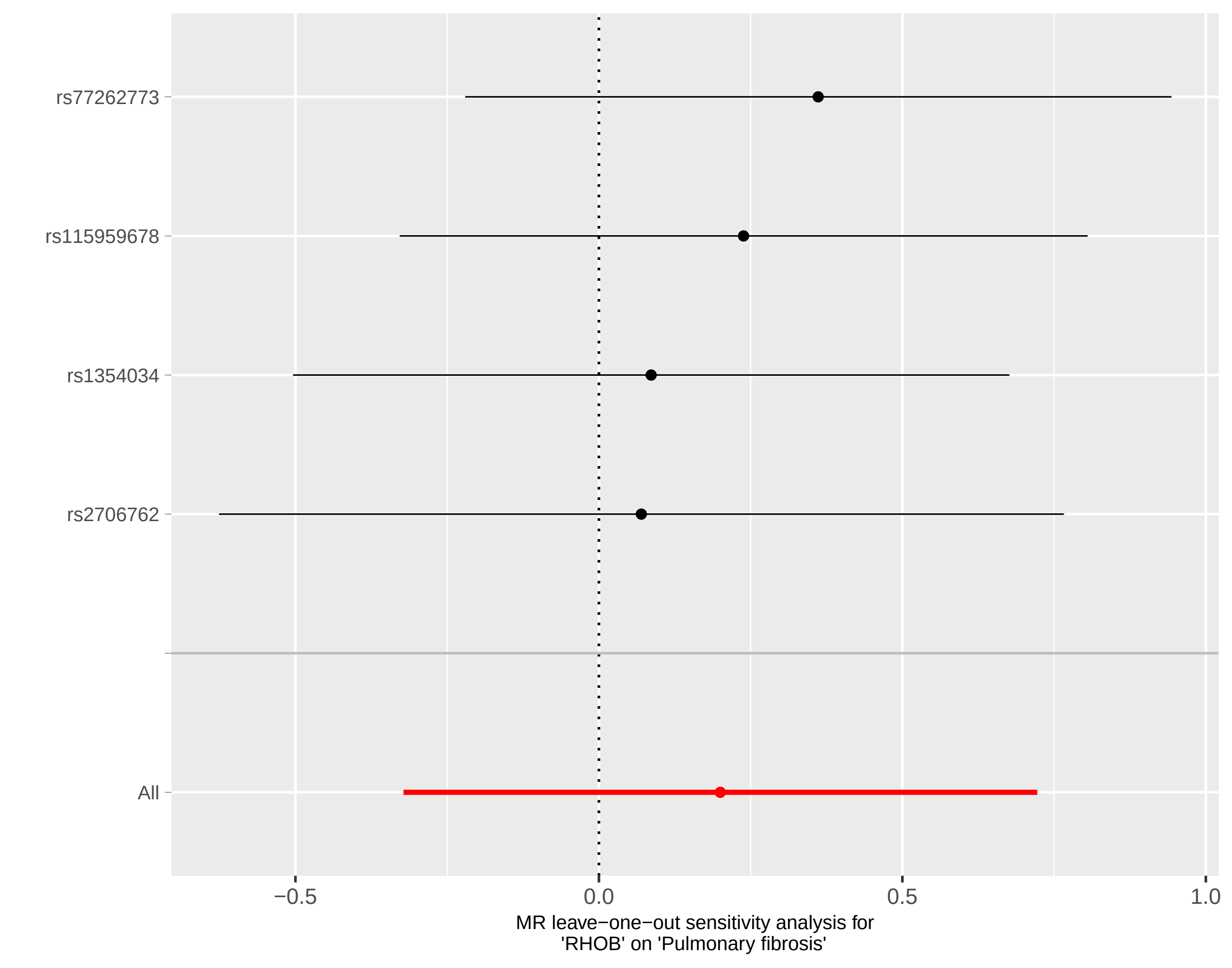

rs77262773
rs115959678
rs1354034
rs2706762
All
−0.5
0.0
0.5
1.0
MR leave−one−out sensitivity analysis for
'RHOB' on 'Pulmonary fibrosis'

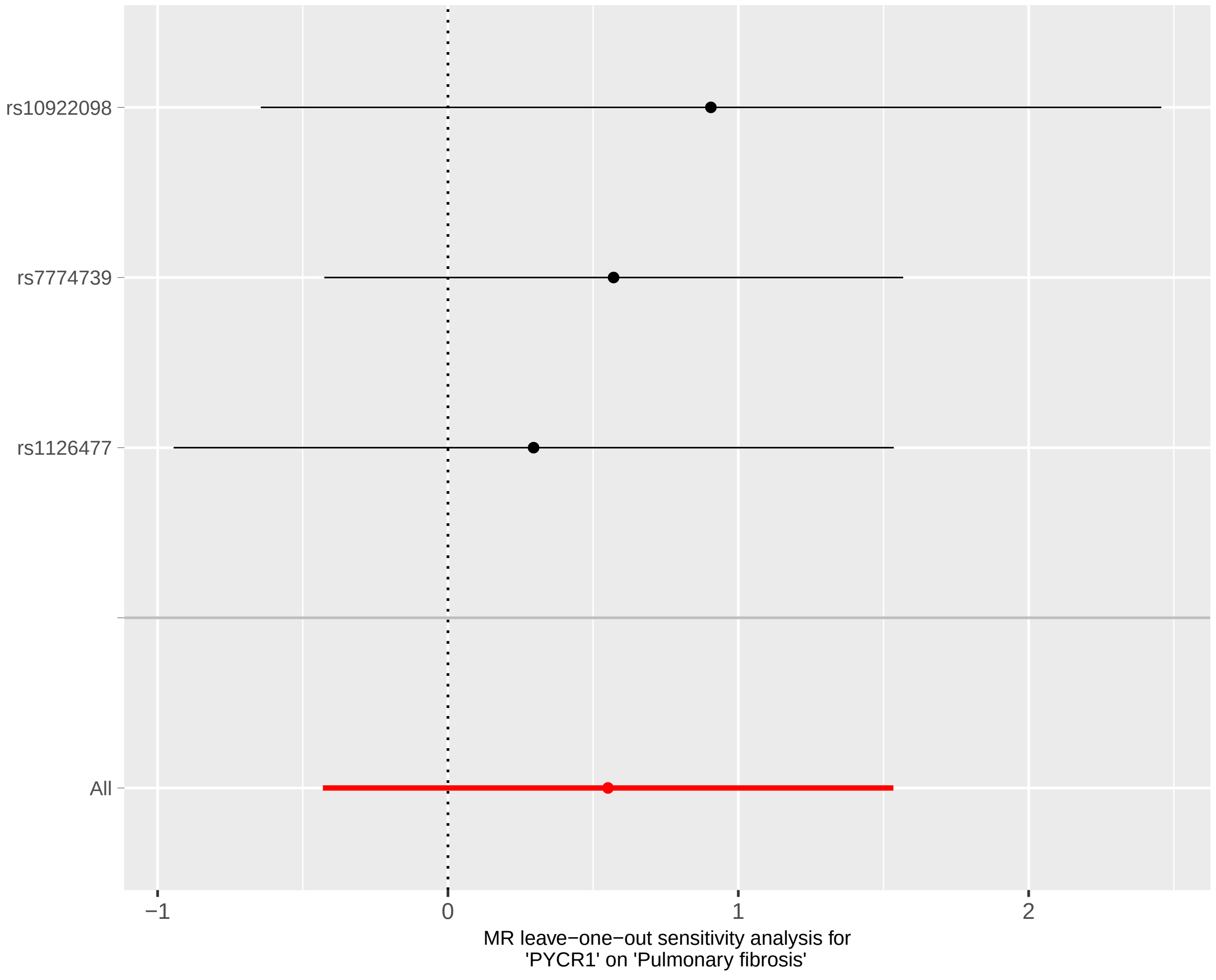
rs10922098
rs7774739
rs1126477
All
−1
0
1
2
MR leave−one−out sensitivity analysis for
'PYCR1' on 'Pulmonary fibrosis'

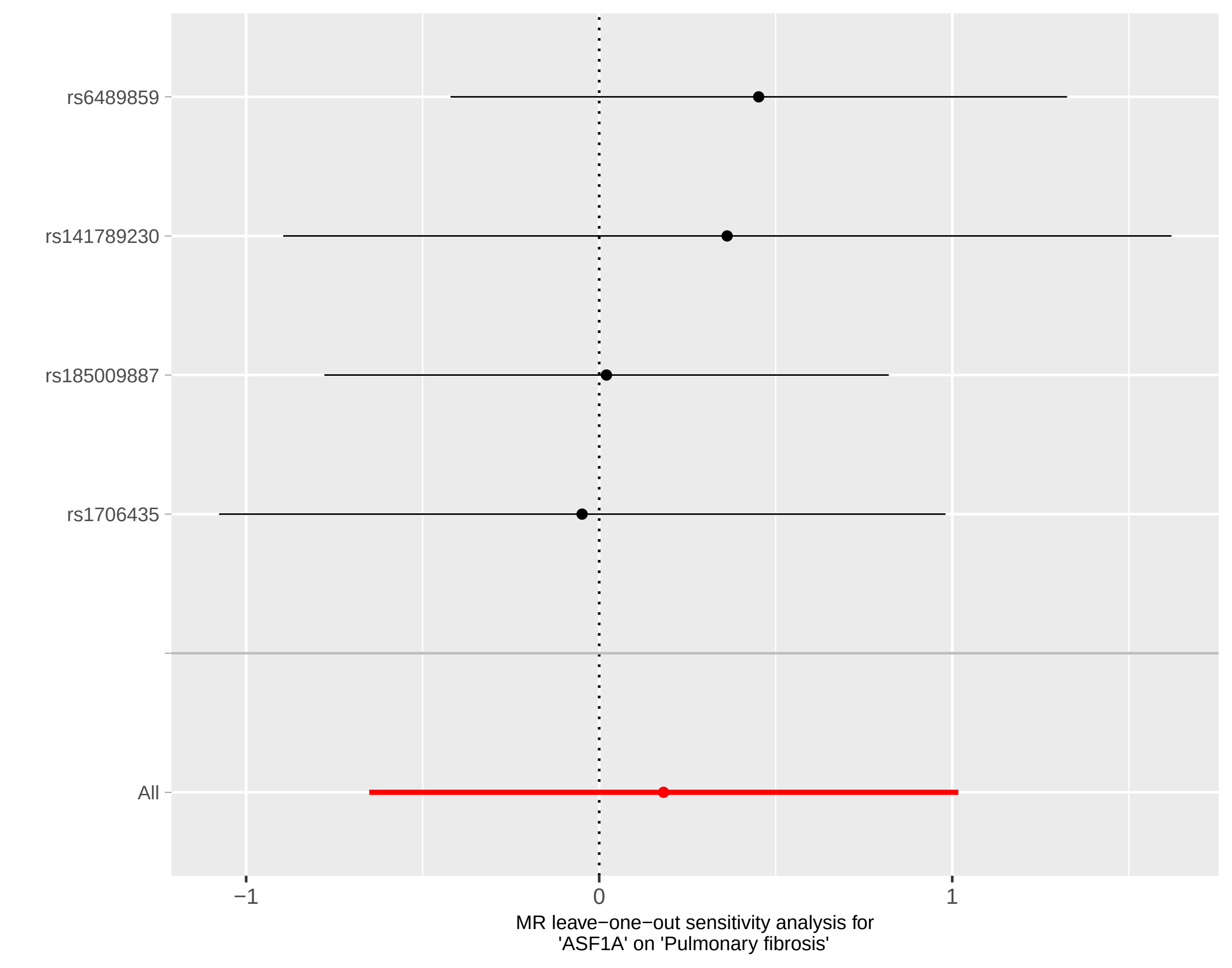

rs6489859
rs141789230
rs185009887
rs1706435
All
−1
0
1
MR leave−one−out sensitivity analysis for
'ASF1A' on 'Pulmonary fibrosis'

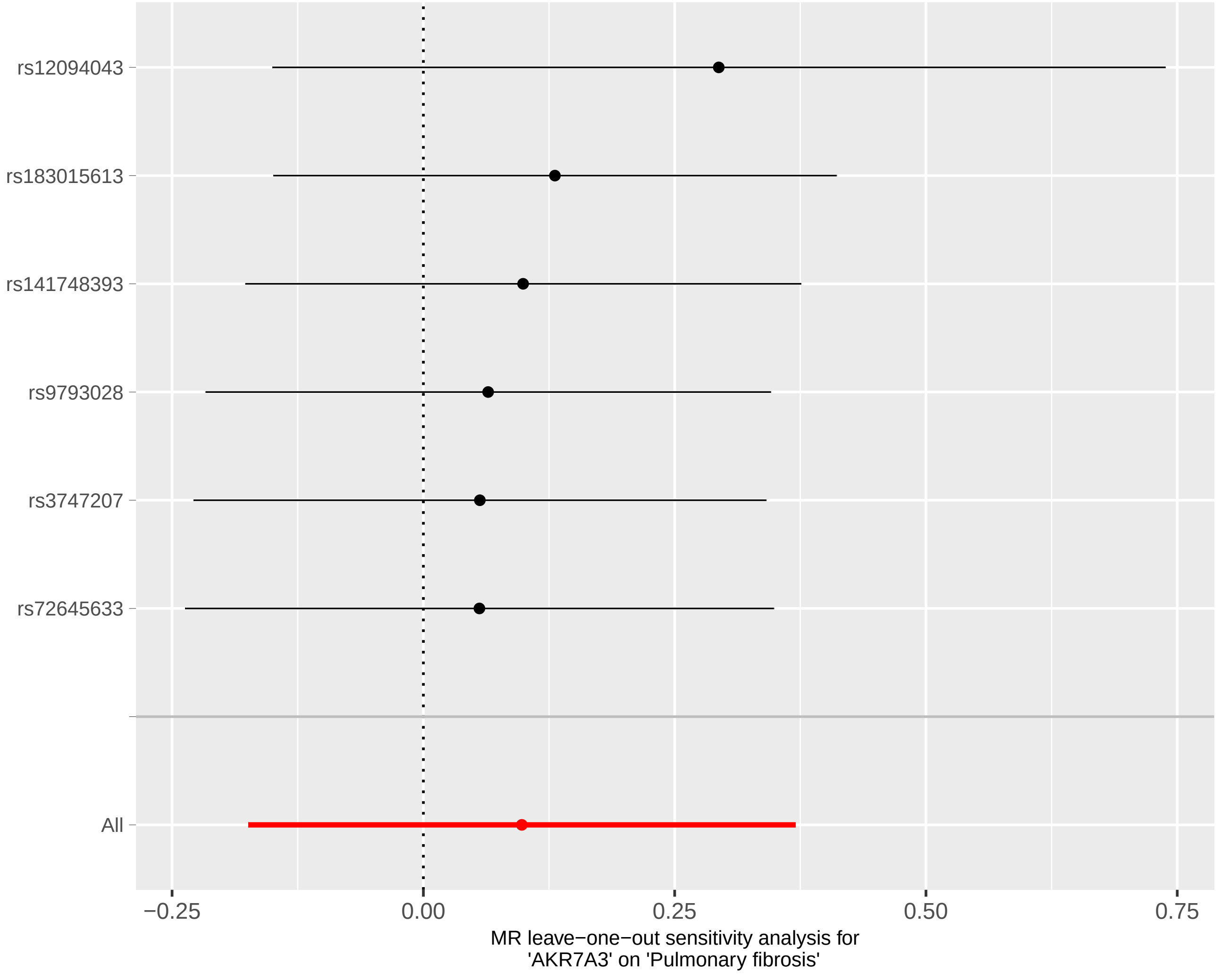
rs12094043
rs183015613
rs141748393
rs9793028
rs3747207
rs72645633
All
−0.25
0.00
0.25
0.50
0.75
MR leave−one−out sensitivity analysis for
'AKR7A3' on 'Pulmonary fibrosis'

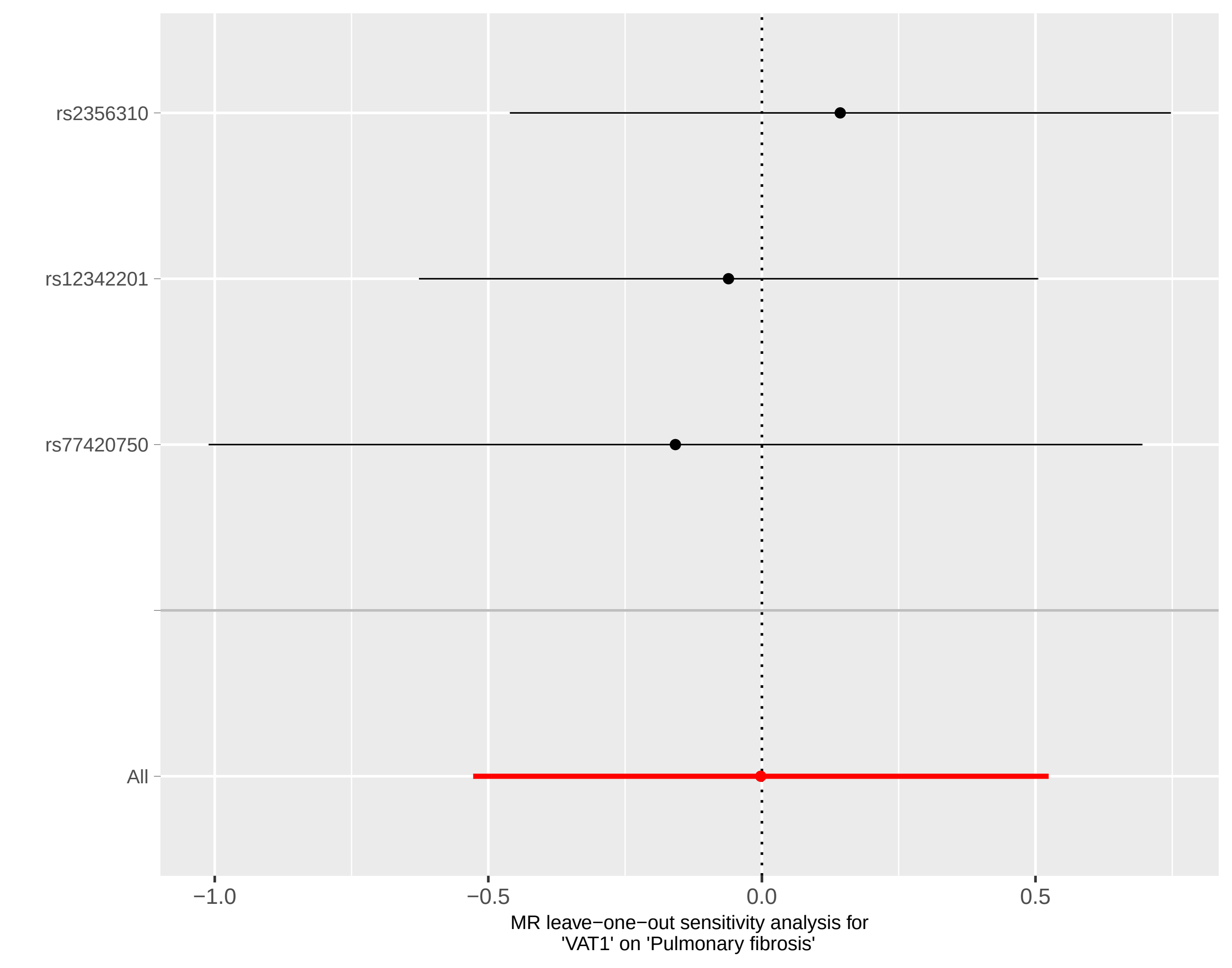
rs2356310
rs12342201
rs77420750
All
−1.0
−0.5
0.0
0.5
MR leave−one−out sensitivity analysis for
'VAT1' on 'Pulmonary fibrosis'

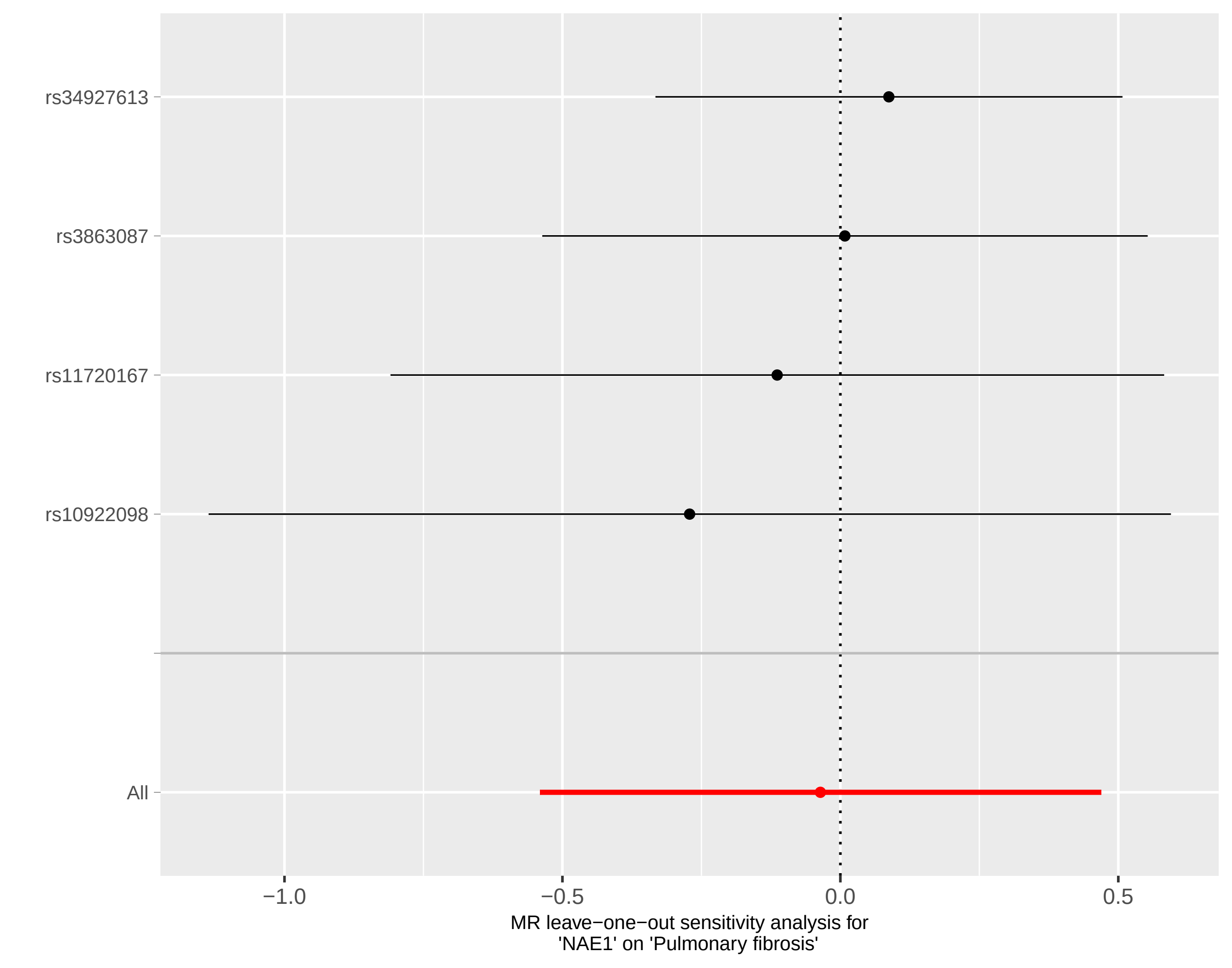
rs34927613
rs3863087
rs11720167
rs10922098
All
−1.0
−0.5
0.0
0.5
MR leave−one−out sensitivity analysis for
'NAE1' on 'Pulmonary fibrosis'

Insufficient number of SNPs

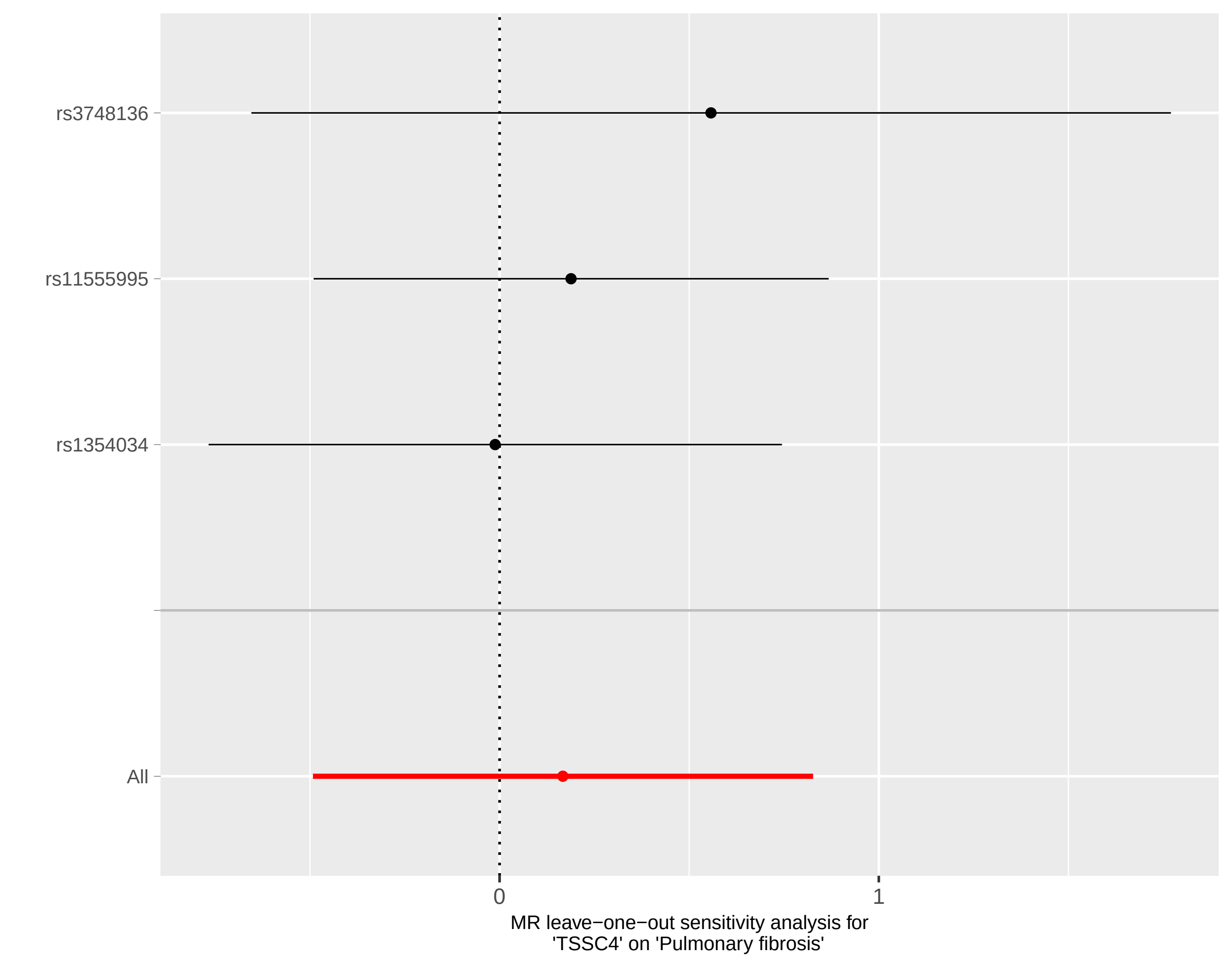
rs3748136
rs11555995
rs1354034
All
0
1
MR leave−one−out sensitivity analysis for
'TSSC4' on 'Pulmonary fibrosis'

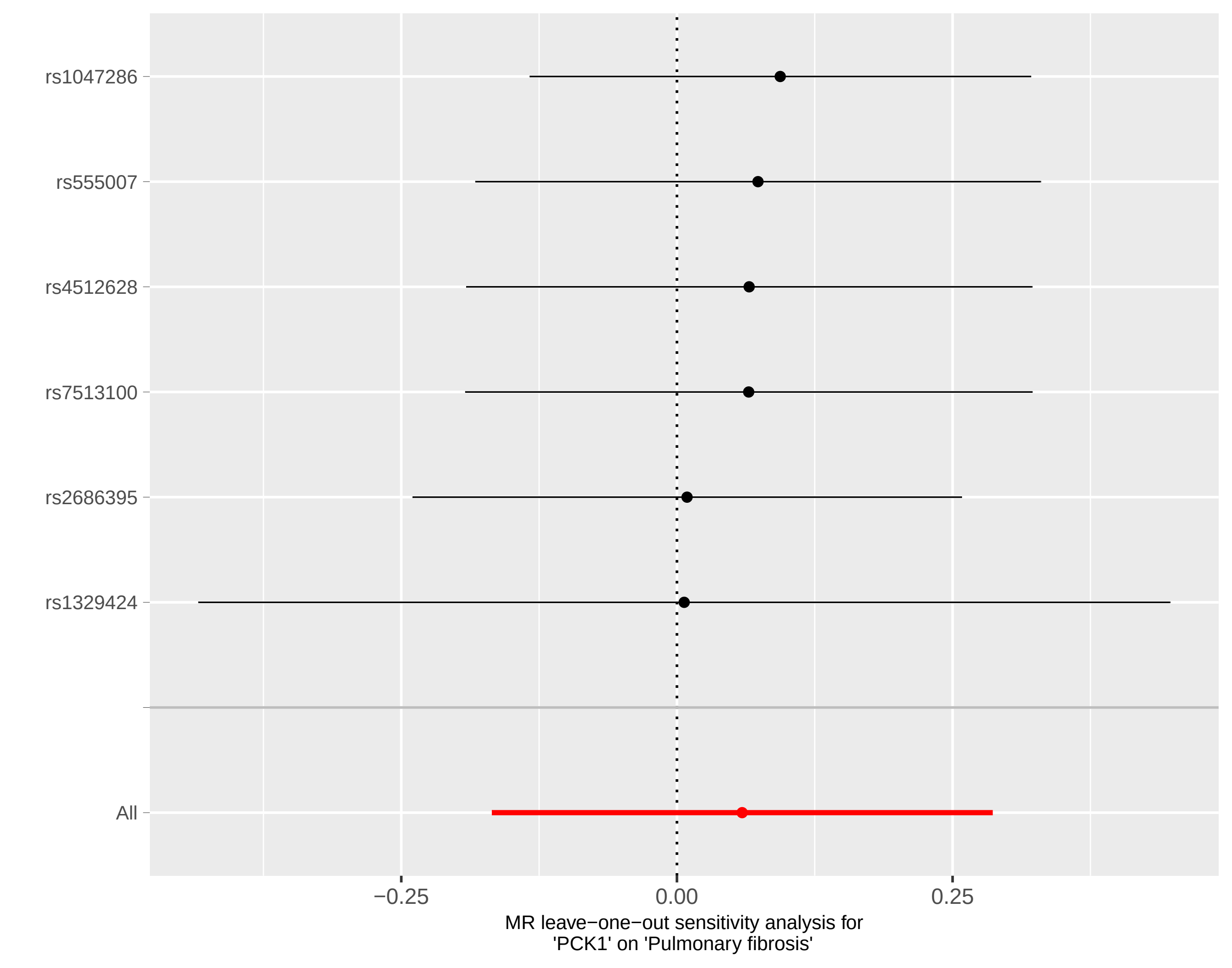
rs1047286
rs555007
rs4512628
rs7513100
rs2686395
rs1329424
All
−0.25
0.00
0.25
MR leave−one−out sensitivity analysis for
'PCK1' on 'Pulmonary fibrosis'

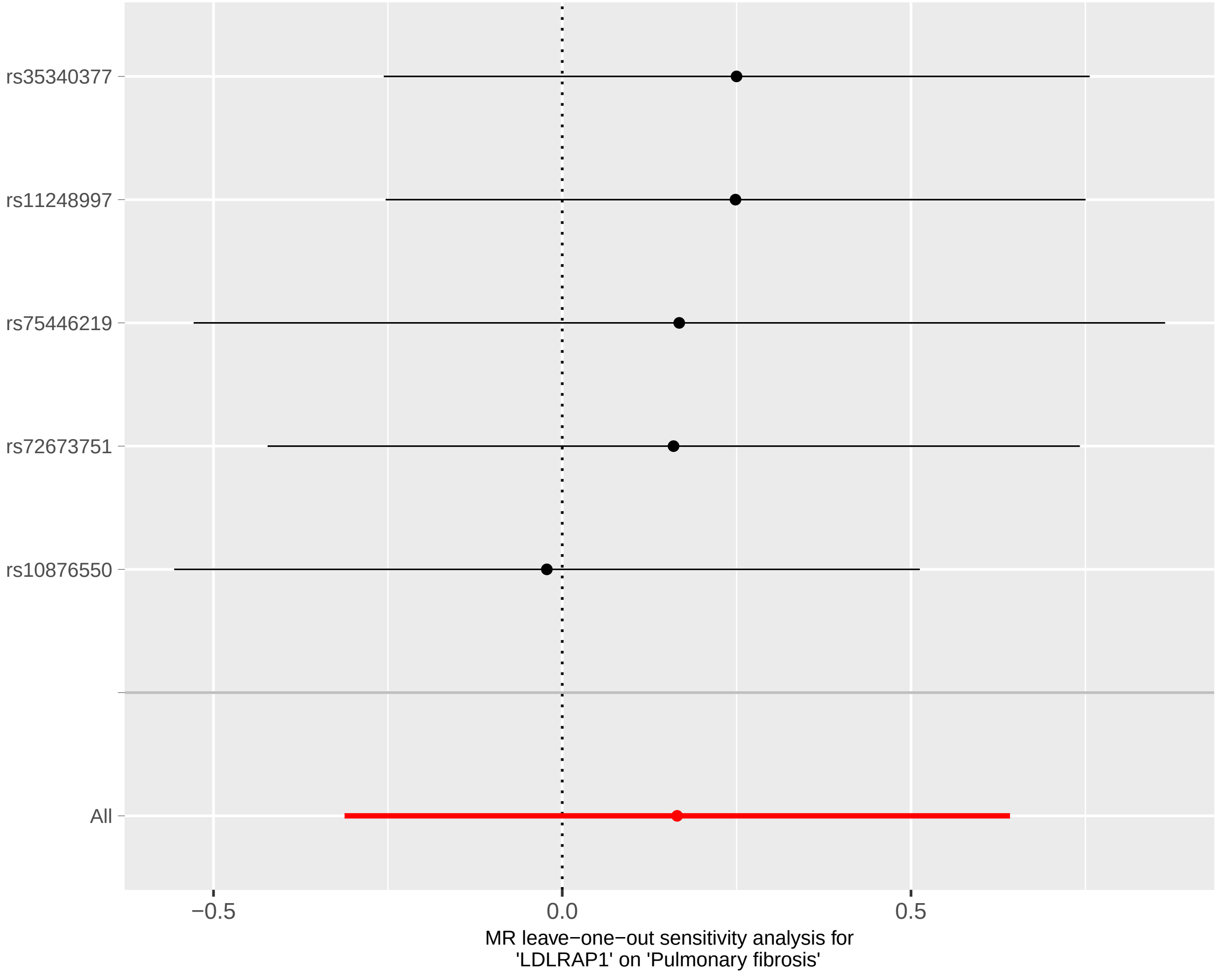
rs35340377
rs11248997
rs75446219
rs72673751
rs10876550
All
−0.5
0.0
0.5
MR leave−one−out sensitivity analysis for
'LDLRAP1' on 'Pulmonary fibrosis'

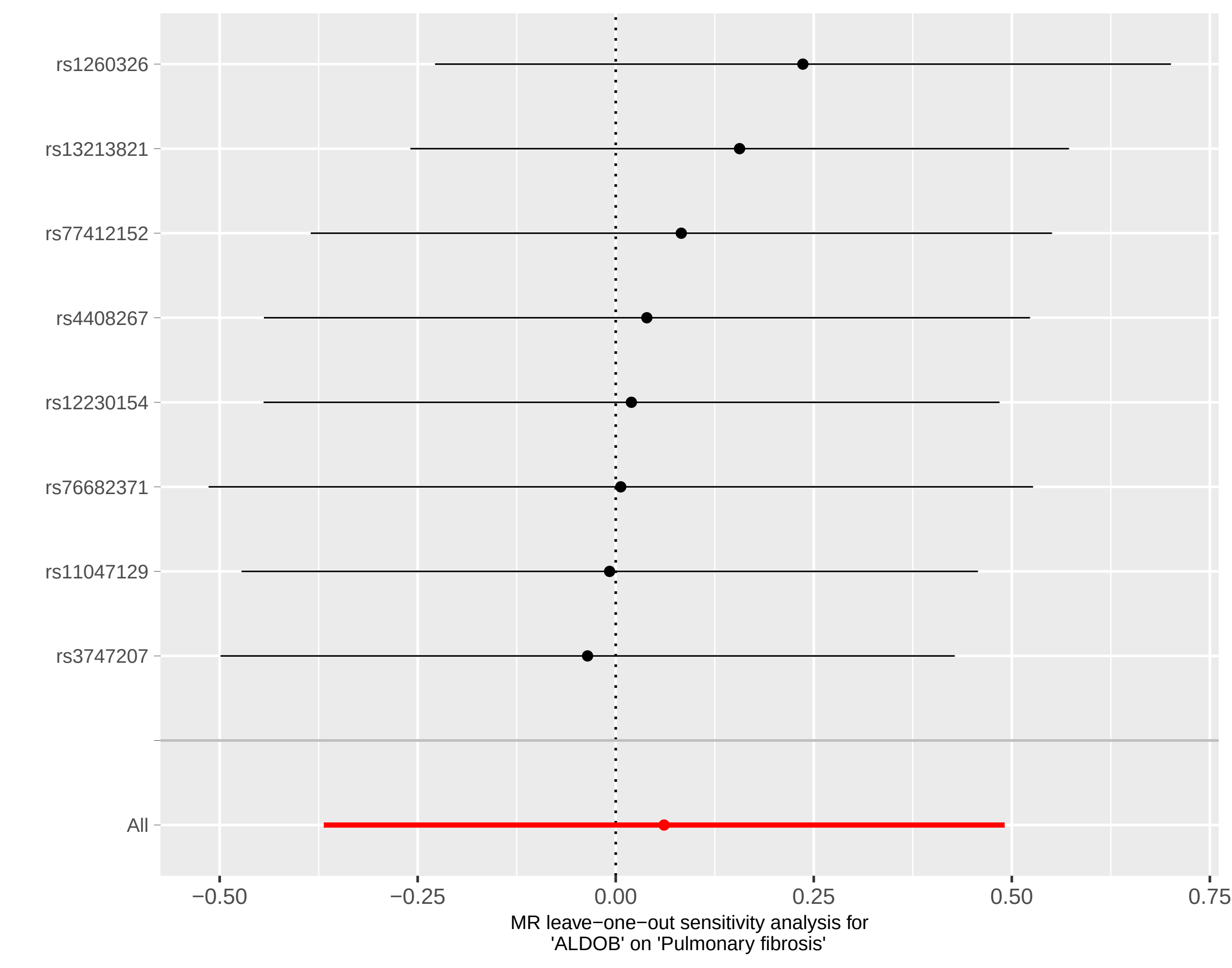
rs1260326
rs13213821
rs77412152
rs4408267
rs12230154
rs76682371
rs11047129
rs3747207
All
−0.50
−0.25
0.00
0.25
0.50
0.75
MR leave−one−out sensitivity analysis for
'ALDOB' on 'Pulmonary fibrosis'

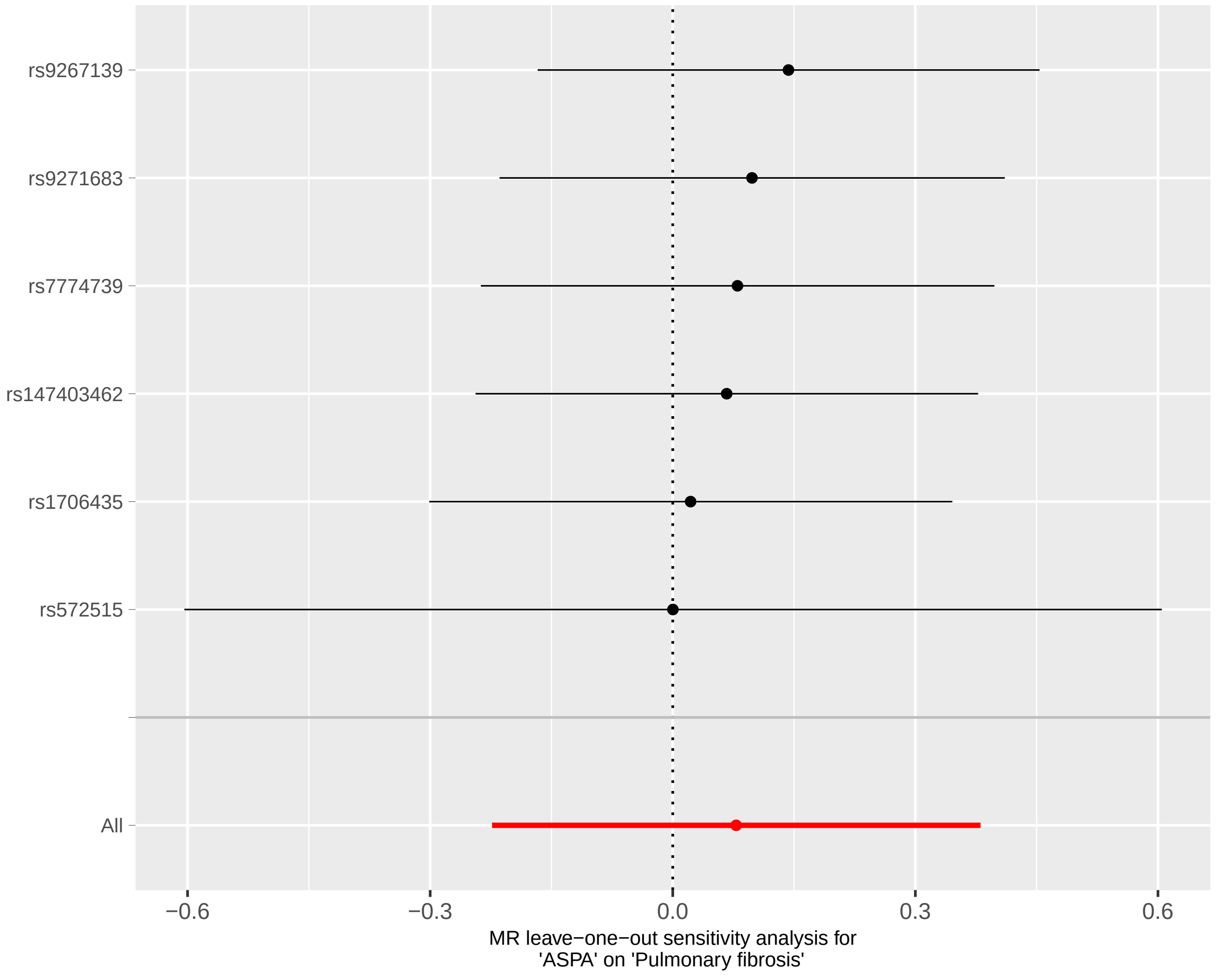
rs9267139
rs9271683
rs7774739
rs147403462
rs1706435
rs572515
All
−0.6
−0.3
0.0
0.3
0.6
MR leave−one−out sensitivity analysis for
'ASPA' on 'Pulmonary fibrosis'

Insufficient number of SNPs

Insufficient number of SNPs

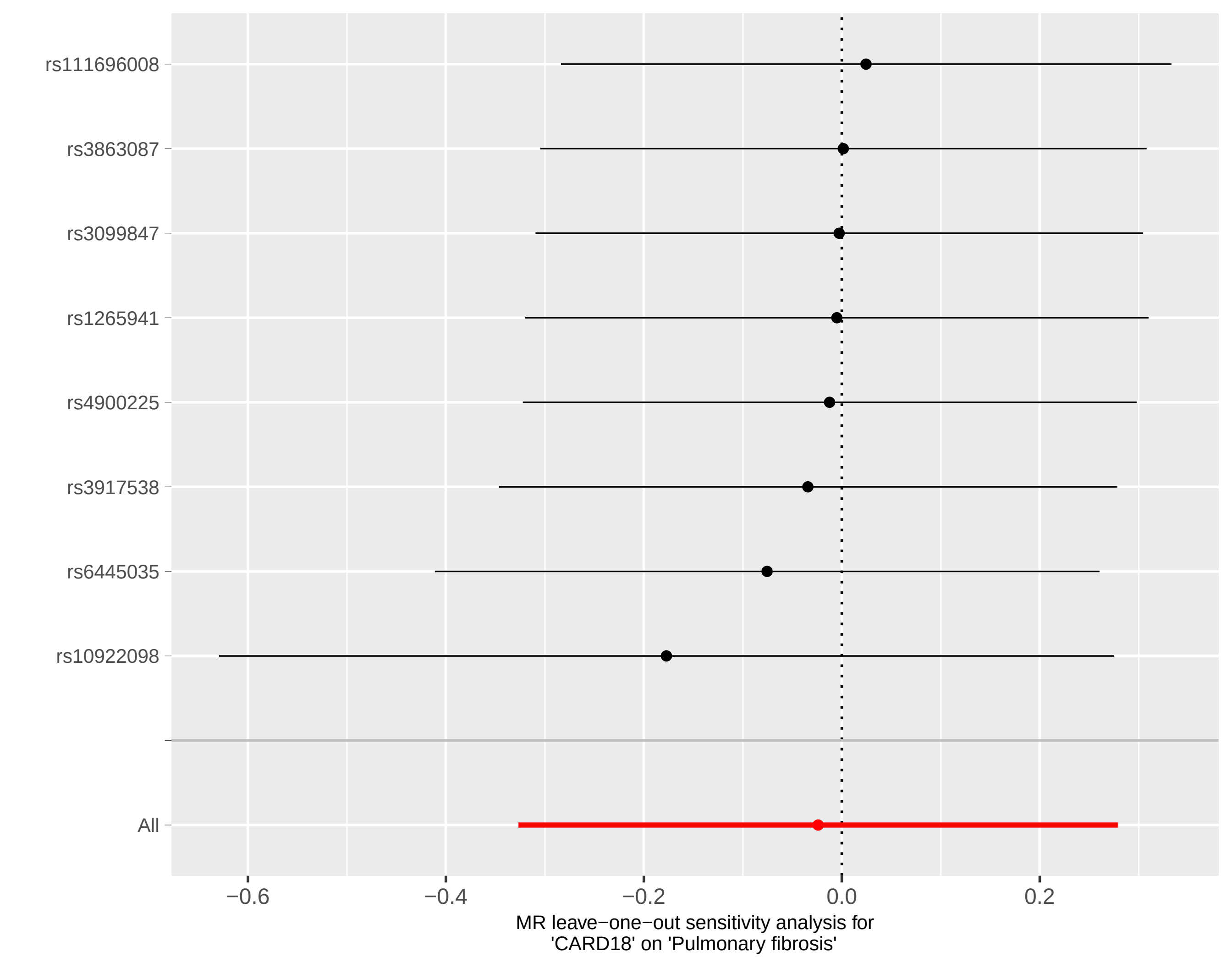

rs111696008
rs3863087
rs3099847
rs1265941
rs4900225
rs3917538
rs6445035
rs10922098
All
−0.6
−0.4
−0.2
0.0
0.2
MR leave−one−out sensitivity analysis for
'CARD18' on 'Pulmonary fibrosis'

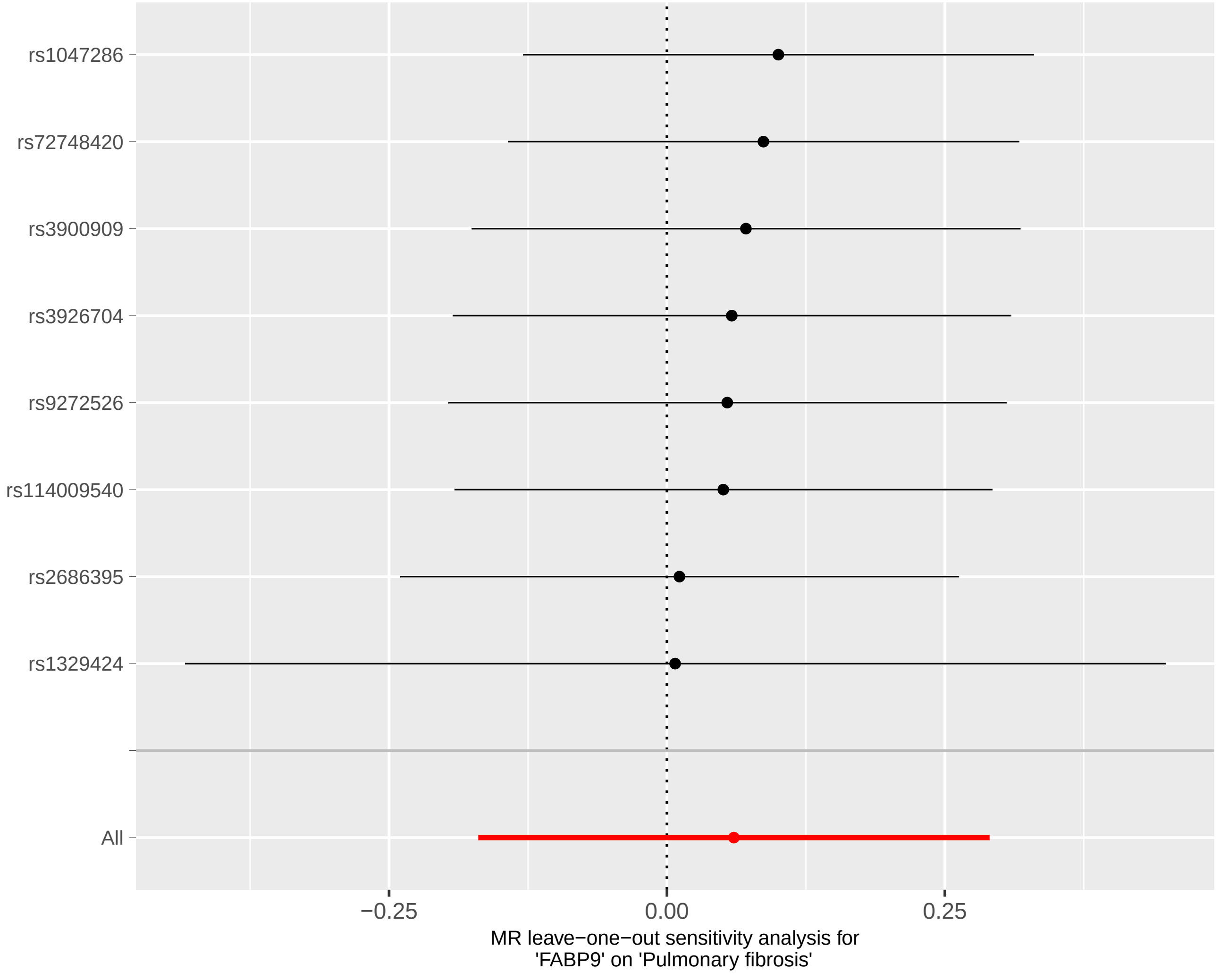
rs1047286
rs72748420
rs3900909
rs3926704
rs9272526
rs114009540
rs2686395
rs1329424
All
−0.25
0.00
0.25
MR leave−one−out sensitivity analysis for
'FABP9' on 'Pulmonary fibrosis'

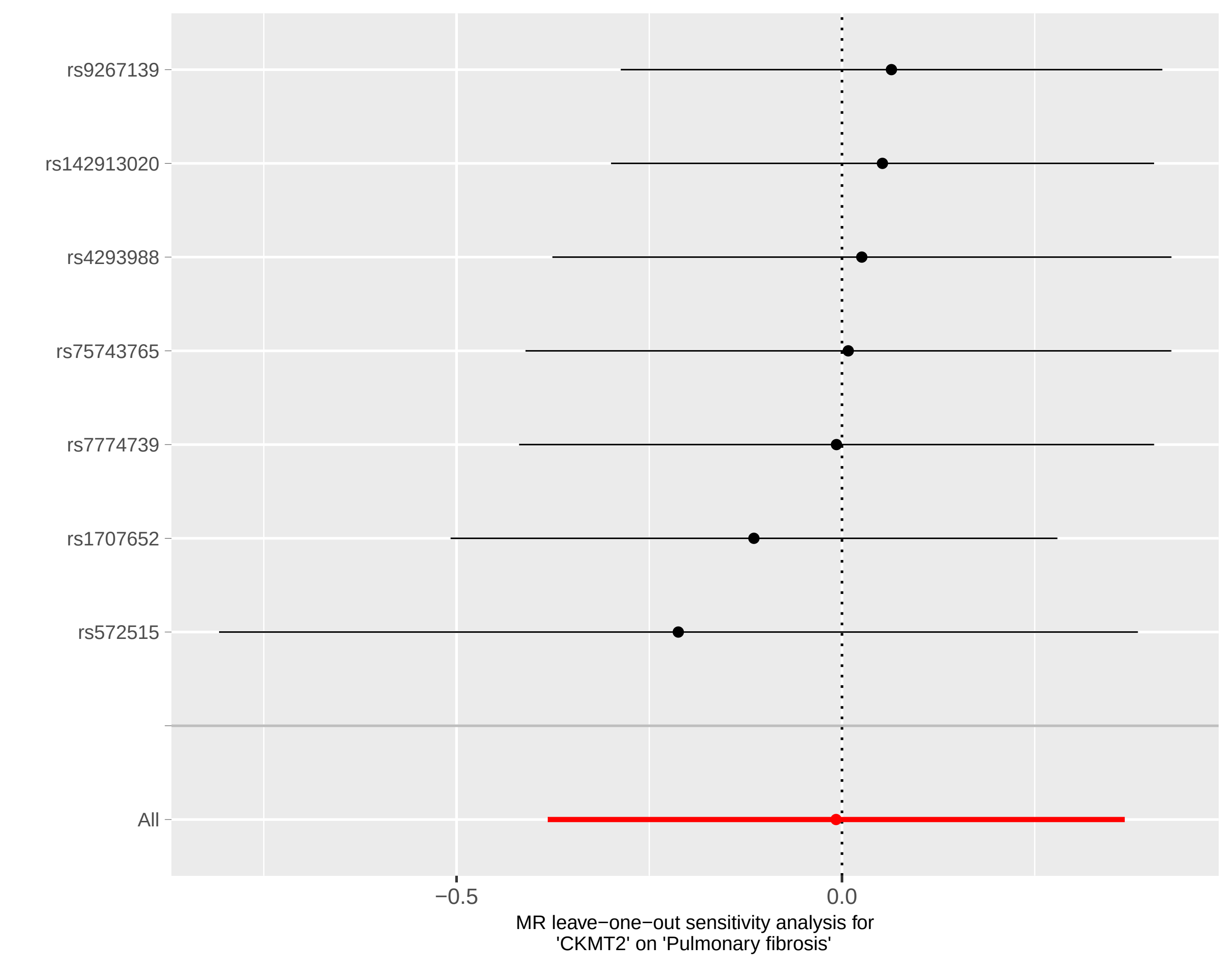
rs9267139
rs142913020
rs4293988
rs75743765
rs7774739
rs1707652
rs572515
All
−0.5
0.0
MR leave−one−out sensitivity analysis for
'CKMT2' on 'Pulmonary fibrosis'

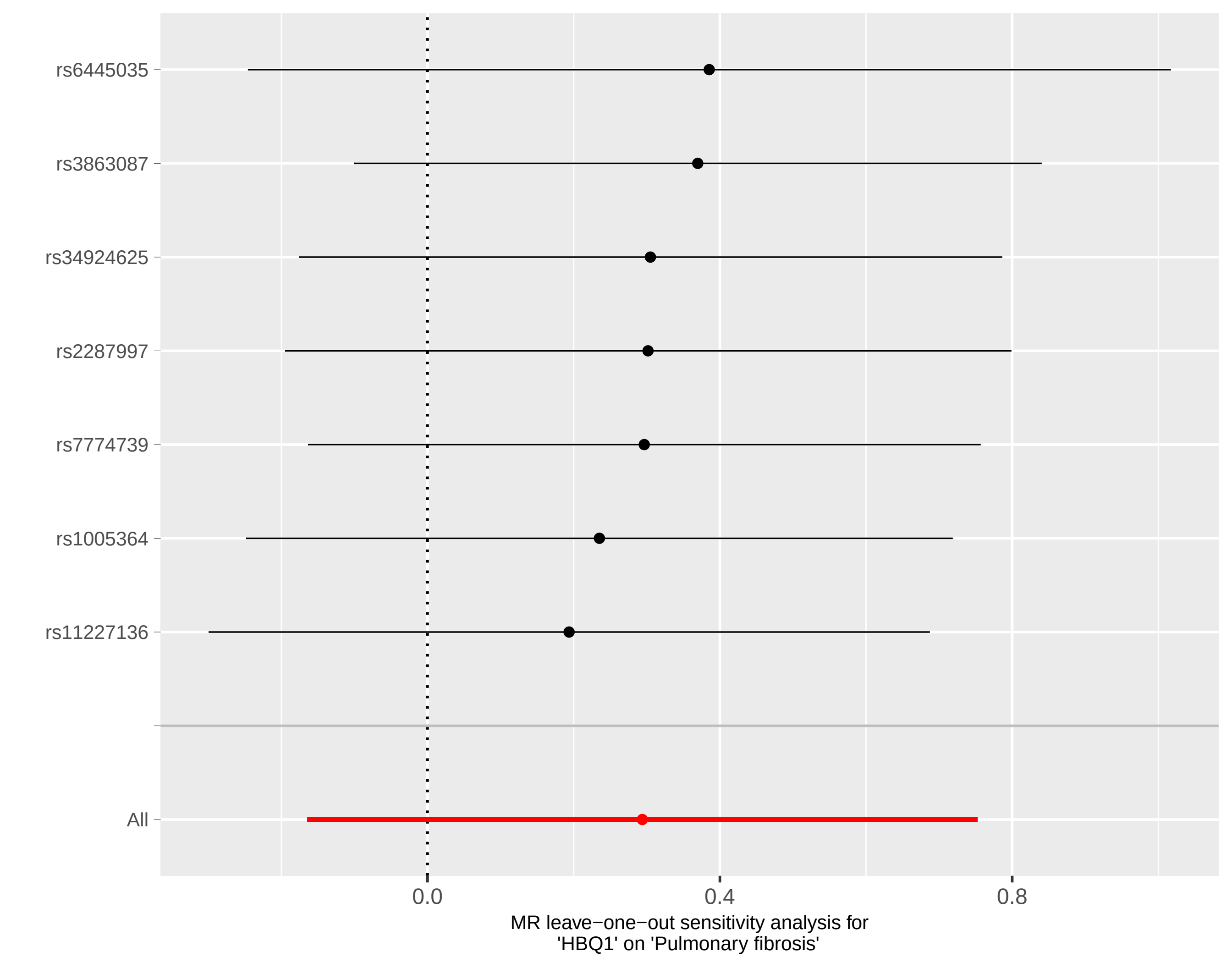
rs6445035
rs3863087
rs34924625
rs2287997
rs7774739
rs1005364
rs11227136
All
0.0
0.4
0.8
MR leave−one−out sensitivity analysis for
'HBQ1' on 'Pulmonary fibrosis'

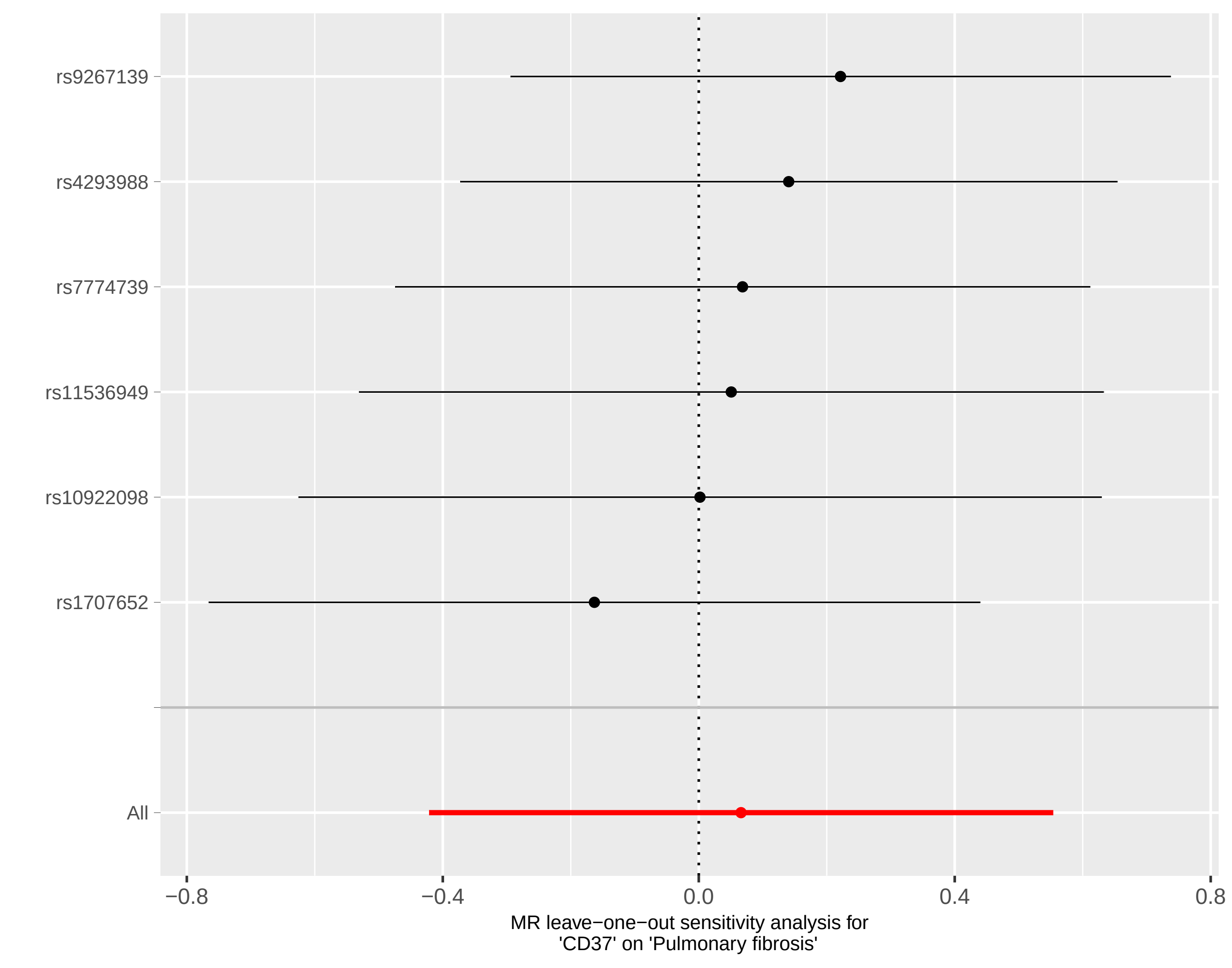
rs9267139
rs4293988
rs7774739
rs11536949
rs10922098
rs1707652
All
−0.8
−0.4
0.0
0.4
0.8
MR leave−one−out sensitivity analysis for
'CD37' on 'Pulmonary fibrosis'

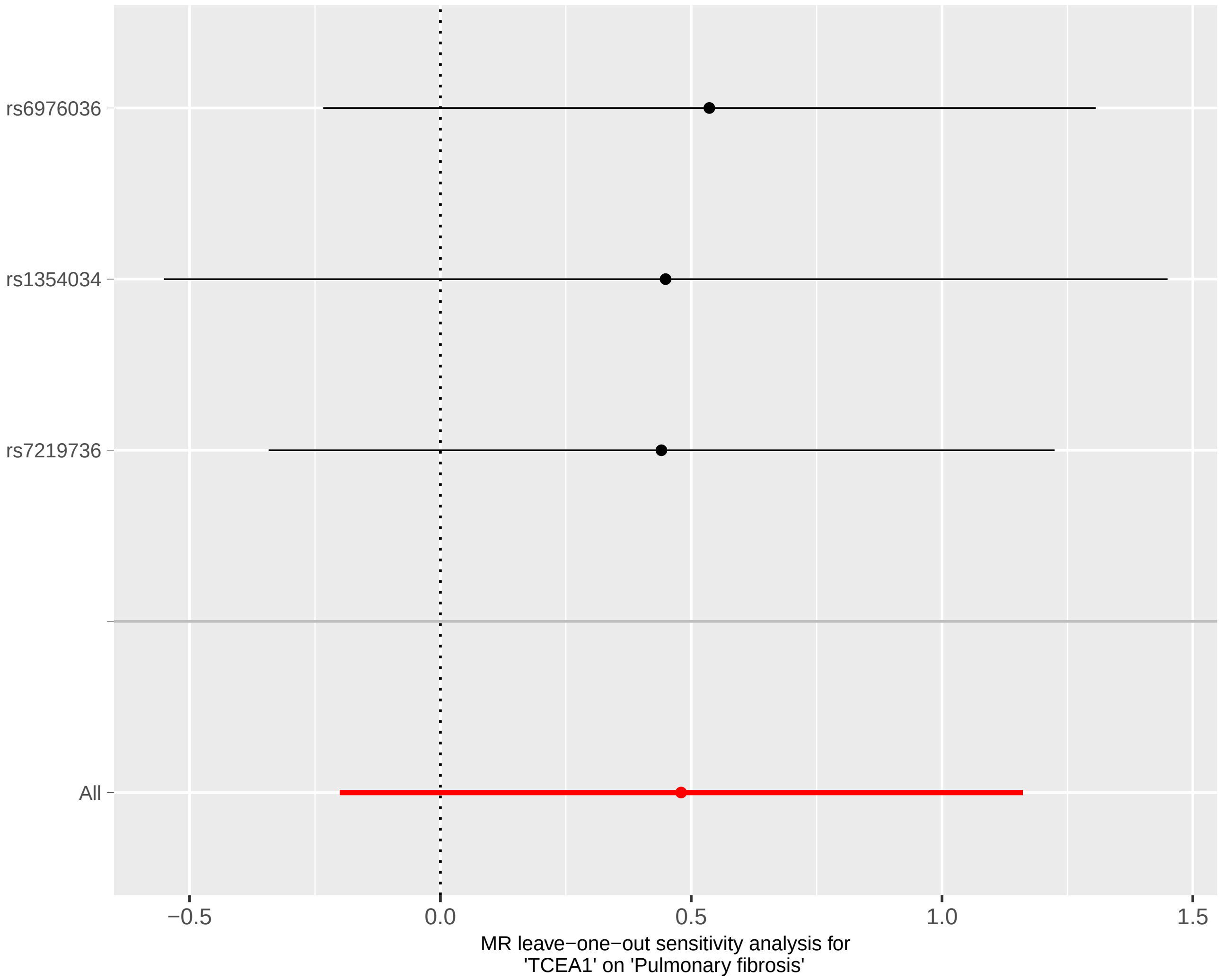

rs6976036
rs1354034
rs7219736
All
−0.5
0.0
0.5
1.0
1.5
MR leave−one−out sensitivity analysis for
'TCEA1' on 'Pulmonary fibrosis'

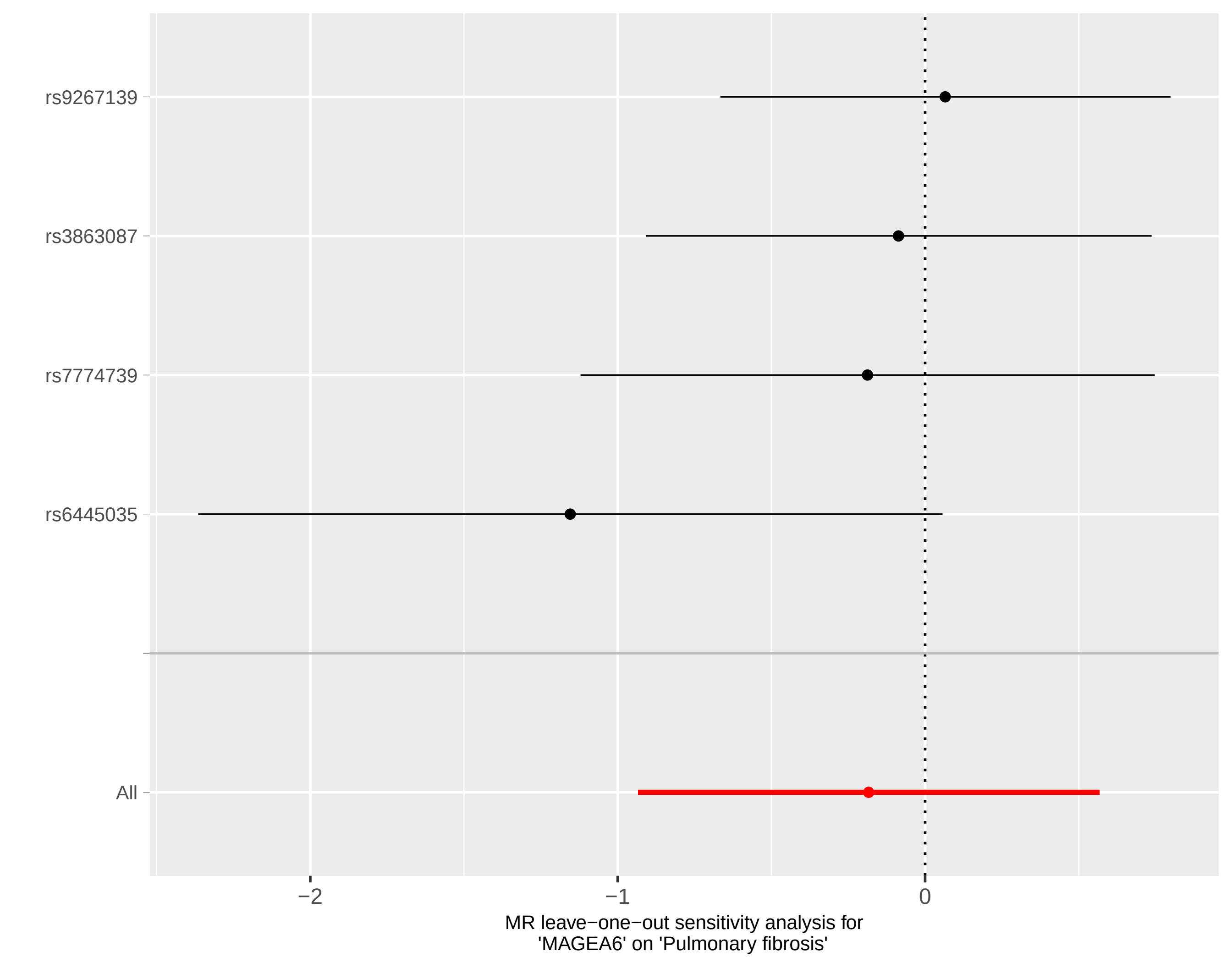
rs9267139
rs3863087
rs7774739
rs6445035
All
−2
−1
0
MR leave−one−out sensitivity analysis for
'MAGEA6' on 'Pulmonary fibrosis'

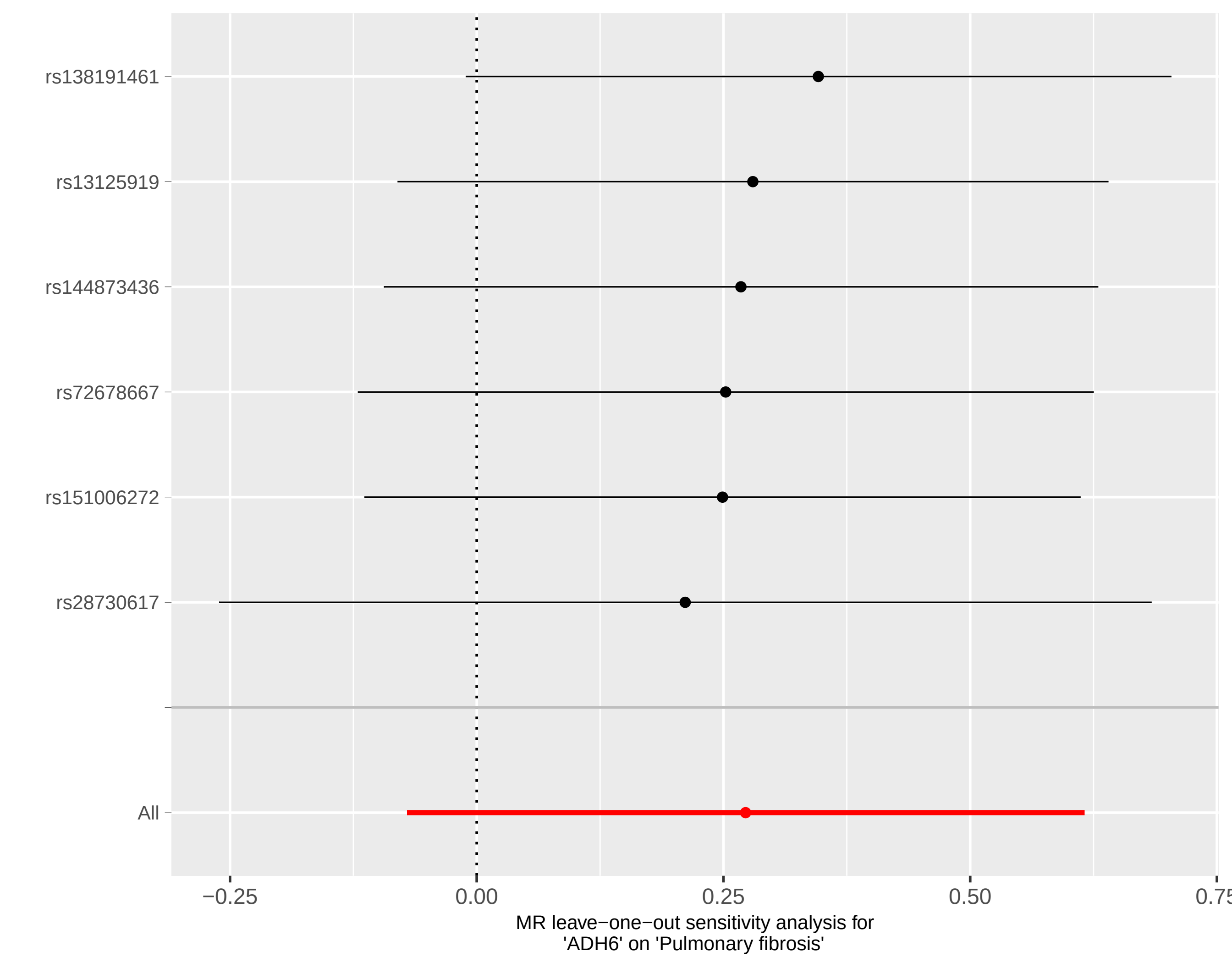

rs138191461
rs13125919
rs144873436
rs72678667
rs151006272
rs28730617
All
−0.25
0.00
0.25
0.50
0.75
MR leave−one−out sensitivity analysis for
'ADH6' on 'Pulmonary fibrosis'

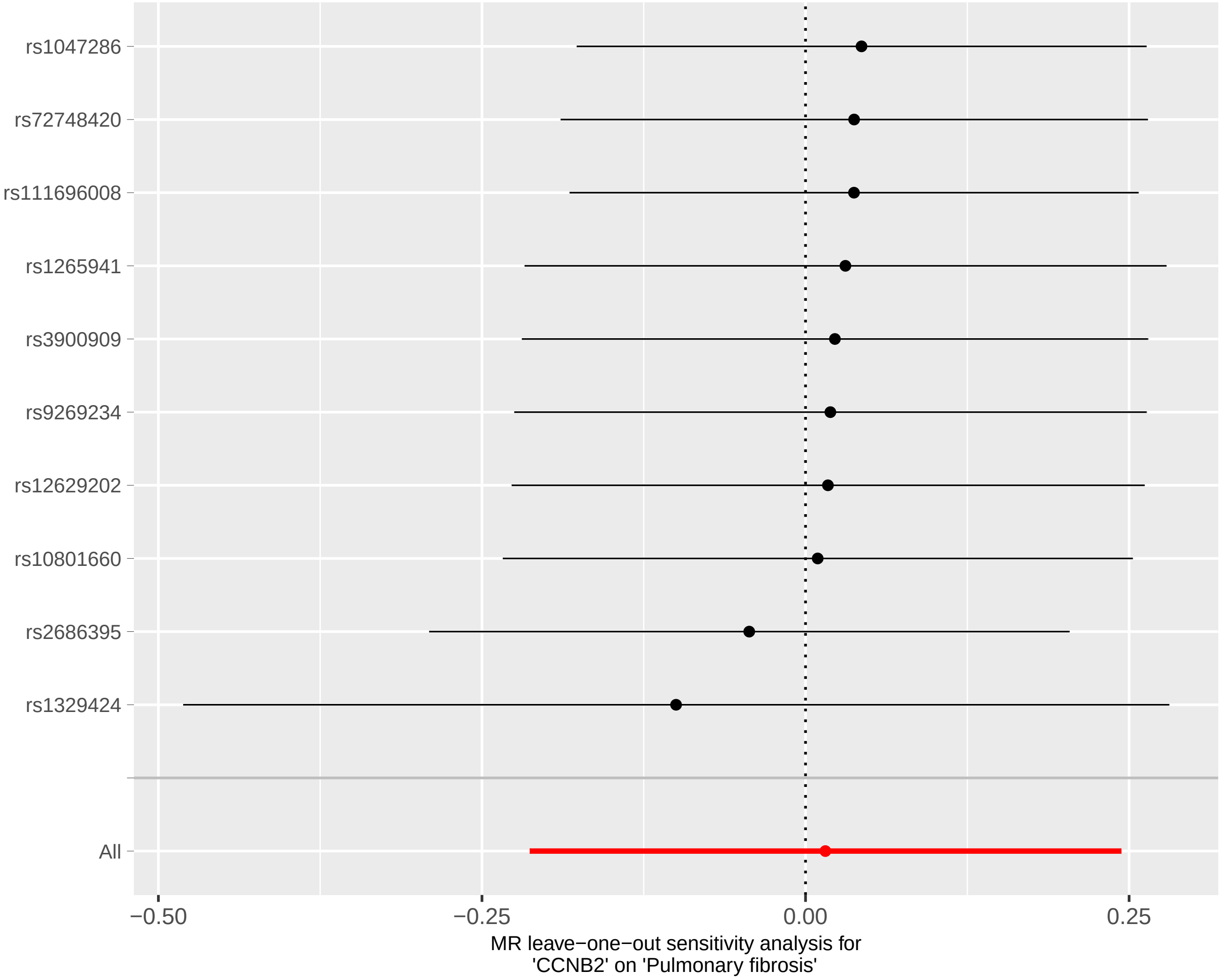

rs1047286
rs72748420
rs111696008
rs1265941
rs3900909
rs9269234
rs12629202
rs10801660
rs2686395
rs1329424
All
−0.50
−0.25
0.00
0.25
MR leave−one−out sensitivity analysis for
'CCNB2' on 'Pulmonary fibrosis'

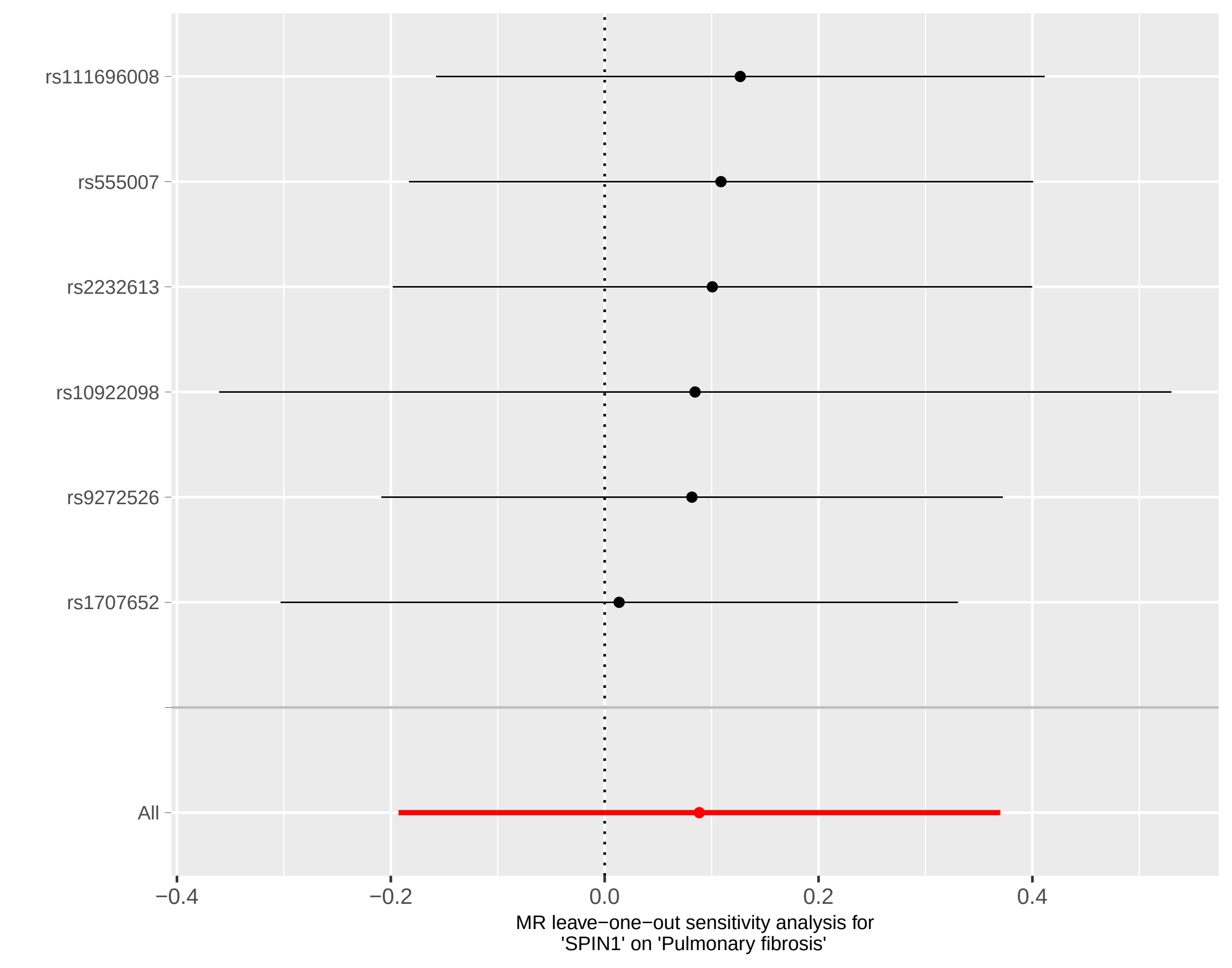
rs111696008
rs555007
rs2232613
rs10922098
rs9272526
rs1707652
All
−0.4
−0.2
0.0
0.2
0.4
MR leave−one−out sensitivity analysis for
'SPIN1' on 'Pulmonary fibrosis'

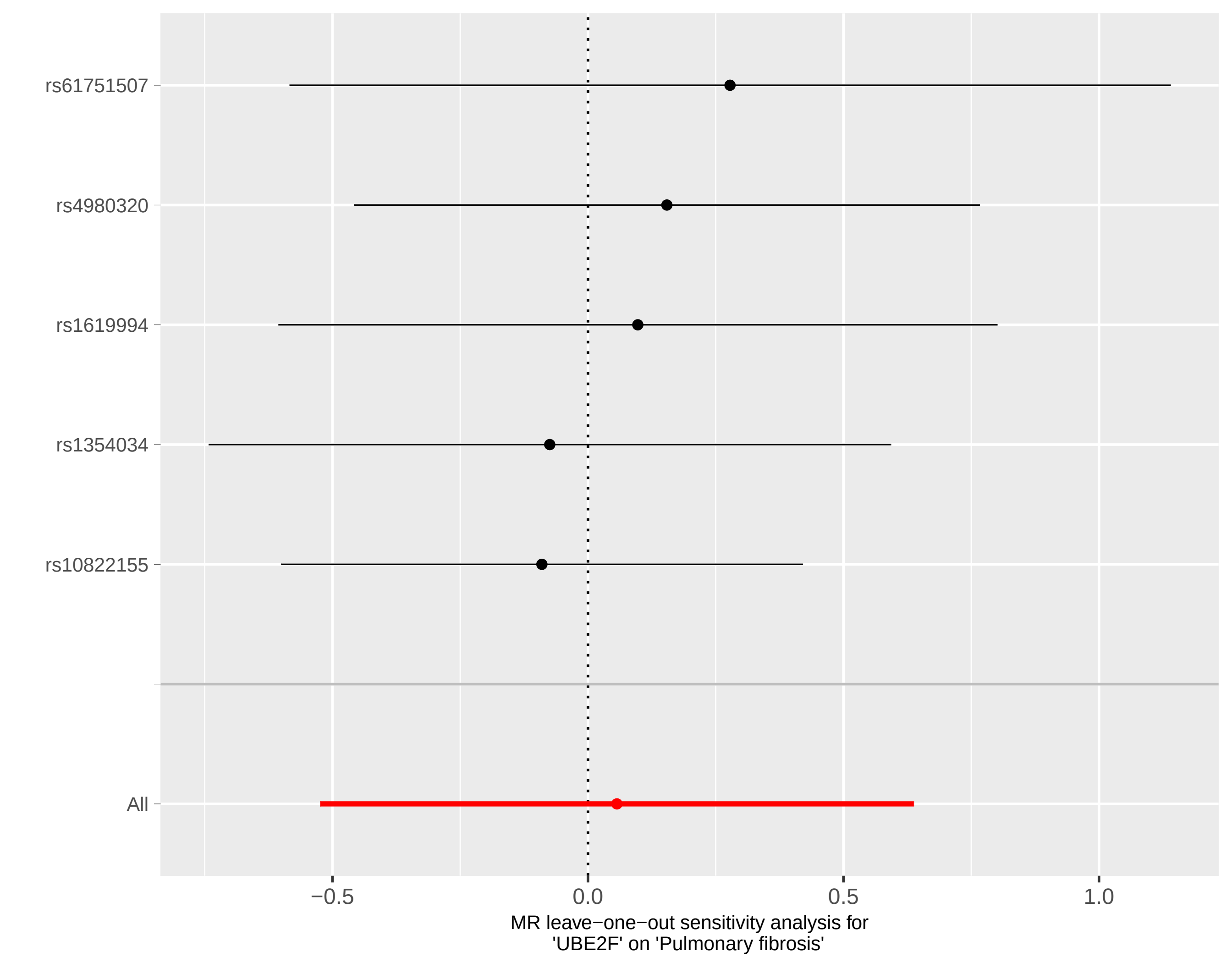

rs61751507
rs4980320
rs1619994
rs1354034
rs10822155
All
−0.5
0.0
0.5
1.0
MR leave−one−out sensitivity analysis for
'UBE2F' on 'Pulmonary fibrosis'

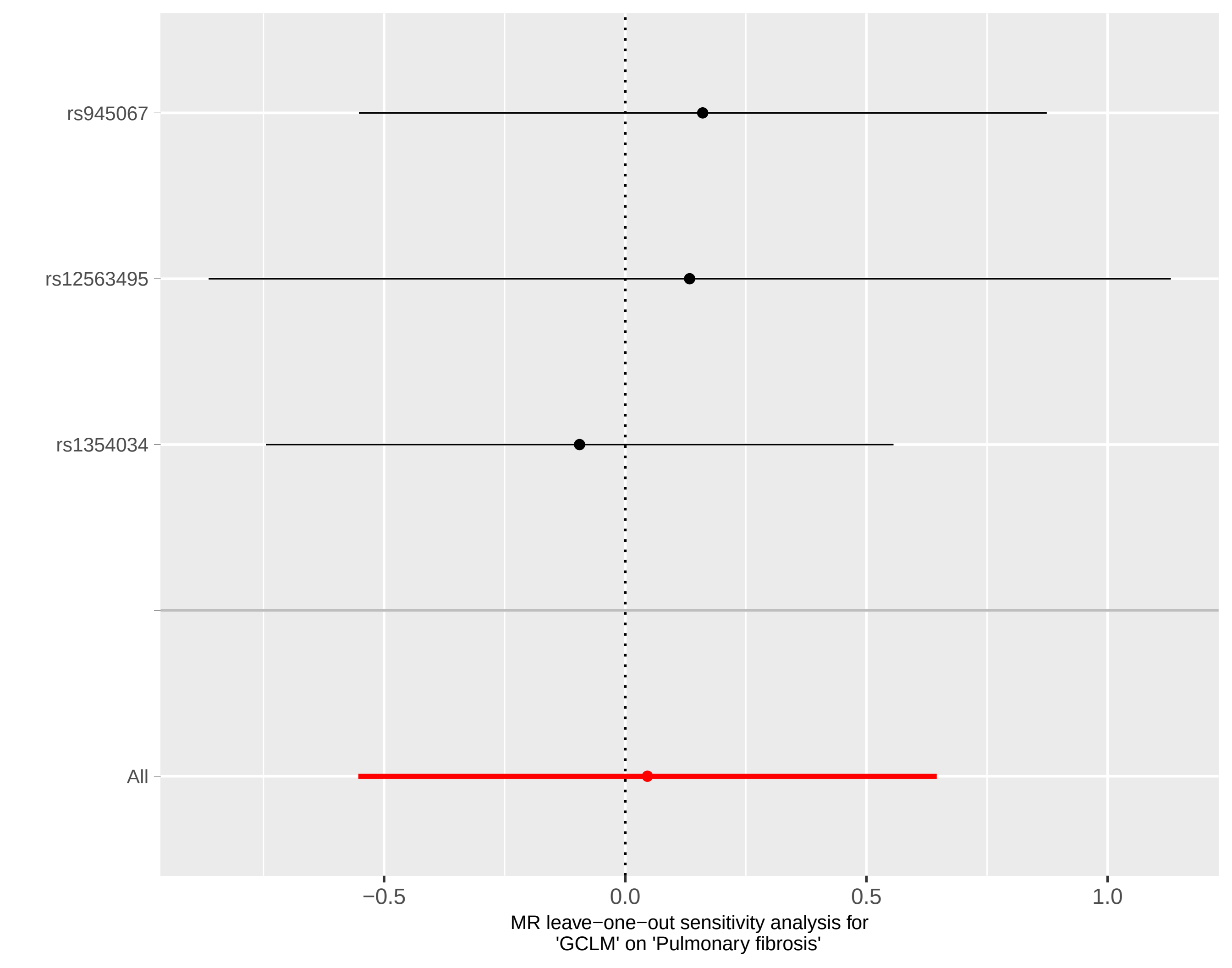
rs945067
rs12563495
rs1354034
All
−0.5
0.0
0.5
1.0
MR leave−one−out sensitivity analysis for
'GCLM' on 'Pulmonary fibrosis'

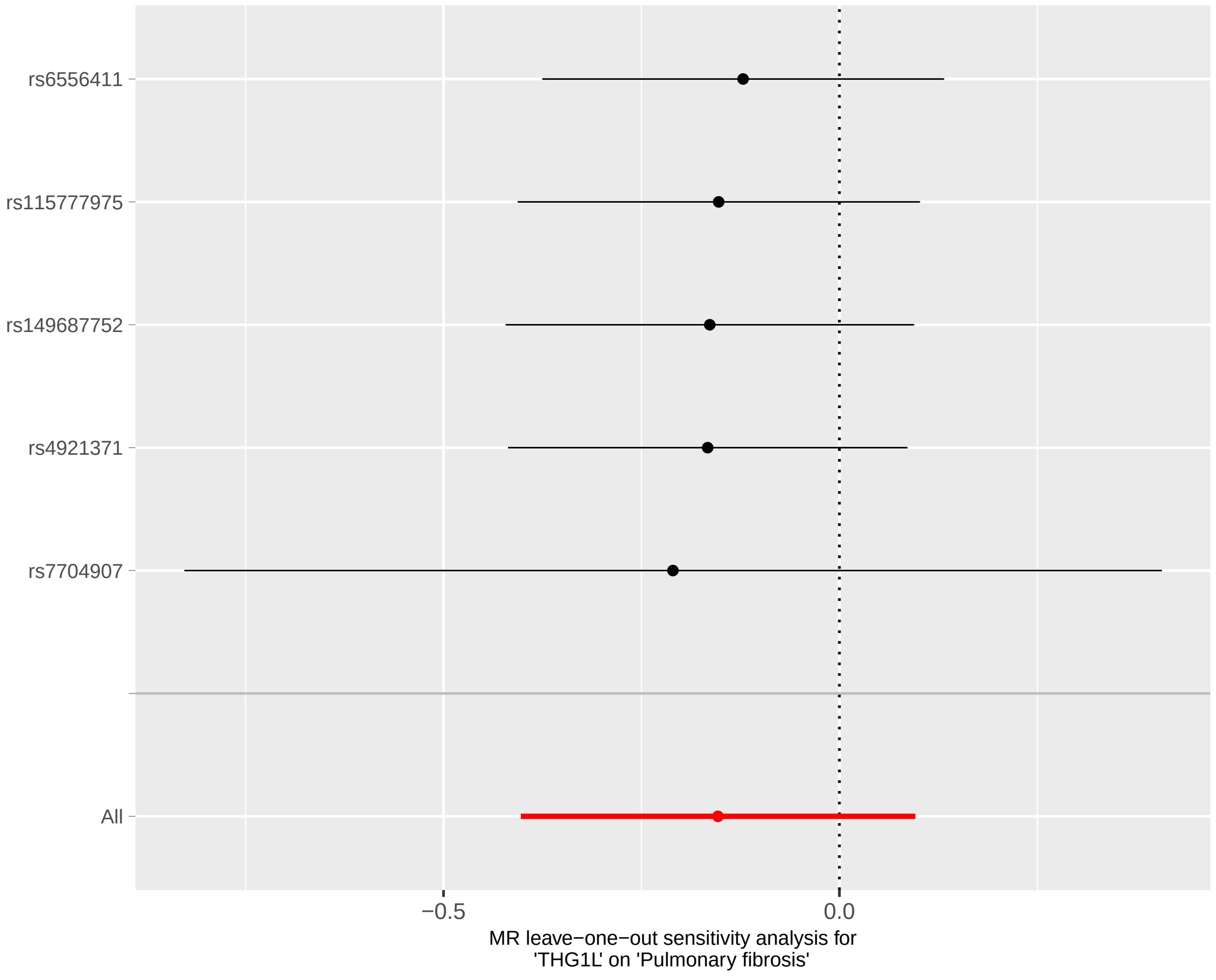

rs6556411
rs115777975
rs149687752
rs4921371
rs7704907
All
−0.5
0.0
MR leave−one−out sensitivity analysis for
'THG1L' on 'Pulmonary fibrosis'

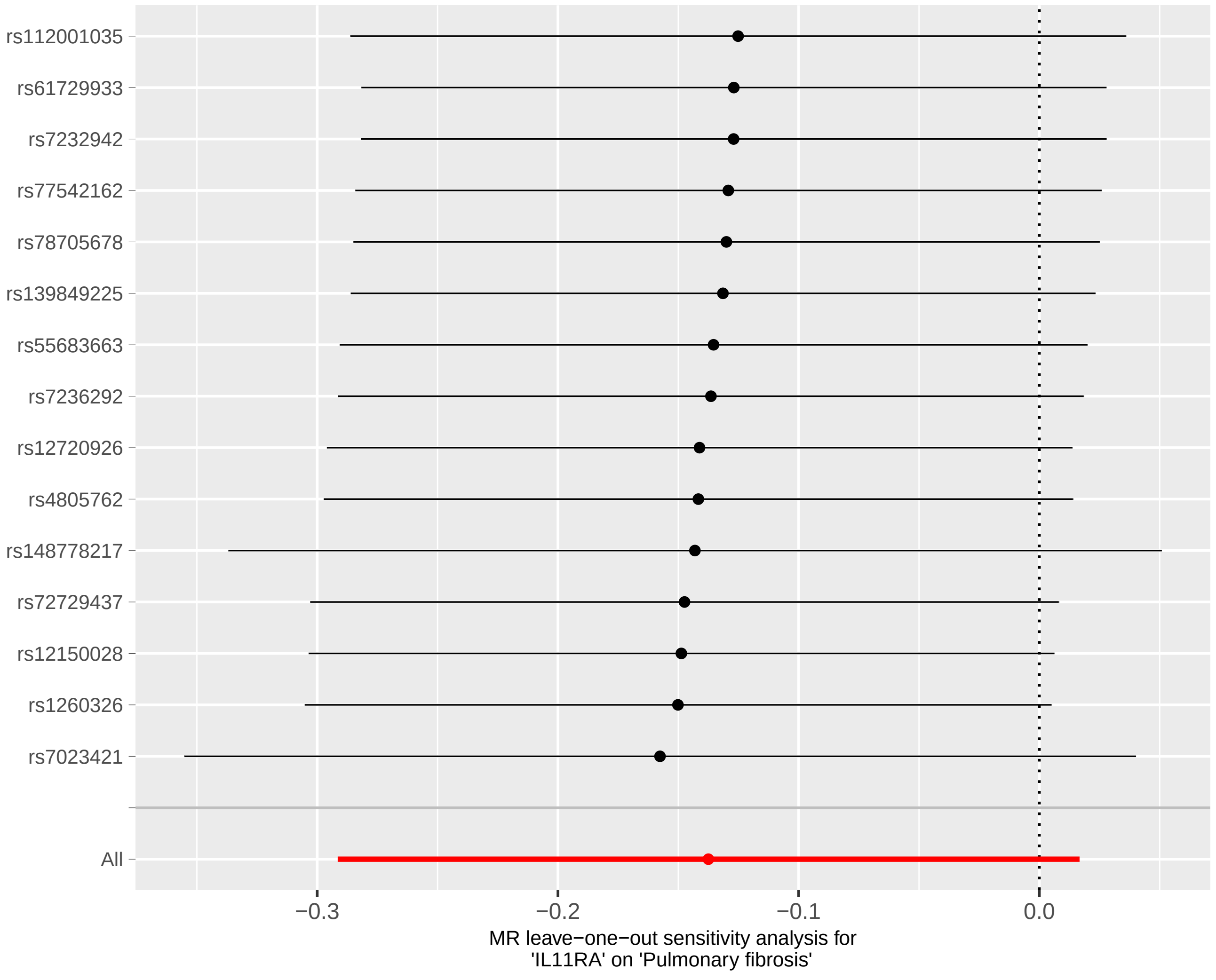

rs112001035
rs61729933
rs7232942
rs77542162
rs78705678
rs139849225
rs55683663
rs7236292
rs12720926
rs4805762
rs148778217
rs72729437
rs12150028
rs1260326
rs7023421
All
−0.3
−0.2
−0.1
0.0
MR leave−one−out sensitivity analysis for
'IL11RA' on 'Pulmonary fibrosis'

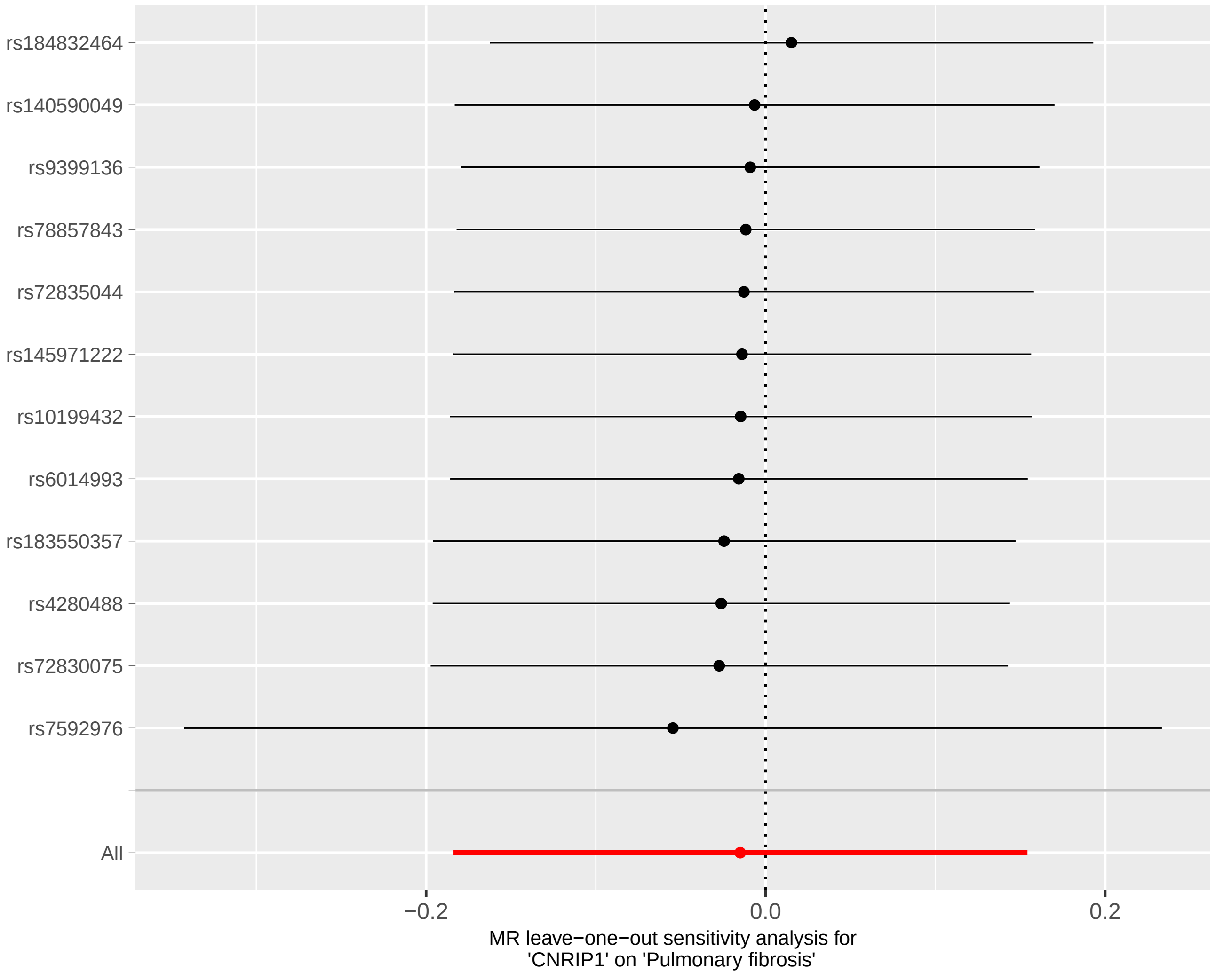

rs184832464
rs140590049
rs9399136
rs78857843
rs72835044
rs145971222
rs10199432
rs6014993
rs183550357
rs4280488
rs72830075
rs7592976
All
−0.2
0.0
0.2
MR leave−one−out sensitivity analysis for
'CNRIP1' on 'Pulmonary fibrosis'

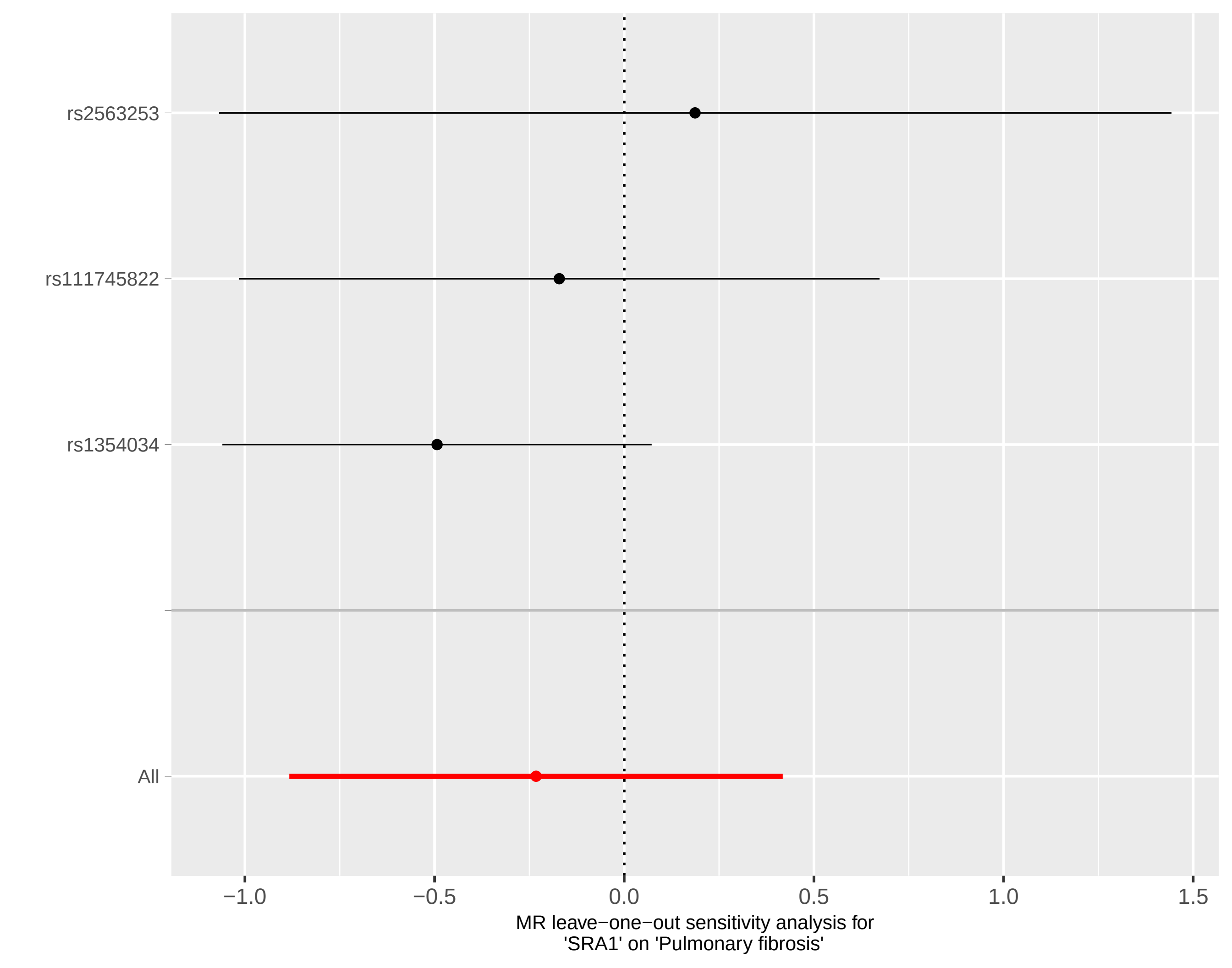
rs2563253
rs111745822
rs1354034
All
−1.0
−0.5
0.0
0.5
1.0
1.5
MR leave−one−out sensitivity analysis for
'SRA1' on 'Pulmonary fibrosis'

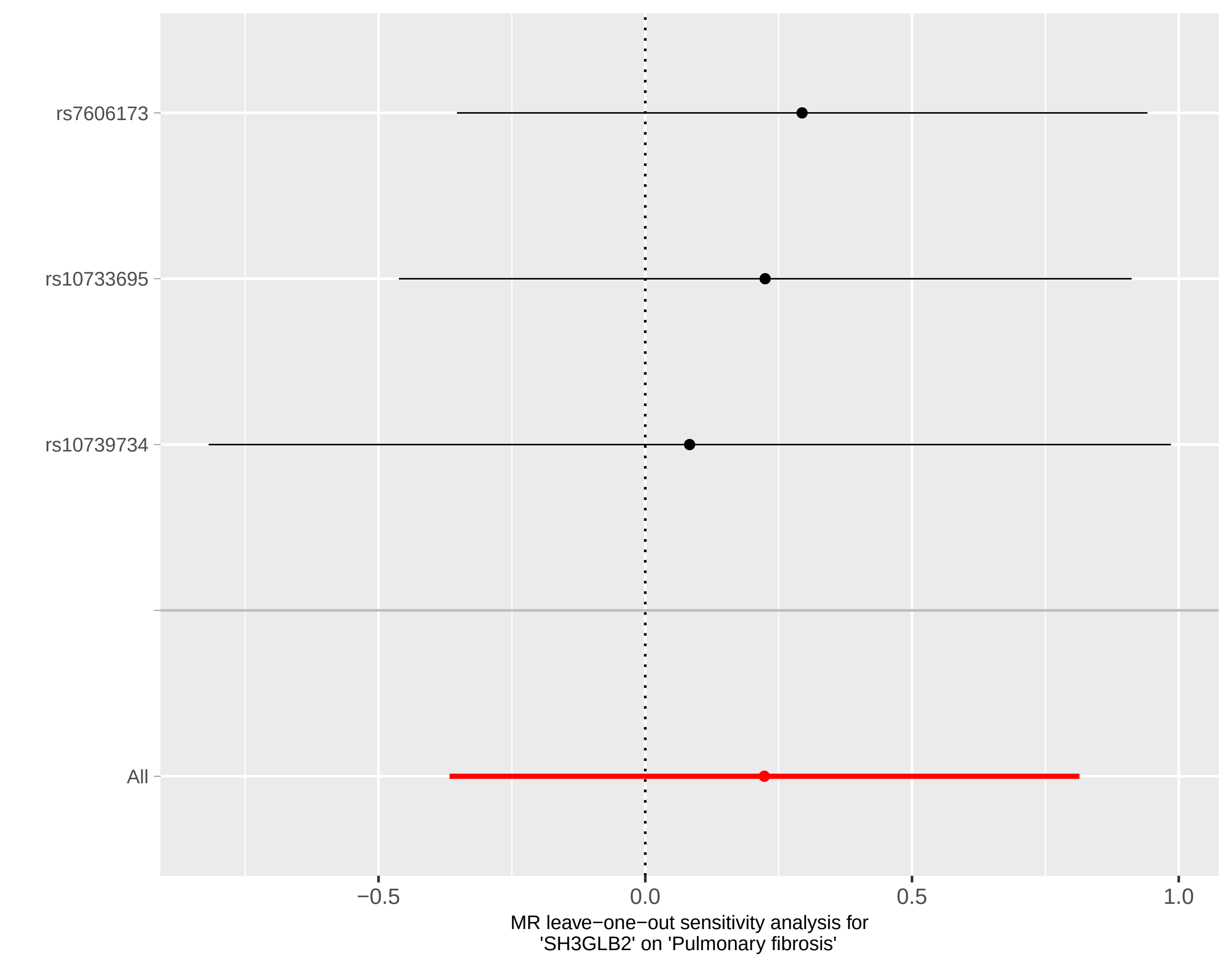
rs7606173
rs10733695
rs10739734
All
−0.5
0.0
0.5
1.0
MR leave−one−out sensitivity analysis for
'SH3GLB2' on 'Pulmonary fibrosis'

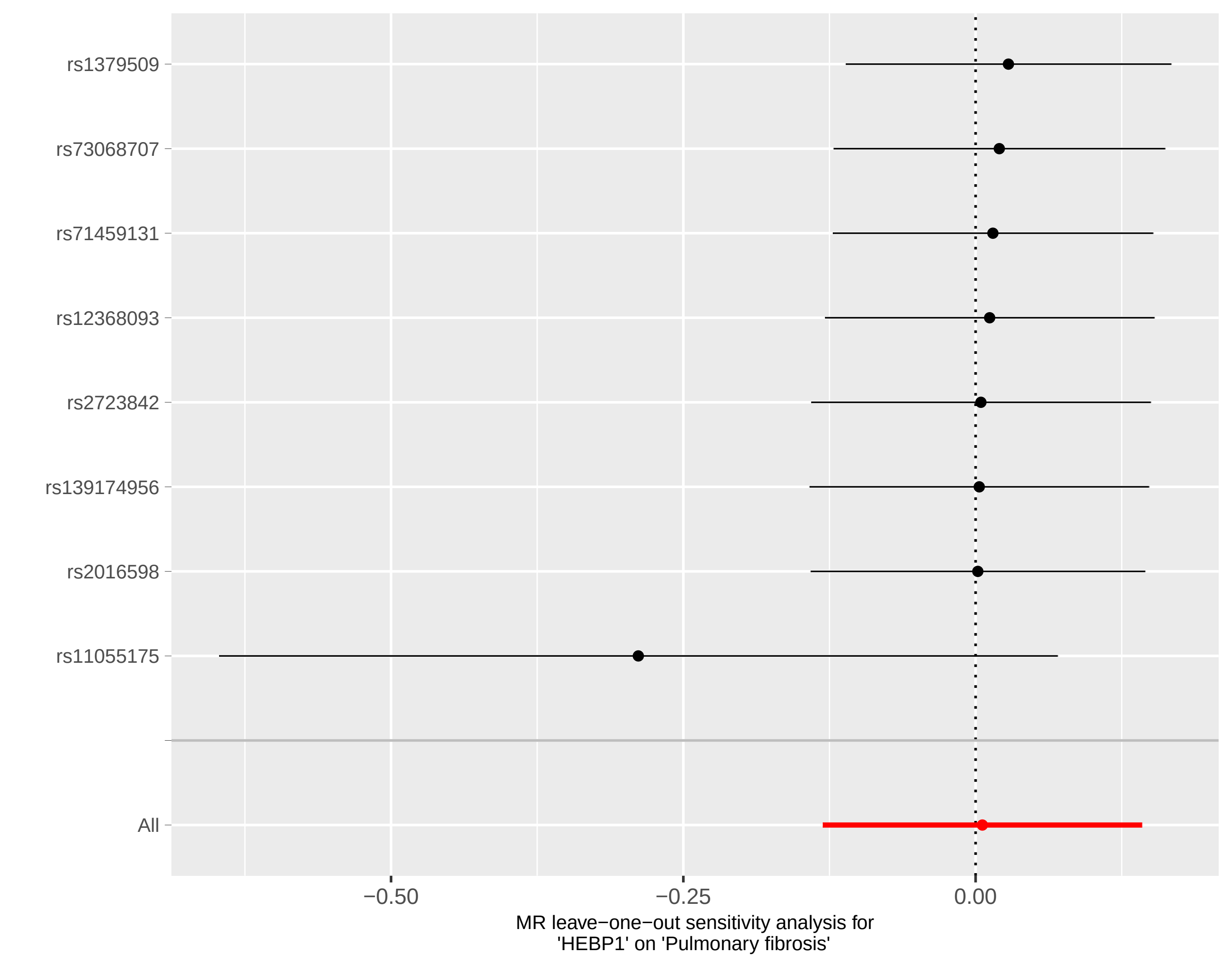
rs1379509
rs73068707
rs71459131
rs12368093
rs2723842
rs139174956
rs2016598
rs11055175
All
−0.50
−0.25
0.00
MR leave−one−out sensitivity analysis for
'HEBP1' on 'Pulmonary fibrosis'

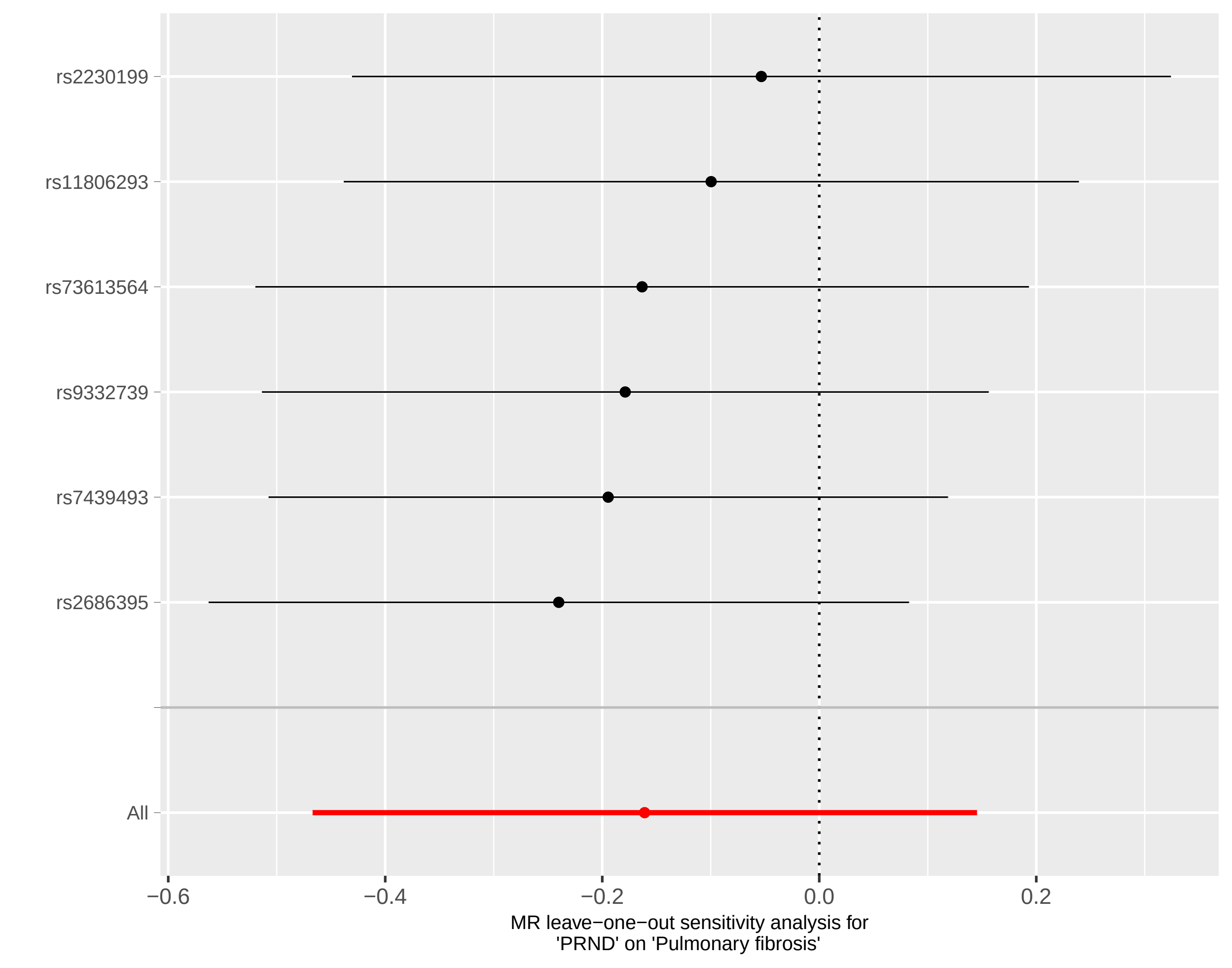

rs2230199
rs11806293
rs73613564
rs9332739
rs7439493
rs2686395
All
−0.6
−0.4
−0.2
0.0
0.2
MR leave−one−out sensitivity analysis for
'PRND' on 'Pulmonary fibrosis'

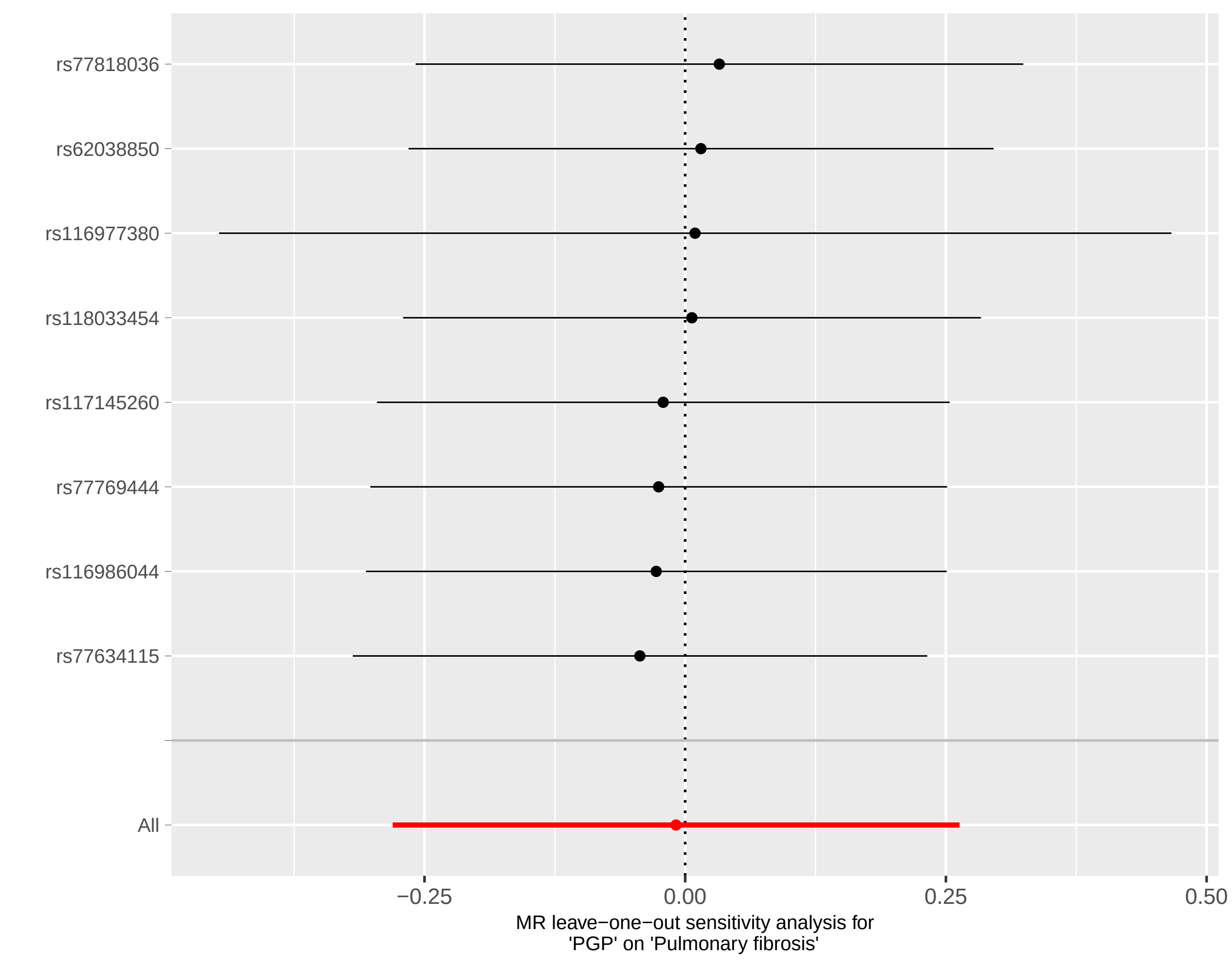
rs77818036
rs62038850
rs116977380
rs118033454
rs117145260
rs77769444
rs116986044
rs77634115
All
−0.25
0.00
0.25
0.50
MR leave−one−out sensitivity analysis for
'PGP' on 'Pulmonary fibrosis'

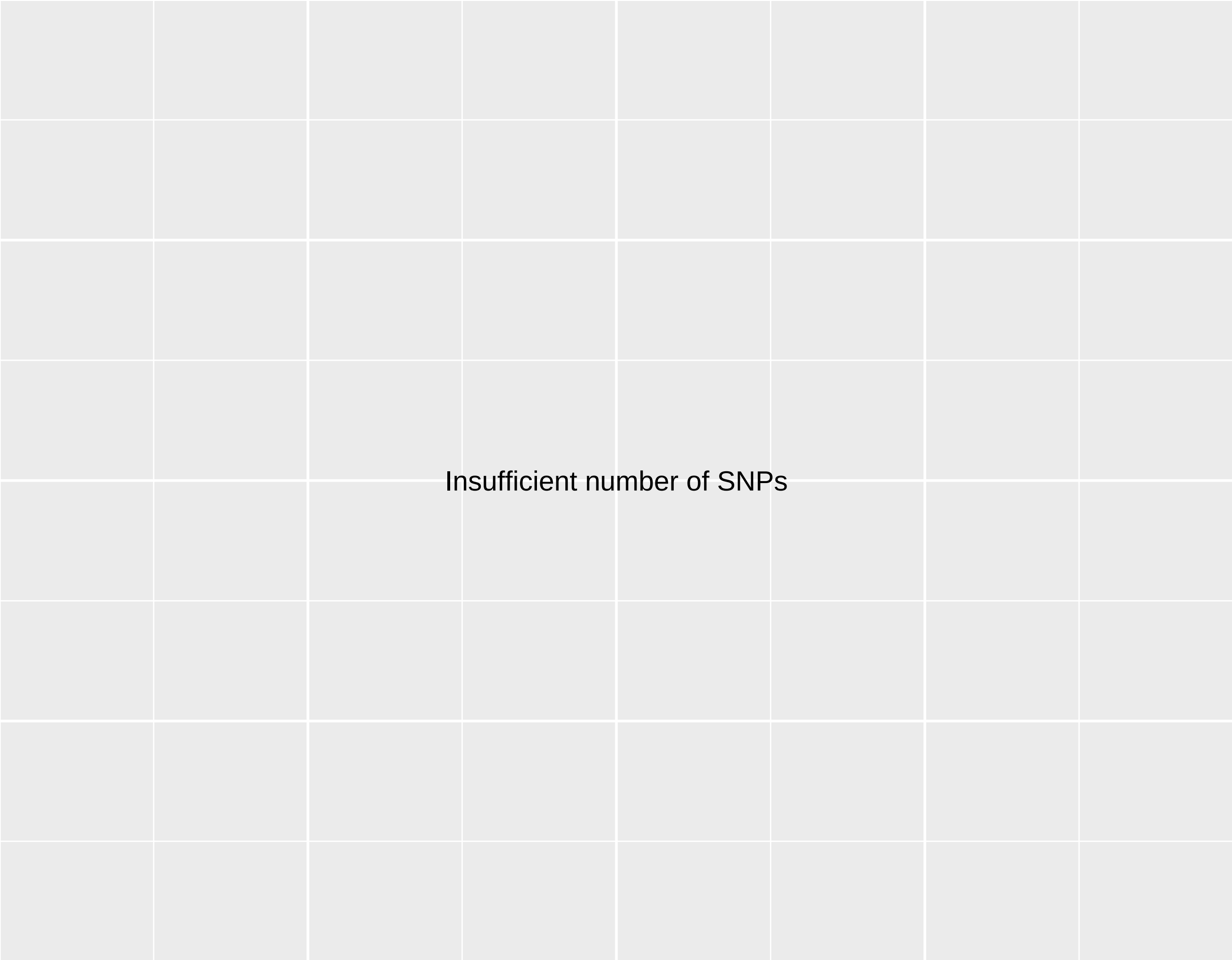
Insufficient number of SNPs

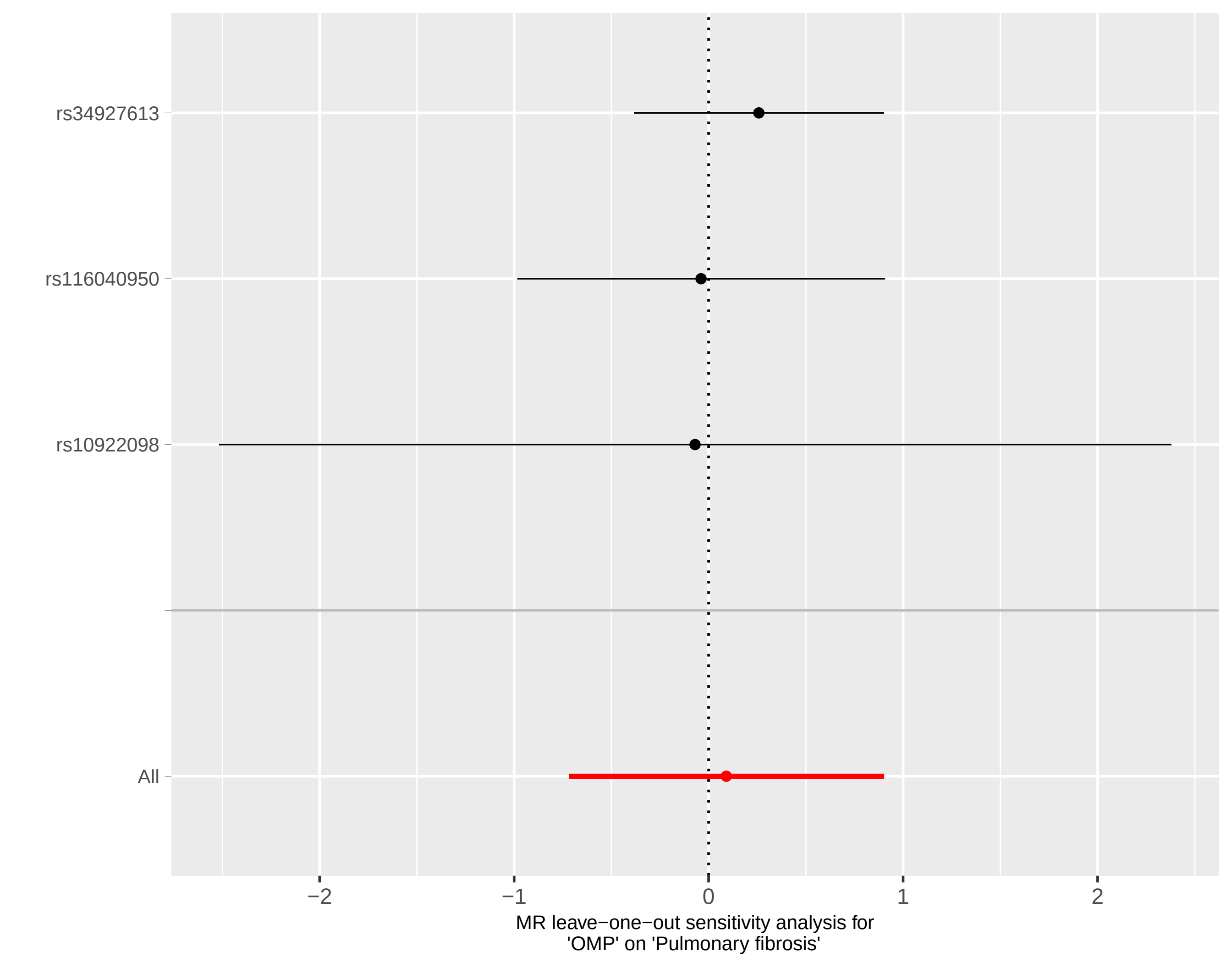
rs34927613
rs116040950
rs10922098
All
−2
−1
0
1
2
MR leave−one−out sensitivity analysis for
'OMP' on 'Pulmonary fibrosis'

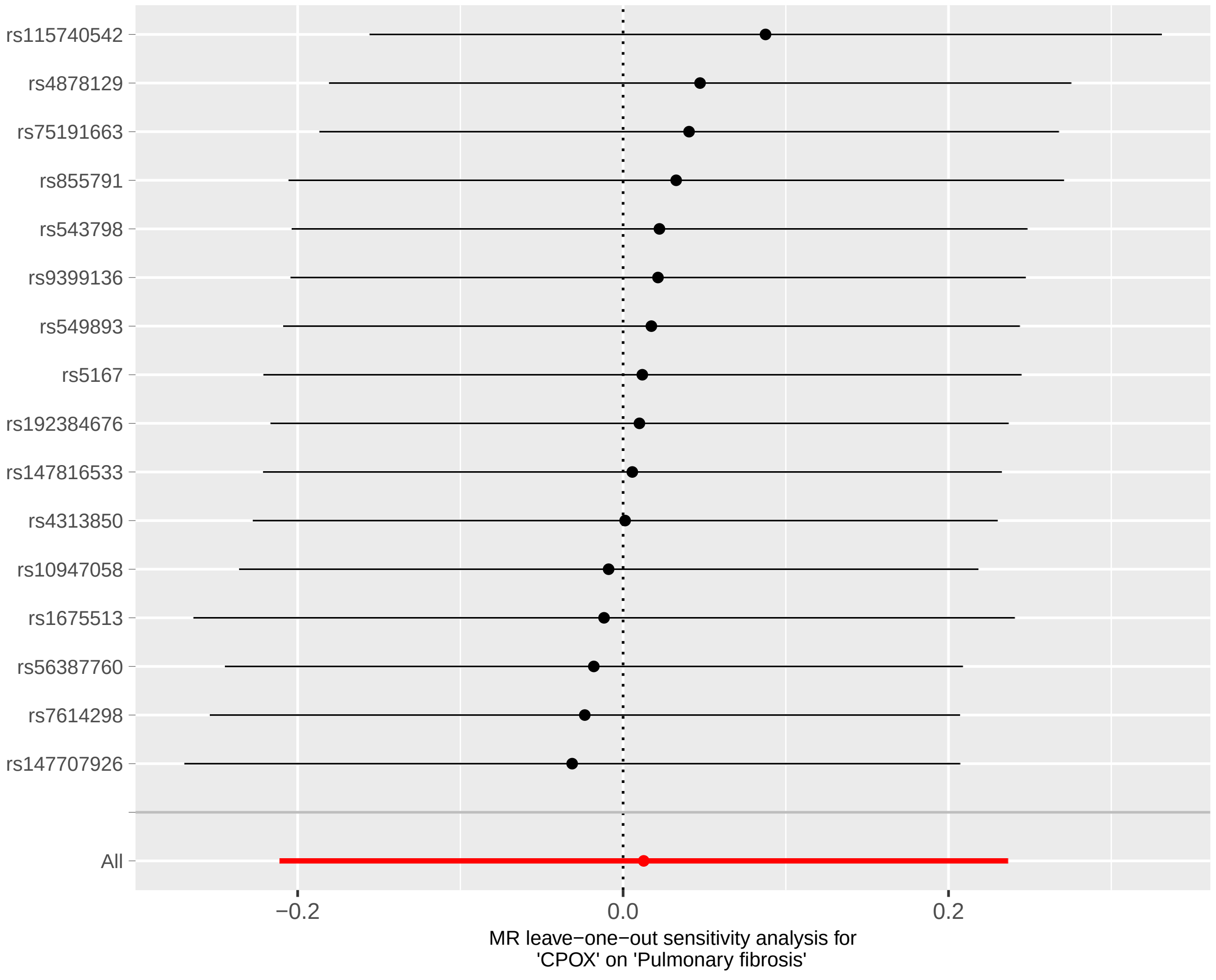

rs115740542
rs4878129
rs75191663
rs855791
rs543798
rs9399136
rs549893
rs5167
rs192384676
rs147816533
rs4313850
rs10947058
rs1675513
rs56387760
rs7614298
rs147707926
All
−0.2
0.0
0.2
MR leave−one−out sensitivity analysis for
'CPOX' on 'Pulmonary fibrosis'

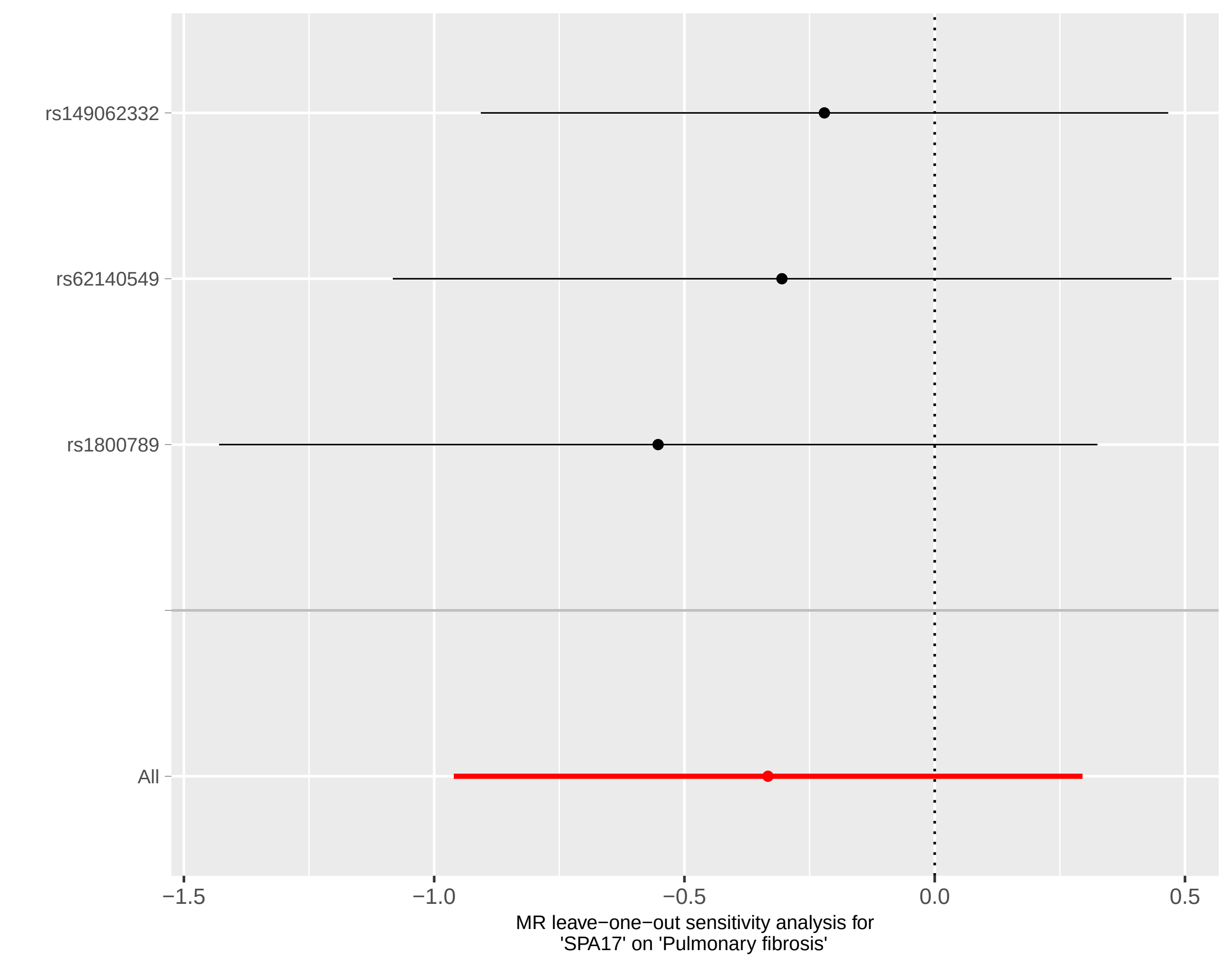
rs149062332
rs62140549
rs1800789
All
−1.5
−1.0
−0.5
0.0
0.5
MR leave−one−out sensitivity analysis for
'SPA17' on 'Pulmonary fibrosis'

Insufficient number of SNPs

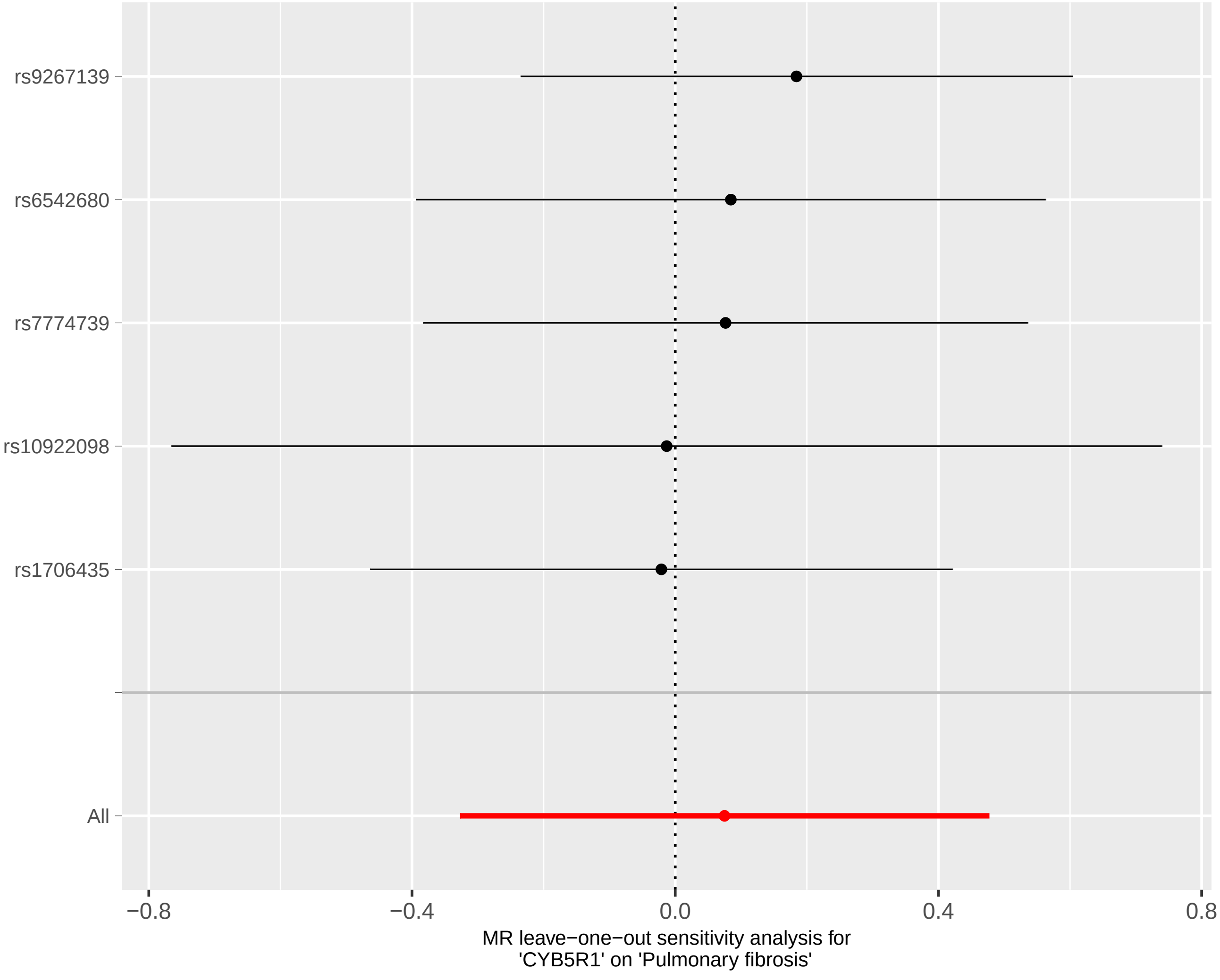

rs9267139
rs6542680
rs7774739
rs10922098
rs1706435
All
−0.8
−0.4
0.0
0.4
0.8
MR leave−one−out sensitivity analysis for
'CYB5R1' on 'Pulmonary fibrosis'

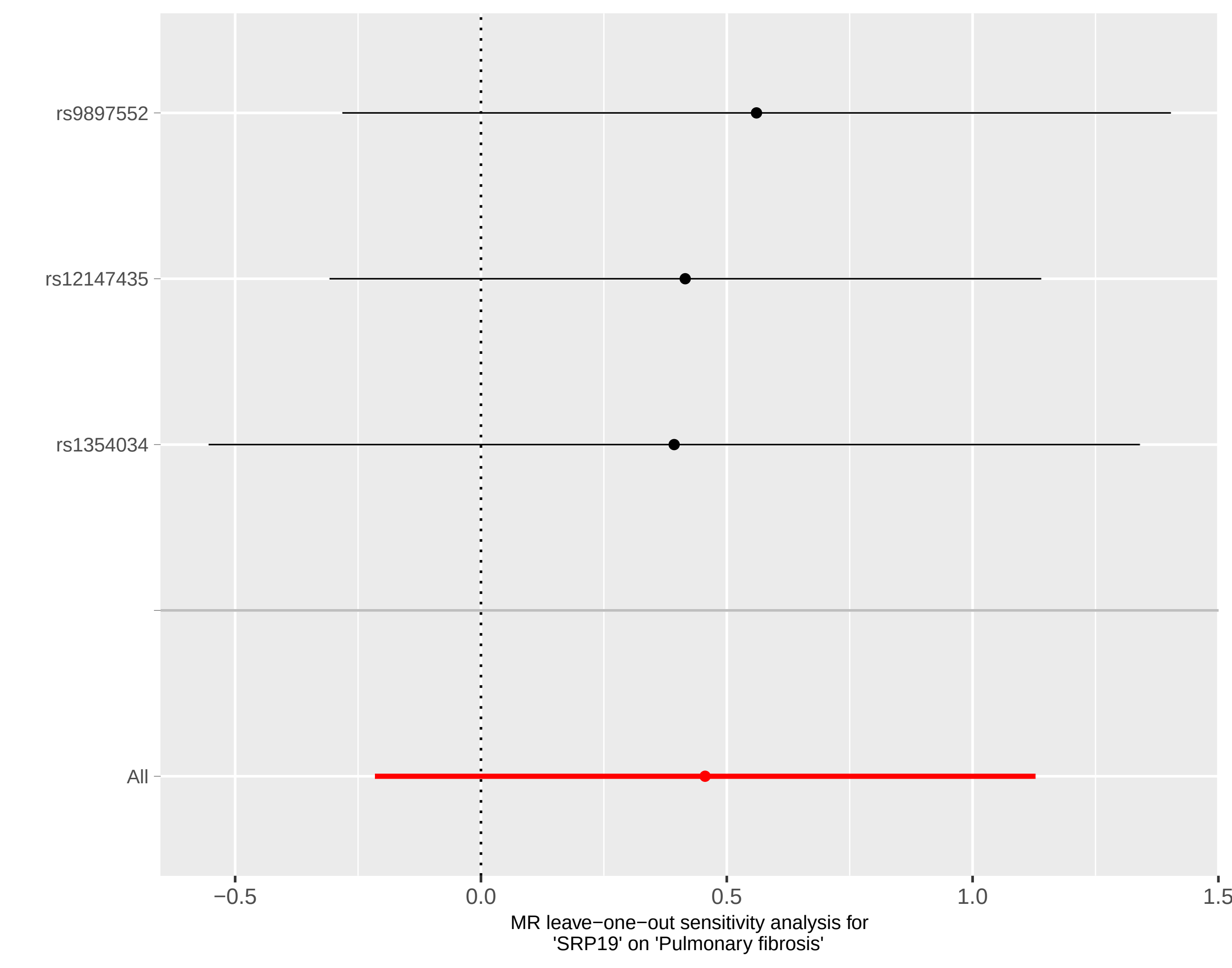
rs9897552
rs12147435
rs1354034
All
−0.5
0.0
0.5
1.0
1.5
MR leave−one−out sensitivity analysis for
'SRP19' on 'Pulmonary fibrosis'

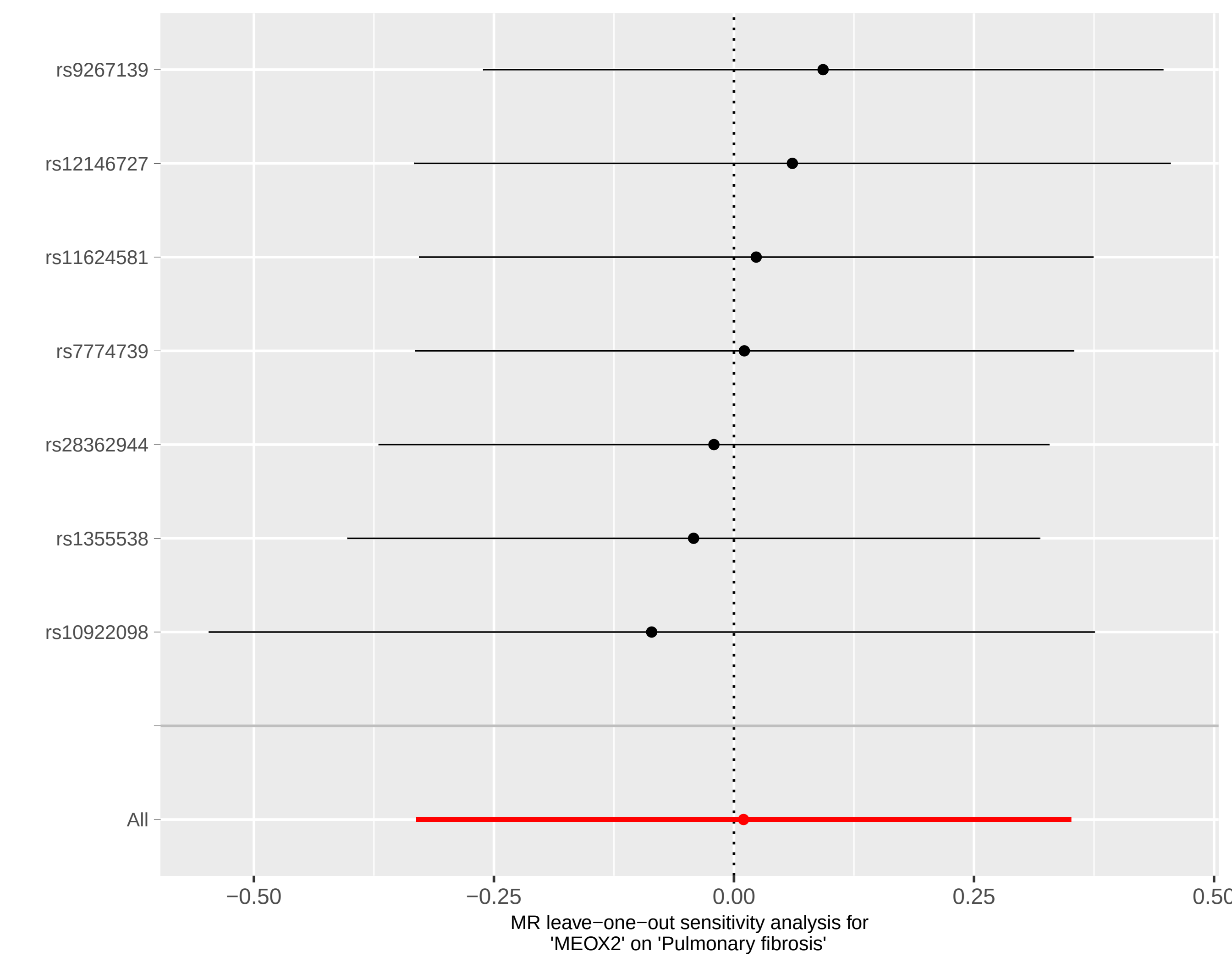

rs9267139
rs12146727
rs11624581
rs7774739
rs28362944
rs1355538
rs10922098
All
−0.50
−0.25
0.00
0.25
0.50
MR leave−one−out sensitivity analysis for
'MEOX2' on 'Pulmonary fibrosis'

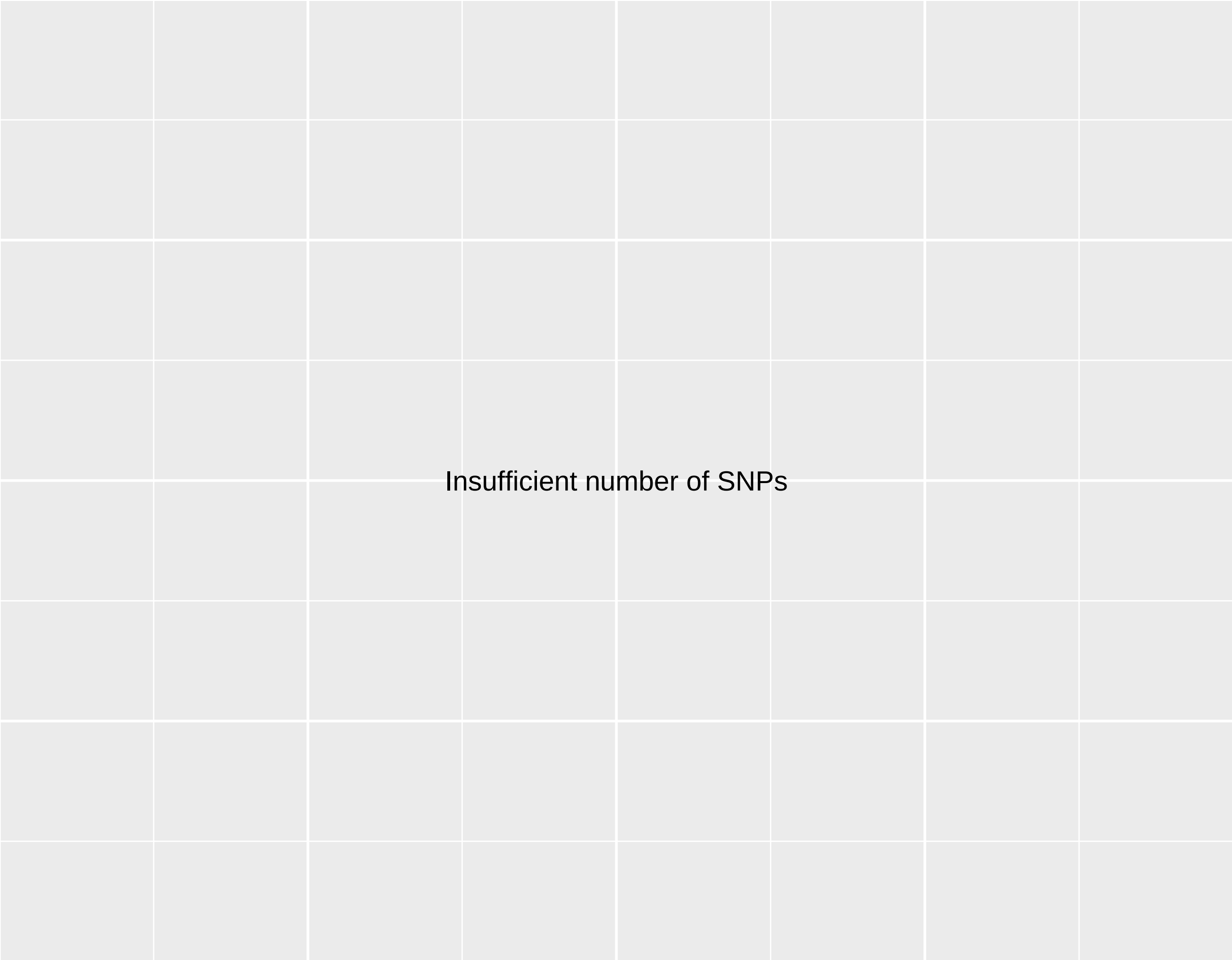
Insufficient number of SNPs

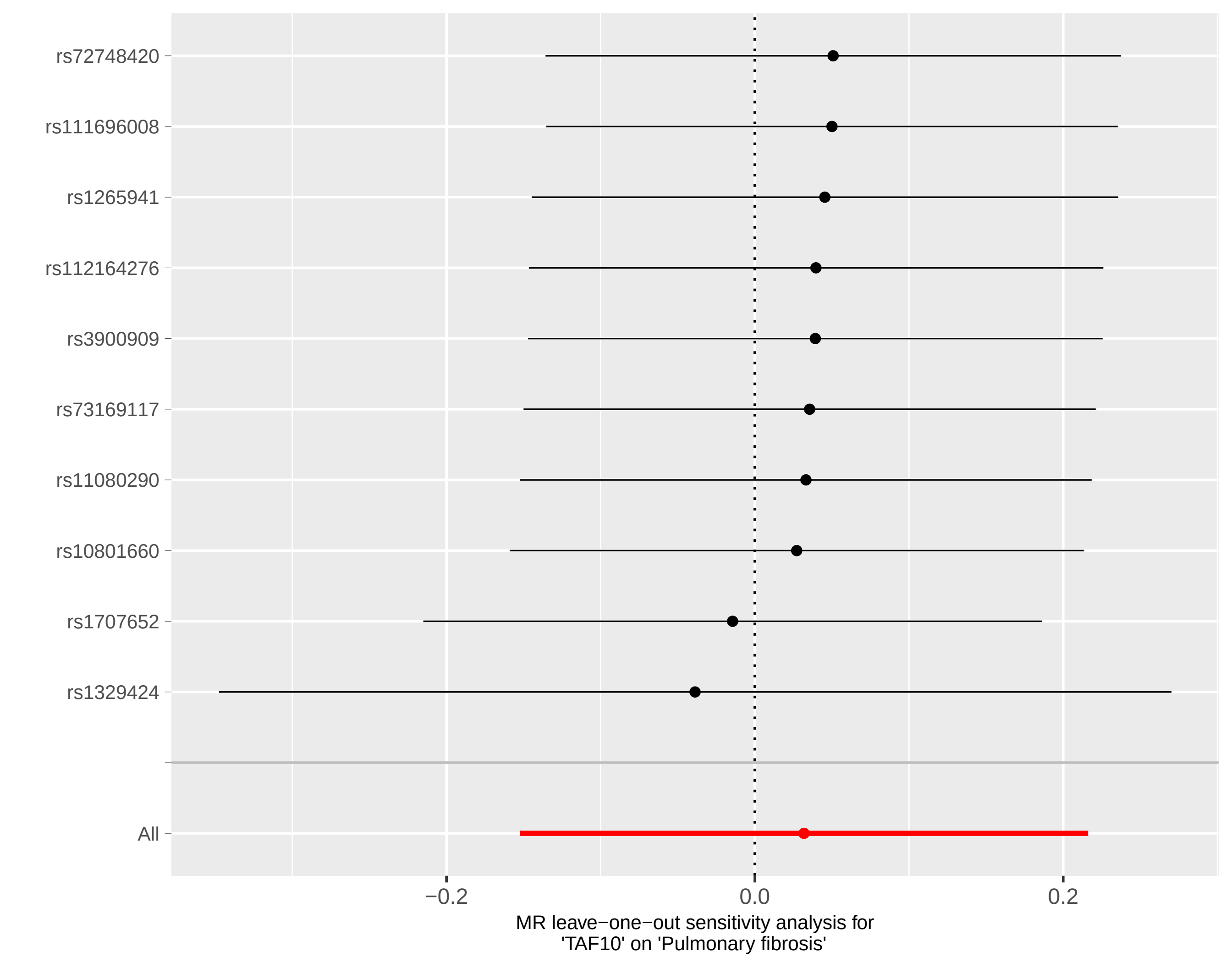
rs72748420
rs111696008
rs1265941
rs112164276
rs3900909
rs73169117
rs11080290
rs10801660
rs1707652
rs1329424
All
−0.2
0.0
0.2
MR leave−one−out sensitivity analysis for
'TAF10' on 'Pulmonary fibrosis'

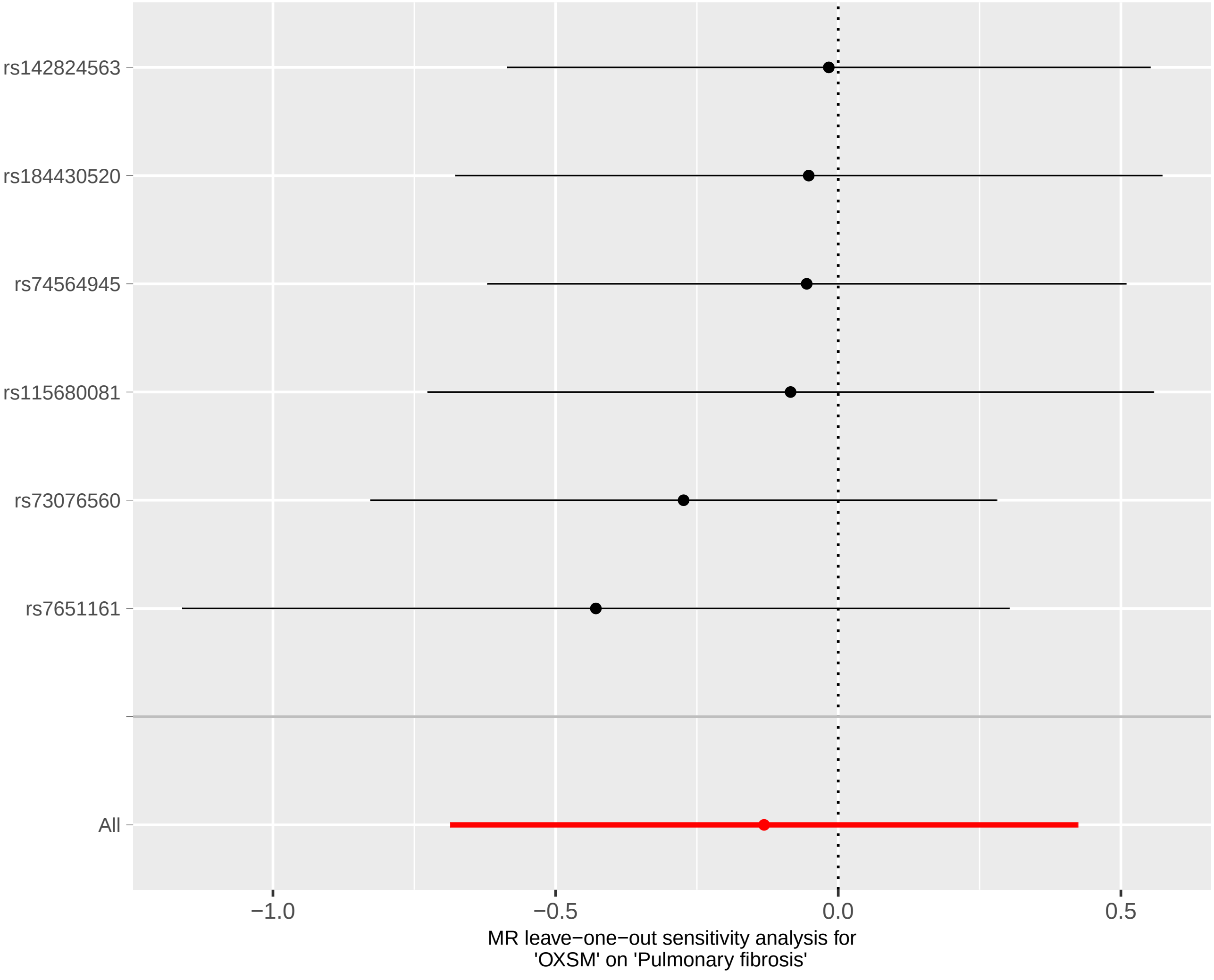
rs142824563
rs184430520
rs74564945
rs115680081
rs73076560
rs7651161
All
−1.0
−0.5
0.0
0.5
MR leave−one−out sensitivity analysis for
'OXSM' on 'Pulmonary fibrosis'

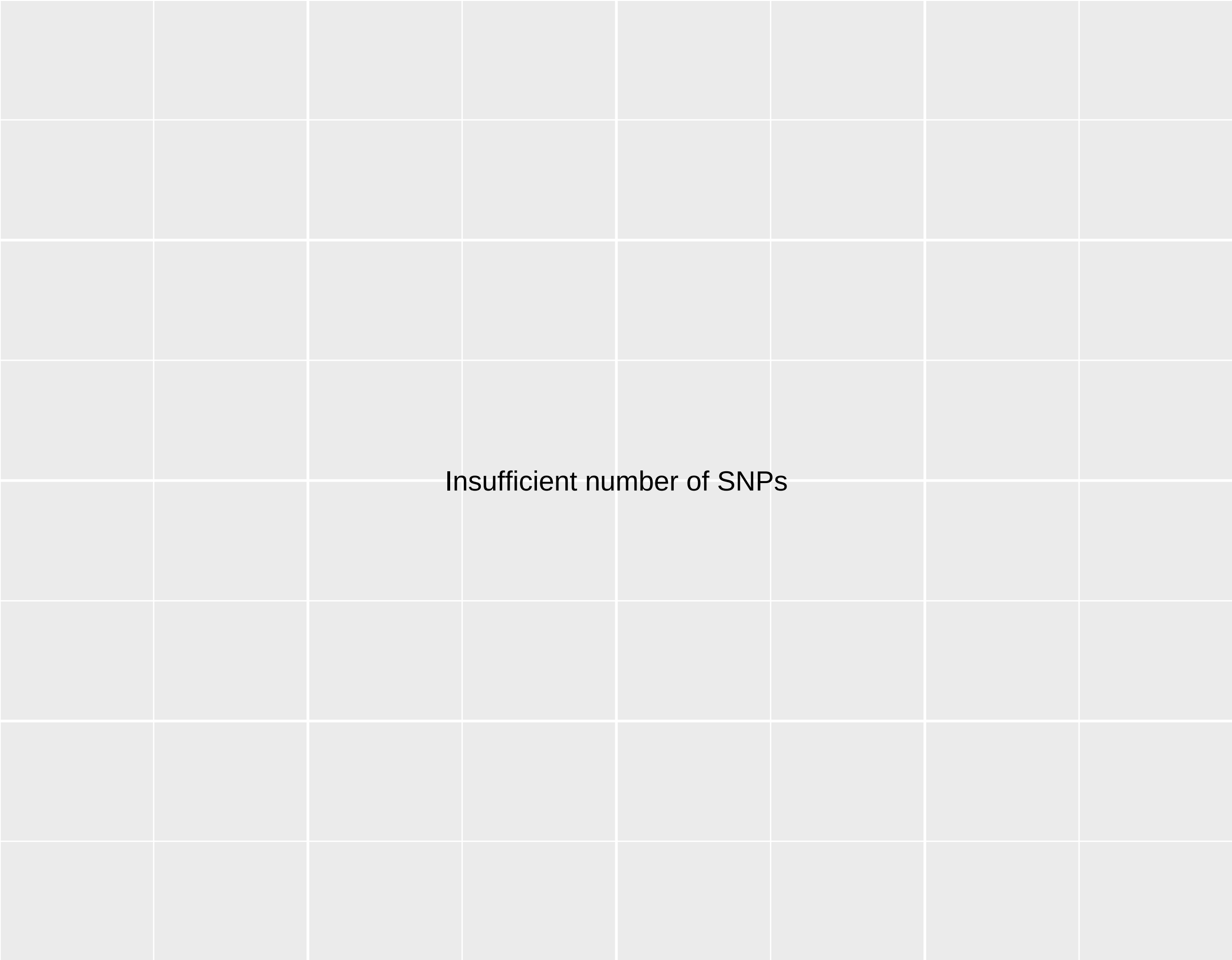
Insufficient number of SNPs

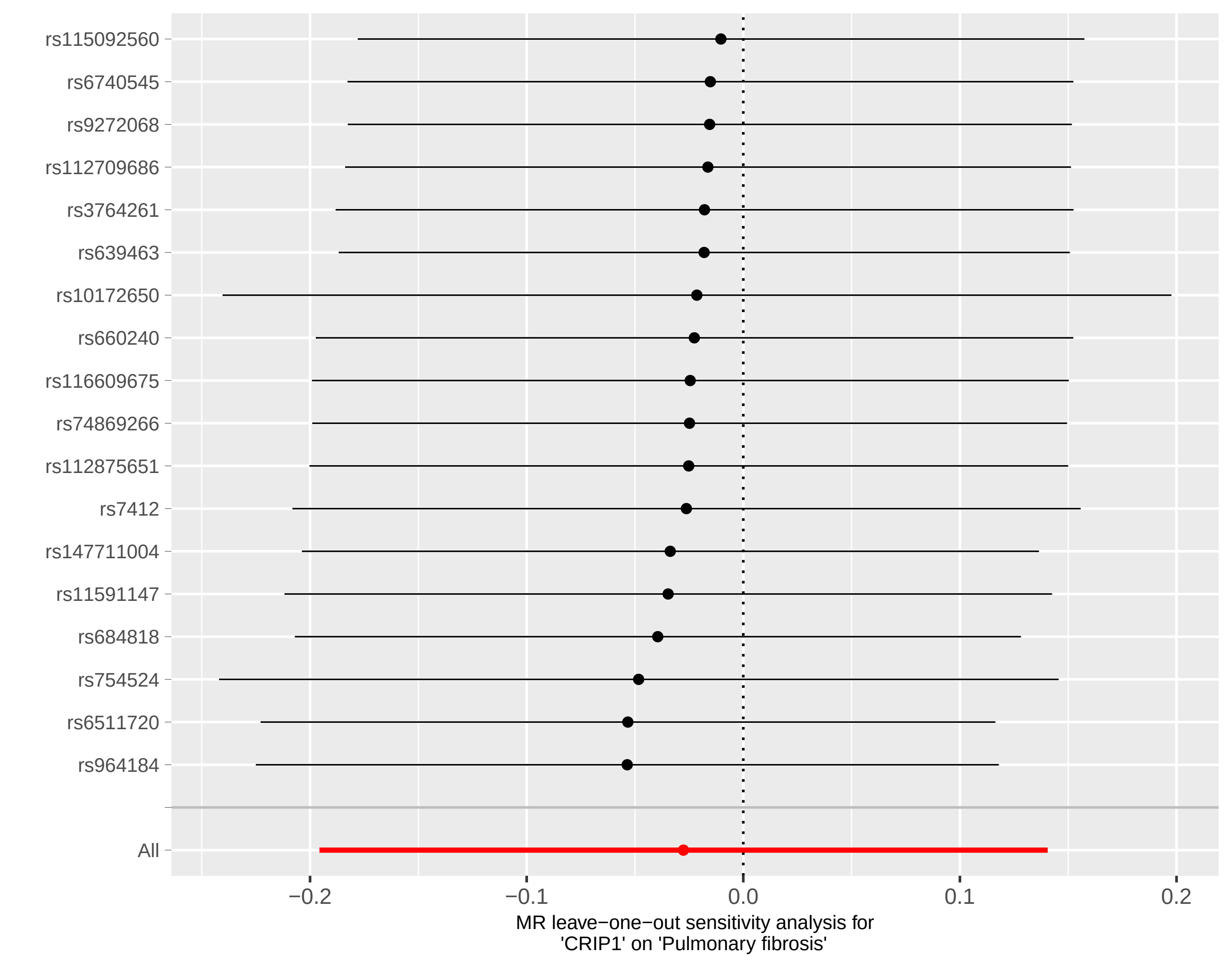
rs115092560
rs6740545
rs9272068
rs112709686
rs3764261
rs639463
rs10172650
rs660240
rs116609675
rs74869266
rs112875651
rs7412
rs147711004
rs11591147
rs684818
rs754524
rs6511720
rs964184
All
−0.2
−0.1
0.0
0.1
0.2
MR leave−one−out sensitivity analysis for
'CRIP1' on 'Pulmonary fibrosis'

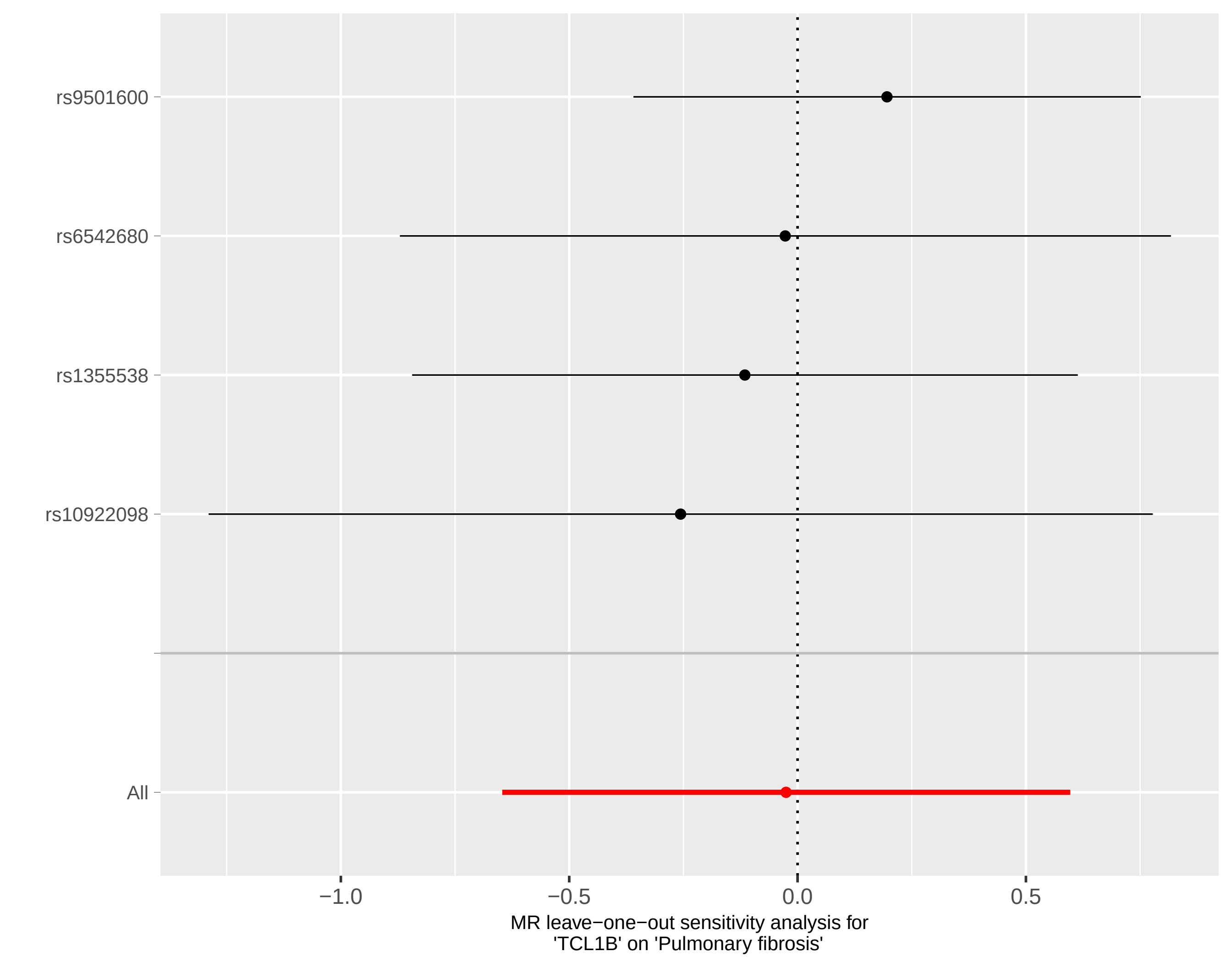
rs9501600
rs6542680
rs1355538
rs10922098
All
−1.0
−0.5
0.0
0.5
MR leave−one−out sensitivity analysis for
'TCL1B' on 'Pulmonary fibrosis'

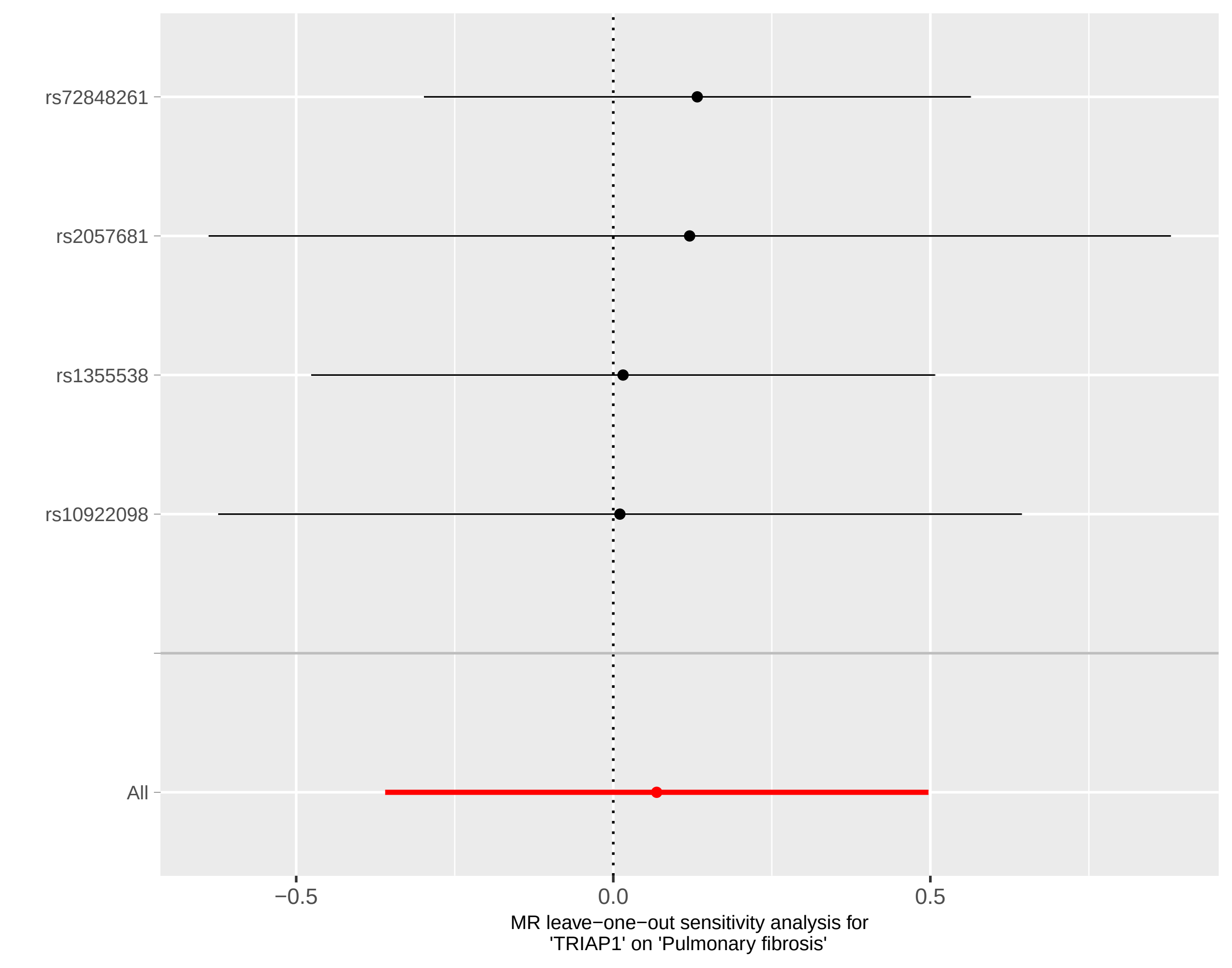

rs72848261
rs2057681
rs1355538
rs10922098
All
−0.5
0.0
0.5
MR leave−one−out sensitivity analysis for
'TRIAP1' on 'Pulmonary fibrosis'

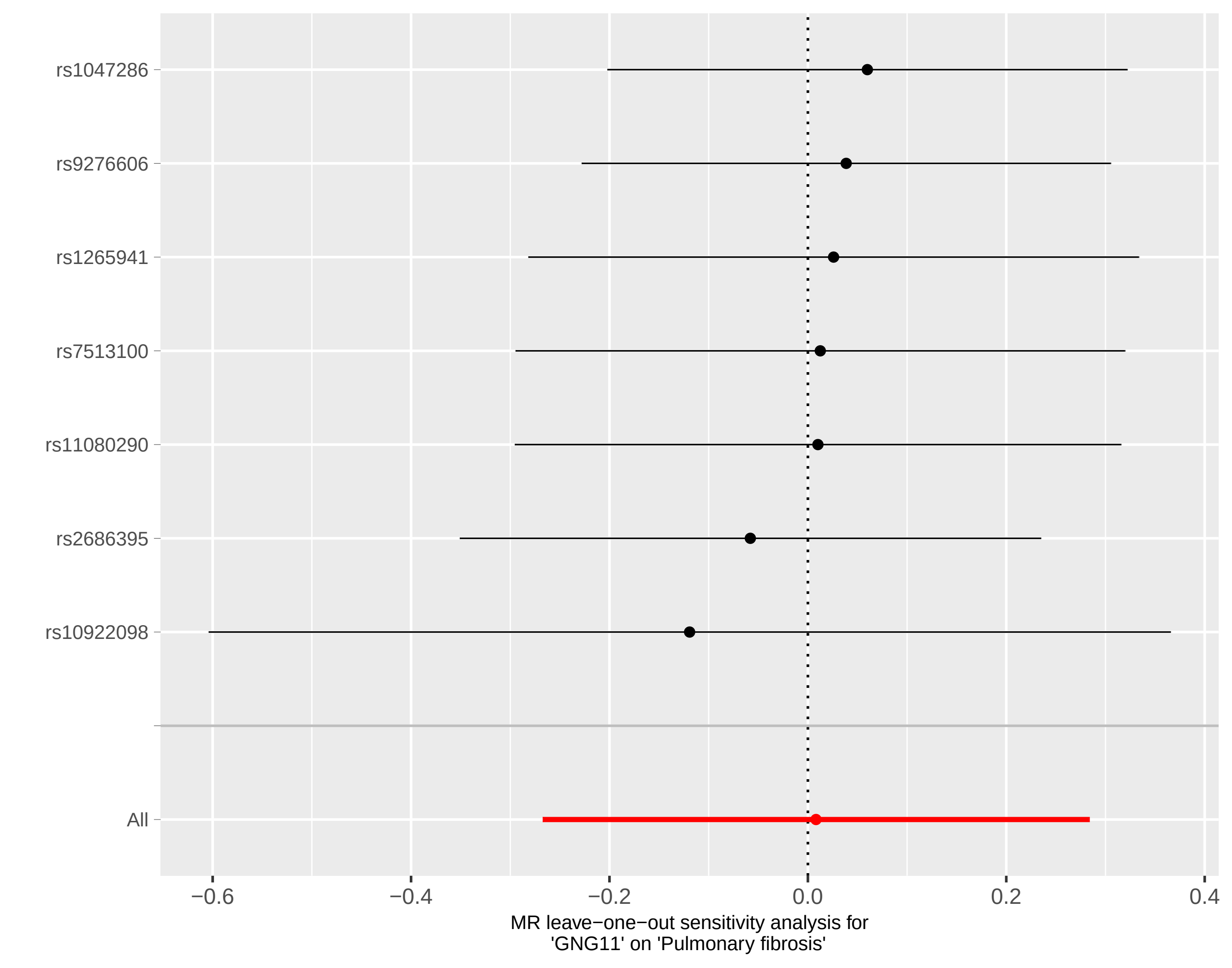
rs1047286
rs9276606
rs1265941
rs7513100
rs11080290
rs2686395
rs10922098
All
−0.6
−0.4
−0.2
0.0
0.2
0.4
MR leave−one−out sensitivity analysis for
'GNG11' on 'Pulmonary fibrosis'

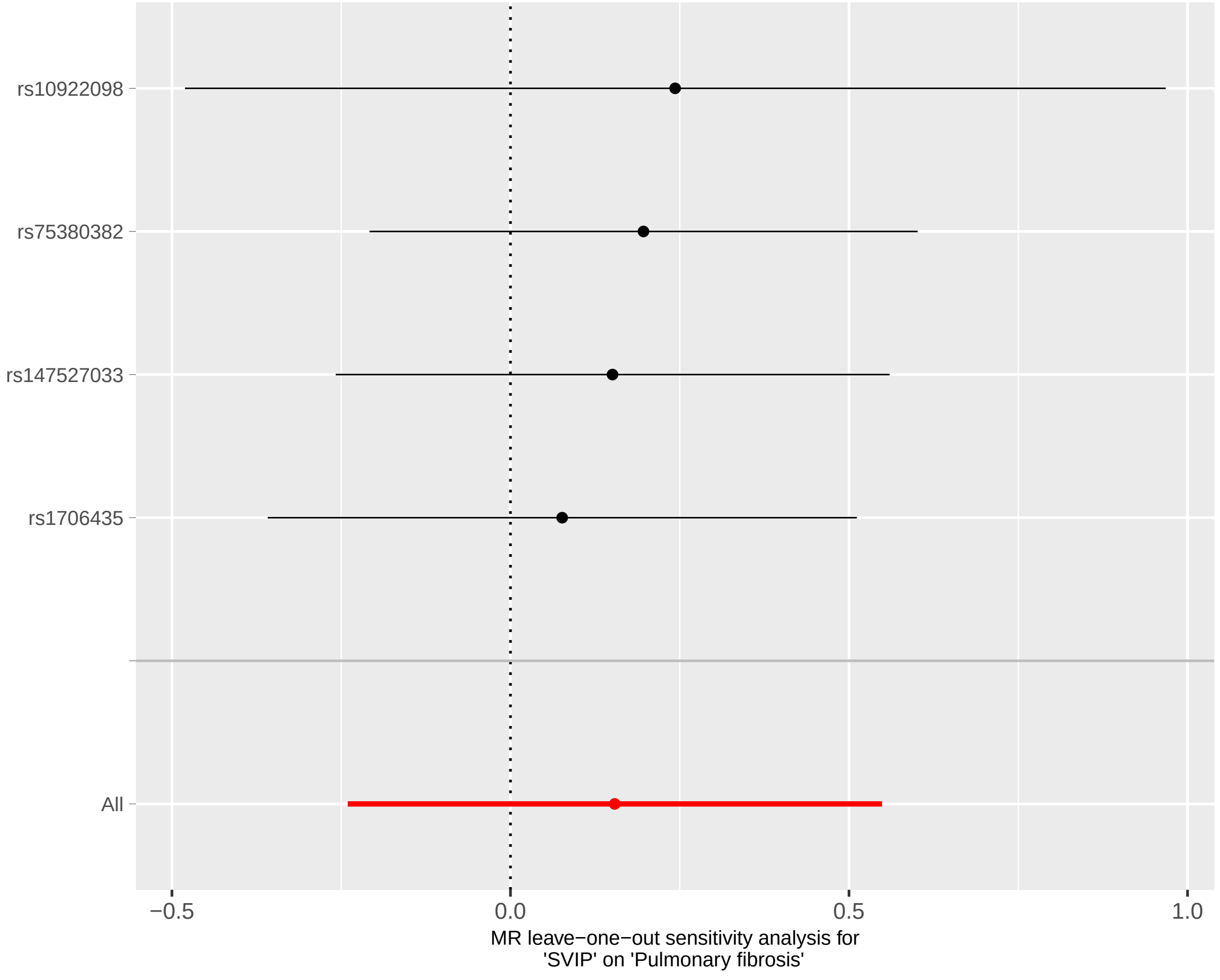
rs10922098
rs75380382
rs147527033
rs1706435
All
−0.5
0.0
0.5
1.0
MR leave−one−out sensitivity analysis for
'SVIP' on 'Pulmonary fibrosis'

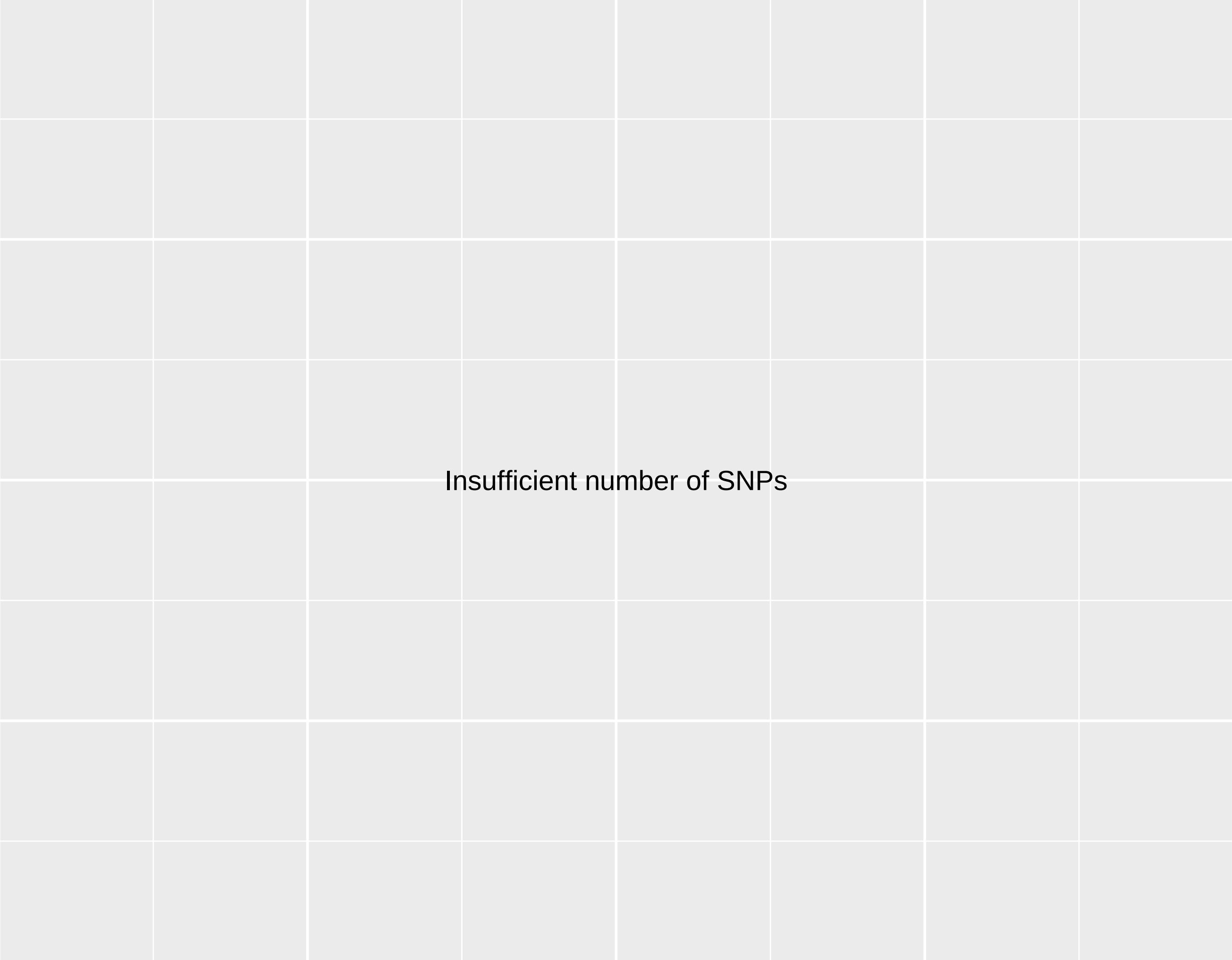
Insufficient number of SNPs

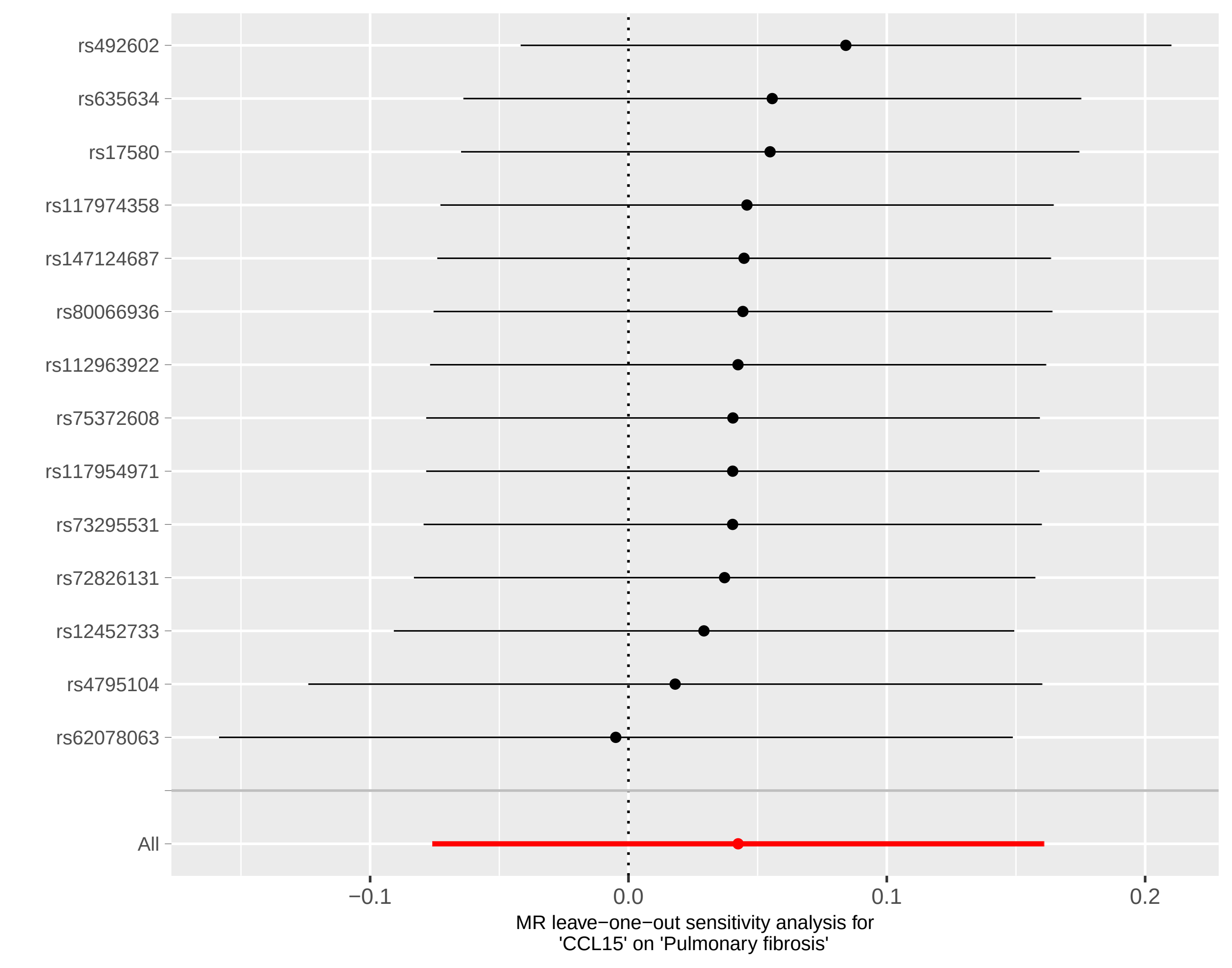
rs492602
rs635634
rs17580
rs117974358
rs147124687
rs80066936
rs112963922
rs75372608
rs117954971
rs73295531
rs72826131
rs12452733
rs4795104
rs62078063
All
−0.1
0.0
0.1
0.2
MR leave−one−out sensitivity analysis for
'CCL15' on 'Pulmonary fibrosis'

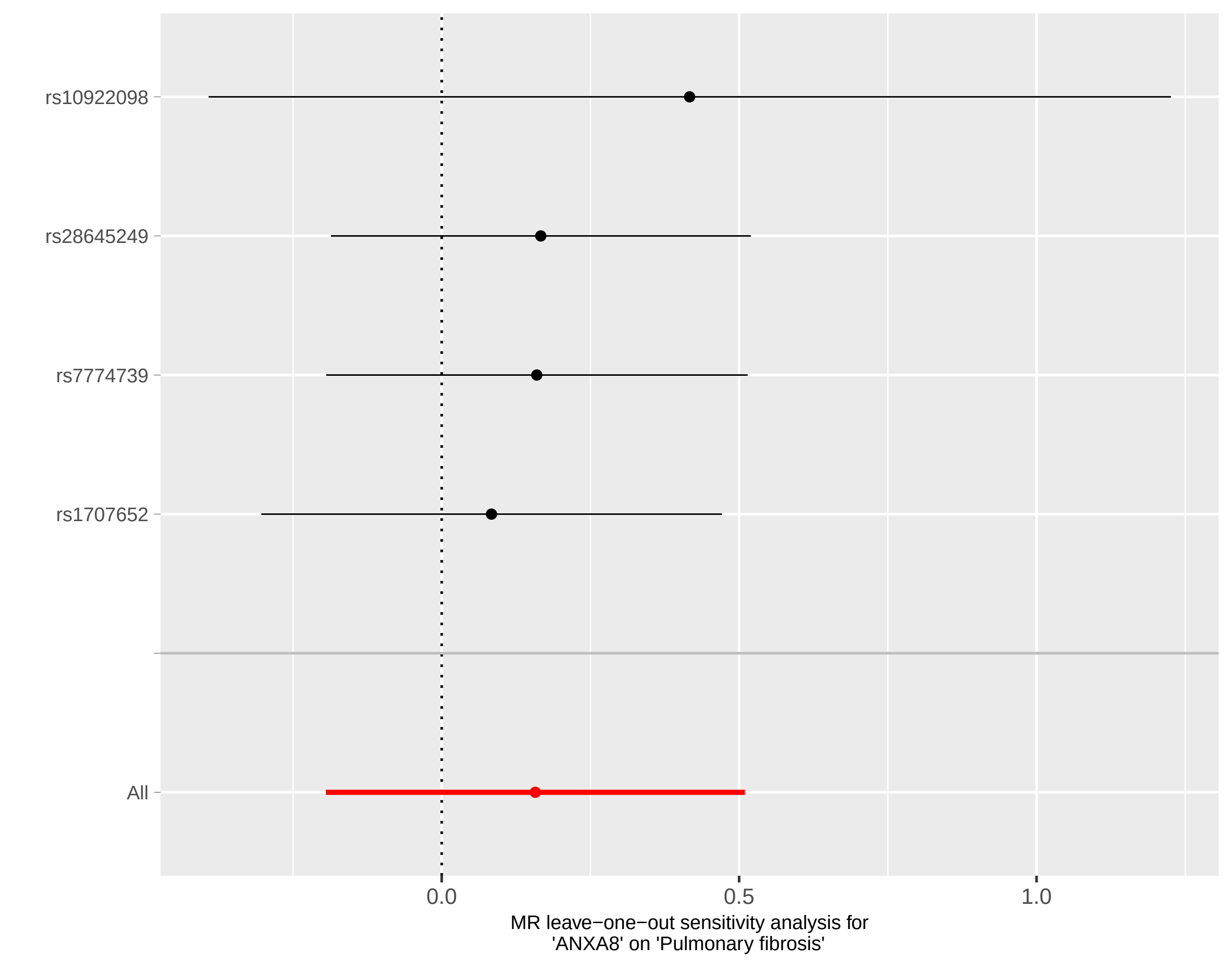
rs10922098
rs28645249
rs7774739
rs1707652
All
0.0
0.5
1.0
MR leave−one−out sensitivity analysis for
'ANXA8' on 'Pulmonary fibrosis'

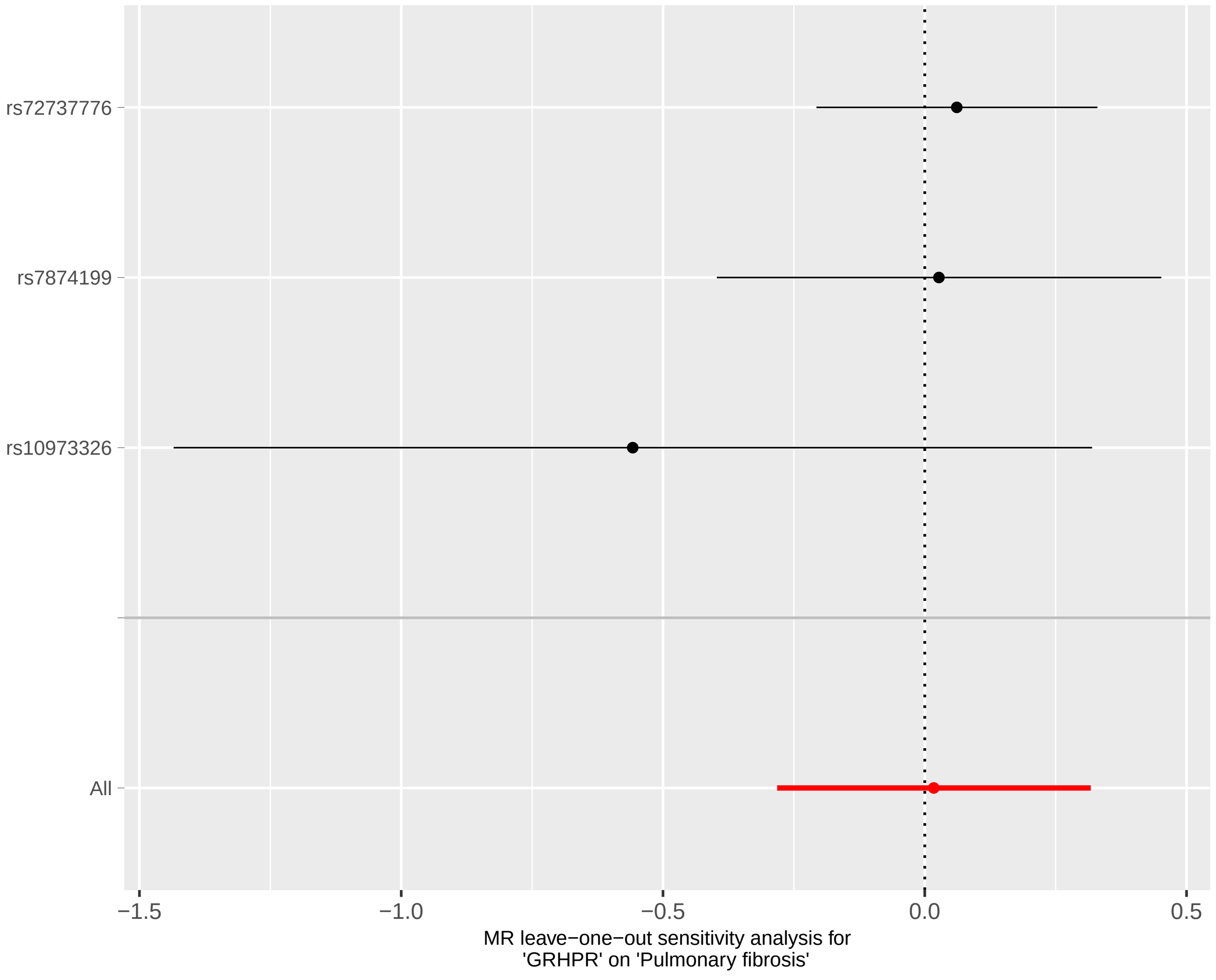

rs72737776
rs7874199
rs10973326
All
−1.5
−1.0
−0.5
0.0
0.5
MR leave−one−out sensitivity analysis for
'GRHPR' on 'Pulmonary fibrosis'

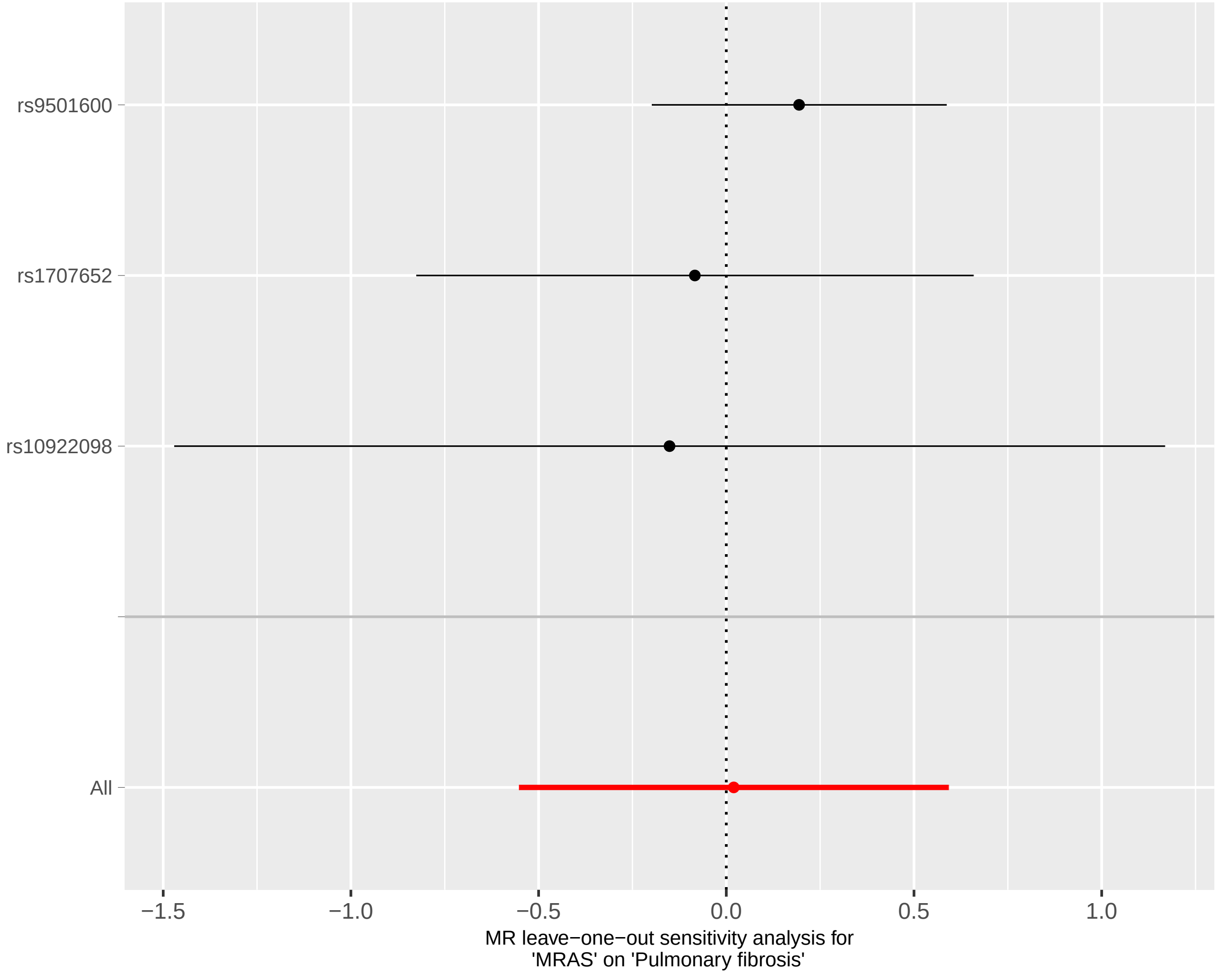
rs9501600
rs1707652
rs10922098
All
−1.5
−1.0
−0.5
0.0
0.5
1.0
MR leave−one−out sensitivity analysis for
'MRAS' on 'Pulmonary fibrosis'

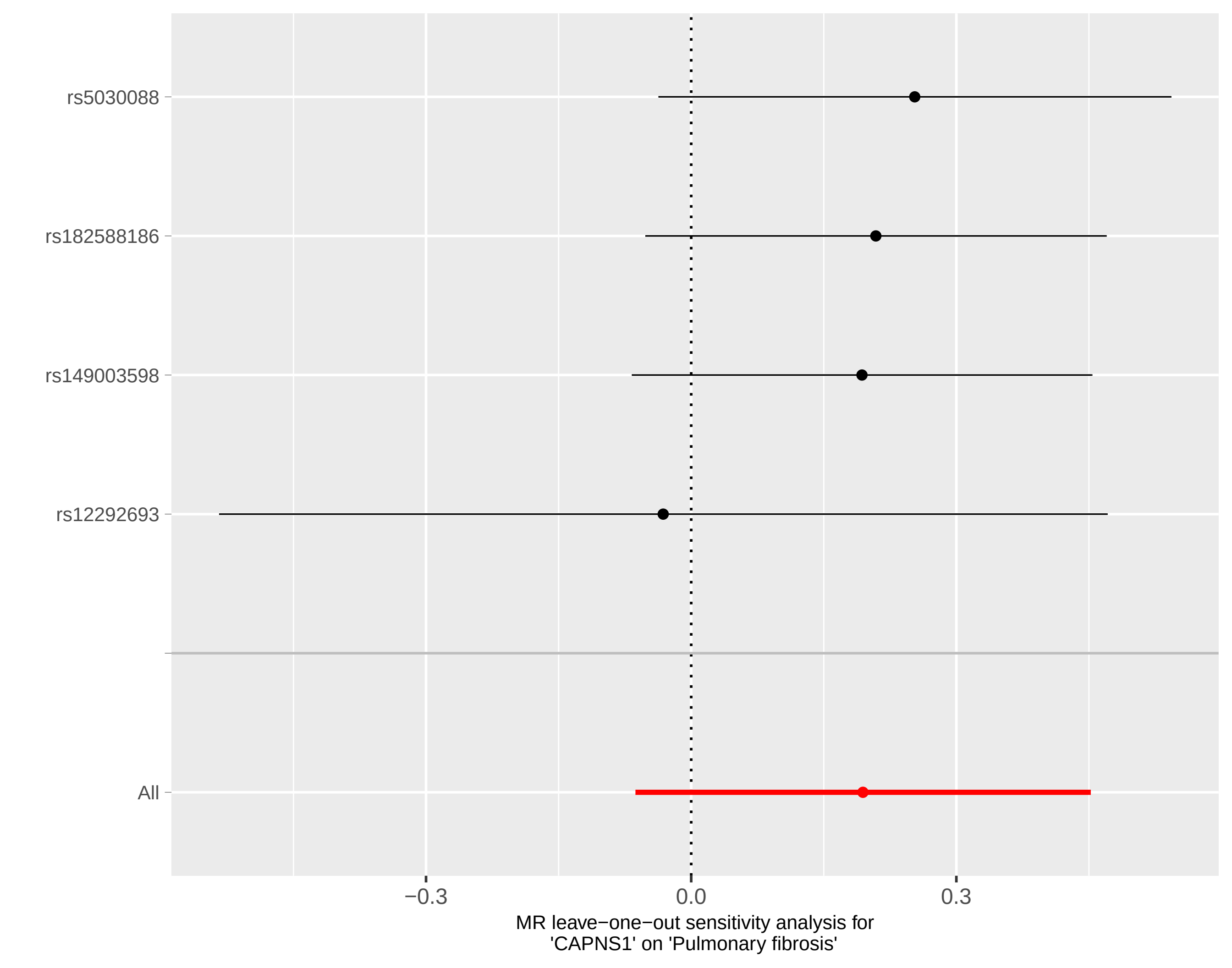
rs5030088
rs182588186
rs149003598
rs12292693
All
−0.3
0.0
0.3
MR leave−one−out sensitivity analysis for
'CAPNS1' on 'Pulmonary fibrosis'

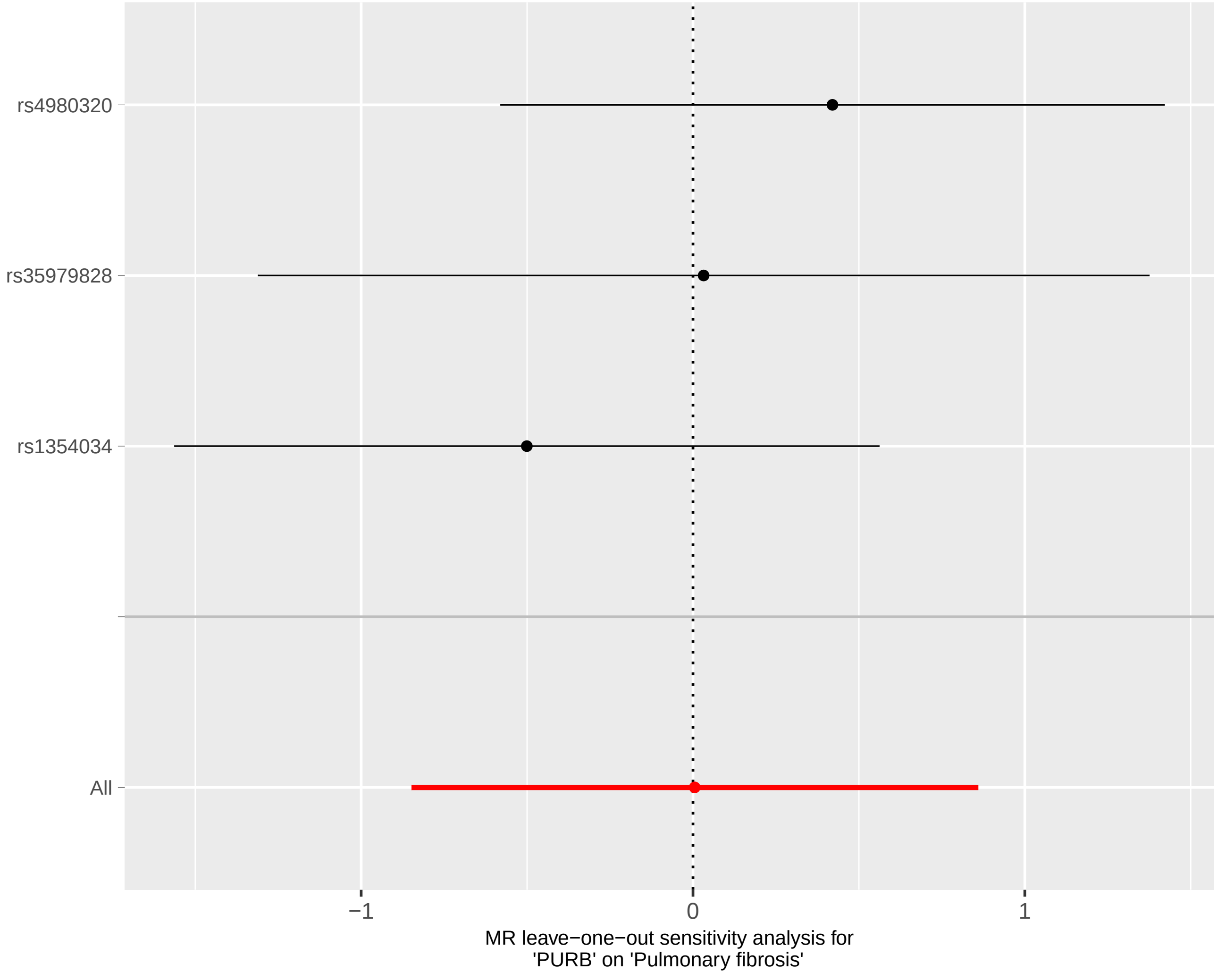
rs4980320
rs35979828
rs1354034
All
−1
0
1
MR leave−one−out sensitivity analysis for
'PURB' on 'Pulmonary fibrosis'

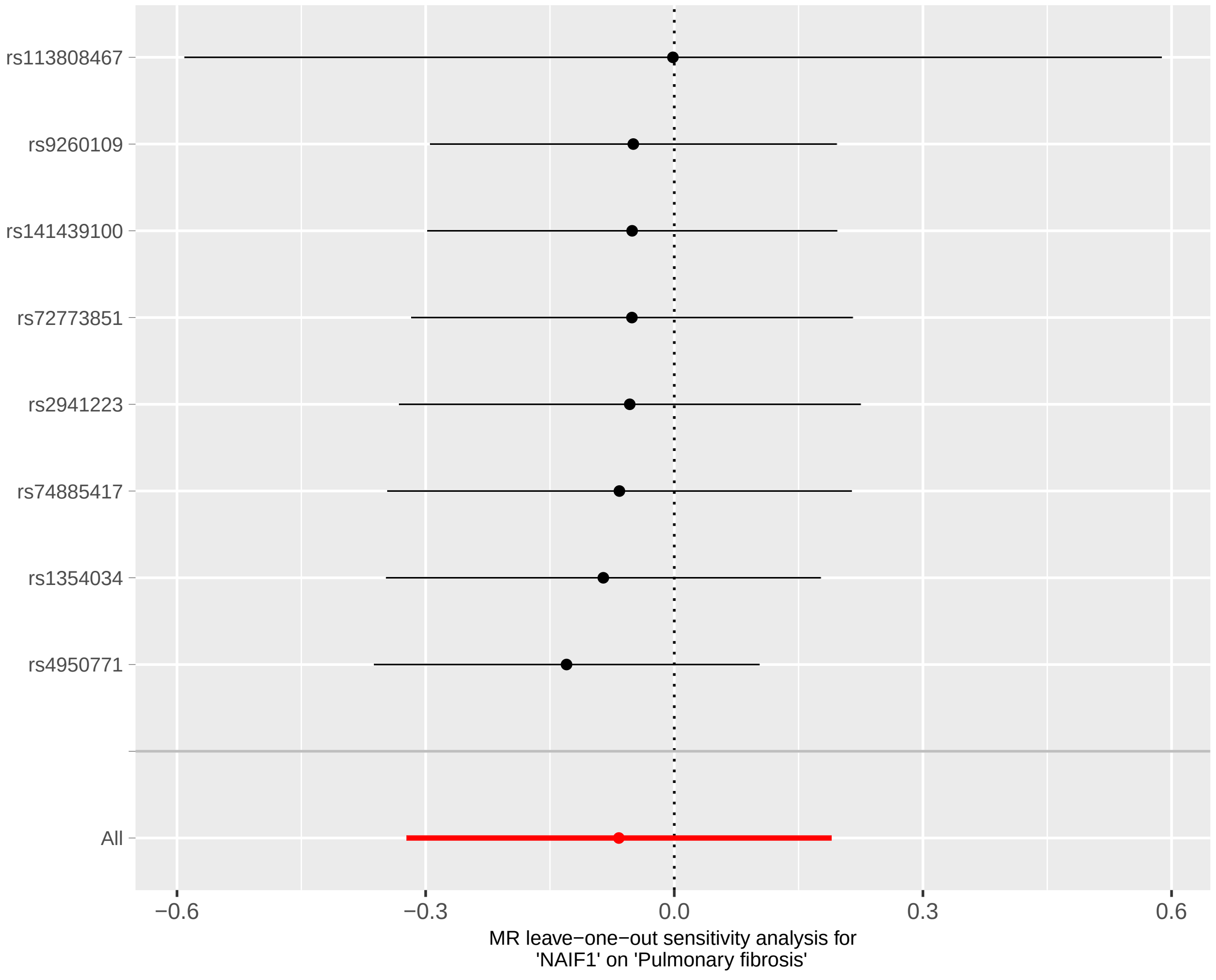

rs113808467
rs9260109
rs141439100
rs72773851
rs2941223
rs74885417
rs1354034
rs4950771
All
−0.6
−0.3
0.0
0.3
0.6
MR leave−one−out sensitivity analysis for
'NAIF1' on 'Pulmonary fibrosis'

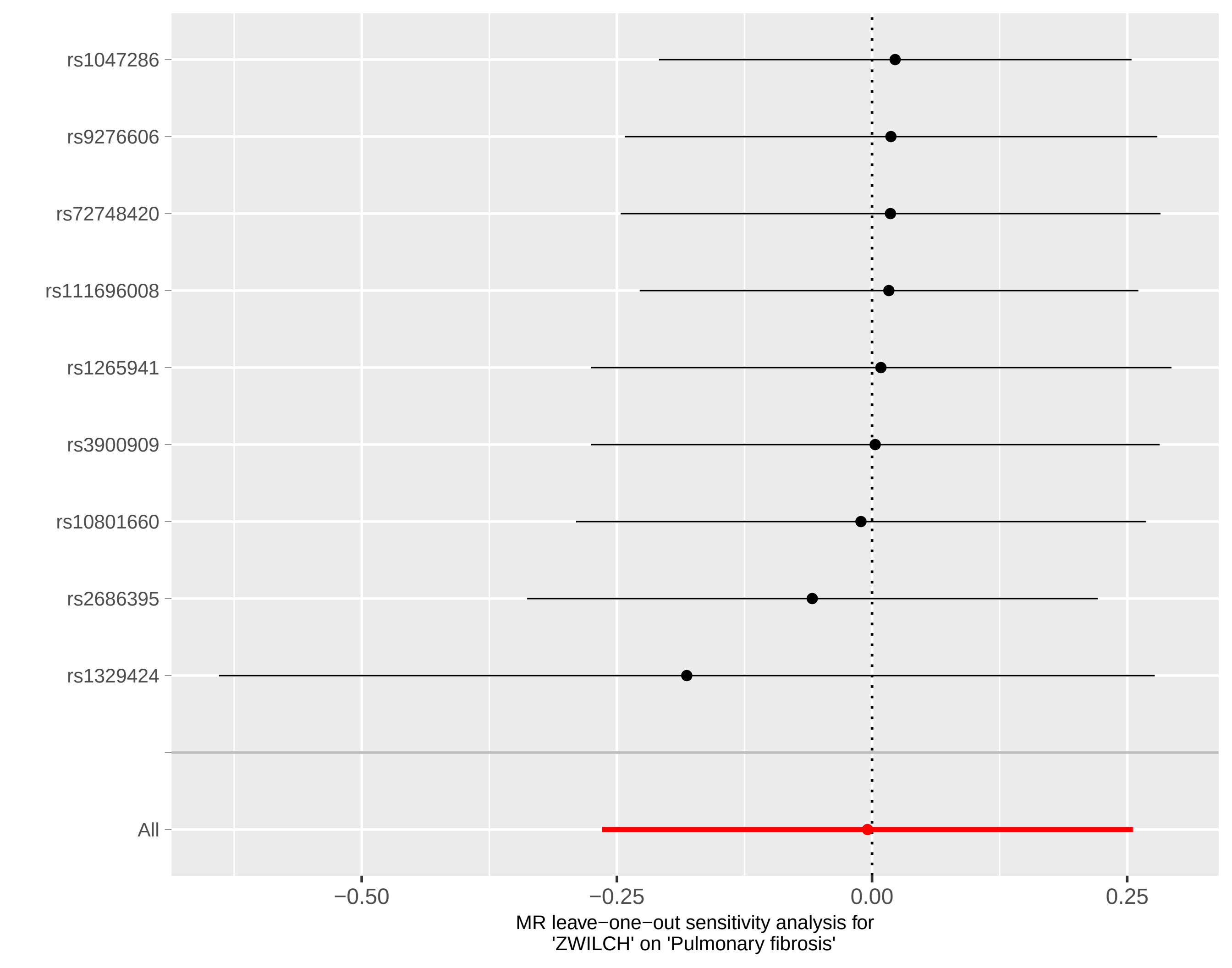
rs1047286
rs9276606
rs72748420
rs111696008
rs1265941
rs3900909
rs10801660
rs2686395
rs1329424
All
−0.50
−0.25
0.00
0.25
MR leave−one−out sensitivity analysis for
'ZWILCH' on 'Pulmonary fibrosis'

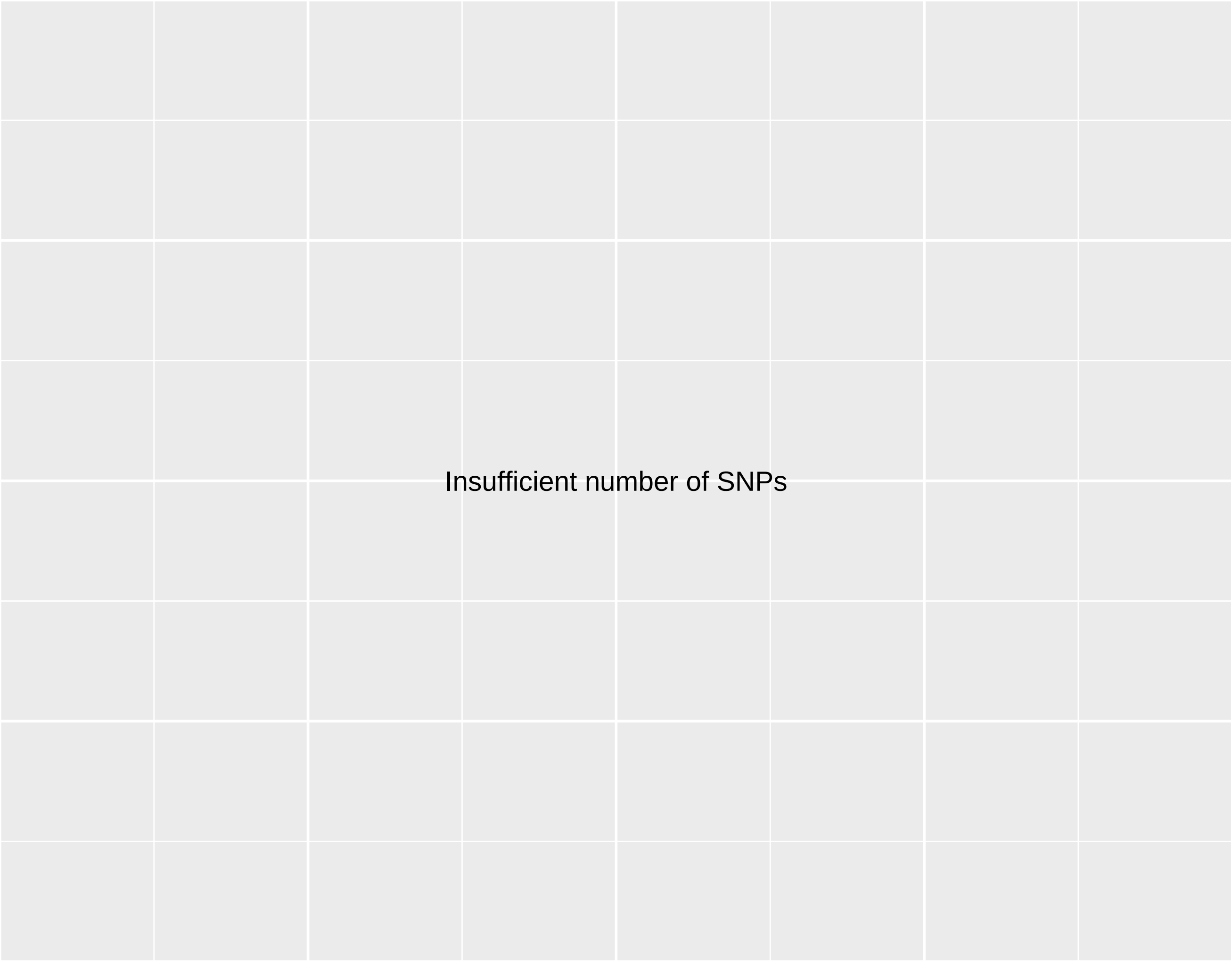
Insufficient number of SNPs

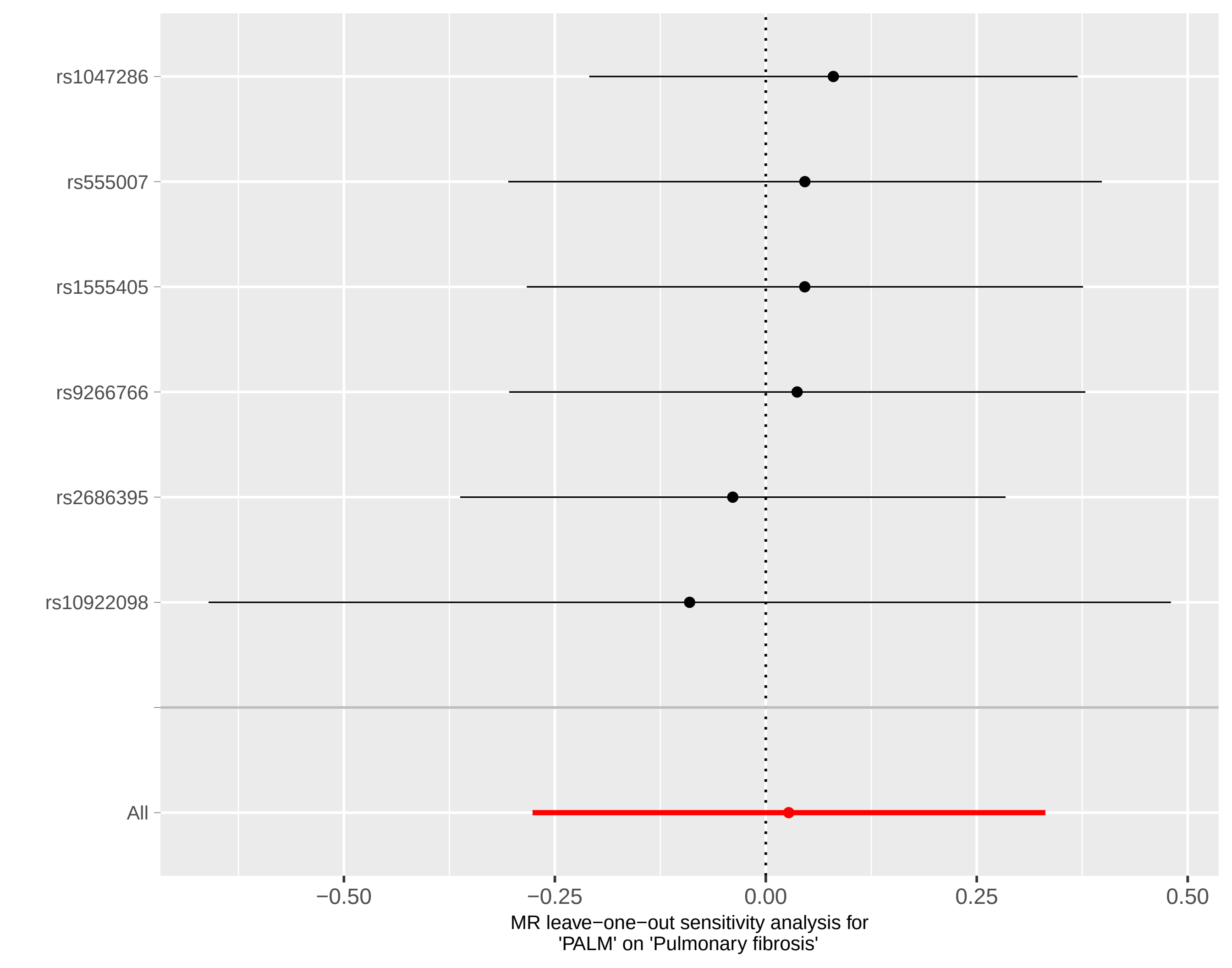
rs1047286
rs555007
rs1555405
rs9266766
rs2686395
rs10922098
All
−0.50
−0.25
0.00
0.25
0.50
MR leave−one−out sensitivity analysis for
'PALM' on 'Pulmonary fibrosis'

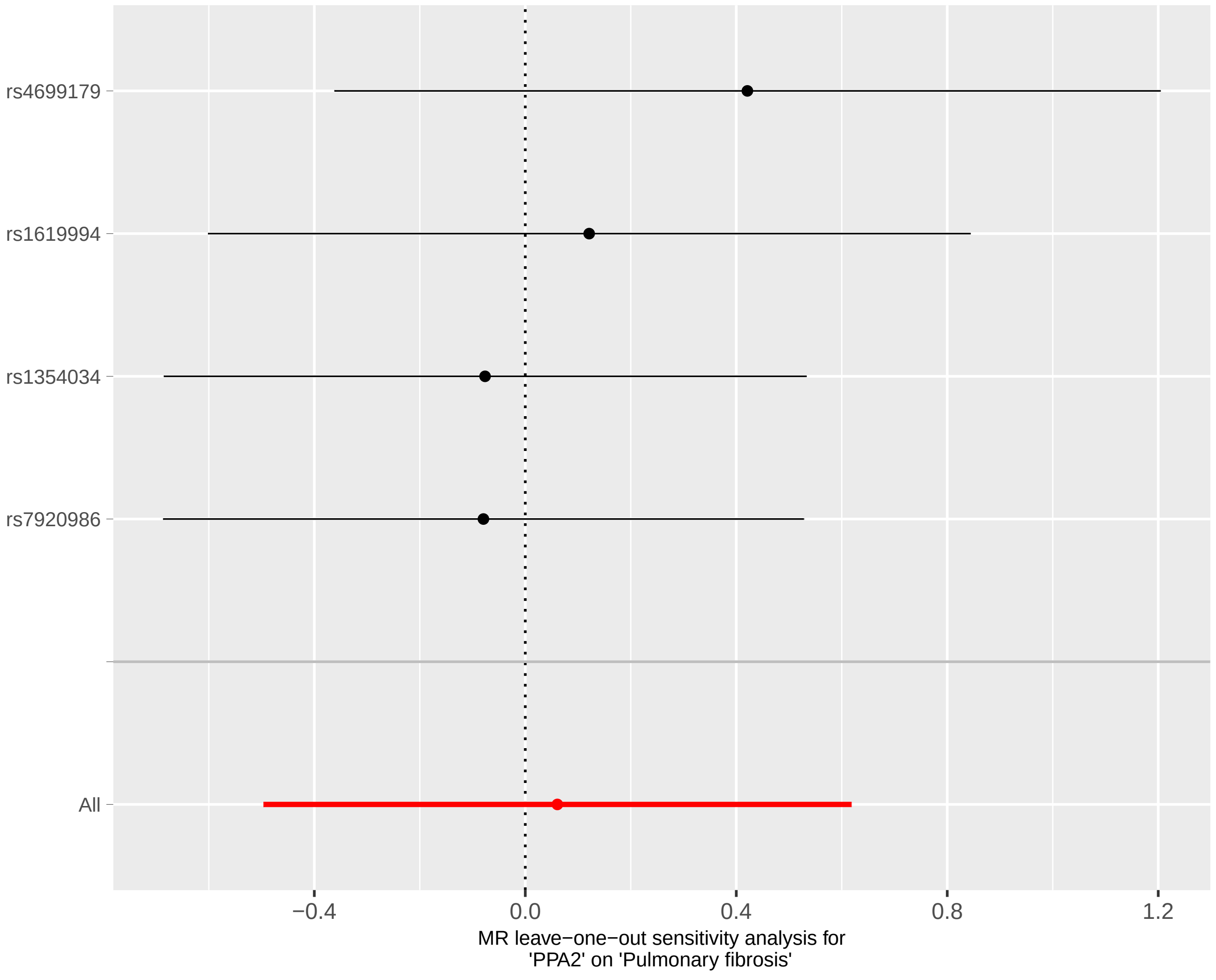
rs4699179
rs1619994
rs1354034
rs7920986
All
−0.4
0.0
0.4
0.8
1.2
MR leave−one−out sensitivity analysis for
'PPA2' on 'Pulmonary fibrosis'

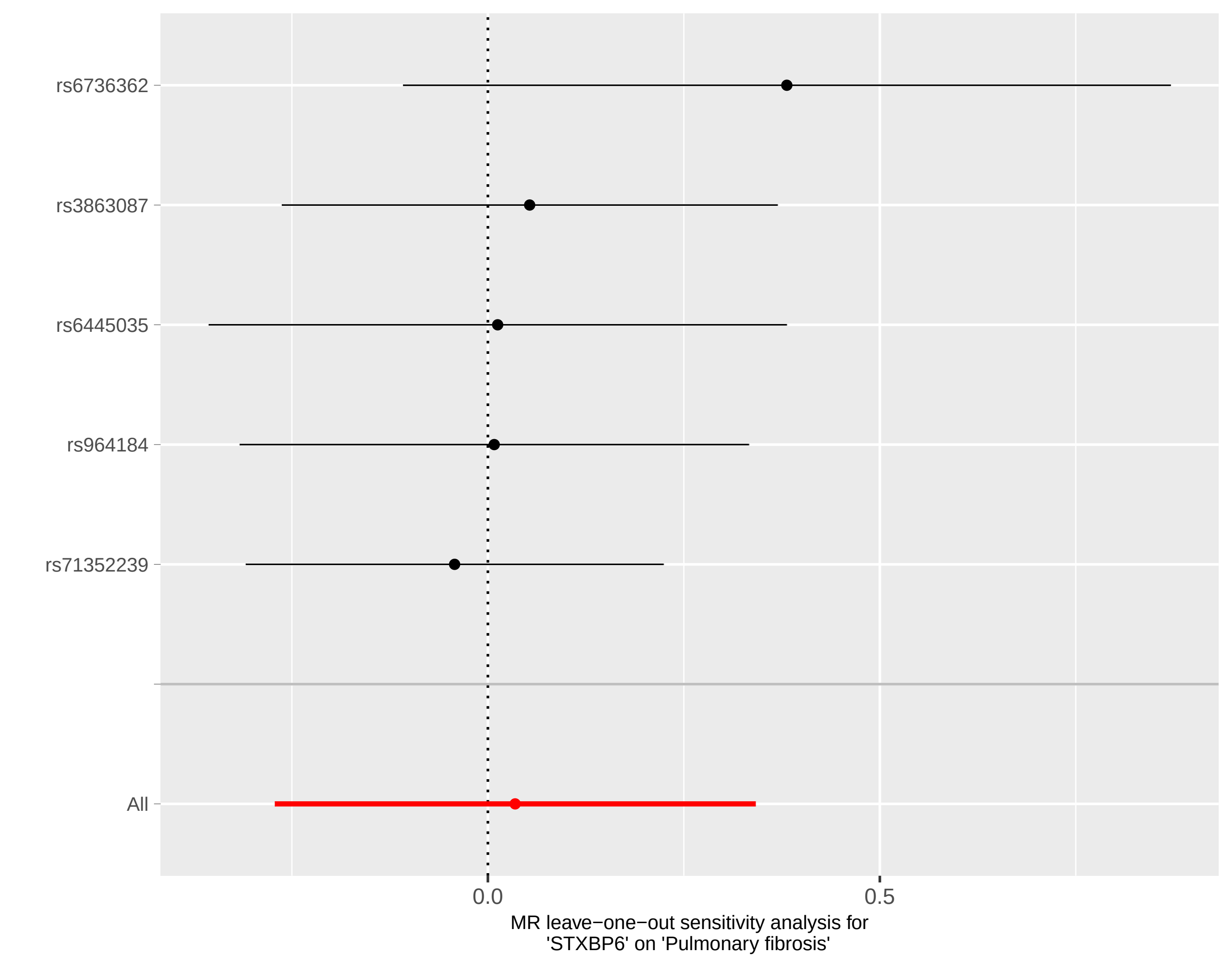
rs6736362
rs3863087
rs6445035
rs964184
rs71352239
All
0.0
0.5
MR leave−one−out sensitivity analysis for
'STXBP6' on 'Pulmonary fibrosis'

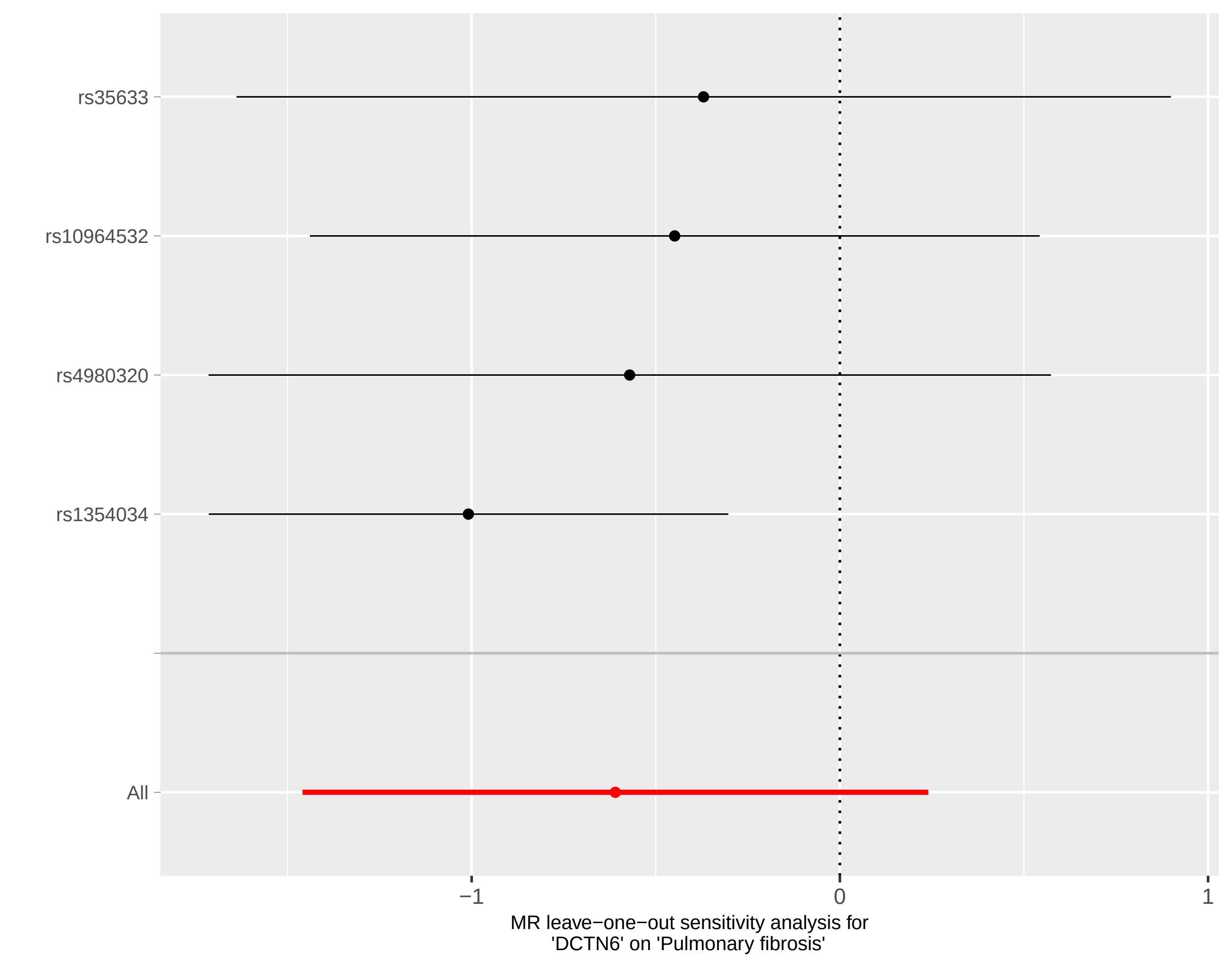
rs35633
rs10964532
rs4980320
rs1354034
All
−1
0
1
MR leave−one−out sensitivity analysis for
'DCTN6' on 'Pulmonary fibrosis'

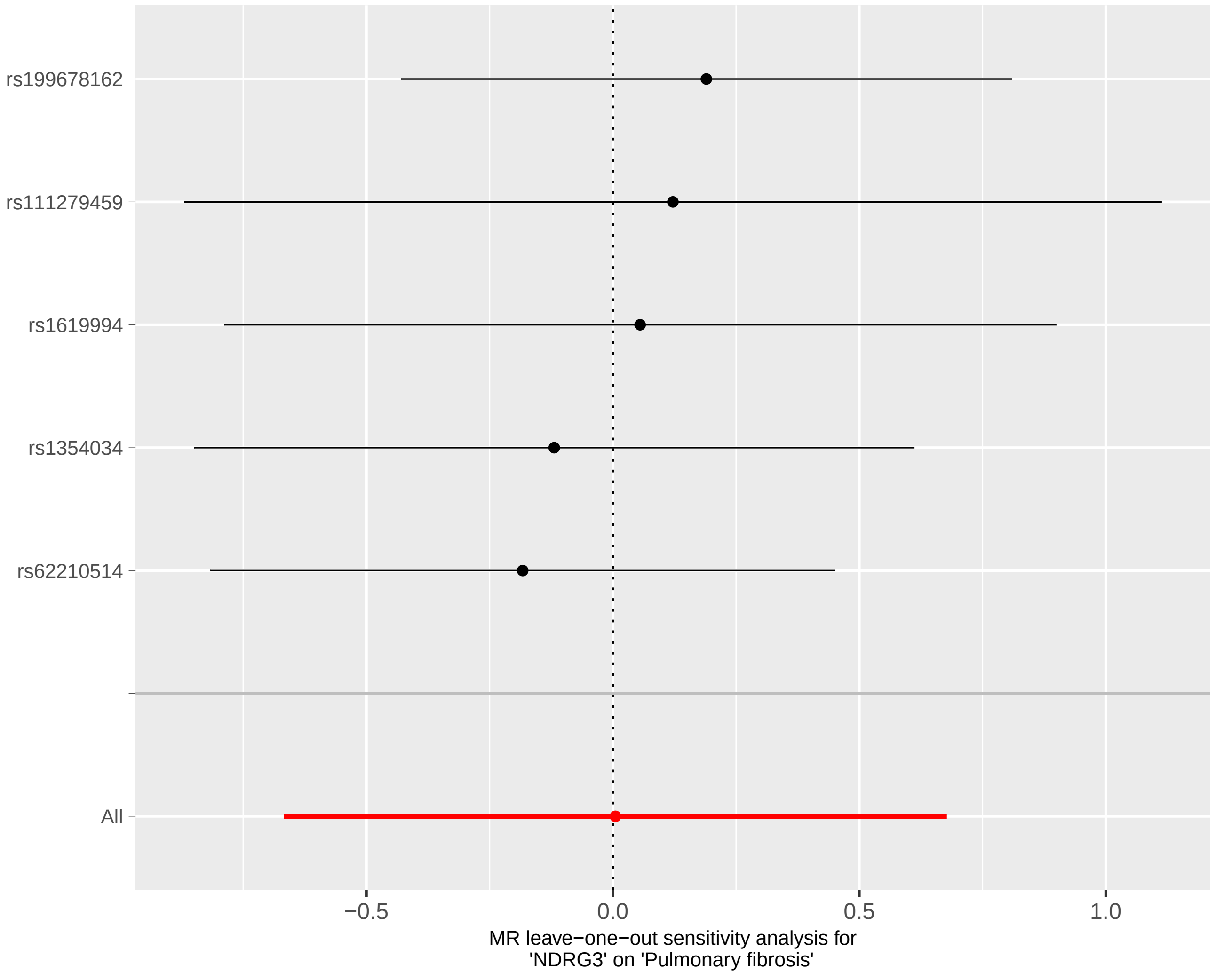
rs199678162
rs111279459
rs1619994
rs1354034
rs62210514
All
−0.5
0.0
0.5
1.0
MR leave−one−out sensitivity analysis for
'NDRG3' on 'Pulmonary fibrosis'

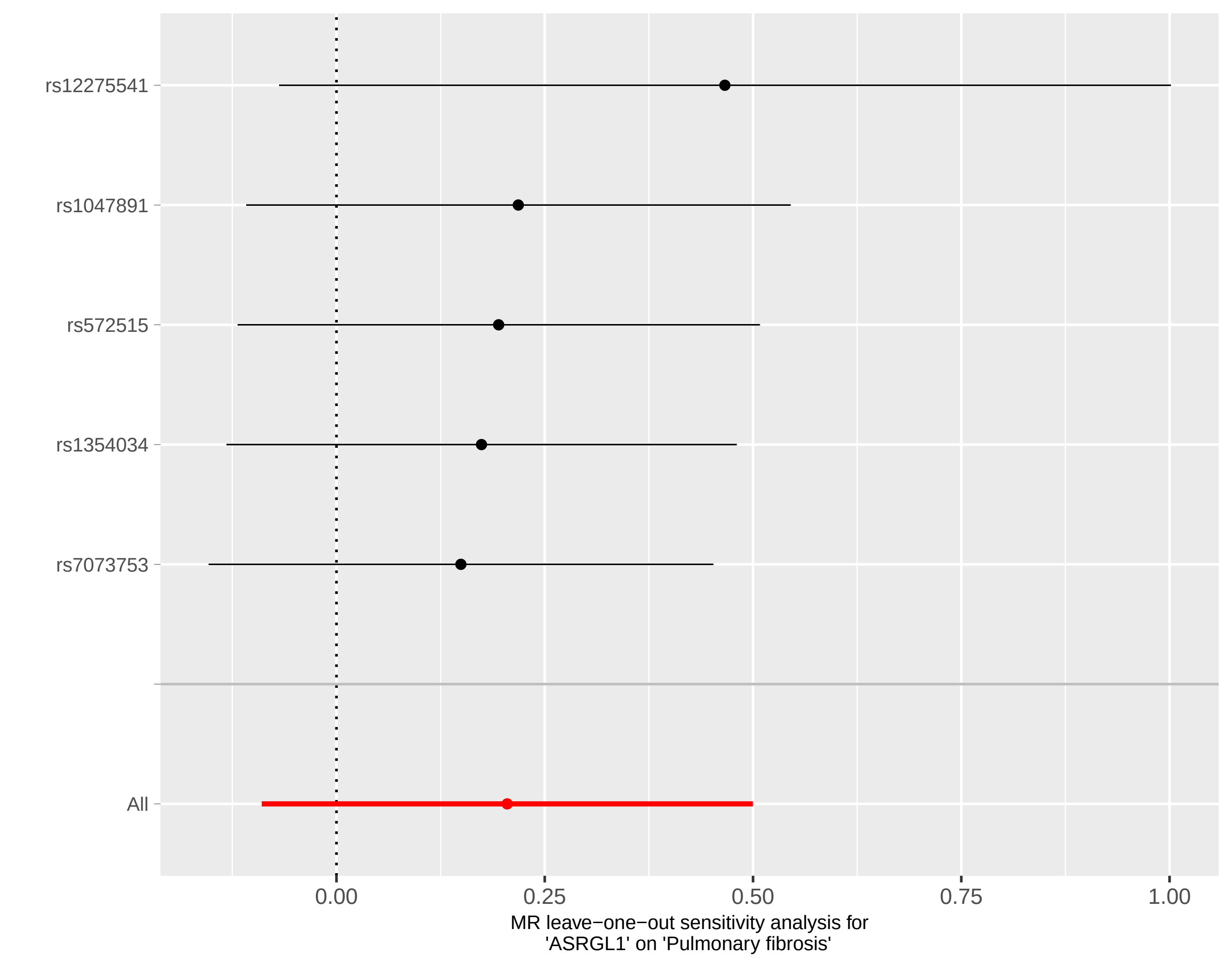
rs12275541
rs1047891
rs572515
rs1354034
rs7073753
All
0.00
0.25
0.50
0.75
1.00
MR leave−one−out sensitivity analysis for
'ASRGL1' on 'Pulmonary fibrosis'

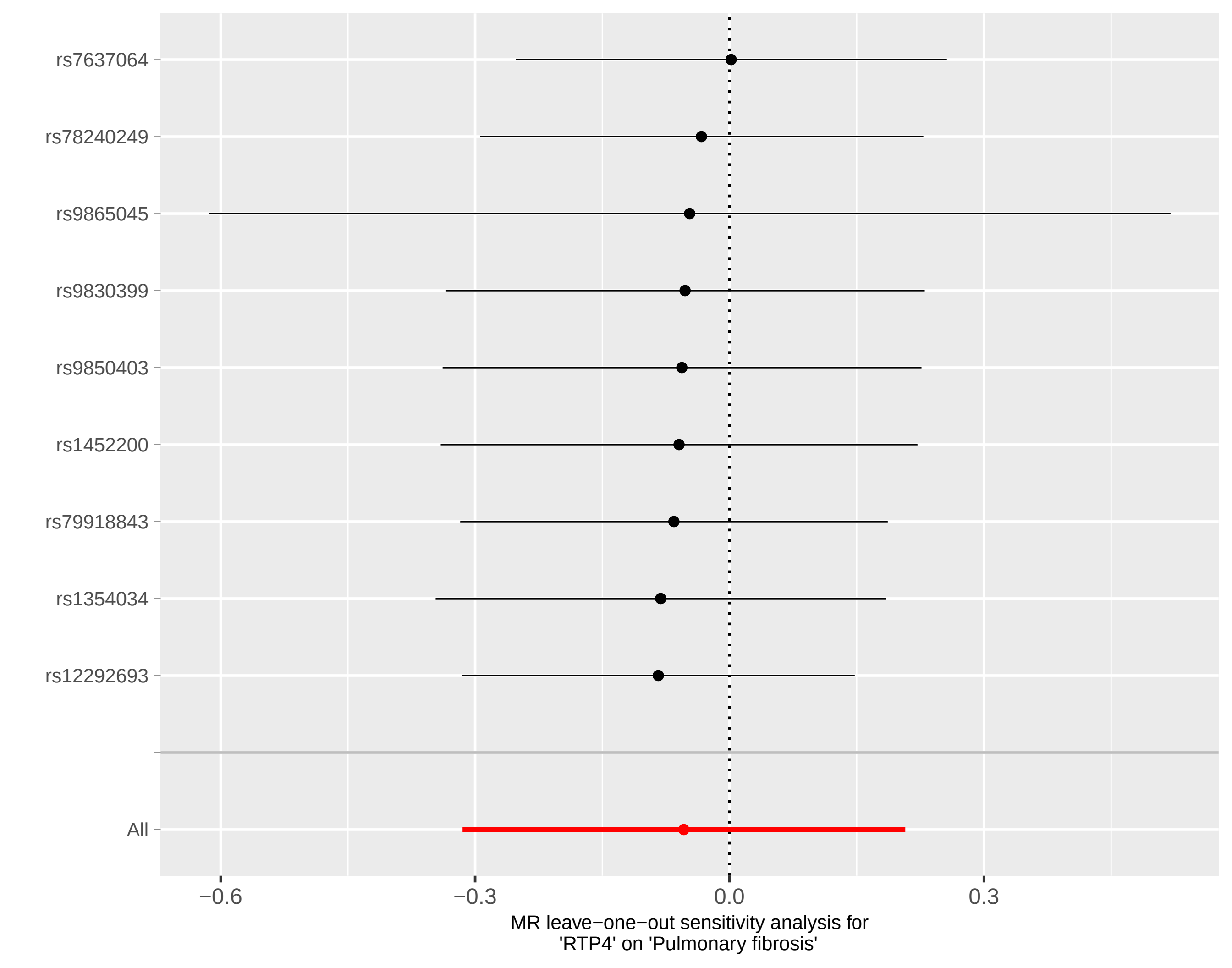

rs7637064
rs78240249
rs9865045
rs9830399
rs9850403
rs1452200
rs79918843
rs1354034
rs12292693
All
−0.6
−0.3
0.0
0.3
MR leave−one−out sensitivity analysis for
'RTP4' on 'Pulmonary fibrosis'

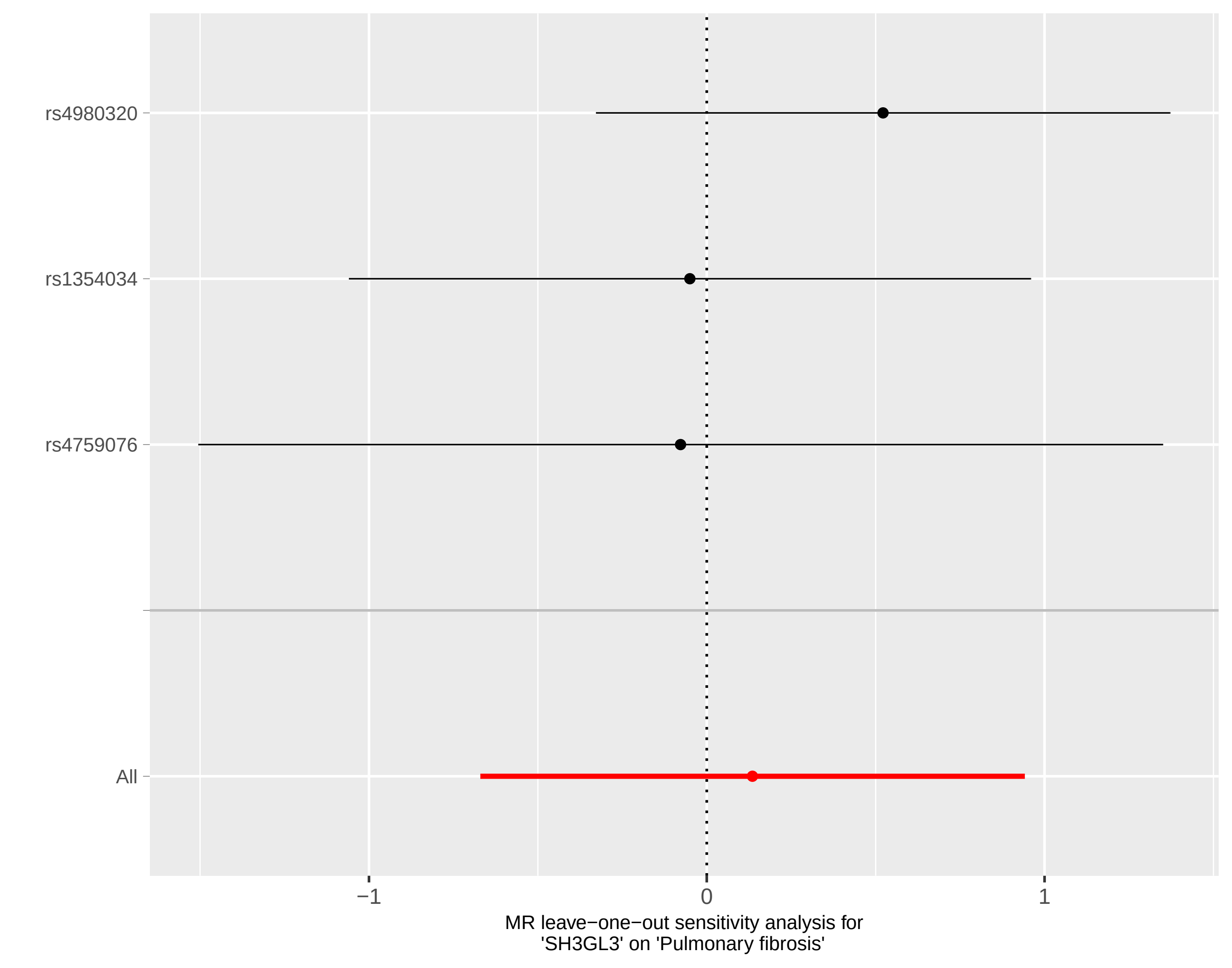
rs4980320
rs1354034
rs4759076
All
−1
0
1
MR leave−one−out sensitivity analysis for
'SH3GL3' on 'Pulmonary fibrosis'

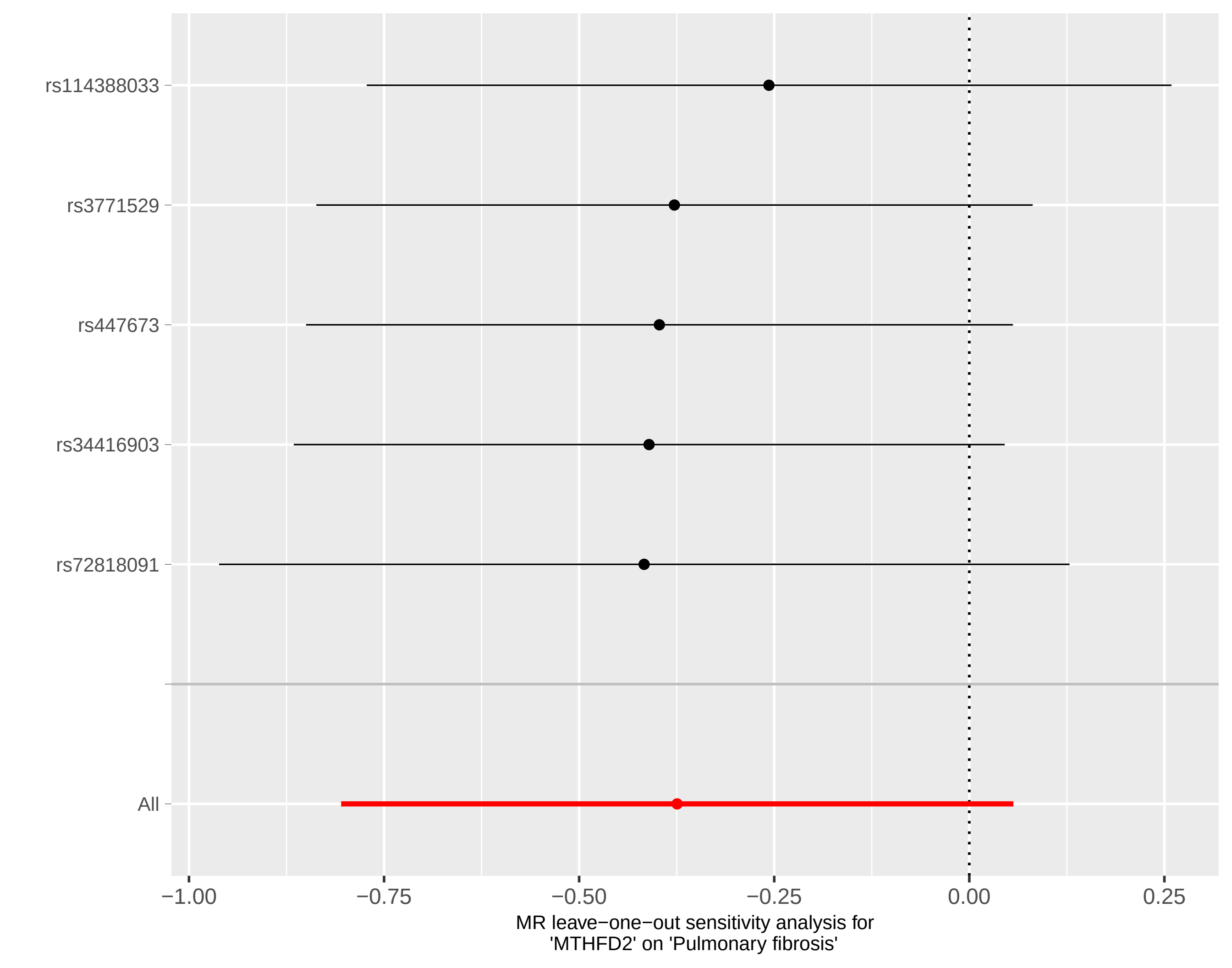
rs114388033
rs3771529
rs447673
rs34416903
rs72818091
All
−1.00
−0.75
−0.50
−0.25
0.00
0.25
MR leave−one−out sensitivity analysis for
'MTHFD2' on 'Pulmonary fibrosis'

Insufficient number of SNPs

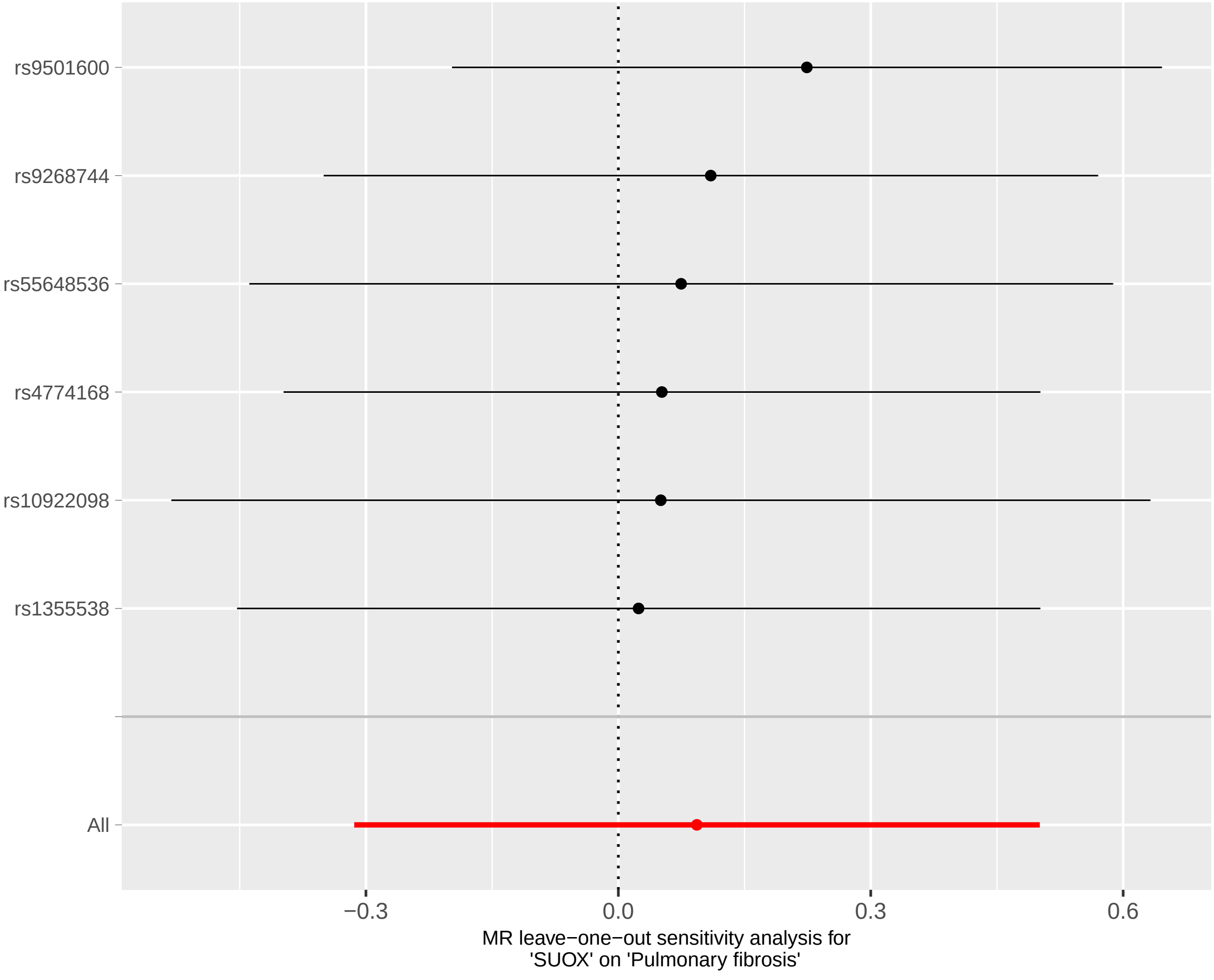

rs9501600
rs9268744
rs55648536
rs4774168
rs10922098
rs1355538
All
−0.3
0.0
0.3
0.6
MR leave−one−out sensitivity analysis for
'SUOX' on 'Pulmonary fibrosis'

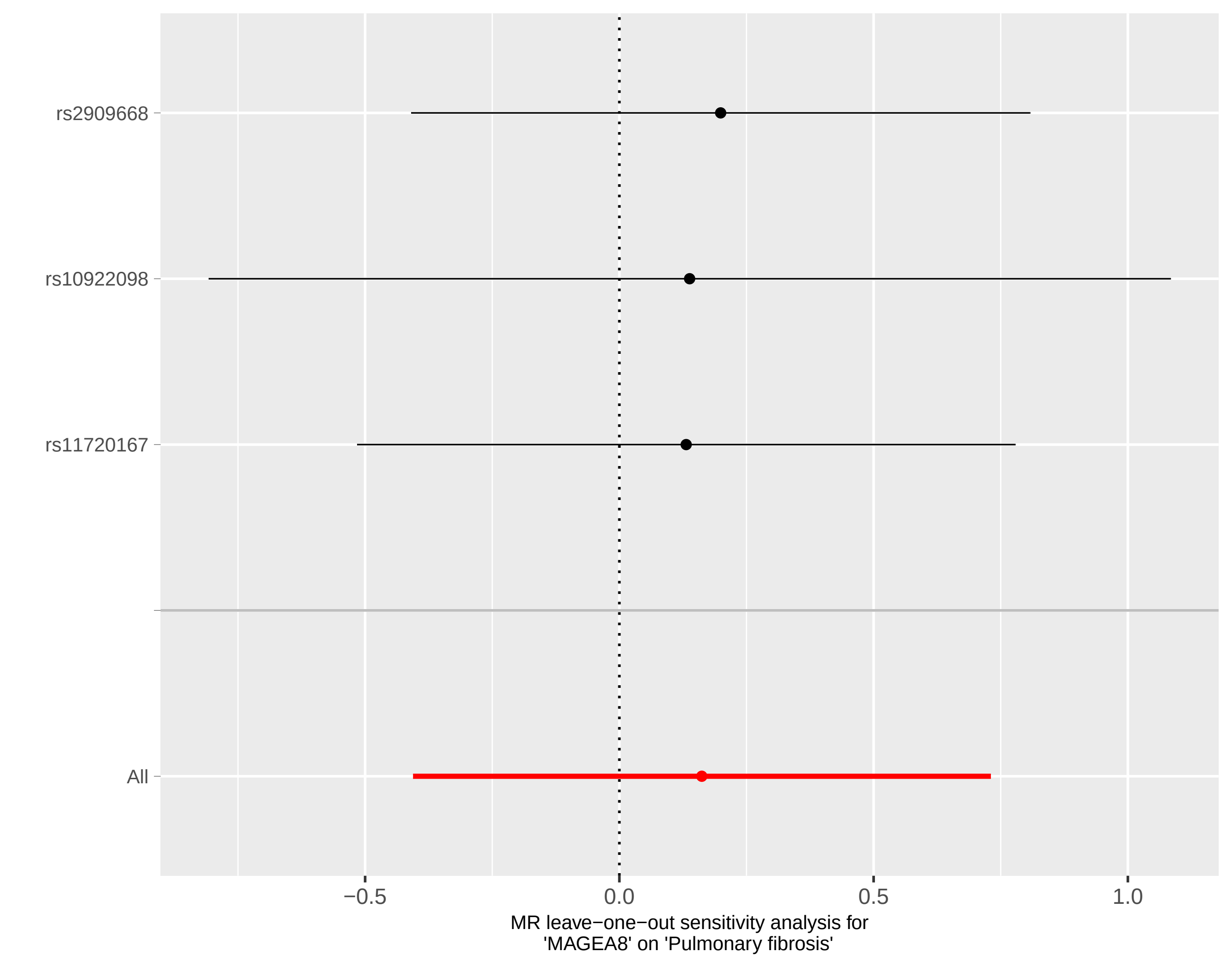
rs2909668
rs10922098
rs11720167
All
−0.5
0.0
0.5
1.0
MR leave−one−out sensitivity analysis for
'MAGEA8' on 'Pulmonary fibrosis'

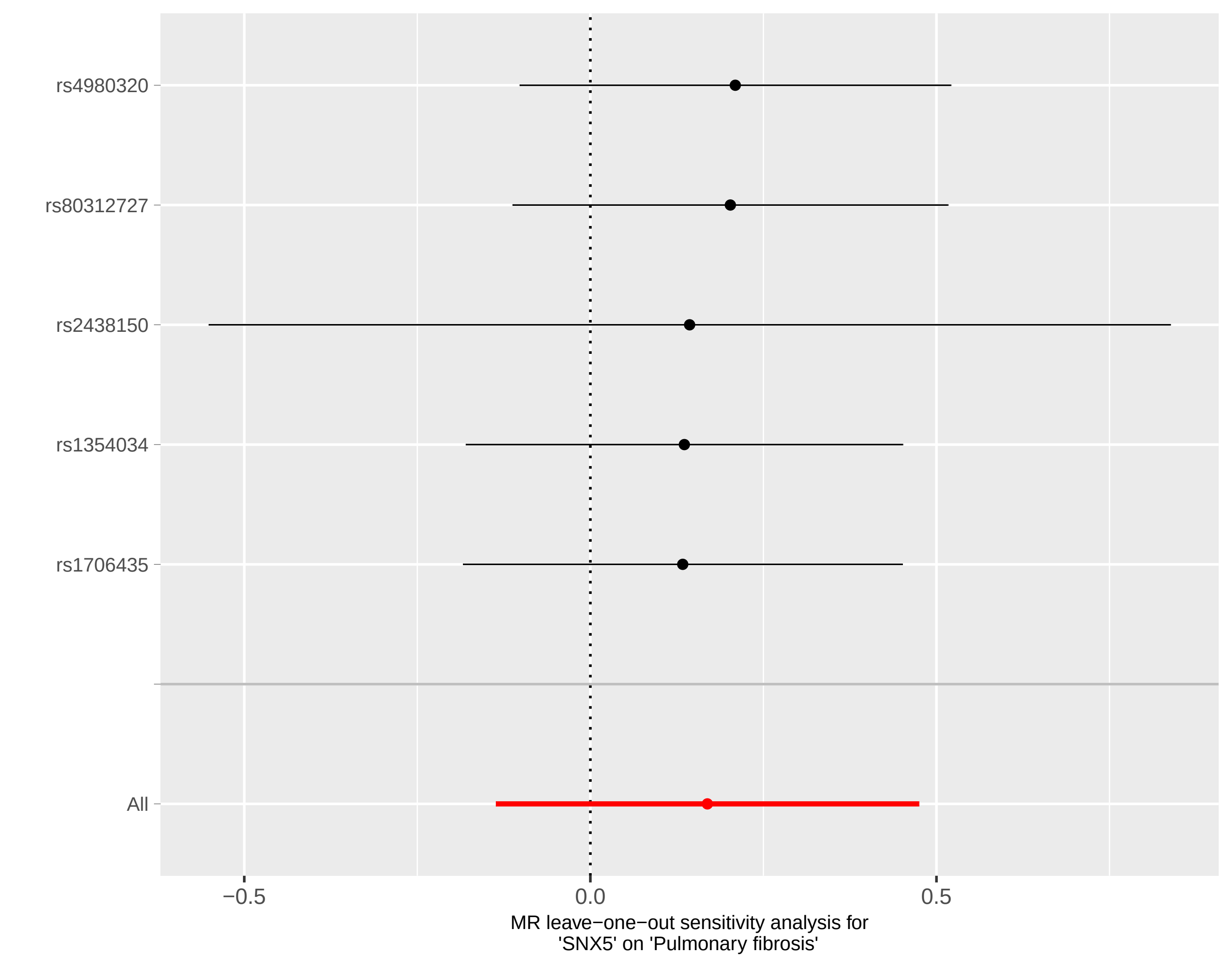

rs4980320
rs80312727
rs2438150
rs1354034
rs1706435
All
−0.5
0.0
0.5
MR leave−one−out sensitivity analysis for
'SNX5' on 'Pulmonary fibrosis'

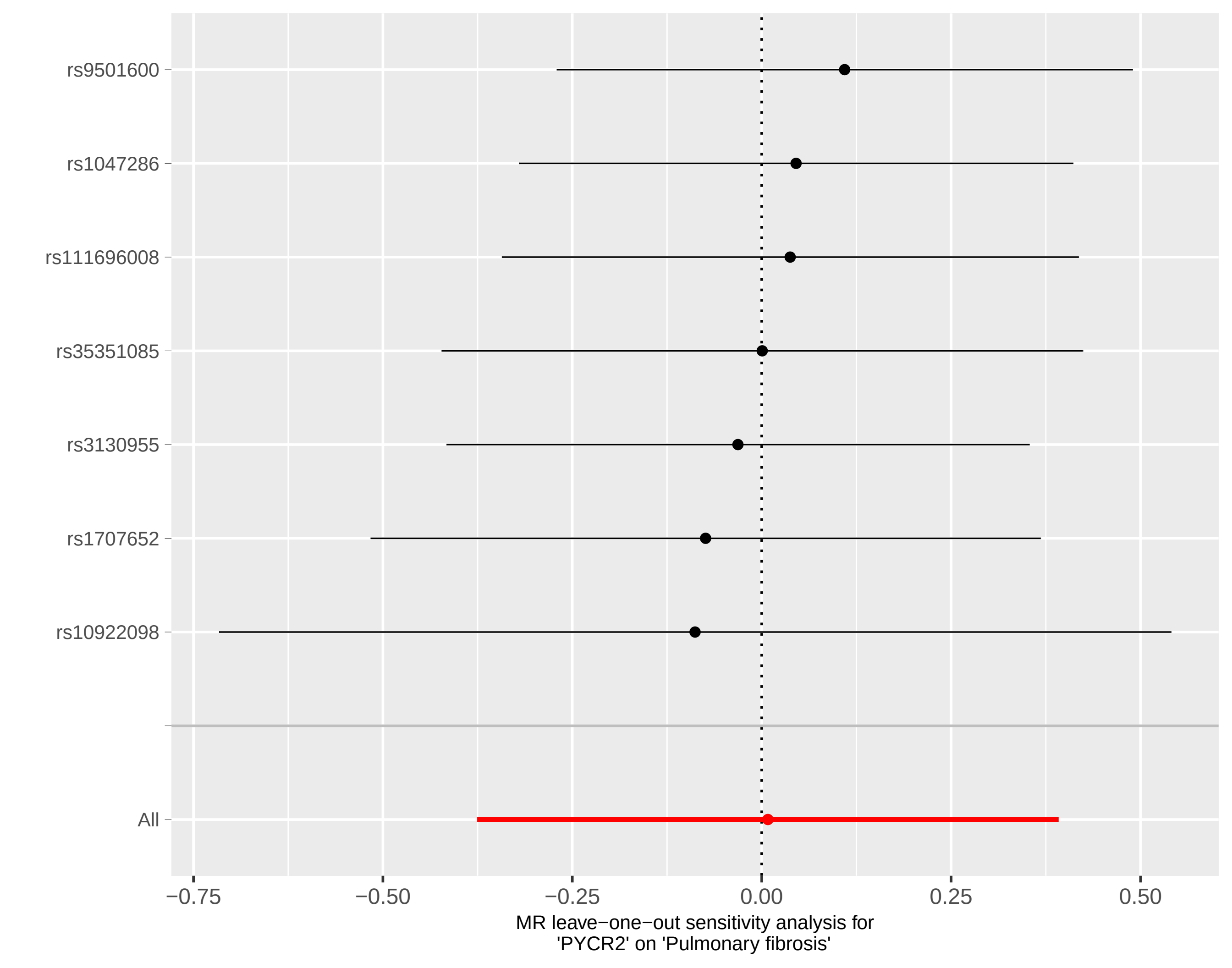
rs9501600
rs1047286
rs111696008
rs35351085
rs3130955
rs1707652
rs10922098
All
−0.75
−0.50
−0.25
0.00
0.25
0.50
MR leave−one−out sensitivity analysis for
'PYCR2' on 'Pulmonary fibrosis'

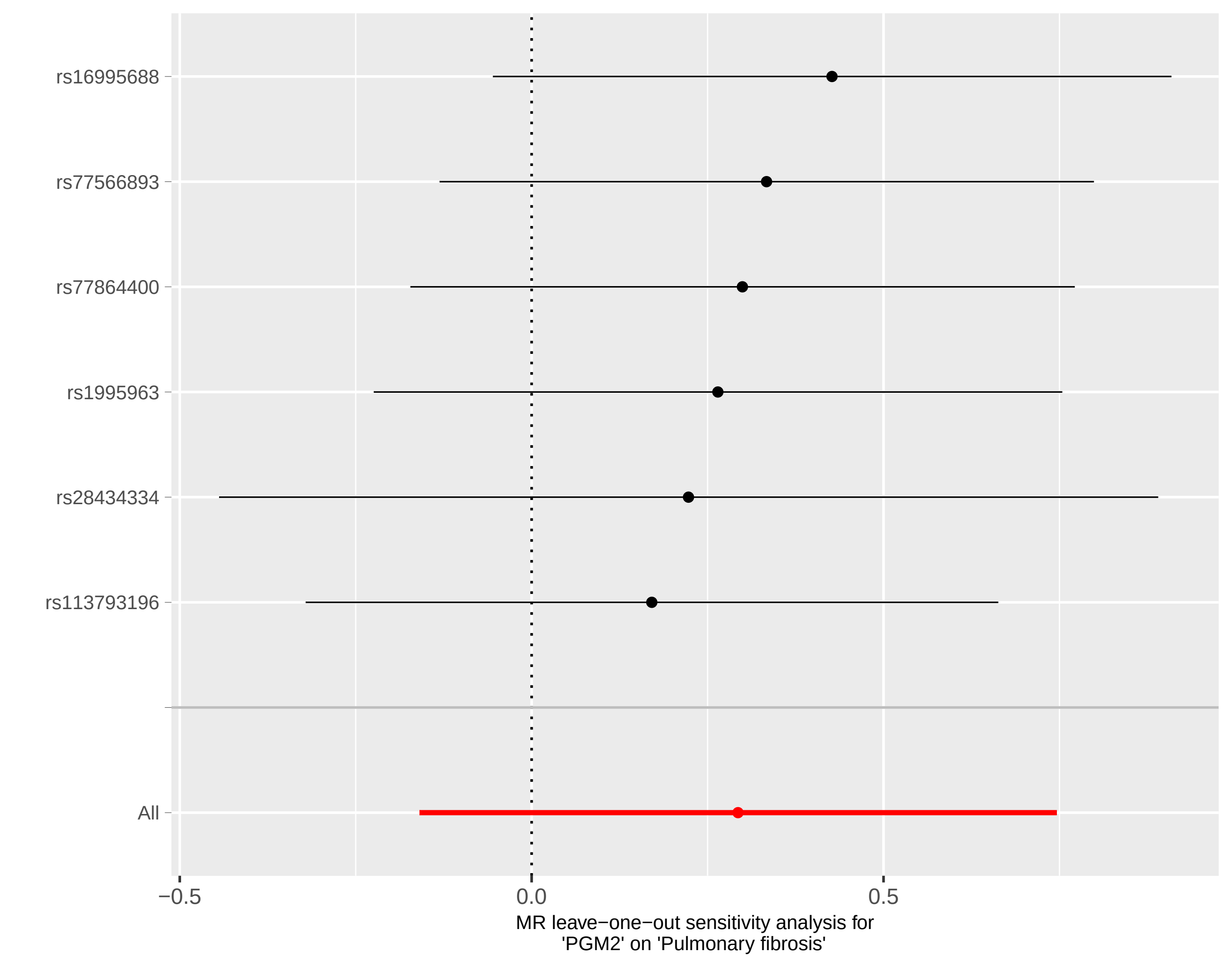
rs16995688
rs77566893
rs77864400
rs1995963
rs28434334
rs113793196
All
−0.5
0.0
0.5
MR leave−one−out sensitivity analysis for
'PGM2' on 'Pulmonary fibrosis'

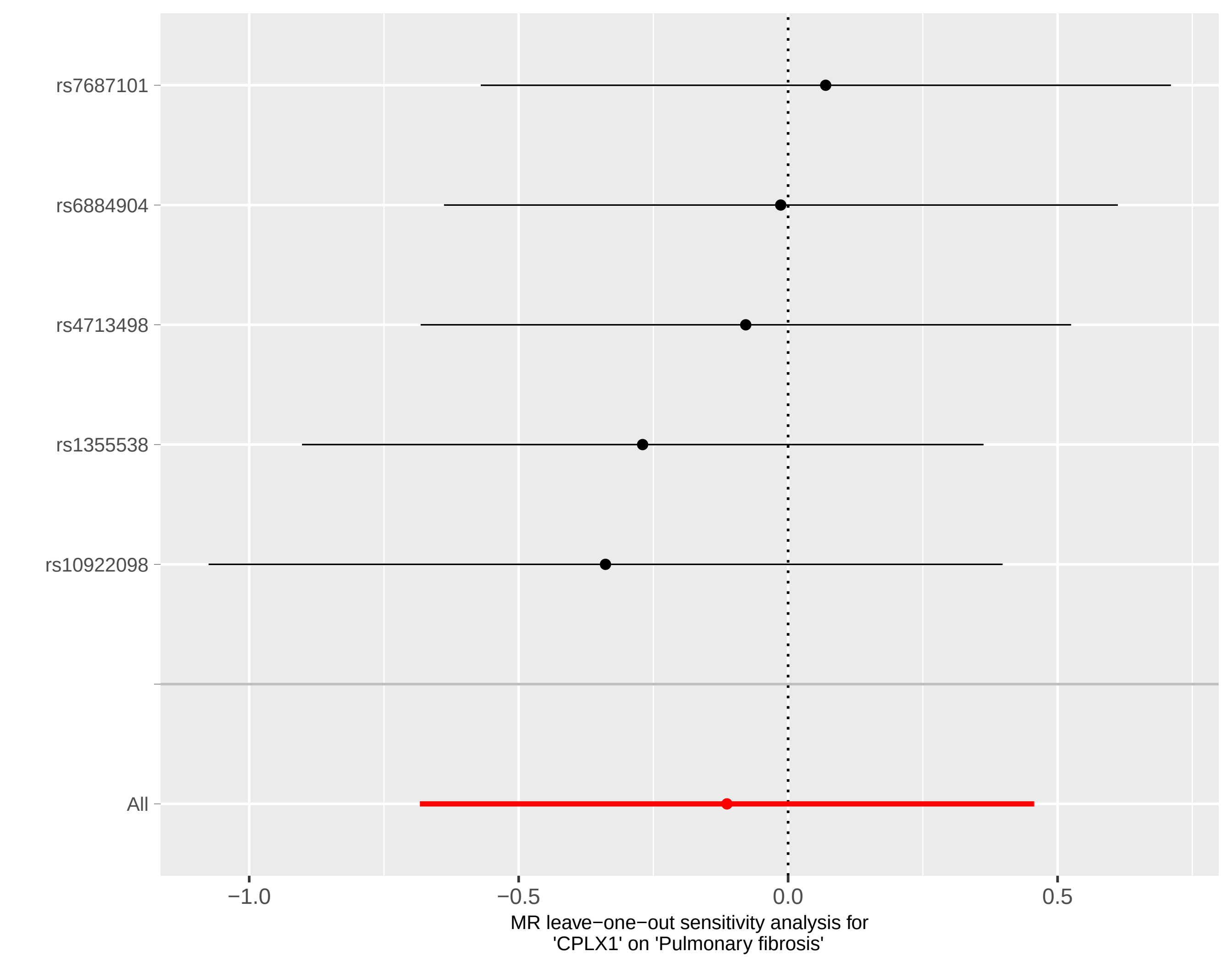
rs7687101
rs6884904
rs4713498
rs1355538
rs10922098
All
−1.0
−0.5
0.0
0.5
MR leave−one−out sensitivity analysis for
'CPLX1' on 'Pulmonary fibrosis'

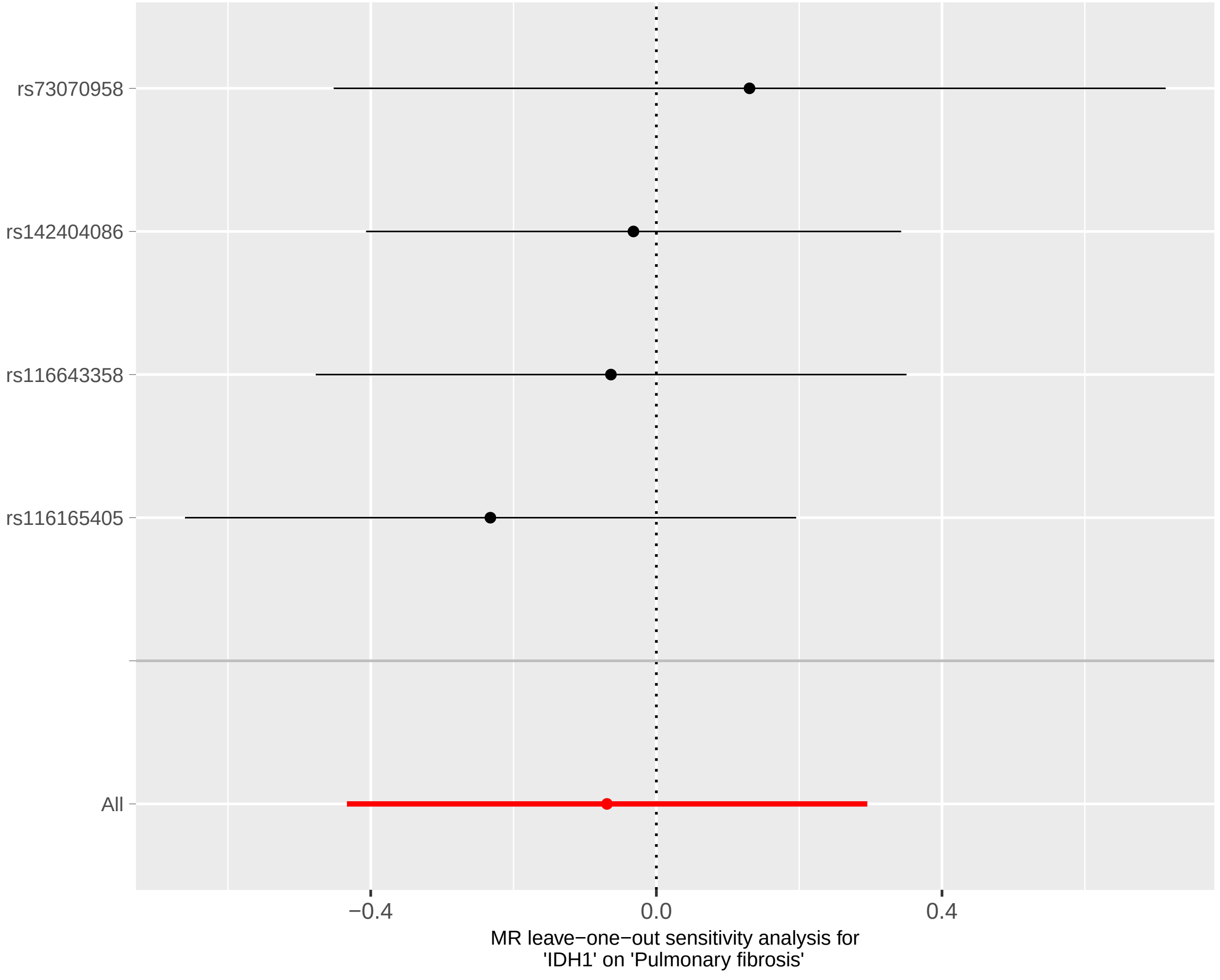
rs73070958
rs142404086
rs116643358
rs116165405
All
−0.4
0.0
0.4
MR leave−one−out sensitivity analysis for
'IDH1' on 'Pulmonary fibrosis'

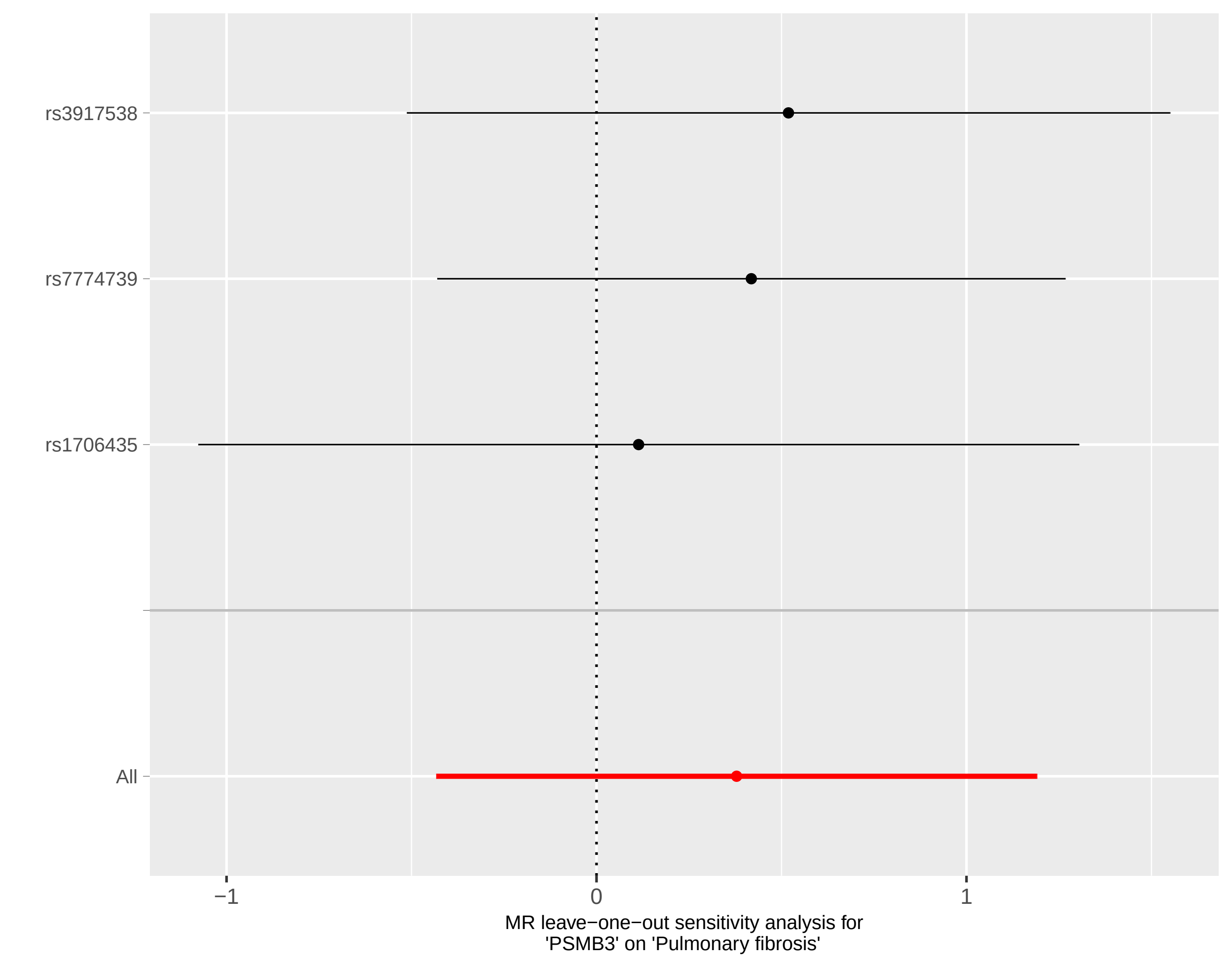
rs3917538
rs7774739
rs1706435
All
−1
0
1
MR leave−one−out sensitivity analysis for
'PSMB3' on 'Pulmonary fibrosis'

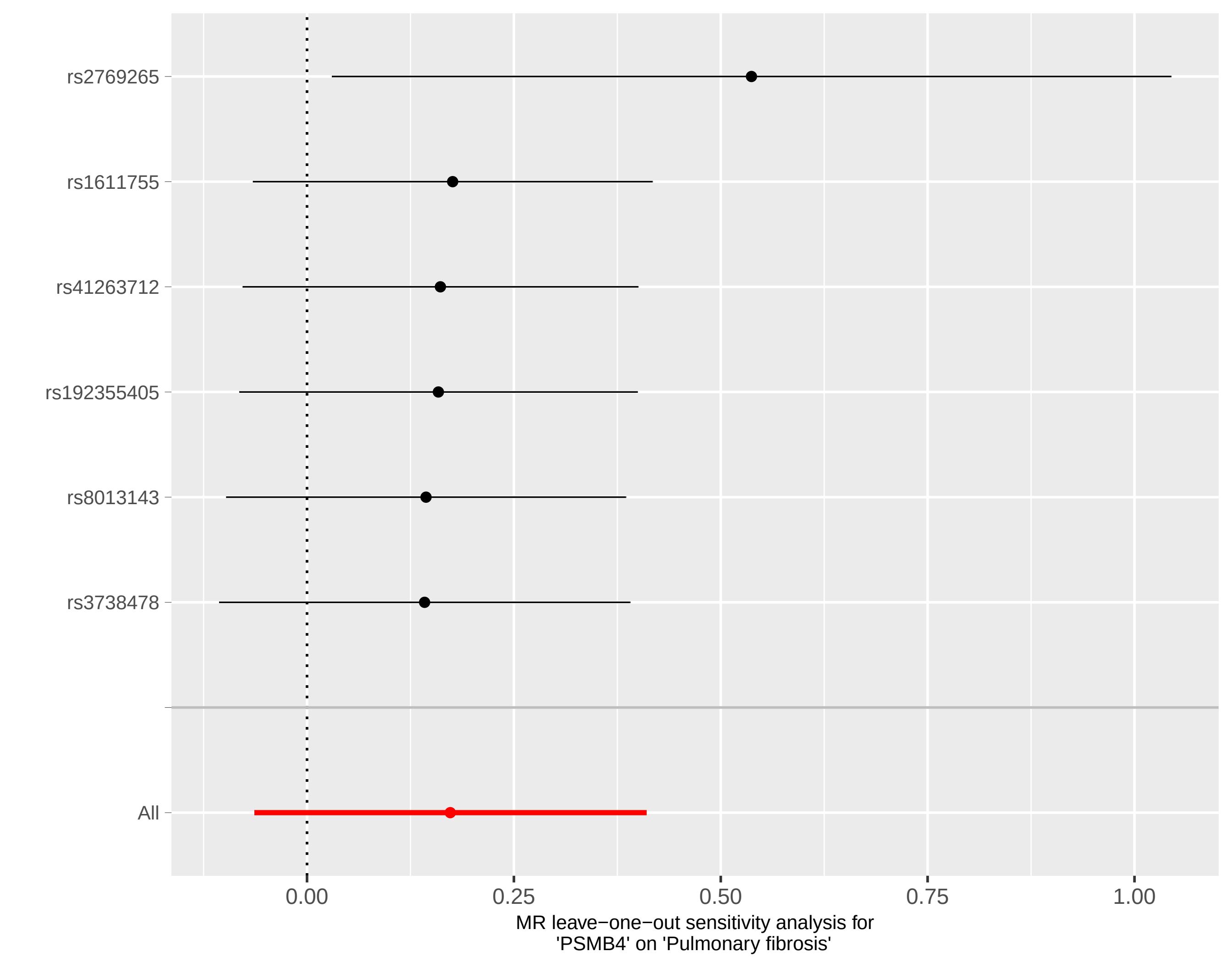
rs2769265
rs1611755
rs41263712
rs192355405
rs8013143
rs3738478
All
0.00
0.25
0.50
0.75
1.00
MR leave−one−out sensitivity analysis for
'PSMB4' on 'Pulmonary fibrosis'

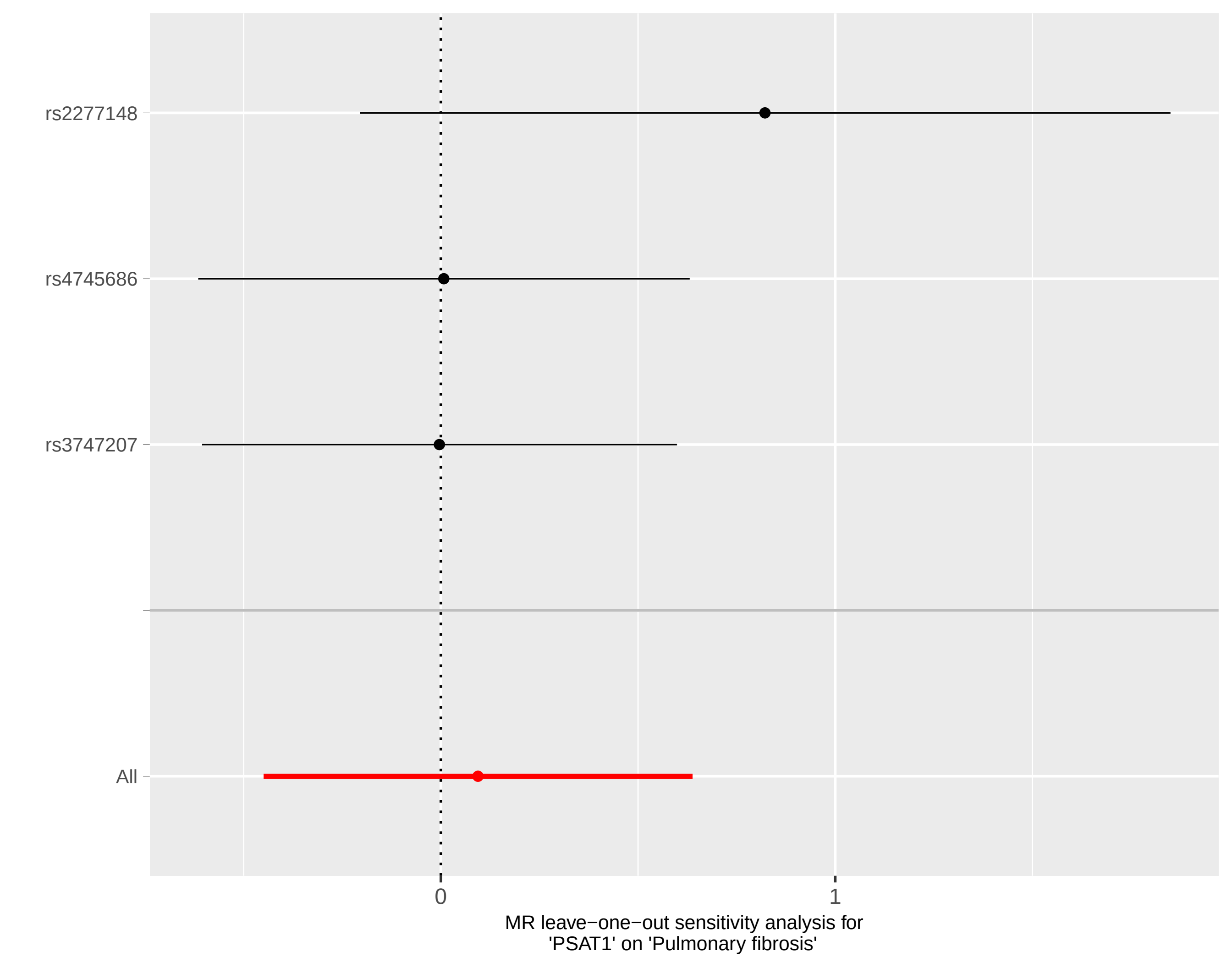
rs2277148
rs4745686
rs3747207
All
0
1
MR leave−one−out sensitivity analysis for
'PSAT1' on 'Pulmonary fibrosis'

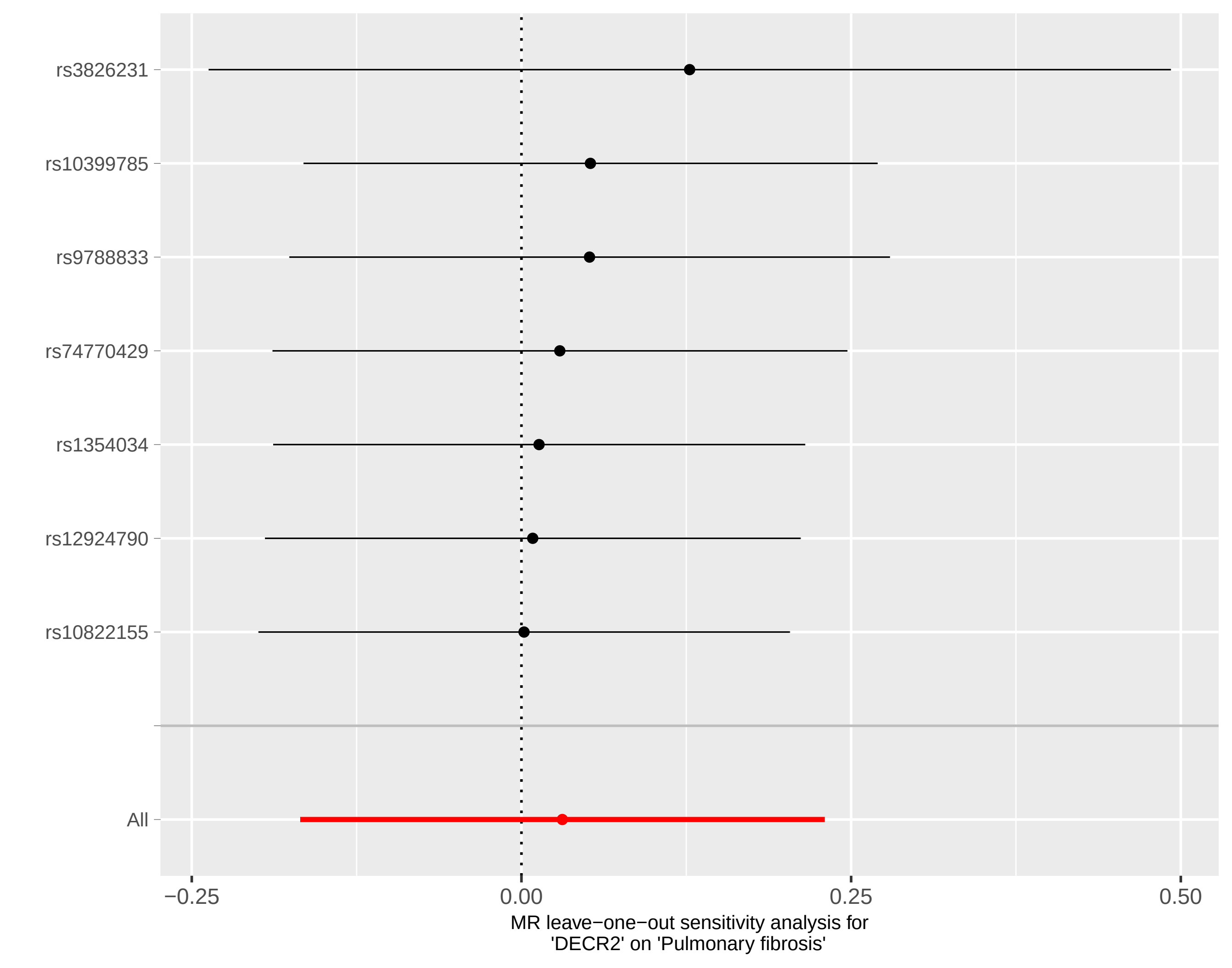
rs3826231
rs10399785
rs9788833
rs74770429
rs1354034
rs12924790
rs10822155
All
−0.25
0.00
0.25
0.50
MR leave−one−out sensitivity analysis for
'DECR2' on 'Pulmonary fibrosis'

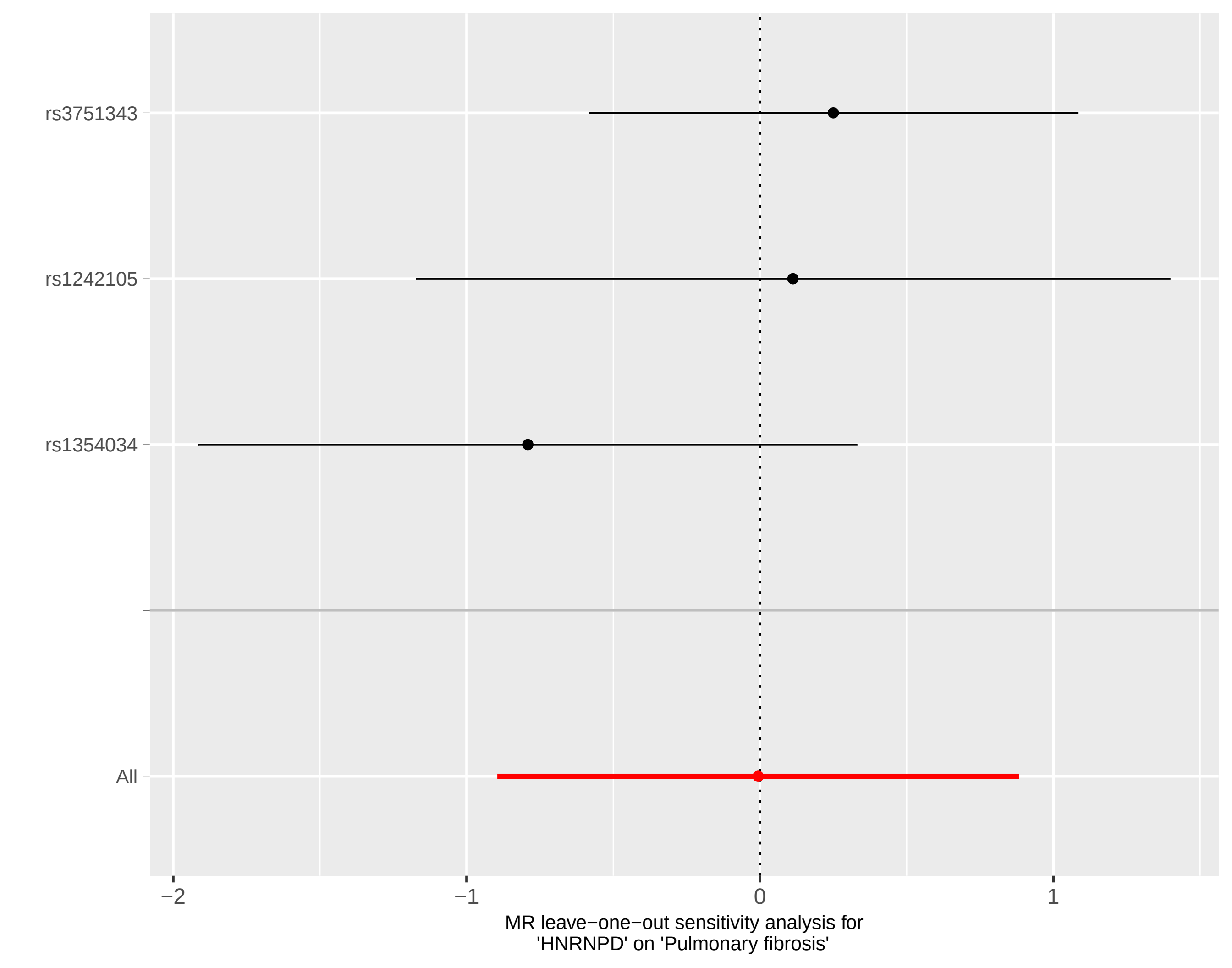
rs3751343
rs1242105
rs1354034
All
−2
−1
0
1
MR leave−one−out sensitivity analysis for
'HNRNPD' on 'Pulmonary fibrosis'

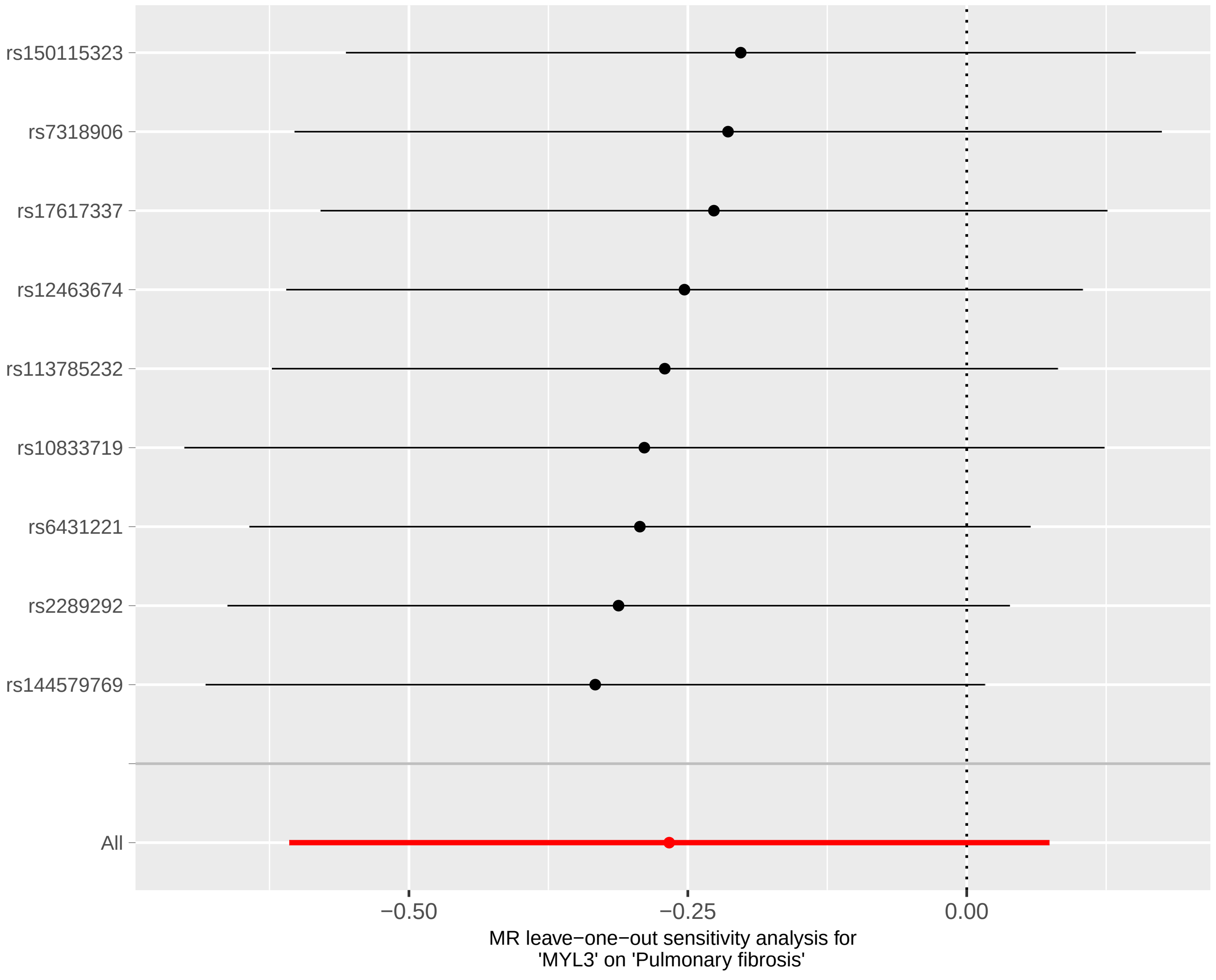

rs150115323
rs7318906
rs17617337
rs12463674
rs113785232
rs10833719
rs6431221
rs2289292
rs144579769
All
−0.50
−0.25
0.00
MR leave−one−out sensitivity analysis for
'MYL3' on 'Pulmonary fibrosis'

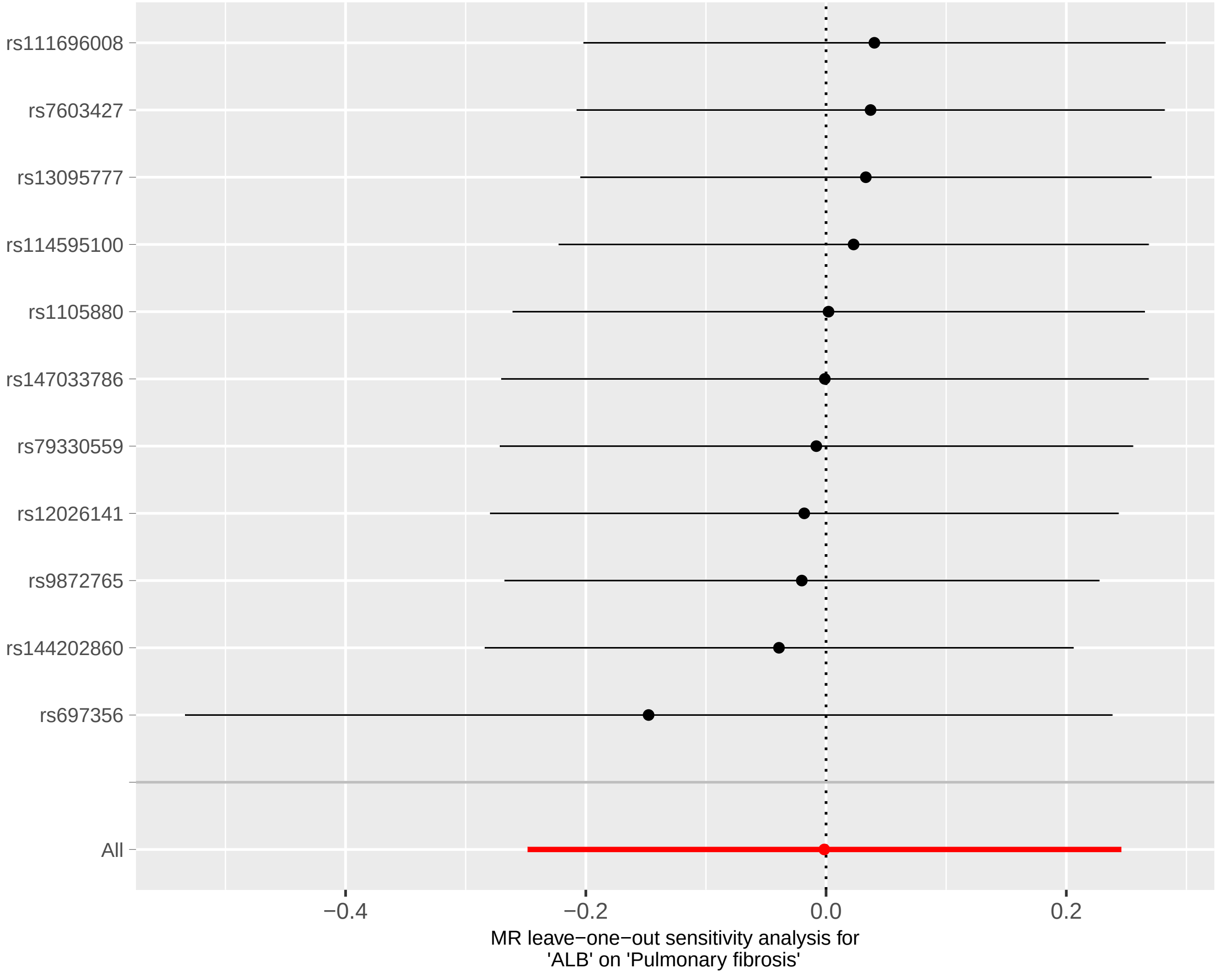

rs111696008
rs7603427
rs13095777
rs114595100
rs1105880
rs147033786
rs79330559
rs12026141
rs9872765
rs144202860
rs697356
All
−0.4
−0.2
0.0
0.2
MR leave−one−out sensitivity analysis for
'ALB' on 'Pulmonary fibrosis'

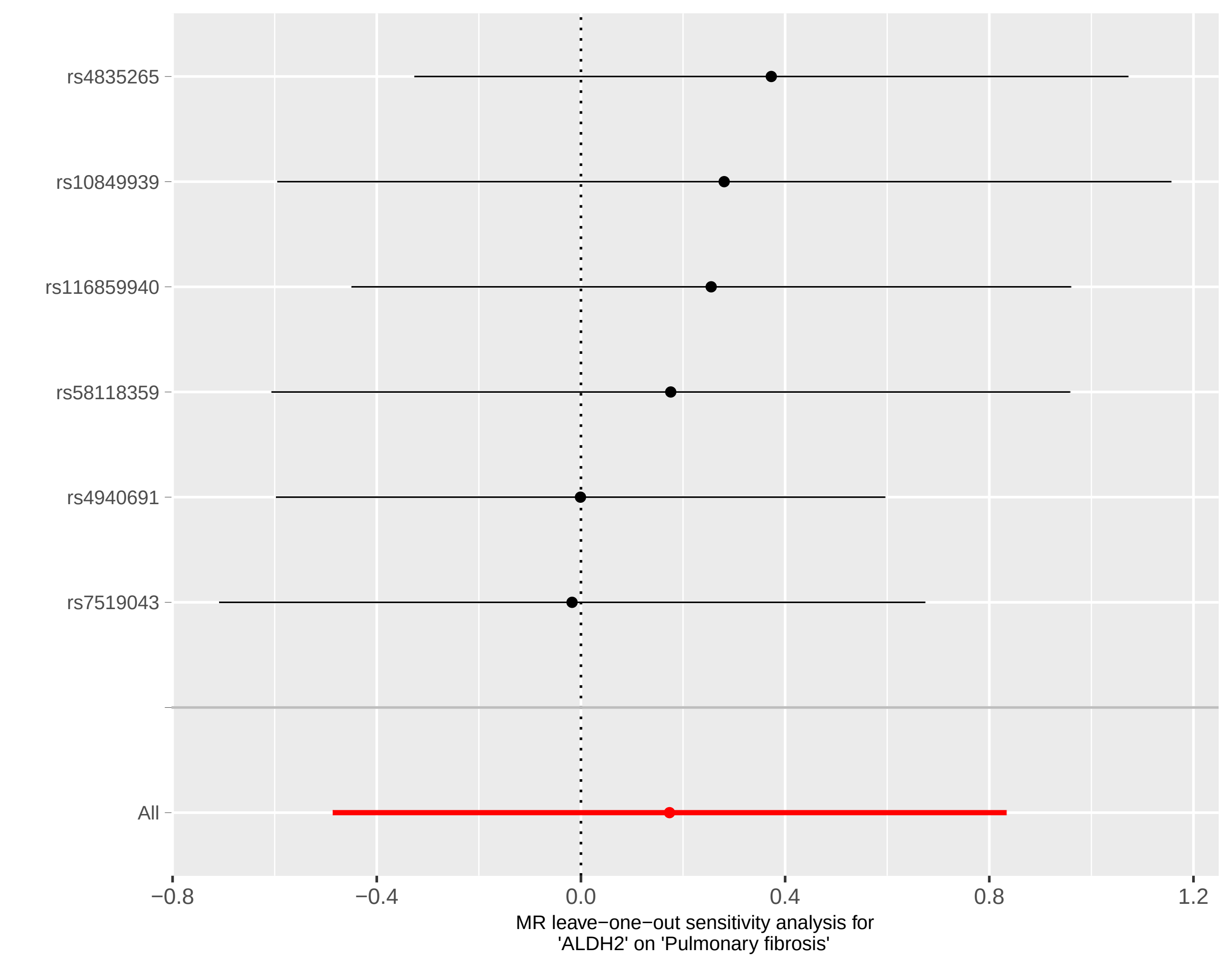

rs4835265
rs10849939
rs116859940
rs58118359
rs4940691
rs7519043
All
−0.8
−0.4
0.0
0.4
0.8
1.2
MR leave−one−out sensitivity analysis for
'ALDH2' on 'Pulmonary fibrosis'

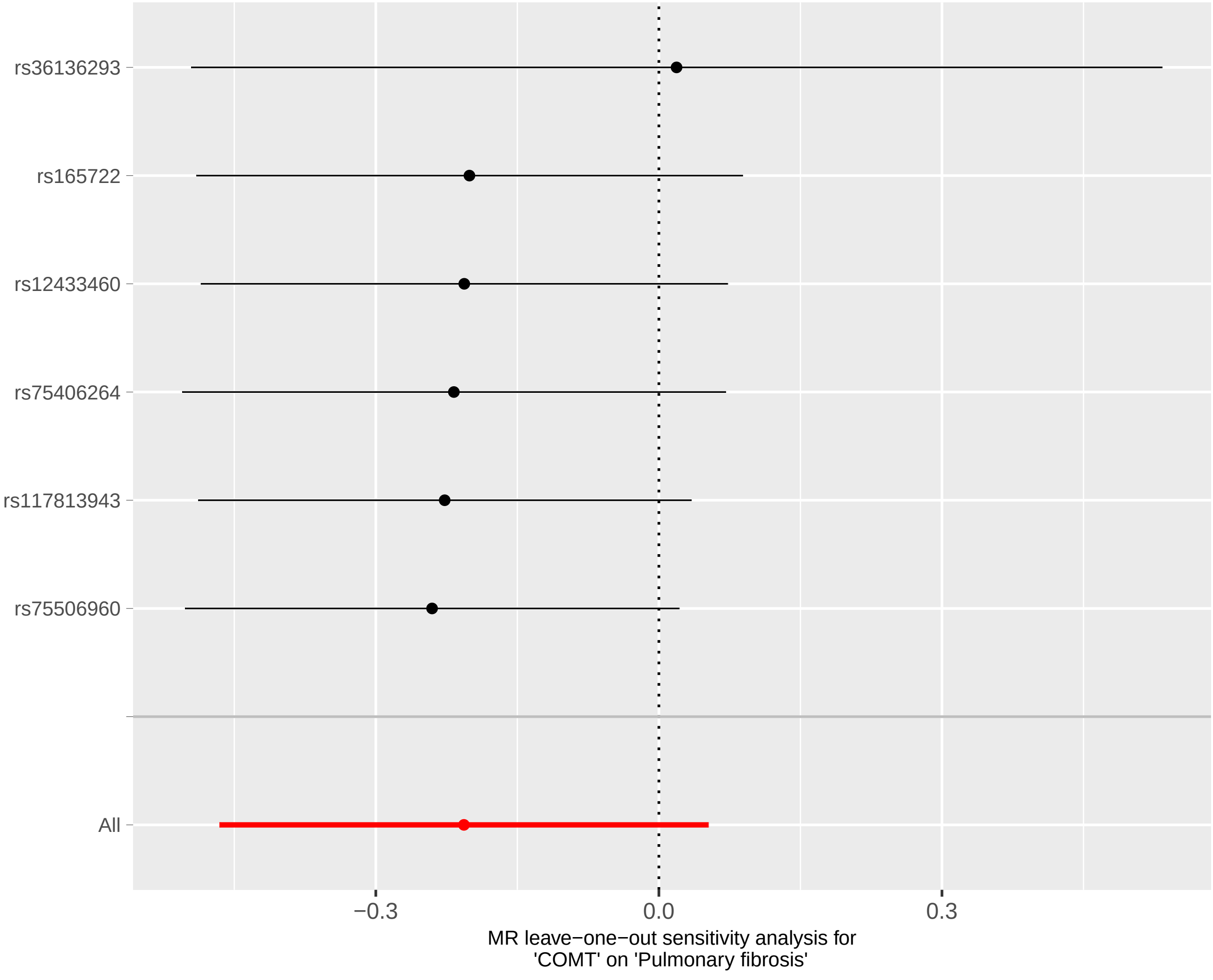
rs36136293
rs165722
rs12433460
rs75406264
rs117813943
rs75506960
All
−0.3
0.0
0.3
MR leave−one−out sensitivity analysis for
'COMT' on 'Pulmonary fibrosis'

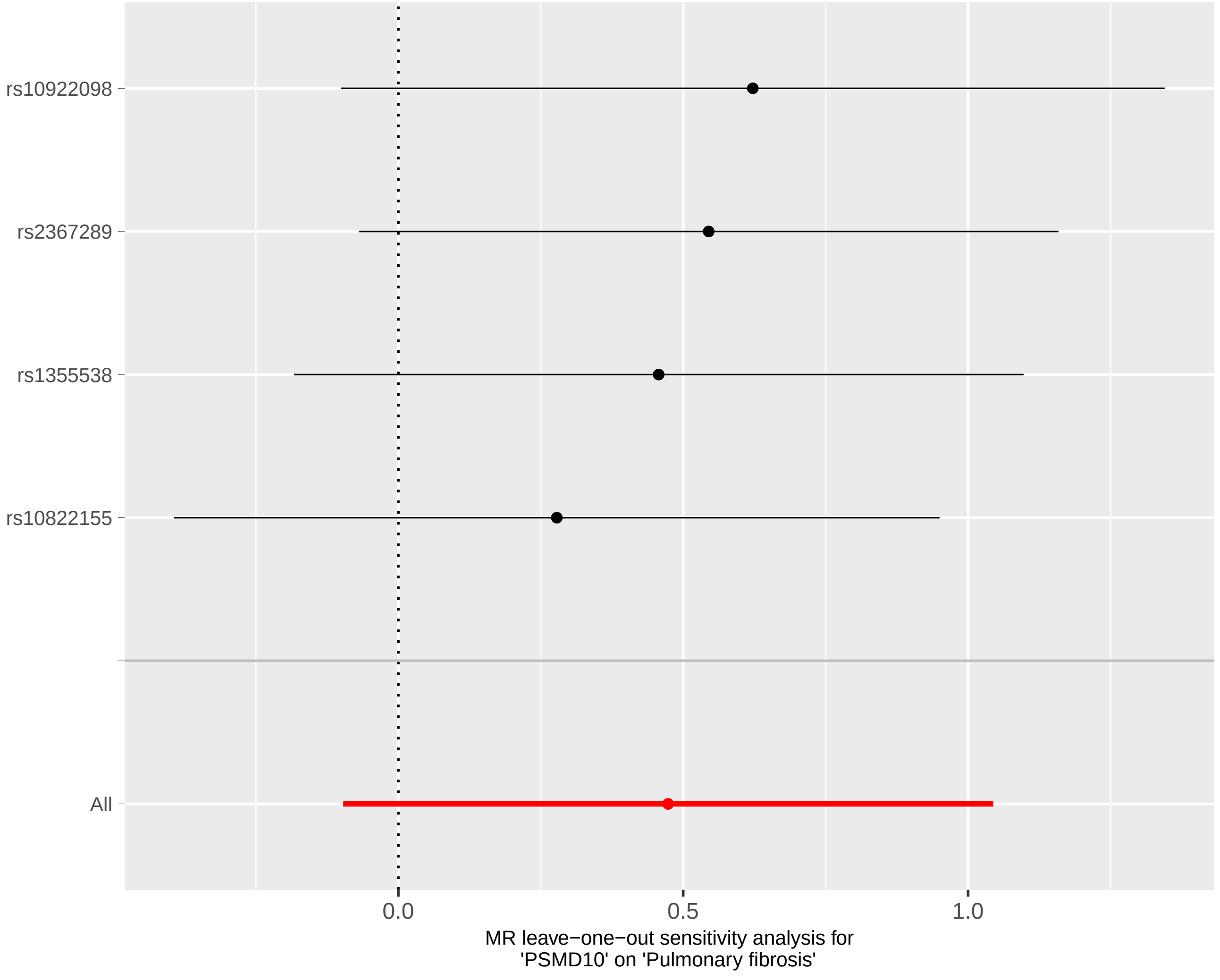
rs10922098
rs2367289
rs1355538
rs10822155
All
0.0
0.5
1.0
MR leave−one−out sensitivity analysis for
'PSMD10' on 'Pulmonary fibrosis'

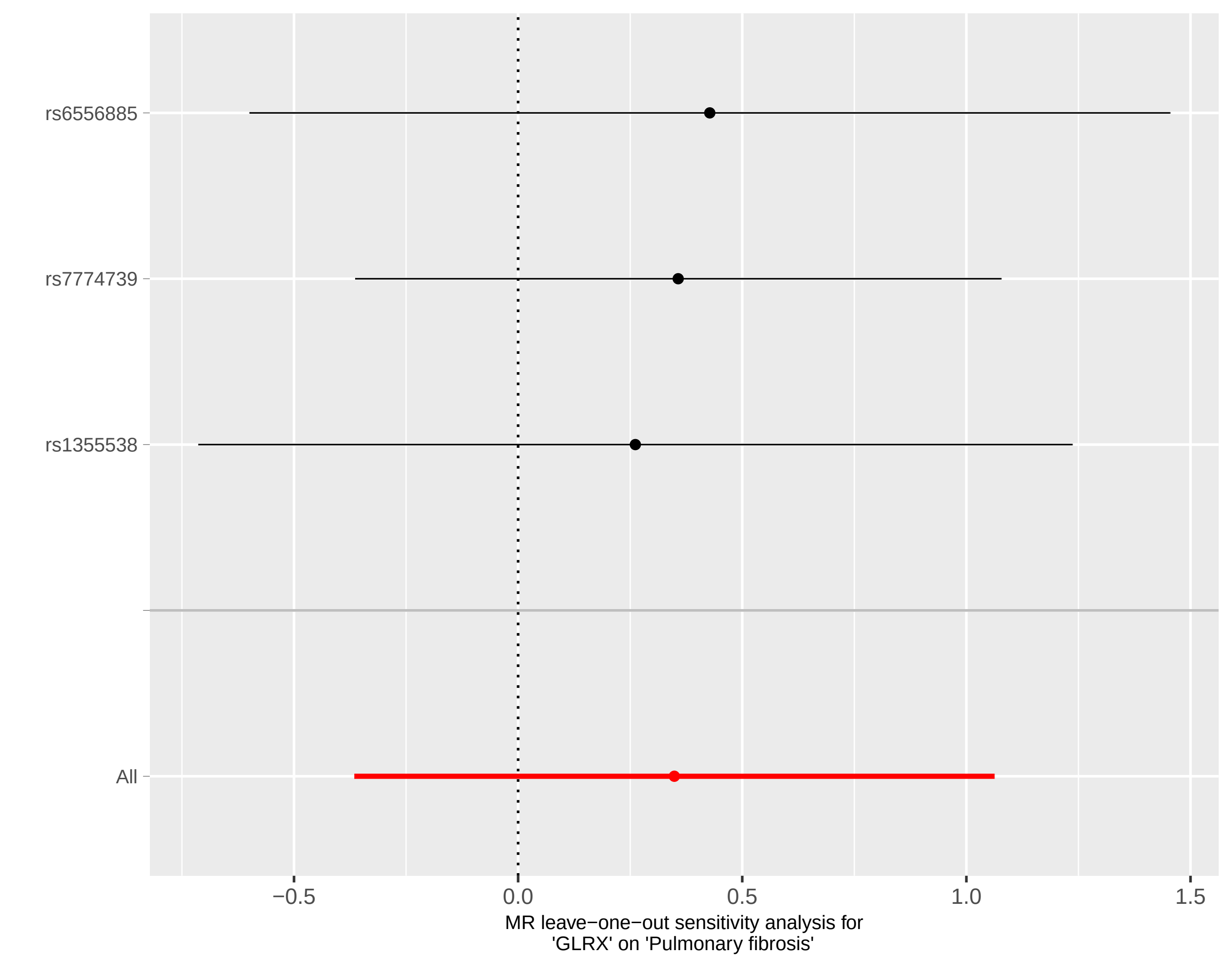
rs6556885
rs7774739
rs1355538
All
−0.5
0.0
0.5
1.0
1.5
MR leave−one−out sensitivity analysis for
'GLRX' on 'Pulmonary fibrosis'

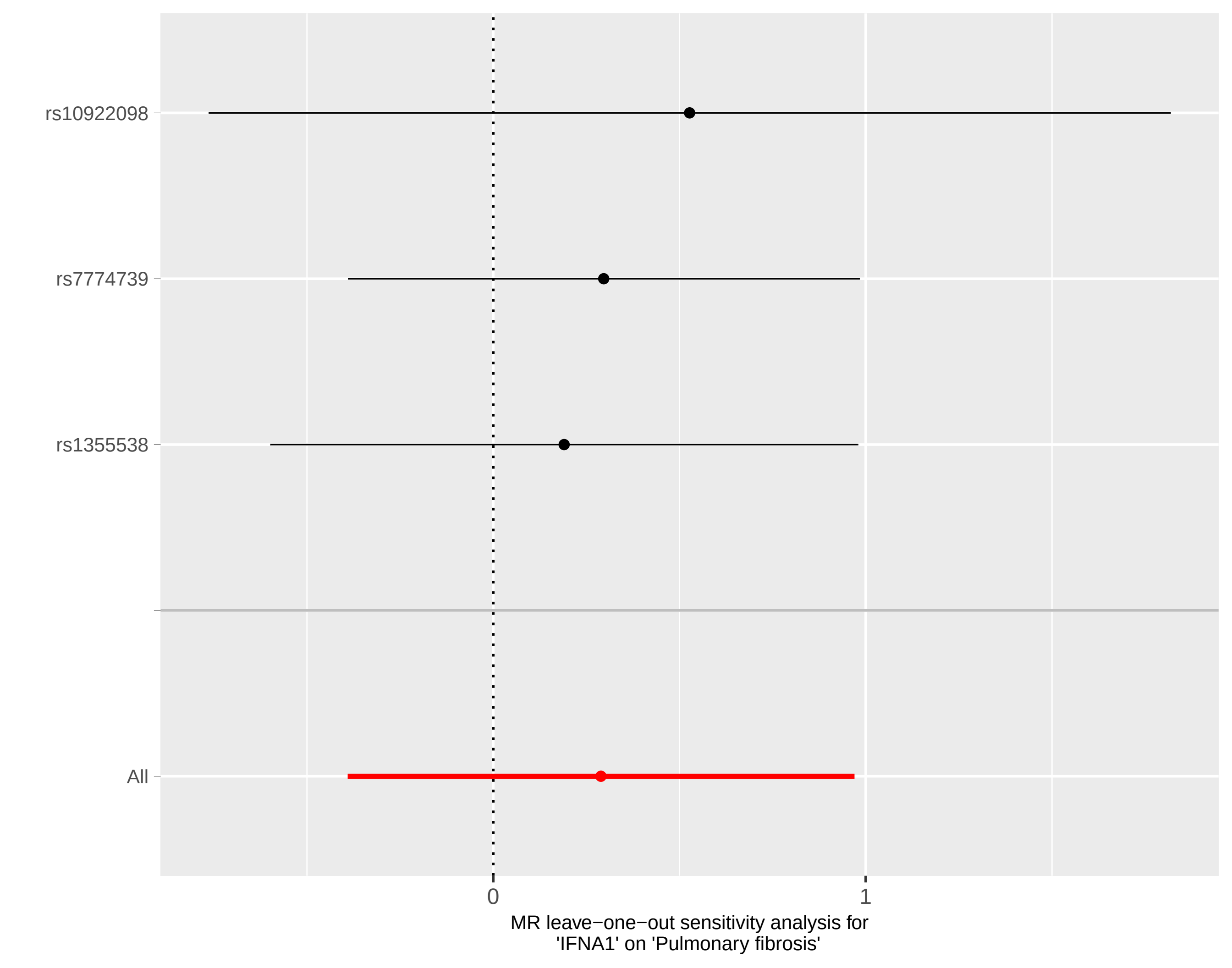
rs10922098
rs7774739
rs1355538
All
0
1
MR leave−one−out sensitivity analysis for
'IFNA1' on 'Pulmonary fibrosis'

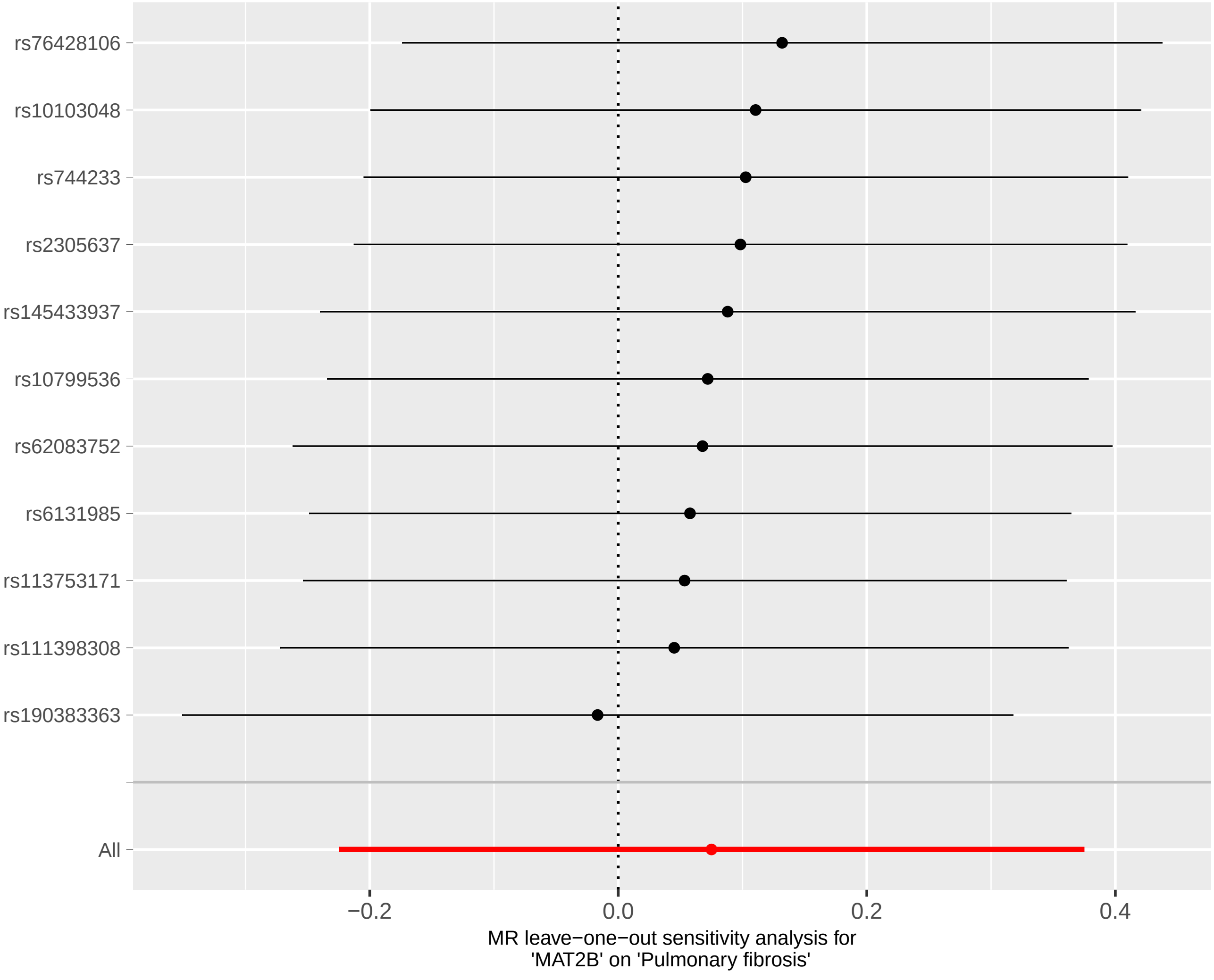
rs76428106
rs10103048
rs744233
rs2305637
rs145433937
rs10799536
rs62083752
rs6131985
rs113753171
rs111398308
rs190383363
All
−0.2
0.0
0.2
0.4
MR leave−one−out sensitivity analysis for
'MAT2B' on 'Pulmonary fibrosis'

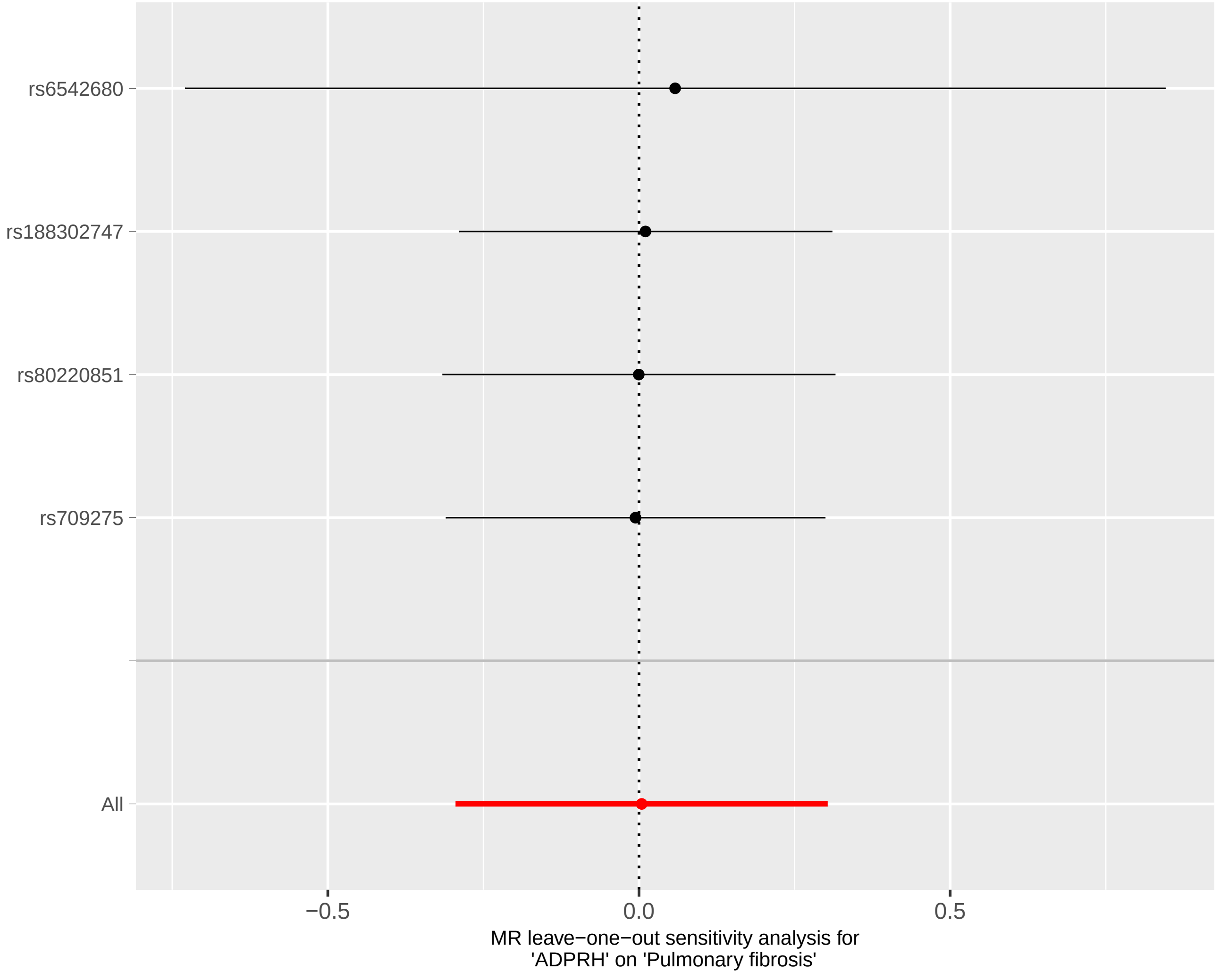

rs6542680
rs188302747
rs80220851
rs709275
All
−0.5
0.0
0.5
MR leave−one−out sensitivity analysis for
'ADPRH' on 'Pulmonary fibrosis'

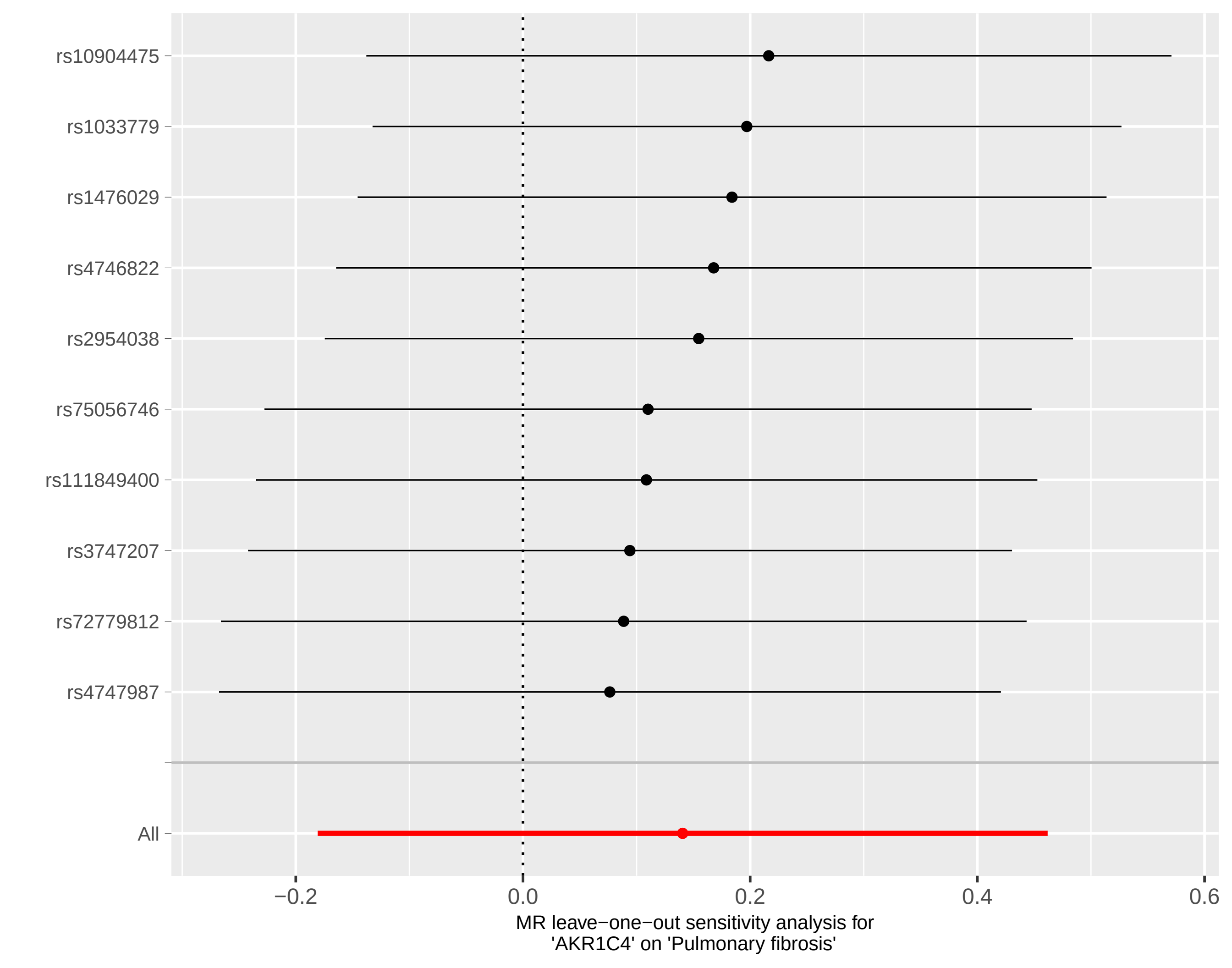

rs10904475
rs1033779
rs1476029
rs4746822
rs2954038
rs75056746
rs111849400
rs3747207
rs72779812
rs4747987
All
−0.2
0.0
0.2
0.4
0.6
MR leave−one−out sensitivity analysis for
'AKR1C4' on 'Pulmonary fibrosis'

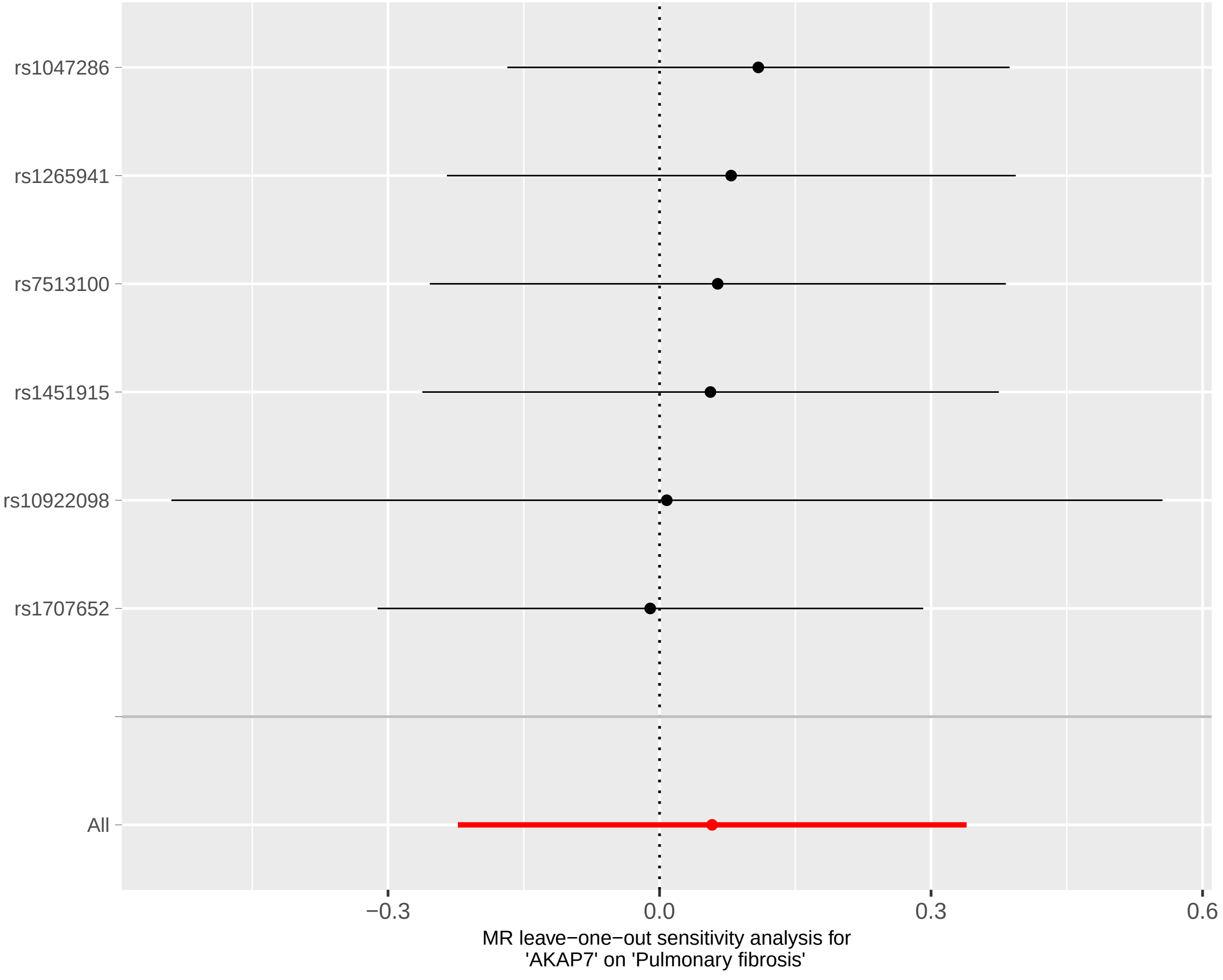
rs1047286
rs1265941
rs7513100
rs1451915
rs10922098
rs1707652
All
−0.3
0.0
0.3
0.6
MR leave−one−out sensitivity analysis for
'AKAP7' on 'Pulmonary fibrosis'

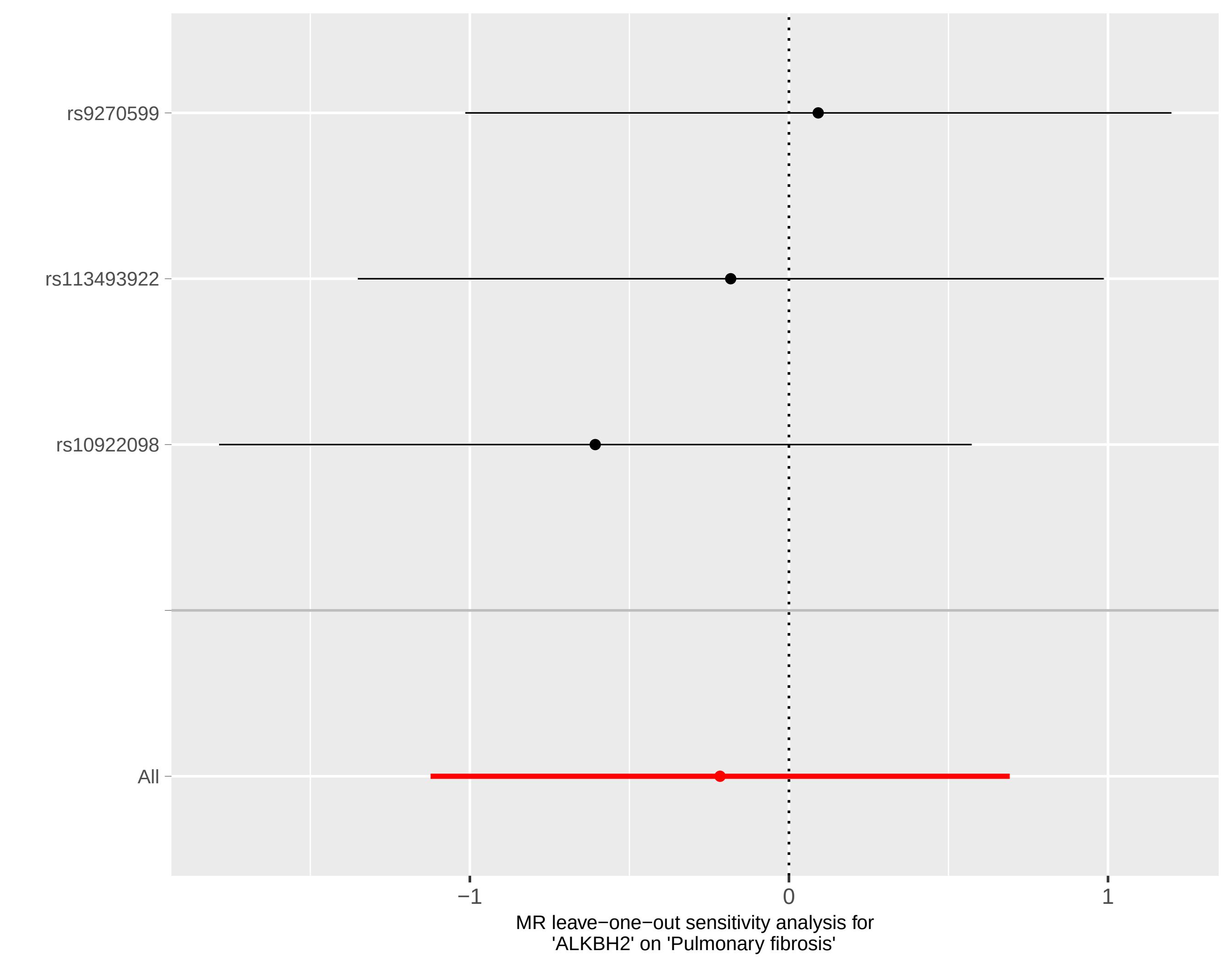

rs9270599
rs113493922
rs10922098
All
−1
0
1
MR leave−one−out sensitivity analysis for
'ALKBH2' on 'Pulmonary fibrosis'

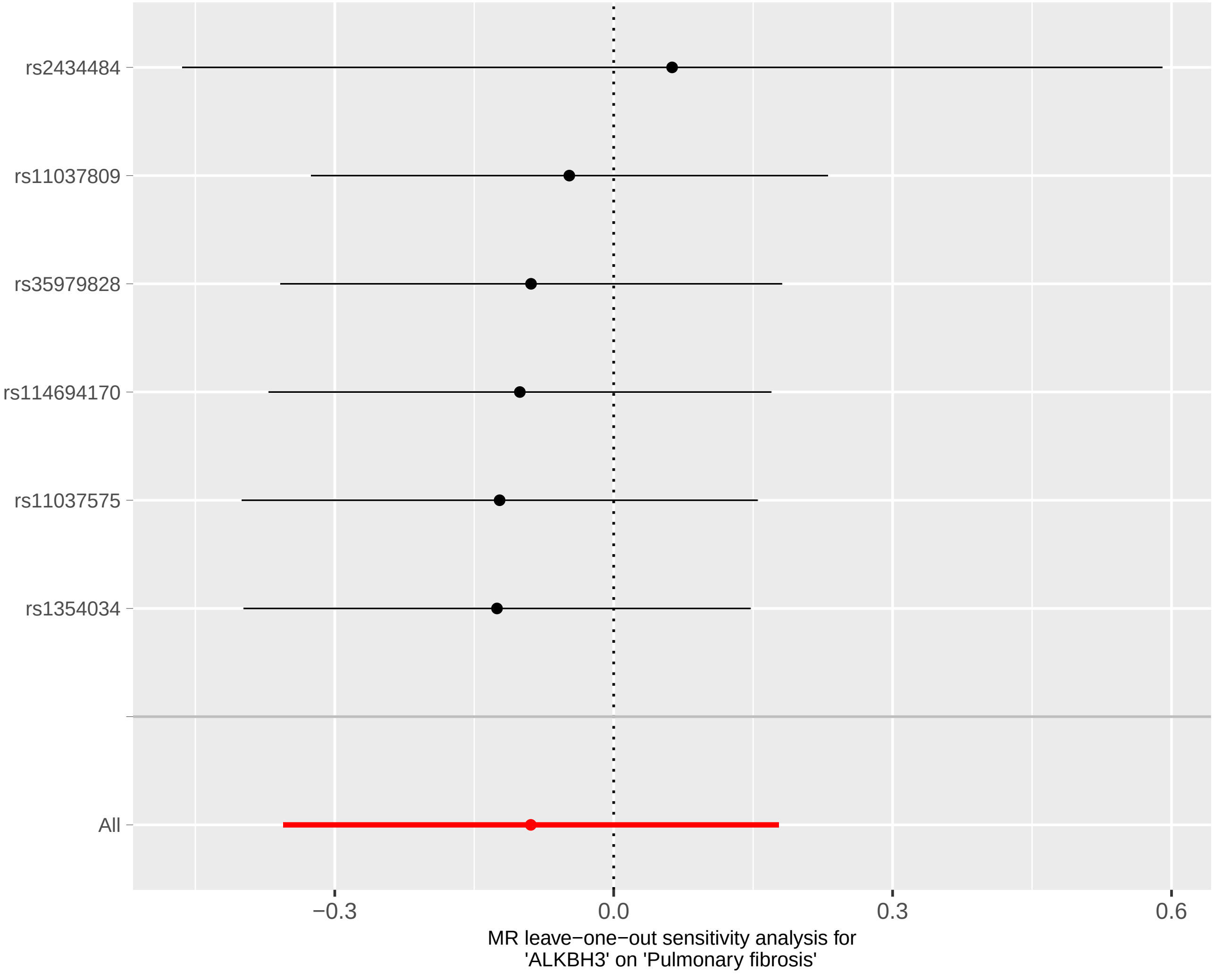
rs2434484
rs11037809
rs35979828
rs114694170
rs11037575
rs1354034
All
−0.3
0.0
0.3
0.6
MR leave−one−out sensitivity analysis for
'ALKBH3' on 'Pulmonary fibrosis'

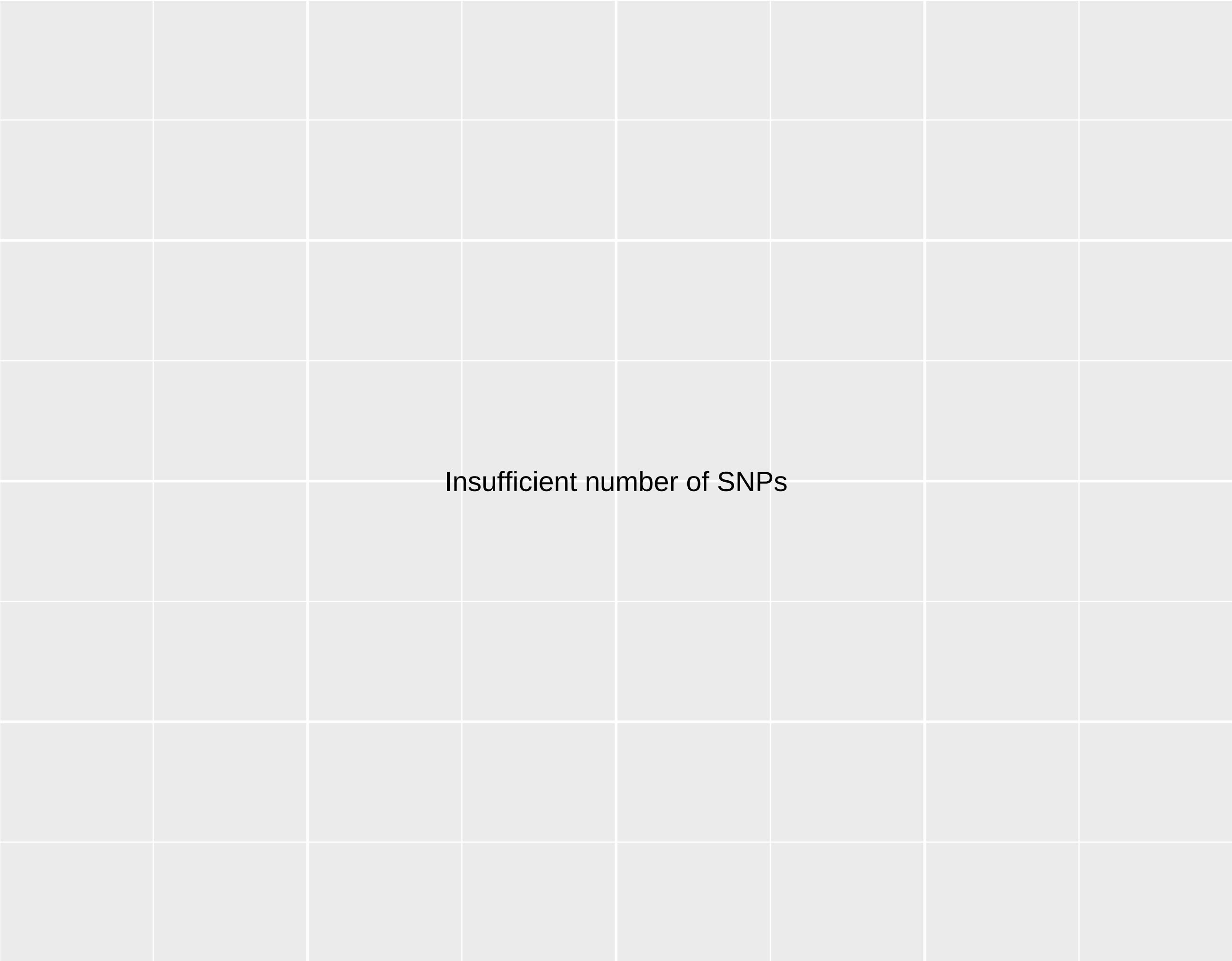
Insufficient number of SNPs

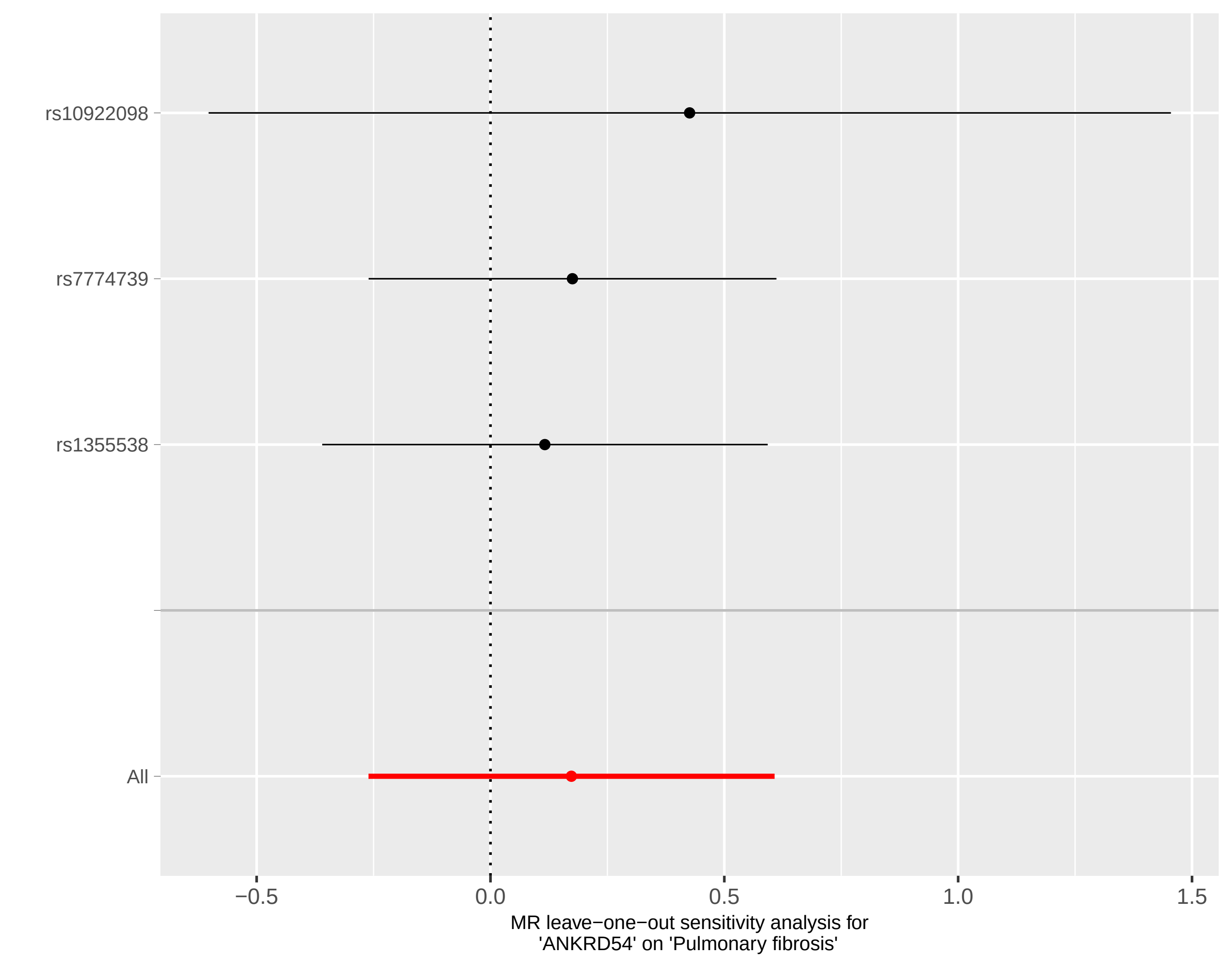

rs10922098
rs7774739
rs1355538
All
−0.5
0.0
0.5
1.0
1.5
MR leave−one−out sensitivity analysis for
'ANKRD54' on 'Pulmonary fibrosis'

Insufficient number of SNPs

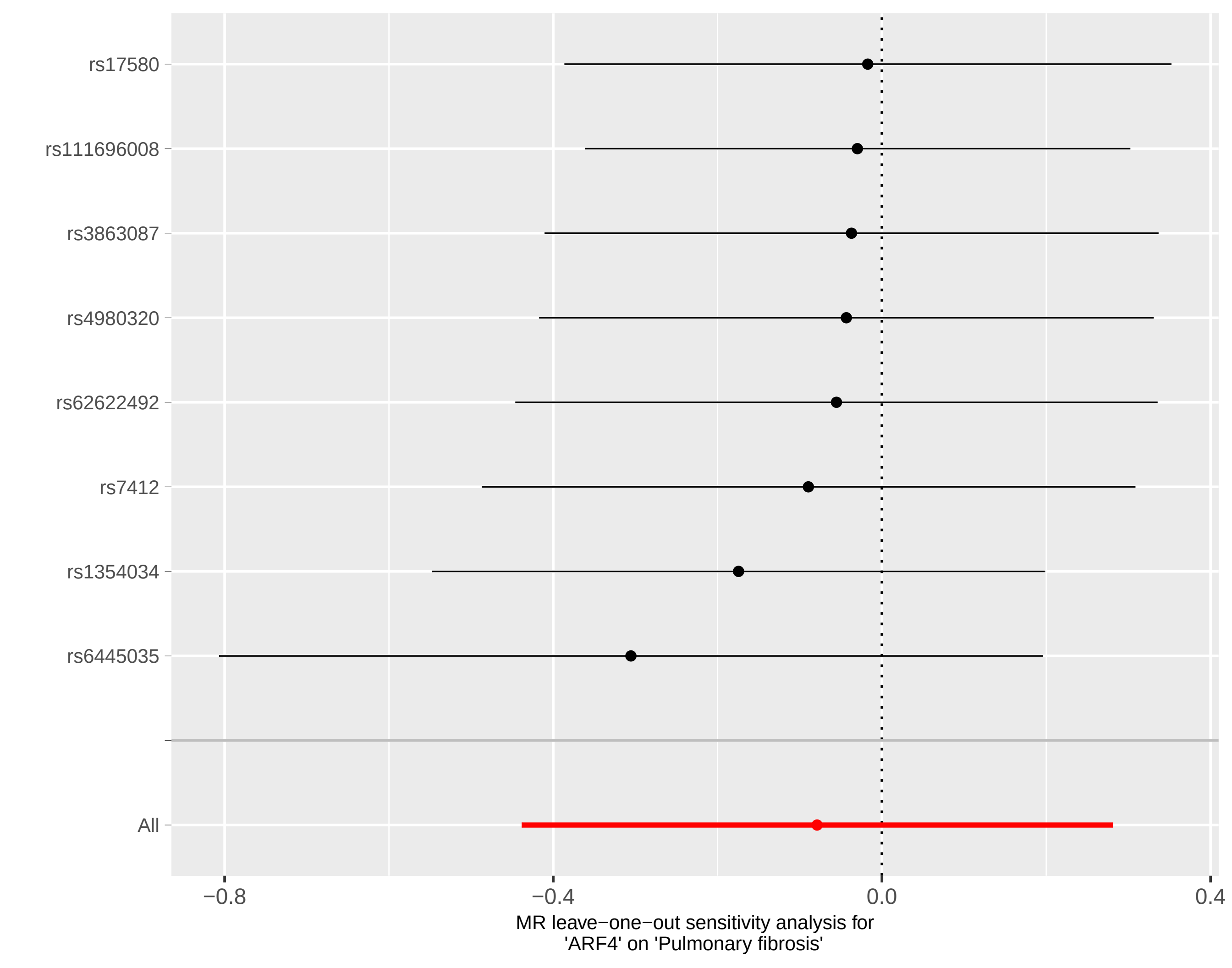
rs17580
rs111696008
rs3863087
rs4980320
rs62622492
rs7412
rs1354034
rs6445035
All
−0.8
−0.4
0.0
0.4
MR leave−one−out sensitivity analysis for
'ARF4' on 'Pulmonary fibrosis'

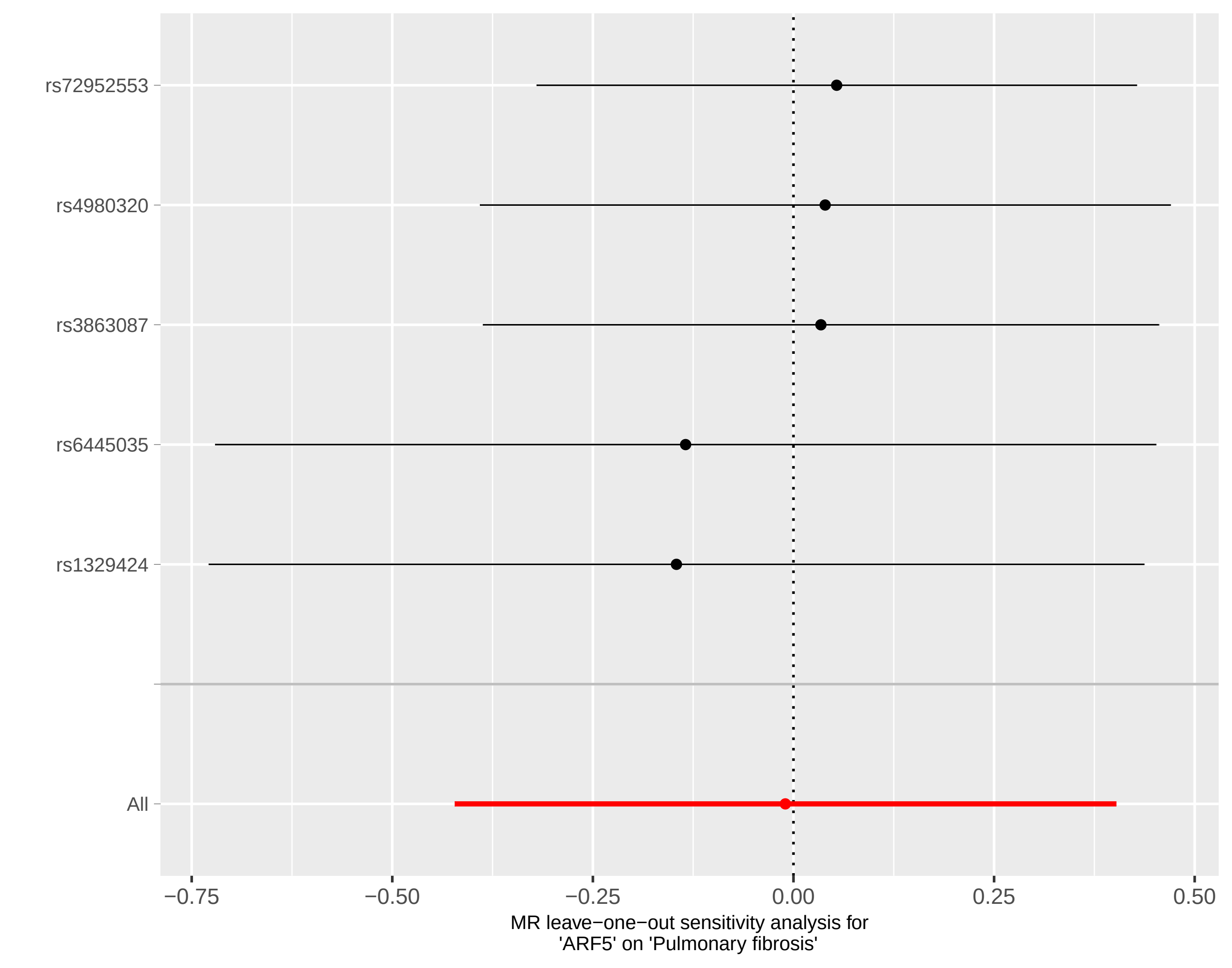
rs72952553
rs4980320
rs3863087
rs6445035
rs1329424
All
−0.75
−0.50
−0.25
0.00
0.25
0.50
MR leave−one−out sensitivity analysis for
'ARF5' on 'Pulmonary fibrosis'

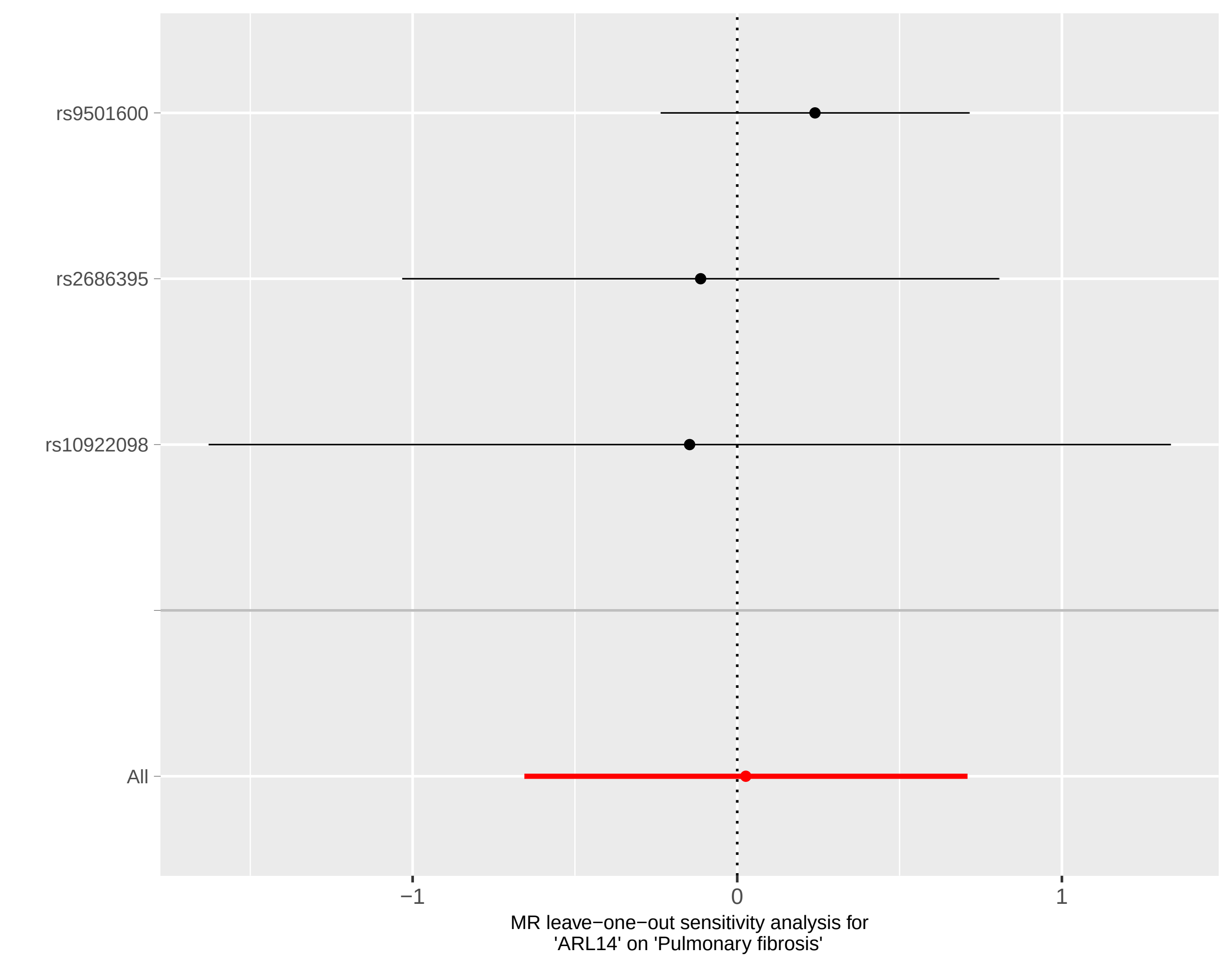
rs9501600
rs2686395
rs10922098
All
−1
0
1
MR leave−one−out sensitivity analysis for
'ARL14' on 'Pulmonary fibrosis'

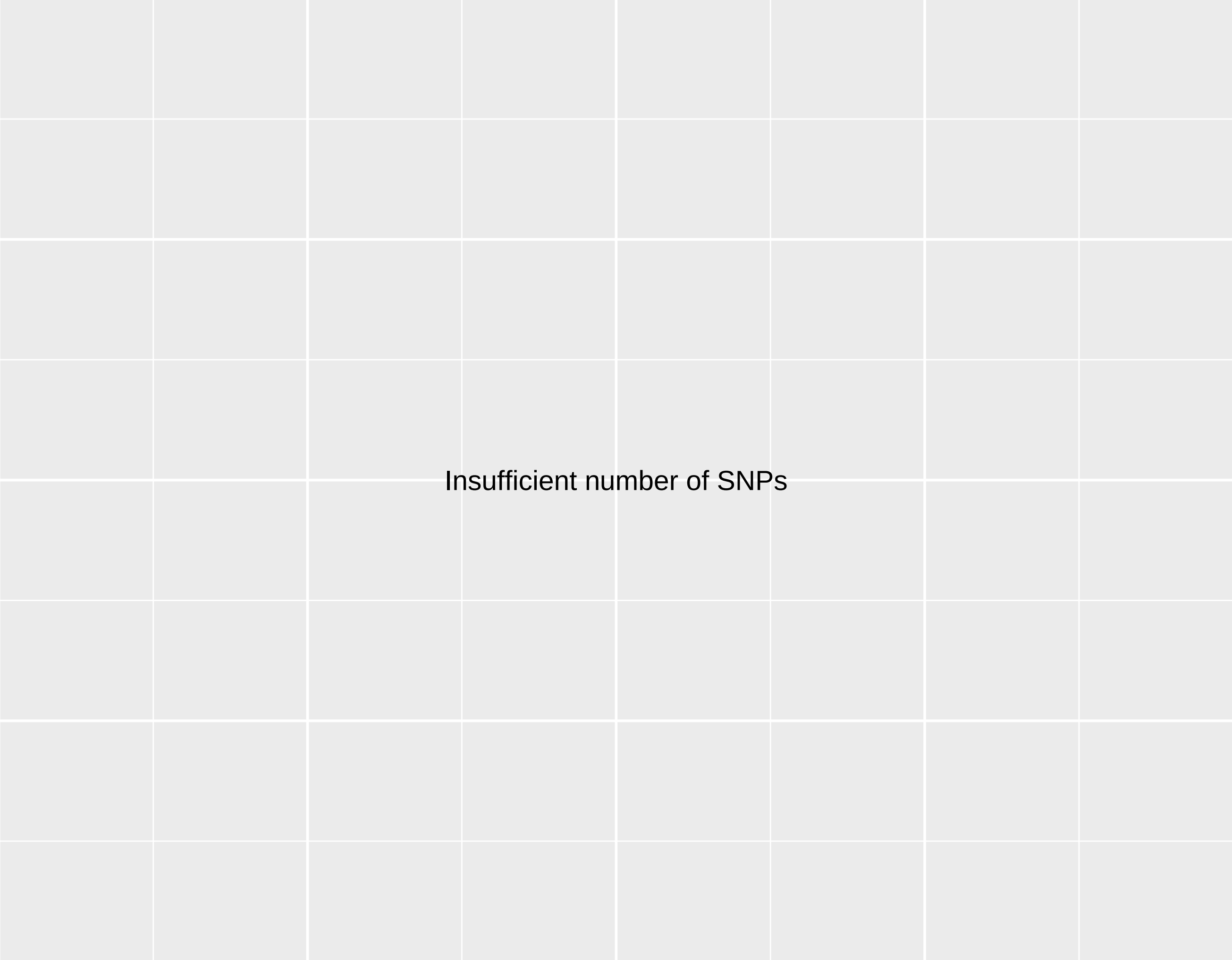
Insufficient number of SNPs

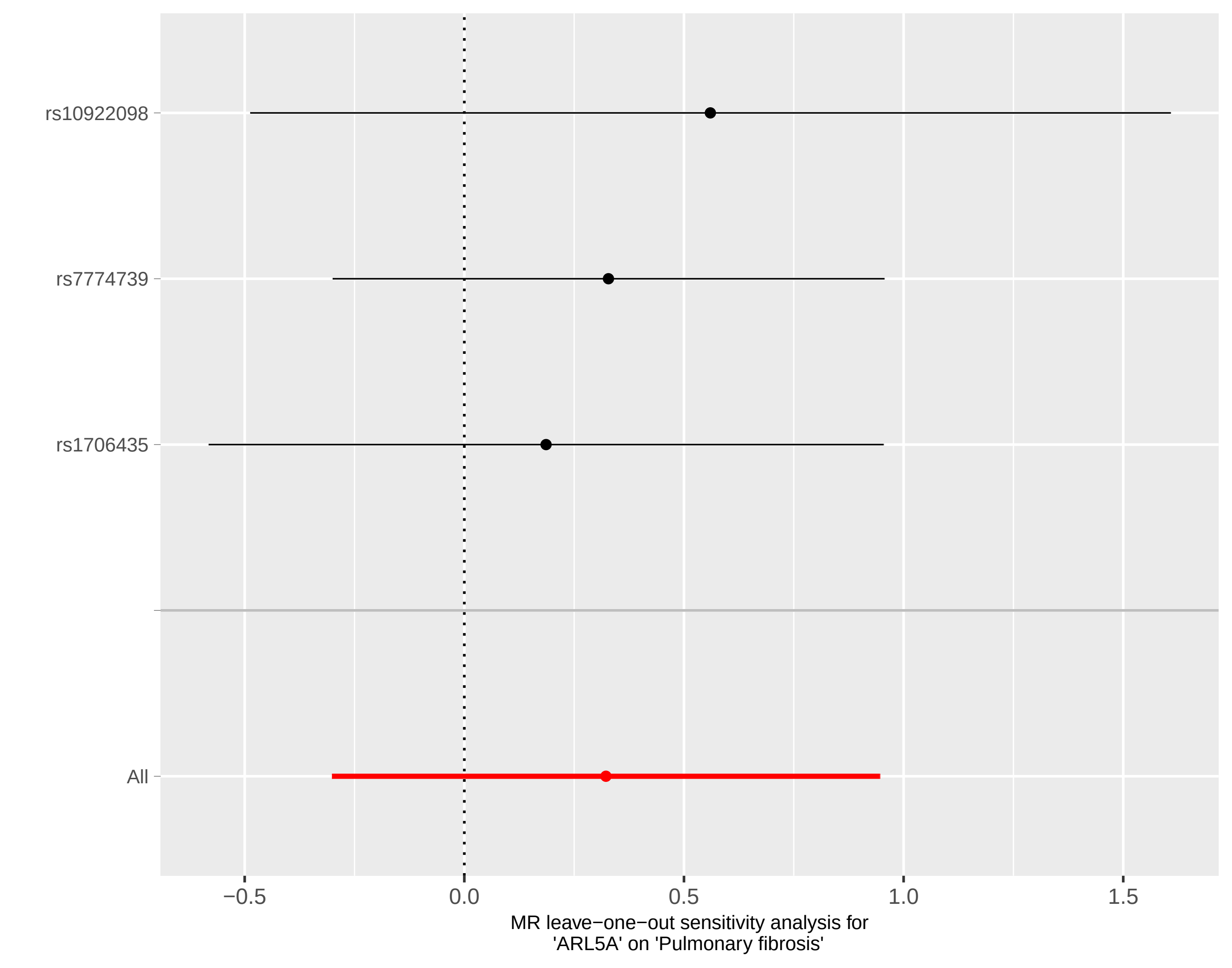

rs10922098
rs7774739
rs1706435
All
−0.5
0.0
0.5
1.0
1.5
MR leave−one−out sensitivity analysis for
'ARL5A' on 'Pulmonary fibrosis'

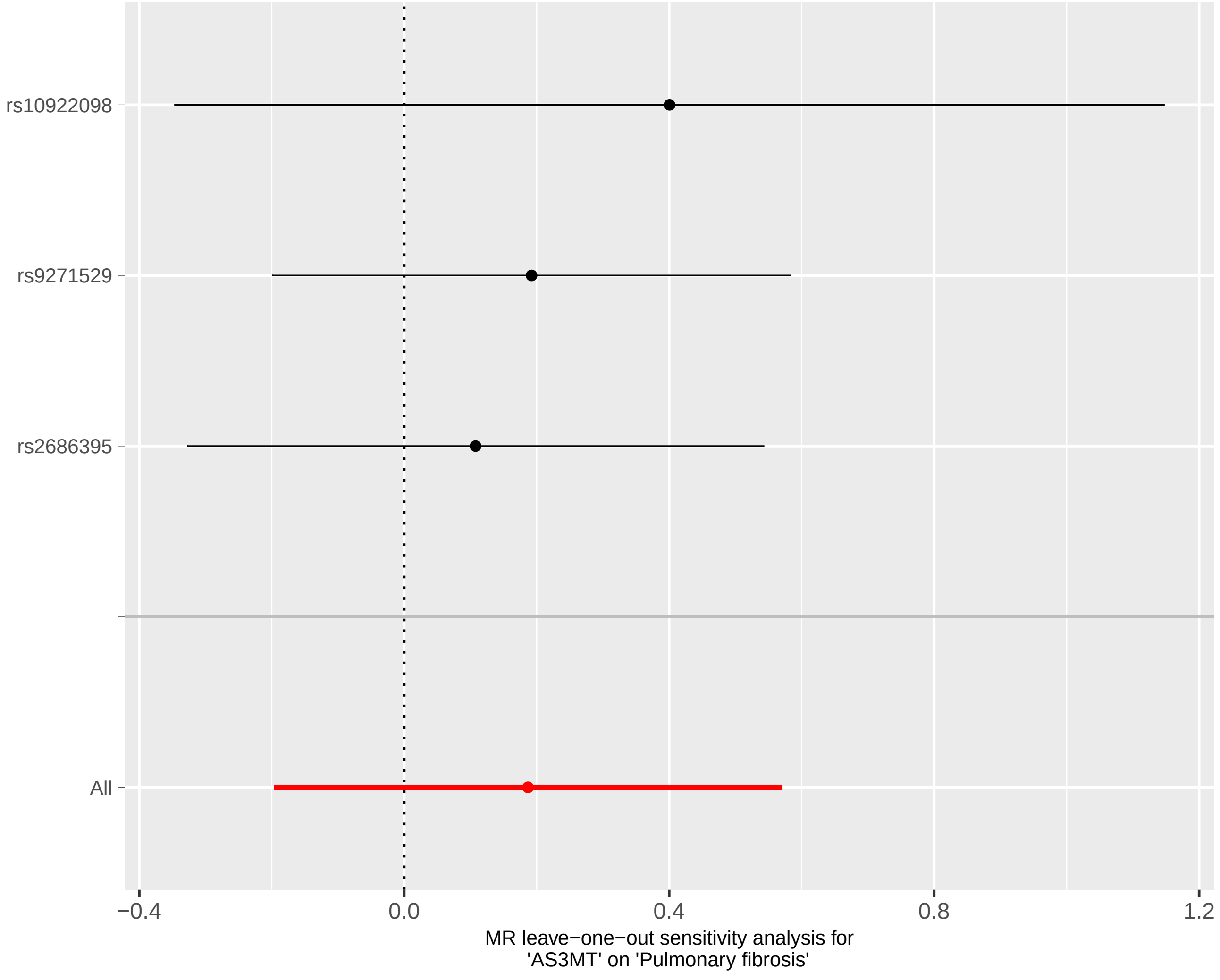
rs10922098
rs9271529
rs2686395
All
−0.4
0.0
0.4
0.8
1.2
MR leave−one−out sensitivity analysis for
'AS3MT' on 'Pulmonary fibrosis'

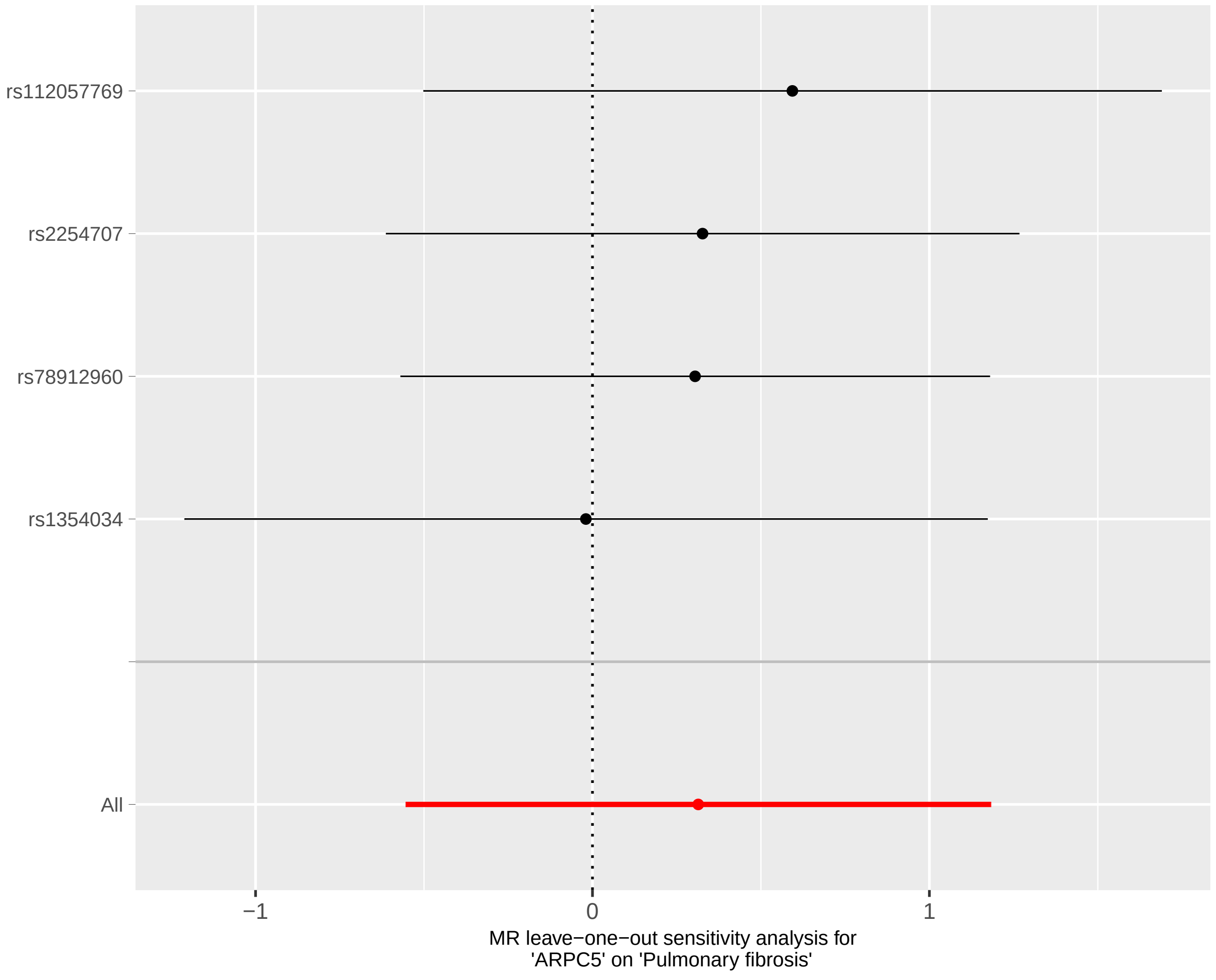
rs112057769
rs2254707
rs78912960
rs1354034
All
−1
0
1
MR leave−one−out sensitivity analysis for
'ARPC5' on 'Pulmonary fibrosis'

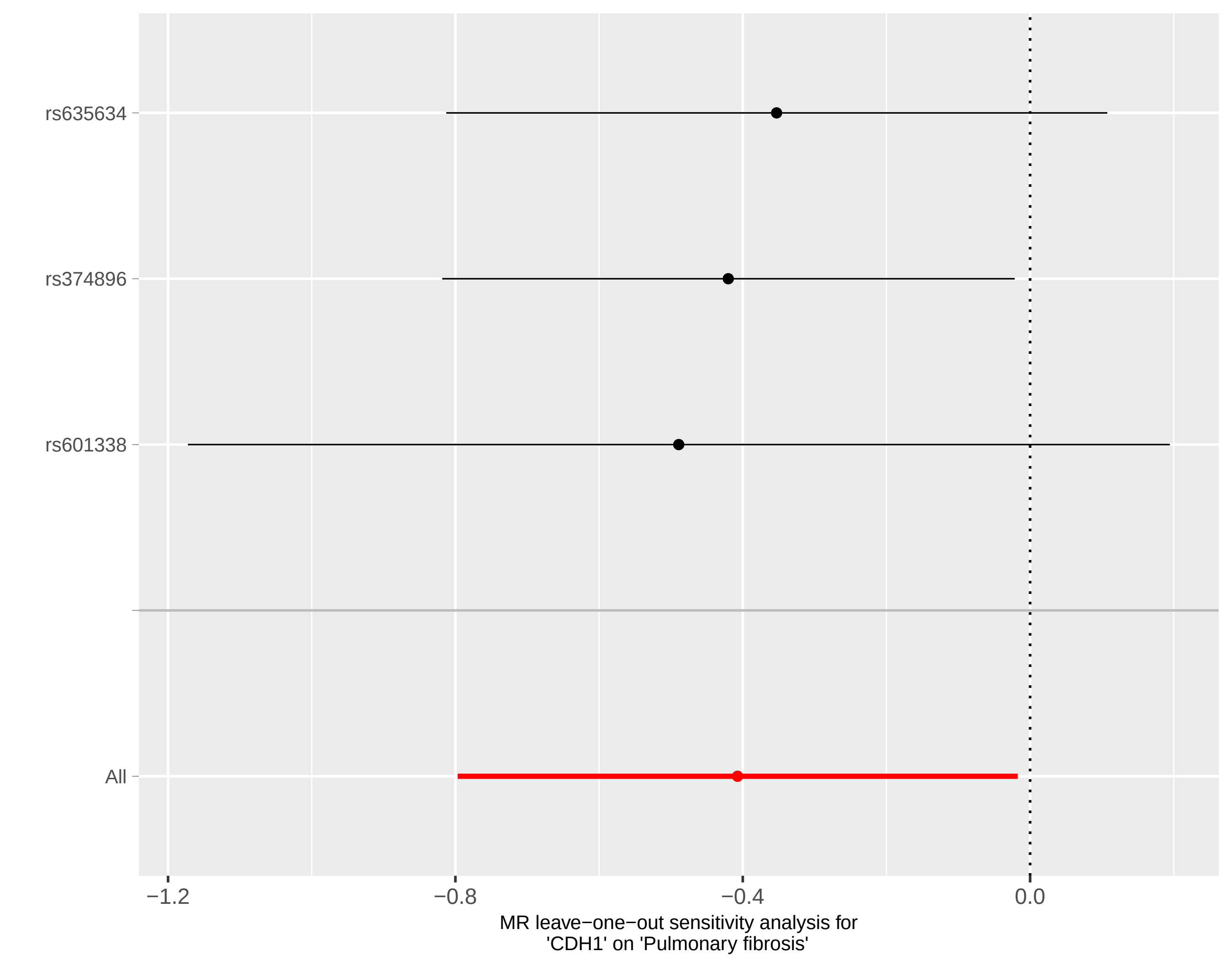
rs635634
rs374896
rs601338
All
−1.2
−0.8
−0.4
0.0
MR leave−one−out sensitivity analysis for
'CDH1' on 'Pulmonary fibrosis'

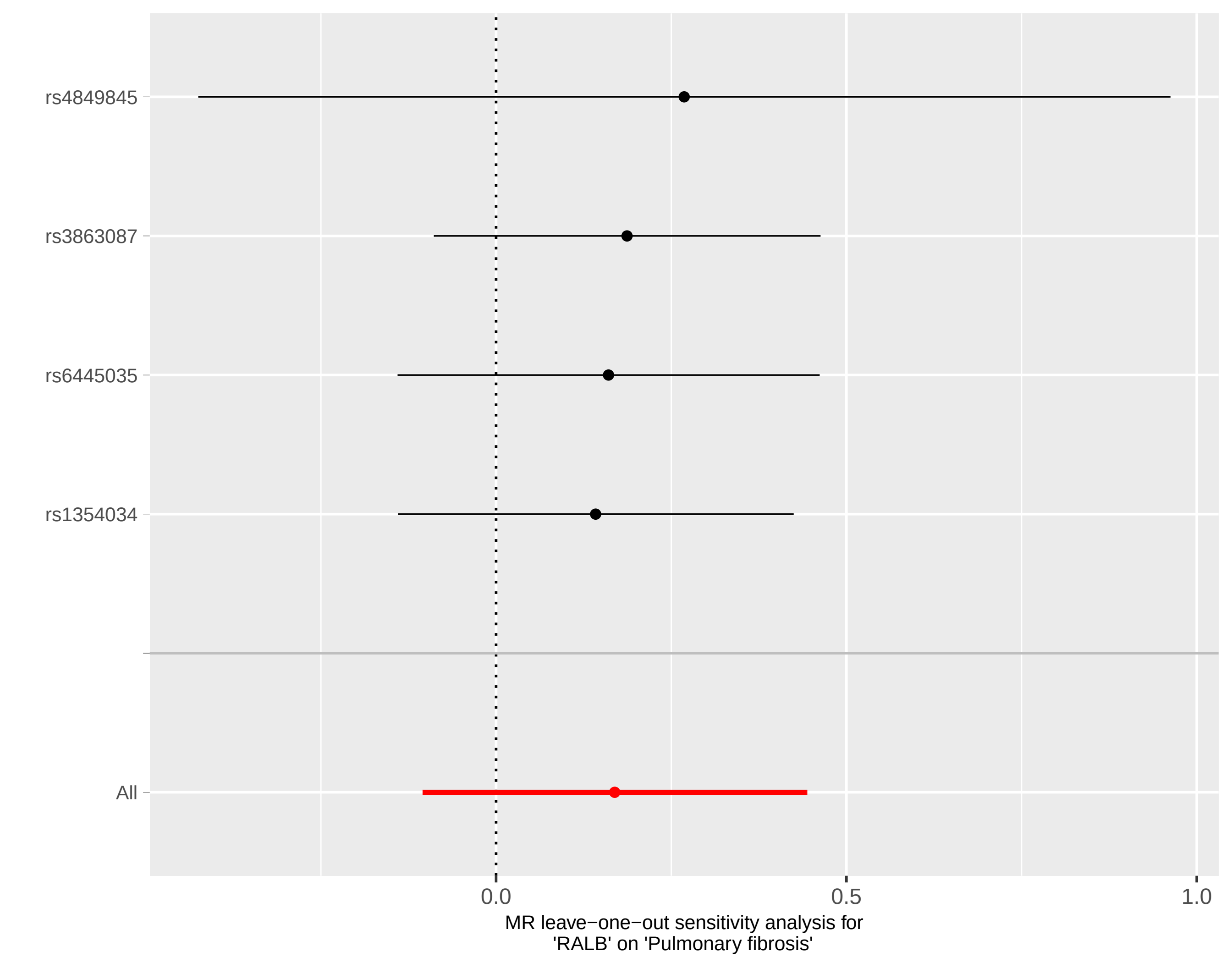
rs4849845
rs3863087
rs6445035
rs1354034
All
0.0
0.5
1.0
MR leave−one−out sensitivity analysis for
'RALB' on 'Pulmonary fibrosis'

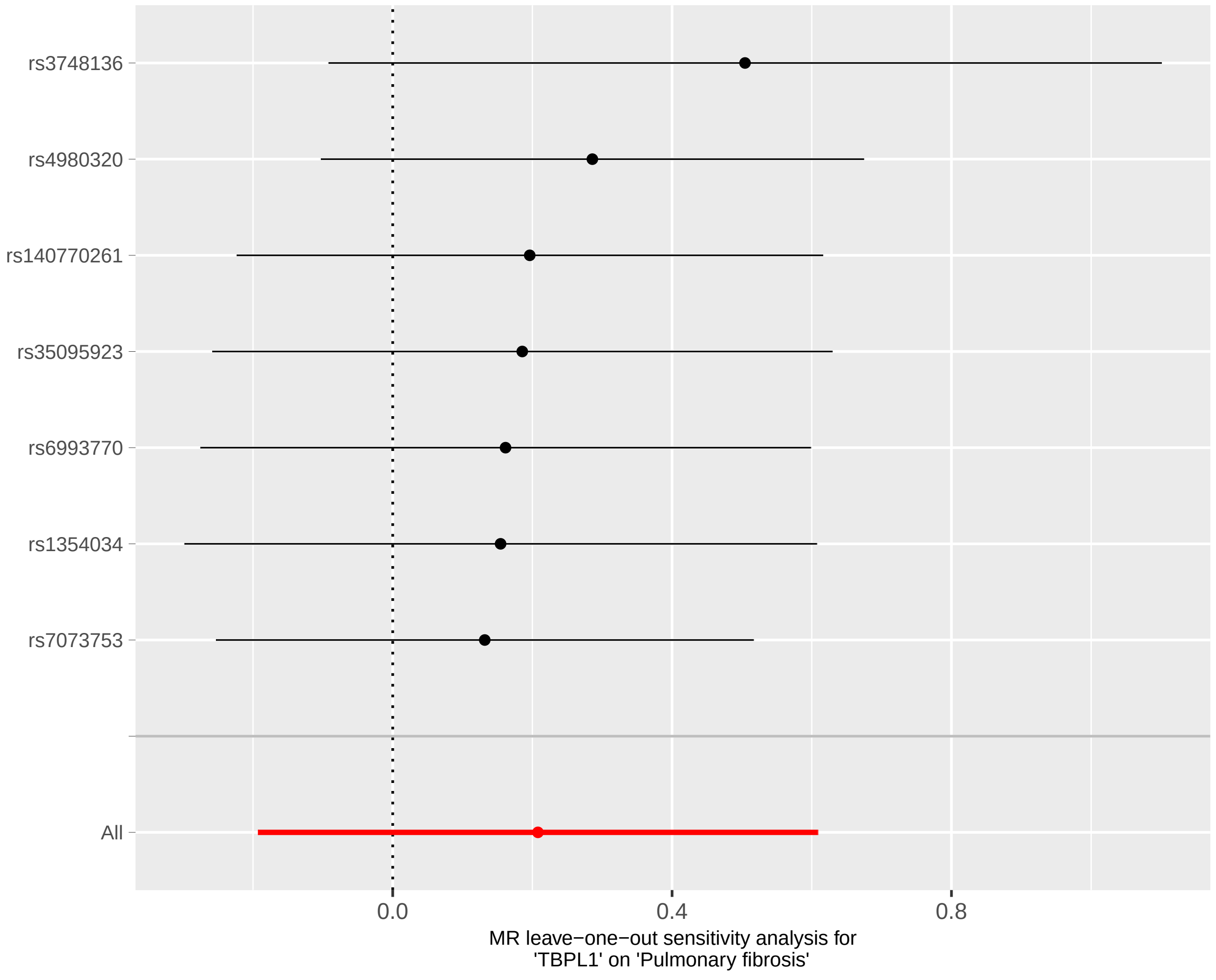
rs3748136
rs4980320
rs140770261
rs35095923
rs6993770
rs1354034
rs7073753
All
0.0
0.4
0.8
MR leave−one−out sensitivity analysis for
'TBPL1' on 'Pulmonary fibrosis'

Insufficient number of SNPs

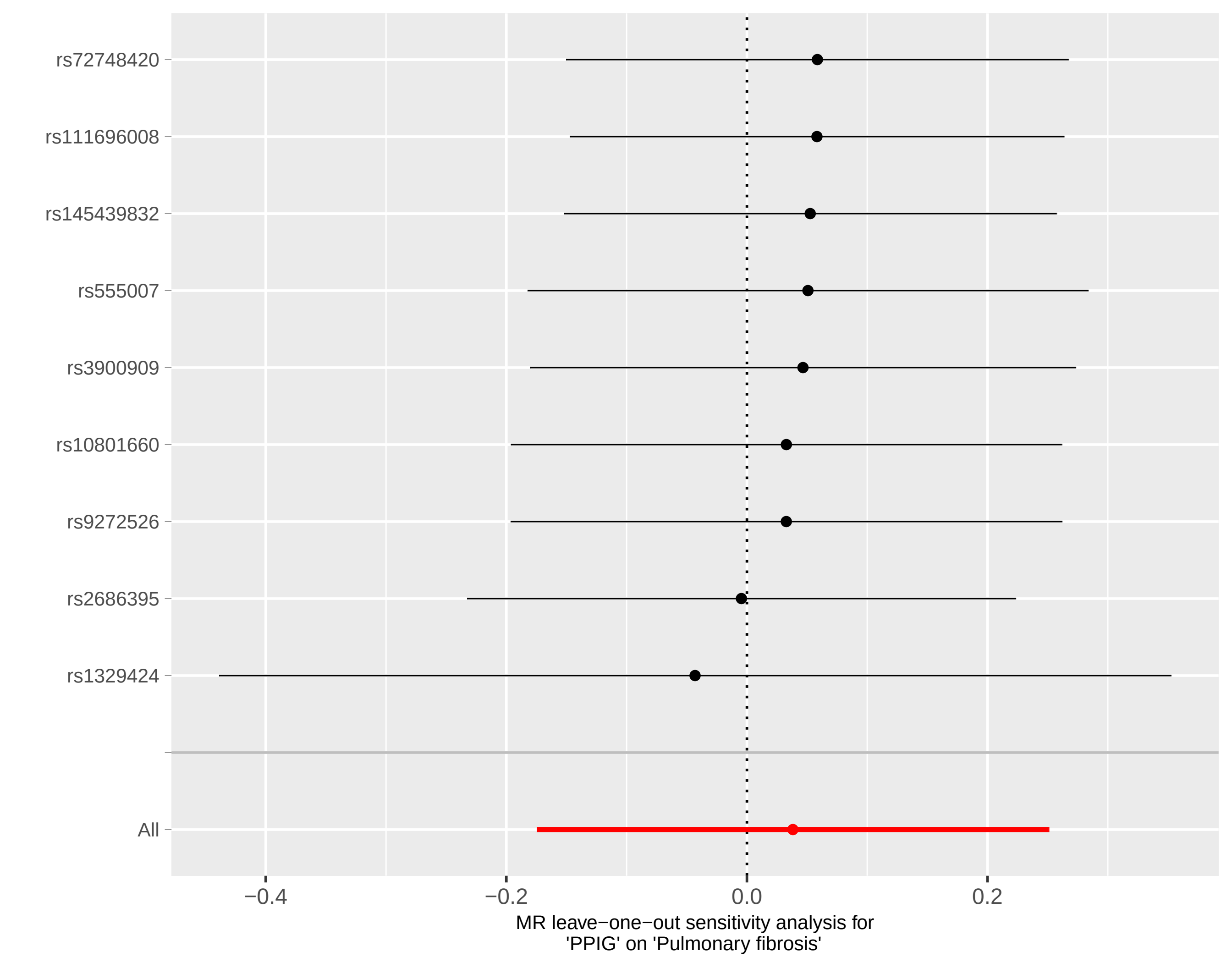
rs72748420
rs111696008
rs145439832
rs555007
rs3900909
rs10801660
rs9272526
rs2686395
rs1329424
All
−0.4
−0.2
0.0
0.2
MR leave−one−out sensitivity analysis for
'PPIG' on 'Pulmonary fibrosis'

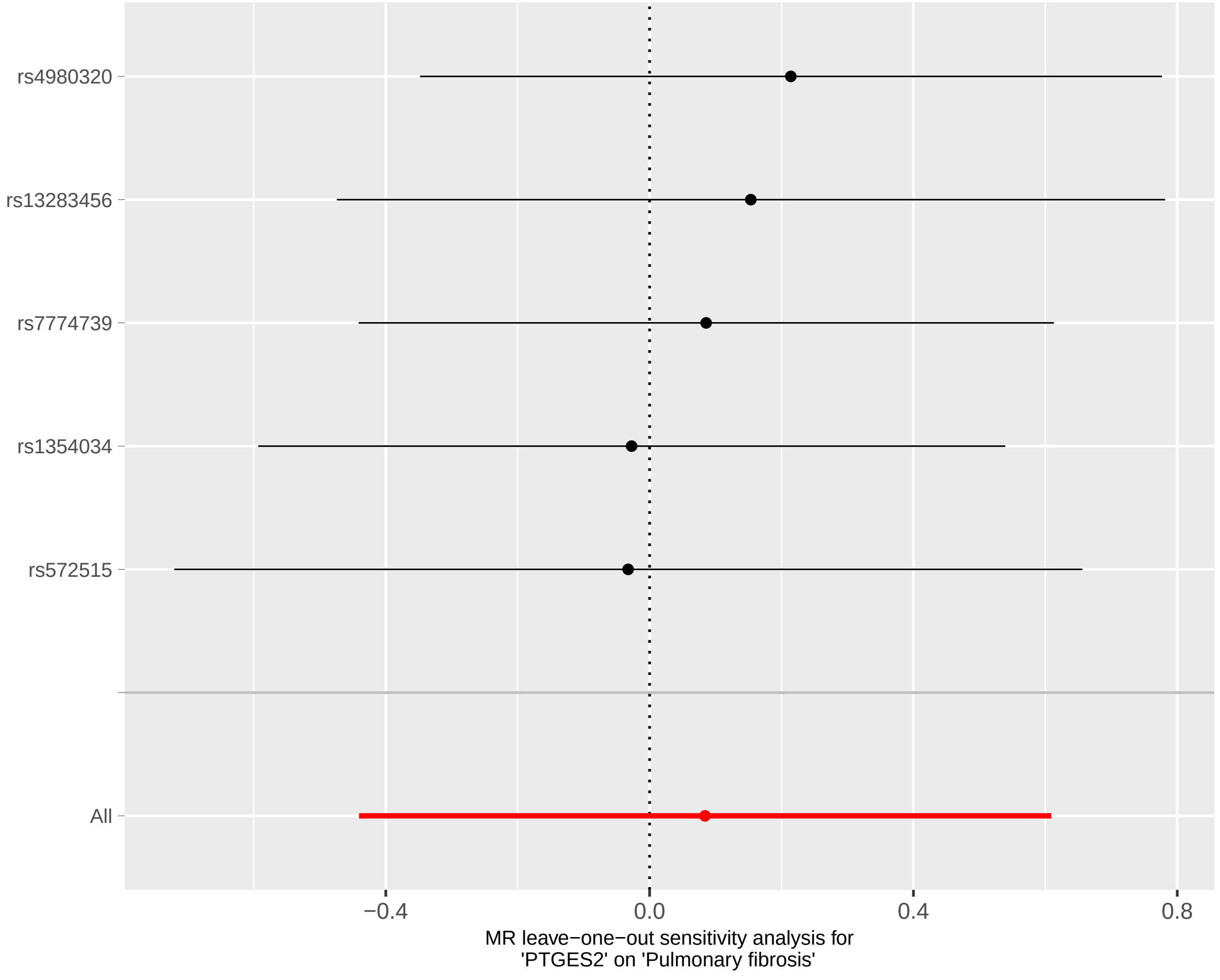
rs4980320
rs13283456
rs7774739
rs1354034
rs572515
All
−0.4
0.0
0.4
0.8
MR leave−one−out sensitivity analysis for
'PTGES2' on 'Pulmonary fibrosis'

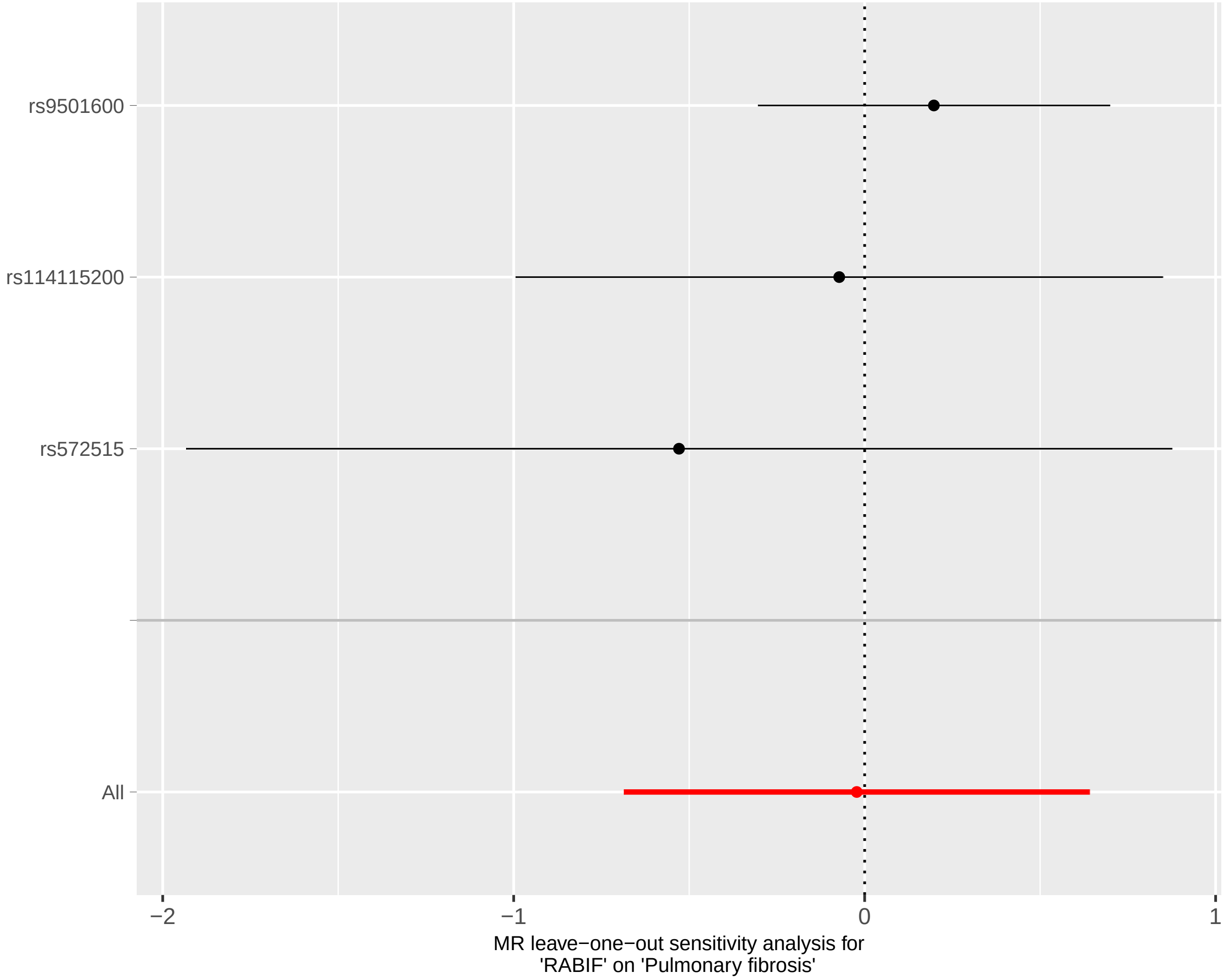
rs9501600
rs114115200
rs572515
All
−2
−1
0
1
MR leave−one−out sensitivity analysis for
'RABIF' on 'Pulmonary fibrosis'

Insufficient number of SNPs

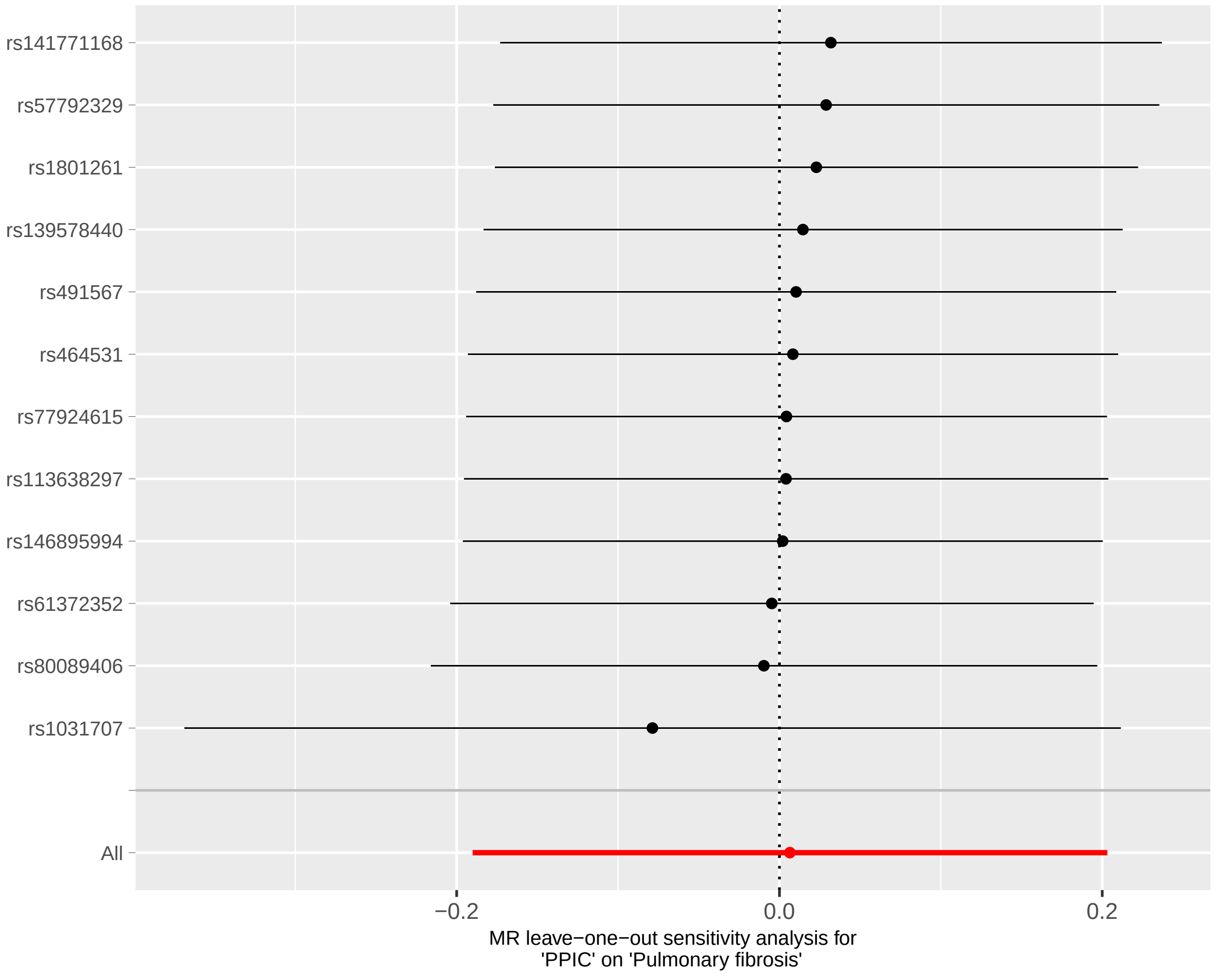

rs141771168
rs57792329
rs1801261
rs139578440
rs491567
rs464531
rs77924615
rs113638297
rs146895994
rs61372352
rs80089406
rs1031707
All
−0.2
0.0
0.2
MR leave−one−out sensitivity analysis for
'PPIC' on 'Pulmonary fibrosis'

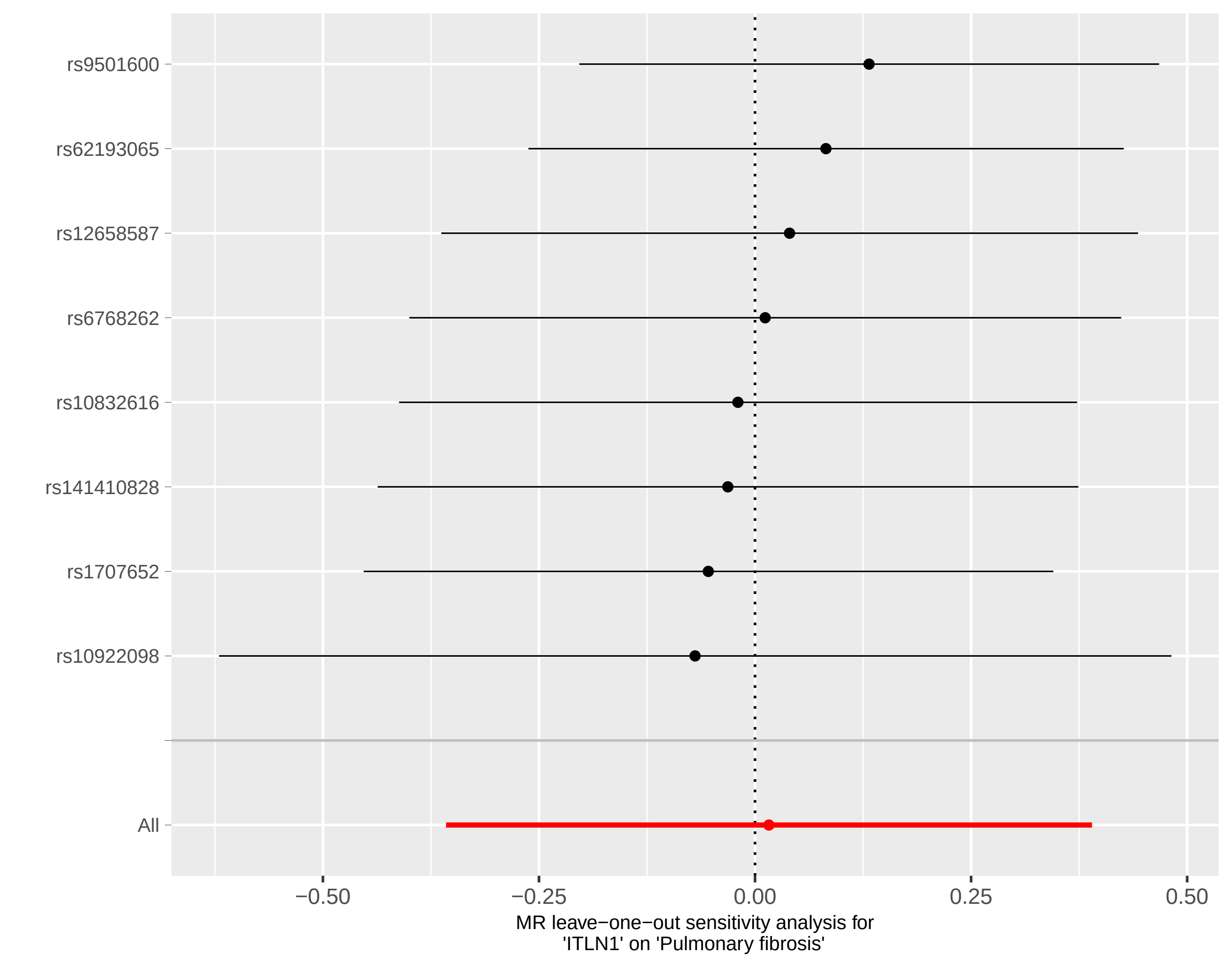
rs9501600
rs62193065
rs12658587
rs6768262
rs10832616
rs141410828
rs1707652
rs10922098
All
−0.50
−0.25
0.00
0.25
0.50
MR leave−one−out sensitivity analysis for
'ITLN1' on 'Pulmonary fibrosis'

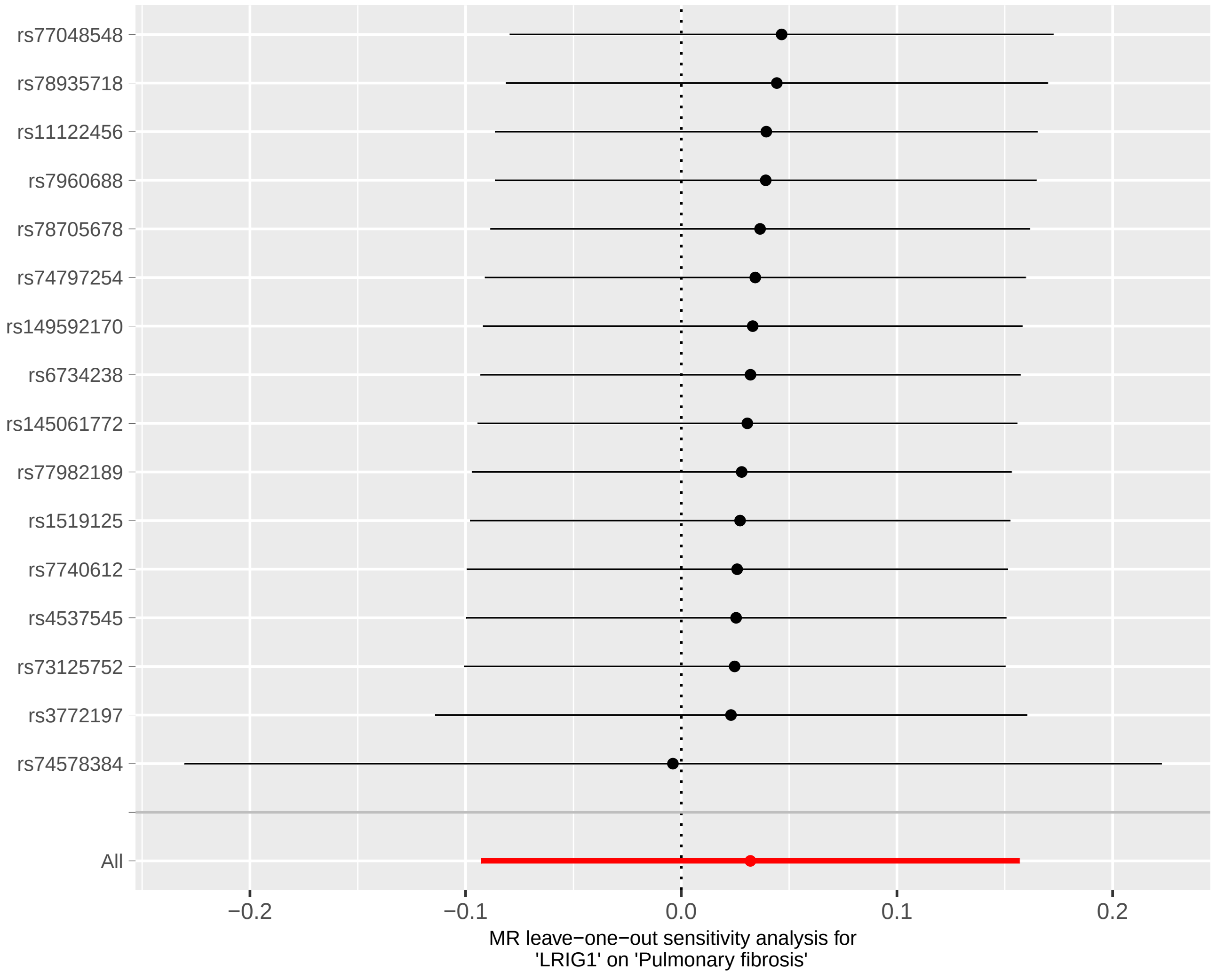
rs77048548
rs78935718
rs11122456
rs7960688
rs78705678
rs74797254
rs149592170
rs6734238
rs145061772
rs77982189
rs1519125
rs7740612
rs4537545
rs73125752
rs3772197
rs74578384
All
−0.2
−0.1
0.0
0.1
0.2
MR leave−one−out sensitivity analysis for
'LRIG1' on 'Pulmonary fibrosis'

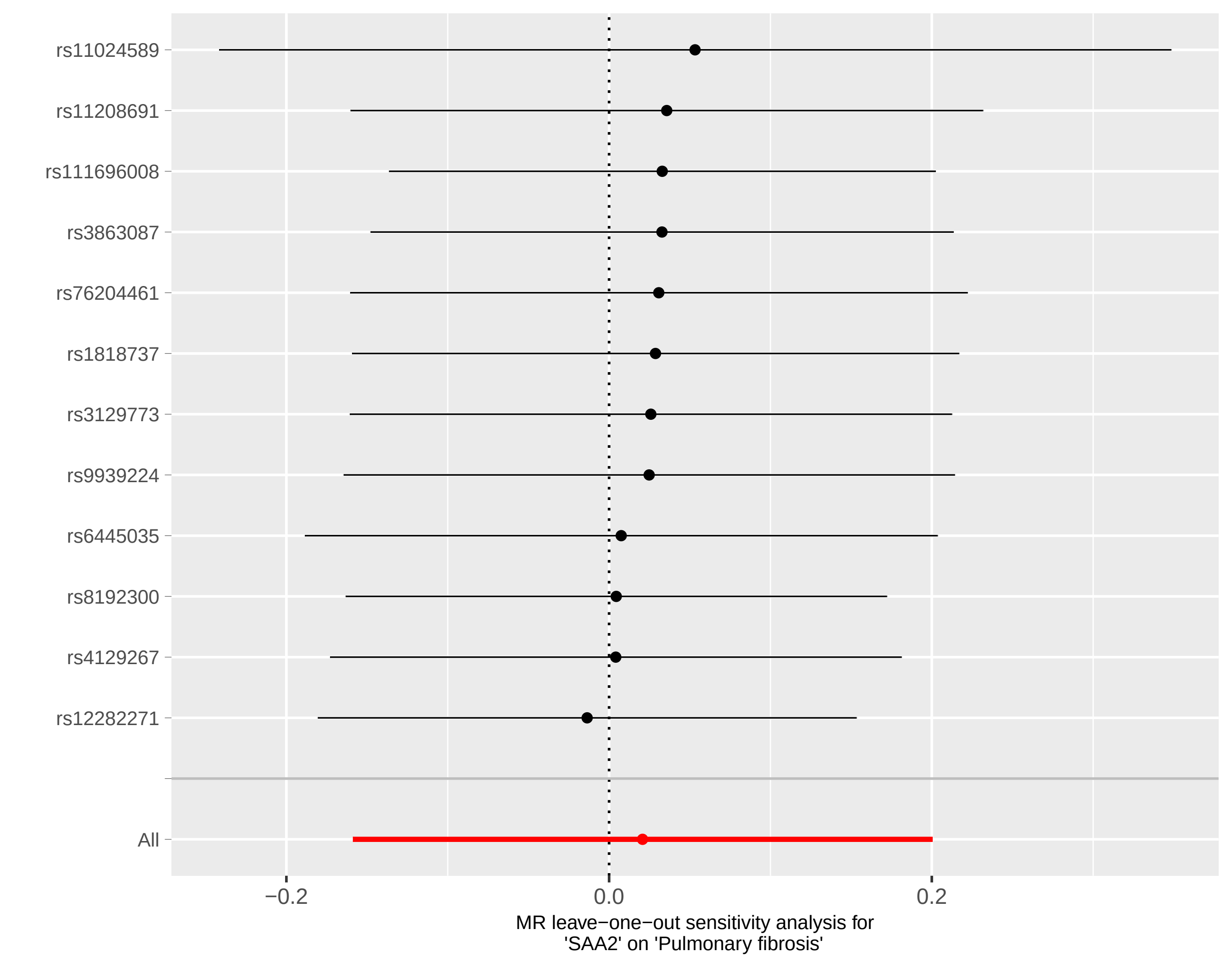

rs11024589
rs11208691
rs111696008
rs3863087
rs76204461
rs1818737
rs3129773
rs9939224
rs6445035
rs8192300
rs4129267
rs12282271
All
−0.2
0.0
0.2
MR leave−one−out sensitivity analysis for
'SAA2' on 'Pulmonary fibrosis'

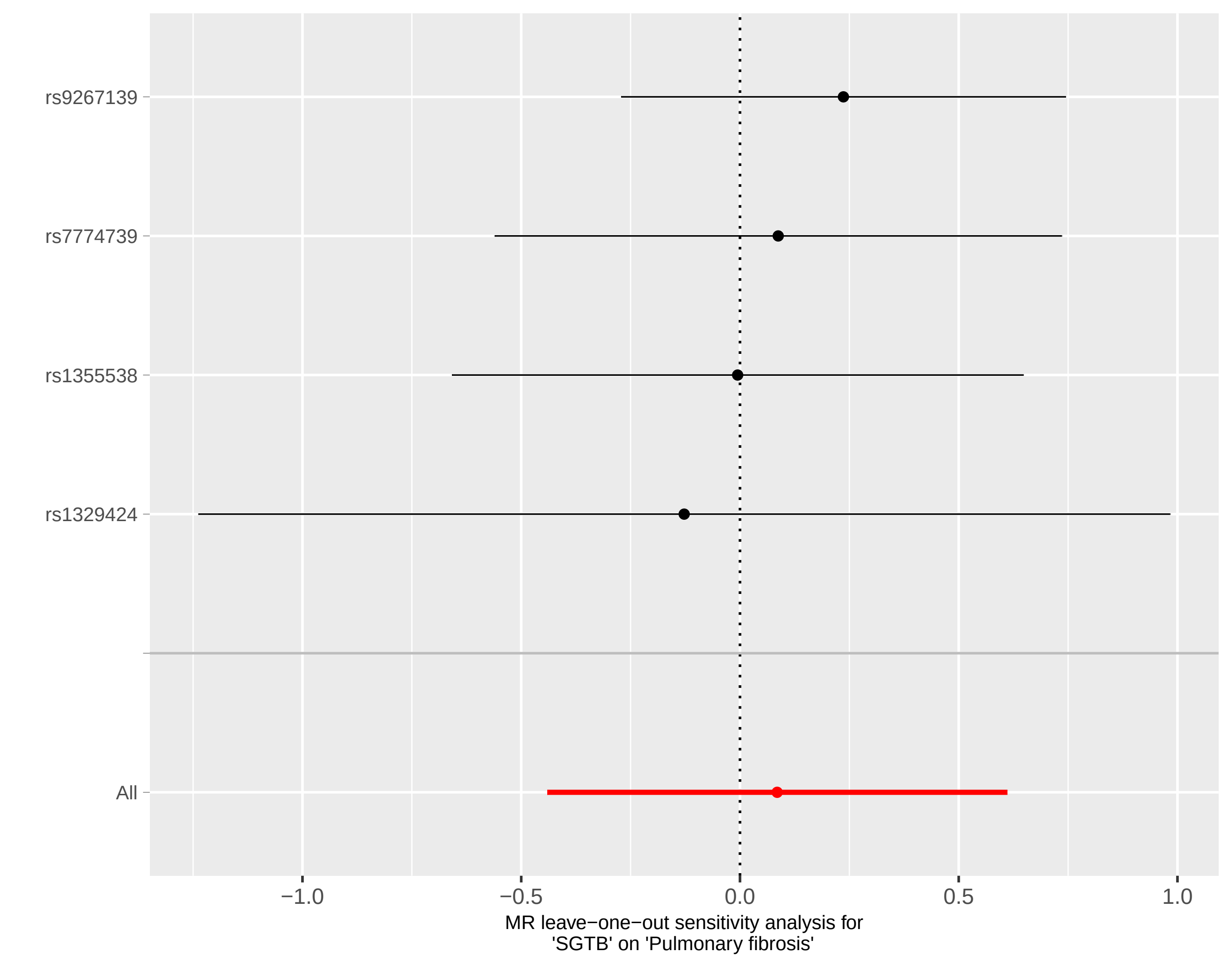
rs9267139
rs7774739
rs1355538
rs1329424
All
−1.0
−0.5
0.0
0.5
1.0
MR leave−one−out sensitivity analysis for
'SGTB' on 'Pulmonary fibrosis'

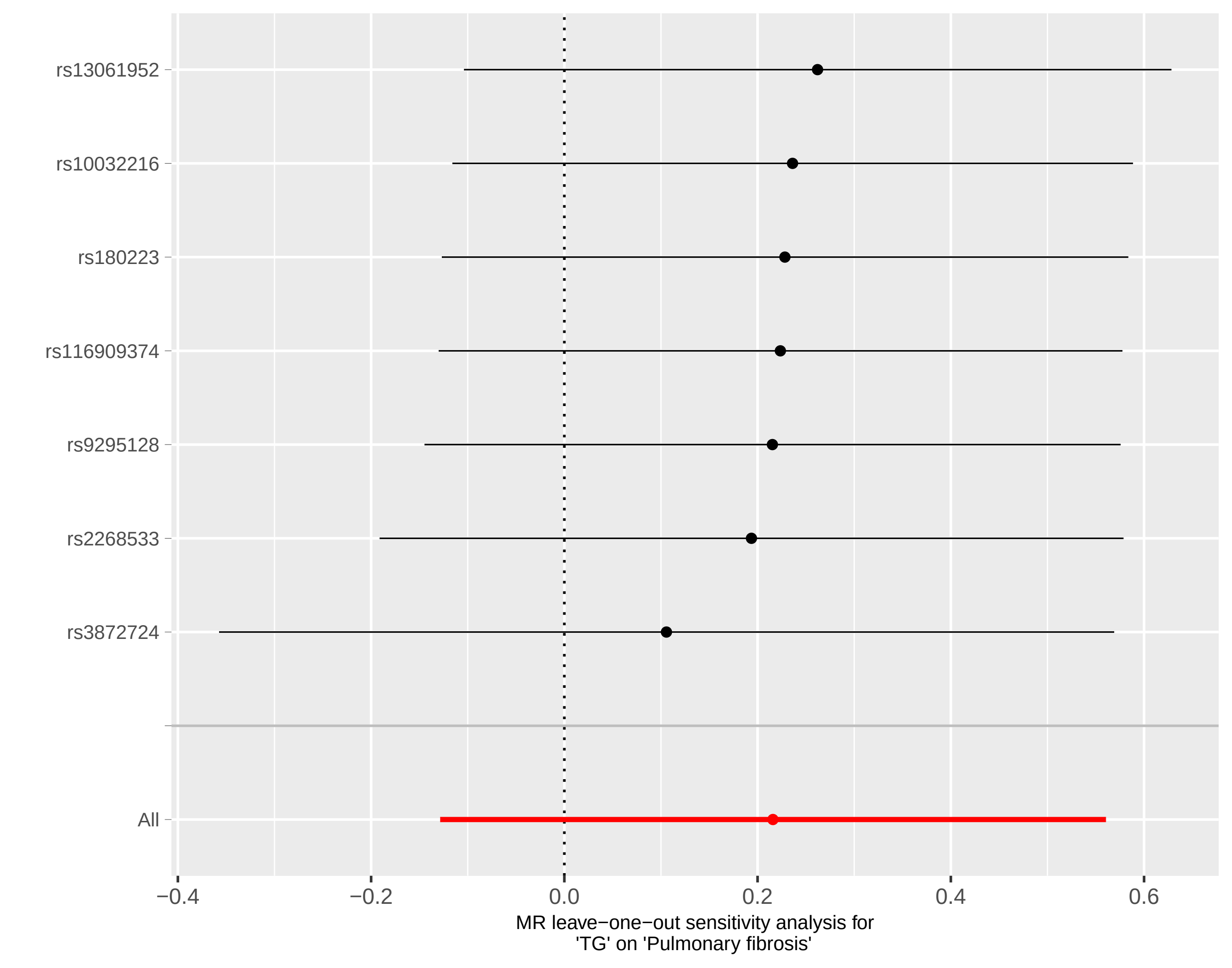
rs13061952
rs10032216
rs180223
rs116909374
rs9295128
rs2268533
rs3872724
All
−0.4
−0.2
0.0
0.2
0.4
0.6
MR leave−one−out sensitivity analysis for
'TG' on 'Pulmonary fibrosis'

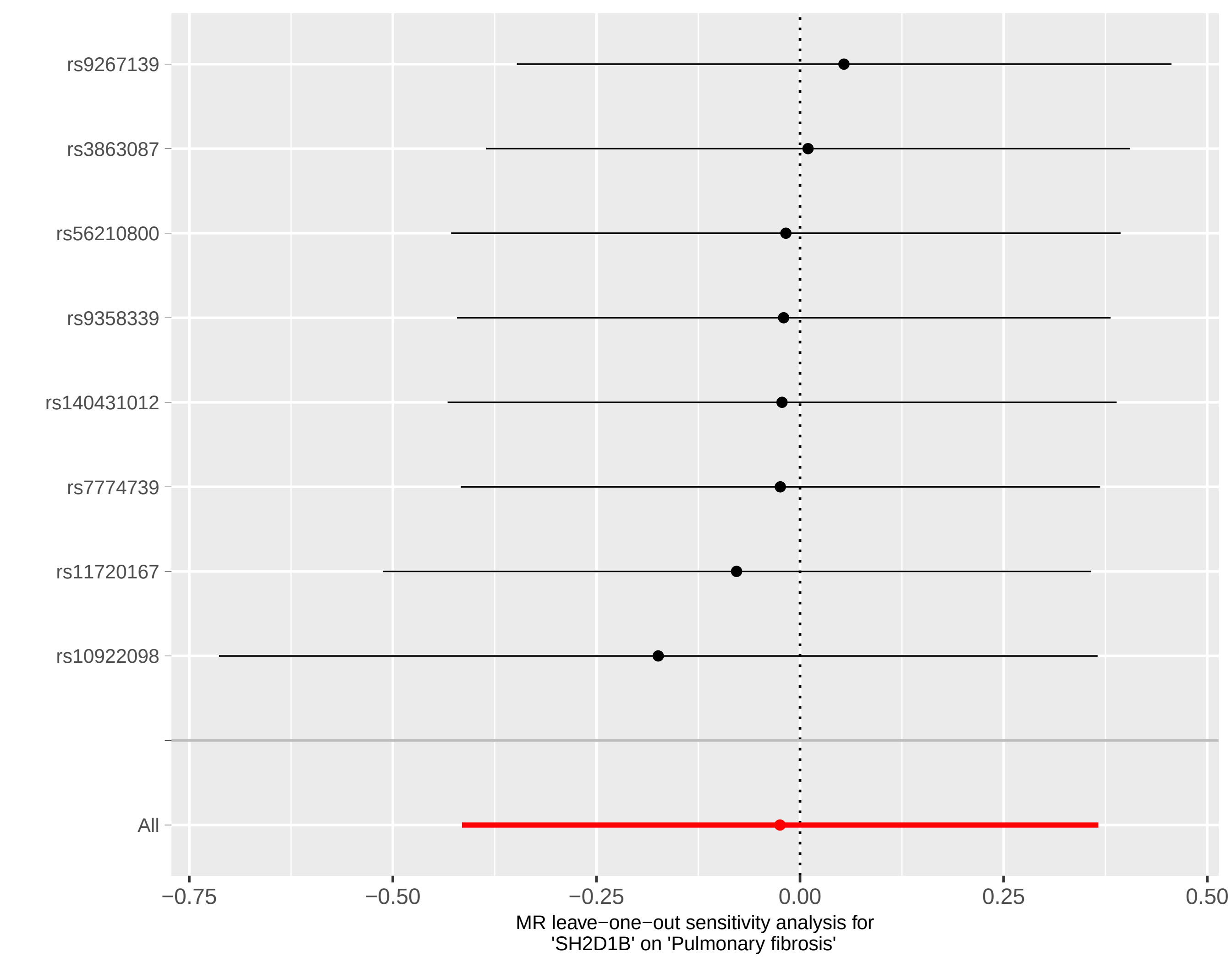
rs9267139
rs3863087
rs56210800
rs9358339
rs140431012
rs7774739
rs11720167
rs10922098
All
−0.75
−0.50
−0.25
0.00
0.25
0.50
MR leave−one−out sensitivity analysis for
'SH2D1B' on 'Pulmonary fibrosis'

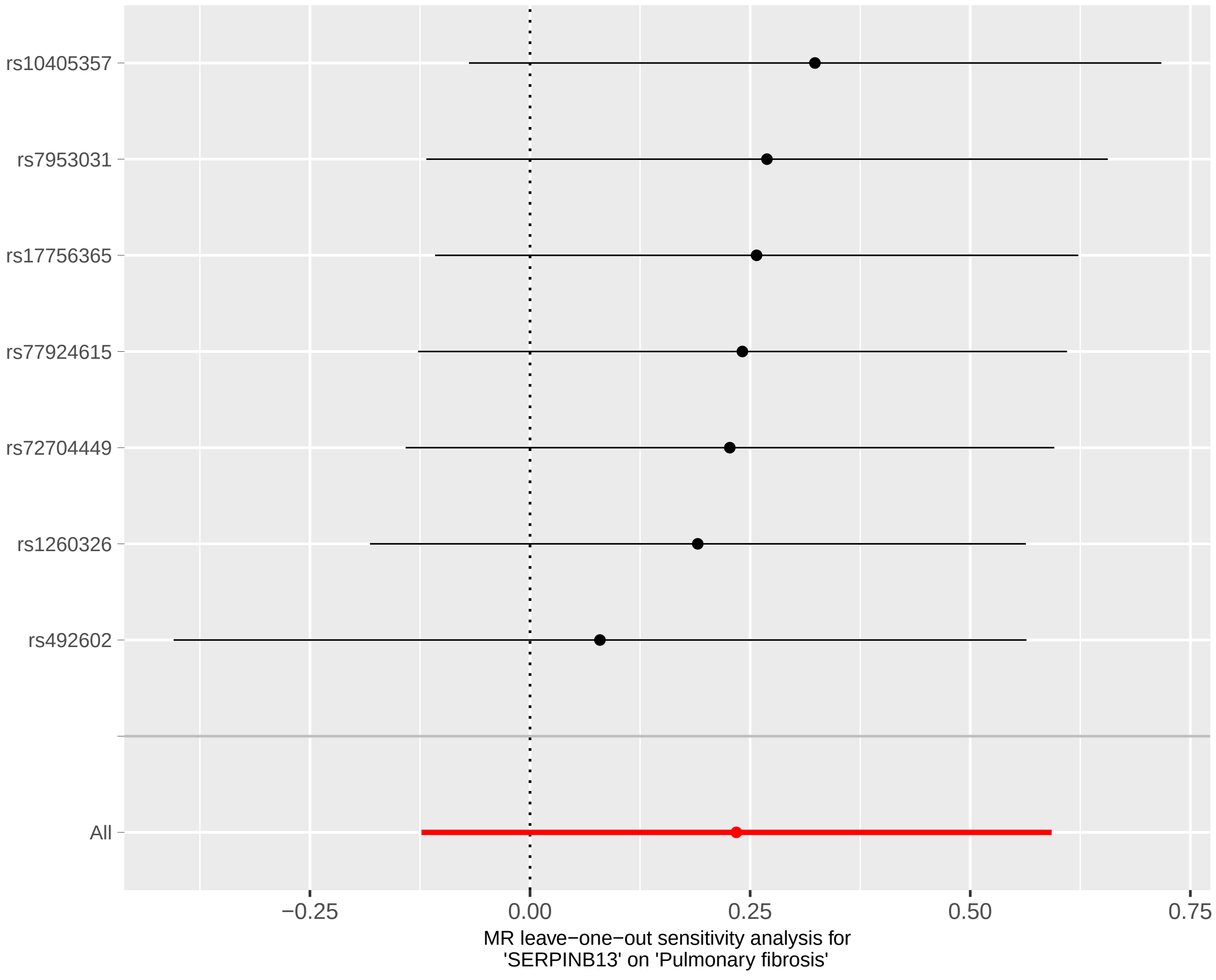

rs10405357
rs7953031
rs17756365
rs77924615
rs72704449
rs1260326
rs492602
All
−0.25
0.00
0.25
0.50
0.75
MR leave−one−out sensitivity analysis for
'SERPINB13' on 'Pulmonary fibrosis'

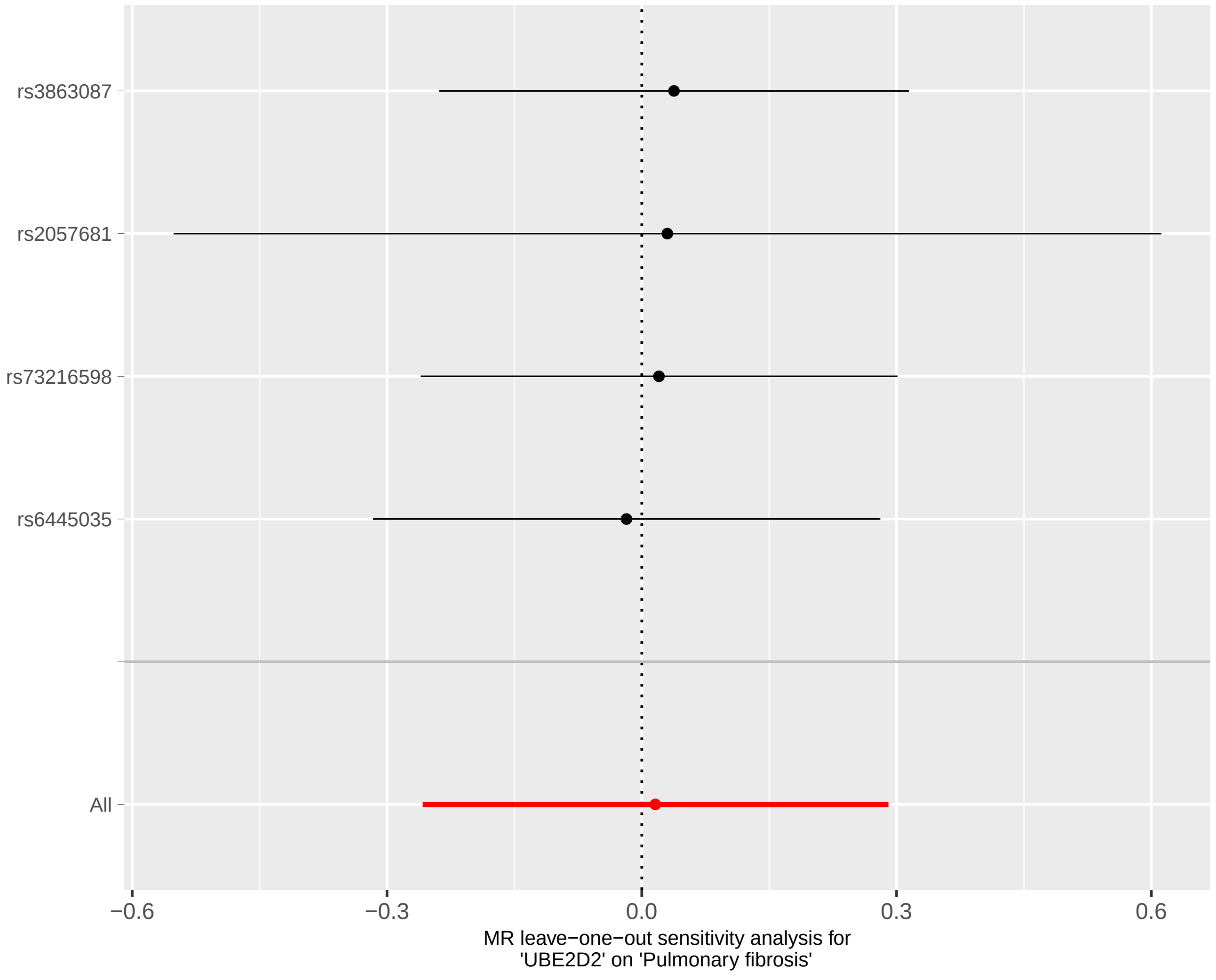
rs3863087
rs2057681
rs73216598
rs6445035
All
−0.6
−0.3
0.0
0.3
0.6
MR leave−one−out sensitivity analysis for
'UBE2D2' on 'Pulmonary fibrosis'

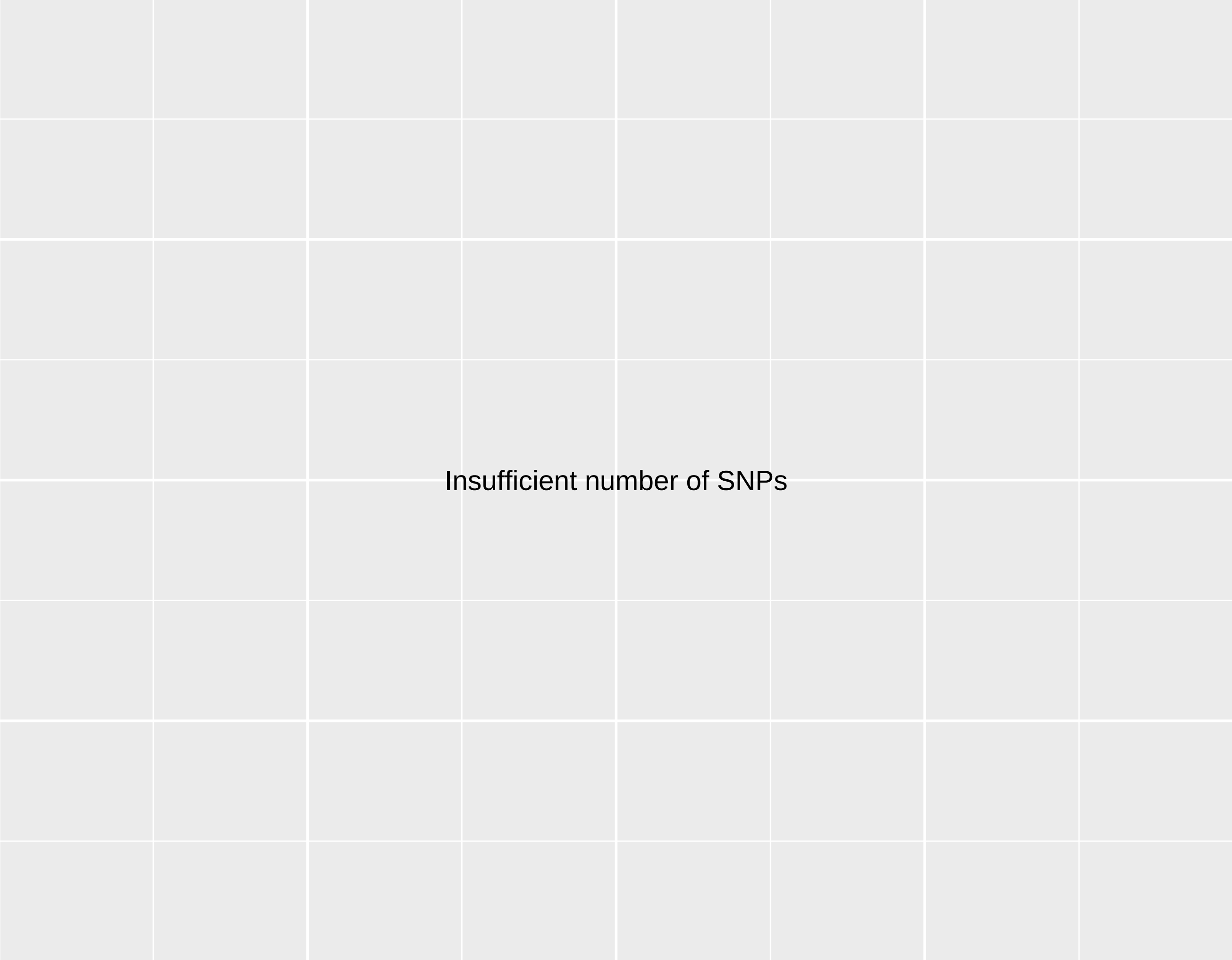
Insufficient number of SNPs

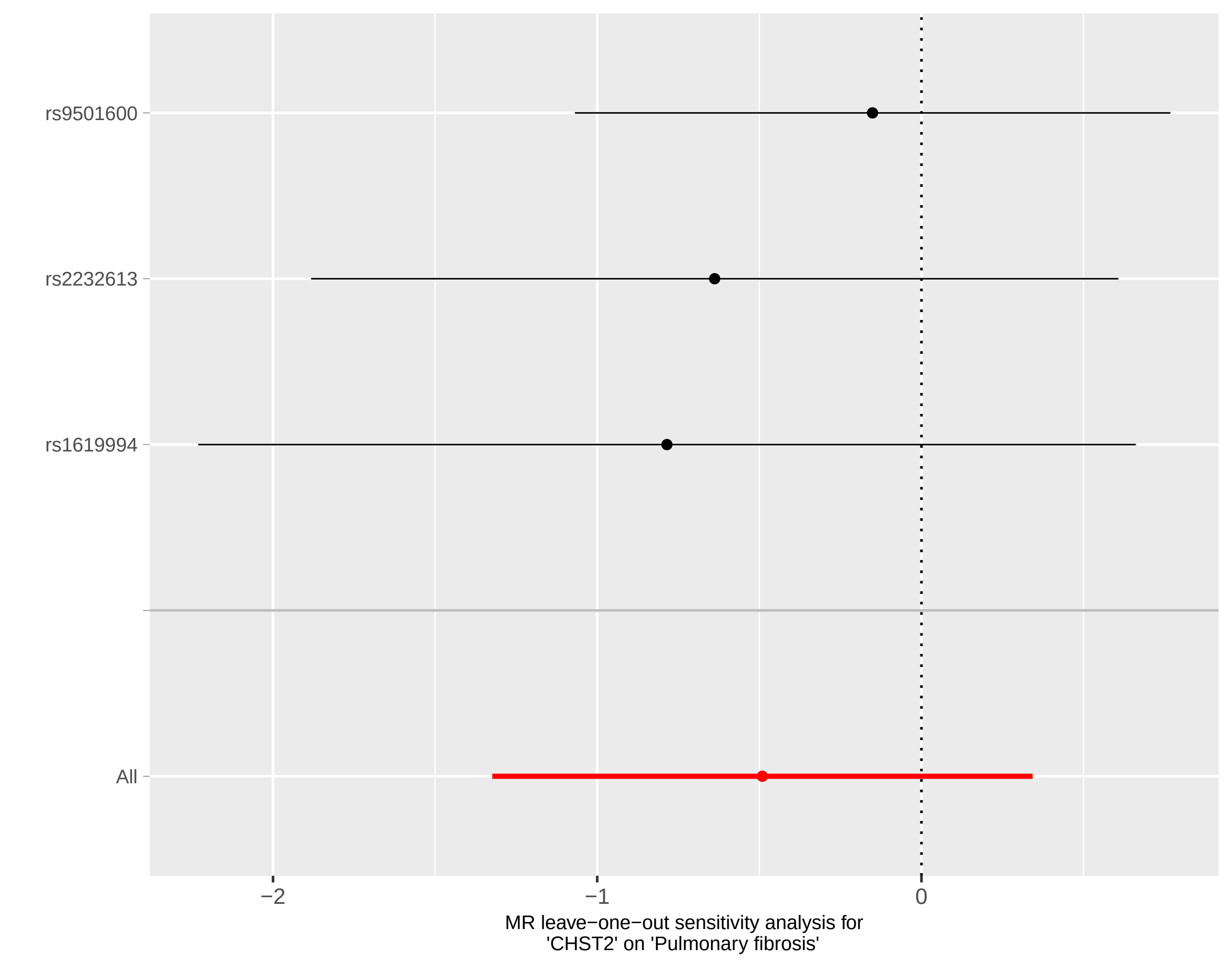
rs9501600
rs2232613
rs1619994
All
−2
−1
0
MR leave−one−out sensitivity analysis for
'CHST2' on 'Pulmonary fibrosis'

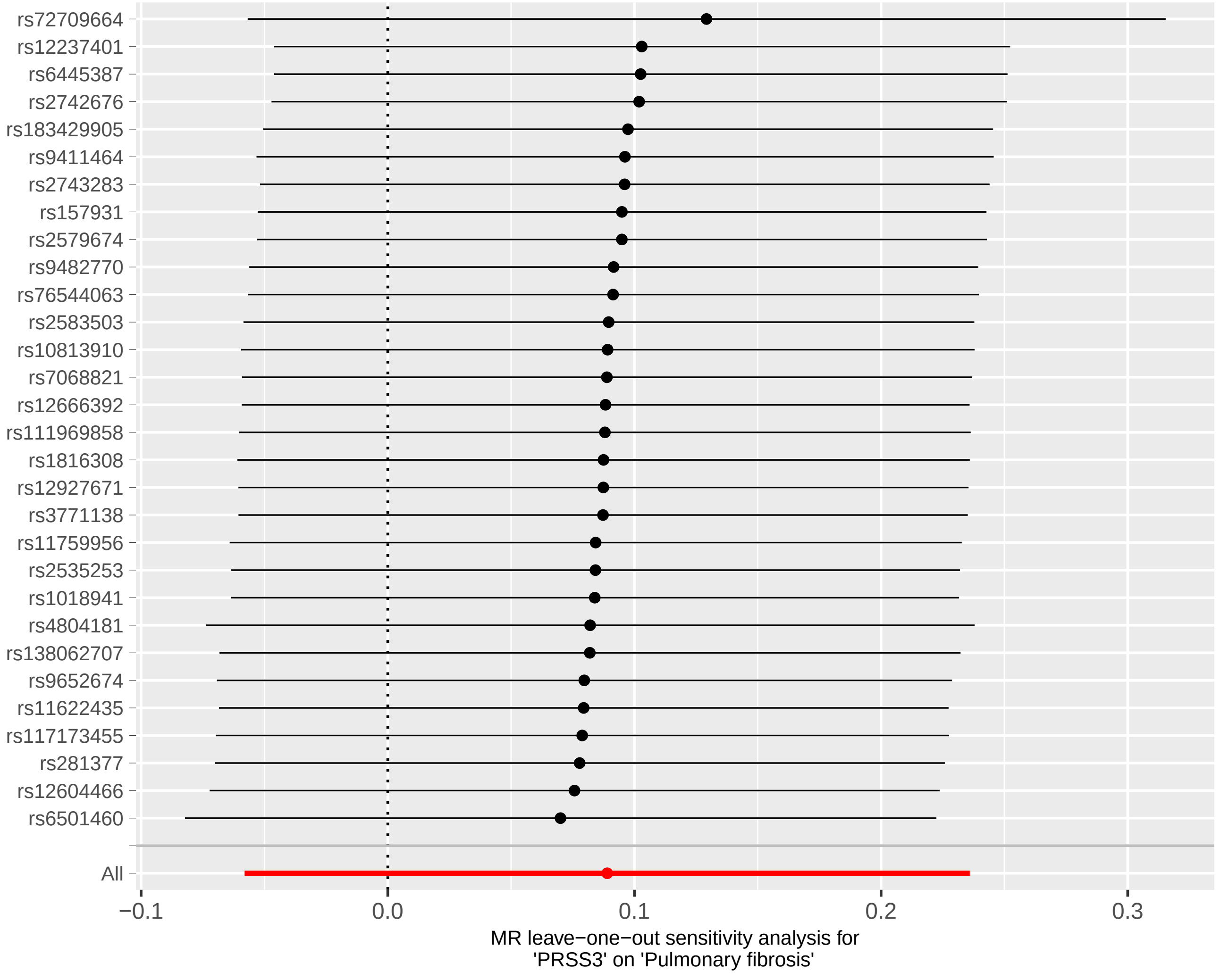

rs72709664
rs12237401
rs6445387
rs2742676
rs183429905
rs9411464
rs2743283
rs157931
rs2579674
rs9482770
rs76544063
rs2583503
rs10813910
rs7068821
rs12666392
rs111969858
rs1816308
rs12927671
rs3771138
rs11759956
rs2535253
rs1018941
rs4804181
rs138062707
rs9652674
rs11622435
rs117173455
rs281377
rs12604466
rs6501460
All
−0.1
0.0
0.1
0.2
0.3
MR leave−one−out sensitivity analysis for
'PRSS3' on 'Pulmonary fibrosis'

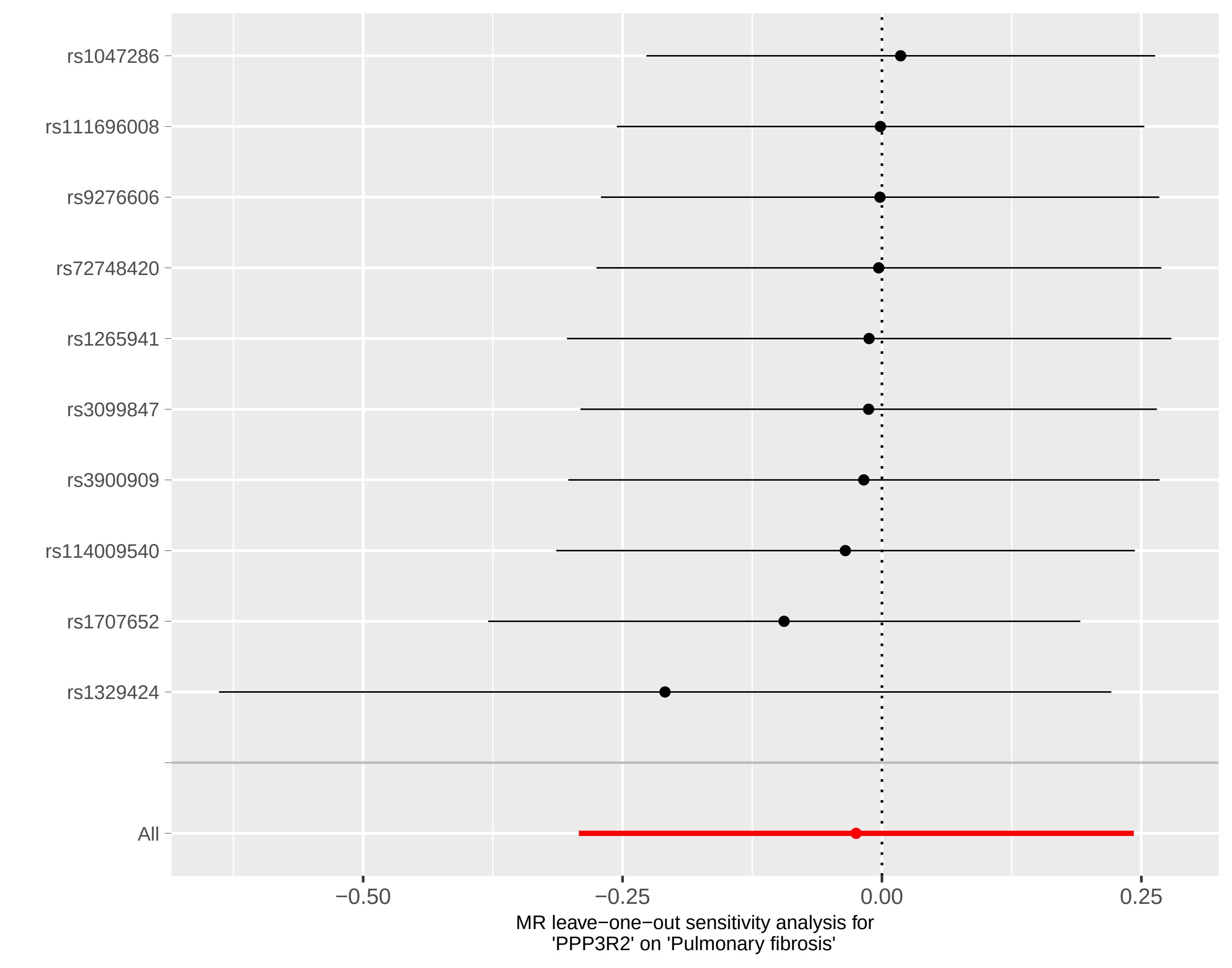

rs1047286
rs111696008
rs9276606
rs72748420
rs1265941
rs3099847
rs3900909
rs114009540
rs1707652
rs1329424
All
−0.50
−0.25
0.00
0.25
MR leave−one−out sensitivity analysis for
'PPP3R2' on 'Pulmonary fibrosis'

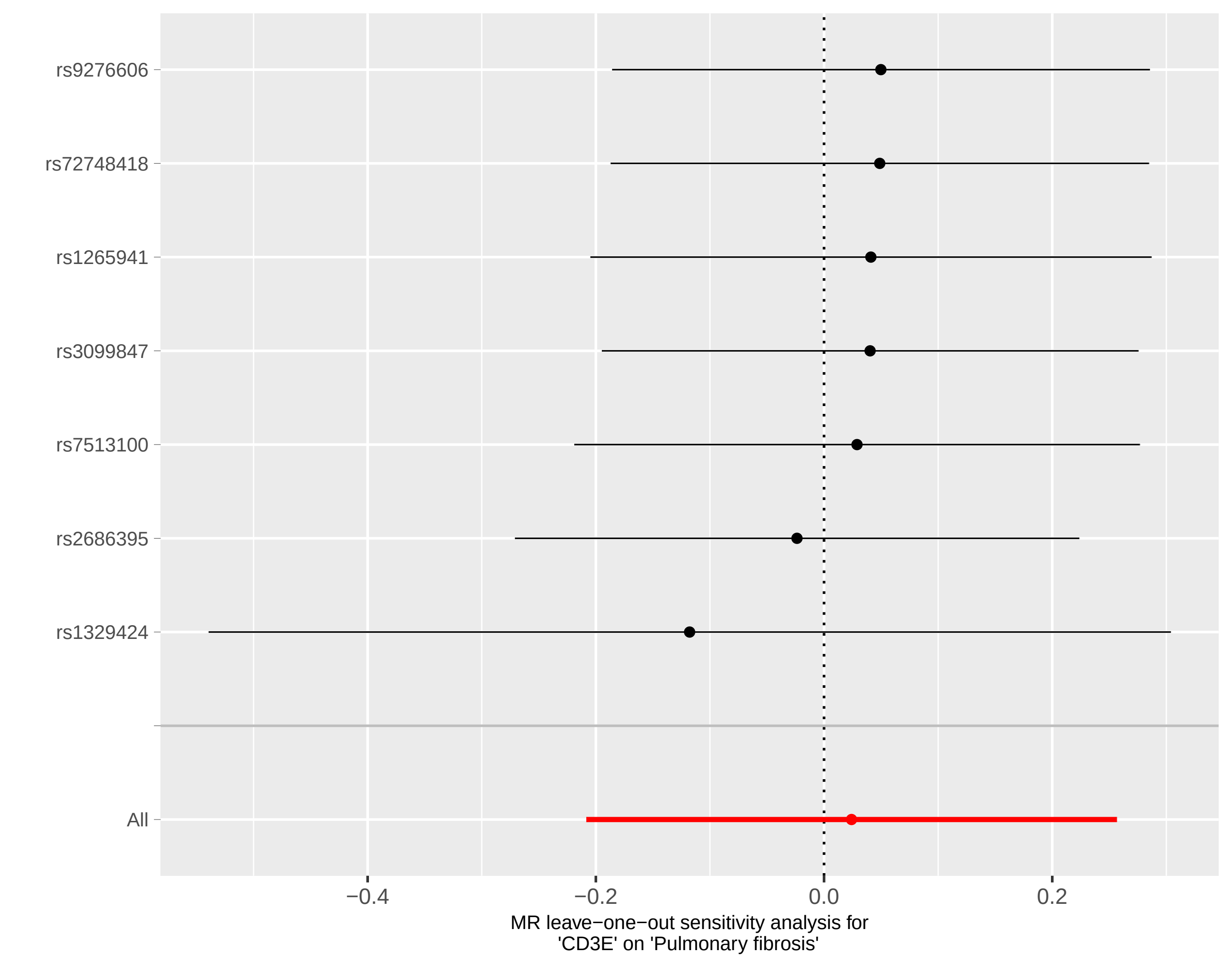
rs9276606
rs72748418
rs1265941
rs3099847
rs7513100
rs2686395
rs1329424
All
−0.4
−0.2
0.0
0.2
MR leave−one−out sensitivity analysis for
'CD3E' on 'Pulmonary fibrosis'

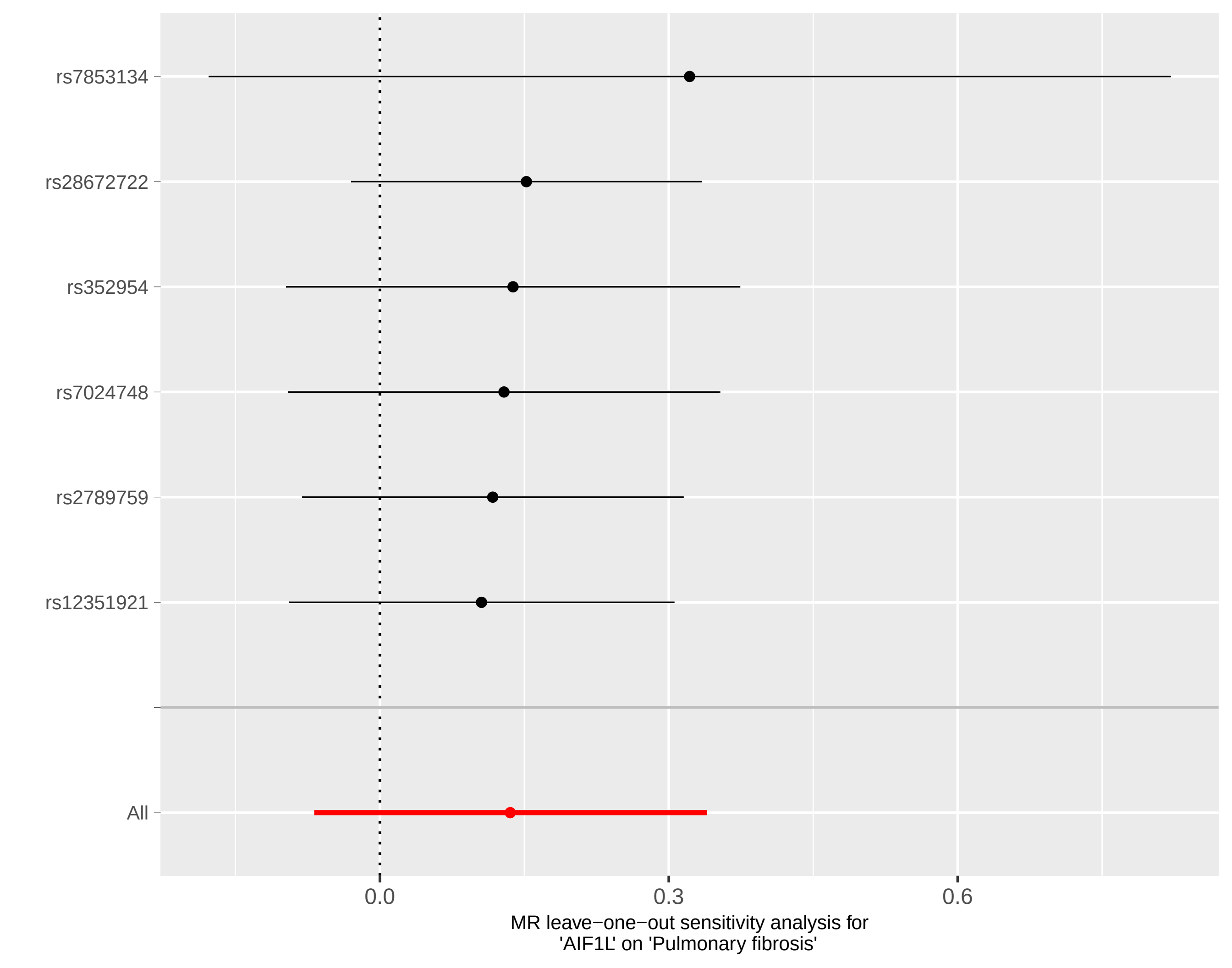
rs7853134
rs28672722
rs352954
rs7024748
rs2789759
rs12351921
All
0.0
0.3
0.6
MR leave−one−out sensitivity analysis for
'AIF1L' on 'Pulmonary fibrosis'

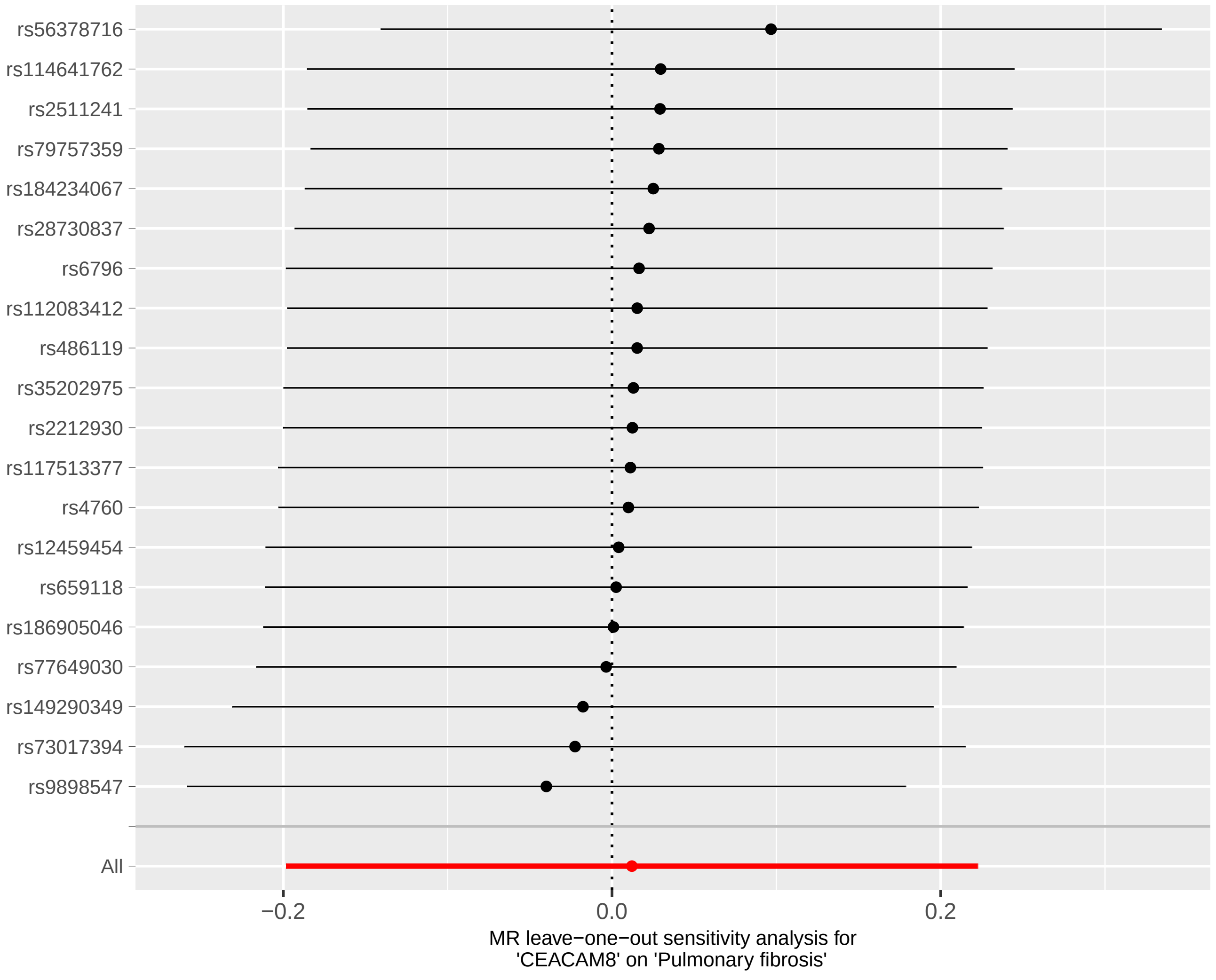
rs56378716
rs114641762
rs2511241
rs79757359
rs184234067
rs28730837
rs6796
rs112083412
rs486119
rs35202975
rs2212930
rs117513377
rs4760
rs12459454
rs659118
rs186905046
rs77649030
rs149290349
rs73017394
rs9898547
All
−0.2
0.0
0.2
MR leave−one−out sensitivity analysis for
'CEACAM8' on 'Pulmonary fibrosis'

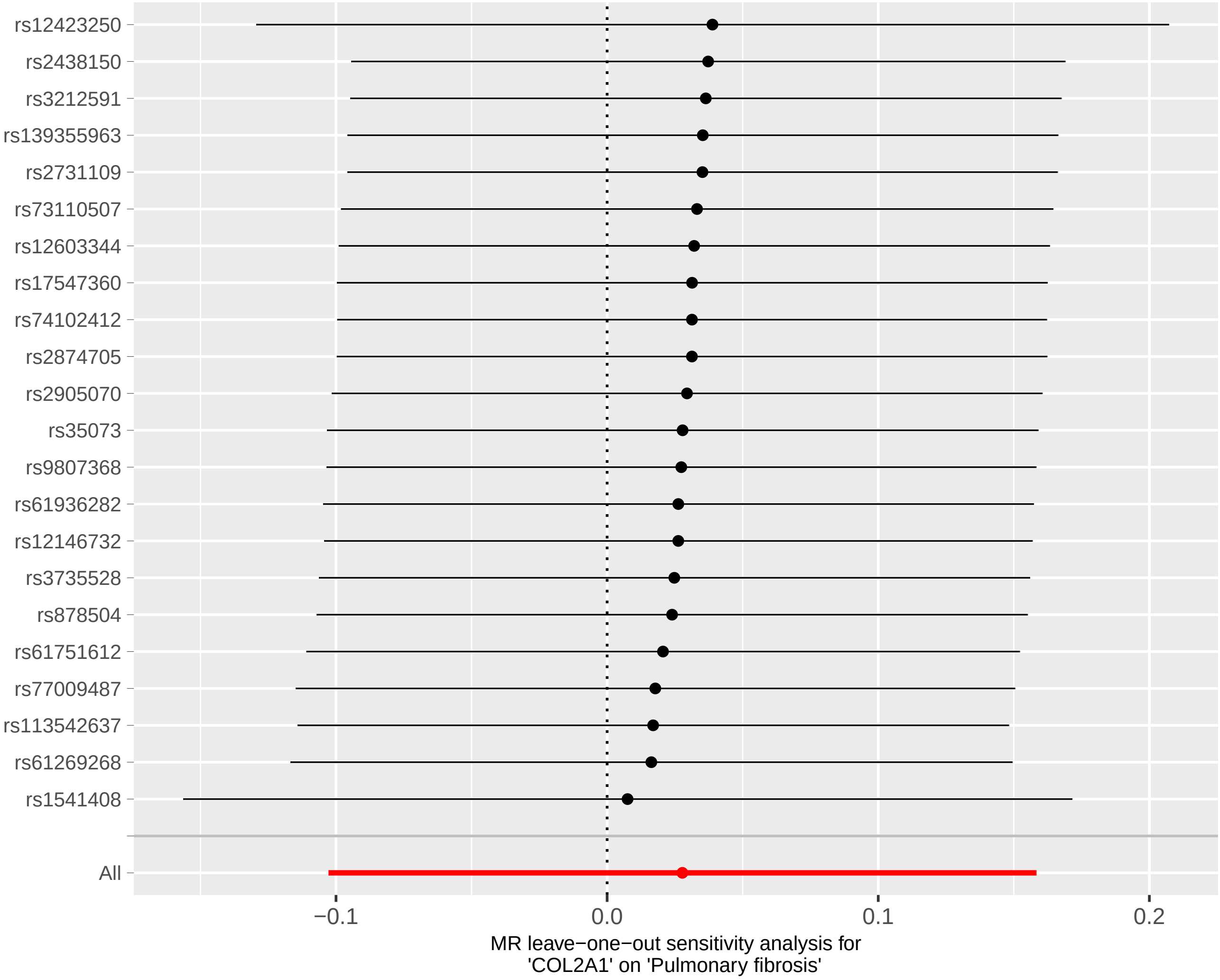

rs12423250
rs2438150
rs3212591
rs139355963
rs2731109
rs73110507
rs12603344
rs17547360
rs74102412
rs2874705
rs2905070
rs35073
rs9807368
rs61936282
rs12146732
rs3735528
rs878504
rs61751612
rs77009487
rs113542637
rs61269268
rs1541408
All
−0.1
0.0
0.1
0.2
MR leave−one−out sensitivity analysis for
'COL2A1' on 'Pulmonary fibrosis'

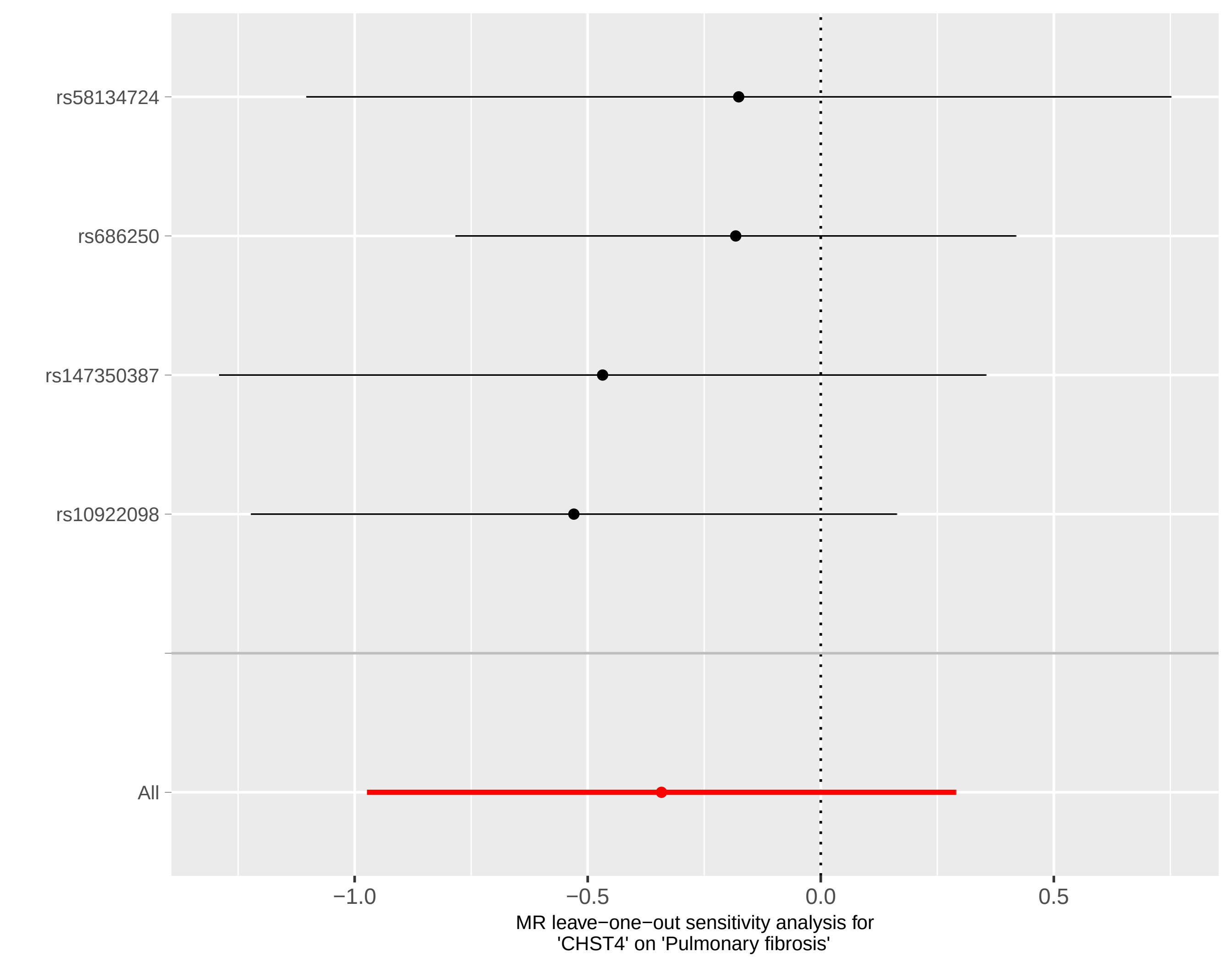
rs58134724
rs686250
rs147350387
rs10922098
All
−1.0
−0.5
0.0
0.5
MR leave−one−out sensitivity analysis for
'CHST4' on 'Pulmonary fibrosis'

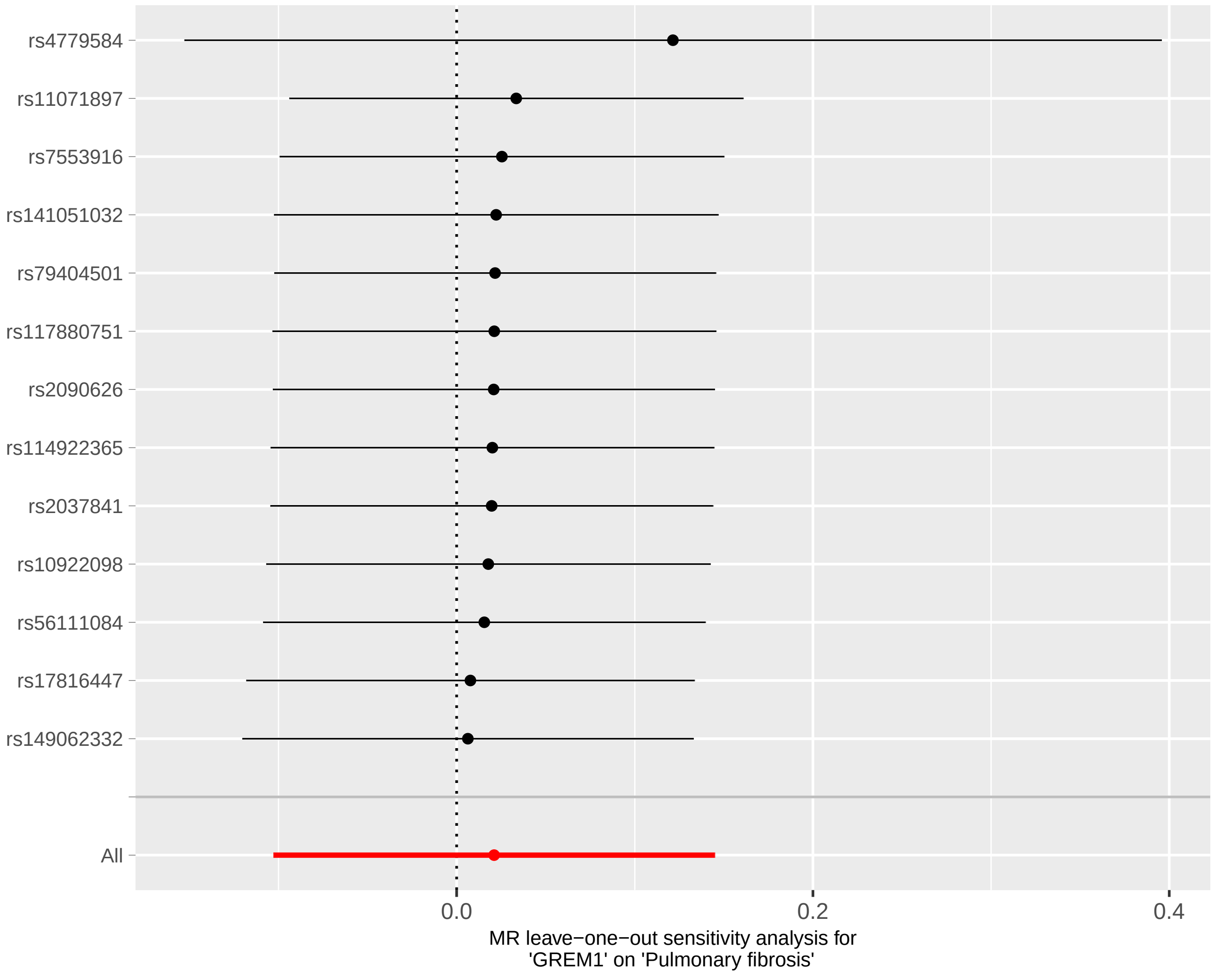
rs4779584
rs11071897
rs7553916
rs141051032
rs79404501
rs117880751
rs2090626
rs114922365
rs2037841
rs10922098
rs56111084
rs17816447
rs149062332
All
0.0
0.2
0.4
MR leave−one−out sensitivity analysis for
'GREM1' on 'Pulmonary fibrosis'

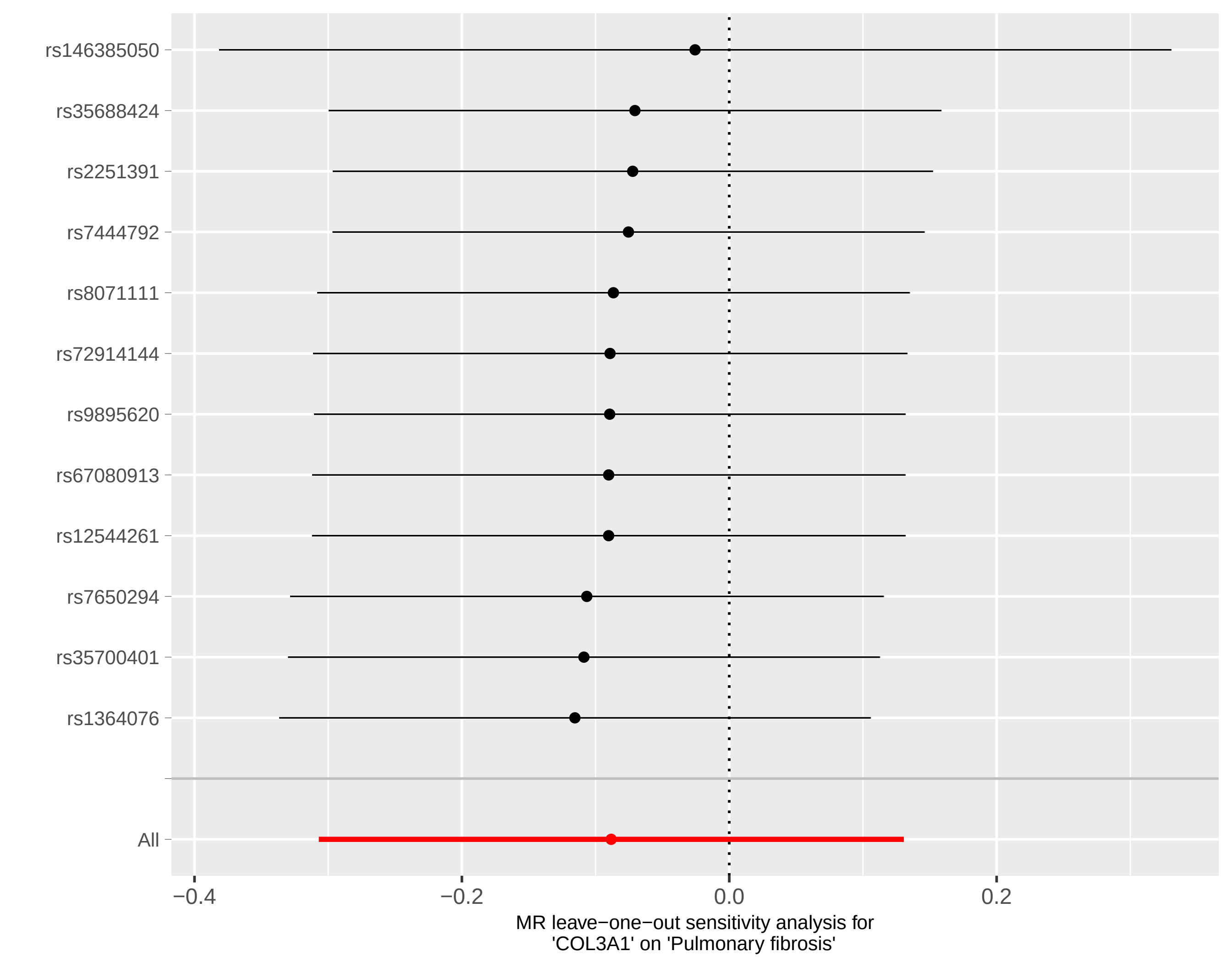
rs146385050
rs35688424
rs2251391
rs7444792
rs8071111
rs72914144
rs9895620
rs67080913
rs12544261
rs7650294
rs35700401
rs1364076
All
−0.4
−0.2
0.0
0.2
MR leave−one−out sensitivity analysis for
'COL3A1' on 'Pulmonary fibrosis'

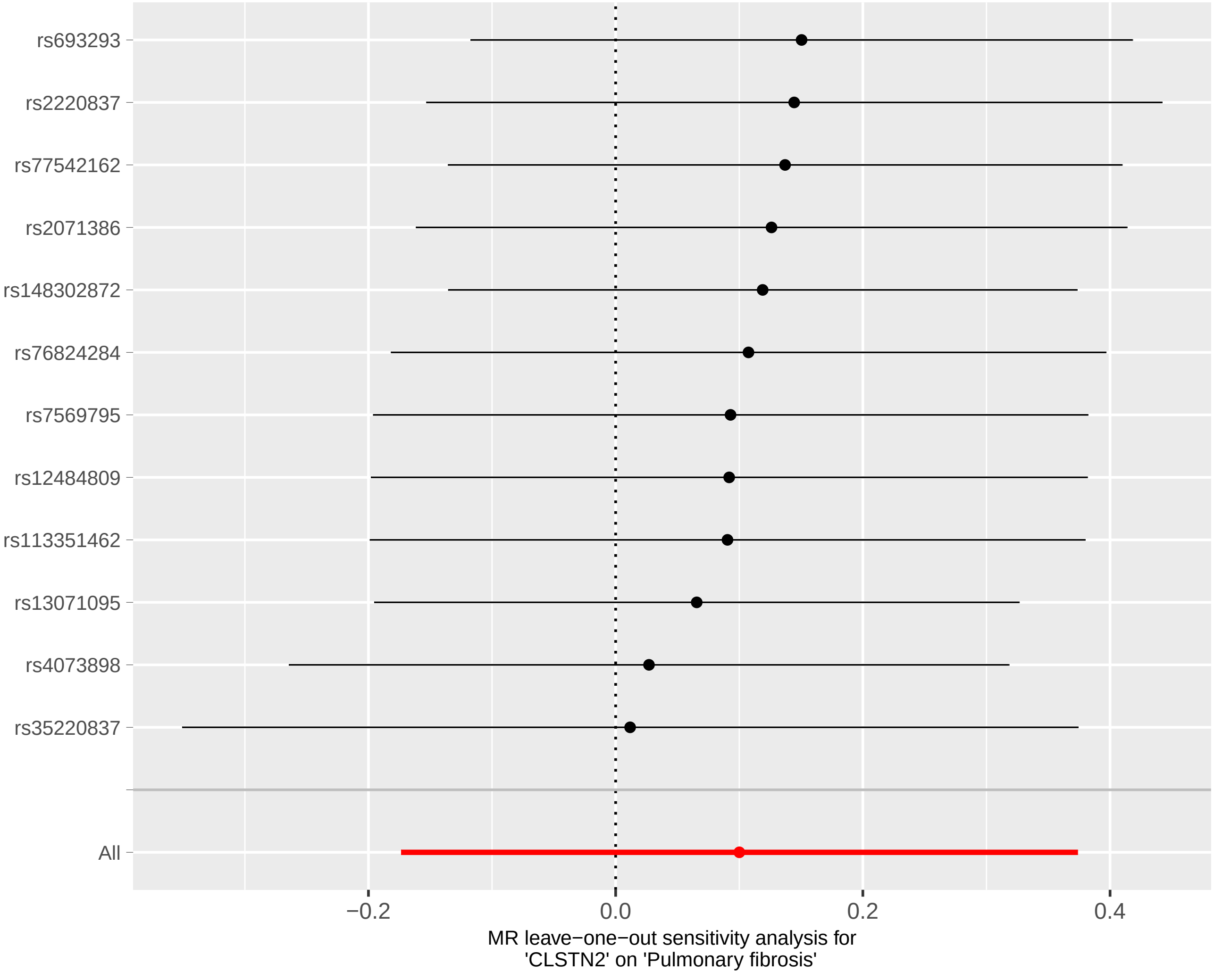
rs693293
rs2220837
rs77542162
rs2071386
rs148302872
rs76824284
rs7569795
rs12484809
rs113351462
rs13071095
rs4073898
rs35220837
All
−0.2
0.0
0.2
0.4
MR leave−one−out sensitivity analysis for
'CLSTN2' on 'Pulmonary fibrosis'

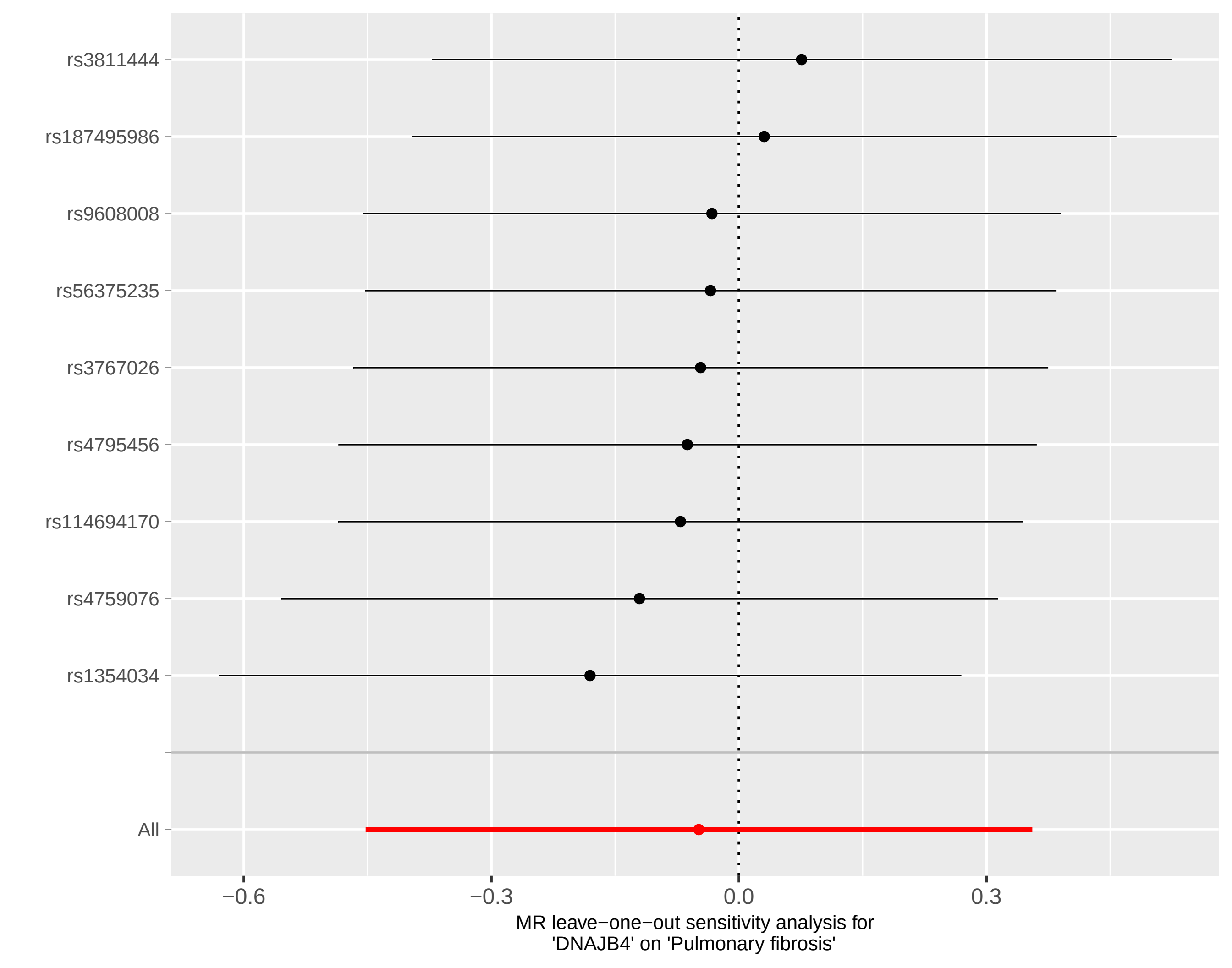
rs3811444
rs187495986
rs9608008
rs56375235
rs3767026
rs4795456
rs114694170
rs4759076
rs1354034
All
−0.6
−0.3
0.0
0.3
MR leave−one−out sensitivity analysis for
'DNAJB4' on 'Pulmonary fibrosis'

Insufficient number of SNPs

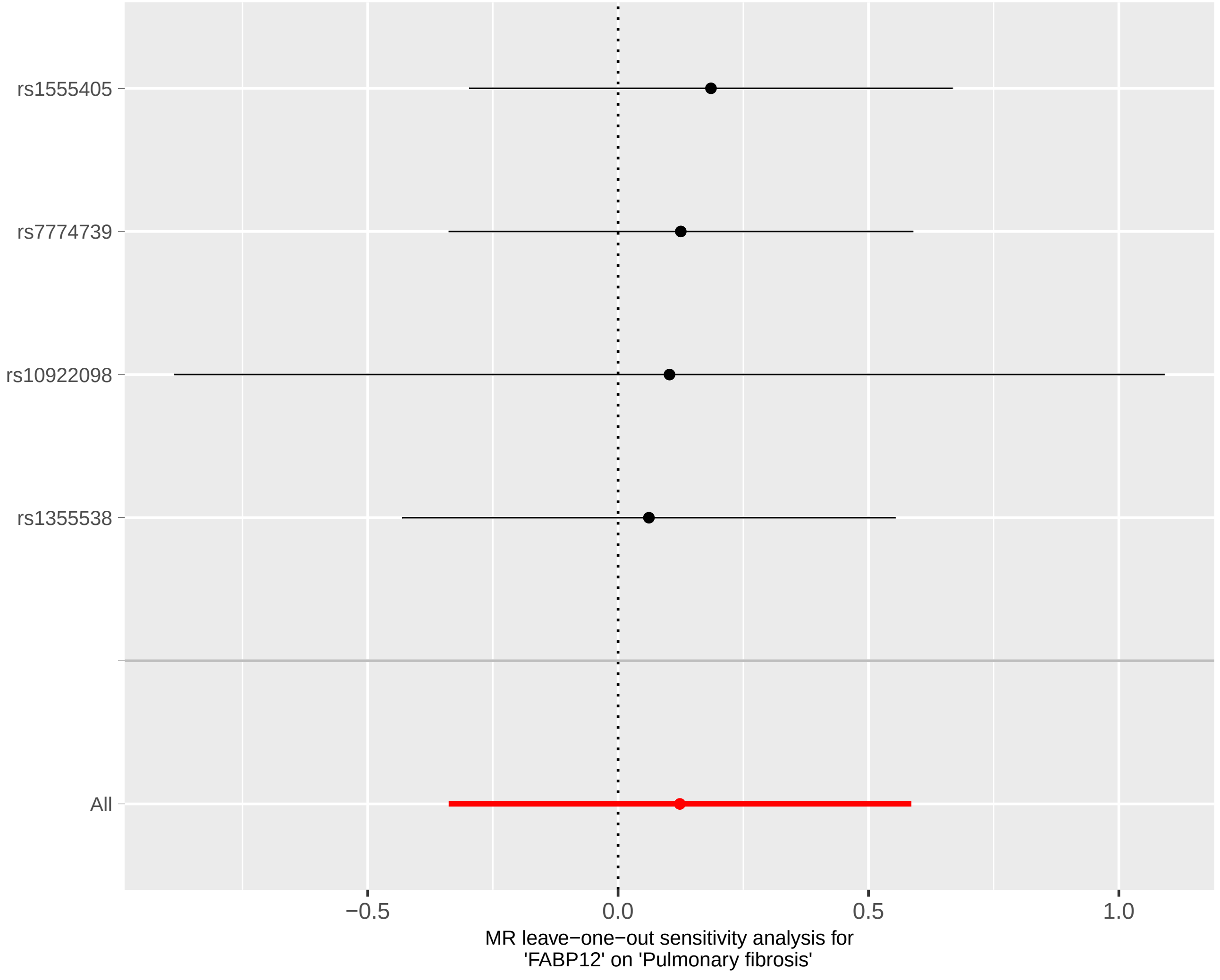
rs1555405
rs7774739
rs10922098
rs1355538
All
−0.5
0.0
0.5
1.0
MR leave−one−out sensitivity analysis for
'FABP12' on 'Pulmonary fibrosis'

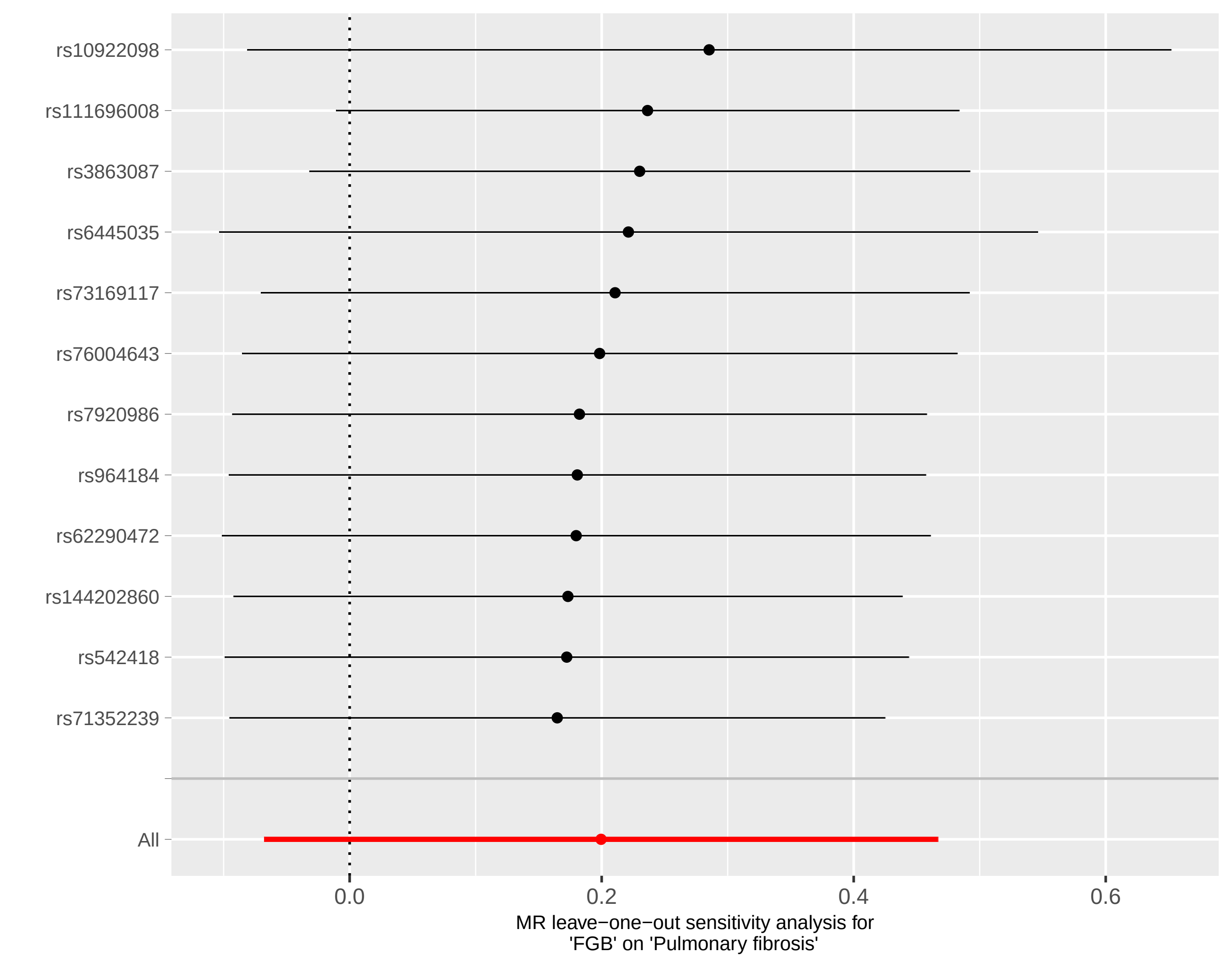
rs10922098
rs111696008
rs3863087
rs6445035
rs73169117
rs76004643
rs7920986
rs964184
rs62290472
rs144202860
rs542418
rs71352239
All
0.0
0.2
0.4
0.6
MR leave−one−out sensitivity analysis for
'FGB' on 'Pulmonary fibrosis'

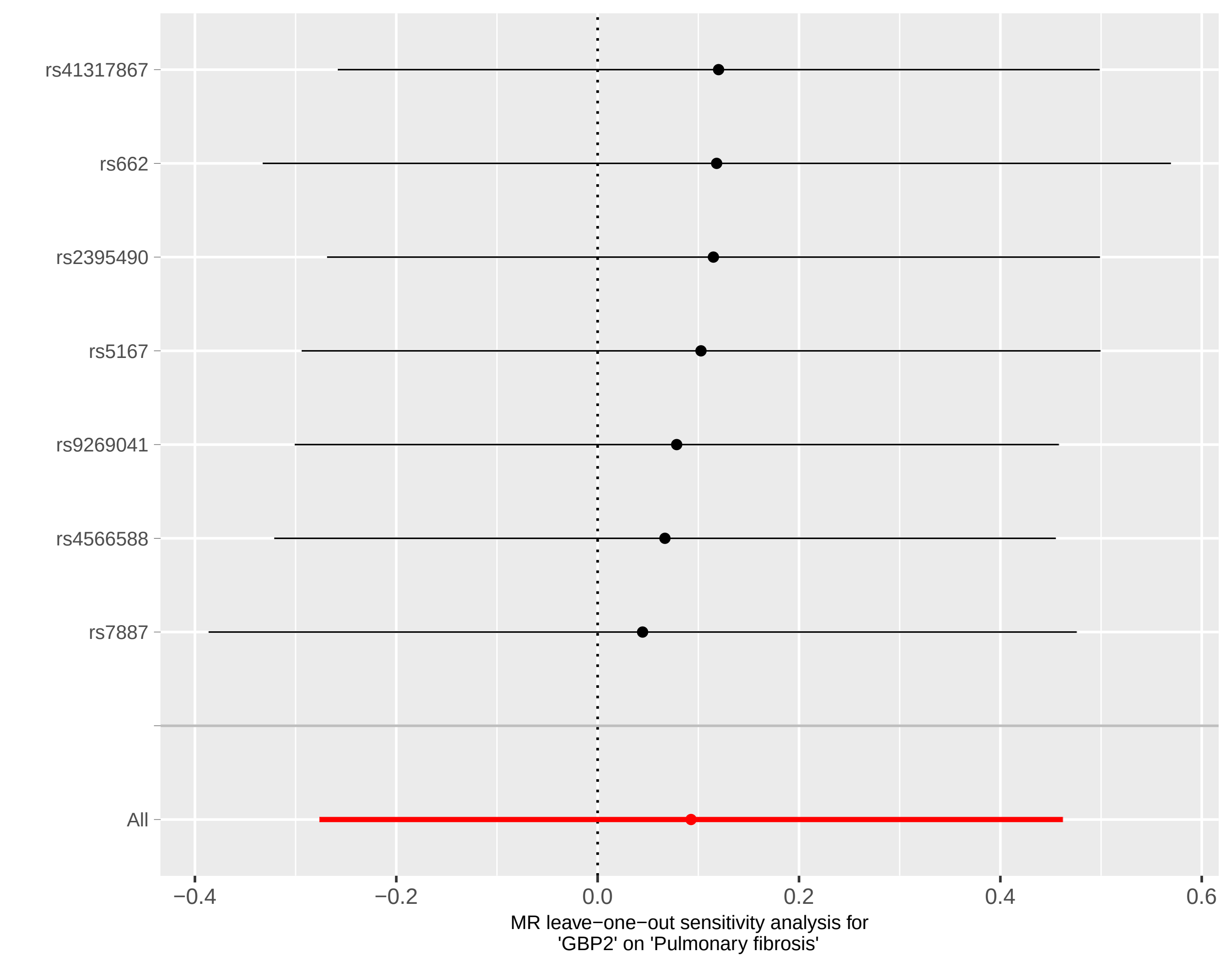
rs41317867
rs662
rs2395490
rs5167
rs9269041
rs4566588
rs7887
All
−0.4
−0.2
0.0
0.2
0.4
0.6
MR leave−one−out sensitivity analysis for
'GBP2' on 'Pulmonary fibrosis'

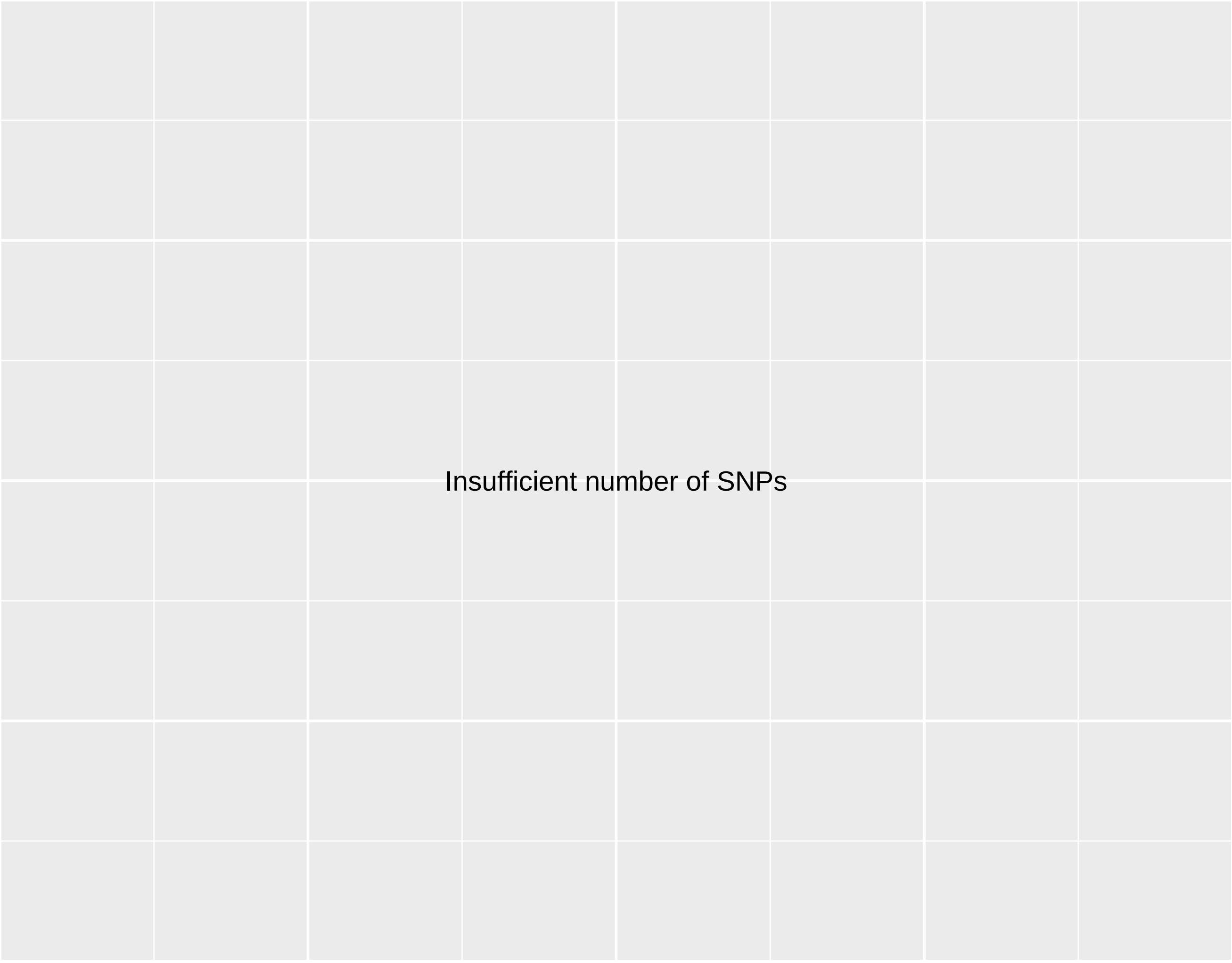
Insufficient number of SNPs

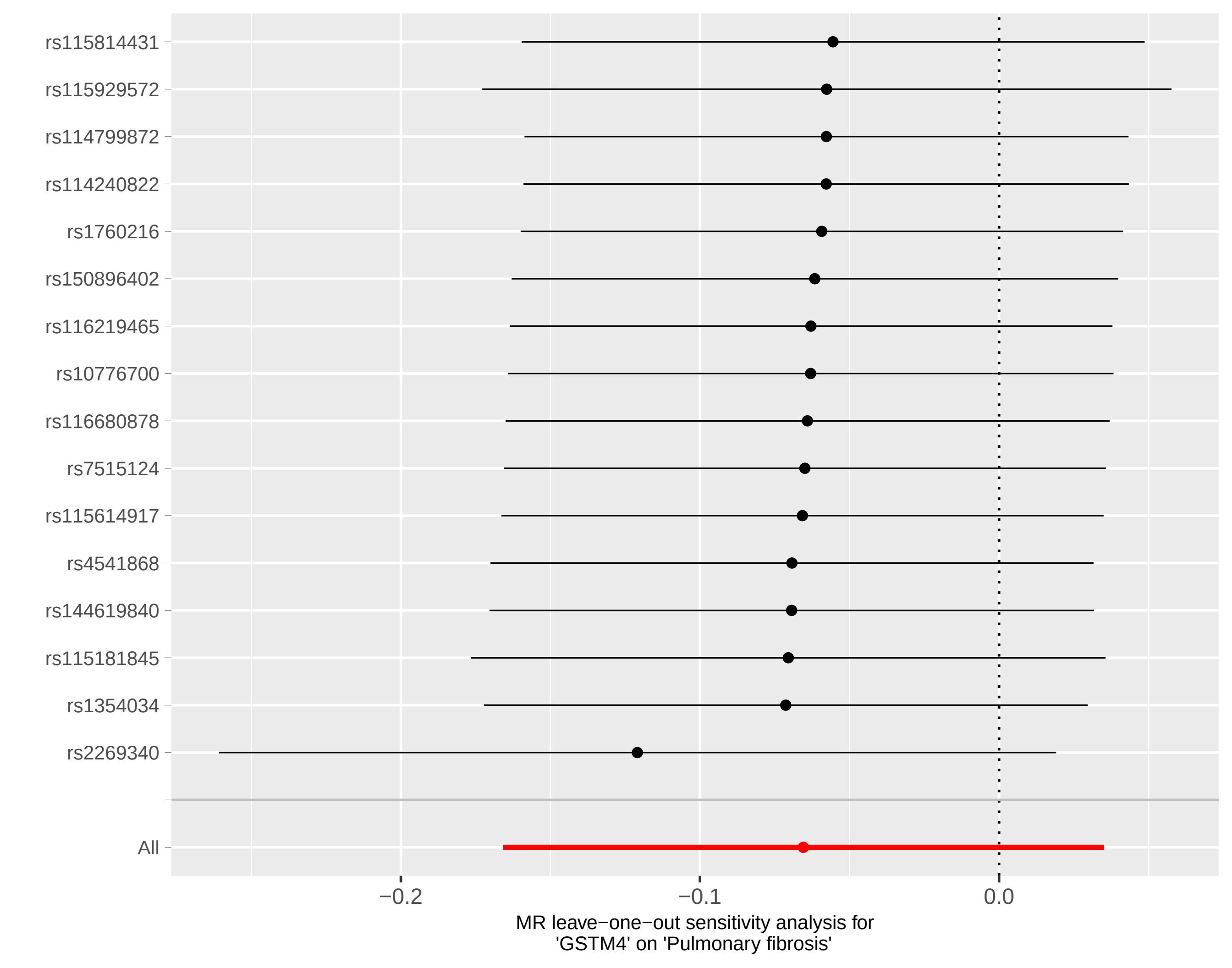

rs115814431
rs115929572
rs114799872
rs114240822
rs1760216
rs150896402
rs116219465
rs10776700
rs116680878
rs7515124
rs115614917
rs4541868
rs144619840
rs115181845
rs1354034
rs2269340
All
−0.2
−0.1
0.0
MR leave−one−out sensitivity analysis for
'GSTM4' on 'Pulmonary fibrosis'

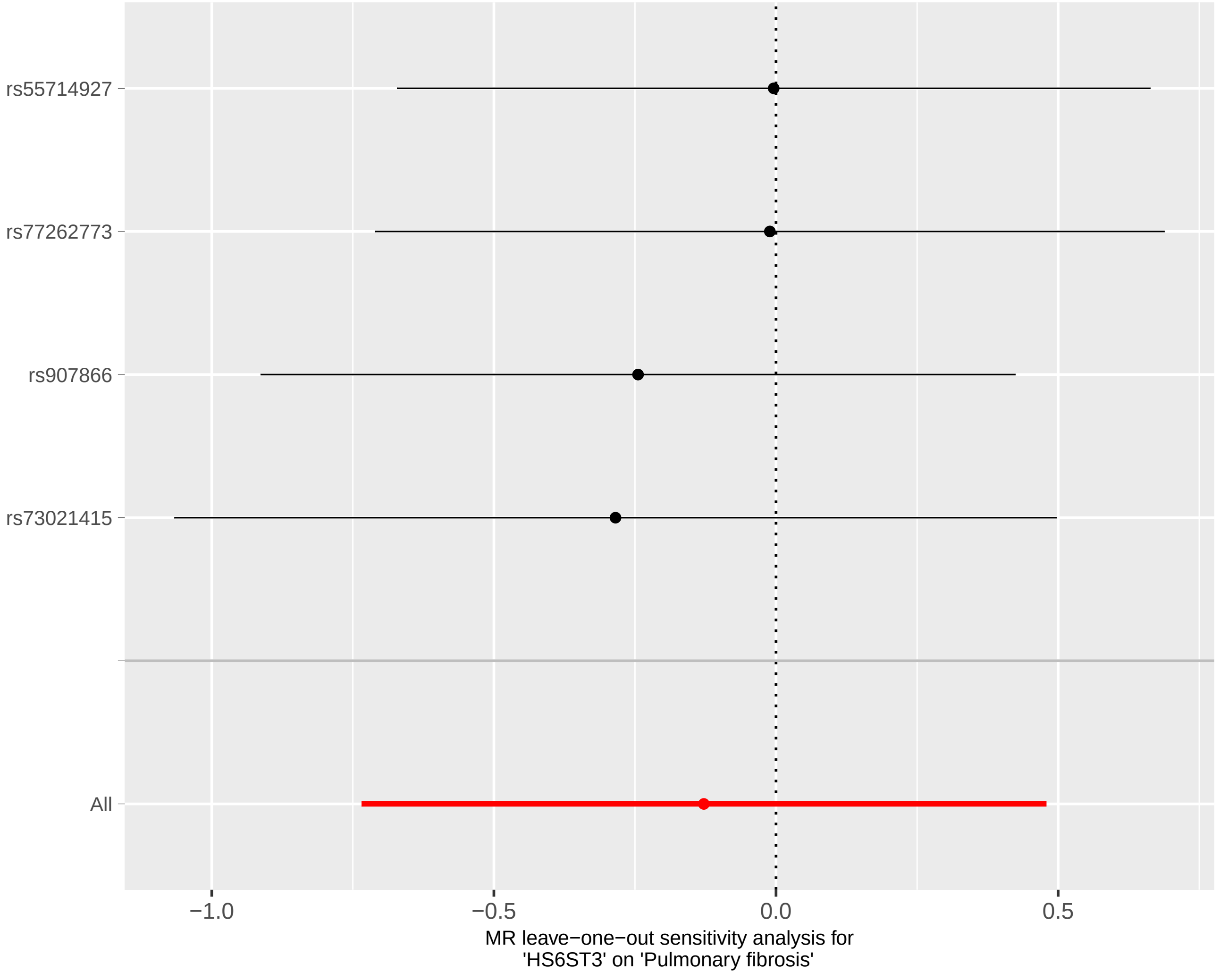

rs55714927
rs77262773
rs907866
rs73021415
All
−1.0
−0.5
0.0
0.5
MR leave−one−out sensitivity analysis for
'HS6ST3' on 'Pulmonary fibrosis'

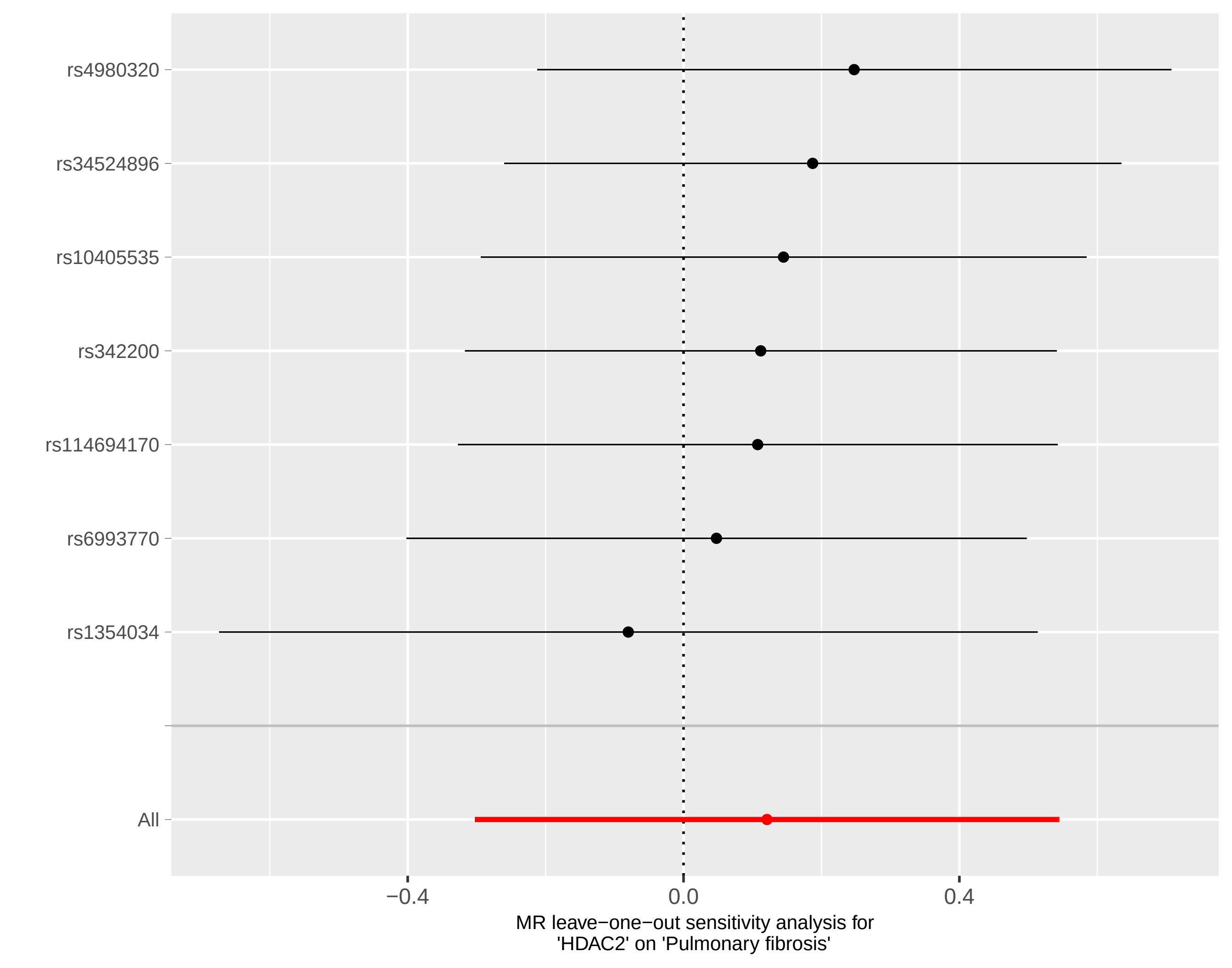
rs4980320
rs34524896
rs10405535
rs342200
rs114694170
rs6993770
rs1354034
All
−0.4
0.0
0.4
MR leave−one−out sensitivity analysis for
'HDAC2' on 'Pulmonary fibrosis'

Insufficient number of SNPs

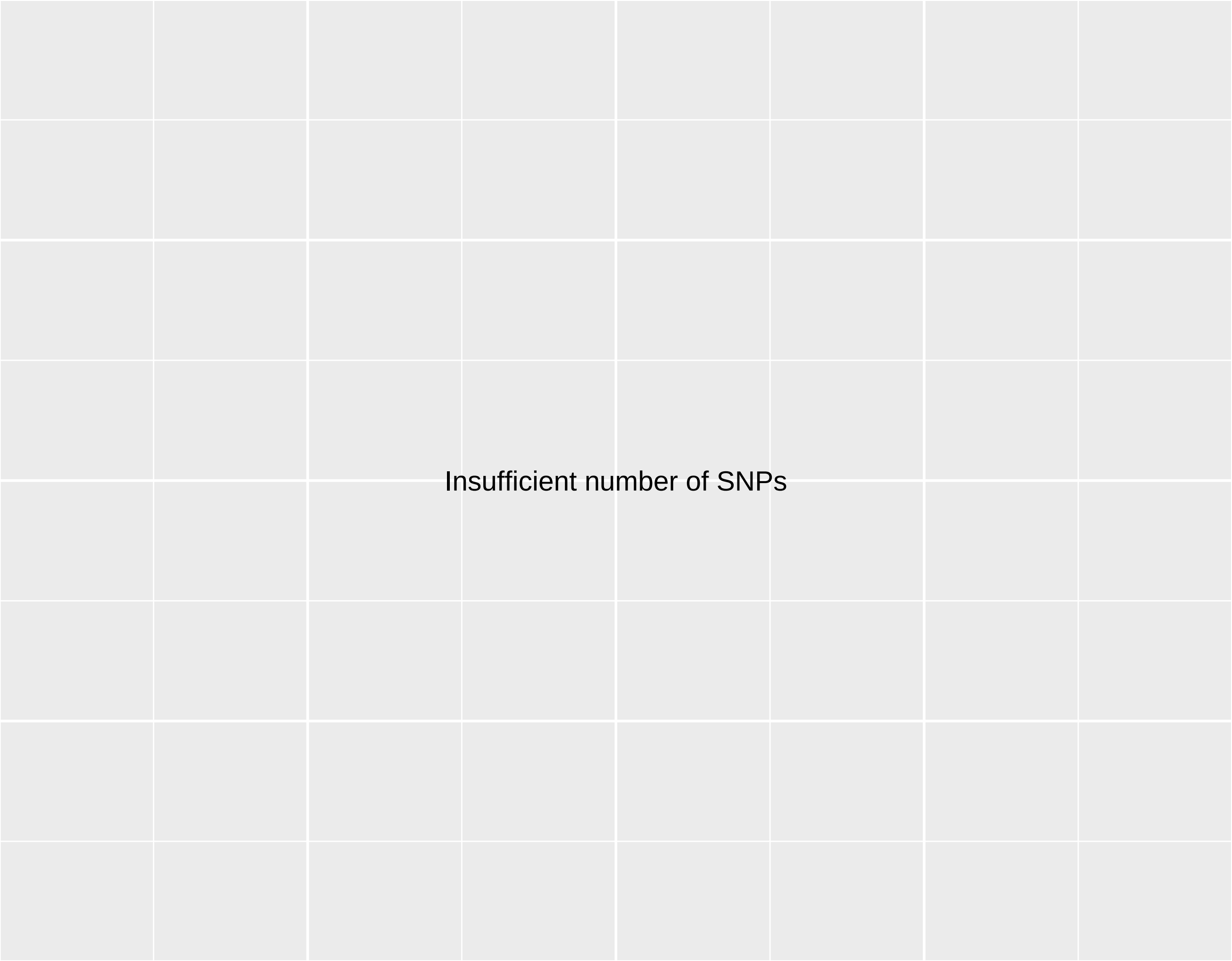
Insufficient number of SNPs

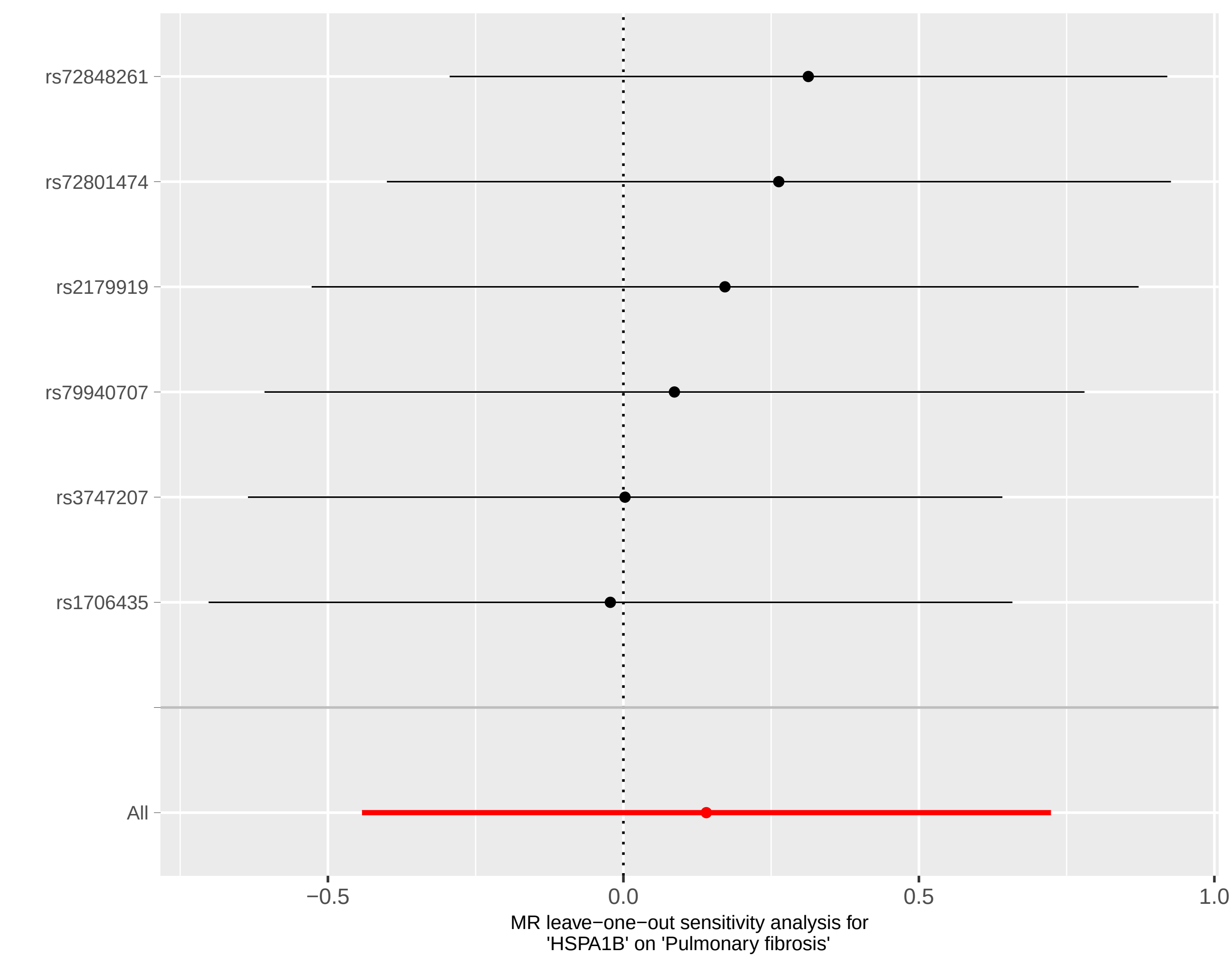
rs72848261
rs72801474
rs2179919
rs79940707
rs3747207
rs1706435
All
−0.5
0.0
0.5
1.0
MR leave−one−out sensitivity analysis for
'HSPA1B' on 'Pulmonary fibrosis'

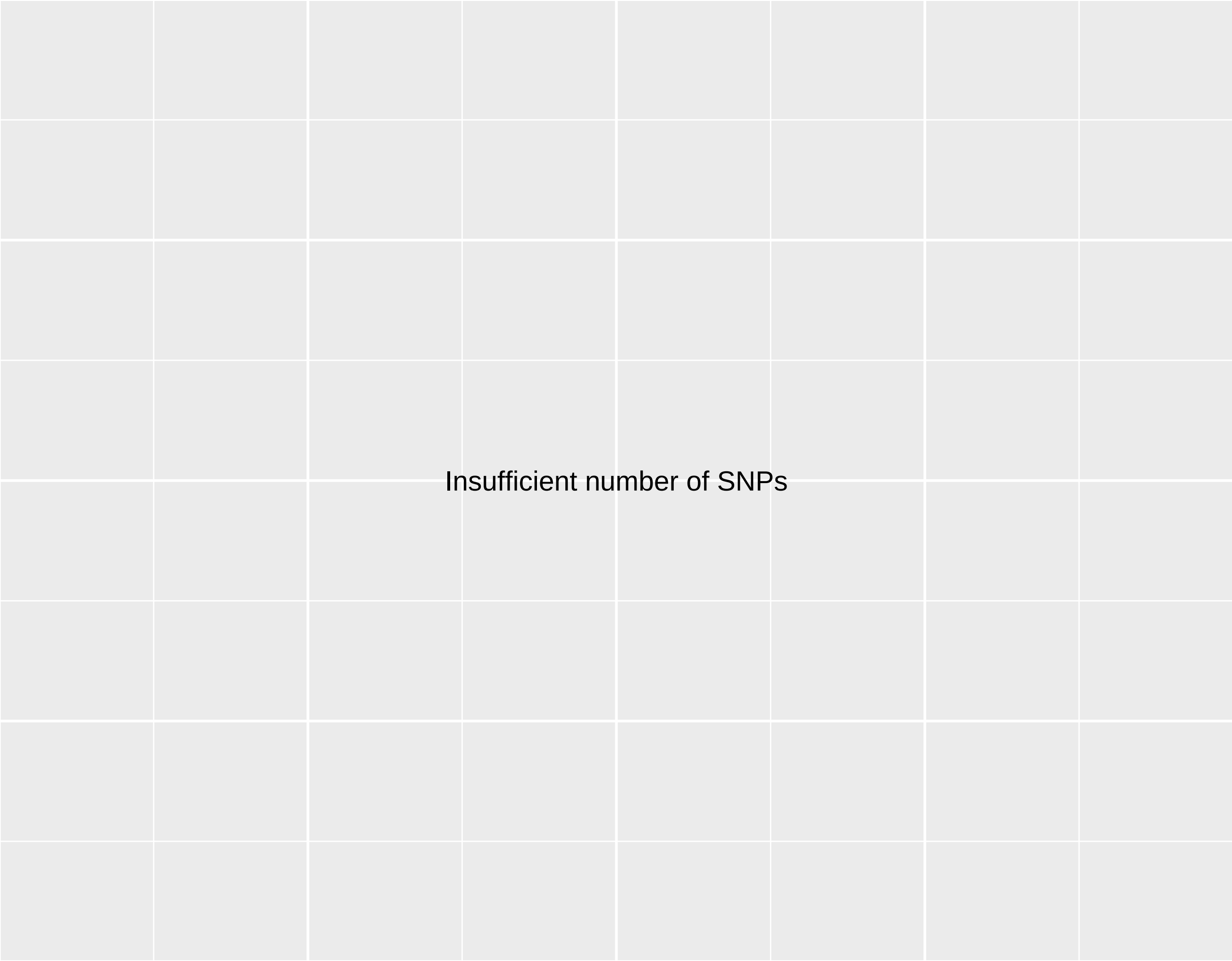
Insufficient number of SNPs

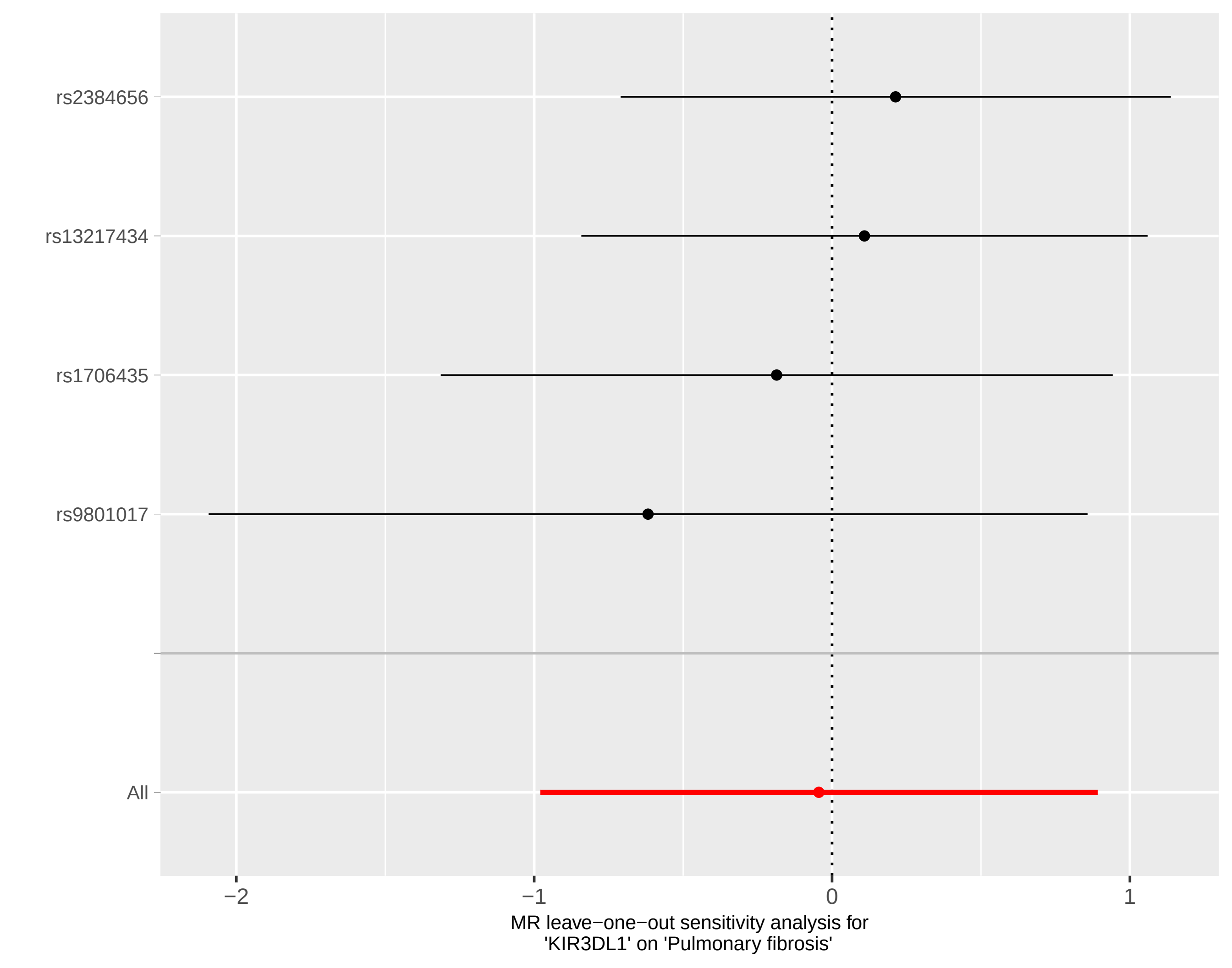
rs2384656
rs13217434
rs1706435
rs9801017
All
−2
−1
0
1
MR leave−one−out sensitivity analysis for
'KIR3DL1' on 'Pulmonary fibrosis'

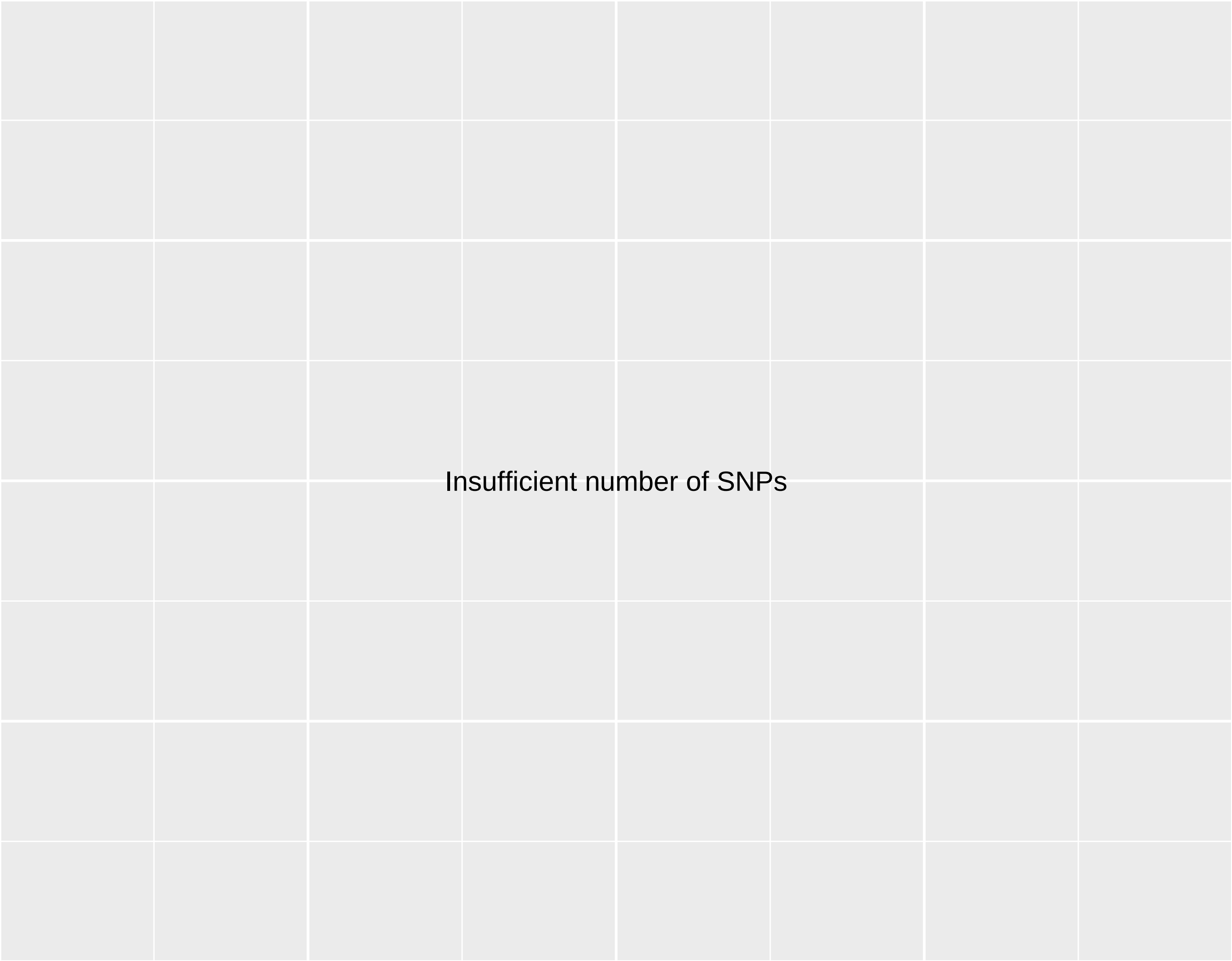
Insufficient number of SNPs

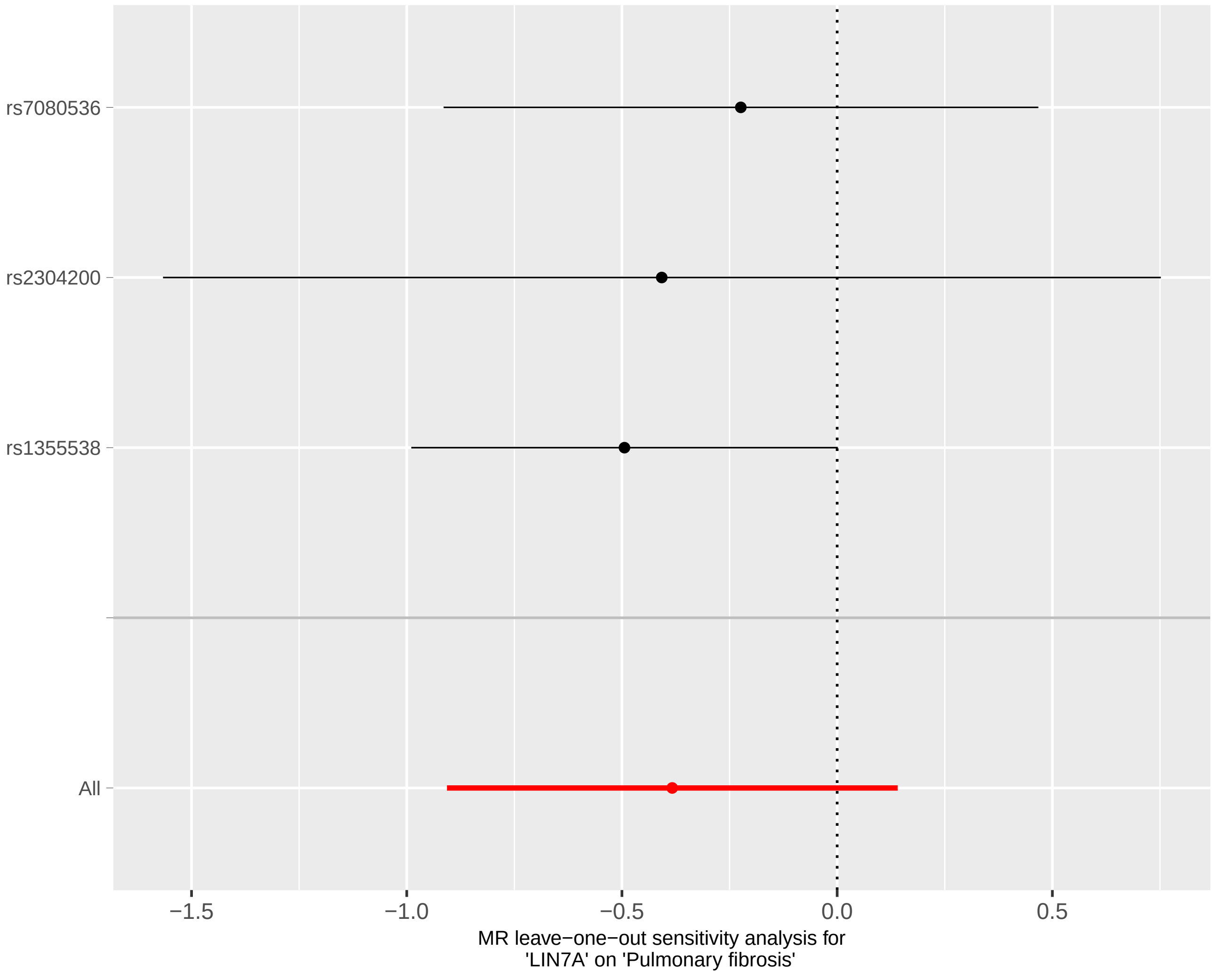
rs7080536
rs2304200
rs1355538
All
−1.5
−1.0
−0.5
0.0
0.5
MR leave−one−out sensitivity analysis for
'LIN7A' on 'Pulmonary fibrosis'

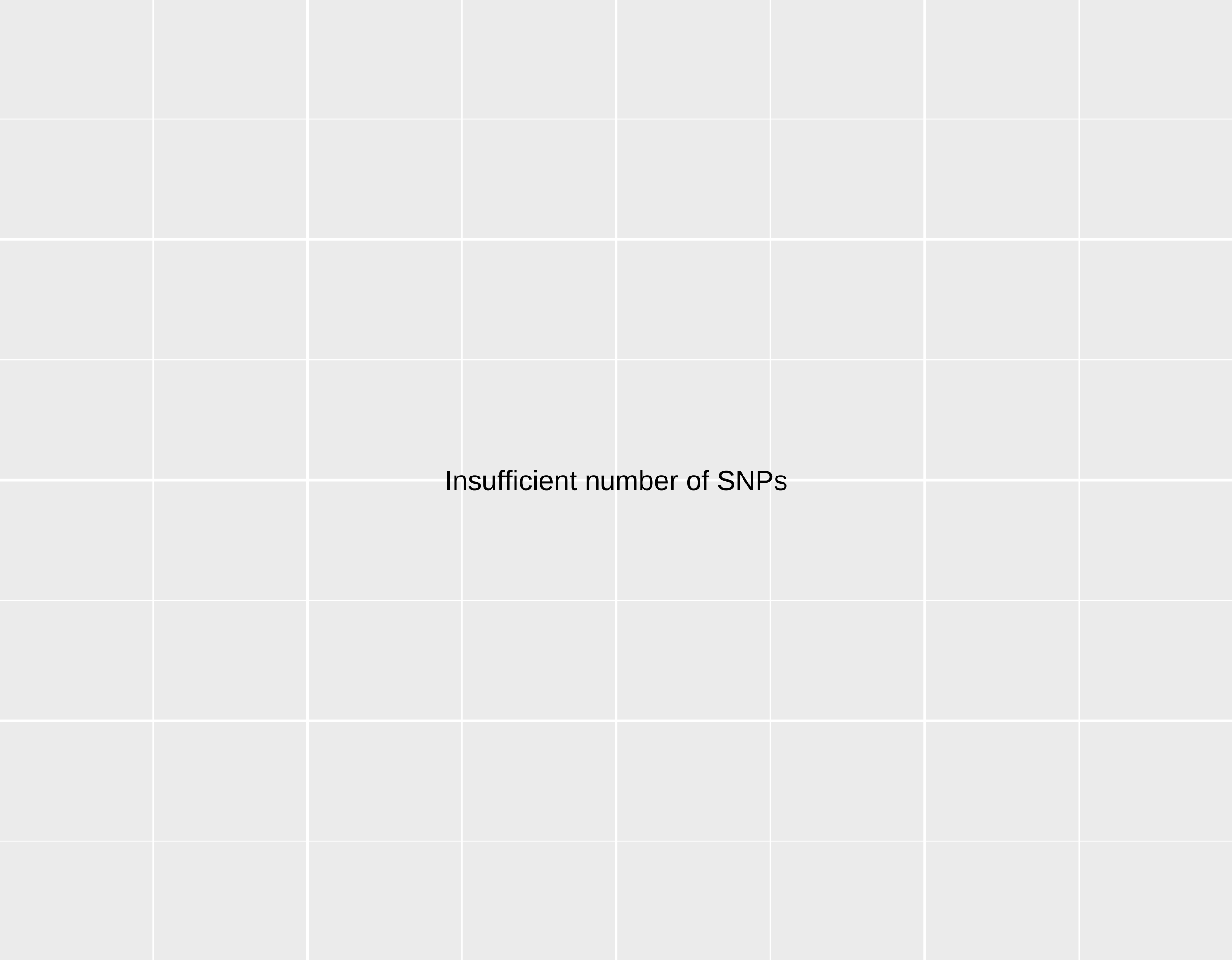
Insufficient number of SNPs

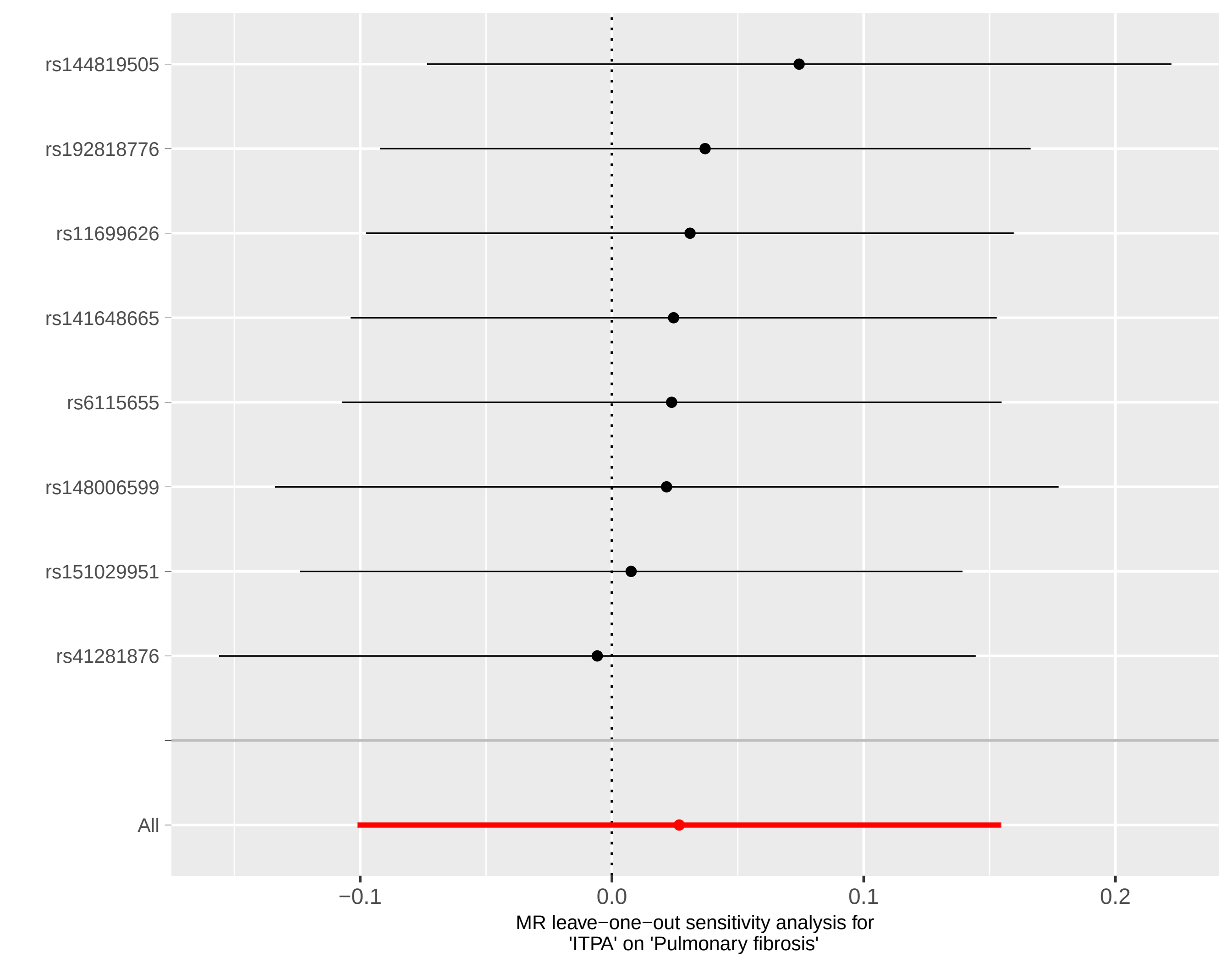
rs144819505
rs192818776
rs11699626
rs141648665
rs6115655
rs148006599
rs151029951
rs41281876
All
−0.1
0.0
0.1
0.2
MR leave−one−out sensitivity analysis for
'ITPA' on 'Pulmonary fibrosis'

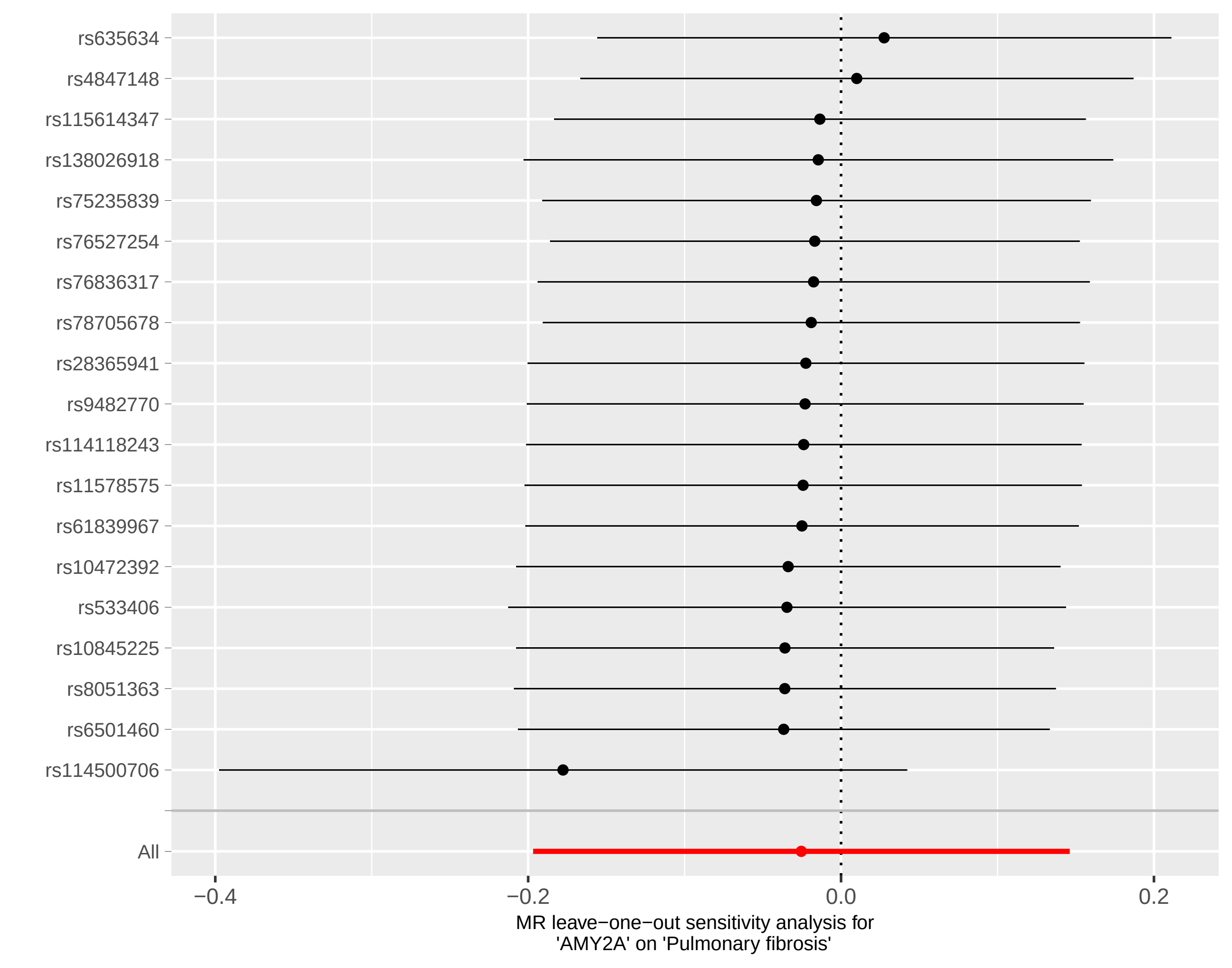

rs635634
rs4847148
rs115614347
rs138026918
rs75235839
rs76527254
rs76836317
rs78705678
rs28365941
rs9482770
rs114118243
rs11578575
rs61839967
rs10472392
rs533406
rs10845225
rs8051363
rs6501460
rs114500706
All
−0.4
−0.2
0.0
0.2
MR leave−one−out sensitivity analysis for
'AMY2A' on 'Pulmonary fibrosis'

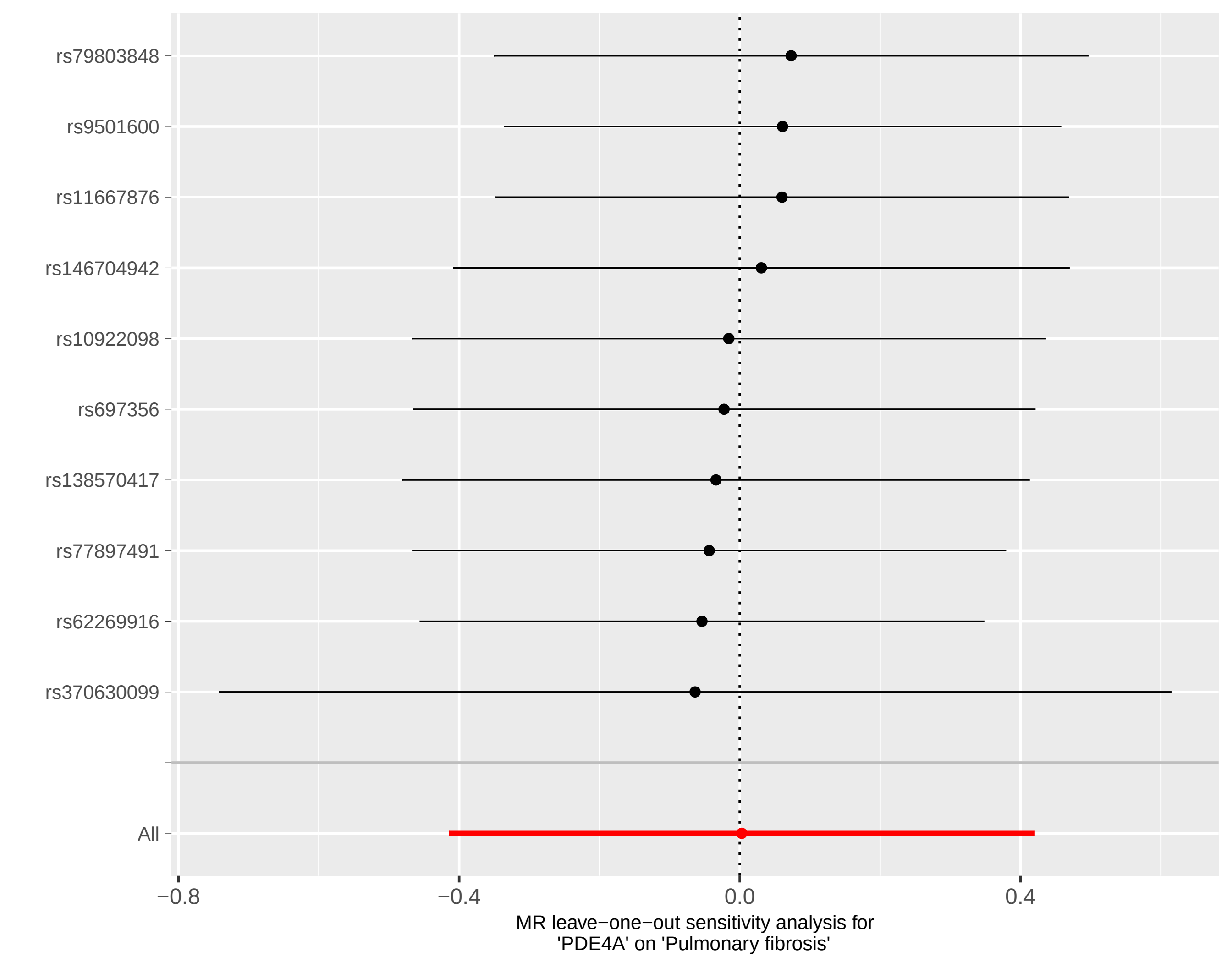
rs79803848
rs9501600
rs11667876
rs146704942
rs10922098
rs697356
rs138570417
rs77897491
rs62269916
rs370630099
All
−0.8
−0.4
0.0
0.4
MR leave−one−out sensitivity analysis for
'PDE4A' on 'Pulmonary fibrosis'

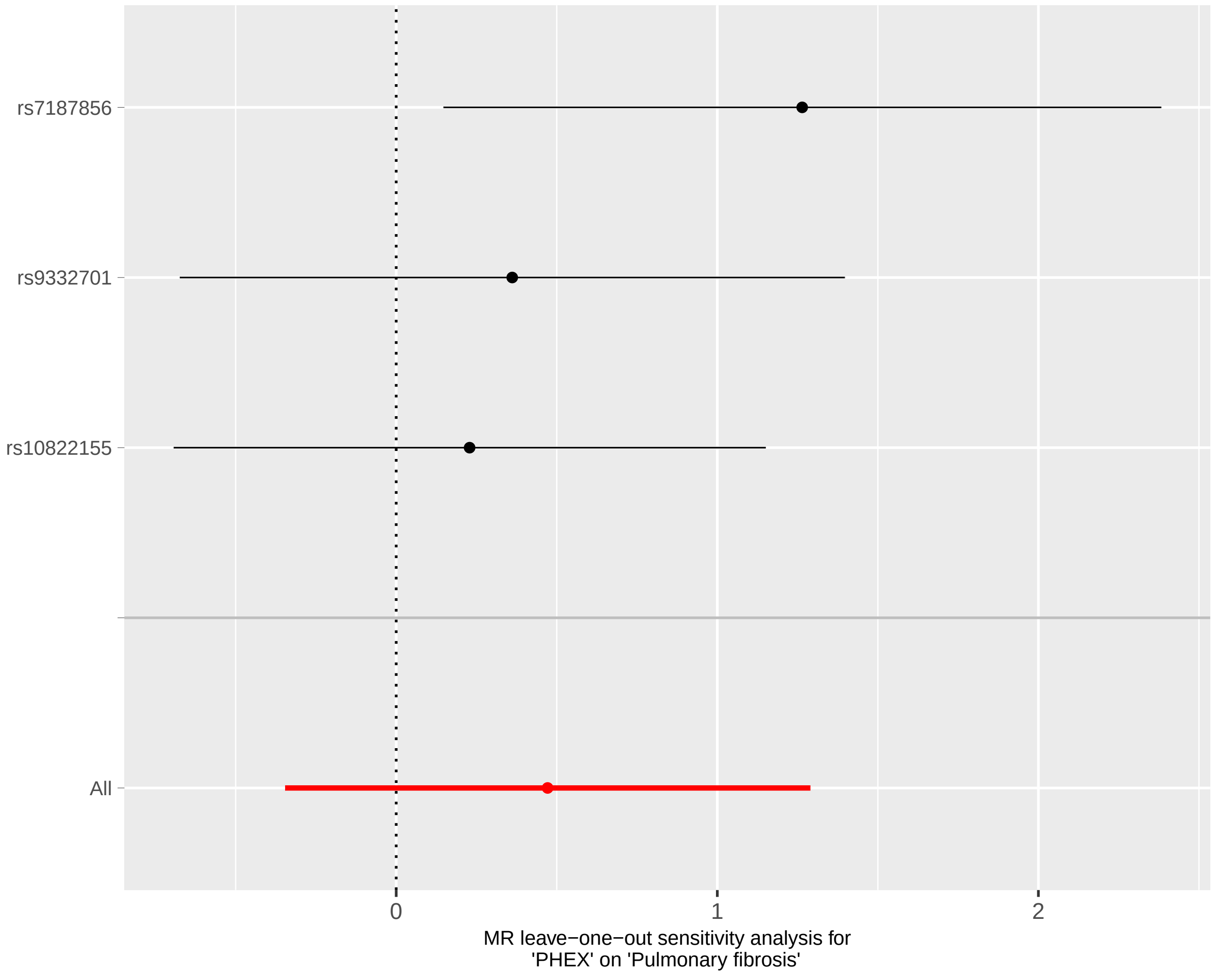
rs7187856
rs9332701
rs10822155
All
0
1
2
MR leave−one−out sensitivity analysis for
'PHEX' on 'Pulmonary fibrosis'

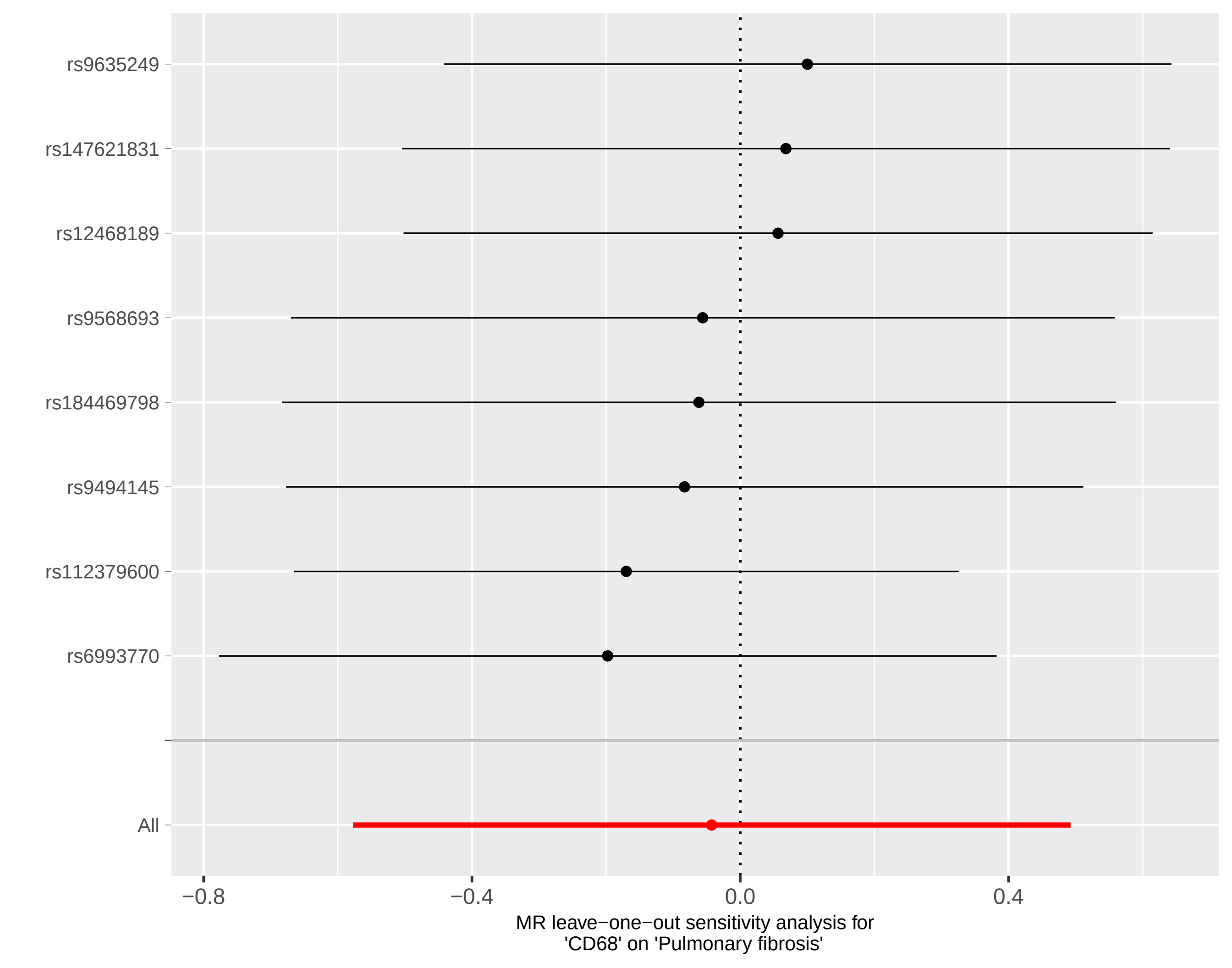
rs9635249
rs147621831
rs12468189
rs9568693
rs184469798
rs9494145
rs112379600
rs6993770
All
−0.8
−0.4
0.0
0.4
MR leave−one−out sensitivity analysis for
'CD68' on 'Pulmonary fibrosis'

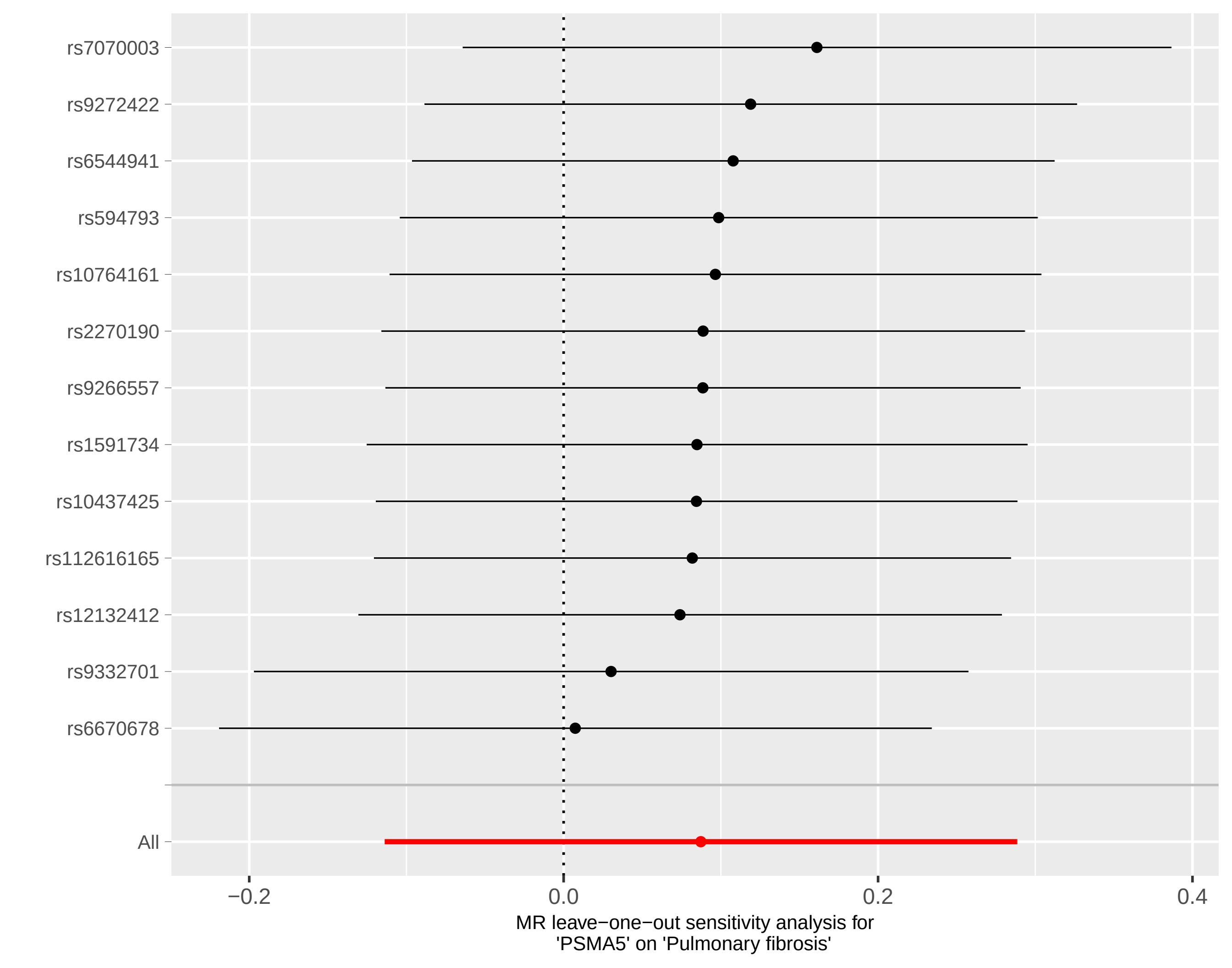
rs7070003
rs9272422
rs6544941
rs594793
rs10764161
rs2270190
rs9266557
rs1591734
rs10437425
rs112616165
rs12132412
rs9332701
rs6670678
All
−0.2
0.0
0.2
0.4
MR leave−one−out sensitivity analysis for
'PSMA5' on 'Pulmonary fibrosis'

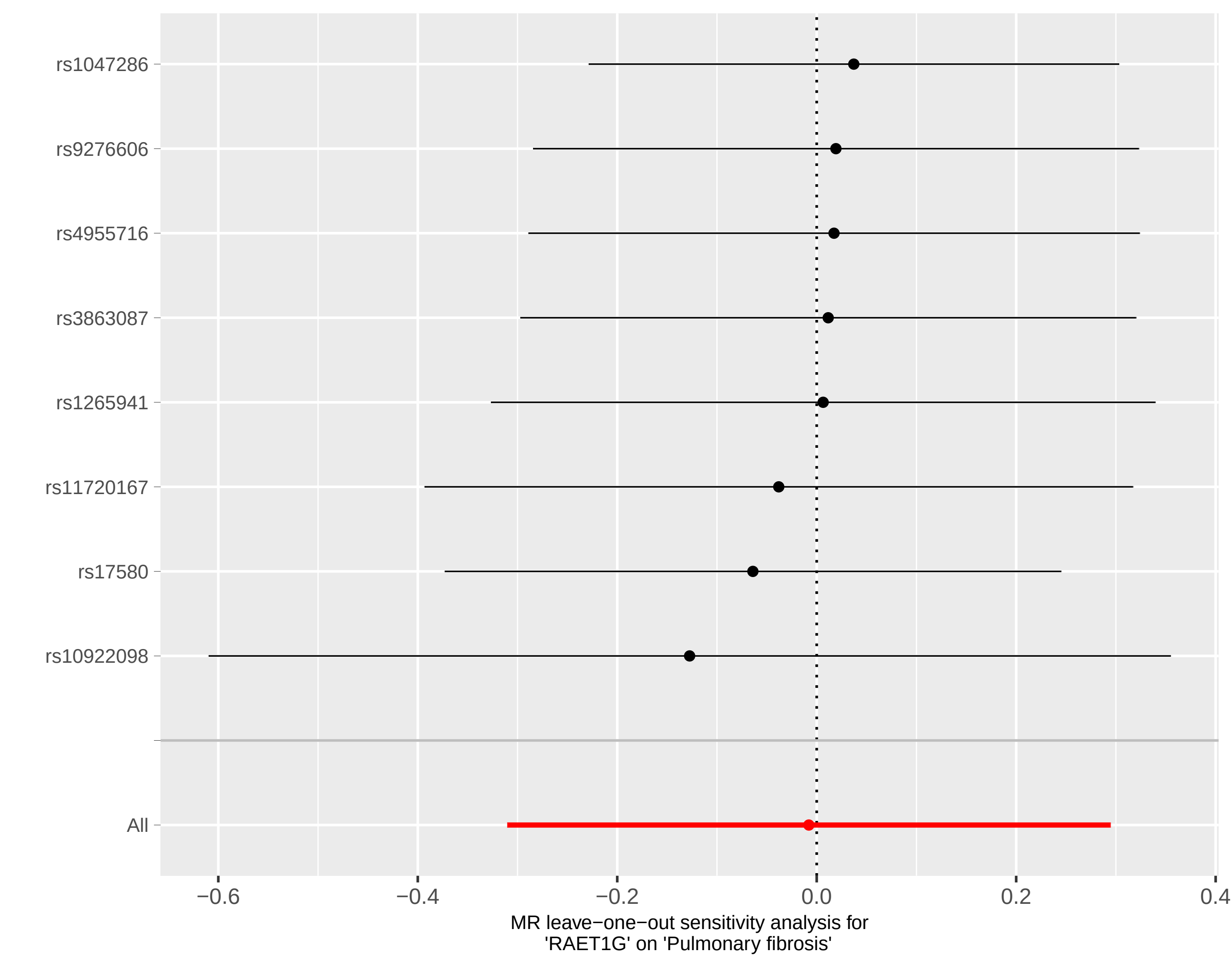
rs1047286
rs9276606
rs4955716
rs3863087
rs1265941
rs11720167
rs17580
rs10922098
All
−0.6
−0.4
−0.2
0.0
0.2
0.4
MR leave−one−out sensitivity analysis for
'RAET1G' on 'Pulmonary fibrosis'

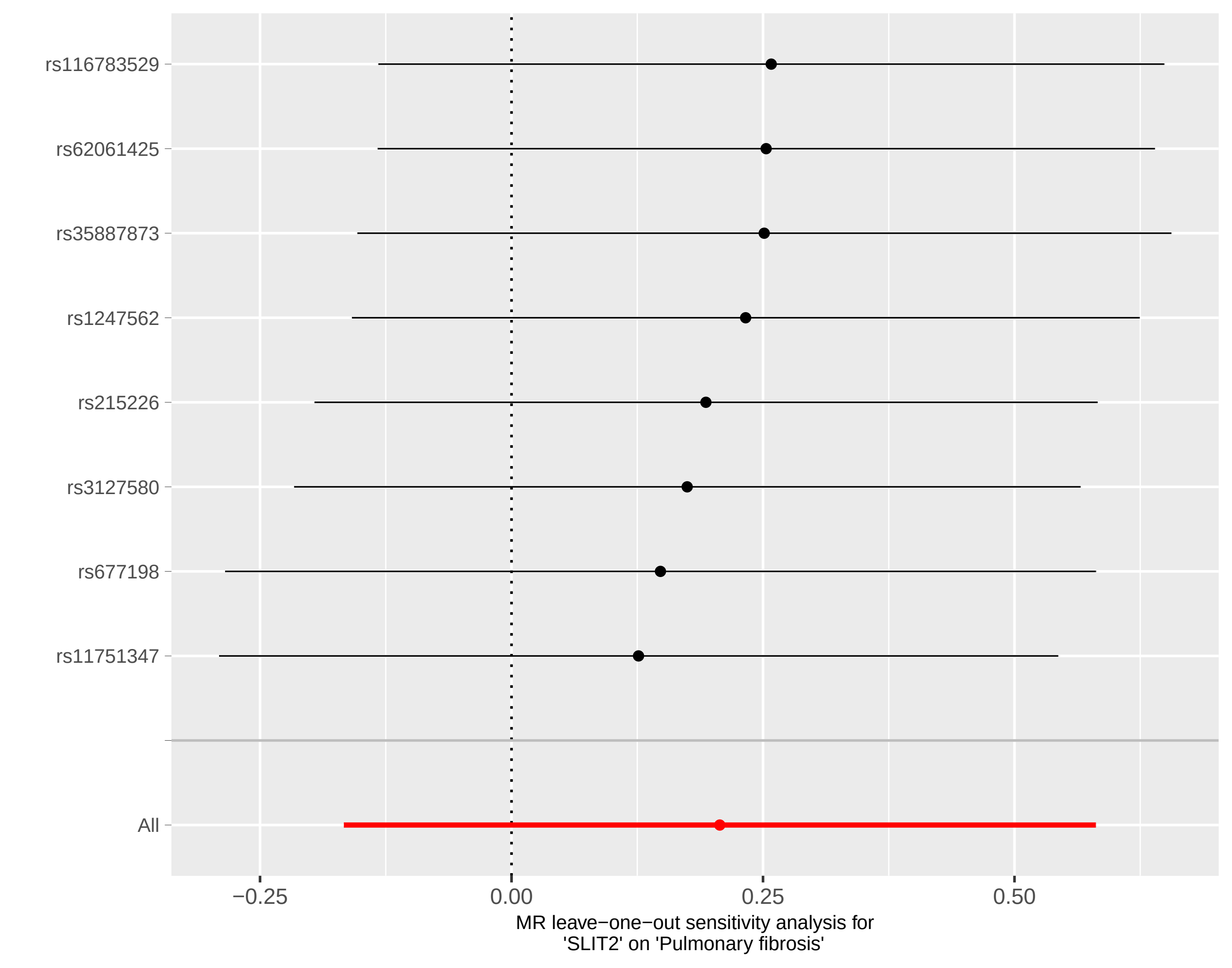
rs116783529
rs62061425
rs35887873
rs1247562
rs215226
rs3127580
rs677198
rs11751347
All
−0.25
0.00
0.25
0.50
MR leave−one−out sensitivity analysis for
'SLIT2' on 'Pulmonary fibrosis'

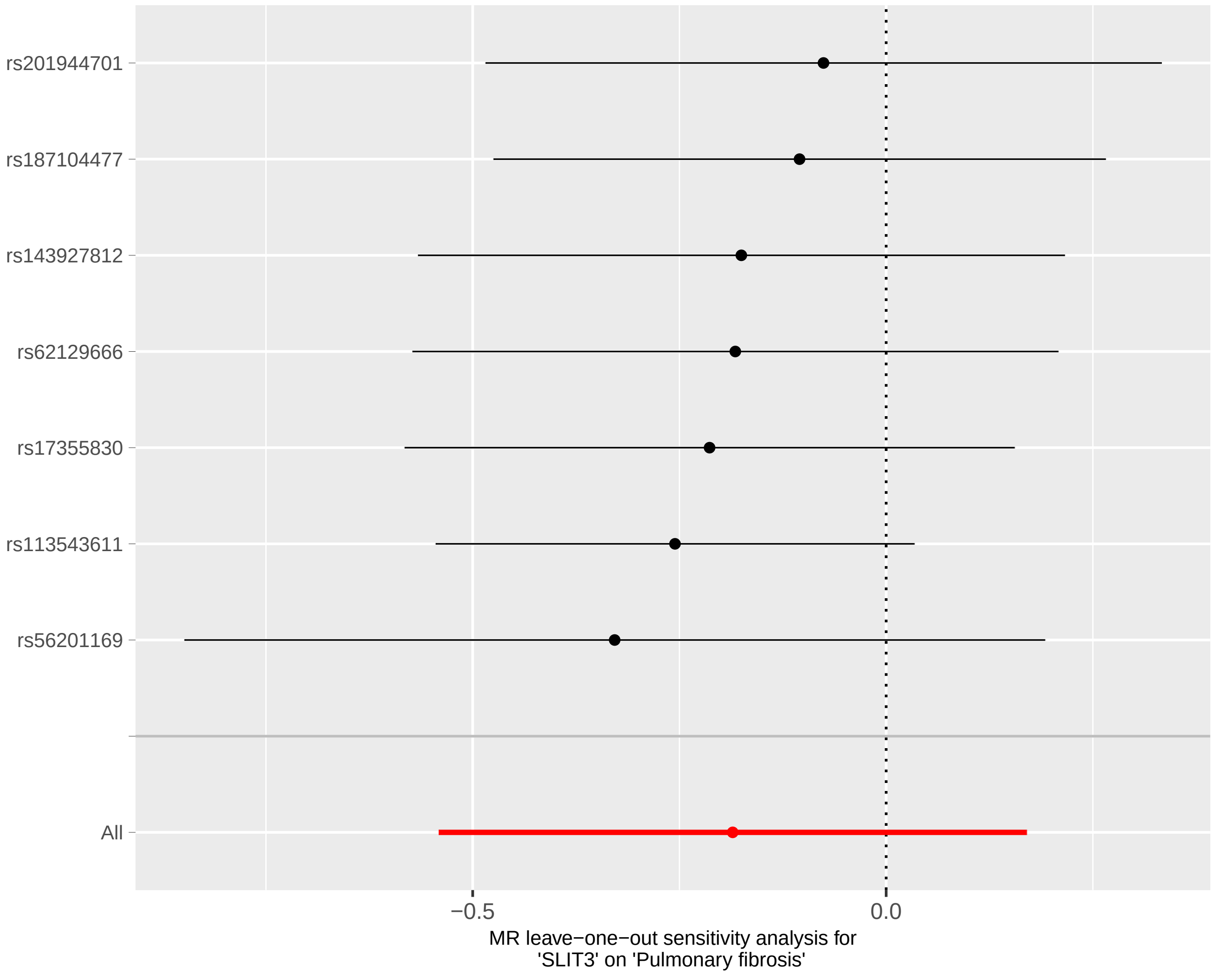
rs201944701
rs187104477
rs143927812
rs62129666
rs17355830
rs113543611
rs56201169
All
−0.5
0.0
MR leave−one−out sensitivity analysis for
'SLIT3' on 'Pulmonary fibrosis'

Insufficient number of SNPs

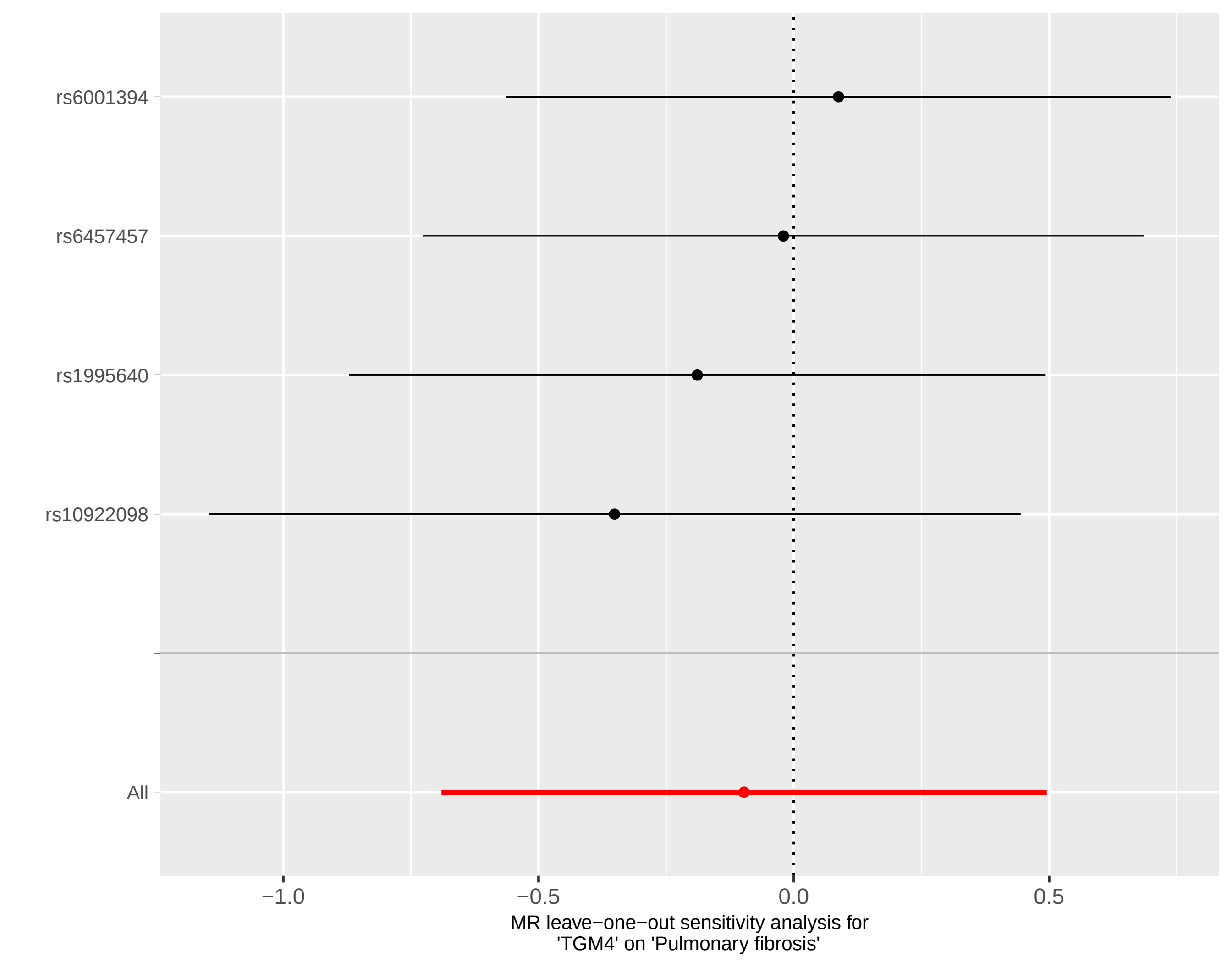
rs6001394
rs6457457
rs1995640
rs10922098
All
−1.0
−0.5
0.0
0.5
MR leave−one−out sensitivity analysis for
'TGM4' on 'Pulmonary fibrosis'

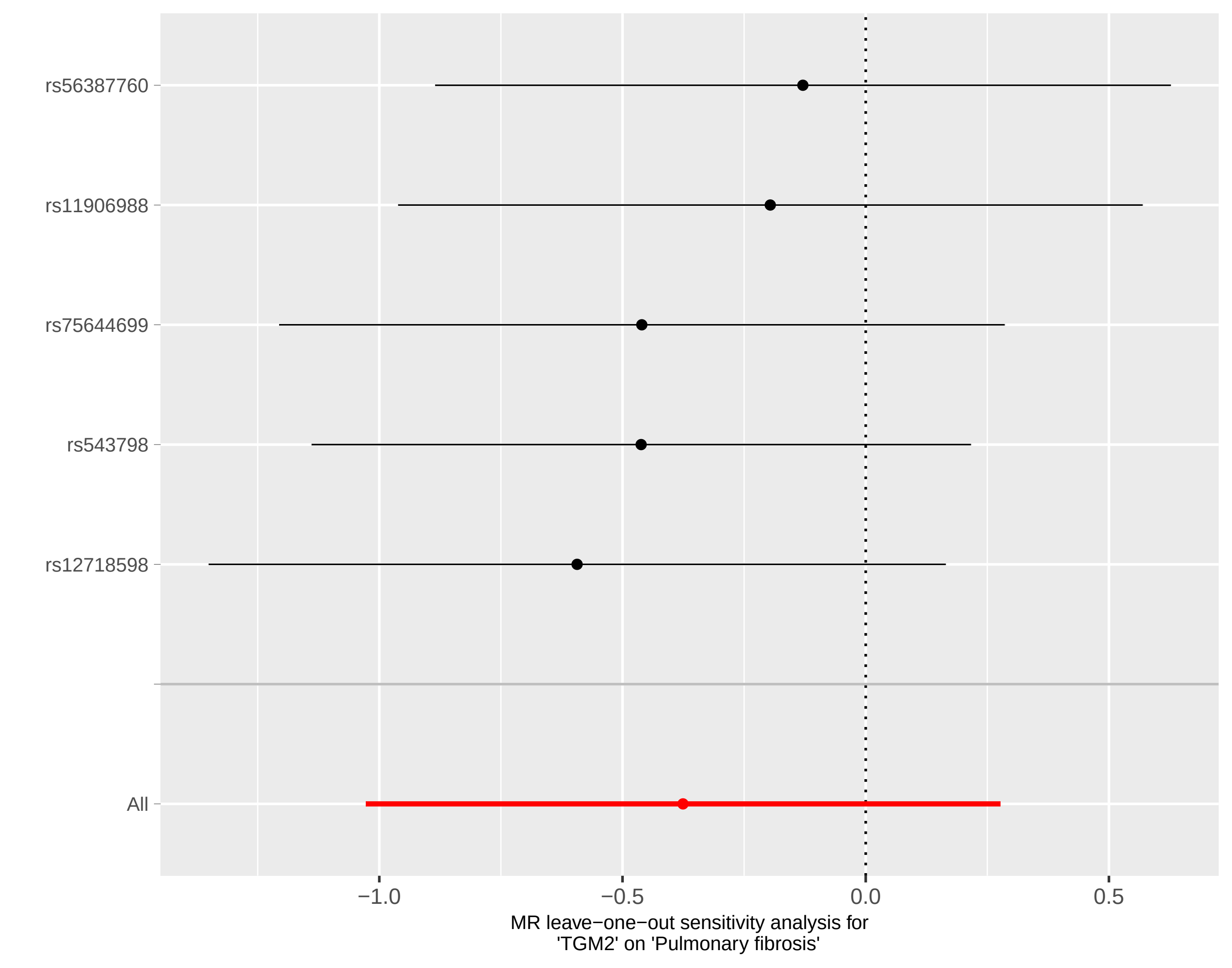
rs56387760
rs11906988
rs75644699
rs543798
rs12718598
All
−1.0
−0.5
0.0
0.5
MR leave−one−out sensitivity analysis for
'TGM2' on 'Pulmonary fibrosis'

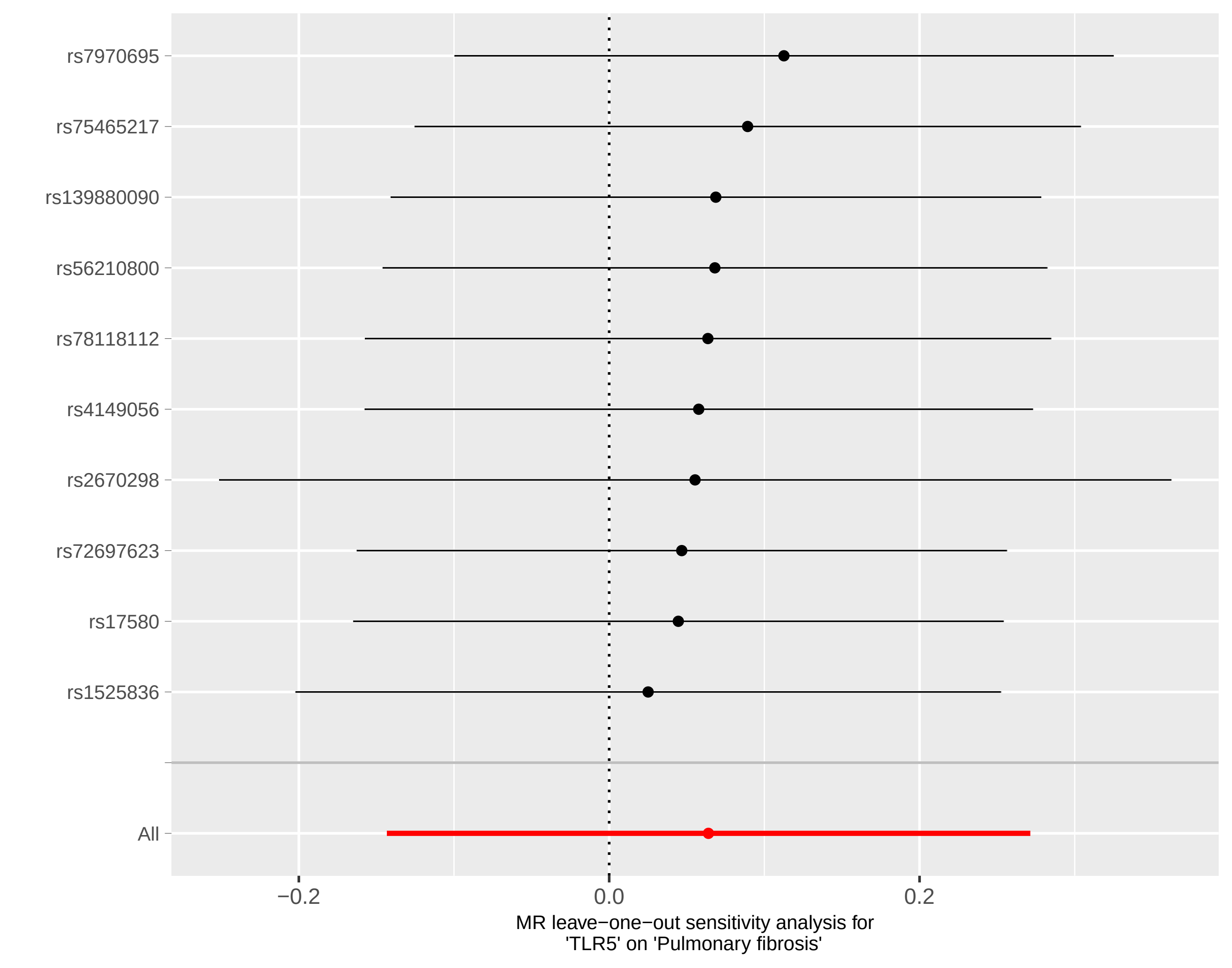
rs7970695
rs75465217
rs139880090
rs56210800
rs78118112
rs4149056
rs2670298
rs72697623
rs17580
rs1525836
All
−0.2
0.0
0.2
MR leave−one−out sensitivity analysis for
'TLR5' on 'Pulmonary fibrosis'

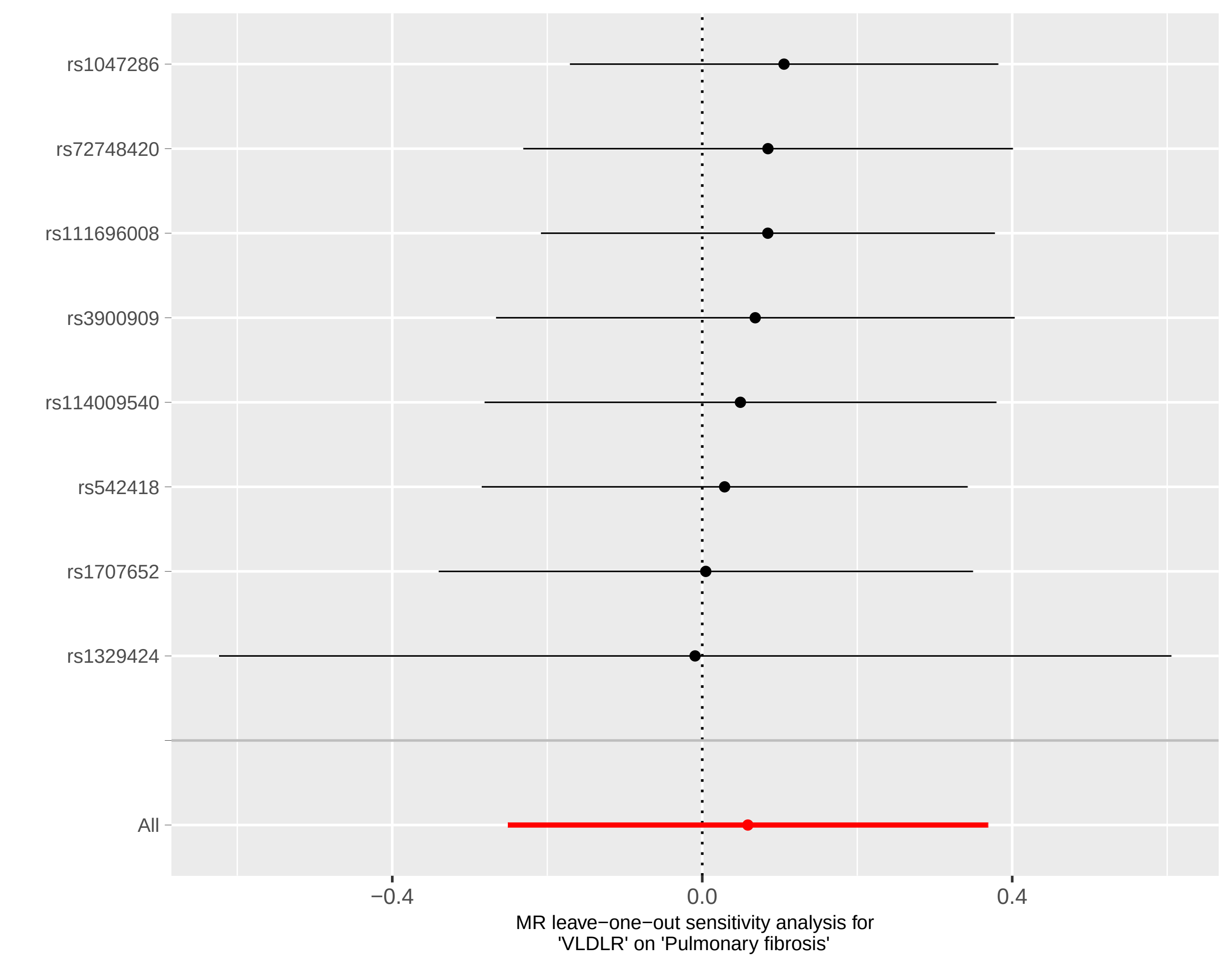
rs1047286
rs72748420
rs111696008
rs3900909
rs114009540
rs542418
rs1707652
rs1329424
All
−0.4
0.0
0.4
MR leave−one−out sensitivity analysis for
'VLDLR' on 'Pulmonary fibrosis'

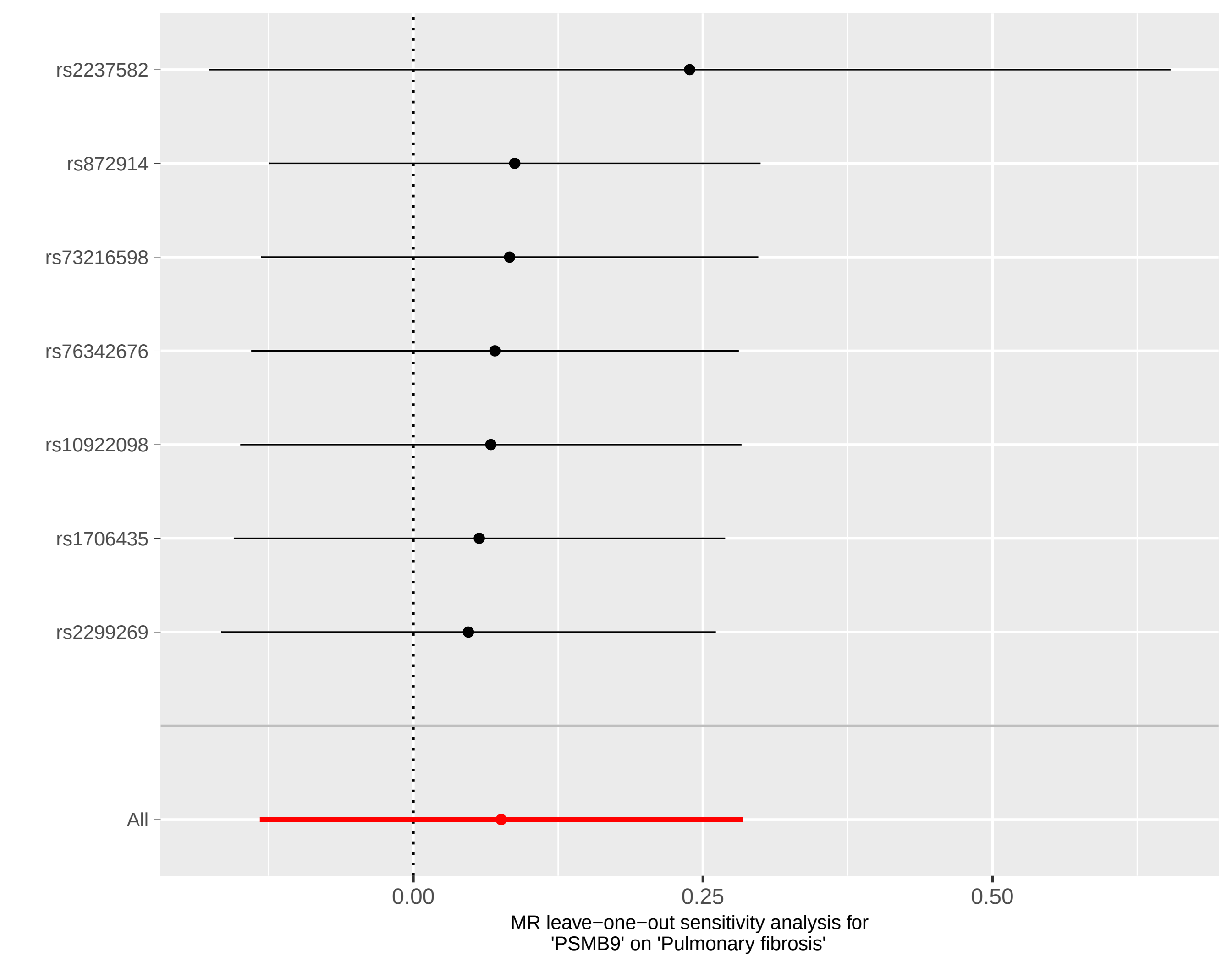
rs2237582
rs872914
rs73216598
rs76342676
rs10922098
rs1706435
rs2299269
All
0.00
0.25
0.50
MR leave−one−out sensitivity analysis for
'PSMB9' on 'Pulmonary fibrosis'

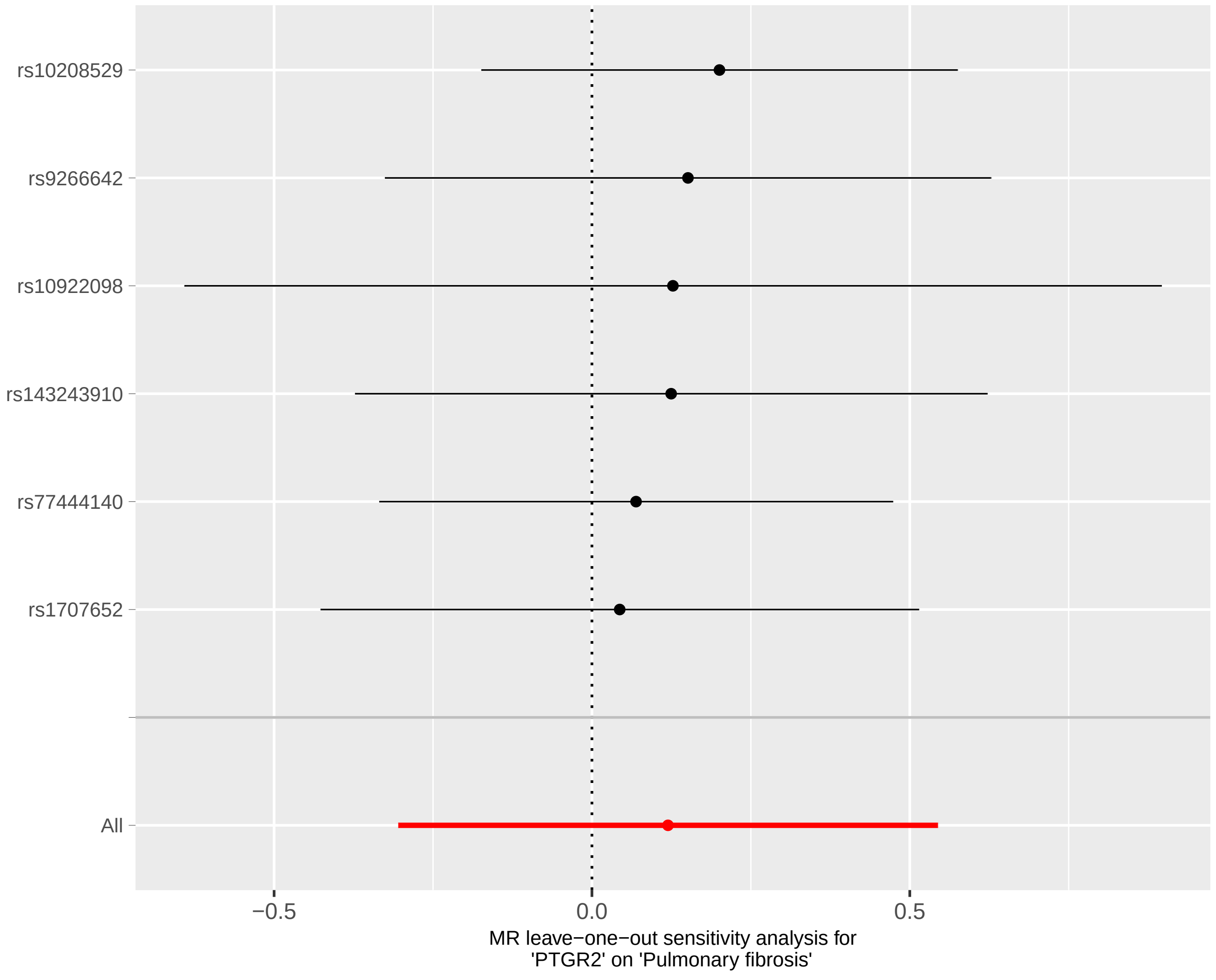
rs10208529
rs9266642
rs10922098
rs143243910
rs77444140
rs1707652
All
−0.5
0.0
0.5
MR leave−one−out sensitivity analysis for
'PTGR2' on 'Pulmonary fibrosis'

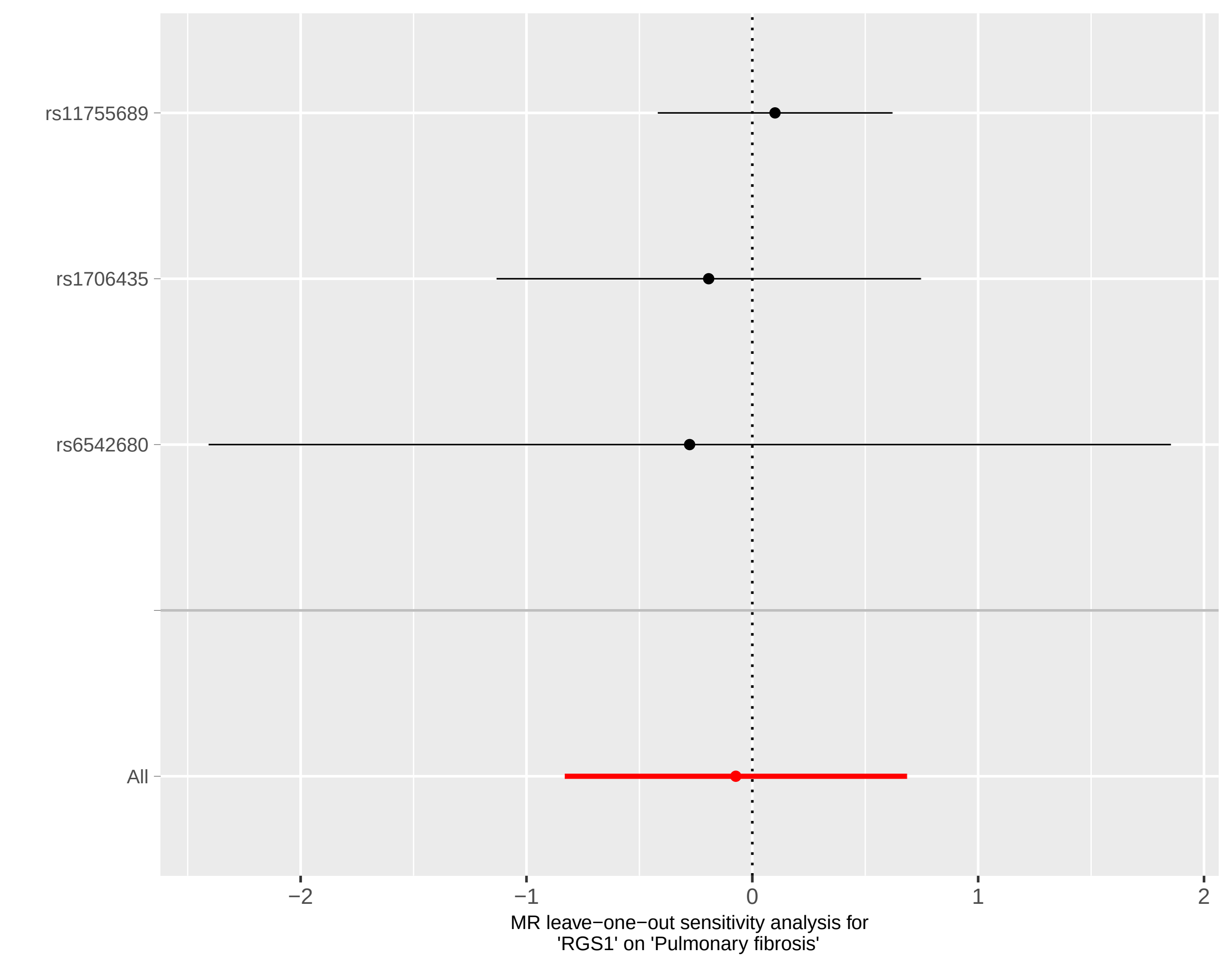
rs11755689
rs1706435
rs6542680
All
−2
−1
0
1
2
MR leave−one−out sensitivity analysis for
'RGS1' on 'Pulmonary fibrosis'

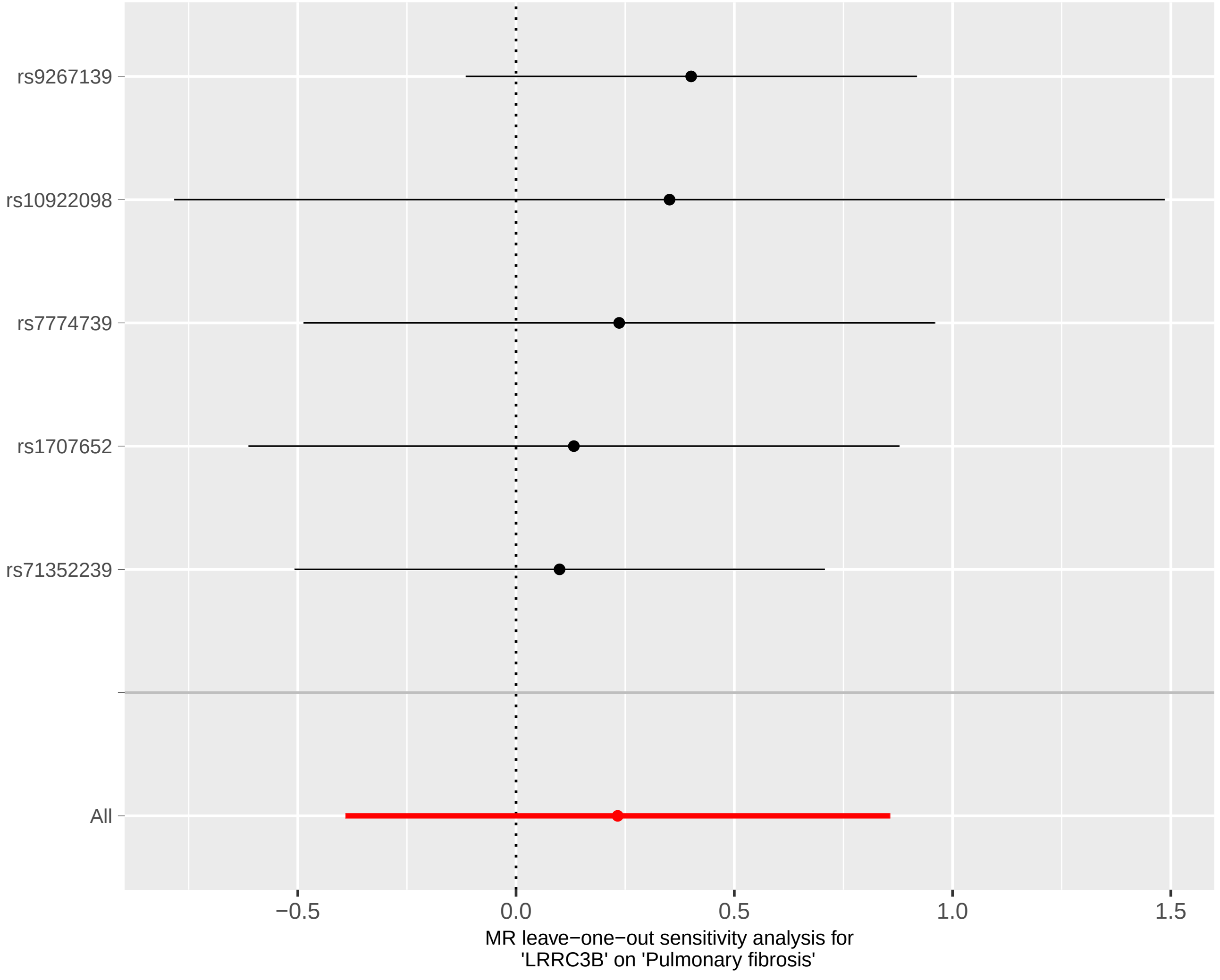
rs9267139
rs10922098
rs7774739
rs1707652
rs71352239
All
−0.5
0.0
0.5
1.0
1.5
MR leave−one−out sensitivity analysis for
'LRRC3B' on 'Pulmonary fibrosis'

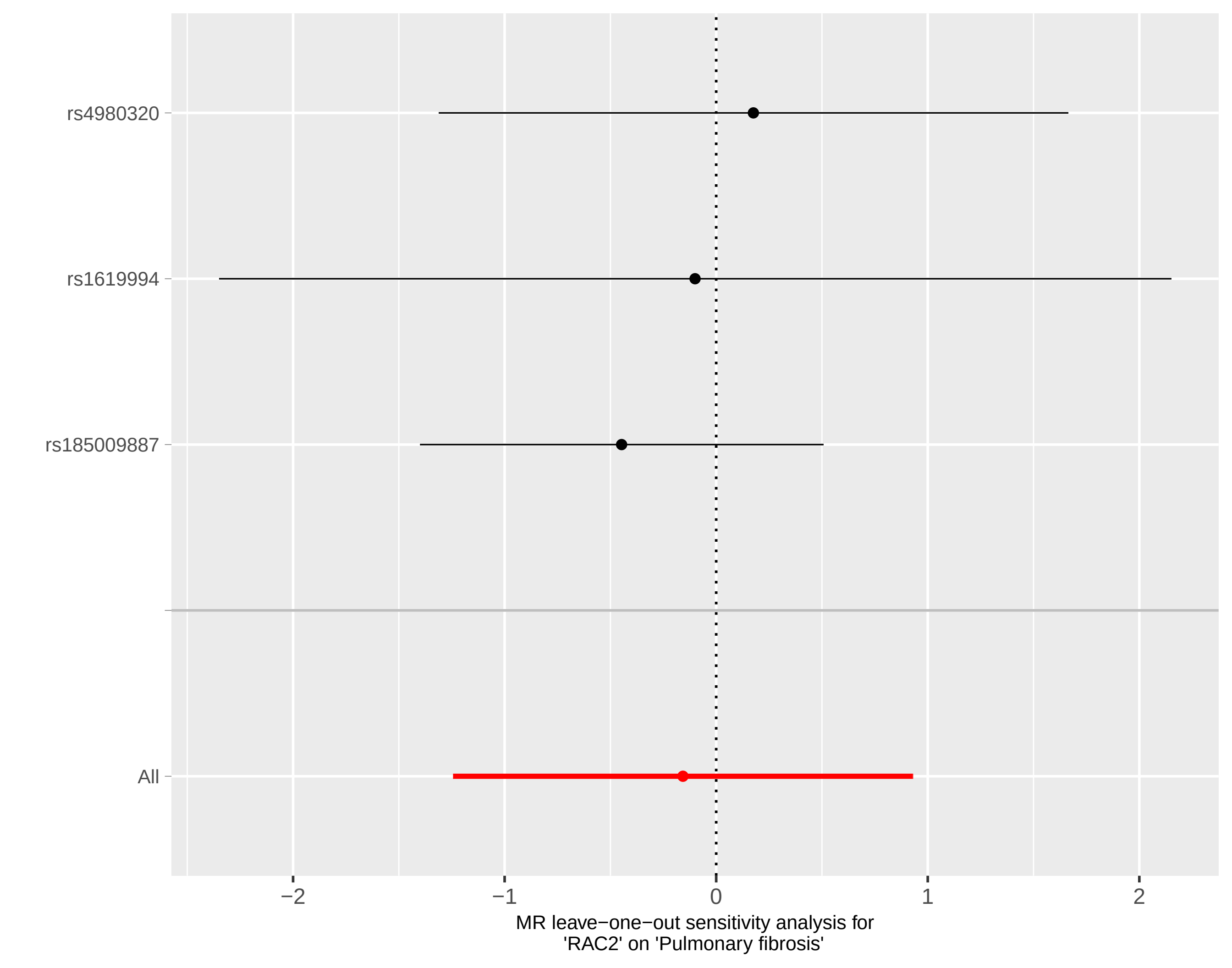
rs4980320
rs1619994
rs185009887
All
−2
−1
0
1
2
MR leave−one−out sensitivity analysis for
'RAC2' on 'Pulmonary fibrosis'

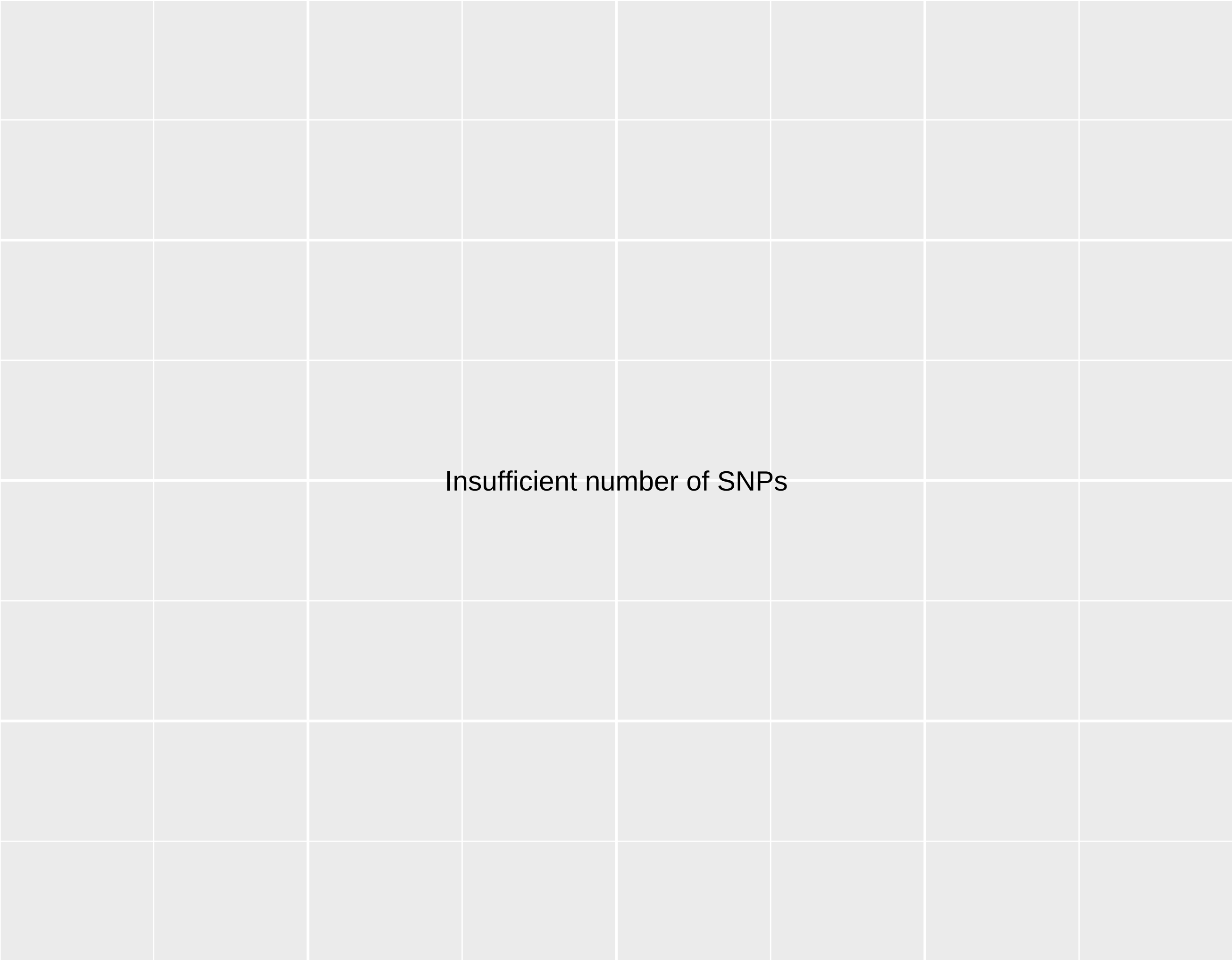
Insufficient number of SNPs

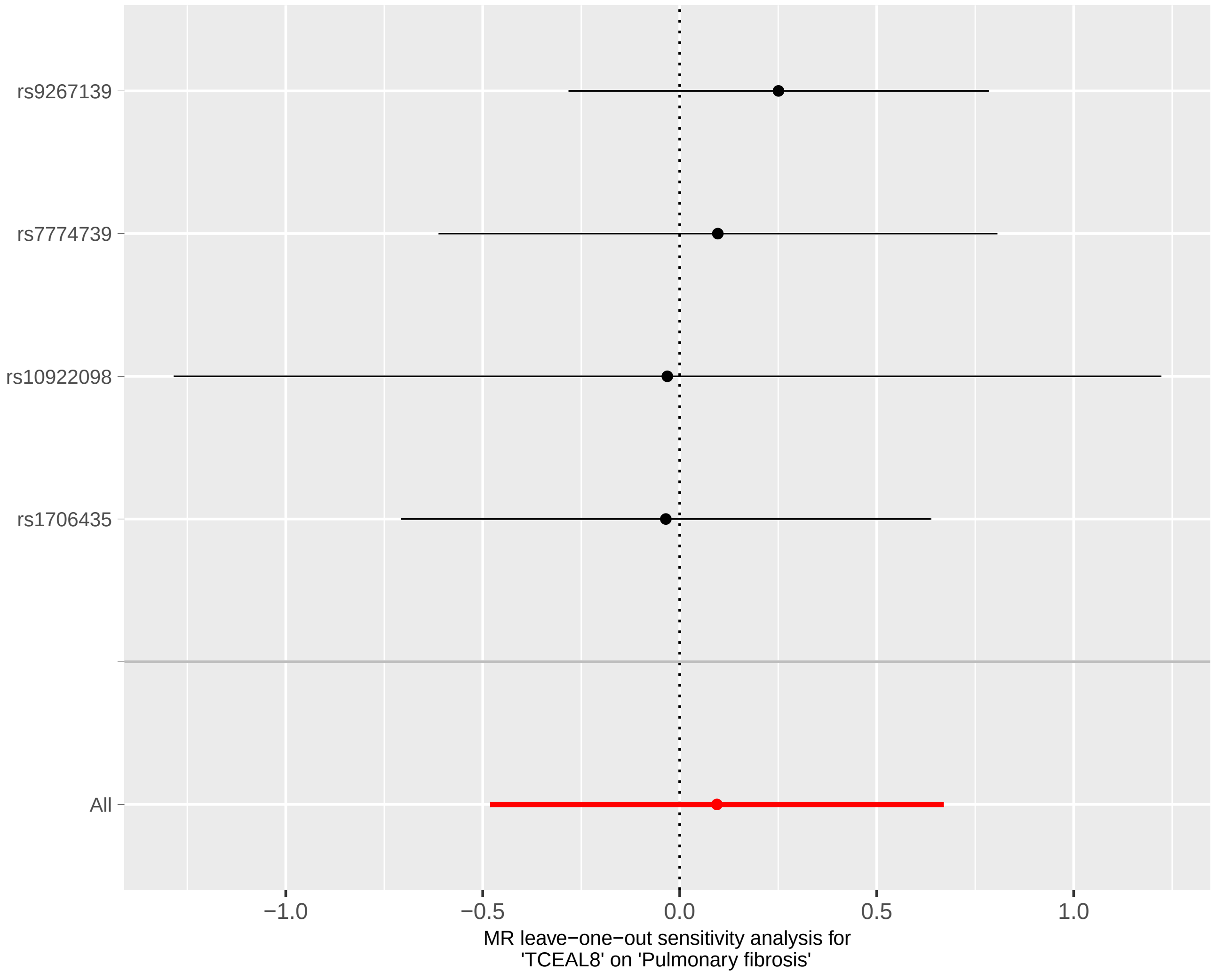

rs9267139
rs7774739
rs10922098
rs1706435
All
−1.0
−0.5
0.0
0.5
1.0
MR leave−one−out sensitivity analysis for
'TCEAL8' on 'Pulmonary fibrosis'

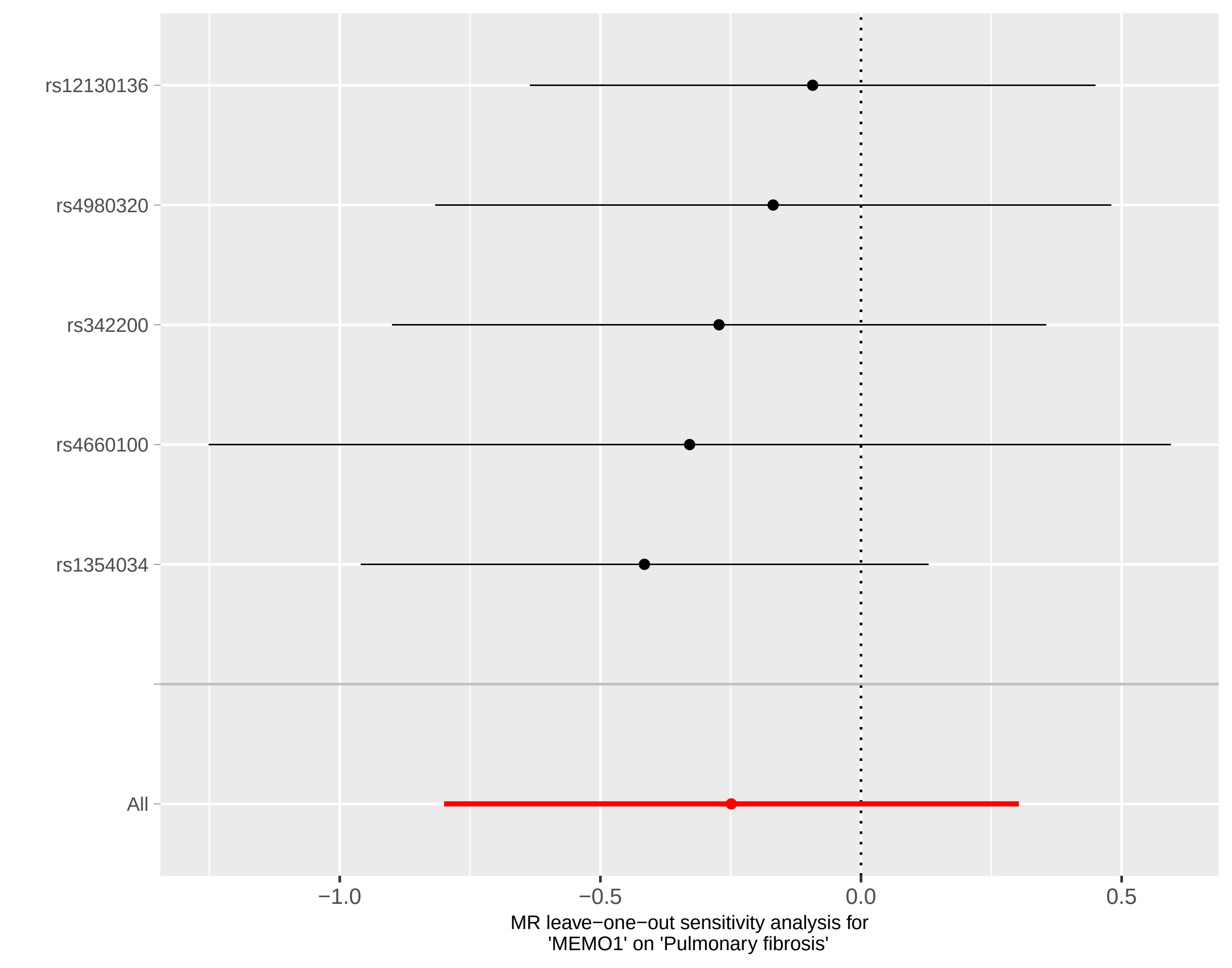

rs12130136
rs4980320
rs342200
rs4660100
rs1354034
All
−1.0
−0.5
0.0
0.5
MR leave−one−out sensitivity analysis for
'MEMO1' on 'Pulmonary fibrosis'

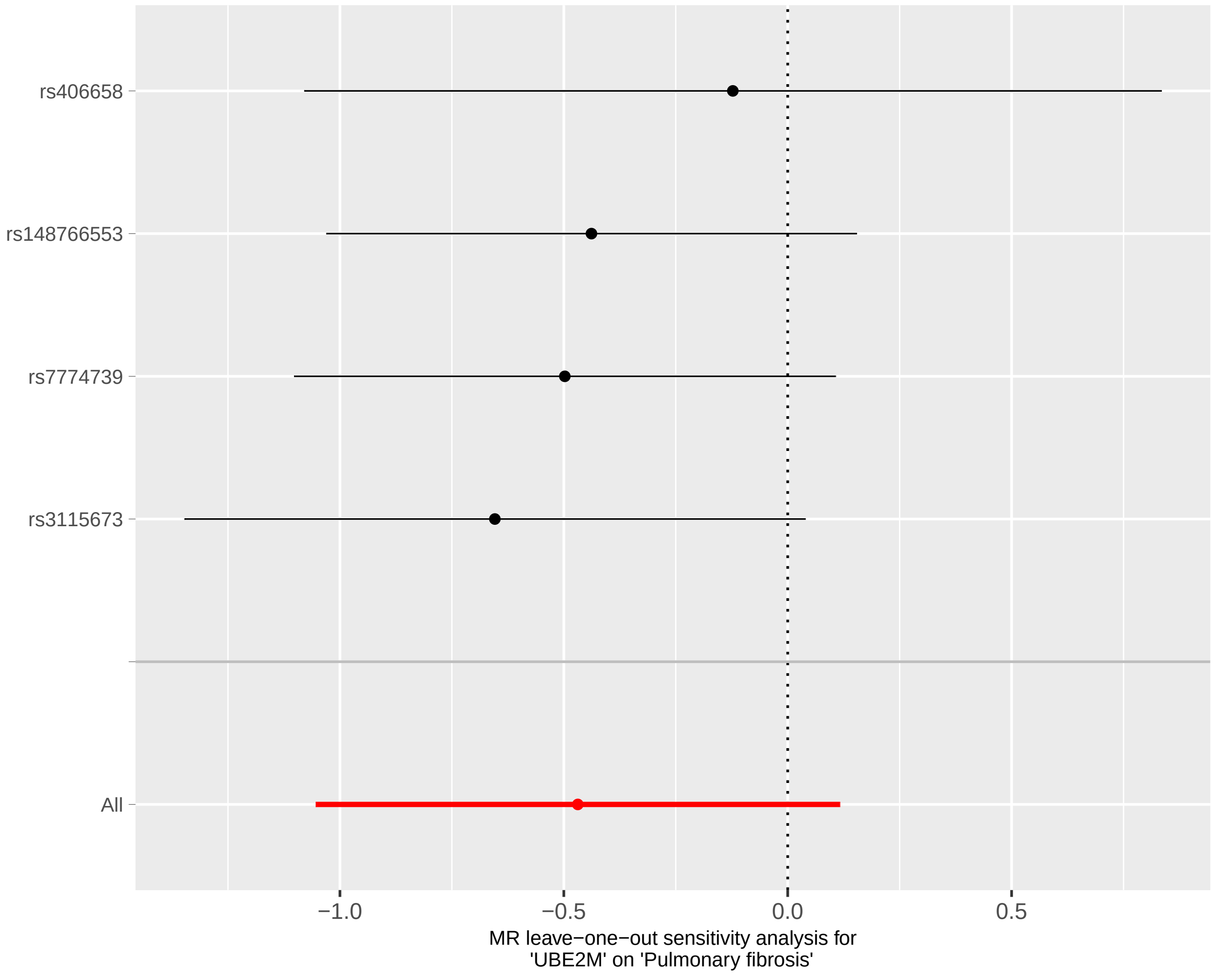

rs406658
rs148766553
rs7774739
rs3115673
All
−1.0
−0.5
0.0
0.5
MR leave−one−out sensitivity analysis for
'UBE2M' on 'Pulmonary fibrosis'

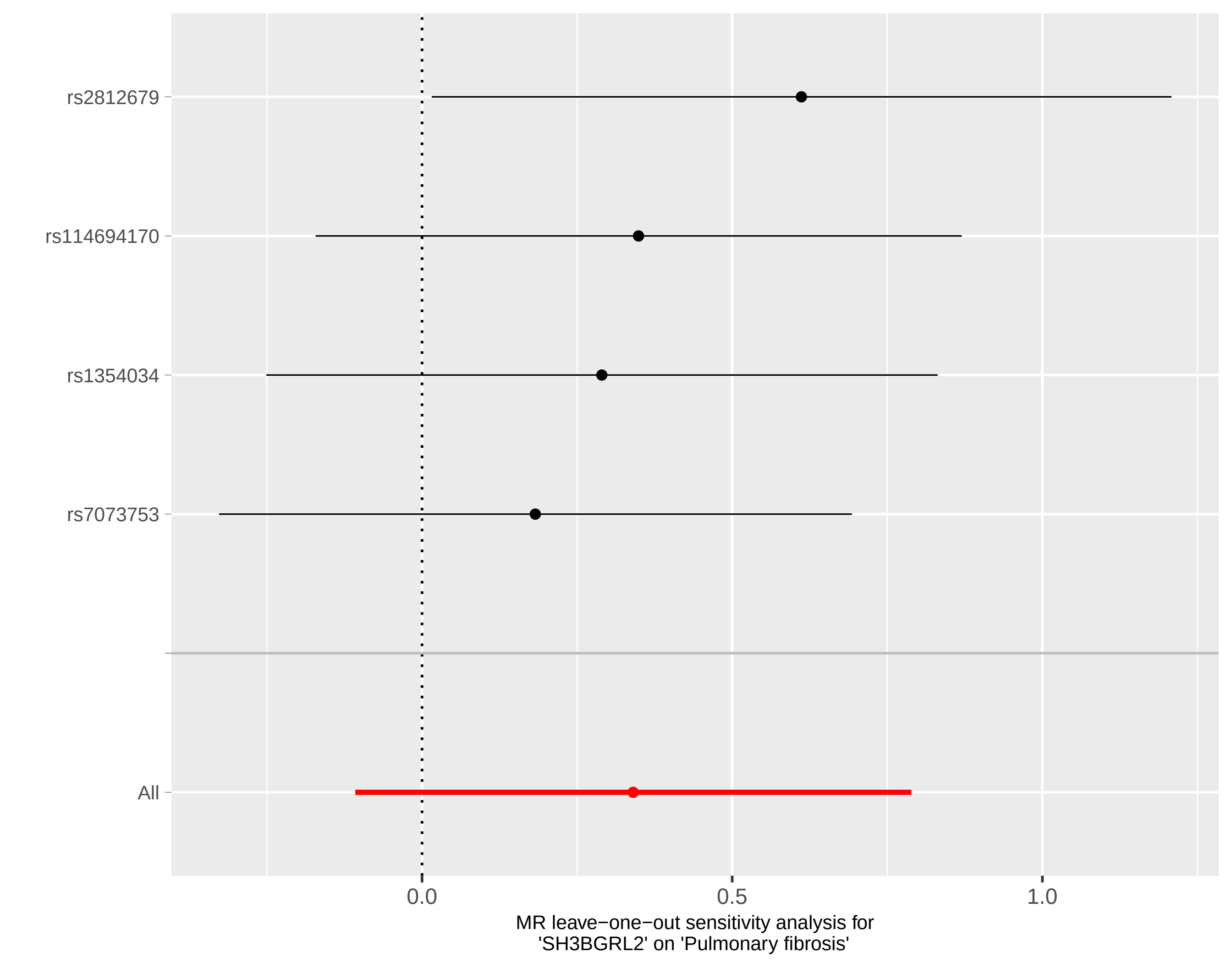

rs2812679
rs114694170
rs1354034
rs7073753
All
0.0
0.5
1.0
MR leave−one−out sensitivity analysis for
'SH3BGRL2' on 'Pulmonary fibrosis'

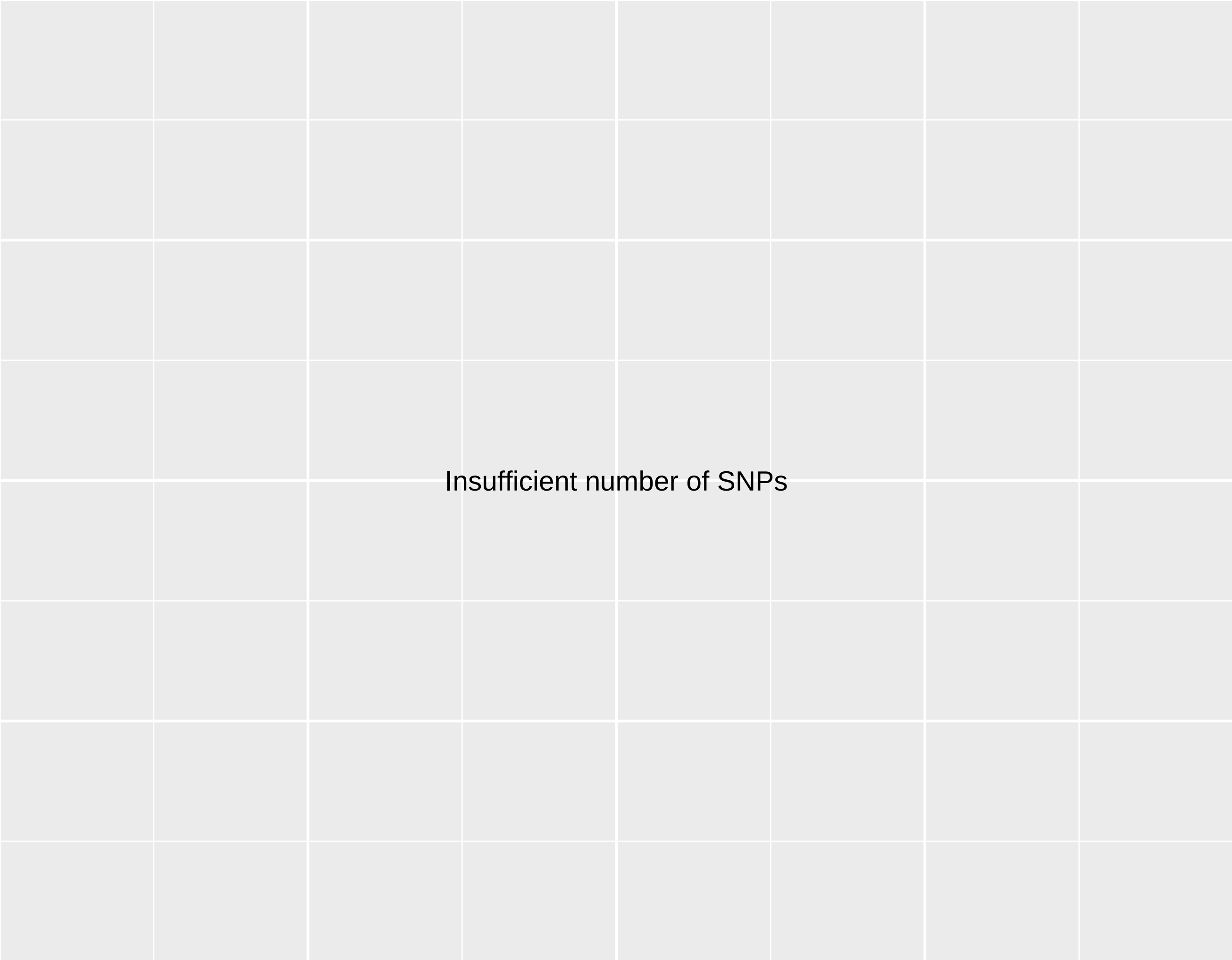
Insufficient number of SNPs

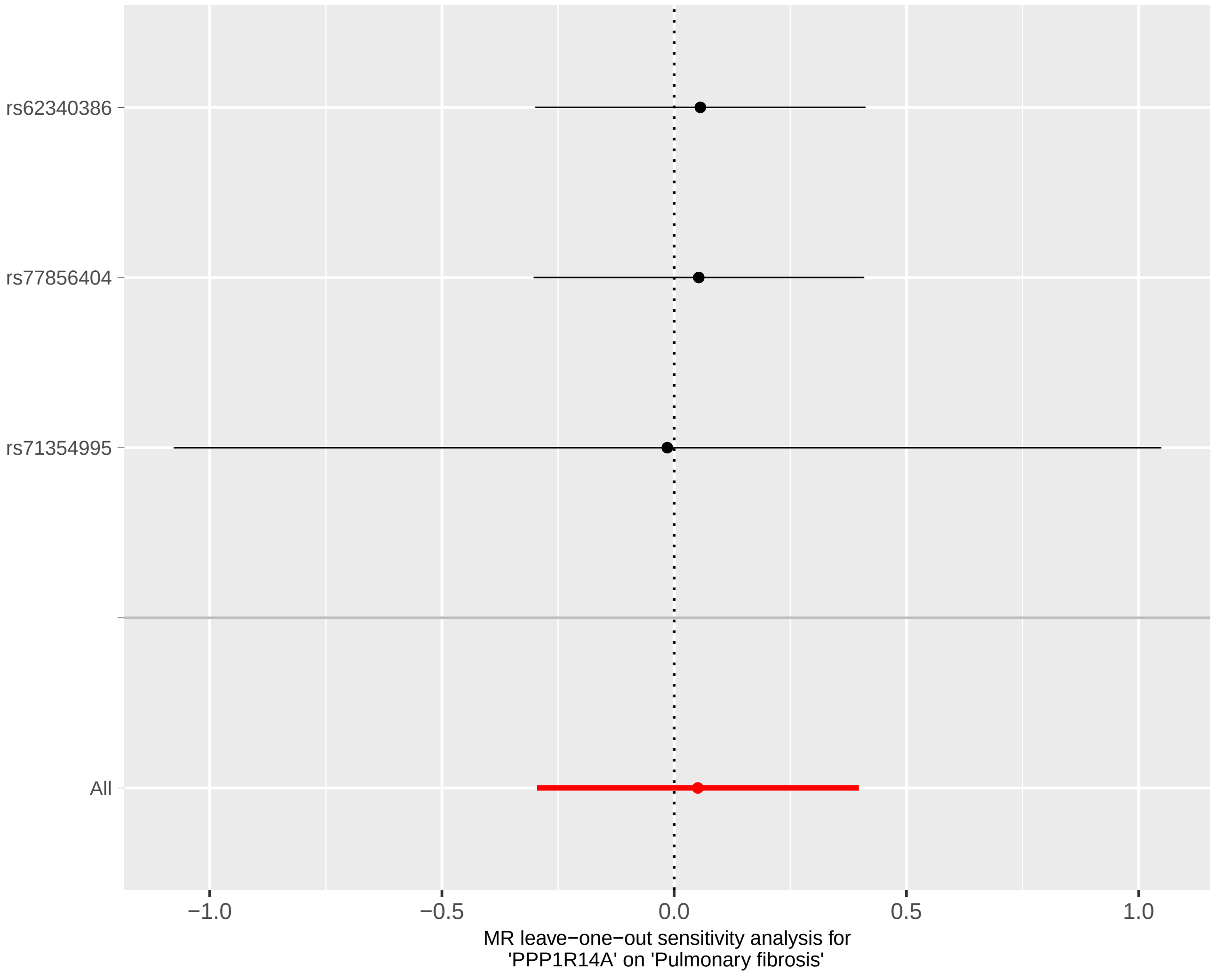
rs62340386
rs77856404
rs71354995
All
−1.0
−0.5
0.0
0.5
1.0
MR leave−one−out sensitivity analysis for
'PPP1R14A' on 'Pulmonary fibrosis'

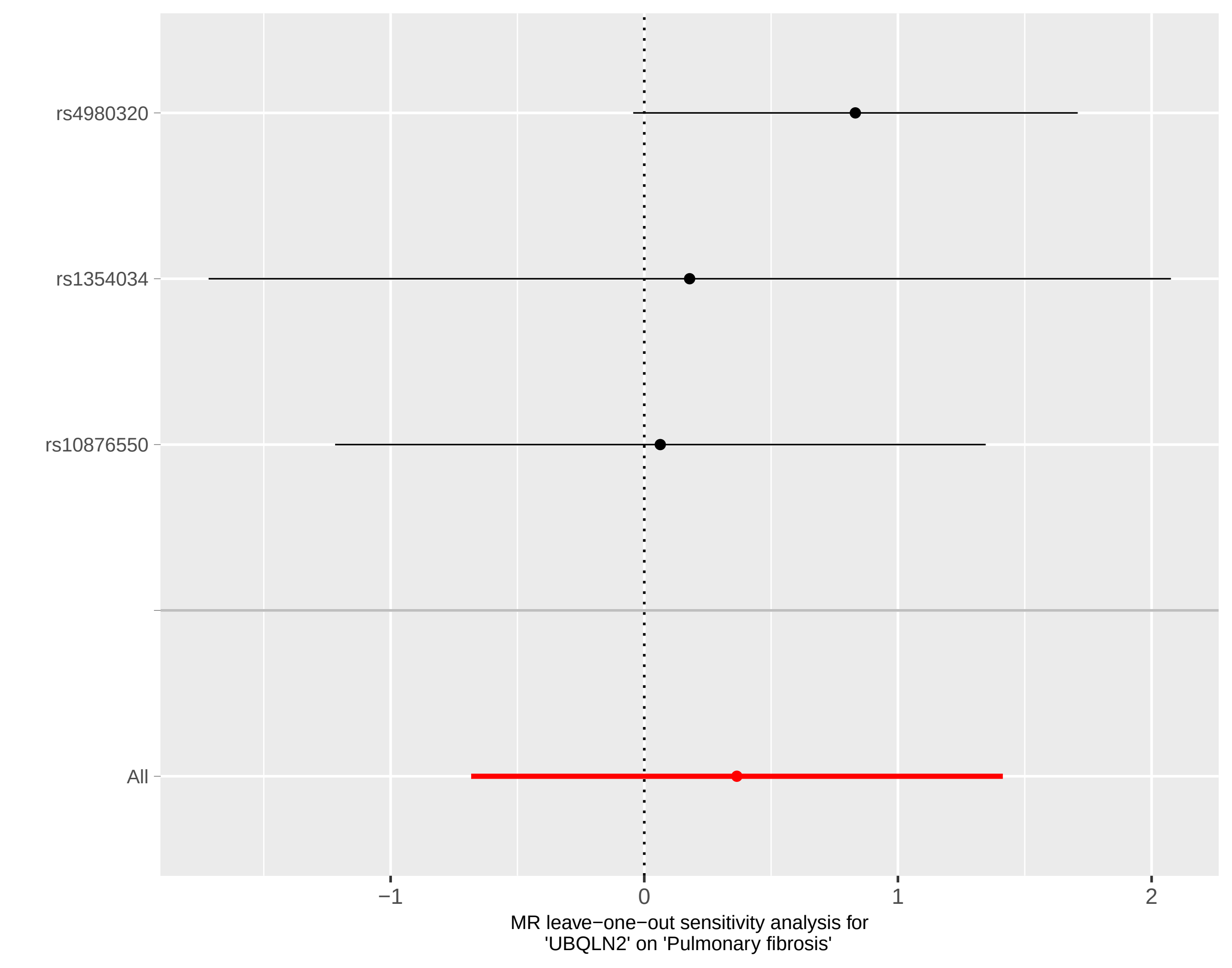
rs4980320
rs1354034
rs10876550
All
−1
0
1
2
MR leave−one−out sensitivity analysis for
'UBQLN2' on 'Pulmonary fibrosis'

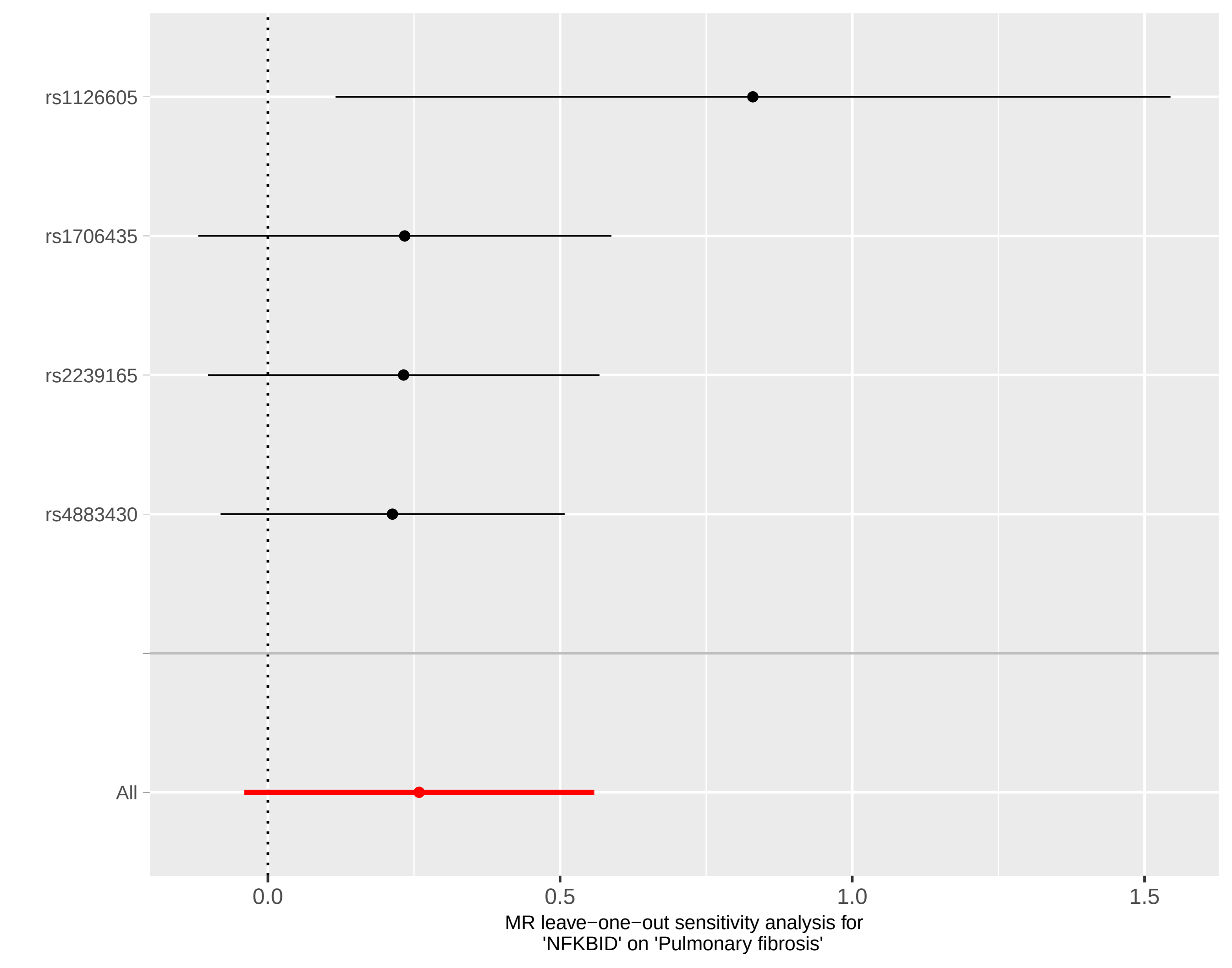
rs1126605
rs1706435
rs2239165
rs4883430
All
0.0
0.5
1.0
1.5
MR leave−one−out sensitivity analysis for
'NFKBID' on 'Pulmonary fibrosis'

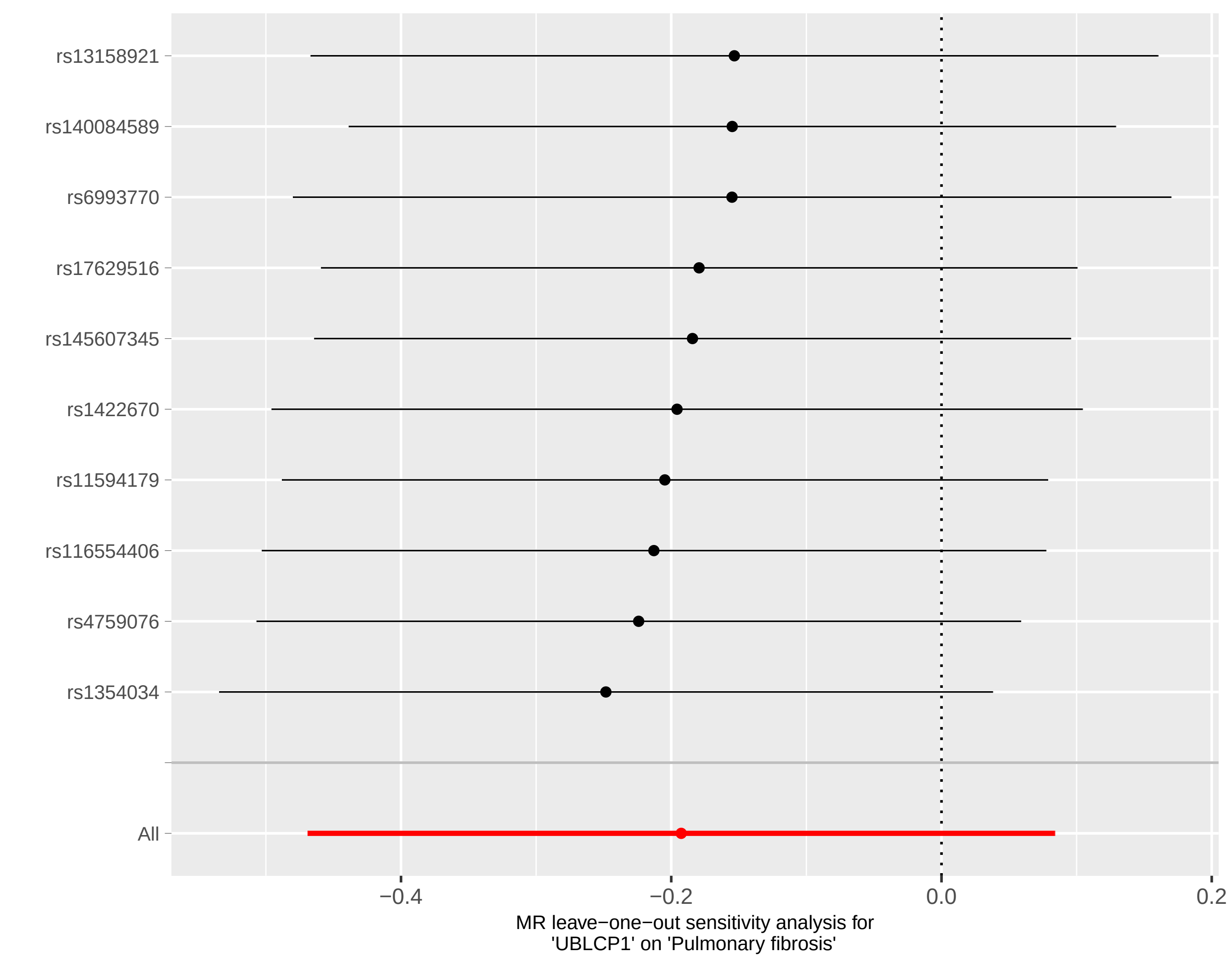
rs13158921
rs140084589
rs6993770
rs17629516
rs145607345
rs1422670
rs11594179
rs116554406
rs4759076
rs1354034
All
−0.4
−0.2
0.0
0.2
MR leave−one−out sensitivity analysis for
'UBLCP1' on 'Pulmonary fibrosis'

Insufficient number of SNPs

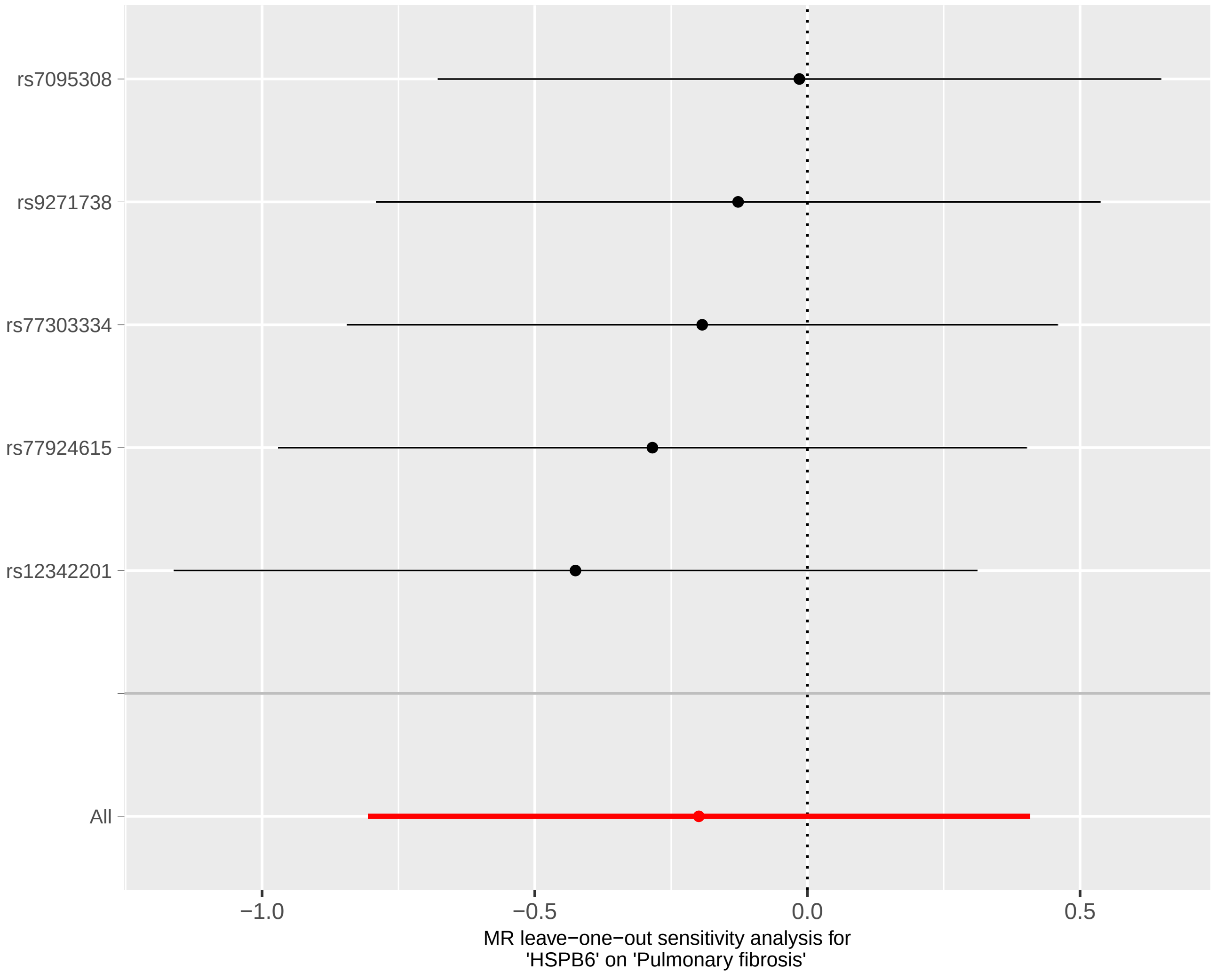
rs7095308
rs9271738
rs77303334
rs77924615
rs12342201
All
−1.0
−0.5
0.0
0.5
MR leave−one−out sensitivity analysis for
'HSPB6' on 'Pulmonary fibrosis'

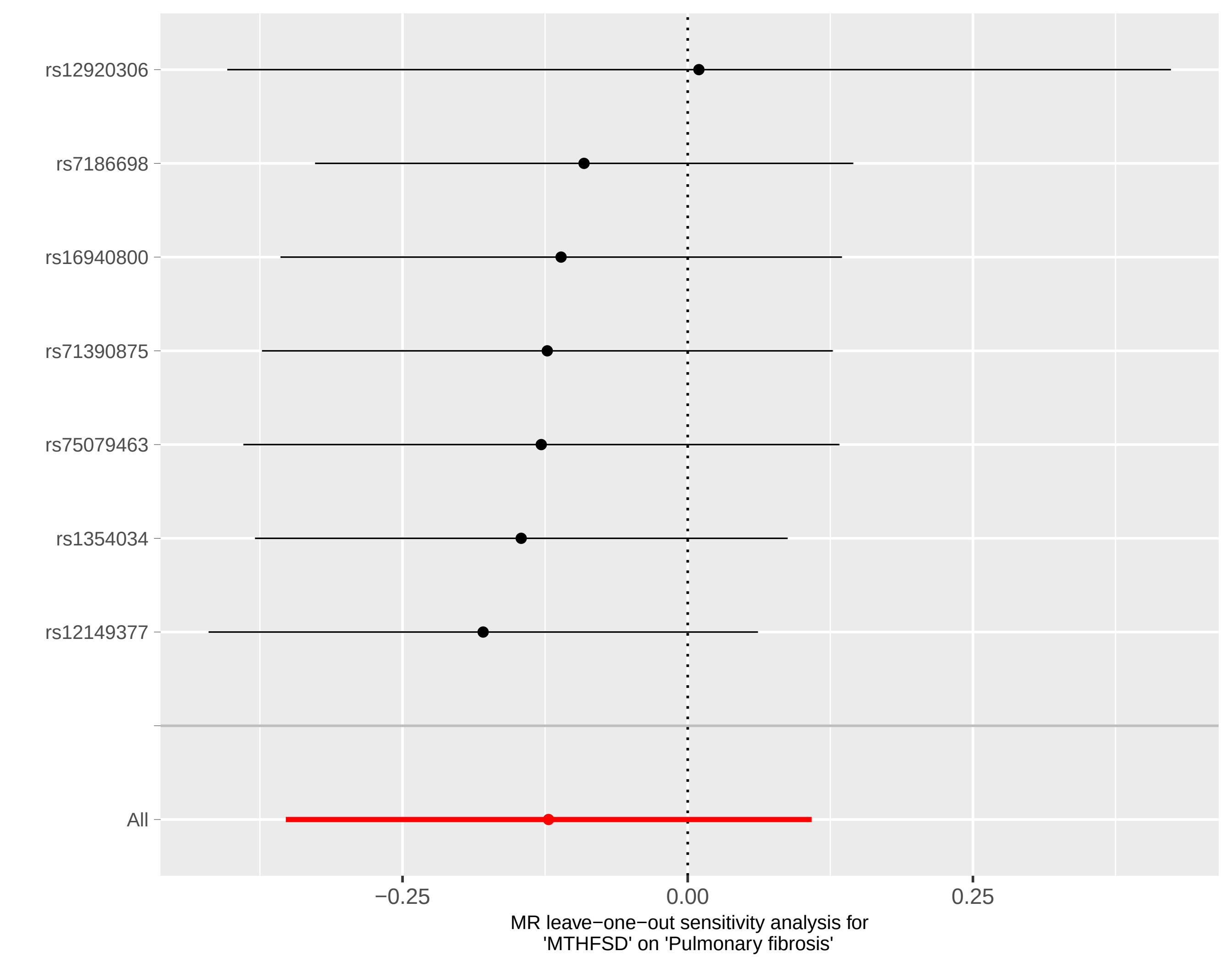
rs12920306
rs7186698
rs16940800
rs71390875
rs75079463
rs1354034
rs12149377
All
−0.25
0.00
0.25
MR leave−one−out sensitivity analysis for
'MTHFSD' on 'Pulmonary fibrosis'

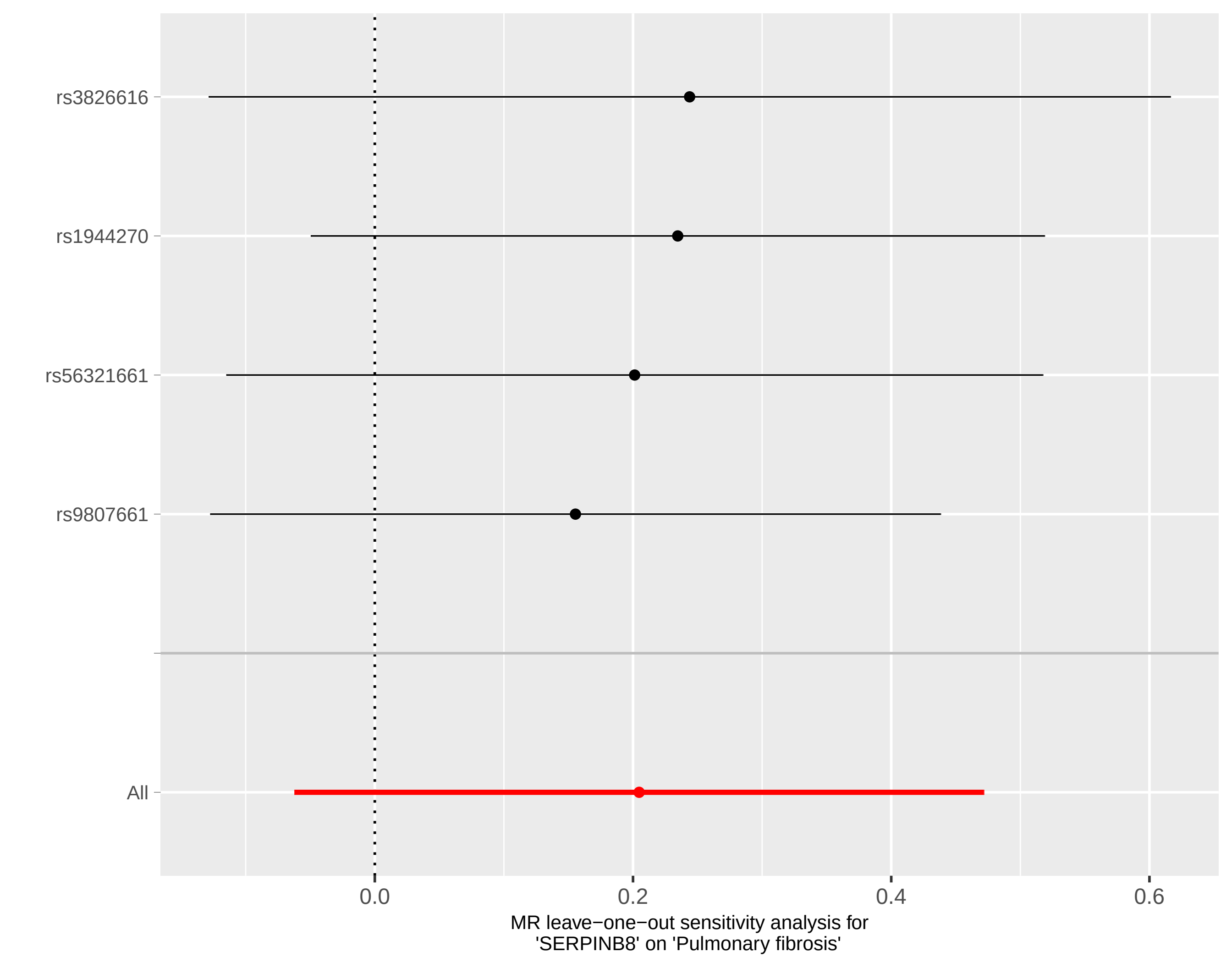
rs3826616
rs1944270
rs56321661
rs9807661
All
0.0
0.2
0.4
0.6
MR leave−one−out sensitivity analysis for
'SERPINB8' on 'Pulmonary fibrosis'

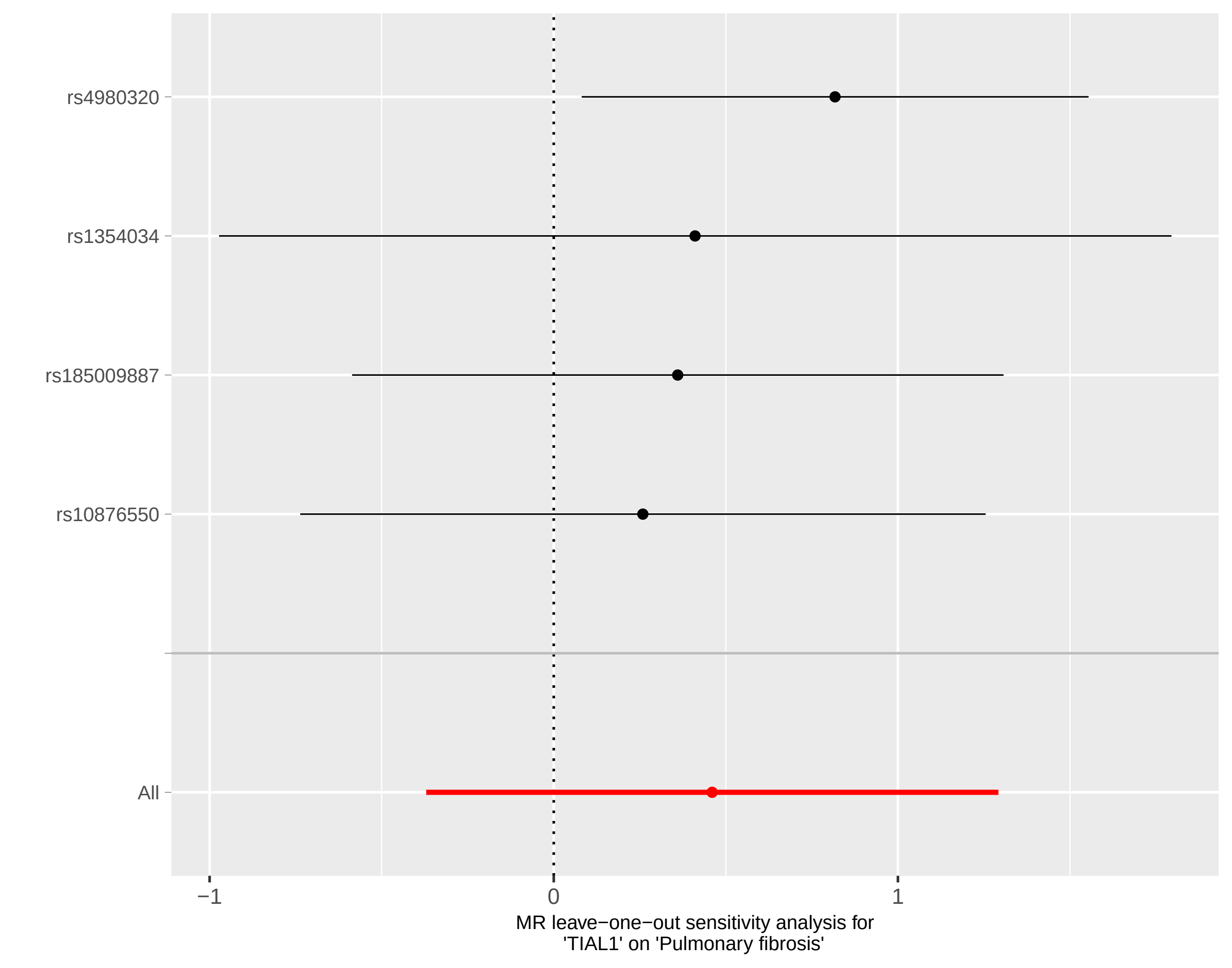
rs4980320
rs1354034
rs185009887
rs10876550
All
−1
0
1
MR leave−one−out sensitivity analysis for
'TIAL1' on 'Pulmonary fibrosis'

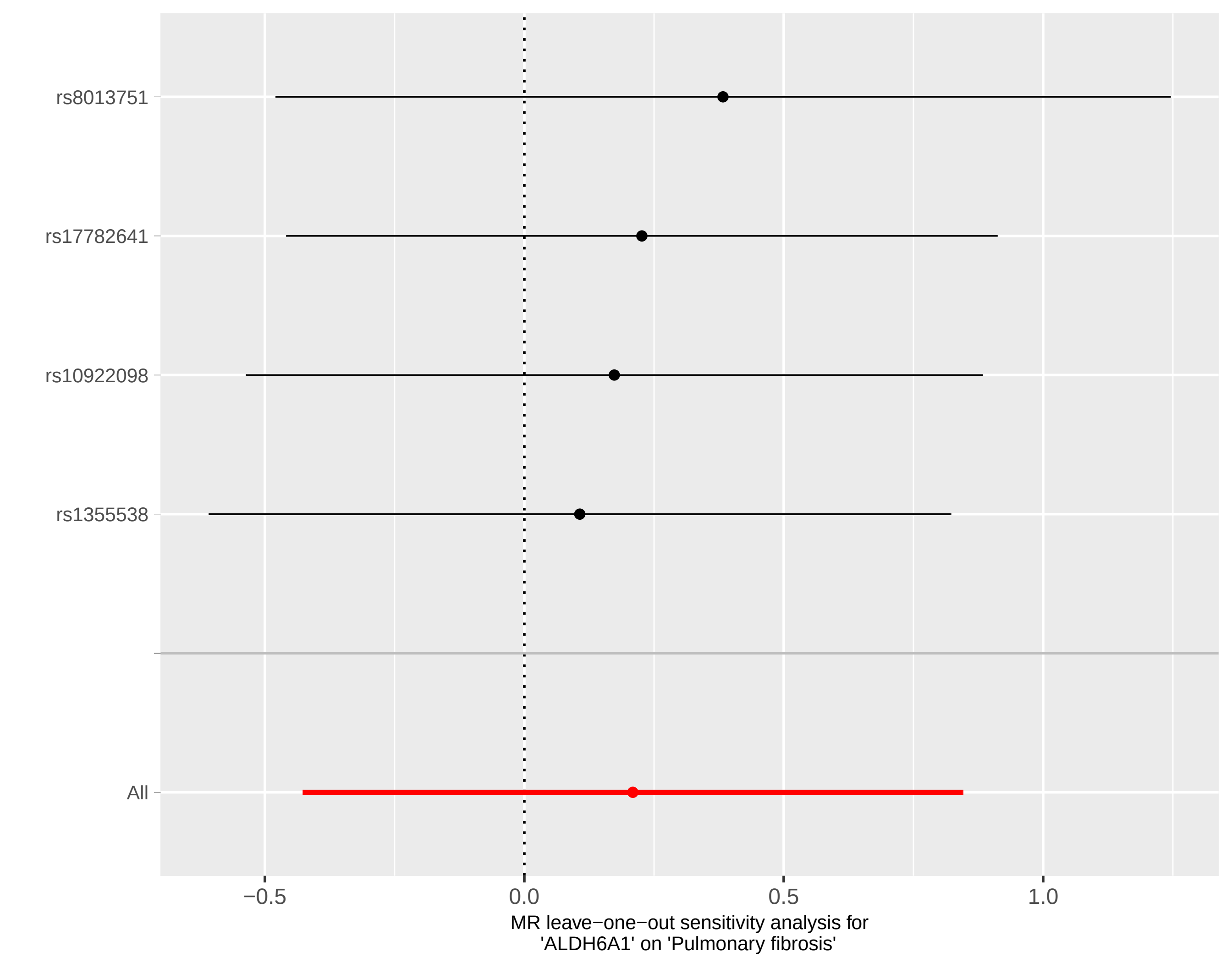
rs8013751
rs17782641
rs10922098
rs1355538
All
−0.5
0.0
0.5
1.0
MR leave−one−out sensitivity analysis for
'ALDH6A1' on 'Pulmonary fibrosis'

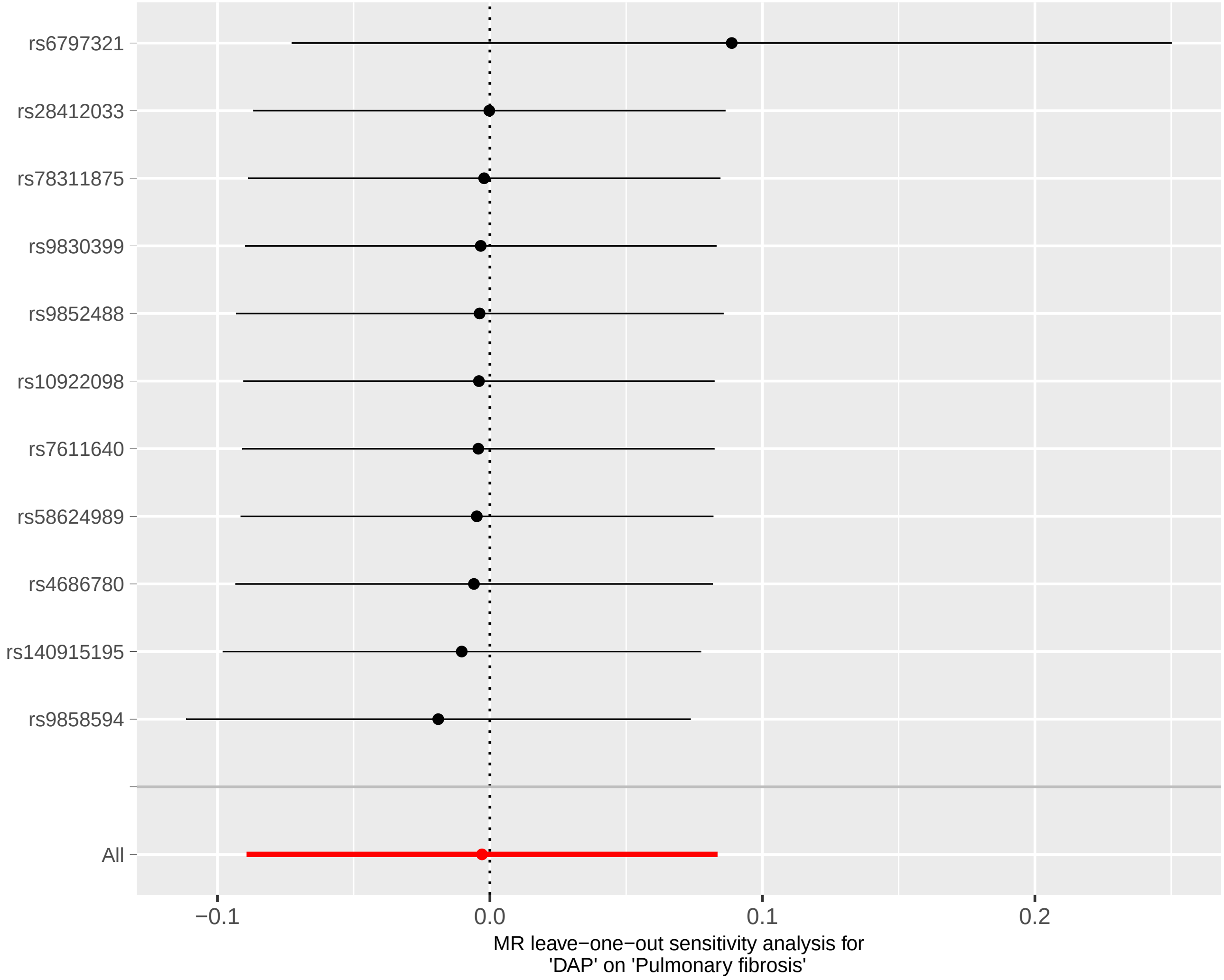
rs6797321
rs28412033
rs78311875
rs9830399
rs9852488
rs10922098
rs7611640
rs58624989
rs4686780
rs140915195
rs9858594
All
−0.1
0.0
0.1
0.2
MR leave−one−out sensitivity analysis for
'DAP' on 'Pulmonary fibrosis'

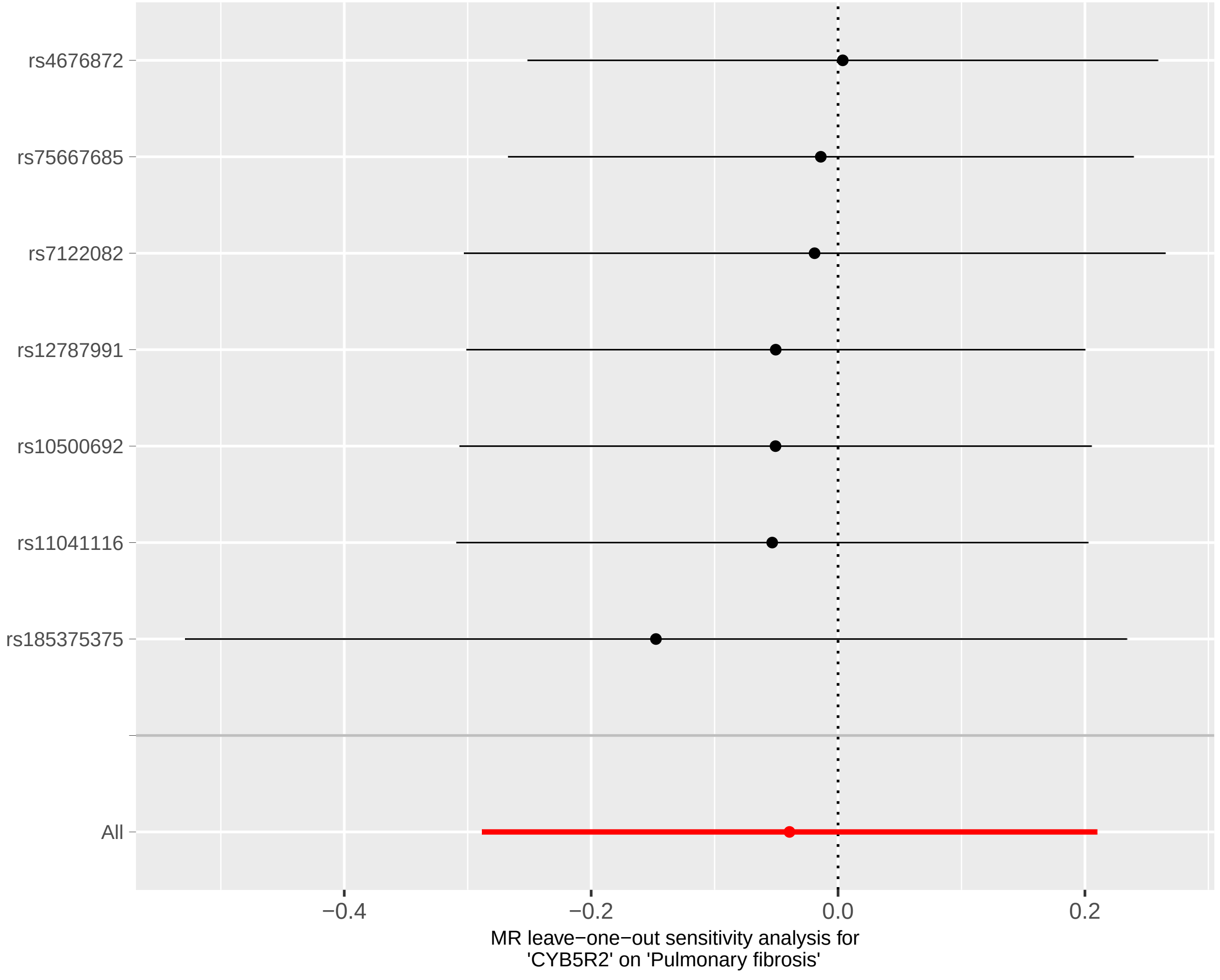
rs4676872
rs75667685
rs7122082
rs12787991
rs10500692
rs11041116
rs185375375
All
−0.4
−0.2
0.0
0.2
MR leave−one−out sensitivity analysis for
'CYB5R2' on 'Pulmonary fibrosis'

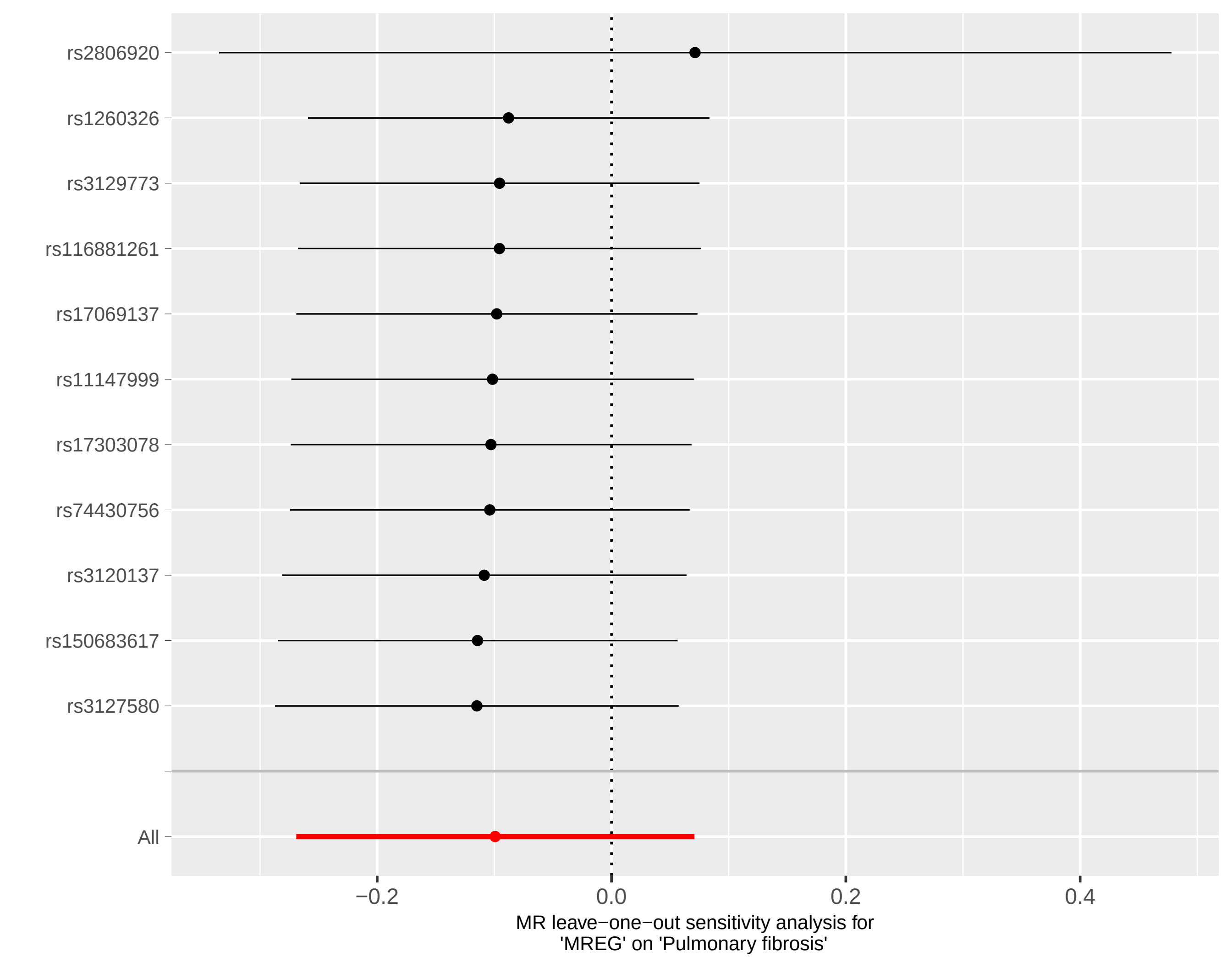
rs2806920
rs1260326
rs3129773
rs116881261
rs17069137
rs11147999
rs17303078
rs74430756
rs3120137
rs150683617
rs3127580
All
−0.2
0.0
0.2
0.4
MR leave−one−out sensitivity analysis for
'MREG' on 'Pulmonary fibrosis'

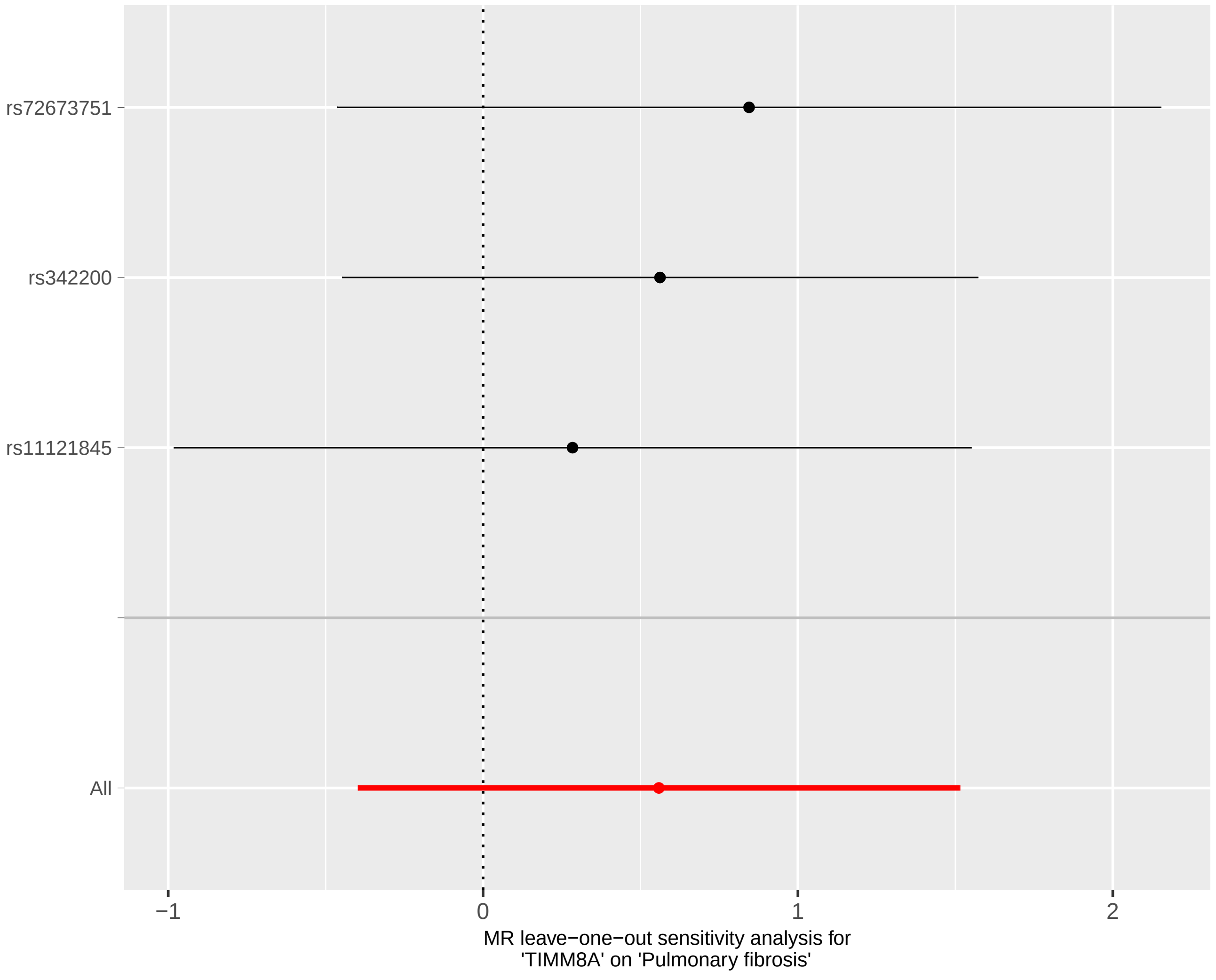

rs72673751
rs342200
rs11121845
All
−1
0
1
2
MR leave−one−out sensitivity analysis for
'TIMM8A' on 'Pulmonary fibrosis'

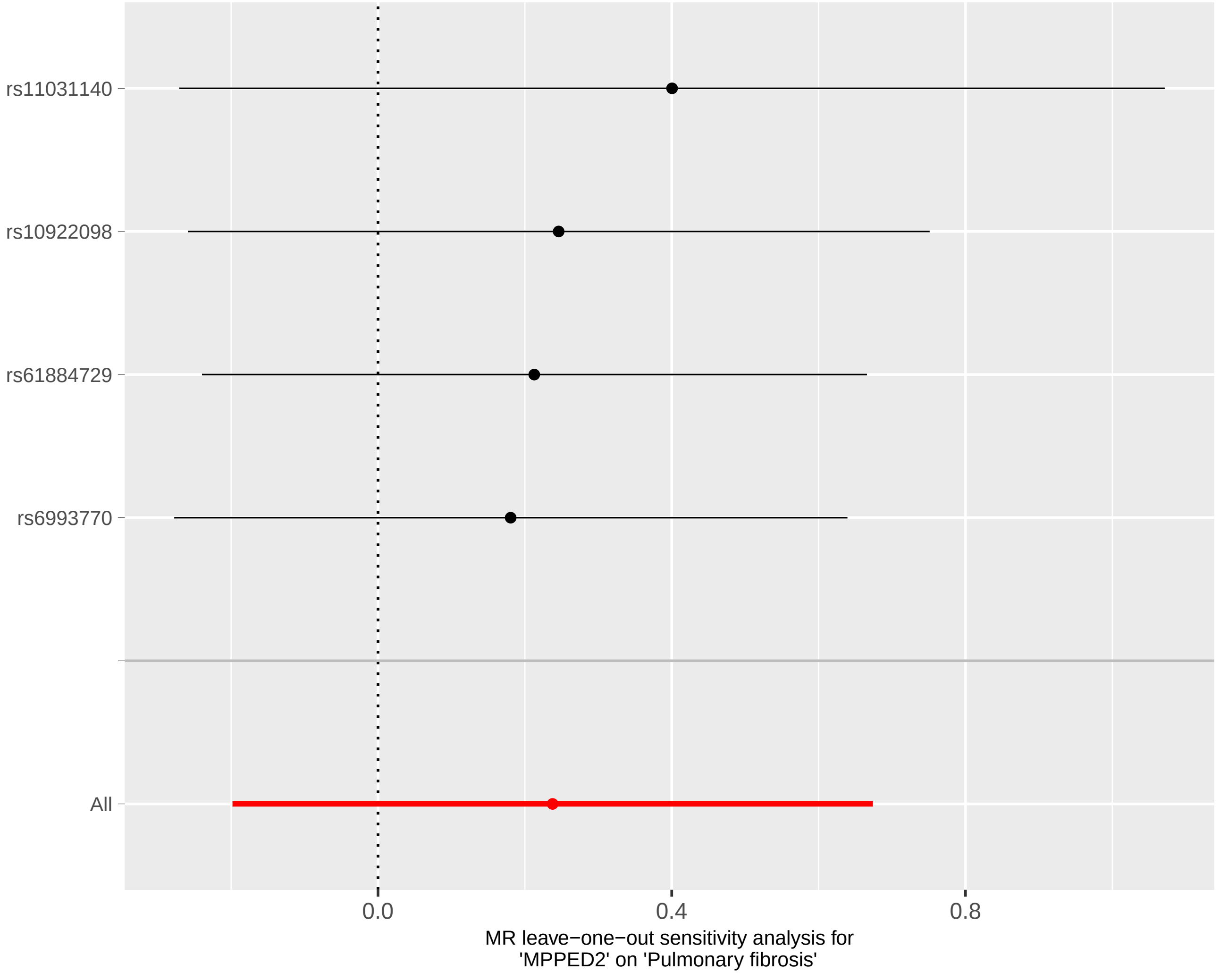
rs11031140
rs10922098
rs61884729
rs6993770
All
0.0
0.4
0.8
MR leave−one−out sensitivity analysis for
'MPPED2' on 'Pulmonary fibrosis'

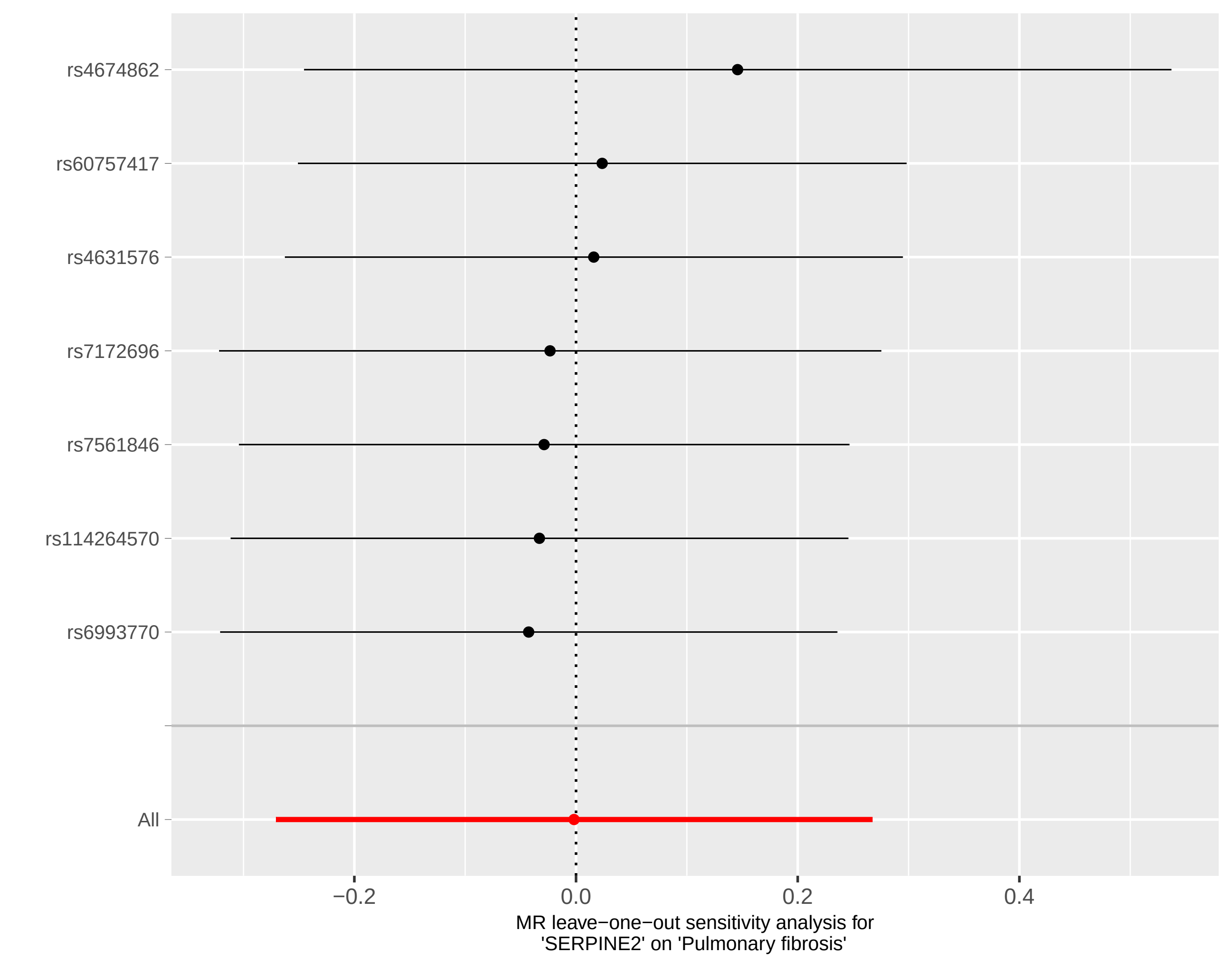
rs4674862
rs60757417
rs4631576
rs7172696
rs7561846
rs114264570
rs6993770
All
−0.2
0.0
0.2
0.4
MR leave−one−out sensitivity analysis for
'SERPINE2' on 'Pulmonary fibrosis'

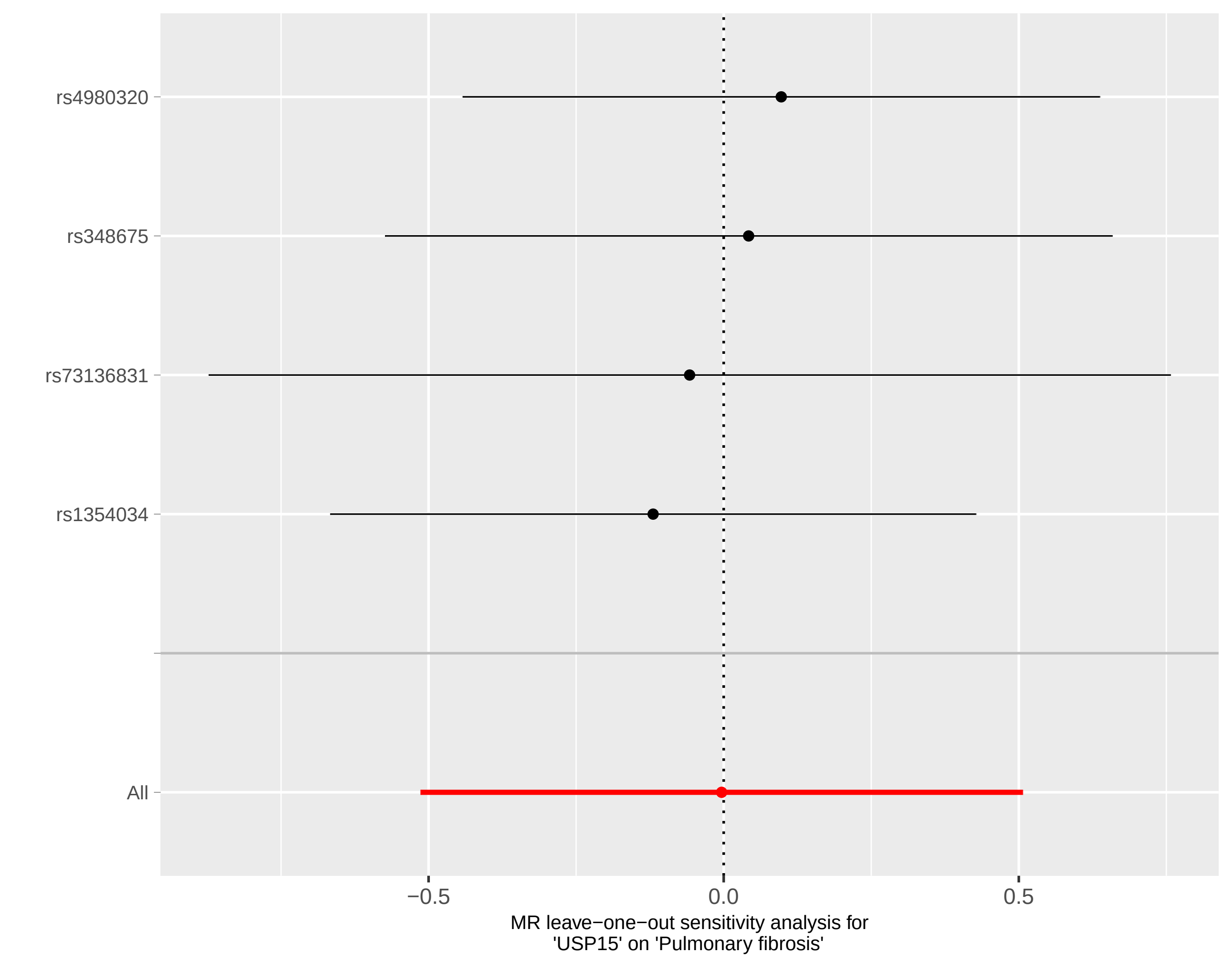
rs4980320
rs348675
rs73136831
rs1354034
All
−0.5
0.0
0.5
MR leave−one−out sensitivity analysis for
'USP15' on 'Pulmonary fibrosis'

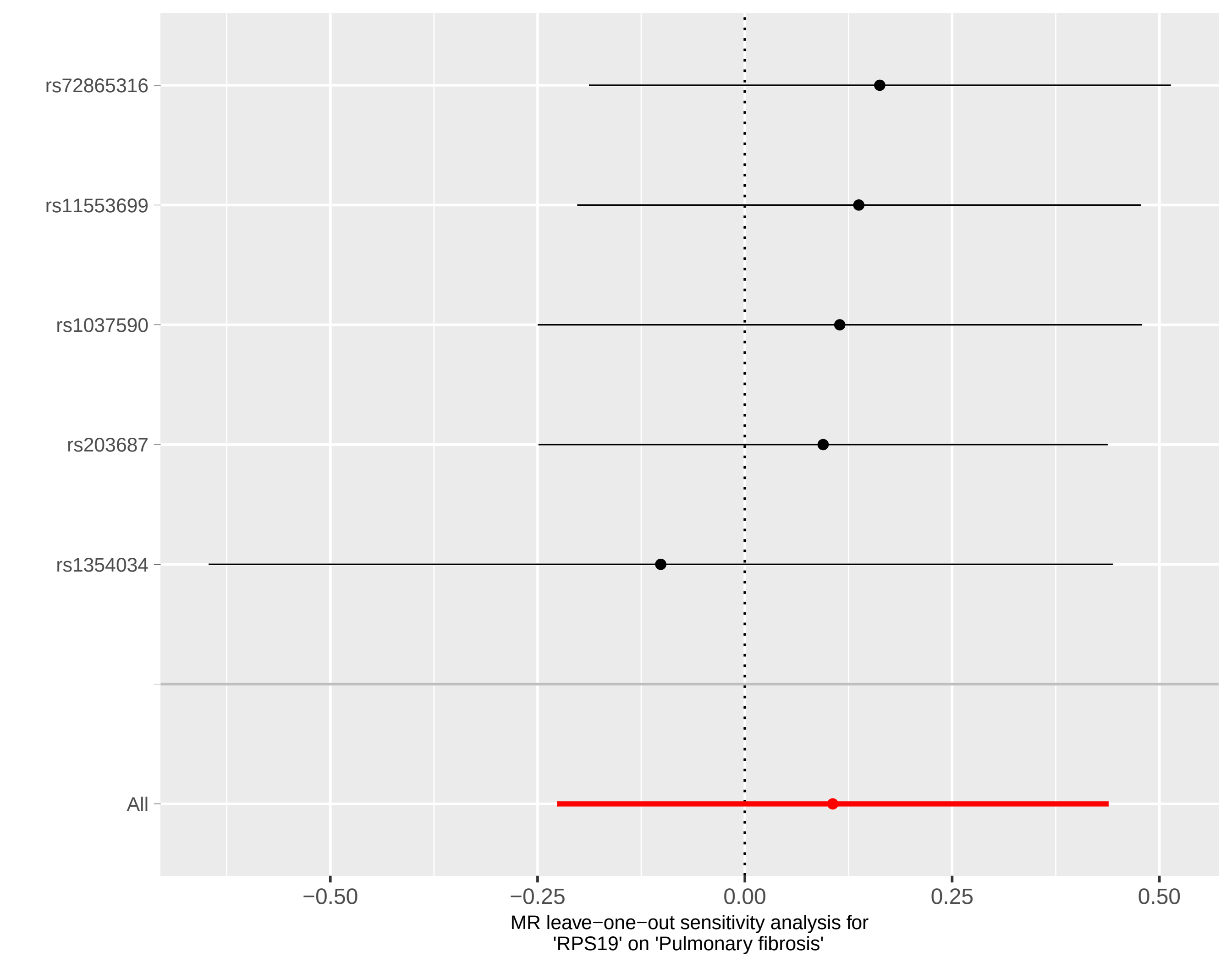
rs72865316
rs11553699
rs1037590
rs203687
rs1354034
All
−0.50
−0.25
0.00
0.25
0.50
MR leave−one−out sensitivity analysis for
'RPS19' on 'Pulmonary fibrosis'

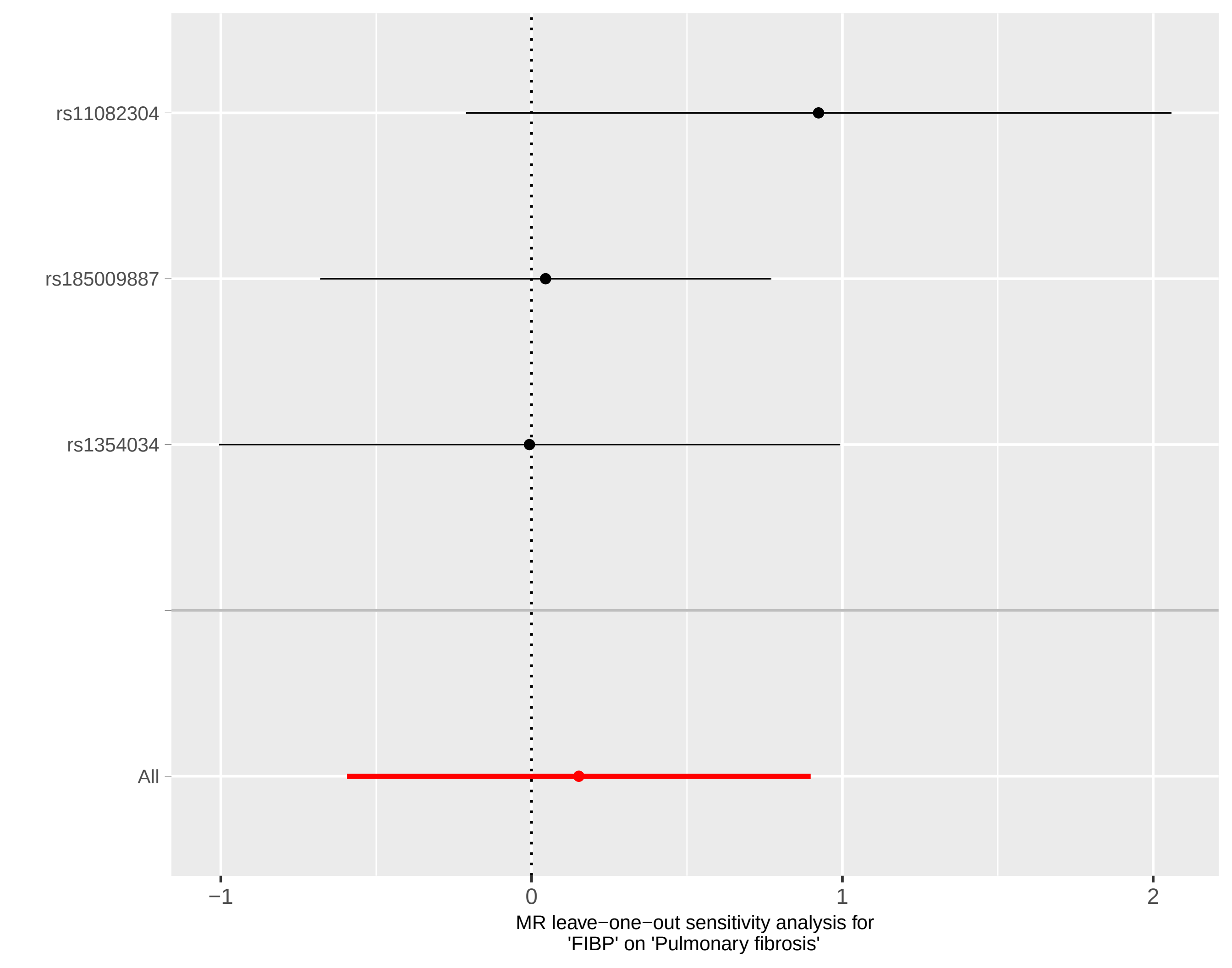

rs11082304
rs185009887
rs1354034
All
−1
0
1
2
MR leave−one−out sensitivity analysis for
'FIBP' on 'Pulmonary fibrosis'

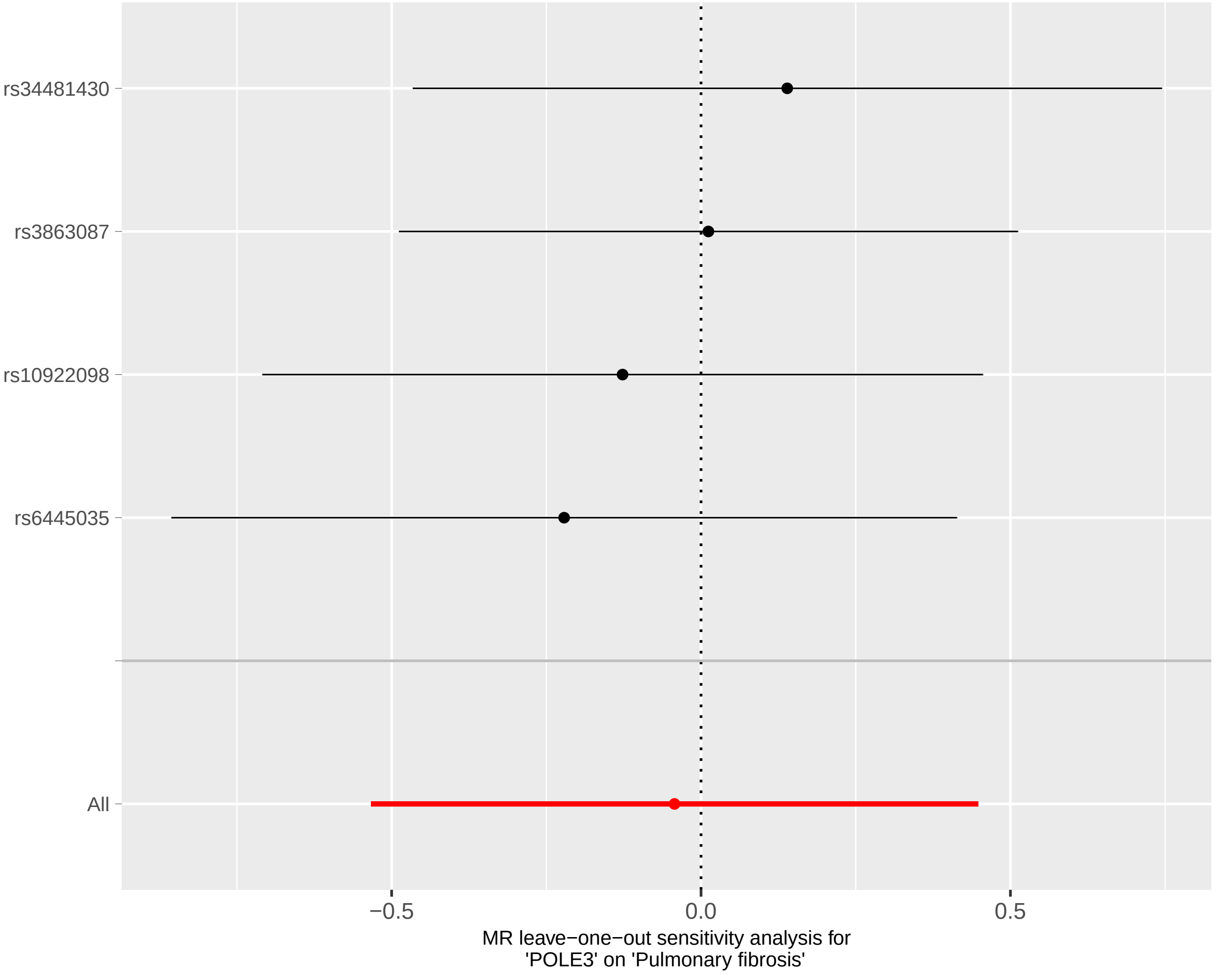
rs34481430
rs3863087
rs10922098
rs6445035
All
−0.5
0.0
0.5
MR leave−one−out sensitivity analysis for
'POLE3' on 'Pulmonary fibrosis'

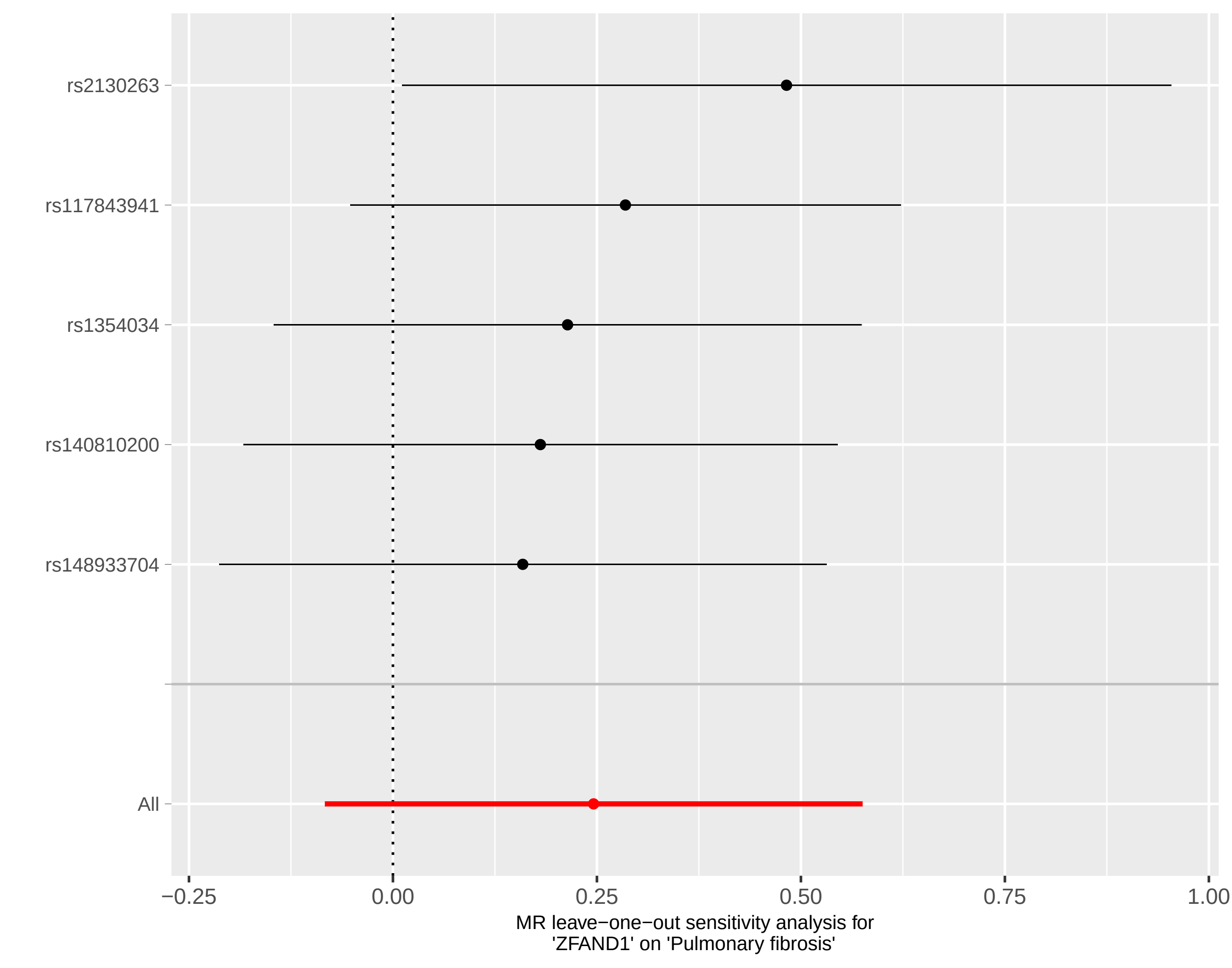
rs2130263
rs117843941
rs1354034
rs140810200
rs148933704
All
−0.25
0.00
0.25
0.50
0.75
1.00
MR leave−one−out sensitivity analysis for
'ZFAND1' on 'Pulmonary fibrosis'

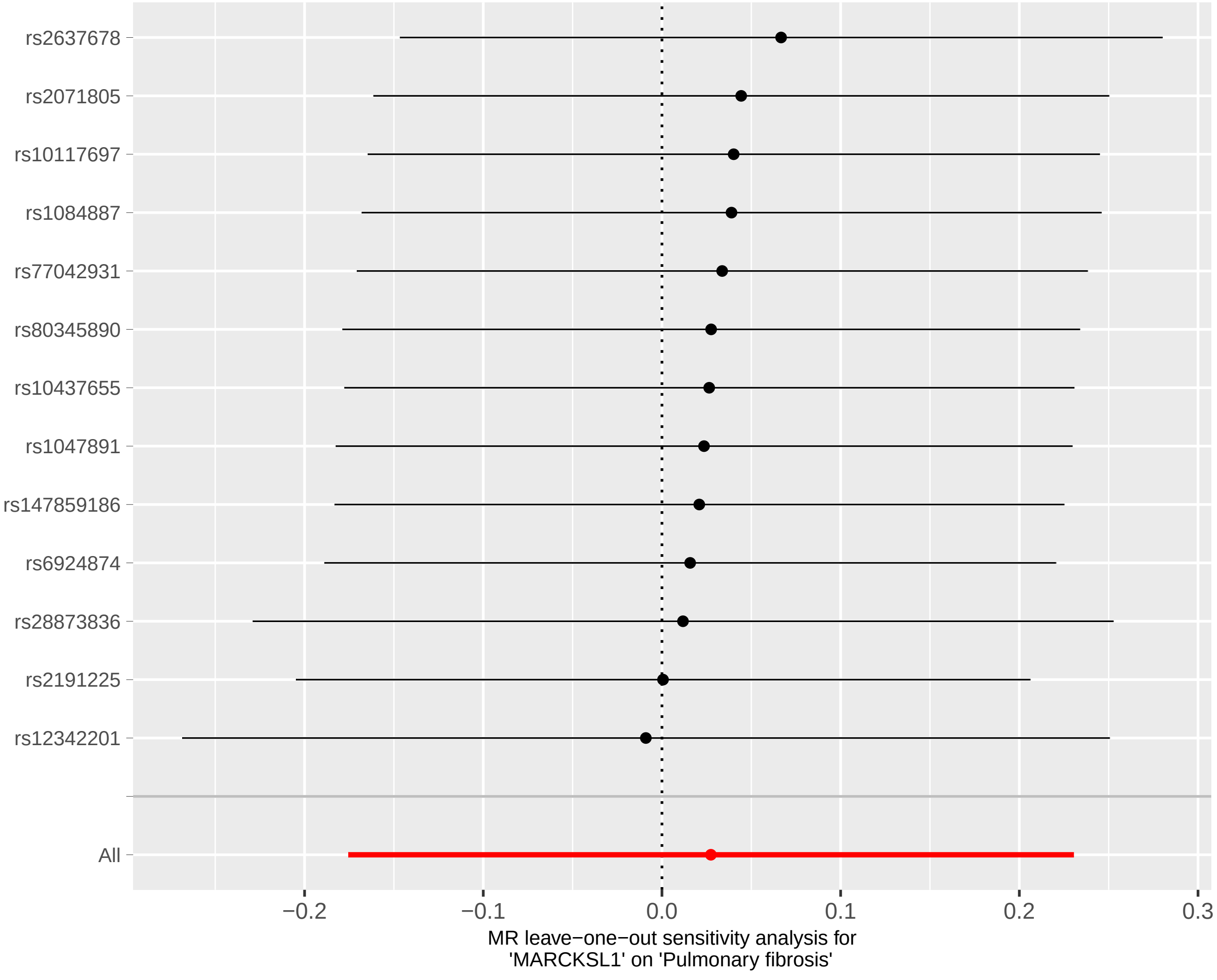
rs2637678
rs2071805
rs10117697
rs1084887
rs77042931
rs80345890
rs10437655
rs1047891
rs147859186
rs6924874
rs28873836
rs2191225
rs12342201
All
−0.2
−0.1
0.0
0.1
0.2
0.3
MR leave−one−out sensitivity analysis for
'MARCKSL1' on 'Pulmonary fibrosis'

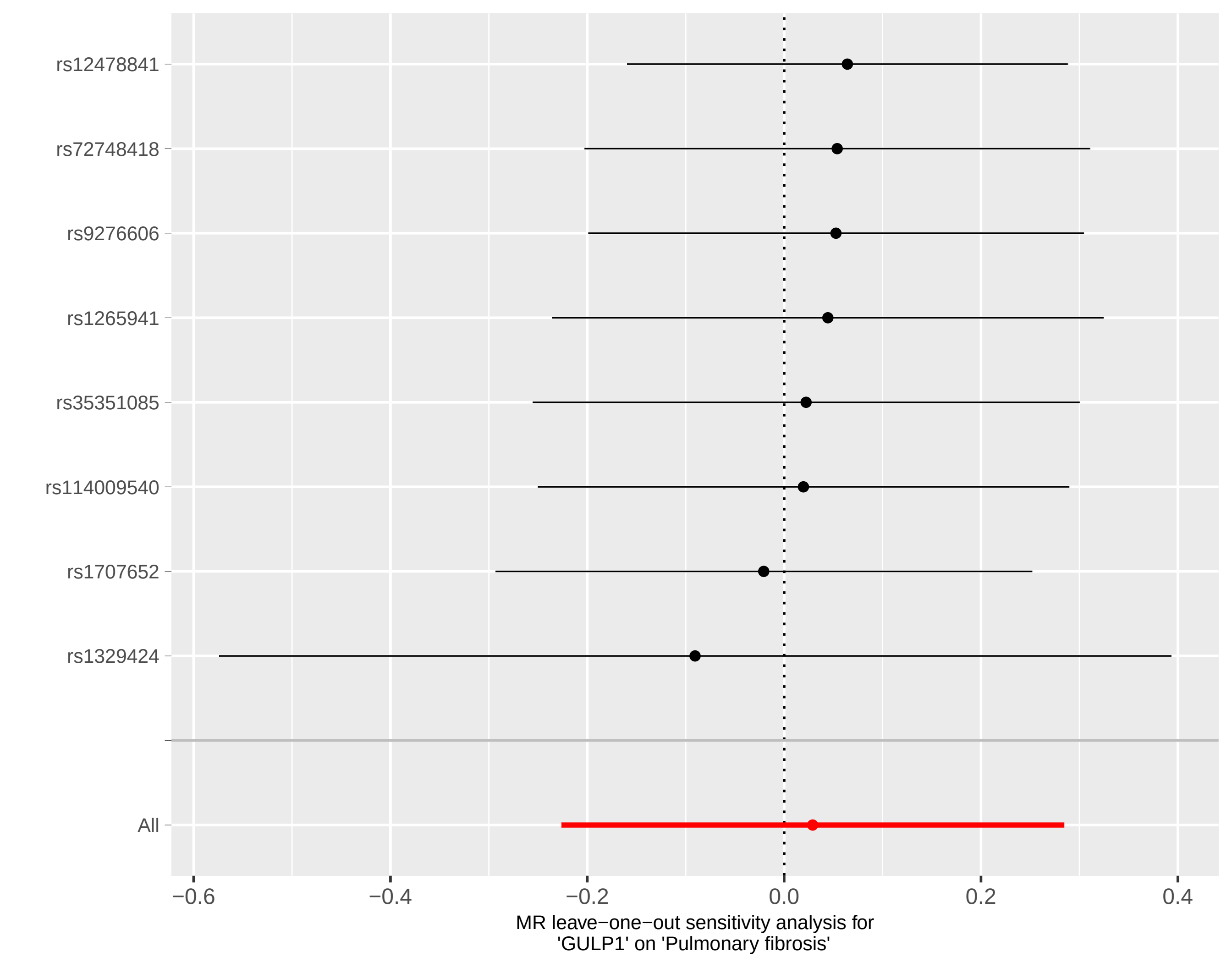
rs12478841
rs72748418
rs9276606
rs1265941
rs35351085
rs114009540
rs1707652
rs1329424
All
−0.6
−0.4
−0.2
0.0
0.2
0.4
MR leave−one−out sensitivity analysis for
'GULP1' on 'Pulmonary fibrosis'

Insufficient number of SNPs

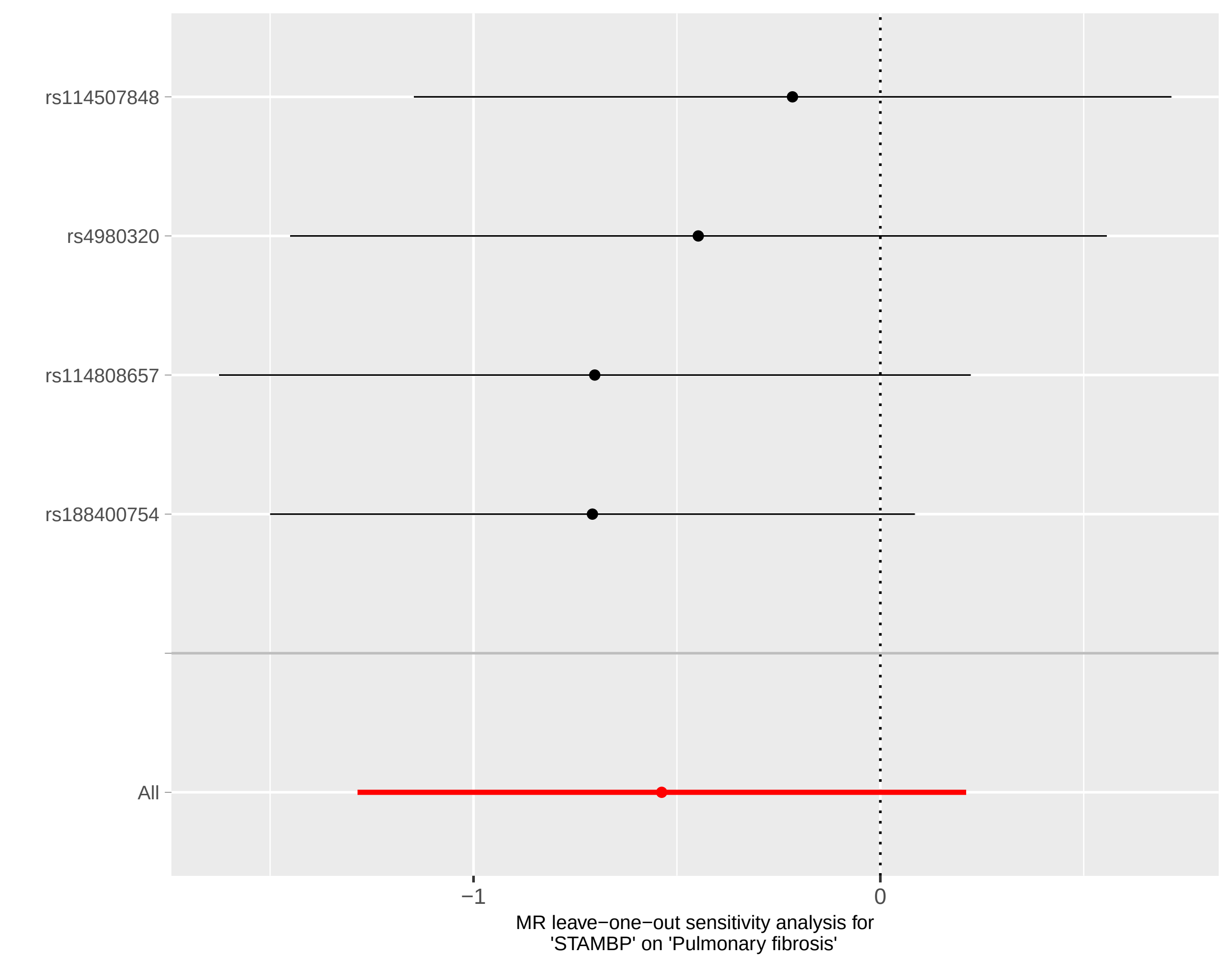
rs114507848
rs4980320
rs114808657
rs188400754
All
−1
0
MR leave−one−out sensitivity analysis for
'STAMBP' on 'Pulmonary fibrosis'

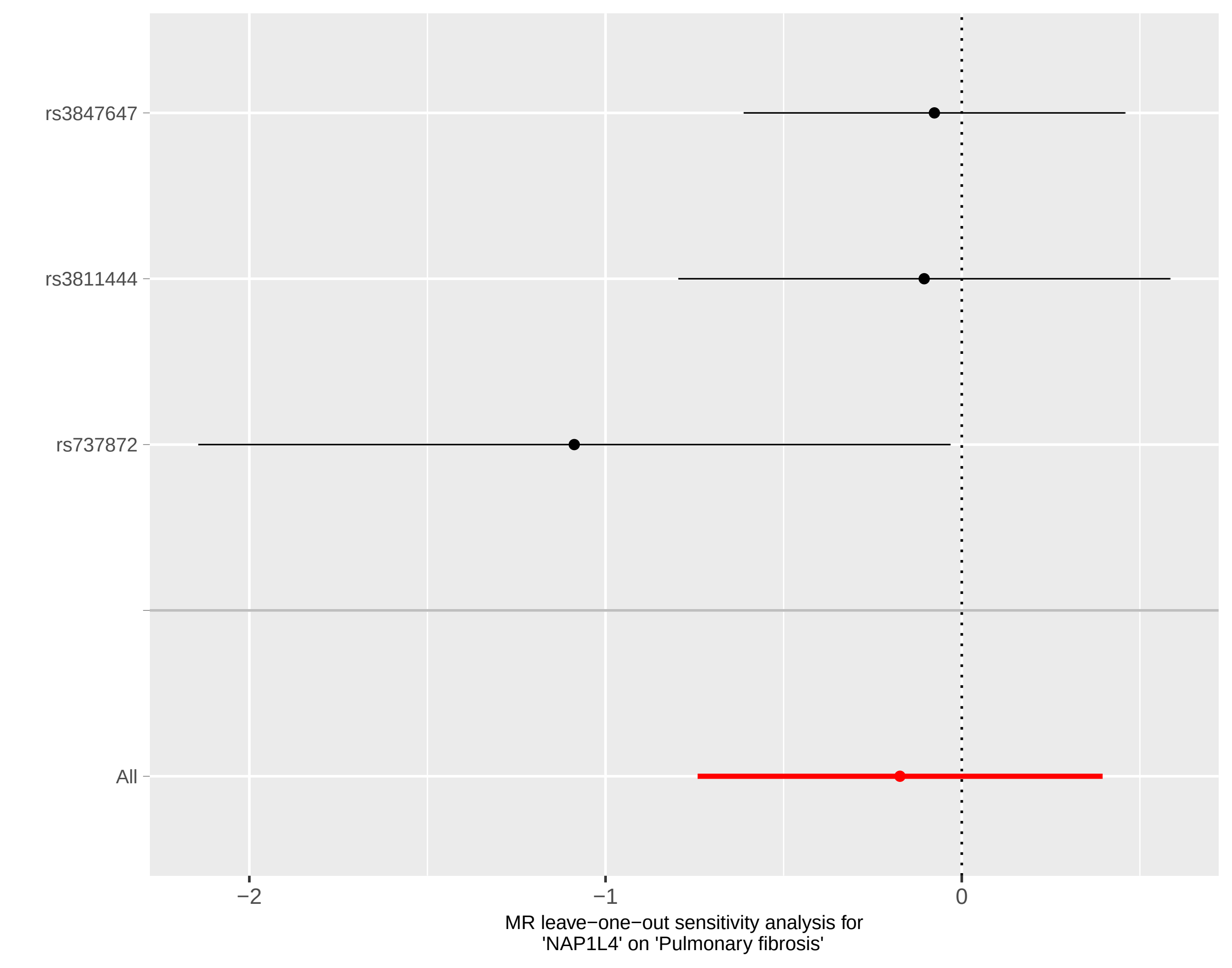
rs3847647
rs3811444
rs737872
All
−2
−1
0
MR leave−one−out sensitivity analysis for
'NAP1L4' on 'Pulmonary fibrosis'

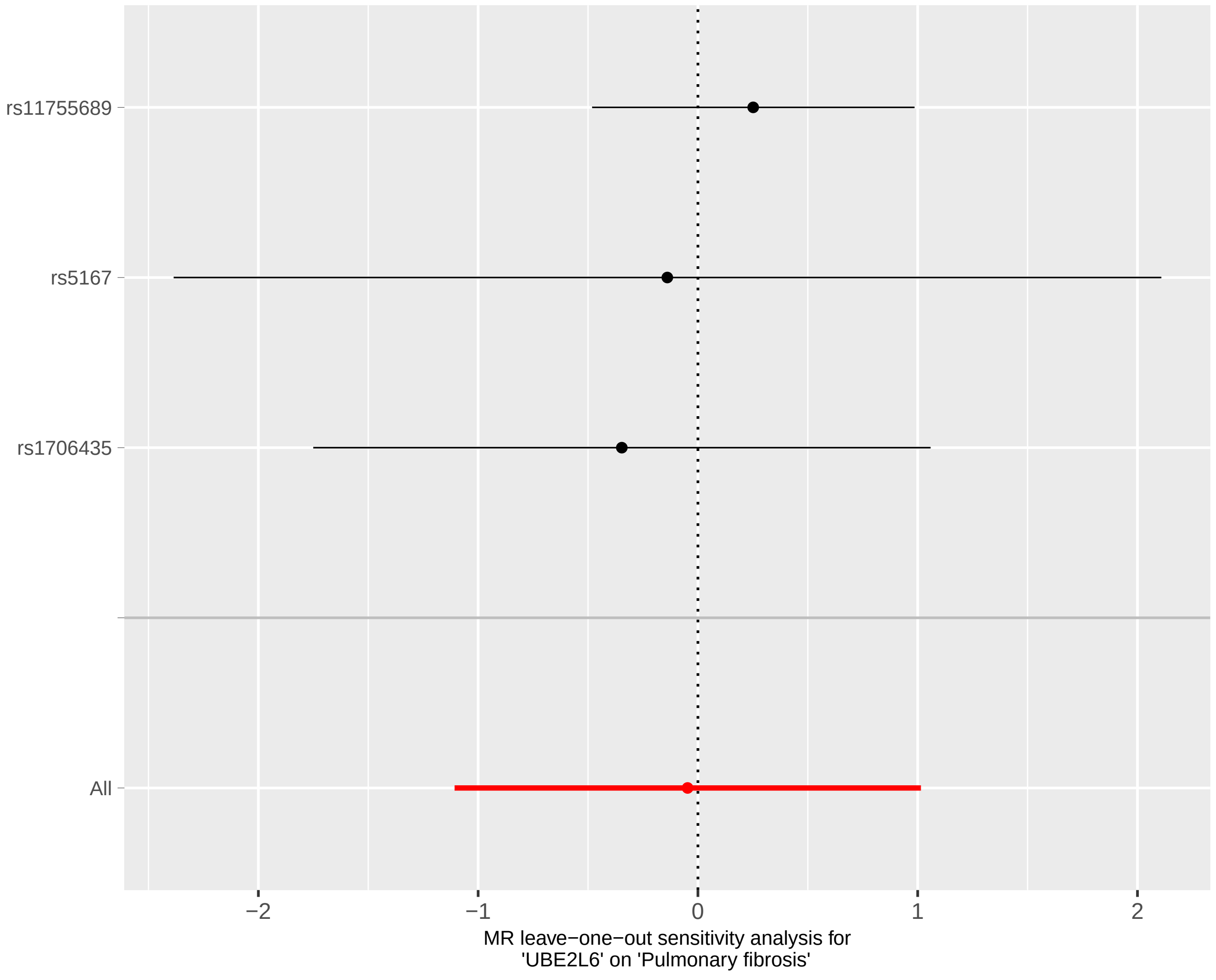
rs11755689
rs5167
rs1706435
All
−2
−1
0
1
2
MR leave−one−out sensitivity analysis for
'UBE2L6' on 'Pulmonary fibrosis'

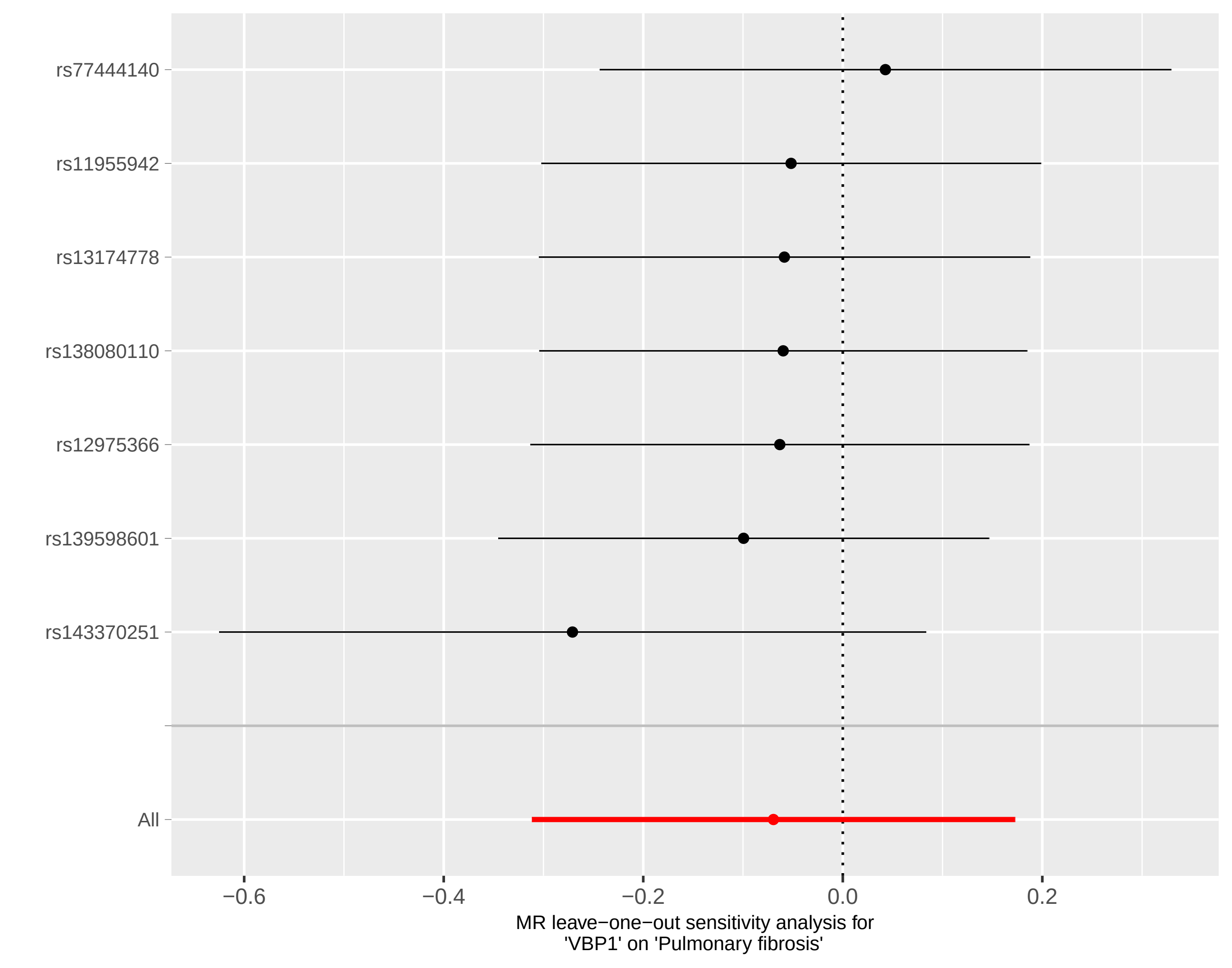
rs77444140
rs11955942
rs13174778
rs138080110
rs12975366
rs139598601
rs143370251
All
−0.6
−0.4
−0.2
0.0
0.2
MR leave−one−out sensitivity analysis for
'VBP1' on 'Pulmonary fibrosis'

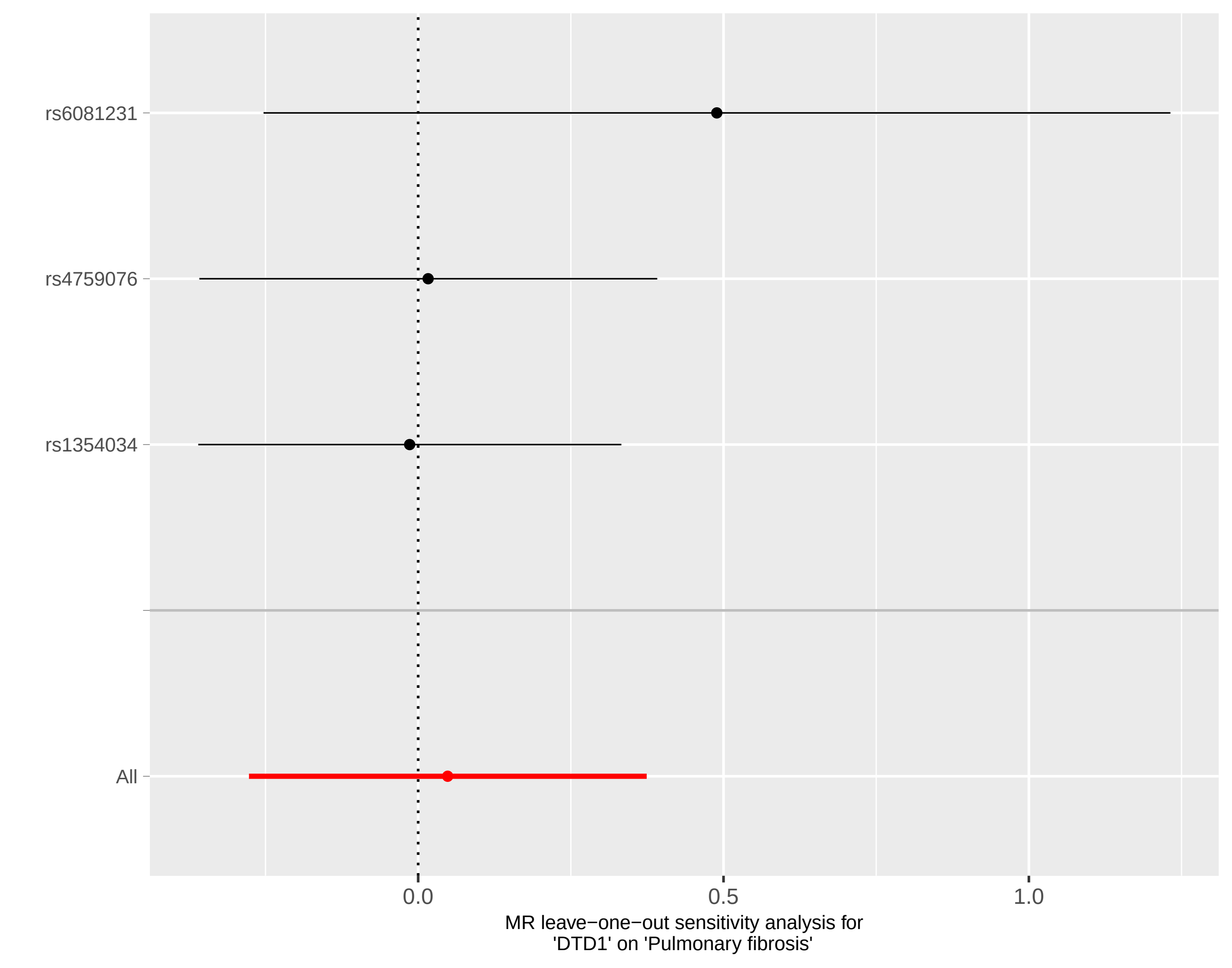
rs6081231
rs4759076
rs1354034
All
0.0
0.5
1.0
MR leave−one−out sensitivity analysis for
'DTD1' on 'Pulmonary fibrosis'

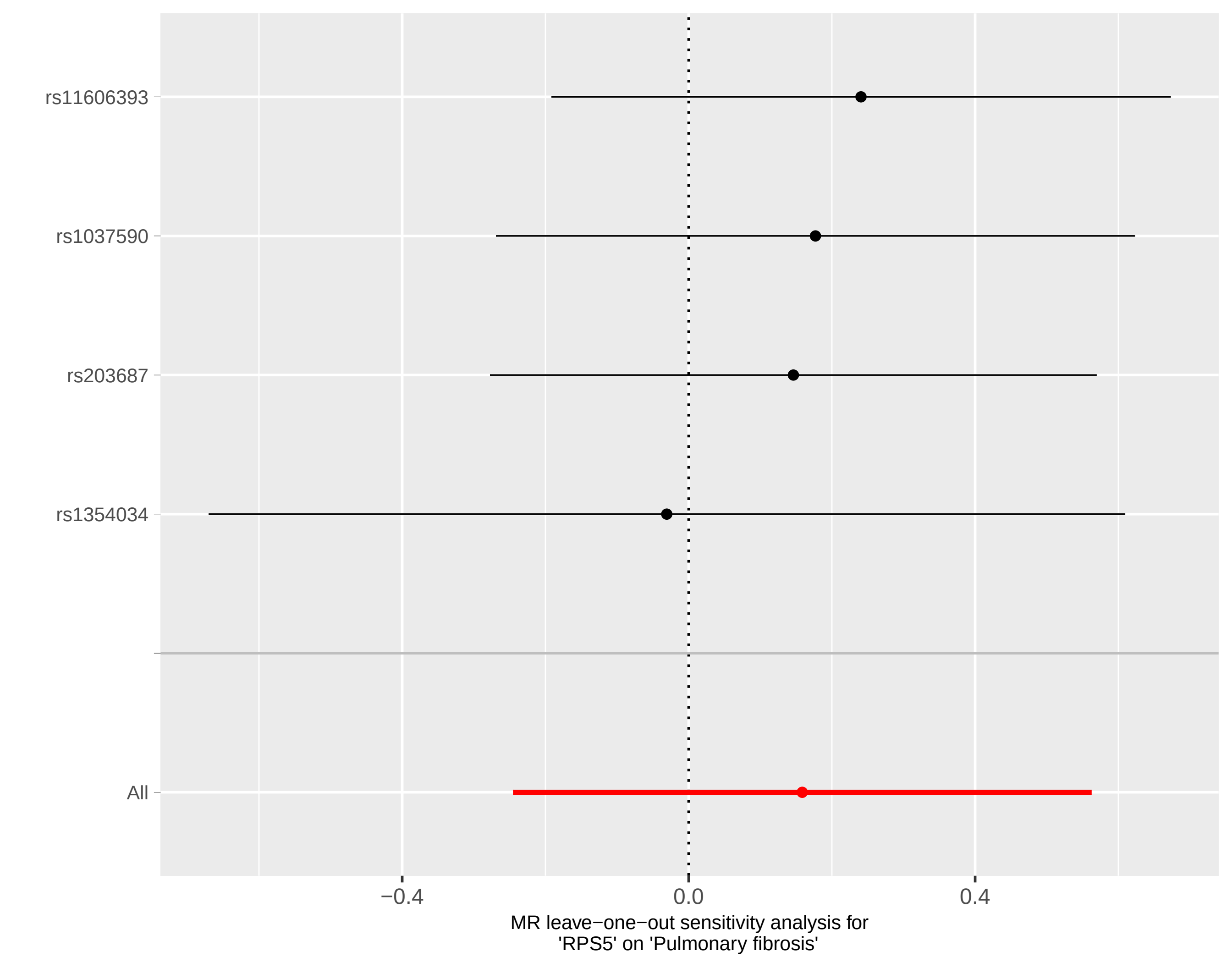
rs11606393
rs1037590
rs203687
rs1354034
All
−0.4
0.0
0.4
MR leave−one−out sensitivity analysis for
'RPS5' on 'Pulmonary fibrosis'

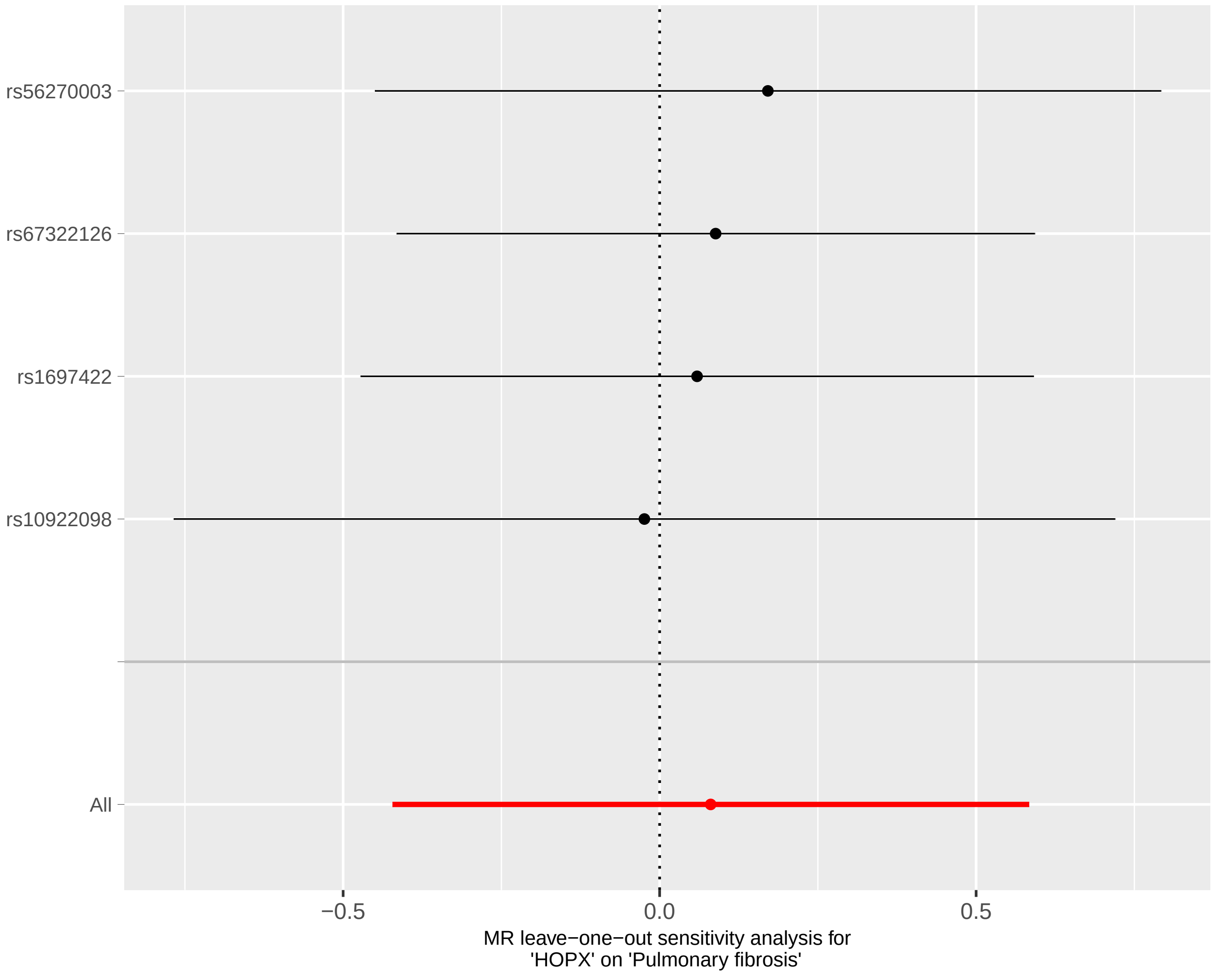

rs56270003
rs67322126
rs1697422
rs10922098
All
−0.5
0.0
0.5
MR leave−one−out sensitivity analysis for
'HOPX' on 'Pulmonary fibrosis'

Insufficient number of SNPs

Insufficient number of SNPs

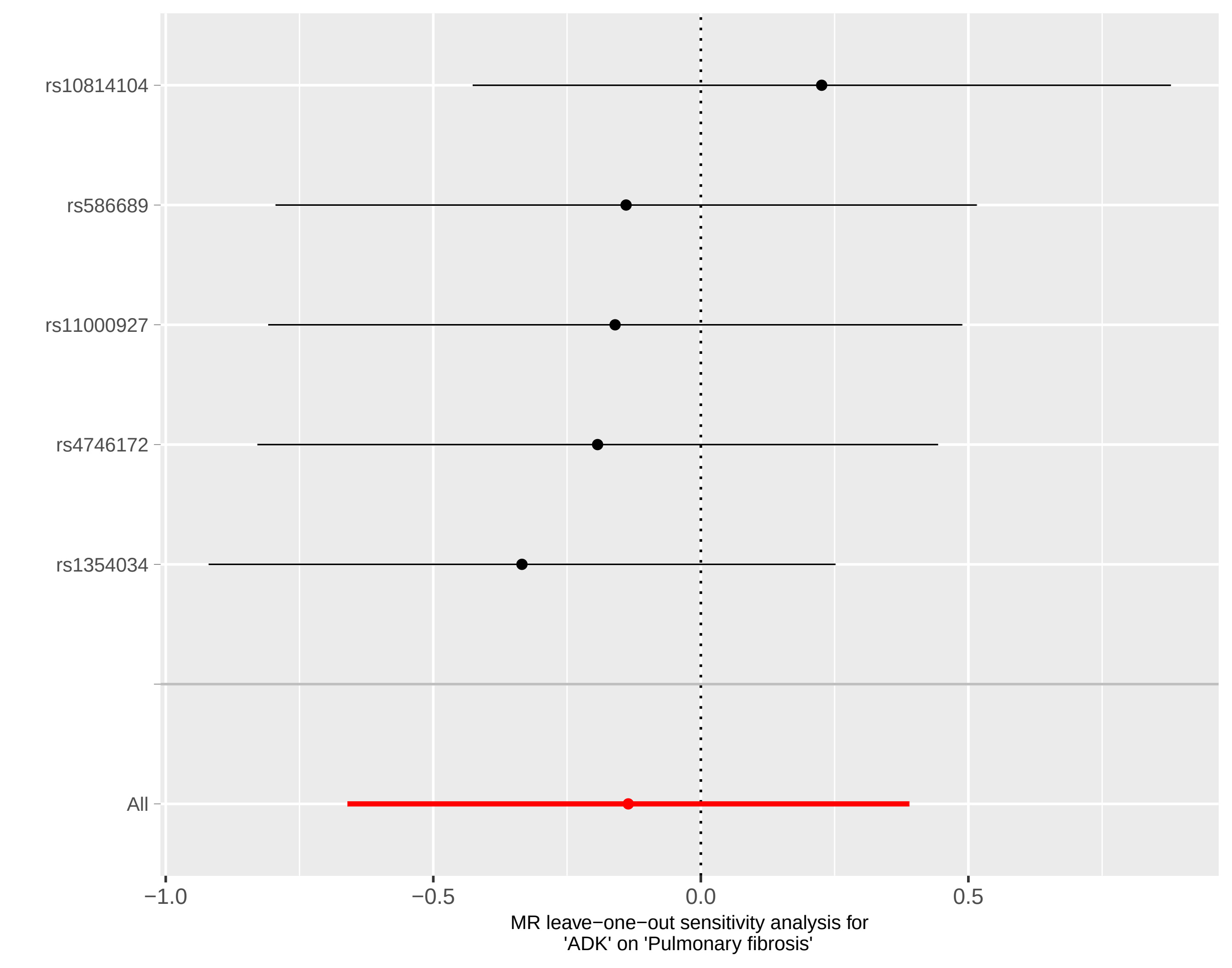

rs10814104
rs586689
rs11000927
rs4746172
rs1354034
All
−1.0
−0.5
0.0
0.5
MR leave−one−out sensitivity analysis for
'ADK' on 'Pulmonary fibrosis'

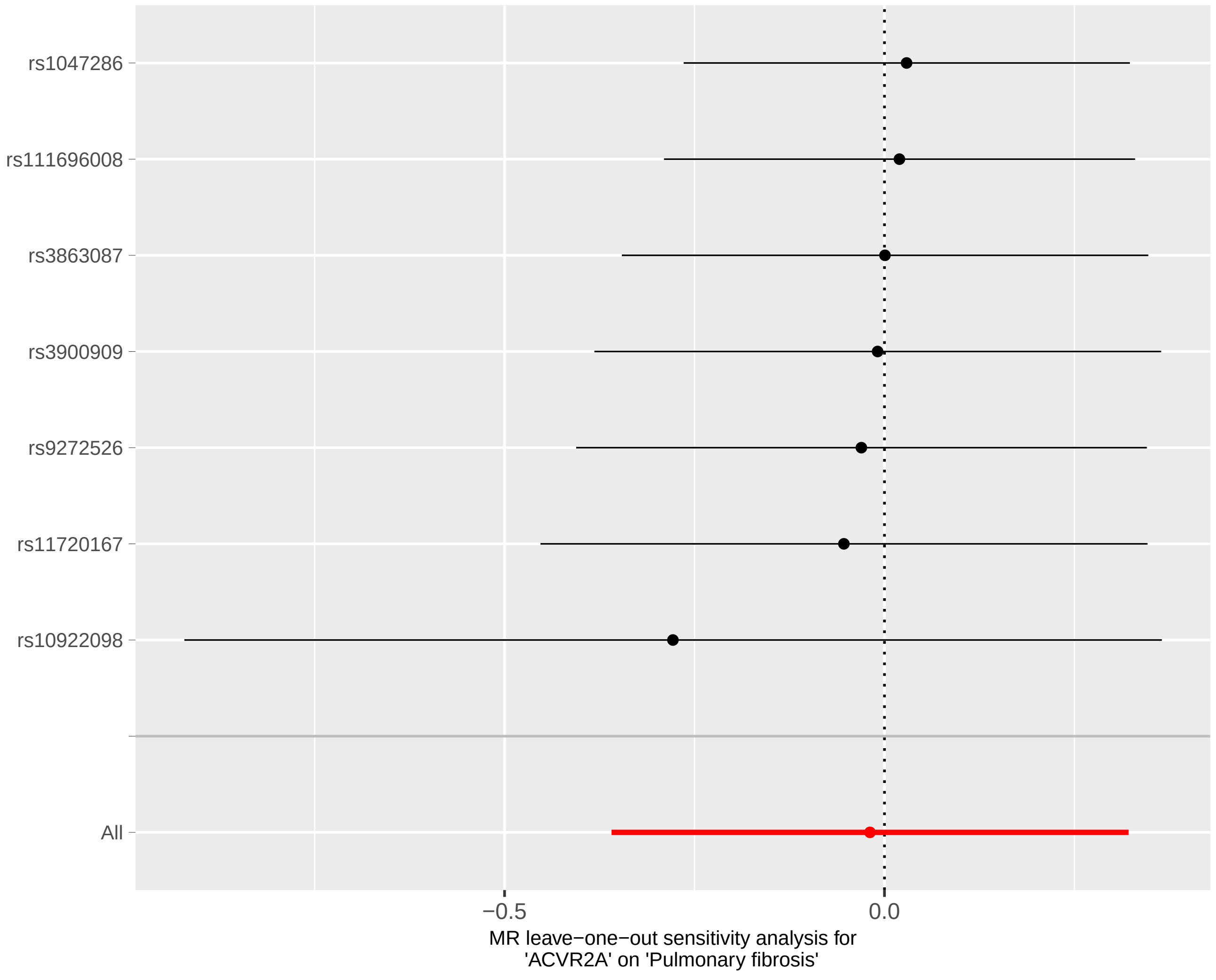
rs1047286
rs111696008
rs3863087
rs3900909
rs9272526
rs11720167
rs10922098
All
−0.5
0.0
MR leave−one−out sensitivity analysis for
'ACVR2A' on 'Pulmonary fibrosis'

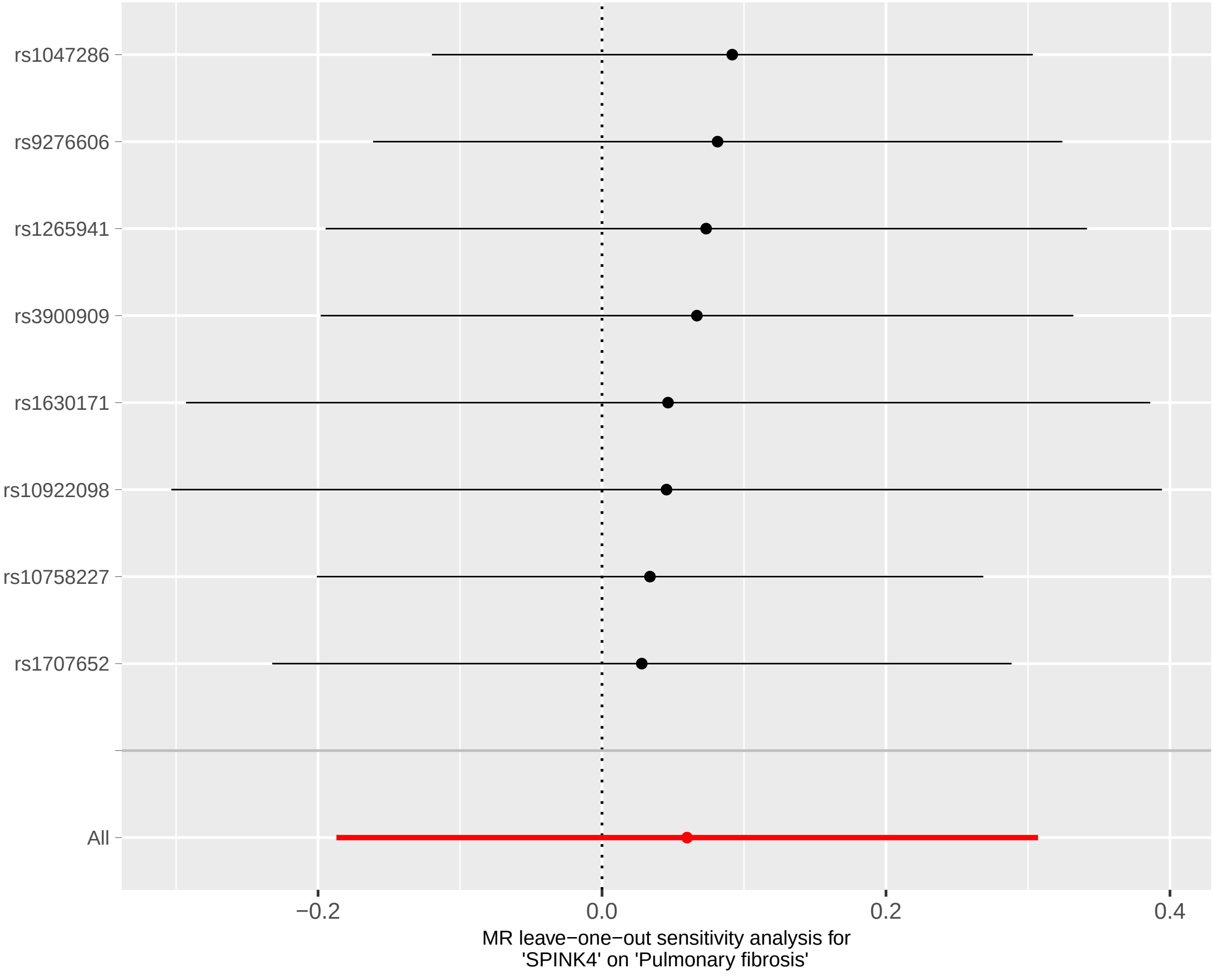
rs1047286
rs9276606
rs1265941
rs3900909
rs1630171
rs10922098
rs10758227
rs1707652
All
−0.2
0.0
0.2
0.4
MR leave−one−out sensitivity analysis for
'SPINK4' on 'Pulmonary fibrosis'

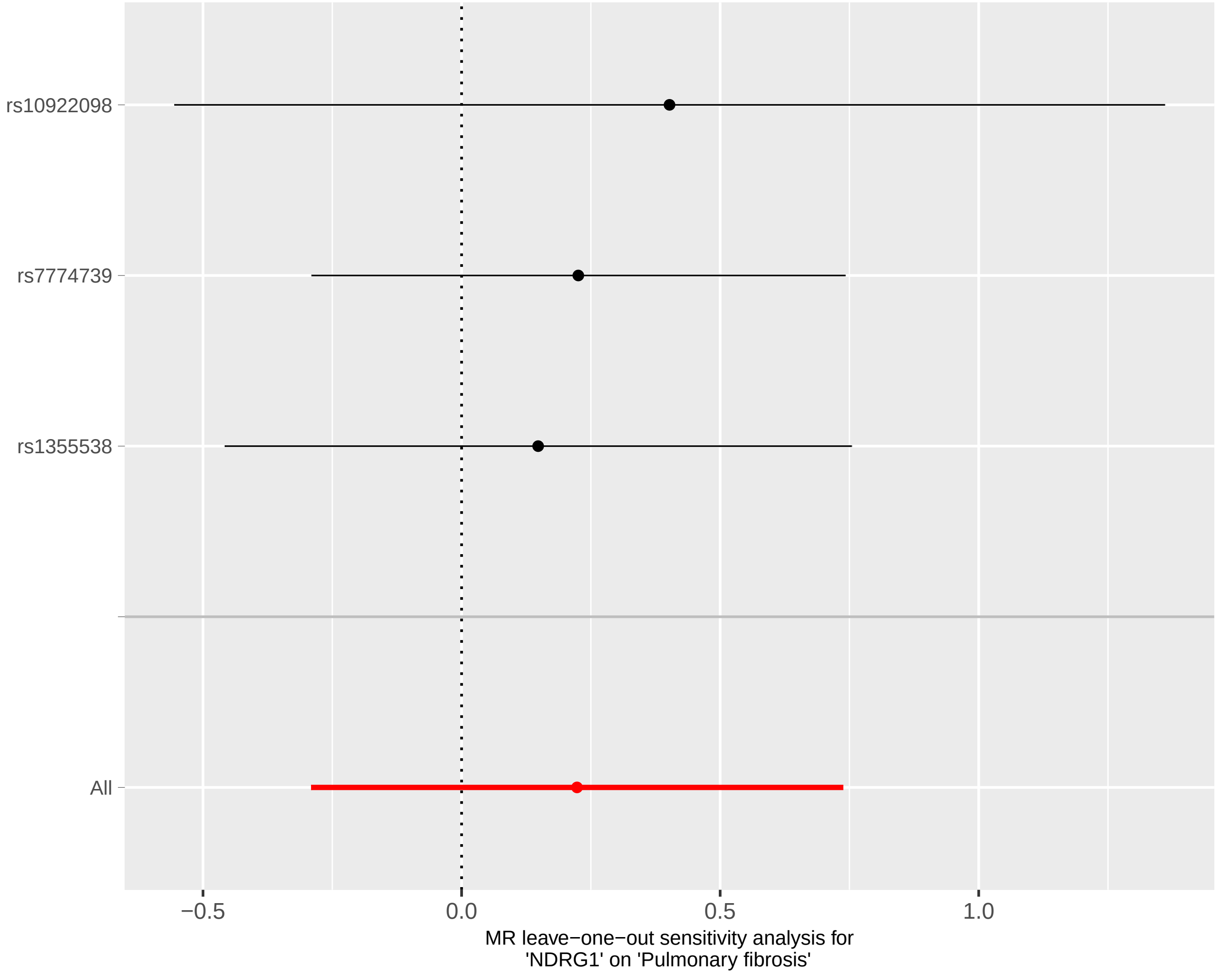
rs10922098
rs7774739
rs1355538
All
−0.5
0.0
0.5
1.0
MR leave−one−out sensitivity analysis for
'NDRG1' on 'Pulmonary fibrosis'

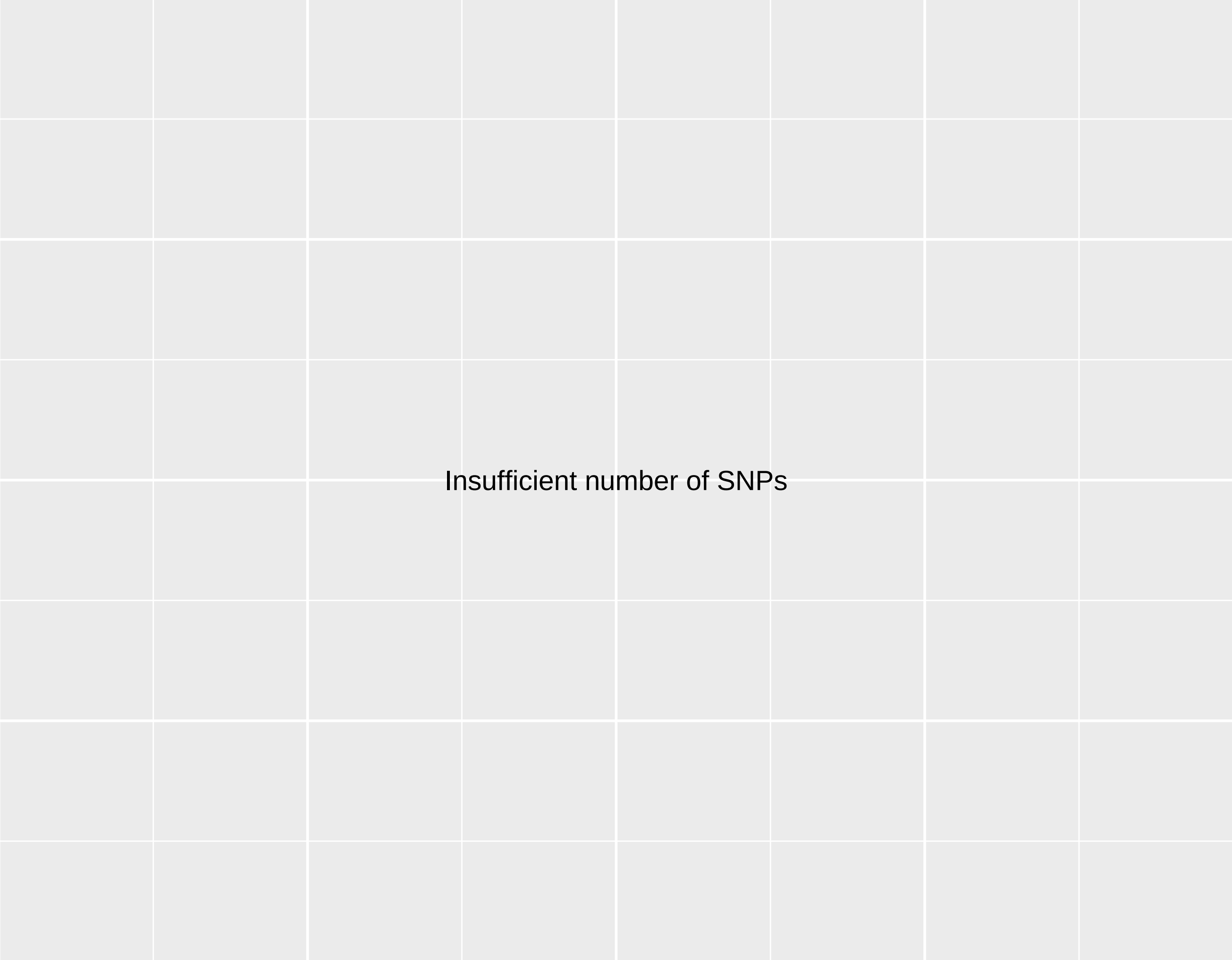
Insufficient number of SNPs

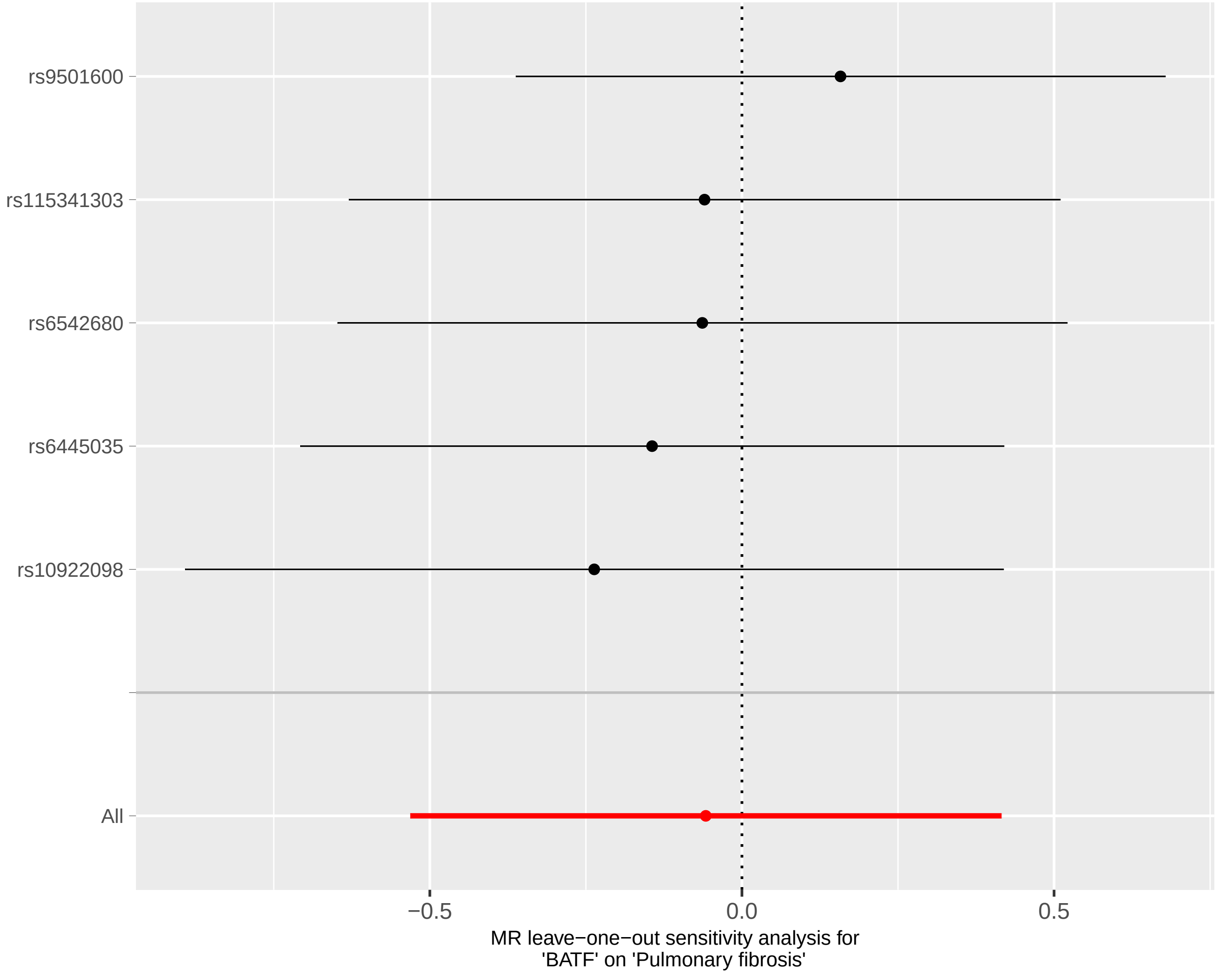
rs9501600
rs115341303
rs6542680
rs6445035
rs10922098
All
−0.5
0.0
0.5
MR leave−one−out sensitivity analysis for
'BATF' on 'Pulmonary fibrosis'

Insufficient number of SNPs

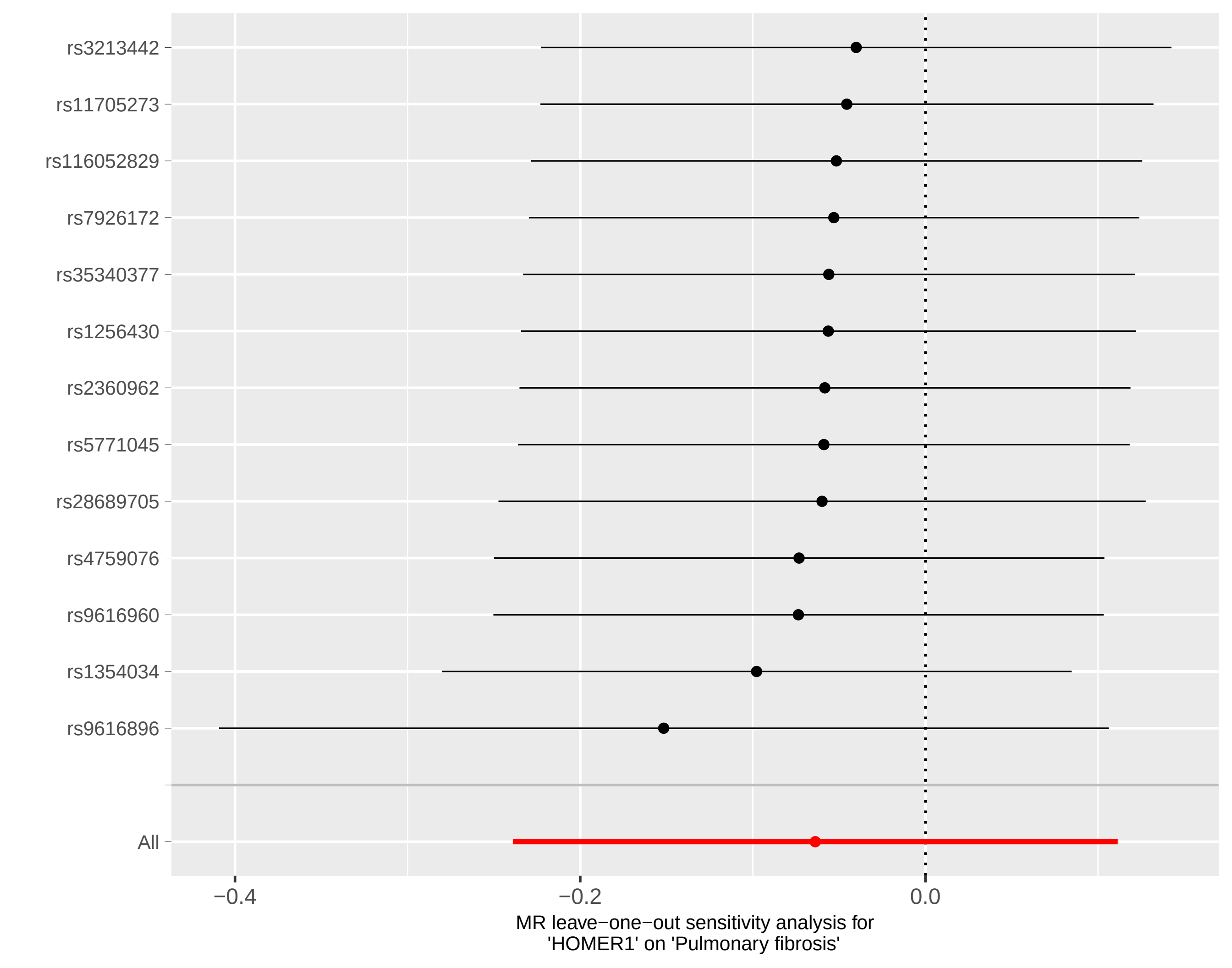
rs3213442
rs11705273
rs116052829
rs7926172
rs35340377
rs1256430
rs2360962
rs5771045
rs28689705
rs4759076
rs9616960
rs1354034
rs9616896
All
−0.4
−0.2
0.0
MR leave−one−out sensitivity analysis for
'HOMER1' on 'Pulmonary fibrosis'

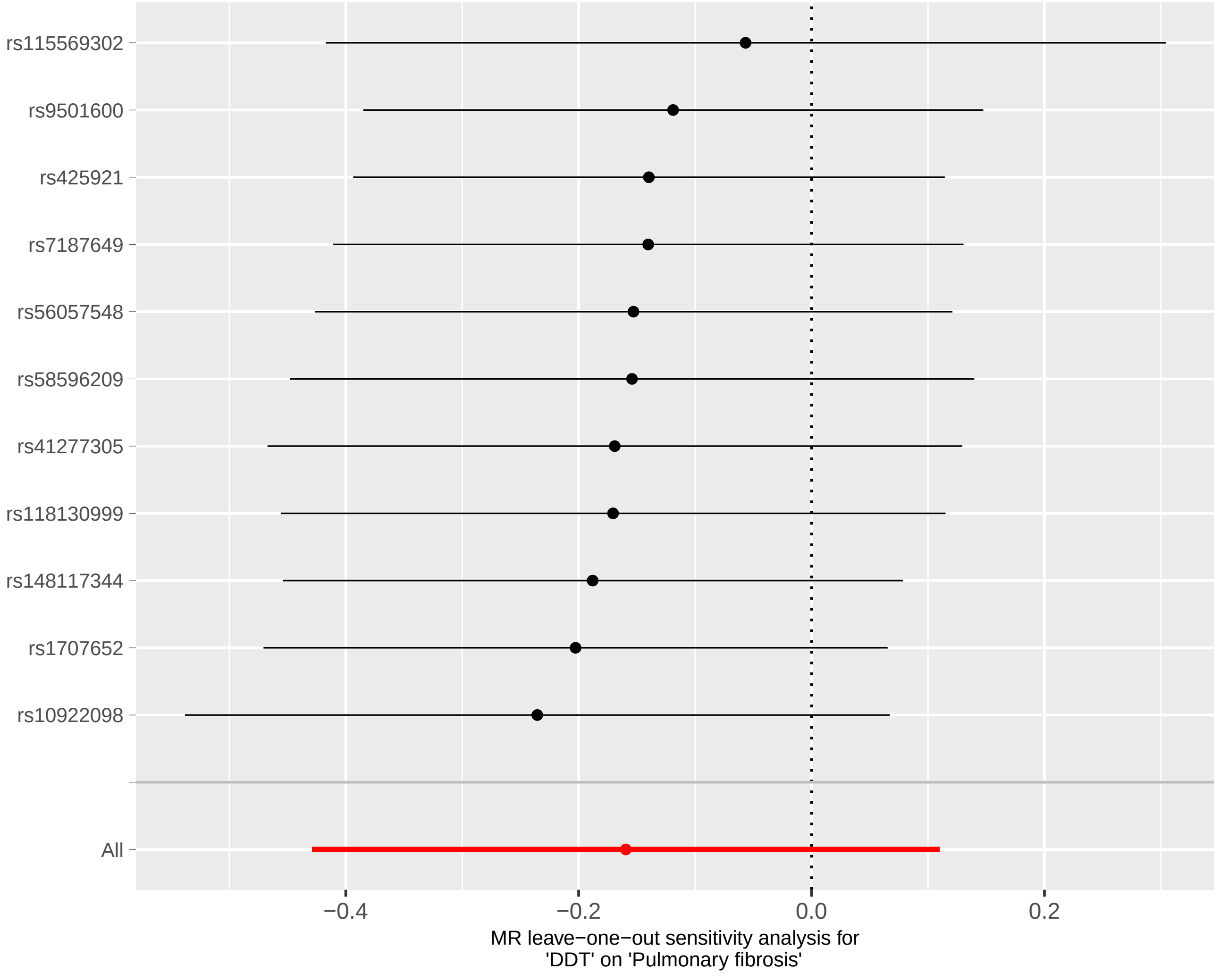

rs115569302
rs9501600
rs425921
rs7187649
rs56057548
rs58596209
rs41277305
rs118130999
rs148117344
rs1707652
rs10922098
All
−0.4
−0.2
0.0
0.2
MR leave−one−out sensitivity analysis for
'DDT' on 'Pulmonary fibrosis'

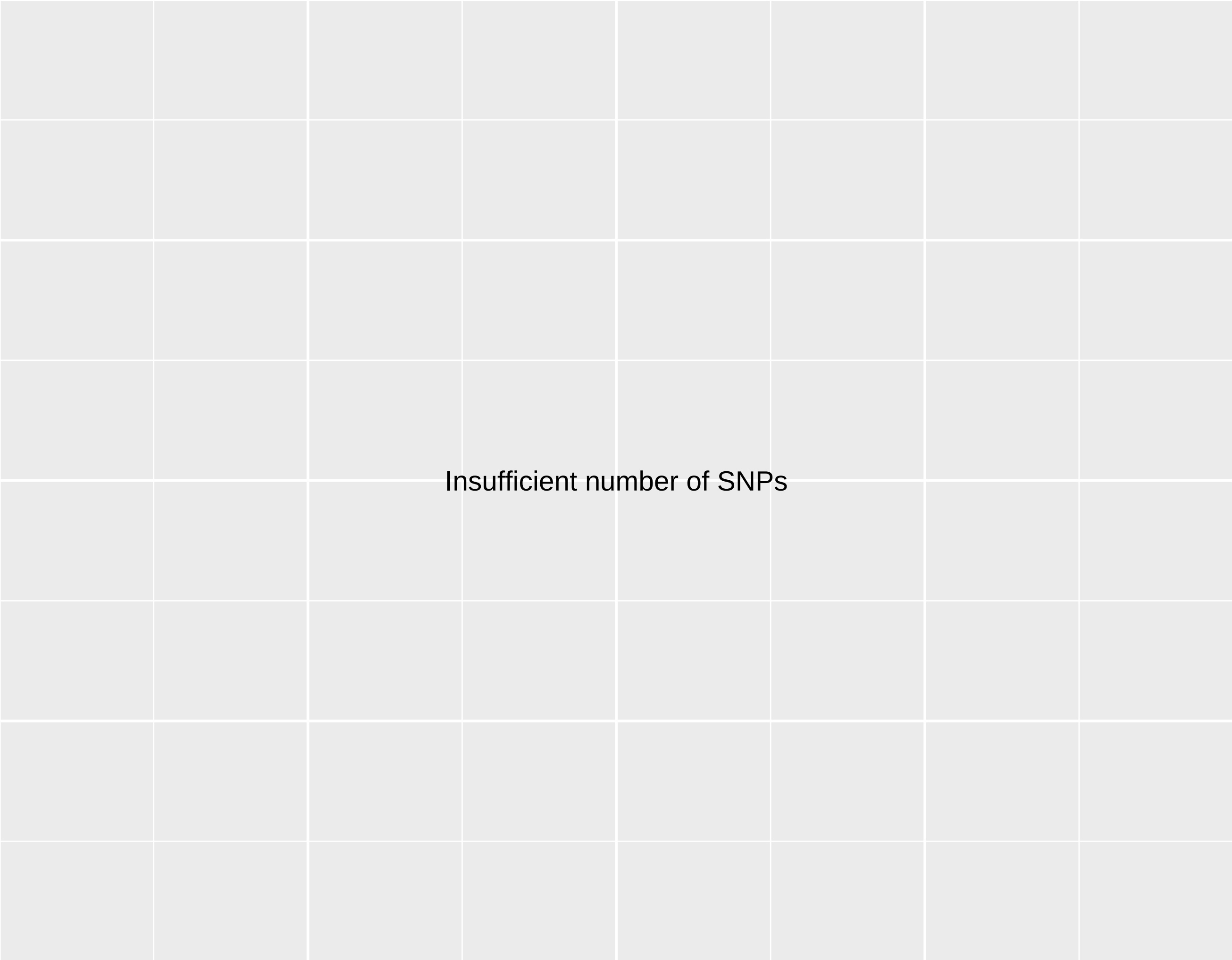
Insufficient number of SNPs

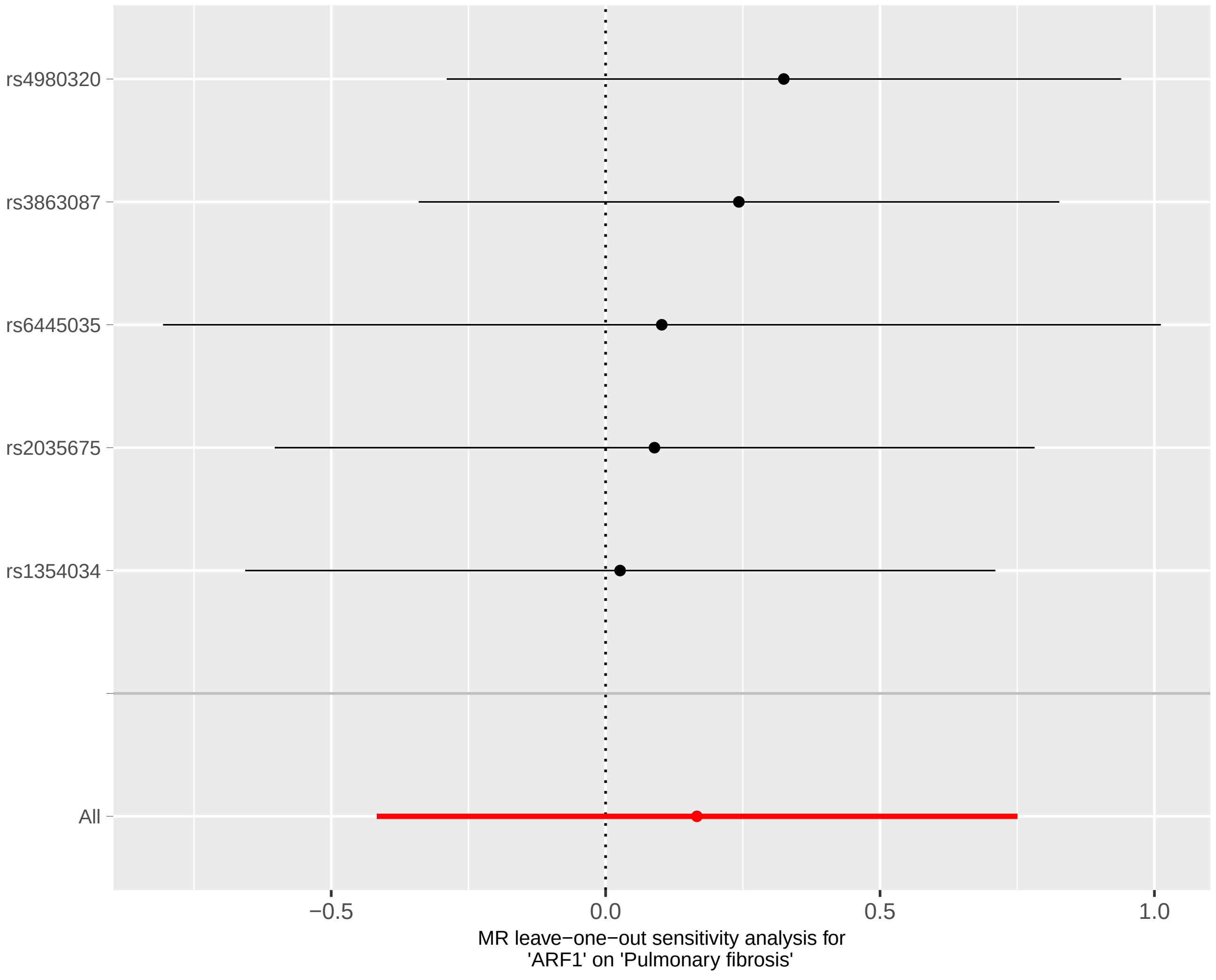

rs4980320
rs3863087
rs6445035
rs2035675
rs1354034
All
−0.5
0.0
0.5
1.0
MR leave−one−out sensitivity analysis for
'ARF1' on 'Pulmonary fibrosis'

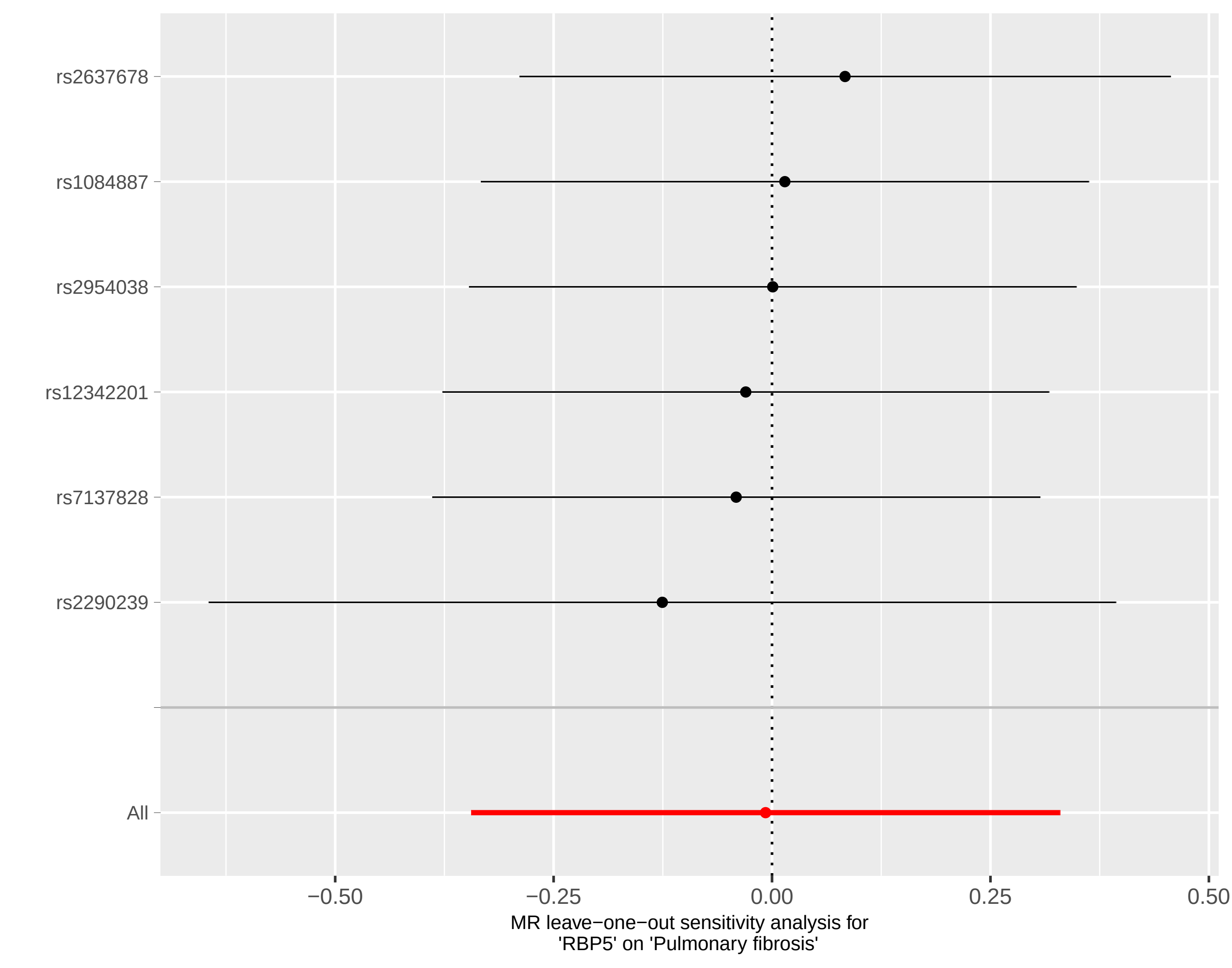
rs2637678
rs1084887
rs2954038
rs12342201
rs7137828
rs2290239
All
−0.50
−0.25
0.00
0.25
0.50
MR leave−one−out sensitivity analysis for
'RBP5' on 'Pulmonary fibrosis'

Insufficient number of SNPs

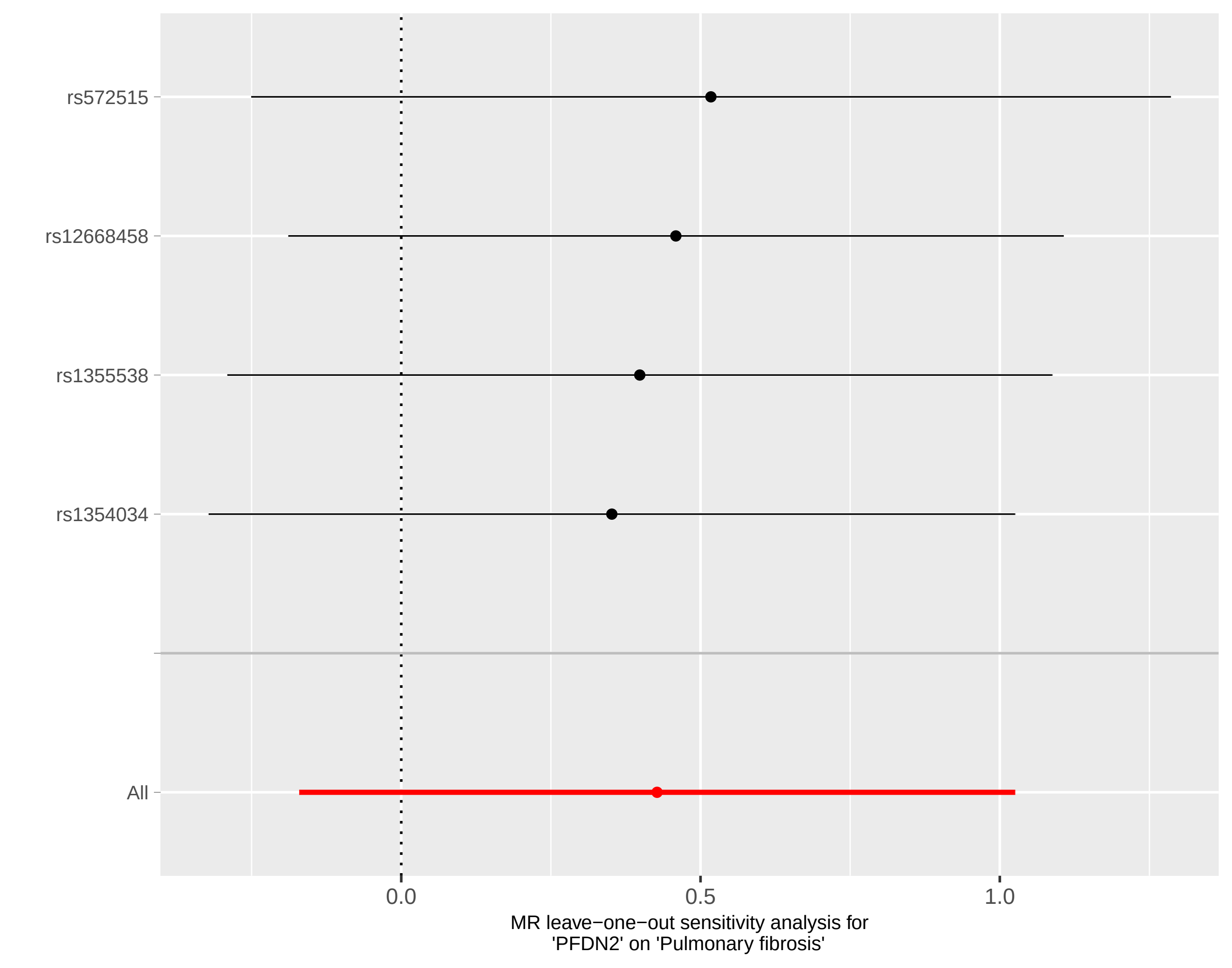
rs572515
rs12668458
rs1355538
rs1354034
All
0.0
0.5
1.0
MR leave−one−out sensitivity analysis for
'PFDN2' on 'Pulmonary fibrosis'

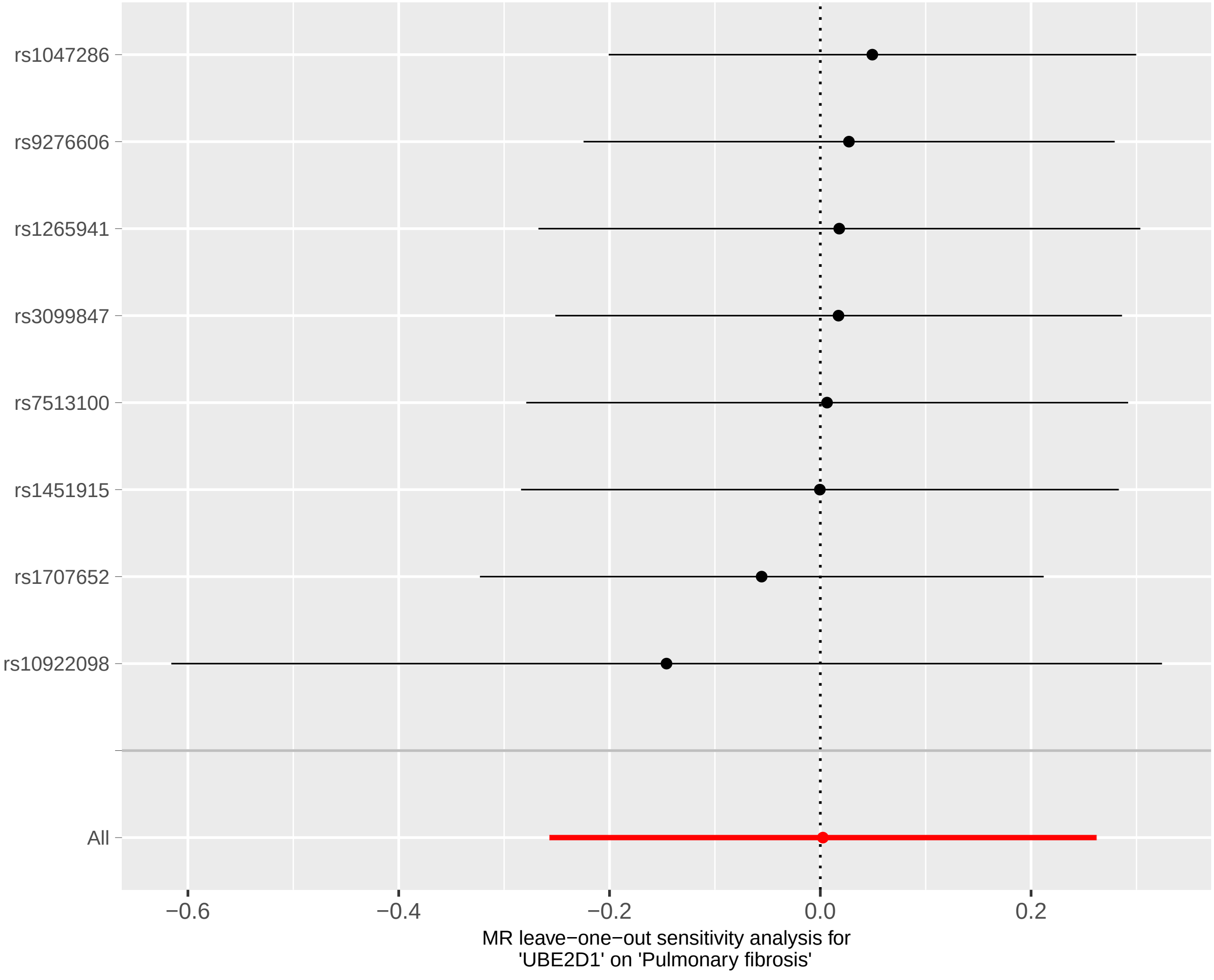
rs1047286
rs9276606
rs1265941
rs3099847
rs7513100
rs1451915
rs1707652
rs10922098
All
−0.6
−0.4
−0.2
0.0
0.2
MR leave−one−out sensitivity analysis for
'UBE2D1' on 'Pulmonary fibrosis'

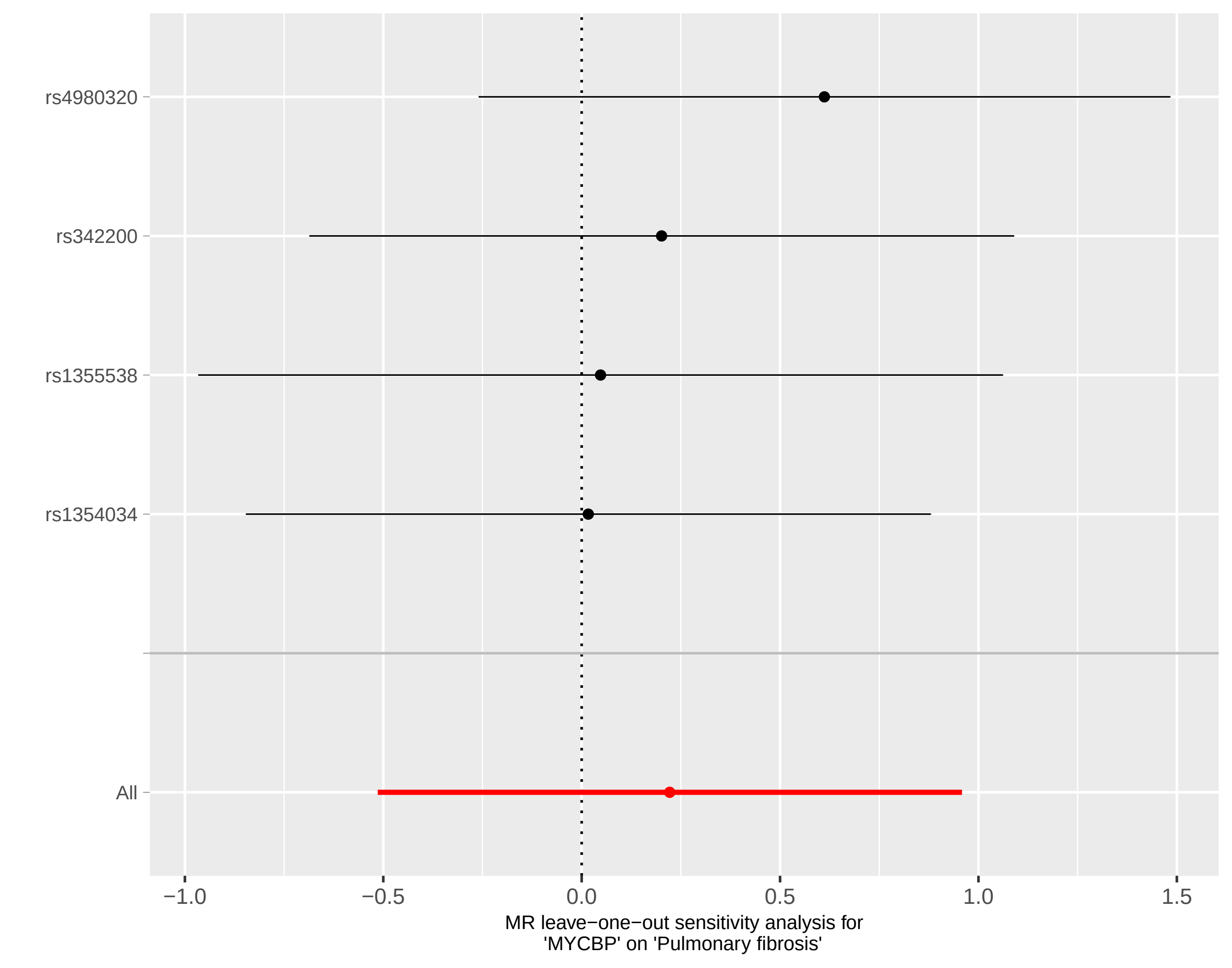
rs4980320
rs342200
rs1355538
rs1354034
All
−1.0
−0.5
0.0
0.5
1.0
1.5
MR leave−one−out sensitivity analysis for
'MYCBP' on 'Pulmonary fibrosis'

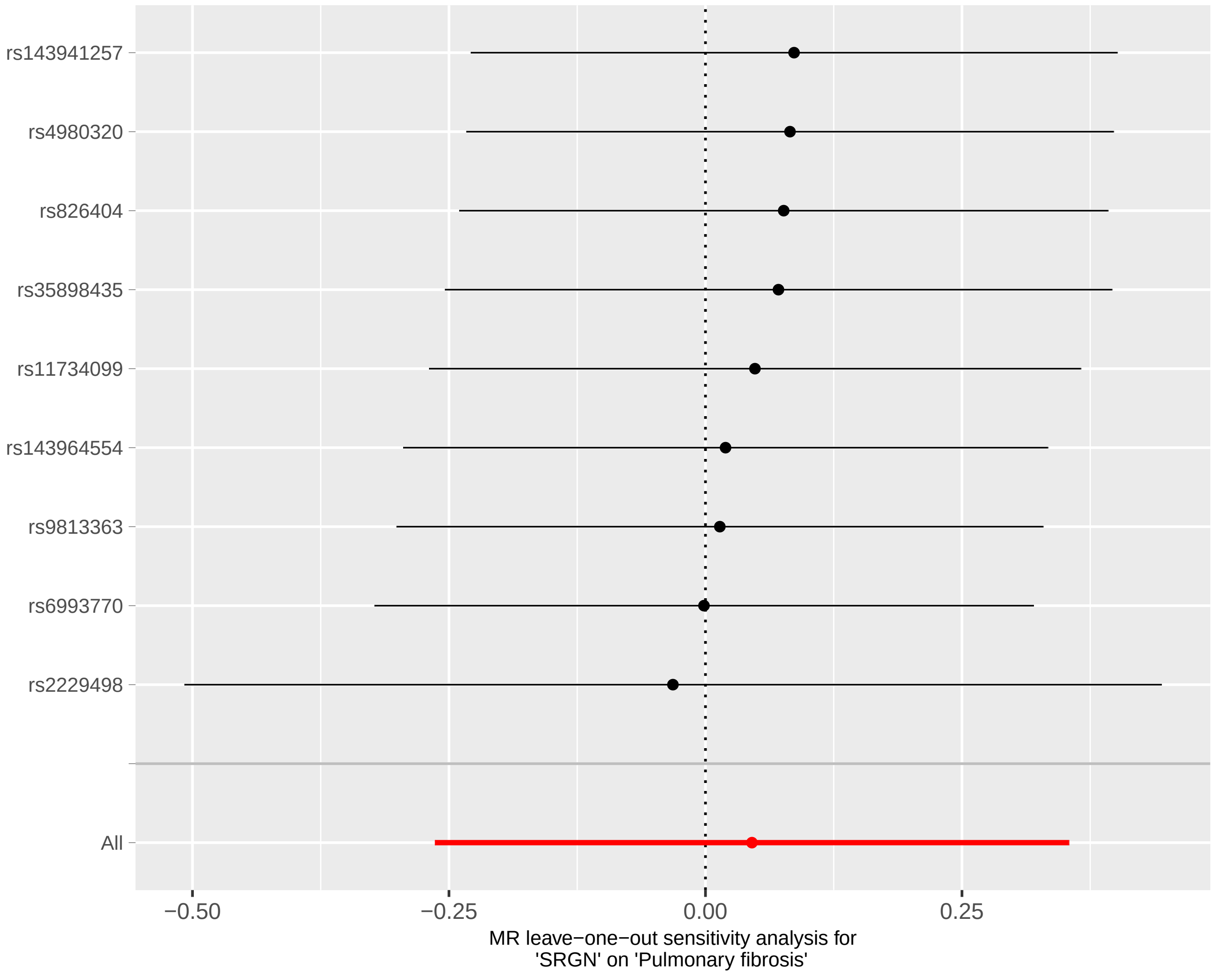
rs143941257
rs4980320
rs826404
rs35898435
rs11734099
rs143964554
rs9813363
rs6993770
rs2229498
All
−0.50
−0.25
0.00
0.25
MR leave−one−out sensitivity analysis for
'SRGN' on 'Pulmonary fibrosis'

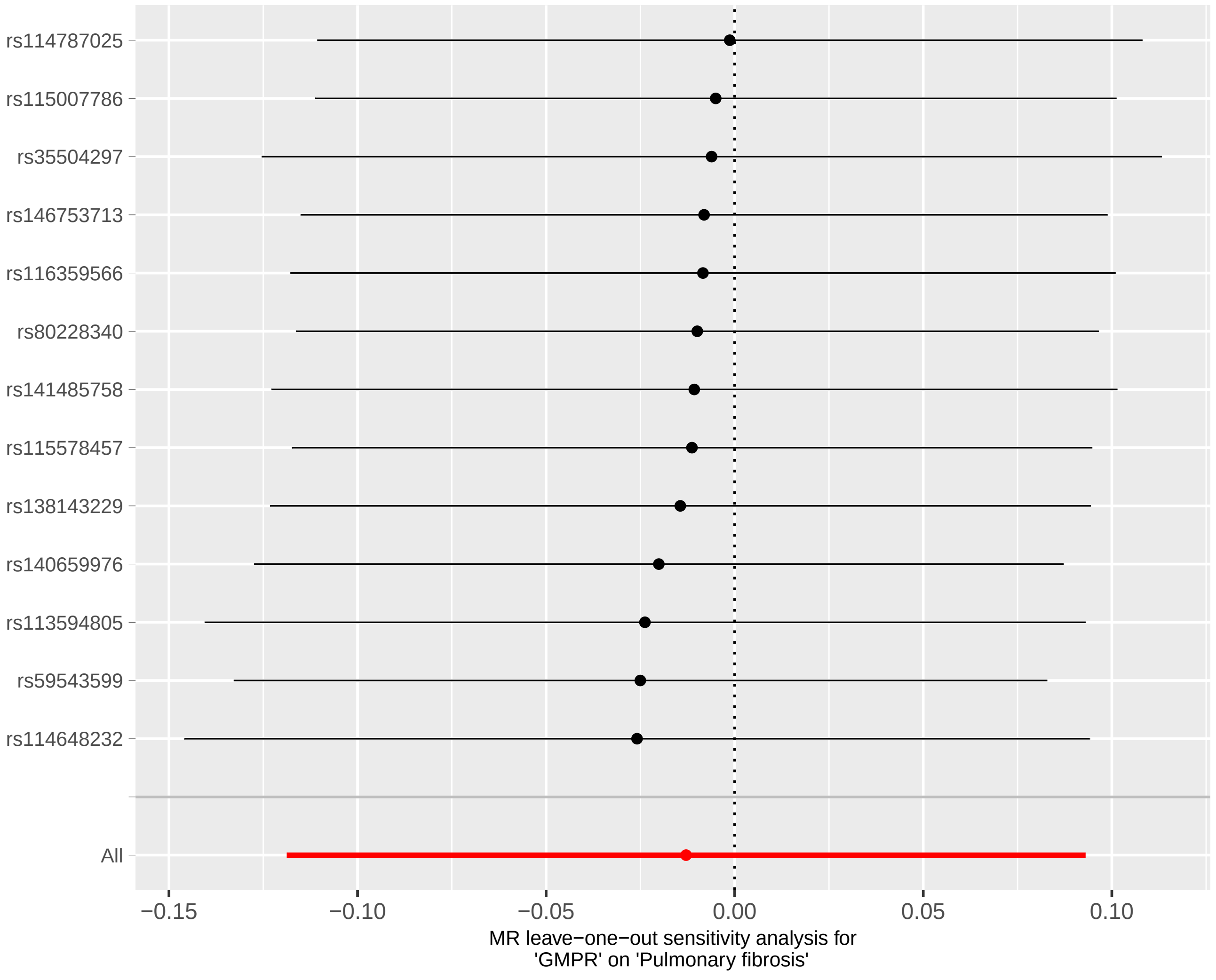

rs114787025
rs115007786
rs35504297
rs146753713
rs116359566
rs80228340
rs141485758
rs115578457
rs138143229
rs140659976
rs113594805
rs59543599
rs114648232
All
−0.15
−0.10
−0.05
0.00
0.05
0.10
MR leave−one−out sensitivity analysis for
'GMPR' on 'Pulmonary fibrosis'

Insufficient number of SNPs

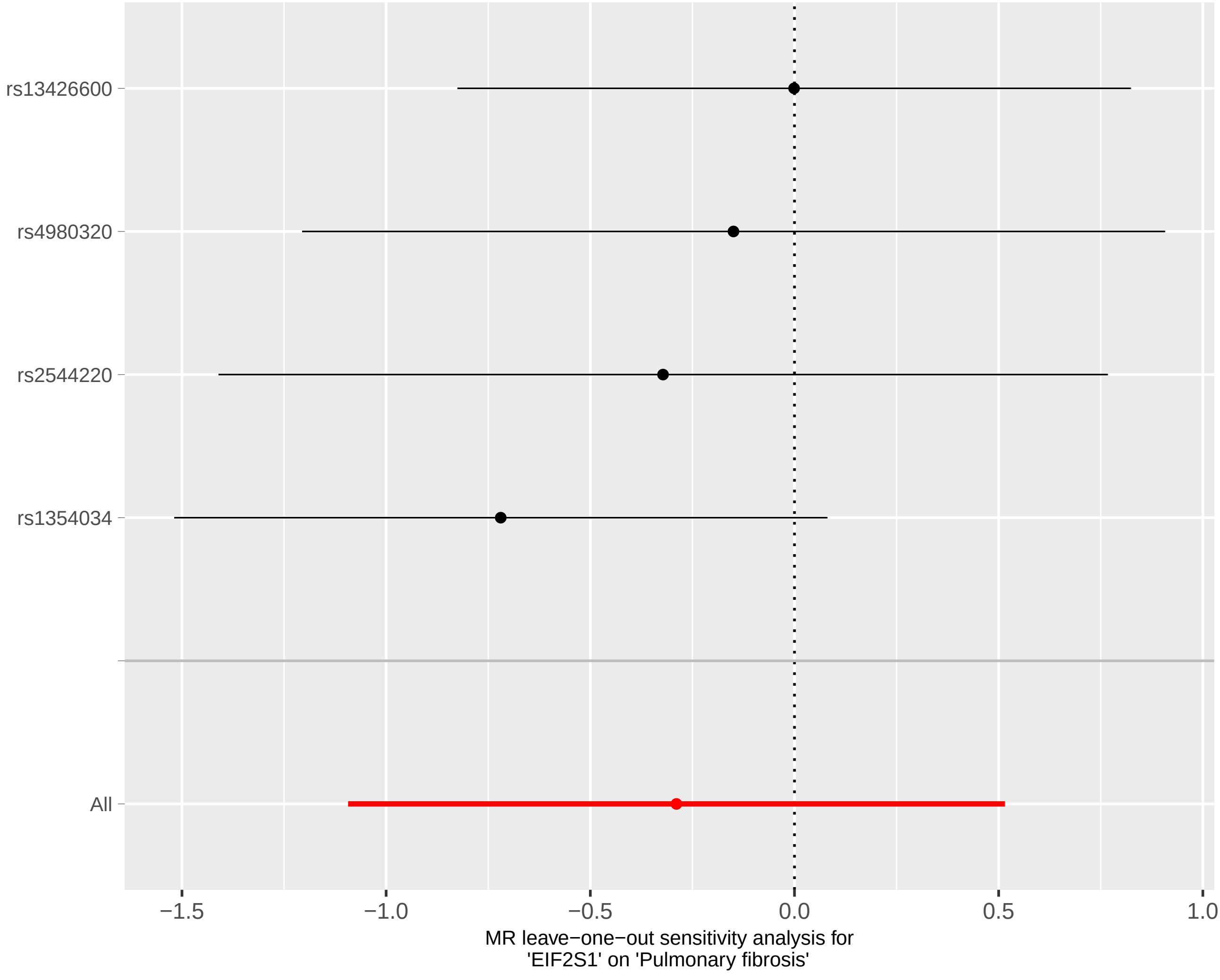
rs13426600
rs4980320
rs2544220
rs1354034
All
−1.5
−1.0
−0.5
0.0
0.5
1.0
MR leave−one−out sensitivity analysis for
'EIF2S1' on 'Pulmonary fibrosis'

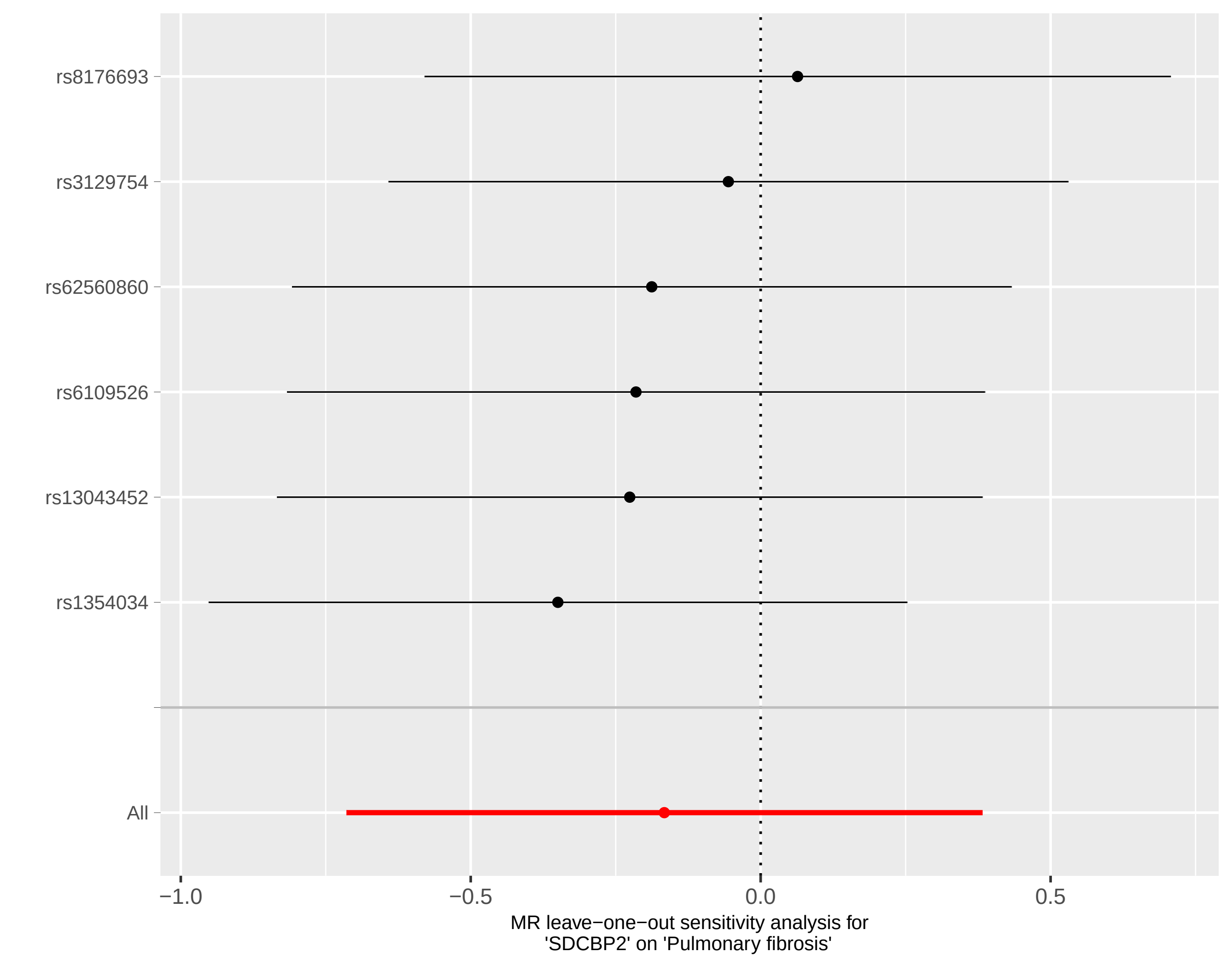
rs8176693
rs3129754
rs62560860
rs6109526
rs13043452
rs1354034
All
−1.0
−0.5
0.0
0.5
MR leave−one−out sensitivity analysis for
'SDCBP2' on 'Pulmonary fibrosis'

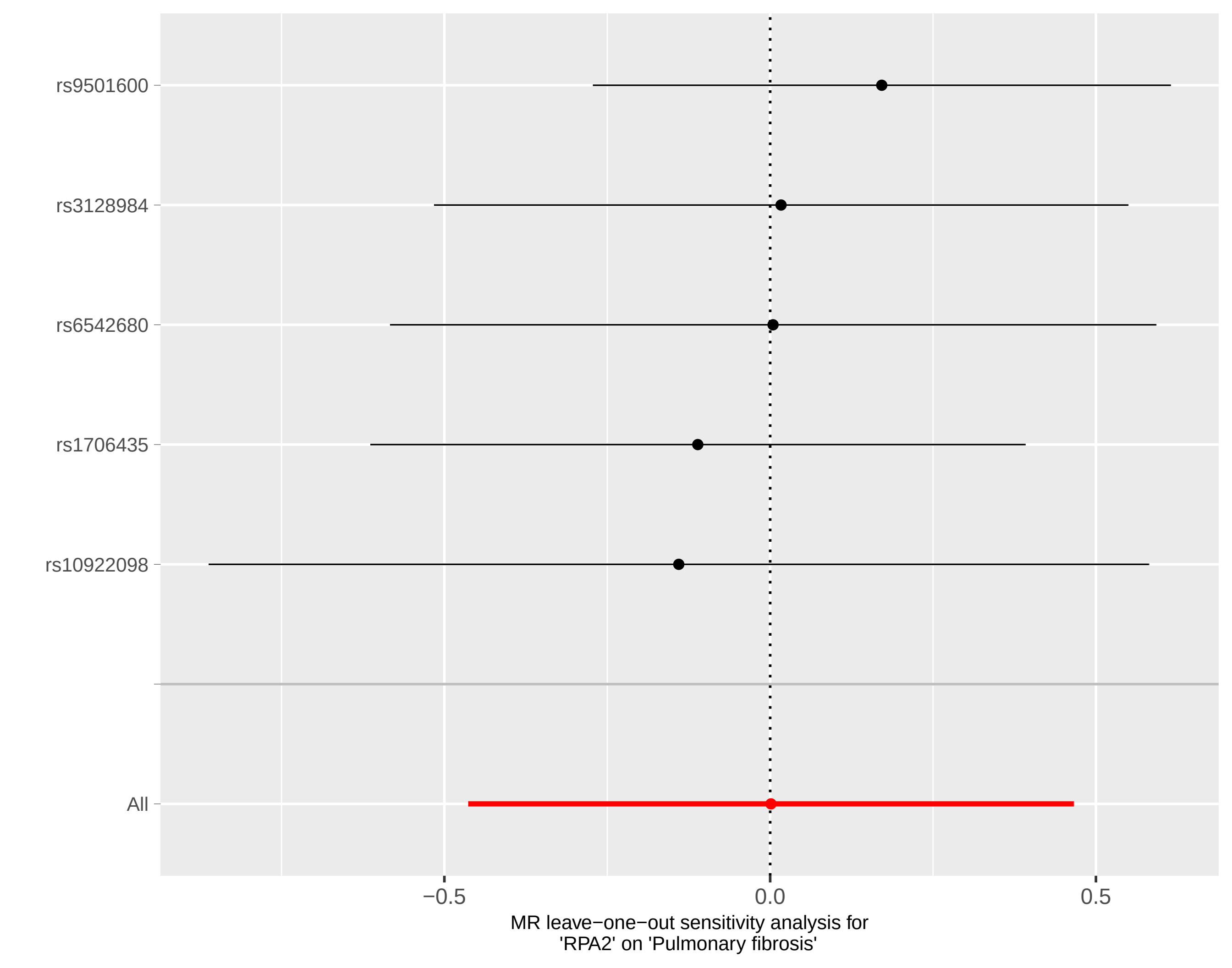
rs9501600
rs3128984
rs6542680
rs1706435
rs10922098
All
−0.5
0.0
0.5
MR leave−one−out sensitivity analysis for
'RPA2' on 'Pulmonary fibrosis'

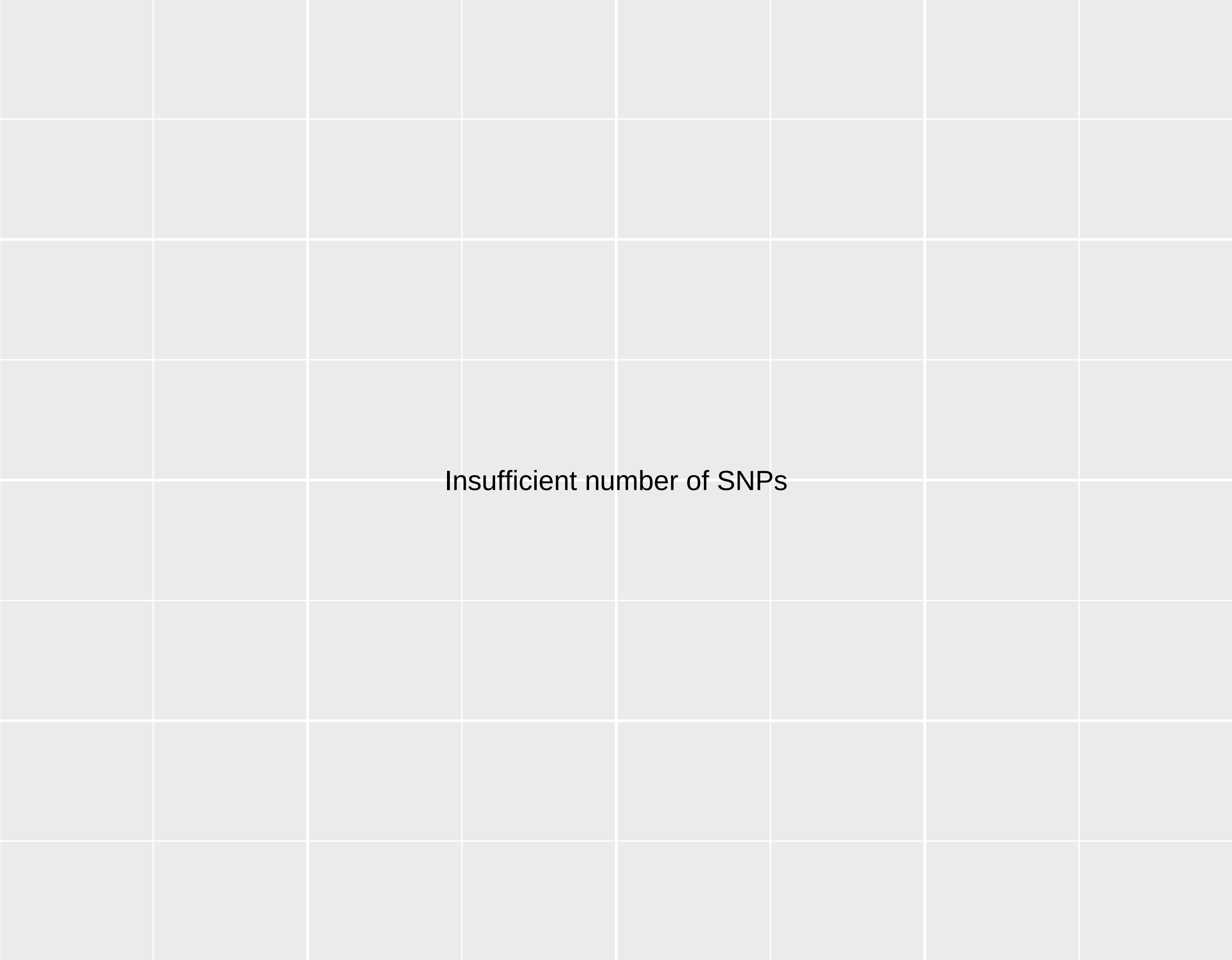
Insufficient number of SNPs

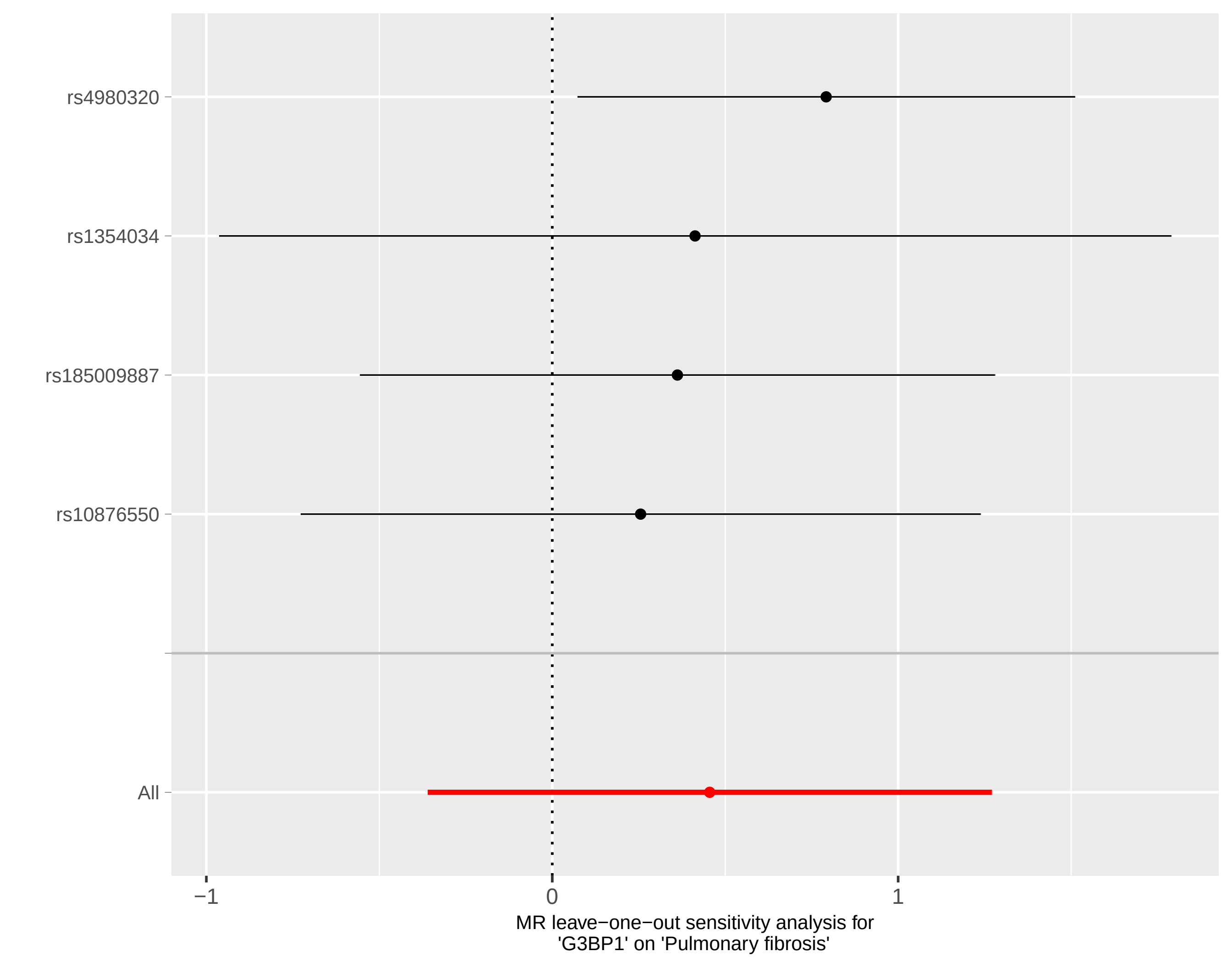
rs4980320
rs1354034
rs185009887
rs10876550
All
−1
0
1
MR leave−one−out sensitivity analysis for
'G3BP1' on 'Pulmonary fibrosis'

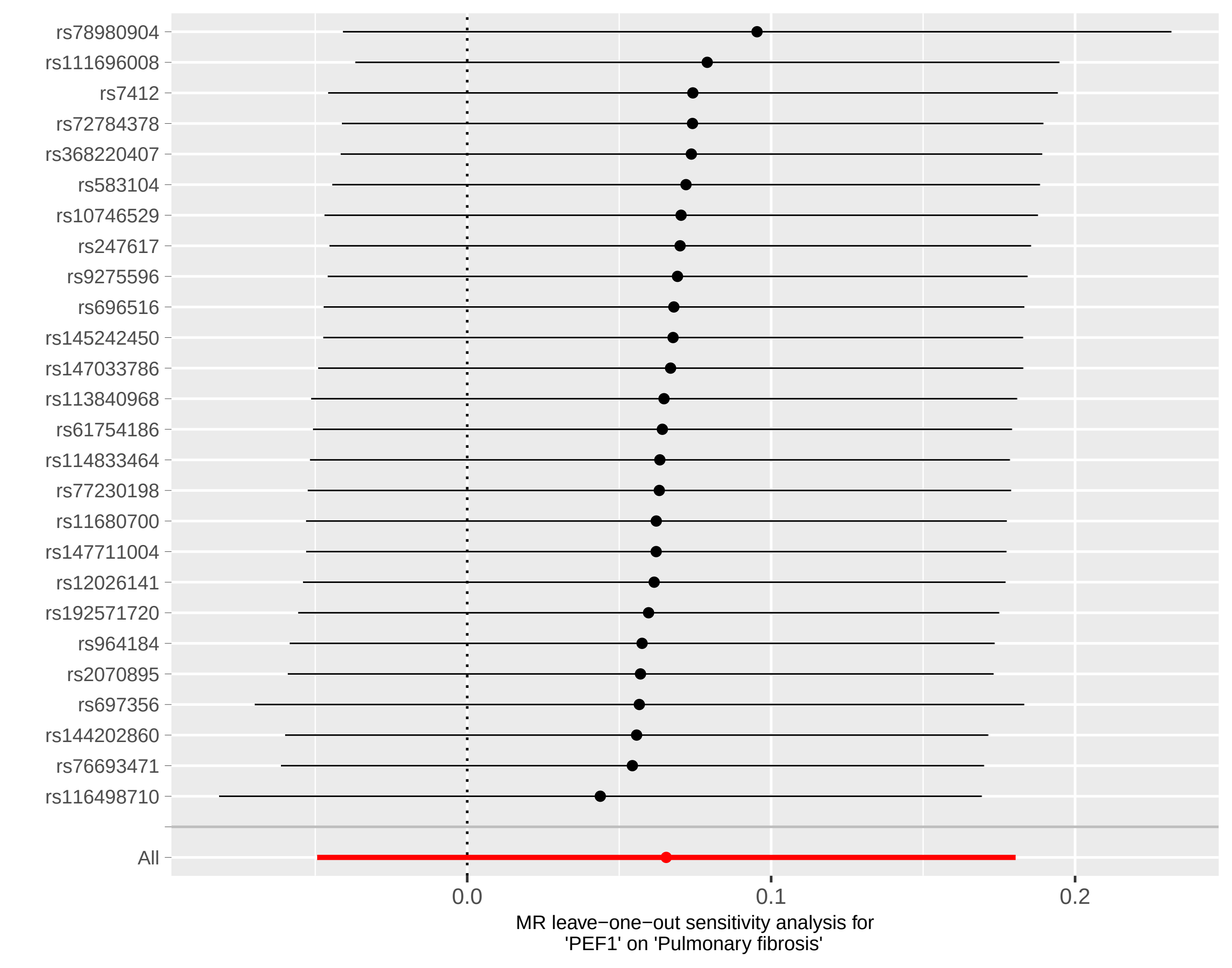
rs78980904
rs111696008
rs7412
rs72784378
rs368220407
rs583104
rs10746529
rs247617
rs9275596
rs696516
rs145242450
rs147033786
rs113840968
rs61754186
rs114833464
rs77230198
rs11680700
rs147711004
rs12026141
rs192571720
rs964184
rs2070895
rs697356
rs144202860
rs76693471
rs116498710
All
0.0
0.1
0.2
MR leave−one−out sensitivity analysis for
'PEF1' on 'Pulmonary fibrosis'

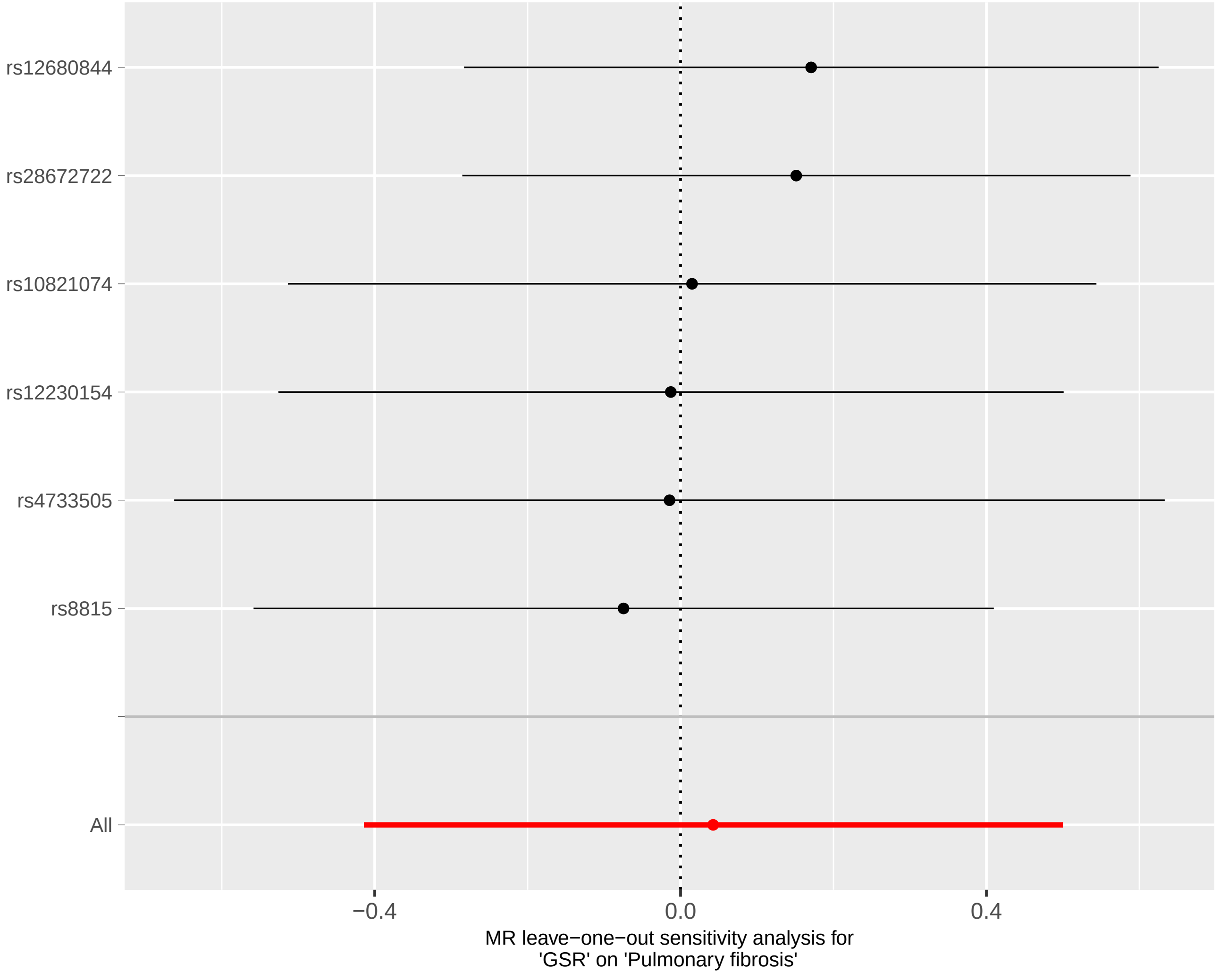

rs12680844
rs28672722
rs10821074
rs12230154
rs4733505
rs8815
All
−0.4
0.0
0.4
MR leave−one−out sensitivity analysis for
'GSR' on 'Pulmonary fibrosis'

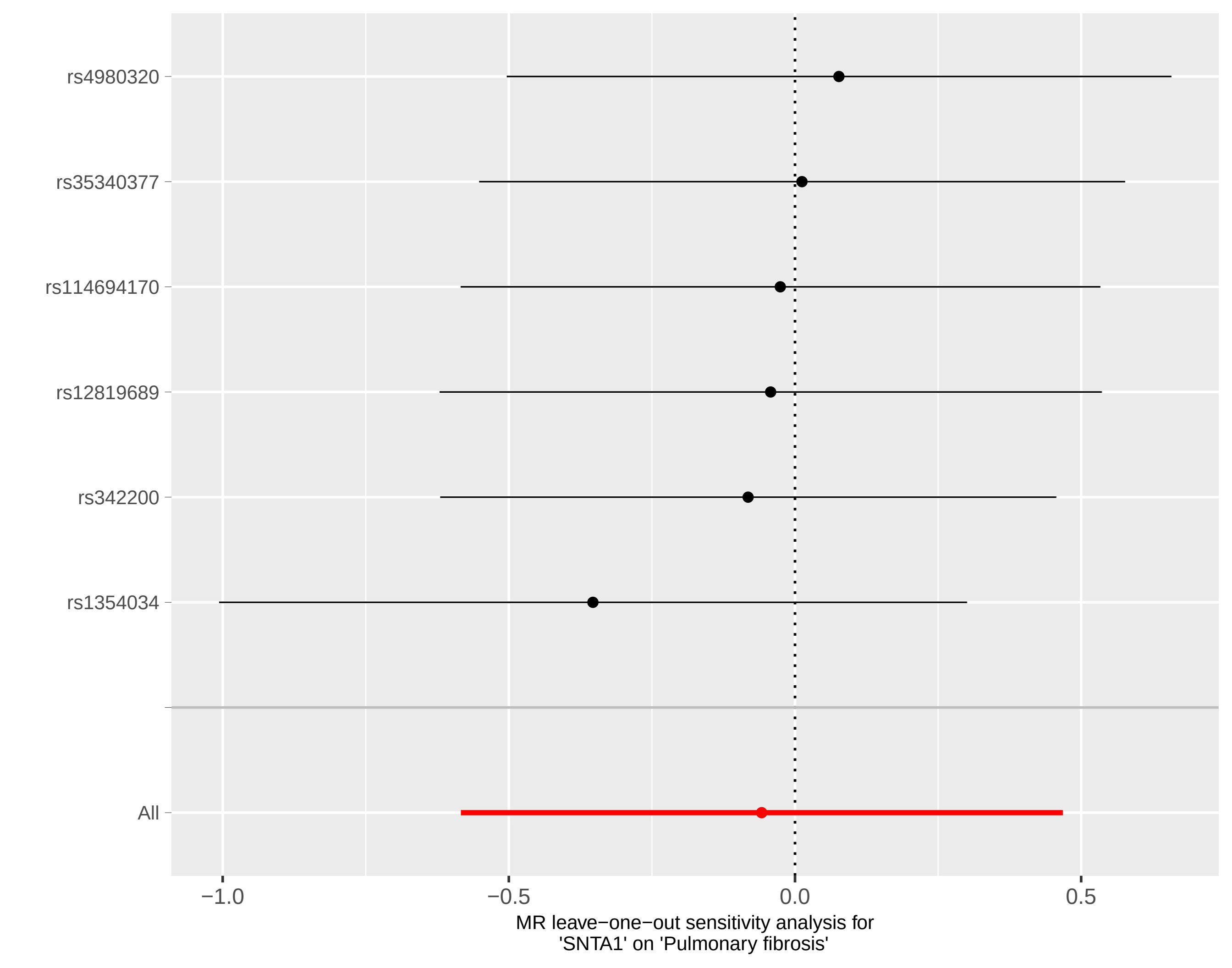
rs4980320
rs35340377
rs114694170
rs12819689
rs342200
rs1354034
All
−1.0
−0.5
0.0
0.5
MR leave−one−out sensitivity analysis for
'SNTA1' on 'Pulmonary fibrosis'

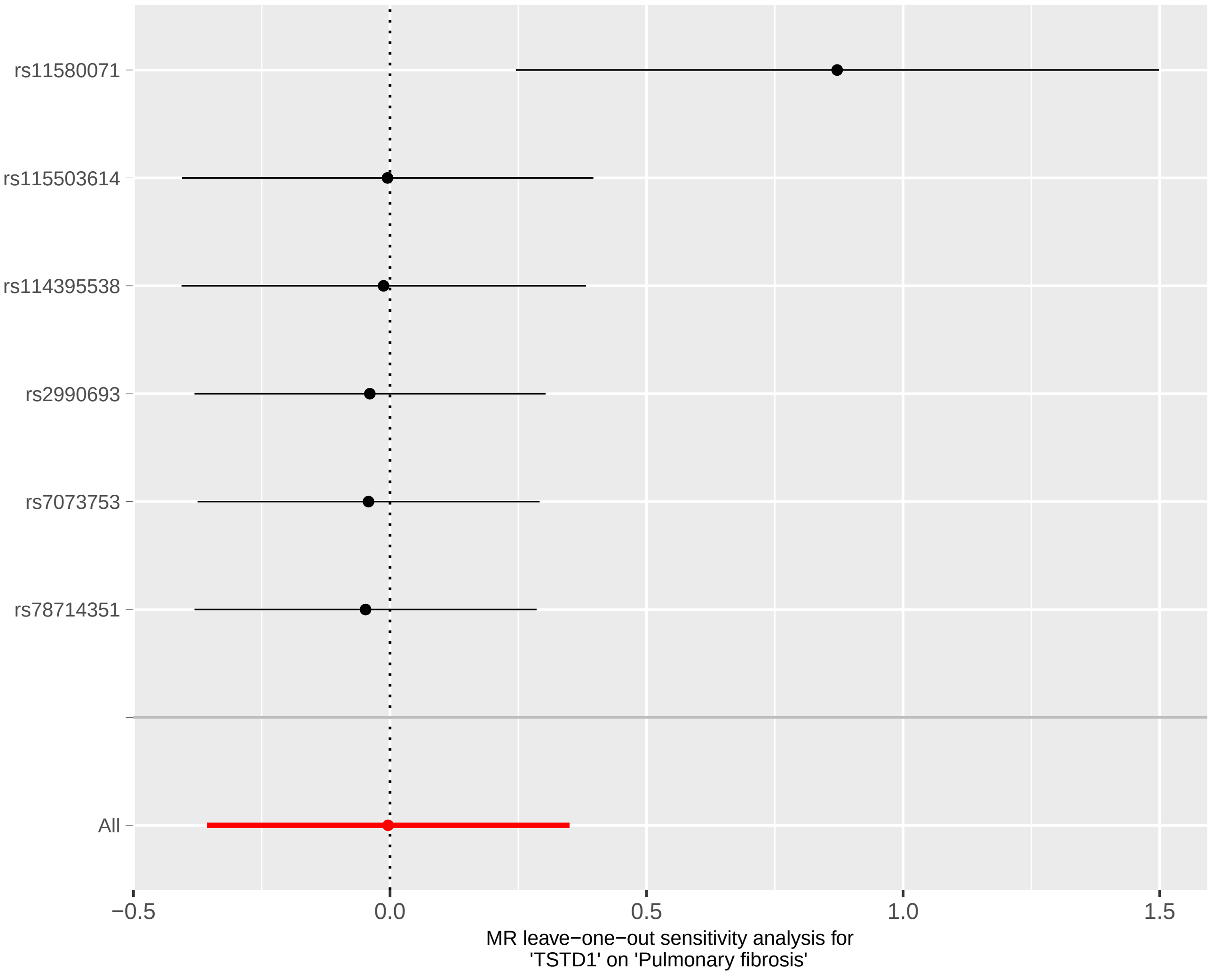
rs11580071
rs115503614
rs114395538
rs2990693
rs7073753
rs78714351
All
−0.5
0.0
0.5
1.0
1.5
MR leave−one−out sensitivity analysis for
'TSTD1' on 'Pulmonary fibrosis'

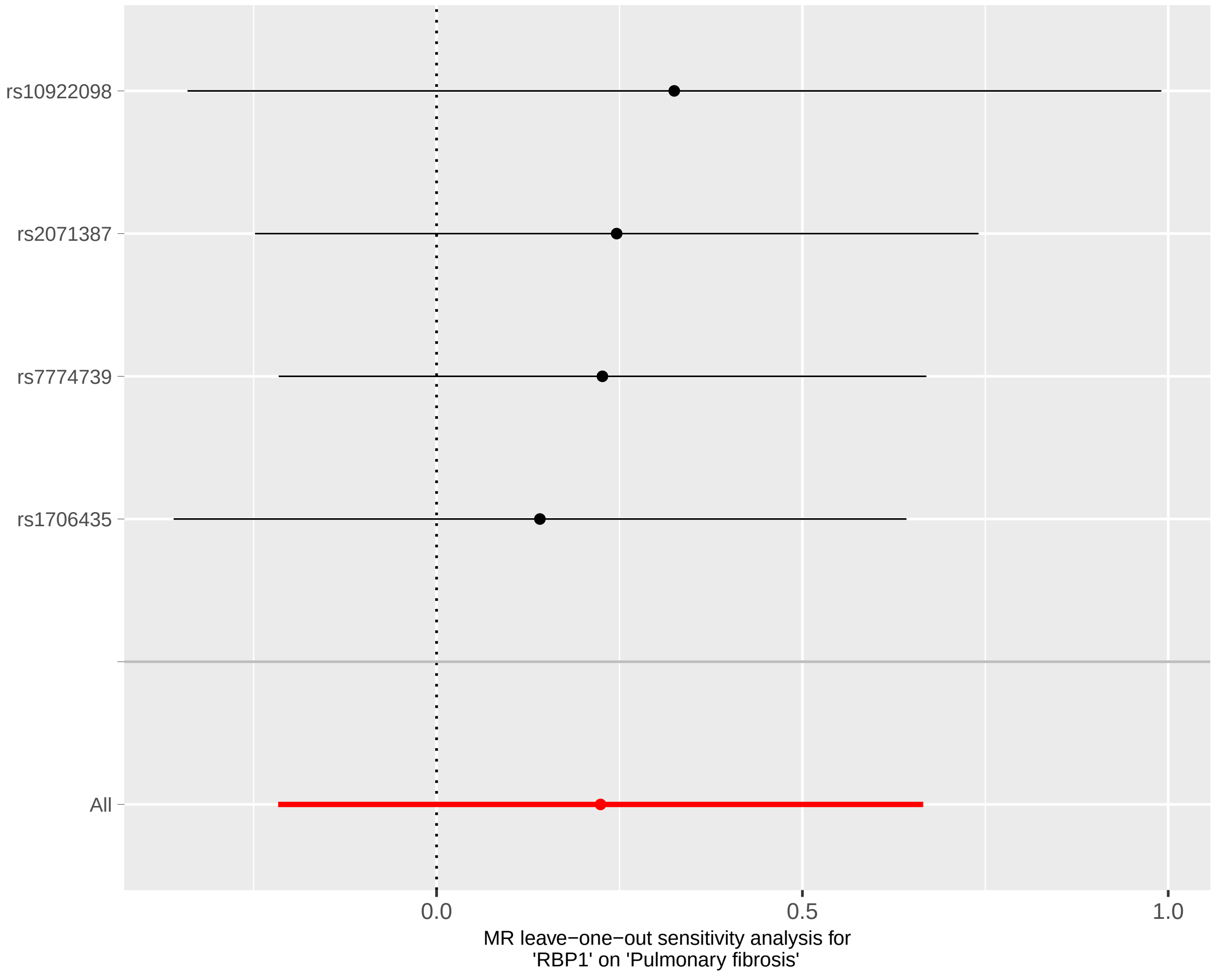
rs10922098
rs2071387
rs7774739
rs1706435
All
0.0
0.5
1.0
MR leave−one−out sensitivity analysis for
'RBP1' on 'Pulmonary fibrosis'

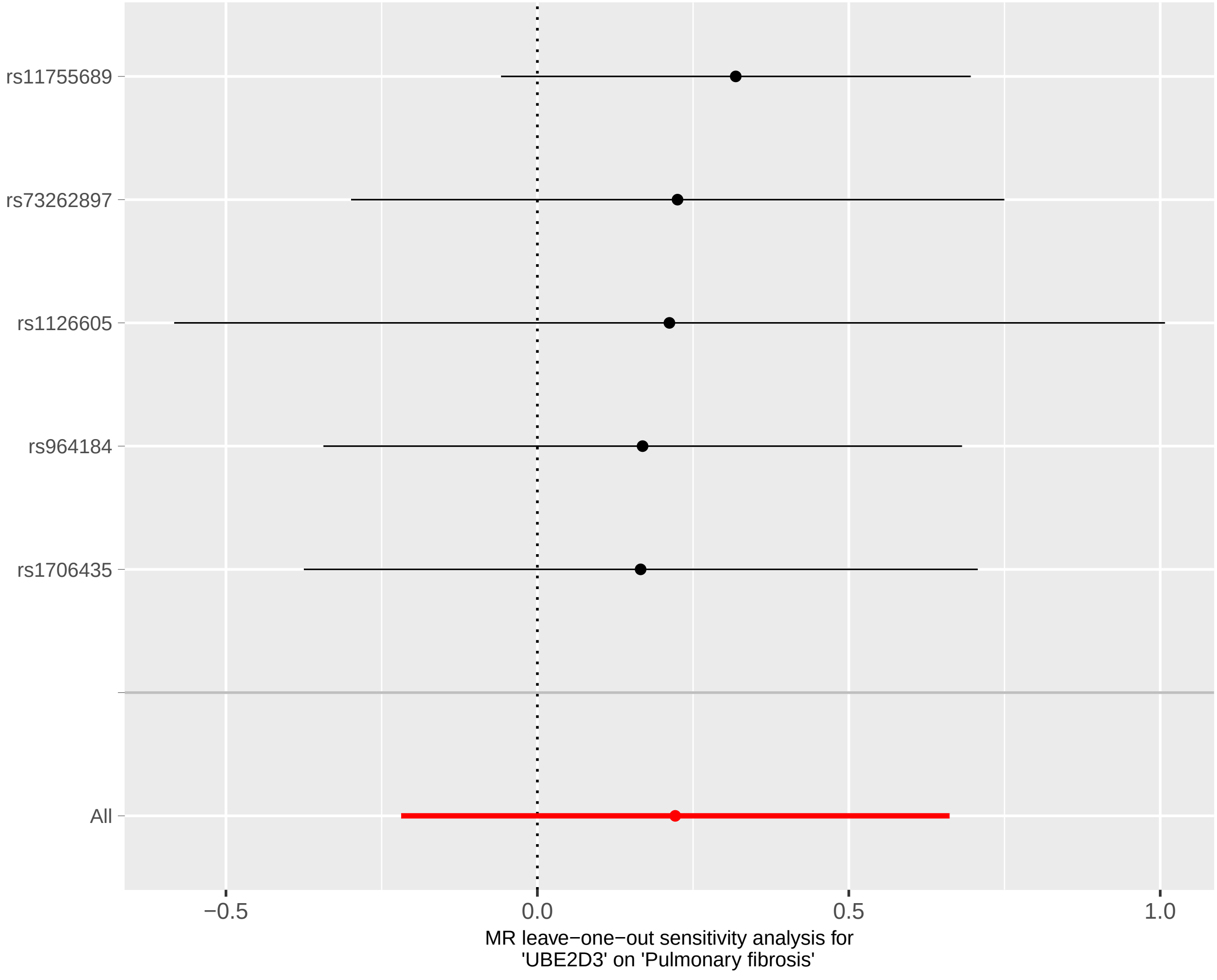
rs11755689
rs73262897
rs1126605
rs964184
rs1706435
All
−0.5
0.0
0.5
1.0
MR leave−one−out sensitivity analysis for
'UBE2D3' on 'Pulmonary fibrosis'

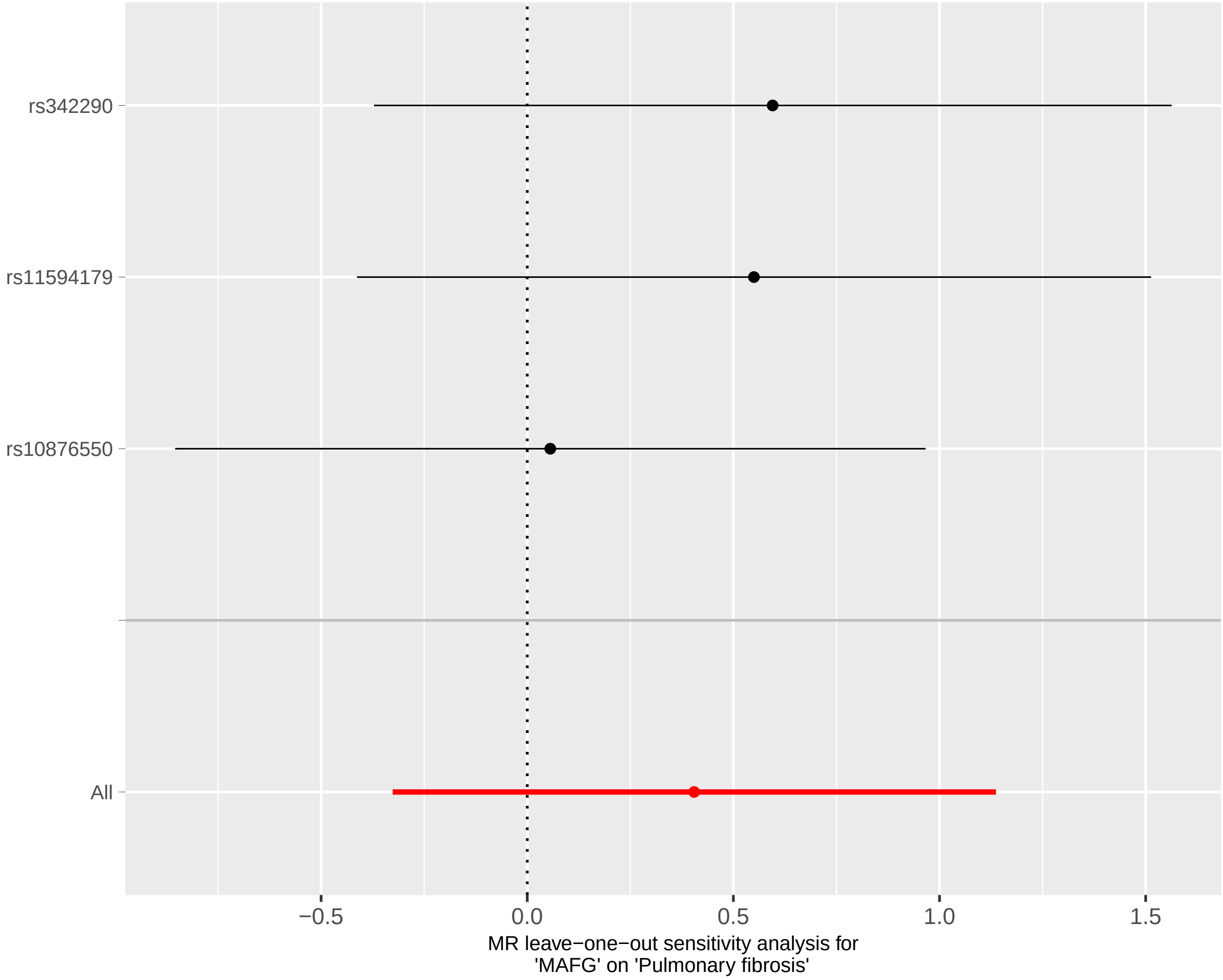
rs342290
rs11594179
rs10876550
All
−0.5
0.0
0.5
1.0
1.5
MR leave−one−out sensitivity analysis for
'MAFG' on 'Pulmonary fibrosis'

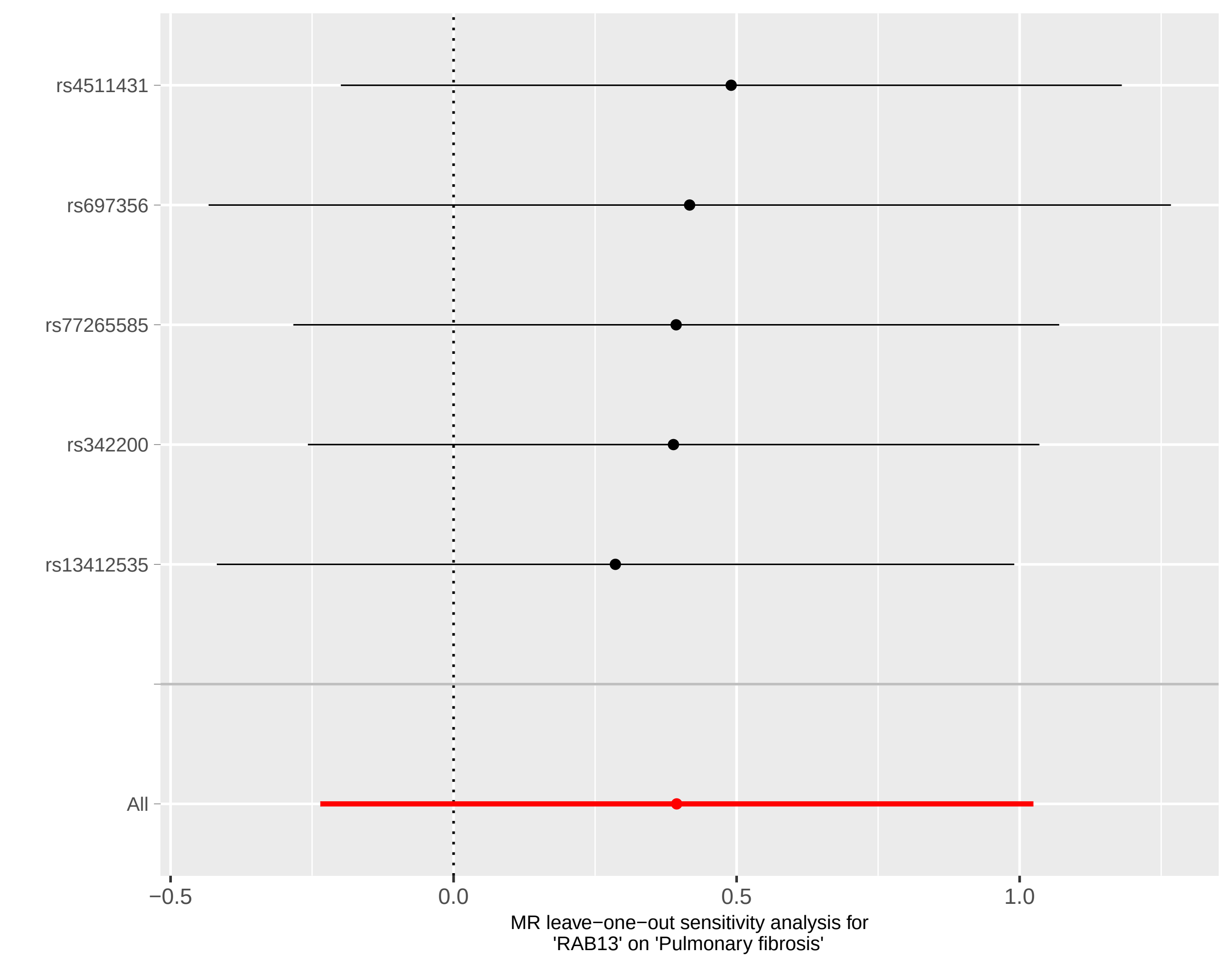
rs4511431
rs697356
rs77265585
rs342200
rs13412535
All
−0.5
0.0
0.5
1.0
MR leave−one−out sensitivity analysis for
'RAB13' on 'Pulmonary fibrosis'

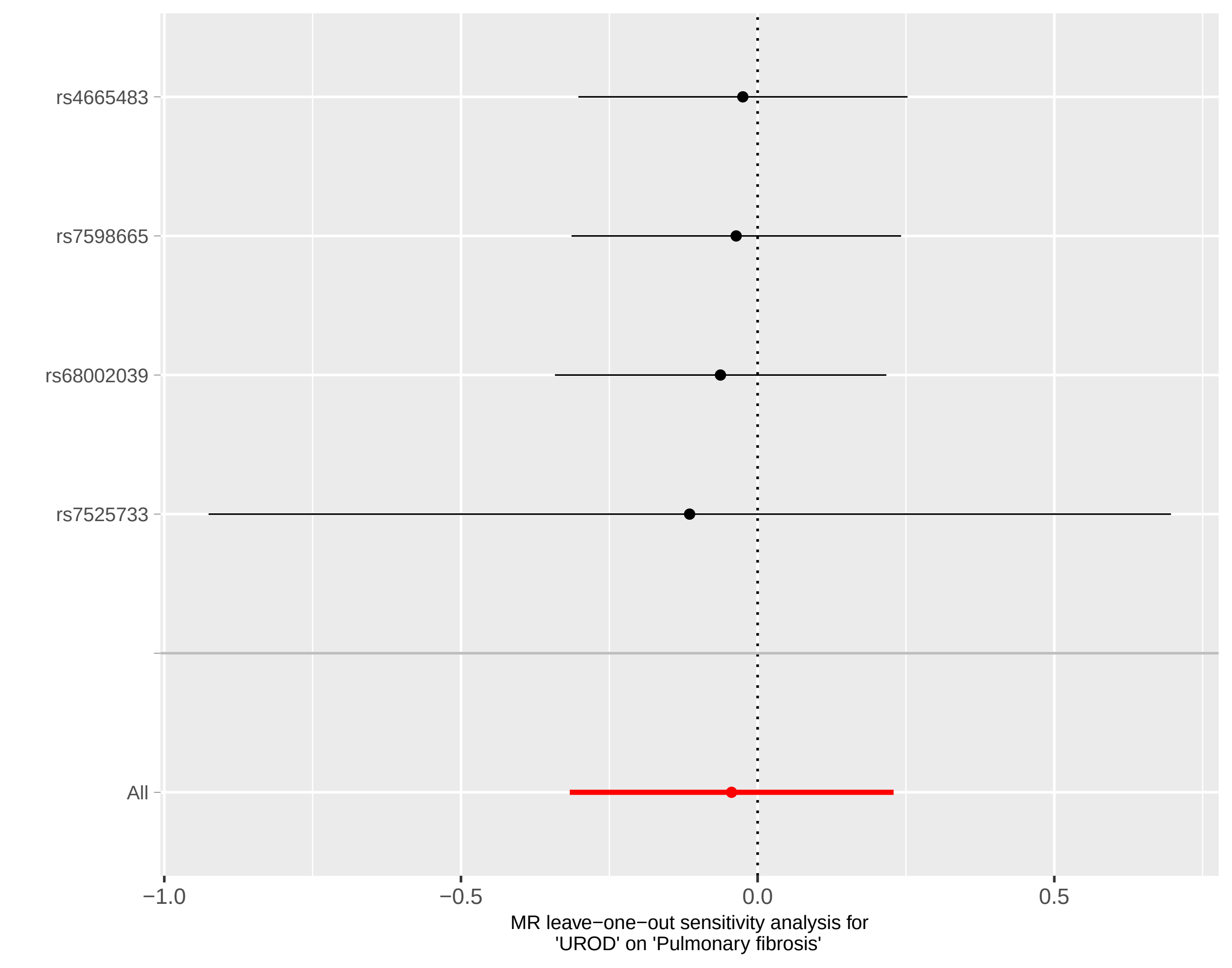

rs4665483
rs7598665
rs68002039
rs7525733
All
−1.0
−0.5
0.0
0.5
MR leave−one−out sensitivity analysis for
'UROD' on 'Pulmonary fibrosis'

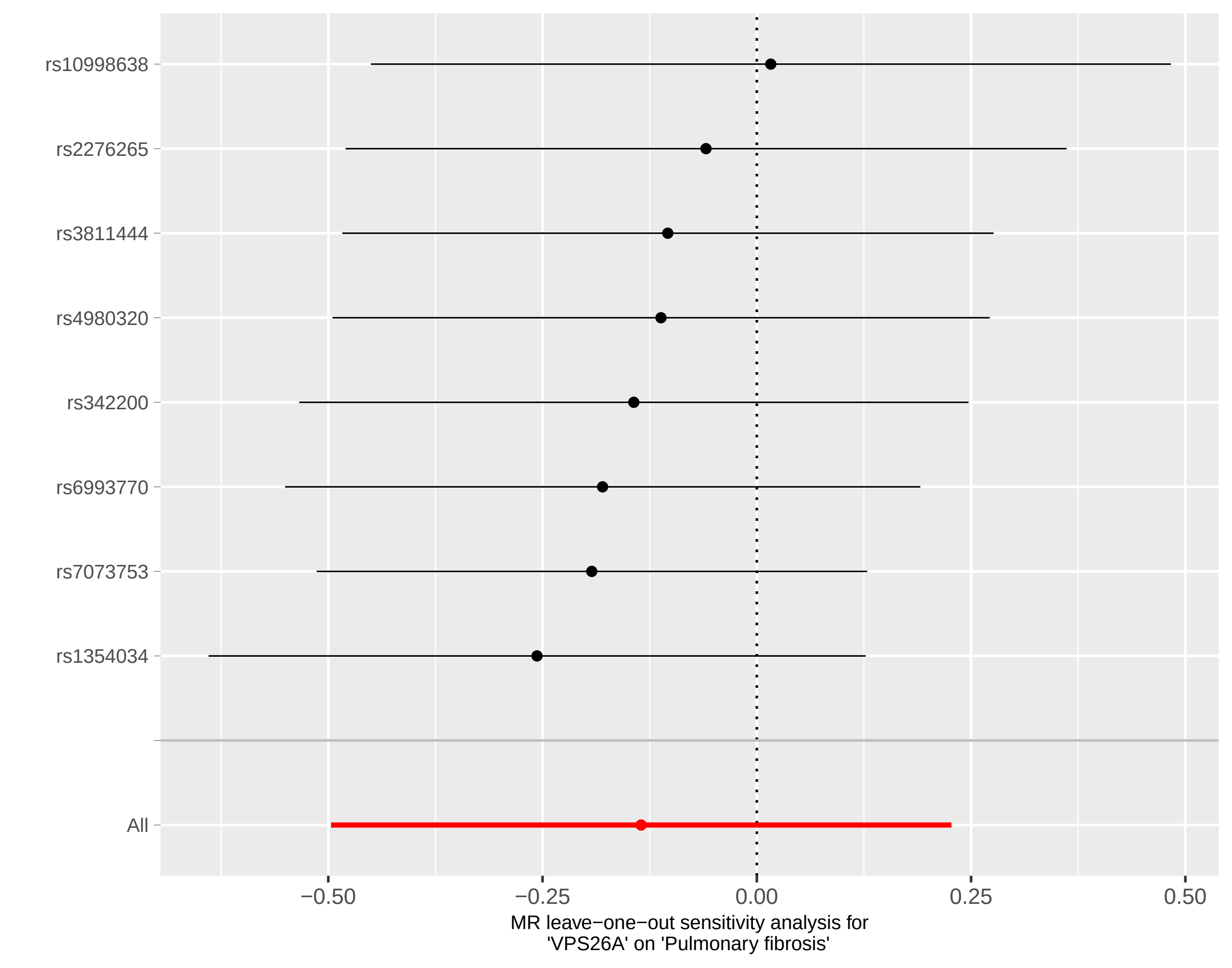
rs10998638
rs2276265
rs3811444
rs4980320
rs342200
rs6993770
rs7073753
rs1354034
All
−0.50
−0.25
0.00
0.25
0.50
MR leave−one−out sensitivity analysis for
'VPS26A' on 'Pulmonary fibrosis'

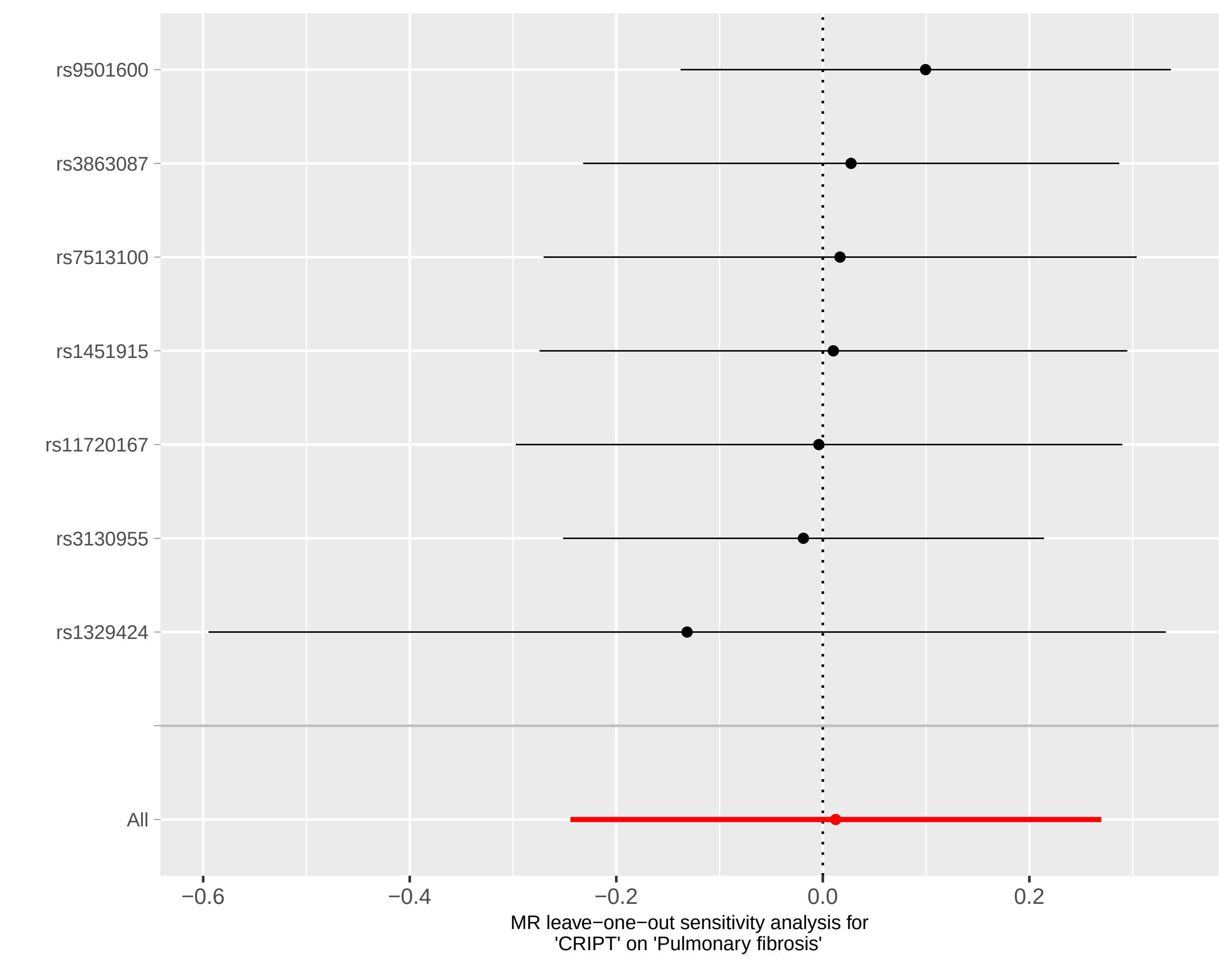
rs9501600
rs3863087
rs7513100
rs1451915
rs11720167
rs3130955
rs1329424
All
−0.6
−0.4
−0.2
0.0
0.2
MR leave−one−out sensitivity analysis for
'CRIPT' on 'Pulmonary fibrosis'

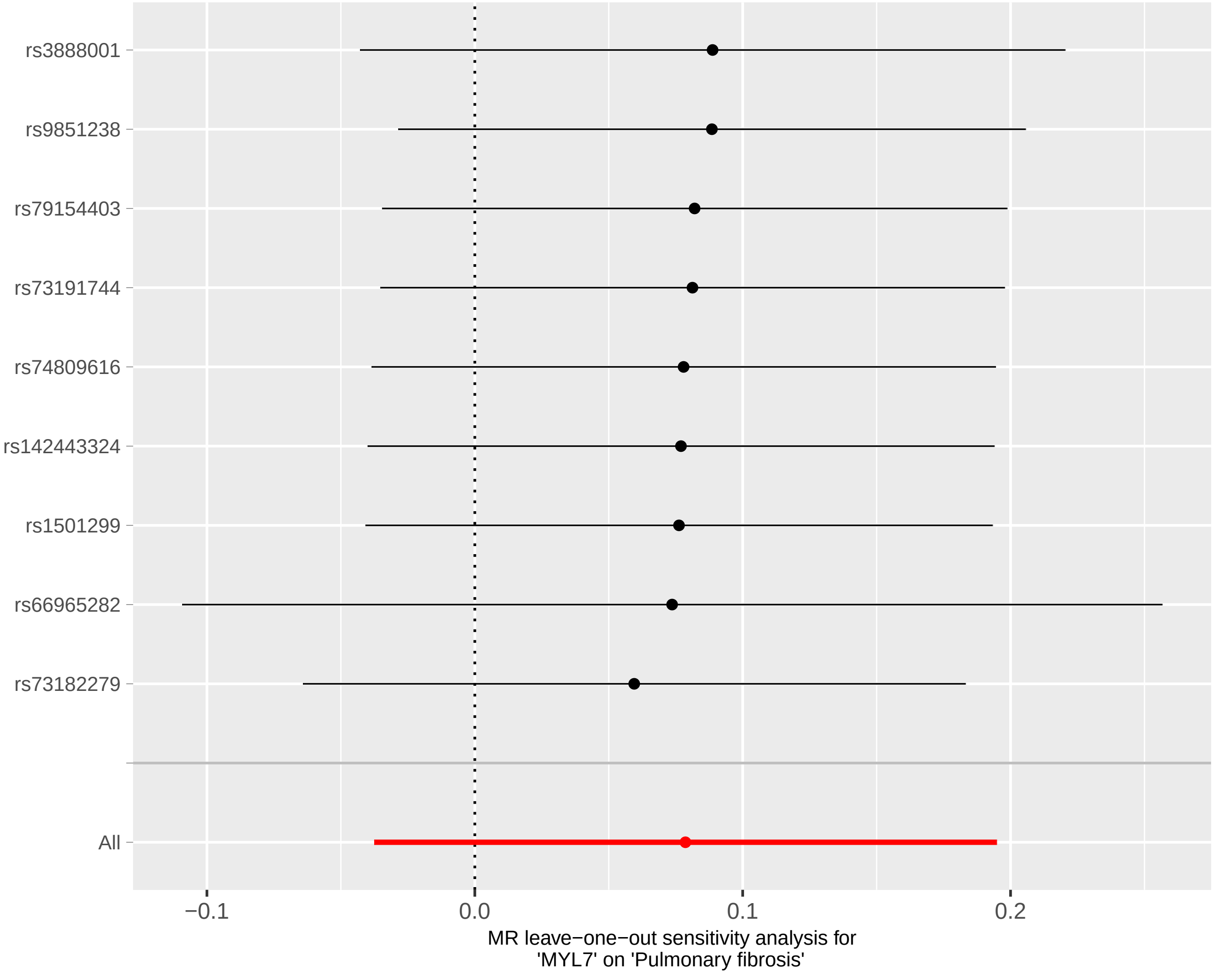
rs3888001
rs9851238
rs79154403
rs73191744
rs74809616
rs142443324
rs1501299
rs66965282
rs73182279
All
−0.1
0.0
0.1
0.2
MR leave−one−out sensitivity analysis for
'MYL7' on 'Pulmonary fibrosis'

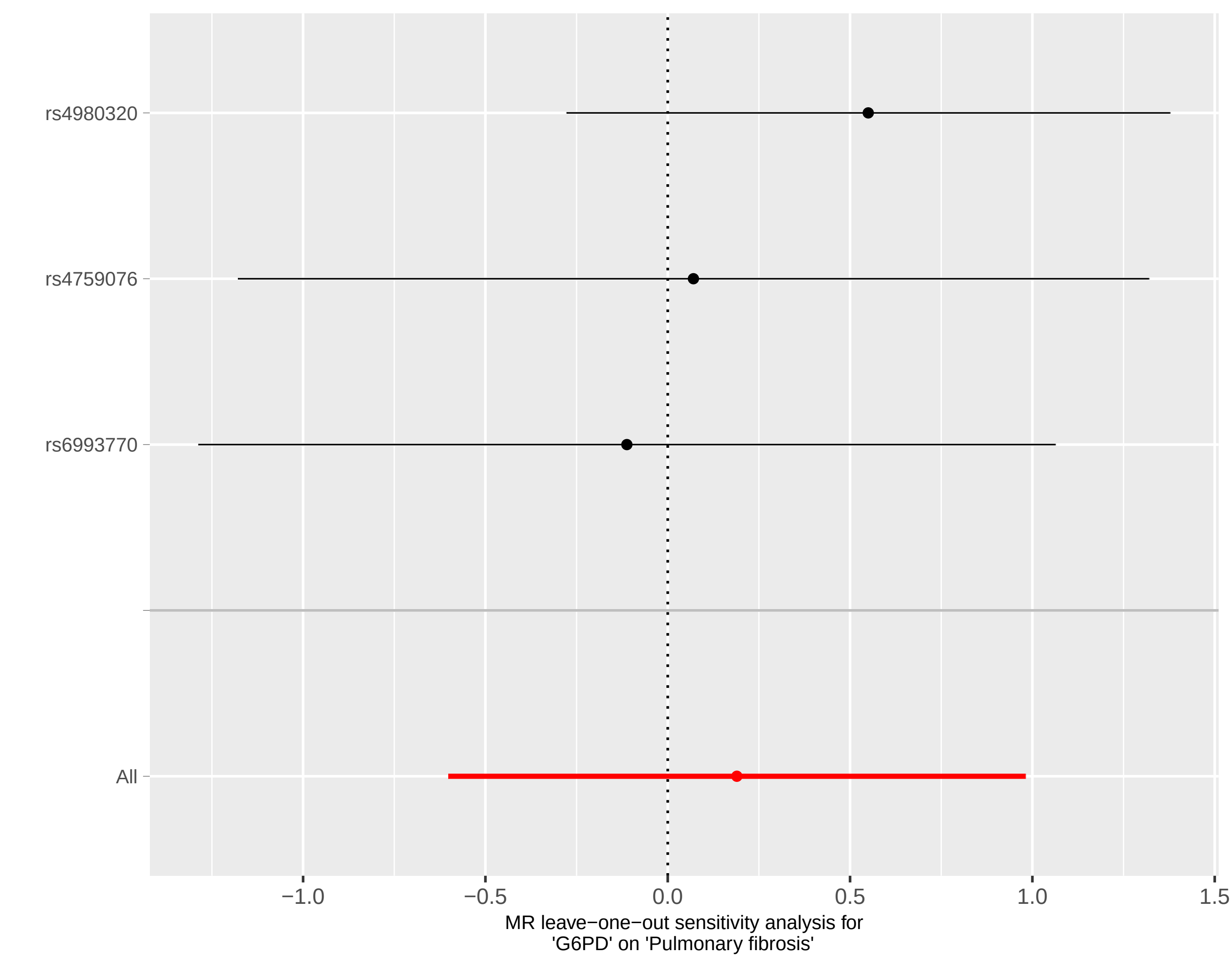
rs4980320
rs4759076
rs6993770
All
−1.0
−0.5
0.0
0.5
1.0
1.5
MR leave−one−out sensitivity analysis for
'G6PD' on 'Pulmonary fibrosis'

Insufficient number of SNPs

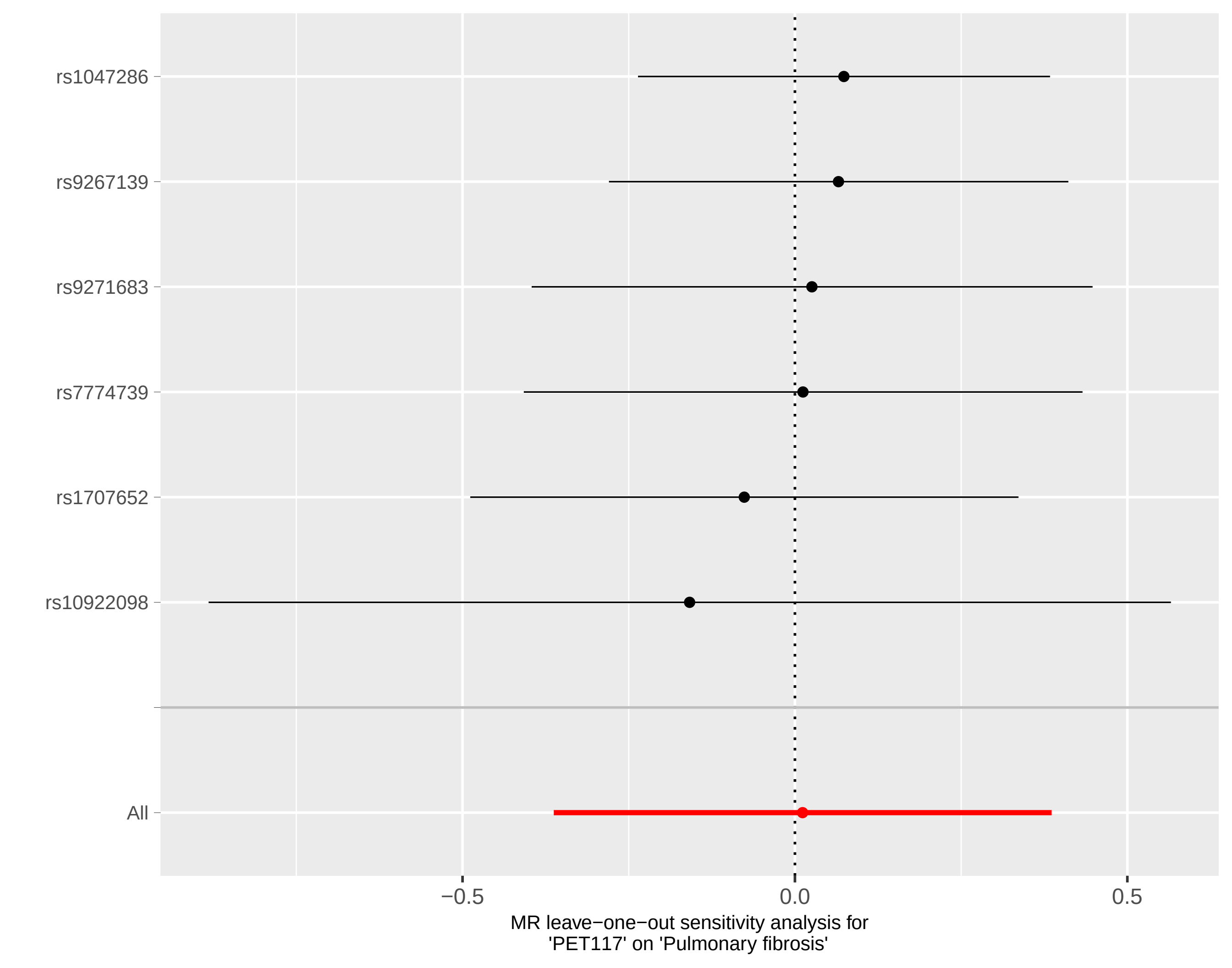
rs1047286
rs9267139
rs9271683
rs7774739
rs1707652
rs10922098
All
−0.5
0.0
0.5
MR leave−one−out sensitivity analysis for
'PET117' on 'Pulmonary fibrosis'

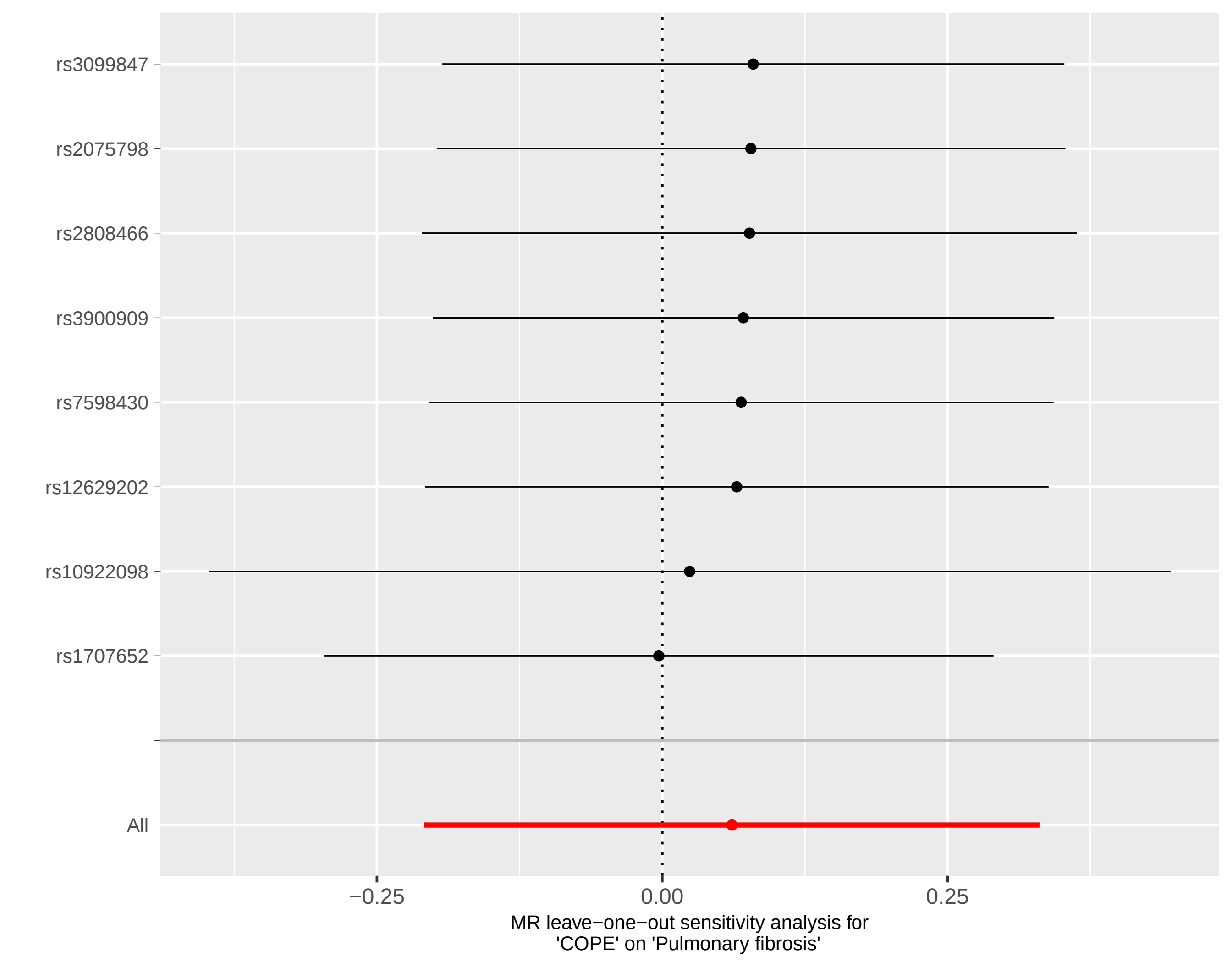

rs3099847
rs2075798
rs2808466
rs3900909
rs7598430
rs12629202
rs10922098
rs1707652
All
−0.25
0.00
0.25
MR leave−one−out sensitivity analysis for
'COPE' on 'Pulmonary fibrosis'

Insufficient number of SNPs

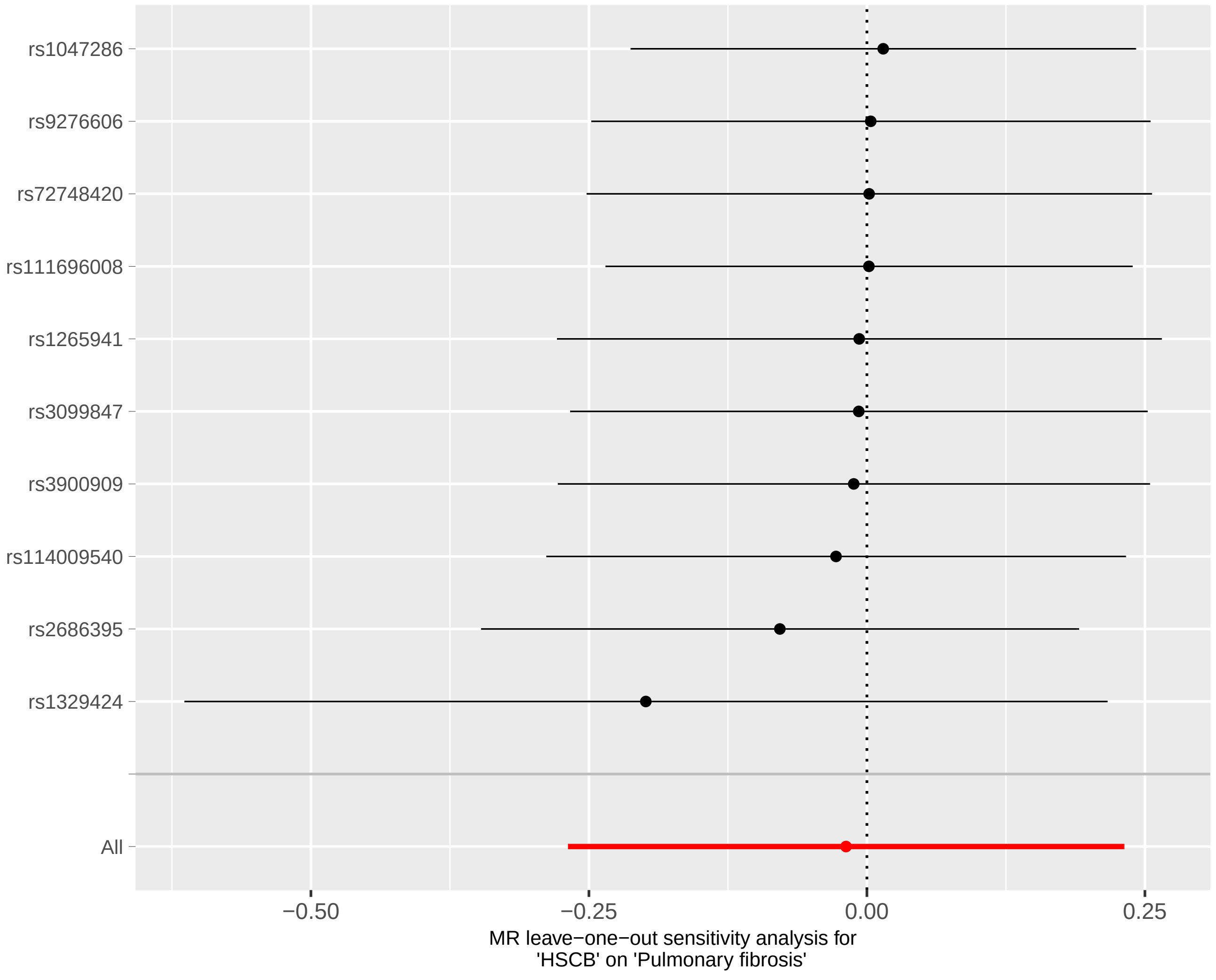
rs1047286
rs9276606
rs72748420
rs111696008
rs1265941
rs3099847
rs3900909
rs114009540
rs2686395
rs1329424
All
−0.50
−0.25
0.00
0.25
MR leave−one−out sensitivity analysis for
'HSCB' on 'Pulmonary fibrosis'

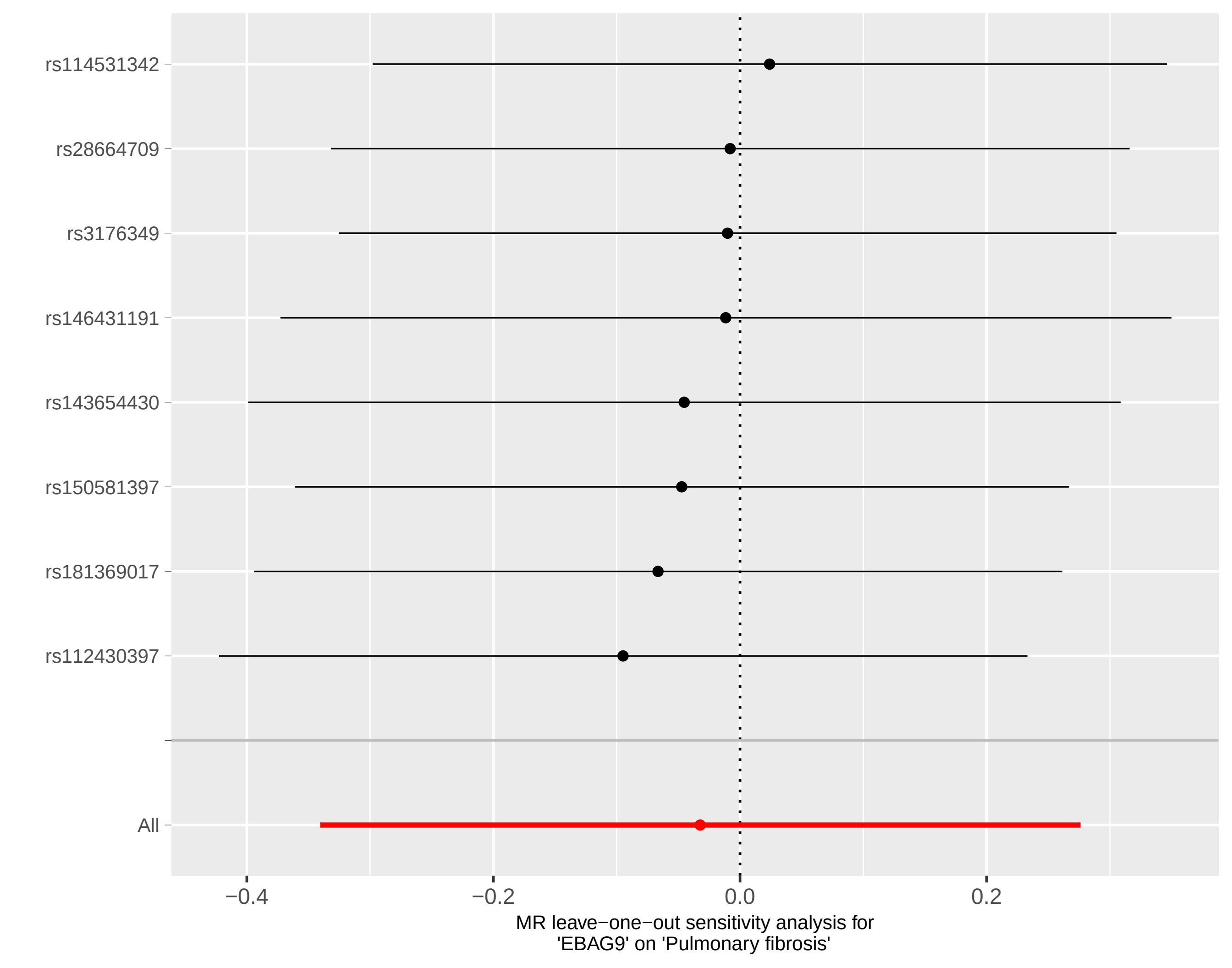
rs114531342
rs28664709
rs3176349
rs146431191
rs143654430
rs150581397
rs181369017
rs112430397
All
−0.4
−0.2
0.0
0.2
MR leave−one−out sensitivity analysis for
'EBAG9' on 'Pulmonary fibrosis'

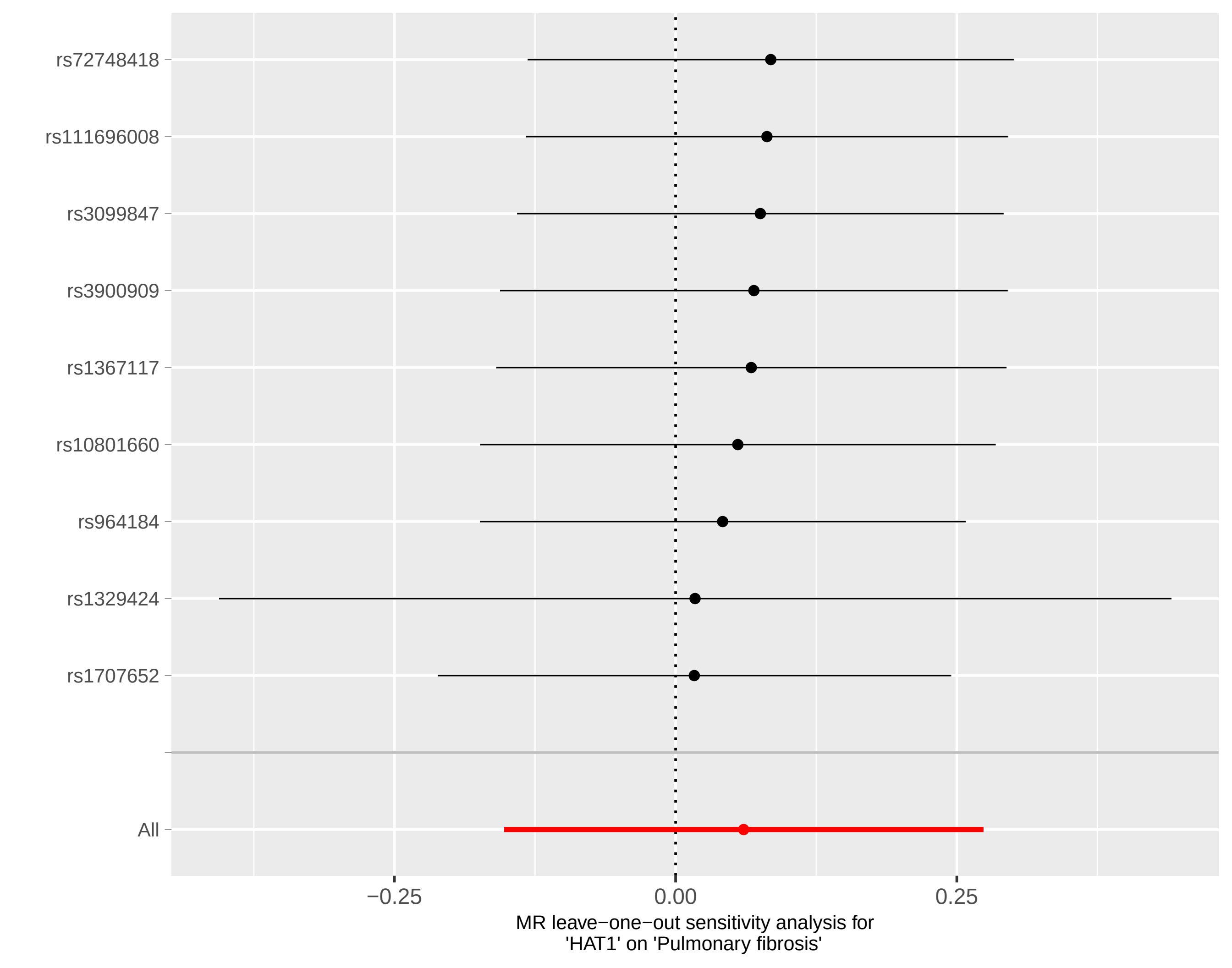

rs72748418
rs111696008
rs3099847
rs3900909
rs1367117
rs10801660
rs964184
rs1329424
rs1707652
All
−0.25
0.00
0.25
MR leave−one−out sensitivity analysis for
'HAT1' on 'Pulmonary fibrosis'

Insufficient number of SNPs

Insufficient number of SNPs

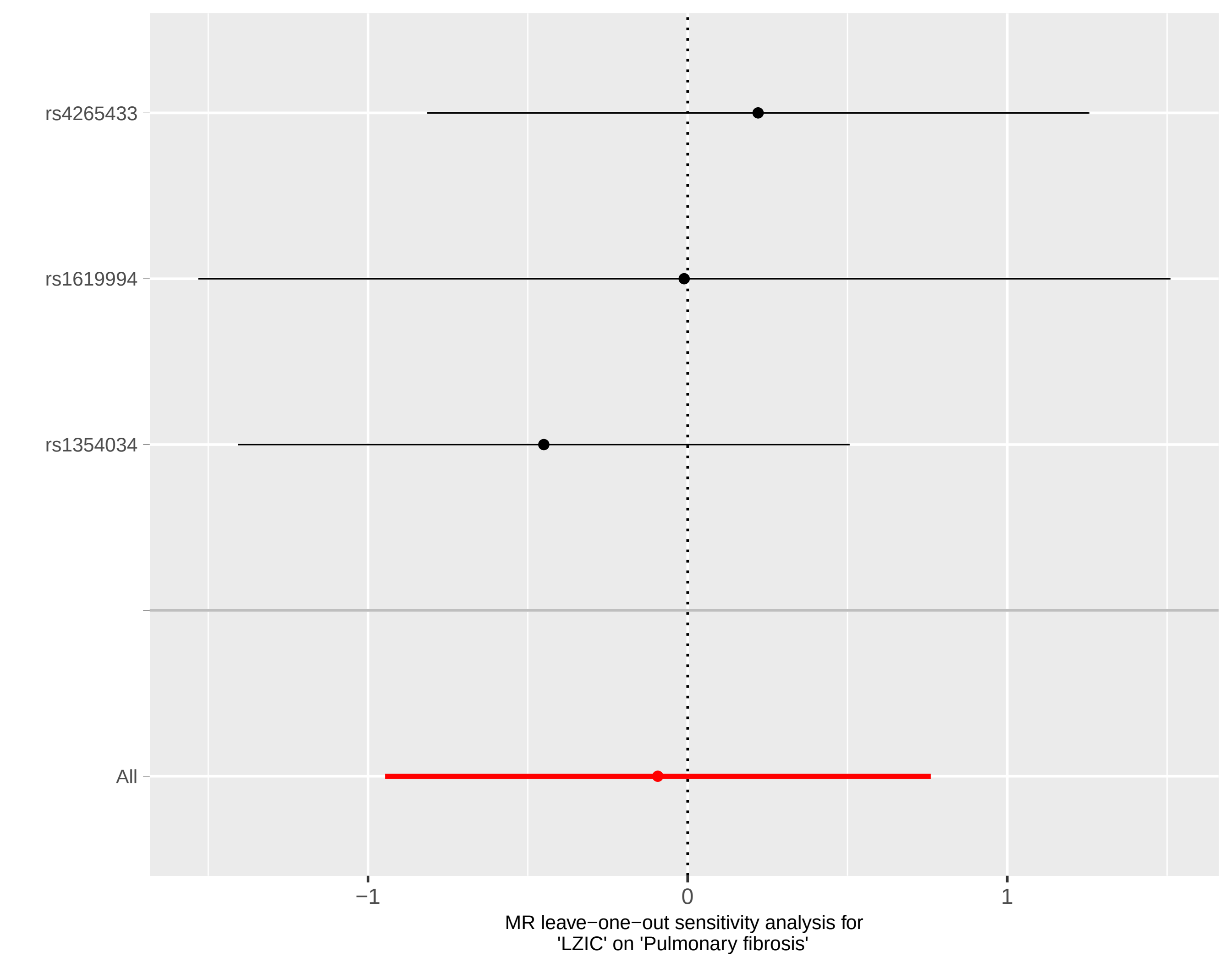

rs4265433
rs1619994
rs1354034
All
−1
0
1
MR leave−one−out sensitivity analysis for
'LZIC' on 'Pulmonary fibrosis'

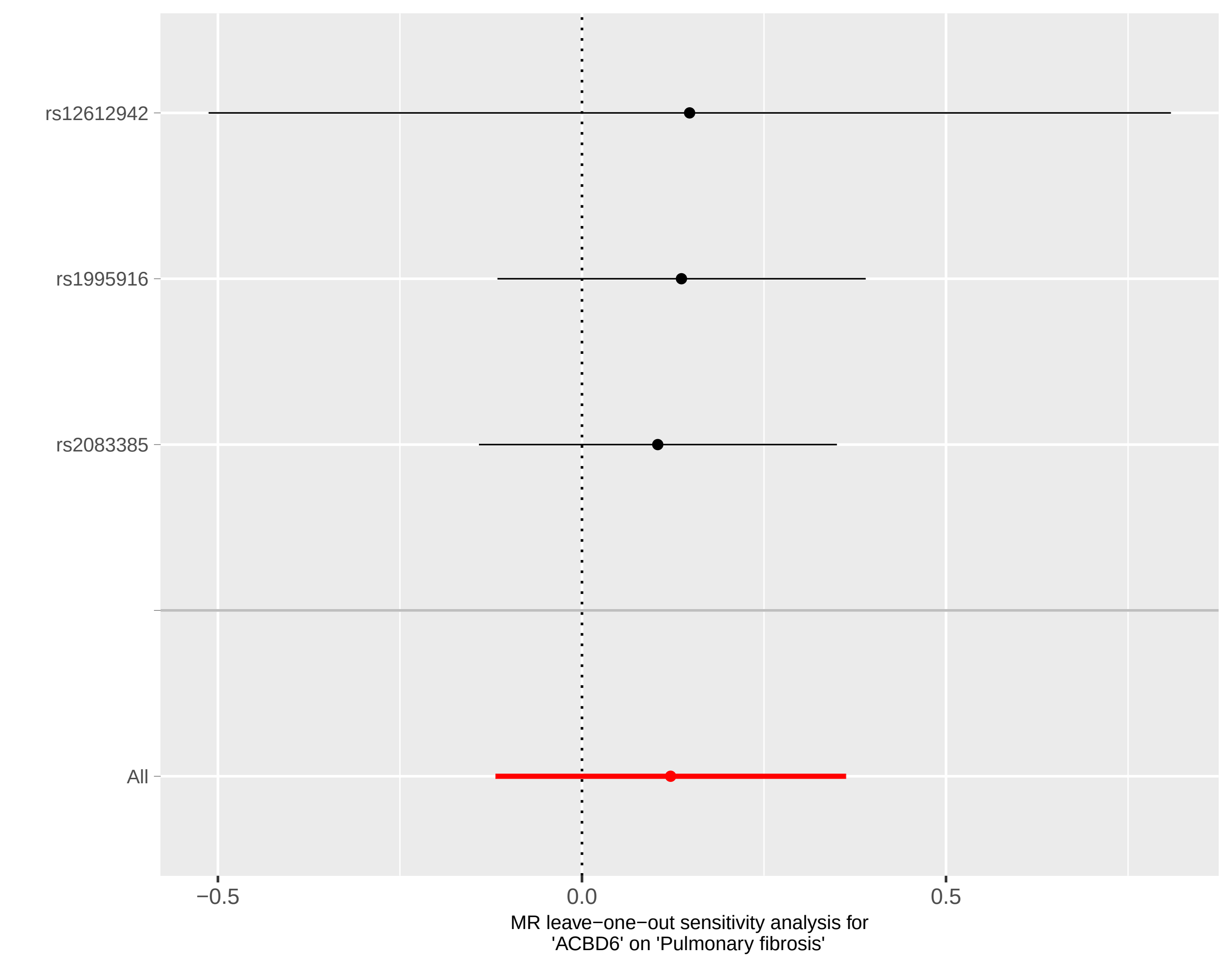
rs12612942
rs1995916
rs2083385
All
−0.5
0.0
0.5
MR leave−one−out sensitivity analysis for
'ACBD6' on 'Pulmonary fibrosis'

Insufficient number of SNPs

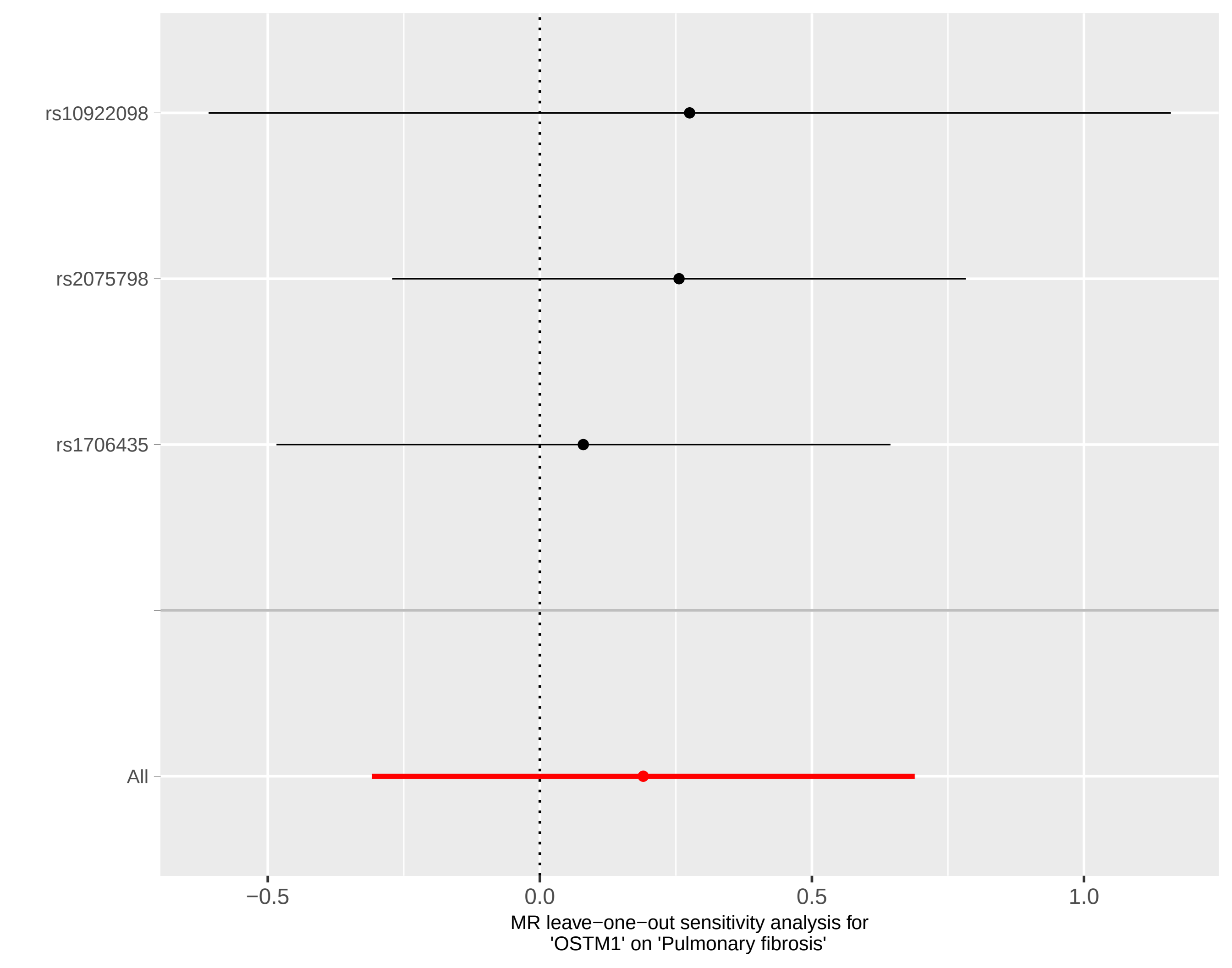
rs10922098
rs2075798
rs1706435
All
−0.5
0.0
0.5
1.0
MR leave−one−out sensitivity analysis for
'OSTM1' on 'Pulmonary fibrosis'

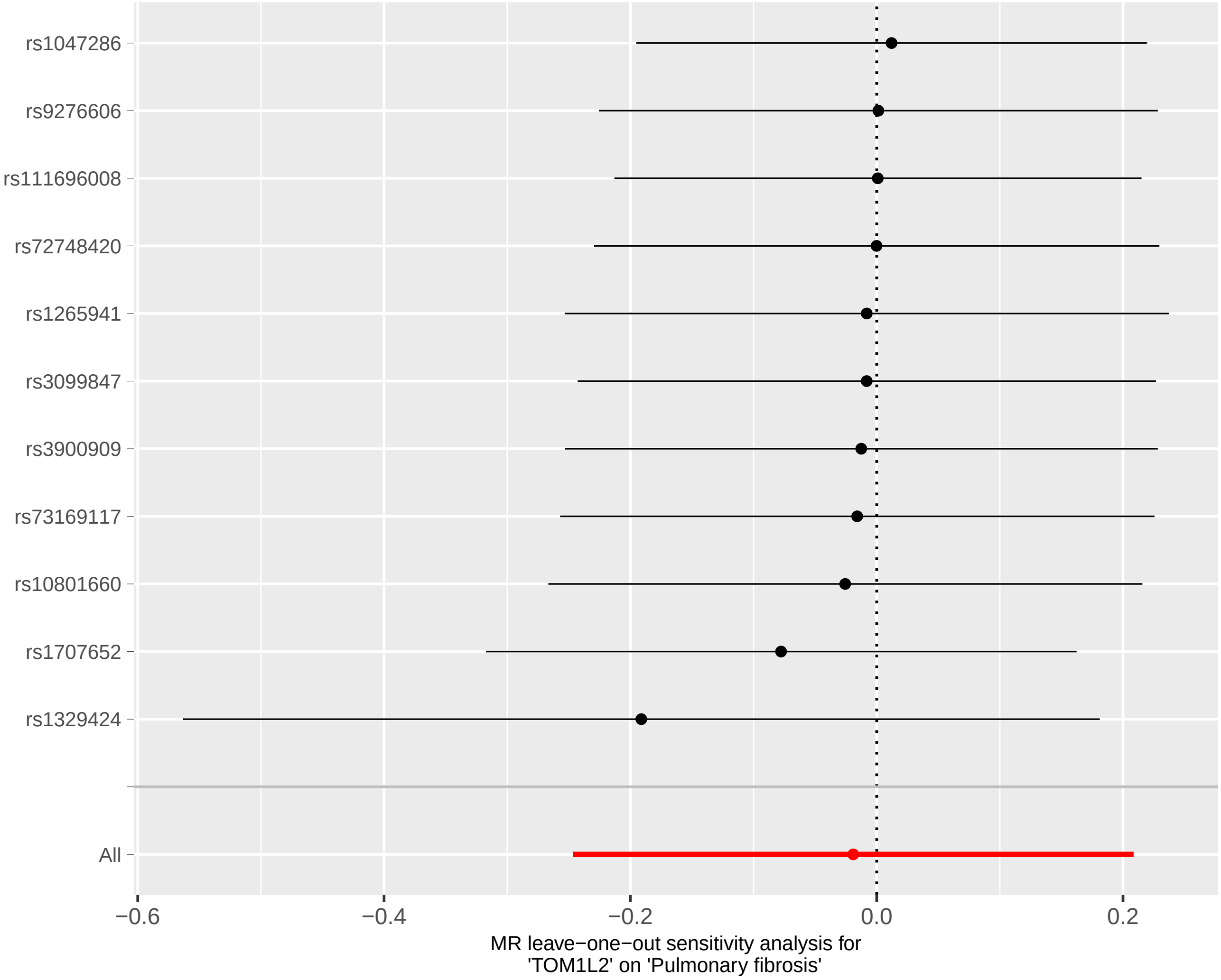
rs1047286
rs9276606
rs111696008
rs72748420
rs1265941
rs3099847
rs3900909
rs73169117
rs10801660
rs1707652
rs1329424
All
−0.6
−0.4
−0.2
0.0
0.2
MR leave−one−out sensitivity analysis for
'TOM1L2' on 'Pulmonary fibrosis'

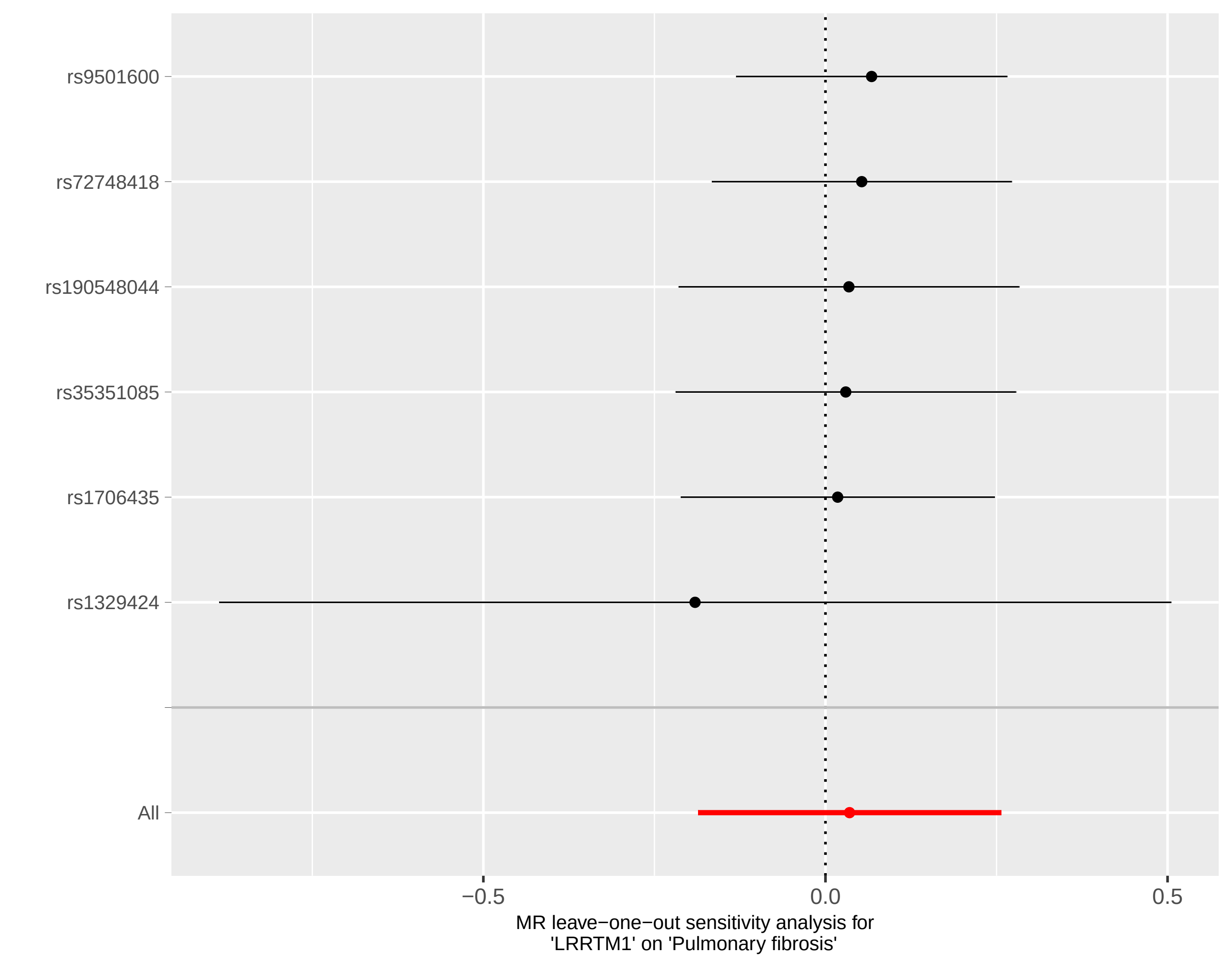
rs9501600
rs72748418
rs190548044
rs35351085
rs1706435
rs1329424
All
−0.5
0.0
0.5
MR leave−one−out sensitivity analysis for
'LRRTM1' on 'Pulmonary fibrosis'

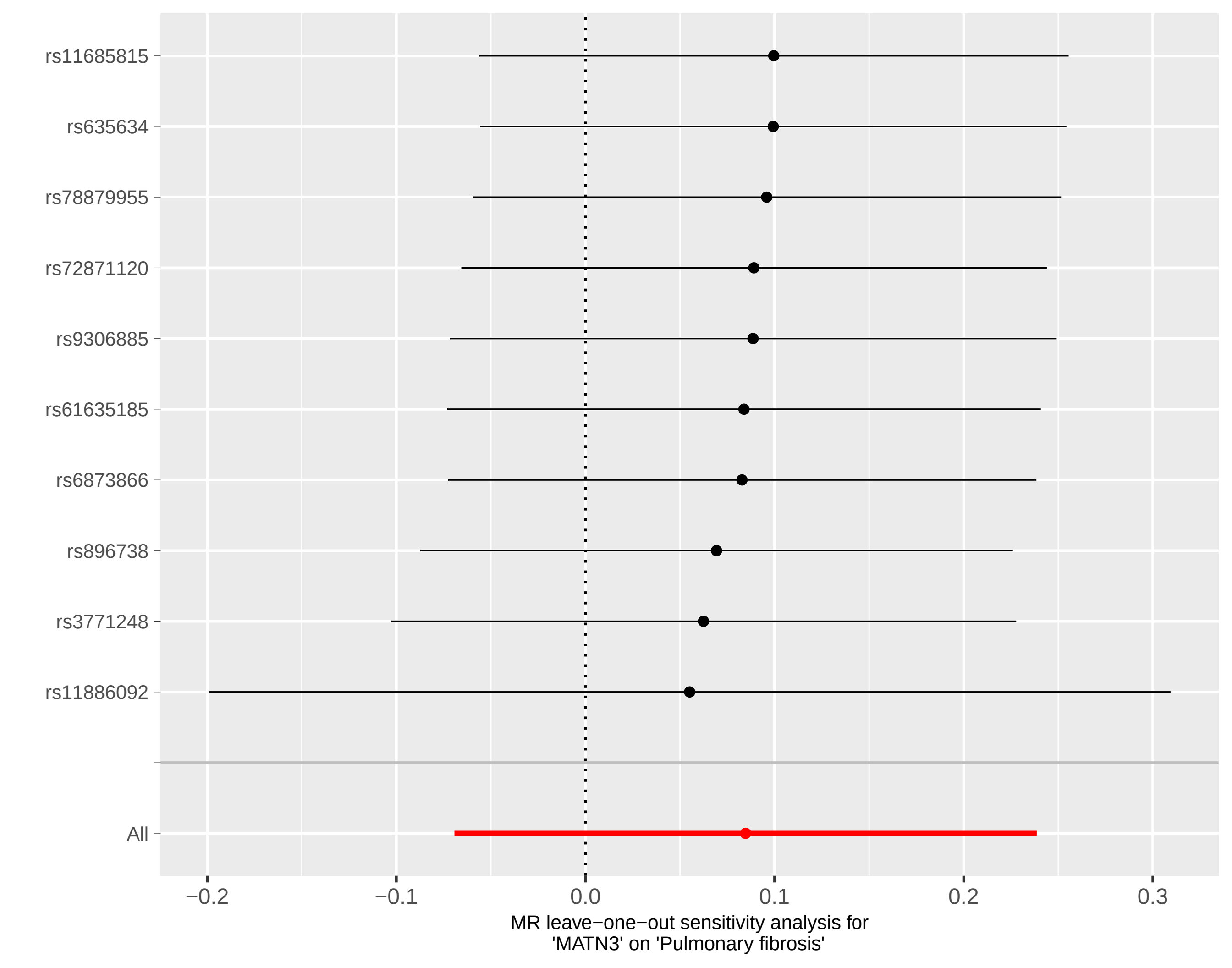

rs11685815
rs635634
rs78879955
rs72871120
rs9306885
rs61635185
rs6873866
rs896738
rs3771248
rs11886092
All
−0.2
−0.1
0.0
0.1
0.2
0.3
MR leave−one−out sensitivity analysis for
'MATN3' on 'Pulmonary fibrosis'

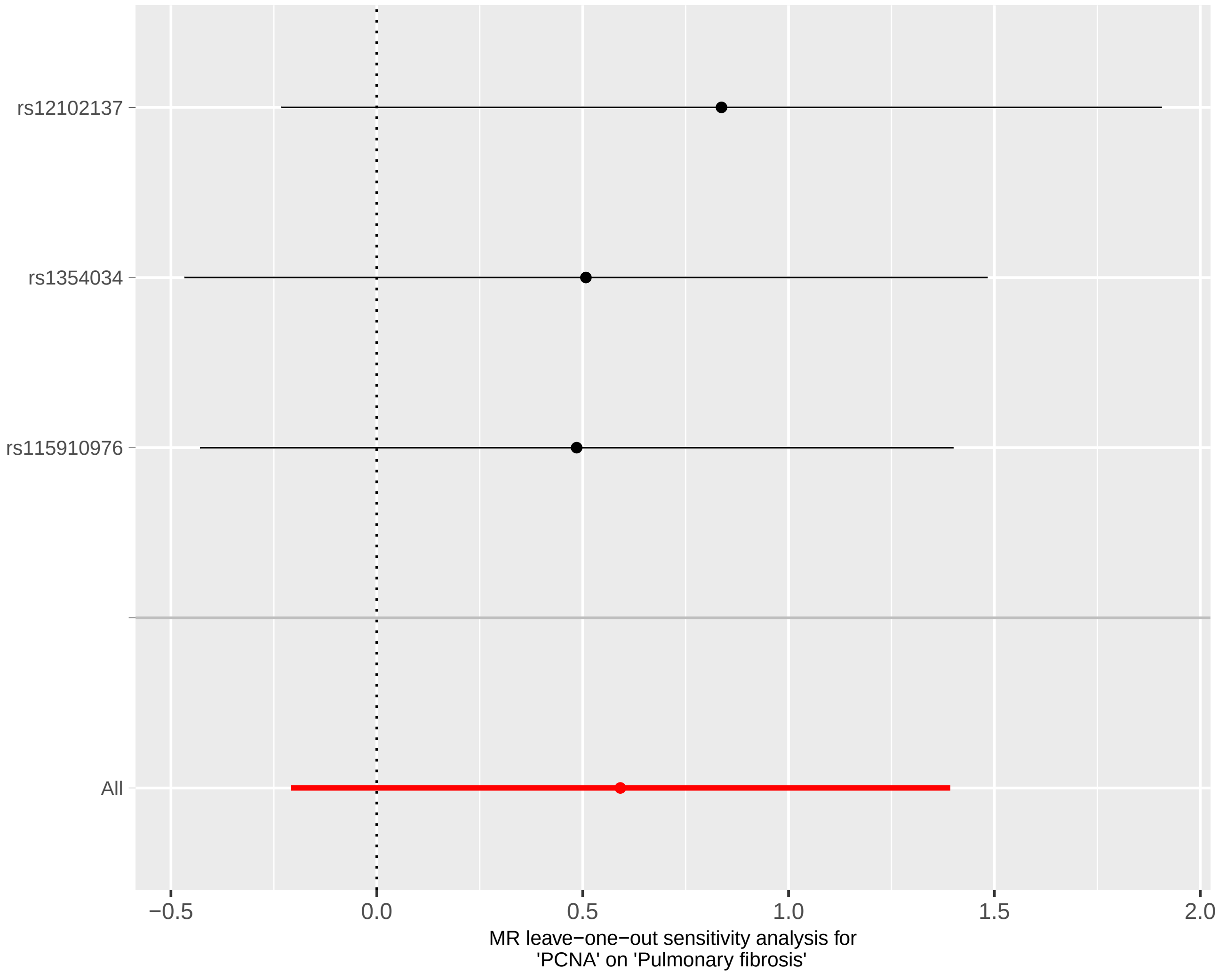
rs12102137
rs1354034
rs115910976
All
−0.5
0.0
0.5
1.0
1.5
2.0
MR leave−one−out sensitivity analysis for
'PCNA' on 'Pulmonary fibrosis'

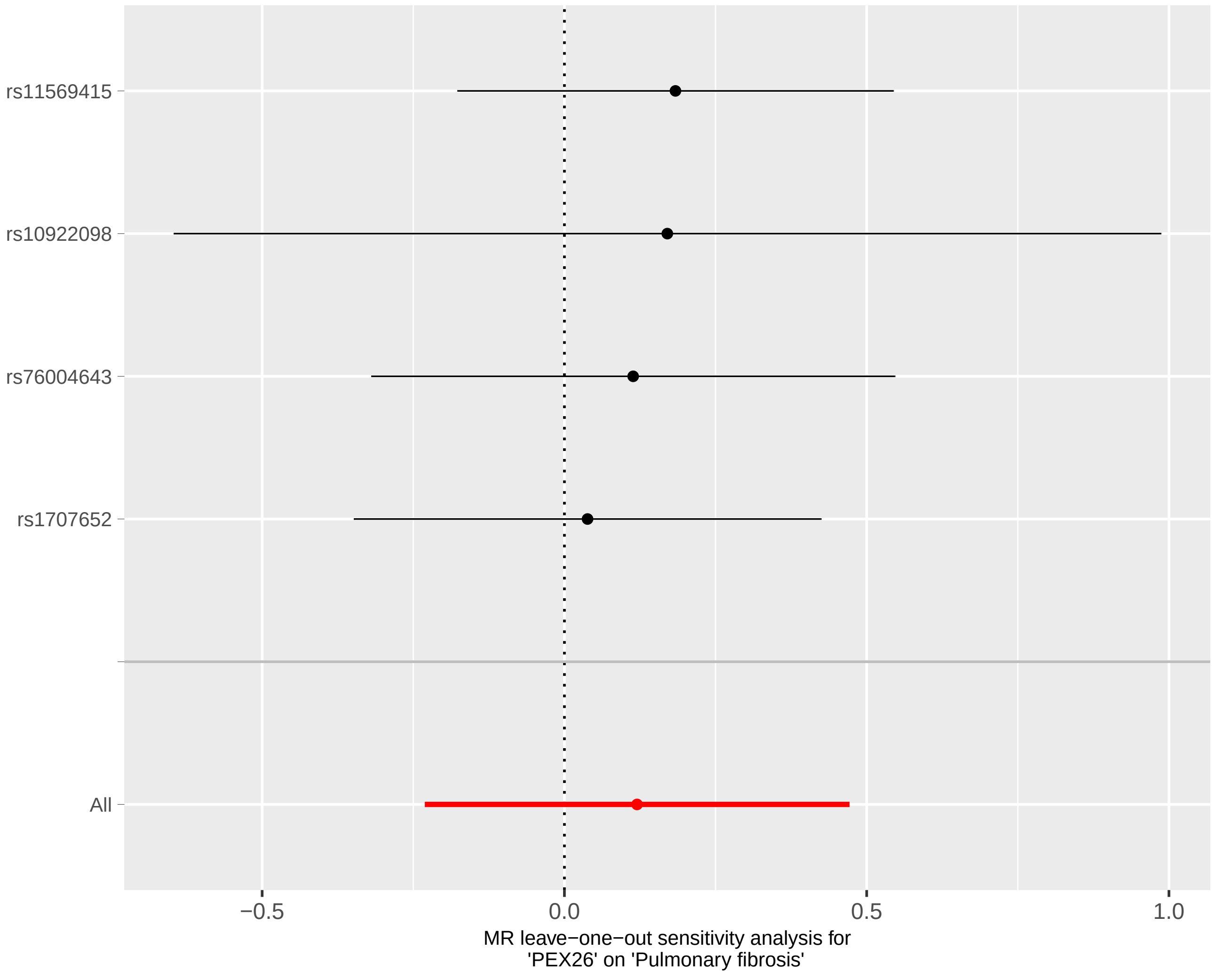
rs11569415
rs10922098
rs76004643
rs1707652
All
−0.5
0.0
0.5
1.0
MR leave−one−out sensitivity analysis for
'PEX26' on 'Pulmonary fibrosis'

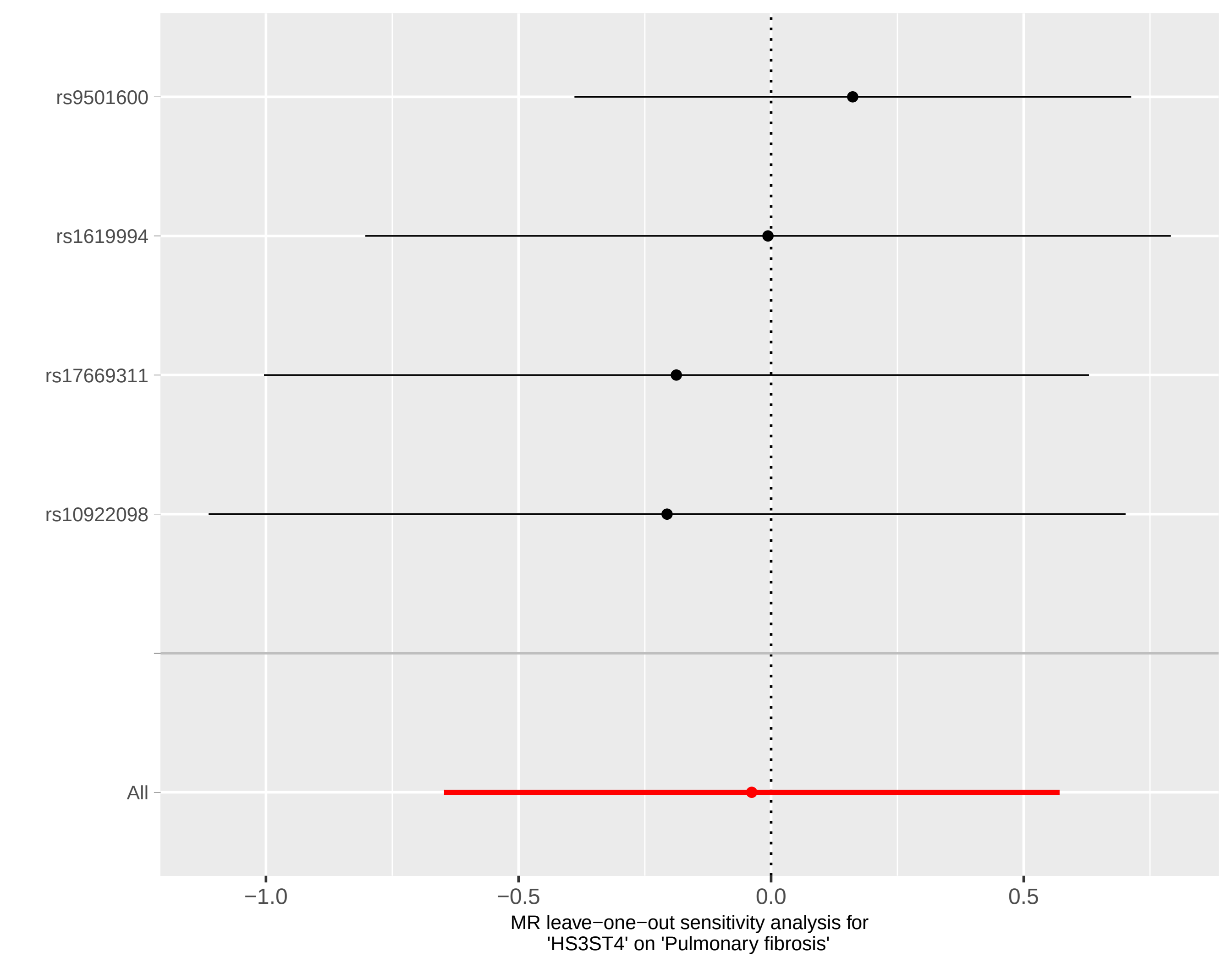
rs9501600
rs1619994
rs17669311
rs10922098
All
−1.0
−0.5
0.0
0.5
MR leave−one−out sensitivity analysis for
'HS3ST4' on 'Pulmonary fibrosis'

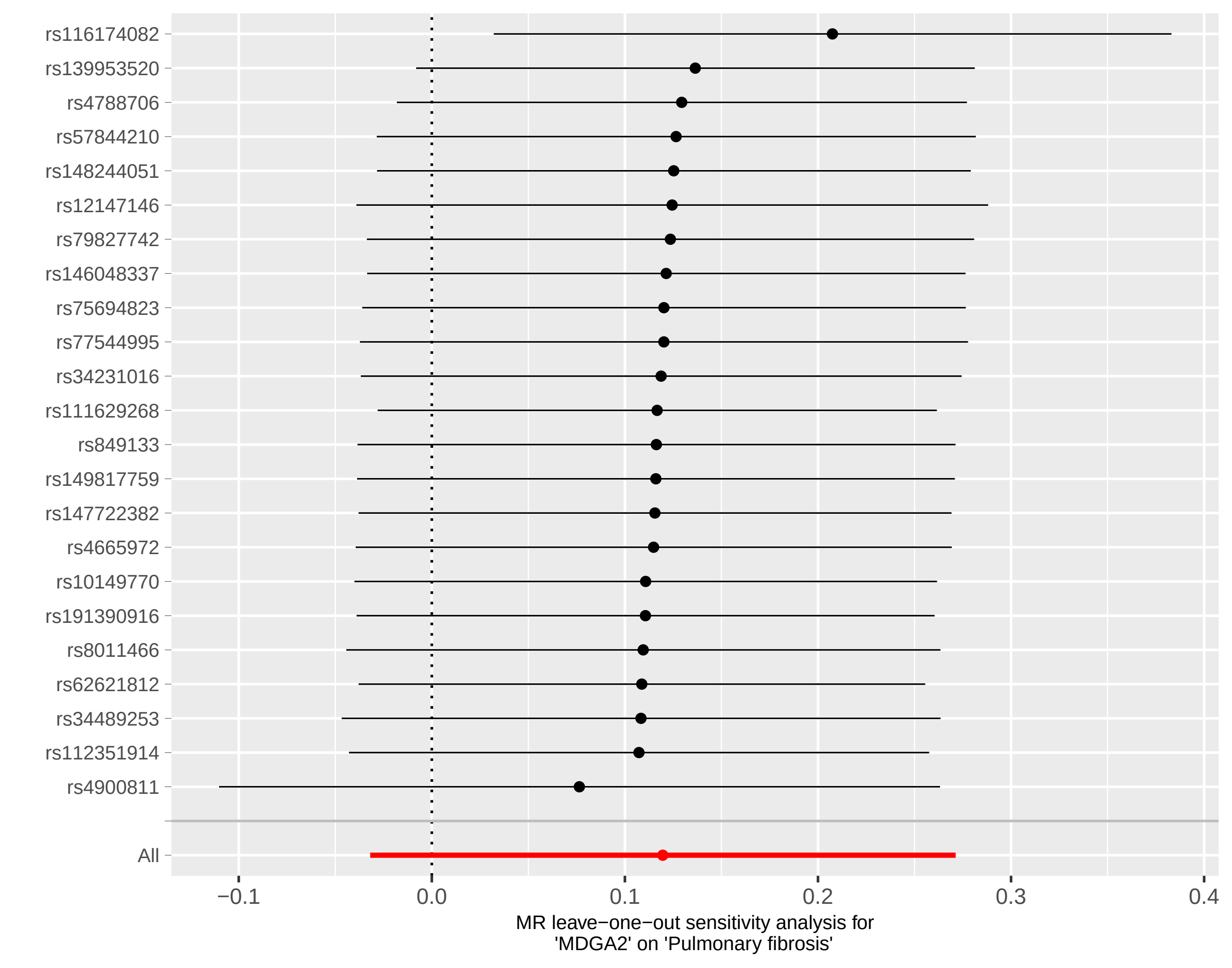

rs116174082
rs139953520
rs4788706
rs57844210
rs148244051
rs12147146
rs79827742
rs146048337
rs75694823
rs77544995
rs34231016
rs111629268
rs849133
rs149817759
rs147722382
rs4665972
rs10149770
rs191390916
rs8011466
rs62621812
rs34489253
rs112351914
rs4900811
All
−0.1
0.0
0.1
0.2
0.3
0.4
MR leave−one−out sensitivity analysis for
'MDGA2' on 'Pulmonary fibrosis'

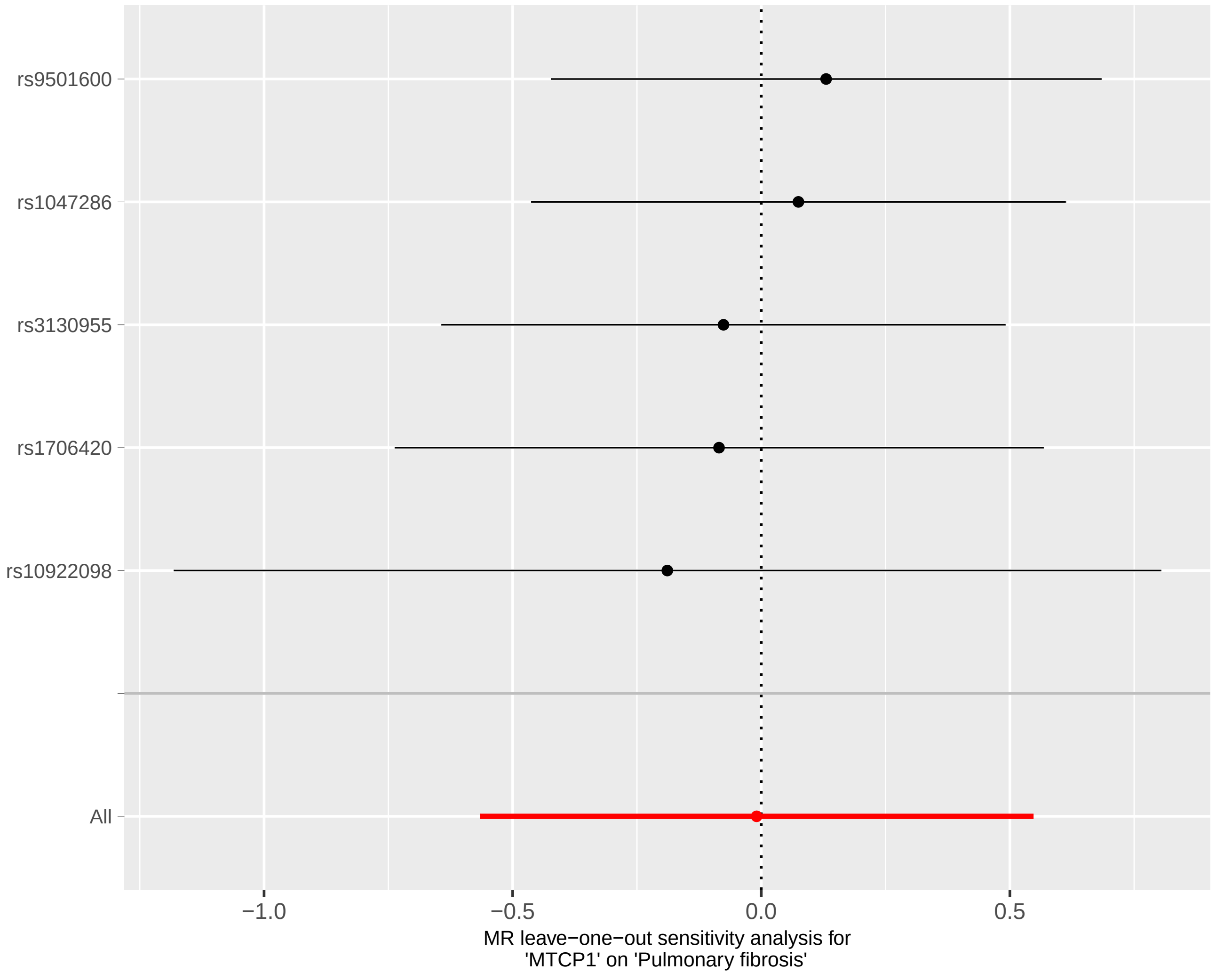
rs9501600
rs1047286
rs3130955
rs1706420
rs10922098
All
−1.0
−0.5
0.0
0.5
MR leave−one−out sensitivity analysis for
'MTCP1' on 'Pulmonary fibrosis'

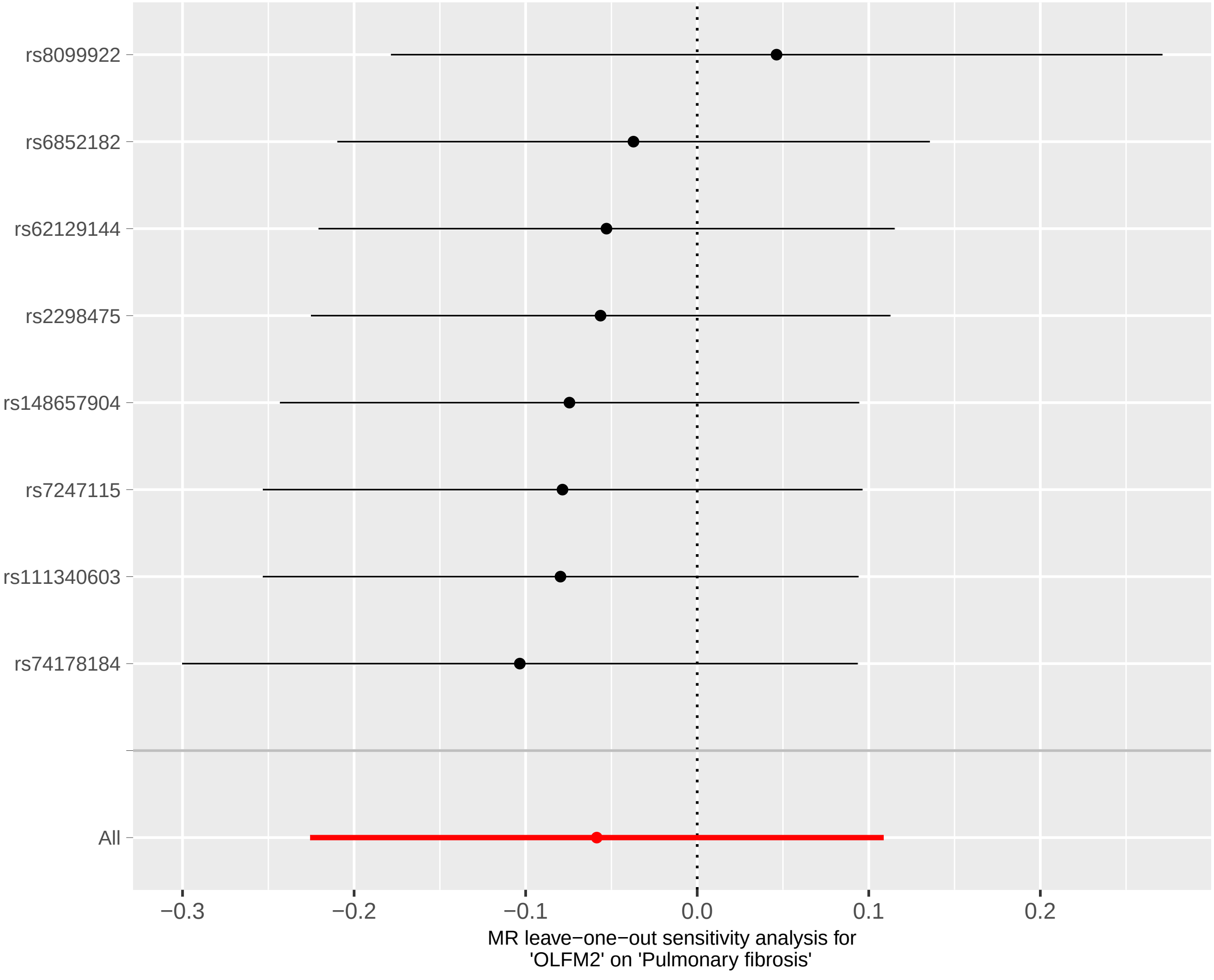
rs8099922
rs6852182
rs62129144
rs2298475
rs148657904
rs7247115
rs111340603
rs74178184
All
−0.3
−0.2
−0.1
0.0
0.1
0.2
MR leave−one−out sensitivity analysis for
'OLFM2' on 'Pulmonary fibrosis'

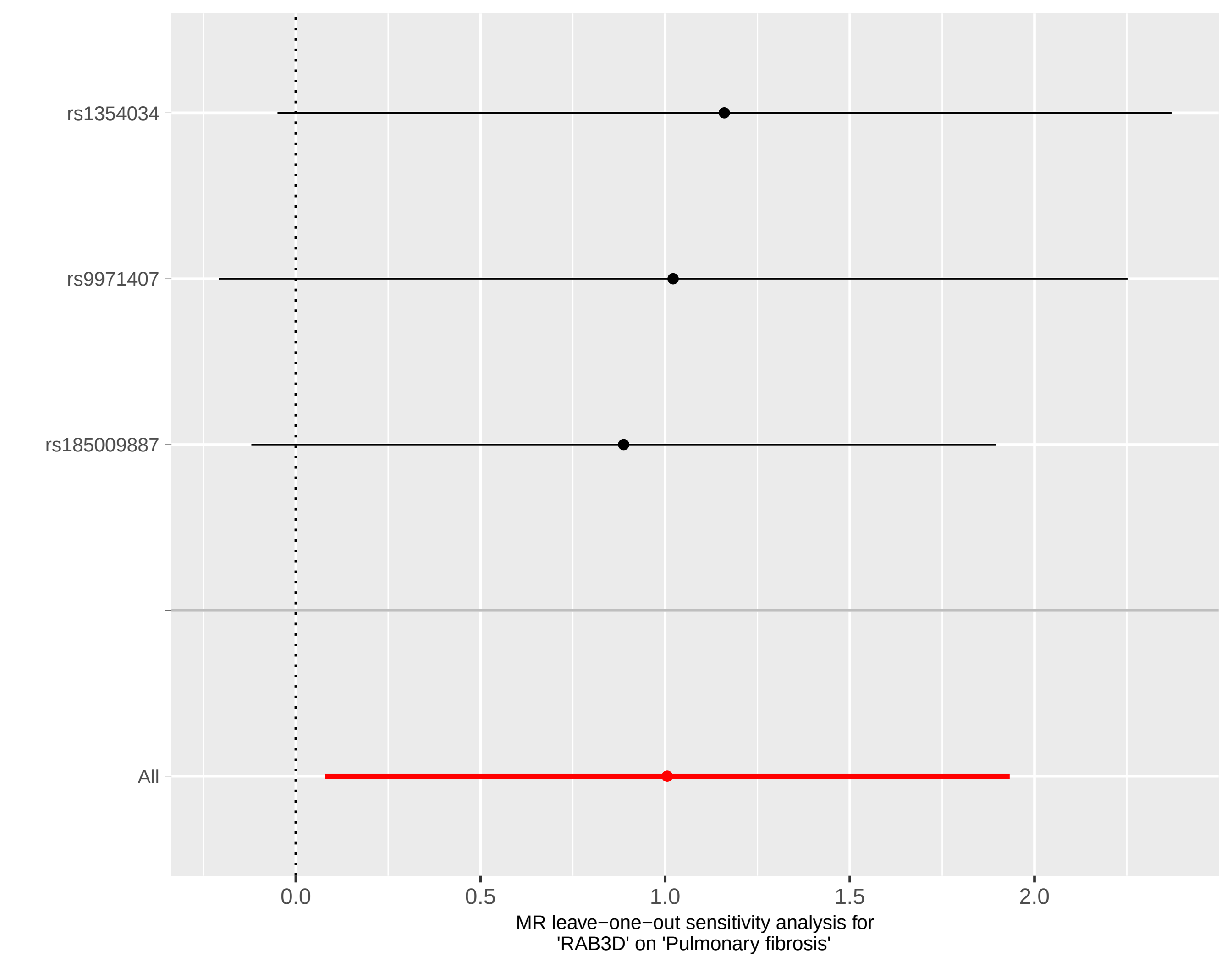

rs1354034
rs9971407
rs185009887
All
0.0
0.5
1.0
1.5
2.0
MR leave−one−out sensitivity analysis for
'RAB3D' on 'Pulmonary fibrosis'

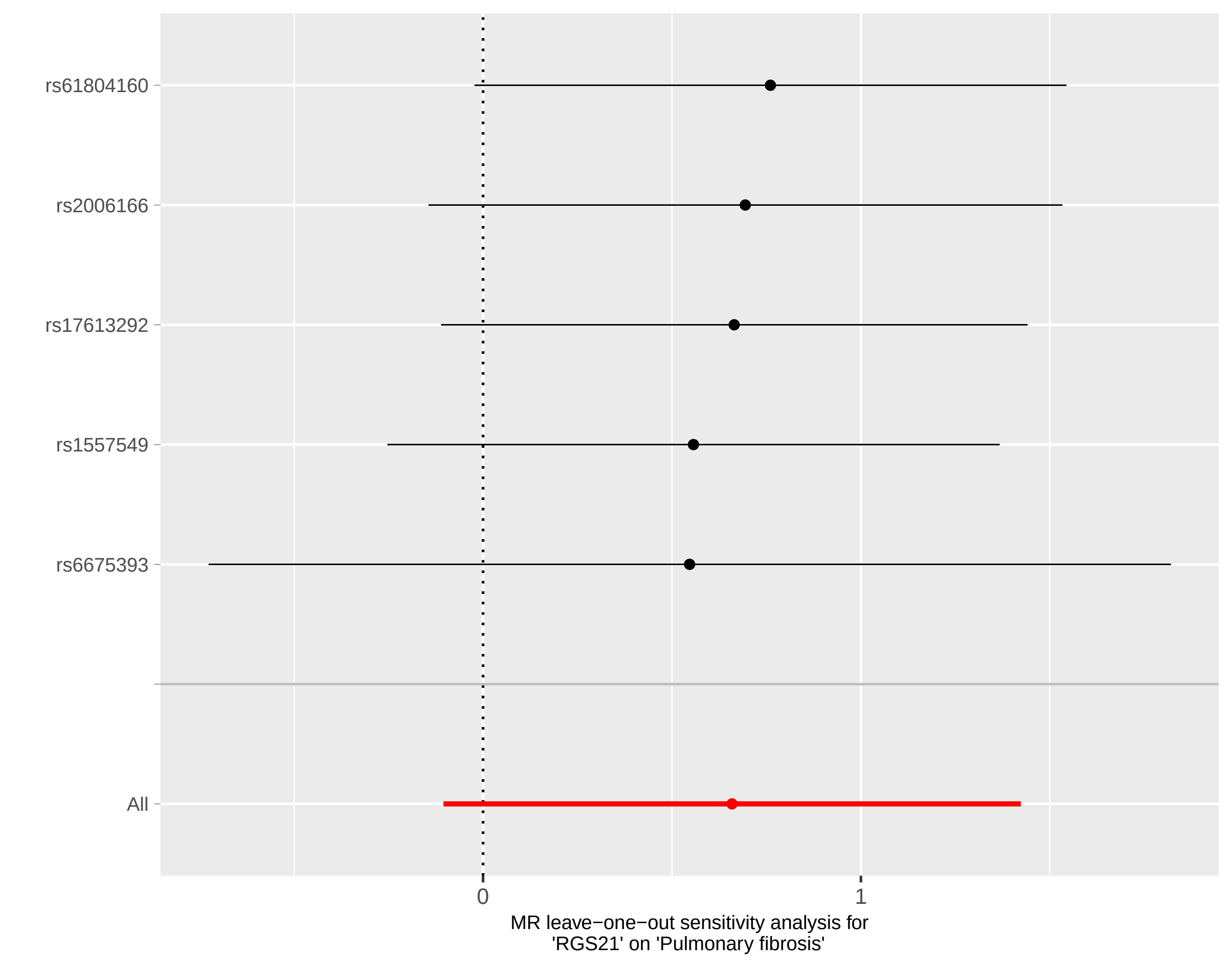
rs61804160
rs2006166
rs17613292
rs1557549
rs6675393
All
0
1
MR leave−one−out sensitivity analysis for
'RGS21' on 'Pulmonary fibrosis'

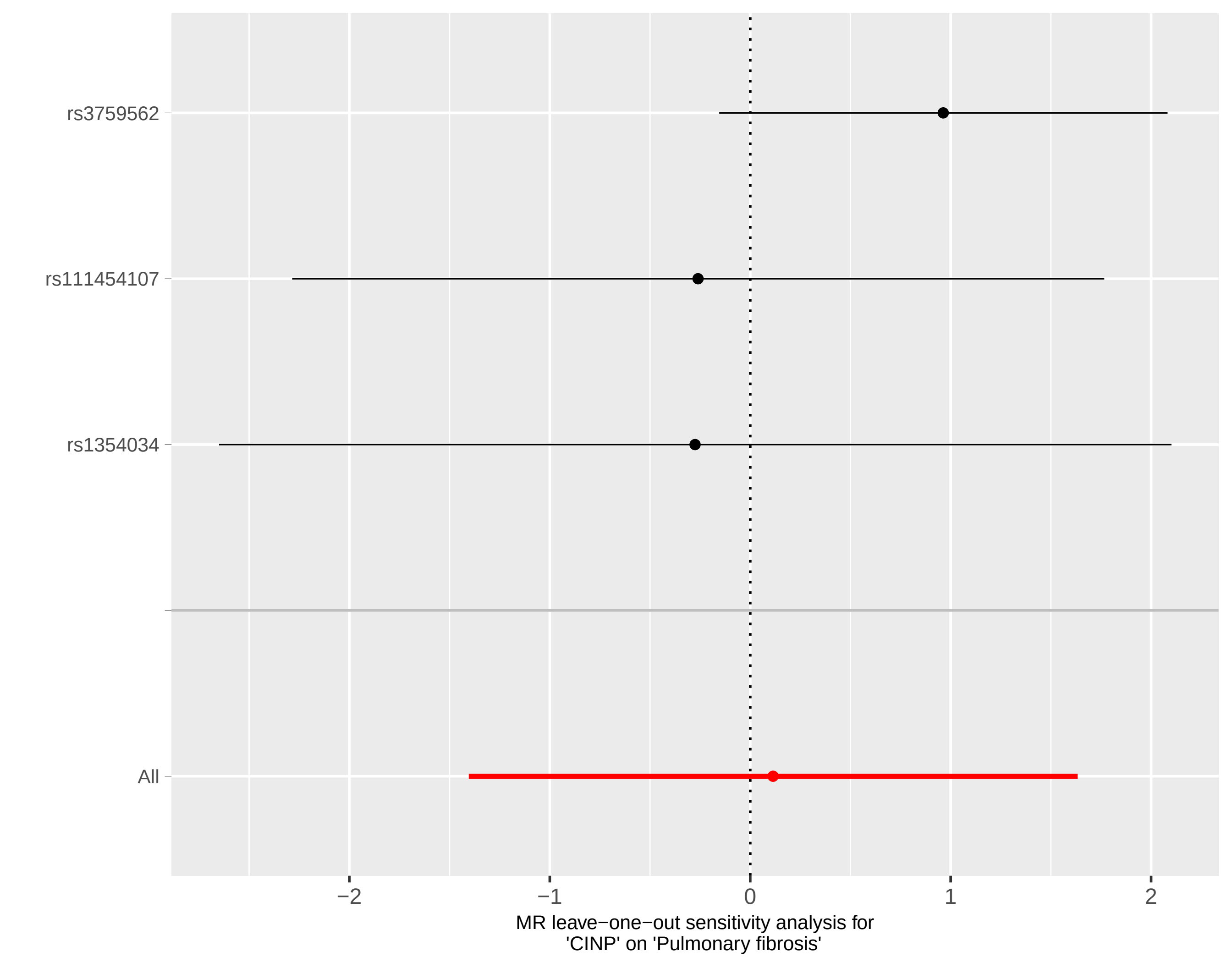

rs3759562
rs111454107
rs1354034
All
−2
−1
0
1
2
MR leave−one−out sensitivity analysis for
'CINP' on 'Pulmonary fibrosis'

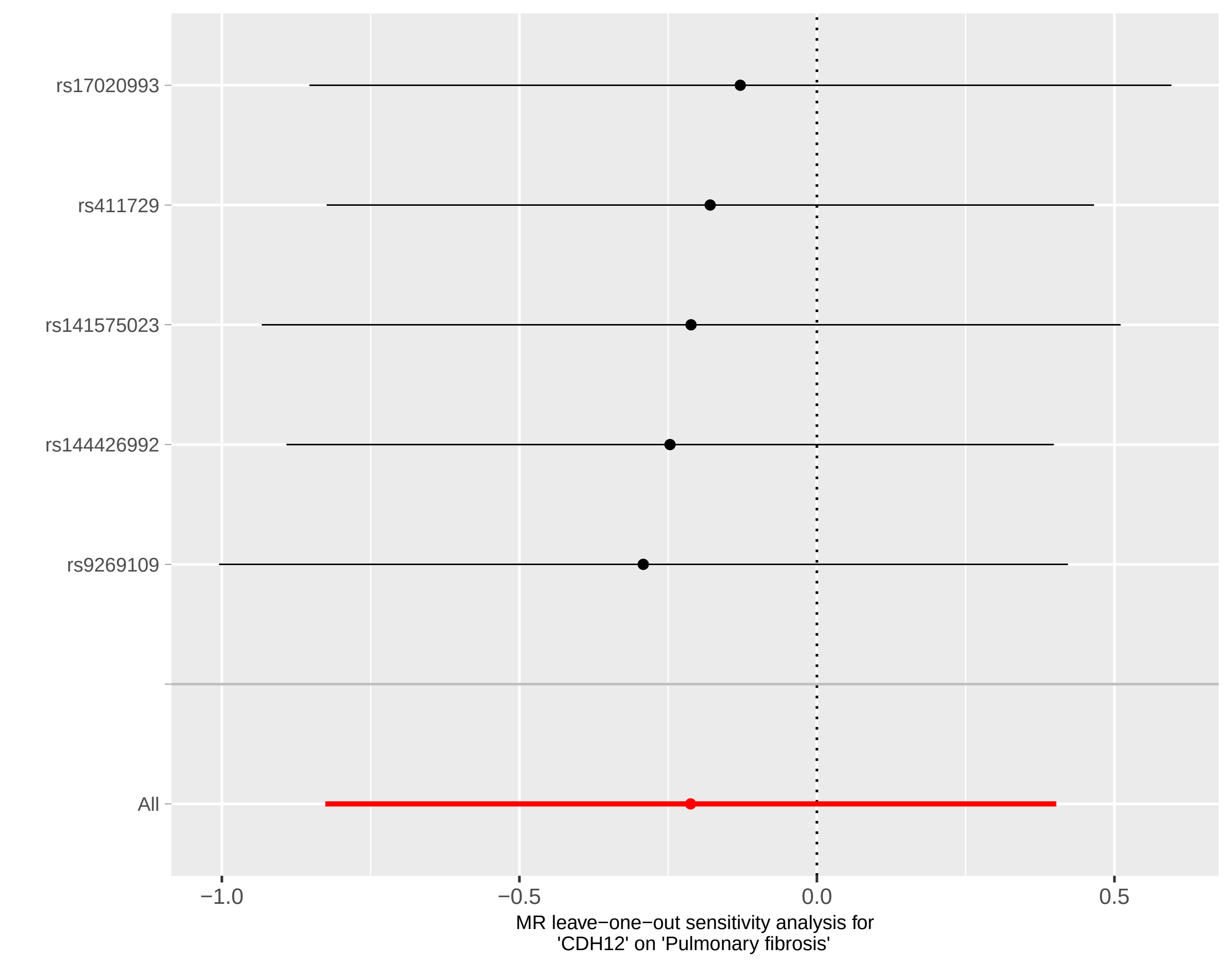
rs17020993
rs411729
rs141575023
rs144426992
rs9269109
All
−1.0
−0.5
0.0
0.5
MR leave−one−out sensitivity analysis for
'CDH12' on 'Pulmonary fibrosis'

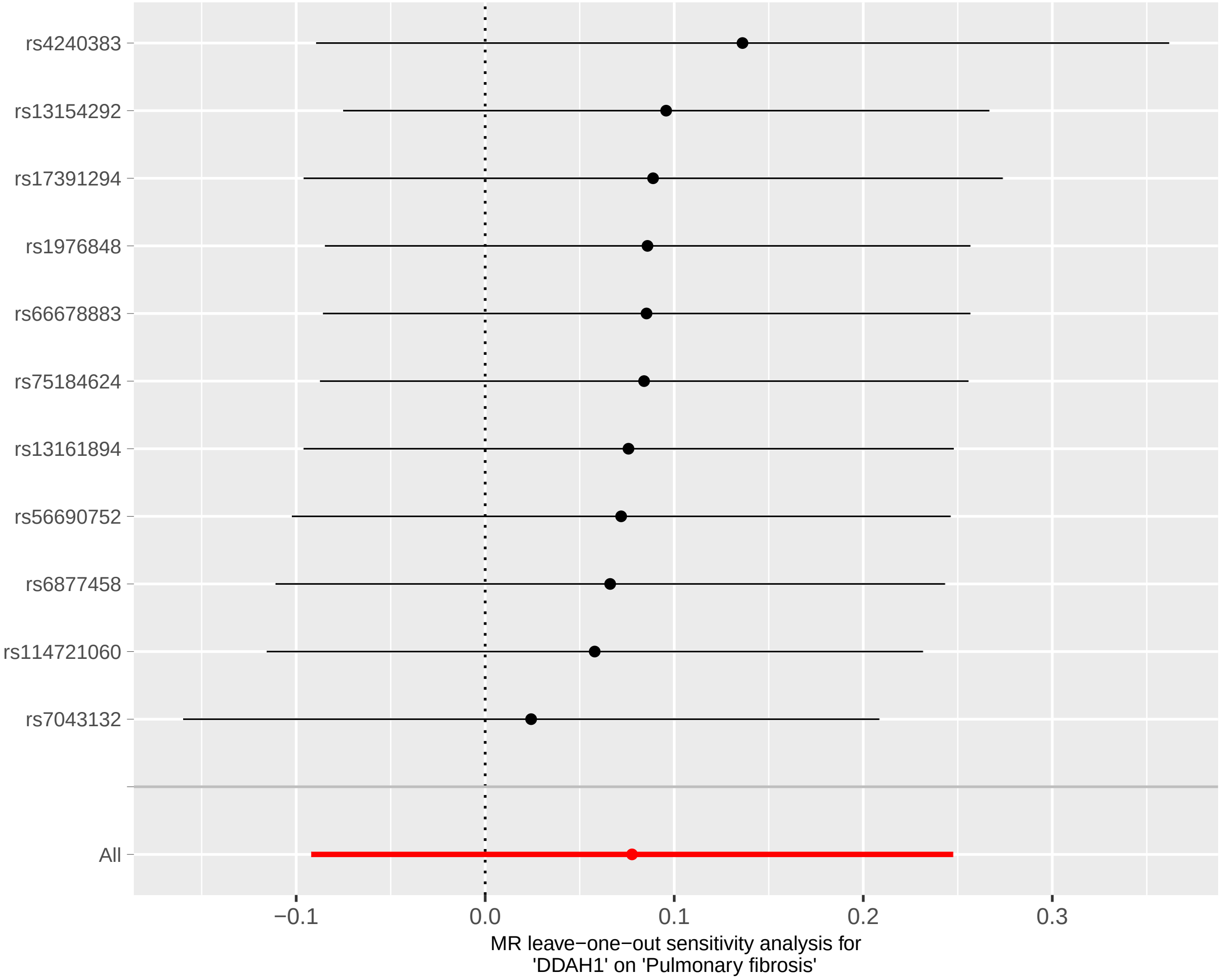
rs4240383
rs13154292
rs17391294
rs1976848
rs66678883
rs75184624
rs13161894
rs56690752
rs6877458
rs114721060
rs7043132
All
−0.1
0.0
0.1
0.2
0.3
MR leave−one−out sensitivity analysis for
'DDAH1' on 'Pulmonary fibrosis'

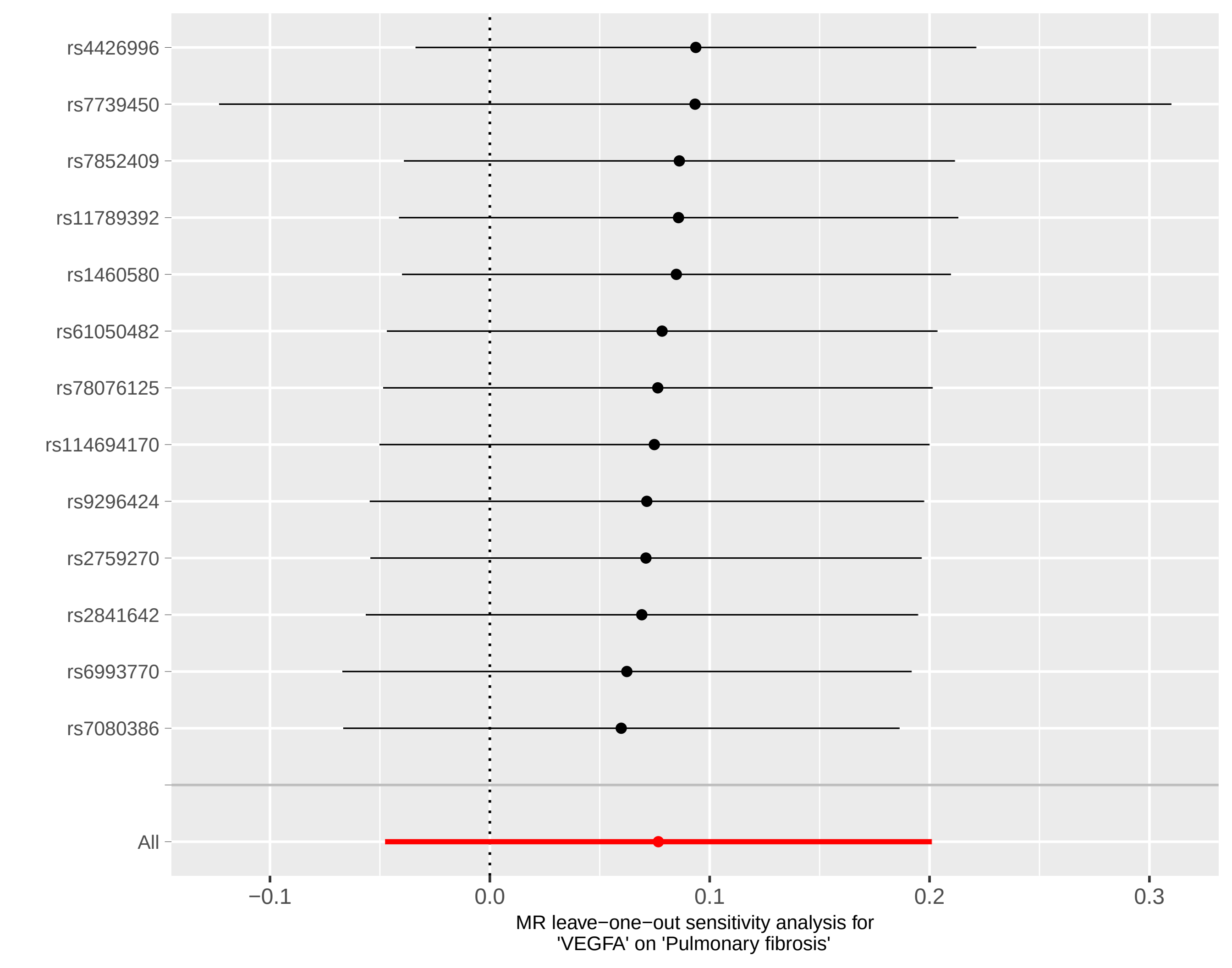

rs4426996
rs7739450
rs7852409
rs11789392
rs1460580
rs61050482
rs78076125
rs114694170
rs9296424
rs2759270
rs2841642
rs6993770
rs7080386
All
−0.1
0.0
0.1
0.2
0.3
MR leave−one−out sensitivity analysis for
'VEGFA' on 'Pulmonary fibrosis'

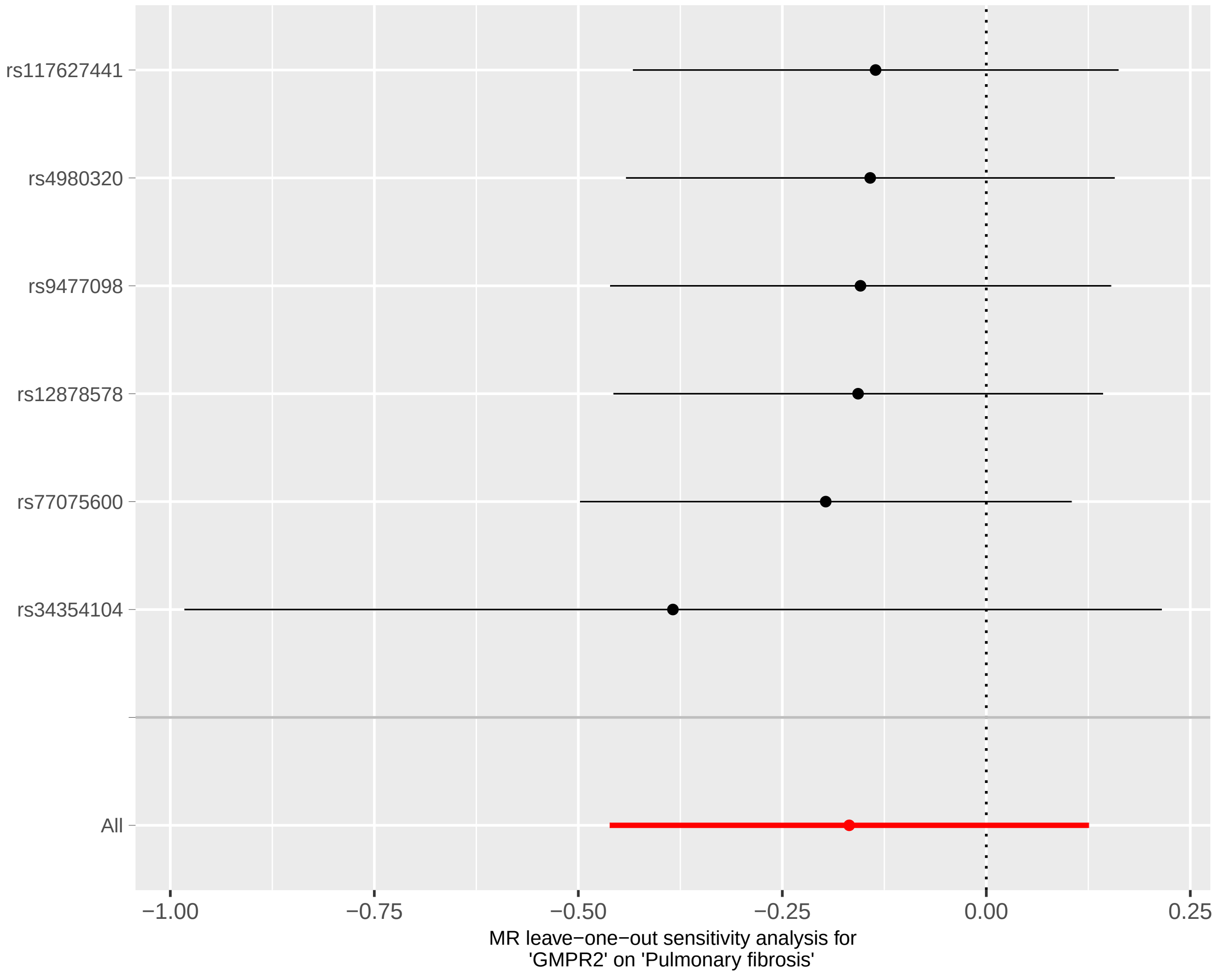

rs117627441
rs4980320
rs9477098
rs12878578
rs77075600
rs34354104
All
−1.00
−0.75
−0.50
−0.25
0.00
0.25
MR leave−one−out sensitivity analysis for
'GMPR2' on 'Pulmonary fibrosis'

Insufficient number of SNPs

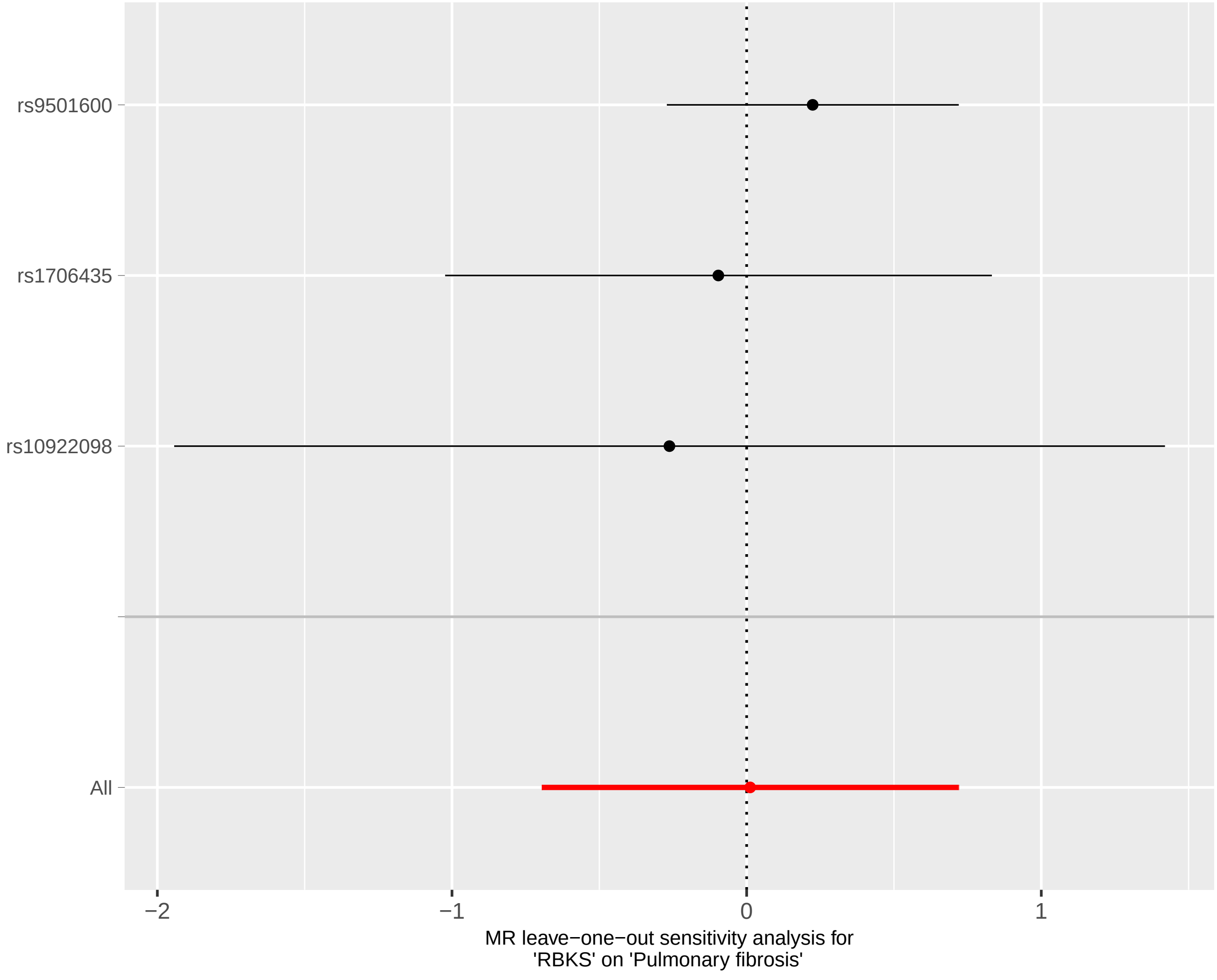
rs9501600
rs1706435
rs10922098
All
−2
−1
0
1
MR leave−one−out sensitivity analysis for
'RBKS' on 'Pulmonary fibrosis'

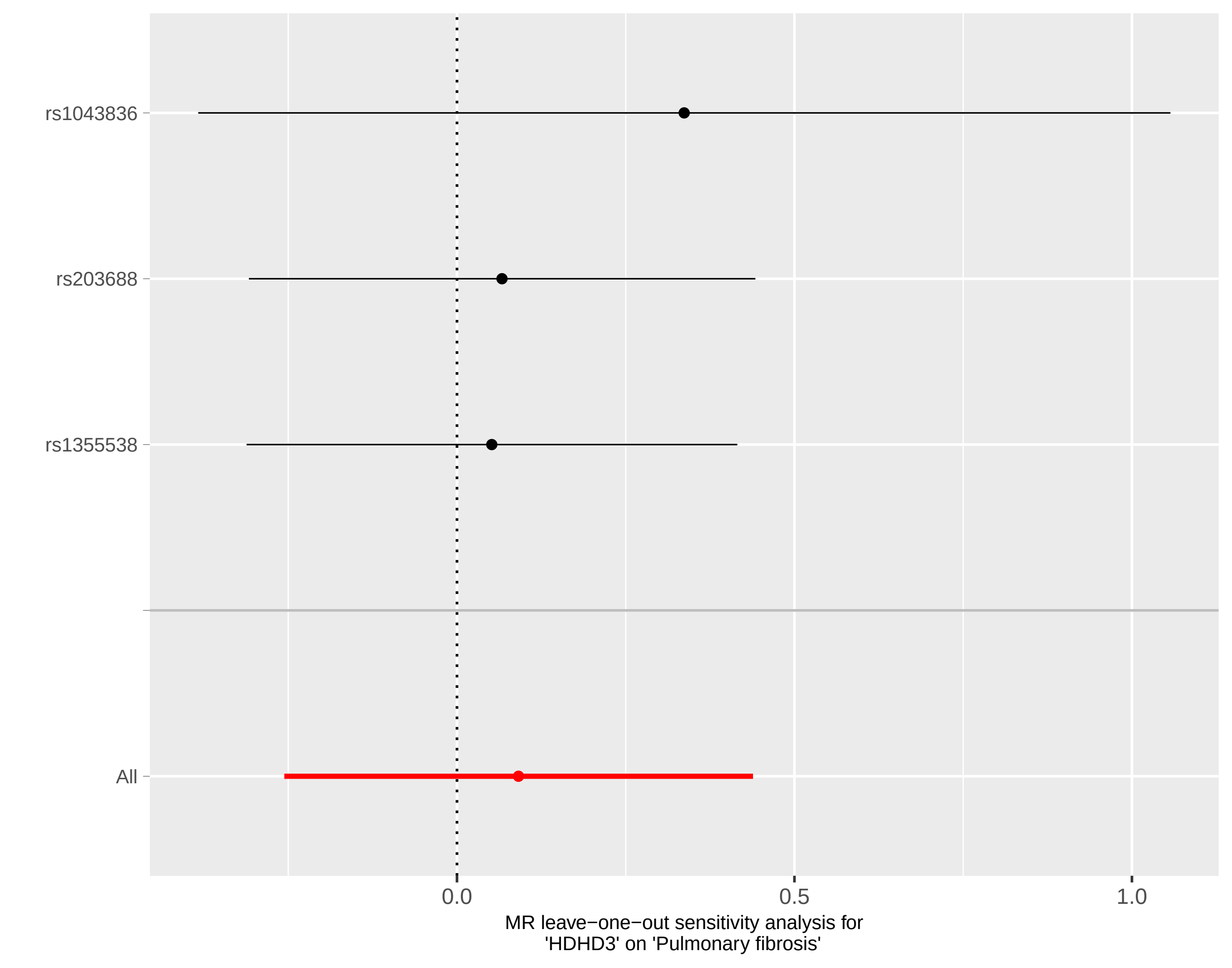
rs1043836
rs203688
rs1355538
All
0.0
0.5
1.0
MR leave−one−out sensitivity analysis for
'HDHD3' on 'Pulmonary fibrosis'

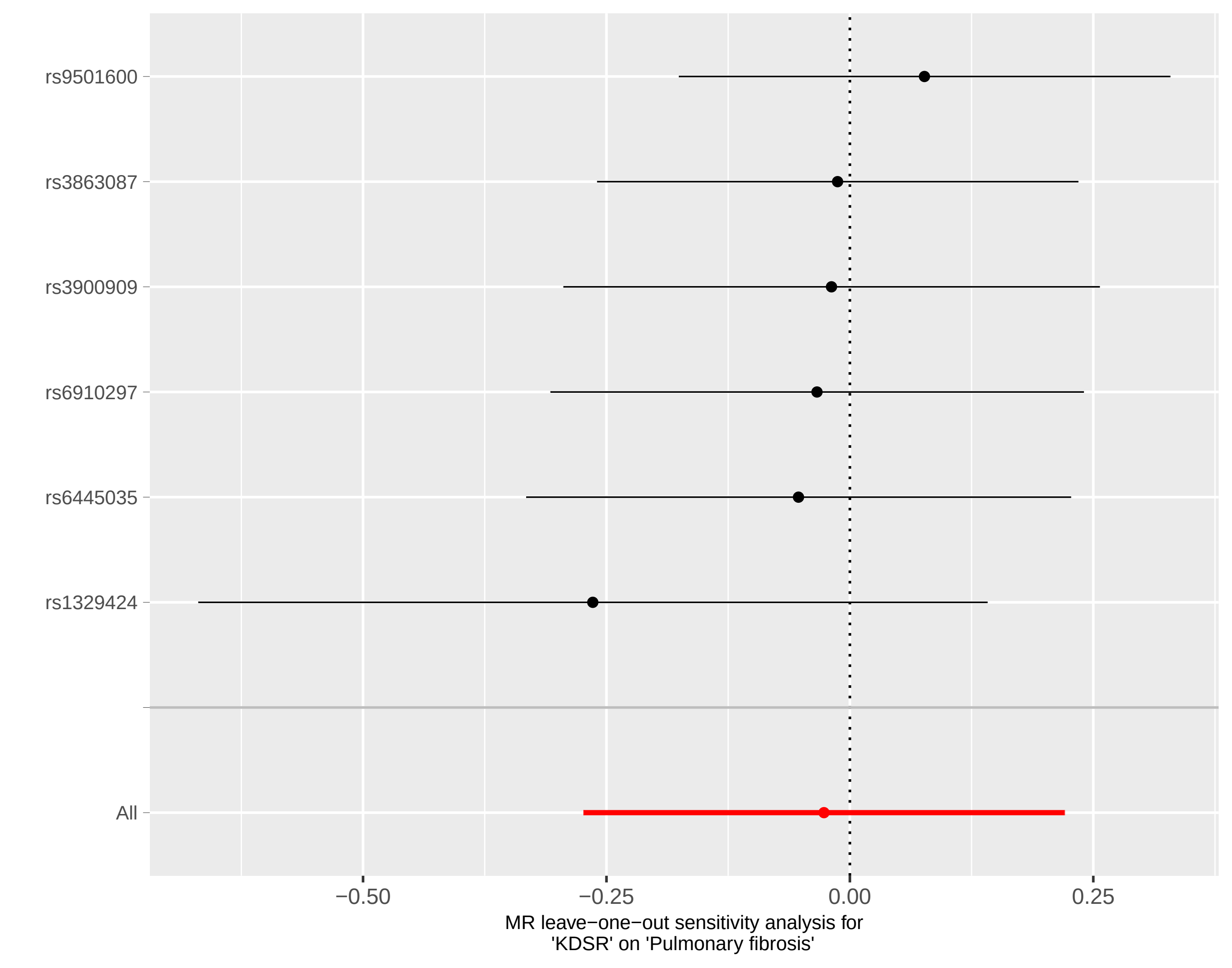
rs9501600
rs3863087
rs3900909
rs6910297
rs6445035
rs1329424
All
−0.50
−0.25
0.00
0.25
MR leave−one−out sensitivity analysis for
'KDSR' on 'Pulmonary fibrosis'

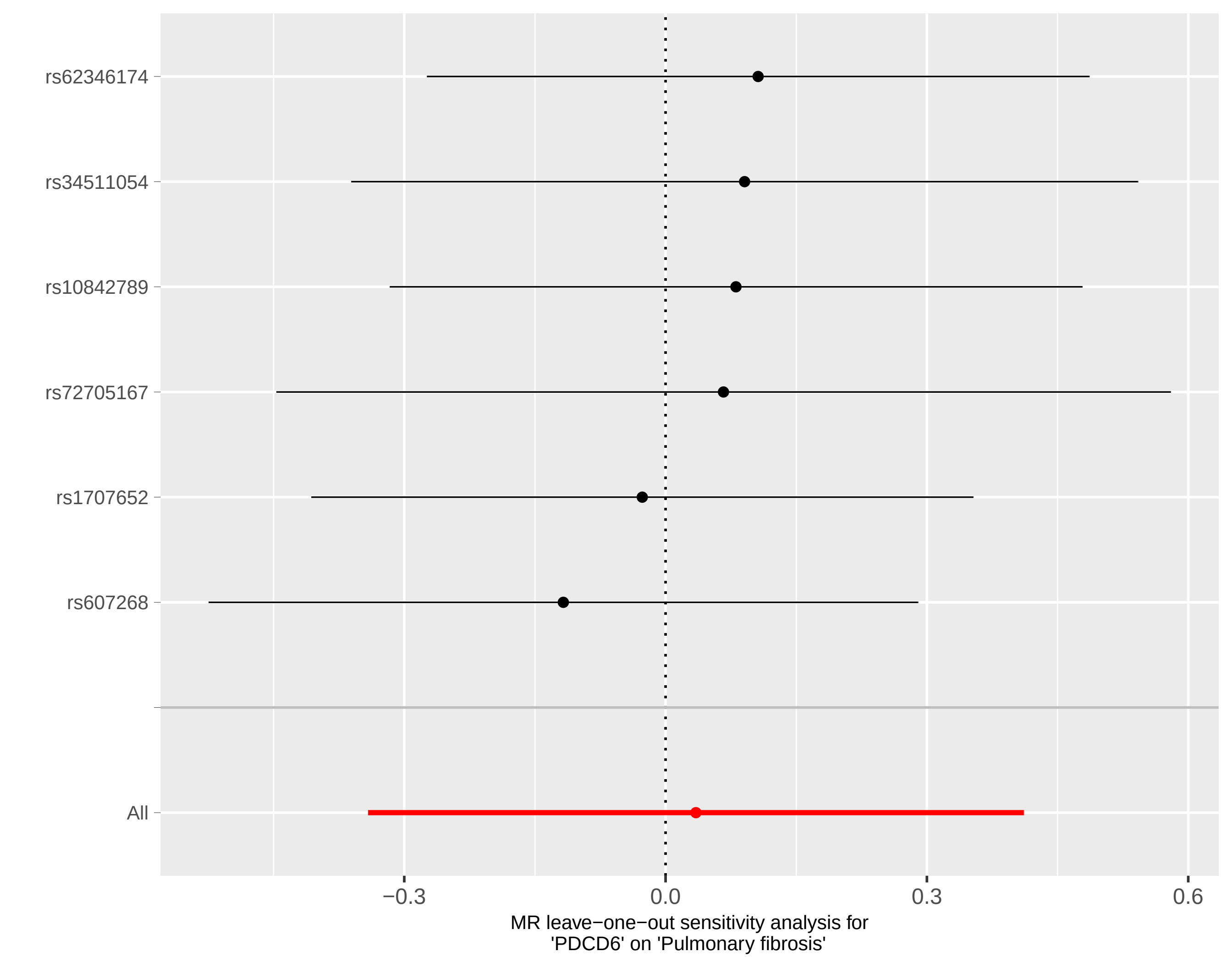

rs62346174
rs34511054
rs10842789
rs72705167
rs1707652
rs607268
All
−0.3
0.0
0.3
0.6
MR leave−one−out sensitivity analysis for
'PDCD6' on 'Pulmonary fibrosis'

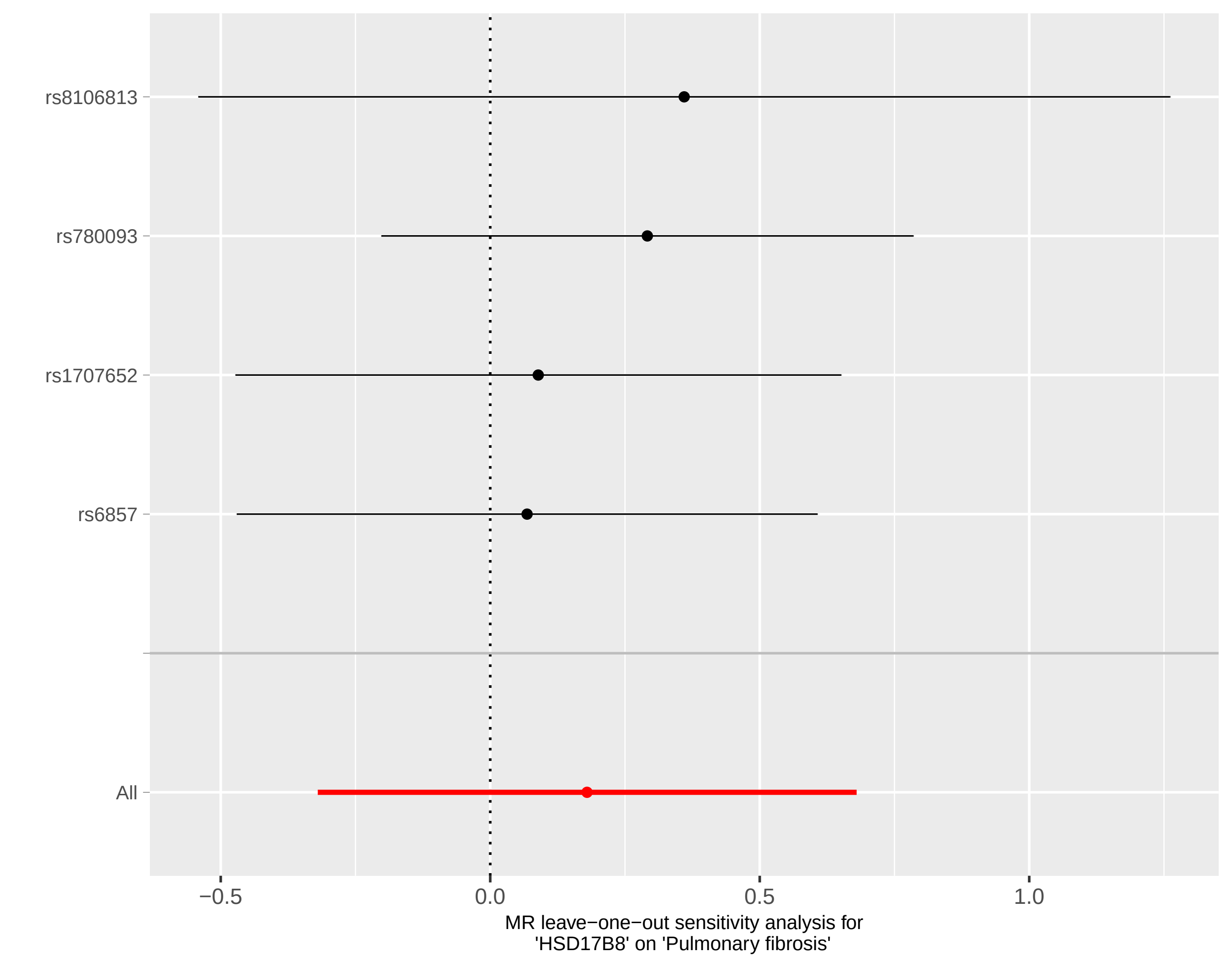

rs8106813
rs780093
rs1707652
rs6857
All
−0.5
0.0
0.5
1.0
MR leave−one−out sensitivity analysis for
'HSD17B8' on 'Pulmonary fibrosis'

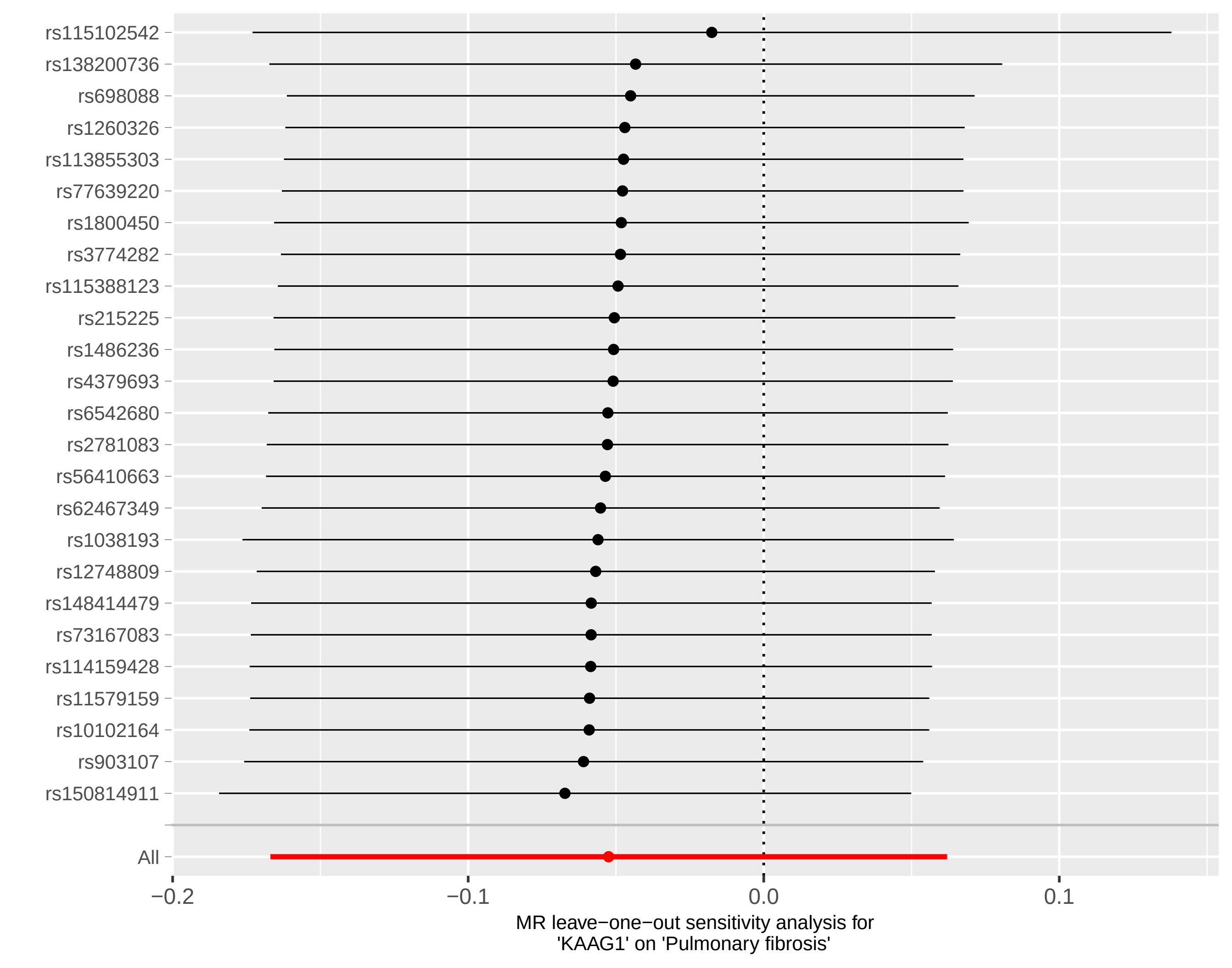

rs115102542
rs138200736
rs698088
rs1260326
rs113855303
rs77639220
rs1800450
rs3774282
rs115388123
rs215225
rs1486236
rs4379693
rs6542680
rs2781083
rs56410663
rs62467349
rs1038193
rs12748809
rs148414479
rs73167083
rs114159428
rs11579159
rs10102164
rs903107
rs150814911
All
−0.2
−0.1
0.0
0.1
MR leave−one−out sensitivity analysis for
'KAAG1' on 'Pulmonary fibrosis'

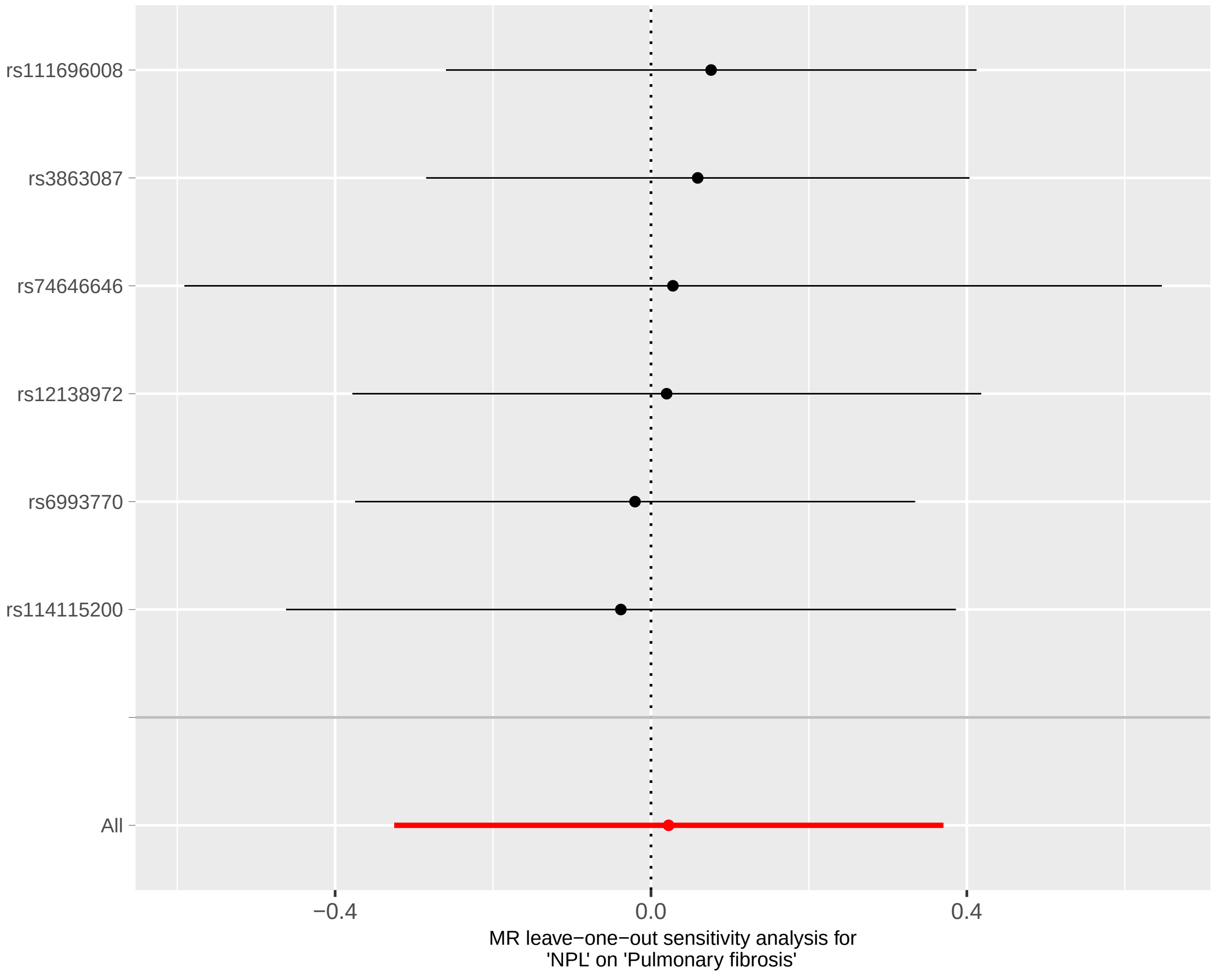
rs111696008
rs3863087
rs74646646
rs12138972
rs6993770
rs114115200
All
−0.4
0.0
0.4
MR leave−one−out sensitivity analysis for
'NPL' on 'Pulmonary fibrosis'

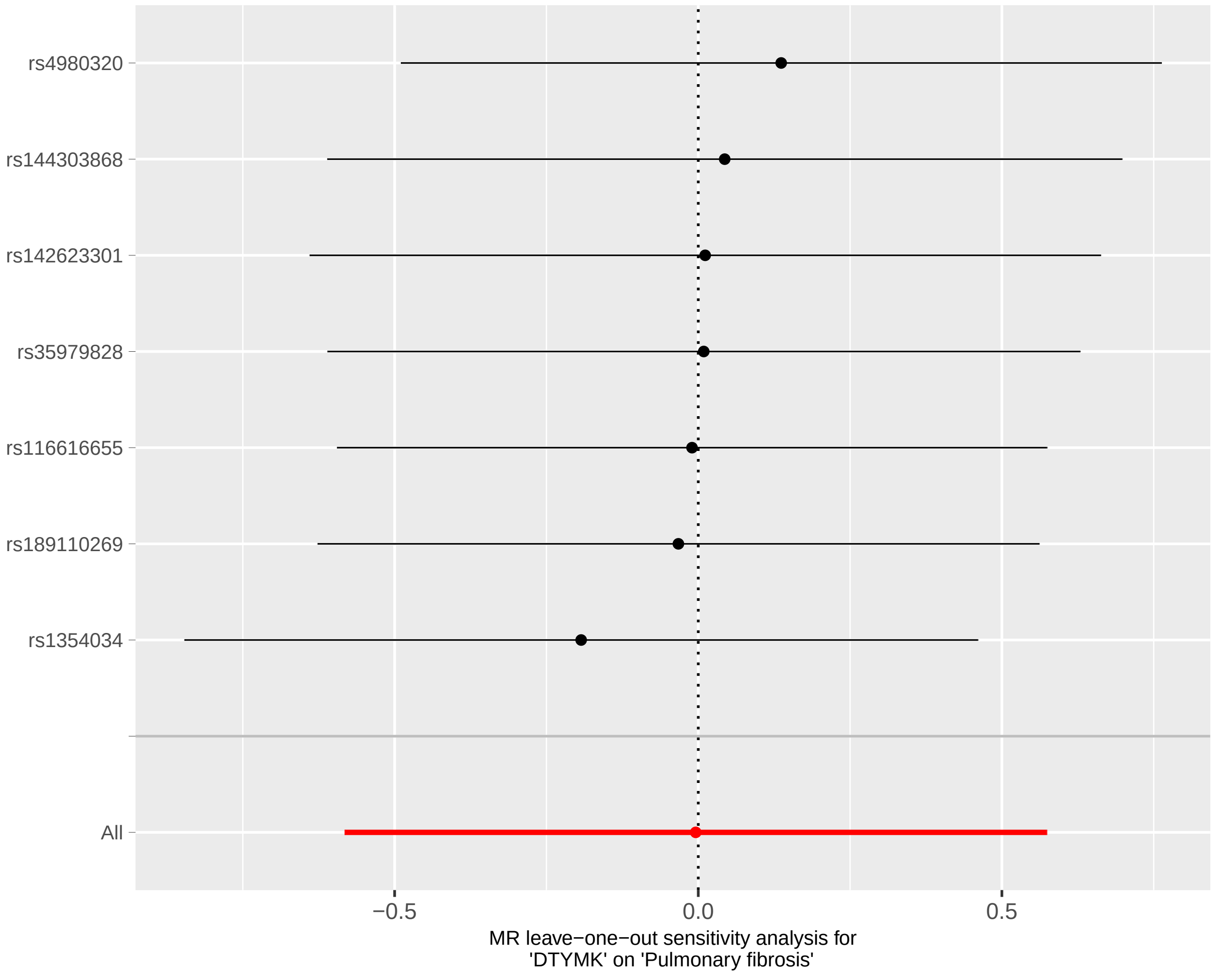

rs4980320
rs144303868
rs142623301
rs35979828
rs116616655
rs189110269
rs1354034
All
−0.5
0.0
0.5
MR leave−one−out sensitivity analysis for
'DTYMK' on 'Pulmonary fibrosis'

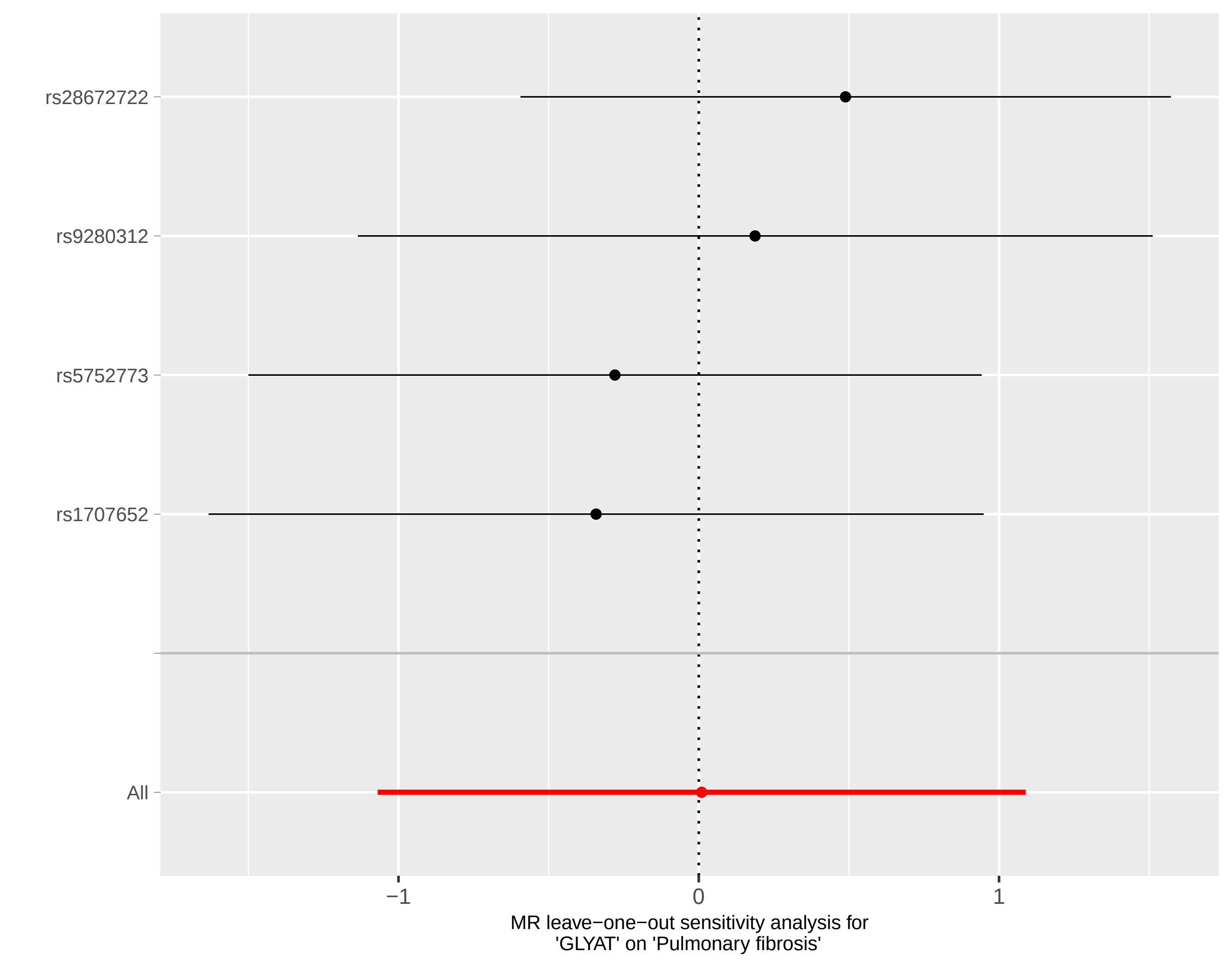

rs28672722
rs9280312
rs5752773
rs1707652
All
−1
0
1
MR leave−one−out sensitivity analysis for
'GLYAT' on 'Pulmonary fibrosis'

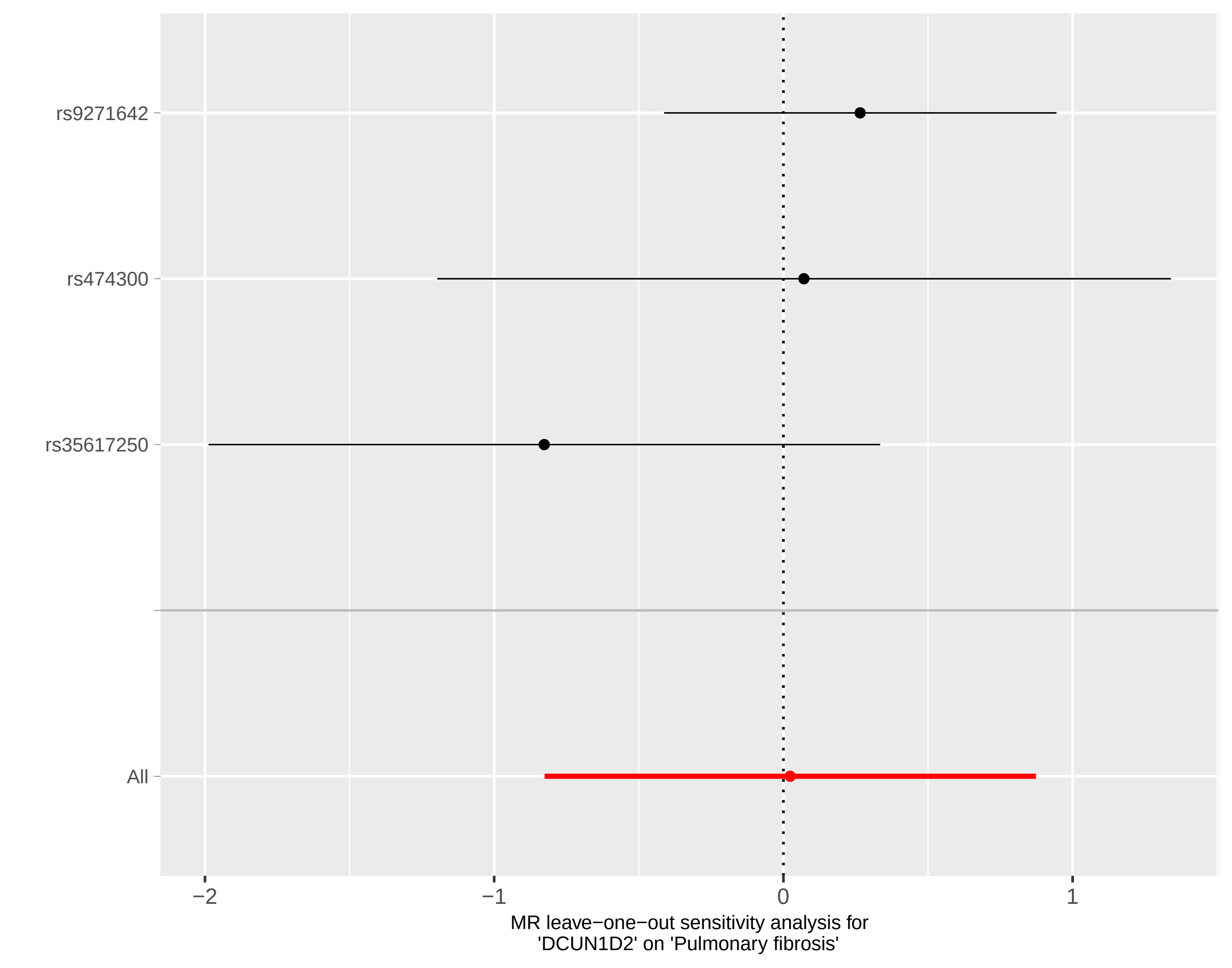
rs9271642
rs474300
rs35617250
All
−2
−1
0
1
MR leave−one−out sensitivity analysis for
'DCUN1D2' on 'Pulmonary fibrosis'

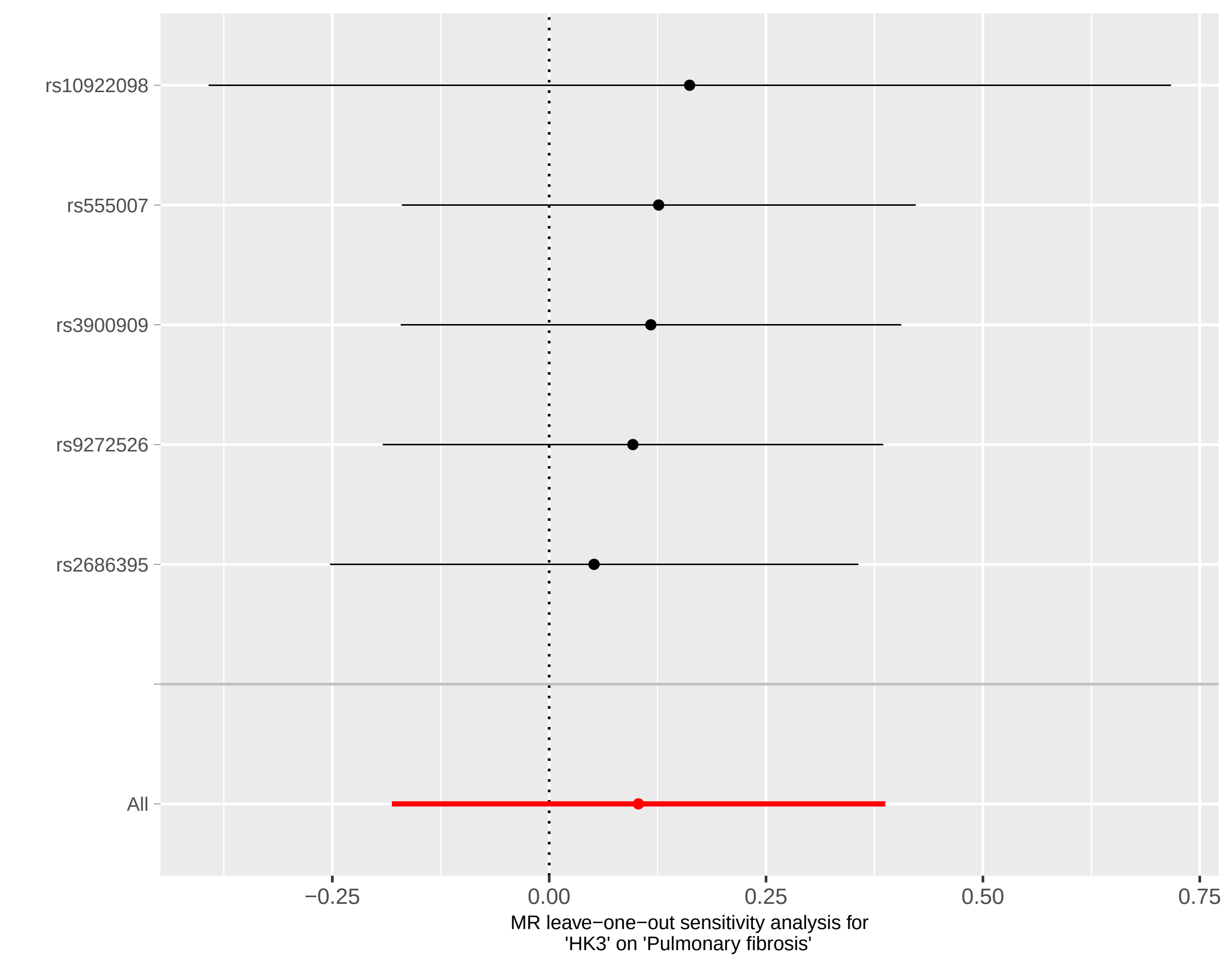

rs10922098
rs555007
rs3900909
rs9272526
rs2686395
All
−0.25
0.00
0.25
0.50
0.75
MR leave−one−out sensitivity analysis for
'HK3' on 'Pulmonary fibrosis'

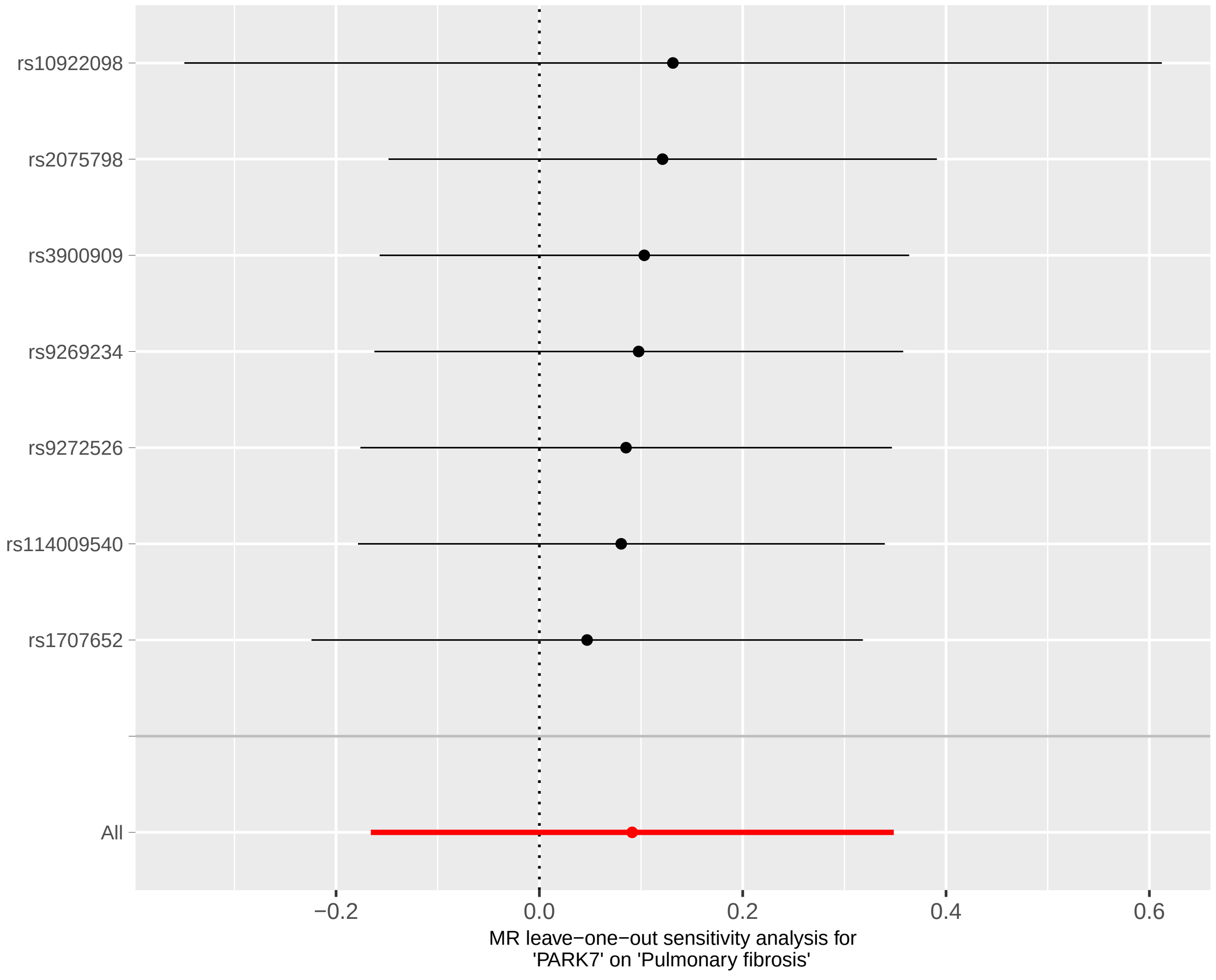
rs10922098
rs2075798
rs3900909
rs9269234
rs9272526
rs114009540
rs1707652
All
−0.2
0.0
0.2
0.4
0.6
MR leave−one−out sensitivity analysis for
'PARK7' on 'Pulmonary fibrosis'

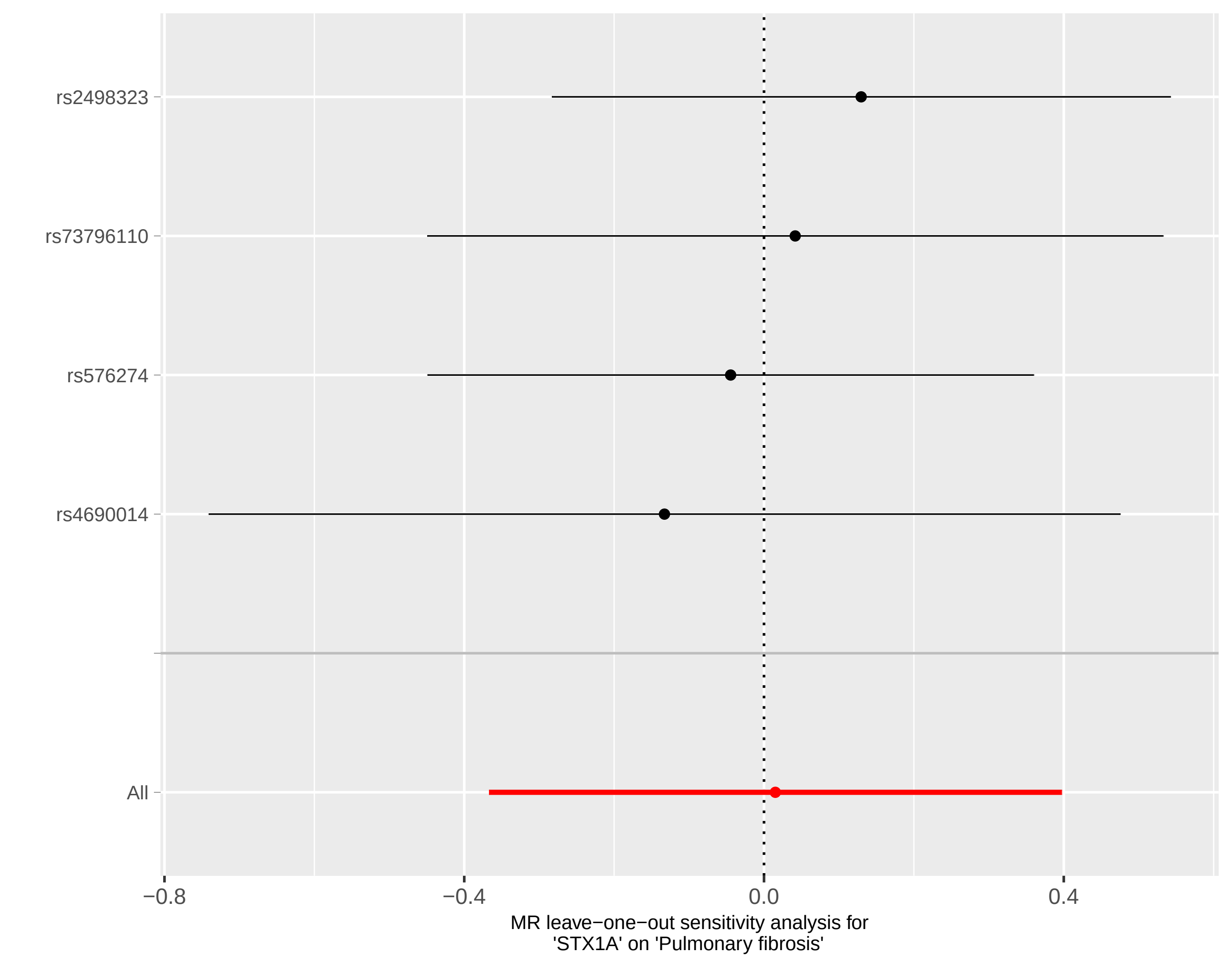

rs2498323
rs73796110
rs576274
rs4690014
All
−0.8
−0.4
0.0
0.4
MR leave−one−out sensitivity analysis for
'STX1A' on 'Pulmonary fibrosis'

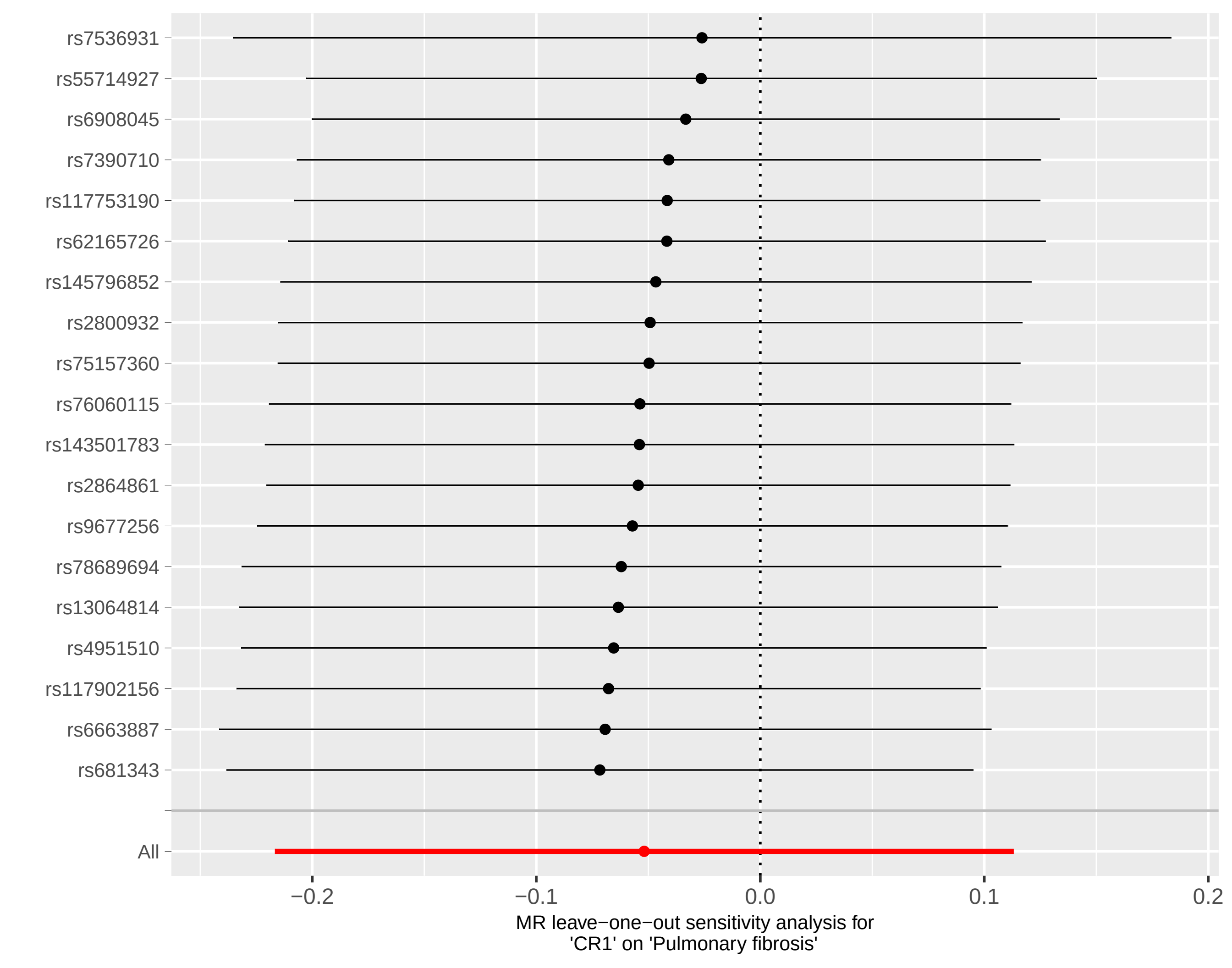

rs7536931
rs55714927
rs6908045
rs7390710
rs117753190
rs62165726
rs145796852
rs2800932
rs75157360
rs76060115
rs143501783
rs2864861
rs9677256
rs78689694
rs13064814
rs4951510
rs117902156
rs6663887
rs681343
All
−0.2
−0.1
0.0
0.1
0.2
MR leave−one−out sensitivity analysis for
'CR1' on 'Pulmonary fibrosis'

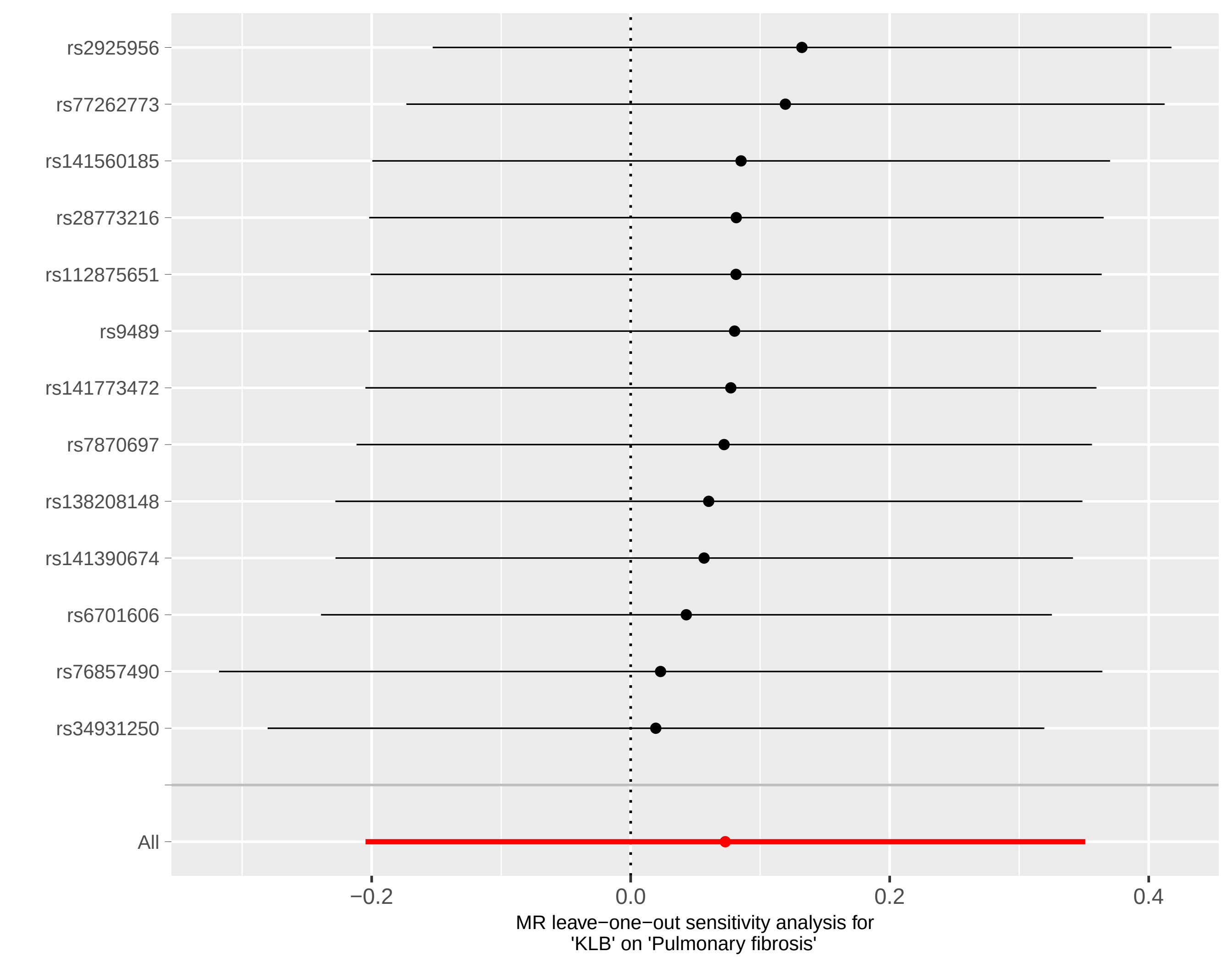

rs2925956
rs77262773
rs141560185
rs28773216
rs112875651
rs9489
rs141773472
rs7870697
rs138208148
rs141390674
rs6701606
rs76857490
rs34931250
All
−0.2
0.0
0.2
0.4
MR leave−one−out sensitivity analysis for
'KLB' on 'Pulmonary fibrosis'

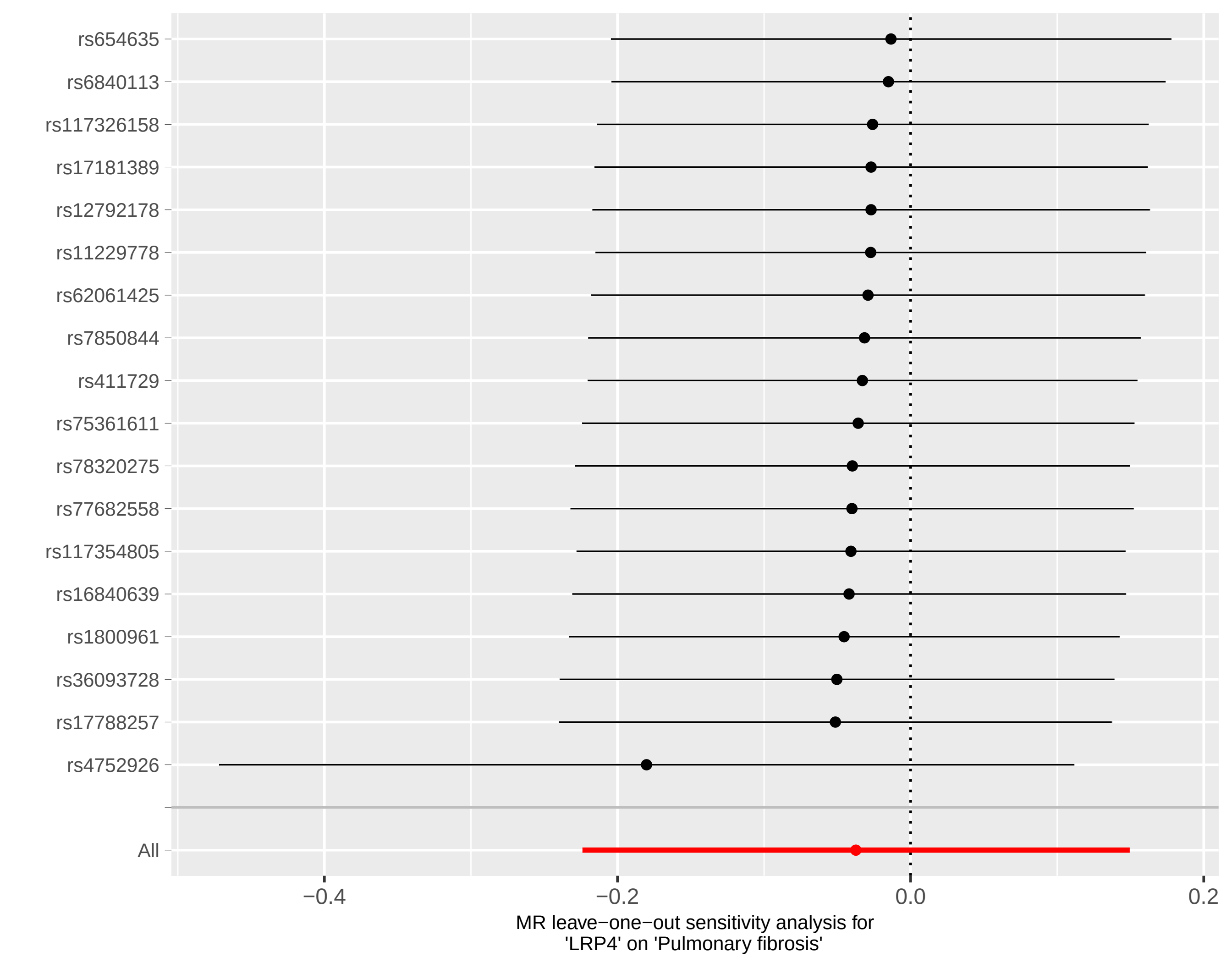
rs654635
rs6840113
rs117326158
rs17181389
rs12792178
rs11229778
rs62061425
rs7850844
rs411729
rs75361611
rs78320275
rs77682558
rs117354805
rs16840639
rs1800961
rs36093728
rs17788257
rs4752926
All
−0.4
−0.2
0.0
0.2
MR leave−one−out sensitivity analysis for
'LRP4' on 'Pulmonary fibrosis'

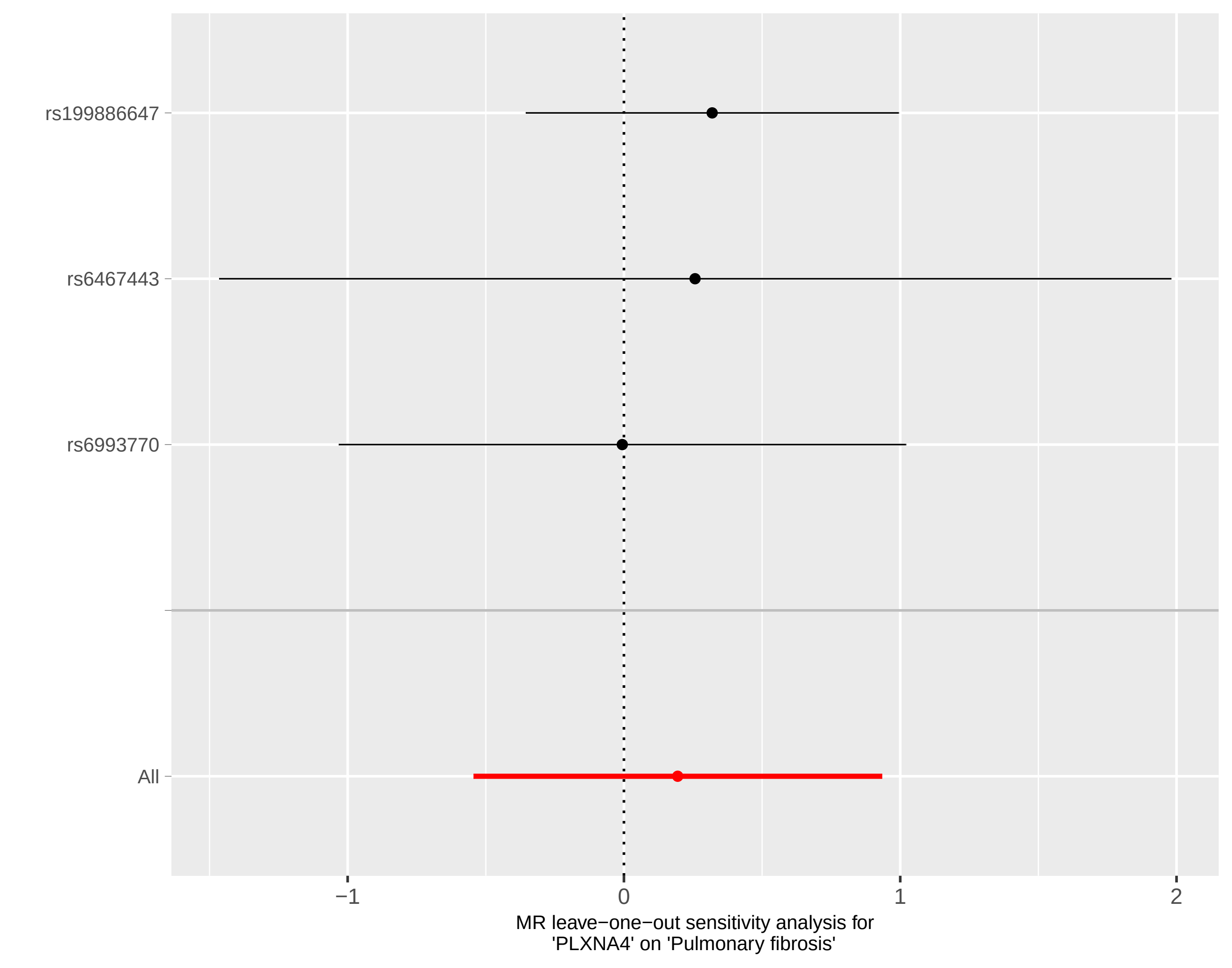
rs199886647
rs6467443
rs6993770
All
−1
0
1
2
MR leave−one−out sensitivity analysis for
'PLXNA4' on 'Pulmonary fibrosis'

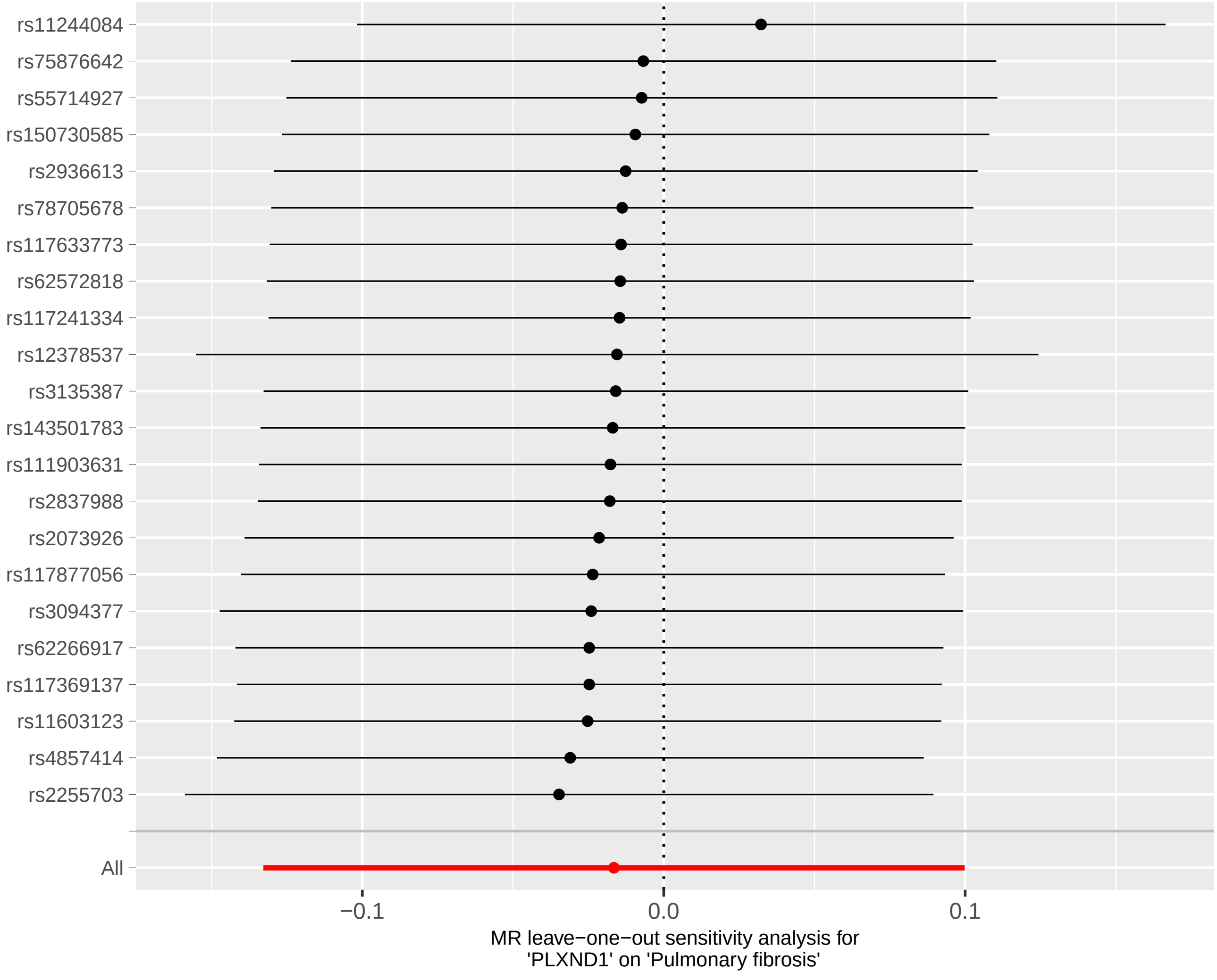
rs11244084
rs75876642
rs55714927
rs150730585
rs2936613
rs78705678
rs117633773
rs62572818
rs117241334
rs12378537
rs3135387
rs143501783
rs111903631
rs2837988
rs2073926
rs117877056
rs3094377
rs62266917
rs117369137
rs11603123
rs4857414
rs2255703
All
−0.1
0.0
0.1
MR leave−one−out sensitivity analysis for
'PLXND1' on 'Pulmonary fibrosis'

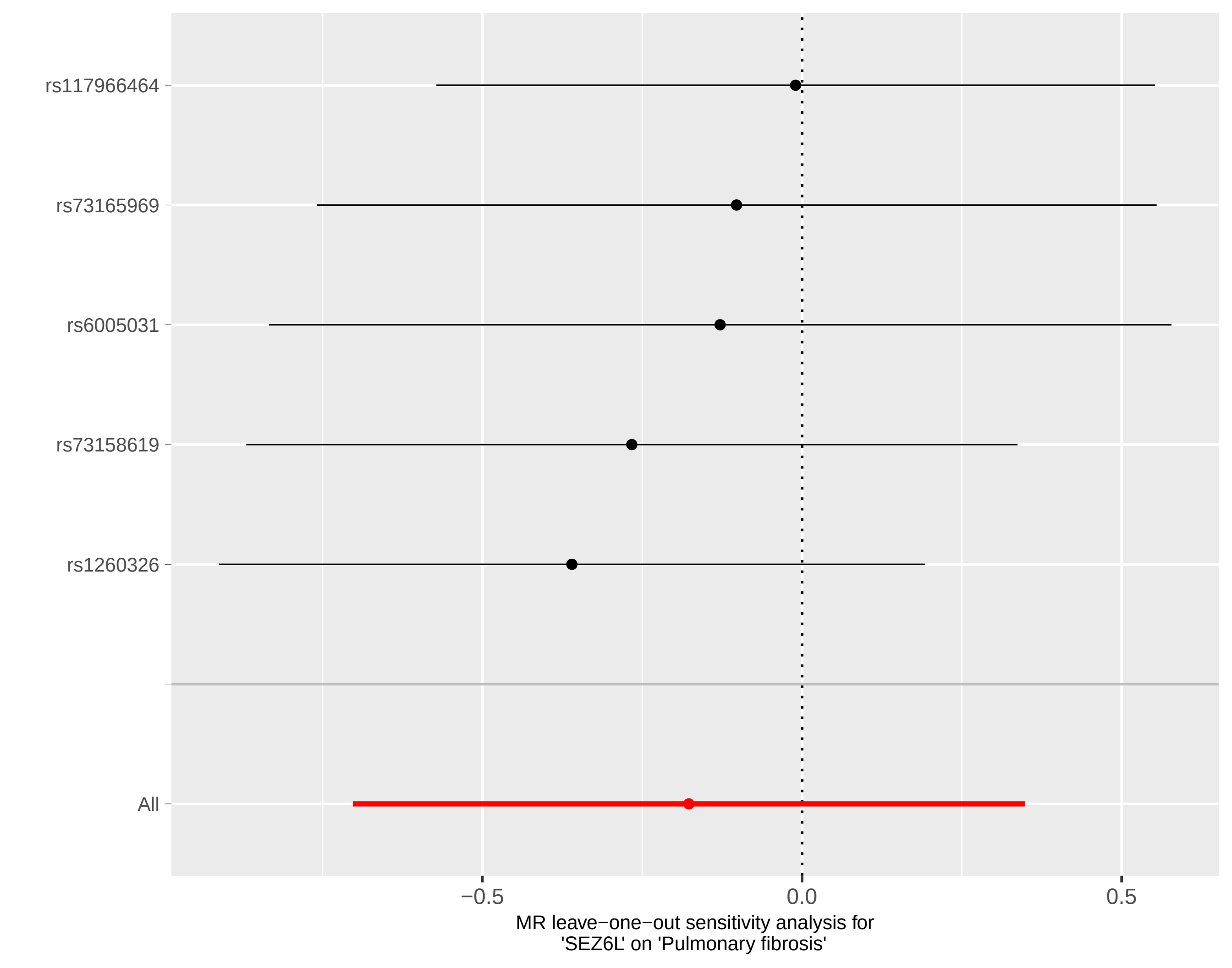
rs117966464
rs73165969
rs6005031
rs73158619
rs1260326
All
−0.5
0.0
0.5
MR leave−one−out sensitivity analysis for
'SEZ6L' on 'Pulmonary fibrosis'

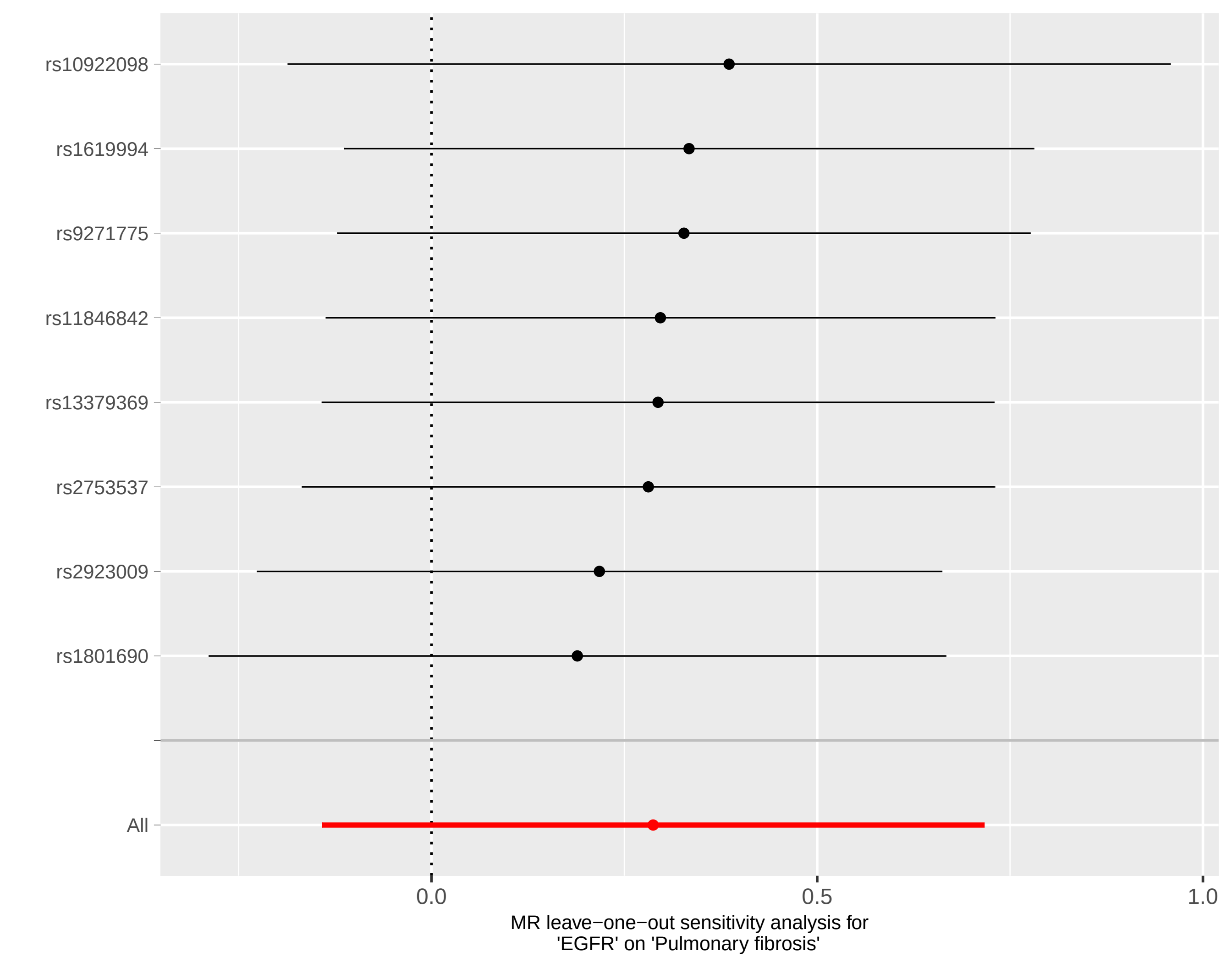
rs10922098
rs1619994
rs9271775
rs11846842
rs13379369
rs2753537
rs2923009
rs1801690
All
0.0
0.5
1.0
MR leave−one−out sensitivity analysis for
'EGFR' on 'Pulmonary fibrosis'

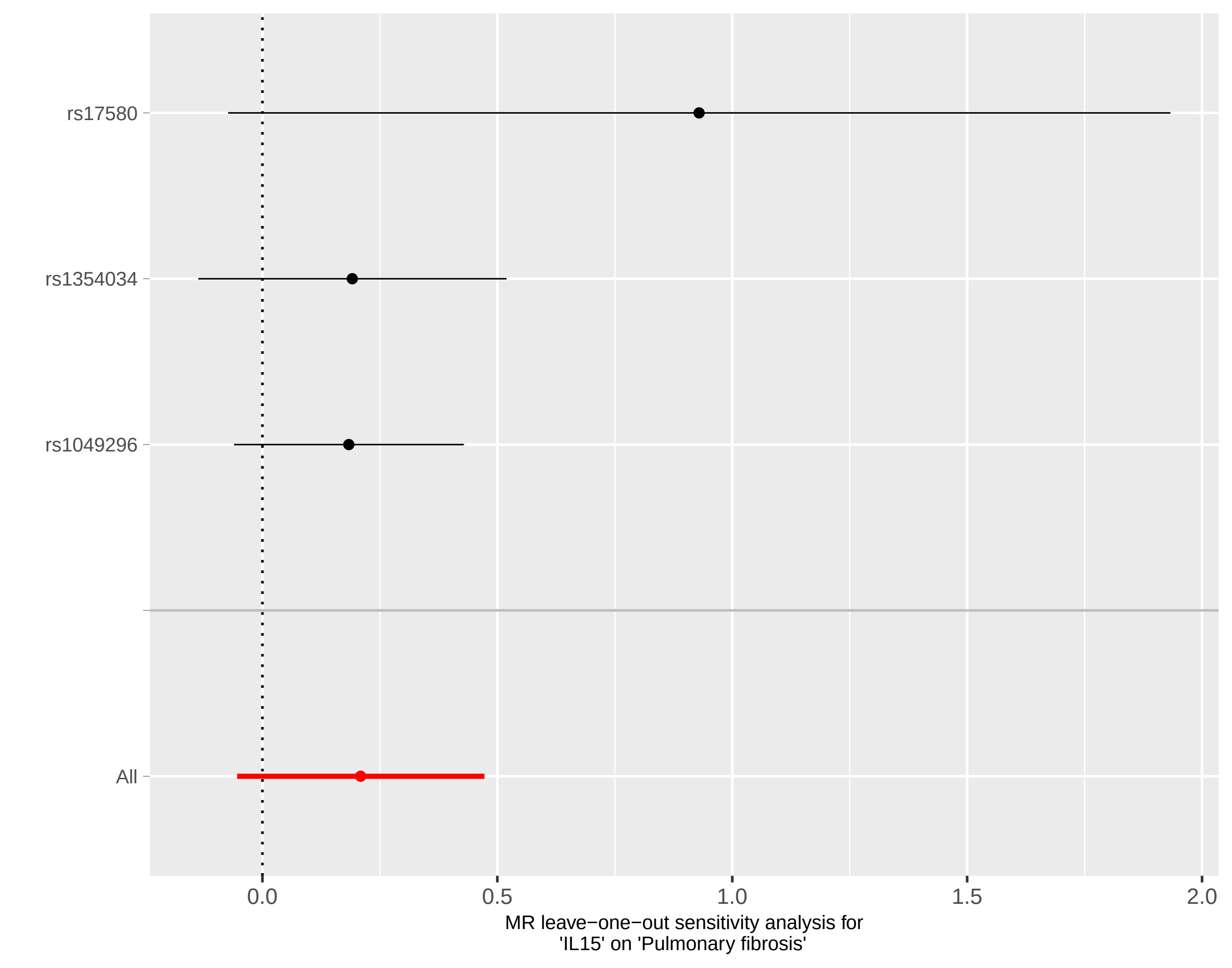
rs17580
rs1354034
rs1049296
All
0.0
0.5
1.0
1.5
2.0
MR leave−one−out sensitivity analysis for
'IL15' on 'Pulmonary fibrosis'

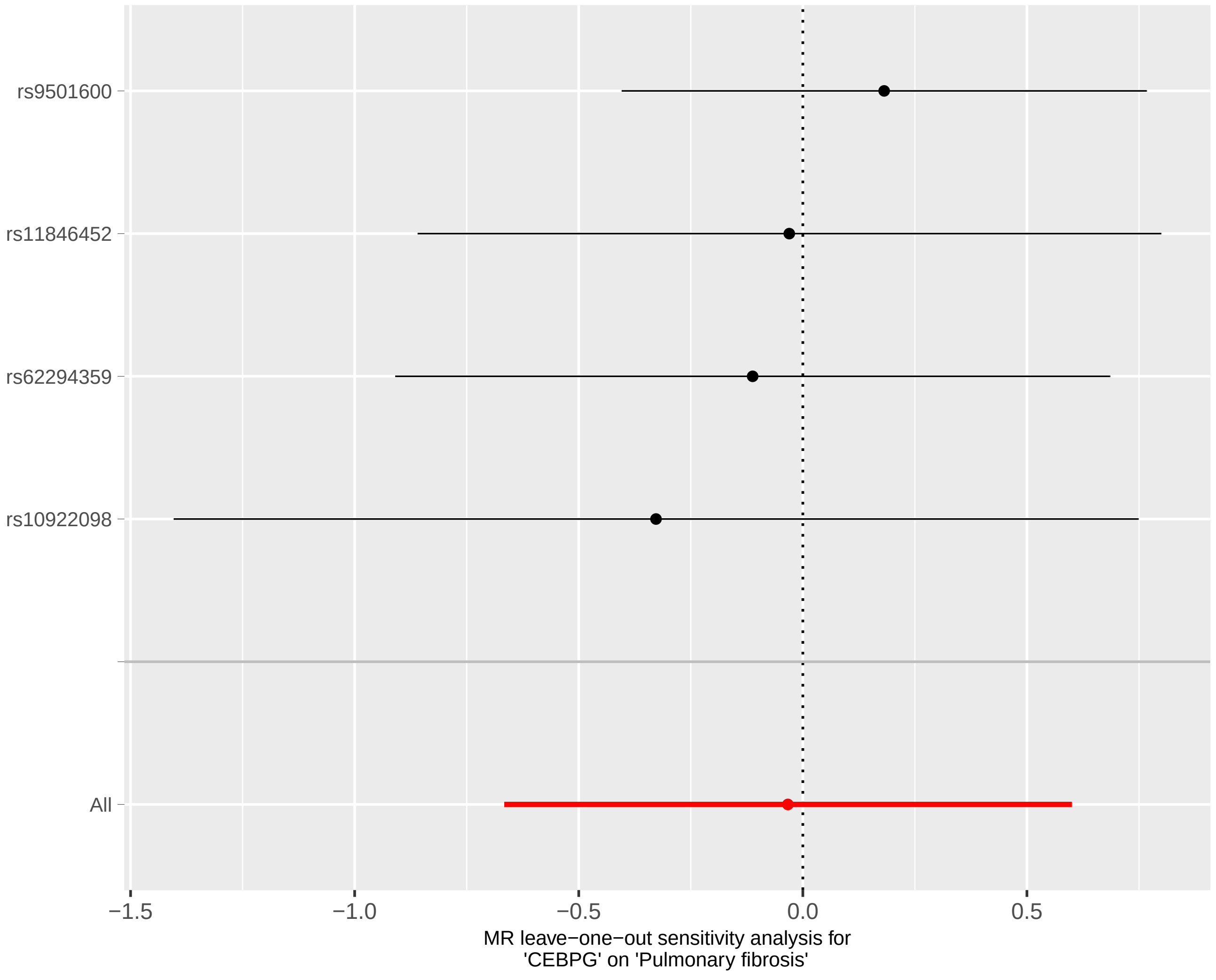
rs9501600
rs11846452
rs62294359
rs10922098
All
−1.5
−1.0
−0.5
0.0
0.5
MR leave−one−out sensitivity analysis for
'CEBPG' on 'Pulmonary fibrosis'

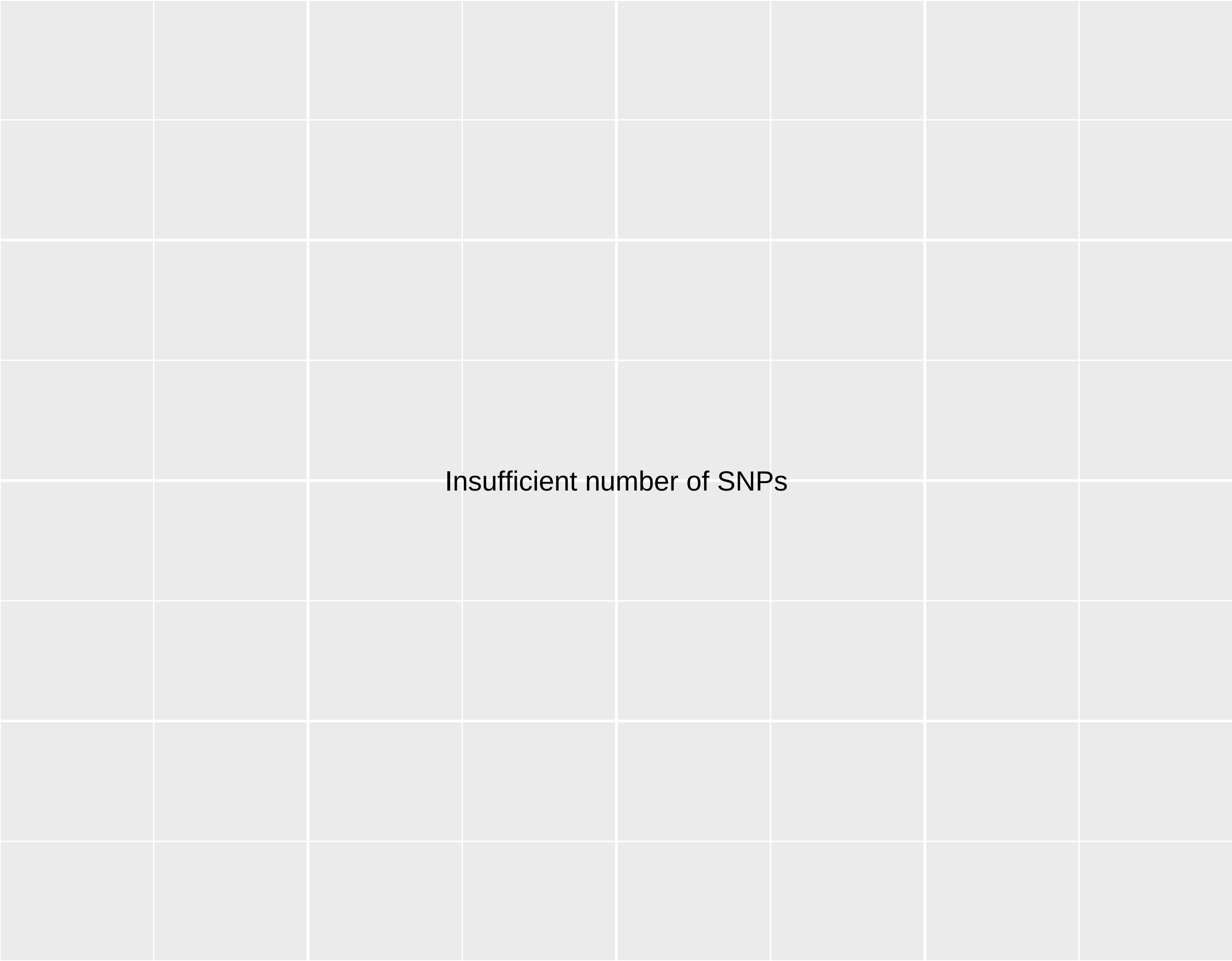
Insufficient number of SNPs

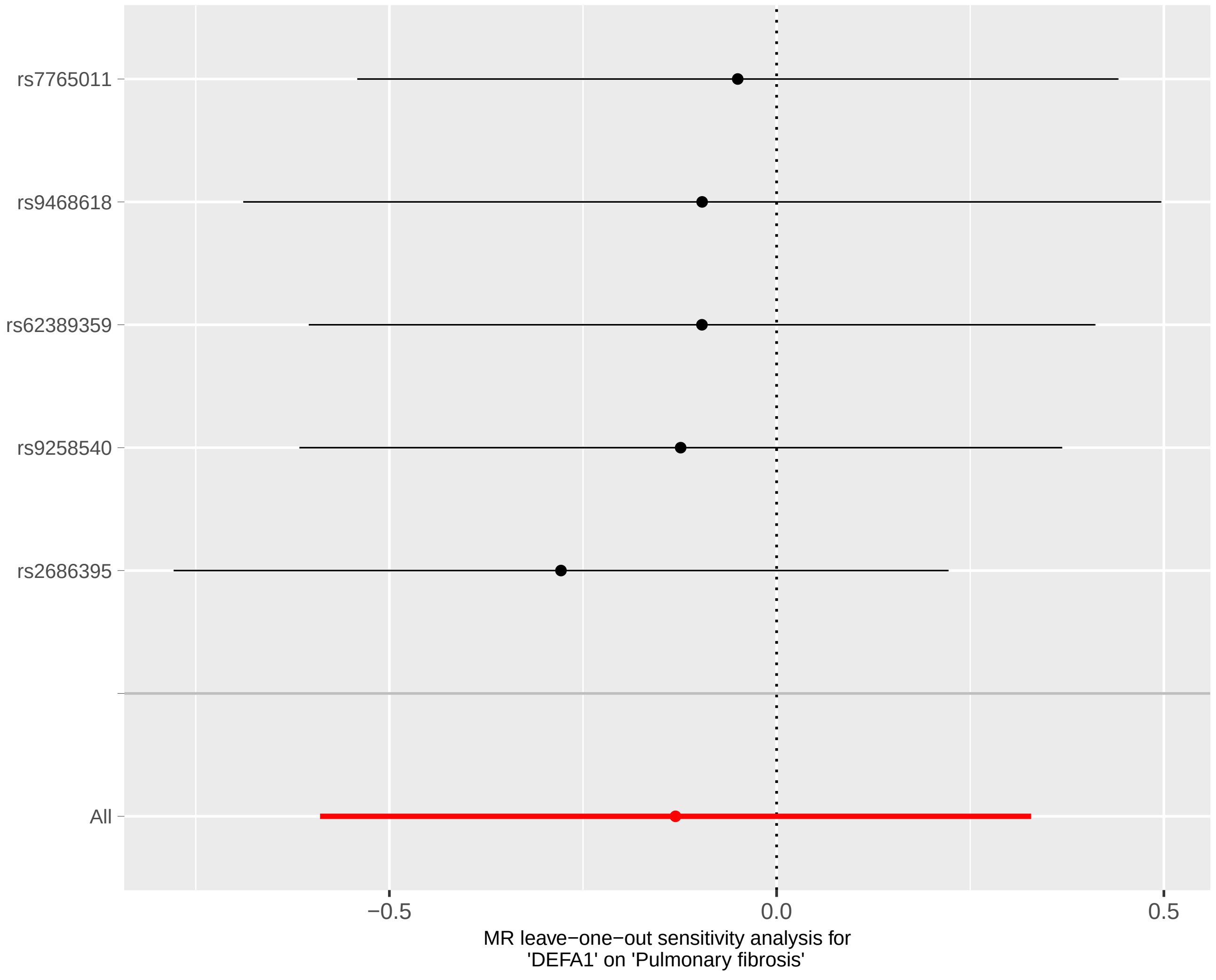
rs7765011
rs9468618
rs62389359
rs9258540
rs2686395
All
−0.5
0.0
0.5
MR leave−one−out sensitivity analysis for
'DEFA1' on 'Pulmonary fibrosis'

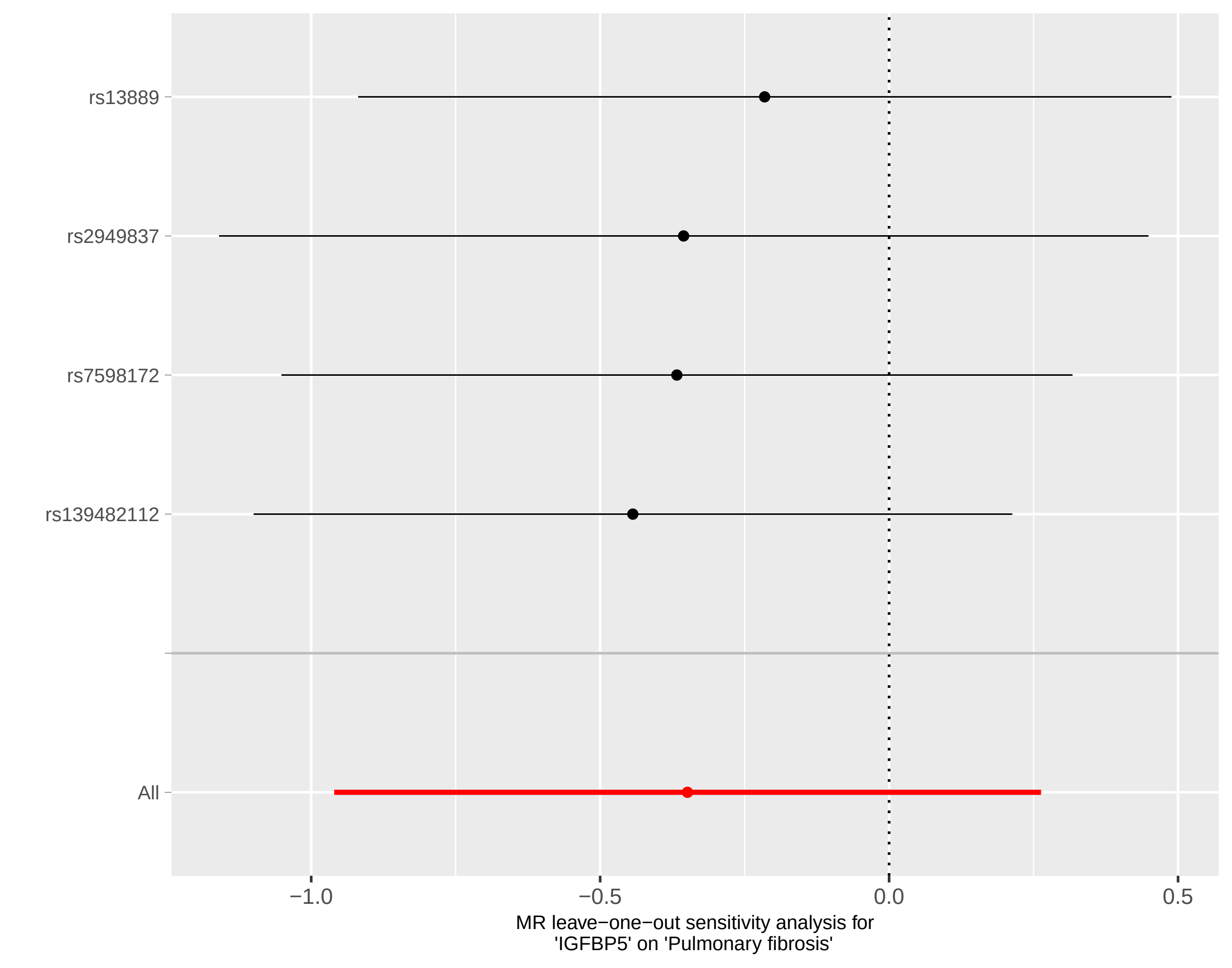

rs13889
rs2949837
rs7598172
rs139482112
All
−1.0
−0.5
0.0
0.5
MR leave−one−out sensitivity analysis for
'IGFBP5' on 'Pulmonary fibrosis'

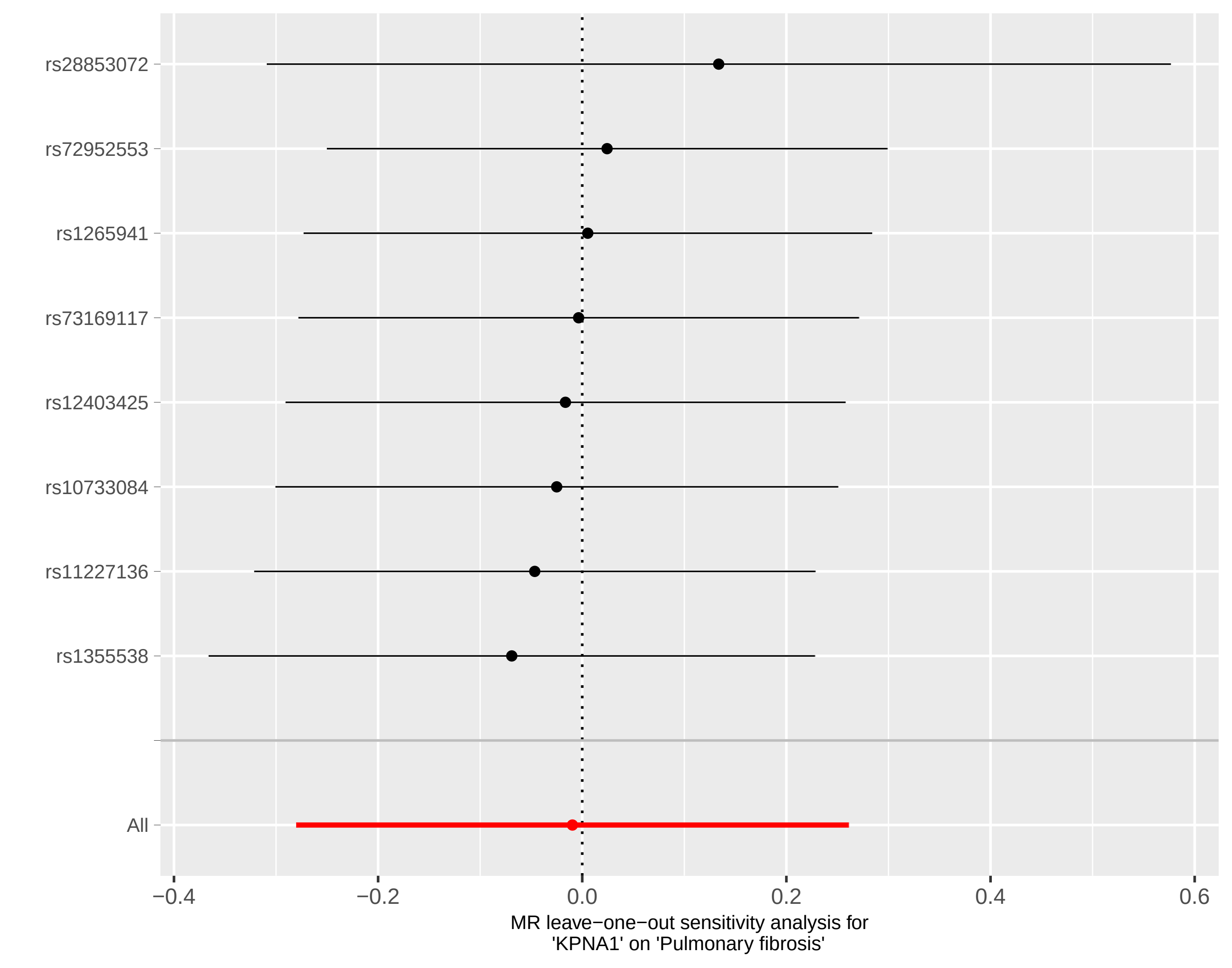

rs28853072
rs72952553
rs1265941
rs73169117
rs12403425
rs10733084
rs11227136
rs1355538
All
−0.4
−0.2
0.0
0.2
0.4
0.6
MR leave−one−out sensitivity analysis for
'KPNA1' on 'Pulmonary fibrosis'

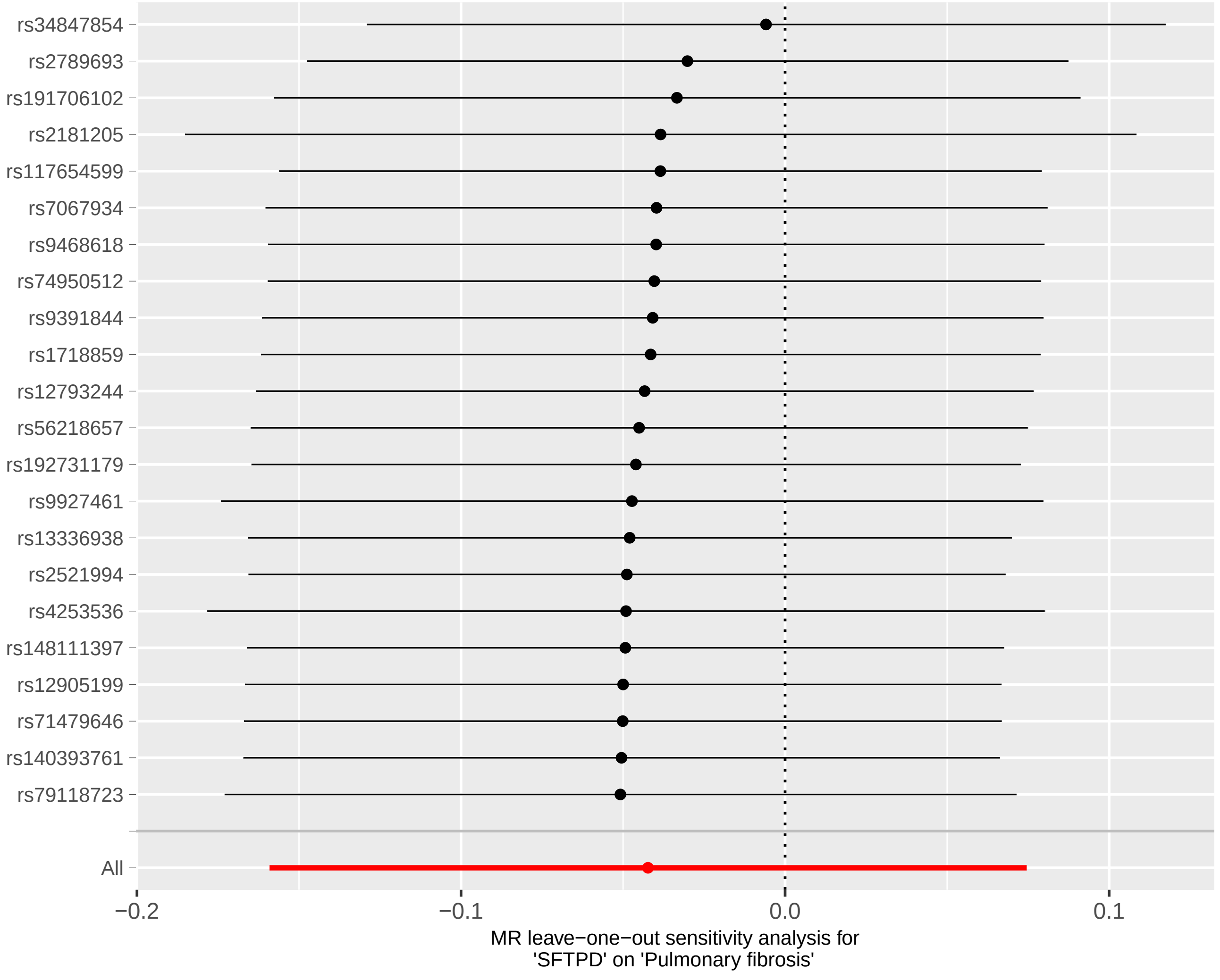
rs34847854
rs2789693
rs191706102
rs2181205
rs117654599
rs7067934
rs9468618
rs74950512
rs9391844
rs1718859
rs12793244
rs56218657
rs192731179
rs9927461
rs13336938
rs2521994
rs4253536
rs148111397
rs12905199
rs71479646
rs140393761
rs79118723
All
−0.2
−0.1
0.0
0.1
MR leave−one−out sensitivity analysis for
'SFTPD' on 'Pulmonary fibrosis'

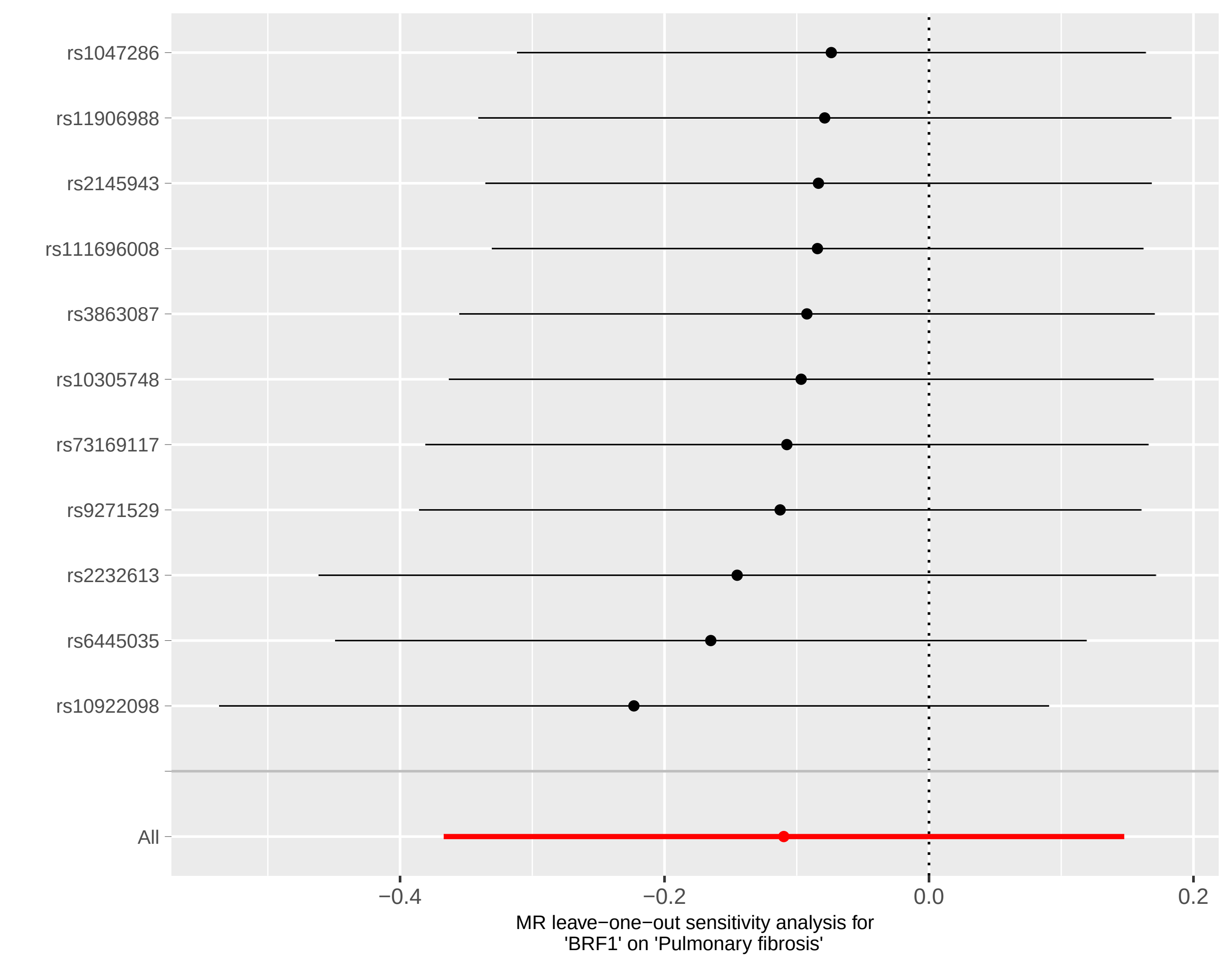
rs1047286
rs11906988
rs2145943
rs111696008
rs3863087
rs10305748
rs73169117
rs9271529
rs2232613
rs6445035
rs10922098
All
−0.4
−0.2
0.0
0.2
MR leave−one−out sensitivity analysis for
'BRF1' on 'Pulmonary fibrosis'

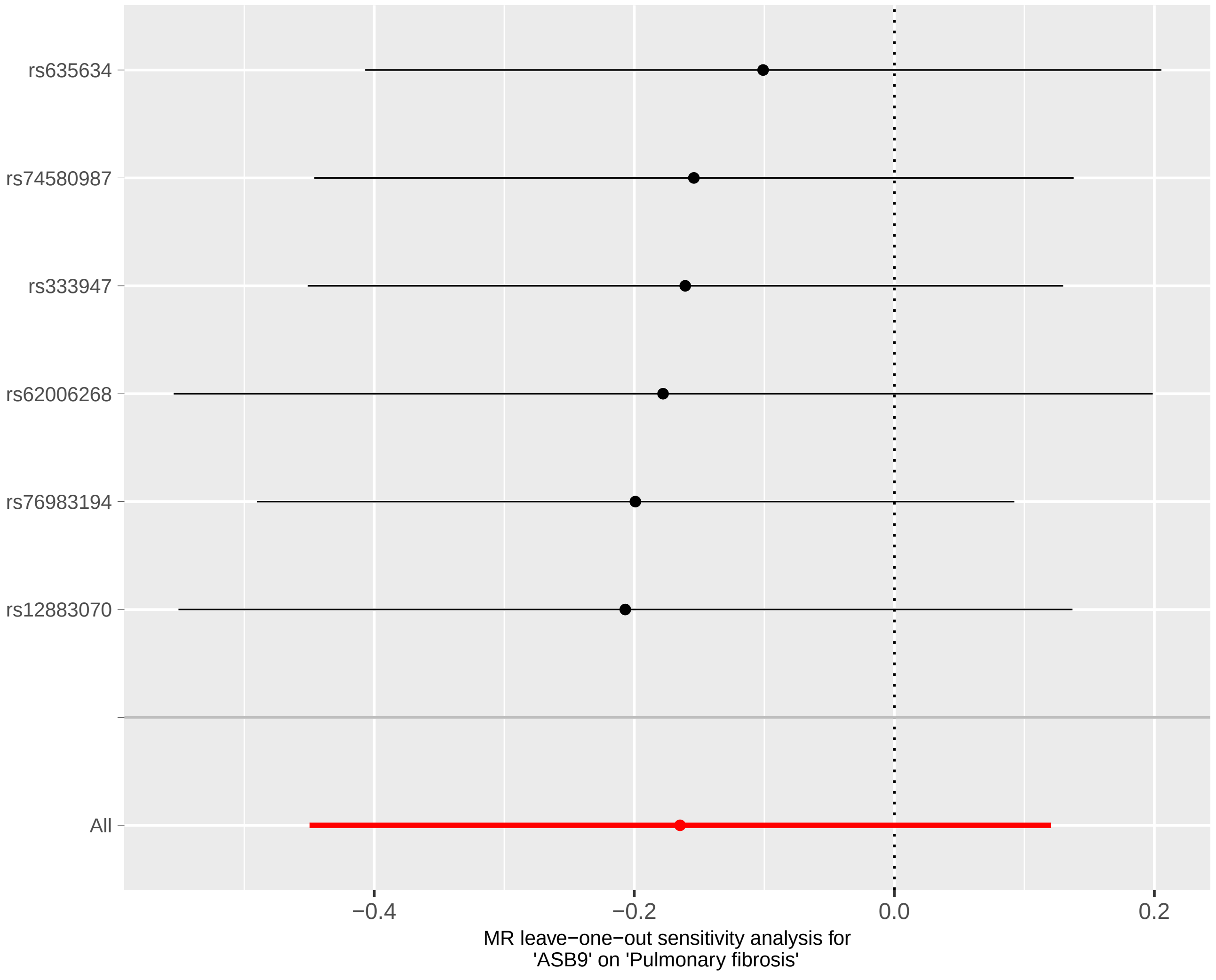

rs635634
rs74580987
rs333947
rs62006268
rs76983194
rs12883070
All
−0.4
−0.2
0.0
0.2
MR leave−one−out sensitivity analysis for
'ASB9' on 'Pulmonary fibrosis'

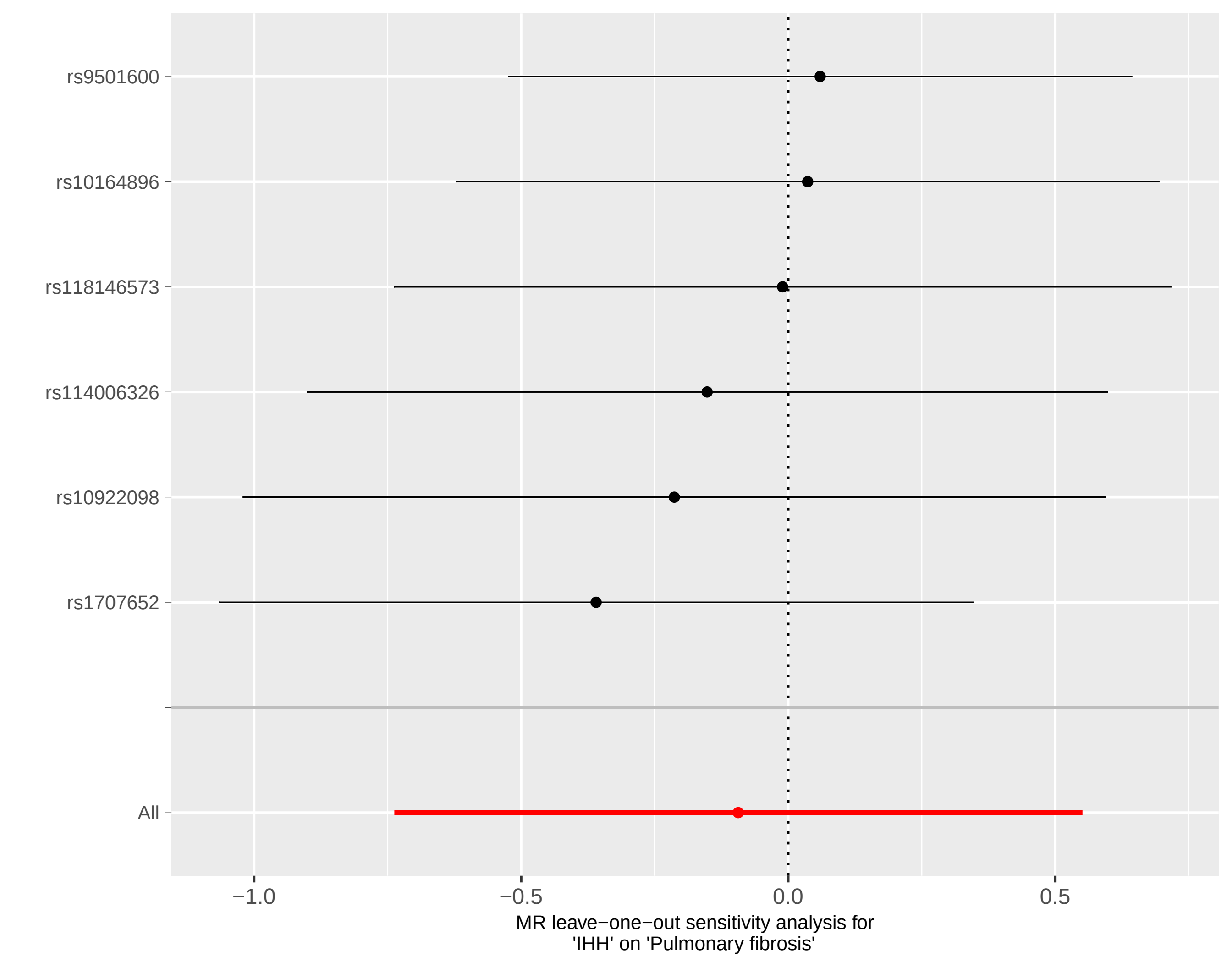
rs9501600
rs10164896
rs118146573
rs114006326
rs10922098
rs1707652
All
−1.0
−0.5
0.0
0.5
MR leave−one−out sensitivity analysis for
'IHH' on 'Pulmonary fibrosis'

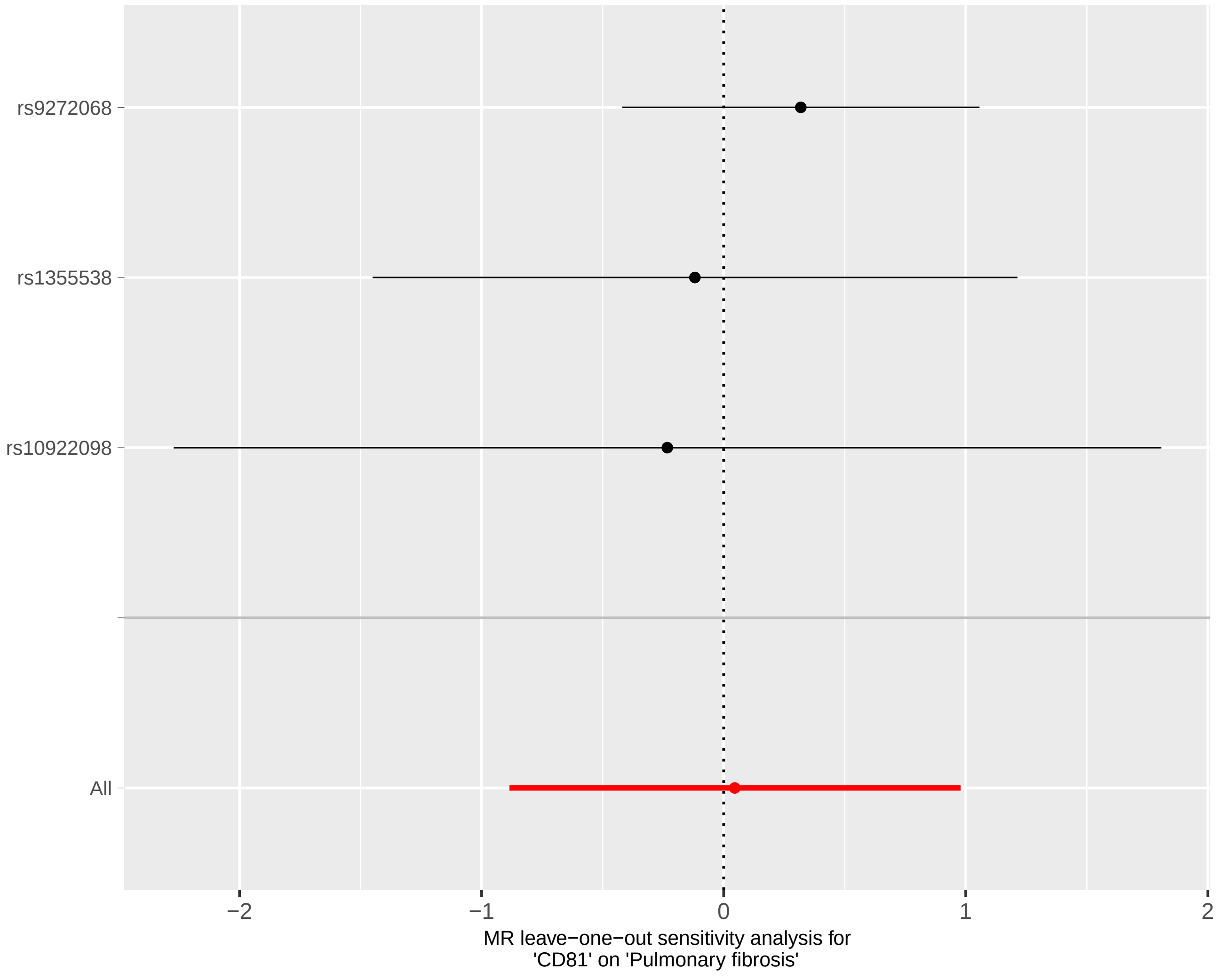
rs9272068
rs1355538
rs10922098
All
−2
−1
0
1
2
MR leave−one−out sensitivity analysis for
'CD81' on 'Pulmonary fibrosis'

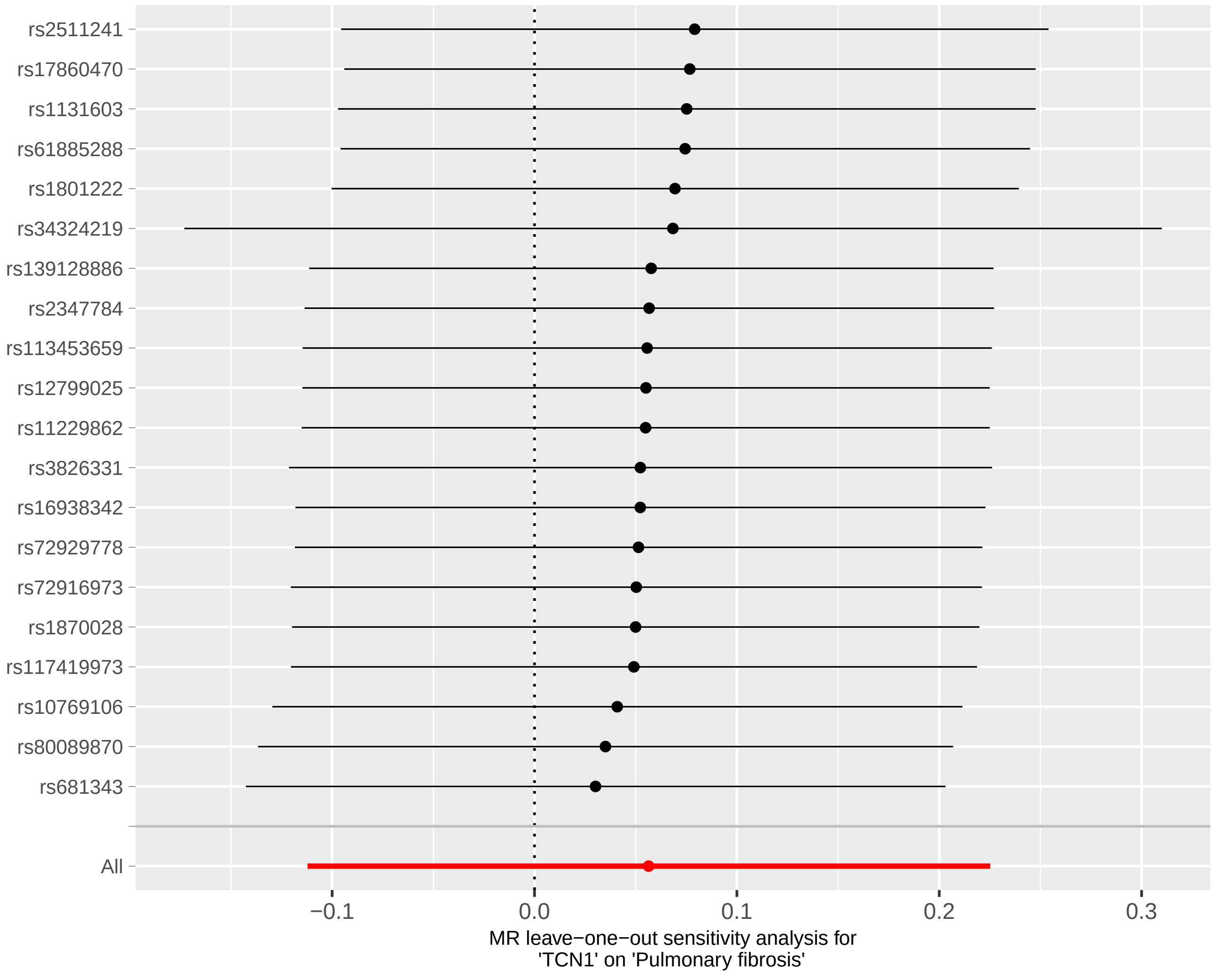

rs2511241
rs17860470
rs1131603
rs61885288
rs1801222
rs34324219
rs139128886
rs2347784
rs113453659
rs12799025
rs11229862
rs3826331
rs16938342
rs72929778
rs72916973
rs1870028
rs117419973
rs10769106
rs80089870
rs681343
All
−0.1
0.0
0.1
0.2
0.3
MR leave−one−out sensitivity analysis for
'TCN1' on 'Pulmonary fibrosis'

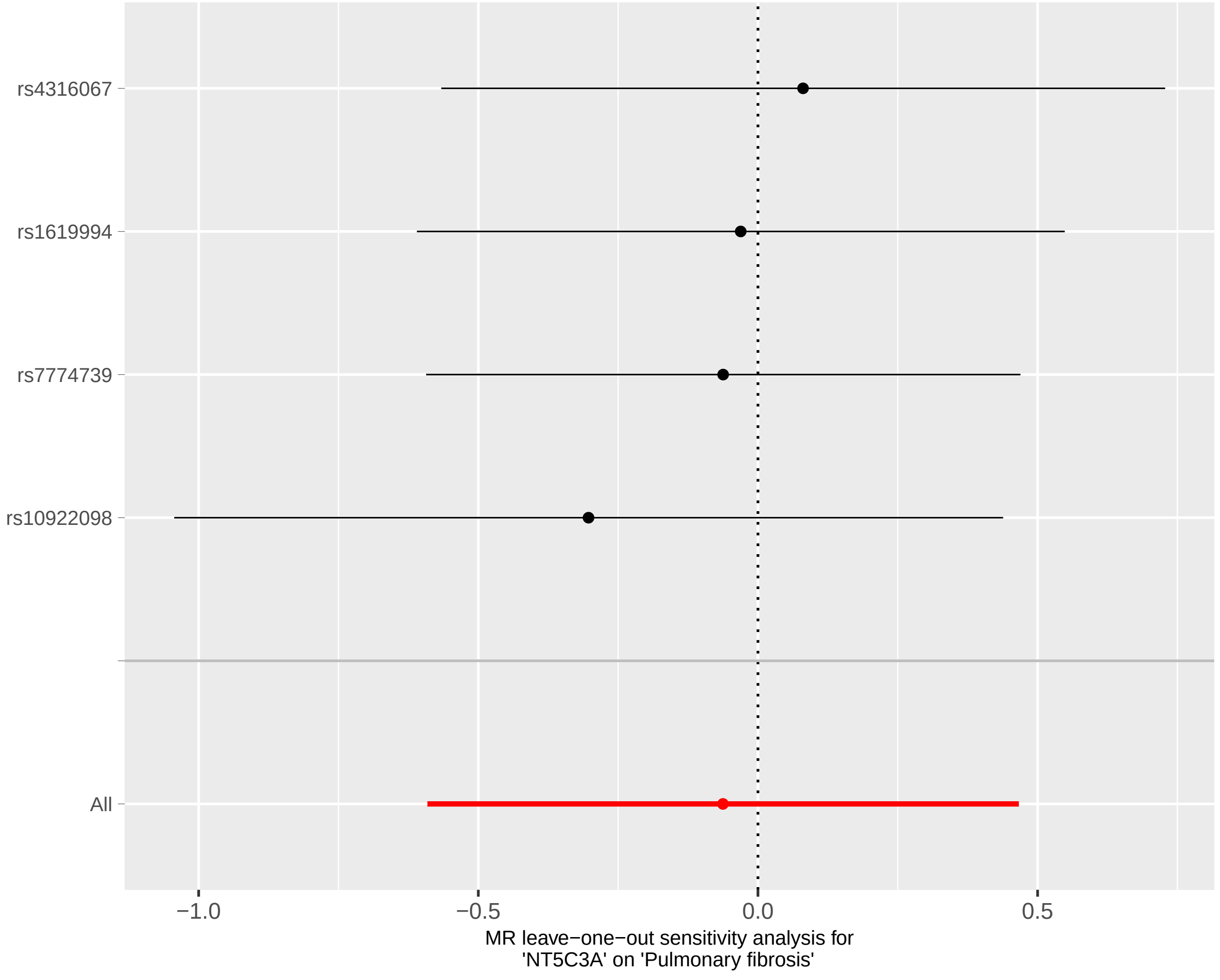
rs4316067
rs1619994
rs7774739
rs10922098
All
−1.0
−0.5
0.0
0.5
MR leave−one−out sensitivity analysis for
'NT5C3A' on 'Pulmonary fibrosis'

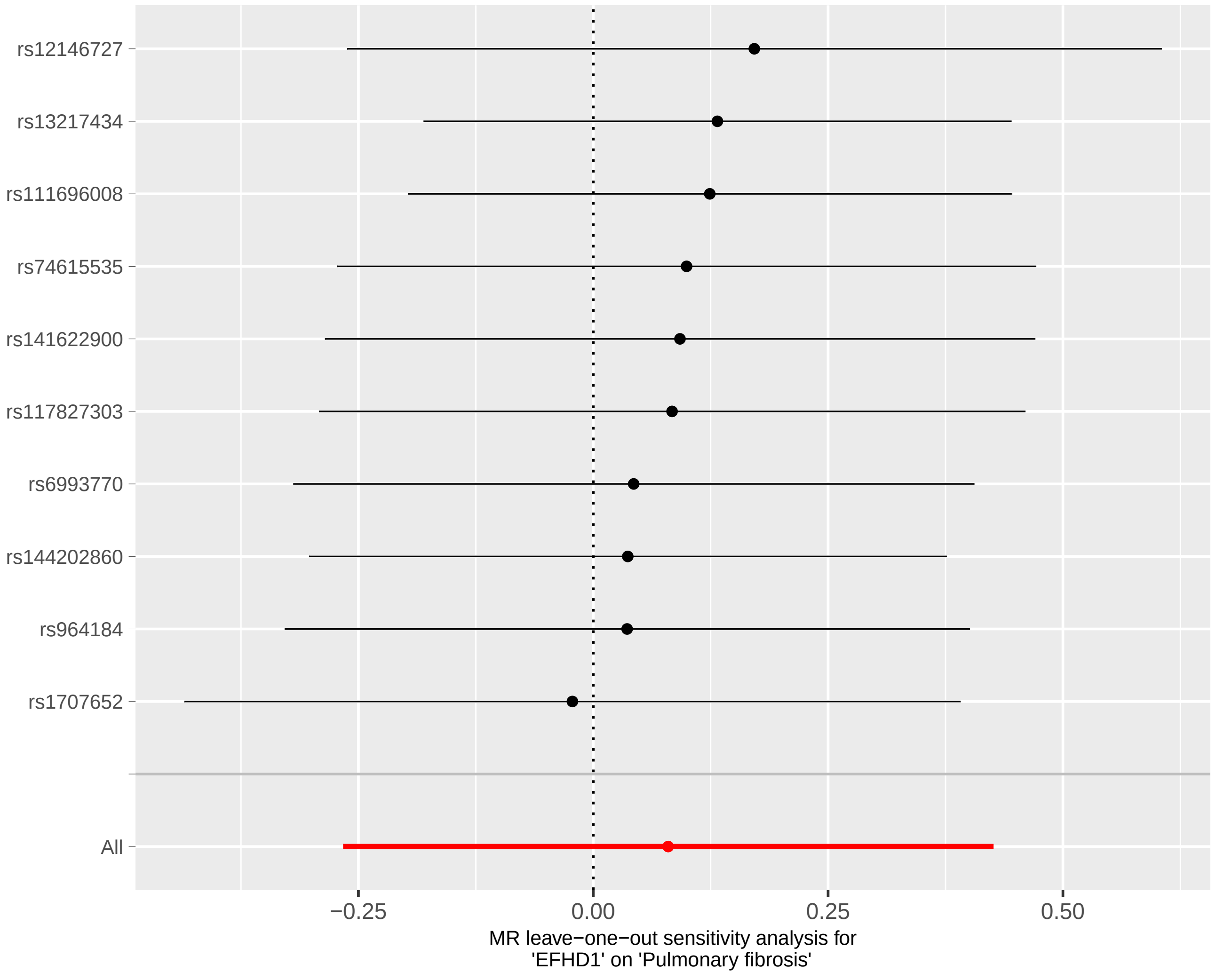
rs12146727
rs13217434
rs111696008
rs74615535
rs141622900
rs117827303
rs6993770
rs144202860
rs964184
rs1707652
All
−0.25
0.00
0.25
0.50
MR leave−one−out sensitivity analysis for
'EFHD1' on 'Pulmonary fibrosis'

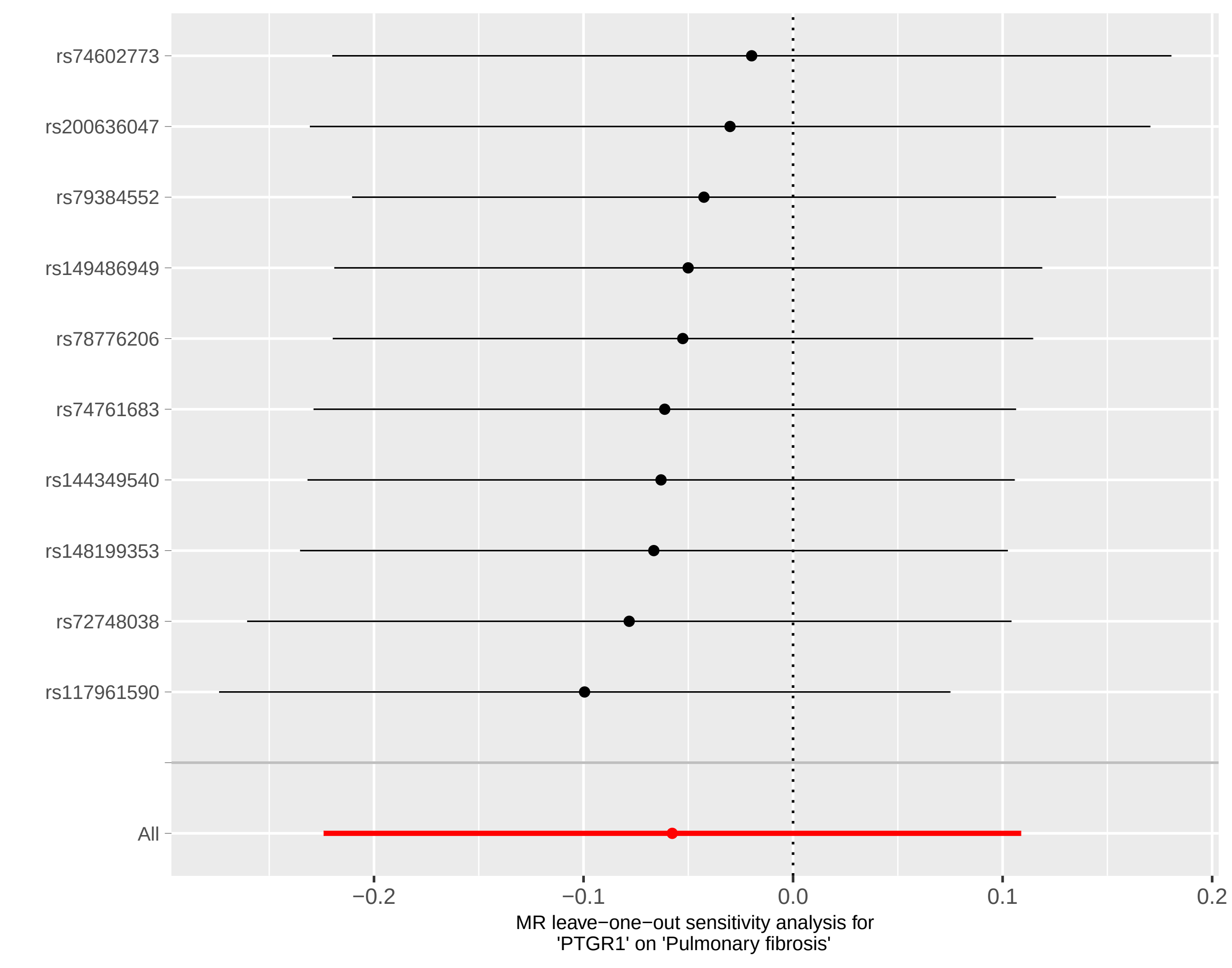
rs74602773
rs200636047
rs79384552
rs149486949
rs78776206
rs74761683
rs144349540
rs148199353
rs72748038
rs117961590
All
−0.2
−0.1
0.0
0.1
0.2
MR leave−one−out sensitivity analysis for
'PTGR1' on 'Pulmonary fibrosis'

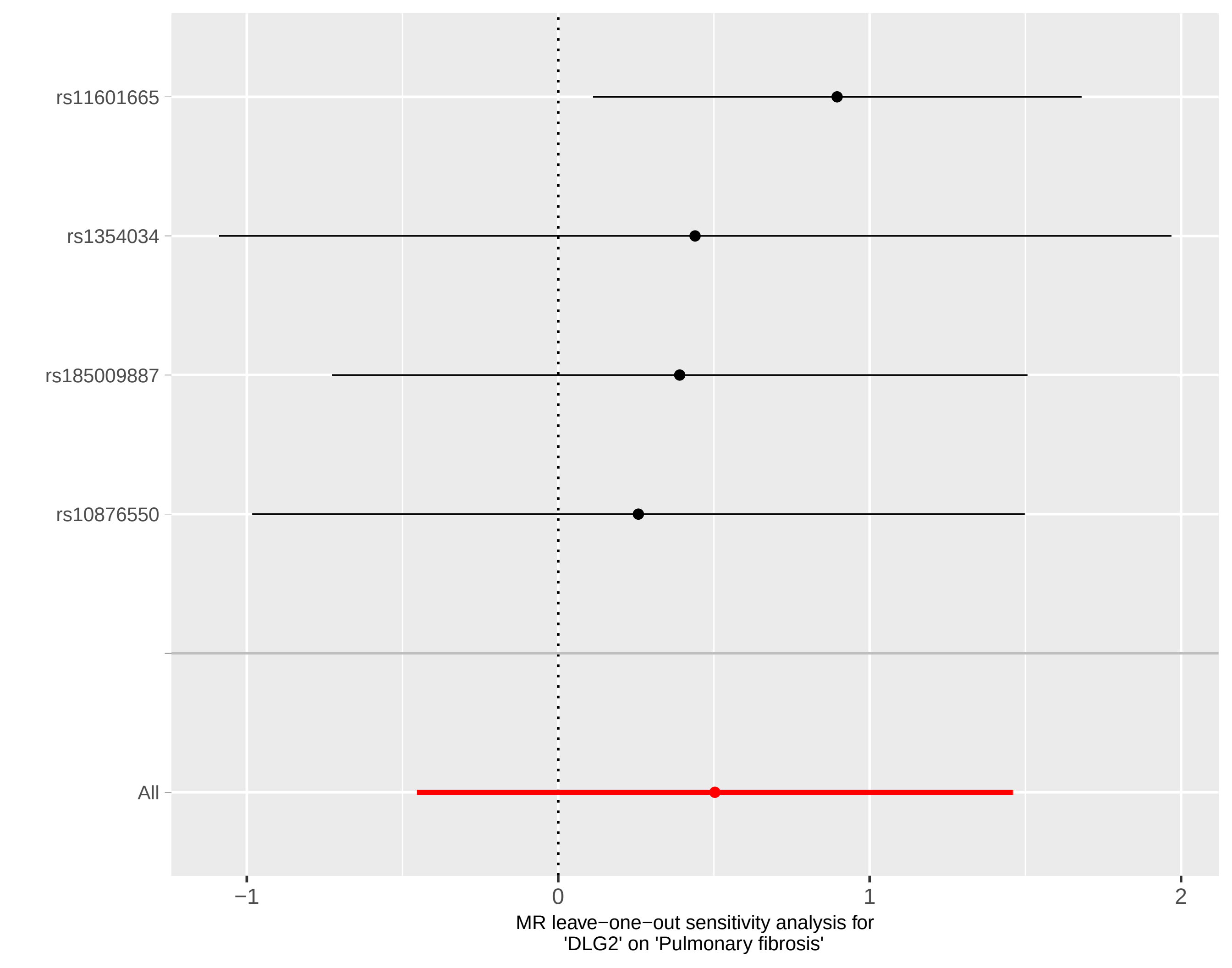
rs11601665
rs1354034
rs185009887
rs10876550
All
−1
0
1
2
MR leave−one−out sensitivity analysis for
'DLG2' on 'Pulmonary fibrosis'

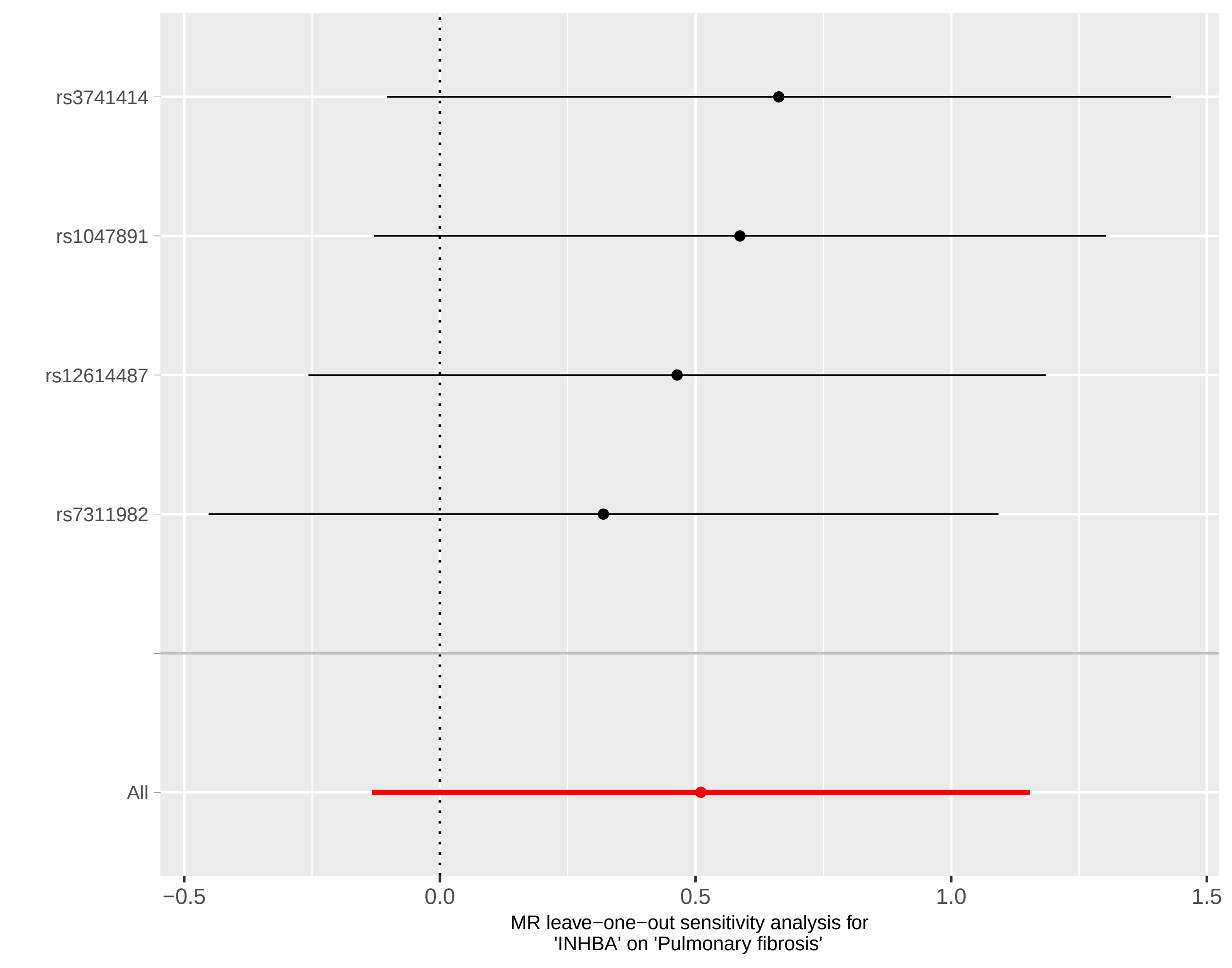
rs3741414
rs1047891
rs12614487
rs7311982
All
−0.5
0.0
0.5
1.0
1.5
MR leave−one−out sensitivity analysis for
'INHBA' on 'Pulmonary fibrosis'

Insufficient number of SNPs

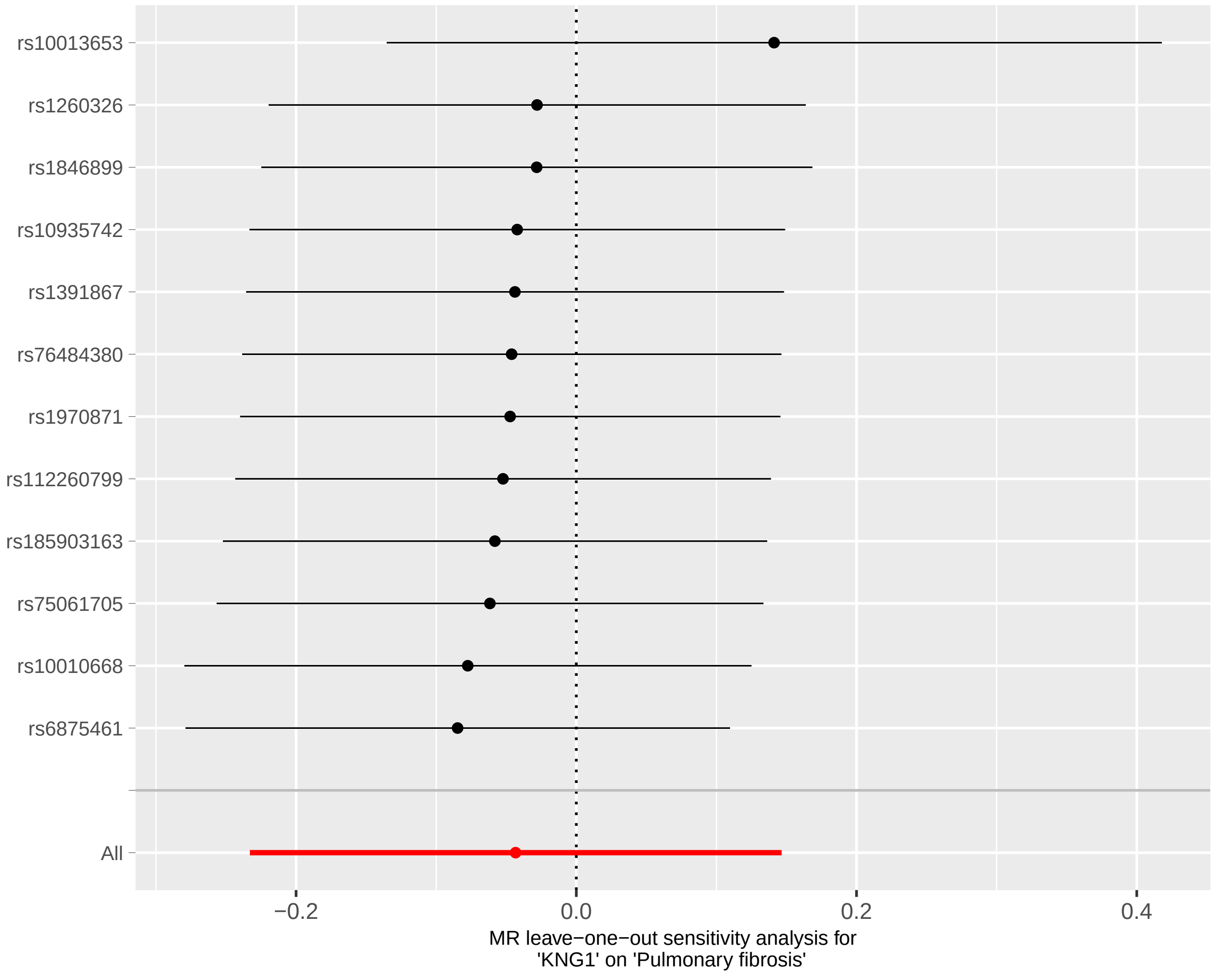
rs10013653
rs1260326
rs1846899
rs10935742
rs1391867
rs76484380
rs1970871
rs112260799
rs185903163
rs75061705
rs10010668
rs6875461
All
−0.2
0.0
0.2
0.4
MR leave−one−out sensitivity analysis for
'KNG1' on 'Pulmonary fibrosis'

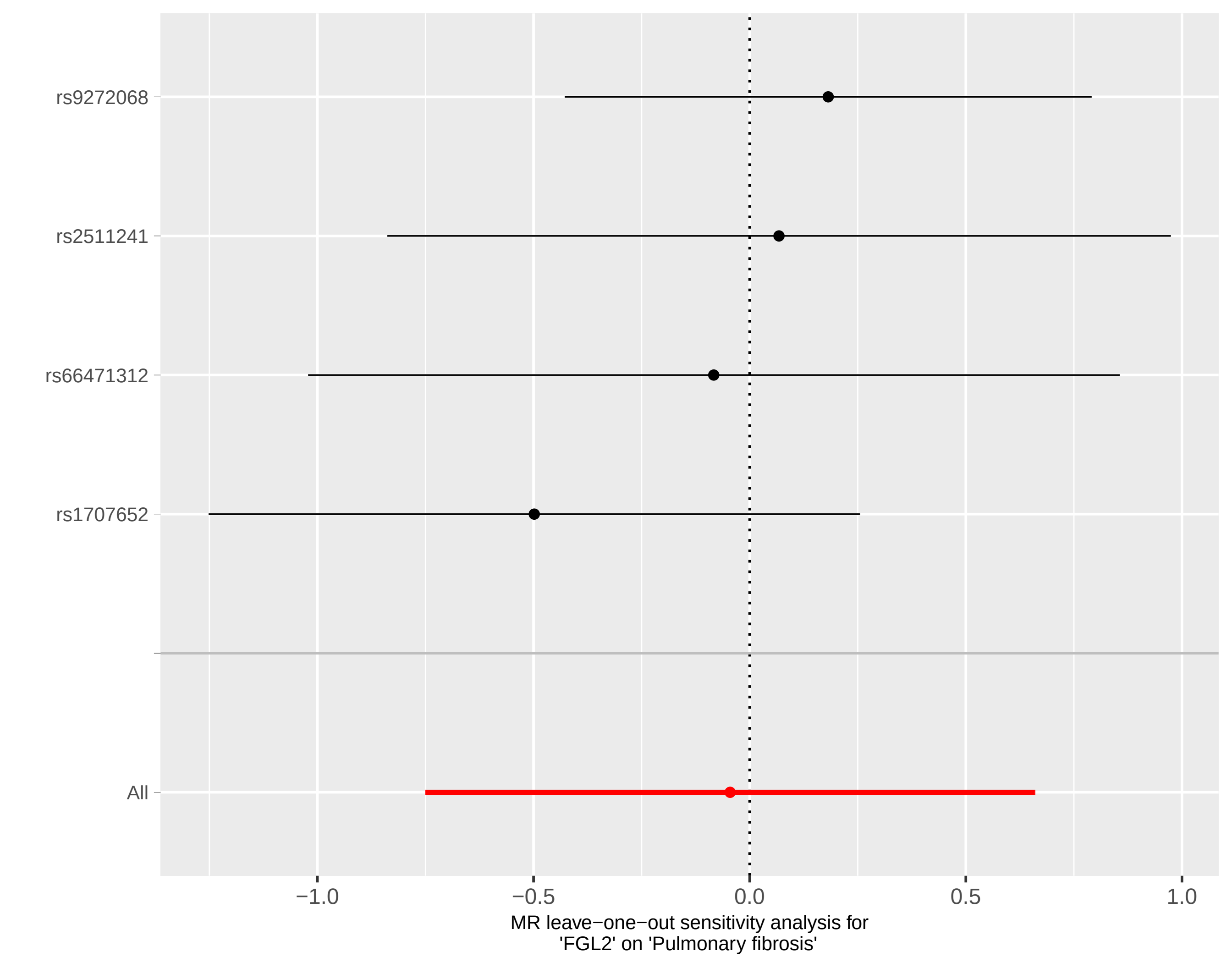
rs9272068
rs2511241
rs66471312
rs1707652
All
−1.0
−0.5
0.0
0.5
1.0
MR leave−one−out sensitivity analysis for
'FGL2' on 'Pulmonary fibrosis'

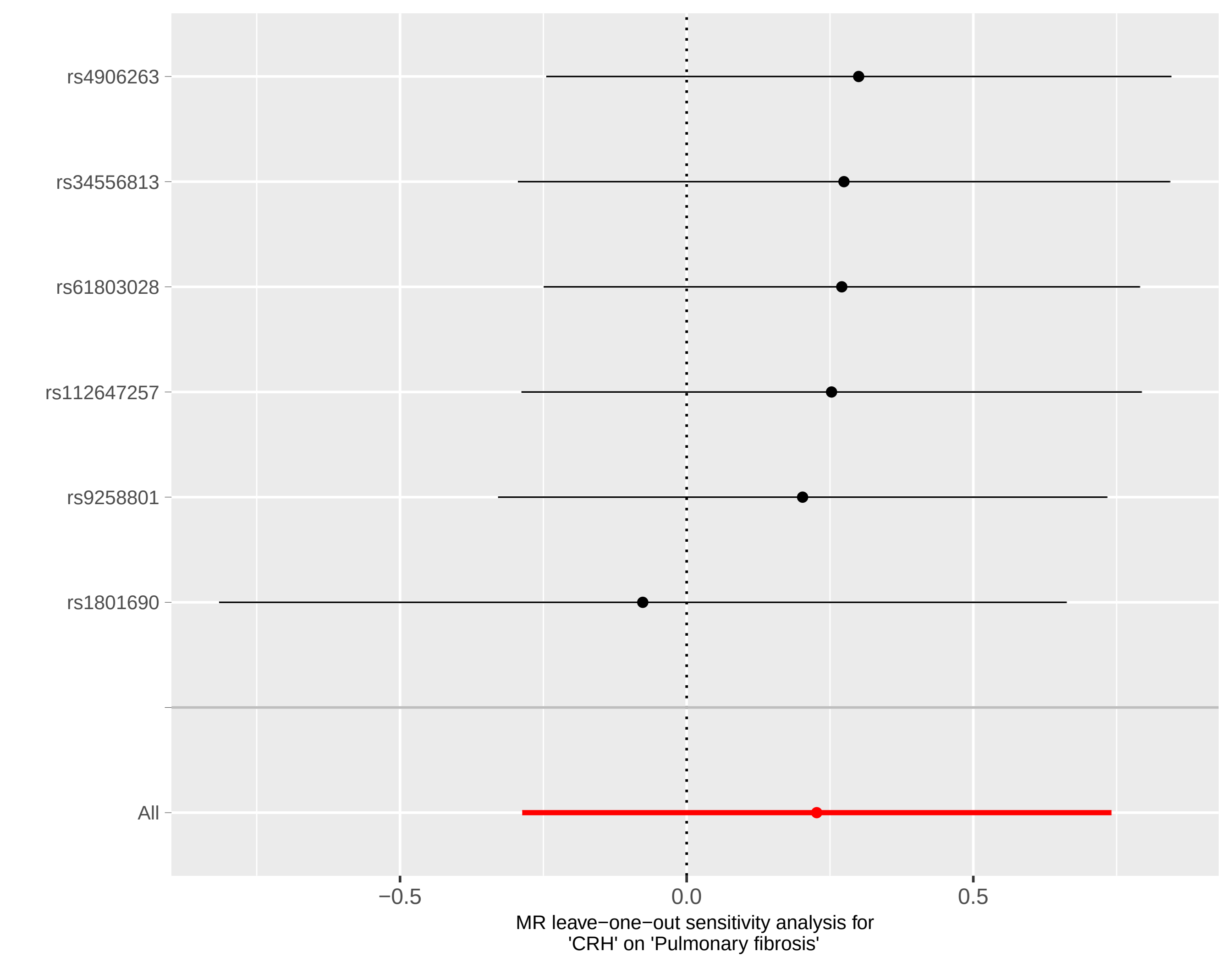
rs4906263
rs34556813
rs61803028
rs112647257
rs9258801
rs1801690
All
−0.5
0.0
0.5
MR leave−one−out sensitivity analysis for
'CRH' on 'Pulmonary fibrosis'

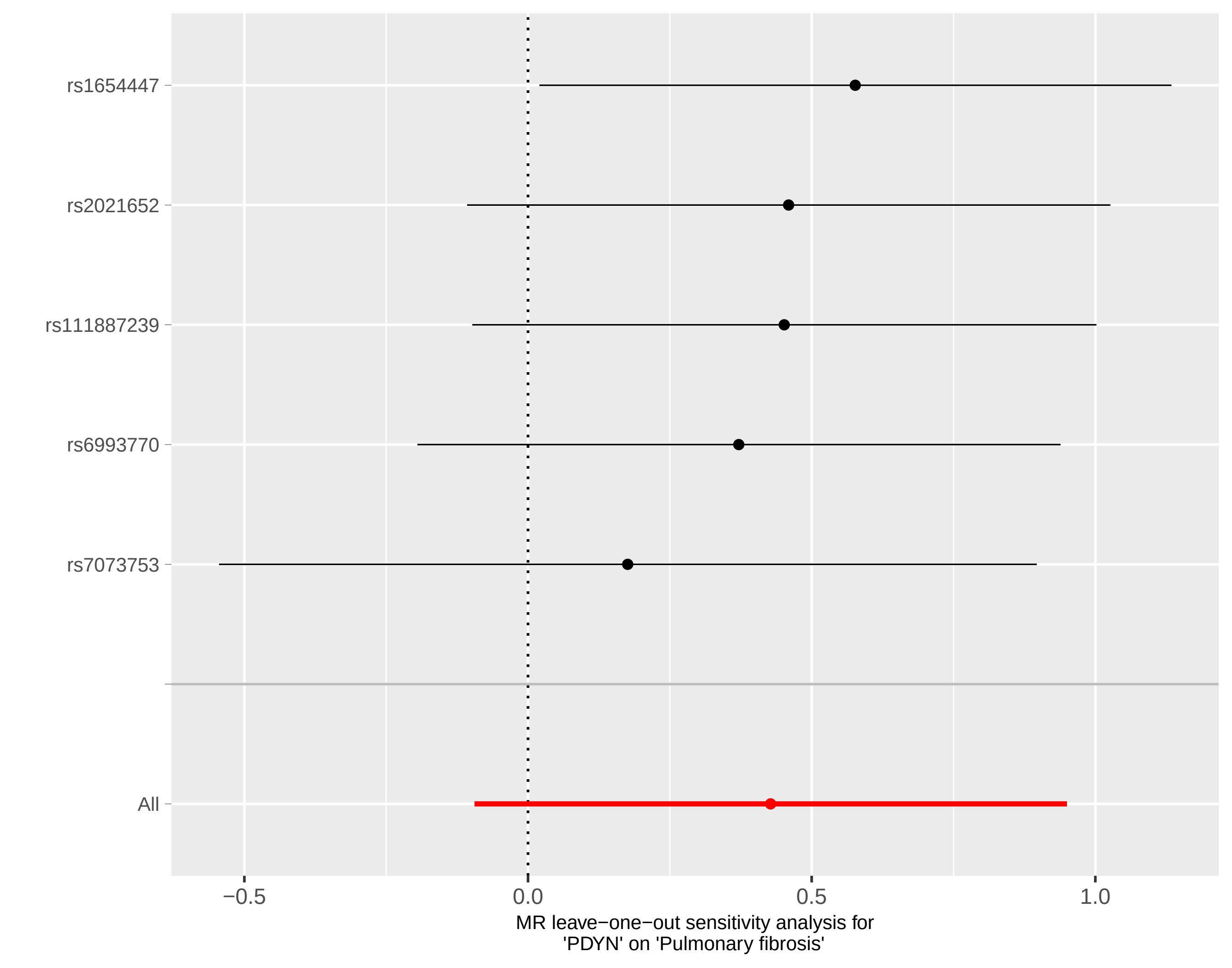
rs1654447
rs2021652
rs111887239
rs6993770
rs7073753
All
−0.5
0.0
0.5
1.0
MR leave−one−out sensitivity analysis for
'PDYN' on 'Pulmonary fibrosis'

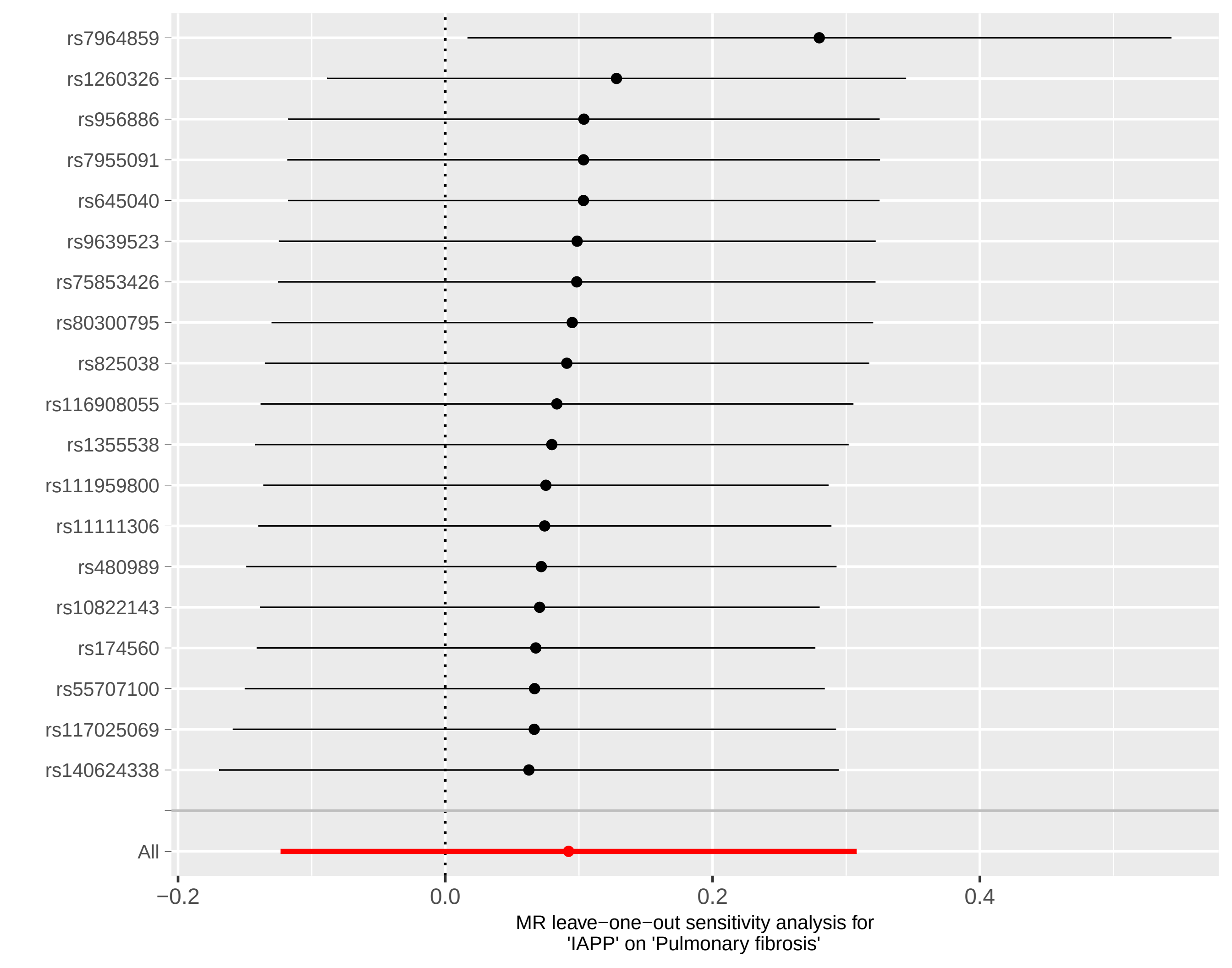
rs7964859
rs1260326
rs956886
rs7955091
rs645040
rs9639523
rs75853426
rs80300795
rs825038
rs116908055
rs1355538
rs111959800
rs11111306
rs480989
rs10822143
rs174560
rs55707100
rs117025069
rs140624338
All
−0.2
0.0
0.2
0.4
MR leave−one−out sensitivity analysis for
'IAPP' on 'Pulmonary fibrosis'

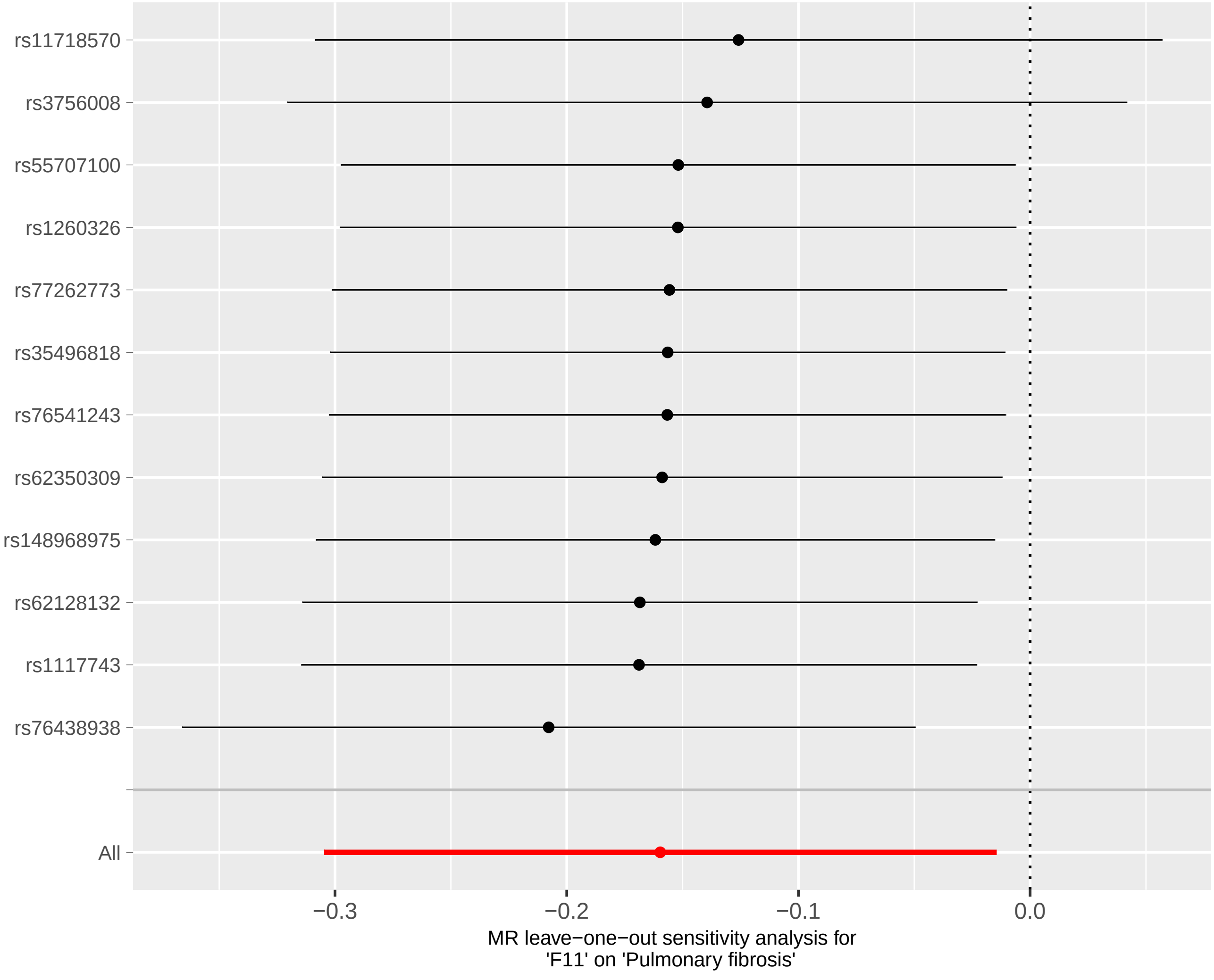

rs11718570
rs3756008
rs55707100
rs1260326
rs77262773
rs35496818
rs76541243
rs62350309
rs148968975
rs62128132
rs1117743
rs76438938
All
−0.3
−0.2
−0.1
0.0
MR leave−one−out sensitivity analysis for
'F11' on 'Pulmonary fibrosis'

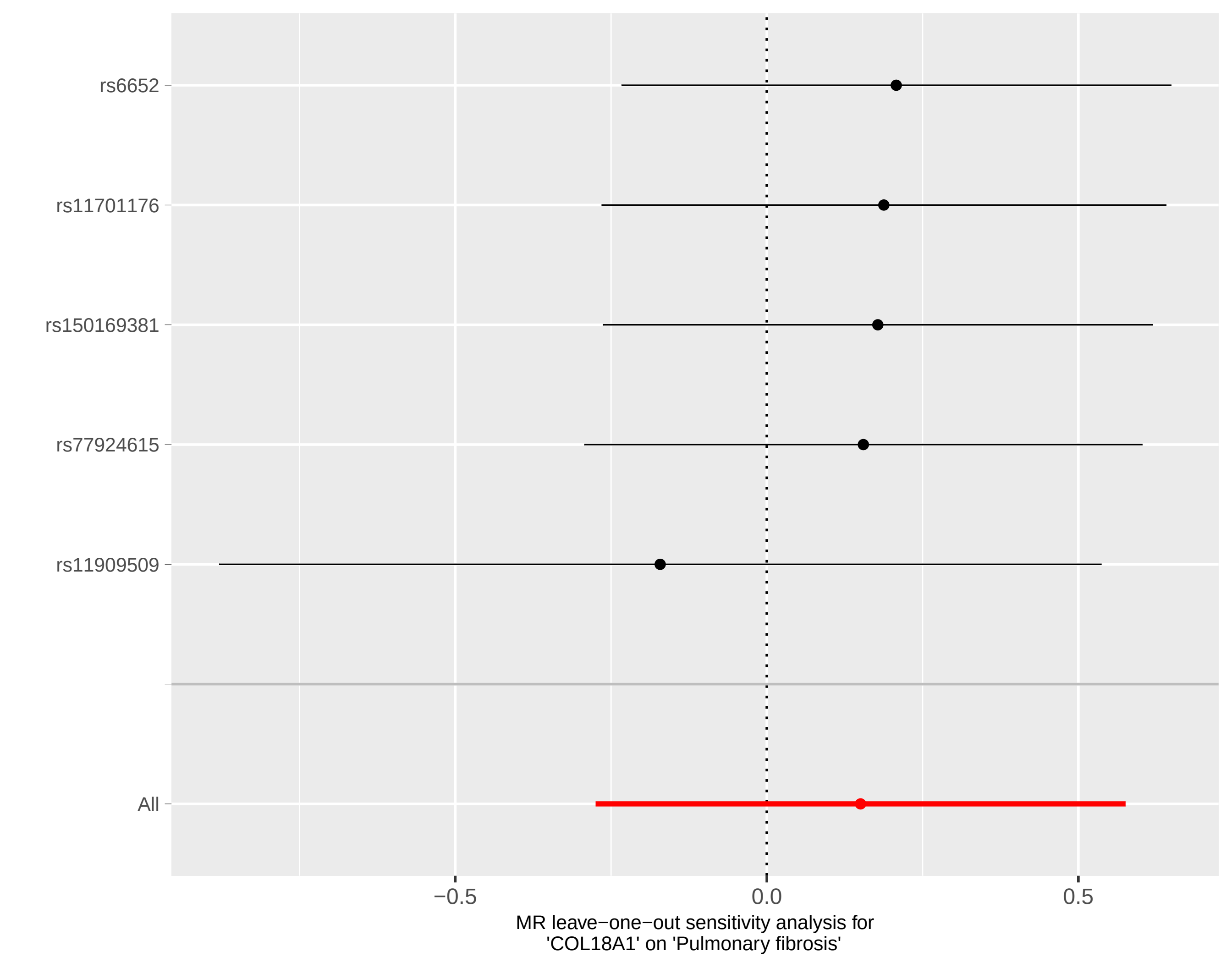
rs6652
rs11701176
rs150169381
rs77924615
rs11909509
All
−0.5
0.0
0.5
MR leave−one−out sensitivity analysis for
'COL18A1' on 'Pulmonary fibrosis'

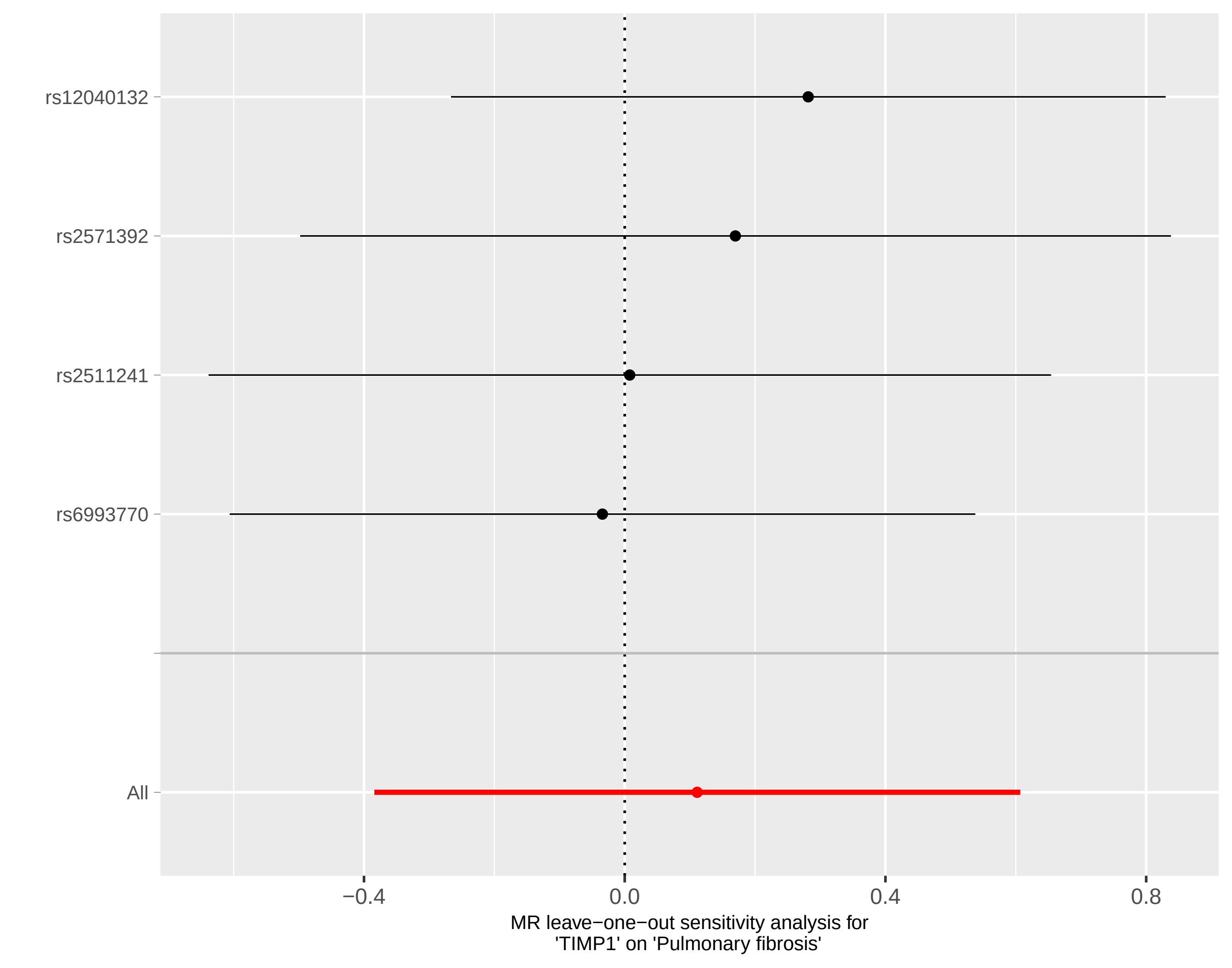
rs12040132
rs2571392
rs2511241
rs6993770
All
−0.4
0.0
0.4
0.8
MR leave−one−out sensitivity analysis for
'TIMP1' on 'Pulmonary fibrosis'

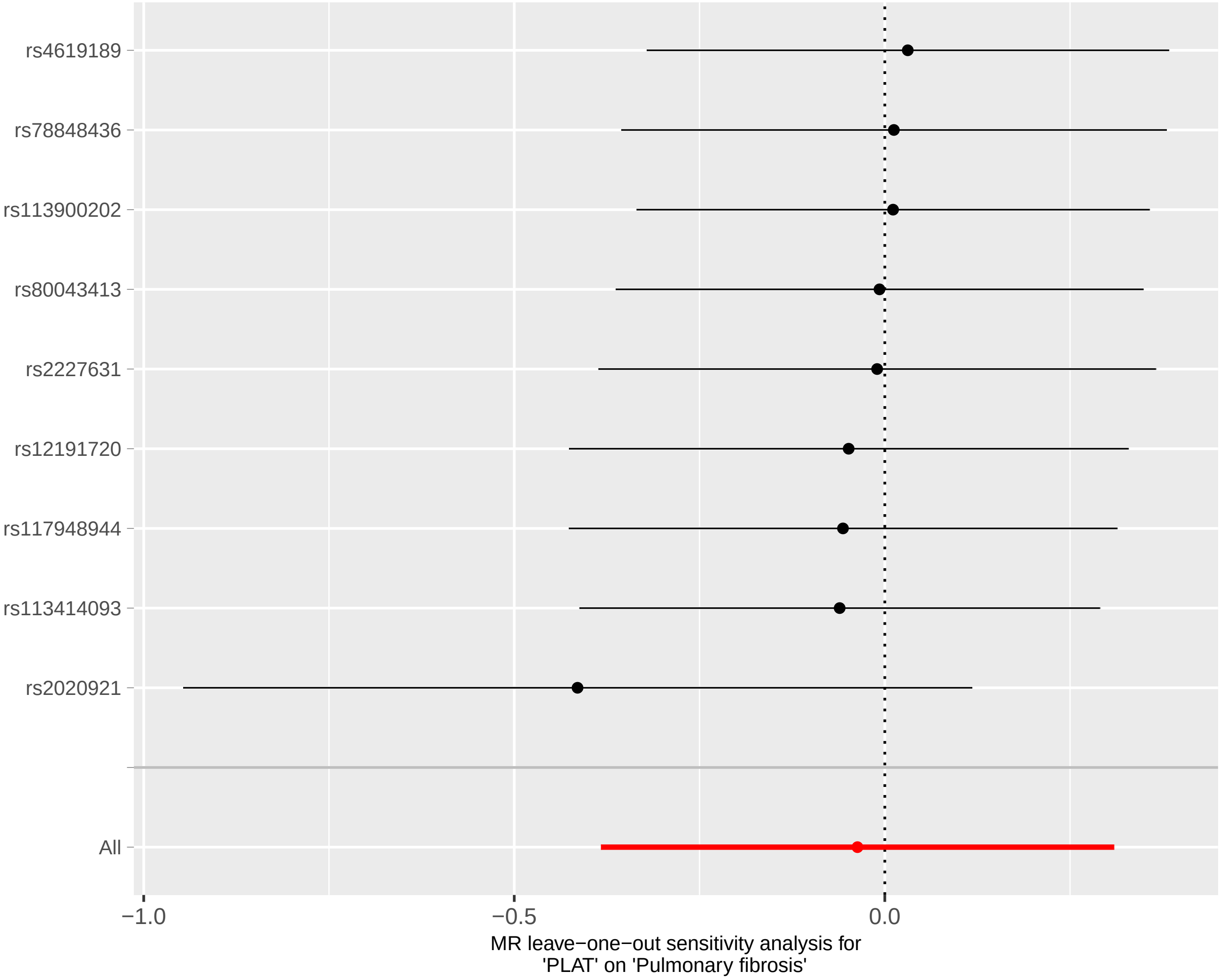
rs4619189
rs78848436
rs113900202
rs80043413
rs2227631
rs12191720
rs117948944
rs113414093
rs2020921
All
−1.0
−0.5
0.0
MR leave−one−out sensitivity analysis for
'PLAT' on 'Pulmonary fibrosis'

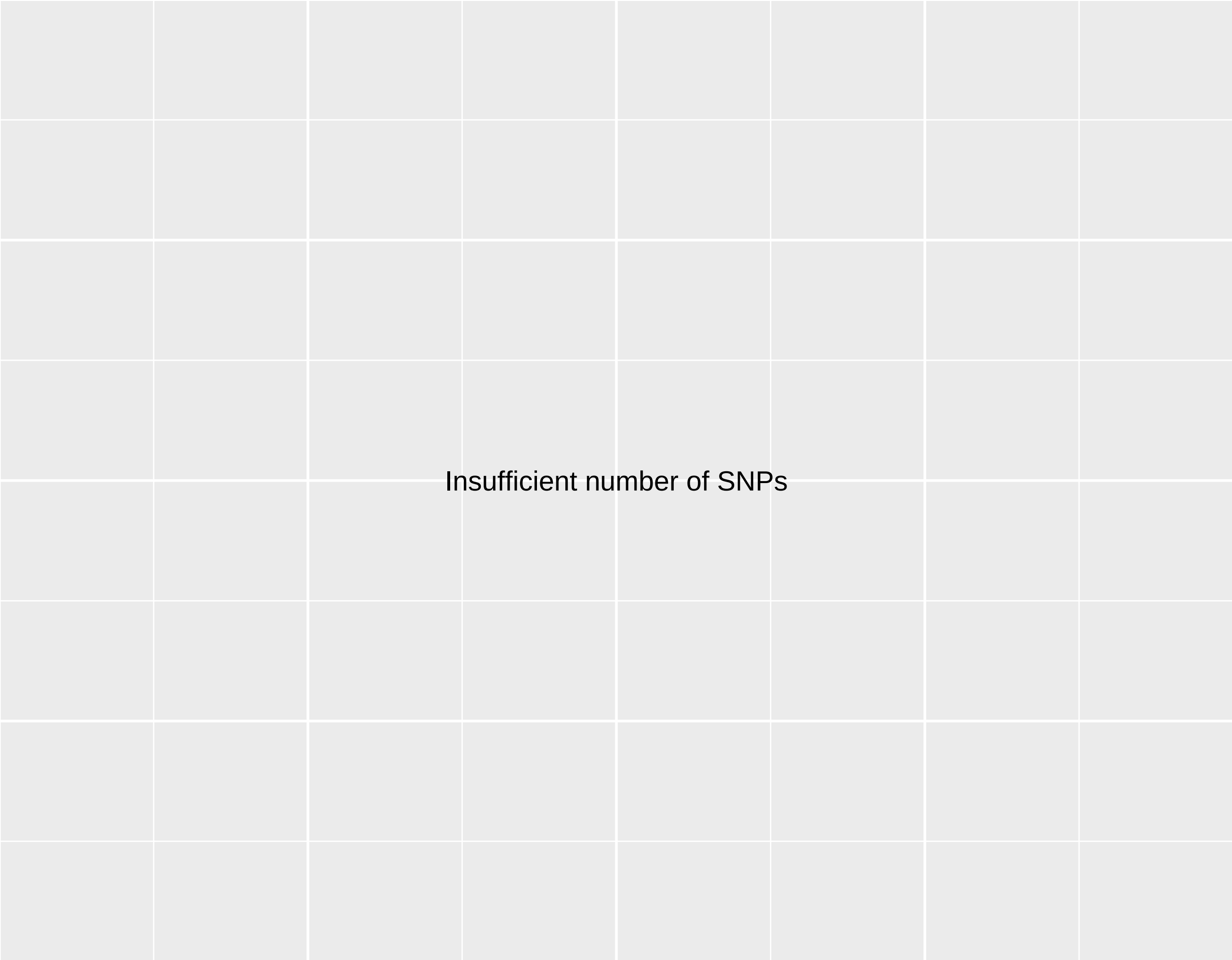
Insufficient number of SNPs

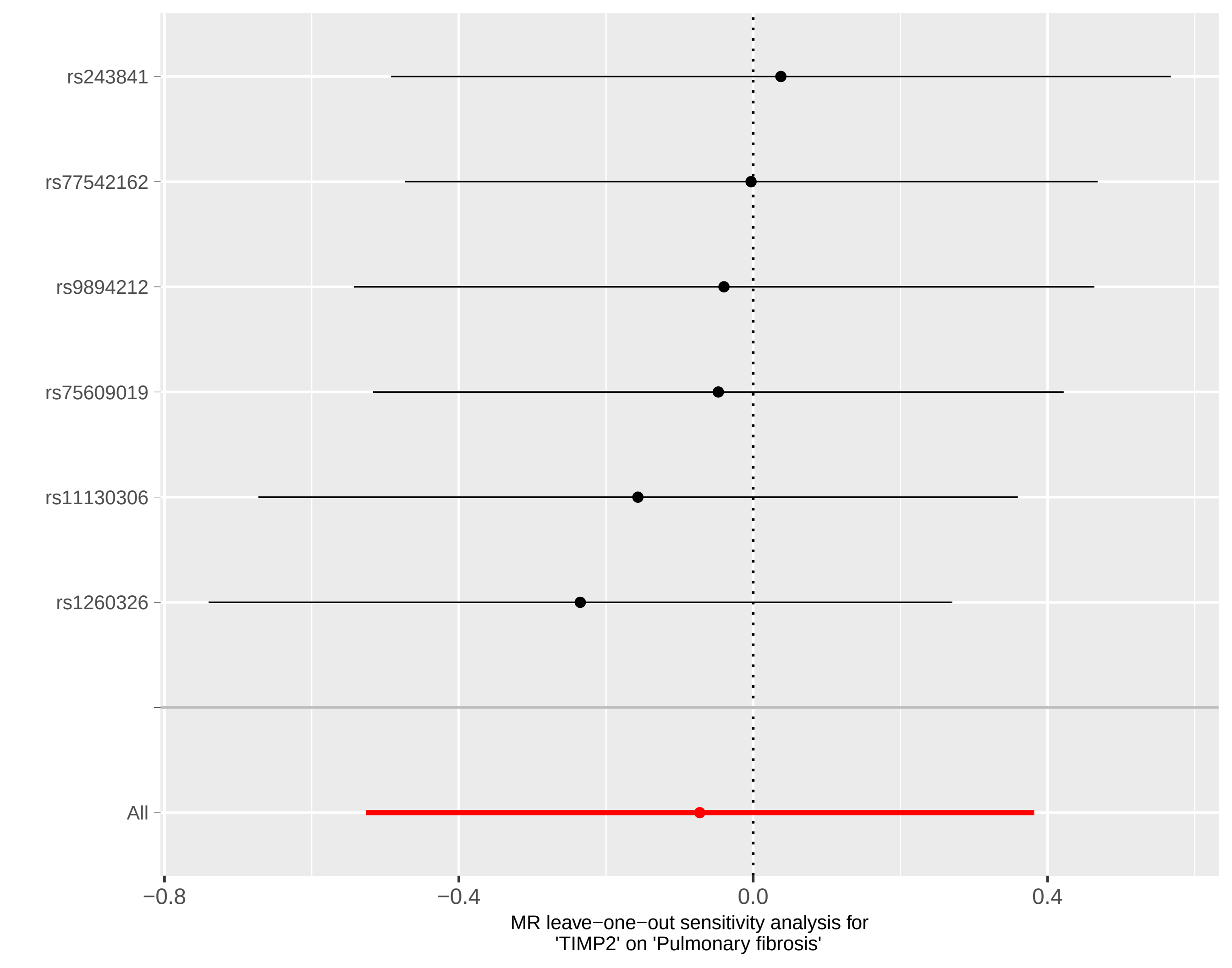
rs243841
rs77542162
rs9894212
rs75609019
rs11130306
rs1260326
All
−0.8
−0.4
0.0
0.4
MR leave−one−out sensitivity analysis for
'TIMP2' on 'Pulmonary fibrosis'

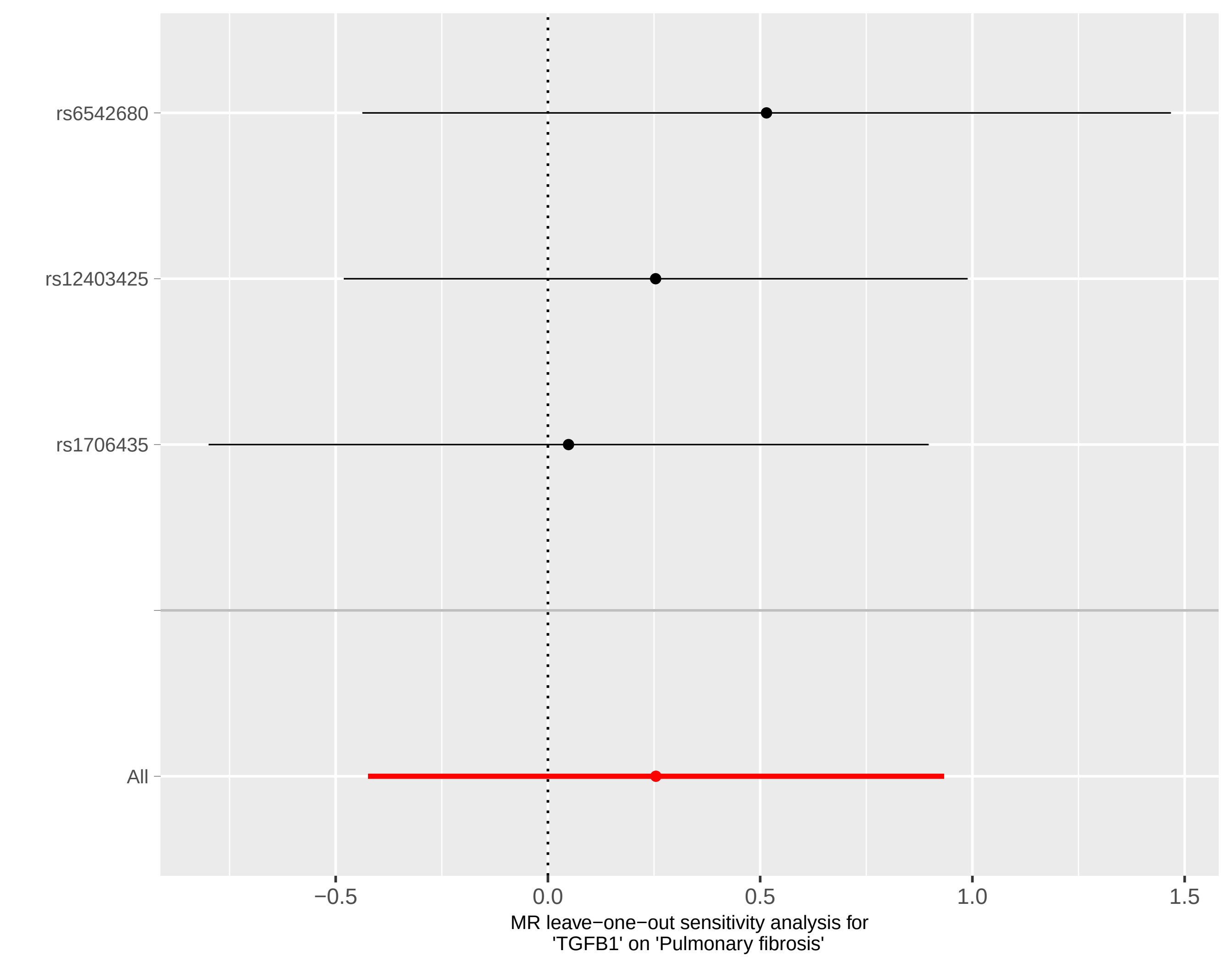
rs6542680
rs12403425
rs1706435
All
−0.5
0.0
0.5
1.0
1.5
MR leave−one−out sensitivity analysis for
'TGFB1' on 'Pulmonary fibrosis'

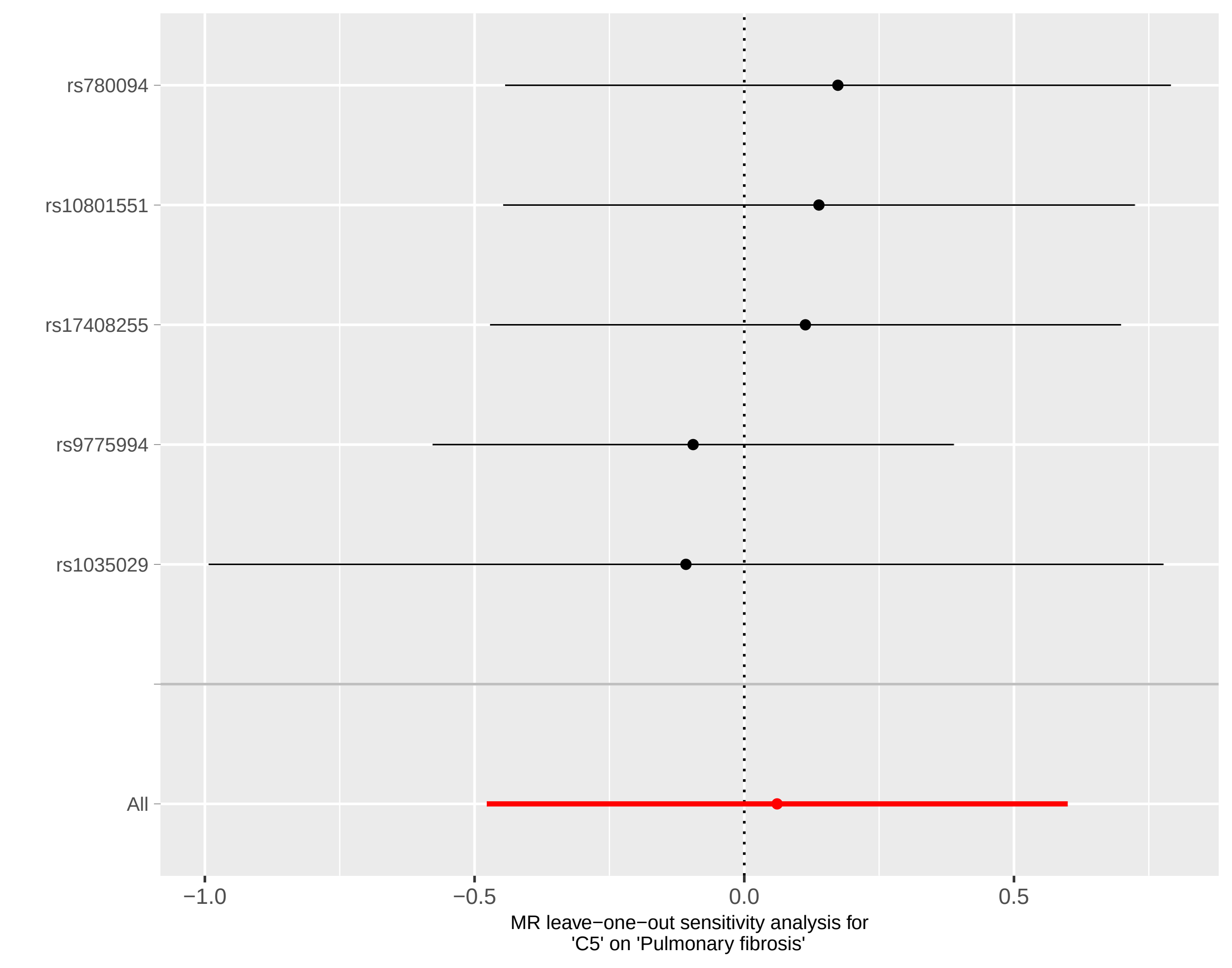
rs780094
rs10801551
rs17408255
rs9775994
rs1035029
All
−1.0
−0.5
0.0
0.5
MR leave−one−out sensitivity analysis for
'C5' on 'Pulmonary fibrosis'

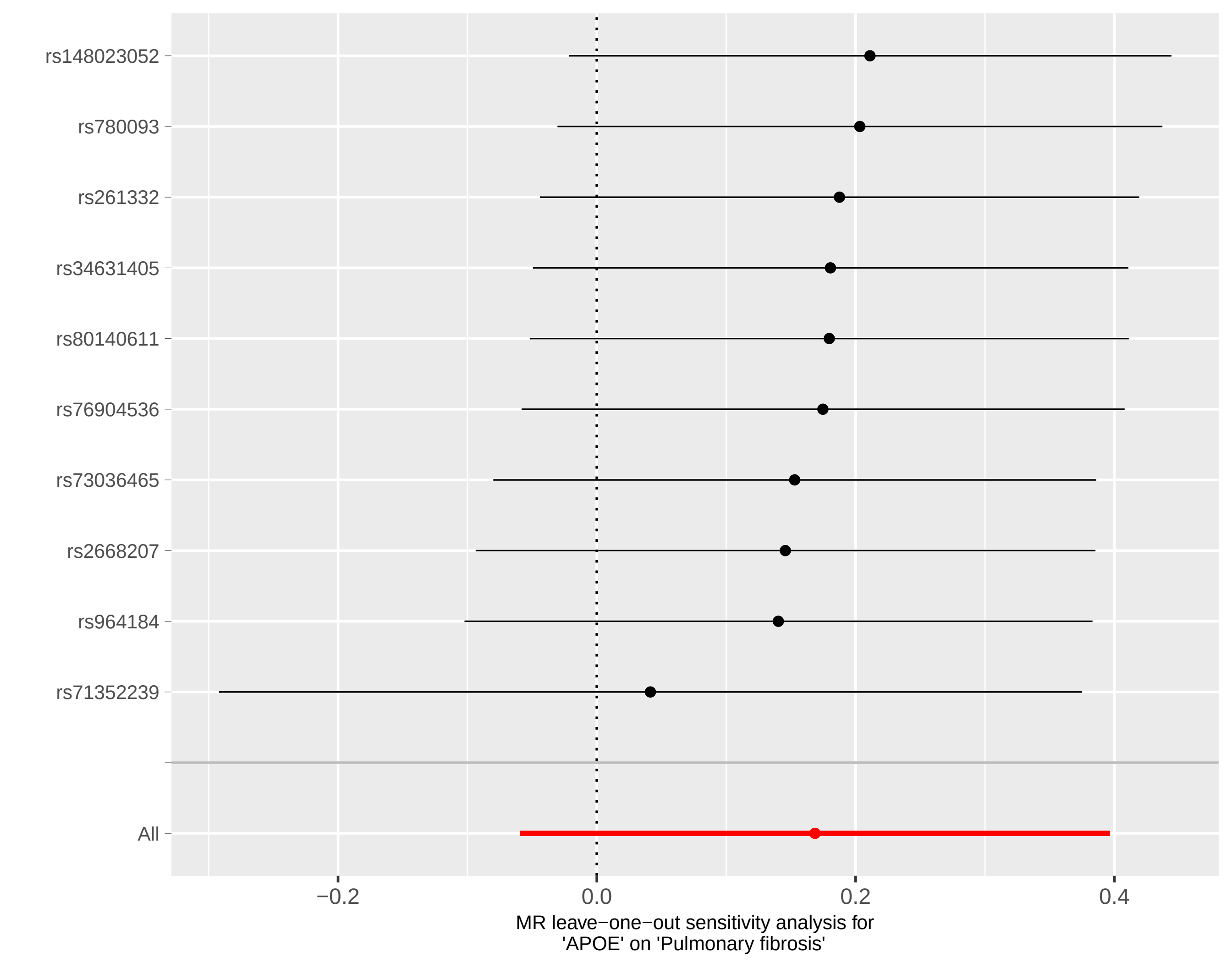
rs148023052
rs780093
rs261332
rs34631405
rs80140611
rs76904536
rs73036465
rs2668207
rs964184
rs71352239
All
−0.2
0.0
0.2
0.4
MR leave−one−out sensitivity analysis for
'APOE' on 'Pulmonary fibrosis'

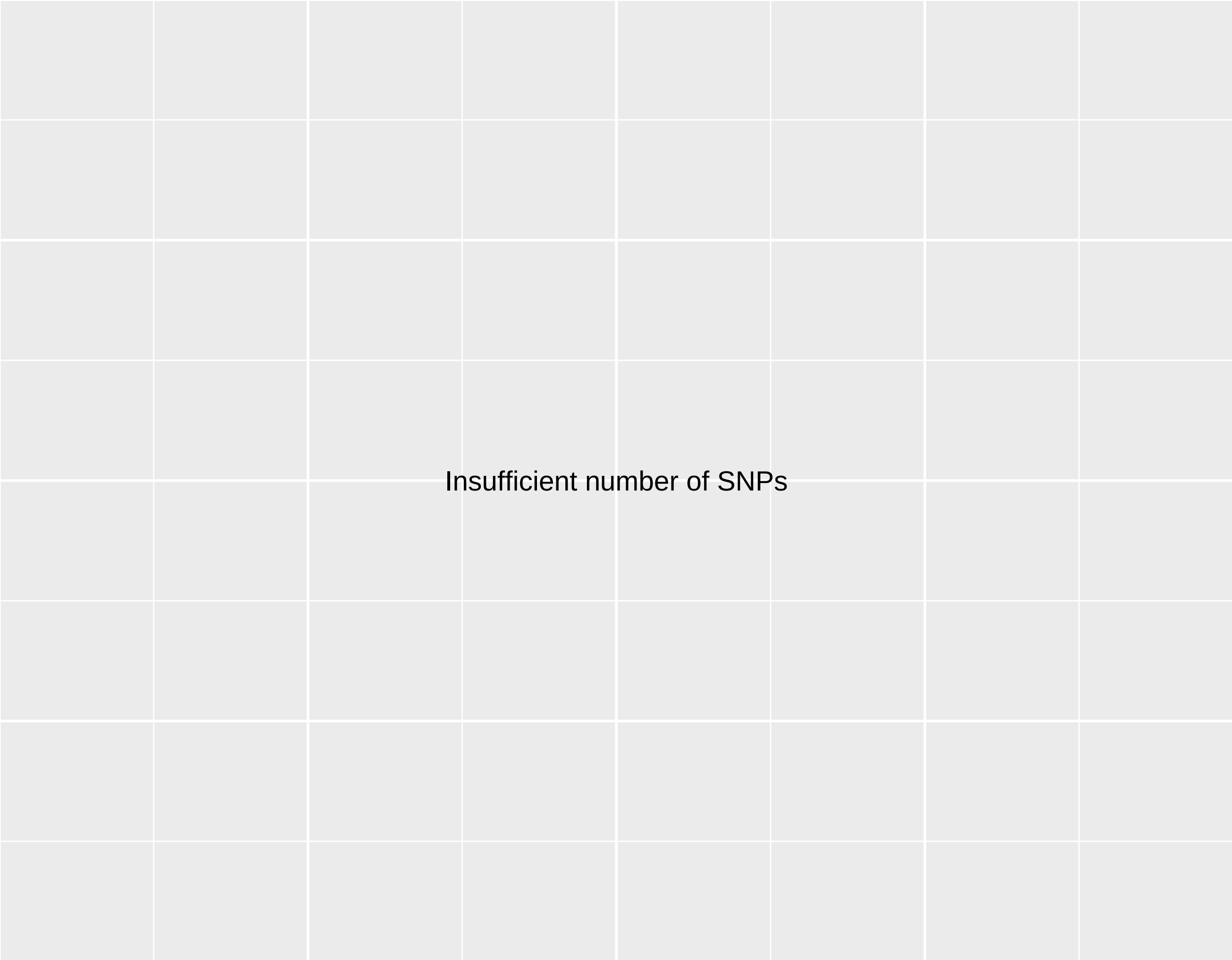
Insufficient number of SNPs

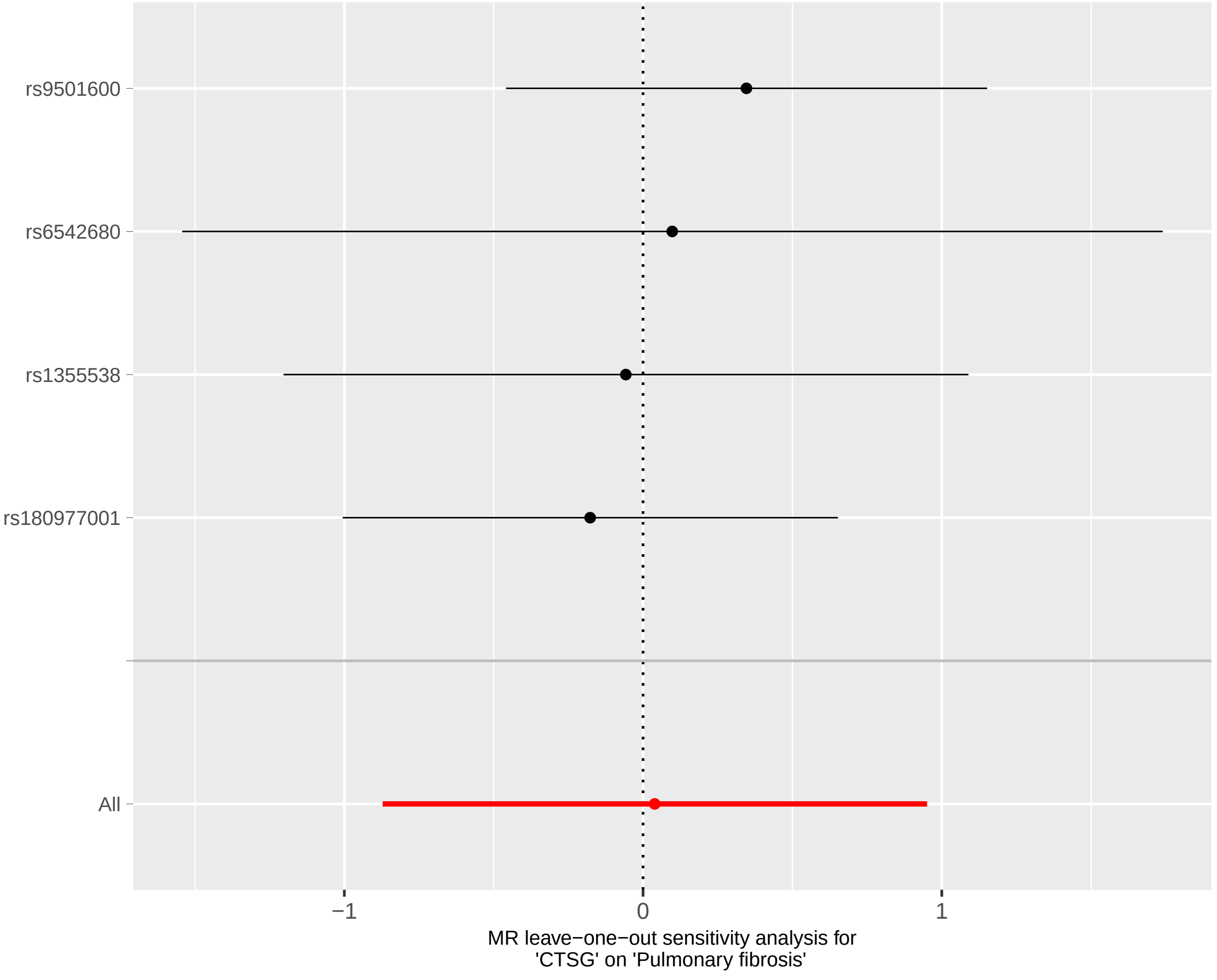
rs9501600
rs6542680
rs1355538
rs180977001
All
−1
0
1
MR leave−one−out sensitivity analysis for
'CTSG' on 'Pulmonary fibrosis'

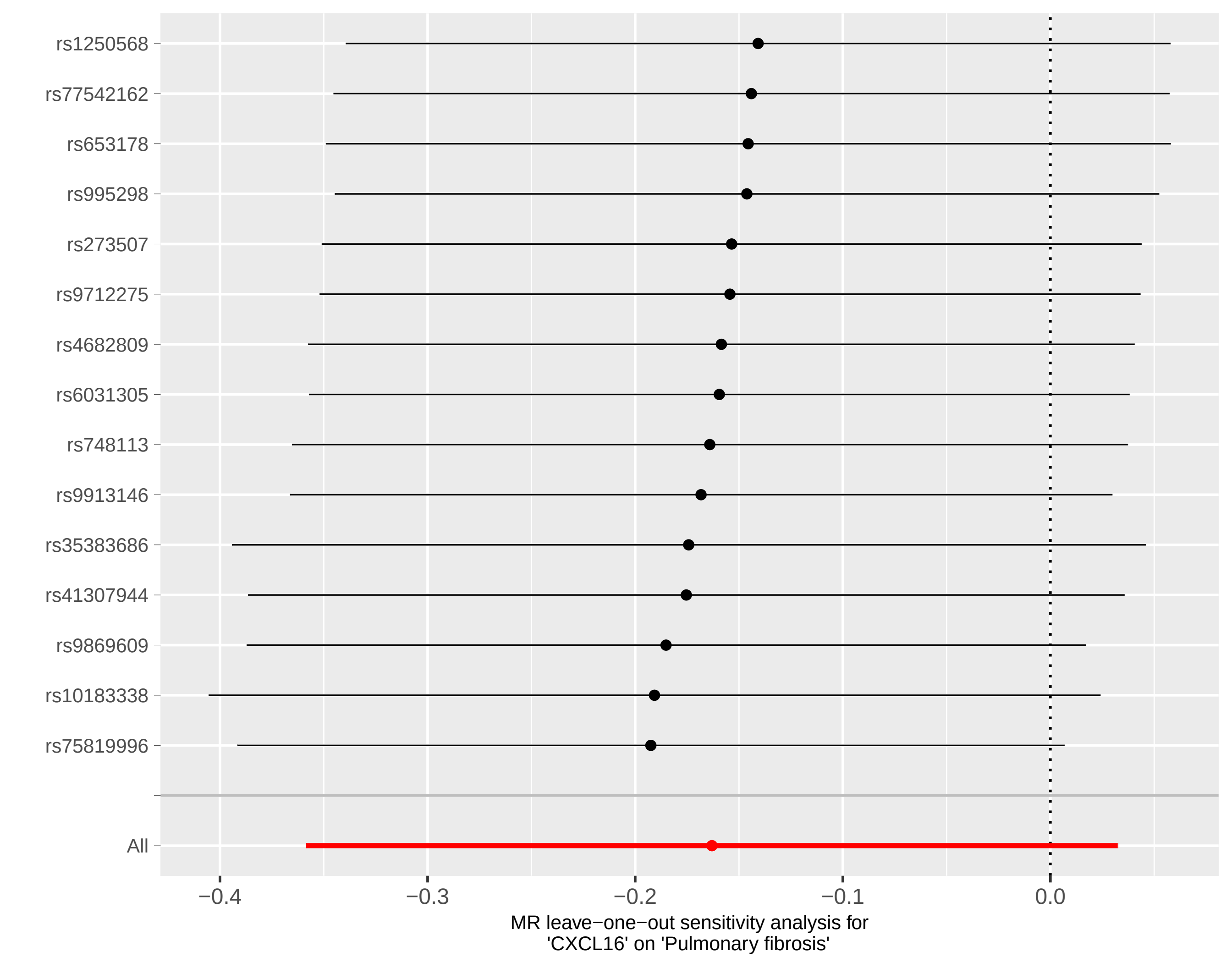
rs1250568
rs77542162
rs653178
rs995298
rs273507
rs9712275
rs4682809
rs6031305
rs748113
rs9913146
rs35383686
rs41307944
rs9869609
rs10183338
rs75819996
All
−0.4
−0.3
−0.2
−0.1
0.0
MR leave−one−out sensitivity analysis for
'CXCL16' on 'Pulmonary fibrosis'

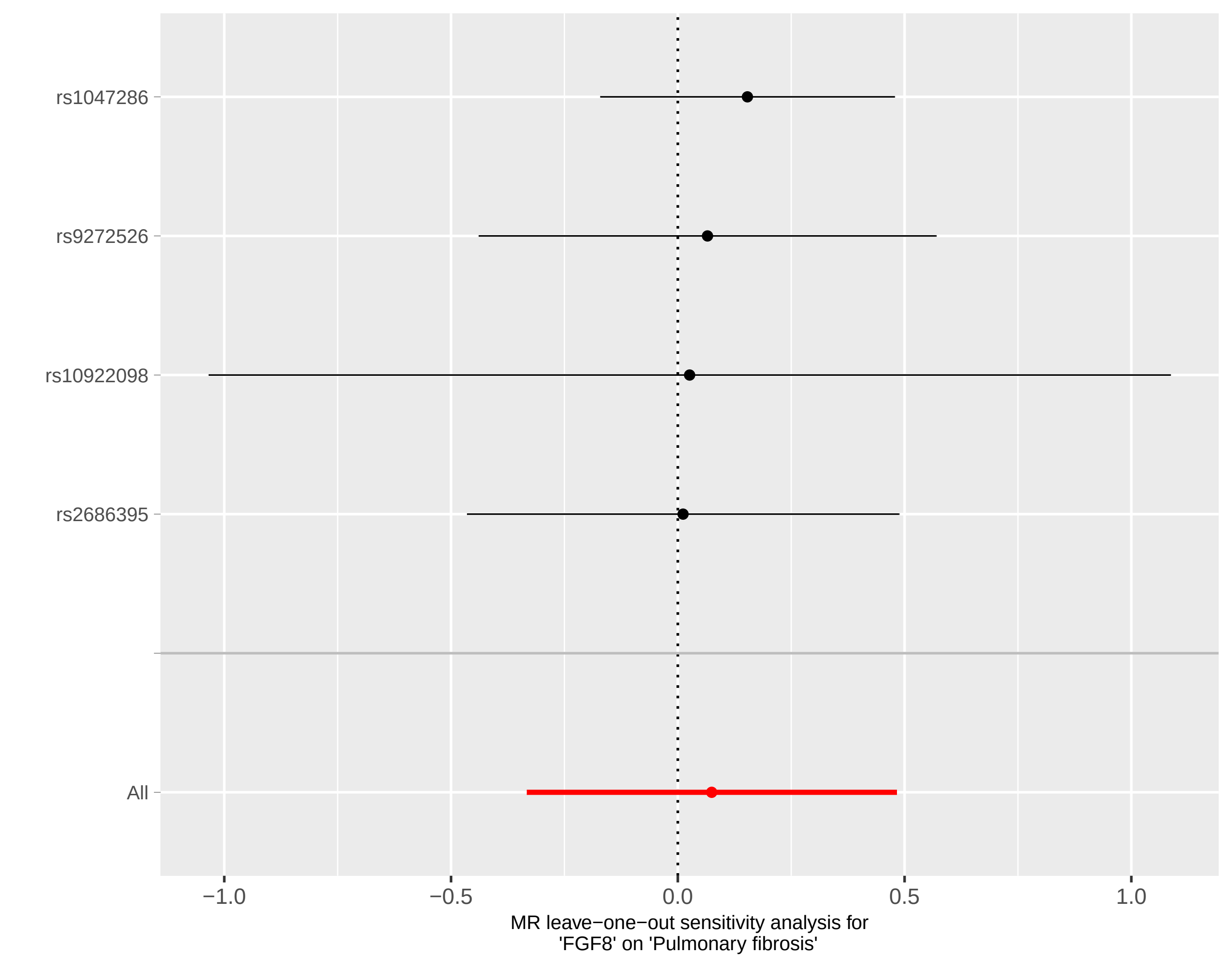
rs1047286
rs9272526
rs10922098
rs2686395
All
−1.0
−0.5
0.0
0.5
1.0
MR leave−one−out sensitivity analysis for
'FGF8' on 'Pulmonary fibrosis'

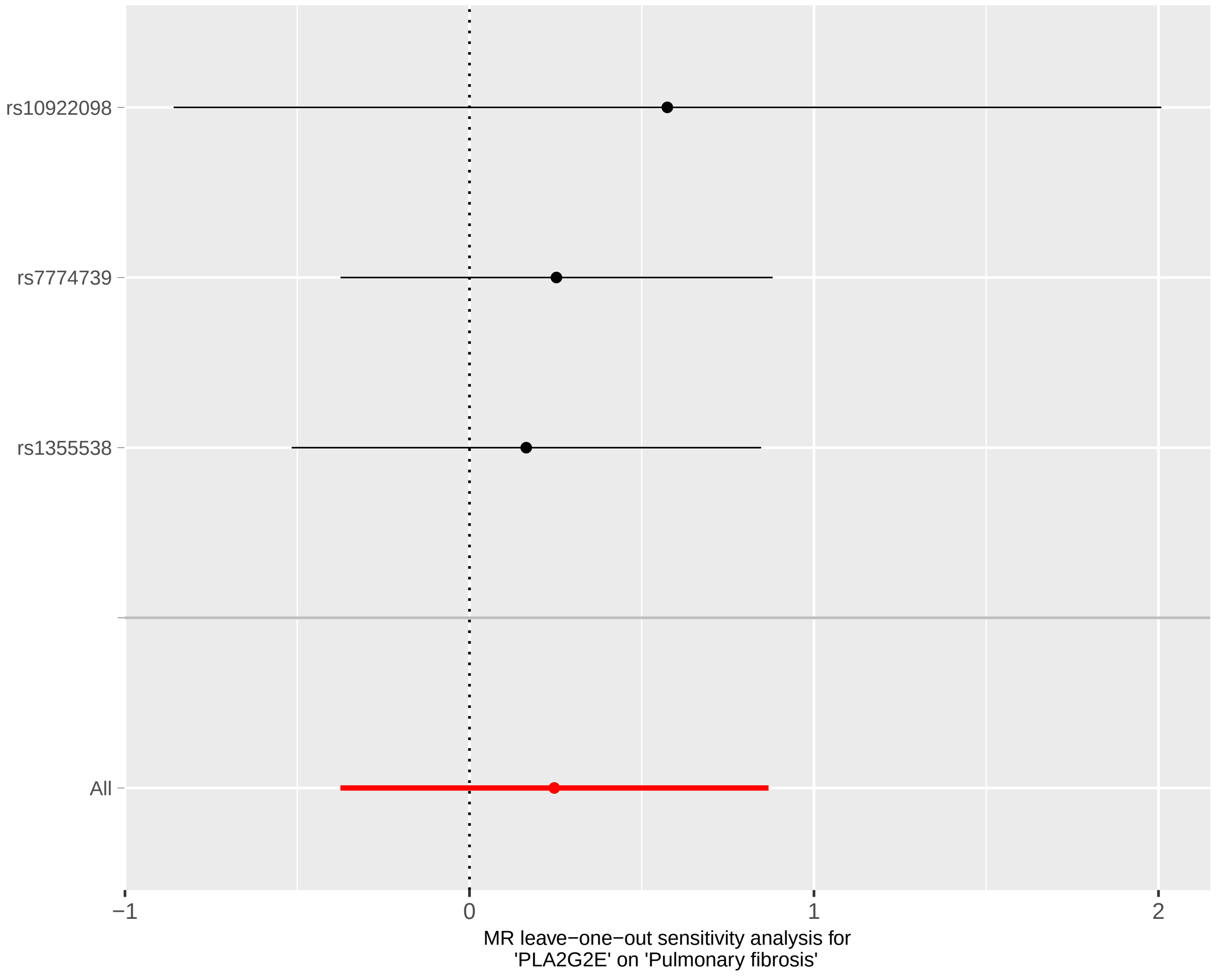
rs10922098
rs7774739
rs1355538
All
−1
0
1
2
MR leave−one−out sensitivity analysis for
'PLA2G2E' on 'Pulmonary fibrosis'

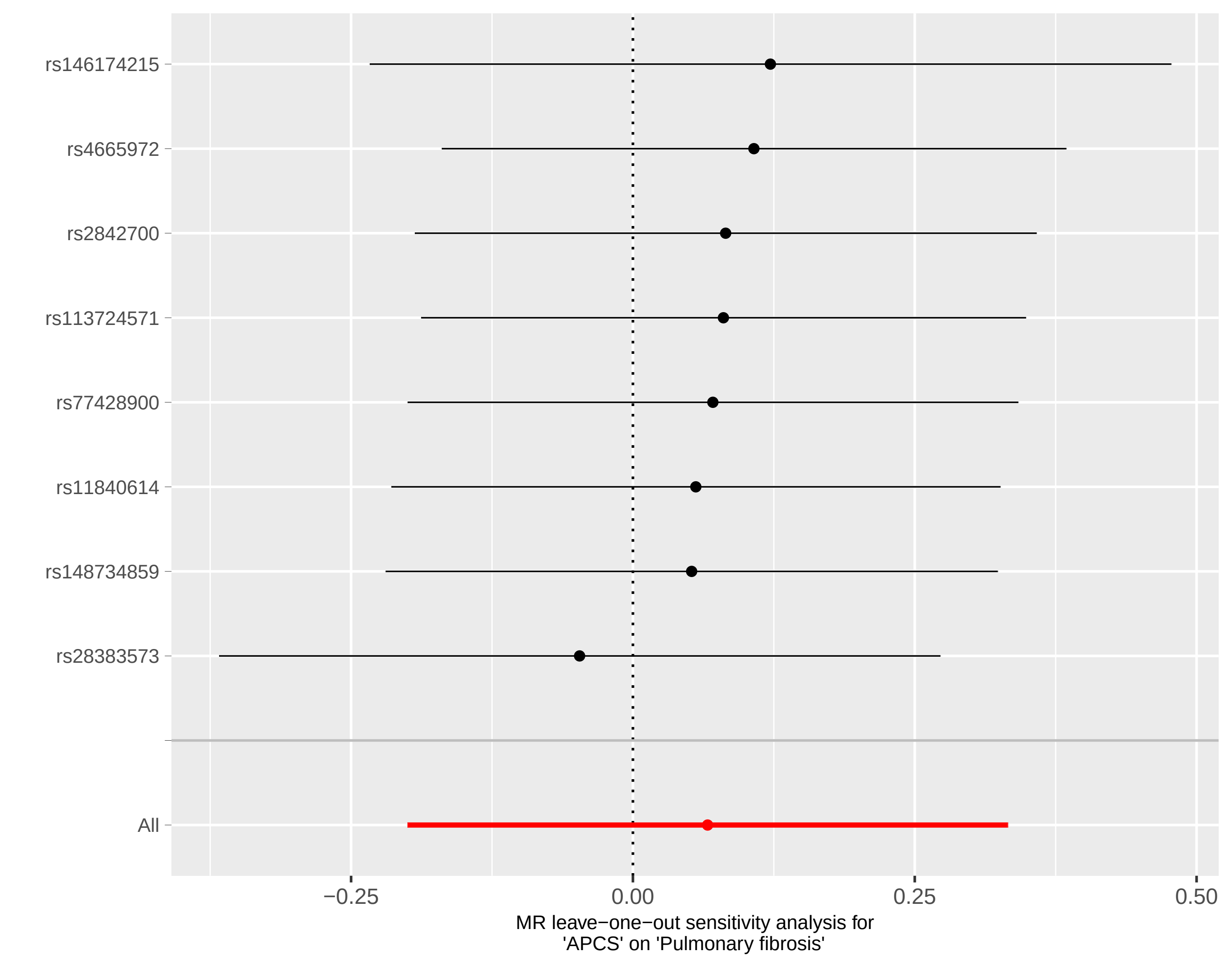
rs146174215
rs4665972
rs2842700
rs113724571
rs77428900
rs11840614
rs148734859
rs28383573
All
−0.25
0.00
0.25
0.50
MR leave−one−out sensitivity analysis for
'APCS' on 'Pulmonary fibrosis'

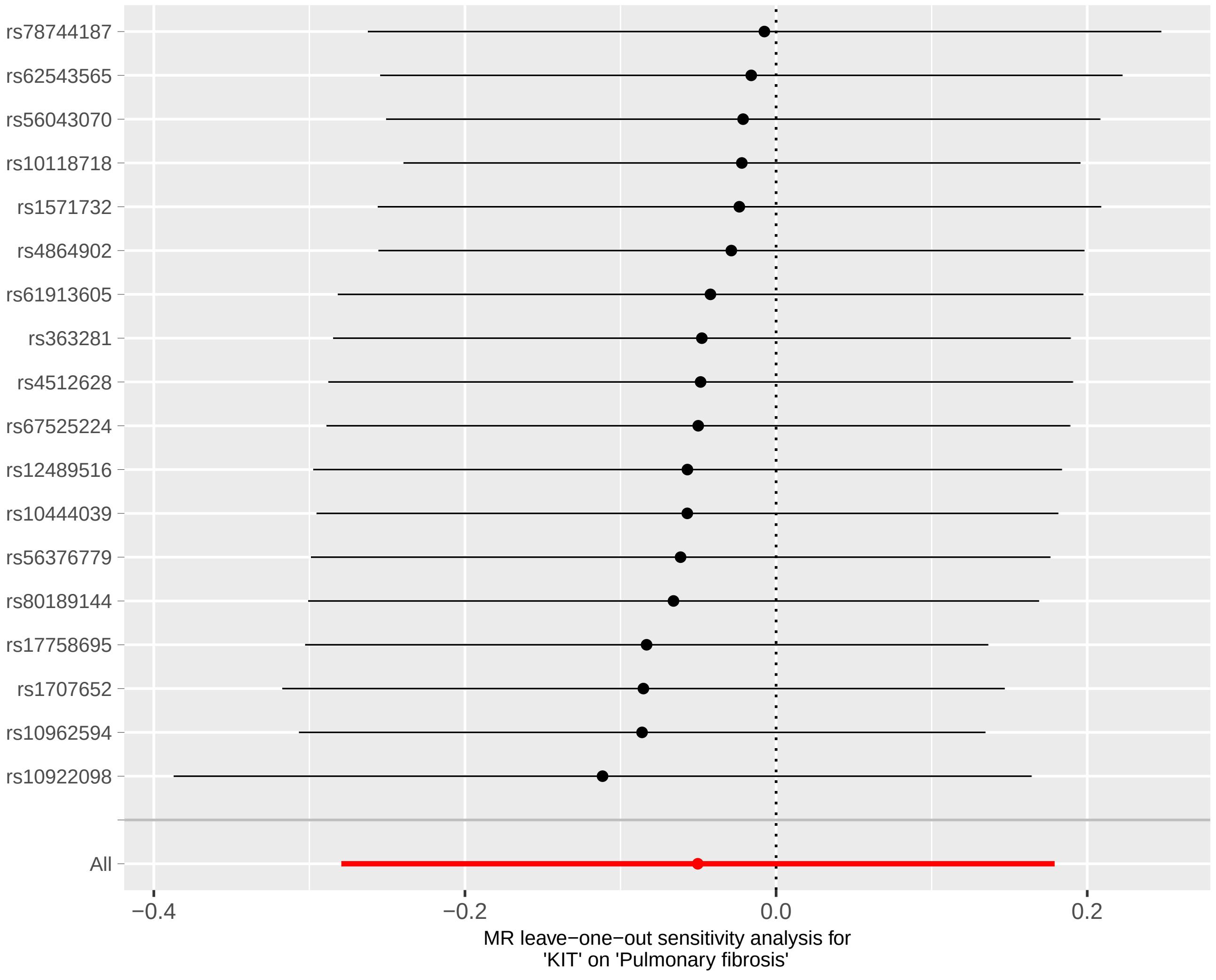
rs78744187
rs62543565
rs56043070
rs10118718
rs1571732
rs4864902
rs61913605
rs363281
rs4512628
rs67525224
rs12489516
rs10444039
rs56376779
rs80189144
rs17758695
rs1707652
rs10962594
rs10922098
All
−0.4
−0.2
0.0
0.2
MR leave−one−out sensitivity analysis for
'KIT' on 'Pulmonary fibrosis'

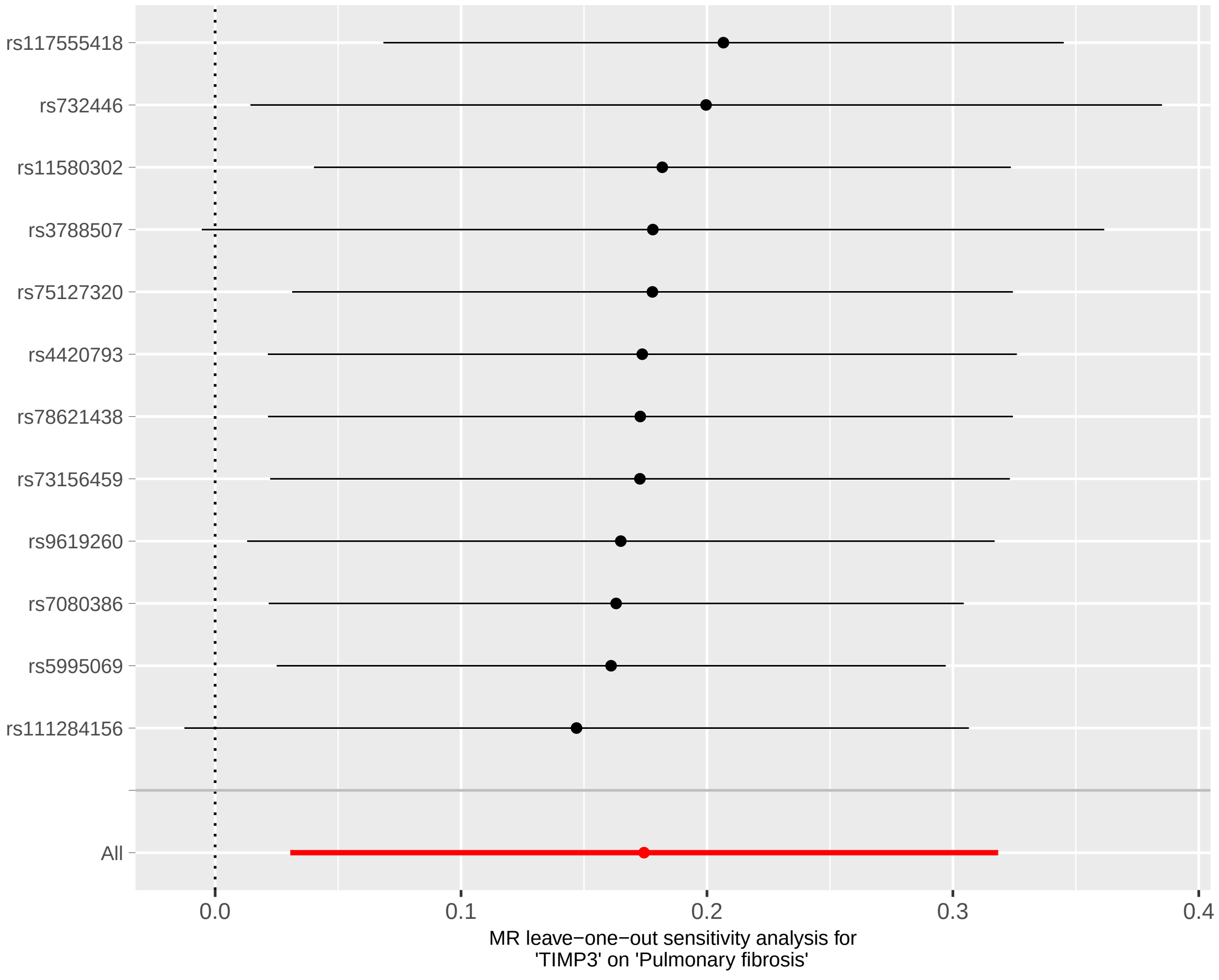

rs117555418
rs732446
rs11580302
rs3788507
rs75127320
rs4420793
rs78621438
rs73156459
rs9619260
rs7080386
rs5995069
rs111284156
All
0.0
0.1
0.2
0.3
0.4
MR leave−one−out sensitivity analysis for
'TIMP3' on 'Pulmonary fibrosis'

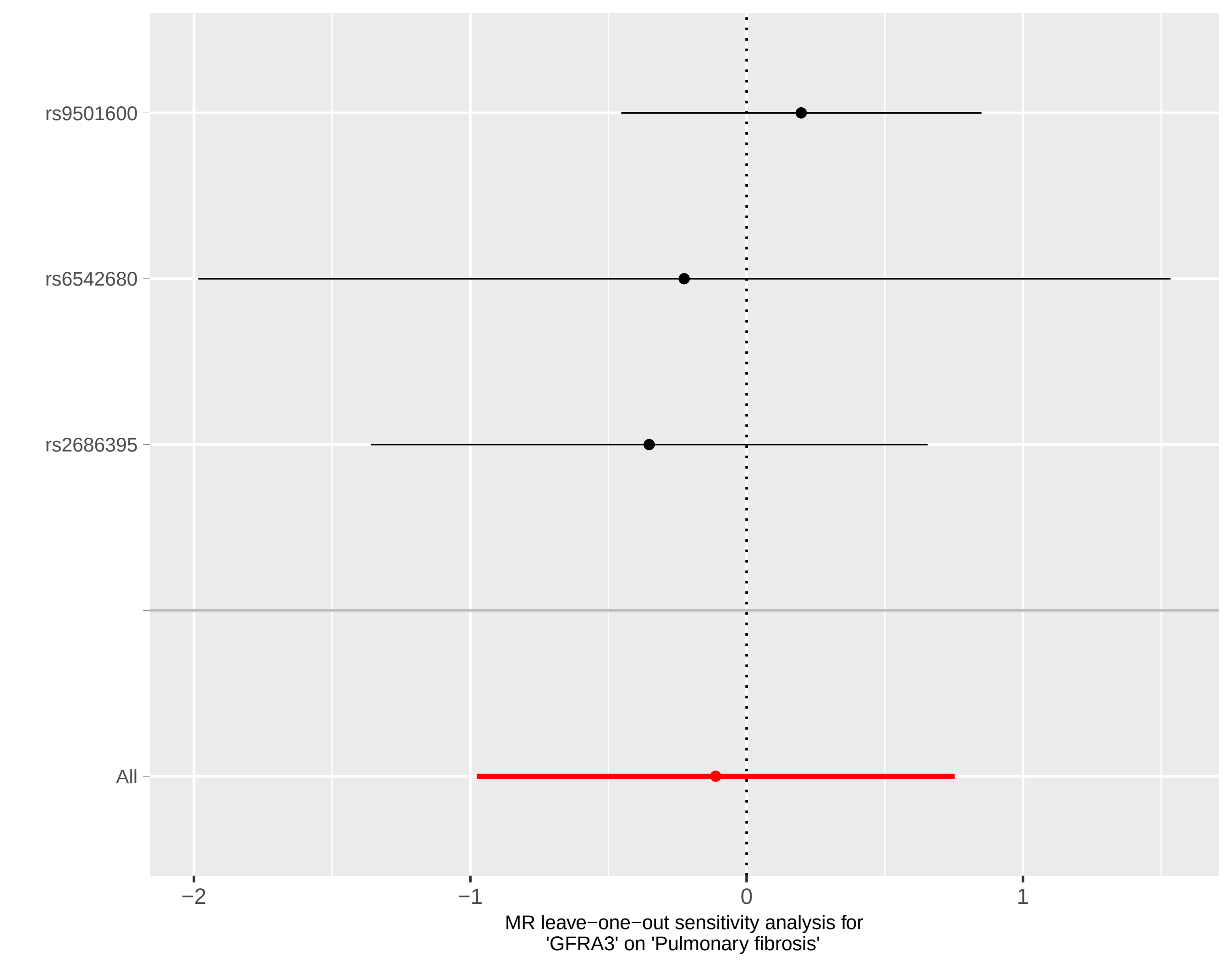
rs9501600
rs6542680
rs2686395
All
−2
−1
0
1
MR leave−one−out sensitivity analysis for
'GFRA3' on 'Pulmonary fibrosis'

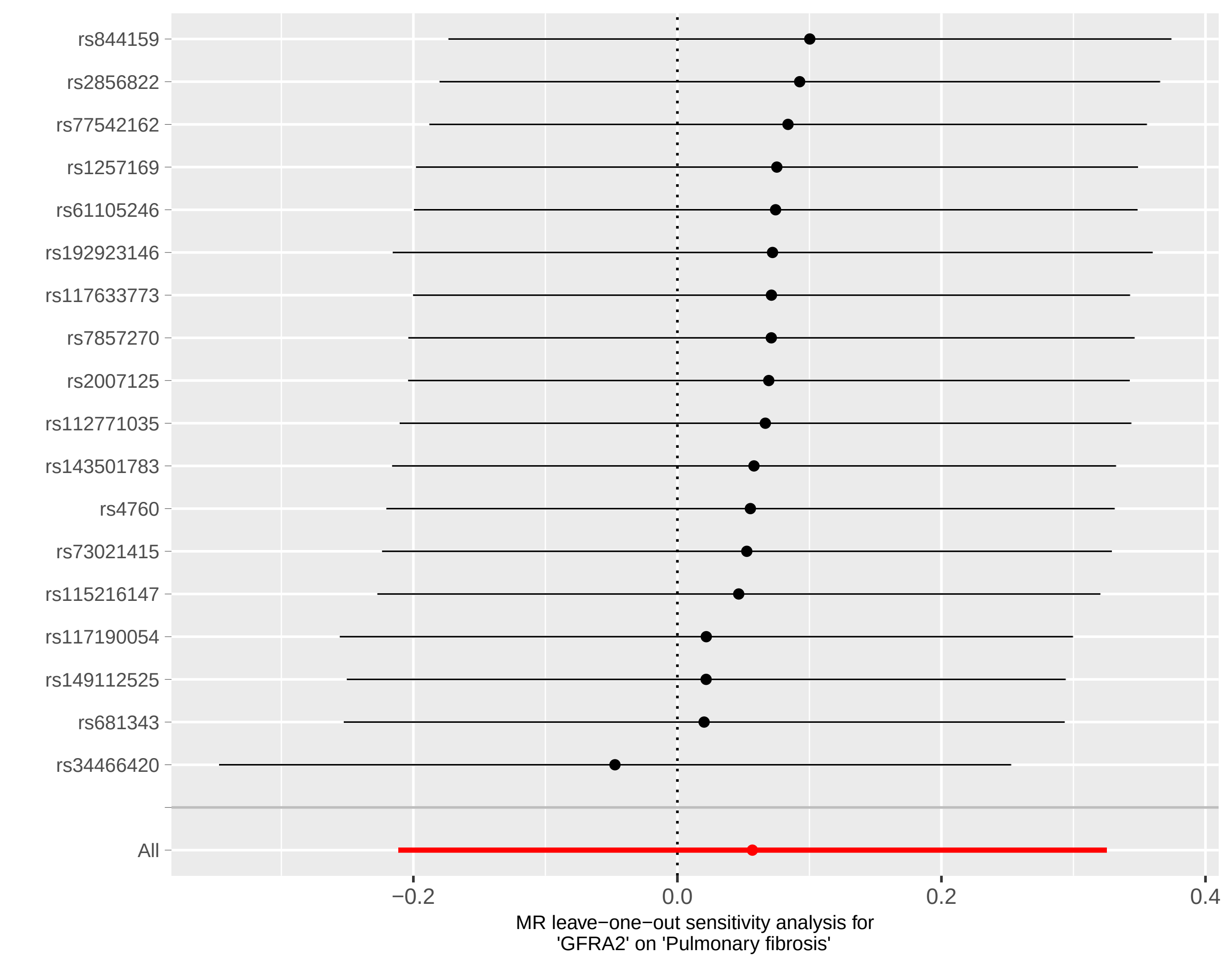
rs844159
rs2856822
rs77542162
rs1257169
rs61105246
rs192923146
rs117633773
rs7857270
rs2007125
rs112771035
rs143501783
rs4760
rs73021415
rs115216147
rs117190054
rs149112525
rs681343
rs34466420
All
−0.2
0.0
0.2
0.4
MR leave−one−out sensitivity analysis for
'GFRA2' on 'Pulmonary fibrosis'

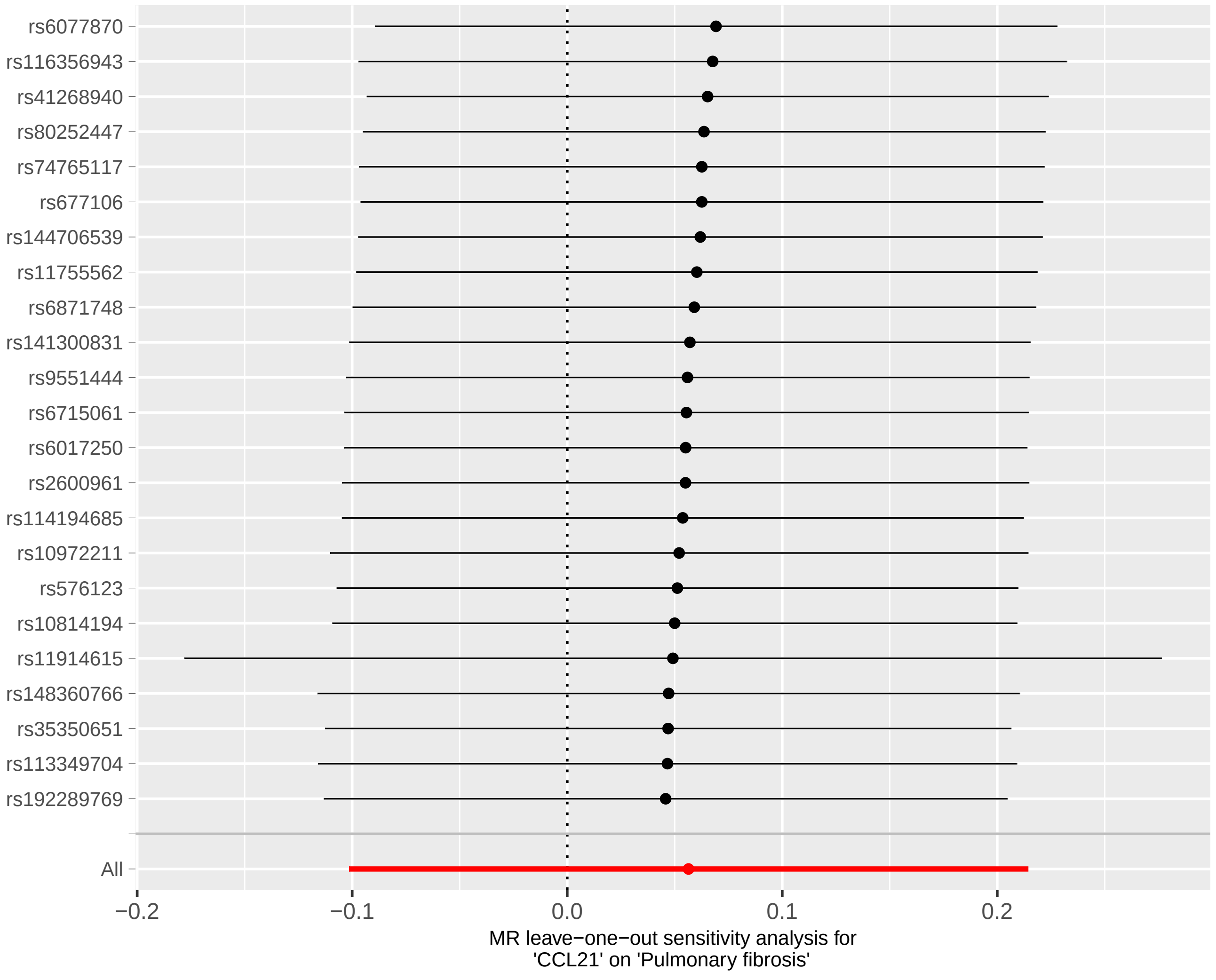
rs6077870
rs116356943
rs41268940
rs80252447
rs74765117
rs677106
rs144706539
rs11755562
rs6871748
rs141300831
rs9551444
rs6715061
rs6017250
rs2600961
rs114194685
rs10972211
rs576123
rs10814194
rs11914615
rs148360766
rs35350651
rs113349704
rs192289769
All
−0.2
−0.1
0.0
0.1
0.2
MR leave−one−out sensitivity analysis for
'CCL21' on 'Pulmonary fibrosis'

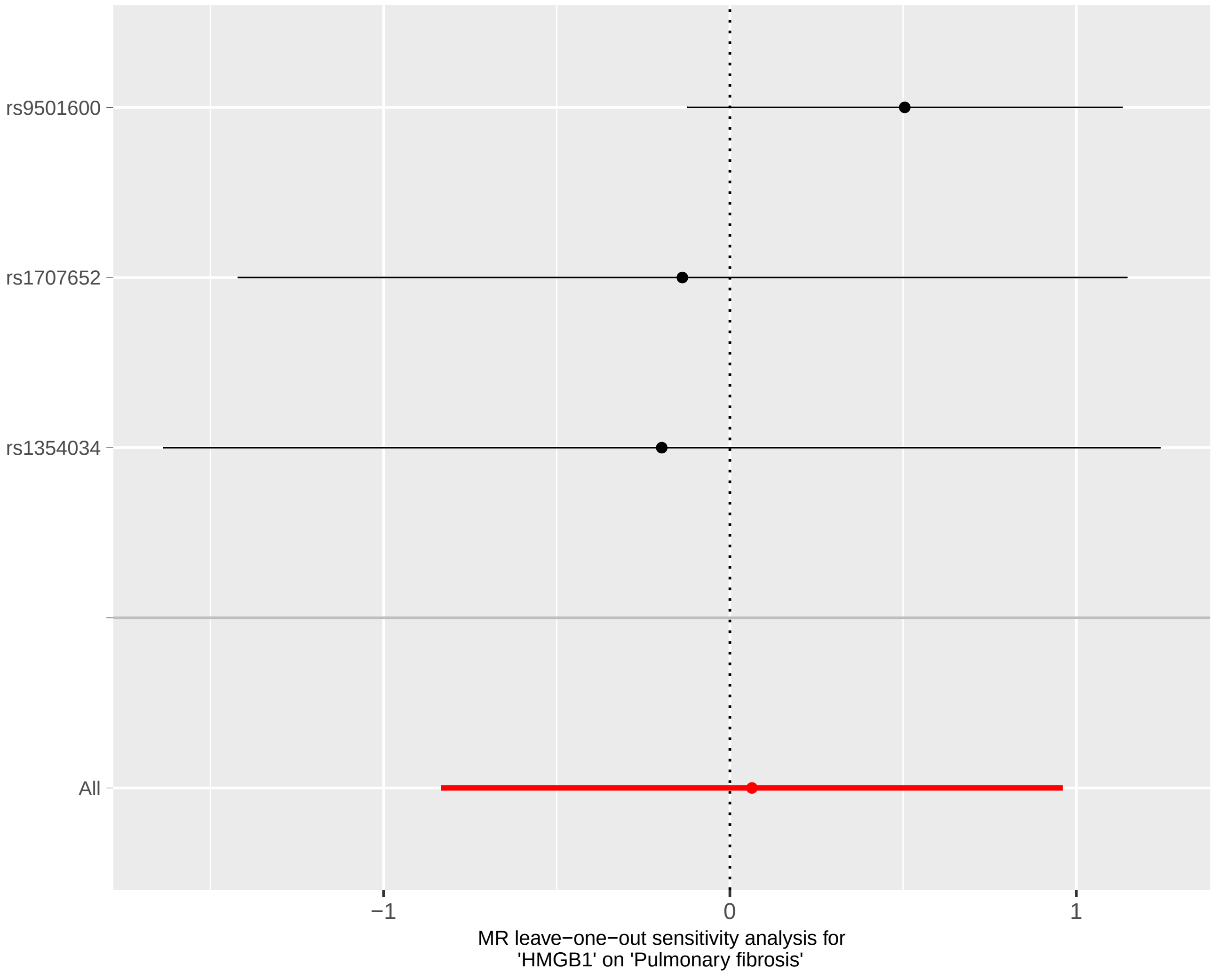
rs9501600
rs1707652
rs1354034
All
−1
0
1
MR leave−one−out sensitivity analysis for
'HMGB1' on 'Pulmonary fibrosis'

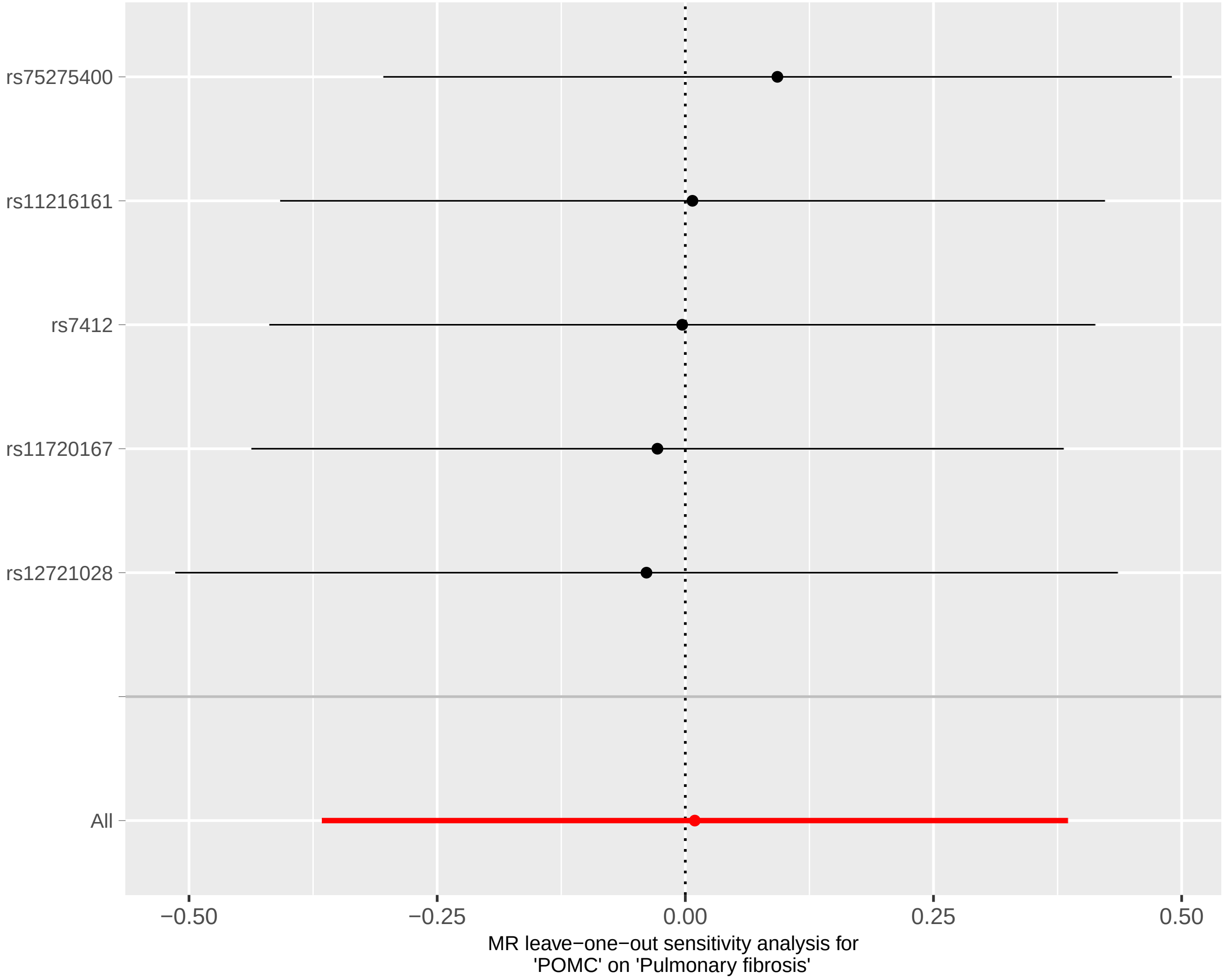
rs75275400
rs11216161
rs7412
rs11720167
rs12721028
All
−0.50
−0.25
0.00
0.25
0.50
MR leave−one−out sensitivity analysis for
'POMC' on 'Pulmonary fibrosis'

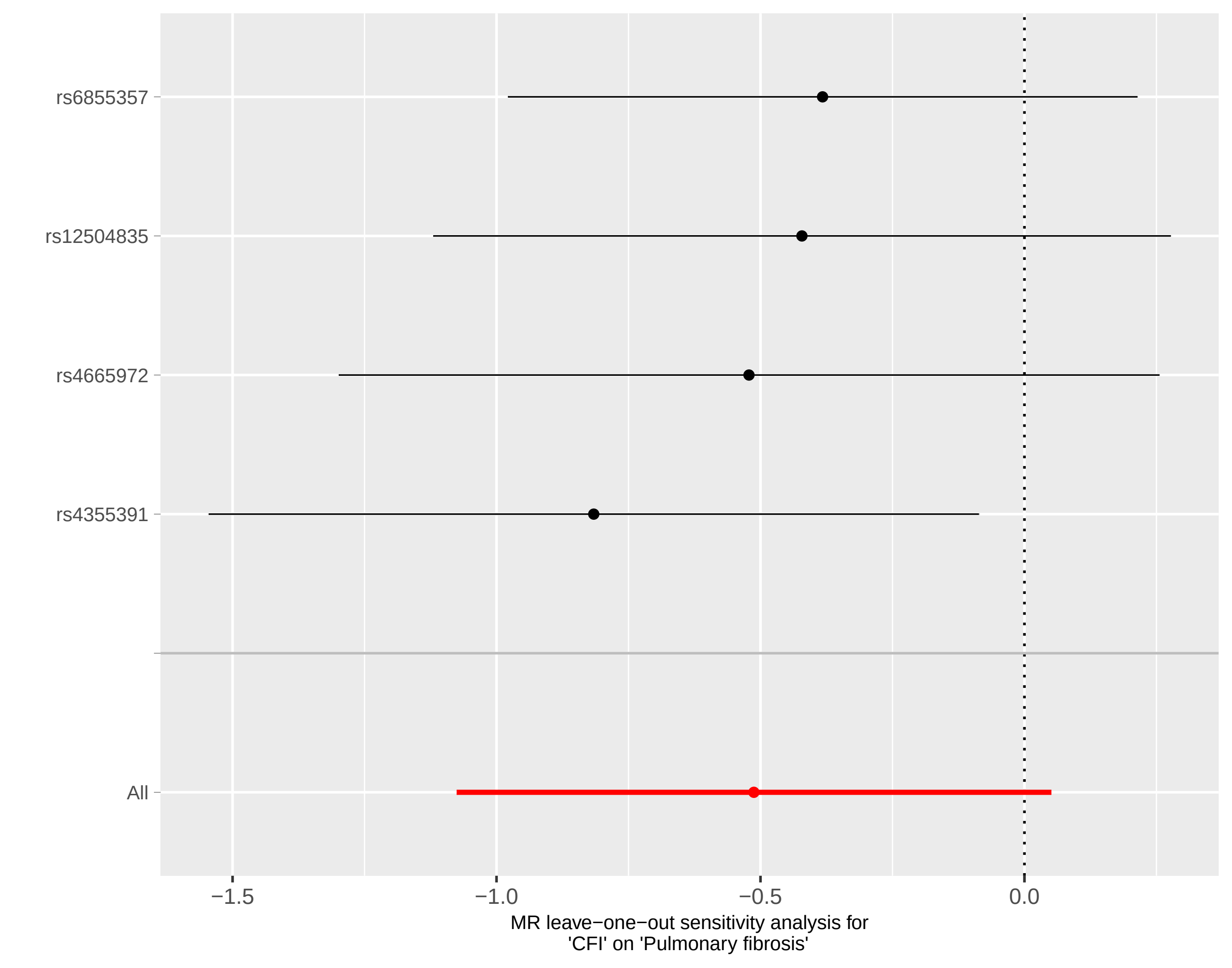

rs6855357
rs12504835
rs4665972
rs4355391
All
−1.5
−1.0
−0.5
0.0
MR leave−one−out sensitivity analysis for
'CFI' on 'Pulmonary fibrosis'

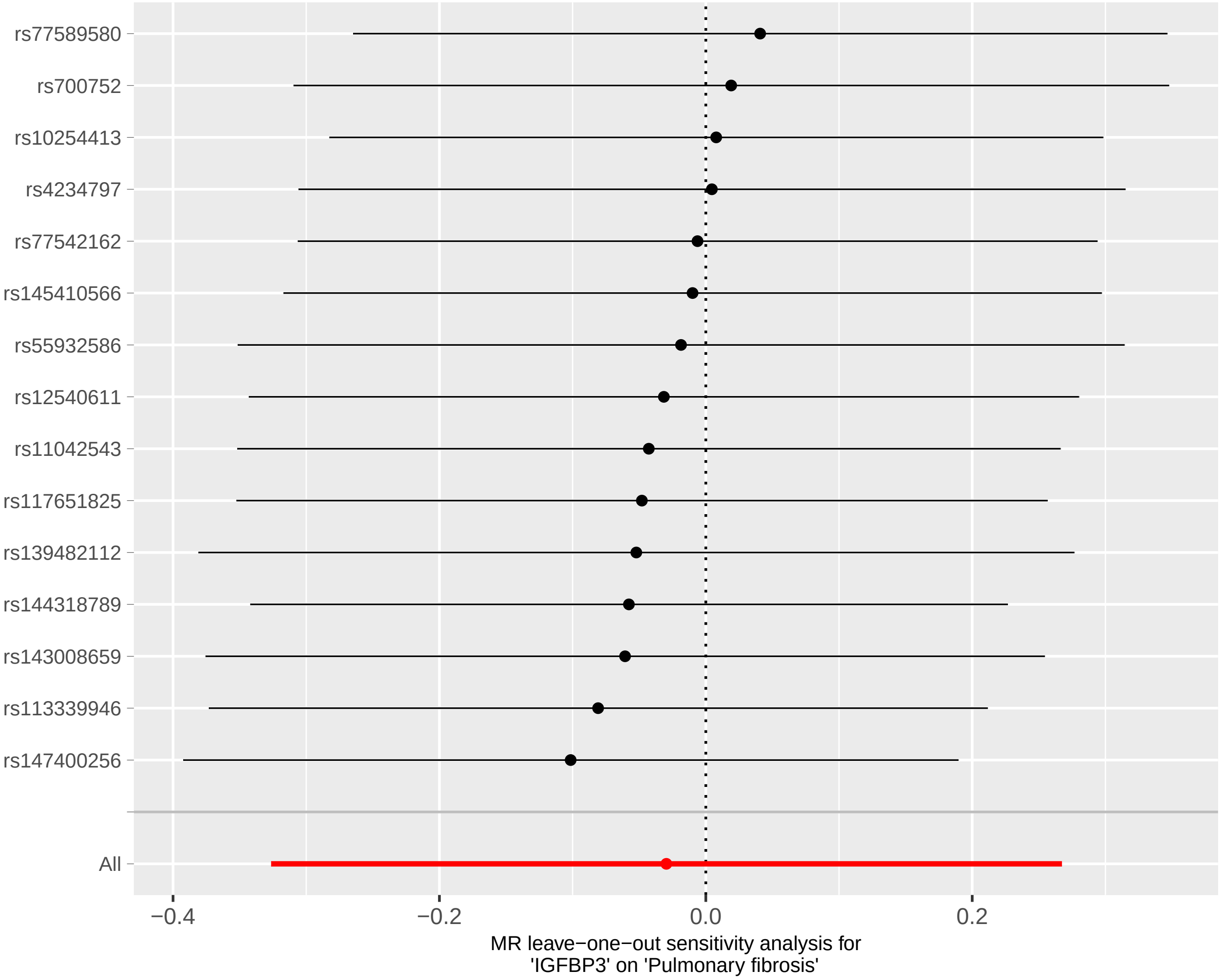
rs77589580
rs700752
rs10254413
rs4234797
rs77542162
rs145410566
rs55932586
rs12540611
rs11042543
rs117651825
rs139482112
rs144318789
rs143008659
rs113339946
rs147400256
All
−0.4
−0.2
0.0
0.2
MR leave−one−out sensitivity analysis for
'IGFBP3' on 'Pulmonary fibrosis'

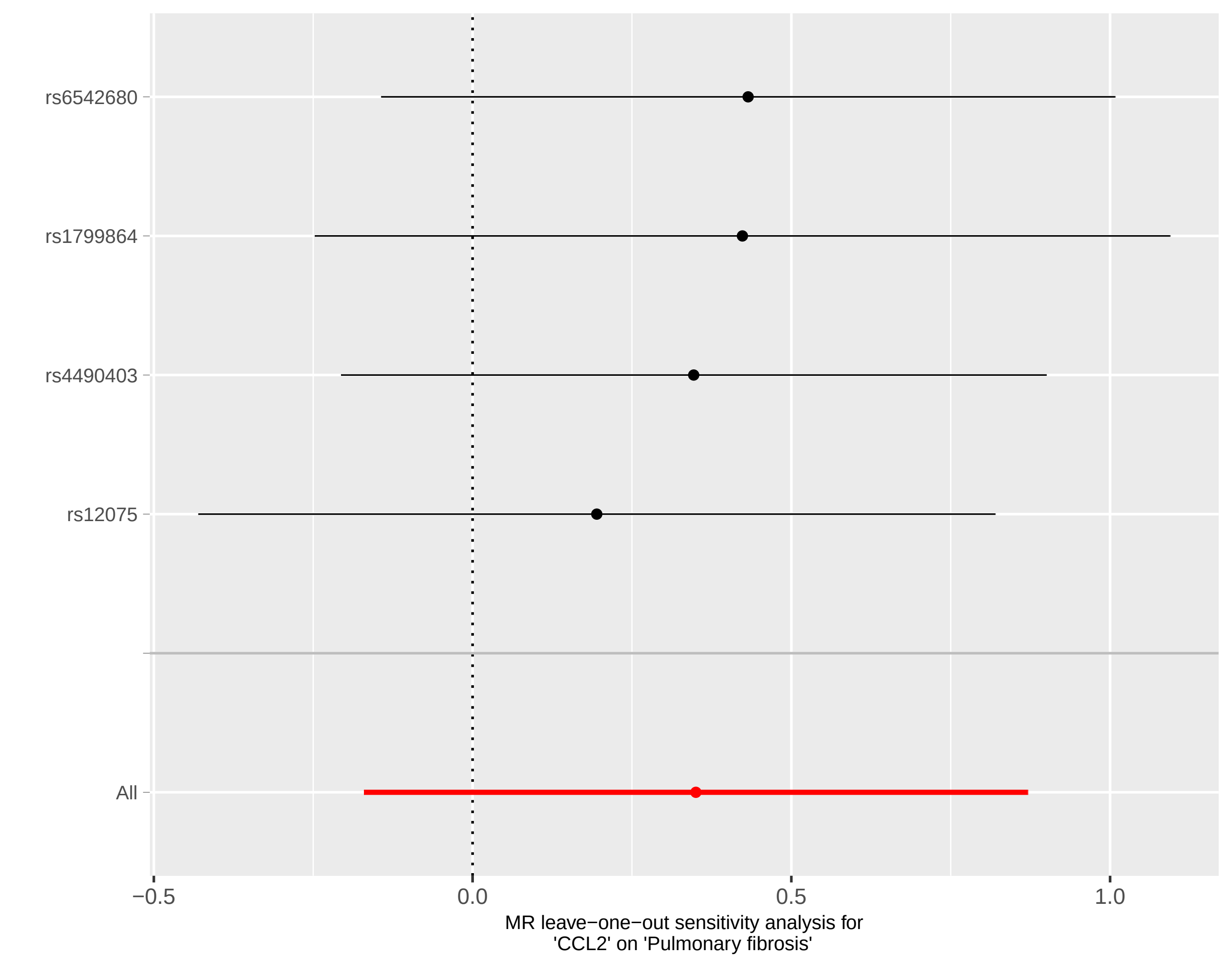
rs6542680
rs1799864
rs4490403
rs12075
All
−0.5
0.0
0.5
1.0
MR leave−one−out sensitivity analysis for
'CCL2' on 'Pulmonary fibrosis'

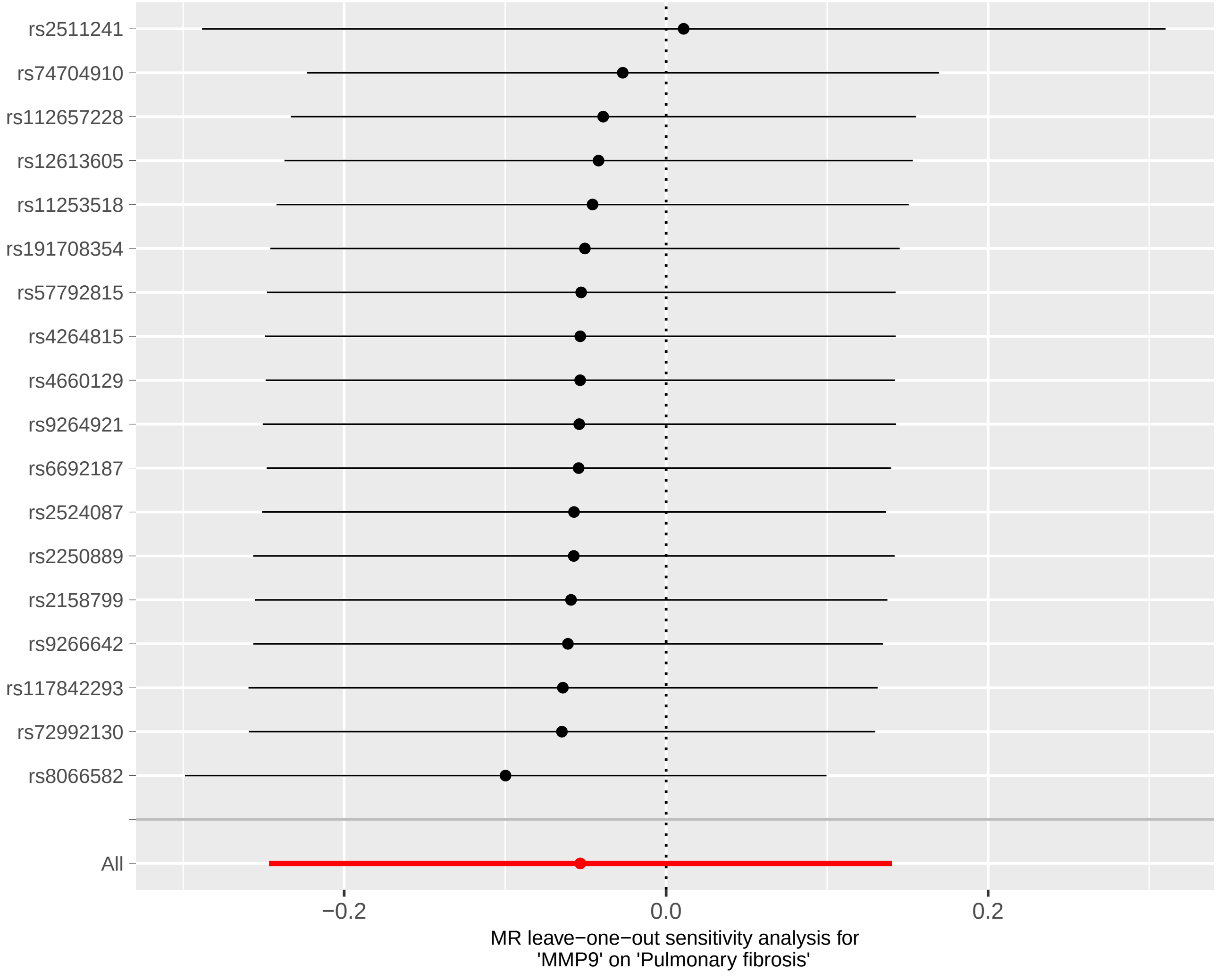
rs2511241
rs74704910
rs112657228
rs12613605
rs11253518
rs191708354
rs57792815
rs4264815
rs4660129
rs9264921
rs6692187
rs2524087
rs2250889
rs2158799
rs9266642
rs117842293
rs72992130
rs8066582
All
−0.2
0.0
0.2
MR leave−one−out sensitivity analysis for
'MMP9' on 'Pulmonary fibrosis'

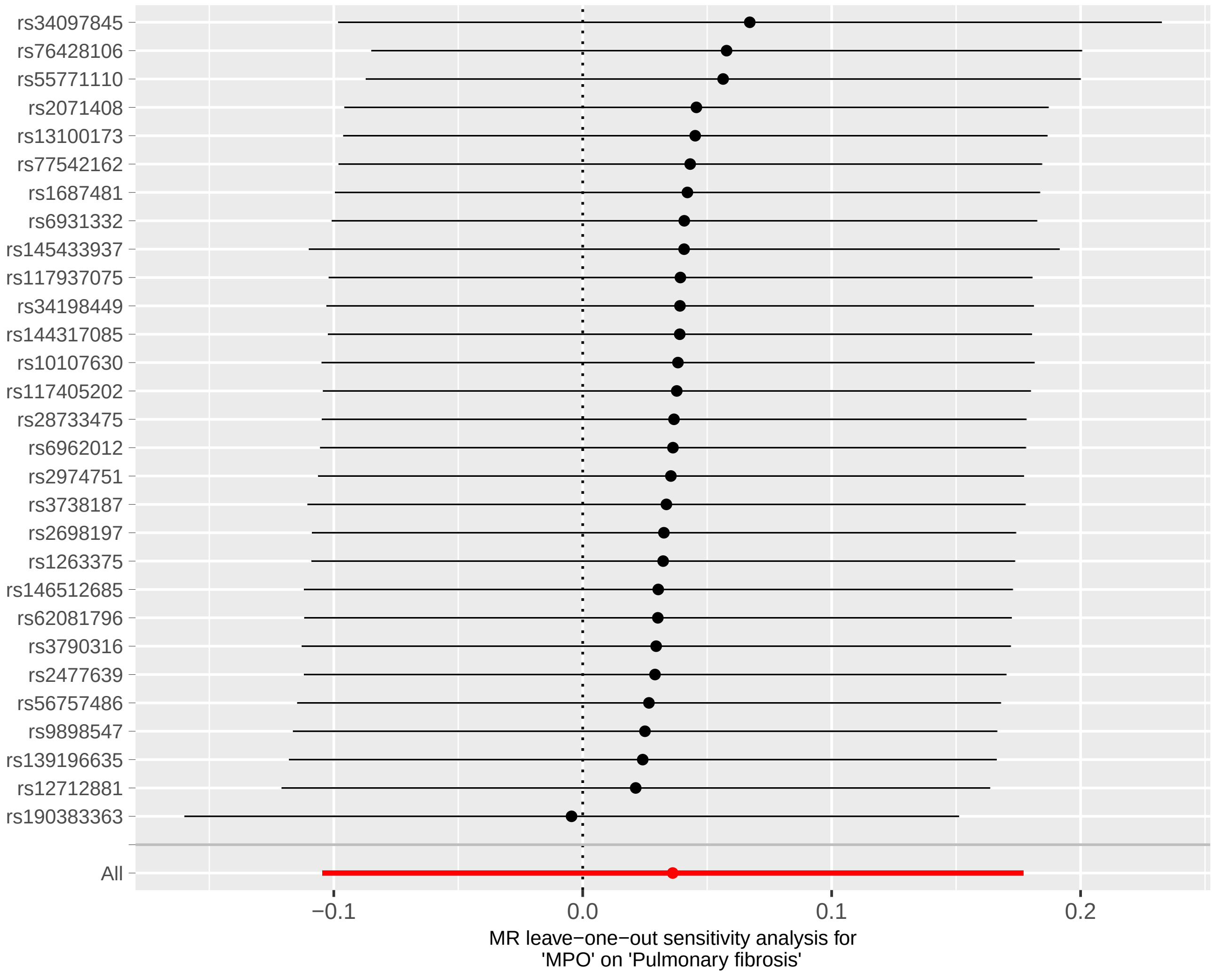
rs34097845
rs76428106
rs55771110
rs2071408
rs13100173
rs77542162
rs1687481
rs6931332
rs145433937
rs117937075
rs34198449
rs144317085
rs10107630
rs117405202
rs28733475
rs6962012
rs2974751
rs3738187
rs2698197
rs1263375
rs146512685
rs62081796
rs3790316
rs2477639
rs56757486
rs9898547
rs139196635
rs12712881
rs190383363
All
−0.1
0.0
0.1
0.2
MR leave−one−out sensitivity analysis for
'MPO' on 'Pulmonary fibrosis'

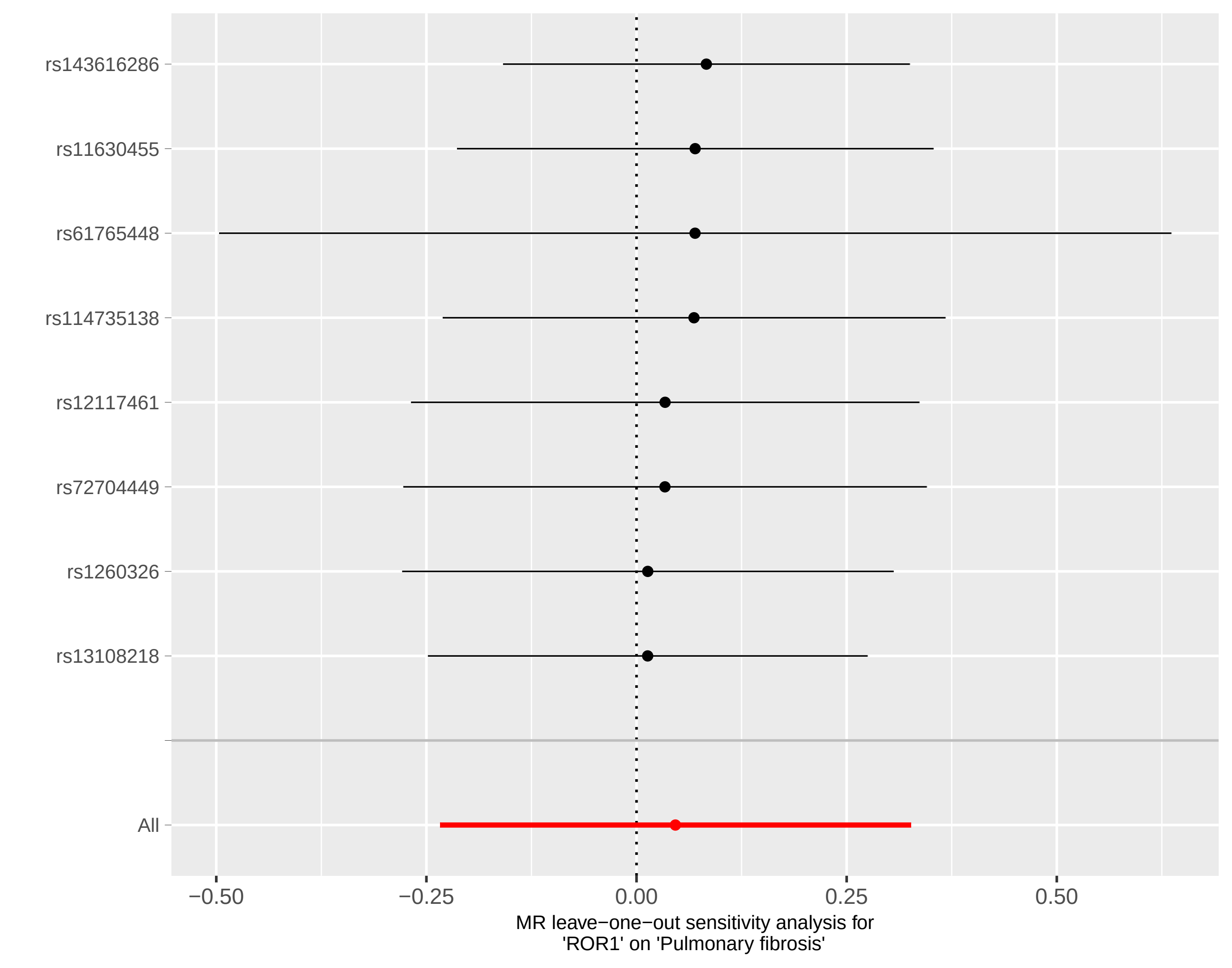
rs143616286
rs11630455
rs61765448
rs114735138
rs12117461
rs72704449
rs1260326
rs13108218
All
−0.50
−0.25
0.00
0.25
0.50
MR leave−one−out sensitivity analysis for
'ROR1' on 'Pulmonary fibrosis'

Insufficient number of SNPs

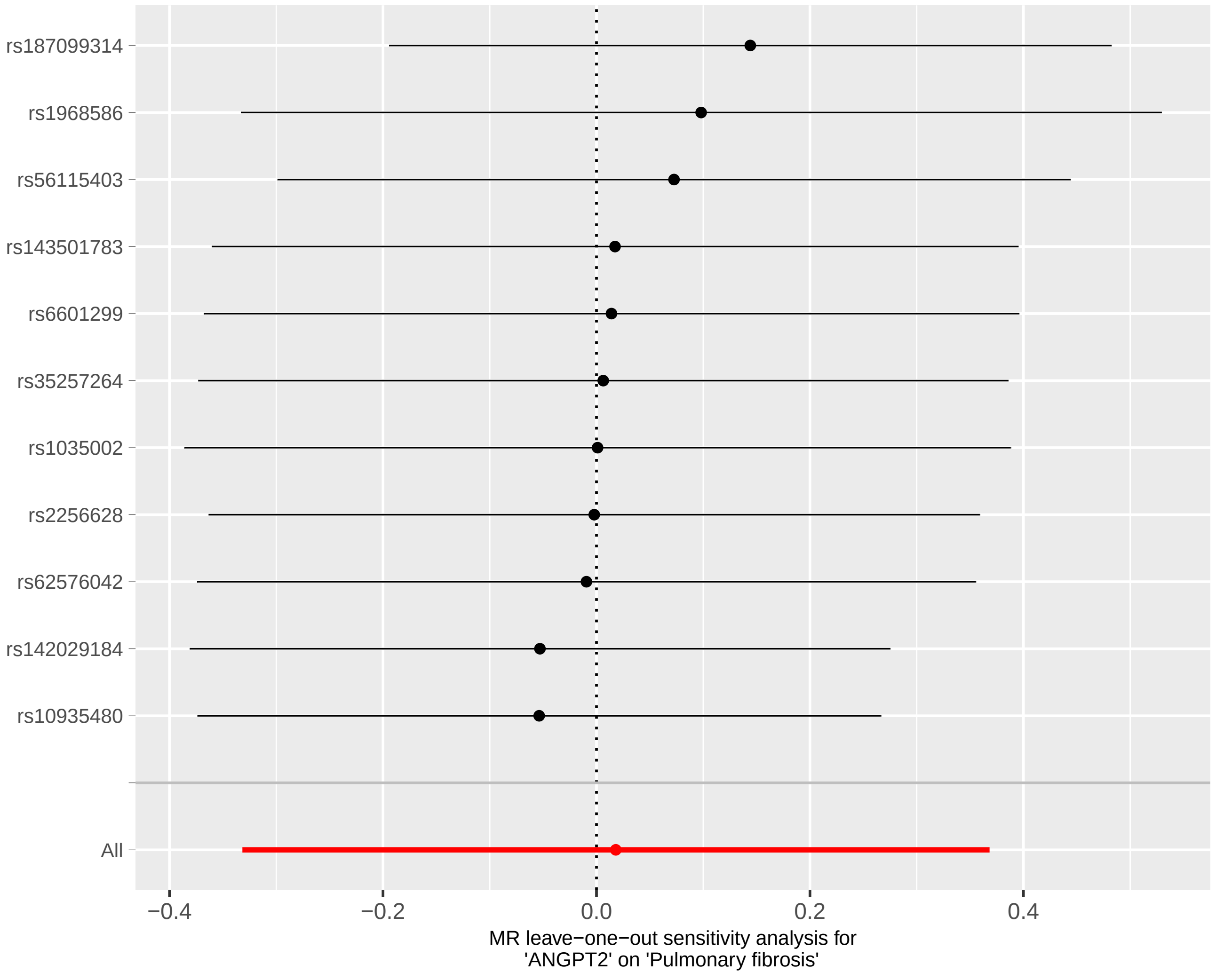
rs187099314
rs1968586
rs56115403
rs143501783
rs6601299
rs35257264
rs1035002
rs2256628
rs62576042
rs142029184
rs10935480
All
−0.4
−0.2
0.0
0.2
0.4
MR leave−one−out sensitivity analysis for
'ANGPT2' on 'Pulmonary fibrosis'

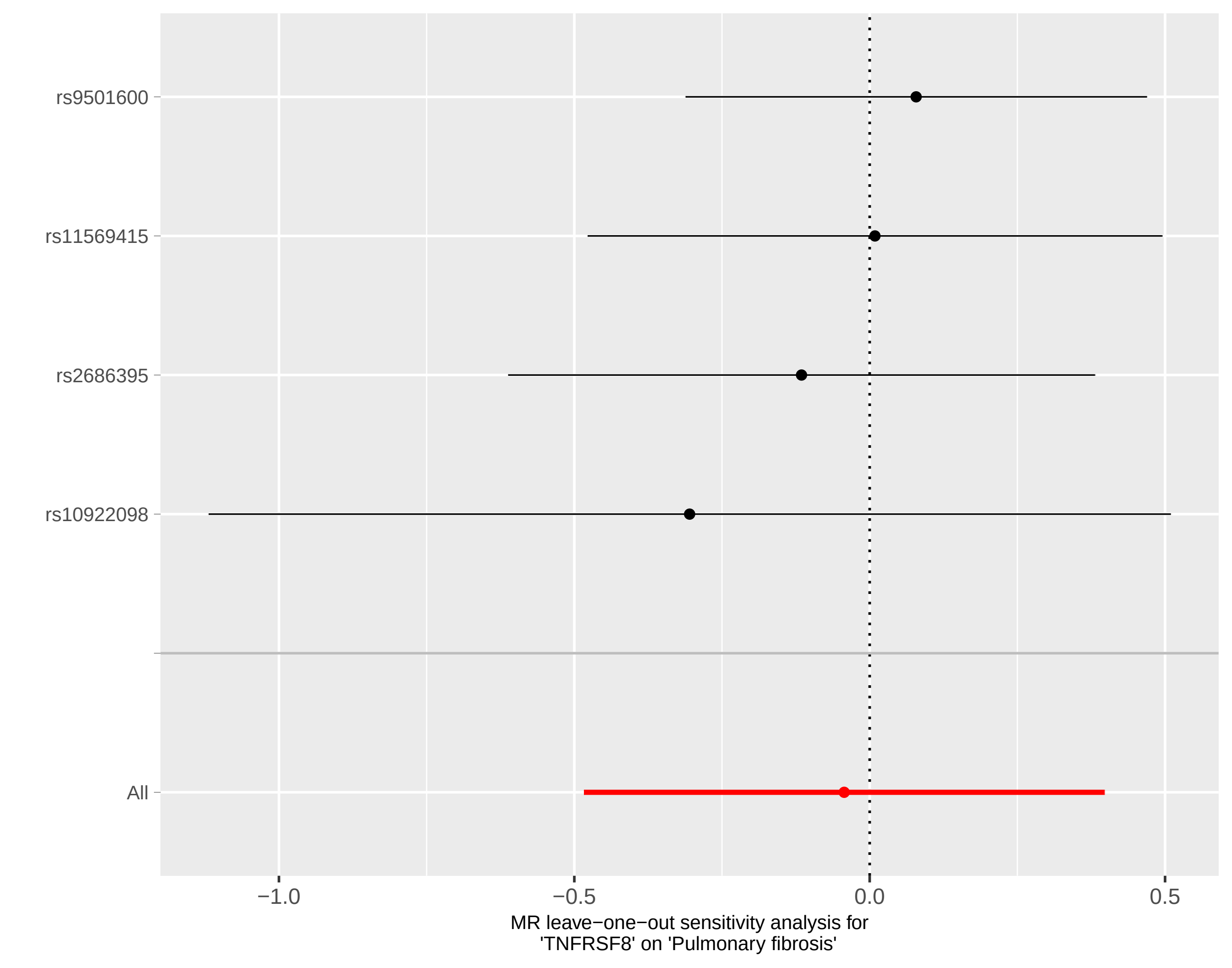
rs9501600
rs11569415
rs2686395
rs10922098
All
−1.0
−0.5
0.0
0.5
MR leave−one−out sensitivity analysis for
'TNFRSF8' on 'Pulmonary fibrosis'

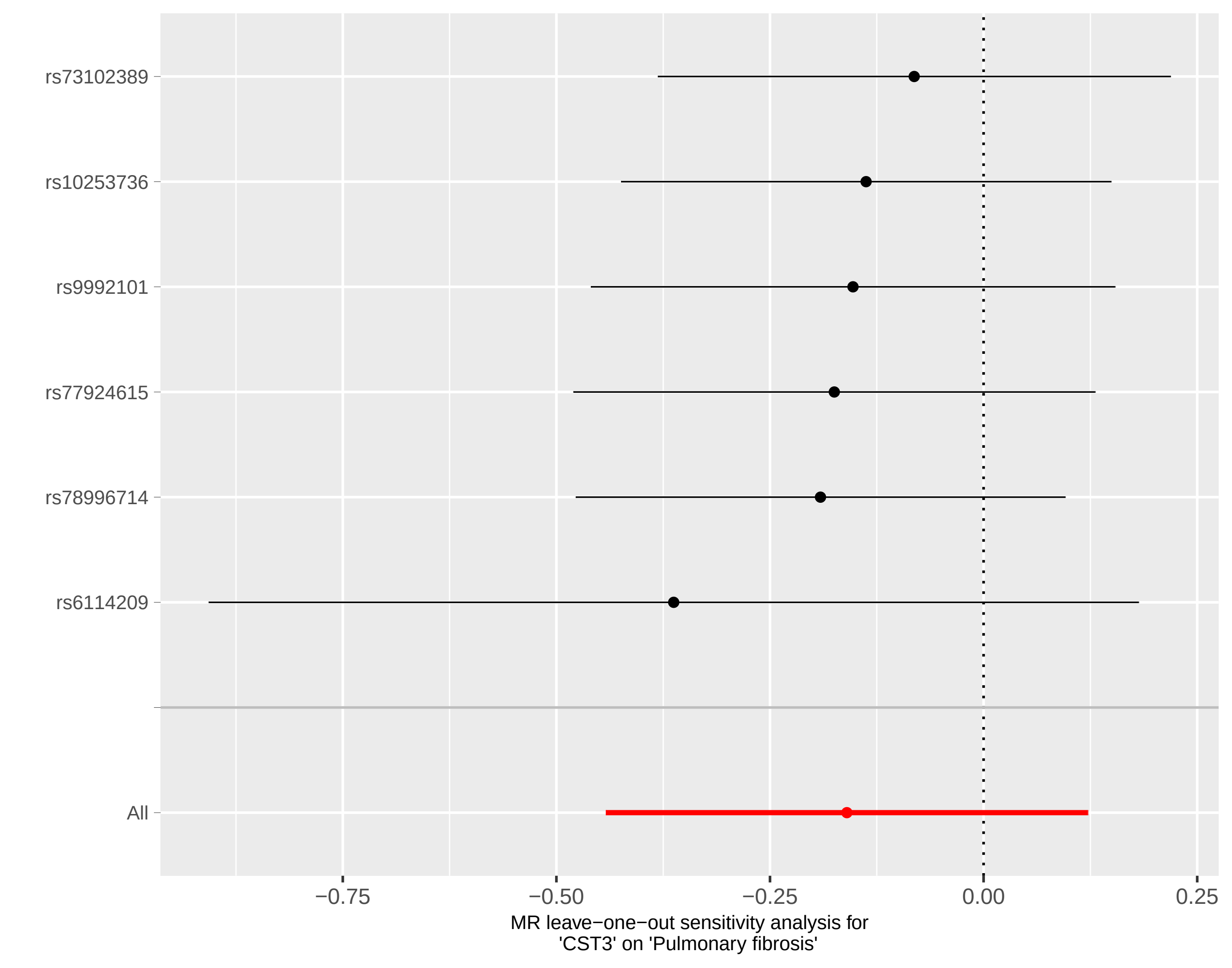
rs73102389
rs10253736
rs9992101
rs77924615
rs78996714
rs6114209
All
−0.75
−0.50
−0.25
0.00
0.25
MR leave−one−out sensitivity analysis for
'CST3' on 'Pulmonary fibrosis'

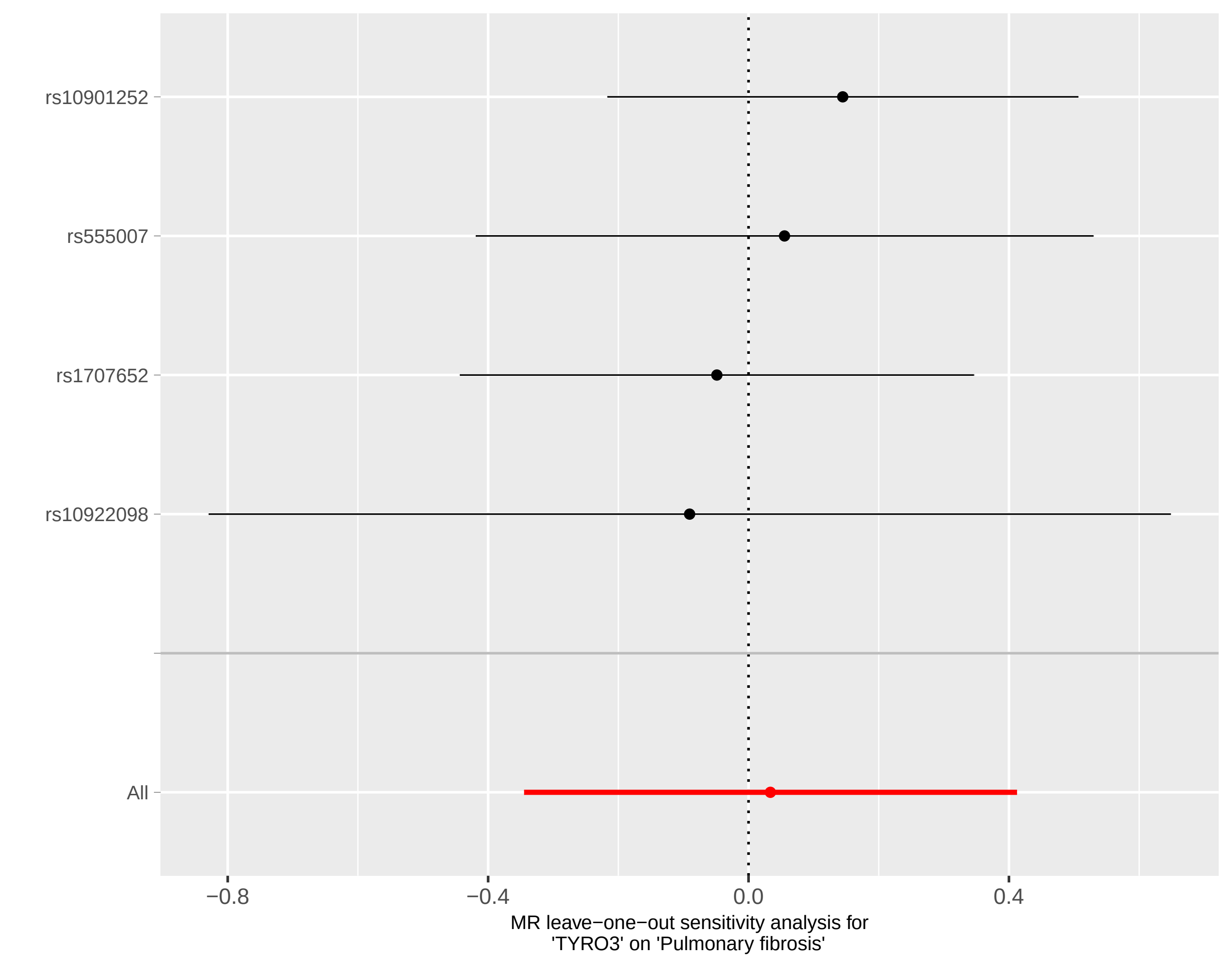

rs10901252
rs555007
rs1707652
rs10922098
All
−0.8
−0.4
0.0
0.4
MR leave−one−out sensitivity analysis for
'TYRO3' on 'Pulmonary fibrosis'

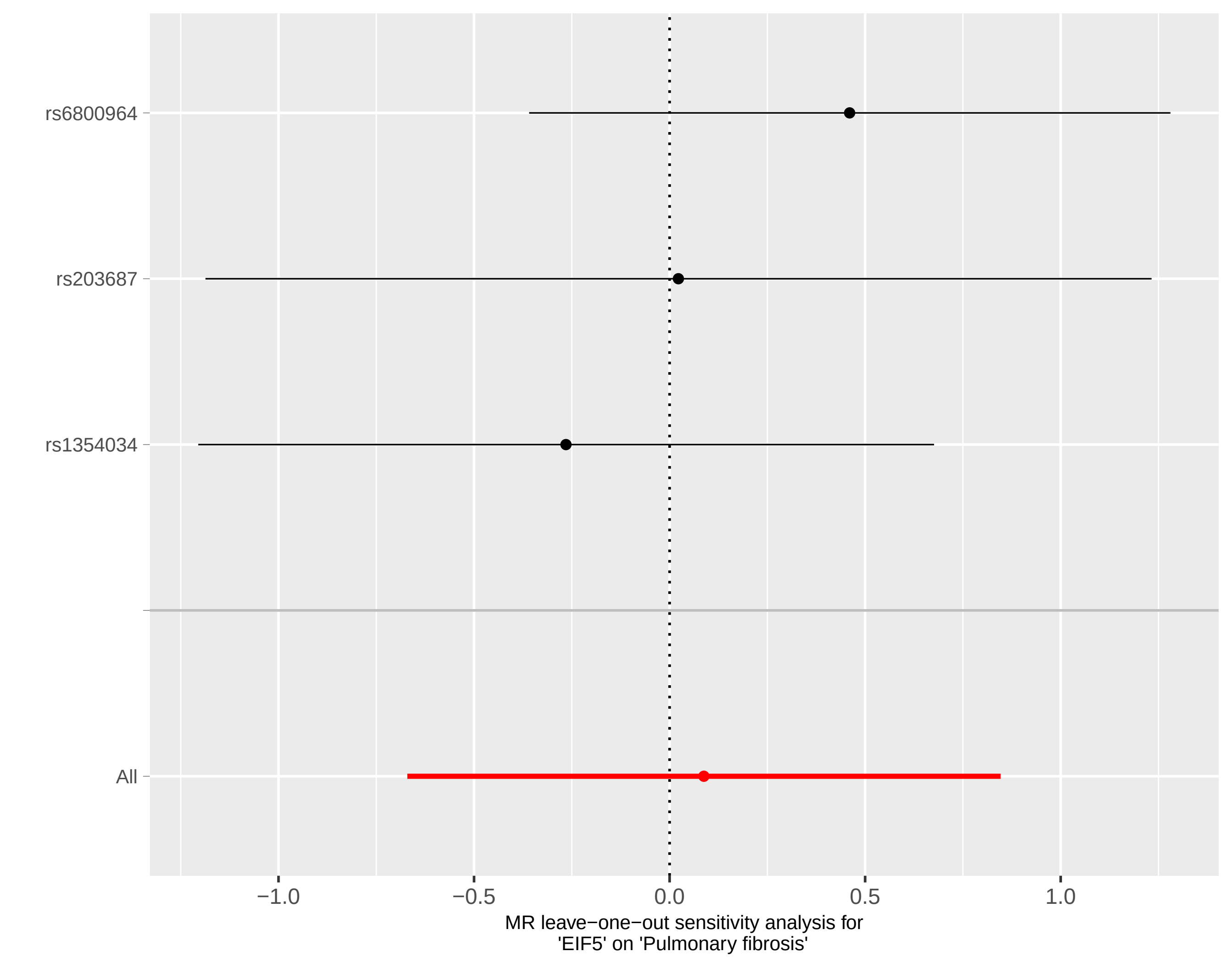
rs6800964
rs203687
rs1354034
All
−1.0
−0.5
0.0
0.5
1.0
MR leave−one−out sensitivity analysis for
'EIF5' on 'Pulmonary fibrosis'

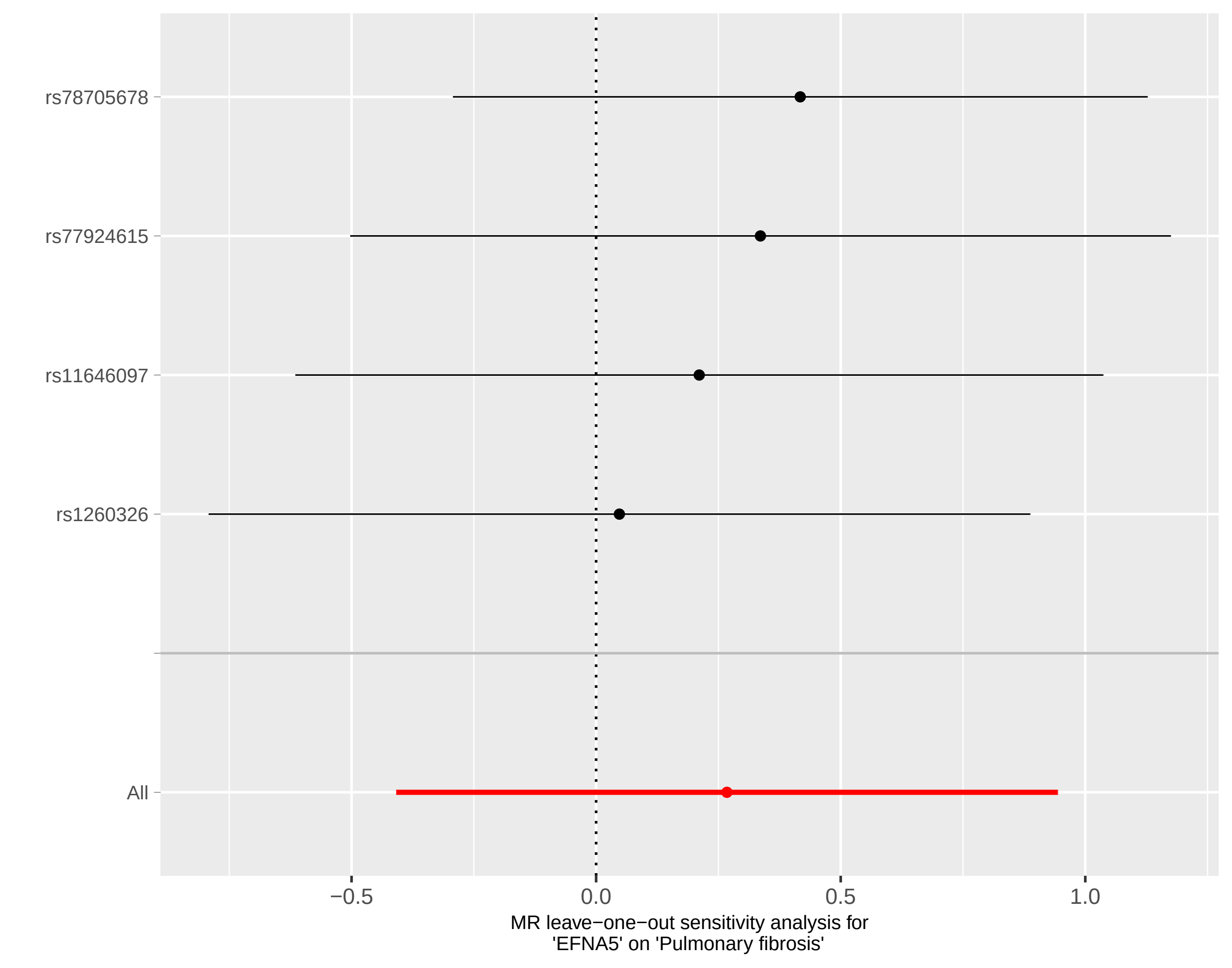

rs78705678
rs77924615
rs11646097
rs1260326
All
−0.5
0.0
0.5
1.0
MR leave−one−out sensitivity analysis for
'EFNA5' on 'Pulmonary fibrosis'

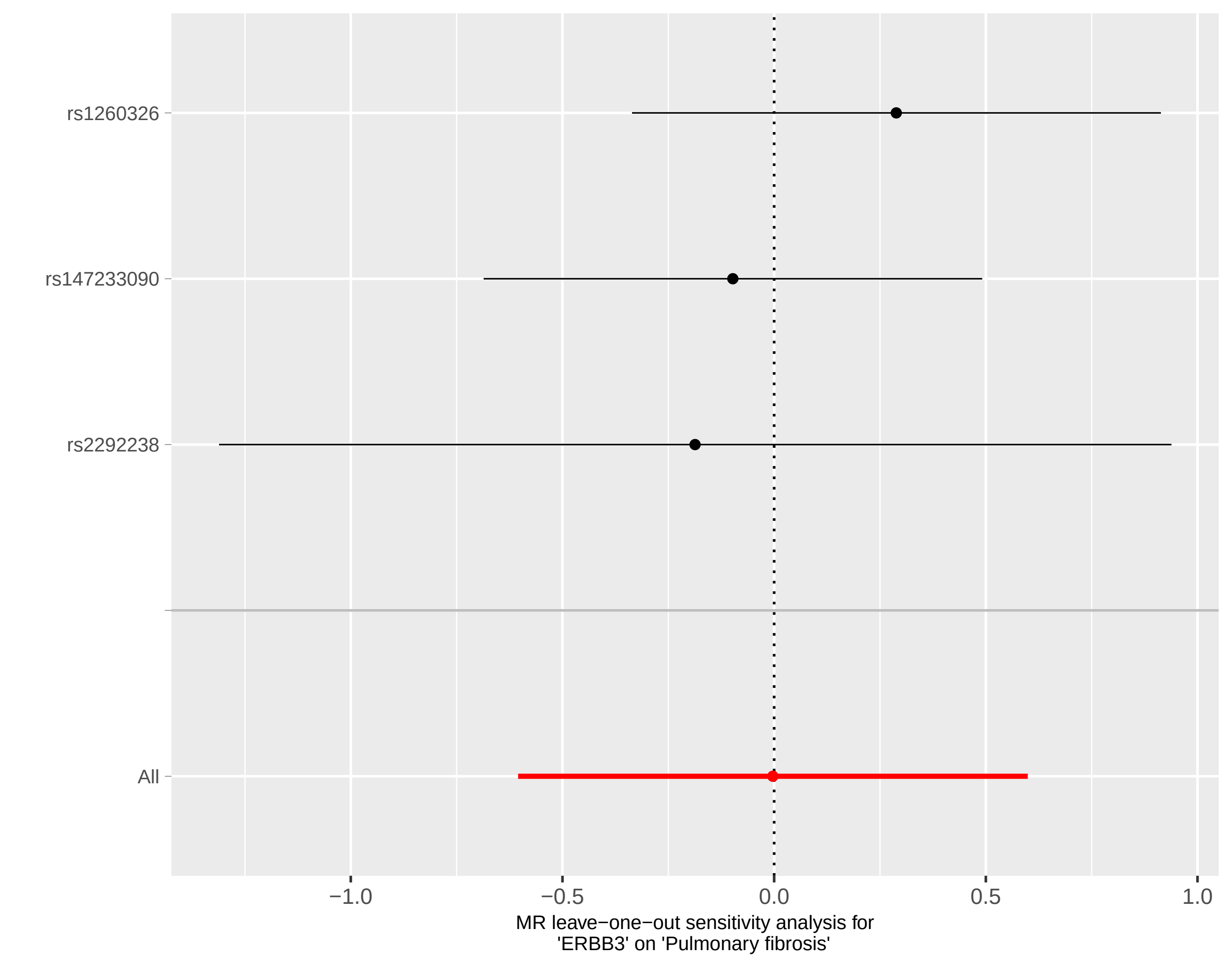
rs1260326
rs147233090
rs2292238
All
−1.0
−0.5
0.0
0.5
1.0
MR leave−one−out sensitivity analysis for
'ERBB3' on 'Pulmonary fibrosis'

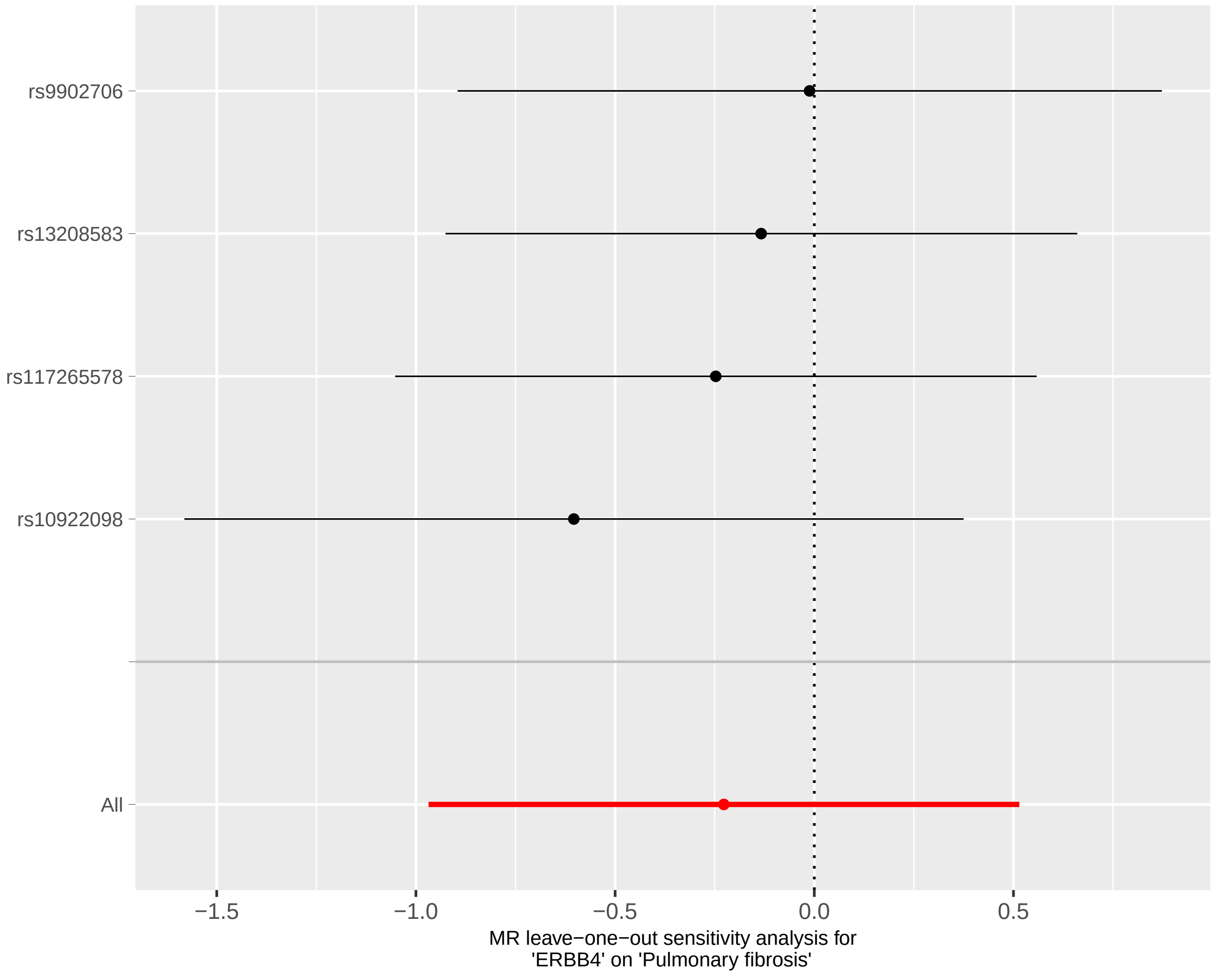
rs9902706
rs13208583
rs117265578
rs10922098
All
−1.5
−1.0
−0.5
0.0
0.5
MR leave−one−out sensitivity analysis for
'ERBB4' on 'Pulmonary fibrosis'

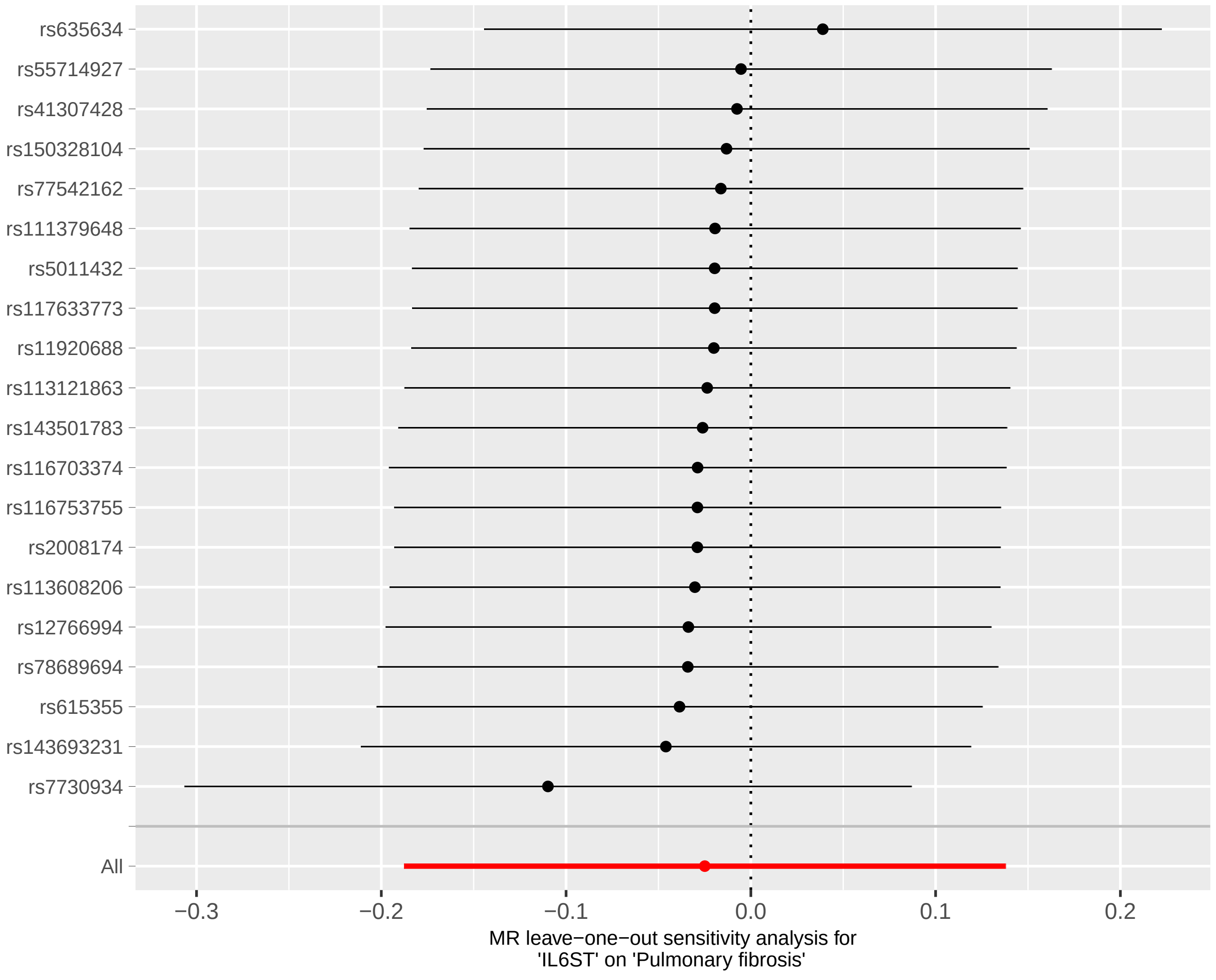
rs635634
rs55714927
rs41307428
rs150328104
rs77542162
rs111379648
rs5011432
rs117633773
rs11920688
rs113121863
rs143501783
rs116703374
rs116753755
rs2008174
rs113608206
rs12766994
rs78689694
rs615355
rs143693231
rs7730934
All
−0.3
−0.2
−0.1
0.0
0.1
0.2
MR leave−one−out sensitivity analysis for
'IL6ST' on 'Pulmonary fibrosis'

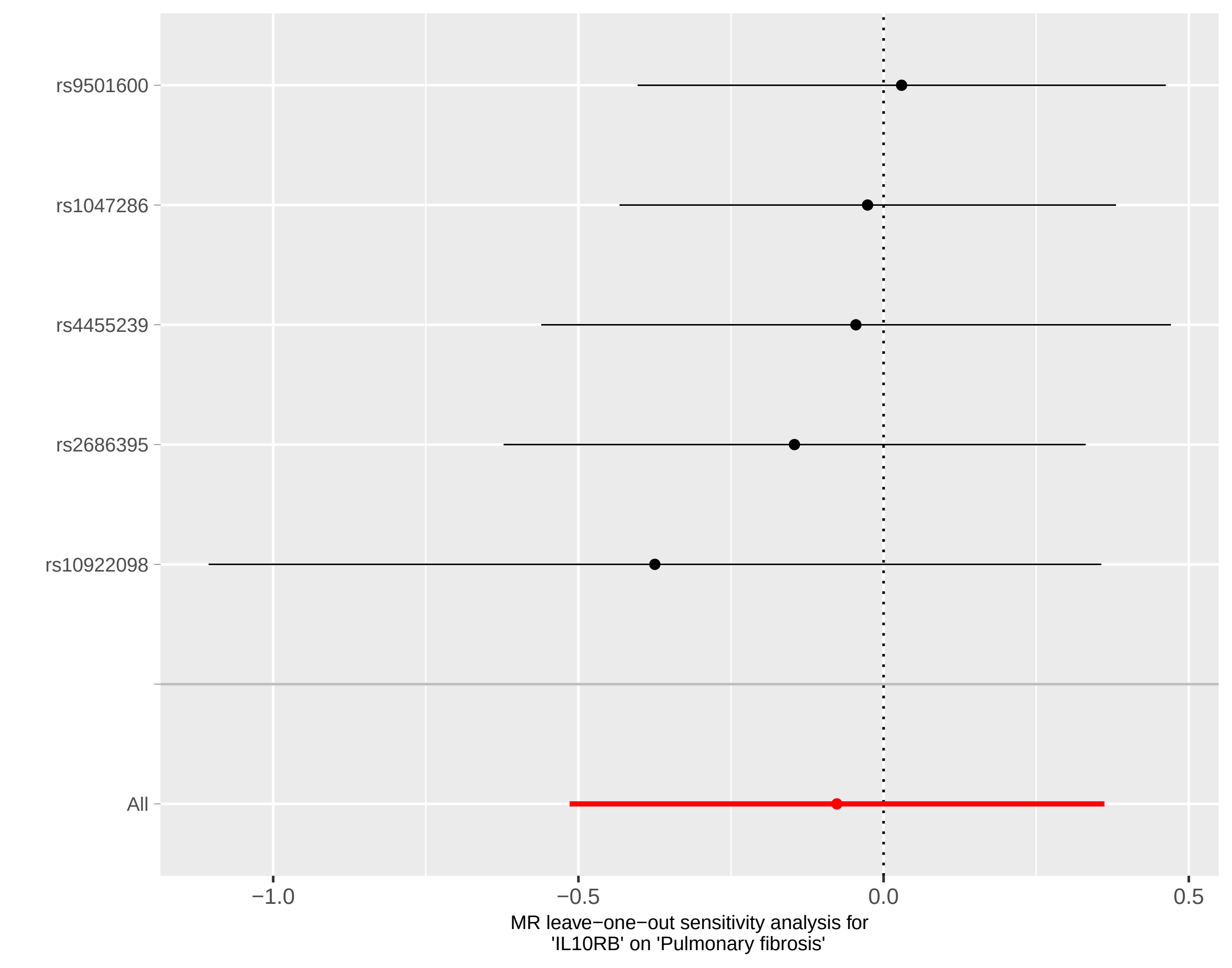
rs9501600
rs1047286
rs4455239
rs2686395
rs10922098
All
−1.0
−0.5
0.0
0.5
MR leave−one−out sensitivity analysis for
'IL10RB' on 'Pulmonary fibrosis'

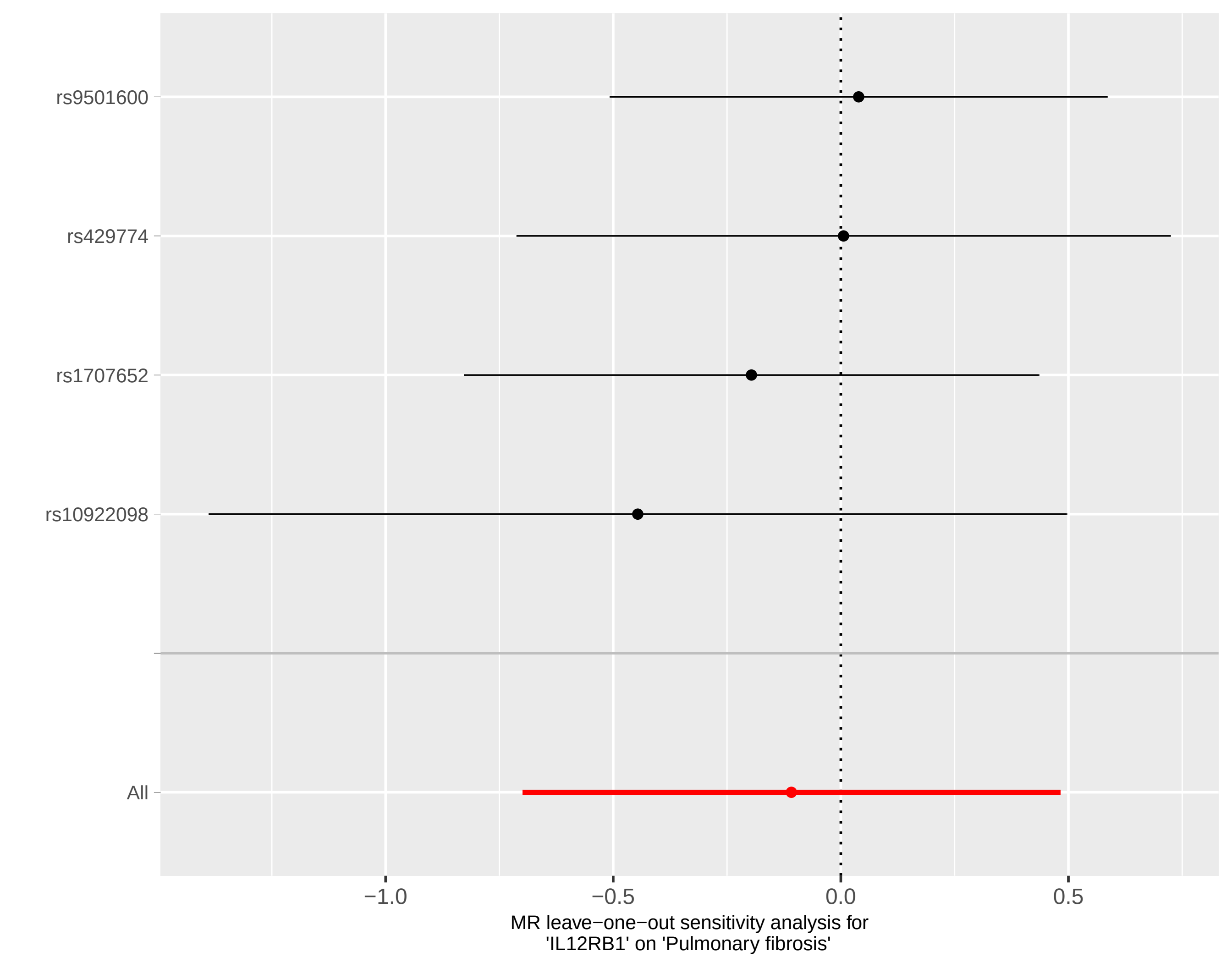
rs9501600
rs429774
rs1707652
rs10922098
All
−1.0
−0.5
0.0
0.5
MR leave−one−out sensitivity analysis for
'IL12RB1' on 'Pulmonary fibrosis'

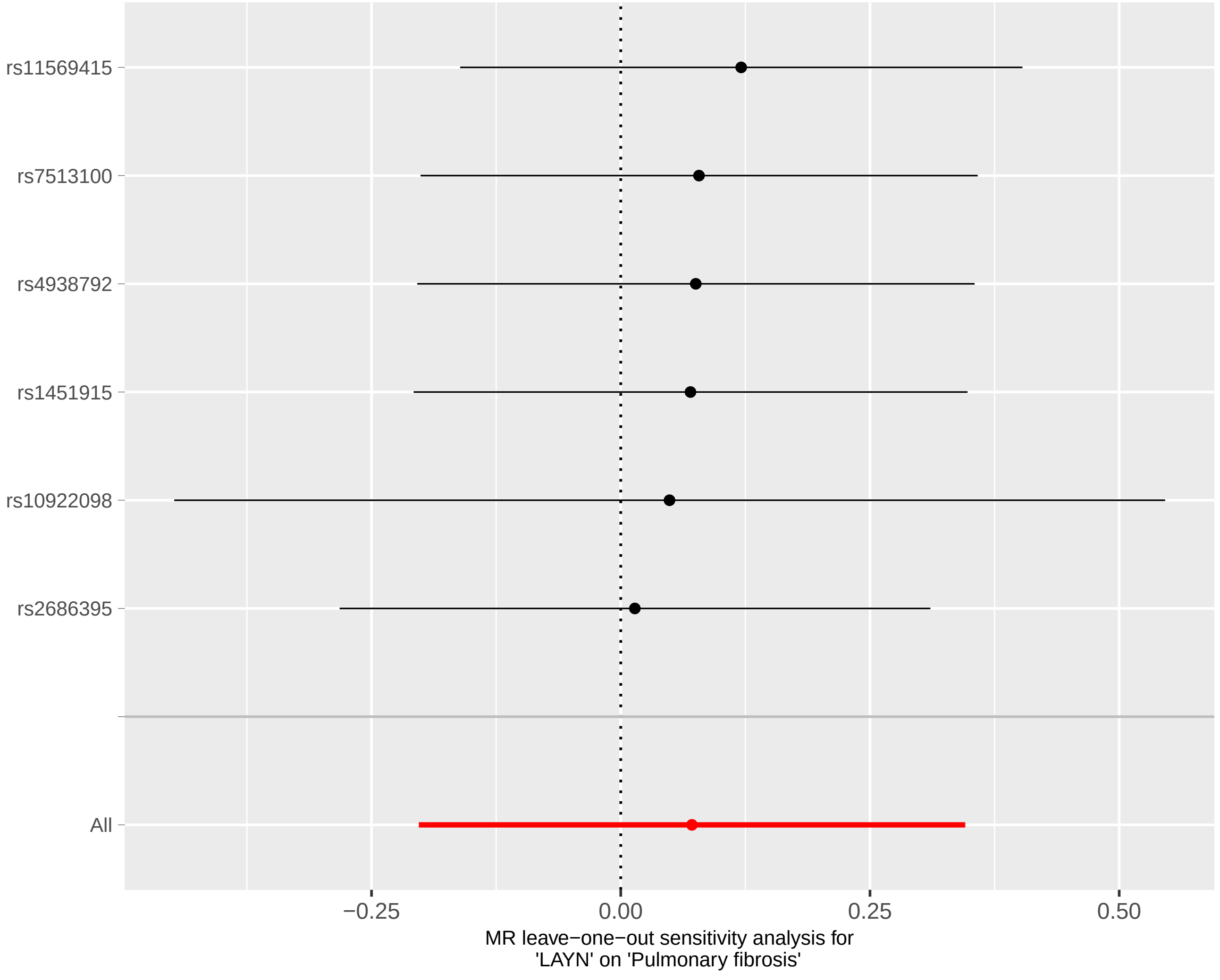
rs11569415
rs7513100
rs4938792
rs1451915
rs10922098
rs2686395
All
−0.25
0.00
0.25
0.50
MR leave−one−out sensitivity analysis for
'LAYN' on 'Pulmonary fibrosis'

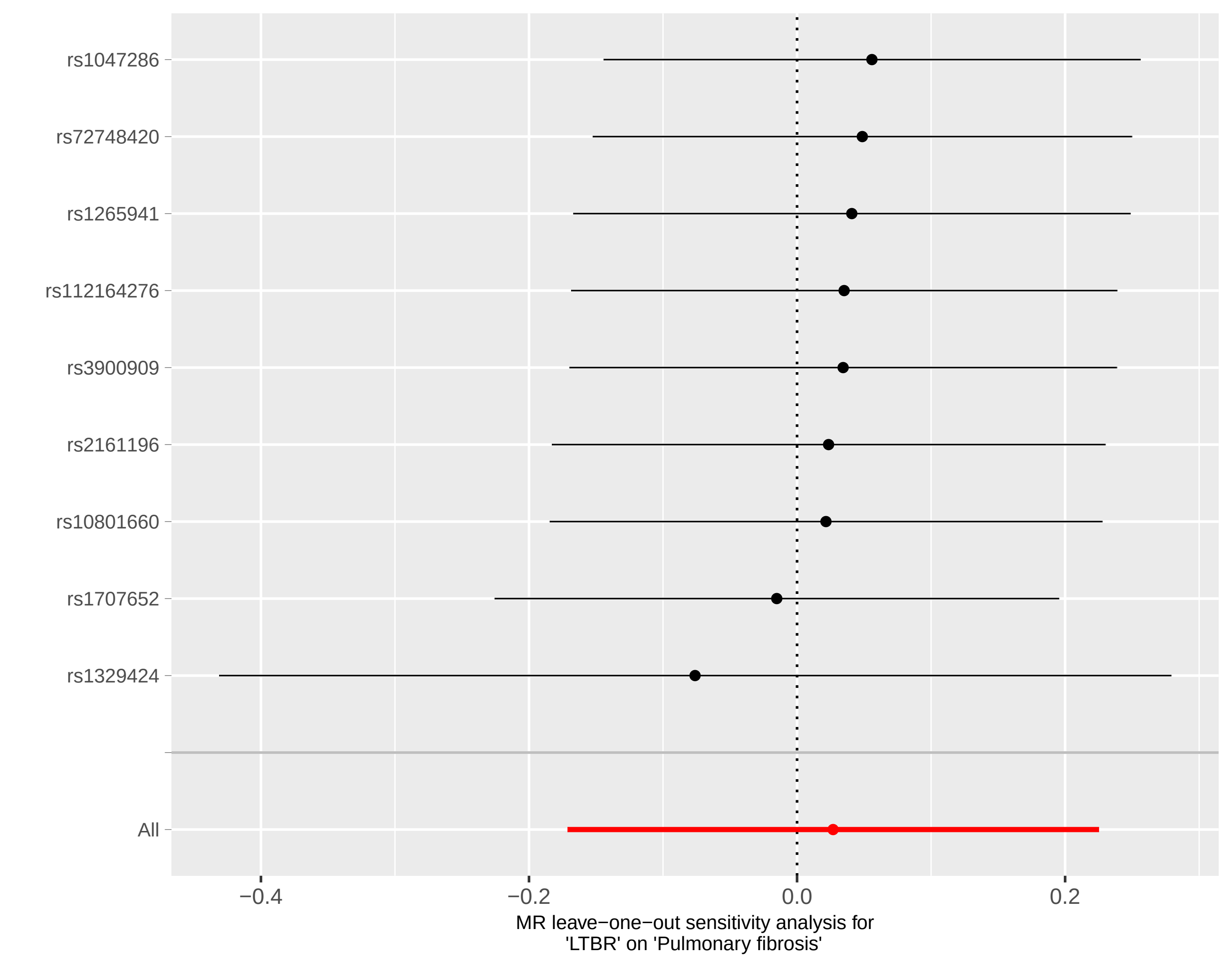
rs1047286
rs72748420
rs1265941
rs112164276
rs3900909
rs2161196
rs10801660
rs1707652
rs1329424
All
−0.4
−0.2
0.0
0.2
MR leave−one−out sensitivity analysis for
'LTBR' on 'Pulmonary fibrosis'

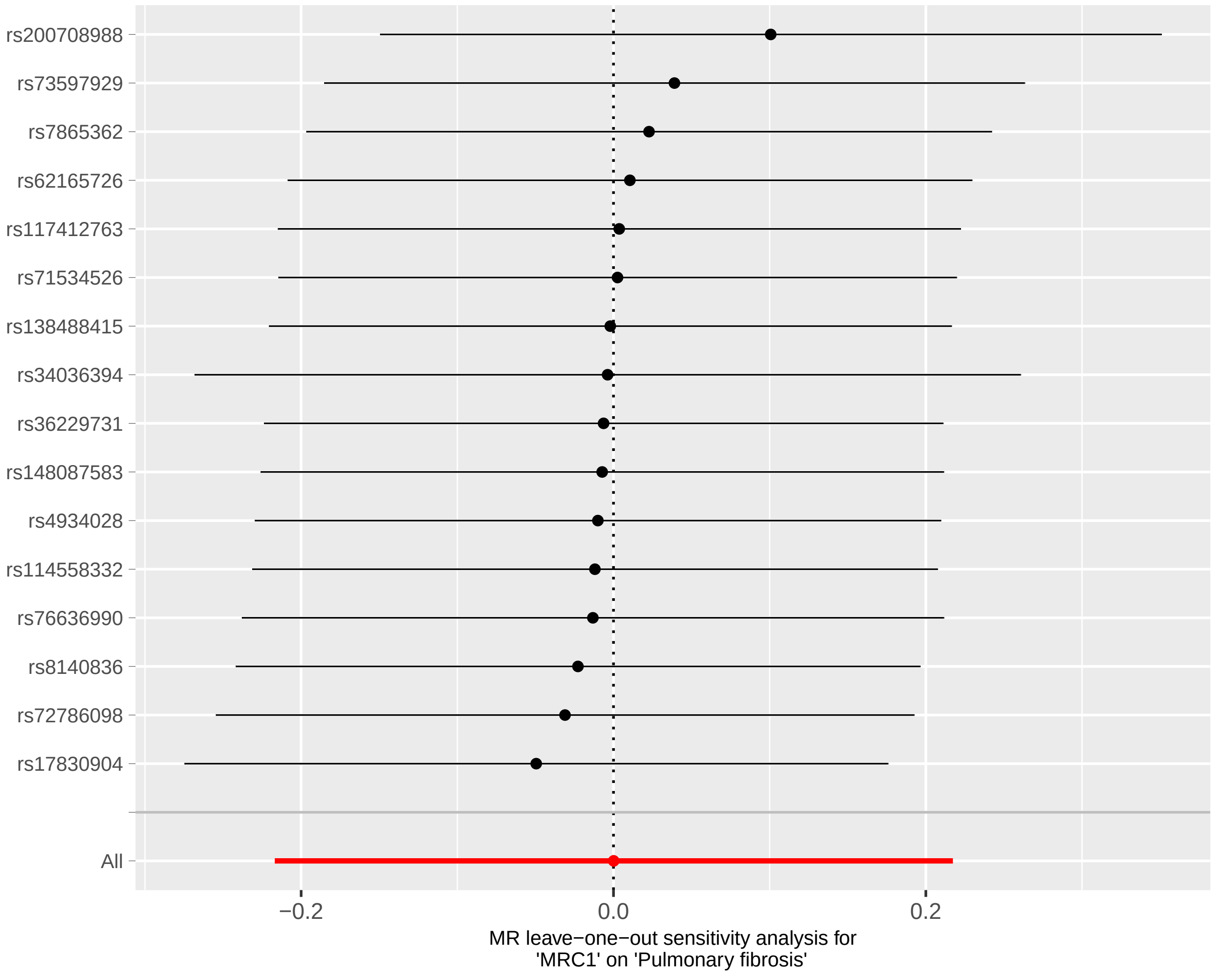
rs200708988
rs73597929
rs7865362
rs62165726
rs117412763
rs71534526
rs138488415
rs34036394
rs36229731
rs148087583
rs4934028
rs114558332
rs76636990
rs8140836
rs72786098
rs17830904
All
−0.2
0.0
0.2
MR leave−one−out sensitivity analysis for
'MRC1' on 'Pulmonary fibrosis'

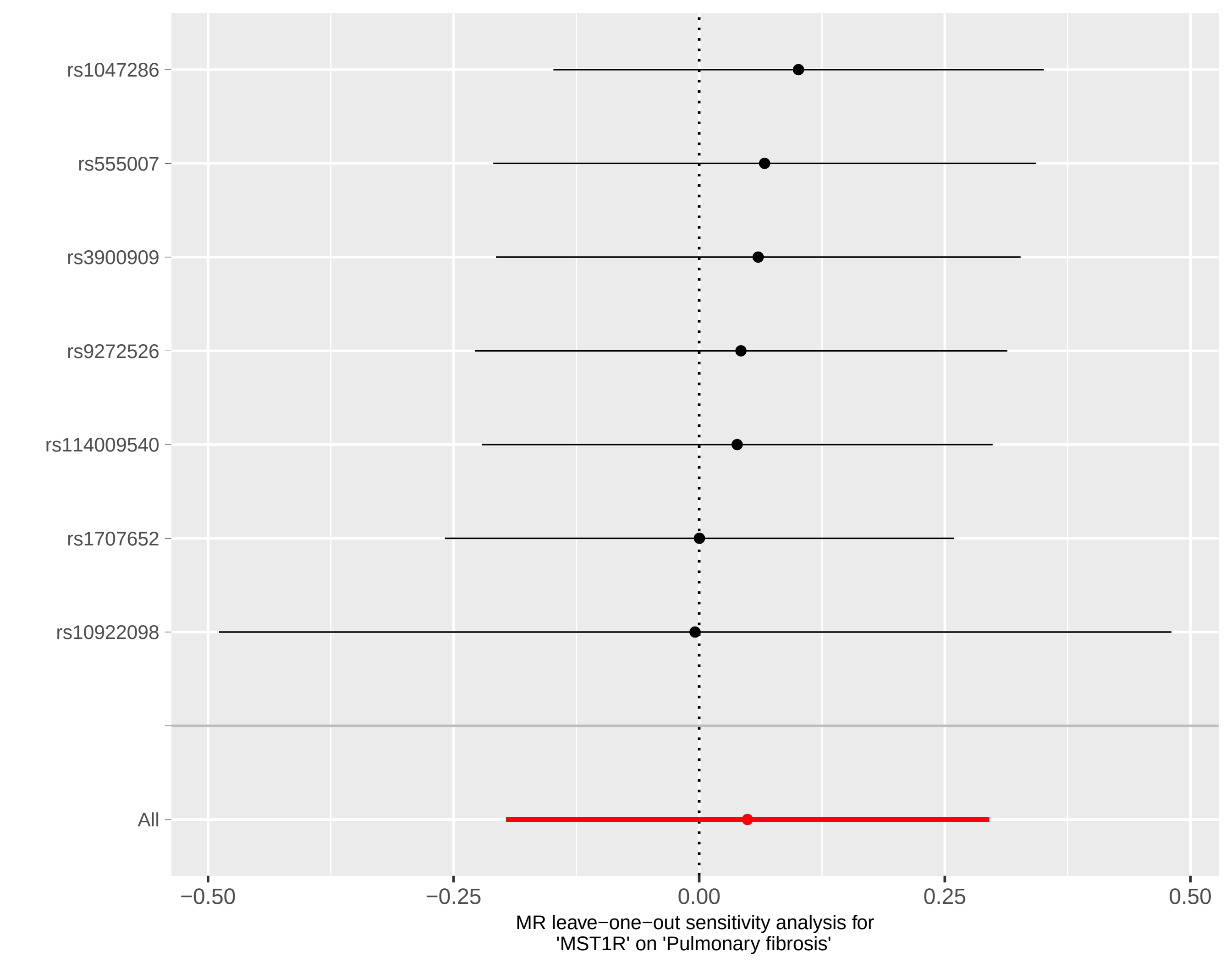
rs1047286
rs555007
rs3900909
rs9272526
rs114009540
rs1707652
rs10922098
All
−0.50
−0.25
0.00
0.25
0.50
MR leave−one−out sensitivity analysis for
'MST1R' on 'Pulmonary fibrosis'

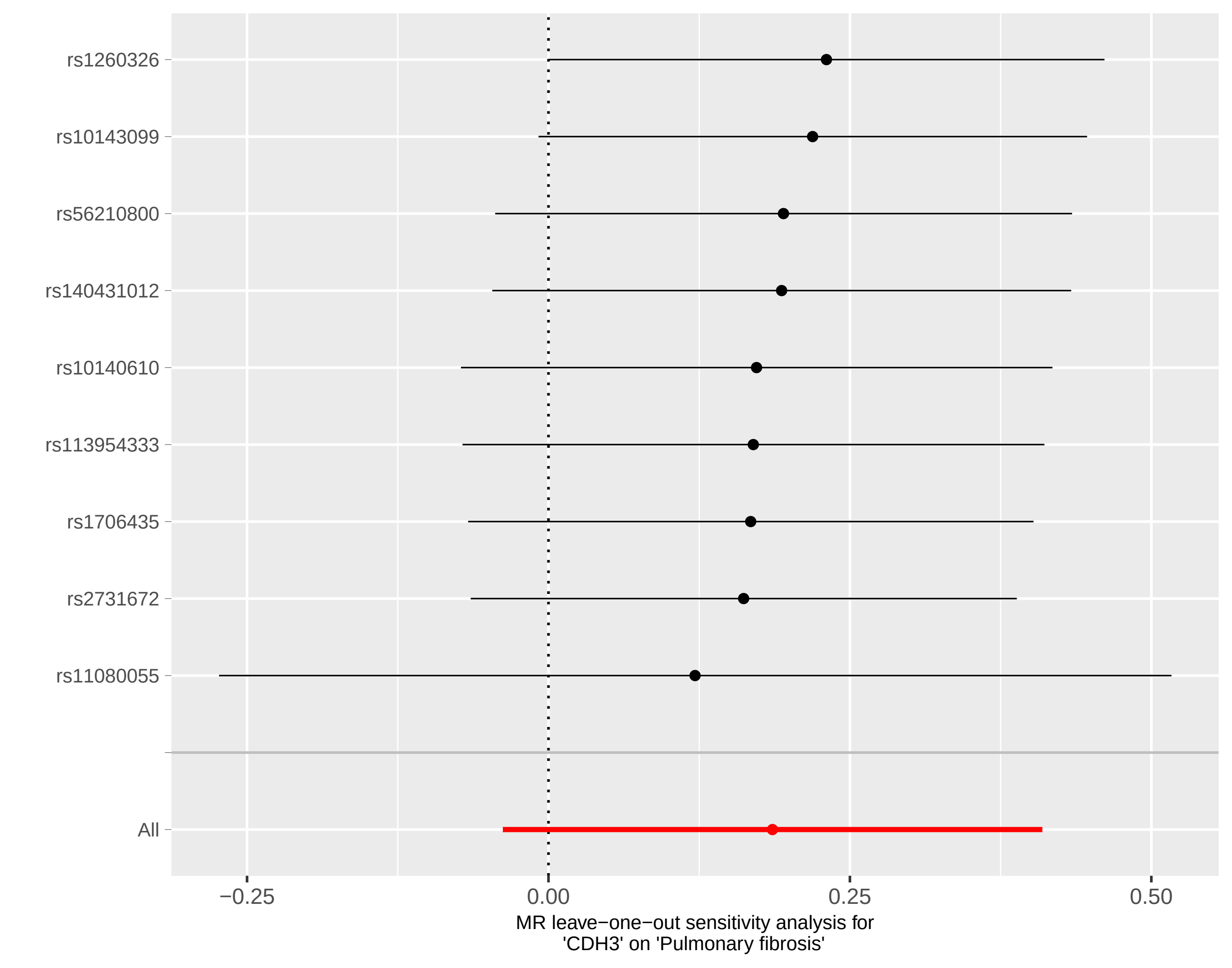
rs1260326
rs10143099
rs56210800
rs140431012
rs10140610
rs113954333
rs1706435
rs2731672
rs11080055
All
−0.25
0.00
0.25
0.50
MR leave−one−out sensitivity analysis for
'CDH3' on 'Pulmonary fibrosis'

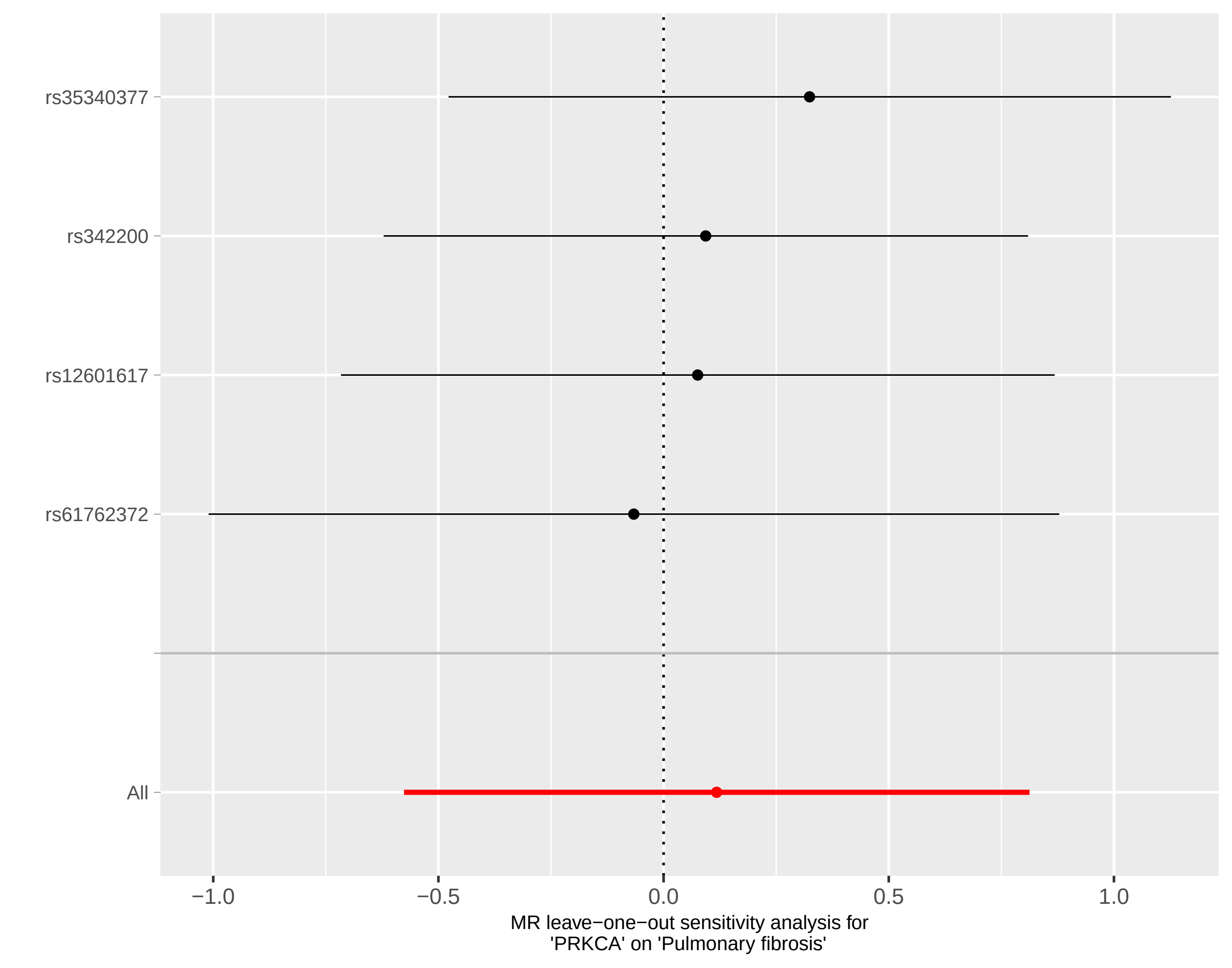
rs35340377
rs342200
rs12601617
rs61762372
All
−1.0
−0.5
0.0
0.5
1.0
MR leave−one−out sensitivity analysis for
'PRKCA' on 'Pulmonary fibrosis'

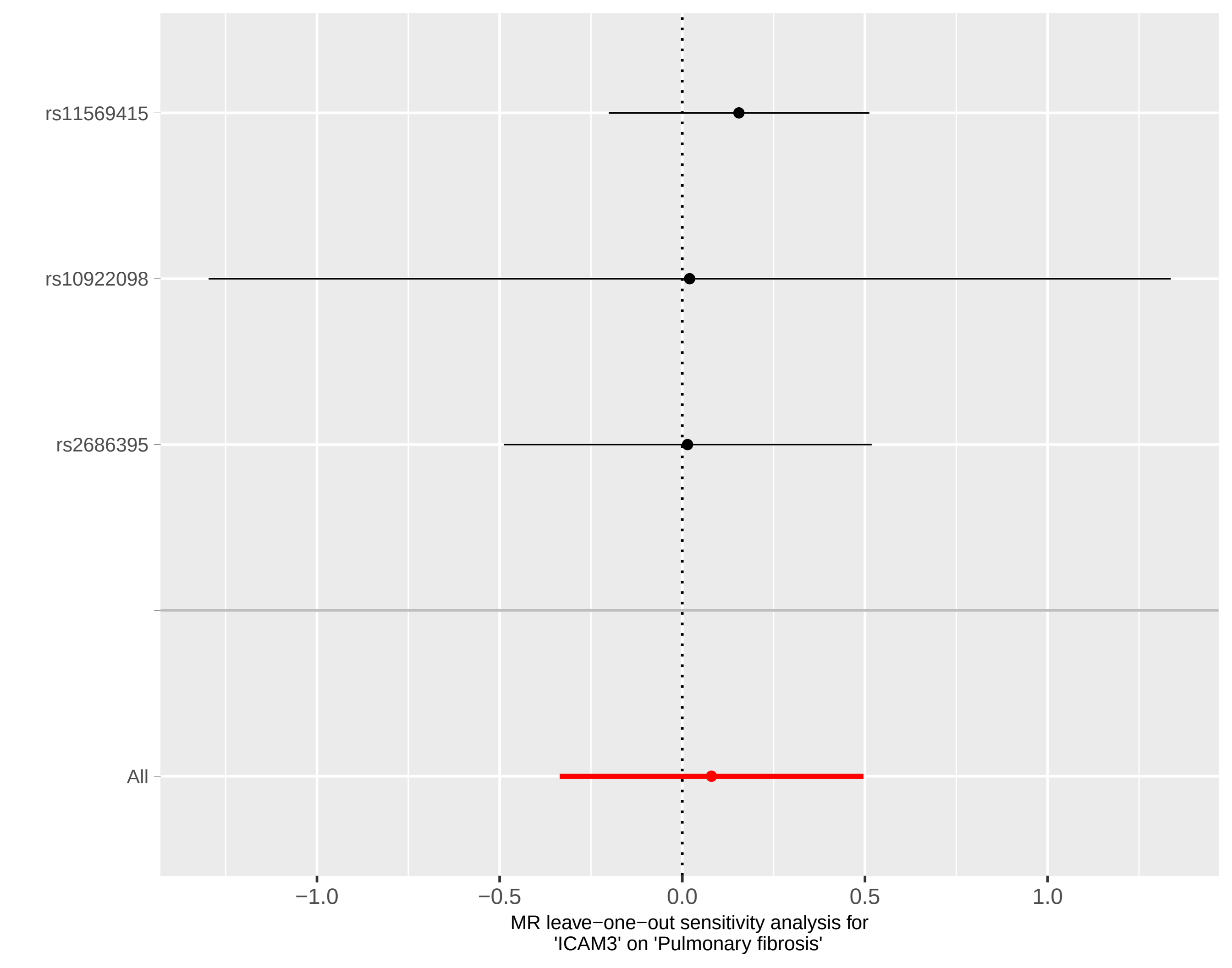
rs11569415
rs10922098
rs2686395
All
−1.0
−0.5
0.0
0.5
1.0
MR leave−one−out sensitivity analysis for
'ICAM3' on 'Pulmonary fibrosis'

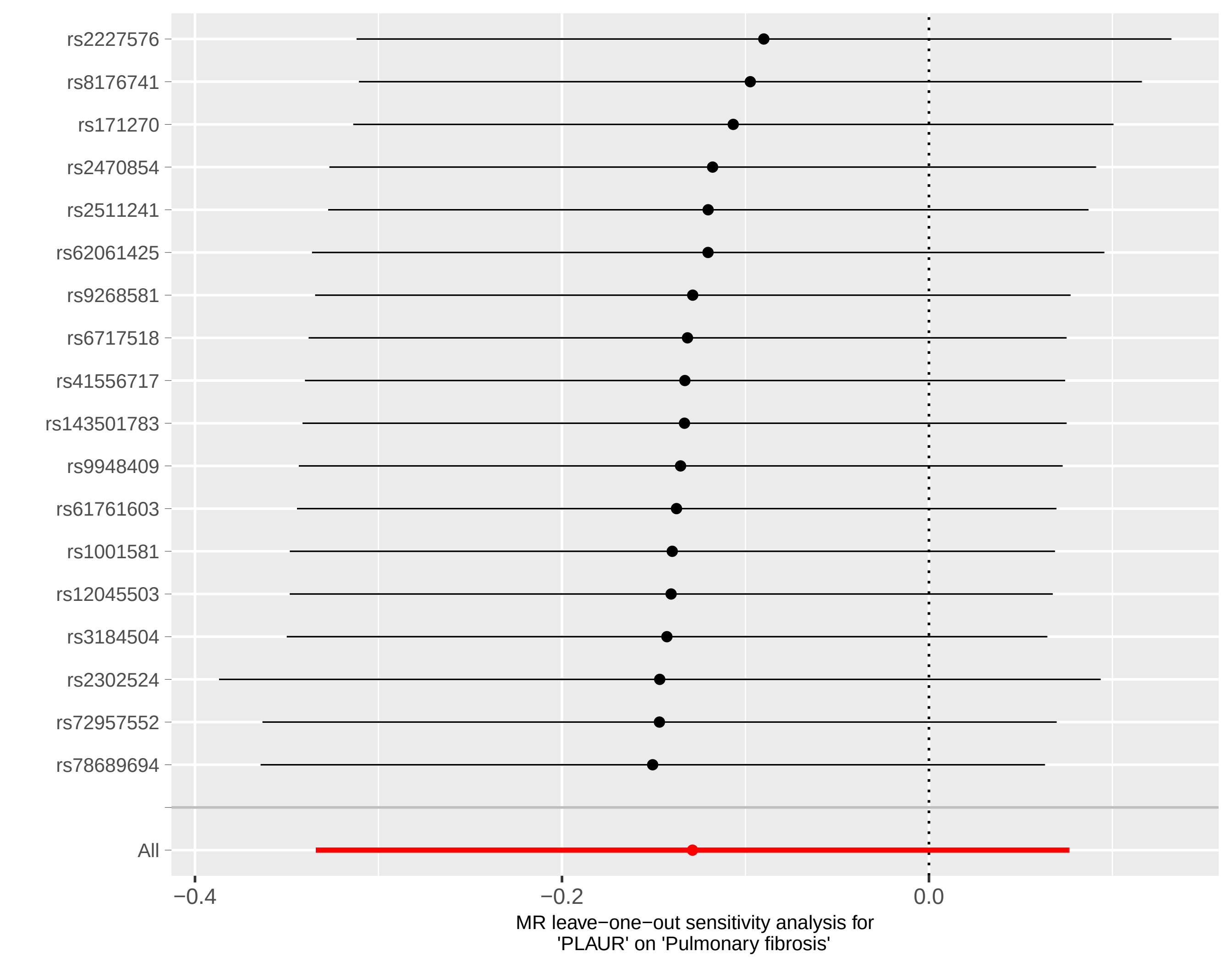
rs2227576
rs8176741
rs171270
rs2470854
rs2511241
rs62061425
rs9268581
rs6717518
rs41556717
rs143501783
rs9948409
rs61761603
rs1001581
rs12045503
rs3184504
rs2302524
rs72957552
rs78689694
All
−0.4
−0.2
0.0
MR leave−one−out sensitivity analysis for
'PLAUR' on 'Pulmonary fibrosis'

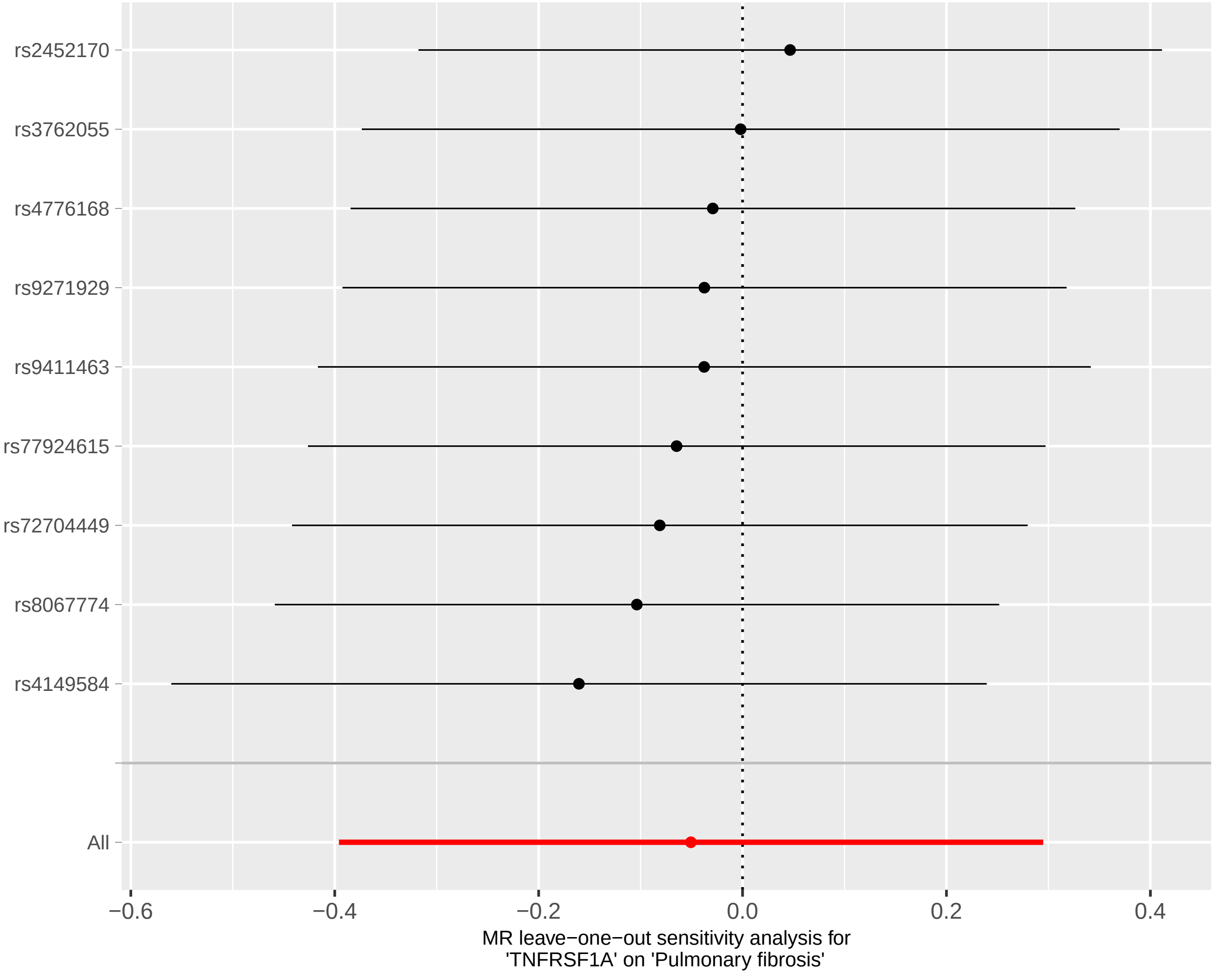
rs2452170
rs3762055
rs4776168
rs9271929
rs9411463
rs77924615
rs72704449
rs8067774
rs4149584
All
−0.6
−0.4
−0.2
0.0
0.2
0.4
MR leave−one−out sensitivity analysis for
'TNFRSF1A' on 'Pulmonary fibrosis'

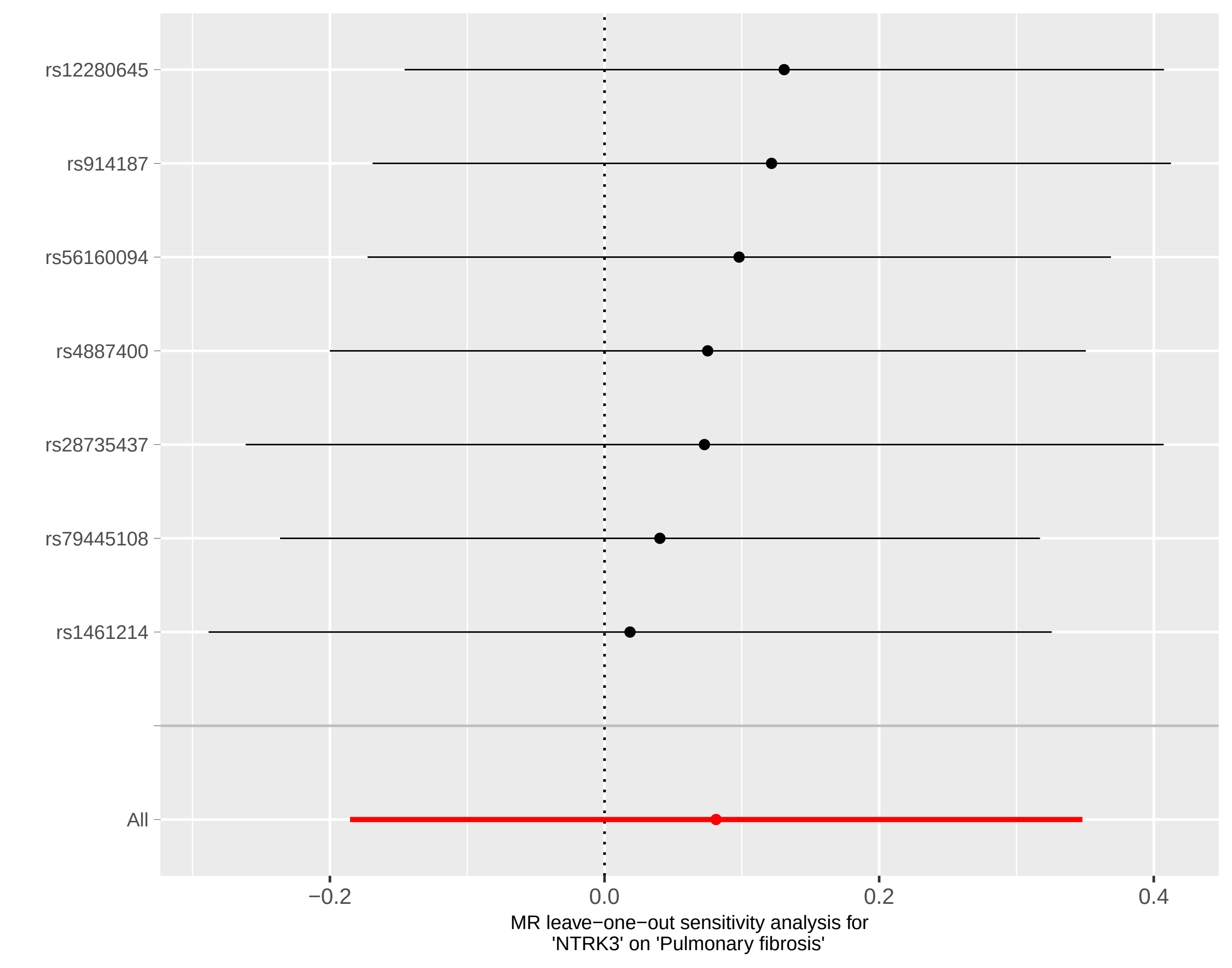
rs12280645
rs914187
rs56160094
rs4887400
rs28735437
rs79445108
rs1461214
All
−0.2
0.0
0.2
0.4
MR leave−one−out sensitivity analysis for
'NTRK3' on 'Pulmonary fibrosis'

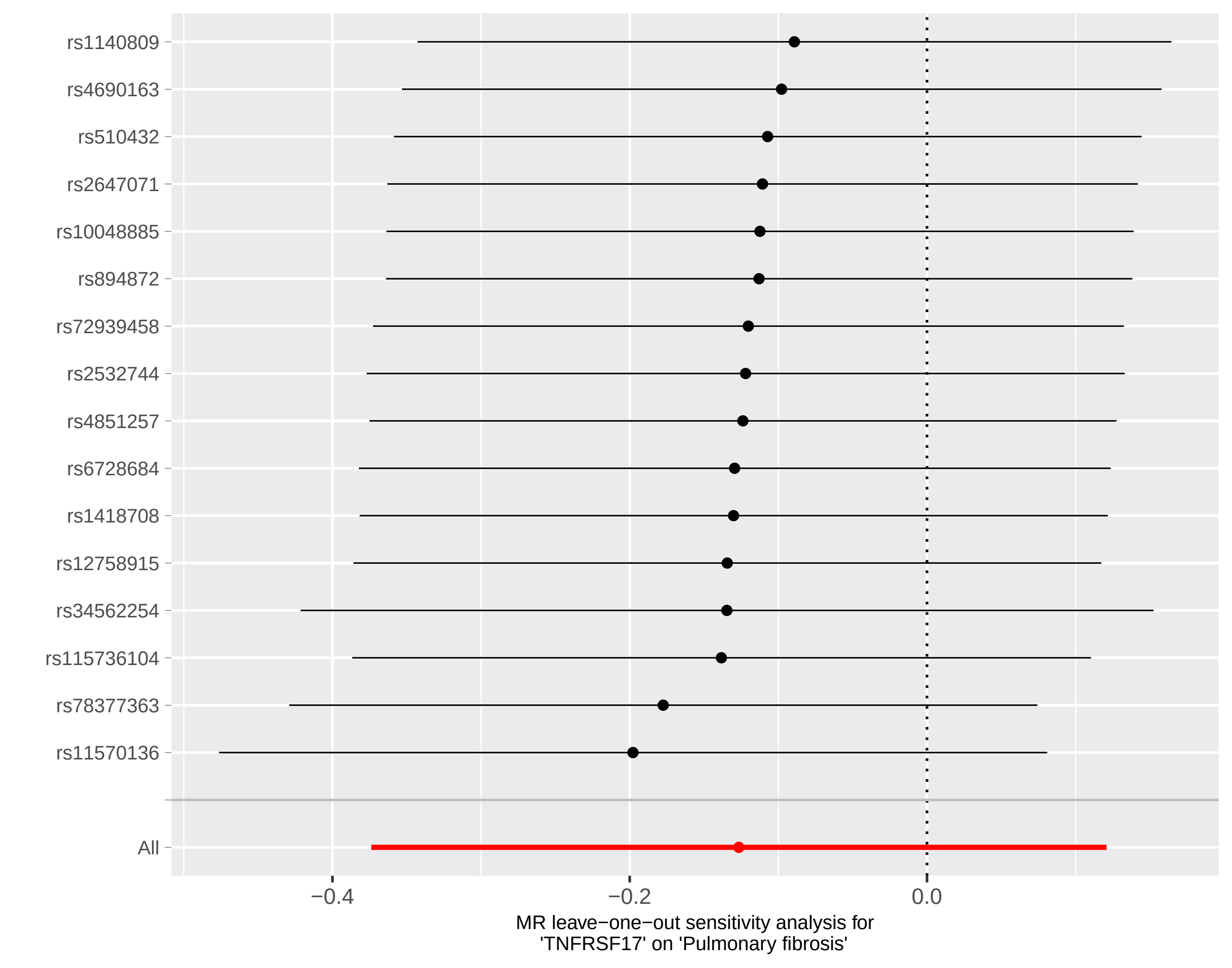
rs1140809
rs4690163
rs510432
rs2647071
rs10048885
rs894872
rs72939458
rs2532744
rs4851257
rs6728684
rs1418708
rs12758915
rs34562254
rs115736104
rs78377363
rs11570136
All
−0.4
−0.2
0.0
MR leave−one−out sensitivity analysis for
'TNFRSF17' on 'Pulmonary fibrosis'

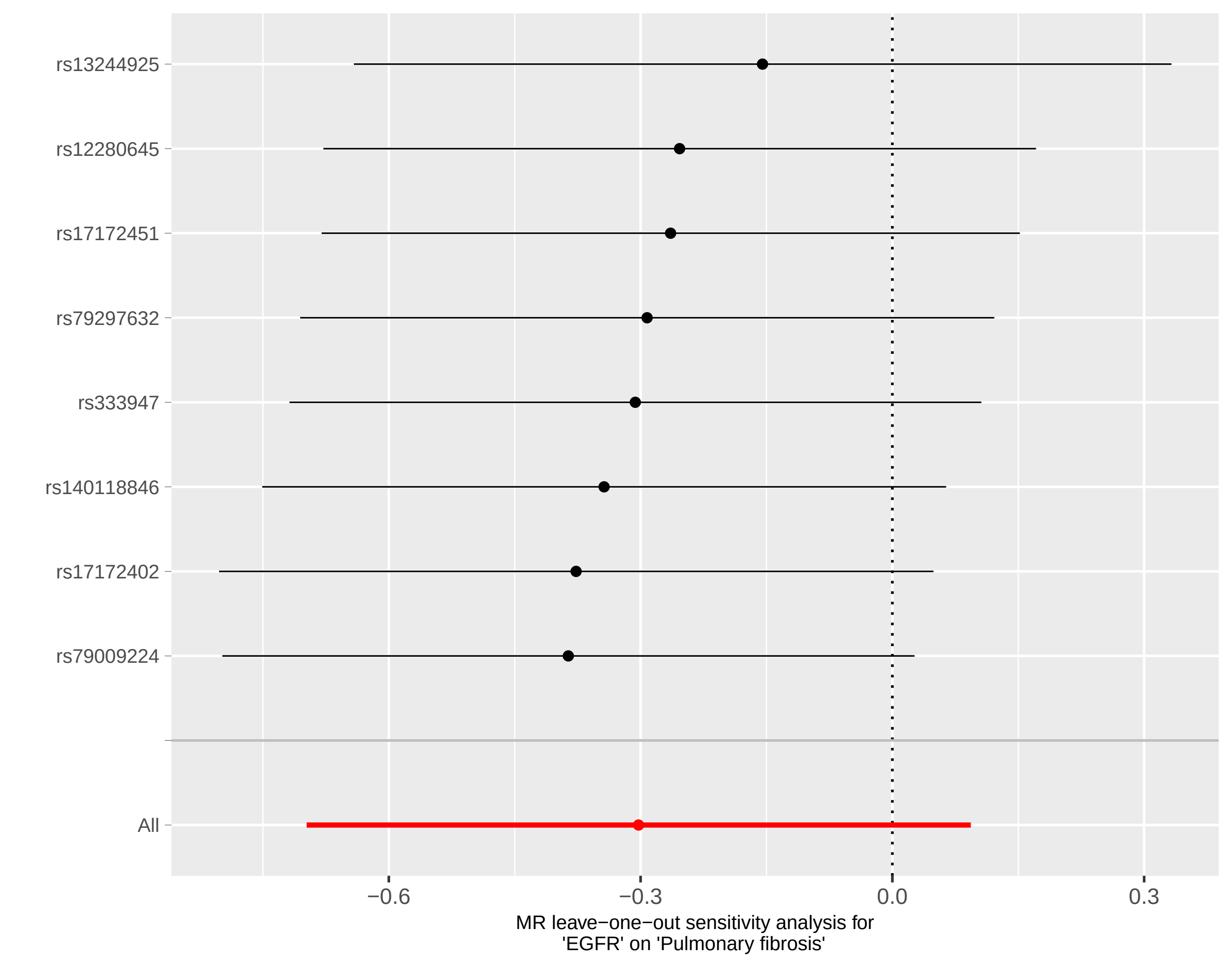
rs13244925
rs12280645
rs17172451
rs79297632
rs333947
rs140118846
rs17172402
rs79009224
All
−0.6
−0.3
0.0
0.3
MR leave−one−out sensitivity analysis for
'EGFR' on 'Pulmonary fibrosis'

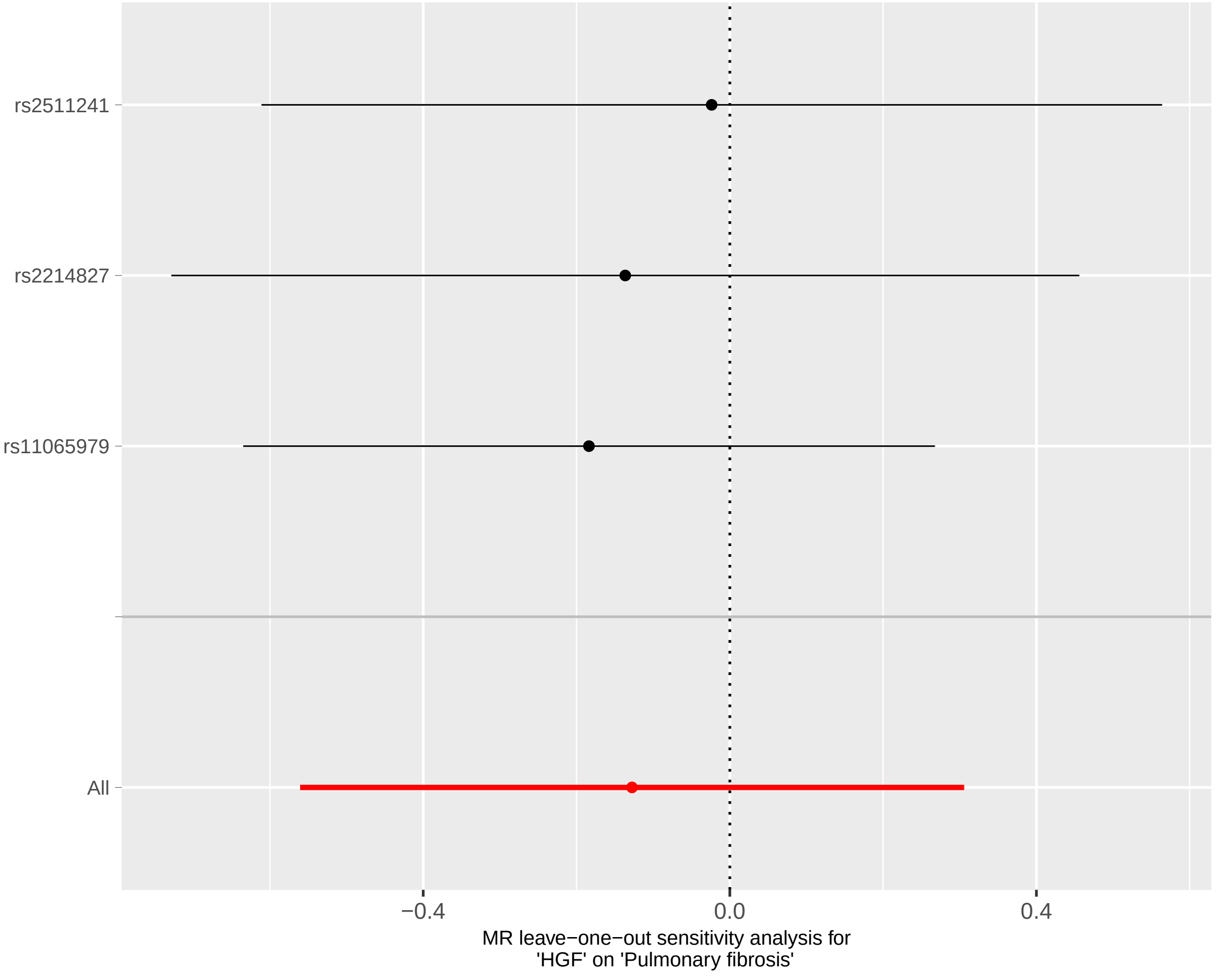
rs2511241
rs2214827
rs11065979
All
−0.4
0.0
0.4
MR leave−one−out sensitivity analysis for
'HGF' on 'Pulmonary fibrosis'

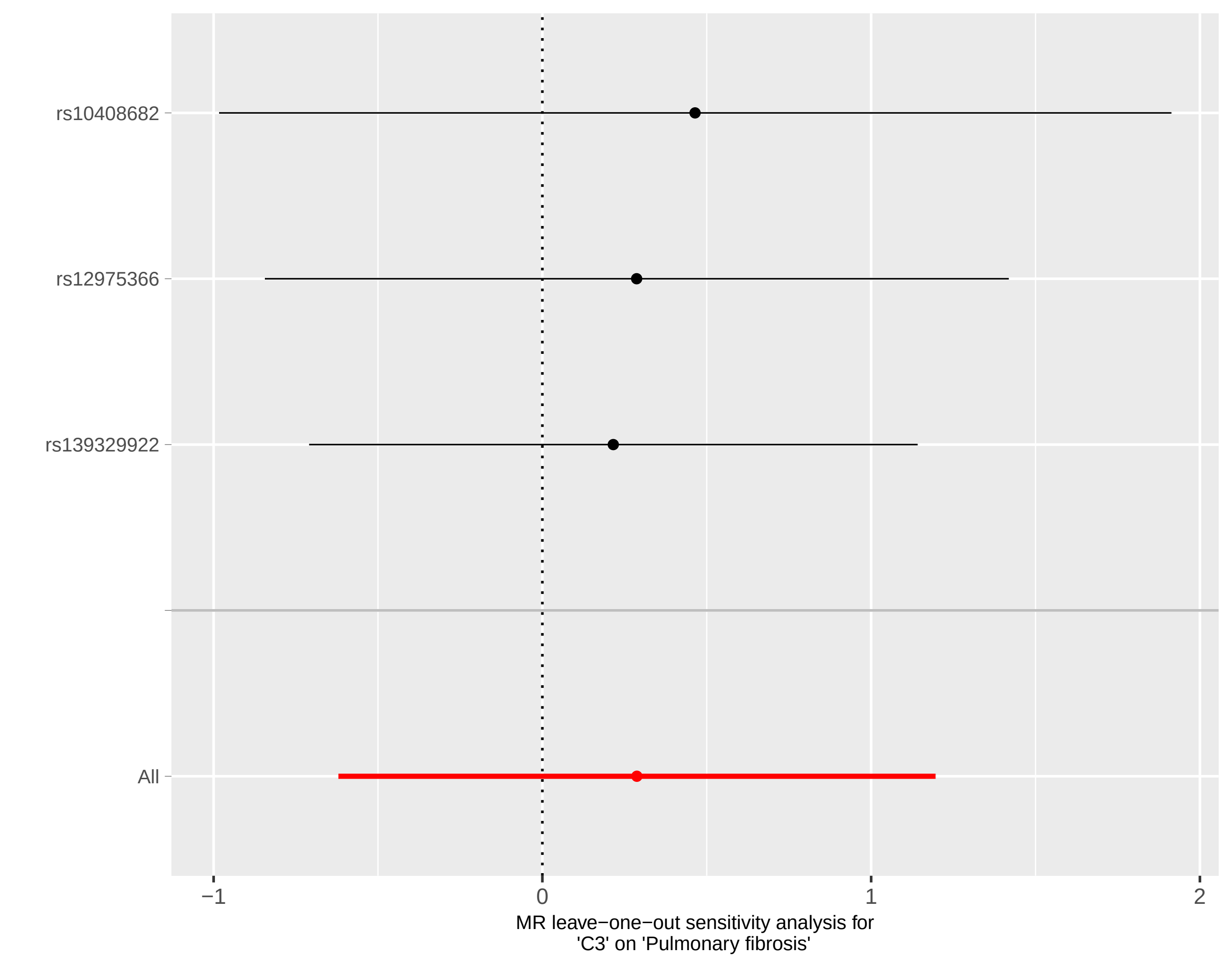

rs10408682
rs12975366
rs139329922
All
−1
0
1
2
MR leave−one−out sensitivity analysis for
'C3' on 'Pulmonary fibrosis'

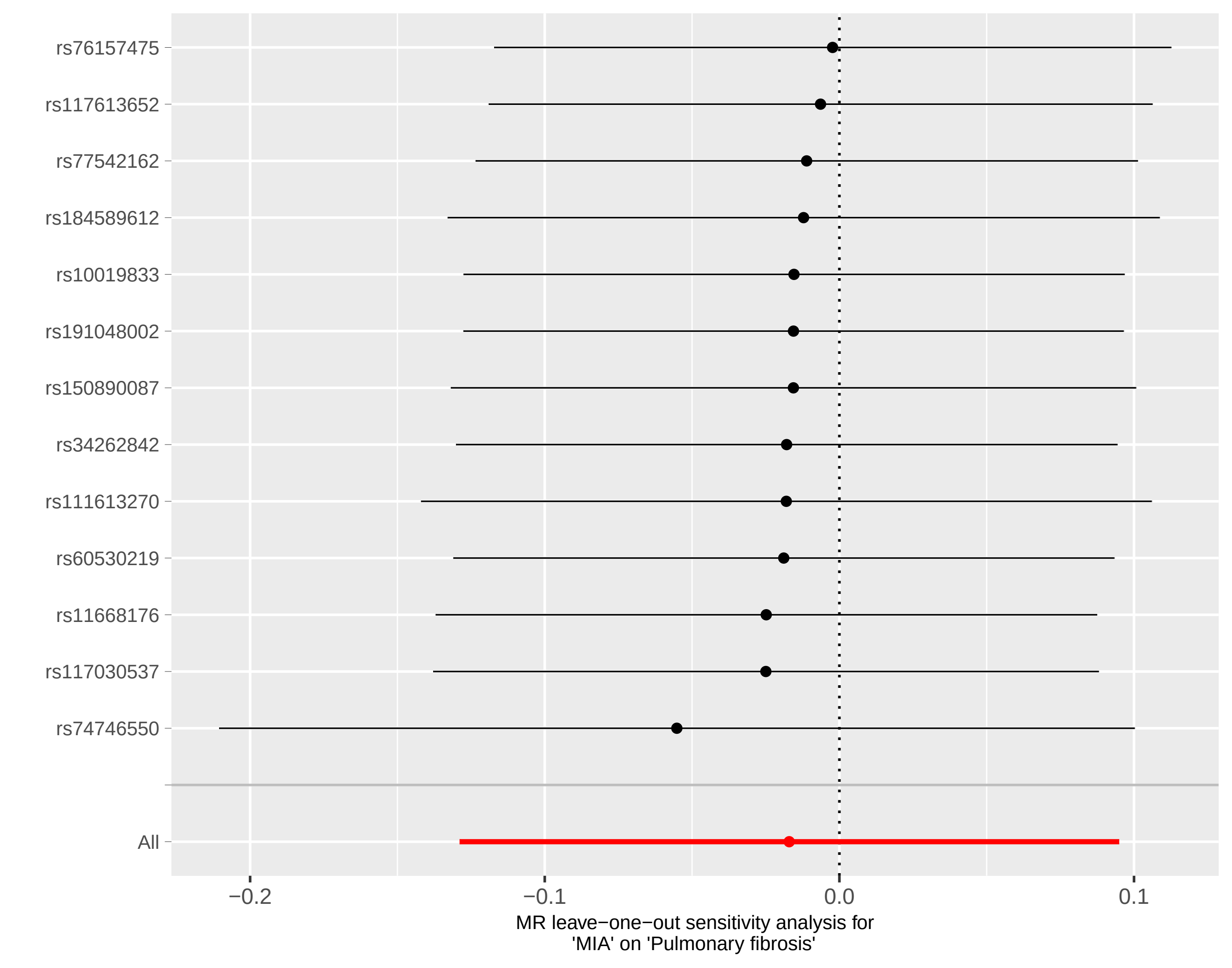
rs76157475
rs117613652
rs77542162
rs184589612
rs10019833
rs191048002
rs150890087
rs34262842
rs111613270
rs60530219
rs11668176
rs117030537
rs74746550
All
−0.2
−0.1
0.0
0.1
MR leave−one−out sensitivity analysis for
'MIA' on 'Pulmonary fibrosis'

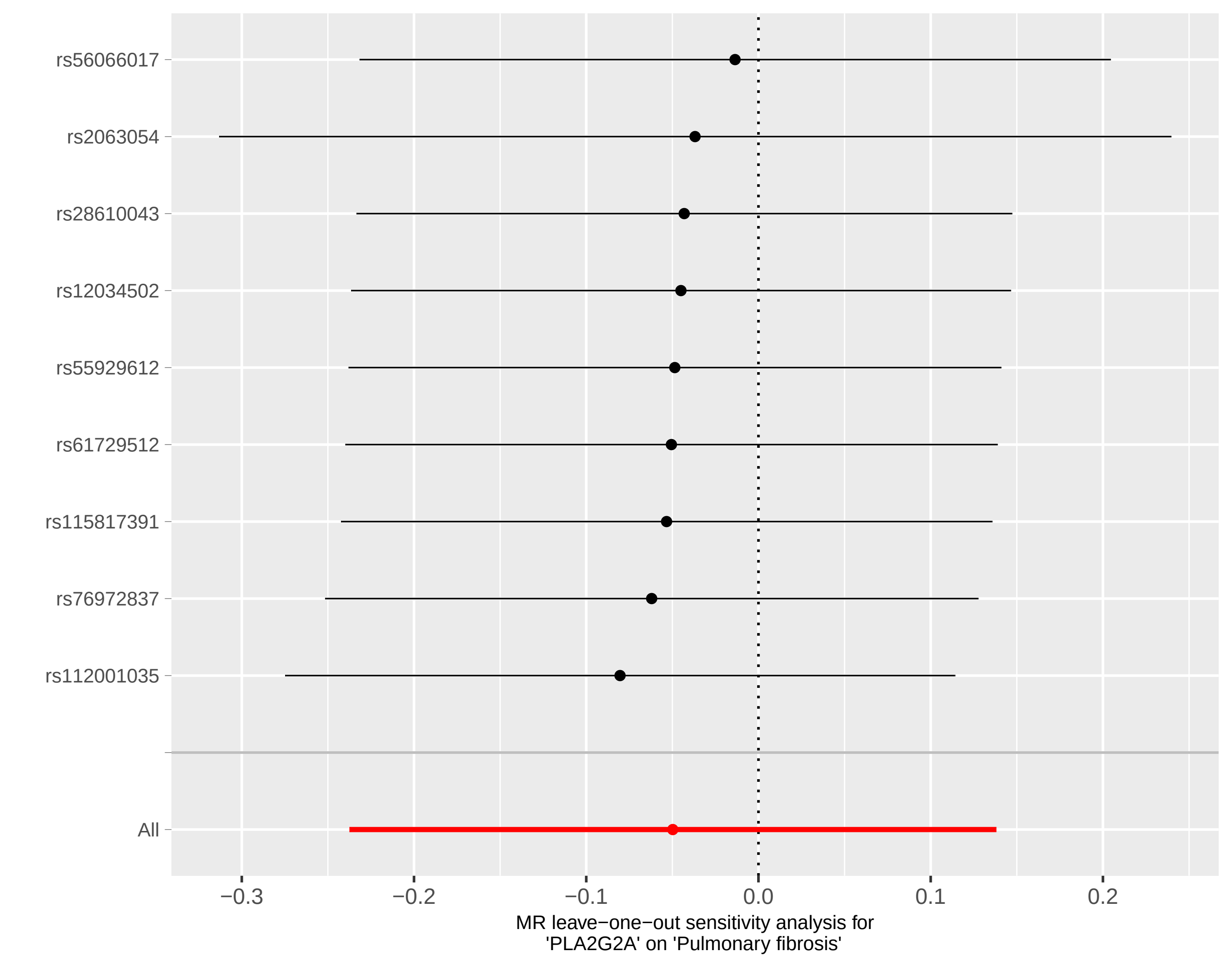

rs56066017
rs2063054
rs28610043
rs12034502
rs55929612
rs61729512
rs115817391
rs76972837
rs112001035
All
−0.3
−0.2
−0.1
0.0
0.1
0.2
MR leave−one−out sensitivity analysis for
'PLA2G2A' on 'Pulmonary fibrosis'

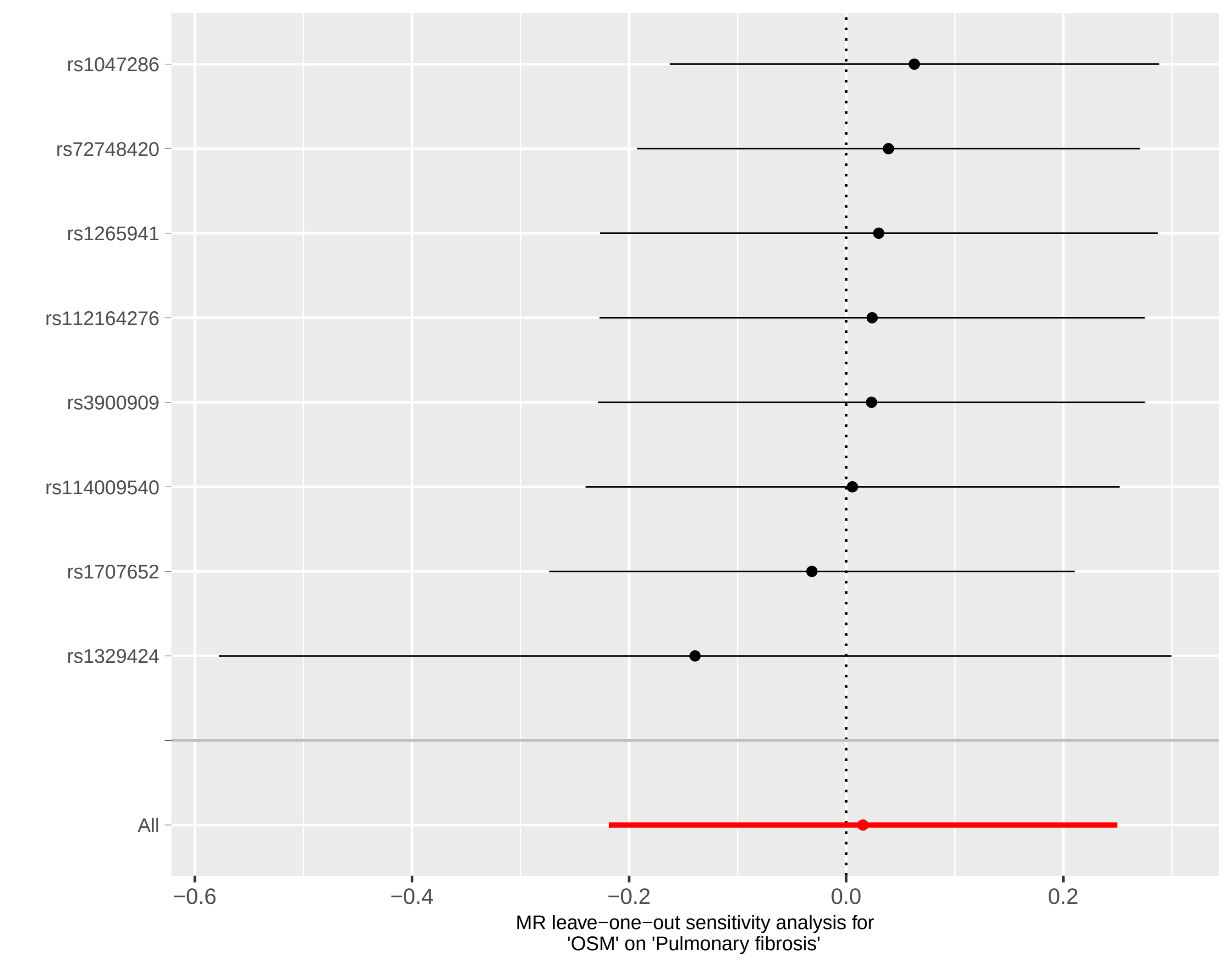
rs1047286
rs72748420
rs1265941
rs112164276
rs3900909
rs114009540
rs1707652
rs1329424
All
−0.6
−0.4
−0.2
0.0
0.2
MR leave−one−out sensitivity analysis for
'OSM' on 'Pulmonary fibrosis'

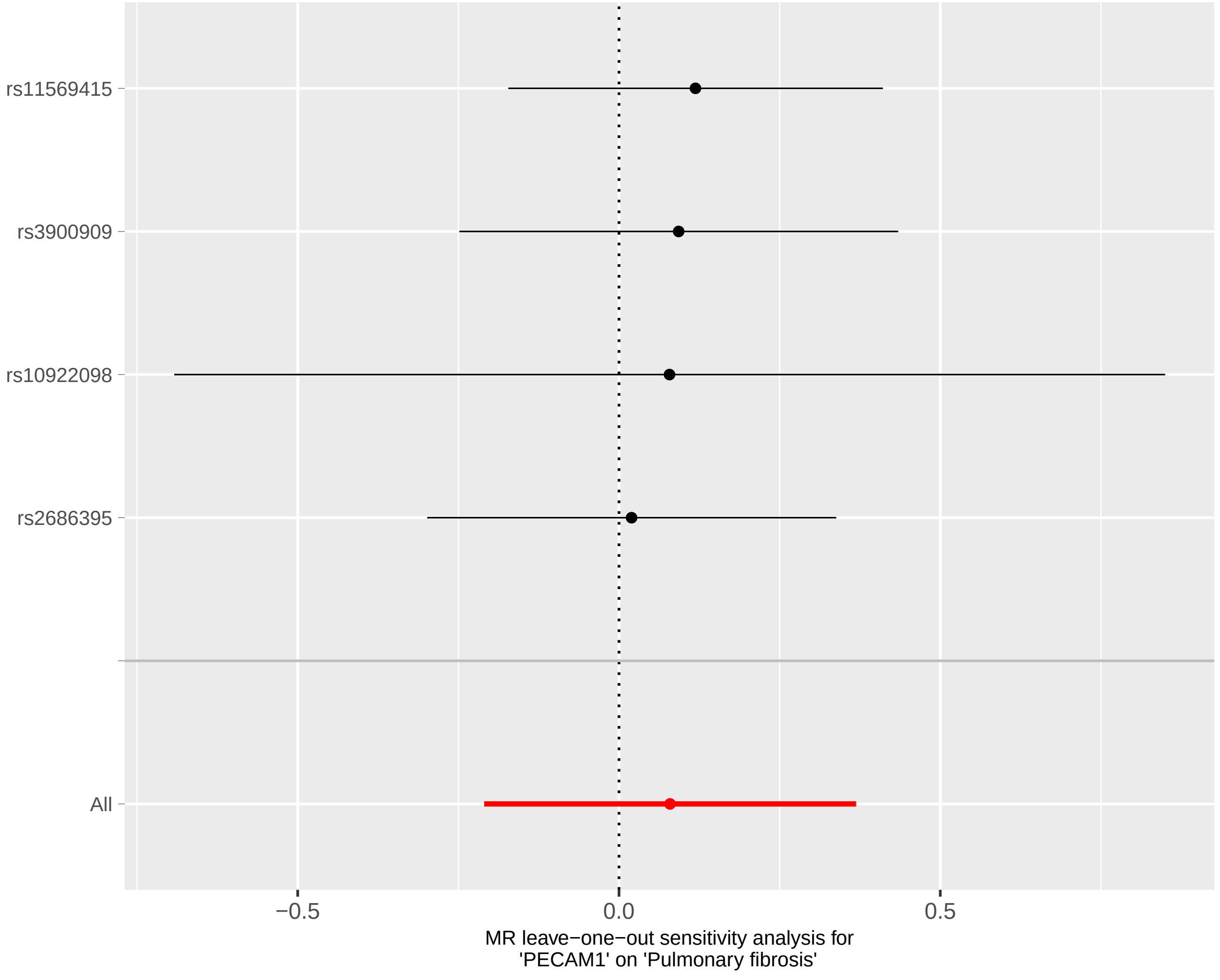
rs11569415
rs3900909
rs10922098
rs2686395
All
−0.5
0.0
0.5
MR leave−one−out sensitivity analysis for
'PECAM1' on 'Pulmonary fibrosis'

Insufficient number of SNPs

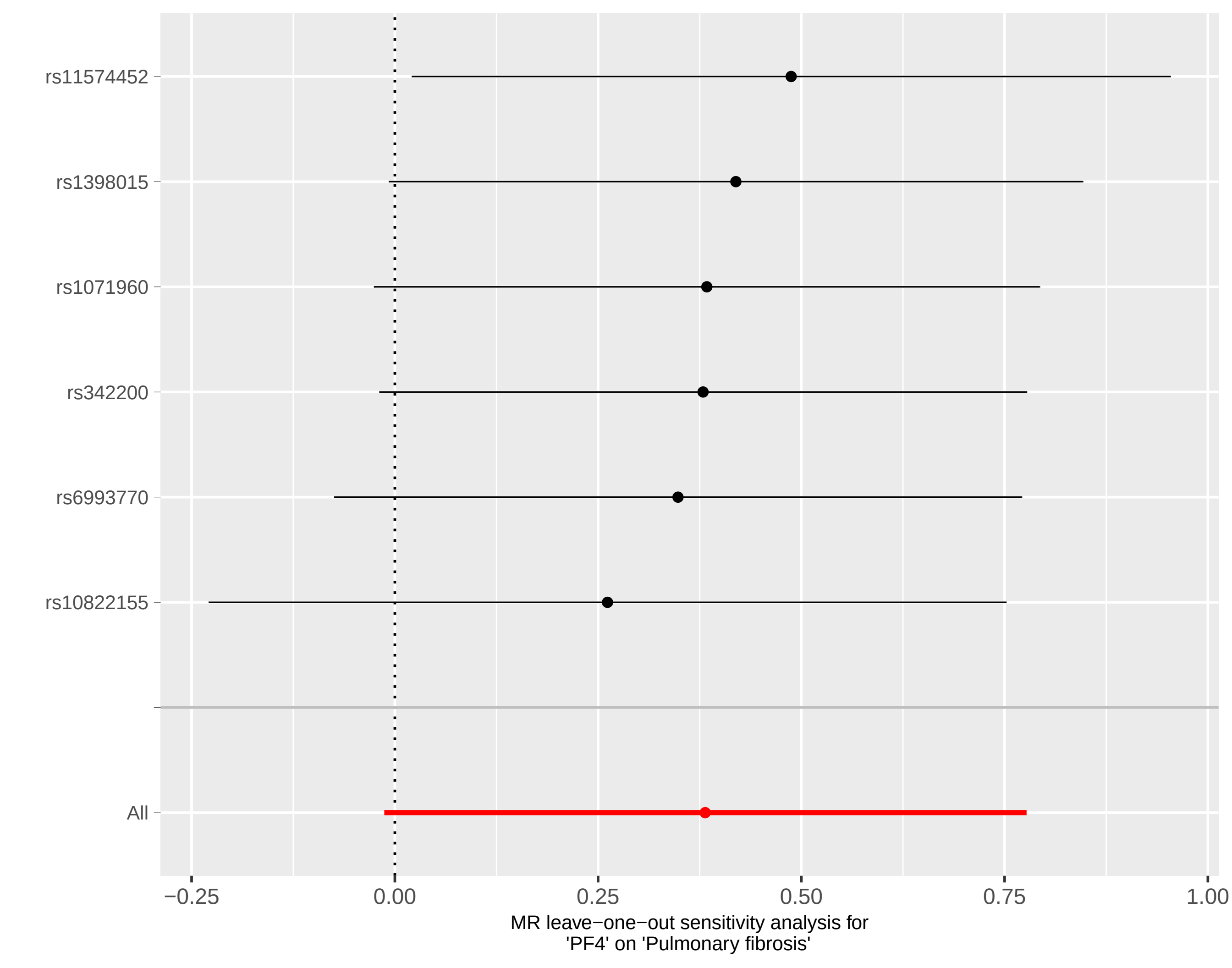

rs11574452
rs1398015
rs1071960
rs342200
rs6993770
rs10822155
All
−0.25
0.00
0.25
0.50
0.75
1.00
MR leave−one−out sensitivity analysis for
'PF4' on 'Pulmonary fibrosis'

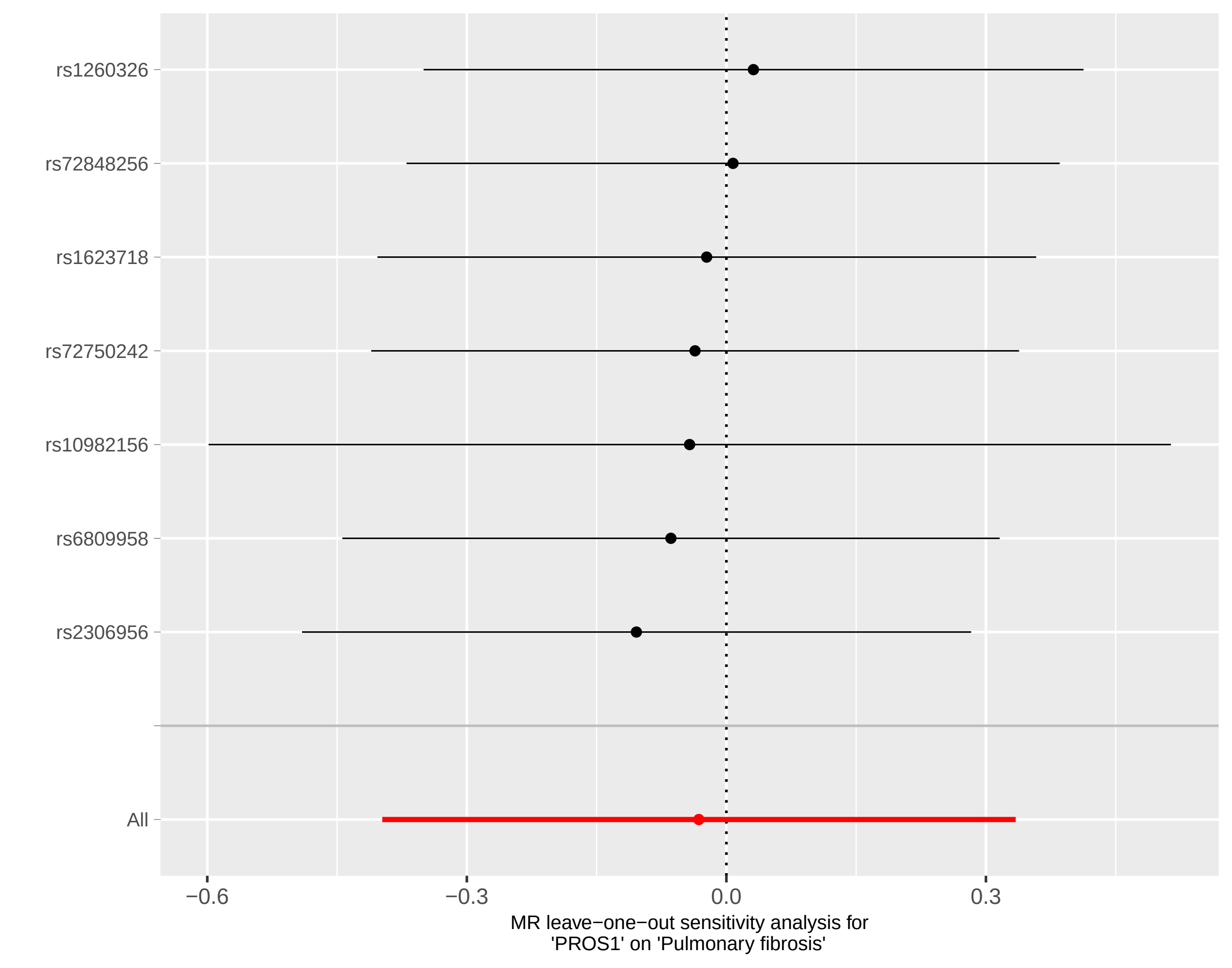

rs1260326
rs72848256
rs1623718
rs72750242
rs10982156
rs6809958
rs2306956
All
−0.6
−0.3
0.0
0.3
MR leave−one−out sensitivity analysis for
'PROS1' on 'Pulmonary fibrosis'

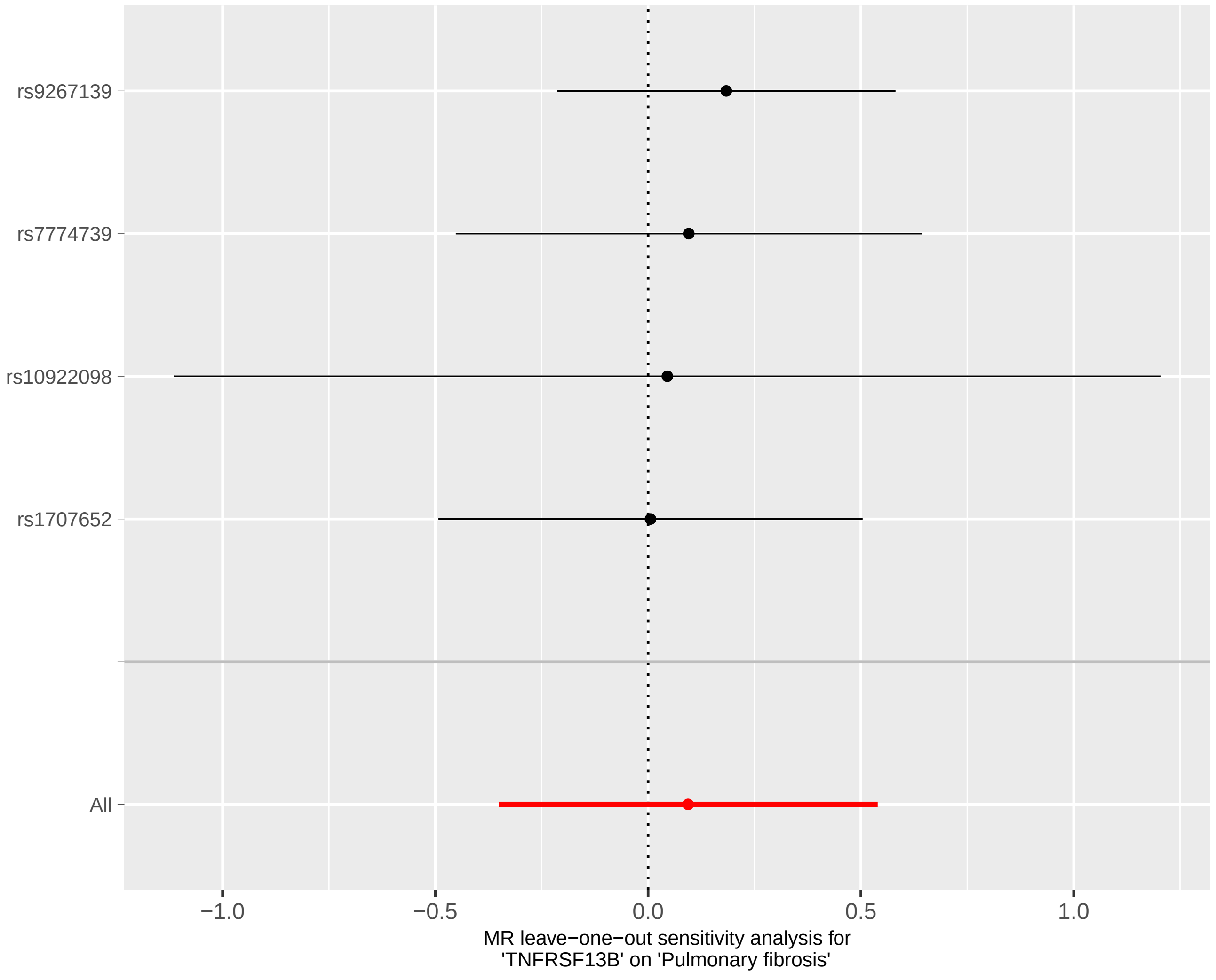
rs9267139
rs7774739
rs10922098
rs1707652
All
−1.0
−0.5
0.0
0.5
1.0
MR leave−one−out sensitivity analysis for
'TNFRSF13B' on 'Pulmonary fibrosis'

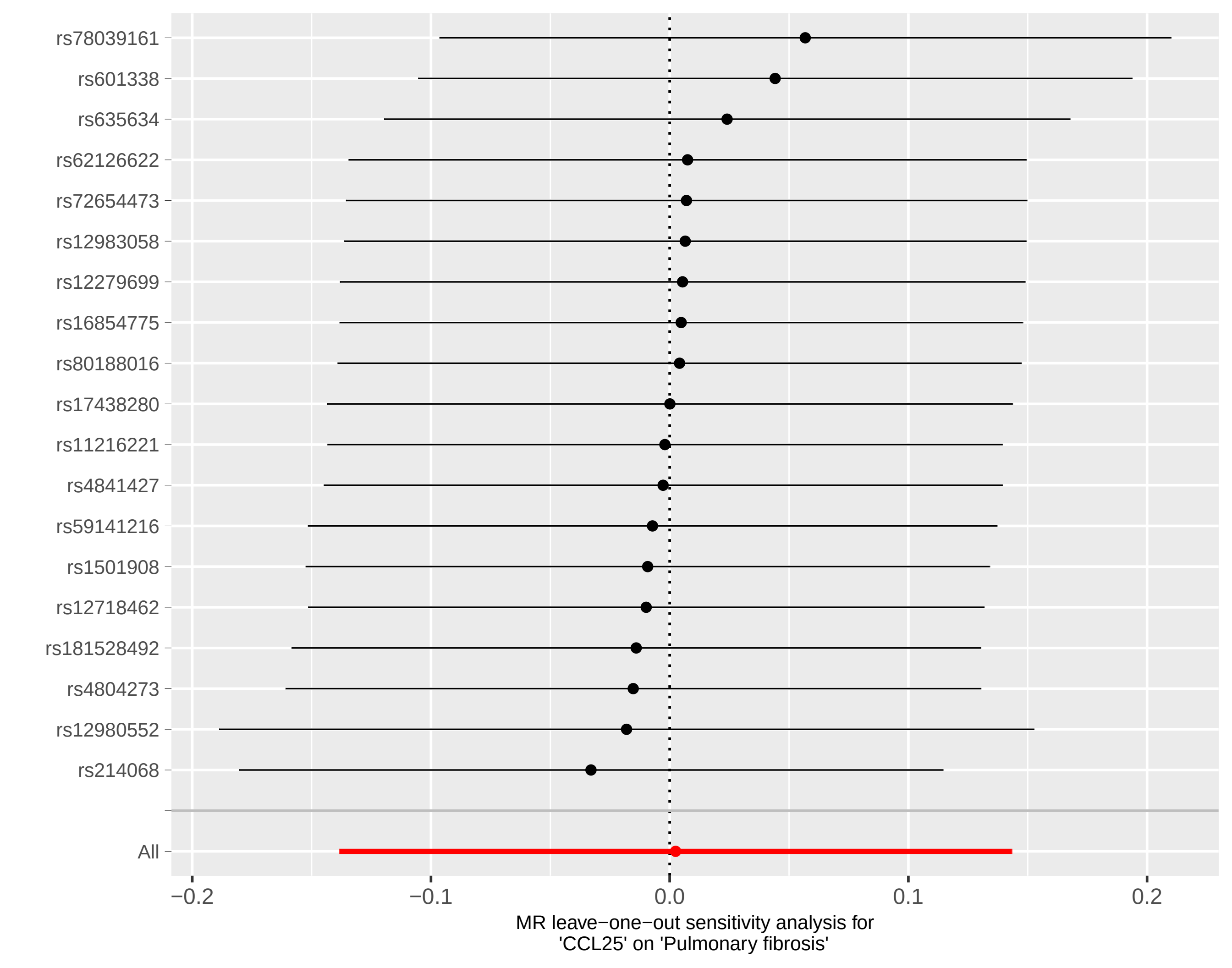
rs78039161
rs601338
rs635634
rs62126622
rs72654473
rs12983058
rs12279699
rs16854775
rs80188016
rs17438280
rs11216221
rs4841427
rs59141216
rs1501908
rs12718462
rs181528492
rs4804273
rs12980552
rs214068
All
−0.2
−0.1
0.0
0.1
0.2
MR leave−one−out sensitivity analysis for
'CCL25' on 'Pulmonary fibrosis'

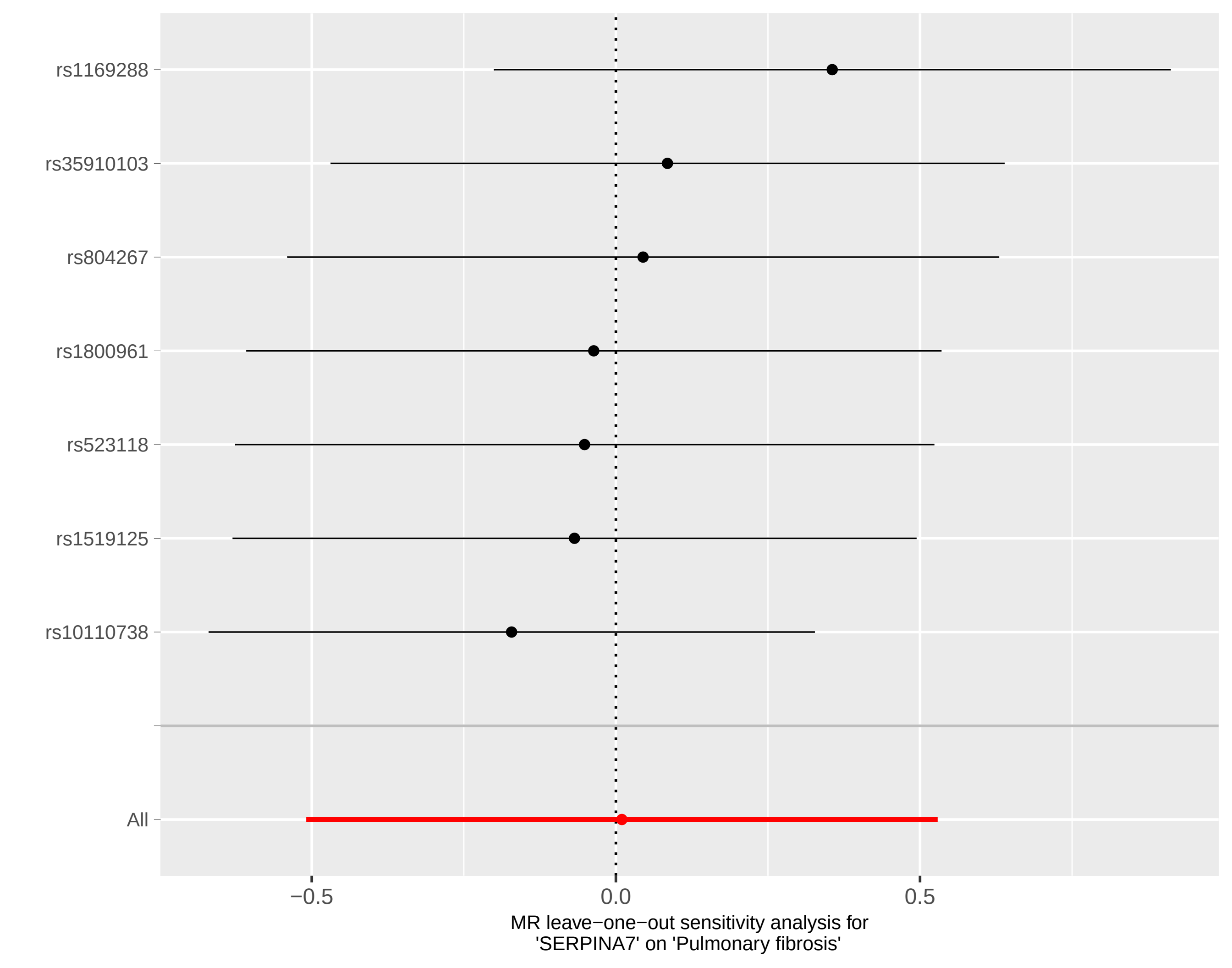
rs1169288
rs35910103
rs804267
rs1800961
rs523118
rs1519125
rs10110738
All
−0.5
0.0
0.5
MR leave−one−out sensitivity analysis for
'SERPINA7' on 'Pulmonary fibrosis'

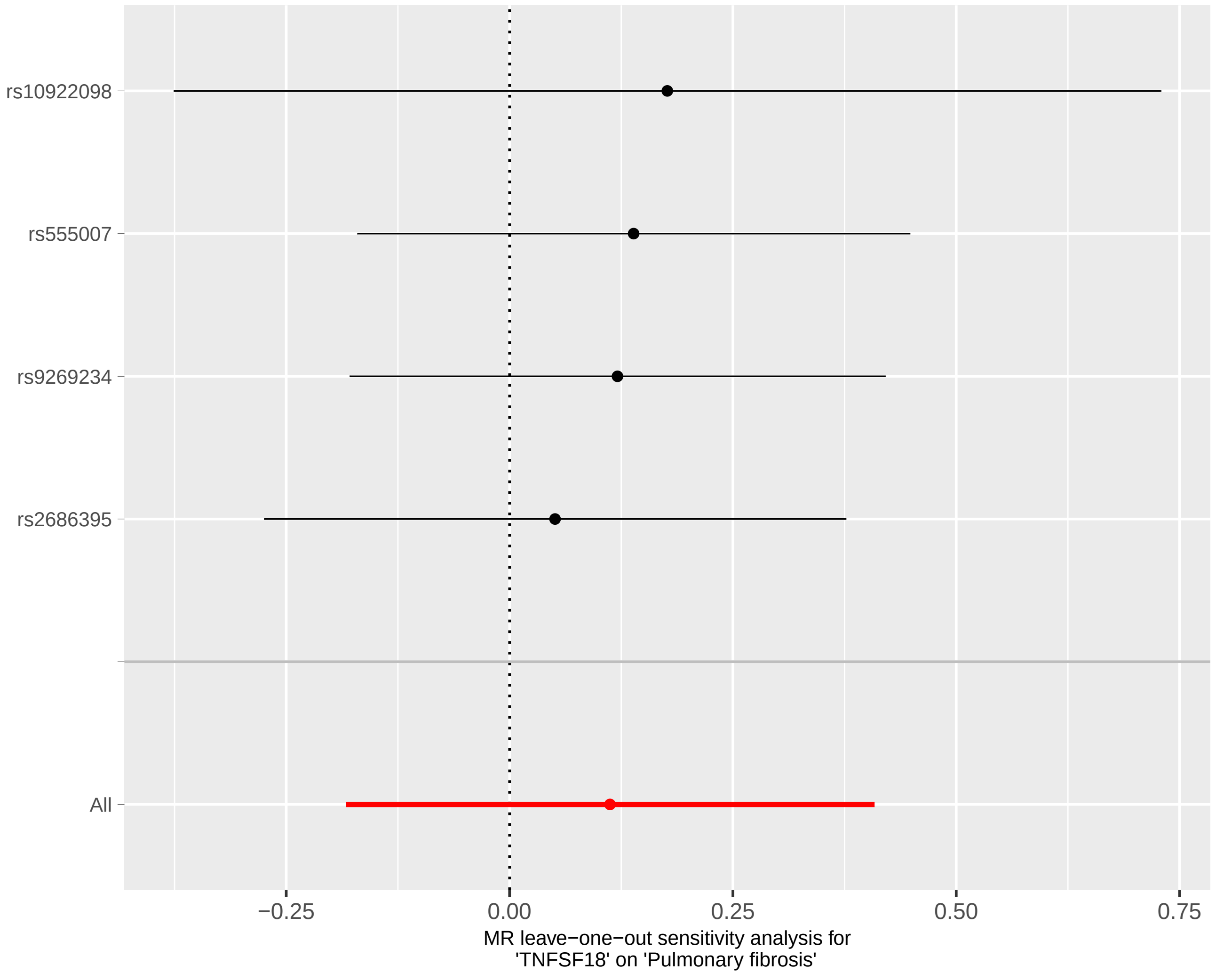

rs10922098
rs555007
rs9269234
rs2686395
All
−0.25
0.00
0.25
0.50
0.75
MR leave−one−out sensitivity analysis for
'TNFSF18' on 'Pulmonary fibrosis'

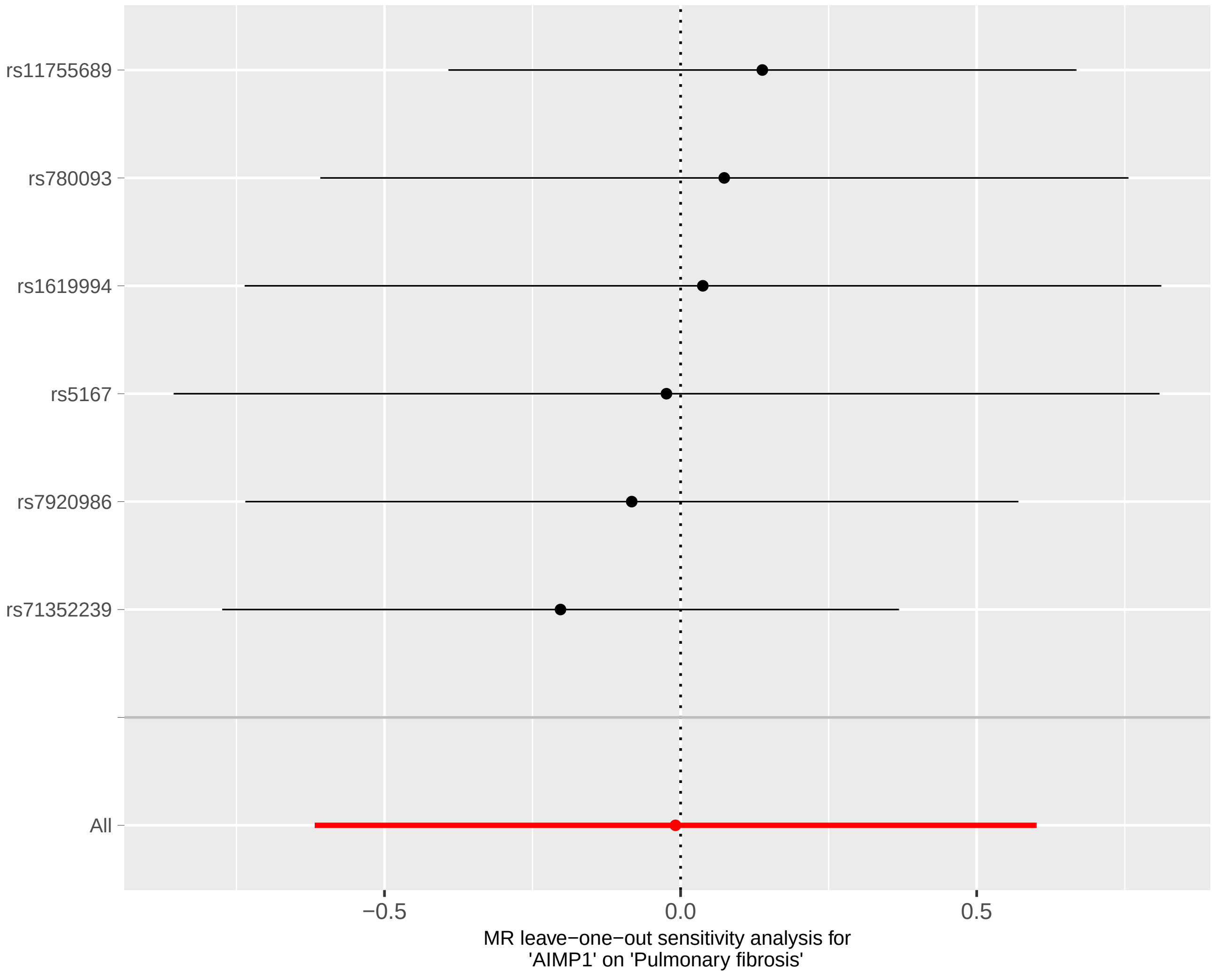
rs11755689
rs780093
rs1619994
rs5167
rs7920986
rs71352239
All
−0.5
0.0
0.5
MR leave−one−out sensitivity analysis for
'AIMP1' on 'Pulmonary fibrosis'

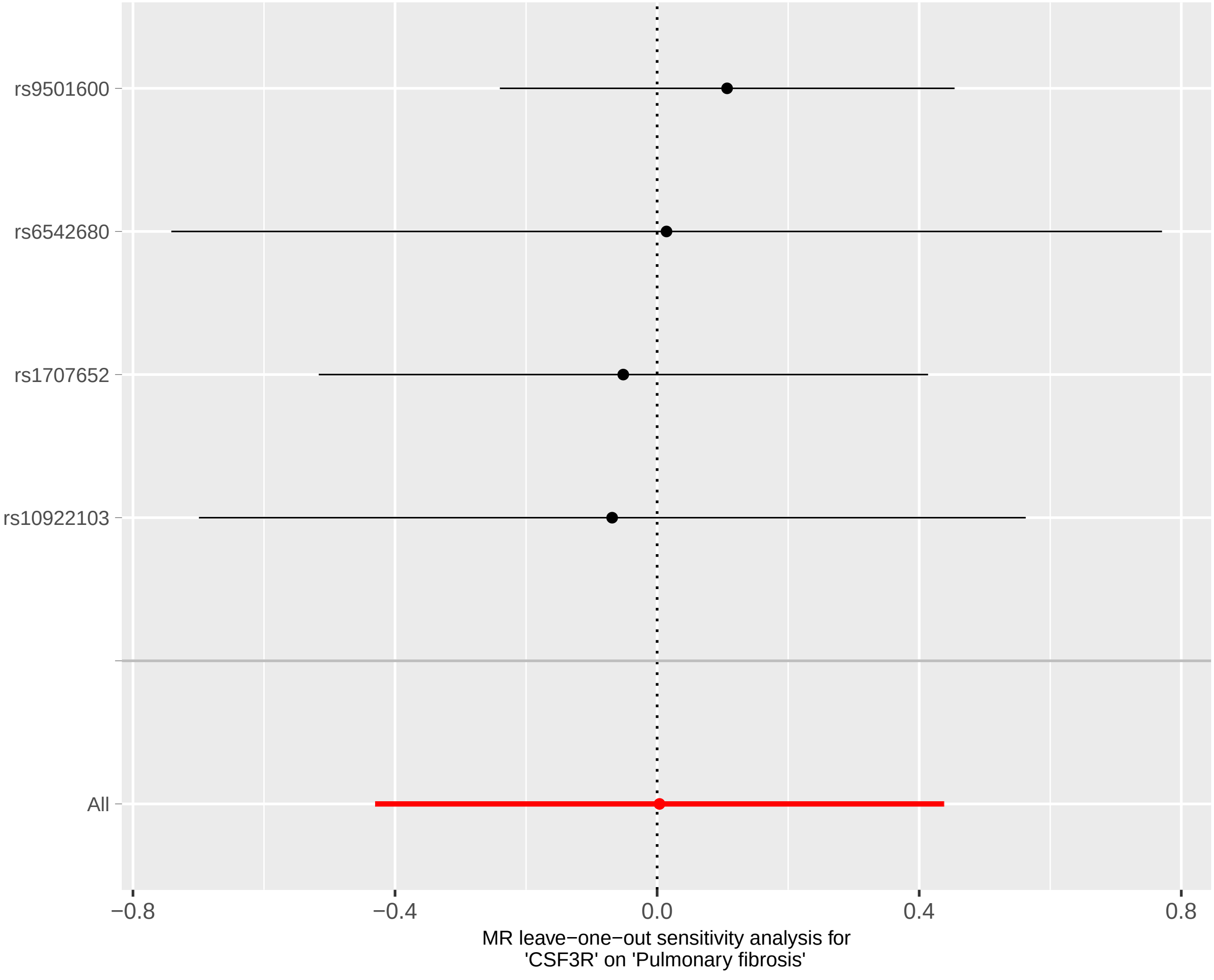
rs9501600
rs6542680
rs1707652
rs10922103
All
−0.8
−0.4
0.0
0.4
0.8
MR leave−one−out sensitivity analysis for
'CSF3R' on 'Pulmonary fibrosis'

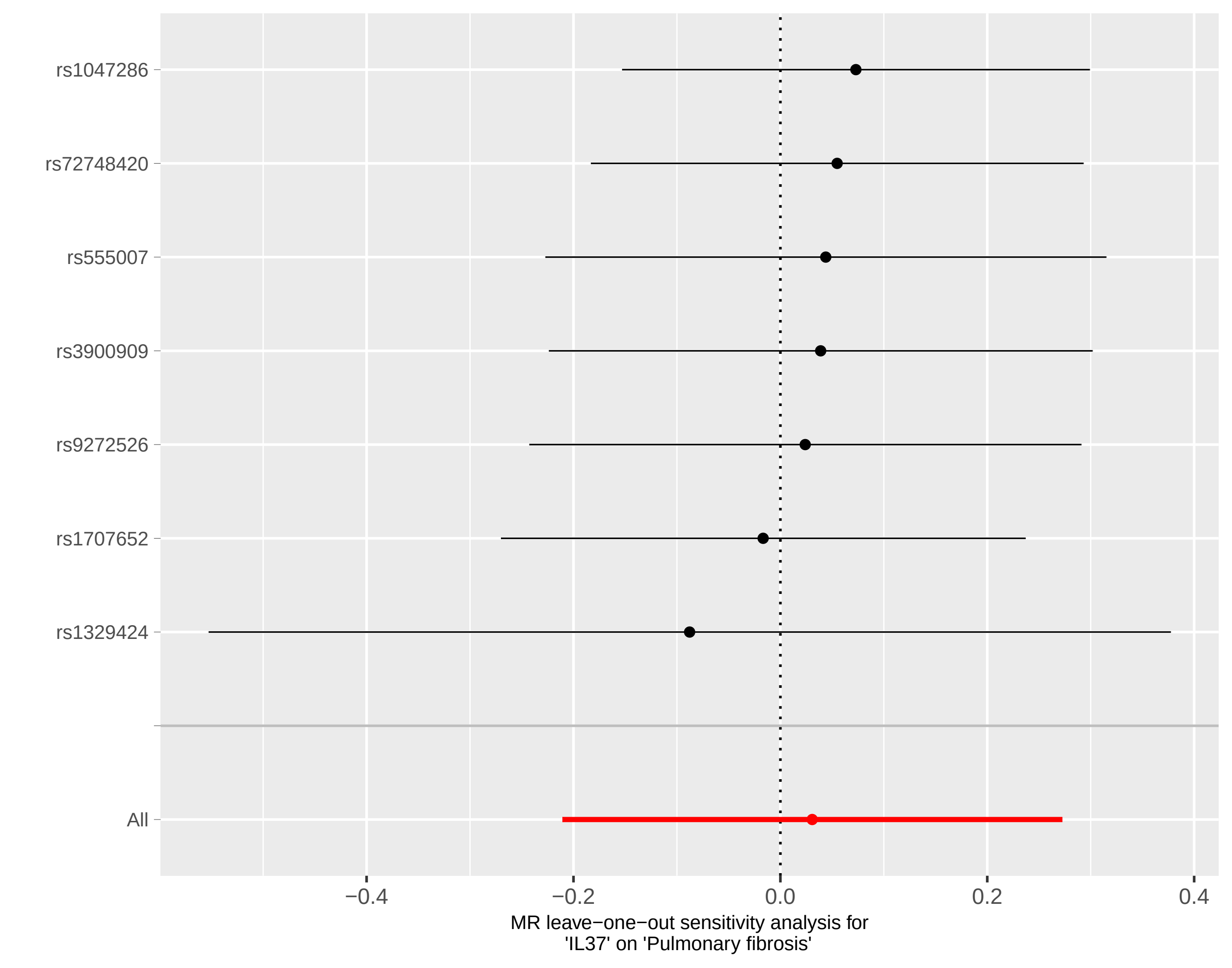
rs1047286
rs72748420
rs555007
rs3900909
rs9272526
rs1707652
rs1329424
All
−0.4
−0.2
0.0
0.2
0.4
MR leave−one−out sensitivity analysis for
'IL37' on 'Pulmonary fibrosis'

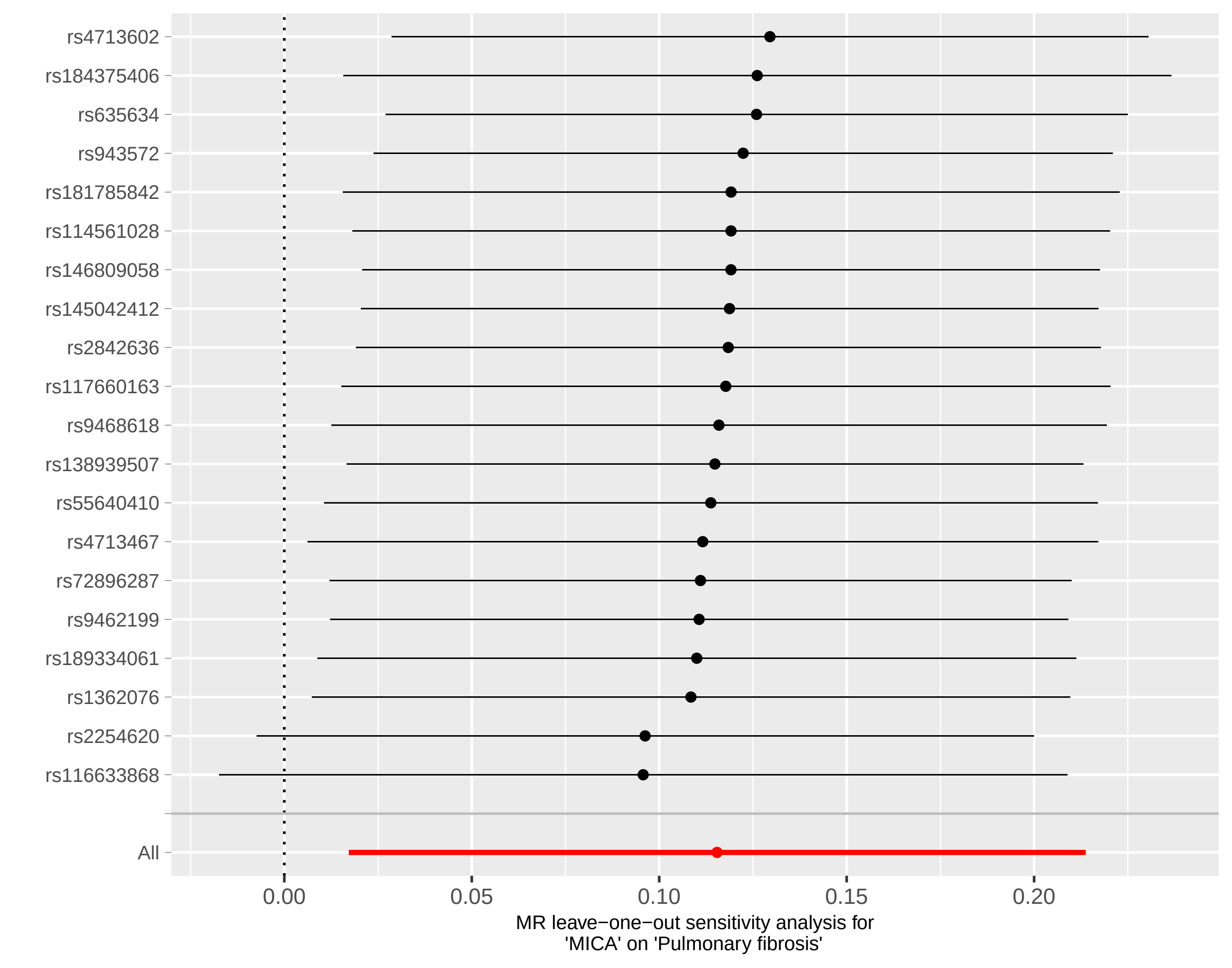
rs4713602
rs184375406
rs635634
rs943572
rs181785842
rs114561028
rs146809058
rs145042412
rs2842636
rs117660163
rs9468618
rs138939507
rs55640410
rs4713467
rs72896287
rs9462199
rs189334061
rs1362076
rs2254620
rs116633868
All
0.00
0.05
0.10
0.15
0.20
MR leave−one−out sensitivity analysis for
'MICA' on 'Pulmonary fibrosis'

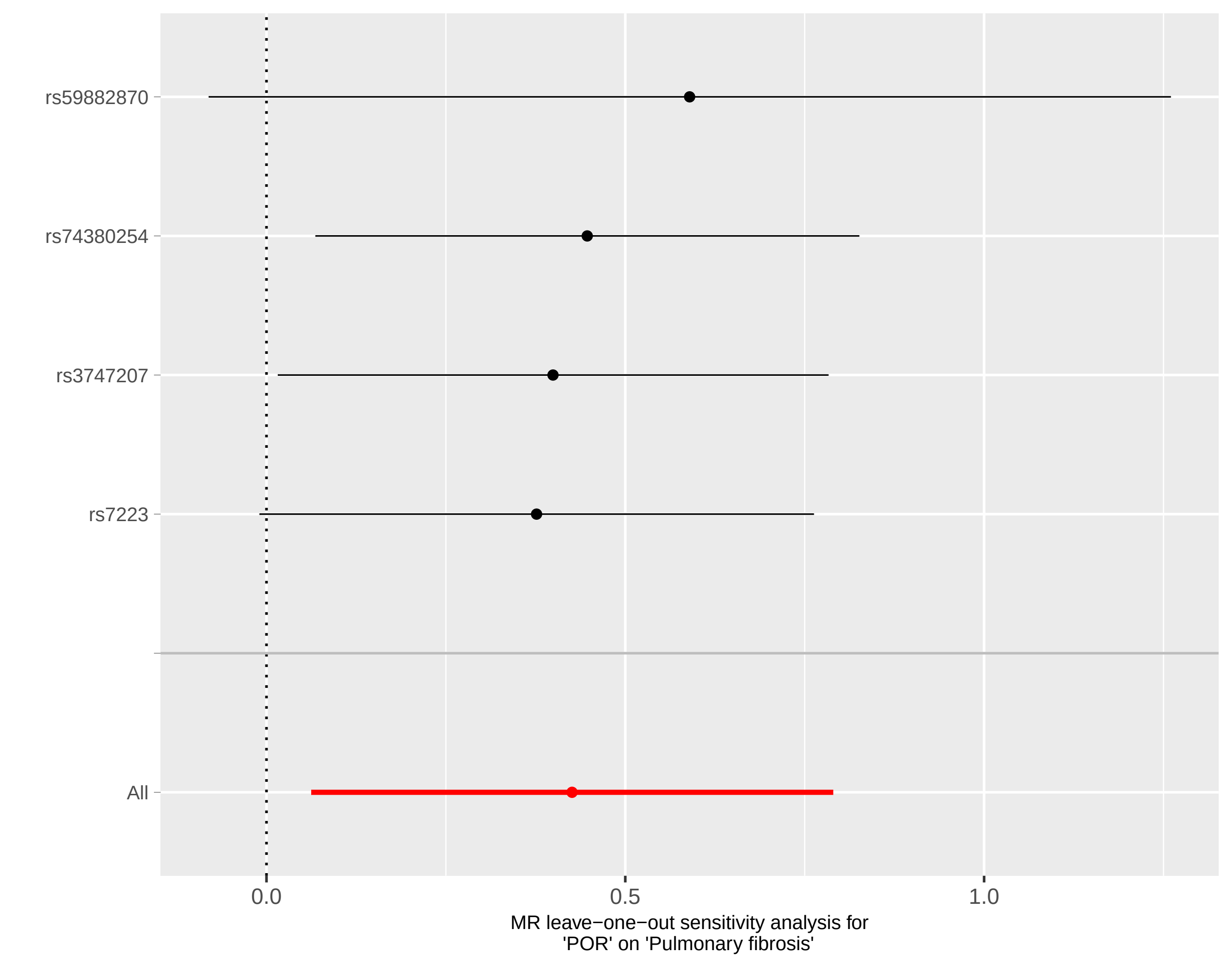
rs59882870
rs74380254
rs3747207
rs7223
All
0.0
0.5
1.0
MR leave−one−out sensitivity analysis for
'POR' on 'Pulmonary fibrosis'

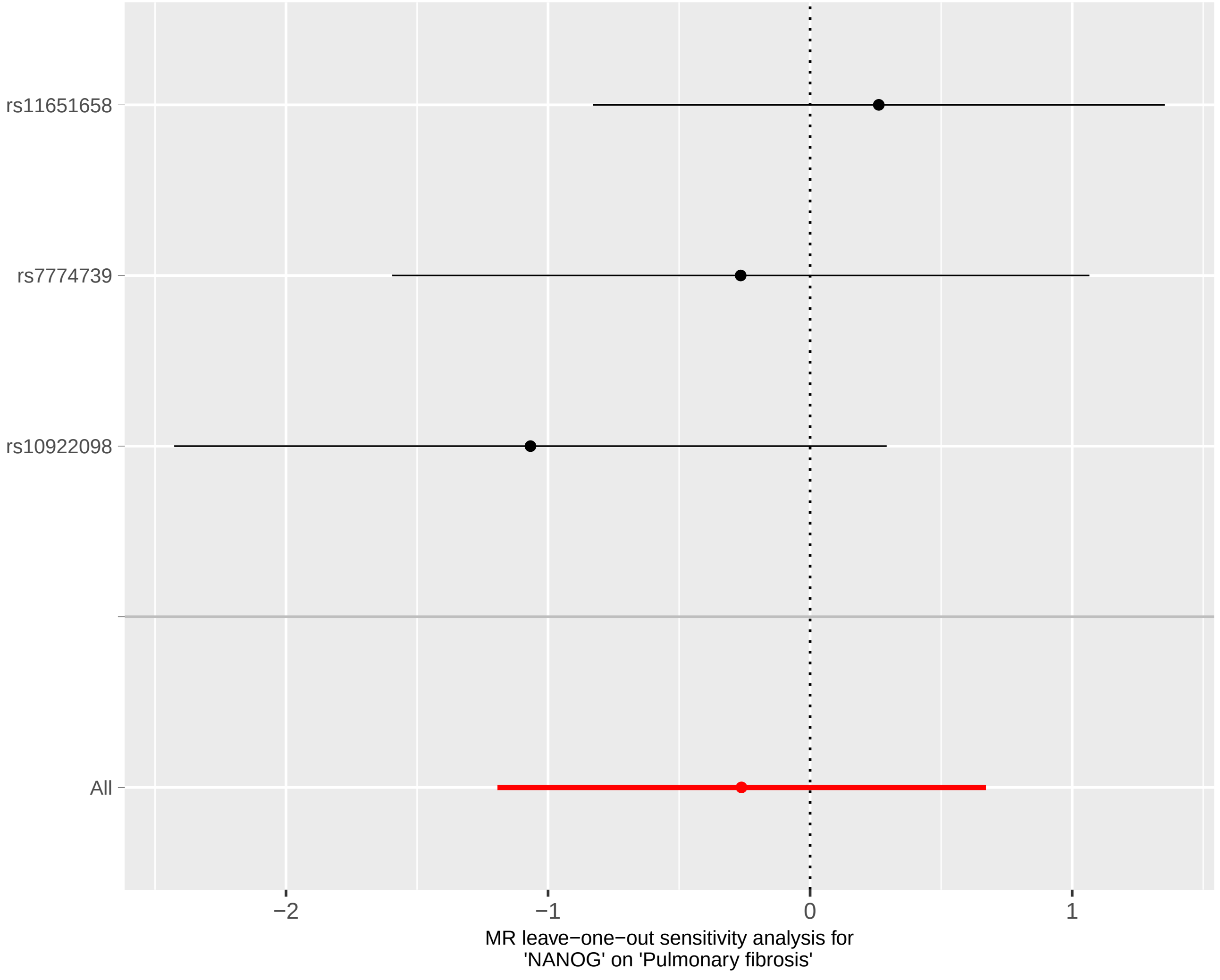
rs11651658
rs7774739
rs10922098
All
−2
−1
0
1
MR leave−one−out sensitivity analysis for
'NANOG' on 'Pulmonary fibrosis'

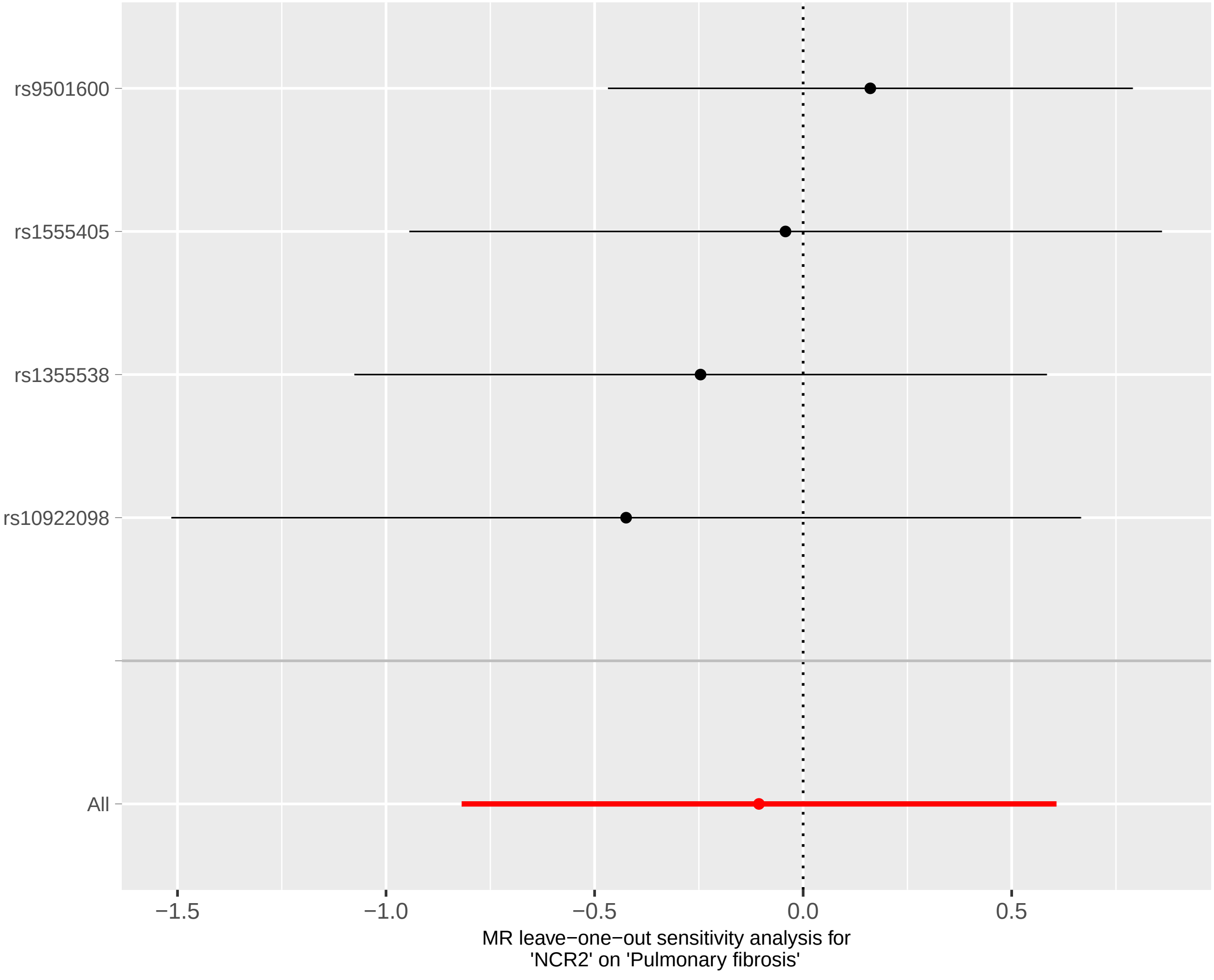
rs9501600
rs1555405
rs1355538
rs10922098
All
−1.5
−1.0
−0.5
0.0
0.5
MR leave−one−out sensitivity analysis for
'NCR2' on 'Pulmonary fibrosis'

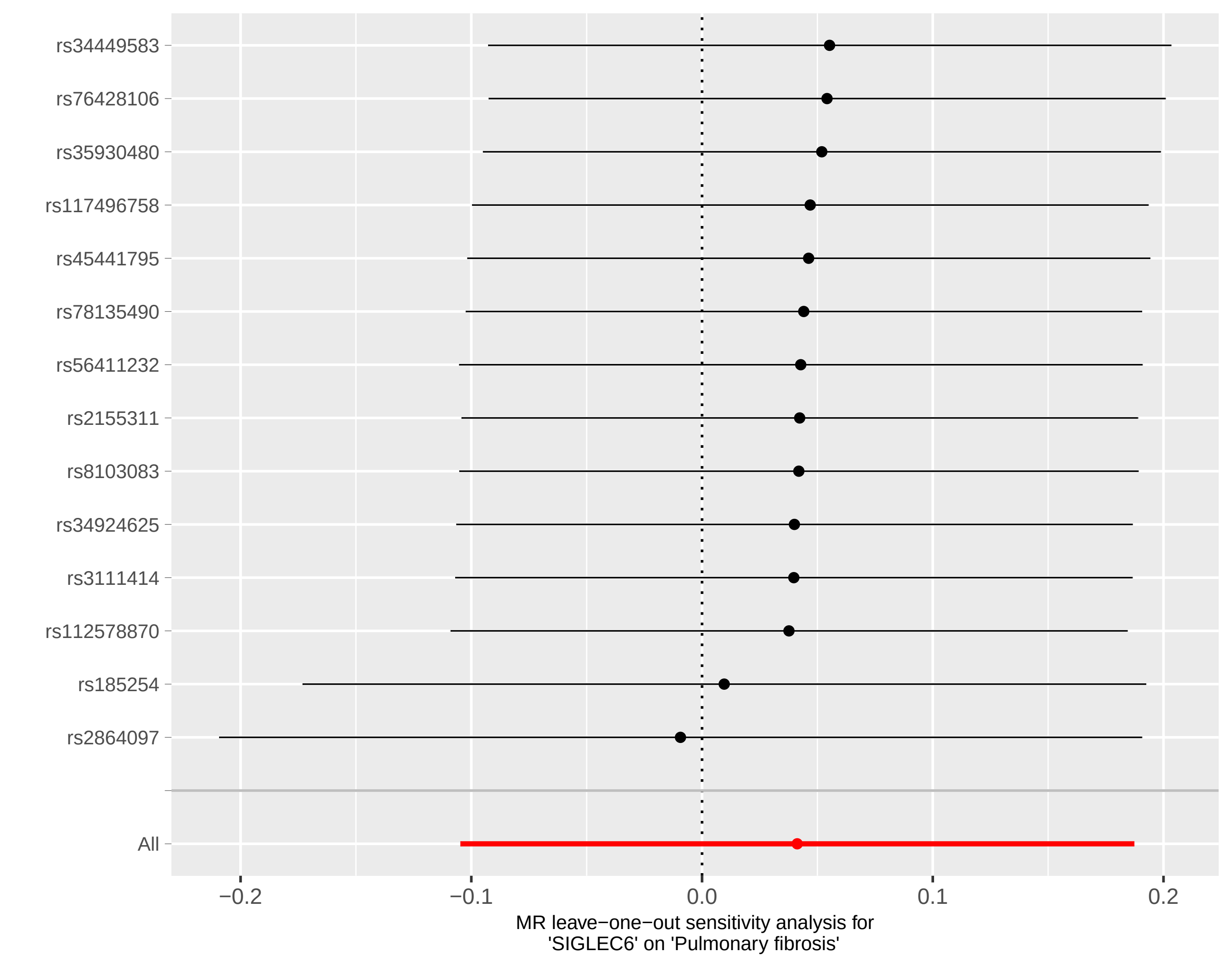

rs34449583
rs76428106
rs35930480
rs117496758
rs45441795
rs78135490
rs56411232
rs2155311
rs8103083
rs34924625
rs3111414
rs112578870
rs185254
rs2864097
All
−0.2
−0.1
0.0
0.1
0.2
MR leave−one−out sensitivity analysis for
'SIGLEC6' on 'Pulmonary fibrosis'

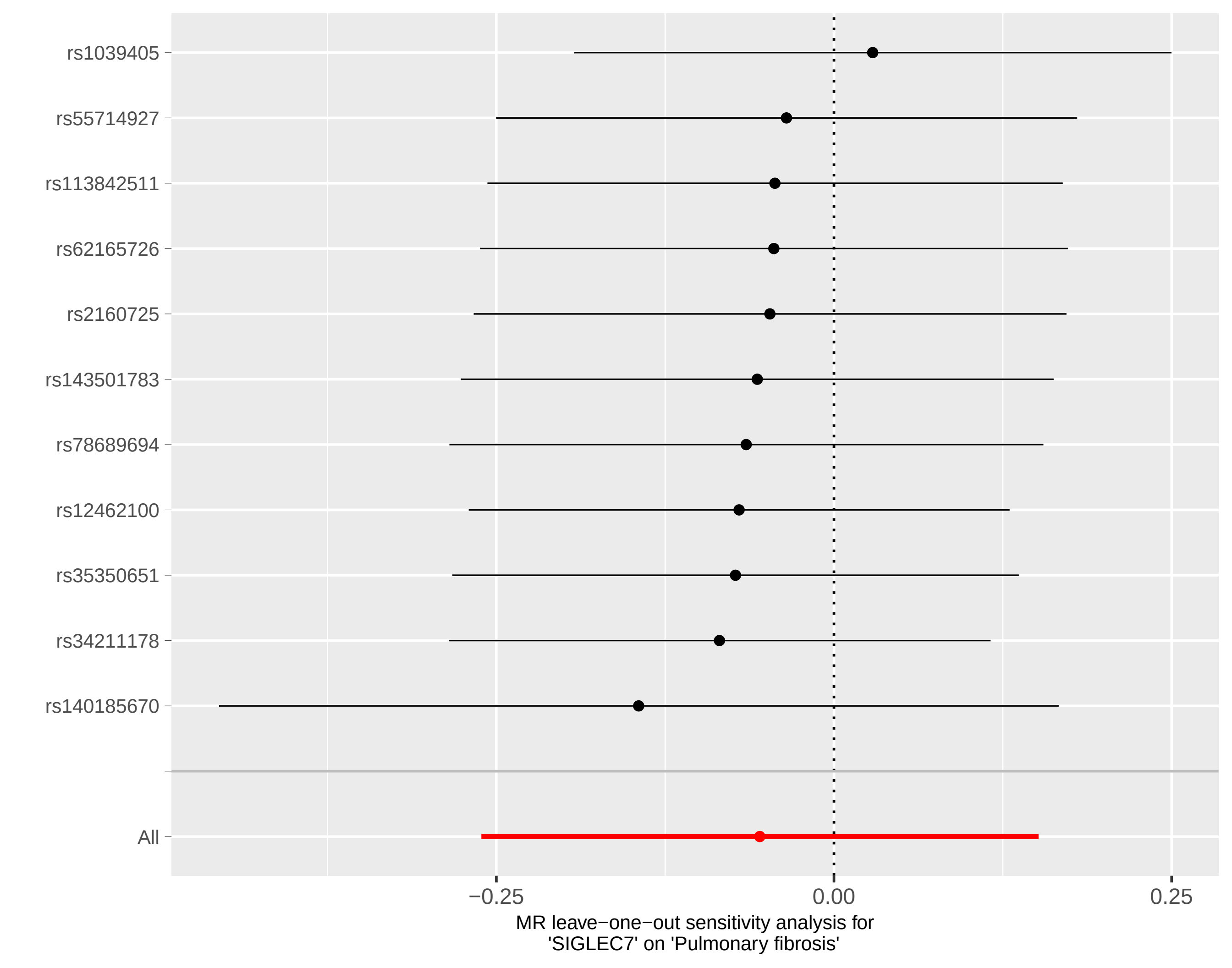
rs1039405
rs55714927
rs113842511
rs62165726
rs2160725
rs143501783
rs78689694
rs12462100
rs35350651
rs34211178
rs140185670
All
−0.25
0.00
0.25
MR leave−one−out sensitivity analysis for
'SIGLEC7' on 'Pulmonary fibrosis'

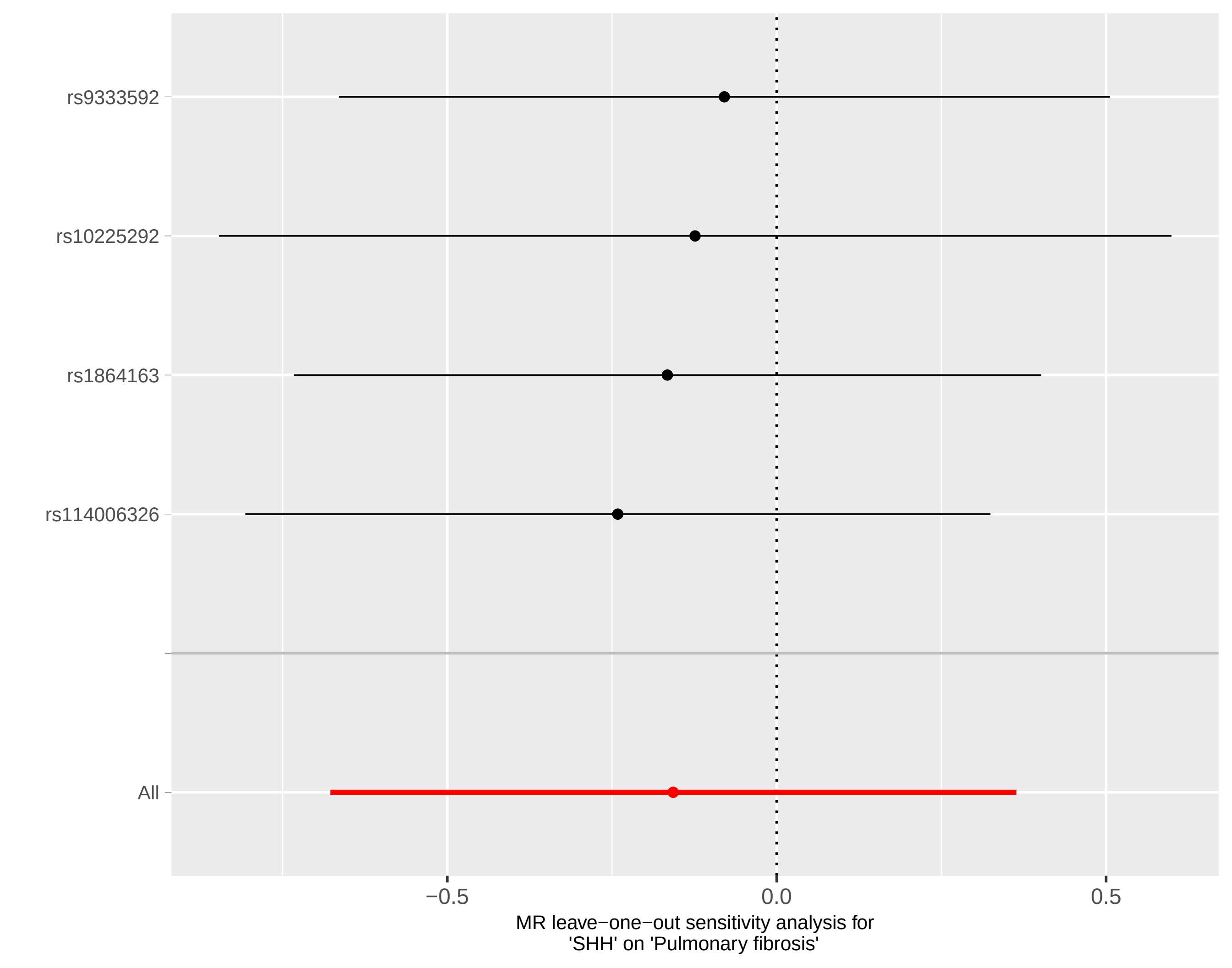
rs9333592
rs10225292
rs1864163
rs114006326
All
−0.5
0.0
0.5
MR leave−one−out sensitivity analysis for
'SHH' on 'Pulmonary fibrosis'

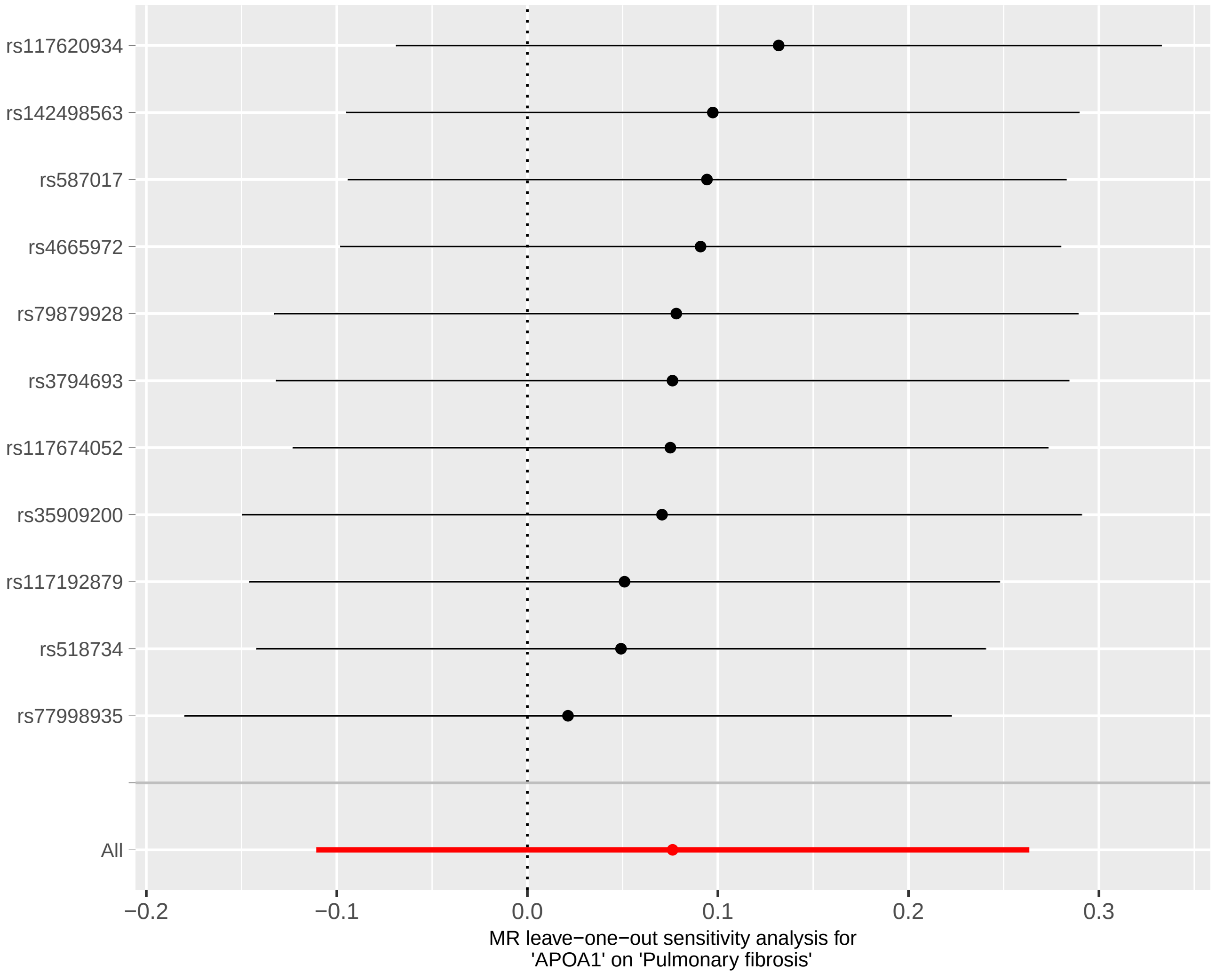
rs117620934
rs142498563
rs587017
rs4665972
rs79879928
rs3794693
rs117674052
rs35909200
rs117192879
rs518734
rs77998935
All
−0.2
−0.1
0.0
0.1
0.2
0.3
MR leave−one−out sensitivity analysis for
'APOA1' on 'Pulmonary fibrosis'

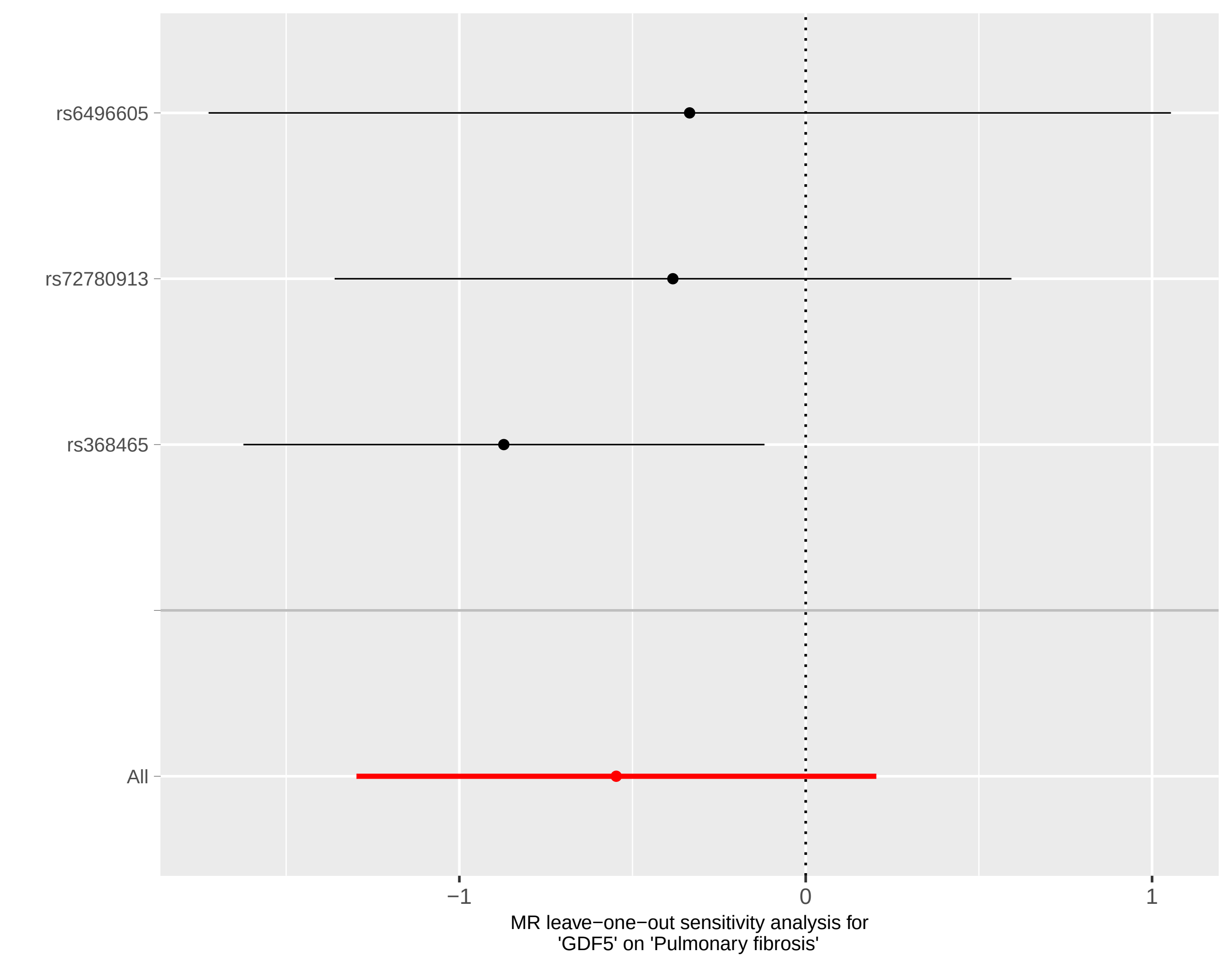
rs6496605
rs72780913
rs368465
All
−1
0
1
MR leave−one−out sensitivity analysis for
'GDF5' on 'Pulmonary fibrosis'

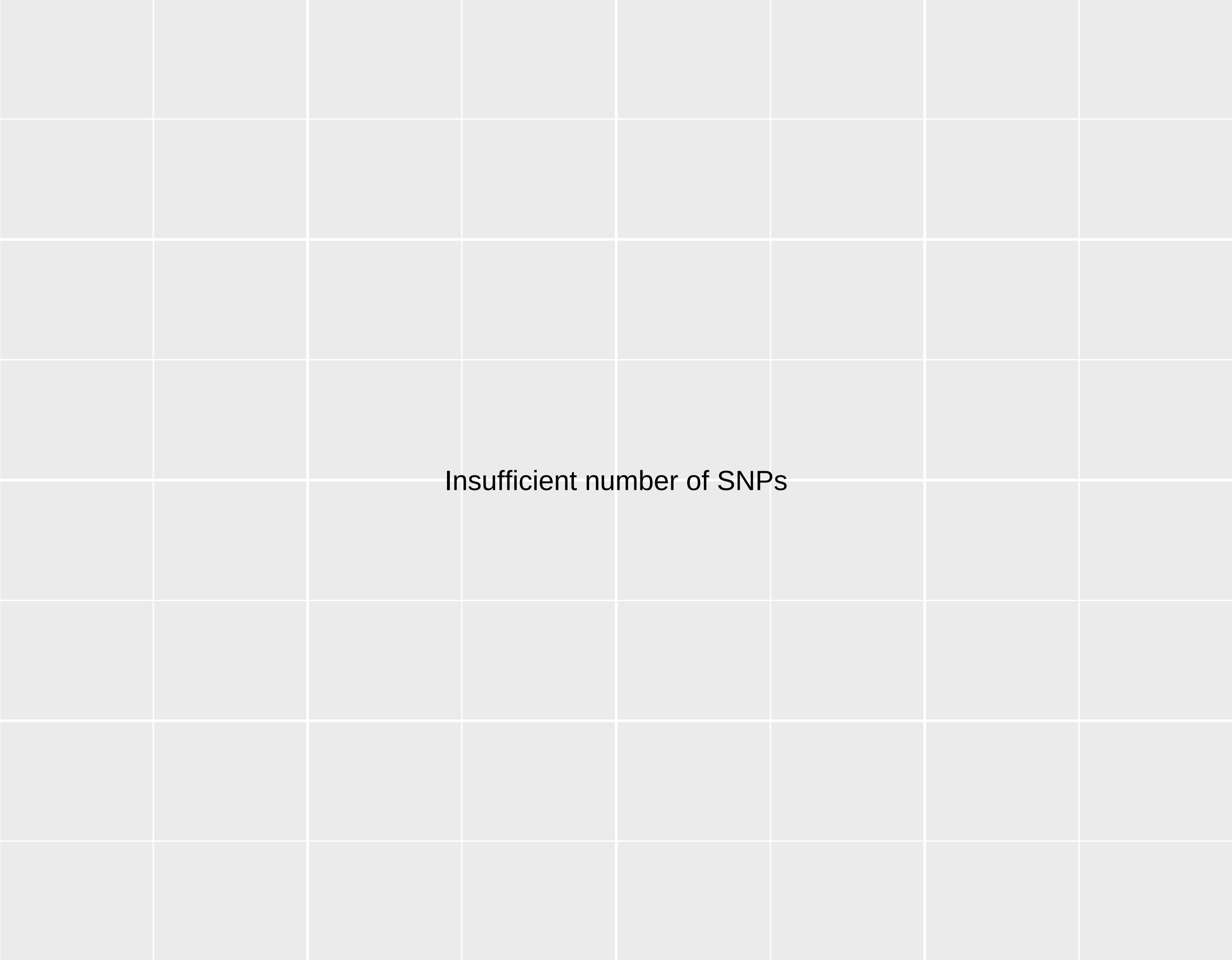
Insufficient number of SNPs

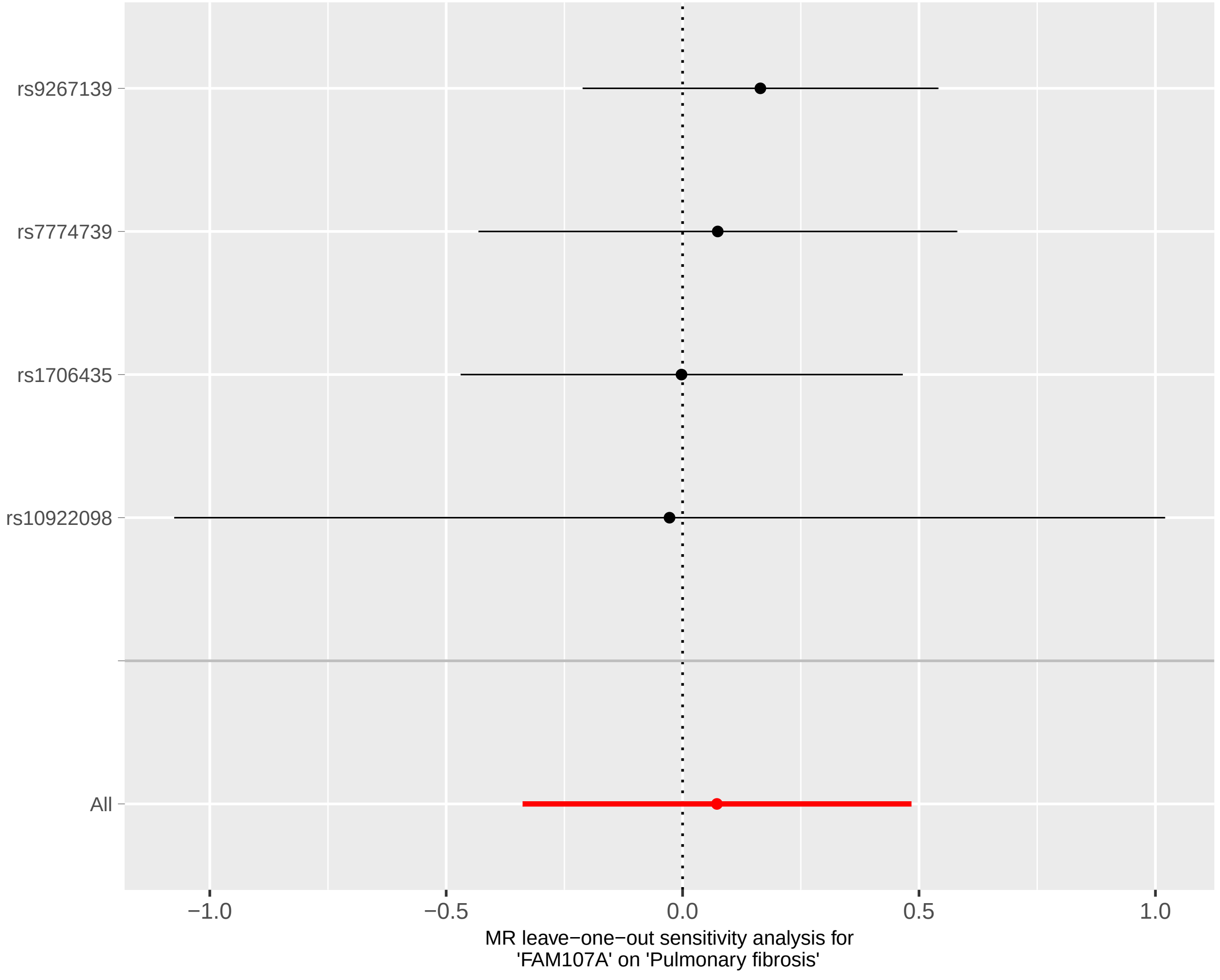
rs9267139
rs7774739
rs1706435
rs10922098
All
−1.0
−0.5
0.0
0.5
1.0
MR leave−one−out sensitivity analysis for
'FAM107A' on 'Pulmonary fibrosis'

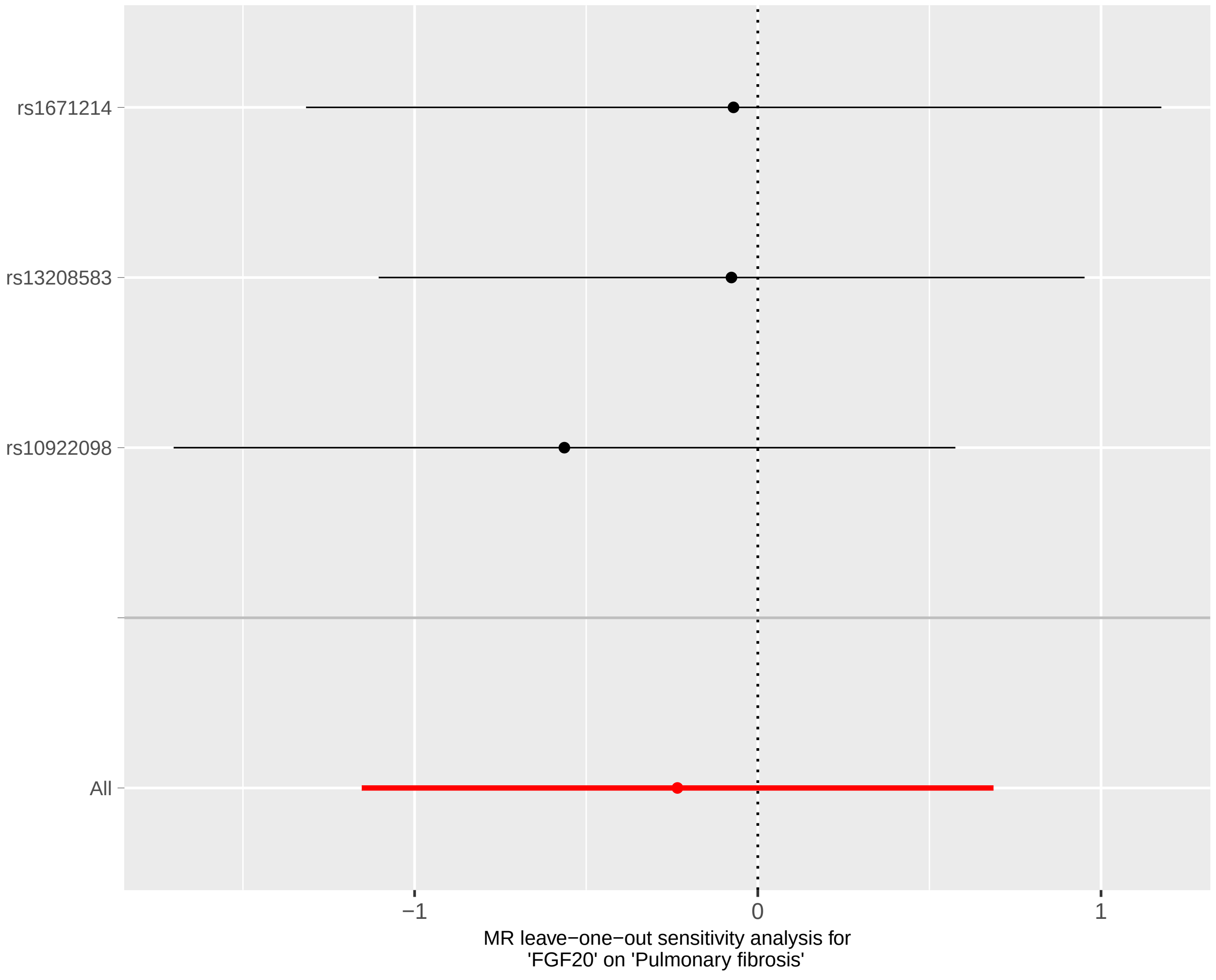
rs1671214
rs13208583
rs10922098
All
−1
0
1
MR leave−one−out sensitivity analysis for
'FGF20' on 'Pulmonary fibrosis'

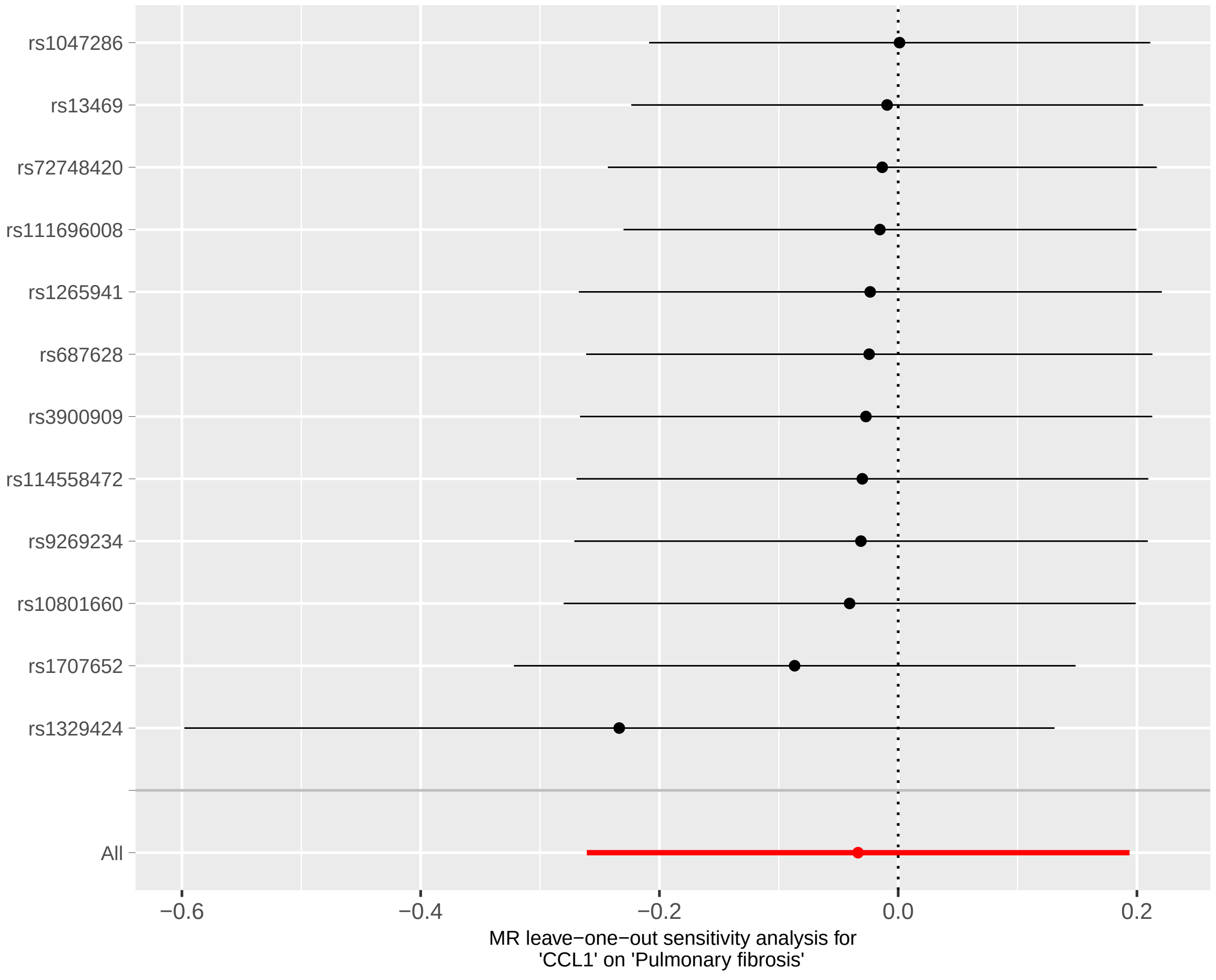
rs1047286
rs13469
rs72748420
rs111696008
rs1265941
rs687628
rs3900909
rs114558472
rs9269234
rs10801660
rs1707652
rs1329424
All
−0.6
−0.4
−0.2
0.0
0.2
MR leave−one−out sensitivity analysis for
'CCL1' on 'Pulmonary fibrosis'

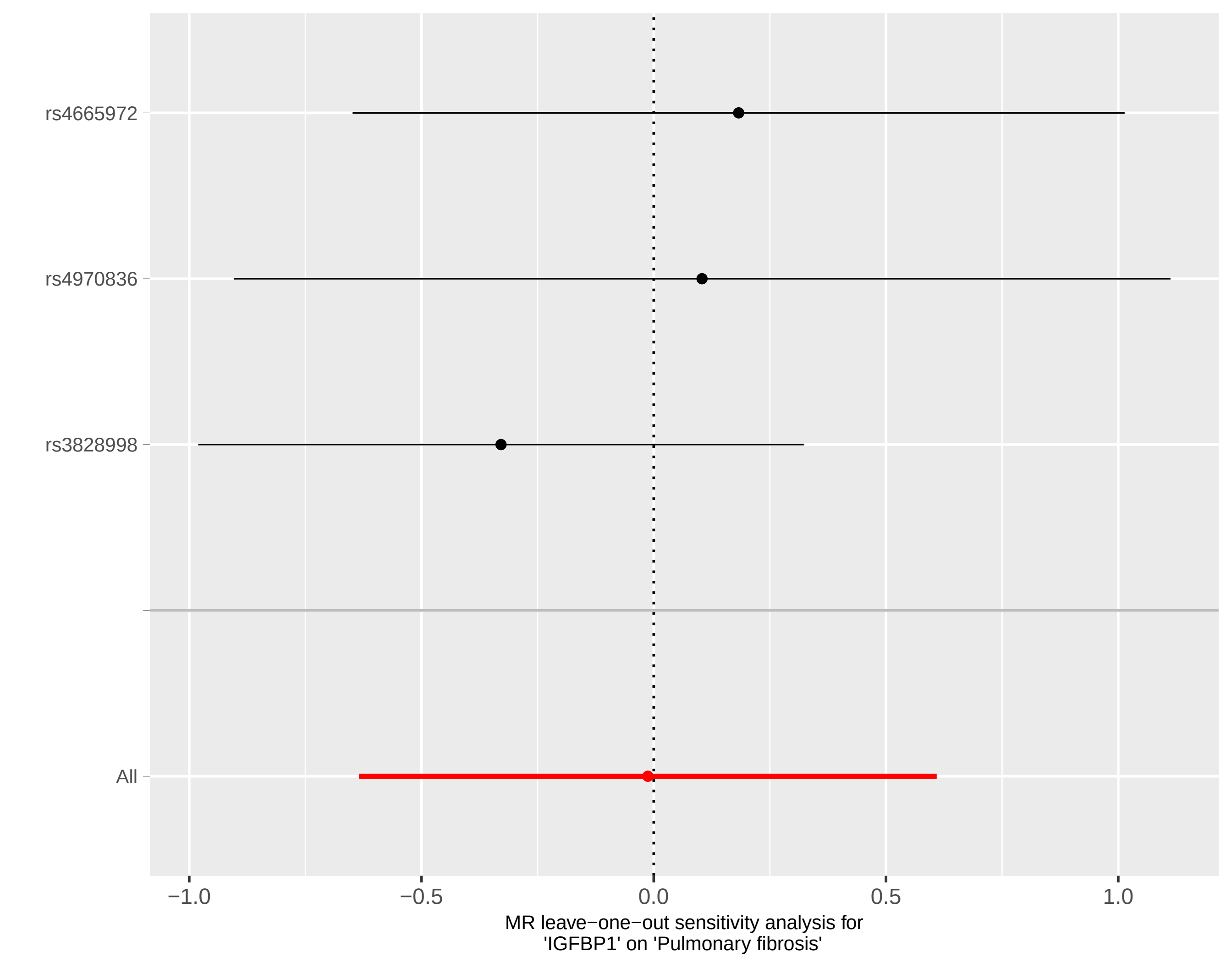
rs4665972
rs4970836
rs3828998
All
−1.0
−0.5
0.0
0.5
1.0
MR leave−one−out sensitivity analysis for
'IGFBP1' on 'Pulmonary fibrosis'

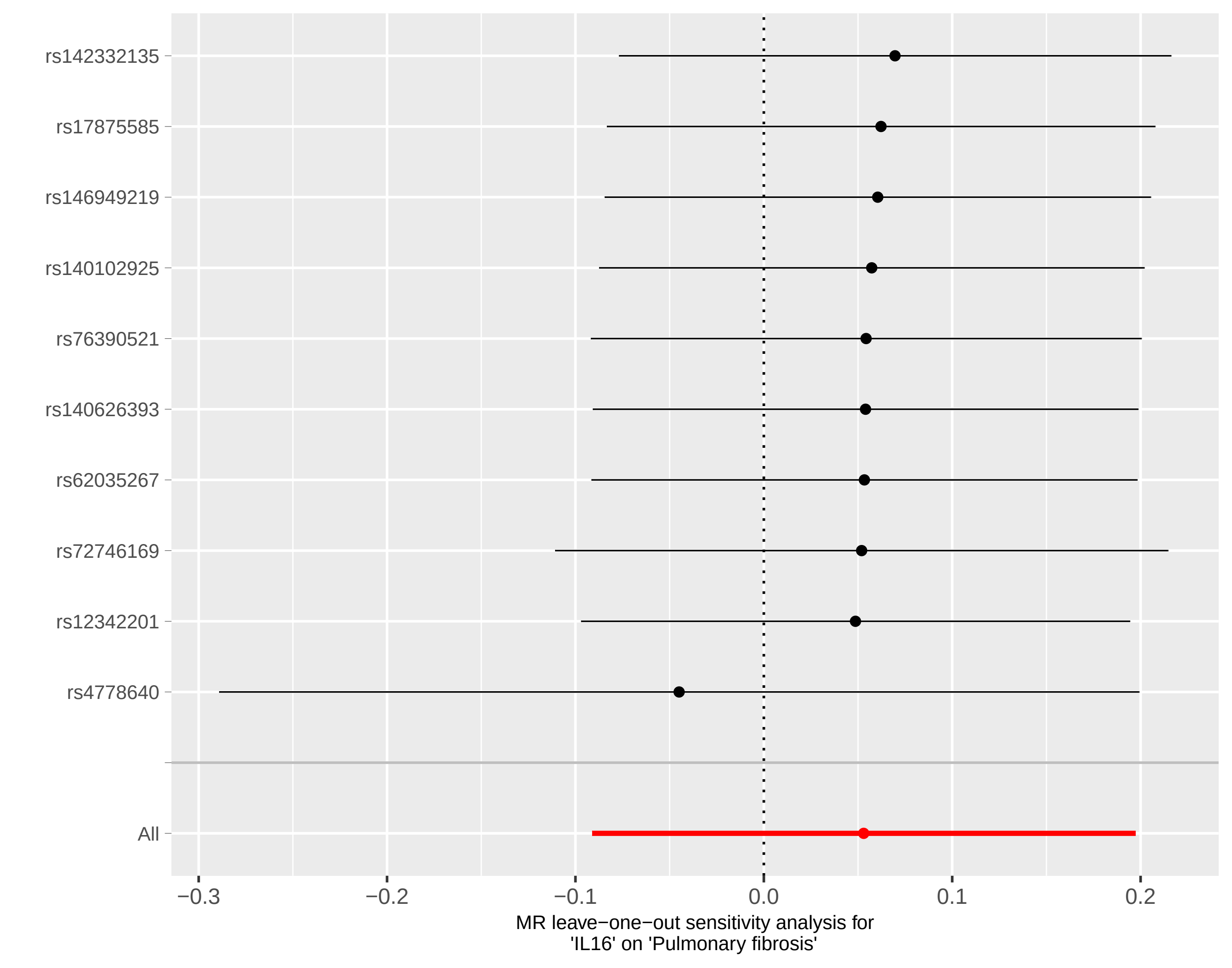
rs142332135
rs17875585
rs146949219
rs140102925
rs76390521
rs140626393
rs62035267
rs72746169
rs12342201
rs4778640
All
−0.3
−0.2
−0.1
0.0
0.1
0.2
MR leave−one−out sensitivity analysis for
'IL16' on 'Pulmonary fibrosis'

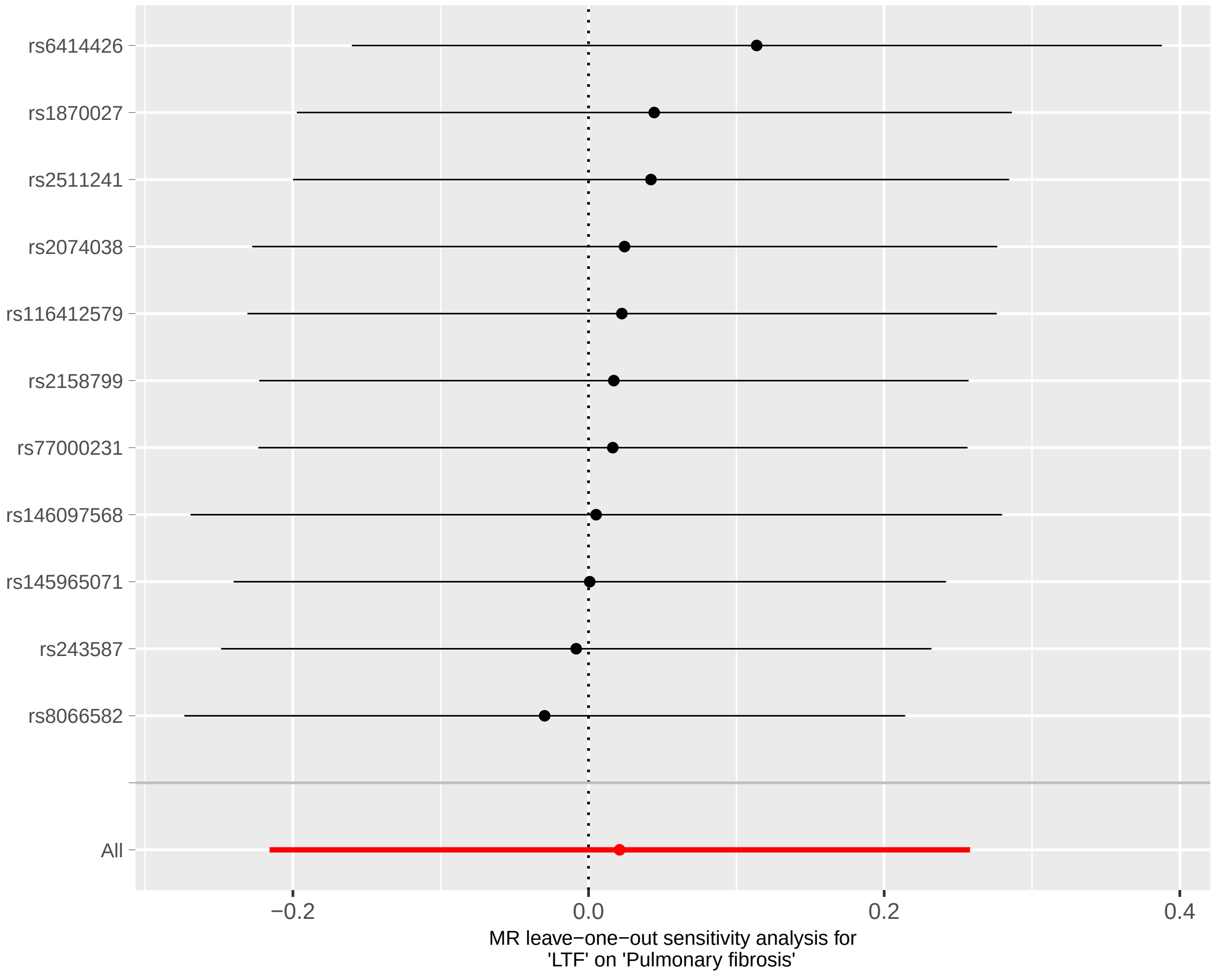
rs6414426
rs1870027
rs2511241
rs2074038
rs116412579
rs2158799
rs77000231
rs146097568
rs145965071
rs243587
rs8066582
All
−0.2
0.0
0.2
0.4
MR leave−one−out sensitivity analysis for
'LTF' on 'Pulmonary fibrosis'

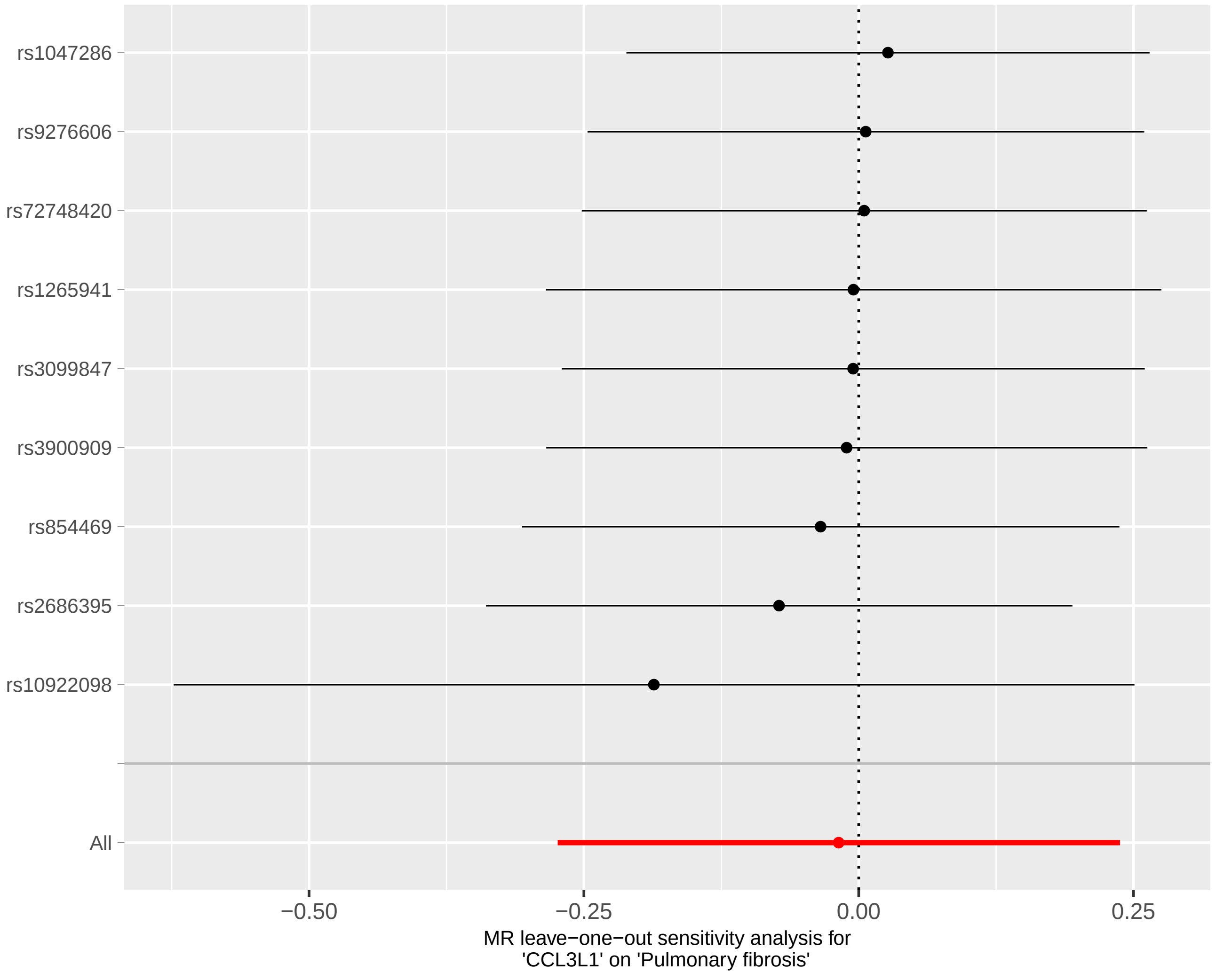
rs1047286
rs9276606
rs72748420
rs1265941
rs3099847
rs3900909
rs854469
rs2686395
rs10922098
All
−0.50
−0.25
0.00
0.25
MR leave−one−out sensitivity analysis for
'CCL3L1' on 'Pulmonary fibrosis'

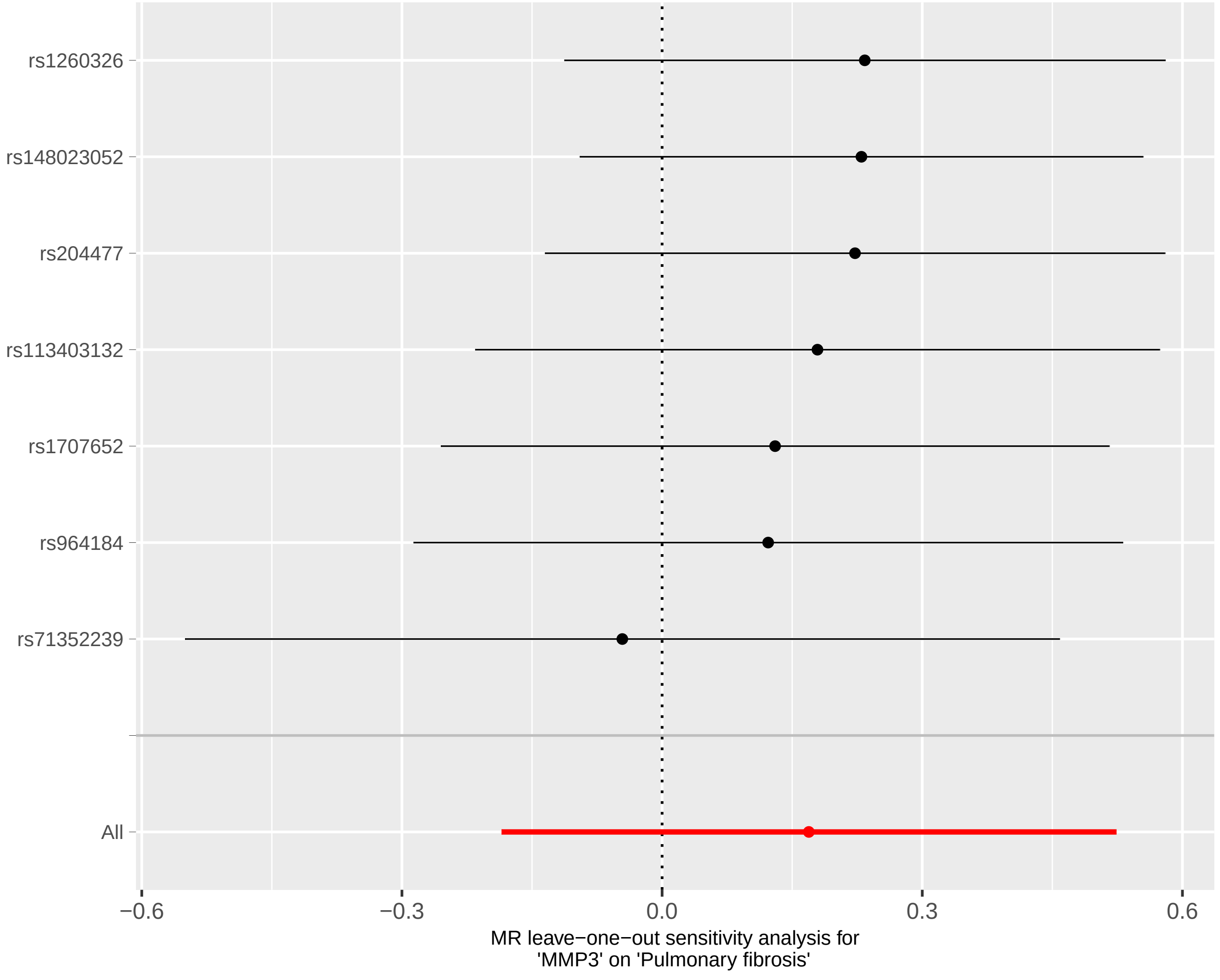
rs1260326
rs148023052
rs204477
rs113403132
rs1707652
rs964184
rs71352239
All
−0.6
−0.3
0.0
0.3
0.6
MR leave−one−out sensitivity analysis for
'MMP3' on 'Pulmonary fibrosis'

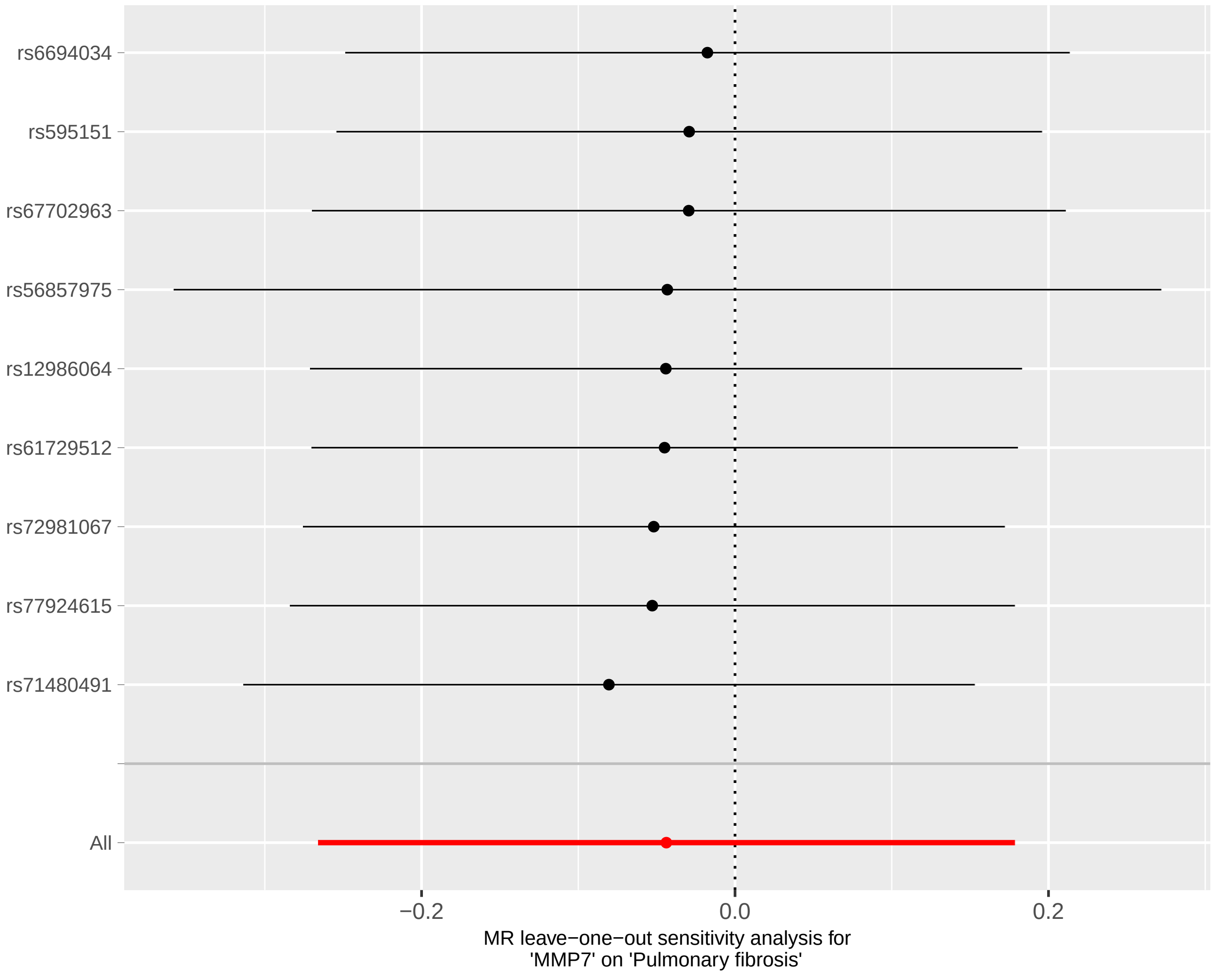
rs6694034
rs595151
rs67702963
rs56857975
rs12986064
rs61729512
rs72981067
rs77924615
rs71480491
All
−0.2
0.0
0.2
MR leave−one−out sensitivity analysis for
'MMP7' on 'Pulmonary fibrosis'

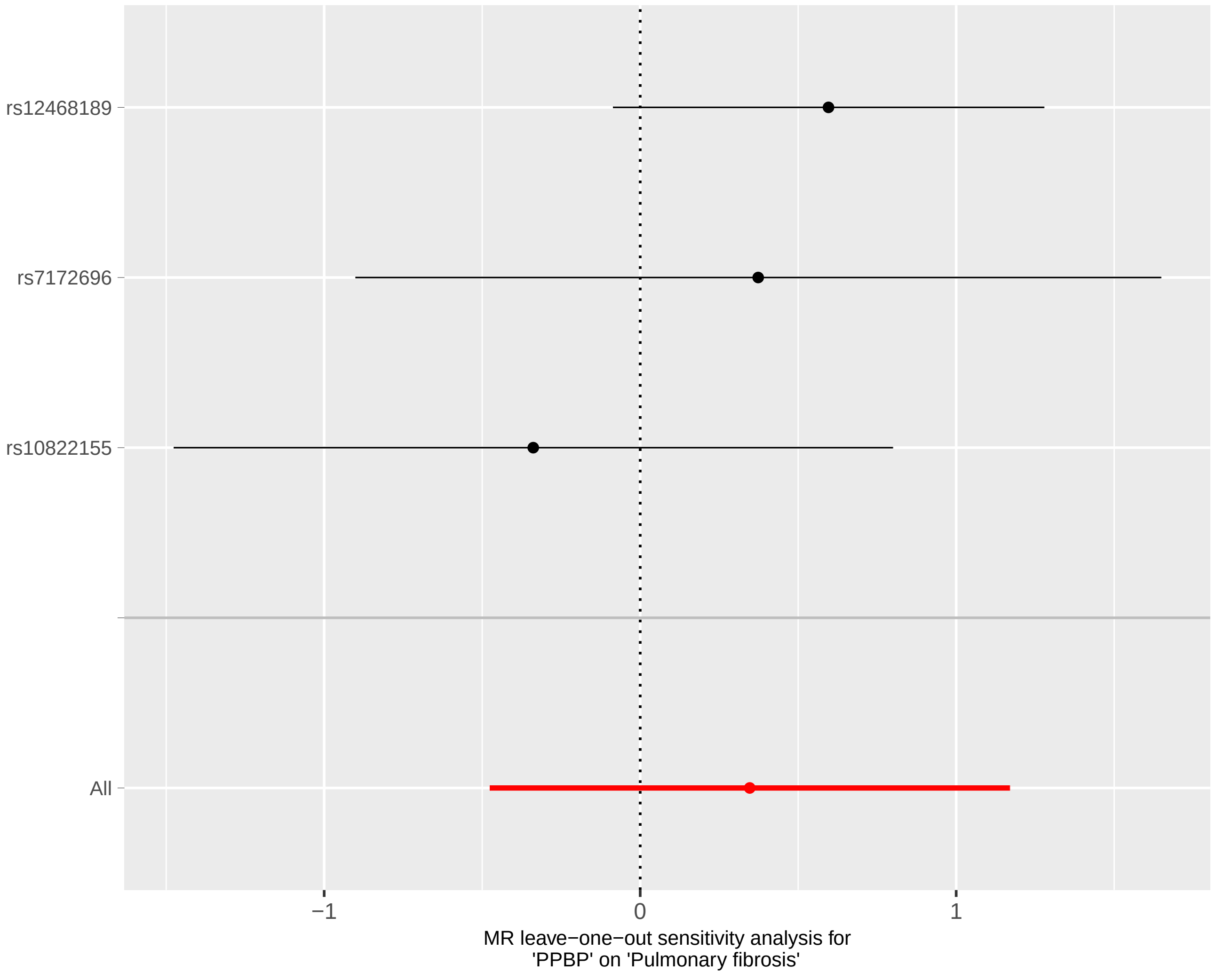

rs12468189
rs7172696
rs10822155
All
−1
0
1
MR leave−one−out sensitivity analysis for
'PPBP' on 'Pulmonary fibrosis'

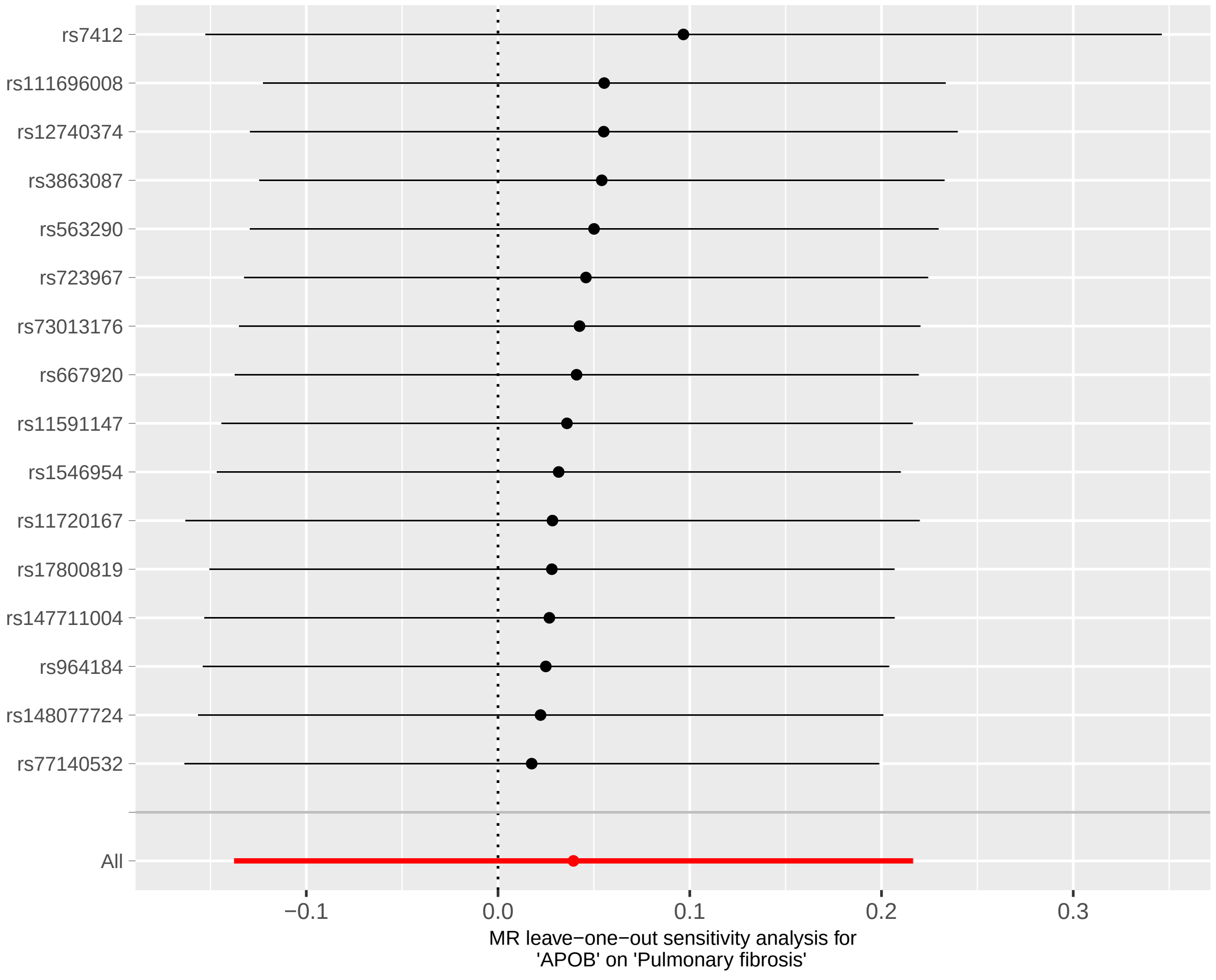

rs7412
rs111696008
rs12740374
rs3863087
rs563290
rs723967
rs73013176
rs667920
rs11591147
rs1546954
rs11720167
rs17800819
rs147711004
rs964184
rs148077724
rs77140532
All
−0.1
0.0
0.1
0.2
0.3
MR leave−one−out sensitivity analysis for
'APOB' on 'Pulmonary fibrosis'

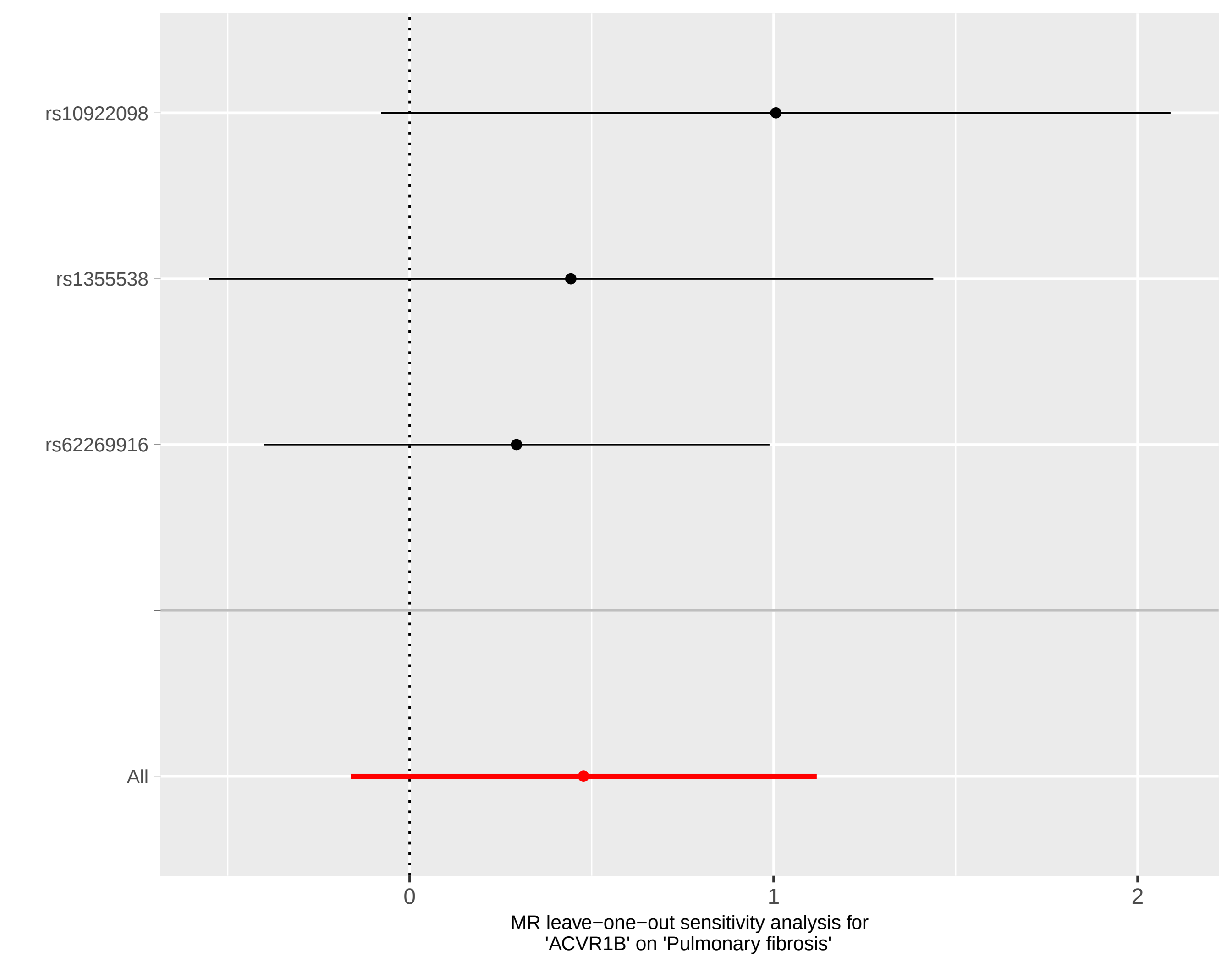
rs10922098
rs1355538
rs62269916
All
0
1
2
MR leave−one−out sensitivity analysis for
'ACVR1B' on 'Pulmonary fibrosis'

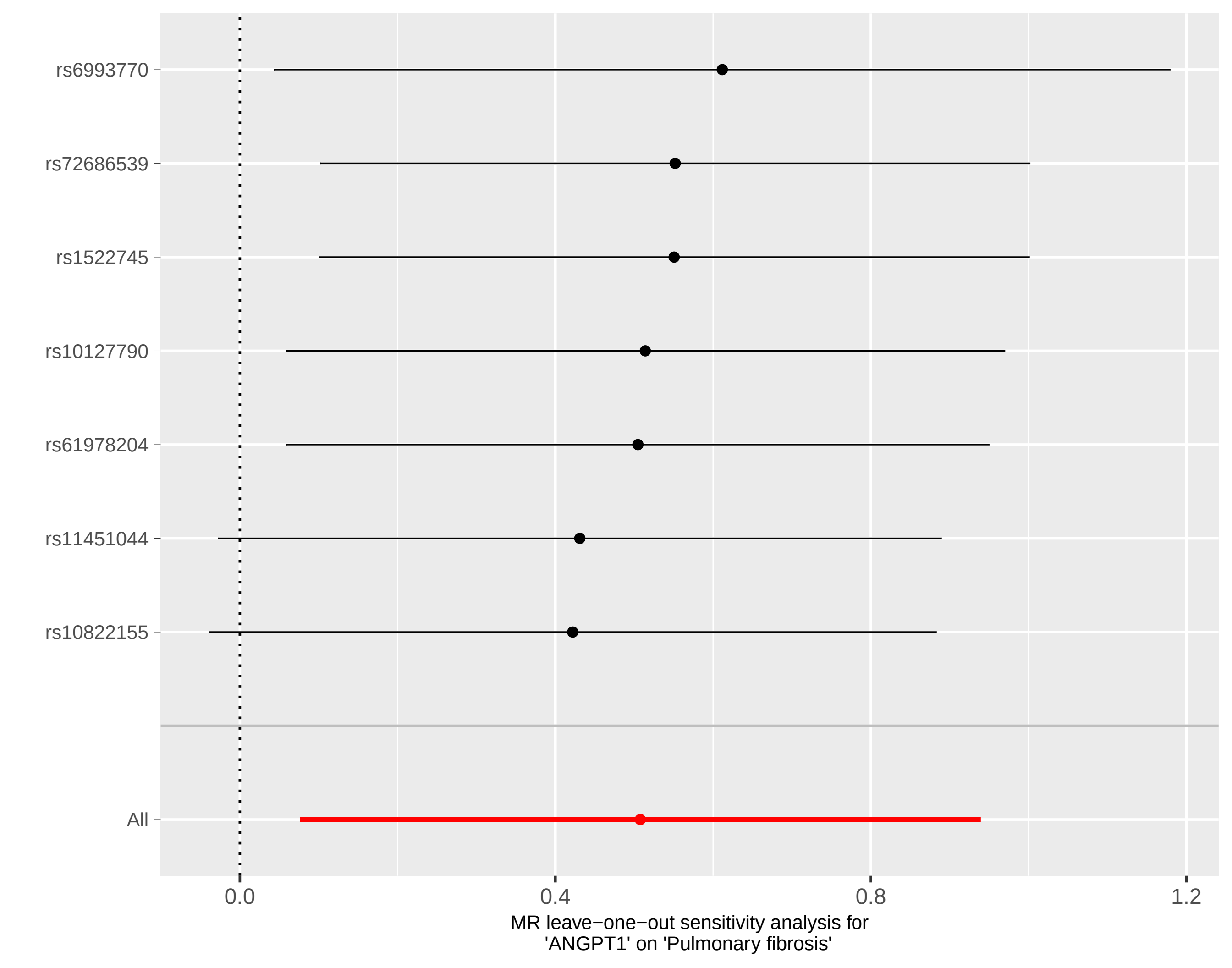
rs6993770
rs72686539
rs1522745
rs10127790
rs61978204
rs11451044
rs10822155
All
0.0
0.4
0.8
1.2
MR leave−one−out sensitivity analysis for
'ANGPT1' on 'Pulmonary fibrosis'

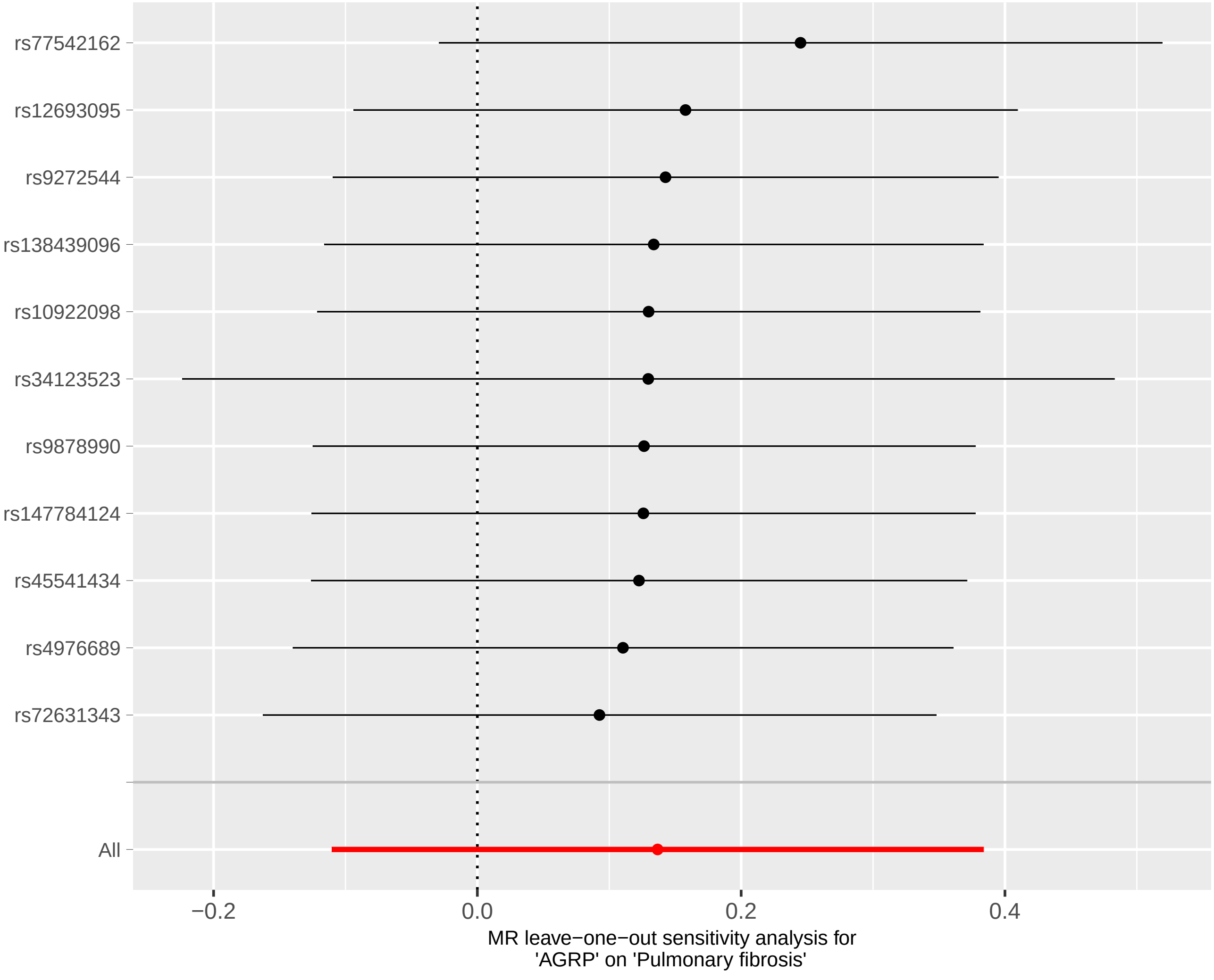
rs77542162
rs12693095
rs9272544
rs138439096
rs10922098
rs34123523
rs9878990
rs147784124
rs45541434
rs4976689
rs72631343
All
−0.2
0.0
0.2
0.4
MR leave−one−out sensitivity analysis for
'AGRP' on 'Pulmonary fibrosis'

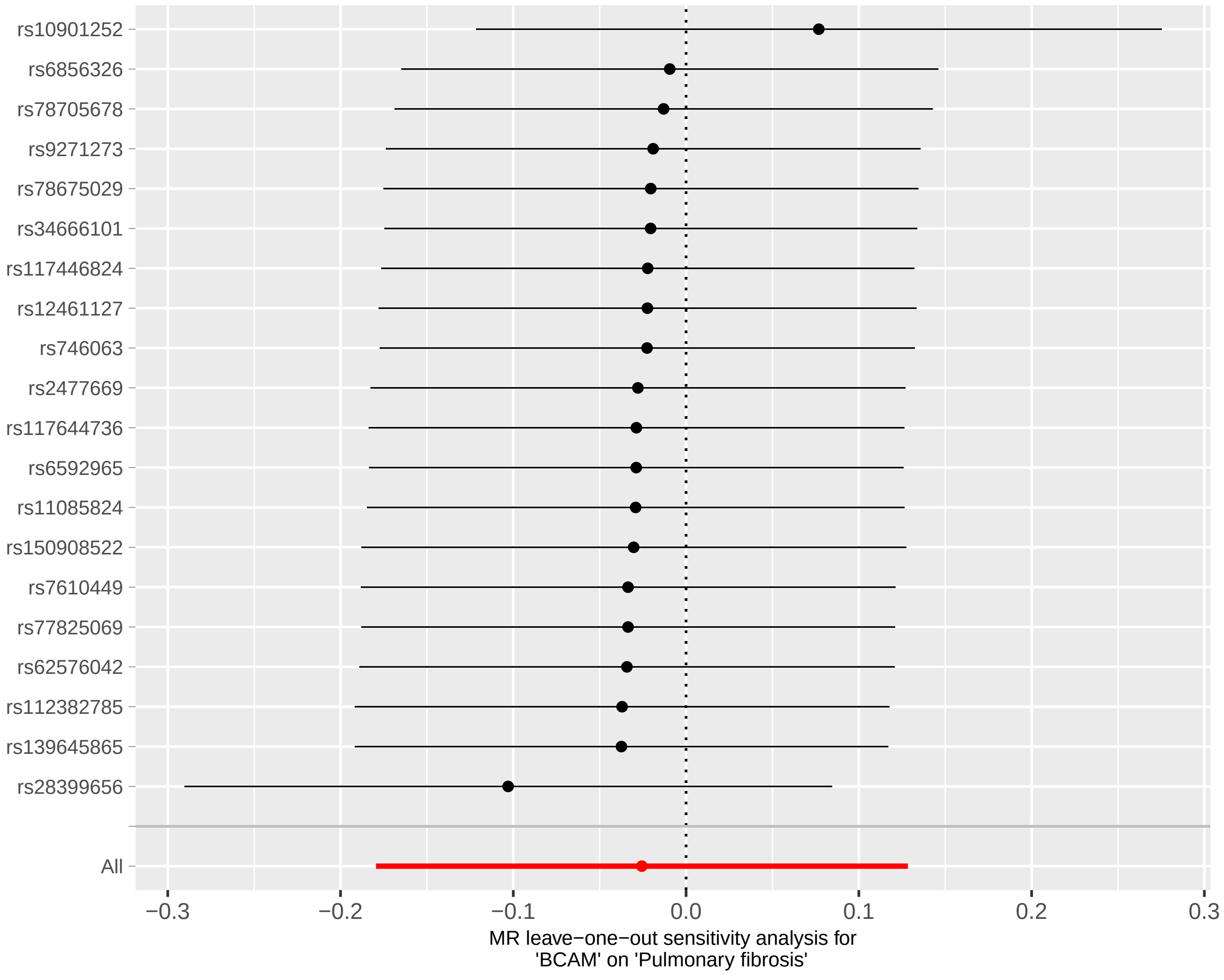

rs10901252
rs6856326
rs78705678
rs9271273
rs78675029
rs34666101
rs117446824
rs12461127
rs746063
rs2477669
rs117644736
rs6592965
rs11085824
rs150908522
rs7610449
rs77825069
rs62576042
rs112382785
rs139645865
rs28399656
All
−0.3
−0.2
−0.1
0.0
0.1
0.2
0.3
MR leave−one−out sensitivity analysis for
'BCAM' on 'Pulmonary fibrosis'

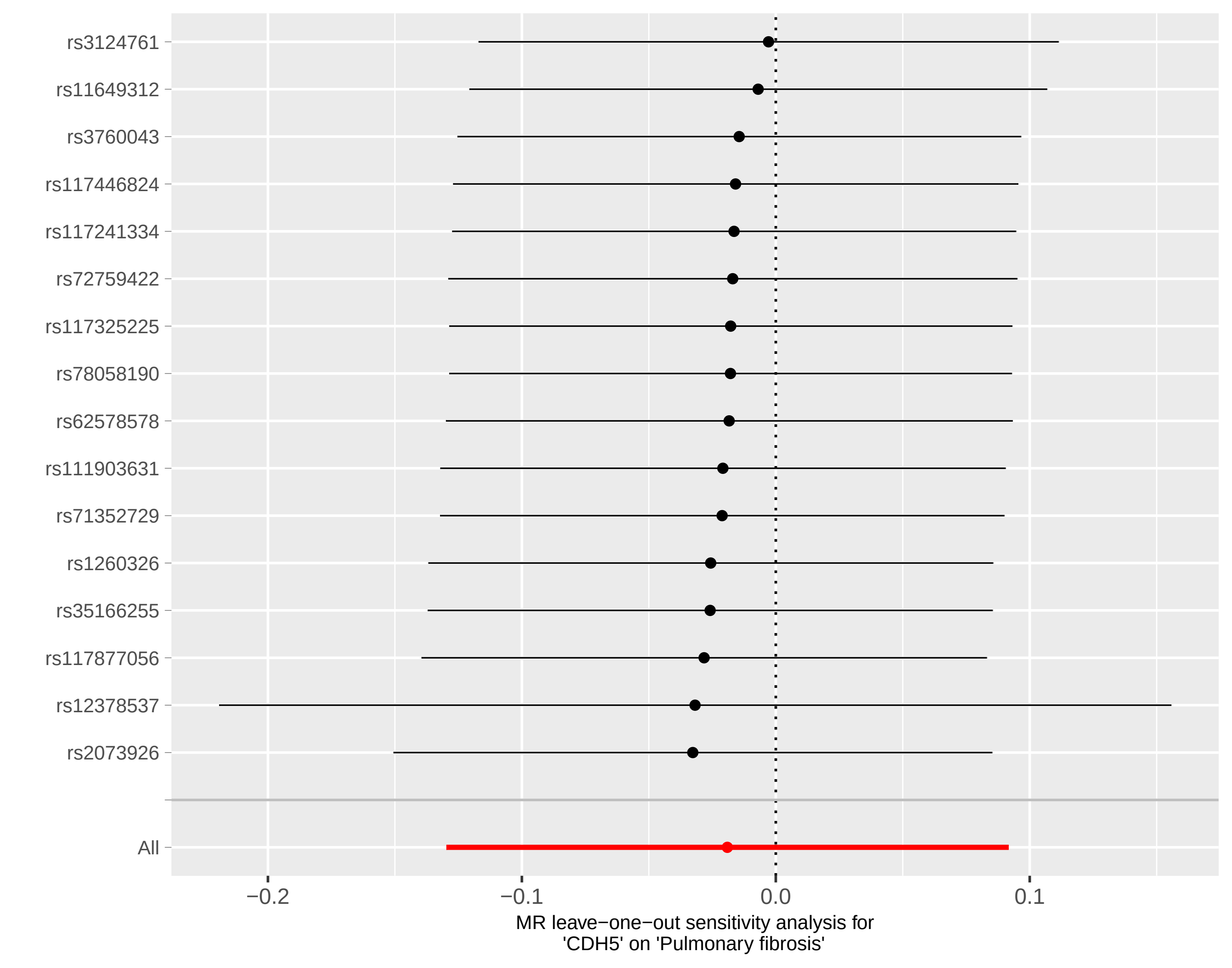
rs3124761
rs11649312
rs3760043
rs117446824
rs117241334
rs72759422
rs117325225
rs78058190
rs62578578
rs111903631
rs71352729
rs1260326
rs35166255
rs117877056
rs12378537
rs2073926
All
−0.2
−0.1
0.0
0.1
MR leave−one−out sensitivity analysis for
'CDH5' on 'Pulmonary fibrosis'

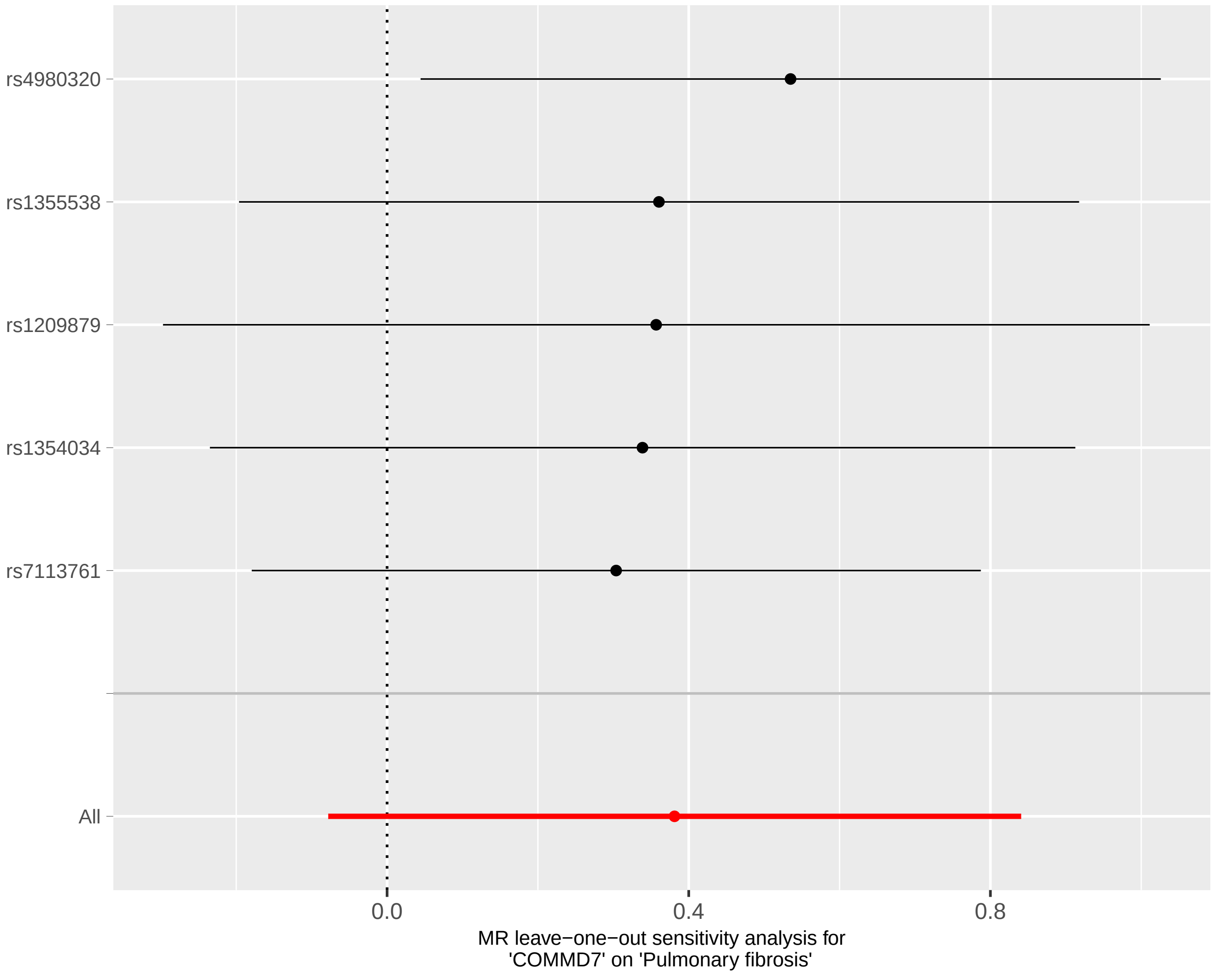
rs4980320
rs1355538
rs1209879
rs1354034
rs7113761
All
0.0
0.4
0.8
MR leave−one−out sensitivity analysis for
'COMMD7' on 'Pulmonary fibrosis'

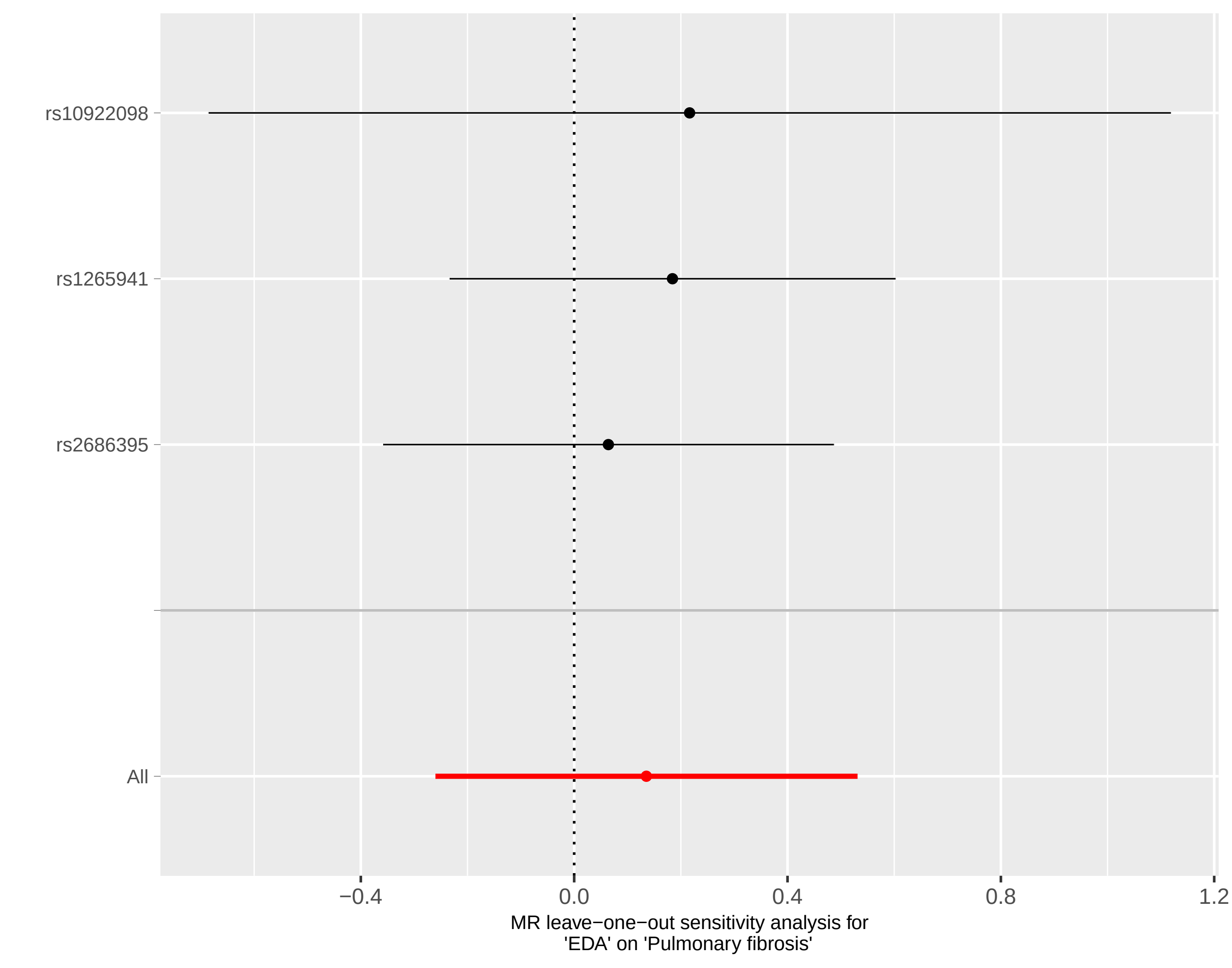
rs10922098
rs1265941
rs2686395
All
−0.4
0.0
0.4
0.8
1.2
MR leave−one−out sensitivity analysis for
'EDA' on 'Pulmonary fibrosis'

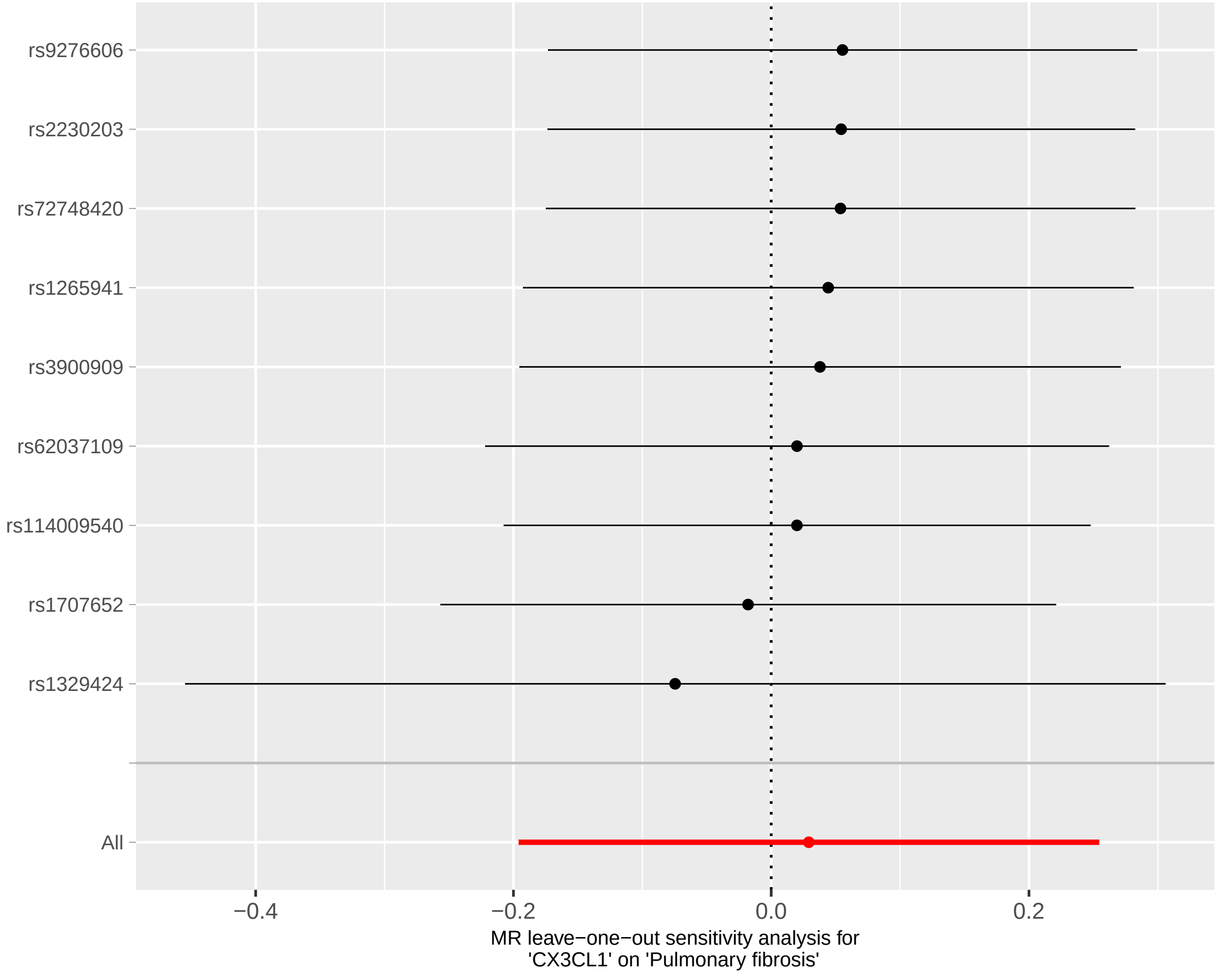
rs9276606
rs2230203
rs72748420
rs1265941
rs3900909
rs62037109
rs114009540
rs1707652
rs1329424
All
−0.4
−0.2
0.0
0.2
MR leave−one−out sensitivity analysis for
'CX3CL1' on 'Pulmonary fibrosis'

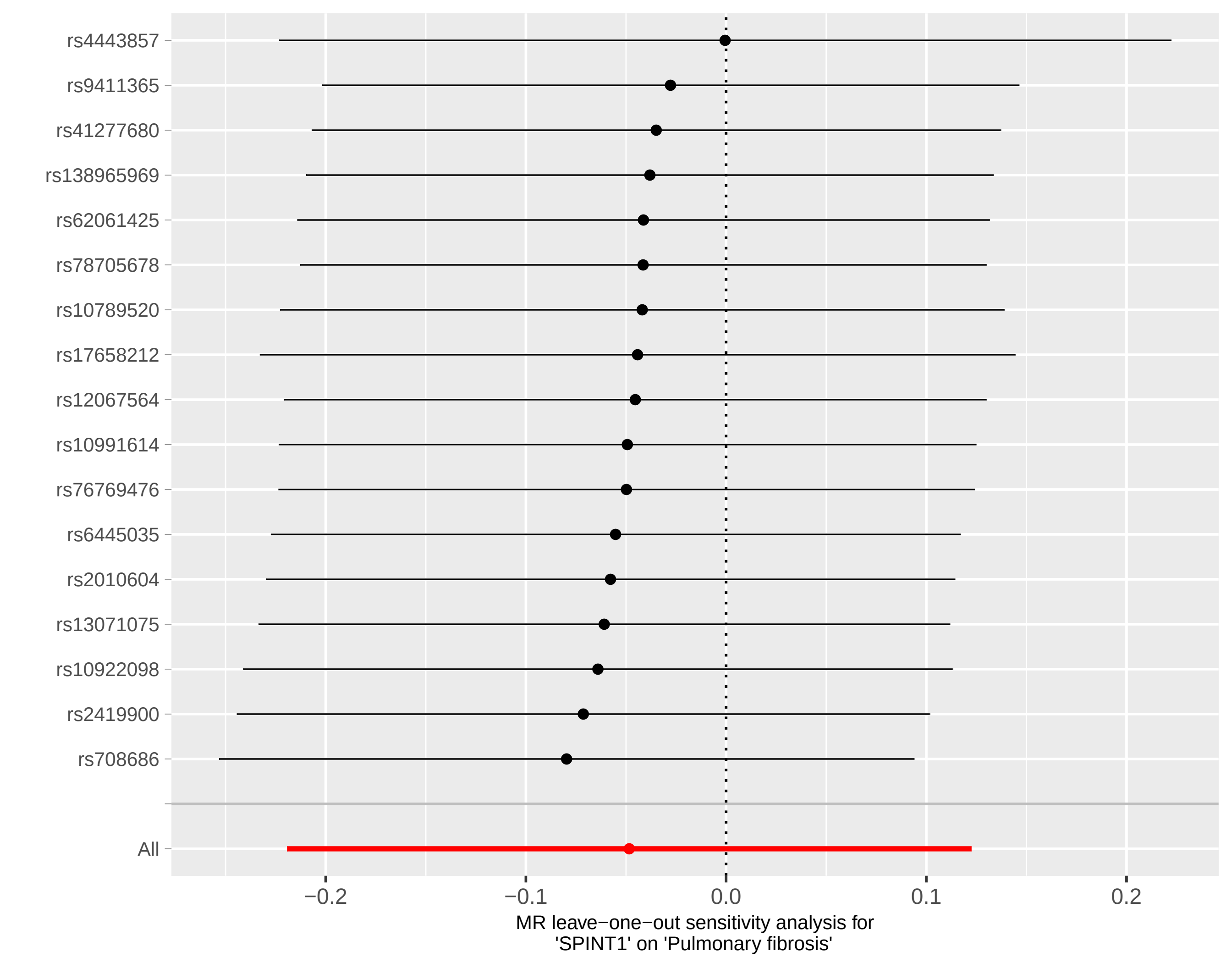
rs4443857
rs9411365
rs41277680
rs138965969
rs62061425
rs78705678
rs10789520
rs17658212
rs12067564
rs10991614
rs76769476
rs6445035
rs2010604
rs13071075
rs10922098
rs2419900
rs708686
All
−0.2
−0.1
0.0
0.1
0.2
MR leave−one−out sensitivity analysis for
'SPINT1' on 'Pulmonary fibrosis'

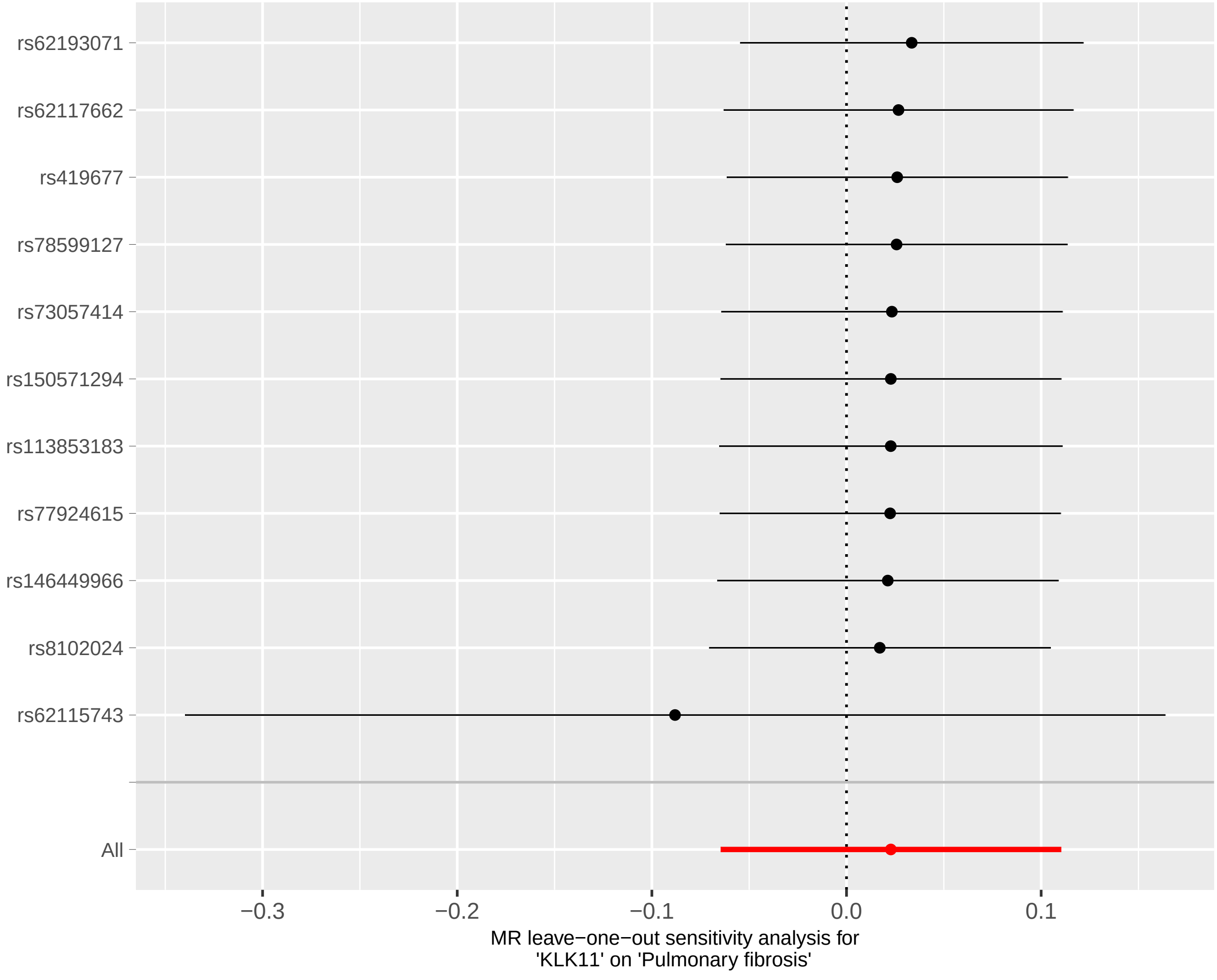
rs62193071
rs62117662
rs419677
rs78599127
rs73057414
rs150571294
rs113853183
rs77924615
rs146449966
rs8102024
rs62115743
All
−0.3
−0.2
−0.1
0.0
0.1
MR leave−one−out sensitivity analysis for
'KLK11' on 'Pulmonary fibrosis'

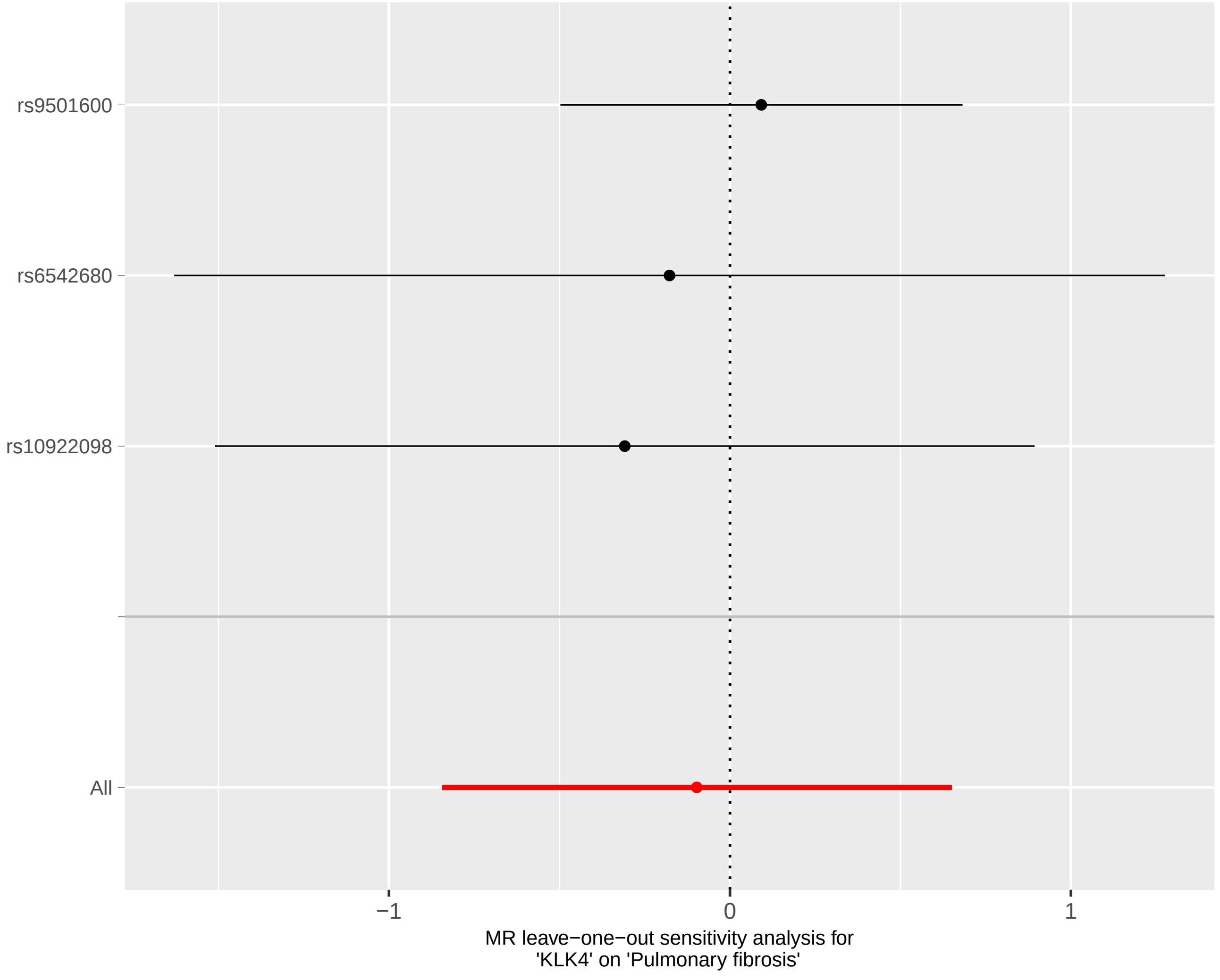
rs9501600
rs6542680
rs10922098
All
−1
0
1
MR leave−one−out sensitivity analysis for
'KLK4' on 'Pulmonary fibrosis'

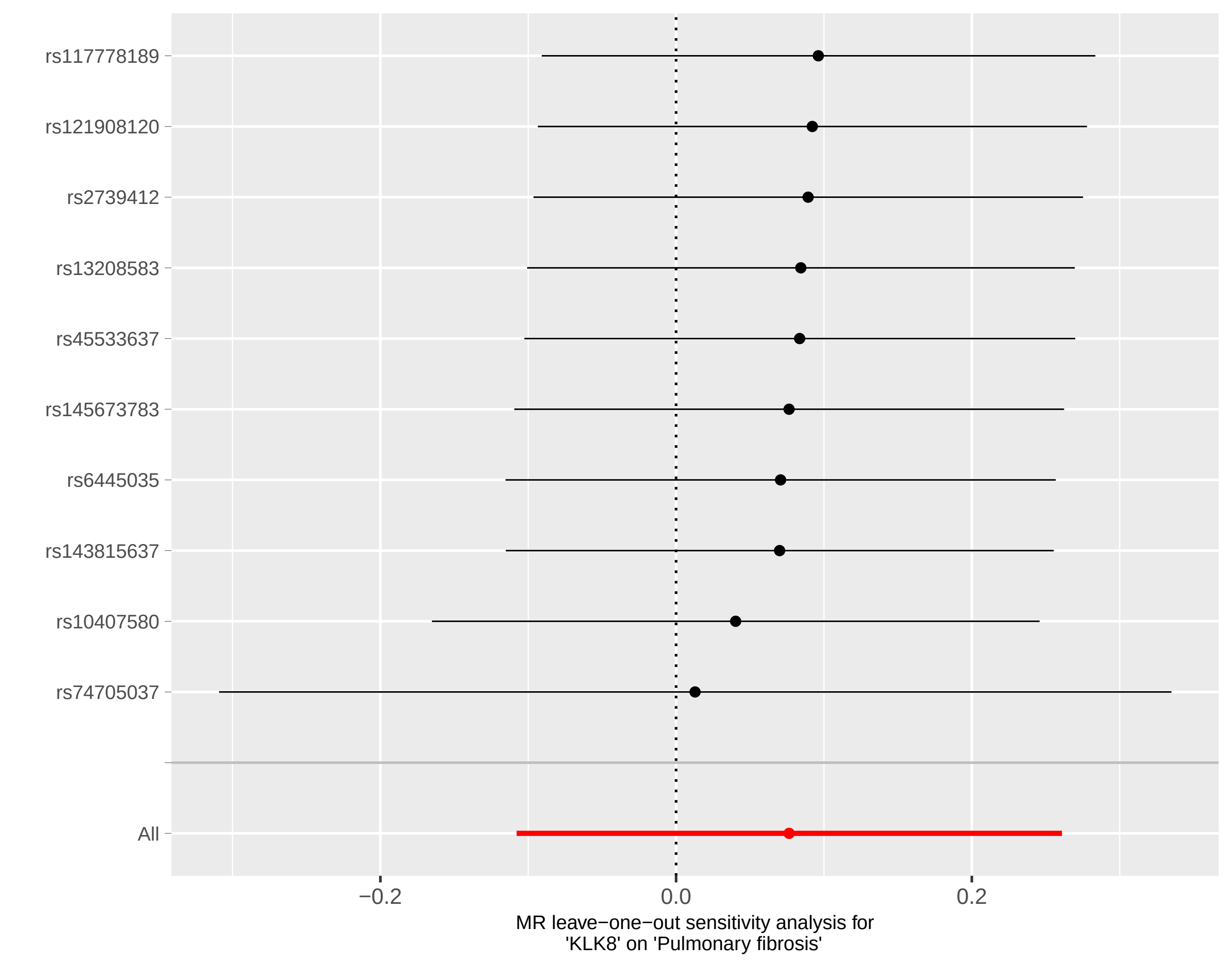
rs117778189
rs121908120
rs2739412
rs13208583
rs45533637
rs145673783
rs6445035
rs143815637
rs10407580
rs74705037
All
−0.2
0.0
0.2
MR leave−one−out sensitivity analysis for
'KLK8' on 'Pulmonary fibrosis'

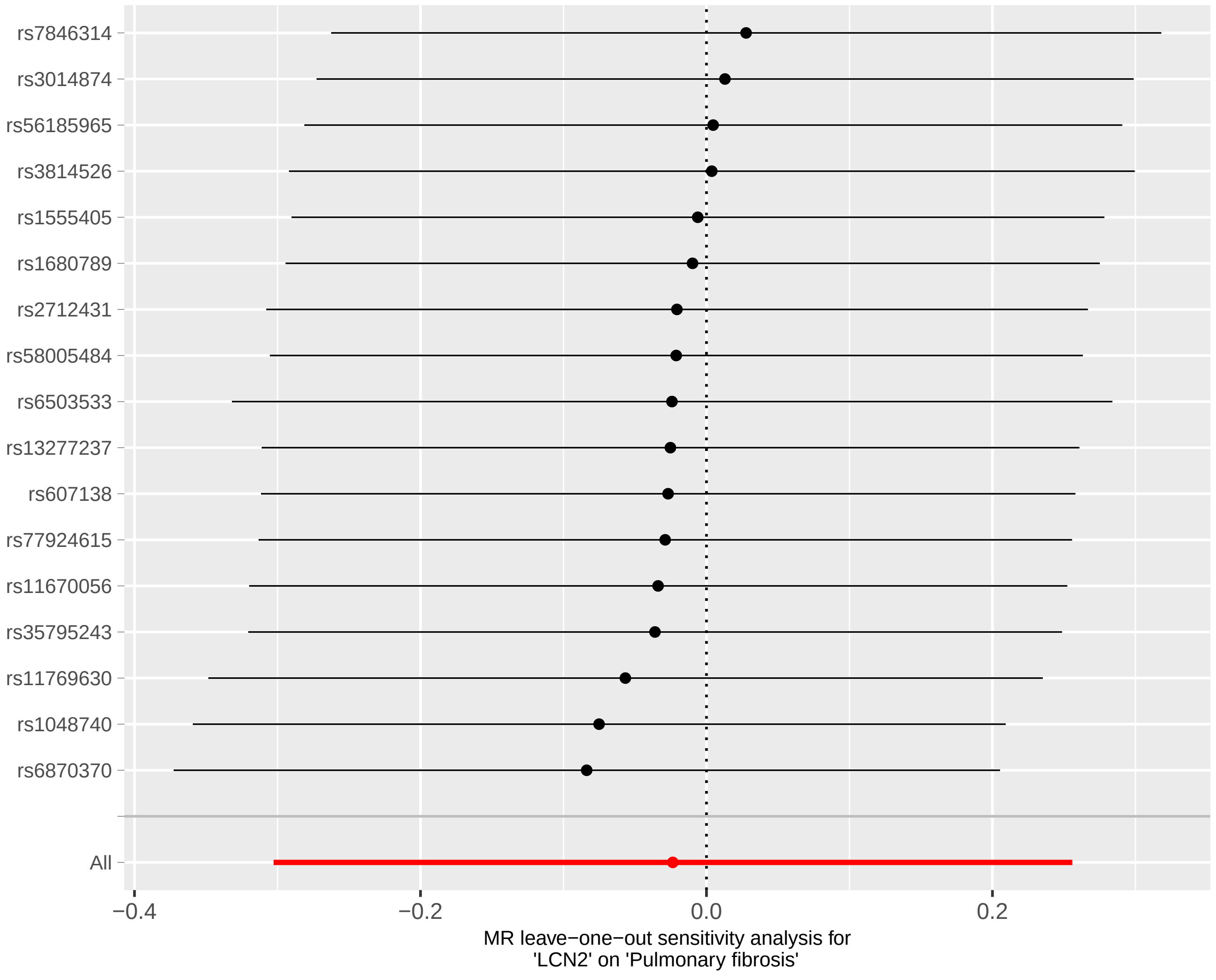

rs7846314
rs3014874
rs56185965
rs3814526
rs1555405
rs1680789
rs2712431
rs58005484
rs6503533
rs13277237
rs607138
rs77924615
rs11670056
rs35795243
rs11769630
rs1048740
rs6870370
All
−0.4
−0.2
0.0
0.2
MR leave−one−out sensitivity analysis for
'LCN2' on 'Pulmonary fibrosis'

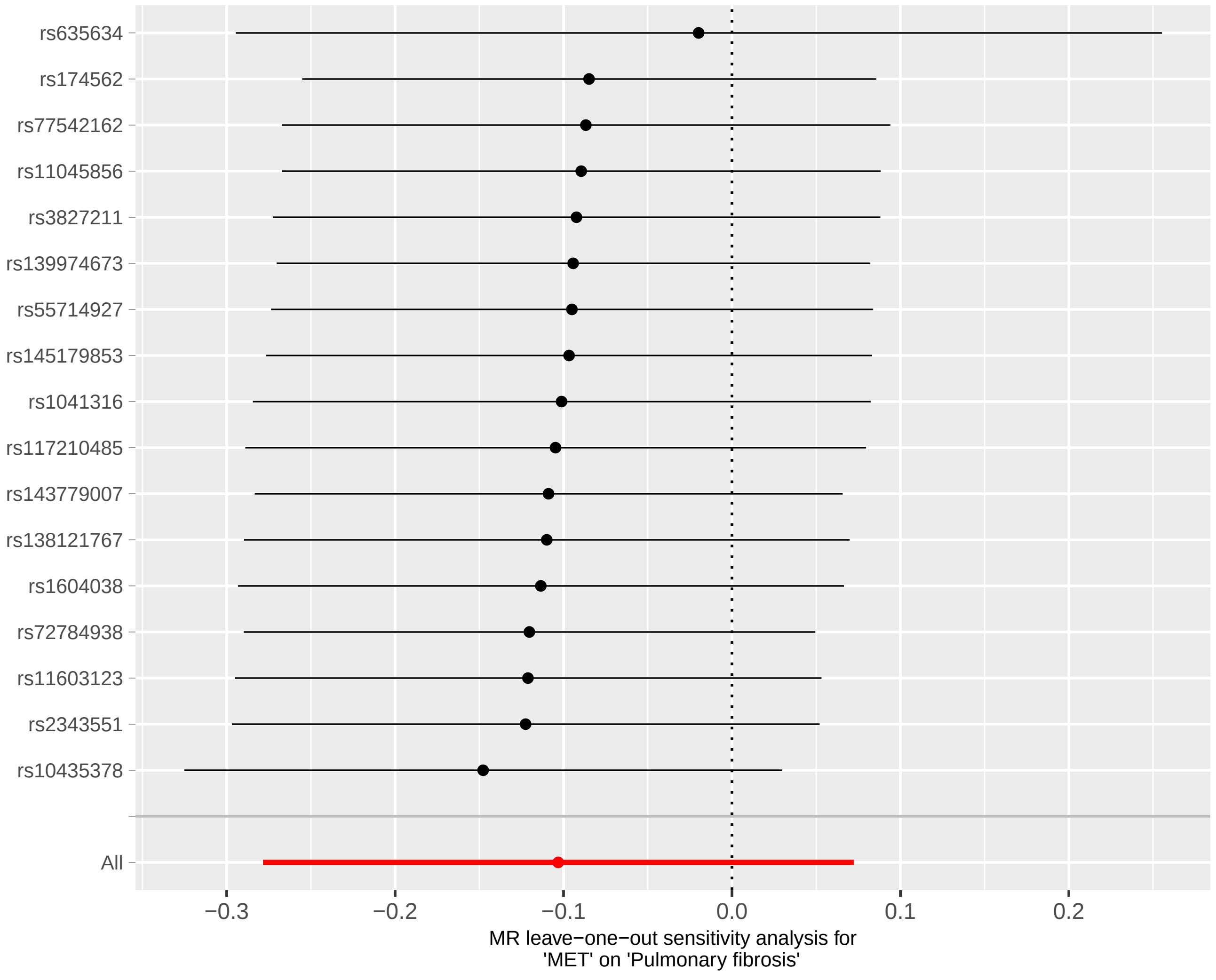
rs635634
rs174562
rs77542162
rs11045856
rs3827211
rs139974673
rs55714927
rs145179853
rs1041316
rs117210485
rs143779007
rs138121767
rs1604038
rs72784938
rs11603123
rs2343551
rs10435378
All
−0.3
−0.2
−0.1
0.0
0.1
0.2
MR leave−one−out sensitivity analysis for
'MET' on 'Pulmonary fibrosis'

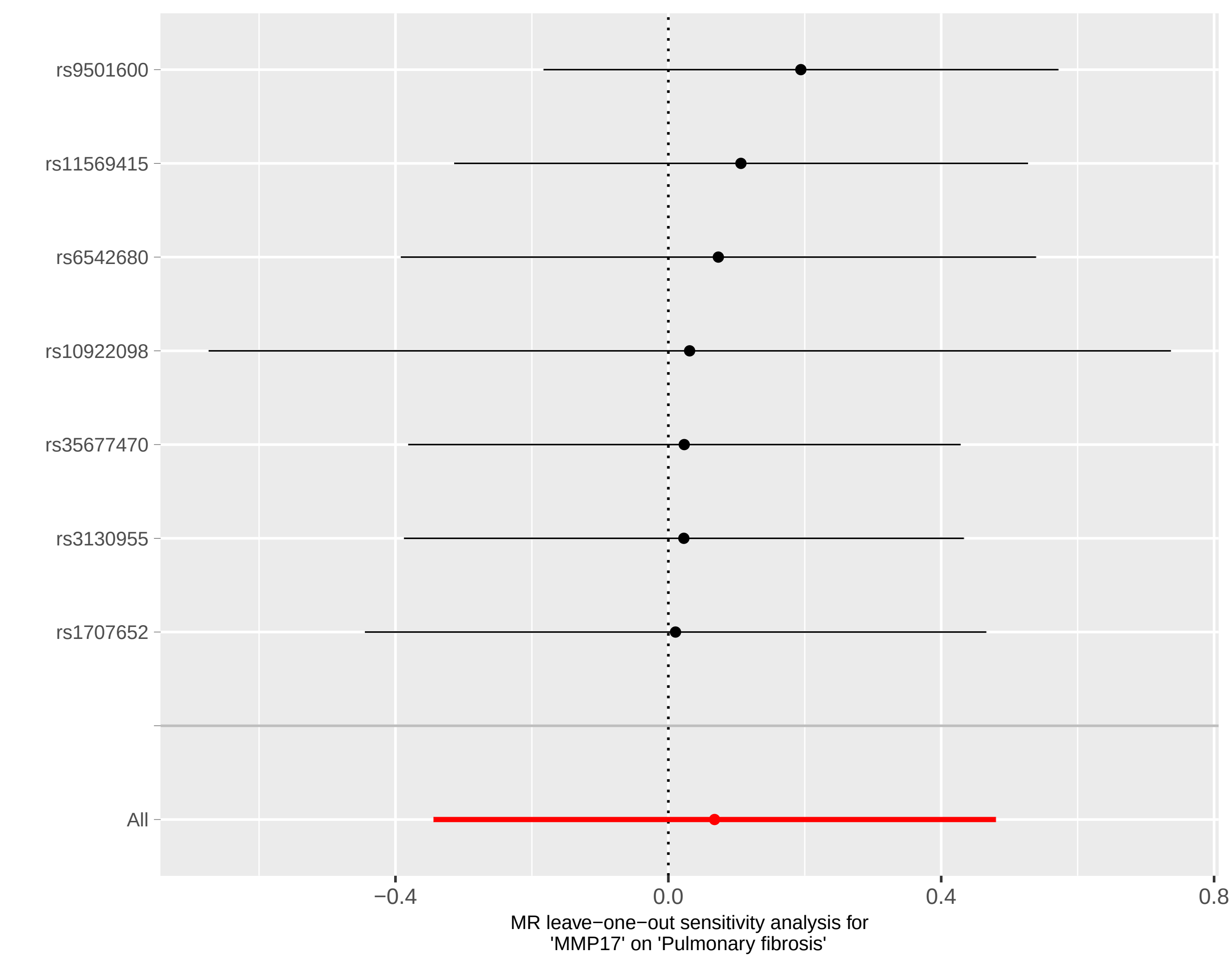

rs9501600
rs11569415
rs6542680
rs10922098
rs35677470
rs3130955
rs1707652
All
−0.4
0.0
0.4
0.8
MR leave−one−out sensitivity analysis for
'MMP17' on 'Pulmonary fibrosis'

Insufficient number of SNPs

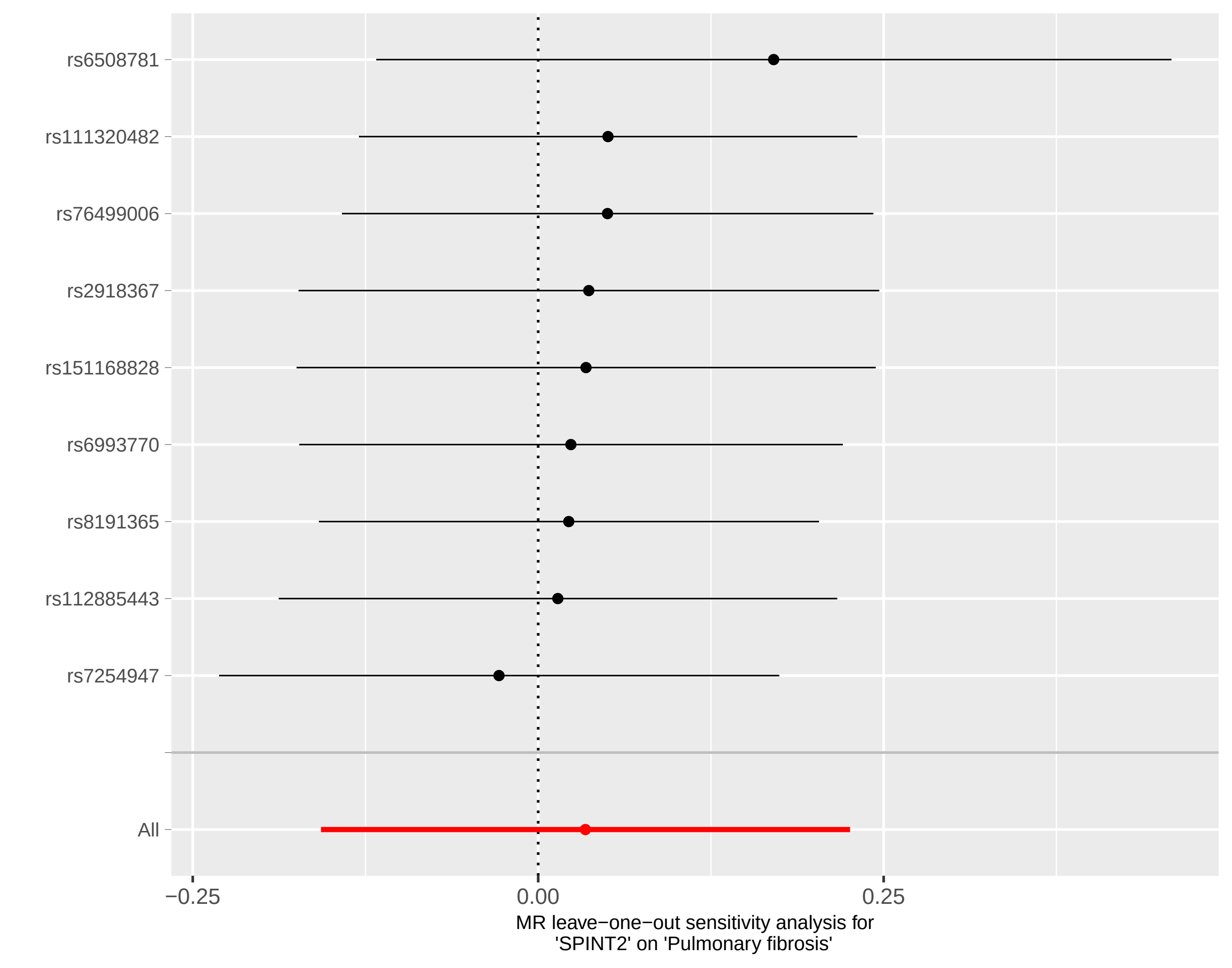

rs6508781
rs111320482
rs76499006
rs2918367
rs151168828
rs6993770
rs8191365
rs112885443
rs7254947
All
−0.25
0.00
0.25
MR leave−one−out sensitivity analysis for
'SPINT2' on 'Pulmonary fibrosis'

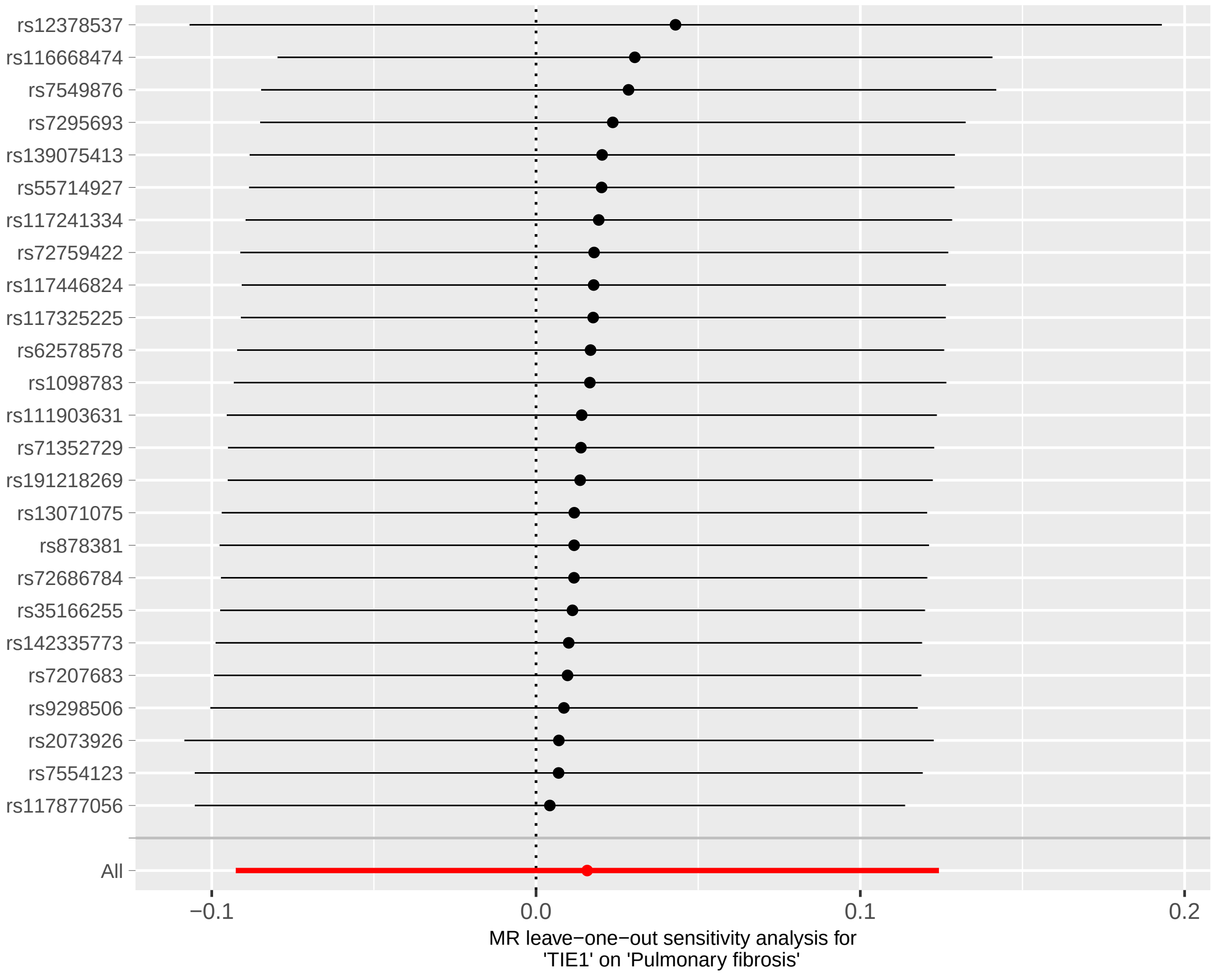

rs12378537
rs116668474
rs7549876
rs7295693
rs139075413
rs55714927
rs117241334
rs72759422
rs117446824
rs117325225
rs62578578
rs1098783
rs111903631
rs71352729
rs191218269
rs13071075
rs878381
rs72686784
rs35166255
rs142335773
rs7207683
rs9298506
rs2073926
rs7554123
rs117877056
All
−0.1
0.0
0.1
0.2
MR leave−one−out sensitivity analysis for
'TIE1' on 'Pulmonary fibrosis'

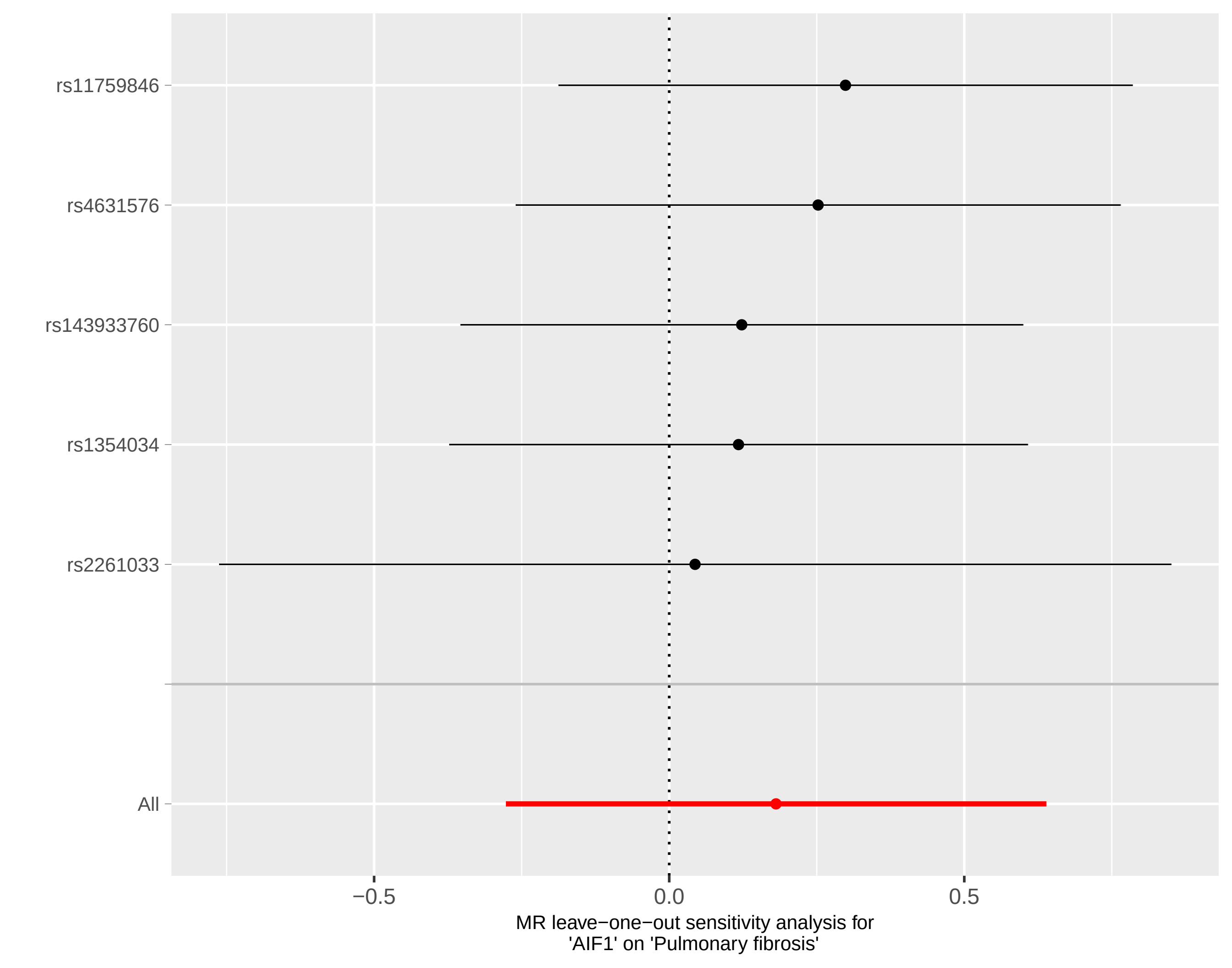
rs11759846
rs4631576
rs143933760
rs1354034
rs2261033
All
−0.5
0.0
0.5
MR leave−one−out sensitivity analysis for
'AIF1' on 'Pulmonary fibrosis'

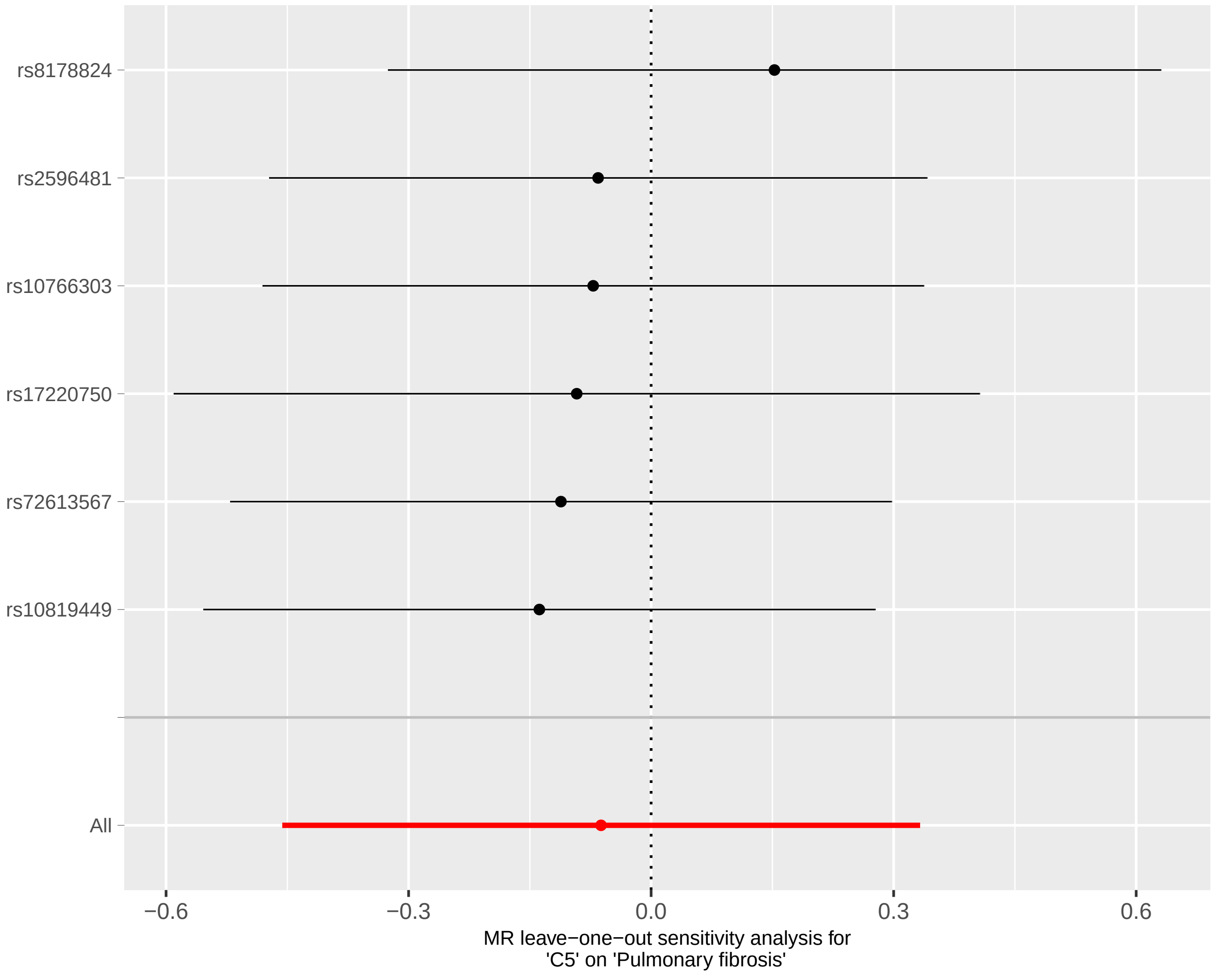

rs8178824
rs2596481
rs10766303
rs17220750
rs72613567
rs10819449
All
−0.6
−0.3
0.0
0.3
0.6
MR leave−one−out sensitivity analysis for
'C5' on 'Pulmonary fibrosis'

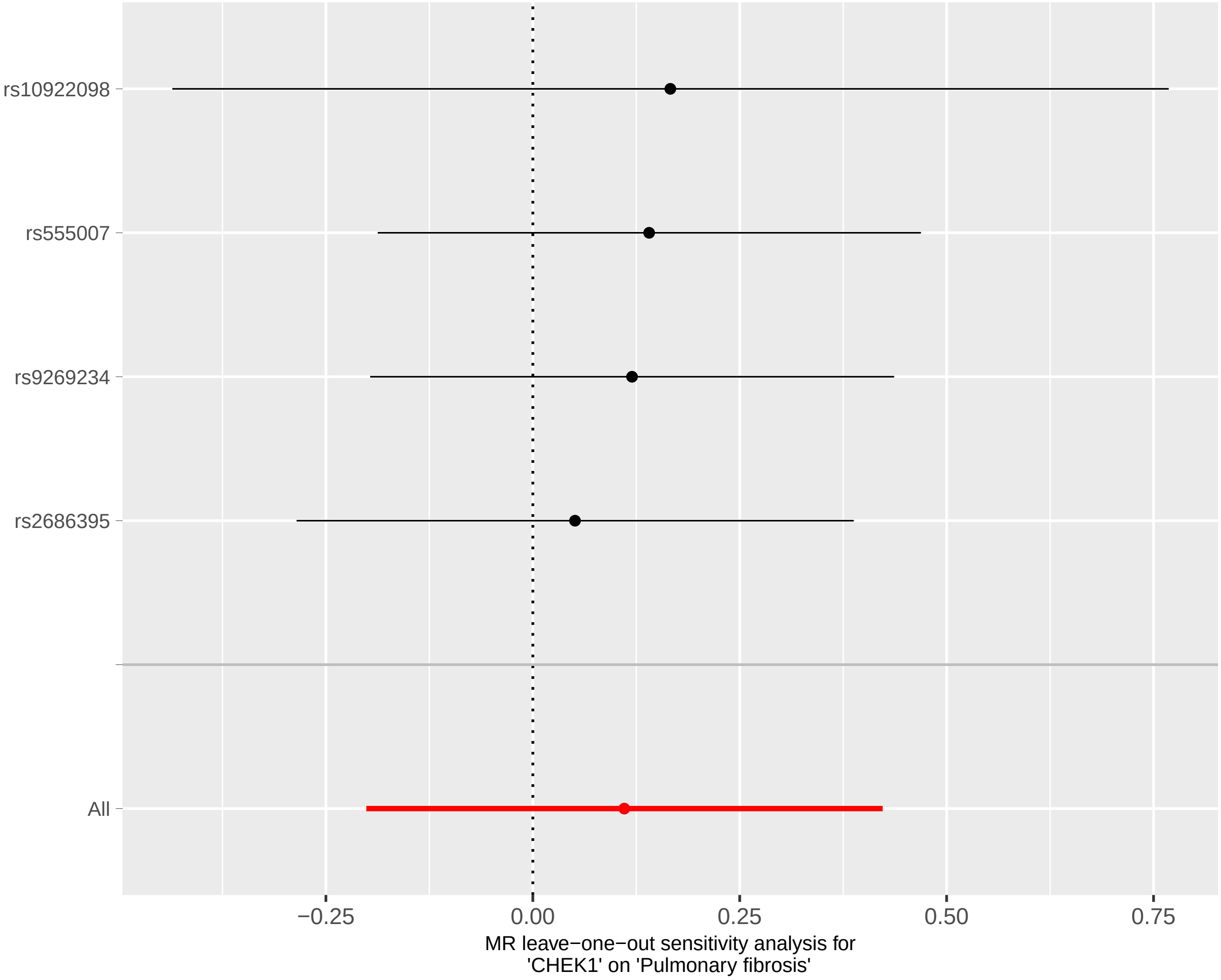
rs10922098
rs555007
rs9269234
rs2686395
All
−0.25
0.00
0.25
0.50
0.75
MR leave−one−out sensitivity analysis for
'CHEK1' on 'Pulmonary fibrosis'

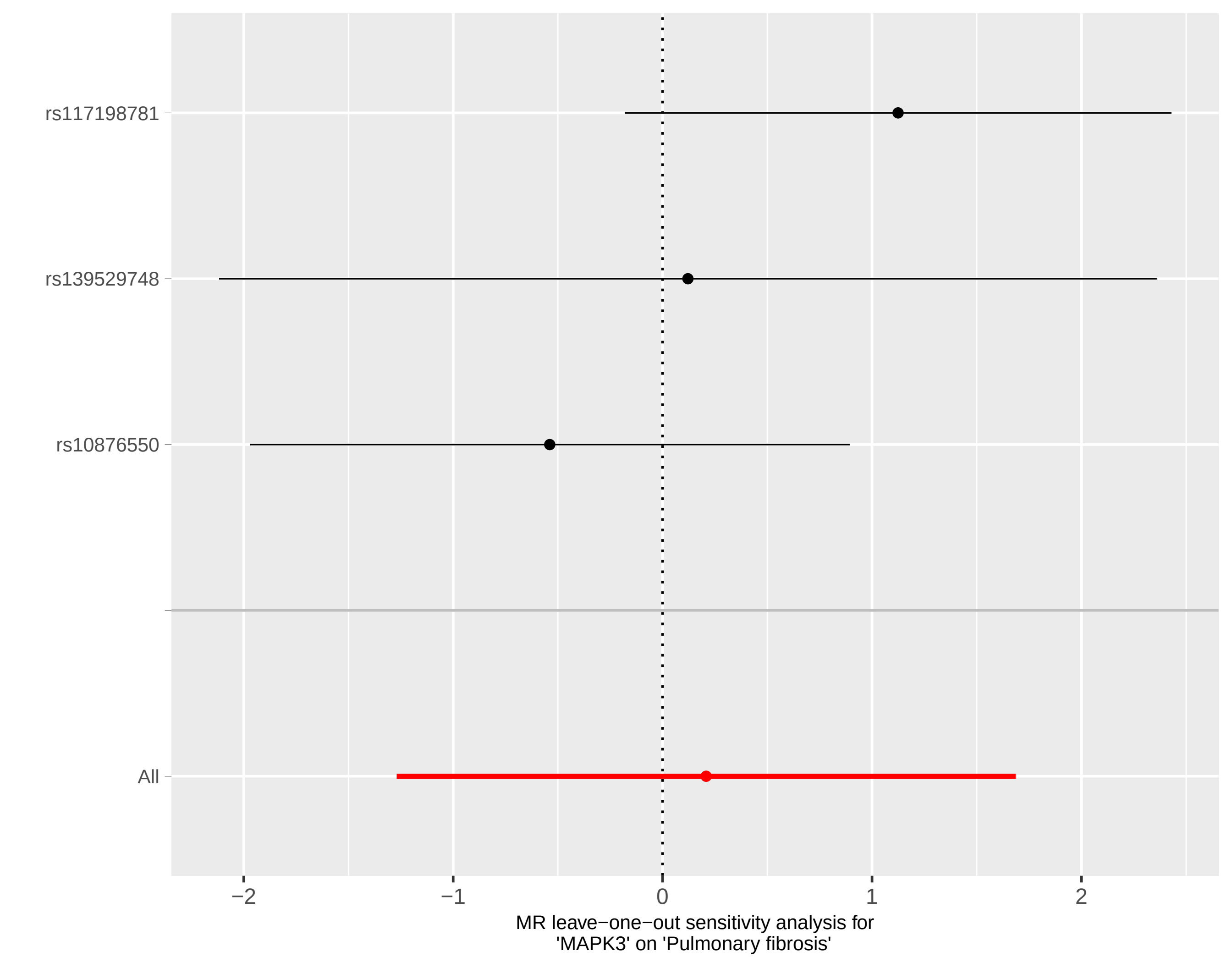
rs117198781
rs139529748
rs10876550
All
−2
−1
0
1
2
MR leave−one−out sensitivity analysis for
'MAPK3' on 'Pulmonary fibrosis'

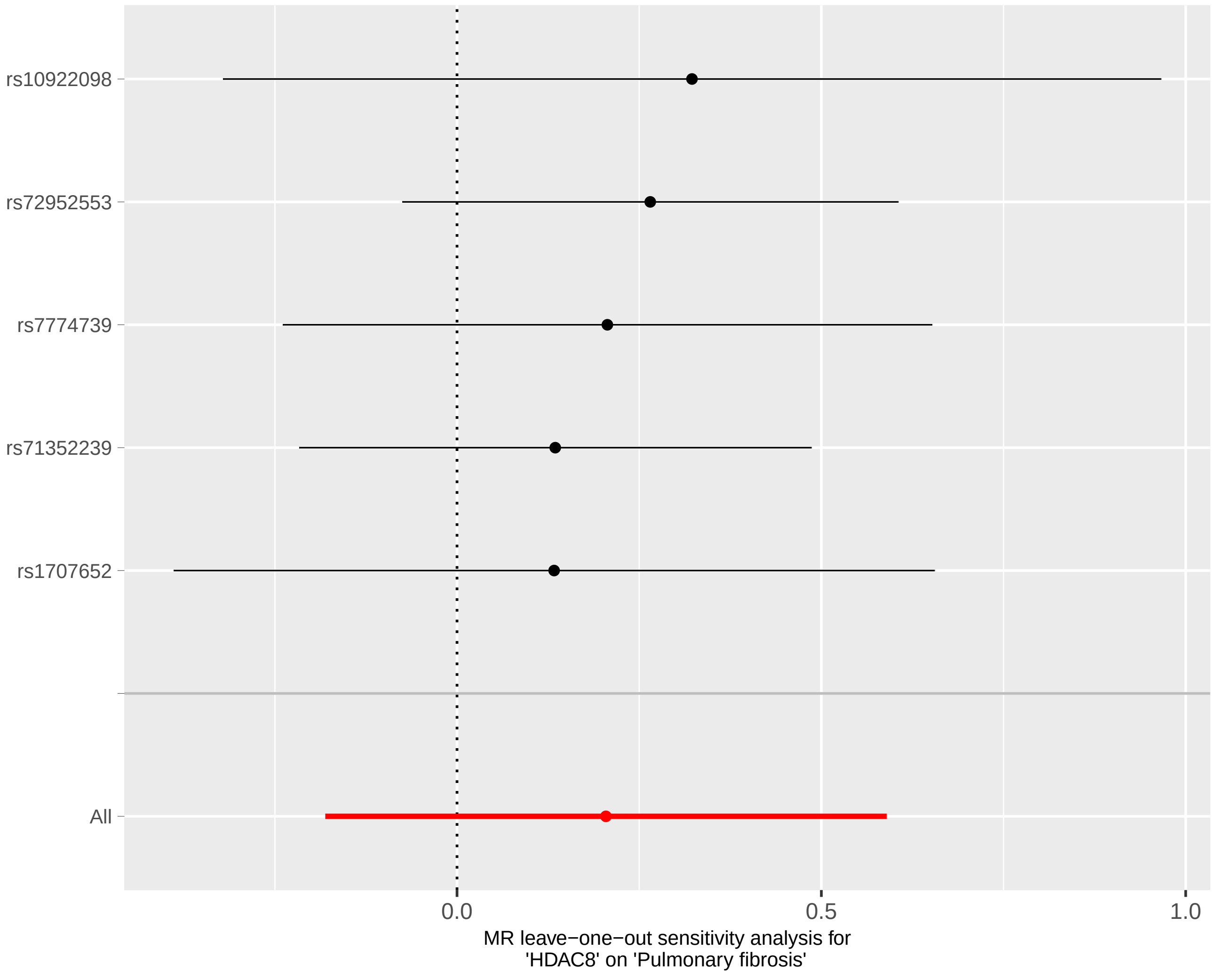
rs10922098
rs72952553
rs7774739
rs71352239
rs1707652
All
0.0
0.5
1.0
MR leave−one−out sensitivity analysis for
'HDAC8' on 'Pulmonary fibrosis'

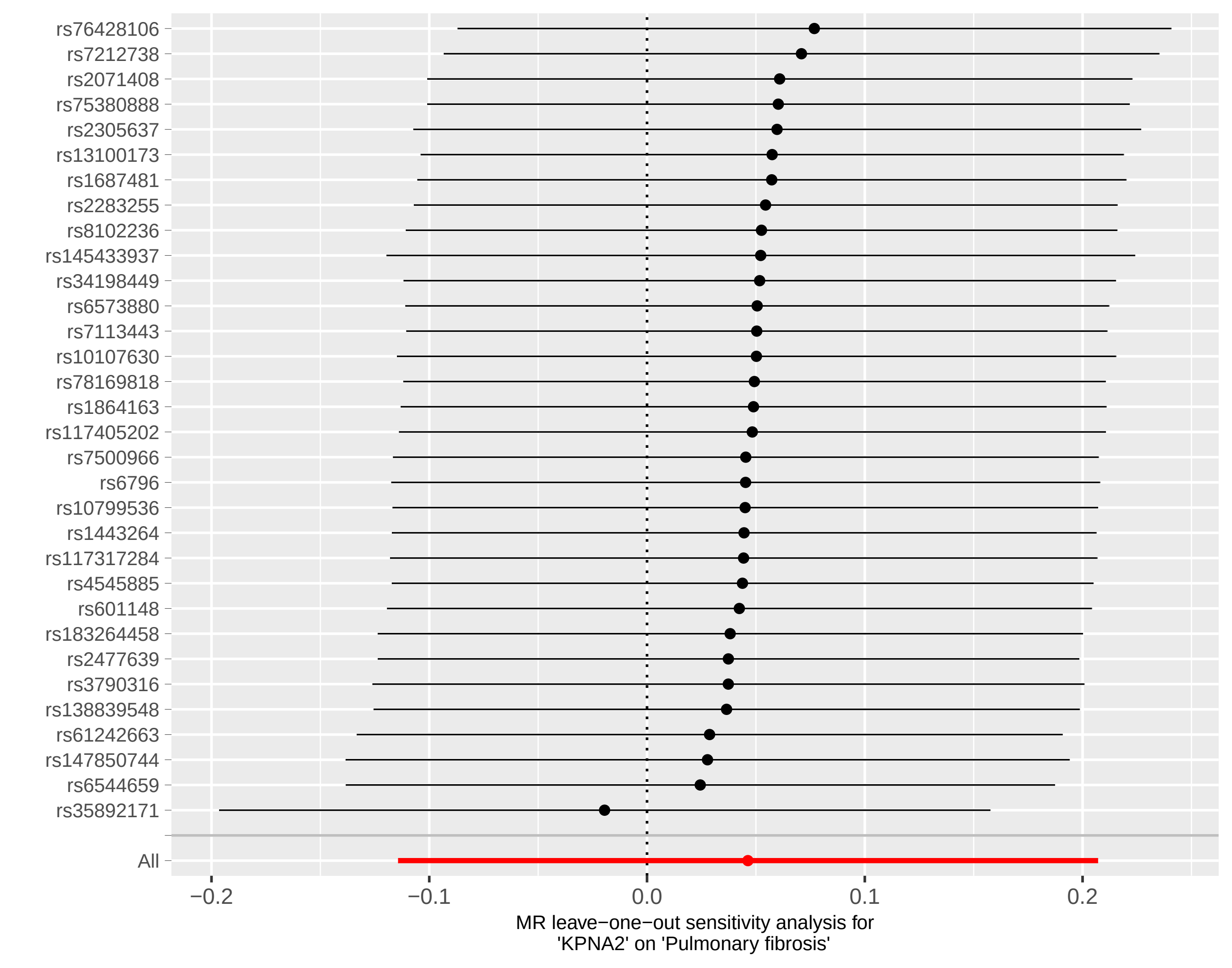
rs76428106
rs7212738
rs2071408
rs75380888
rs2305637
rs13100173
rs1687481
rs2283255
rs8102236
rs145433937
rs34198449
rs6573880
rs7113443
rs10107630
rs78169818
rs1864163
rs117405202
rs7500966
rs6796
rs10799536
rs1443264
rs117317284
rs4545885
rs601148
rs183264458
rs2477639
rs3790316
rs138839548
rs61242663
rs147850744
rs6544659
rs35892171
All
−0.2
−0.1
0.0
0.1
0.2
MR leave−one−out sensitivity analysis for
'KPNA2' on 'Pulmonary fibrosis'

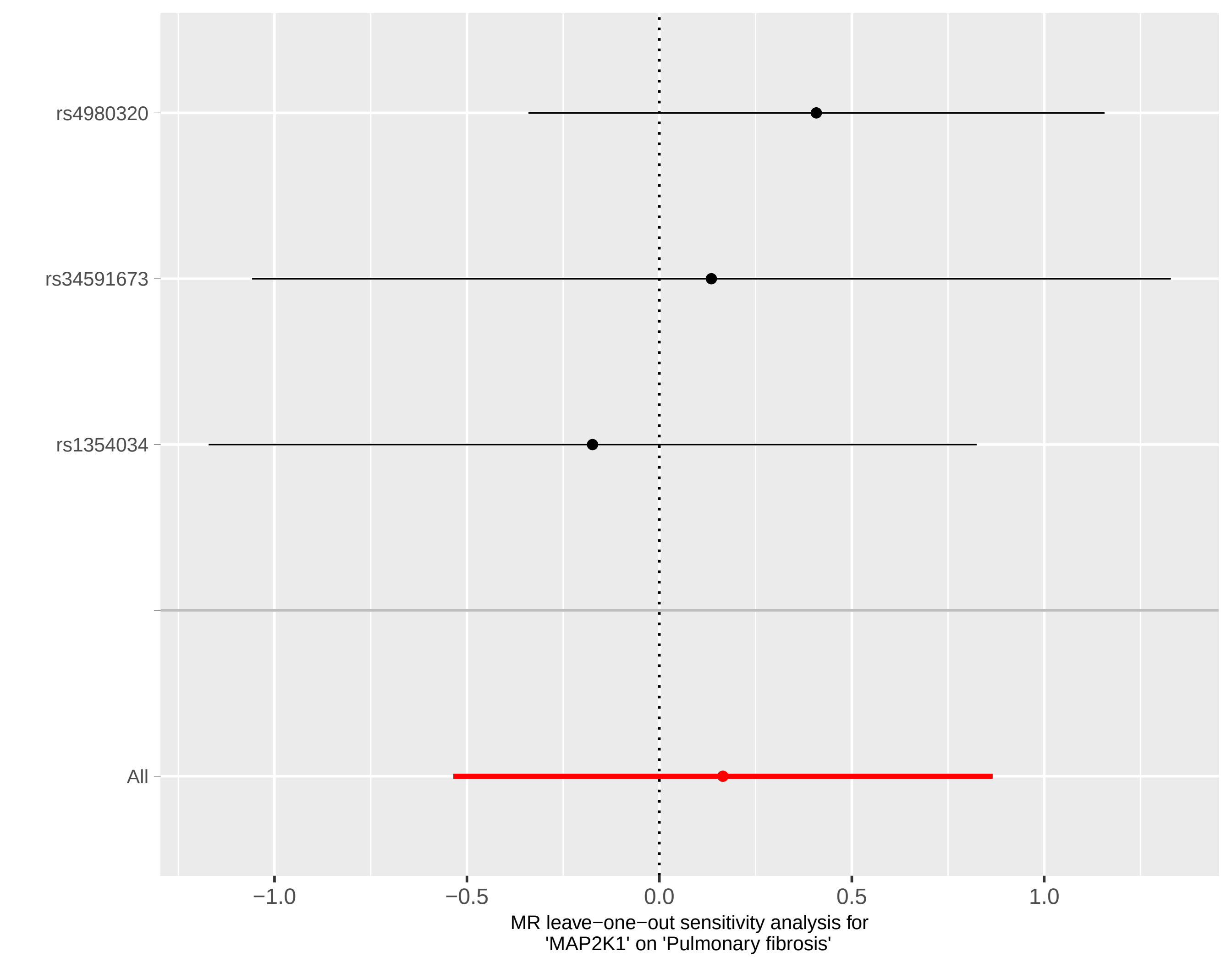
rs4980320
rs34591673
rs1354034
All
−1.0
−0.5
0.0
0.5
1.0
MR leave−one−out sensitivity analysis for
'MAP2K1' on 'Pulmonary fibrosis'

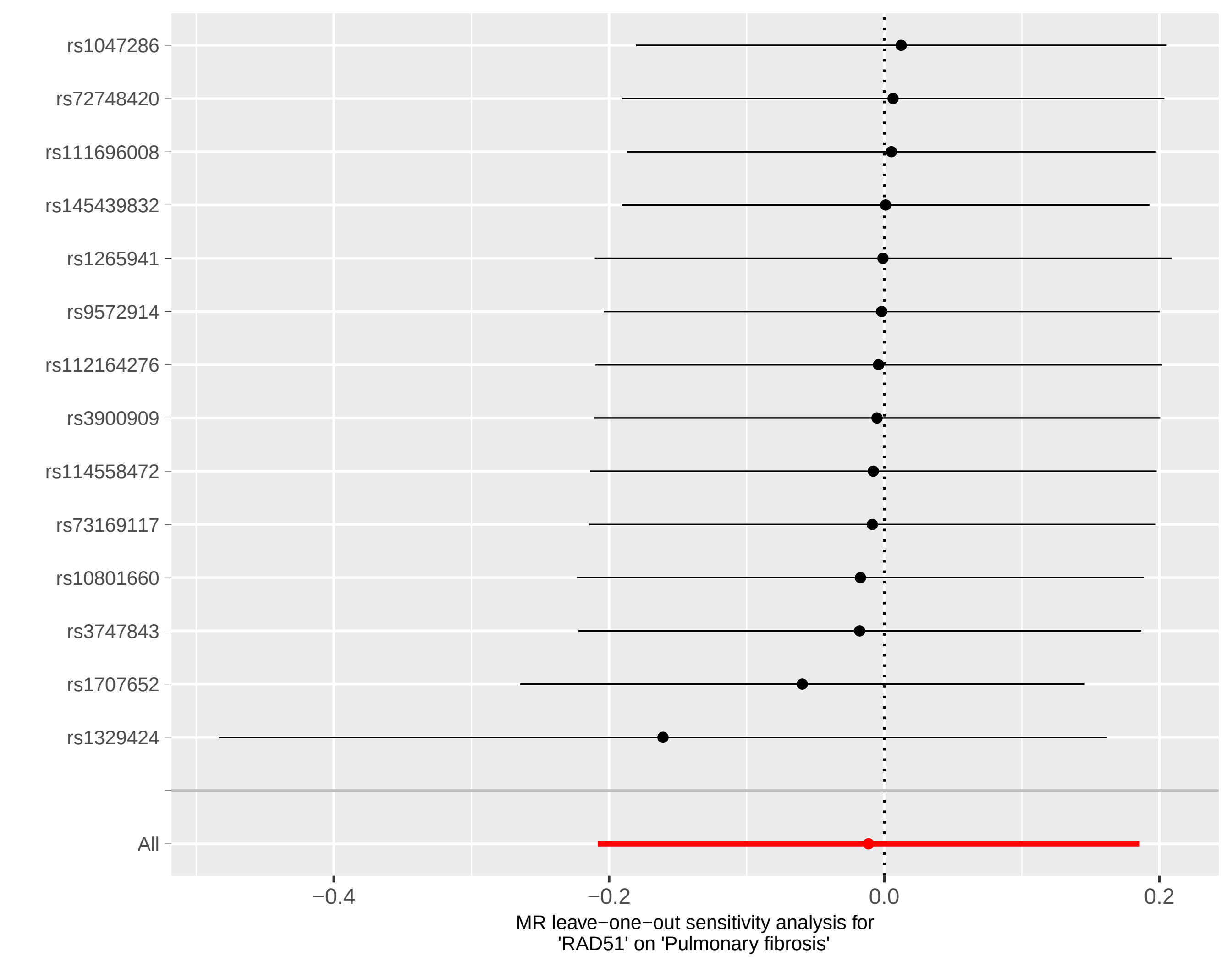

rs1047286
rs72748420
rs111696008
rs145439832
rs1265941
rs9572914
rs112164276
rs3900909
rs114558472
rs73169117
rs10801660
rs3747843
rs1707652
rs1329424
All
−0.4
−0.2
0.0
0.2
MR leave−one−out sensitivity analysis for
'RAD51' on 'Pulmonary fibrosis'

Insufficient number of SNPs

Insufficient number of SNPs

Insufficient number of SNPs

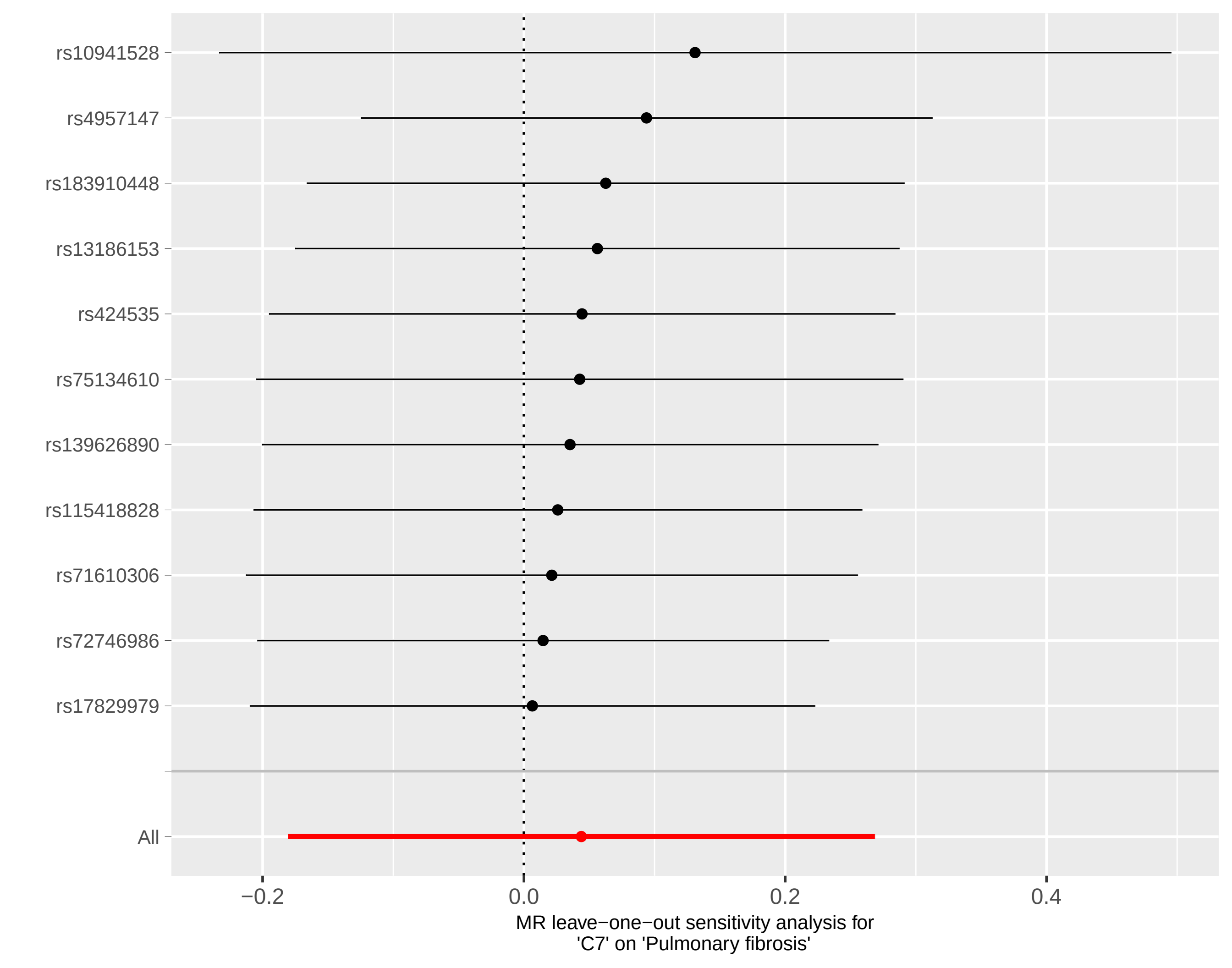
rs10941528
rs4957147
rs183910448
rs13186153
rs424535
rs75134610
rs139626890
rs115418828
rs71610306
rs72746986
rs17829979
All
−0.2
0.0
0.2
0.4
MR leave−one−out sensitivity analysis for
'C7' on 'Pulmonary fibrosis'

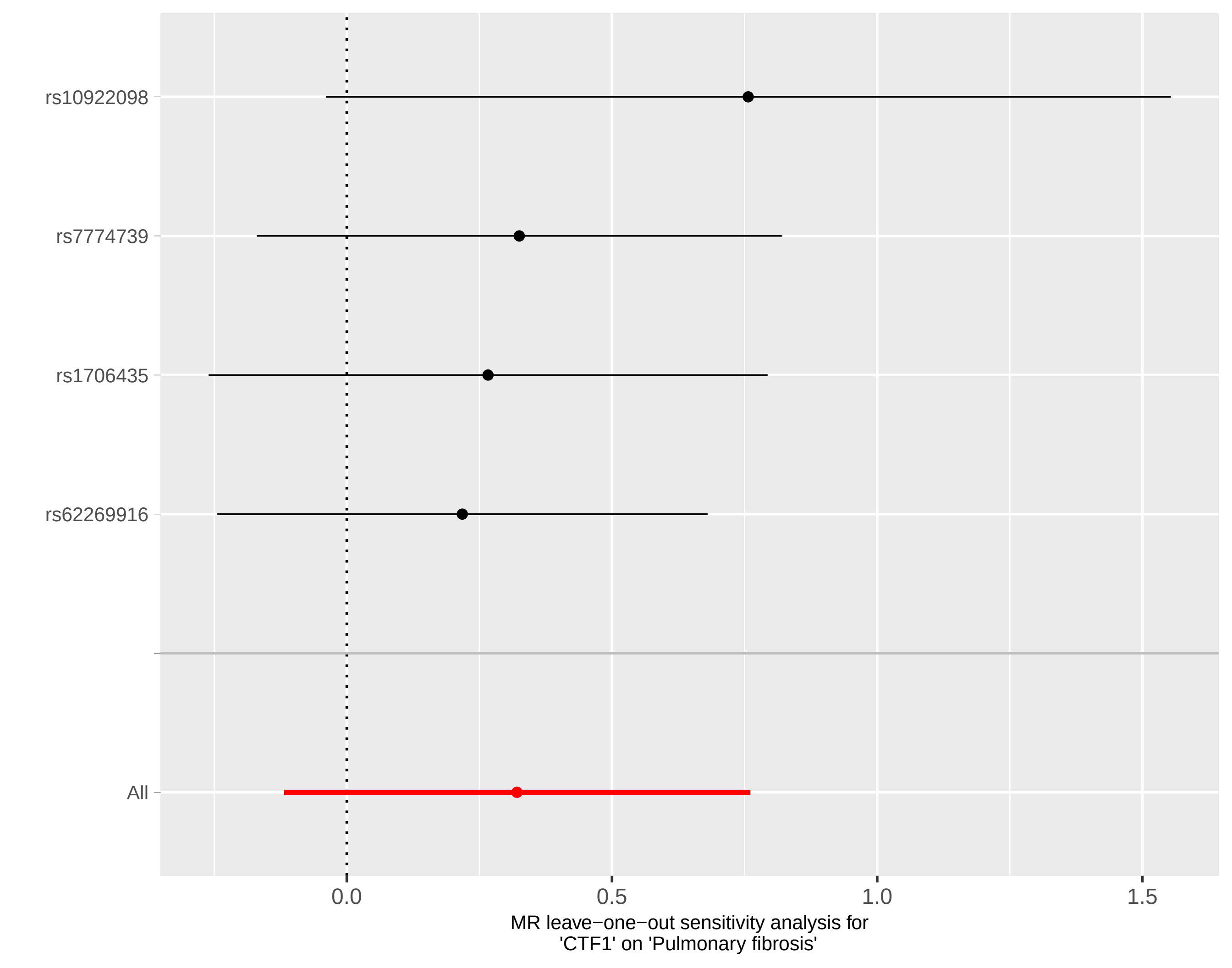
rs10922098
rs7774739
rs1706435
rs62269916
All
0.0
0.5
1.0
1.5
MR leave−one−out sensitivity analysis for
'CTF1' on 'Pulmonary fibrosis'

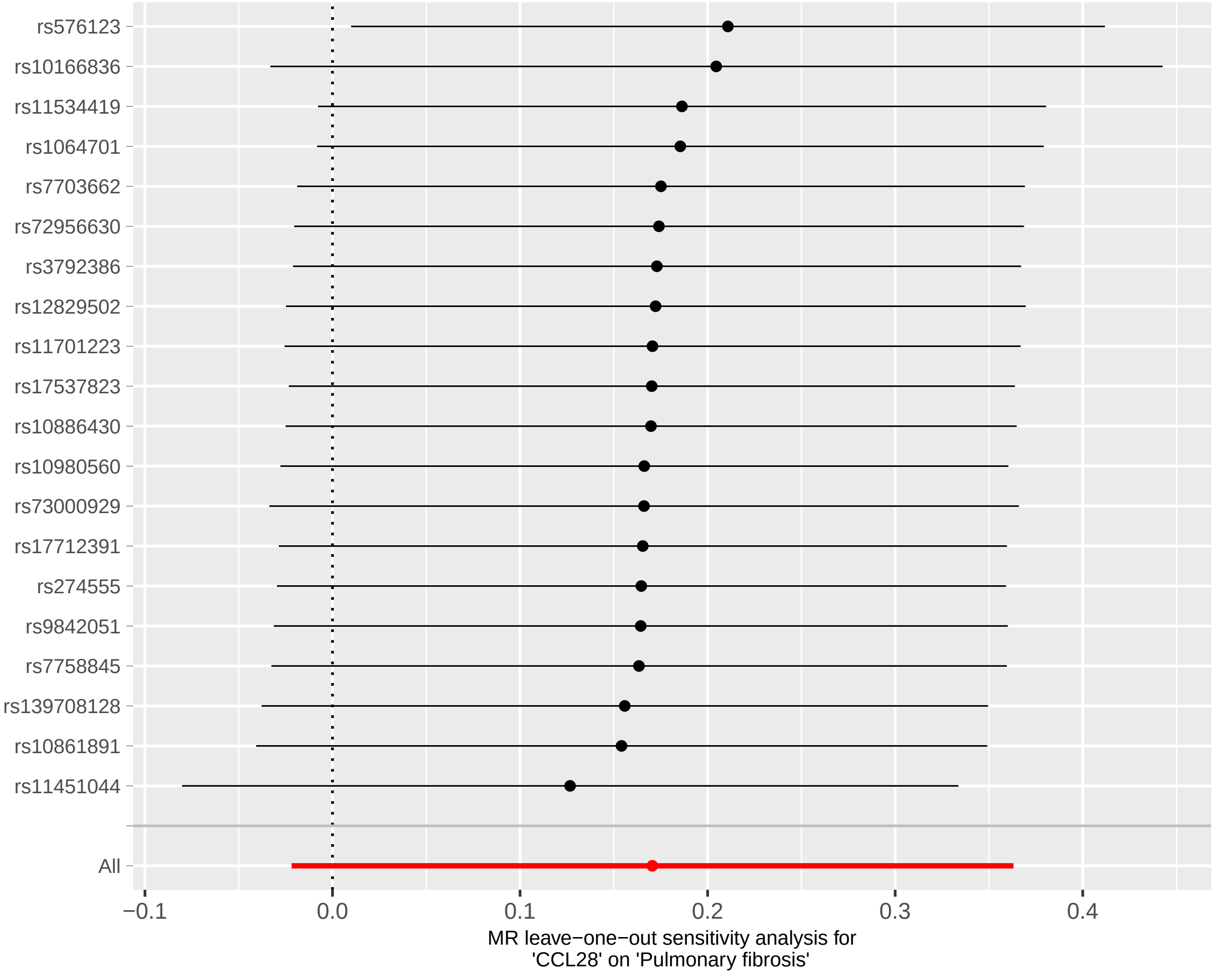
rs576123
rs10166836
rs11534419
rs1064701
rs7703662
rs72956630
rs3792386
rs12829502
rs11701223
rs17537823
rs10886430
rs10980560
rs73000929
rs17712391
rs274555
rs9842051
rs7758845
rs139708128
rs10861891
rs11451044
All
−0.1
0.0
0.1
0.2
0.3
0.4
MR leave−one−out sensitivity analysis for
'CCL28' on 'Pulmonary fibrosis'

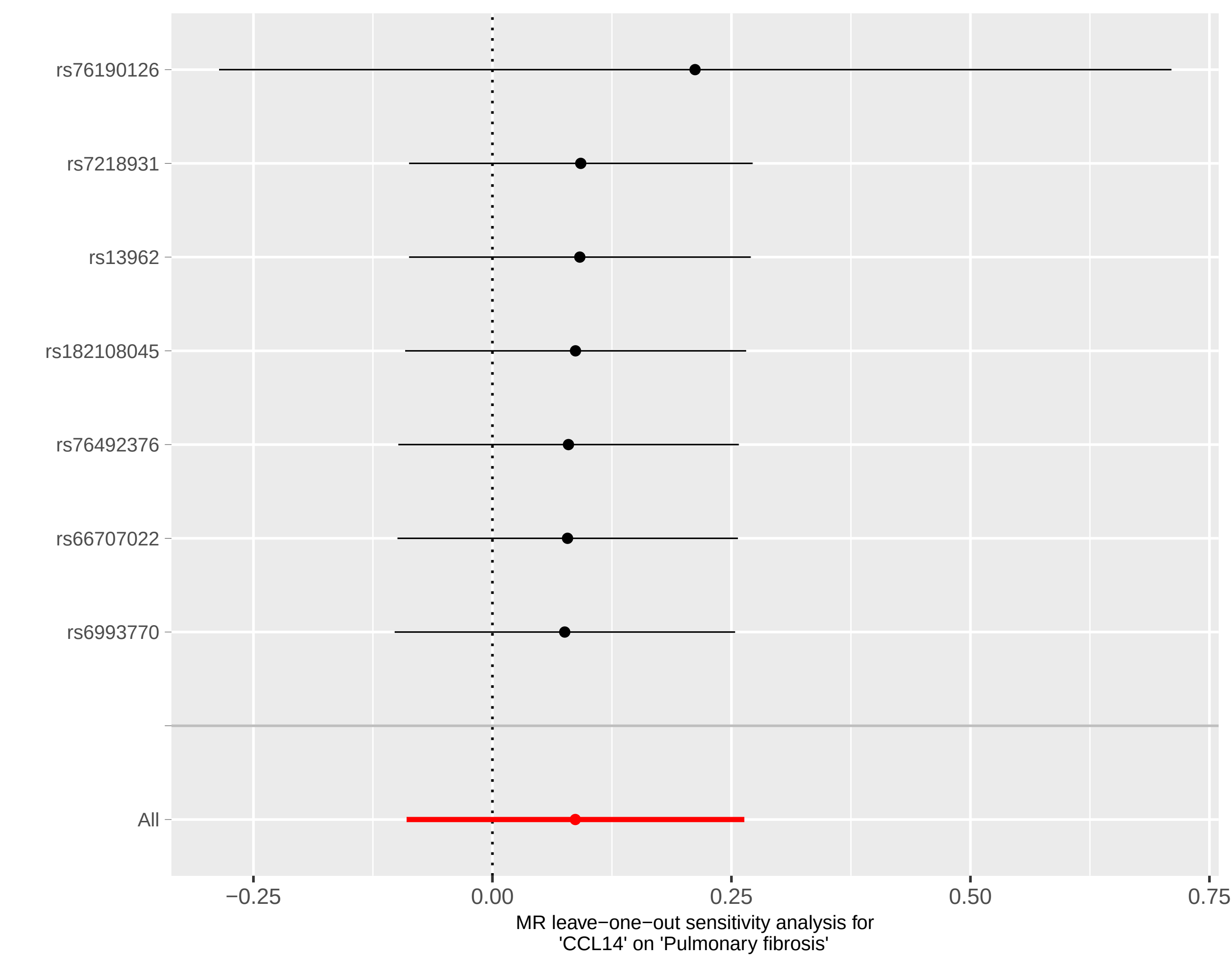
rs76190126
rs7218931
rs13962
rs182108045
rs76492376
rs66707022
rs6993770
All
−0.25
0.00
0.25
0.50
0.75
MR leave−one−out sensitivity analysis for
'CCL14' on 'Pulmonary fibrosis'

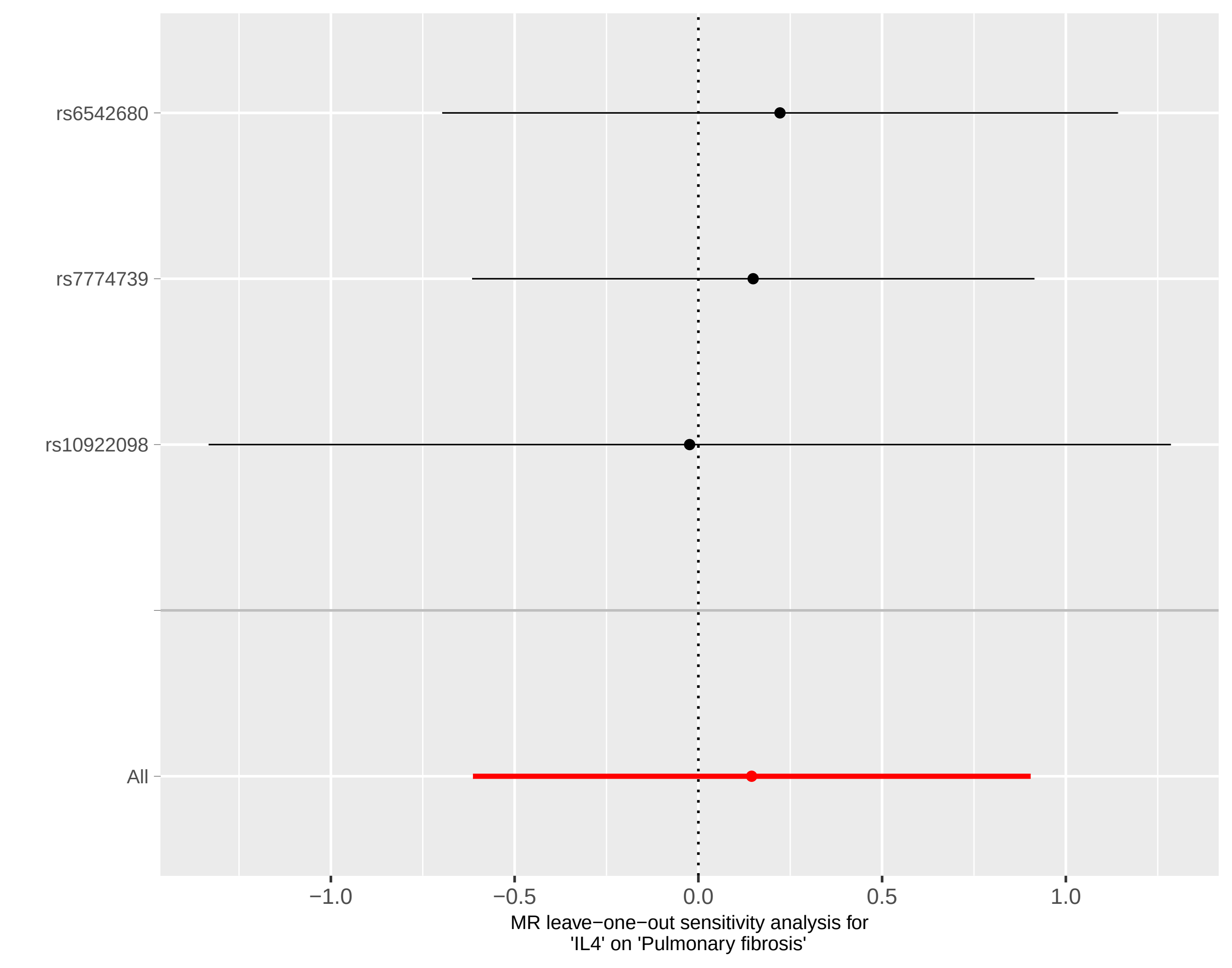

rs6542680
rs7774739
rs10922098
All
−1.0
−0.5
0.0
0.5
1.0
MR leave−one−out sensitivity analysis for
'IL4' on 'Pulmonary fibrosis'

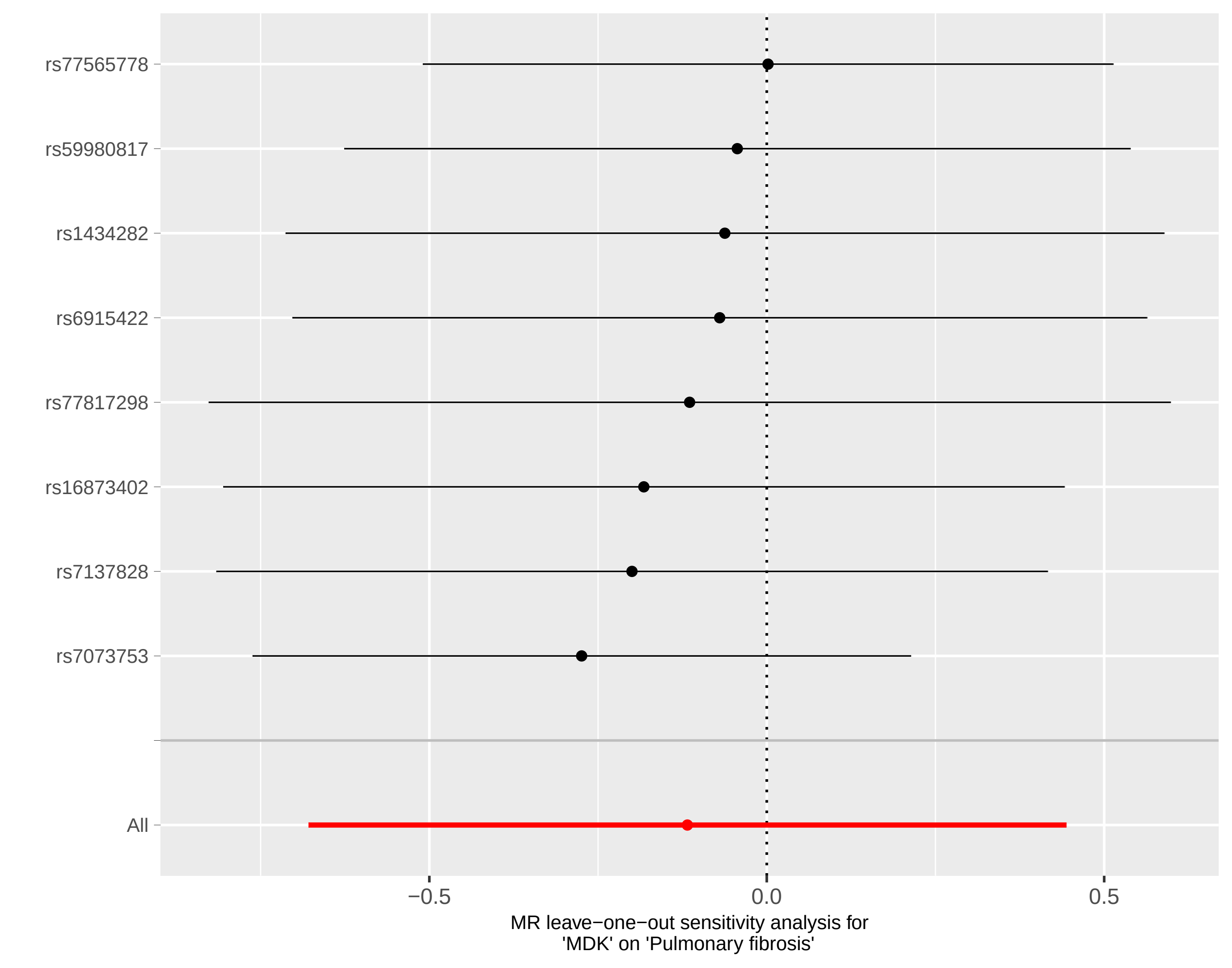
rs77565778
rs59980817
rs1434282
rs6915422
rs77817298
rs16873402
rs7137828
rs7073753
All
−0.5
0.0
0.5
MR leave−one−out sensitivity analysis for
'MDK' on 'Pulmonary fibrosis'

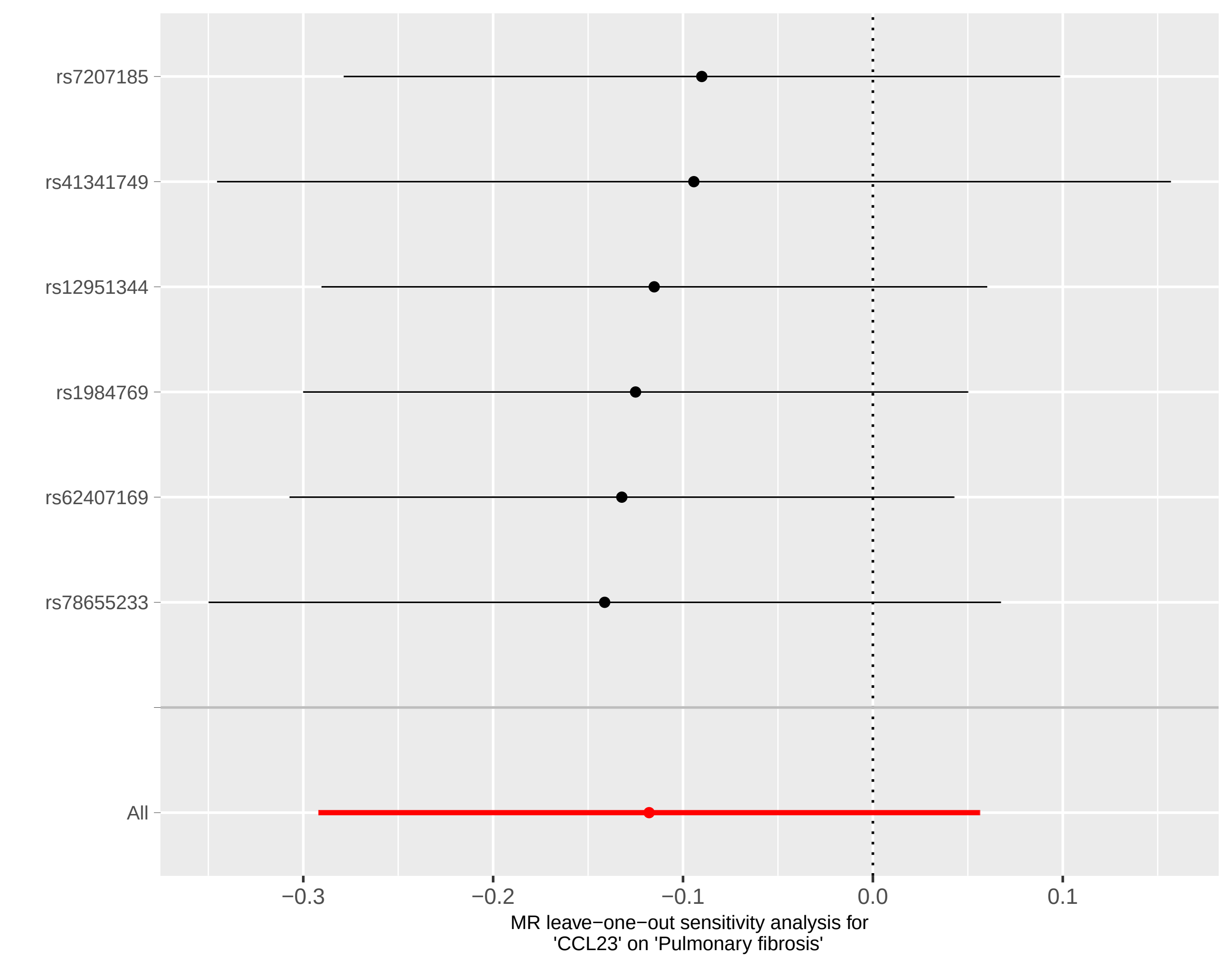
rs7207185
rs41341749
rs12951344
rs1984769
rs62407169
rs78655233
All
−0.3
−0.2
−0.1
0.0
0.1
MR leave−one−out sensitivity analysis for
'CCL23' on 'Pulmonary fibrosis'

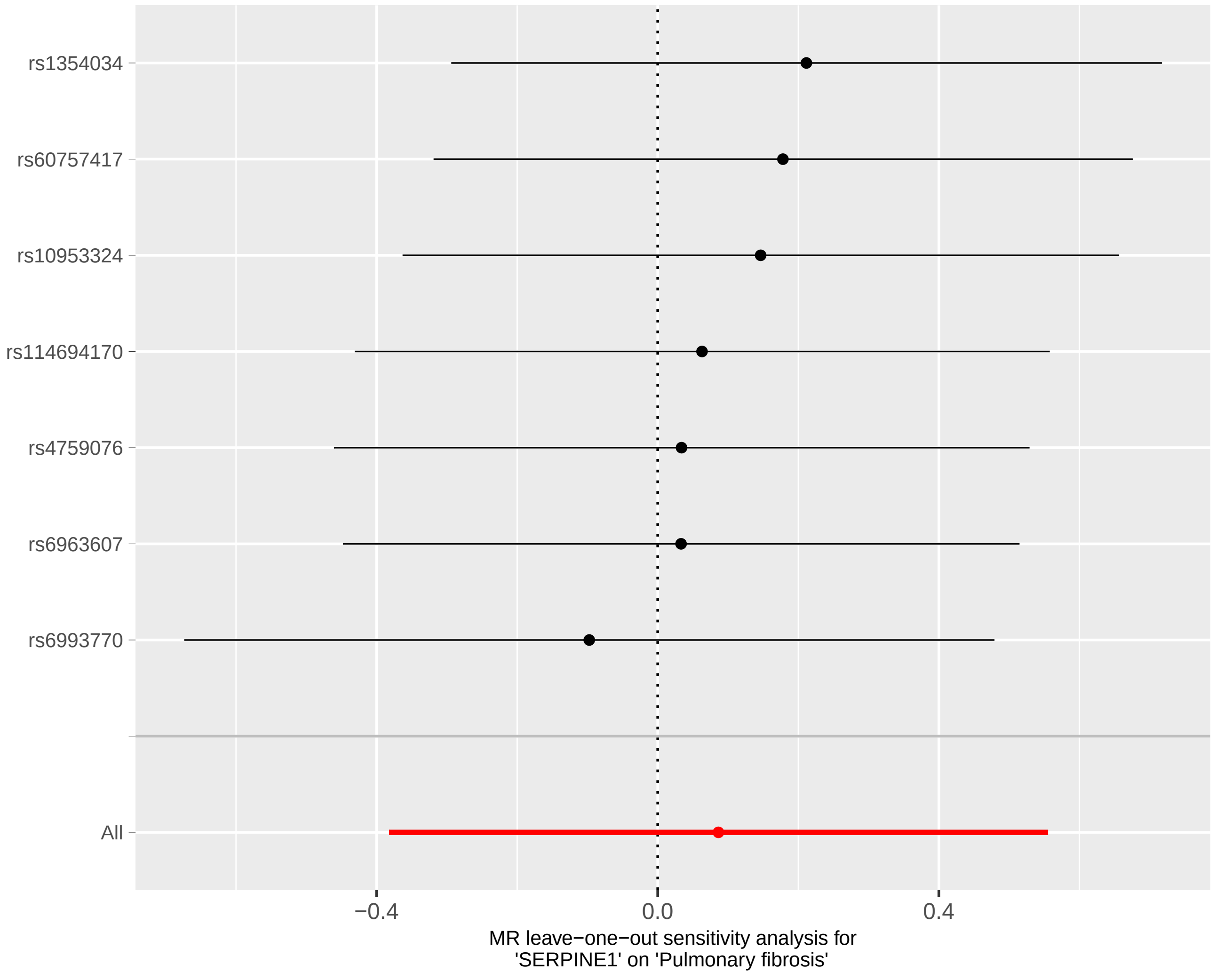
rs1354034
rs60757417
rs10953324
rs114694170
rs4759076
rs6963607
rs6993770
All
−0.4
0.0
0.4
MR leave−one−out sensitivity analysis for
'SERPINE1' on 'Pulmonary fibrosis'

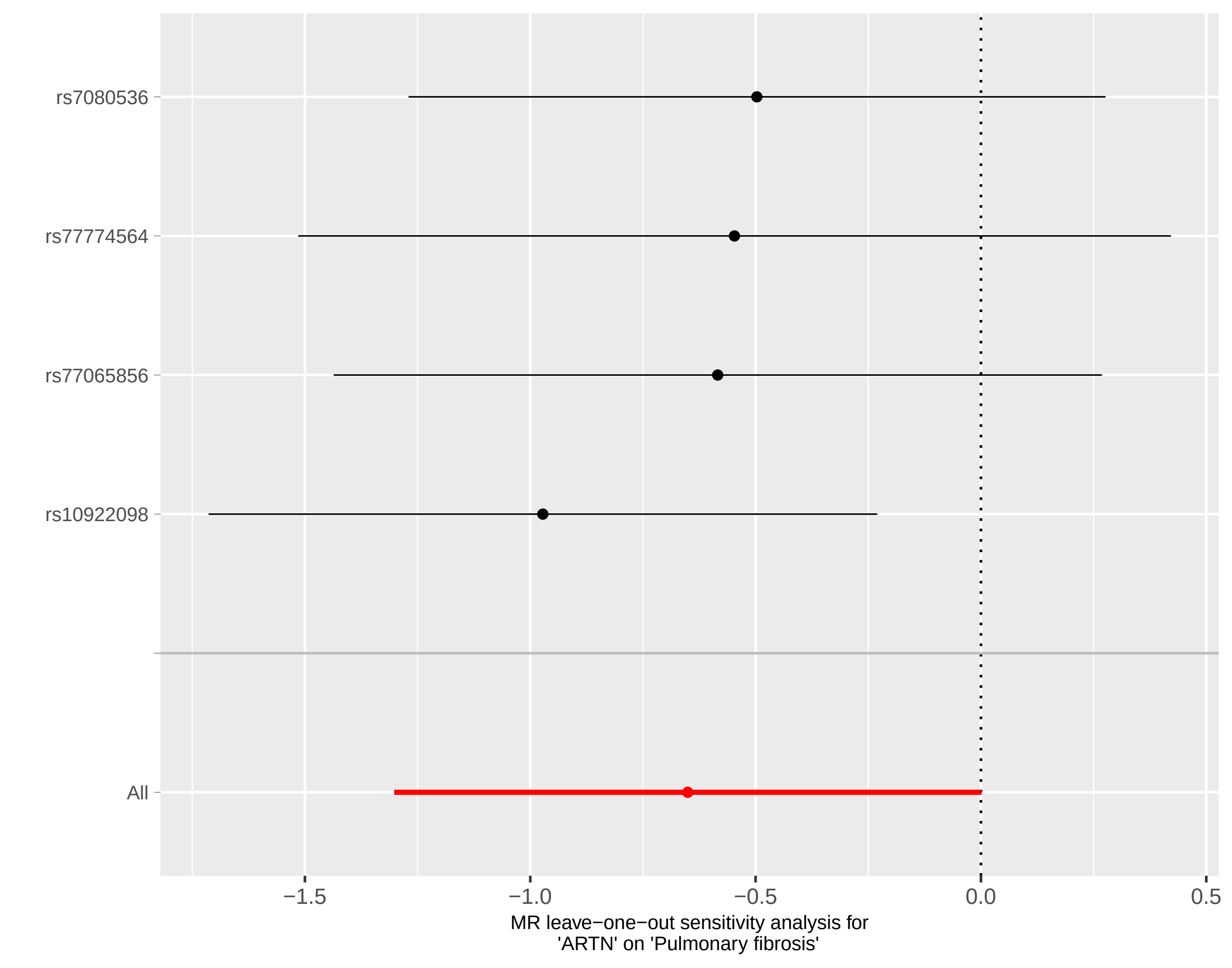
rs7080536
rs77774564
rs77065856
rs10922098
All
−1.5
−1.0
−0.5
0.0
0.5
MR leave−one−out sensitivity analysis for
'ARTN' on 'Pulmonary fibrosis'

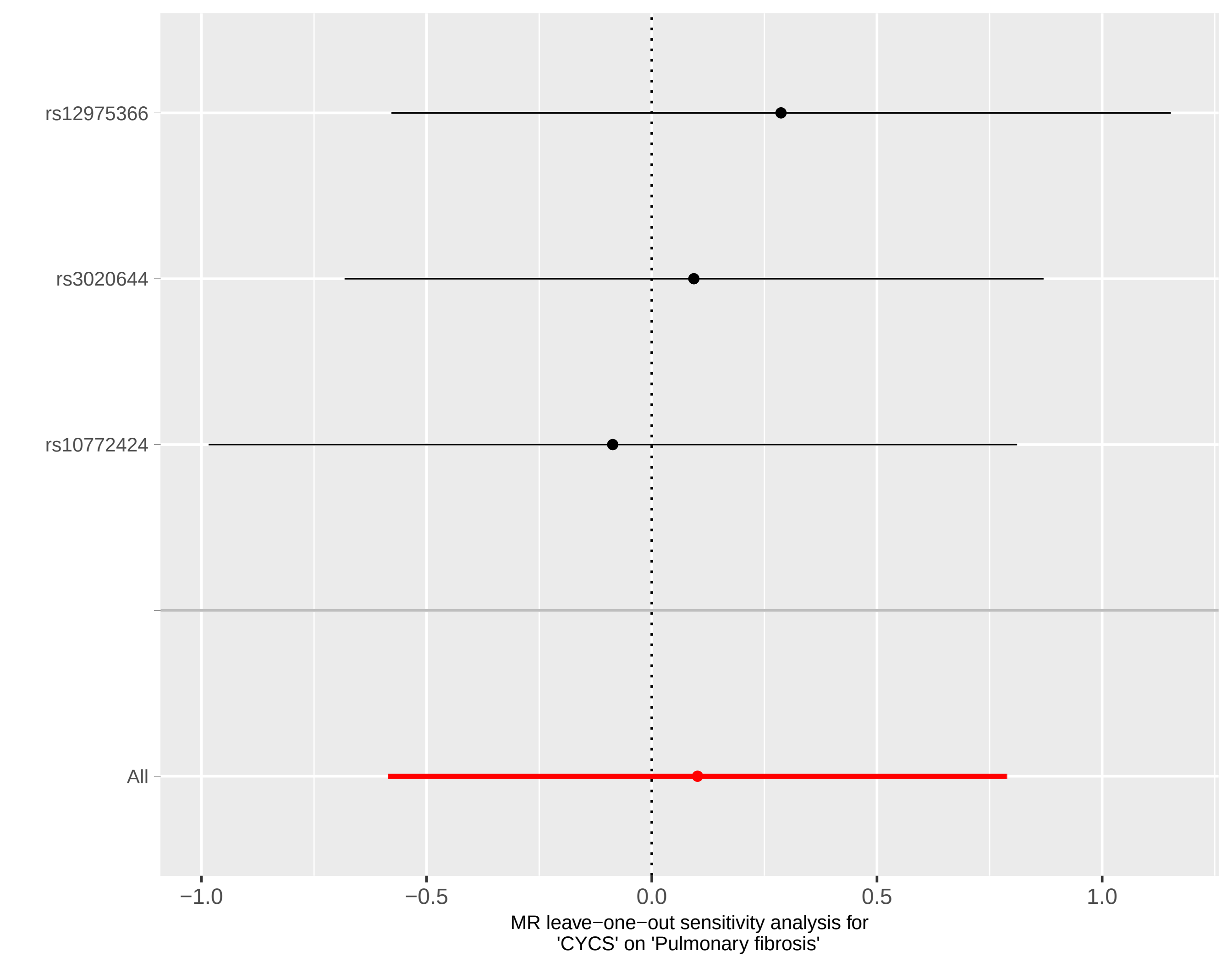
rs12975366
rs3020644
rs10772424
All
−1.0
−0.5
0.0
0.5
1.0
MR leave−one−out sensitivity analysis for
'CYCS' on 'Pulmonary fibrosis'

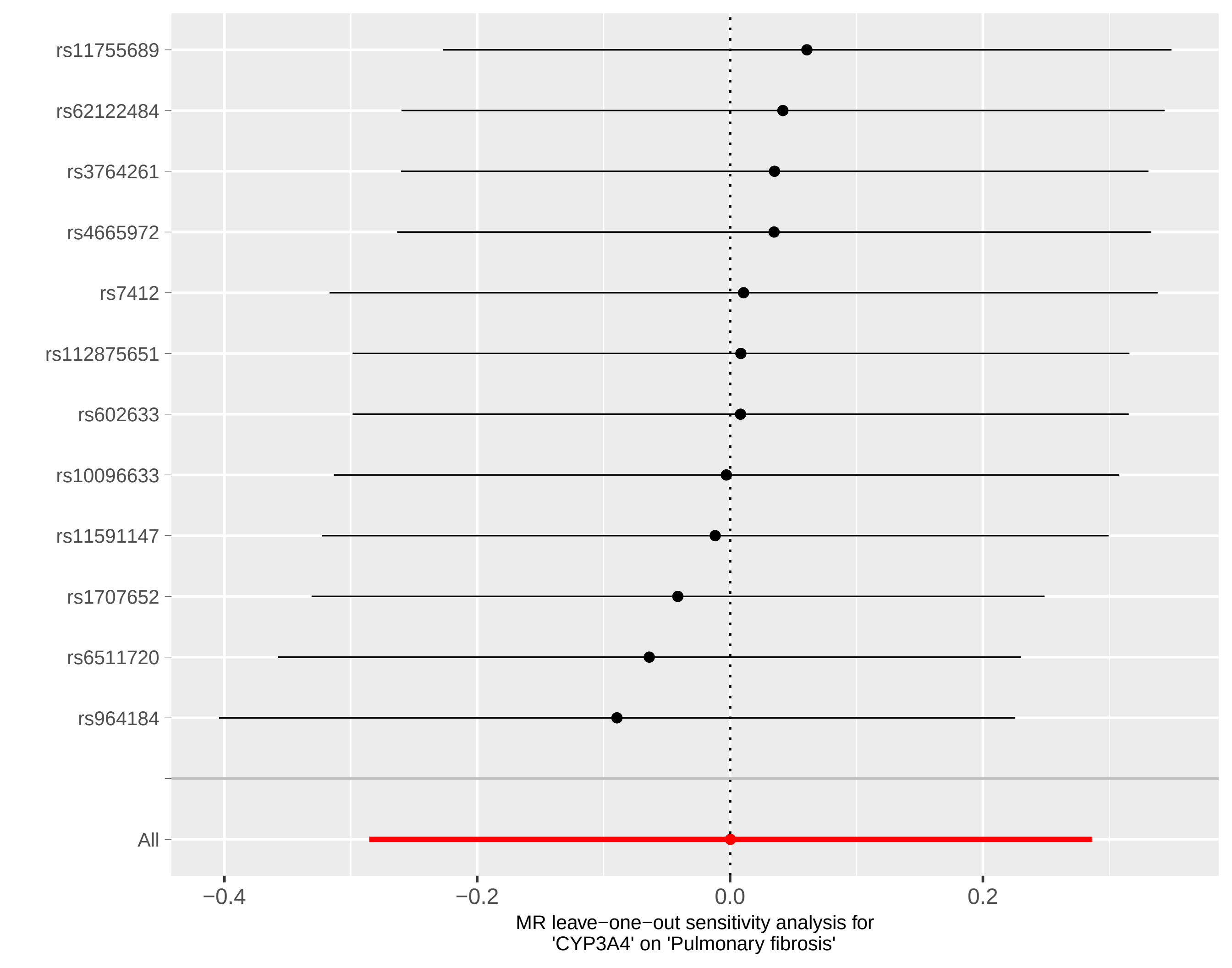
rs11755689
rs62122484
rs3764261
rs4665972
rs7412
rs112875651
rs602633
rs10096633
rs11591147
rs1707652
rs6511720
rs964184
All
−0.4
−0.2
0.0
0.2
MR leave−one−out sensitivity analysis for
'CYP3A4' on 'Pulmonary fibrosis'

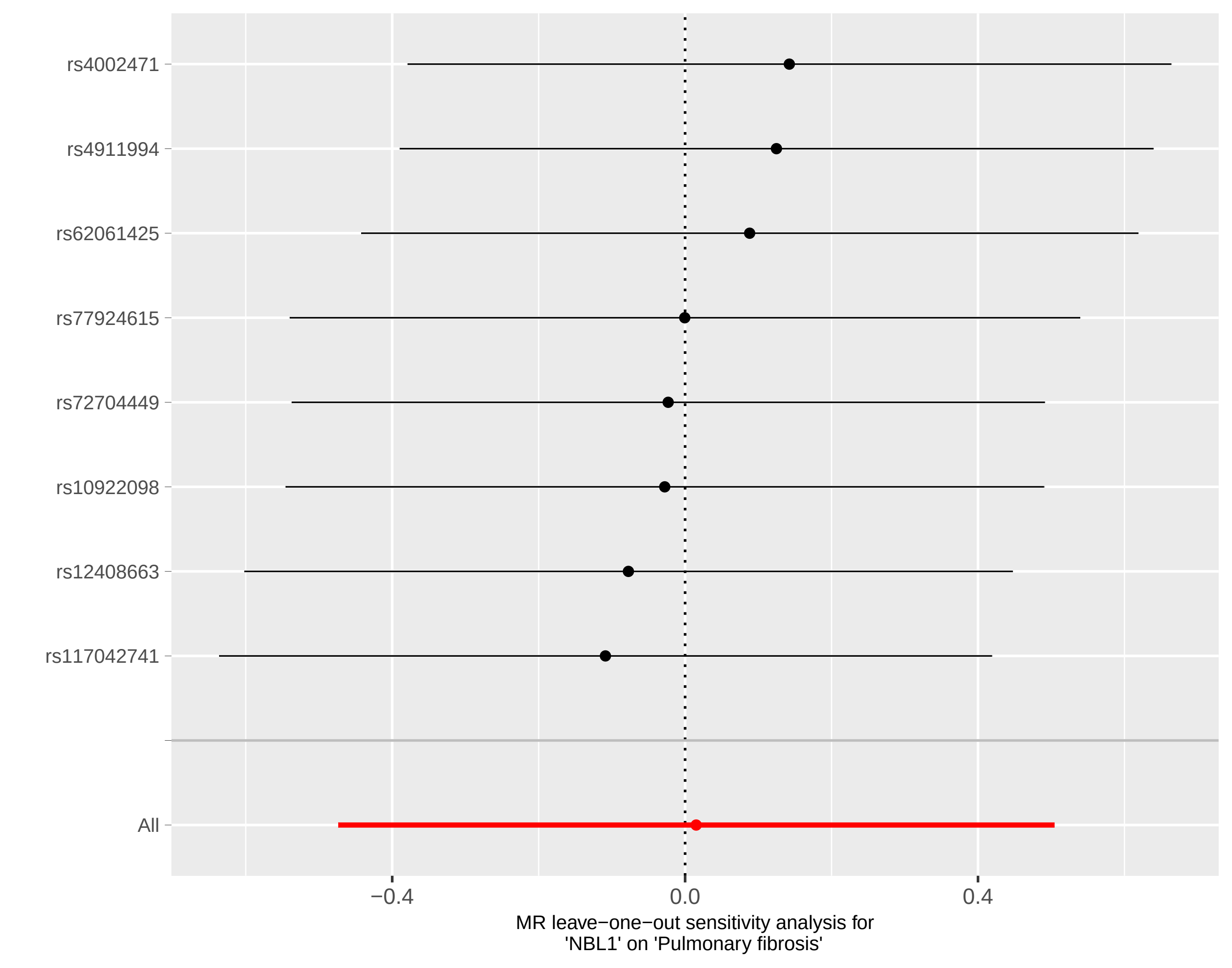

rs4002471
rs4911994
rs62061425
rs77924615
rs72704449
rs10922098
rs12408663
rs117042741
All
−0.4
0.0
0.4
MR leave−one−out sensitivity analysis for
'NBL1' on 'Pulmonary fibrosis'

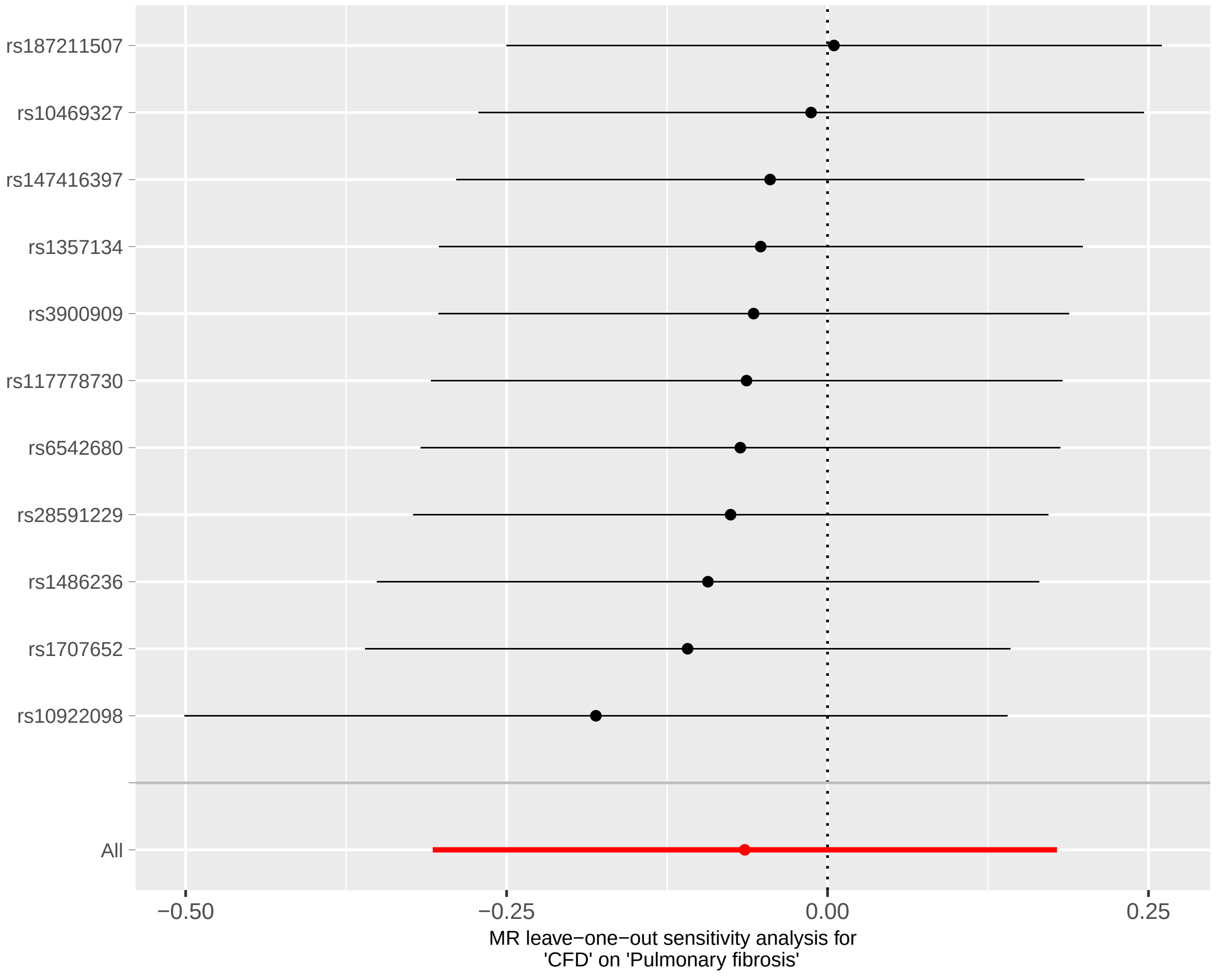
rs187211507
rs10469327
rs147416397
rs1357134
rs3900909
rs117778730
rs6542680
rs28591229
rs1486236
rs1707652
rs10922098
All
−0.50
−0.25
0.00
0.25
MR leave−one−out sensitivity analysis for
'CFD' on 'Pulmonary fibrosis'

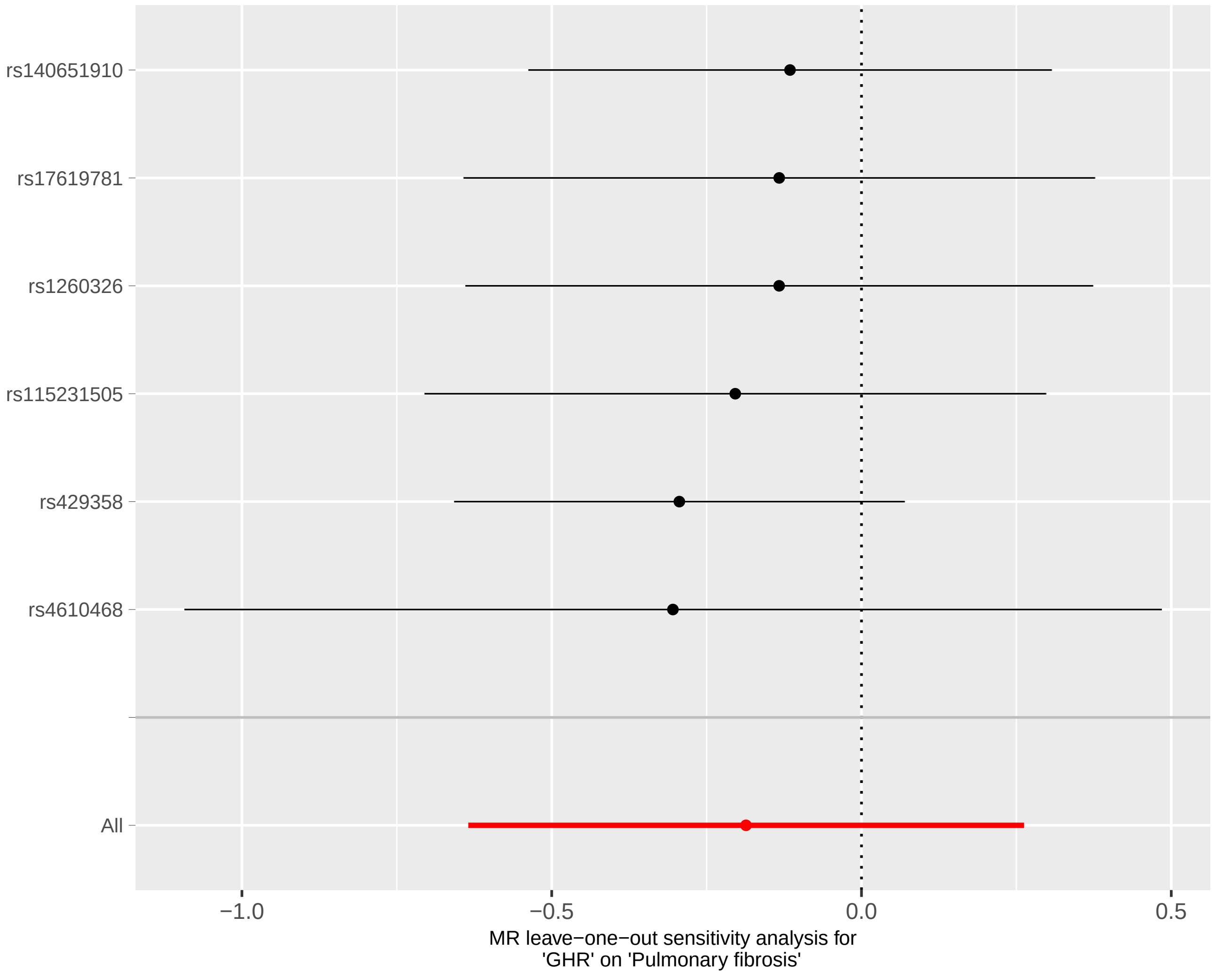
rs140651910
rs17619781
rs1260326
rs115231505
rs429358
rs4610468
All
−1.0
−0.5
0.0
0.5
MR leave−one−out sensitivity analysis for
'GHR' on 'Pulmonary fibrosis'

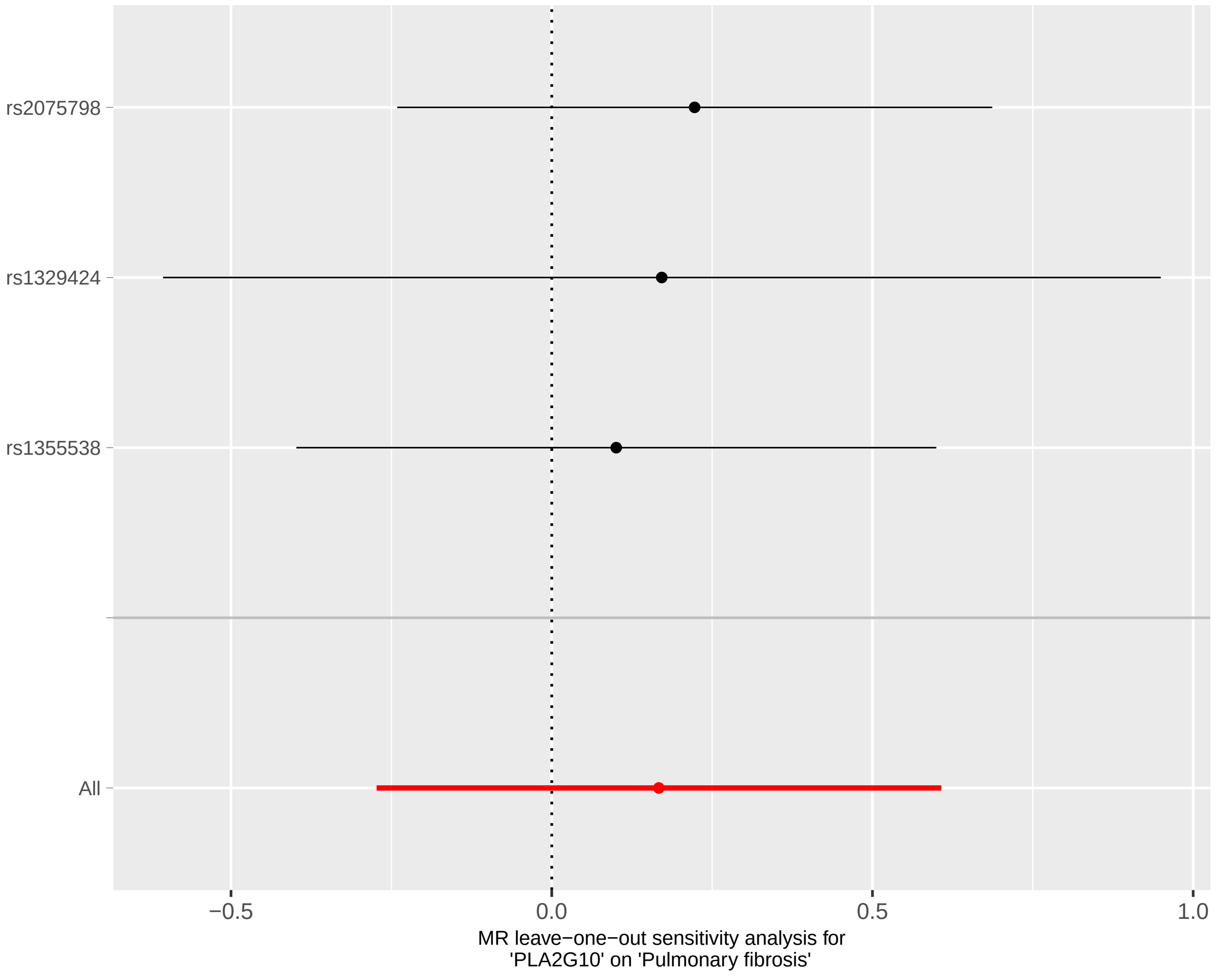
rs2075798
rs1329424
rs1355538
All
−0.5
0.0
0.5
1.0
MR leave−one−out sensitivity analysis for
'PLA2G10' on 'Pulmonary fibrosis'

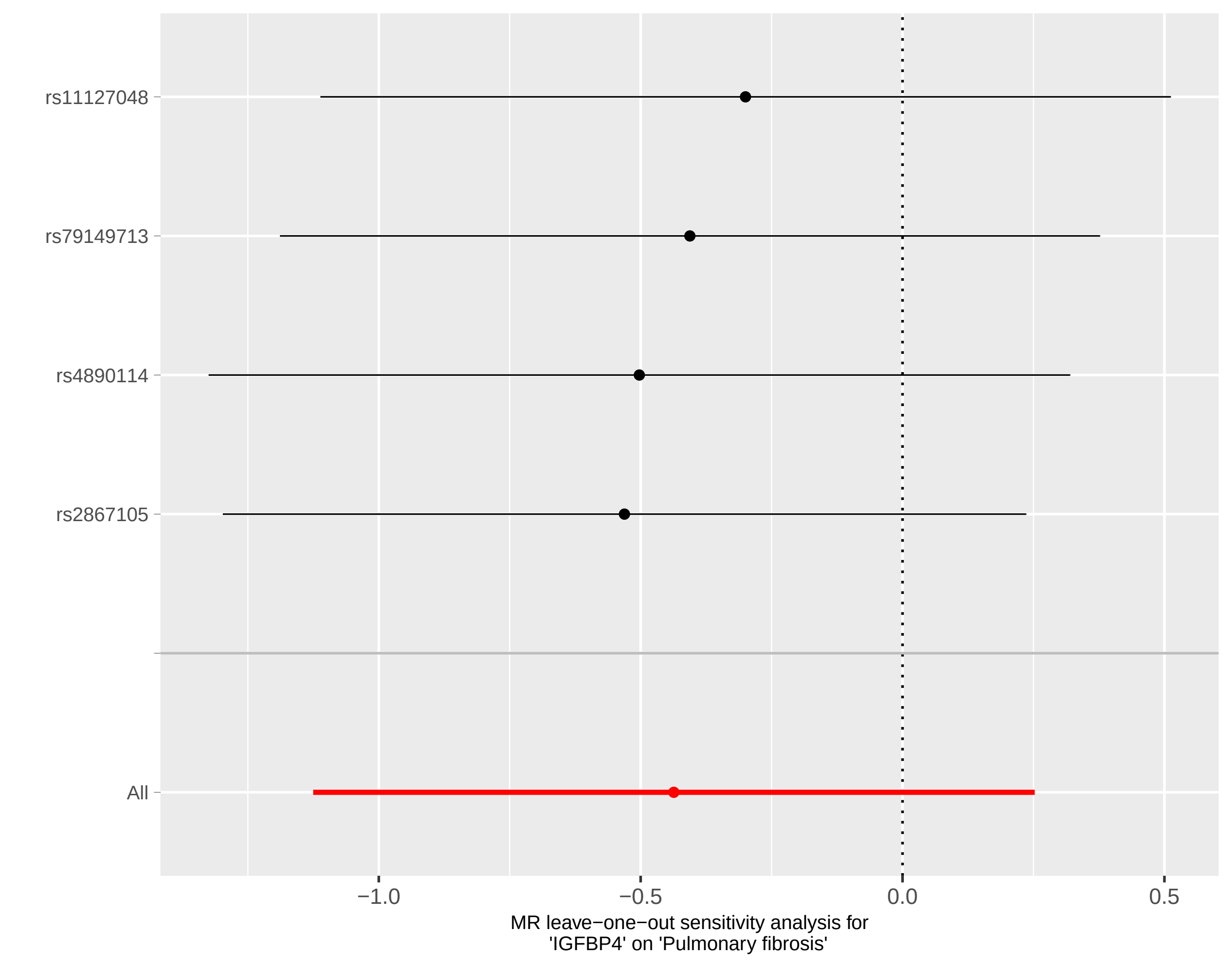
rs11127048
rs79149713
rs4890114
rs2867105
All
−1.0
−0.5
0.0
0.5
MR leave−one−out sensitivity analysis for
'IGFBP4' on 'Pulmonary fibrosis'

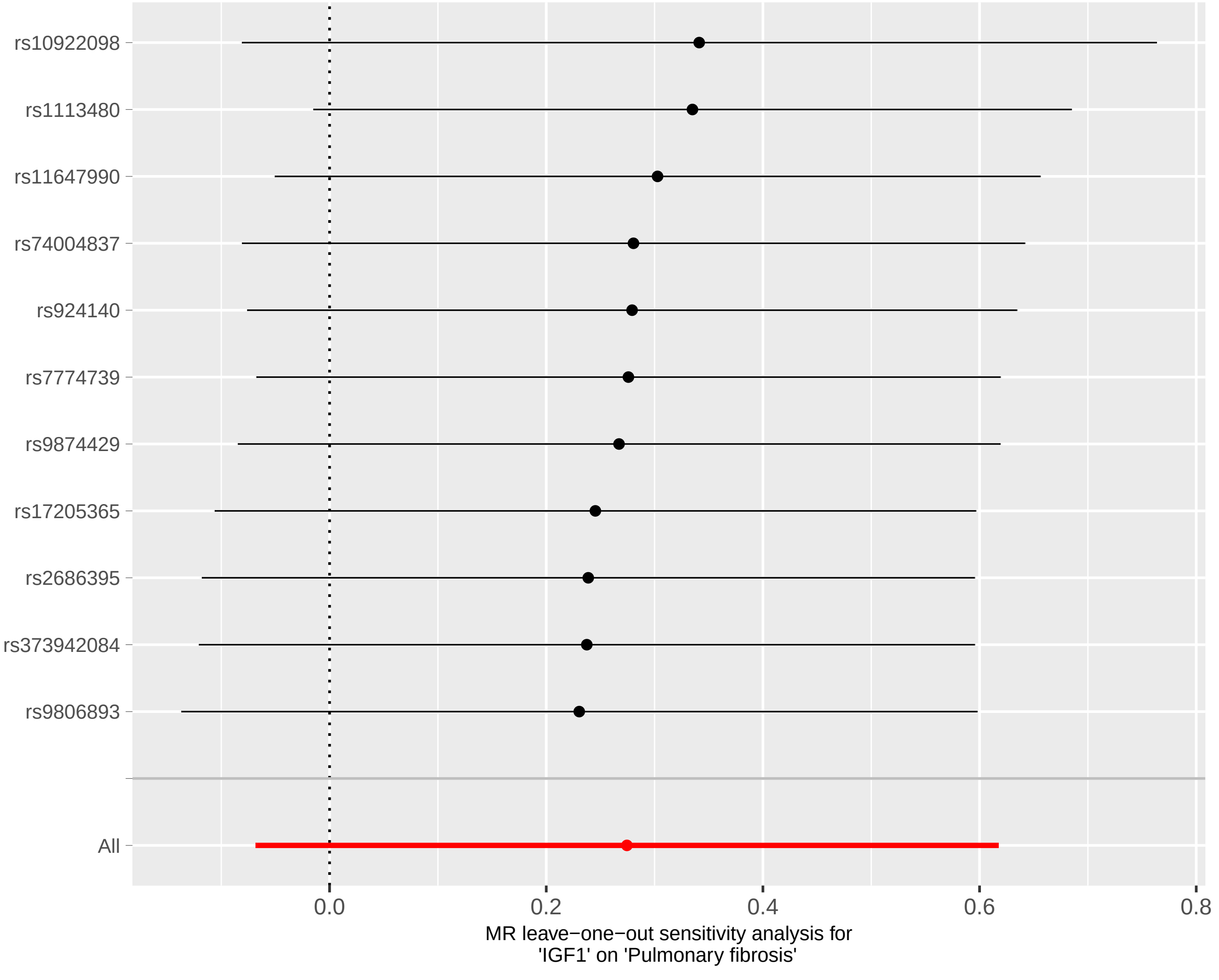
rs10922098
rs1113480
rs11647990
rs74004837
rs924140
rs7774739
rs9874429
rs17205365
rs2686395
rs373942084
rs9806893
All
0.0
0.2
0.4
0.6
0.8
MR leave−one−out sensitivity analysis for
'IGF1' on 'Pulmonary fibrosis'

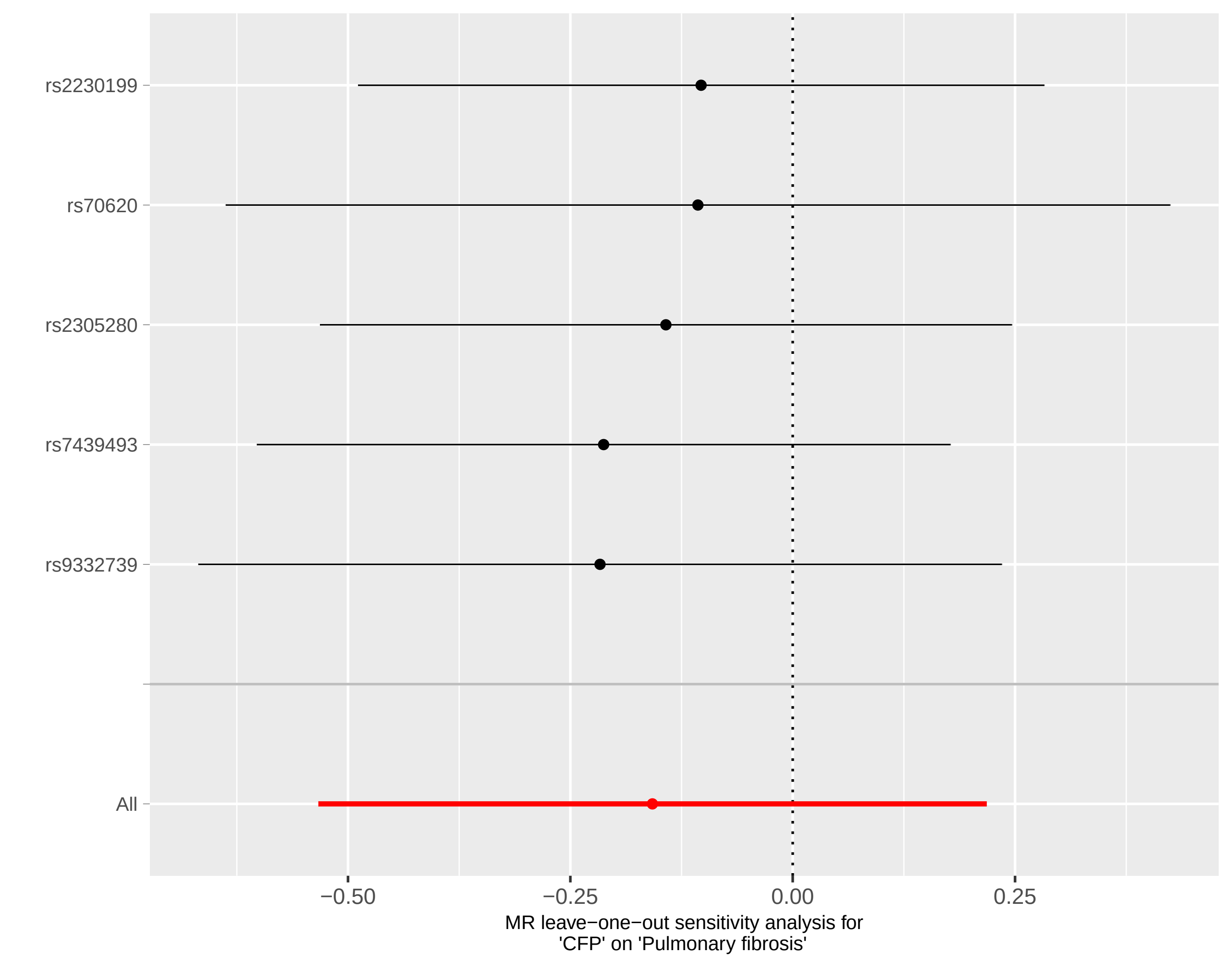
rs2230199
rs70620
rs2305280
rs7439493
rs9332739
All
−0.50
−0.25
0.00
0.25
MR leave−one−out sensitivity analysis for
'CFP' on 'Pulmonary fibrosis'

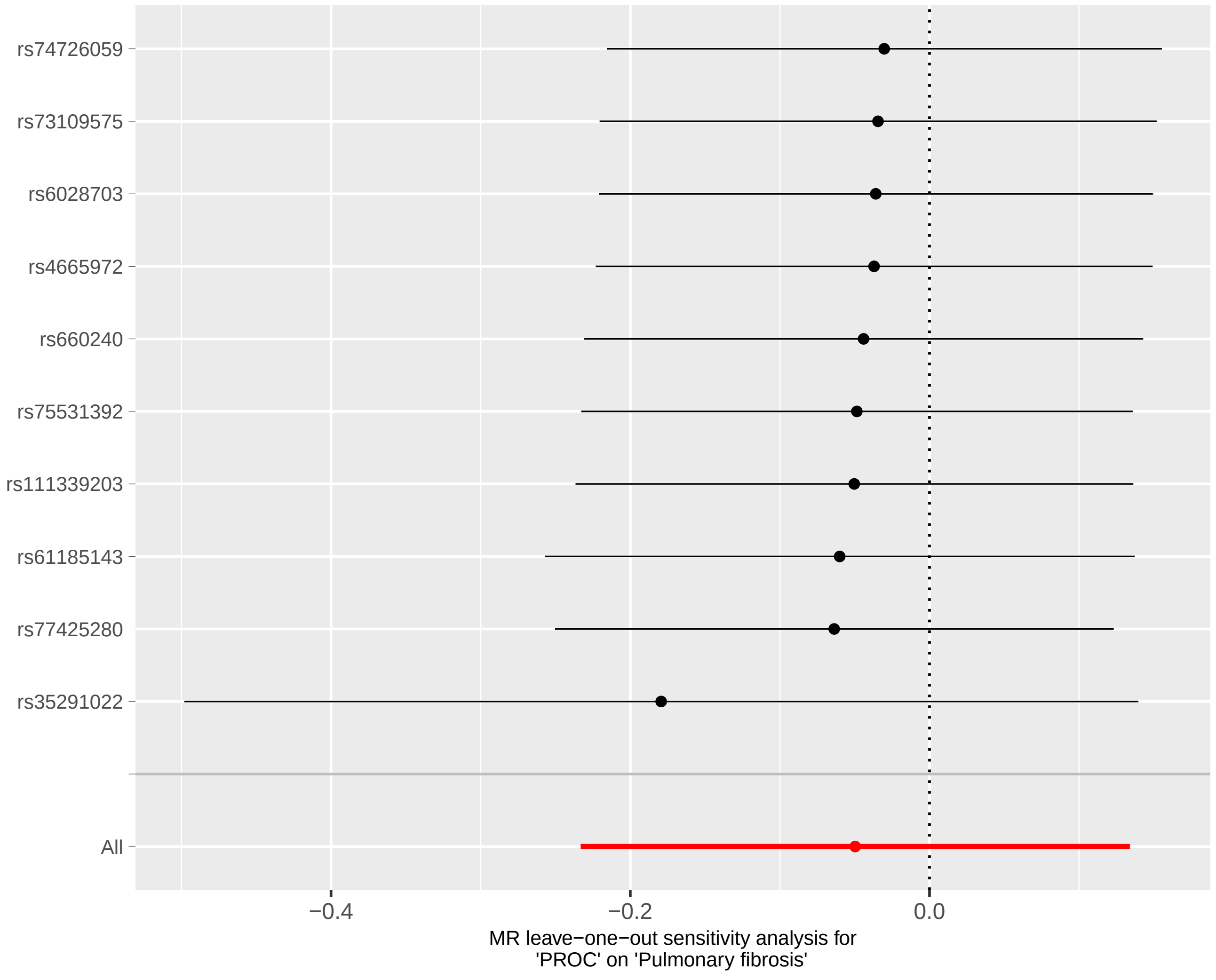
rs74726059
rs73109575
rs6028703
rs4665972
rs660240
rs75531392
rs111339203
rs61185143
rs77425280
rs35291022
All
−0.4
−0.2
0.0
MR leave−one−out sensitivity analysis for
'PROC' on 'Pulmonary fibrosis'

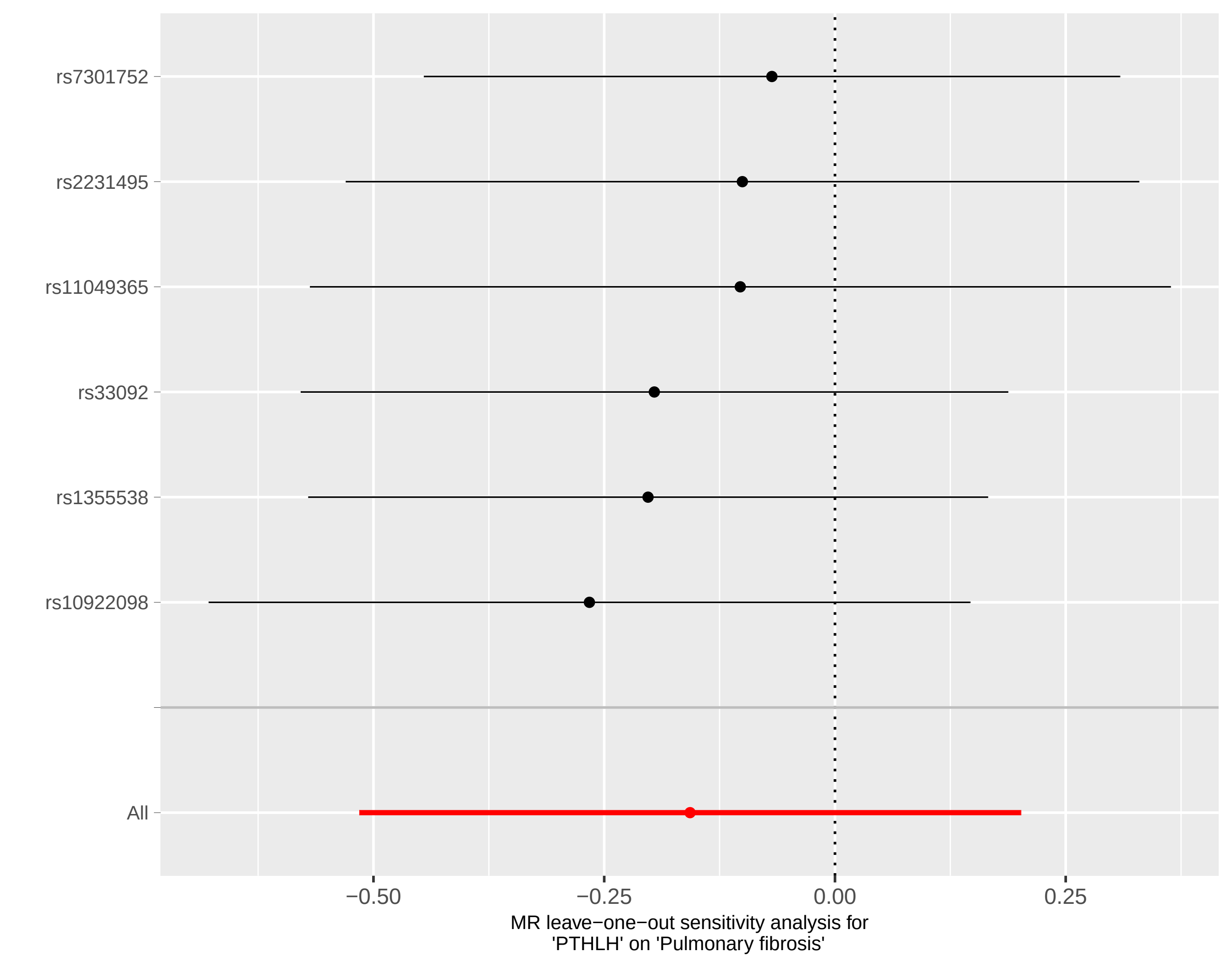
rs7301752
rs2231495
rs11049365
rs33092
rs1355538
rs10922098
All
−0.50
−0.25
0.00
0.25
MR leave−one−out sensitivity analysis for
'PTHLH' on 'Pulmonary fibrosis'

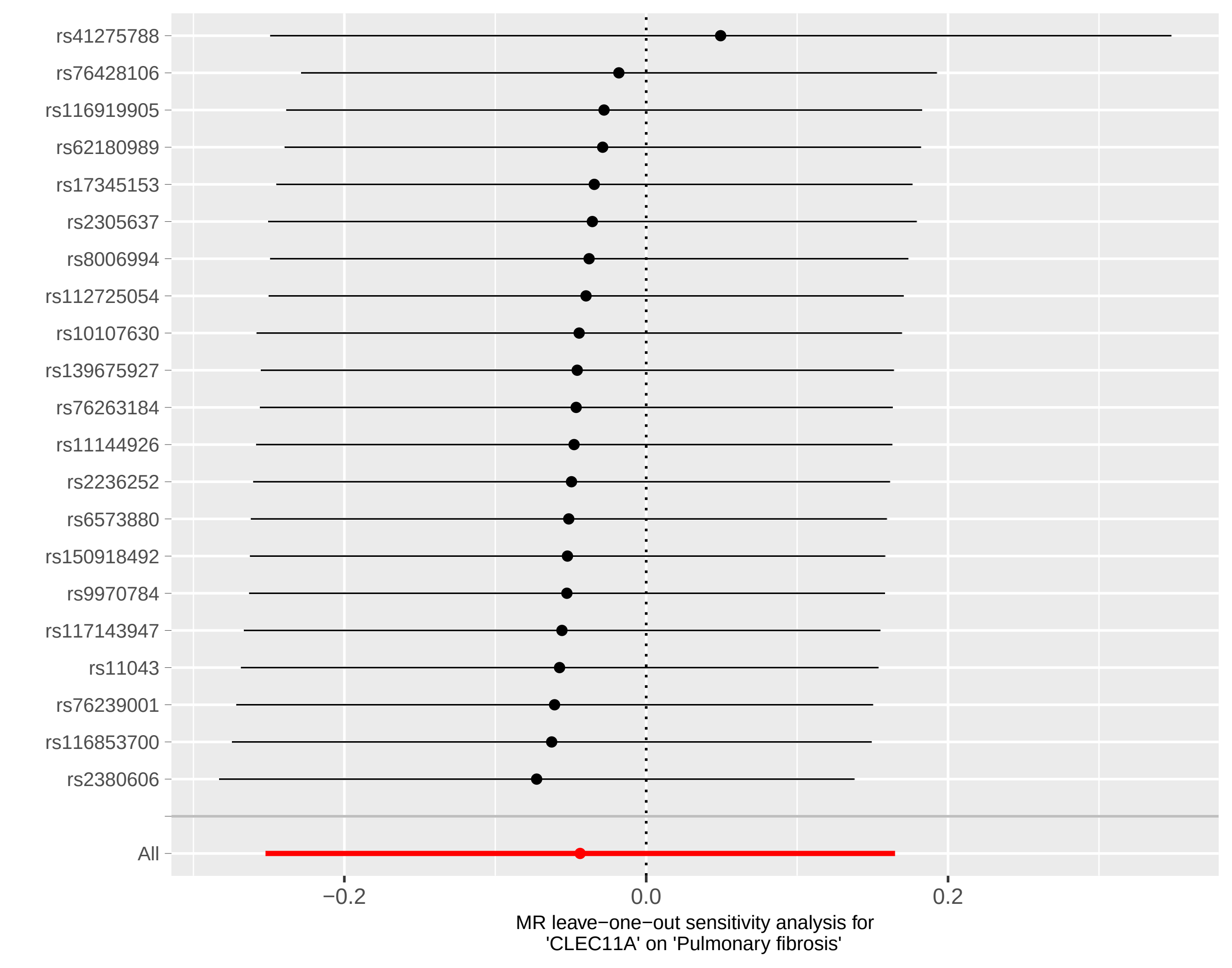
rs41275788
rs76428106
rs116919905
rs62180989
rs17345153
rs2305637
rs8006994
rs112725054
rs10107630
rs139675927
rs76263184
rs11144926
rs2236252
rs6573880
rs150918492
rs9970784
rs117143947
rs11043
rs76239001
rs116853700
rs2380606
All
−0.2
0.0
0.2
MR leave−one−out sensitivity analysis for
'CLEC11A' on 'Pulmonary fibrosis'

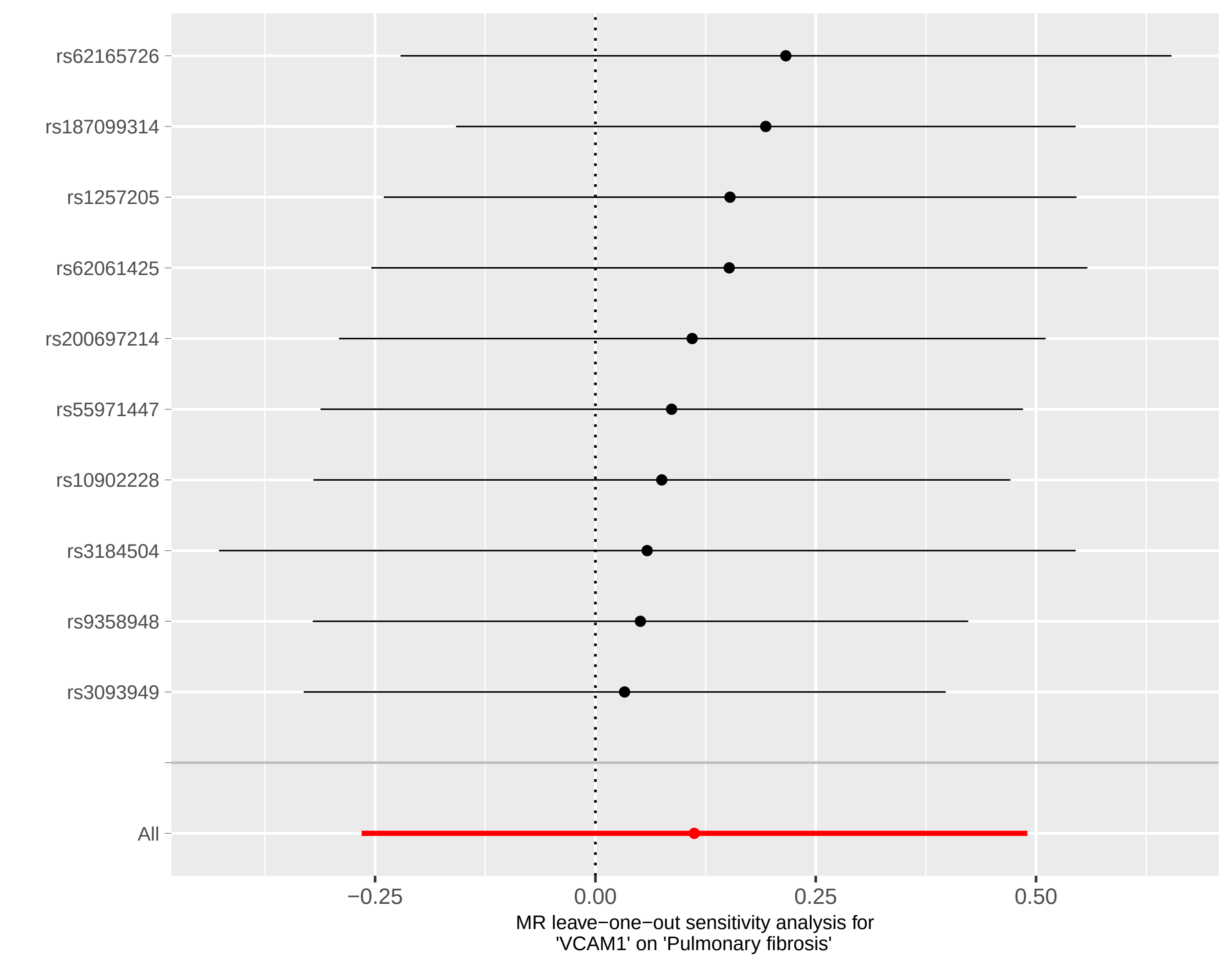

rs62165726
rs187099314
rs1257205
rs62061425
rs200697214
rs55971447
rs10902228
rs3184504
rs9358948
rs3093949
All
−0.25
0.00
0.25
0.50
MR leave−one−out sensitivity analysis for
'VCAM1' on 'Pulmonary fibrosis'

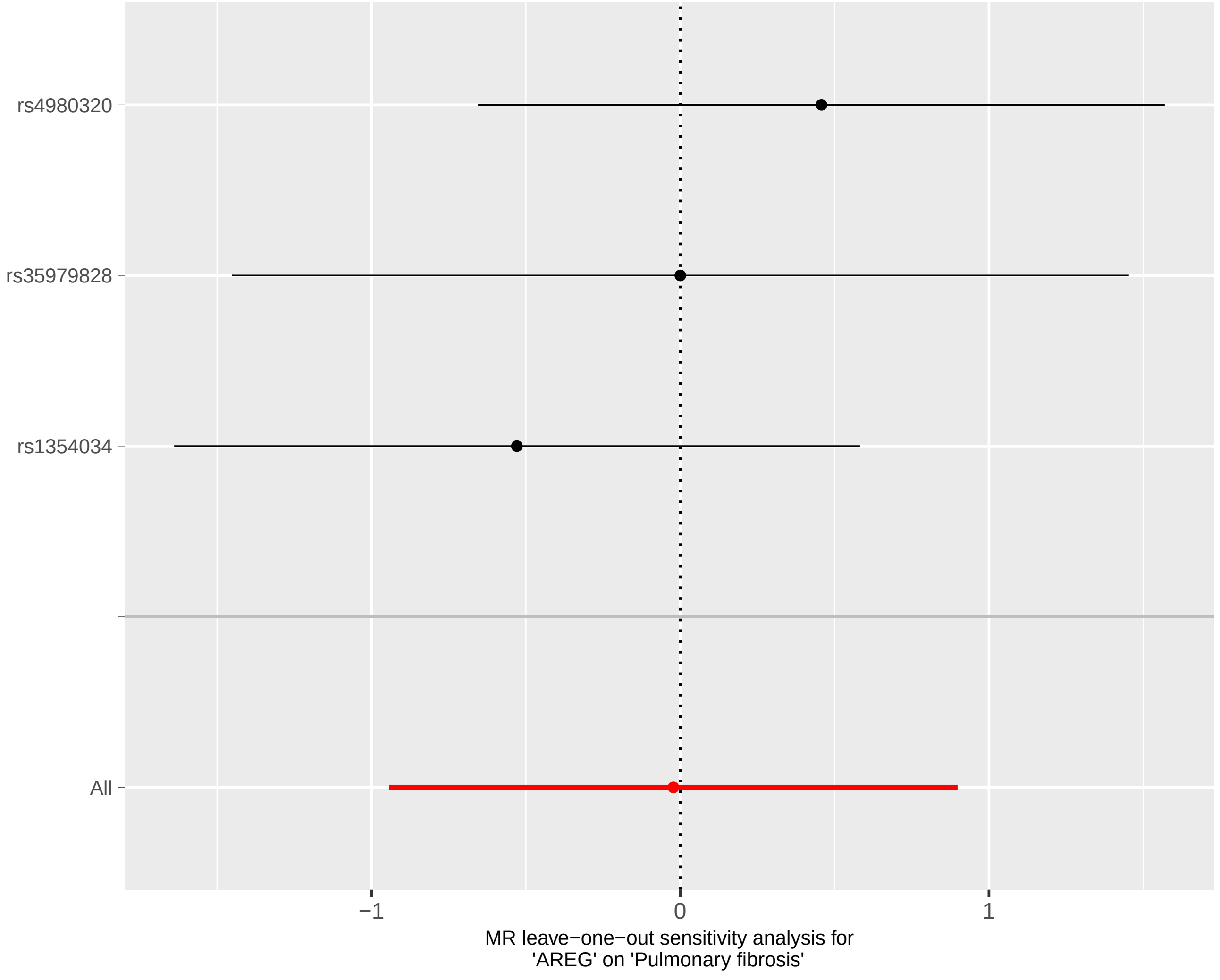
rs4980320
rs35979828
rs1354034
All
−1
0
1
MR leave−one−out sensitivity analysis for
'AREG' on 'Pulmonary fibrosis'

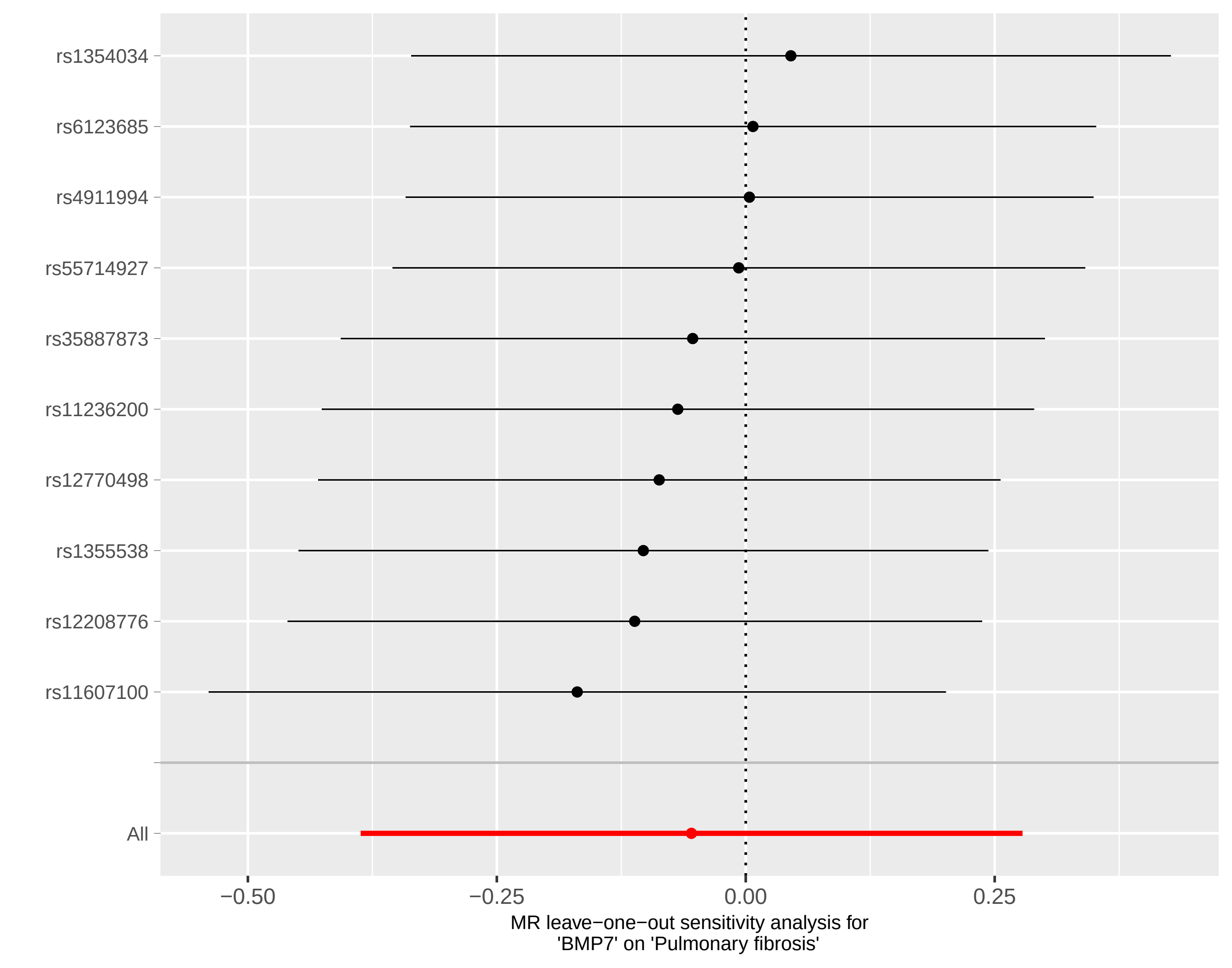

rs1354034
rs6123685
rs4911994
rs55714927
rs35887873
rs11236200
rs12770498
rs1355538
rs12208776
rs11607100
All
−0.50
−0.25
0.00
0.25
MR leave−one−out sensitivity analysis for
'BMP7' on 'Pulmonary fibrosis'

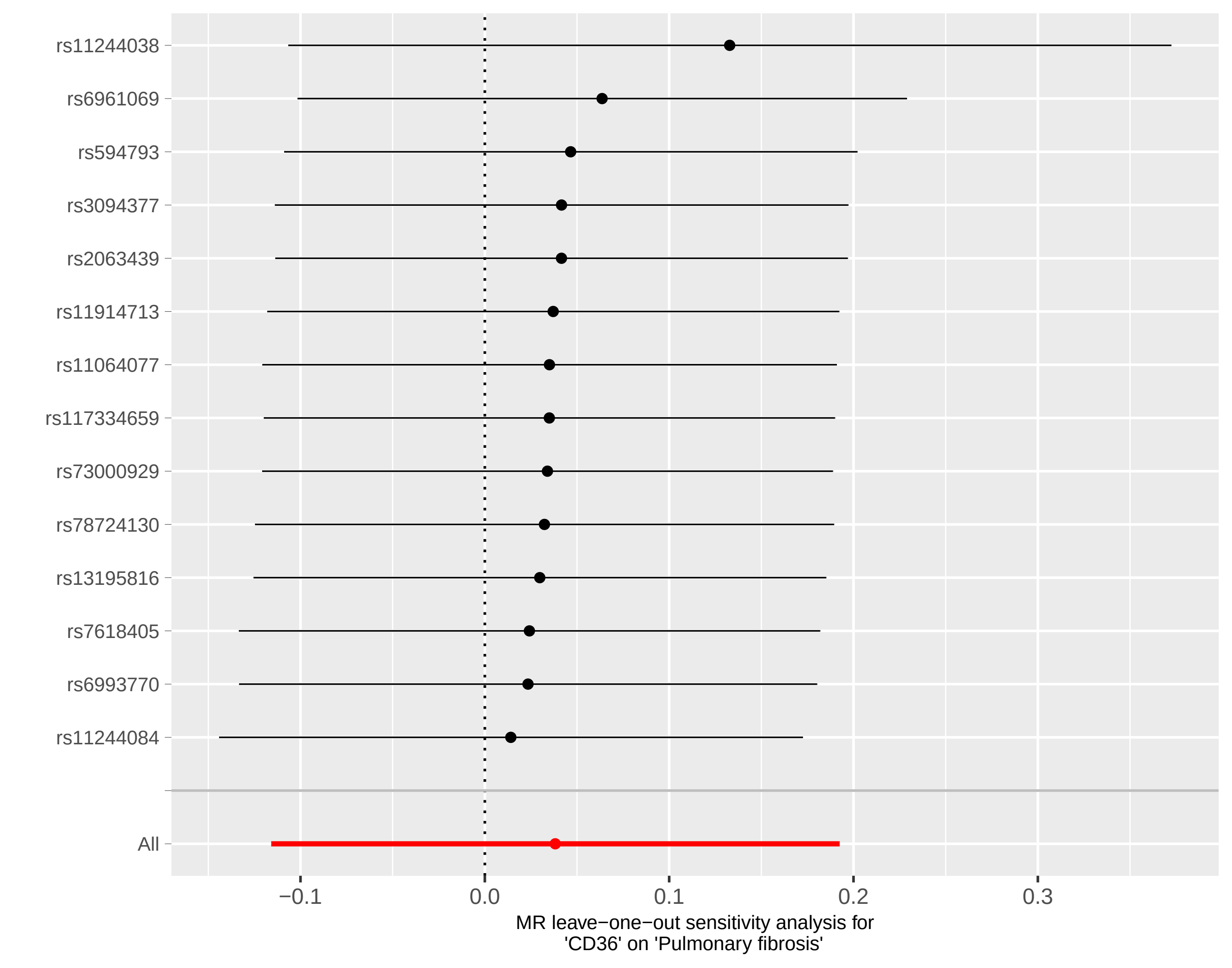
rs11244038
rs6961069
rs594793
rs3094377
rs2063439
rs11914713
rs11064077
rs117334659
rs73000929
rs78724130
rs13195816
rs7618405
rs6993770
rs11244084
All
−0.1
0.0
0.1
0.2
0.3
MR leave−one−out sensitivity analysis for
'CD36' on 'Pulmonary fibrosis'

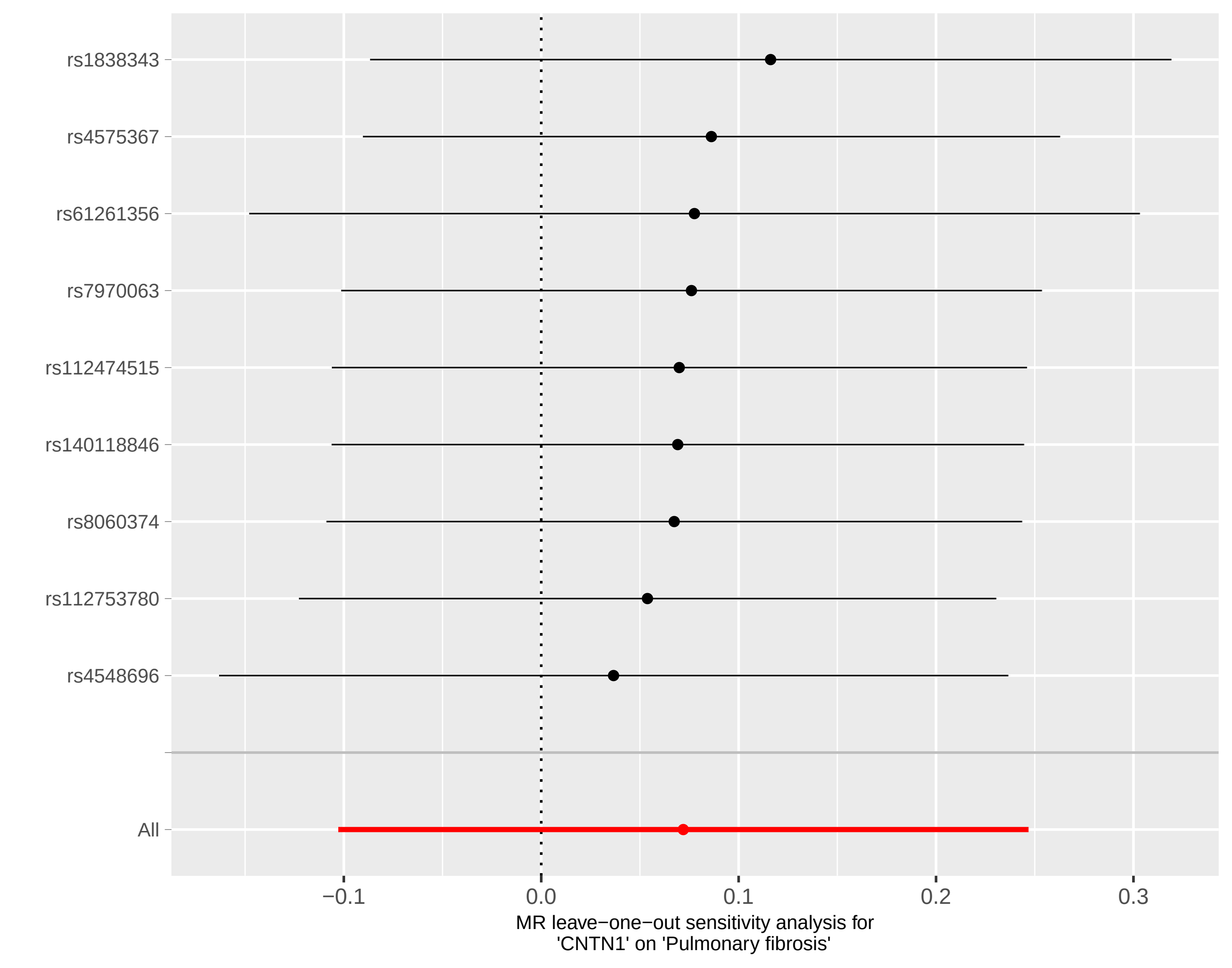

rs1838343
rs4575367
rs61261356
rs7970063
rs112474515
rs140118846
rs8060374
rs112753780
rs4548696
All
−0.1
0.0
0.1
0.2
0.3
MR leave−one−out sensitivity analysis for
'CNTN1' on 'Pulmonary fibrosis'

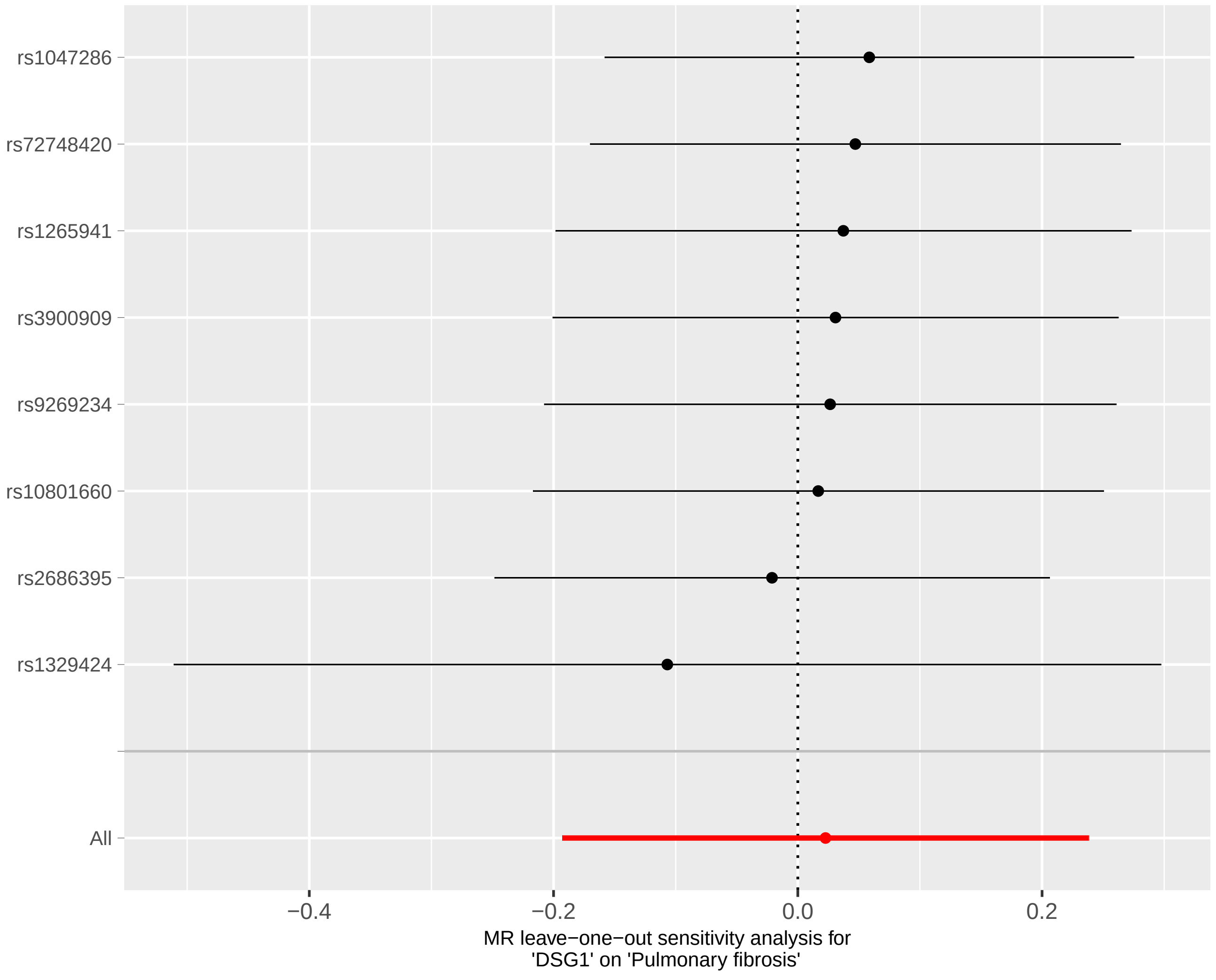
rs1047286
rs72748420
rs1265941
rs3900909
rs9269234
rs10801660
rs2686395
rs1329424
All
−0.4
−0.2
0.0
0.2
MR leave−one−out sensitivity analysis for
'DSG1' on 'Pulmonary fibrosis'

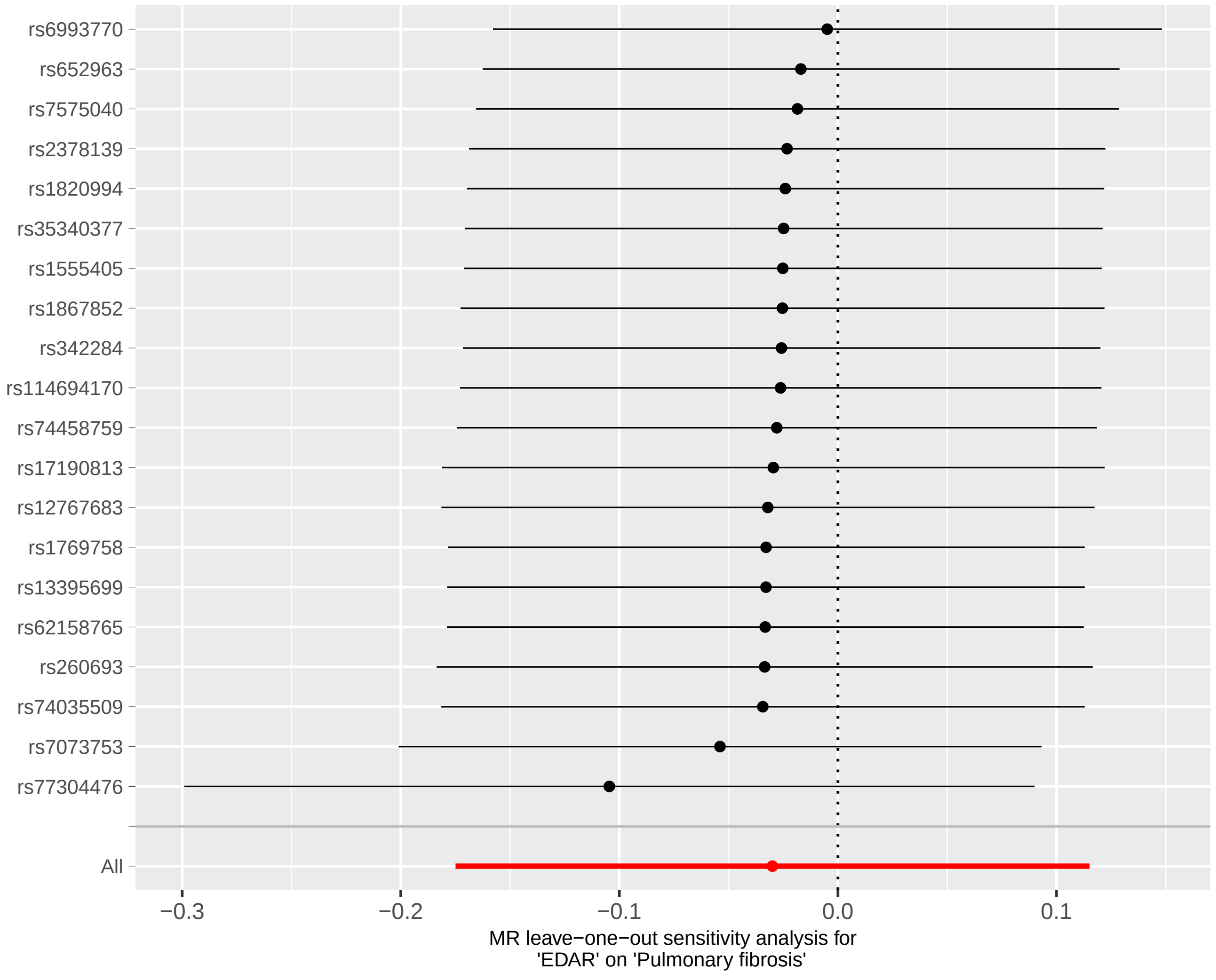
rs6993770
rs652963
rs7575040
rs2378139
rs1820994
rs35340377
rs1555405
rs1867852
rs342284
rs114694170
rs74458759
rs17190813
rs12767683
rs1769758
rs13395699
rs62158765
rs260693
rs74035509
rs7073753
rs77304476
All
−0.3
−0.2
−0.1
0.0
0.1
MR leave−one−out sensitivity analysis for
'EDAR' on 'Pulmonary fibrosis'

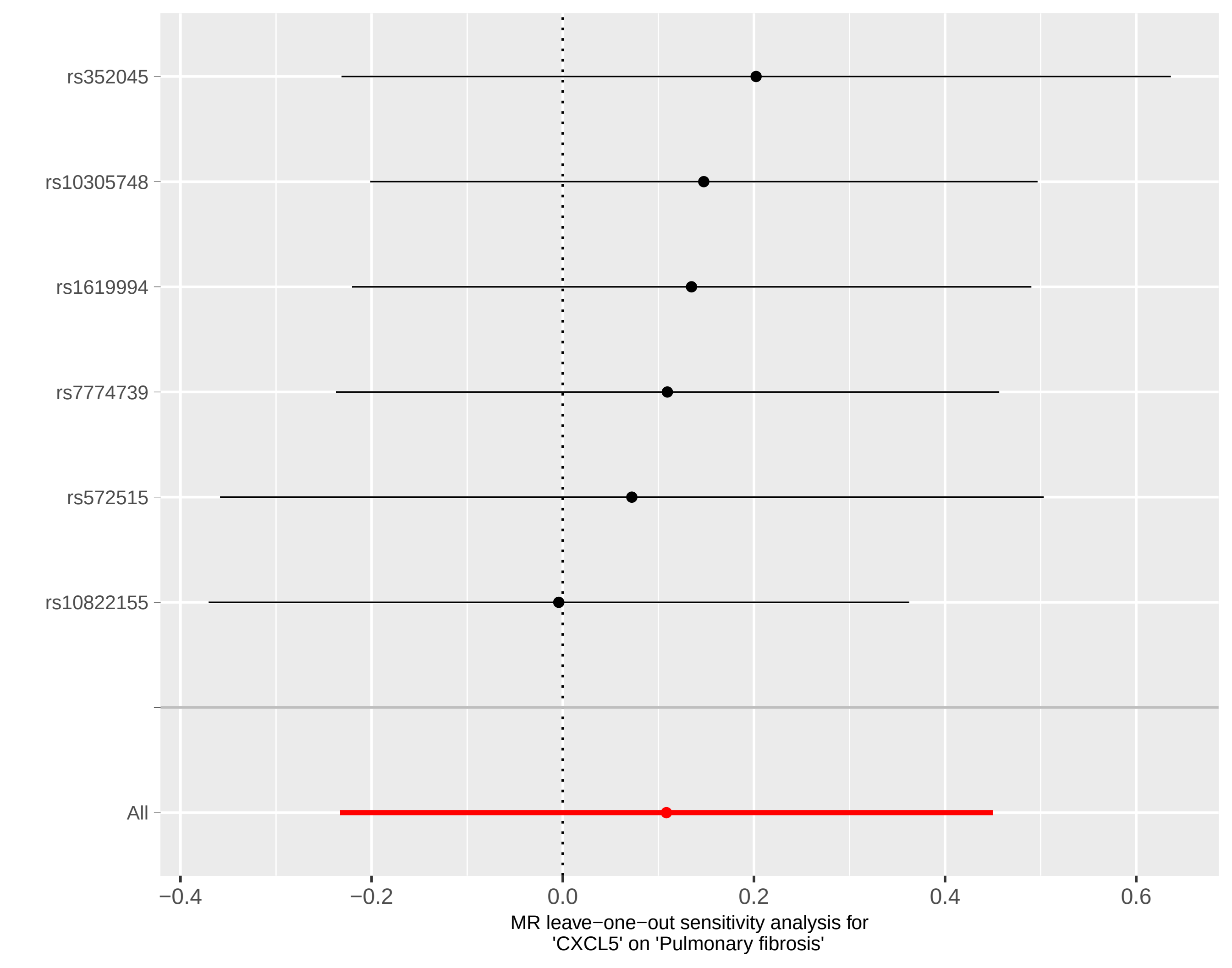
rs352045
rs10305748
rs1619994
rs7774739
rs572515
rs10822155
All
−0.4
−0.2
0.0
0.2
0.4
0.6
MR leave−one−out sensitivity analysis for
'CXCL5' on 'Pulmonary fibrosis'

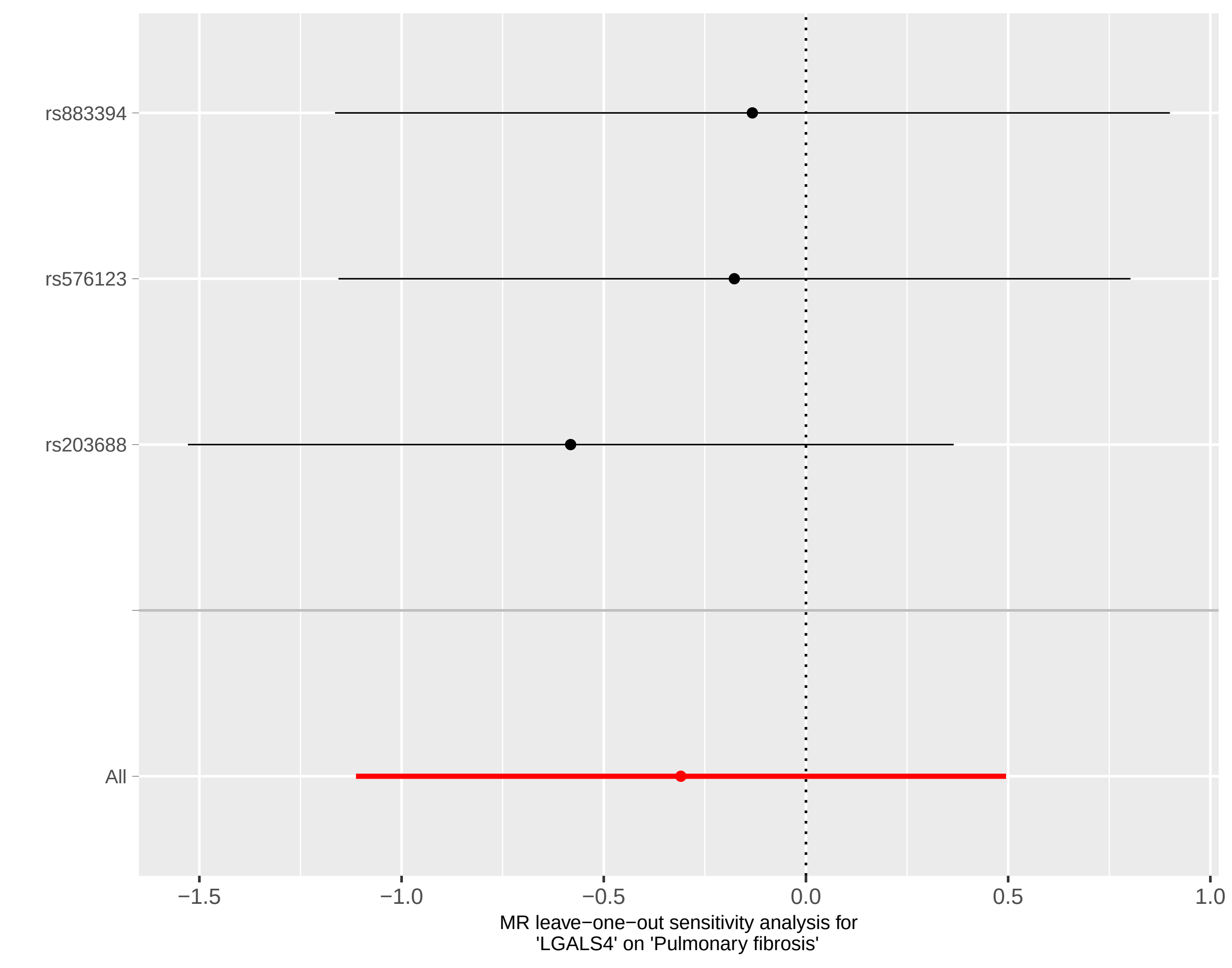
rs883394
rs576123
rs203688
All
−1.5
−1.0
−0.5
0.0
0.5
1.0
MR leave−one−out sensitivity analysis for
'LGALS4' on 'Pulmonary fibrosis'

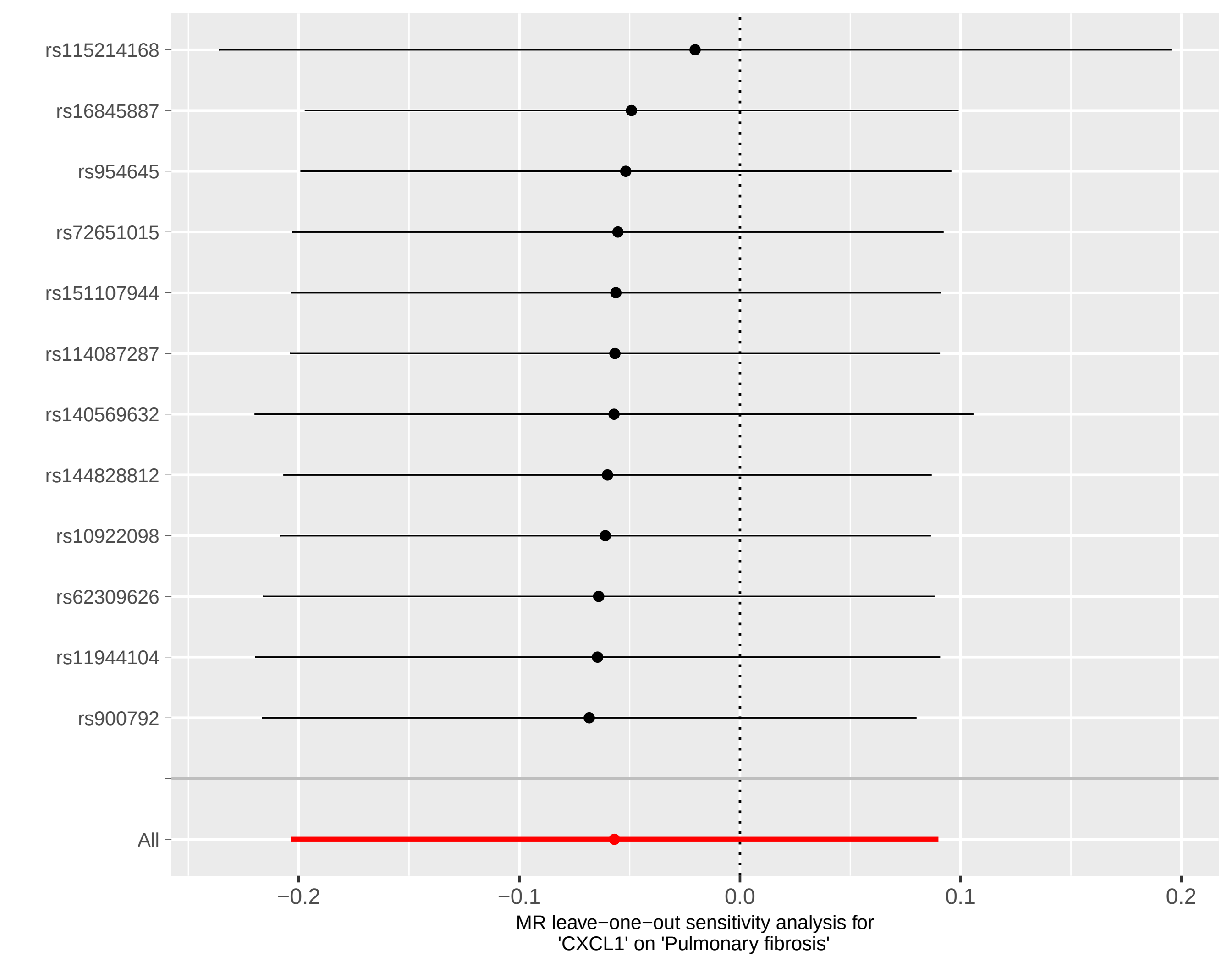
rs115214168
rs16845887
rs954645
rs72651015
rs151107944
rs114087287
rs140569632
rs144828812
rs10922098
rs62309626
rs11944104
rs900792
All
−0.2
−0.1
0.0
0.1
0.2
MR leave−one−out sensitivity analysis for
'CXCL1' on 'Pulmonary fibrosis'

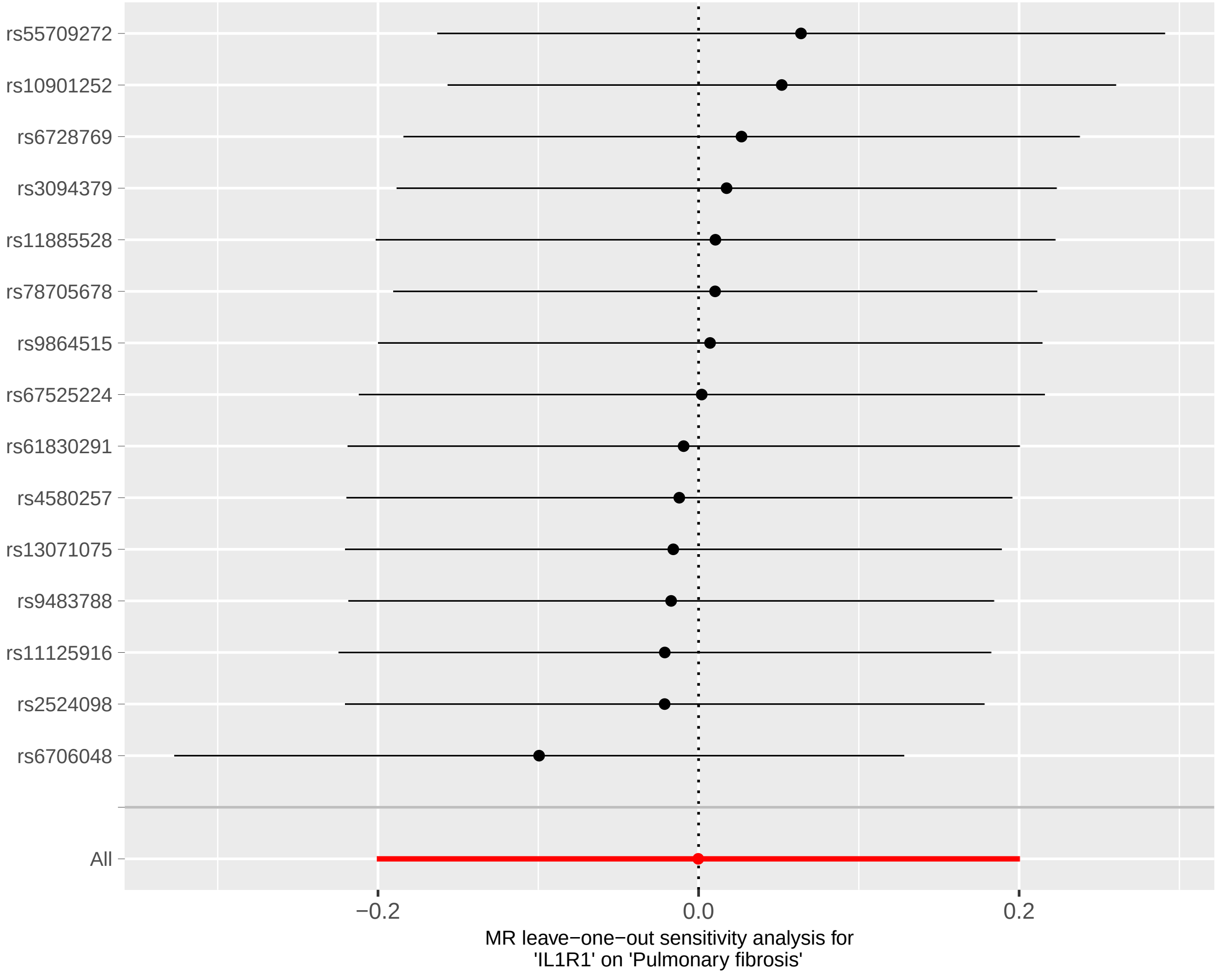
rs55709272
rs10901252
rs6728769
rs3094379
rs11885528
rs78705678
rs9864515
rs67525224
rs61830291
rs4580257
rs13071075
rs9483788
rs11125916
rs2524098
rs6706048
All
−0.2
0.0
0.2
MR leave−one−out sensitivity analysis for
'IL1R1' on 'Pulmonary fibrosis'

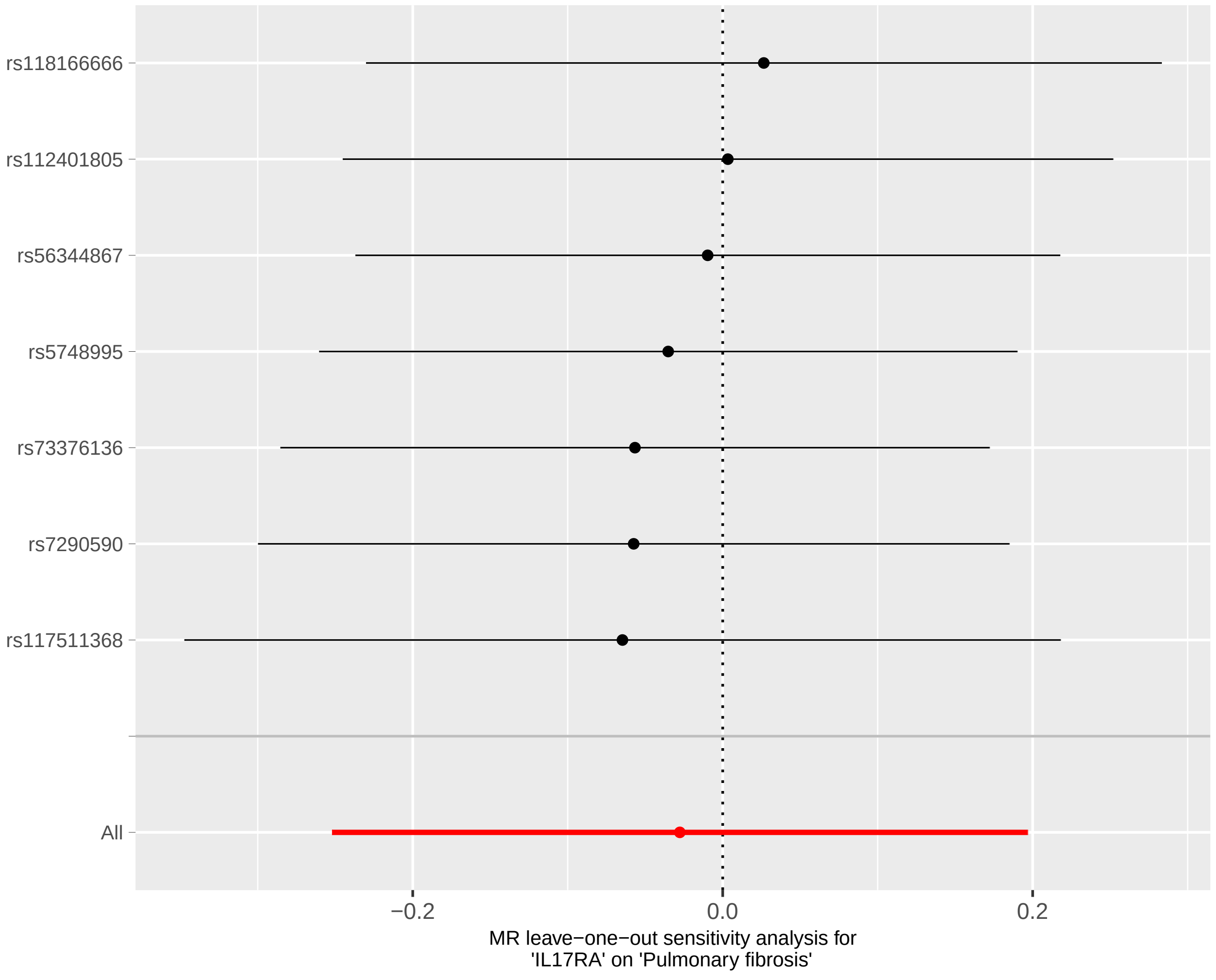
rs118166666
rs112401805
rs56344867
rs5748995
rs73376136
rs7290590
rs117511368
All
−0.2
0.0
0.2
MR leave−one−out sensitivity analysis for
'IL17RA' on 'Pulmonary fibrosis'

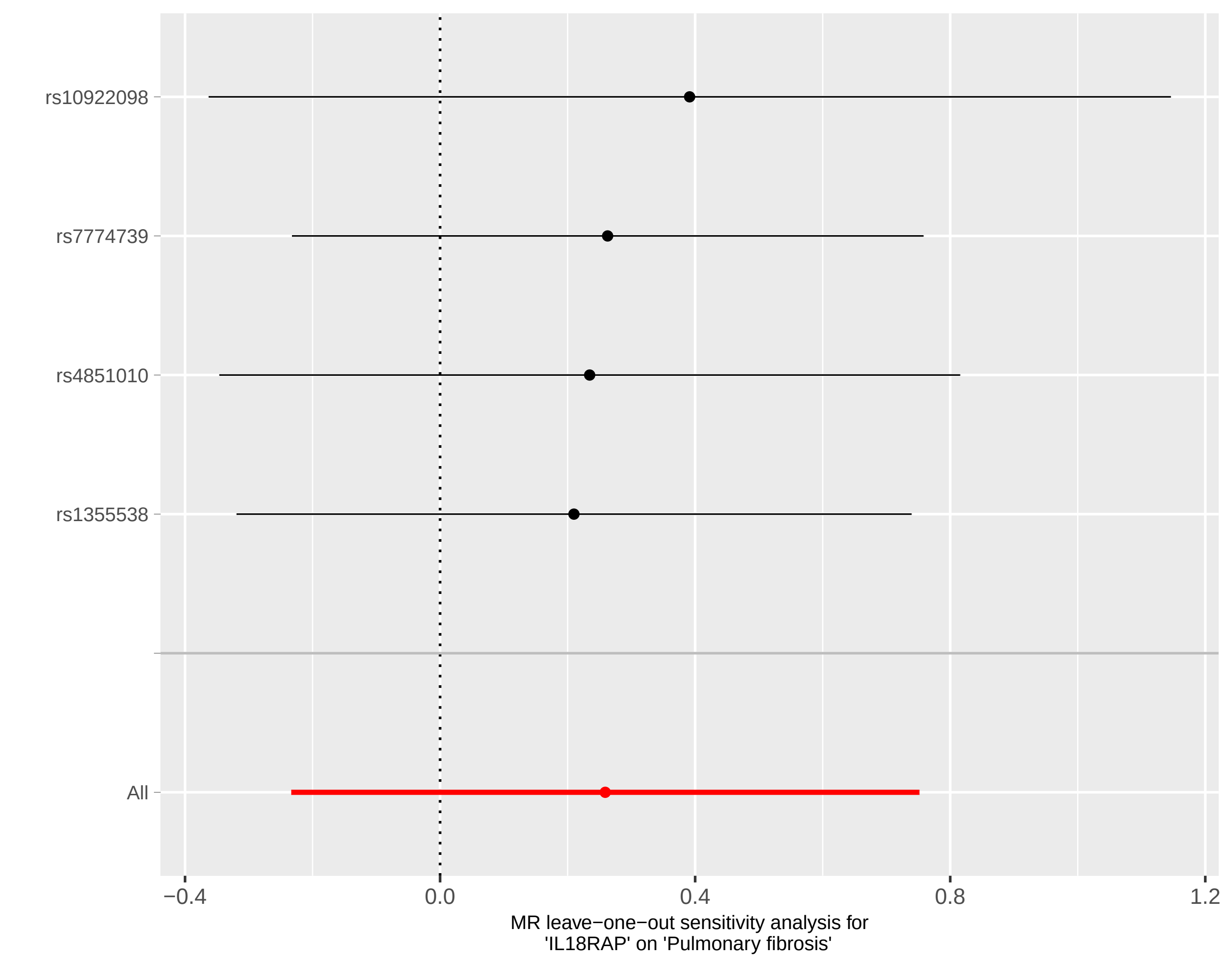
rs10922098
rs7774739
rs4851010
rs1355538
All
−0.4
0.0
0.4
0.8
1.2
MR leave−one−out sensitivity analysis for
'IL18RAP' on 'Pulmonary fibrosis'

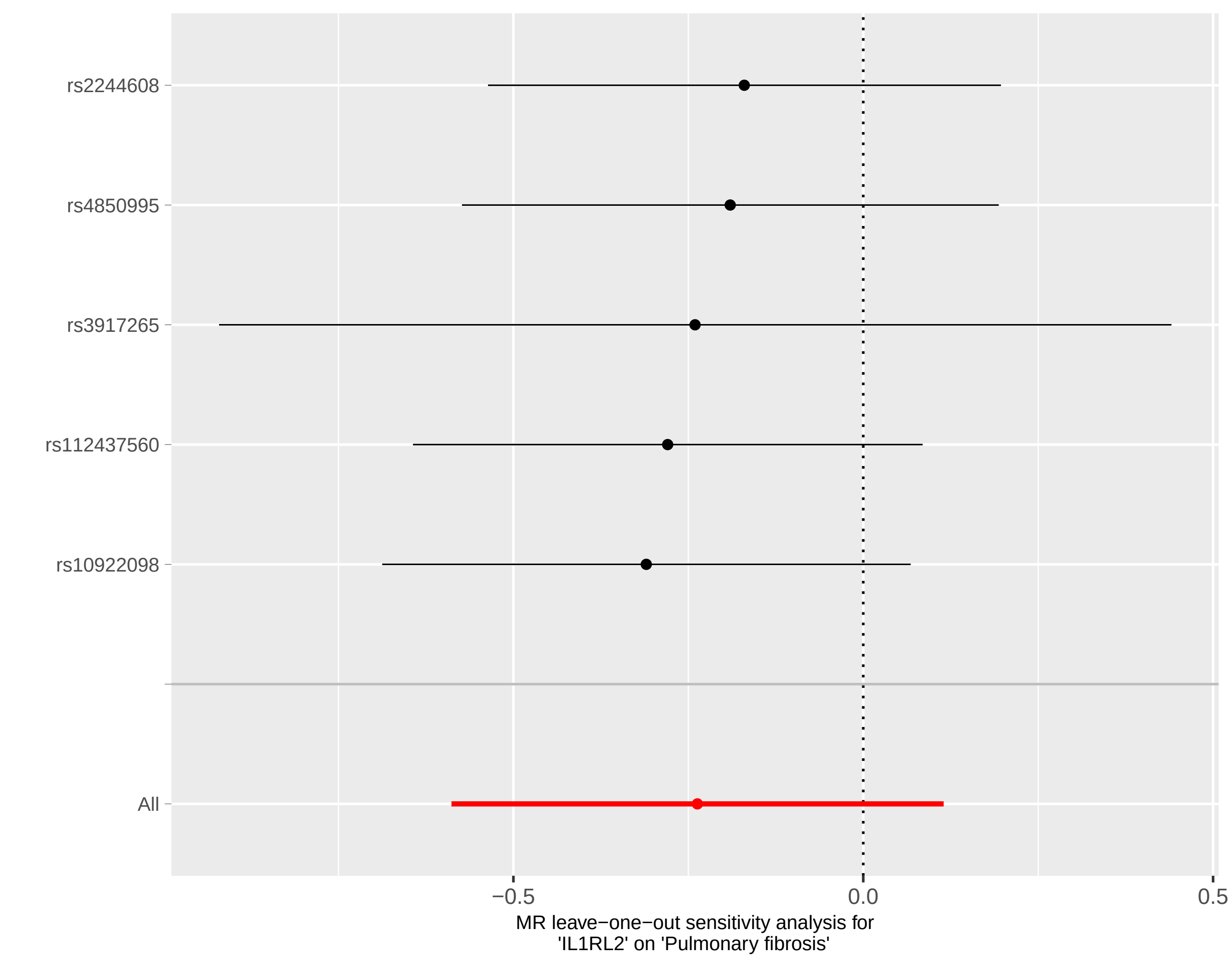
rs2244608
rs4850995
rs3917265
rs112437560
rs10922098
All
−0.5
0.0
0.5
MR leave−one−out sensitivity analysis for
'IL1RL2' on 'Pulmonary fibrosis'

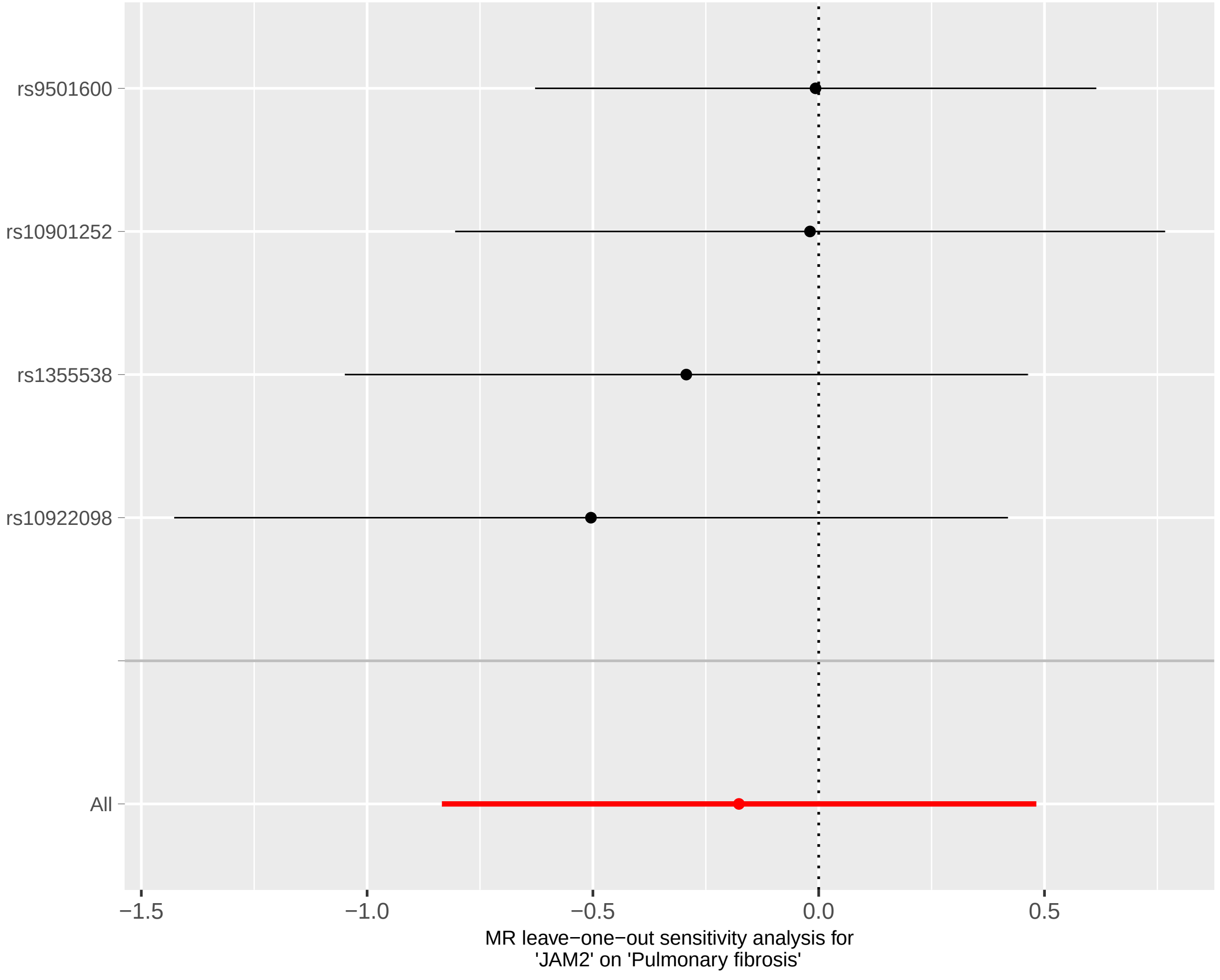
rs9501600
rs10901252
rs1355538
rs10922098
All
−1.5
−1.0
−0.5
0.0
0.5
MR leave−one−out sensitivity analysis for
'JAM2' on 'Pulmonary fibrosis'

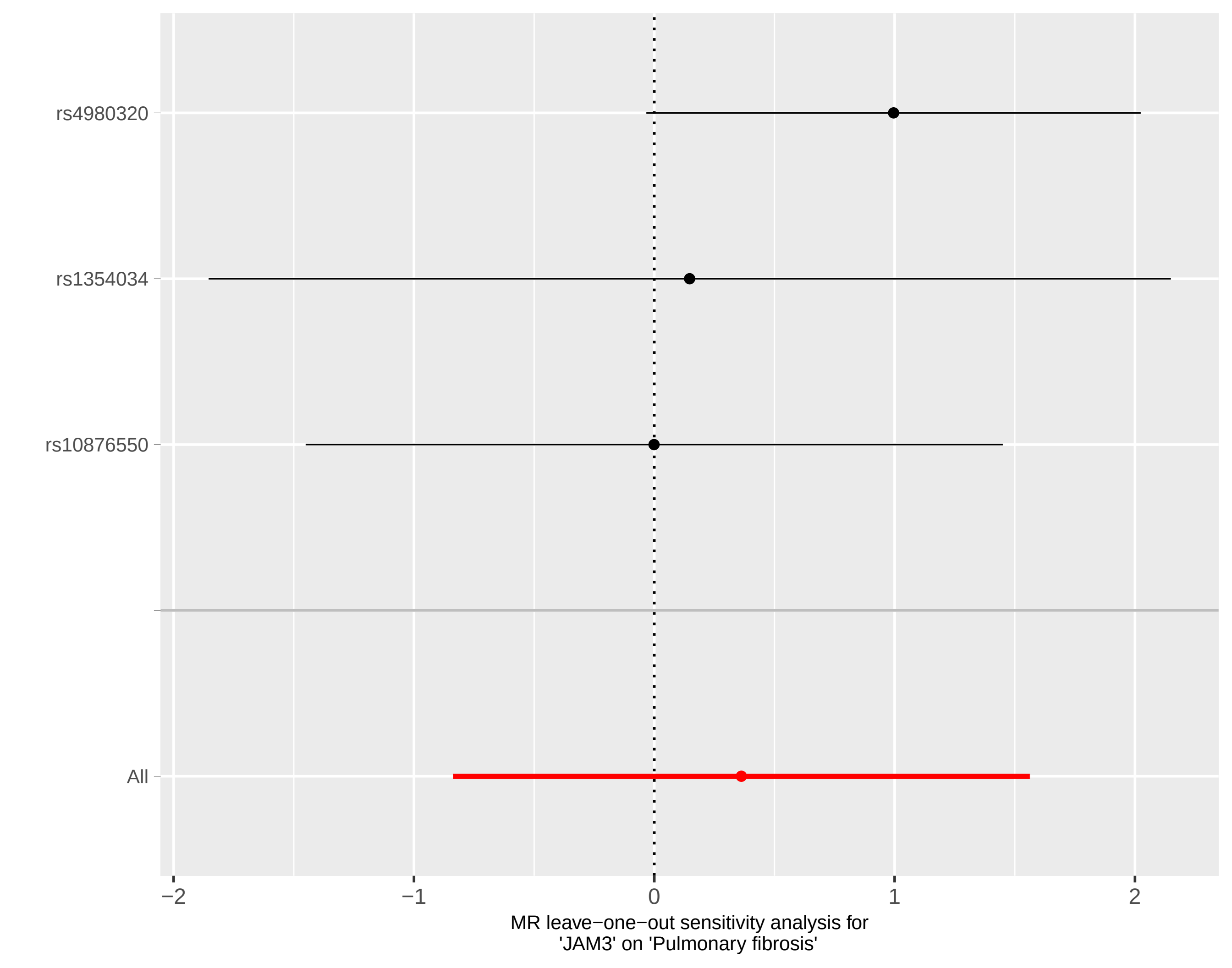
rs4980320
rs1354034
rs10876550
All
−2
−1
0
1
2
MR leave−one−out sensitivity analysis for
'JAM3' on 'Pulmonary fibrosis'

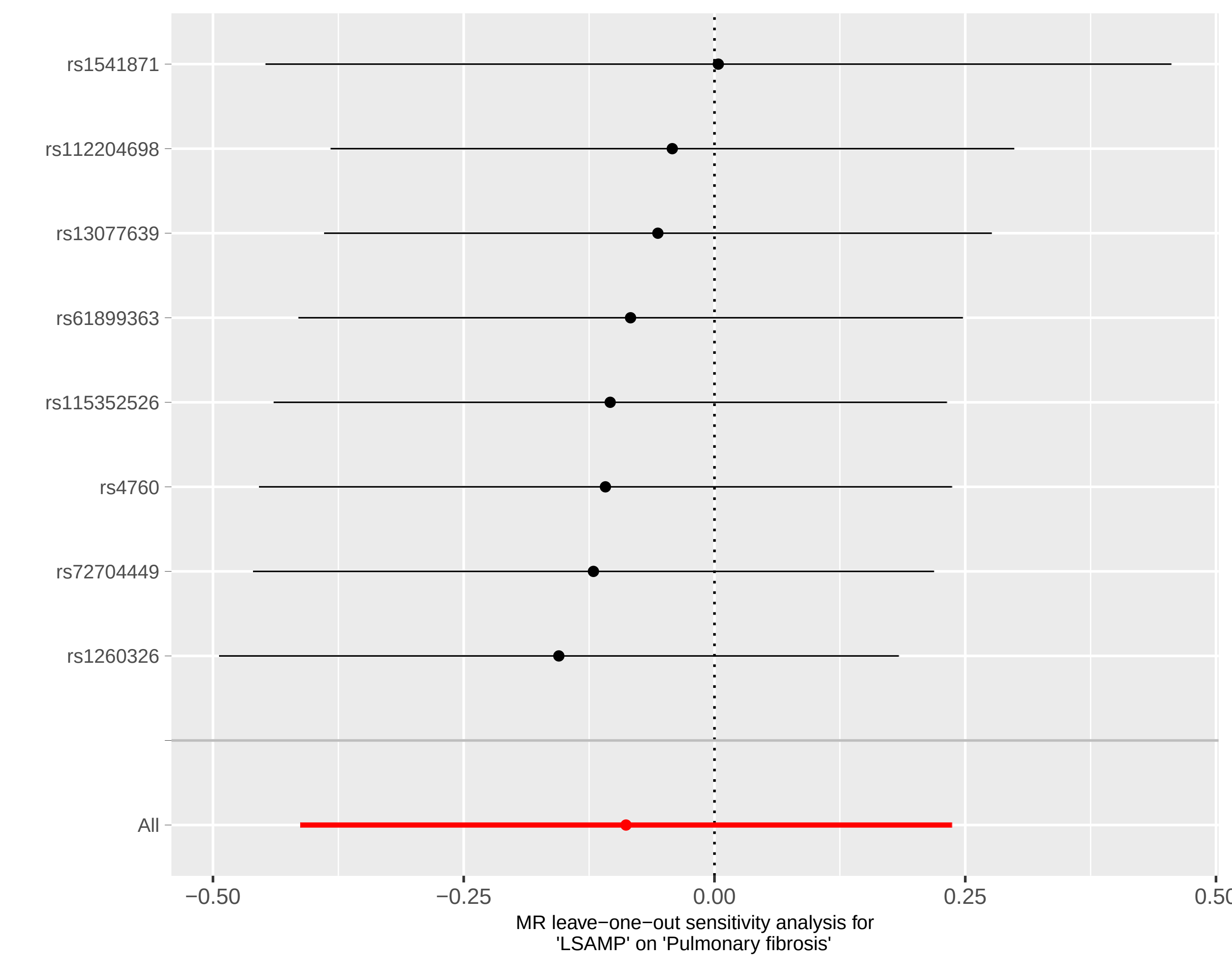
rs1541871
rs112204698
rs13077639
rs61899363
rs115352526
rs4760
rs72704449
rs1260326
All
−0.50
−0.25
0.00
0.25
0.50
MR leave−one−out sensitivity analysis for
'LSAMP' on 'Pulmonary fibrosis'

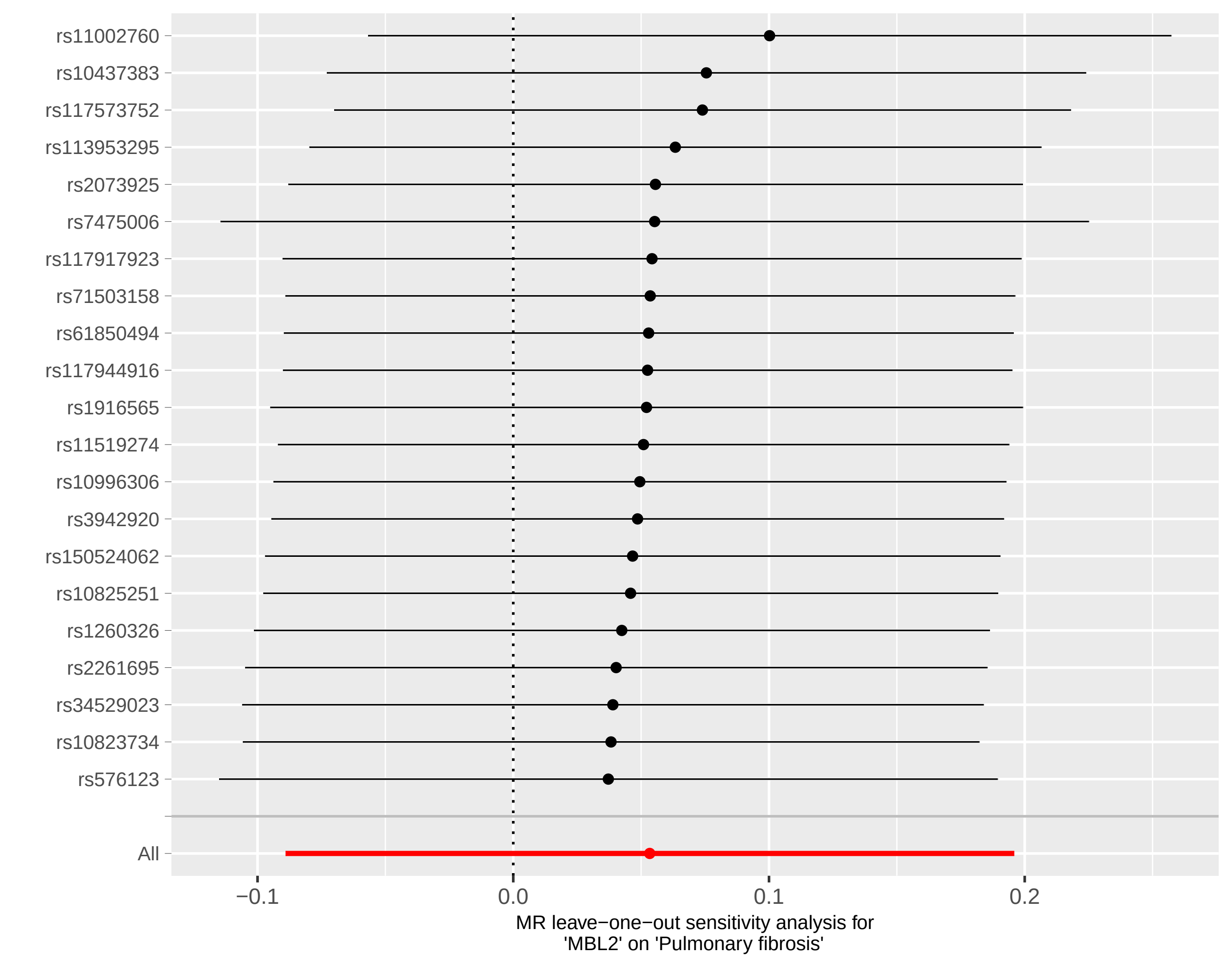
rs11002760
rs10437383
rs117573752
rs113953295
rs2073925
rs7475006
rs117917923
rs71503158
rs61850494
rs117944916
rs1916565
rs11519274
rs10996306
rs3942920
rs150524062
rs10825251
rs1260326
rs2261695
rs34529023
rs10823734
rs576123
All
−0.1
0.0
0.1
0.2
MR leave−one−out sensitivity analysis for
'MBL2' on 'Pulmonary fibrosis'

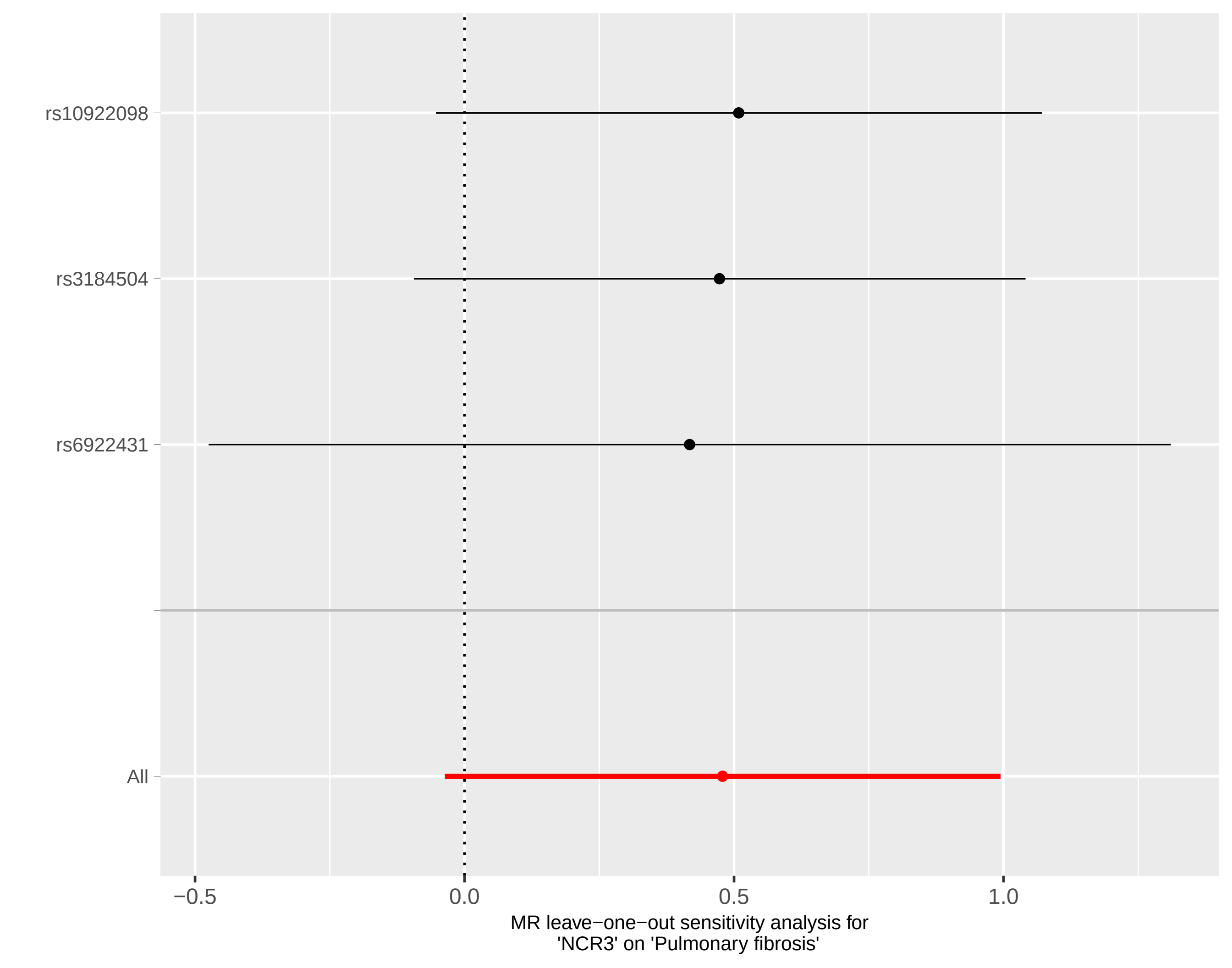
rs10922098
rs3184504
rs6922431
All
−0.5
0.0
0.5
1.0
MR leave−one−out sensitivity analysis for
'NCR3' on 'Pulmonary fibrosis'

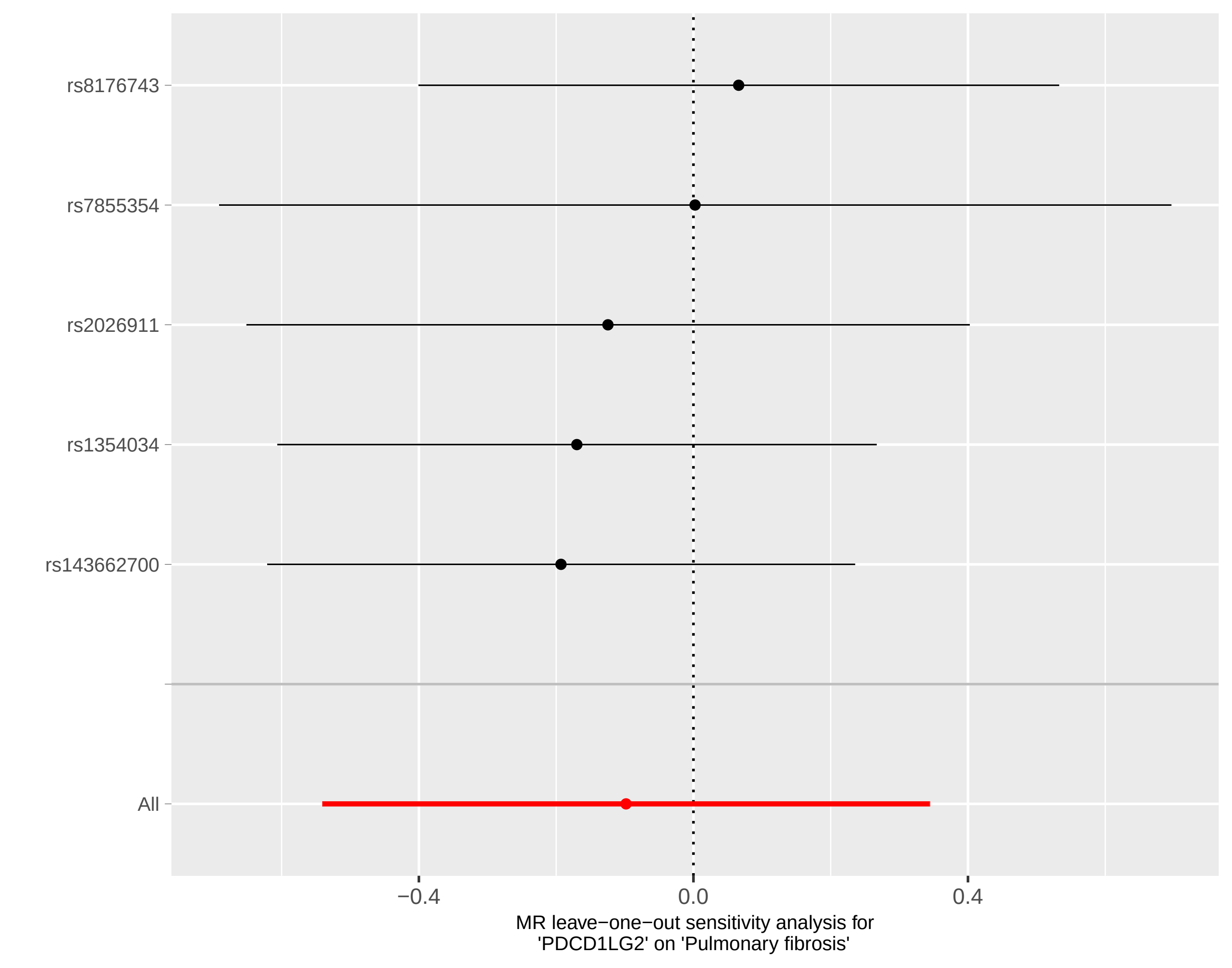
rs8176743
rs7855354
rs2026911
rs1354034
rs143662700
All
−0.4
0.0
0.4
MR leave−one−out sensitivity analysis for
'PDCD1LG2' on 'Pulmonary fibrosis'

Insufficient number of SNPs

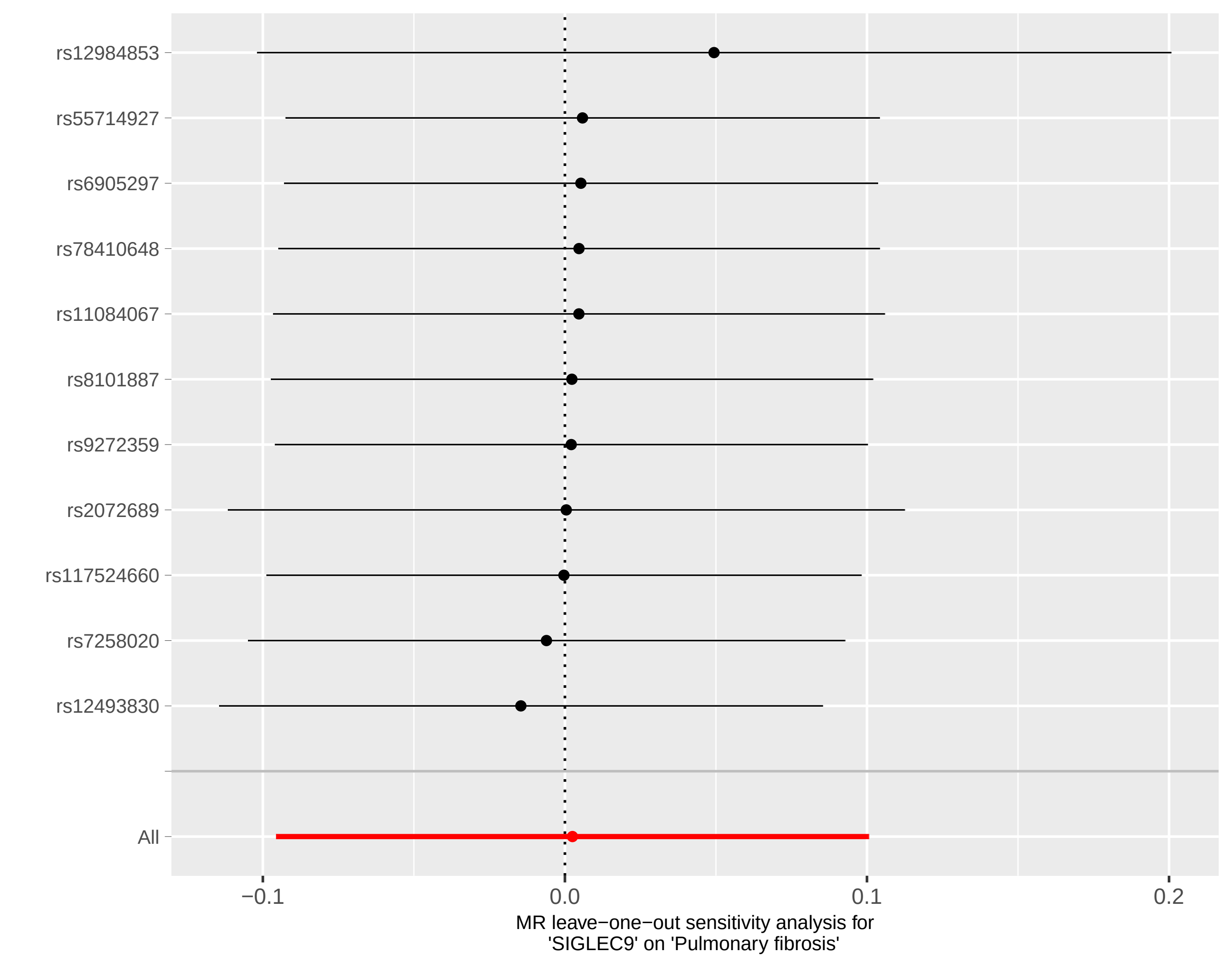
rs12984853
rs55714927
rs6905297
rs78410648
rs11084067
rs8101887
rs9272359
rs2072689
rs117524660
rs7258020
rs12493830
All
−0.1
0.0
0.1
0.2
MR leave−one−out sensitivity analysis for
'SIGLEC9' on 'Pulmonary fibrosis'

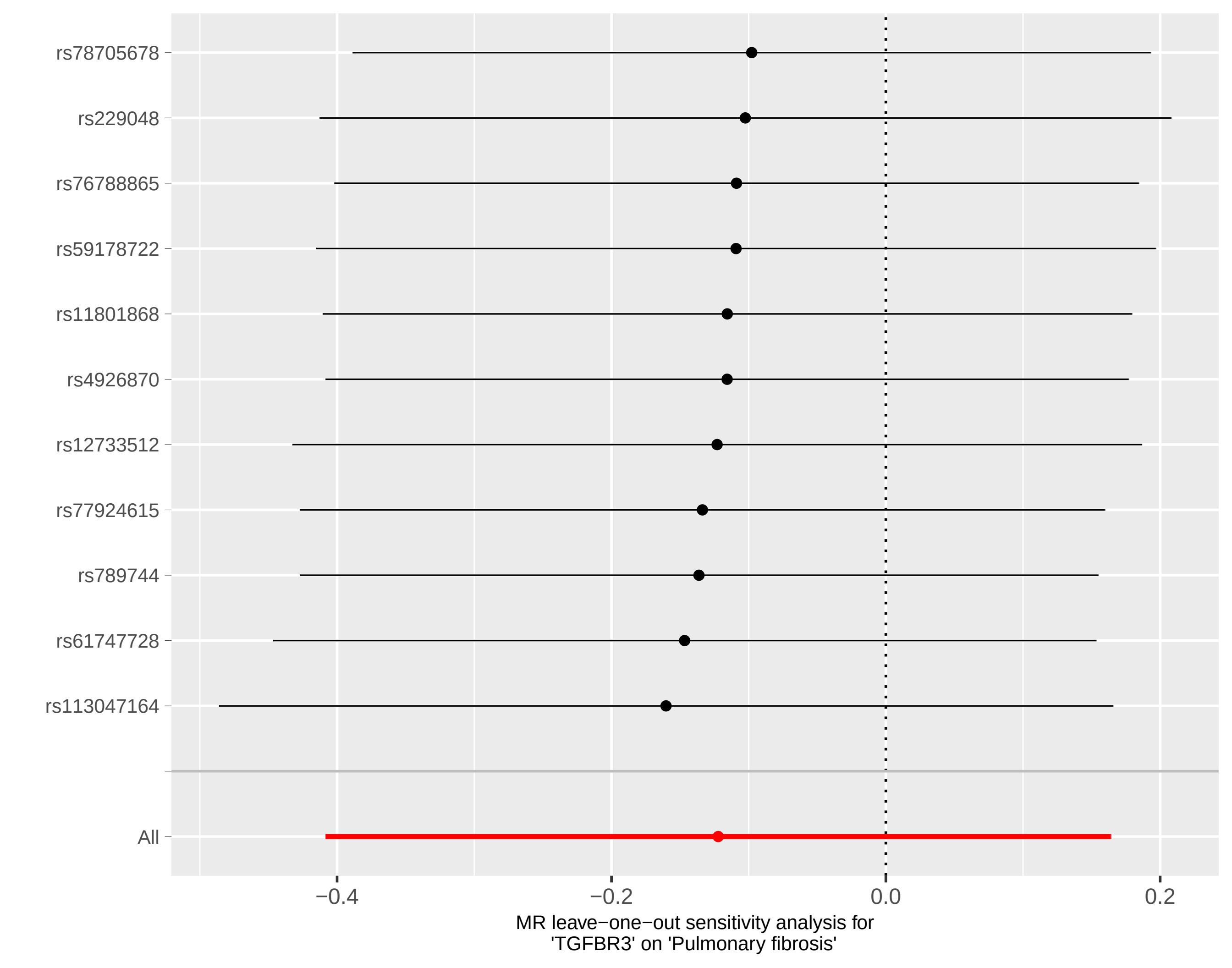
rs78705678
rs229048
rs76788865
rs59178722
rs11801868
rs4926870
rs12733512
rs77924615
rs789744
rs61747728
rs113047164
All
−0.4
−0.2
0.0
0.2
MR leave−one−out sensitivity analysis for
'TGFBR3' on 'Pulmonary fibrosis'

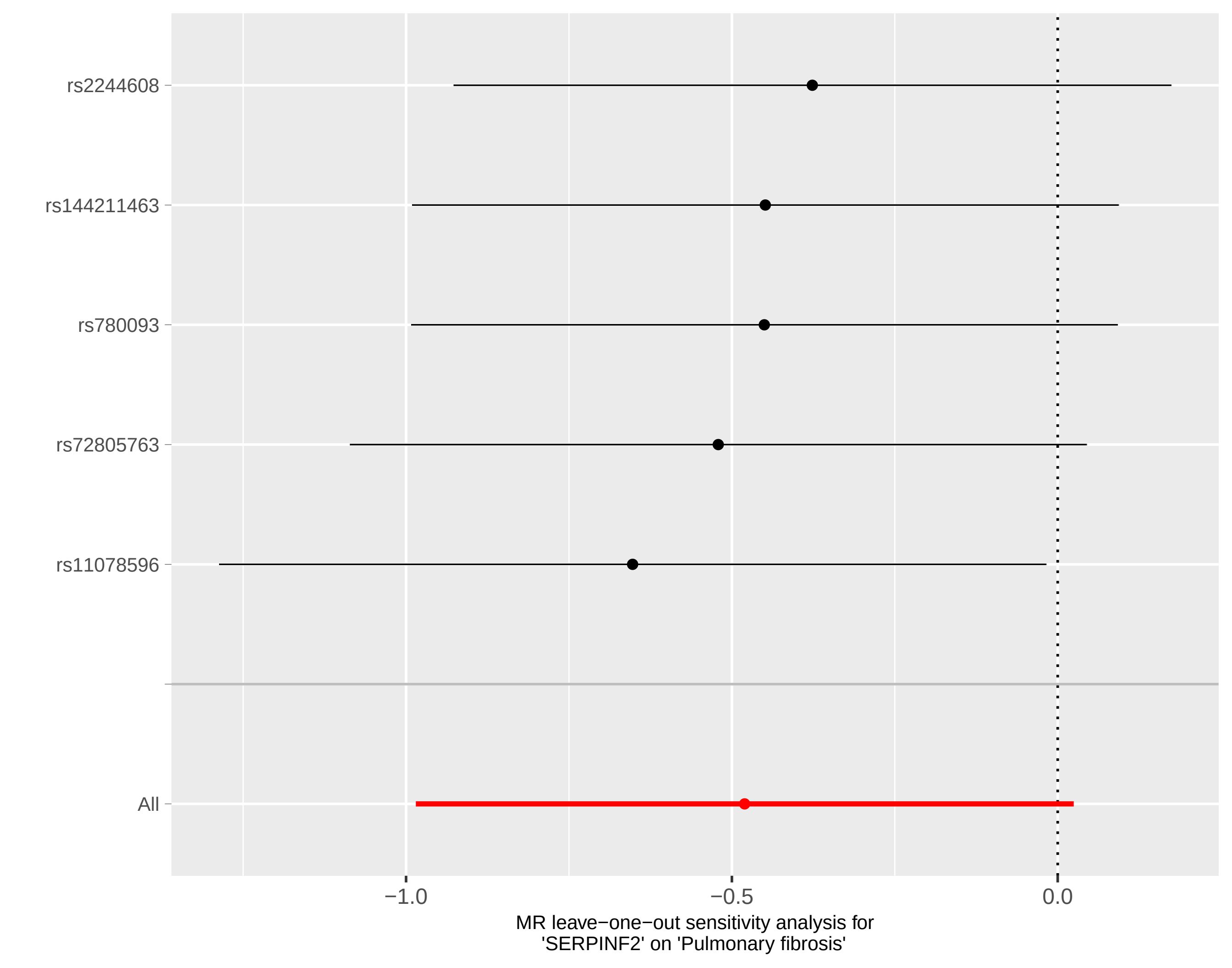
rs2244608
rs144211463
rs780093
rs72805763
rs11078596
All
−1.0
−0.5
0.0
MR leave−one−out sensitivity analysis for
'SERPINF2' on 'Pulmonary fibrosis'

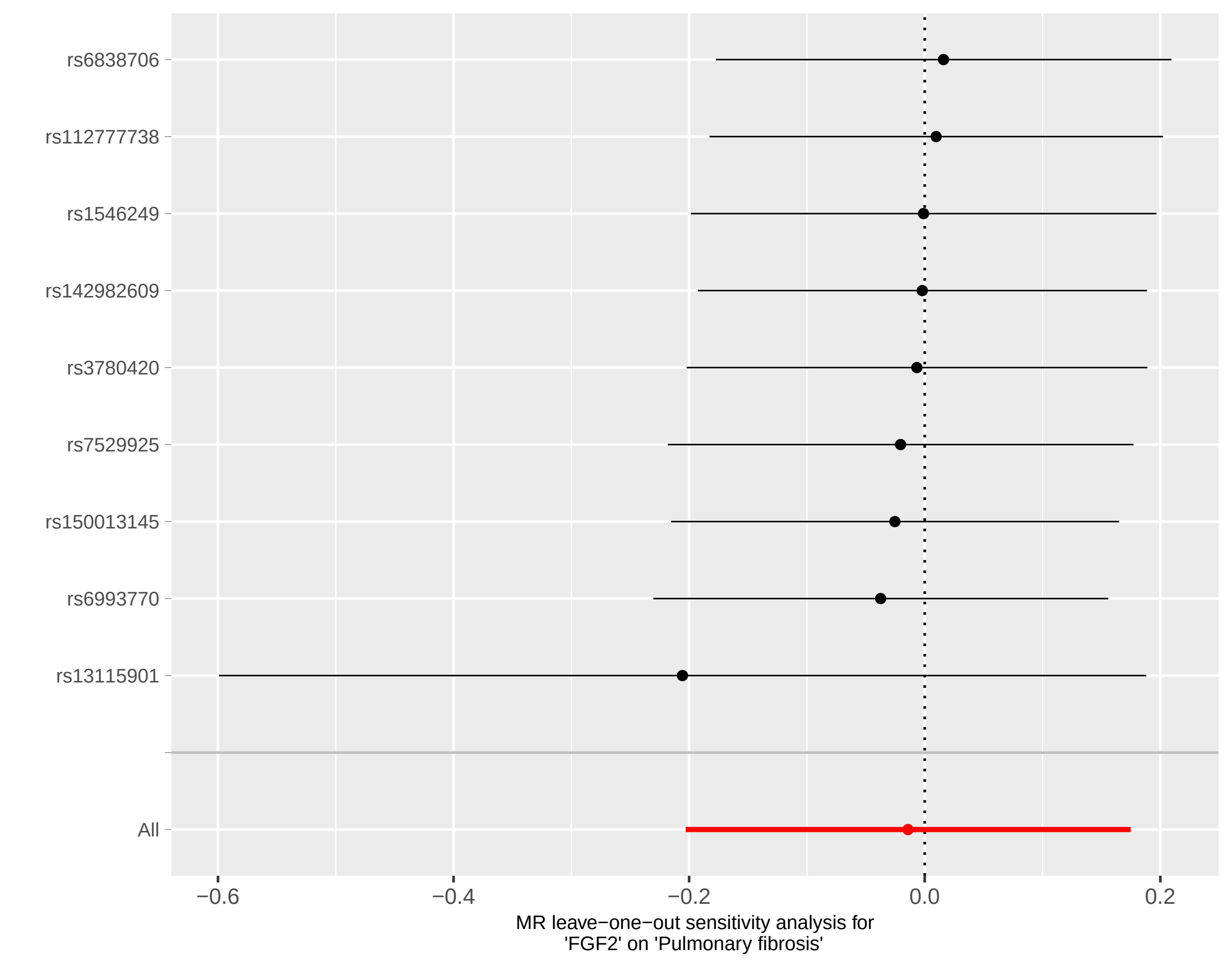

rs6838706
rs112777738
rs1546249
rs142982609
rs3780420
rs7529925
rs150013145
rs6993770
rs13115901
All
−0.6
−0.4
−0.2
0.0
0.2
MR leave−one−out sensitivity analysis for
'FGF2' on 'Pulmonary fibrosis'

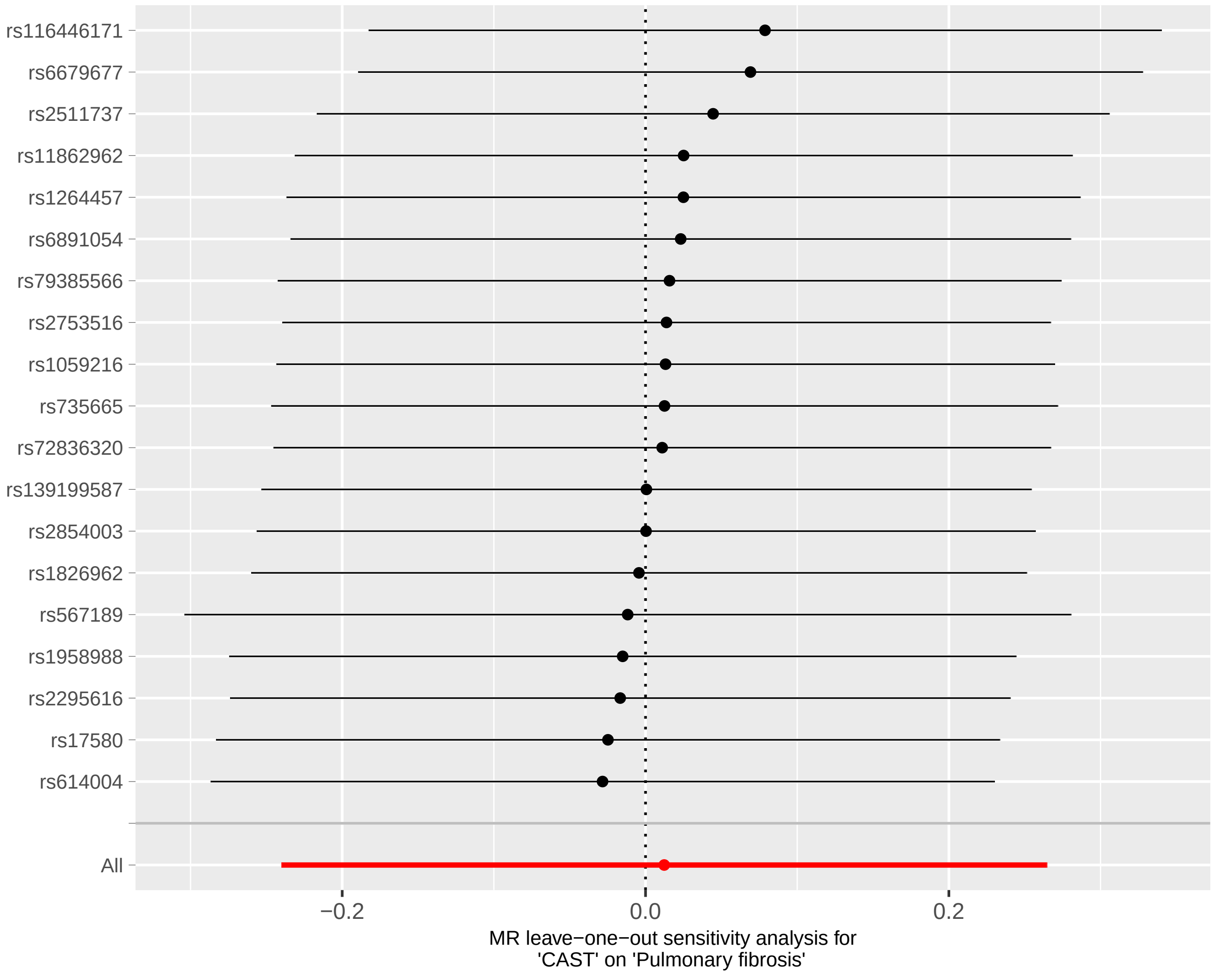
rs116446171
rs6679677
rs2511737
rs11862962
rs1264457
rs6891054
rs79385566
rs2753516
rs1059216
rs735665
rs72836320
rs139199587
rs2854003
rs1826962
rs567189
rs1958988
rs2295616
rs17580
rs614004
All
−0.2
0.0
0.2
MR leave−one−out sensitivity analysis for
'CAST' on 'Pulmonary fibrosis'

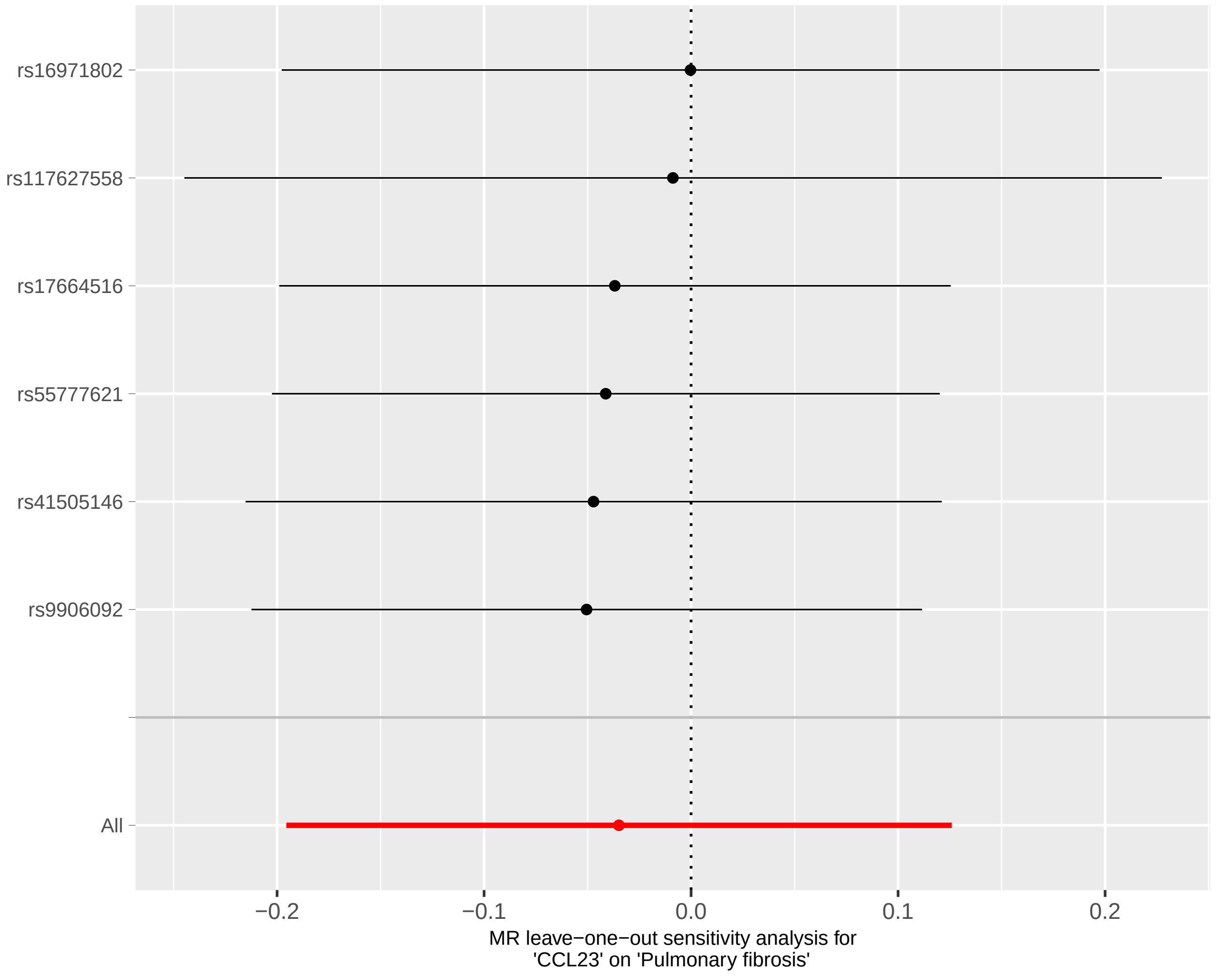
rs16971802
rs117627558
rs17664516
rs55777621
rs41505146
rs9906092
All
−0.2
−0.1
0.0
0.1
0.2
MR leave−one−out sensitivity analysis for
'CCL23' on 'Pulmonary fibrosis'

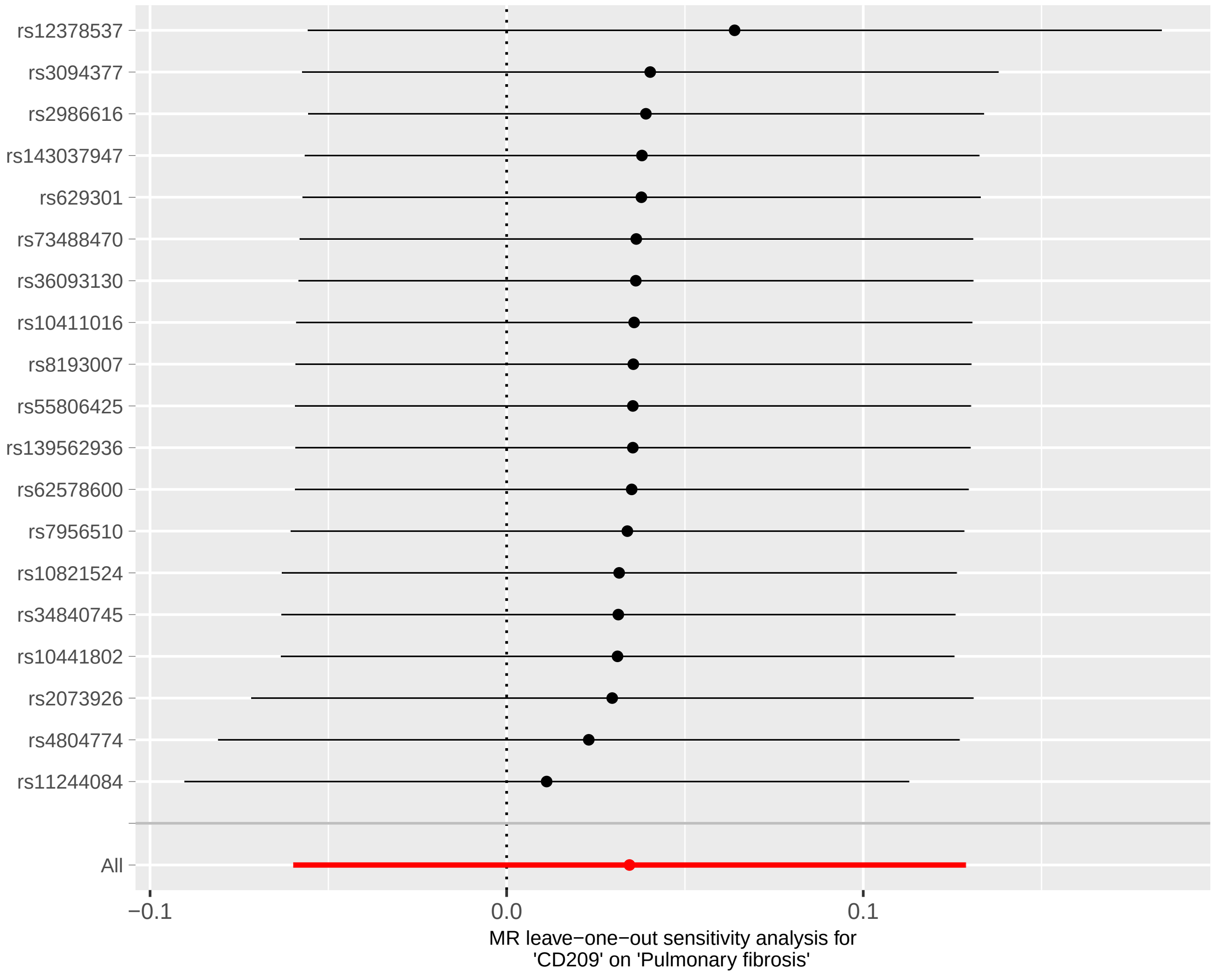
rs12378537
rs3094377
rs2986616
rs143037947
rs629301
rs73488470
rs36093130
rs10411016
rs8193007
rs55806425
rs139562936
rs62578600
rs7956510
rs10821524
rs34840745
rs10441802
rs2073926
rs4804774
rs11244084
All
−0.1
0.0
0.1
MR leave−one−out sensitivity analysis for
'CD209' on 'Pulmonary fibrosis'

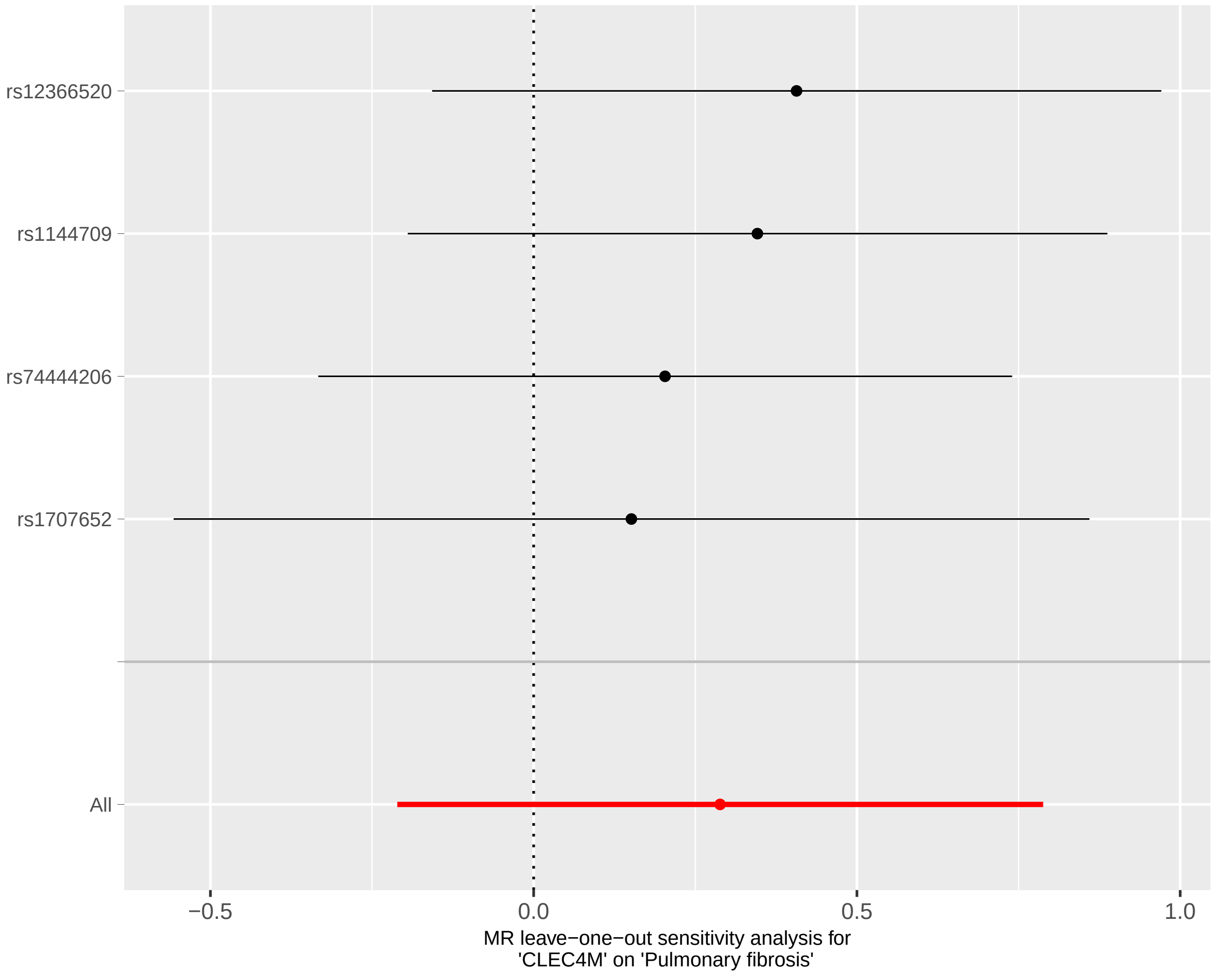

rs12366520
rs1144709
rs74444206
rs1707652
All
−0.5
0.0
0.5
1.0
MR leave−one−out sensitivity analysis for
'CLEC4M' on 'Pulmonary fibrosis'

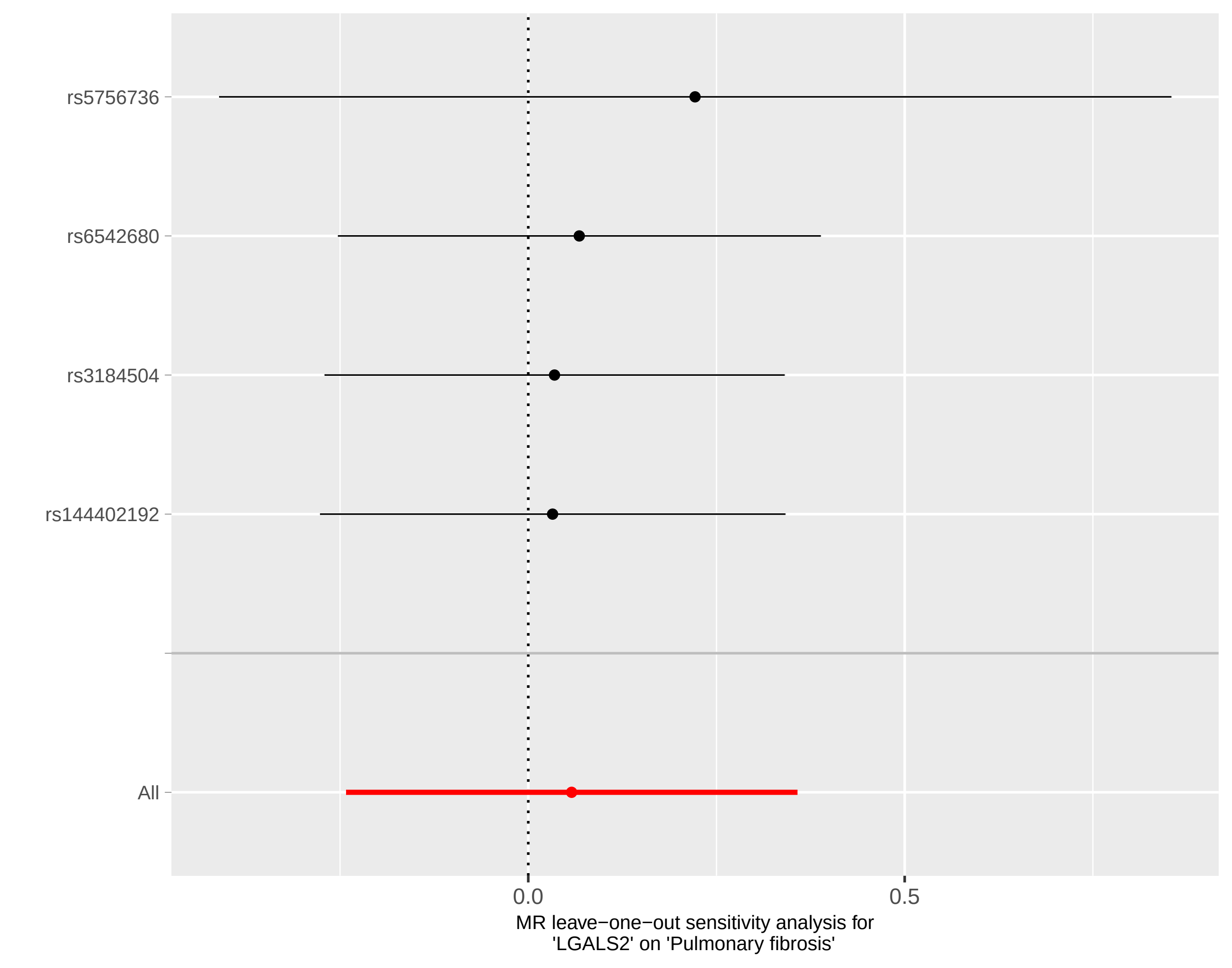

rs5756736
rs6542680
rs3184504
rs144402192
All
0.0
0.5
MR leave−one−out sensitivity analysis for
'LGALS2' on 'Pulmonary fibrosis'

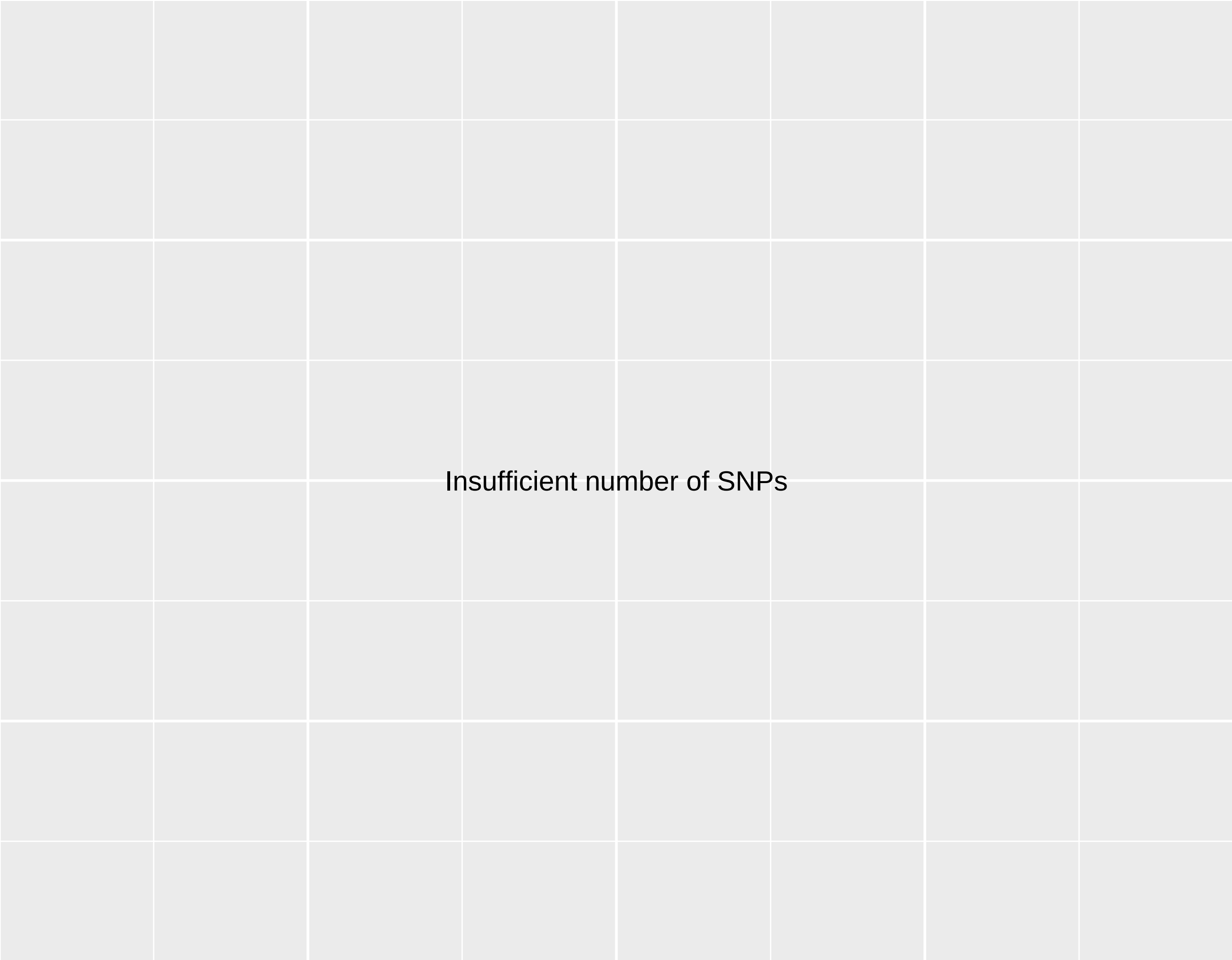
Insufficient number of SNPs

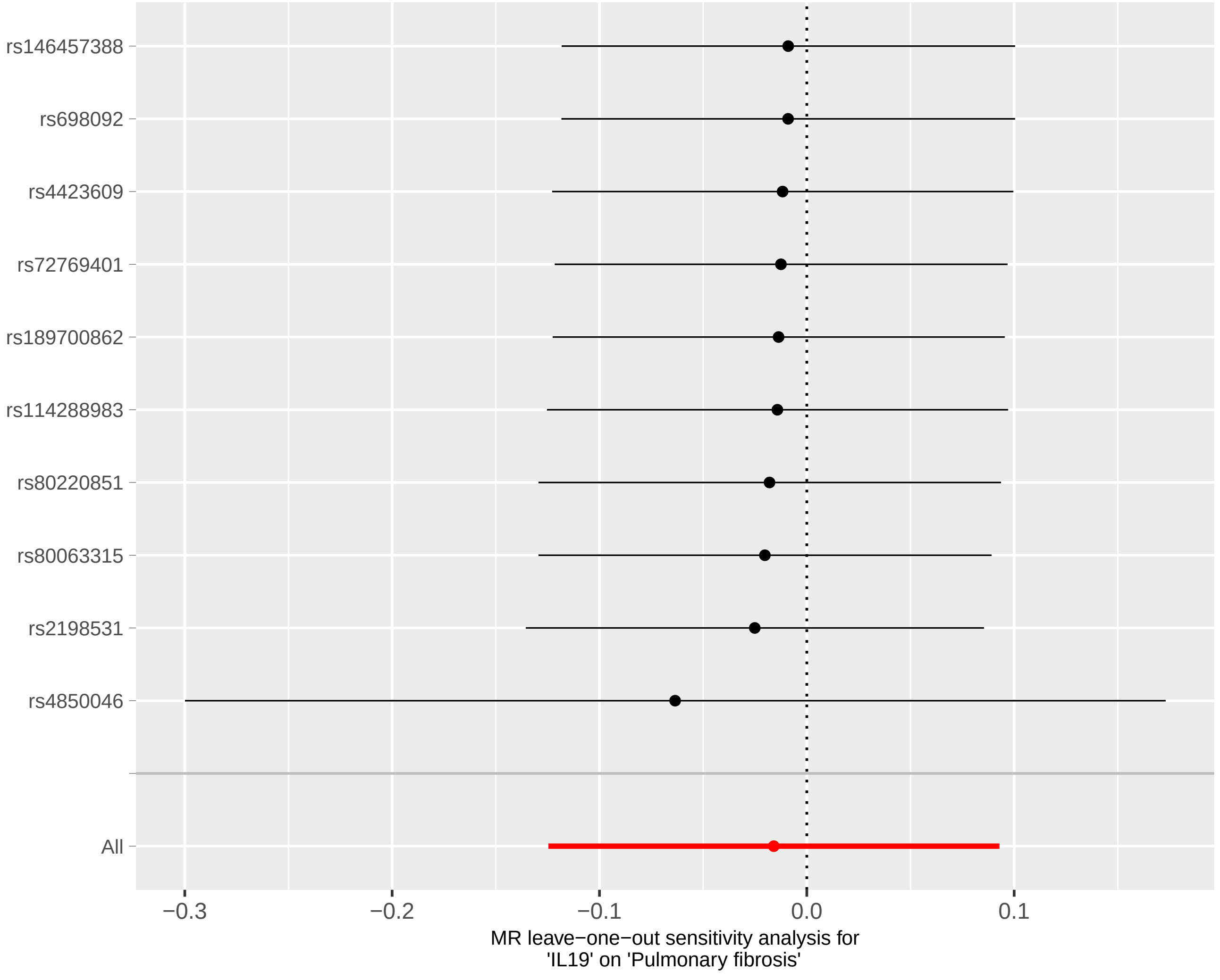
rs146457388
rs698092
rs4423609
rs72769401
rs189700862
rs114288983
rs80220851
rs80063315
rs2198531
rs4850046
All
−0.3
−0.2
−0.1
0.0
0.1
MR leave−one−out sensitivity analysis for
'IL19' on 'Pulmonary fibrosis'

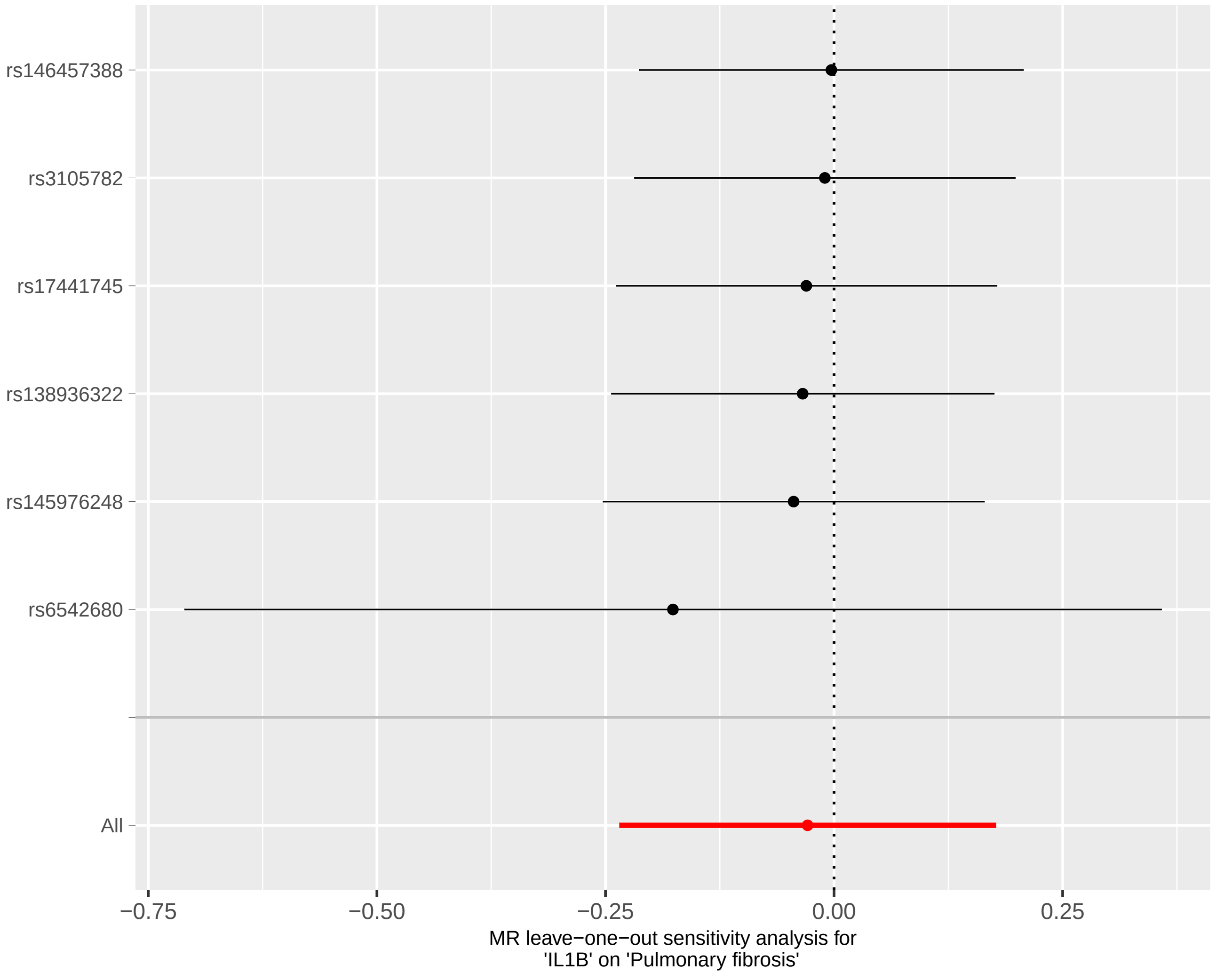
rs146457388
rs3105782
rs17441745
rs138936322
rs145976248
rs6542680
All
−0.75
−0.50
−0.25
0.00
0.25
MR leave−one−out sensitivity analysis for
'IL1B' on 'Pulmonary fibrosis'

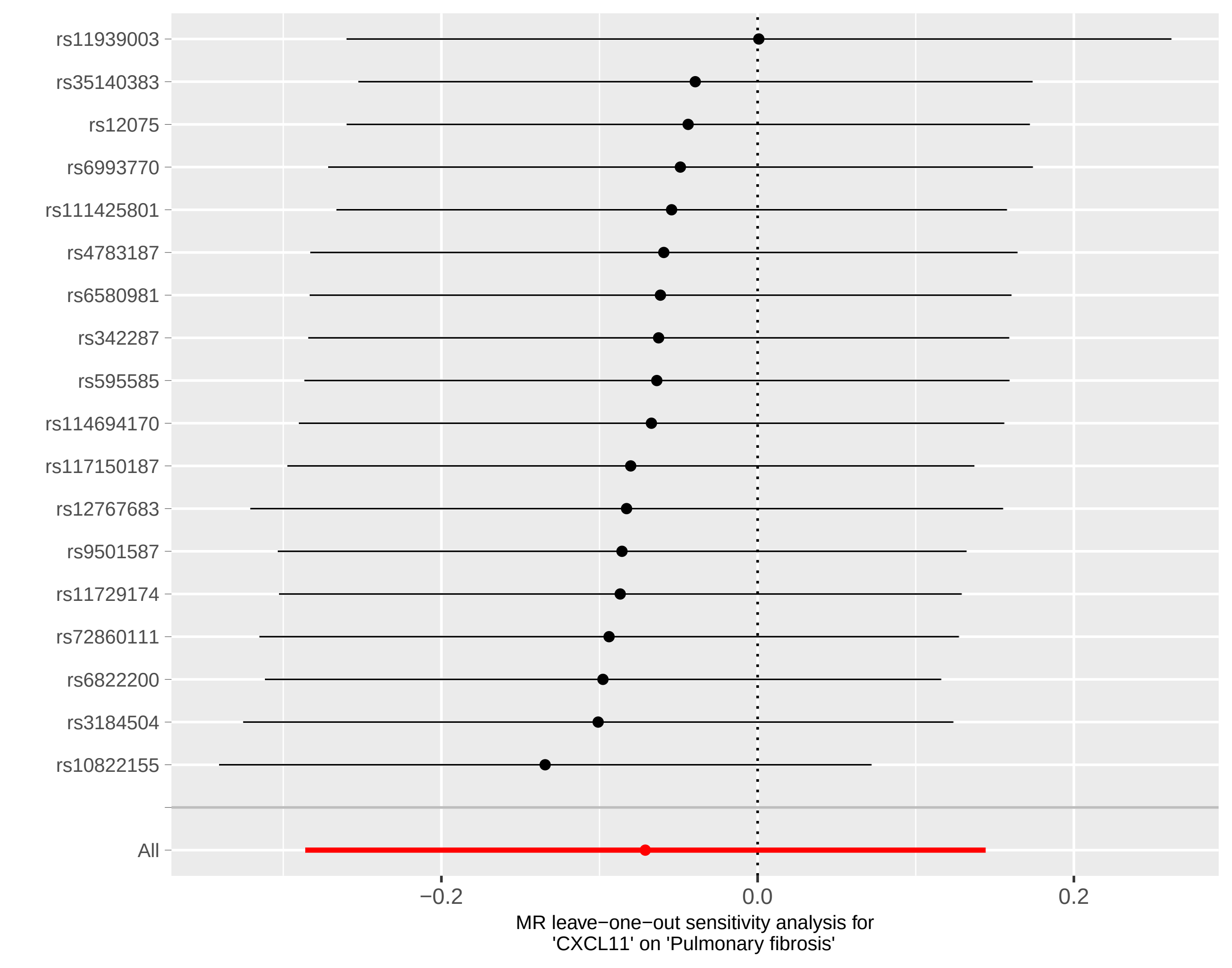
rs11939003
rs35140383
rs12075
rs6993770
rs111425801
rs4783187
rs6580981
rs342287
rs595585
rs114694170
rs117150187
rs12767683
rs9501587
rs11729174
rs72860111
rs6822200
rs3184504
rs10822155
All
−0.2
0.0
0.2
MR leave−one−out sensitivity analysis for
'CXCL11' on 'Pulmonary fibrosis'

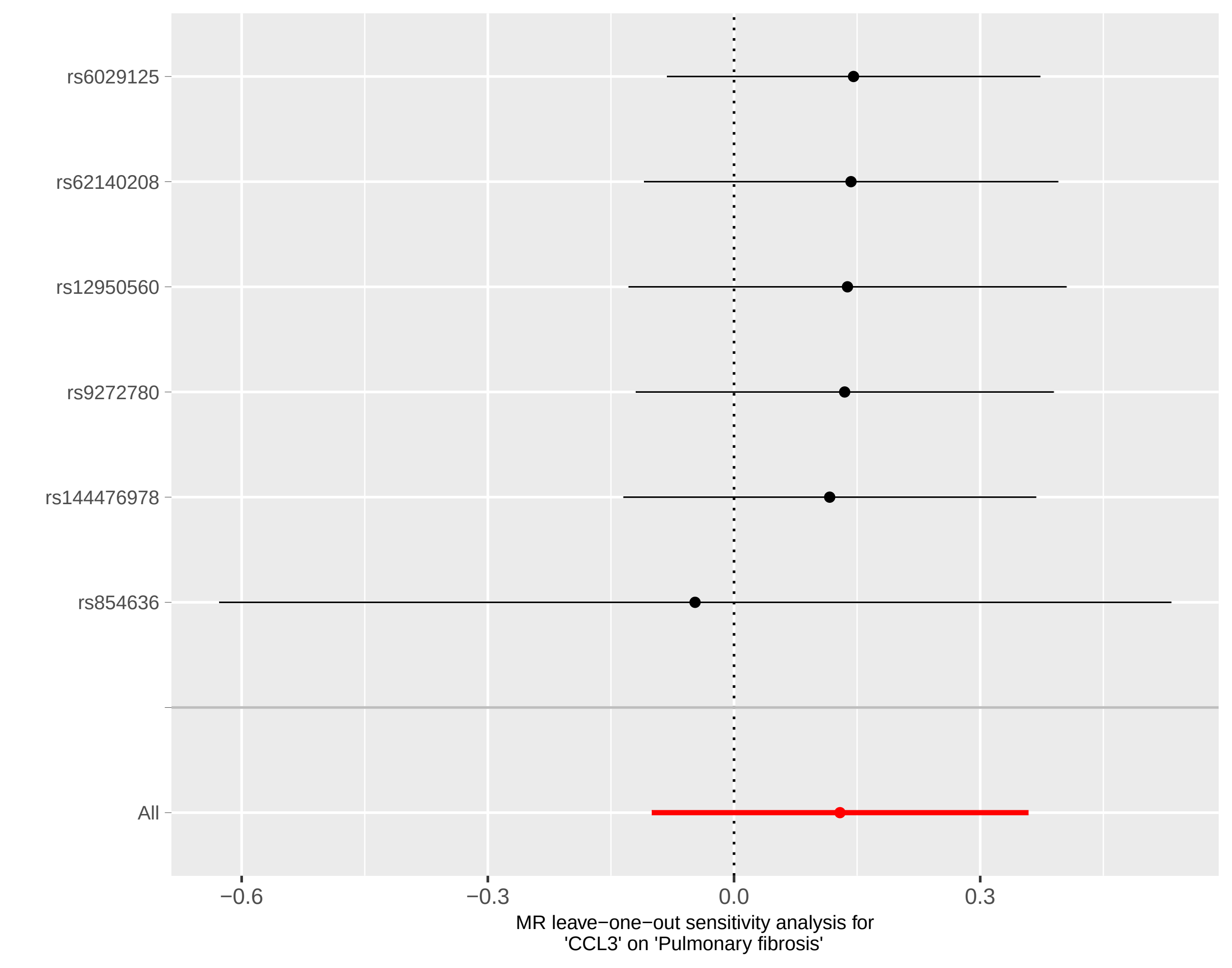
rs6029125
rs62140208
rs12950560
rs9272780
rs144476978
rs854636
All
−0.6
−0.3
0.0
0.3
MR leave−one−out sensitivity analysis for
'CCL3' on 'Pulmonary fibrosis'

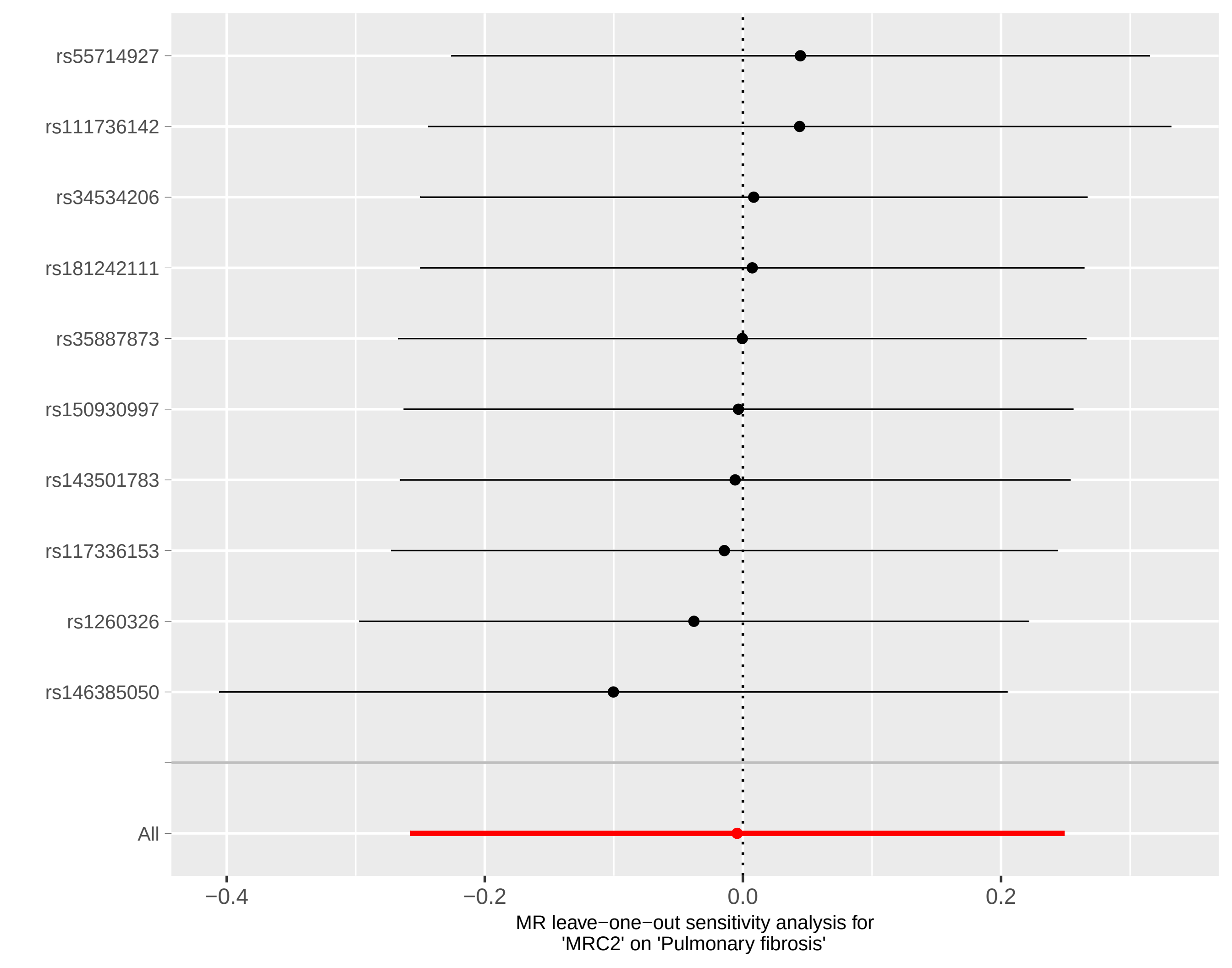
rs55714927
rs111736142
rs34534206
rs181242111
rs35887873
rs150930997
rs143501783
rs117336153
rs1260326
rs146385050
All
−0.4
−0.2
0.0
0.2
MR leave−one−out sensitivity analysis for
'MRC2' on 'Pulmonary fibrosis'

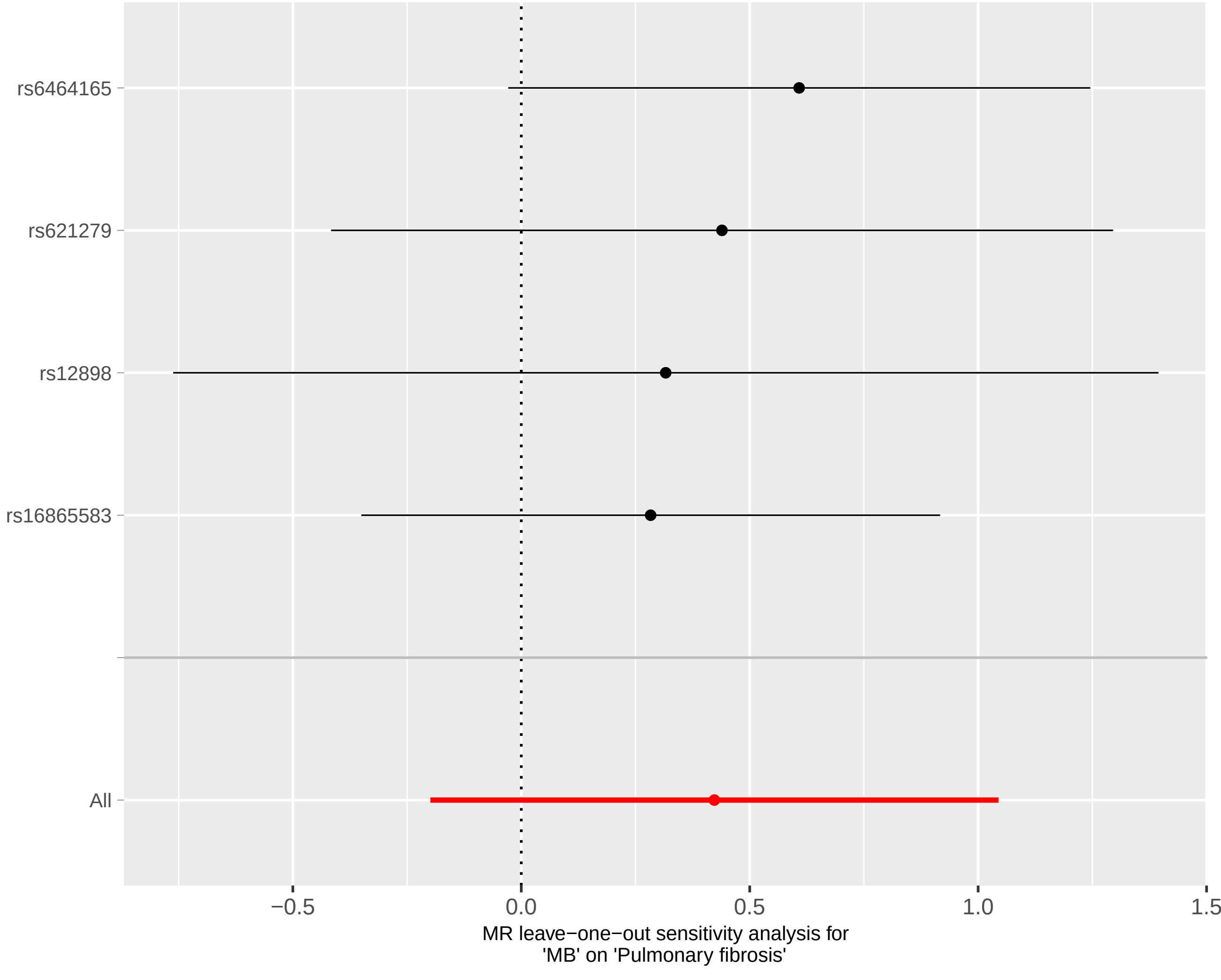
rs6464165
rs621279
rs12898
rs16865583
All
−0.5
0.0
0.5
1.0
1.5
MR leave−one−out sensitivity analysis for
'MB' on 'Pulmonary fibrosis'

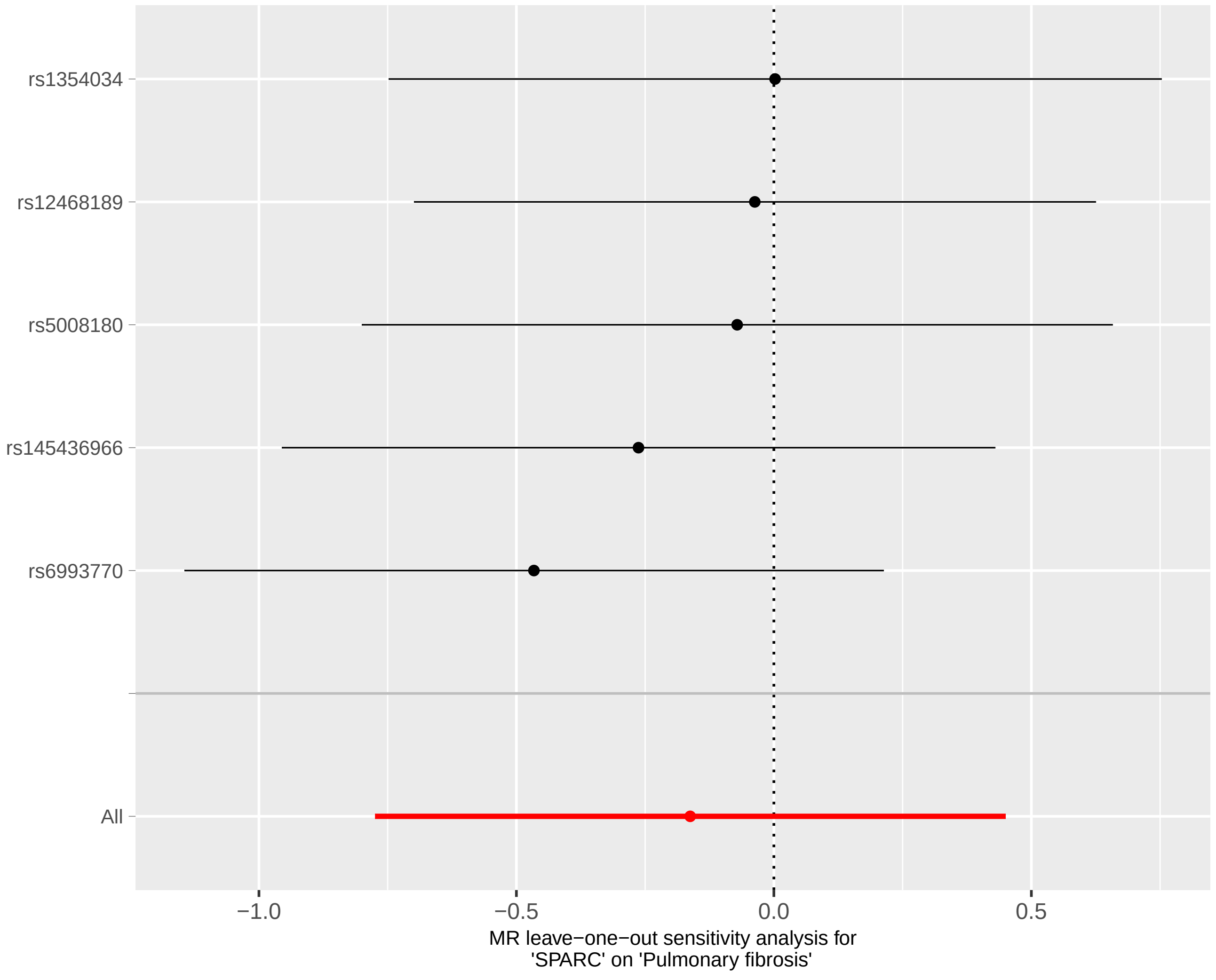
rs1354034
rs12468189
rs5008180
rs145436966
rs6993770
All
−1.0
−0.5
0.0
0.5
MR leave−one−out sensitivity analysis for
'SPARC' on 'Pulmonary fibrosis'

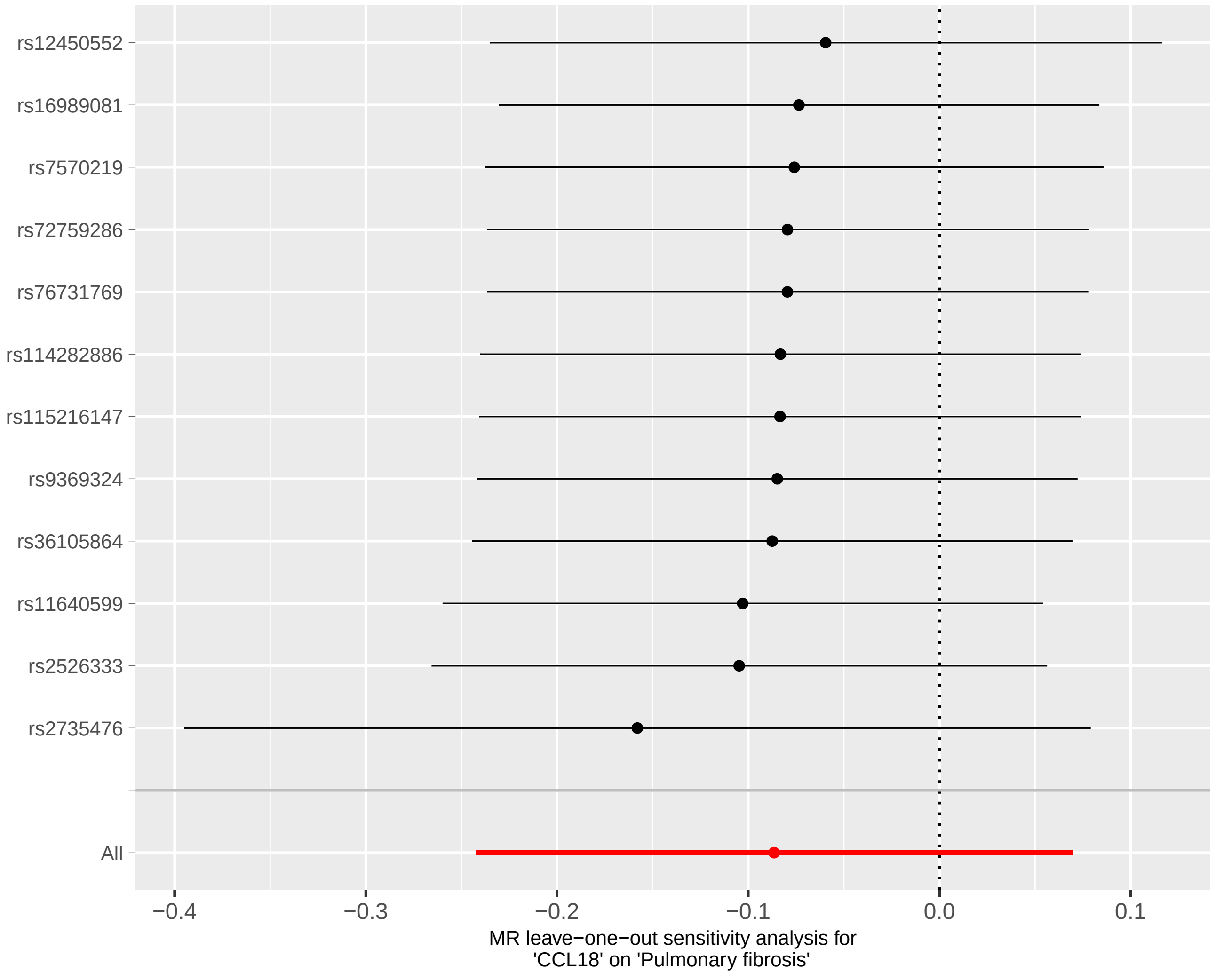
rs12450552
rs16989081
rs7570219
rs72759286
rs76731769
rs114282886
rs115216147
rs9369324
rs36105864
rs11640599
rs2526333
rs2735476
All
−0.4
−0.3
−0.2
−0.1
0.0
0.1
MR leave−one−out sensitivity analysis for
'CCL18' on 'Pulmonary fibrosis'

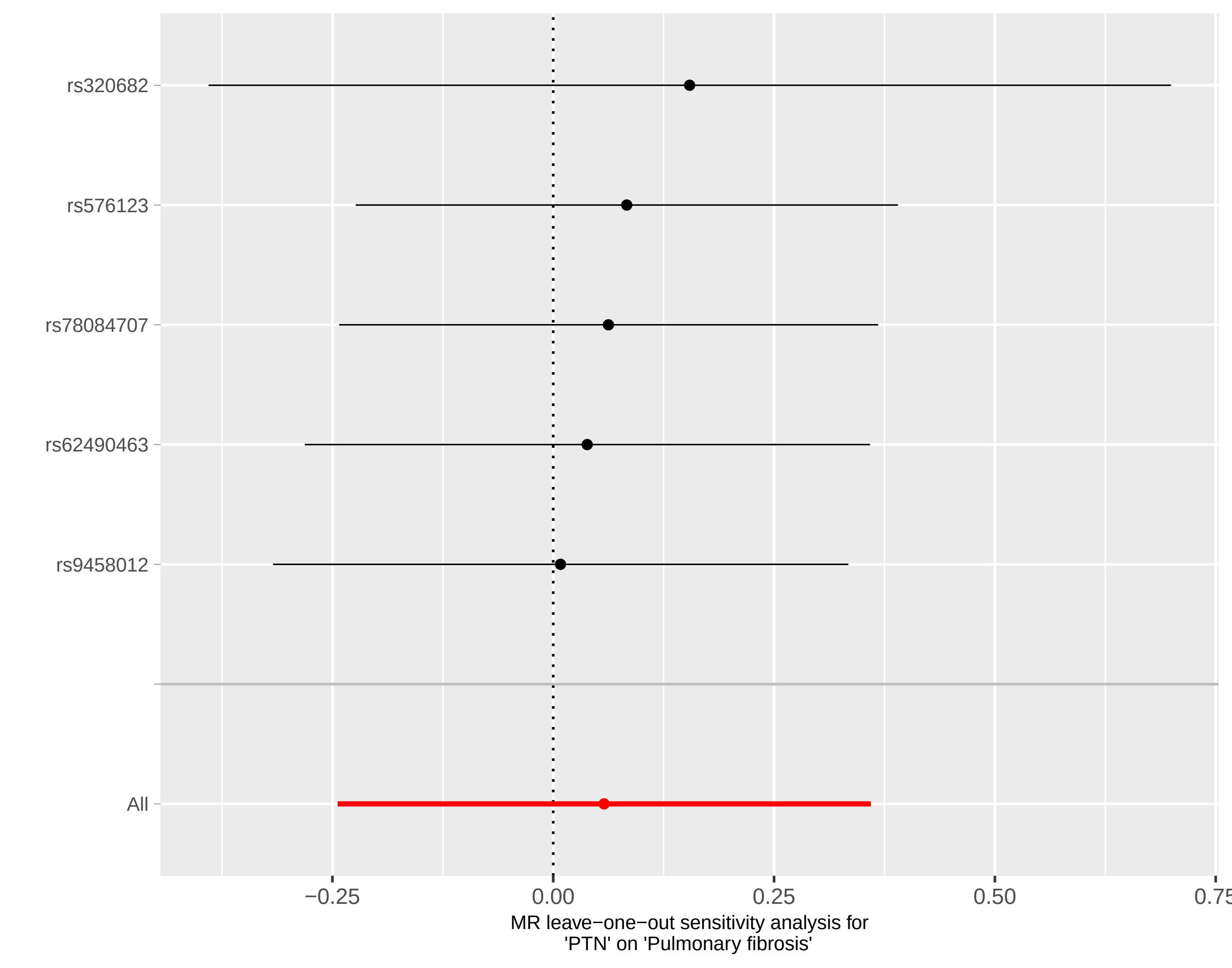
rs320682
rs576123
rs78084707
rs62490463
rs9458012
All
−0.25
0.00
0.25
0.50
MR leave−one−out sensitivity analysis for
'PTN' on 'Pulmonary fibrosis'

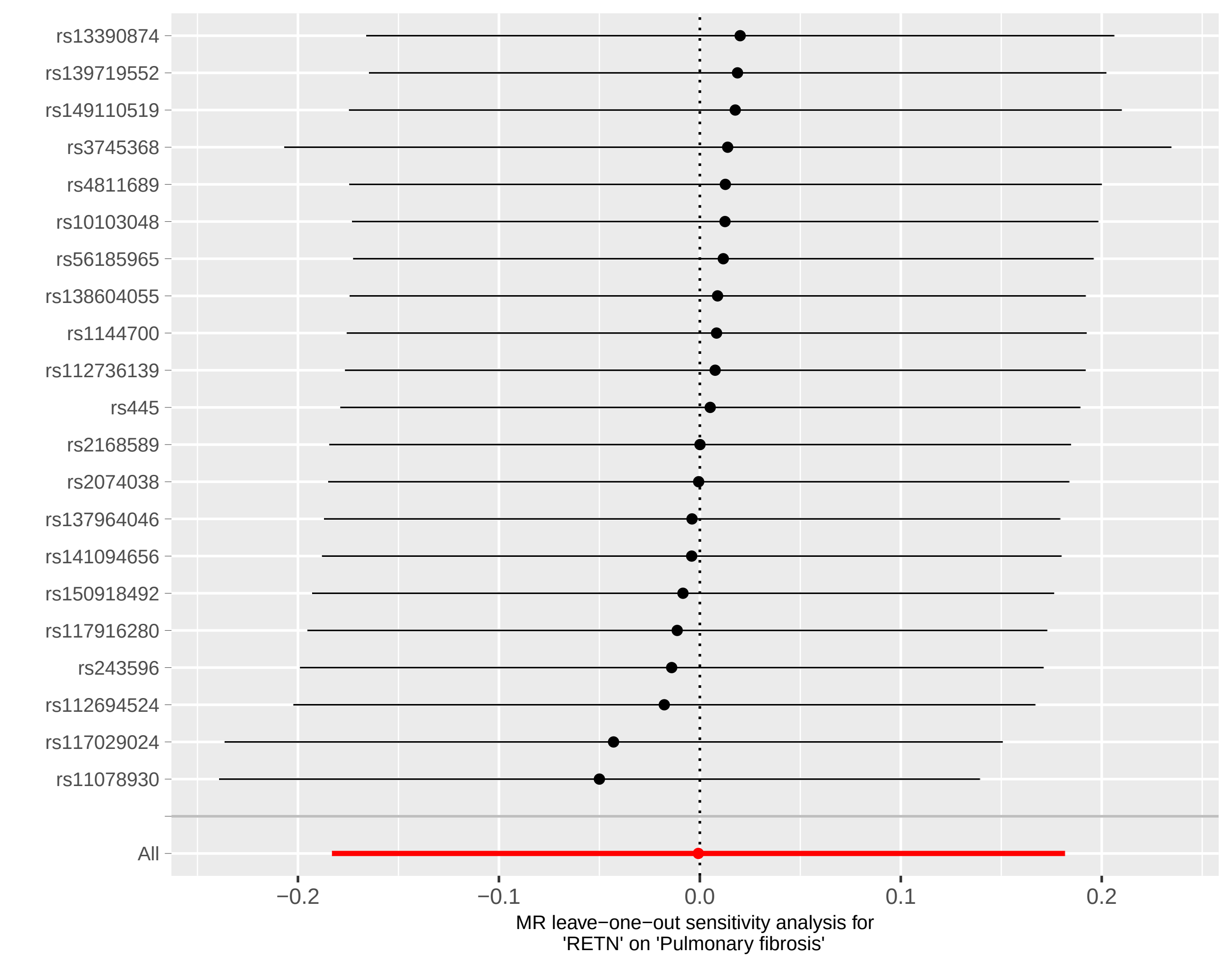
rs13390874
rs139719552
rs149110519
rs3745368
rs4811689
rs10103048
rs56185965
rs138604055
rs1144700
rs112736139
rs445
rs2168589
rs2074038
rs137964046
rs141094656
rs150918492
rs117916280
rs243596
rs112694524
rs117029024
rs11078930
All
−0.2
−0.1
0.0
0.1
0.2
MR leave−one−out sensitivity analysis for
'RETN' on 'Pulmonary fibrosis'

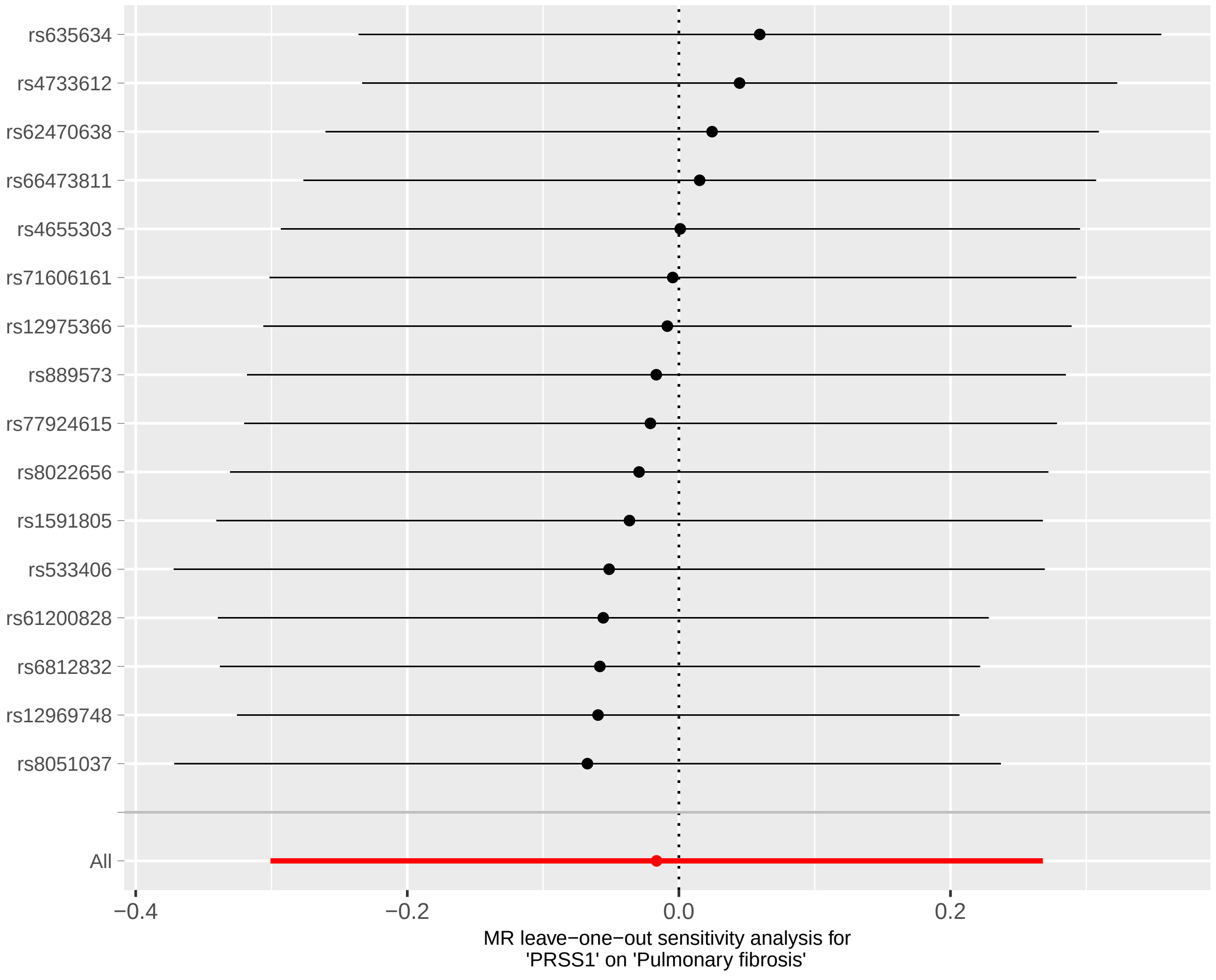
rs635634
rs4733612
rs62470638
rs66473811
rs4655303
rs71606161
rs12975366
rs889573
rs77924615
rs8022656
rs1591805
rs533406
rs61200828
rs6812832
rs12969748
rs8051037
All
−0.4
−0.2
0.0
0.2
MR leave−one−out sensitivity analysis for
'PRSS1' on 'Pulmonary fibrosis'

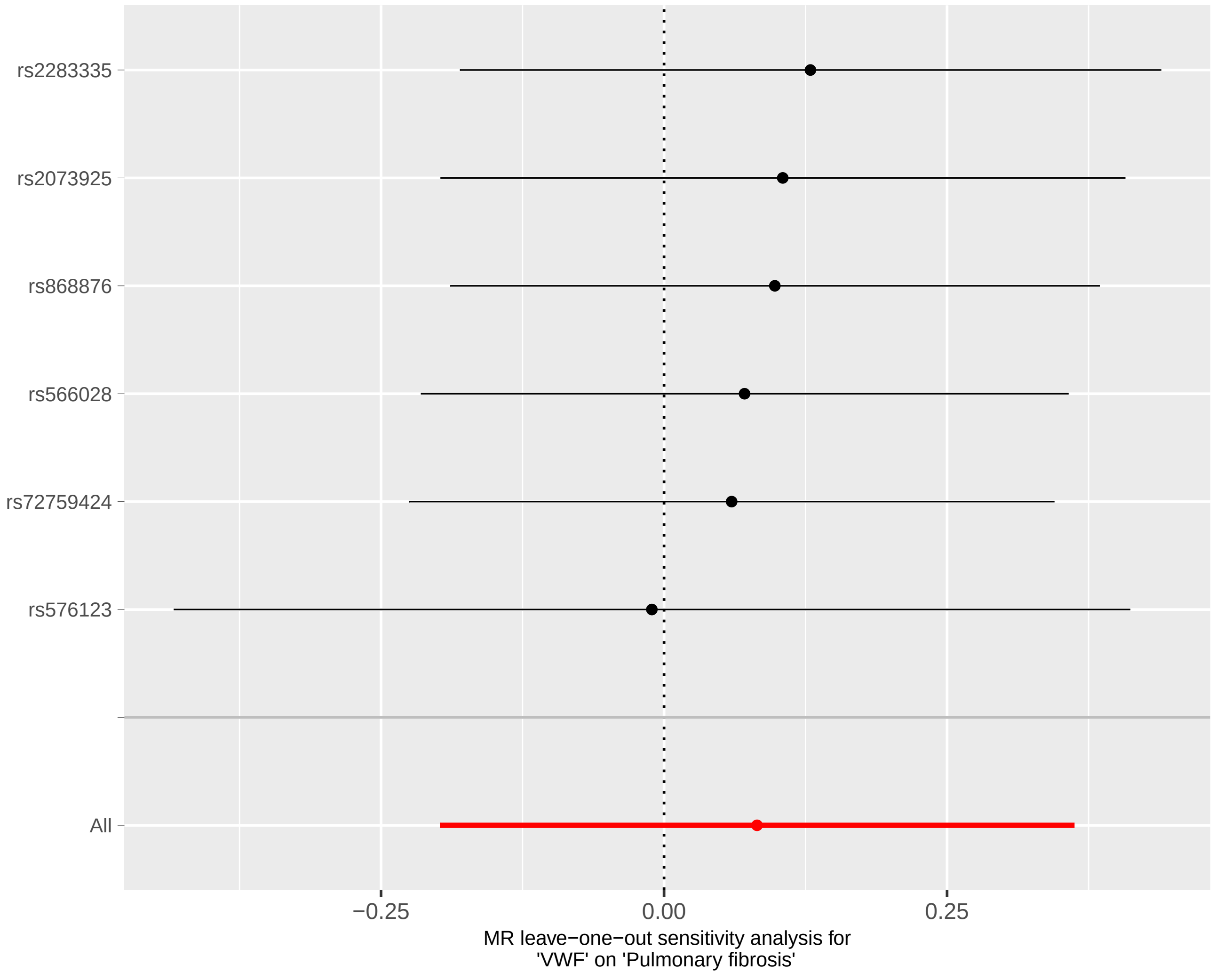

rs2283335
rs2073925
rs868876
rs566028
rs72759424
rs576123
All
−0.25
0.00
0.25
MR leave−one−out sensitivity analysis for
'VWF' on 'Pulmonary fibrosis'

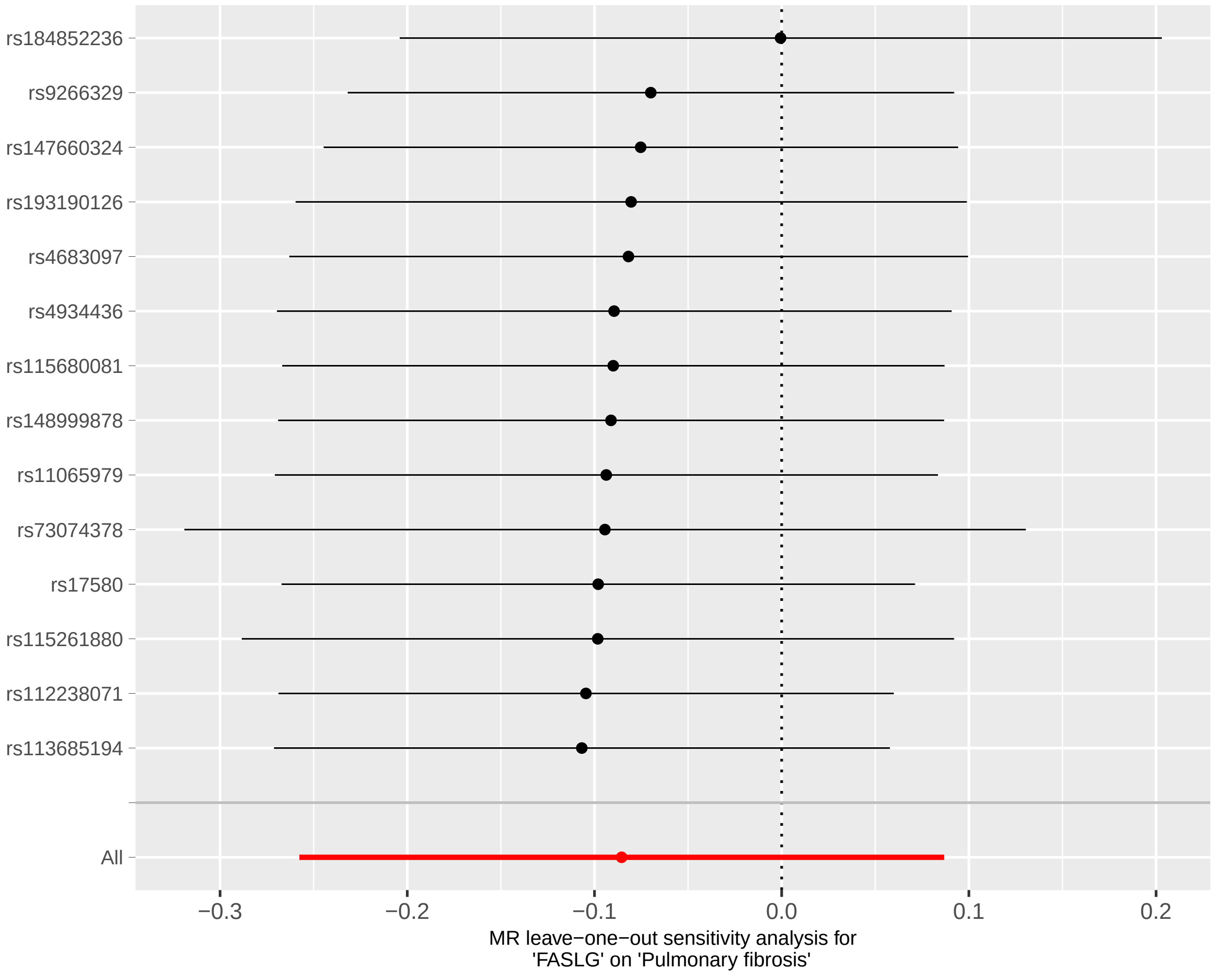
rs184852236
rs9266329
rs147660324
rs193190126
rs4683097
rs4934436
rs115680081
rs148999878
rs11065979
rs73074378
rs17580
rs115261880
rs112238071
rs113685194
All
−0.3
−0.2
−0.1
0.0
0.1
0.2
MR leave−one−out sensitivity analysis for
'FASLG' on 'Pulmonary fibrosis'

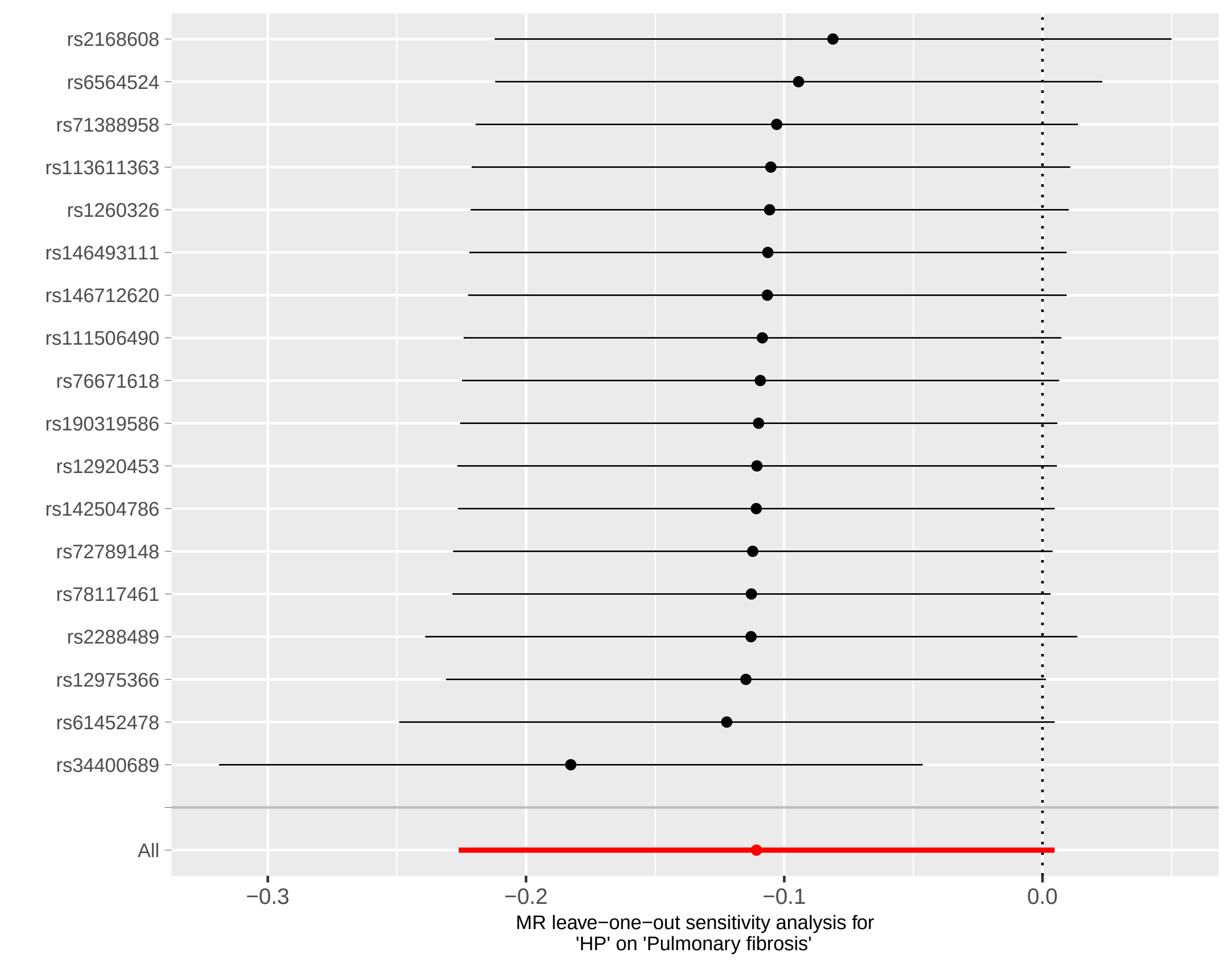
rs2168608
rs6564524
rs71388958
rs113611363
rs1260326
rs146493111
rs146712620
rs111506490
rs76671618
rs190319586
rs12920453
rs142504786
rs72789148
rs78117461
rs2288489
rs12975366
rs61452478
rs34400689
All
−0.3
−0.2
−0.1
0.0
MR leave−one−out sensitivity analysis for
'HP' on 'Pulmonary fibrosis'

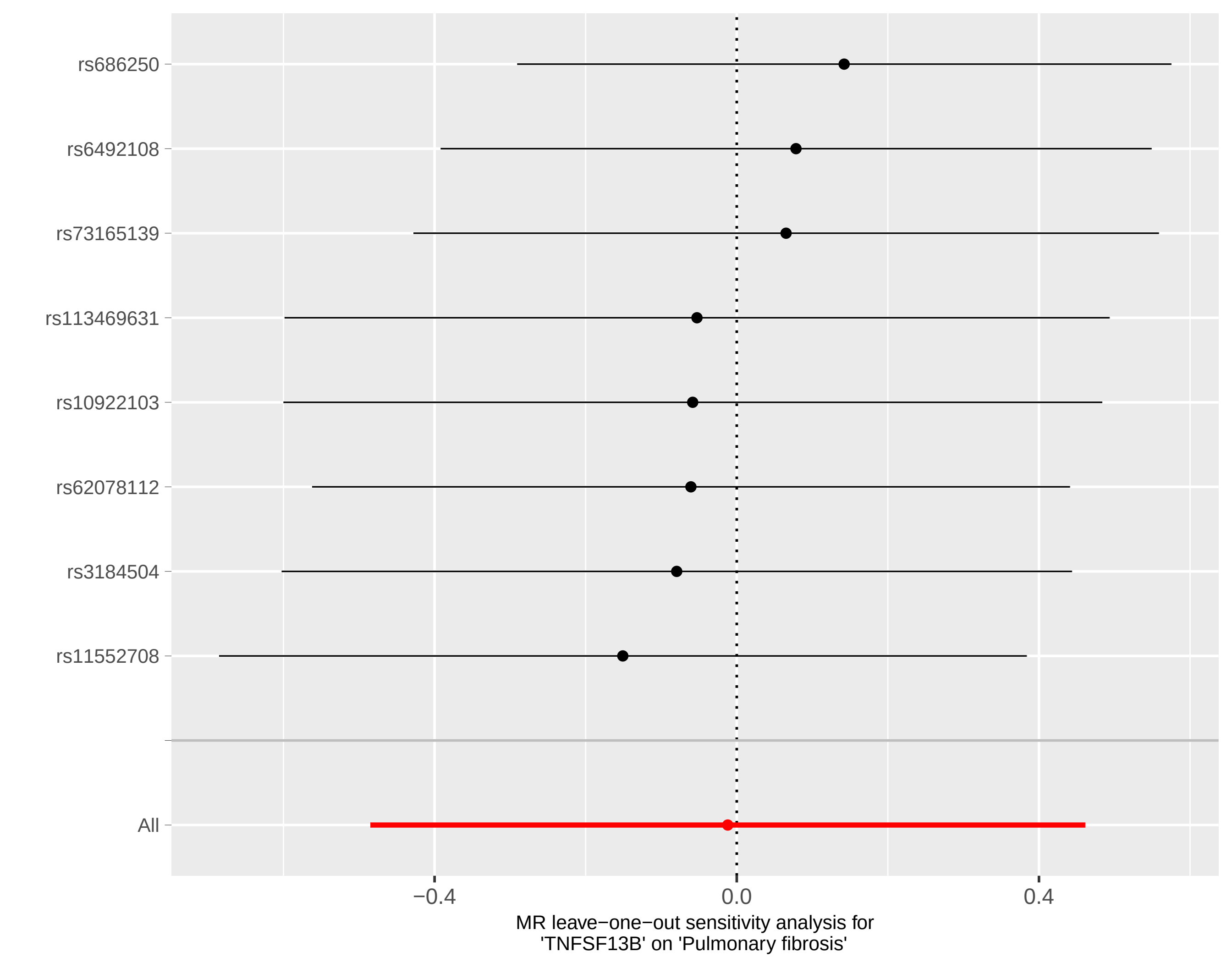
rs686250
rs6492108
rs73165139
rs113469631
rs10922103
rs62078112
rs3184504
rs11552708
All
−0.4
0.0
0.4
MR leave−one−out sensitivity analysis for
'TNFSF13B' on 'Pulmonary fibrosis'

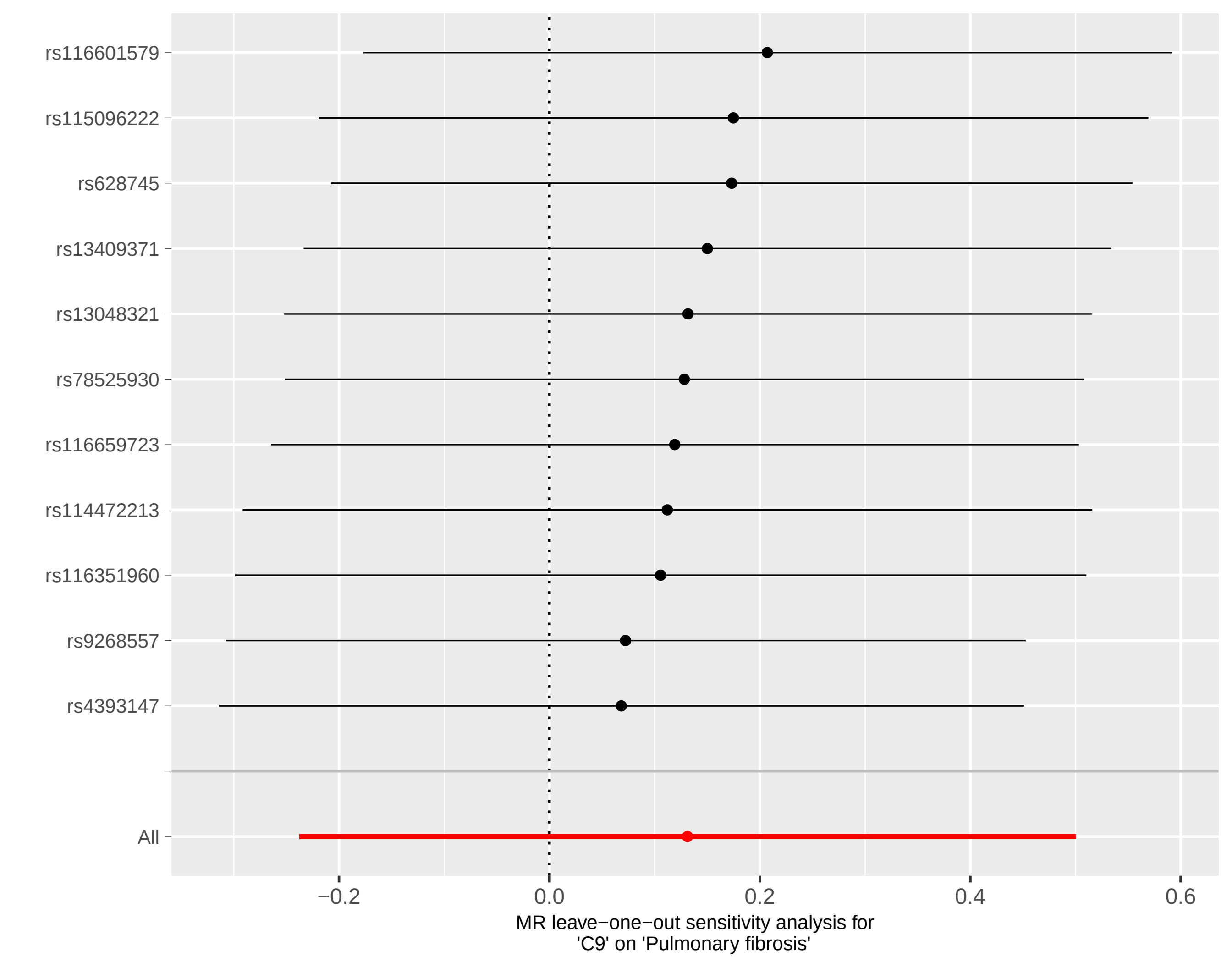
rs116601579
rs115096222
rs628745
rs13409371
rs13048321
rs78525930
rs116659723
rs114472213
rs116351960
rs9268557
rs4393147
All
−0.2
0.0
0.2
0.4
0.6
MR leave−one−out sensitivity analysis for
'C9' on 'Pulmonary fibrosis'

Insufficient number of SNPs

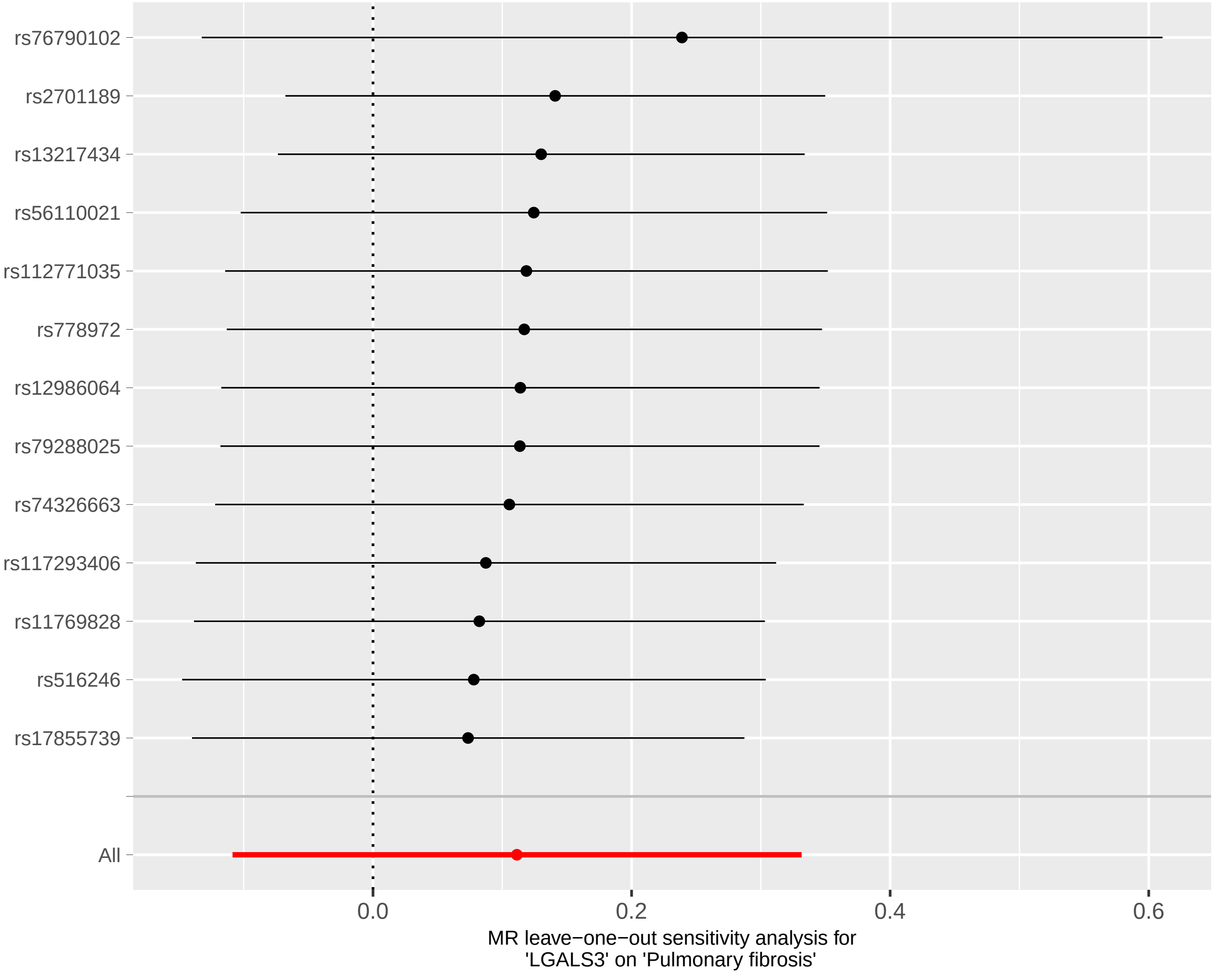
rs76790102
rs2701189
rs13217434
rs56110021
rs112771035
rs778972
rs12986064
rs79288025
rs74326663
rs117293406
rs11769828
rs516246
rs17855739
All
0.0
0.2
0.4
0.6
MR leave−one−out sensitivity analysis for
'LGALS3' on 'Pulmonary fibrosis'

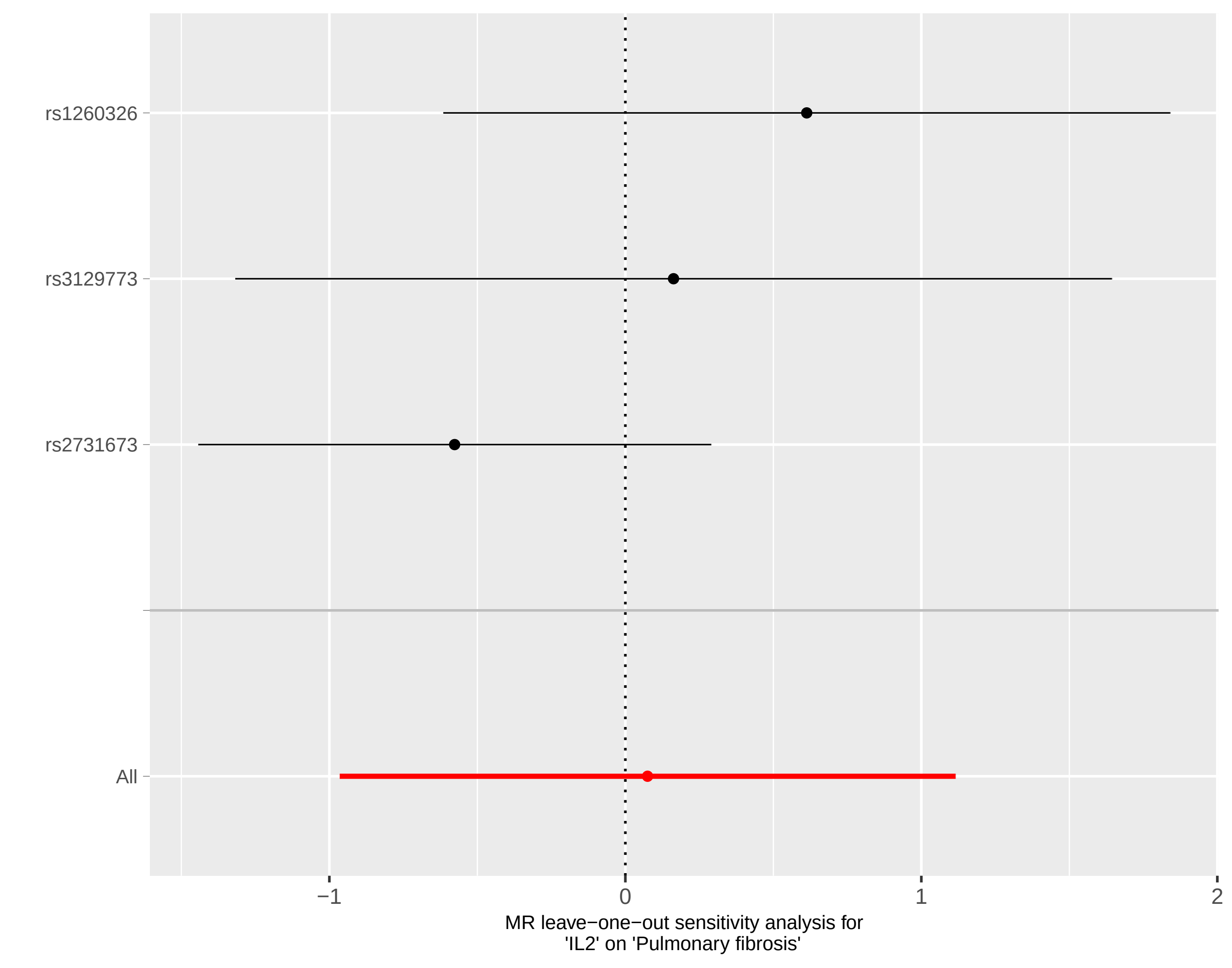
rs1260326
rs3129773
rs2731673
All
−1
0
1
2
MR leave−one−out sensitivity analysis for
'IL2' on 'Pulmonary fibrosis'

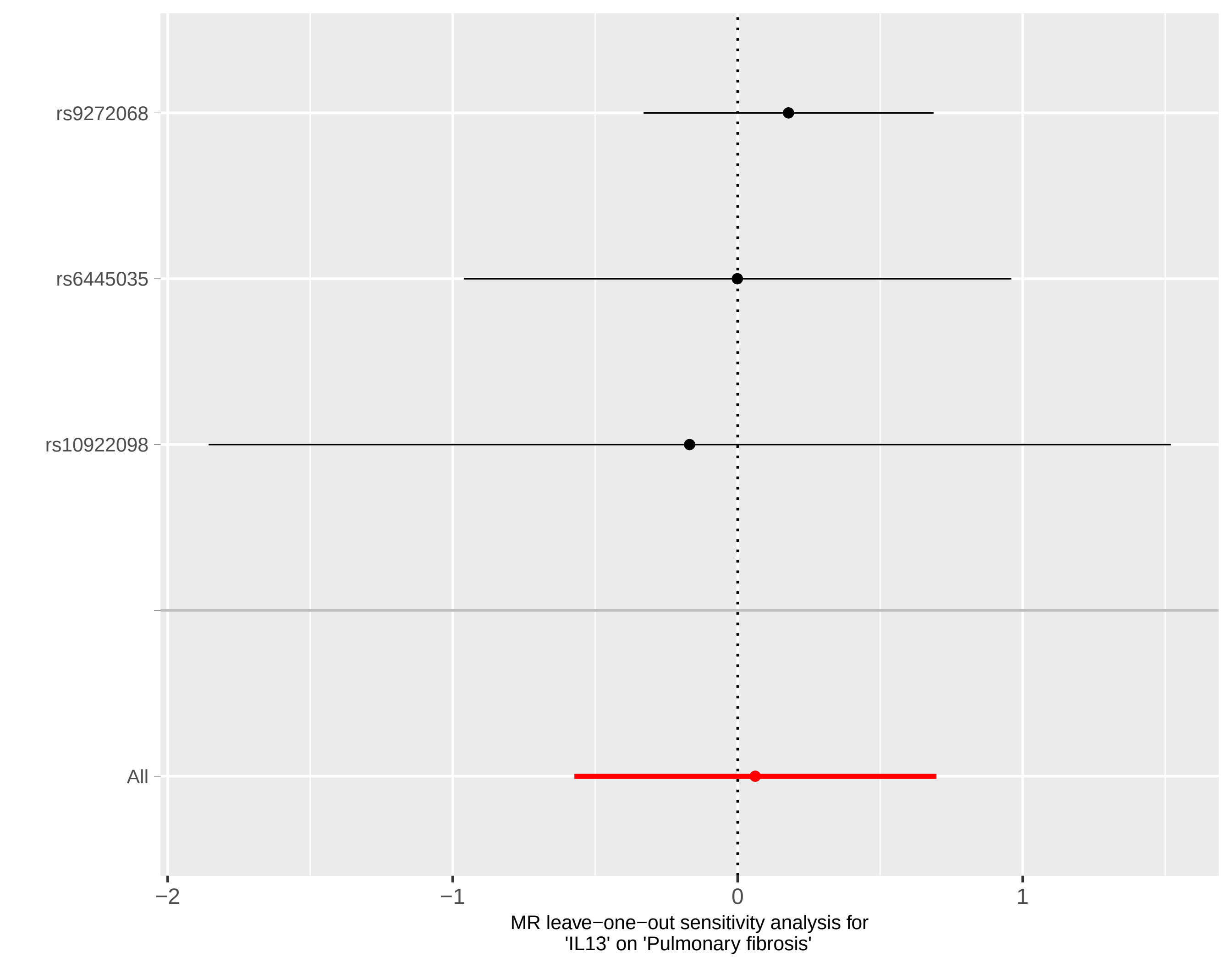
rs9272068
rs6445035
rs10922098
All
−2
−1
0
1
MR leave−one−out sensitivity analysis for
'IL13' on 'Pulmonary fibrosis'

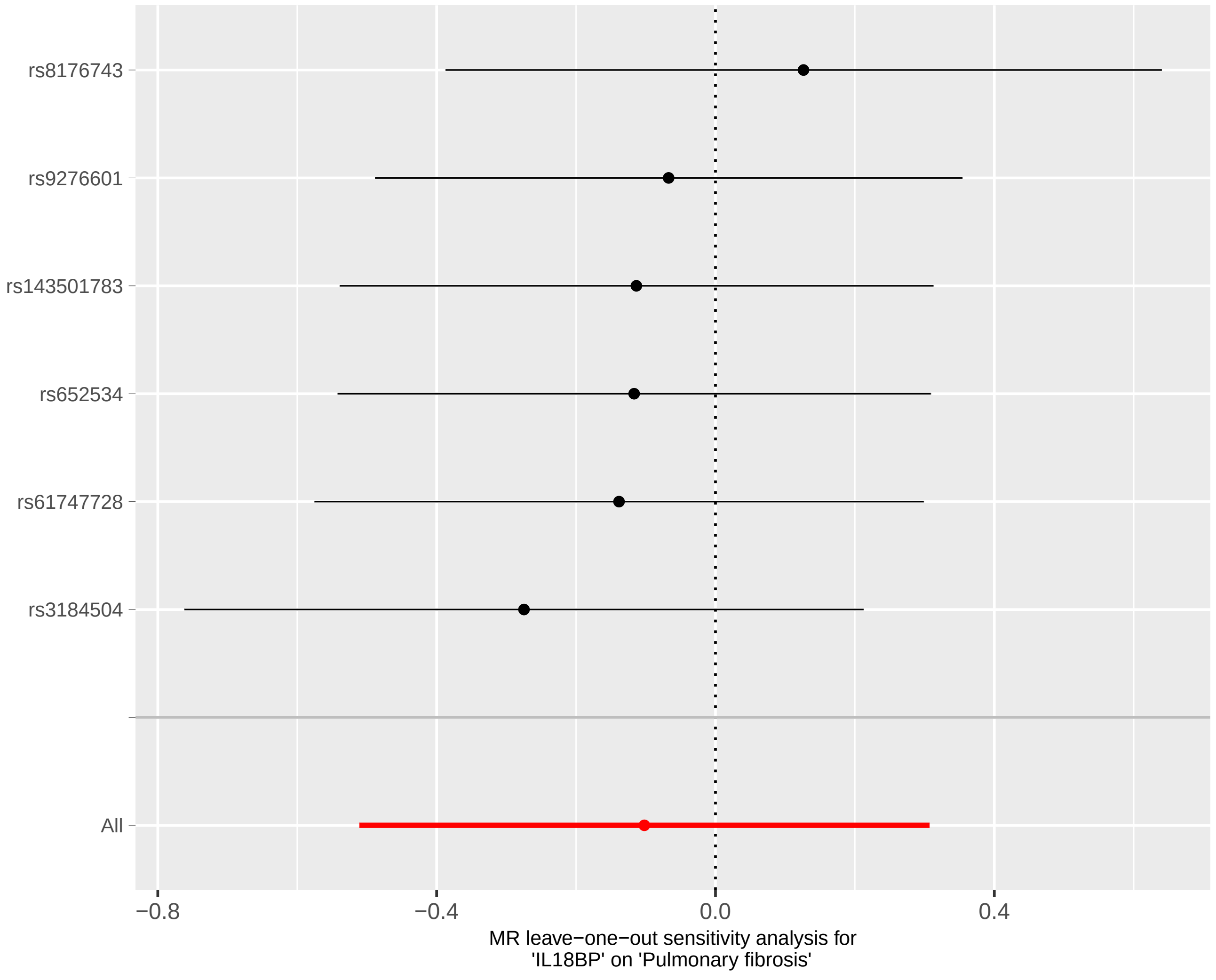

rs8176743
rs9276601
rs143501783
rs652534
rs61747728
rs3184504
All
−0.8
−0.4
0.0
0.4
MR leave−one−out sensitivity analysis for
'IL18BP' on 'Pulmonary fibrosis'

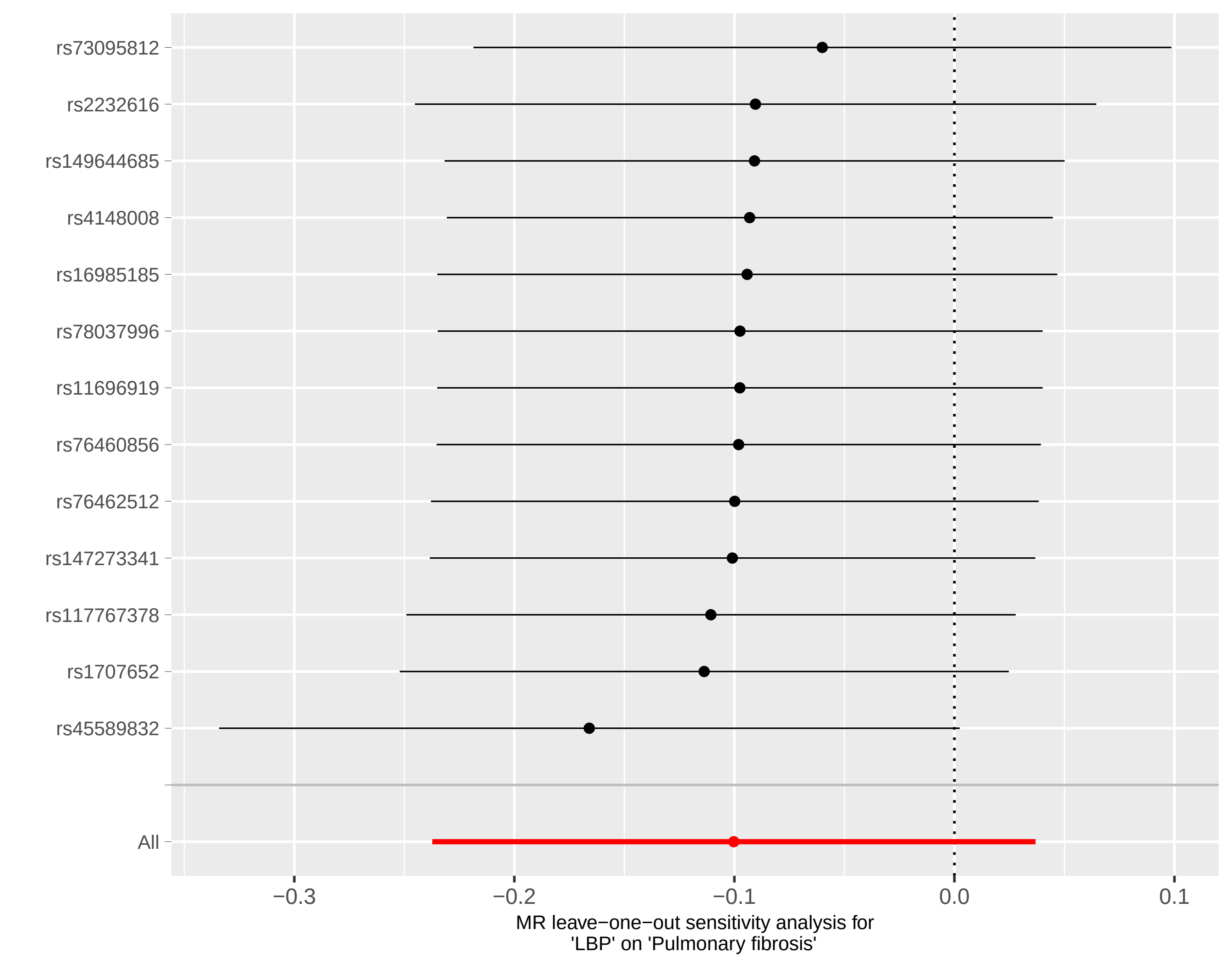

rs73095812
rs2232616
rs149644685
rs4148008
rs16985185
rs78037996
rs11696919
rs76460856
rs76462512
rs147273341
rs117767378
rs1707652
rs45589832
All
−0.3
−0.2
−0.1
0.0
0.1
MR leave−one−out sensitivity analysis for
'LBP' on 'Pulmonary fibrosis'

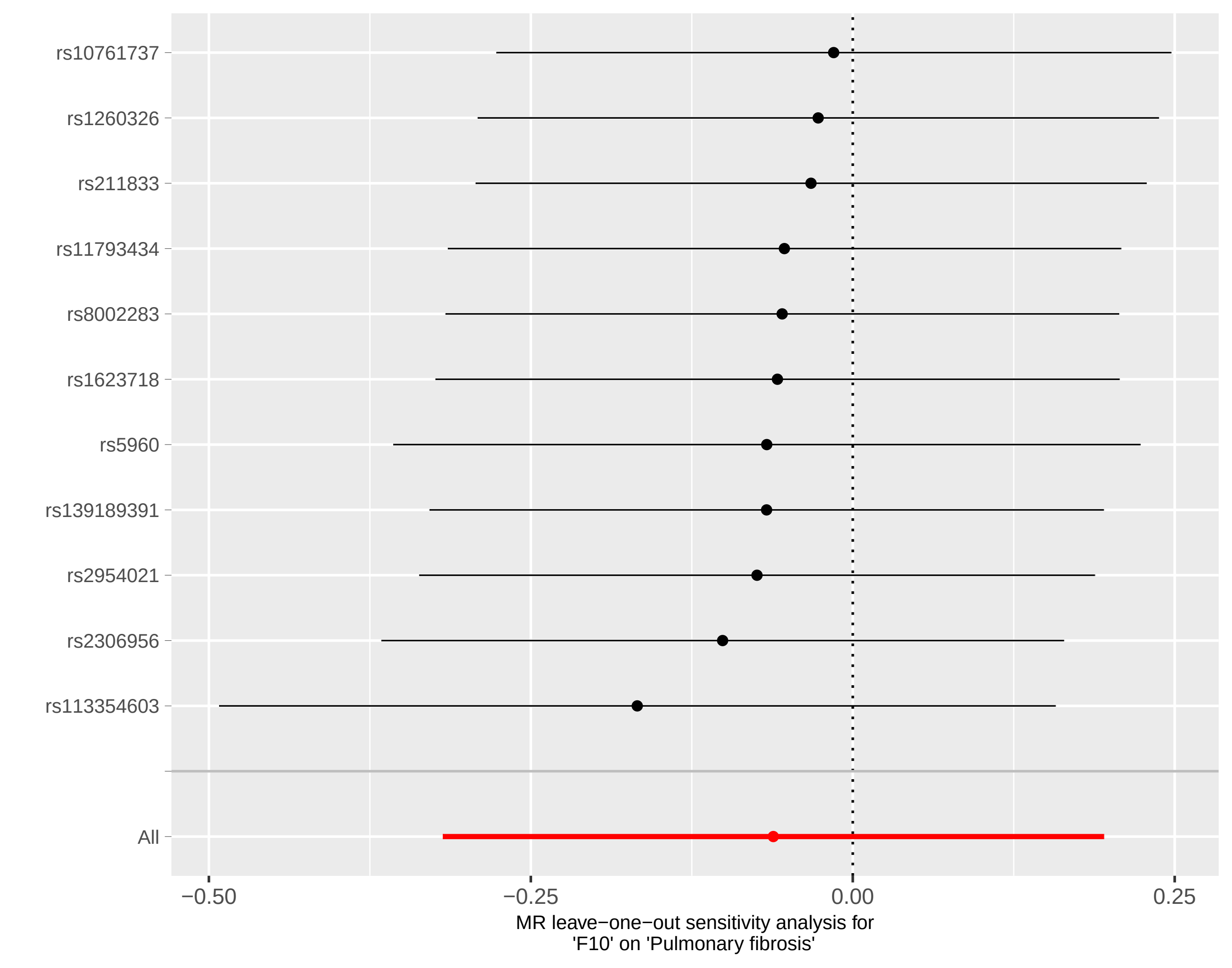
rs10761737
rs1260326
rs211833
rs11793434
rs8002283
rs1623718
rs5960
rs139189391
rs2954021
rs2306956
rs113354603
All
−0.50
−0.25
0.00
0.25
MR leave−one−out sensitivity analysis for
'F10' on 'Pulmonary fibrosis'

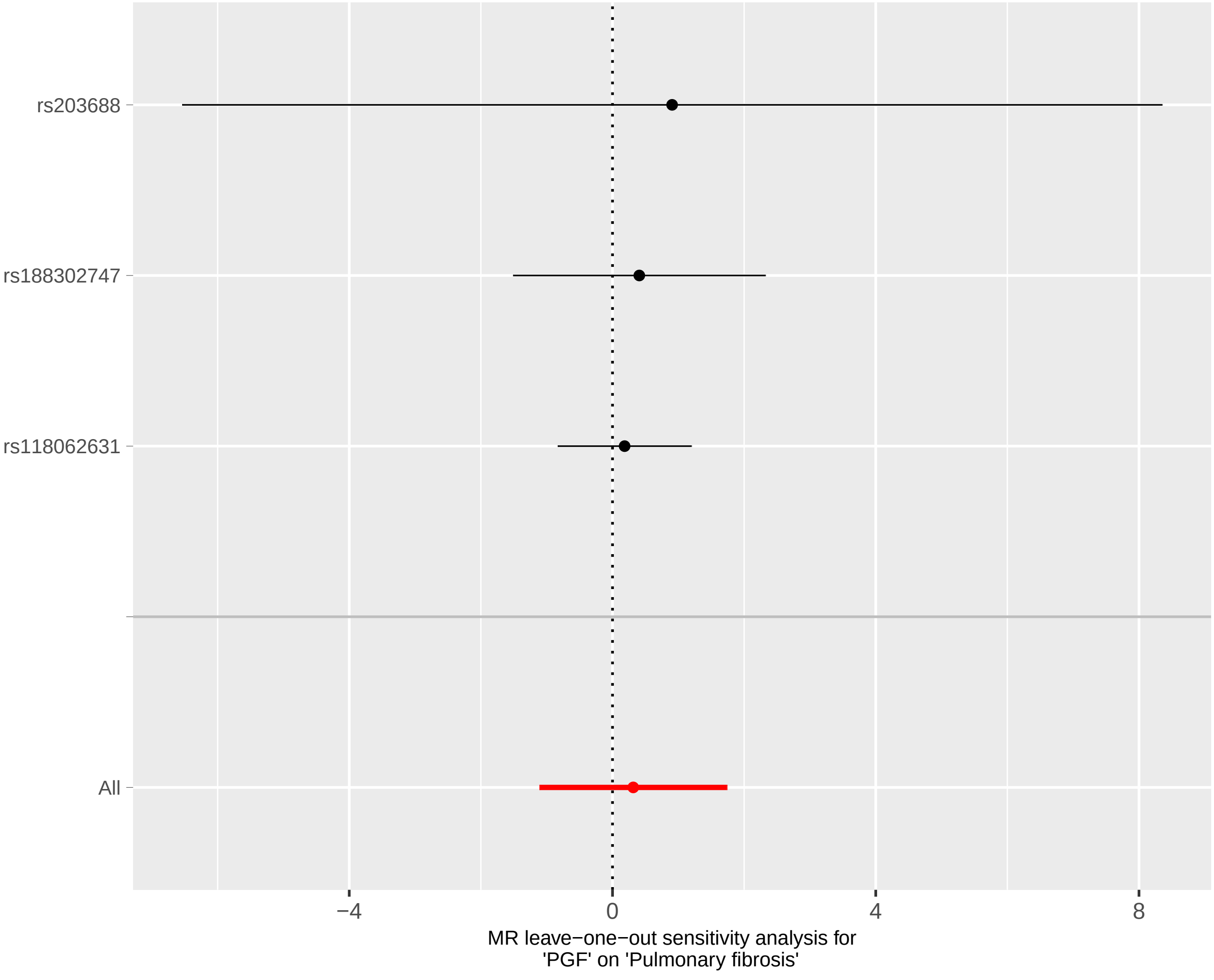
rs203688
rs188302747
rs118062631
All
−4
0
4
8
MR leave−one−out sensitivity analysis for
'PGF' on 'Pulmonary fibrosis'

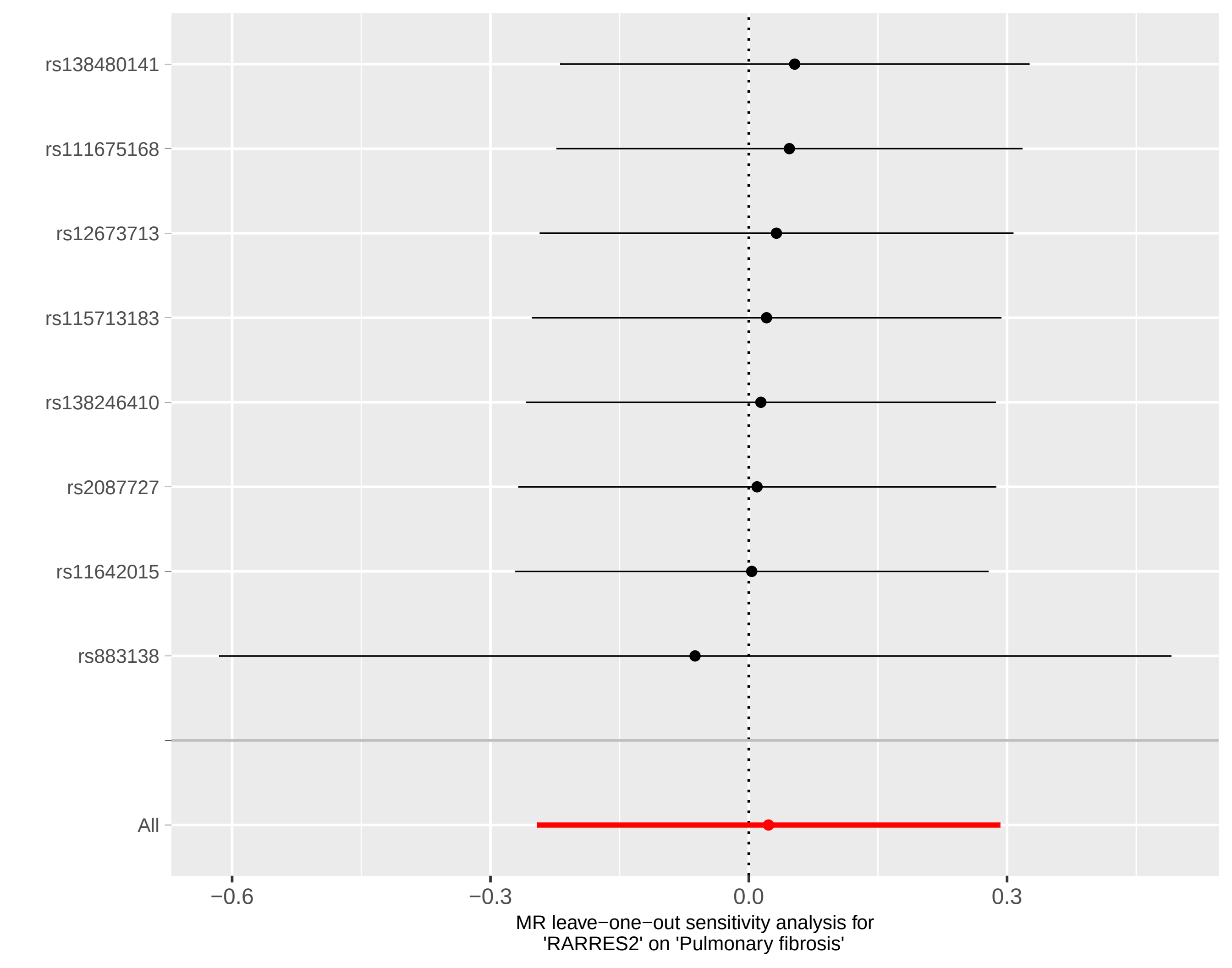

rs138480141
rs111675168
rs12673713
rs115713183
rs138246410
rs2087727
rs11642015
rs883138
All
−0.6
−0.3
0.0
0.3
MR leave−one−out sensitivity analysis for
'RARRES2' on 'Pulmonary fibrosis'

Insufficient number of SNPs

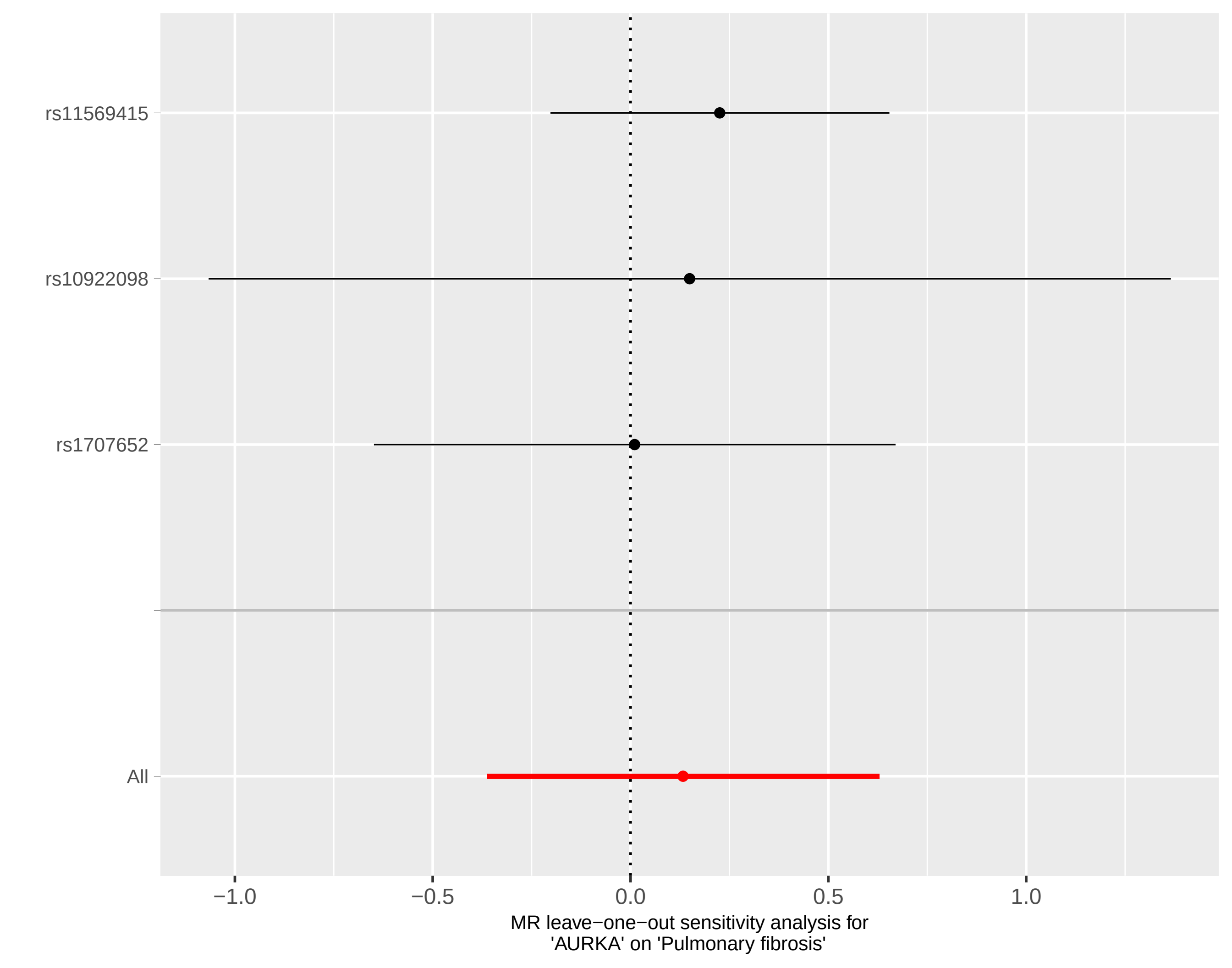
rs11569415
rs10922098
rs1707652
All
−1.0
−0.5
0.0
0.5
1.0
MR leave−one−out sensitivity analysis for
'AURKA' on 'Pulmonary fibrosis'

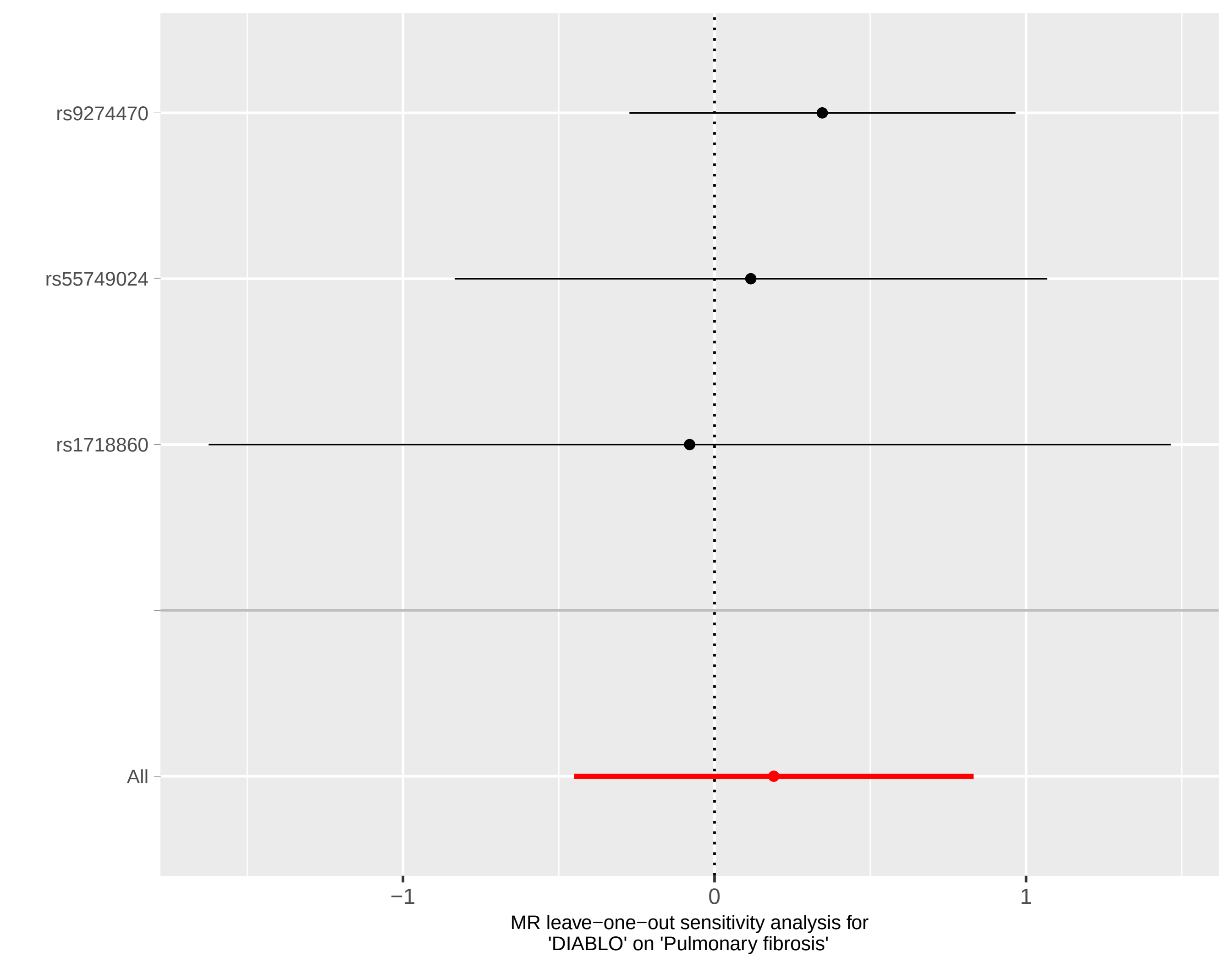
rs9274470
rs55749024
rs1718860
All
−1
0
1
MR leave−one−out sensitivity analysis for
'DIABLO' on 'Pulmonary fibrosis'

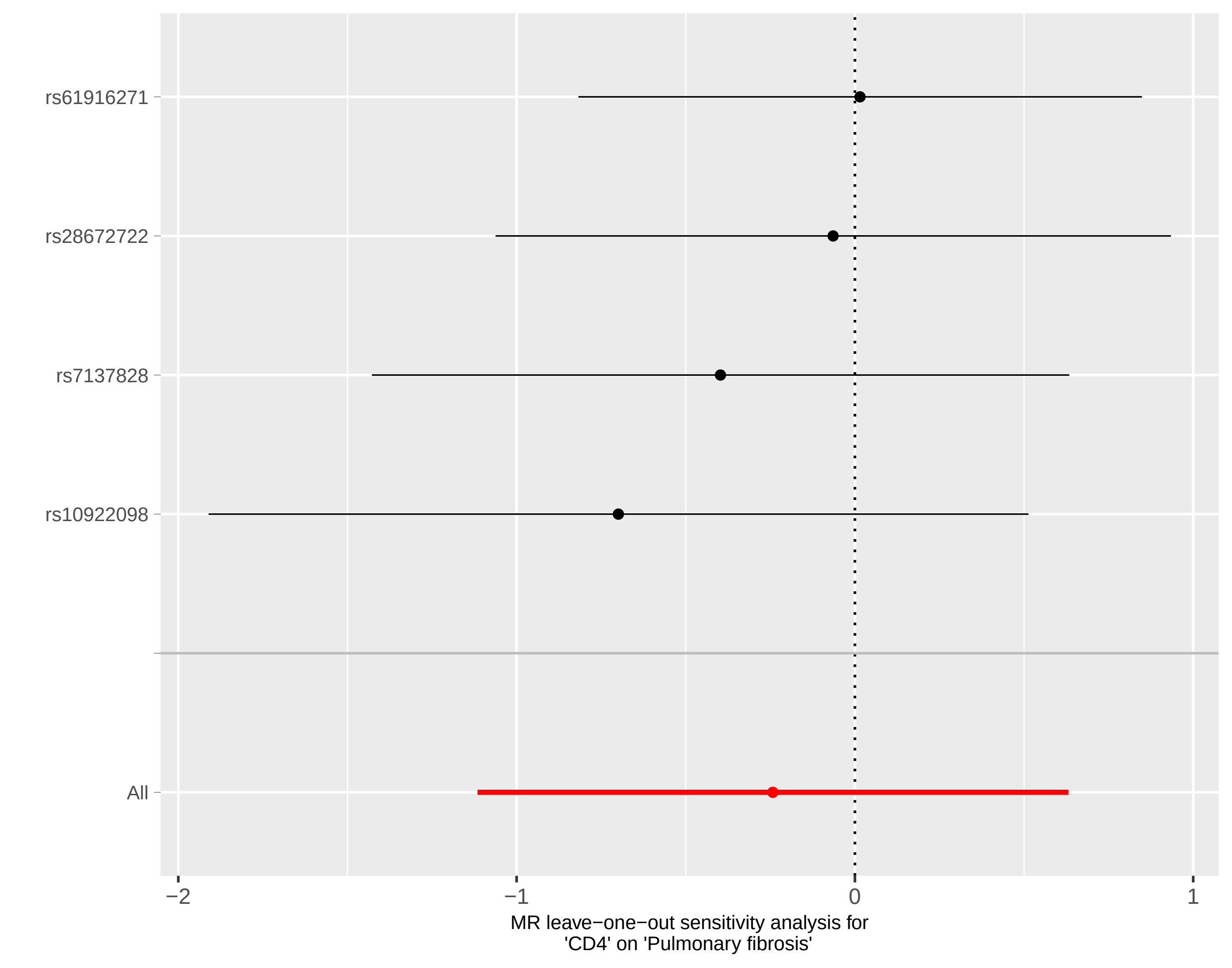
rs61916271
rs28672722
rs7137828
rs10922098
All
−2
−1
0
1
MR leave−one−out sensitivity analysis for
'CD4' on 'Pulmonary fibrosis'

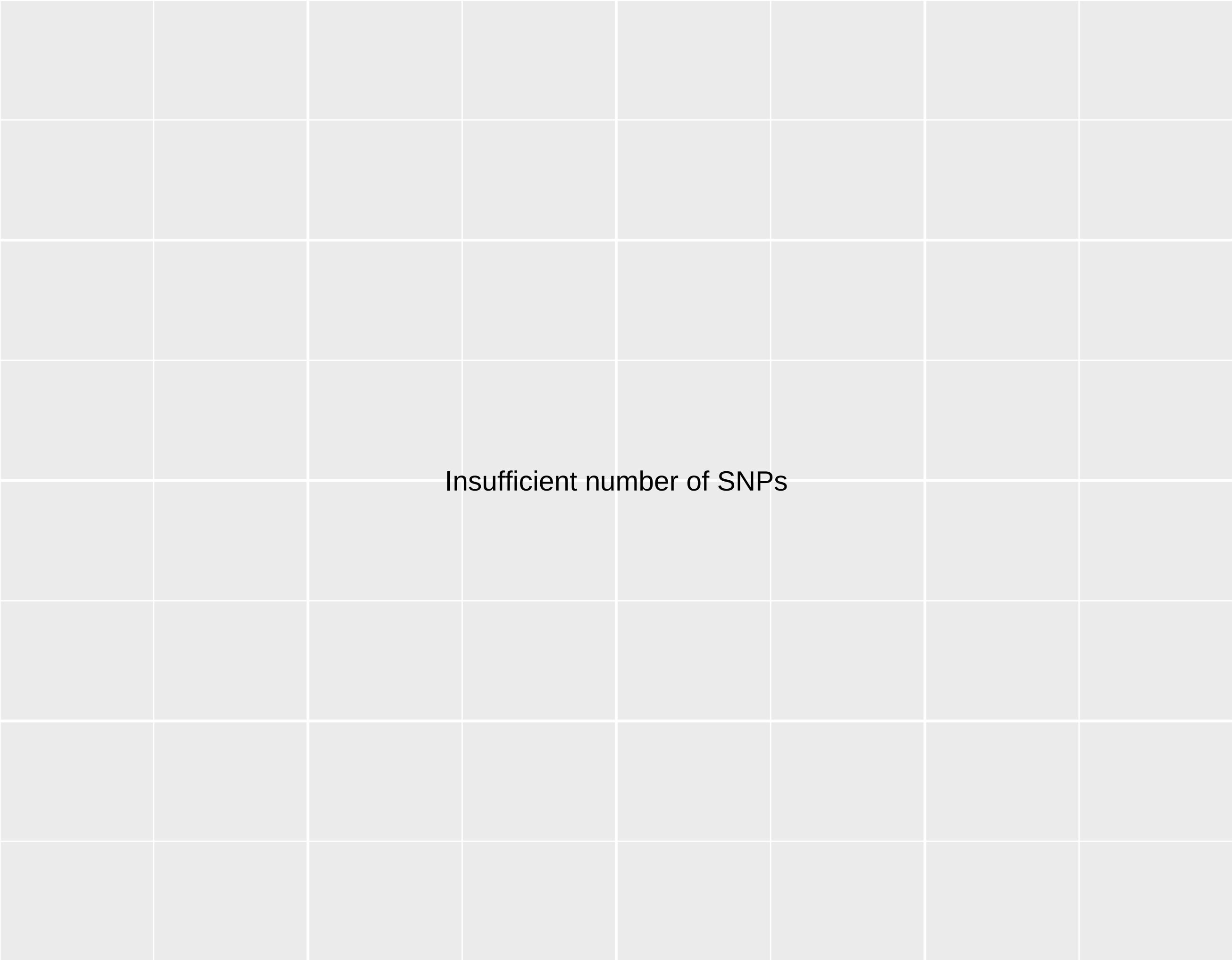
Insufficient number of SNPs

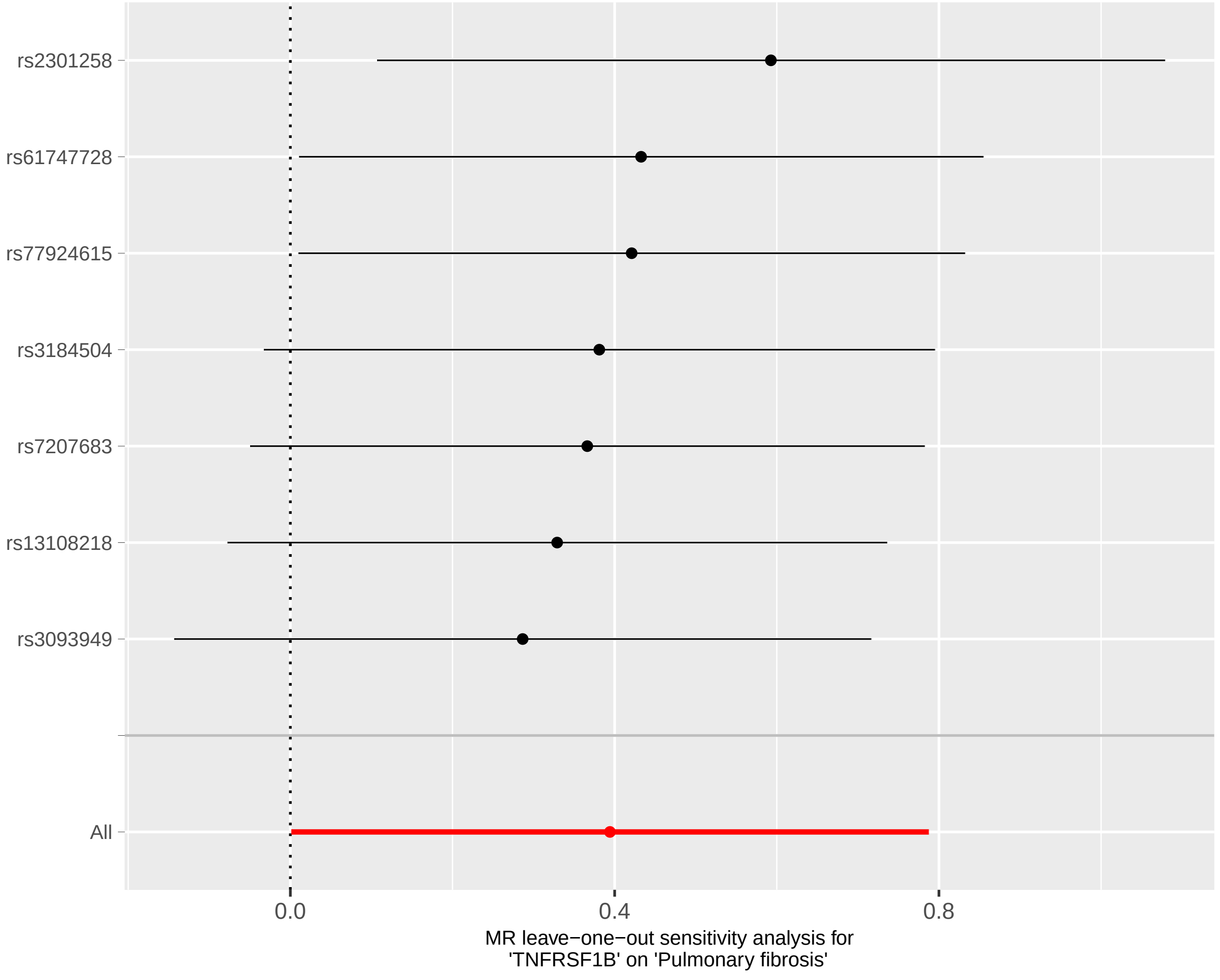
rs2301258
rs61747728
rs77924615
rs3184504
rs7207683
rs13108218
rs3093949
All
0.0
0.4
0.8
MR leave−one−out sensitivity analysis for
'TNFRSF1B' on 'Pulmonary fibrosis'

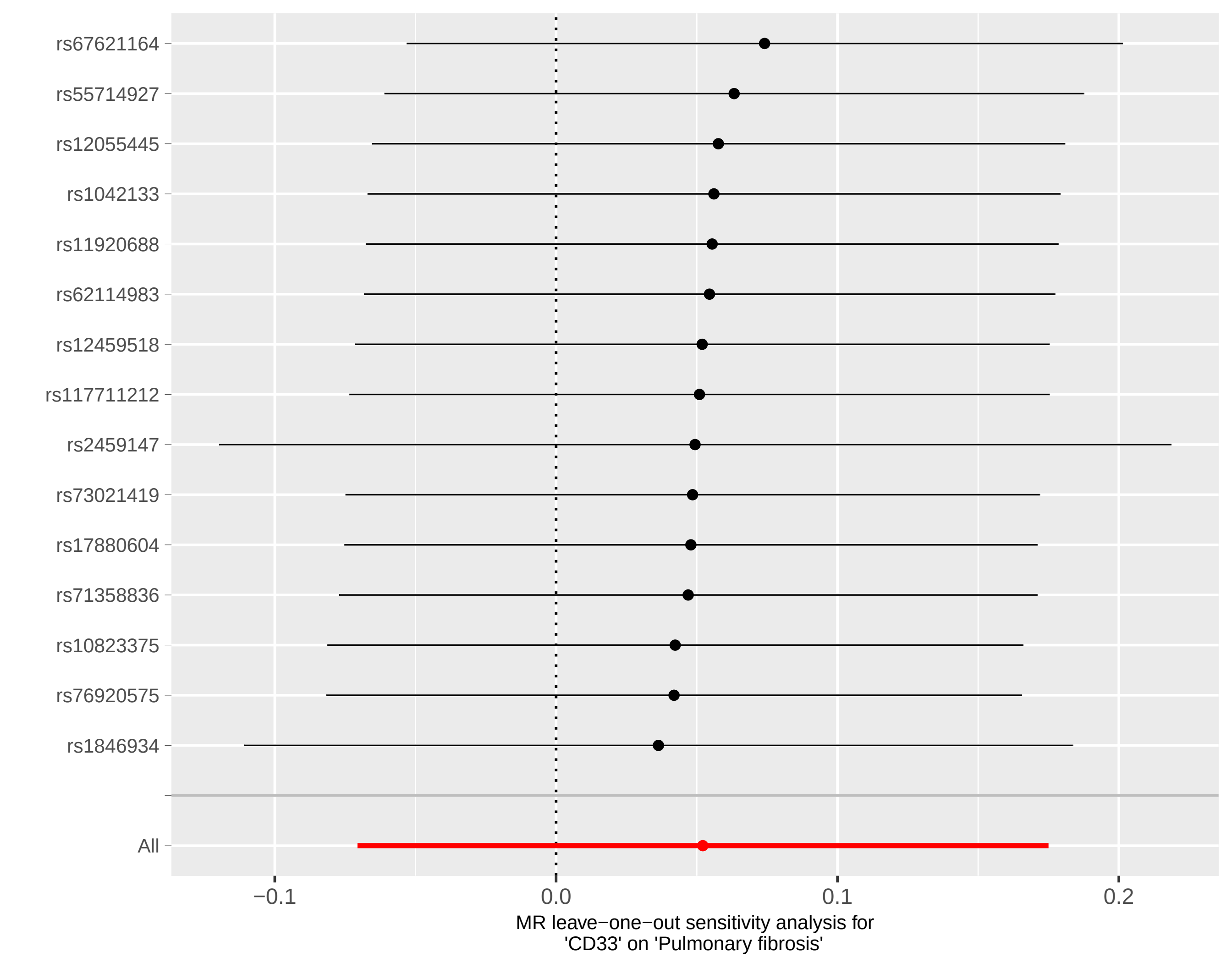
rs67621164
rs55714927
rs12055445
rs1042133
rs11920688
rs62114983
rs12459518
rs117711212
rs2459147
rs73021419
rs17880604
rs71358836
rs10823375
rs76920575
rs1846934
All
−0.1
0.0
0.1
0.2
MR leave−one−out sensitivity analysis for
'CD33' on 'Pulmonary fibrosis'

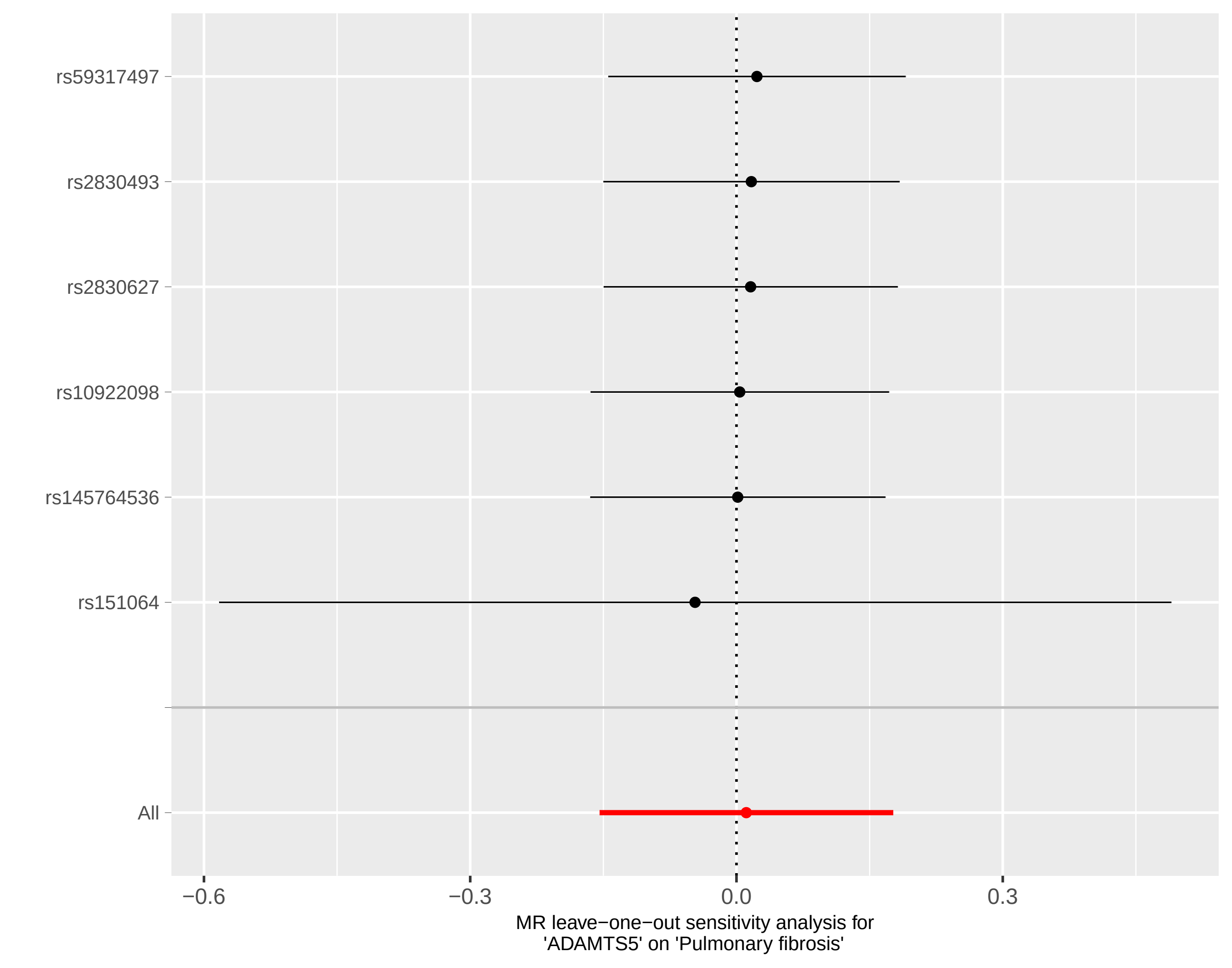
rs59317497
rs2830493
rs2830627
rs10922098
rs145764536
rs151064
All
−0.6
−0.3
0.0
0.3
MR leave−one−out sensitivity analysis for
'ADAMTS5' on 'Pulmonary fibrosis'

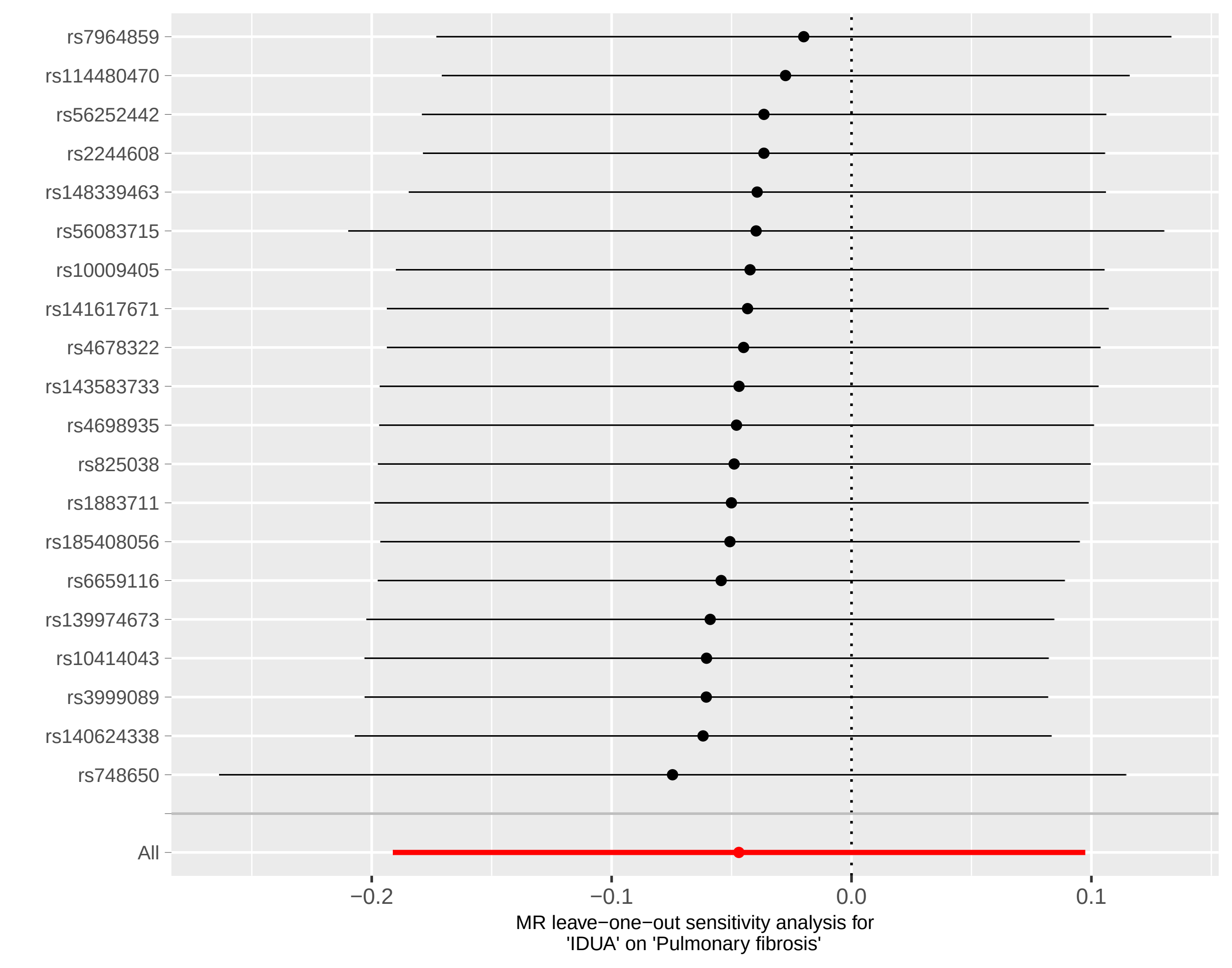
rs7964859
rs114480470
rs56252442
rs2244608
rs148339463
rs56083715
rs10009405
rs141617671
rs4678322
rs143583733
rs4698935
rs825038
rs1883711
rs185408056
rs6659116
rs139974673
rs10414043
rs3999089
rs140624338
rs748650
All
−0.2
−0.1
0.0
0.1
MR leave−one−out sensitivity analysis for
'IDUA' on 'Pulmonary fibrosis'

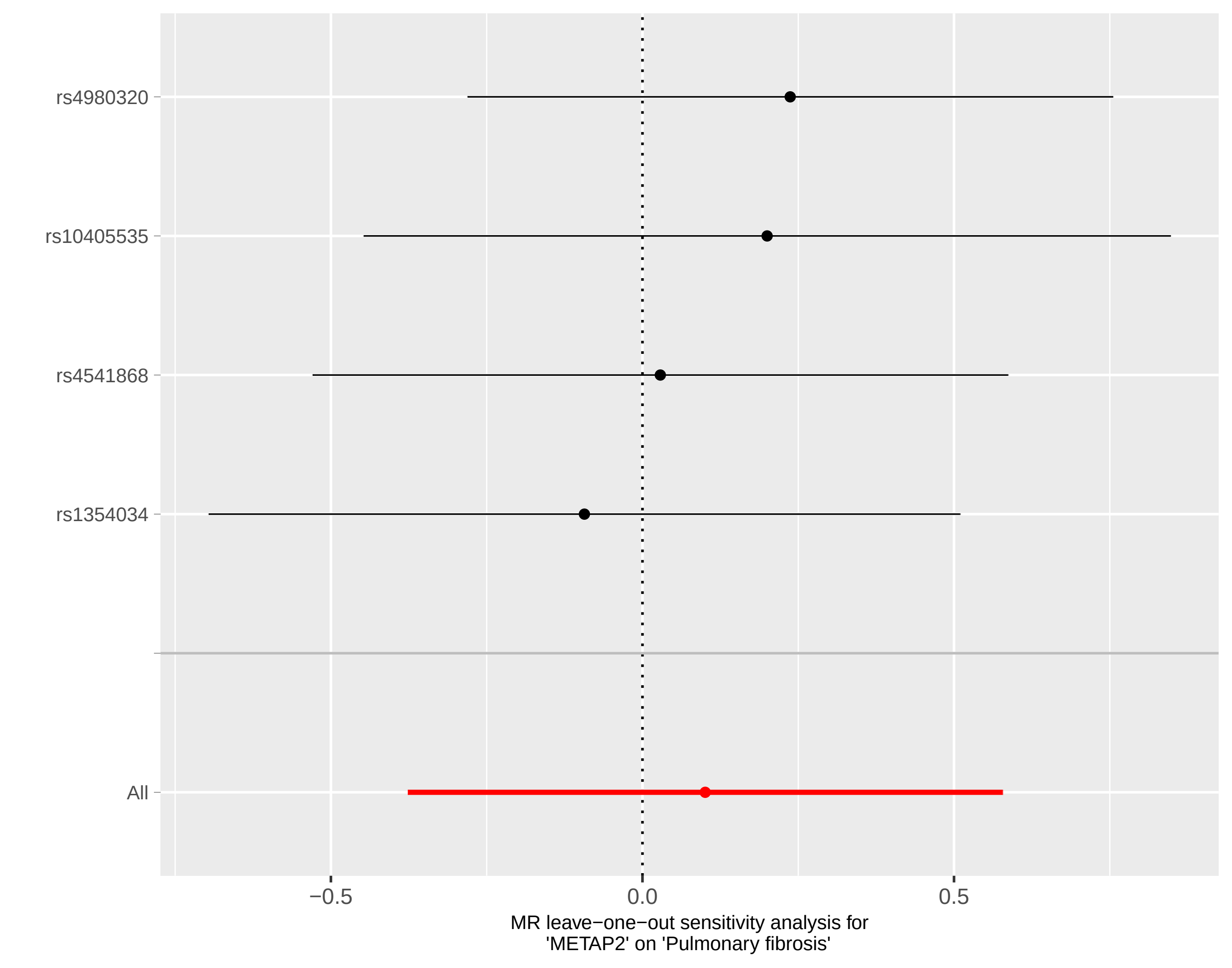
rs4980320
rs10405535
rs4541868
rs1354034
All
−0.5
0.0
0.5
MR leave−one−out sensitivity analysis for
'METAP2' on 'Pulmonary fibrosis'

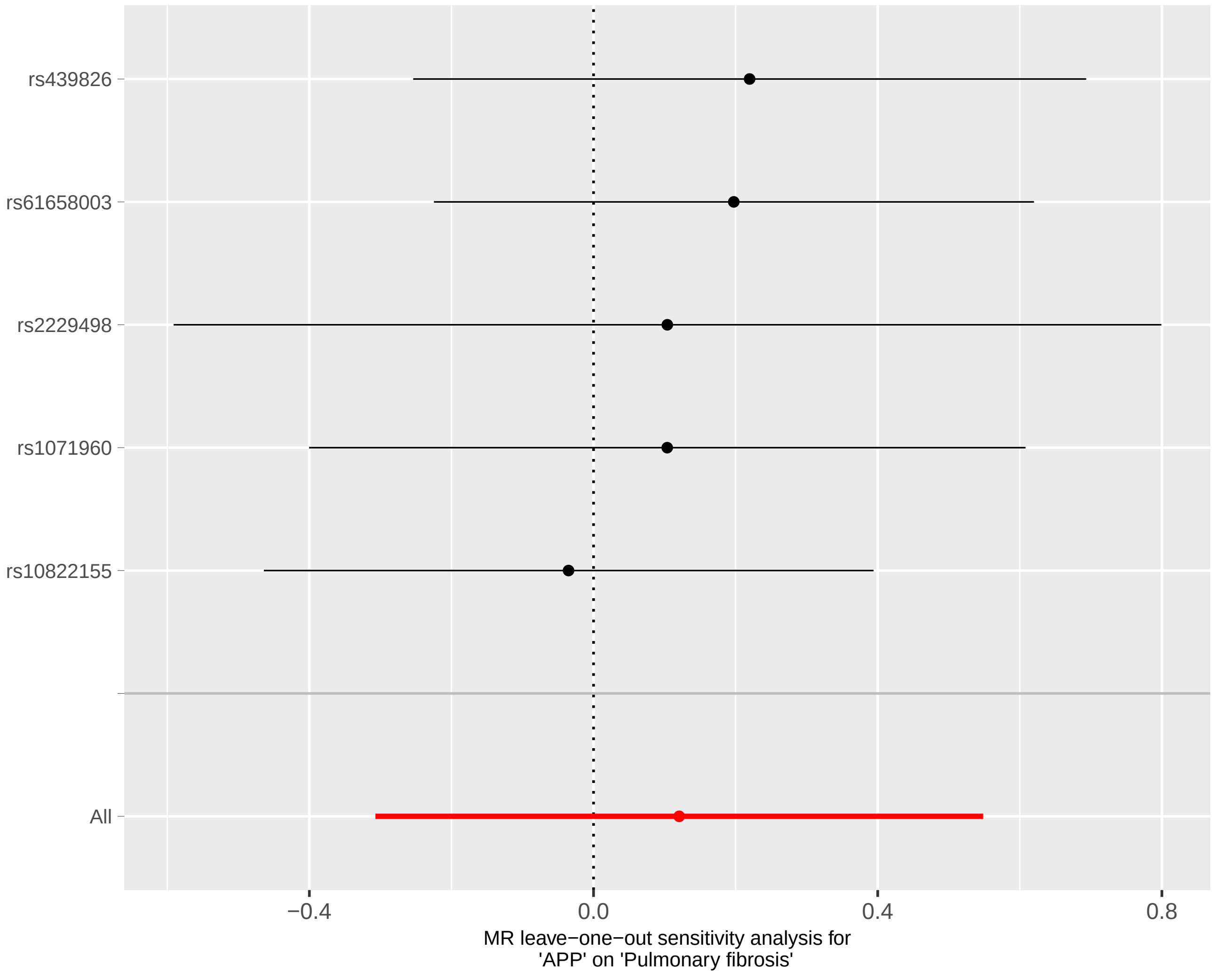
rs439826
rs61658003
rs2229498
rs1071960
rs10822155
All
−0.4
0.0
0.4
0.8
MR leave−one−out sensitivity analysis for
'APP' on 'Pulmonary fibrosis'

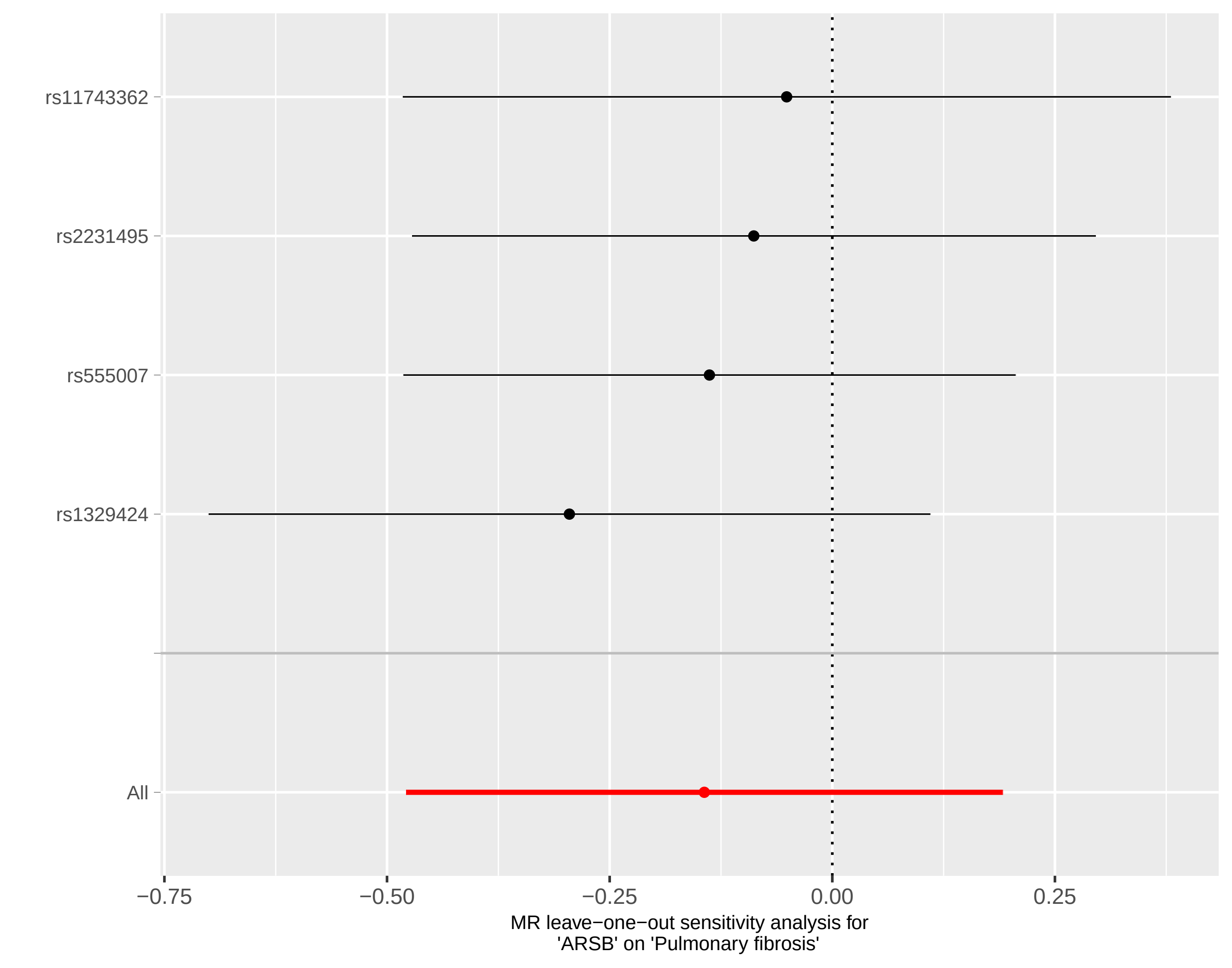
rs11743362
rs2231495
rs555007
rs1329424
All
−0.75
−0.50
−0.25
0.00
0.25
MR leave−one−out sensitivity analysis for
'ARSB' on 'Pulmonary fibrosis'

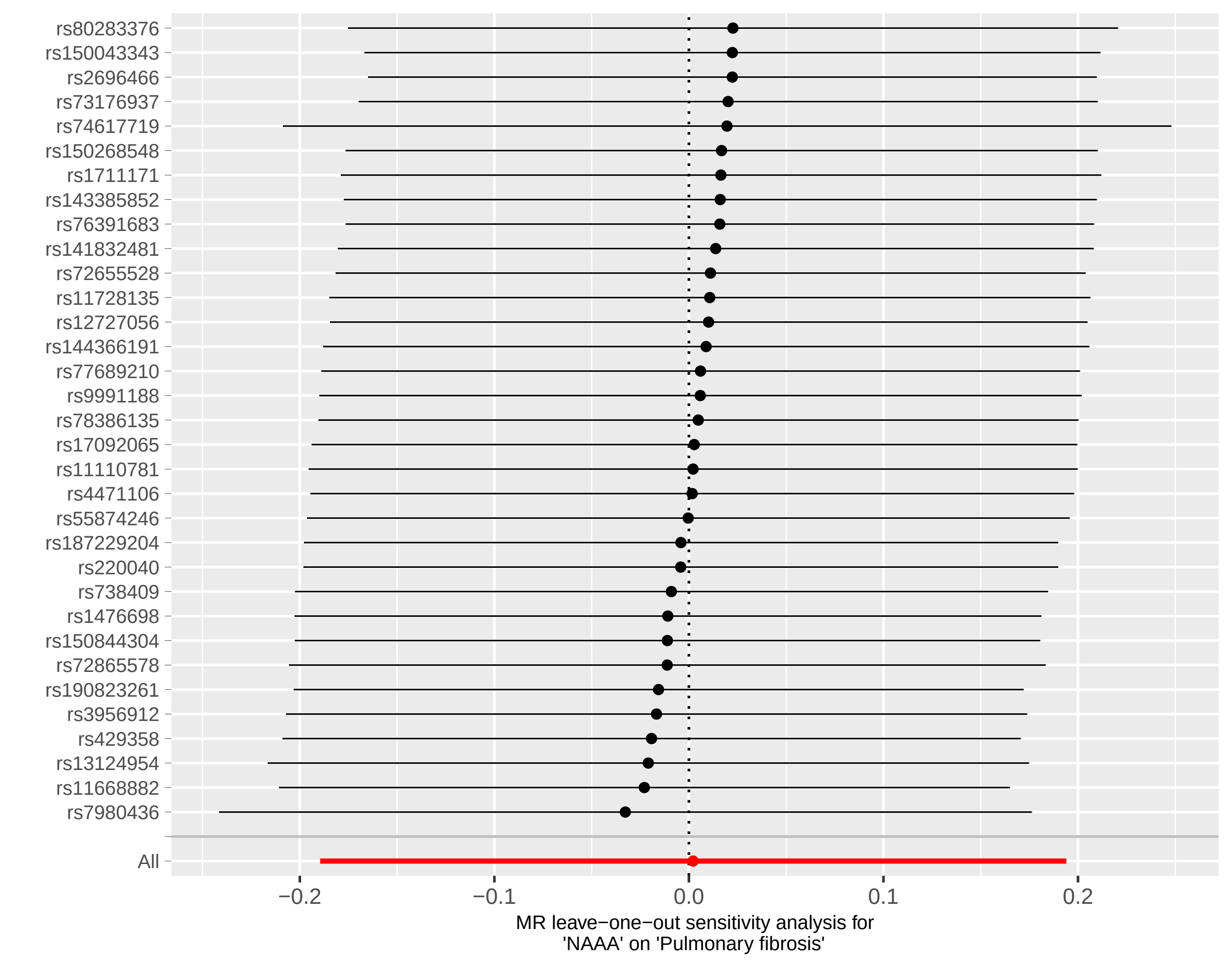
rs80283376
rs150043343
rs2696466
rs73176937
rs74617719
rs150268548
rs1711171
rs143385852
rs76391683
rs141832481
rs72655528
rs11728135
rs12727056
rs144366191
rs77689210
rs9991188
rs78386135
rs17092065
rs11110781
rs4471106
rs55874246
rs187229204
rs220040
rs738409
rs1476698
rs150844304
rs72865578
rs190823261
rs3956912
rs429358
rs13124954
rs11668882
rs7980436
All
−0.2
−0.1
0.0
0.1
0.2
MR leave−one−out sensitivity analysis for
'NAAA' on 'Pulmonary fibrosis'

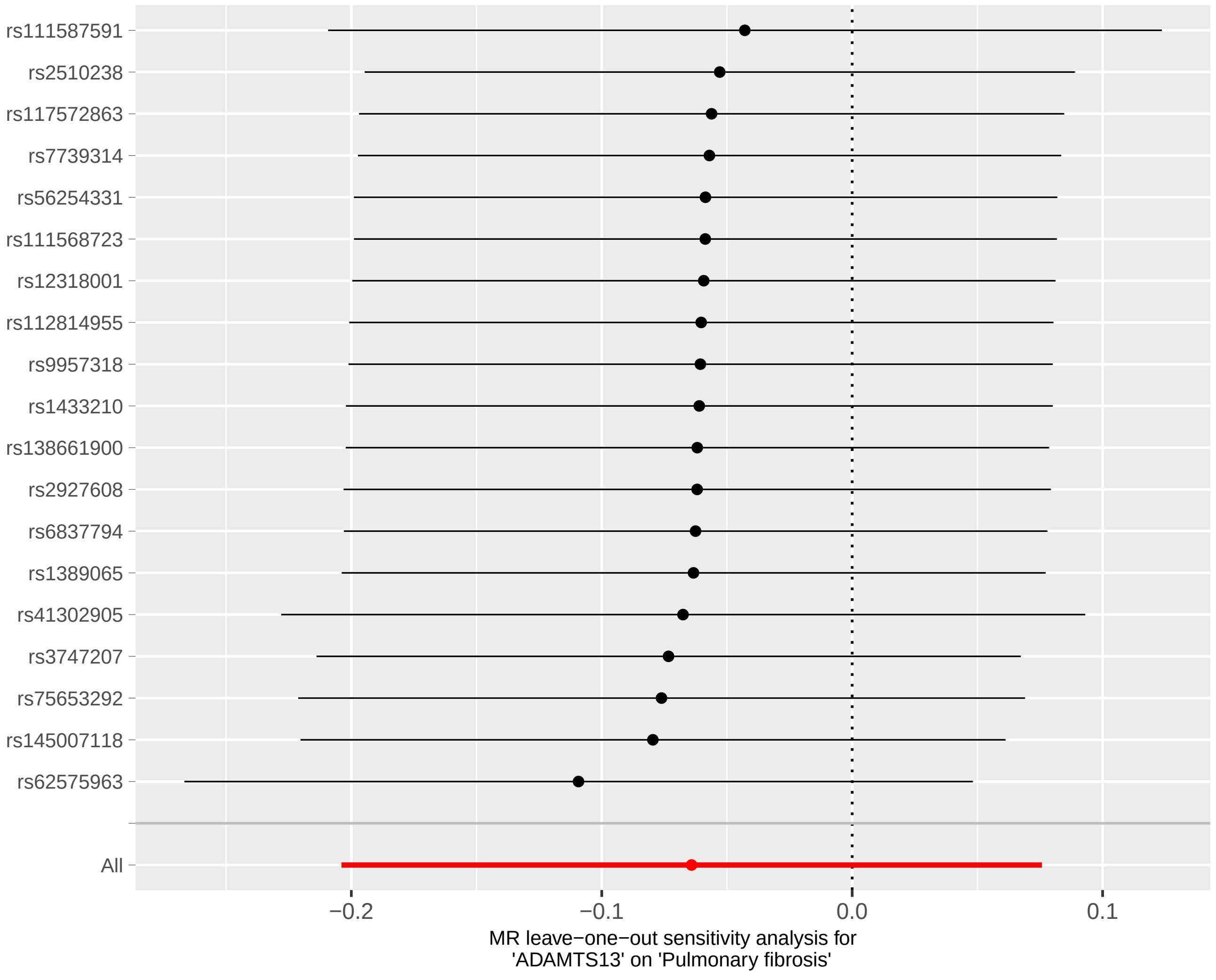

rs111587591
rs2510238
rs117572863
rs7739314
rs56254331
rs111568723
rs12318001
rs112814955
rs9957318
rs1433210
rs138661900
rs2927608
rs6837794
rs1389065
rs41302905
rs3747207
rs75653292
rs145007118
rs62575963
All
−0.2
−0.1
0.0
0.1
MR leave−one−out sensitivity analysis for
'ADAMTS13' on 'Pulmonary fibrosis'

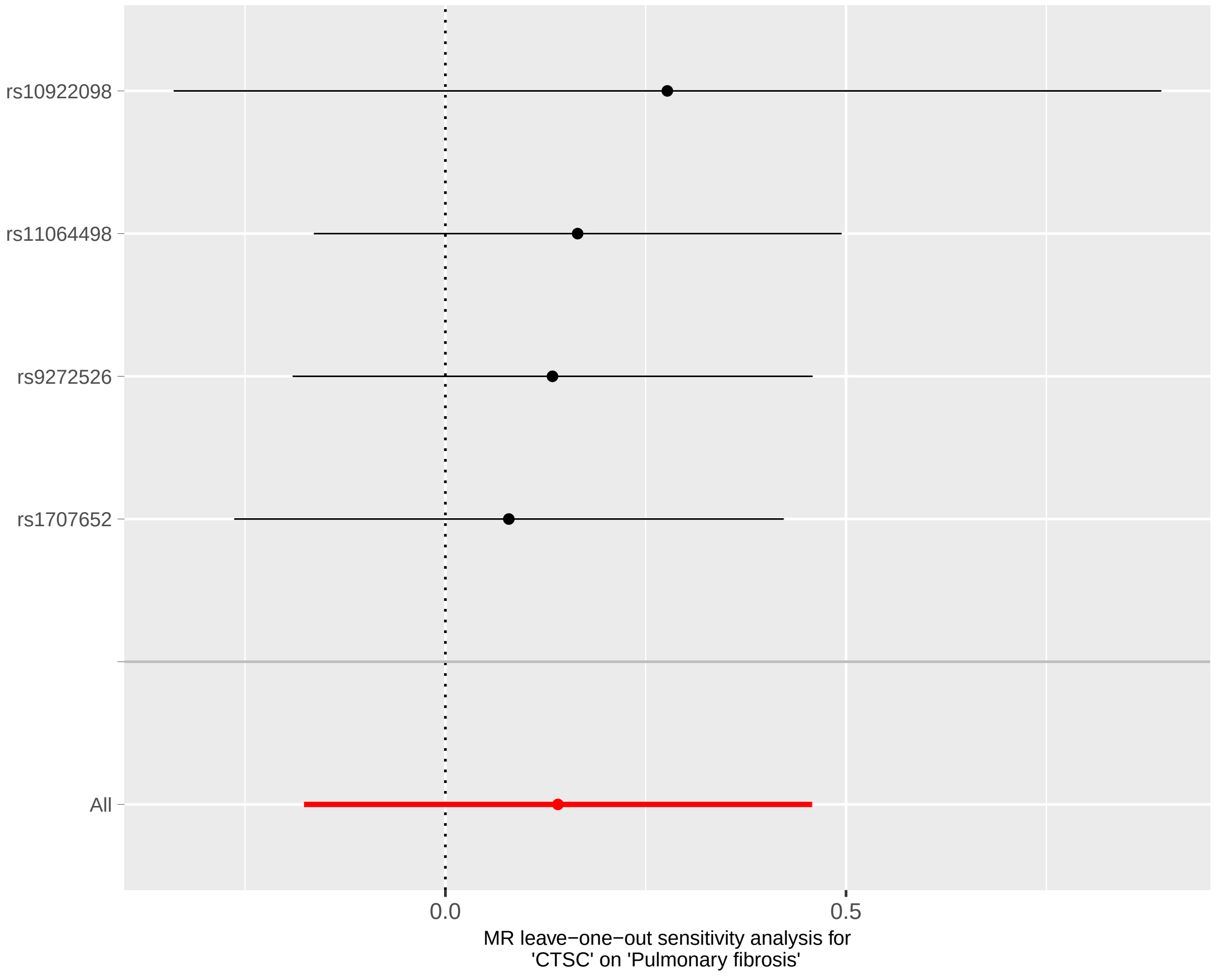
rs10922098
rs11064498
rs9272526
rs1707652
All
0.0
0.5
MR leave−one−out sensitivity analysis for
'CTSC' on 'Pulmonary fibrosis'

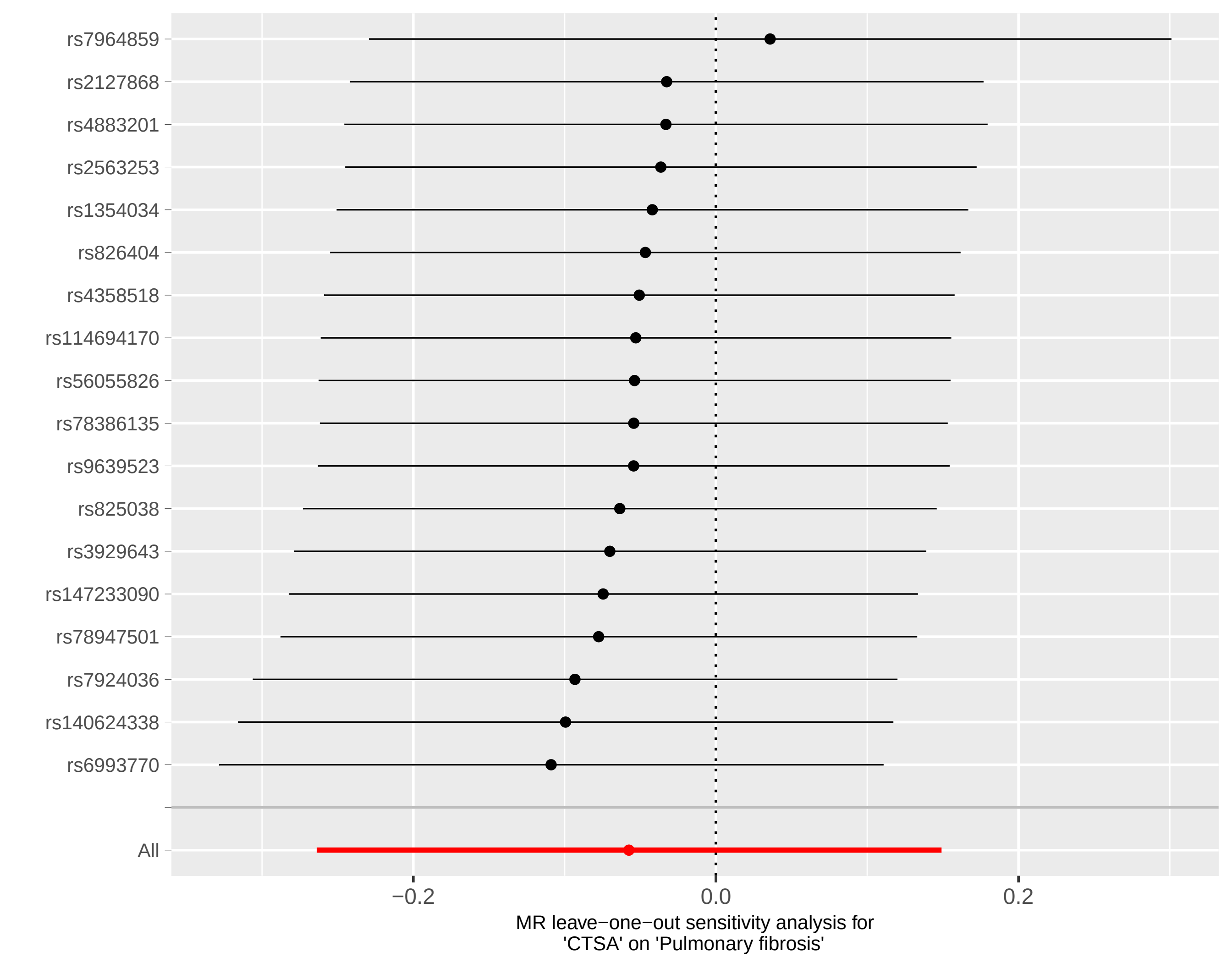
rs7964859
rs2127868
rs4883201
rs2563253
rs1354034
rs826404
rs4358518
rs114694170
rs56055826
rs78386135
rs9639523
rs825038
rs3929643
rs147233090
rs78947501
rs7924036
rs140624338
rs6993770
All
−0.2
0.0
0.2
MR leave−one−out sensitivity analysis for
'CTSA' on 'Pulmonary fibrosis'

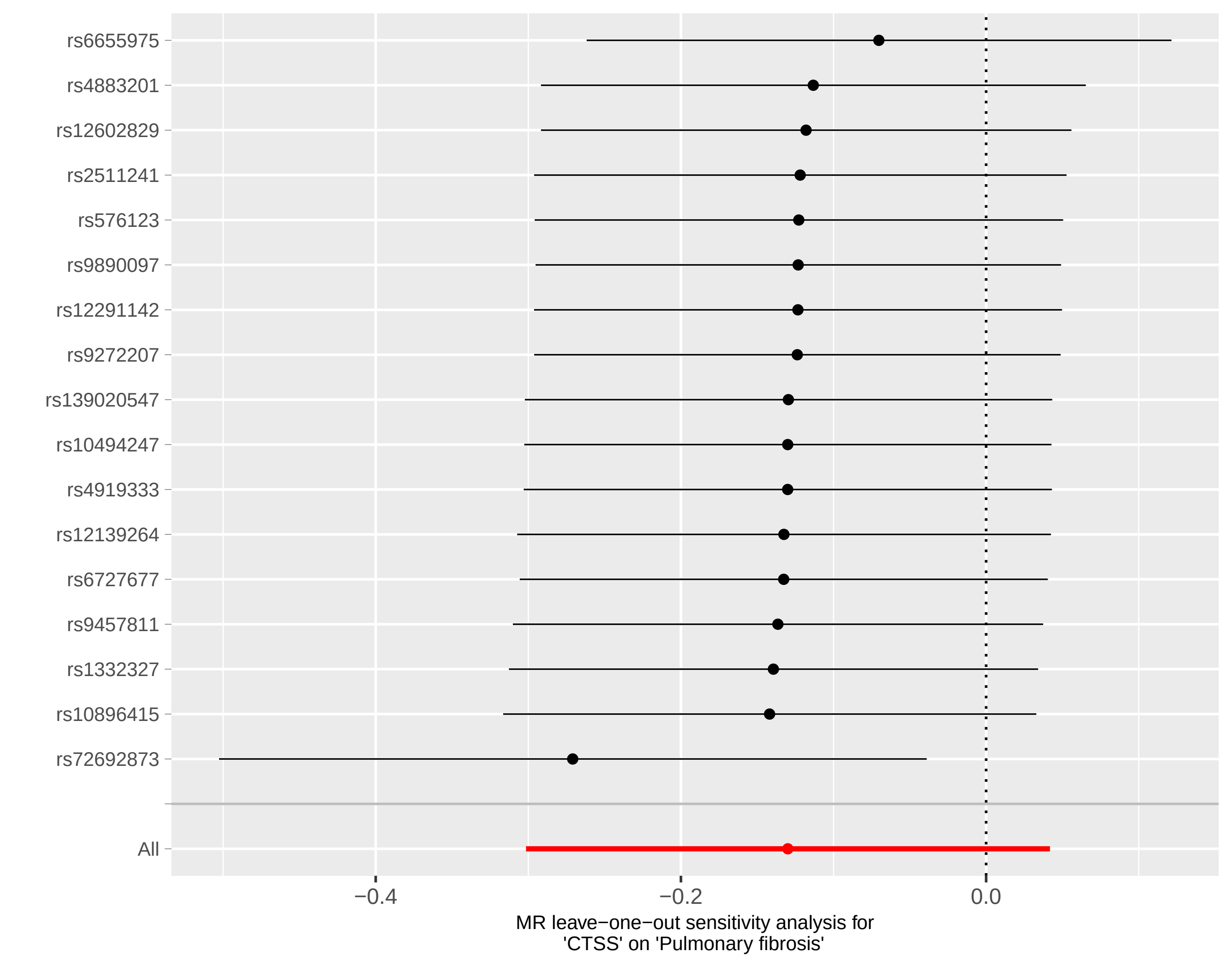
rs6655975
rs4883201
rs12602829
rs2511241
rs576123
rs9890097
rs12291142
rs9272207
rs139020547
rs10494247
rs4919333
rs12139264
rs6727677
rs9457811
rs1332327
rs10896415
rs72692873
All
−0.4
−0.2
0.0
MR leave−one−out sensitivity analysis for
'CTSS' on 'Pulmonary fibrosis'

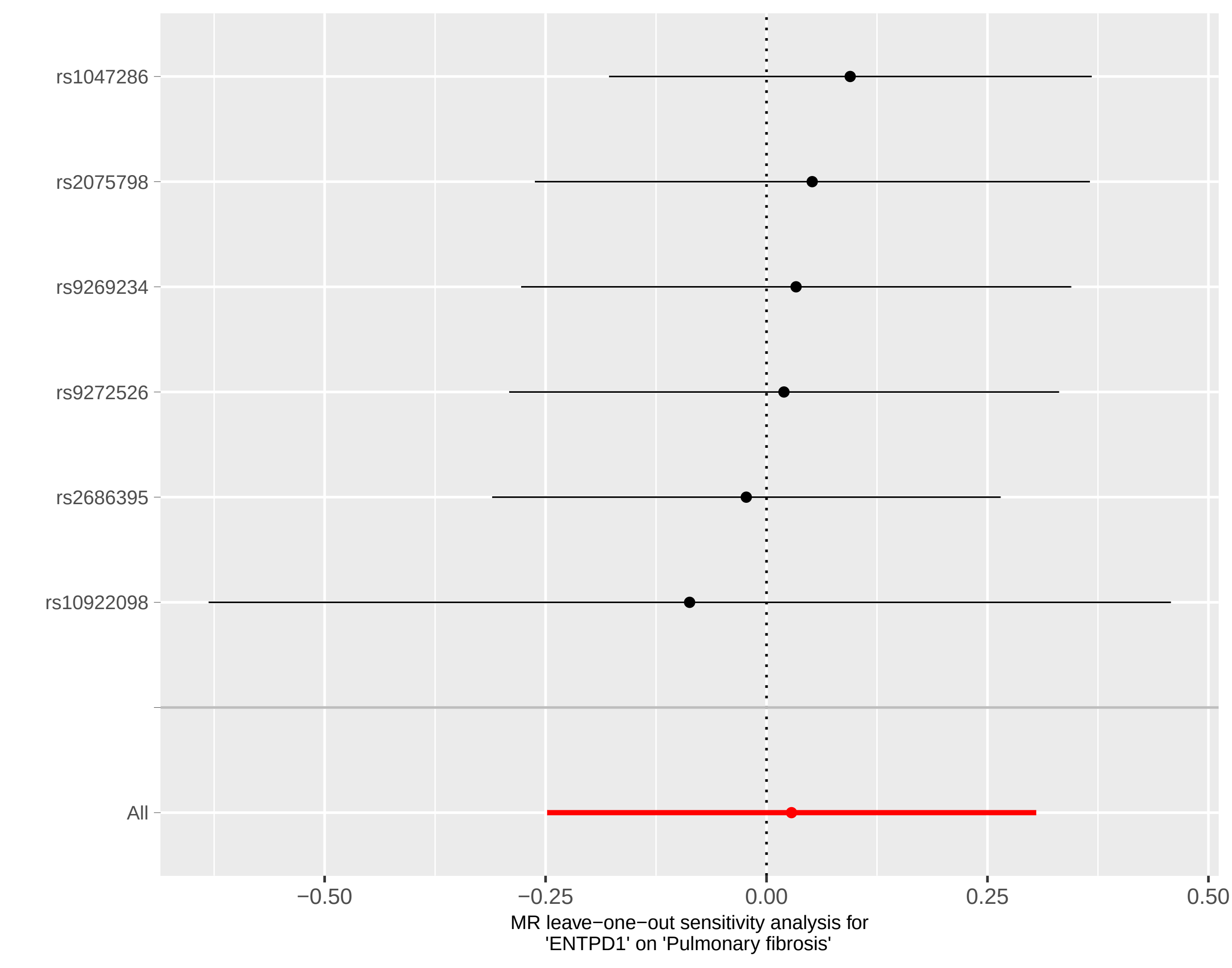
rs1047286
rs2075798
rs9269234
rs9272526
rs2686395
rs10922098
All
−0.50
−0.25
0.00
0.25
0.50
MR leave−one−out sensitivity analysis for
'ENTPD1' on 'Pulmonary fibrosis'

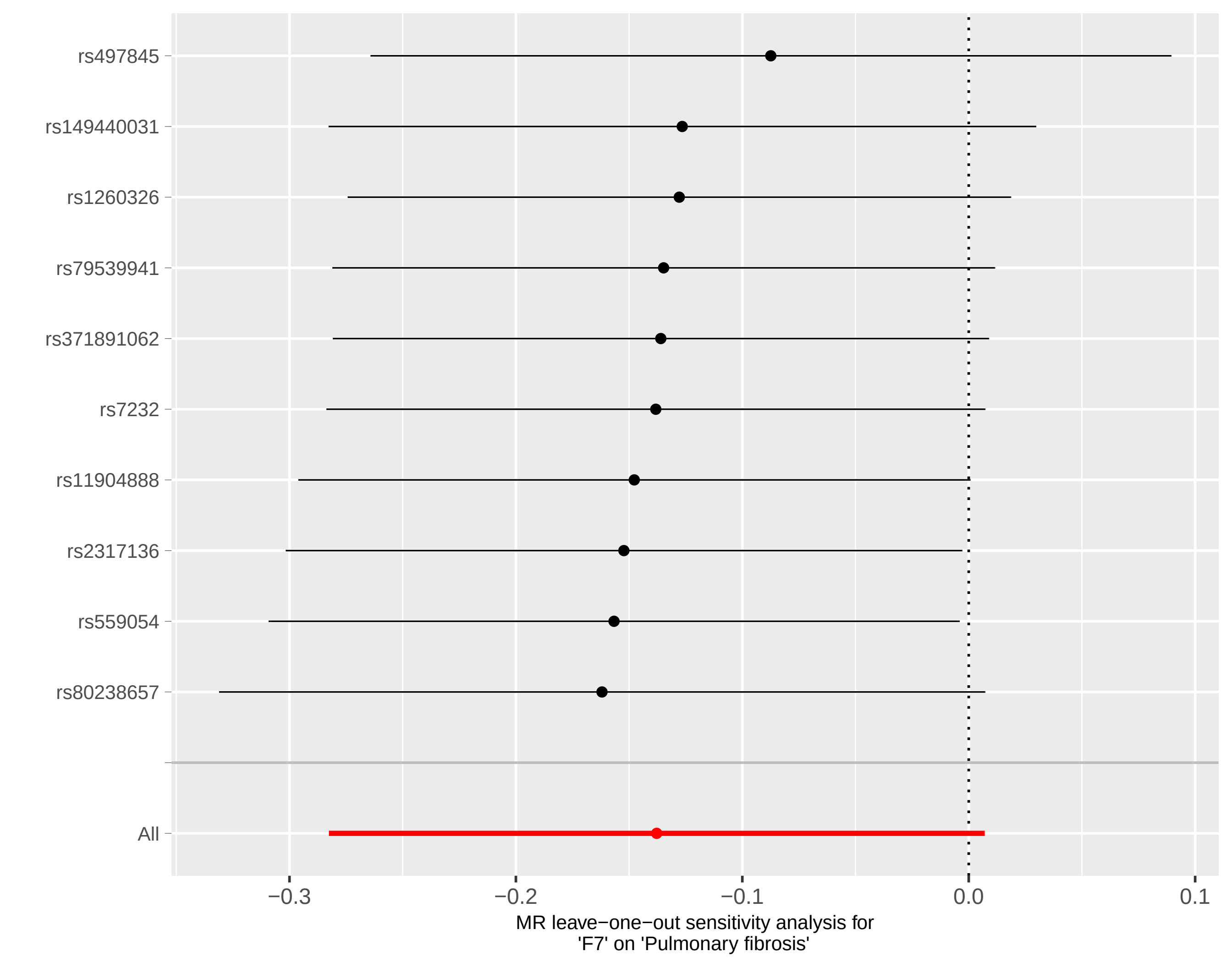
rs497845
rs149440031
rs1260326
rs79539941
rs371891062
rs7232
rs11904888
rs2317136
rs559054
rs80238657
All
−0.3
−0.2
−0.1
0.0
0.1
MR leave−one−out sensitivity analysis for
'F7' on 'Pulmonary fibrosis'

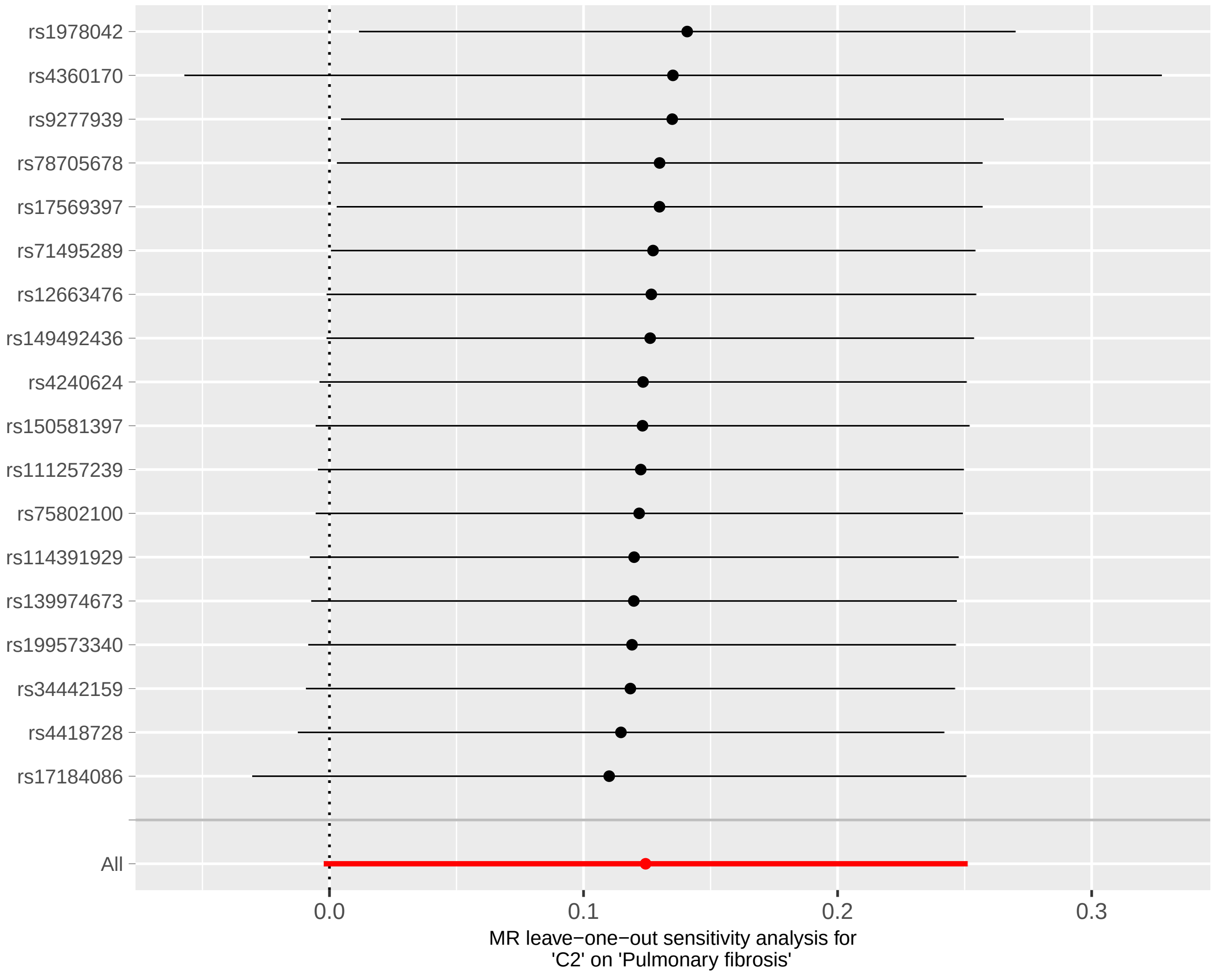

rs1978042
rs4360170
rs9277939
rs78705678
rs17569397
rs71495289
rs12663476
rs149492436
rs4240624
rs150581397
rs111257239
rs75802100
rs114391929
rs139974673
rs199573340
rs34442159
rs4418728
rs17184086
All
0.0
0.1
0.2
0.3
MR leave−one−out sensitivity analysis for
'C2' on 'Pulmonary fibrosis'

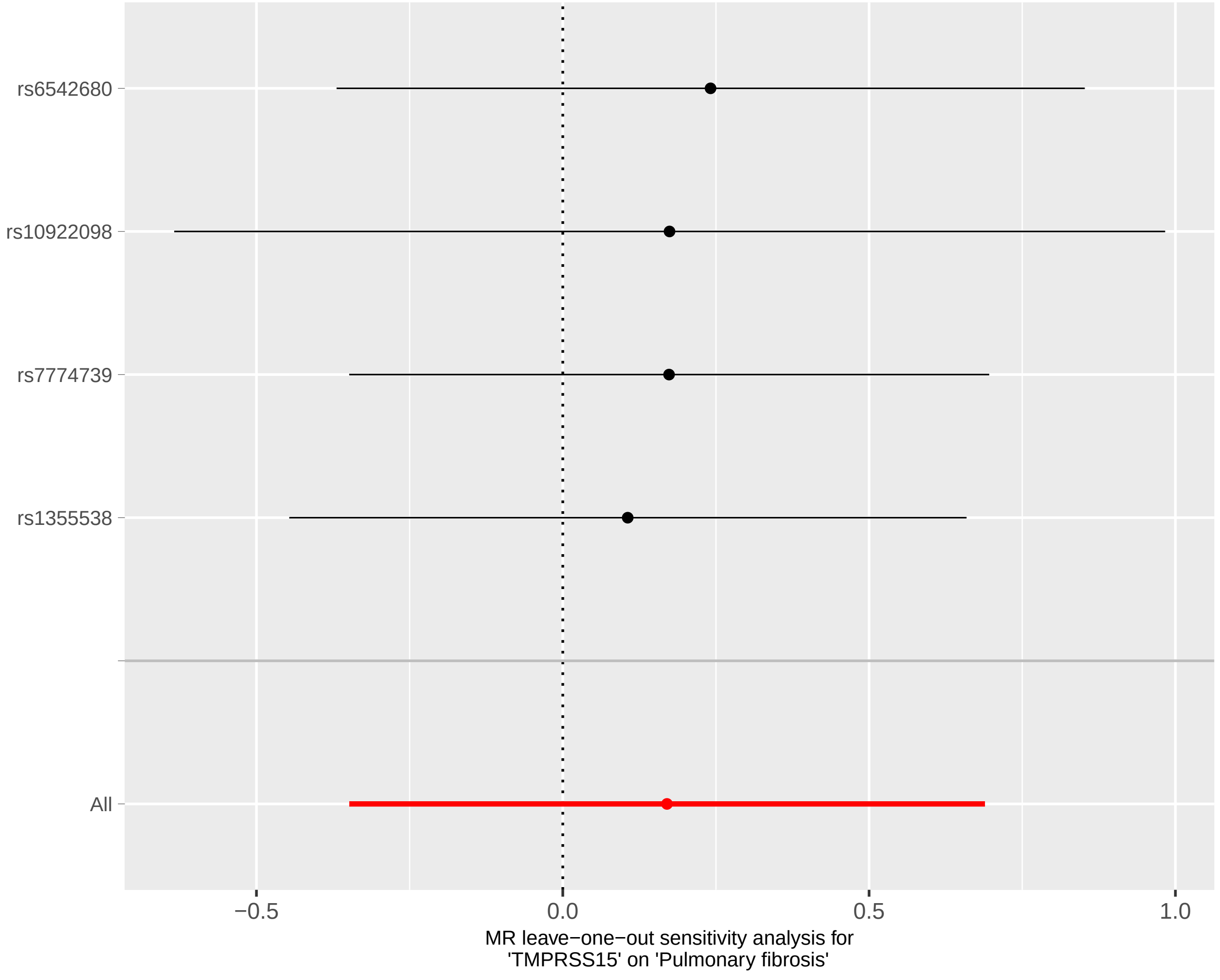
rs6542680
rs10922098
rs7774739
rs1355538
All
−0.5
0.0
0.5
1.0
MR leave−one−out sensitivity analysis for
'TMPRSS15' on 'Pulmonary fibrosis'

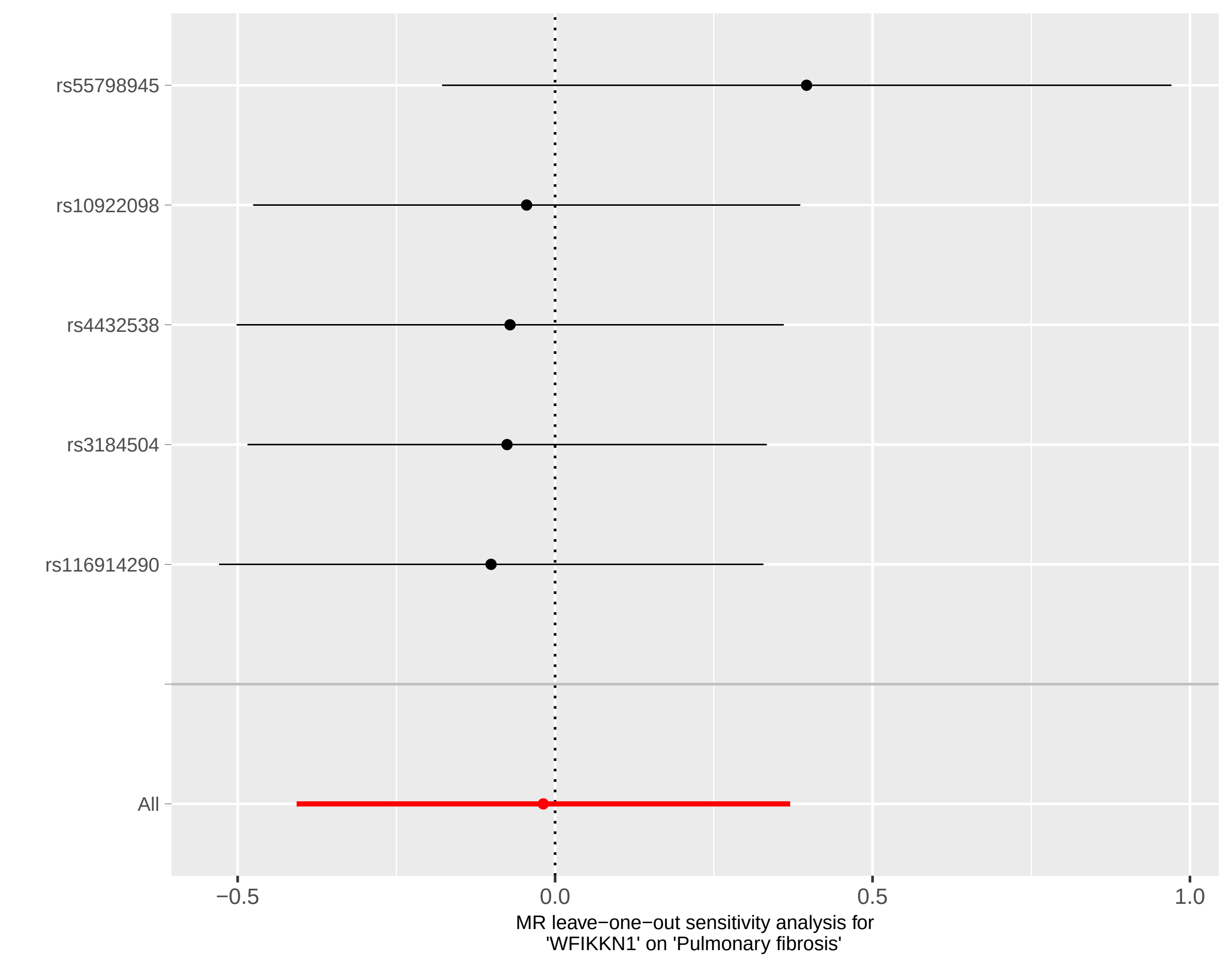

rs55798945
rs10922098
rs4432538
rs3184504
rs116914290
All
−0.5
0.0
0.5
1.0
MR leave−one−out sensitivity analysis for
'WFIKKN1' on 'Pulmonary fibrosis'

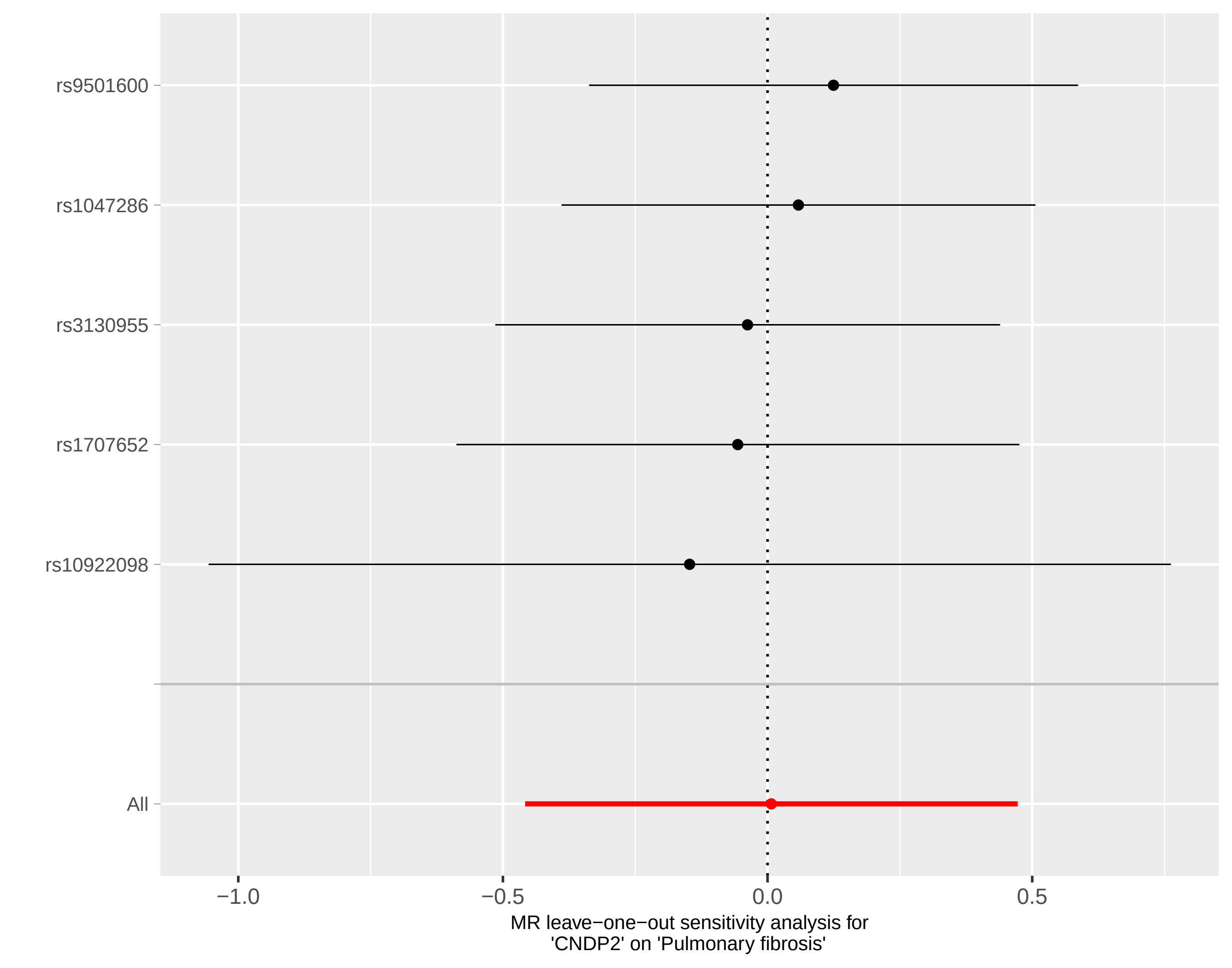
rs9501600
rs1047286
rs3130955
rs1707652
rs10922098
All
−1.0
−0.5
0.0
0.5
MR leave−one−out sensitivity analysis for
'CNDP2' on 'Pulmonary fibrosis'

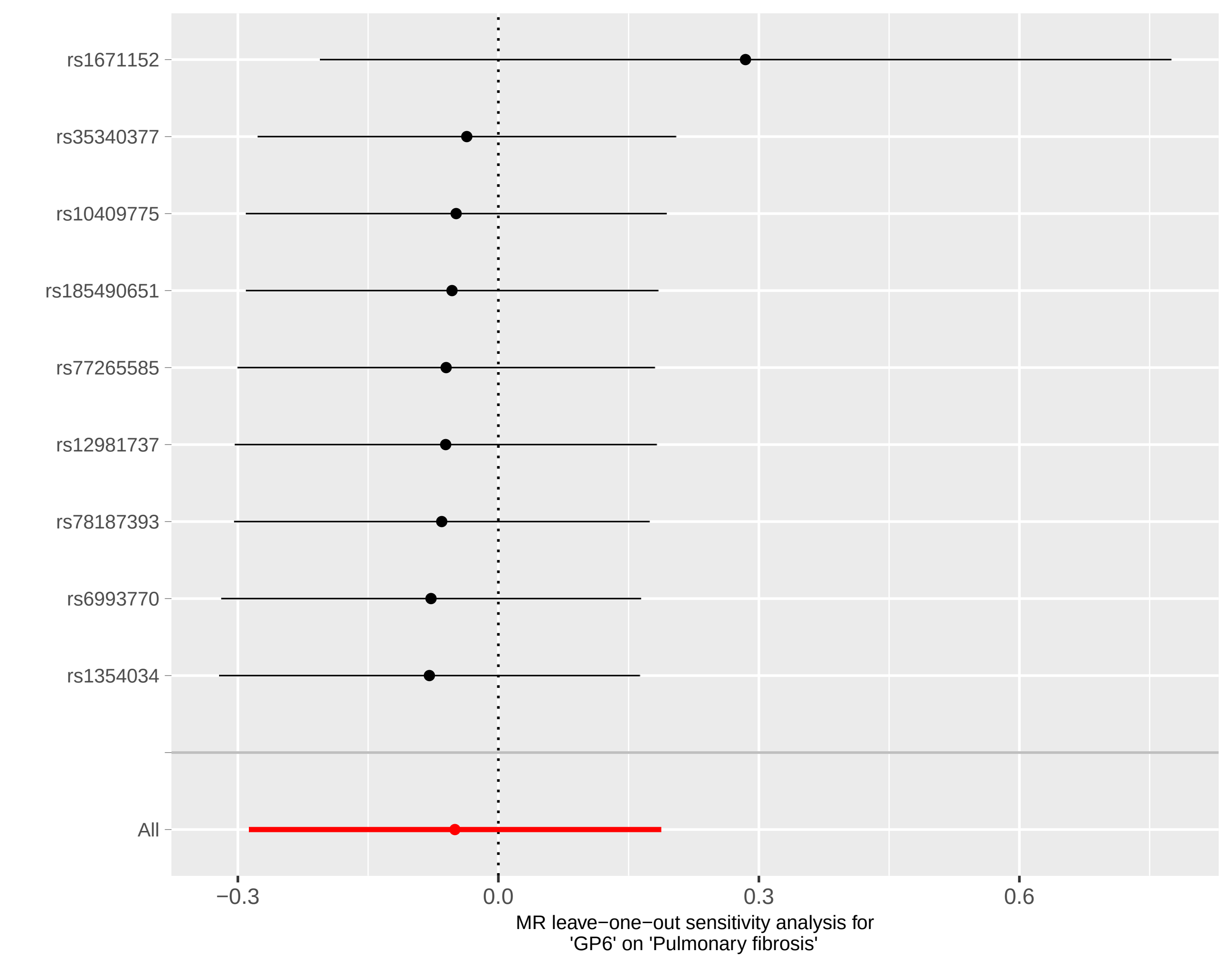
rs1671152
rs35340377
rs10409775
rs185490651
rs77265585
rs12981737
rs78187393
rs6993770
rs1354034
All
−0.3
0.0
0.3
0.6
MR leave−one−out sensitivity analysis for
'GP6' on 'Pulmonary fibrosis'

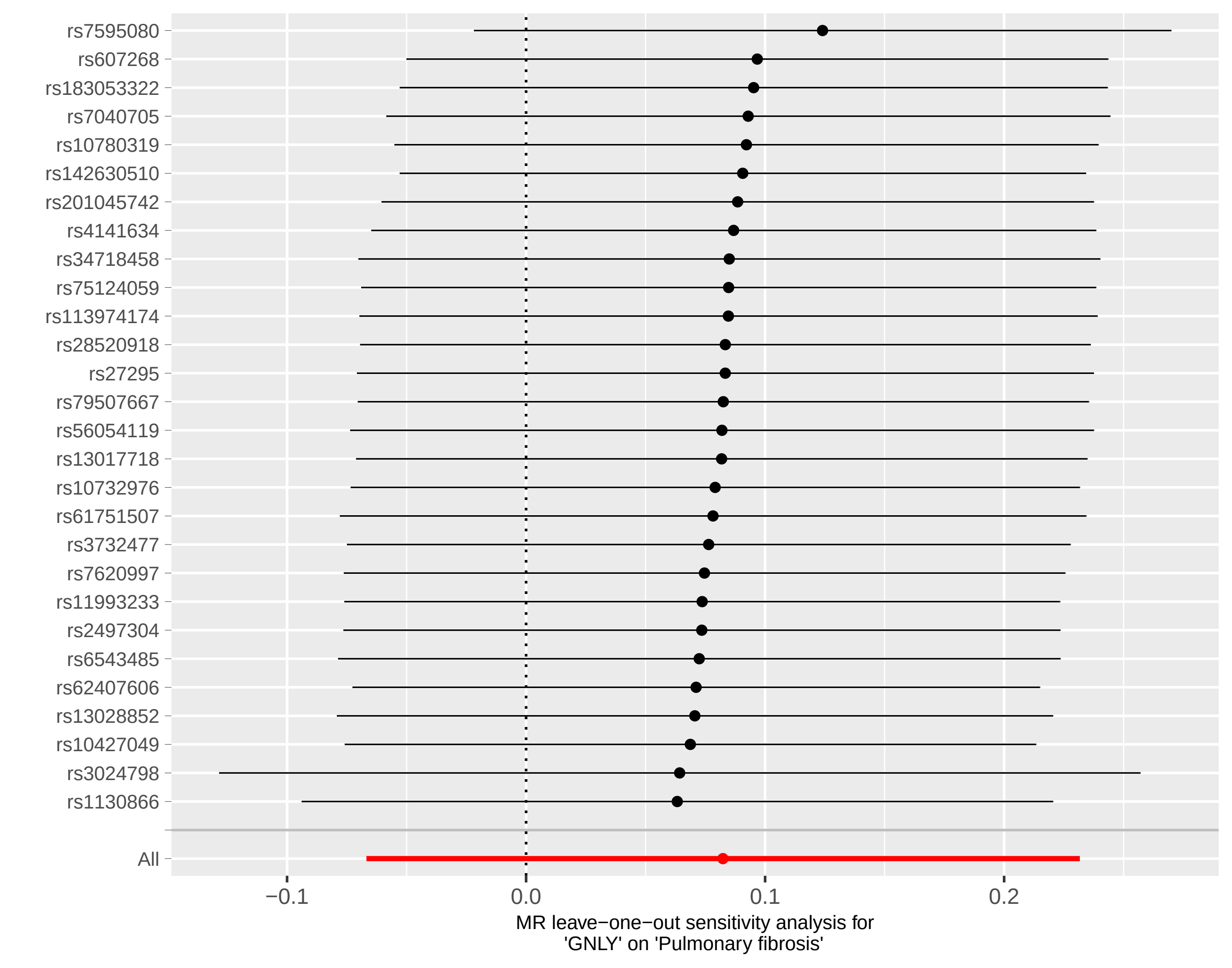
rs7595080
rs607268
rs183053322
rs7040705
rs10780319
rs142630510
rs201045742
rs4141634
rs34718458
rs75124059
rs113974174
rs28520918
rs27295
rs79507667
rs56054119
rs13017718
rs10732976
rs61751507
rs3732477
rs7620997
rs11993233
rs2497304
rs6543485
rs62407606
rs13028852
rs10427049
rs3024798
rs1130866
All
−0.1
0.0
0.1
0.2
MR leave−one−out sensitivity analysis for
'GNLY' on 'Pulmonary fibrosis'

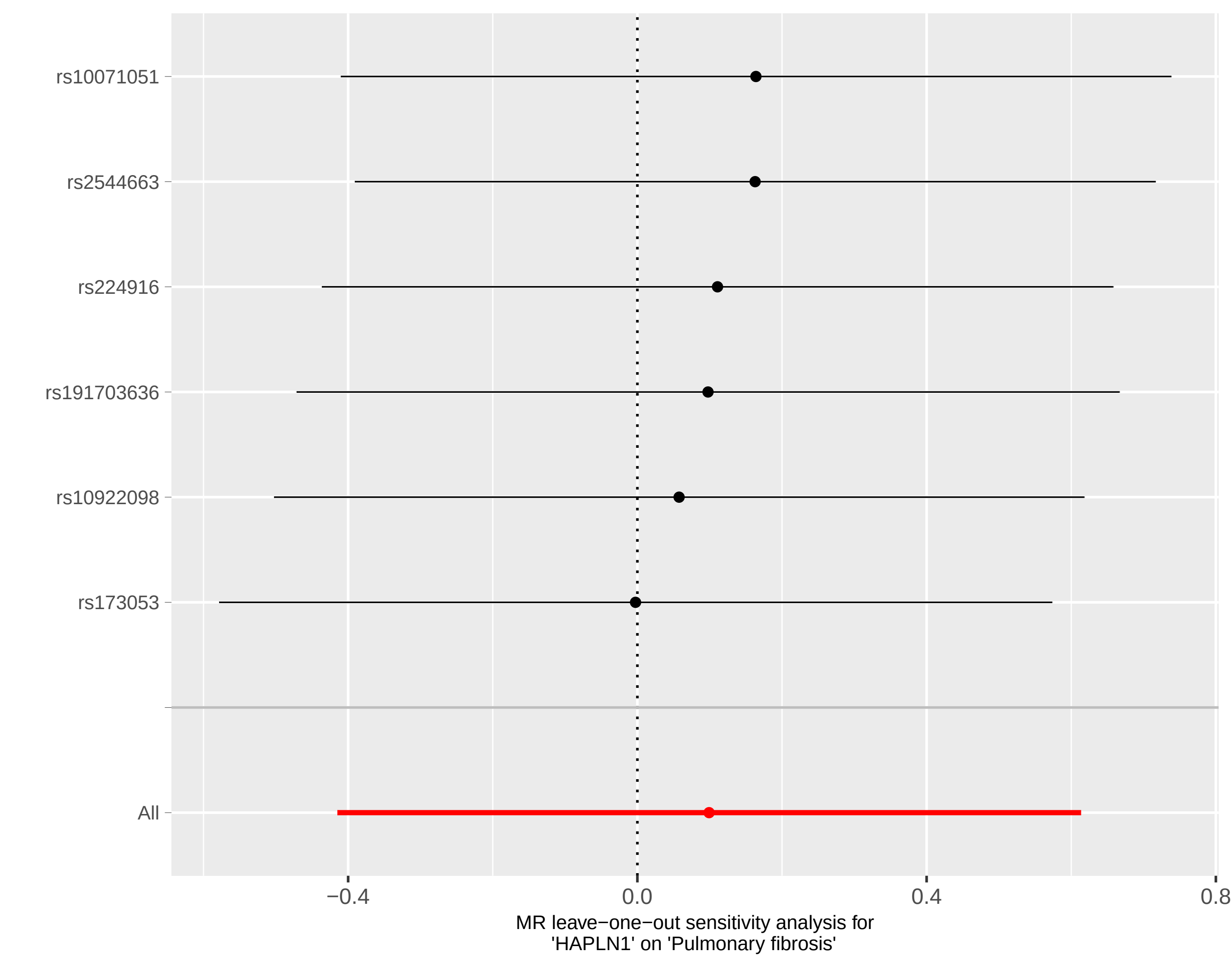
rs10071051
rs2544663
rs224916
rs191703636
rs10922098
rs173053
All
−0.4
0.0
0.4
0.8
MR leave−one−out sensitivity analysis for
'HAPLN1' on 'Pulmonary fibrosis'

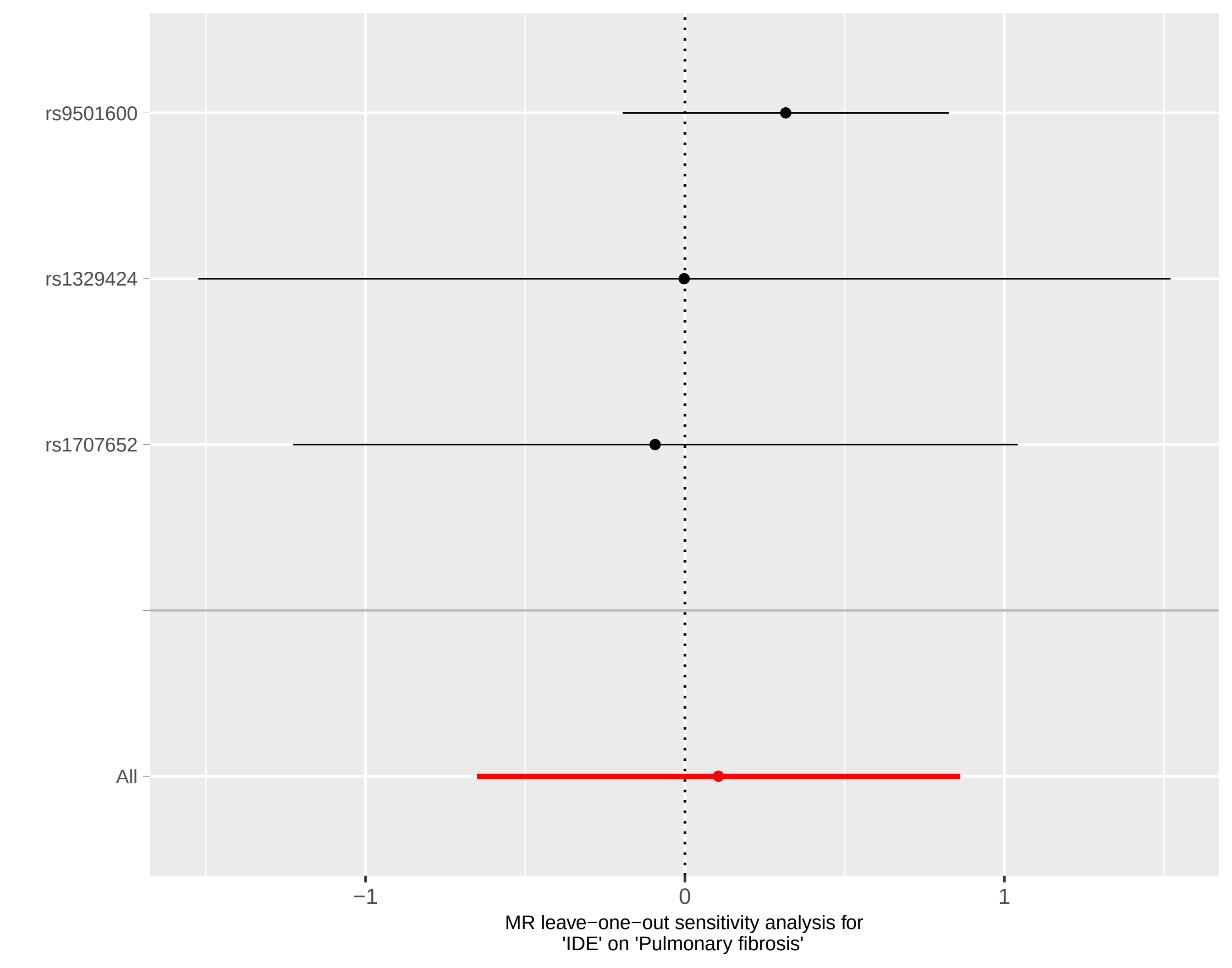
rs9501600
rs1329424
rs1707652
All
−1
0
1
MR leave−one−out sensitivity analysis for
'IDE' on 'Pulmonary fibrosis'

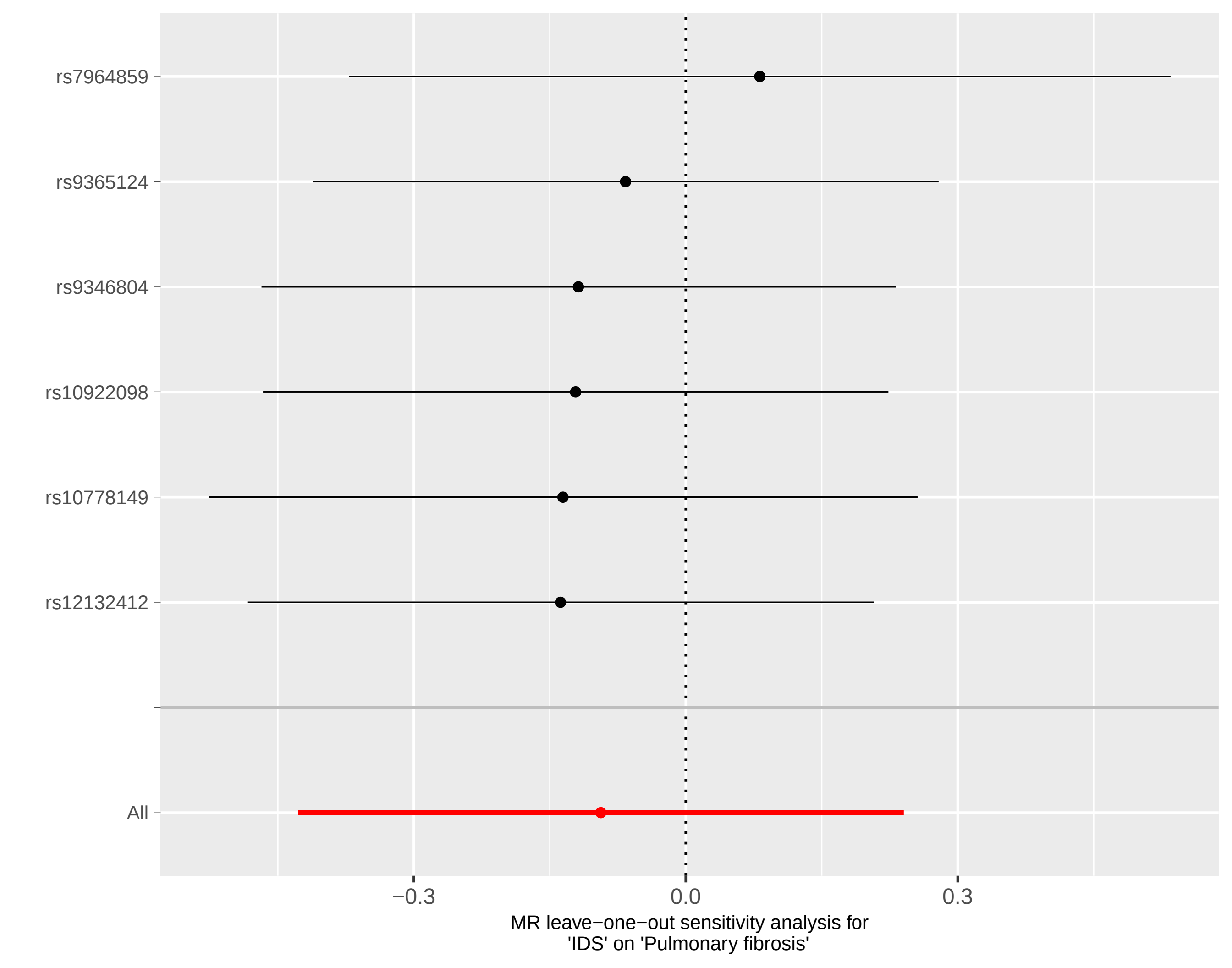
rs7964859
rs9365124
rs9346804
rs10922098
rs10778149
rs12132412
All
−0.3
0.0
0.3
MR leave−one−out sensitivity analysis for
'IDS' on 'Pulmonary fibrosis'

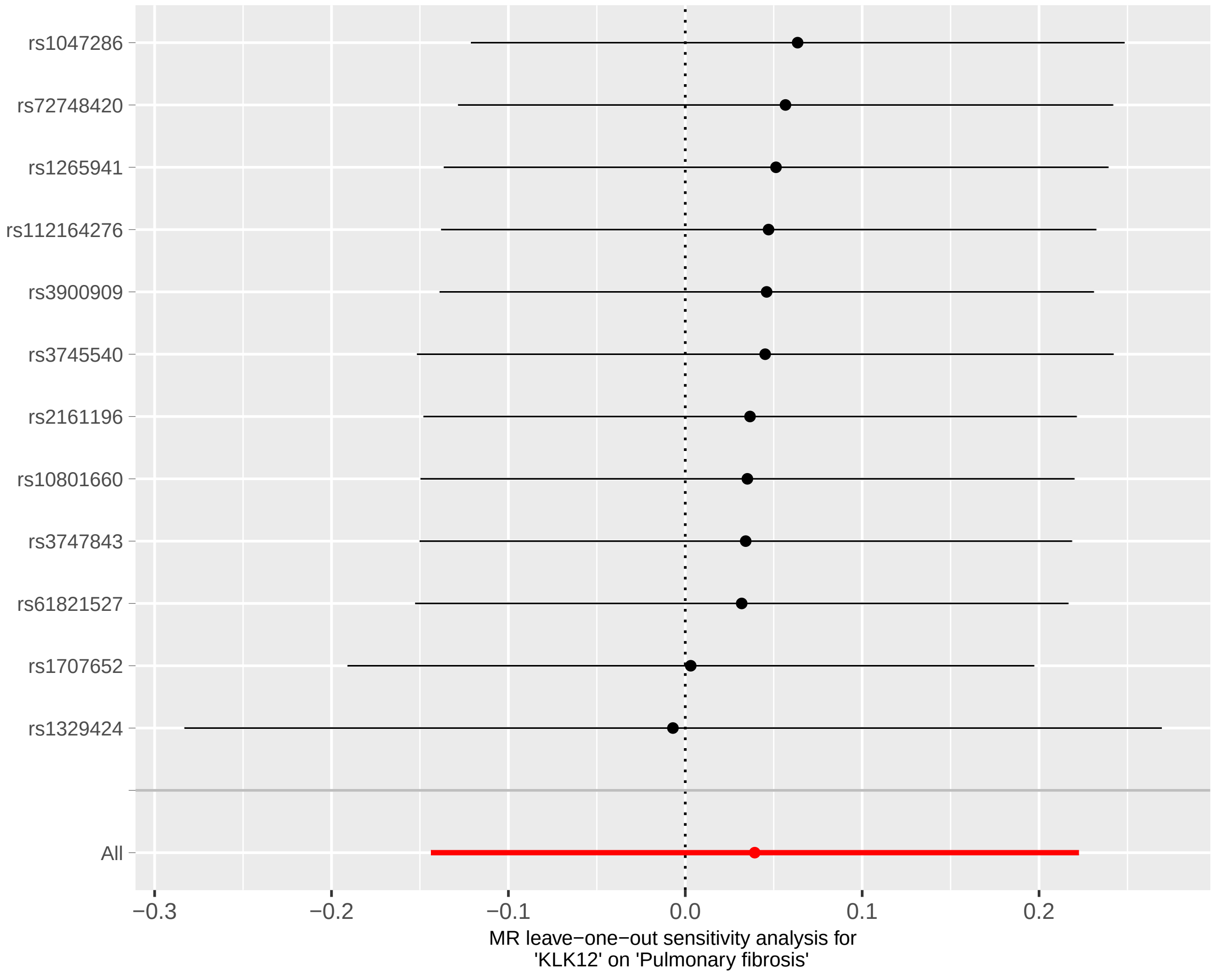
rs1047286
rs72748420
rs1265941
rs112164276
rs3900909
rs3745540
rs2161196
rs10801660
rs3747843
rs61821527
rs1707652
rs1329424
All
−0.3
−0.2
−0.1
0.0
0.1
0.2
MR leave−one−out sensitivity analysis for
'KLK12' on 'Pulmonary fibrosis'

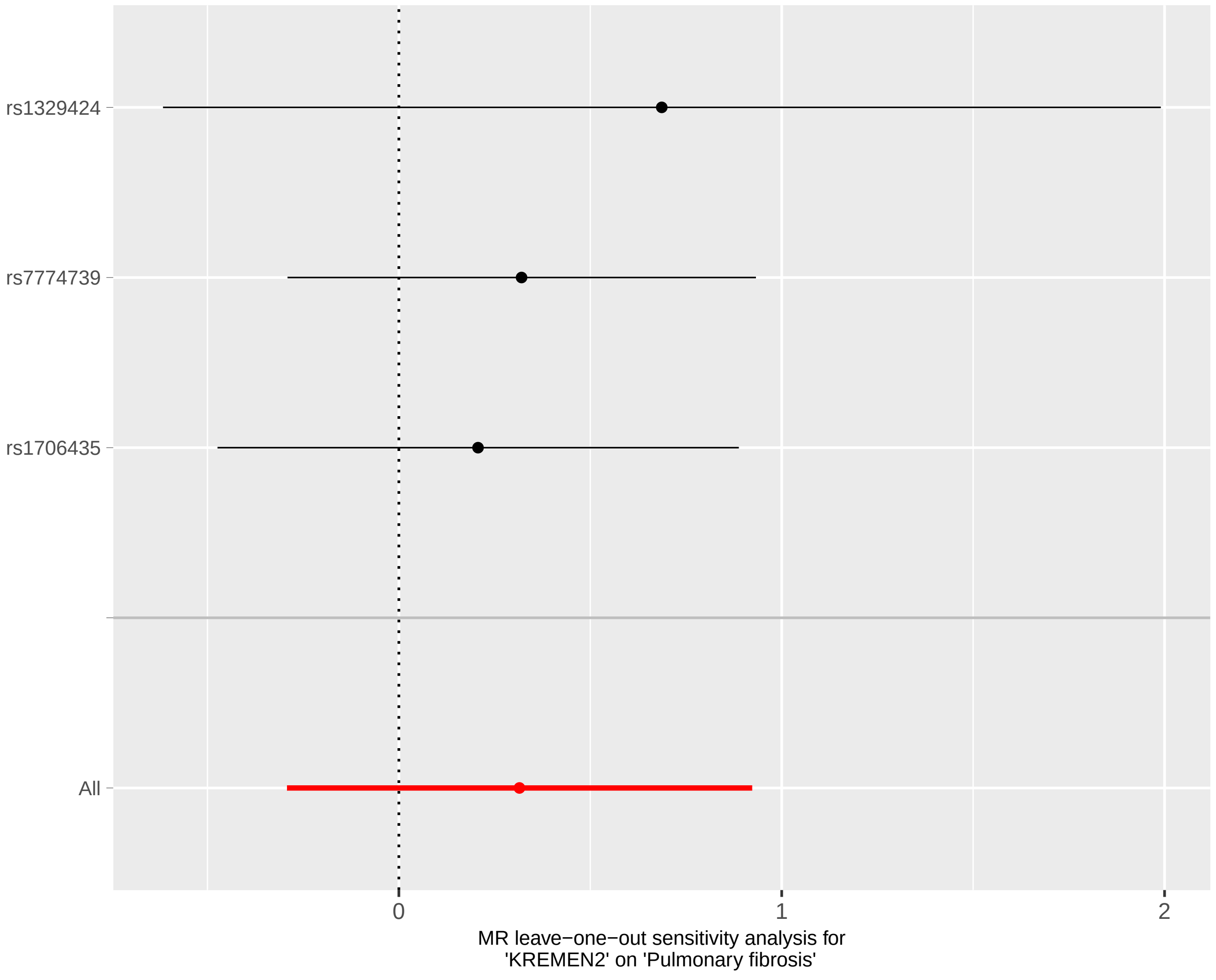
rs1329424
rs7774739
rs1706435
All
0
1
2
MR leave−one−out sensitivity analysis for
'KREMEN2' on 'Pulmonary fibrosis'

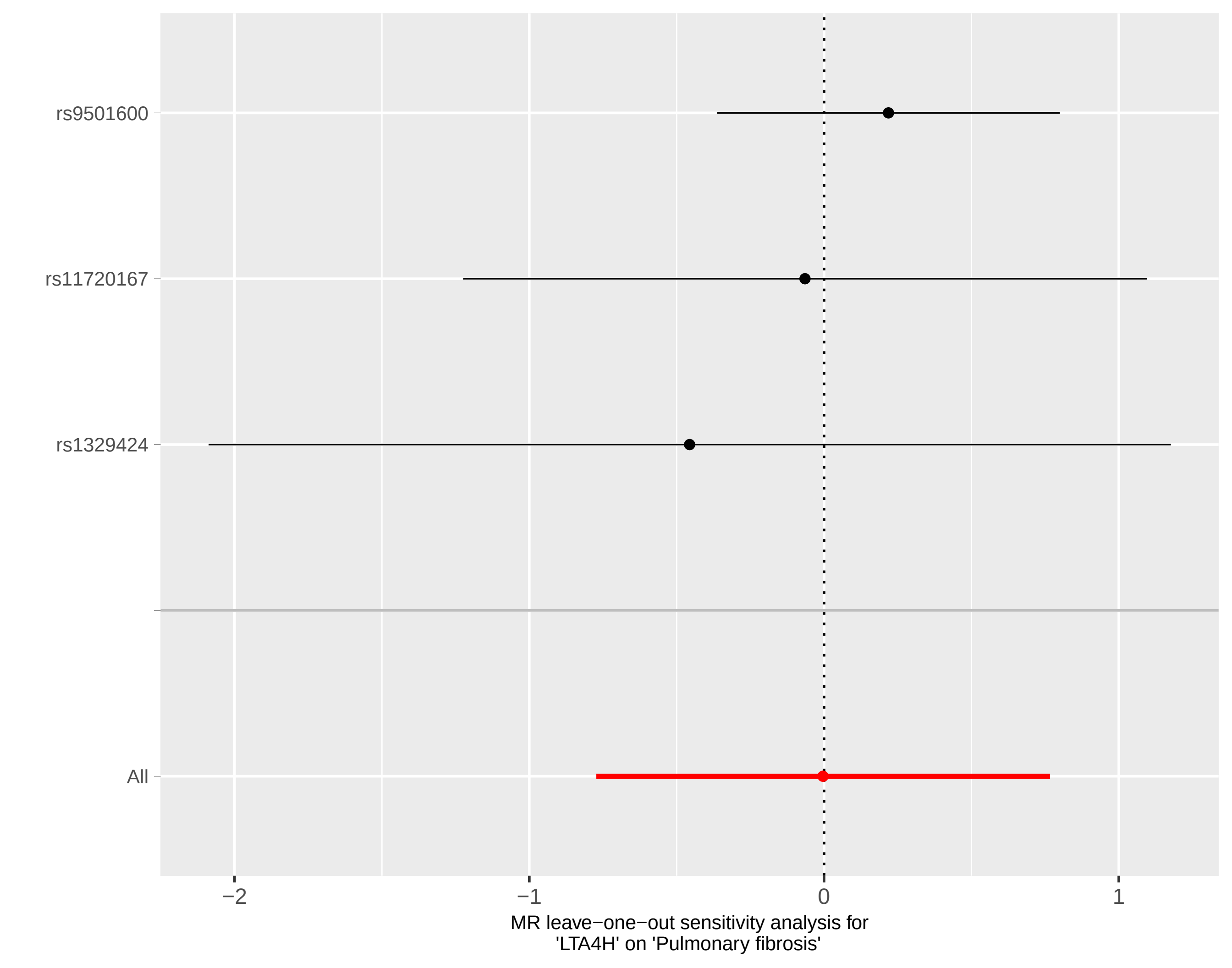

rs9501600
rs11720167
rs1329424
All
−2
−1
0
1
MR leave−one−out sensitivity analysis for
'LTA4H' on 'Pulmonary fibrosis'

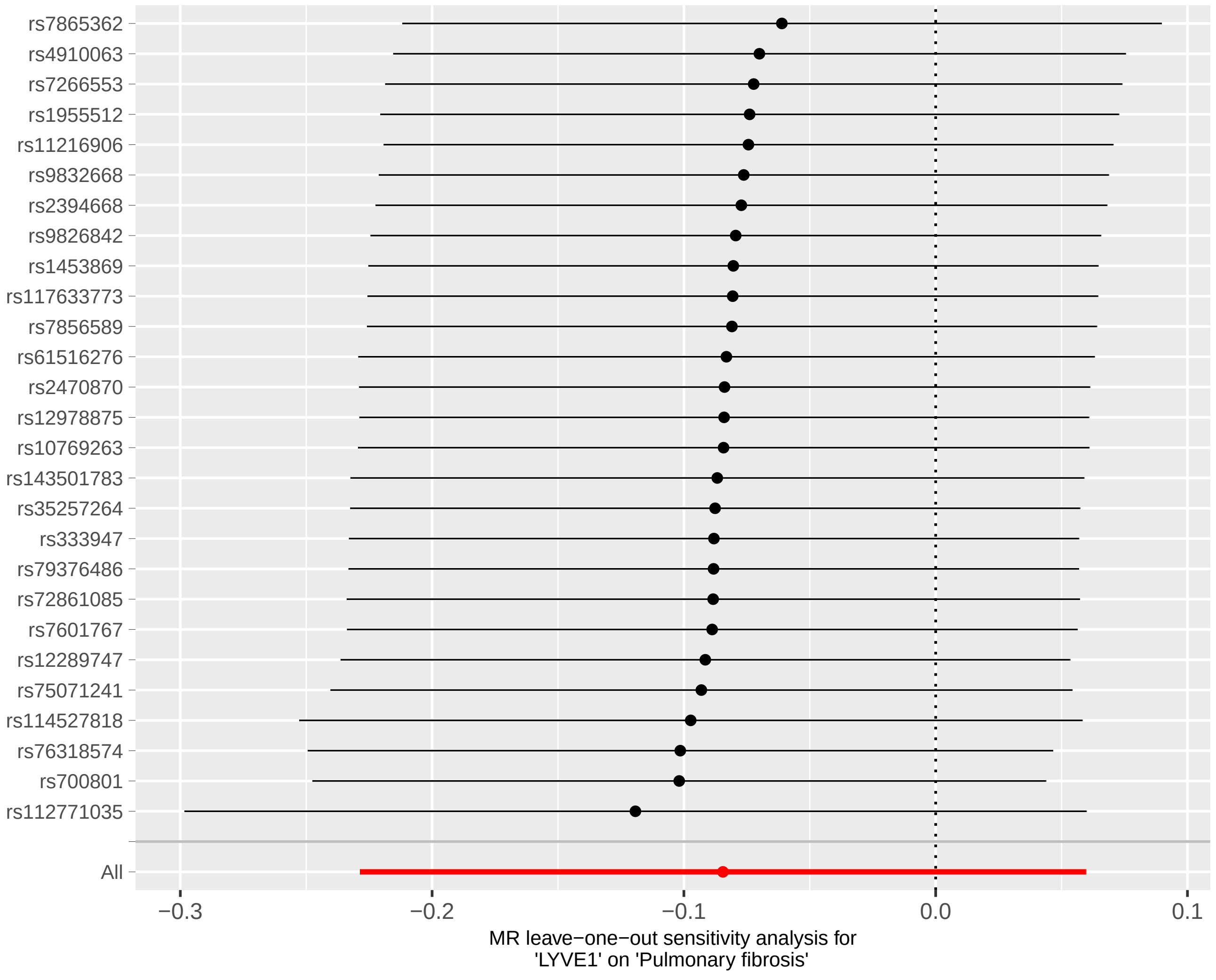
rs7865362
rs4910063
rs7266553
rs1955512
rs11216906
rs9832668
rs2394668
rs9826842
rs1453869
rs117633773
rs7856589
rs61516276
rs2470870
rs12978875
rs10769263
rs143501783
rs35257264
rs333947
rs79376486
rs72861085
rs7601767
rs12289747
rs75071241
rs114527818
rs76318574
rs700801
rs112771035
All
−0.3
−0.2
−0.1
0.0
0.1
MR leave−one−out sensitivity analysis for
'LYVE1' on 'Pulmonary fibrosis'

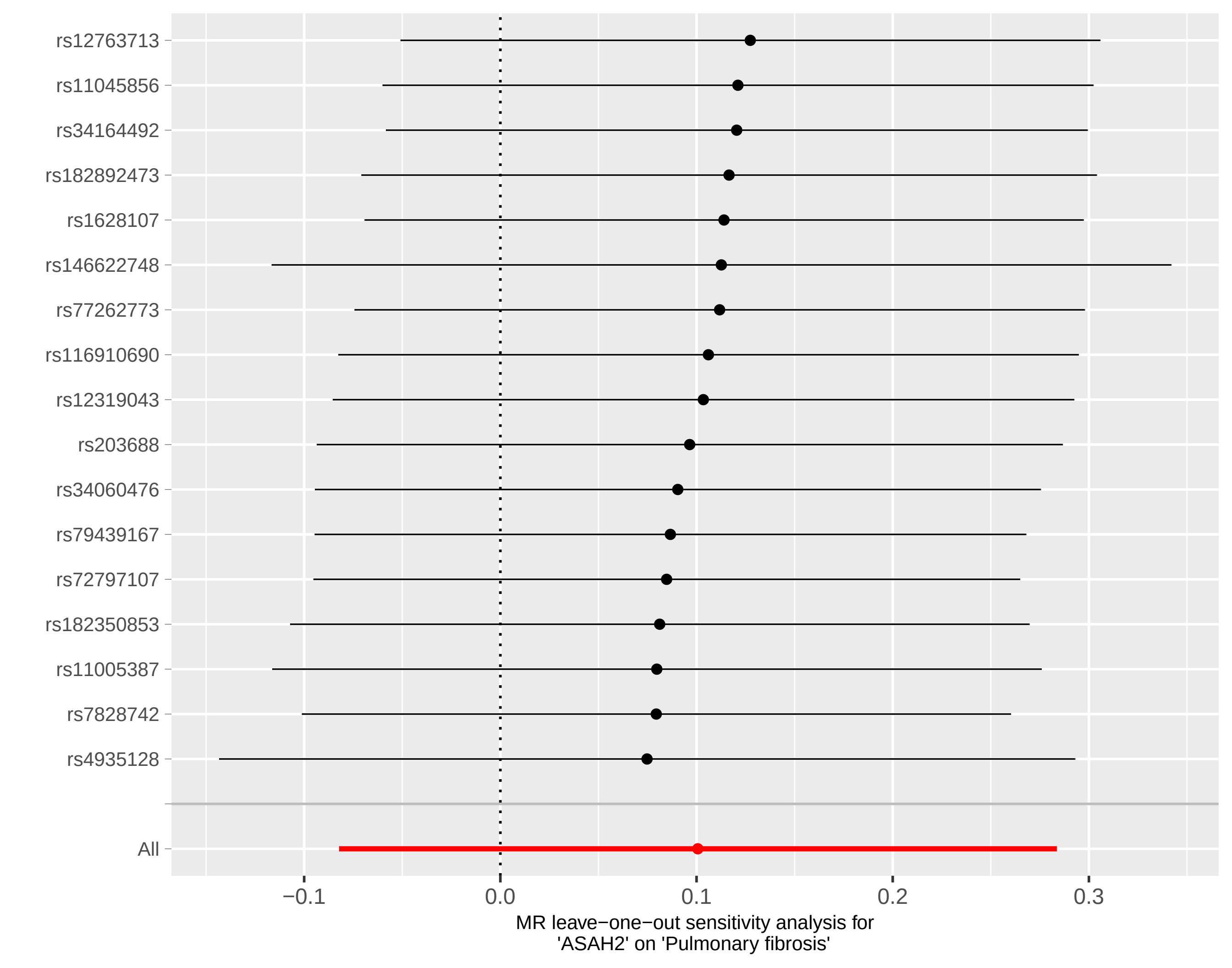
rs12763713
rs11045856
rs34164492
rs182892473
rs1628107
rs146622748
rs77262773
rs116910690
rs12319043
rs203688
rs34060476
rs79439167
rs72797107
rs182350853
rs11005387
rs7828742
rs4935128
All
−0.1
0.0
0.1
0.2
0.3
MR leave−one−out sensitivity analysis for
'ASAH2' on 'Pulmonary fibrosis'

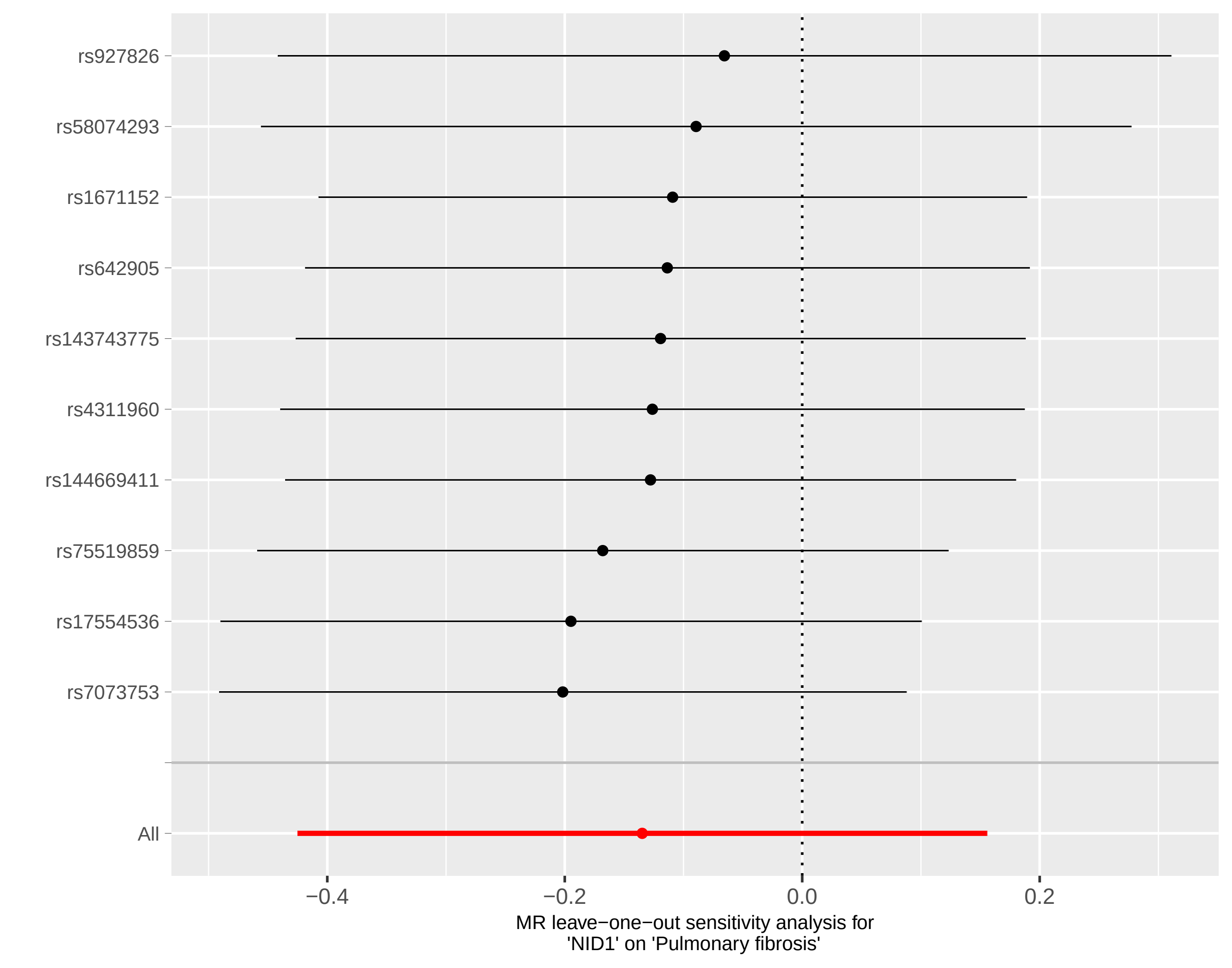
rs927826
rs58074293
rs1671152
rs642905
rs143743775
rs4311960
rs144669411
rs75519859
rs17554536
rs7073753
All
−0.4
−0.2
0.0
0.2
MR leave−one−out sensitivity analysis for
'NID1' on 'Pulmonary fibrosis'

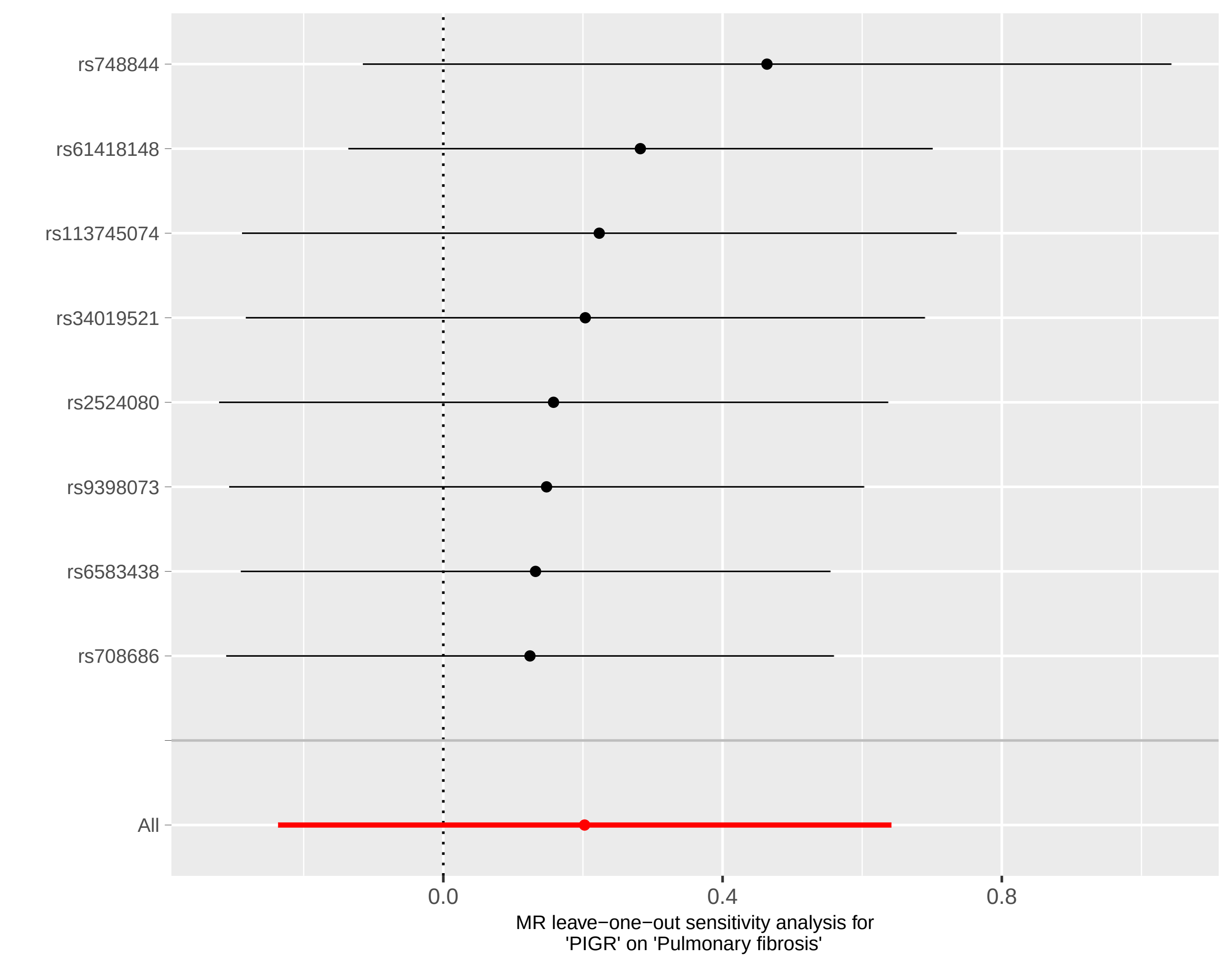
rs748844
rs61418148
rs113745074
rs34019521
rs2524080
rs9398073
rs6583438
rs708686
All
0.0
0.4
0.8
MR leave−one−out sensitivity analysis for
'PIGR' on 'Pulmonary fibrosis'

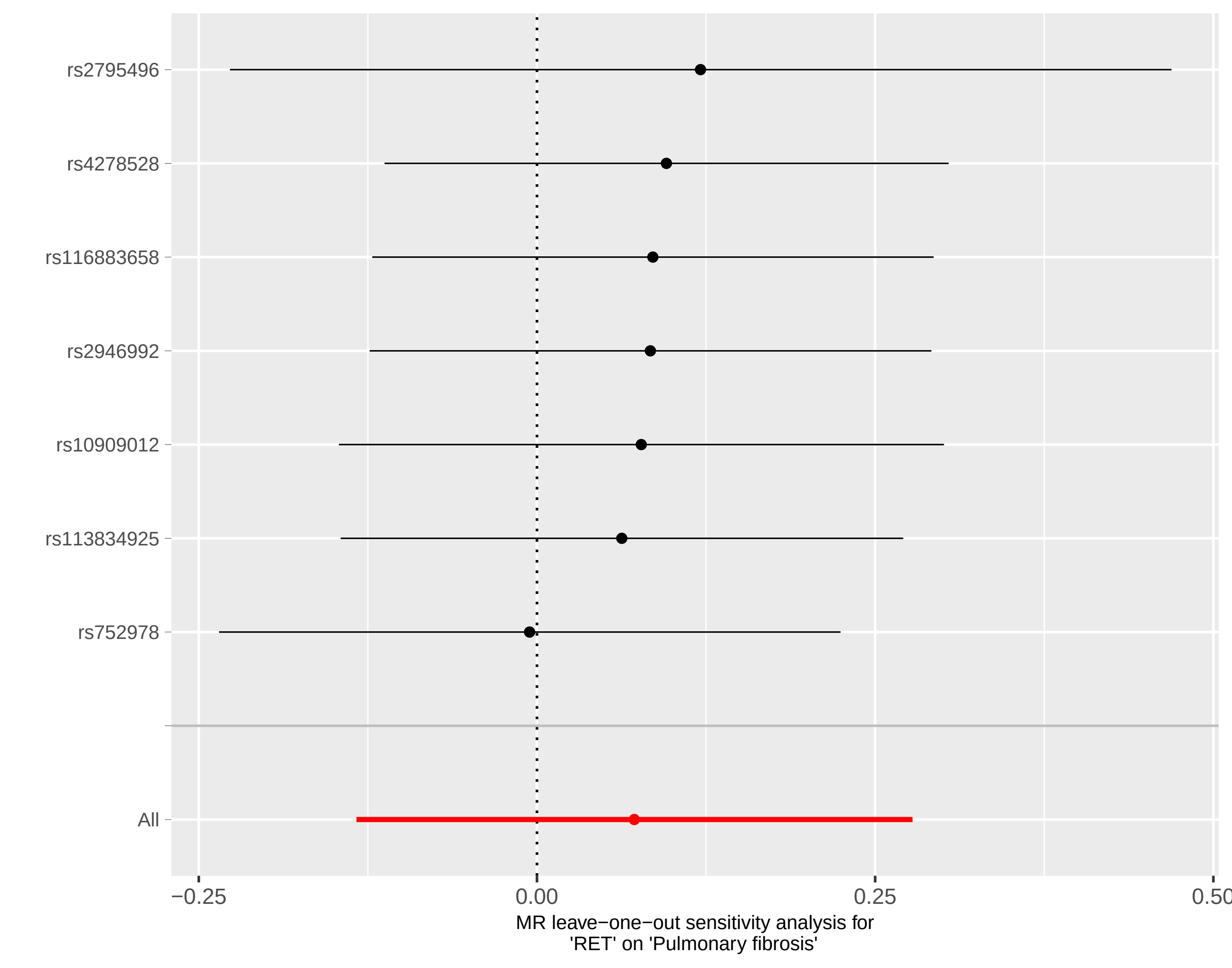
rs2795496
rs4278528
rs116883658
rs2946992
rs10909012
rs113834925
rs752978
All
−0.25
0.00
0.25
0.50
MR leave−one−out sensitivity analysis for
'RET' on 'Pulmonary fibrosis'

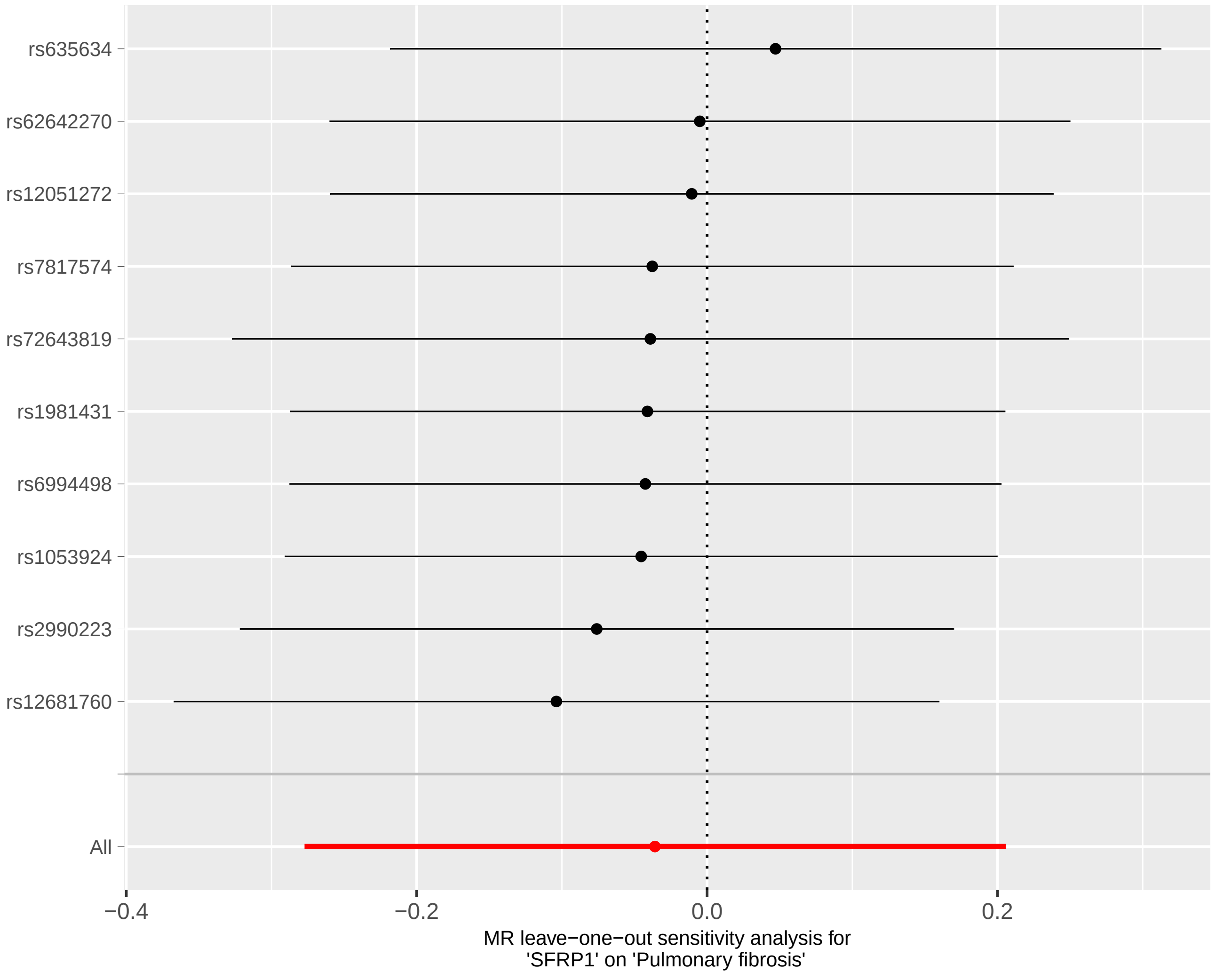

rs635634
rs62642270
rs12051272
rs7817574
rs72643819
rs1981431
rs6994498
rs1053924
rs2990223
rs12681760
All
−0.4
−0.2
0.0
0.2
MR leave−one−out sensitivity analysis for
'SFRP1' on 'Pulmonary fibrosis'

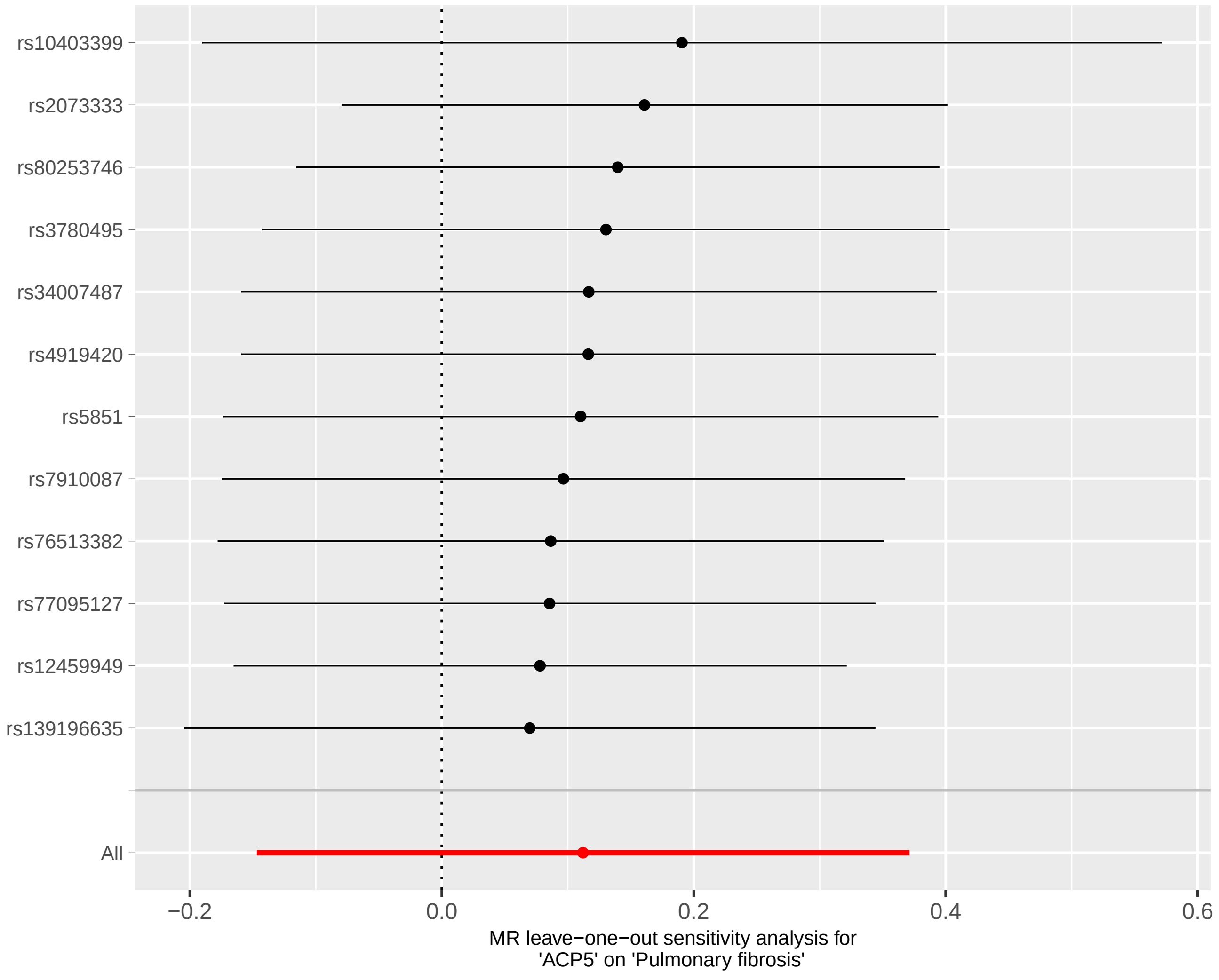

rs10403399
rs2073333
rs80253746
rs3780495
rs34007487
rs4919420
rs5851
rs7910087
rs76513382
rs77095127
rs12459949
rs139196635
All
−0.2
0.0
0.2
0.4
0.6
MR leave−one−out sensitivity analysis for
'ACP5' on 'Pulmonary fibrosis'

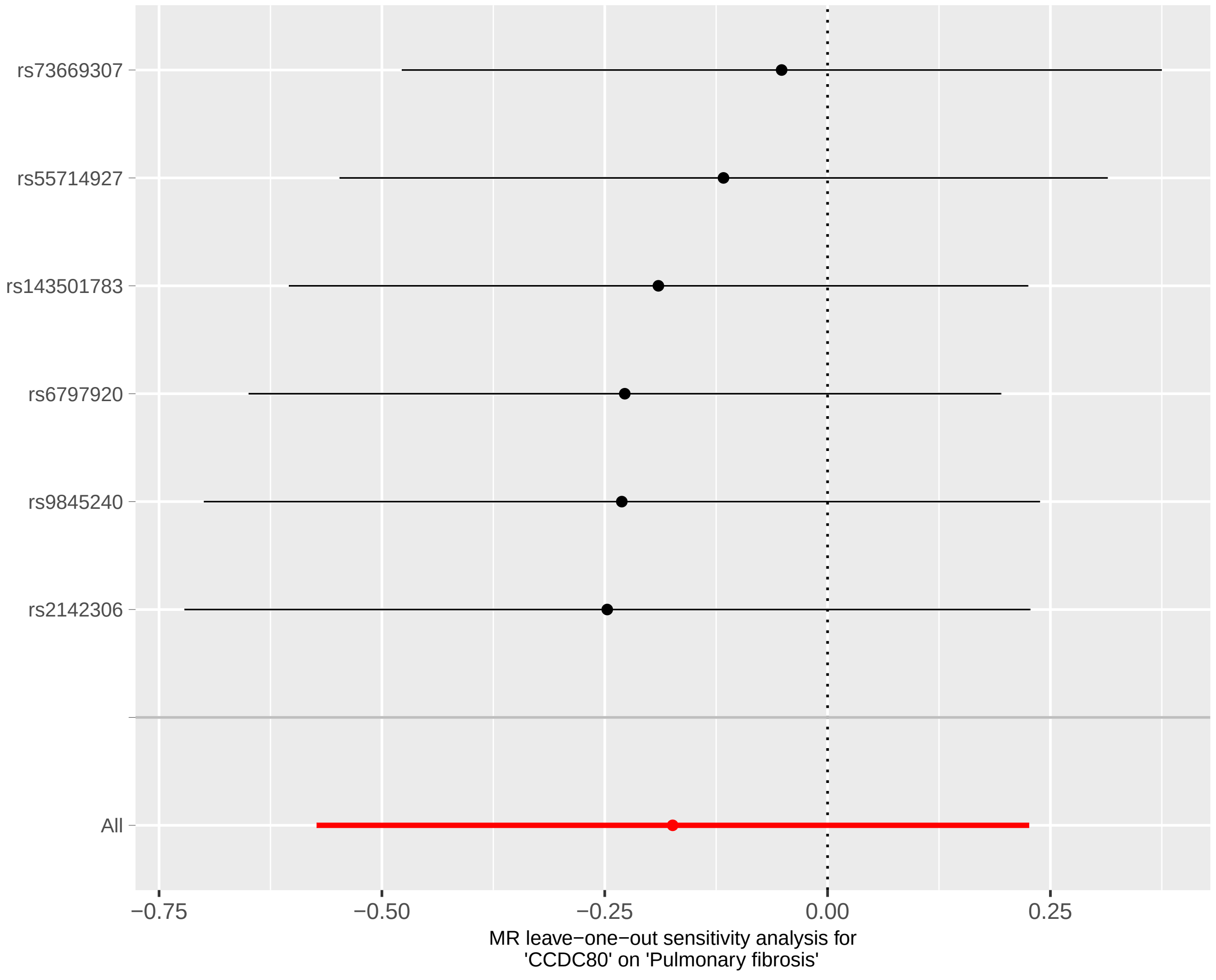
rs73669307
rs55714927
rs143501783
rs6797920
rs9845240
rs2142306
All
−0.75
−0.50
−0.25
0.00
0.25
MR leave−one−out sensitivity analysis for
'CCDC80' on 'Pulmonary fibrosis'

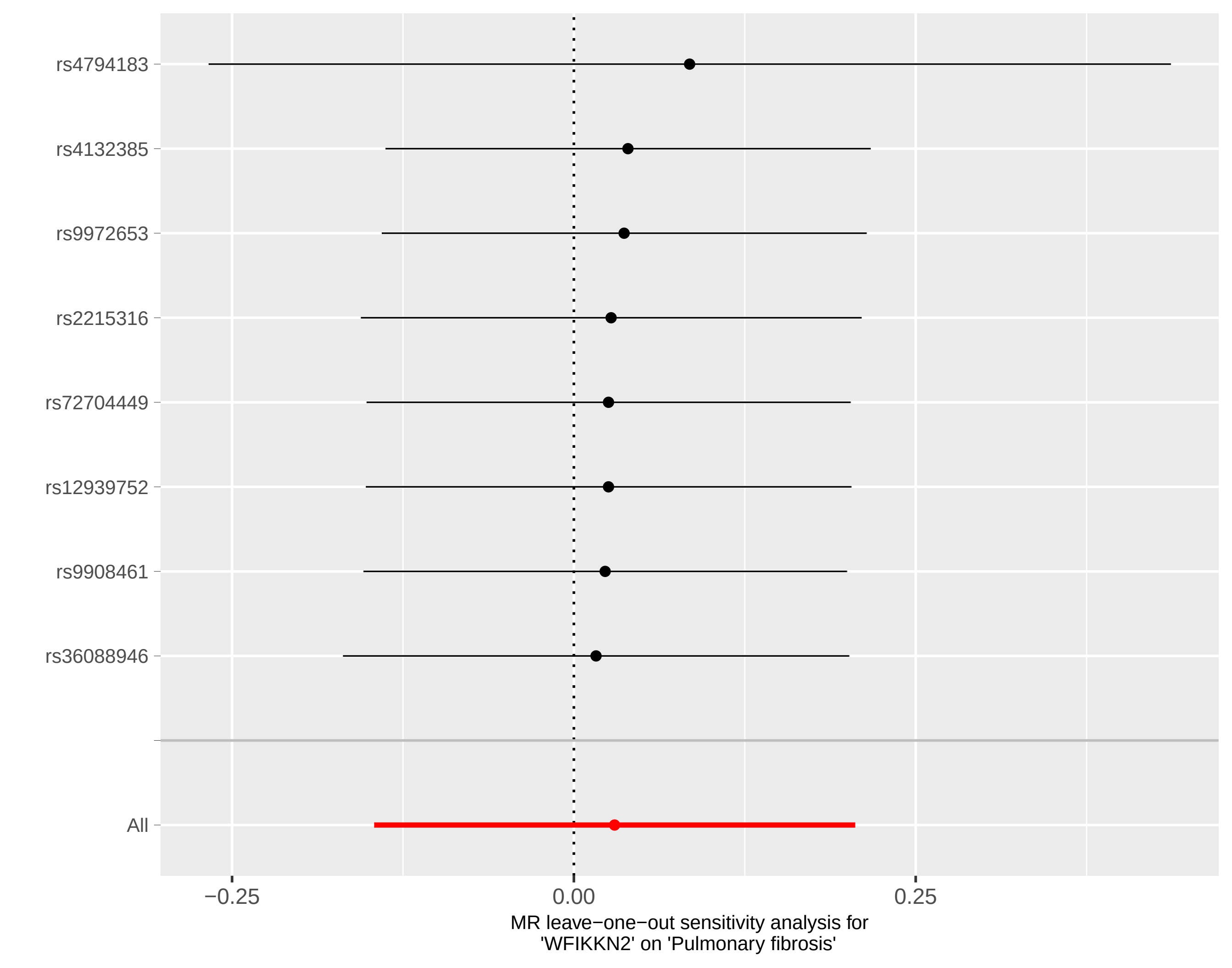
rs4794183
rs4132385
rs9972653
rs2215316
rs72704449
rs12939752
rs9908461
rs36088946
All
−0.25
0.00
0.25
MR leave−one−out sensitivity analysis for
'WFIKKN2' on 'Pulmonary fibrosis'

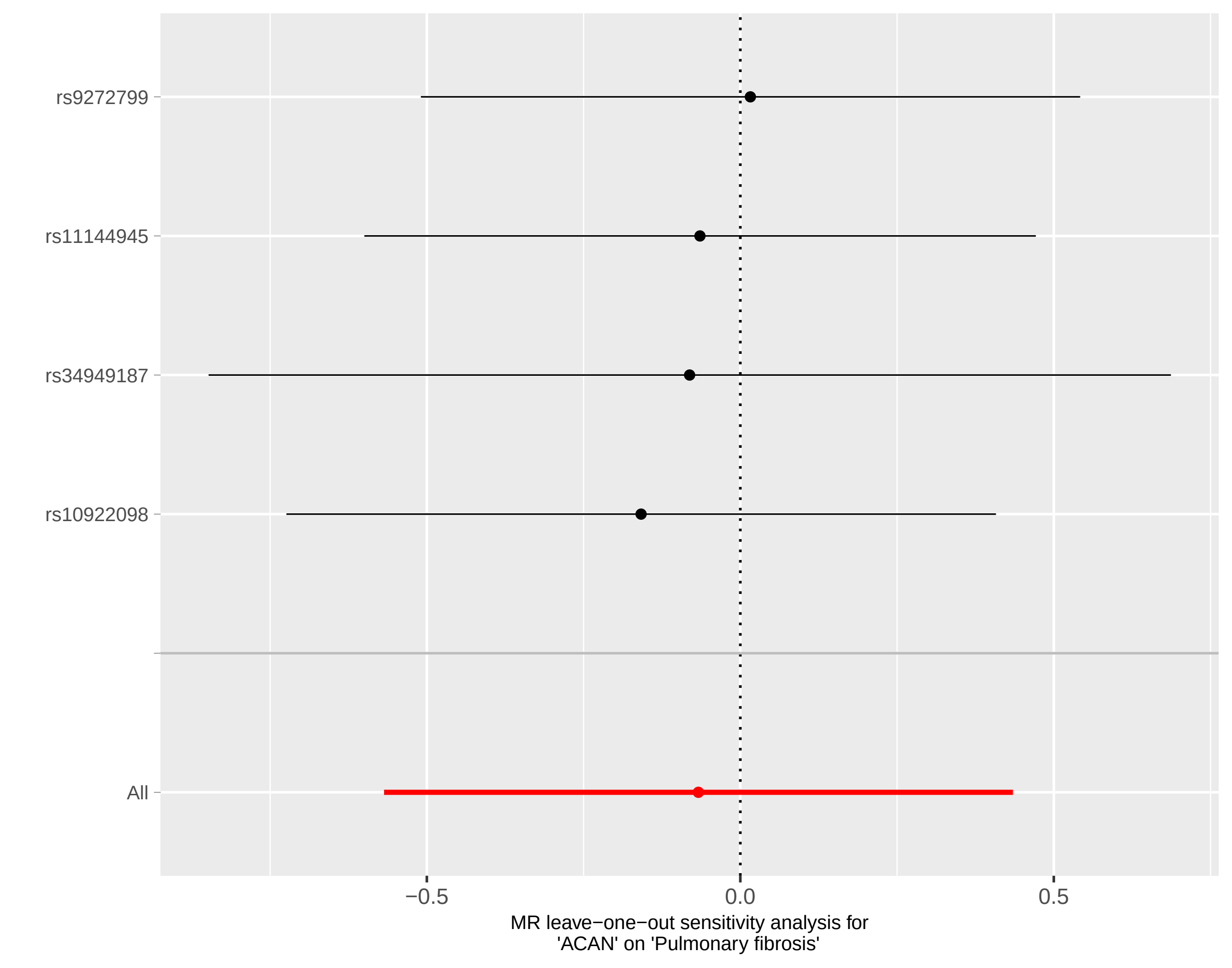

rs9272799
rs11144945
rs34949187
rs10922098
All
−0.5
0.0
0.5
MR leave−one−out sensitivity analysis for
'ACAN' on 'Pulmonary fibrosis'

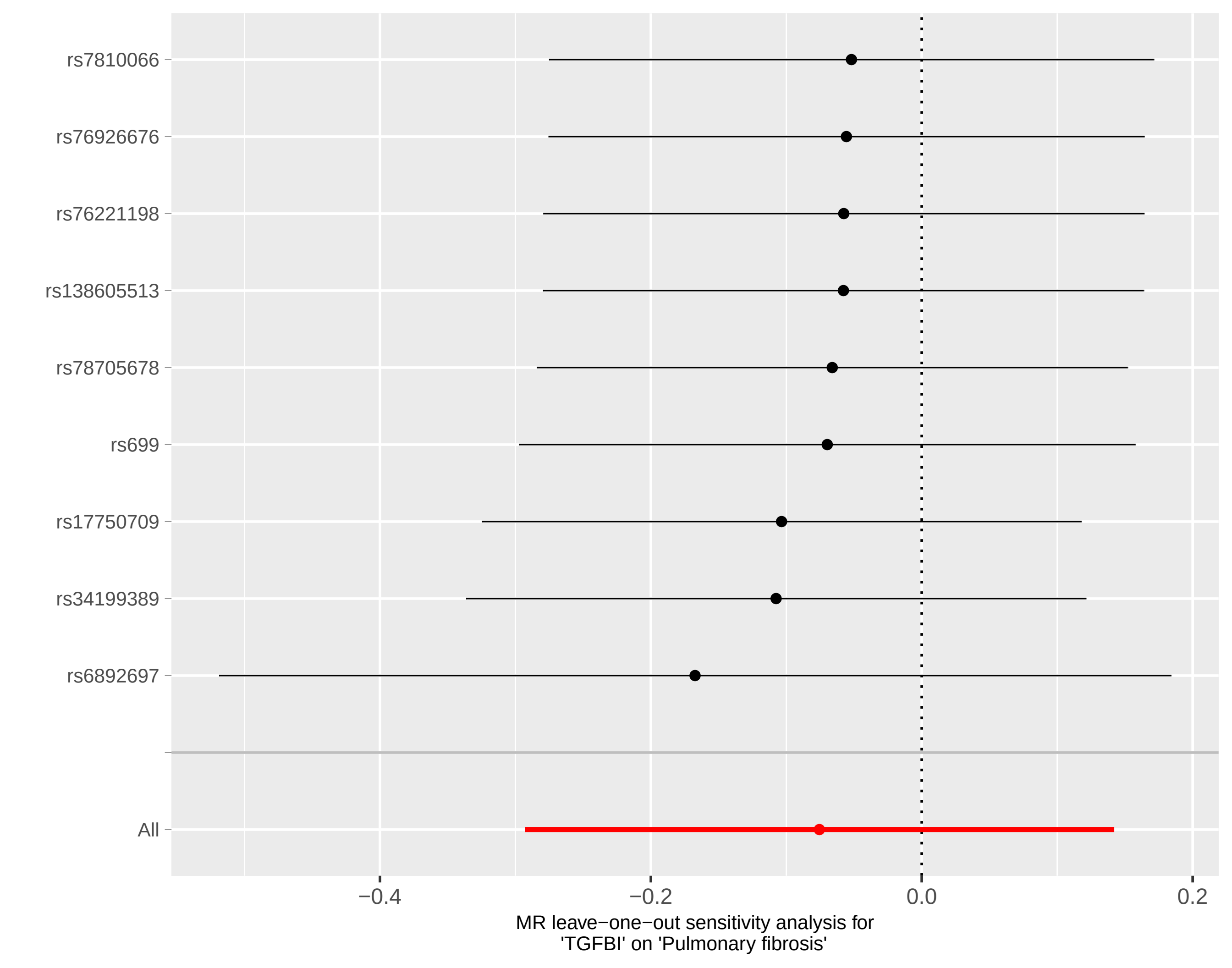
rs7810066
rs76926676
rs76221198
rs138605513
rs78705678
rs699
rs17750709
rs34199389
rs6892697
All
−0.4
−0.2
0.0
0.2
MR leave−one−out sensitivity analysis for
'TGFBI' on 'Pulmonary fibrosis'

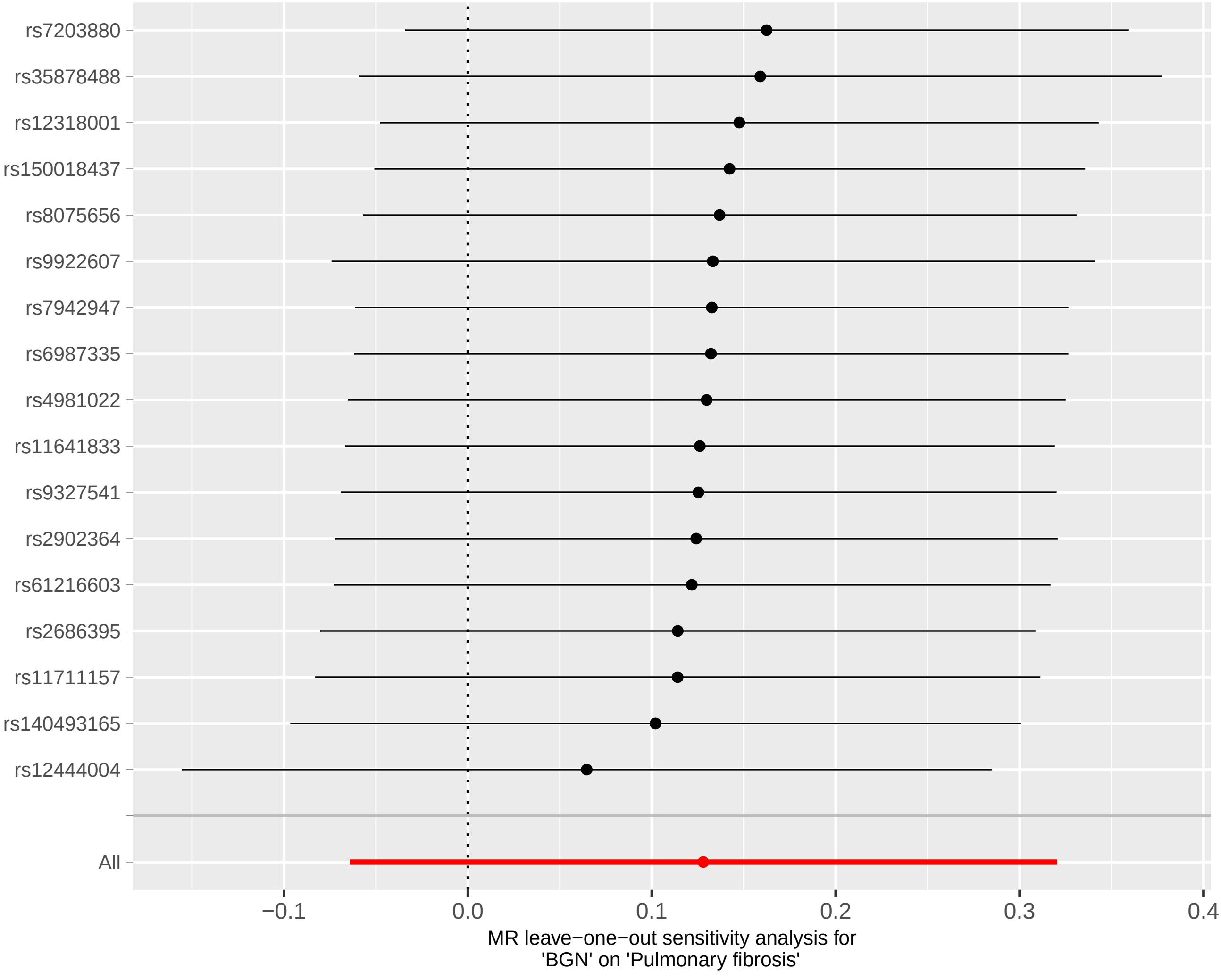
rs7203880
rs35878488
rs12318001
rs150018437
rs8075656
rs9922607
rs7942947
rs6987335
rs4981022
rs11641833
rs9327541
rs2902364
rs61216603
rs2686395
rs11711157
rs140493165
rs12444004
All
−0.1
0.0
0.1
0.2
0.3
0.4
MR leave−one−out sensitivity analysis for
'BGN' on 'Pulmonary fibrosis'

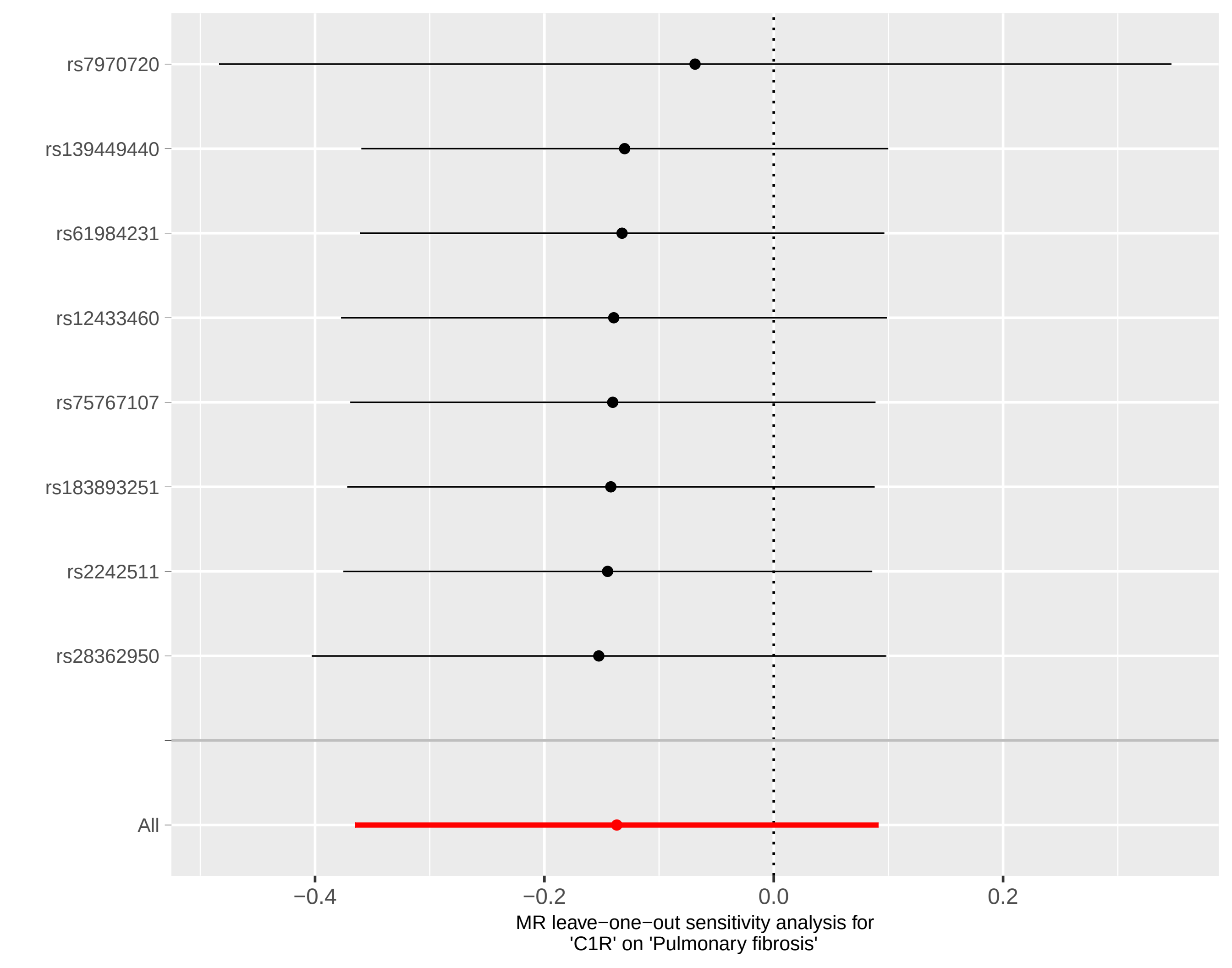
rs7970720
rs139449440
rs61984231
rs12433460
rs75767107
rs183893251
rs2242511
rs28362950
All
−0.4
−0.2
0.0
0.2
MR leave−one−out sensitivity analysis for
'C1R' on 'Pulmonary fibrosis'

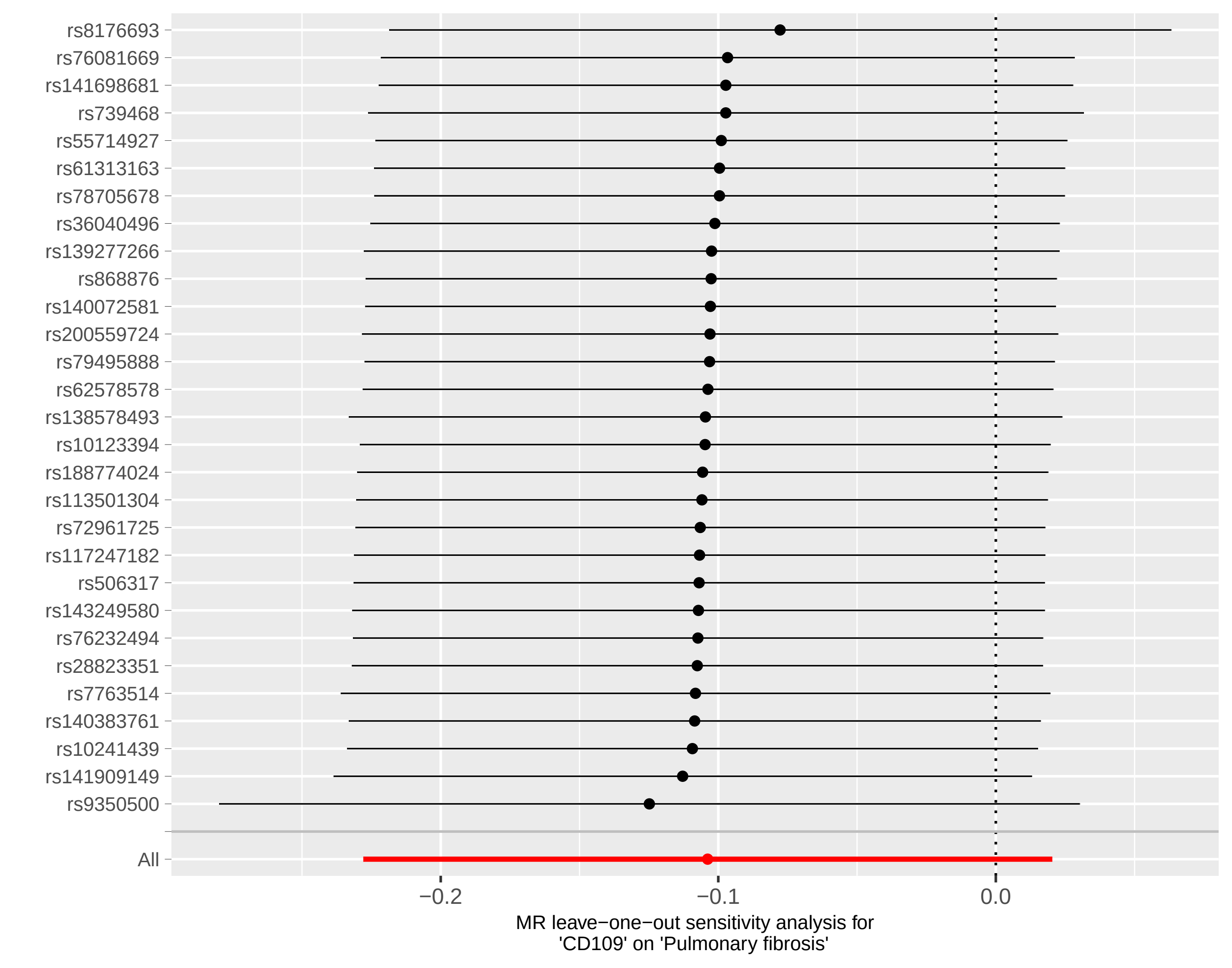
rs8176693
rs76081669
rs141698681
rs739468
rs55714927
rs61313163
rs78705678
rs36040496
rs139277266
rs868876
rs140072581
rs200559724
rs79495888
rs62578578
rs138578493
rs10123394
rs188774024
rs113501304
rs72961725
rs117247182
rs506317
rs143249580
rs76232494
rs28823351
rs7763514
rs140383761
rs10241439
rs141909149
rs9350500
All
−0.2
−0.1
0.0
MR leave−one−out sensitivity analysis for
'CD109' on 'Pulmonary fibrosis'

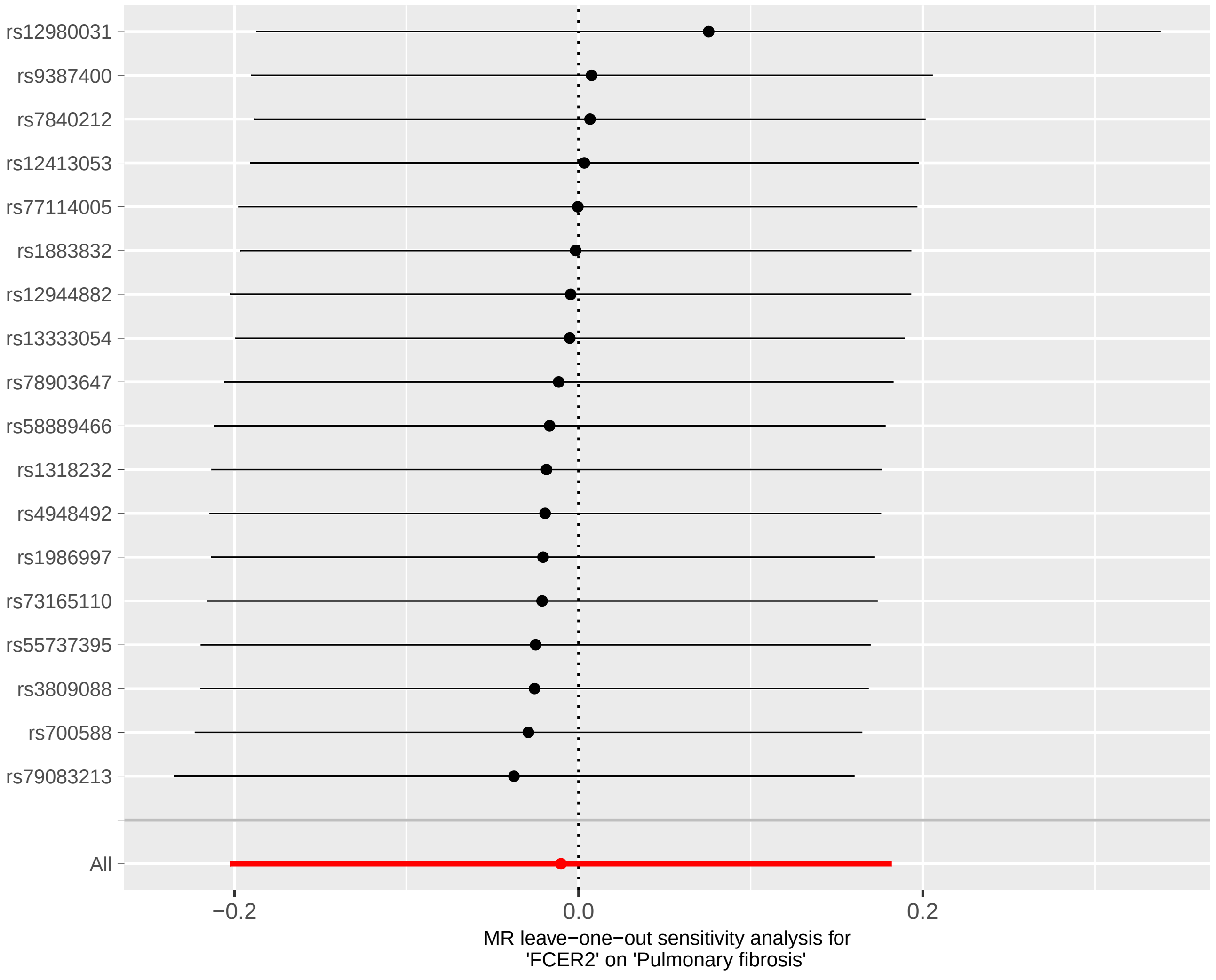
rs12980031
rs9387400
rs7840212
rs12413053
rs77114005
rs1883832
rs12944882
rs13333054
rs78903647
rs58889466
rs1318232
rs4948492
rs1986997
rs73165110
rs55737395
rs3809088
rs700588
rs79083213
All
−0.2
0.0
0.2
MR leave−one−out sensitivity analysis for
'FCER2' on 'Pulmonary fibrosis'

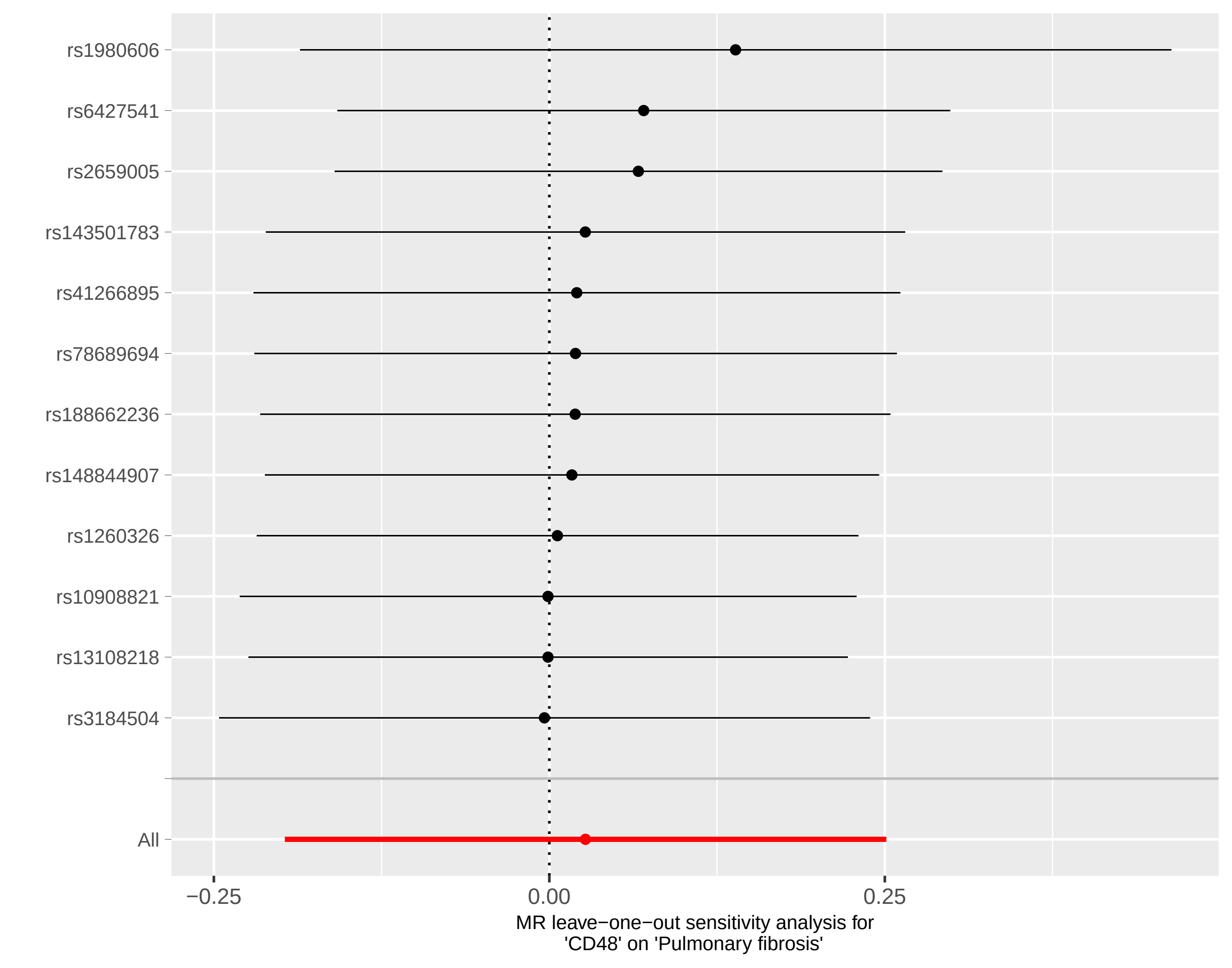
rs1980606
rs6427541
rs2659005
rs143501783
rs41266895
rs78689694
rs188662236
rs148844907
rs1260326
rs10908821
rs13108218
rs3184504
All
−0.25
0.00
0.25
MR leave−one−out sensitivity analysis for
'CD48' on 'Pulmonary fibrosis'

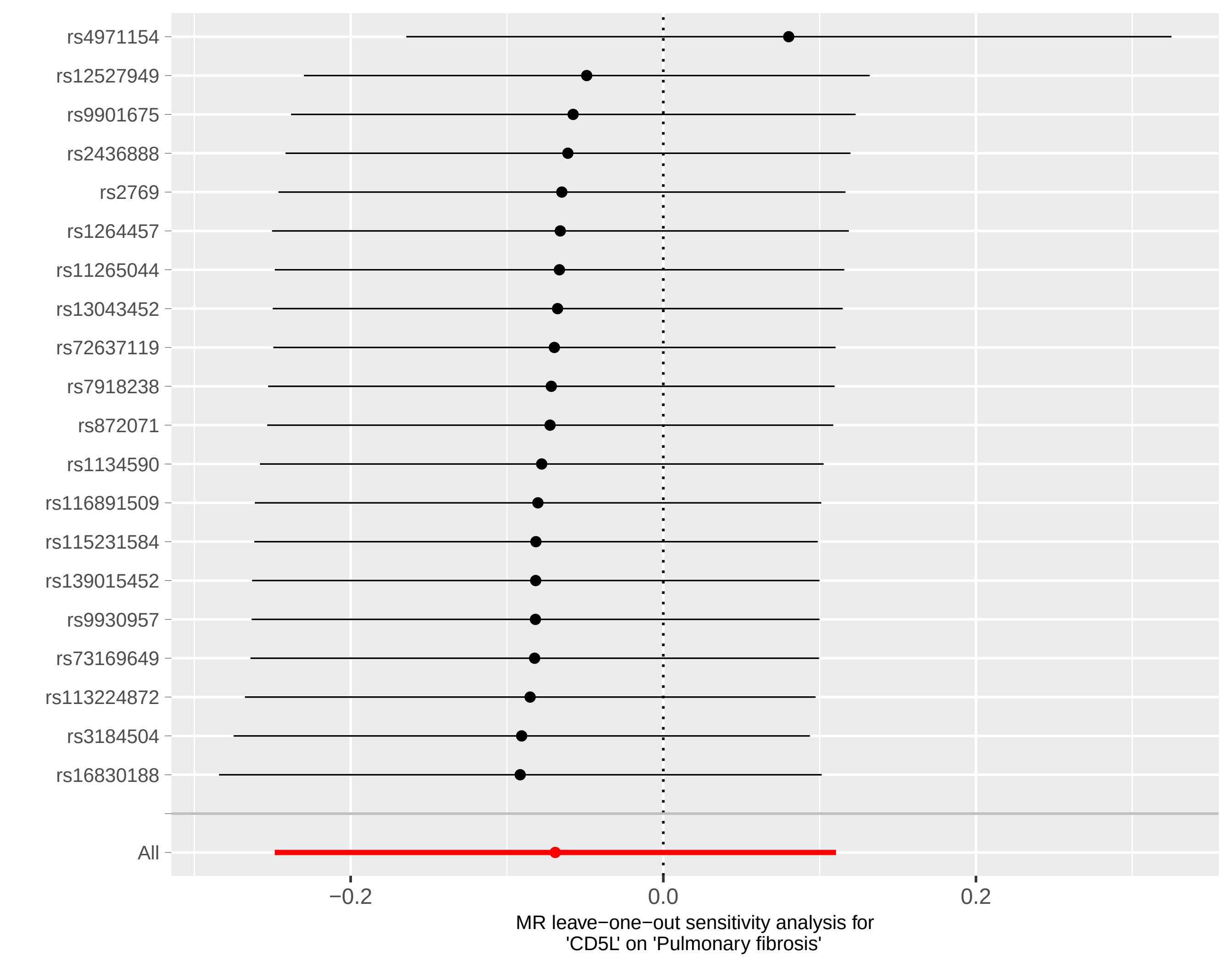
rs4971154
rs12527949
rs9901675
rs2436888
rs2769
rs1264457
rs11265044
rs13043452
rs72637119
rs7918238
rs872071
rs1134590
rs116891509
rs115231584
rs139015452
rs9930957
rs73169649
rs113224872
rs3184504
rs16830188
All
−0.2
0.0
0.2
MR leave−one−out sensitivity analysis for
'CD5L' on 'Pulmonary fibrosis'

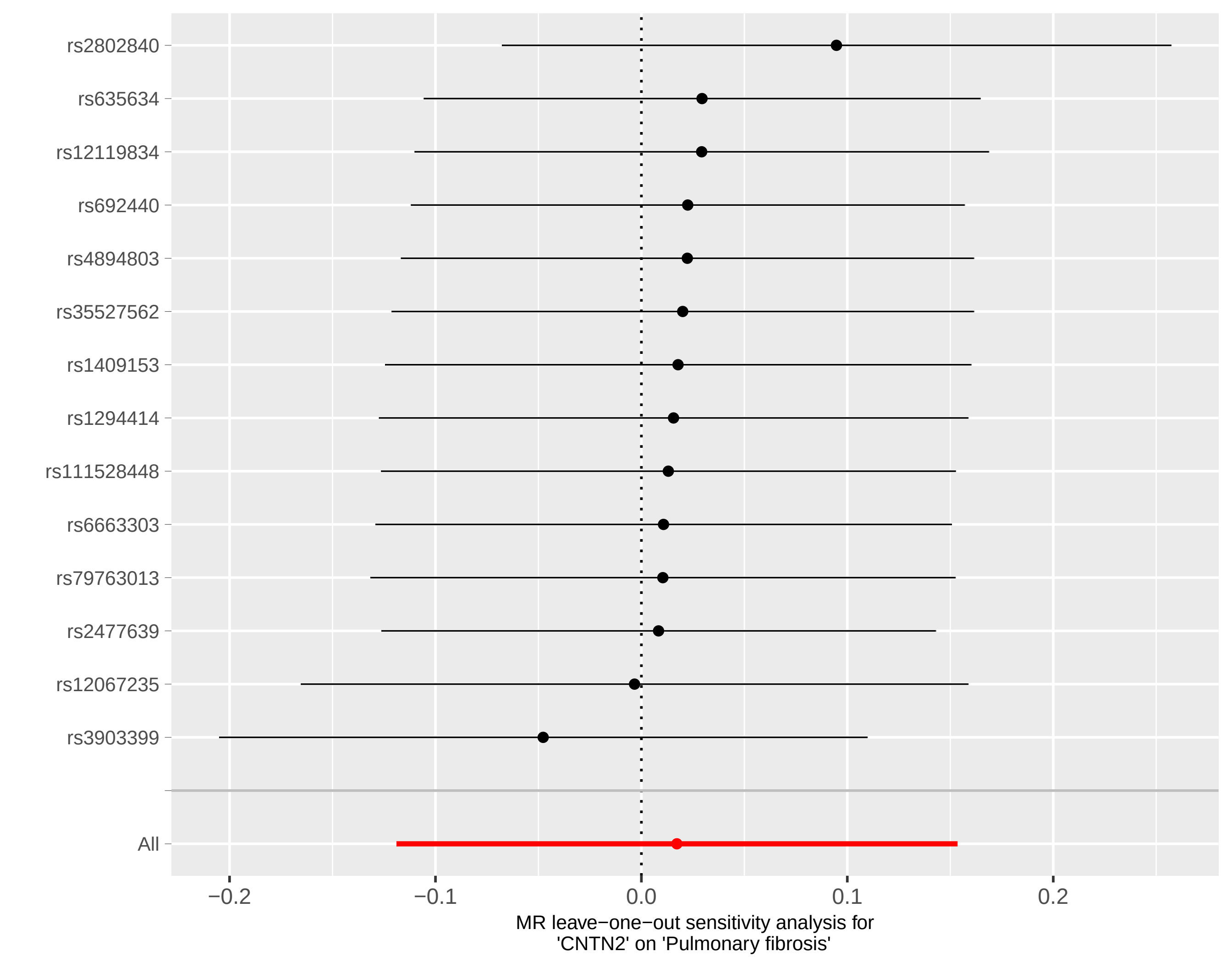

rs2802840
rs635634
rs12119834
rs692440
rs4894803
rs35527562
rs1409153
rs1294414
rs111528448
rs6663303
rs79763013
rs2477639
rs12067235
rs3903399
All
−0.2
−0.1
0.0
0.1
0.2
MR leave−one−out sensitivity analysis for
'CNTN2' on 'Pulmonary fibrosis'

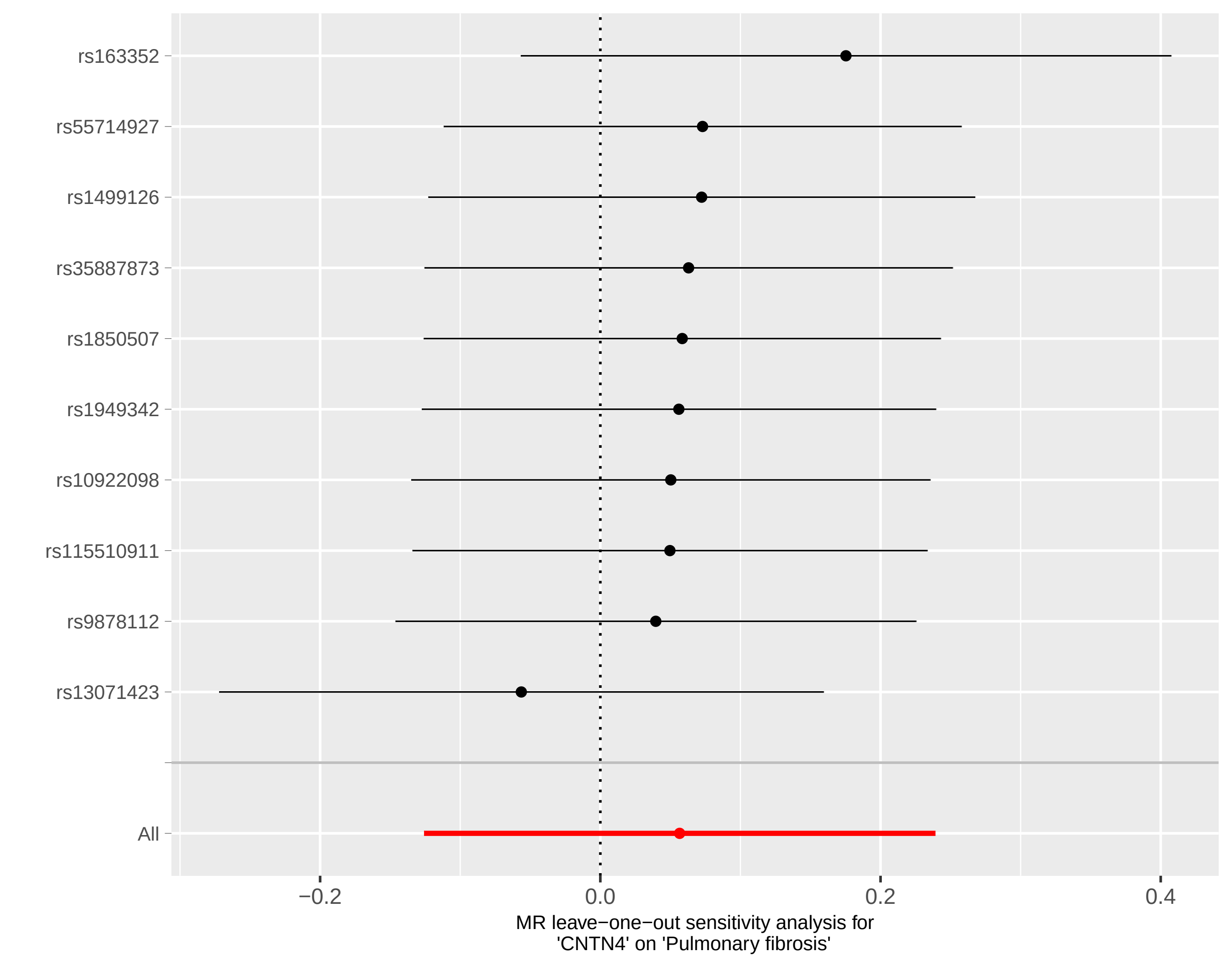
rs163352
rs55714927
rs1499126
rs35887873
rs1850507
rs1949342
rs10922098
rs115510911
rs9878112
rs13071423
All
−0.2
0.0
0.2
0.4
MR leave−one−out sensitivity analysis for
'CNTN4' on 'Pulmonary fibrosis'

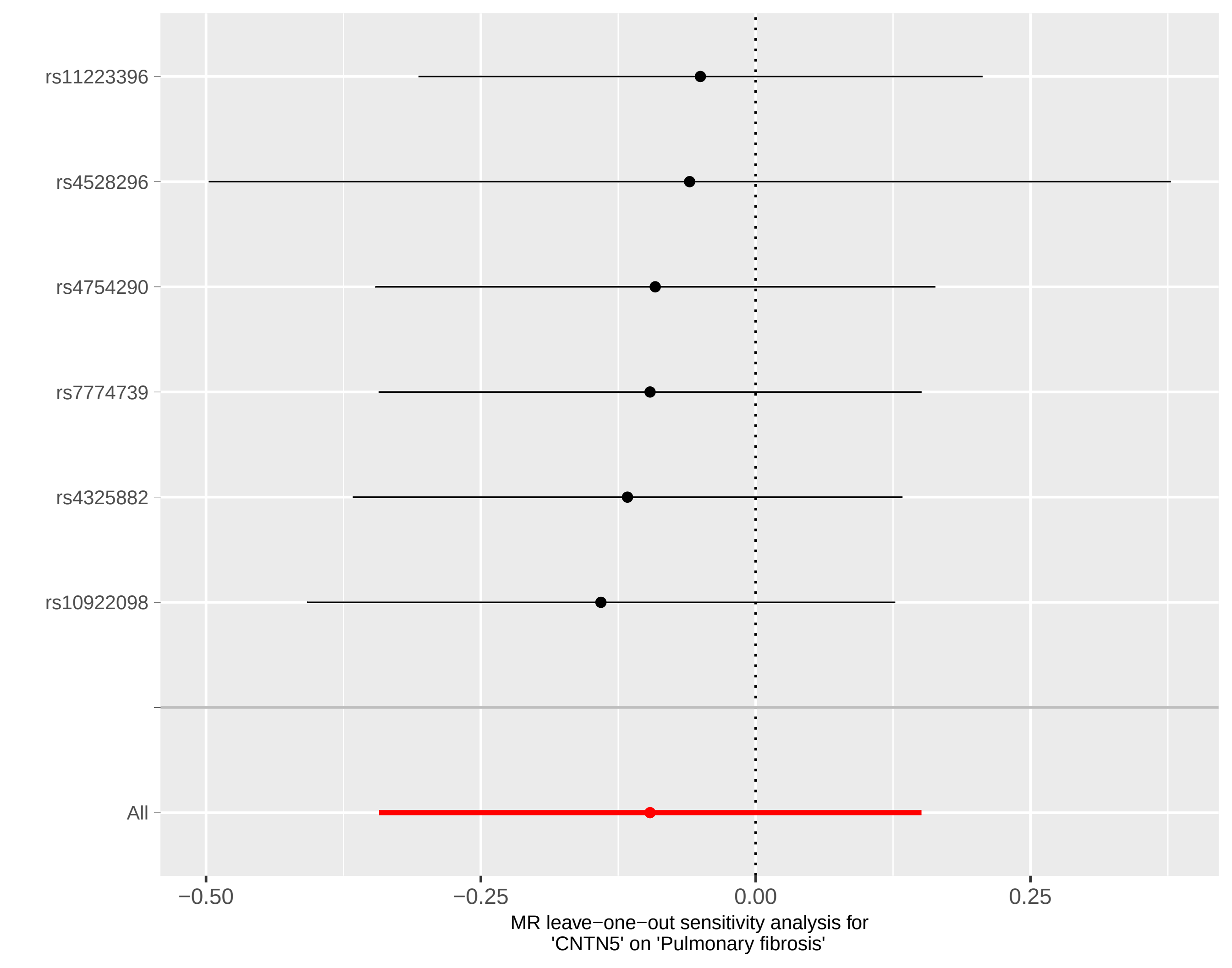

rs11223396
rs4528296
rs4754290
rs7774739
rs4325882
rs10922098
All
−0.50
−0.25
0.00
0.25
MR leave−one−out sensitivity analysis for
'CNTN5' on 'Pulmonary fibrosis'

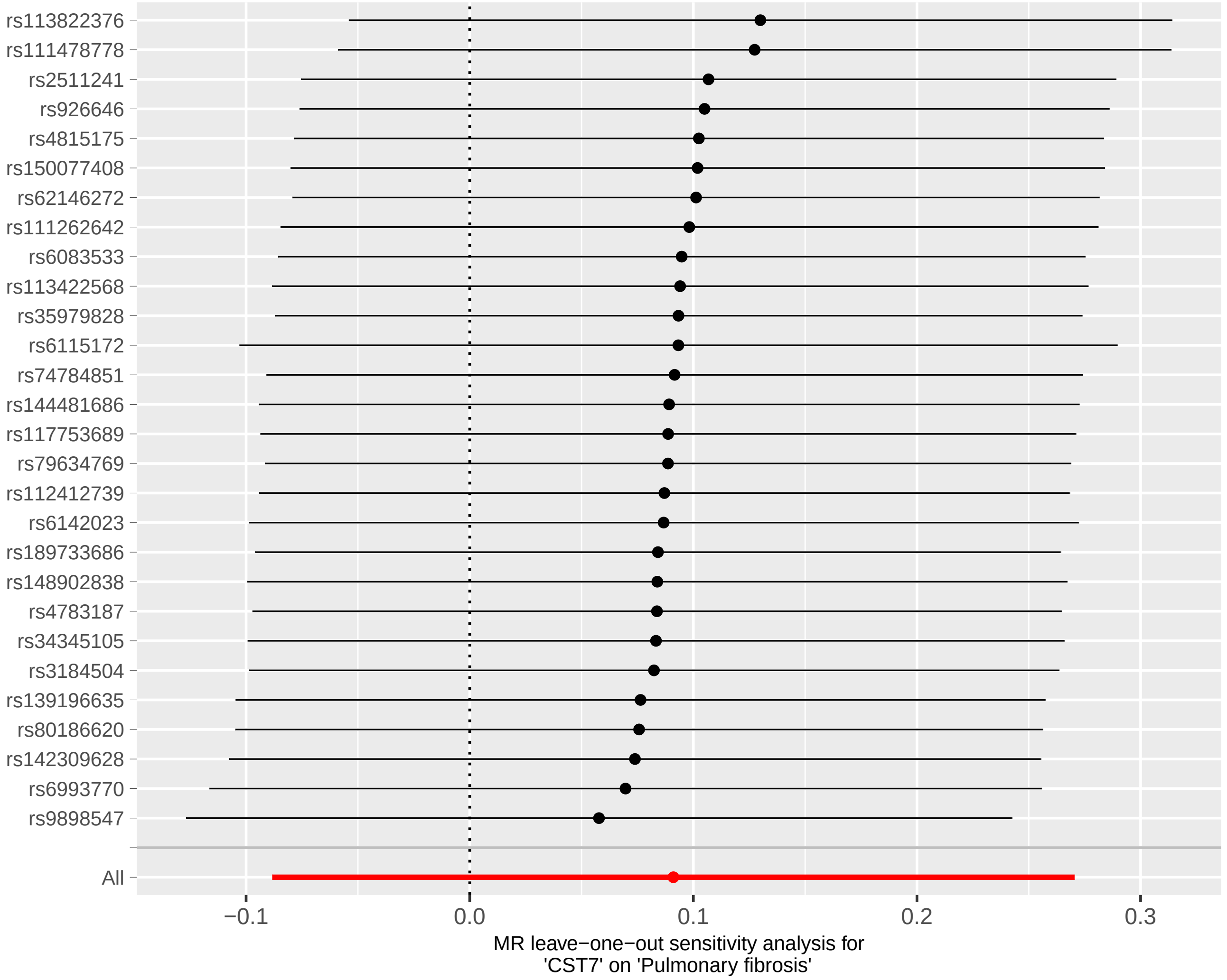
rs113822376
rs111478778
rs2511241
rs926646
rs4815175
rs150077408
rs62146272
rs111262642
rs6083533
rs113422568
rs35979828
rs6115172
rs74784851
rs144481686
rs117753689
rs79634769
rs112412739
rs6142023
rs189733686
rs148902838
rs4783187
rs34345105
rs3184504
rs139196635
rs80186620
rs142309628
rs6993770
rs9898547
All
−0.1
0.0
0.1
0.2
0.3
MR leave−one−out sensitivity analysis for
'CST7' on 'Pulmonary fibrosis'

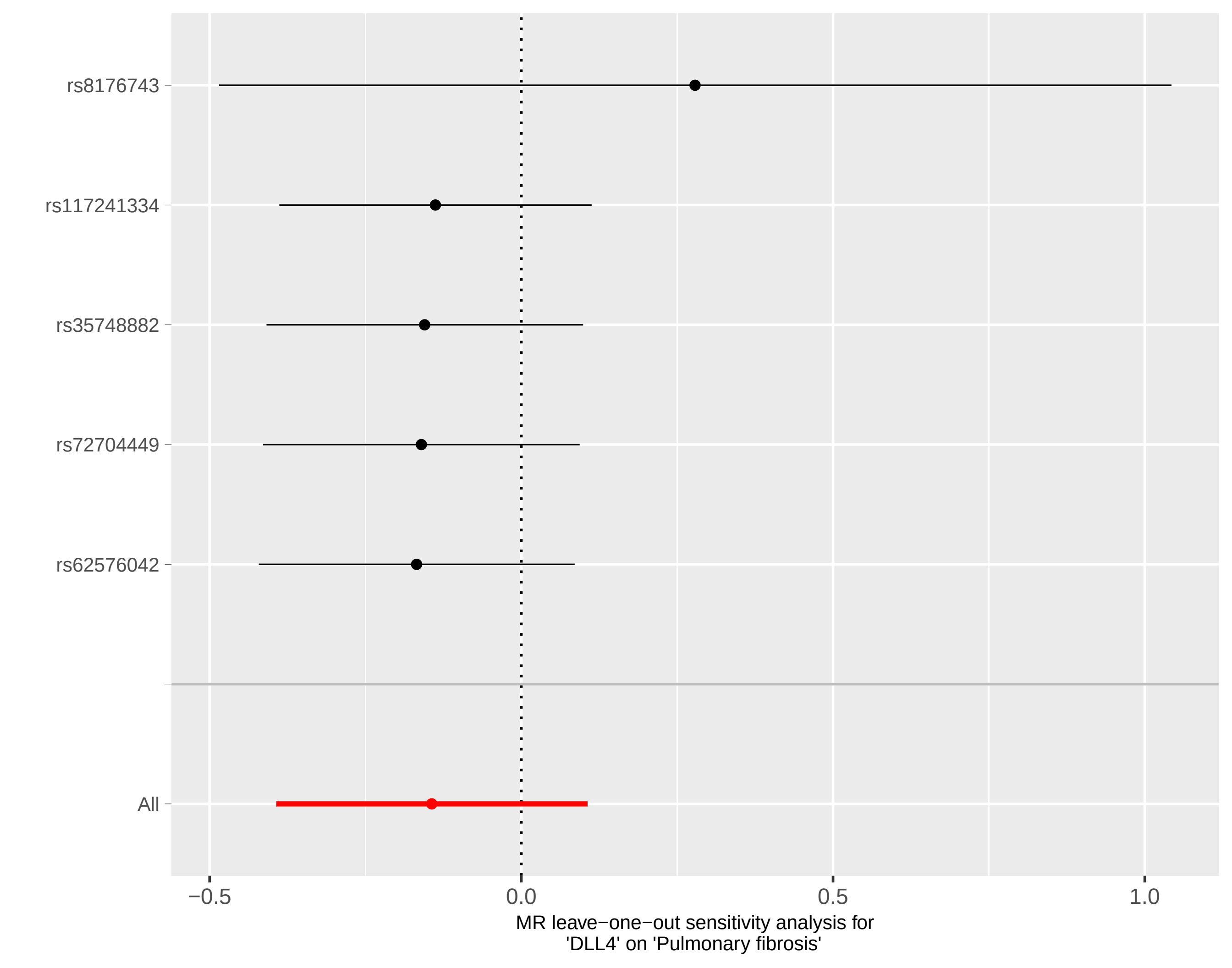
rs8176743
rs117241334
rs35748882
rs72704449
rs62576042
All
−0.5
0.0
0.5
1.0
MR leave−one−out sensitivity analysis for
'DLL4' on 'Pulmonary fibrosis'

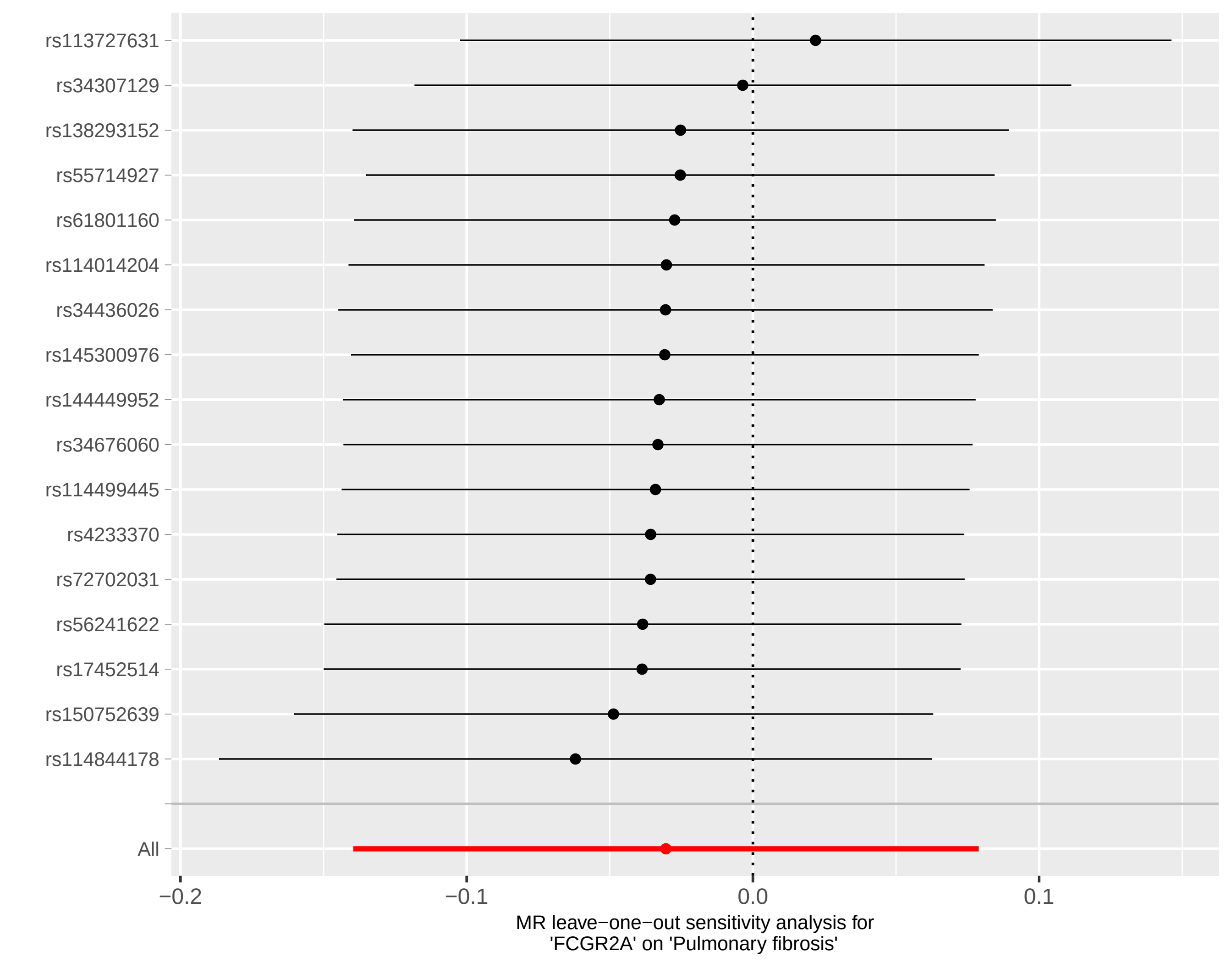

rs113727631
rs34307129
rs138293152
rs55714927
rs61801160
rs114014204
rs34436026
rs145300976
rs144449952
rs34676060
rs114499445
rs4233370
rs72702031
rs56241622
rs17452514
rs150752639
rs114844178
All
−0.2
−0.1
0.0
0.1
MR leave−one−out sensitivity analysis for
'FCGR2A' on 'Pulmonary fibrosis'

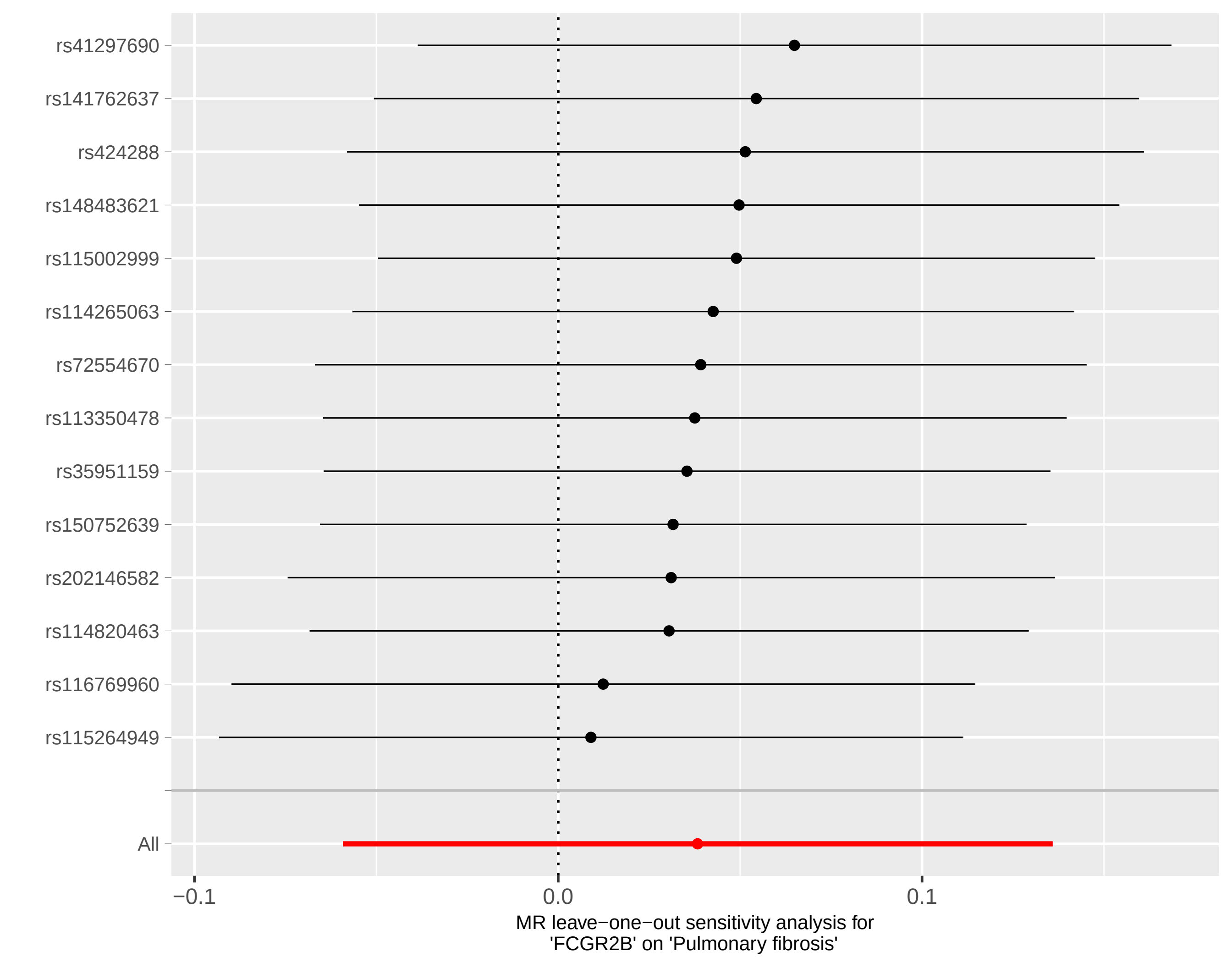
rs41297690
rs141762637
rs424288
rs148483621
rs115002999
rs114265063
rs72554670
rs113350478
rs35951159
rs150752639
rs202146582
rs114820463
rs116769960
rs115264949
All
−0.1
0.0
0.1
MR leave−one−out sensitivity analysis for
'FCGR2B' on 'Pulmonary fibrosis'

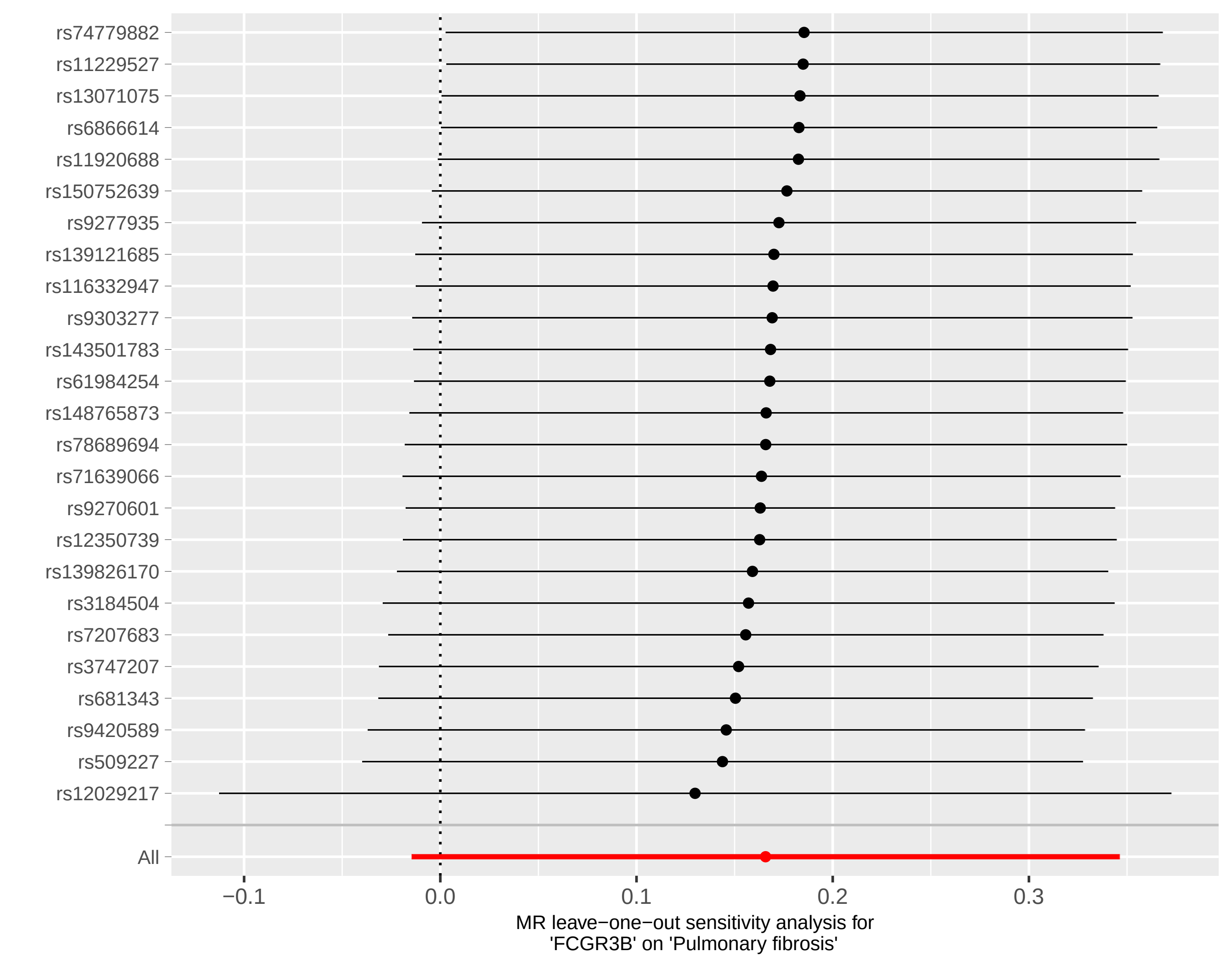
rs74779882
rs11229527
rs13071075
rs6866614
rs11920688
rs150752639
rs9277935
rs139121685
rs116332947
rs9303277
rs143501783
rs61984254
rs148765873
rs78689694
rs71639066
rs9270601
rs12350739
rs139826170
rs3184504
rs7207683
rs3747207
rs681343
rs9420589
rs509227
rs12029217
All
−0.1
0.0
0.1
0.2
0.3
MR leave−one−out sensitivity analysis for
'FCGR3B' on 'Pulmonary fibrosis'

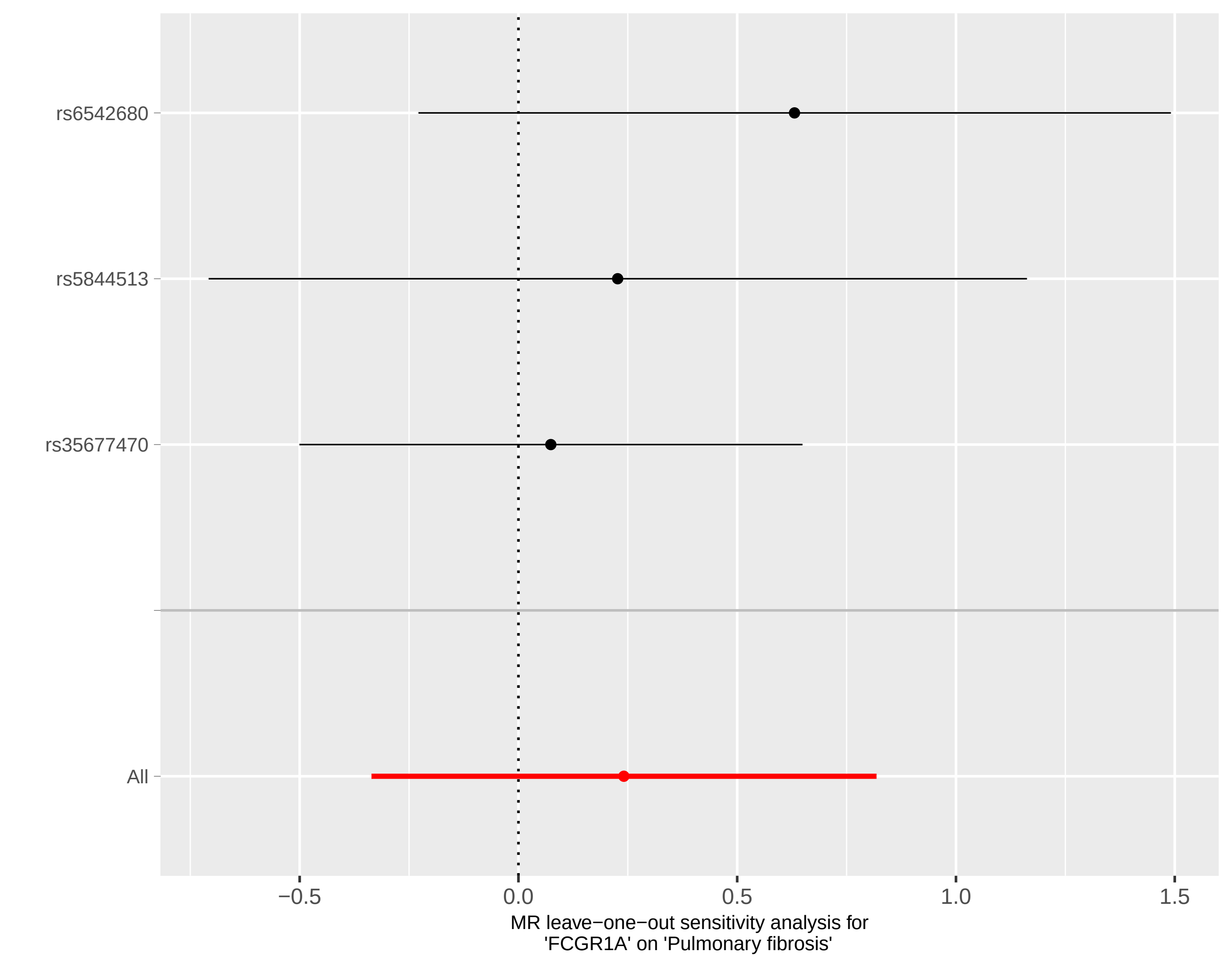
rs6542680
rs5844513
rs35677470
All
−0.5
0.0
0.5
1.0
1.5
MR leave−one−out sensitivity analysis for
'FCGR1A' on 'Pulmonary fibrosis'

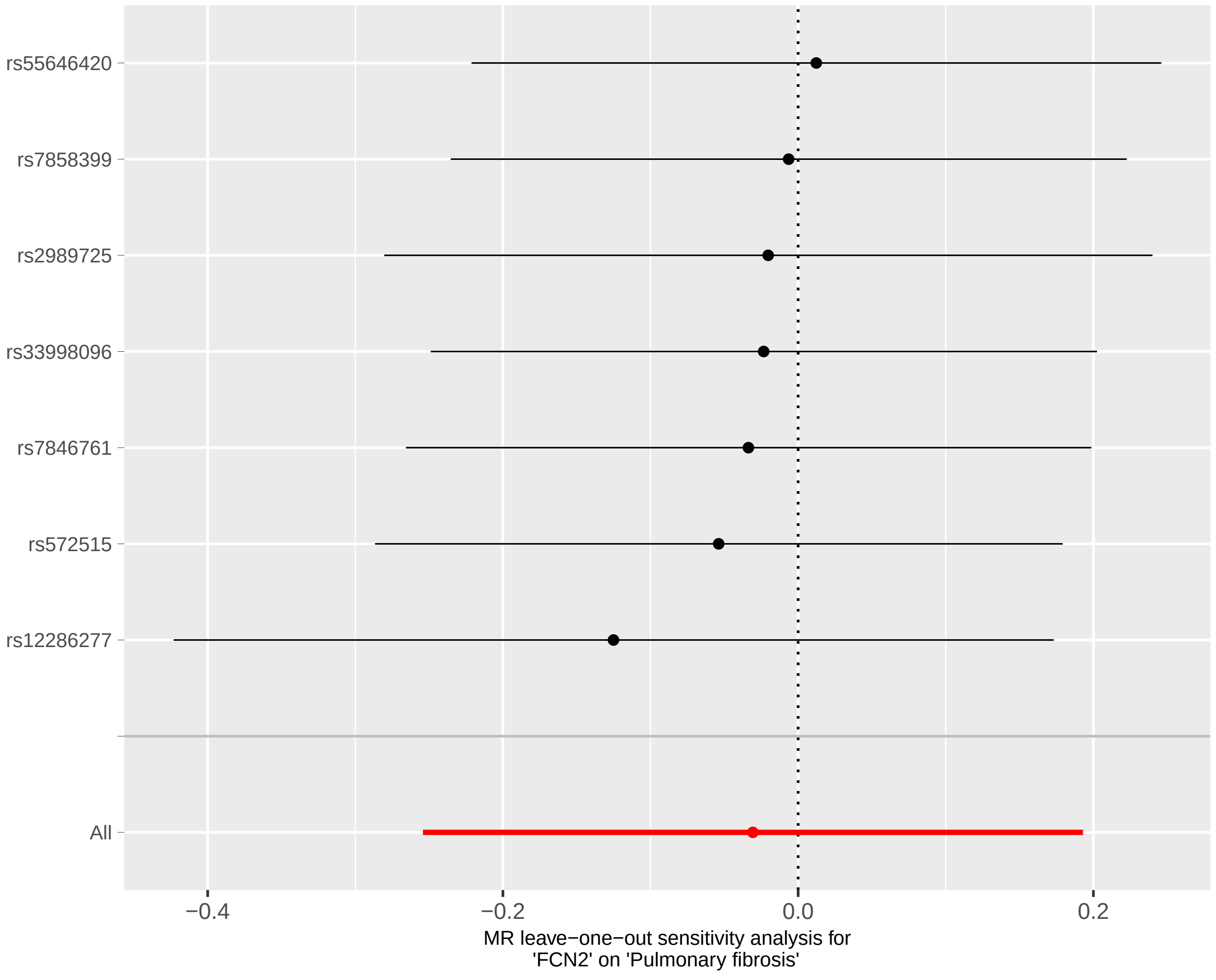

rs55646420
rs7858399
rs2989725
rs33998096
rs7846761
rs572515
rs12286277
All
−0.4
−0.2
0.0
0.2
MR leave−one−out sensitivity analysis for
'FCN2' on 'Pulmonary fibrosis'

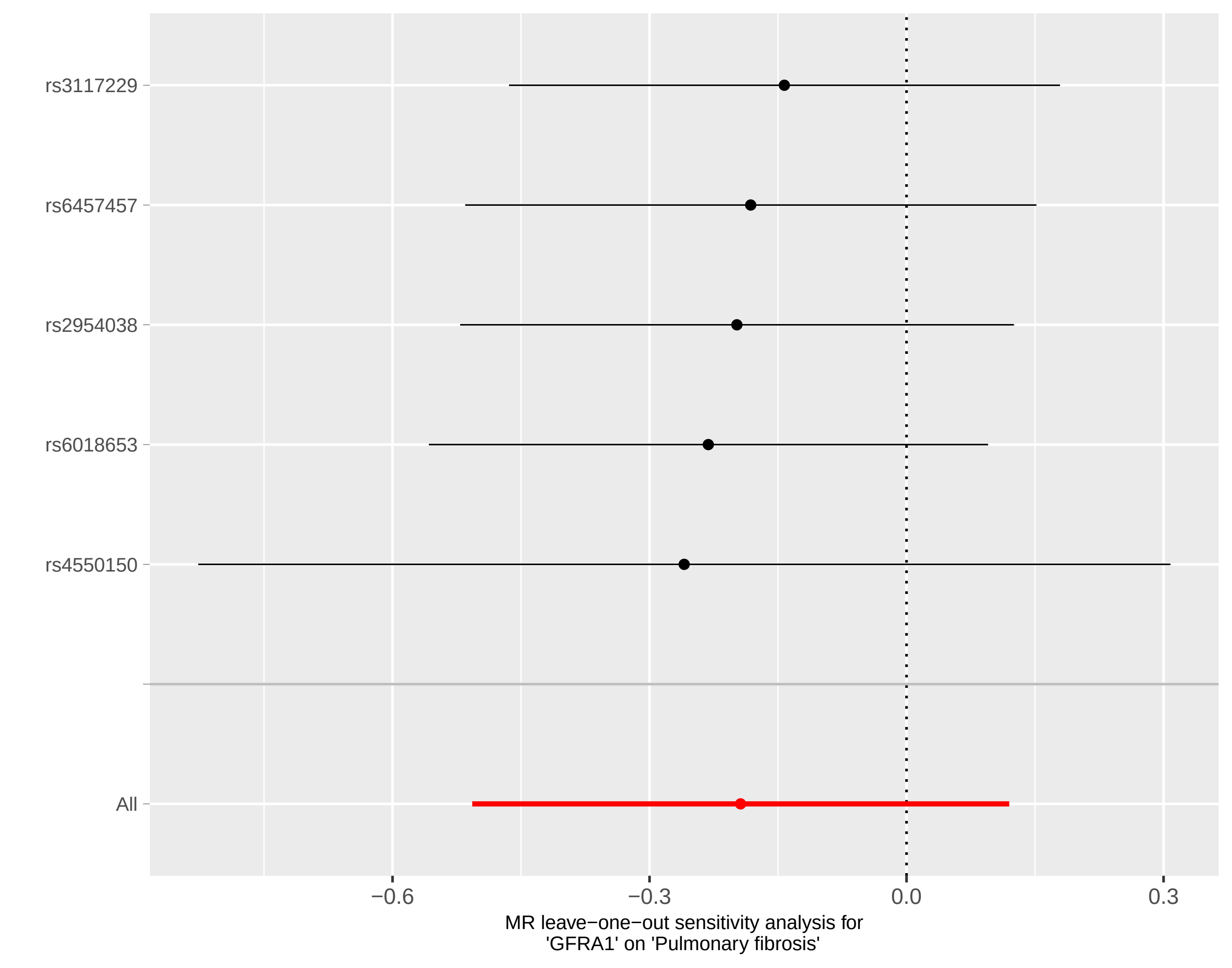

rs3117229
rs6457457
rs2954038
rs6018653
rs4550150
All
−0.6
−0.3
0.0
0.3
MR leave−one−out sensitivity analysis for
'GFRA1' on 'Pulmonary fibrosis'

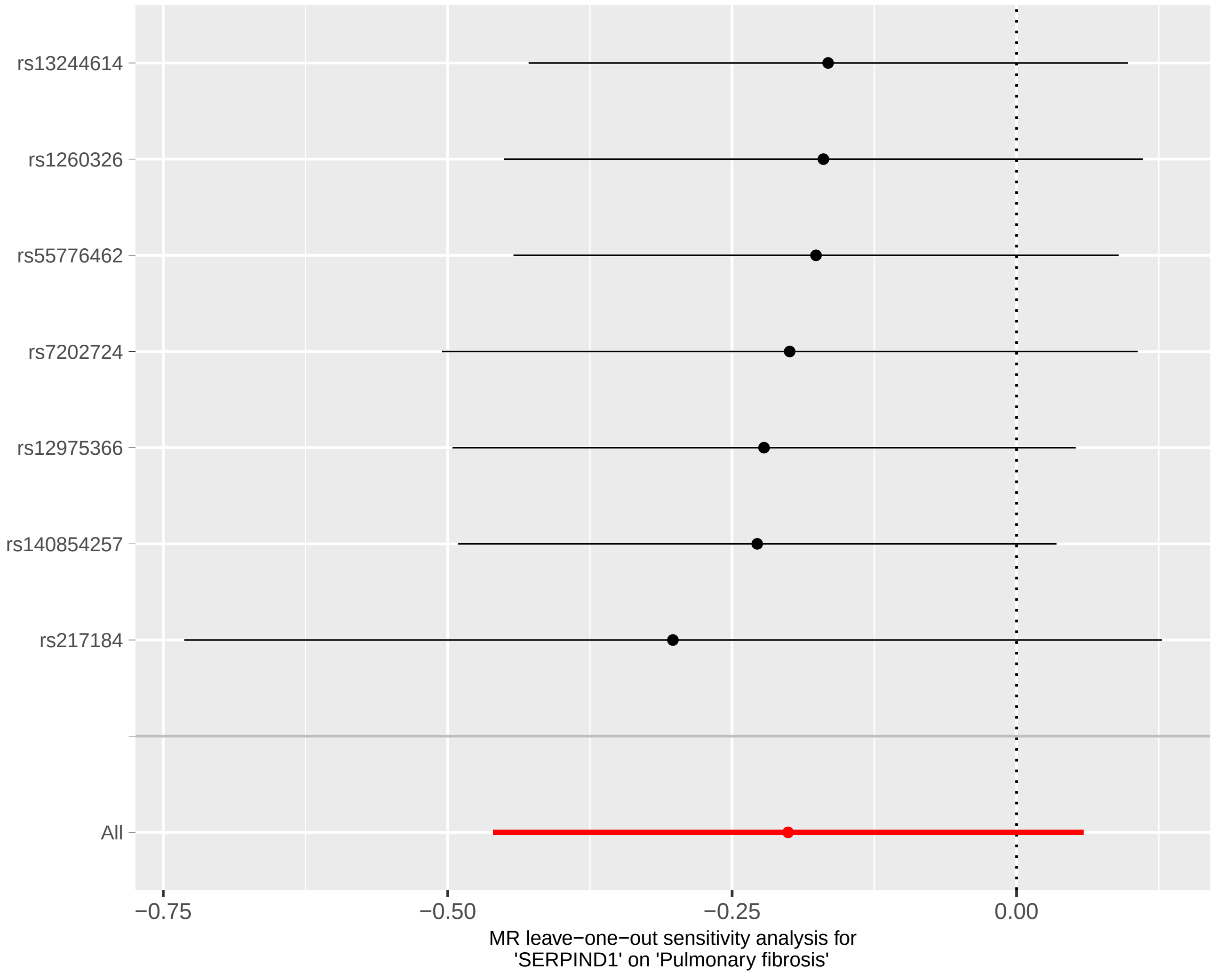
rs13244614
rs1260326
rs55776462
rs7202724
rs12975366
rs140854257
rs217184
All
−0.75
−0.50
−0.25
0.00
MR leave−one−out sensitivity analysis for
'SERPIND1' on 'Pulmonary fibrosis'

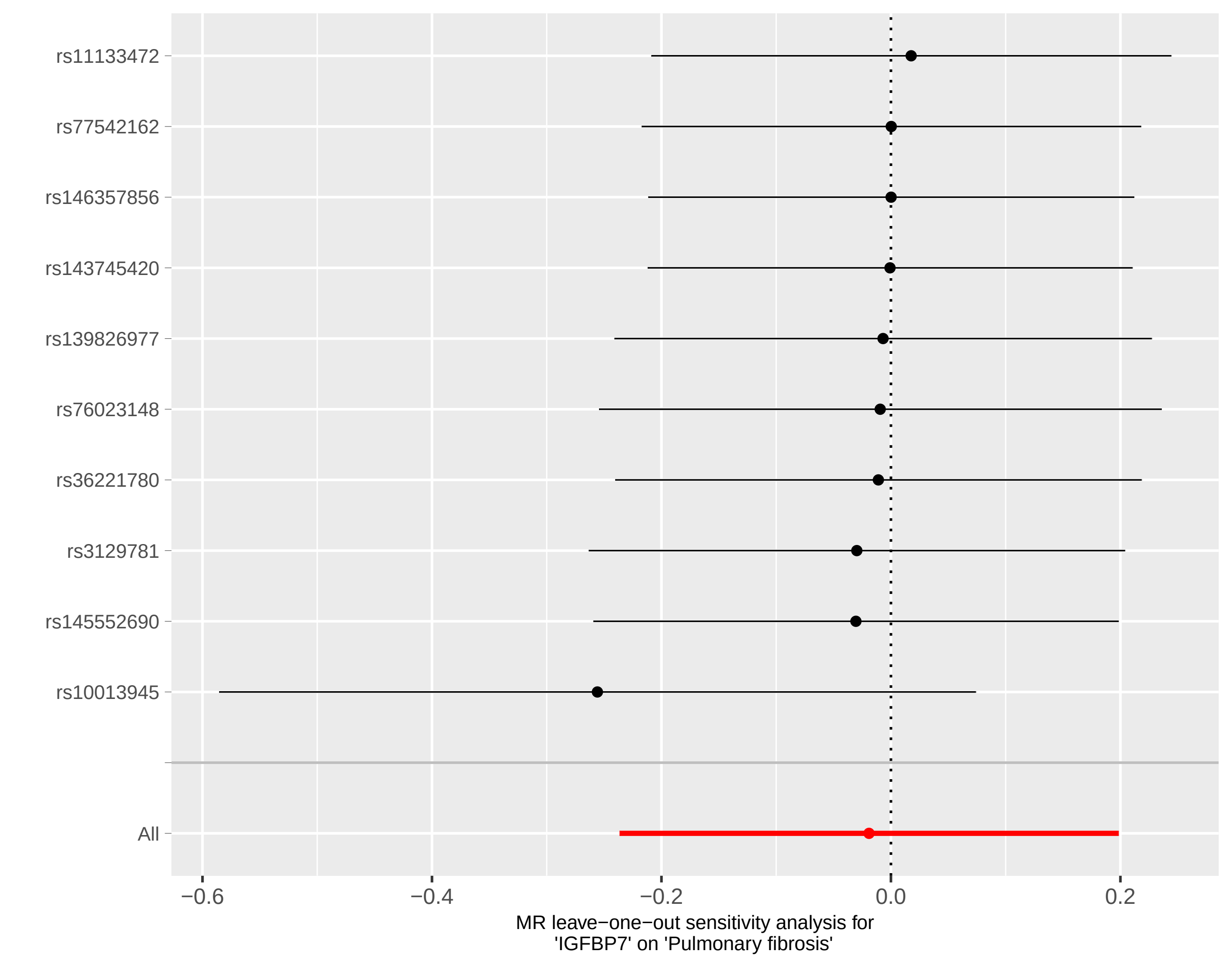
rs11133472
rs77542162
rs146357856
rs143745420
rs139826977
rs76023148
rs36221780
rs3129781
rs145552690
rs10013945
All
−0.6
−0.4
−0.2
0.0
0.2
MR leave−one−out sensitivity analysis for
'IGFBP7' on 'Pulmonary fibrosis'

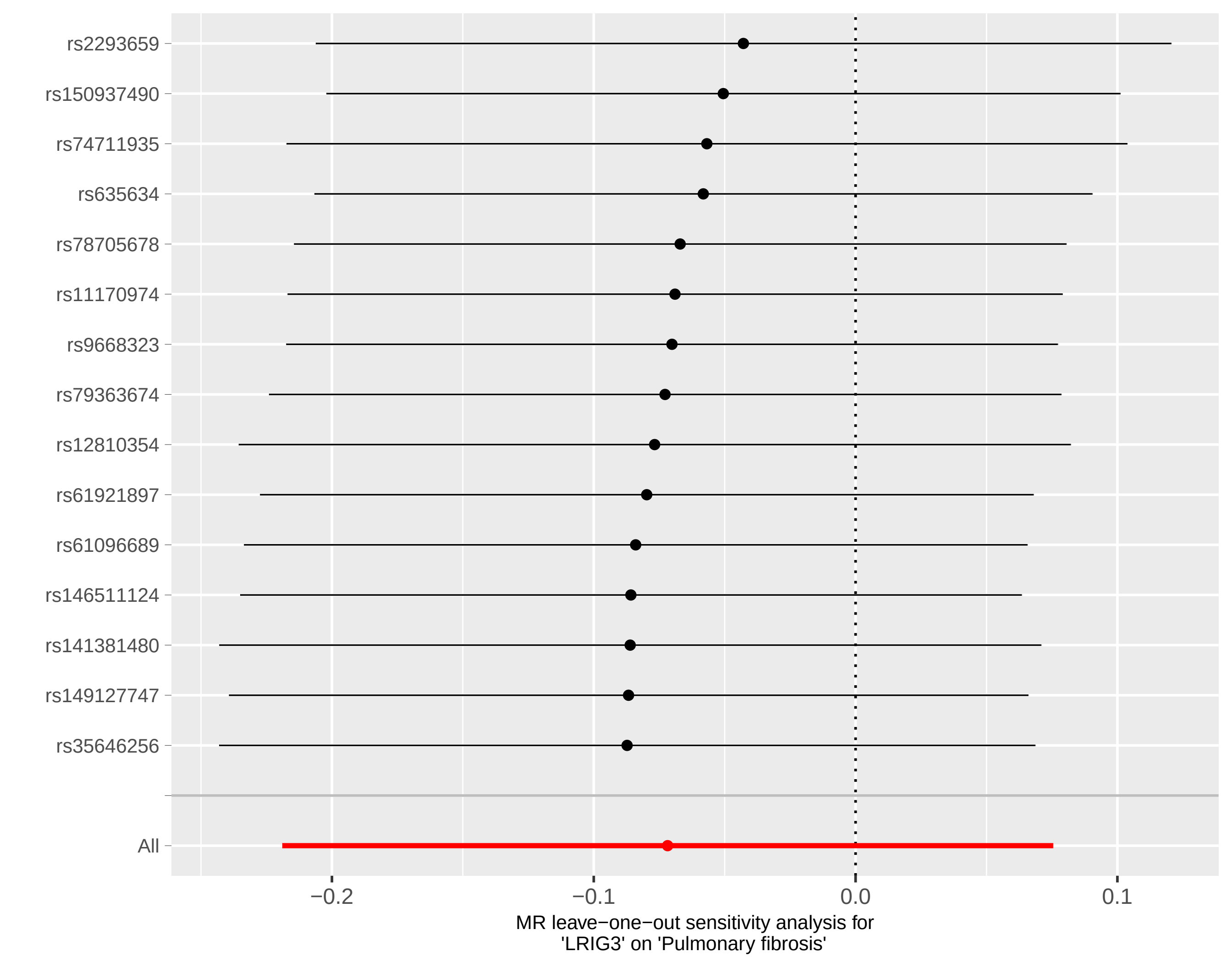
rs2293659
rs150937490
rs74711935
rs635634
rs78705678
rs11170974
rs9668323
rs79363674
rs12810354
rs61921897
rs61096689
rs146511124
rs141381480
rs149127747
rs35646256
All
−0.2
−0.1
0.0
0.1
MR leave−one−out sensitivity analysis for
'LRIG3' on 'Pulmonary fibrosis'

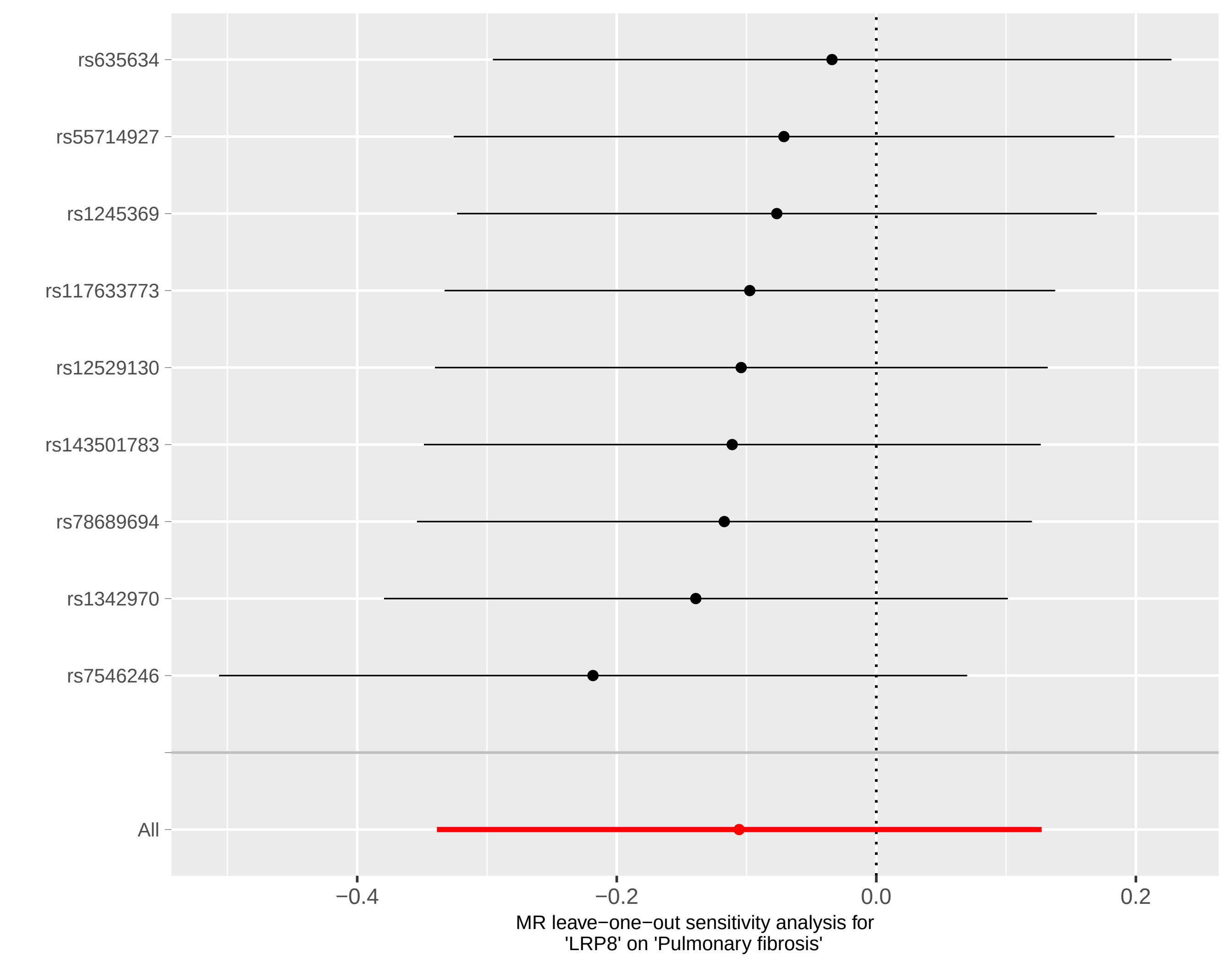
rs635634
rs55714927
rs1245369
rs117633773
rs12529130
rs143501783
rs78689694
rs1342970
rs7546246
All
−0.4
−0.2
0.0
0.2
MR leave−one−out sensitivity analysis for
'LRP8' on 'Pulmonary fibrosis'

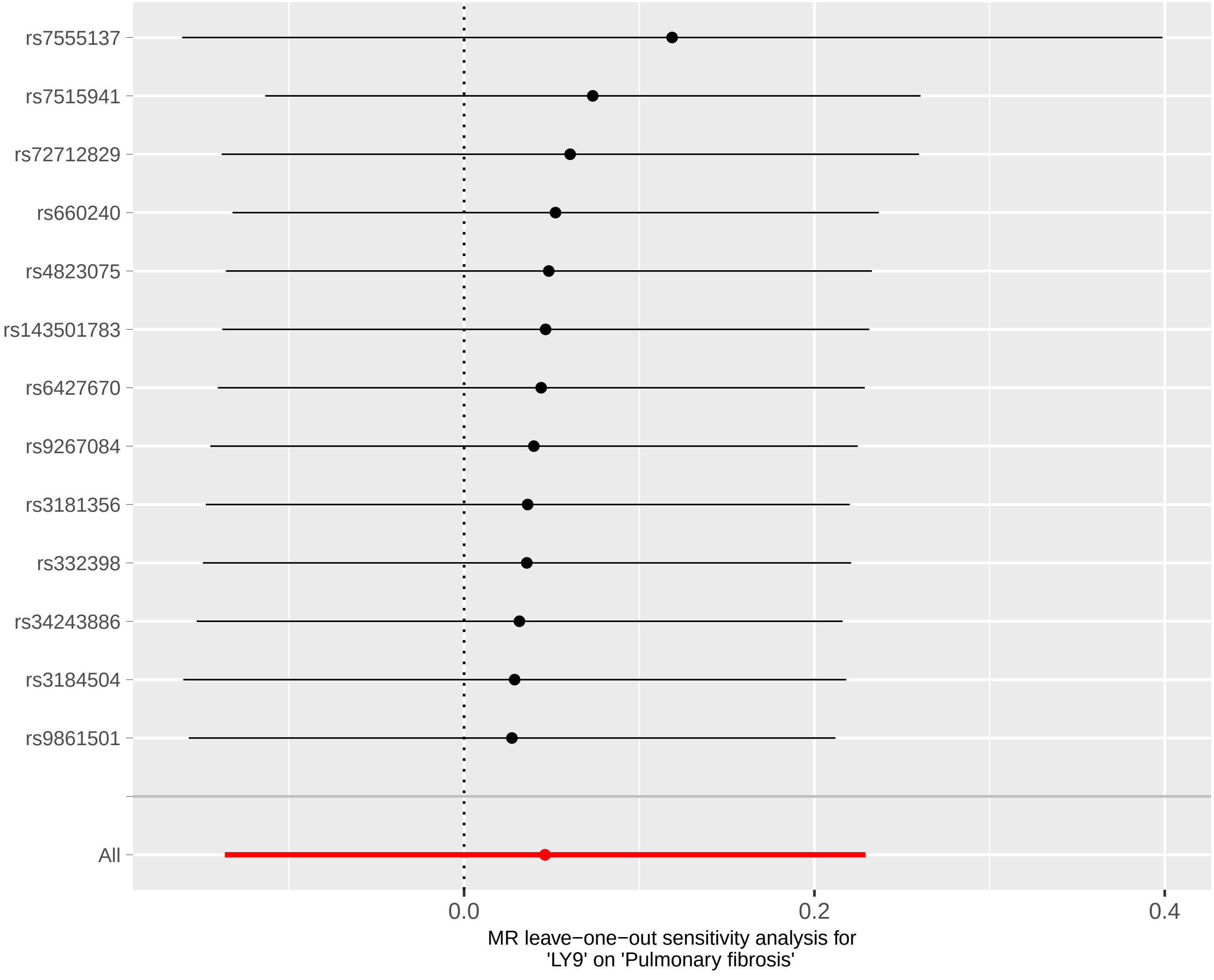
rs7555137
rs7515941
rs72712829
rs660240
rs4823075
rs143501783
rs6427670
rs9267084
rs3181356
rs332398
rs34243886
rs3184504
rs9861501
All
0.0
0.2
0.4
MR leave−one−out sensitivity analysis for
'LY9' on 'Pulmonary fibrosis'

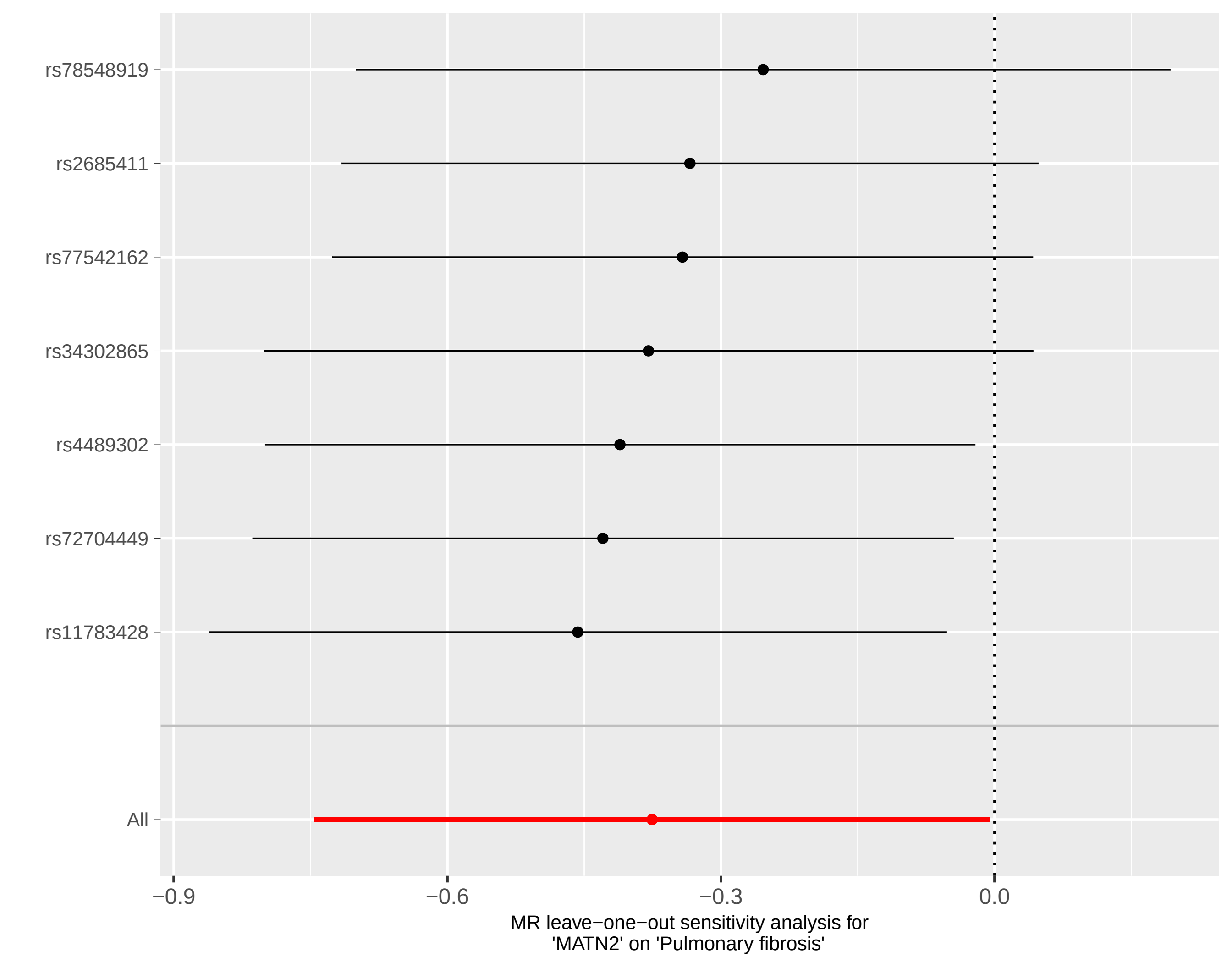
rs78548919
rs2685411
rs77542162
rs34302865
rs4489302
rs72704449
rs11783428
All
−0.9
−0.6
−0.3
0.0
MR leave−one−out sensitivity analysis for
'MATN2' on 'Pulmonary fibrosis'

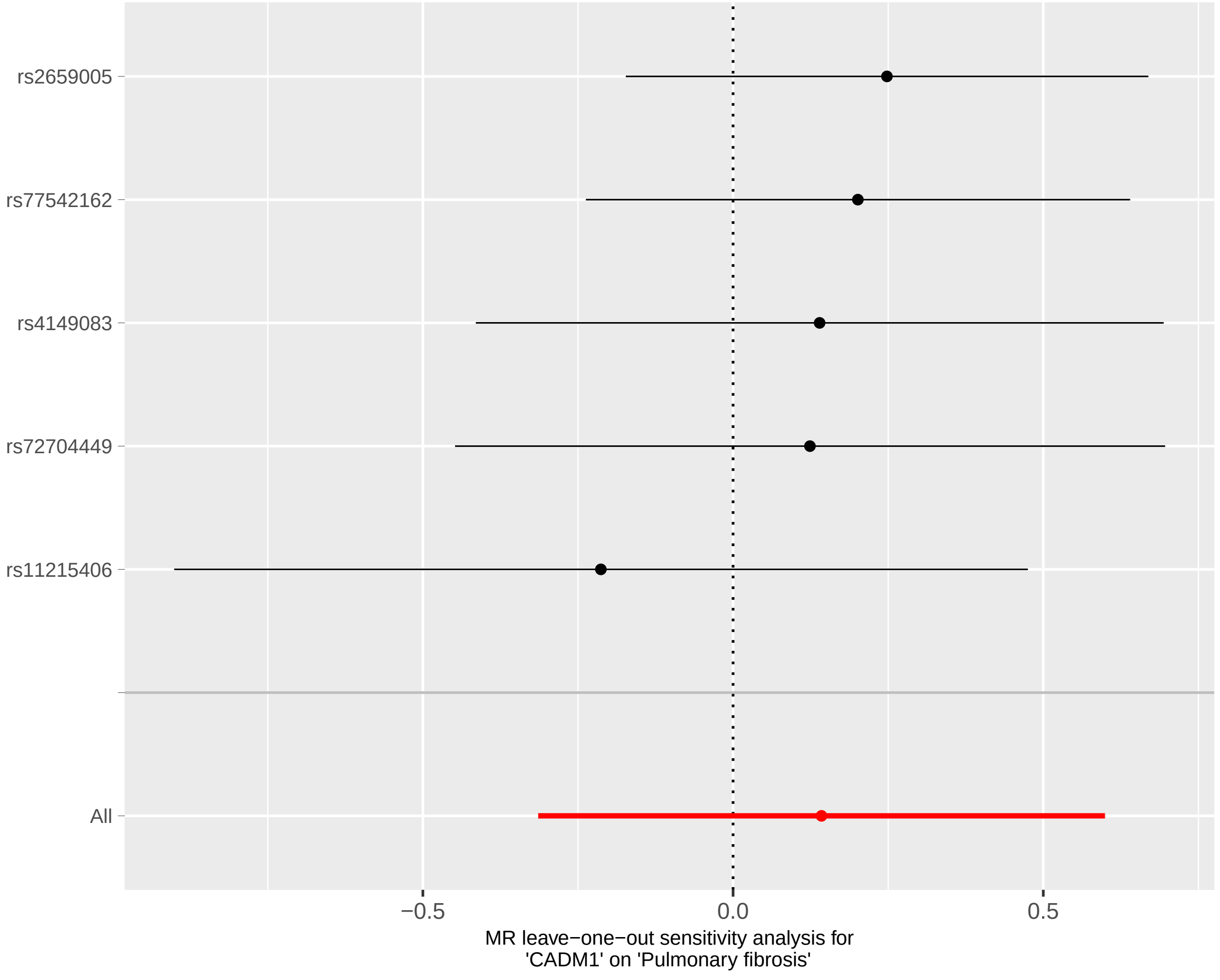
rs2659005
rs77542162
rs4149083
rs72704449
rs11215406
All
−0.5
0.0
0.5
MR leave−one−out sensitivity analysis for
'CADM1' on 'Pulmonary fibrosis'

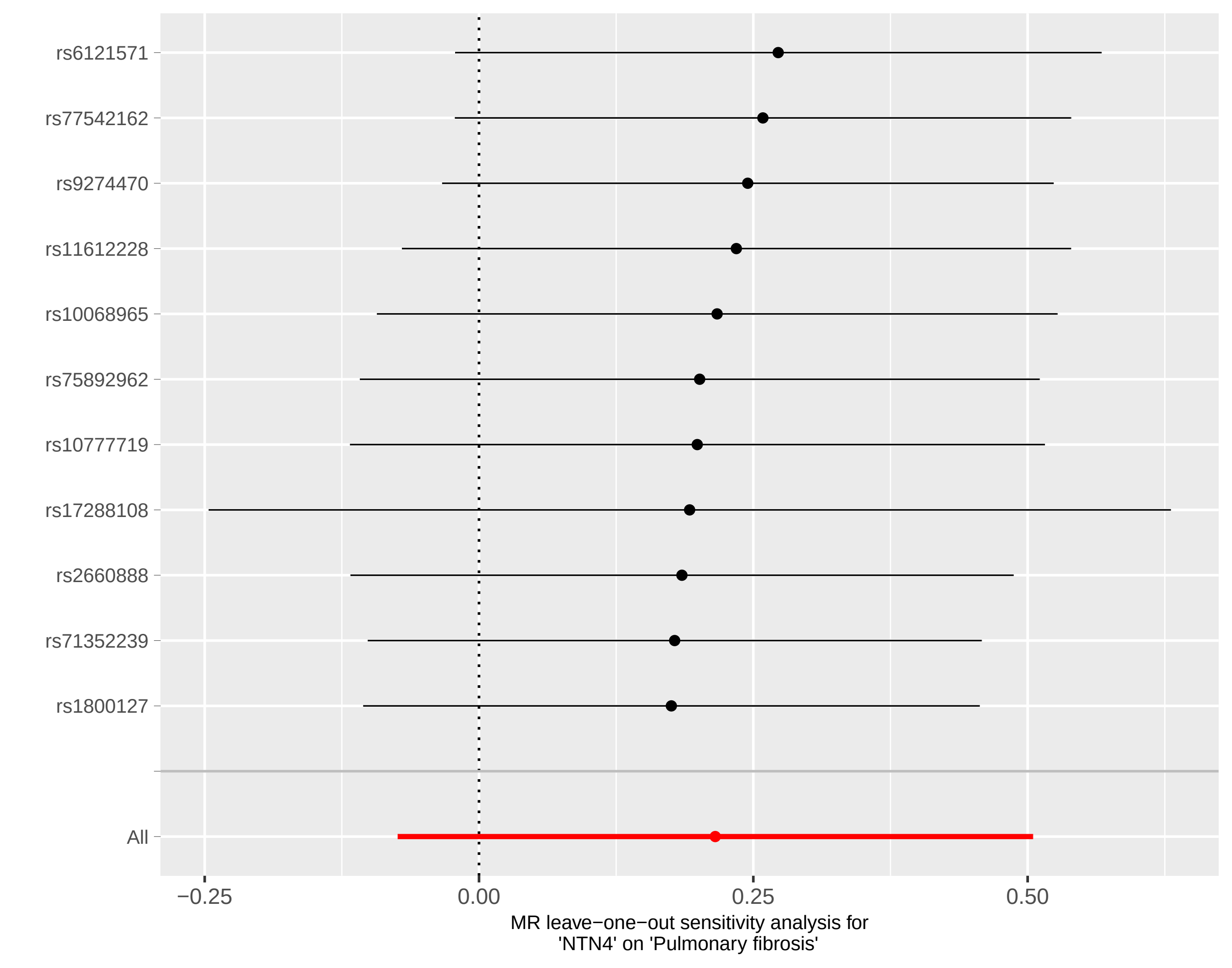
rs6121571
rs77542162
rs9274470
rs11612228
rs10068965
rs75892962
rs10777719
rs17288108
rs2660888
rs71352239
rs1800127
All
−0.25
0.00
0.25
0.50
MR leave−one−out sensitivity analysis for
'NTN4' on 'Pulmonary fibrosis'

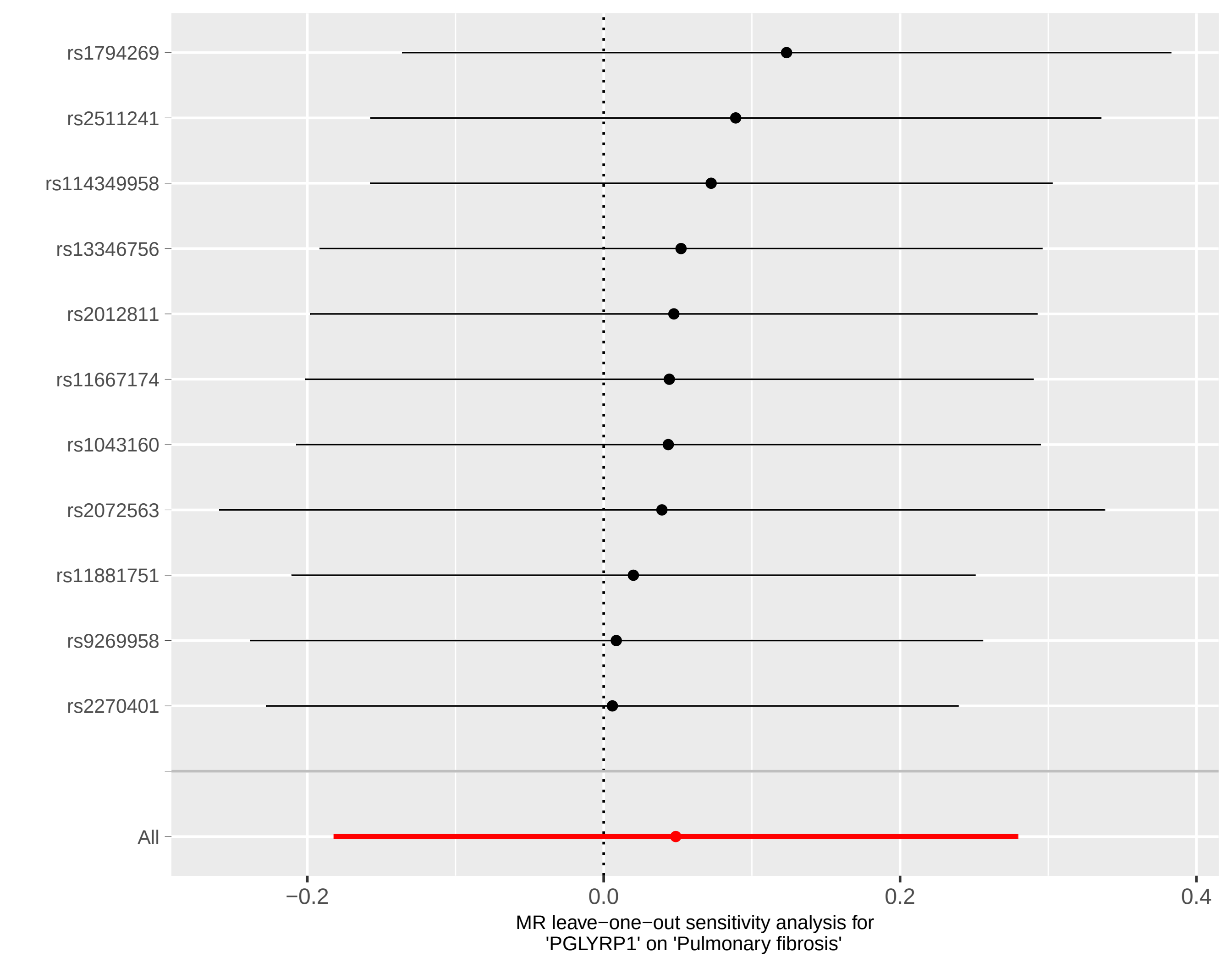
rs1794269
rs2511241
rs114349958
rs13346756
rs2012811
rs11667174
rs1043160
rs2072563
rs11881751
rs9269958
rs2270401
All
−0.2
0.0
0.2
0.4
MR leave−one−out sensitivity analysis for
'PGLYRP1' on 'Pulmonary fibrosis'

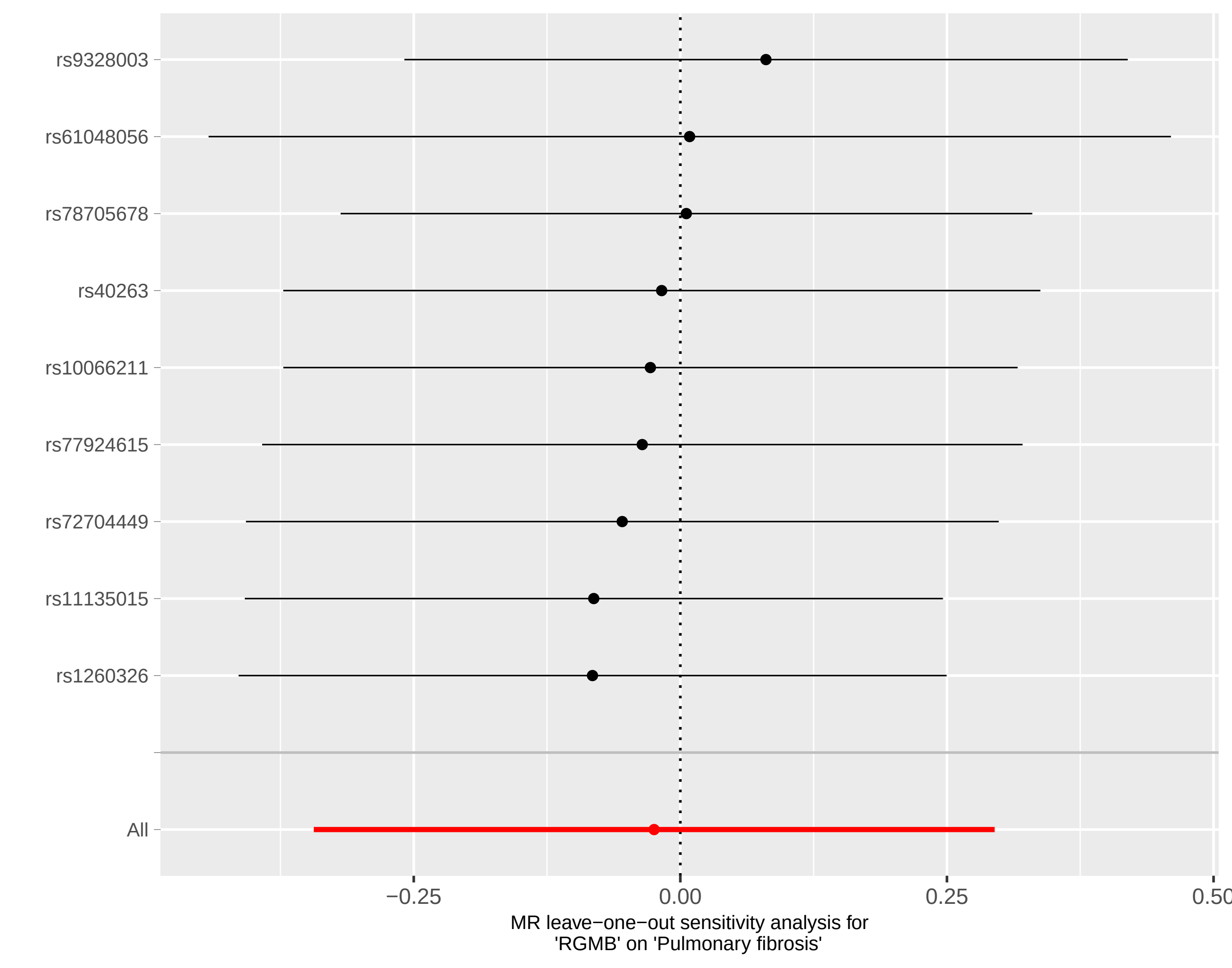
rs9328003
rs61048056
rs78705678
rs40263
rs10066211
rs77924615
rs72704449
rs11135015
rs1260326
All
−0.25
0.00
0.25
0.50
MR leave−one−out sensitivity analysis for
'RGMB' on 'Pulmonary fibrosis'

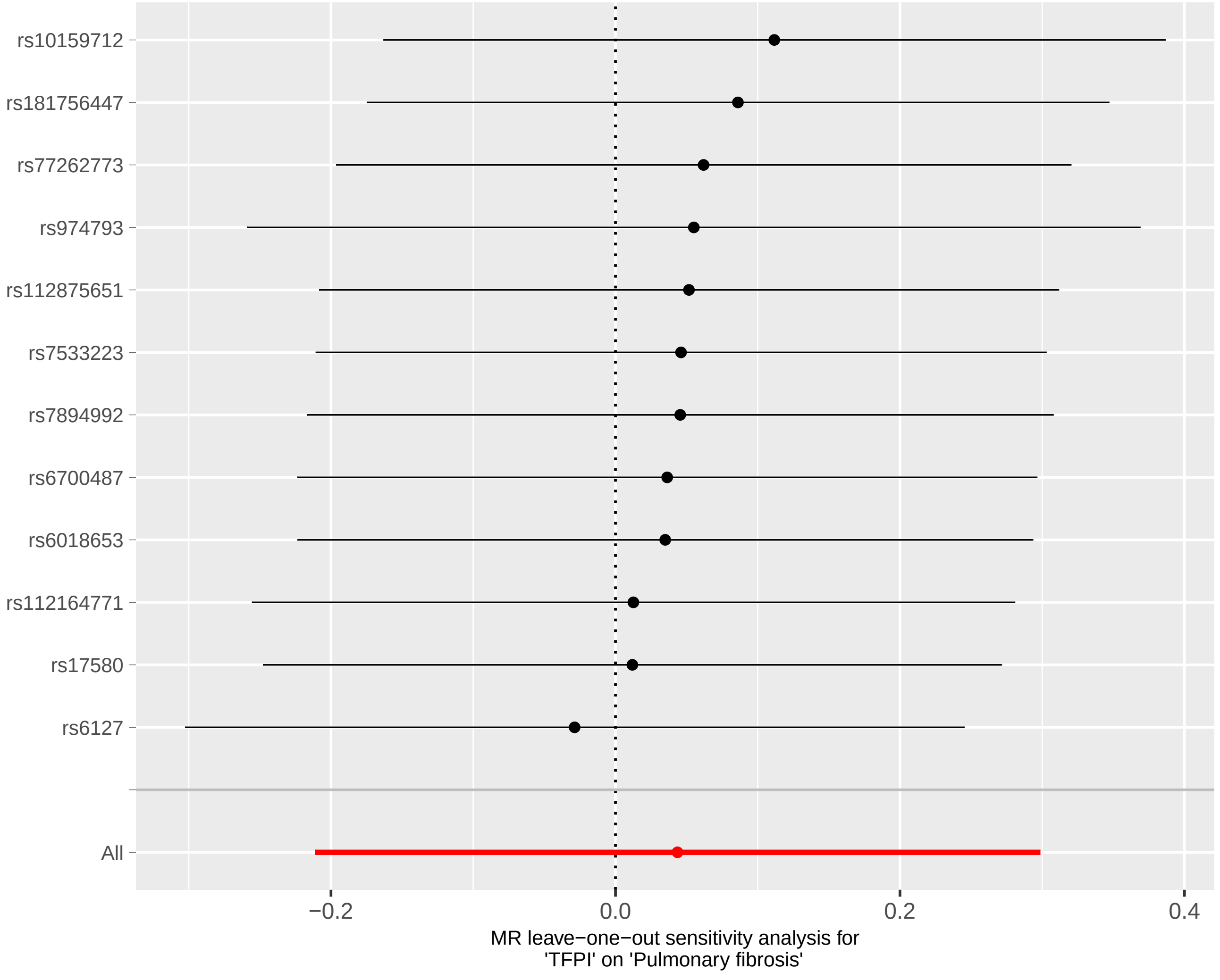
rs10159712
rs181756447
rs77262773
rs974793
rs112875651
rs7533223
rs7894992
rs6700487
rs6018653
rs112164771
rs17580
rs6127
All
−0.2
0.0
0.2
0.4
MR leave−one−out sensitivity analysis for
'TFPI' on 'Pulmonary fibrosis'

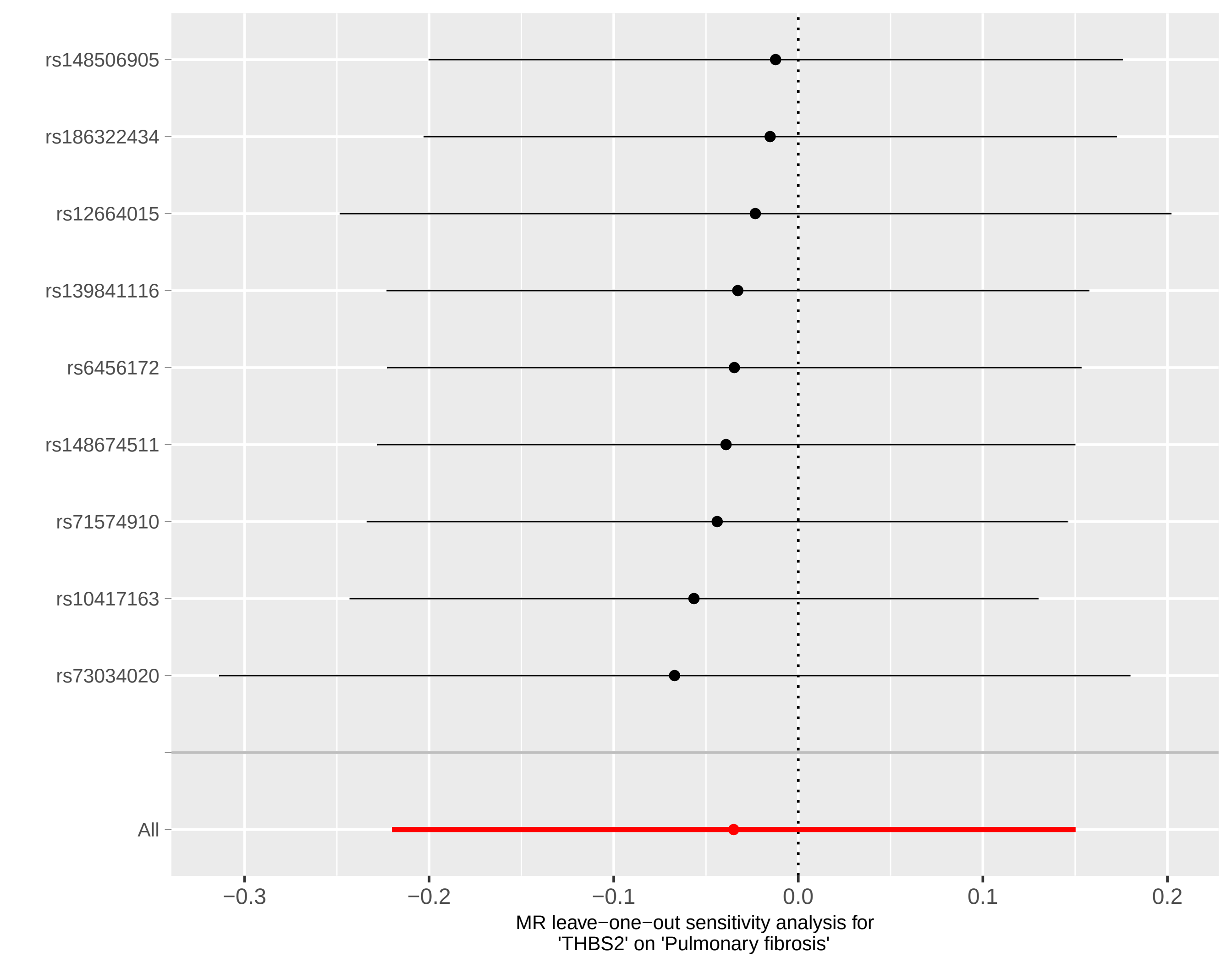
rs148506905
rs186322434
rs12664015
rs139841116
rs6456172
rs148674511
rs71574910
rs10417163
rs73034020
All
−0.3
−0.2
−0.1
0.0
0.1
0.2
MR leave−one−out sensitivity analysis for
'THBS2' on 'Pulmonary fibrosis'

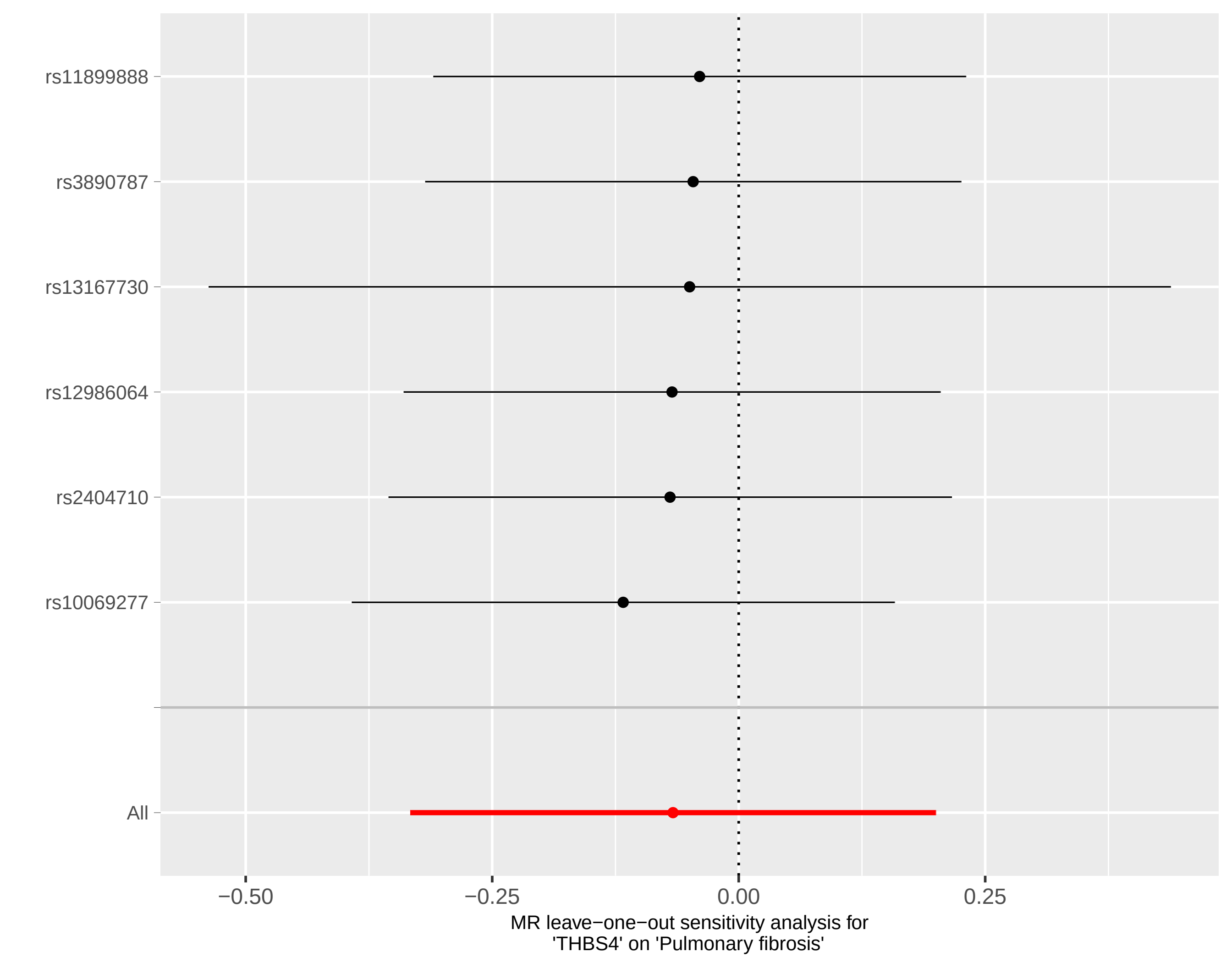

rs11899888
rs3890787
rs13167730
rs12986064
rs2404710
rs10069277
All
−0.50
−0.25
0.00
0.25
MR leave−one−out sensitivity analysis for
'THBS4' on 'Pulmonary fibrosis'

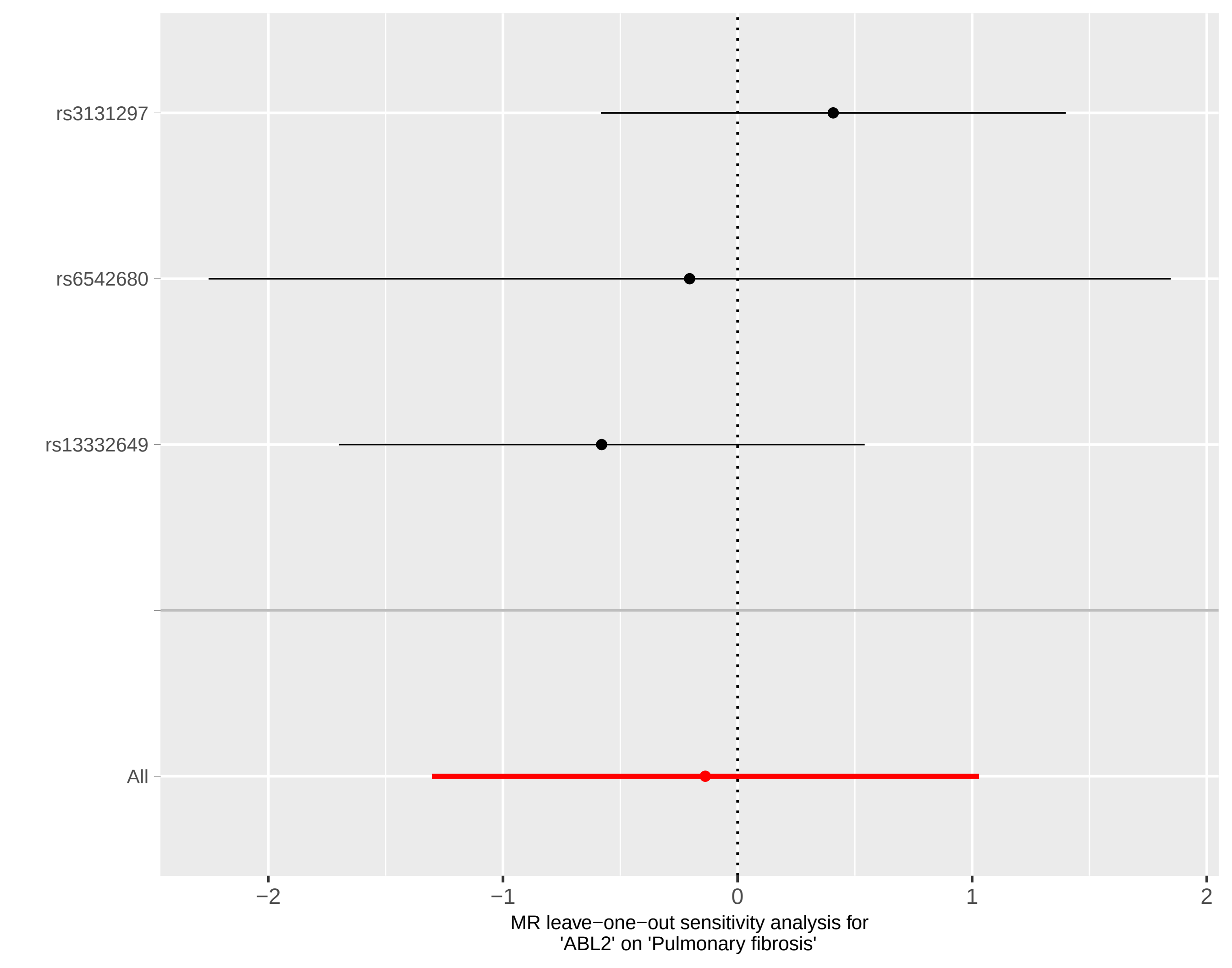
rs3131297
rs6542680
rs13332649
All
−2
−1
0
1
2
MR leave−one−out sensitivity analysis for
'ABL2' on 'Pulmonary fibrosis'

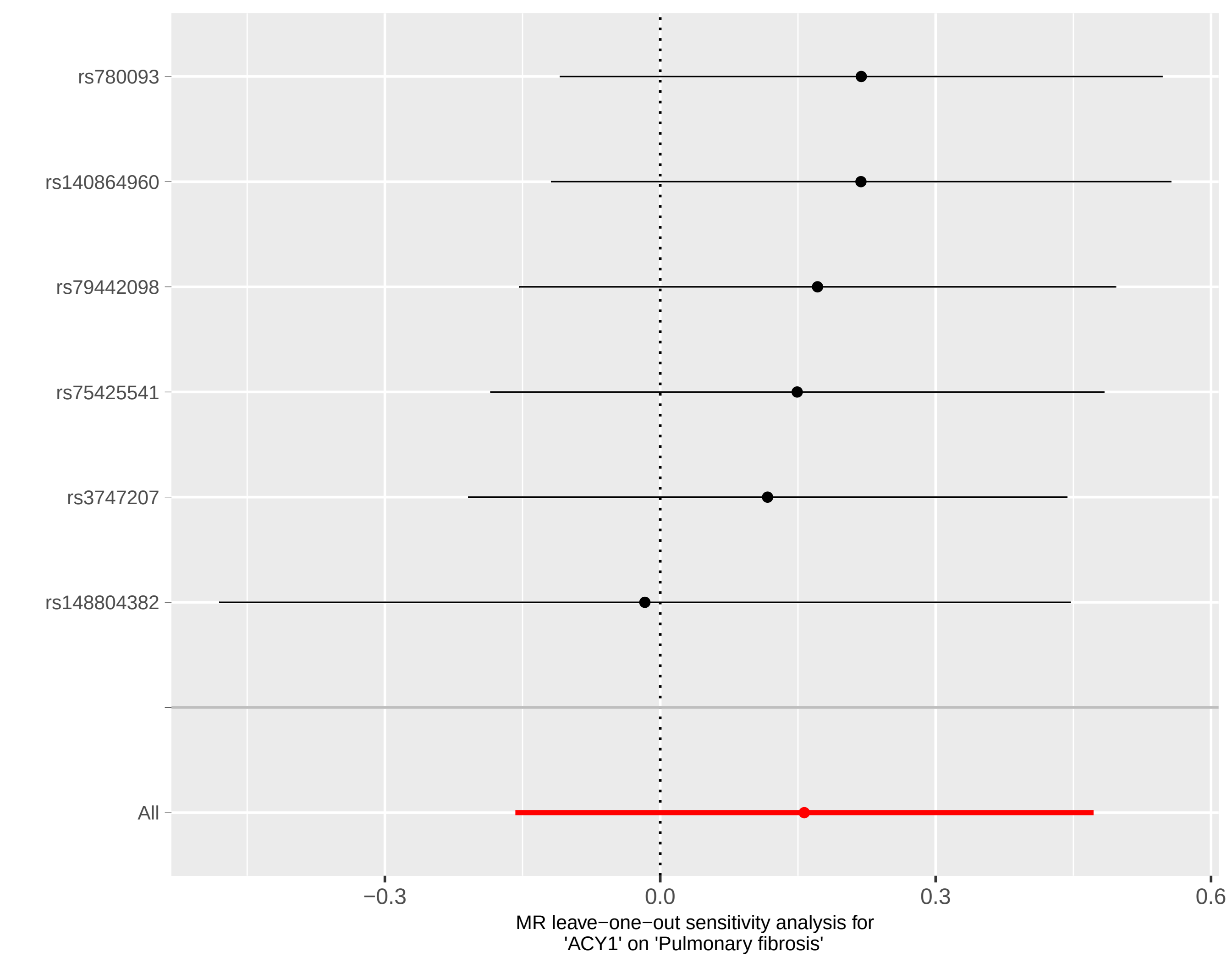
rs780093
rs140864960
rs79442098
rs75425541
rs3747207
rs148804382
All
−0.3
0.0
0.3
0.6
MR leave−one−out sensitivity analysis for
'ACY1' on 'Pulmonary fibrosis'

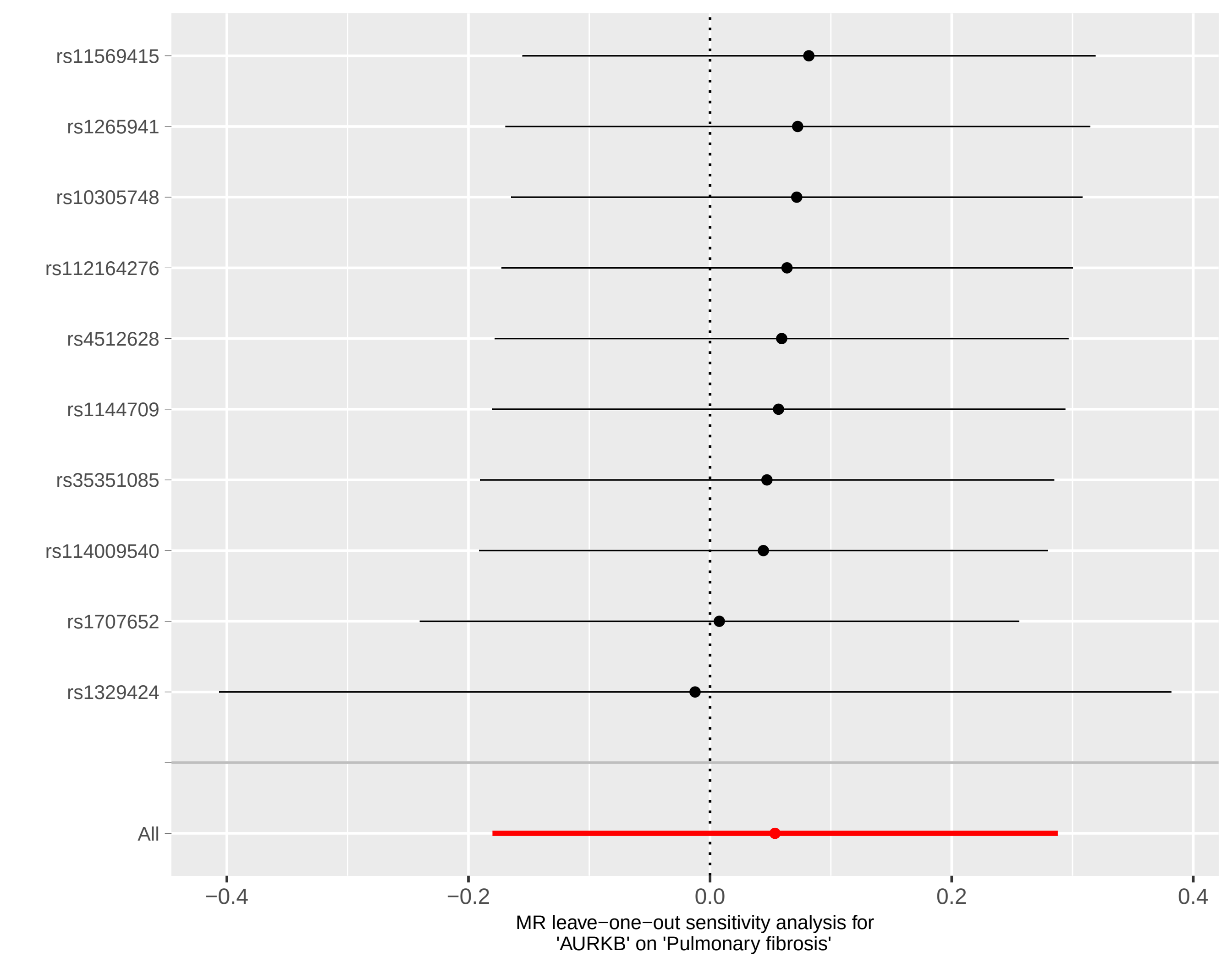
rs11569415
rs1265941
rs10305748
rs112164276
rs4512628
rs1144709
rs35351085
rs114009540
rs1707652
rs1329424
All
−0.4
−0.2
0.0
0.2
0.4
MR leave−one−out sensitivity analysis for
'AURKB' on 'Pulmonary fibrosis'

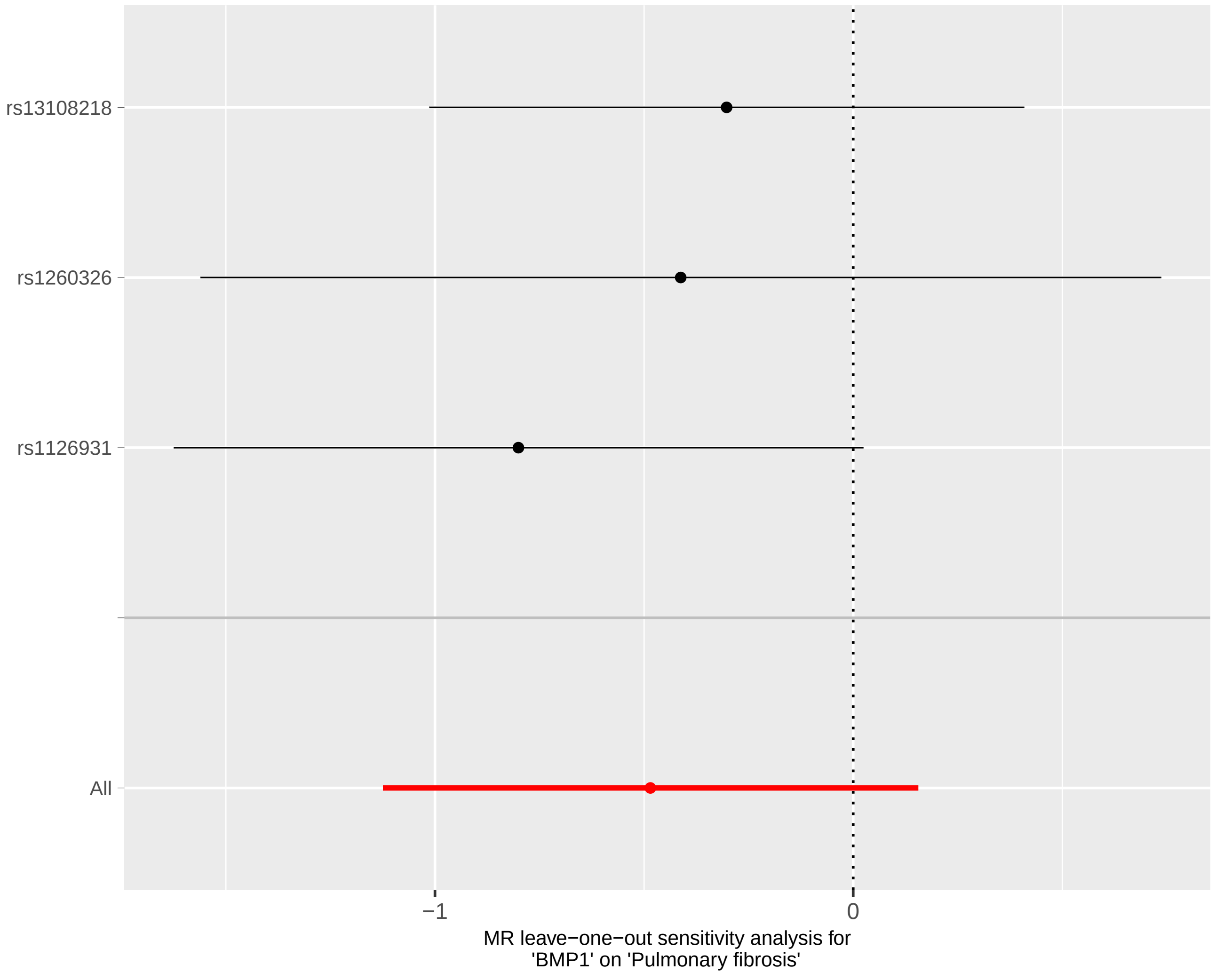
rs13108218
rs1260326
rs1126931
All
−1
0
MR leave−one−out sensitivity analysis for
'BMP1' on 'Pulmonary fibrosis'

Insufficient number of SNPs

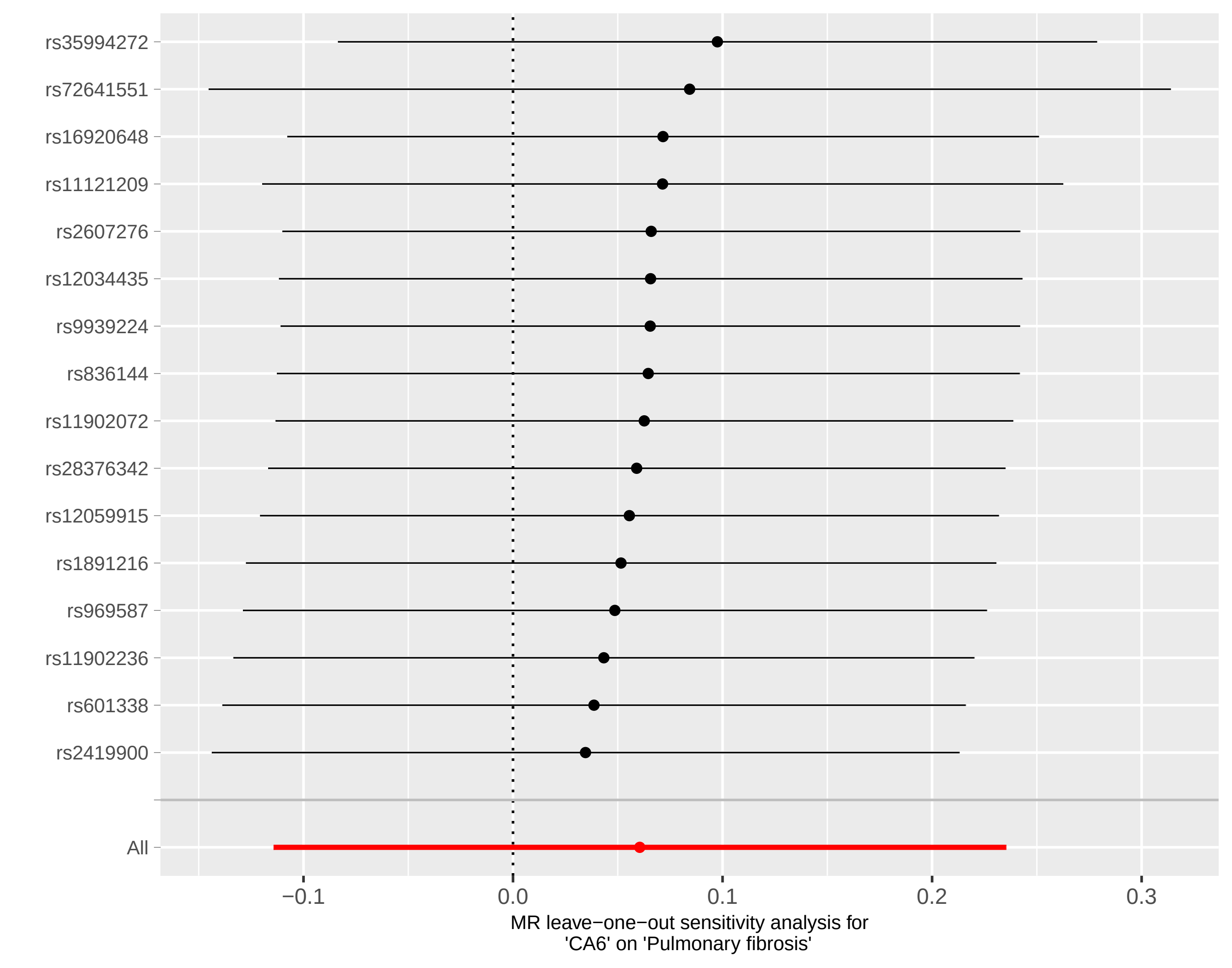

rs35994272
rs72641551
rs16920648
rs11121209
rs2607276
rs12034435
rs9939224
rs836144
rs11902072
rs28376342
rs12059915
rs1891216
rs969587
rs11902236
rs601338
rs2419900
All
−0.1
0.0
0.1
0.2
0.3
MR leave−one−out sensitivity analysis for
'CA6' on 'Pulmonary fibrosis'

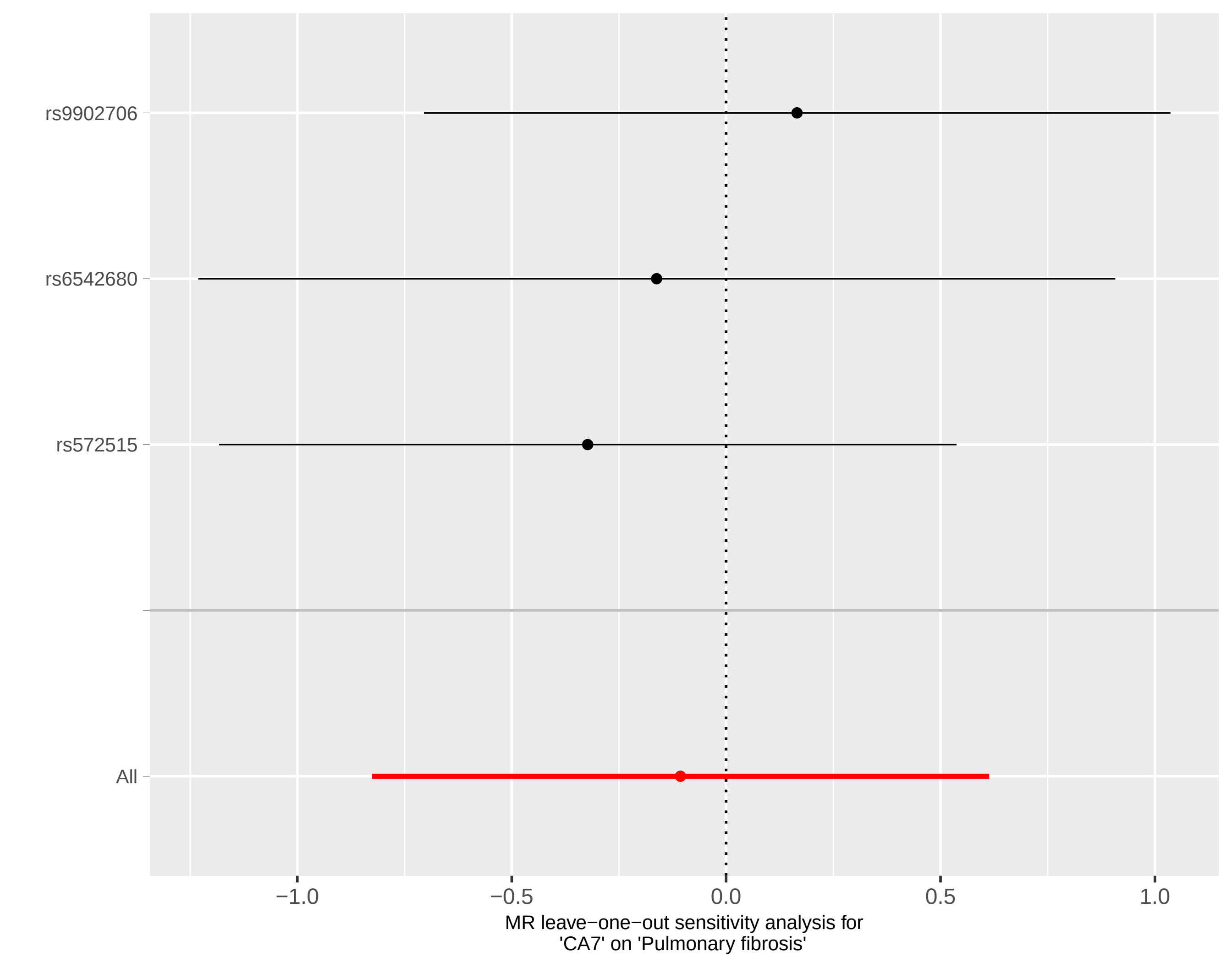
rs9902706
rs6542680
rs572515
All
−1.0
−0.5
0.0
0.5
1.0
MR leave−one−out sensitivity analysis for
'CA7' on 'Pulmonary fibrosis'

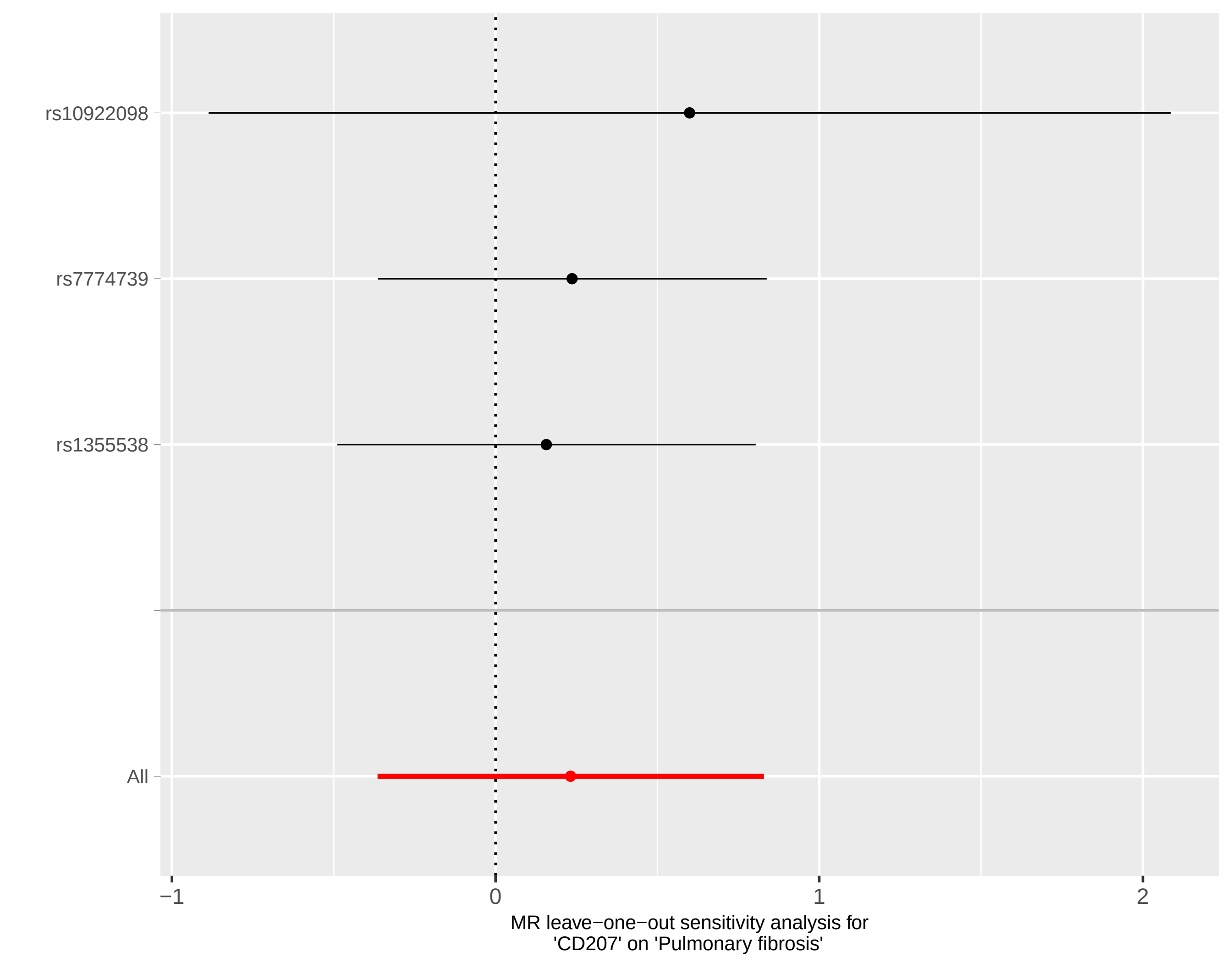
rs10922098
rs7774739
rs1355538
All
−1
0
1
2
MR leave−one−out sensitivity analysis for
'CD207' on 'Pulmonary fibrosis'

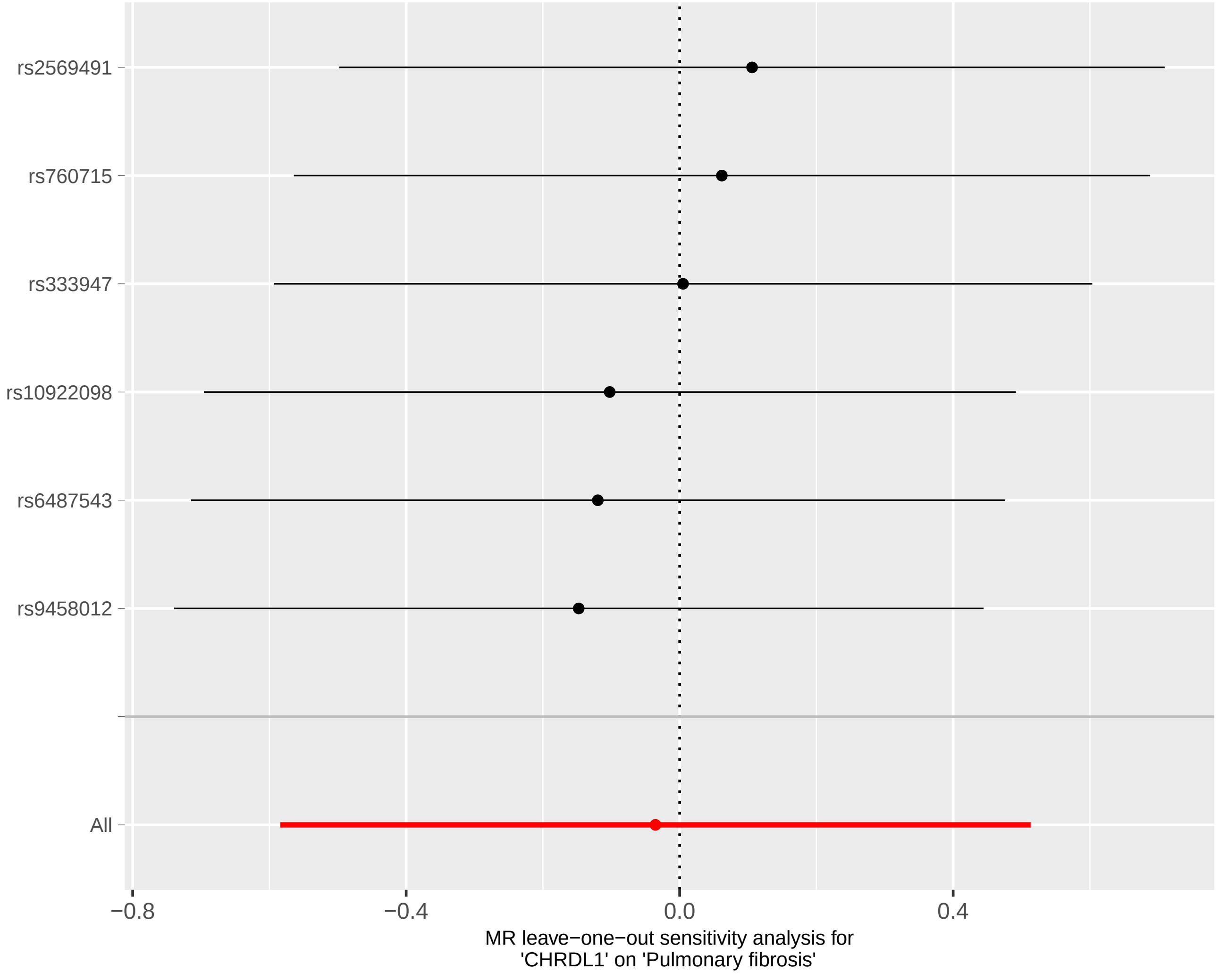
rs2569491
rs760715
rs333947
rs10922098
rs6487543
rs9458012
All
−0.8
−0.4
0.0
0.4
MR leave−one−out sensitivity analysis for
'CHRDL1' on 'Pulmonary fibrosis'

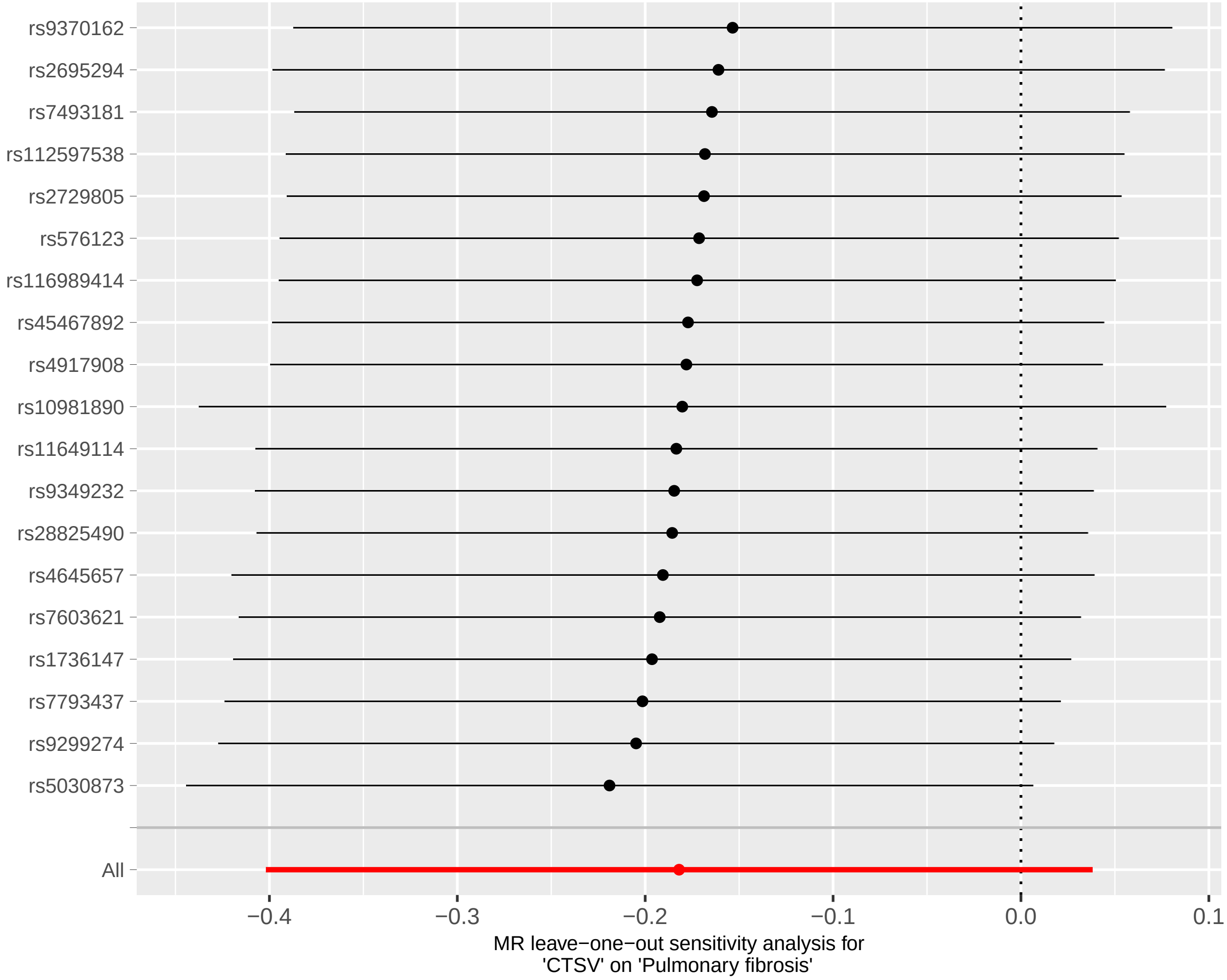
rs9370162
rs2695294
rs7493181
rs112597538
rs2729805
rs576123
rs116989414
rs45467892
rs4917908
rs10981890
rs11649114
rs9349232
rs28825490
rs4645657
rs7603621
rs1736147
rs7793437
rs9299274
rs5030873
All
−0.4
−0.3
−0.2
−0.1
0.0
0.1
MR leave−one−out sensitivity analysis for
'CTSV' on 'Pulmonary fibrosis'

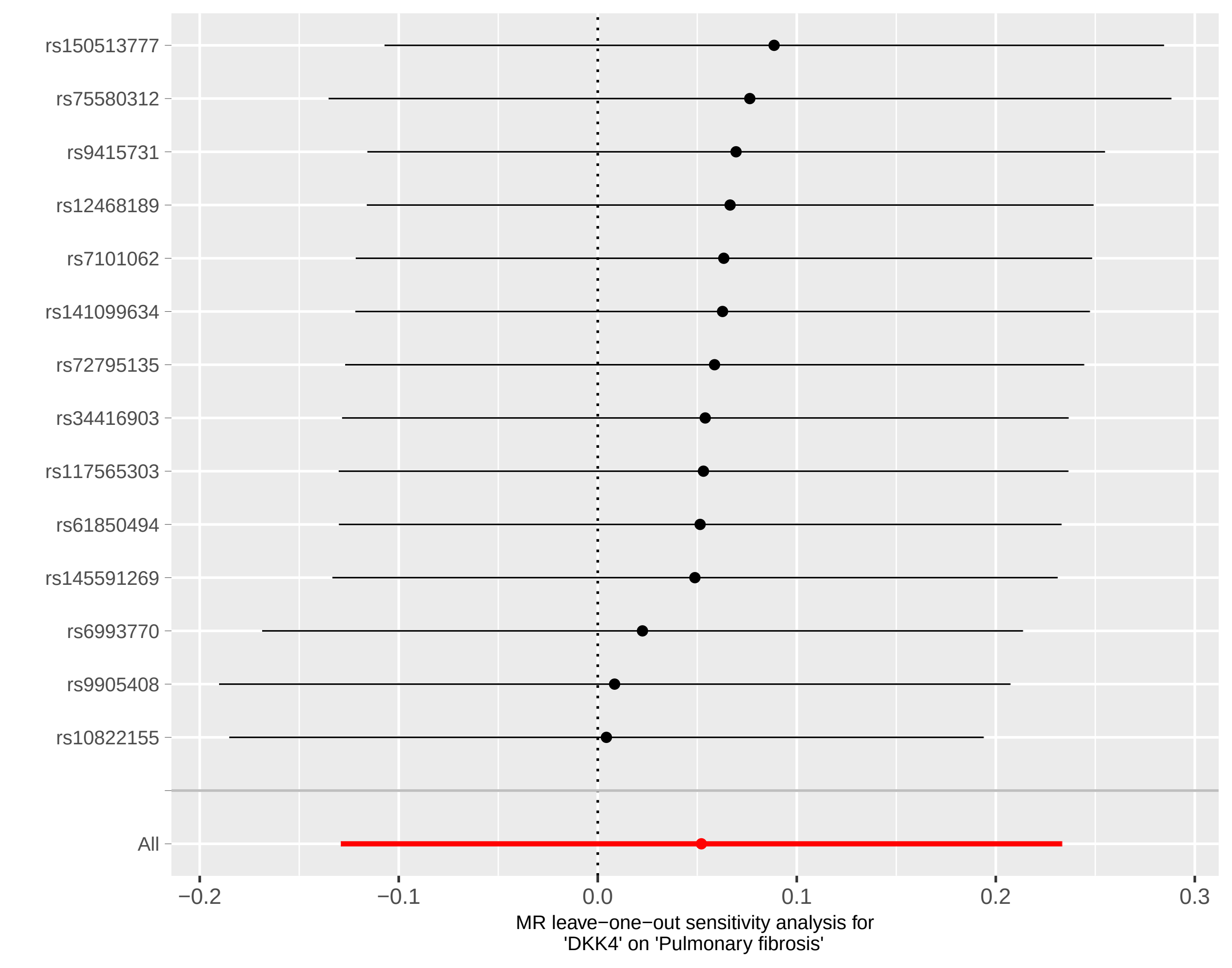

rs150513777
rs75580312
rs9415731
rs12468189
rs7101062
rs141099634
rs72795135
rs34416903
rs117565303
rs61850494
rs145591269
rs6993770
rs9905408
rs10822155
All
−0.2
−0.1
0.0
0.1
0.2
0.3
MR leave−one−out sensitivity analysis for
'DKK4' on 'Pulmonary fibrosis'

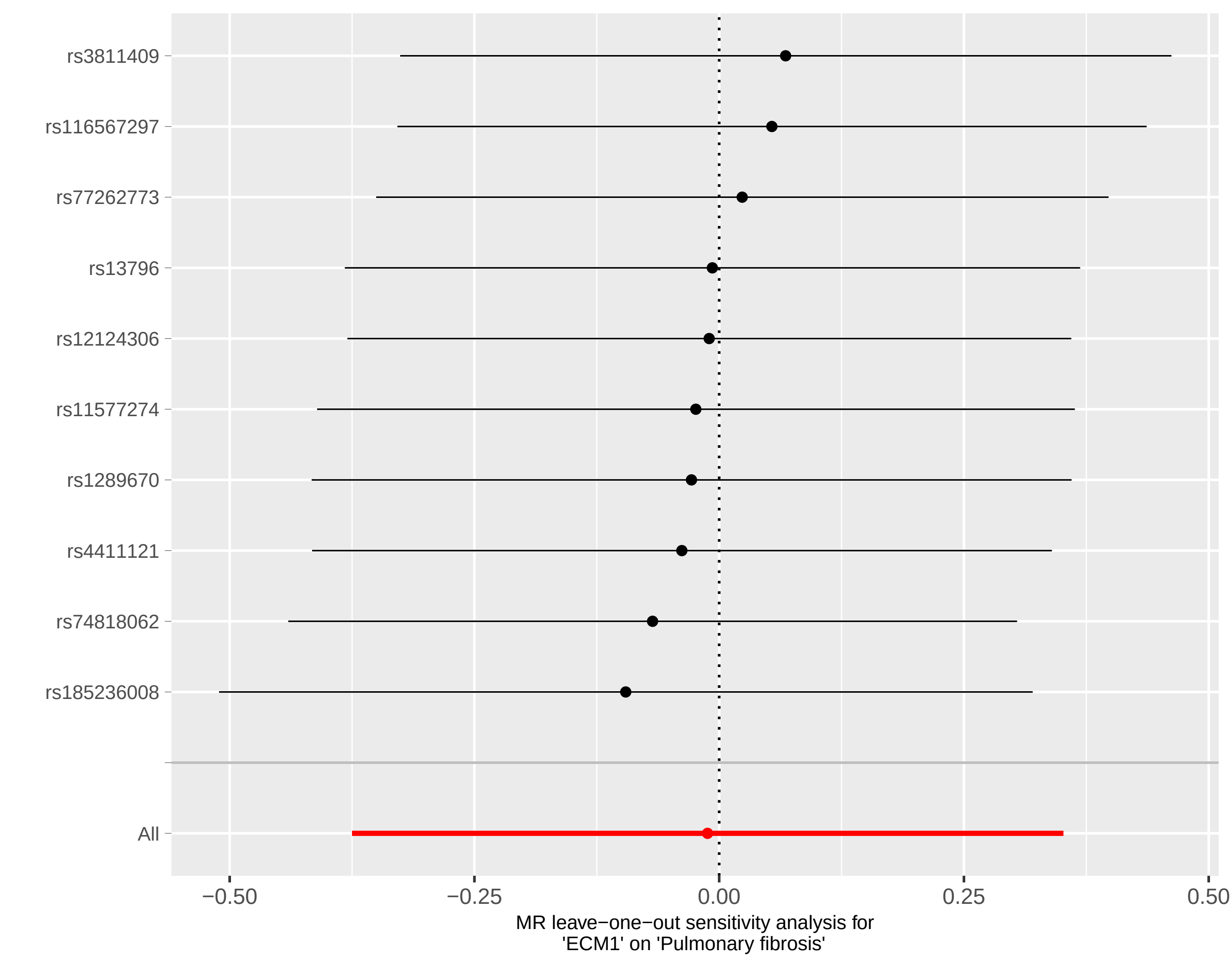
rs3811409
rs116567297
rs77262773
rs13796
rs12124306
rs11577274
rs1289670
rs4411121
rs74818062
rs185236008
All
−0.50
−0.25
0.00
0.25
0.50
MR leave−one−out sensitivity analysis for
'ECM1' on 'Pulmonary fibrosis'

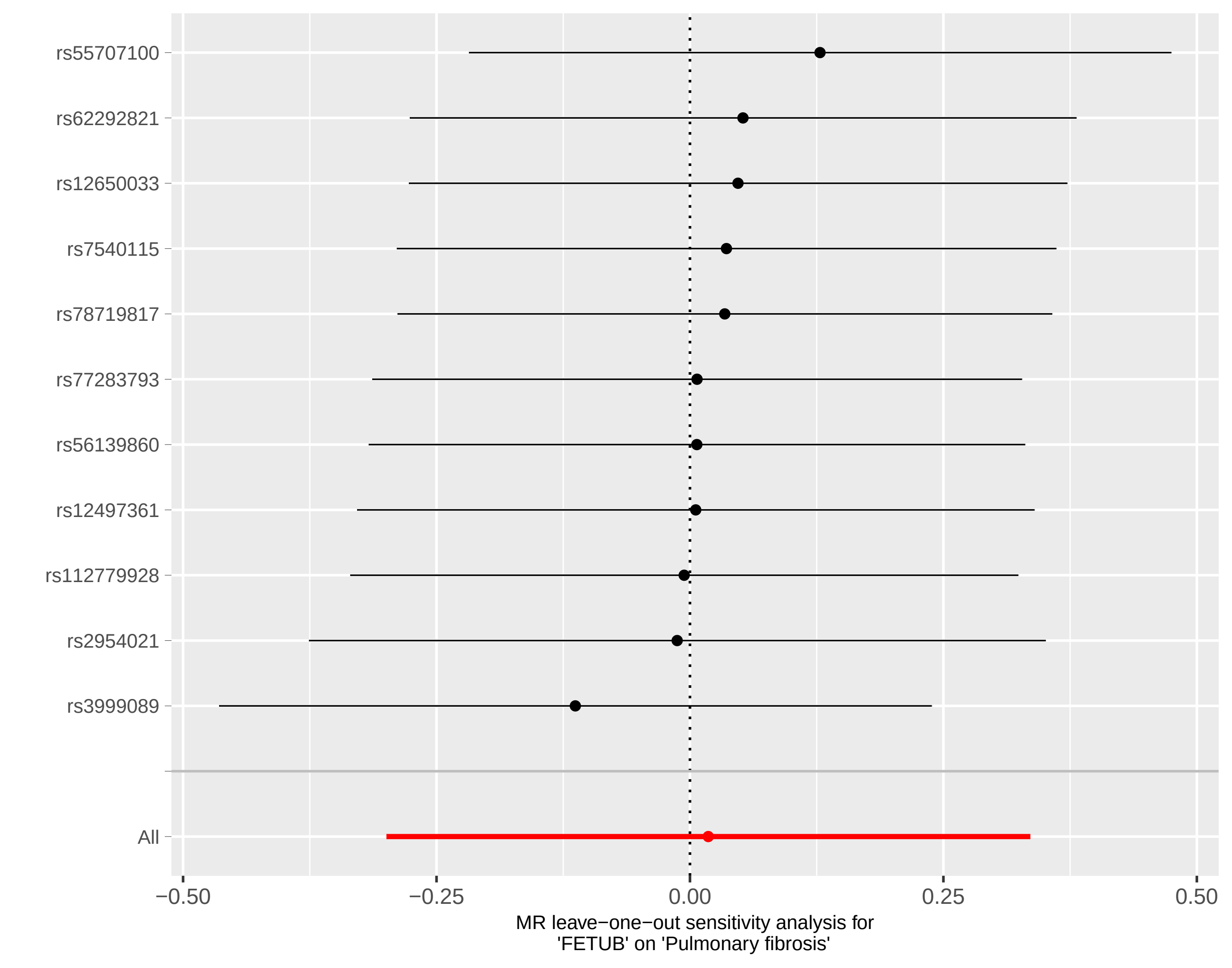

rs55707100
rs62292821
rs12650033
rs7540115
rs78719817
rs77283793
rs56139860
rs12497361
rs112779928
rs2954021
rs3999089
All
−0.50
−0.25
0.00
0.25
0.50
MR leave−one−out sensitivity analysis for
'FETUB' on 'Pulmonary fibrosis'

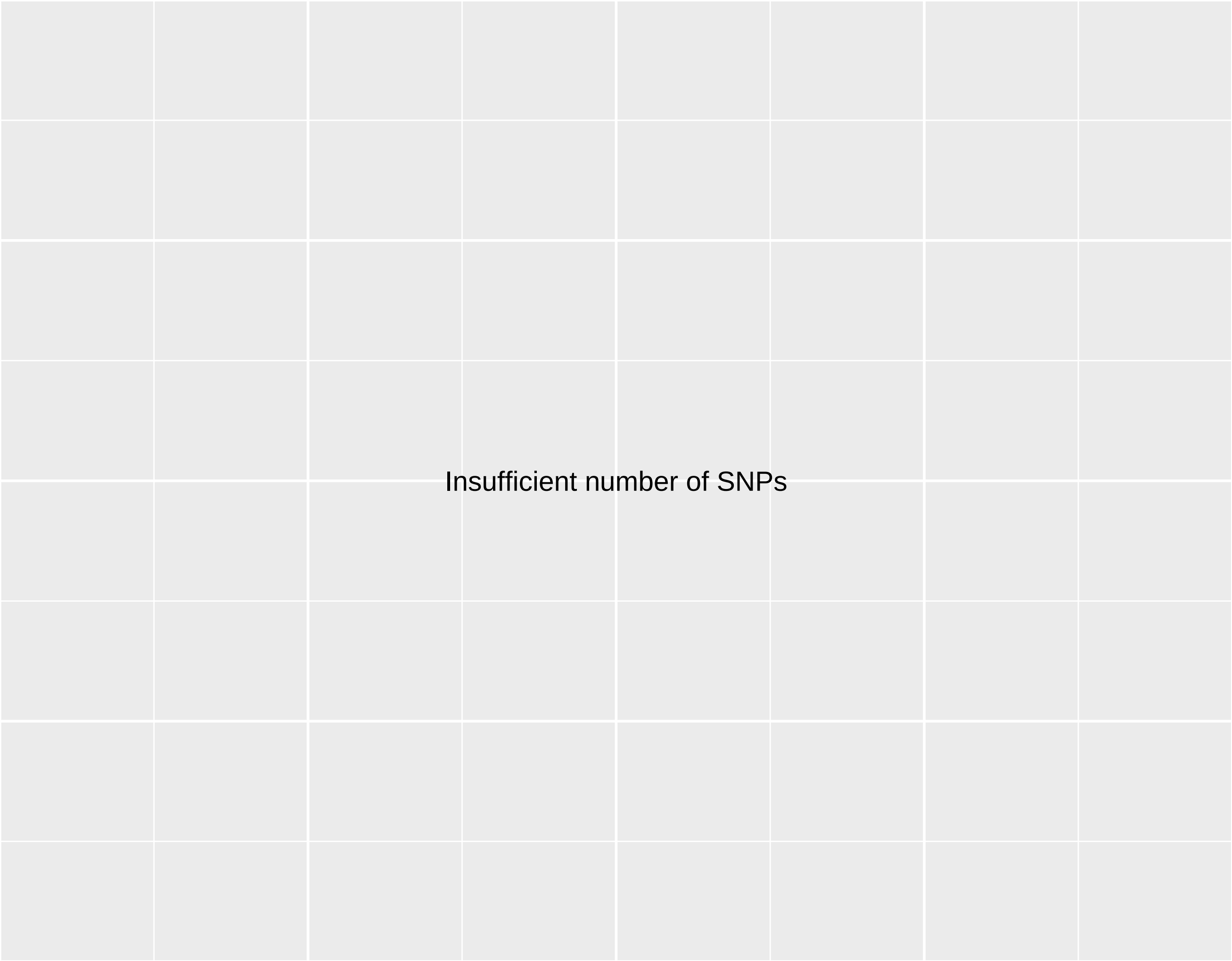
Insufficient number of SNPs

Insufficient number of SNPs

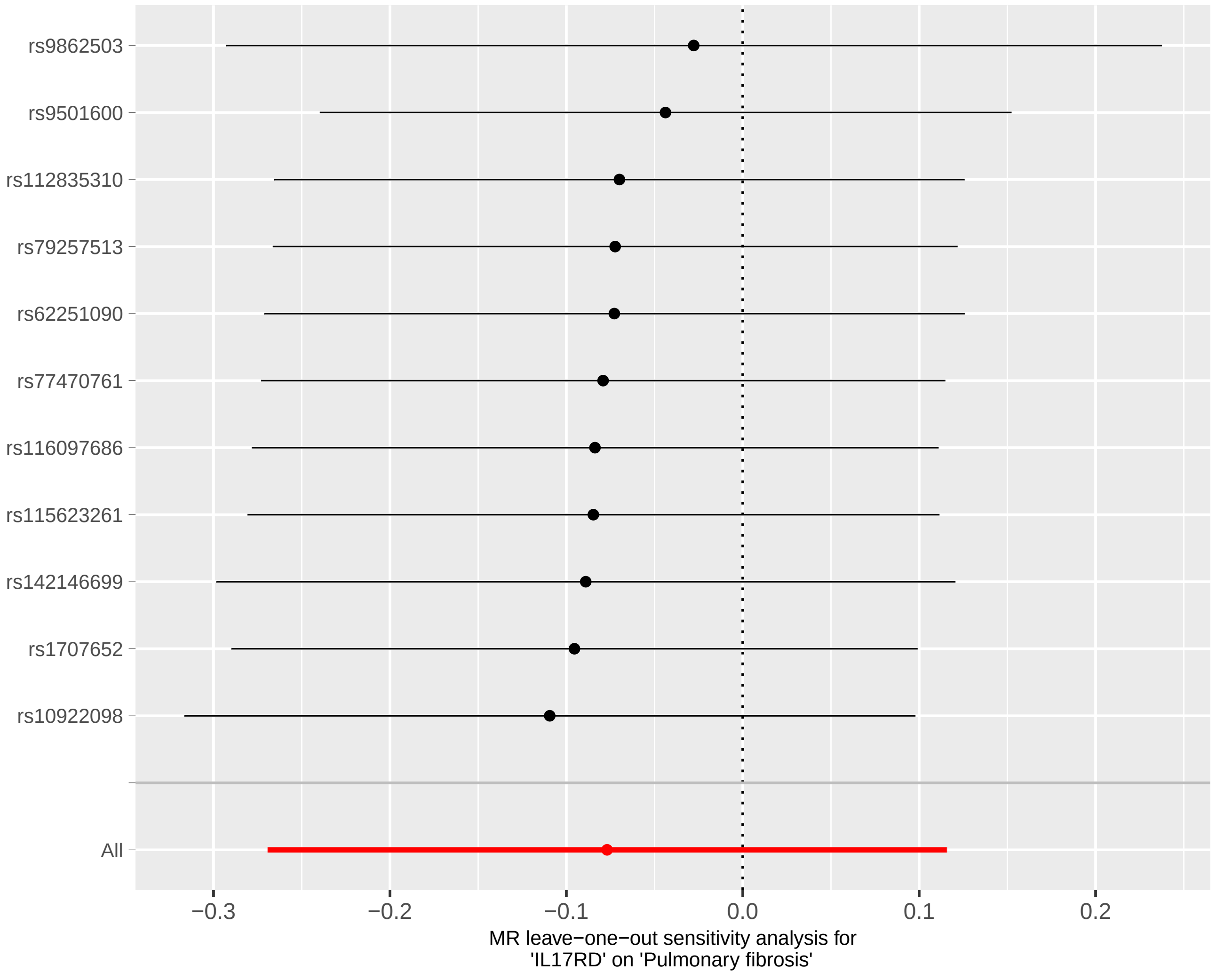
rs9862503
rs9501600
rs112835310
rs79257513
rs62251090
rs77470761
rs116097686
rs115623261
rs142146699
rs1707652
rs10922098
All
−0.3
−0.2
−0.1
0.0
0.1
0.2
MR leave−one−out sensitivity analysis for
'IL17RD' on 'Pulmonary fibrosis'

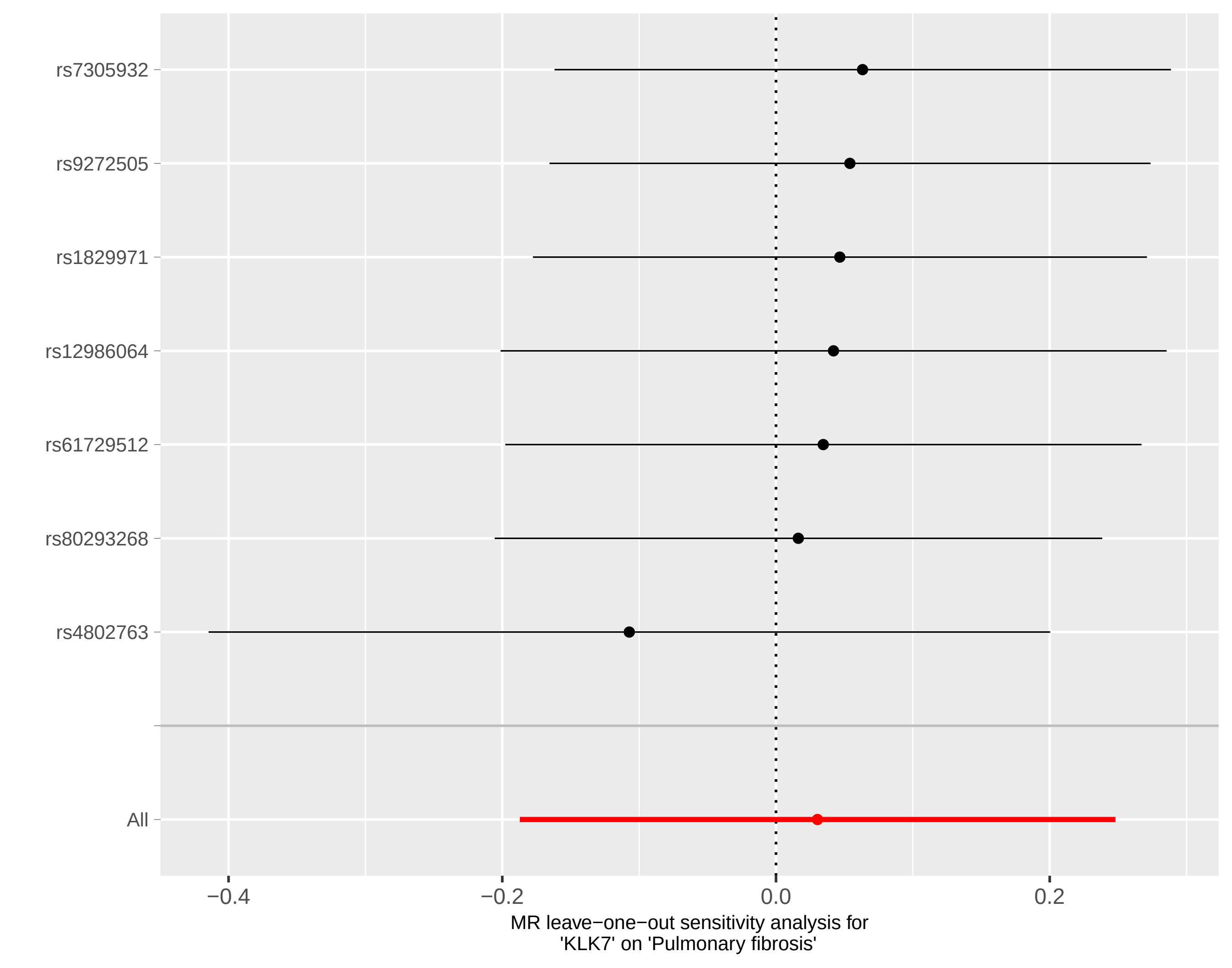
rs7305932
rs9272505
rs1829971
rs12986064
rs61729512
rs80293268
rs4802763
All
−0.4
−0.2
0.0
0.2
MR leave−one−out sensitivity analysis for
'KLK7' on 'Pulmonary fibrosis'

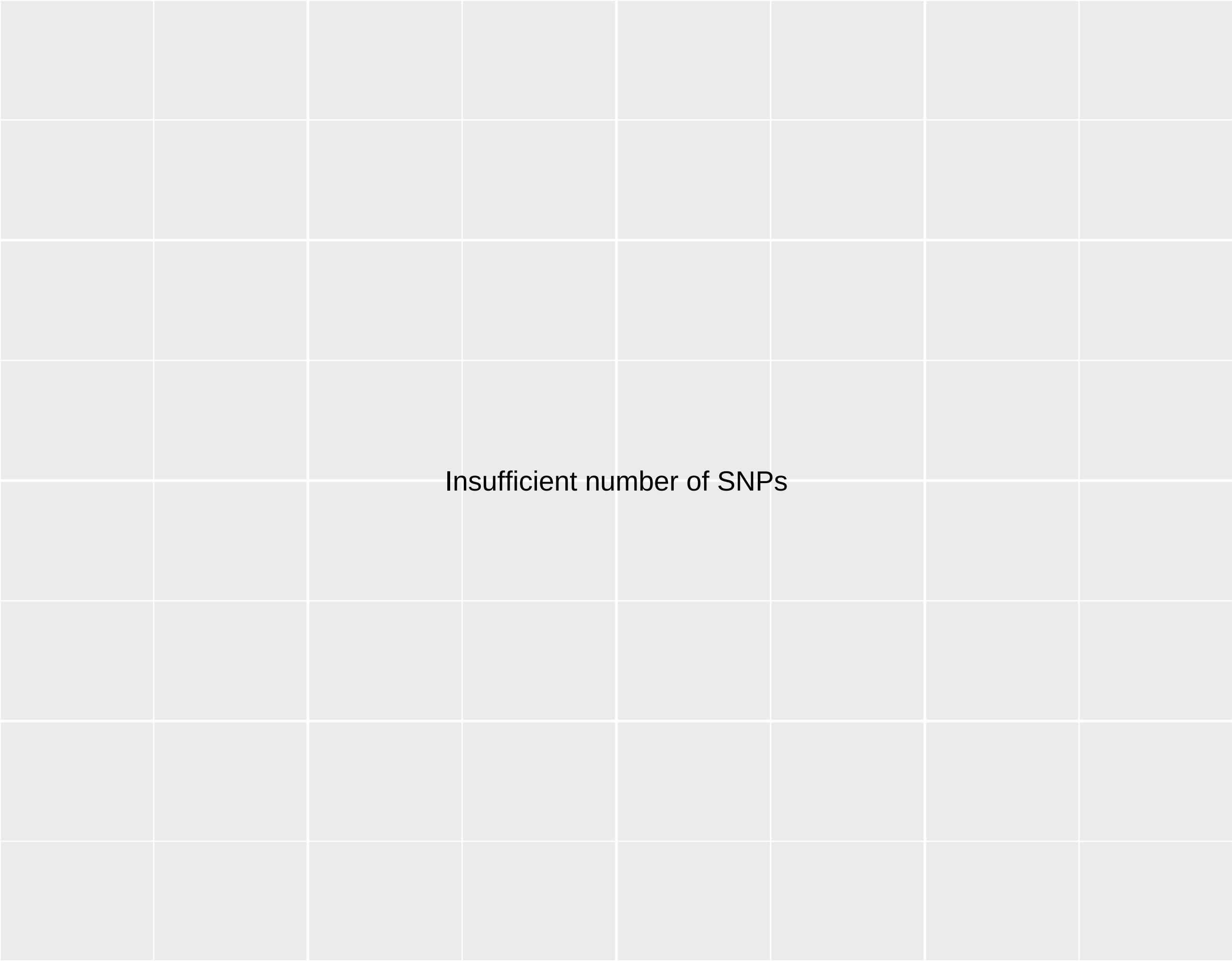
Insufficient number of SNPs

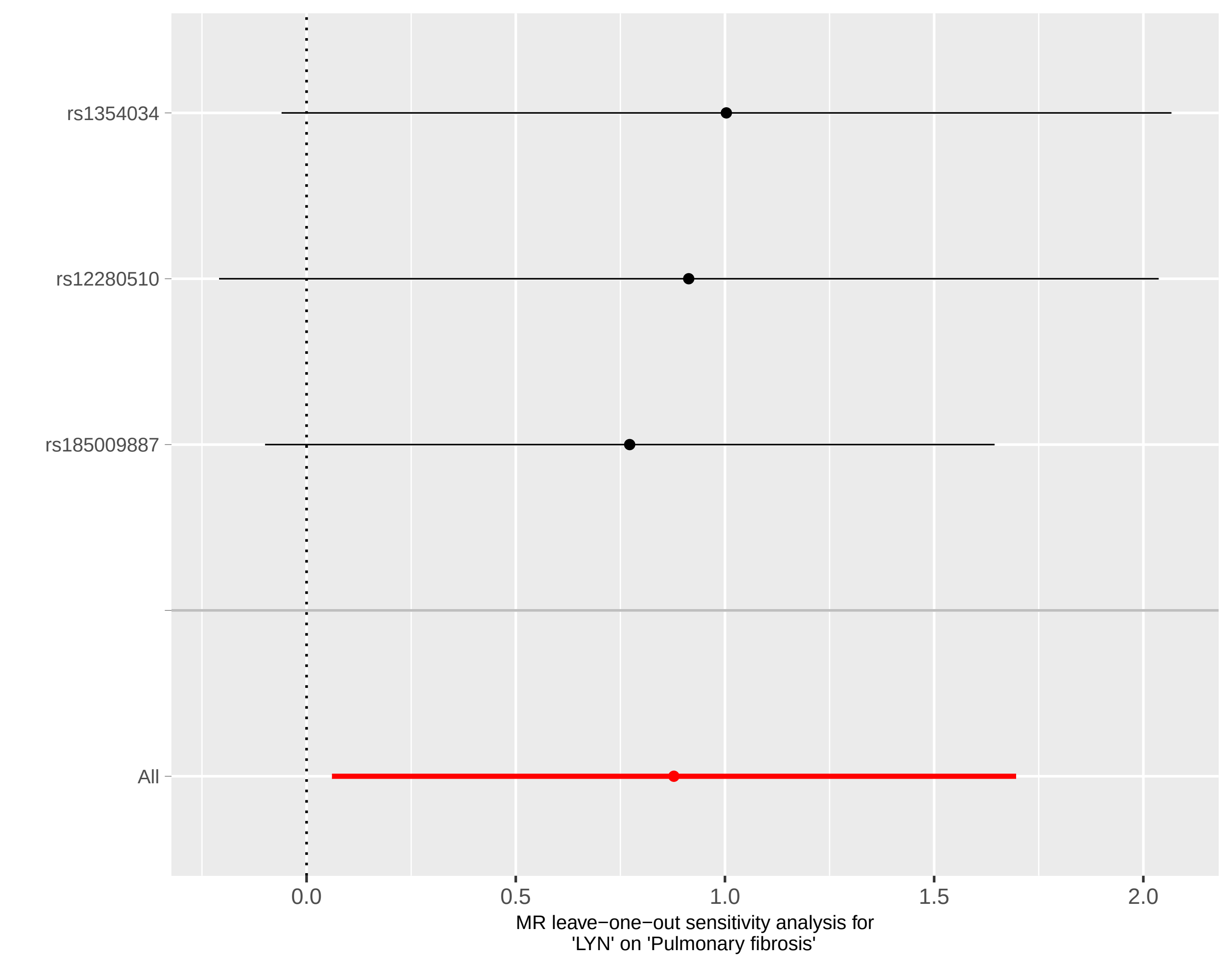
rs1354034
rs12280510
rs185009887
All
0.0
0.5
1.0
1.5
2.0
MR leave−one−out sensitivity analysis for
'LYN' on 'Pulmonary fibrosis'

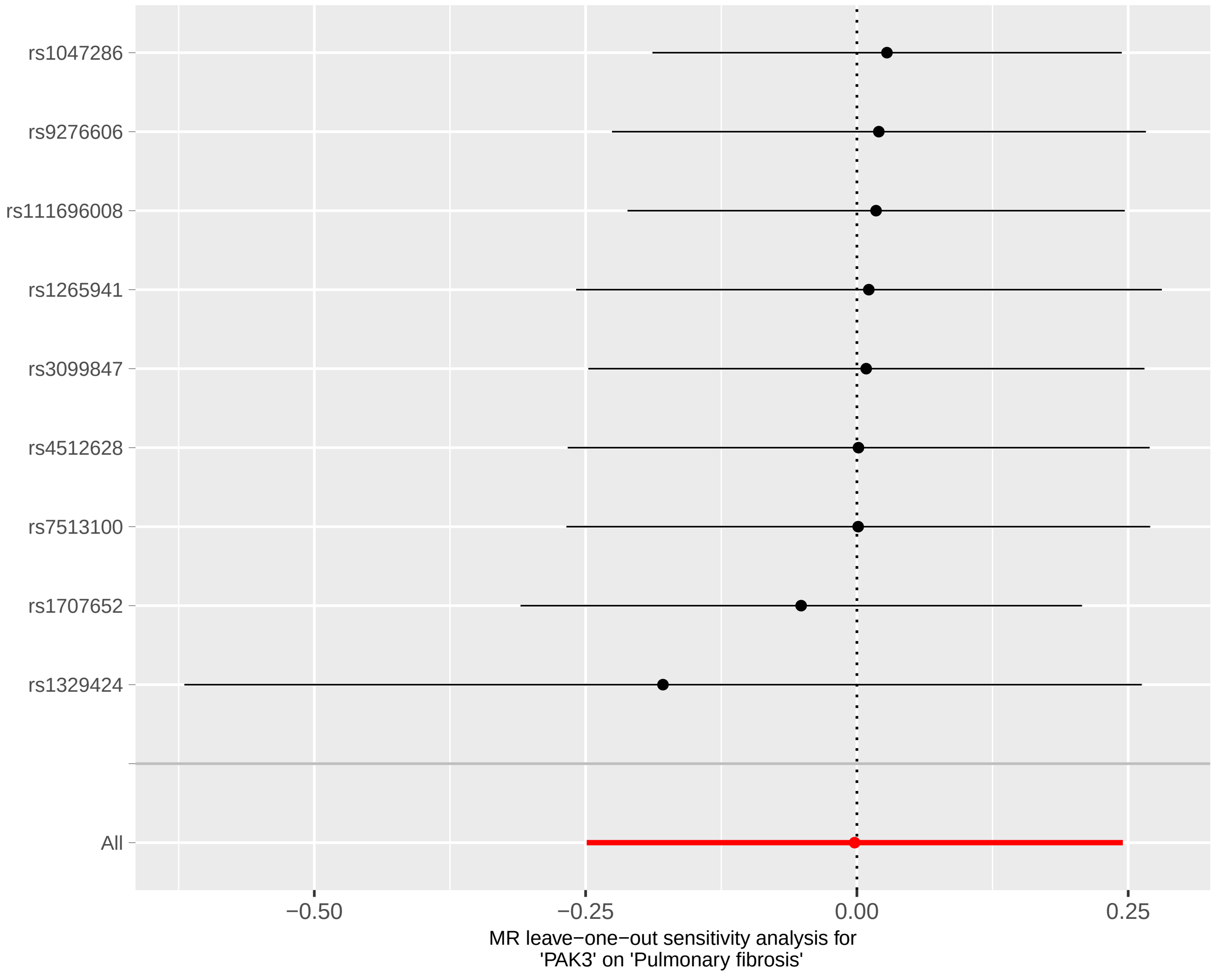

rs1047286
rs9276606
rs111696008
rs1265941
rs3099847
rs4512628
rs7513100
rs1707652
rs1329424
All
−0.50
−0.25
0.00
0.25
MR leave−one−out sensitivity analysis for
'PAK3' on 'Pulmonary fibrosis'

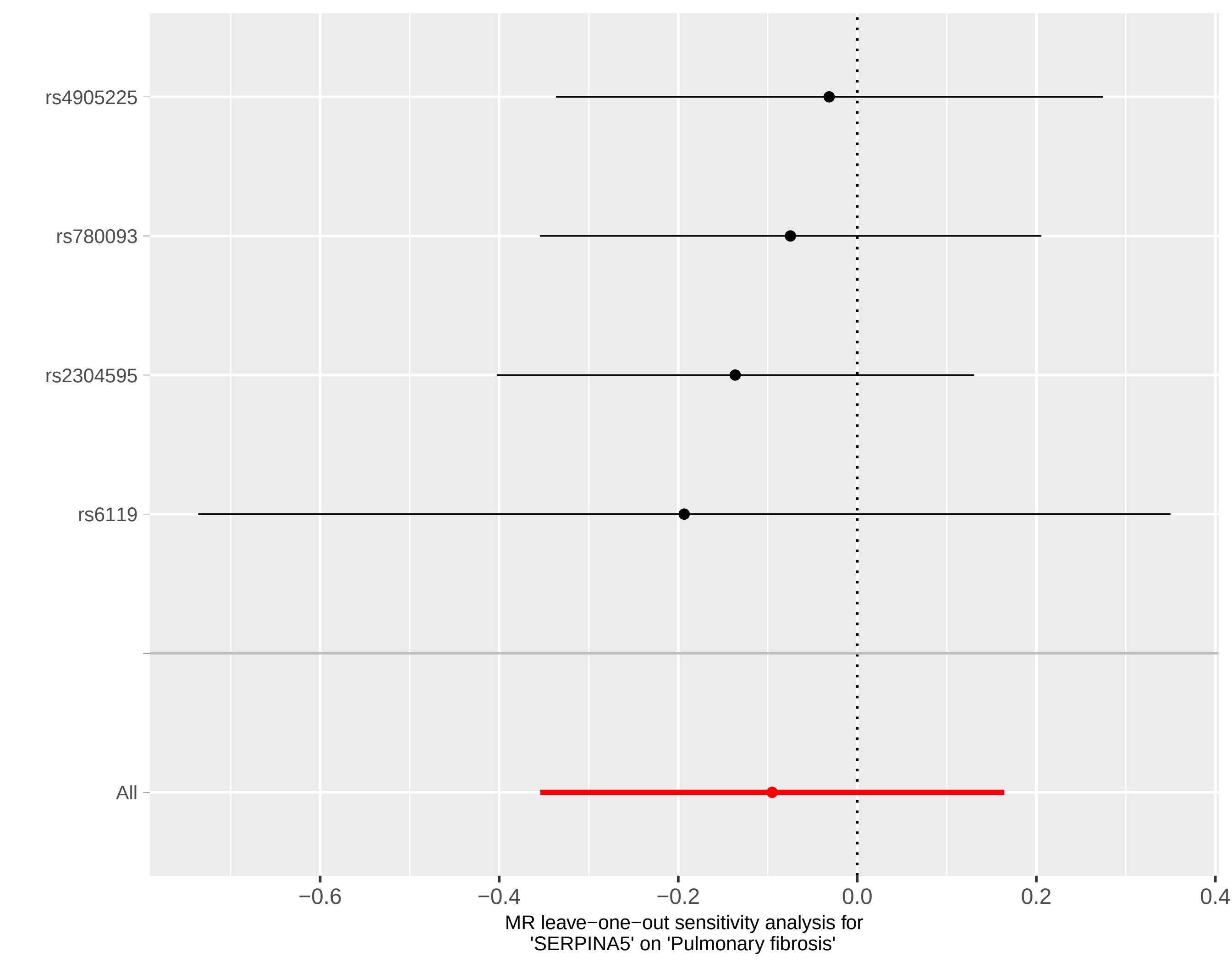
rs4905225
rs780093
rs2304595
rs6119
All
−0.6
−0.4
−0.2
0.0
0.2
0.4
MR leave−one−out sensitivity analysis for
'SERPINA5' on 'Pulmonary fibrosis'

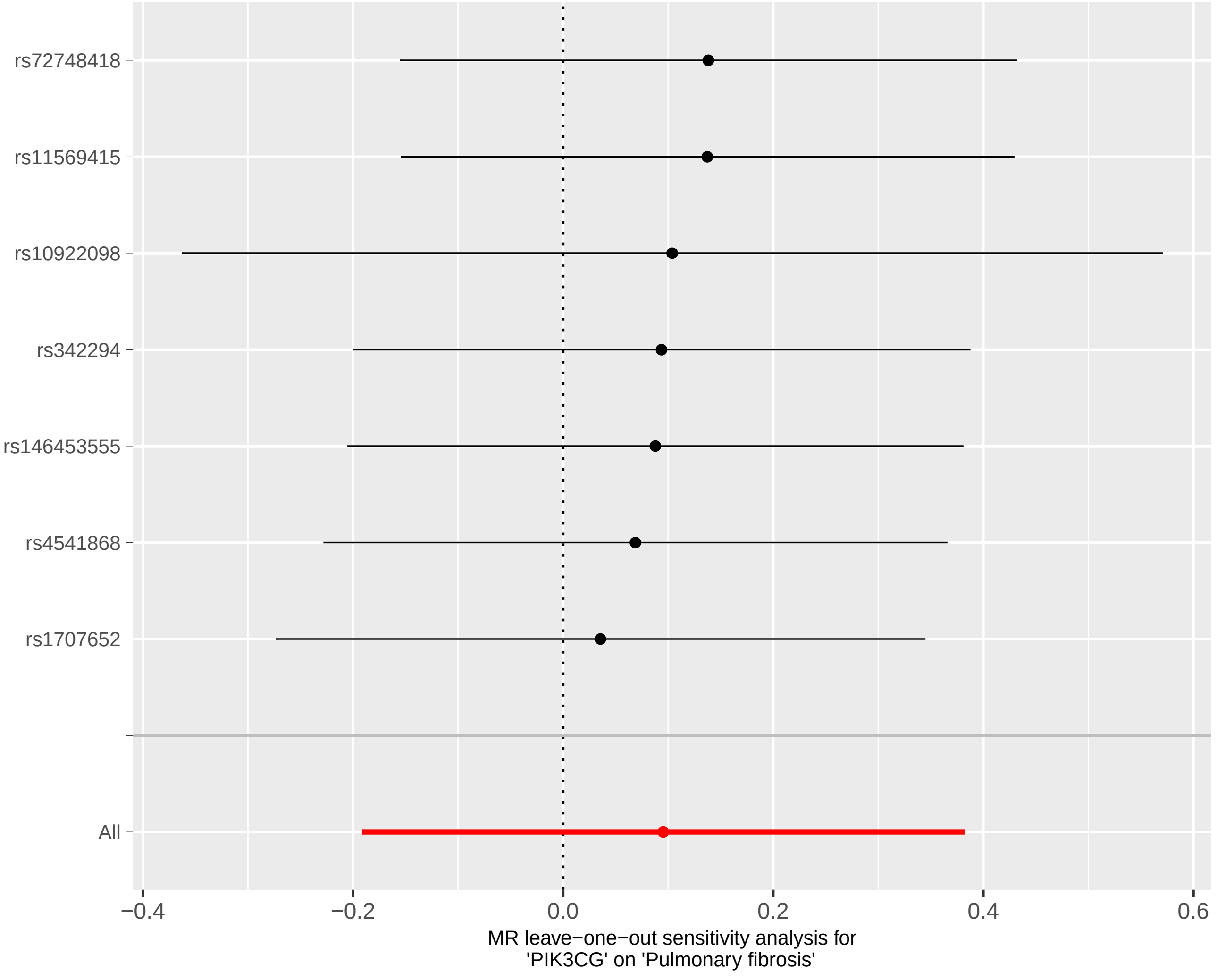
rs72748418
rs11569415
rs10922098
rs342294
rs146453555
rs4541868
rs1707652
All
−0.4
−0.2
0.0
0.2
0.4
0.6
MR leave−one−out sensitivity analysis for
'PIK3CG' on 'Pulmonary fibrosis'

Insufficient number of SNPs

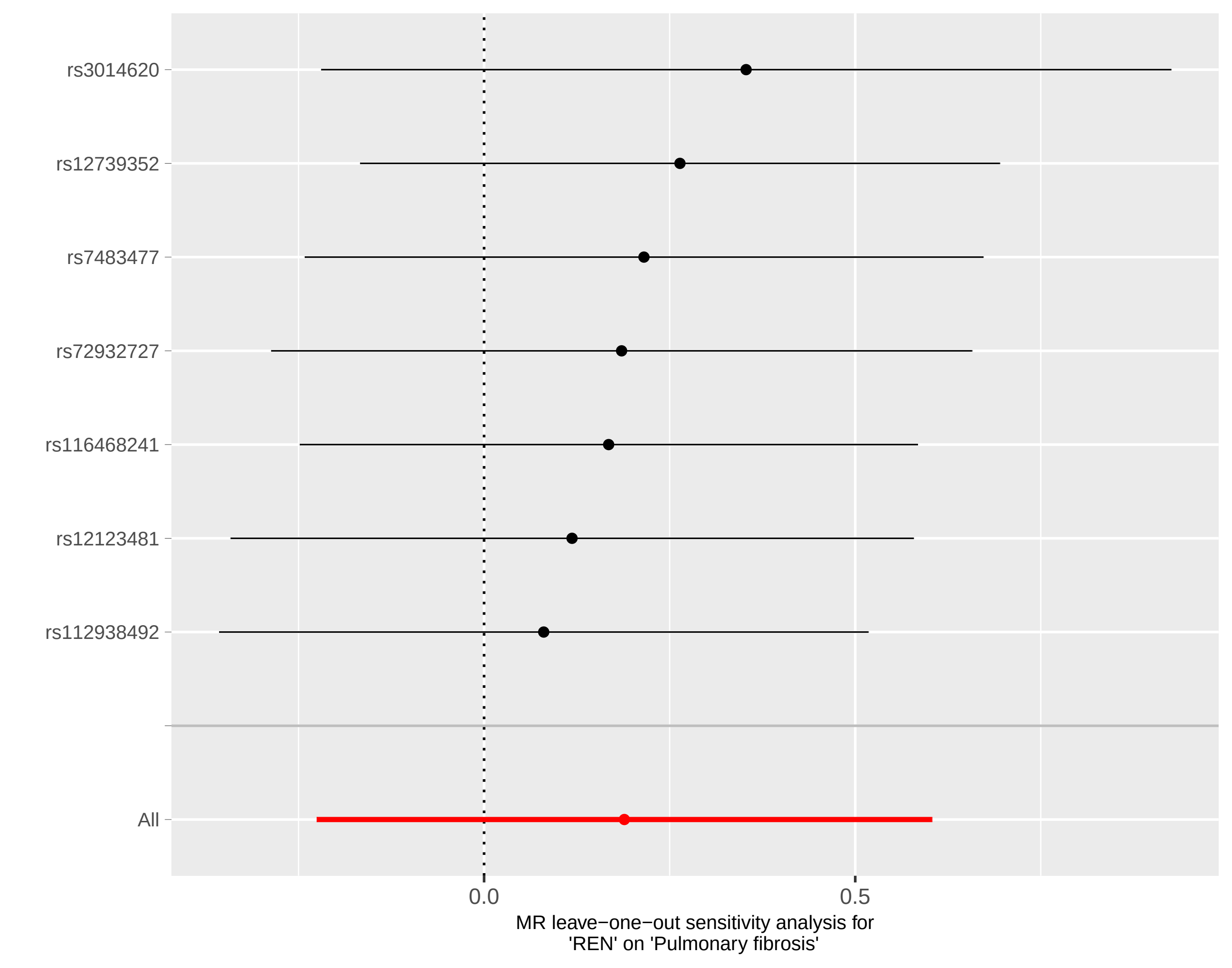

rs3014620
rs12739352
rs7483477
rs72932727
rs116468241
rs12123481
rs112938492
All
0.0
0.5
MR leave−one−out sensitivity analysis for
'REN' on 'Pulmonary fibrosis'

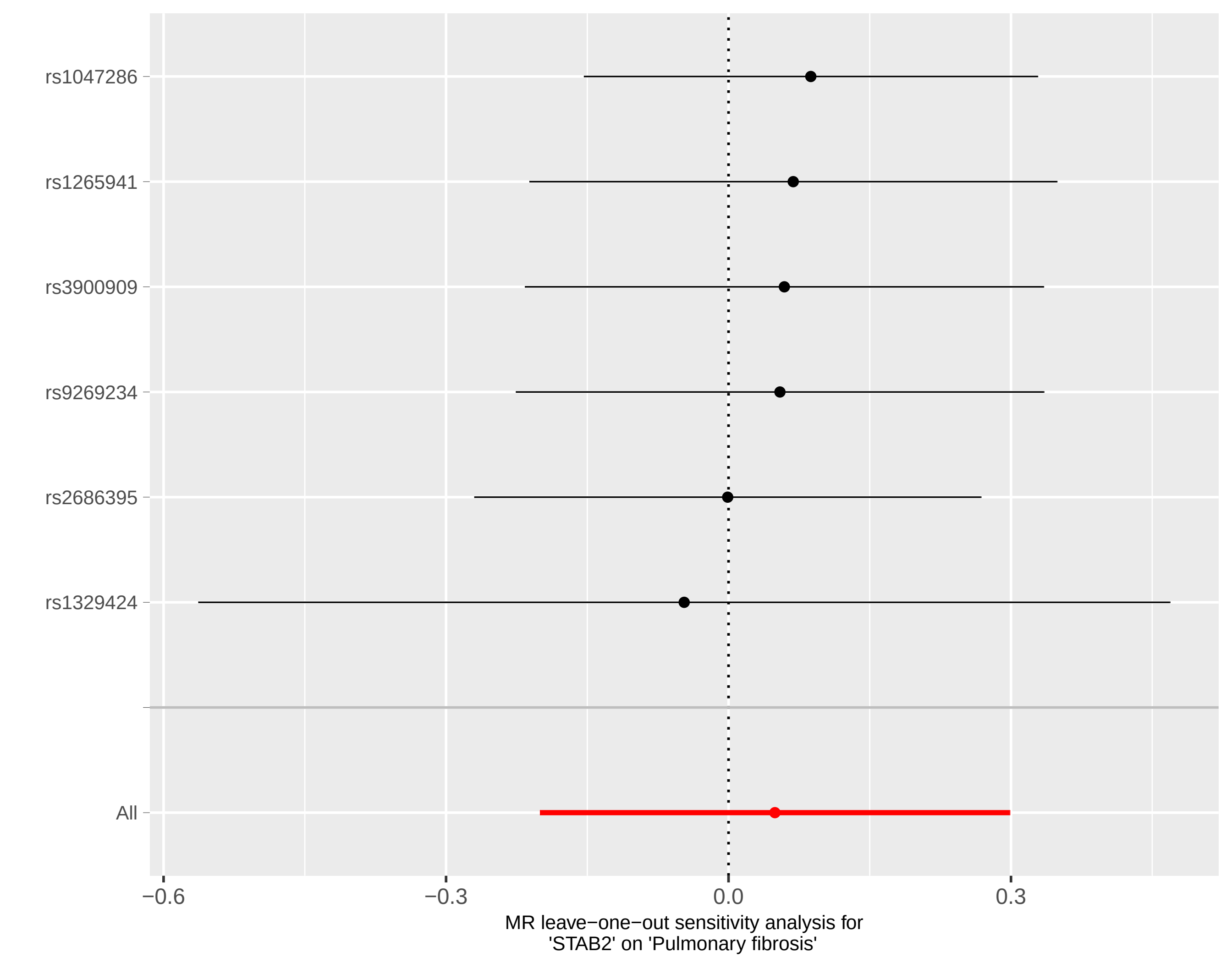

rs1047286
rs1265941
rs3900909
rs9269234
rs2686395
rs1329424
All
−0.6
−0.3
0.0
0.3
MR leave−one−out sensitivity analysis for
'STAB2' on 'Pulmonary fibrosis'

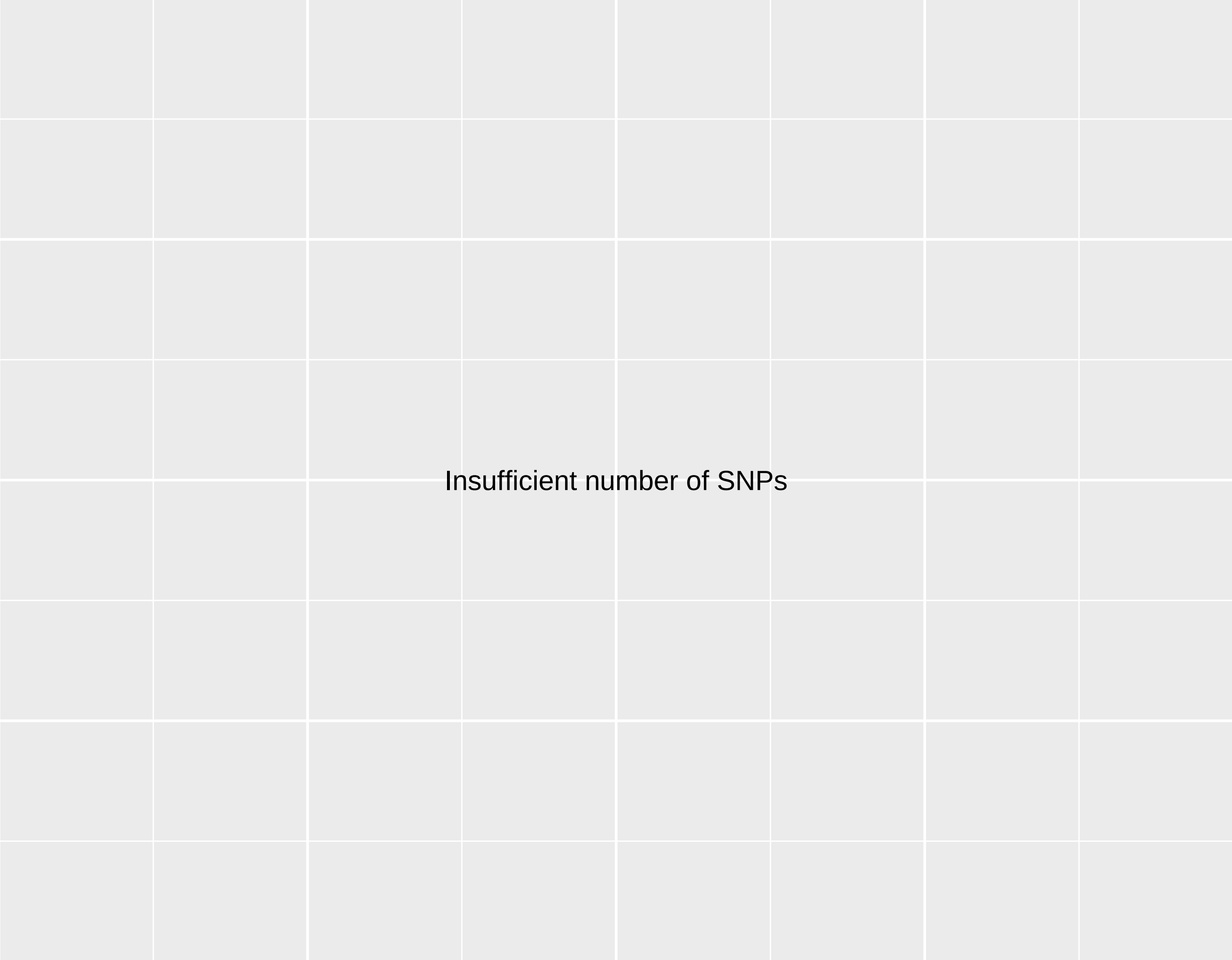
Insufficient number of SNPs

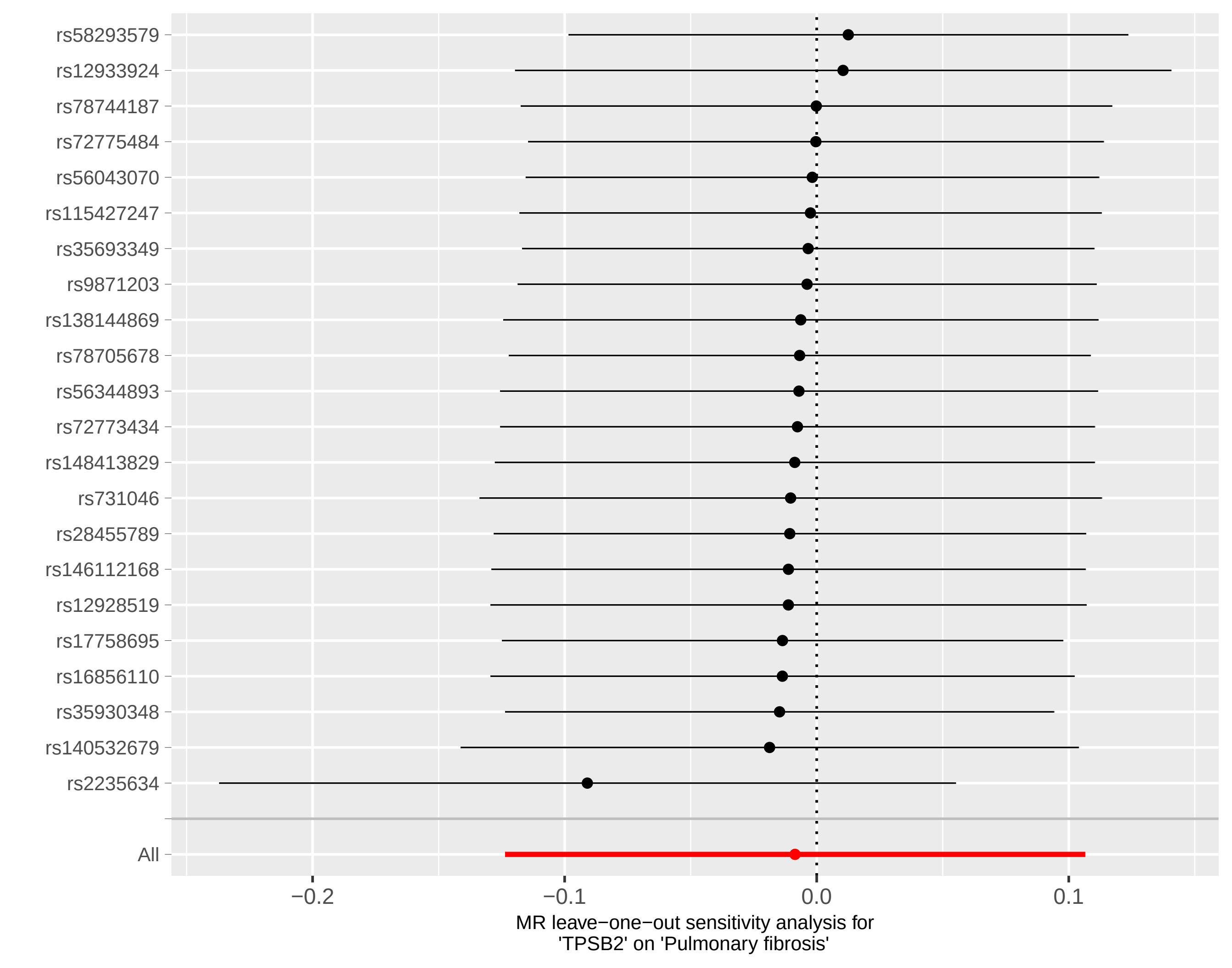
rs58293579
rs12933924
rs78744187
rs72775484
rs56043070
rs115427247
rs35693349
rs9871203
rs138144869
rs78705678
rs56344893
rs72773434
rs148413829
rs731046
rs28455789
rs146112168
rs12928519
rs17758695
rs16856110
rs35930348
rs140532679
rs2235634
All
−0.2
−0.1
0.0
0.1
MR leave−one−out sensitivity analysis for
'TPSB2' on 'Pulmonary fibrosis'

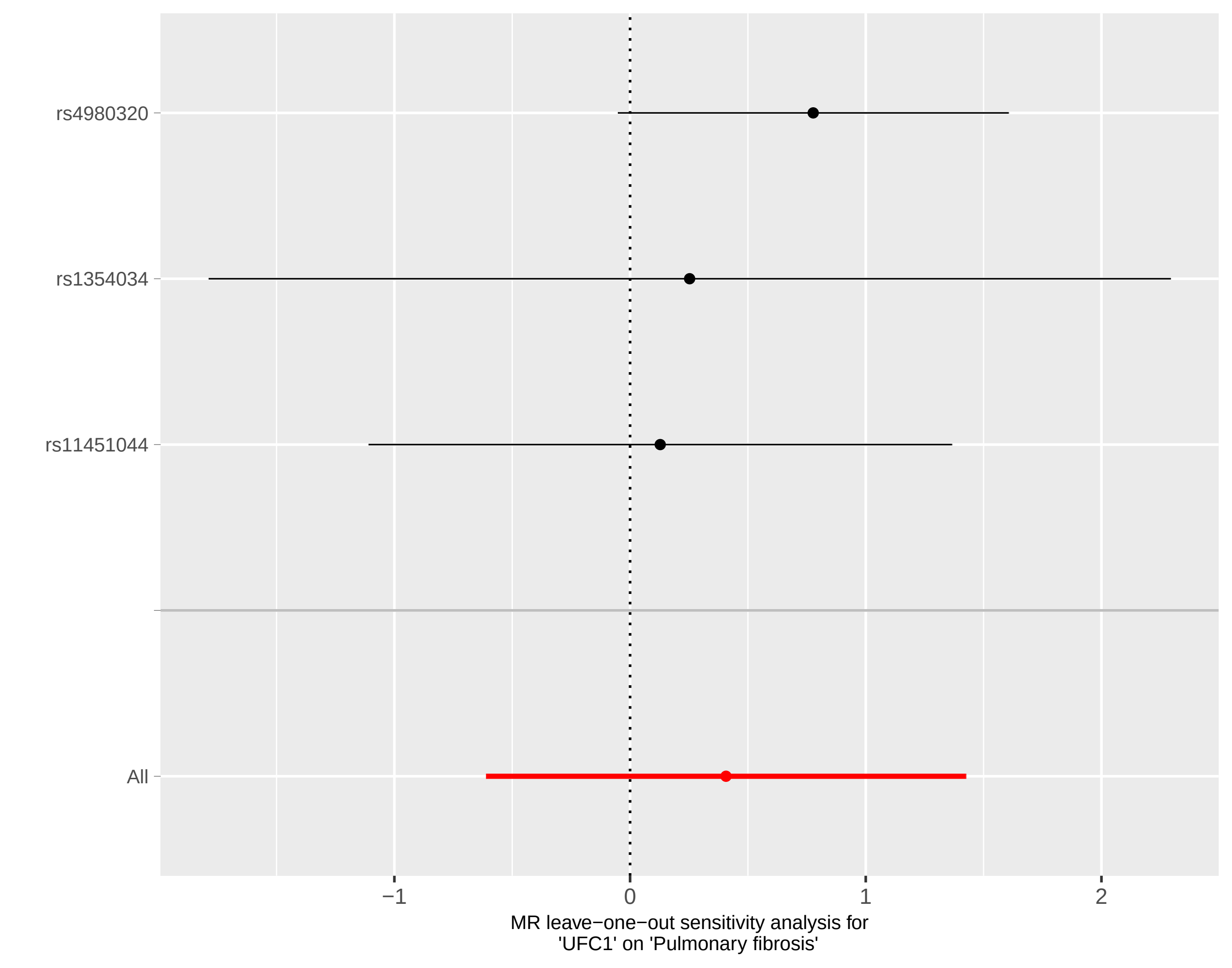
rs4980320
rs1354034
rs11451044
All
−1
0
1
2
MR leave−one−out sensitivity analysis for
'UFC1' on 'Pulmonary fibrosis'

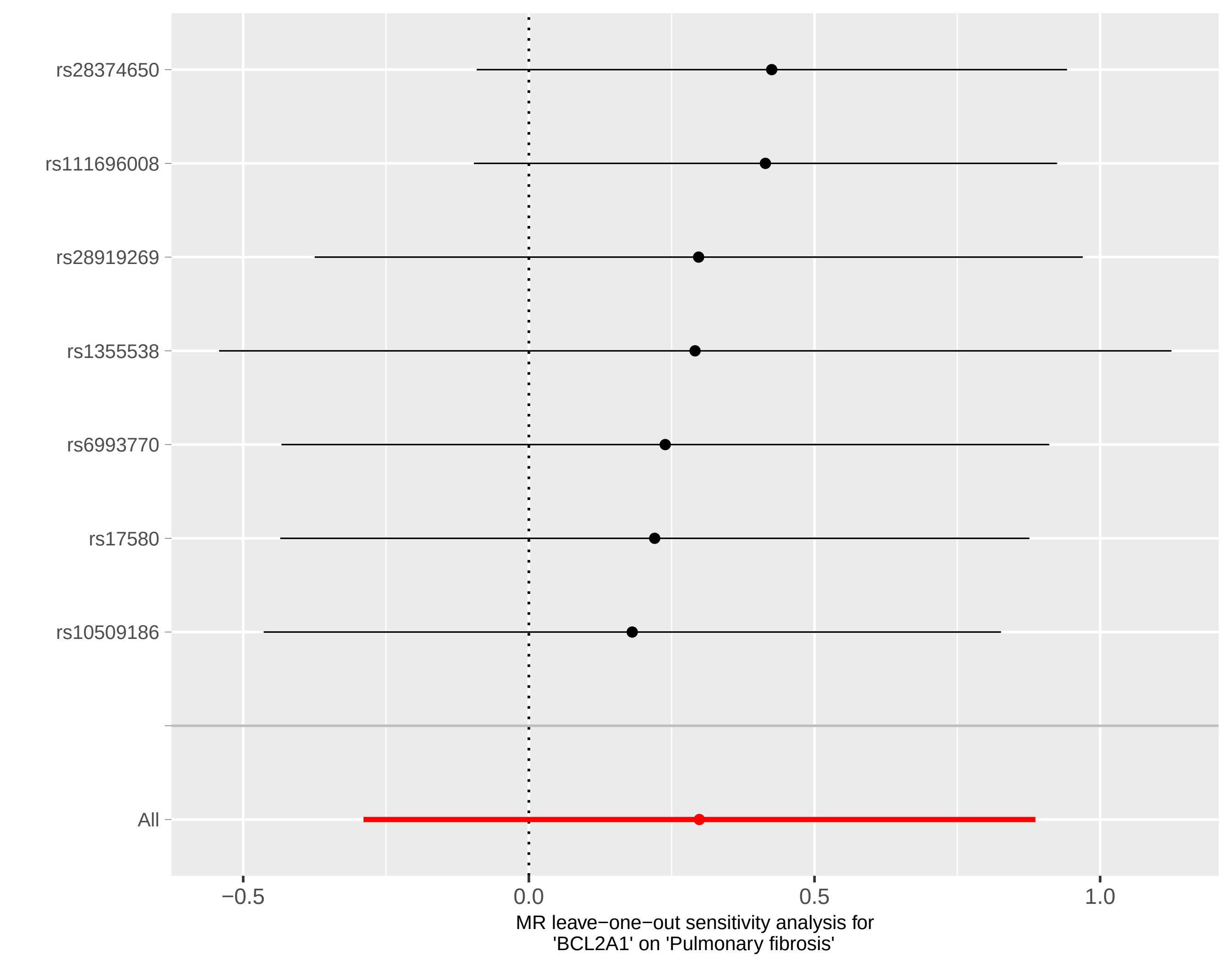
rs28374650
rs111696008
rs28919269
rs1355538
rs6993770
rs17580
rs10509186
All
−0.5
0.0
0.5
1.0
MR leave−one−out sensitivity analysis for
'BCL2A1' on 'Pulmonary fibrosis'

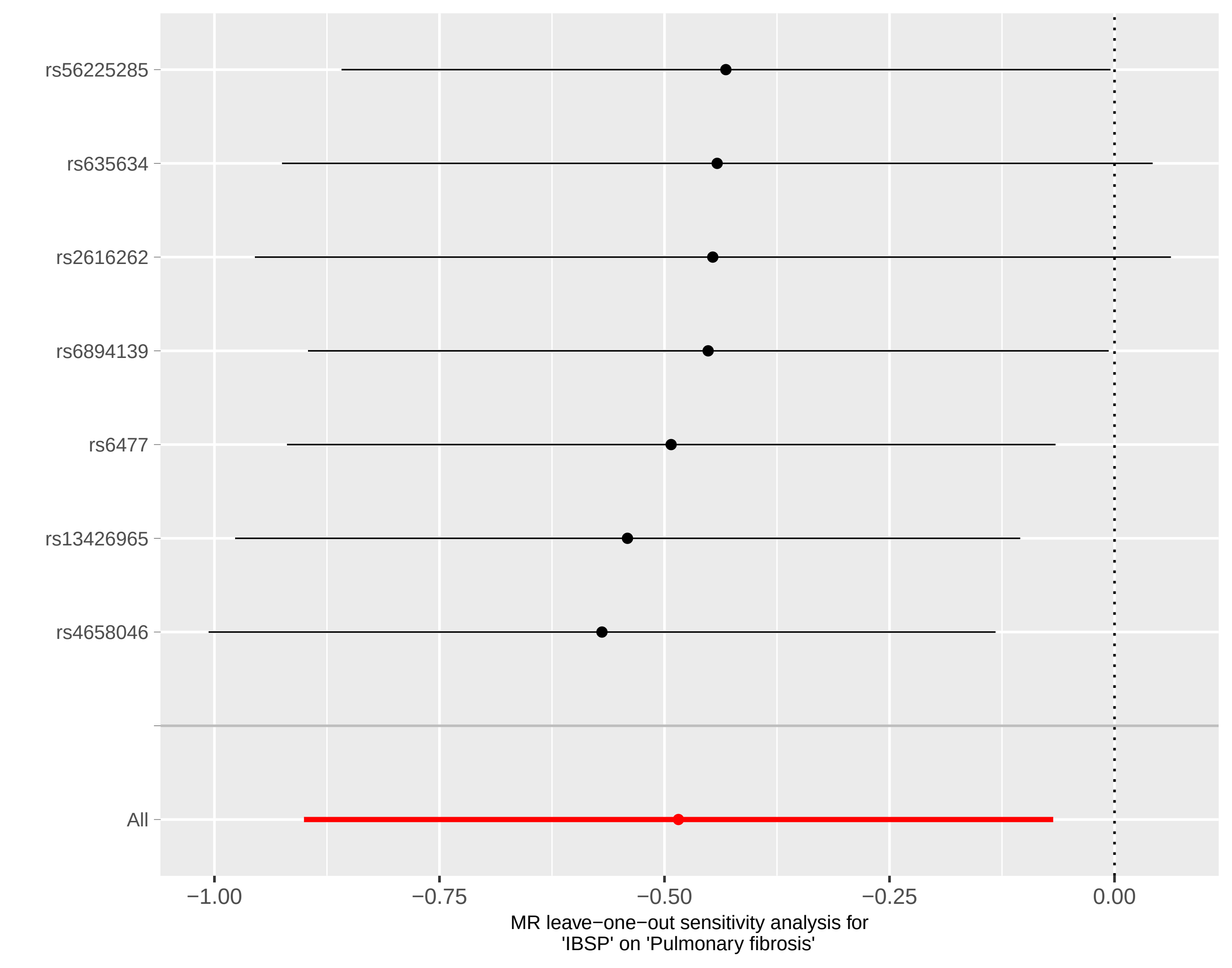

rs56225285
rs635634
rs2616262
rs6894139
rs6477
rs13426965
rs4658046
All
−1.00
−0.75
−0.50
−0.25
0.00
MR leave−one−out sensitivity analysis for
'IBSP' on 'Pulmonary fibrosis'

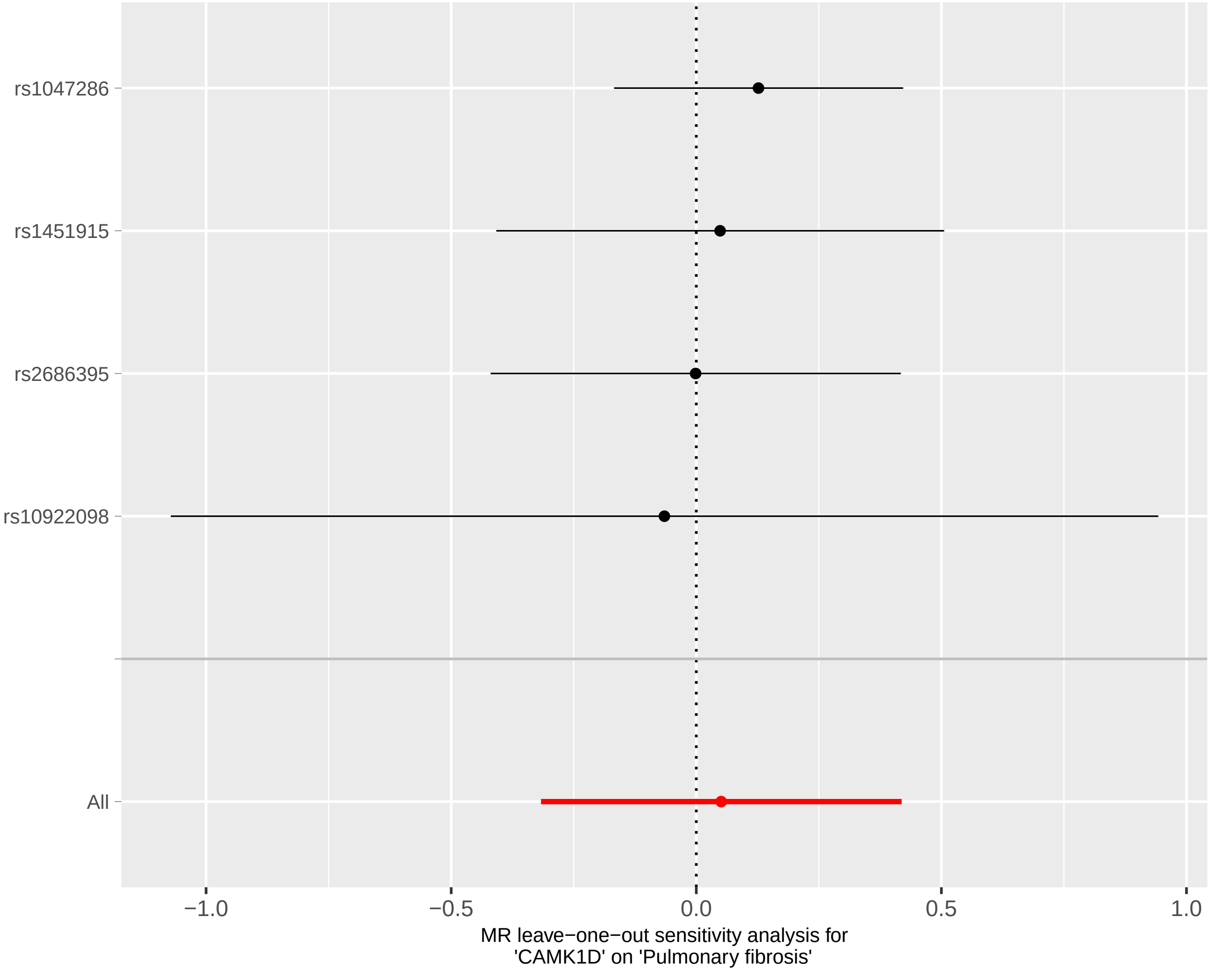
rs1047286
rs1451915
rs2686395
rs10922098
All
−1.0
−0.5
0.0
0.5
1.0
MR leave−one−out sensitivity analysis for
'CAMK1D' on 'Pulmonary fibrosis'

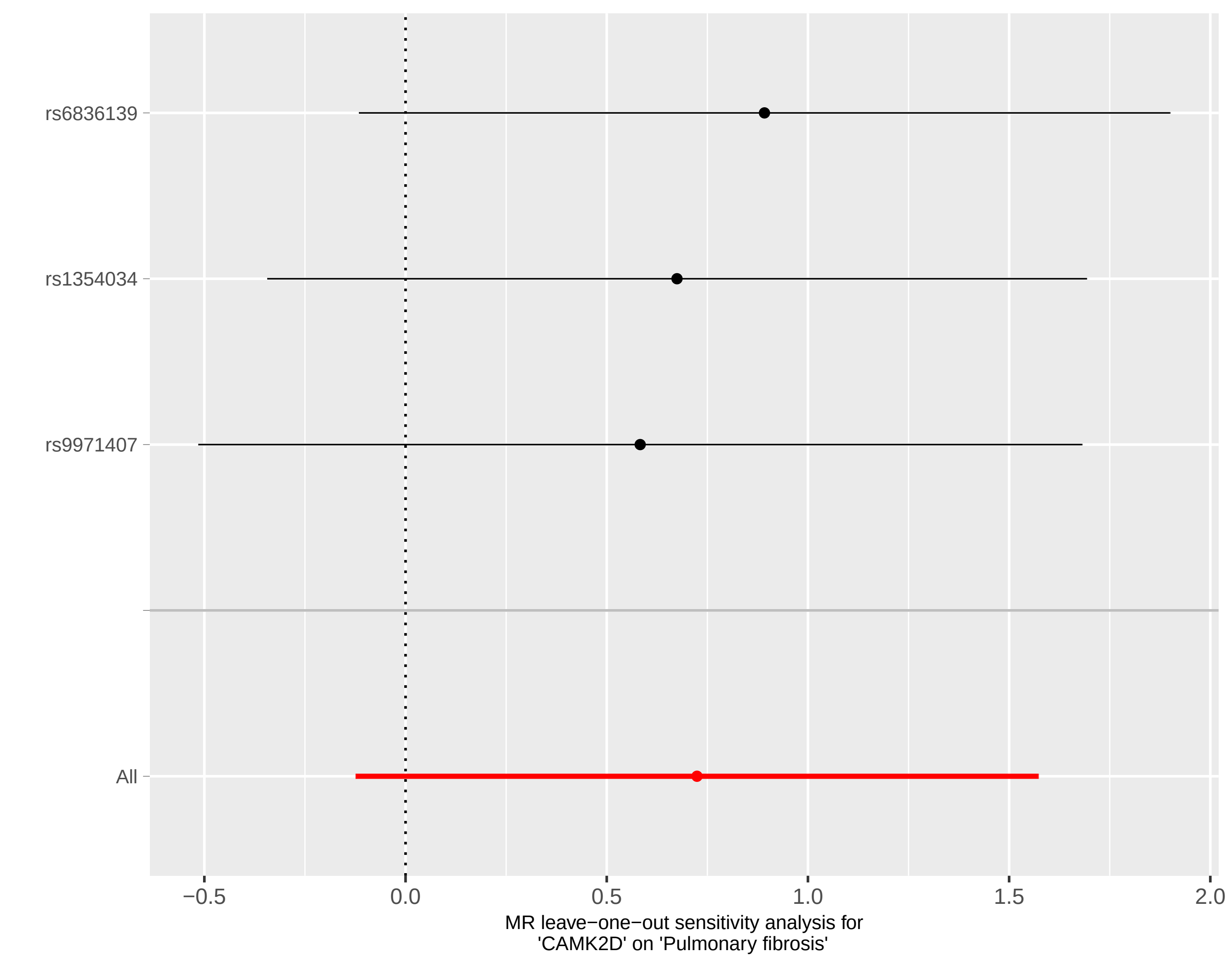
rs6836139
rs1354034
rs9971407
All
−0.5
0.0
0.5
1.0
1.5
2.0
MR leave−one−out sensitivity analysis for
'CAMK2D' on 'Pulmonary fibrosis'

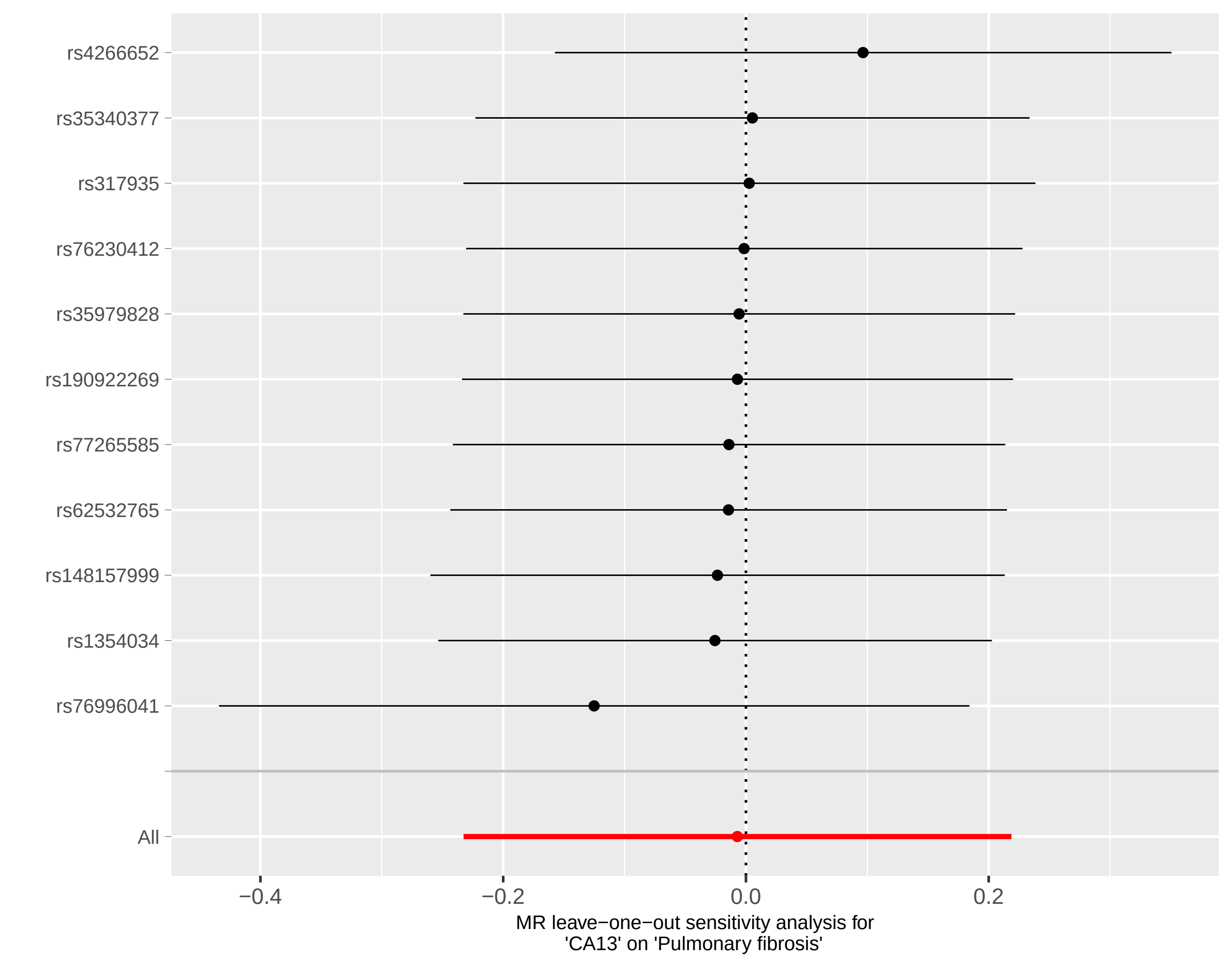
rs4266652
rs35340377
rs317935
rs76230412
rs35979828
rs190922269
rs77265585
rs62532765
rs148157999
rs1354034
rs76996041
All
−0.4
−0.2
0.0
0.2
MR leave−one−out sensitivity analysis for
'CA13' on 'Pulmonary fibrosis'

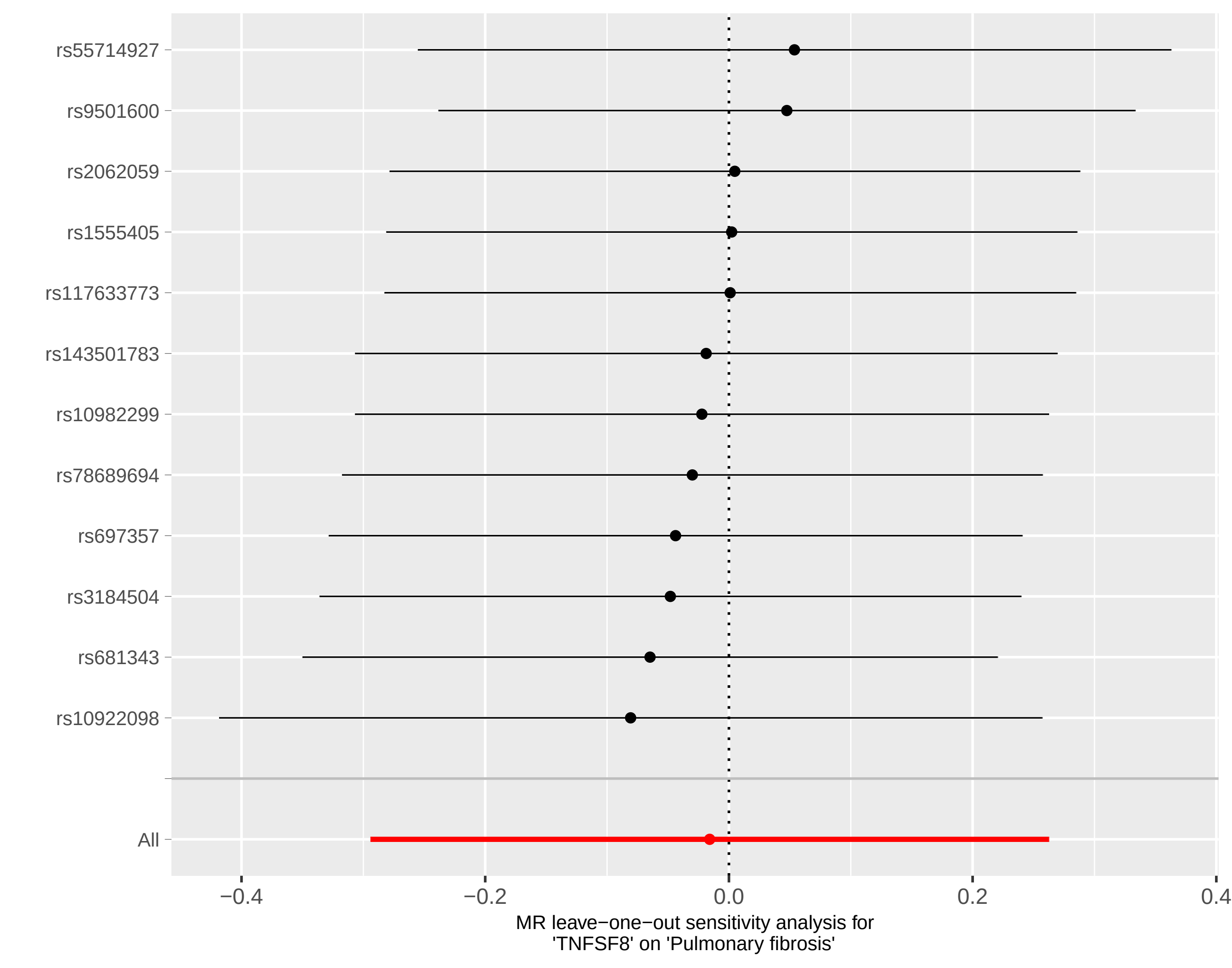
rs55714927
rs9501600
rs2062059
rs1555405
rs117633773
rs143501783
rs10982299
rs78689694
rs697357
rs3184504
rs681343
rs10922098
All
−0.4
−0.2
0.0
0.2
0.4
MR leave−one−out sensitivity analysis for
'TNFSF8' on 'Pulmonary fibrosis'

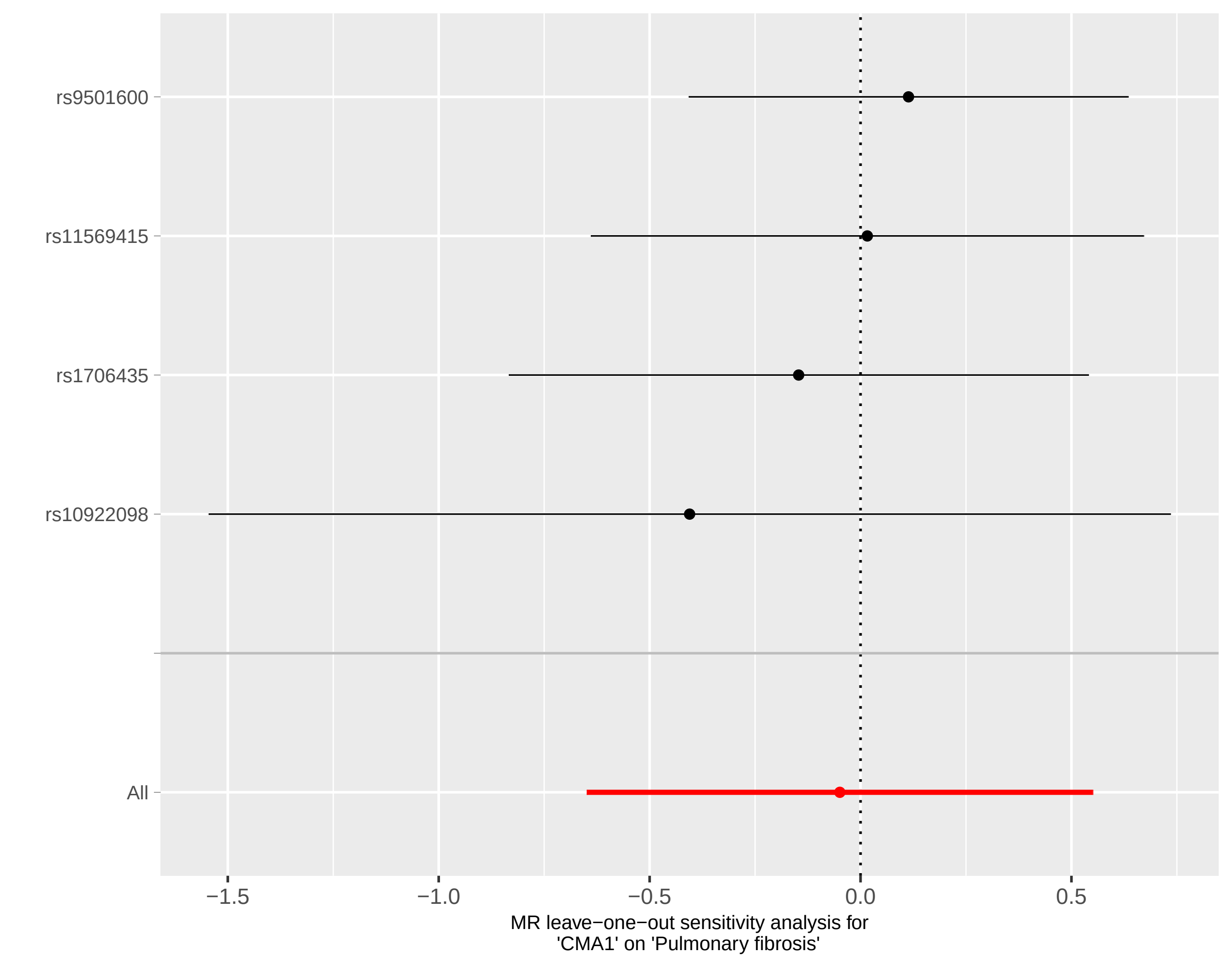

rs9501600
rs11569415
rs1706435
rs10922098
All
−1.5
−1.0
−0.5
0.0
0.5
MR leave−one−out sensitivity analysis for
'CMA1' on 'Pulmonary fibrosis'

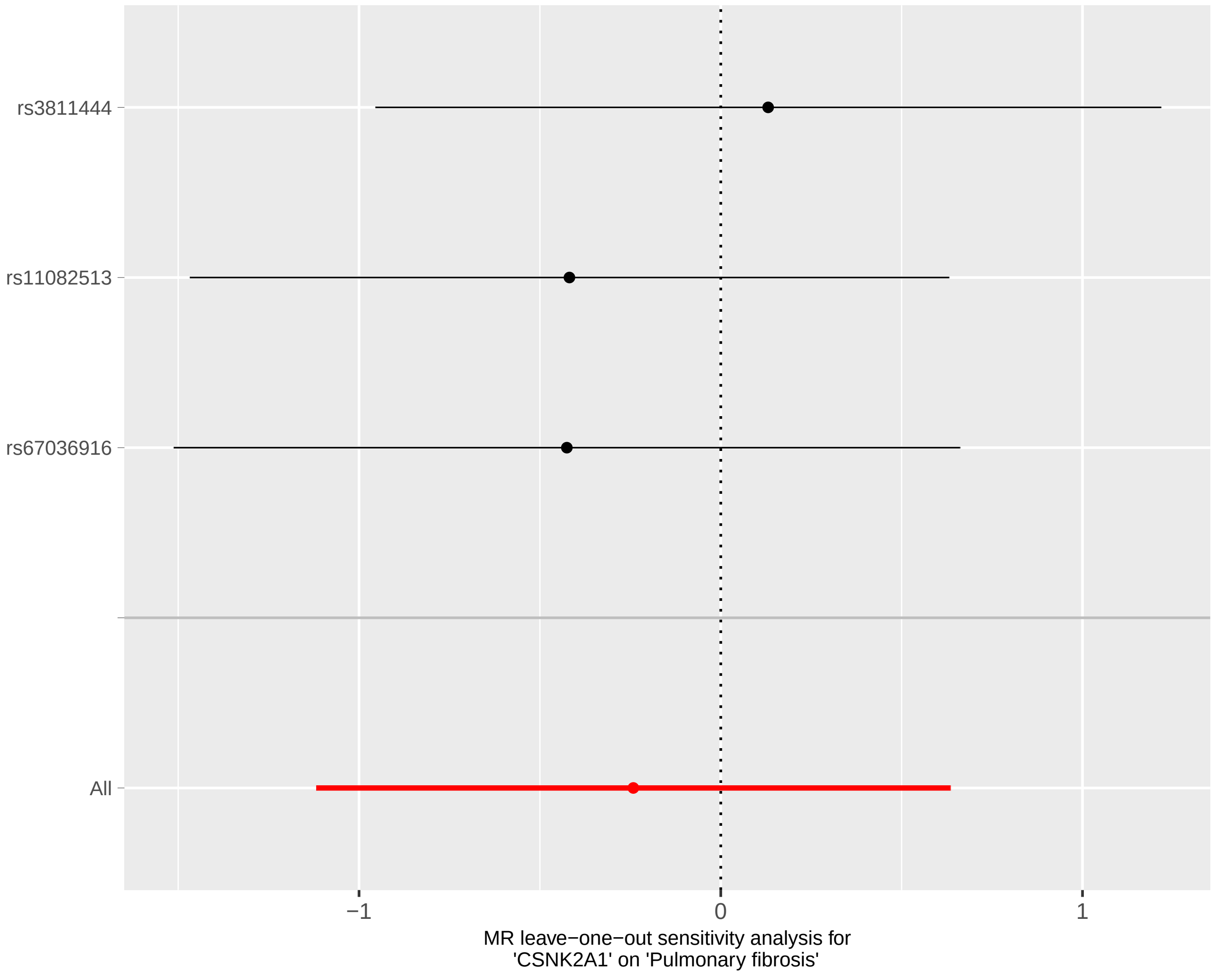
rs3811444
rs11082513
rs67036916
All
−1
0
1
MR leave−one−out sensitivity analysis for
'CSNK2A1' on 'Pulmonary fibrosis'

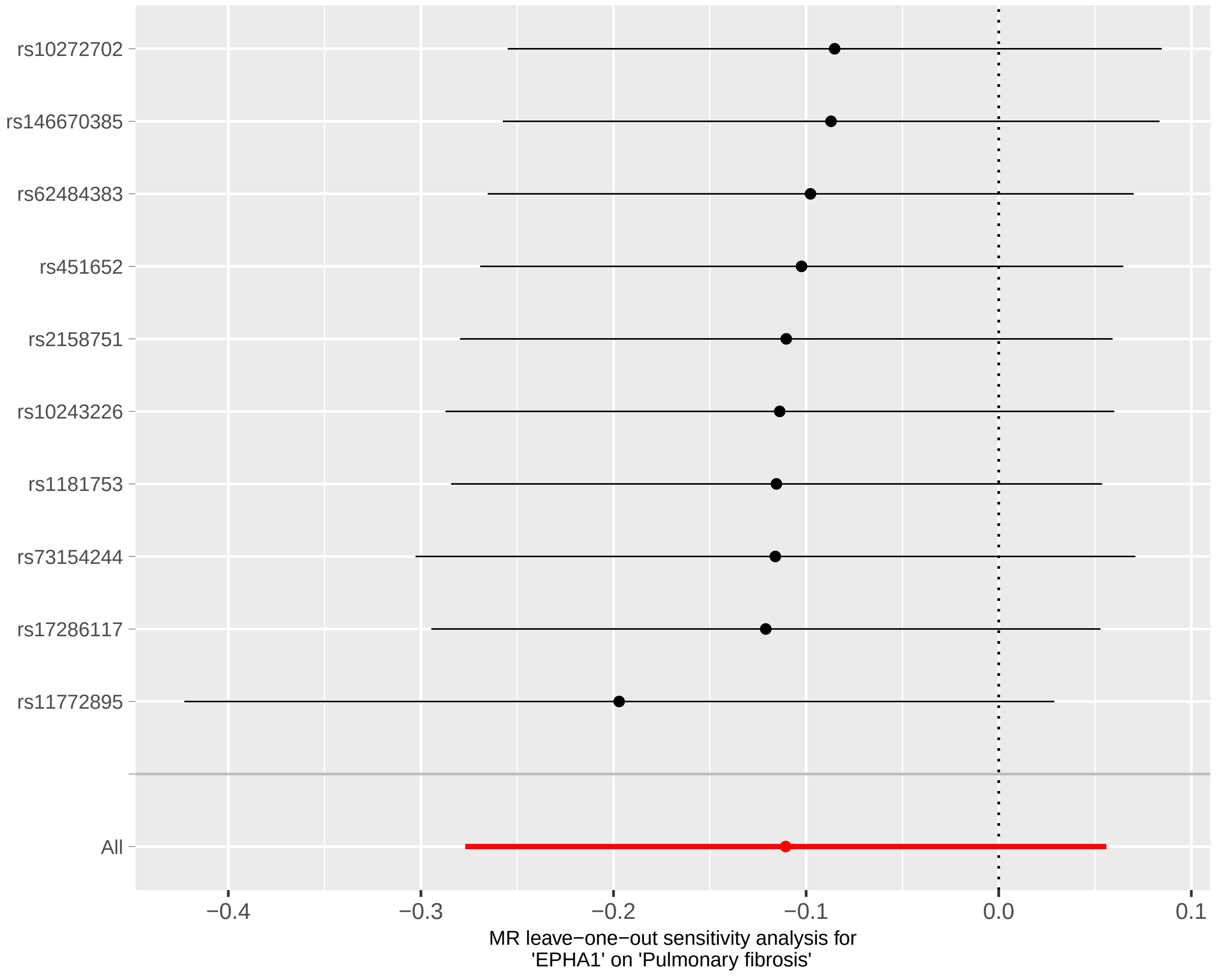

rs10272702
rs146670385
rs62484383
rs451652
rs2158751
rs10243226
rs1181753
rs73154244
rs17286117
rs11772895
All
−0.4
−0.3
−0.2
−0.1
0.0
0.1
MR leave−one−out sensitivity analysis for
'EPHA1' on 'Pulmonary fibrosis'

Insufficient number of SNPs

Insufficient number of SNPs

Insufficient number of SNPs

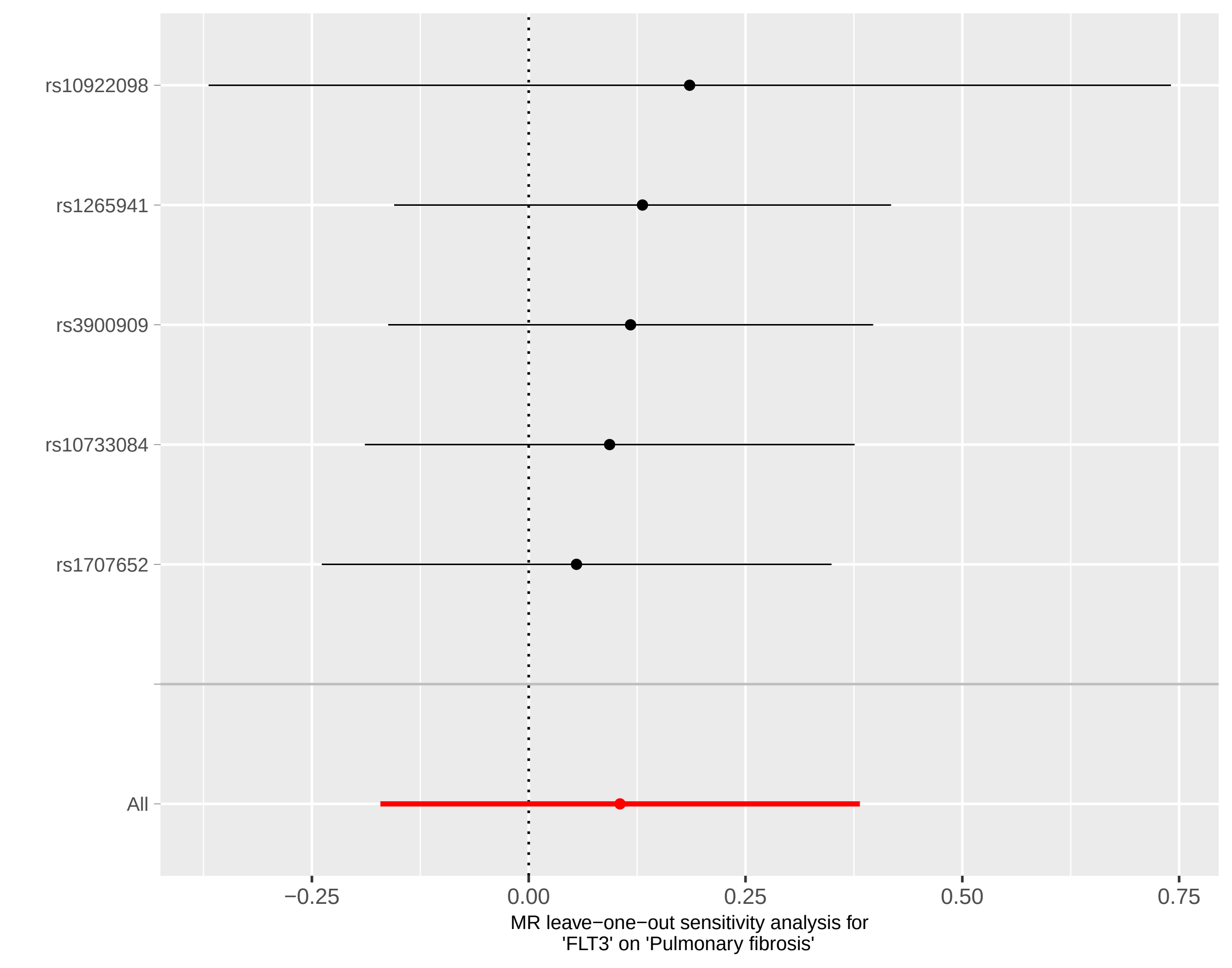
rs10922098
rs1265941
rs3900909
rs10733084
rs1707652
All
−0.25
0.00
0.25
0.50
0.75
MR leave−one−out sensitivity analysis for
'FLT3' on 'Pulmonary fibrosis'

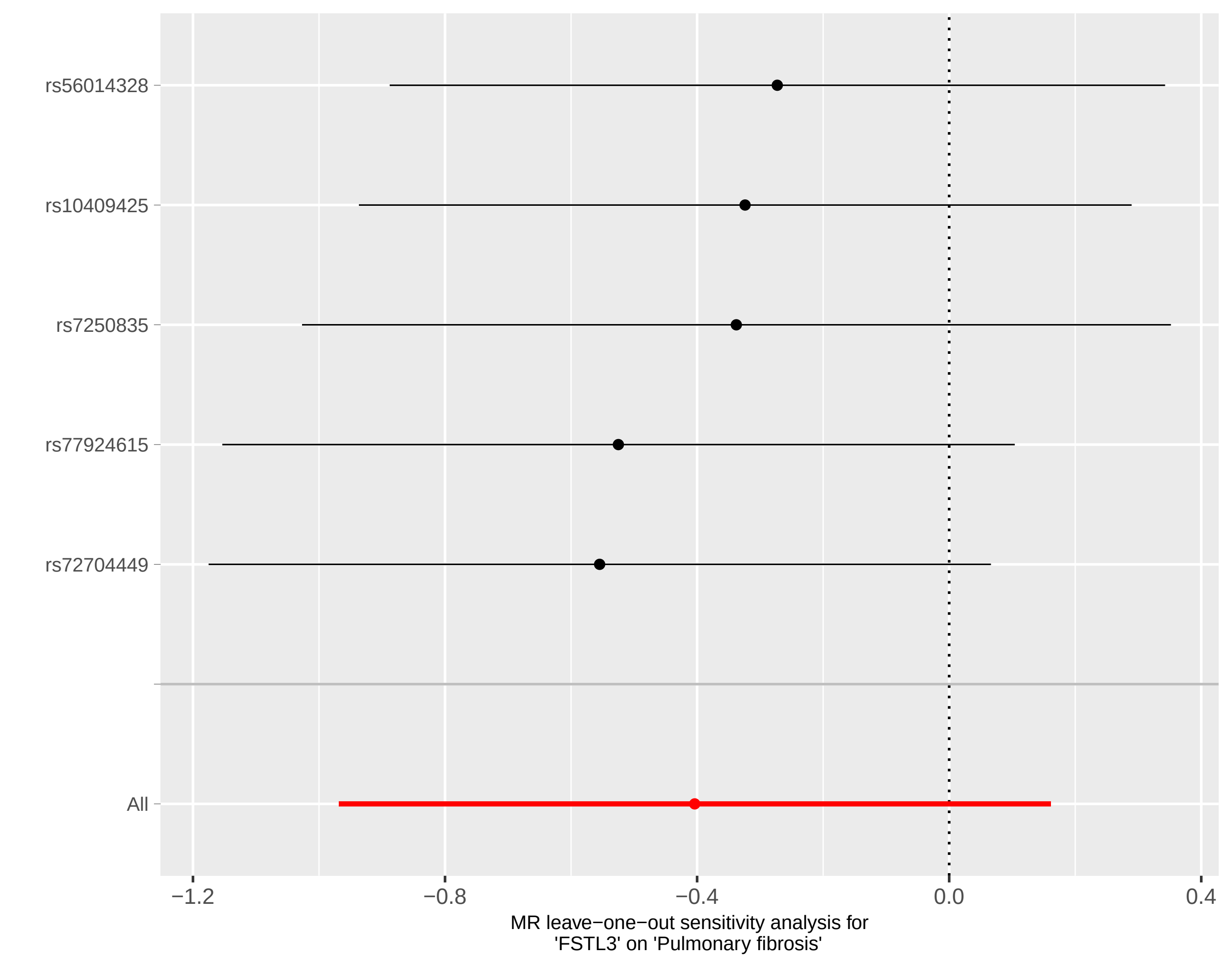
rs56014328
rs10409425
rs7250835
rs77924615
rs72704449
All
−1.2
−0.8
−0.4
0.0
0.4
MR leave−one−out sensitivity analysis for
'FSTL3' on 'Pulmonary fibrosis'

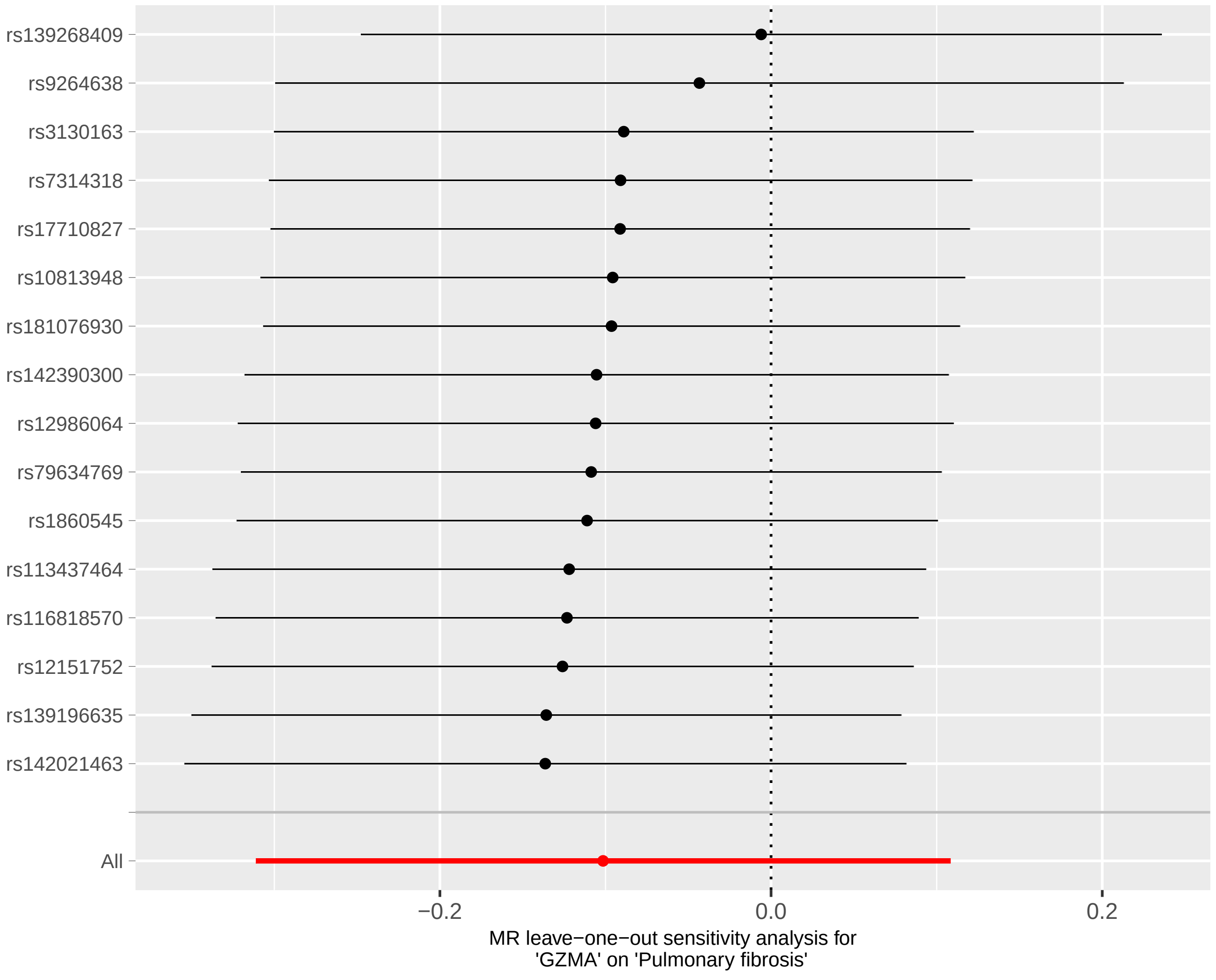
rs139268409
rs9264638
rs3130163
rs7314318
rs17710827
rs10813948
rs181076930
rs142390300
rs12986064
rs79634769
rs1860545
rs113437464
rs116818570
rs12151752
rs139196635
rs142021463
All
−0.2
0.0
0.2
MR leave−one−out sensitivity analysis for
'GZMA' on 'Pulmonary fibrosis'

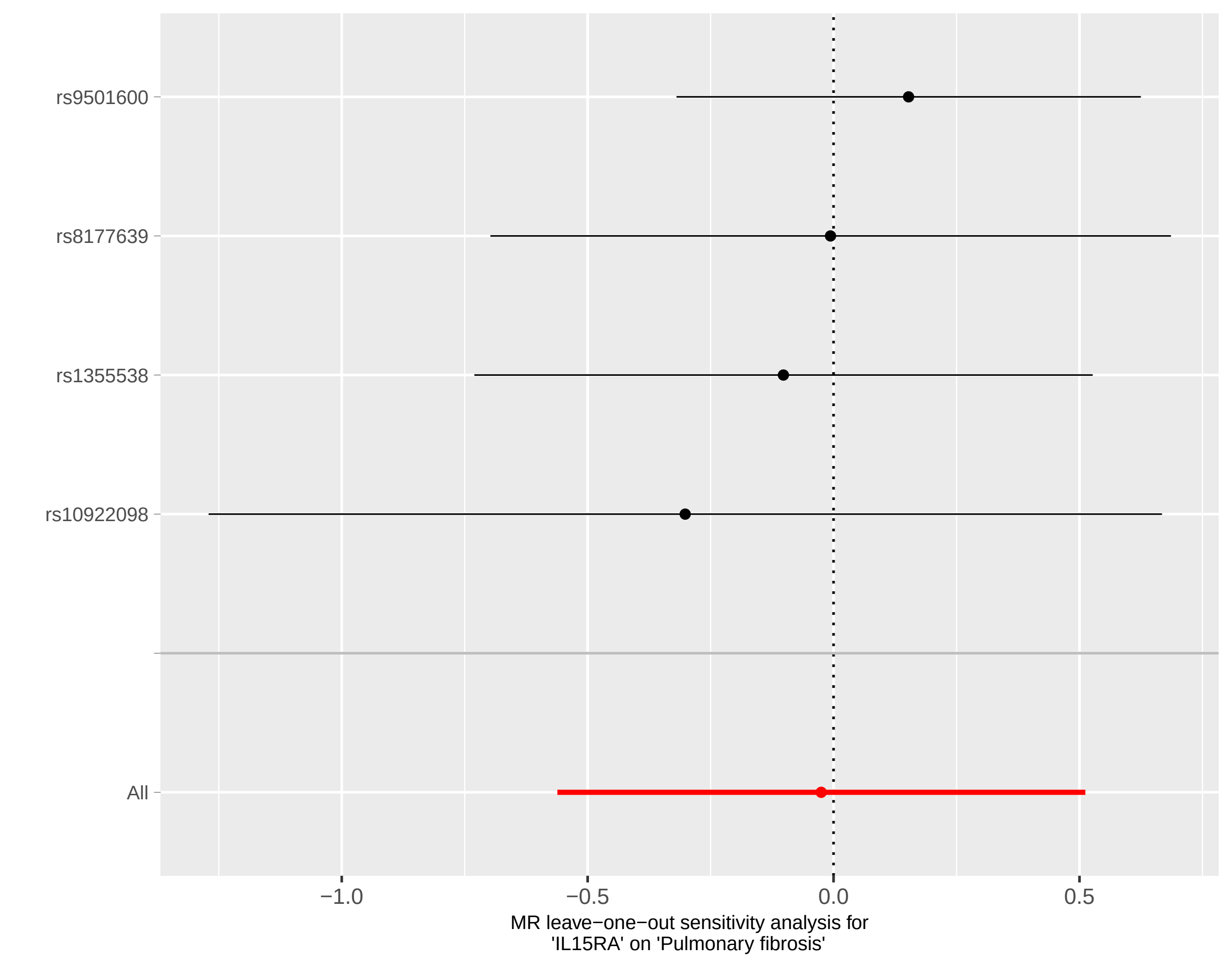

rs9501600
rs8177639
rs1355538
rs10922098
All
−1.0
−0.5
0.0
0.5
MR leave−one−out sensitivity analysis for
'IL15RA' on 'Pulmonary fibrosis'

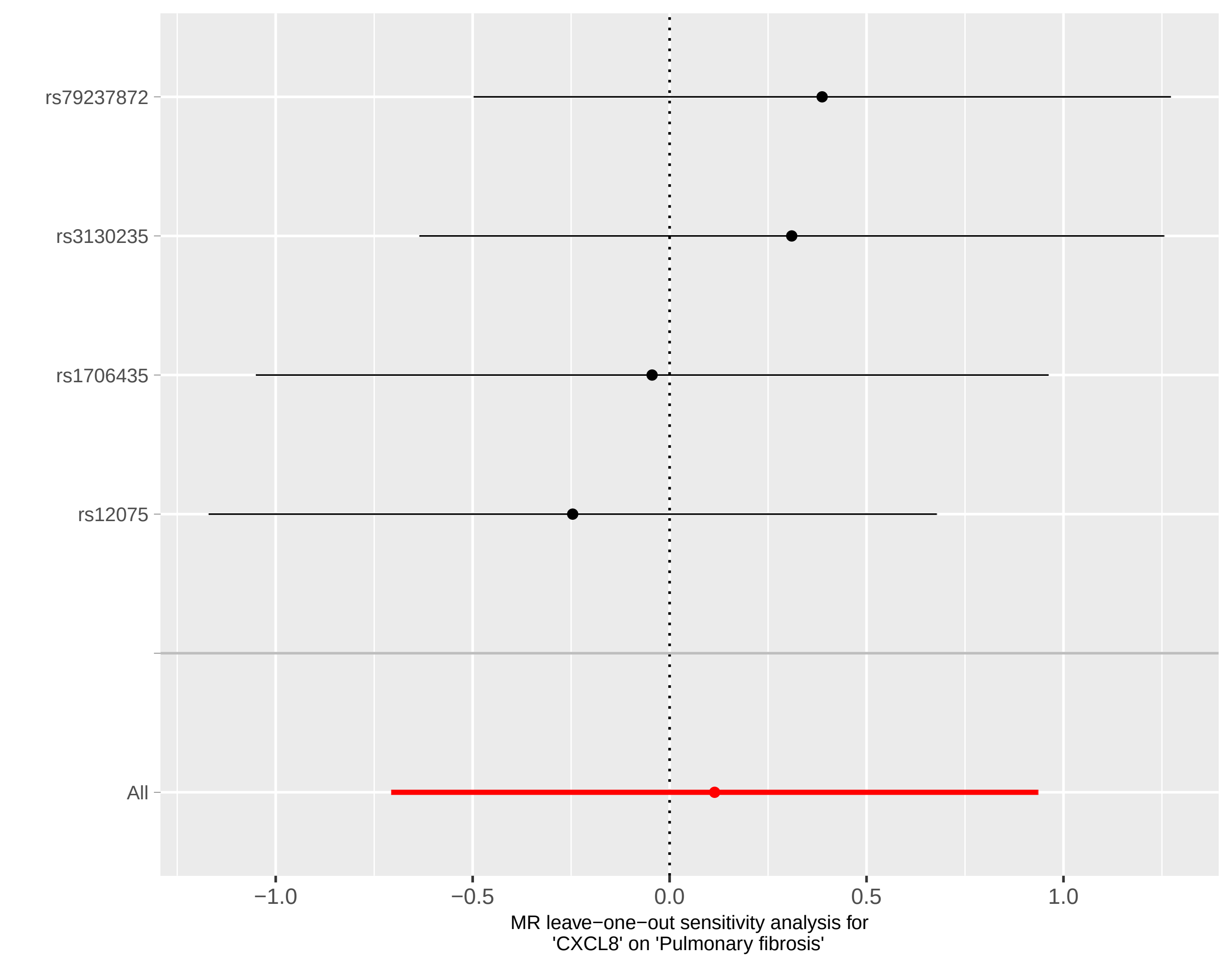
rs79237872
rs3130235
rs1706435
rs12075
All
−1.0
−0.5
0.0
0.5
1.0
MR leave−one−out sensitivity analysis for
'CXCL8' on 'Pulmonary fibrosis'

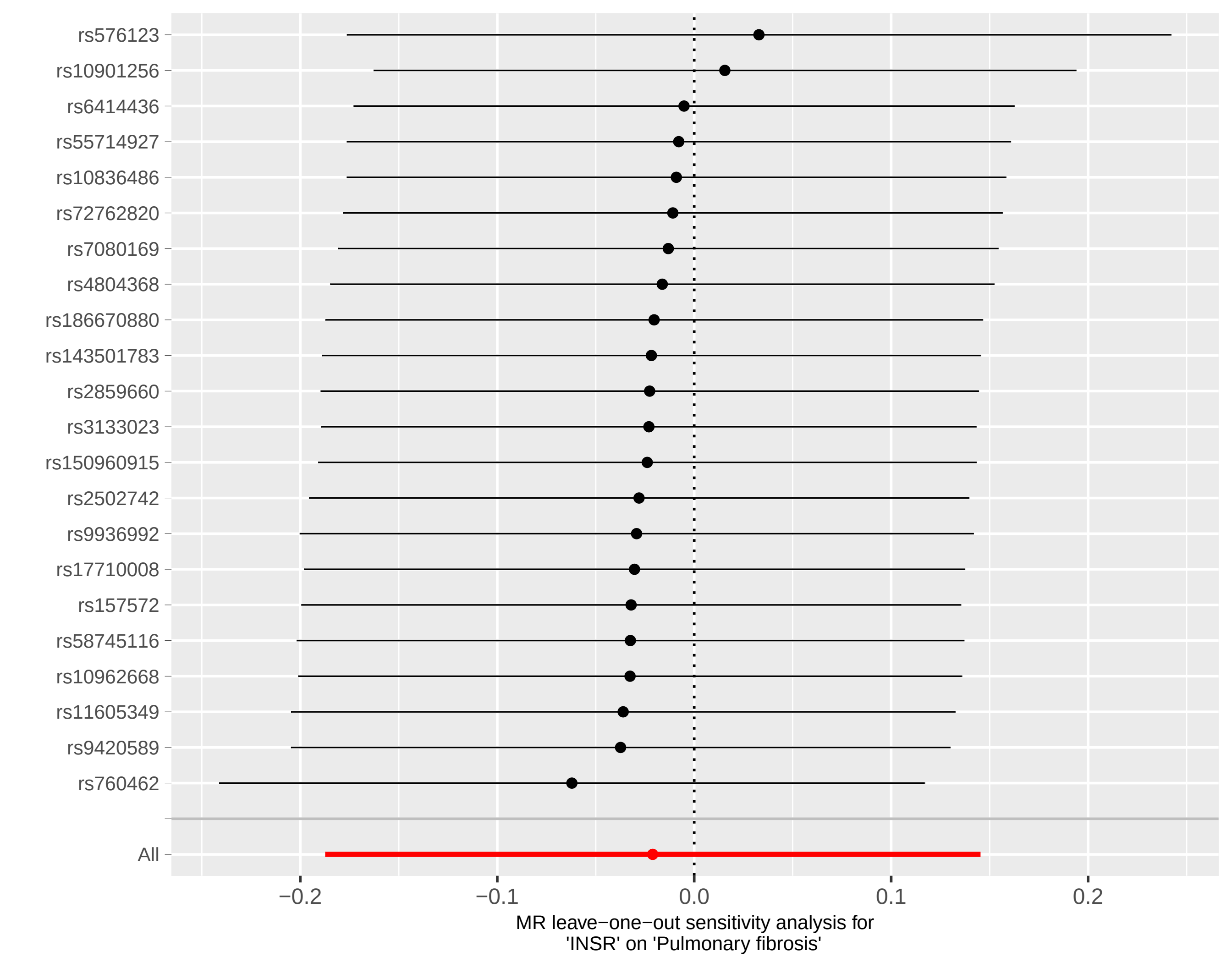

rs576123
rs10901256
rs6414436
rs55714927
rs10836486
rs72762820
rs7080169
rs4804368
rs186670880
rs143501783
rs2859660
rs3133023
rs150960915
rs2502742
rs9936992
rs17710008
rs157572
rs58745116
rs10962668
rs11605349
rs9420589
rs760462
All
−0.2
−0.1
0.0
0.1
0.2
MR leave−one−out sensitivity analysis for
'INSR' on 'Pulmonary fibrosis'

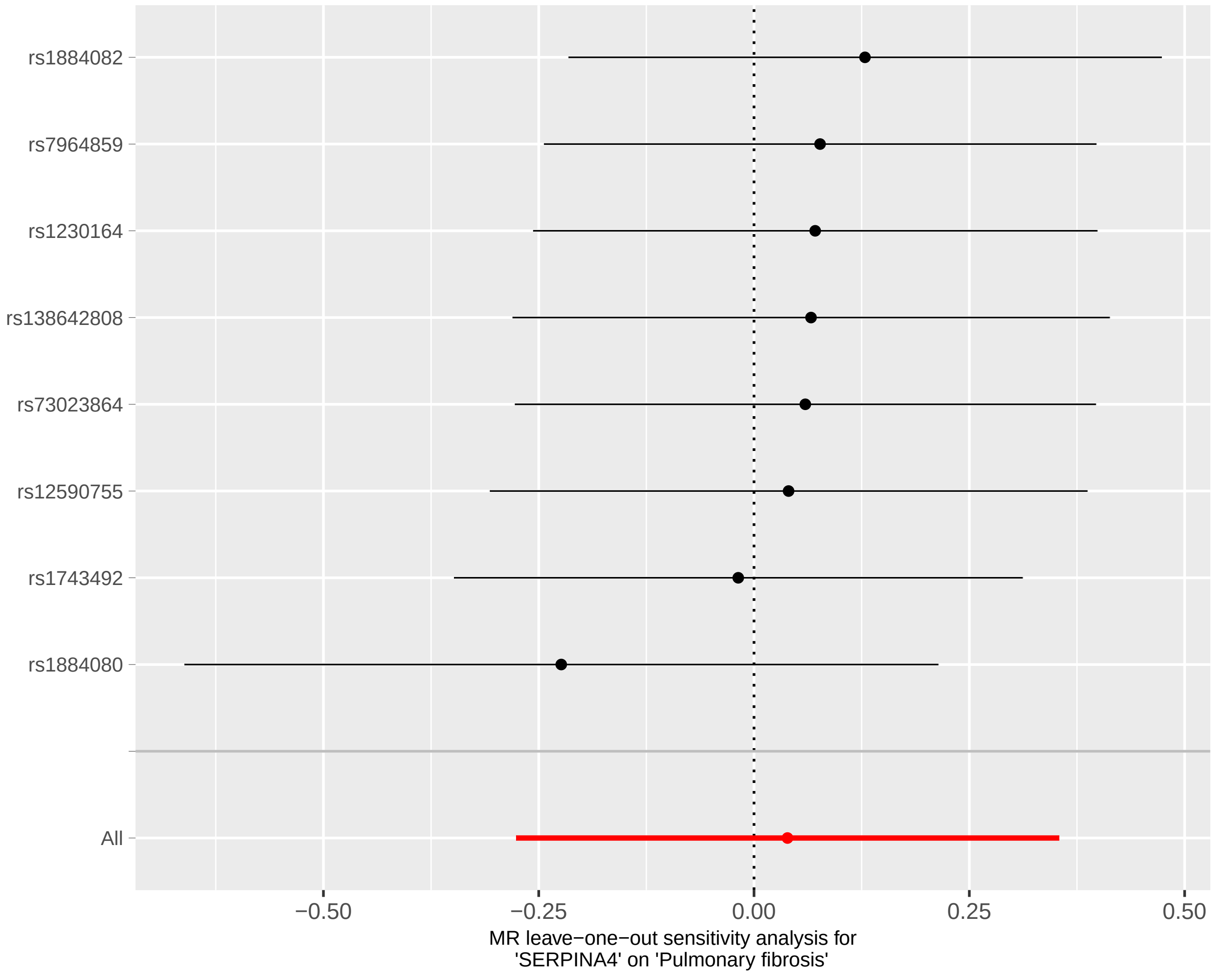

rs1884082
rs7964859
rs1230164
rs138642808
rs73023864
rs12590755
rs1743492
rs1884080
All
−0.50
−0.25
0.00
0.25
0.50
MR leave−one−out sensitivity analysis for
'SERPINA4' on 'Pulmonary fibrosis'

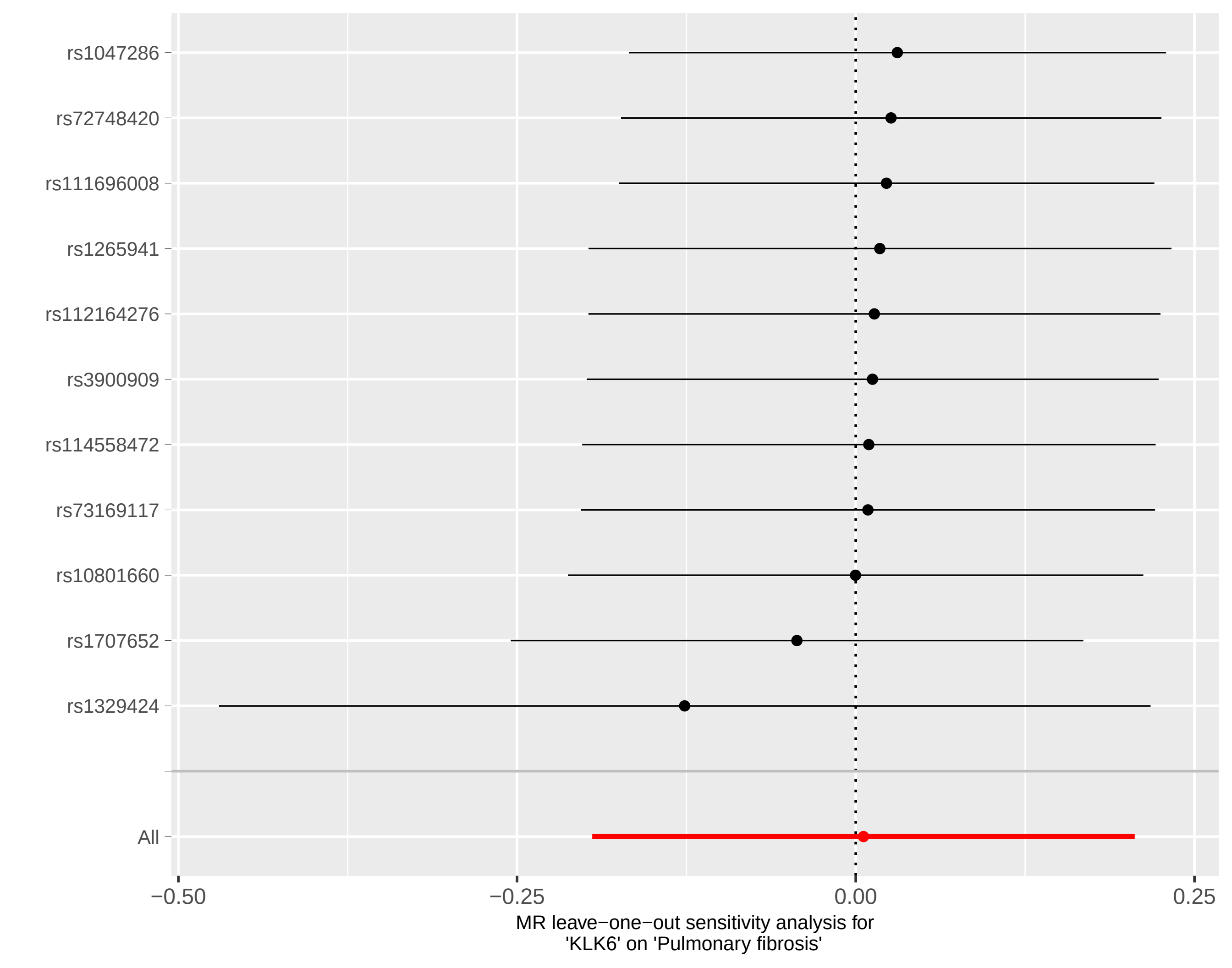
rs1047286
rs72748420
rs111696008
rs1265941
rs112164276
rs3900909
rs114558472
rs73169117
rs10801660
rs1707652
rs1329424
All
−0.50
−0.25
0.00
0.25
MR leave−one−out sensitivity analysis for
'KLK6' on 'Pulmonary fibrosis'

Insufficient number of SNPs

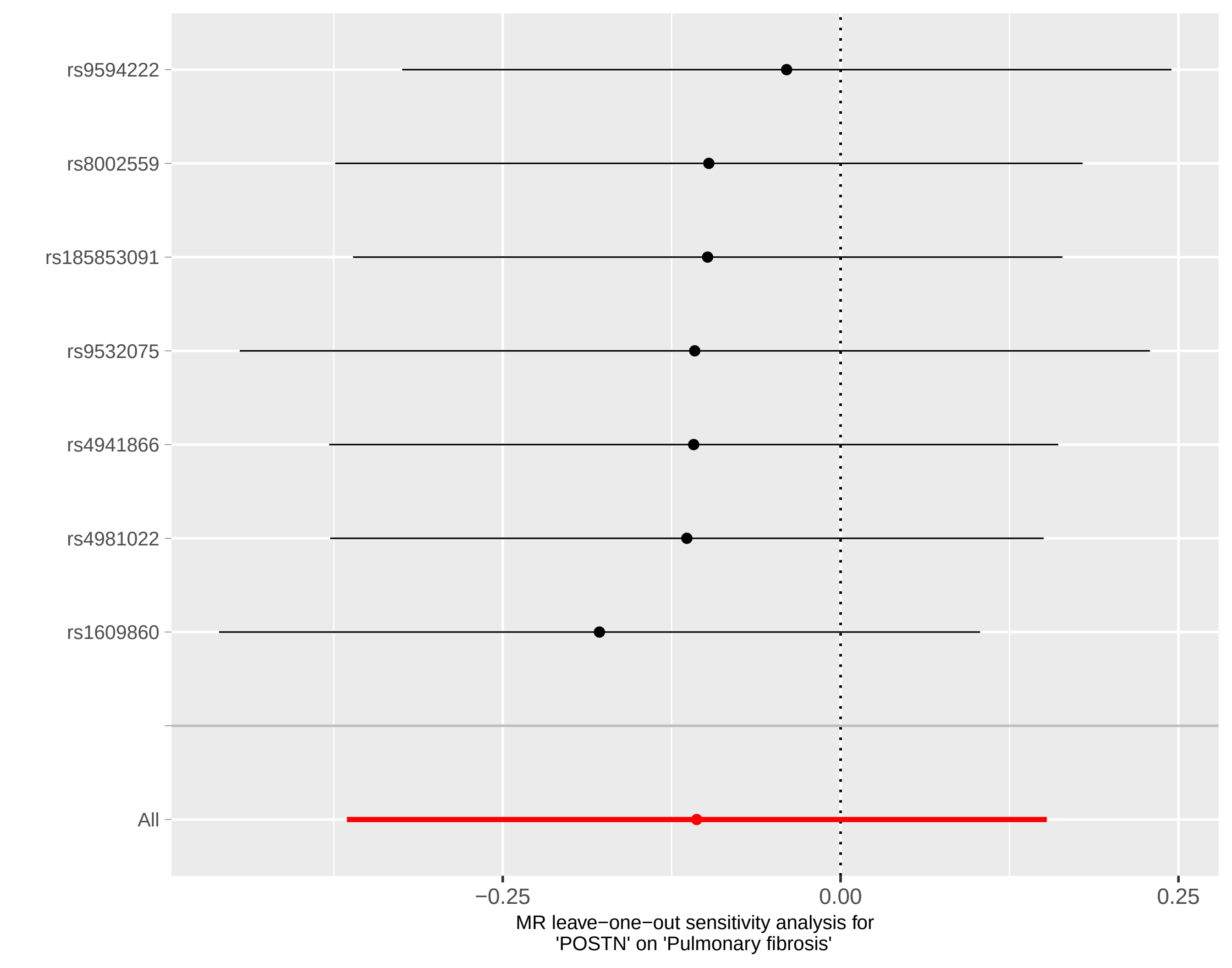
rs9594222
rs8002559
rs185853091
rs9532075
rs4941866
rs4981022
rs1609860
All
−0.25
0.00
0.25
MR leave−one−out sensitivity analysis for
'POSTN' on 'Pulmonary fibrosis'

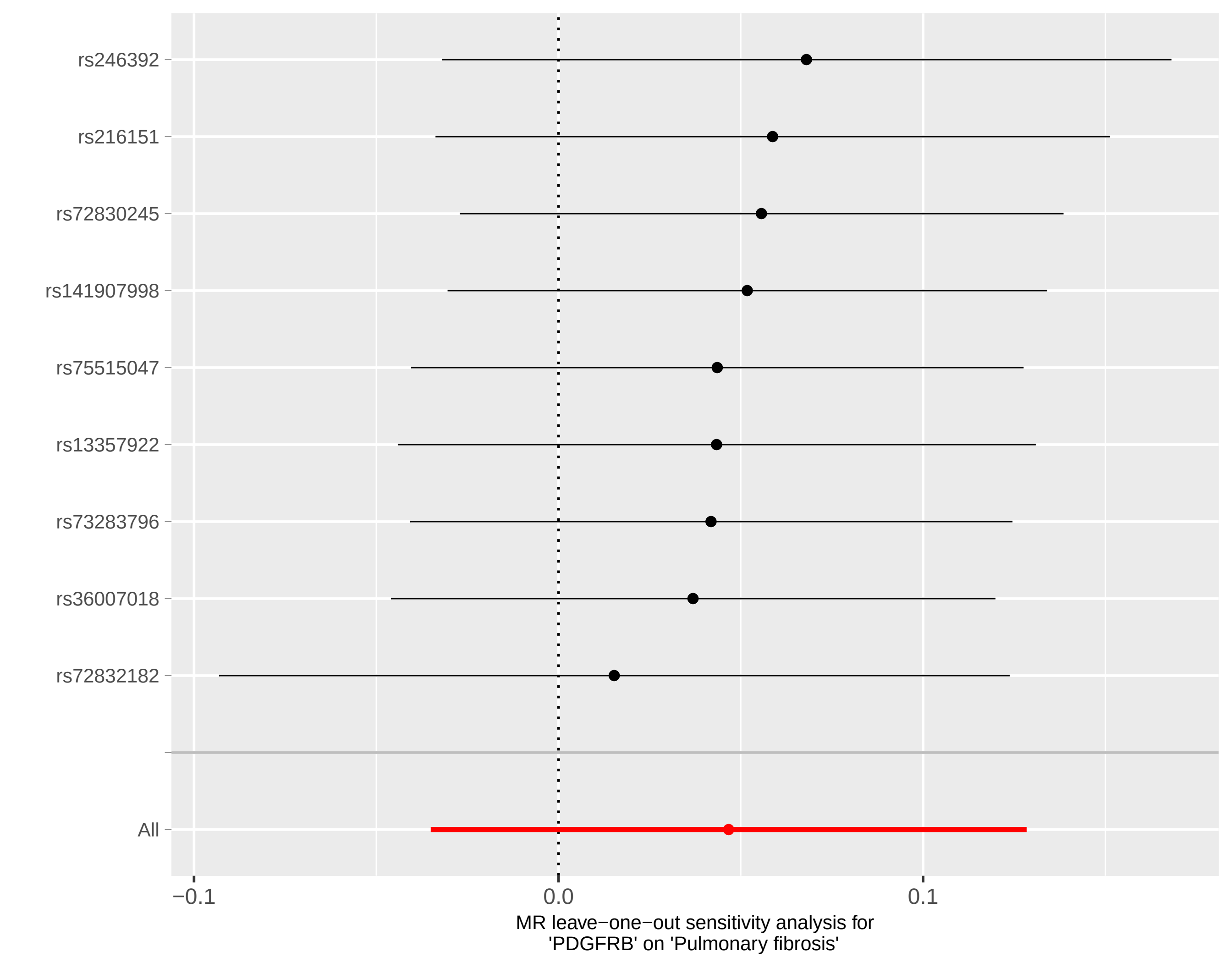

rs246392
rs216151
rs72830245
rs141907998
rs75515047
rs13357922
rs73283796
rs36007018
rs72832182
All
−0.1
0.0
0.1
MR leave−one−out sensitivity analysis for
'PDGFRB' on 'Pulmonary fibrosis'

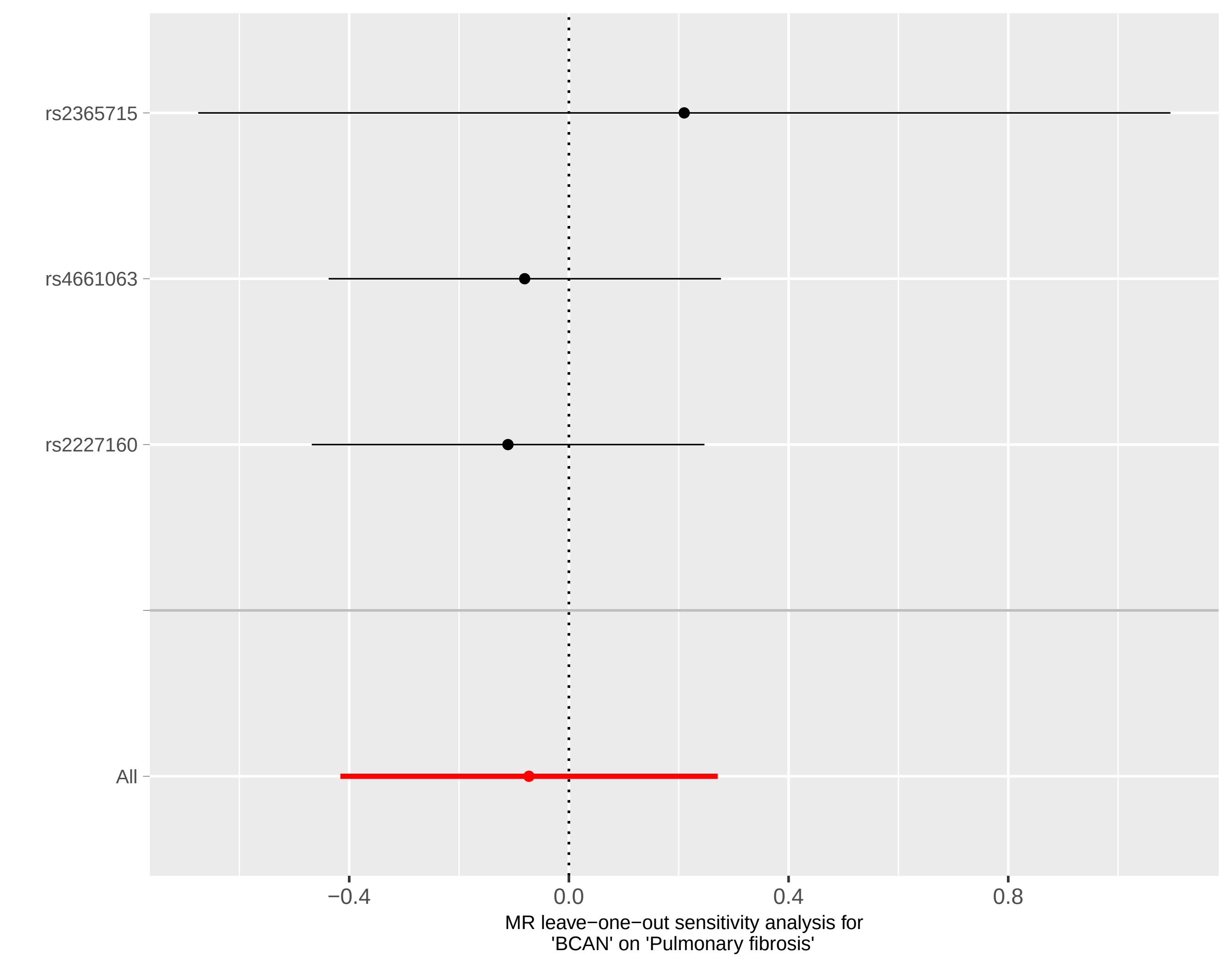
rs2365715
rs4661063
rs2227160
All
−0.4
0.0
0.4
0.8
MR leave−one−out sensitivity analysis for
'BCAN' on 'Pulmonary fibrosis'

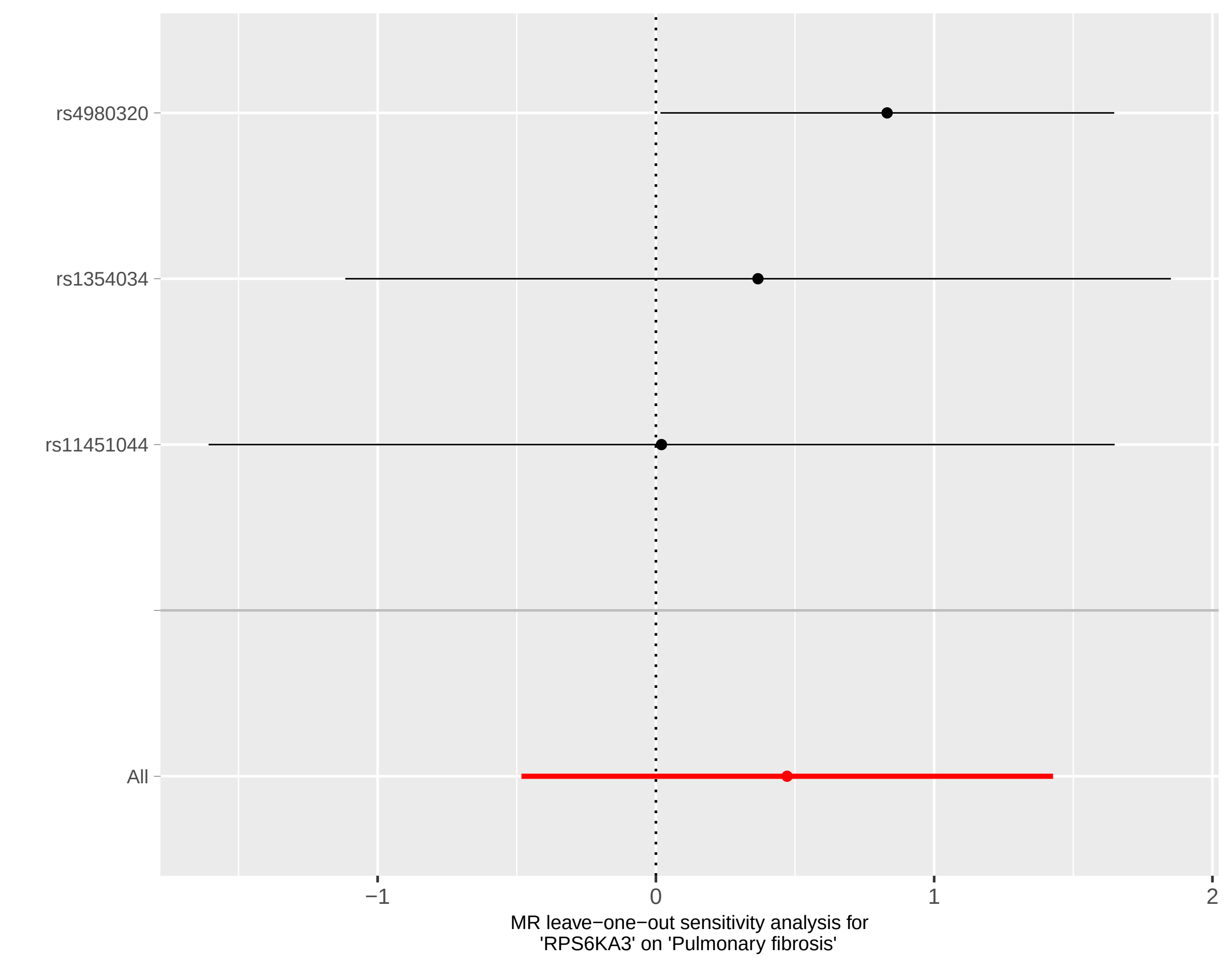
rs4980320
rs1354034
rs11451044
All
−1
0
1
2
MR leave−one−out sensitivity analysis for
'RPS6KA3' on 'Pulmonary fibrosis'

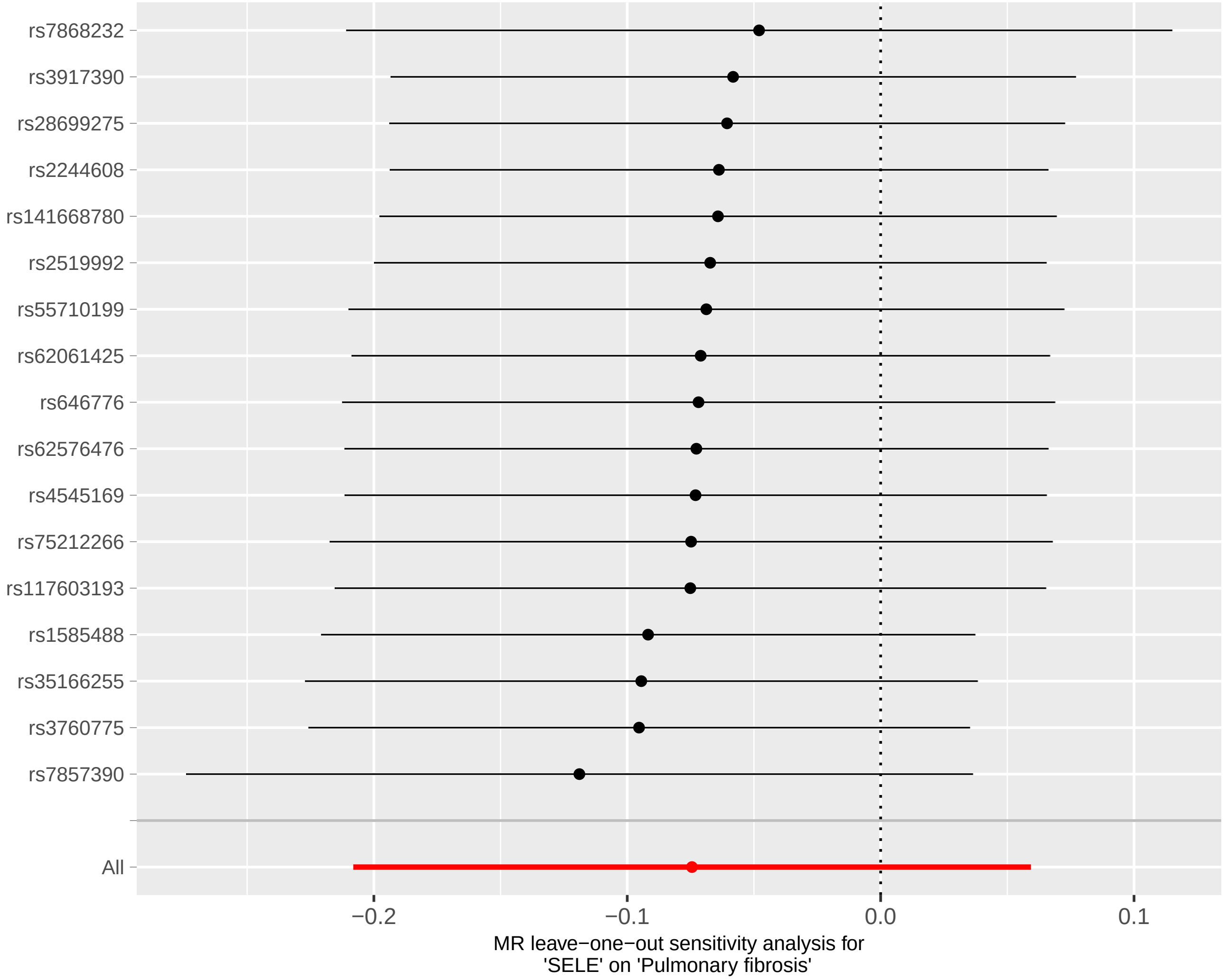
rs7868232
rs3917390
rs28699275
rs2244608
rs141668780
rs2519992
rs55710199
rs62061425
rs646776
rs62576476
rs4545169
rs75212266
rs117603193
rs1585488
rs35166255
rs3760775
rs7857390
All
−0.2
−0.1
0.0
0.1
MR leave−one−out sensitivity analysis for
'SELE' on 'Pulmonary fibrosis'

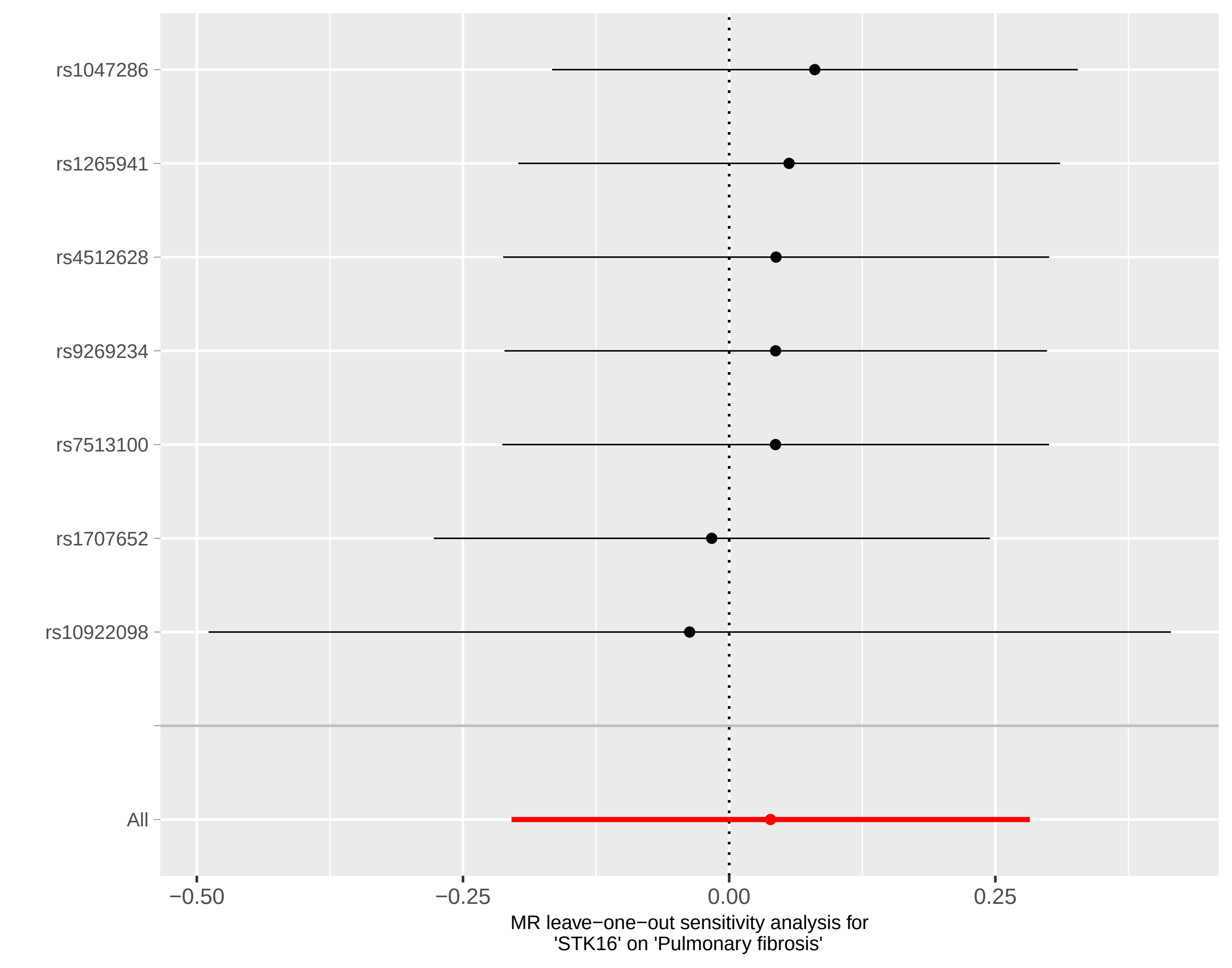
rs1047286
rs1265941
rs4512628
rs9269234
rs7513100
rs1707652
rs10922098
All
−0.50
−0.25
0.00
0.25
MR leave−one−out sensitivity analysis for
'STK16' on 'Pulmonary fibrosis'

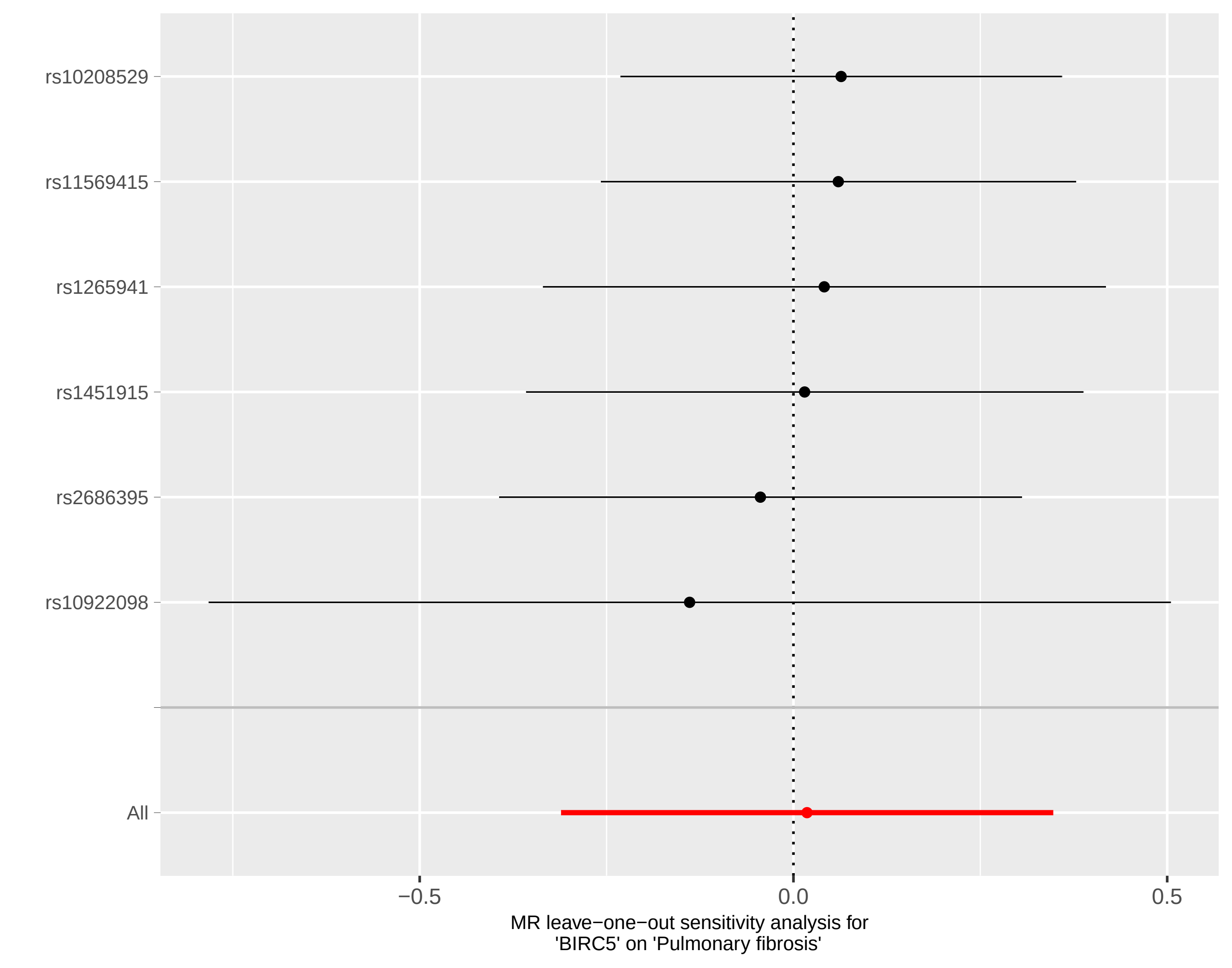
rs10208529
rs11569415
rs1265941
rs1451915
rs2686395
rs10922098
All
−0.5
0.0
0.5
MR leave−one−out sensitivity analysis for
'BIRC5' on 'Pulmonary fibrosis'

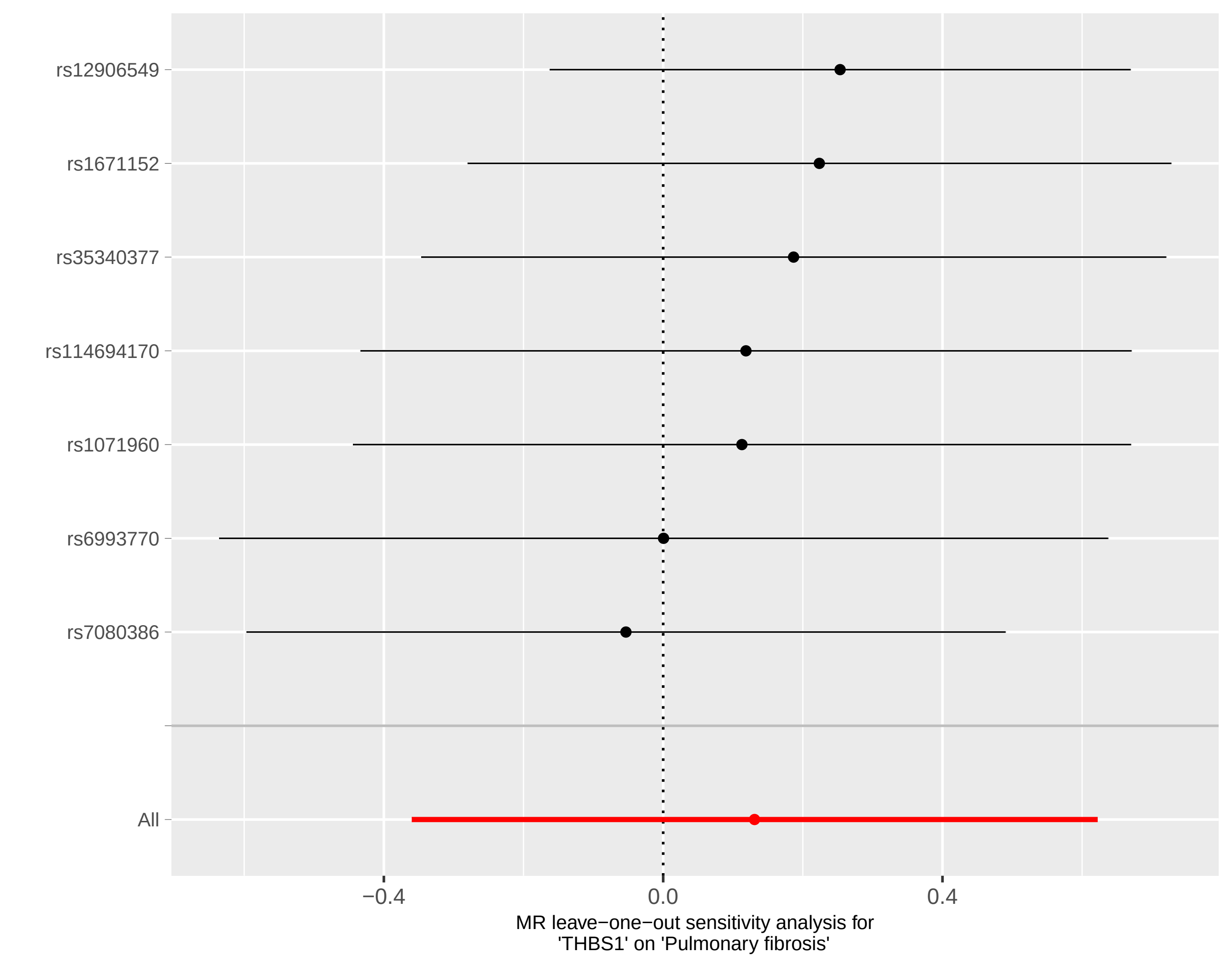
rs12906549
rs1671152
rs35340377
rs114694170
rs1071960
rs6993770
rs7080386
All
−0.4
0.0
0.4
MR leave−one−out sensitivity analysis for
'THBS1' on 'Pulmonary fibrosis'

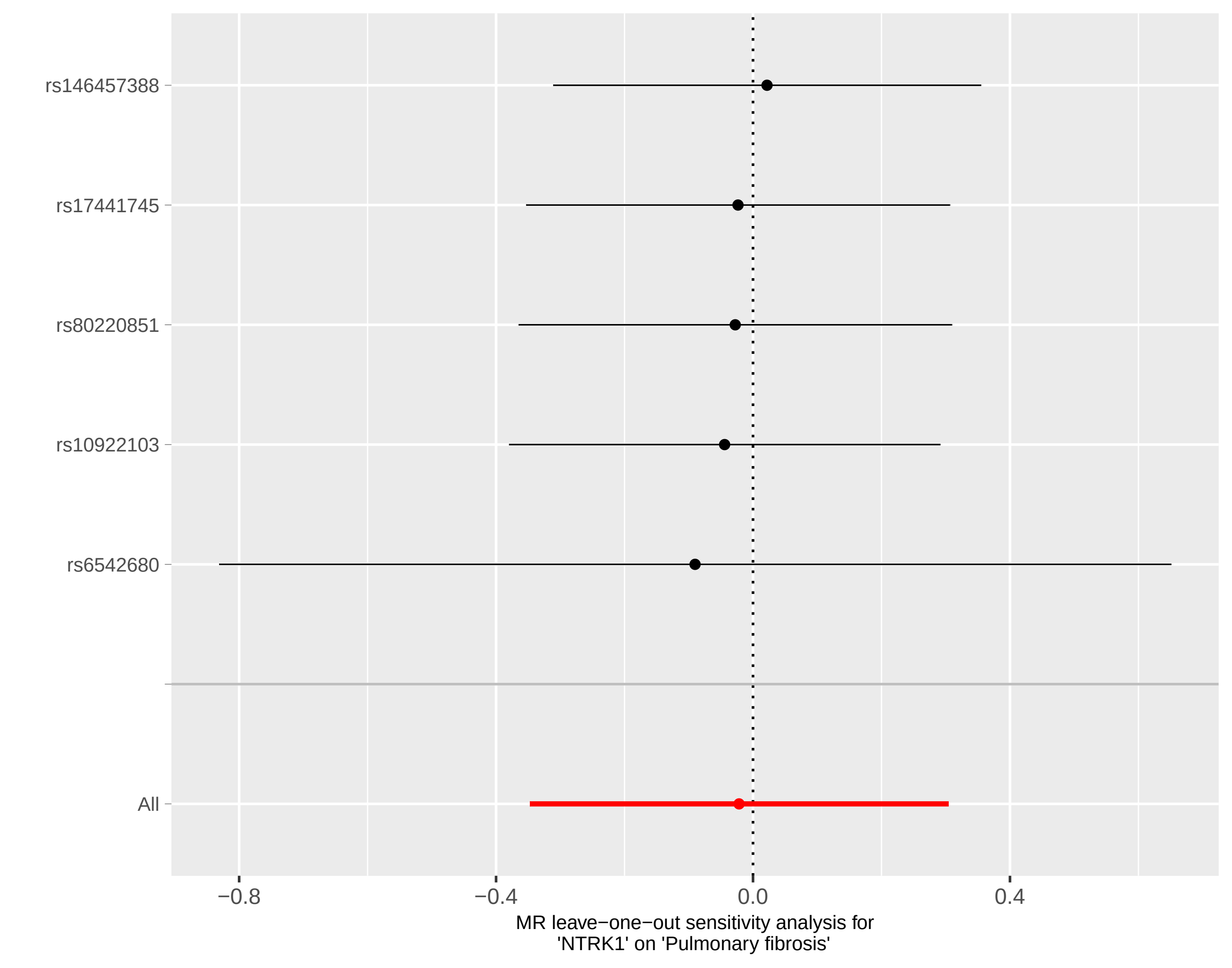
rs146457388
rs17441745
rs80220851
rs10922103
rs6542680
All
−0.8
−0.4
0.0
0.4
MR leave−one−out sensitivity analysis for
'NTRK1' on 'Pulmonary fibrosis'

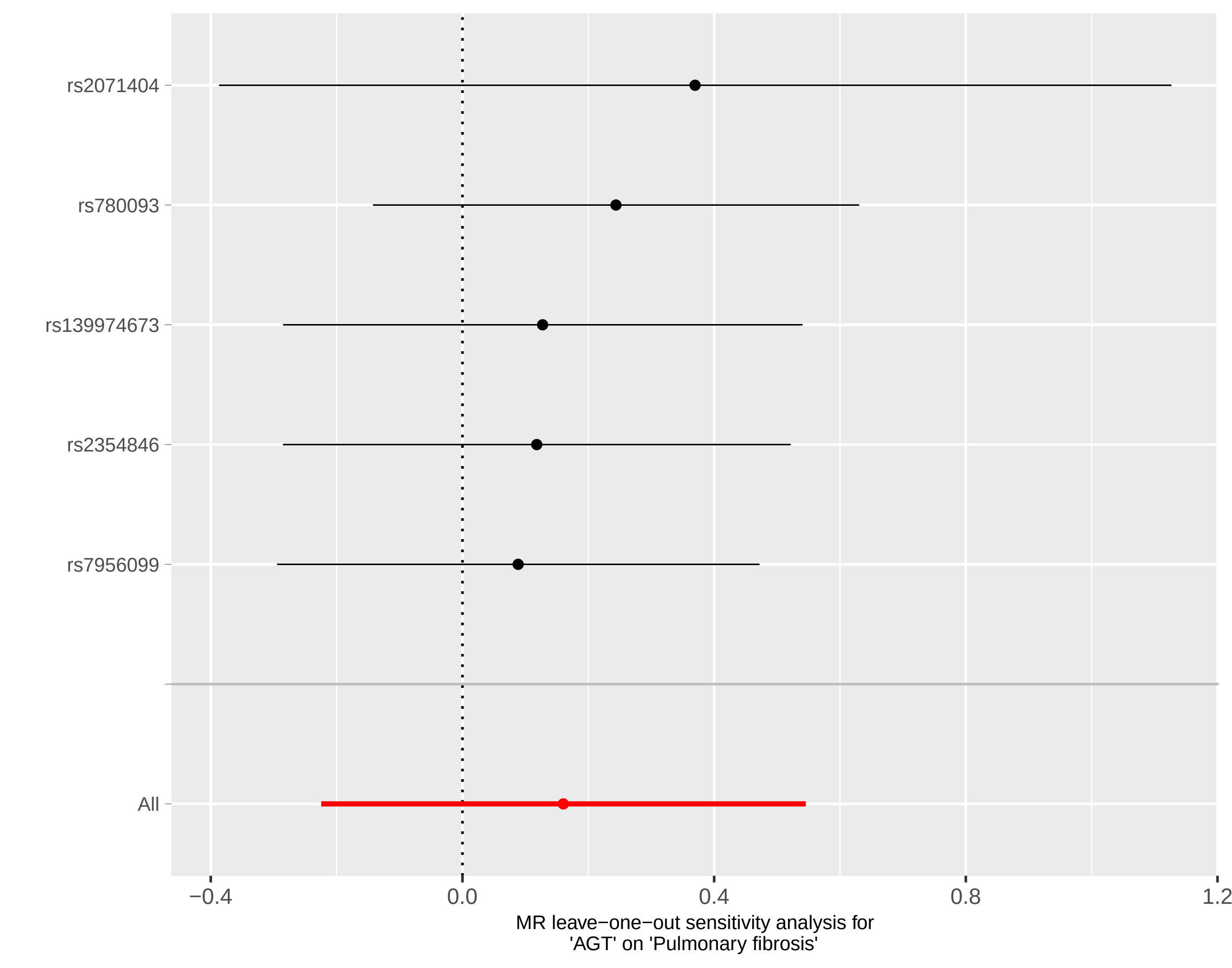

rs2071404
rs780093
rs139974673
rs2354846
rs7956099
All
−0.4
0.0
0.4
0.8
1.2
MR leave−one−out sensitivity analysis for
'AGT' on 'Pulmonary fibrosis'

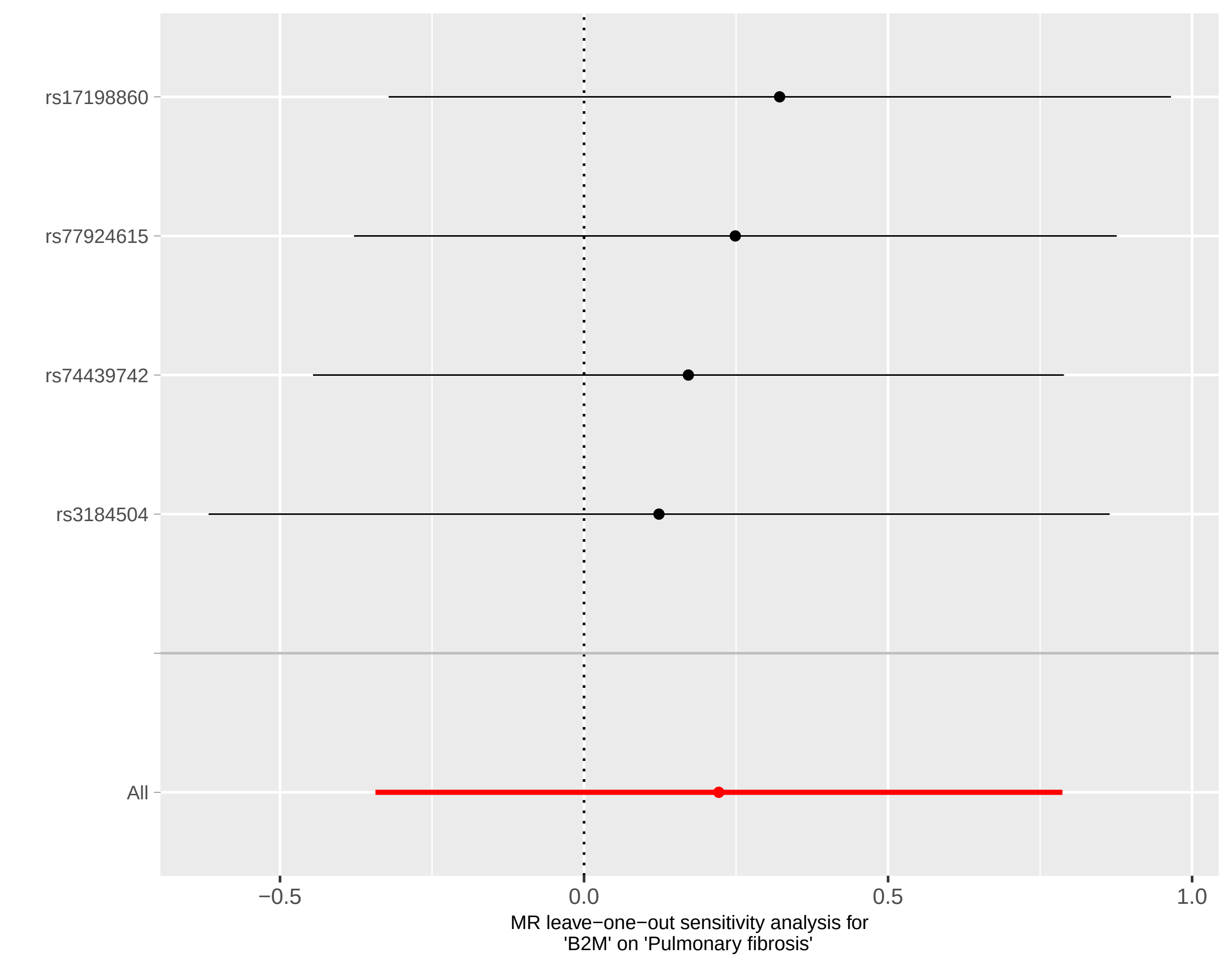

rs17198860
rs77924615
rs74439742
rs3184504
All
−0.5
0.0
0.5
1.0
MR leave−one−out sensitivity analysis for
'B2M' on 'Pulmonary fibrosis'

Insufficient number of SNPs

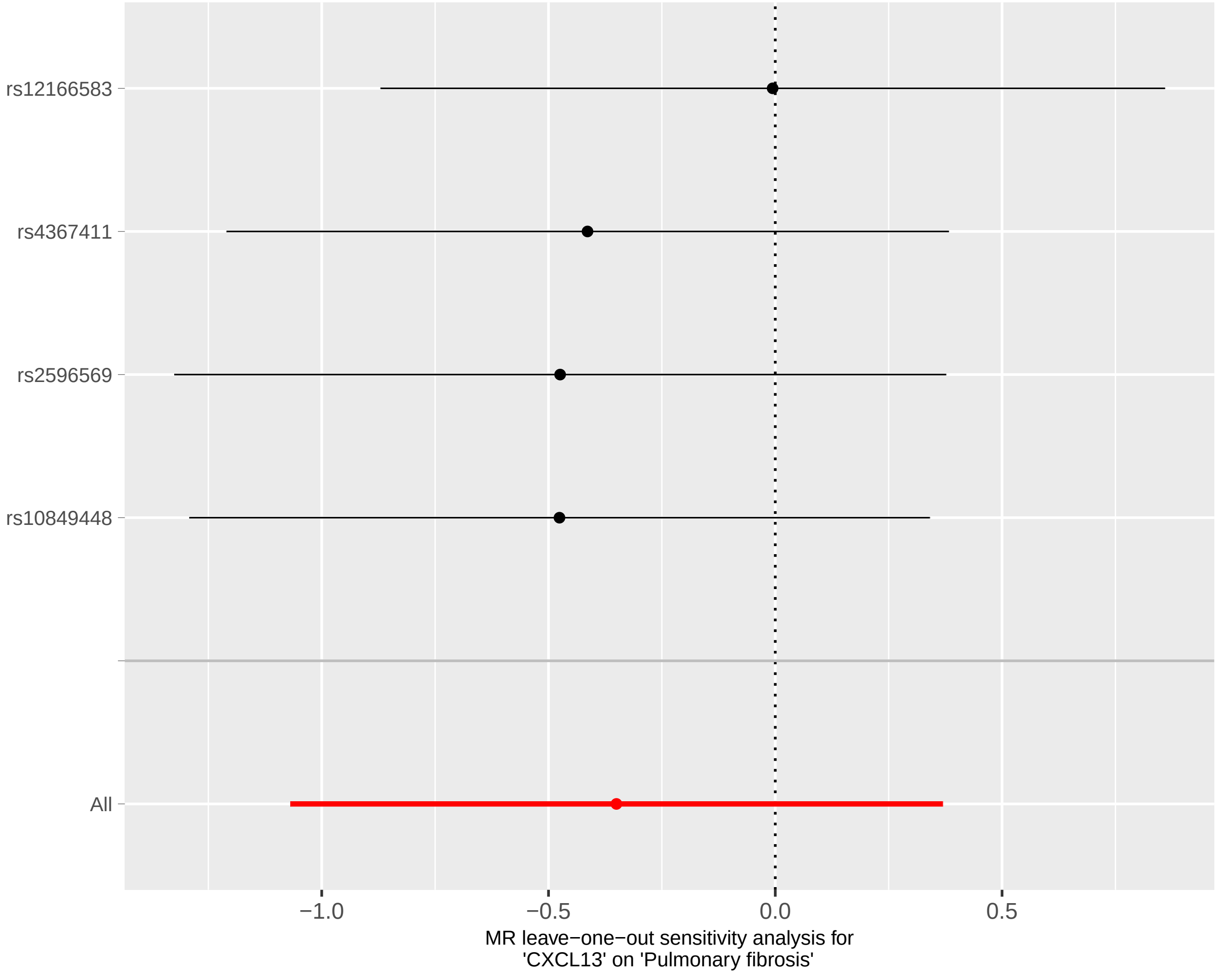

rs12166583
rs4367411
rs2596569
rs10849448
All
−1.0
−0.5
0.0
0.5
MR leave−one−out sensitivity analysis for
'CXCL13' on 'Pulmonary fibrosis'

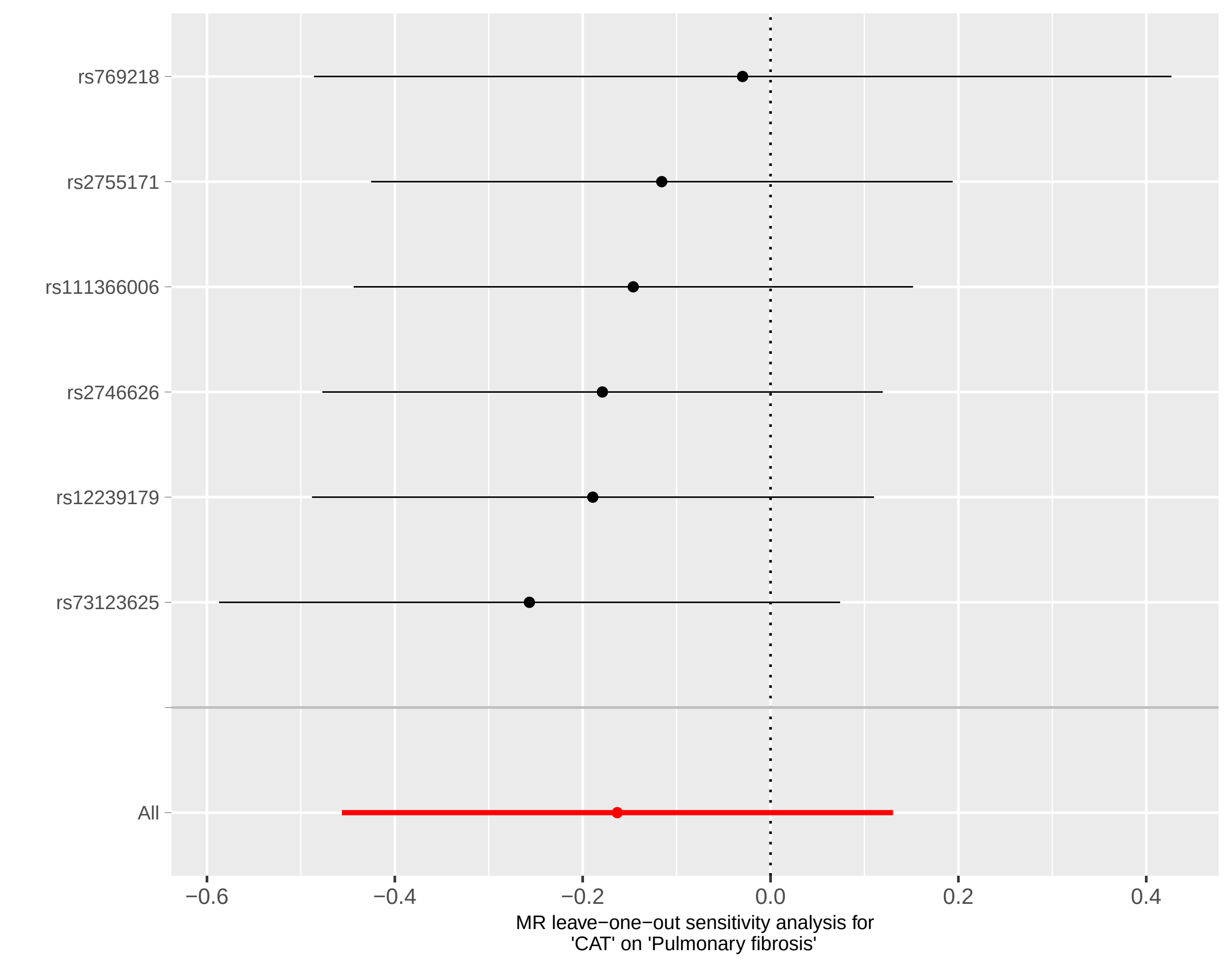
rs769218
rs2755171
rs111366006
rs2746626
rs12239179
rs73123625
All
−0.6
−0.4
−0.2
0.0
0.2
0.4
MR leave−one−out sensitivity analysis for
'CAT' on 'Pulmonary fibrosis'

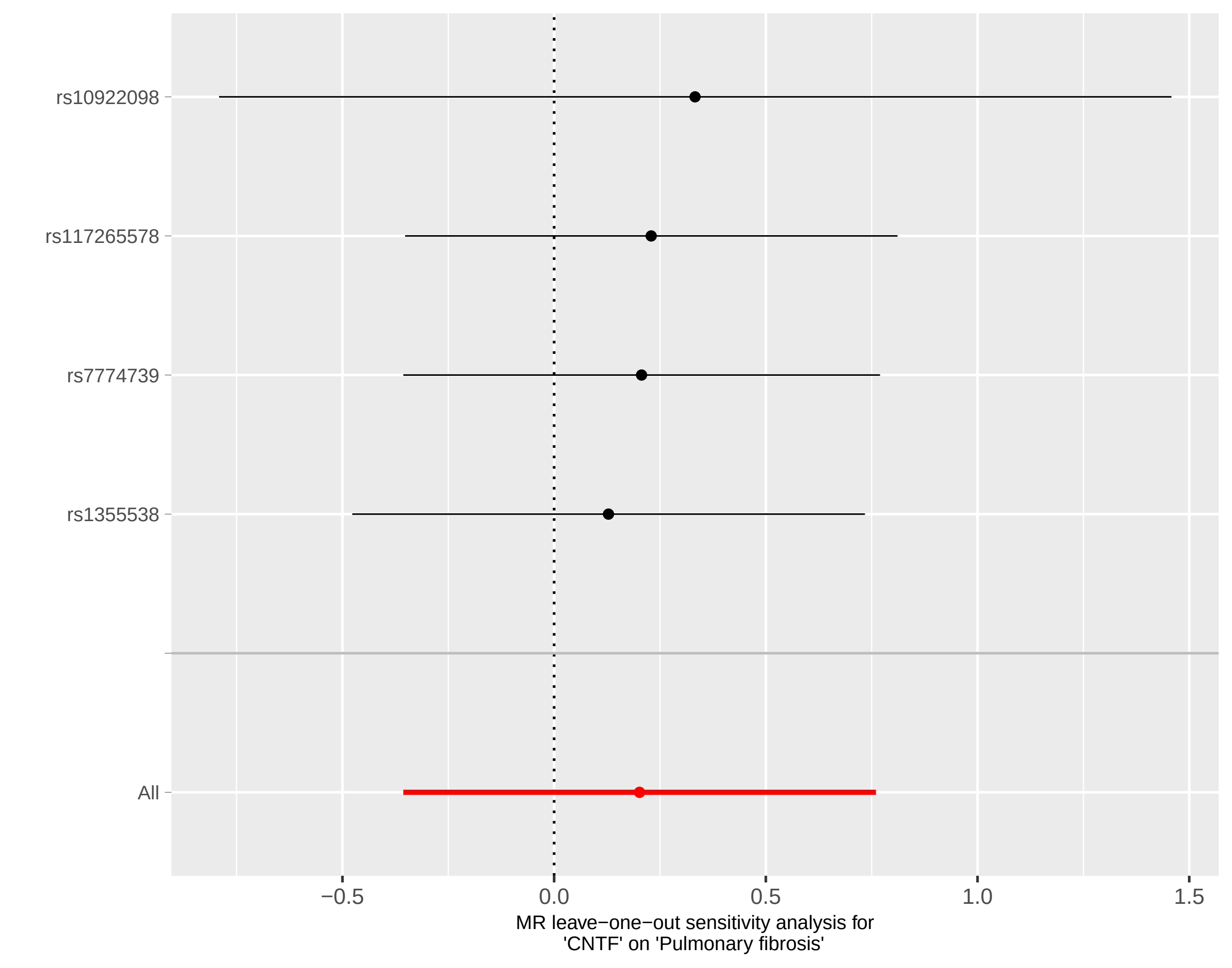
rs10922098
rs117265578
rs7774739
rs1355538
All
−0.5
0.0
0.5
1.0
1.5
MR leave−one−out sensitivity analysis for
'CNTF' on 'Pulmonary fibrosis'

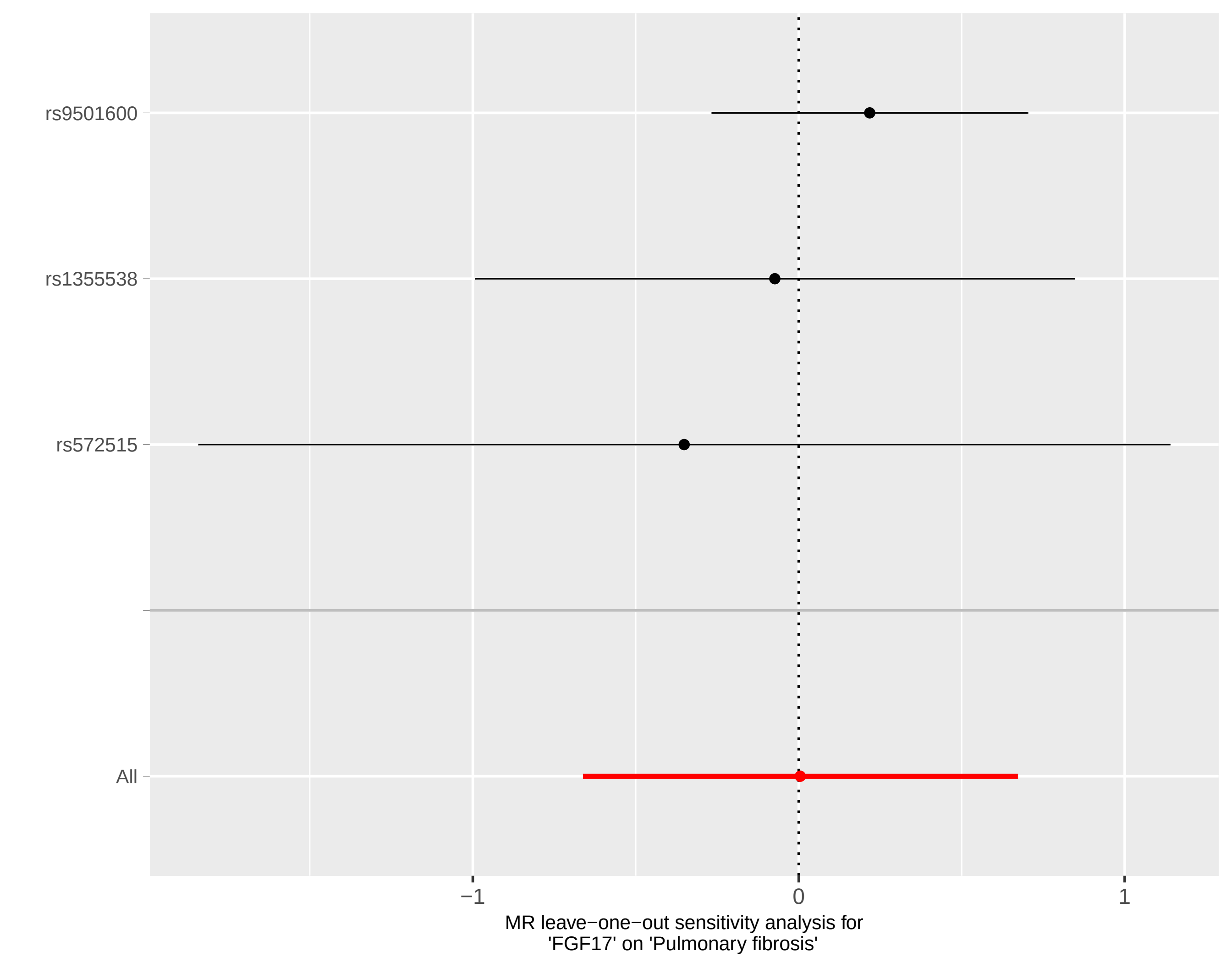
rs9501600
rs1355538
rs572515
All
−1
0
1
MR leave−one−out sensitivity analysis for
'FGF17' on 'Pulmonary fibrosis'

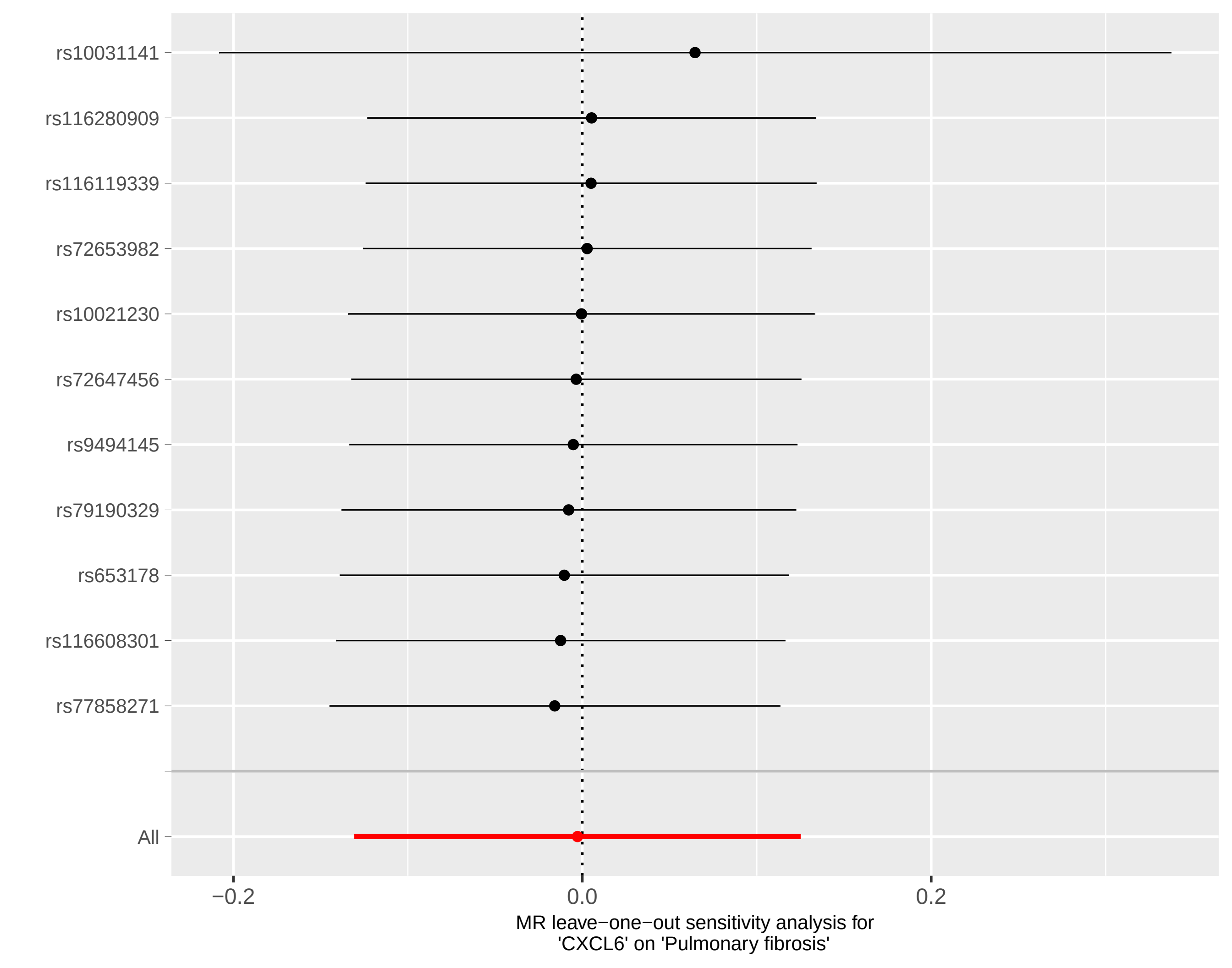
rs10031141
rs116280909
rs116119339
rs72653982
rs10021230
rs72647456
rs9494145
rs79190329
rs653178
rs116608301
rs77858271
All
−0.2
0.0
0.2
MR leave−one−out sensitivity analysis for
'CXCL6' on 'Pulmonary fibrosis'

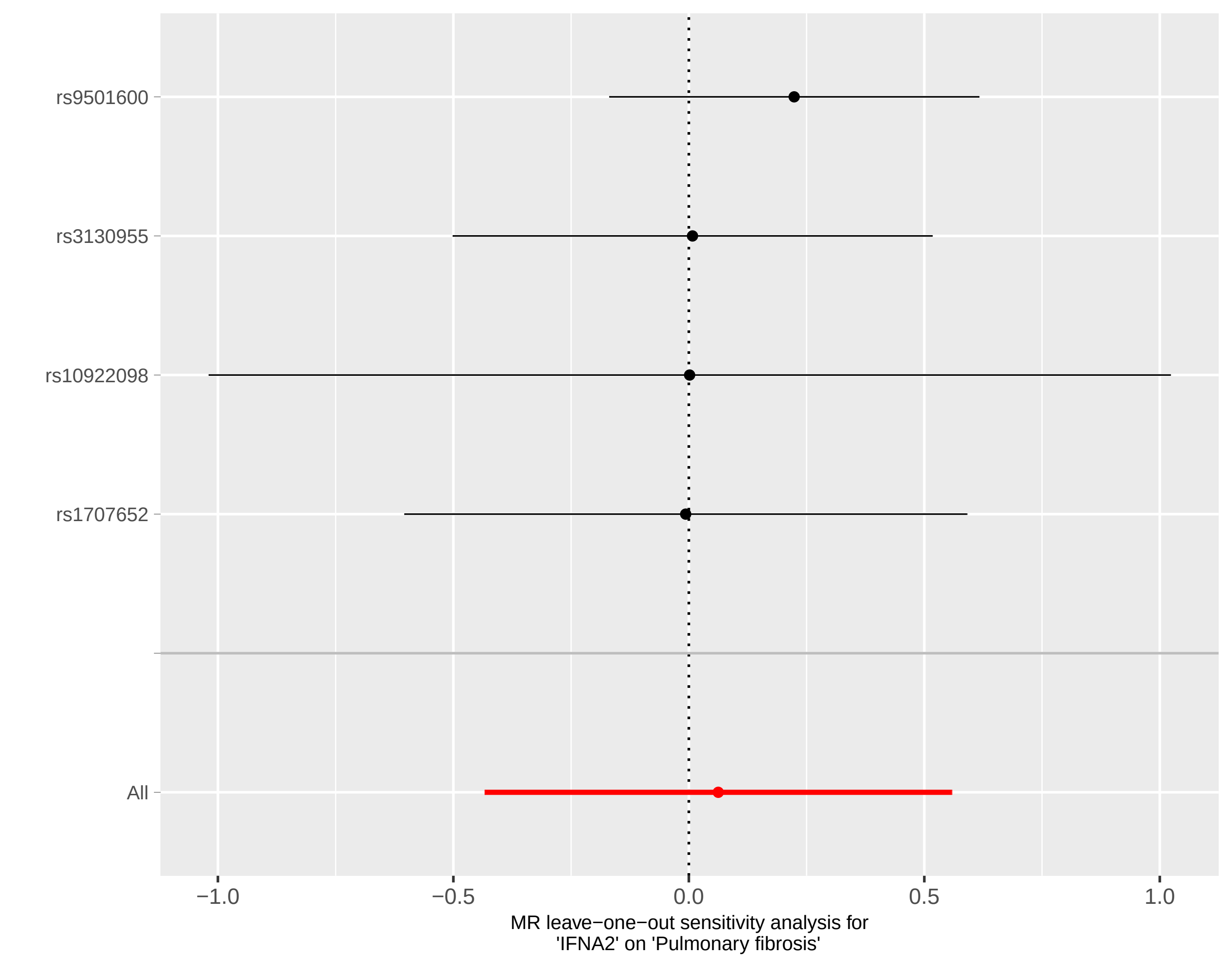
rs9501600
rs3130955
rs10922098
rs1707652
All
−1.0
−0.5
0.0
0.5
1.0
MR leave−one−out sensitivity analysis for
'IFNA2' on 'Pulmonary fibrosis'

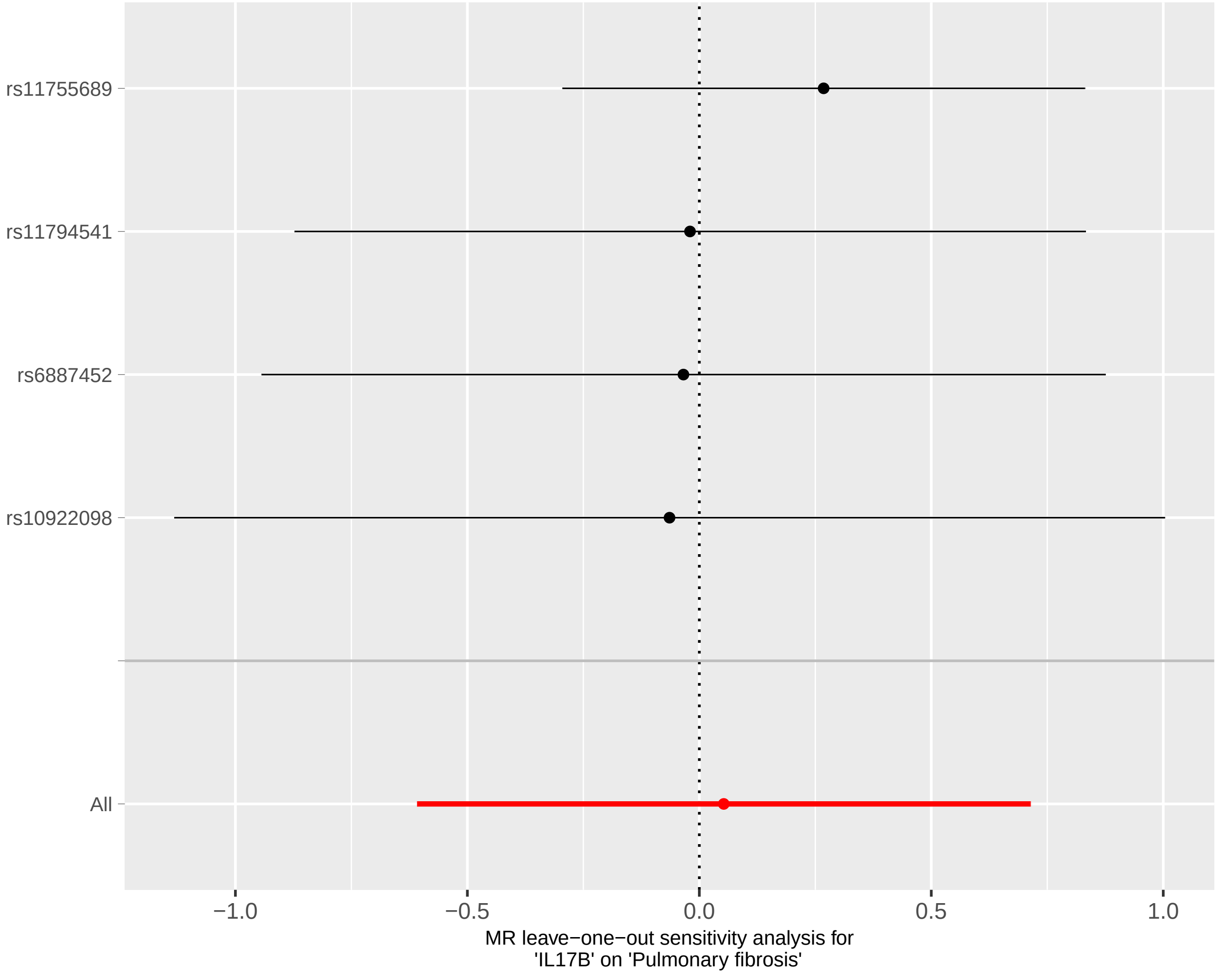

rs11755689
rs11794541
rs6887452
rs10922098
All
−1.0
−0.5
0.0
0.5
1.0
MR leave−one−out sensitivity analysis for
'IL17B' on 'Pulmonary fibrosis'

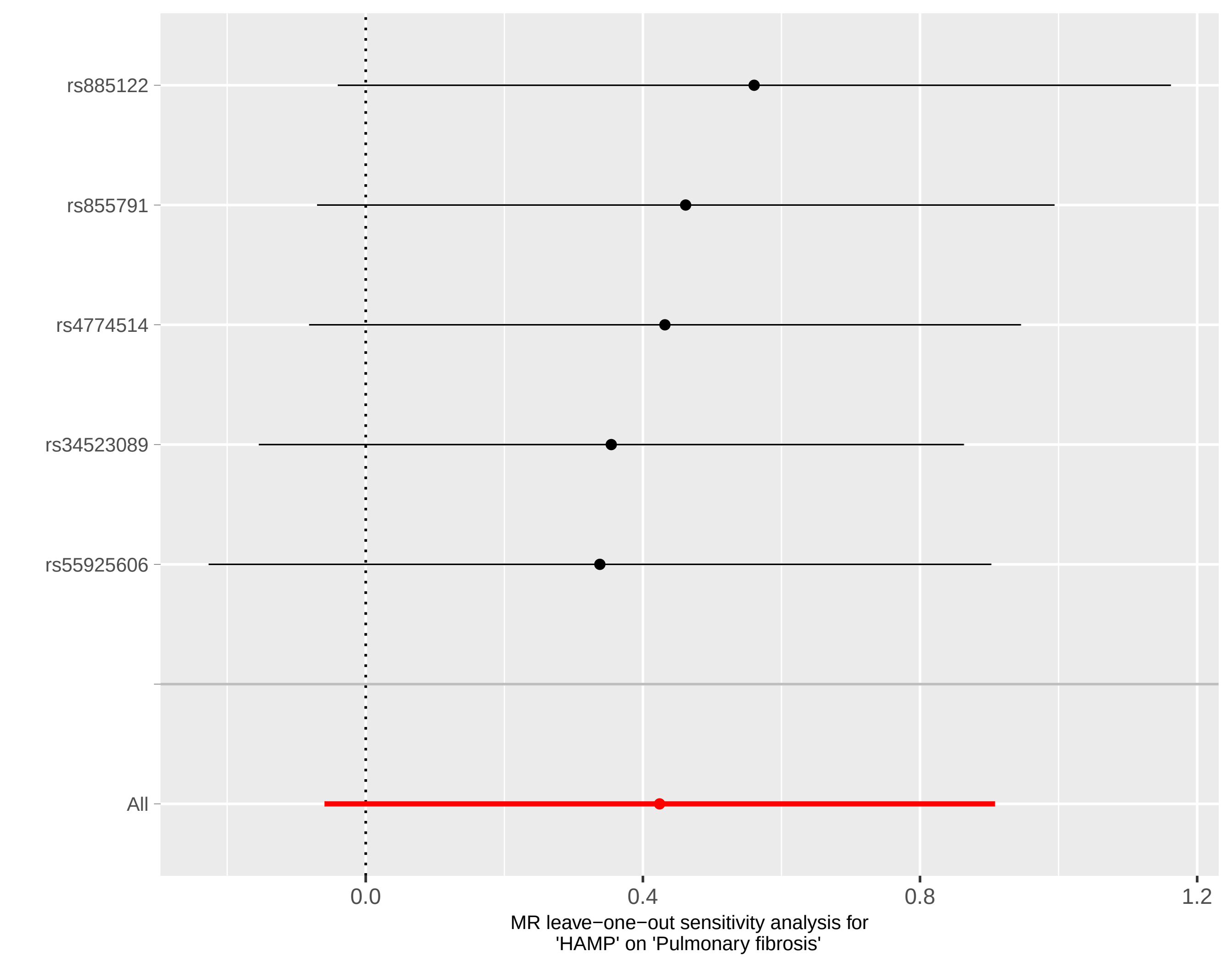
rs885122
rs855791
rs4774514
rs34523089
rs55925606
All
0.0
0.4
0.8
1.2
MR leave−one−out sensitivity analysis for
'HAMP' on 'Pulmonary fibrosis'

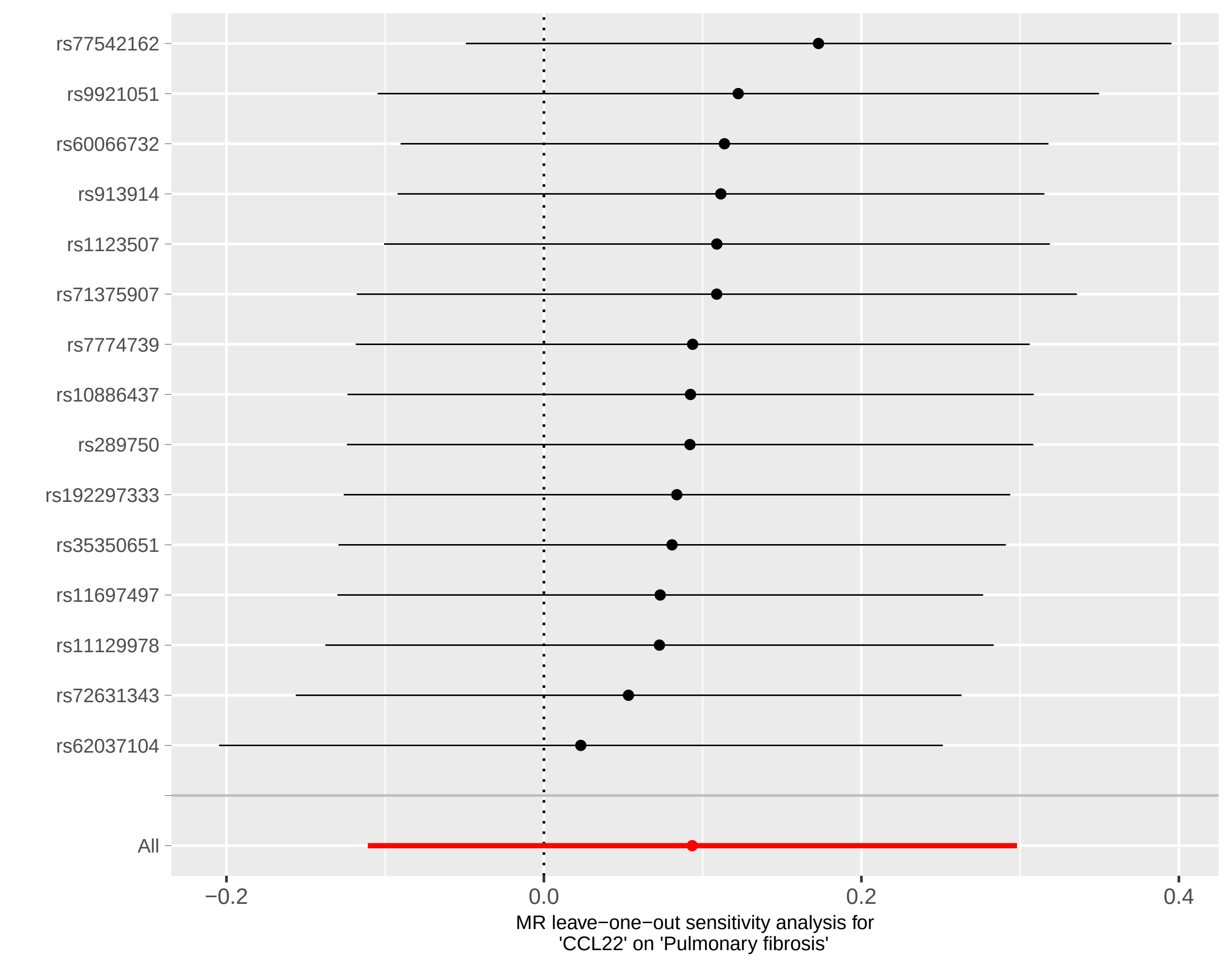
rs77542162
rs9921051
rs60066732
rs913914
rs1123507
rs71375907
rs7774739
rs10886437
rs289750
rs192297333
rs35350651
rs11697497
rs11129978
rs72631343
rs62037104
All
−0.2
0.0
0.2
0.4
MR leave−one−out sensitivity analysis for
'CCL22' on 'Pulmonary fibrosis'

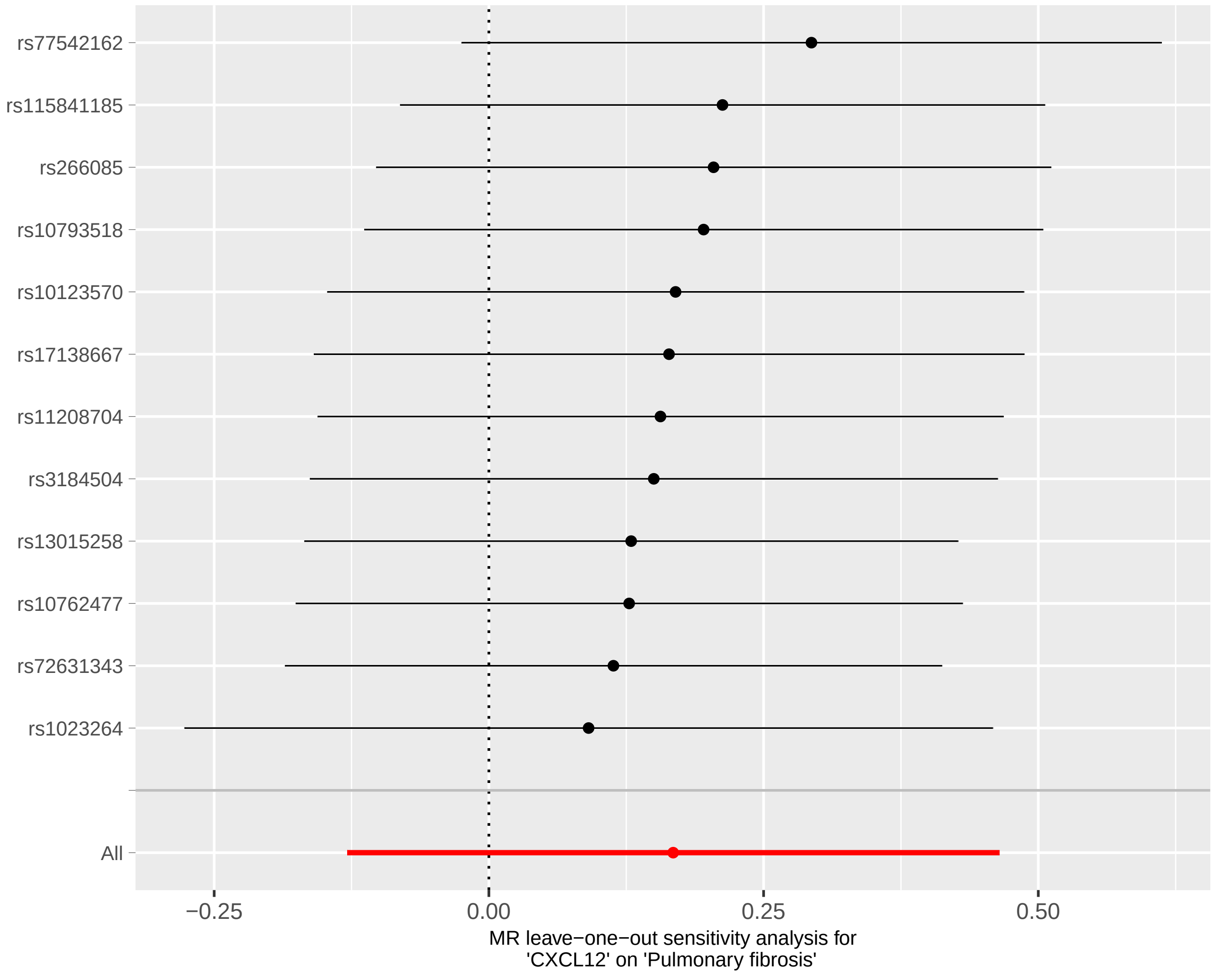
rs77542162
rs115841185
rs266085
rs10793518
rs10123570
rs17138667
rs11208704
rs3184504
rs13015258
rs10762477
rs72631343
rs1023264
All
−0.25
0.00
0.25
0.50
MR leave−one−out sensitivity analysis for
'CXCL12' on 'Pulmonary fibrosis'

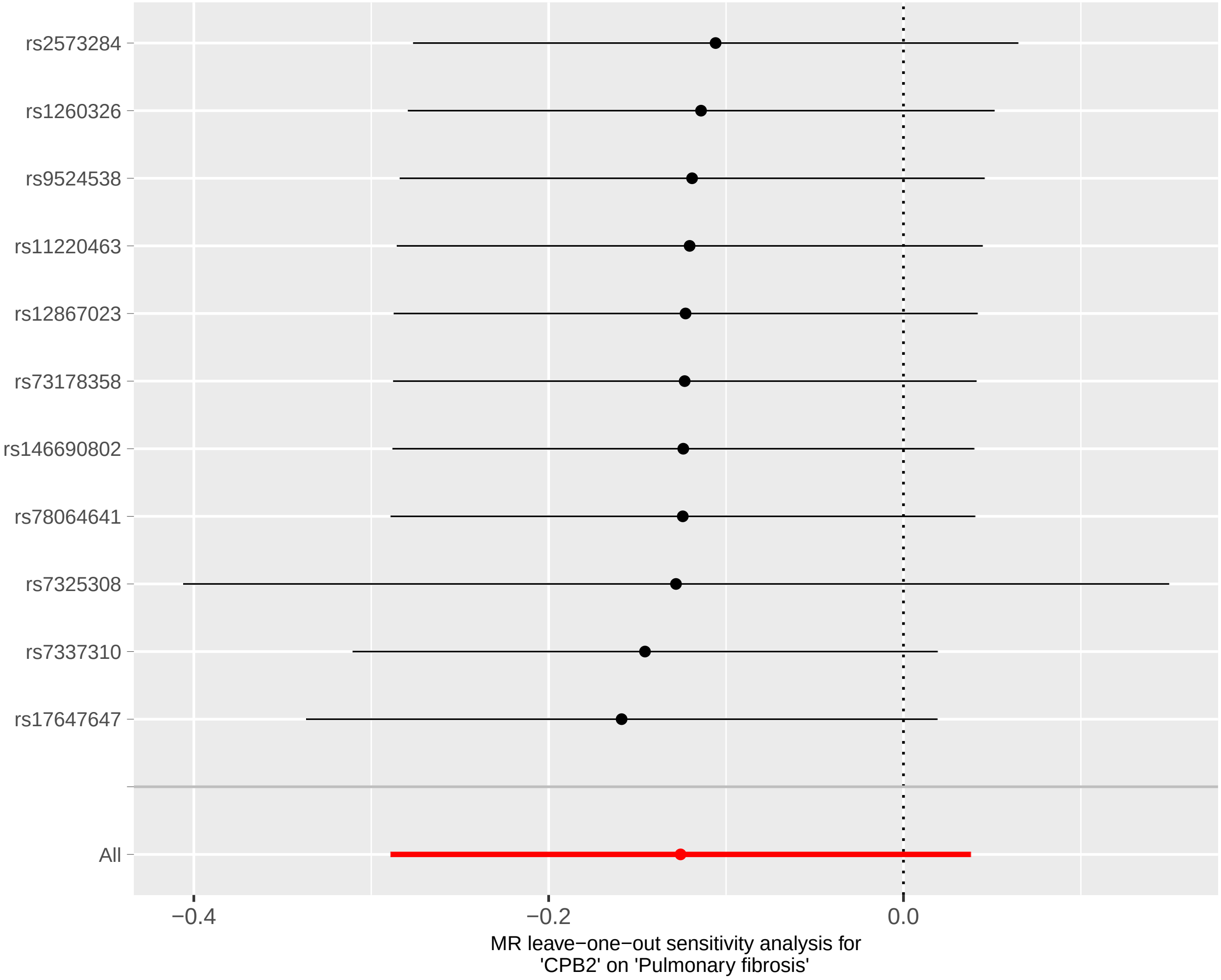
rs2573284
rs1260326
rs9524538
rs11220463
rs12867023
rs73178358
rs146690802
rs78064641
rs7325308
rs7337310
rs17647647
All
−0.4
−0.2
0.0
MR leave−one−out sensitivity analysis for
'CPB2' on 'Pulmonary fibrosis'

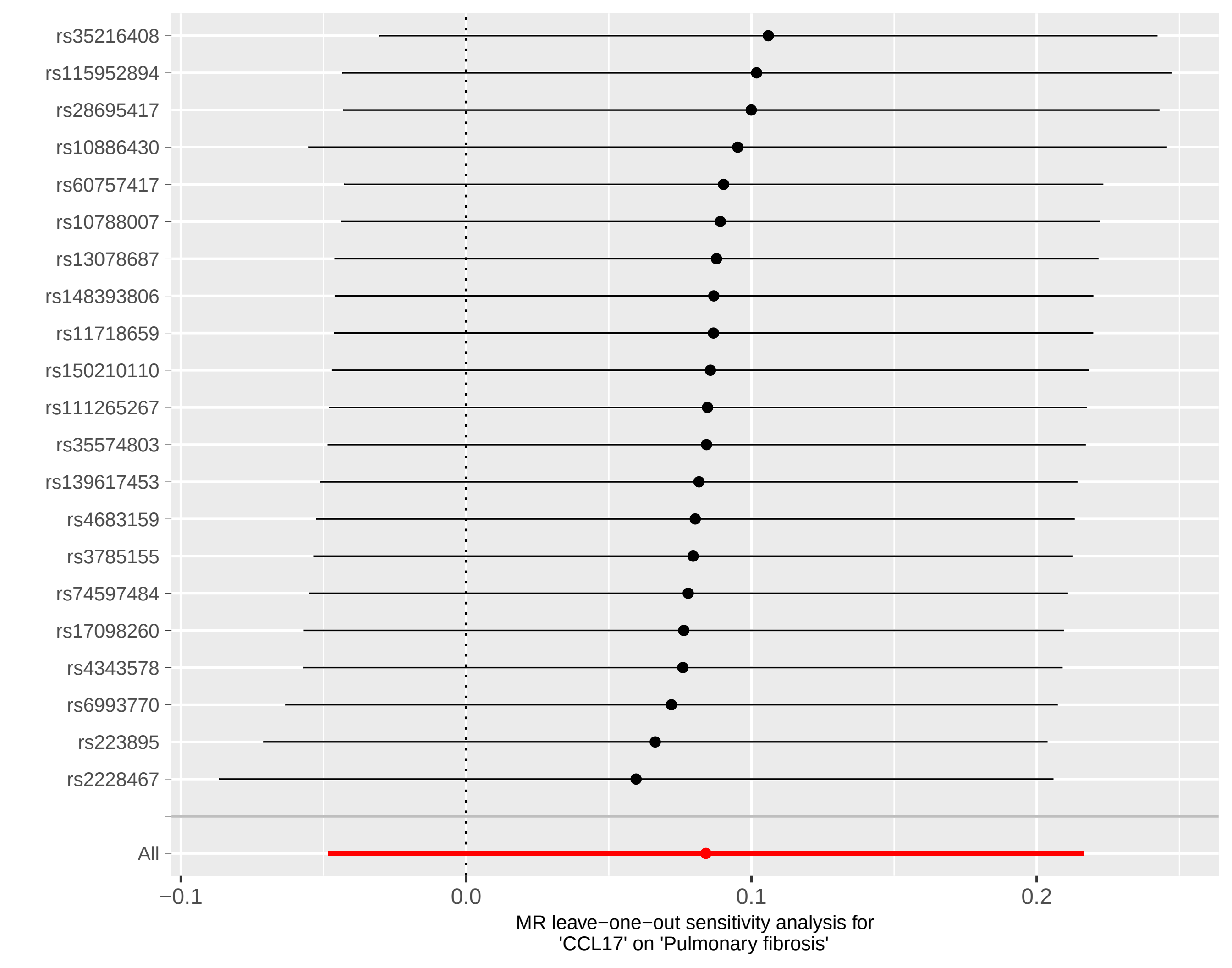

rs35216408
rs115952894
rs28695417
rs10886430
rs60757417
rs10788007
rs13078687
rs148393806
rs11718659
rs150210110
rs111265267
rs35574803
rs139617453
rs4683159
rs3785155
rs74597484
rs17098260
rs4343578
rs6993770
rs223895
rs2228467
All
−0.1
0.0
0.1
0.2
MR leave−one−out sensitivity analysis for
'CCL17' on 'Pulmonary fibrosis'

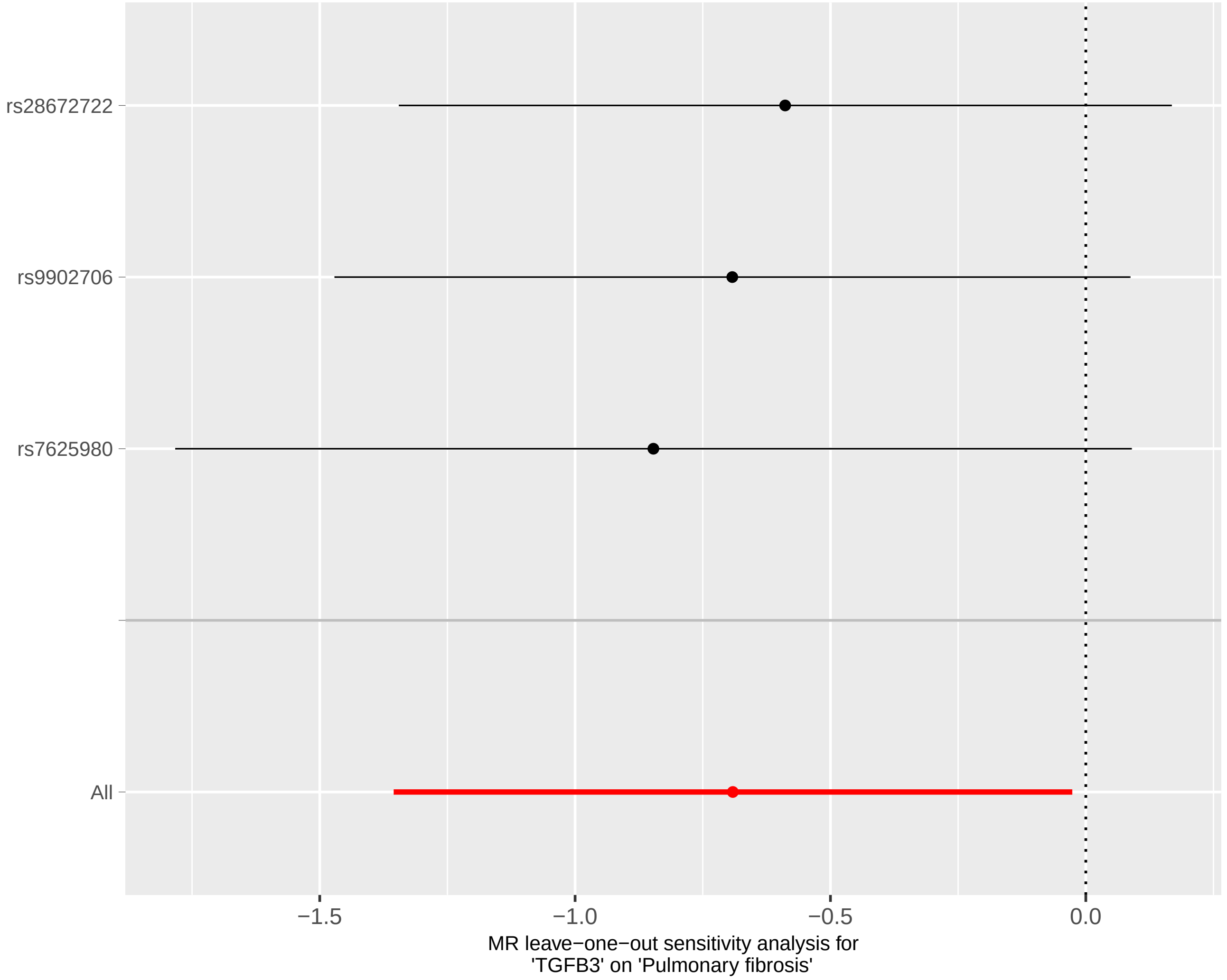

rs28672722
rs9902706
rs7625980
All
−1.5
−1.0
−0.5
0.0
MR leave−one−out sensitivity analysis for
'TGFB3' on 'Pulmonary fibrosis'

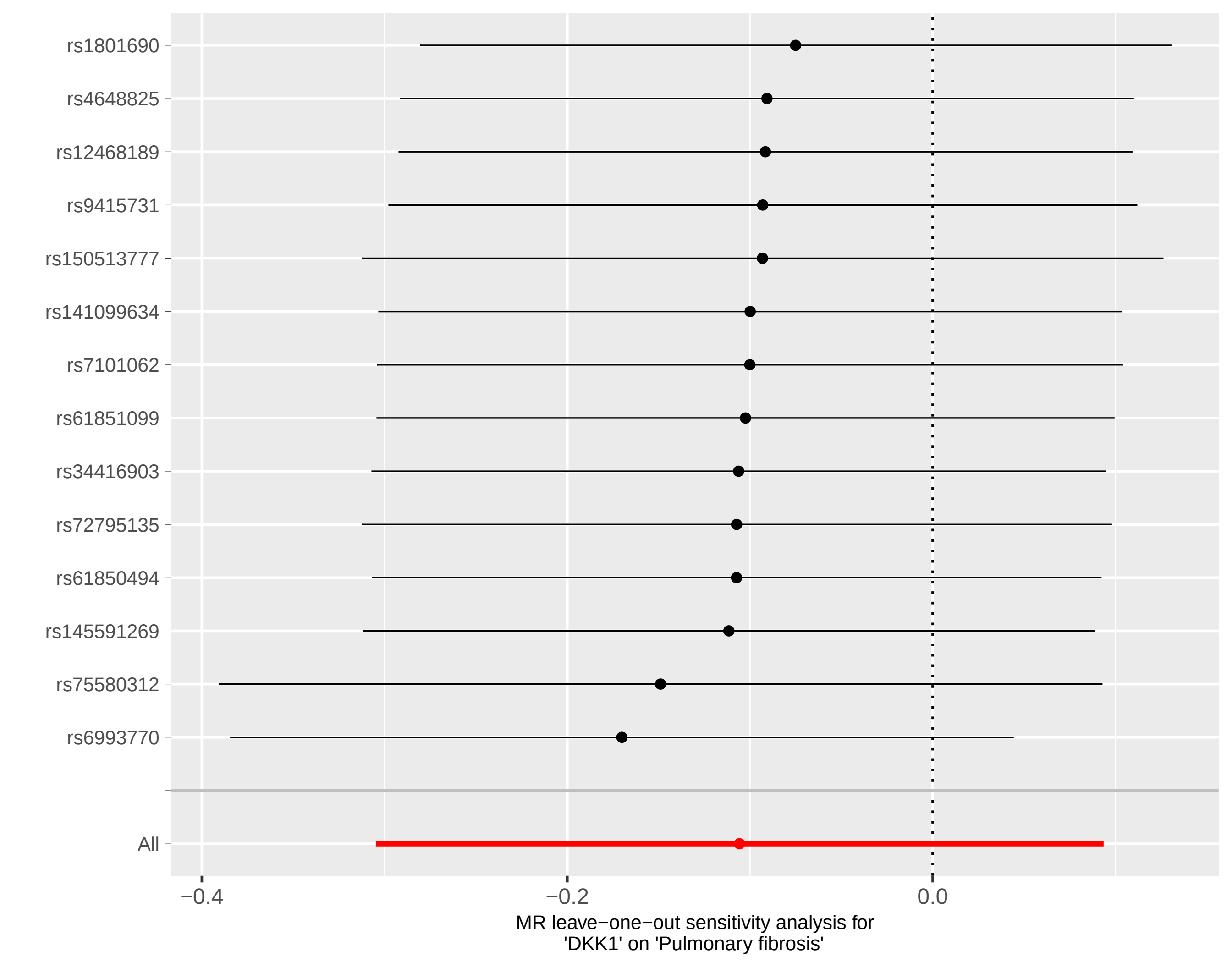
rs1801690
rs4648825
rs12468189
rs9415731
rs150513777
rs141099634
rs7101062
rs61851099
rs34416903
rs72795135
rs61850494
rs145591269
rs75580312
rs6993770
All
−0.4
−0.2
0.0
MR leave−one−out sensitivity analysis for
'DKK1' on 'Pulmonary fibrosis'

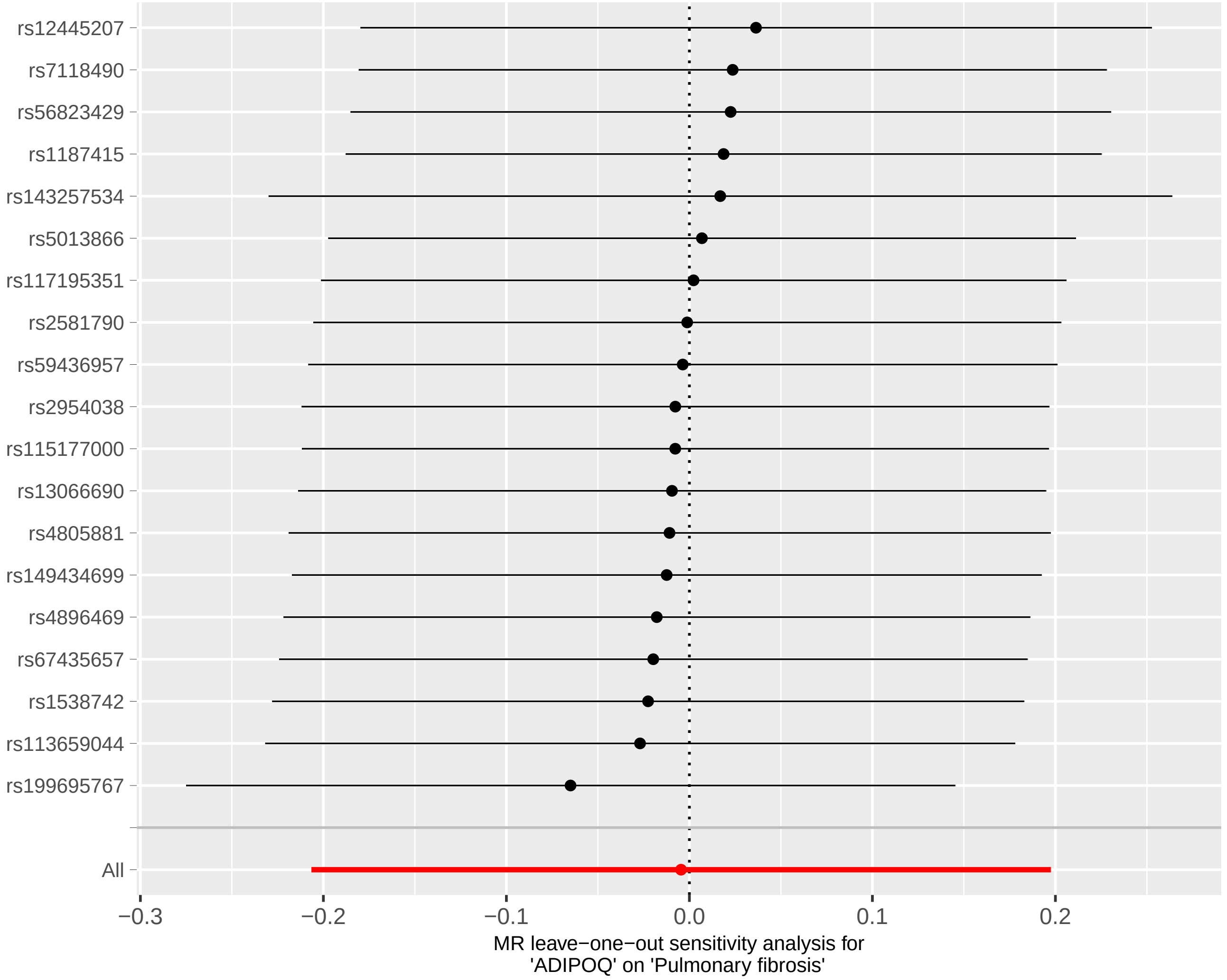
rs12445207
rs7118490
rs56823429
rs1187415
rs143257534
rs5013866
rs117195351
rs2581790
rs59436957
rs2954038
rs115177000
rs13066690
rs4805881
rs149434699
rs4896469
rs67435657
rs1538742
rs113659044
rs199695767
All
−0.3
−0.2
−0.1
0.0
0.1
0.2
MR leave−one−out sensitivity analysis for
'ADIPOQ' on 'Pulmonary fibrosis'

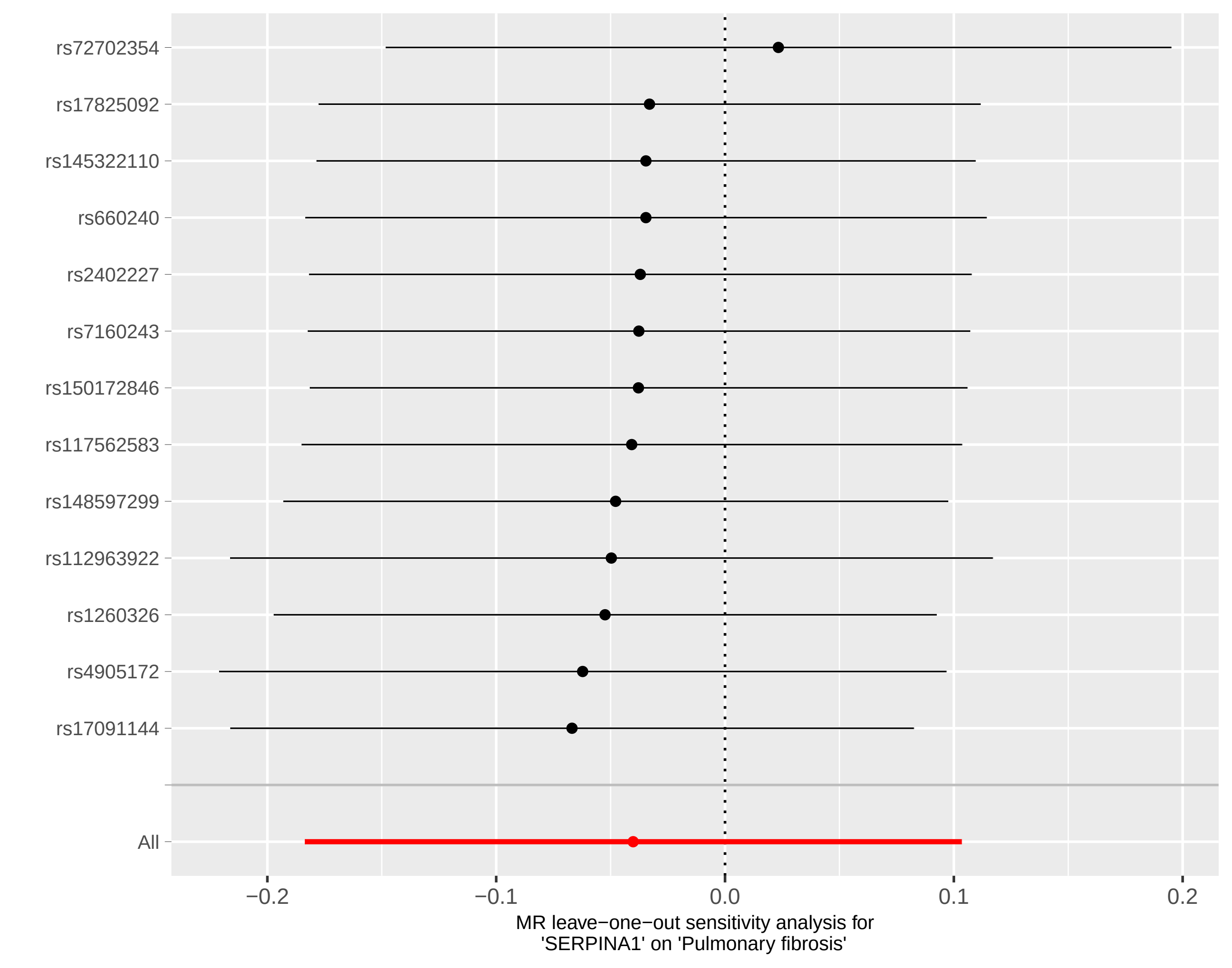
rs72702354
rs17825092
rs145322110
rs660240
rs2402227
rs7160243
rs150172846
rs117562583
rs148597299
rs112963922
rs1260326
rs4905172
rs17091144
All
−0.2
−0.1
0.0
0.1
0.2
MR leave−one−out sensitivity analysis for
'SERPINA1' on 'Pulmonary fibrosis'

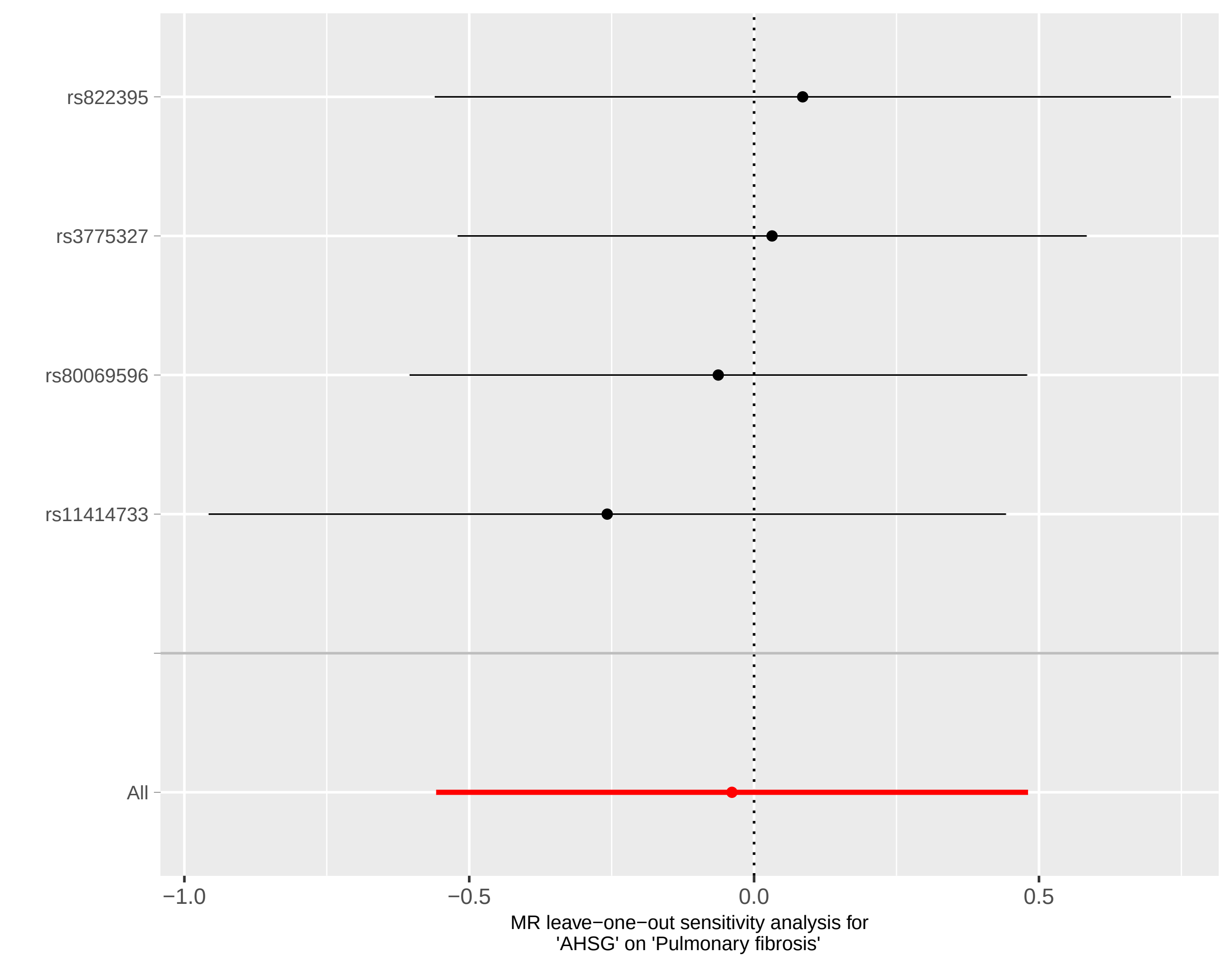
rs822395
rs3775327
rs80069596
rs11414733
All
−1.0
−0.5
0.0
0.5
MR leave−one−out sensitivity analysis for
'AHSG' on 'Pulmonary fibrosis'

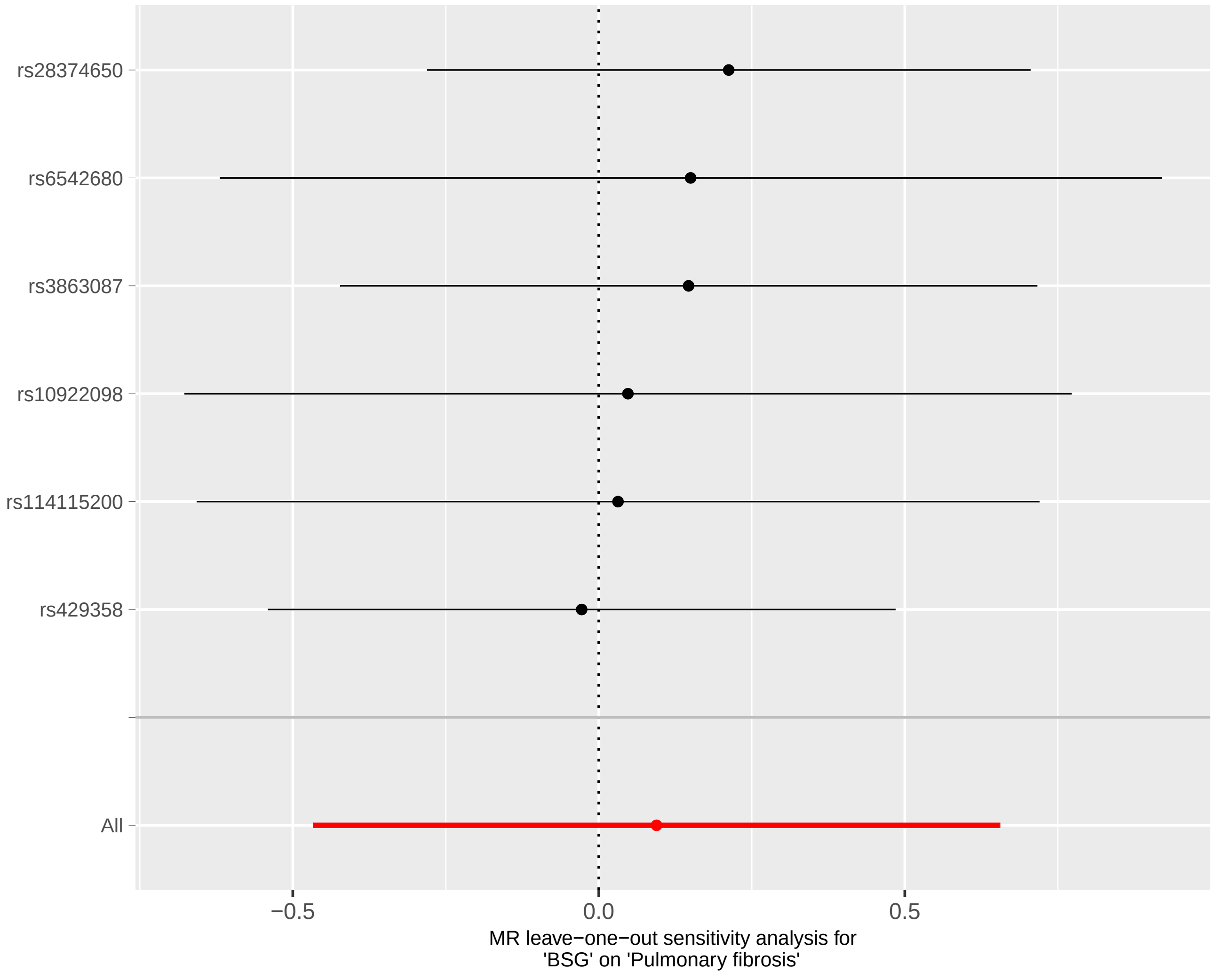
rs28374650
rs6542680
rs3863087
rs10922098
rs114115200
rs429358
All
−0.5
0.0
0.5
MR leave−one−out sensitivity analysis for
'BSG' on 'Pulmonary fibrosis'

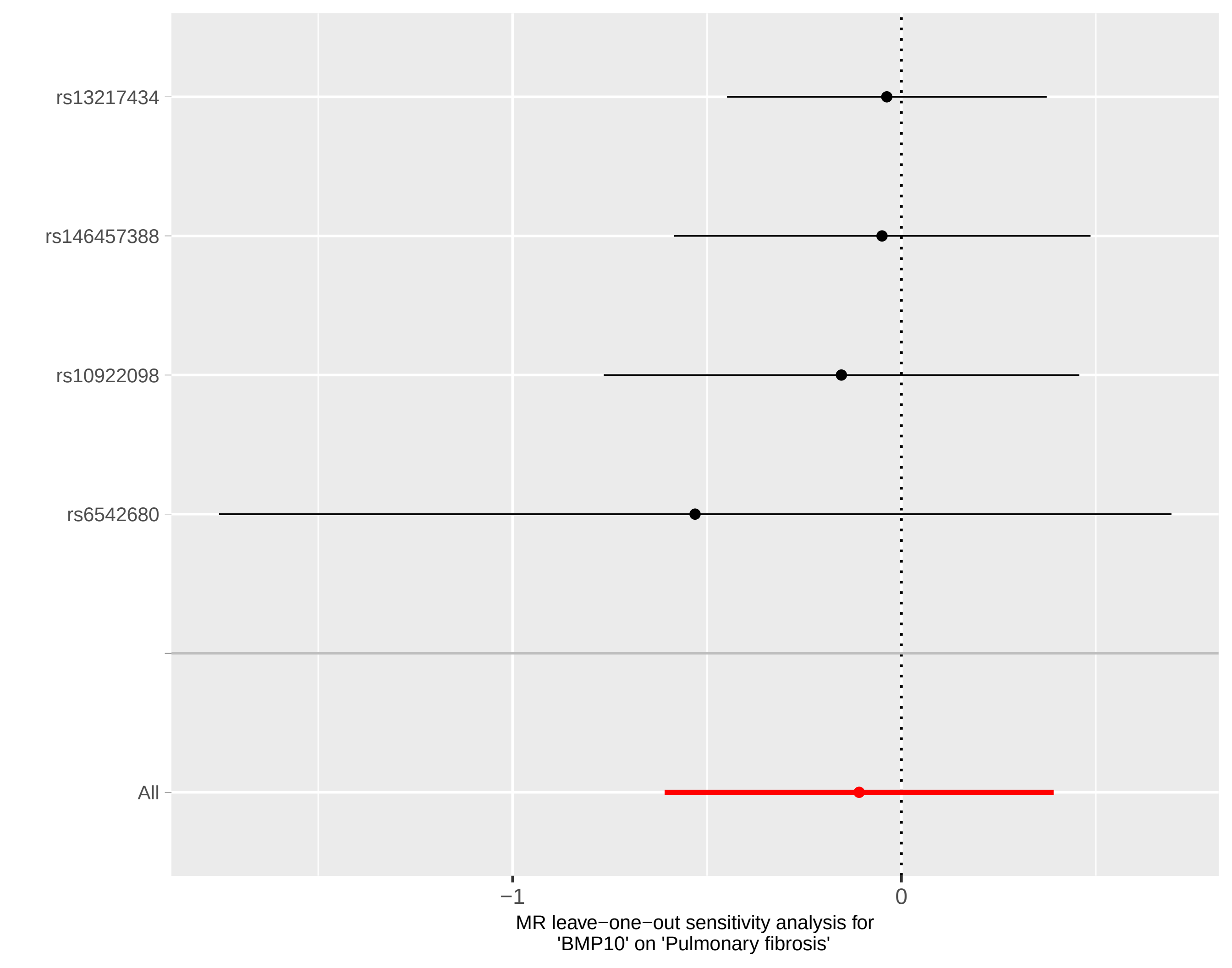
rs13217434
rs146457388
rs10922098
rs6542680
All
−1
0
MR leave−one−out sensitivity analysis for
'BMP10' on 'Pulmonary fibrosis'

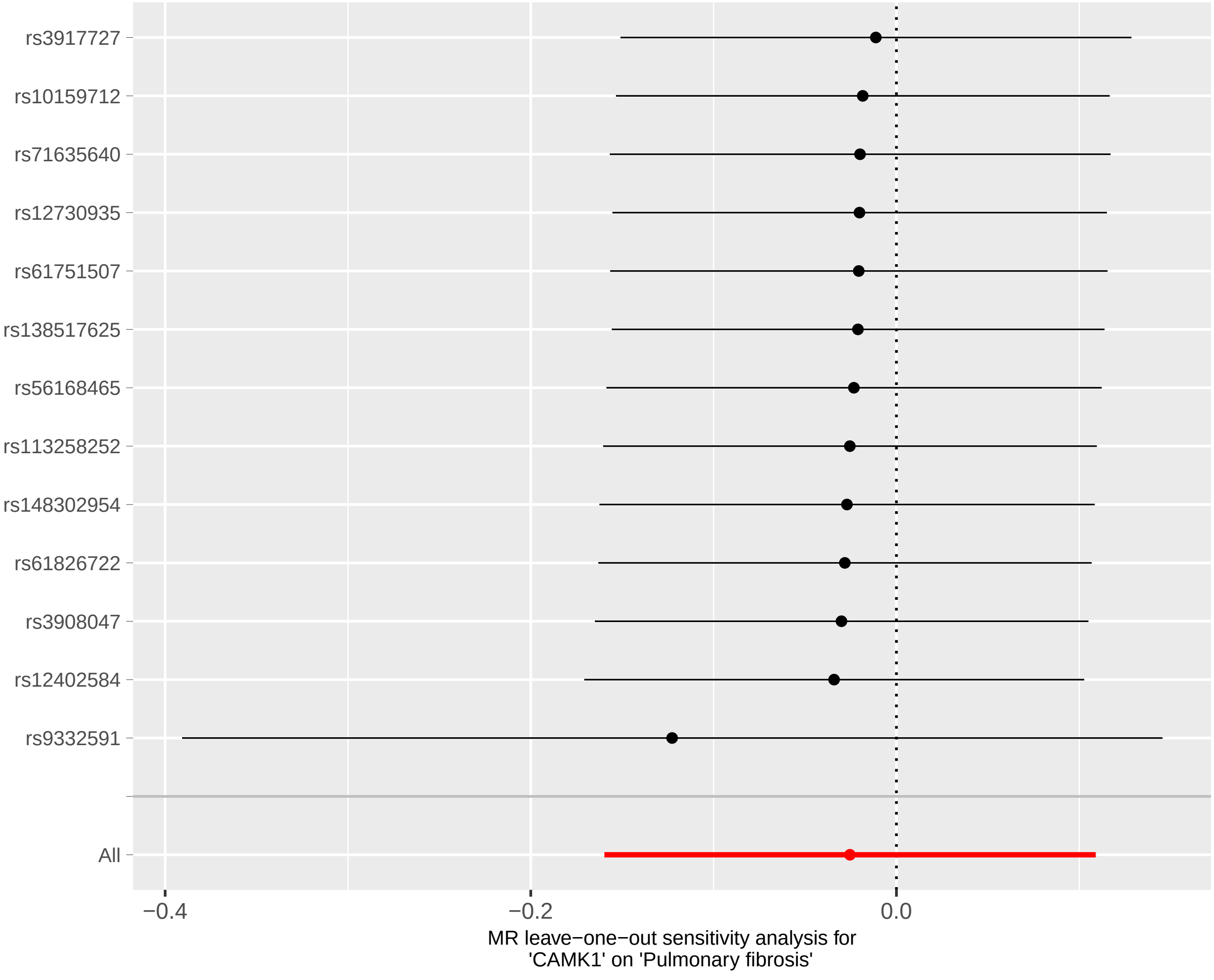
rs3917727
rs10159712
rs71635640
rs12730935
rs61751507
rs138517625
rs56168465
rs113258252
rs148302954
rs61826722
rs3908047
rs12402584
rs9332591
All
−0.4
−0.2
0.0
MR leave−one−out sensitivity analysis for
'CAMK1' on 'Pulmonary fibrosis'

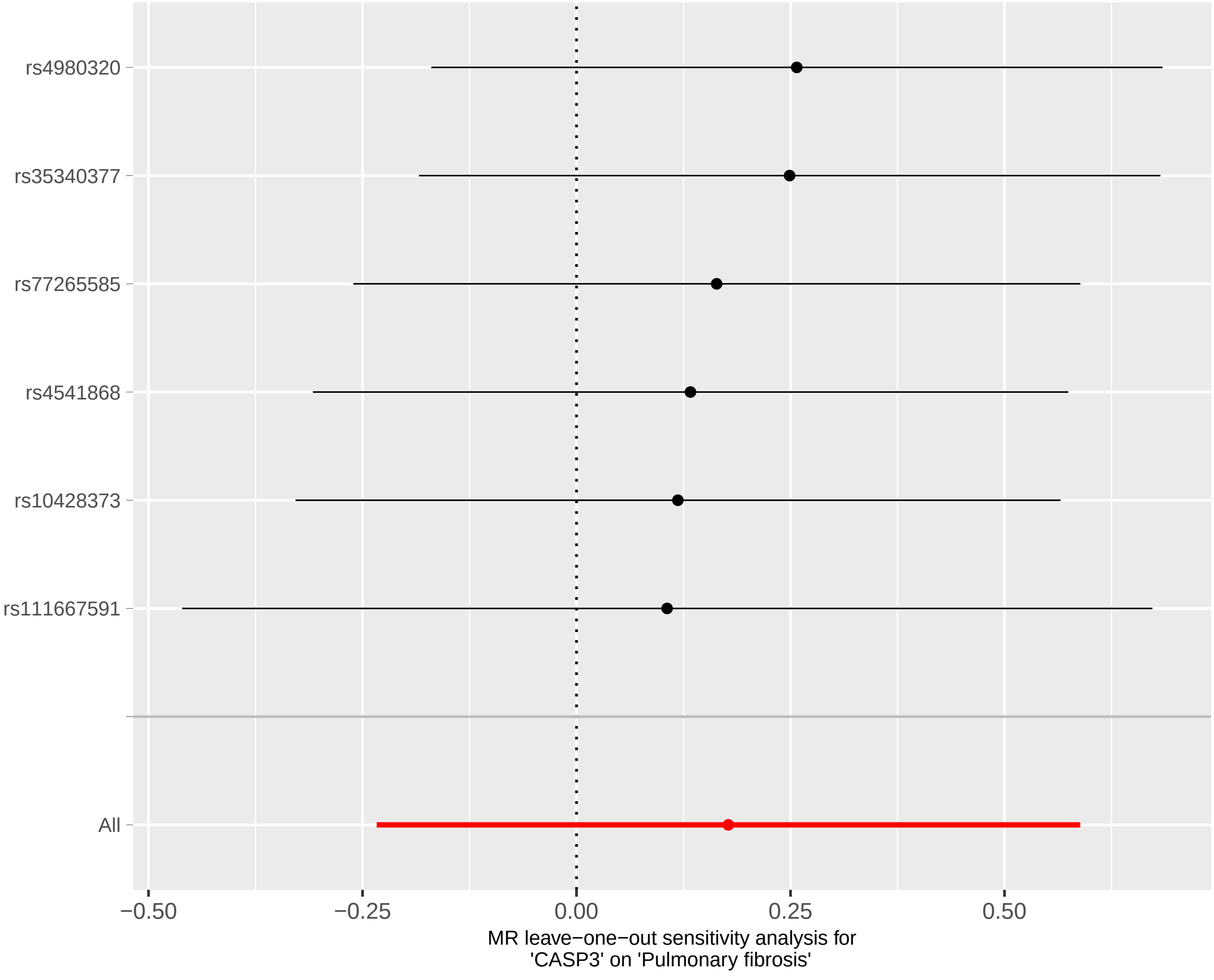

rs4980320
rs35340377
rs77265585
rs4541868
rs10428373
rs111667591
All
−0.50
−0.25
0.00
0.25
0.50
MR leave−one−out sensitivity analysis for
'CASP3' on 'Pulmonary fibrosis'

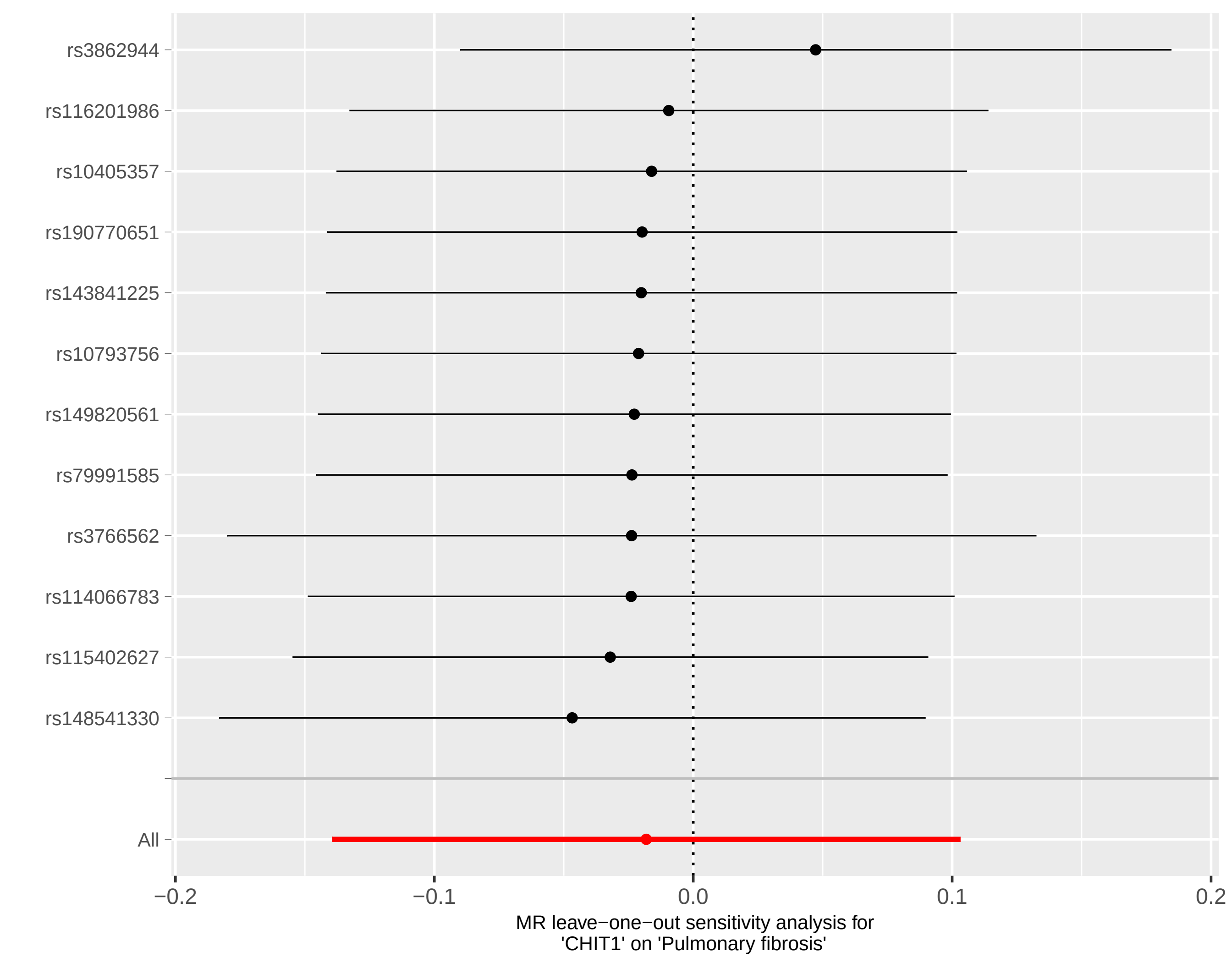
rs3862944
rs116201986
rs10405357
rs190770651
rs143841225
rs10793756
rs149820561
rs79991585
rs3766562
rs114066783
rs115402627
rs148541330
All
−0.2
−0.1
0.0
0.1
0.2
MR leave−one−out sensitivity analysis for
'CHIT1' on 'Pulmonary fibrosis'

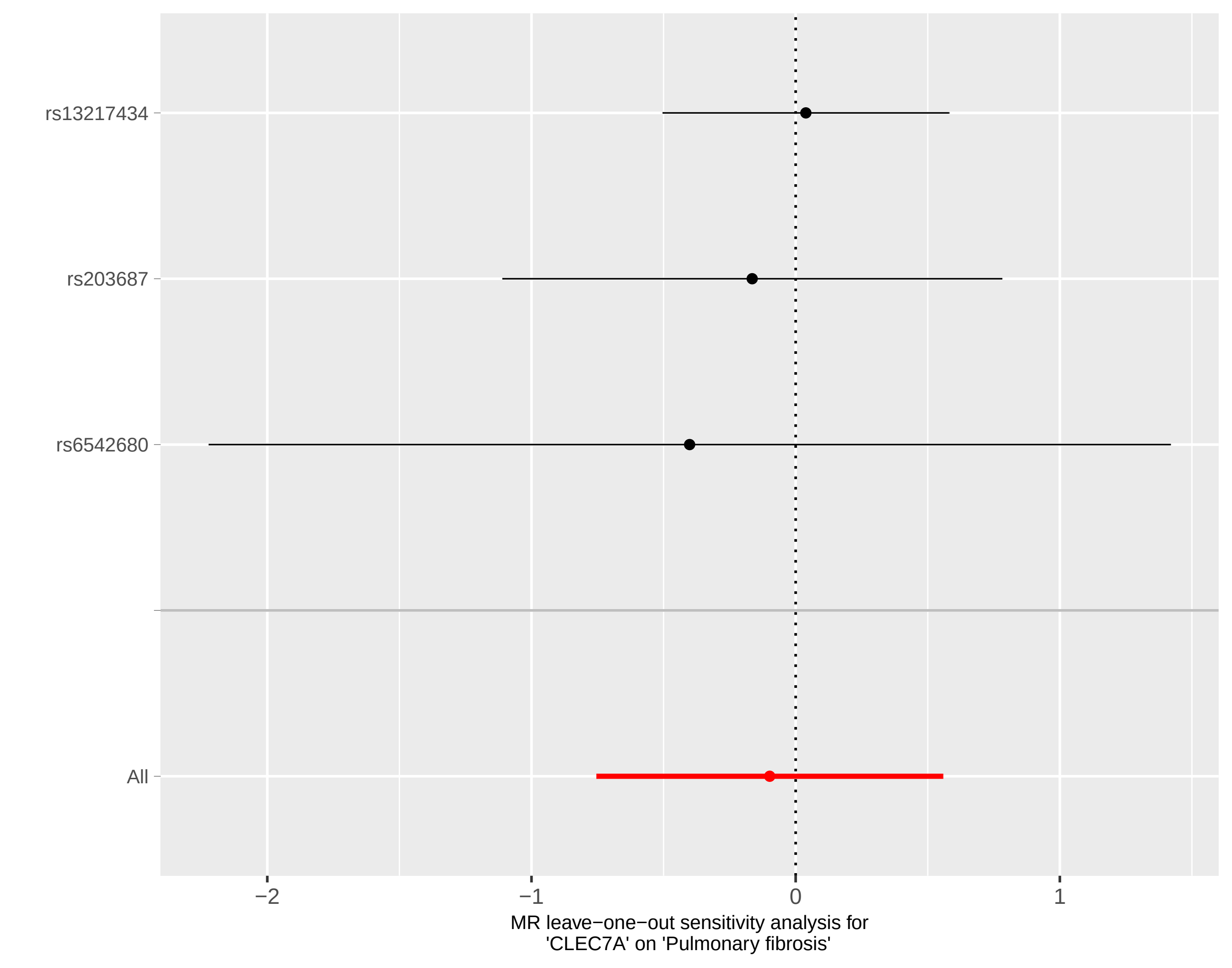
rs13217434
rs203687
rs6542680
All
−2
−1
0
1
MR leave−one−out sensitivity analysis for
'CLEC7A' on 'Pulmonary fibrosis'

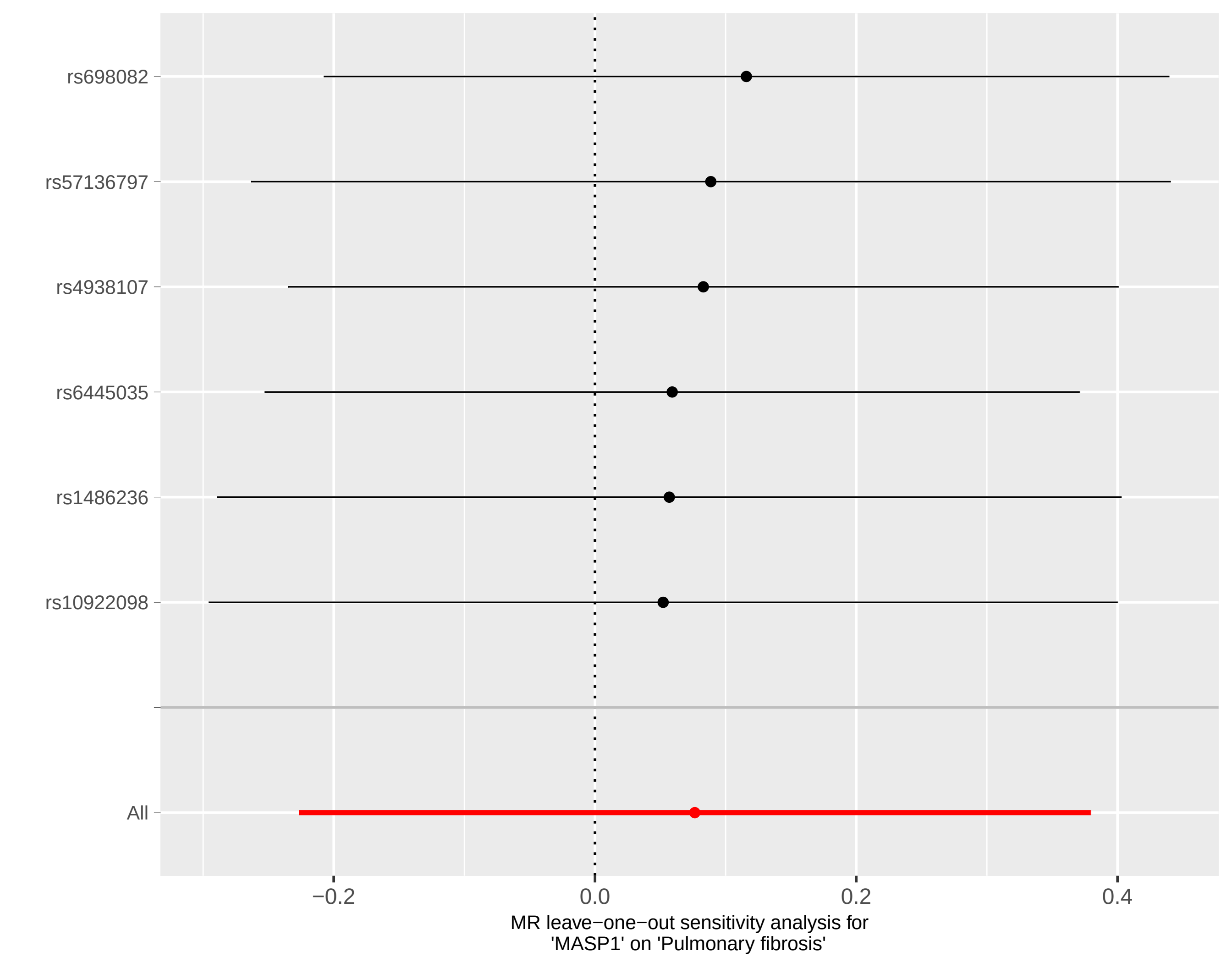
rs698082
rs57136797
rs4938107
rs6445035
rs1486236
rs10922098
All
−0.2
0.0
0.2
0.4
MR leave−one−out sensitivity analysis for
'MASP1' on 'Pulmonary fibrosis'

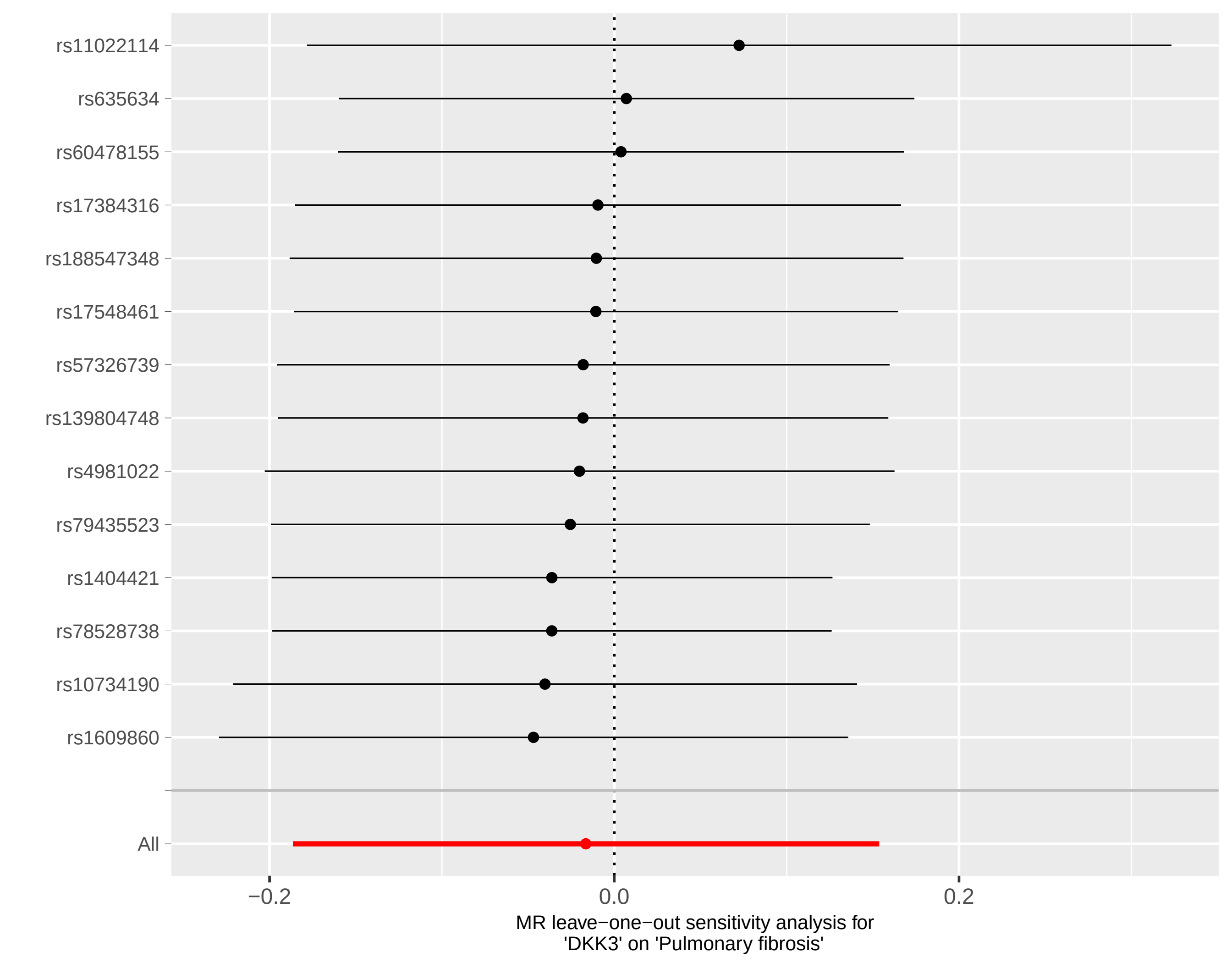
rs11022114
rs635634
rs60478155
rs17384316
rs188547348
rs17548461
rs57326739
rs139804748
rs4981022
rs79435523
rs1404421
rs78528738
rs10734190
rs1609860
All
−0.2
0.0
0.2
MR leave−one−out sensitivity analysis for
'DKK3' on 'Pulmonary fibrosis'

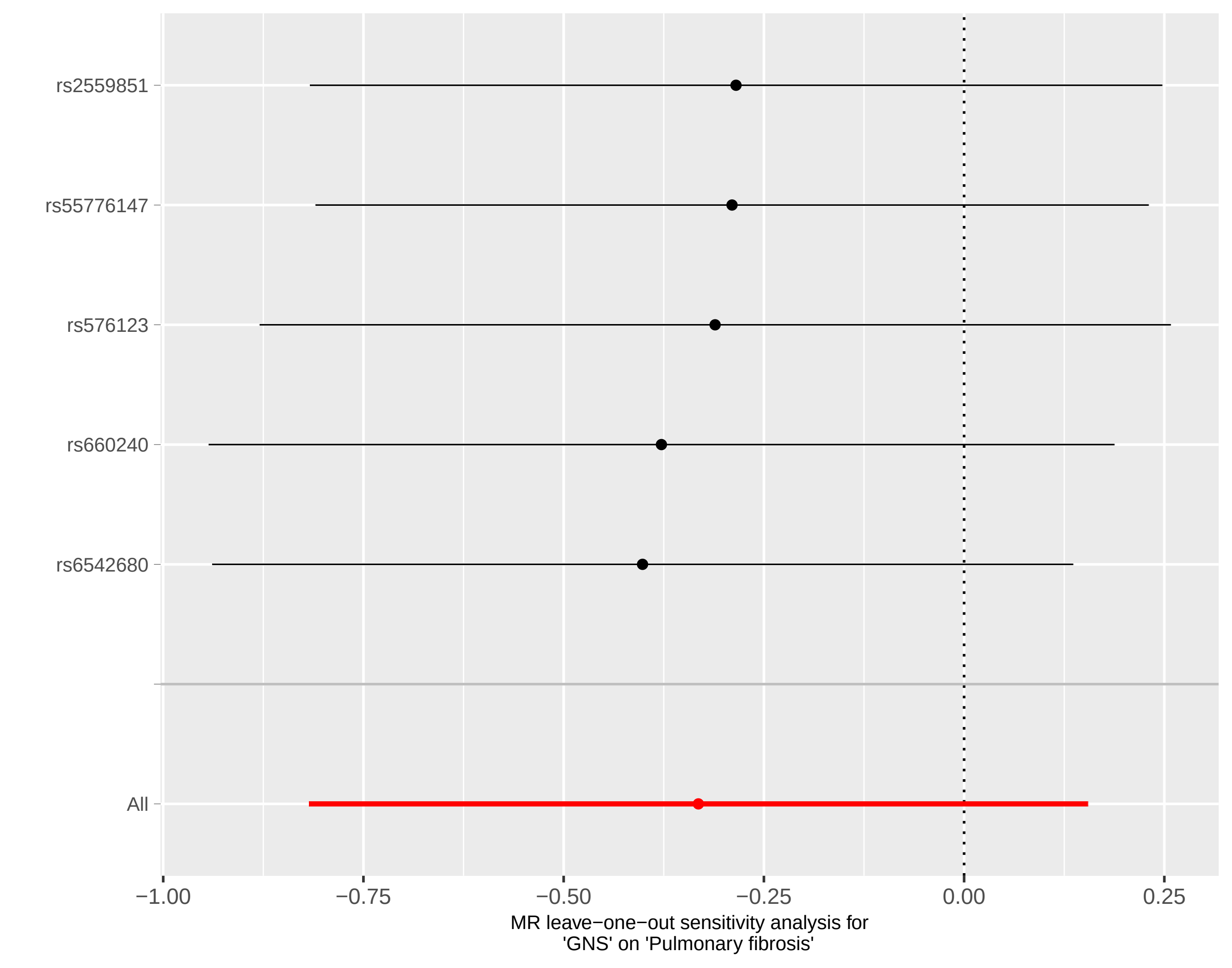
rs2559851
rs55776147
rs576123
rs660240
rs6542680
All
−1.00
−0.75
−0.50
−0.25
0.00
0.25
MR leave−one−out sensitivity analysis for
'GNS' on 'Pulmonary fibrosis'

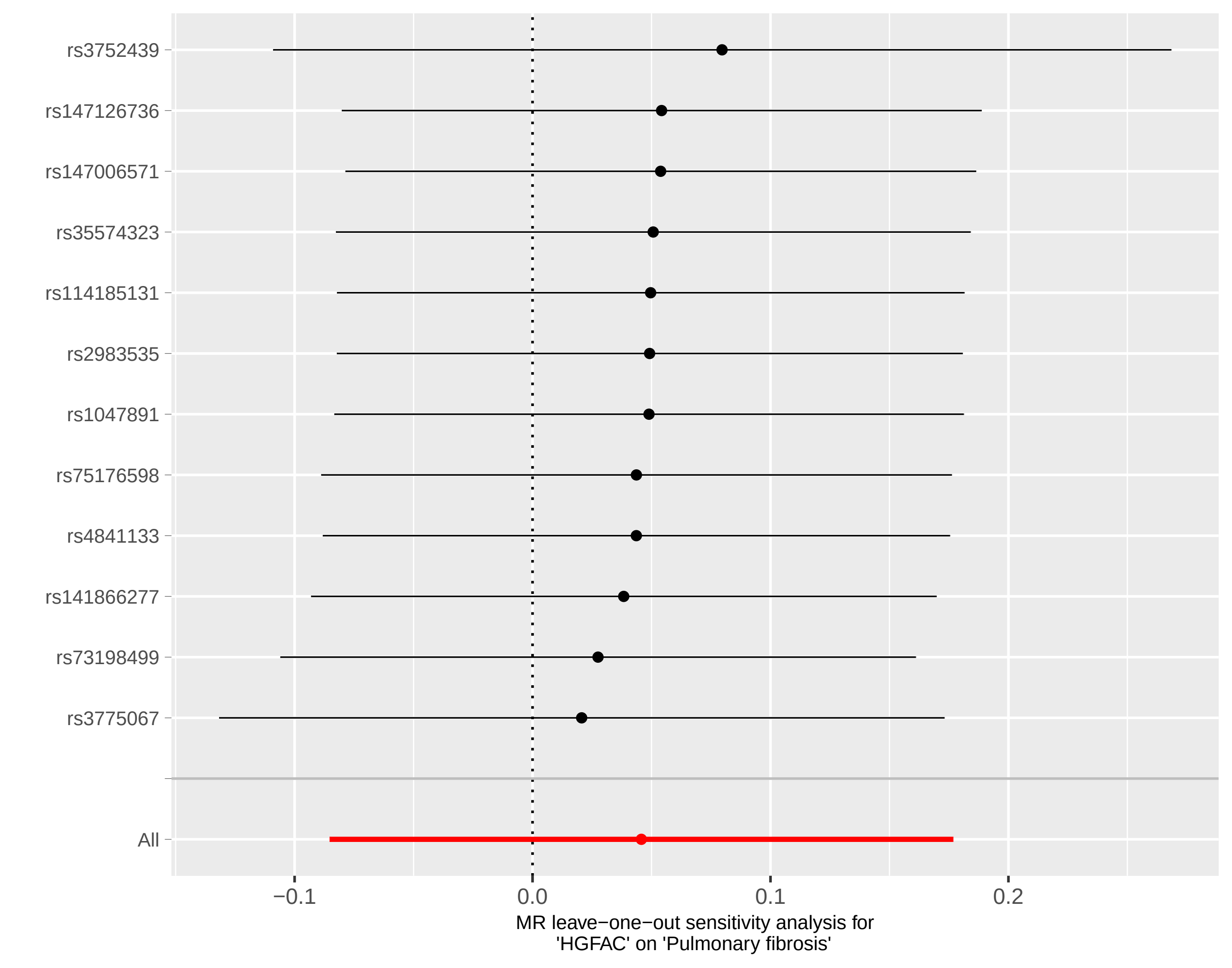
rs3752439
rs147126736
rs147006571
rs35574323
rs114185131
rs2983535
rs1047891
rs75176598
rs4841133
rs141866277
rs73198499
rs3775067
All
−0.1
0.0
0.1
0.2
MR leave−one−out sensitivity analysis for
'HGFAC' on 'Pulmonary fibrosis'

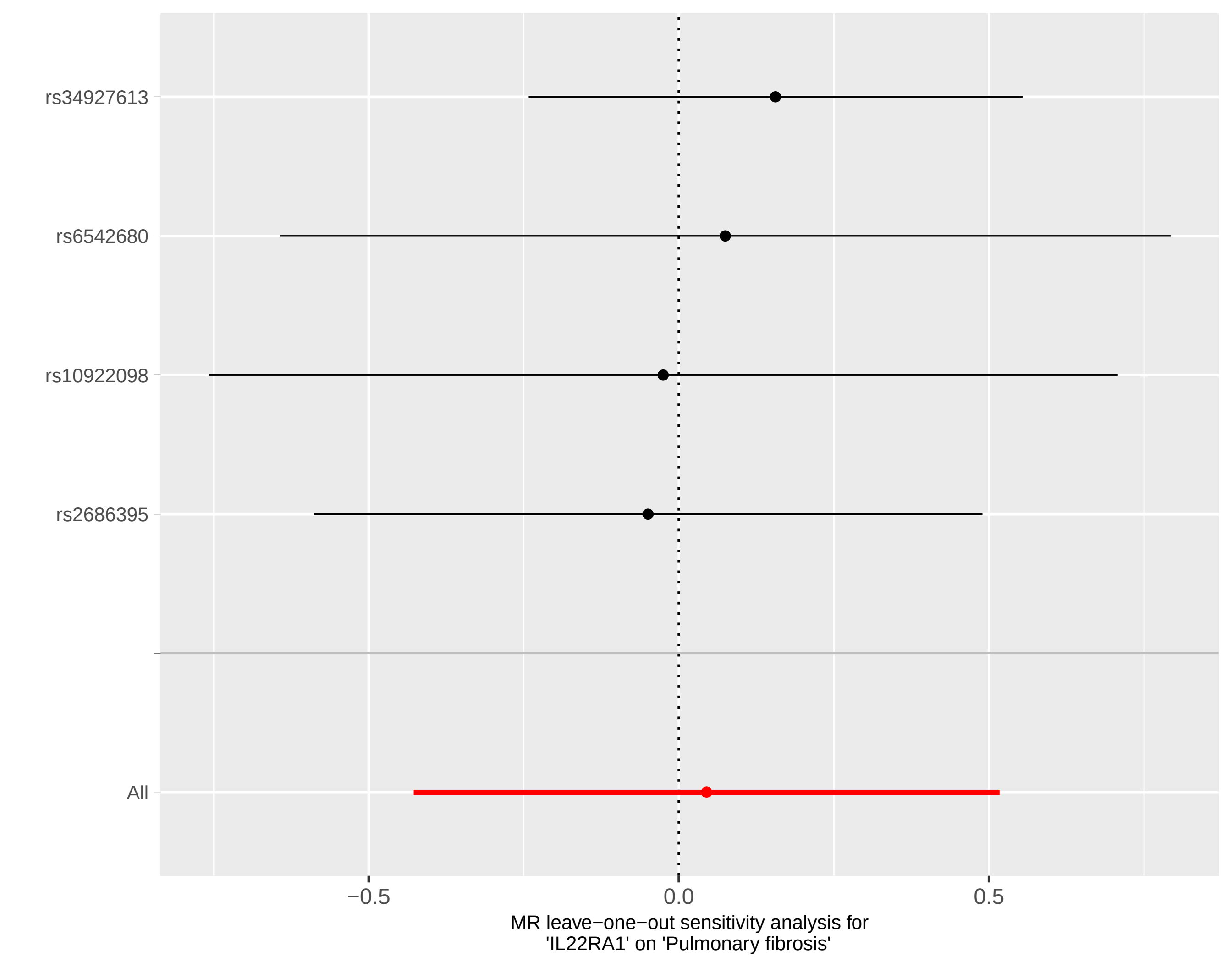
rs34927613
rs6542680
rs10922098
rs2686395
All
−0.5
0.0
0.5
MR leave−one−out sensitivity analysis for
'IL22RA1' on 'Pulmonary fibrosis'

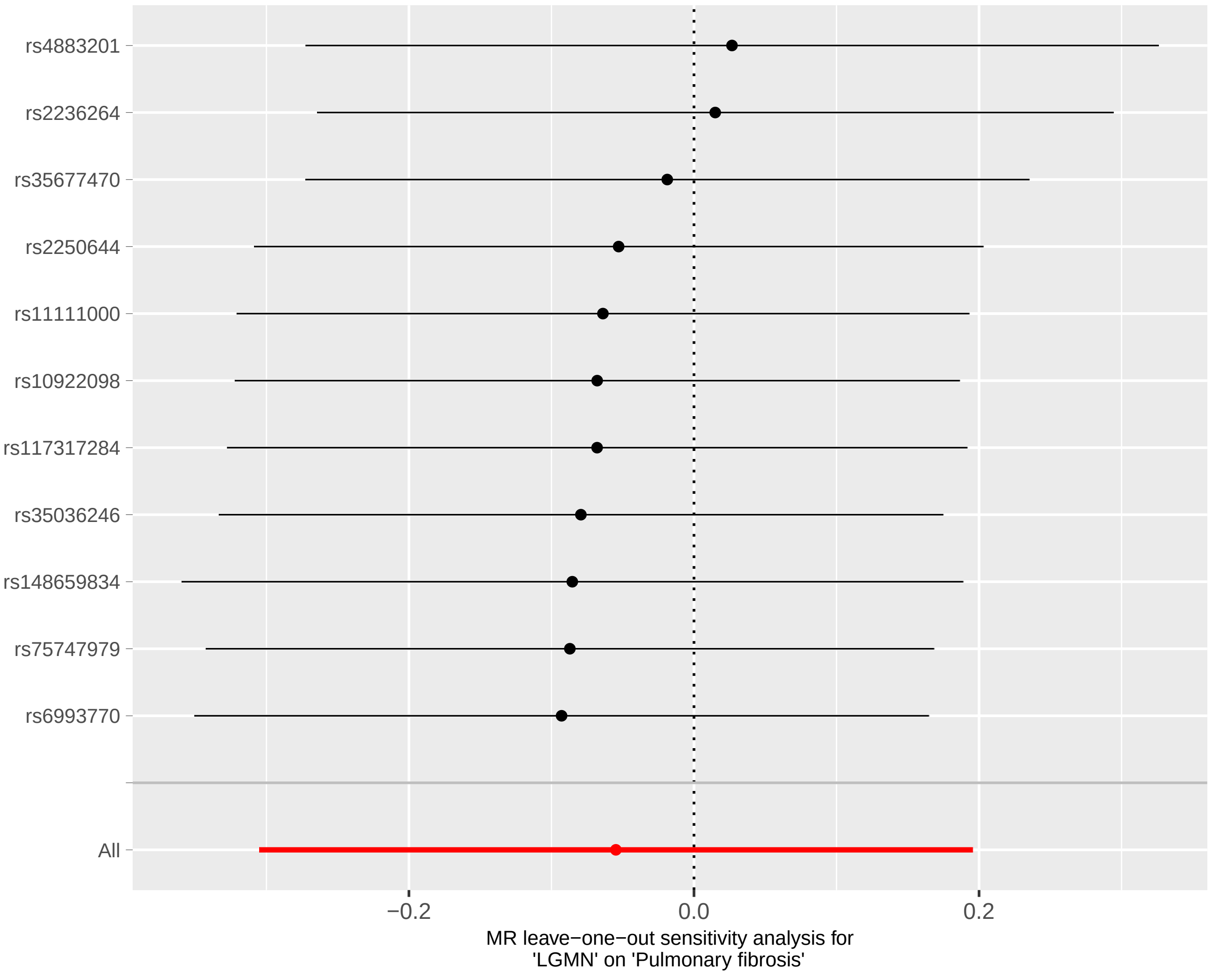
rs4883201
rs2236264
rs35677470
rs2250644
rs11111000
rs10922098
rs117317284
rs35036246
rs148659834
rs75747979
rs6993770
All
−0.2
0.0
0.2
MR leave−one−out sensitivity analysis for
'LGMN' on 'Pulmonary fibrosis'

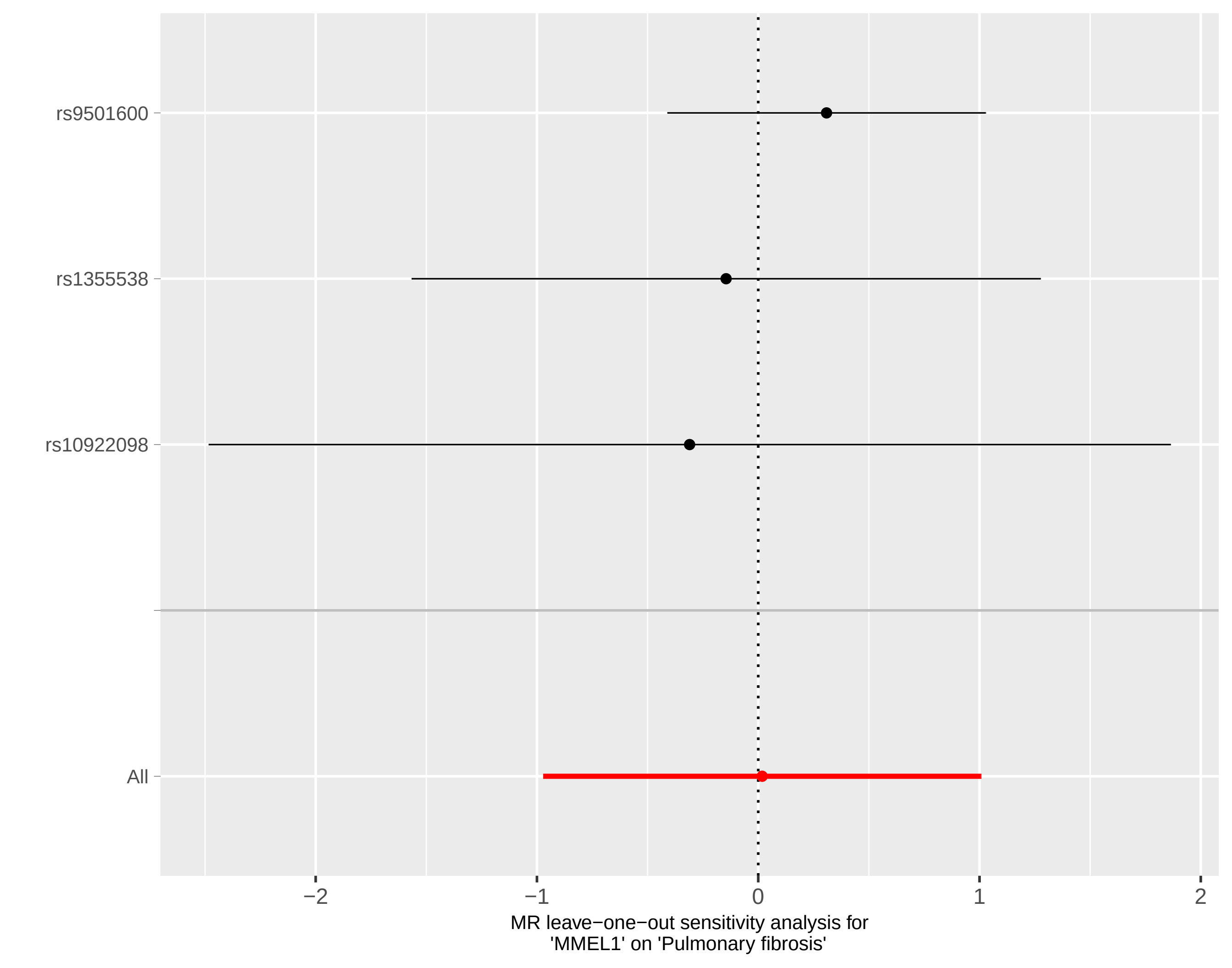

rs9501600
rs1355538
rs10922098
All
−2
−1
0
1
2
MR leave−one−out sensitivity analysis for
'MMEL1' on 'Pulmonary fibrosis'

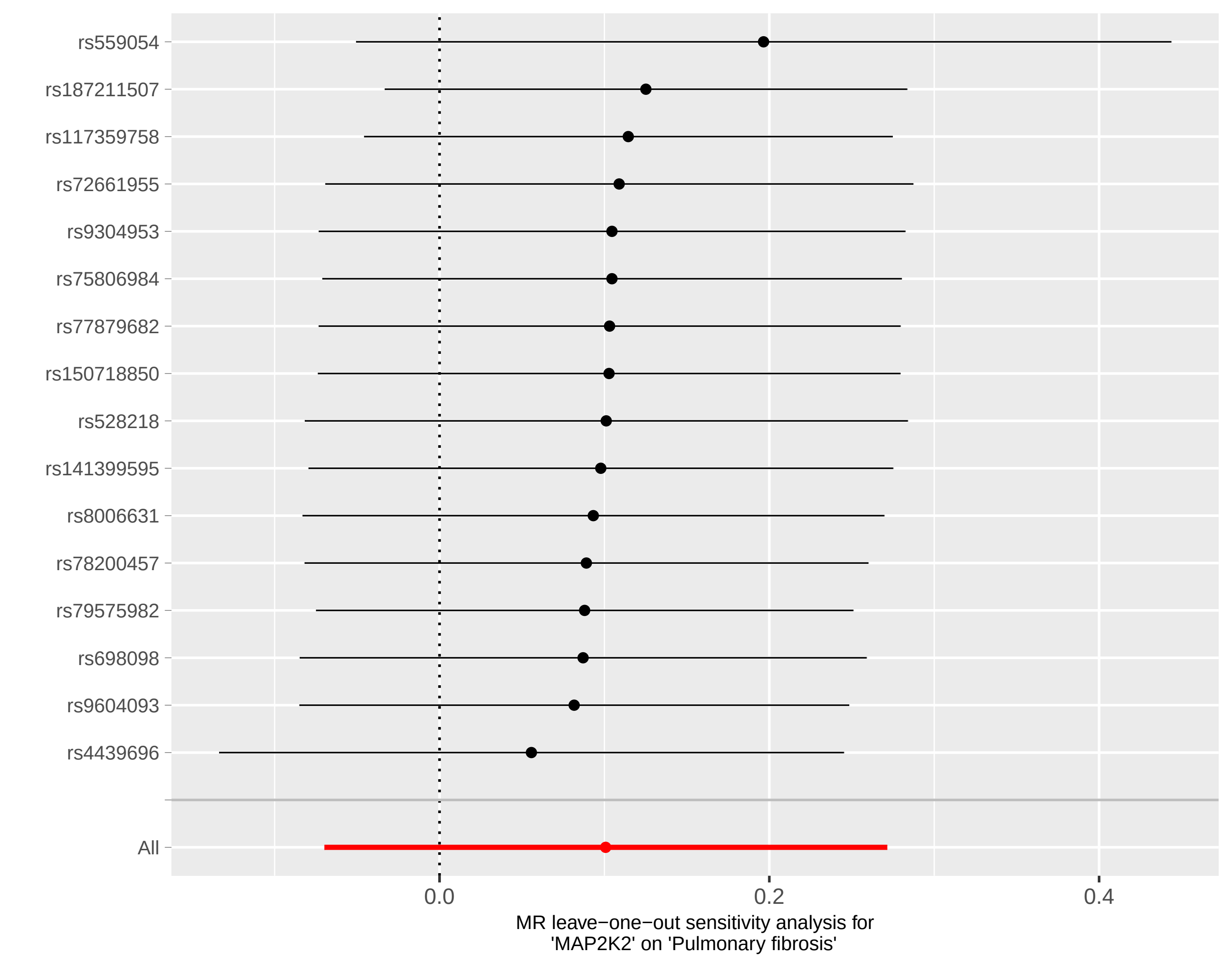

rs559054
rs187211507
rs117359758
rs72661955
rs9304953
rs75806984
rs77879682
rs150718850
rs528218
rs141399595
rs8006631
rs78200457
rs79575982
rs698098
rs9604093
rs4439696
All
0.0
0.2
0.4
MR leave−one−out sensitivity analysis for
'MAP2K2' on 'Pulmonary fibrosis'

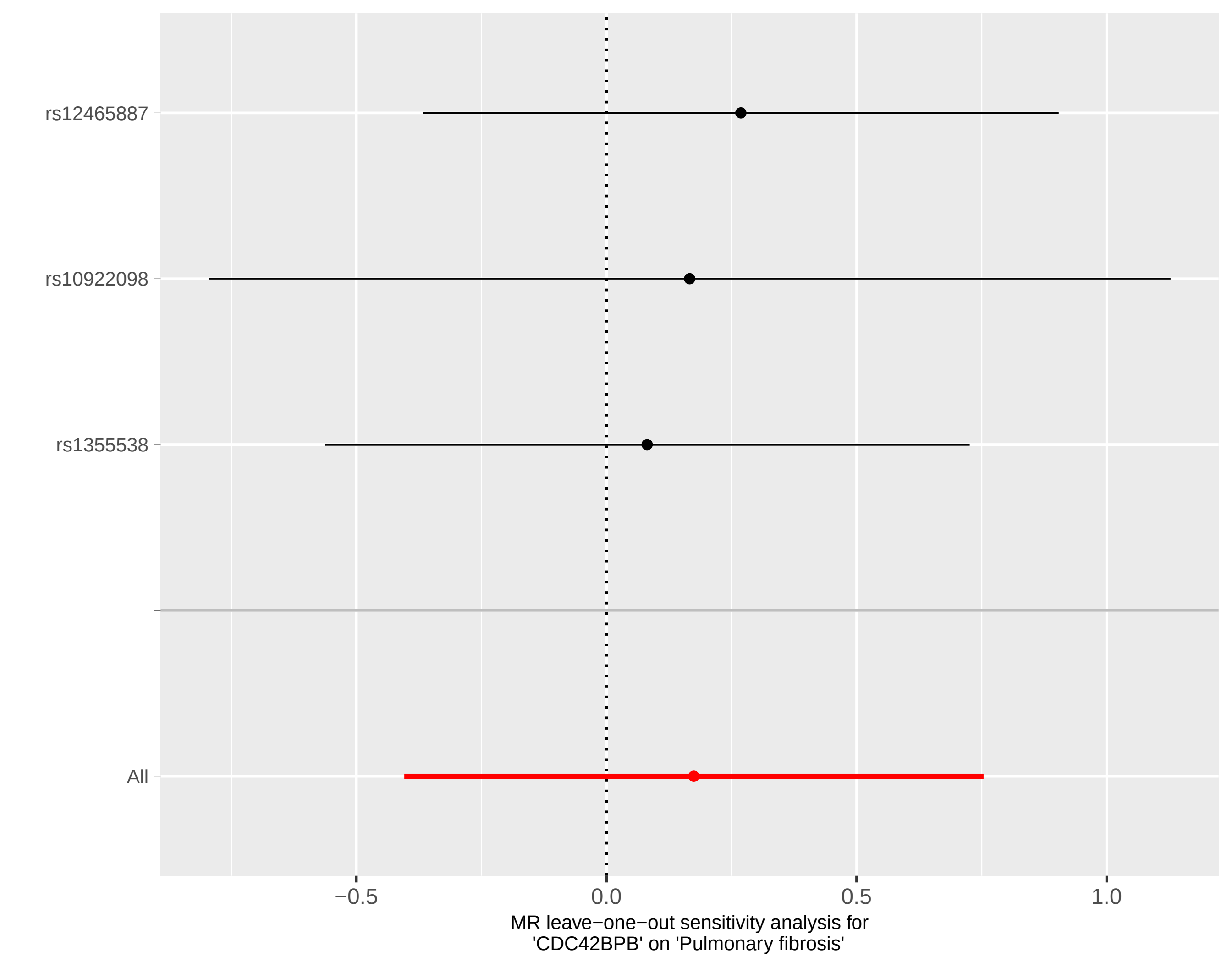
rs12465887
rs10922098
rs1355538
All
−0.5
0.0
0.5
1.0
MR leave−one−out sensitivity analysis for
'CDC42BPB' on 'Pulmonary fibrosis'

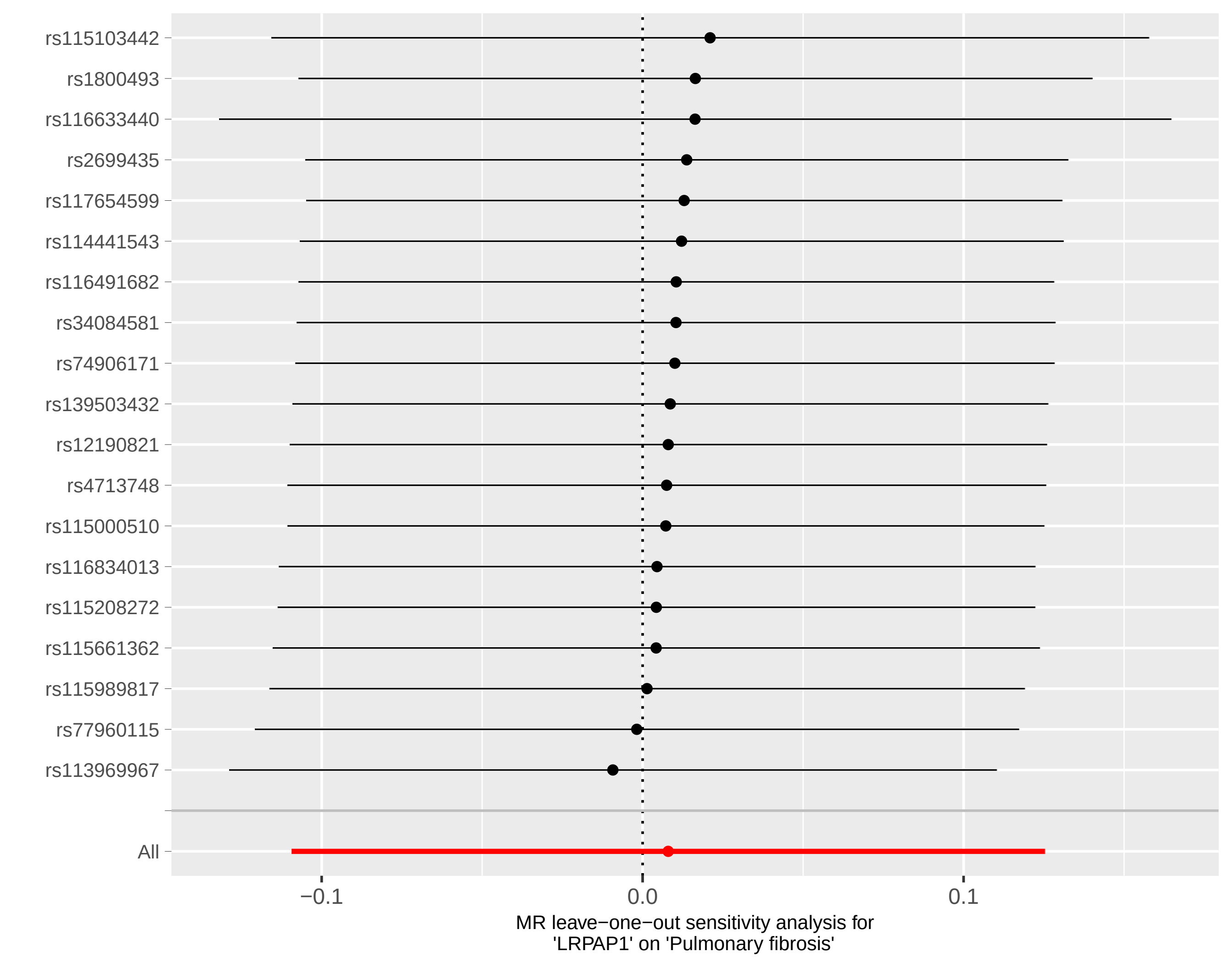
rs115103442
rs1800493
rs116633440
rs2699435
rs117654599
rs114441543
rs116491682
rs34084581
rs74906171
rs139503432
rs12190821
rs4713748
rs115000510
rs116834013
rs115208272
rs115661362
rs115989817
rs77960115
rs113969967
All
−0.1
0.0
0.1
MR leave−one−out sensitivity analysis for
'LRPAP1' on 'Pulmonary fibrosis'

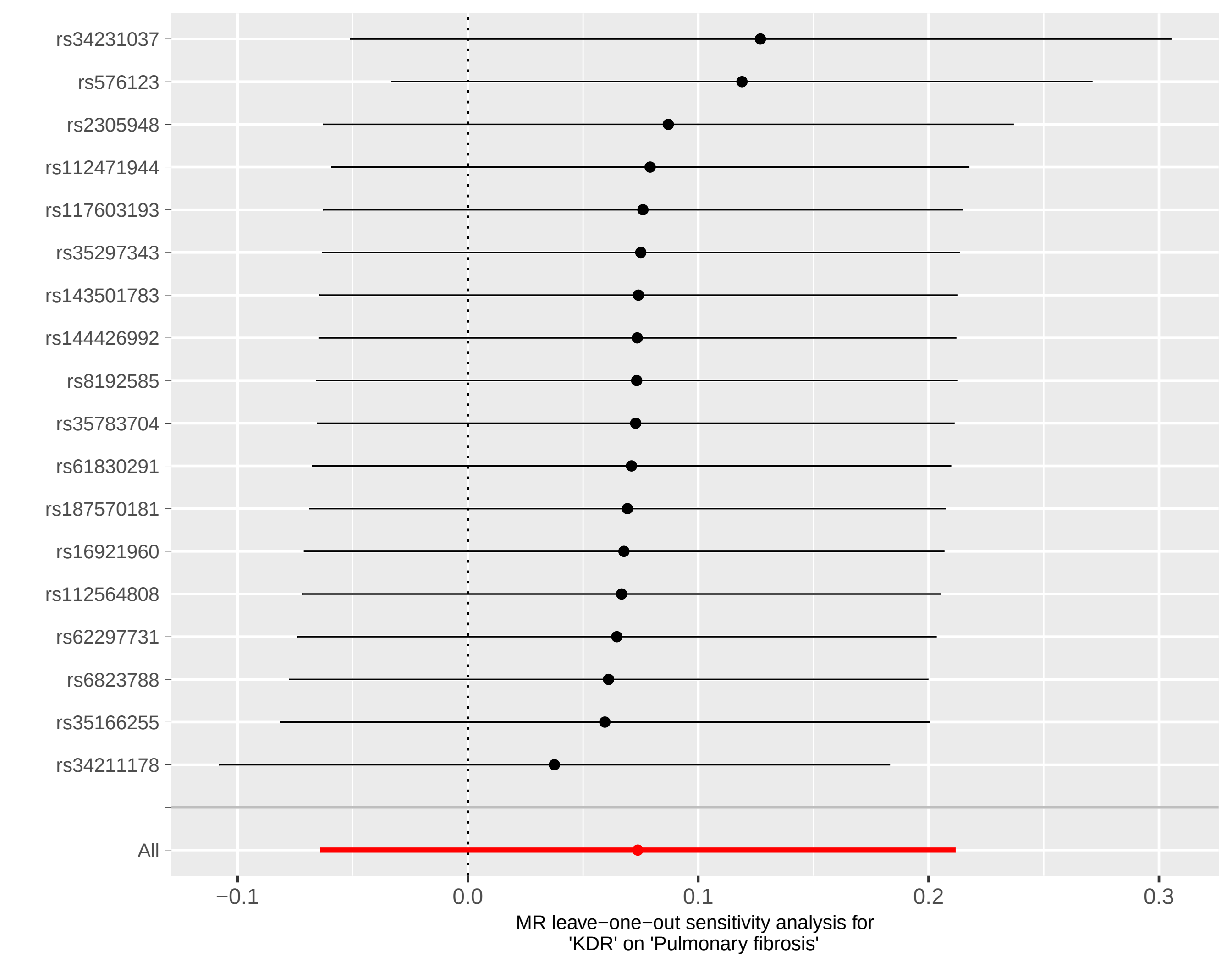
rs34231037
rs576123
rs2305948
rs112471944
rs117603193
rs35297343
rs143501783
rs144426992
rs8192585
rs35783704
rs61830291
rs187570181
rs16921960
rs112564808
rs62297731
rs6823788
rs35166255
rs34211178
All
−0.1
0.0
0.1
0.2
0.3
MR leave−one−out sensitivity analysis for
'KDR' on 'Pulmonary fibrosis'

Insufficient number of SNPs

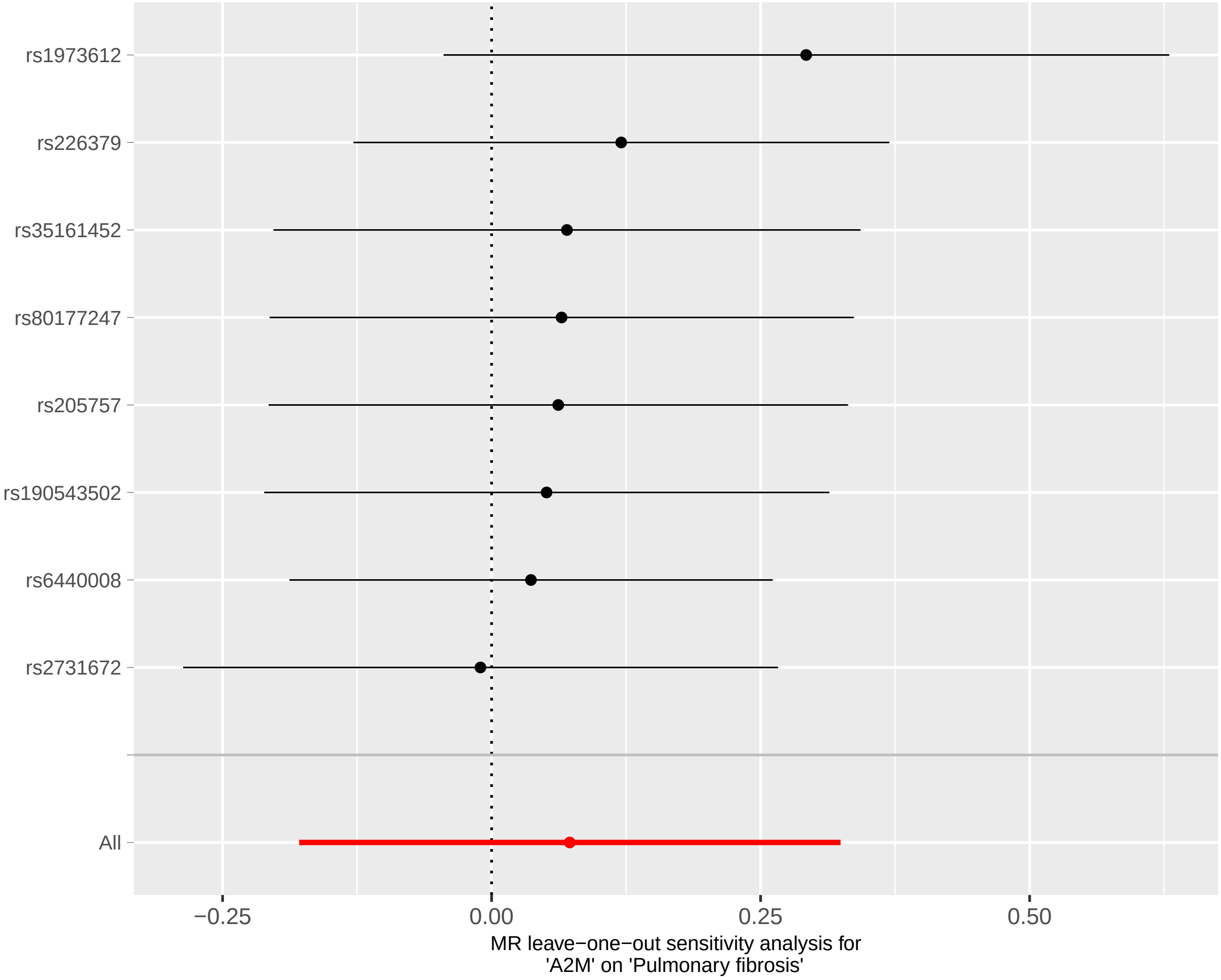

rs1973612
rs226379
rs35161452
rs80177247
rs205757
rs190543502
rs6440008
rs2731672
All
−0.25
0.00
0.25
0.50
MR leave−one−out sensitivity analysis for
'A2M' on 'Pulmonary fibrosis'

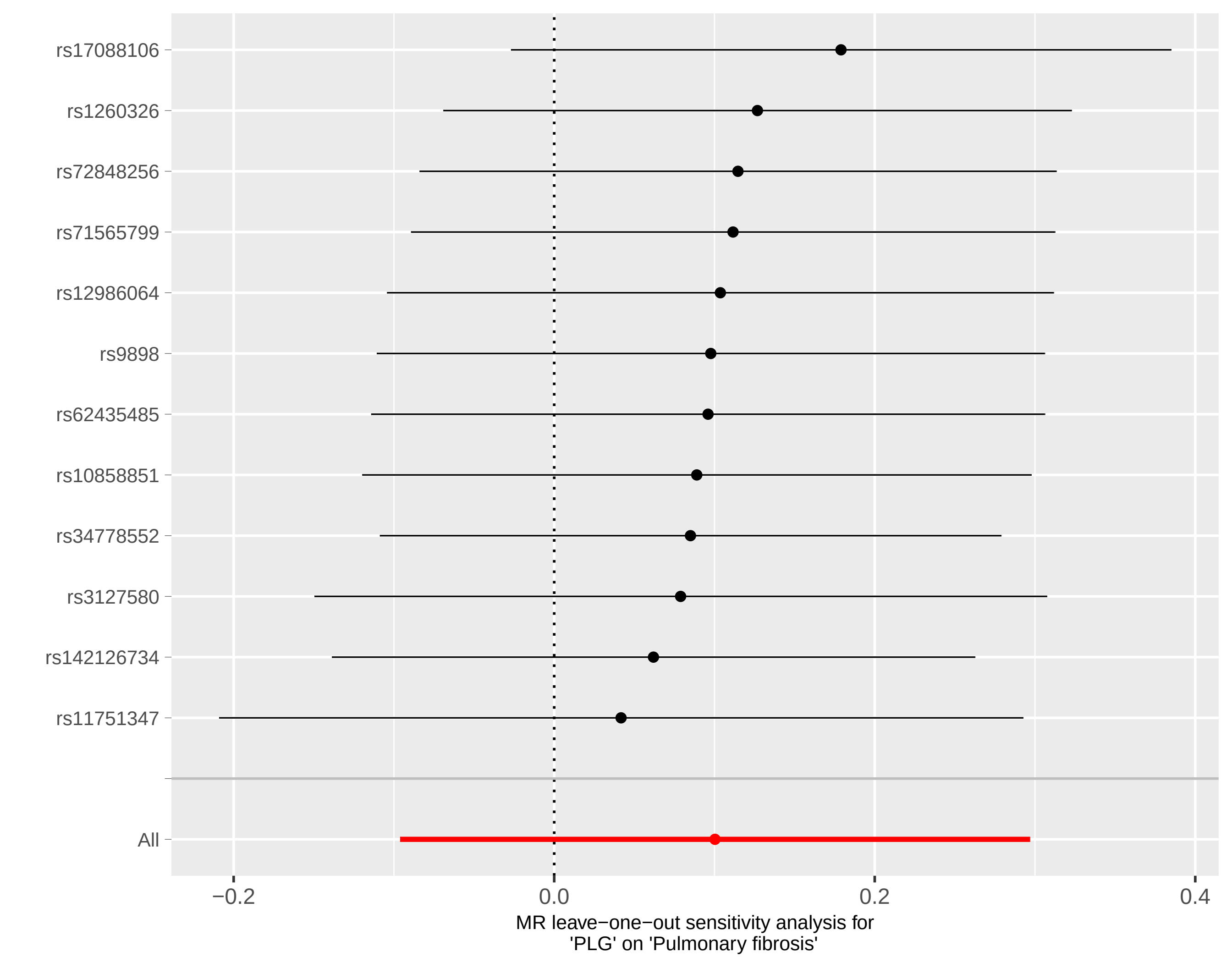

rs17088106
rs1260326
rs72848256
rs71565799
rs12986064
rs9898
rs62435485
rs10858851
rs34778552
rs3127580
rs142126734
rs11751347
All
−0.2
0.0
0.2
0.4
MR leave−one−out sensitivity analysis for
'PLG' on 'Pulmonary fibrosis'

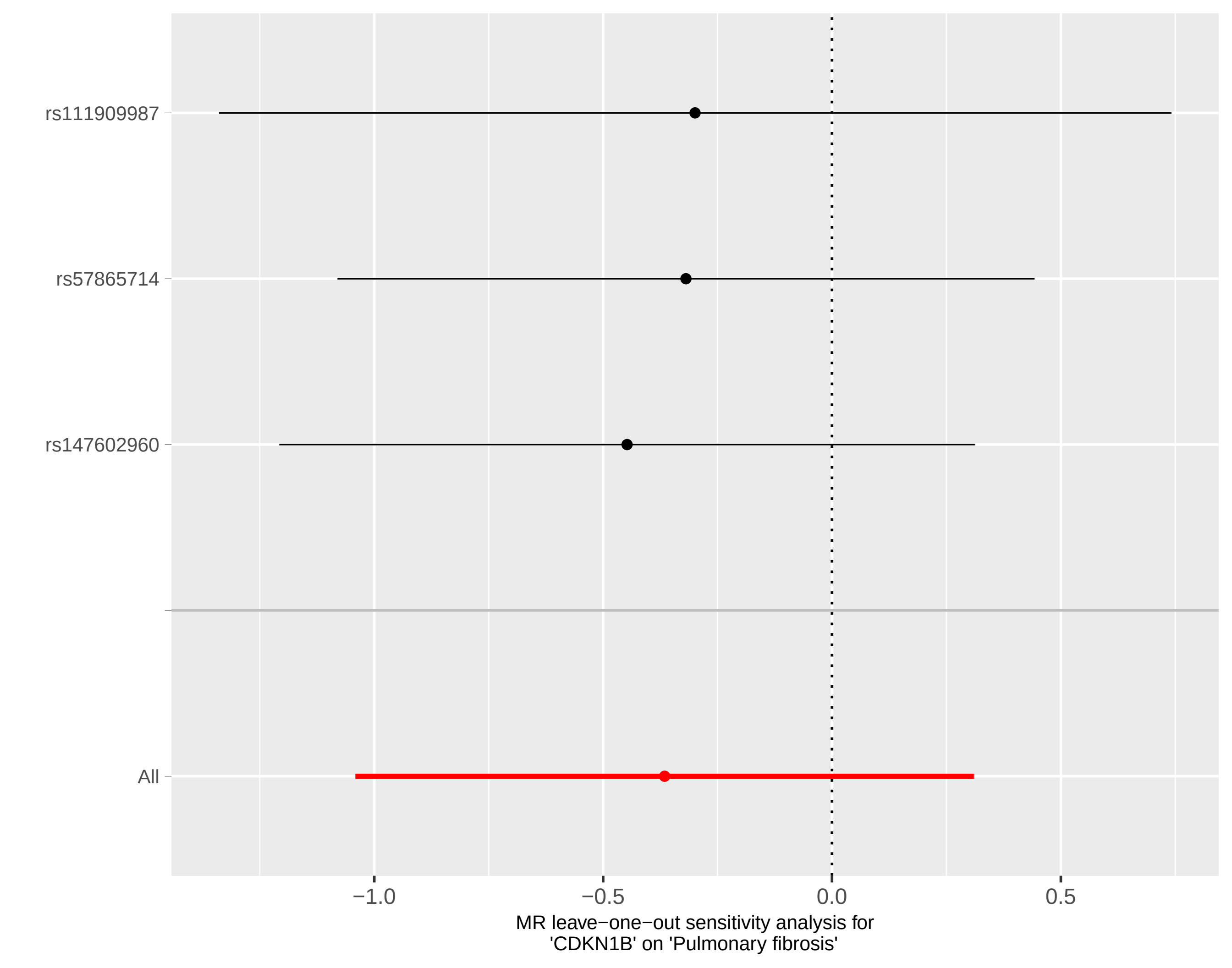
rs111909987
rs57865714
rs147602960
All
−1.0
−0.5
0.0
0.5
MR leave−one−out sensitivity analysis for
'CDKN1B' on 'Pulmonary fibrosis'

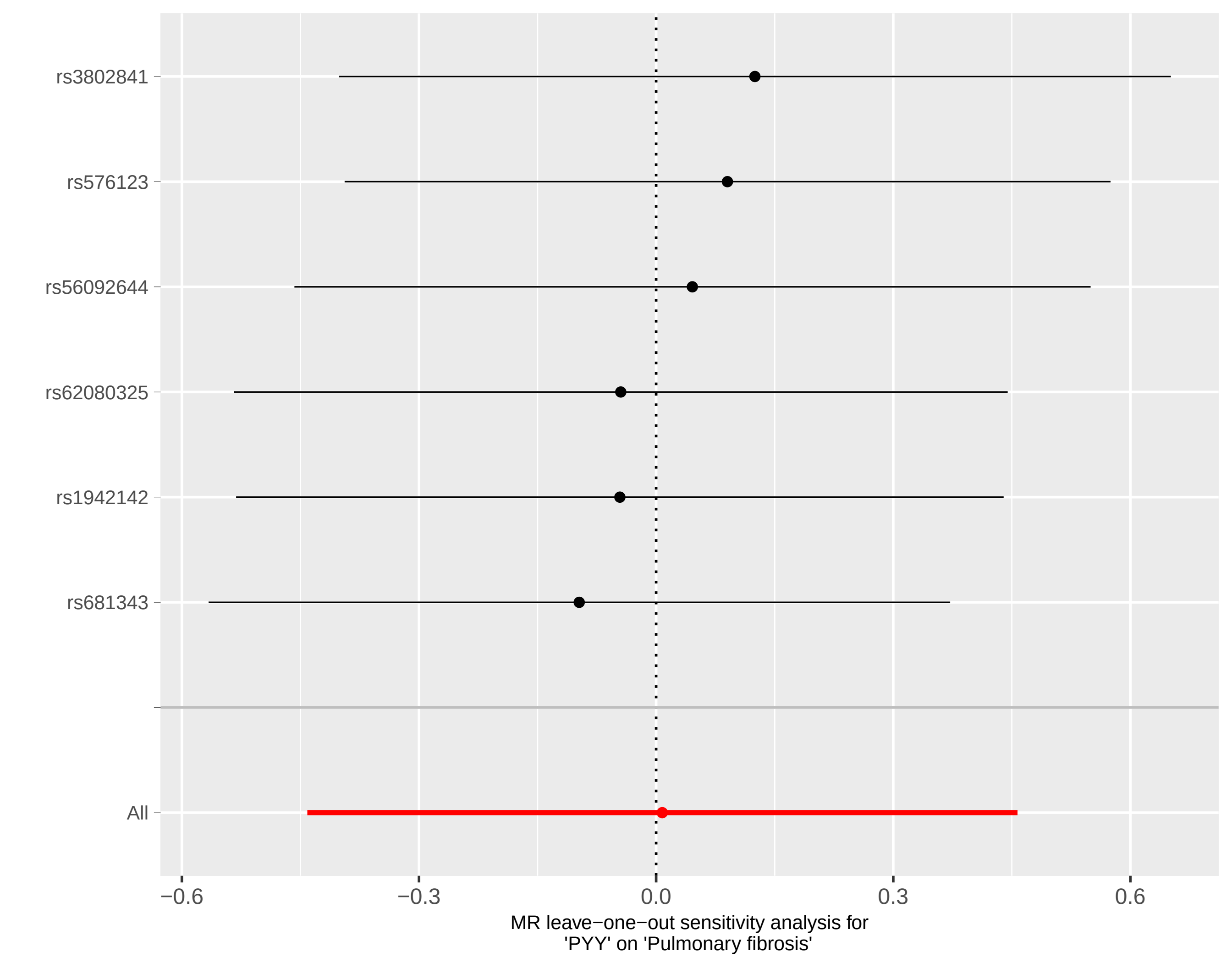
rs3802841
rs576123
rs56092644
rs62080325
rs1942142
rs681343
All
−0.6
−0.3
0.0
0.3
0.6
MR leave−one−out sensitivity analysis for
'PYY' on 'Pulmonary fibrosis'

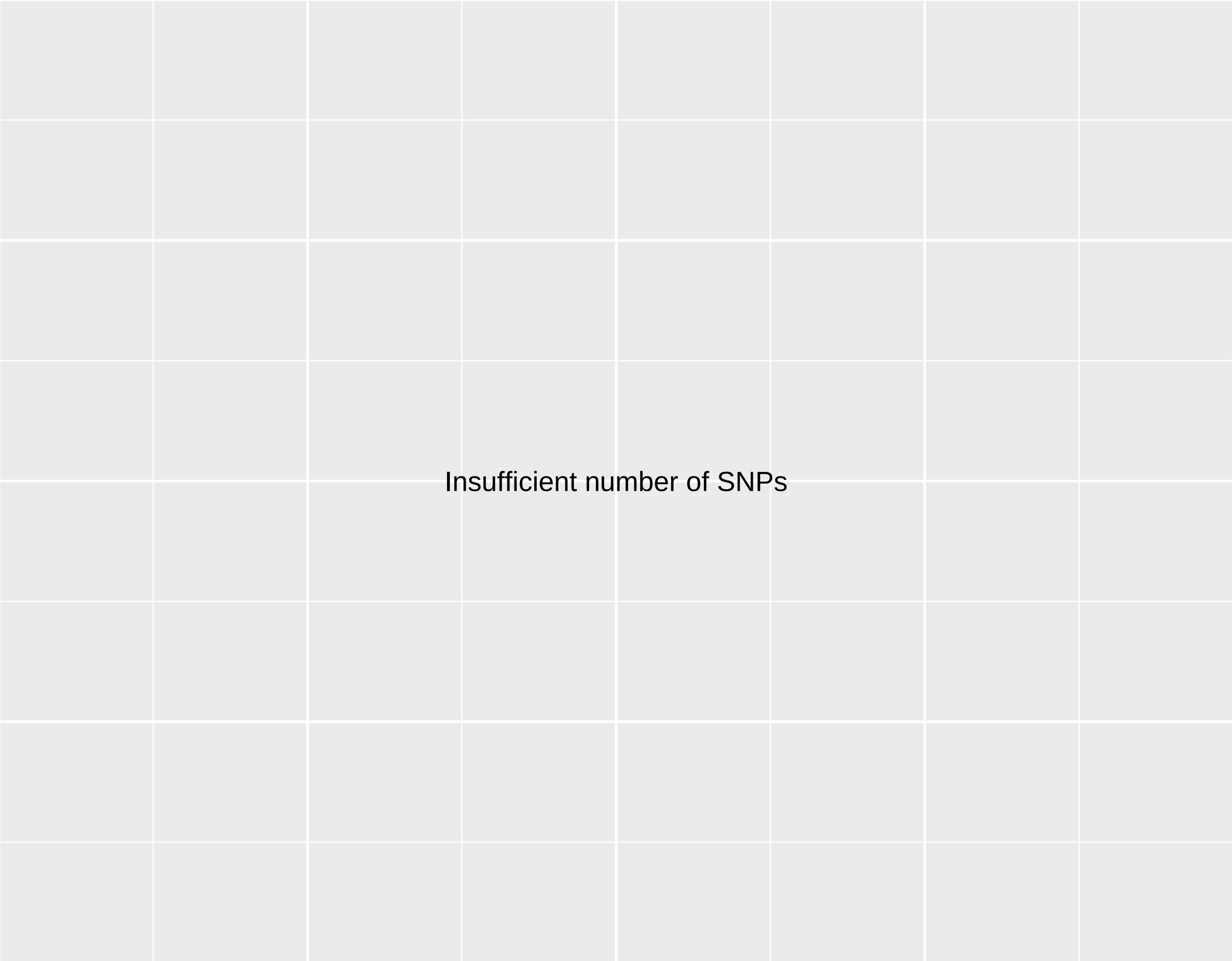
Insufficient number of SNPs

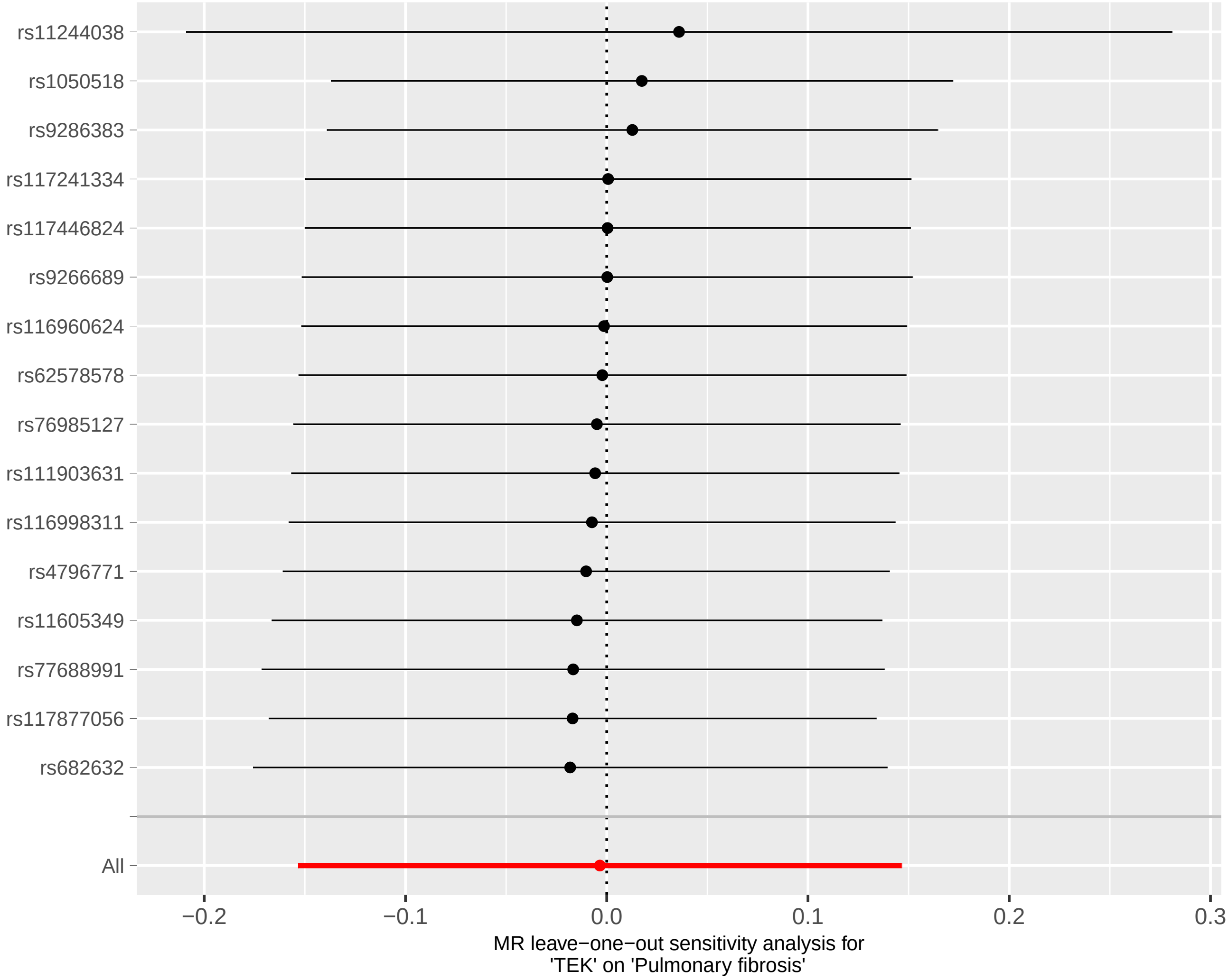
rs11244038
rs1050518
rs9286383
rs117241334
rs117446824
rs9266689
rs116960624
rs62578578
rs76985127
rs111903631
rs116998311
rs4796771
rs11605349
rs77688991
rs117877056
rs682632
All
−0.2
−0.1
0.0
0.1
0.2
0.3
MR leave−one−out sensitivity analysis for
'TEK' on 'Pulmonary fibrosis'

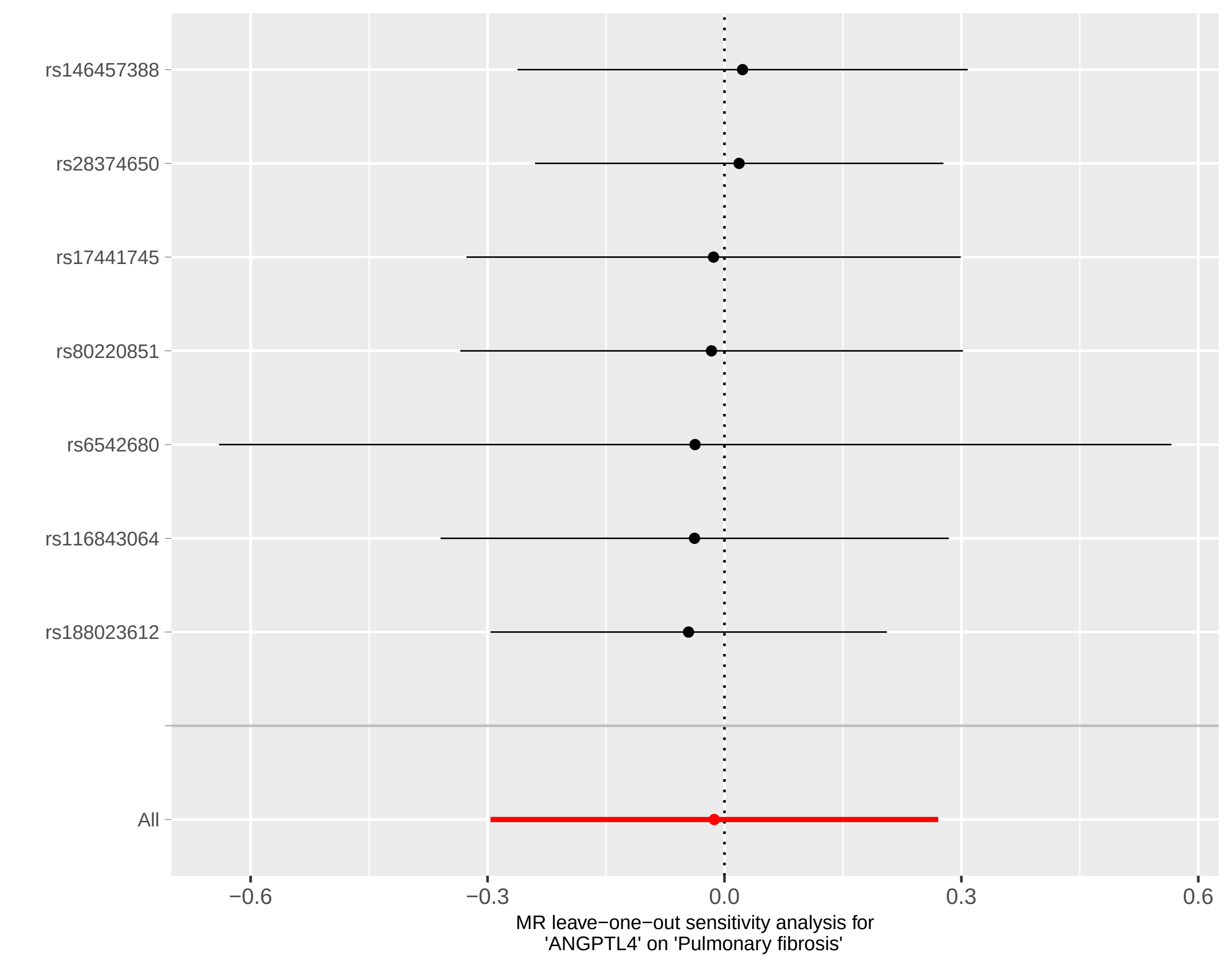
rs146457388
rs28374650
rs17441745
rs80220851
rs6542680
rs116843064
rs188023612
All
−0.6
−0.3
0.0
0.3
0.6
MR leave−one−out sensitivity analysis for
'ANGPTL4' on 'Pulmonary fibrosis'

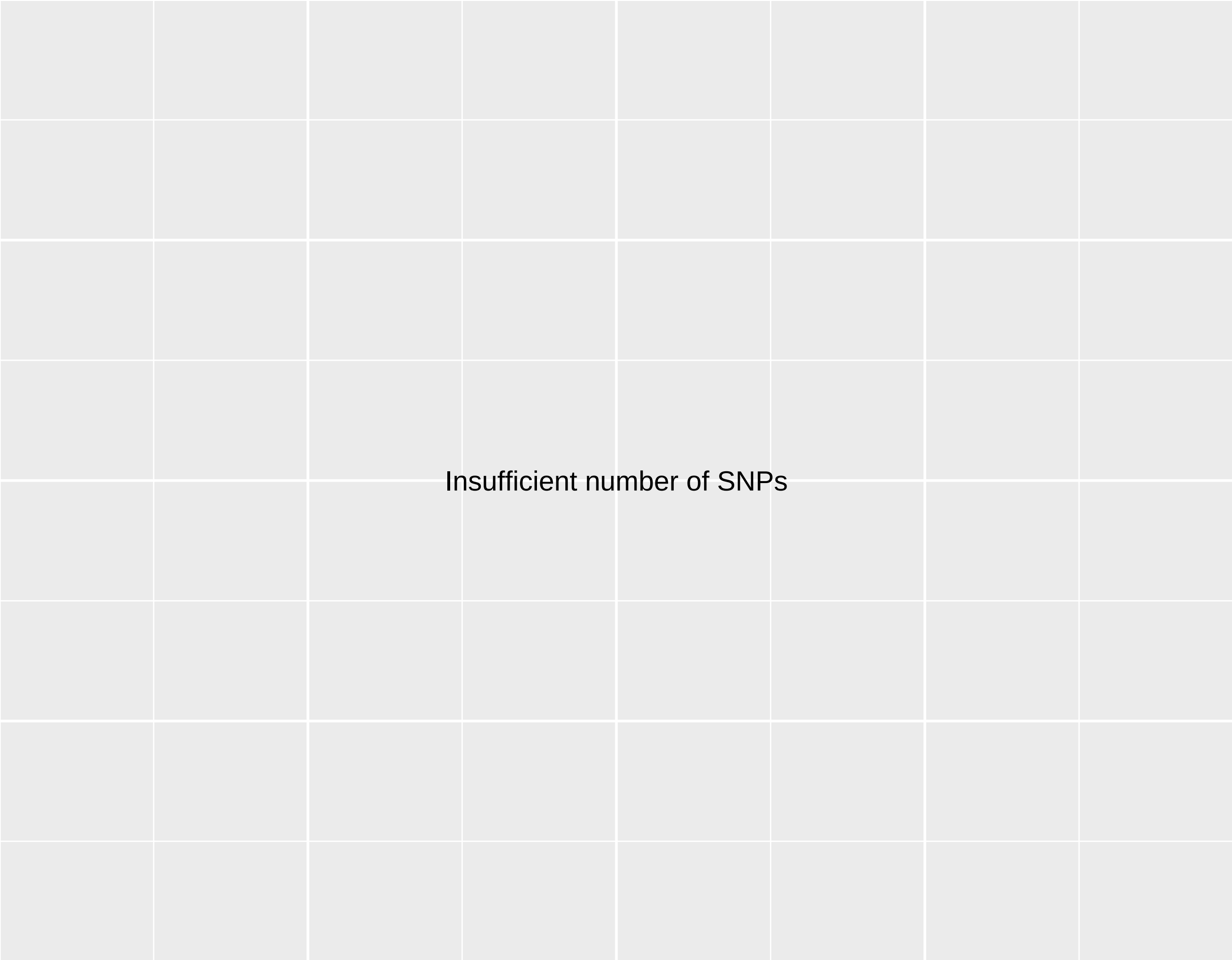
Insufficient number of SNPs

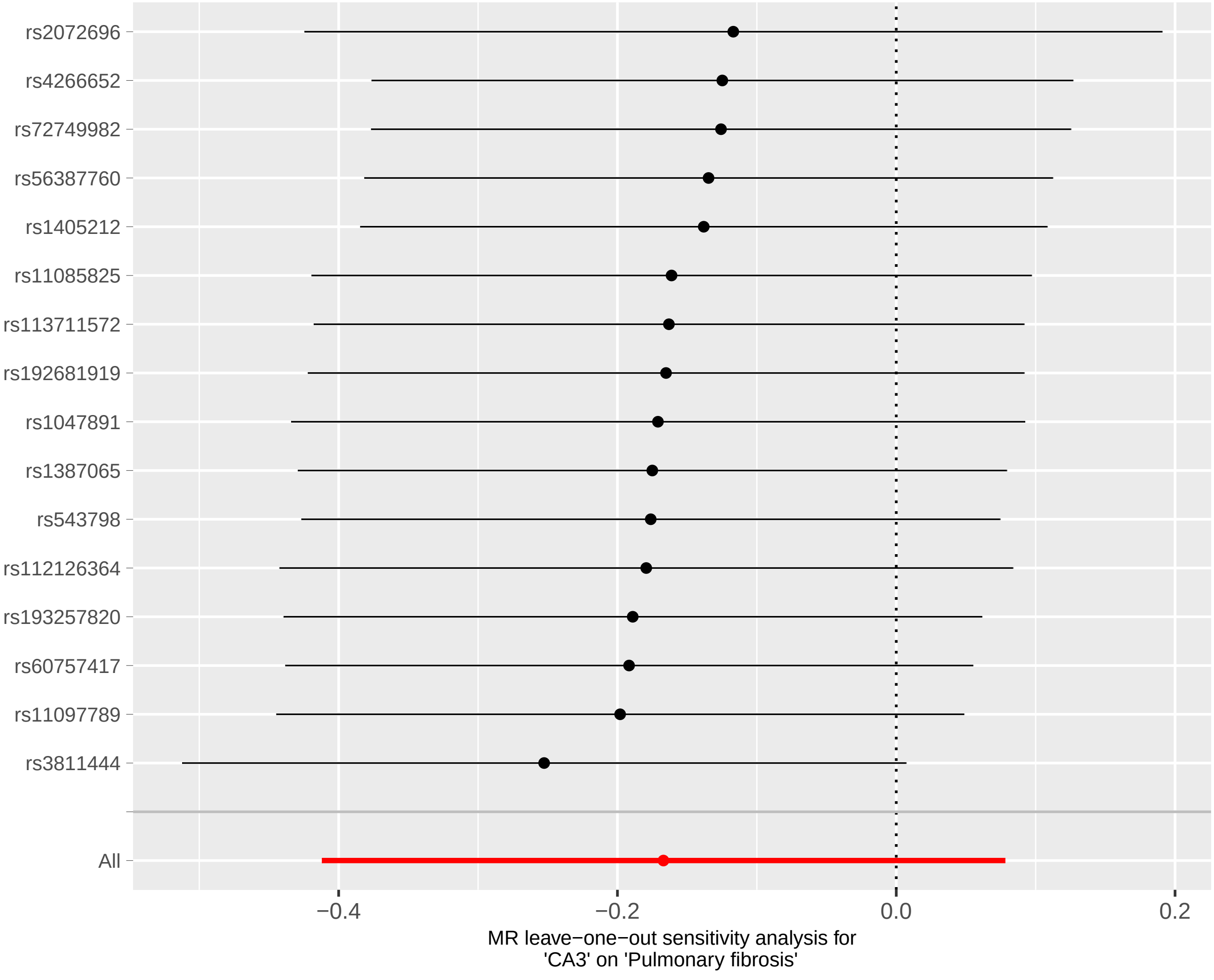
rs2072696
rs4266652
rs72749982
rs56387760
rs1405212
rs11085825
rs113711572
rs192681919
rs1047891
rs1387065
rs543798
rs112126364
rs193257820
rs60757417
rs11097789
rs3811444
All
−0.4
−0.2
0.0
0.2
MR leave−one−out sensitivity analysis for
'CA3' on 'Pulmonary fibrosis'

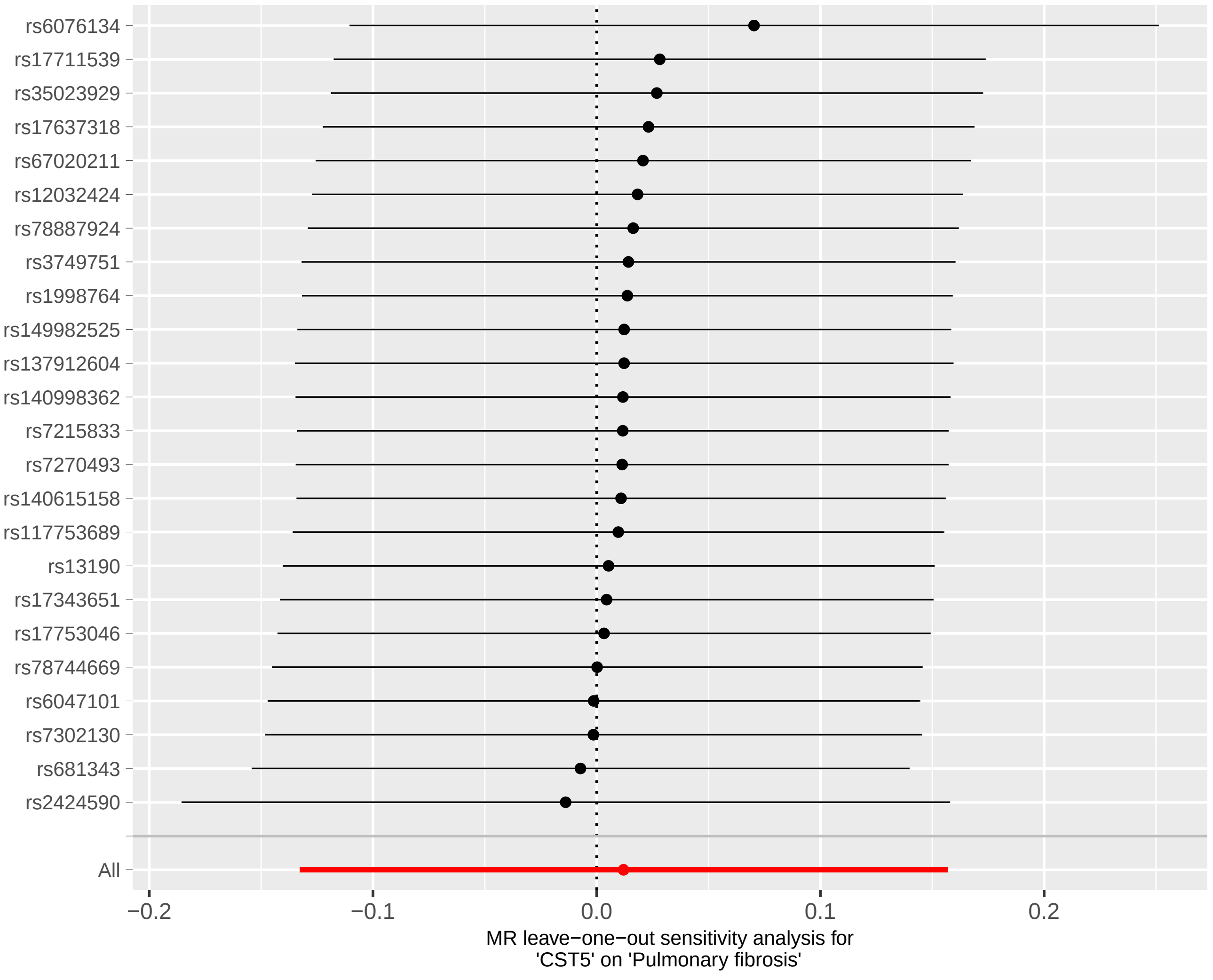
rs6076134
rs17711539
rs35023929
rs17637318
rs67020211
rs12032424
rs78887924
rs3749751
rs1998764
rs149982525
rs137912604
rs140998362
rs7215833
rs7270493
rs140615158
rs117753689
rs13190
rs17343651
rs17753046
rs78744669
rs6047101
rs7302130
rs681343
rs2424590
All
−0.2
−0.1
0.0
0.1
0.2
MR leave−one−out sensitivity analysis for
'CST5' on 'Pulmonary fibrosis'

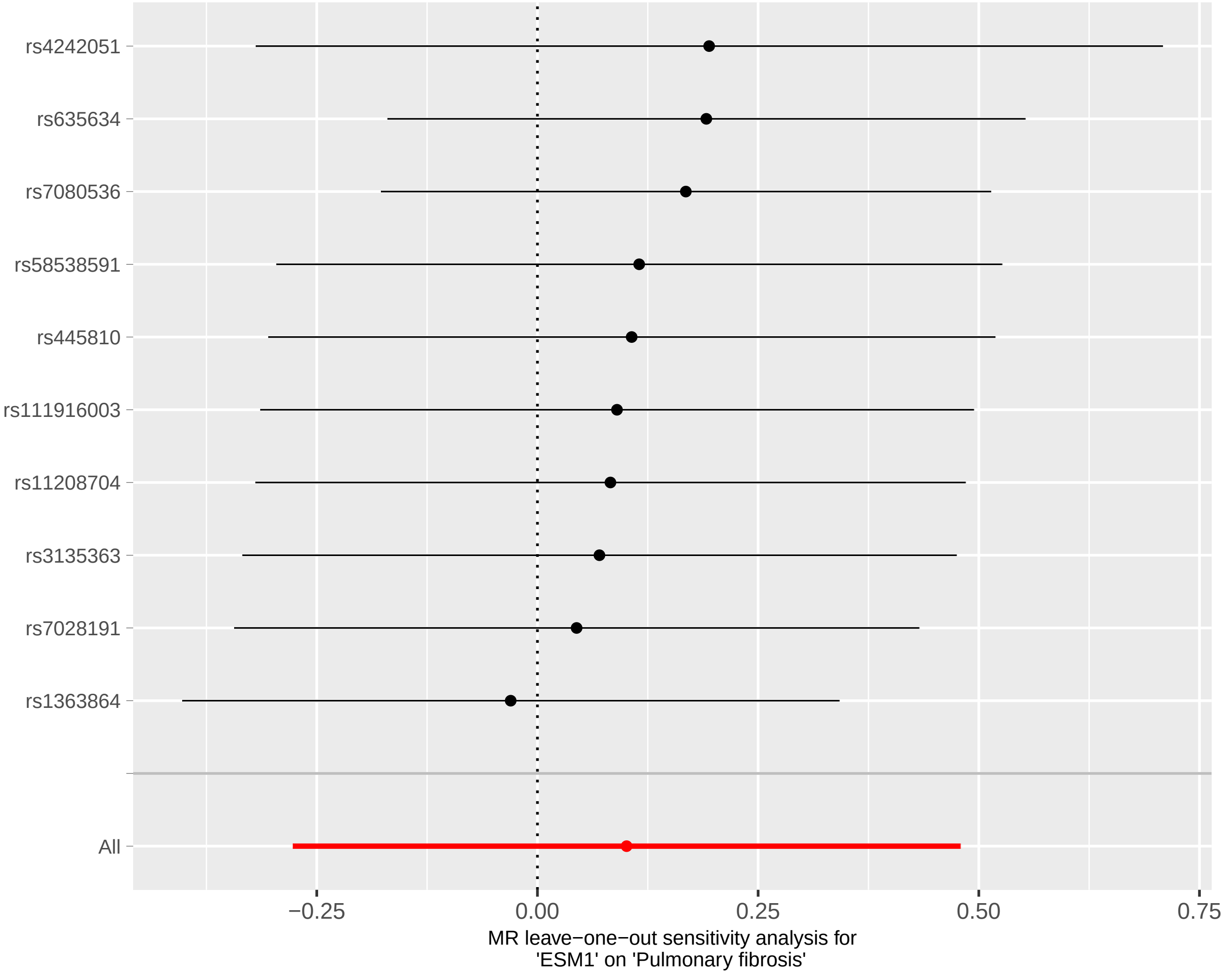
rs4242051
rs635634
rs7080536
rs58538591
rs445810
rs111916003
rs11208704
rs3135363
rs7028191
rs1363864
All
−0.25
0.00
0.25
0.50
0.75
MR leave−one−out sensitivity analysis for
'ESM1' on 'Pulmonary fibrosis'

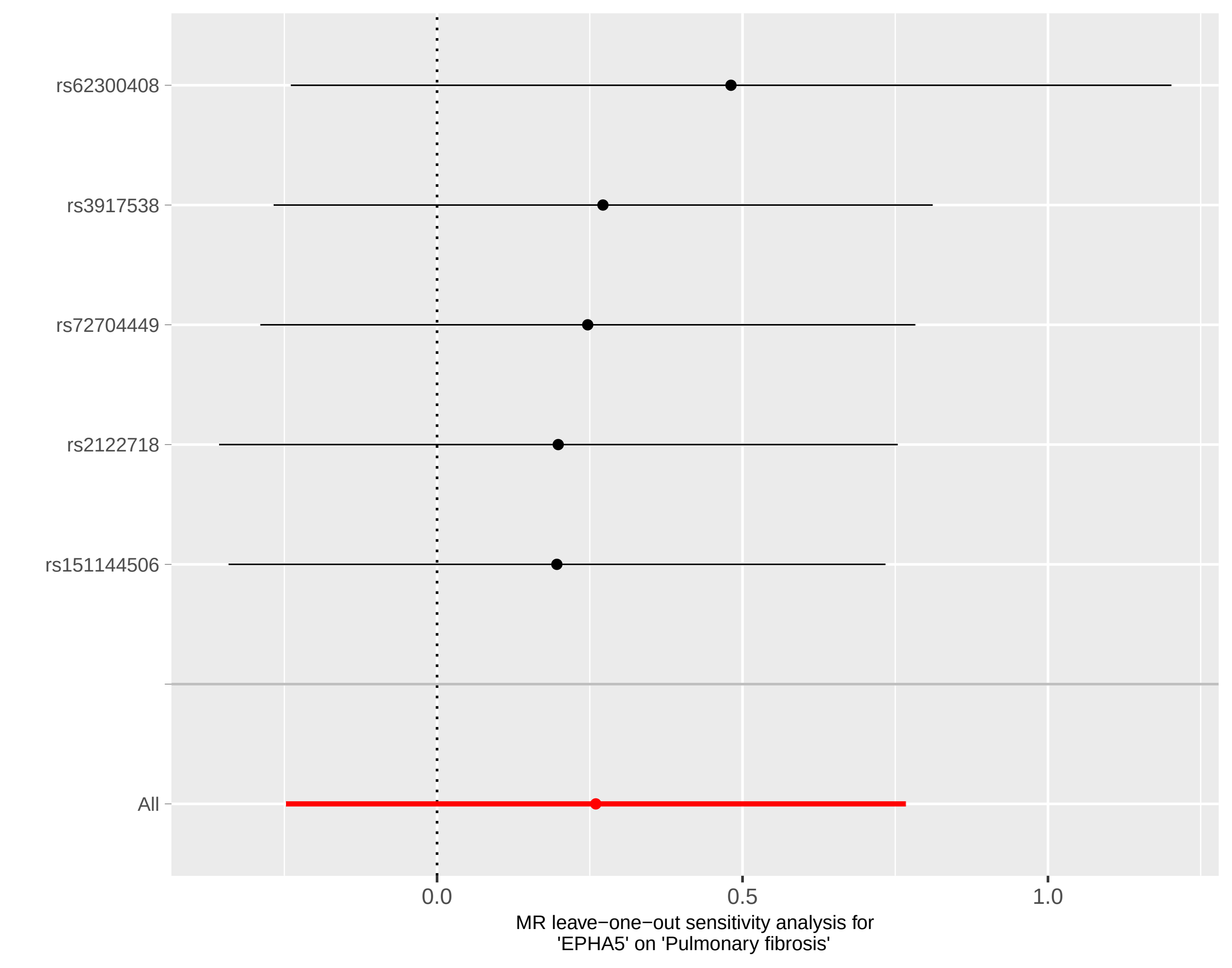
rs62300408
rs3917538
rs72704449
rs2122718
rs151144506
All
0.0
0.5
1.0
MR leave−one−out sensitivity analysis for
'EPHA5' on 'Pulmonary fibrosis'

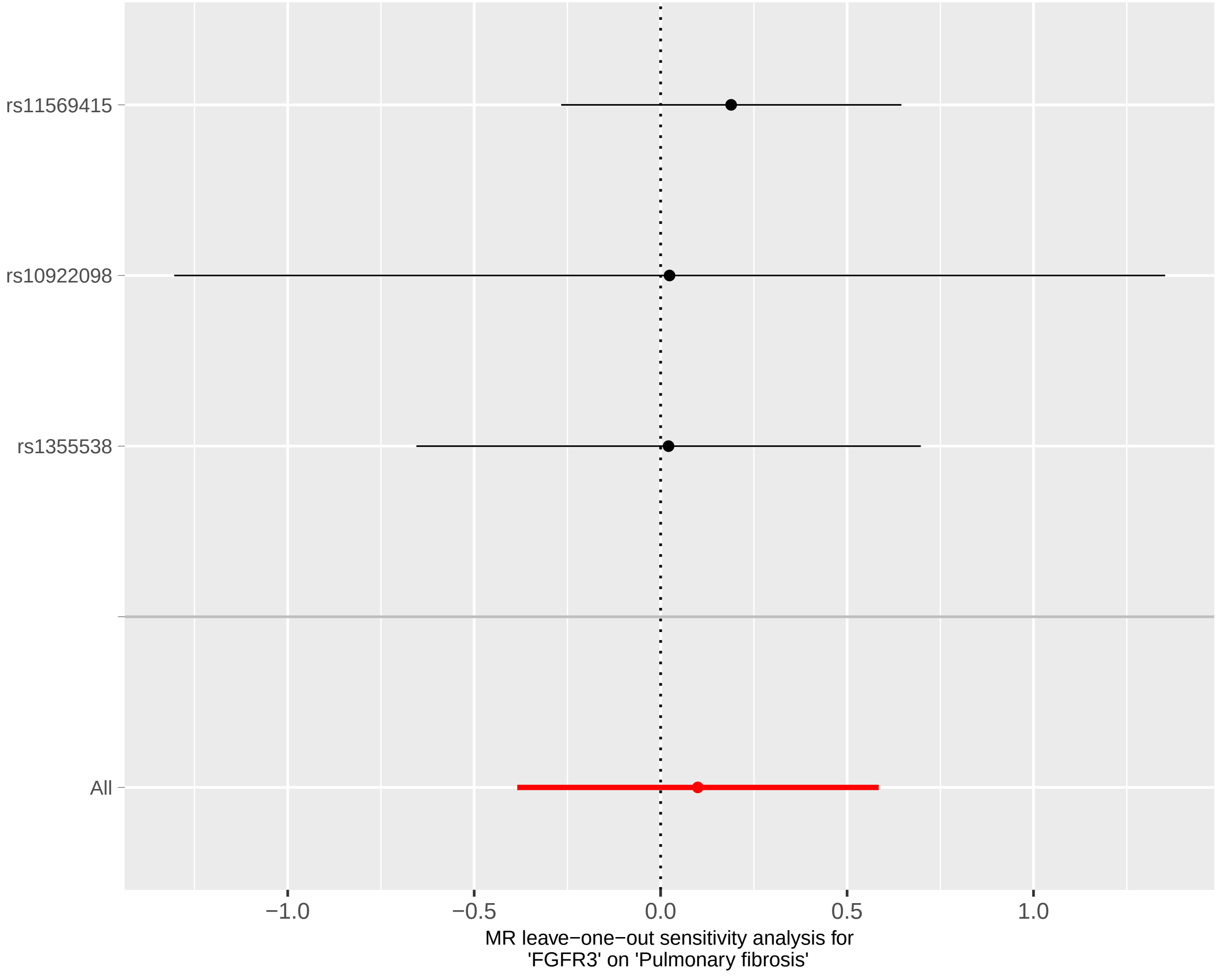
rs11569415
rs10922098
rs1355538
All
−1.0
−0.5
0.0
0.5
1.0
MR leave−one−out sensitivity analysis for
'FGFR3' on 'Pulmonary fibrosis'

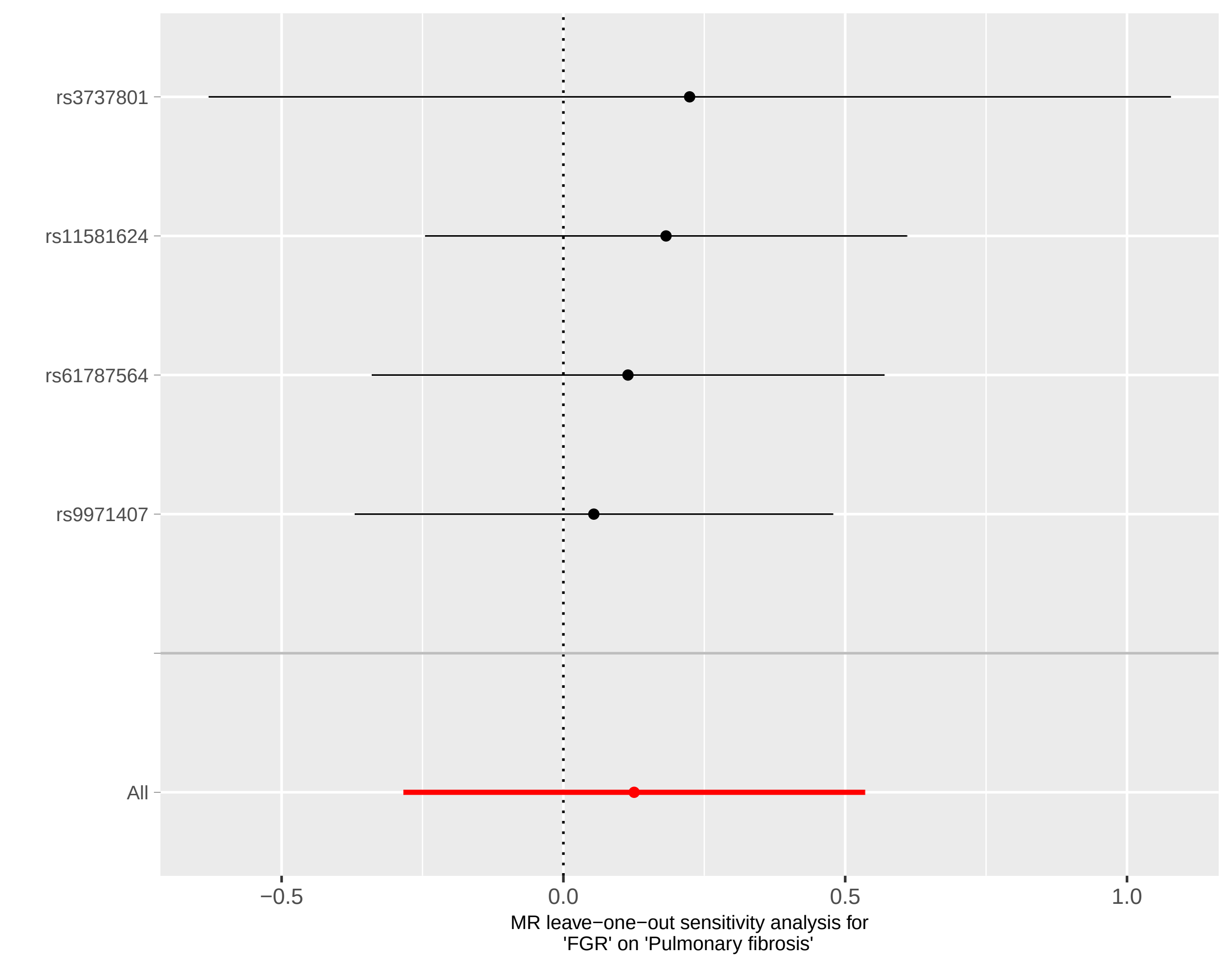
rs3737801
rs11581624
rs61787564
rs9971407
All
−0.5
0.0
0.5
1.0
MR leave−one−out sensitivity analysis for
'FGR' on 'Pulmonary fibrosis'

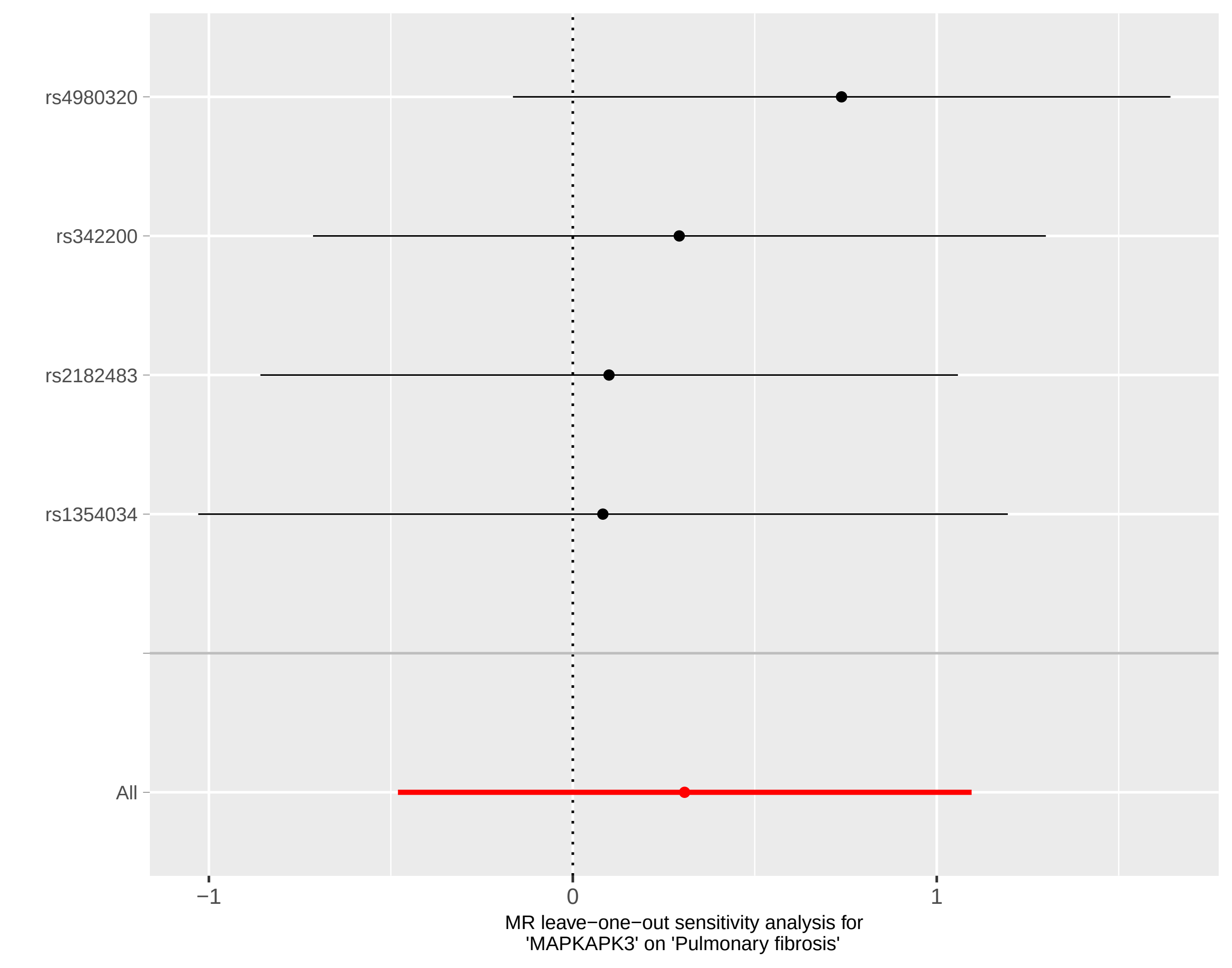
rs4980320
rs342200
rs2182483
rs1354034
All
−1
0
1
MR leave−one−out sensitivity analysis for
'MAPKAPK3' on 'Pulmonary fibrosis'

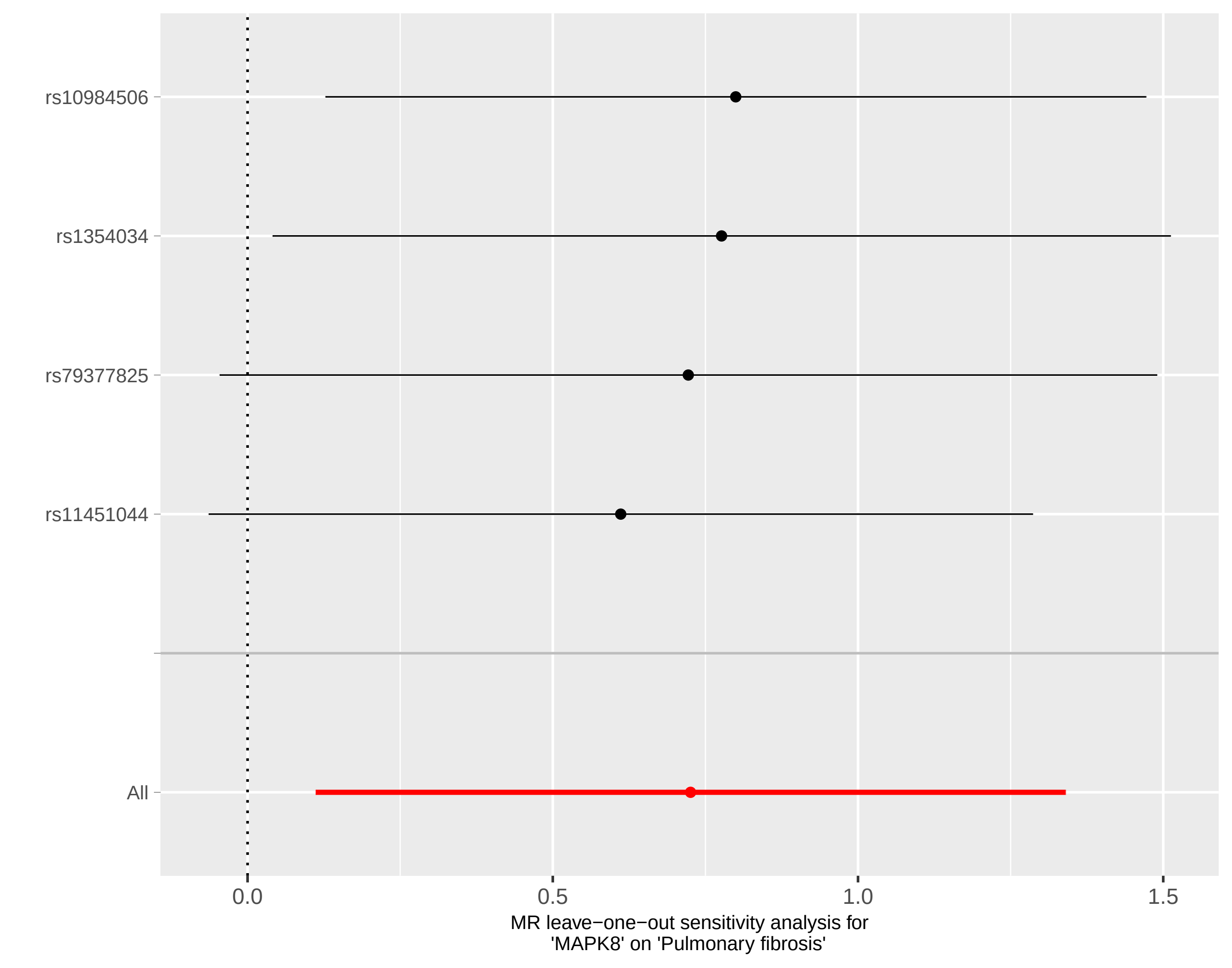

rs10984506
rs1354034
rs79377825
rs11451044
All
0.0
0.5
1.0
1.5
MR leave−one−out sensitivity analysis for
'MAPK8' on 'Pulmonary fibrosis'

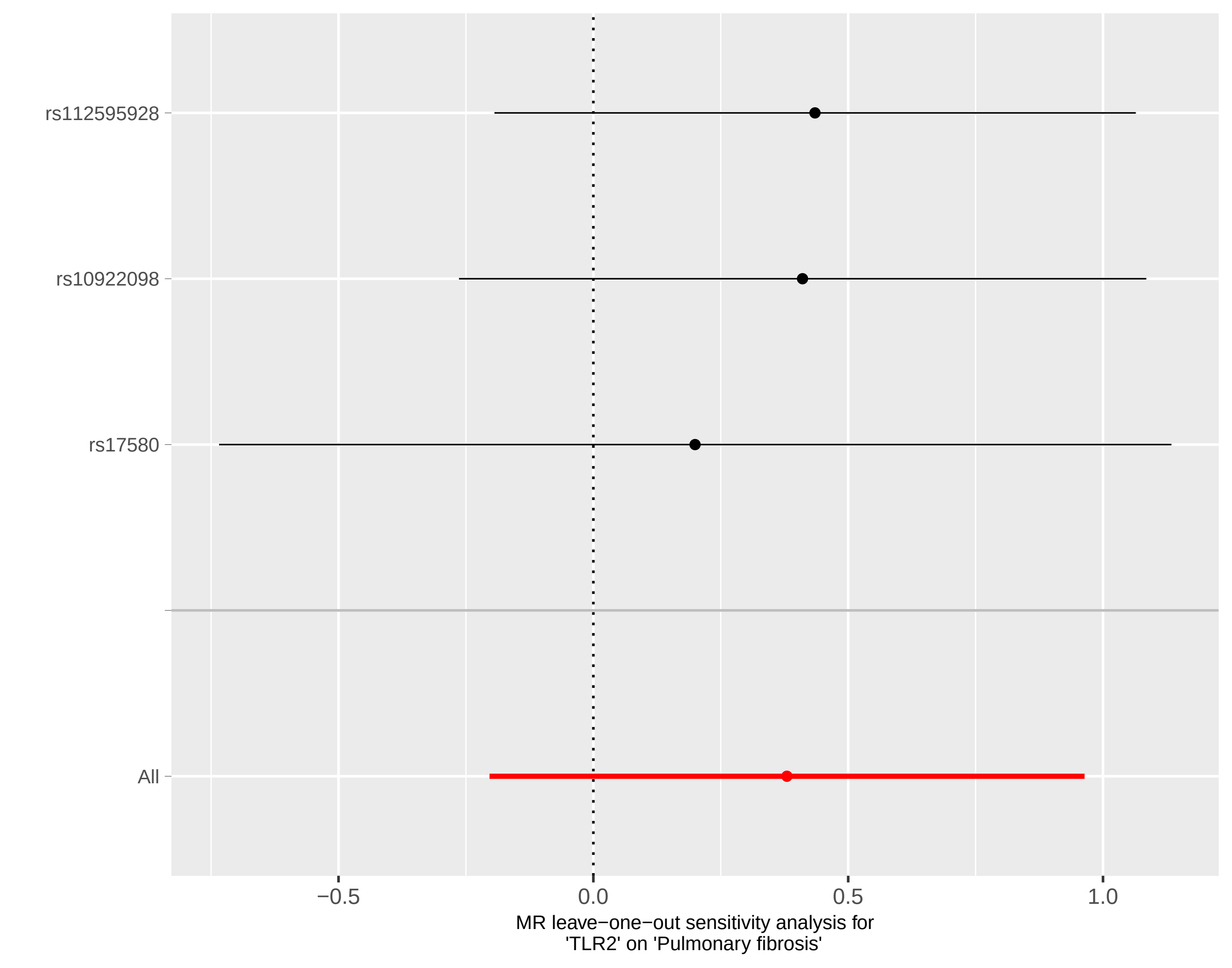
rs112595928
rs10922098
rs17580
All
−0.5
0.0
0.5
1.0
MR leave−one−out sensitivity analysis for
'TLR2' on 'Pulmonary fibrosis'

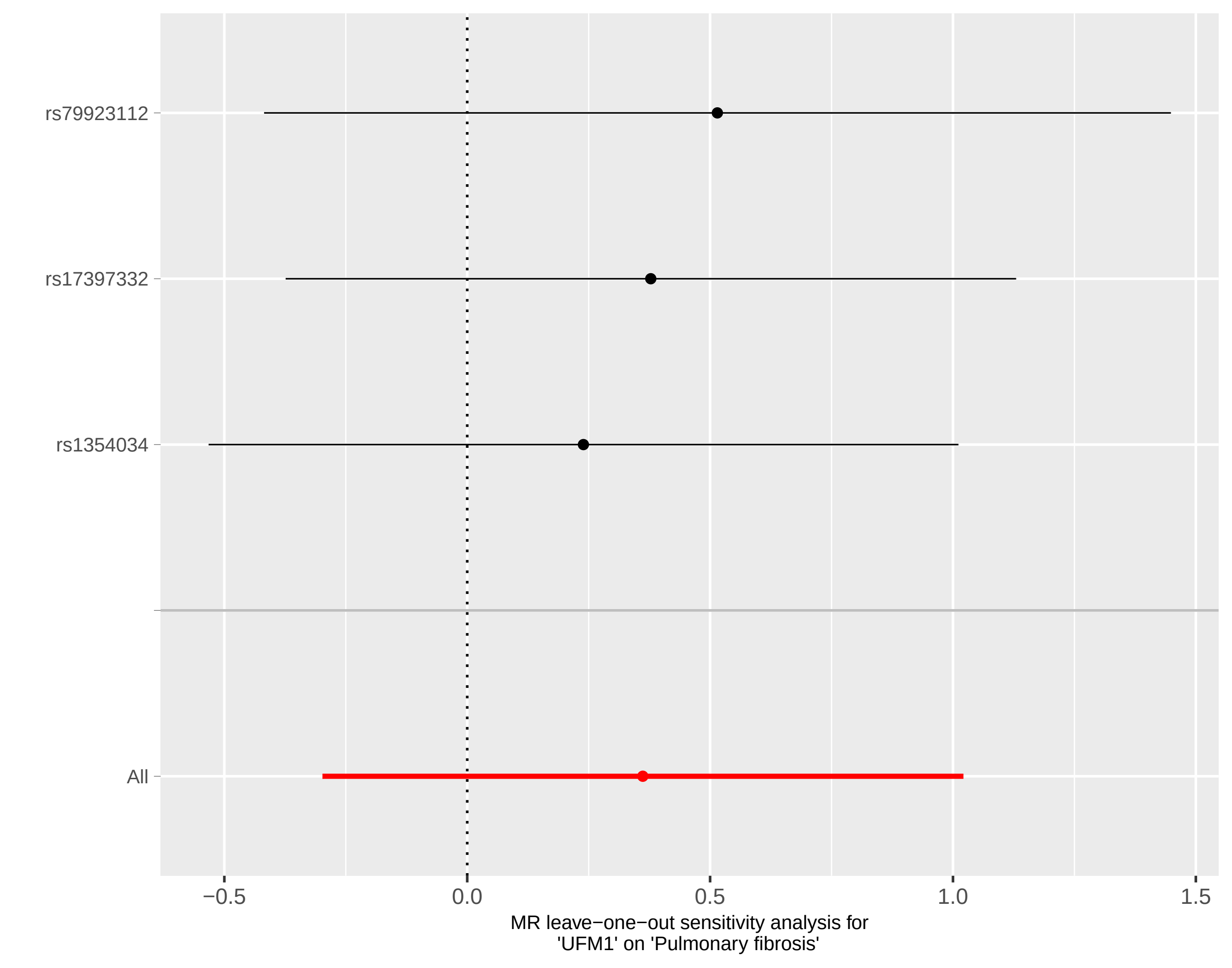

rs79923112
rs17397332
rs1354034
All
−0.5
0.0
0.5
1.0
1.5
MR leave−one−out sensitivity analysis for
'UFM1' on 'Pulmonary fibrosis'

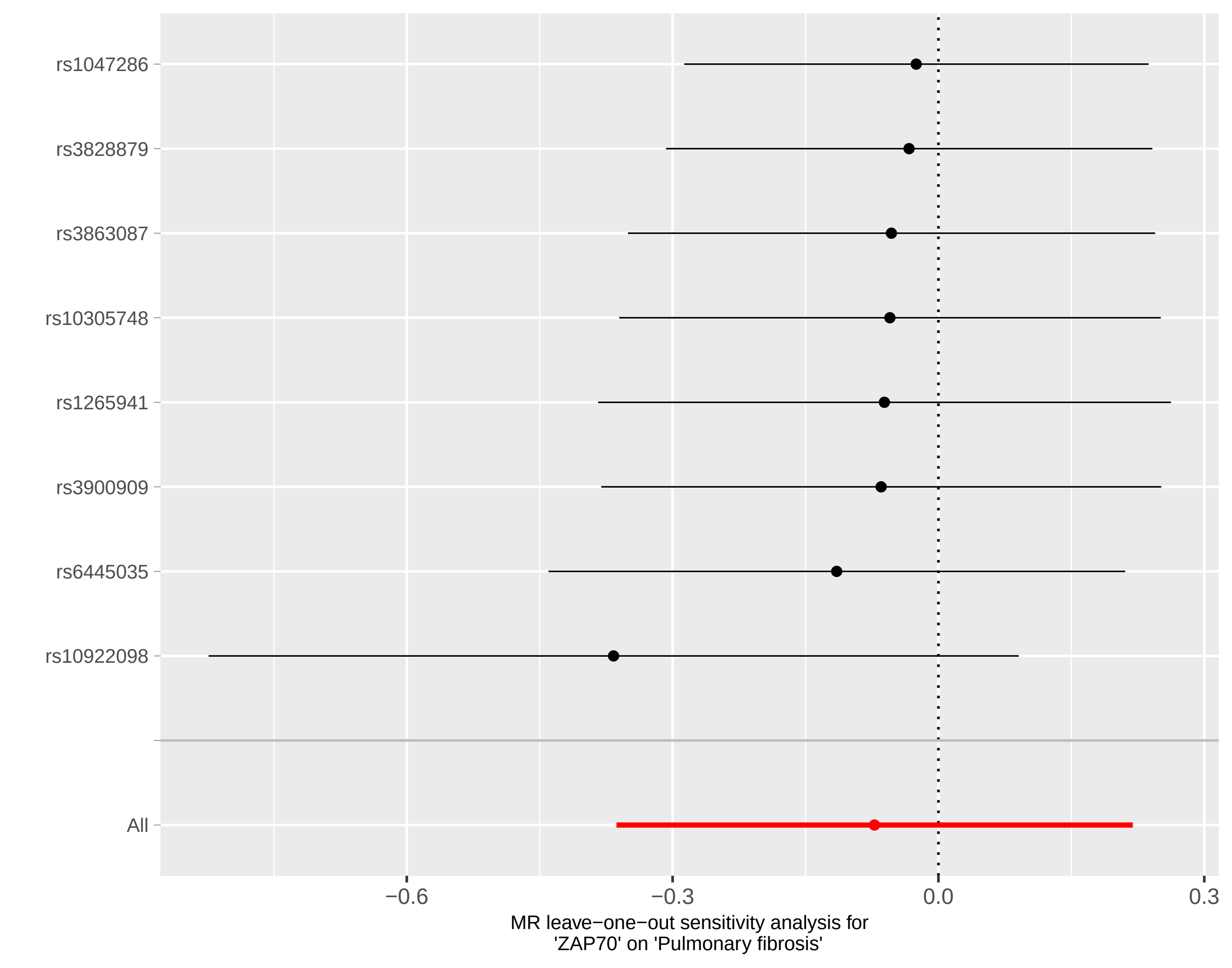
rs1047286
rs3828879
rs3863087
rs10305748
rs1265941
rs3900909
rs6445035
rs10922098
All
−0.6
−0.3
0.0
0.3
MR leave−one−out sensitivity analysis for
'ZAP70' on 'Pulmonary fibrosis'

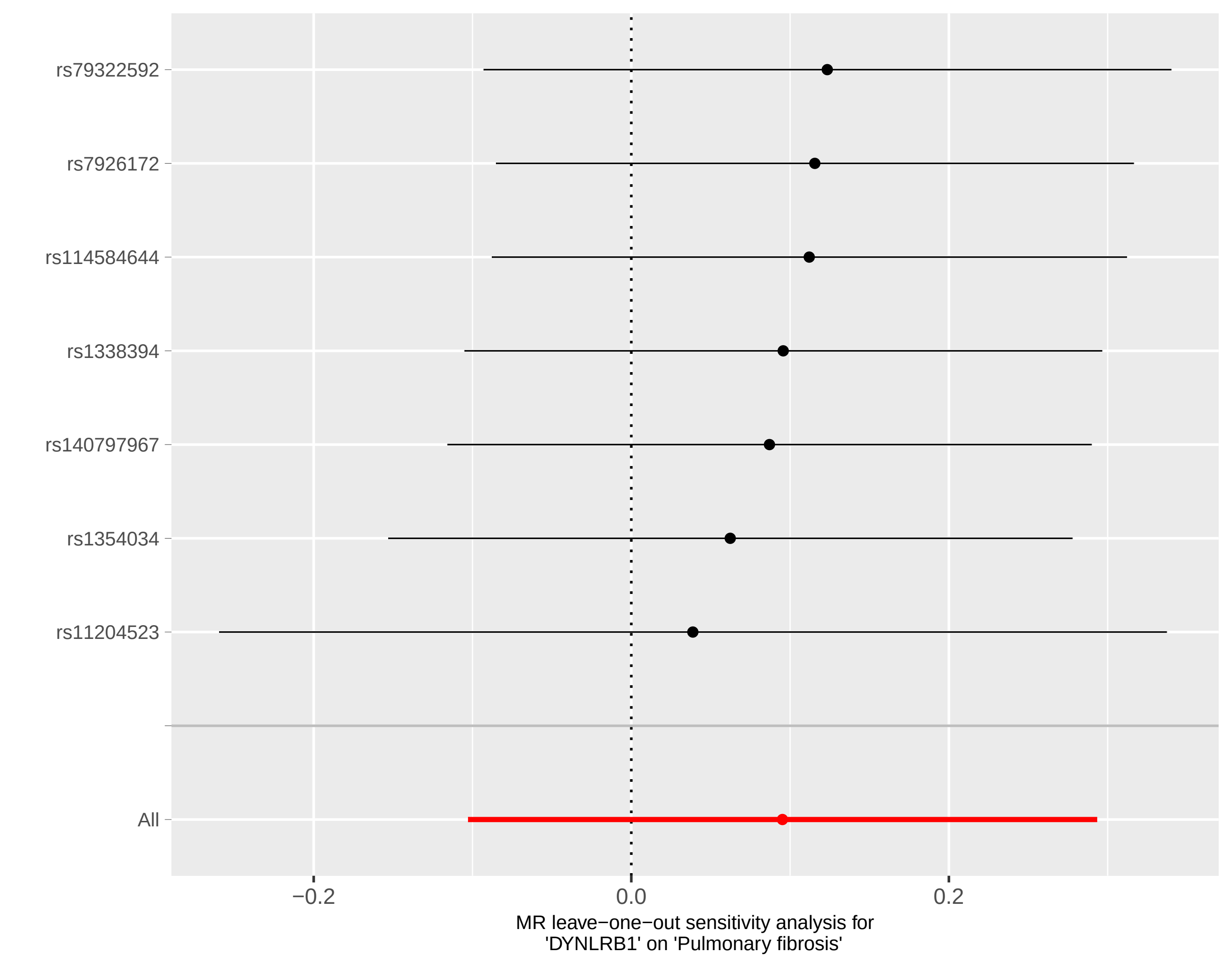
rs79322592
rs7926172
rs114584644
rs1338394
rs140797967
rs1354034
rs11204523
All
−0.2
0.0
0.2
MR leave−one−out sensitivity analysis for
'DYNLRB1' on 'Pulmonary fibrosis'

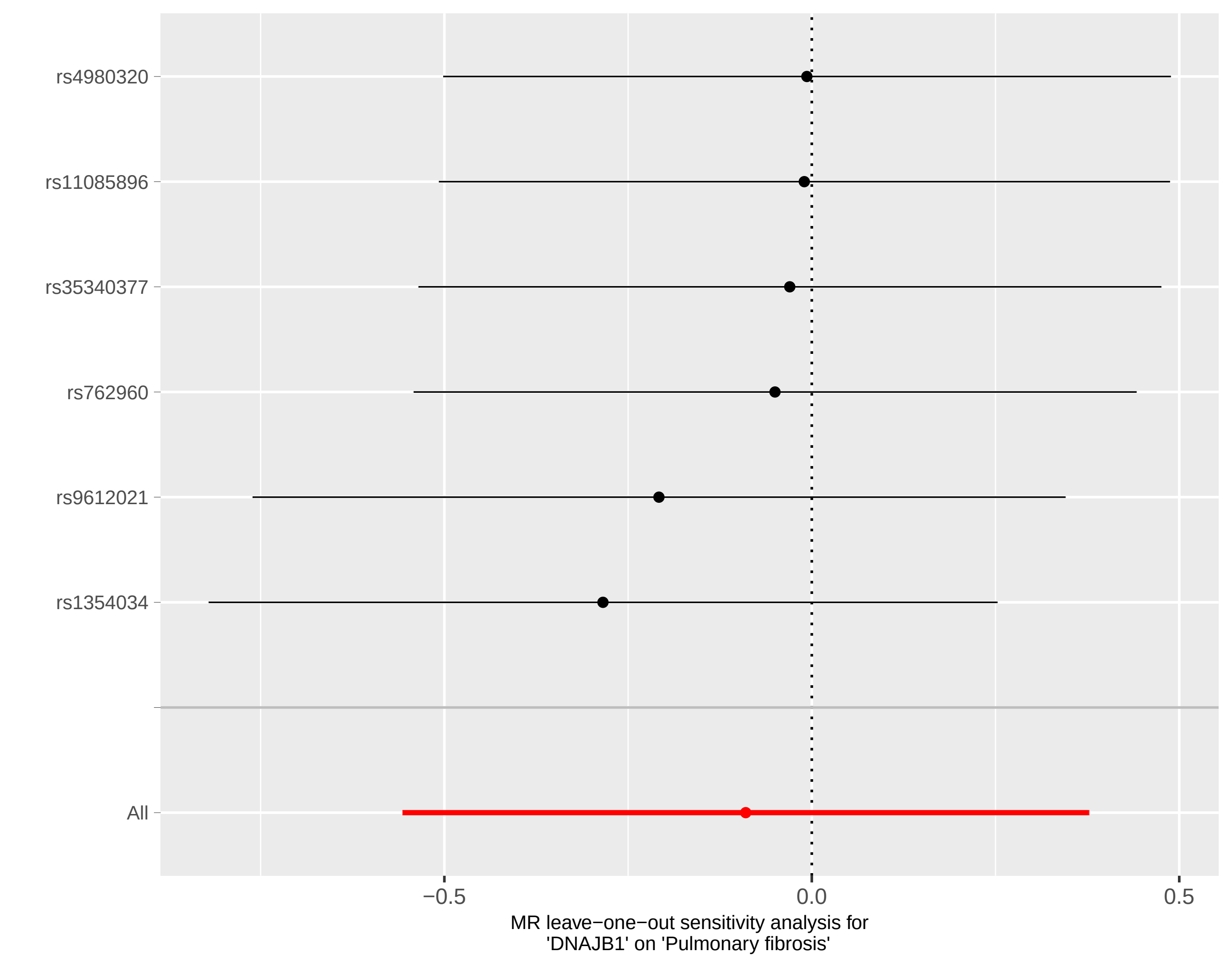
rs4980320
rs11085896
rs35340377
rs762960
rs9612021
rs1354034
All
−0.5
0.0
0.5
MR leave−one−out sensitivity analysis for
'DNAJB1' on 'Pulmonary fibrosis'

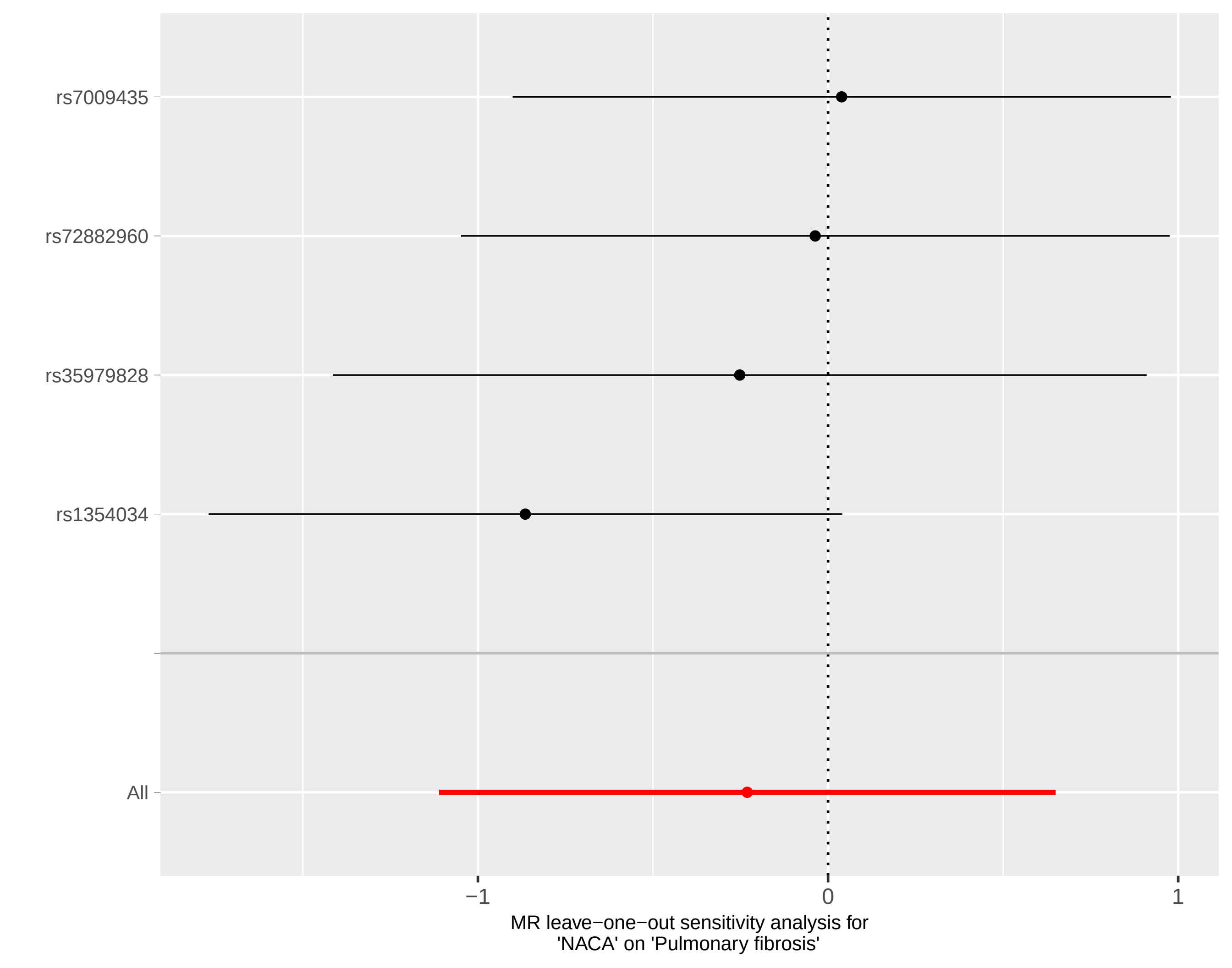
rs7009435
rs72882960
rs35979828
rs1354034
All
−1
0
1
MR leave−one−out sensitivity analysis for
'NACA' on 'Pulmonary fibrosis'

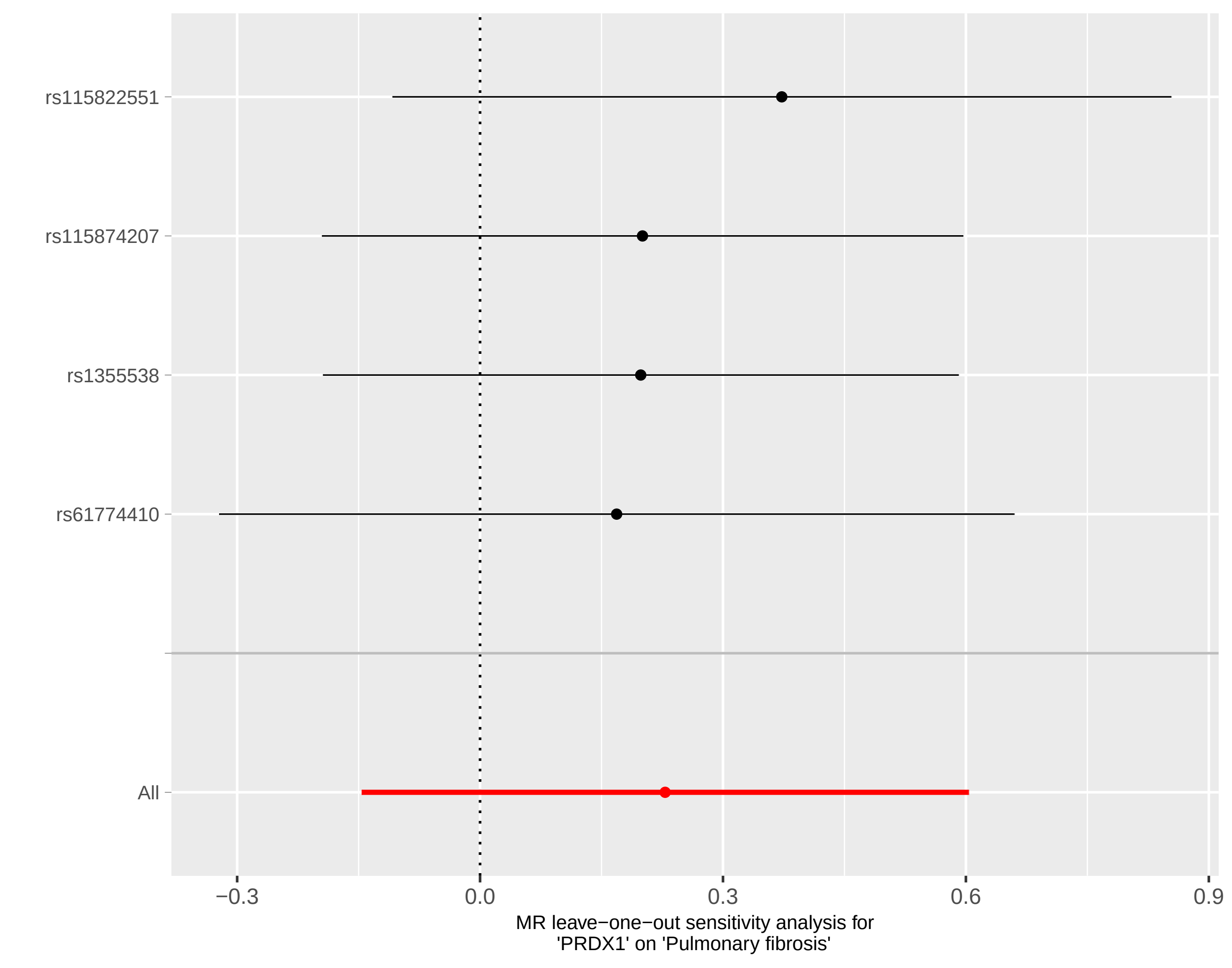
rs115822551
rs115874207
rs1355538
rs61774410
All
−0.3
0.0
0.3
0.6
0.9
MR leave−one−out sensitivity analysis for
'PRDX1' on 'Pulmonary fibrosis'

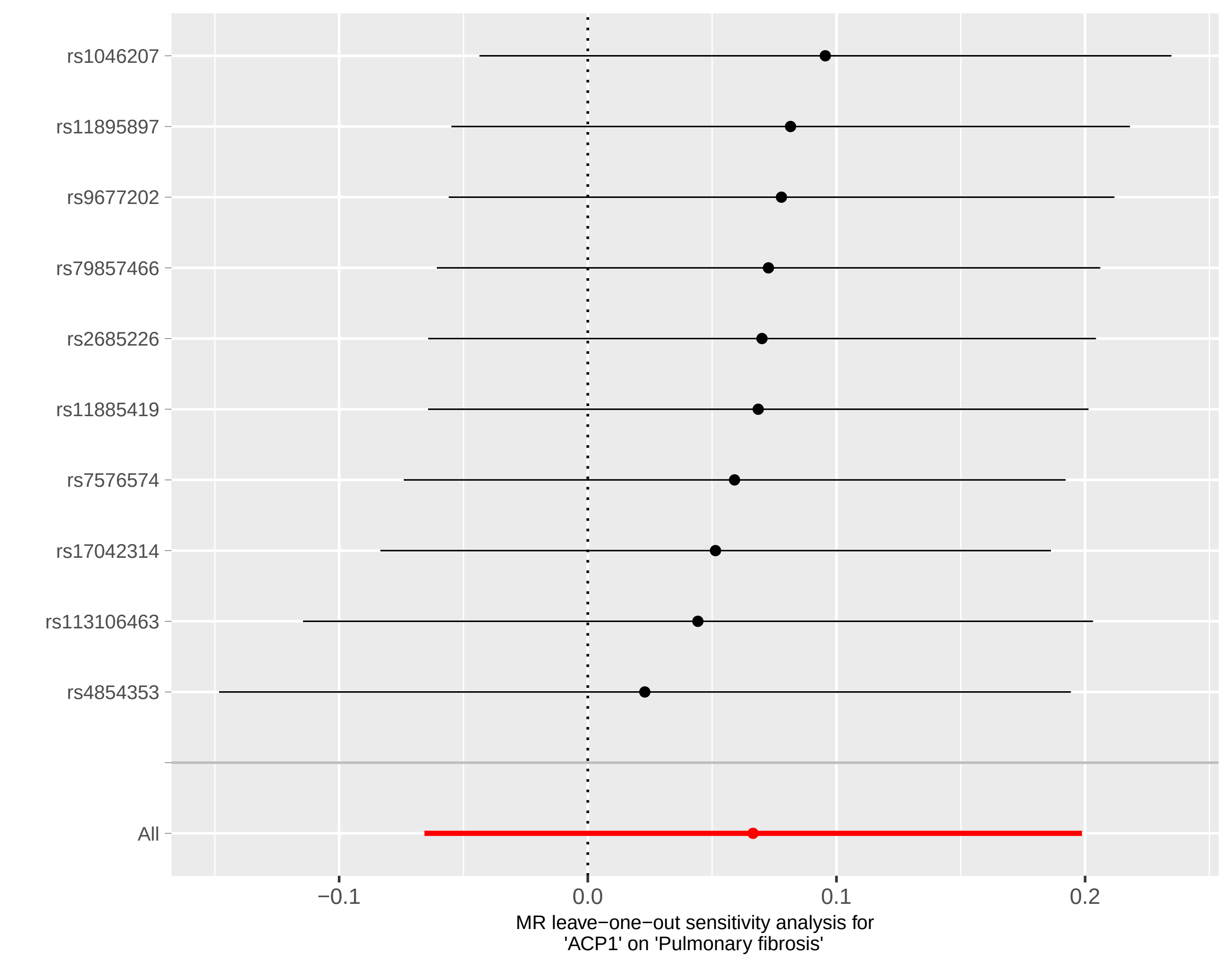
rs1046207
rs11895897
rs9677202
rs79857466
rs2685226
rs11885419
rs7576574
rs17042314
rs113106463
rs4854353
All
−0.1
0.0
0.1
0.2
MR leave−one−out sensitivity analysis for
'ACP1' on 'Pulmonary fibrosis'

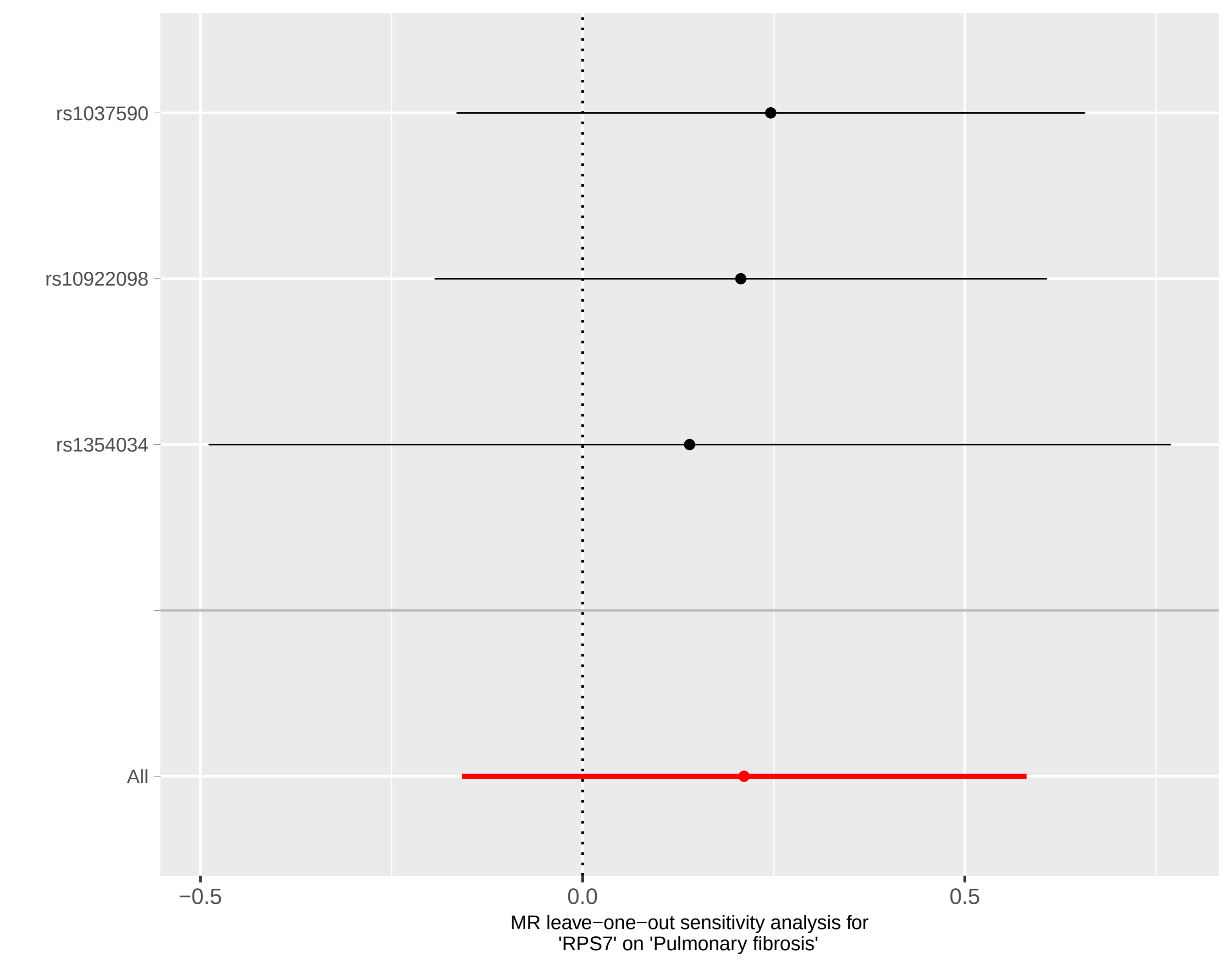
rs1037590
rs10922098
rs1354034
All
−0.5
0.0
0.5
MR leave−one−out sensitivity analysis for
'RPS7' on 'Pulmonary fibrosis'

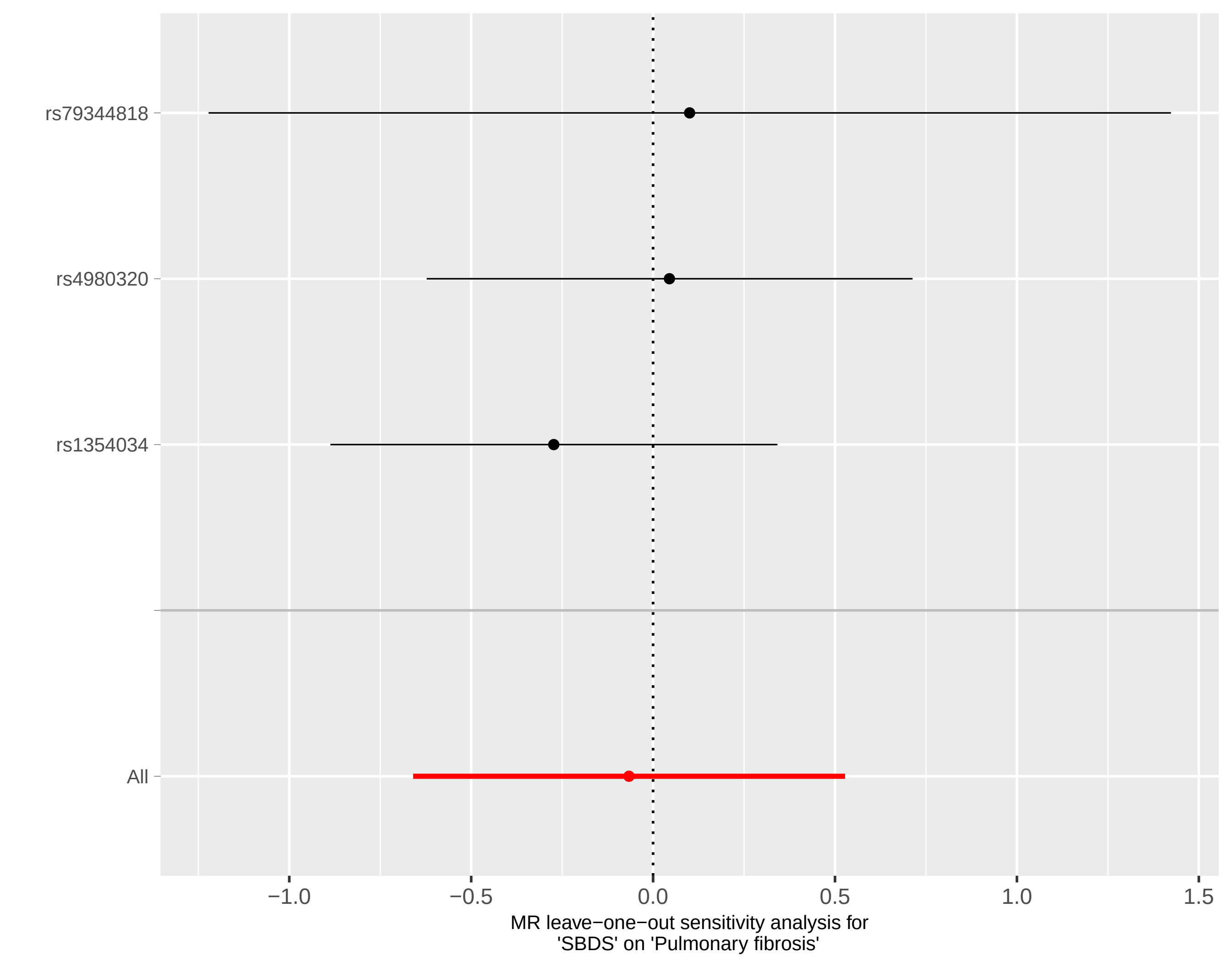

rs79344818
rs4980320
rs1354034
All
−1.0
−0.5
0.0
0.5
1.0
1.5
MR leave−one−out sensitivity analysis for
'SBDS' on 'Pulmonary fibrosis'

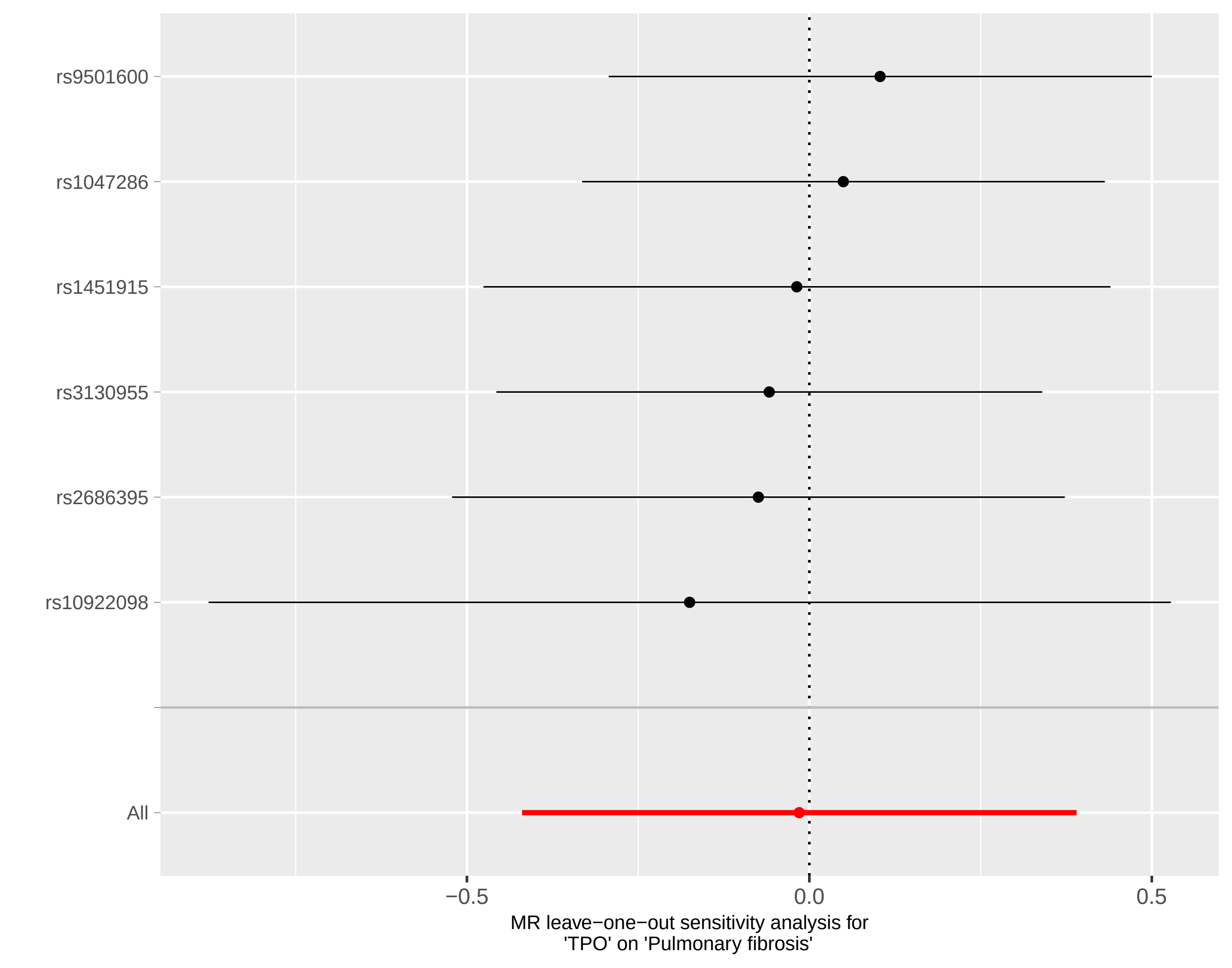
rs9501600
rs1047286
rs1451915
rs3130955
rs2686395
rs10922098
All
−0.5
0.0
0.5
MR leave−one−out sensitivity analysis for
'TPO' on 'Pulmonary fibrosis'

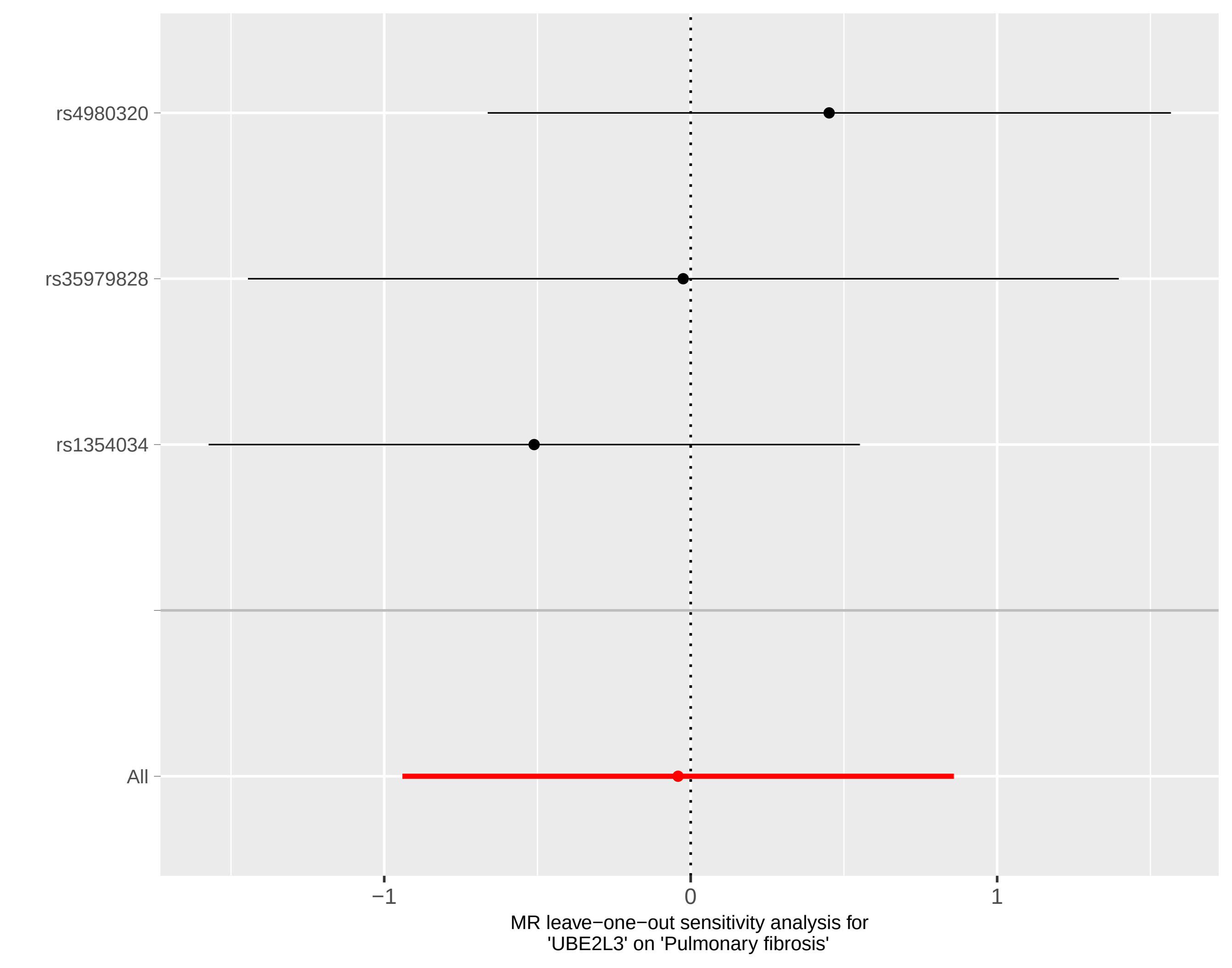
rs4980320
rs35979828
rs1354034
All
−1
0
1
MR leave−one−out sensitivity analysis for
'UBE2L3' on 'Pulmonary fibrosis'

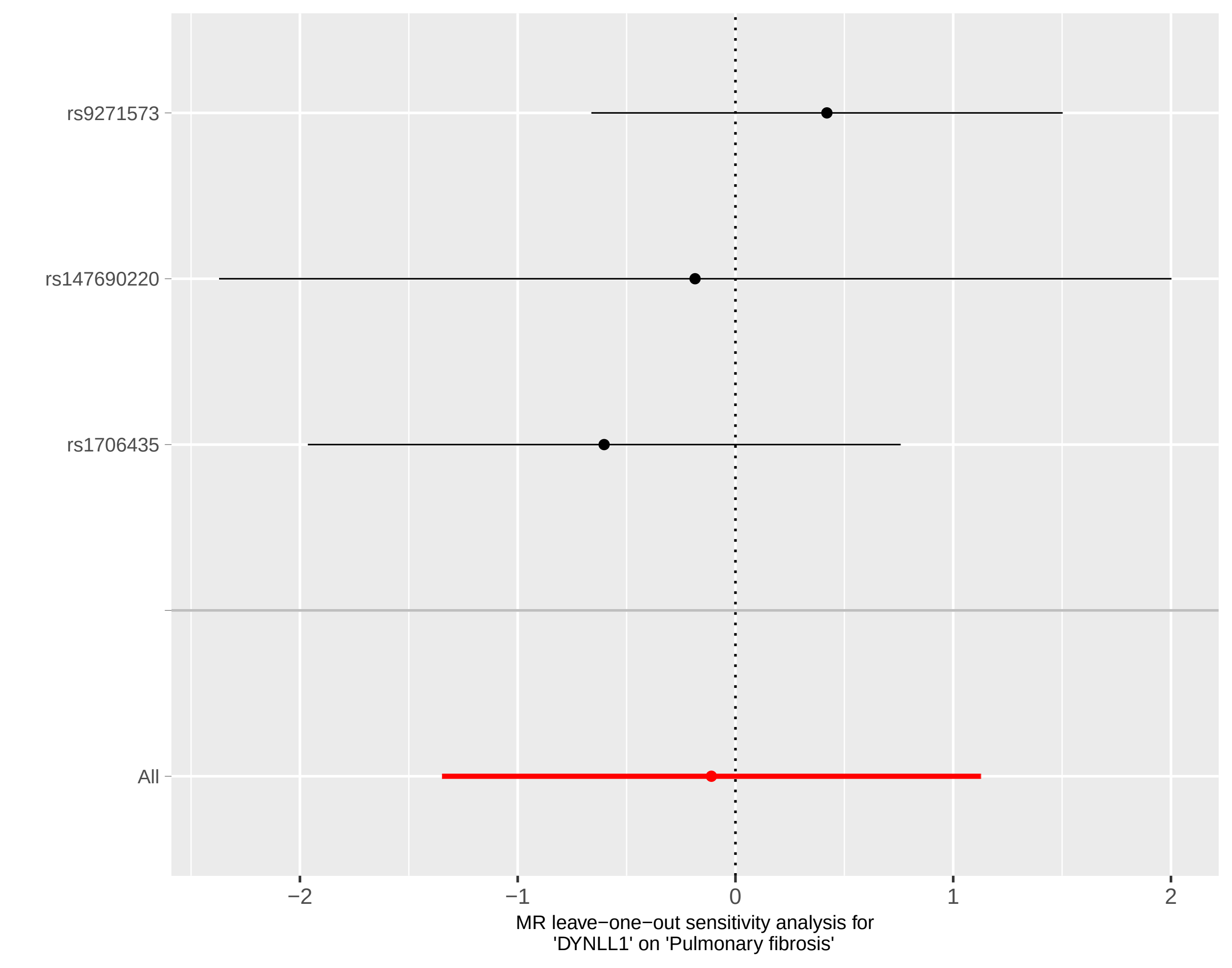
rs9271573
rs147690220
rs1706435
All
−2
−1
0
1
2
MR leave−one−out sensitivity analysis for
'DYNLL1' on 'Pulmonary fibrosis'

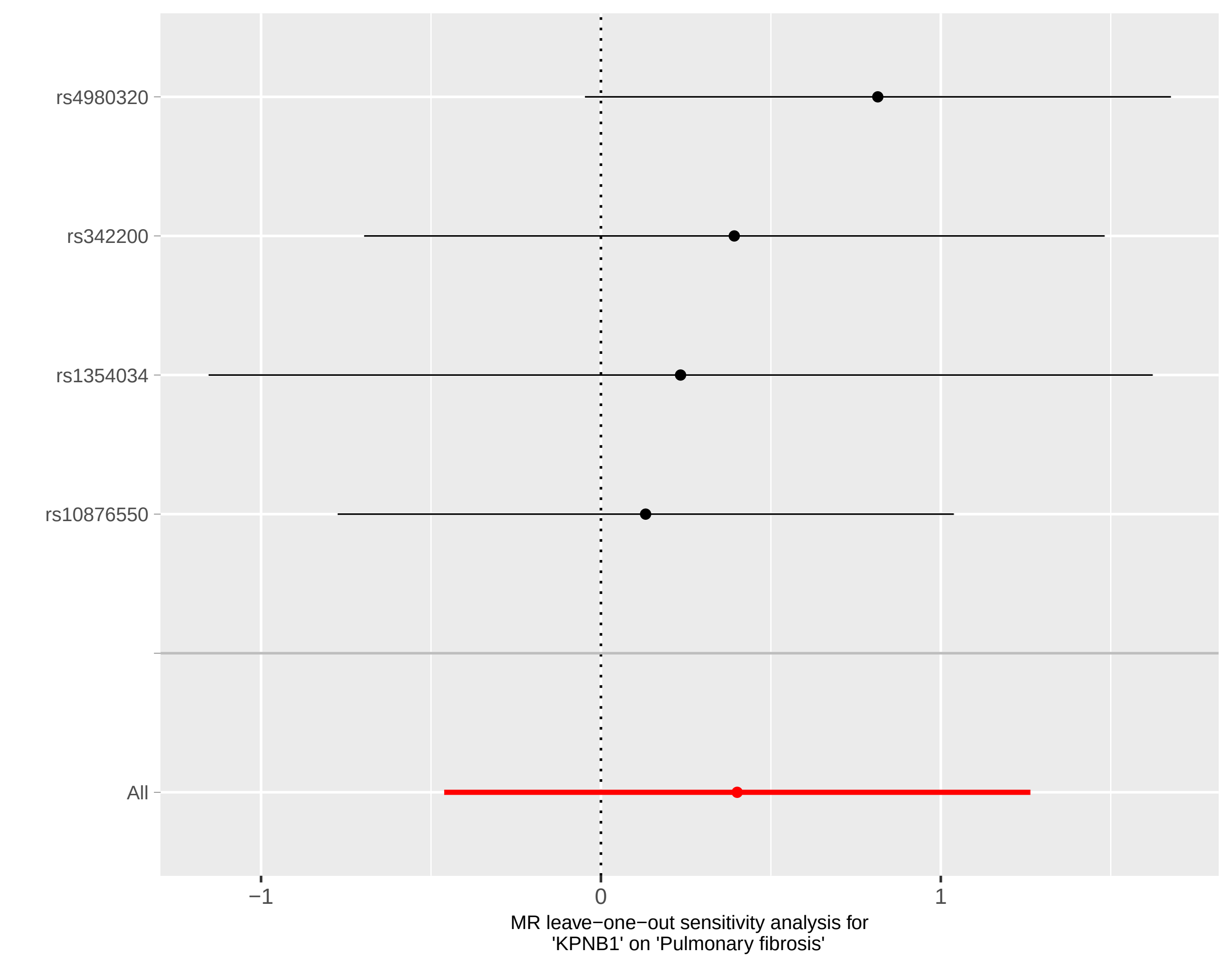
rs4980320
rs342200
rs1354034
rs10876550
All
−1
0
1
MR leave−one−out sensitivity analysis for
'KPNB1' on 'Pulmonary fibrosis'

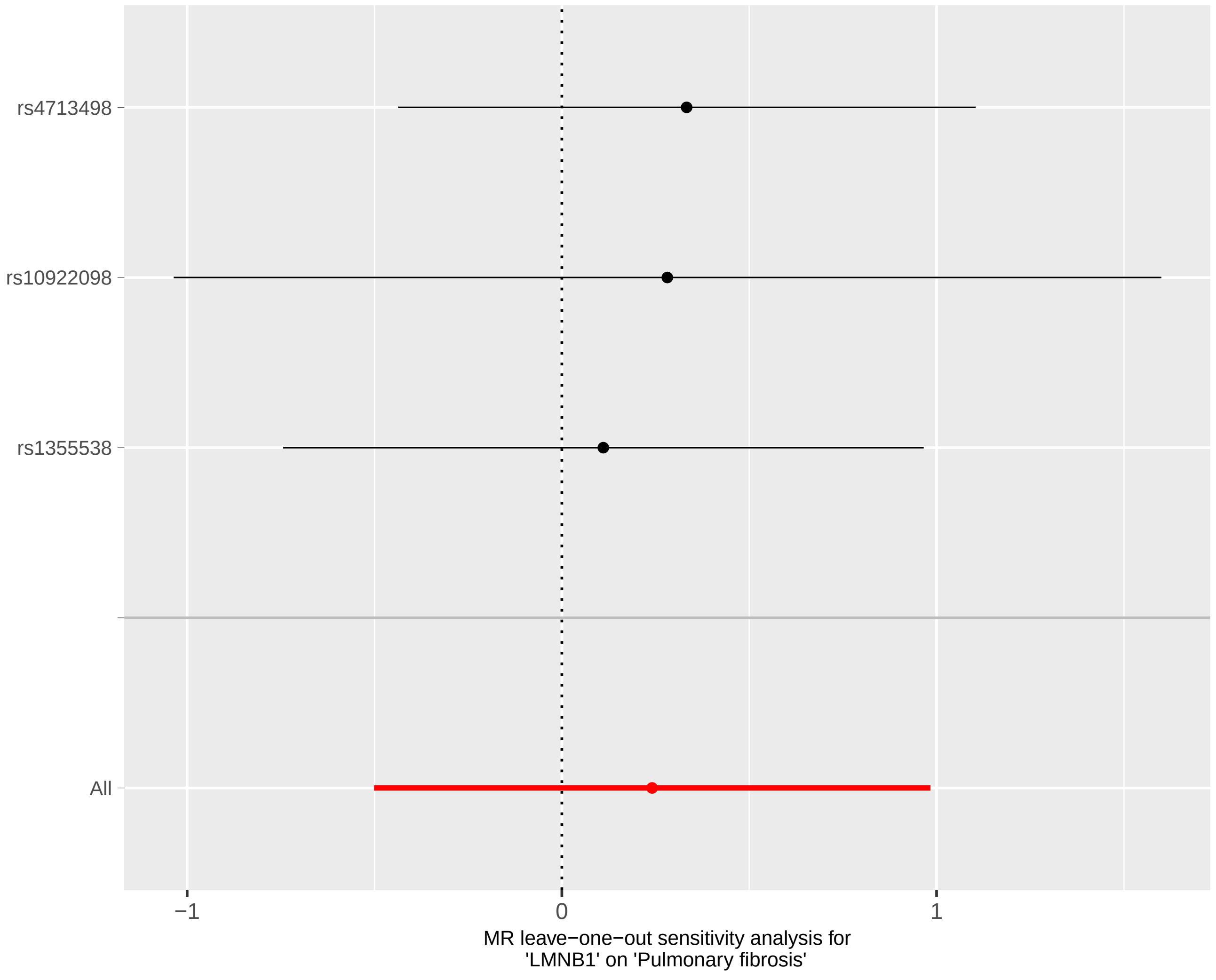
rs4713498
rs10922098
rs1355538
All
−1
0
1
MR leave−one−out sensitivity analysis for
'LMNB1' on 'Pulmonary fibrosis'

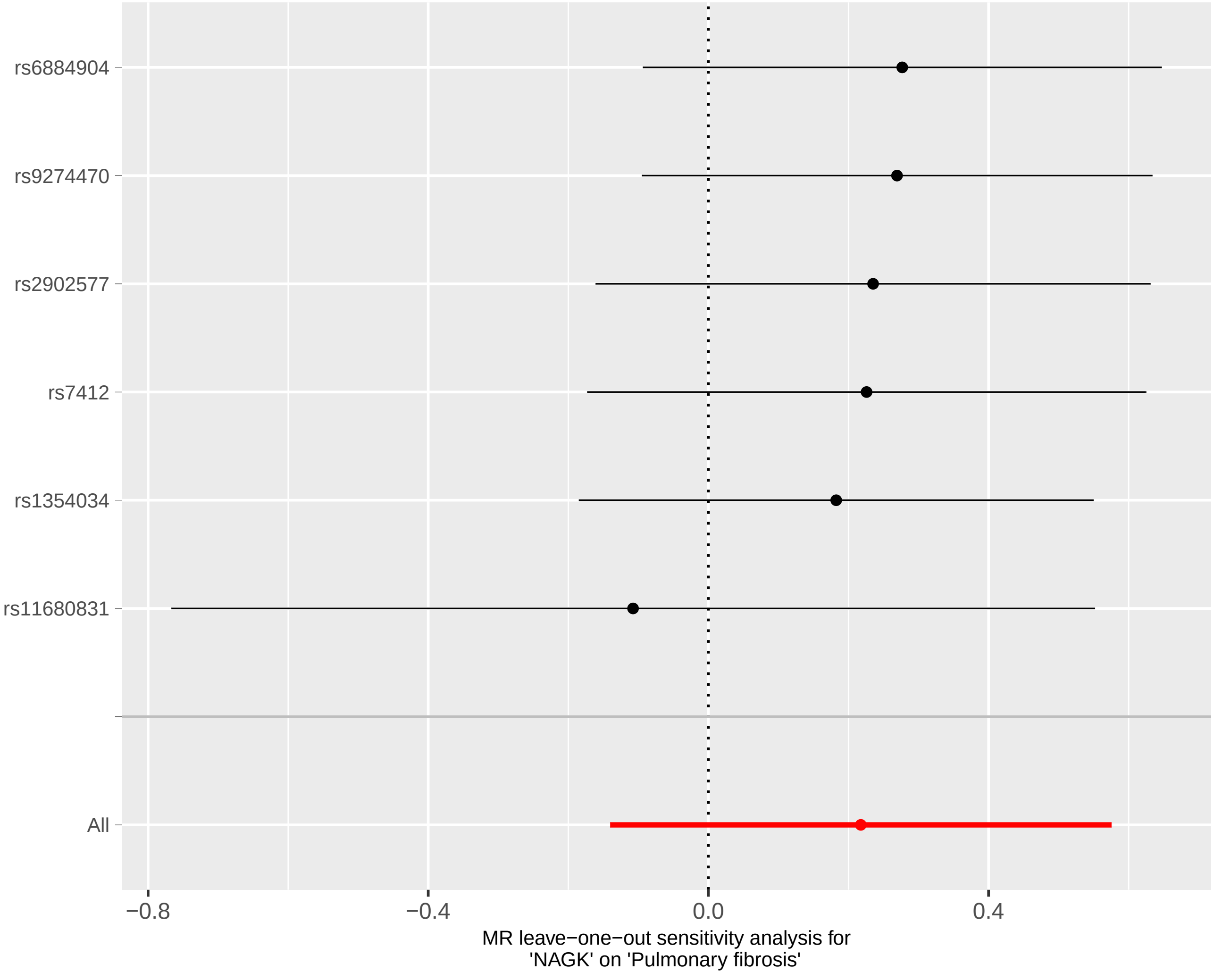
rs6884904
rs9274470
rs2902577
rs7412
rs1354034
rs11680831
All
−0.8
−0.4
0.0
0.4
MR leave−one−out sensitivity analysis for
'NAGK' on 'Pulmonary fibrosis'

Insufficient number of SNPs

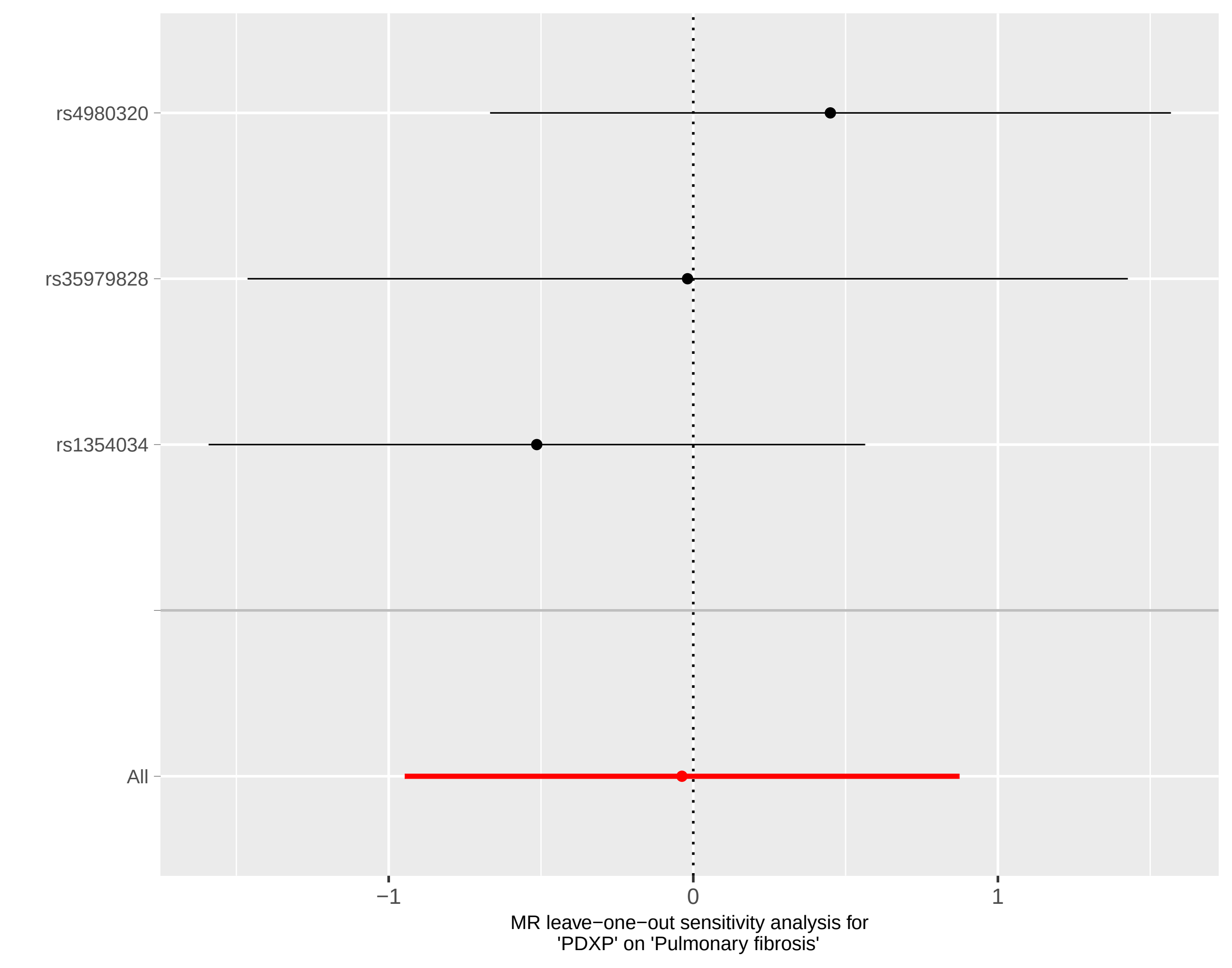
rs4980320
rs35979828
rs1354034
All
−1
0
1
MR leave−one−out sensitivity analysis for
'PDXP' on 'Pulmonary fibrosis'

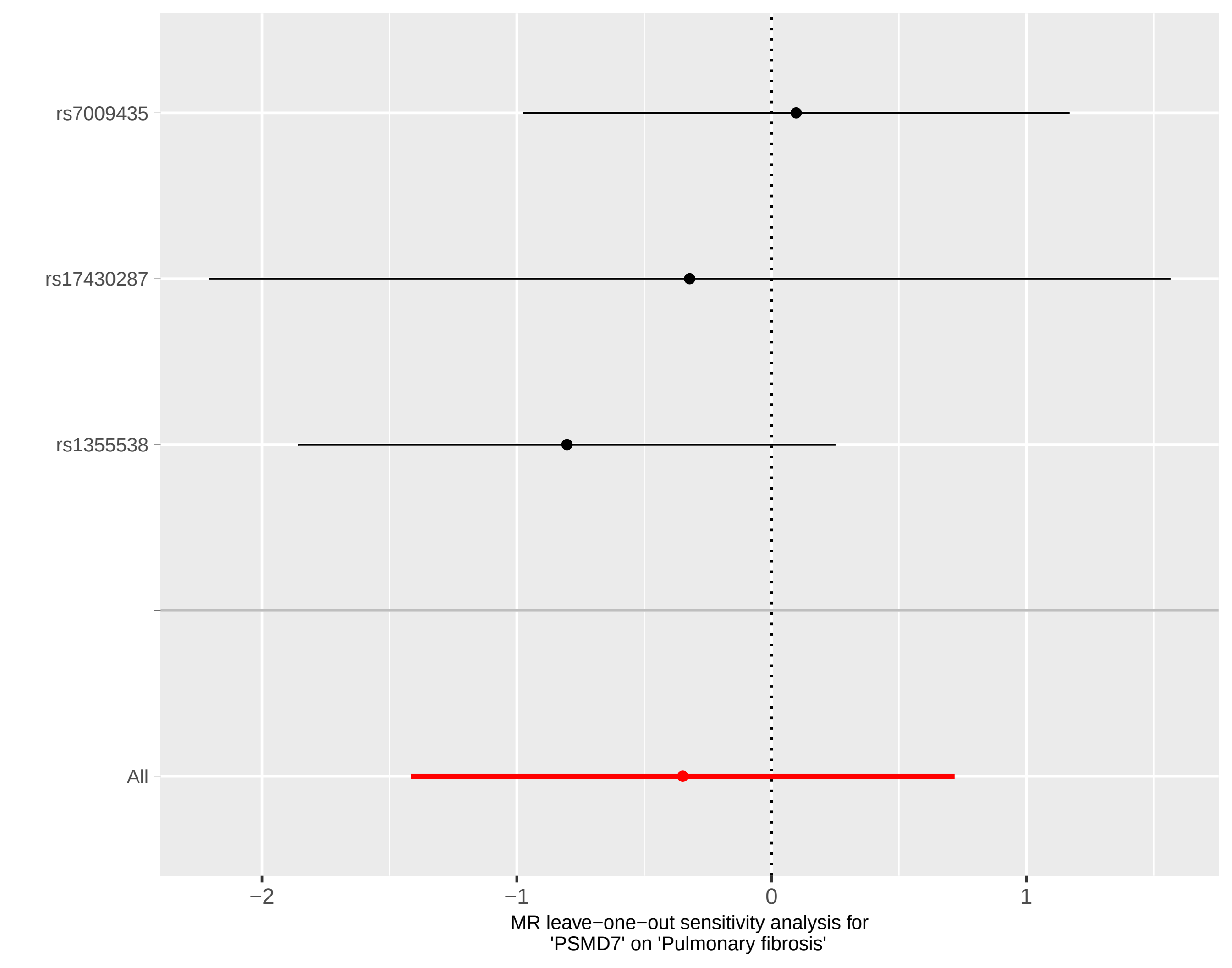
rs7009435
rs17430287
rs1355538
All
−2
−1
0
1
MR leave−one−out sensitivity analysis for
'PSMD7' on 'Pulmonary fibrosis'

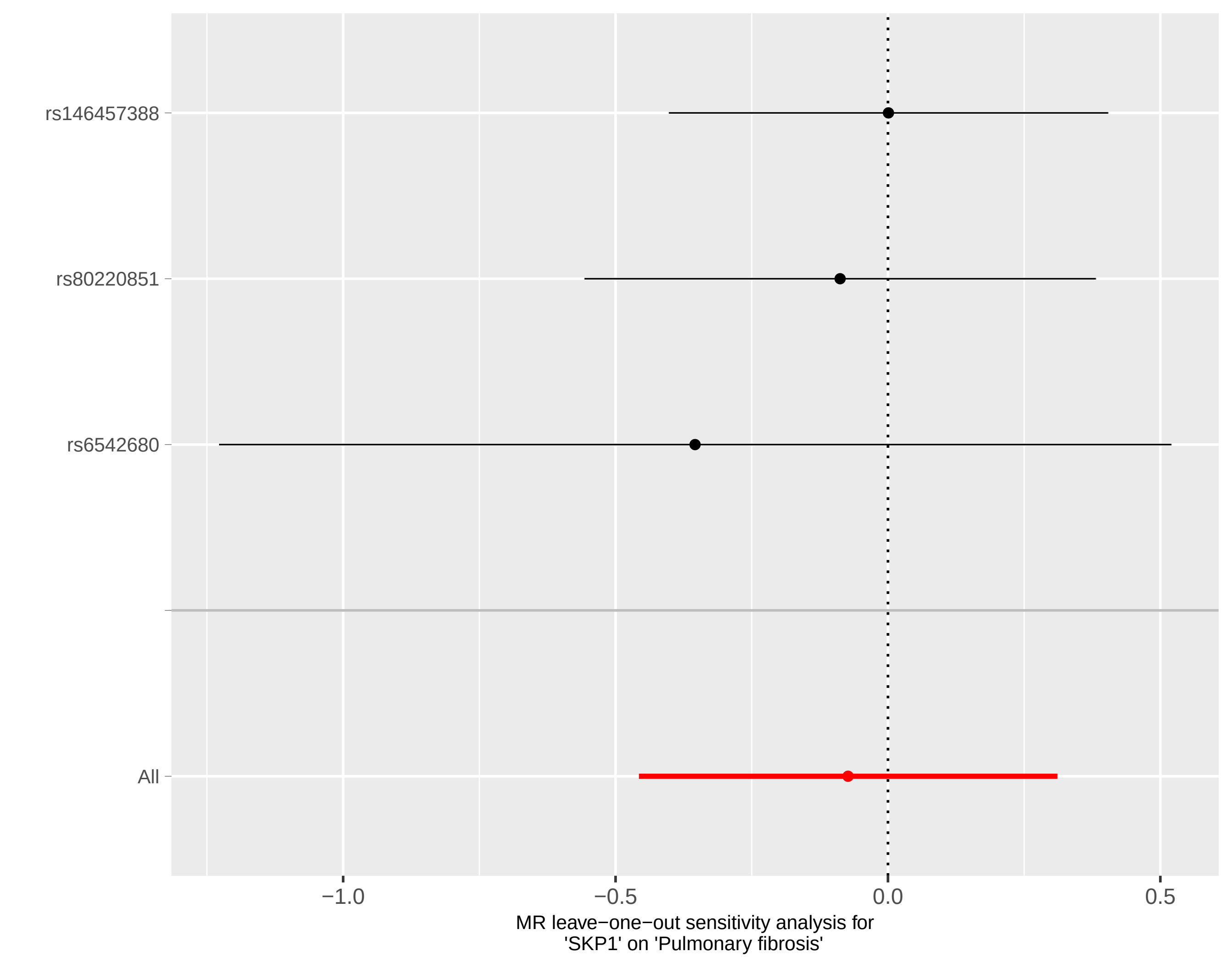
rs146457388
rs80220851
rs6542680
All
−1.0
−0.5
0.0
0.5
MR leave−one−out sensitivity analysis for
'SKP1' on 'Pulmonary fibrosis'

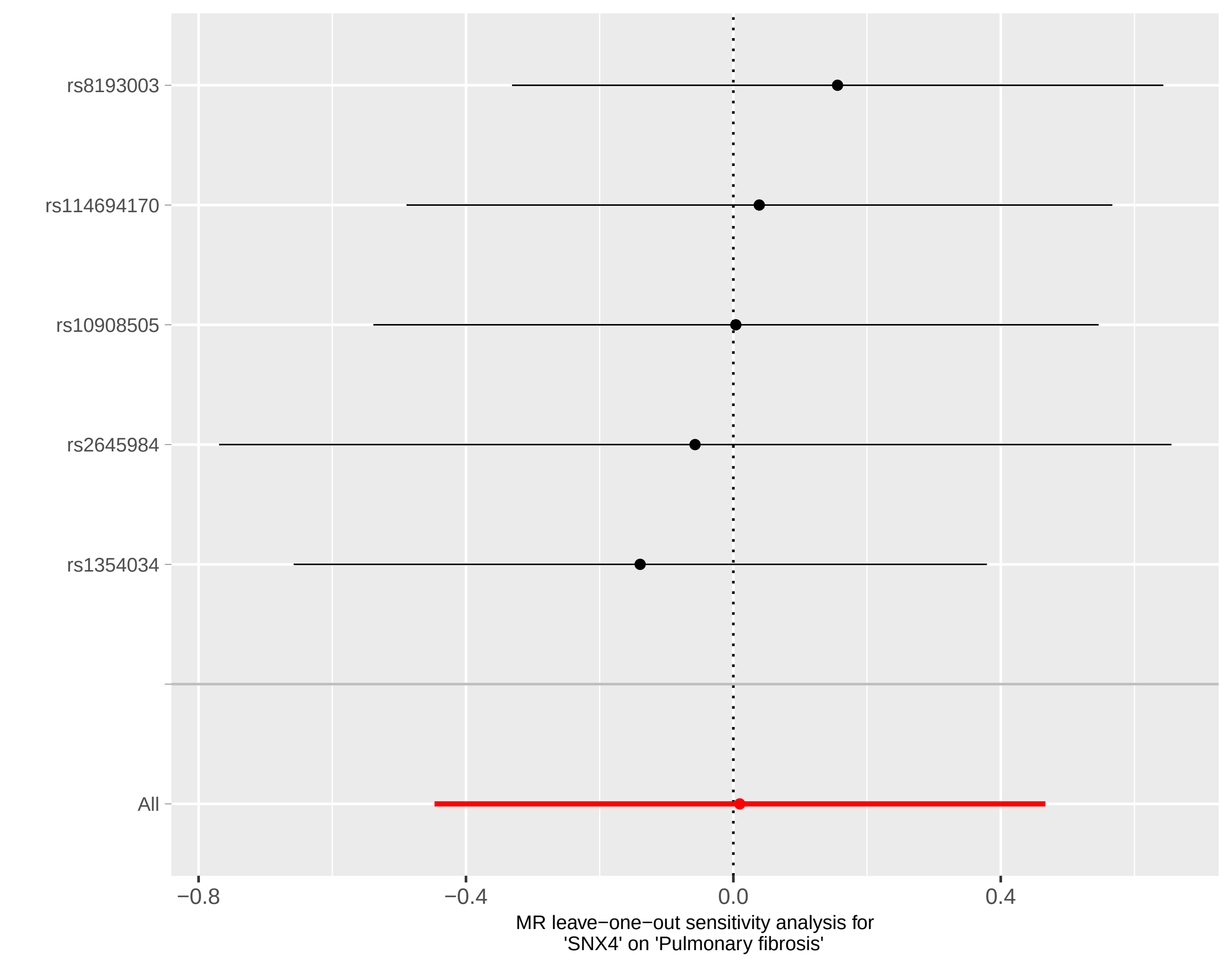
rs8193003
rs114694170
rs10908505
rs2645984
rs1354034
All
−0.8
−0.4
0.0
0.4
MR leave−one−out sensitivity analysis for
'SNX4' on 'Pulmonary fibrosis'

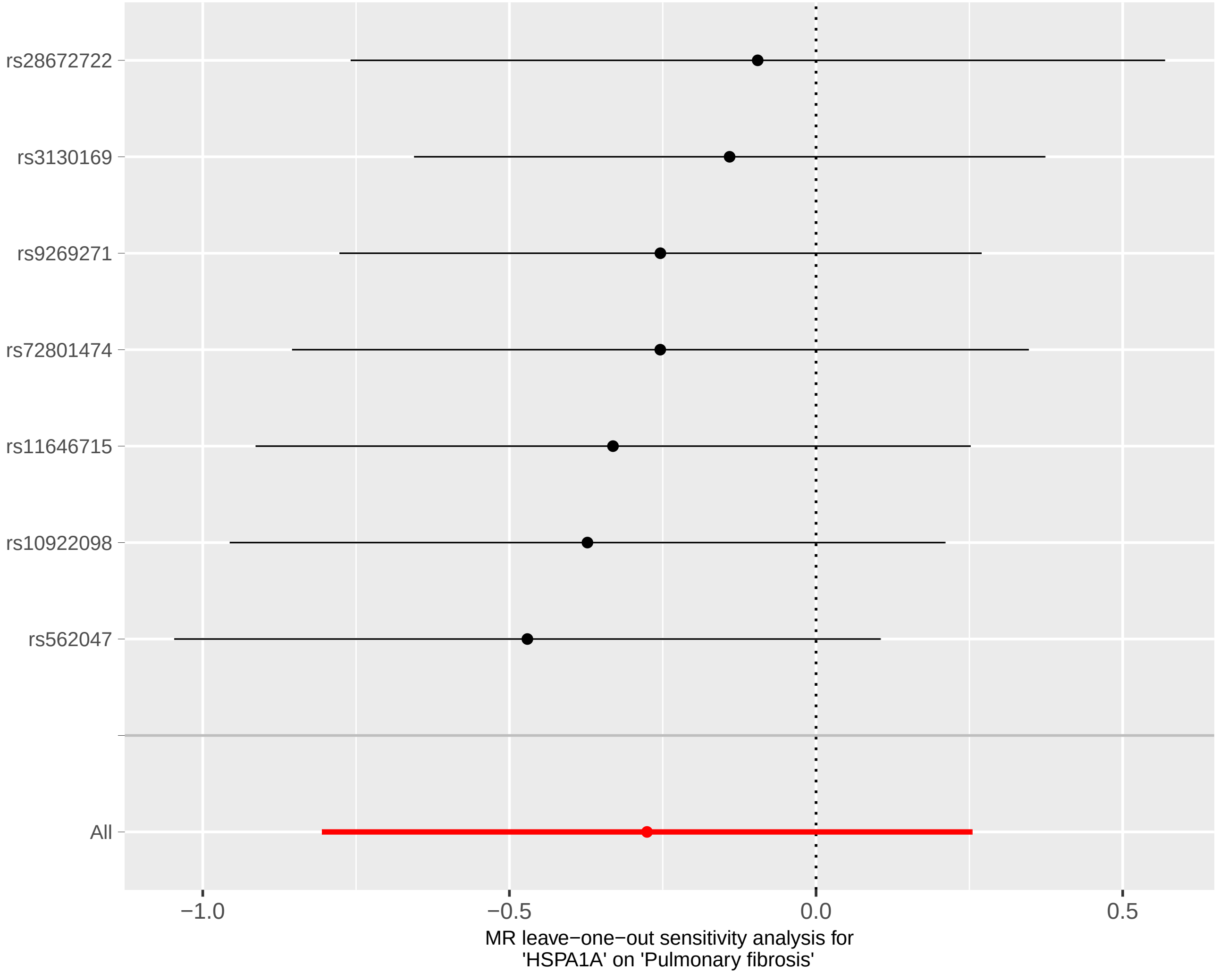
rs28672722
rs3130169
rs9269271
rs72801474
rs11646715
rs10922098
rs562047
All
−1.0
−0.5
0.0
0.5
MR leave−one−out sensitivity analysis for
'HSPA1A' on 'Pulmonary fibrosis'

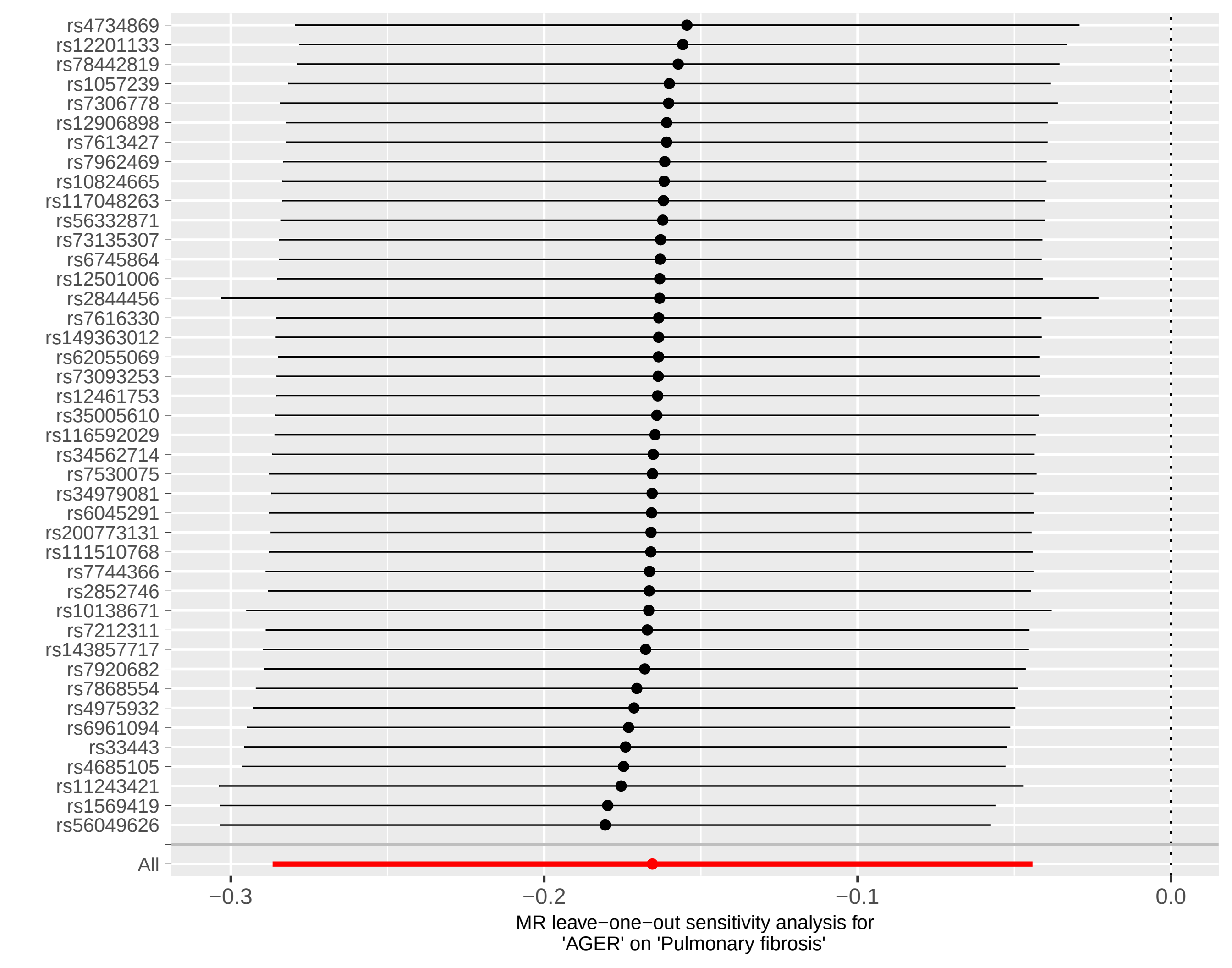
rs4734869
rs12201133
rs78442819
rs1057239
rs7306778
rs12906898
rs7613427
rs7962469
rs10824665
rs117048263
rs56332871
rs73135307
rs6745864
rs12501006
rs2844456
rs7616330
rs149363012
rs62055069
rs73093253
rs12461753
rs35005610
rs116592029
rs34562714
rs7530075
rs34979081
rs6045291
rs200773131
rs111510768
rs7744366
rs2852746
rs10138671
rs7212311
rs143857717
rs7920682
rs7868554
rs4975932
rs6961094
rs33443
rs4685105
rs11243421
rs1569419
rs56049626
All
−0.3
−0.2
−0.1
0.0
MR leave−one−out sensitivity analysis for
'AGER' on 'Pulmonary fibrosis'

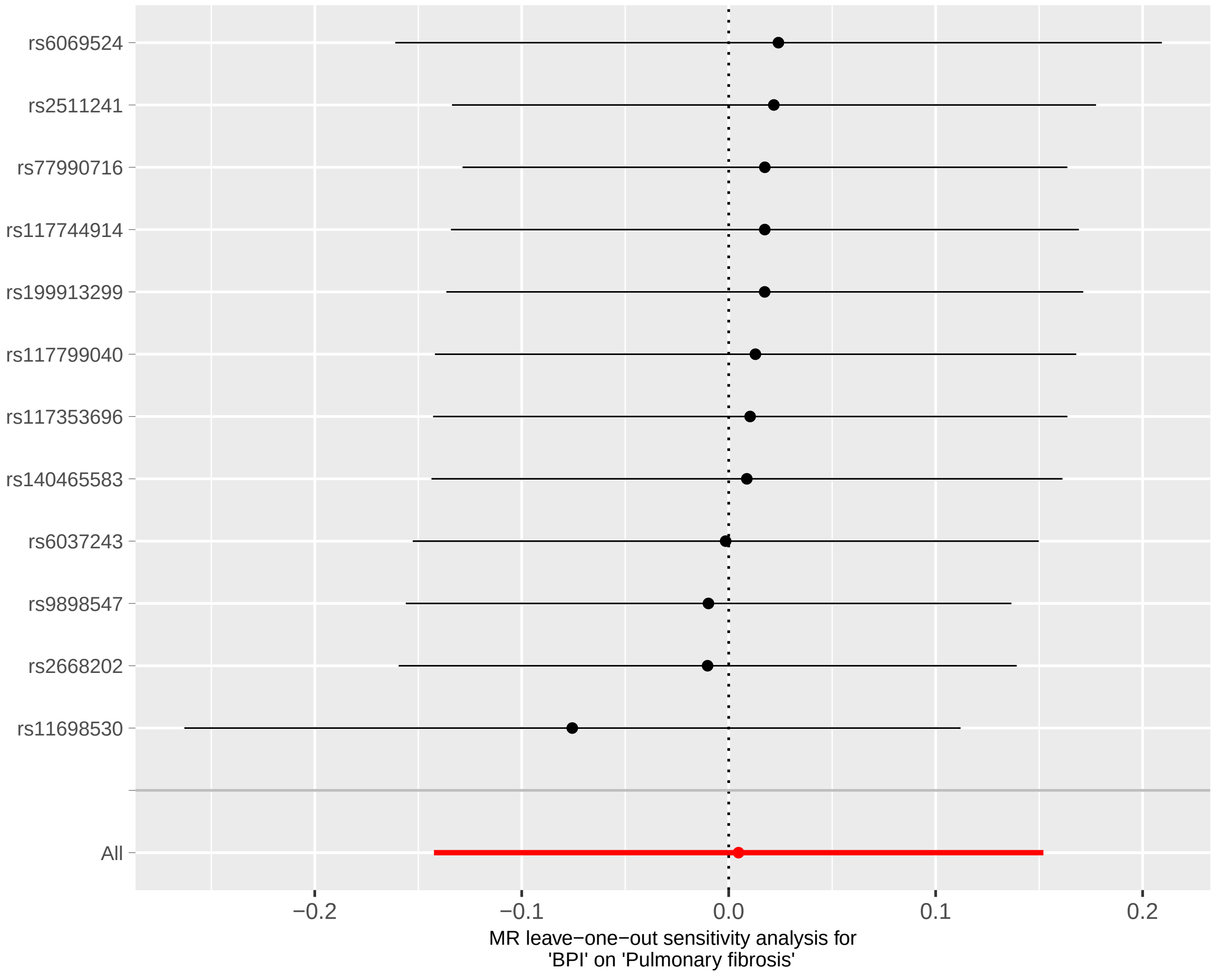
rs6069524
rs2511241
rs77990716
rs117744914
rs199913299
rs117799040
rs117353696
rs140465583
rs6037243
rs9898547
rs2668202
rs11698530
All
−0.2
−0.1
0.0
0.1
0.2
MR leave−one−out sensitivity analysis for
'BPI' on 'Pulmonary fibrosis'

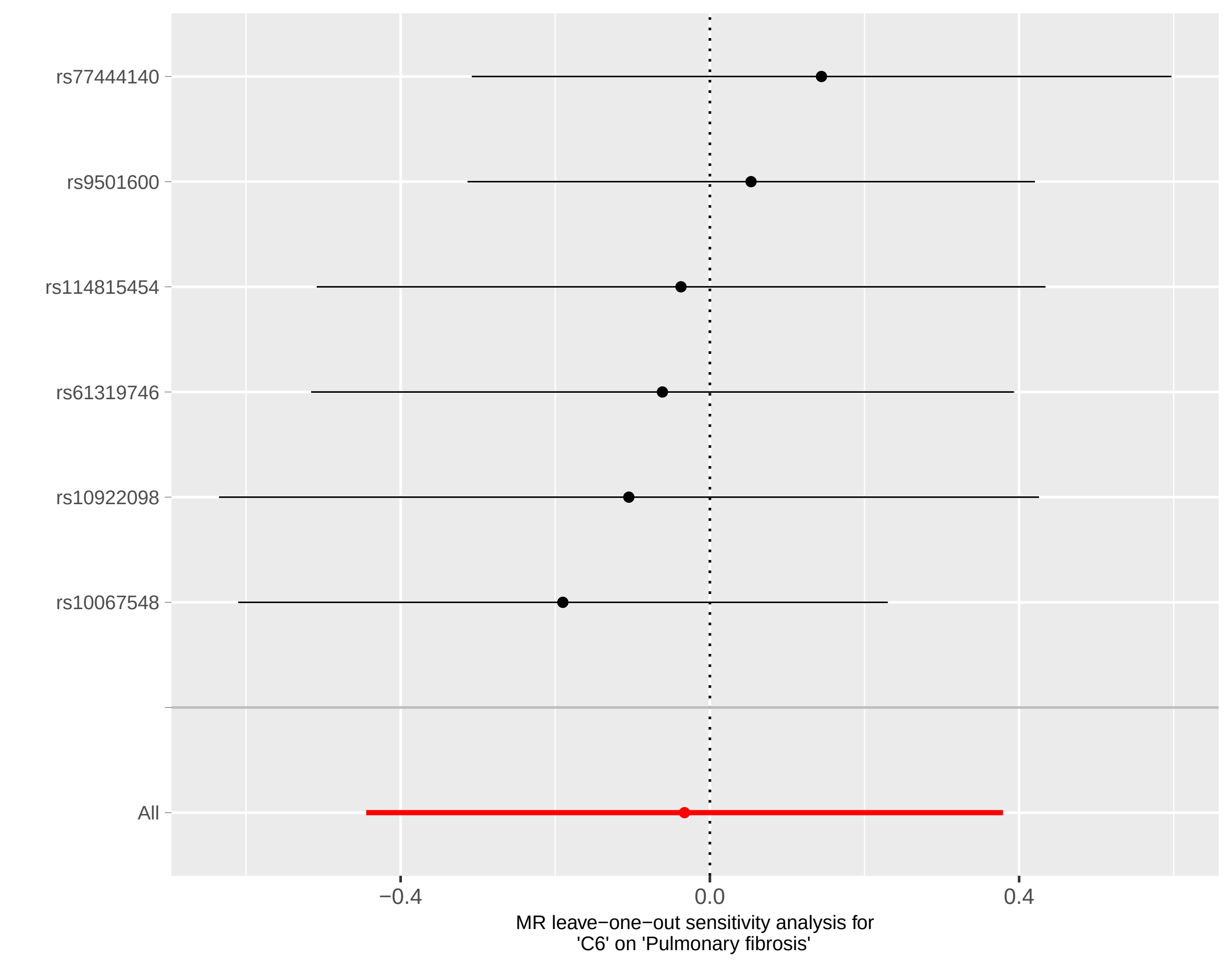
rs77444140
rs9501600
rs114815454
rs61319746
rs10922098
rs10067548
All
−0.4
0.0
0.4
MR leave−one−out sensitivity analysis for
'C6' on 'Pulmonary fibrosis'

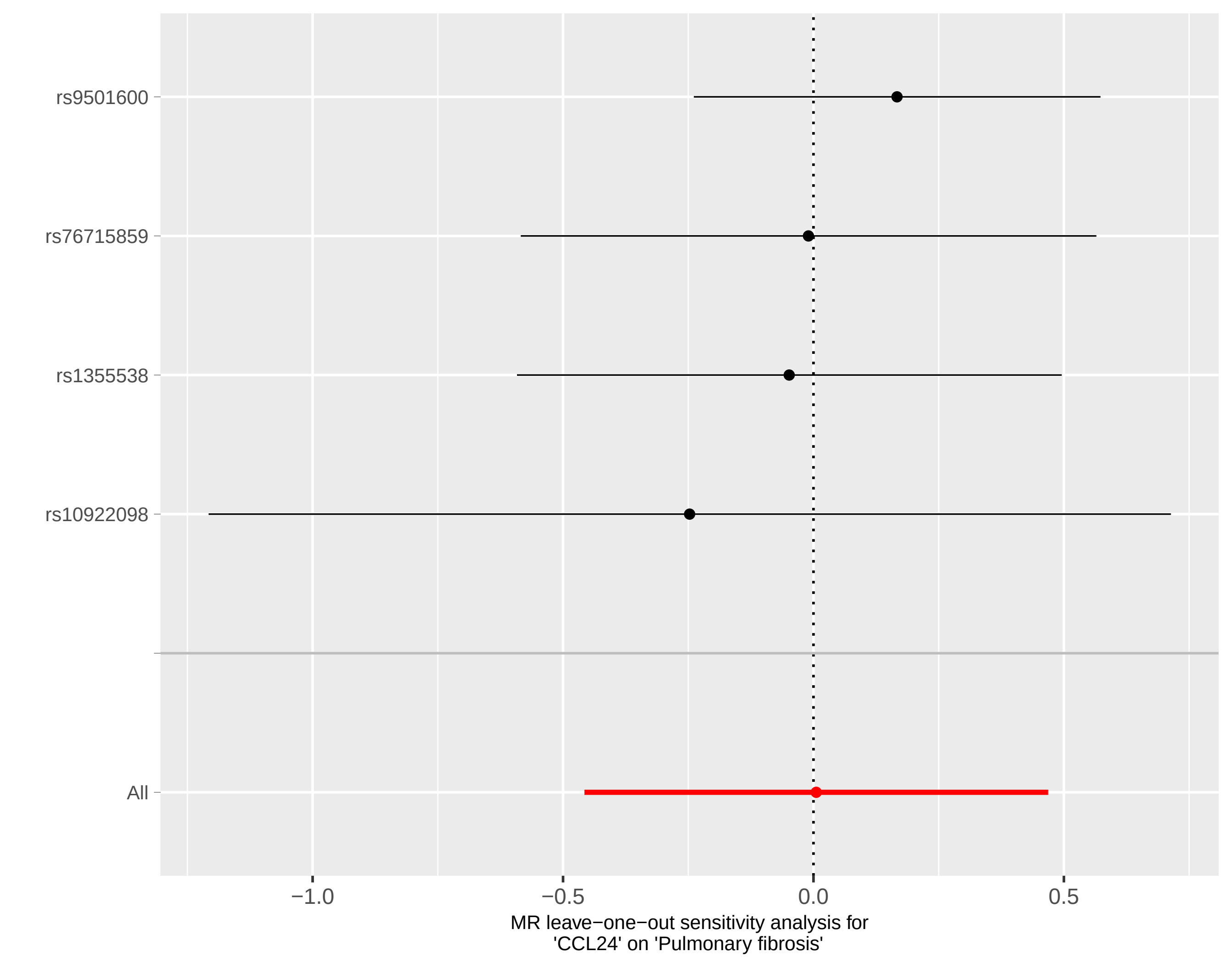
rs9501600
rs76715859
rs1355538
rs10922098
All
−1.0
−0.5
0.0
0.5
MR leave−one−out sensitivity analysis for
'CCL24' on 'Pulmonary fibrosis'

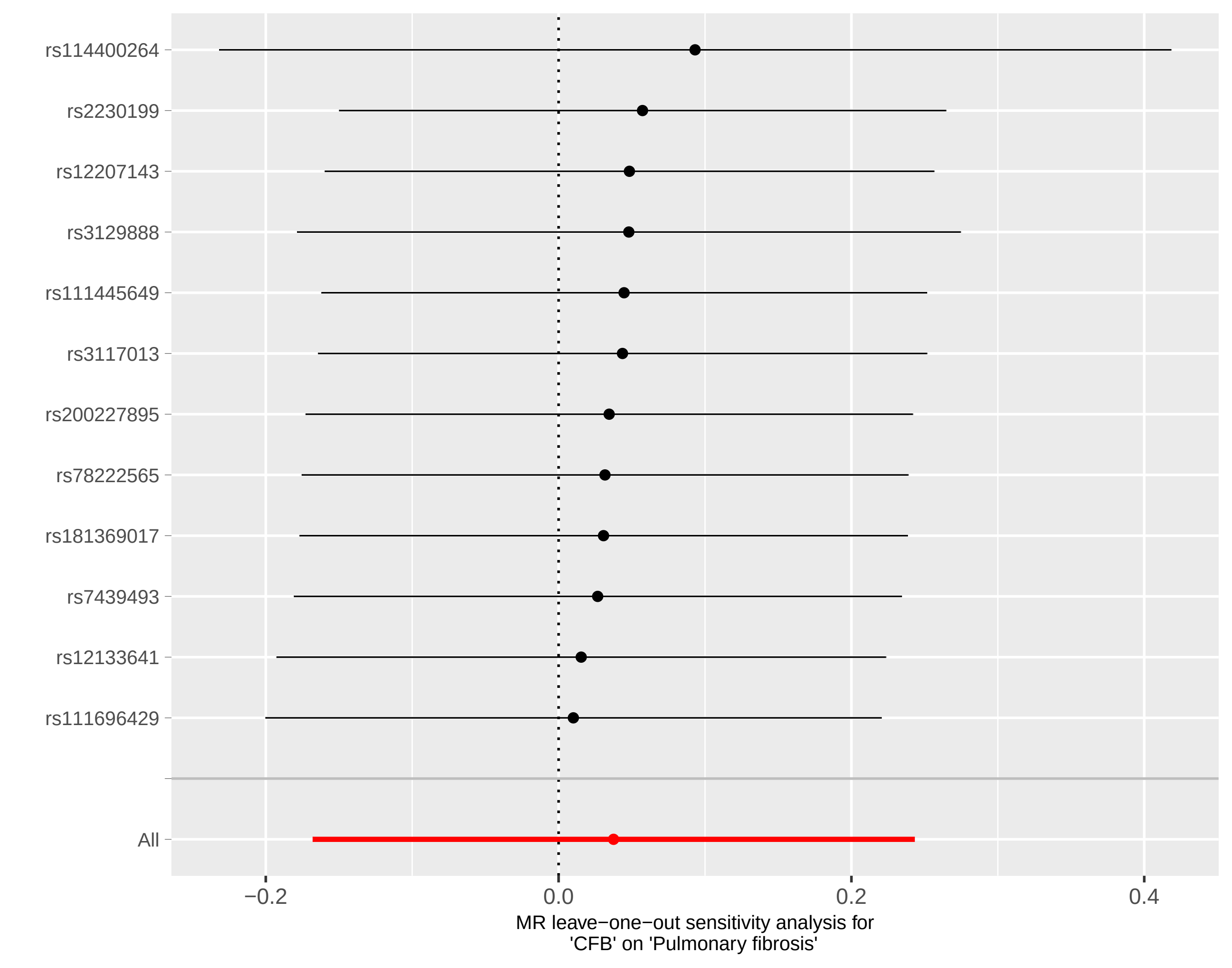
rs114400264
rs2230199
rs12207143
rs3129888
rs111445649
rs3117013
rs200227895
rs78222565
rs181369017
rs7439493
rs12133641
rs111696429
All
−0.2
0.0
0.2
0.4
MR leave−one−out sensitivity analysis for
'CFB' on 'Pulmonary fibrosis'

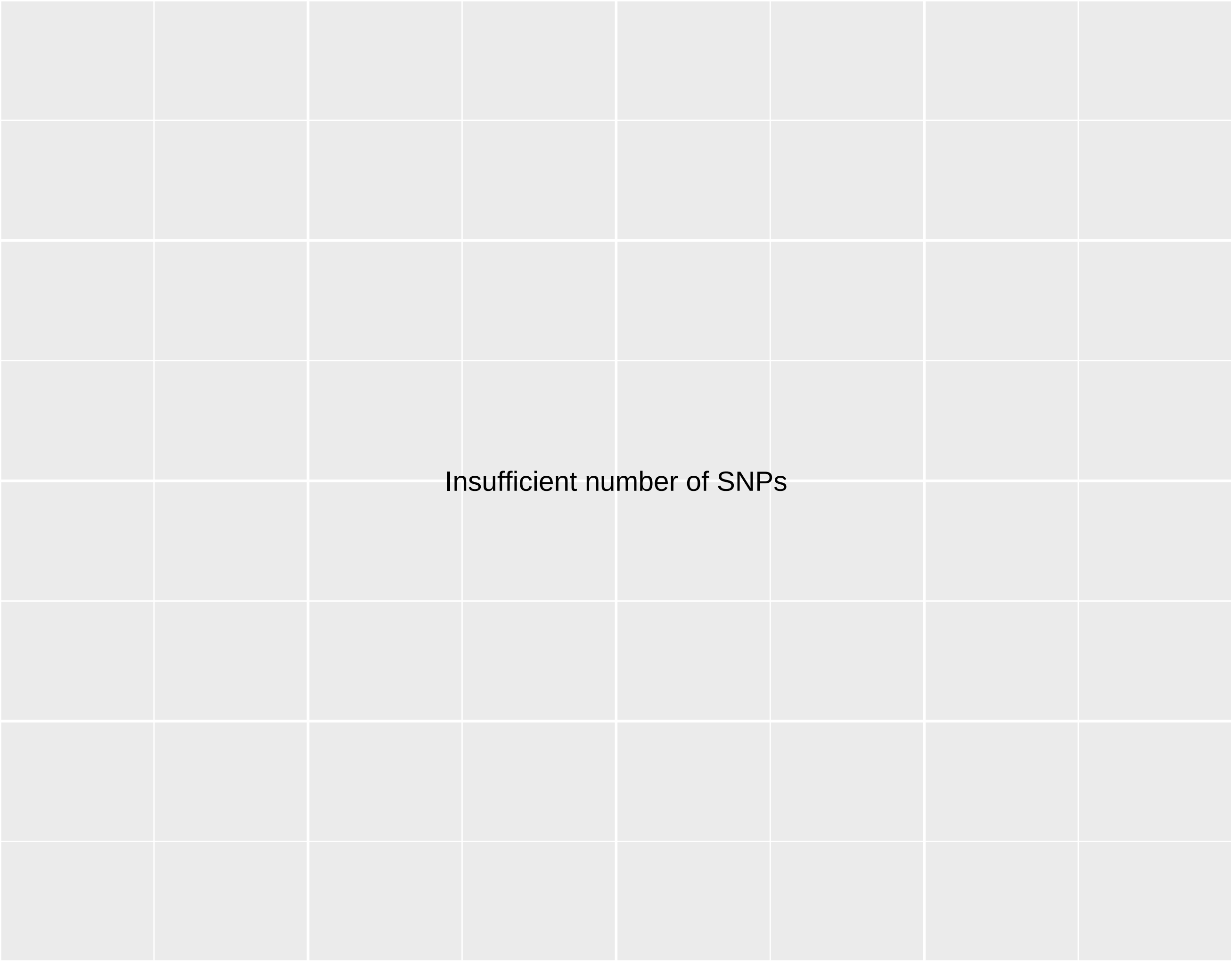
Insufficient number of SNPs

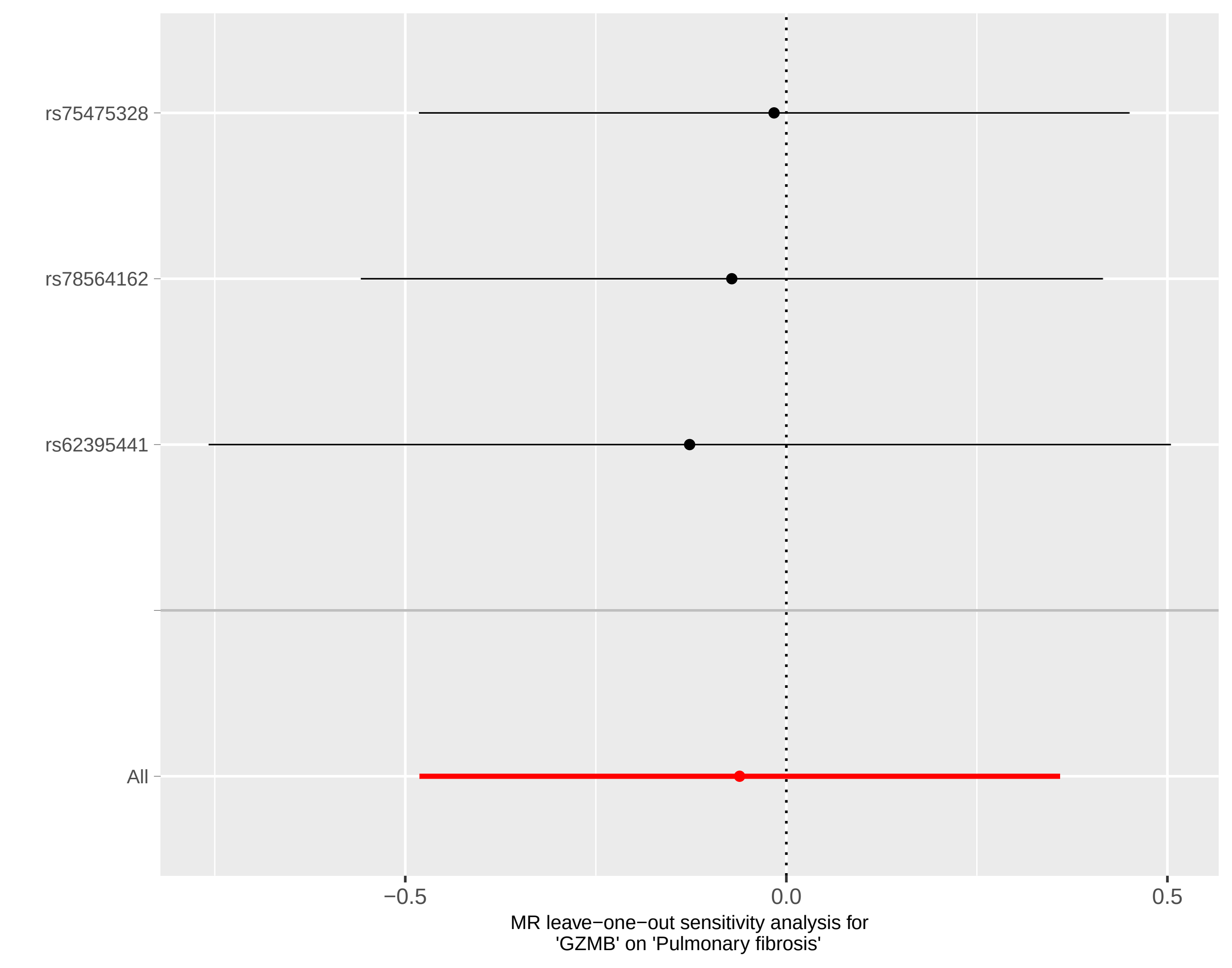
rs75475328
rs78564162
rs62395441
All
−0.5
0.0
0.5
MR leave−one−out sensitivity analysis for
'GZMB' on 'Pulmonary fibrosis'

Insufficient number of SNPs

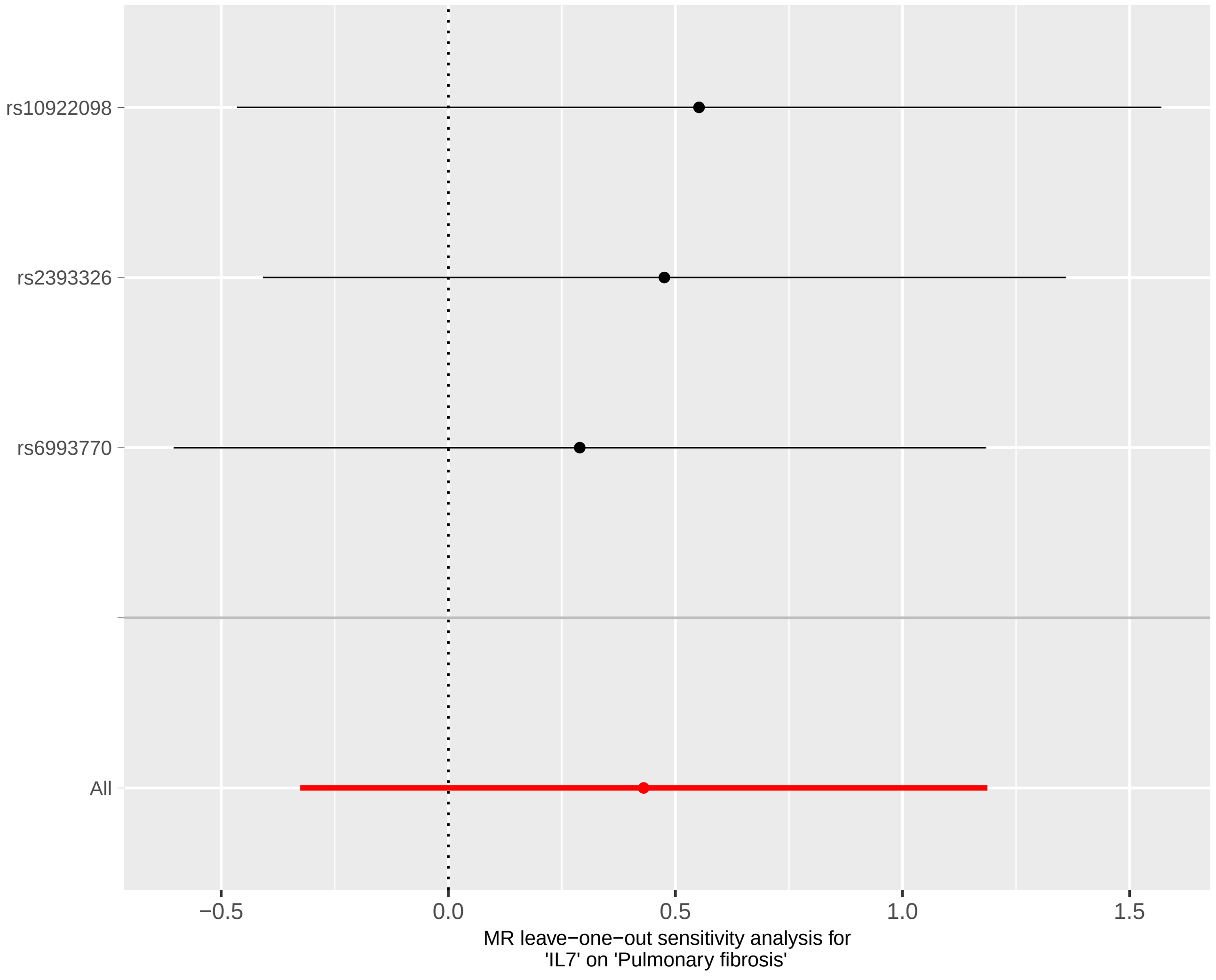
rs10922098
rs2393326
rs6993770
All
−0.5
0.0
0.5
1.0
1.5
MR leave−one−out sensitivity analysis for
'IL7' on 'Pulmonary fibrosis'

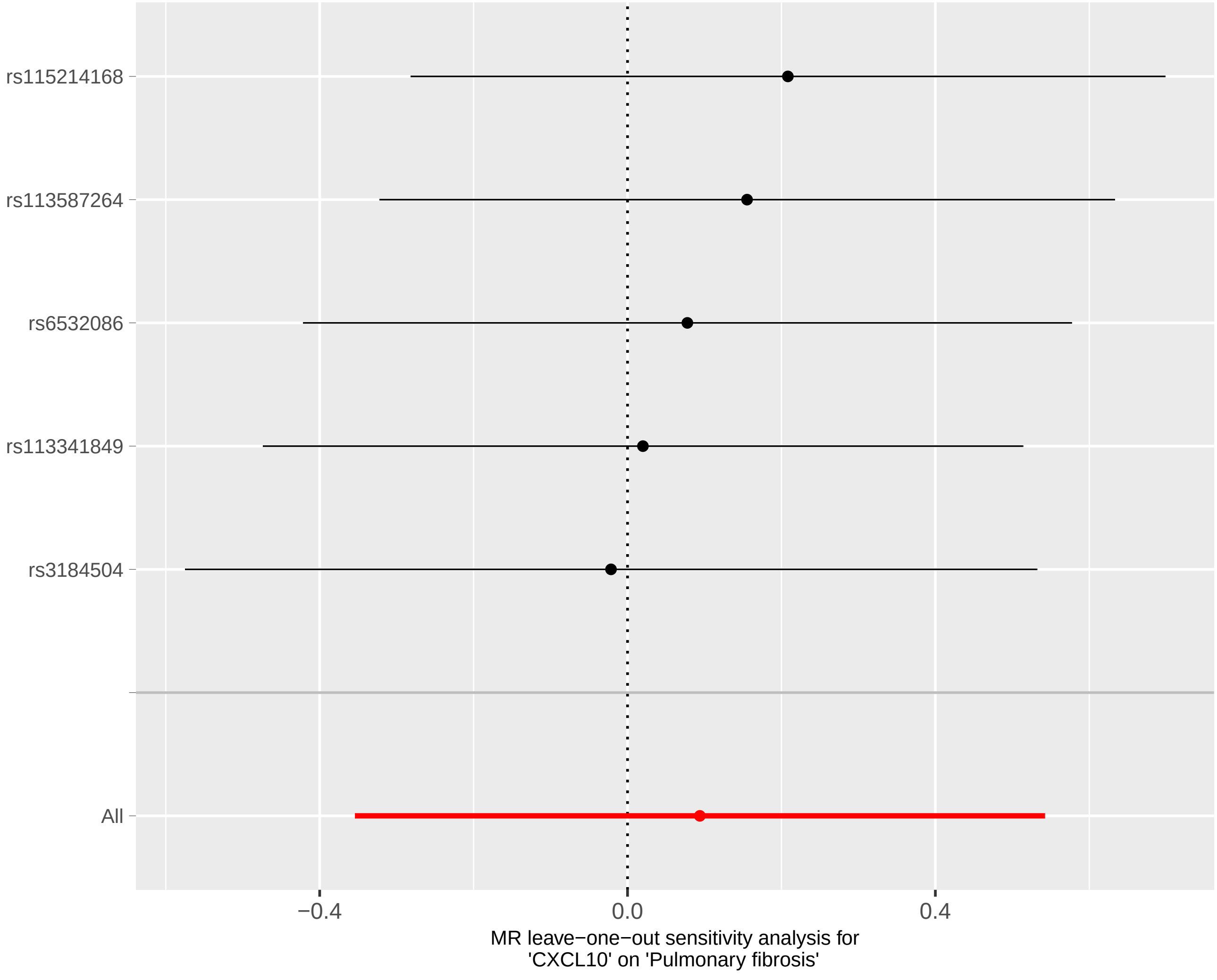
rs115214168
rs113587264
rs6532086
rs113341849
rs3184504
All
−0.4
0.0
0.4
MR leave−one−out sensitivity analysis for
'CXCL10' on 'Pulmonary fibrosis'

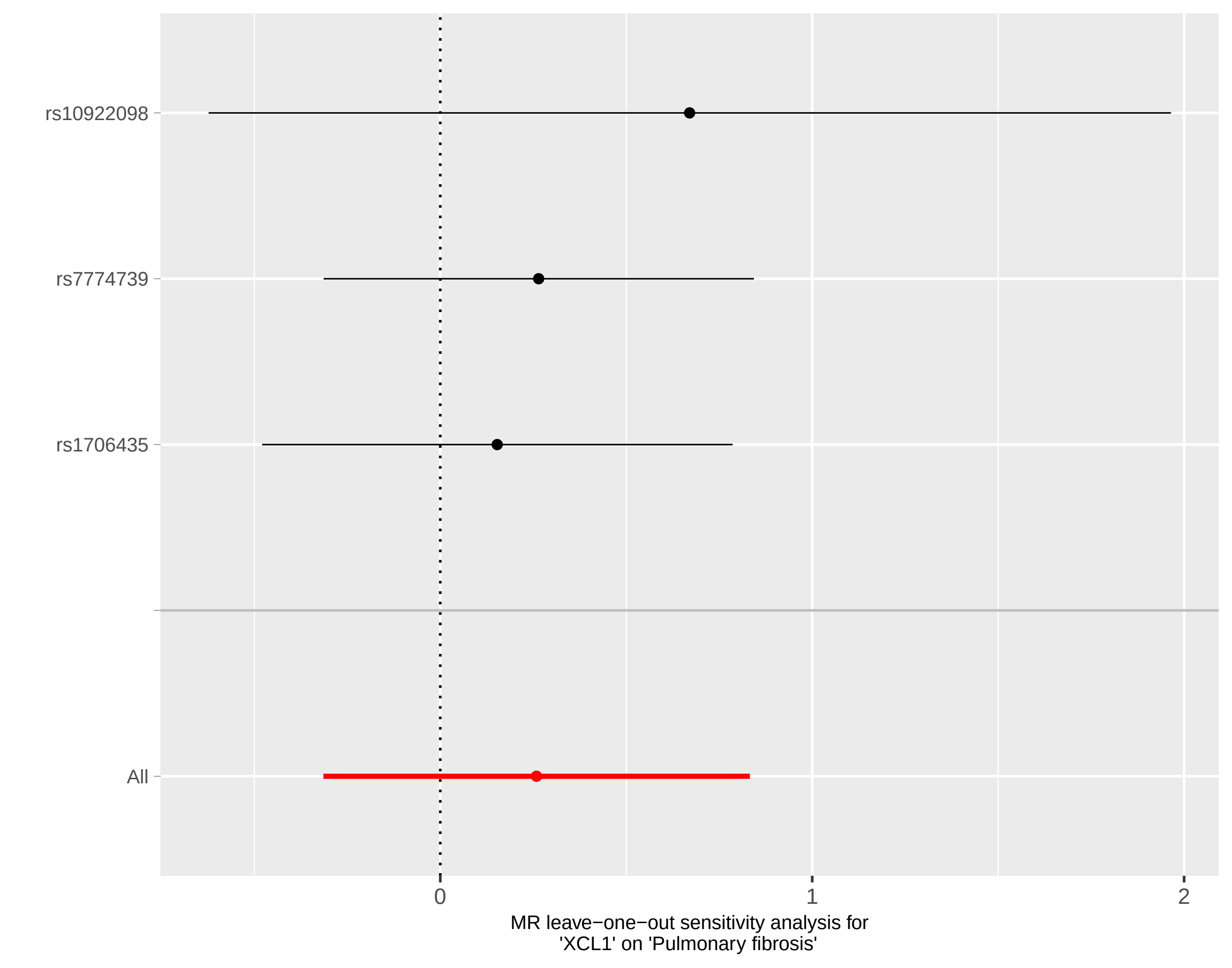
rs10922098
rs7774739
rs1706435
All
0
1
2
MR leave−one−out sensitivity analysis for
'XCL1' on 'Pulmonary fibrosis'

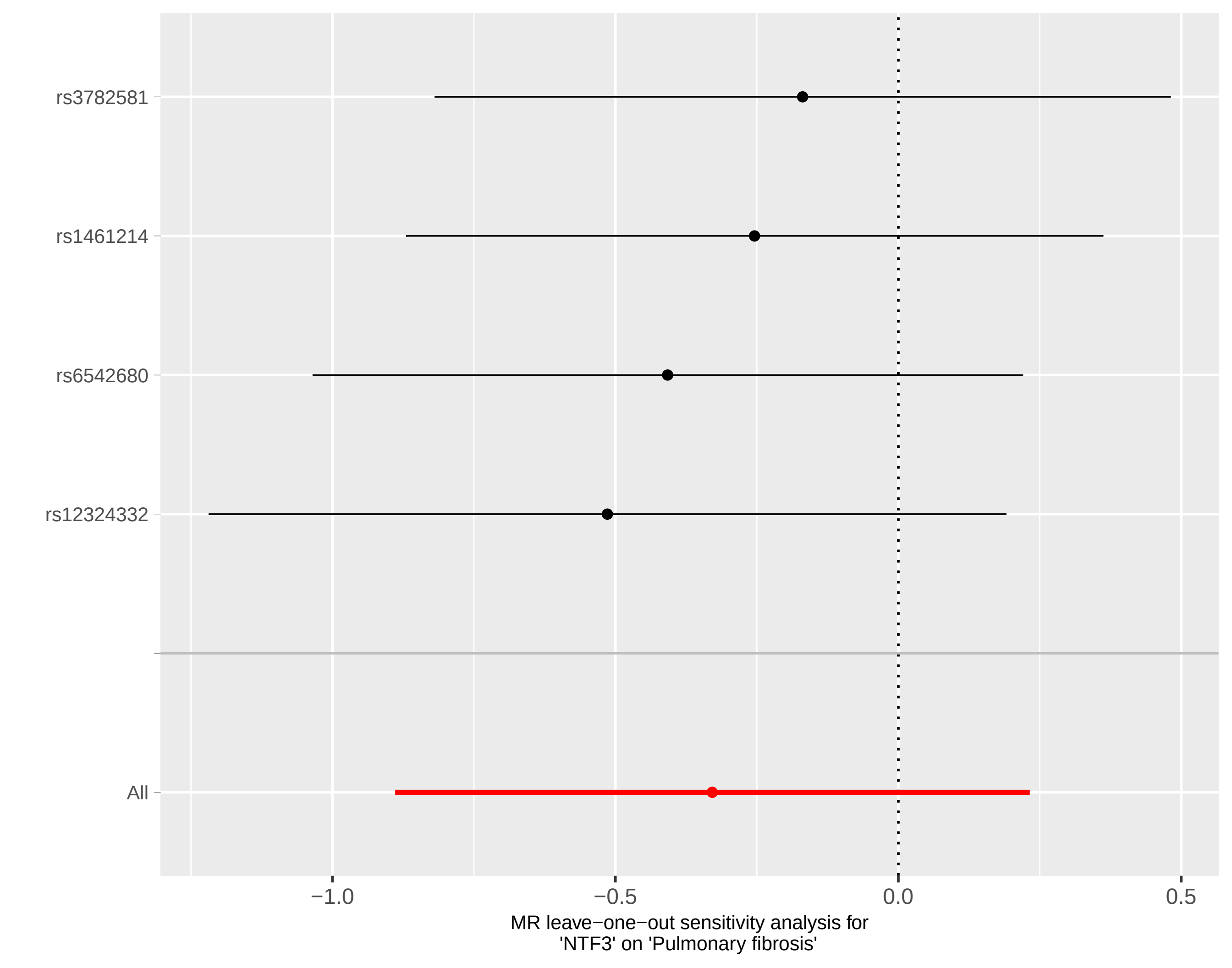

rs3782581
rs1461214
rs6542680
rs12324332
All
−1.0
−0.5
0.0
0.5
MR leave−one−out sensitivity analysis for
'NTF3' on 'Pulmonary fibrosis'

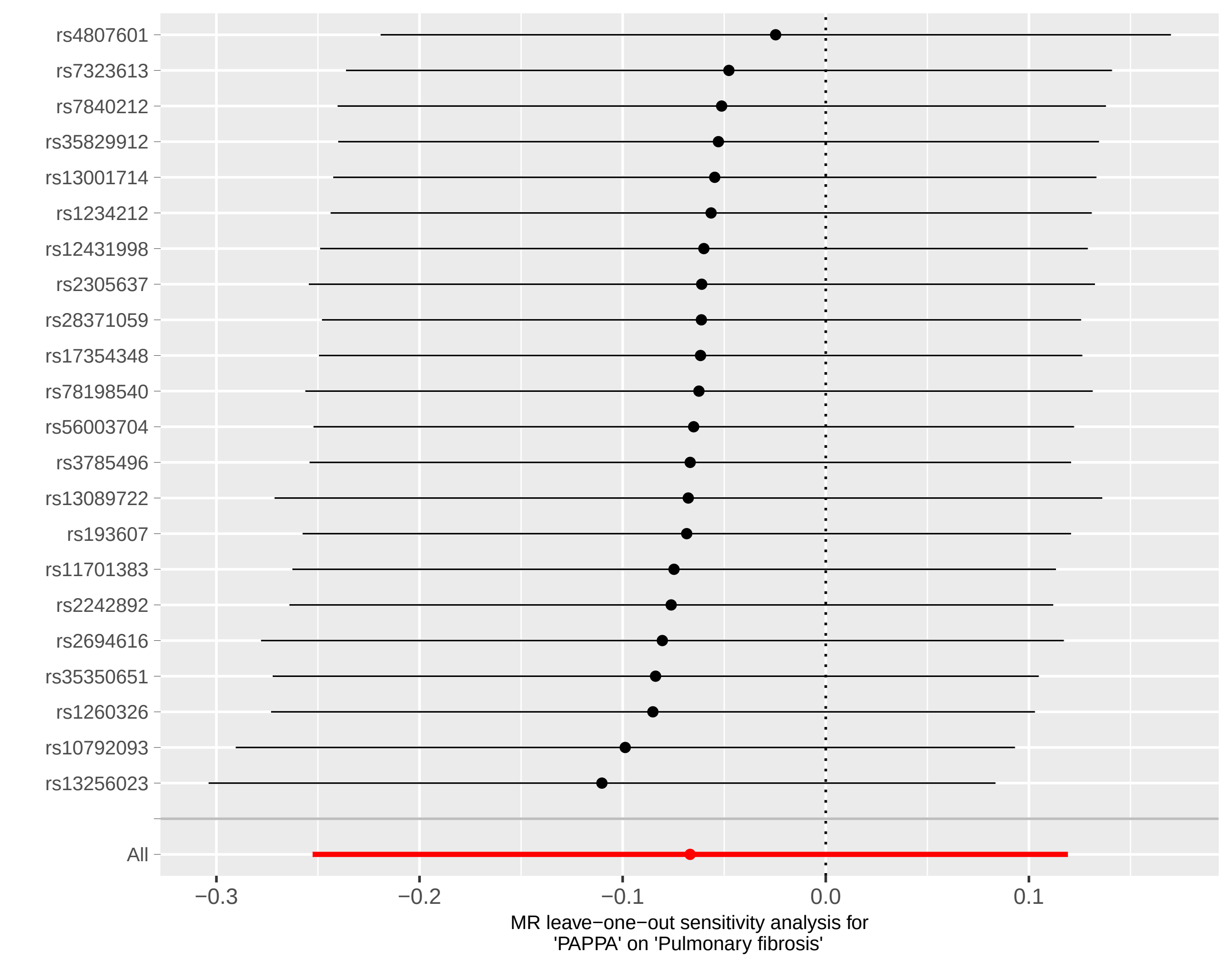
rs4807601
rs7323613
rs7840212
rs35829912
rs13001714
rs1234212
rs12431998
rs2305637
rs28371059
rs17354348
rs78198540
rs56003704
rs3785496
rs13089722
rs193607
rs11701383
rs2242892
rs2694616
rs35350651
rs1260326
rs10792093
rs13256023
All
−0.3
−0.2
−0.1
0.0
0.1
MR leave−one−out sensitivity analysis for
'PAPPA' on 'Pulmonary fibrosis'

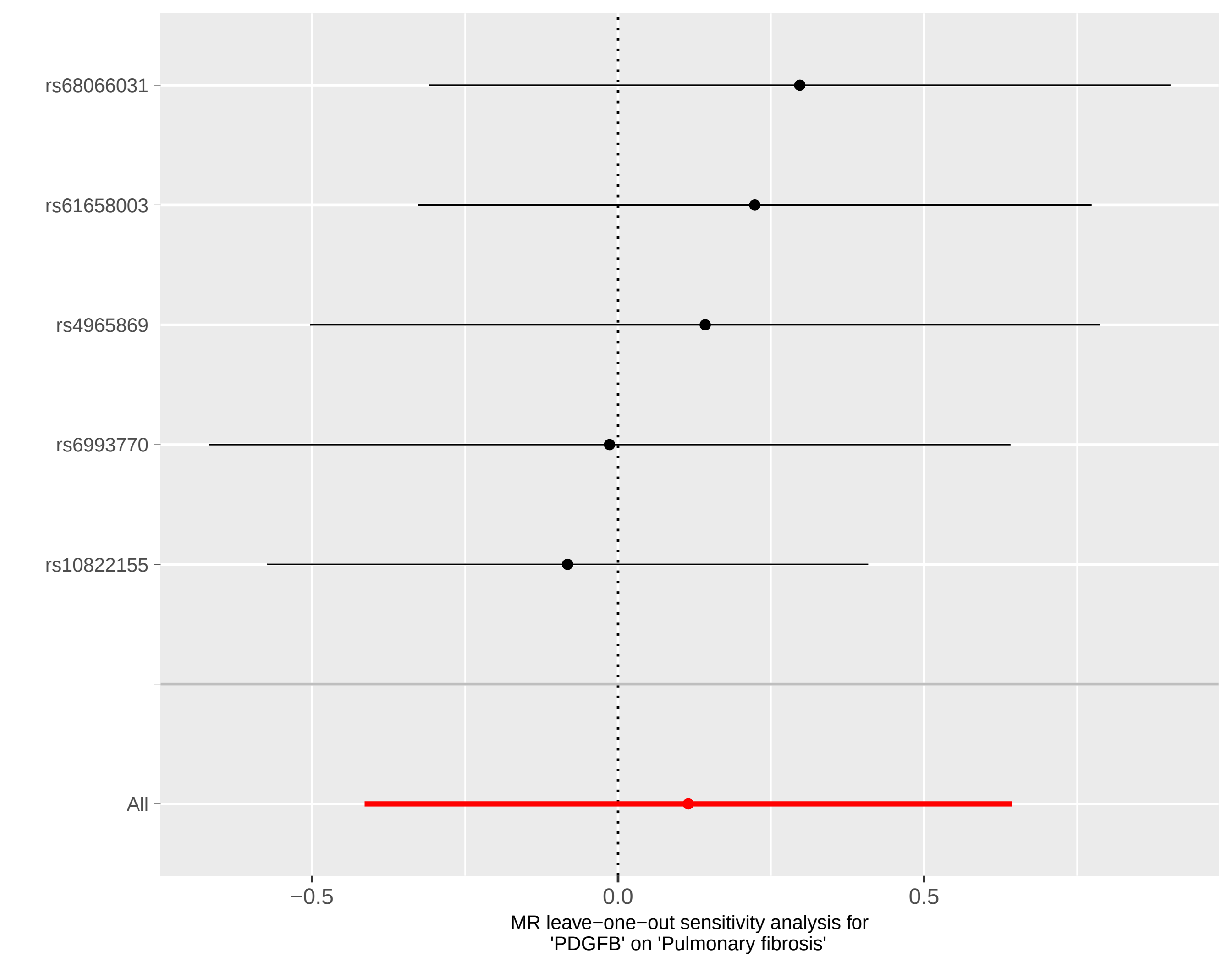
rs68066031
rs61658003
rs4965869
rs6993770
rs10822155
All
−0.5
0.0
0.5
MR leave−one−out sensitivity analysis for
'PDGFB' on 'Pulmonary fibrosis'

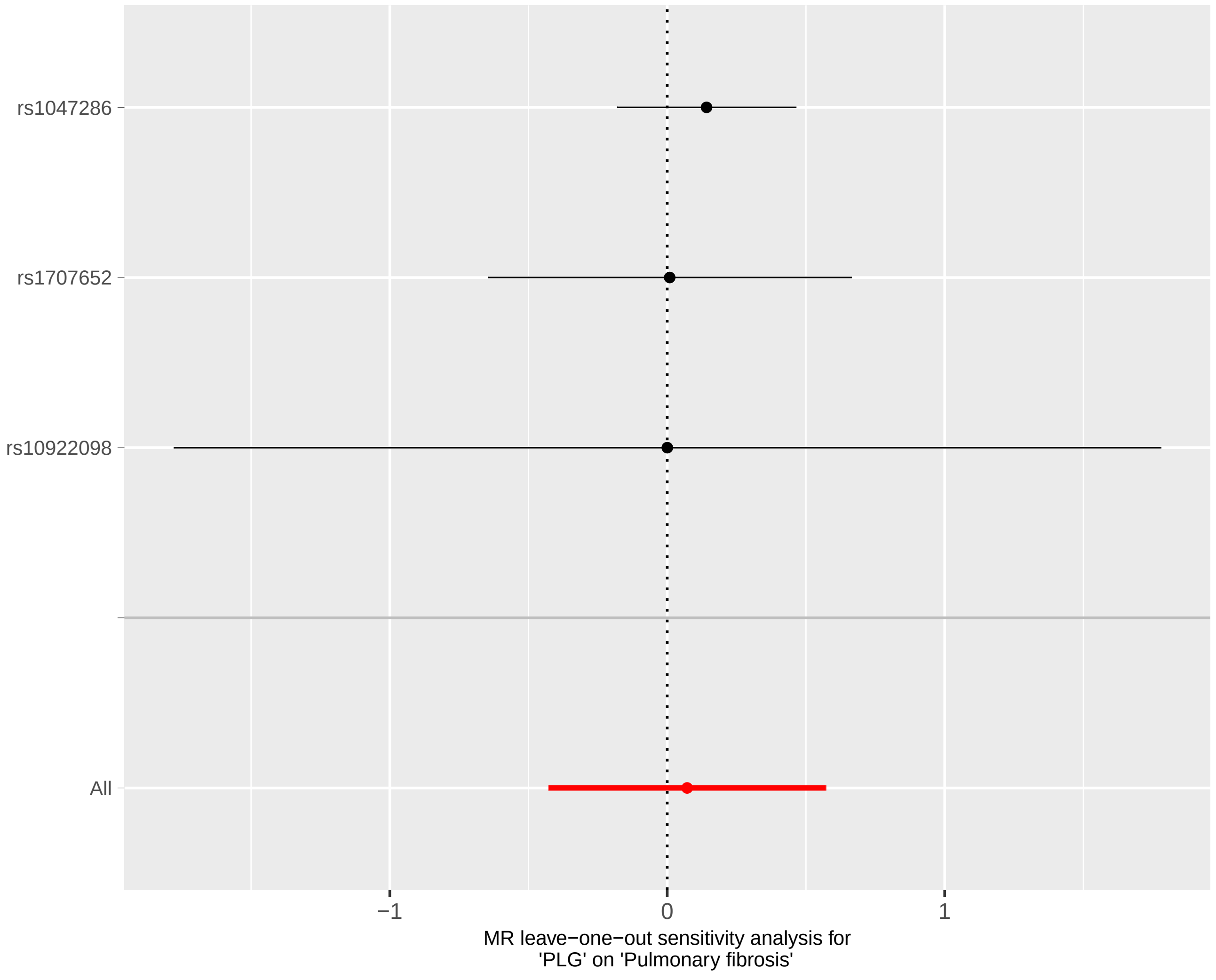
rs1047286
rs1707652
rs10922098
All
−1
0
1
MR leave−one−out sensitivity analysis for
'PLG' on 'Pulmonary fibrosis'

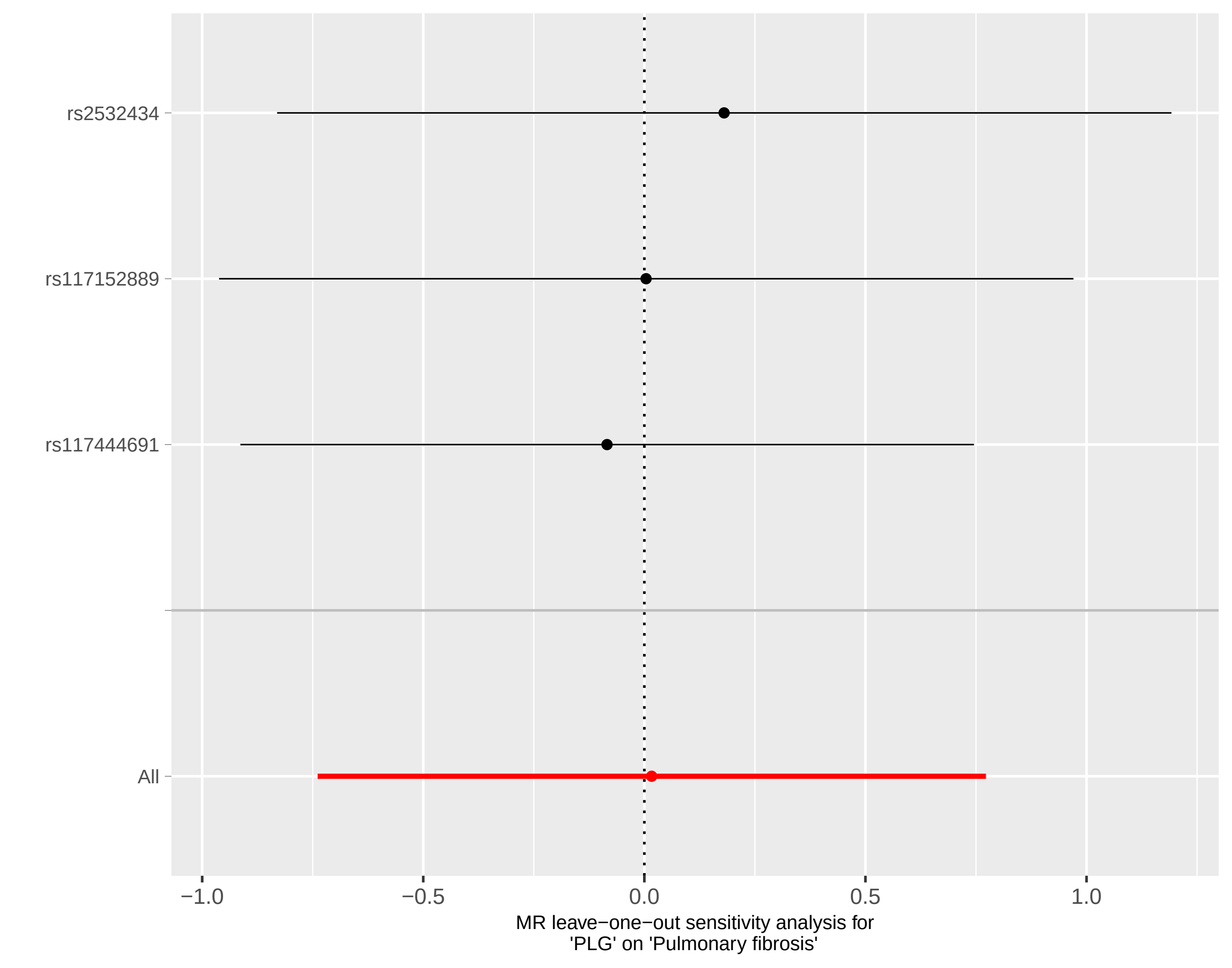
rs2532434
rs117152889
rs117444691
All
−1.0
−0.5
0.0
0.5
1.0
MR leave−one−out sensitivity analysis for
'PLG' on 'Pulmonary fibrosis'

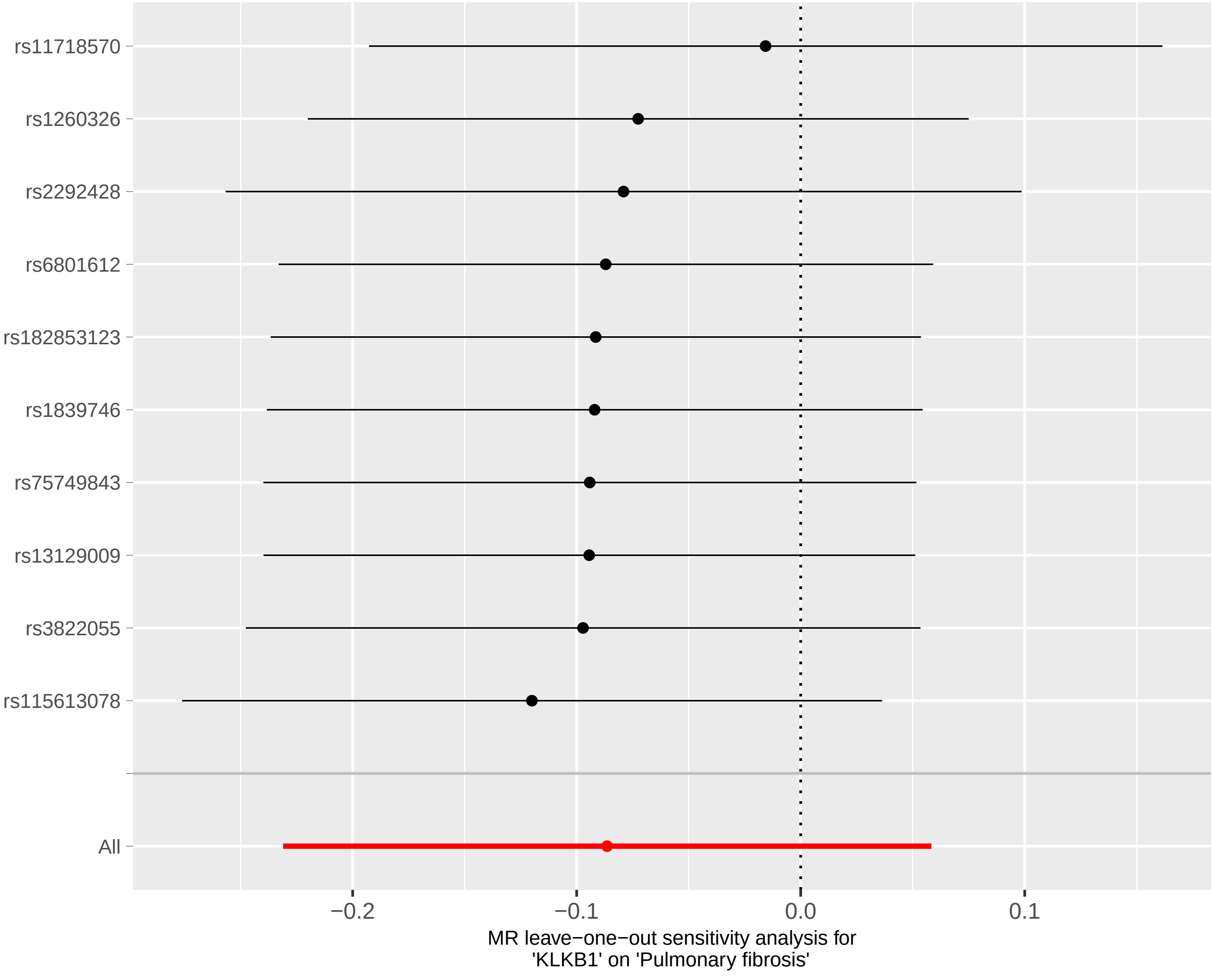
rs11718570
rs1260326
rs2292428
rs6801612
rs182853123
rs1839746
rs75749843
rs13129009
rs3822055
rs115613078
All
−0.2
−0.1
0.0
0.1
MR leave−one−out sensitivity analysis for
'KLKB1' on 'Pulmonary fibrosis'

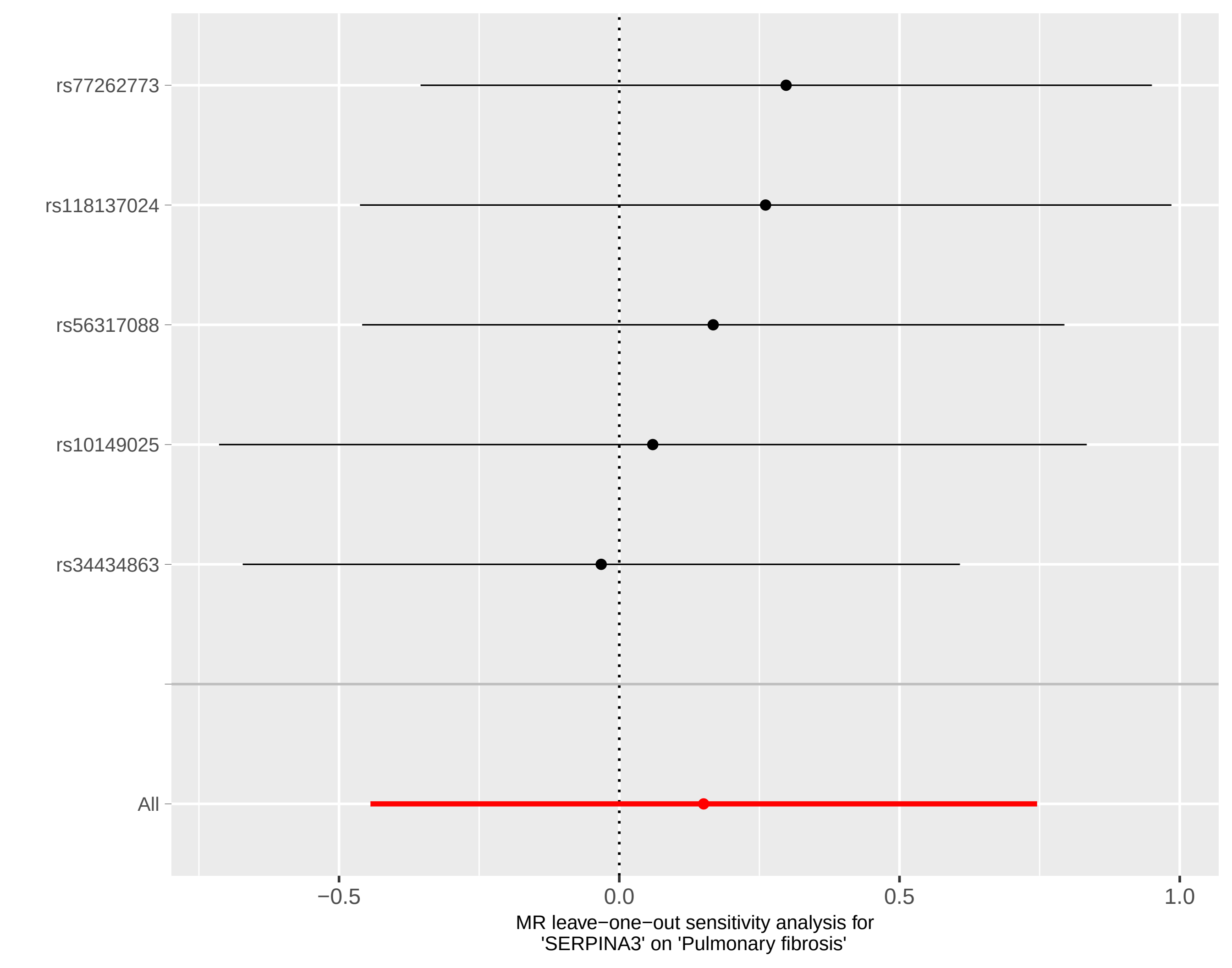
rs77262773
rs118137024
rs56317088
rs10149025
rs34434863
All
−0.5
0.0
0.5
1.0
MR leave−one−out sensitivity analysis for
'SERPINA3' on 'Pulmonary fibrosis'

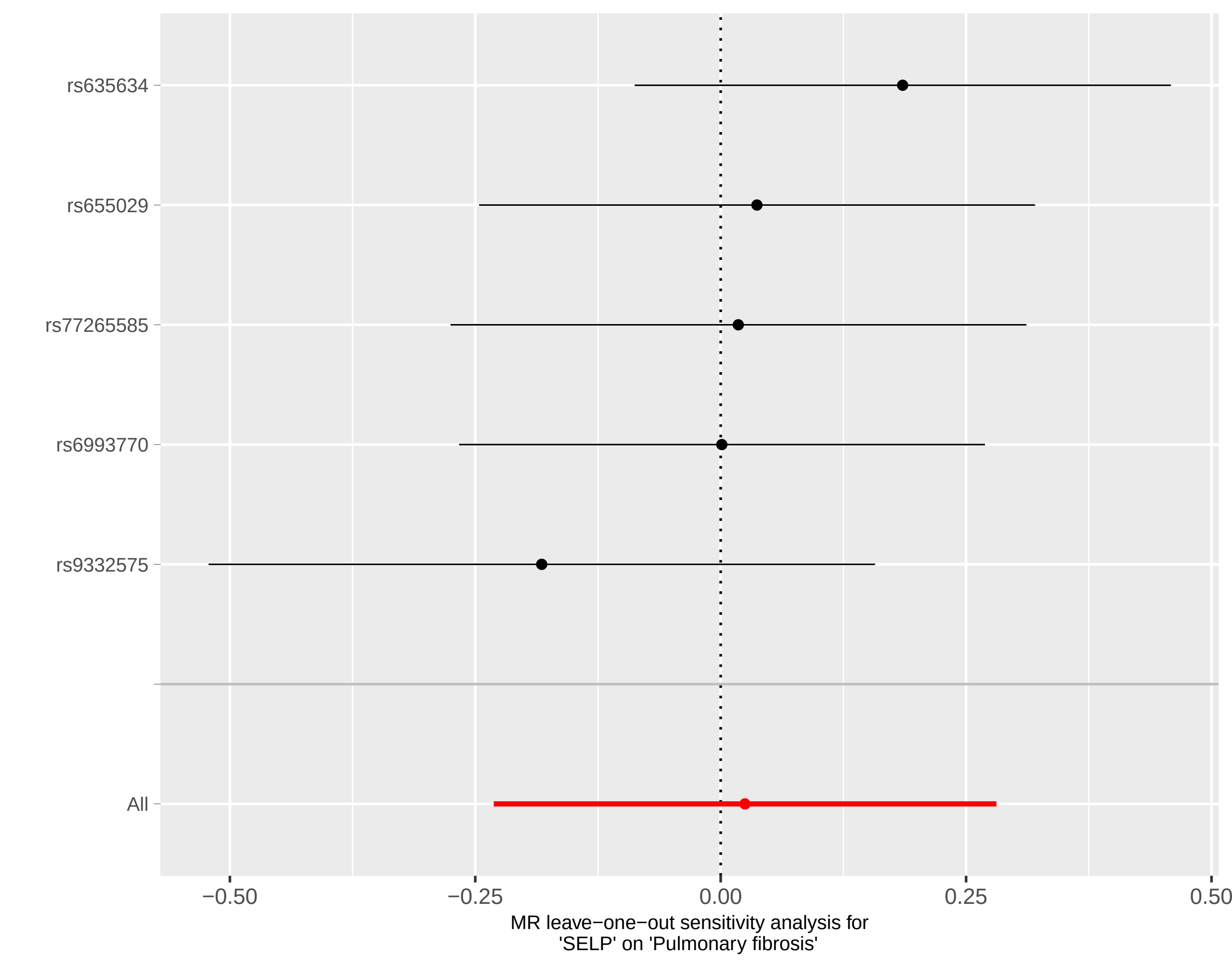
rs635634
rs655029
rs77265585
rs6993770
rs9332575
All
−0.50
−0.25
0.00
0.25
0.50
MR leave−one−out sensitivity analysis for
'SELP' on 'Pulmonary fibrosis'

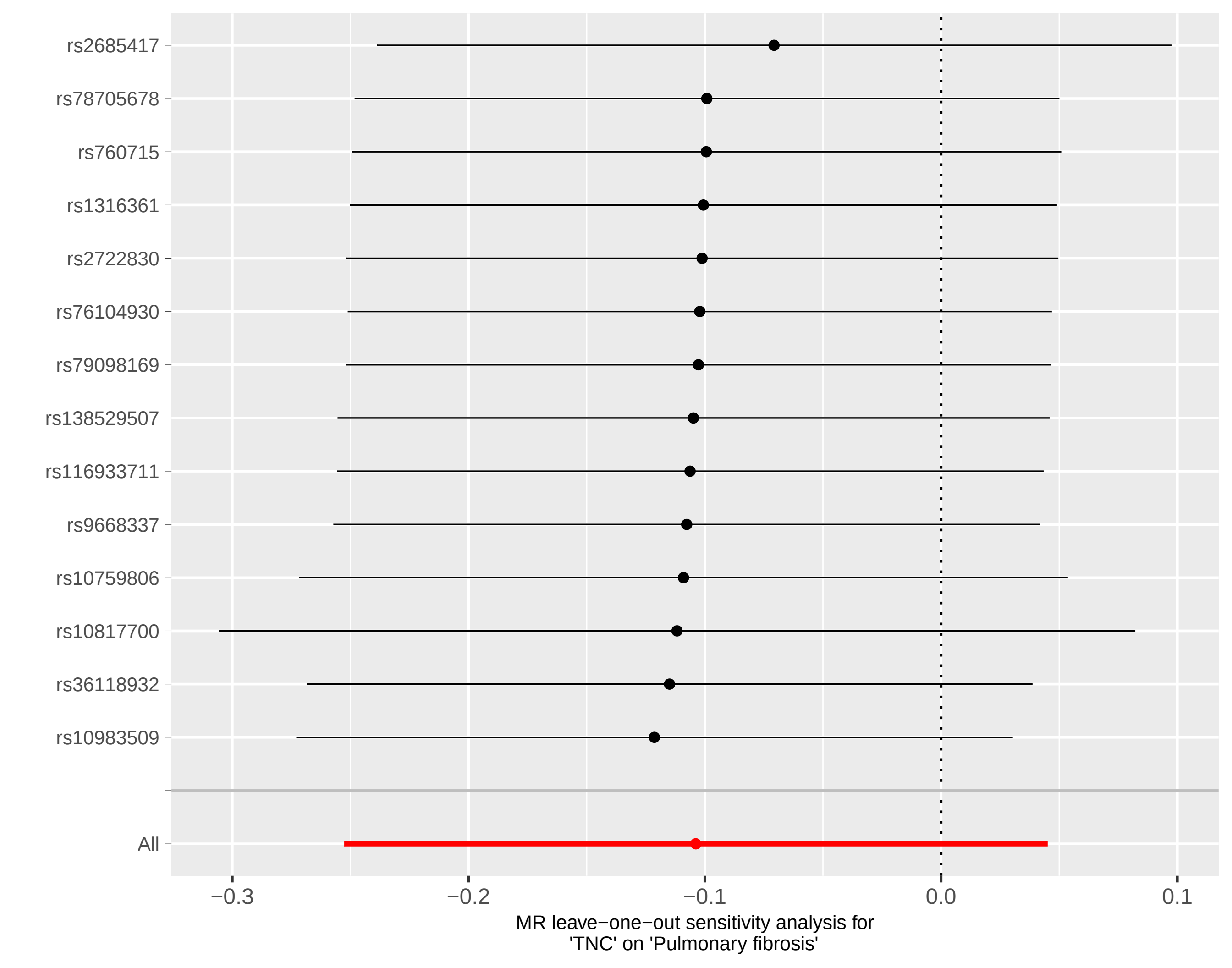

rs2685417
rs78705678
rs760715
rs1316361
rs2722830
rs76104930
rs79098169
rs138529507
rs116933711
rs9668337
rs10759806
rs10817700
rs36118932
rs10983509
All
−0.3
−0.2
−0.1
0.0
0.1
MR leave−one−out sensitivity analysis for
'TNC' on 'Pulmonary fibrosis'

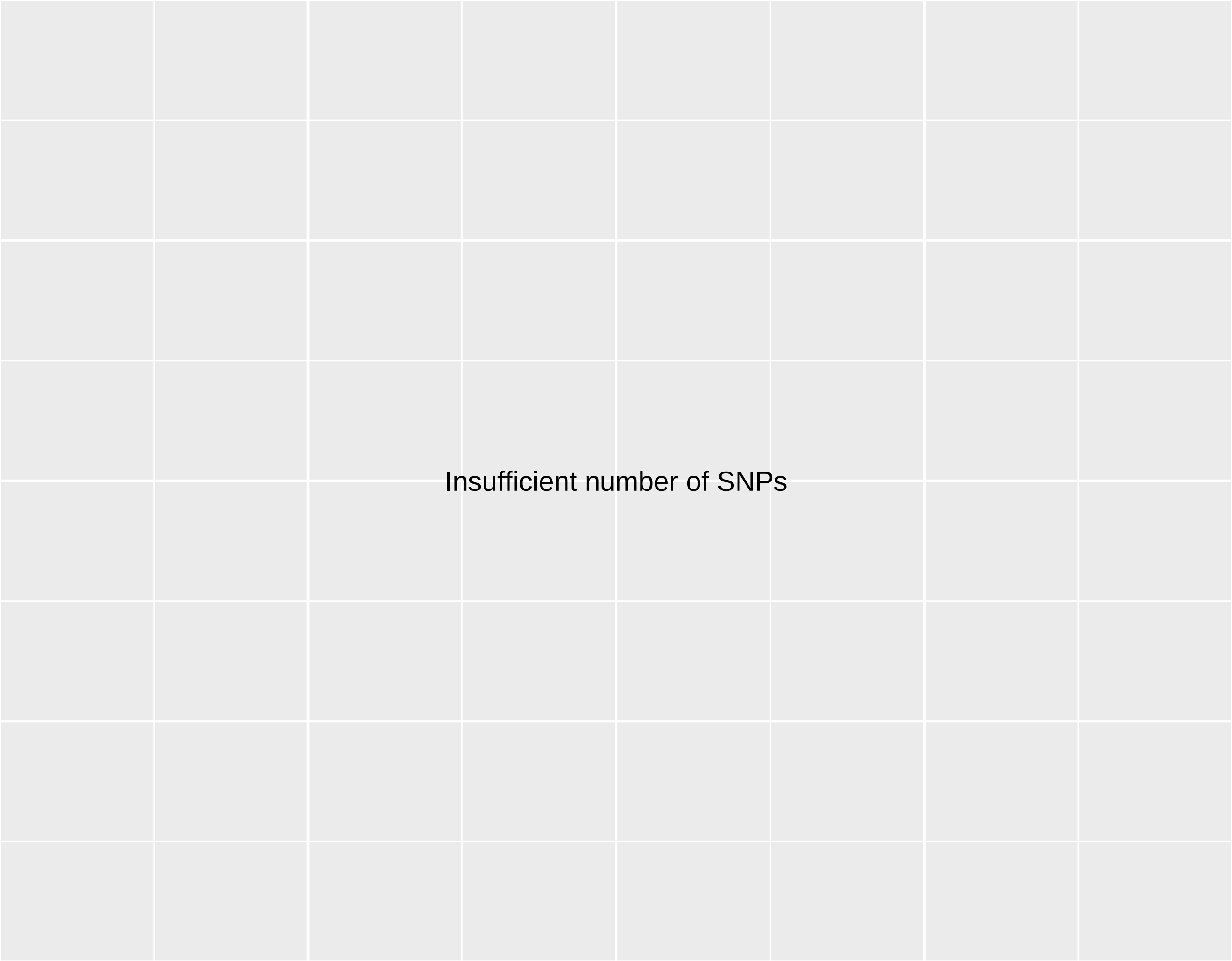
Insufficient number of SNPs

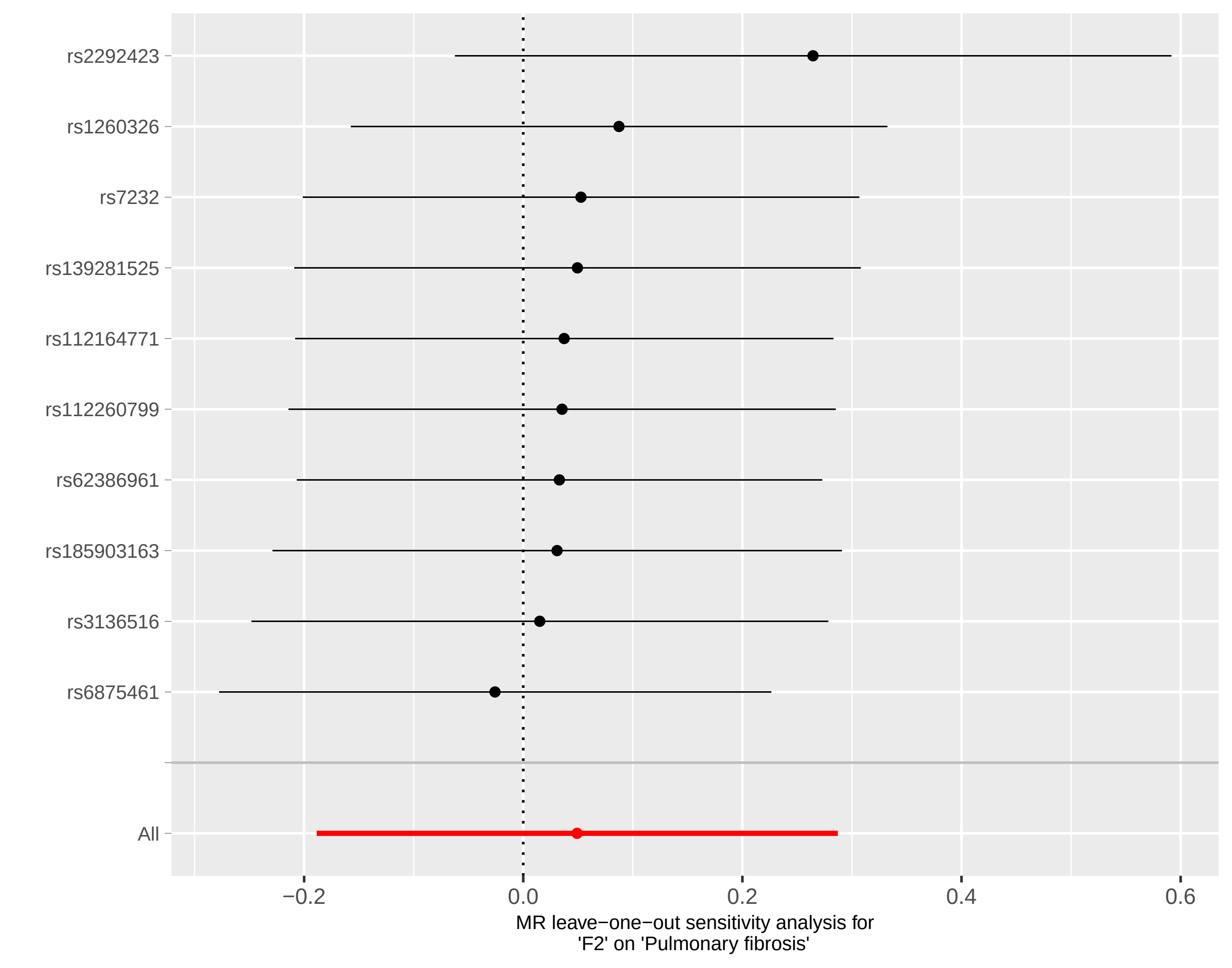
rs2292423
rs1260326
rs7232
rs139281525
rs112164771
rs112260799
rs62386961
rs185903163
rs3136516
rs6875461
All
−0.2
0.0
0.2
0.4
0.6
MR leave−one−out sensitivity analysis for
'F2' on 'Pulmonary fibrosis'

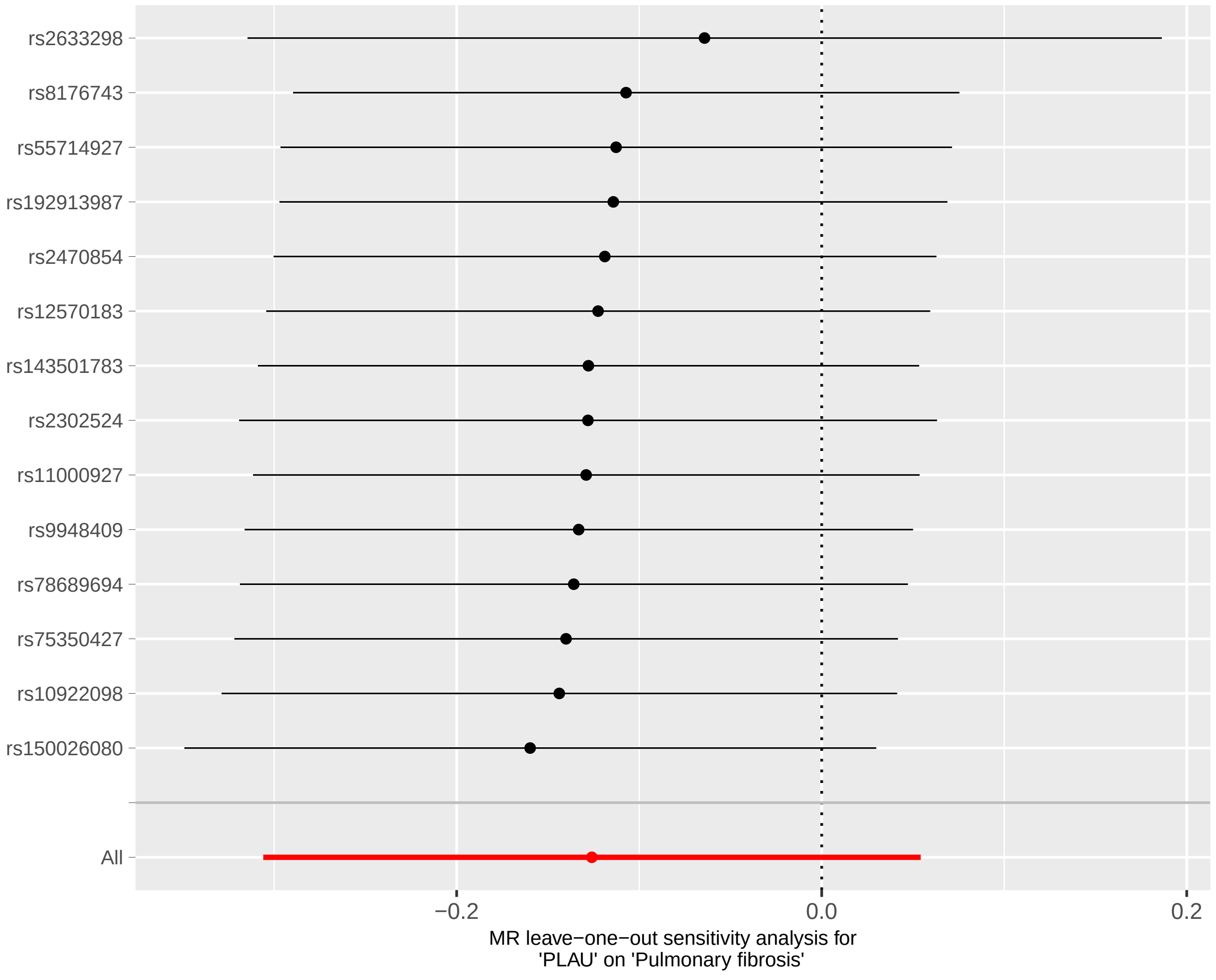

rs2633298
rs8176743
rs55714927
rs192913987
rs2470854
rs12570183
rs143501783
rs2302524
rs11000927
rs9948409
rs78689694
rs75350427
rs10922098
rs150026080
All
−0.2
0.0
0.2
MR leave−one−out sensitivity analysis for
'PLAU' on 'Pulmonary fibrosis'

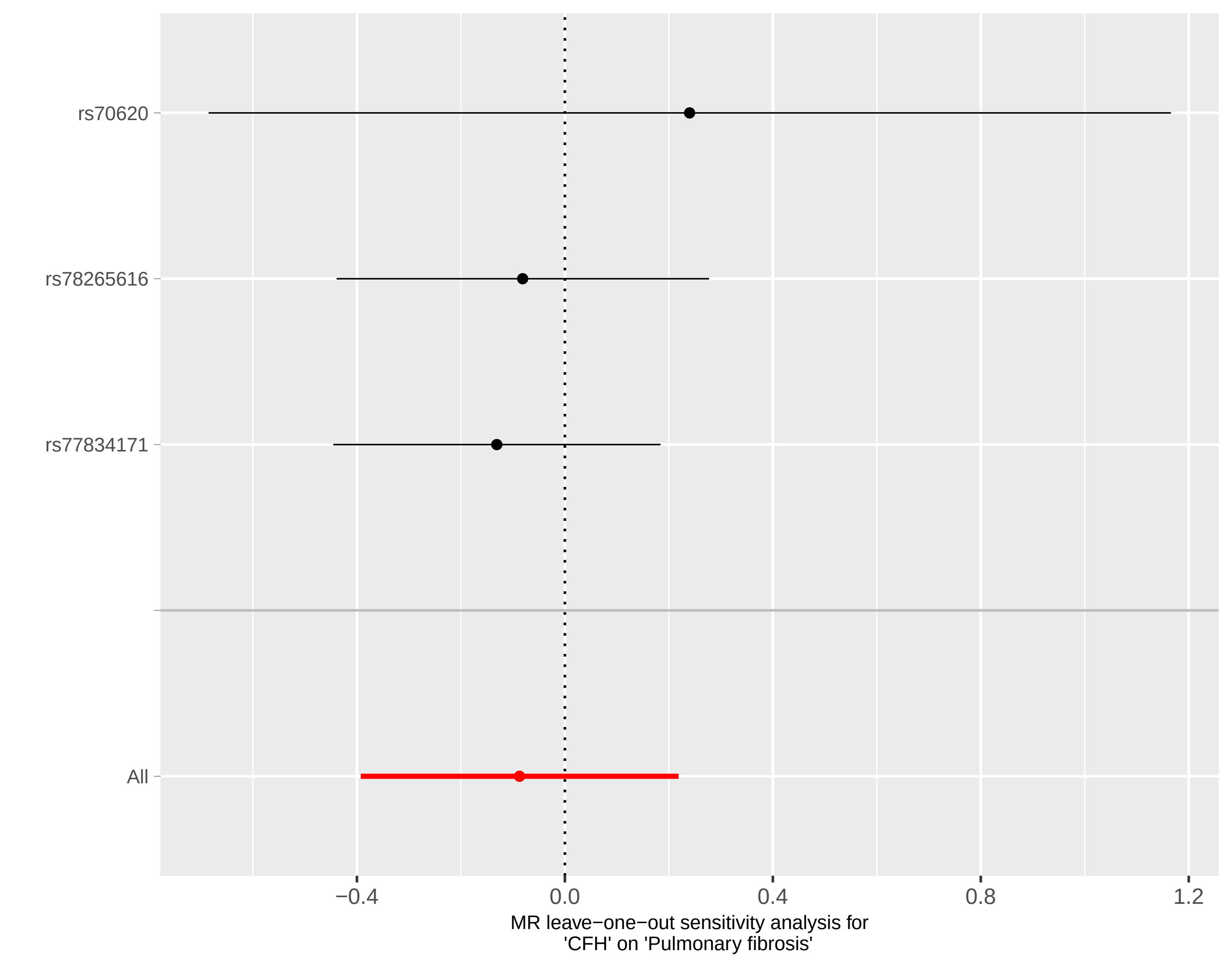
rs70620
rs78265616
rs77834171
All
−0.4
0.0
0.4
0.8
1.2
MR leave−one−out sensitivity analysis for
'CFH' on 'Pulmonary fibrosis'

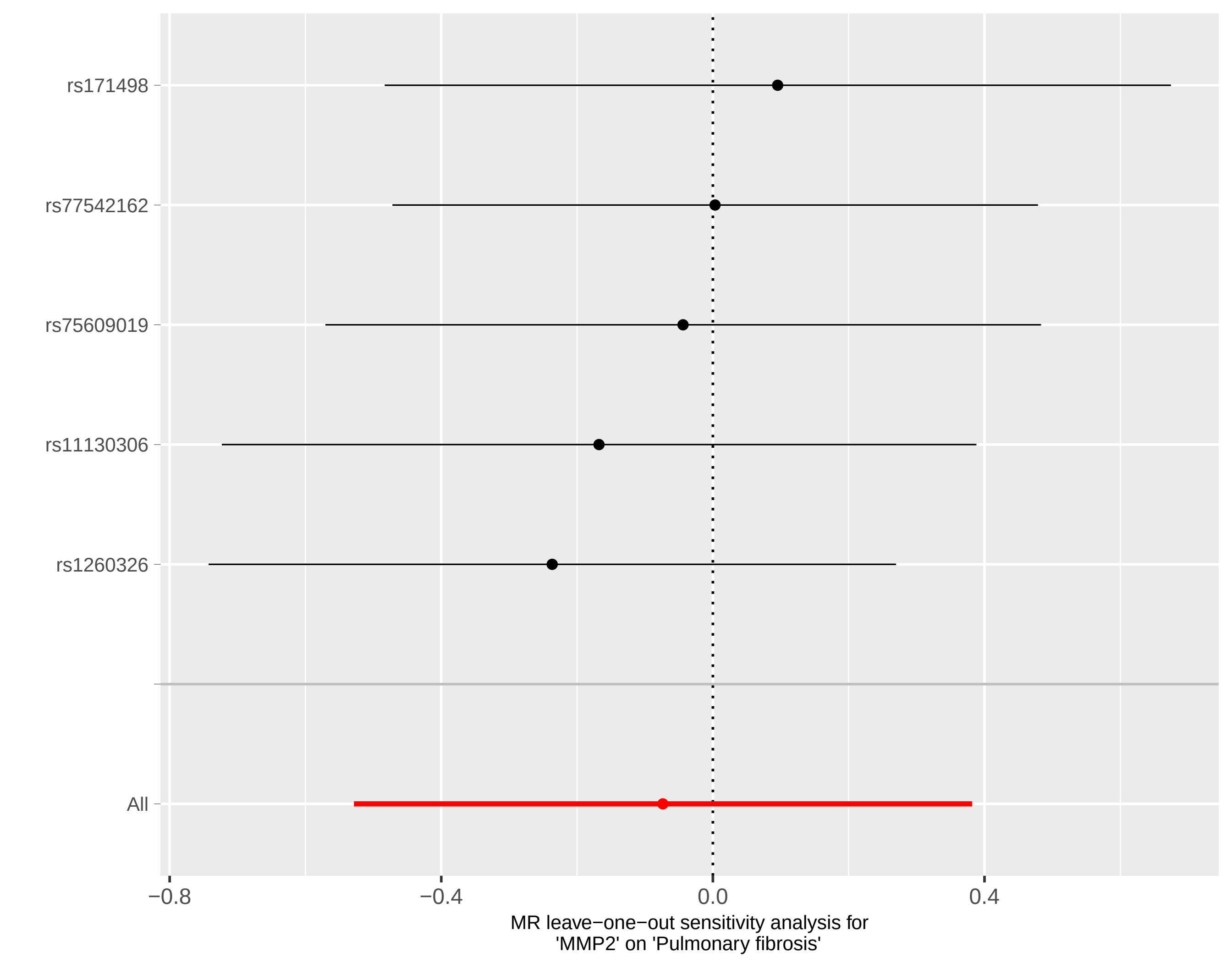
rs171498
rs77542162
rs75609019
rs11130306
rs1260326
All
−0.8
−0.4
0.0
0.4
MR leave−one−out sensitivity analysis for
'MMP2' on 'Pulmonary fibrosis'

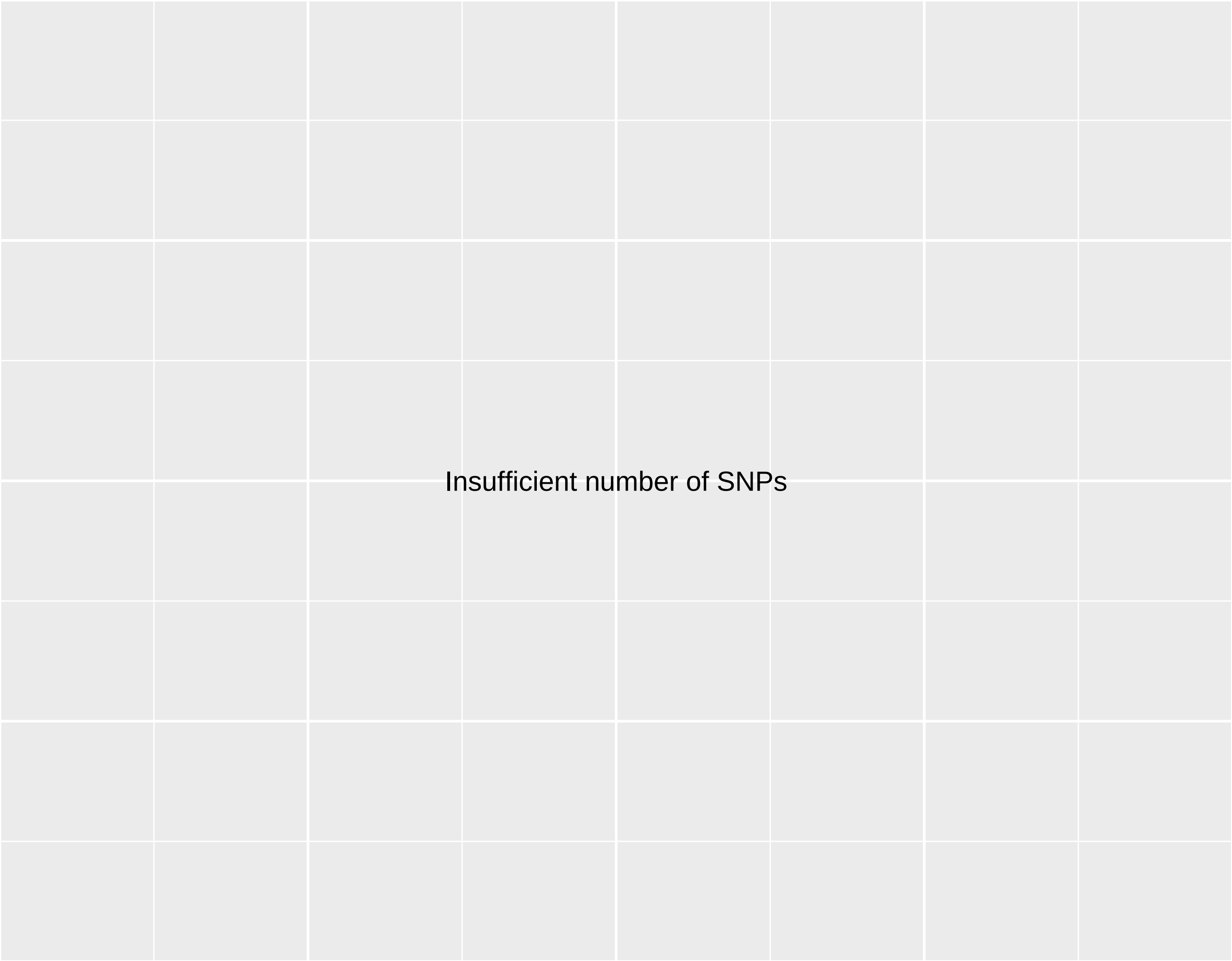
Insufficient number of SNPs

Insufficient number of SNPs

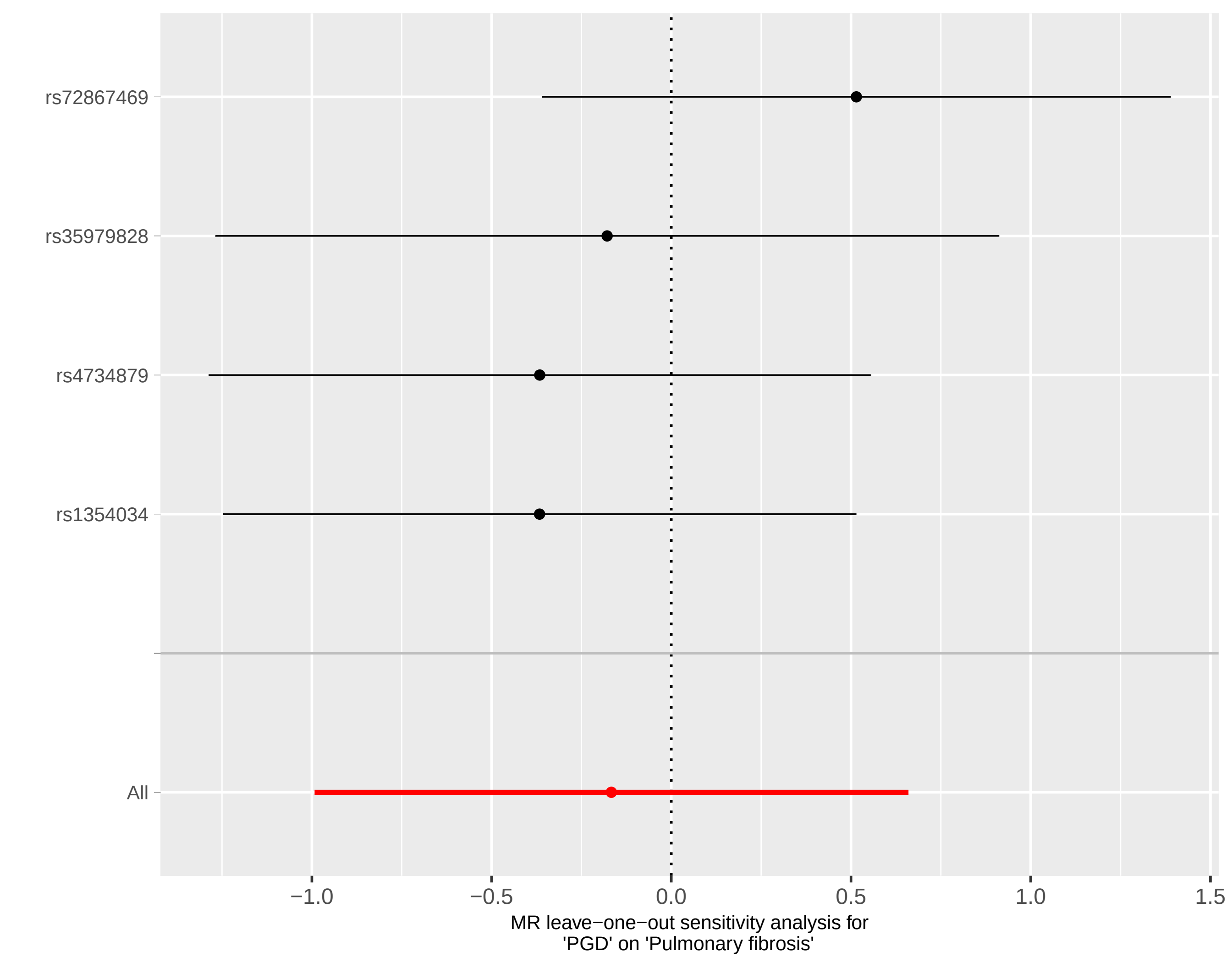
rs72867469
rs35979828
rs4734879
rs1354034
All
−1.0
−0.5
0.0
0.5
1.0
1.5
MR leave−one−out sensitivity analysis for
'PGD' on 'Pulmonary fibrosis'

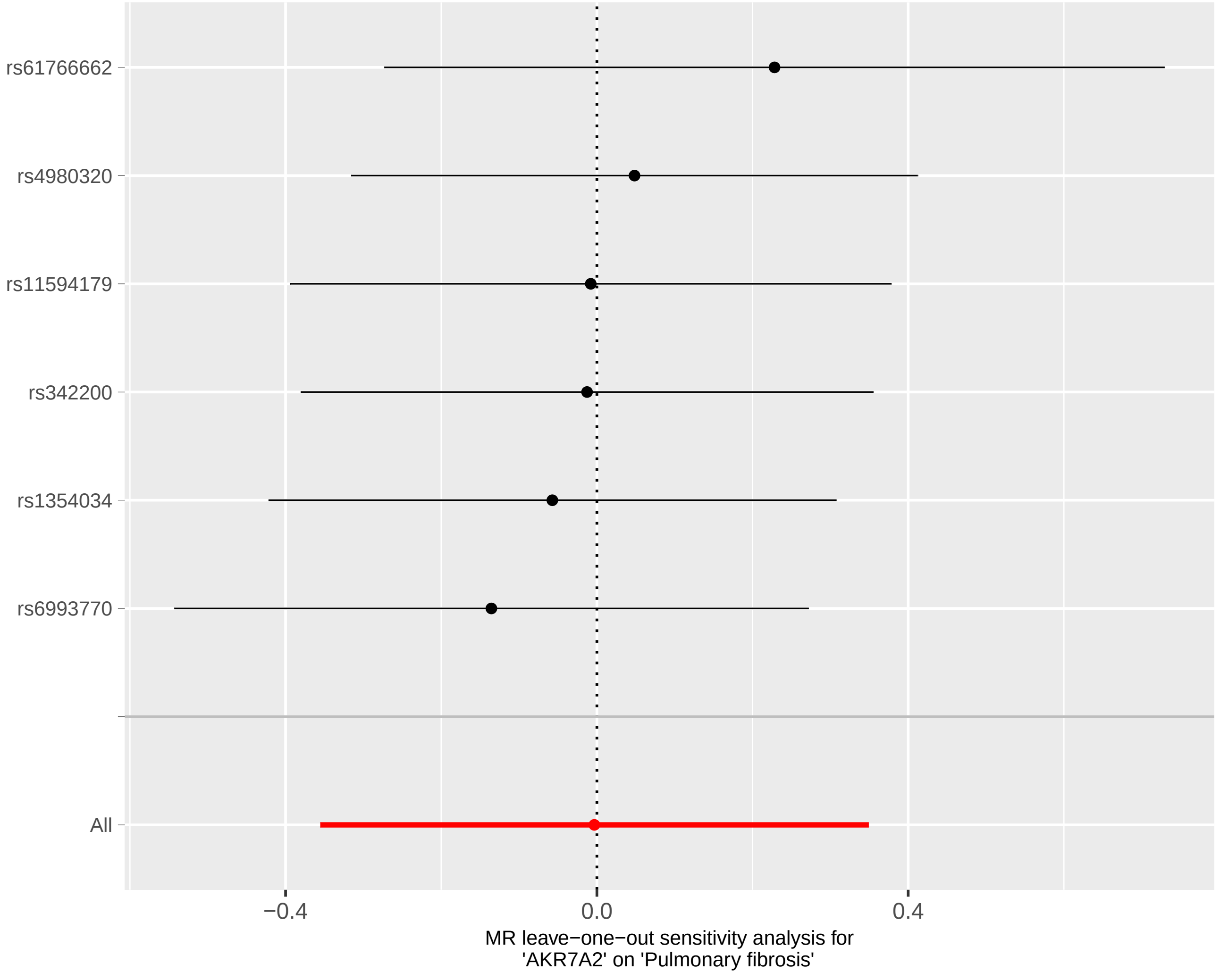
rs61766662
rs4980320
rs11594179
rs342200
rs1354034
rs6993770
All
−0.4
0.0
0.4
MR leave−one−out sensitivity analysis for
'AKR7A2' on 'Pulmonary fibrosis'

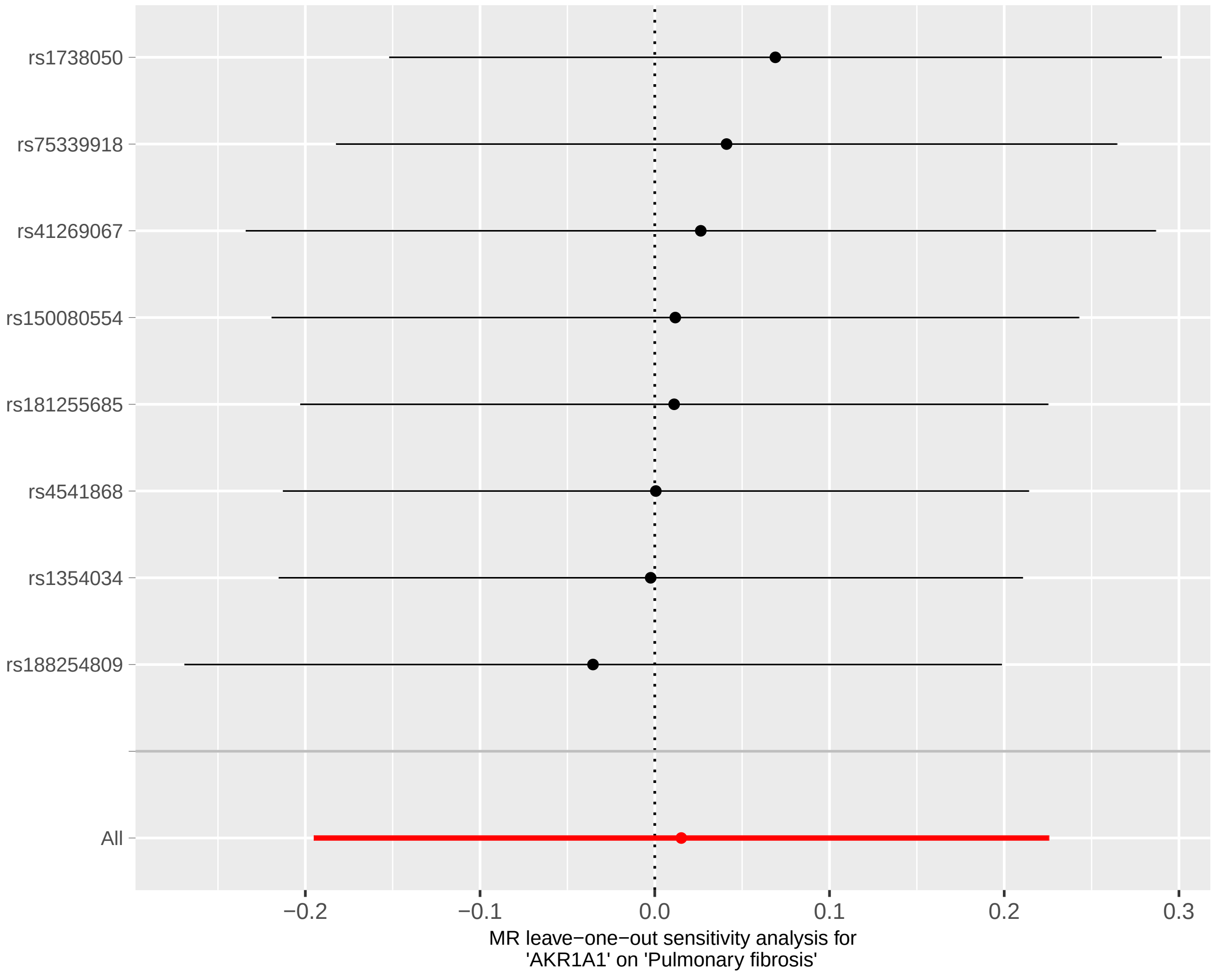
rs1738050
rs75339918
rs41269067
rs150080554
rs181255685
rs4541868
rs1354034
rs188254809
All
−0.2
−0.1
0.0
0.1
0.2
0.3
MR leave−one−out sensitivity analysis for
'AKR1A1' on 'Pulmonary fibrosis'

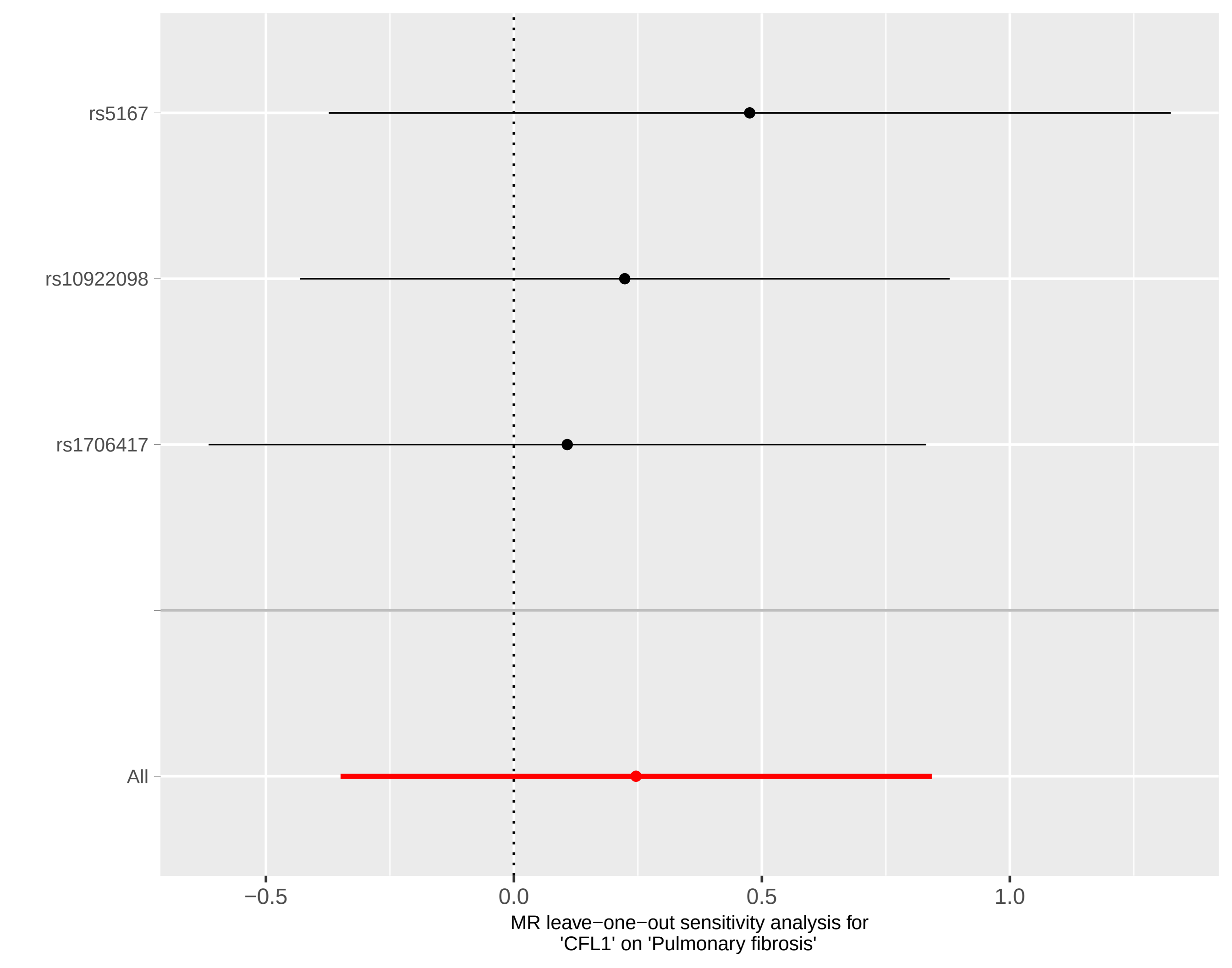
rs5167
rs10922098
rs1706417
All
−0.5
0.0
0.5
1.0
MR leave−one−out sensitivity analysis for
'CFL1' on 'Pulmonary fibrosis'

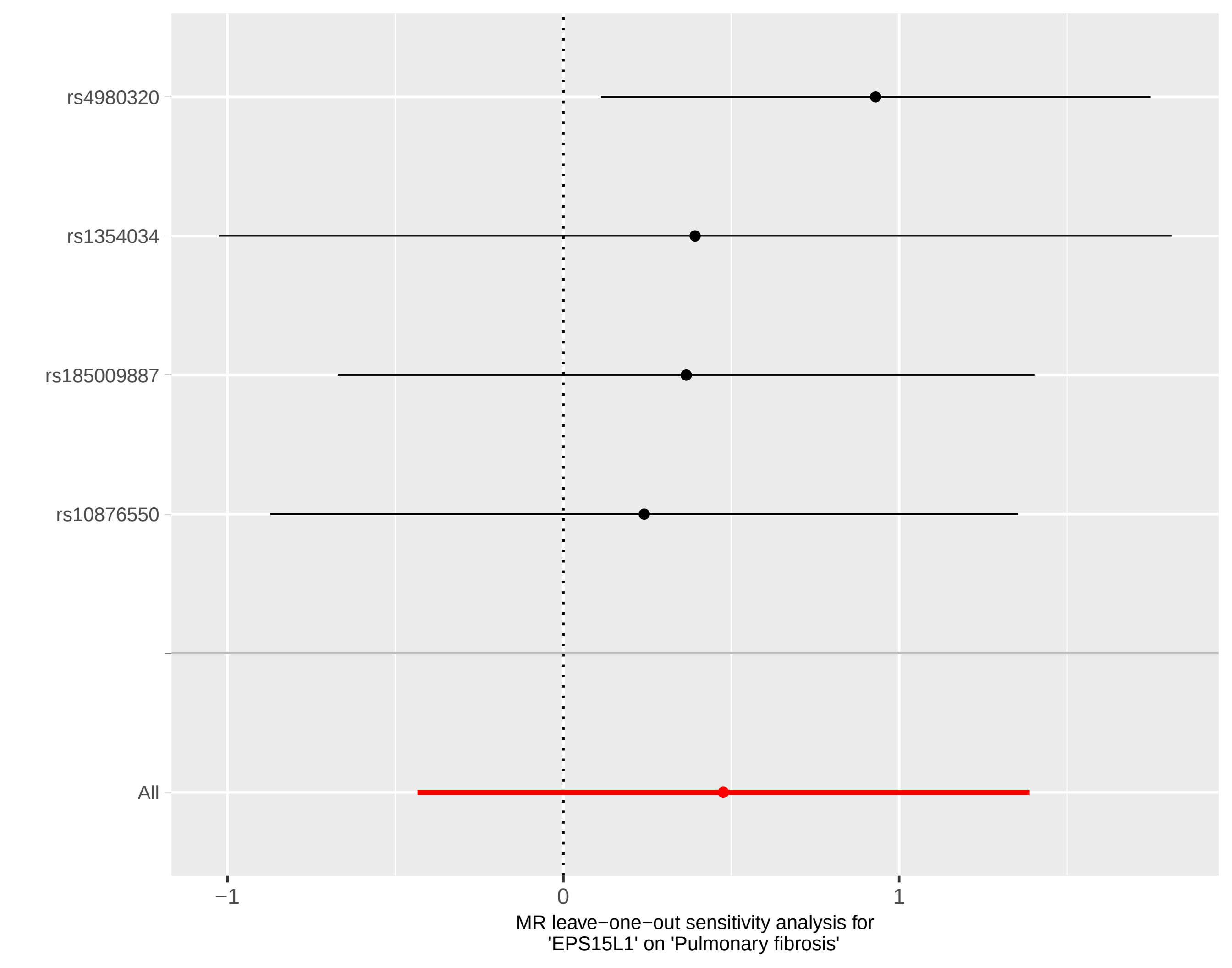
rs4980320
rs1354034
rs185009887
rs10876550
All
−1
0
1
MR leave−one−out sensitivity analysis for
'EPS15L1' on 'Pulmonary fibrosis'

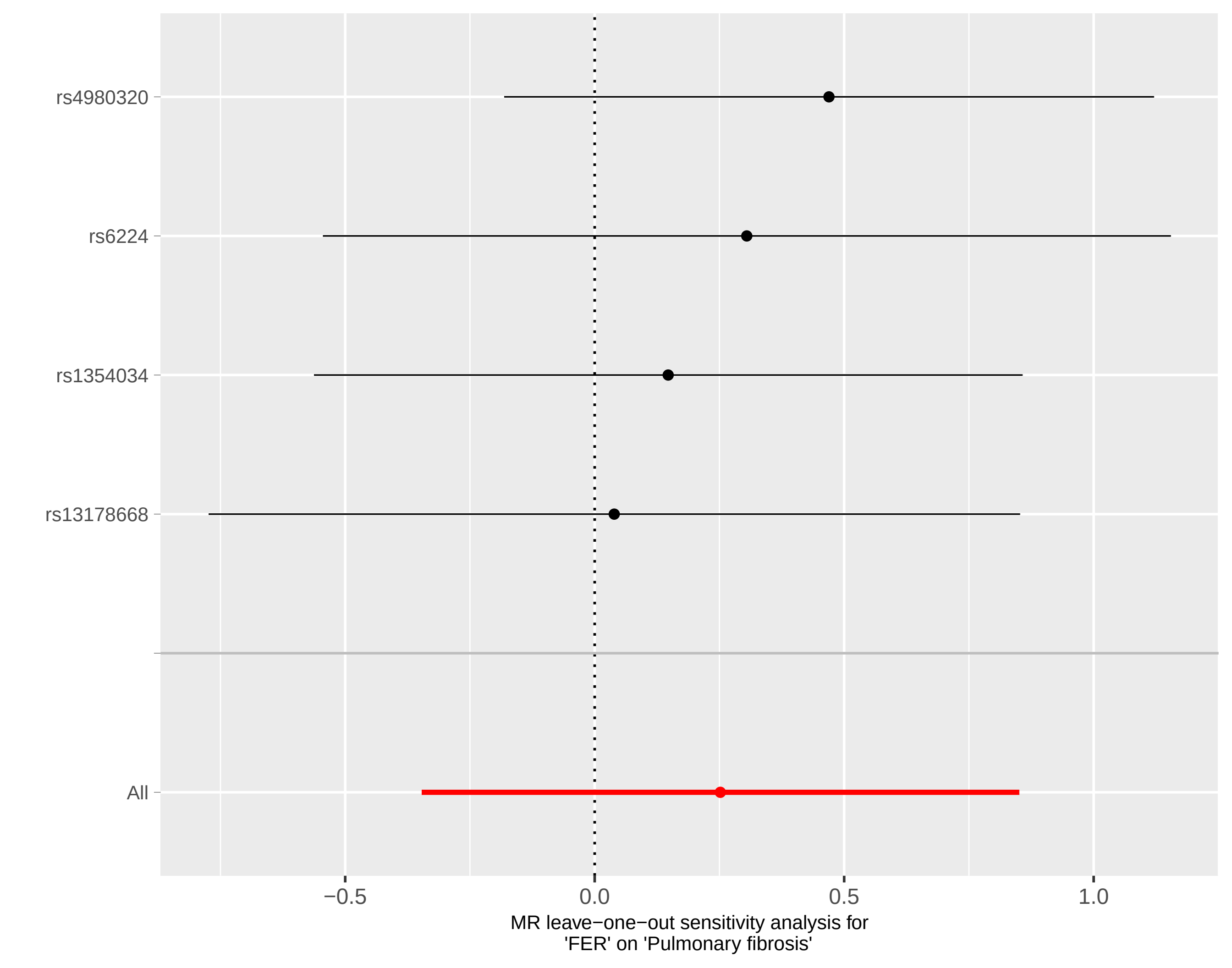
rs4980320
rs6224
rs1354034
rs13178668
All
−0.5
0.0
0.5
1.0
MR leave−one−out sensitivity analysis for
'FER' on 'Pulmonary fibrosis'

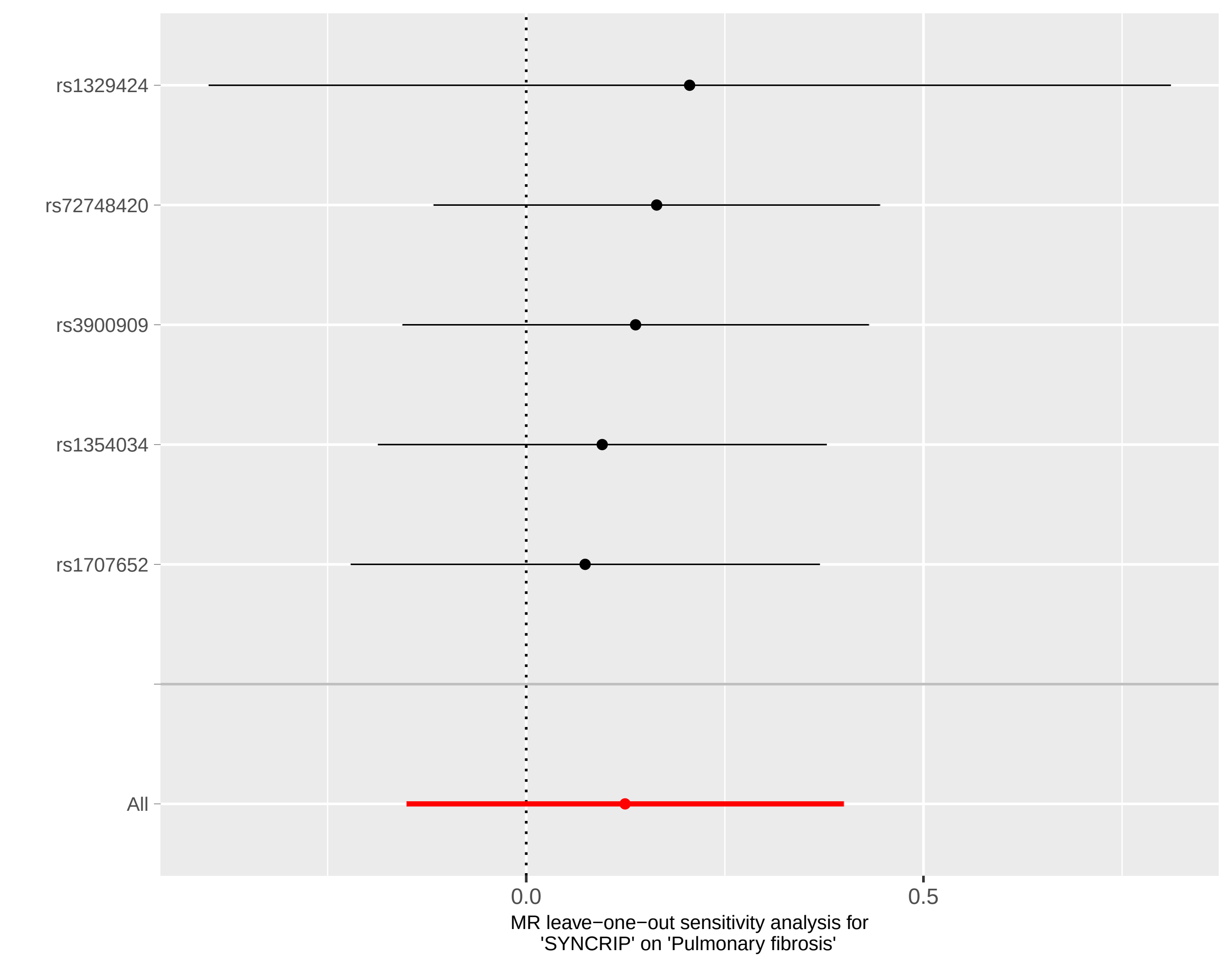
rs1329424
rs72748420
rs3900909
rs1354034
rs1707652
All
0.0
0.5
MR leave−one−out sensitivity analysis for
'SYNCRIP' on 'Pulmonary fibrosis'

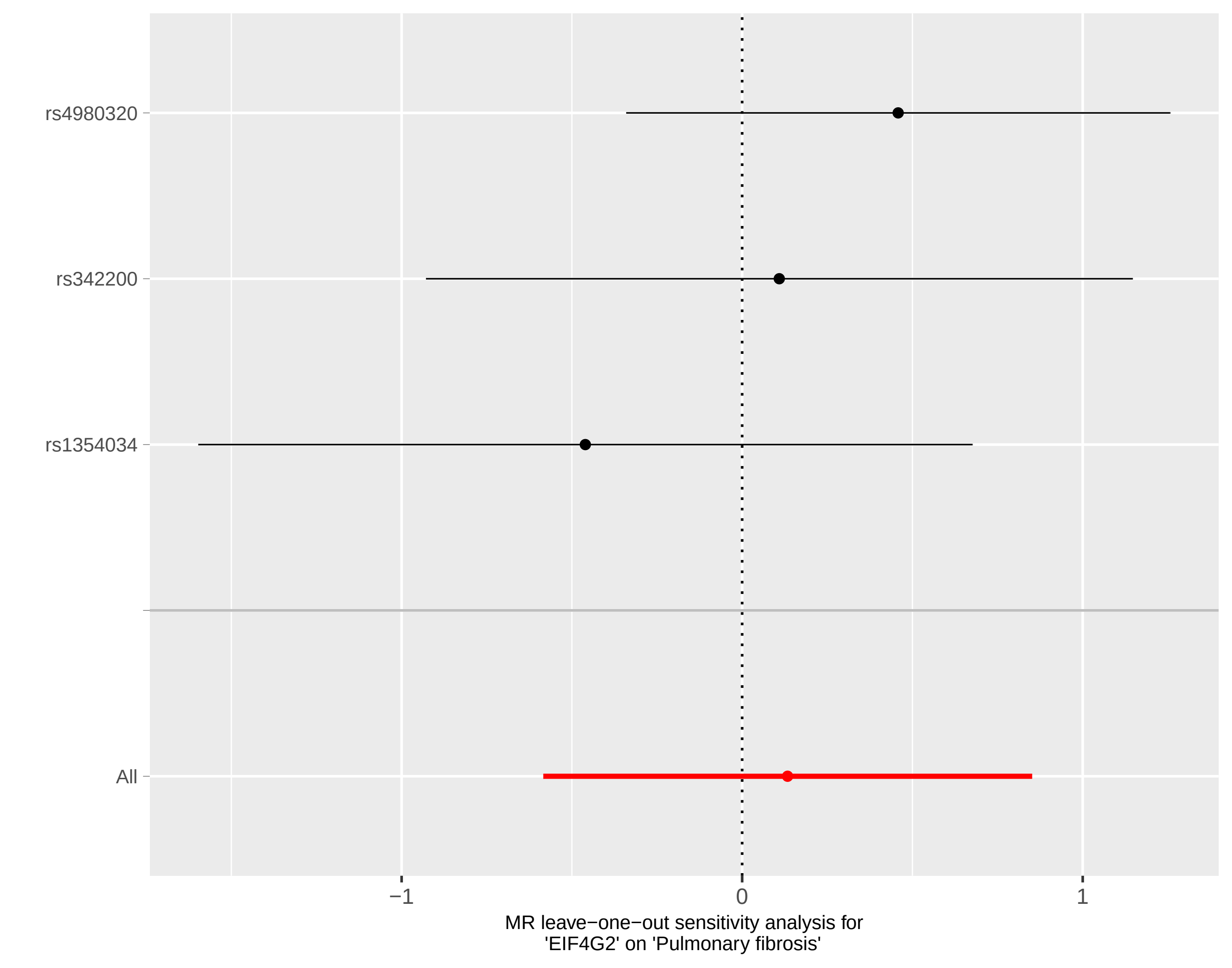
rs4980320
rs342200
rs1354034
All
−1
0
1
MR leave−one−out sensitivity analysis for
'EIF4G2' on 'Pulmonary fibrosis'

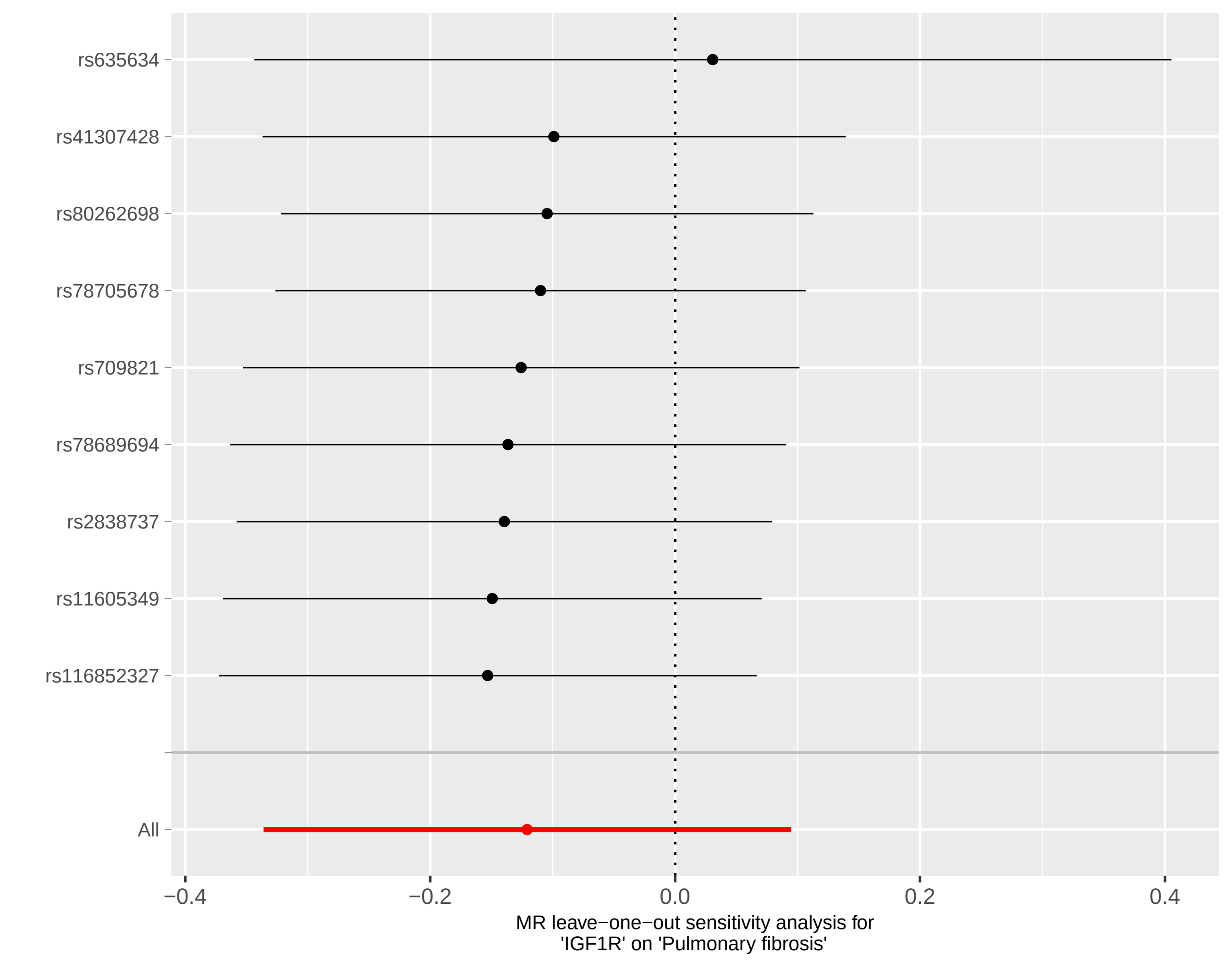
rs635634
rs41307428
rs80262698
rs78705678
rs709821
rs78689694
rs2838737
rs11605349
rs116852327
All
−0.4
−0.2
0.0
0.2
0.4
MR leave−one−out sensitivity analysis for
'IGF1R' on 'Pulmonary fibrosis'

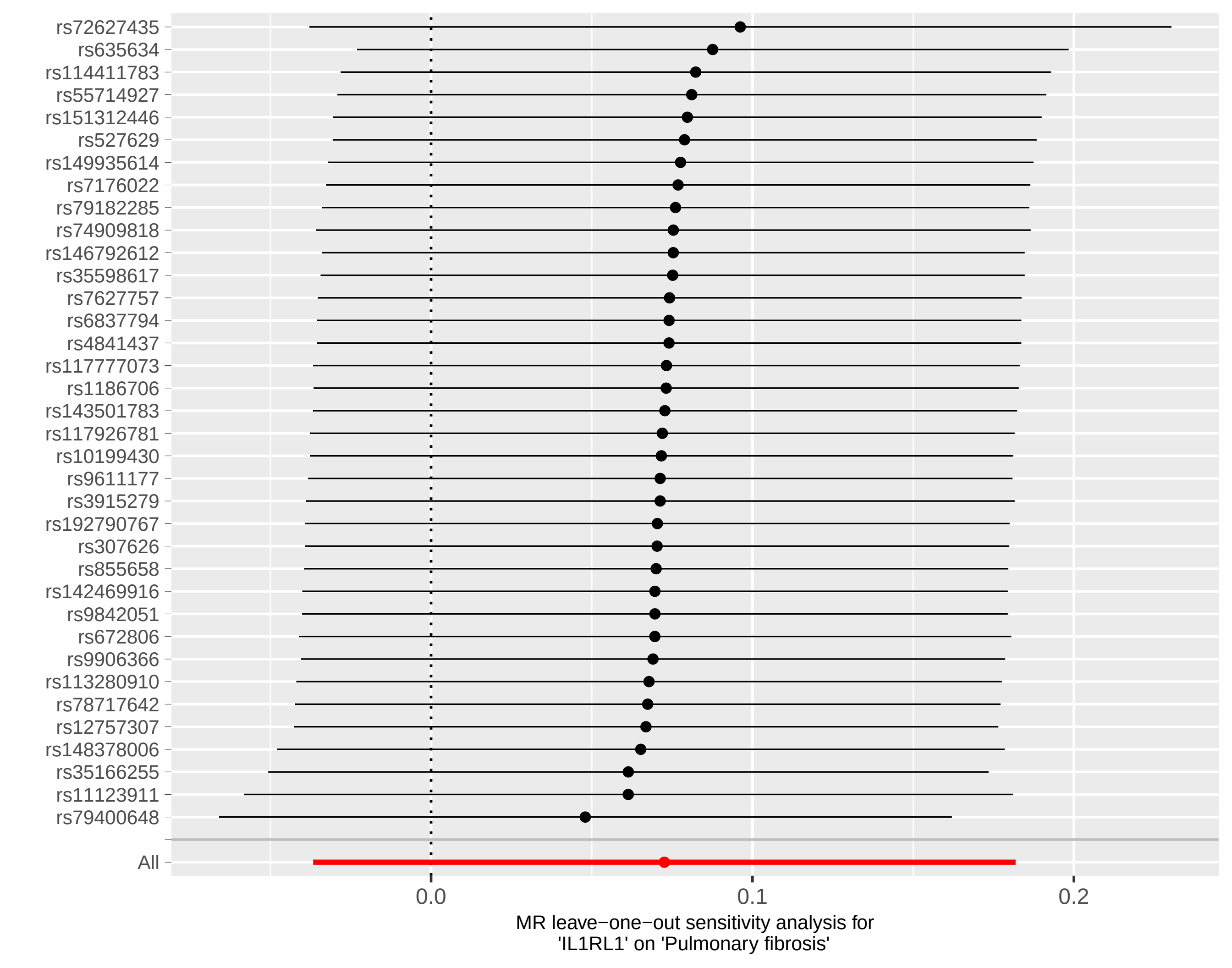
rs72627435
rs635634
rs114411783
rs55714927
rs151312446
rs527629
rs149935614
rs7176022
rs79182285
rs74909818
rs146792612
rs35598617
rs7627757
rs6837794
rs4841437
rs117777073
rs1186706
rs143501783
rs117926781
rs10199430
rs9611177
rs3915279
rs192790767
rs307626
rs855658
rs142469916
rs9842051
rs672806
rs9906366
rs113280910
rs78717642
rs12757307
rs148378006
rs35166255
rs11123911
rs79400648
All
0.0
0.1
0.2
MR leave−one−out sensitivity analysis for
'IL1RL1' on 'Pulmonary fibrosis'

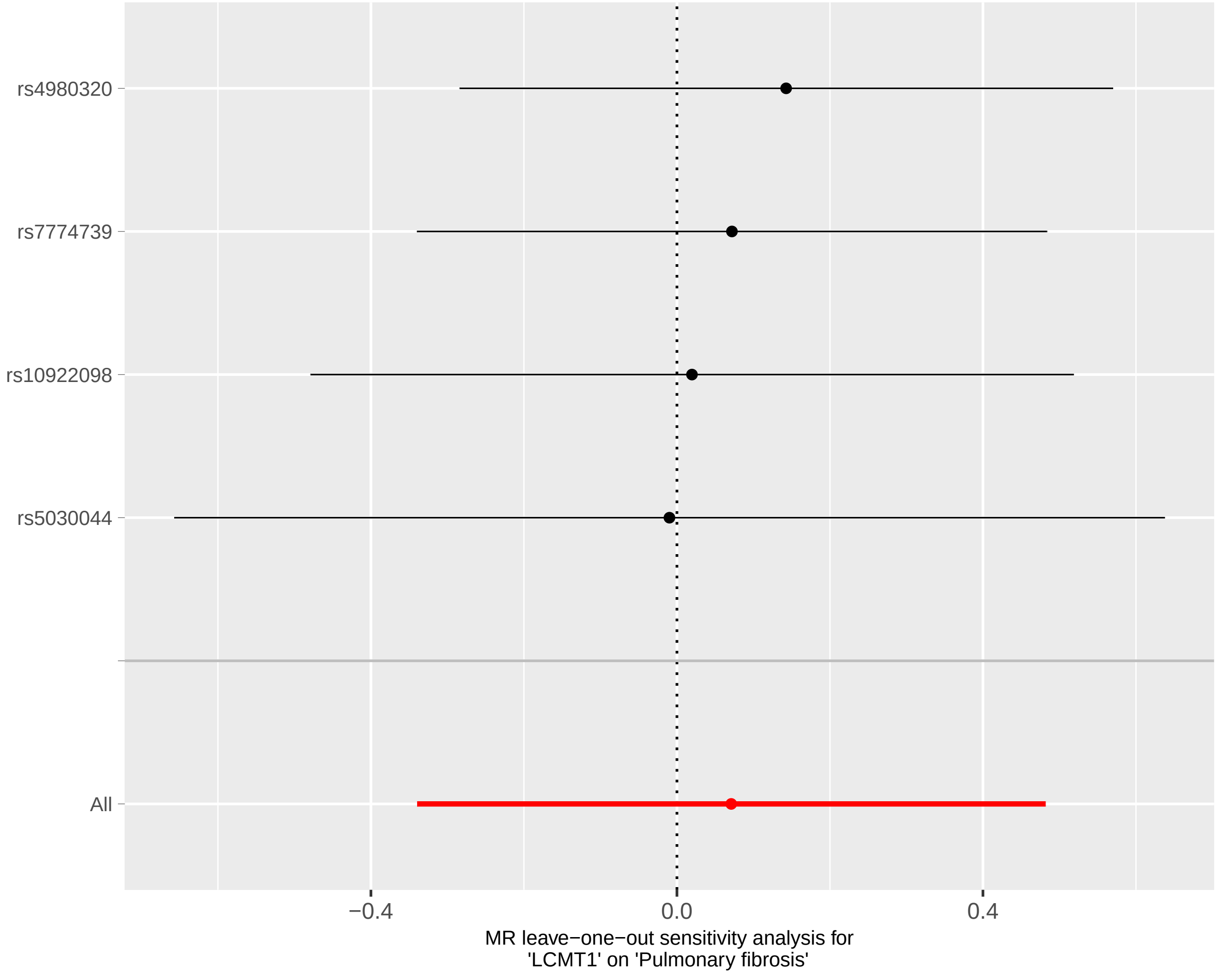
rs4980320
rs7774739
rs10922098
rs5030044
All
−0.4
0.0
0.4
MR leave−one−out sensitivity analysis for
'LCMT1' on 'Pulmonary fibrosis'

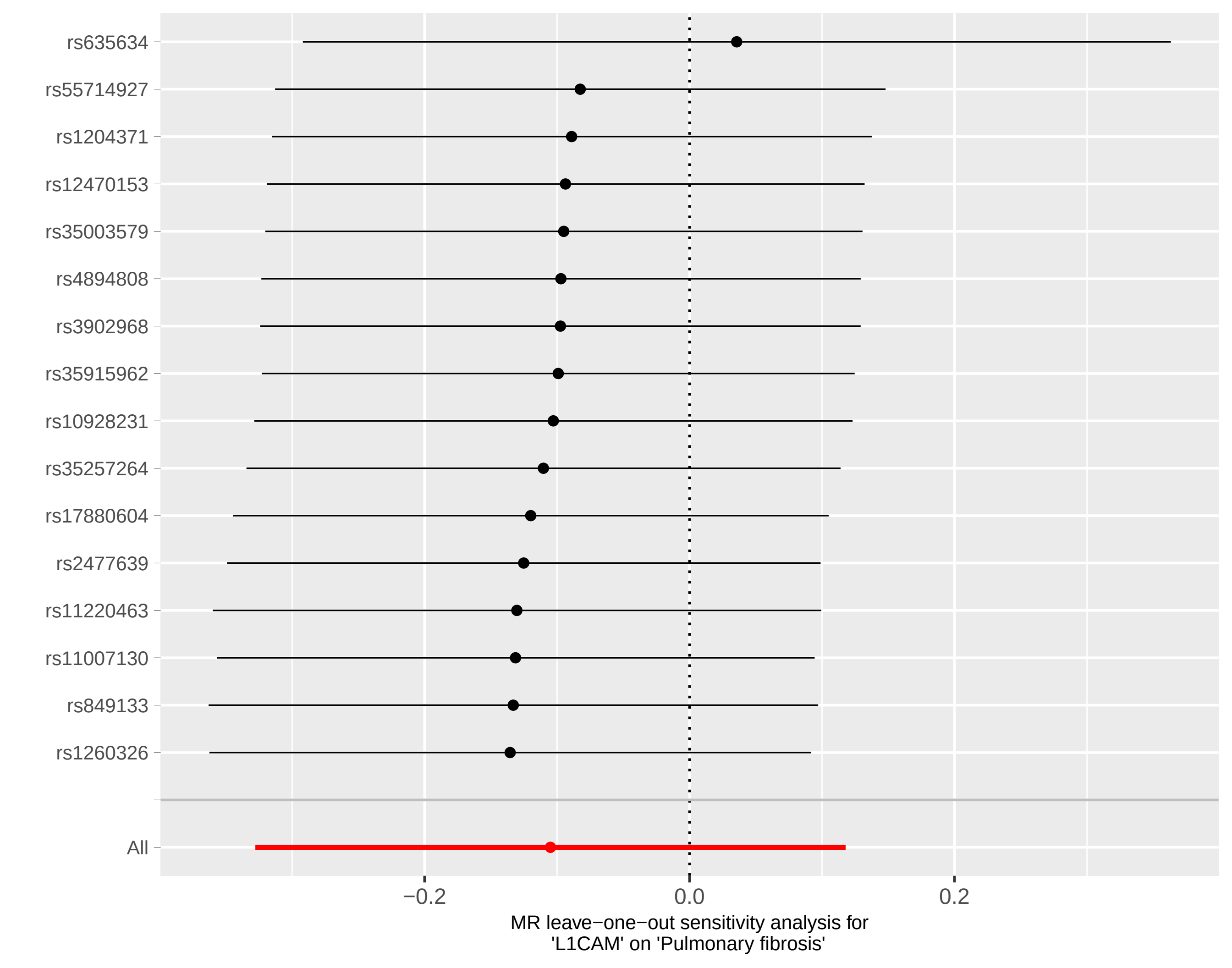

rs635634
rs55714927
rs1204371
rs12470153
rs35003579
rs4894808
rs3902968
rs35915962
rs10928231
rs35257264
rs17880604
rs2477639
rs11220463
rs11007130
rs849133
rs1260326
All
−0.2
0.0
0.2
MR leave−one−out sensitivity analysis for
'L1CAM' on 'Pulmonary fibrosis'

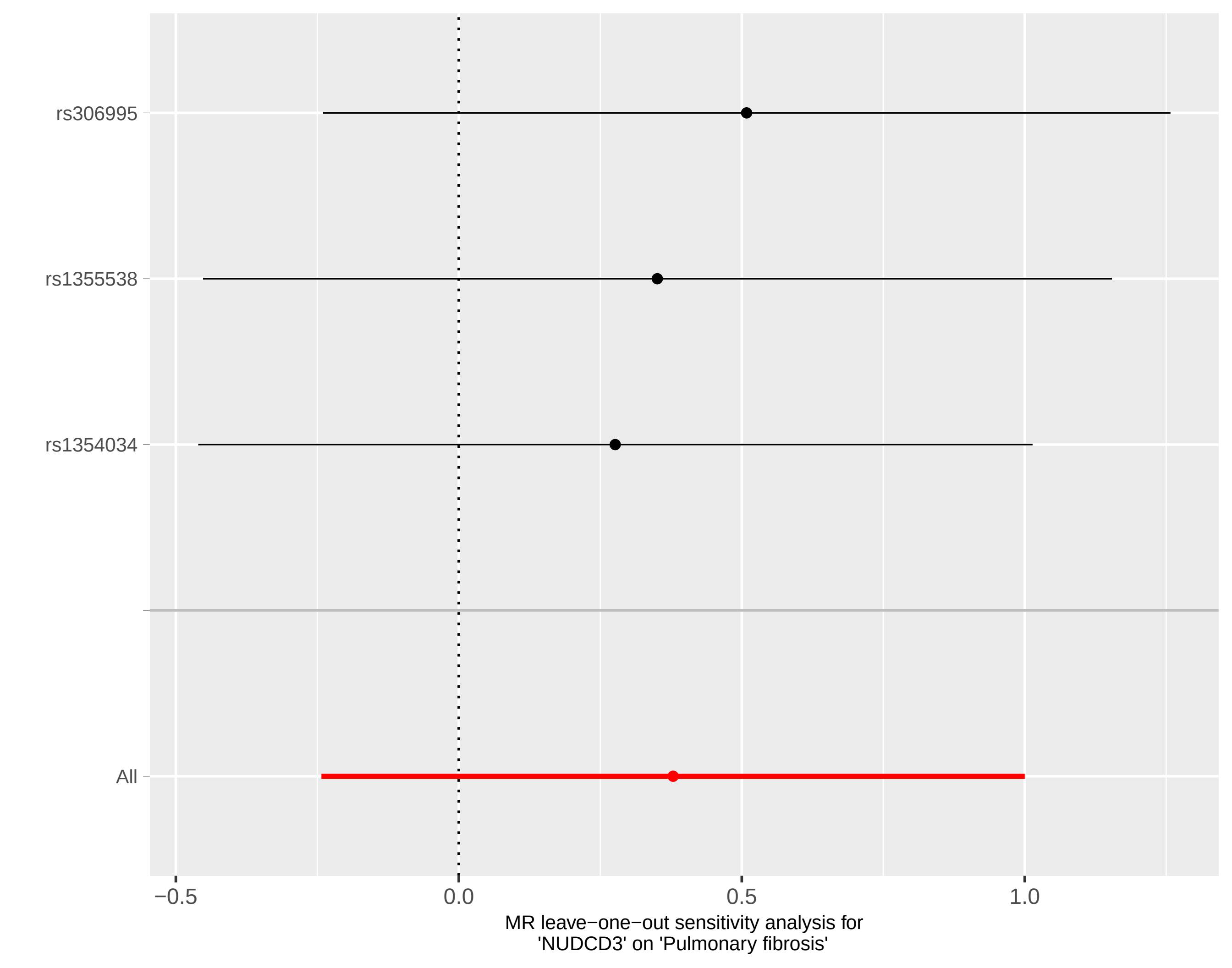
rs306995
rs1355538
rs1354034
All
−0.5
0.0
0.5
1.0
MR leave−one−out sensitivity analysis for
'NUDCD3' on 'Pulmonary fibrosis'

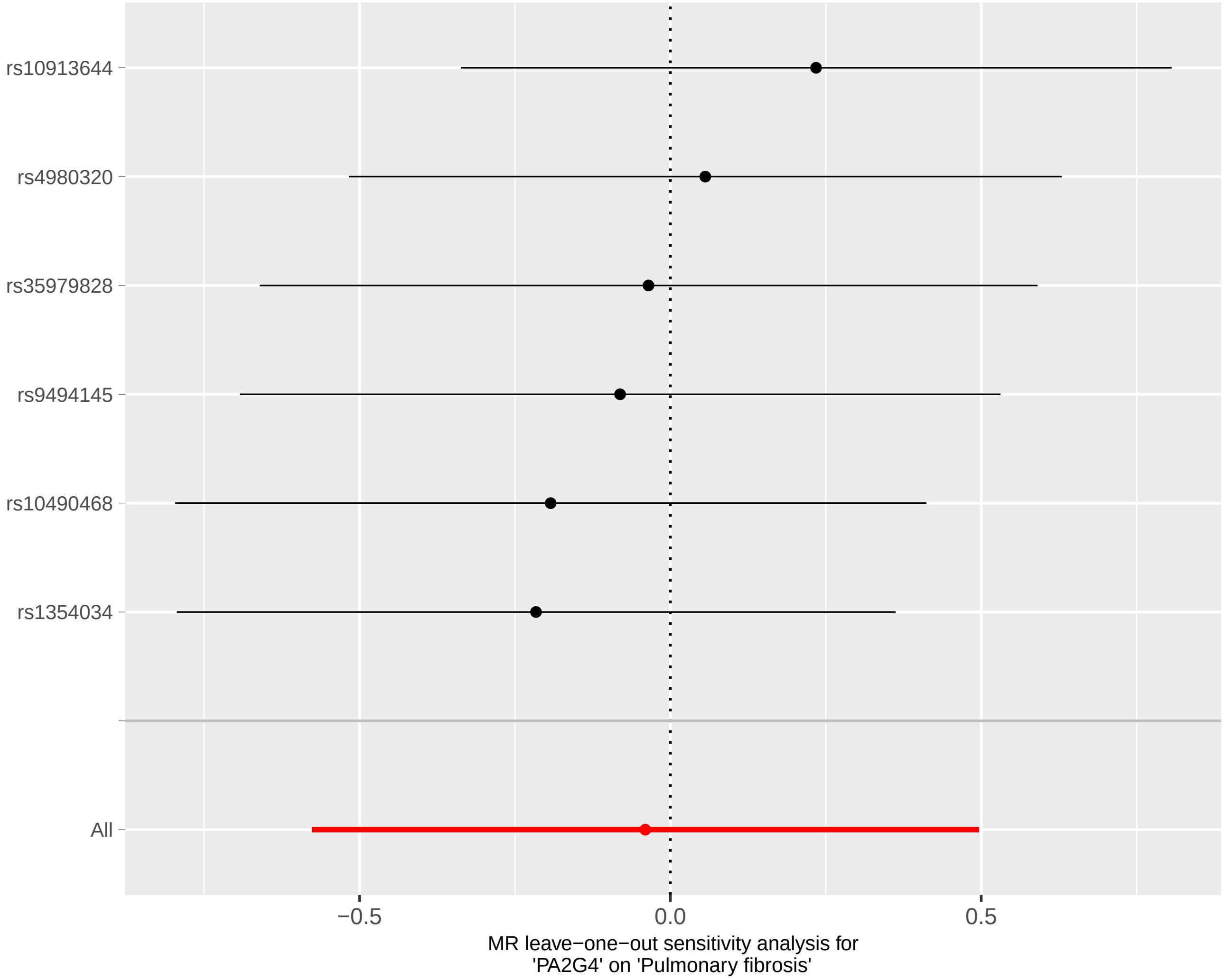

rs10913644
rs4980320
rs35979828
rs9494145
rs10490468
rs1354034
All
−0.5
0.0
0.5
MR leave−one−out sensitivity analysis for
'PA2G4' on 'Pulmonary fibrosis'

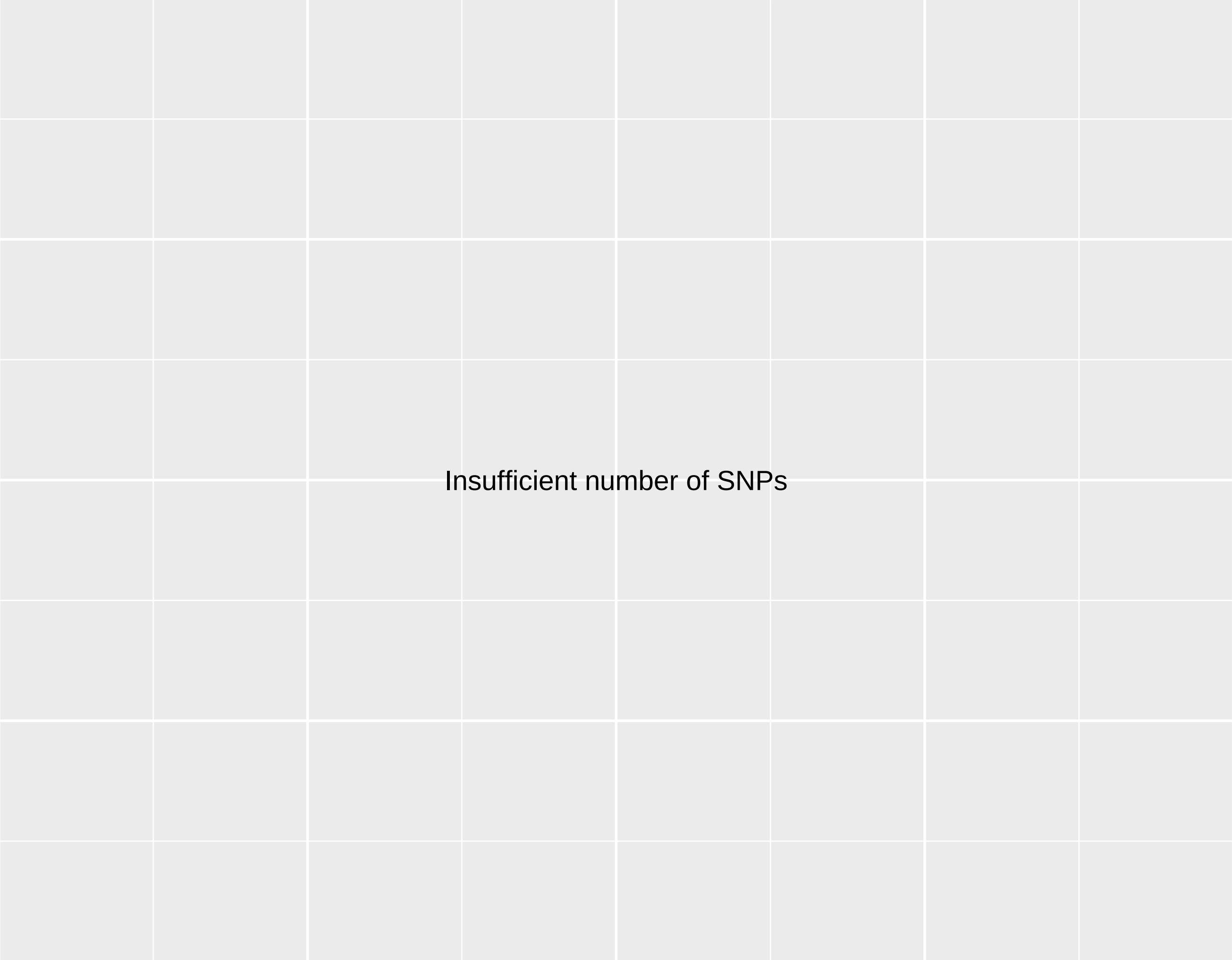
Insufficient number of SNPs

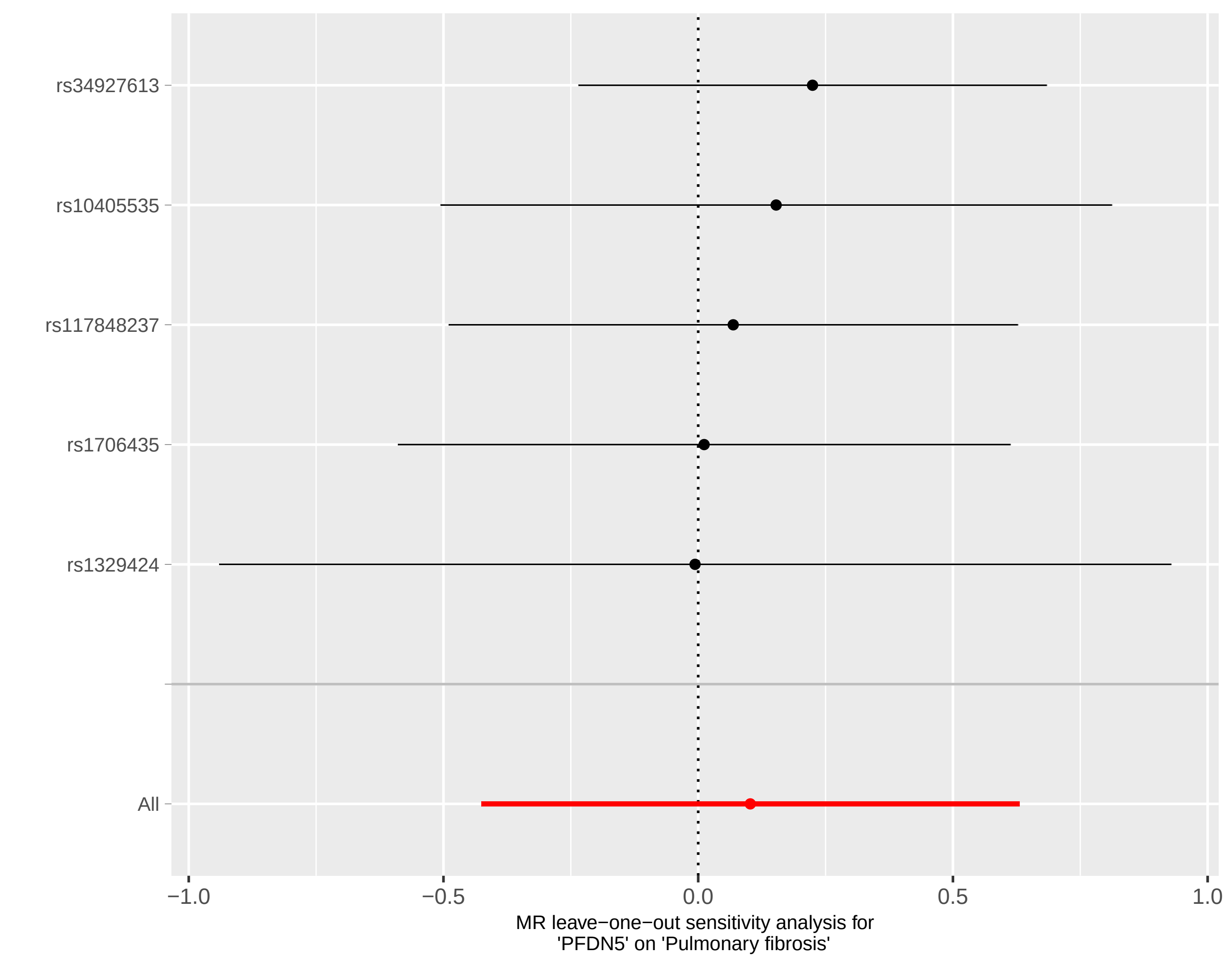

rs34927613
rs10405535
rs117848237
rs1706435
rs1329424
All
−1.0
−0.5
0.0
0.5
1.0
MR leave−one−out sensitivity analysis for
'PFDN5' on 'Pulmonary fibrosis'

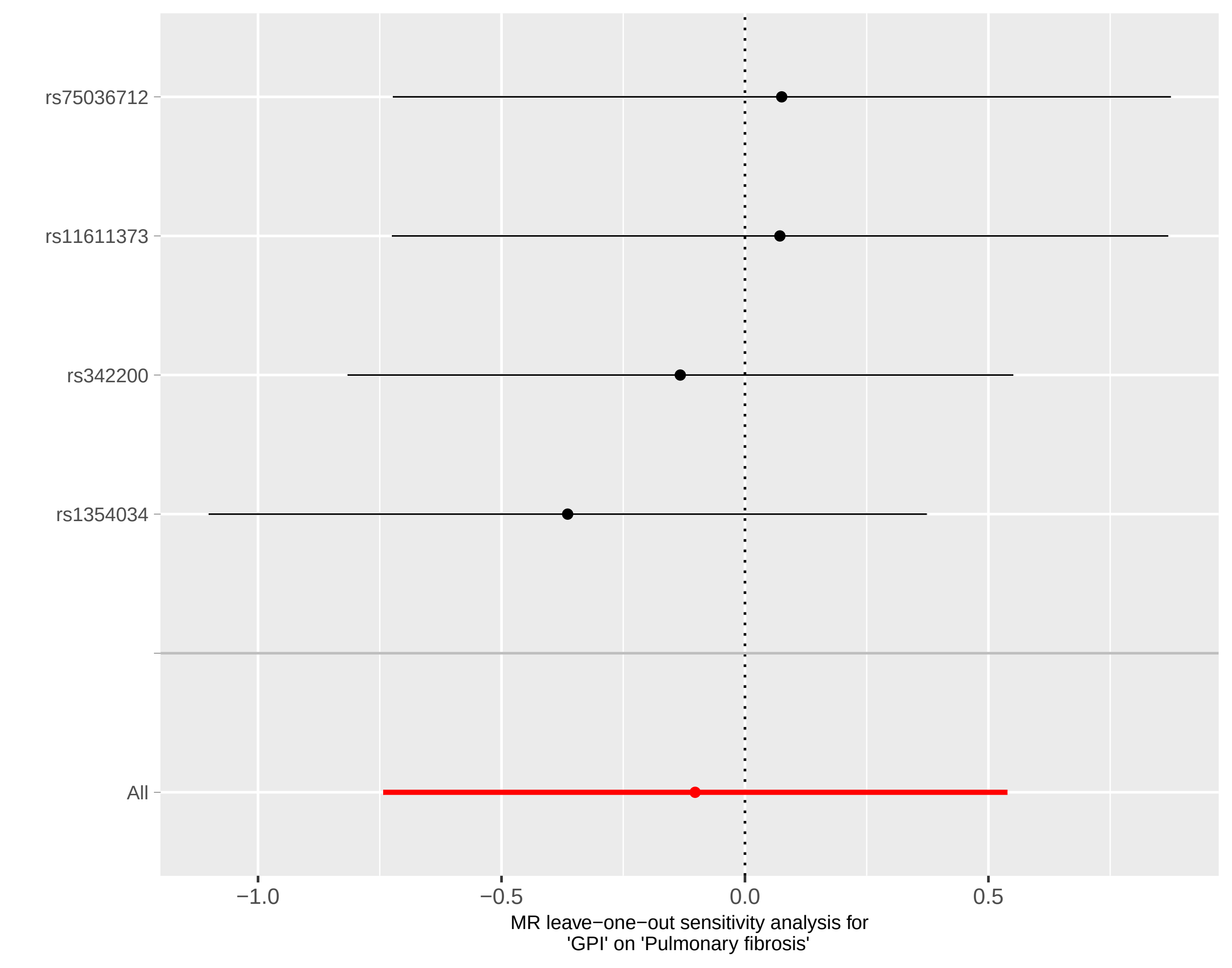
rs75036712
rs11611373
rs342200
rs1354034
All
−1.0
−0.5
0.0
0.5
MR leave−one−out sensitivity analysis for
'GPI' on 'Pulmonary fibrosis'

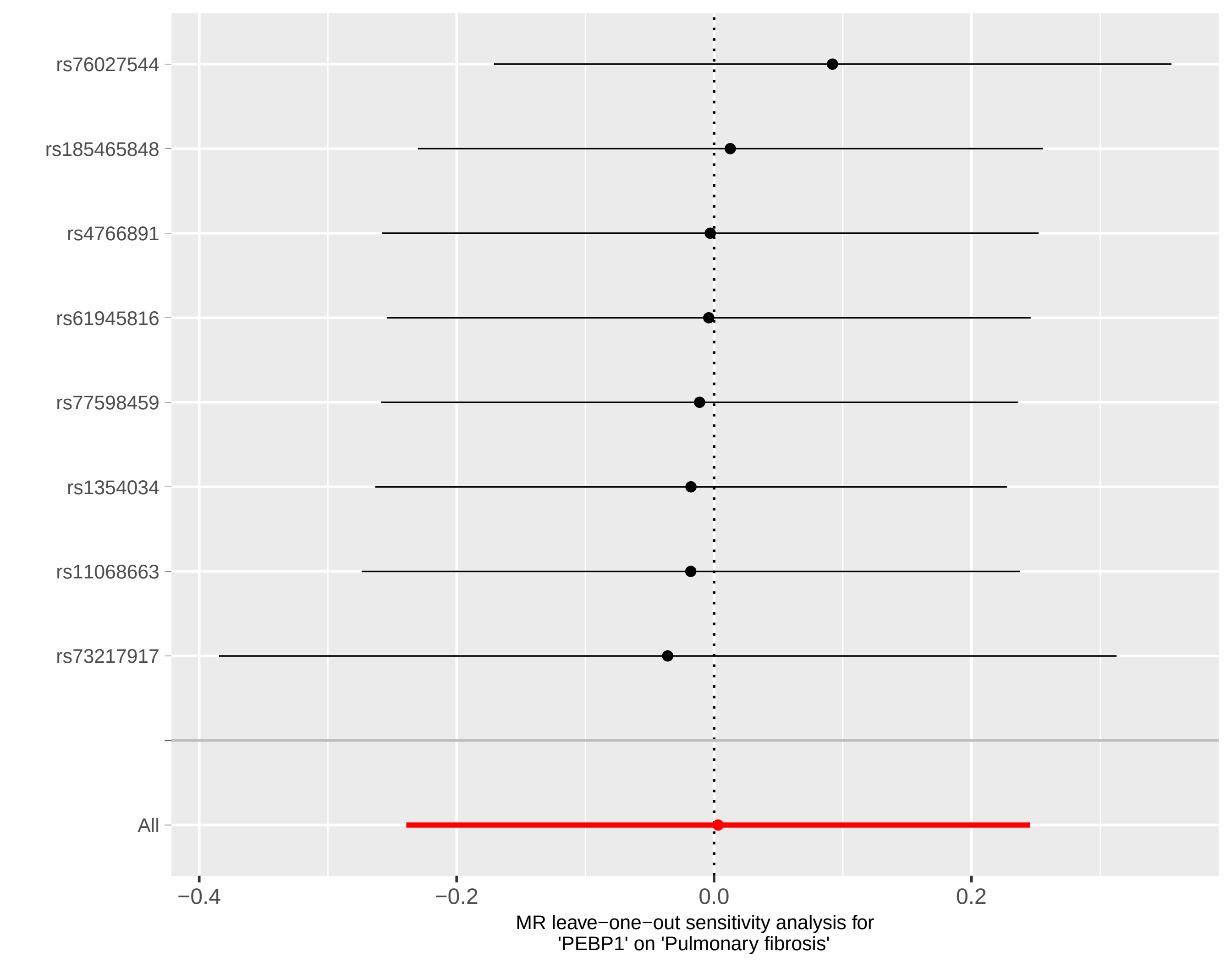

rs76027544
rs185465848
rs4766891
rs61945816
rs77598459
rs1354034
rs11068663
rs73217917
All
−0.4
−0.2
0.0
0.2
MR leave−one−out sensitivity analysis for
'PEBP1' on 'Pulmonary fibrosis'

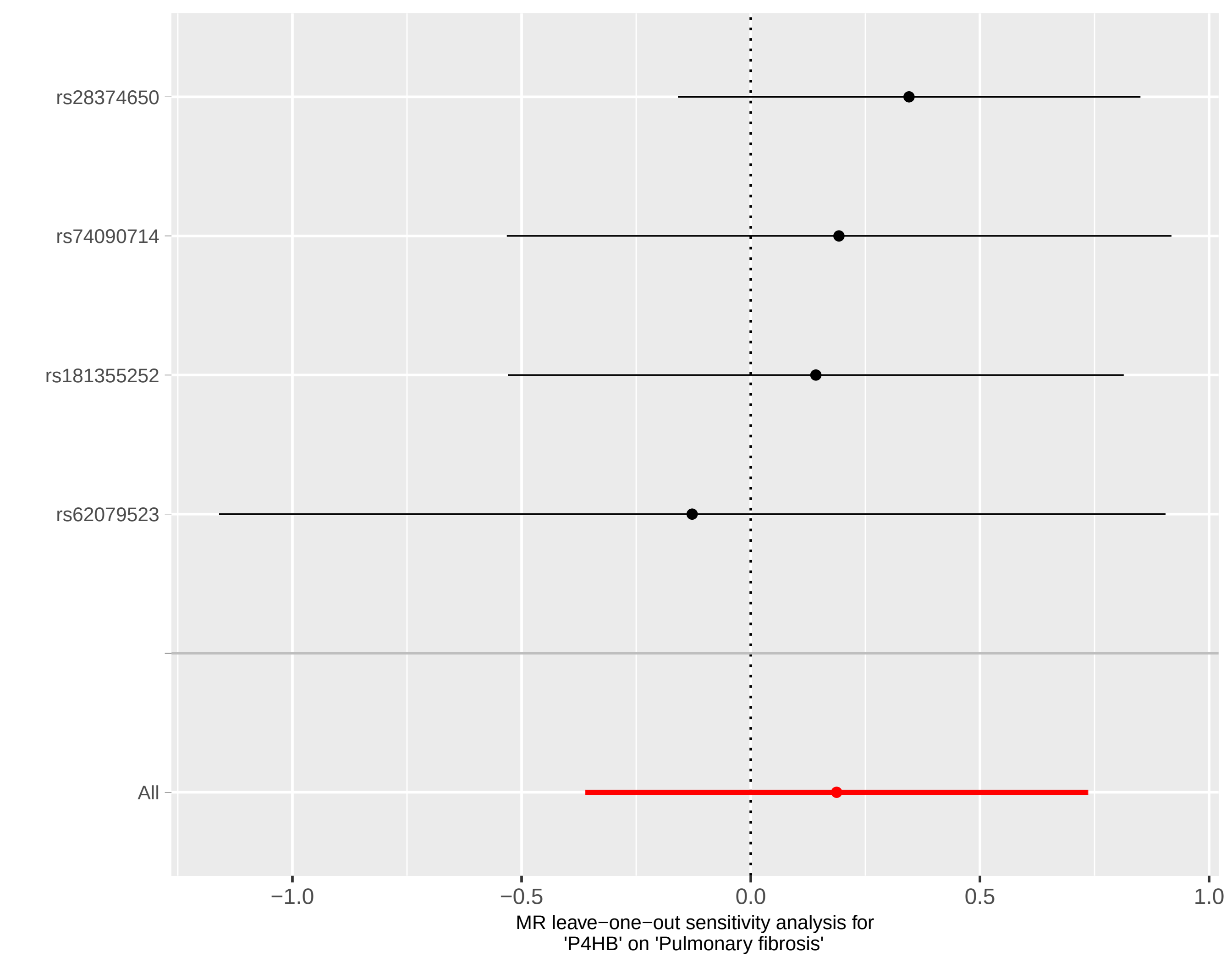
rs28374650
rs74090714
rs181355252
rs62079523
All
−1.0
−0.5
0.0
0.5
1.0
MR leave−one−out sensitivity analysis for
'P4HB' on 'Pulmonary fibrosis'

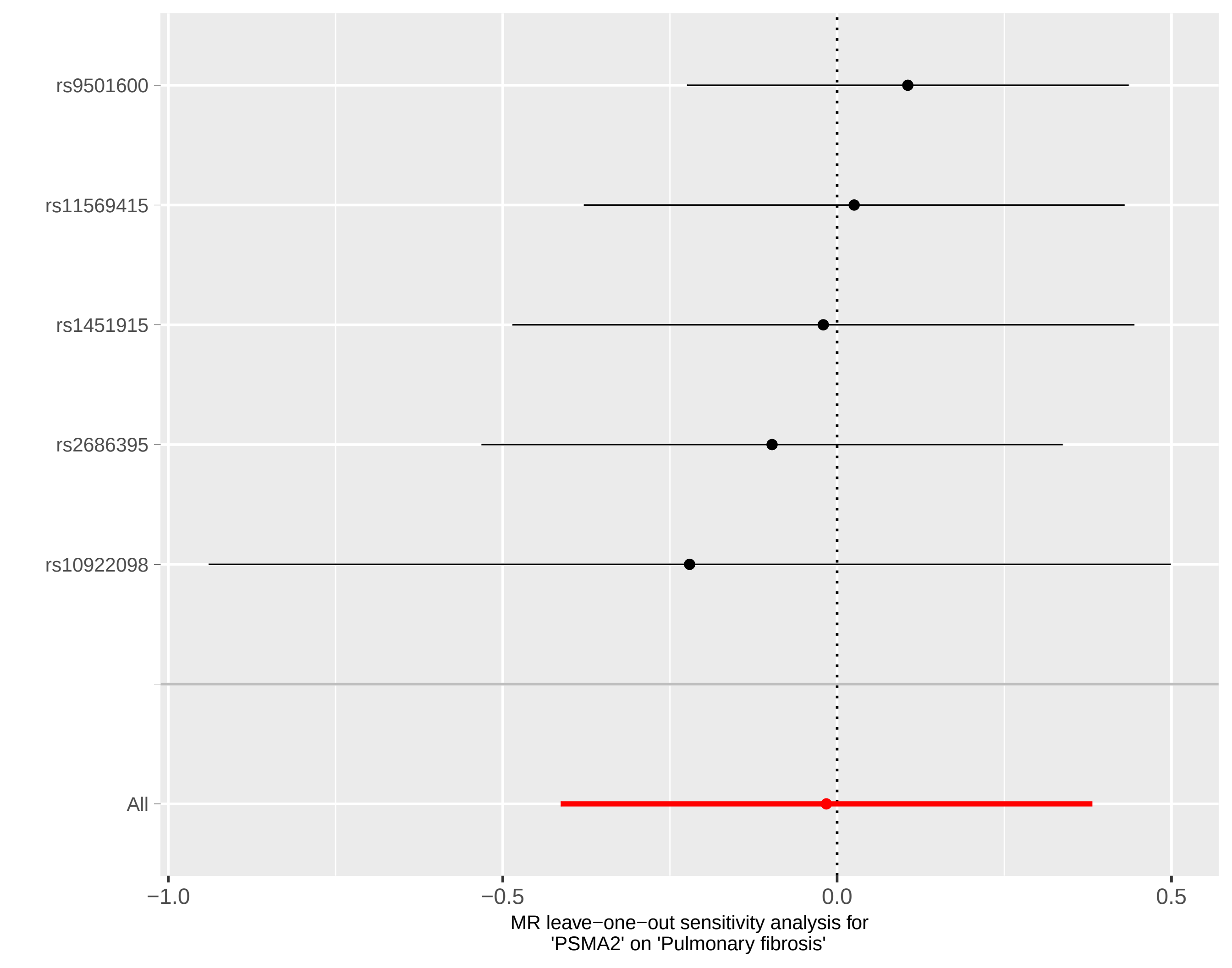
rs9501600
rs11569415
rs1451915
rs2686395
rs10922098
All
−1.0
−0.5
0.0
0.5
MR leave−one−out sensitivity analysis for
'PSMA2' on 'Pulmonary fibrosis'

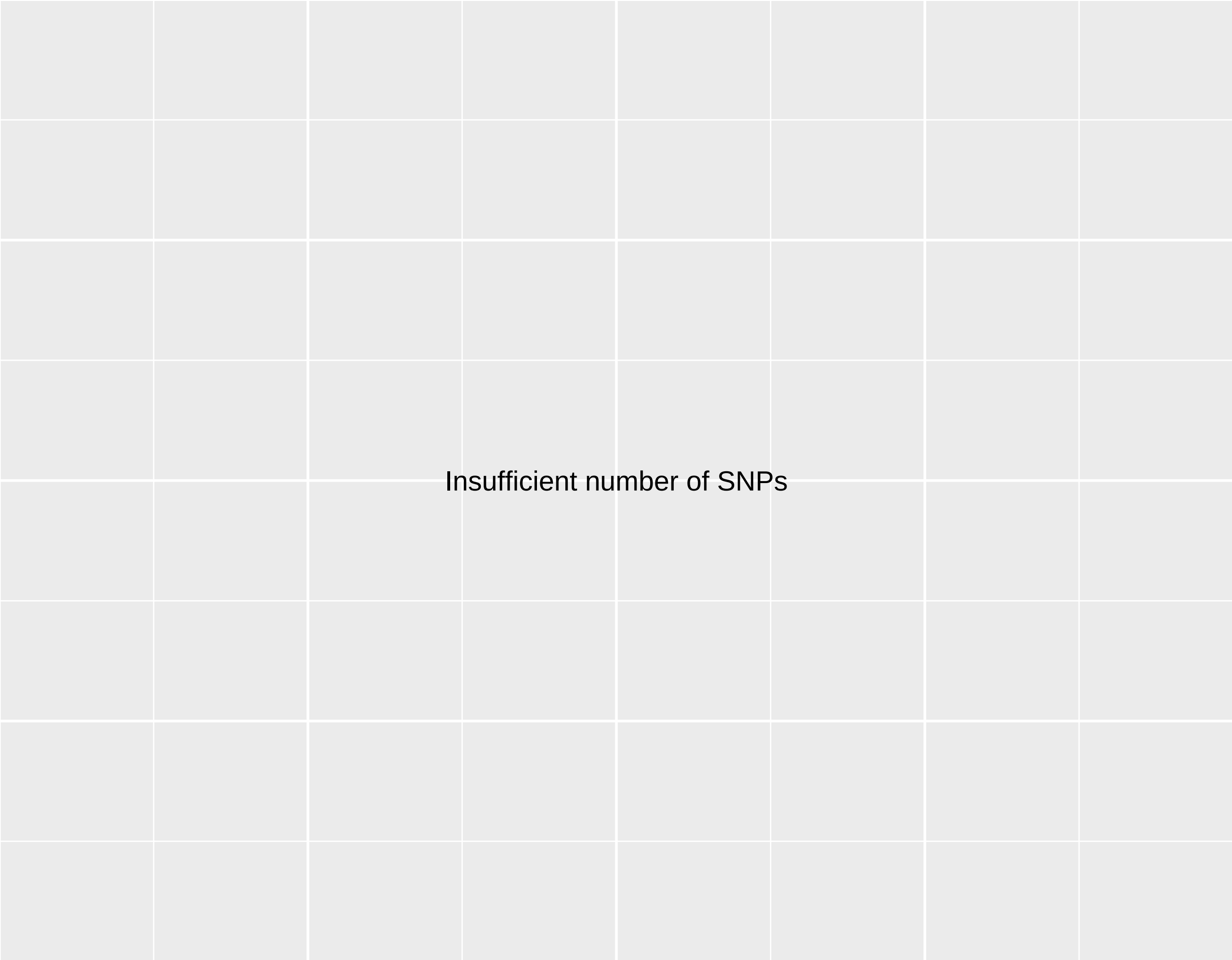
Insufficient number of SNPs

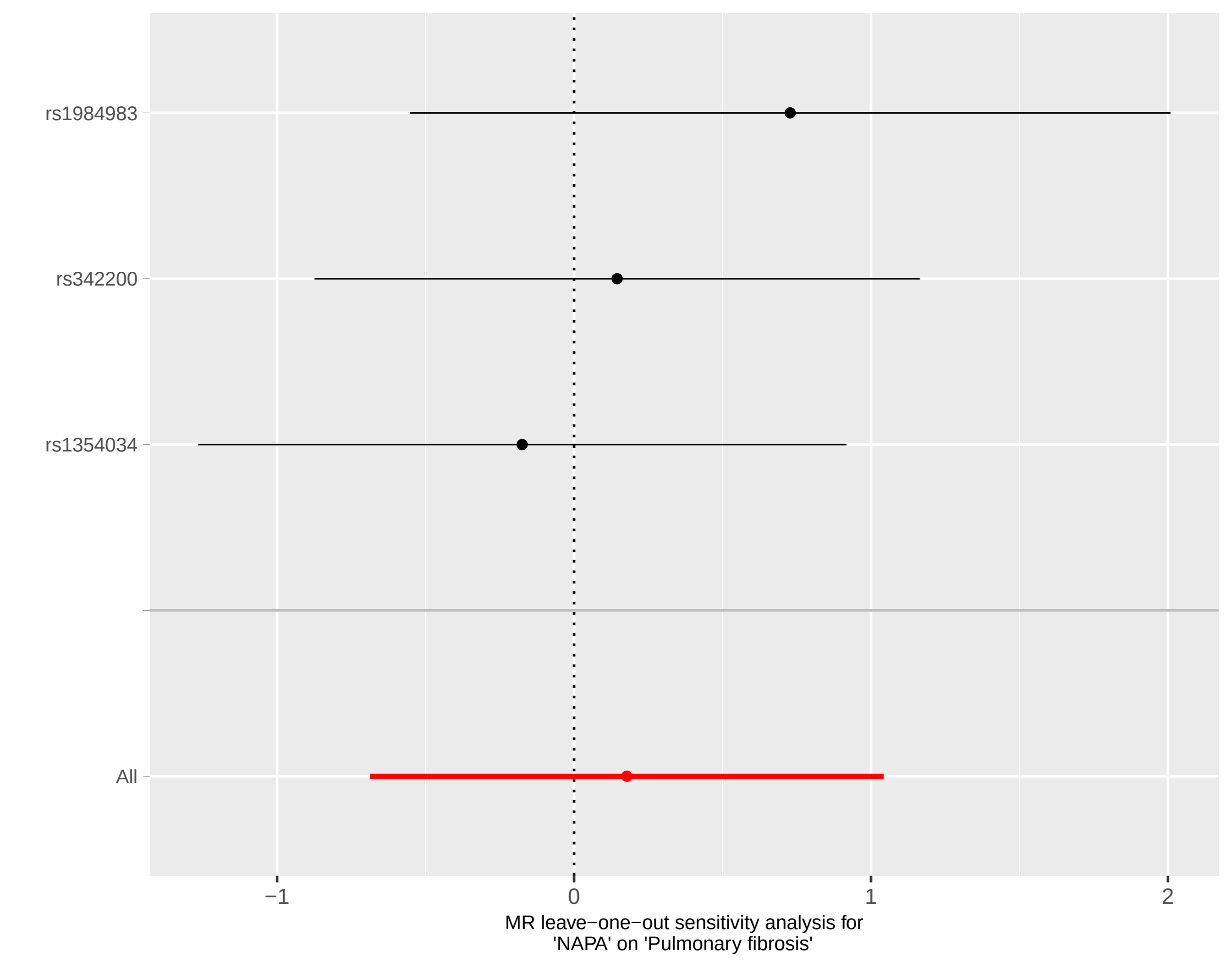
rs1984983
rs342200
rs1354034
All
−1
0
1
2
MR leave−one−out sensitivity analysis for
'NAPA' on 'Pulmonary fibrosis'

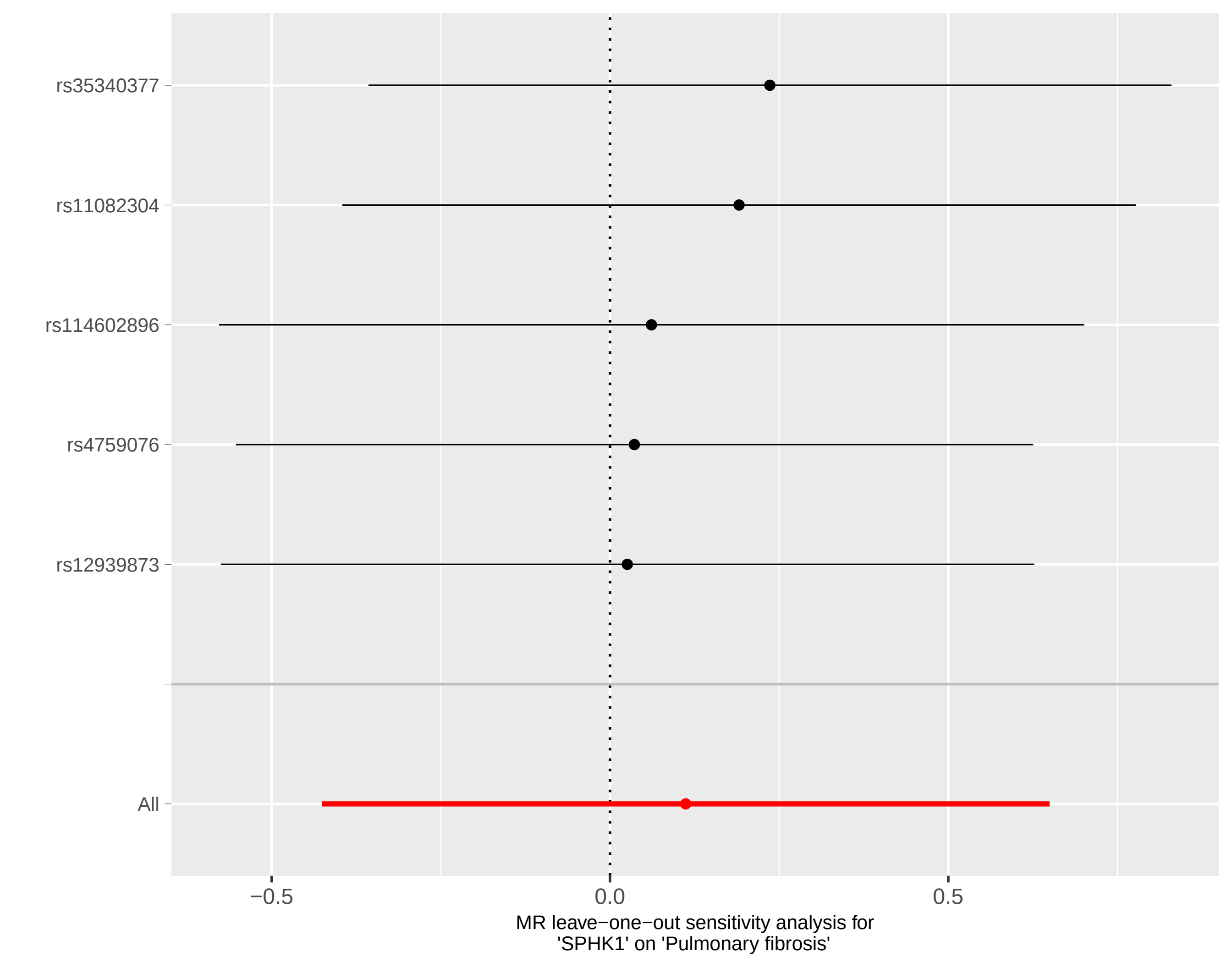
rs35340377
rs11082304
rs114602896
rs4759076
rs12939873
All
−0.5
0.0
0.5
MR leave−one−out sensitivity analysis for
'SPHK1' on 'Pulmonary fibrosis'

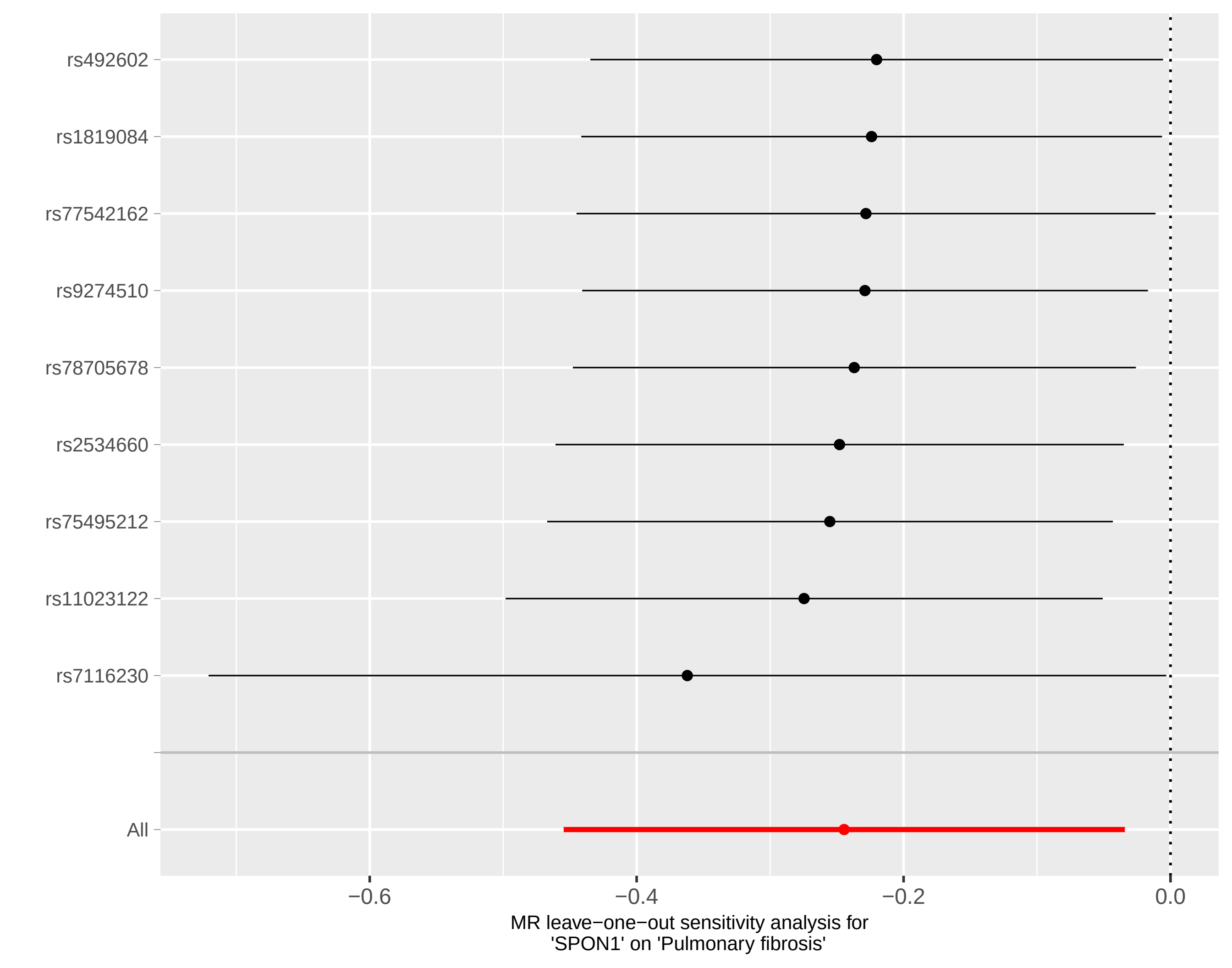
rs492602
rs1819084
rs77542162
rs9274510
rs78705678
rs2534660
rs75495212
rs11023122
rs7116230
All
−0.6
−0.4
−0.2
0.0
MR leave−one−out sensitivity analysis for
'SPON1' on 'Pulmonary fibrosis'

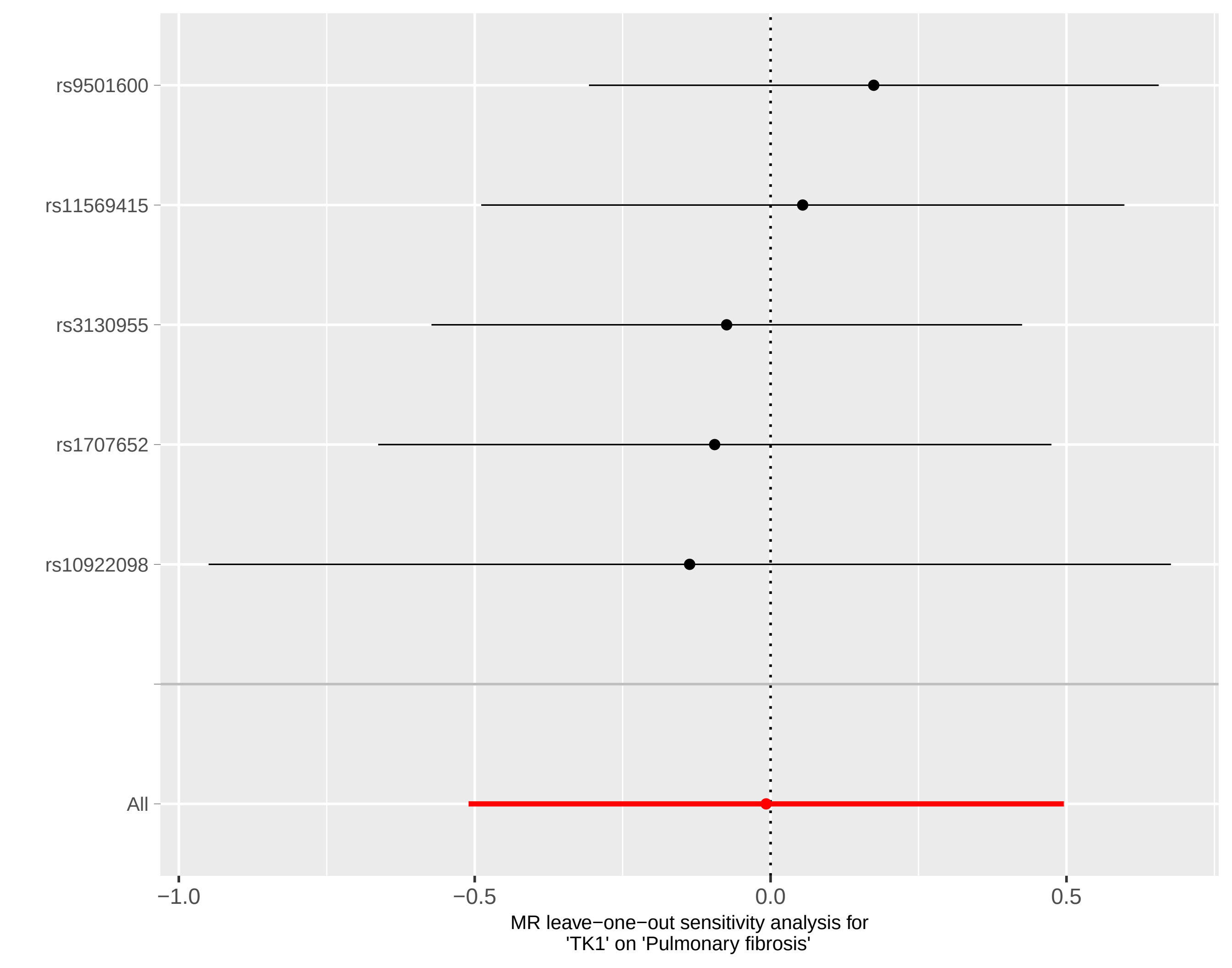

rs9501600
rs11569415
rs3130955
rs1707652
rs10922098
All
−1.0
−0.5
0.0
0.5
MR leave−one−out sensitivity analysis for
'TK1' on 'Pulmonary fibrosis'

Insufficient number of SNPs

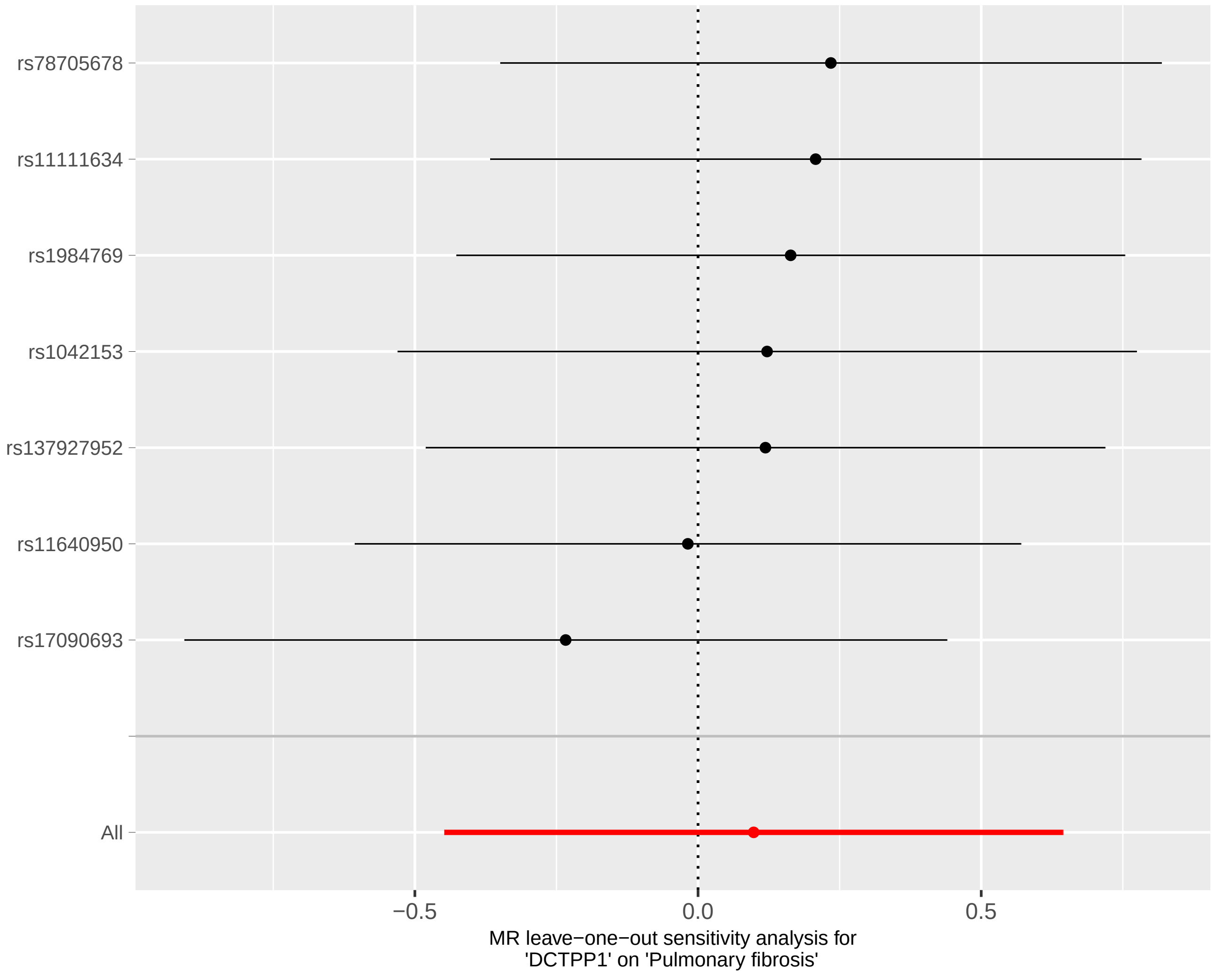
rs78705678
rs11111634
rs1984769
rs1042153
rs137927952
rs11640950
rs17090693
All
−0.5
0.0
0.5
MR leave−one−out sensitivity analysis for
'DCTPP1' on 'Pulmonary fibrosis'

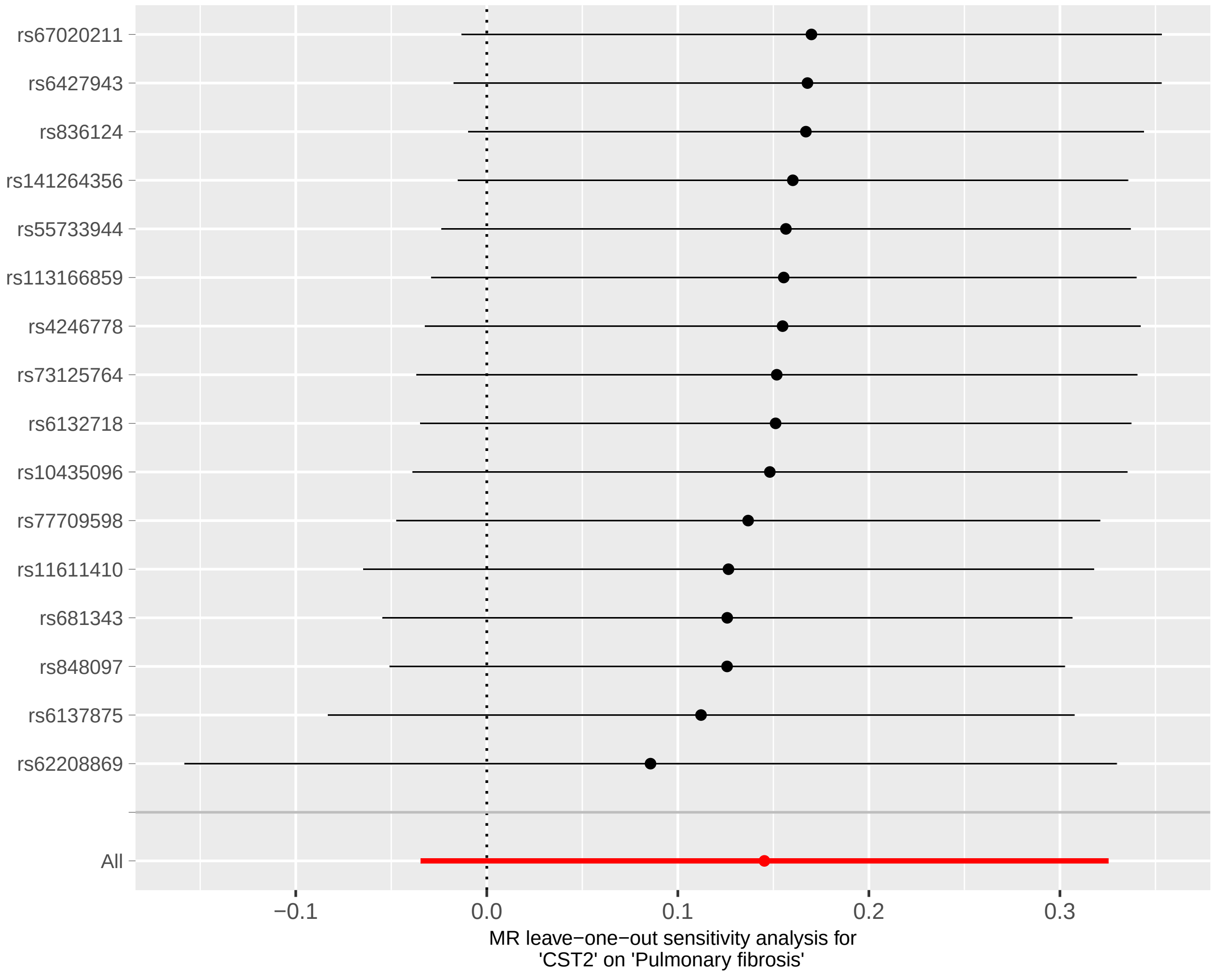
rs67020211
rs6427943
rs836124
rs141264356
rs55733944
rs113166859
rs4246778
rs73125764
rs6132718
rs10435096
rs77709598
rs11611410
rs681343
rs848097
rs6137875
rs62208869
All
−0.1
0.0
0.1
0.2
0.3
MR leave−one−out sensitivity analysis for
'CST2' on 'Pulmonary fibrosis'

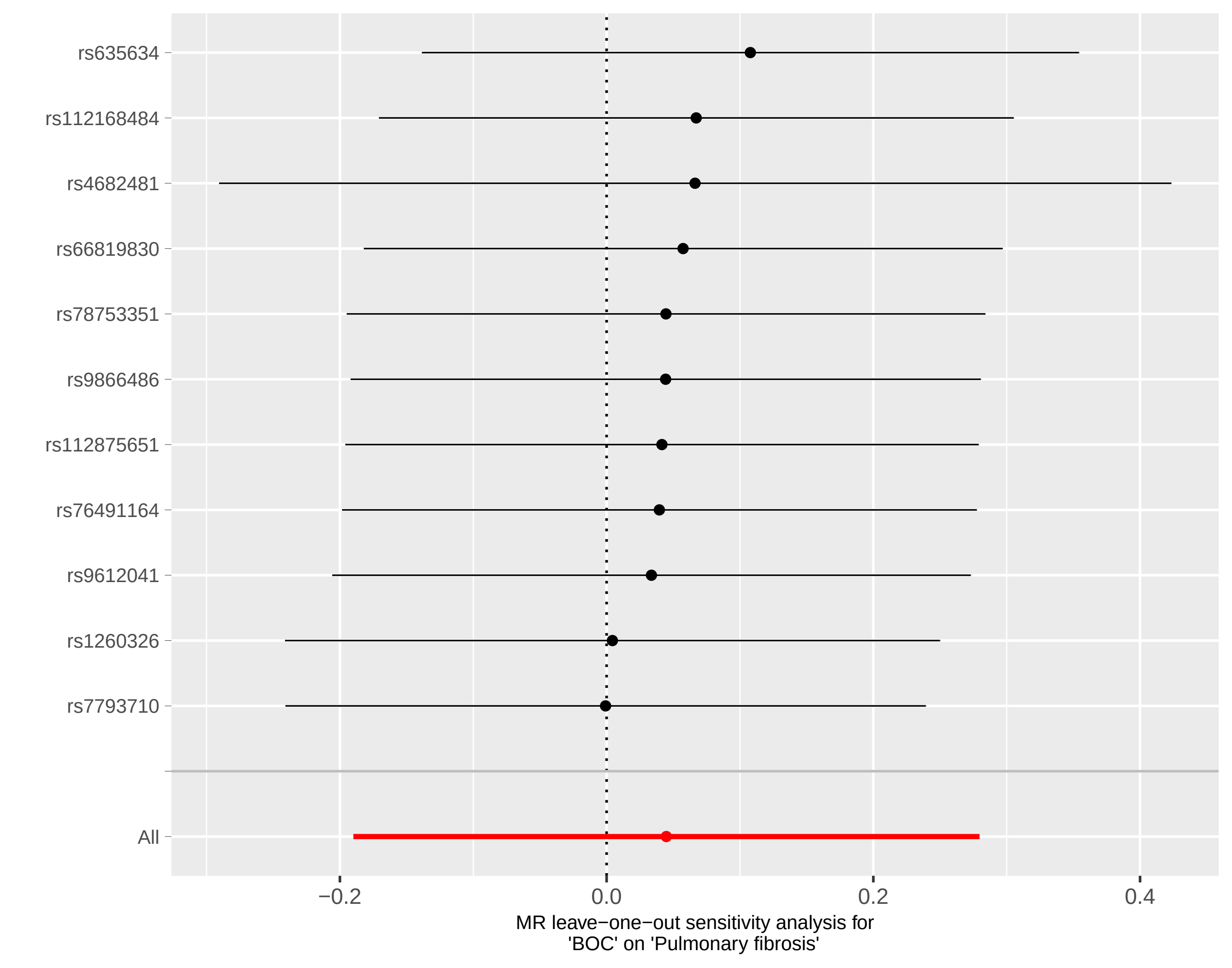
rs635634
rs112168484
rs4682481
rs66819830
rs78753351
rs9866486
rs112875651
rs76491164
rs9612041
rs1260326
rs7793710
All
−0.2
0.0
0.2
0.4
MR leave−one−out sensitivity analysis for
'BOC' on 'Pulmonary fibrosis'

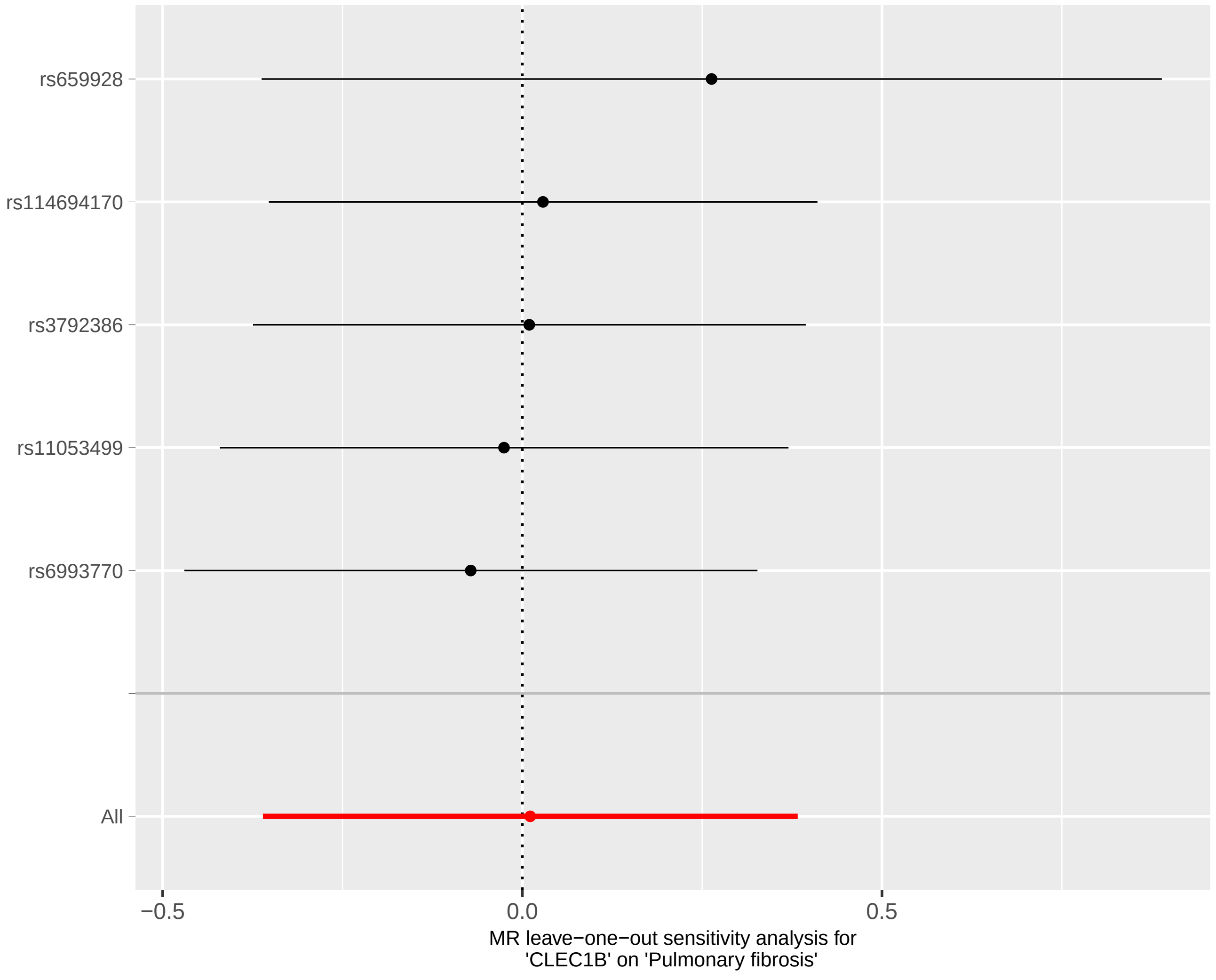
rs659928
rs114694170
rs3792386
rs11053499
rs6993770
All
−0.5
0.0
0.5
MR leave−one−out sensitivity analysis for
'CLEC1B' on 'Pulmonary fibrosis'

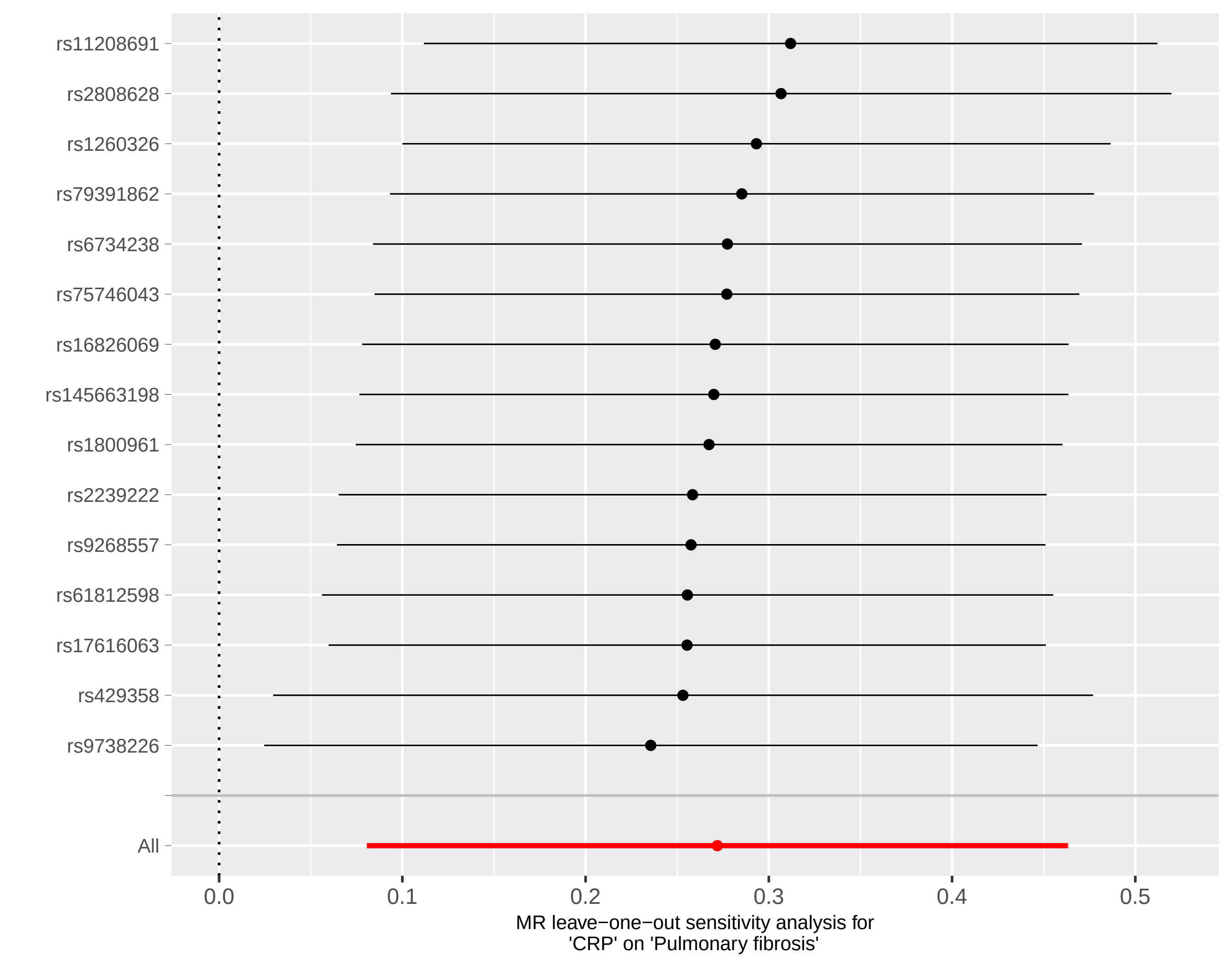

rs11208691
rs2808628
rs1260326
rs79391862
rs6734238
rs75746043
rs16826069
rs145663198
rs1800961
rs2239222
rs9268557
rs61812598
rs17616063
rs429358
rs9738226
All
0.0
0.1
0.2
0.3
0.4
0.5
MR leave−one−out sensitivity analysis for
'CRP' on 'Pulmonary fibrosis'

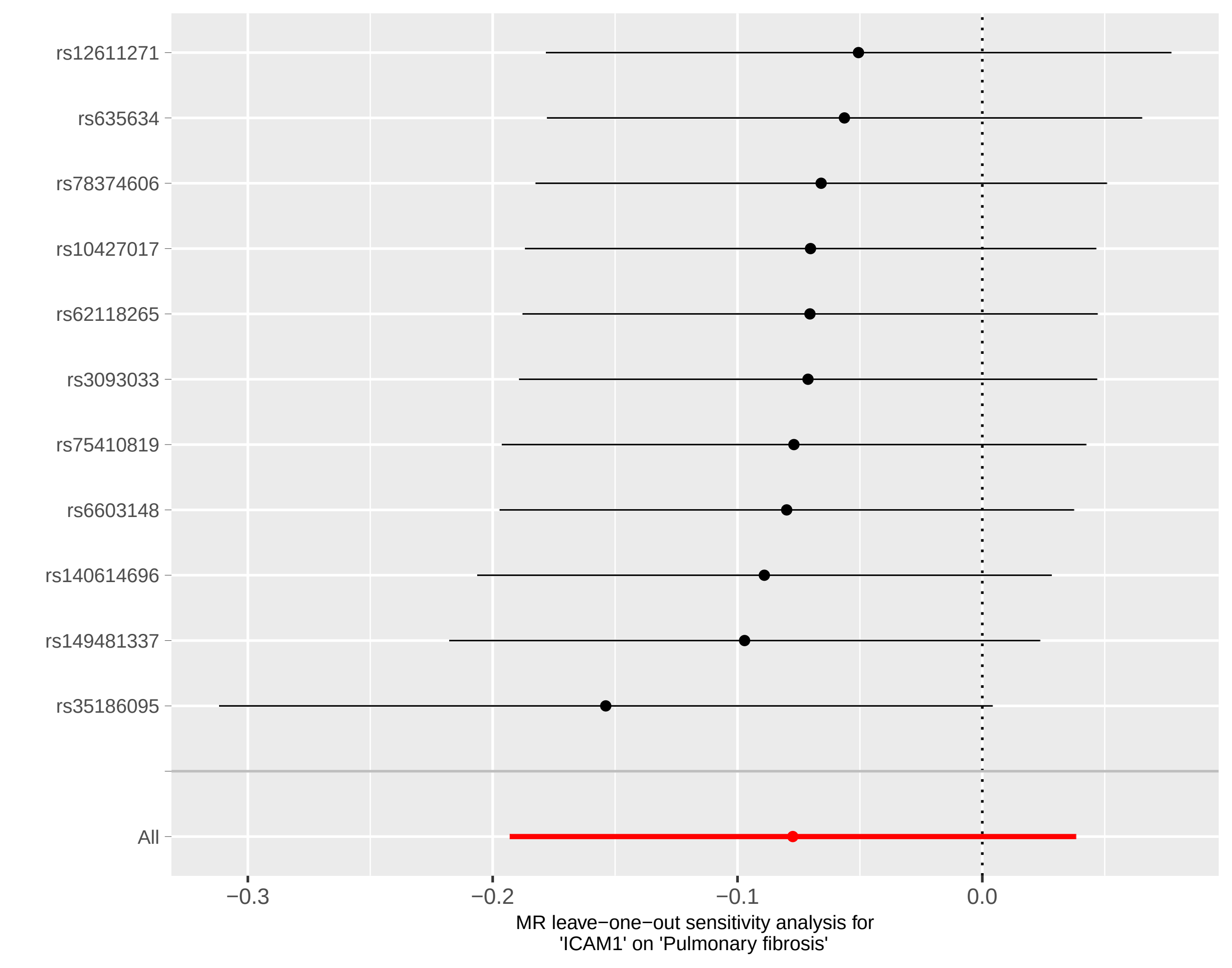
rs12611271
rs635634
rs78374606
rs10427017
rs62118265
rs3093033
rs75410819
rs6603148
rs140614696
rs149481337
rs35186095
All
−0.3
−0.2
−0.1
0.0
MR leave−one−out sensitivity analysis for
'ICAM1' on 'Pulmonary fibrosis'

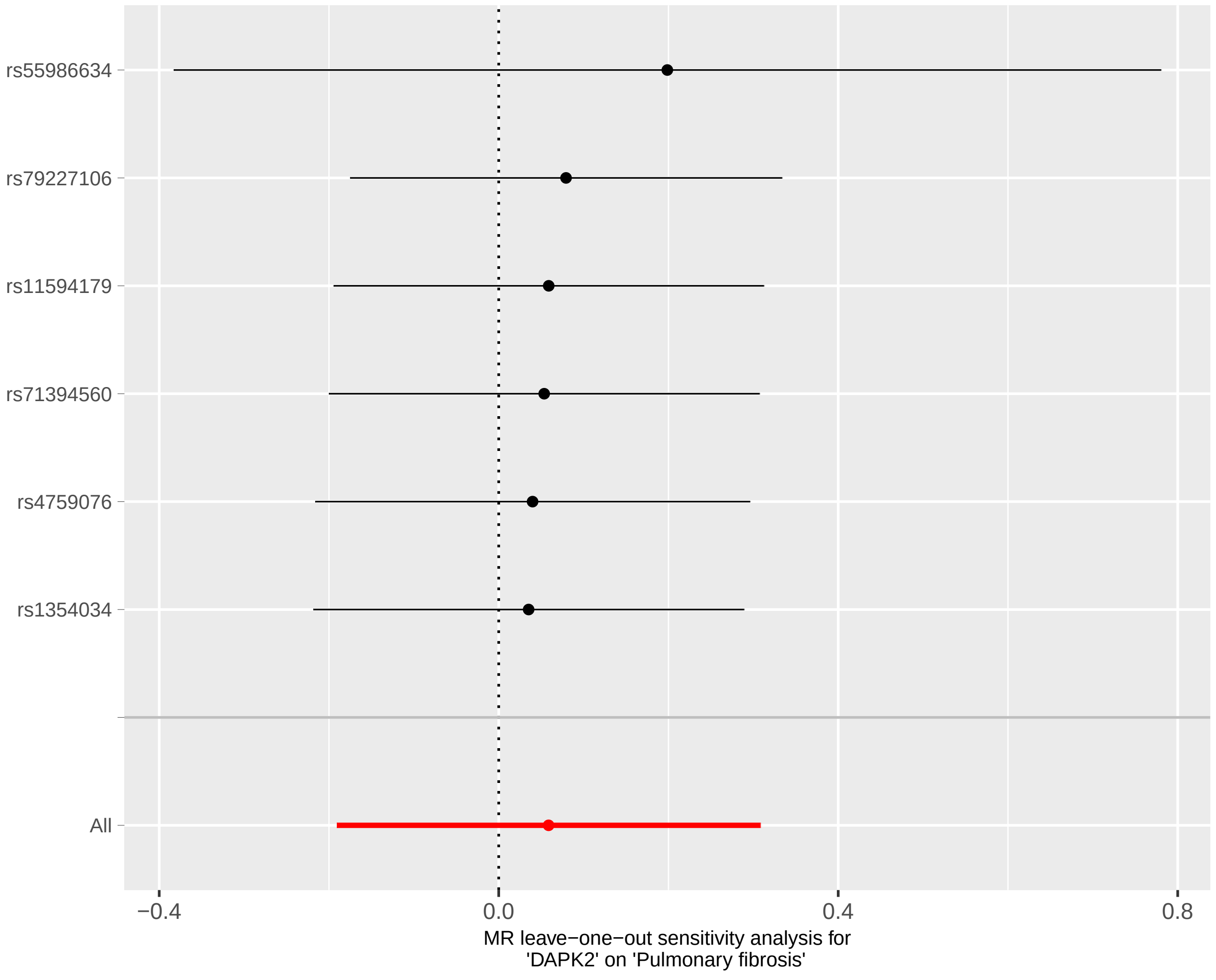
rs55986634
rs79227106
rs11594179
rs71394560
rs4759076
rs1354034
All
−0.4
0.0
0.4
0.8
MR leave−one−out sensitivity analysis for
'DAPK2' on 'Pulmonary fibrosis'

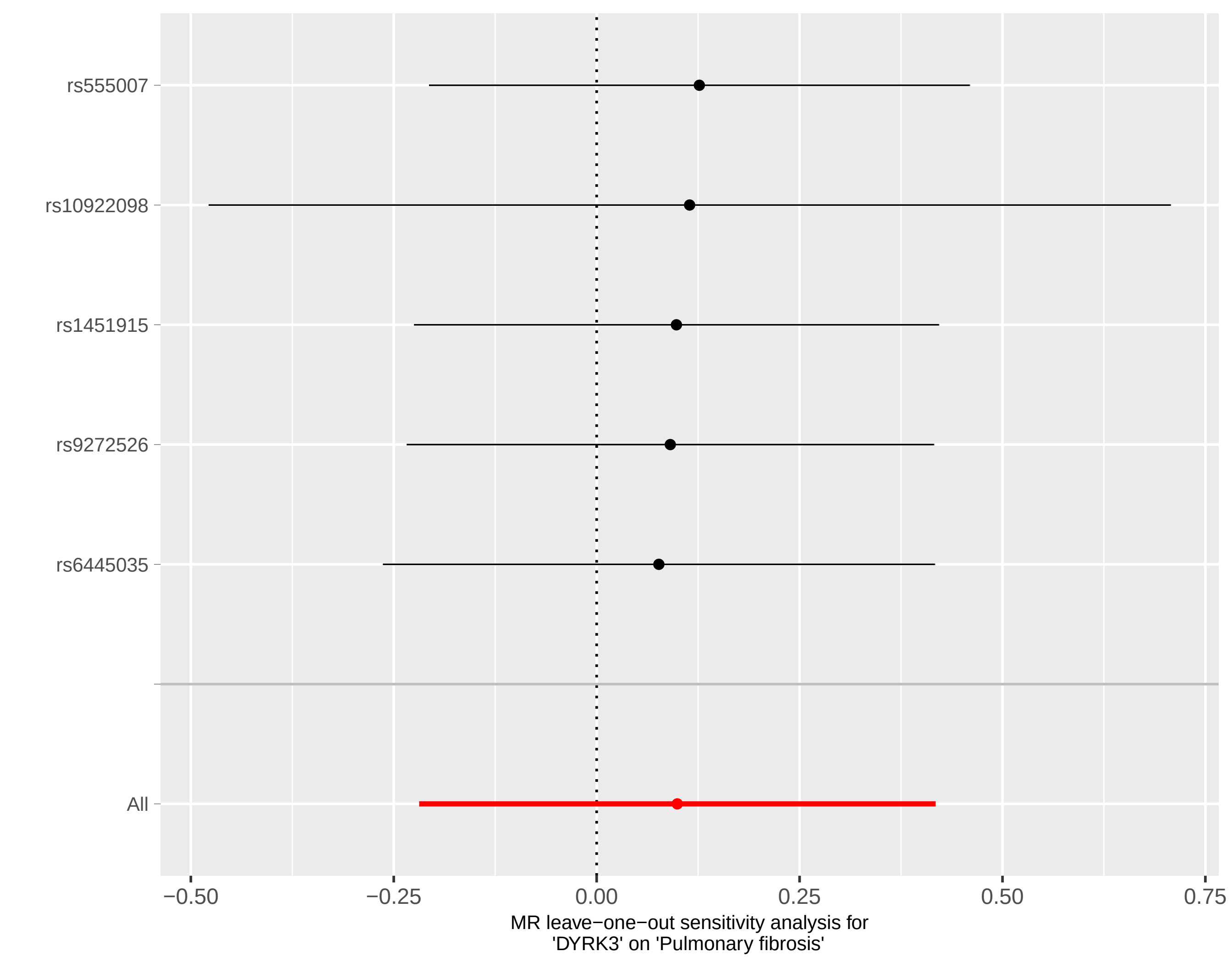
rs555007
rs10922098
rs1451915
rs9272526
rs6445035
All
−0.50
−0.25
0.00
0.25
0.50
0.75
MR leave−one−out sensitivity analysis for
'DYRK3' on 'Pulmonary fibrosis'

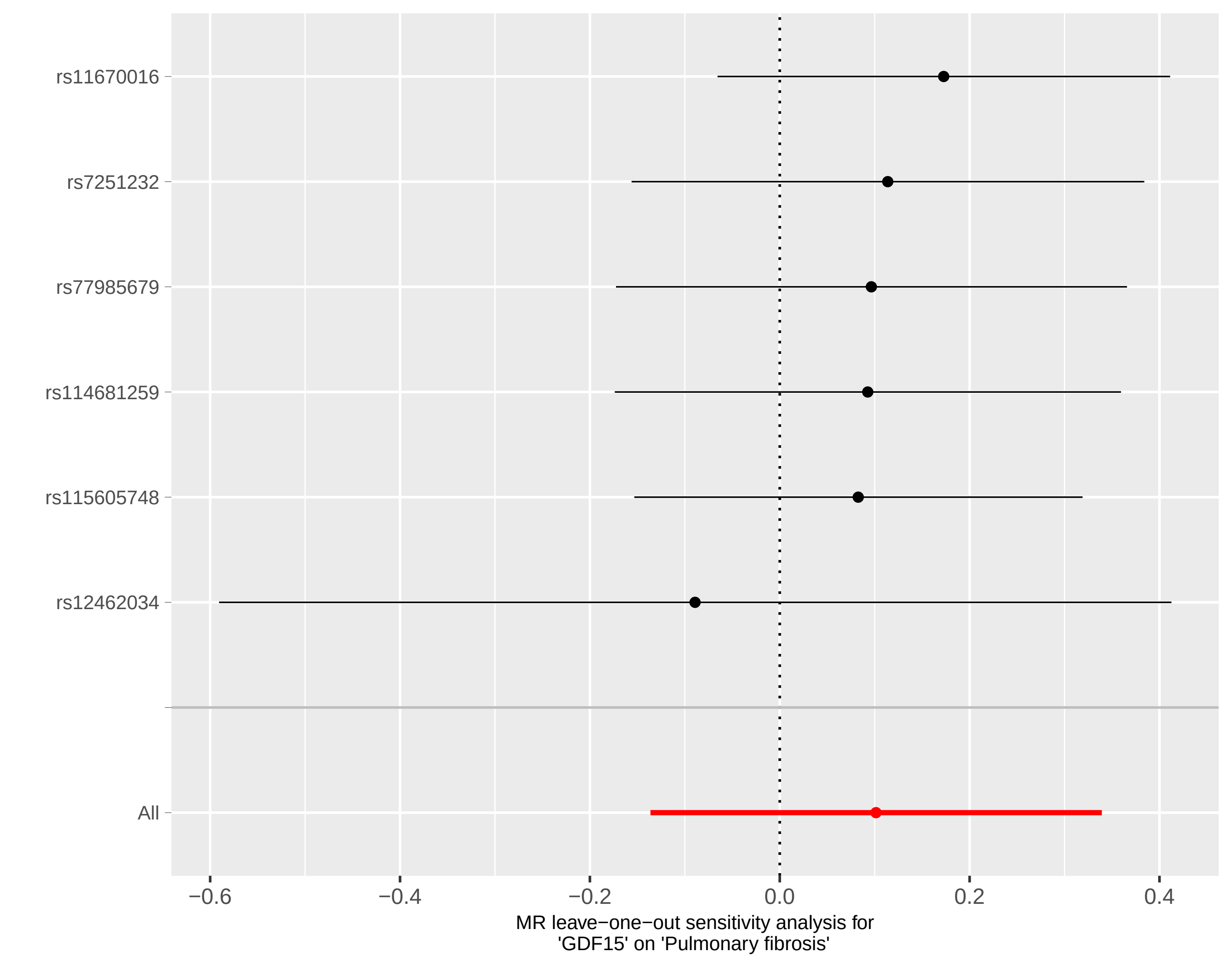

rs11670016
rs7251232
rs77985679
rs114681259
rs115605748
rs12462034
All
−0.6
−0.4
−0.2
0.0
0.2
0.4
MR leave−one−out sensitivity analysis for
'GDF15' on 'Pulmonary fibrosis'

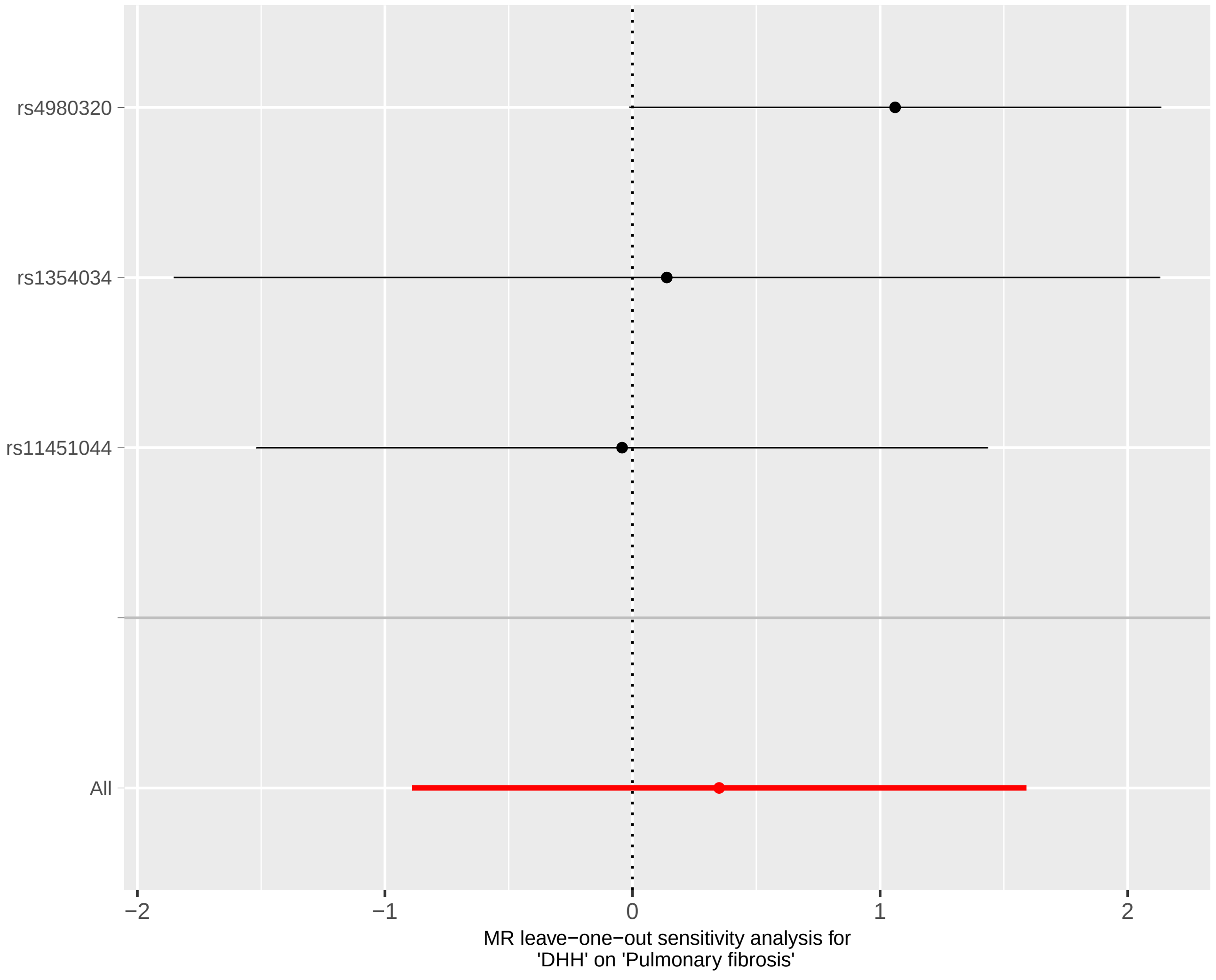
rs4980320
rs1354034
rs11451044
All
−2
−1
0
1
2
MR leave−one−out sensitivity analysis for
'DHH' on 'Pulmonary fibrosis'

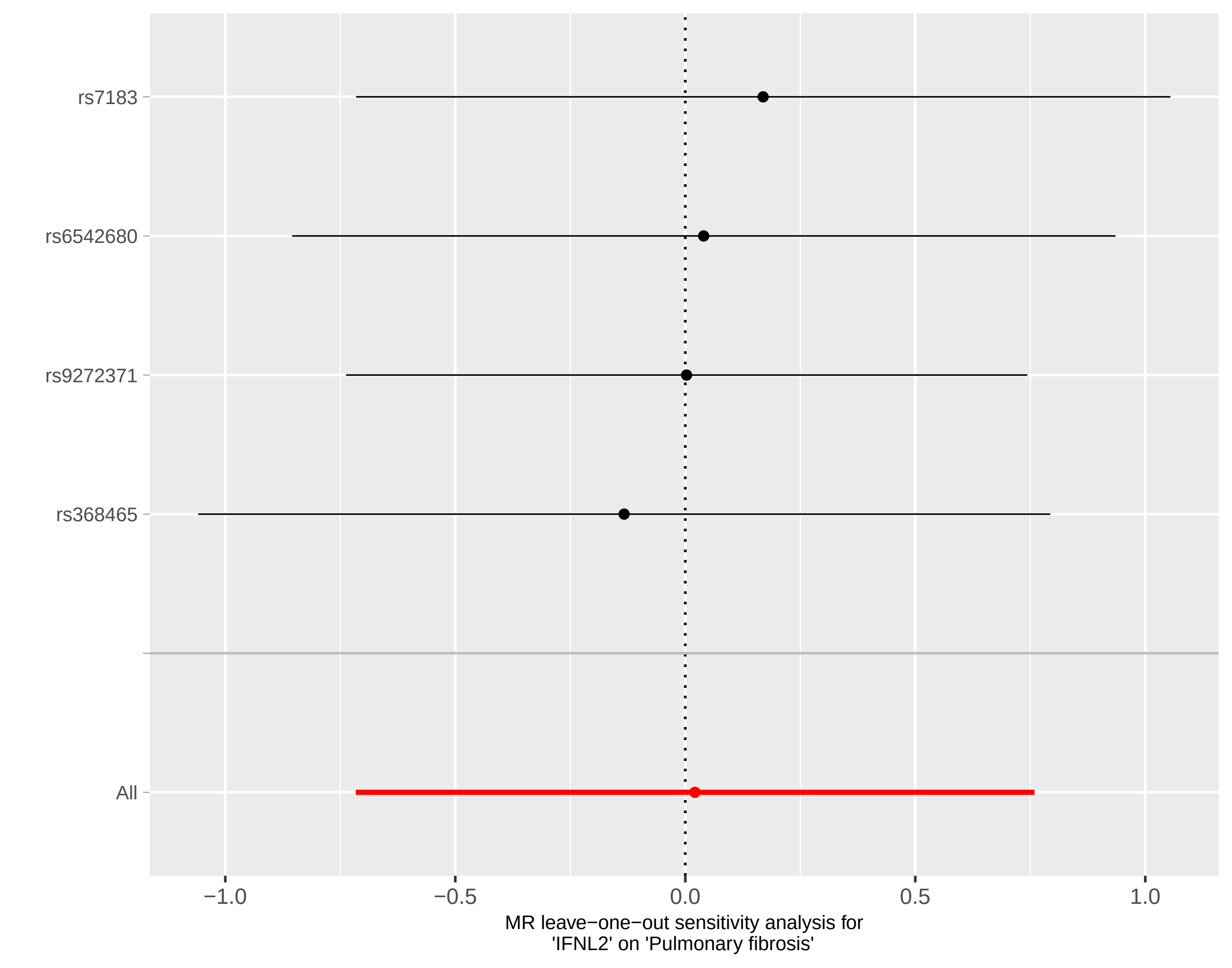

rs7183
rs6542680
rs9272371
rs368465
All
−1.0
−0.5
0.0
0.5
1.0
MR leave−one−out sensitivity analysis for
'IFNL2' on 'Pulmonary fibrosis'

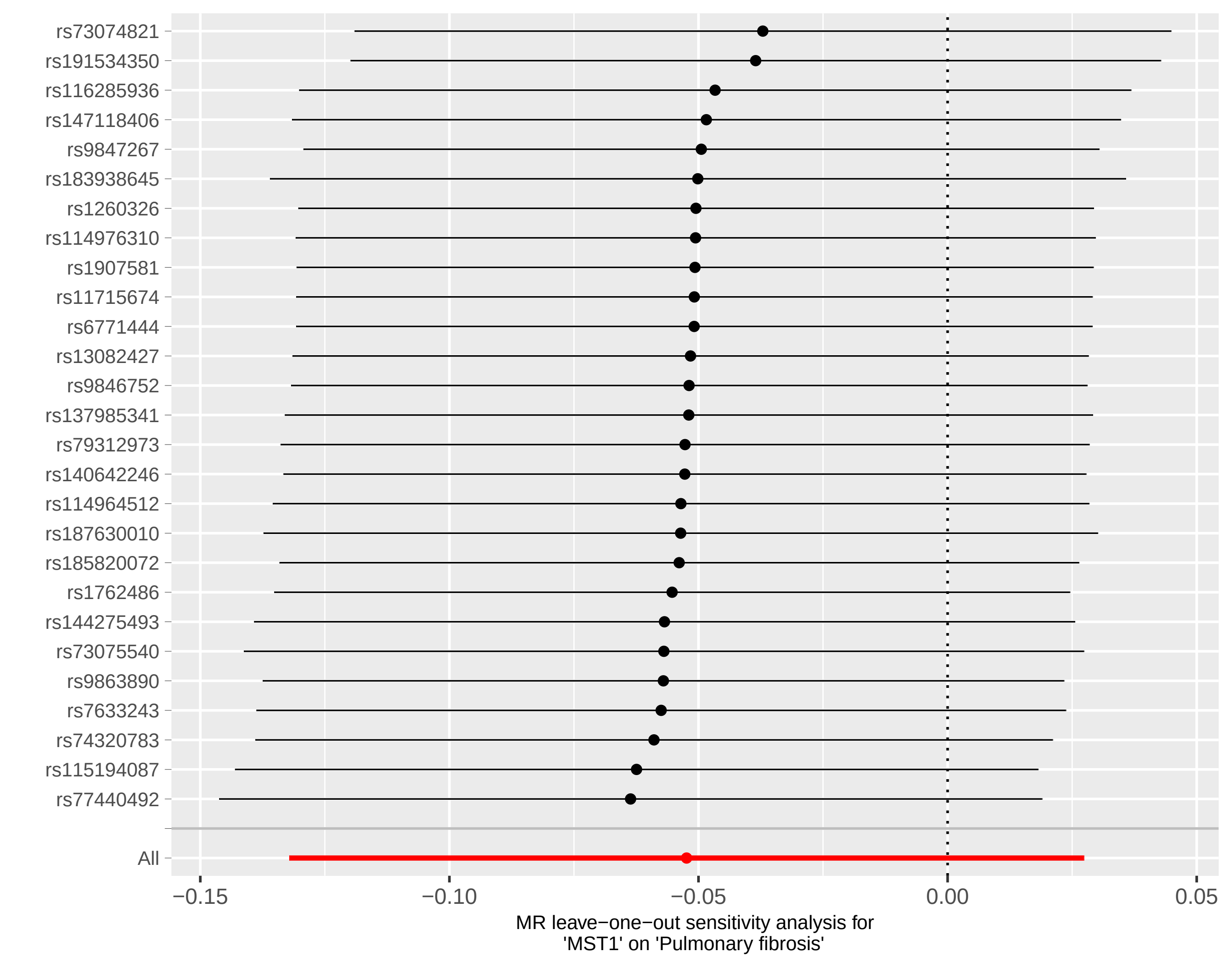
rs73074821
rs191534350
rs116285936
rs147118406
rs9847267
rs183938645
rs1260326
rs114976310
rs1907581
rs11715674
rs6771444
rs13082427
rs9846752
rs137985341
rs79312973
rs140642246
rs114964512
rs187630010
rs185820072
rs1762486
rs144275493
rs73075540
rs9863890
rs7633243
rs74320783
rs115194087
rs77440492
All
−0.15
−0.10
−0.05
0.00
0.05
MR leave−one−out sensitivity analysis for
'MST1' on 'Pulmonary fibrosis'

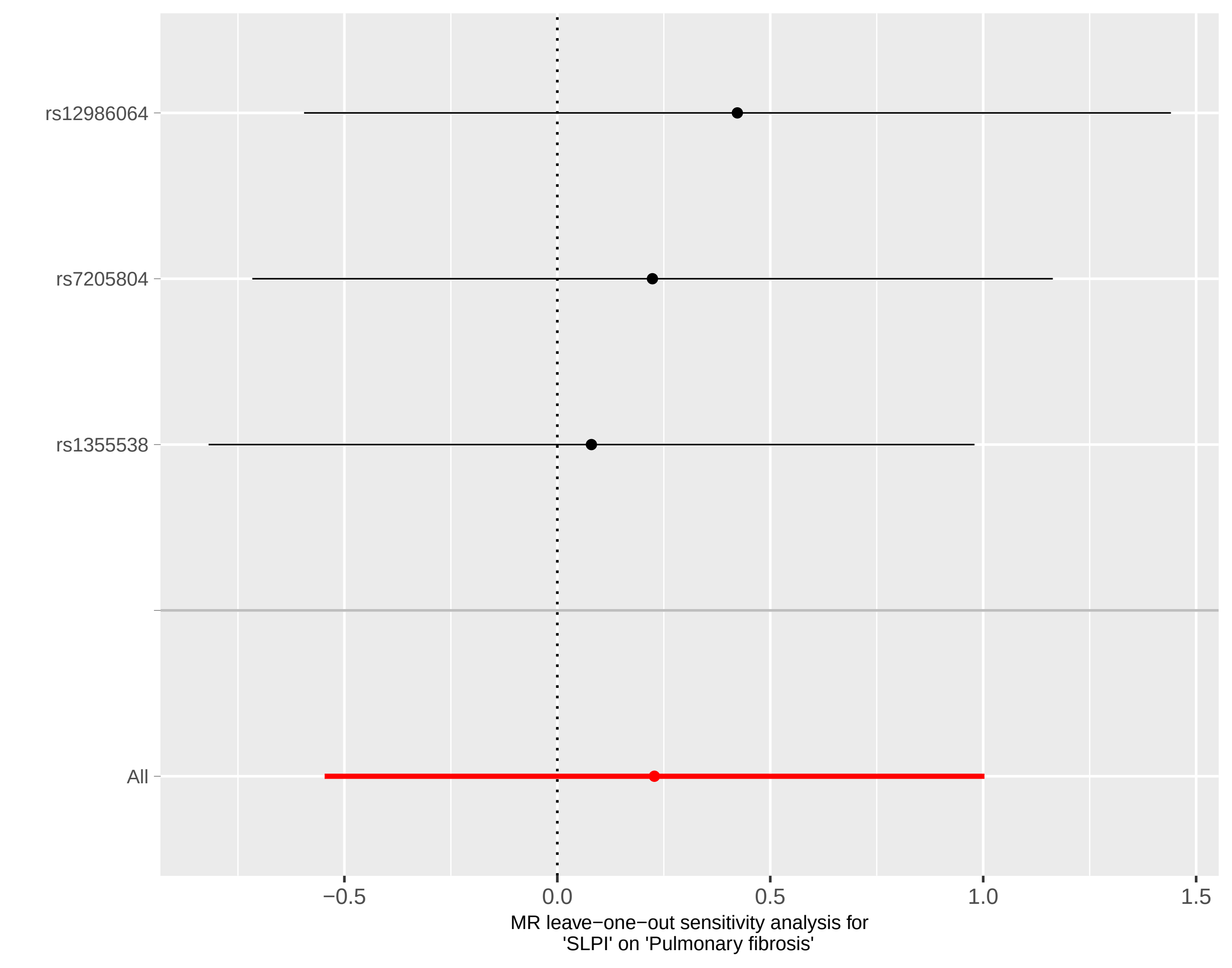
rs12986064
rs7205804
rs1355538
All
−0.5
0.0
0.5
1.0
1.5
MR leave−one−out sensitivity analysis for
'SLPI' on 'Pulmonary fibrosis'

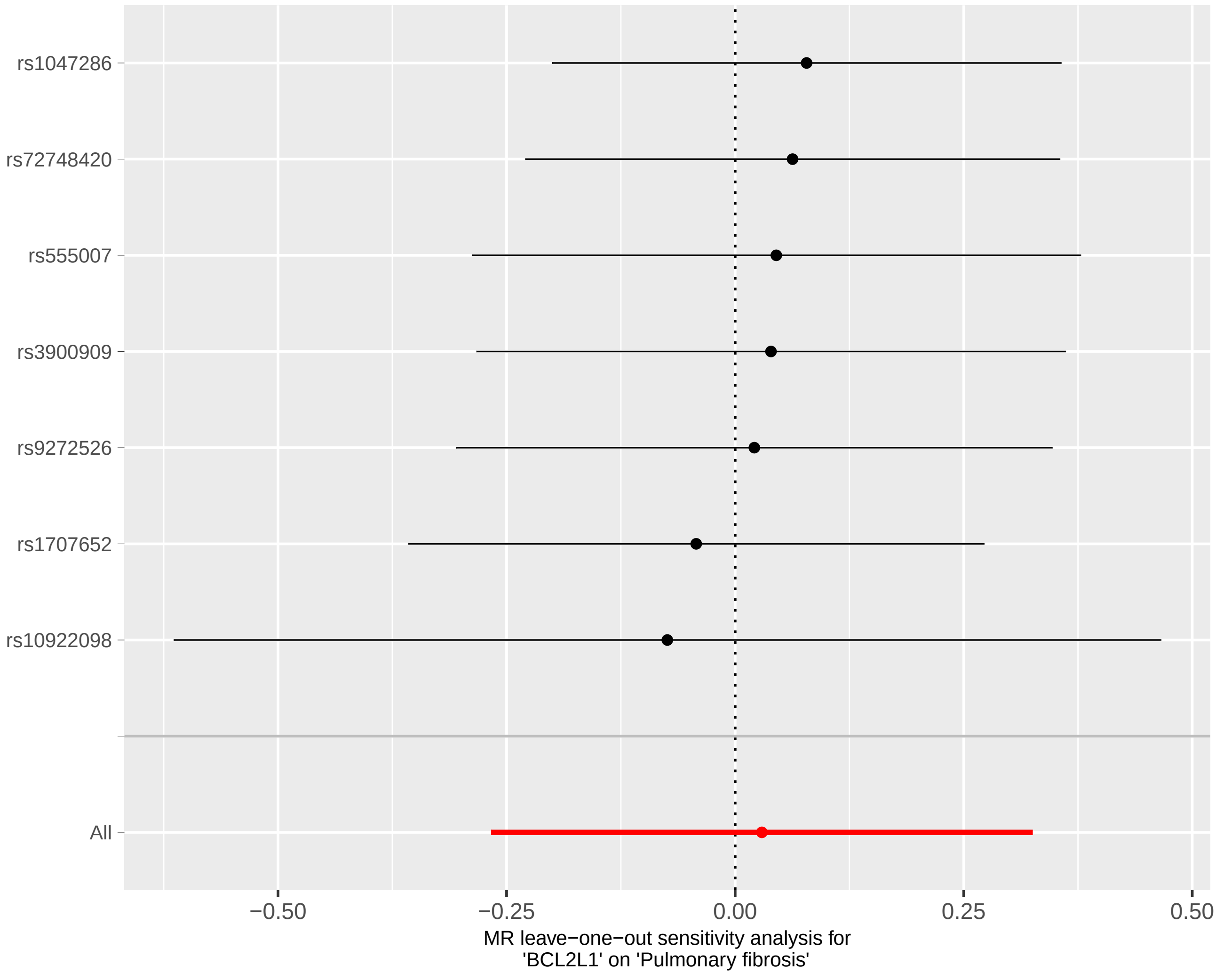
rs1047286
rs72748420
rs555007
rs3900909
rs9272526
rs1707652
rs10922098
All
−0.50
−0.25
0.00
0.25
0.50
MR leave−one−out sensitivity analysis for
'BCL2L1' on 'Pulmonary fibrosis'

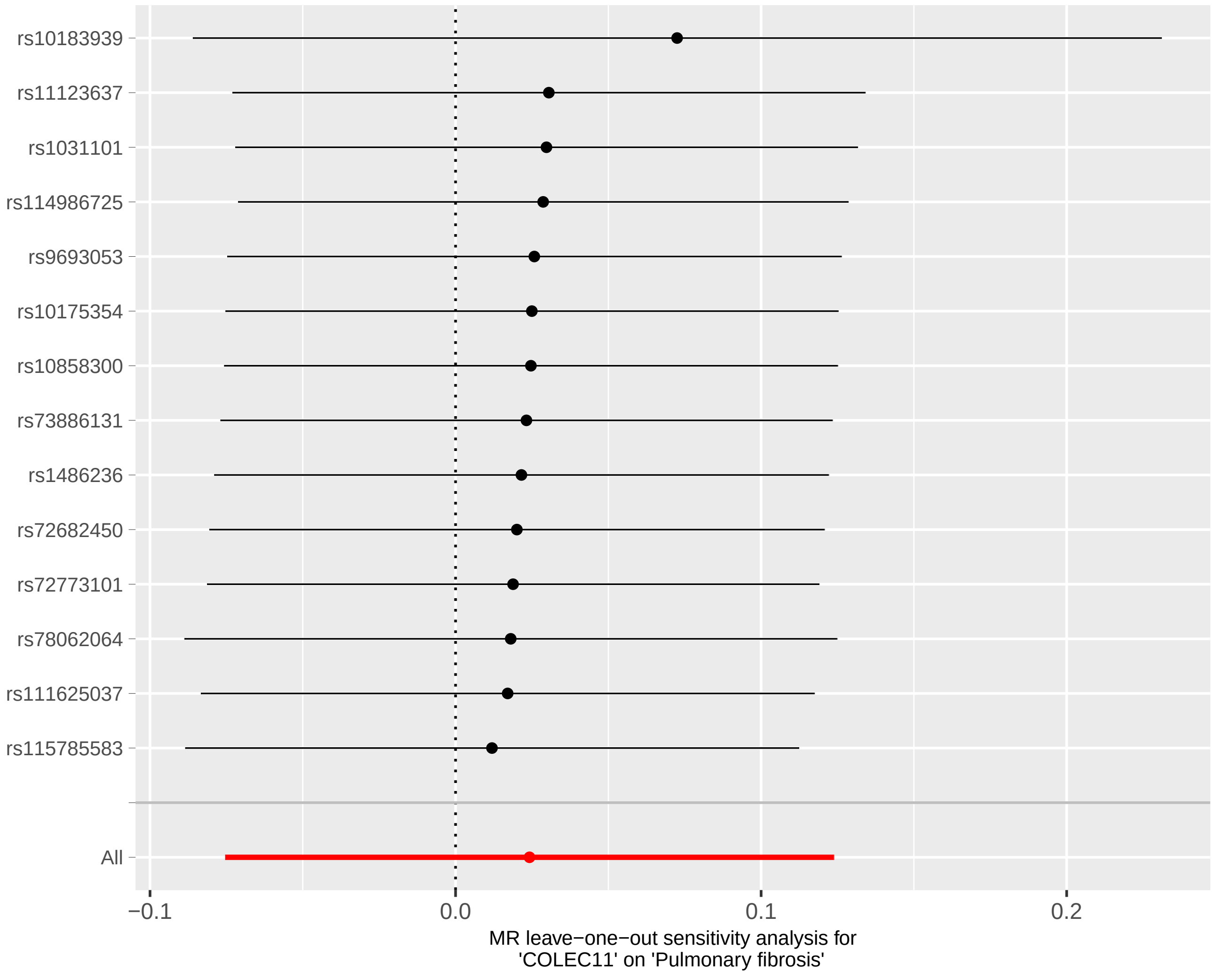
rs10183939
rs11123637
rs1031101
rs114986725
rs9693053
rs10175354
rs10858300
rs73886131
rs1486236
rs72682450
rs72773101
rs78062064
rs111625037
rs115785583
All
−0.1
0.0
0.1
0.2
MR leave−one−out sensitivity analysis for
'COLEC11' on 'Pulmonary fibrosis'

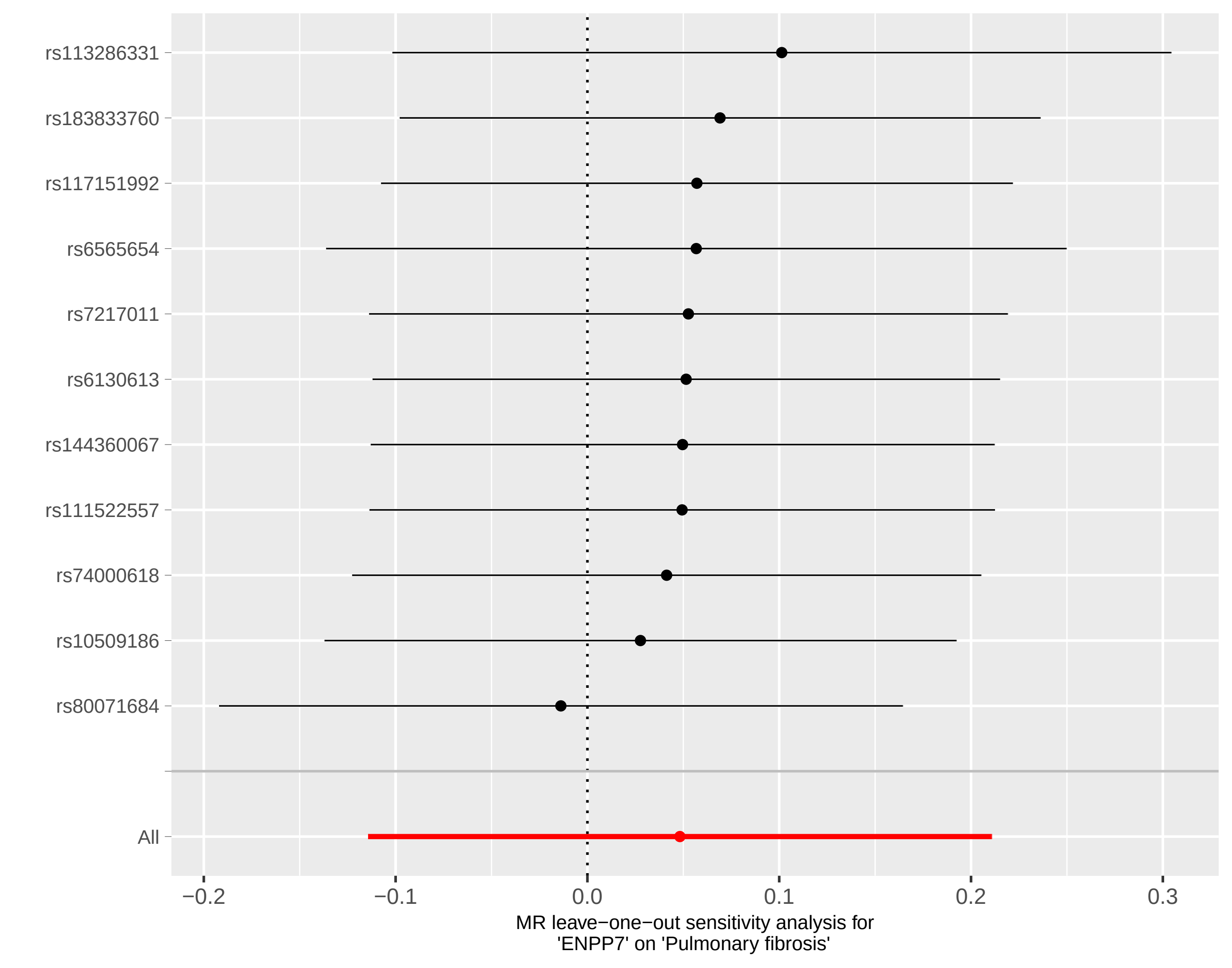
rs113286331
rs183833760
rs117151992
rs6565654
rs7217011
rs6130613
rs144360067
rs111522557
rs74000618
rs10509186
rs80071684
All
−0.2
−0.1
0.0
0.1
0.2
0.3
MR leave−one−out sensitivity analysis for
'ENPP7' on 'Pulmonary fibrosis'

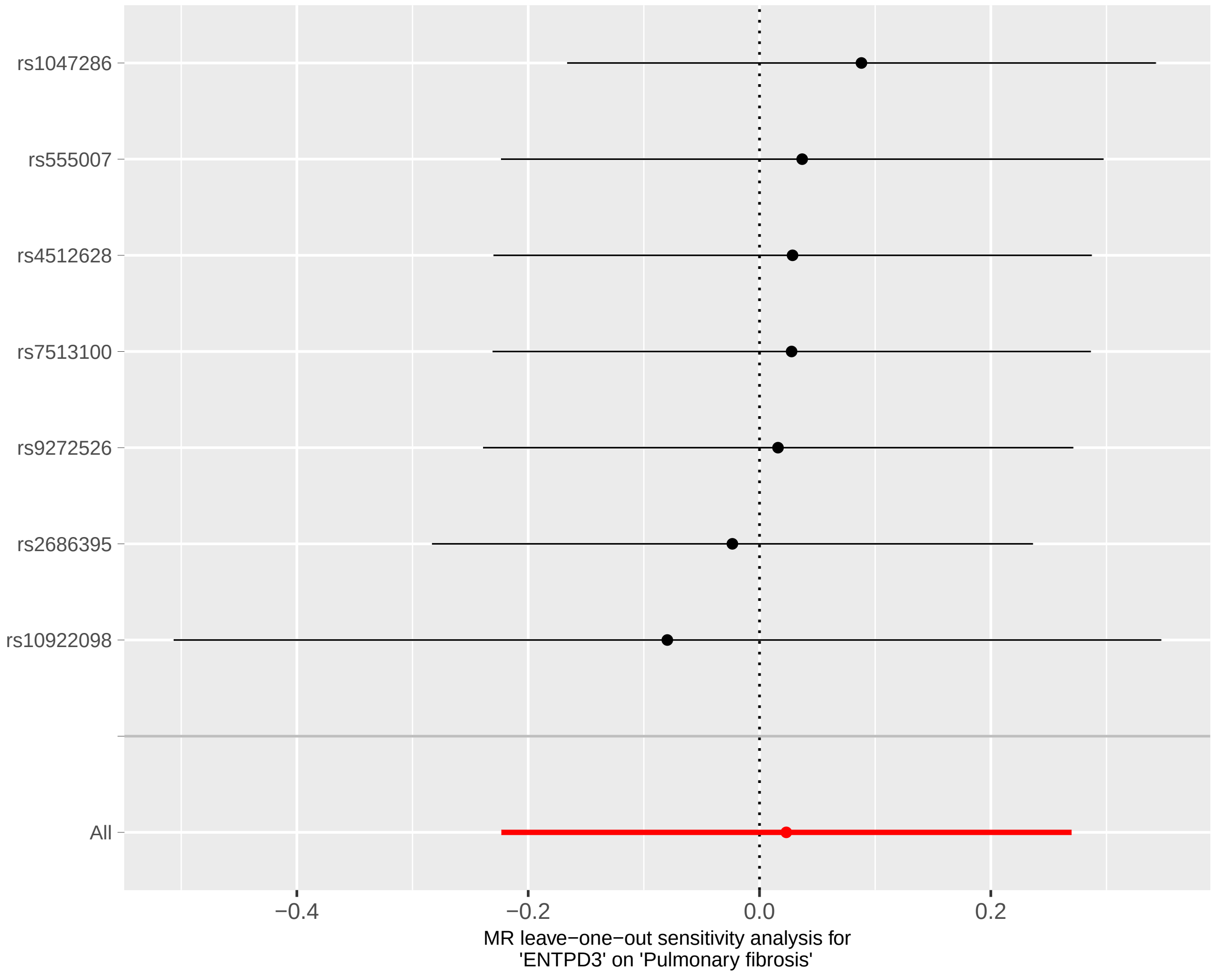
rs1047286
rs555007
rs4512628
rs7513100
rs9272526
rs2686395
rs10922098
All
−0.4
−0.2
0.0
0.2
MR leave−one−out sensitivity analysis for
'ENTPD3' on 'Pulmonary fibrosis'

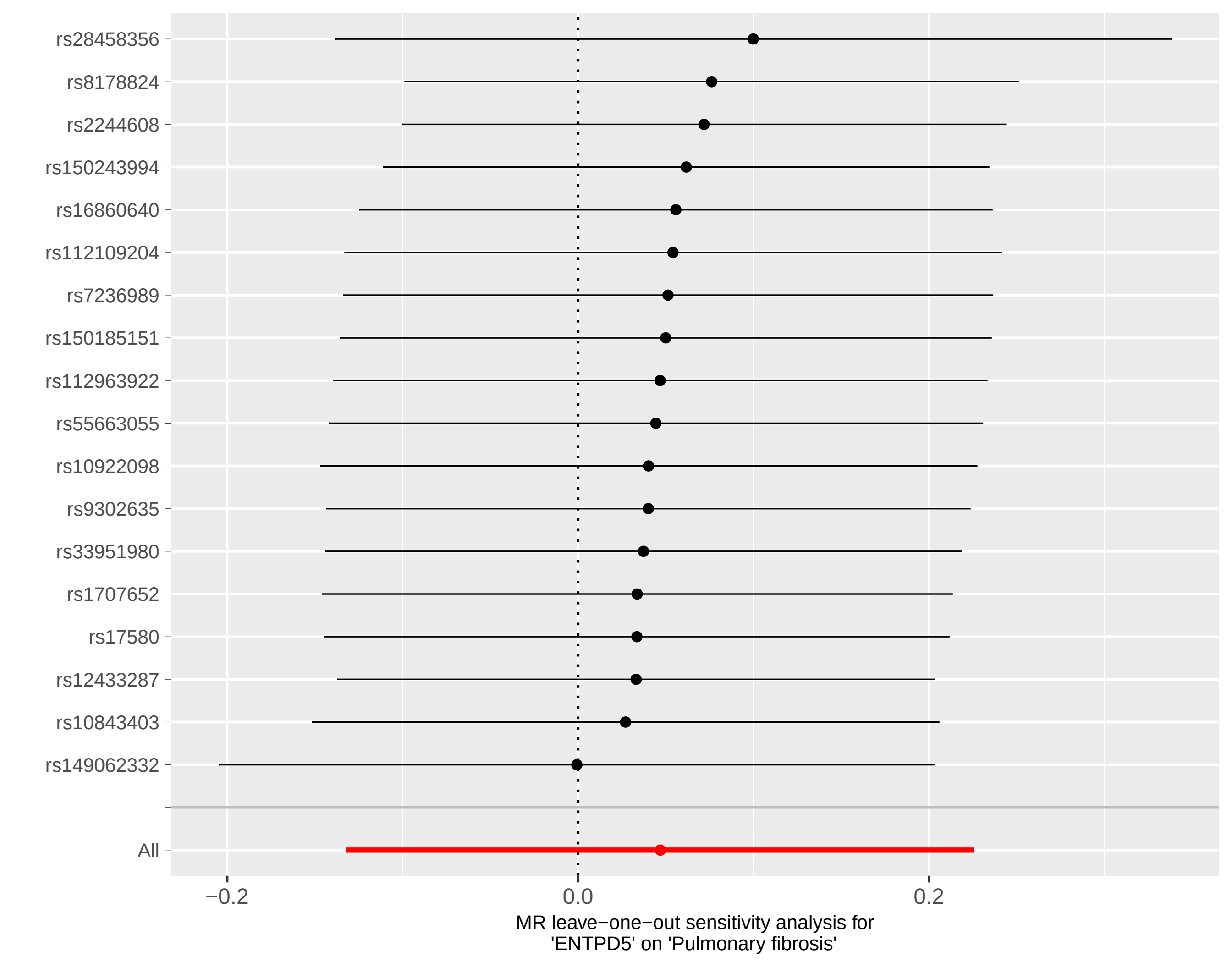
rs28458356
rs8178824
rs2244608
rs150243994
rs16860640
rs112109204
rs7236989
rs150185151
rs112963922
rs55663055
rs10922098
rs9302635
rs33951980
rs1707652
rs17580
rs12433287
rs10843403
rs149062332
All
−0.2
0.0
0.2
MR leave−one−out sensitivity analysis for
'ENTPD5' on 'Pulmonary fibrosis'

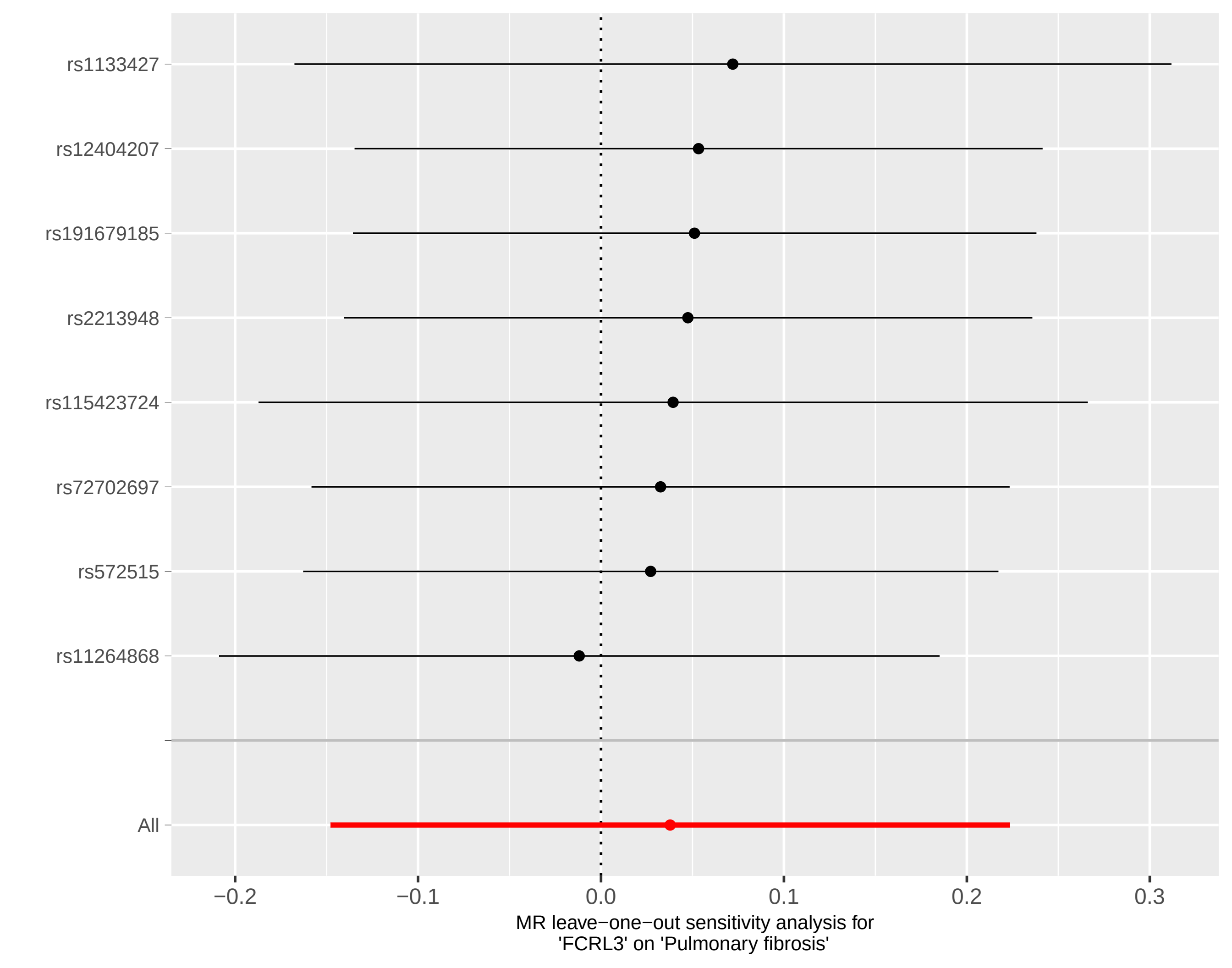
rs1133427
rs12404207
rs191679185
rs2213948
rs115423724
rs72702697
rs572515
rs11264868
All
−0.2
−0.1
0.0
0.1
0.2
0.3
MR leave−one−out sensitivity analysis for
'FCRL3' on 'Pulmonary fibrosis'

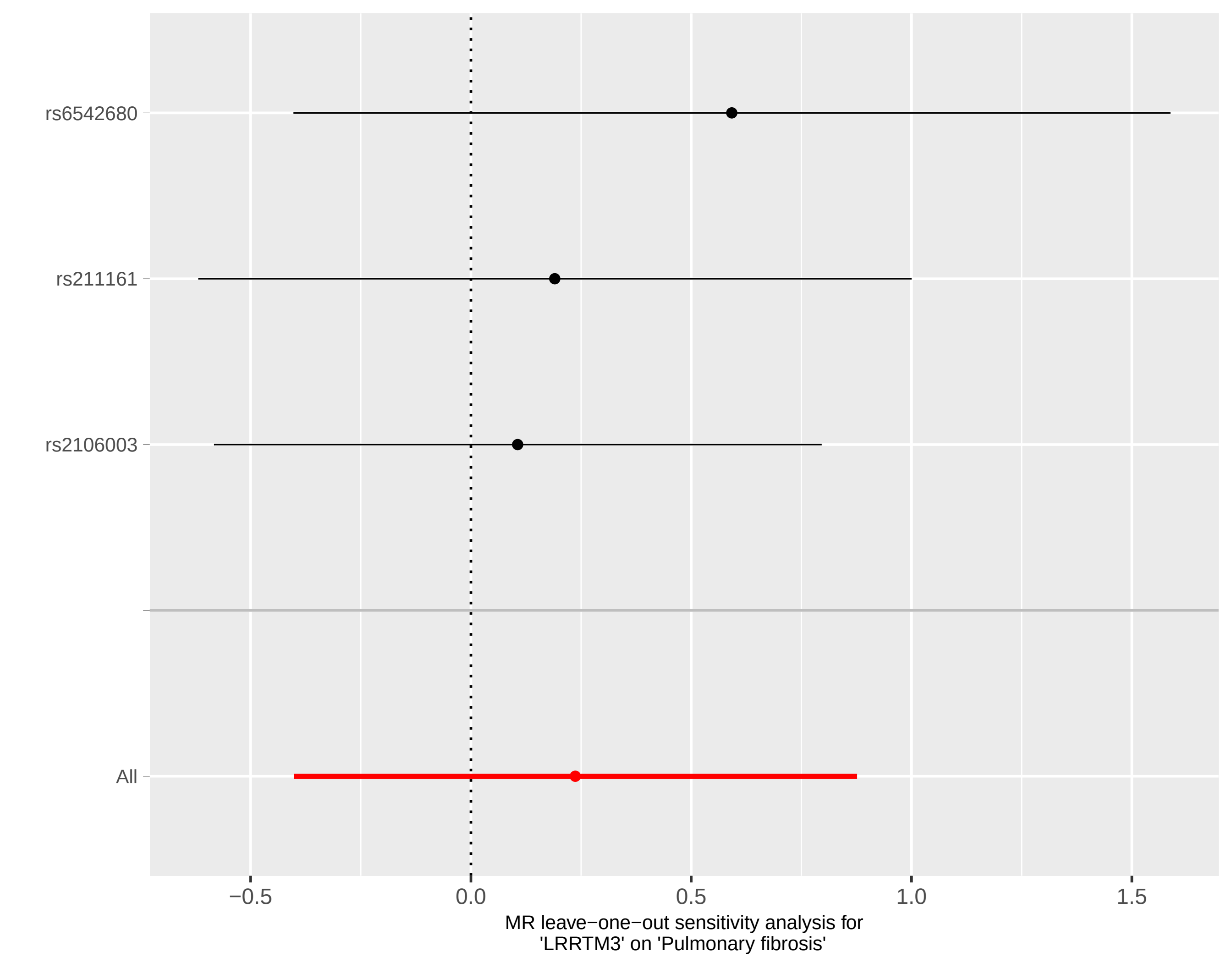
rs6542680
rs211161
rs2106003
All
−0.5
0.0
0.5
1.0
1.5
MR leave−one−out sensitivity analysis for
'LRRTM3' on 'Pulmonary fibrosis'

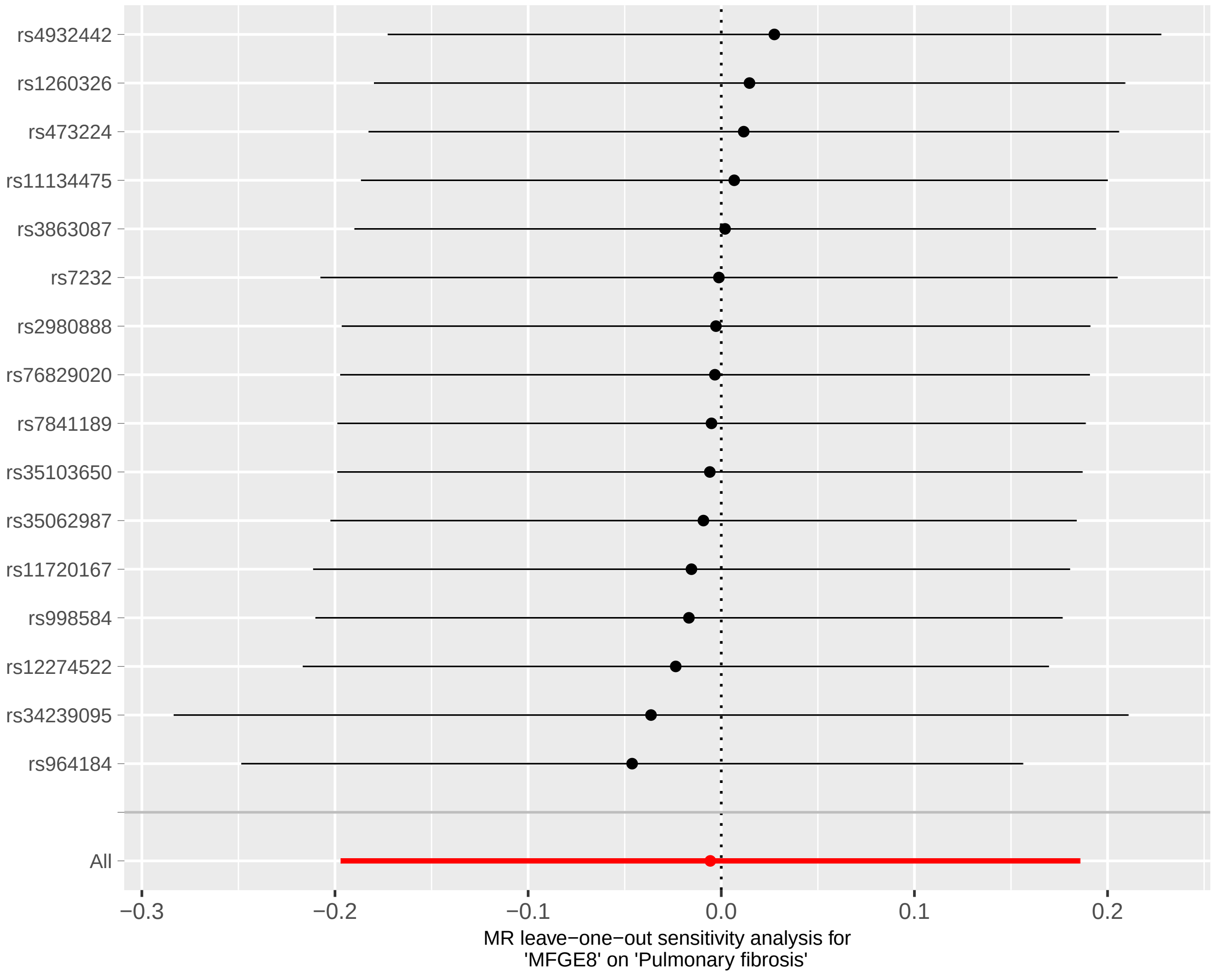
rs4932442
rs1260326
rs473224
rs11134475
rs3863087
rs7232
rs2980888
rs76829020
rs7841189
rs35103650
rs35062987
rs11720167
rs998584
rs12274522
rs34239095
rs964184
All
−0.3
−0.2
−0.1
0.0
0.1
0.2
MR leave−one−out sensitivity analysis for
'MFGE8' on 'Pulmonary fibrosis'

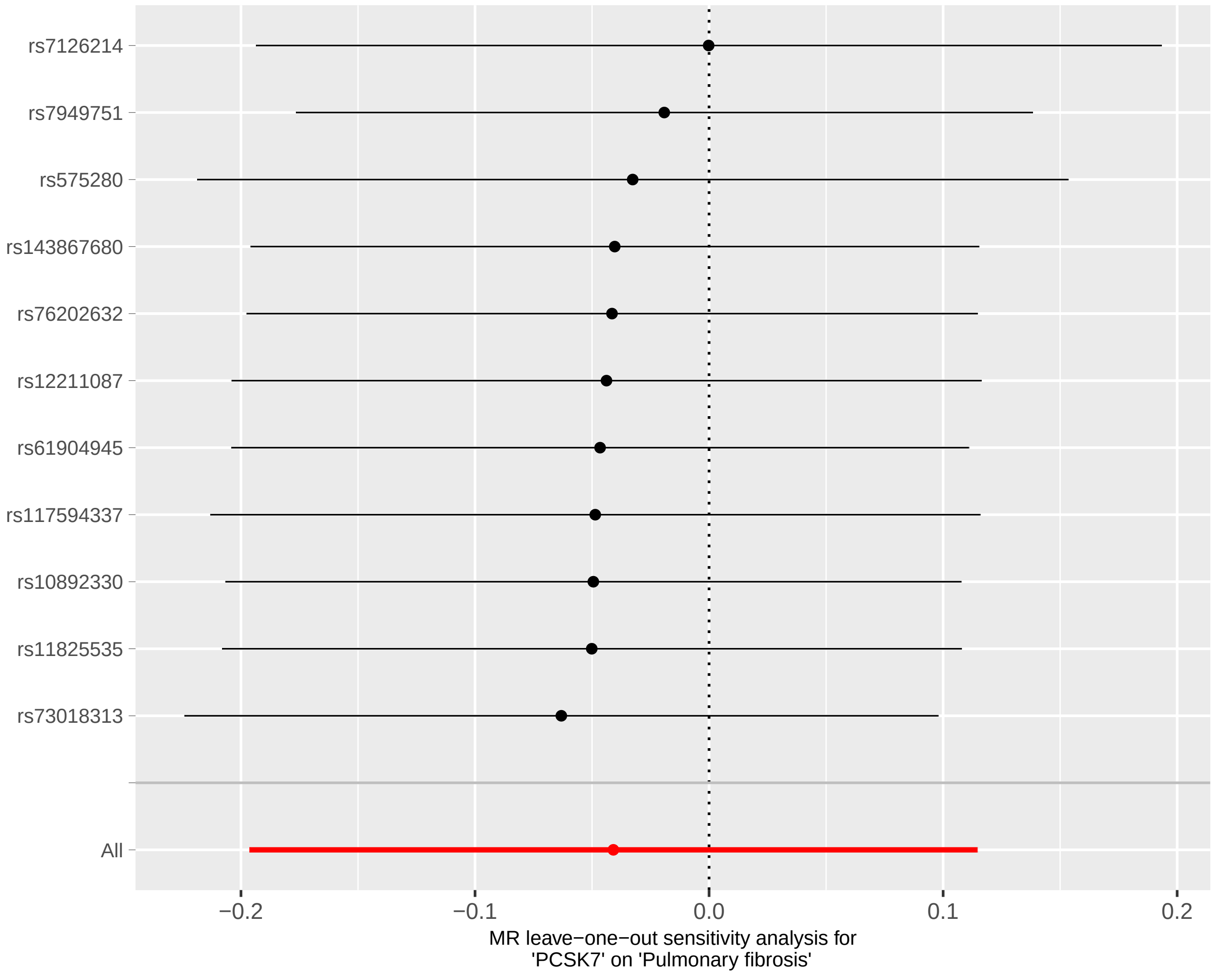
rs7126214
rs7949751
rs575280
rs143867680
rs76202632
rs12211087
rs61904945
rs117594337
rs10892330
rs11825535
rs73018313
All
−0.2
−0.1
0.0
0.1
0.2
MR leave−one−out sensitivity analysis for
'PCSK7' on 'Pulmonary fibrosis'

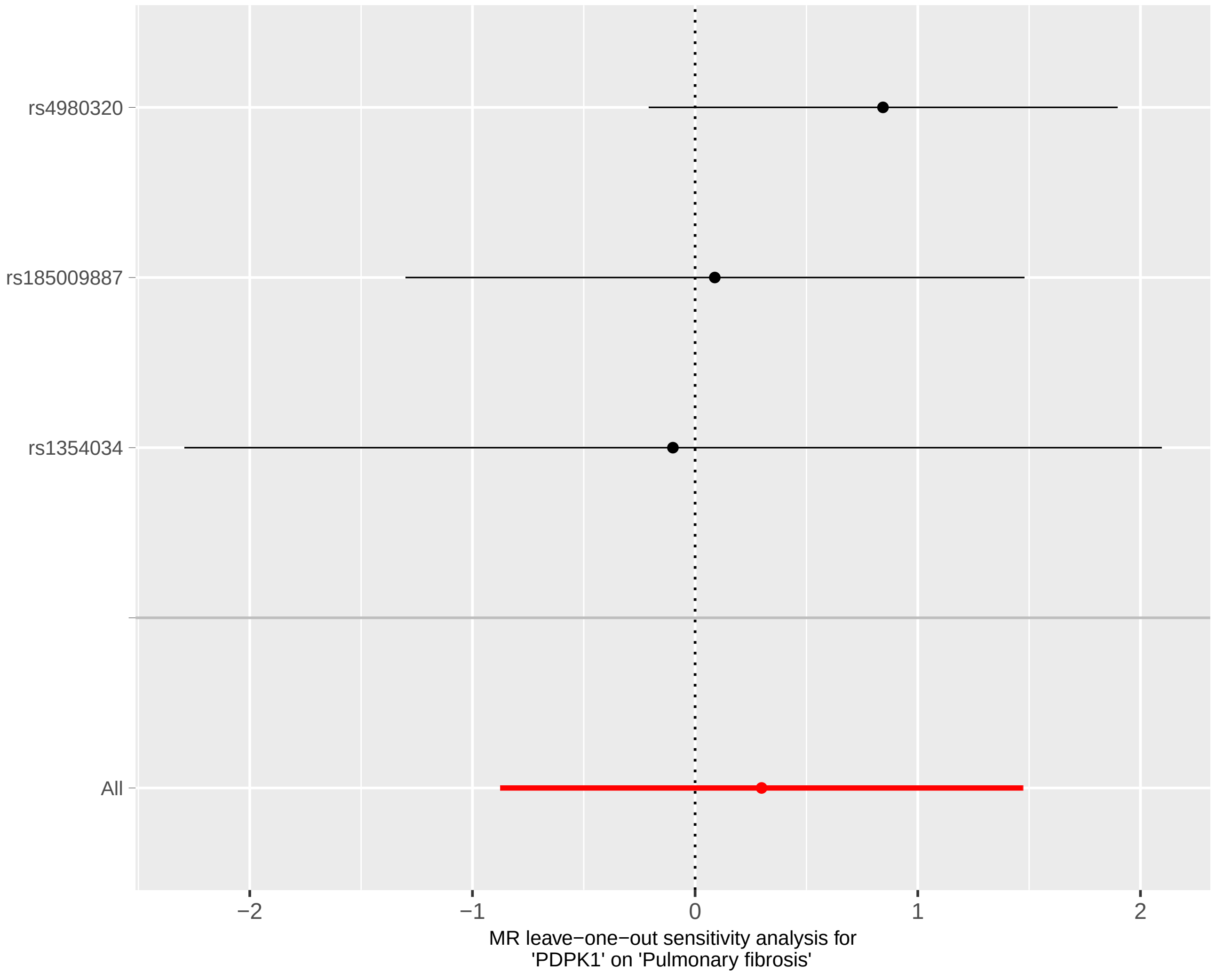
rs4980320
rs185009887
rs1354034
All
−2
−1
0
1
2
MR leave−one−out sensitivity analysis for
'PDPK1' on 'Pulmonary fibrosis'

Insufficient number of SNPs

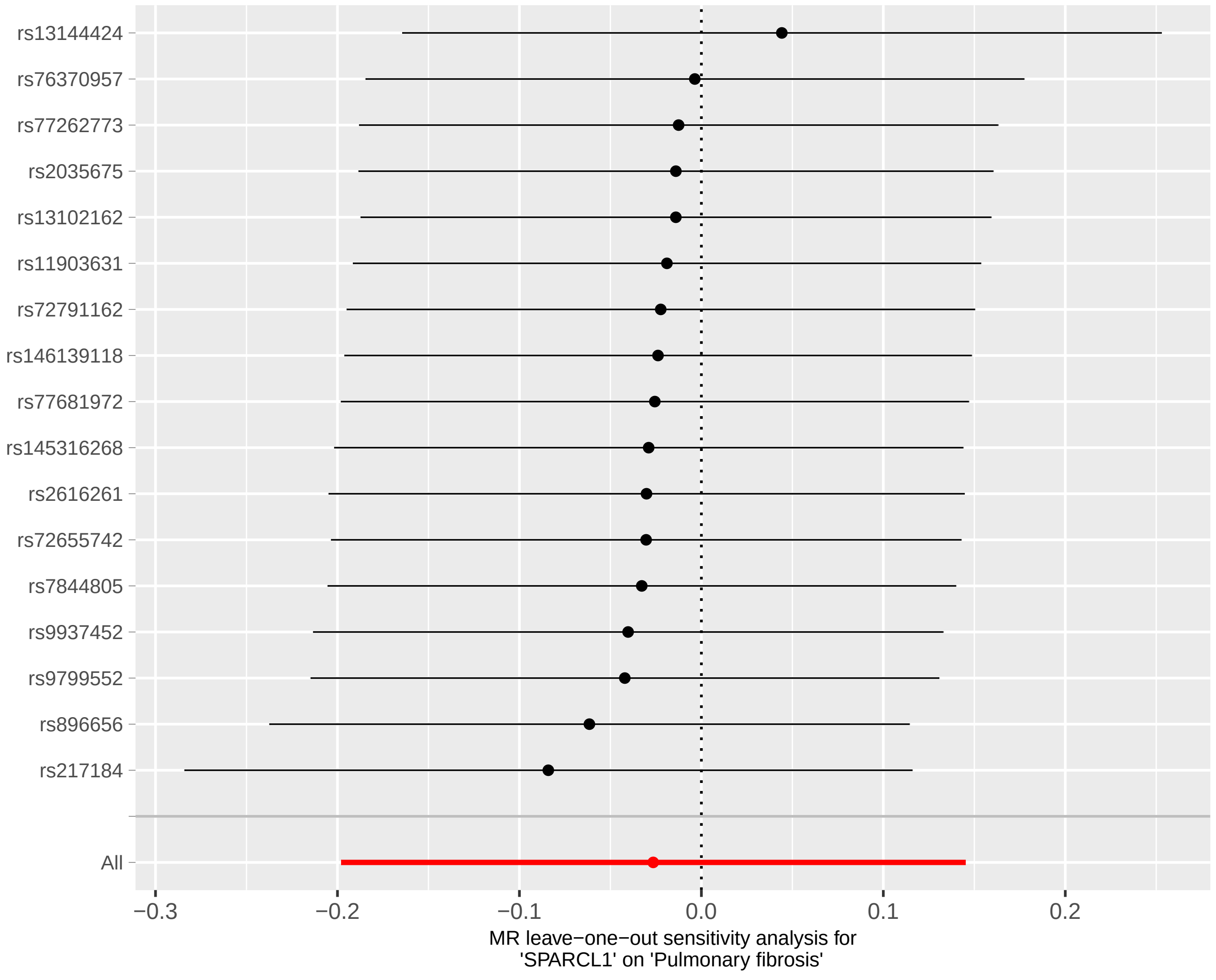
rs13144424
rs76370957
rs77262773
rs2035675
rs13102162
rs11903631
rs72791162
rs146139118
rs77681972
rs145316268
rs2616261
rs72655742
rs7844805
rs9937452
rs9799552
rs896656
rs217184
All
−0.3
−0.2
−0.1
0.0
0.1
0.2
MR leave−one−out sensitivity analysis for
'SPARCL1' on 'Pulmonary fibrosis'

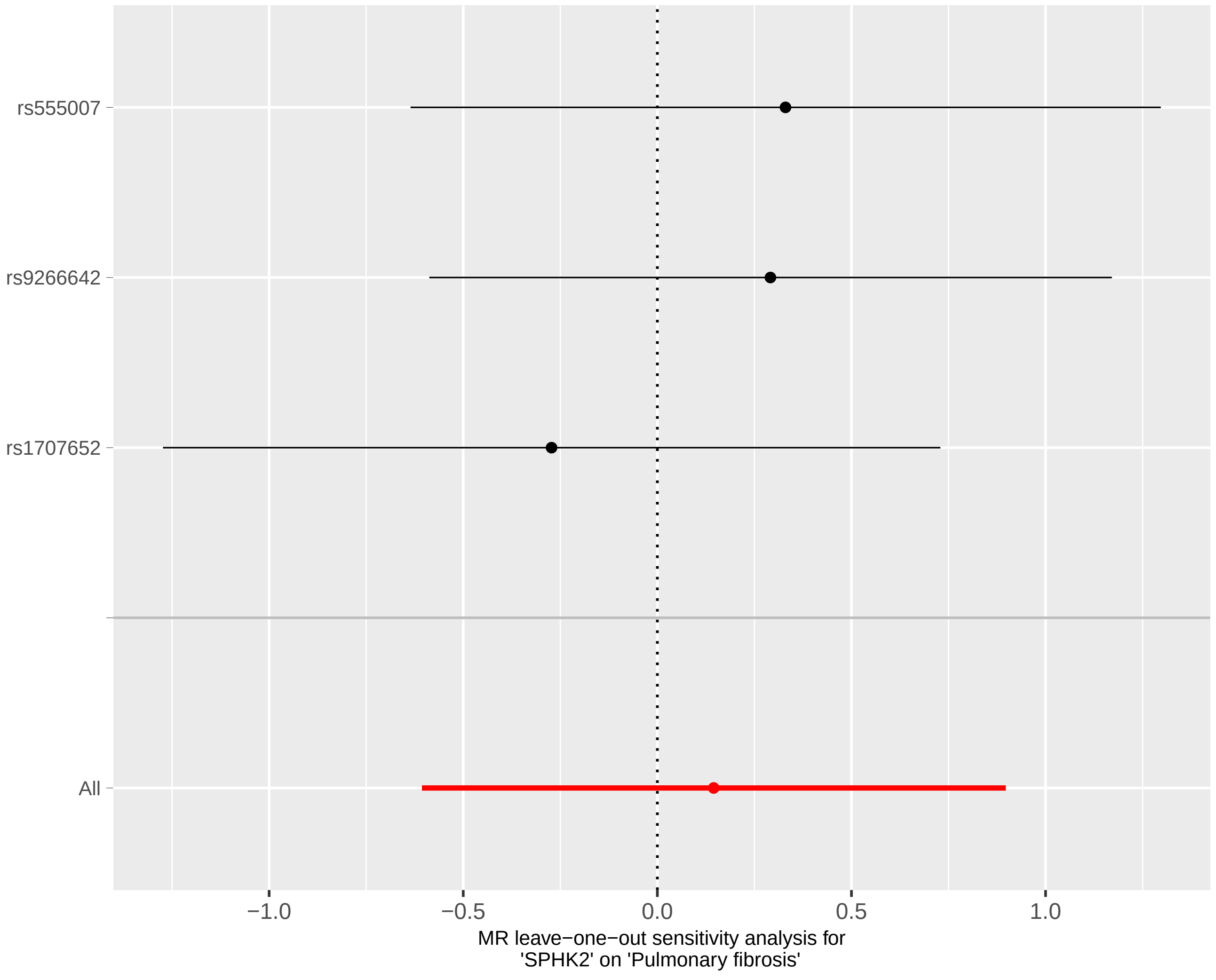
rs555007
rs9266642
rs1707652
All
−1.0
−0.5
0.0
0.5
1.0
MR leave−one−out sensitivity analysis for
'SPHK2' on 'Pulmonary fibrosis'

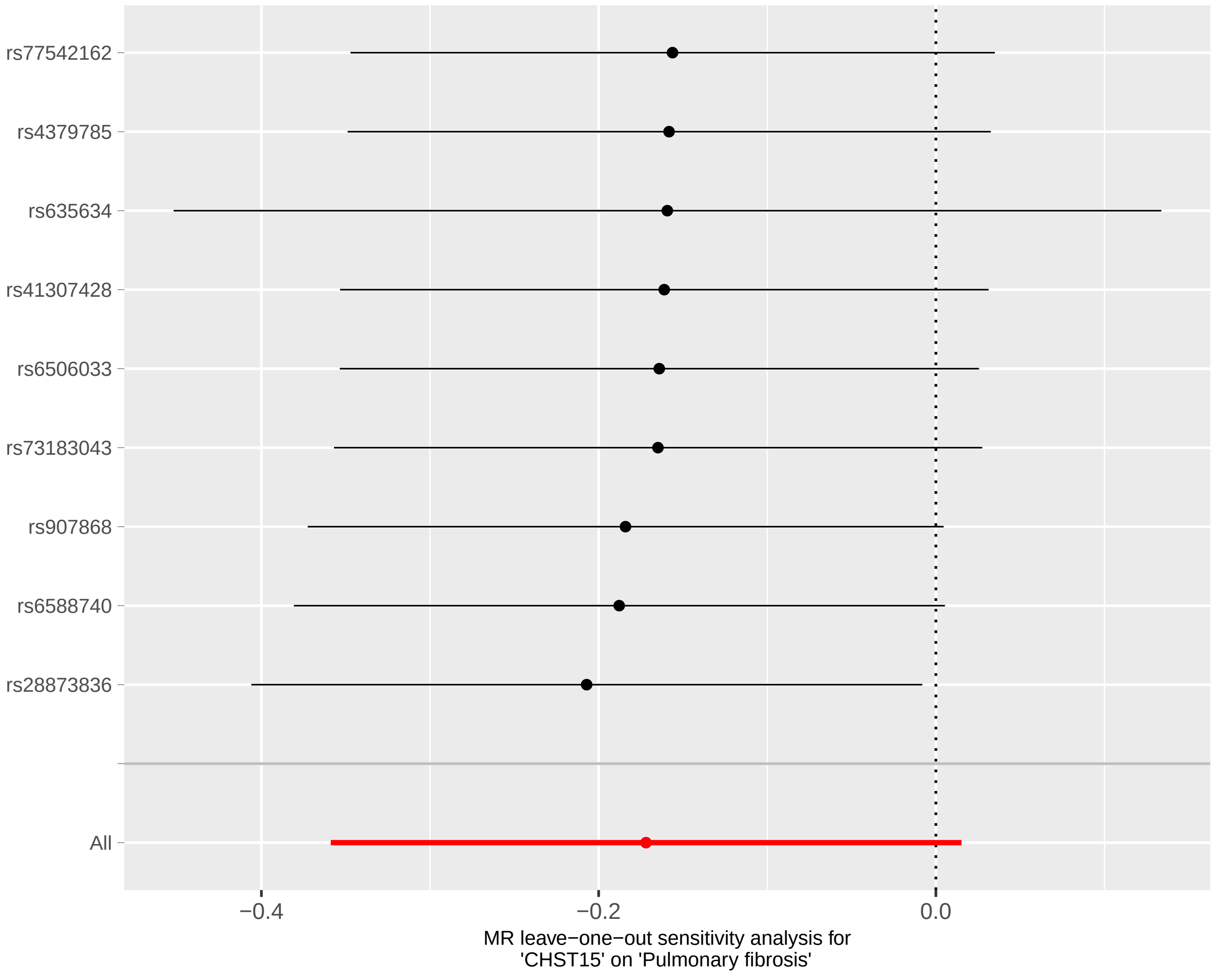
rs77542162
rs4379785
rs635634
rs41307428
rs6506033
rs73183043
rs907868
rs6588740
rs28873836
All
−0.4
−0.2
0.0
MR leave−one−out sensitivity analysis for
'CHST15' on 'Pulmonary fibrosis'

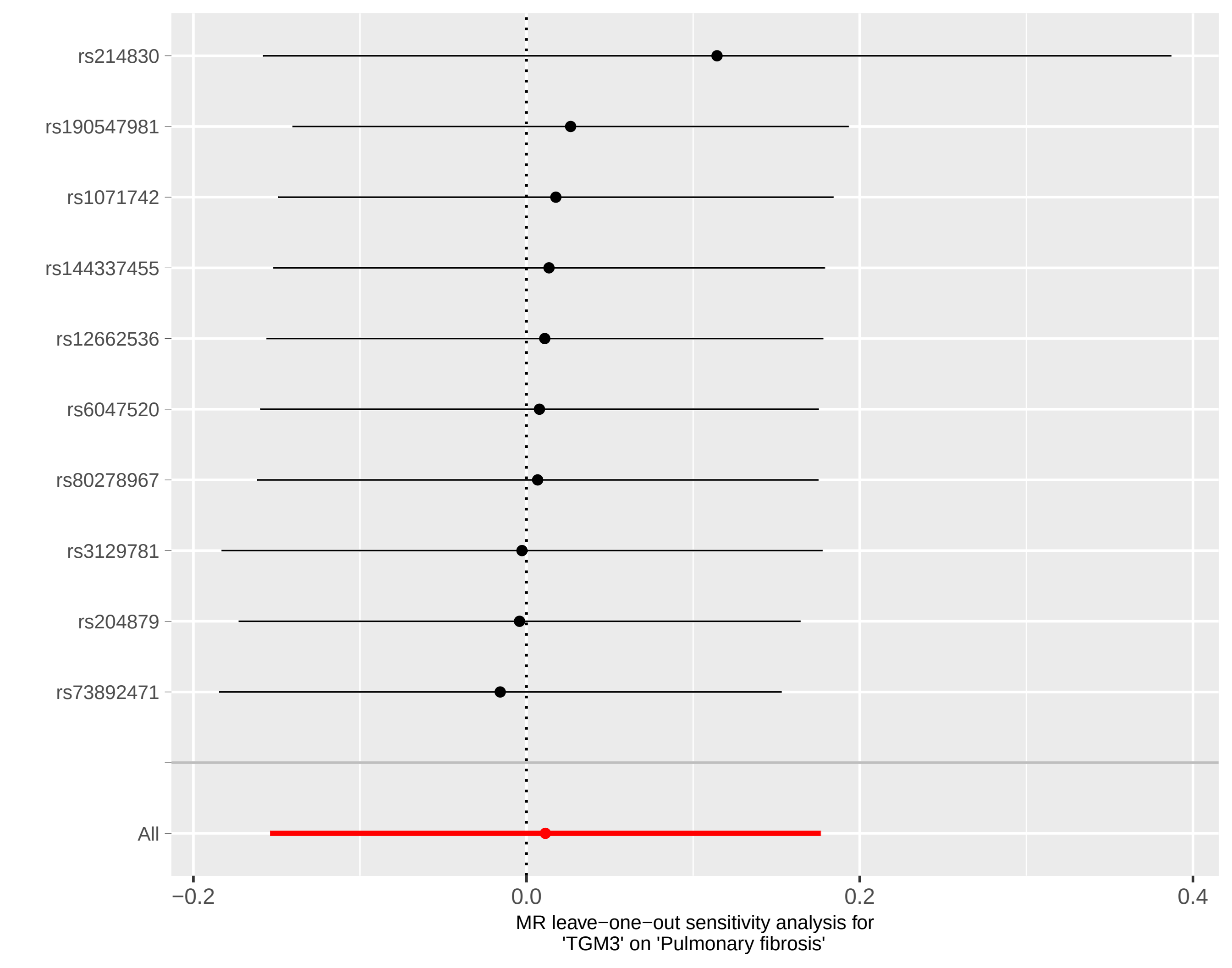

rs214830
rs190547981
rs1071742
rs144337455
rs12662536
rs6047520
rs80278967
rs3129781
rs204879
rs73892471
All
−0.2
0.0
0.2
0.4
MR leave−one−out sensitivity analysis for
'TGM3' on 'Pulmonary fibrosis'

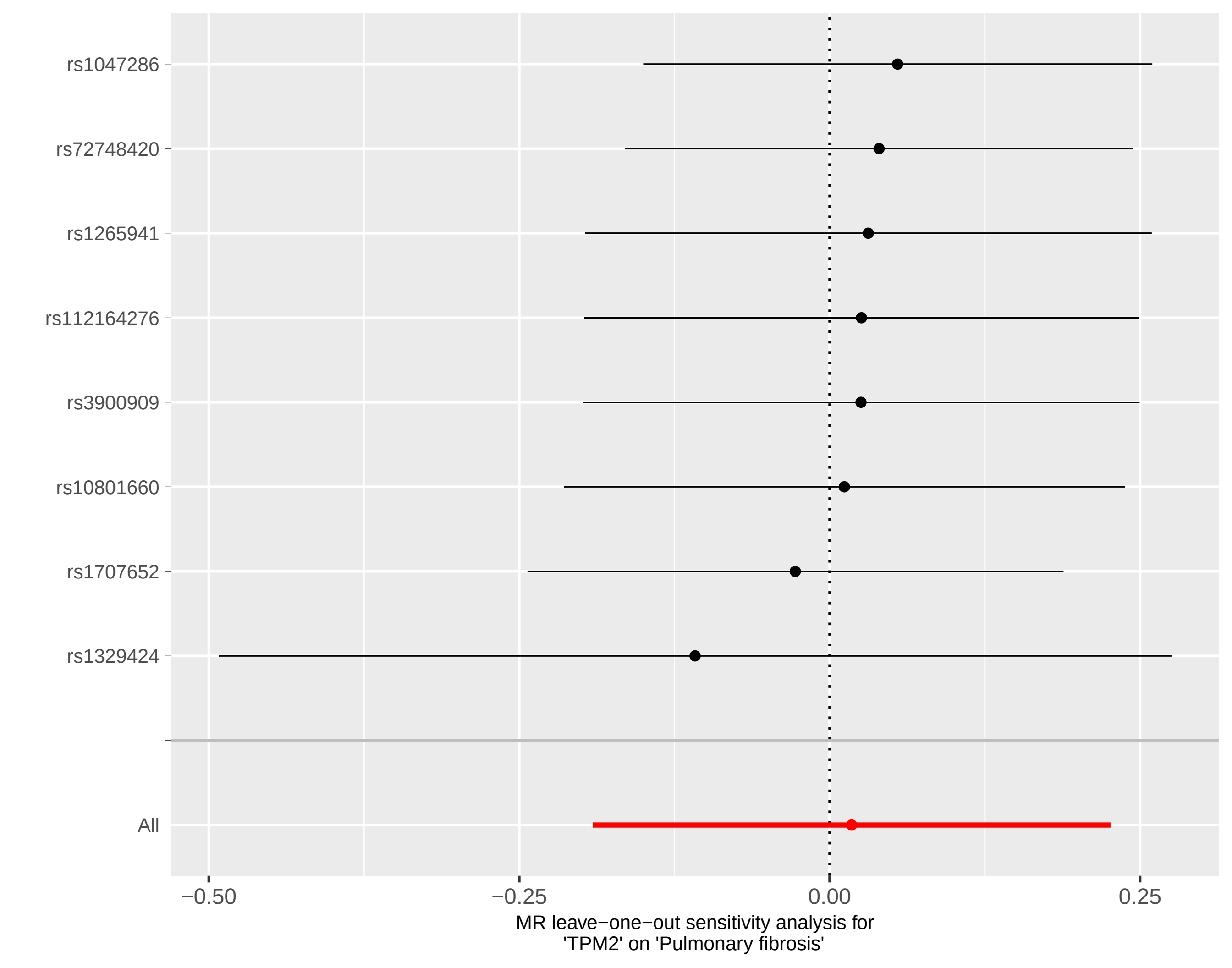

rs1047286
rs72748420
rs1265941
rs112164276
rs3900909
rs10801660
rs1707652
rs1329424
All
−0.50
−0.25
0.00
0.25
MR leave−one−out sensitivity analysis for
'TPM2' on 'Pulmonary fibrosis'

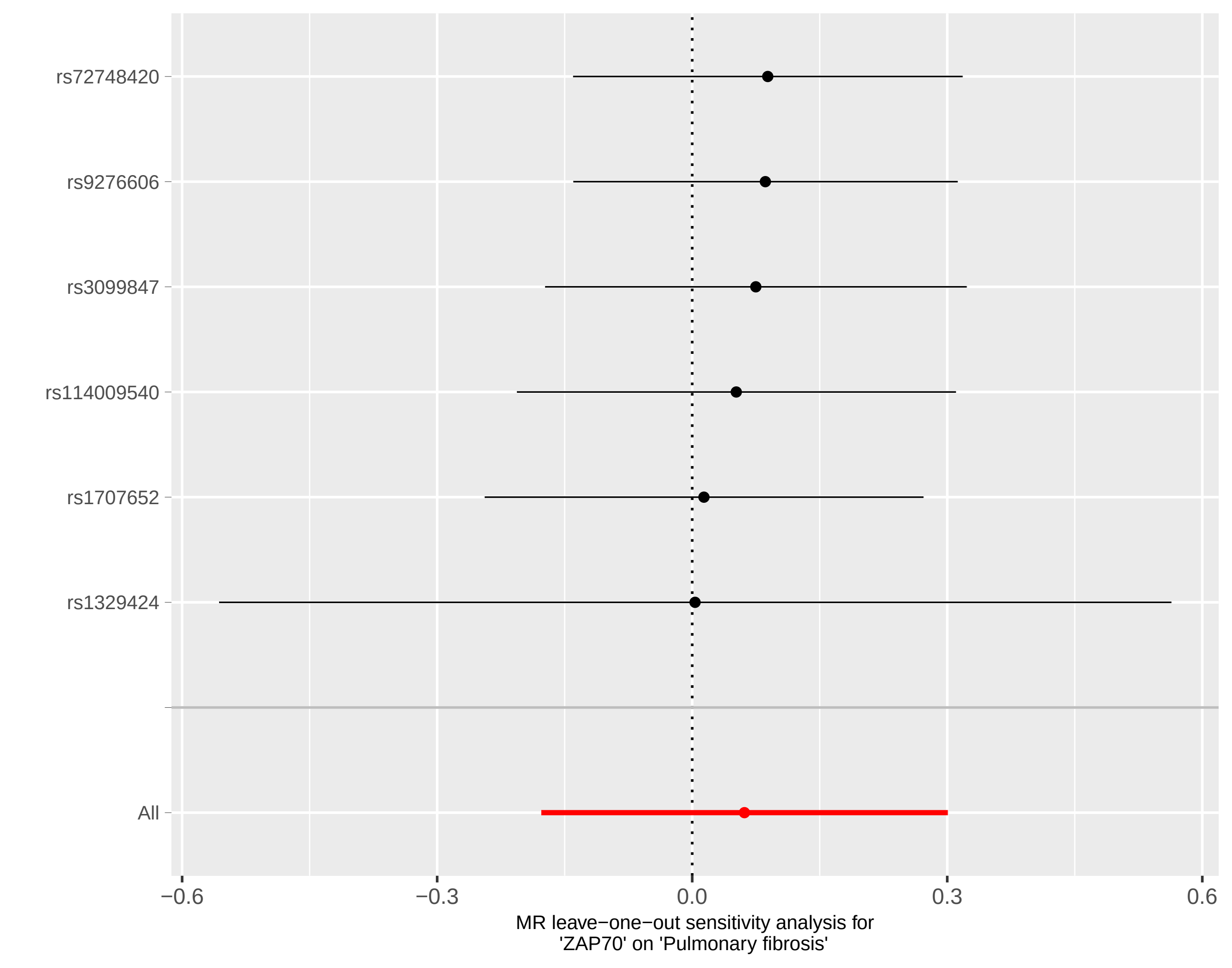
rs72748420
rs9276606
rs3099847
rs114009540
rs1707652
rs1329424
All
−0.6
−0.3
0.0
0.3
0.6
MR leave−one−out sensitivity analysis for
'ZAP70' on 'Pulmonary fibrosis'

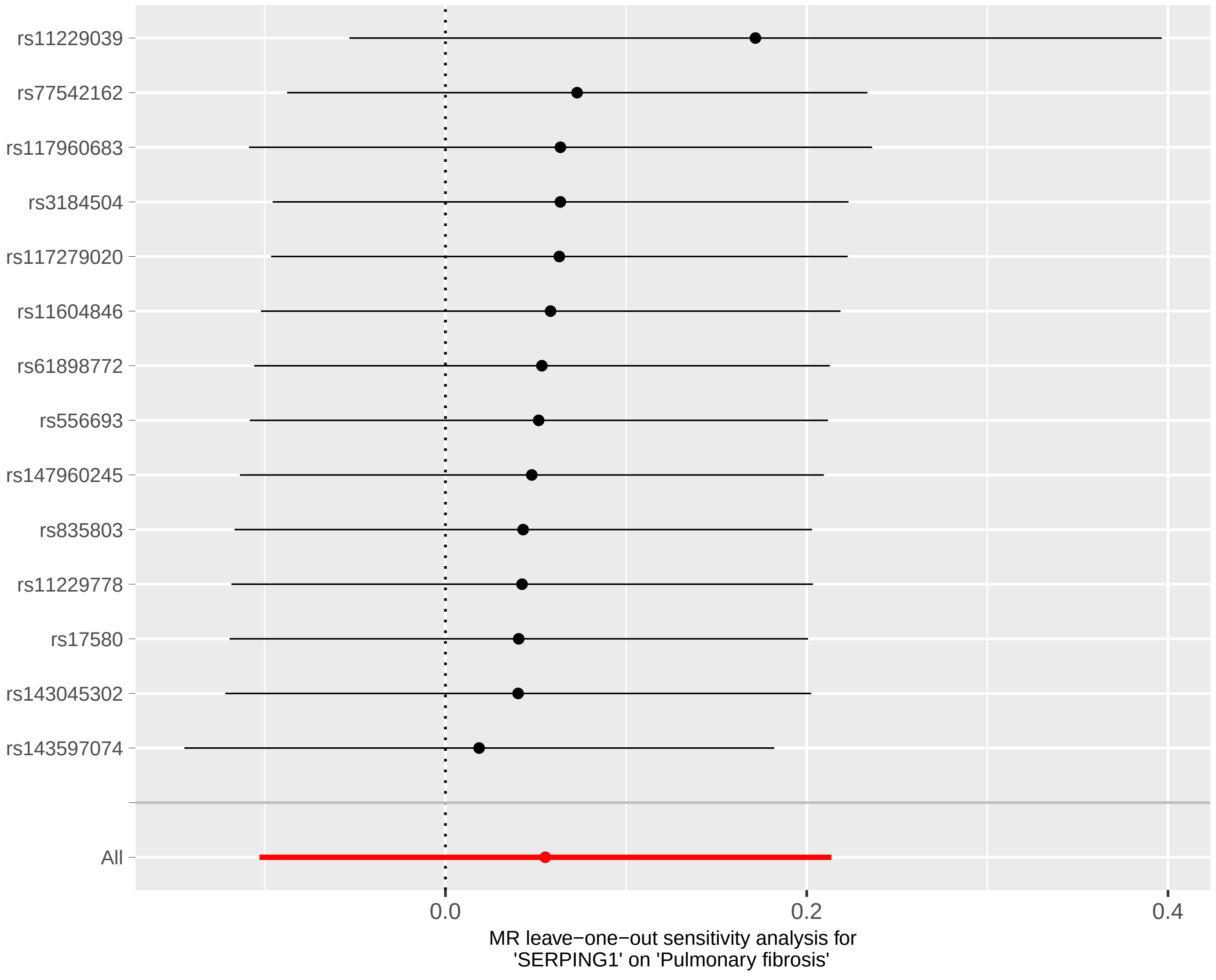
rs11229039
rs77542162
rs117960683
rs3184504
rs117279020
rs11604846
rs61898772
rs556693
rs147960245
rs835803
rs11229778
rs17580
rs143045302
rs143597074
All
0.0
0.2
0.4
MR leave−one−out sensitivity analysis for
'SERPING1' on 'Pulmonary fibrosis'

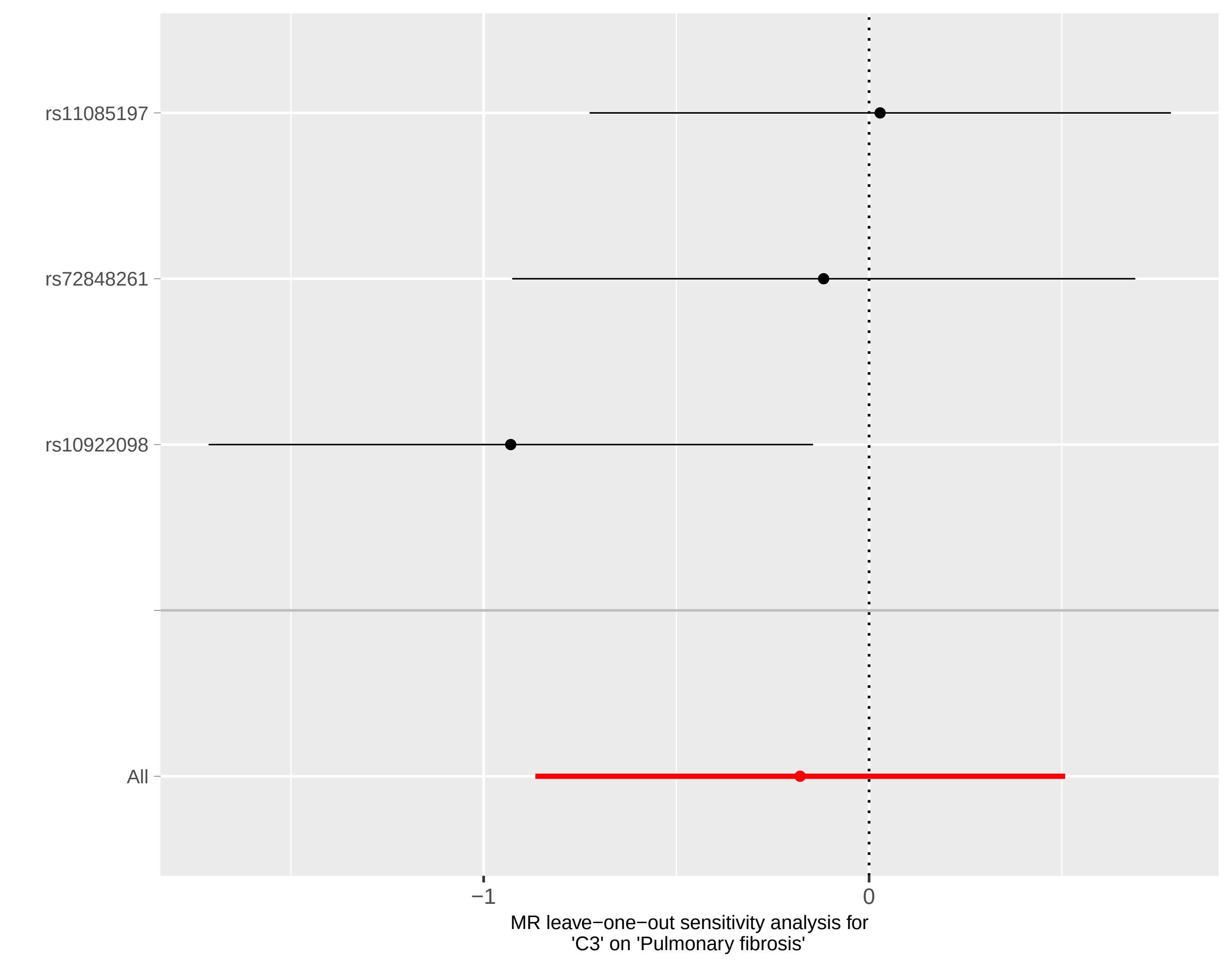

rs11085197
rs72848261
rs10922098
All
−1
0
MR leave−one−out sensitivity analysis for
'C3' on 'Pulmonary fibrosis'

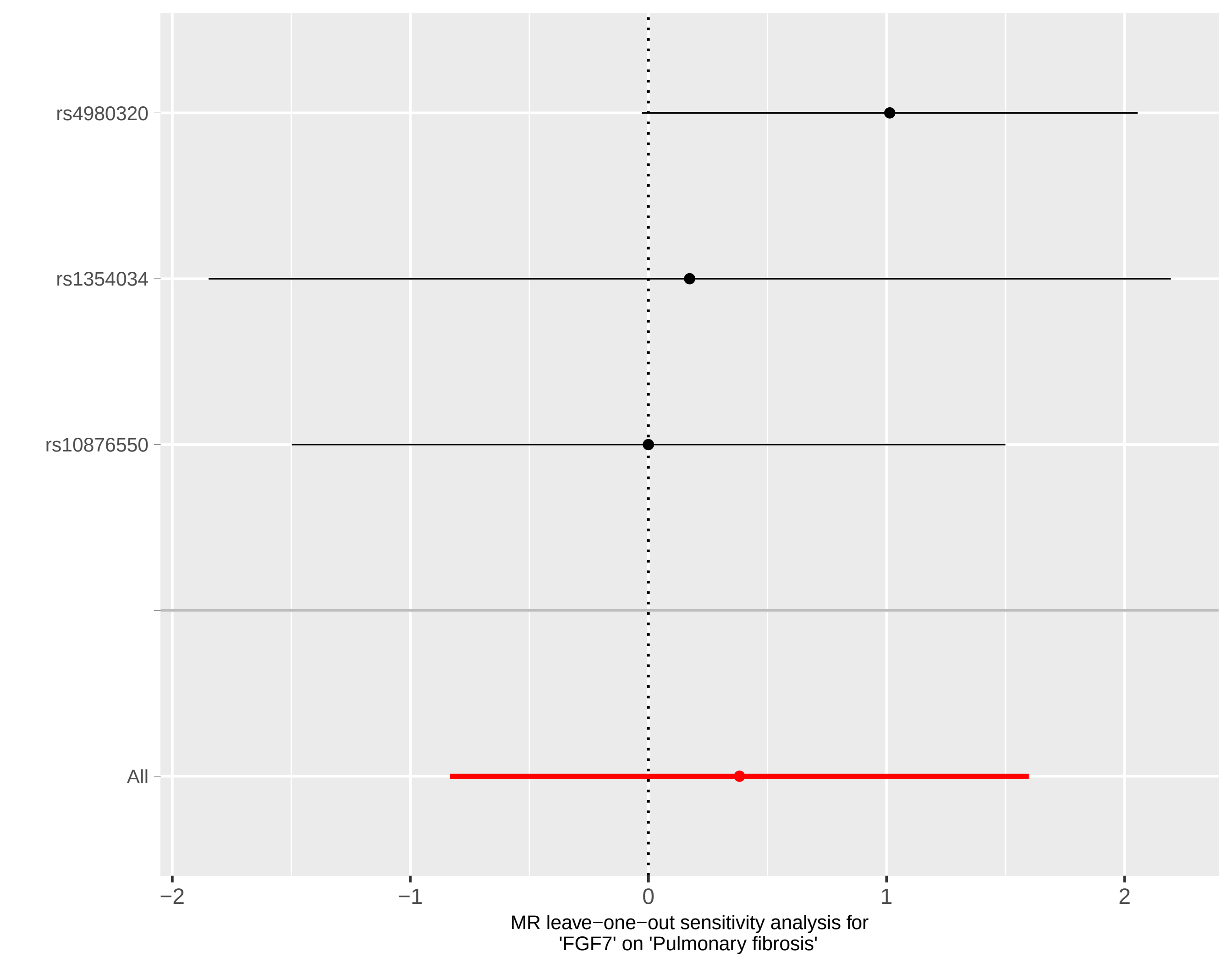
rs4980320
rs1354034
rs10876550
All
−2
−1
0
1
2
MR leave−one−out sensitivity analysis for
'FGF7' on 'Pulmonary fibrosis'

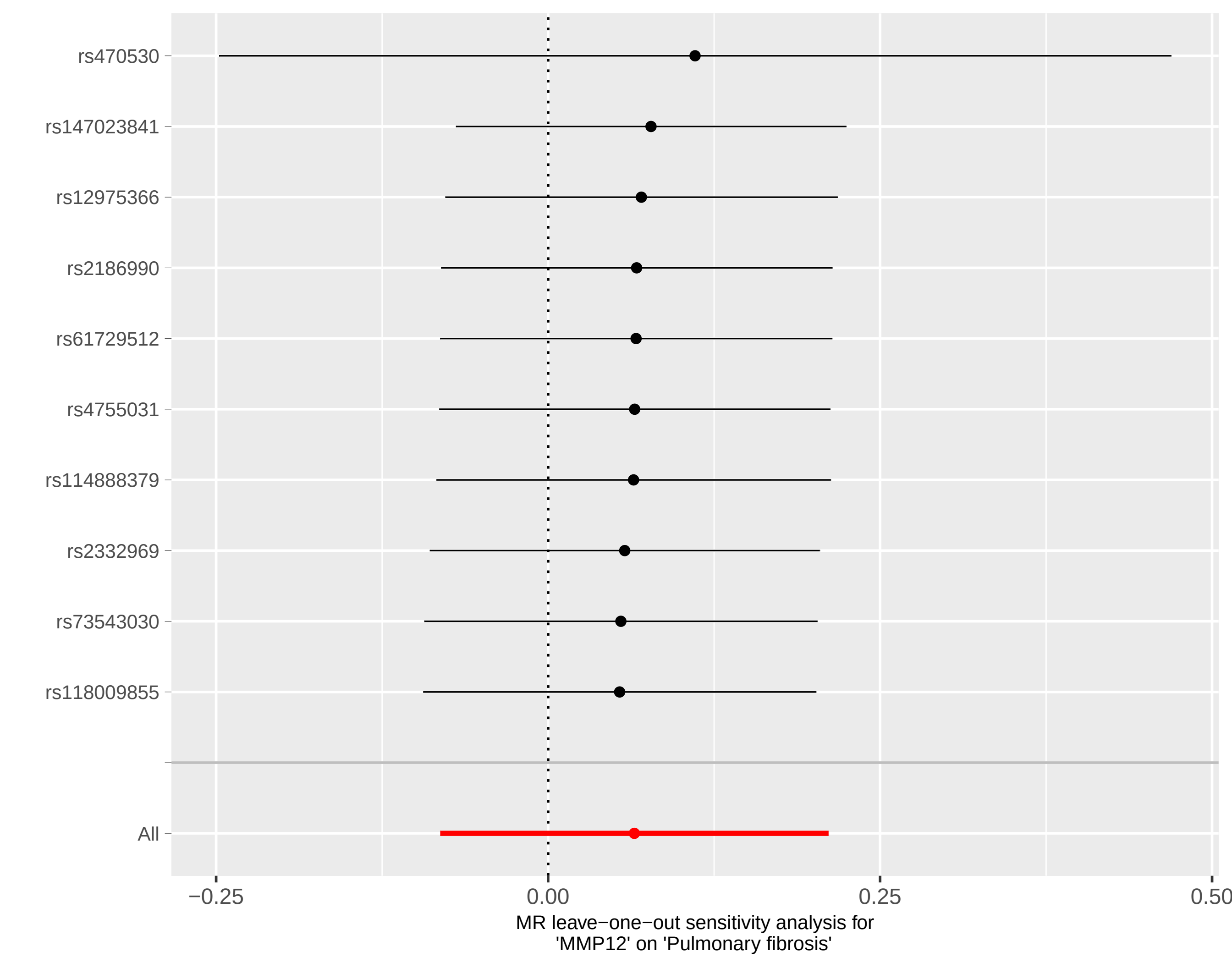
rs470530
rs147023841
rs12975366
rs2186990
rs61729512
rs4755031
rs114888379
rs2332969
rs73543030
rs118009855
All
−0.25
0.00
0.25
0.50
MR leave−one−out sensitivity analysis for
'MMP12' on 'Pulmonary fibrosis'

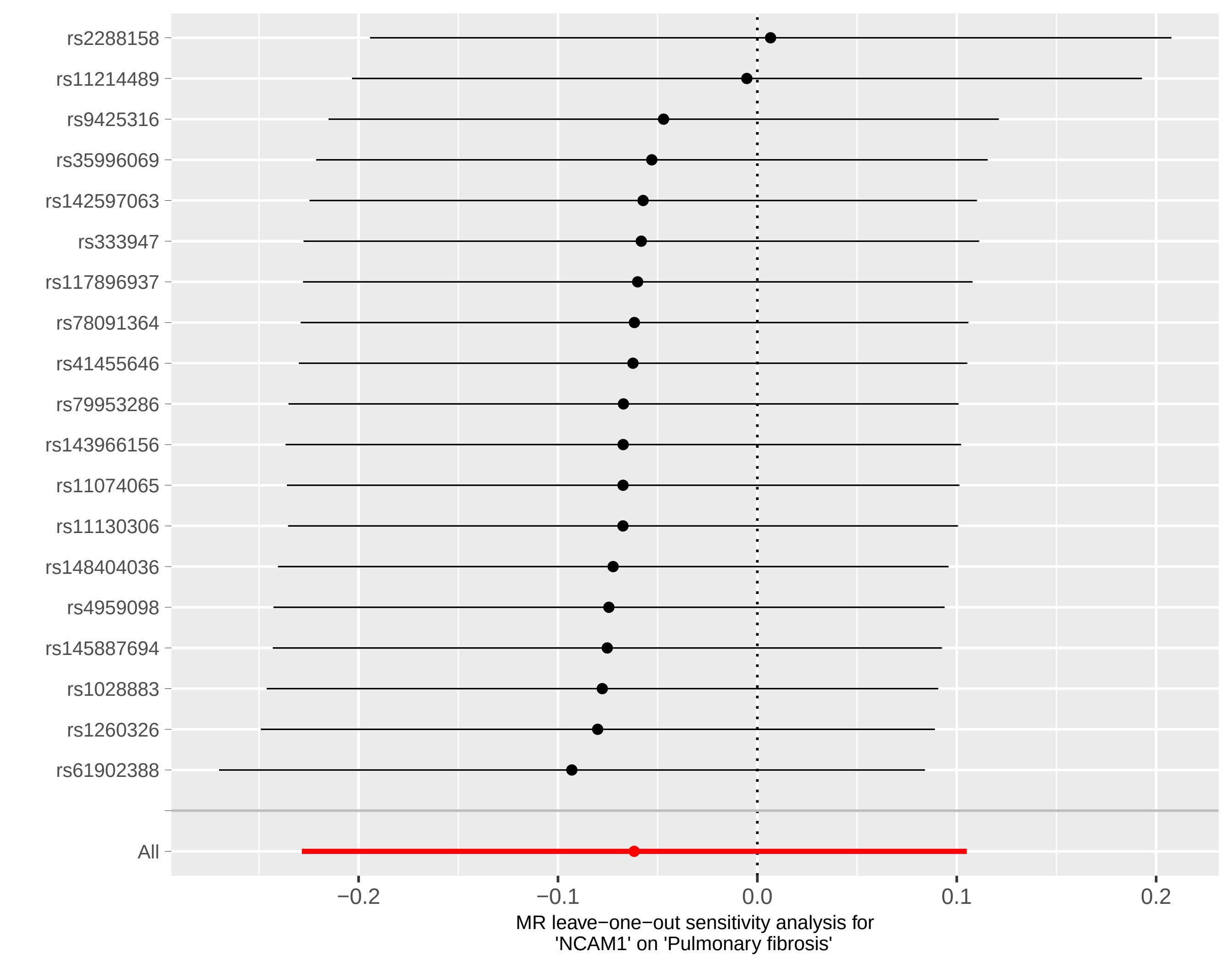
rs2288158
rs11214489
rs9425316
rs35996069
rs142597063
rs333947
rs117896937
rs78091364
rs41455646
rs79953286
rs143966156
rs11074065
rs11130306
rs148404036
rs4959098
rs145887694
rs1028883
rs1260326
rs61902388
All
−0.2
−0.1
0.0
0.1
0.2
MR leave−one−out sensitivity analysis for
'NCAM1' on 'Pulmonary fibrosis'

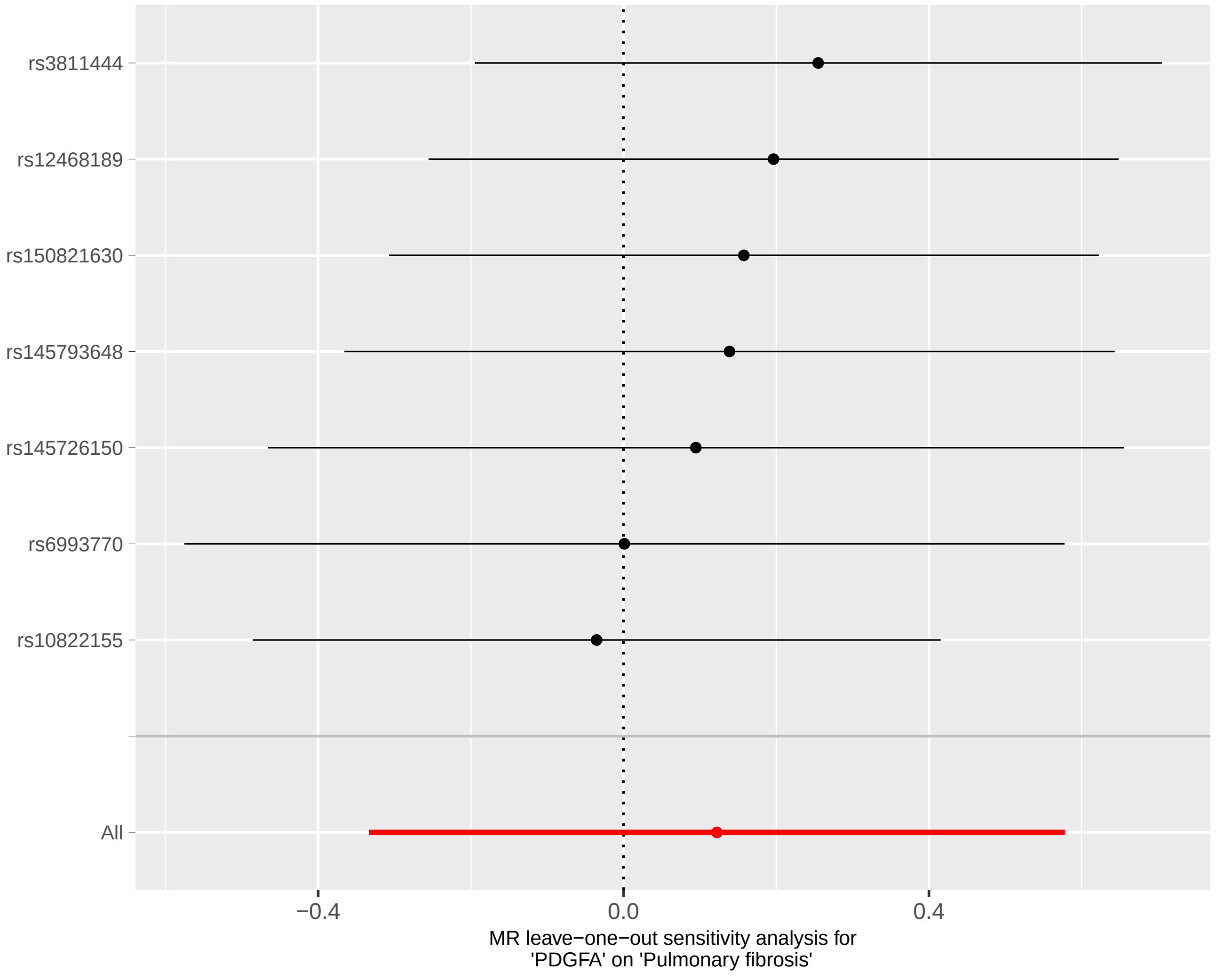
rs3811444
rs12468189
rs150821630
rs145793648
rs145726150
rs6993770
rs10822155
All
−0.4
0.0
0.4
MR leave−one−out sensitivity analysis for
'PDGFA' on 'Pulmonary fibrosis'

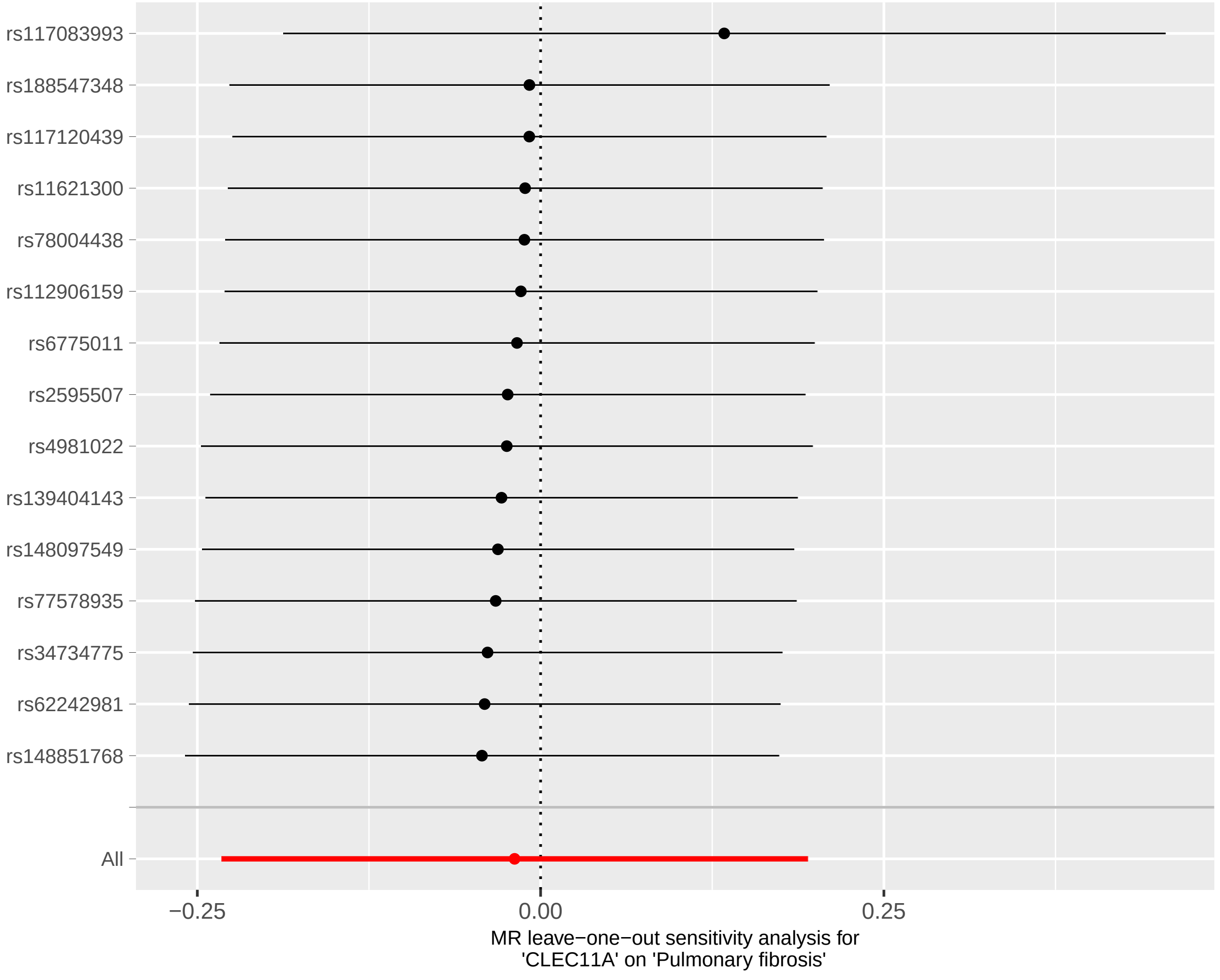
rs117083993
rs188547348
rs117120439
rs11621300
rs78004438
rs112906159
rs6775011
rs2595507
rs4981022
rs139404143
rs148097549
rs77578935
rs34734775
rs62242981
rs148851768
All
−0.25
0.00
0.25
MR leave−one−out sensitivity analysis for
'CLEC11A' on 'Pulmonary fibrosis'

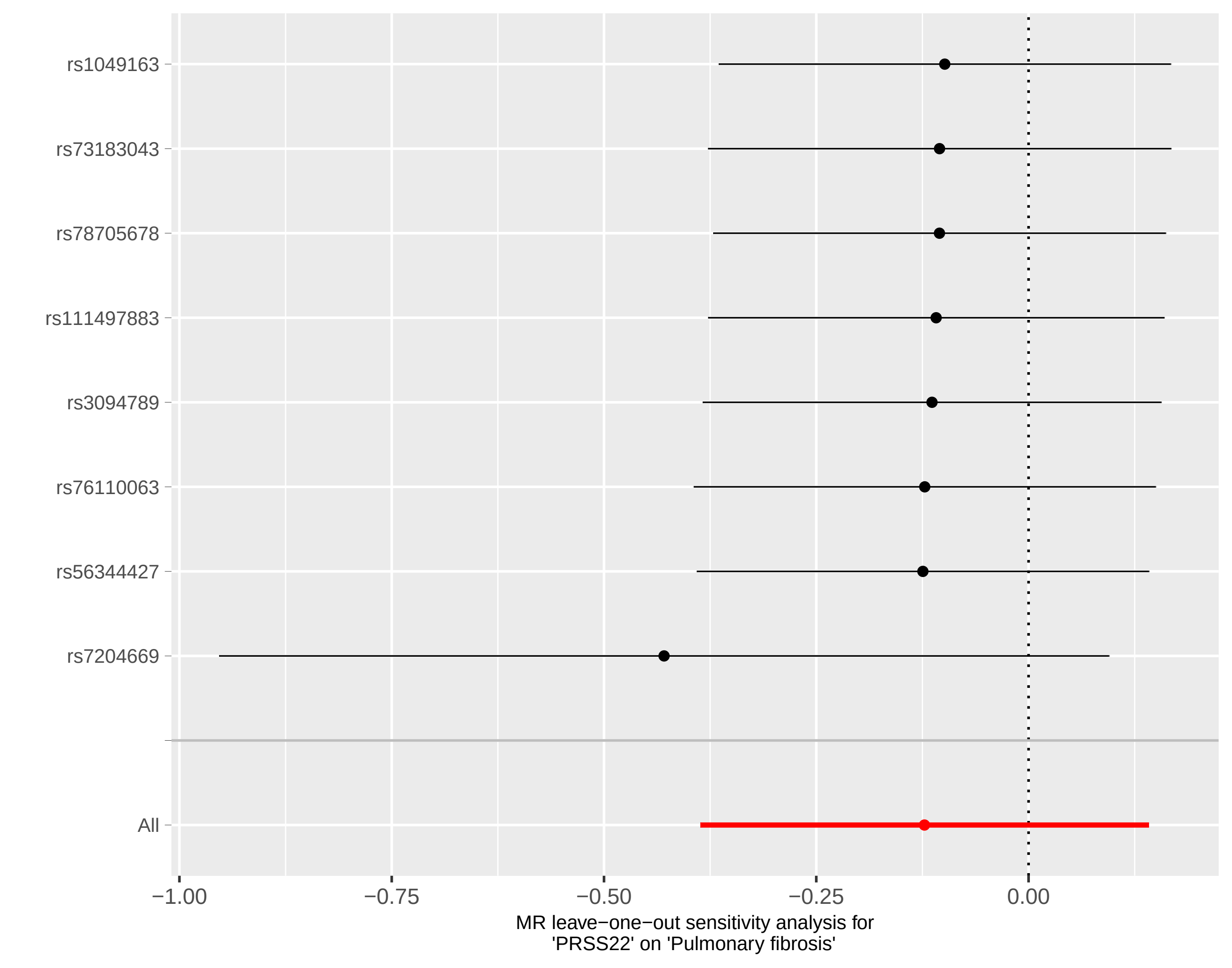
rs1049163
rs73183043
rs78705678
rs111497883
rs3094789
rs76110063
rs56344427
rs7204669
All
−1.00
−0.75
−0.50
−0.25
0.00
MR leave−one−out sensitivity analysis for
'PRSS22' on 'Pulmonary fibrosis'

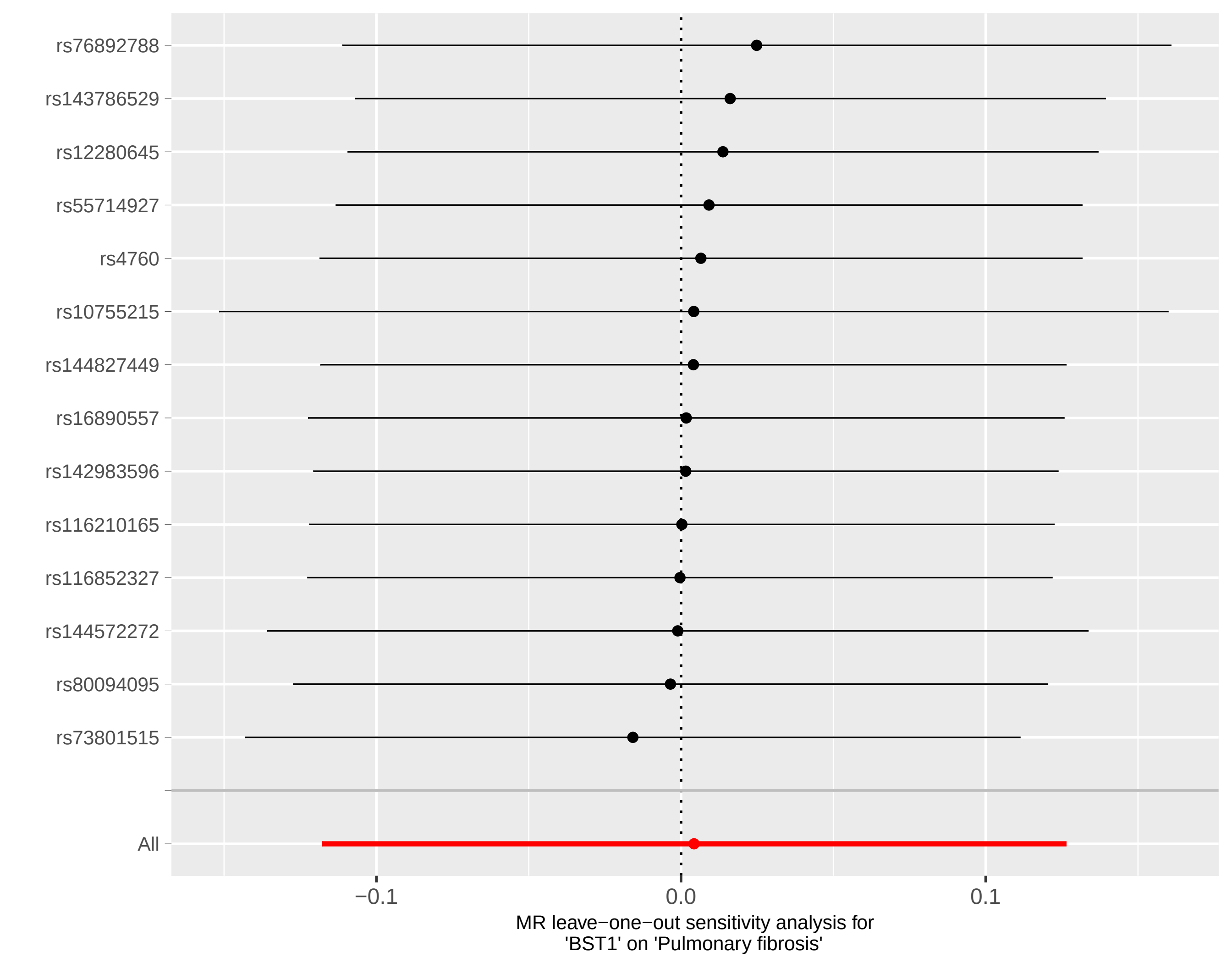
rs76892788
rs143786529
rs12280645
rs55714927
rs4760
rs10755215
rs144827449
rs16890557
rs142983596
rs116210165
rs116852327
rs144572272
rs80094095
rs73801515
All
−0.1
0.0
0.1
MR leave−one−out sensitivity analysis for
'BST1' on 'Pulmonary fibrosis'

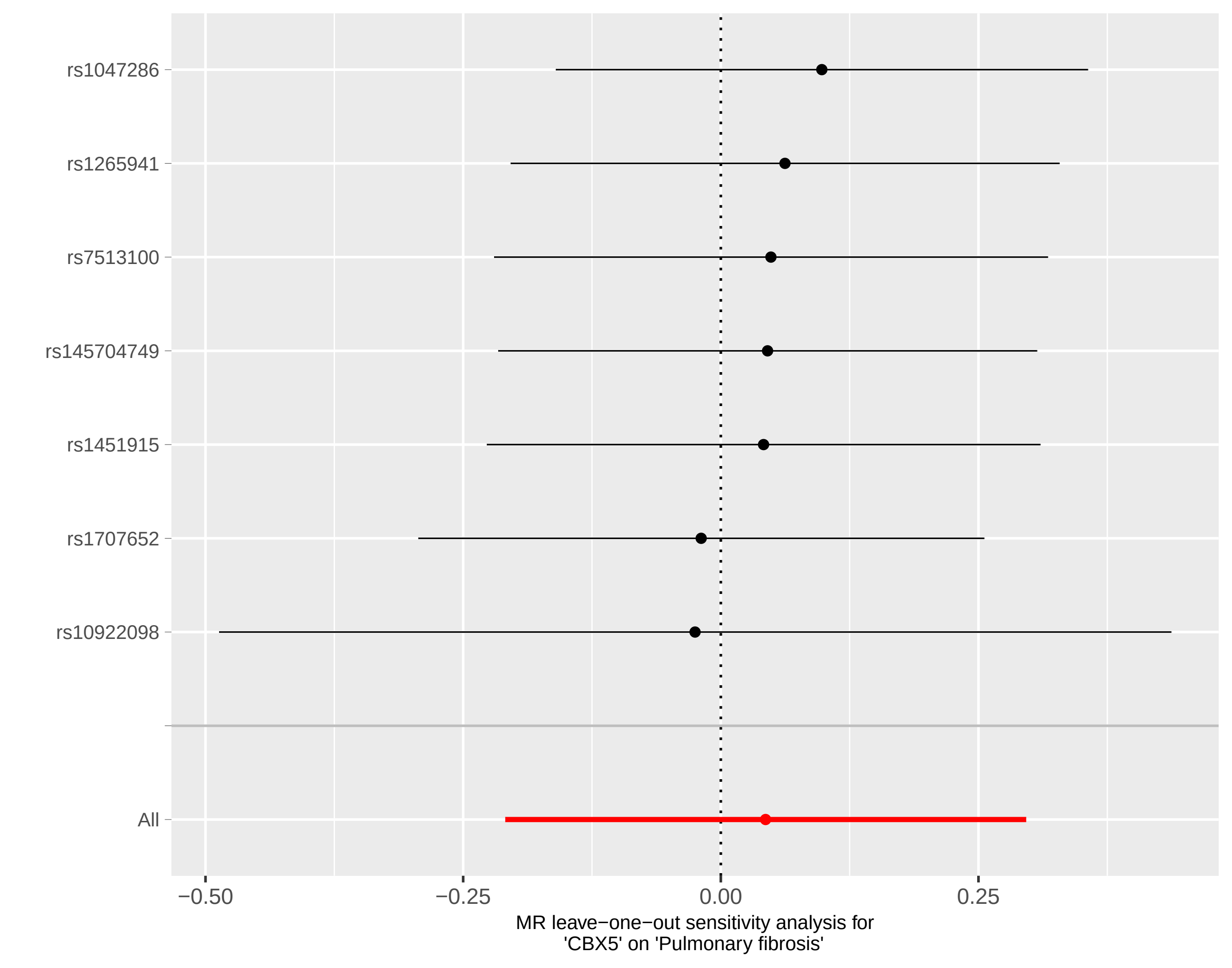
rs1047286
rs1265941
rs7513100
rs145704749
rs1451915
rs1707652
rs10922098
All
−0.50
−0.25
0.00
0.25
MR leave−one−out sensitivity analysis for
'CBX5' on 'Pulmonary fibrosis'

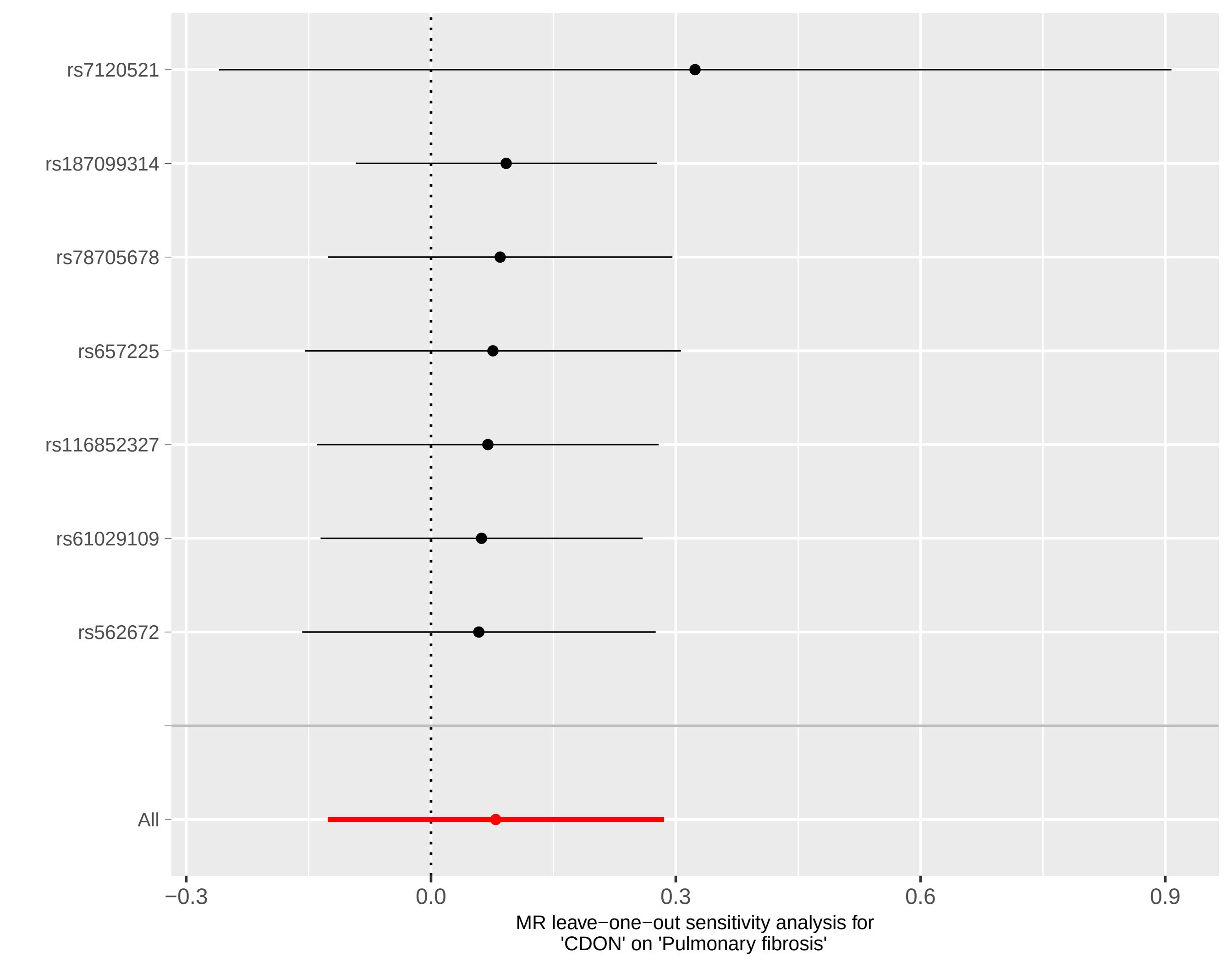
rs7120521
rs187099314
rs78705678
rs657225
rs116852327
rs61029109
rs562672
All
−0.3
0.0
0.3
0.6
0.9
MR leave−one−out sensitivity analysis for
'CDON' on 'Pulmonary fibrosis'

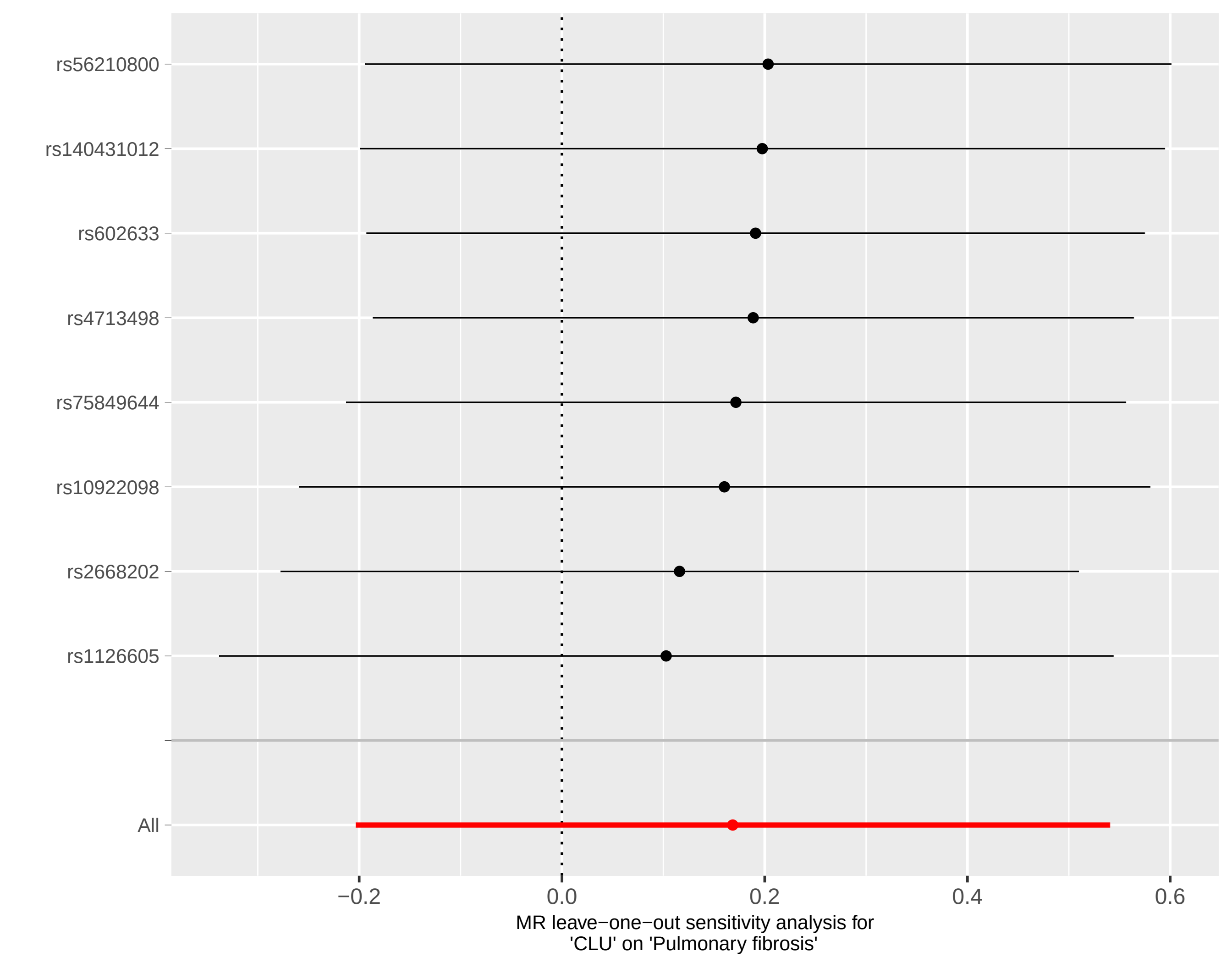

rs56210800
rs140431012
rs602633
rs4713498
rs75849644
rs10922098
rs2668202
rs1126605
All
−0.2
0.0
0.2
0.4
0.6
MR leave−one−out sensitivity analysis for
'CLU' on 'Pulmonary fibrosis'

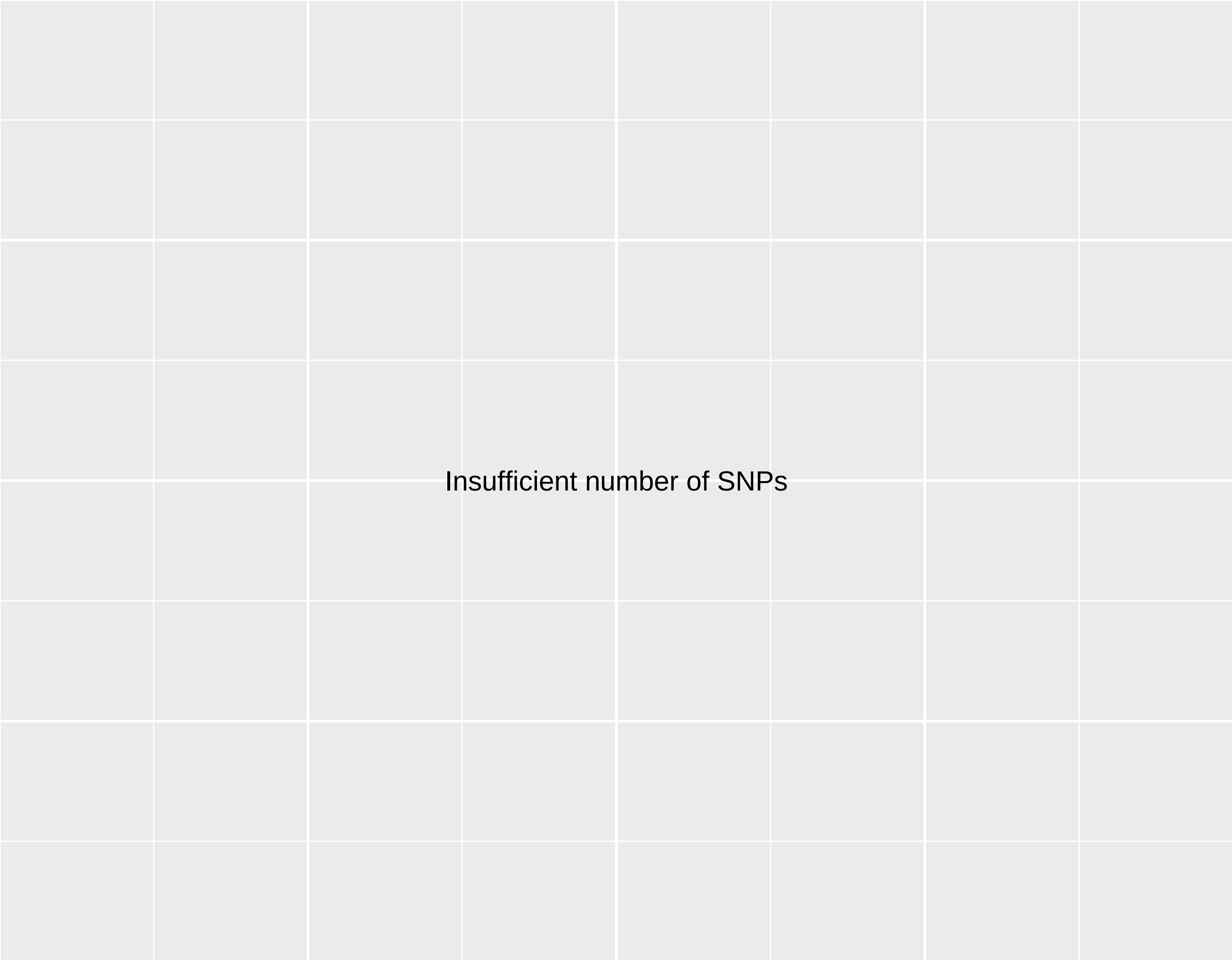
Insufficient number of SNPs

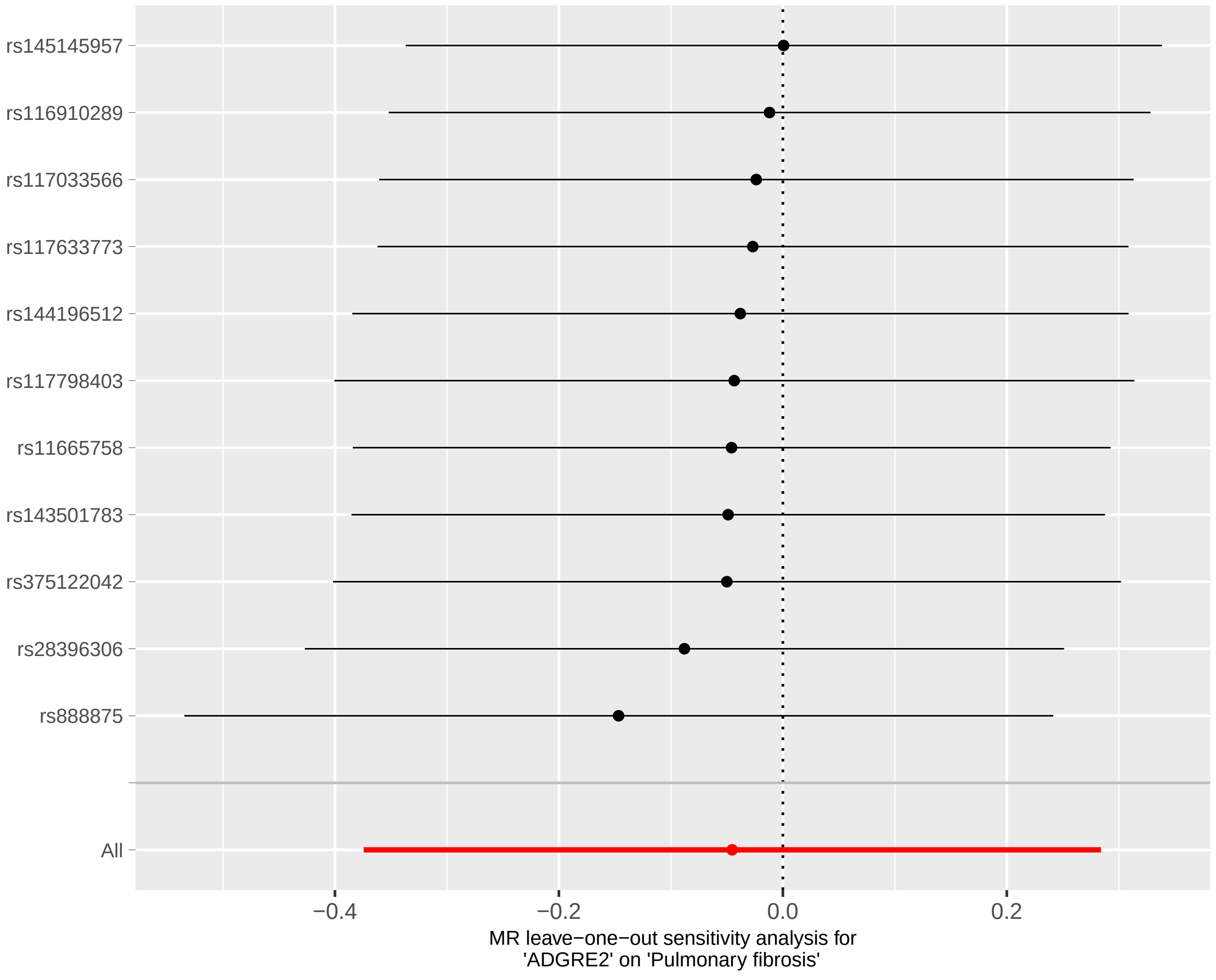

rs145145957
rs116910289
rs117033566
rs117633773
rs144196512
rs117798403
rs11665758
rs143501783
rs375122042
rs28396306
rs888875
All
−0.4
−0.2
0.0
0.2
MR leave−one−out sensitivity analysis for
'ADGRE2' on 'Pulmonary fibrosis'

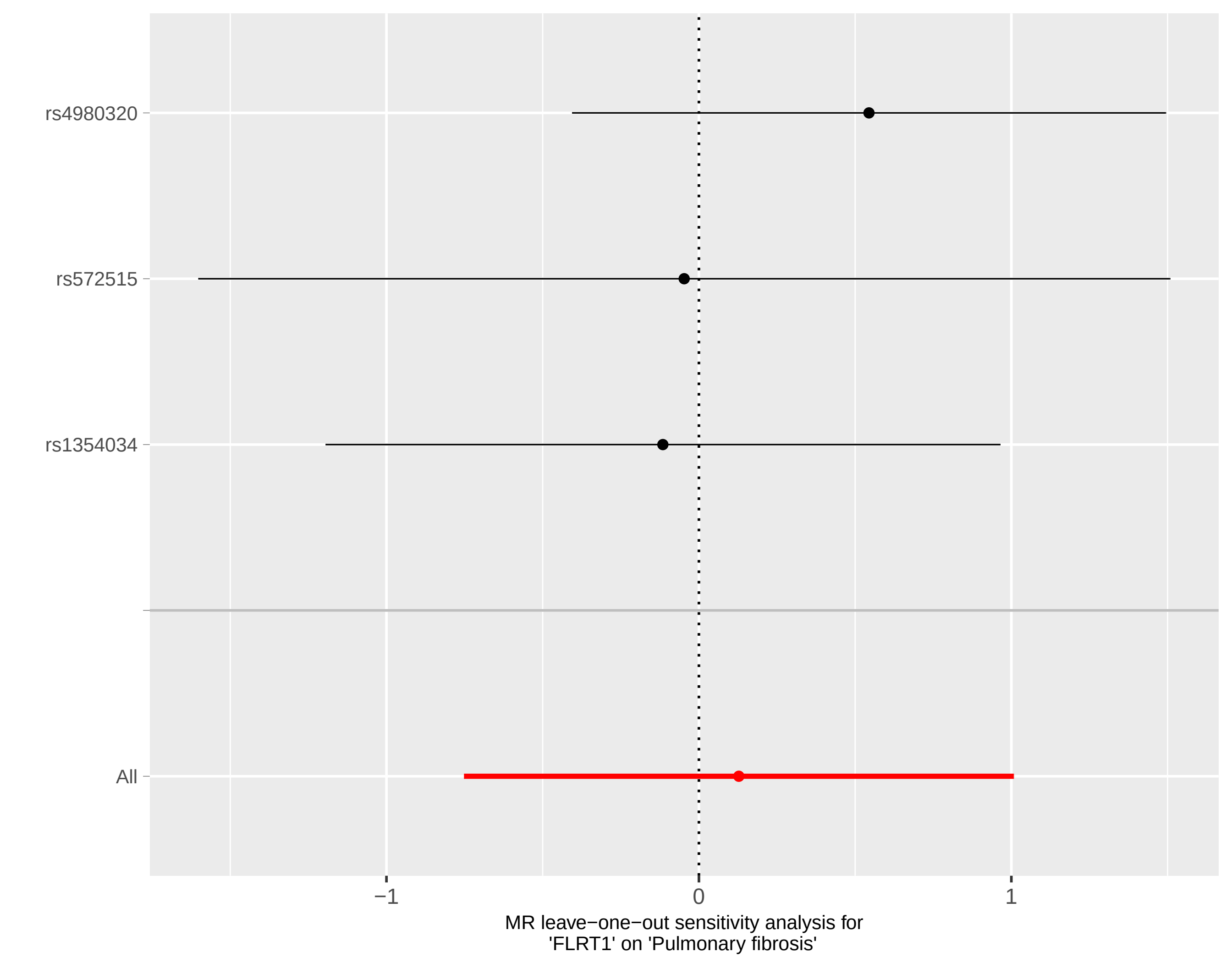

rs4980320
rs572515
rs1354034
All
−1
0
1
MR leave−one−out sensitivity analysis for
'FLRT1' on 'Pulmonary fibrosis'

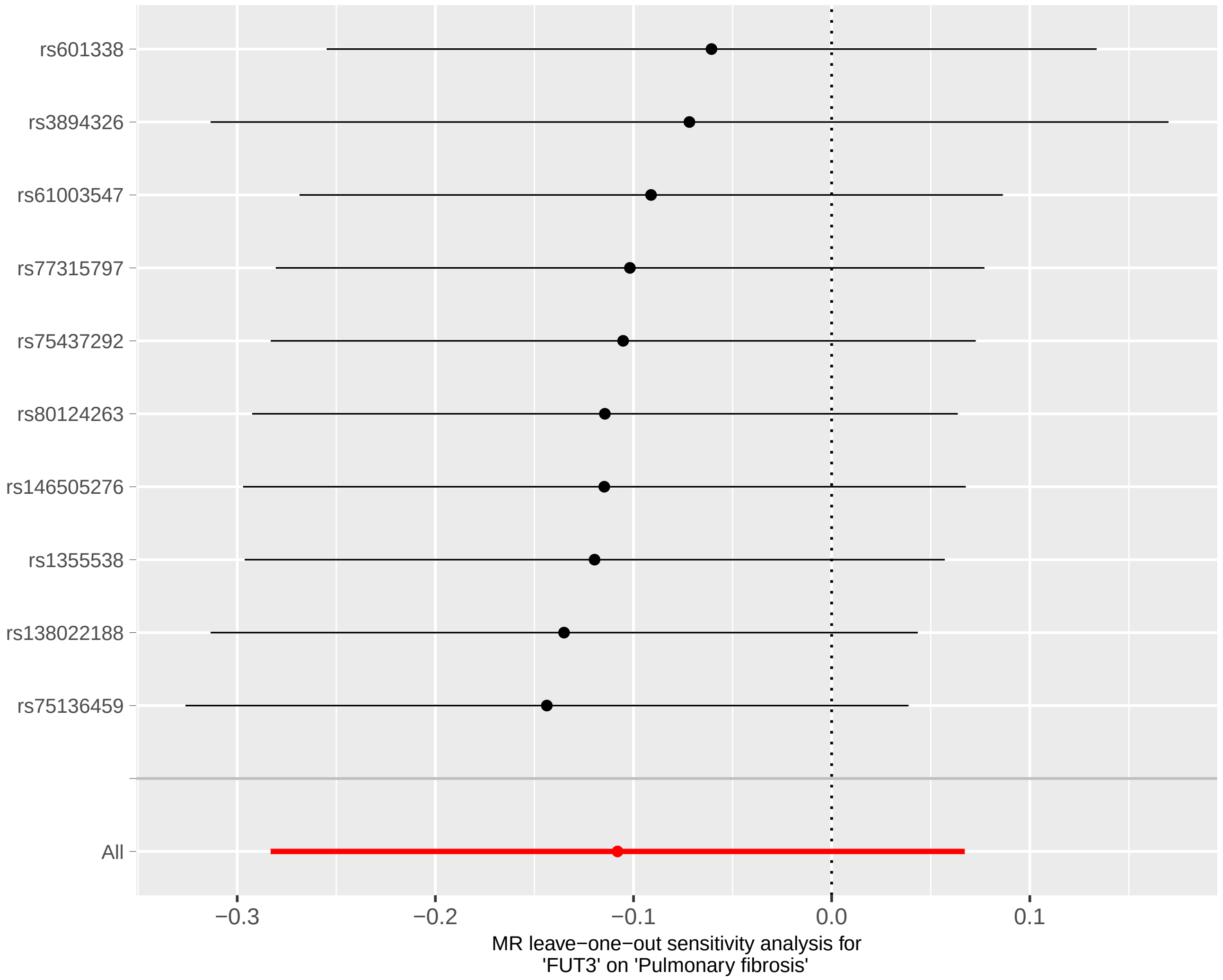
rs601338
rs3894326
rs61003547
rs77315797
rs75437292
rs80124263
rs146505276
rs1355538
rs138022188
rs75136459
All
−0.3
−0.2
−0.1
0.0
0.1
MR leave−one−out sensitivity analysis for
'FUT3' on 'Pulmonary fibrosis'

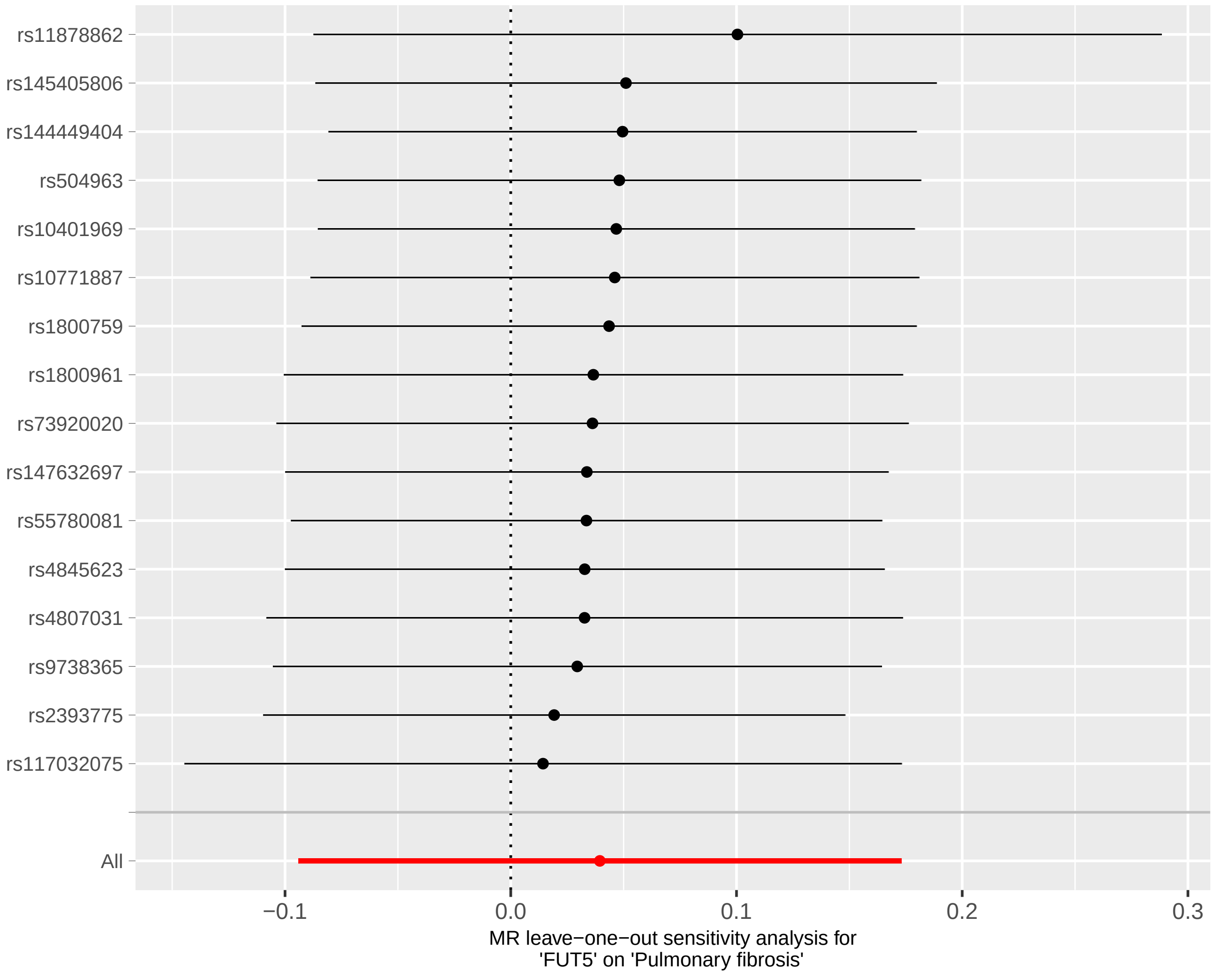

rs11878862
rs145405806
rs144449404
rs504963
rs10401969
rs10771887
rs1800759
rs1800961
rs73920020
rs147632697
rs55780081
rs4845623
rs4807031
rs9738365
rs2393775
rs117032075
All
−0.1
0.0
0.1
0.2
0.3
MR leave−one−out sensitivity analysis for
'FUT5' on 'Pulmonary fibrosis'

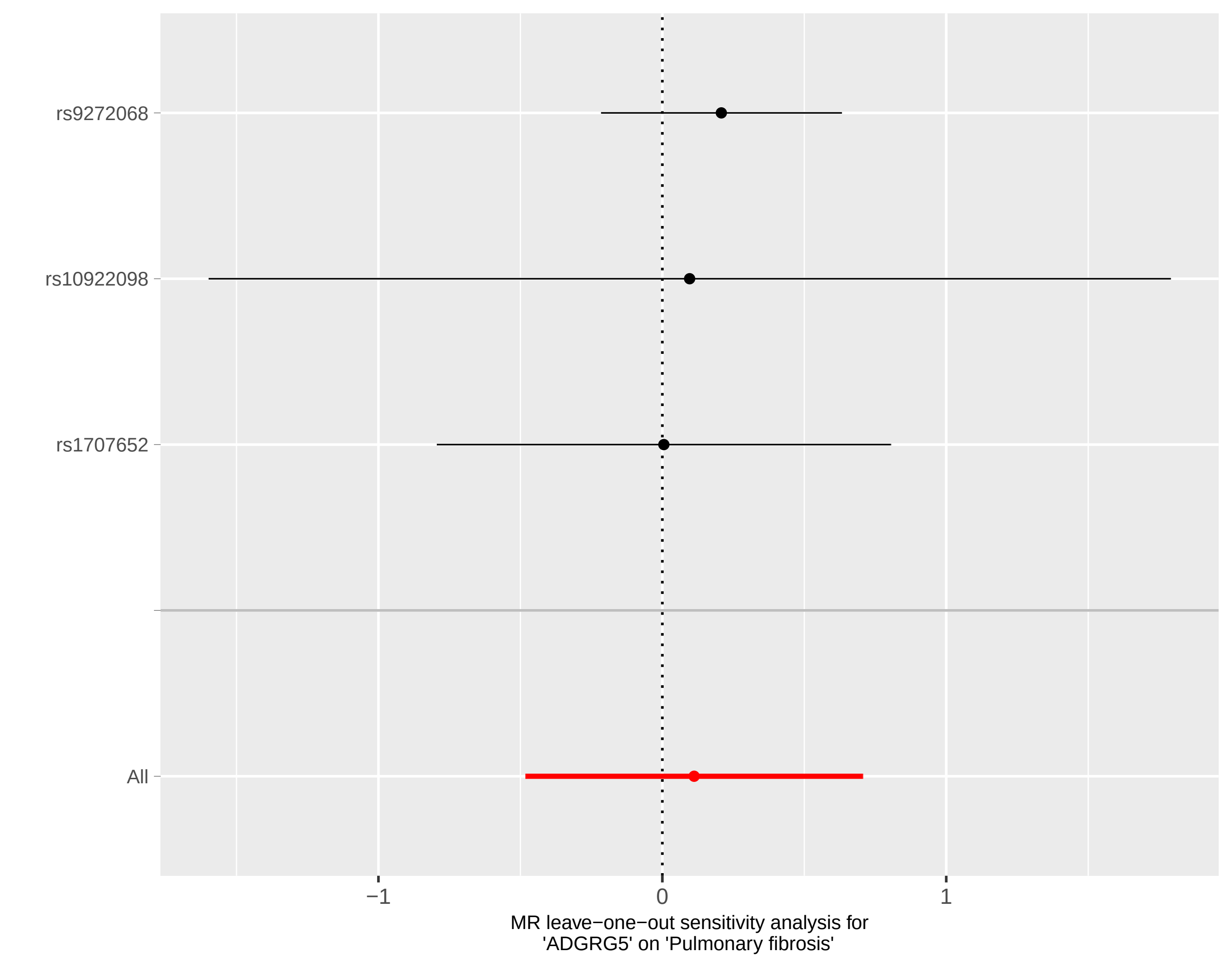
rs9272068
rs10922098
rs1707652
All
−1
0
1
MR leave−one−out sensitivity analysis for
'ADGRG5' on 'Pulmonary fibrosis'

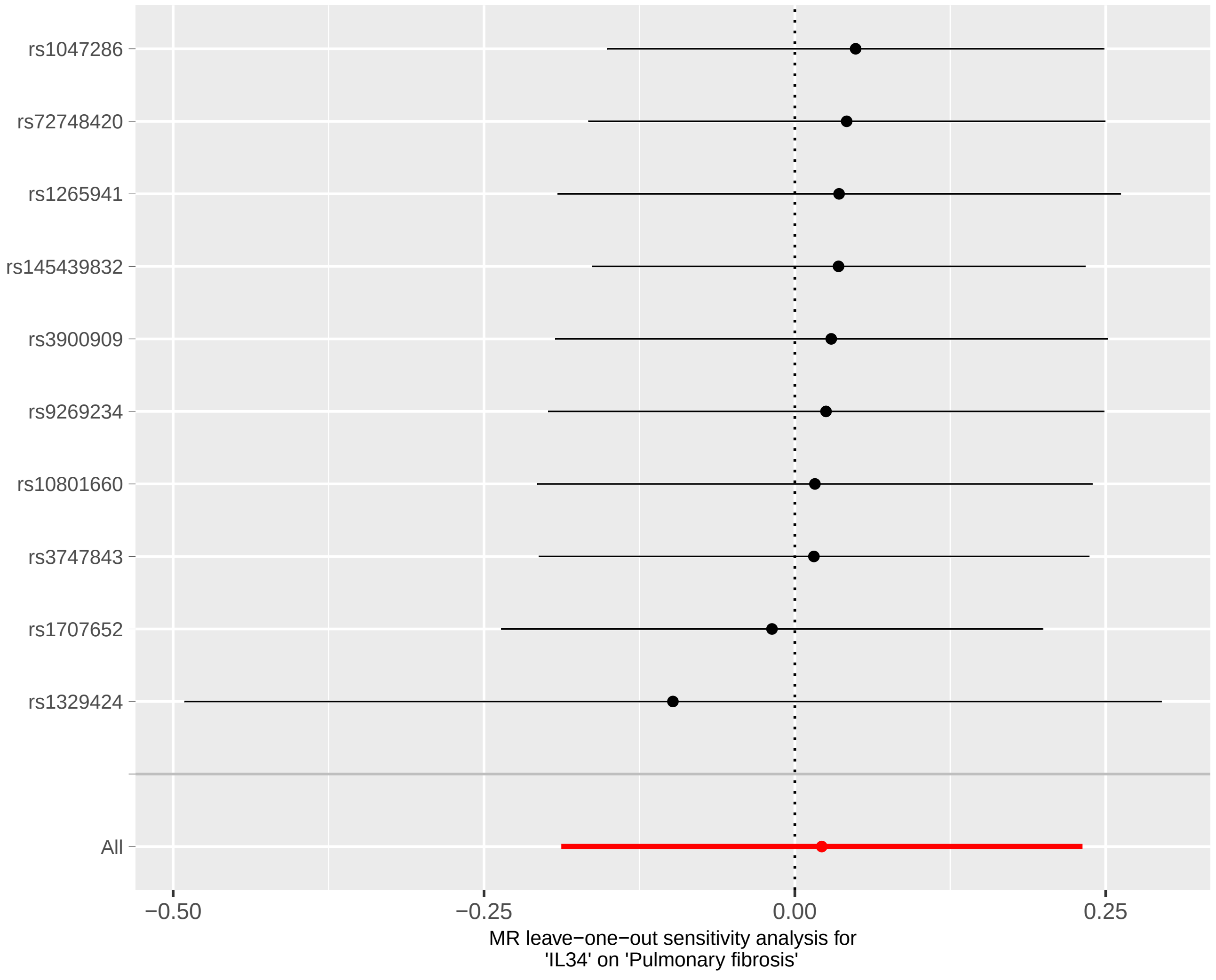

rs1047286
rs72748420
rs1265941
rs145439832
rs3900909
rs9269234
rs10801660
rs3747843
rs1707652
rs1329424
All
−0.50
−0.25
0.00
0.25
MR leave−one−out sensitivity analysis for
'IL34' on 'Pulmonary fibrosis'

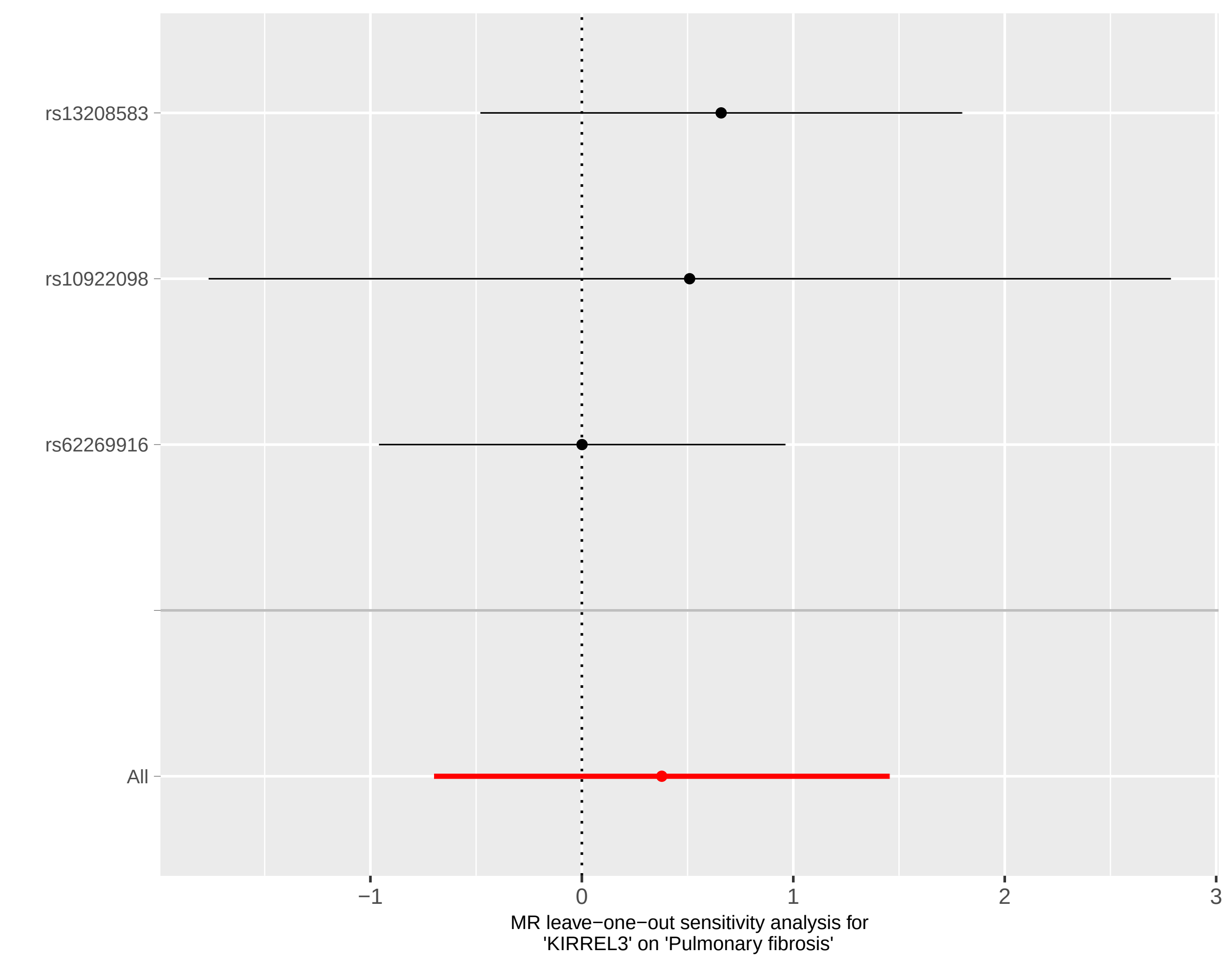

rs13208583
rs10922098
rs62269916
All
−1
0
1
2
3
MR leave−one−out sensitivity analysis for
'KIRREL3' on 'Pulmonary fibrosis'

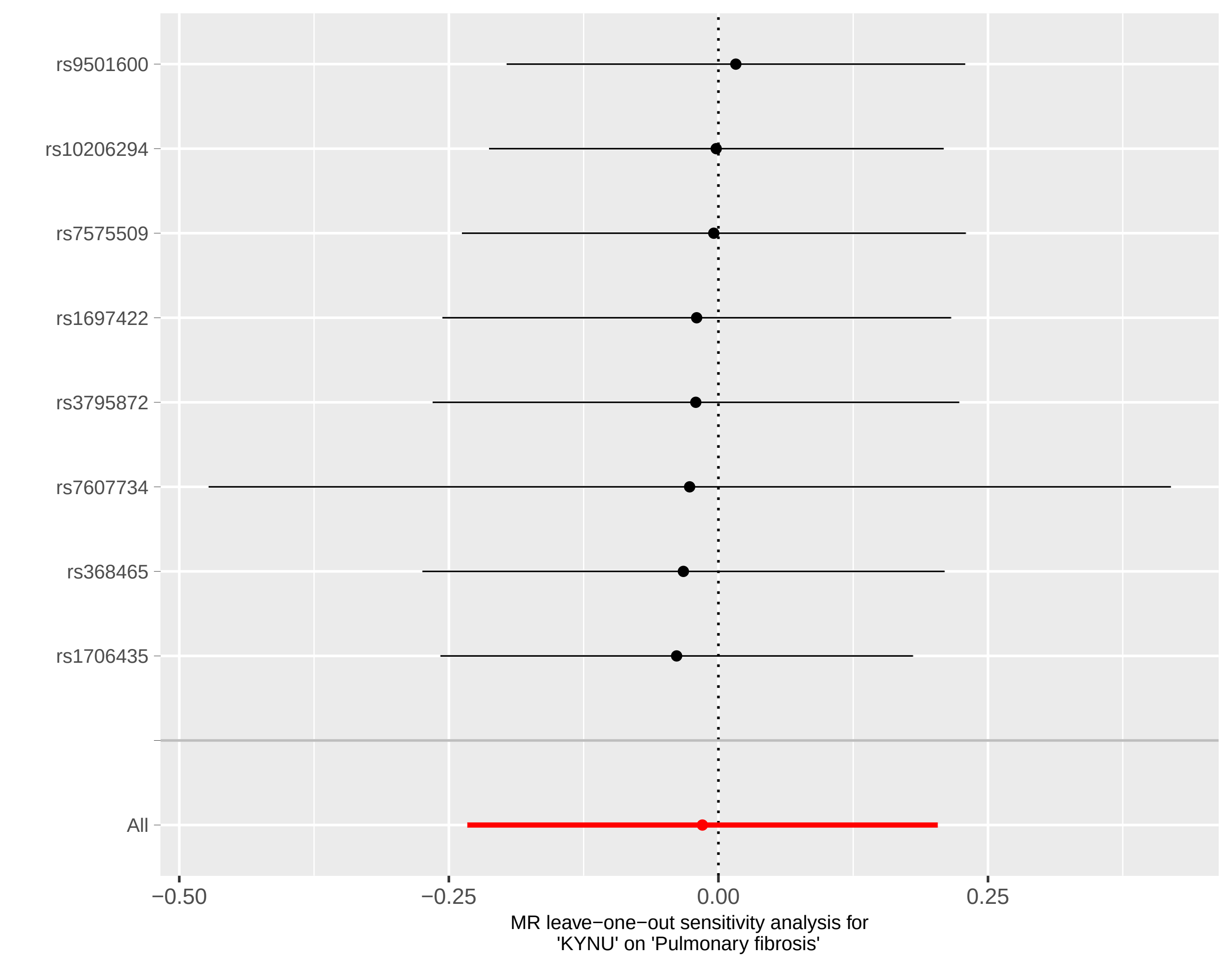
rs9501600
rs10206294
rs7575509
rs1697422
rs3795872
rs7607734
rs368465
rs1706435
All
−0.50
−0.25
0.00
0.25
MR leave−one−out sensitivity analysis for
'KYNU' on 'Pulmonary fibrosis'

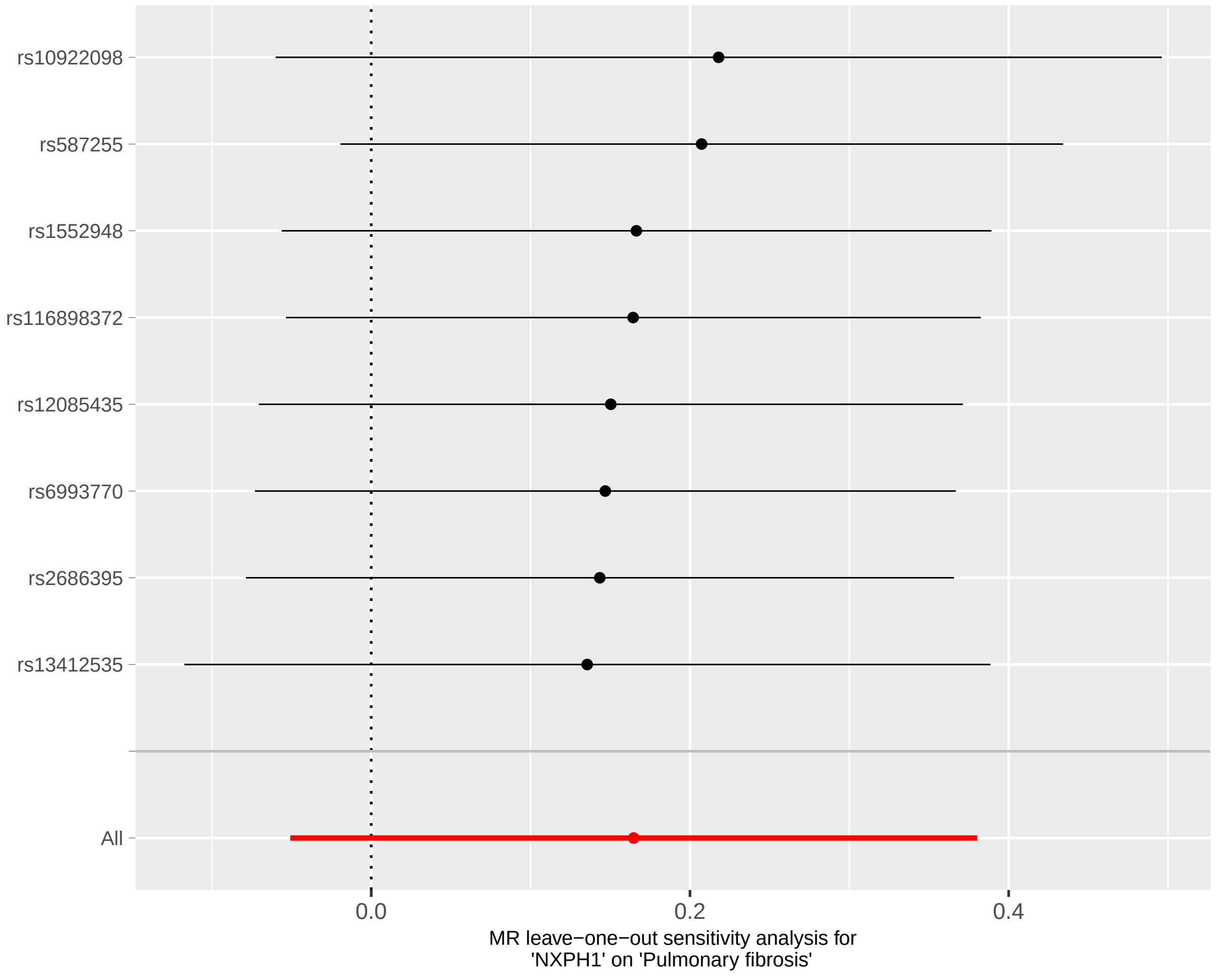
rs10922098
rs587255
rs1552948
rs116898372
rs12085435
rs6993770
rs2686395
rs13412535
All
0.0
0.2
0.4
MR leave−one−out sensitivity analysis for
'NXPH1' on 'Pulmonary fibrosis'

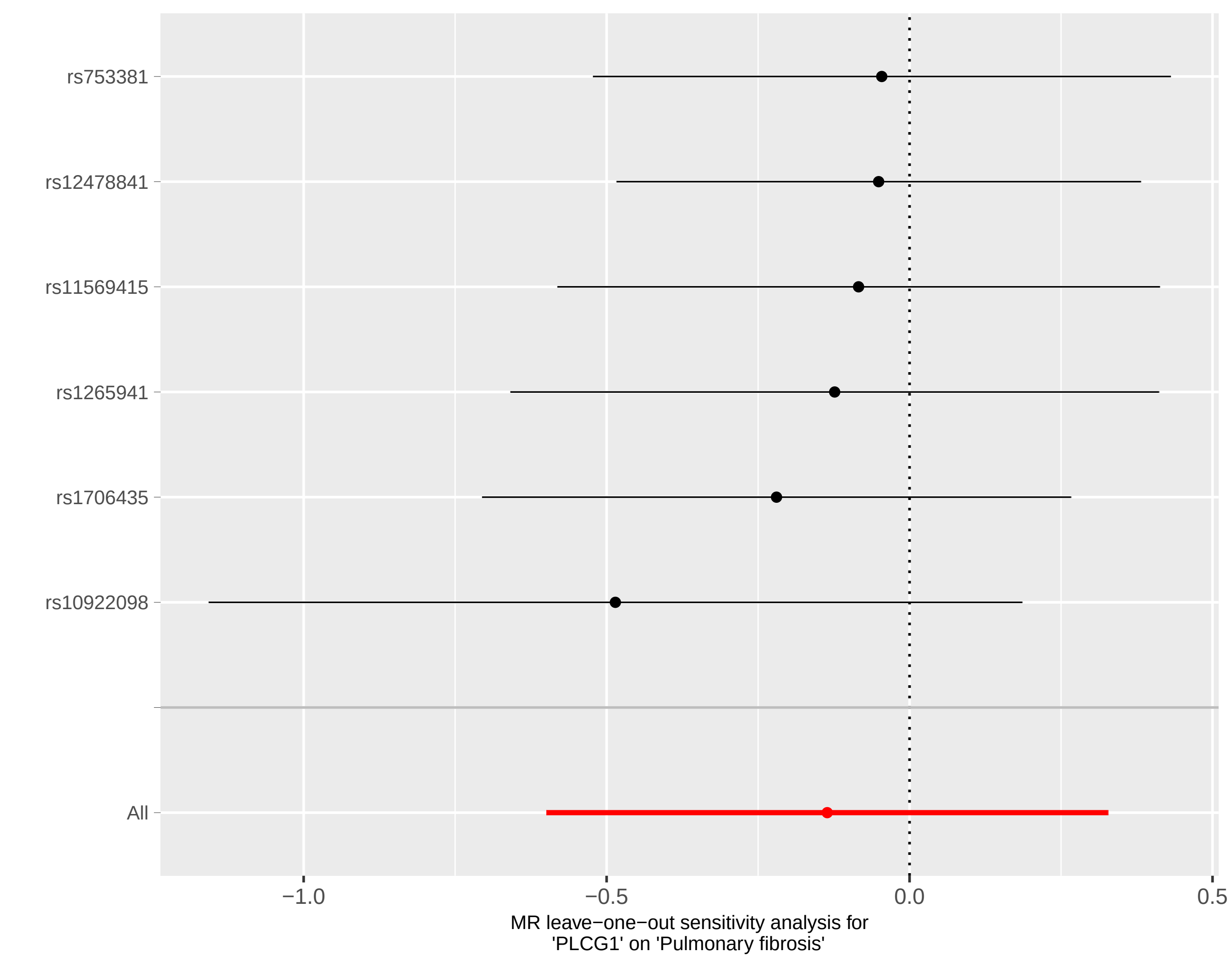
rs753381
rs12478841
rs11569415
rs1265941
rs1706435
rs10922098
All
−1.0
−0.5
0.0
0.5
MR leave−one−out sensitivity analysis for
'PLCG1' on 'Pulmonary fibrosis'

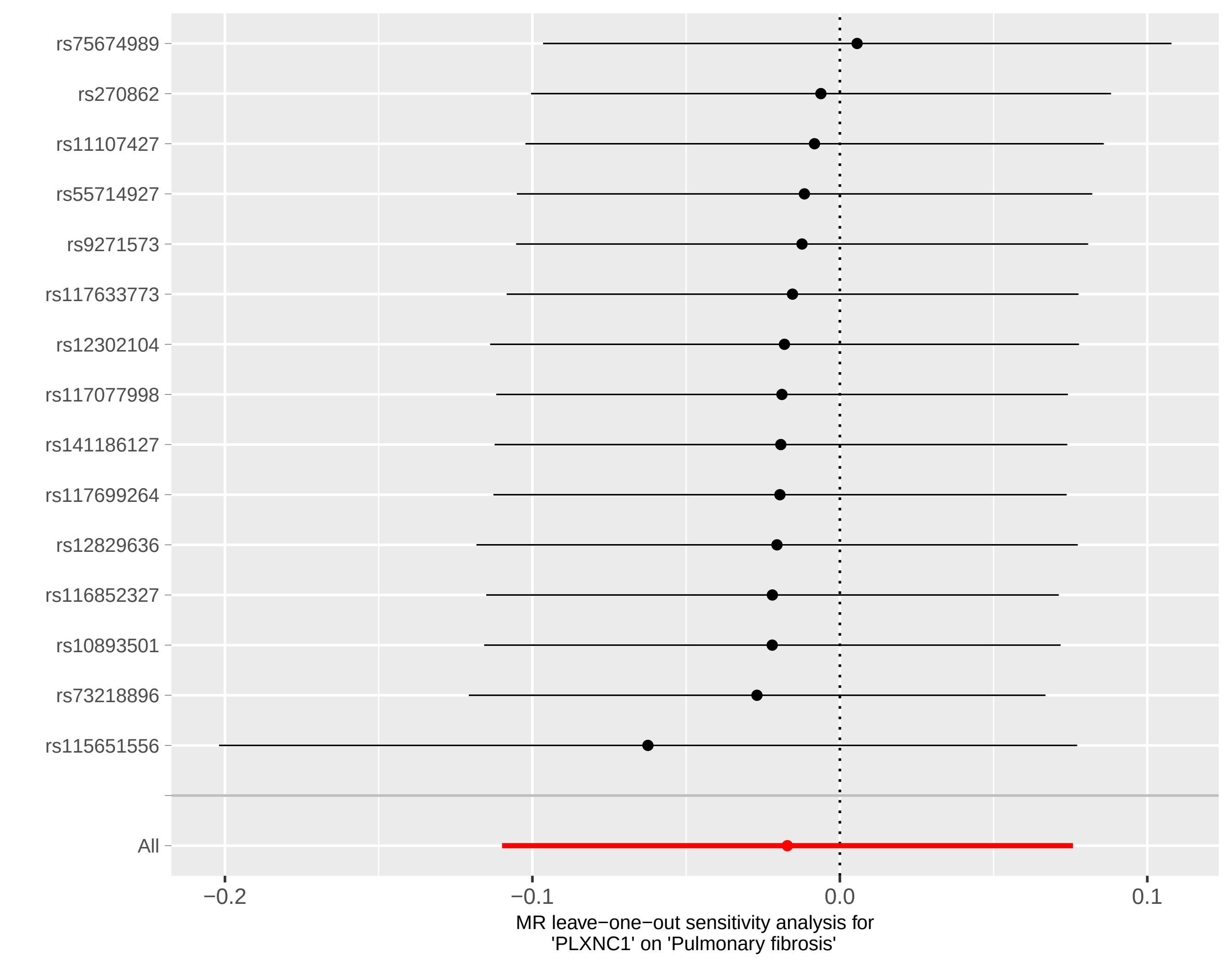
rs75674989
rs270862
rs11107427
rs55714927
rs9271573
rs117633773
rs12302104
rs117077998
rs141186127
rs117699264
rs12829636
rs116852327
rs10893501
rs73218896
rs115651556
All
−0.2
−0.1
0.0
0.1
MR leave−one−out sensitivity analysis for
'PLXNC1' on 'Pulmonary fibrosis'

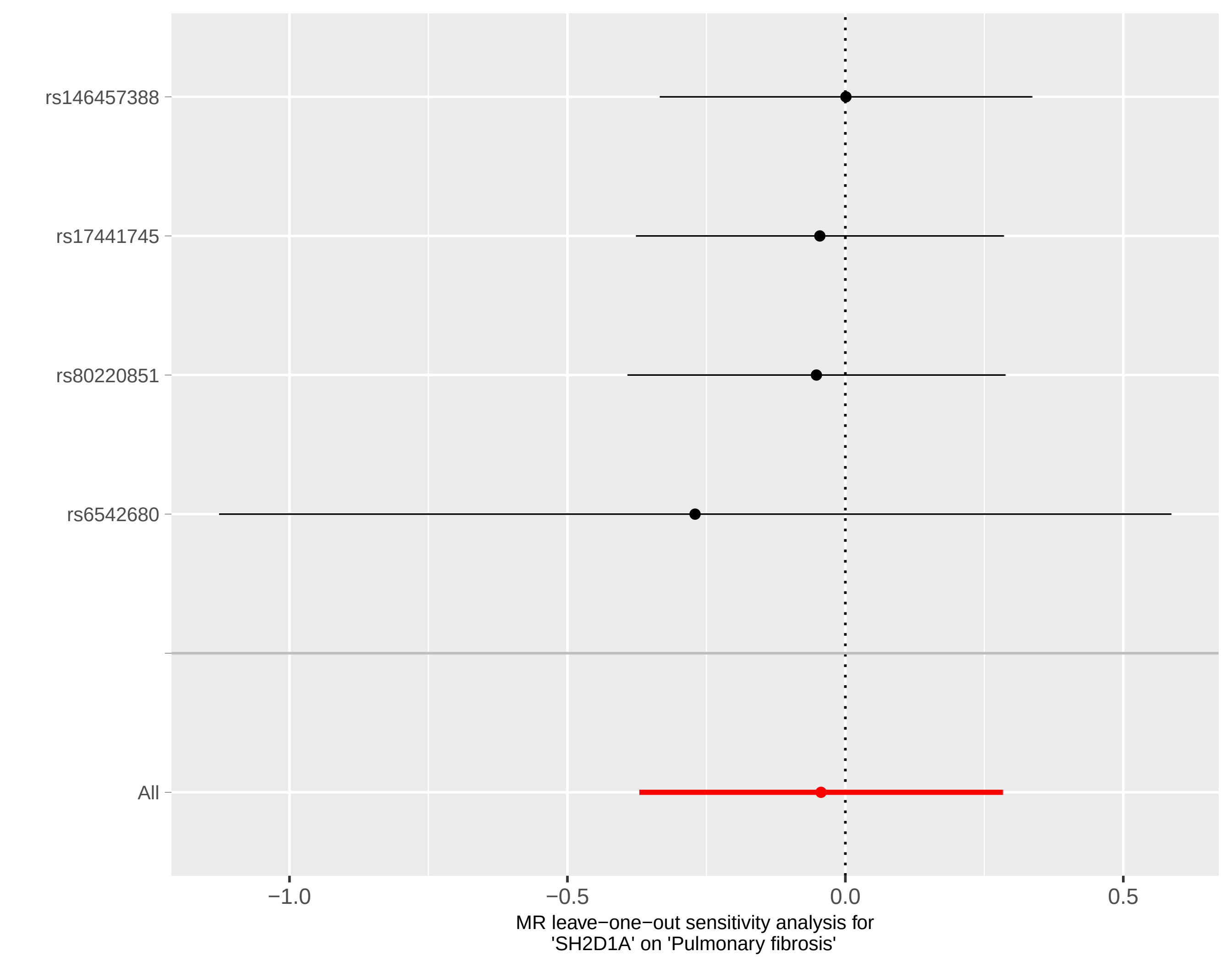
rs146457388
rs17441745
rs80220851
rs6542680
All
−1.0
−0.5
0.0
0.5
MR leave−one−out sensitivity analysis for
'SH2D1A' on 'Pulmonary fibrosis'

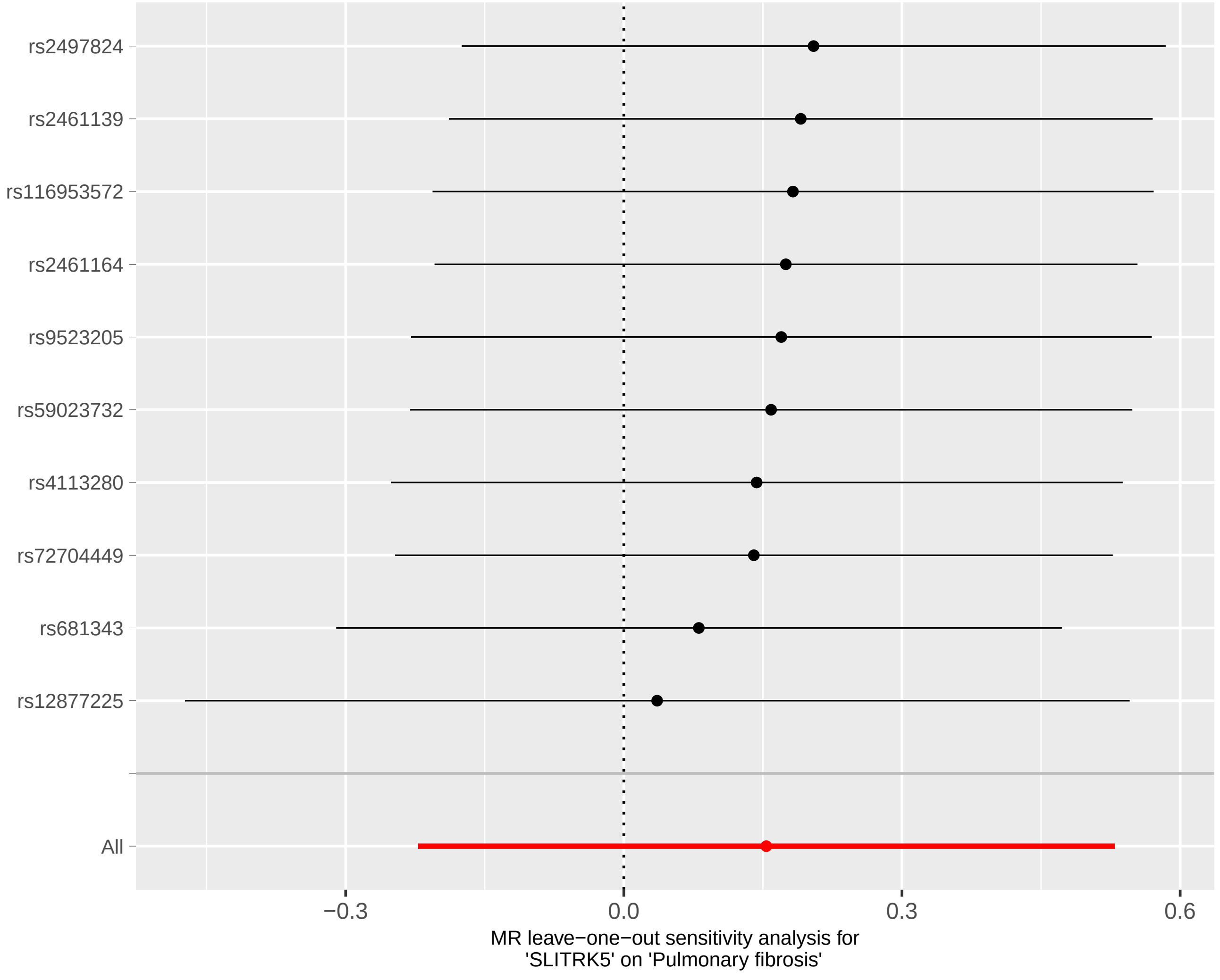

rs2497824
rs2461139
rs116953572
rs2461164
rs9523205
rs59023732
rs4113280
rs72704449
rs681343
rs12877225
All
−0.3
0.0
0.3
0.6
MR leave−one−out sensitivity analysis for
'SLITRK5' on 'Pulmonary fibrosis'

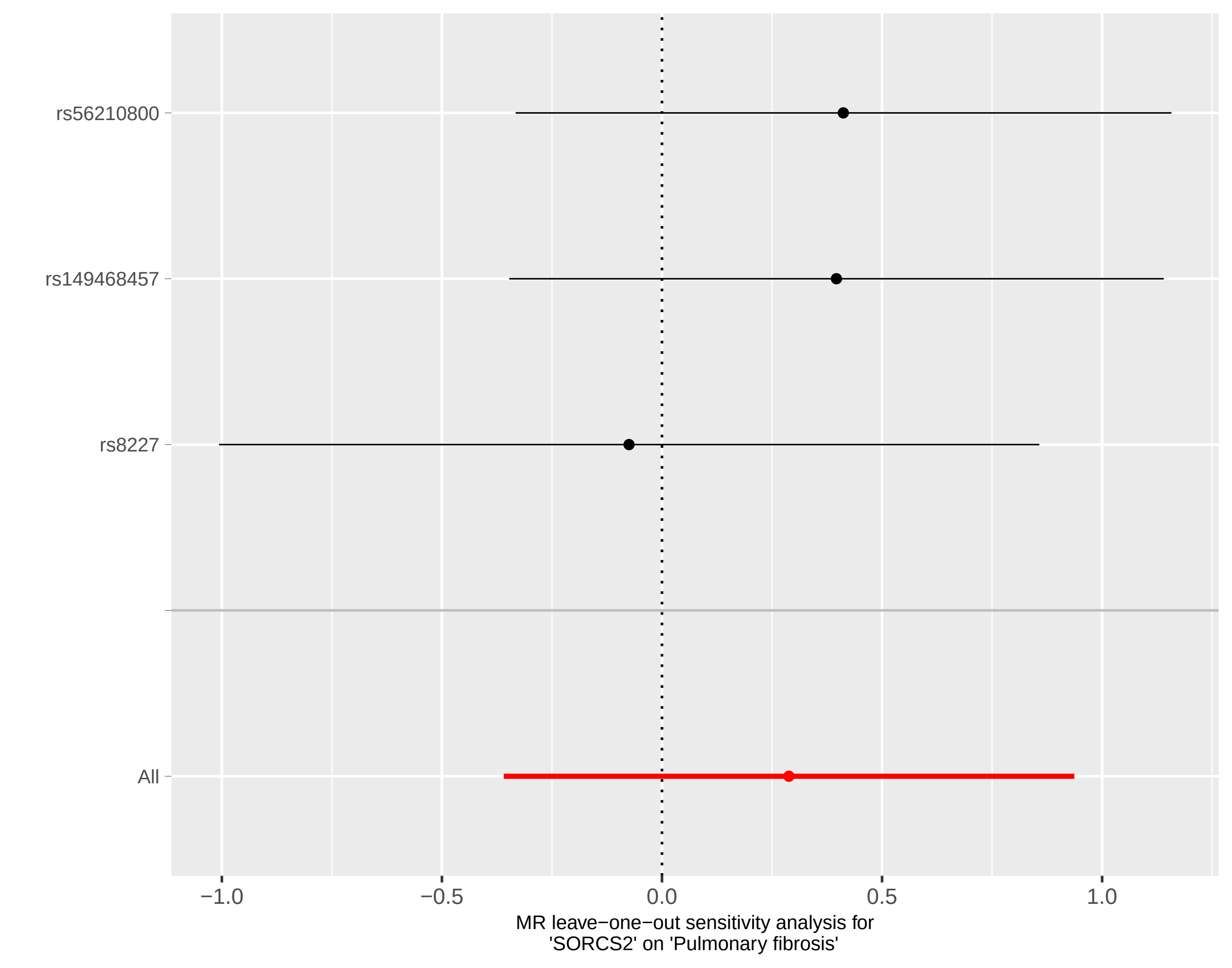
rs56210800
rs149468457
rs8227
All
−1.0
−0.5
0.0
0.5
1.0
MR leave−one−out sensitivity analysis for
'SORCS2' on 'Pulmonary fibrosis'

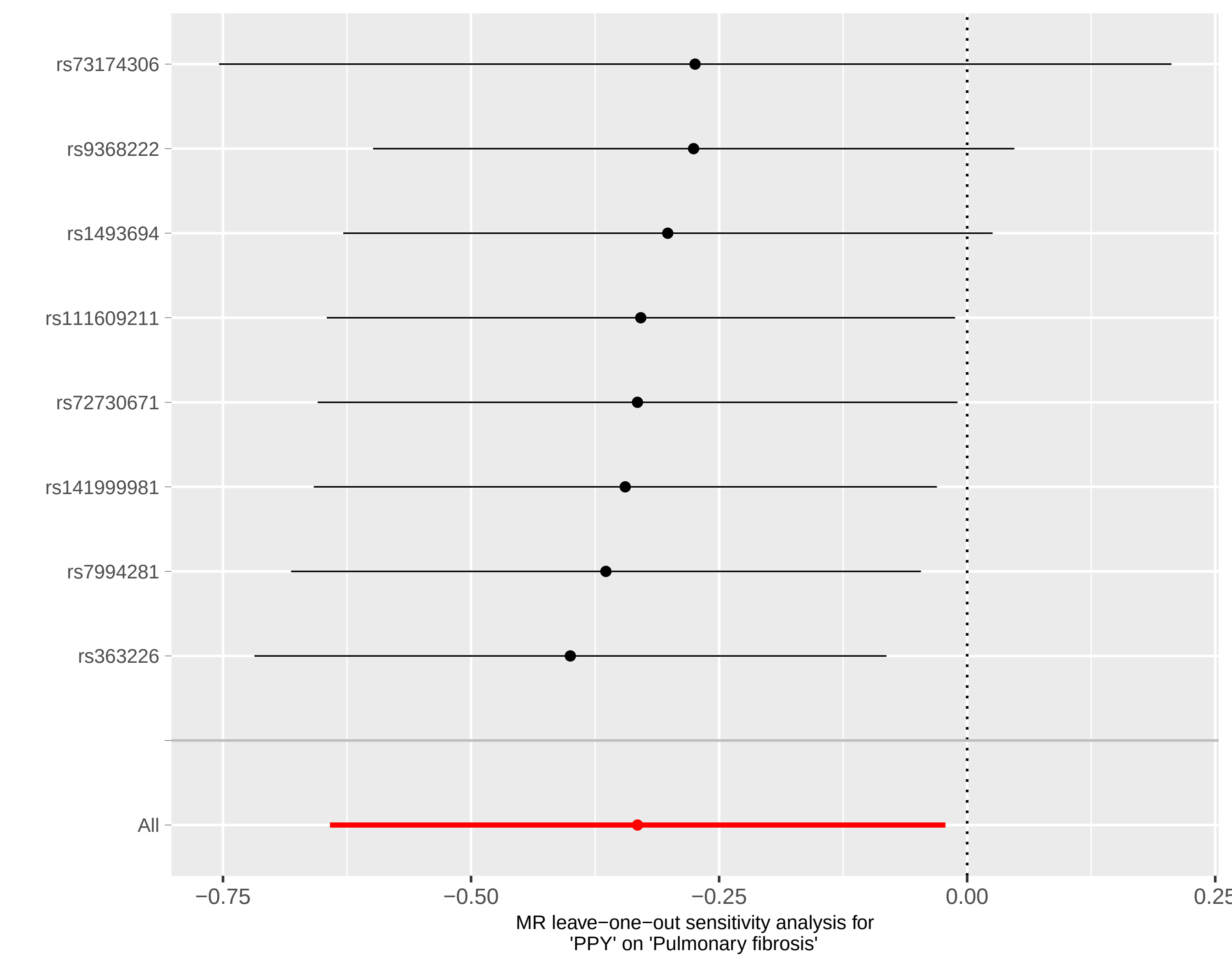
rs73174306
rs9368222
rs1493694
rs111609211
rs72730671
rs141999981
rs7994281
rs363226
All
−0.75
−0.50
−0.25
0.00
0.25
MR leave−one−out sensitivity analysis for
'PPY' on 'Pulmonary fibrosis'

Insufficient number of SNPs

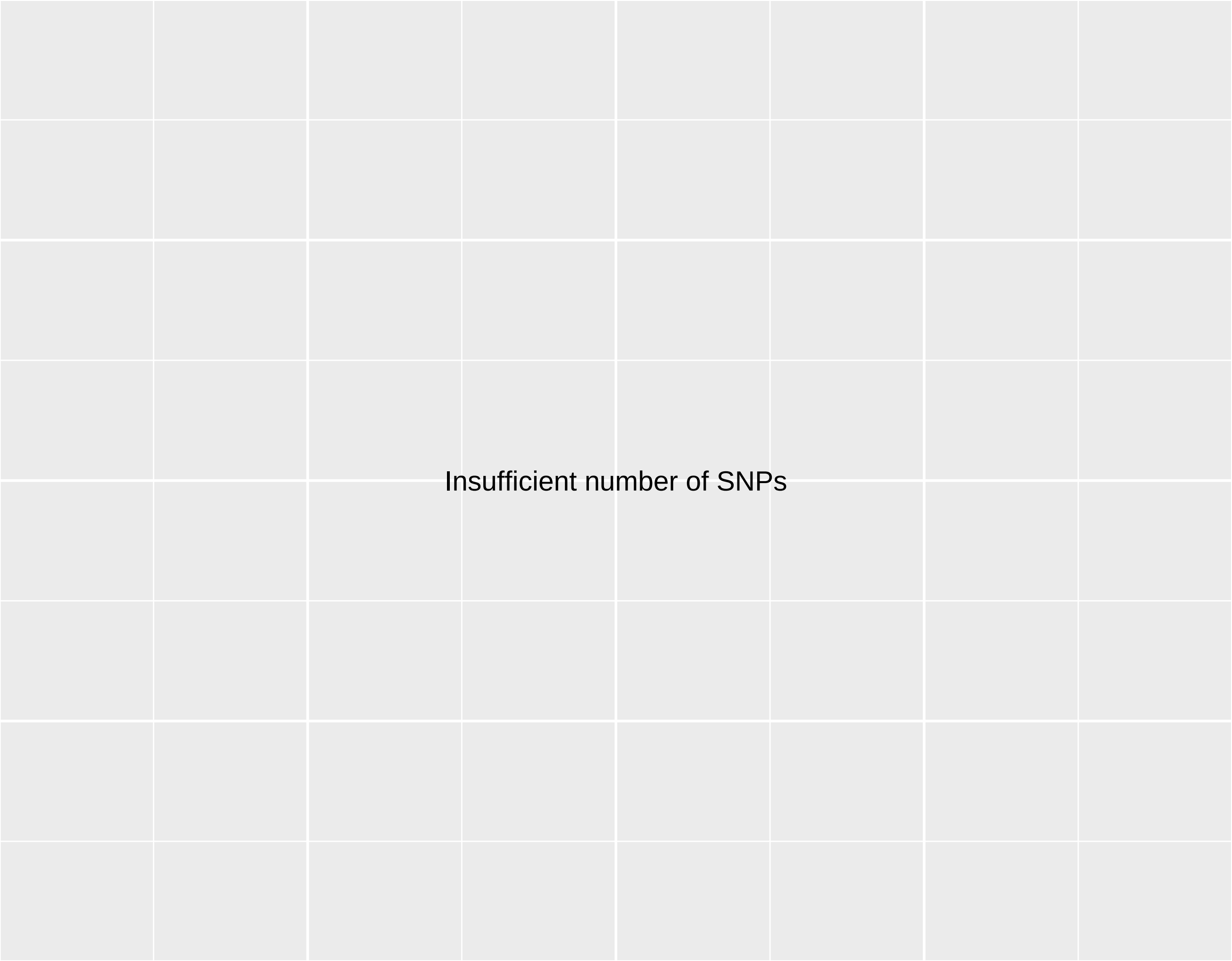
Insufficient number of SNPs

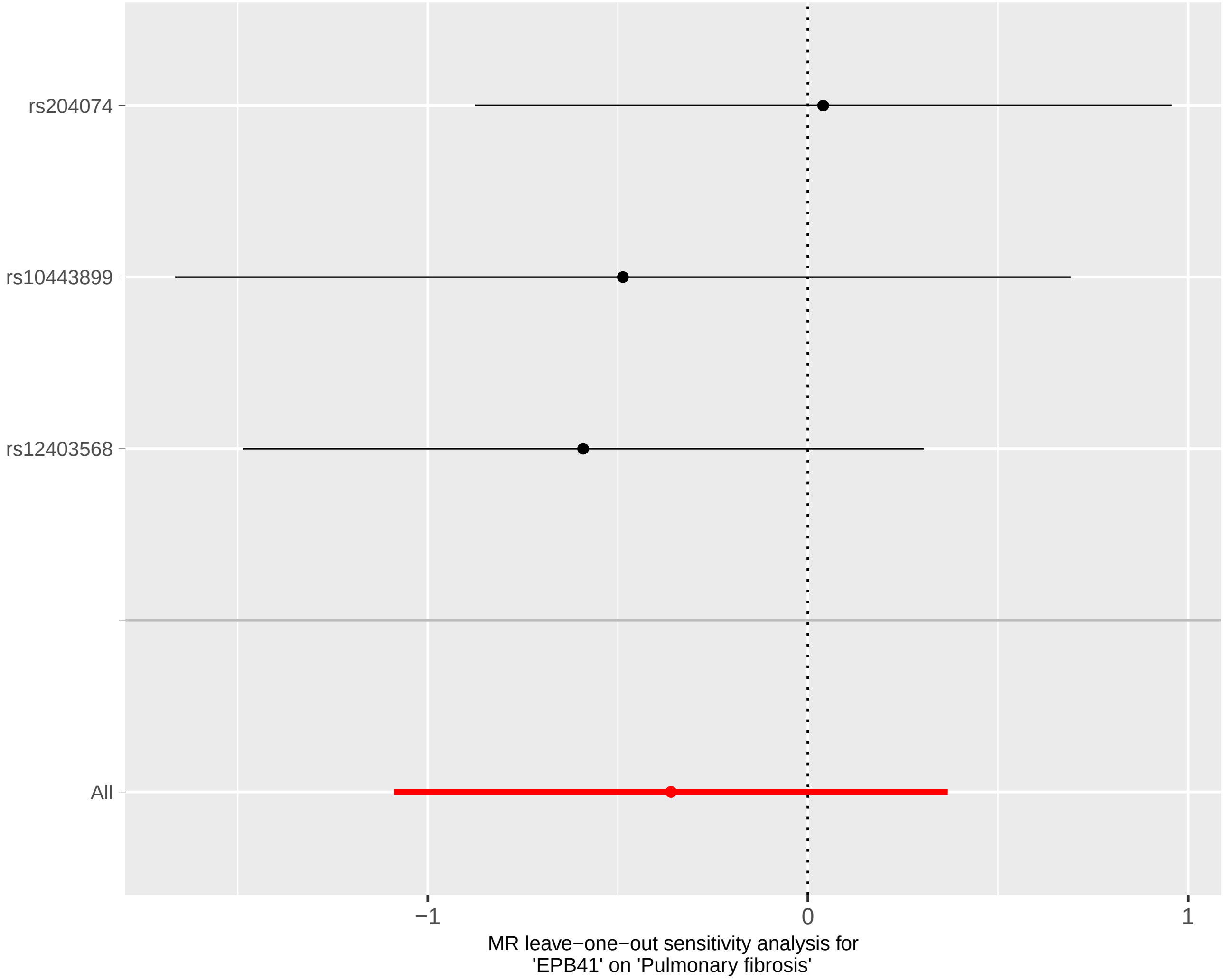
rs204074
rs10443899
rs12403568
All
−1
0
1
MR leave−one−out sensitivity analysis for
'EPB41' on 'Pulmonary fibrosis'

Insufficient number of SNPs

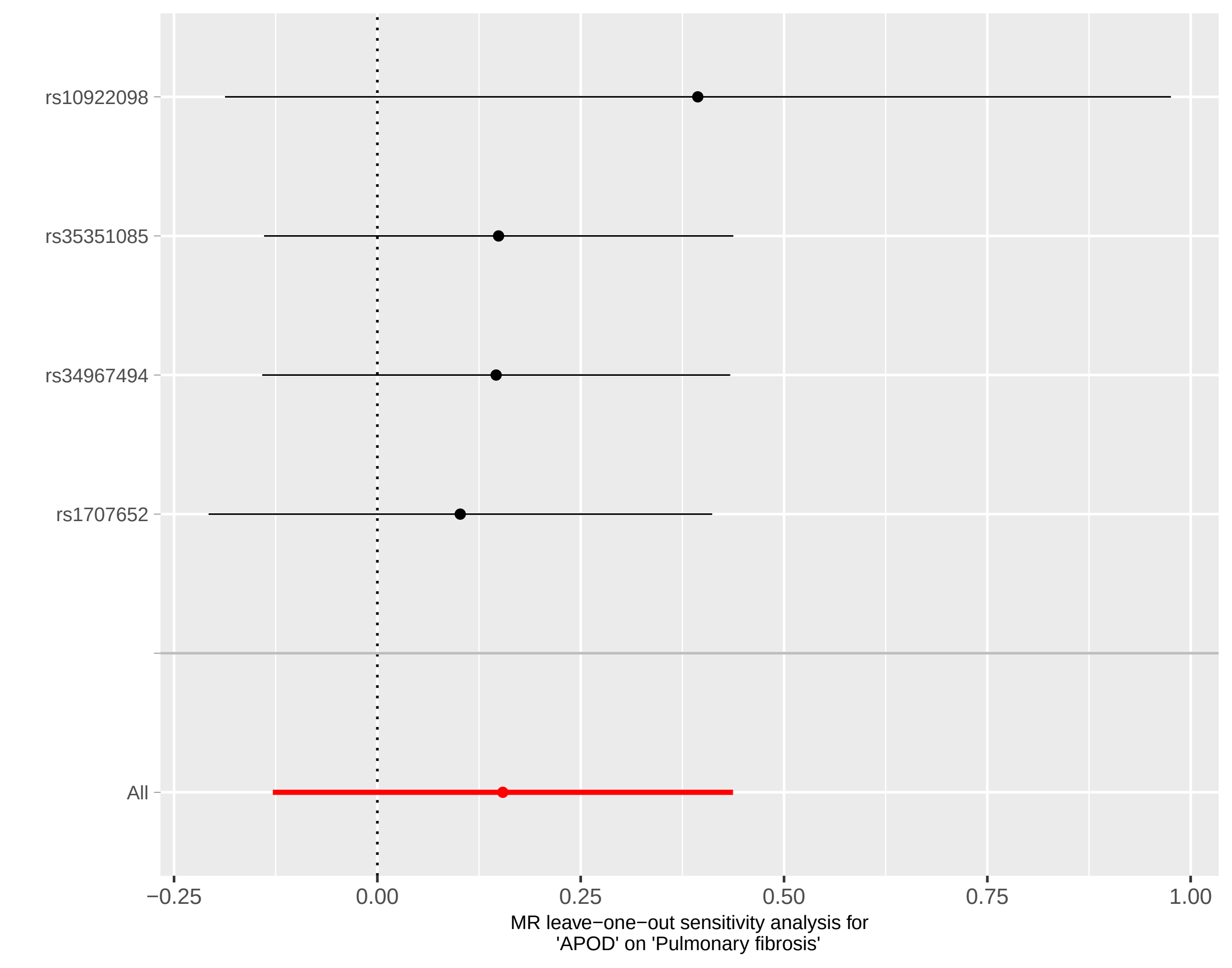
rs10922098
rs35351085
rs34967494
rs1707652
All
−0.25
0.00
0.25
0.50
0.75
1.00
MR leave−one−out sensitivity analysis for
'APOD' on 'Pulmonary fibrosis'

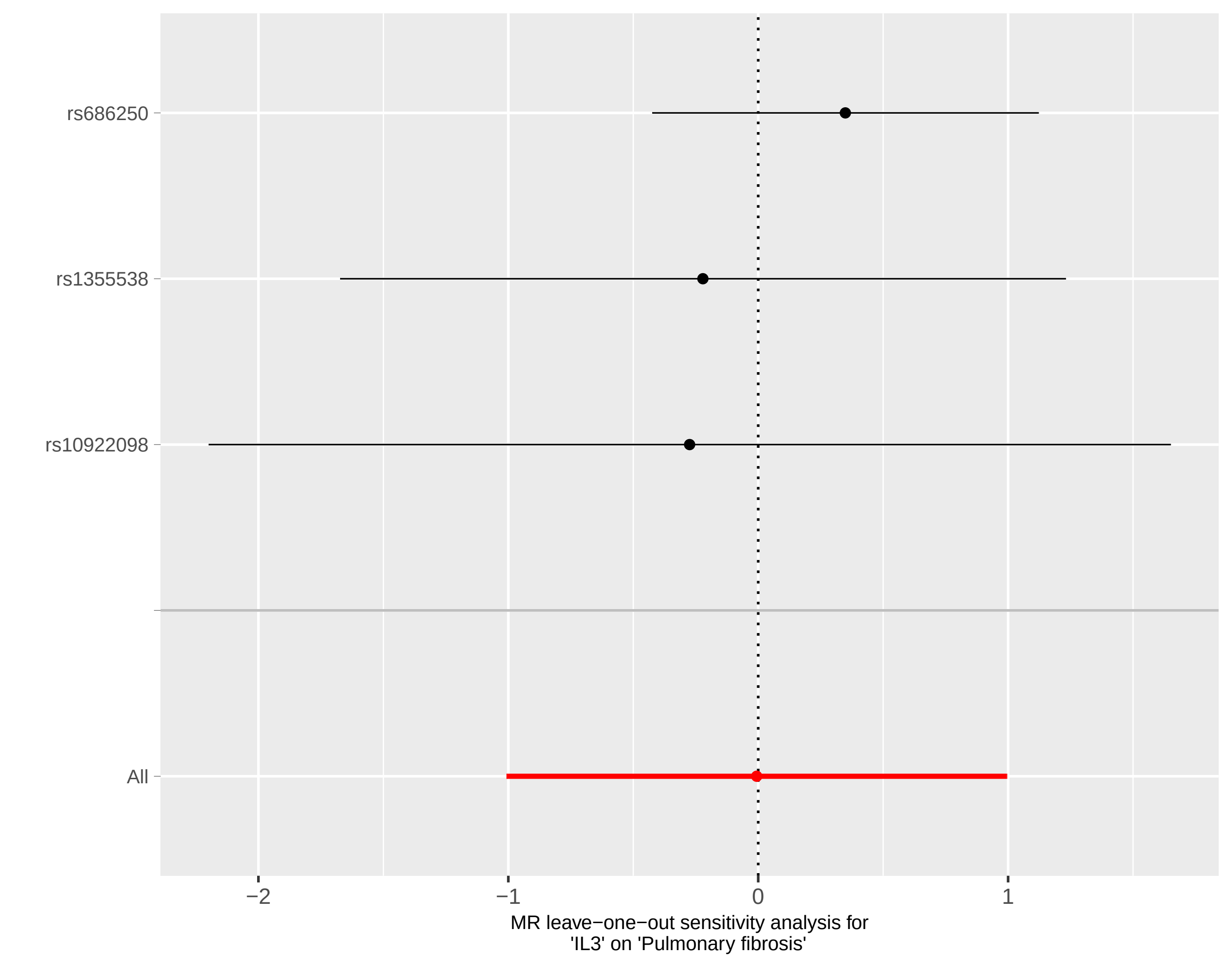

rs686250
rs1355538
rs10922098
All
−2
−1
0
1
MR leave−one−out sensitivity analysis for
'IL3' on 'Pulmonary fibrosis'

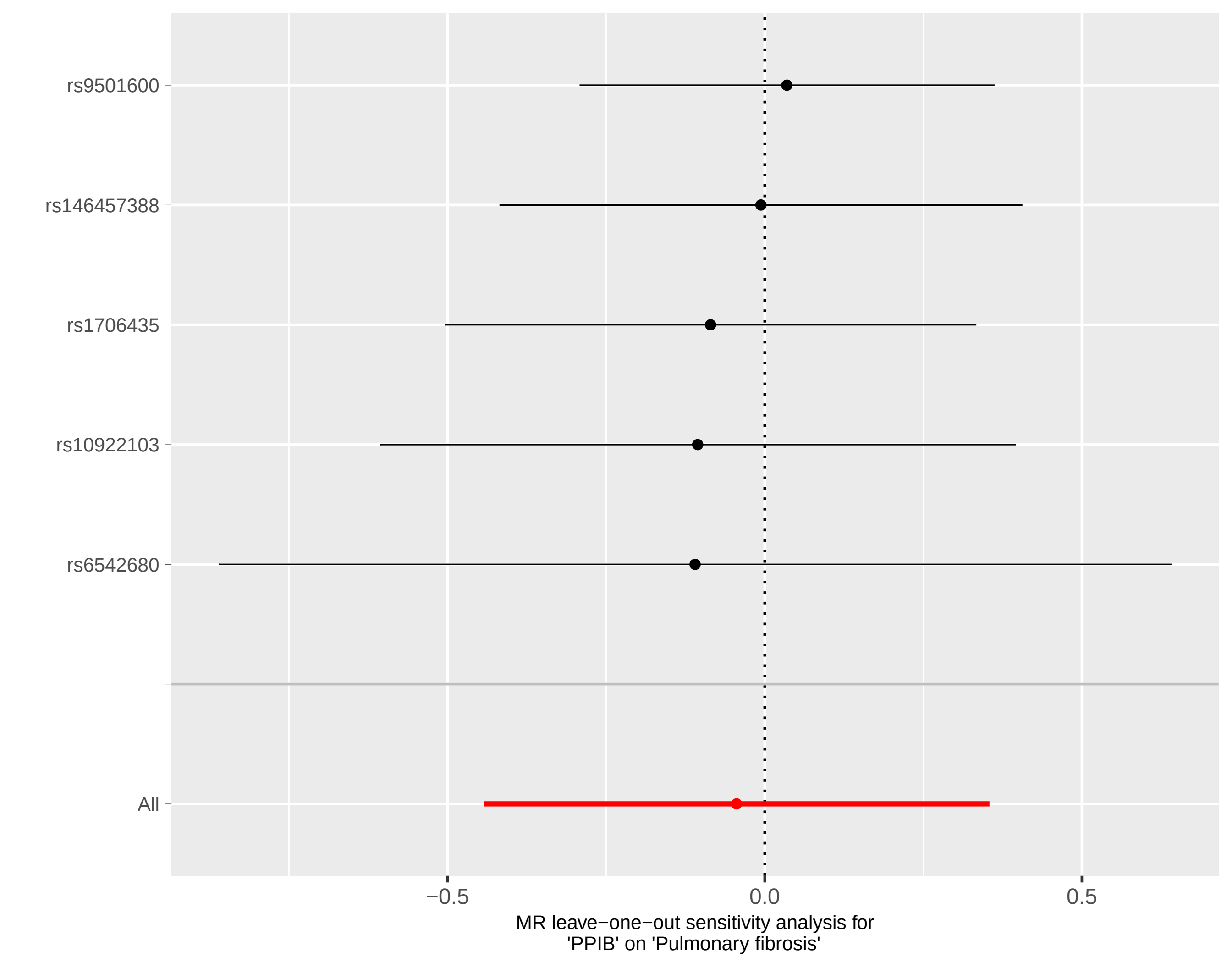
rs9501600
rs146457388
rs1706435
rs10922103
rs6542680
All
−0.5
0.0
0.5
MR leave−one−out sensitivity analysis for
'PPIB' on 'Pulmonary fibrosis'

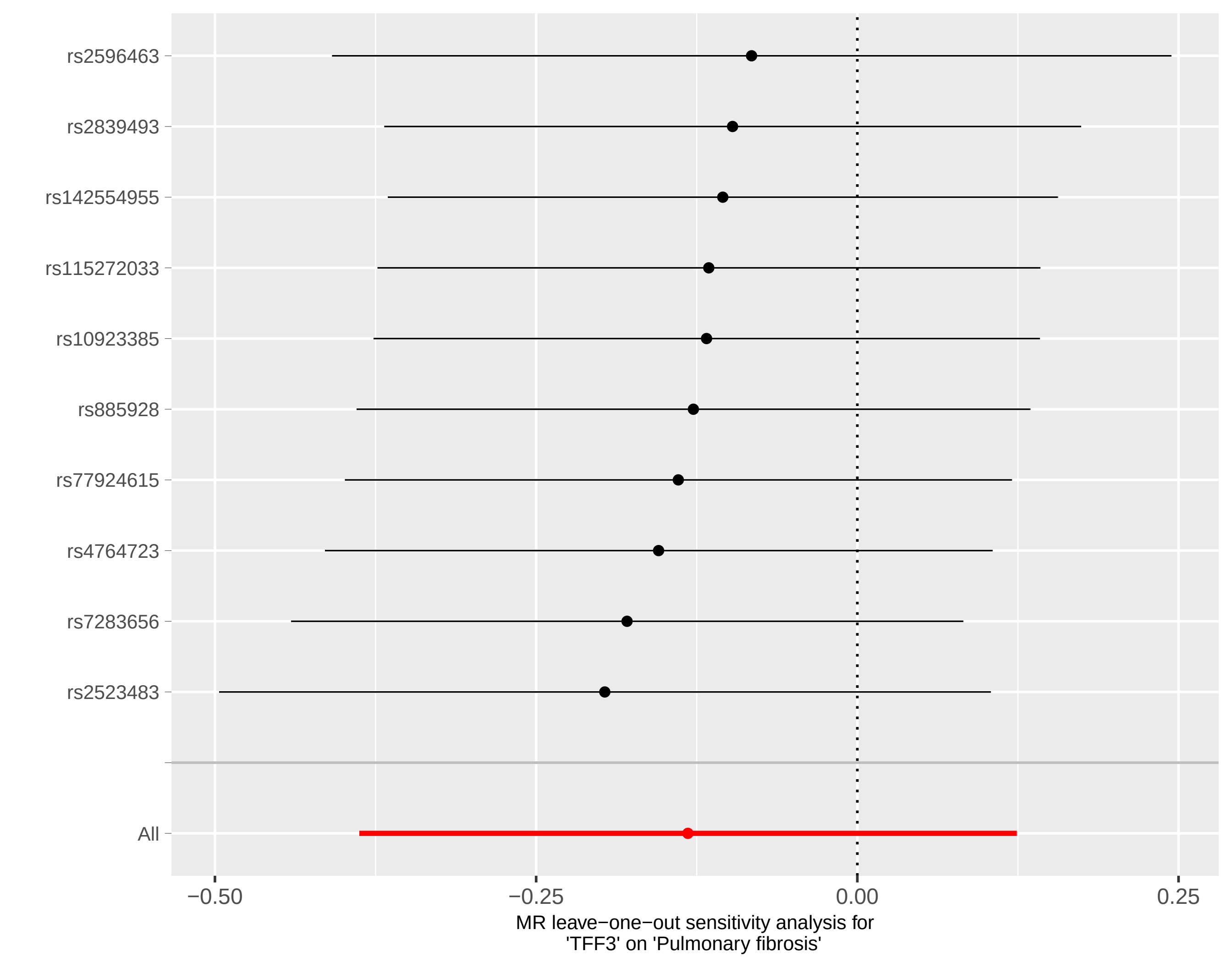
rs2596463
rs2839493
rs142554955
rs115272033
rs10923385
rs885928
rs77924615
rs4764723
rs7283656
rs2523483
All
−0.50
−0.25
0.00
0.25
MR leave−one−out sensitivity analysis for
'TFF3' on 'Pulmonary fibrosis'

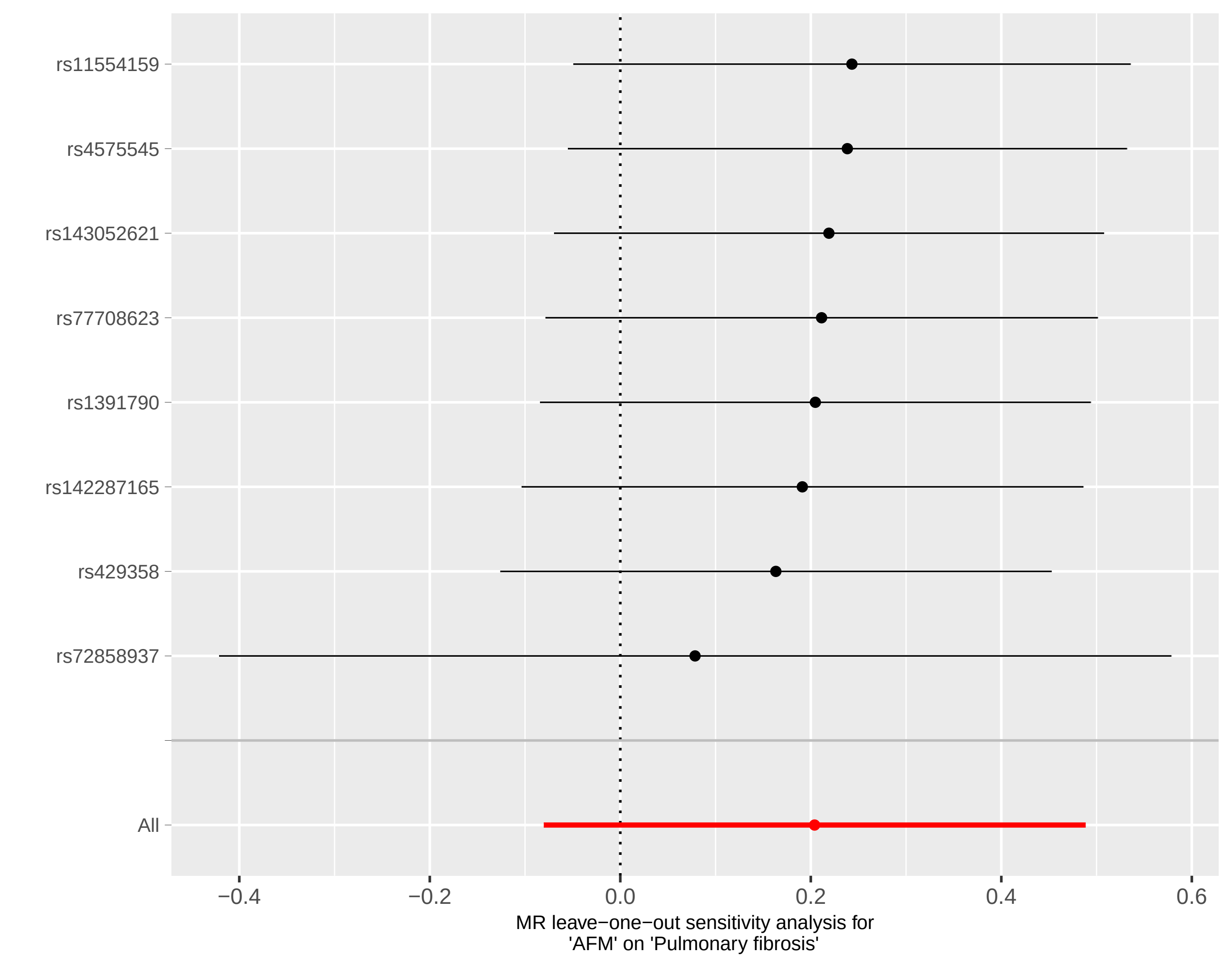
rs11554159
rs4575545
rs143052621
rs77708623
rs1391790
rs142287165
rs429358
rs72858937
All
−0.4
−0.2
0.0
0.2
0.4
0.6
MR leave−one−out sensitivity analysis for
'AFM' on 'Pulmonary fibrosis'

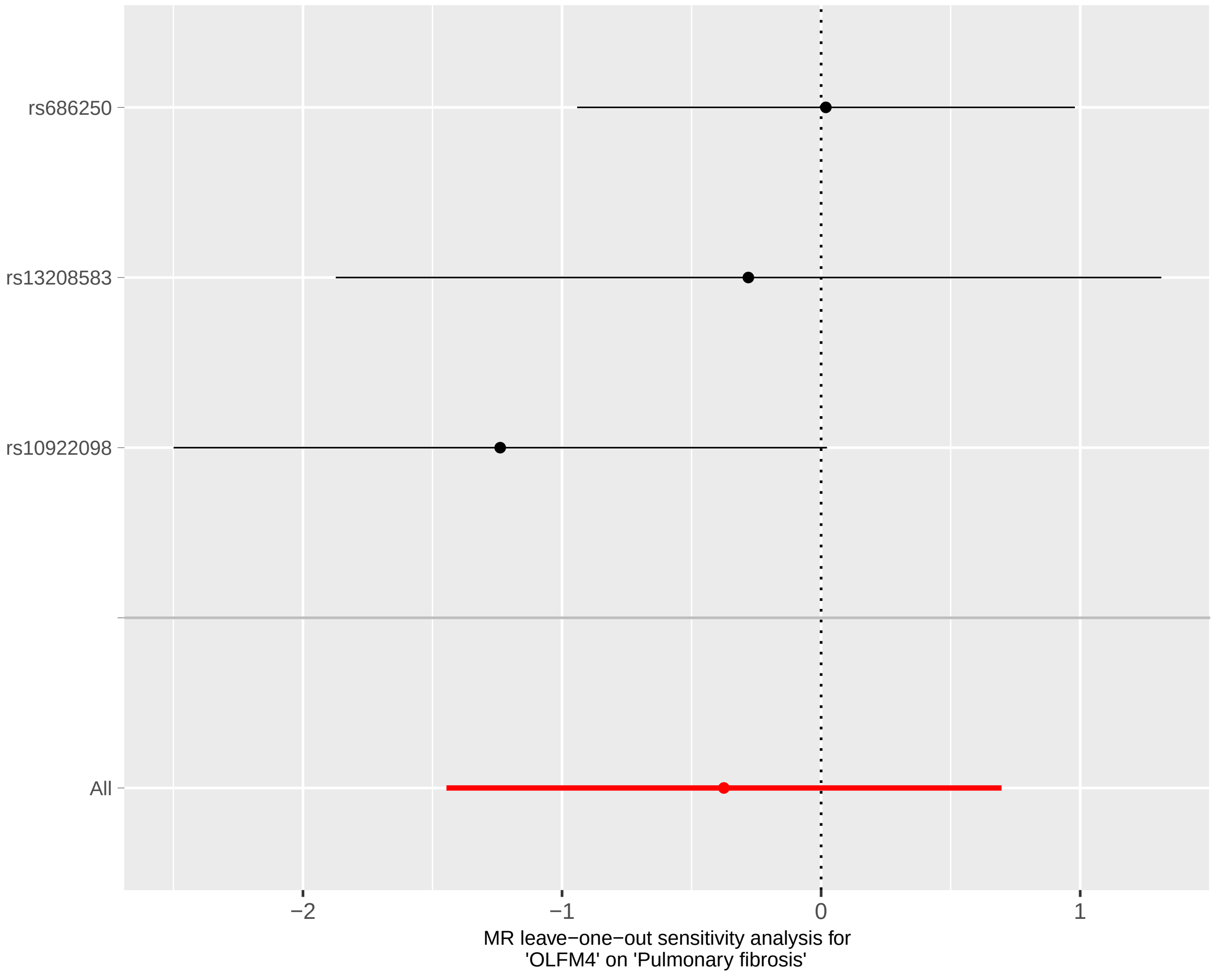
rs686250
rs13208583
rs10922098
All
−2
−1
0
1
MR leave−one−out sensitivity analysis for
'OLFM4' on 'Pulmonary fibrosis'

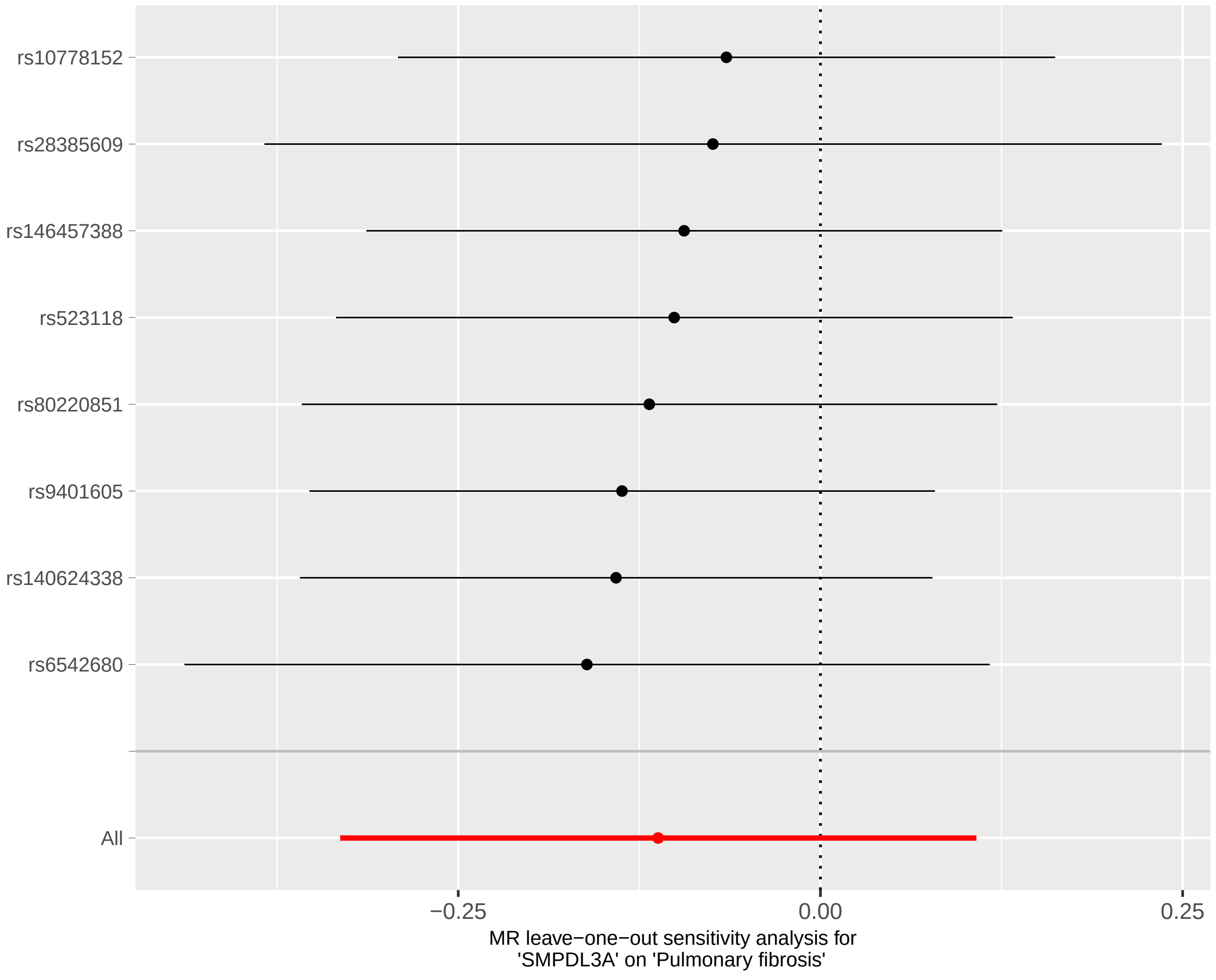
rs10778152
rs28385609
rs146457388
rs523118
rs80220851
rs9401605
rs140624338
rs6542680
All
−0.25
0.00
0.25
MR leave−one−out sensitivity analysis for
'SMPDL3A' on 'Pulmonary fibrosis'

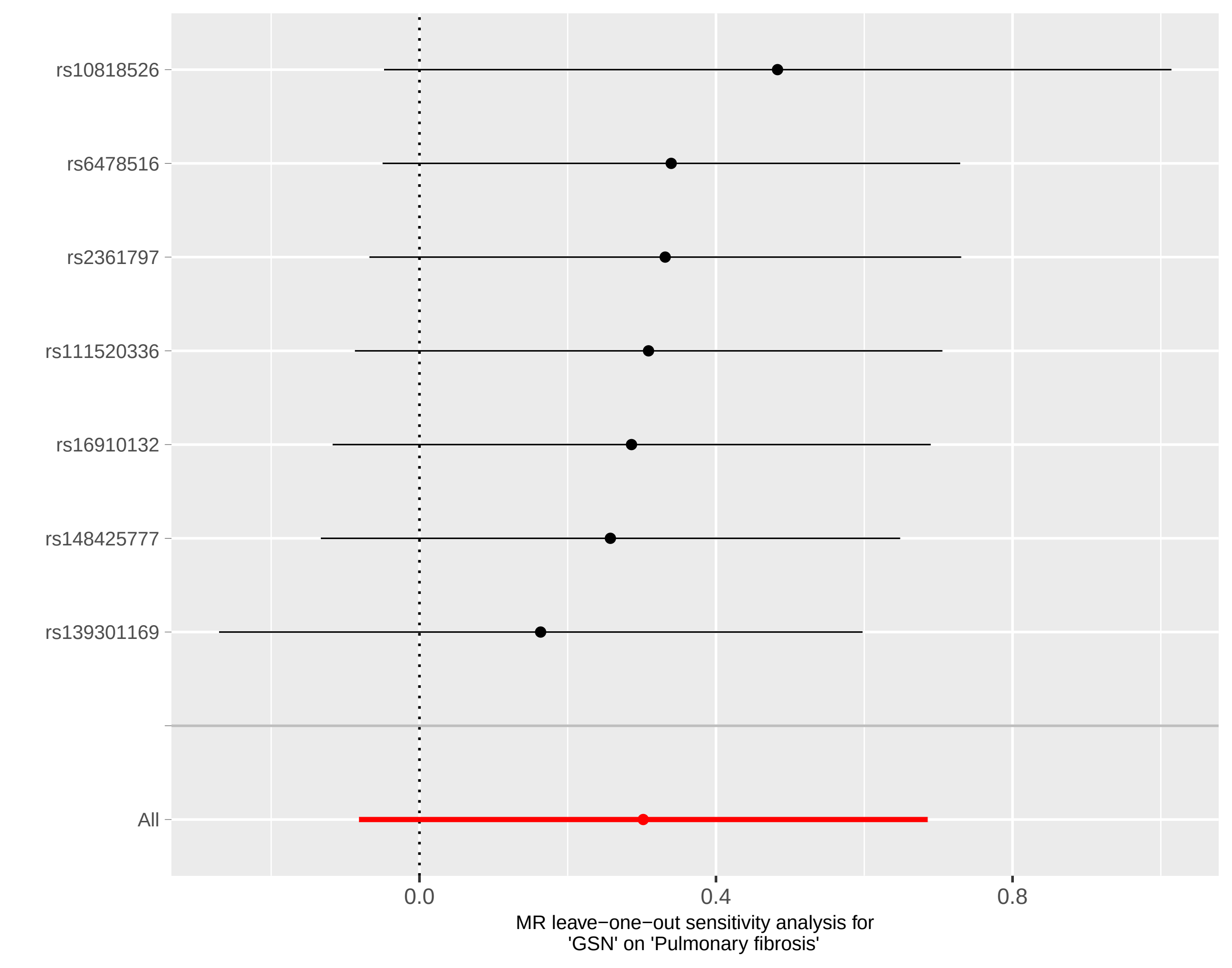
rs10818526
rs6478516
rs2361797
rs111520336
rs16910132
rs148425777
rs139301169
All
0.0
0.4
0.8
MR leave−one−out sensitivity analysis for
'GSN' on 'Pulmonary fibrosis'

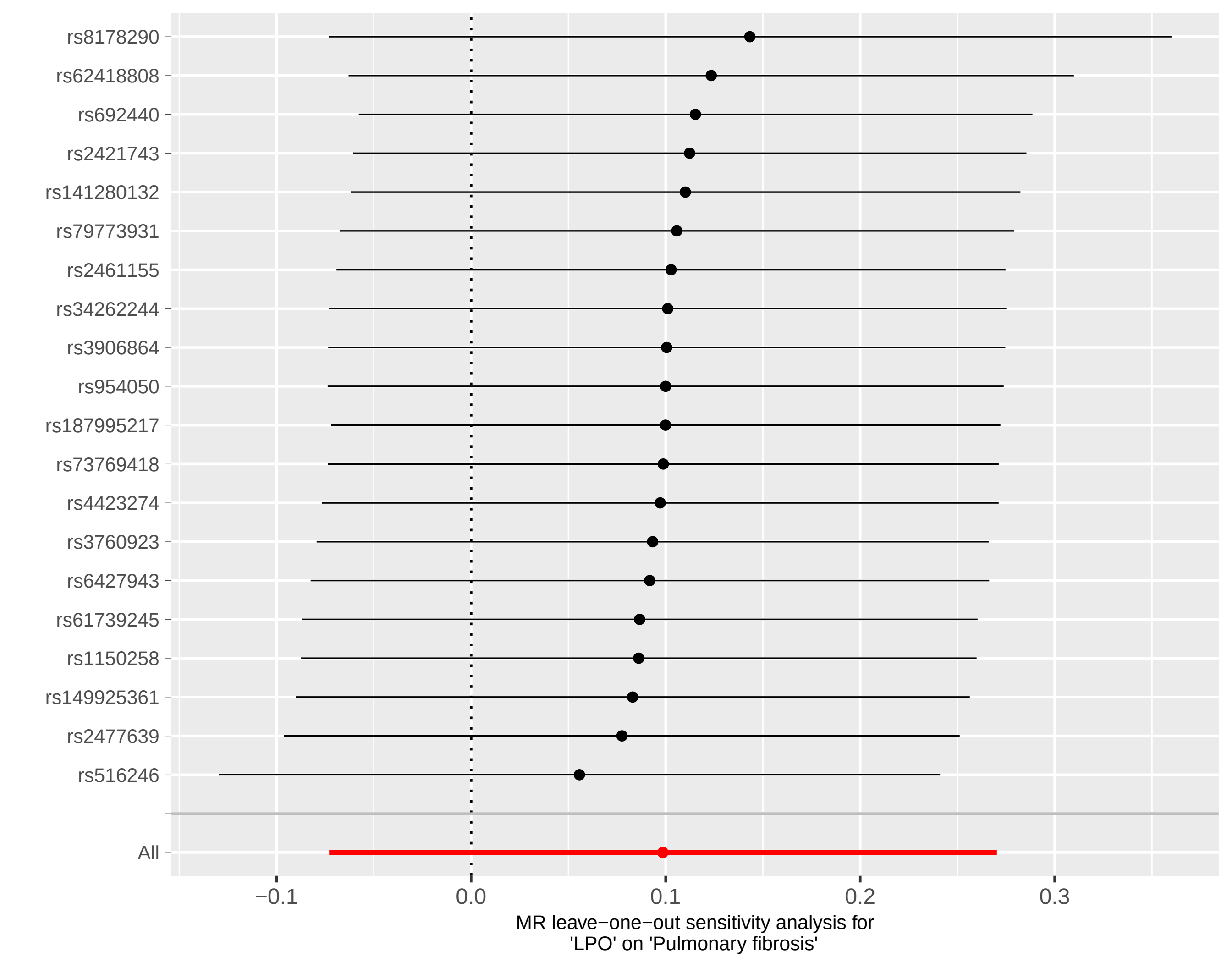
rs8178290
rs62418808
rs692440
rs2421743
rs141280132
rs79773931
rs2461155
rs34262244
rs3906864
rs954050
rs187995217
rs73769418
rs4423274
rs3760923
rs6427943
rs61739245
rs1150258
rs149925361
rs2477639
rs516246
All
−0.1
0.0
0.1
0.2
0.3
MR leave−one−out sensitivity analysis for
'LPO' on 'Pulmonary fibrosis'

Insufficient number of SNPs

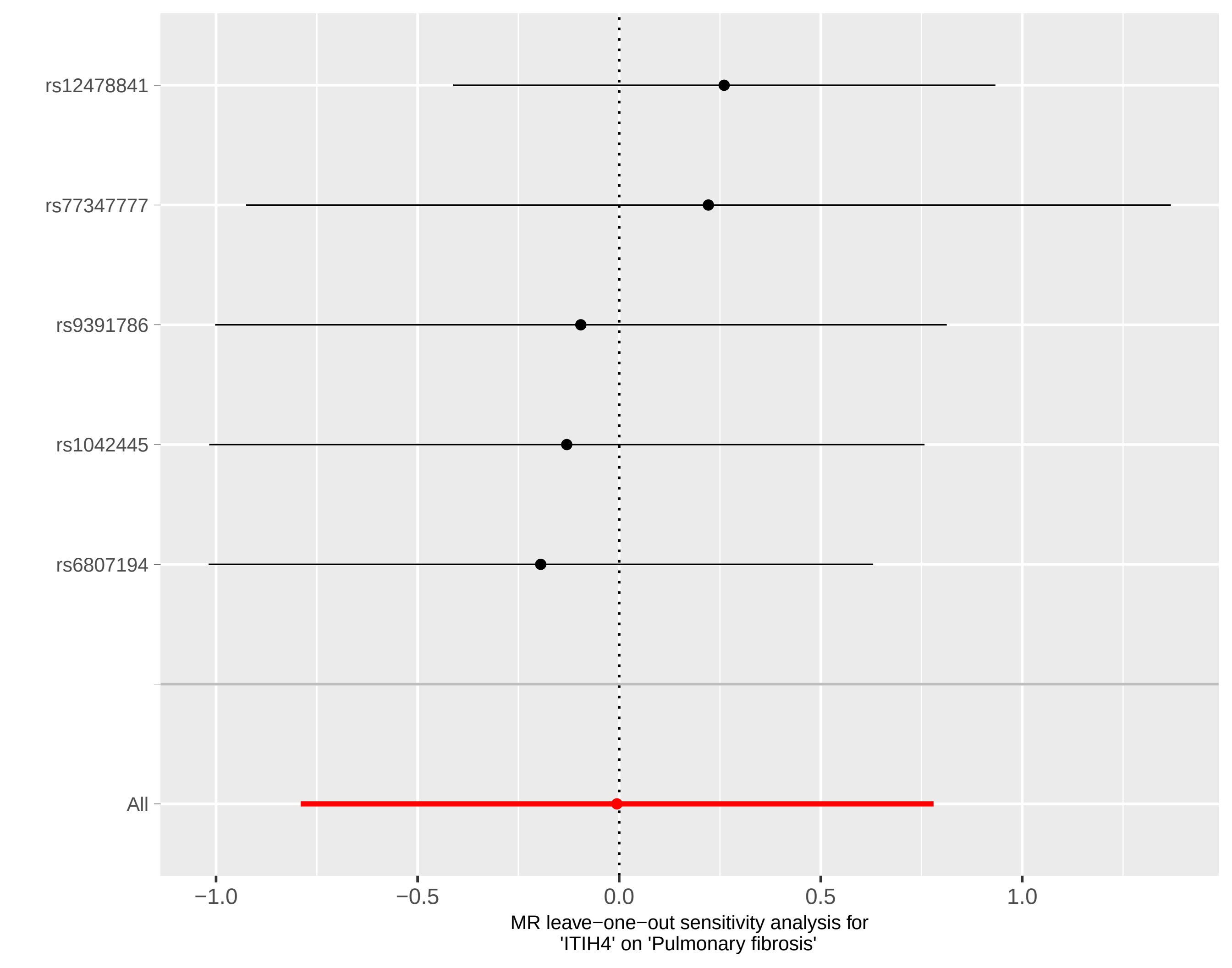
rs12478841
rs77347777
rs9391786
rs1042445
rs6807194
All
−1.0
−0.5
0.0
0.5
1.0
MR leave−one−out sensitivity analysis for
'ITIH4' on 'Pulmonary fibrosis'

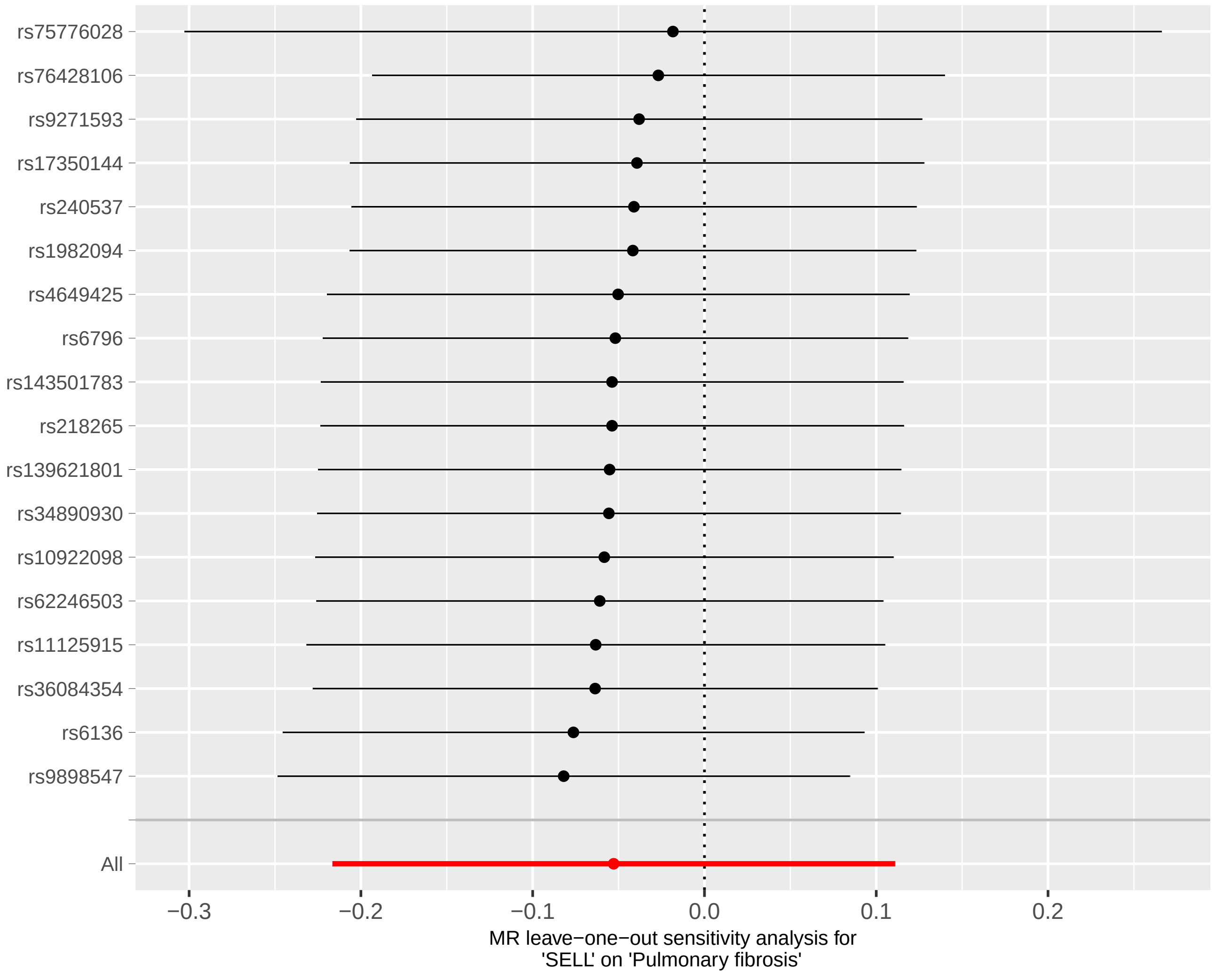
rs75776028
rs76428106
rs9271593
rs17350144
rs240537
rs1982094
rs4649425
rs6796
rs143501783
rs218265
rs139621801
rs34890930
rs10922098
rs62246503
rs11125915
rs36084354
rs6136
rs9898547
All
−0.3
−0.2
−0.1
0.0
0.1
0.2
MR leave−one−out sensitivity analysis for
'SELL' on 'Pulmonary fibrosis'

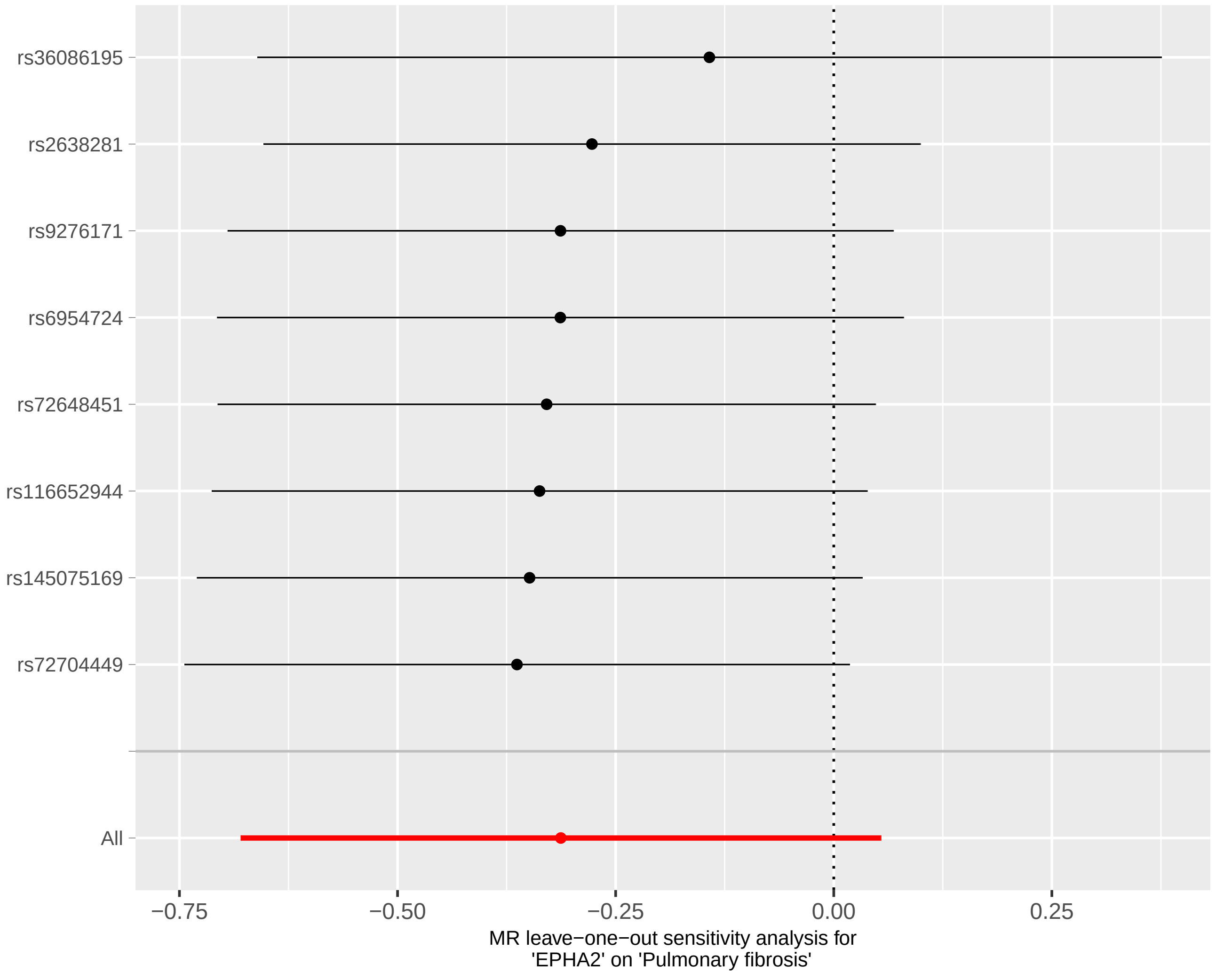
rs36086195
rs2638281
rs9276171
rs6954724
rs72648451
rs116652944
rs145075169
rs72704449
All
−0.75
−0.50
−0.25
0.00
0.25
MR leave−one−out sensitivity analysis for
'EPHA2' on 'Pulmonary fibrosis'

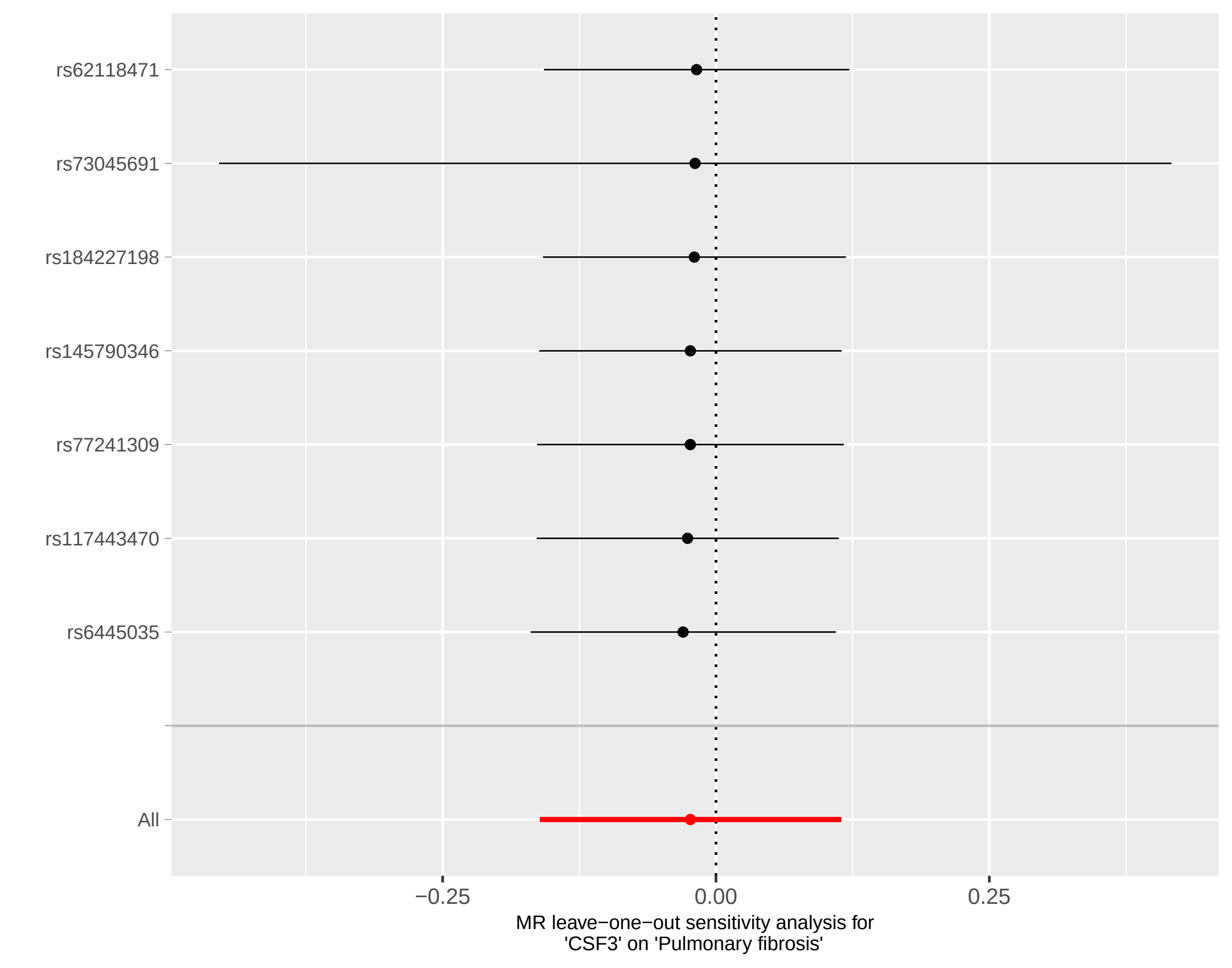

rs62118471
rs73045691
rs184227198
rs145790346
rs77241309
rs117443470
rs6445035
All
−0.25
0.00
0.25
MR leave−one−out sensitivity analysis for
'CSF3' on 'Pulmonary fibrosis'

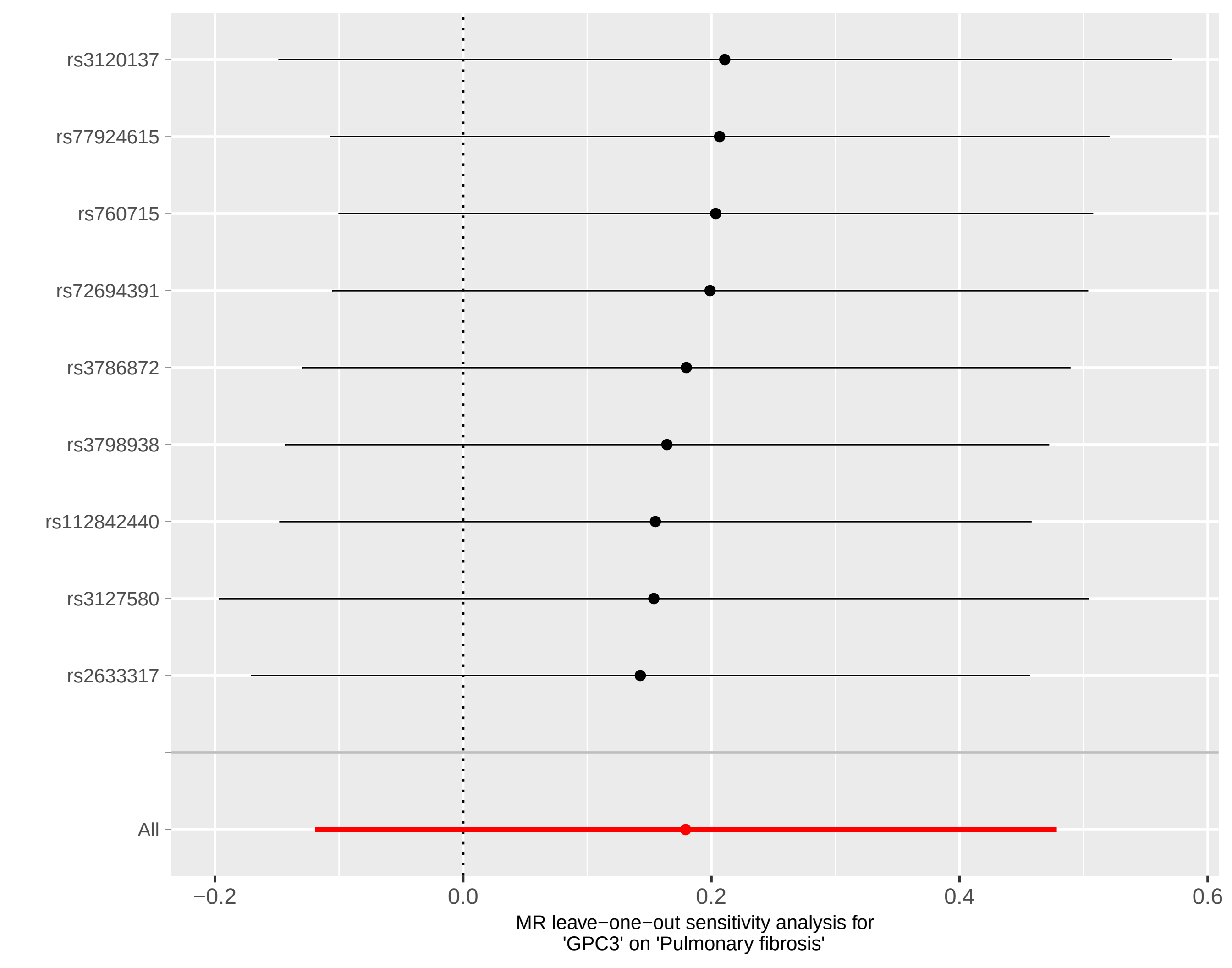
rs3120137
rs77924615
rs760715
rs72694391
rs3786872
rs3798938
rs112842440
rs3127580
rs2633317
All
−0.2
0.0
0.2
0.4
0.6
MR leave−one−out sensitivity analysis for
'GPC3' on 'Pulmonary fibrosis'

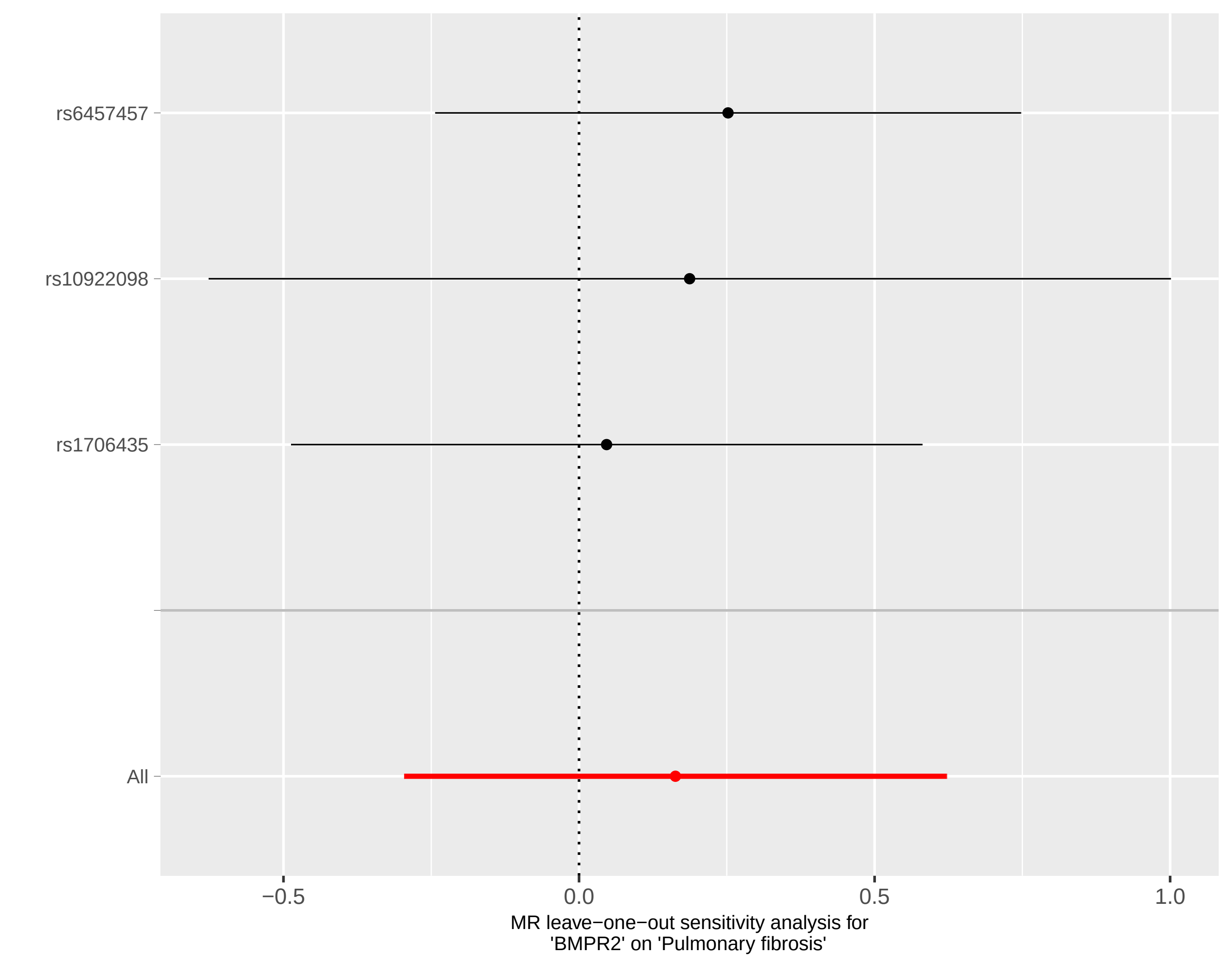
rs6457457
rs10922098
rs1706435
All
−0.5
0.0
0.5
1.0
MR leave−one−out sensitivity analysis for
'BMPR2' on 'Pulmonary fibrosis'

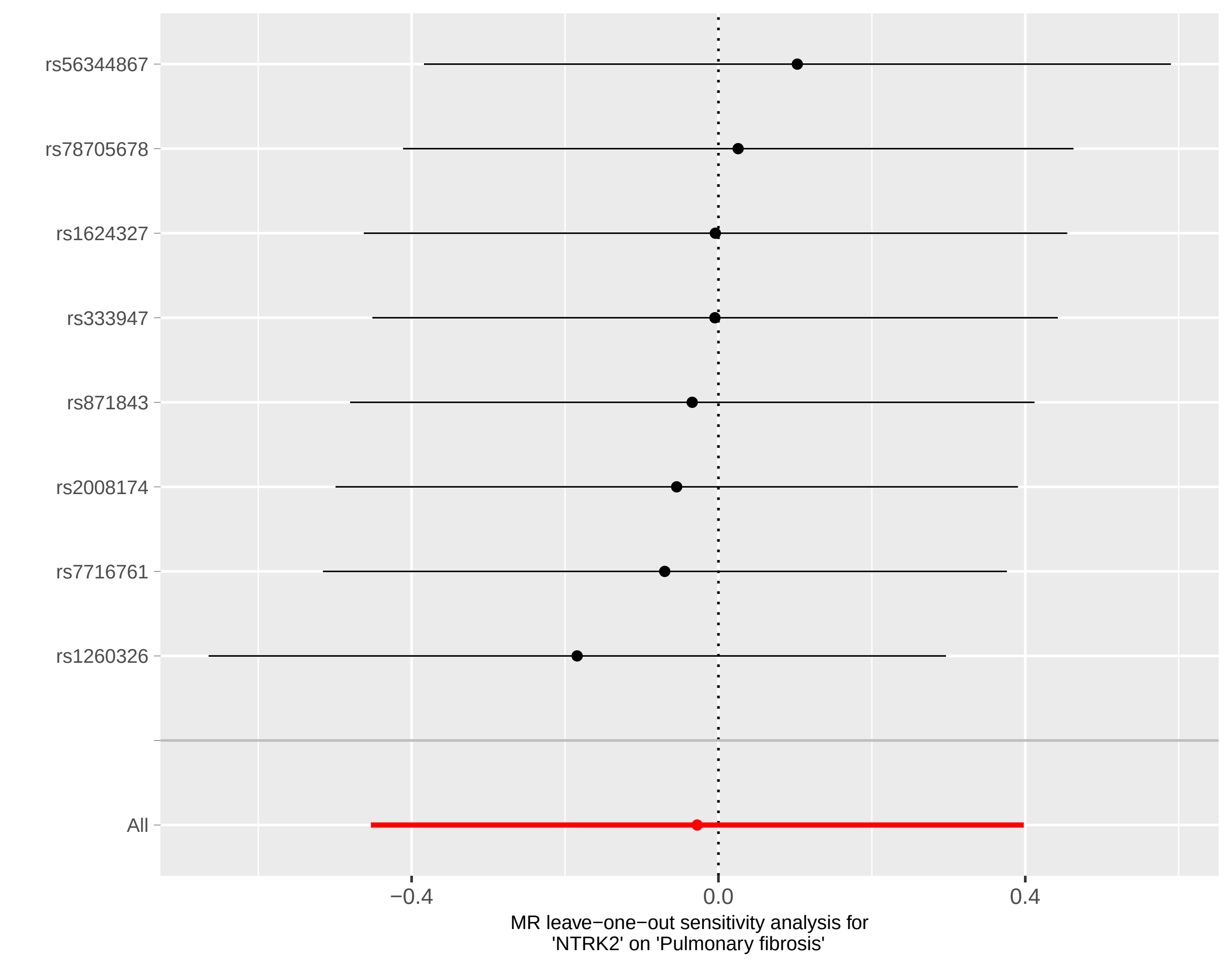
rs56344867
rs78705678
rs1624327
rs333947
rs871843
rs2008174
rs7716761
rs1260326
All
−0.4
0.0
0.4
MR leave−one−out sensitivity analysis for
'NTRK2' on 'Pulmonary fibrosis'

Insufficient number of SNPs

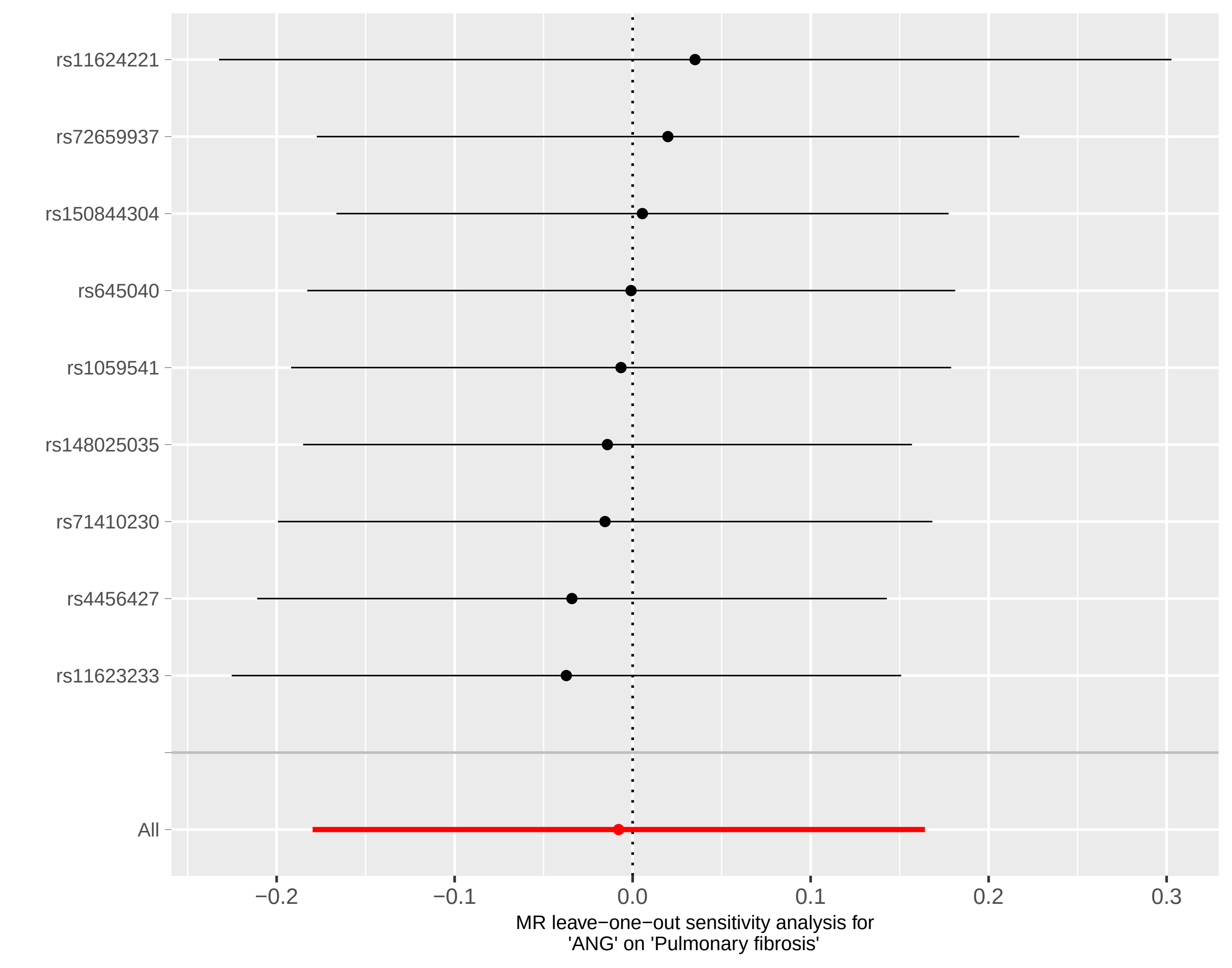
rs11624221
rs72659937
rs150844304
rs645040
rs1059541
rs148025035
rs71410230
rs4456427
rs11623233
All
−0.2
−0.1
0.0
0.1
0.2
0.3
MR leave−one−out sensitivity analysis for
'ANG' on 'Pulmonary fibrosis'

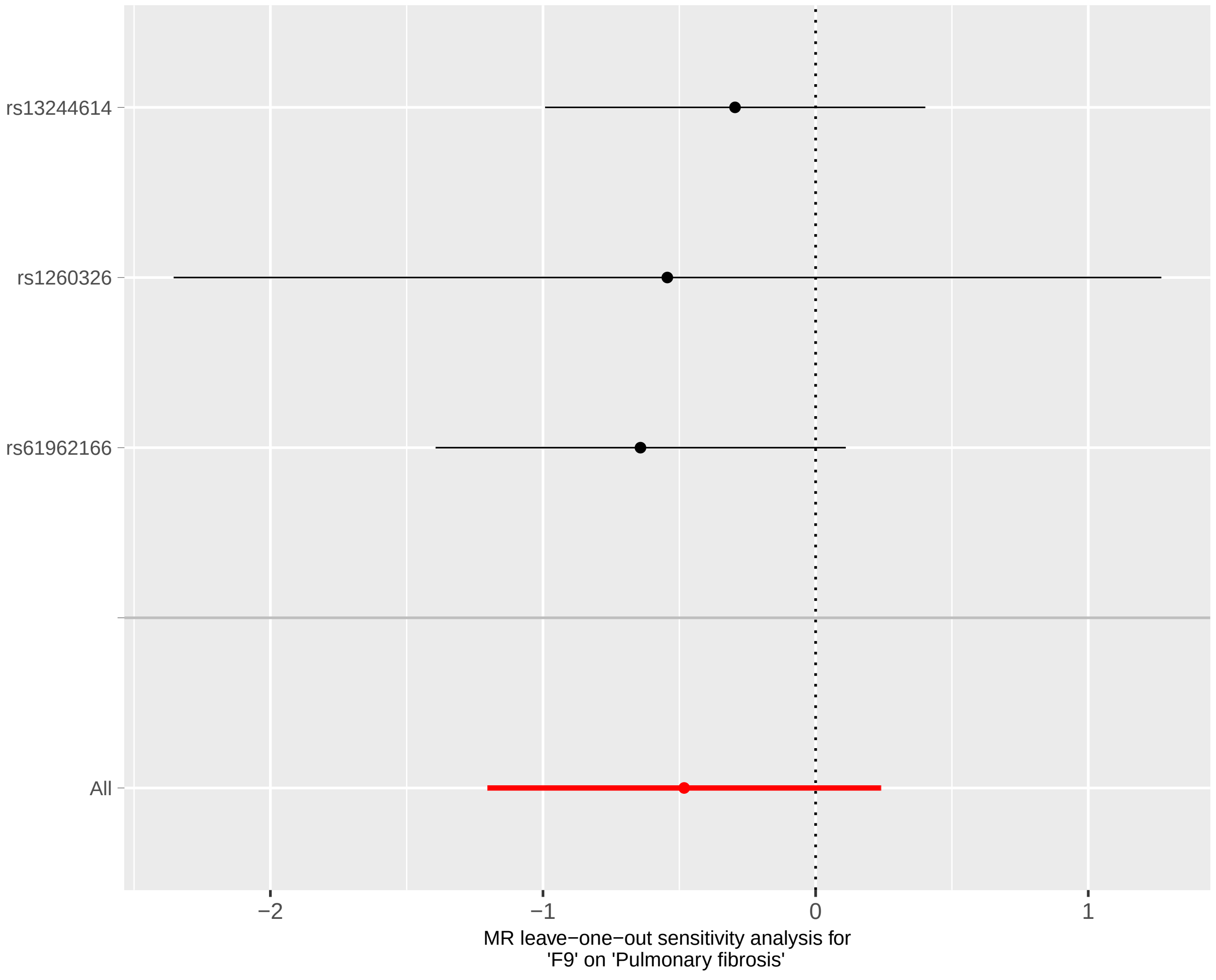

rs13244614
rs1260326
rs61962166
All
−2
−1
0
1
MR leave−one−out sensitivity analysis for
'F9' on 'Pulmonary fibrosis'

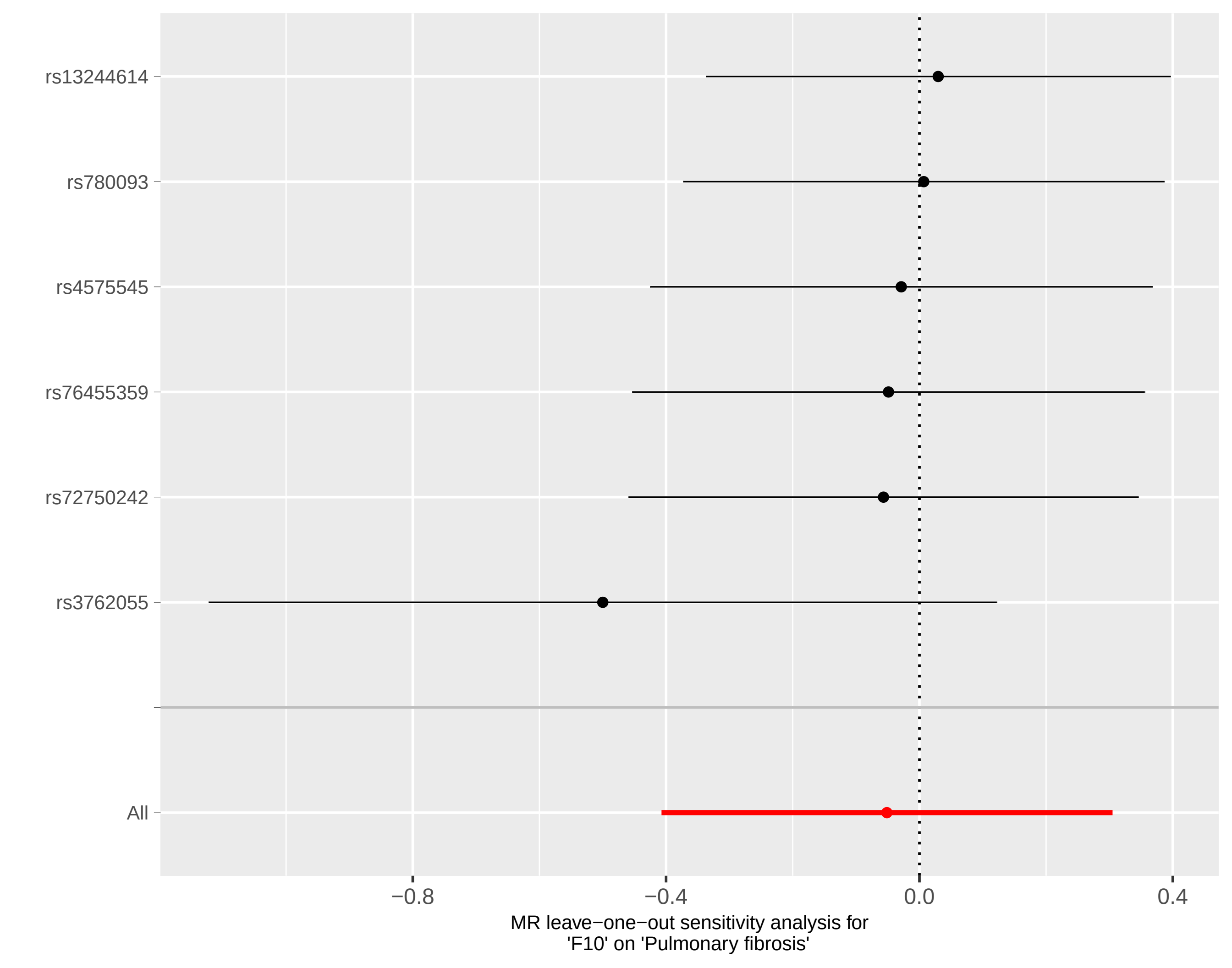
rs13244614
rs780093
rs4575545
rs76455359
rs72750242
rs3762055
All
−0.8
−0.4
0.0
0.4
MR leave−one−out sensitivity analysis for
'F10' on 'Pulmonary fibrosis'

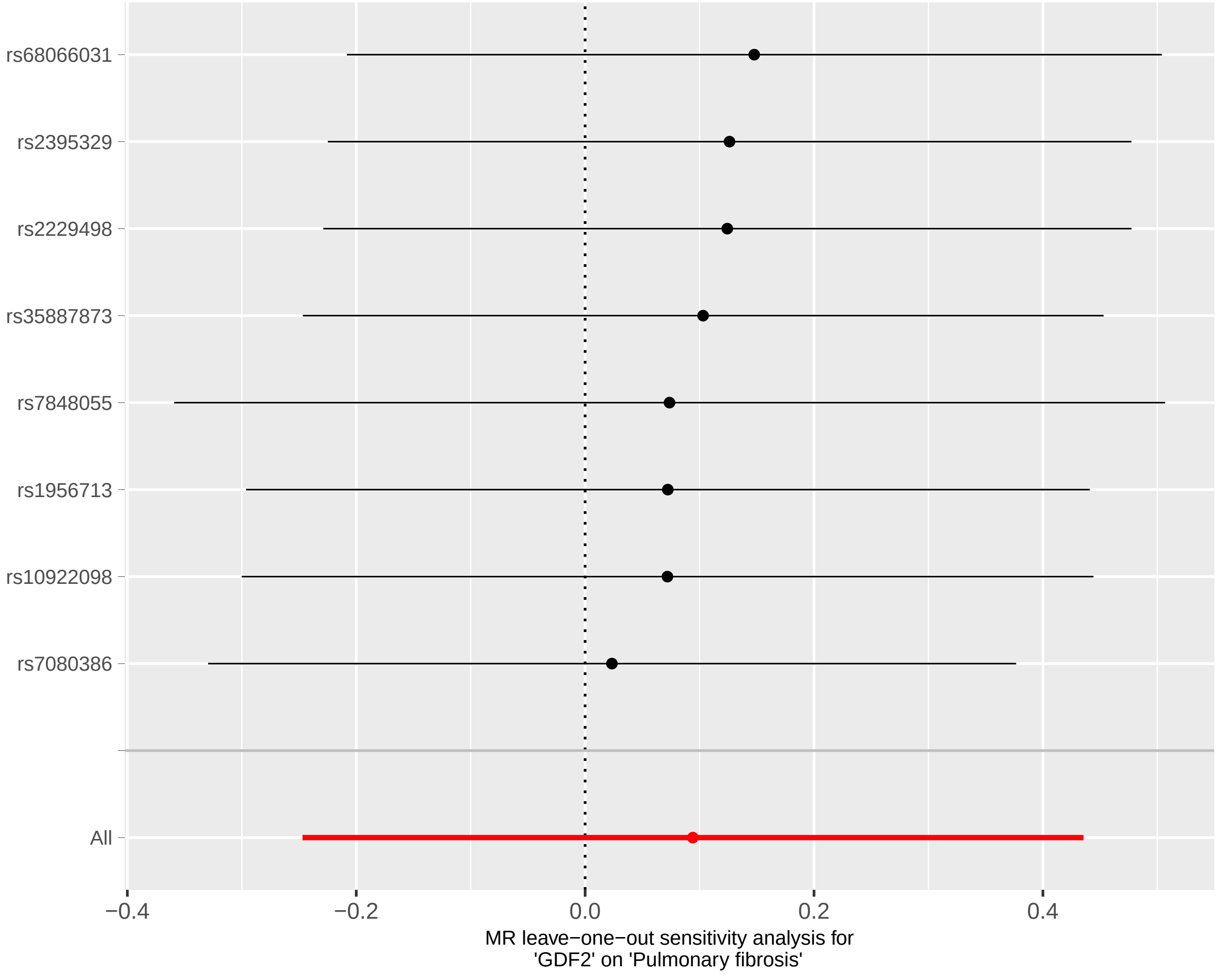
rs68066031
rs2395329
rs2229498
rs35887873
rs7848055
rs1956713
rs10922098
rs7080386
All
−0.4
−0.2
0.0
0.2
0.4
MR leave−one−out sensitivity analysis for
'GDF2' on 'Pulmonary fibrosis'

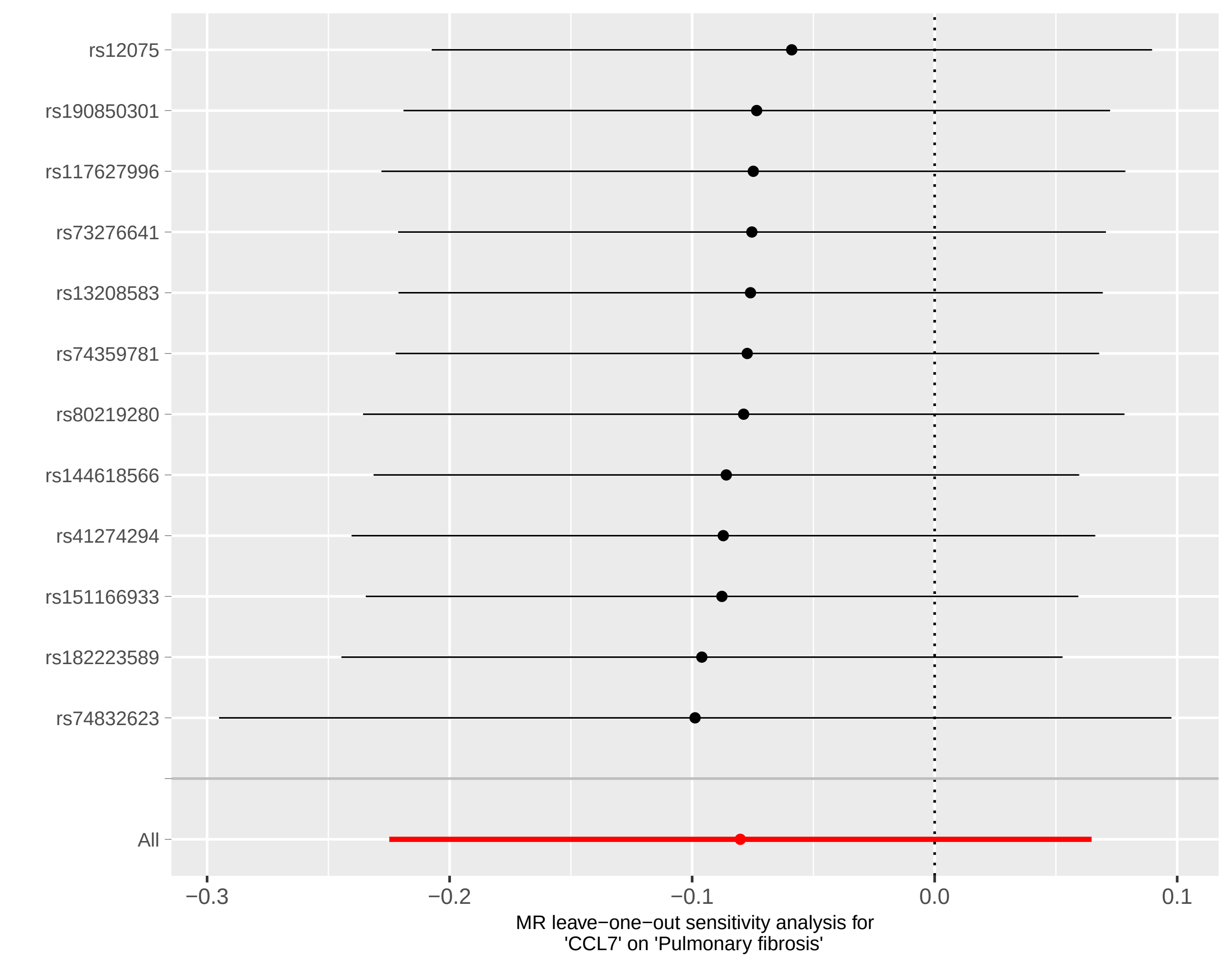
rs12075
rs190850301
rs117627996
rs73276641
rs13208583
rs74359781
rs80219280
rs144618566
rs41274294
rs151166933
rs182223589
rs74832623
All
−0.3
−0.2
−0.1
0.0
0.1
MR leave−one−out sensitivity analysis for
'CCL7' on 'Pulmonary fibrosis'

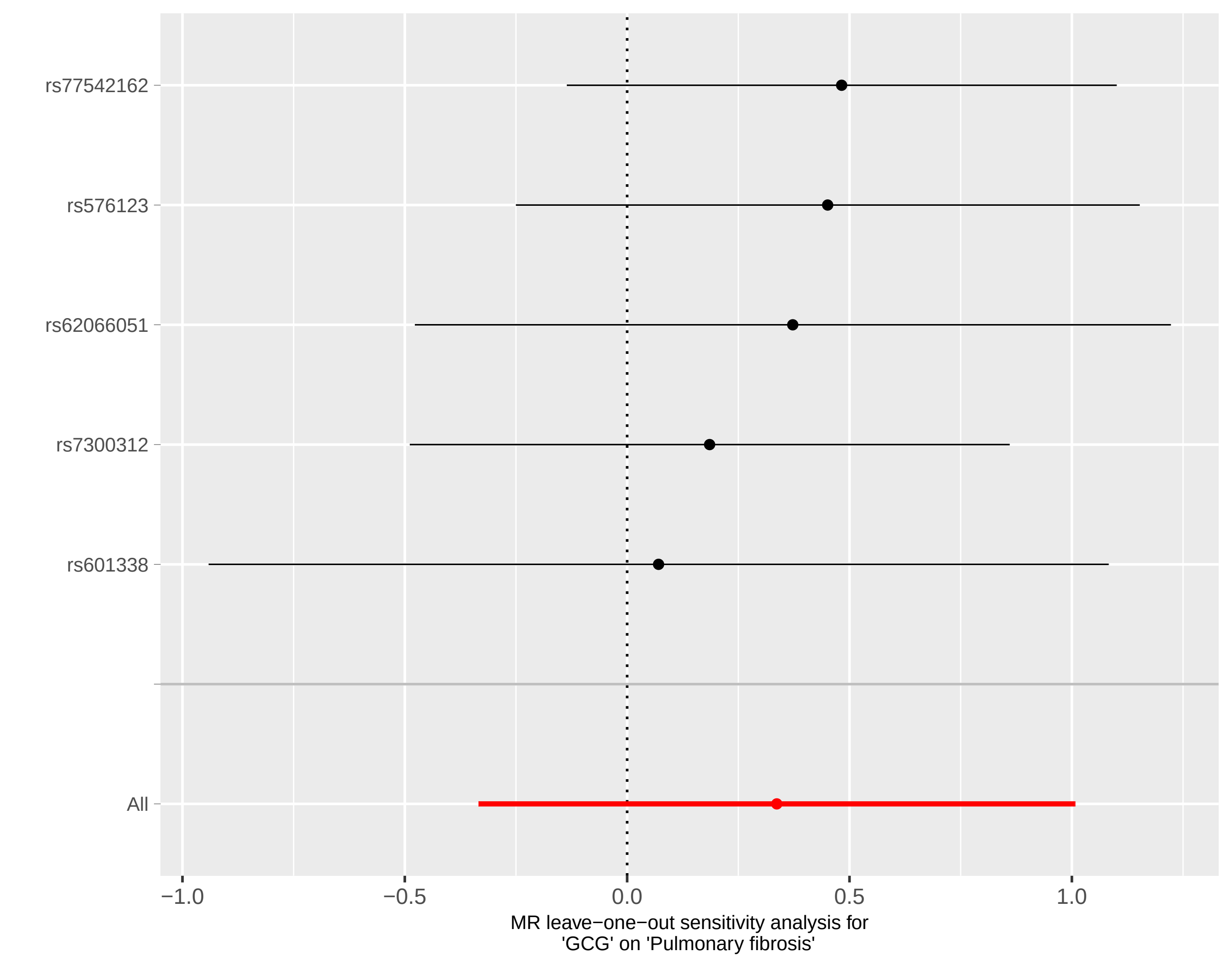
rs77542162
rs576123
rs62066051
rs7300312
rs601338
All
−1.0
−0.5
0.0
0.5
1.0
MR leave−one−out sensitivity analysis for
'GCG' on 'Pulmonary fibrosis'

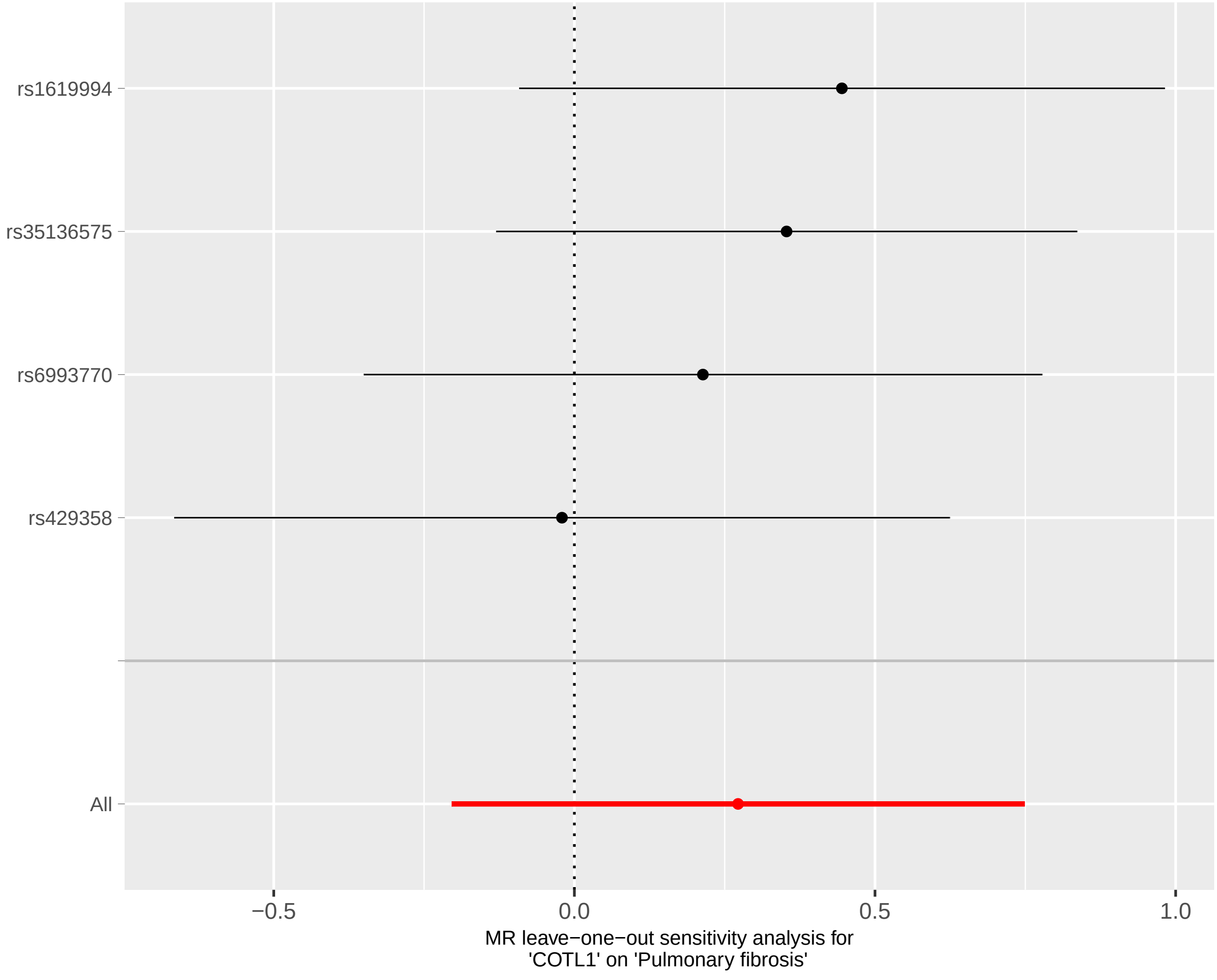

rs1619994
rs35136575
rs6993770
rs429358
All
−0.5
0.0
0.5
1.0
MR leave−one−out sensitivity analysis for
'COTL1' on 'Pulmonary fibrosis'

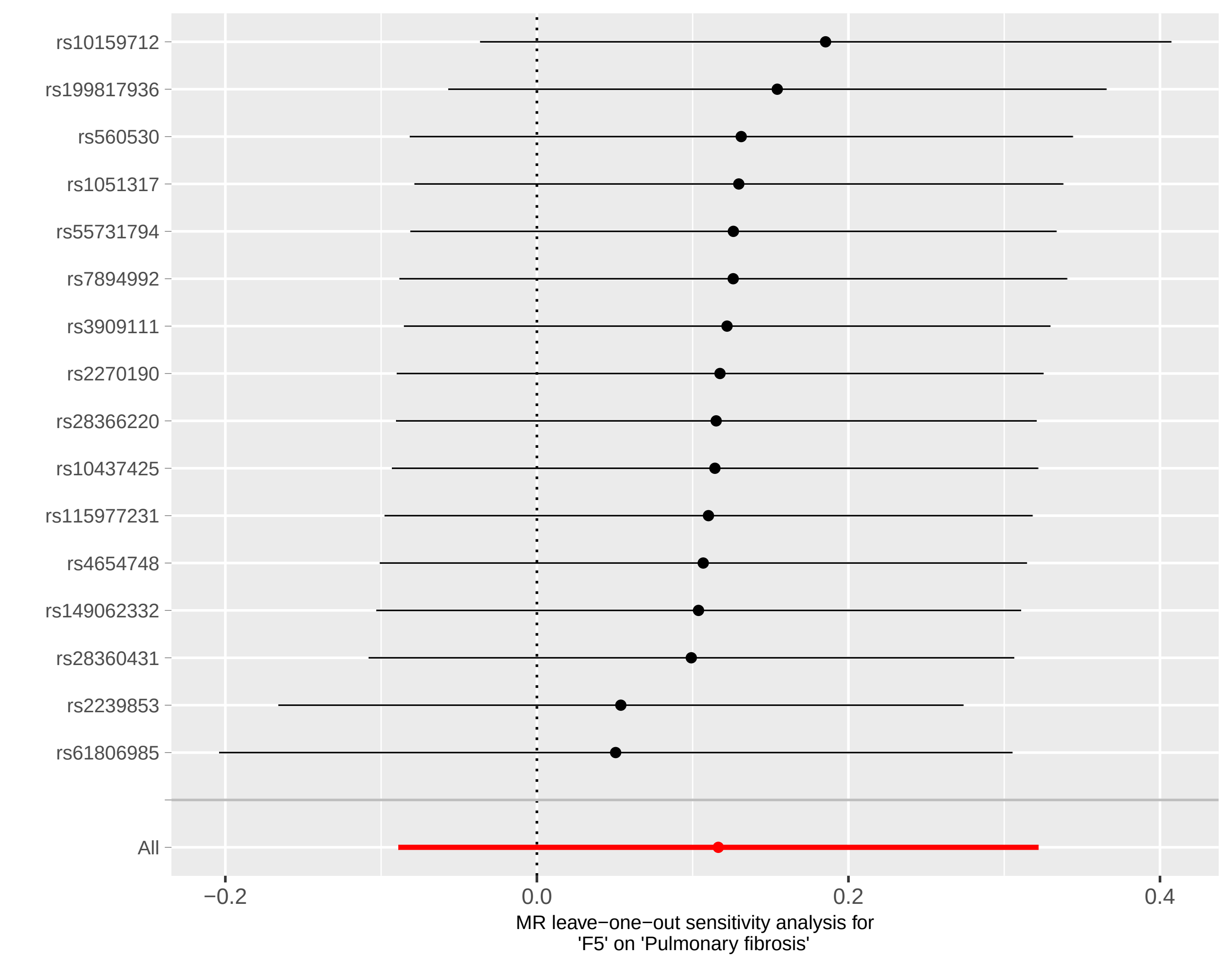

rs10159712
rs199817936
rs560530
rs1051317
rs55731794
rs7894992
rs3909111
rs2270190
rs28366220
rs10437425
rs115977231
rs4654748
rs149062332
rs28360431
rs2239853
rs61806985
All
−0.2
0.0
0.2
0.4
MR leave−one−out sensitivity analysis for
'F5' on 'Pulmonary fibrosis'

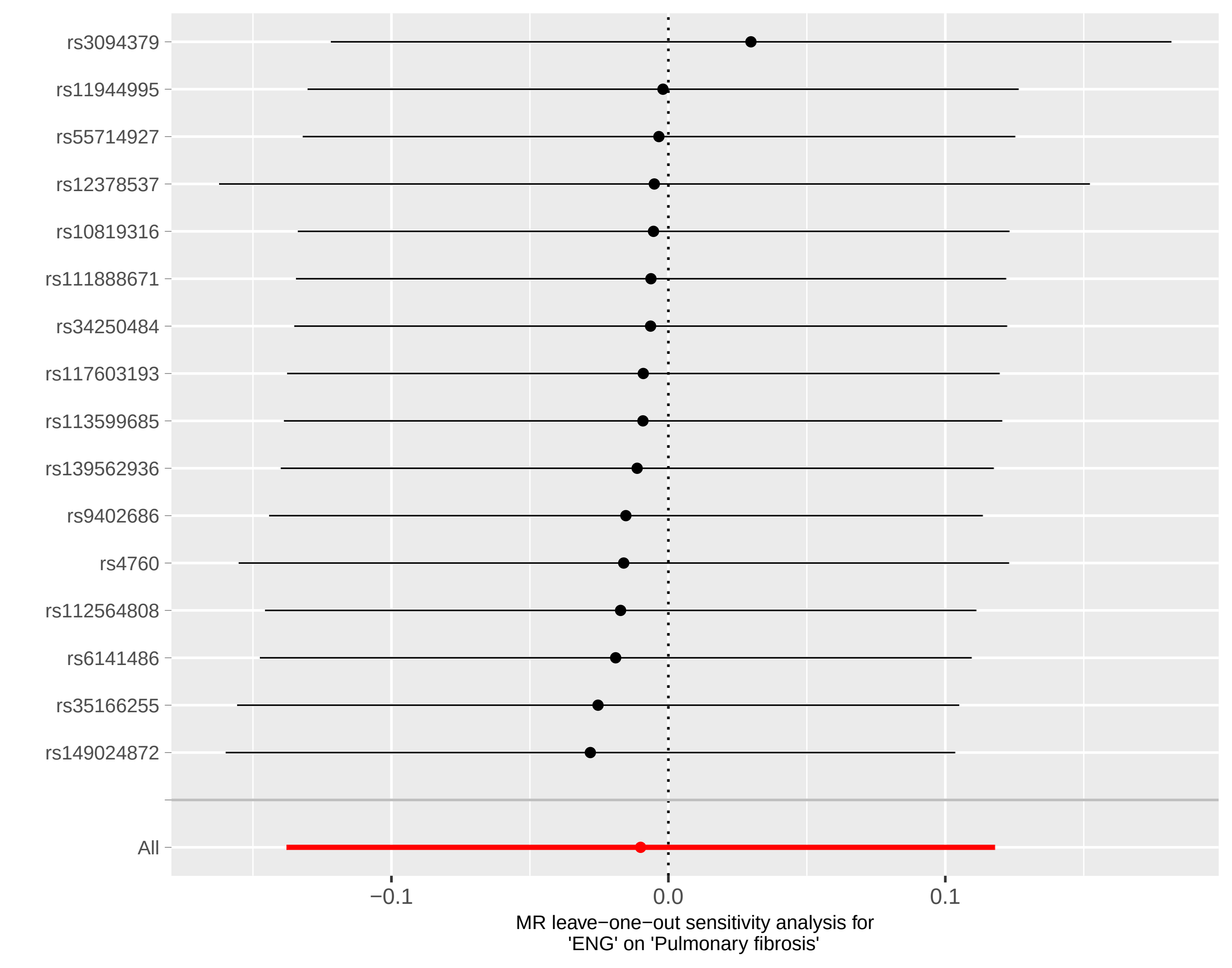

rs3094379
rs11944995
rs55714927
rs12378537
rs10819316
rs111888671
rs34250484
rs117603193
rs113599685
rs139562936
rs9402686
rs4760
rs112564808
rs6141486
rs35166255
rs149024872
All
−0.1
0.0
0.1
MR leave−one−out sensitivity analysis for
'ENG' on 'Pulmonary fibrosis'

Insufficient number of SNPs

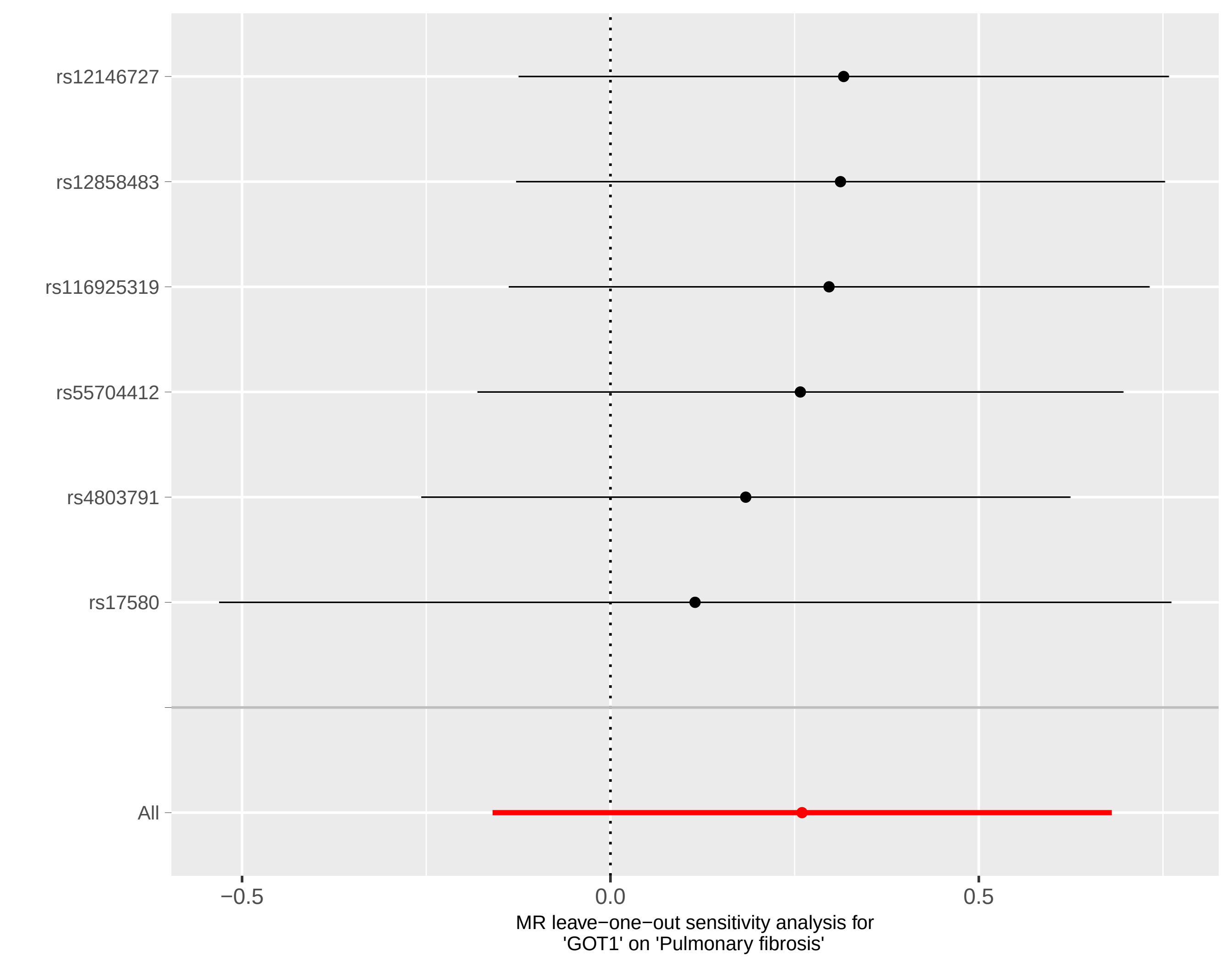

rs12146727
rs12858483
rs116925319
rs55704412
rs4803791
rs17580
All
−0.5
0.0
0.5
MR leave−one−out sensitivity analysis for
'GOT1' on 'Pulmonary fibrosis'

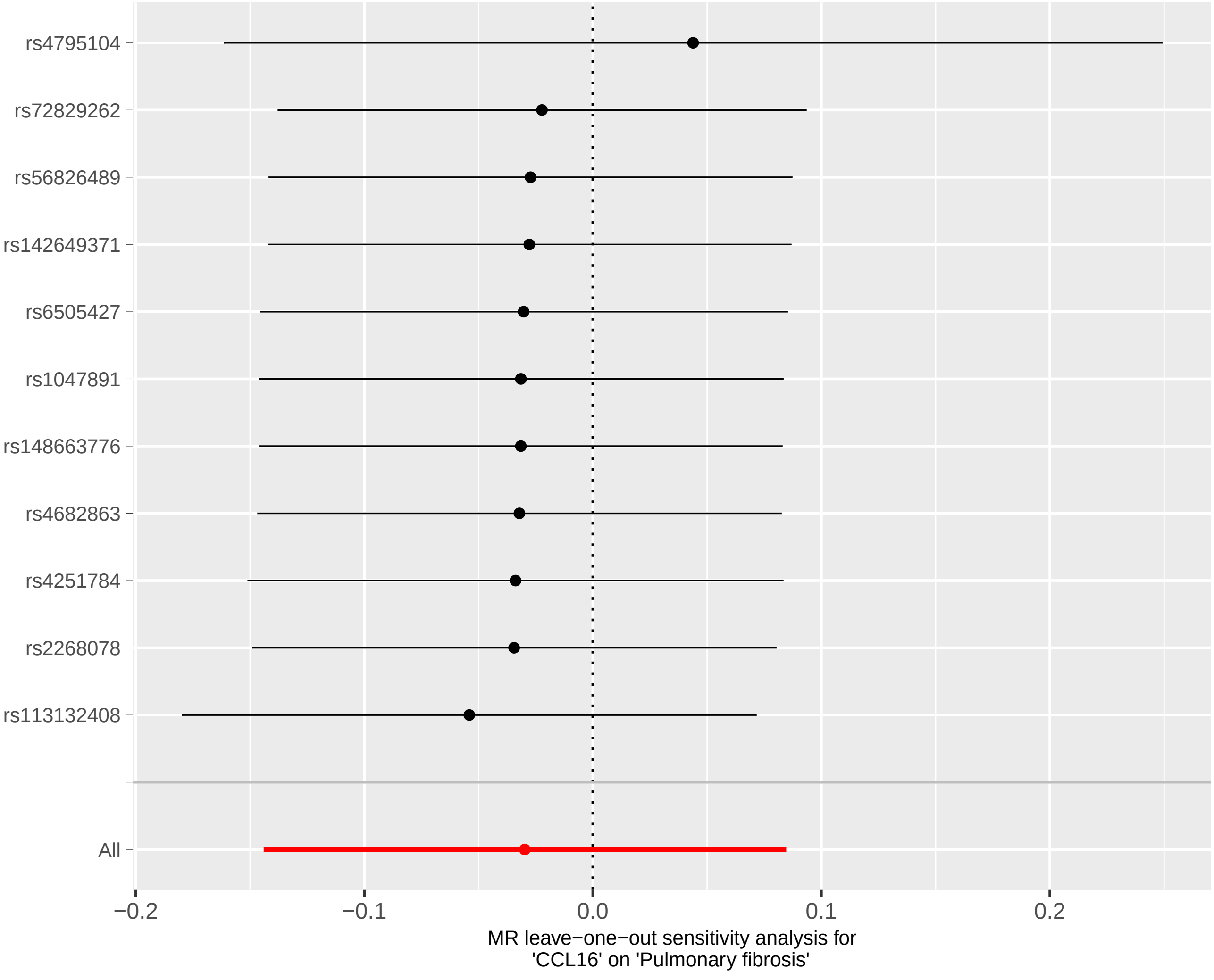
rs4795104
rs72829262
rs56826489
rs142649371
rs6505427
rs1047891
rs148663776
rs4682863
rs4251784
rs2268078
rs113132408
All
−0.2
−0.1
0.0
0.1
0.2
MR leave−one−out sensitivity analysis for
'CCL16' on 'Pulmonary fibrosis'

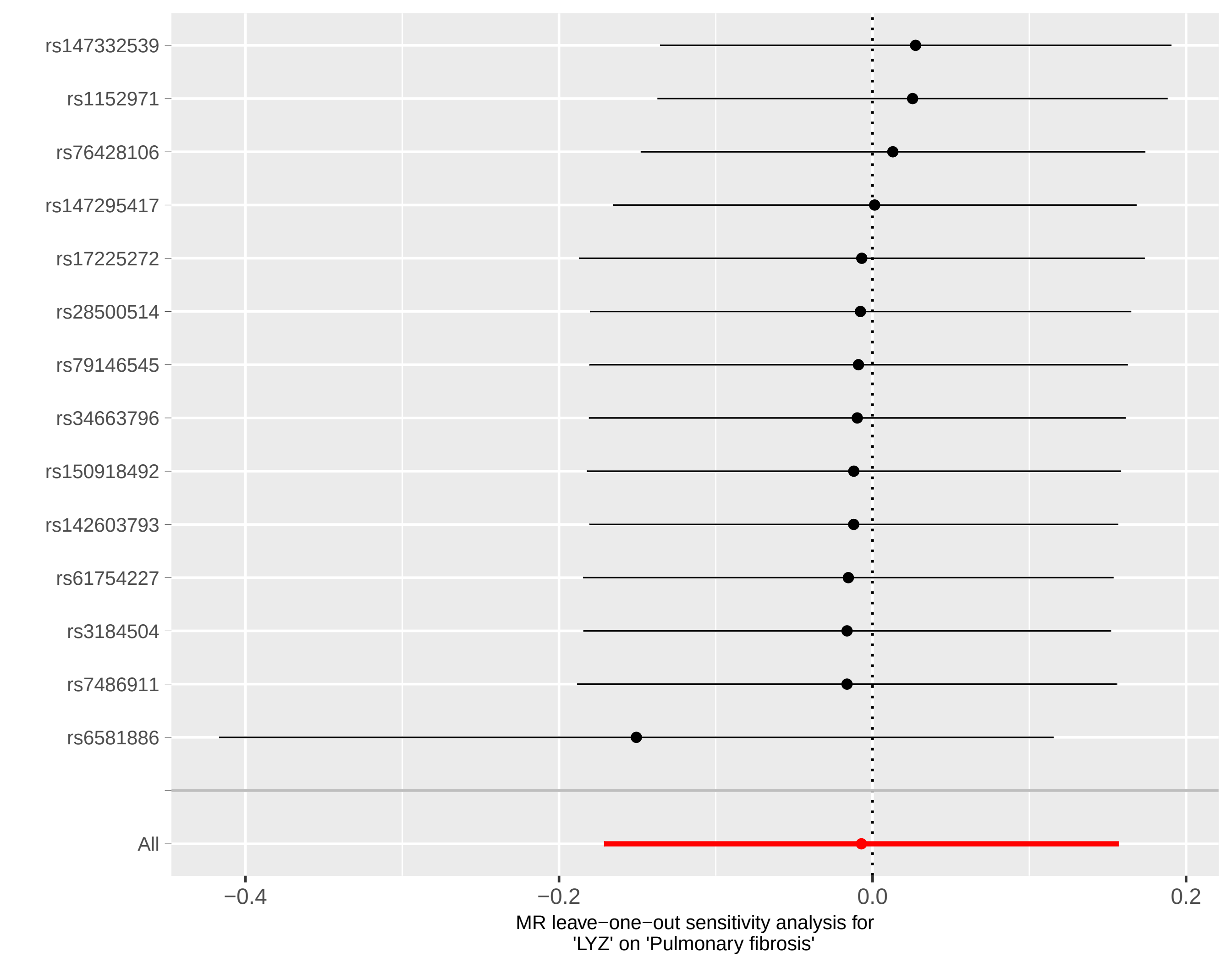
rs147332539
rs1152971
rs76428106
rs147295417
rs17225272
rs28500514
rs79146545
rs34663796
rs150918492
rs142603793
rs61754227
rs3184504
rs7486911
rs6581886
All
−0.4
−0.2
0.0
0.2
MR leave−one−out sensitivity analysis for
'LYZ' on 'Pulmonary fibrosis'

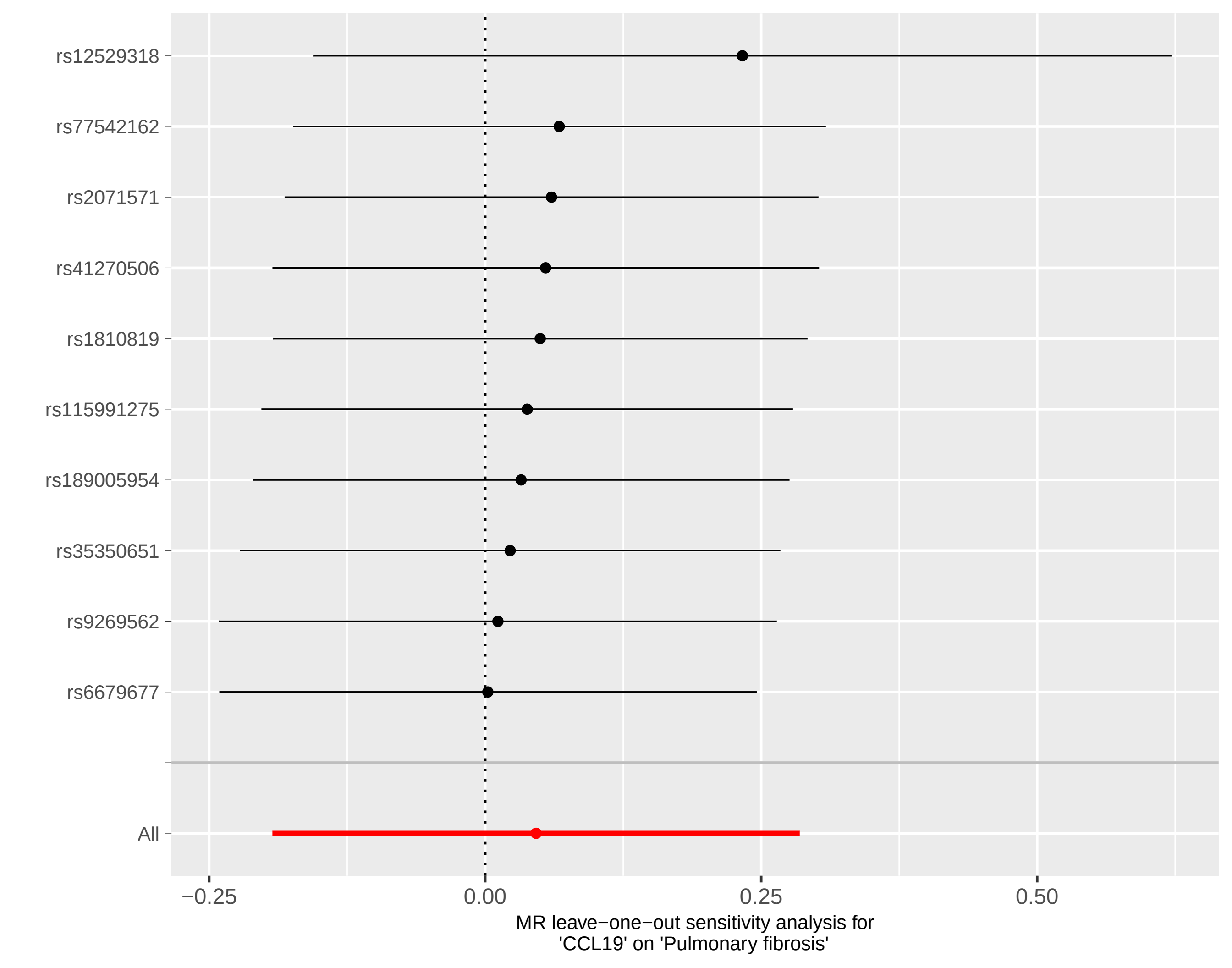
rs12529318
rs77542162
rs2071571
rs41270506
rs1810819
rs115991275
rs189005954
rs35350651
rs9269562
rs6679677
All
−0.25
0.00
0.25
0.50
MR leave−one−out sensitivity analysis for
'CCL19' on 'Pulmonary fibrosis'

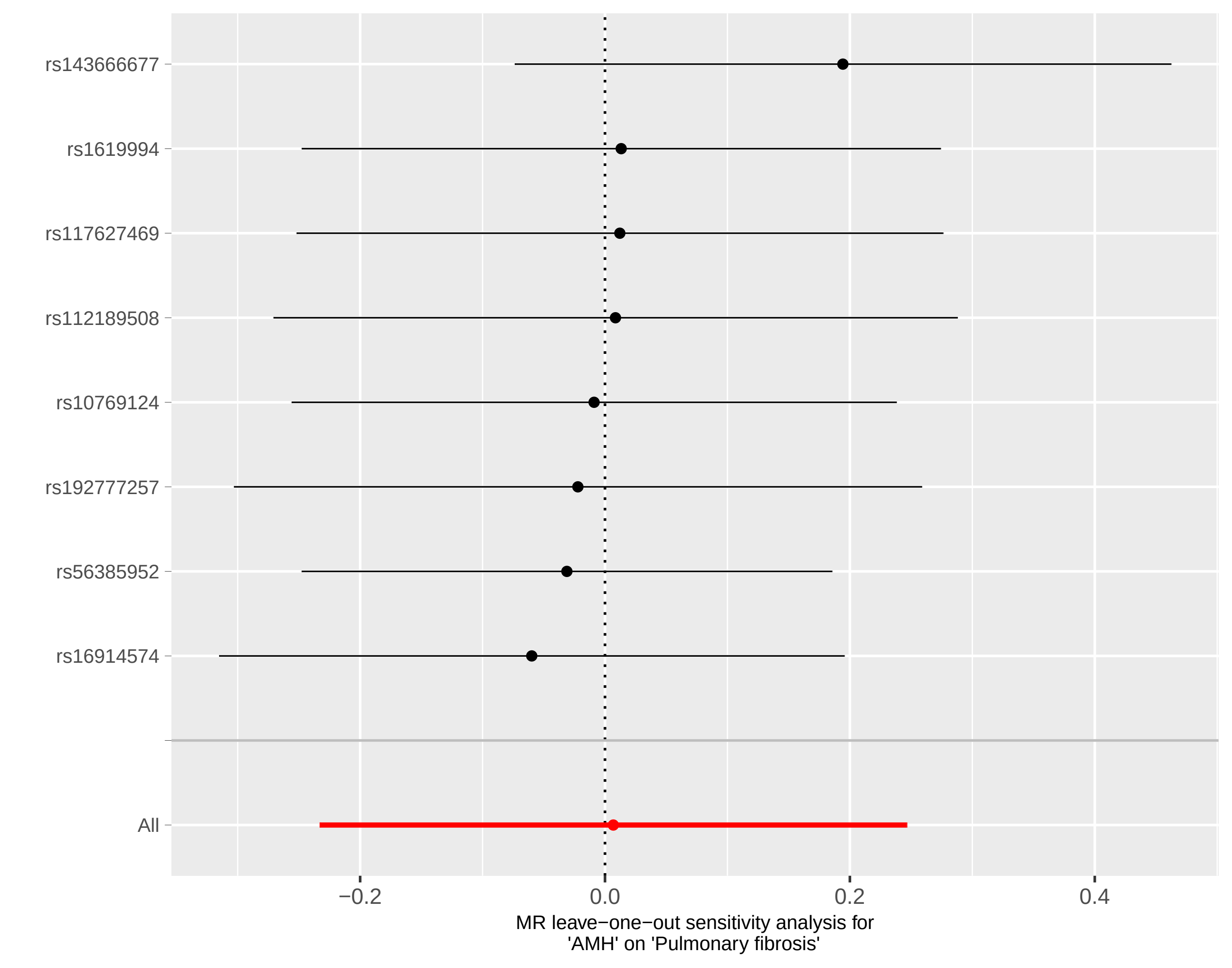

rs143666677
rs1619994
rs117627469
rs112189508
rs10769124
rs192777257
rs56385952
rs16914574
All
−0.2
0.0
0.2
0.4
MR leave−one−out sensitivity analysis for
'AMH' on 'Pulmonary fibrosis'

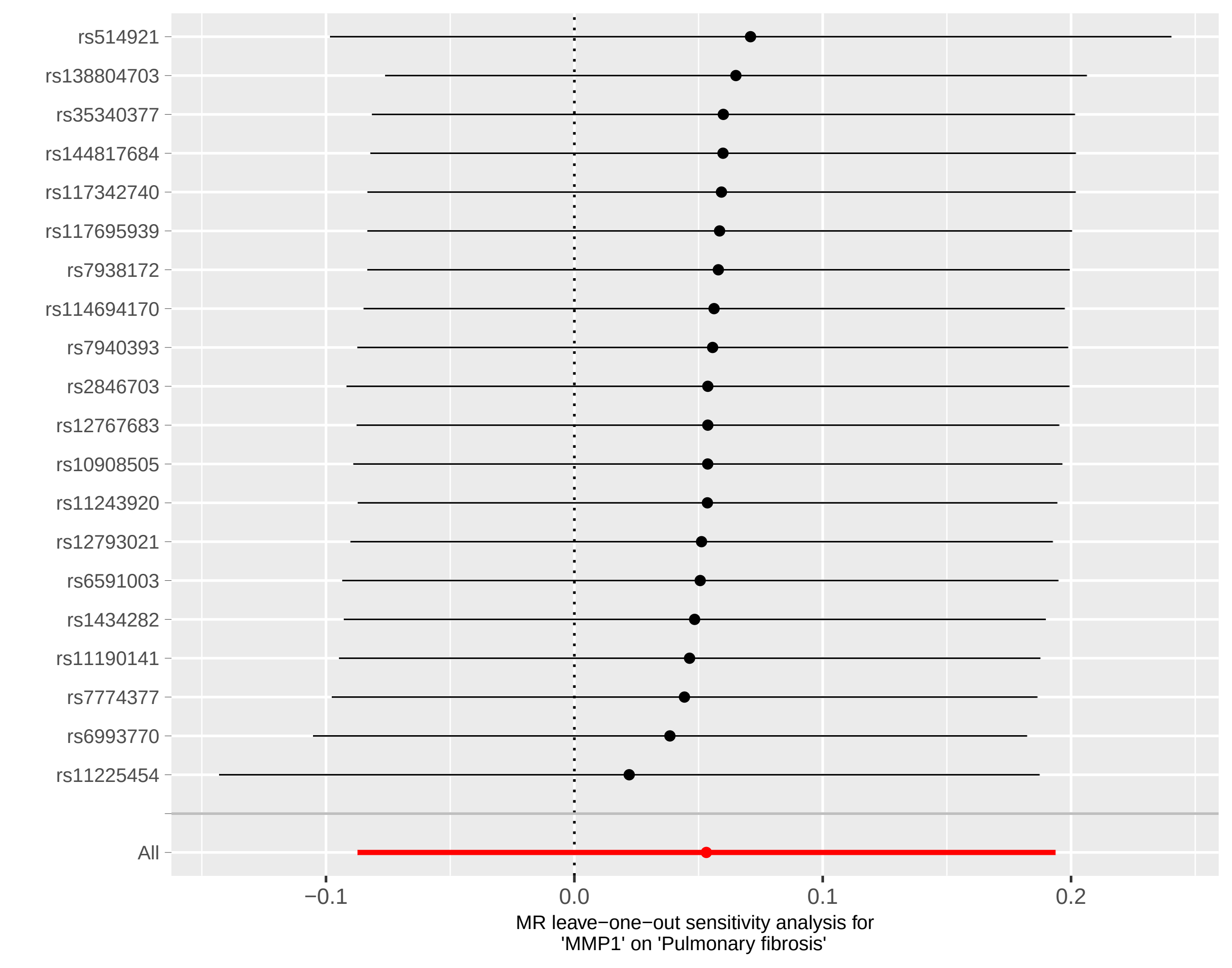
rs514921
rs138804703
rs35340377
rs144817684
rs117342740
rs117695939
rs7938172
rs114694170
rs7940393
rs2846703
rs12767683
rs10908505
rs11243920
rs12793021
rs6591003
rs1434282
rs11190141
rs7774377
rs6993770
rs11225454
All
−0.1
0.0
0.1
0.2
MR leave−one−out sensitivity analysis for
'MMP1' on 'Pulmonary fibrosis'

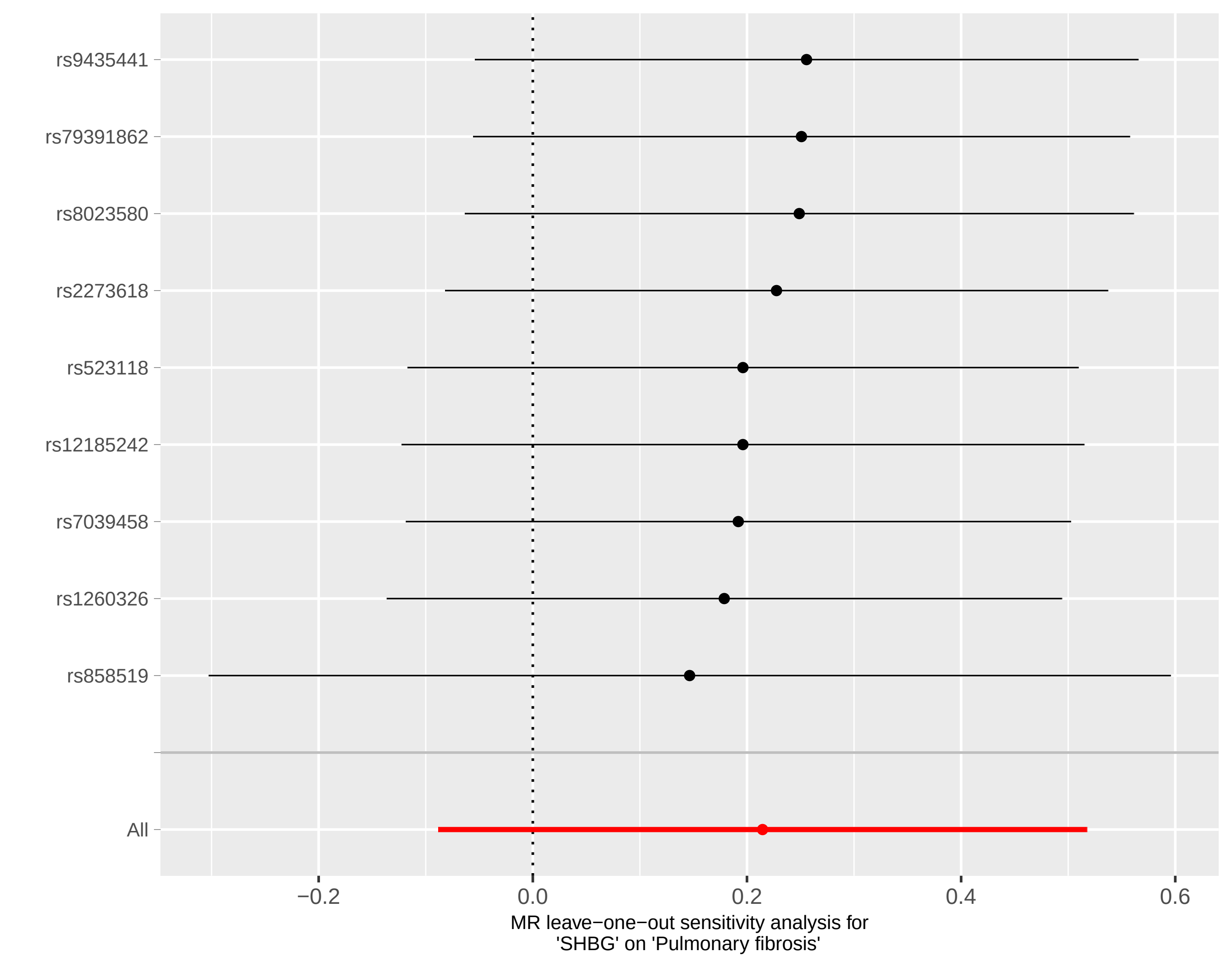
rs9435441
rs79391862
rs8023580
rs2273618
rs523118
rs12185242
rs7039458
rs1260326
rs858519
All
−0.2
0.0
0.2
0.4
0.6
MR leave−one−out sensitivity analysis for
'SHBG' on 'Pulmonary fibrosis'

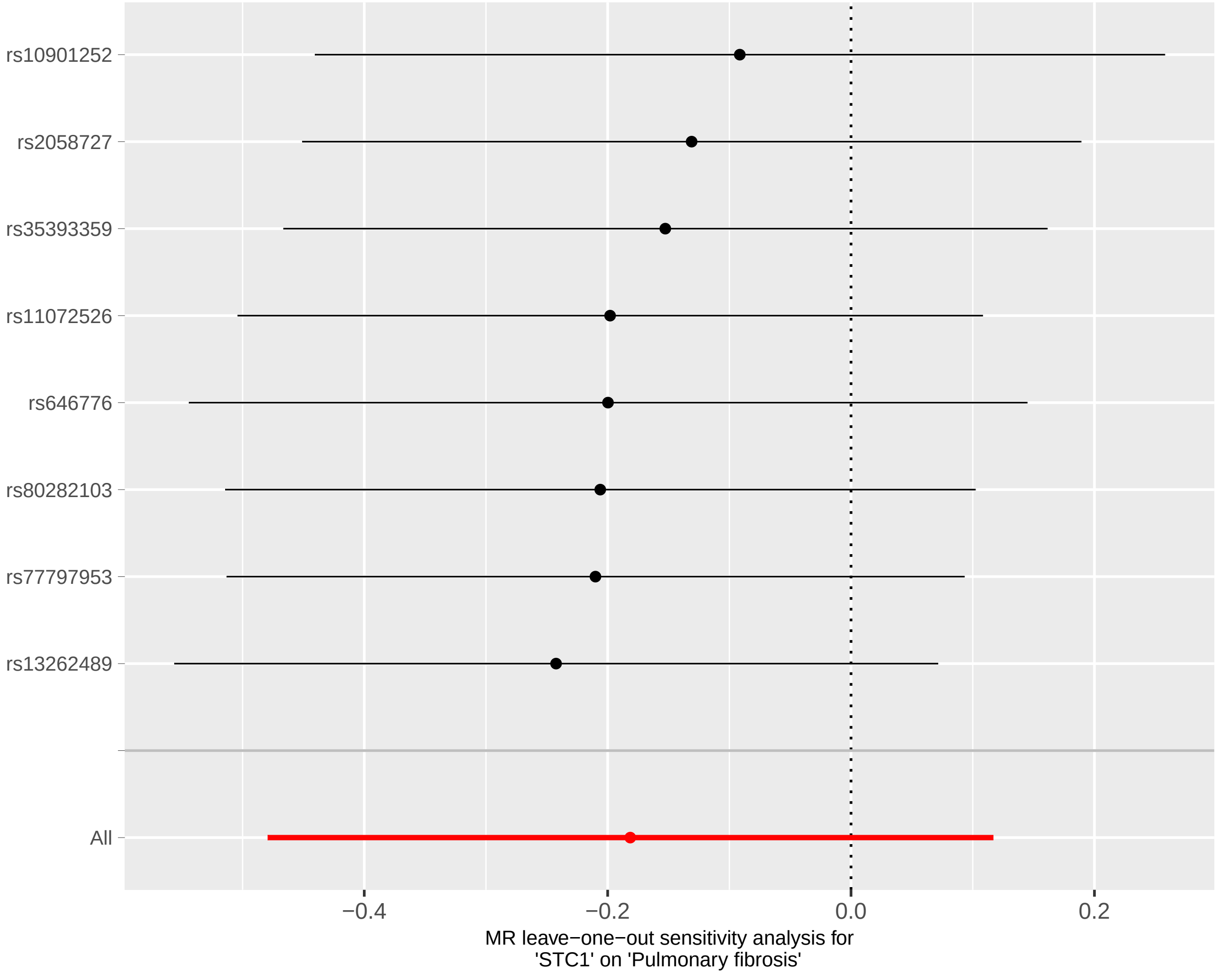
rs10901252
rs2058727
rs35393359
rs11072526
rs646776
rs80282103
rs77797953
rs13262489
All
−0.4
−0.2
0.0
0.2
MR leave−one−out sensitivity analysis for
'STC1' on 'Pulmonary fibrosis'

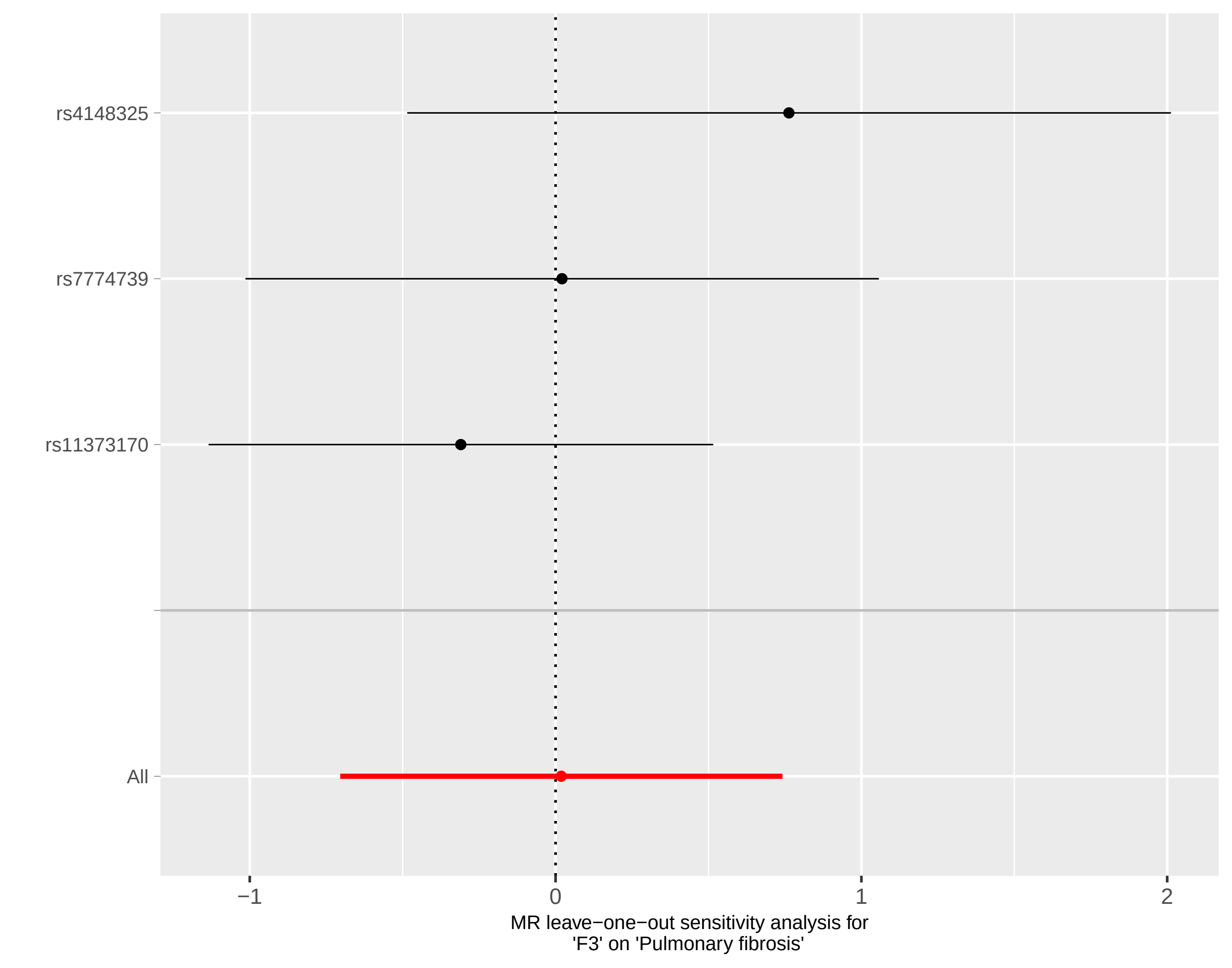
rs4148325
rs7774739
rs11373170
All
−1
0
1
2
MR leave−one−out sensitivity analysis for
'F3' on 'Pulmonary fibrosis'

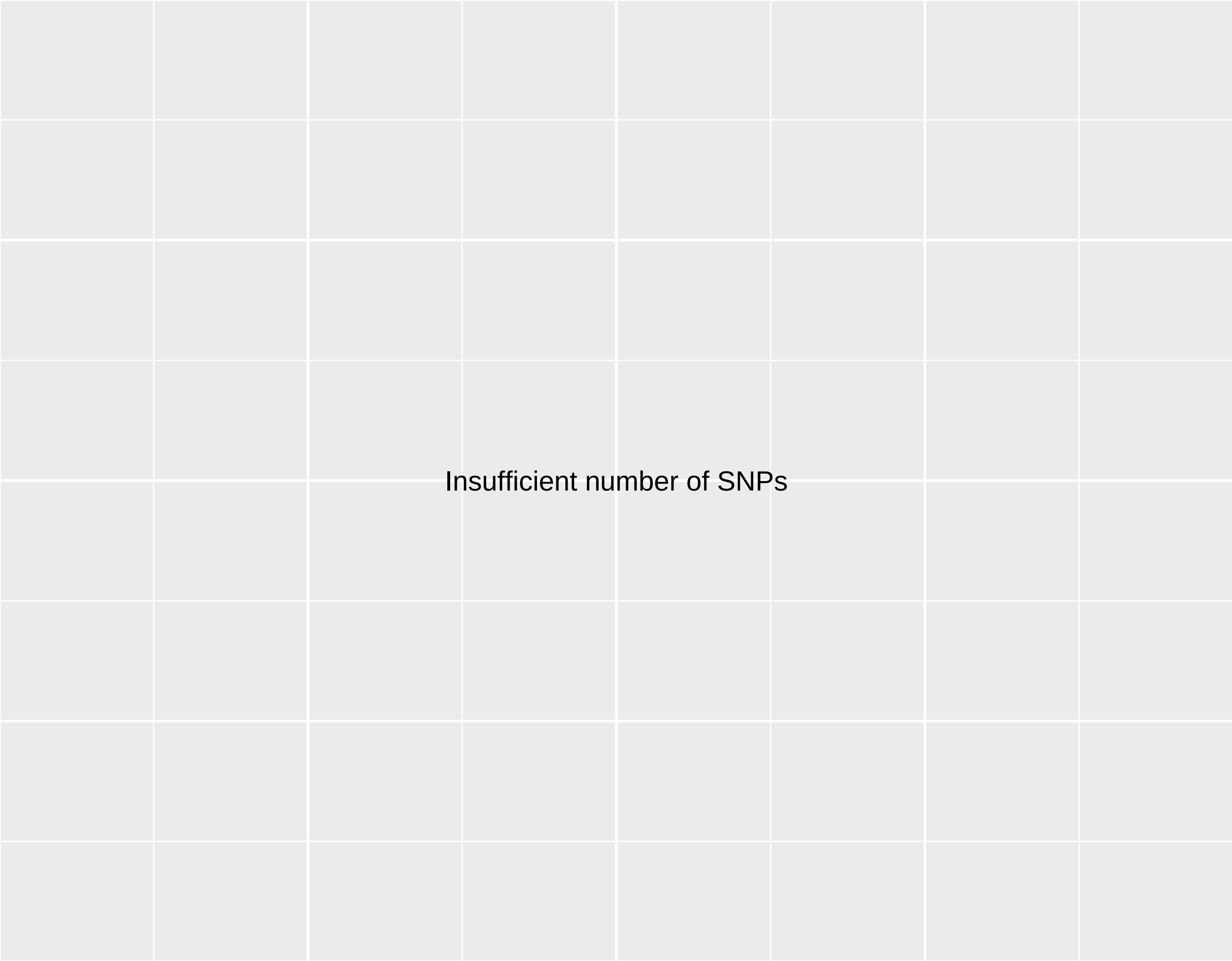
Insufficient number of SNPs

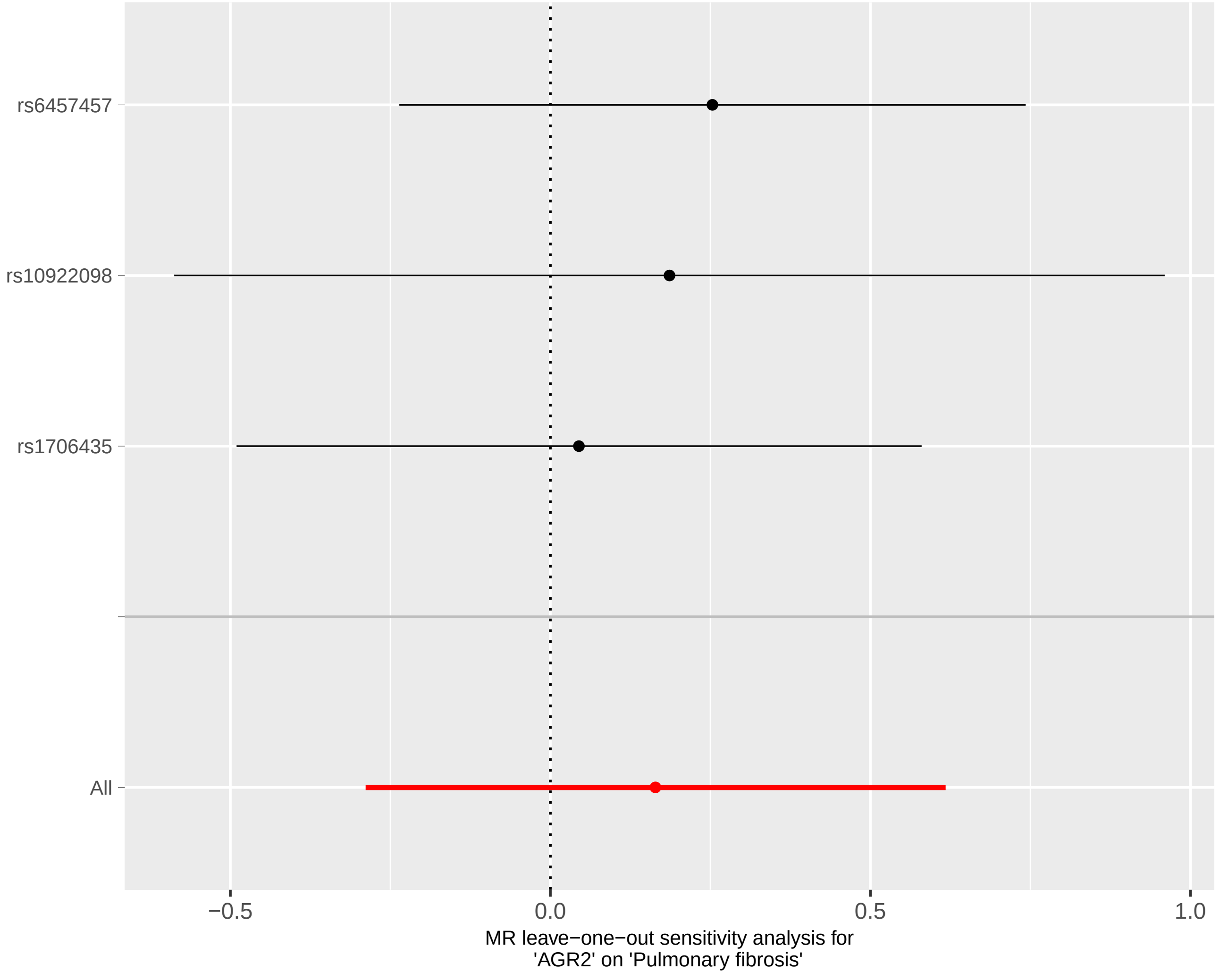

rs6457457
rs10922098
rs1706435
All
−0.5
0.0
0.5
1.0
MR leave−one−out sensitivity analysis for
'AGR2' on 'Pulmonary fibrosis'

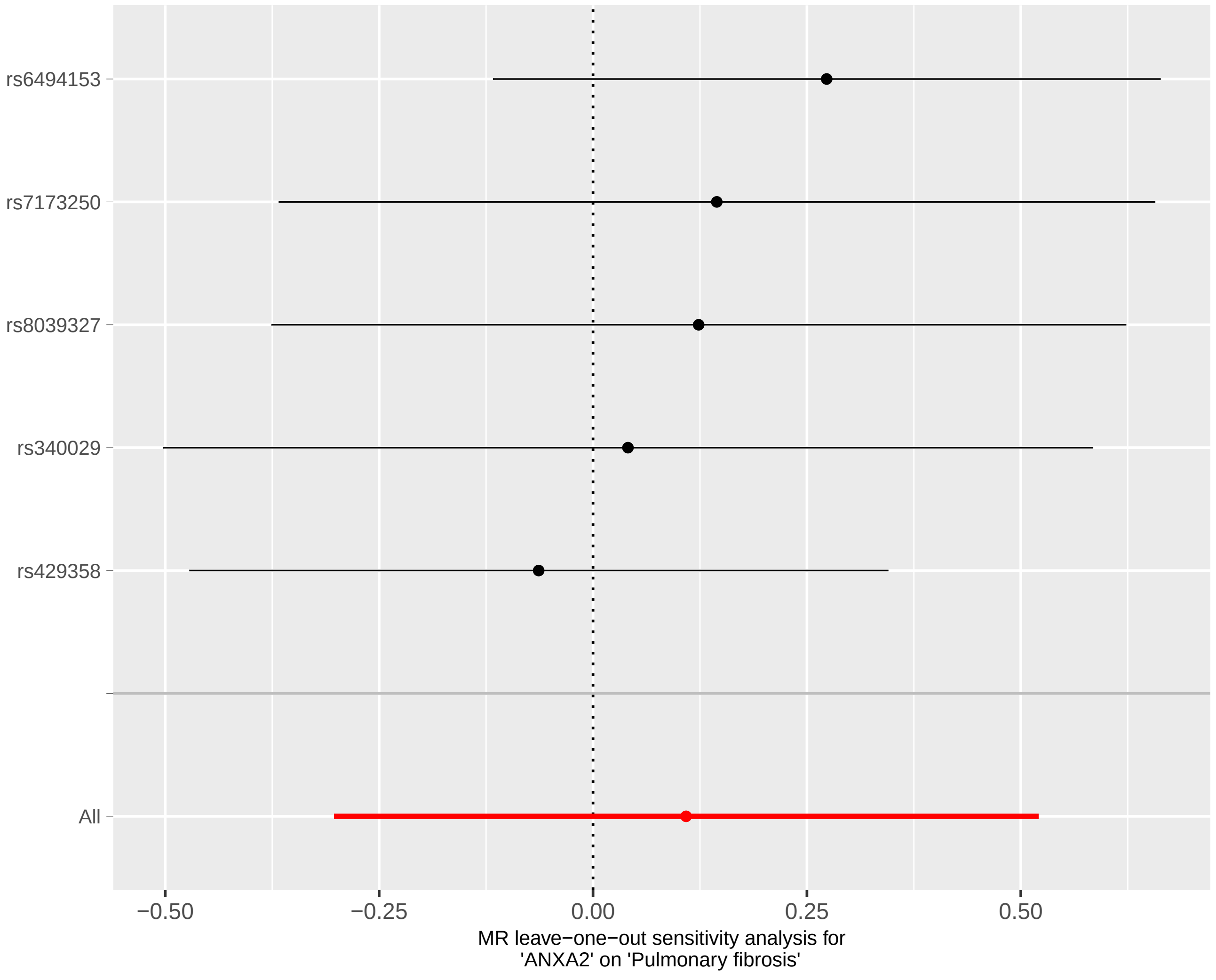
rs6494153
rs7173250
rs8039327
rs340029
rs429358
All
−0.50
−0.25
0.00
0.25
0.50
MR leave−one−out sensitivity analysis for
'ANXA2' on 'Pulmonary fibrosis'

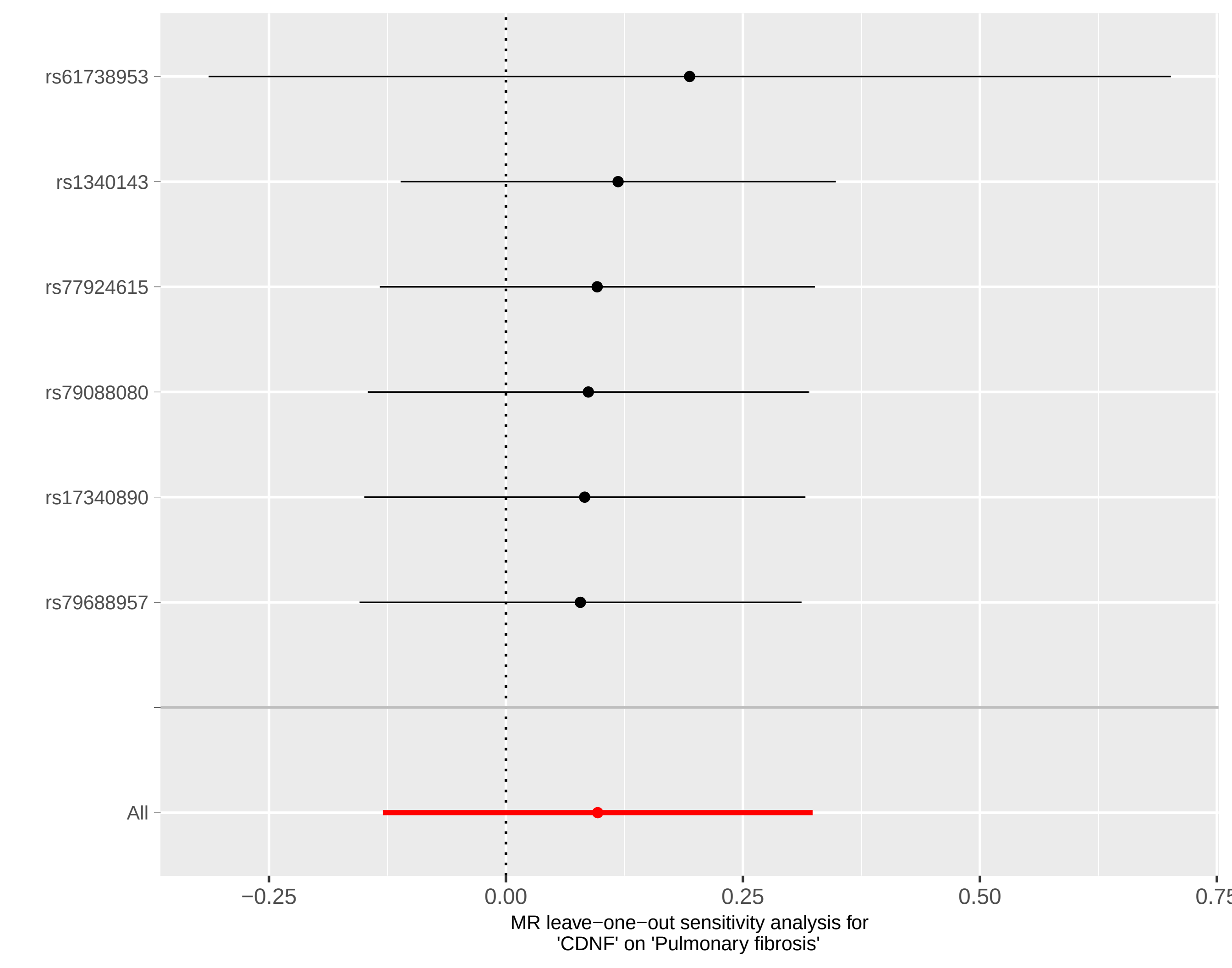
rs61738953
rs1340143
rs77924615
rs79088080
rs17340890
rs79688957
All
−0.25
0.00
0.25
0.50
0.75
MR leave−one−out sensitivity analysis for
'CDNF' on 'Pulmonary fibrosis'

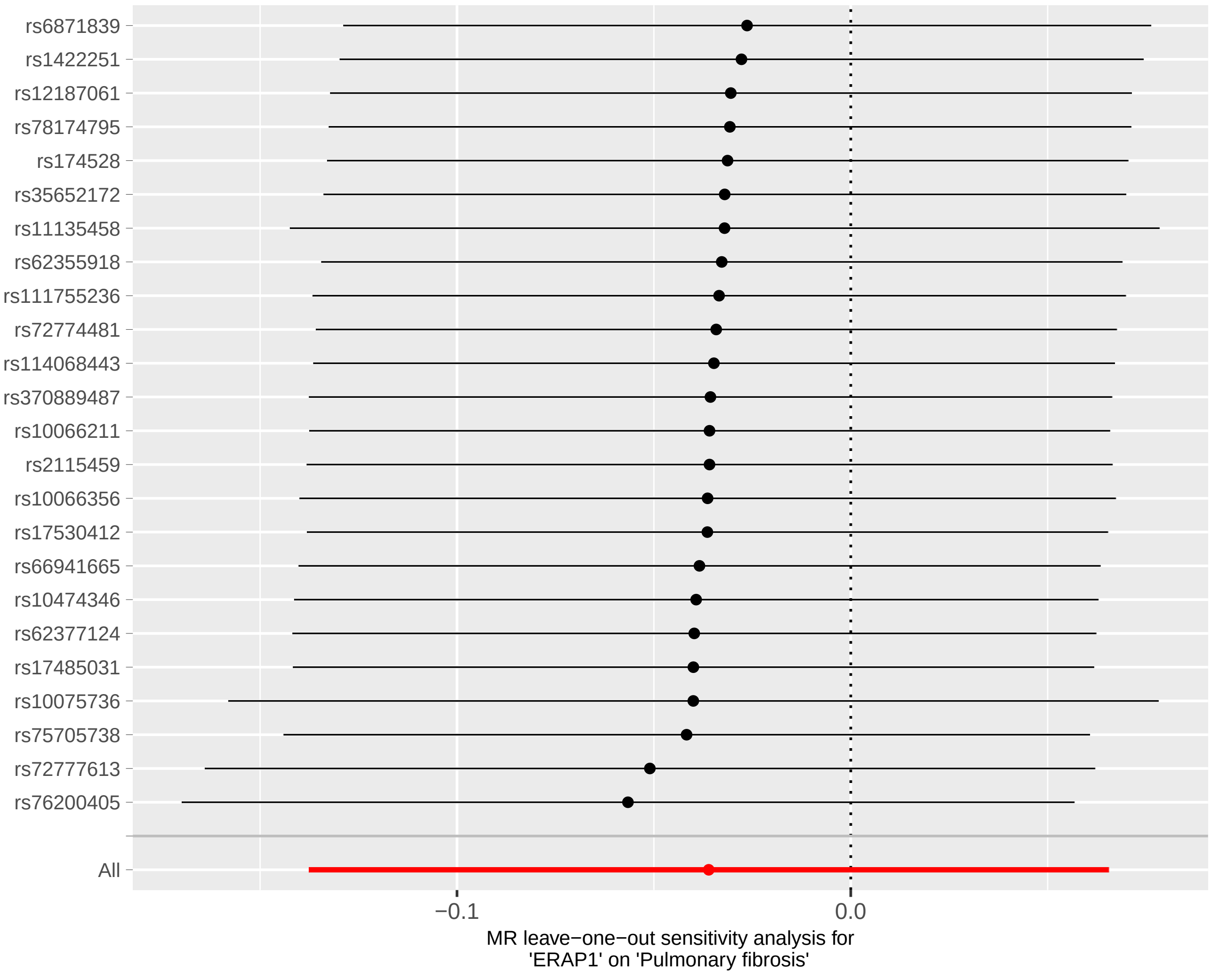

rs6871839
rs1422251
rs12187061
rs78174795
rs174528
rs35652172
rs11135458
rs62355918
rs111755236
rs72774481
rs114068443
rs370889487
rs10066211
rs2115459
rs10066356
rs17530412
rs66941665
rs10474346
rs62377124
rs17485031
rs10075736
rs75705738
rs72777613
rs76200405
All
−0.1
0.0
MR leave−one−out sensitivity analysis for
'ERAP1' on 'Pulmonary fibrosis'

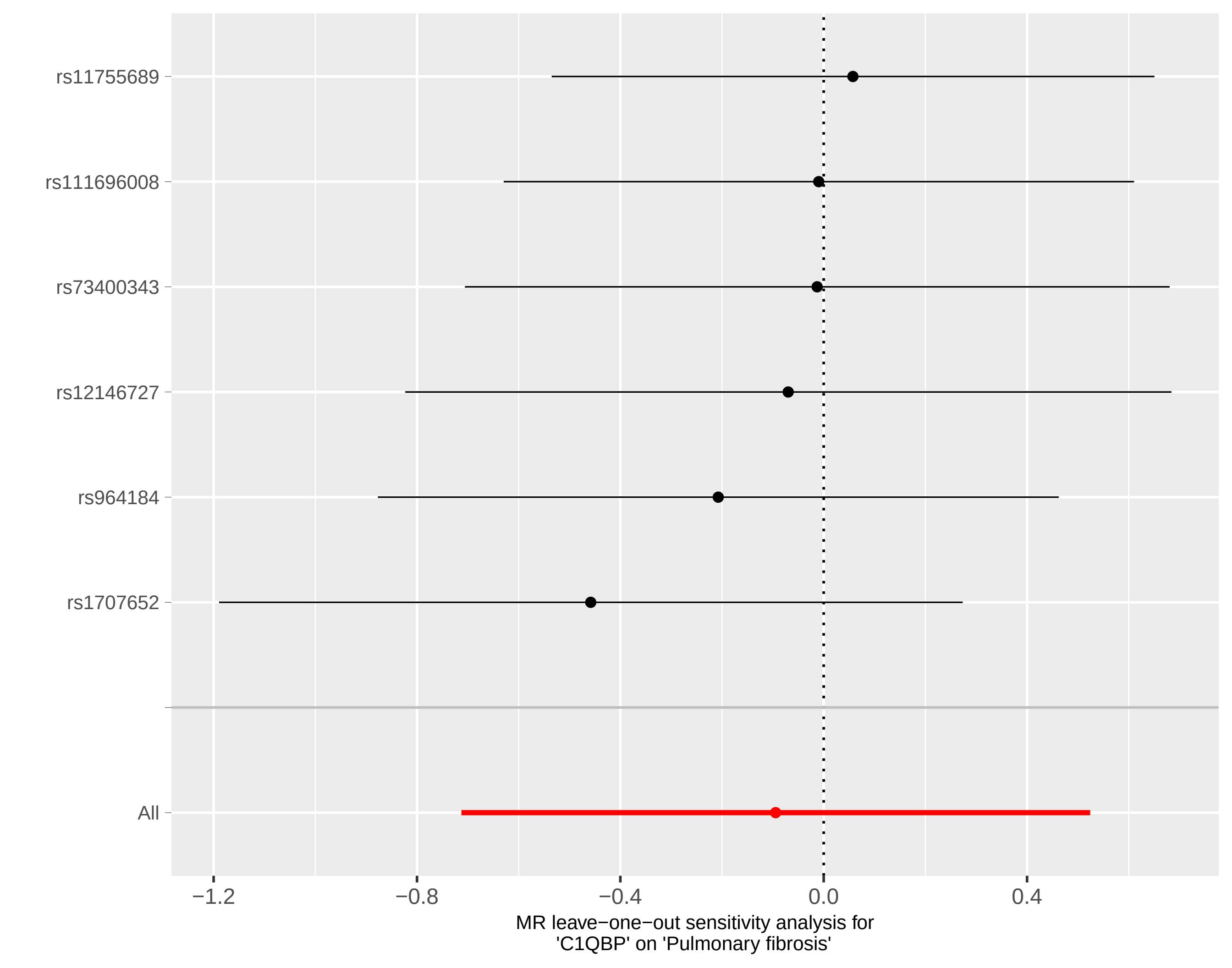
rs11755689
rs111696008
rs73400343
rs12146727
rs964184
rs1707652
All
−1.2
−0.8
−0.4
0.0
0.4
MR leave−one−out sensitivity analysis for
'C1QBP' on 'Pulmonary fibrosis'

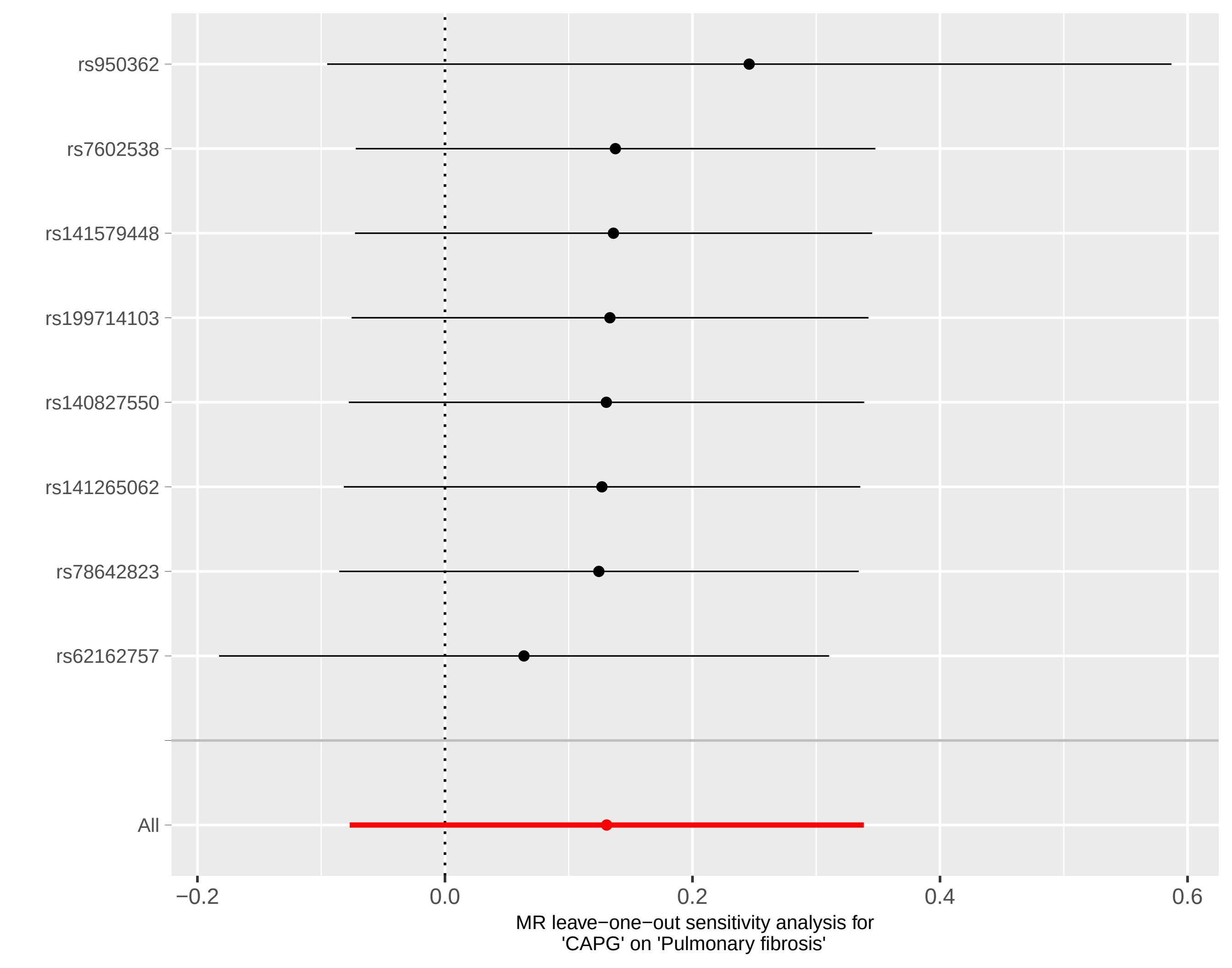
rs950362
rs7602538
rs141579448
rs199714103
rs140827550
rs141265062
rs78642823
rs62162757
All
−0.2
0.0
0.2
0.4
0.6
MR leave−one−out sensitivity analysis for
'CAPG' on 'Pulmonary fibrosis'

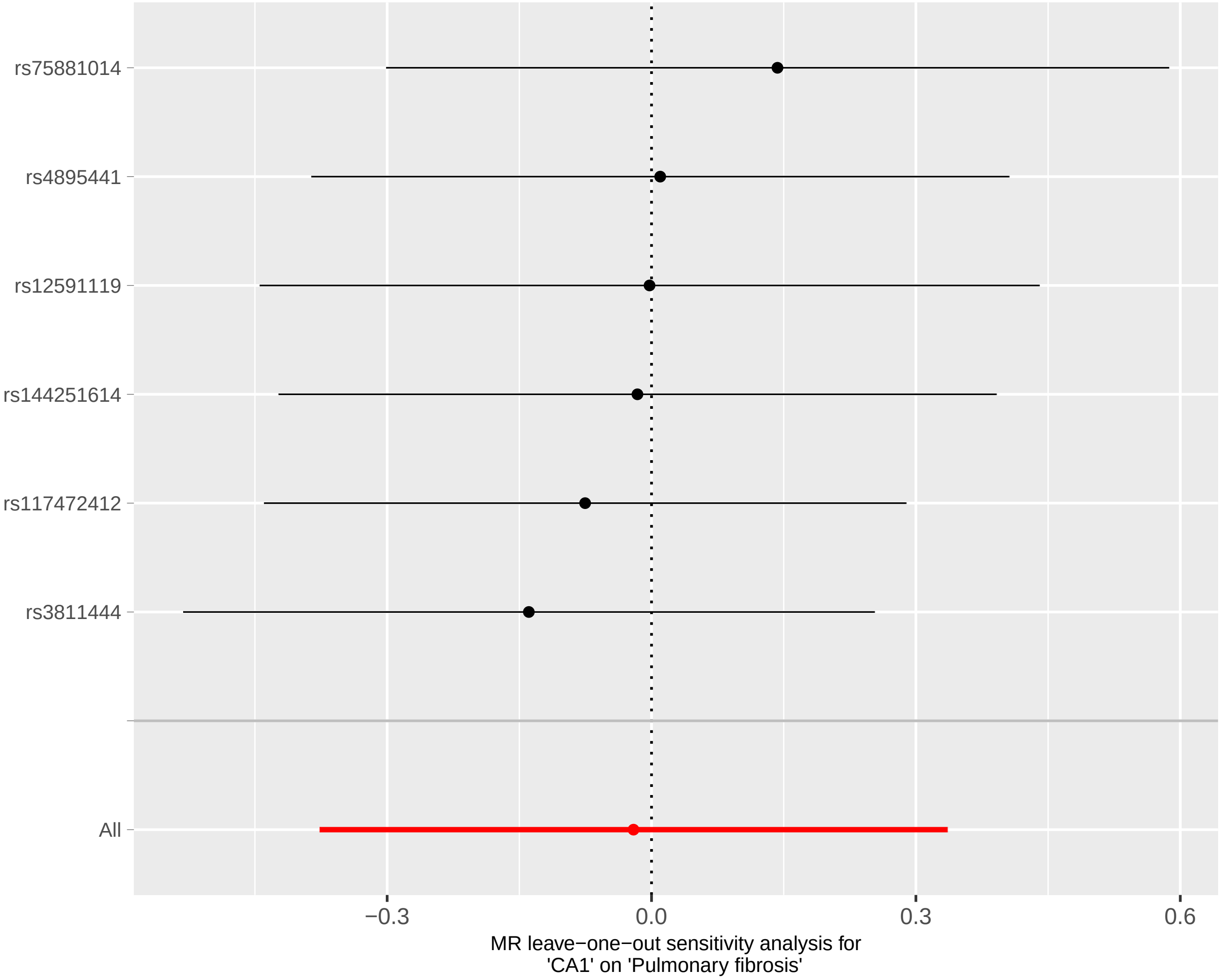
rs75881014
rs4895441
rs12591119
rs144251614
rs117472412
rs3811444
All
−0.3
0.0
0.3
0.6
MR leave−one−out sensitivity analysis for
'CA1' on 'Pulmonary fibrosis'

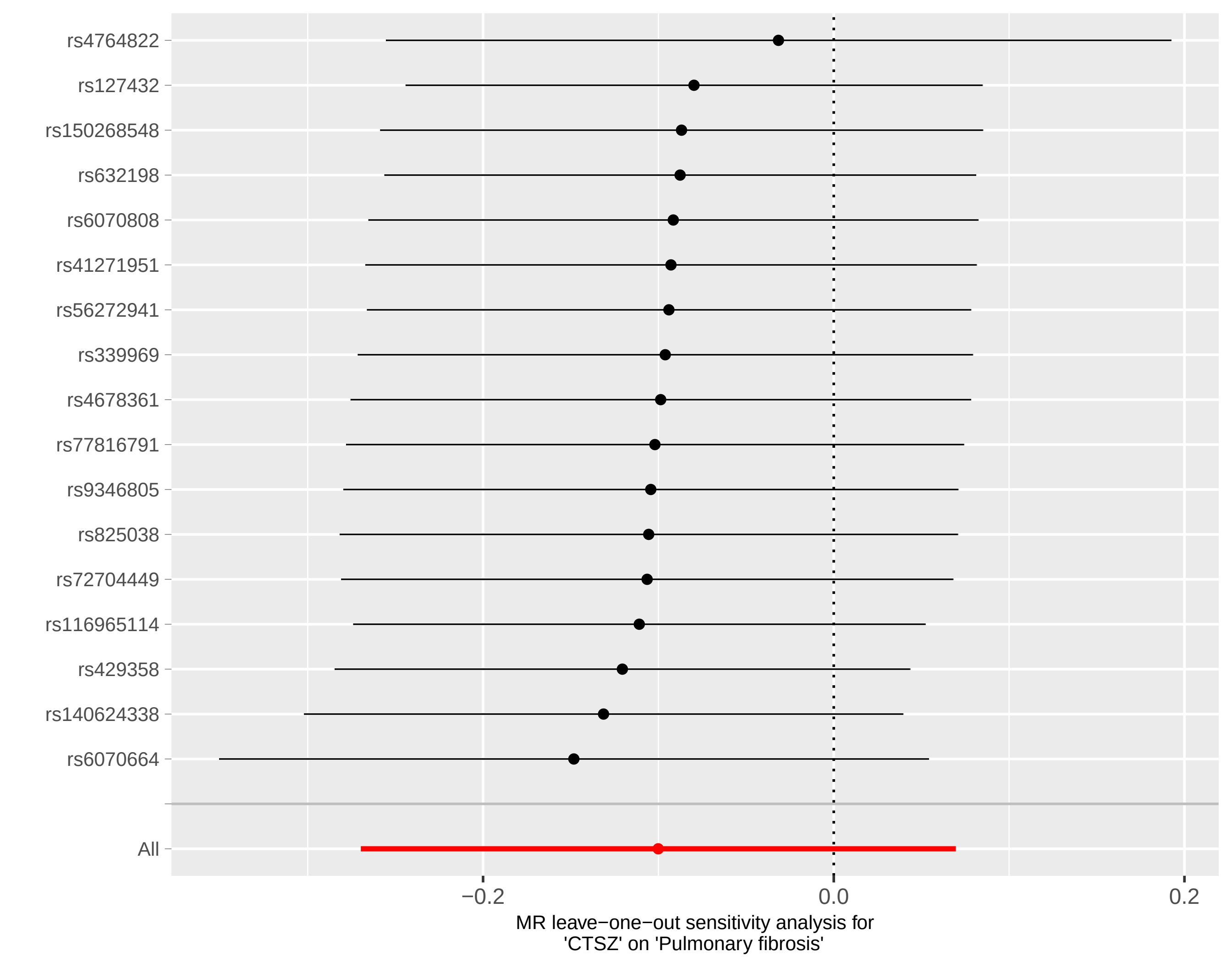
rs4764822
rs127432
rs150268548
rs632198
rs6070808
rs41271951
rs56272941
rs339969
rs4678361
rs77816791
rs9346805
rs825038
rs72704449
rs116965114
rs429358
rs140624338
rs6070664
All
−0.2
0.0
0.2
MR leave−one−out sensitivity analysis for
'CTSZ' on 'Pulmonary fibrosis'

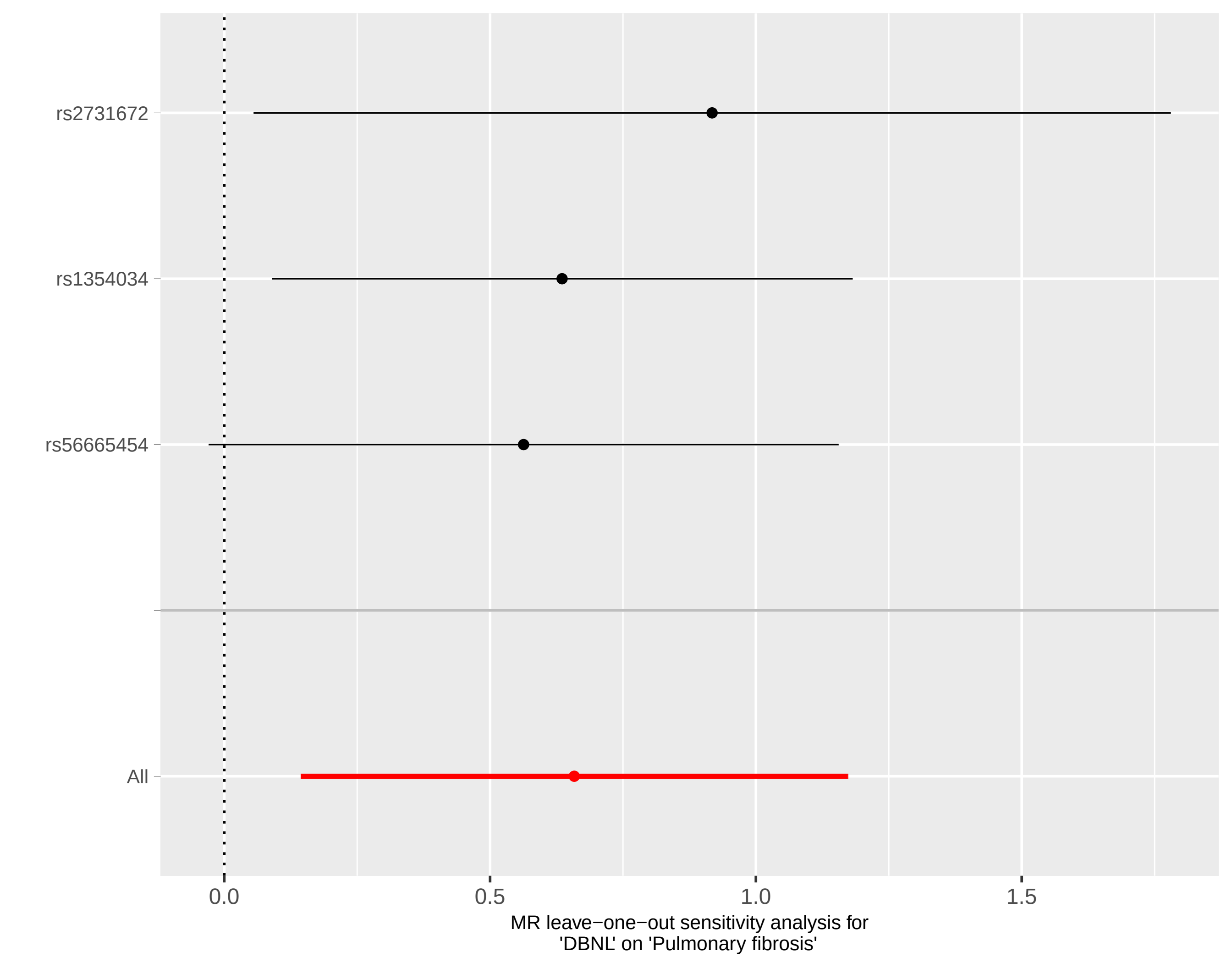

rs2731672
rs1354034
rs56665454
All
0.0
0.5
1.0
1.5
MR leave−one−out sensitivity analysis for
'DBNL' on 'Pulmonary fibrosis'

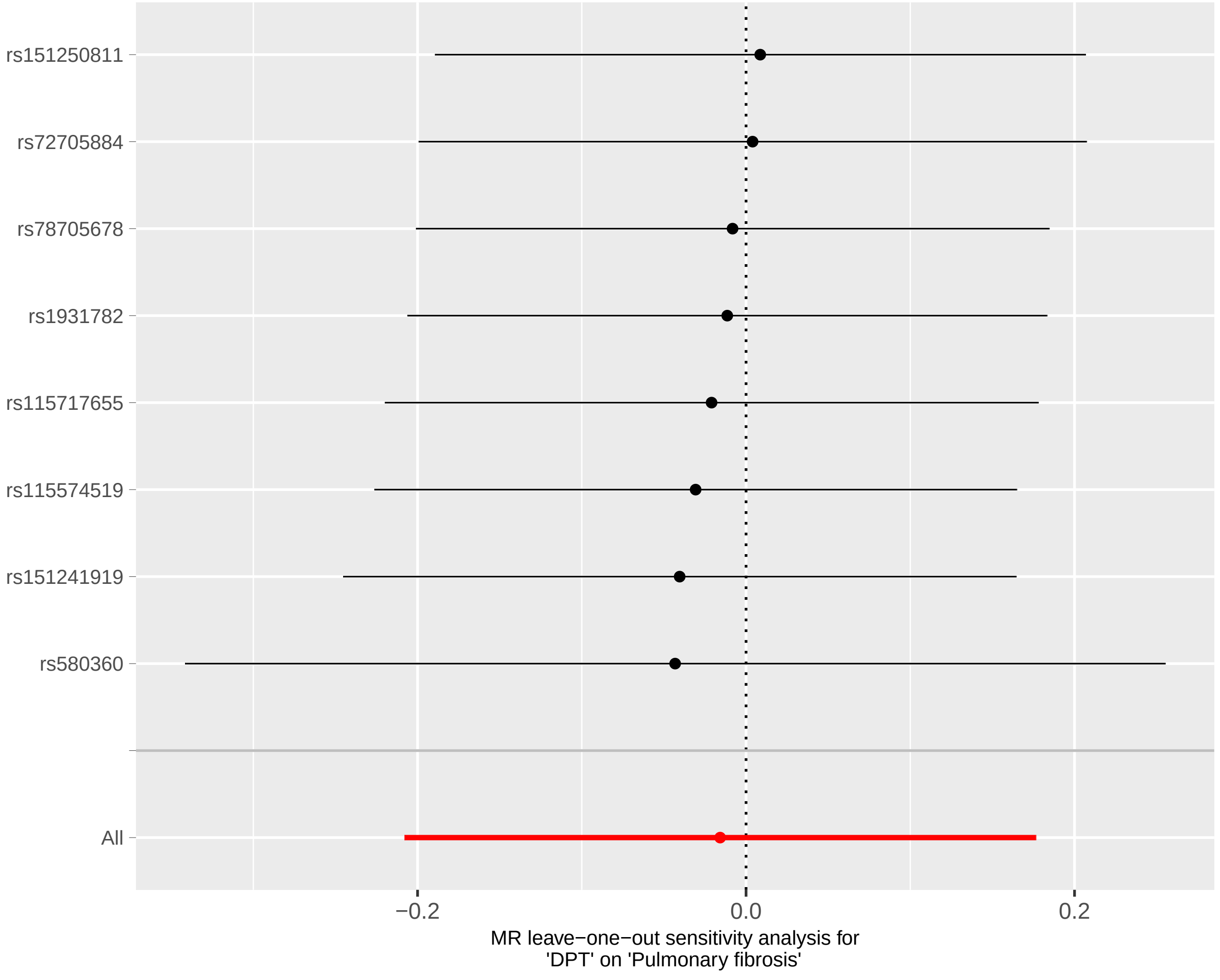
rs151250811
rs72705884
rs78705678
rs1931782
rs115717655
rs115574519
rs151241919
rs580360
All
−0.2
0.0
0.2
MR leave−one−out sensitivity analysis for
'DPT' on 'Pulmonary fibrosis'

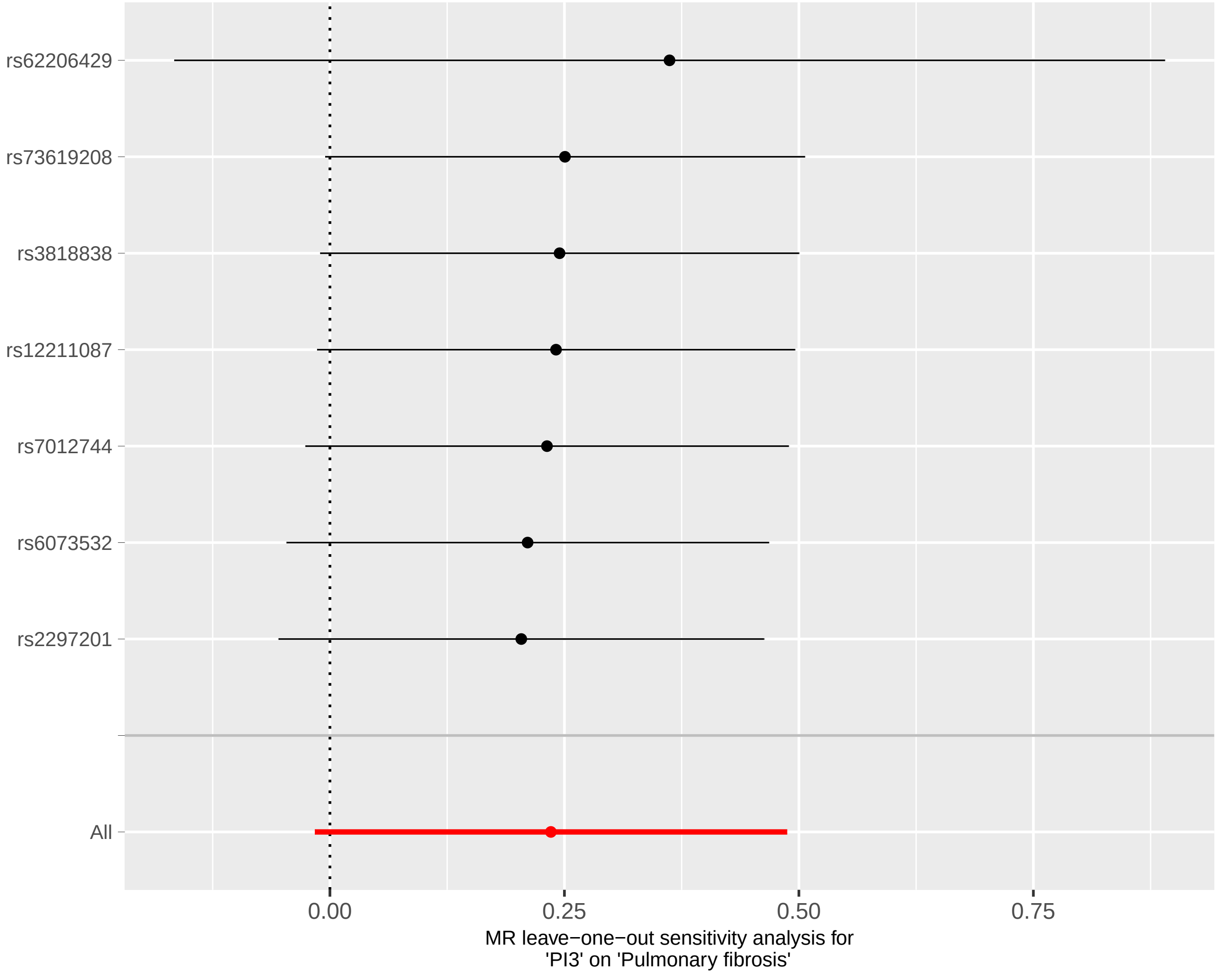
rs62206429
rs73619208
rs3818838
rs12211087
rs7012744
rs6073532
rs2297201
All
0.00
0.25
0.50
0.75
MR leave−one−out sensitivity analysis for
'PI3' on 'Pulmonary fibrosis'

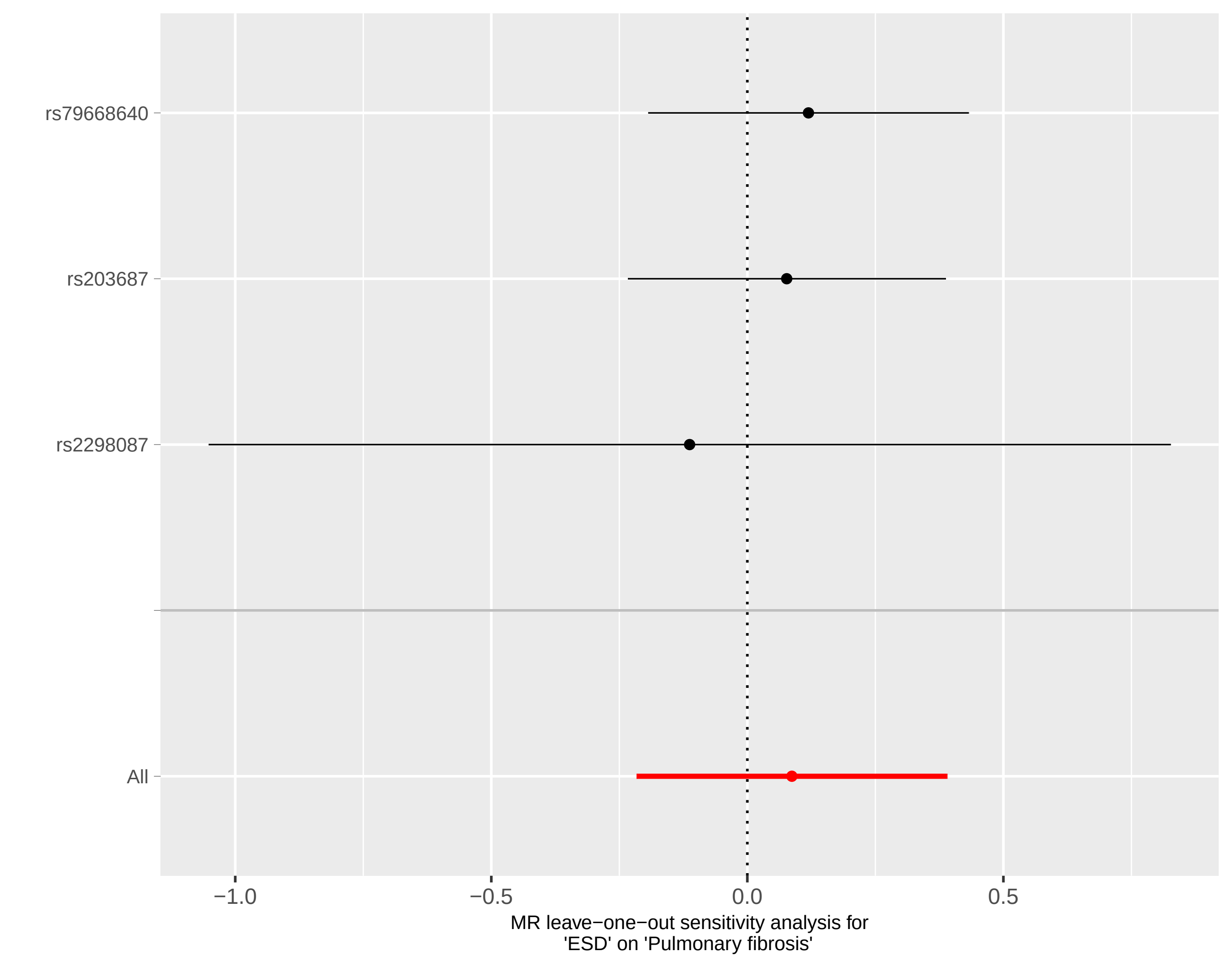
rs79668640
rs203687
rs2298087
All
−1.0
−0.5
0.0
0.5
MR leave−one−out sensitivity analysis for
'ESD' on 'Pulmonary fibrosis'

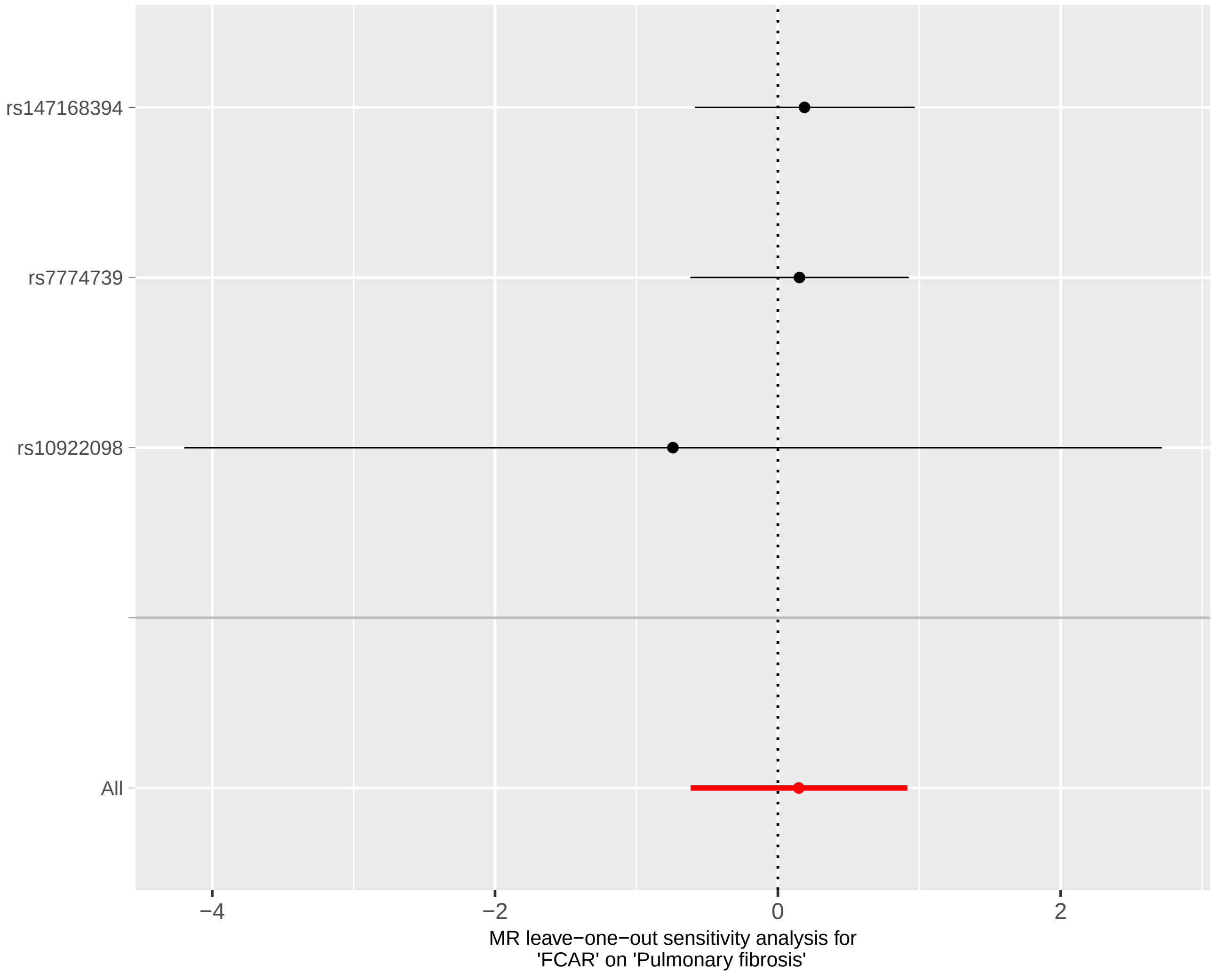
rs147168394
rs7774739
rs10922098
All
−4
−2
0
2
MR leave−one−out sensitivity analysis for
'FCAR' on 'Pulmonary fibrosis'

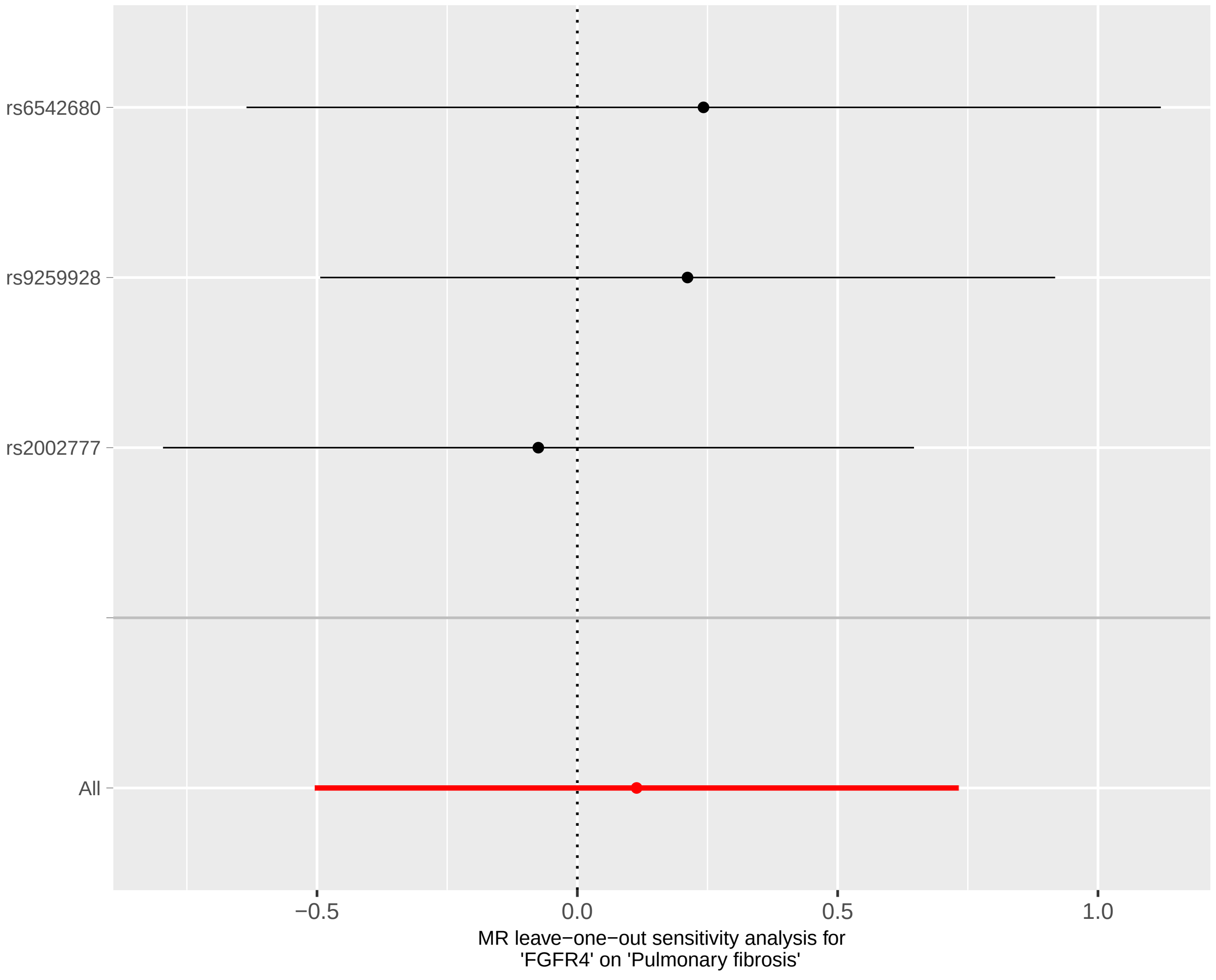
rs6542680
rs9259928
rs2002777
All
−0.5
0.0
0.5
1.0
MR leave−one−out sensitivity analysis for
'FGFR4' on 'Pulmonary fibrosis'

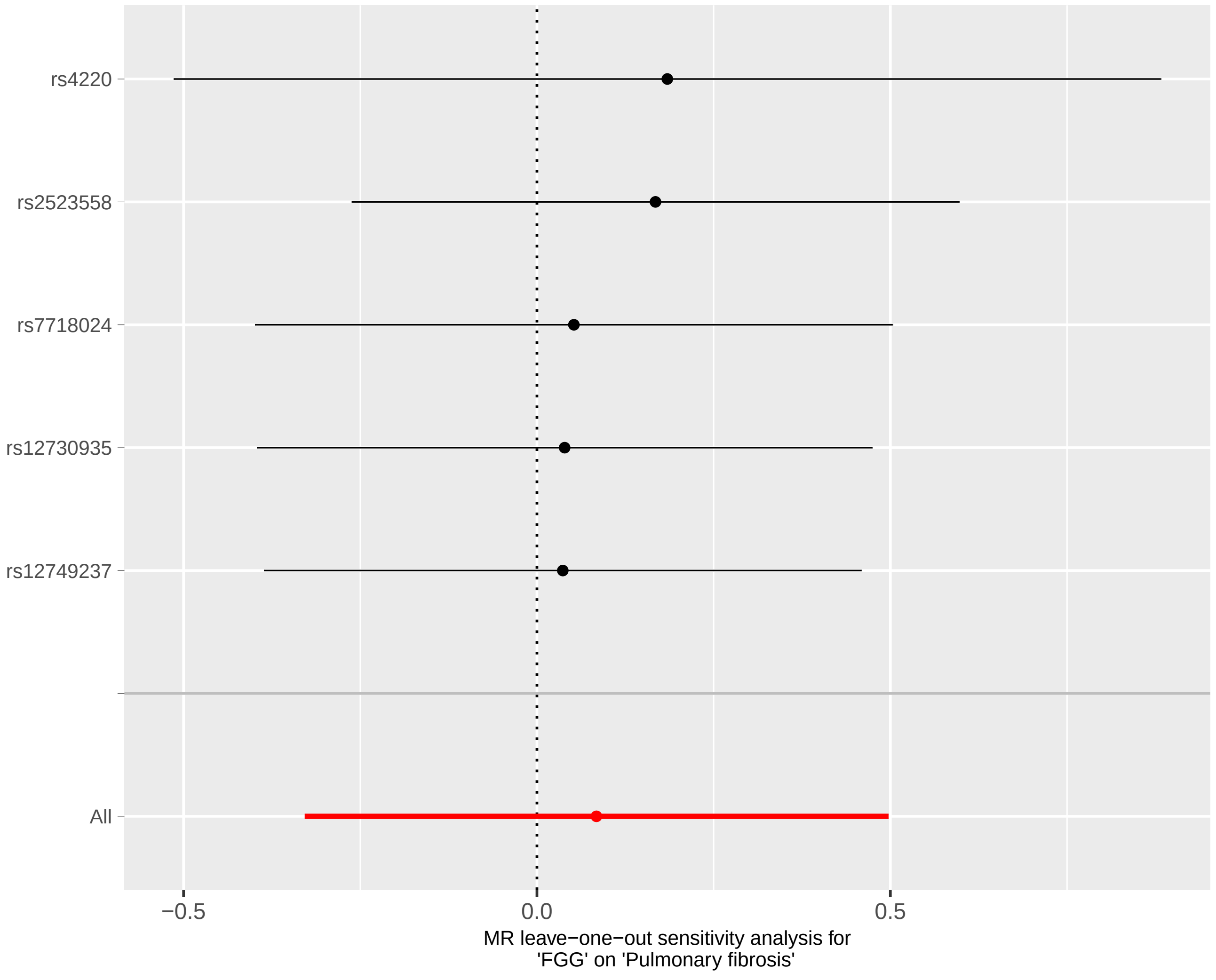
rs4220
rs2523558
rs7718024
rs12730935
rs12749237
All
−0.5
0.0
0.5
MR leave−one−out sensitivity analysis for
'FGG' on 'Pulmonary fibrosis'

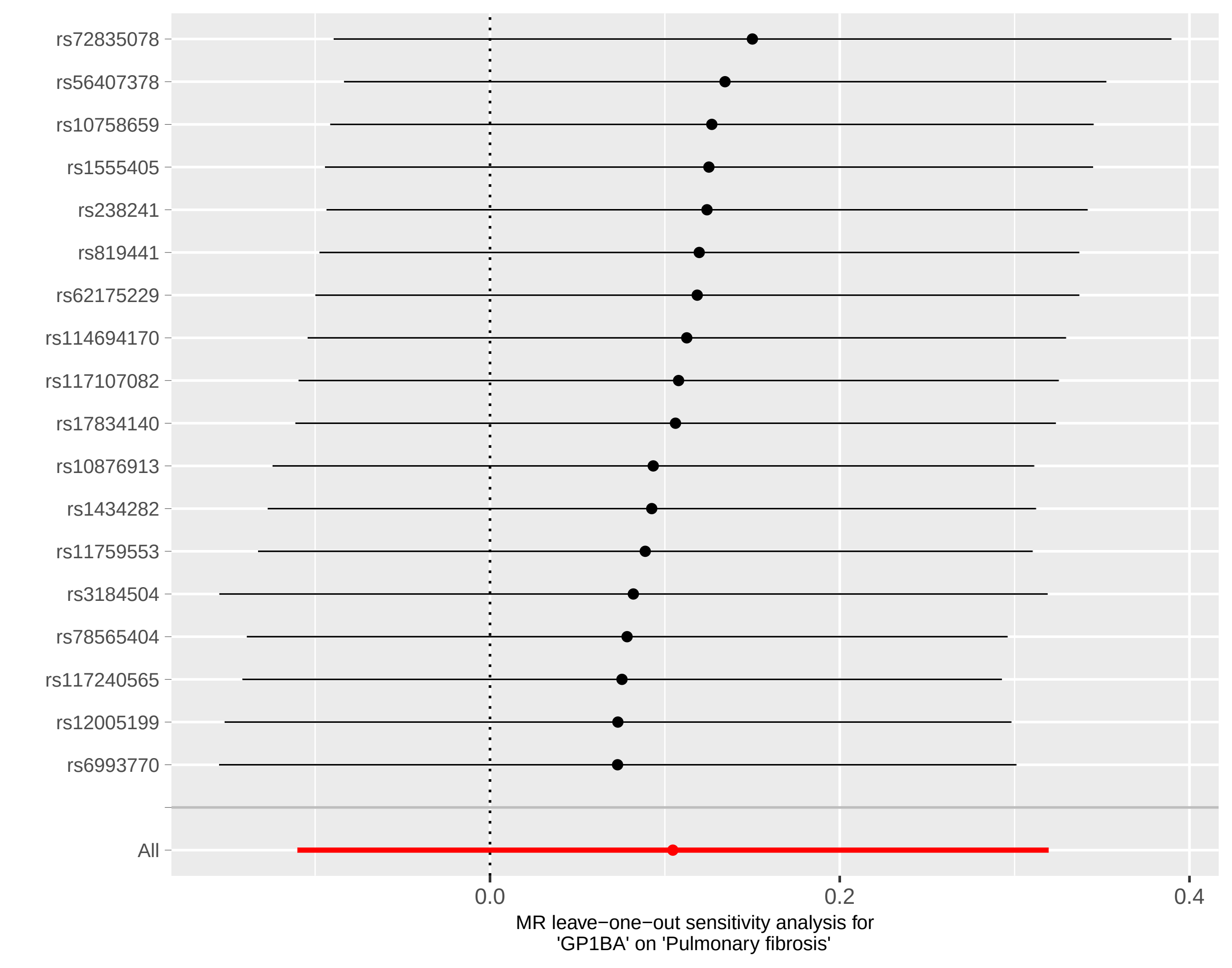
rs72835078
rs56407378
rs10758659
rs1555405
rs238241
rs819441
rs62175229
rs114694170
rs117107082
rs17834140
rs10876913
rs1434282
rs11759553
rs3184504
rs78565404
rs117240565
rs12005199
rs6993770
All
0.0
0.2
0.4
MR leave−one−out sensitivity analysis for
'GP1BA' on 'Pulmonary fibrosis'

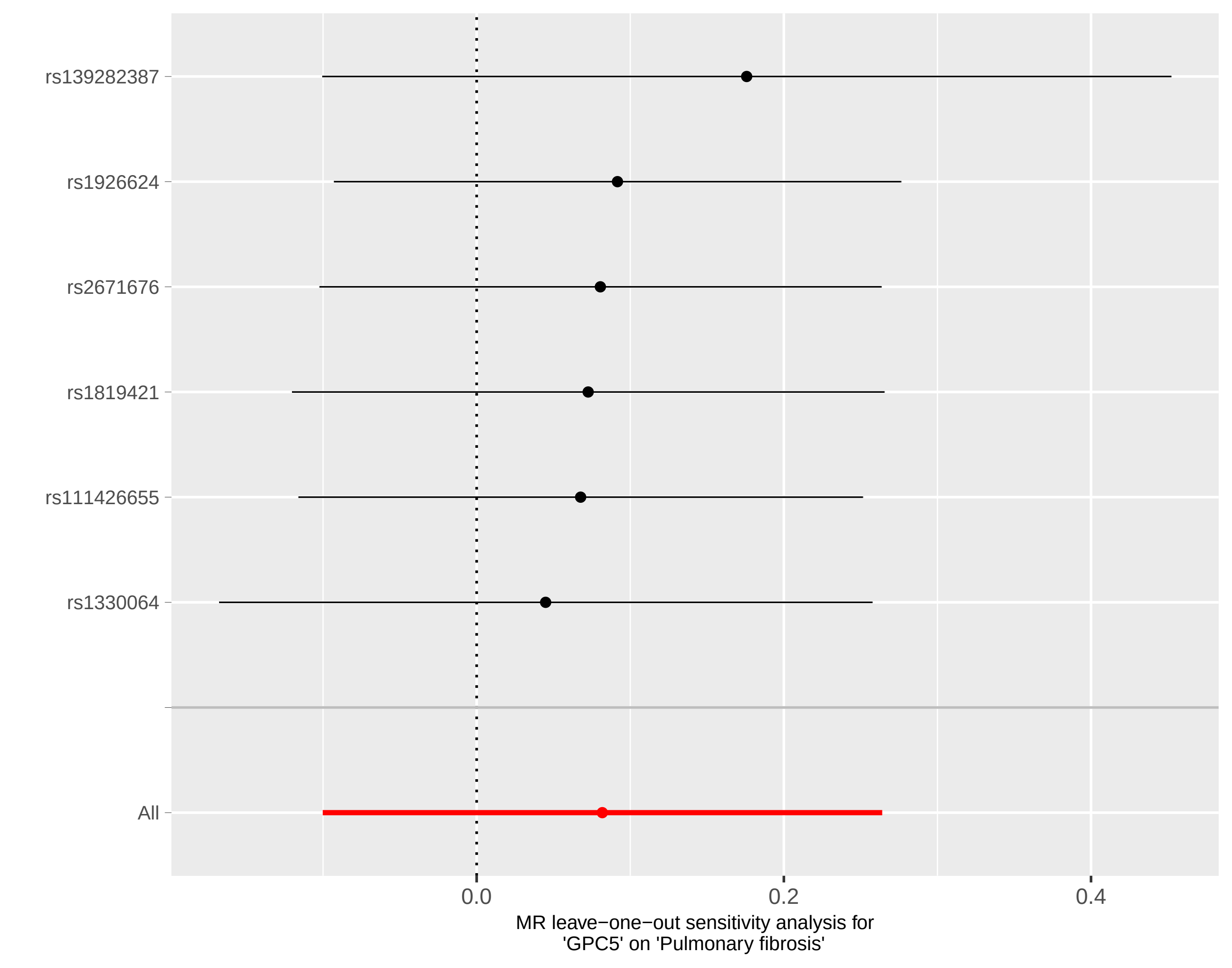
rs139282387
rs1926624
rs2671676
rs1819421
rs111426655
rs1330064
All
0.0
0.2
0.4
MR leave−one−out sensitivity analysis for
'GPC5' on 'Pulmonary fibrosis'

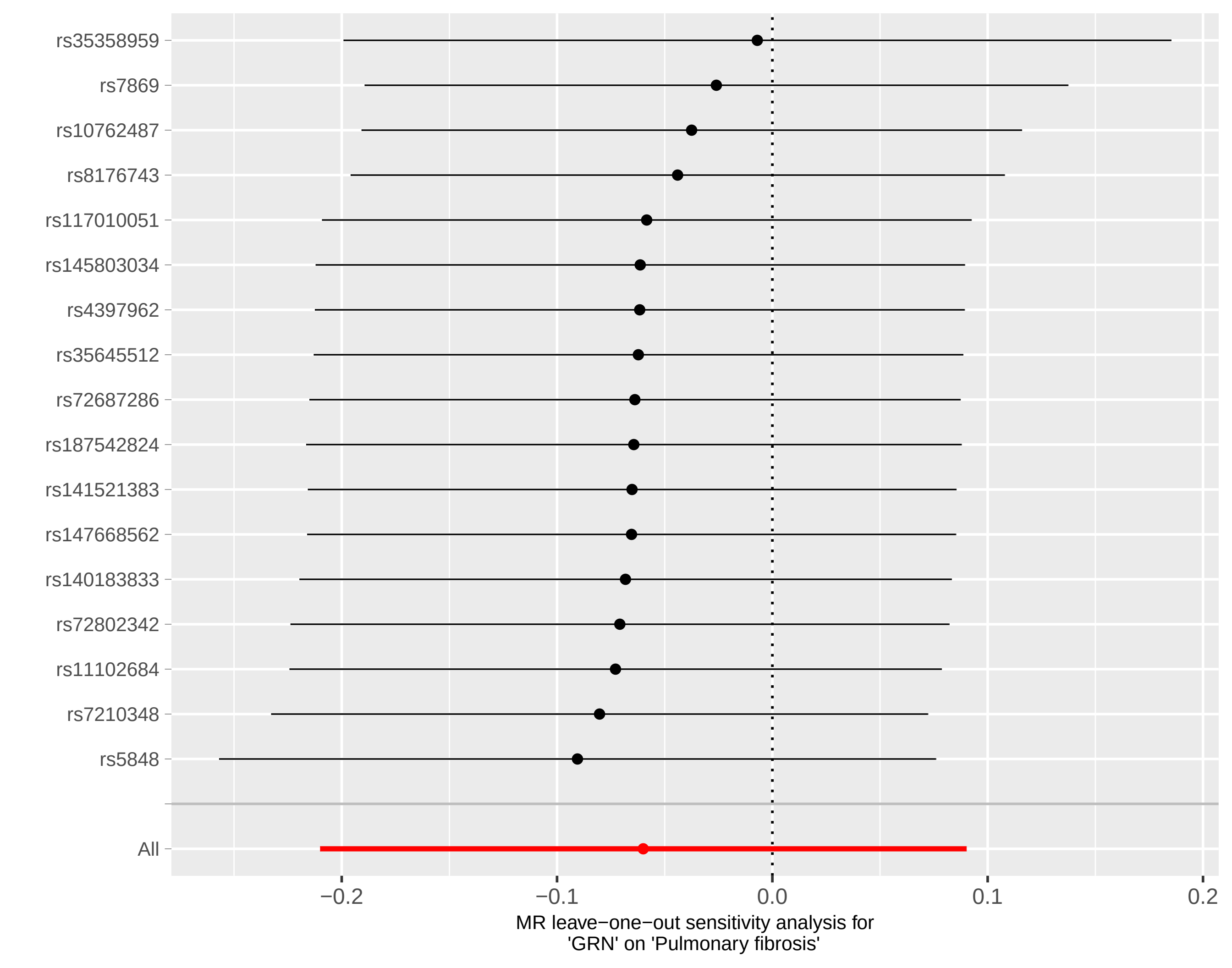
rs35358959
rs7869
rs10762487
rs8176743
rs117010051
rs145803034
rs4397962
rs35645512
rs72687286
rs187542824
rs141521383
rs147668562
rs140183833
rs72802342
rs11102684
rs7210348
rs5848
All
−0.2
−0.1
0.0
0.1
0.2
MR leave−one−out sensitivity analysis for
'GRN' on 'Pulmonary fibrosis'

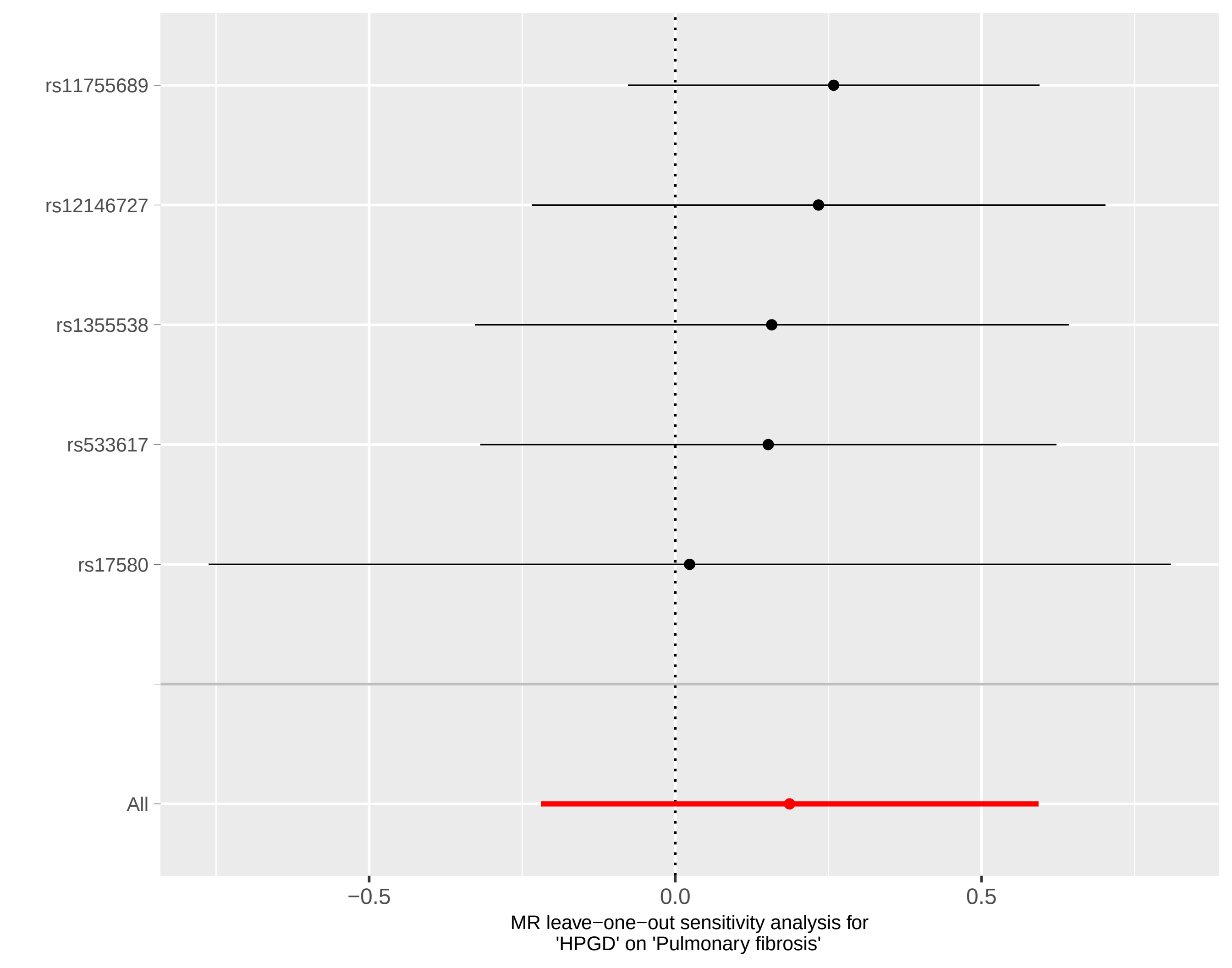
rs11755689
rs12146727
rs1355538
rs533617
rs17580
All
−0.5
0.0
0.5
MR leave−one−out sensitivity analysis for
'HPGD' on 'Pulmonary fibrosis'

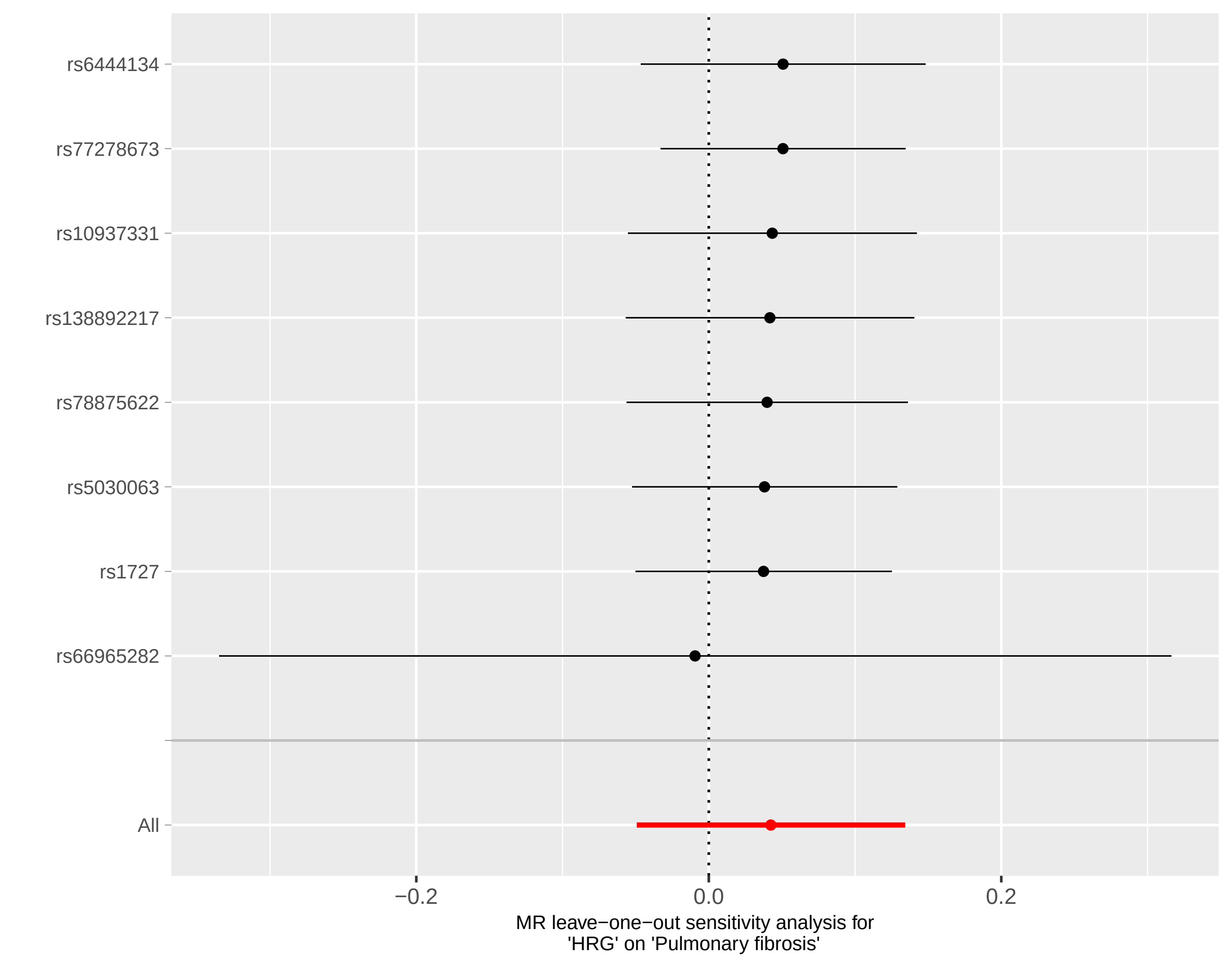
rs6444134
rs77278673
rs10937331
rs138892217
rs78875622
rs5030063
rs1727
rs66965282
All
−0.2
0.0
0.2
MR leave−one−out sensitivity analysis for
'HRG' on 'Pulmonary fibrosis'

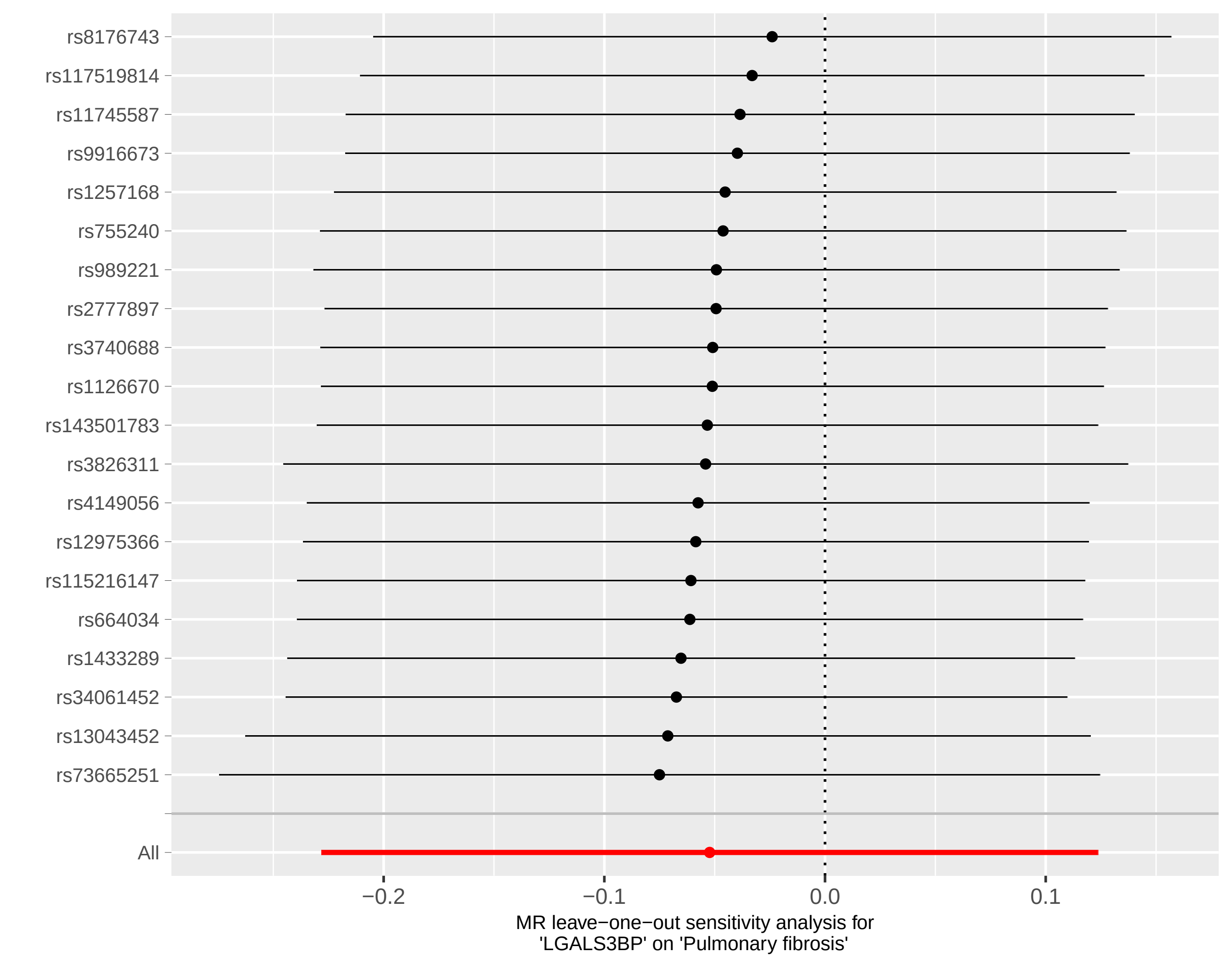
rs8176743
rs117519814
rs11745587
rs9916673
rs1257168
rs755240
rs989221
rs2777897
rs3740688
rs1126670
rs143501783
rs3826311
rs4149056
rs12975366
rs115216147
rs664034
rs1433289
rs34061452
rs13043452
rs73665251
All
−0.2
−0.1
0.0
0.1
MR leave−one−out sensitivity analysis for
'LGALS3BP' on 'Pulmonary fibrosis'

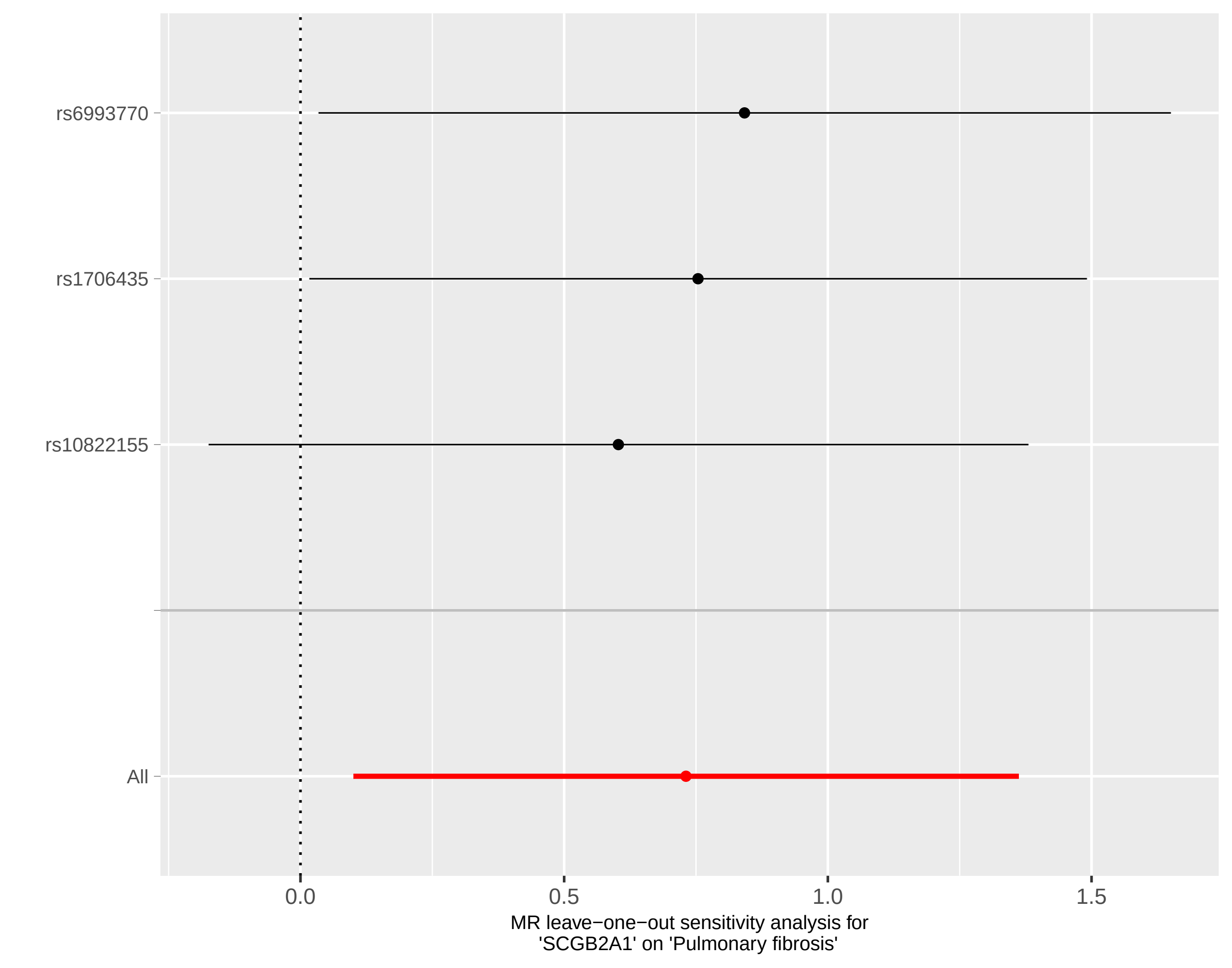
rs6993770
rs1706435
rs10822155
All
0.0
0.5
1.0
1.5
MR leave−one−out sensitivity analysis for
'SCGB2A1' on 'Pulmonary fibrosis'

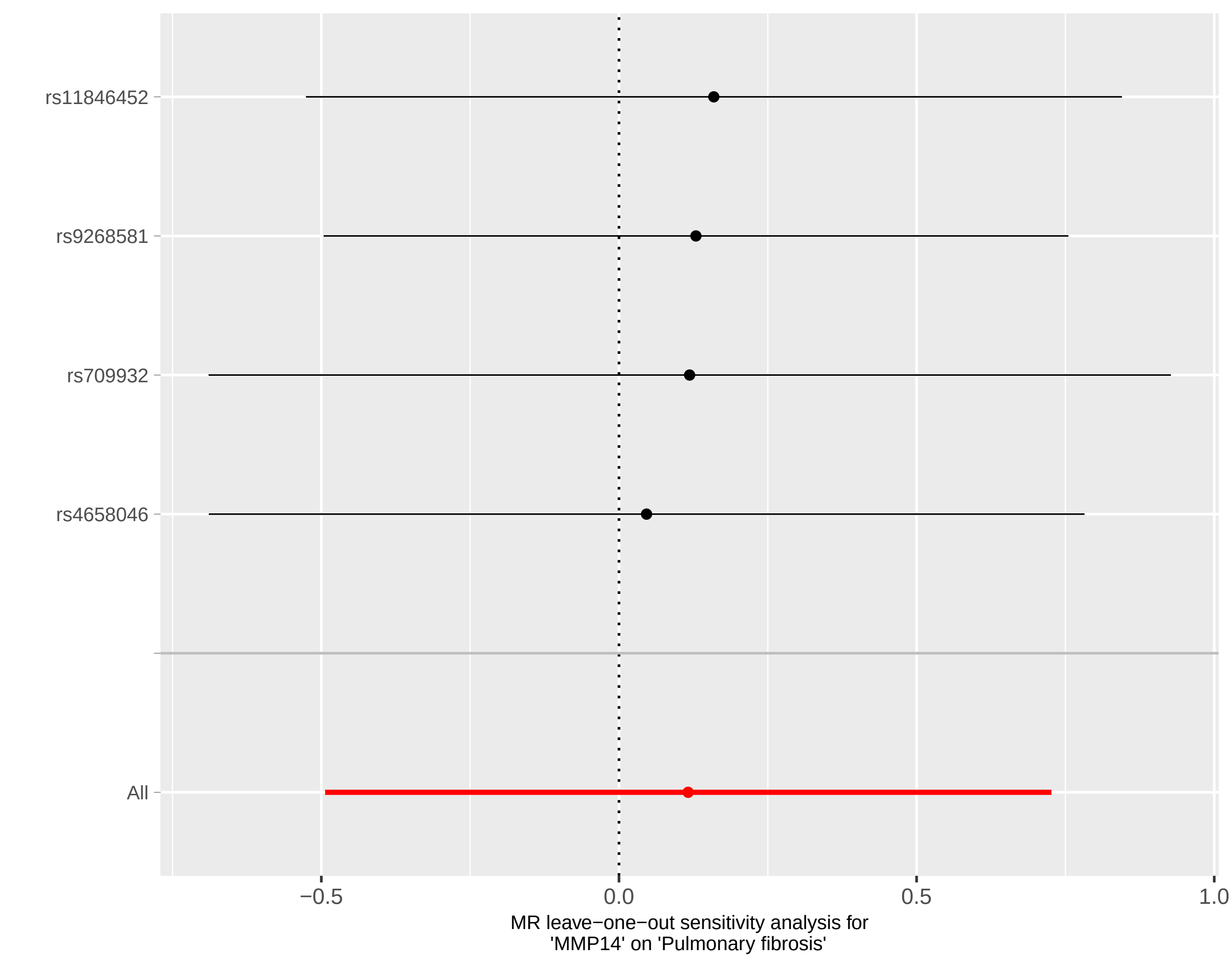

rs11846452
rs9268581
rs709932
rs4658046
All
−0.5
0.0
0.5
1.0
MR leave−one−out sensitivity analysis for
'MMP14' on 'Pulmonary fibrosis'

Insufficient number of SNPs

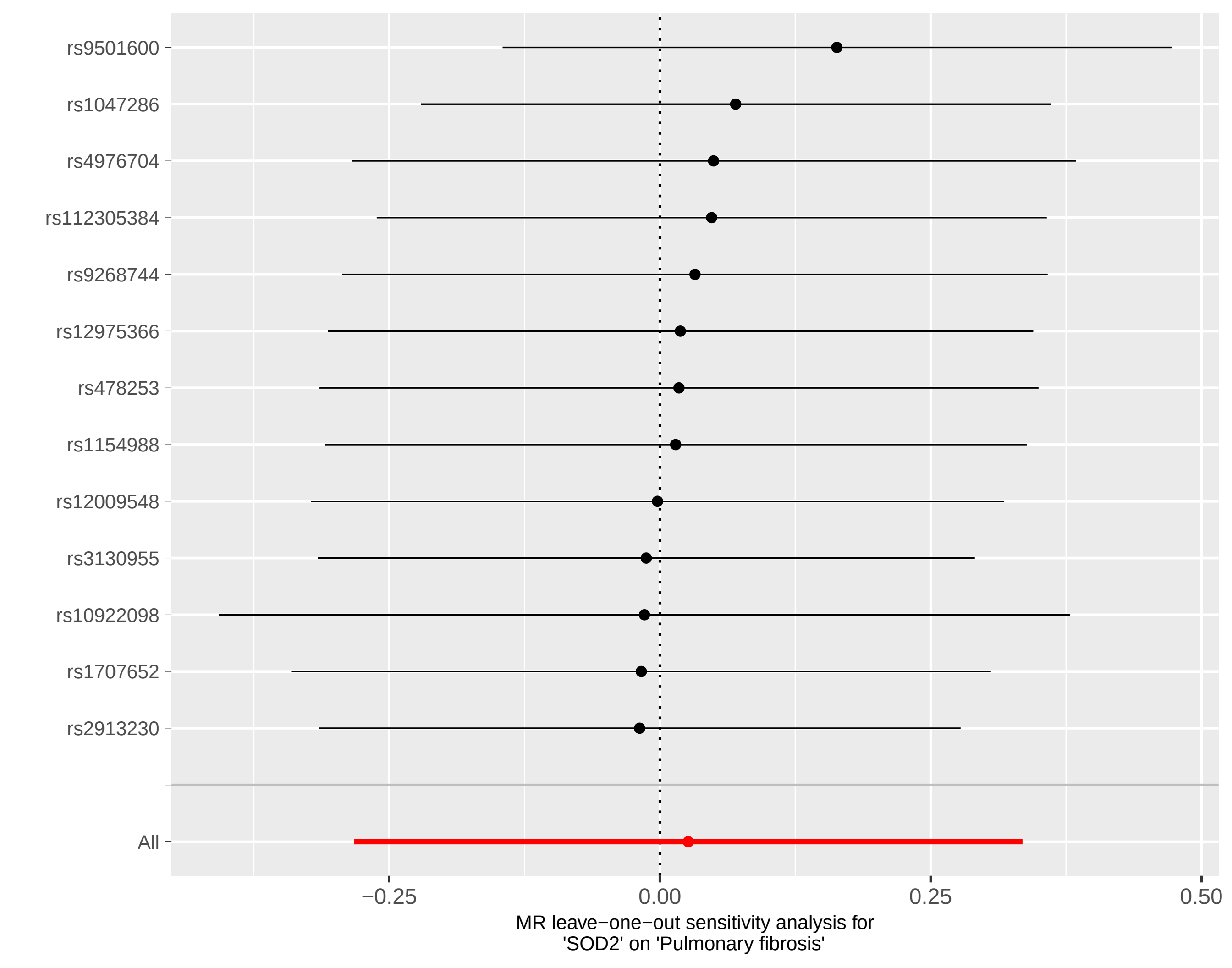
rs9501600
rs1047286
rs4976704
rs112305384
rs9268744
rs12975366
rs478253
rs1154988
rs12009548
rs3130955
rs10922098
rs1707652
rs2913230
All
−0.25
0.00
0.25
0.50
MR leave−one−out sensitivity analysis for
'SOD2' on 'Pulmonary fibrosis'

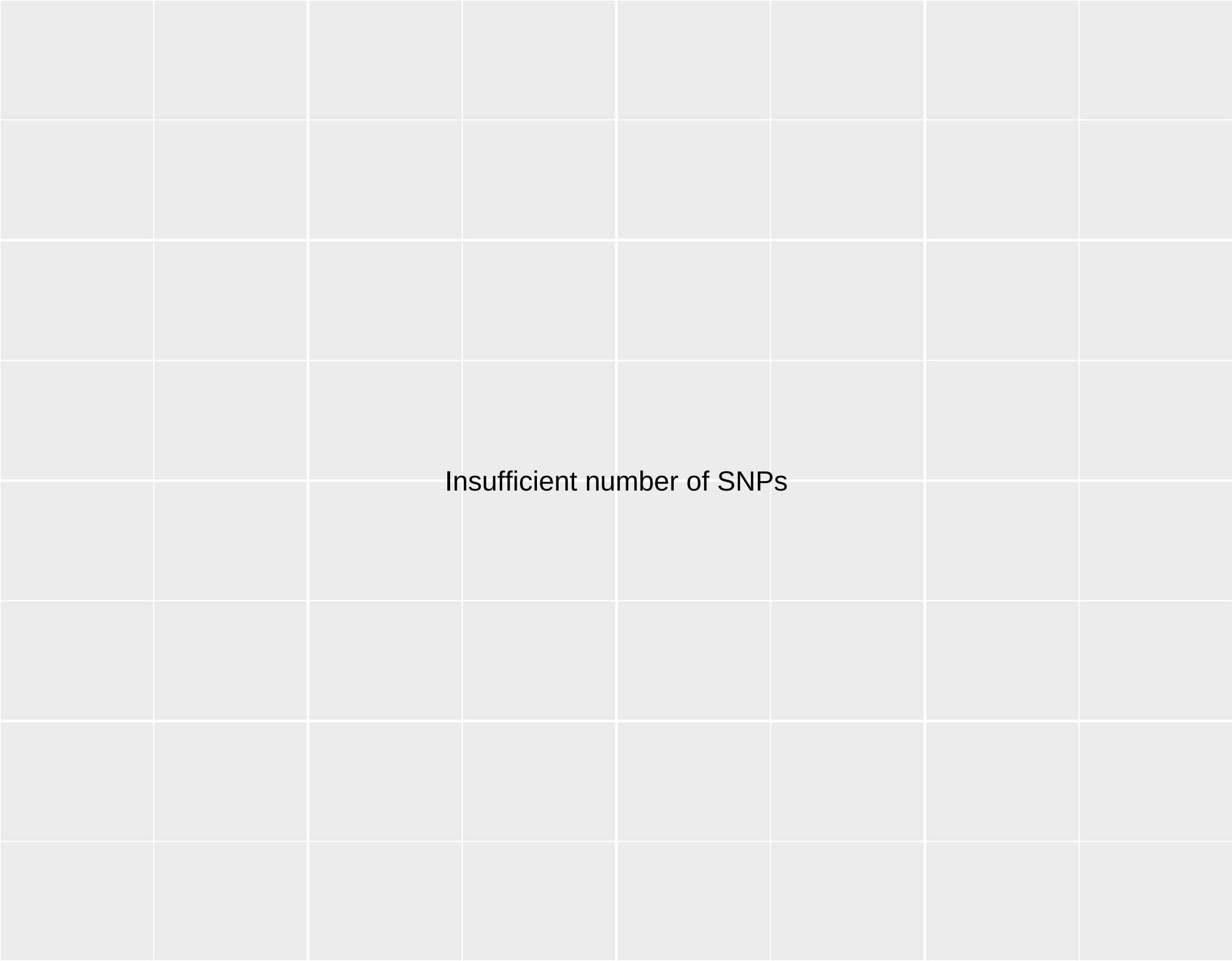
Insufficient number of SNPs

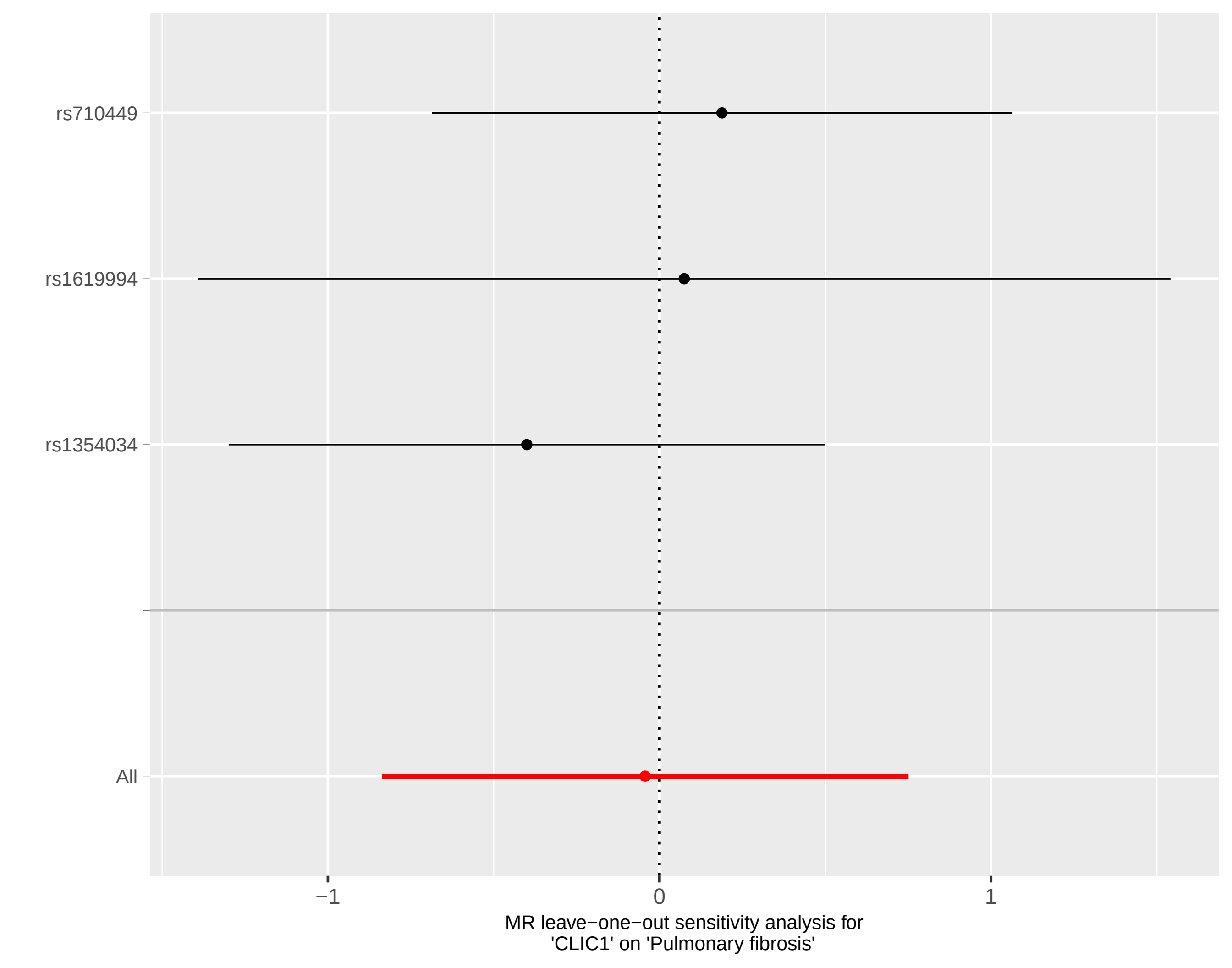

rs710449
rs1619994
rs1354034
All
−1
0
1
MR leave−one−out sensitivity analysis for
'CLIC1' on 'Pulmonary fibrosis'

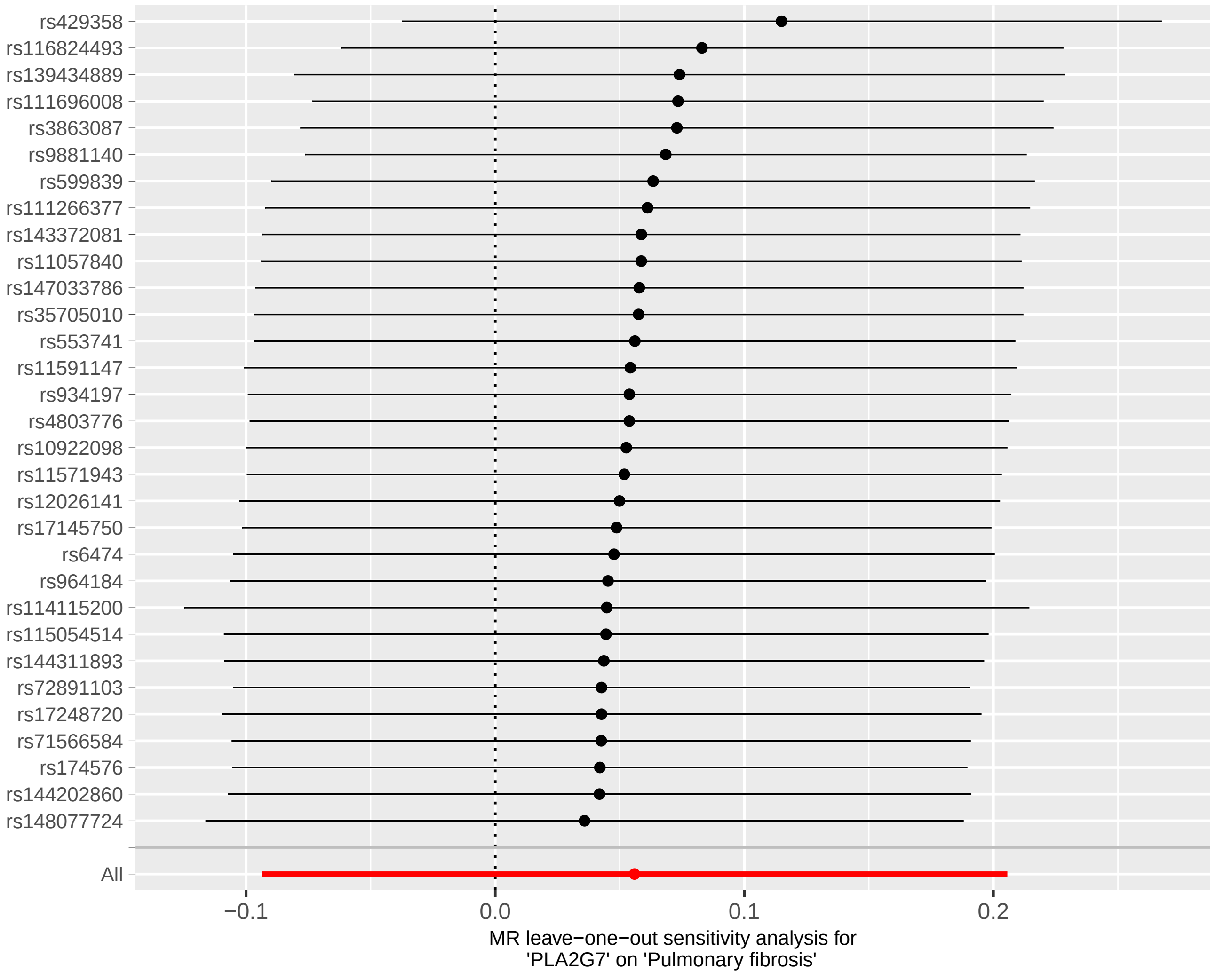

rs429358
rs116824493
rs139434889
rs111696008
rs3863087
rs9881140
rs599839
rs111266377
rs143372081
rs11057840
rs147033786
rs35705010
rs553741
rs11591147
rs934197
rs4803776
rs10922098
rs11571943
rs12026141
rs17145750
rs6474
rs964184
rs114115200
rs115054514
rs144311893
rs72891103
rs17248720
rs71566584
rs174576
rs144202860
rs148077724
All
−0.1
0.0
0.1
0.2
MR leave−one−out sensitivity analysis for
'PLA2G7' on 'Pulmonary fibrosis'

Insufficient number of SNPs

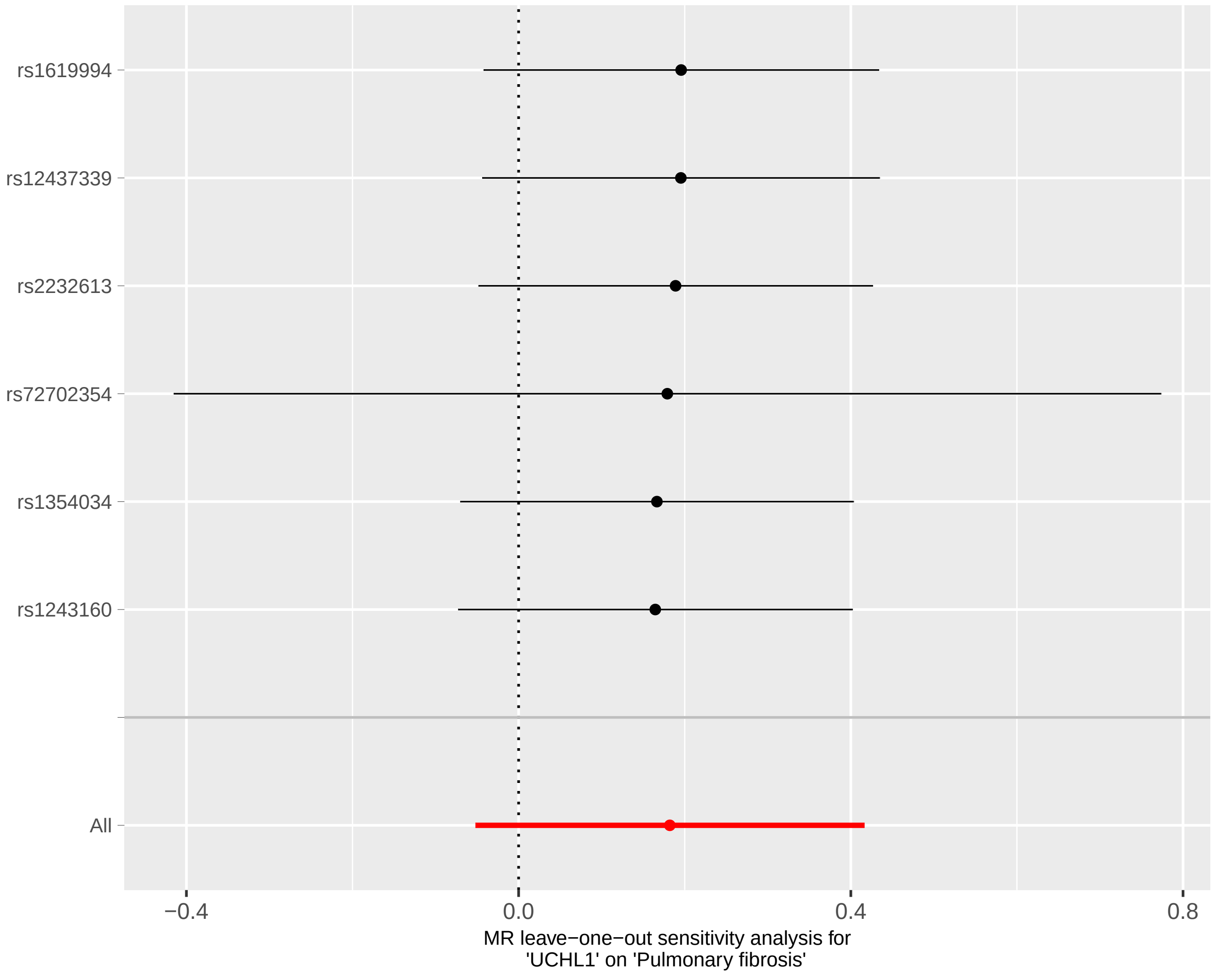
rs1619994
rs12437339
rs2232613
rs72702354
rs1354034
rs1243160
All
−0.4
0.0
0.4
0.8
MR leave−one−out sensitivity analysis for
'UCHL1' on 'Pulmonary fibrosis'

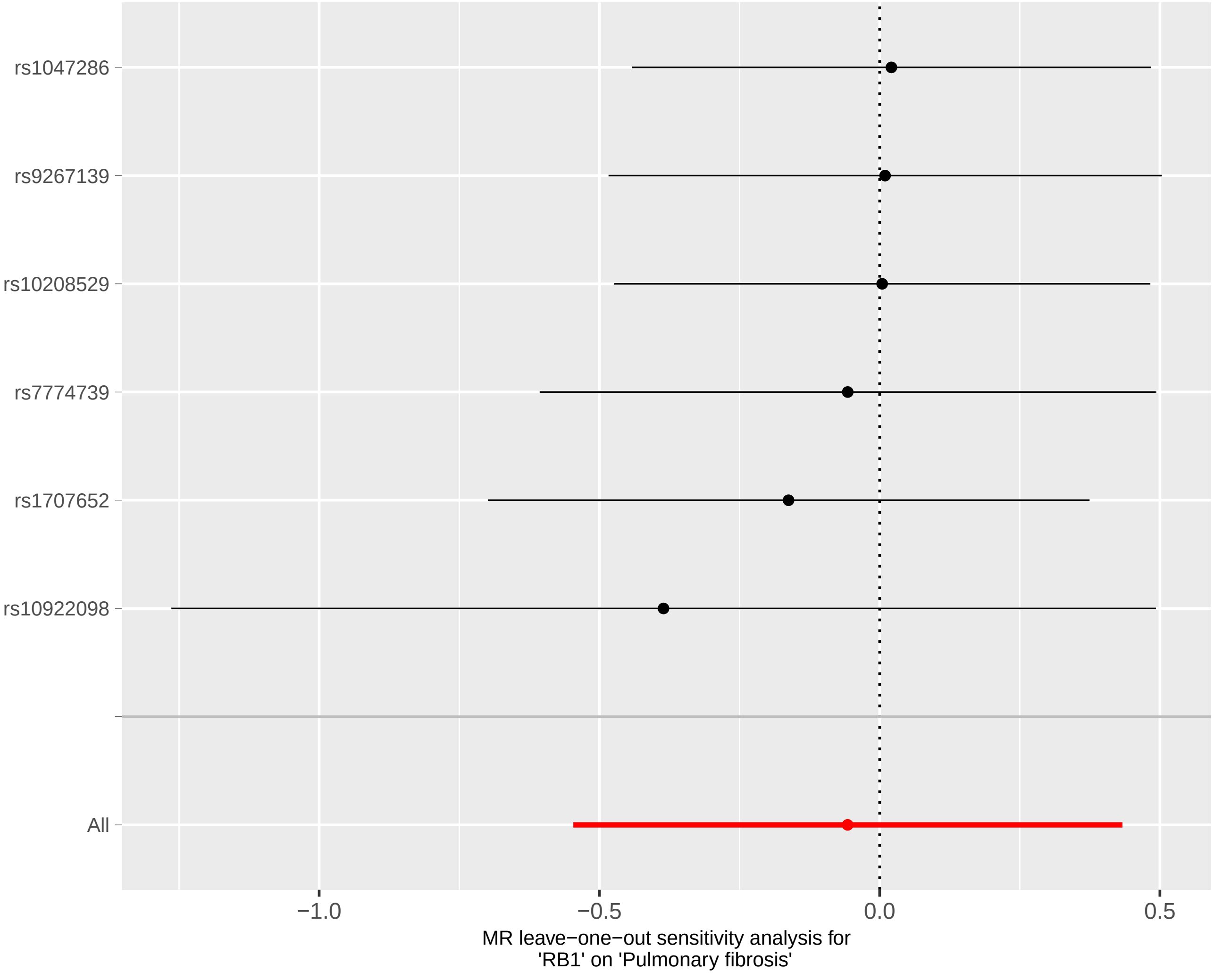
rs1047286
rs9267139
rs10208529
rs7774739
rs1707652
rs10922098
All
−1.0
−0.5
0.0
0.5
MR leave−one−out sensitivity analysis for
'RB1' on 'Pulmonary fibrosis'

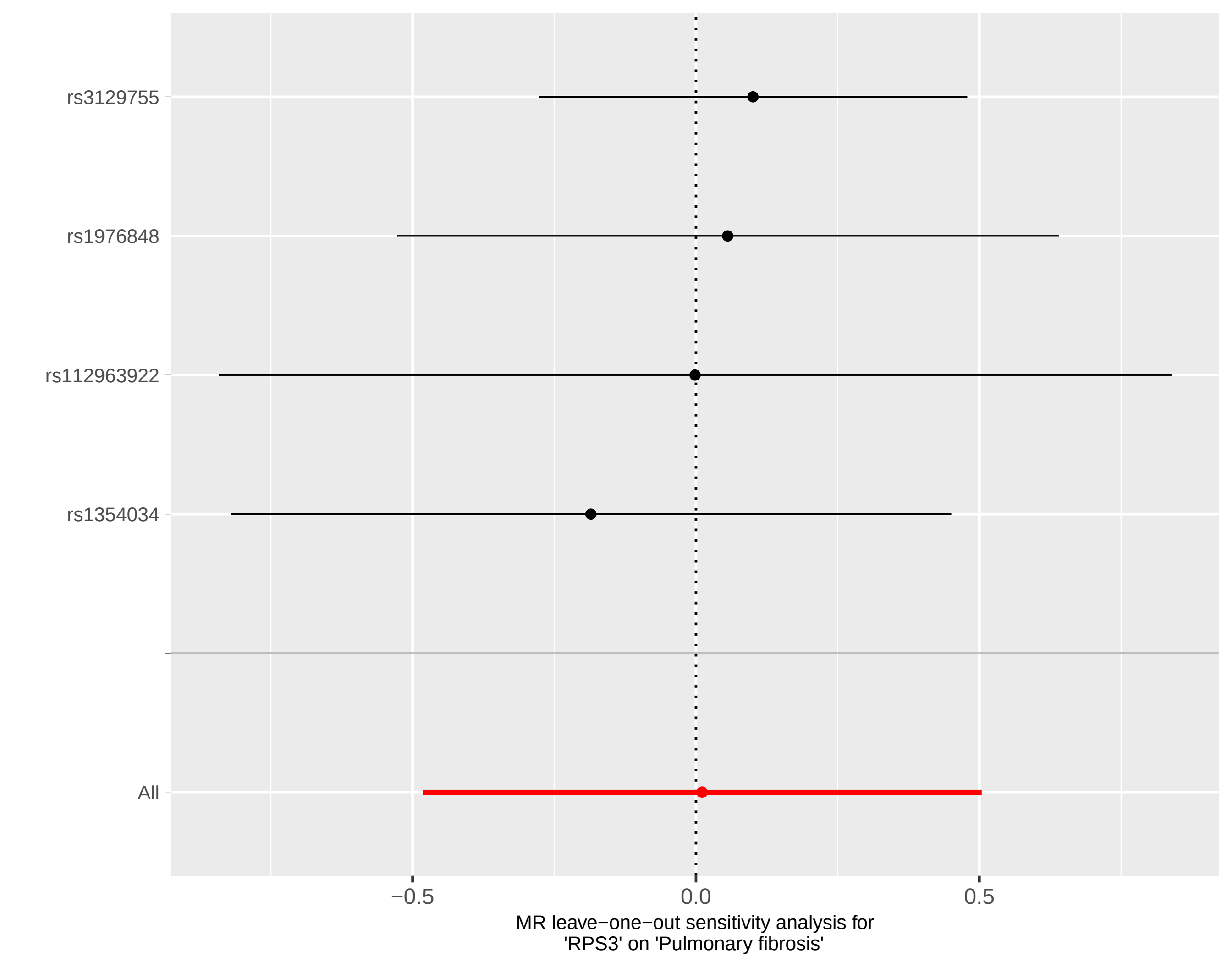
rs3129755
rs1976848
rs112963922
rs1354034
All
−0.5
0.0
0.5
MR leave−one−out sensitivity analysis for
'RPS3' on 'Pulmonary fibrosis'

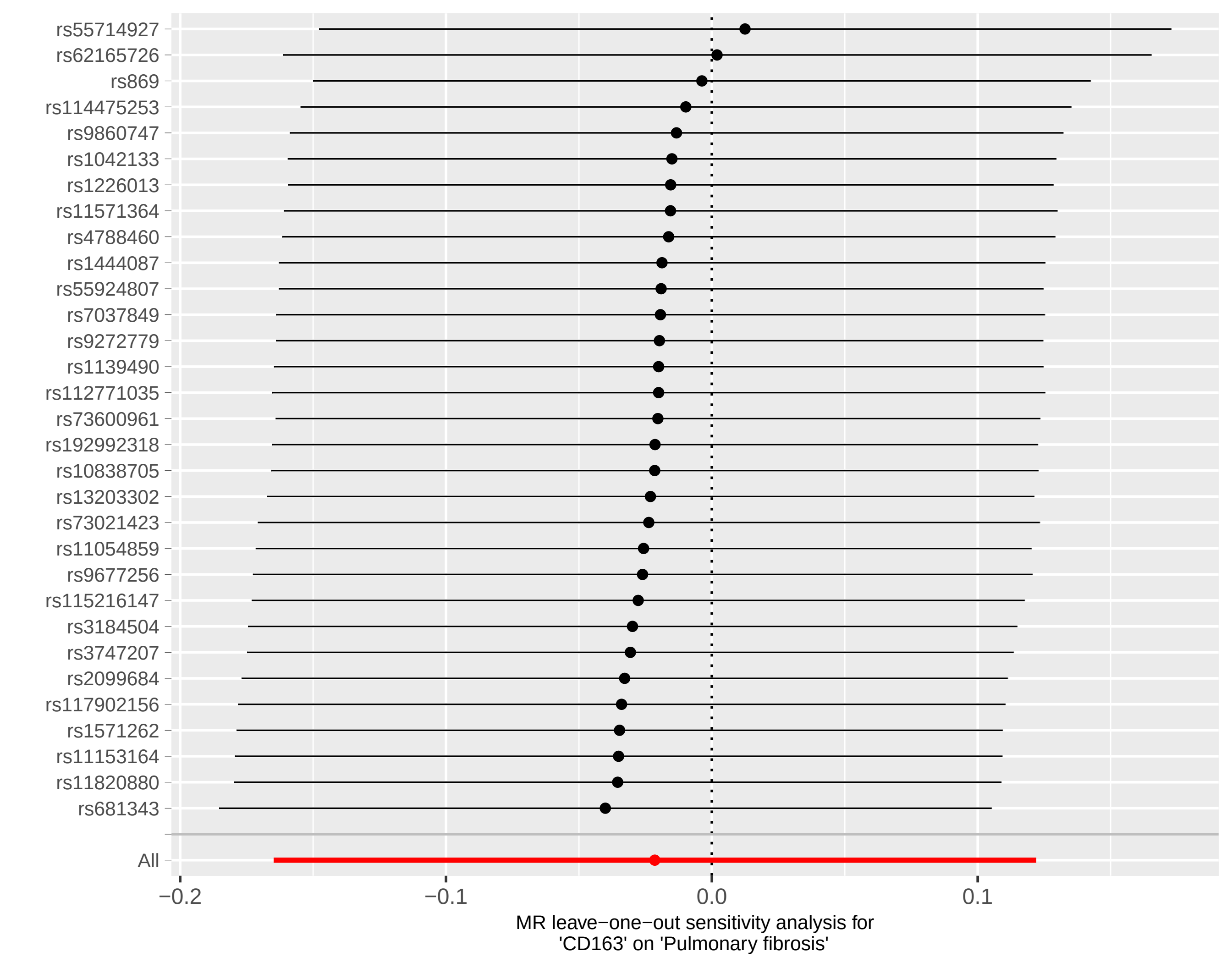
rs55714927
rs62165726
rs869
rs114475253
rs9860747
rs1042133
rs1226013
rs11571364
rs4788460
rs1444087
rs55924807
rs7037849
rs9272779
rs1139490
rs112771035
rs73600961
rs192992318
rs10838705
rs13203302
rs73021423
rs11054859
rs9677256
rs115216147
rs3184504
rs3747207
rs2099684
rs117902156
rs1571262
rs11153164
rs11820880
rs681343
All
−0.2
−0.1
0.0
0.1
MR leave−one−out sensitivity analysis for
'CD163' on 'Pulmonary fibrosis'

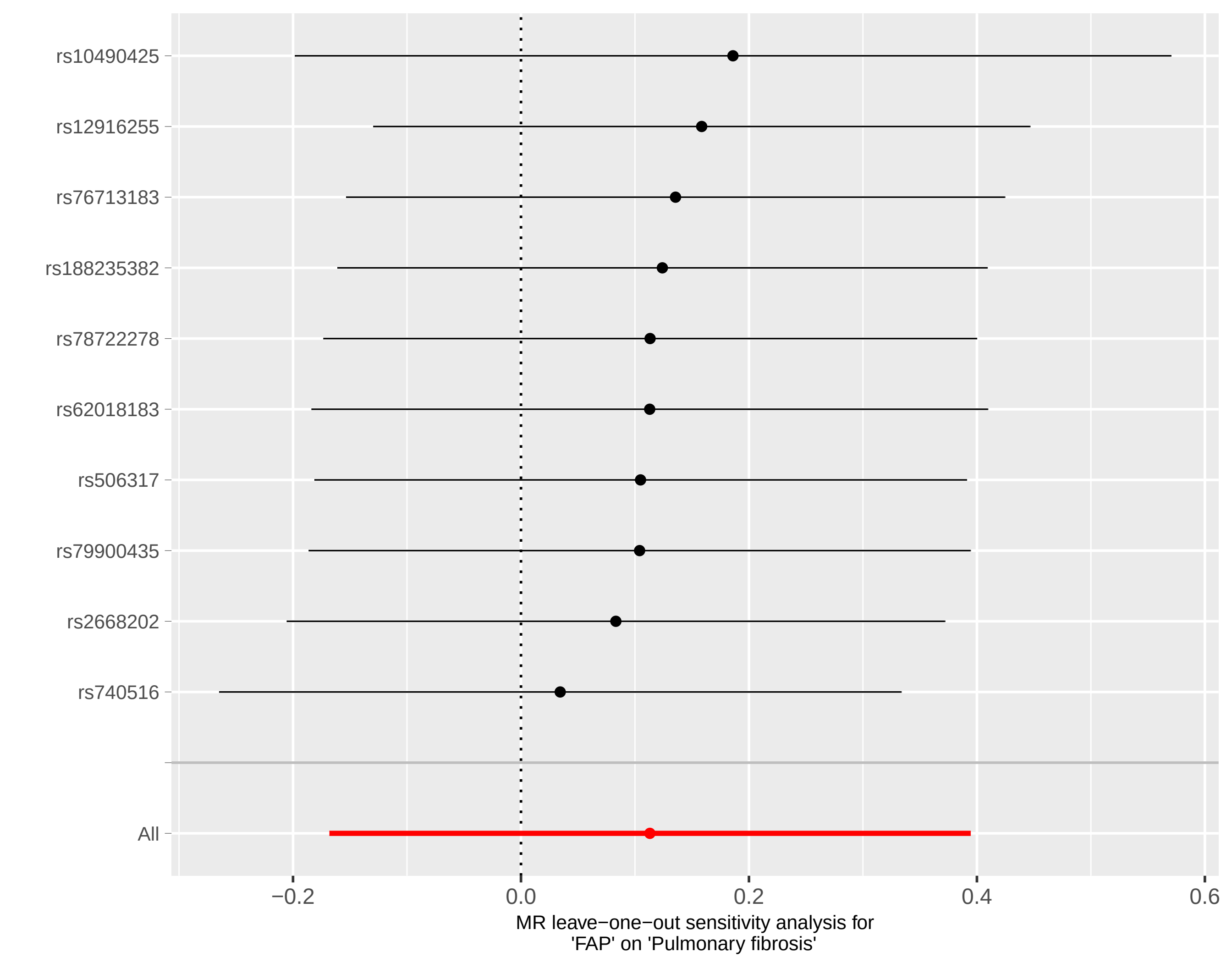
rs10490425
rs12916255
rs76713183
rs188235382
rs78722278
rs62018183
rs506317
rs79900435
rs2668202
rs740516
All
−0.2
0.0
0.2
0.4
0.6
MR leave−one−out sensitivity analysis for
'FAP' on 'Pulmonary fibrosis'

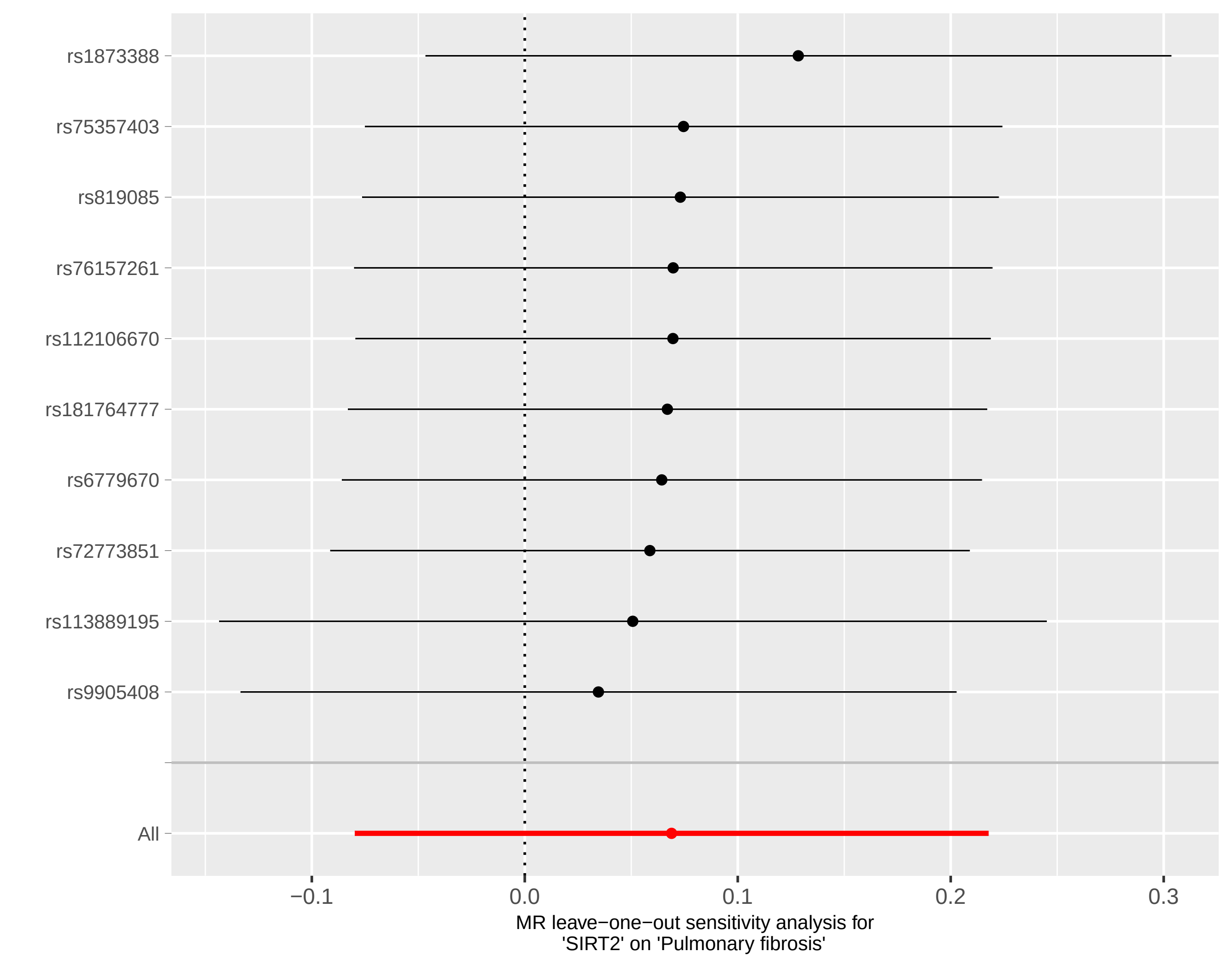
rs1873388
rs75357403
rs819085
rs76157261
rs112106670
rs181764777
rs6779670
rs72773851
rs113889195
rs9905408
All
−0.1
0.0
0.1
0.2
0.3
MR leave−one−out sensitivity analysis for
'SIRT2' on 'Pulmonary fibrosis'

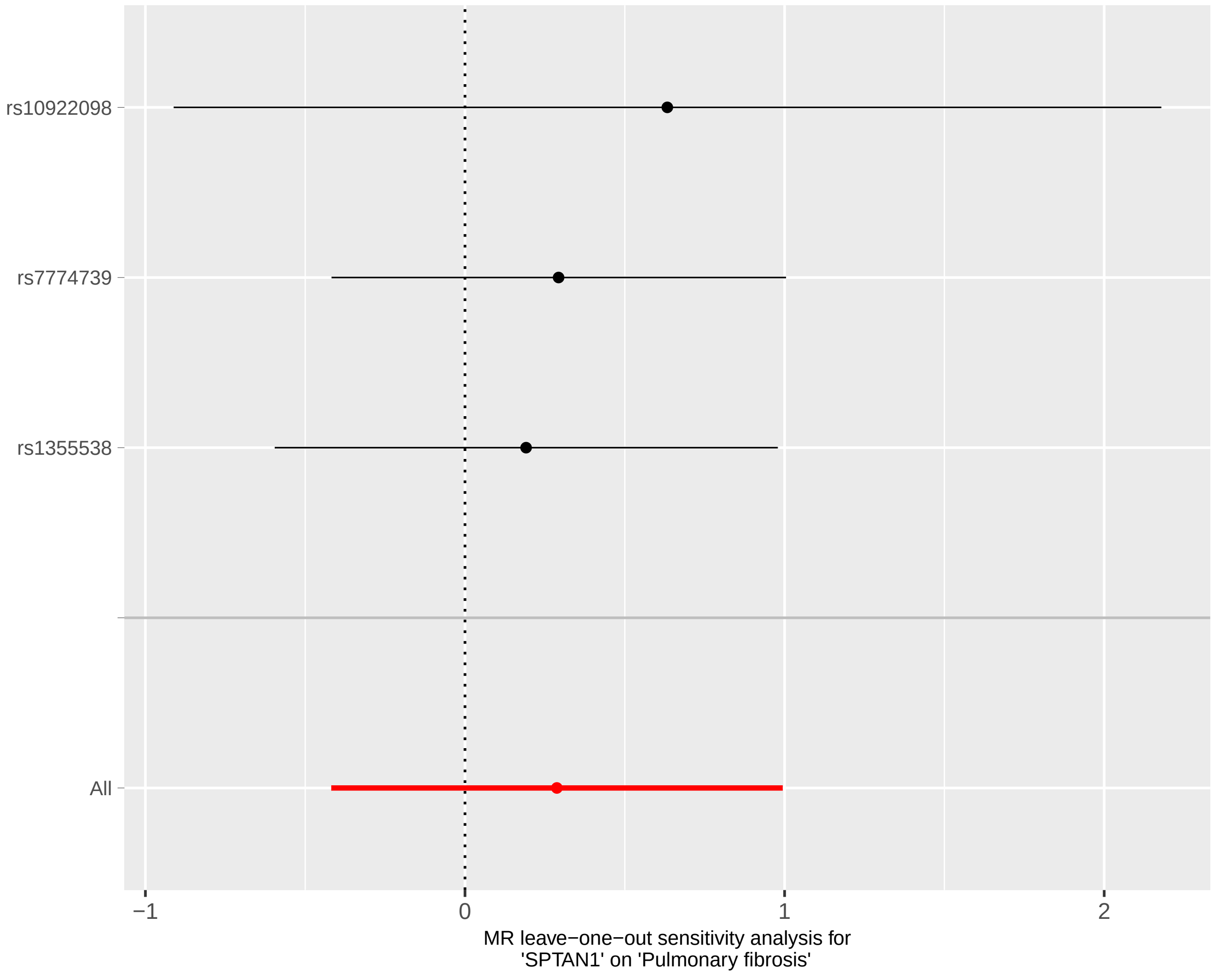
rs10922098
rs7774739
rs1355538
All
−1
0
1
2
MR leave−one−out sensitivity analysis for
'SPTAN1' on 'Pulmonary fibrosis'

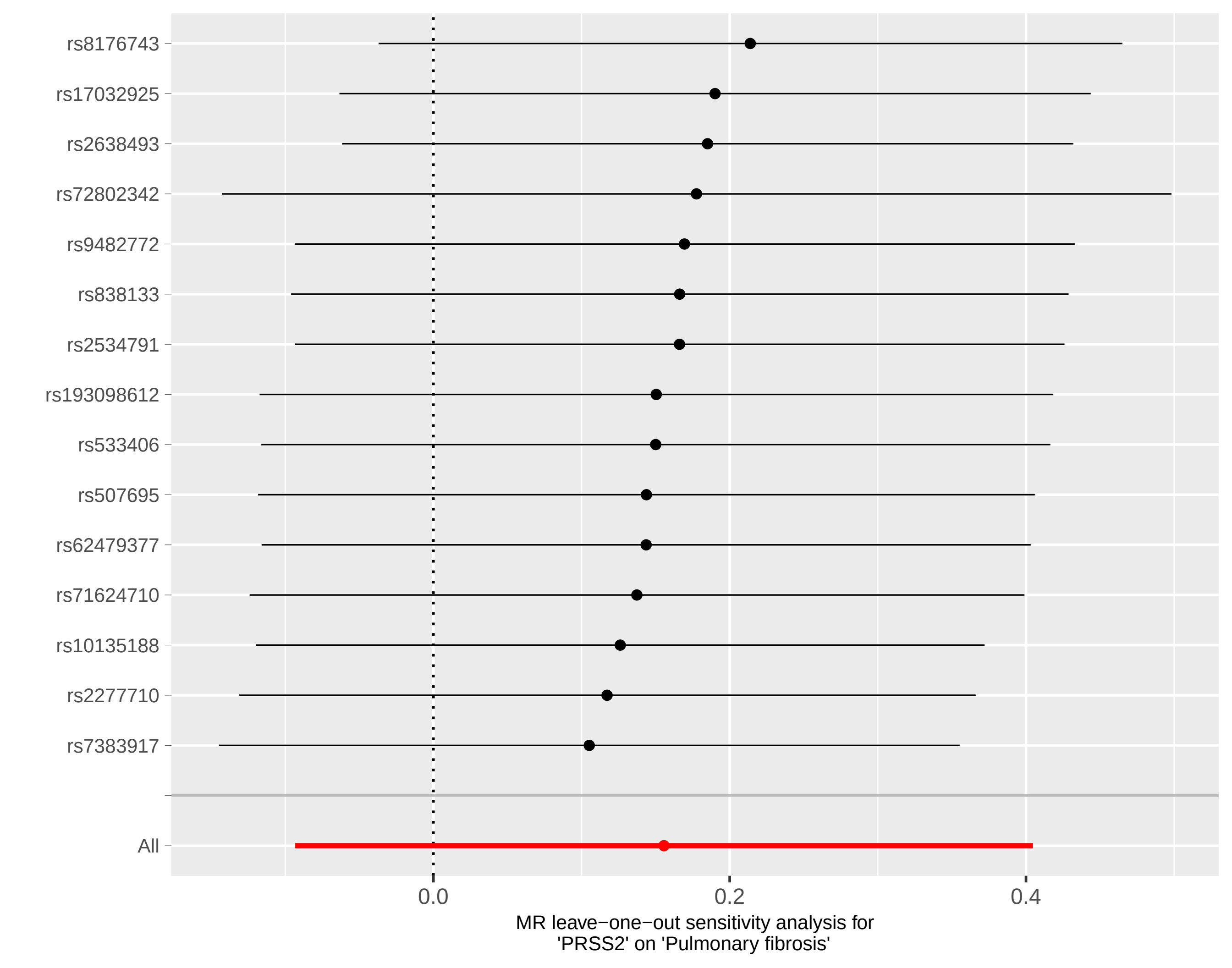

rs8176743
rs17032925
rs2638493
rs72802342
rs9482772
rs838133
rs2534791
rs193098612
rs533406
rs507695
rs62479377
rs71624710
rs10135188
rs2277710
rs7383917
All
0.0
0.2
0.4
MR leave−one−out sensitivity analysis for
'PRSS2' on 'Pulmonary fibrosis'

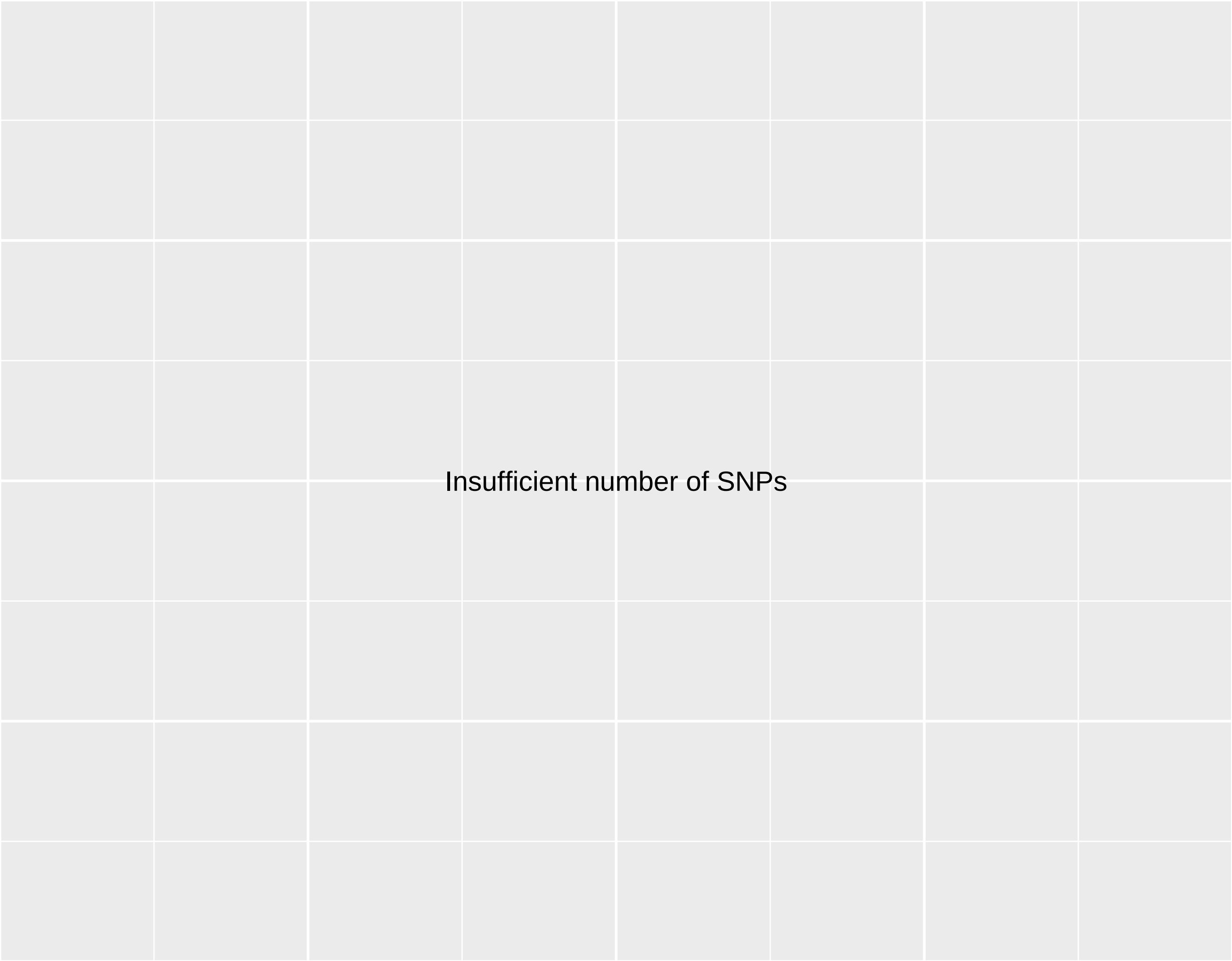
Insufficient number of SNPs

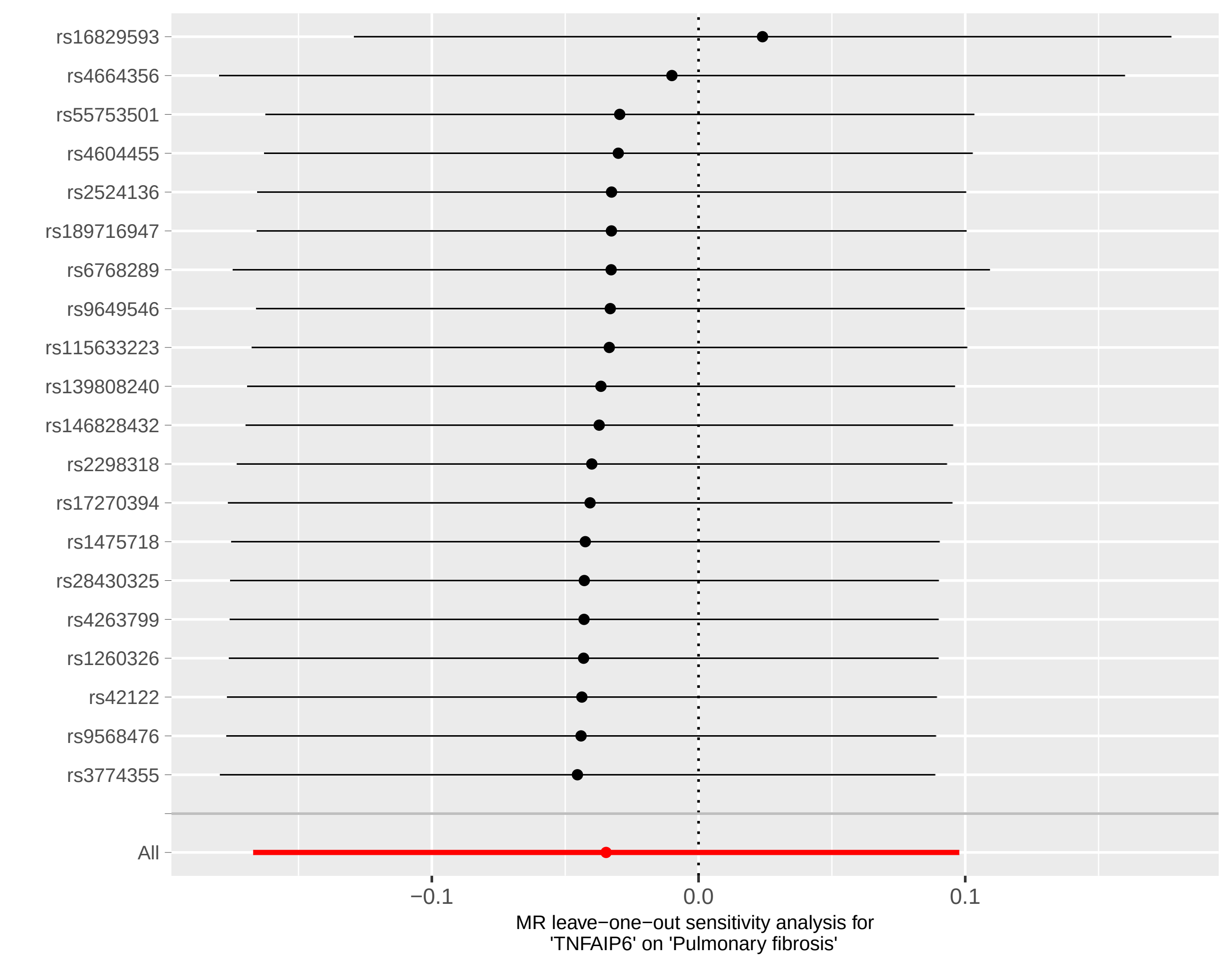

rs16829593
rs4664356
rs55753501
rs4604455
rs2524136
rs189716947
rs6768289
rs9649546
rs115633223
rs139808240
rs146828432
rs2298318
rs17270394
rs1475718
rs28430325
rs4263799
rs1260326
rs42122
rs9568476
rs3774355
All
−0.1
0.0
0.1
MR leave−one−out sensitivity analysis for
'TNFAIP6' on 'Pulmonary fibrosis'

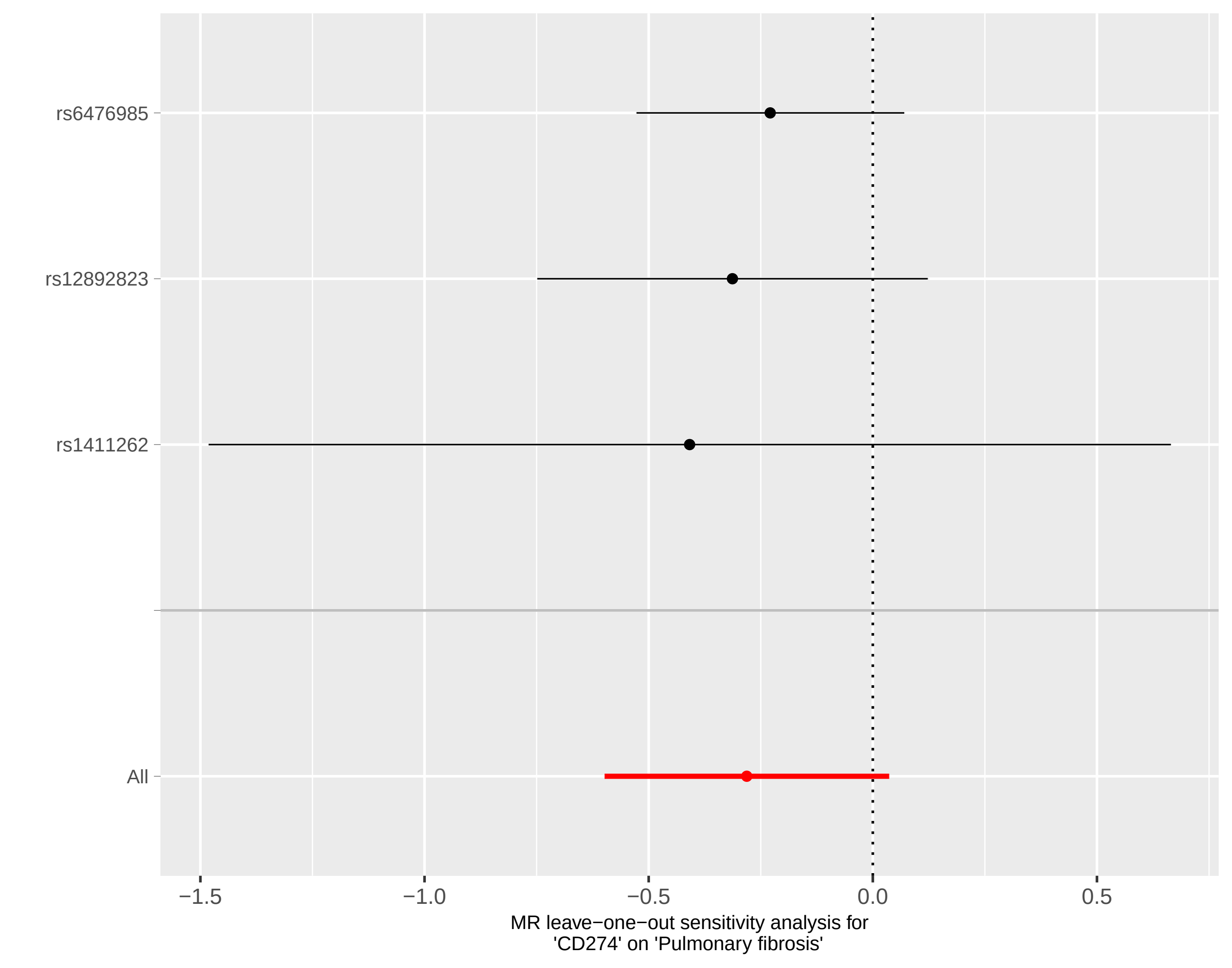
rs6476985
rs12892823
rs1411262
All
−1.5
−1.0
−0.5
0.0
0.5
MR leave−one−out sensitivity analysis for
'CD274' on 'Pulmonary fibrosis'

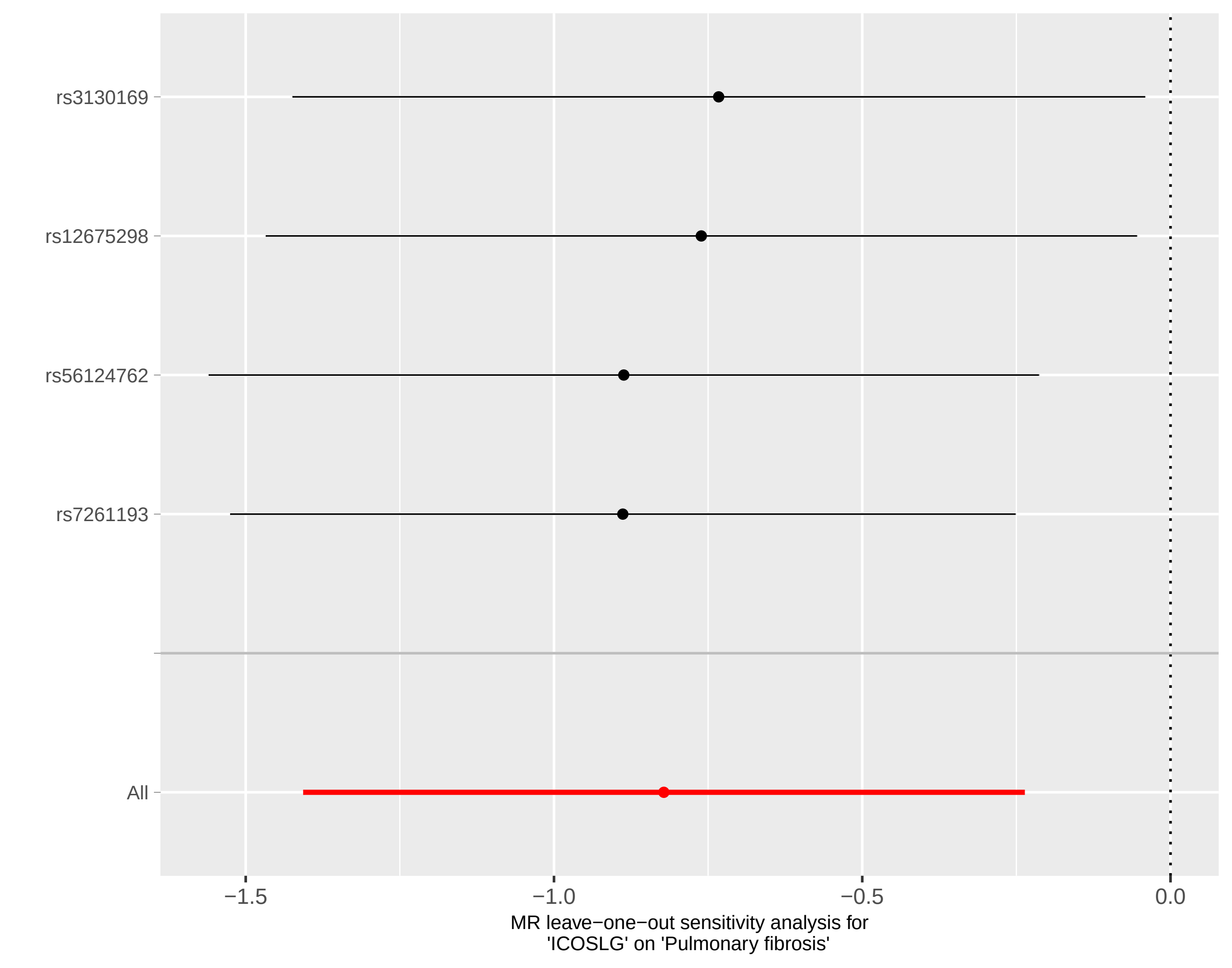

rs3130169
rs12675298
rs56124762
rs7261193
All
−1.5
−1.0
−0.5
0.0
MR leave−one−out sensitivity analysis for
'ICOSLG' on 'Pulmonary fibrosis'

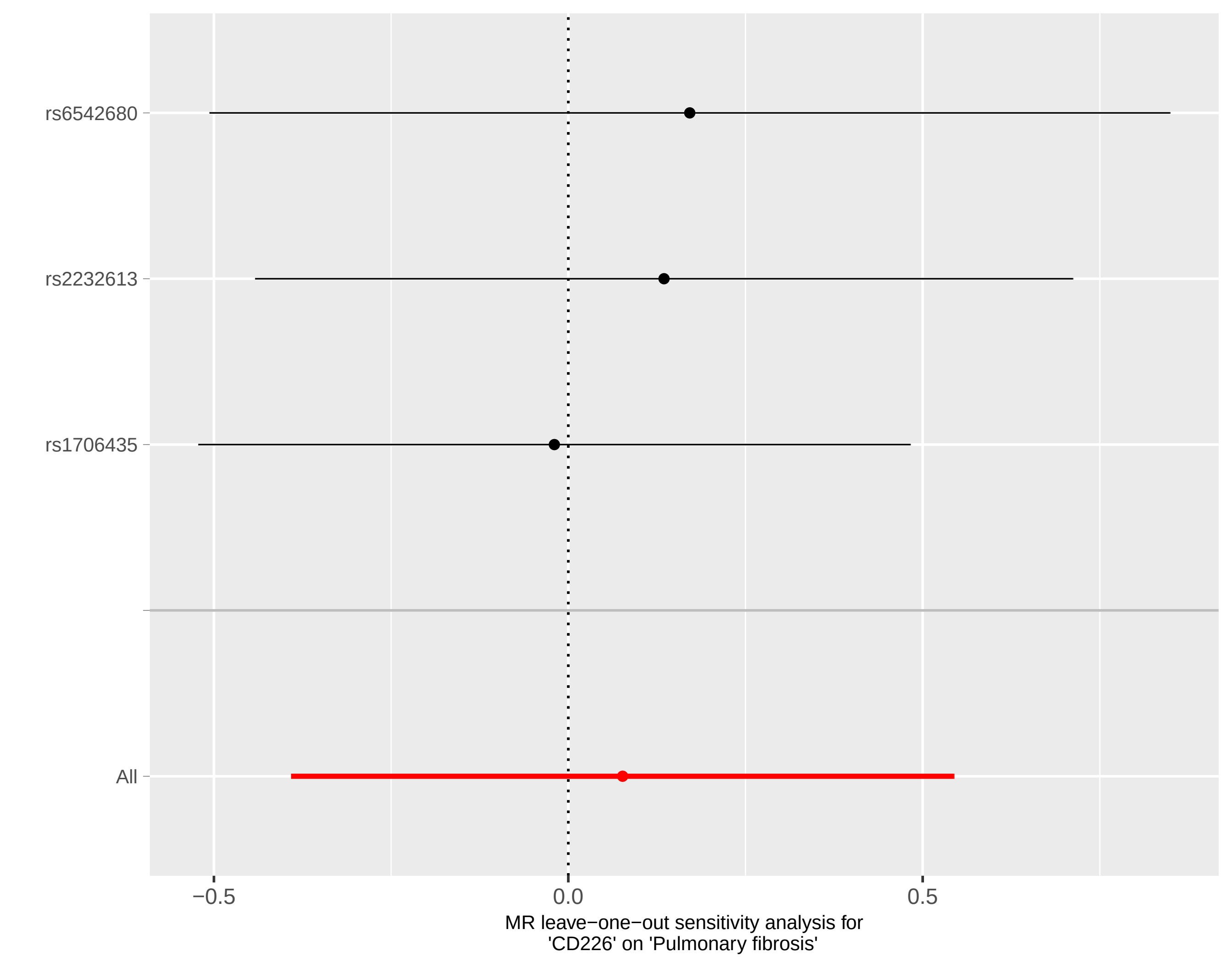
rs6542680
rs2232613
rs1706435
All
−0.5
0.0
0.5
MR leave−one−out sensitivity analysis for
'CD226' on 'Pulmonary fibrosis'

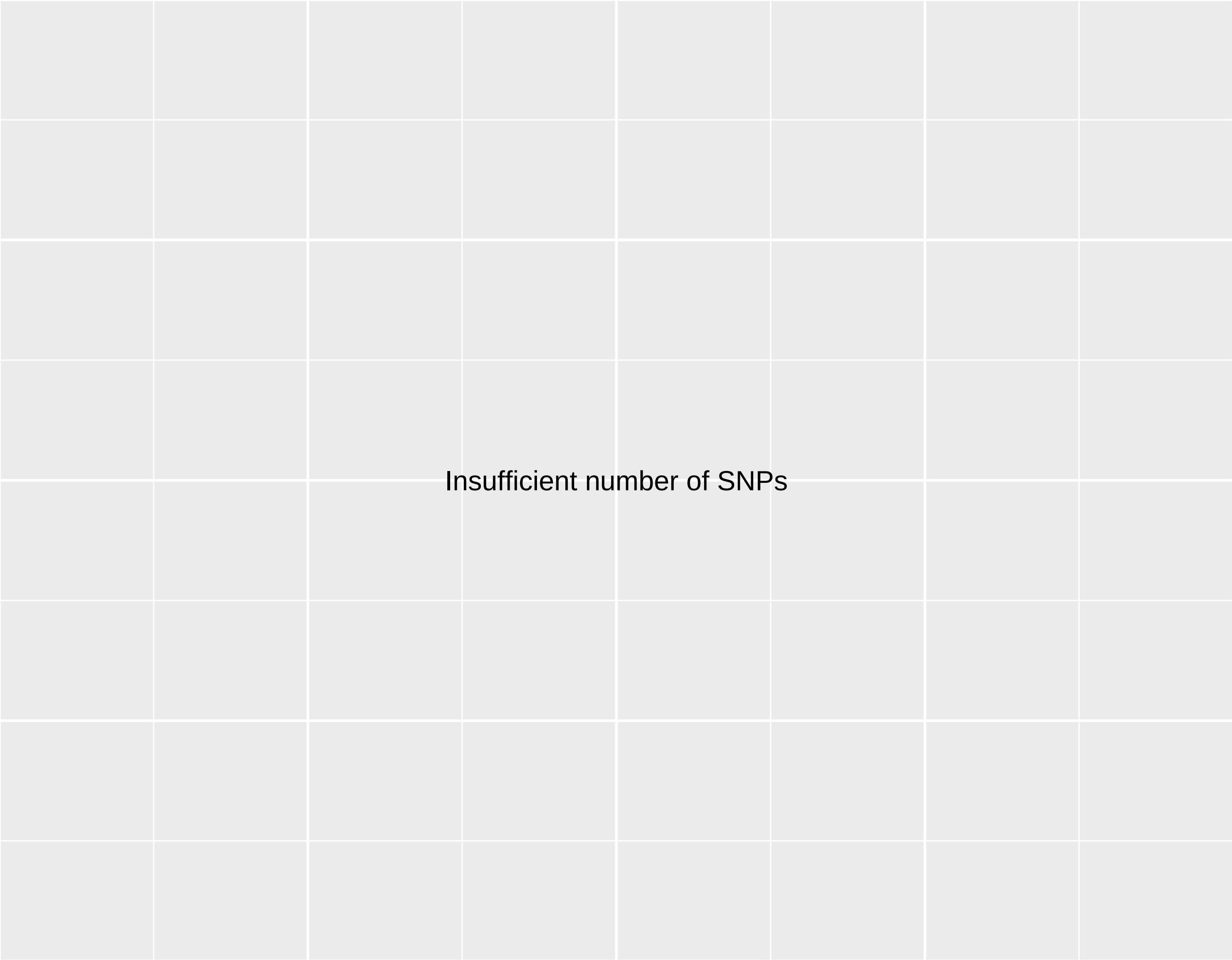
Insufficient number of SNPs

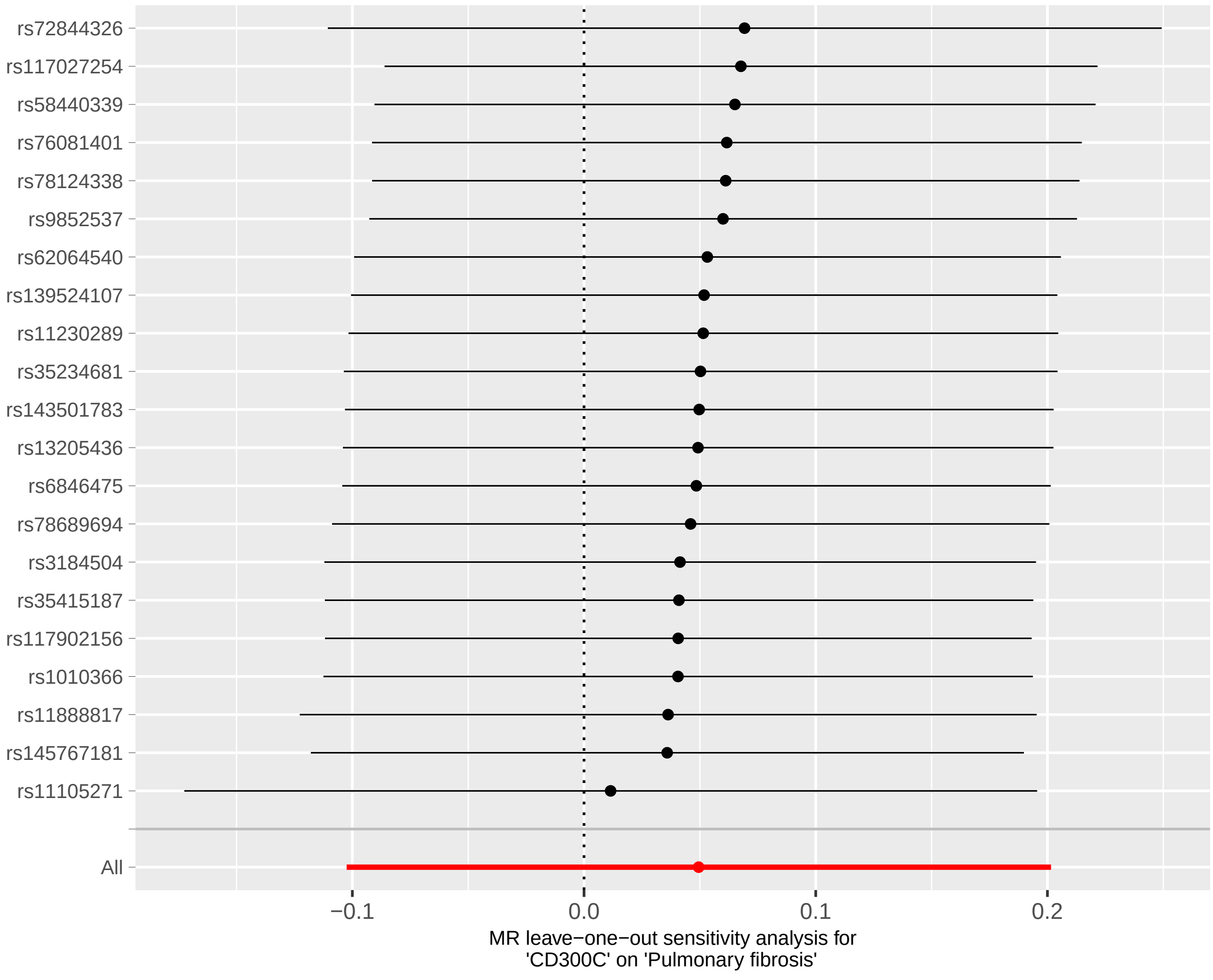

rs72844326
rs117027254
rs58440339
rs76081401
rs78124338
rs9852537
rs62064540
rs139524107
rs11230289
rs35234681
rs143501783
rs13205436
rs6846475
rs78689694
rs3184504
rs35415187
rs117902156
rs1010366
rs11888817
rs145767181
rs11105271
All
−0.1
0.0
0.1
0.2
MR leave−one−out sensitivity analysis for
'CD300C' on 'Pulmonary fibrosis'

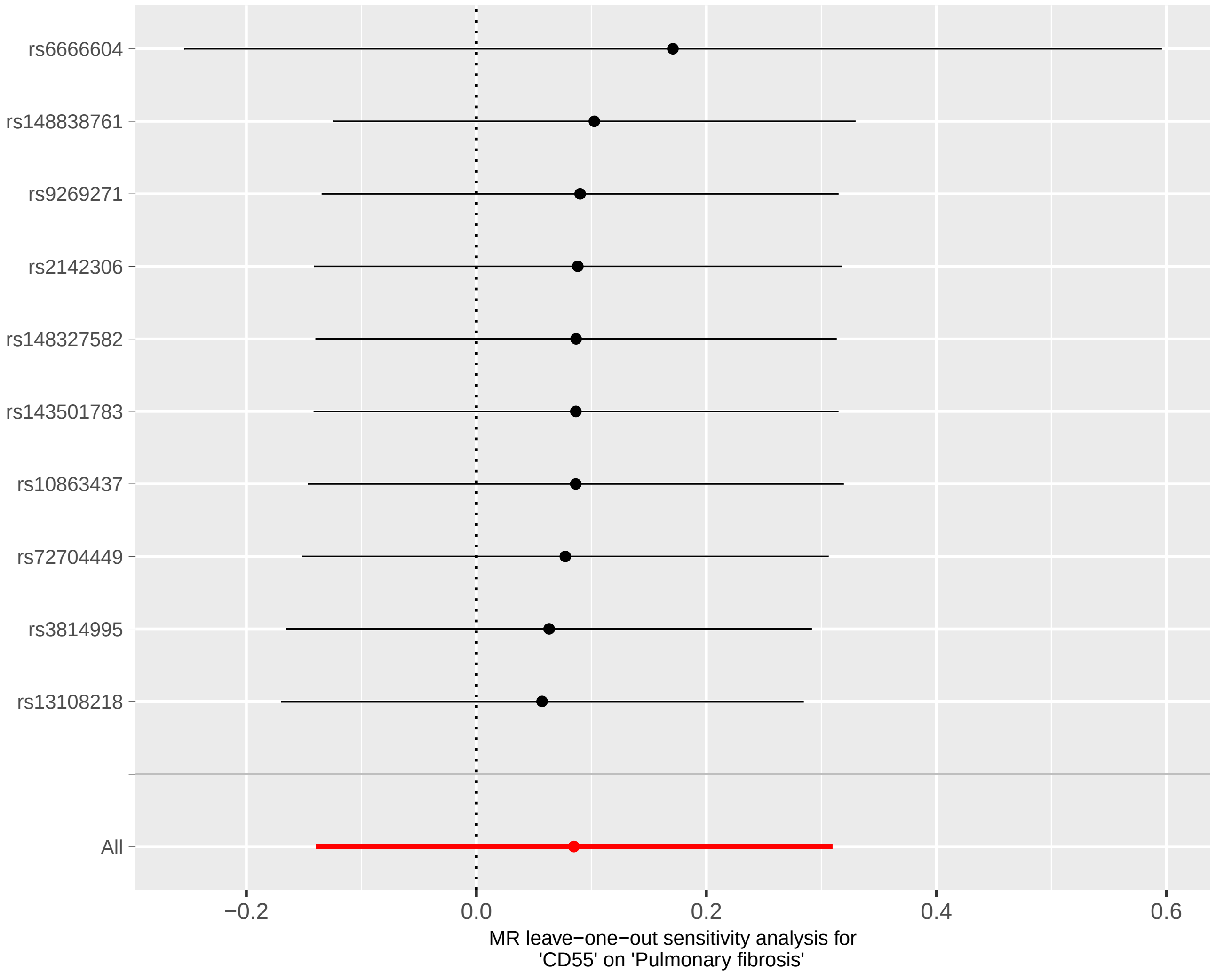
rs6666604
rs148838761
rs9269271
rs2142306
rs148327582
rs143501783
rs10863437
rs72704449
rs3814995
rs13108218
All
−0.2
0.0
0.2
0.4
0.6
MR leave−one−out sensitivity analysis for
'CD55' on 'Pulmonary fibrosis'

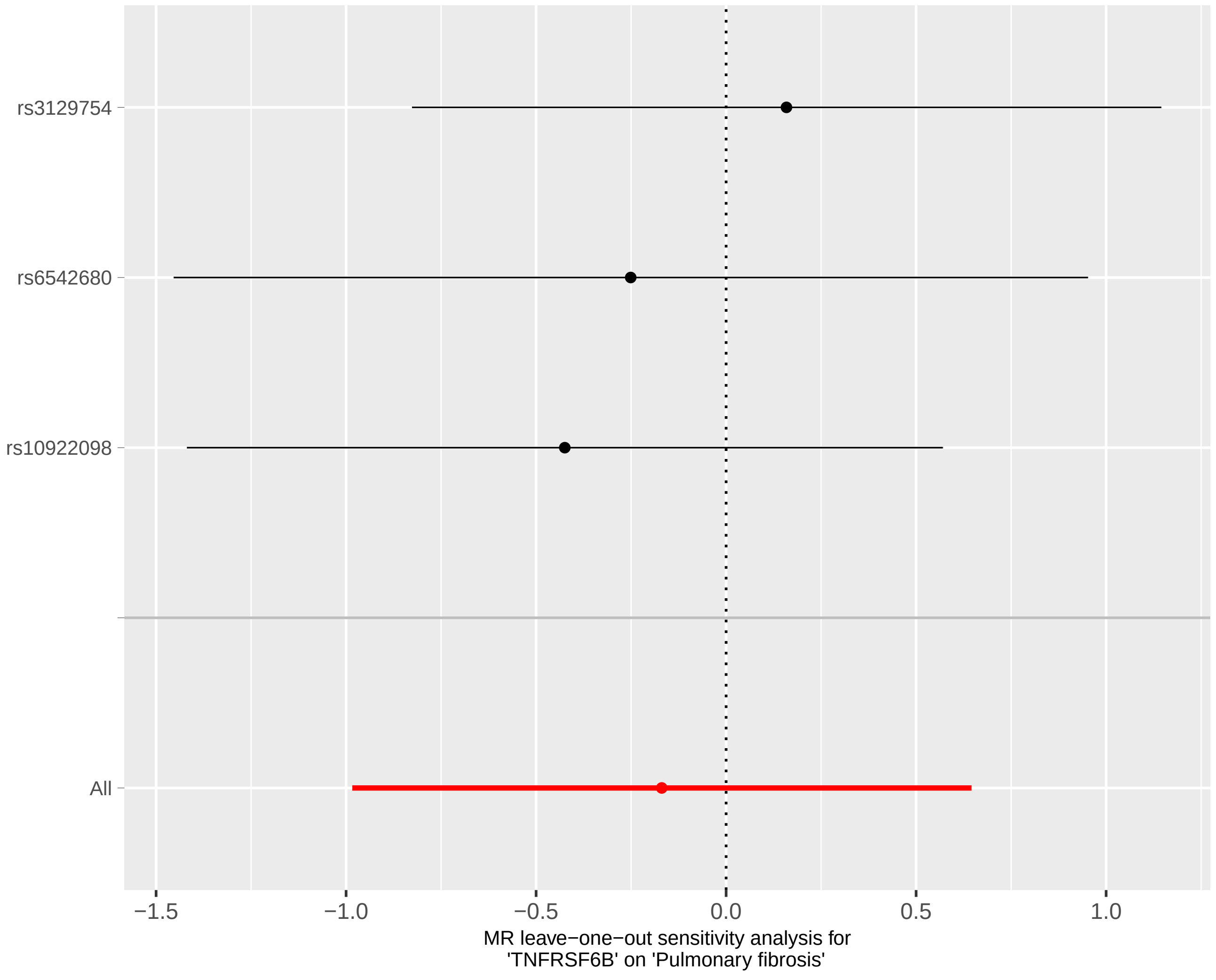
rs3129754
rs6542680
rs10922098
All
−1.5
−1.0
−0.5
0.0
0.5
1.0
MR leave−one−out sensitivity analysis for
'TNFRSF6B' on 'Pulmonary fibrosis'

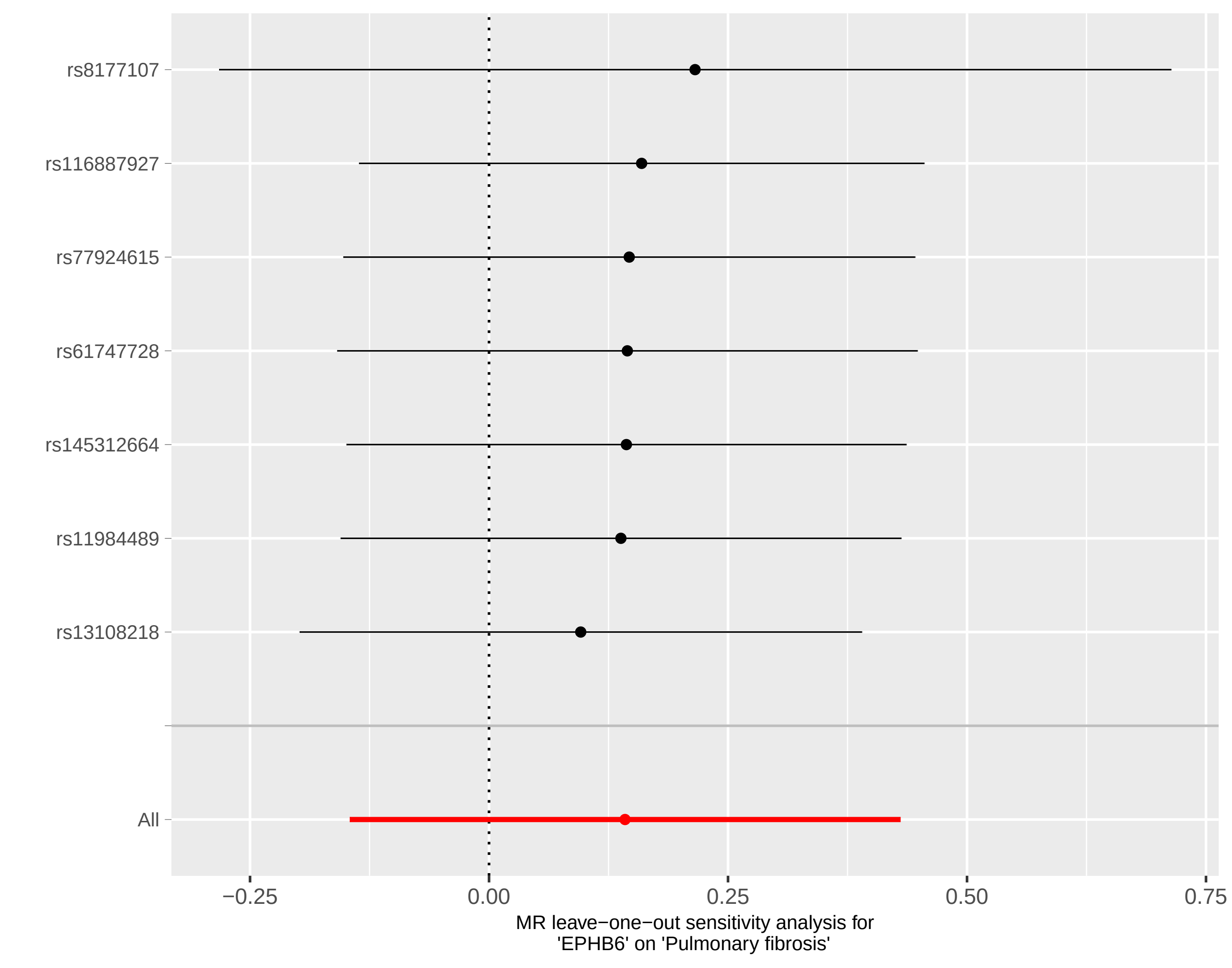
rs8177107
rs116887927
rs77924615
rs61747728
rs145312664
rs11984489
rs13108218
All
−0.25
0.00
0.25
0.50
0.75
MR leave−one−out sensitivity analysis for
'EPHB6' on 'Pulmonary fibrosis'

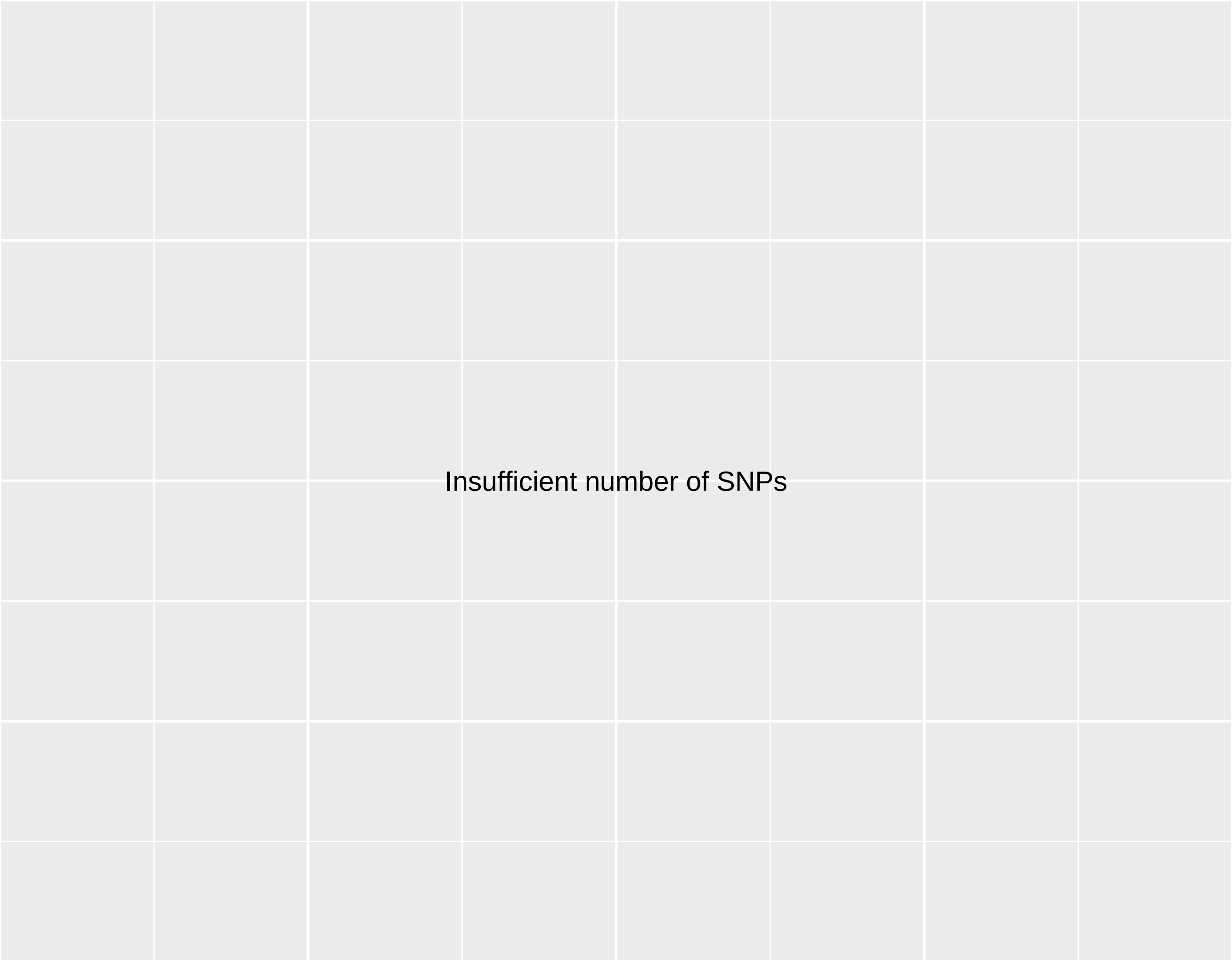
Insufficient number of SNPs

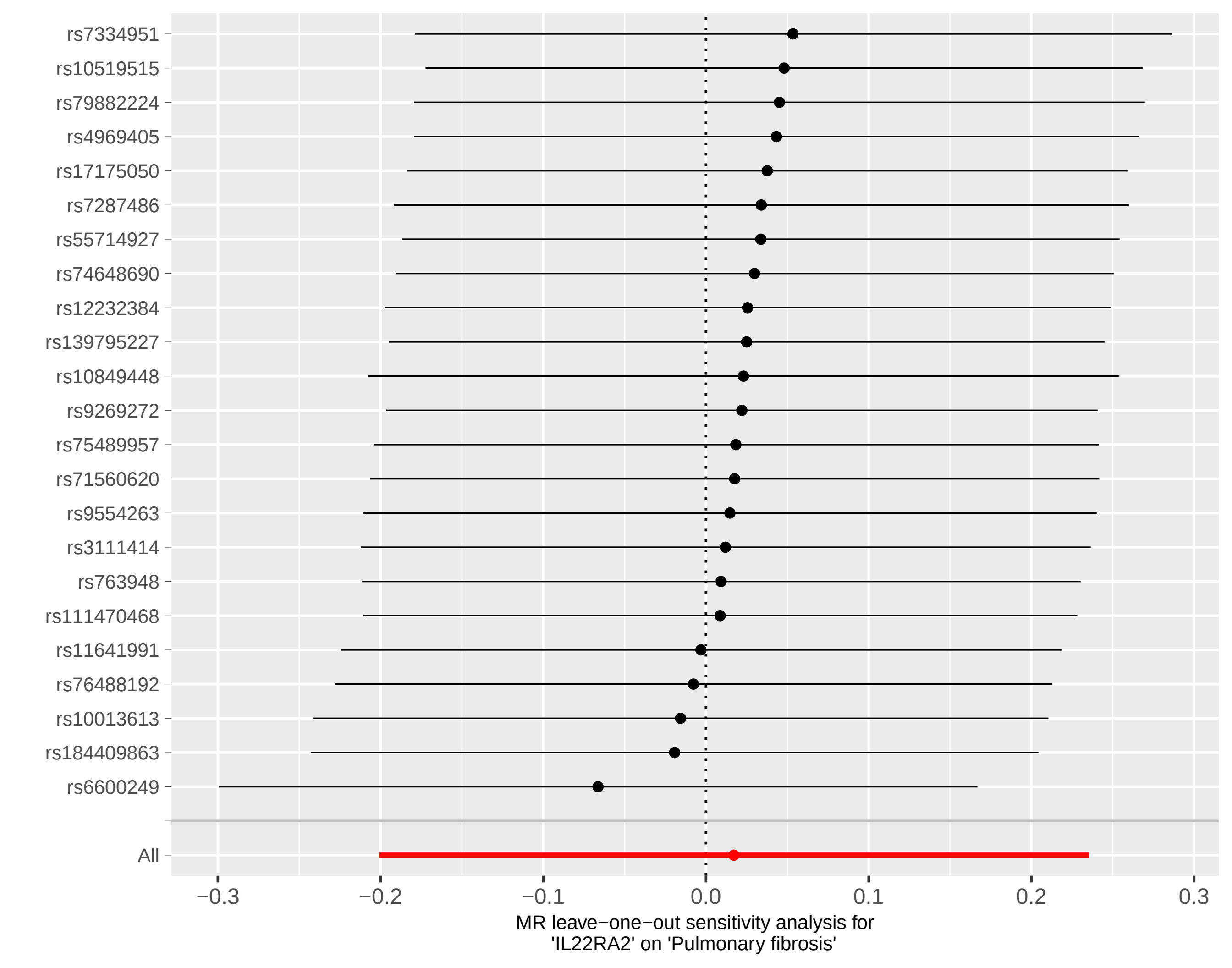
rs7334951
rs10519515
rs79882224
rs4969405
rs17175050
rs7287486
rs55714927
rs74648690
rs12232384
rs139795227
rs10849448
rs9269272
rs75489957
rs71560620
rs9554263
rs3111414
rs763948
rs111470468
rs11641991
rs76488192
rs10013613
rs184409863
rs6600249
All
−0.3
−0.2
−0.1
0.0
0.1
0.2
0.3
MR leave−one−out sensitivity analysis for
'IL22RA2' on 'Pulmonary fibrosis'

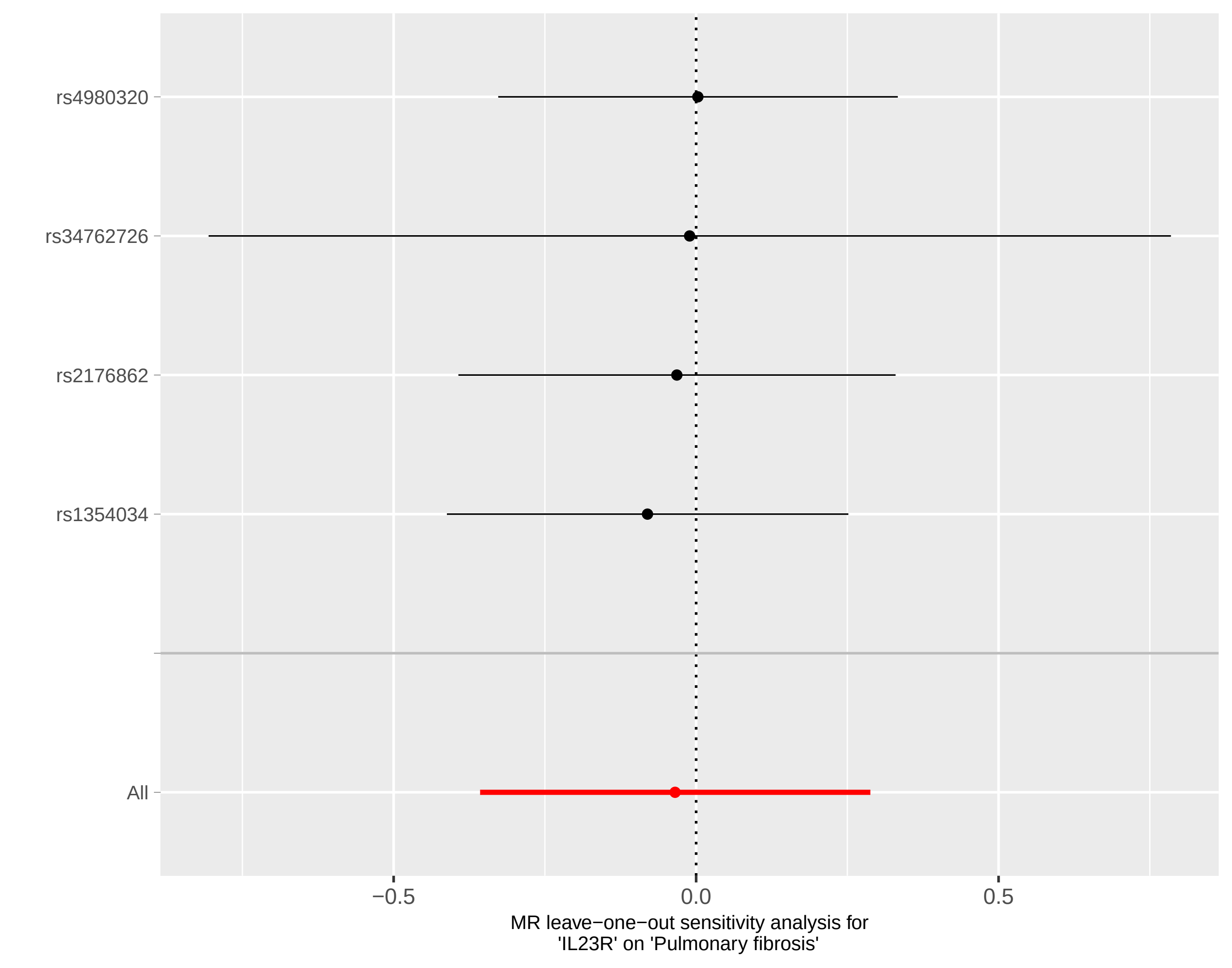
rs4980320
rs34762726
rs2176862
rs1354034
All
−0.5
0.0
0.5
MR leave−one−out sensitivity analysis for
'IL23R' on 'Pulmonary fibrosis'

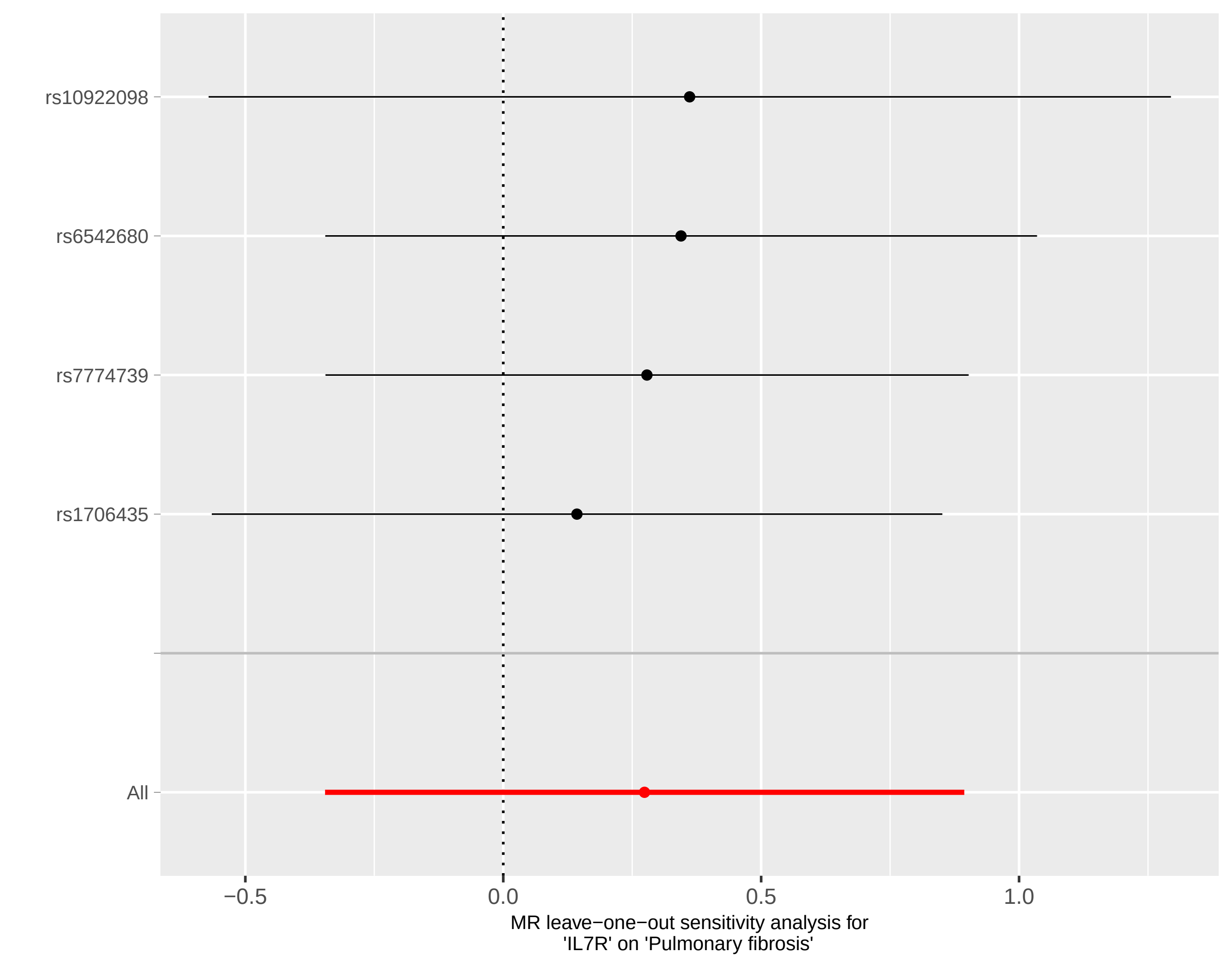
rs10922098
rs6542680
rs7774739
rs1706435
All
−0.5
0.0
0.5
1.0
MR leave−one−out sensitivity analysis for
'IL7R' on 'Pulmonary fibrosis'

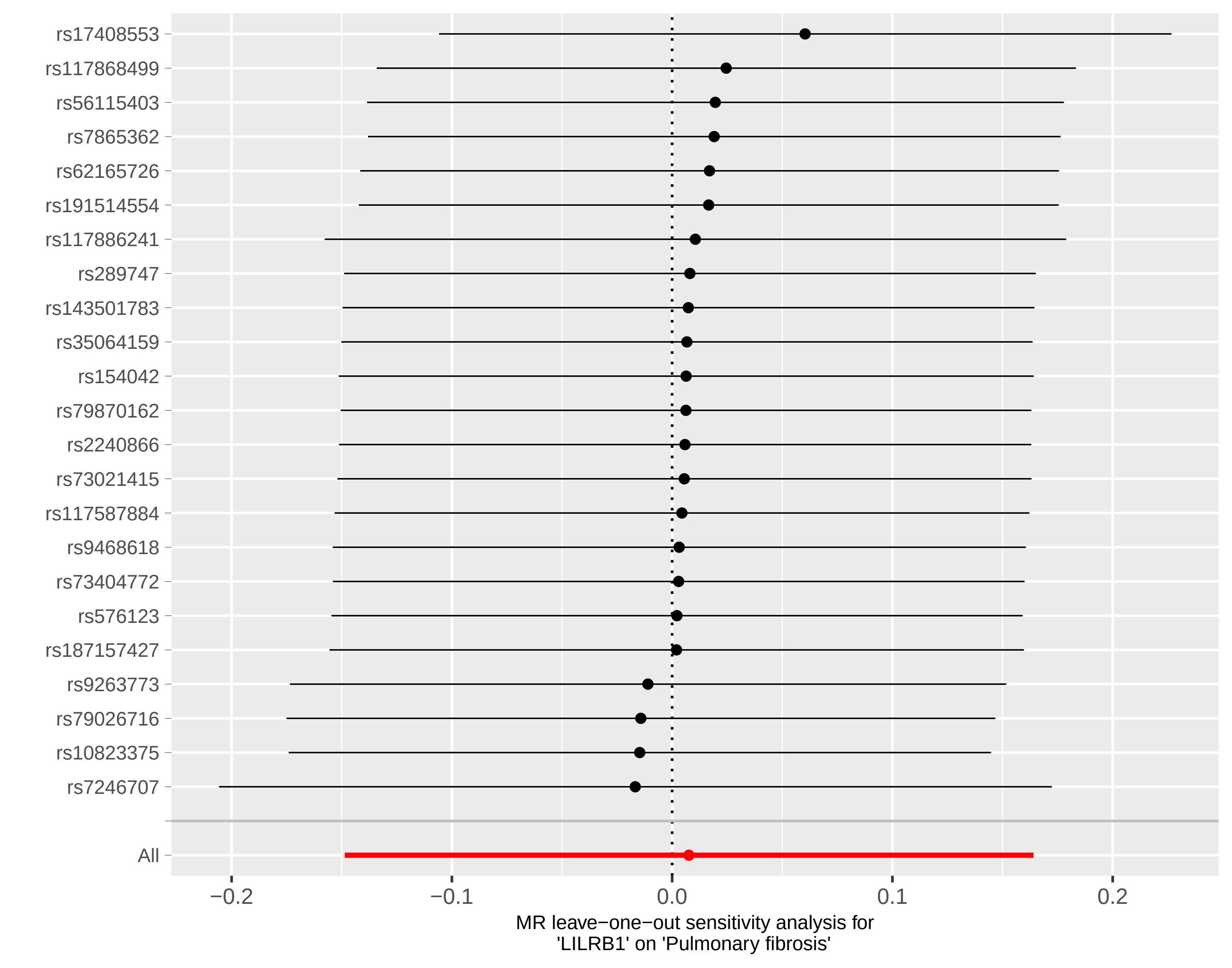
rs17408553
rs117868499
rs56115403
rs7865362
rs62165726
rs191514554
rs117886241
rs289747
rs143501783
rs35064159
rs154042
rs79870162
rs2240866
rs73021415
rs117587884
rs9468618
rs73404772
rs576123
rs187157427
rs9263773
rs79026716
rs10823375
rs7246707
All
−0.2
−0.1
0.0
0.1
0.2
MR leave−one−out sensitivity analysis for
'LILRB1' on 'Pulmonary fibrosis'

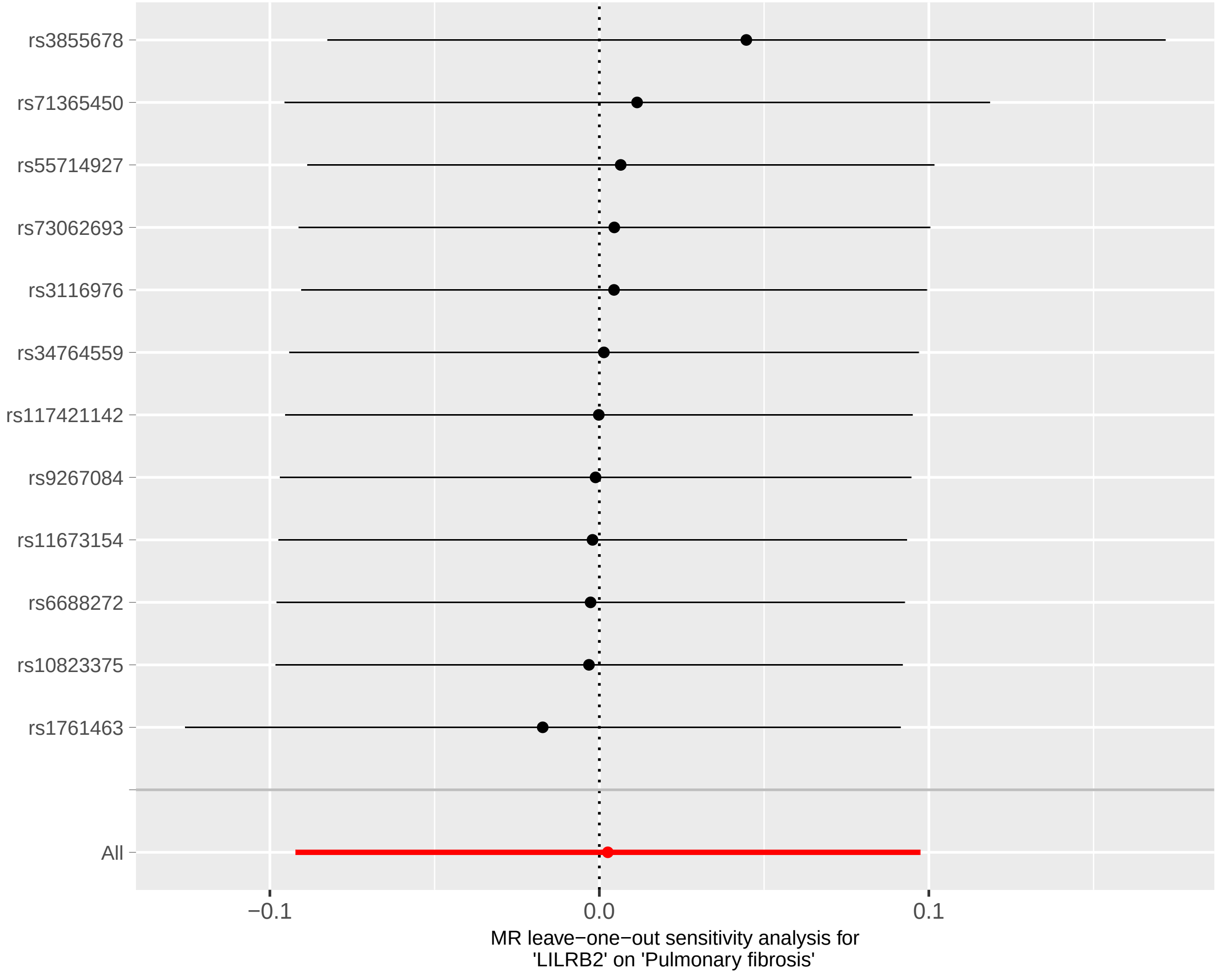

rs3855678
rs71365450
rs55714927
rs73062693
rs3116976
rs34764559
rs117421142
rs9267084
rs11673154
rs6688272
rs10823375
rs1761463
All
−0.1
0.0
0.1
MR leave−one−out sensitivity analysis for
'LILRB2' on 'Pulmonary fibrosis'

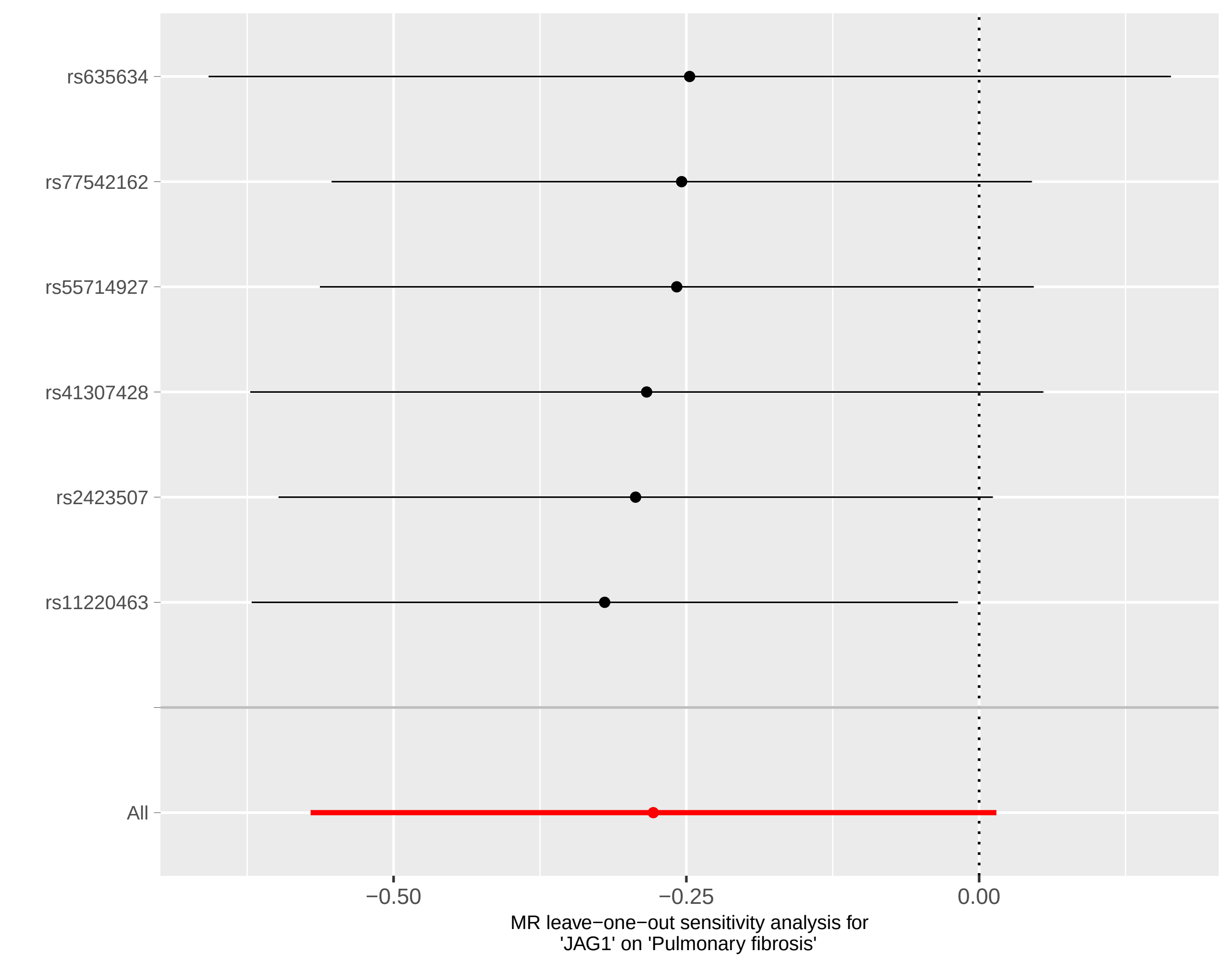
rs635634
rs77542162
rs55714927
rs41307428
rs2423507
rs11220463
All
−0.50
−0.25
0.00
MR leave−one−out sensitivity analysis for
'JAG1' on 'Pulmonary fibrosis'

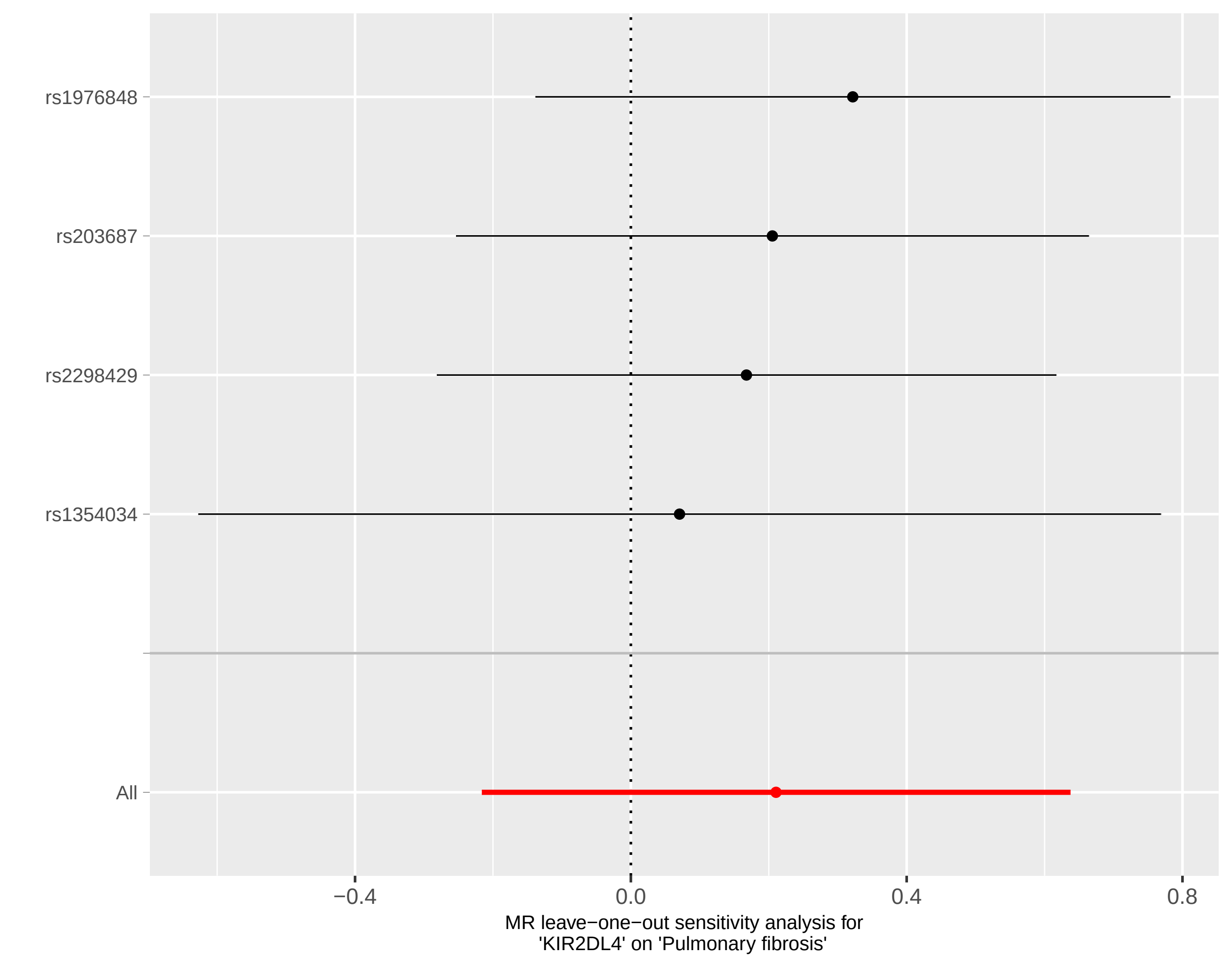
rs1976848
rs203687
rs2298429
rs1354034
All
−0.4
0.0
0.4
0.8
MR leave−one−out sensitivity analysis for
'KIR2DL4' on 'Pulmonary fibrosis'

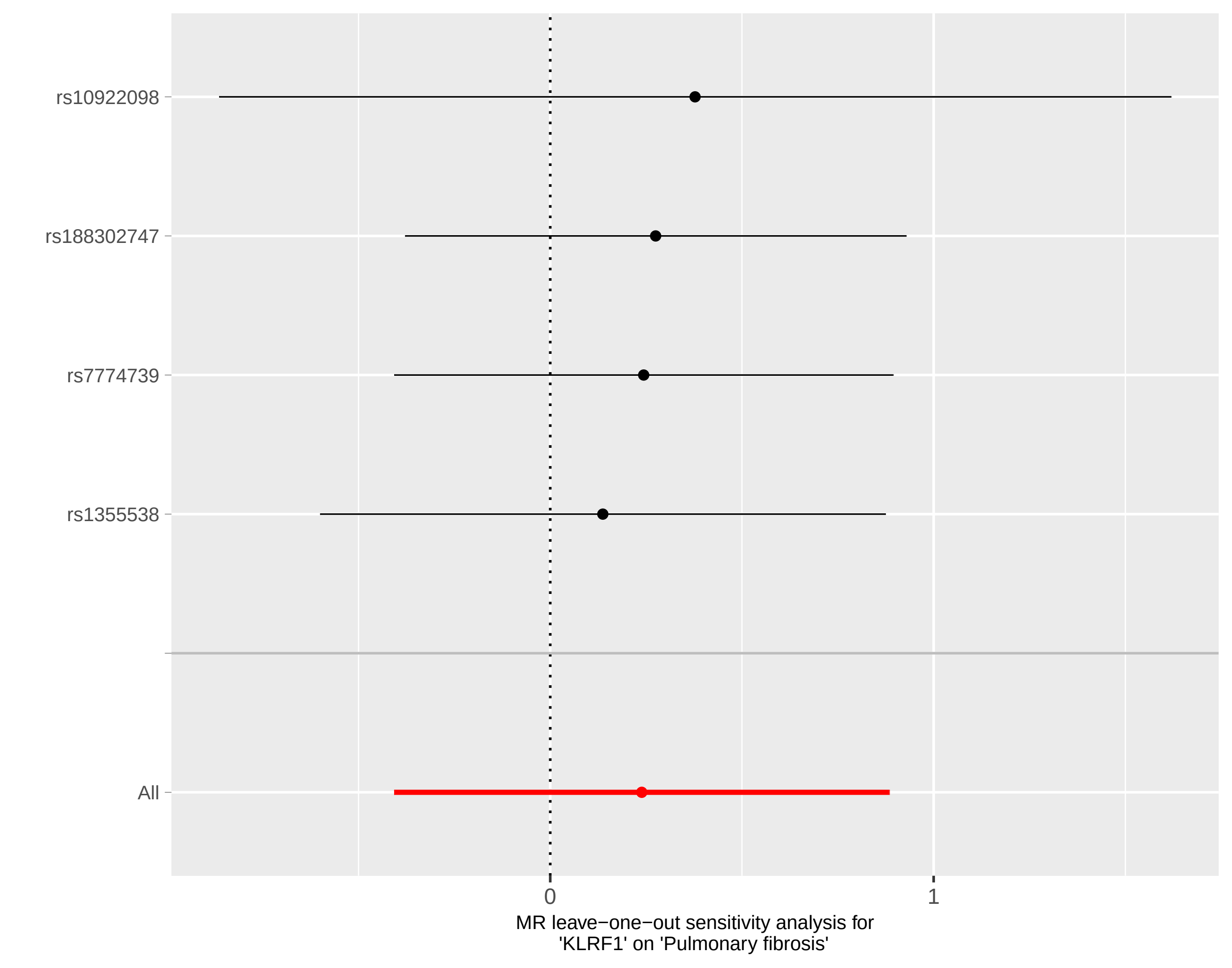
rs10922098
rs188302747
rs7774739
rs1355538
All
0
1
MR leave−one−out sensitivity analysis for
'KLRF1' on 'Pulmonary fibrosis'

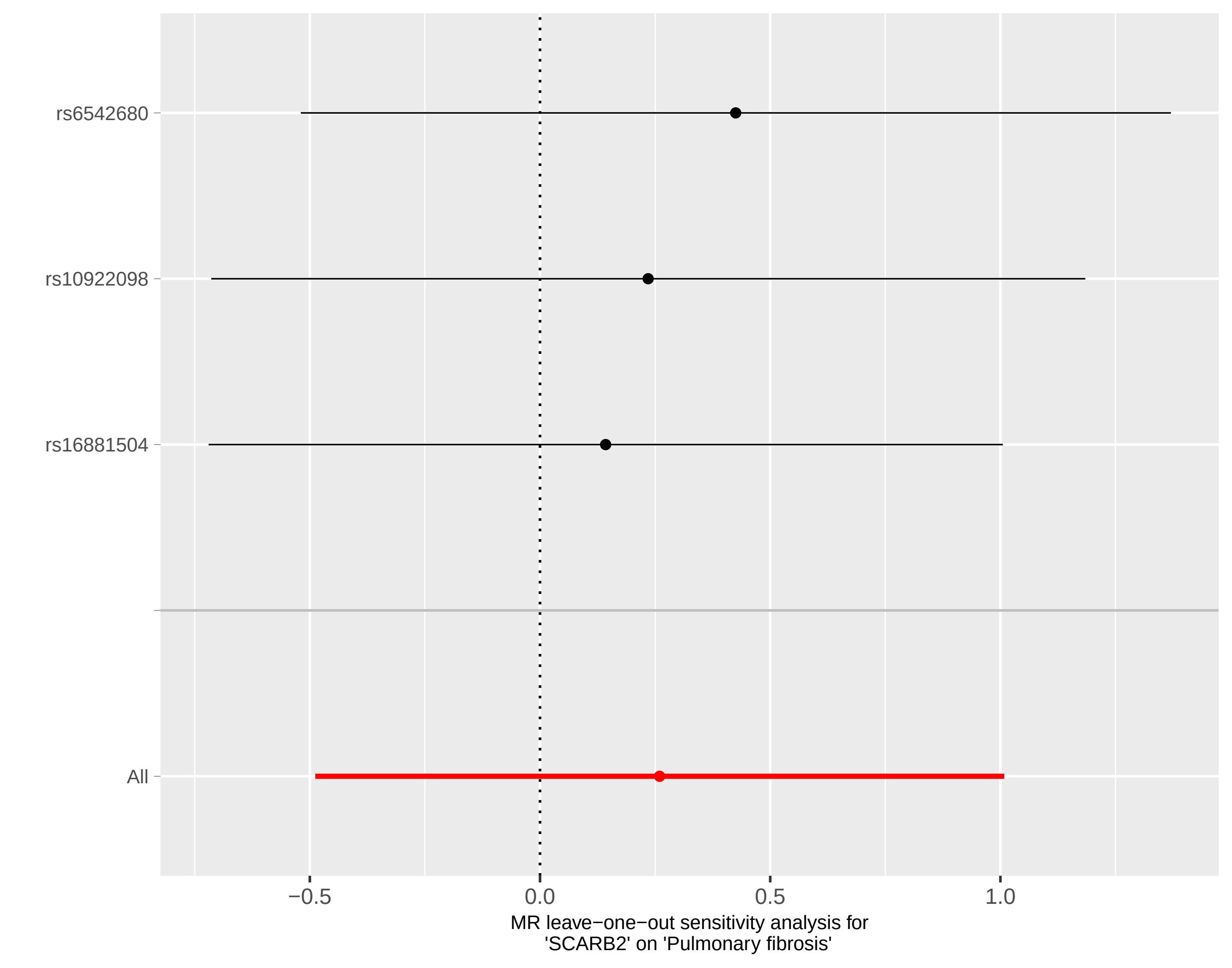
rs6542680
rs10922098
rs16881504
All
−0.5
0.0
0.5
1.0
MR leave−one−out sensitivity analysis for
'SCARB2' on 'Pulmonary fibrosis'

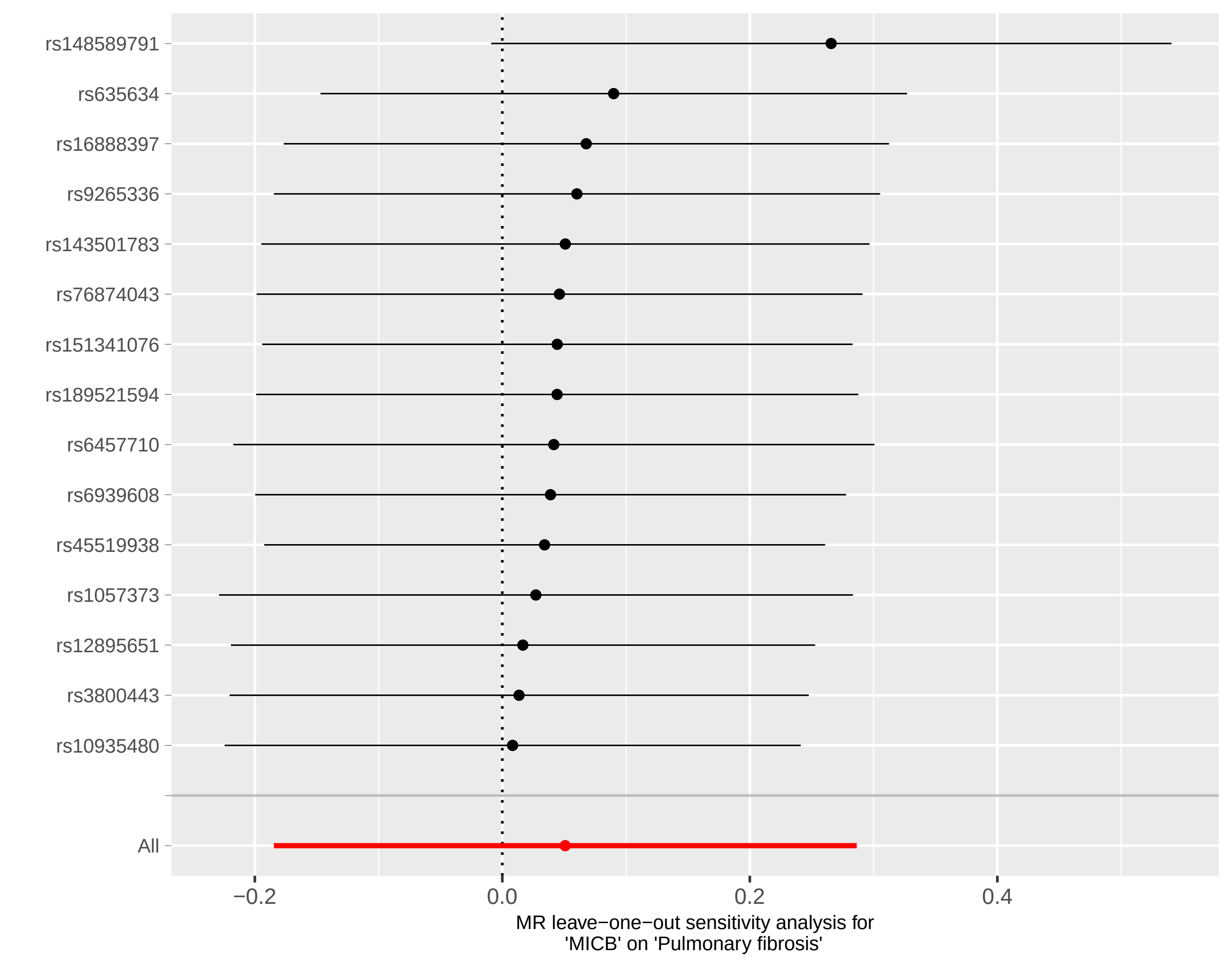
rs148589791
rs635634
rs16888397
rs9265336
rs143501783
rs76874043
rs151341076
rs189521594
rs6457710
rs6939608
rs45519938
rs1057373
rs12895651
rs3800443
rs10935480
All
−0.2
0.0
0.2
0.4
MR leave−one−out sensitivity analysis for
'MICB' on 'Pulmonary fibrosis'

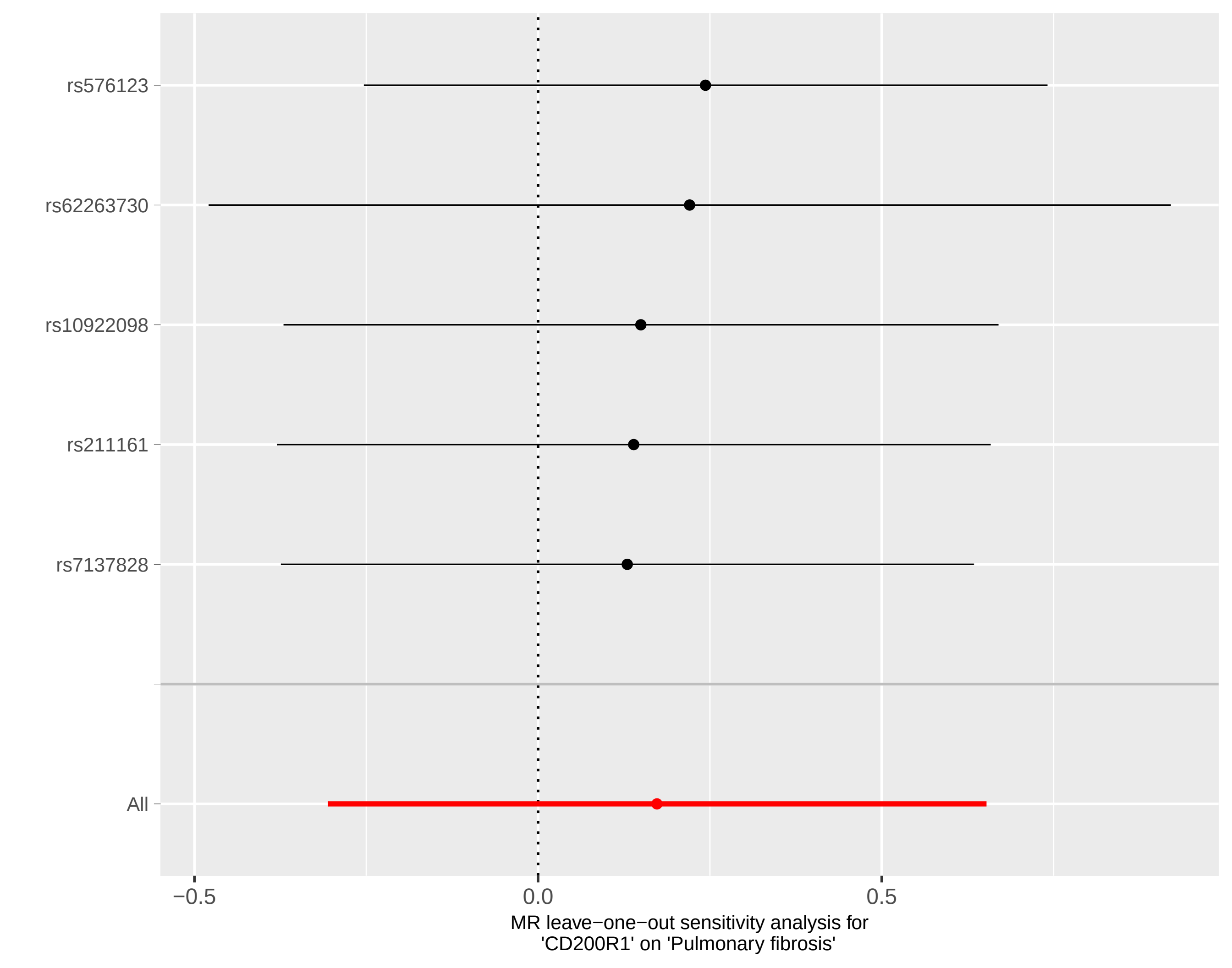

rs576123
rs62263730
rs10922098
rs211161
rs7137828
All
−0.5
0.0
0.5
MR leave−one−out sensitivity analysis for
'CD200R1' on 'Pulmonary fibrosis'

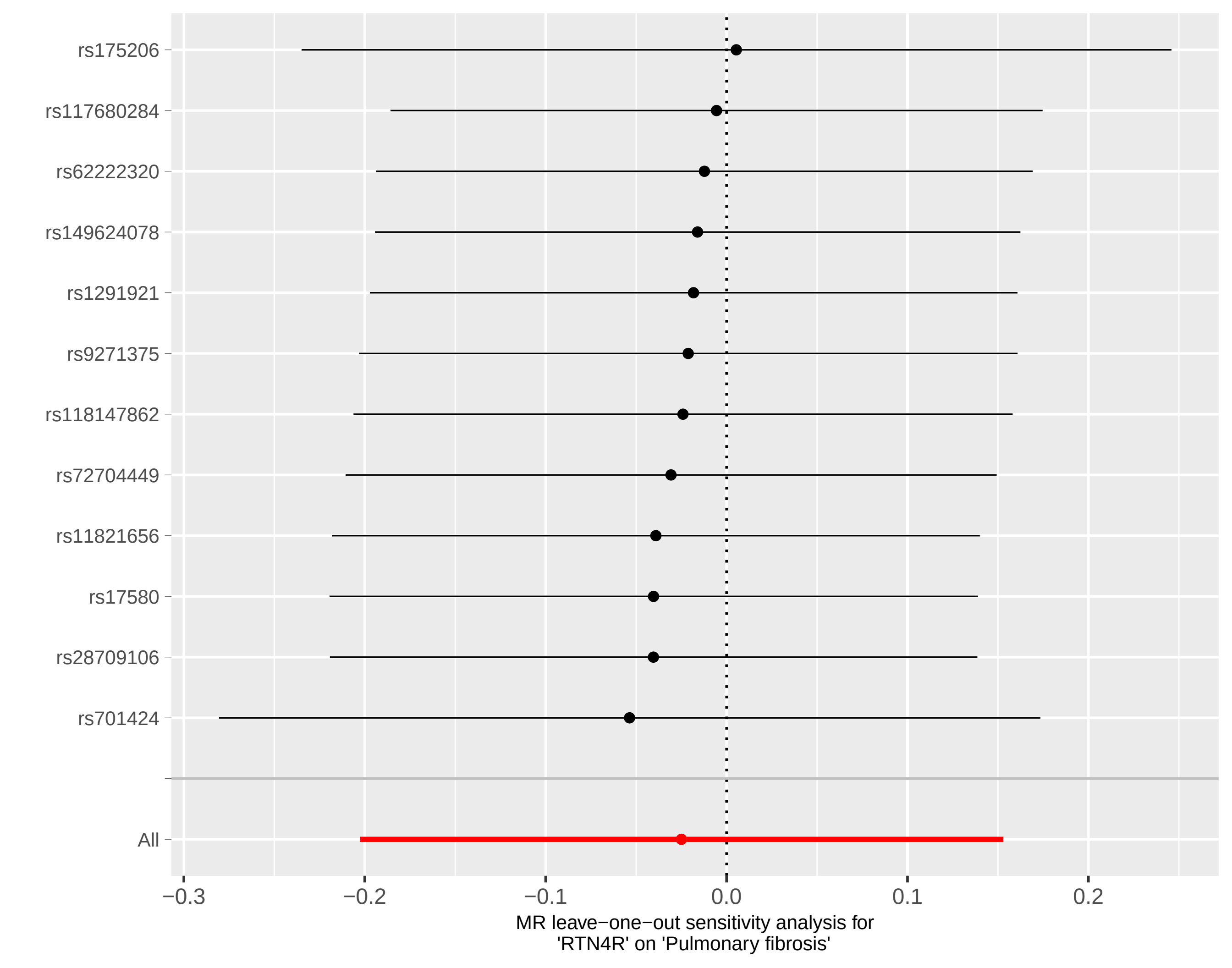
rs175206
rs117680284
rs62222320
rs149624078
rs1291921
rs9271375
rs118147862
rs72704449
rs11821656
rs17580
rs28709106
rs701424
All
−0.3
−0.2
−0.1
0.0
0.1
0.2
MR leave−one−out sensitivity analysis for
'RTN4R' on 'Pulmonary fibrosis'

Insufficient number of SNPs

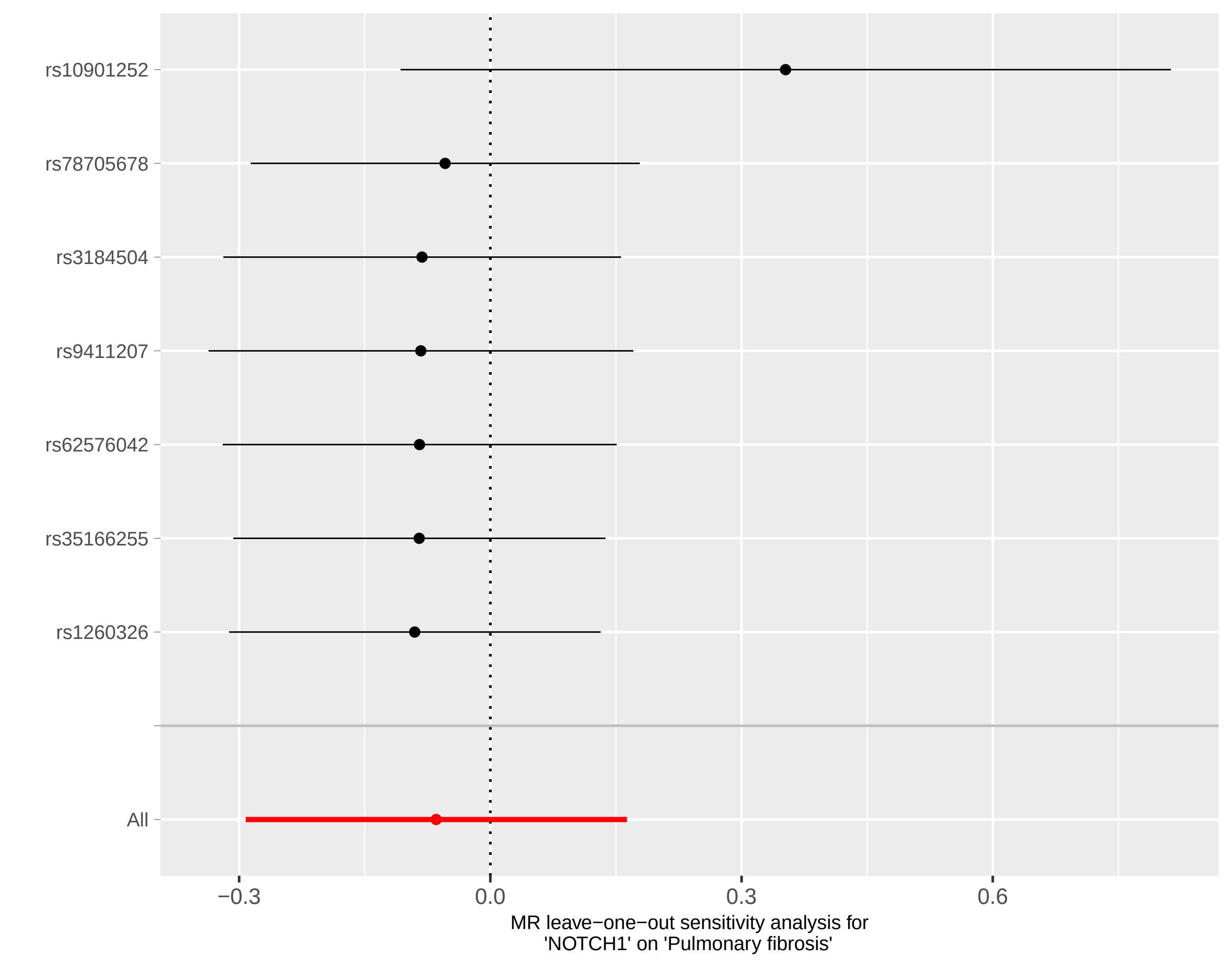
rs10901252
rs78705678
rs3184504
rs9411207
rs62576042
rs35166255
rs1260326
All
−0.3
0.0
0.3
0.6
MR leave−one−out sensitivity analysis for
'NOTCH1' on 'Pulmonary fibrosis'

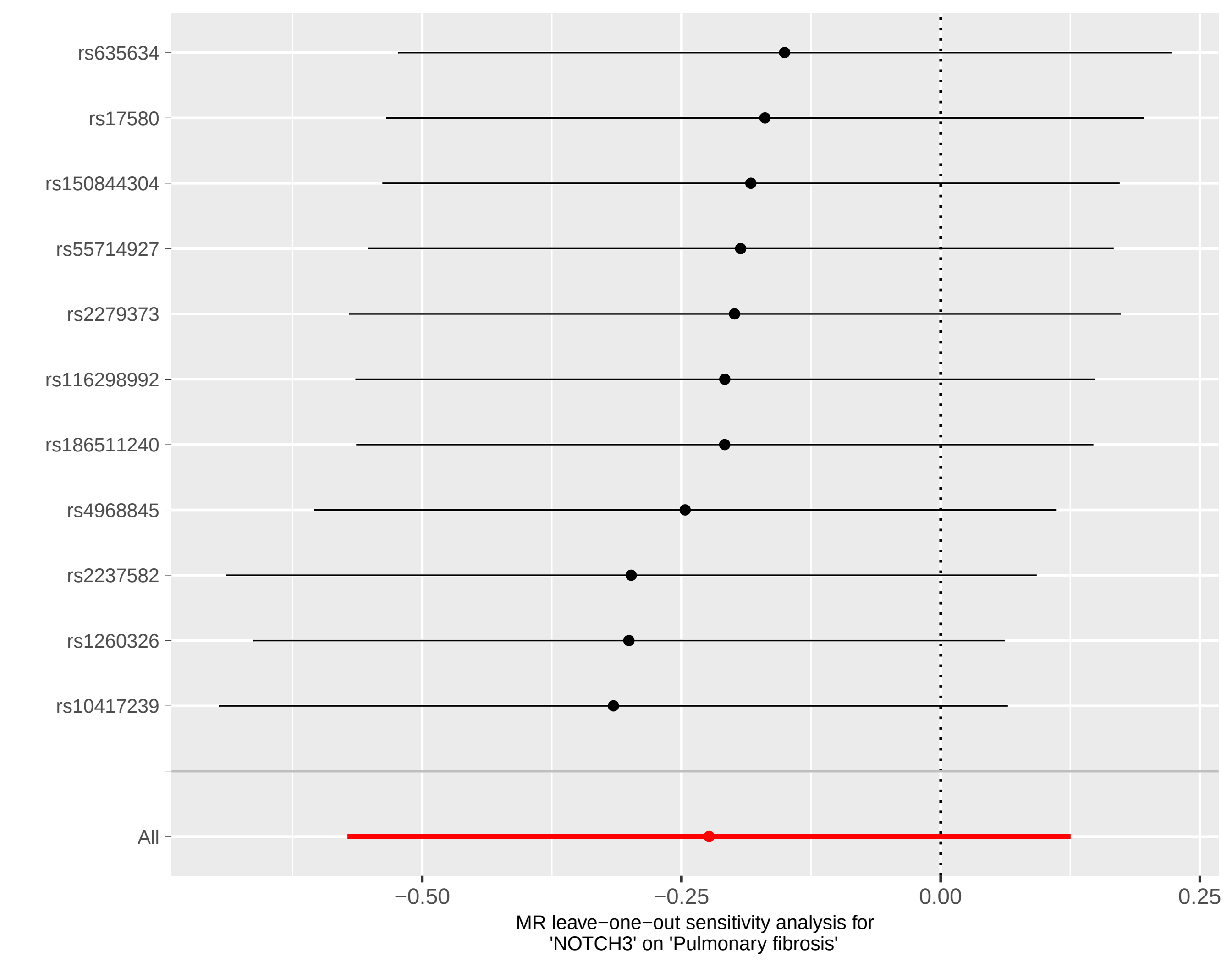
rs635634
rs17580
rs150844304
rs55714927
rs2279373
rs116298992
rs186511240
rs4968845
rs2237582
rs1260326
rs10417239
All
−0.50
−0.25
0.00
0.25
MR leave−one−out sensitivity analysis for
'NOTCH3' on 'Pulmonary fibrosis'

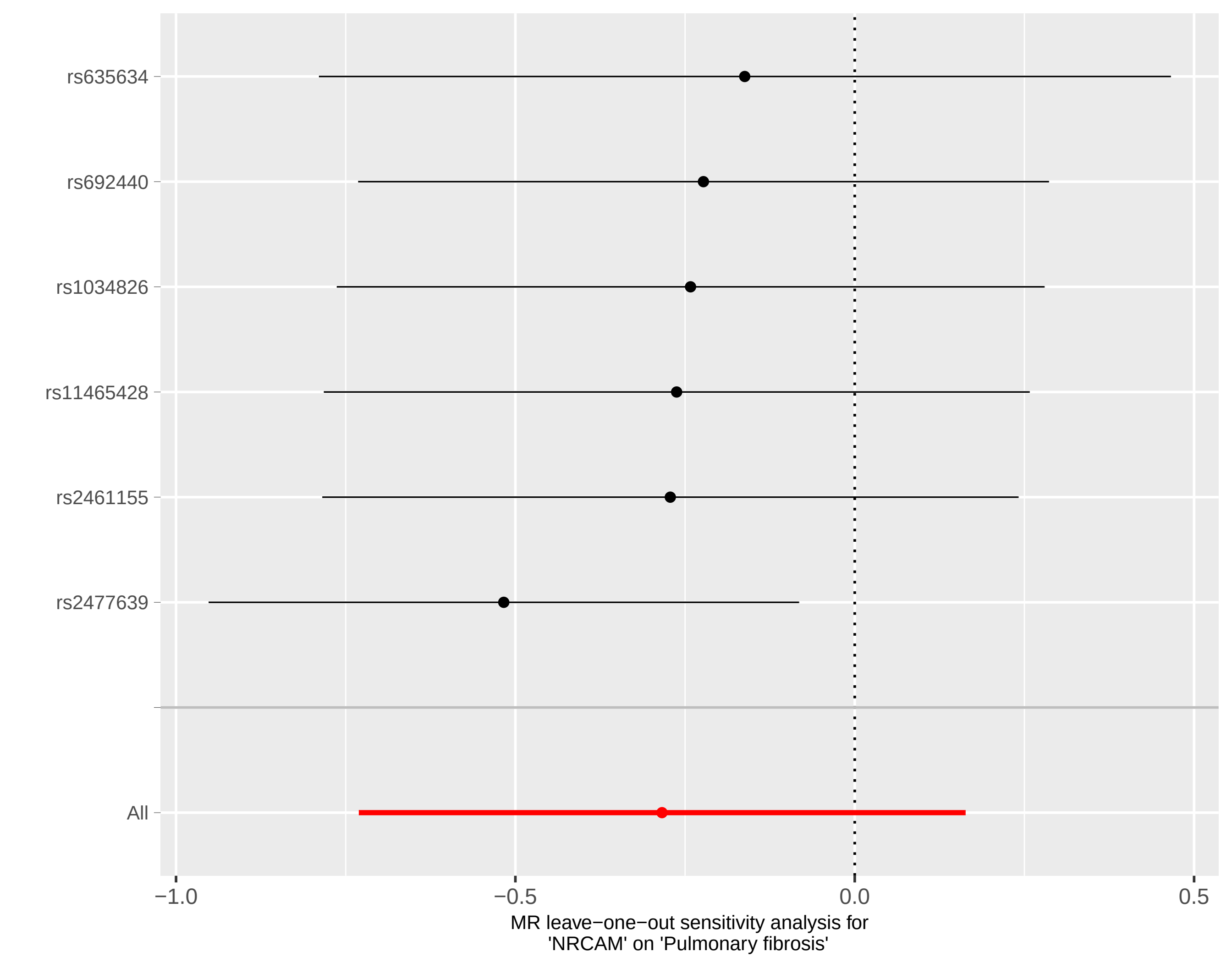

rs635634
rs692440
rs1034826
rs11465428
rs2461155
rs2477639
All
−1.0
−0.5
0.0
0.5
MR leave−one−out sensitivity analysis for
'NRCAM' on 'Pulmonary fibrosis'

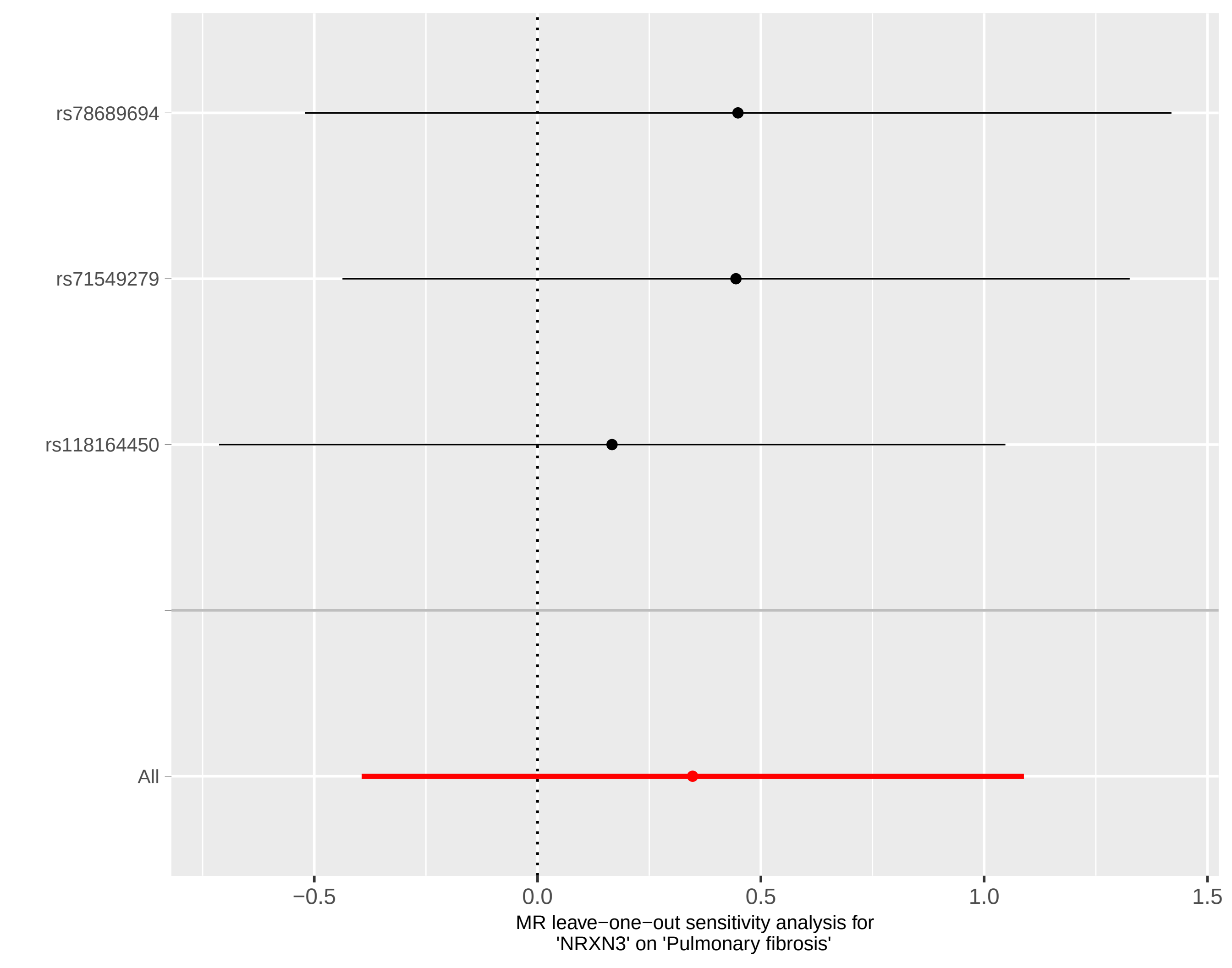

rs78689694
rs71549279
rs118164450
All
−0.5
0.0
0.5
1.0
1.5
MR leave−one−out sensitivity analysis for
'NRXN3' on 'Pulmonary fibrosis'

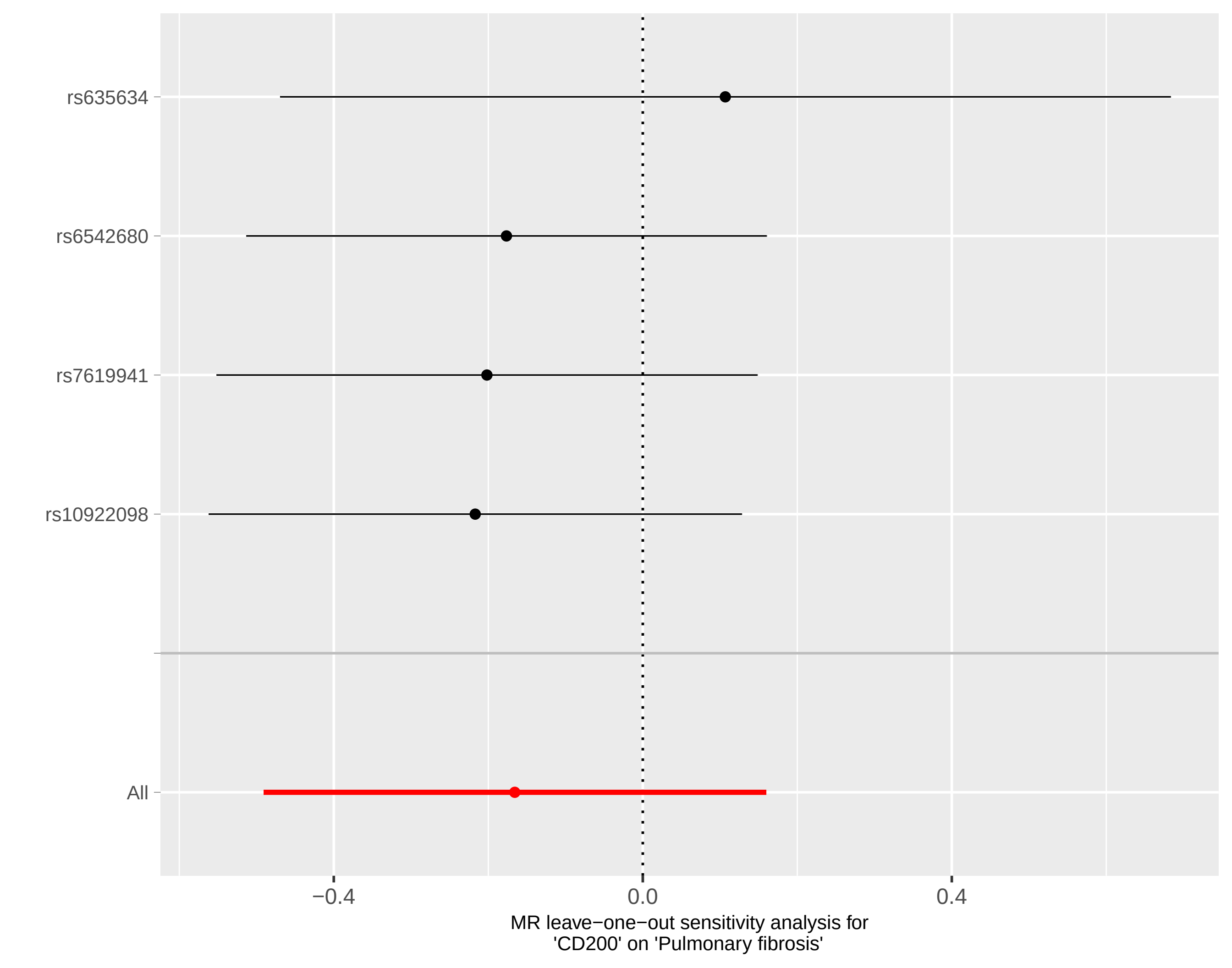
rs635634
rs6542680
rs7619941
rs10922098
All
−0.4
0.0
0.4
MR leave−one−out sensitivity analysis for
'CD200' on 'Pulmonary fibrosis'

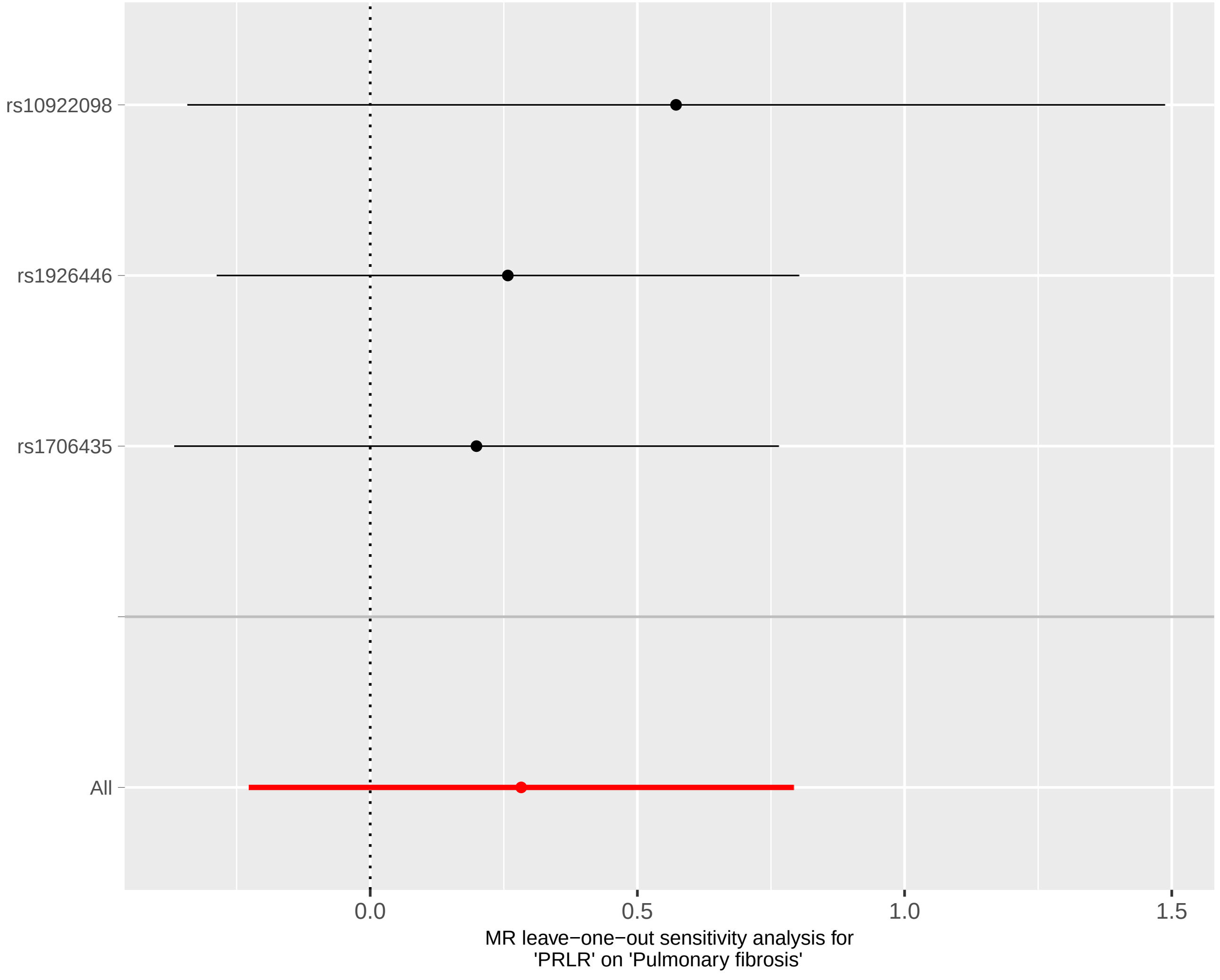

rs10922098
rs1926446
rs1706435
All
0.0
0.5
1.0
1.5
MR leave−one−out sensitivity analysis for
'PRLR' on 'Pulmonary fibrosis'

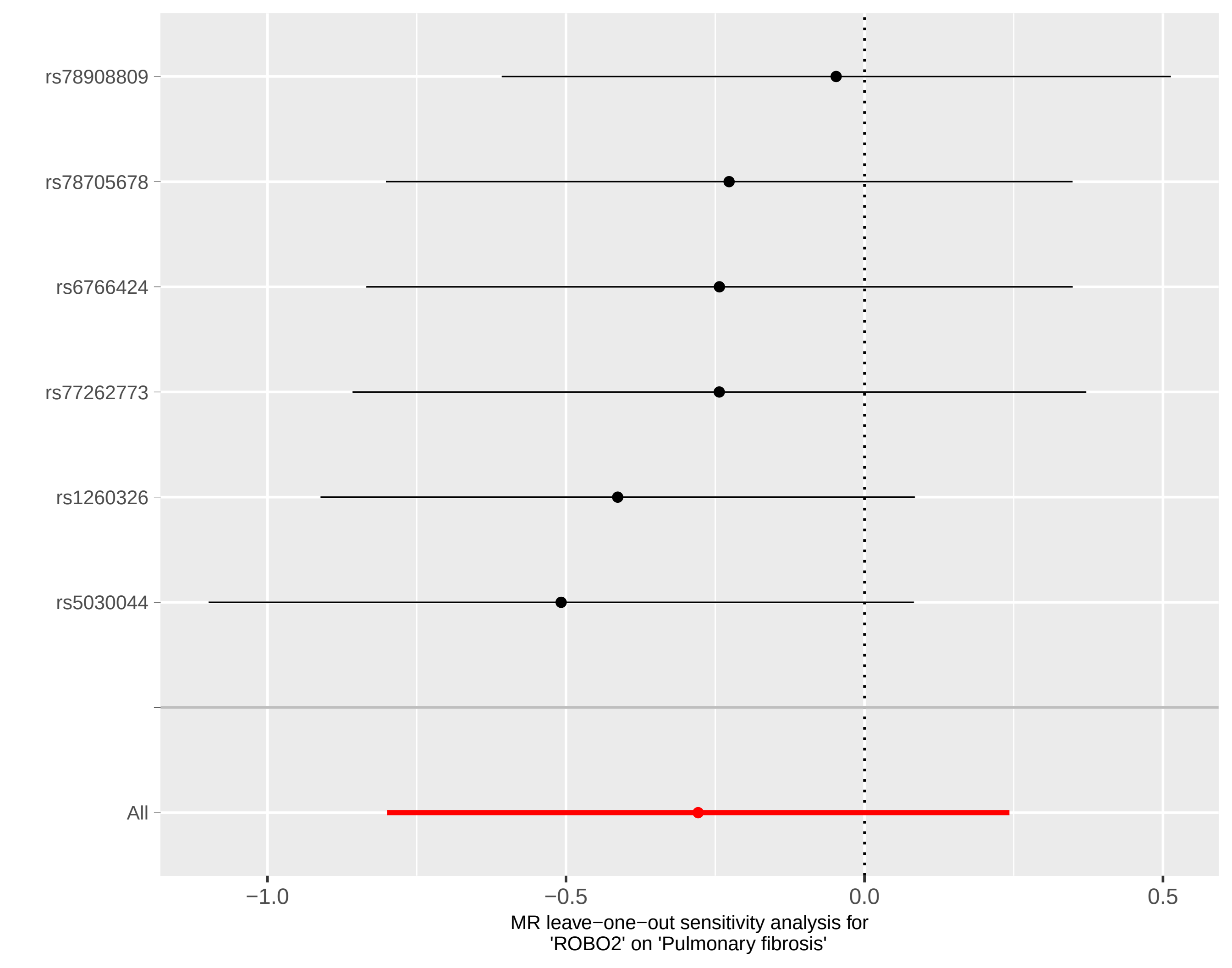
rs78908809
rs78705678
rs6766424
rs77262773
rs1260326
rs5030044
All
−1.0
−0.5
0.0
0.5
MR leave−one−out sensitivity analysis for
'ROBO2' on 'Pulmonary fibrosis'

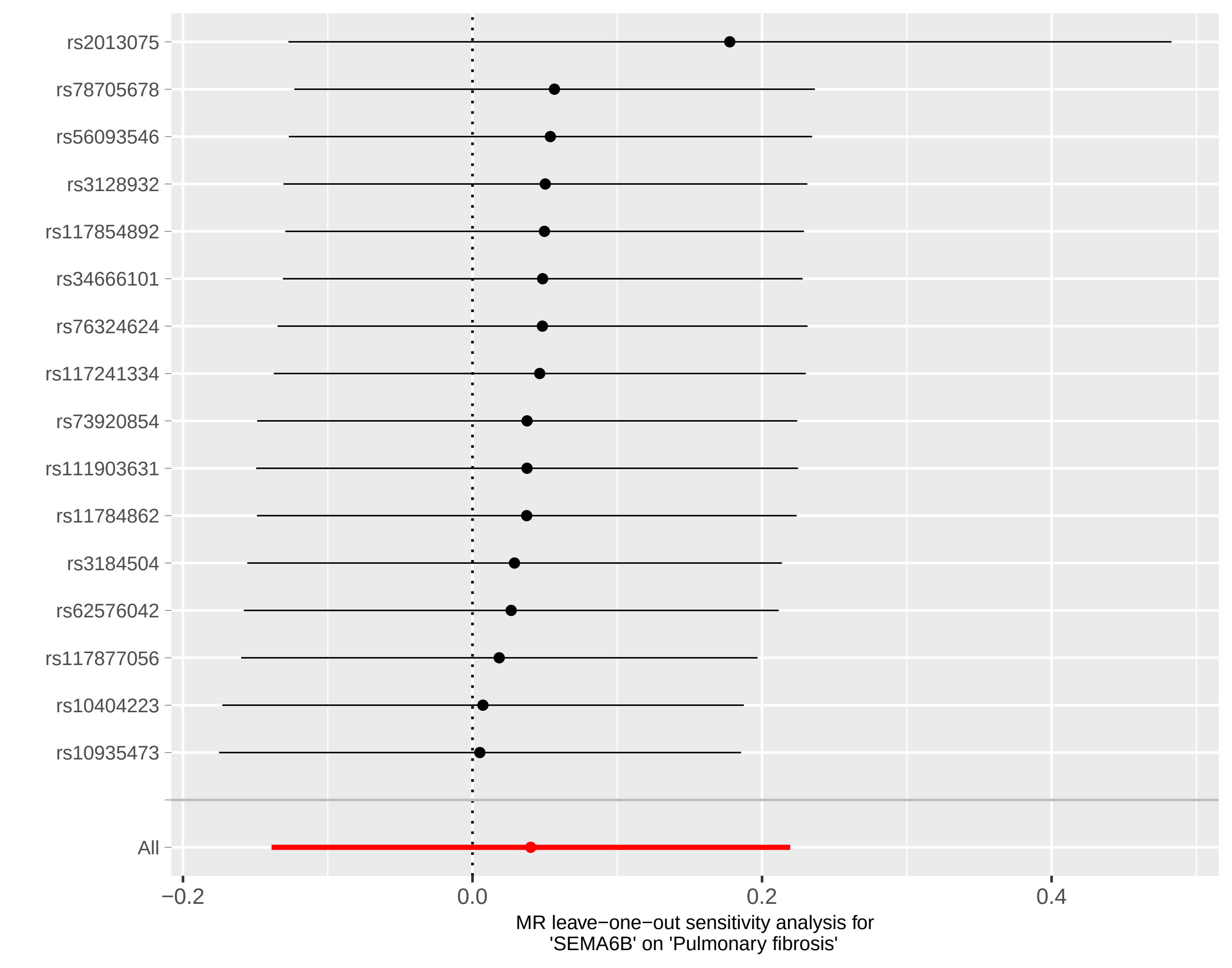

rs2013075
rs78705678
rs56093546
rs3128932
rs117854892
rs34666101
rs76324624
rs117241334
rs73920854
rs111903631
rs11784862
rs3184504
rs62576042
rs117877056
rs10404223
rs10935473
All
−0.2
0.0
0.2
0.4
MR leave−one−out sensitivity analysis for
'SEMA6B' on 'Pulmonary fibrosis'

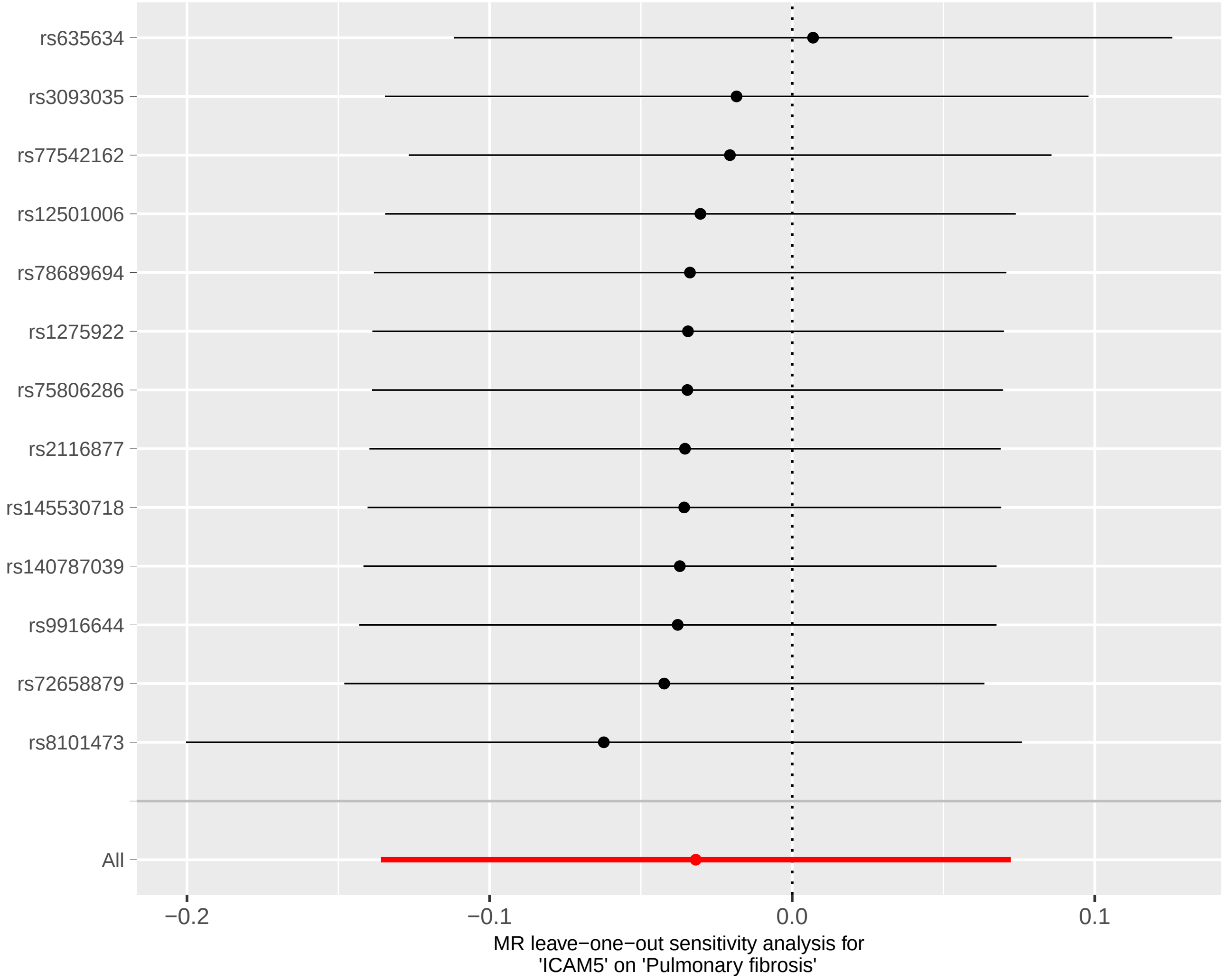
rs635634
rs3093035
rs77542162
rs12501006
rs78689694
rs1275922
rs75806286
rs2116877
rs145530718
rs140787039
rs9916644
rs72658879
rs8101473
All
−0.2
−0.1
0.0
0.1
MR leave−one−out sensitivity analysis for
'ICAM5' on 'Pulmonary fibrosis'

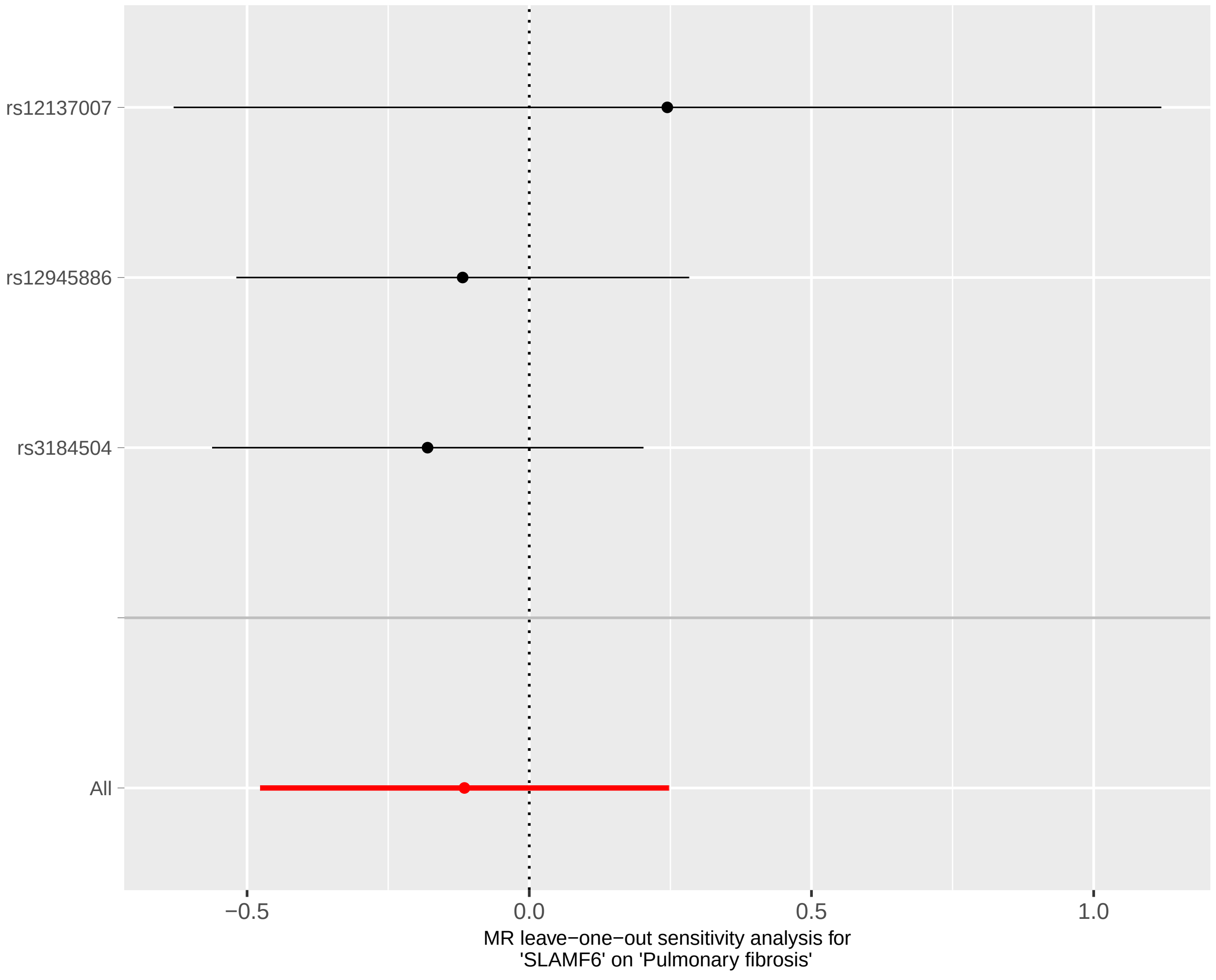
rs12137007
rs12945886
rs3184504
All
−0.5
0.0
0.5
1.0
MR leave−one−out sensitivity analysis for
'SLAMF6' on 'Pulmonary fibrosis'

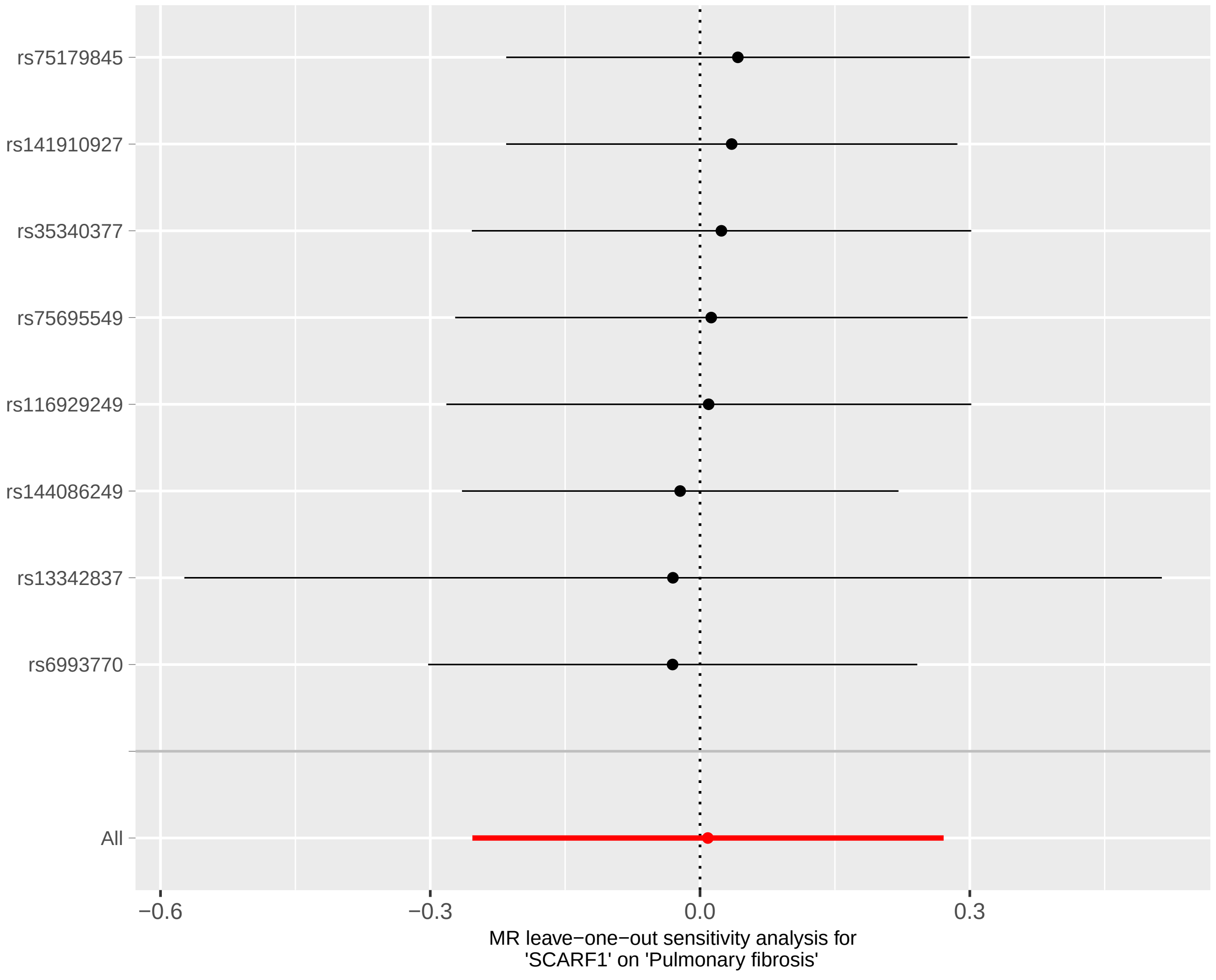
rs75179845
rs141910927
rs35340377
rs75695549
rs116929249
rs144086249
rs13342837
rs6993770
All
−0.6
−0.3
0.0
0.3
MR leave−one−out sensitivity analysis for
'SCARF1' on 'Pulmonary fibrosis'

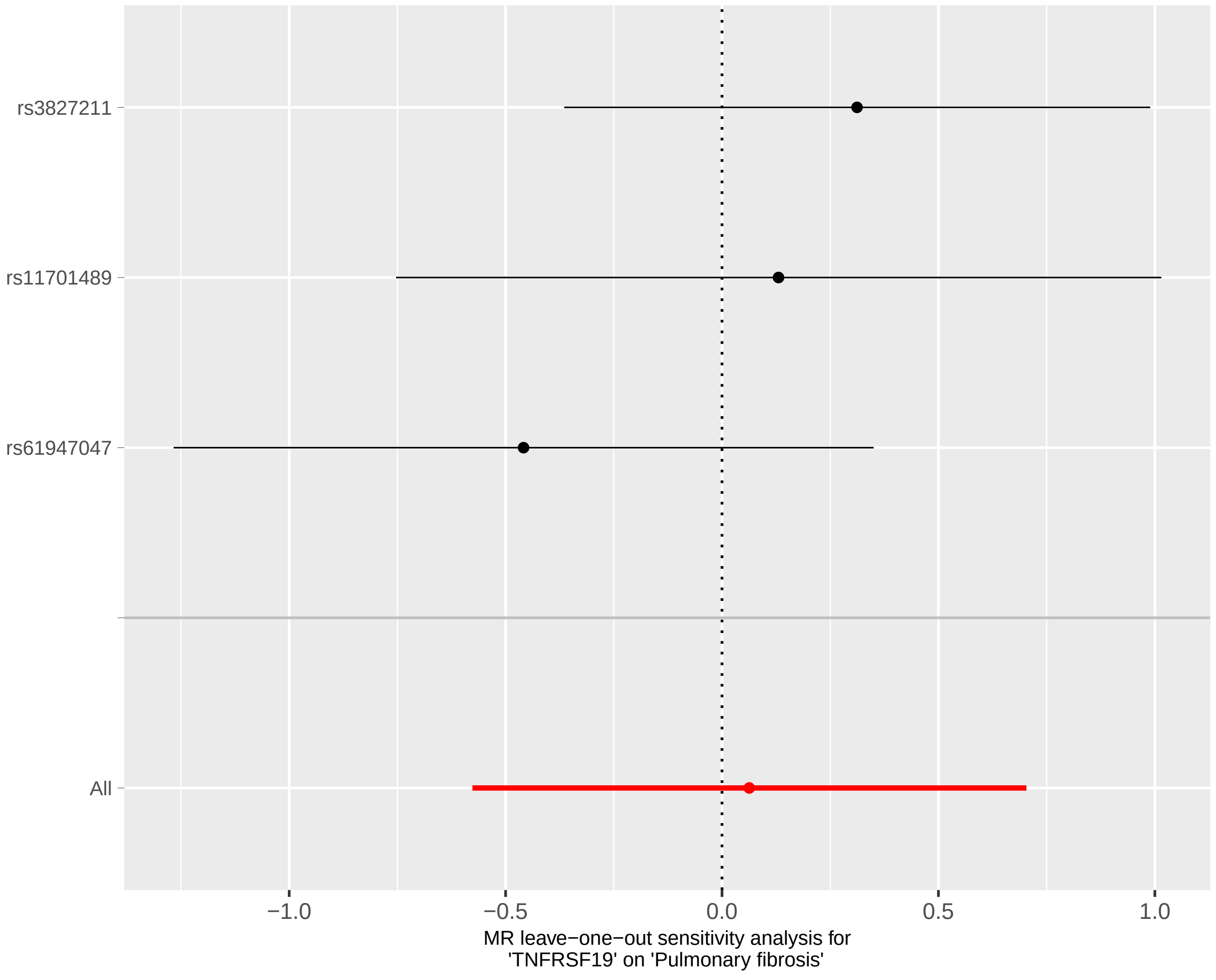
rs3827211
rs11701489
rs61947047
All
−1.0
−0.5
0.0
0.5
1.0
MR leave−one−out sensitivity analysis for
'TNFRSF19' on 'Pulmonary fibrosis'

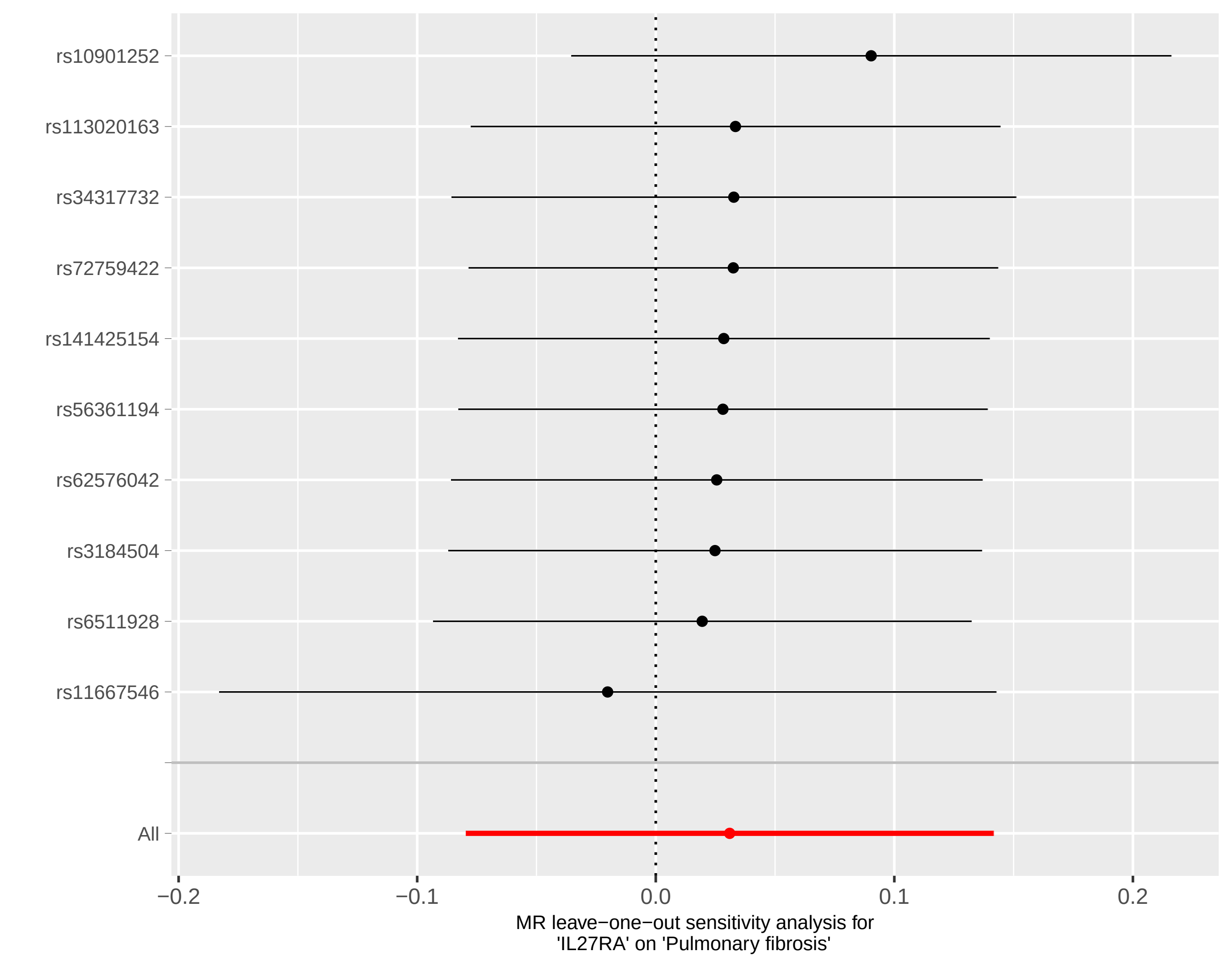
rs10901252
rs113020163
rs34317732
rs72759422
rs141425154
rs56361194
rs62576042
rs3184504
rs6511928
rs11667546
All
−0.2
−0.1
0.0
0.1
0.2
MR leave−one−out sensitivity analysis for
'IL27RA' on 'Pulmonary fibrosis'

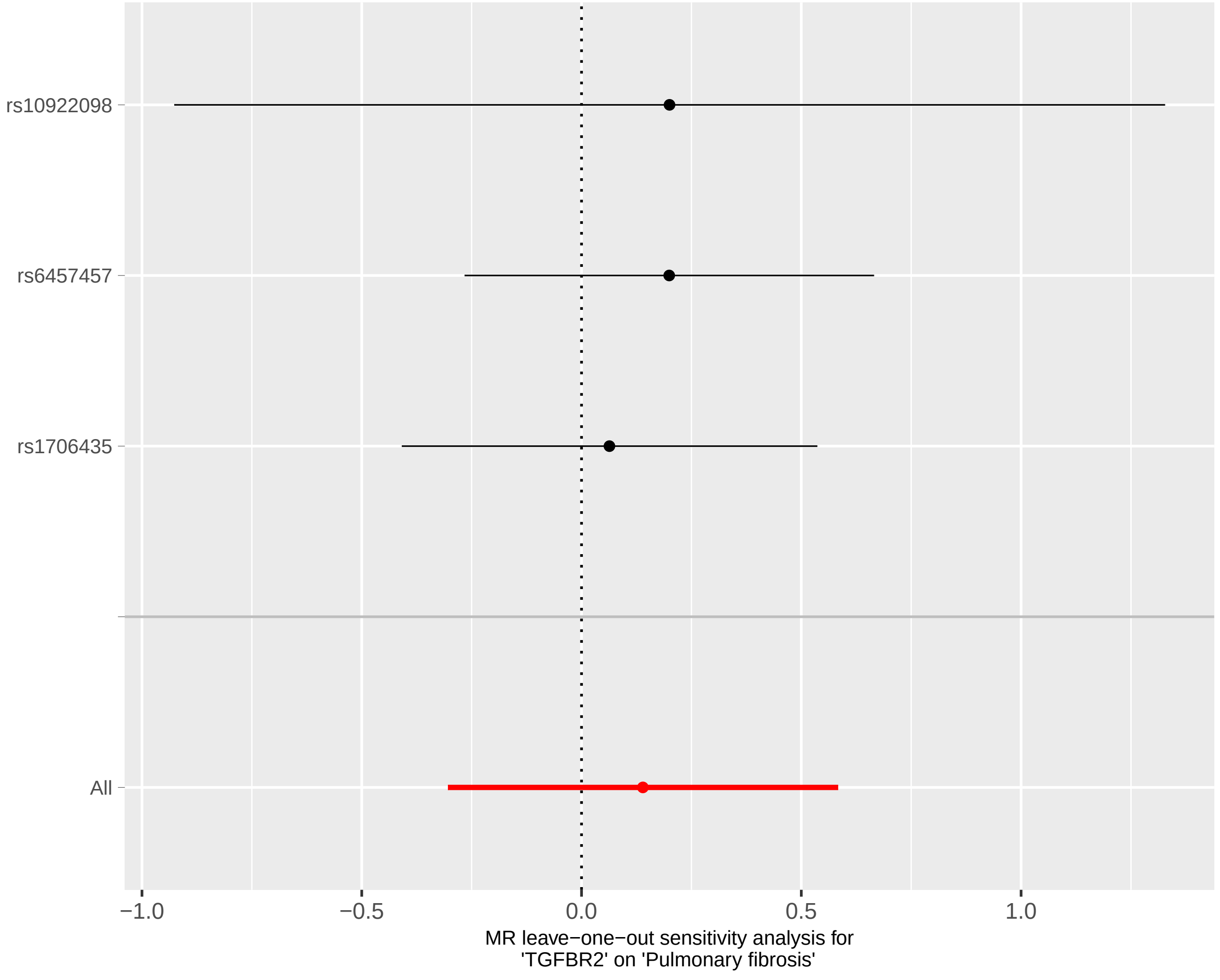
rs10922098
rs6457457
rs1706435
All
−1.0
−0.5
0.0
0.5
1.0
MR leave−one−out sensitivity analysis for
'TGFBR2' on 'Pulmonary fibrosis'

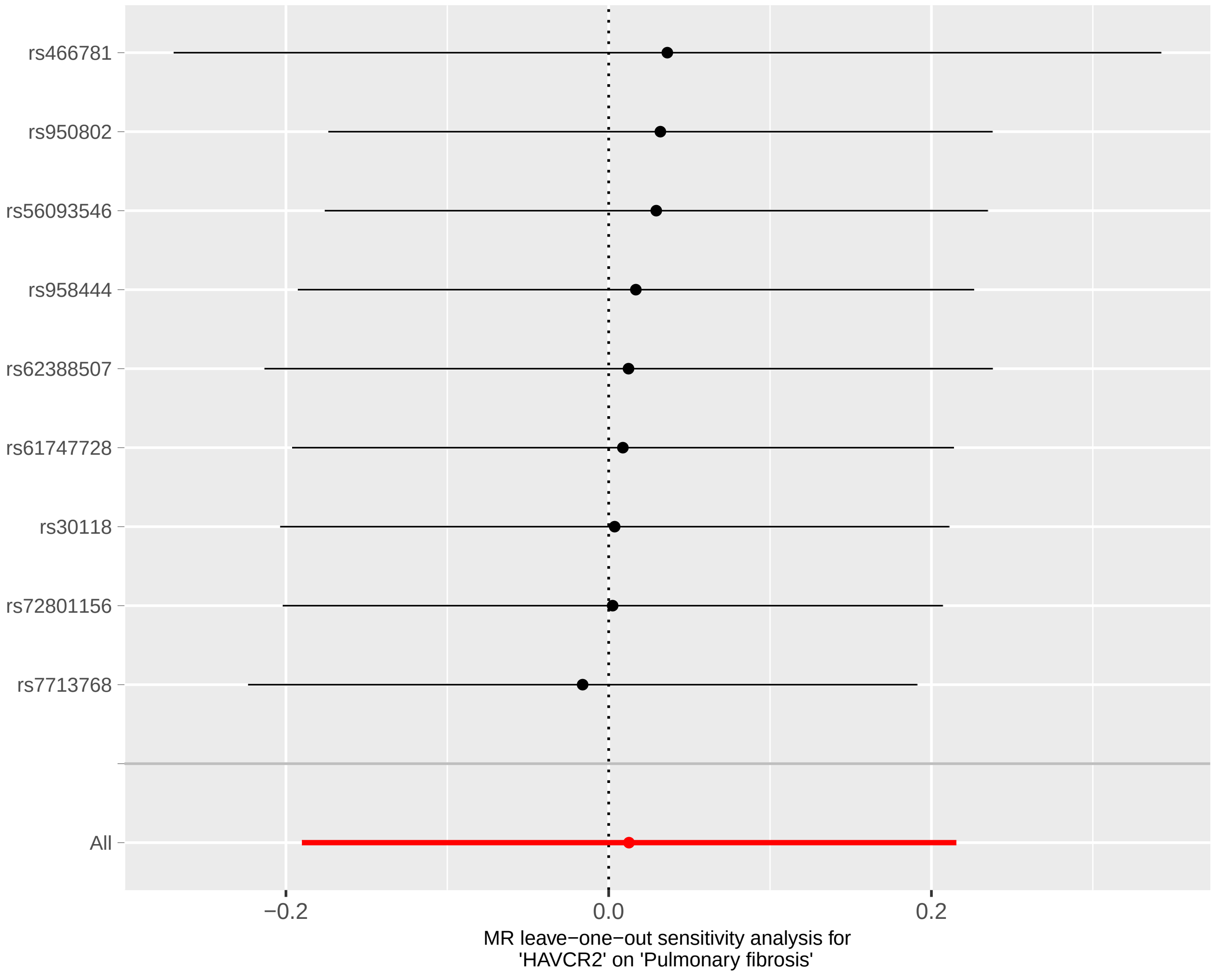
rs466781
rs950802
rs56093546
rs958444
rs62388507
rs61747728
rs30118
rs72801156
rs7713768
All
−0.2
0.0
0.2
MR leave−one−out sensitivity analysis for
'HAVCR2' on 'Pulmonary fibrosis'

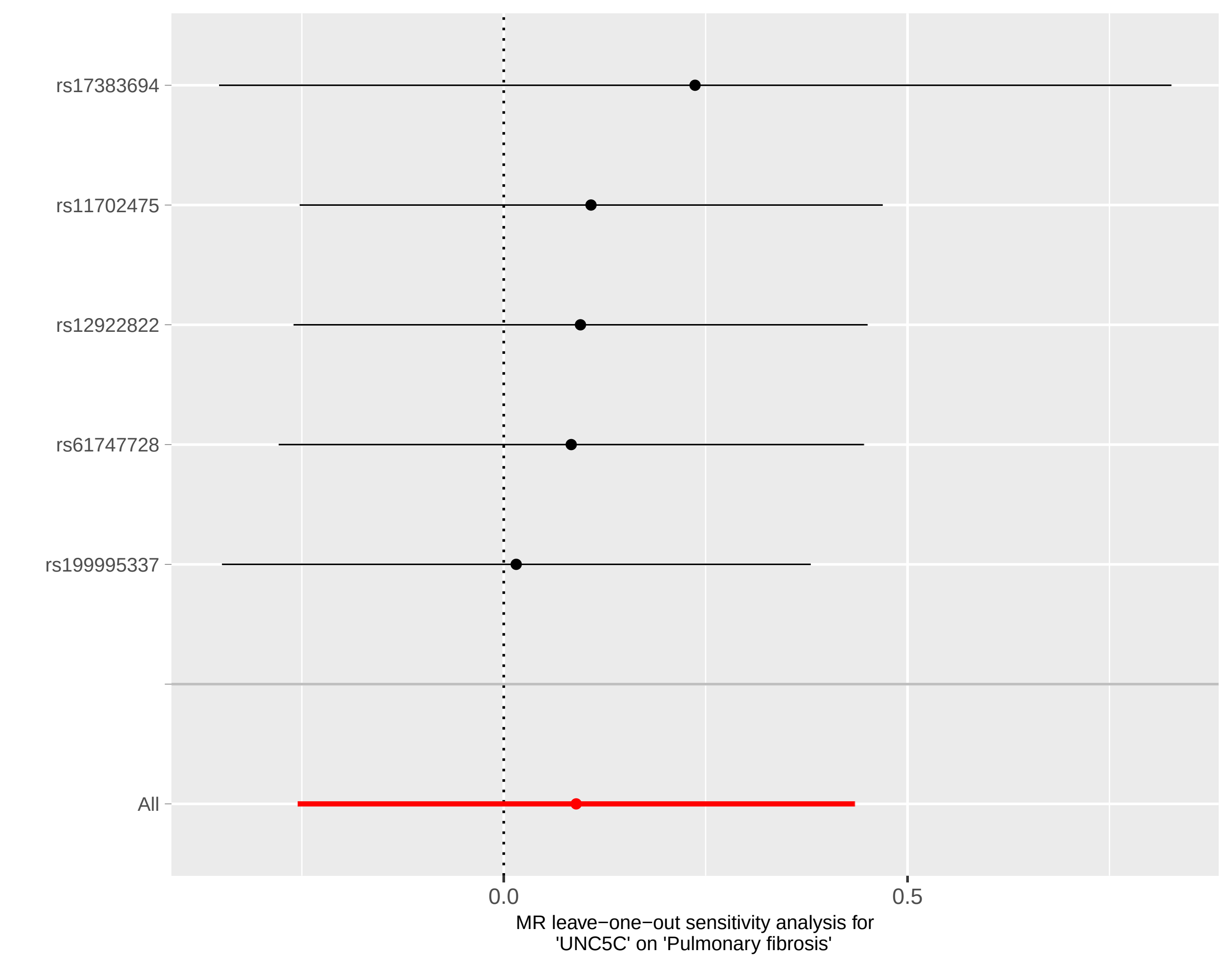
rs17383694
rs11702475
rs12922822
rs61747728
rs199995337
All
0.0
0.5
MR leave−one−out sensitivity analysis for
'UNC5C' on 'Pulmonary fibrosis'

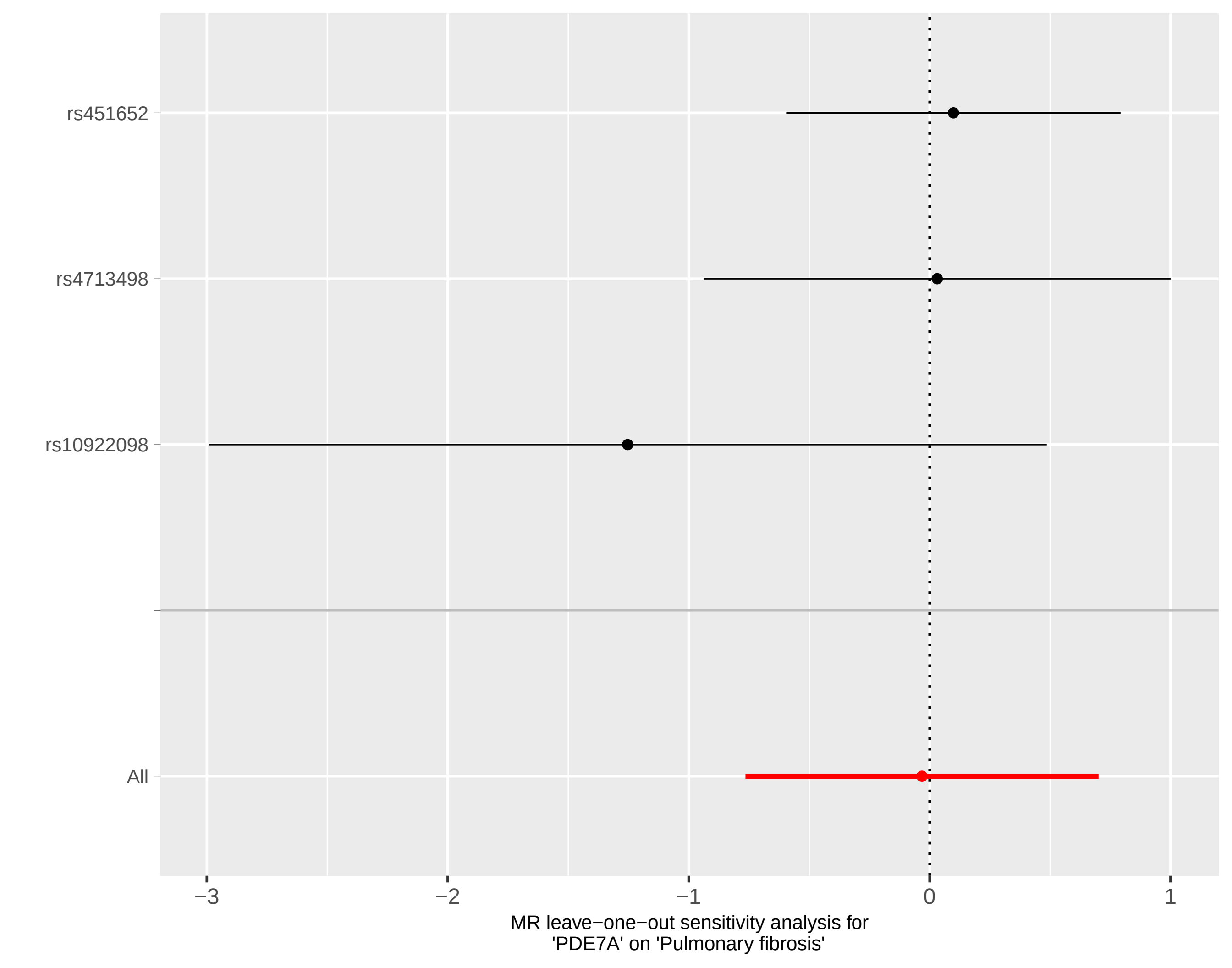
rs451652
rs4713498
rs10922098
All
−3
−2
−1
0
1
MR leave−one−out sensitivity analysis for
'PDE7A' on 'Pulmonary fibrosis'

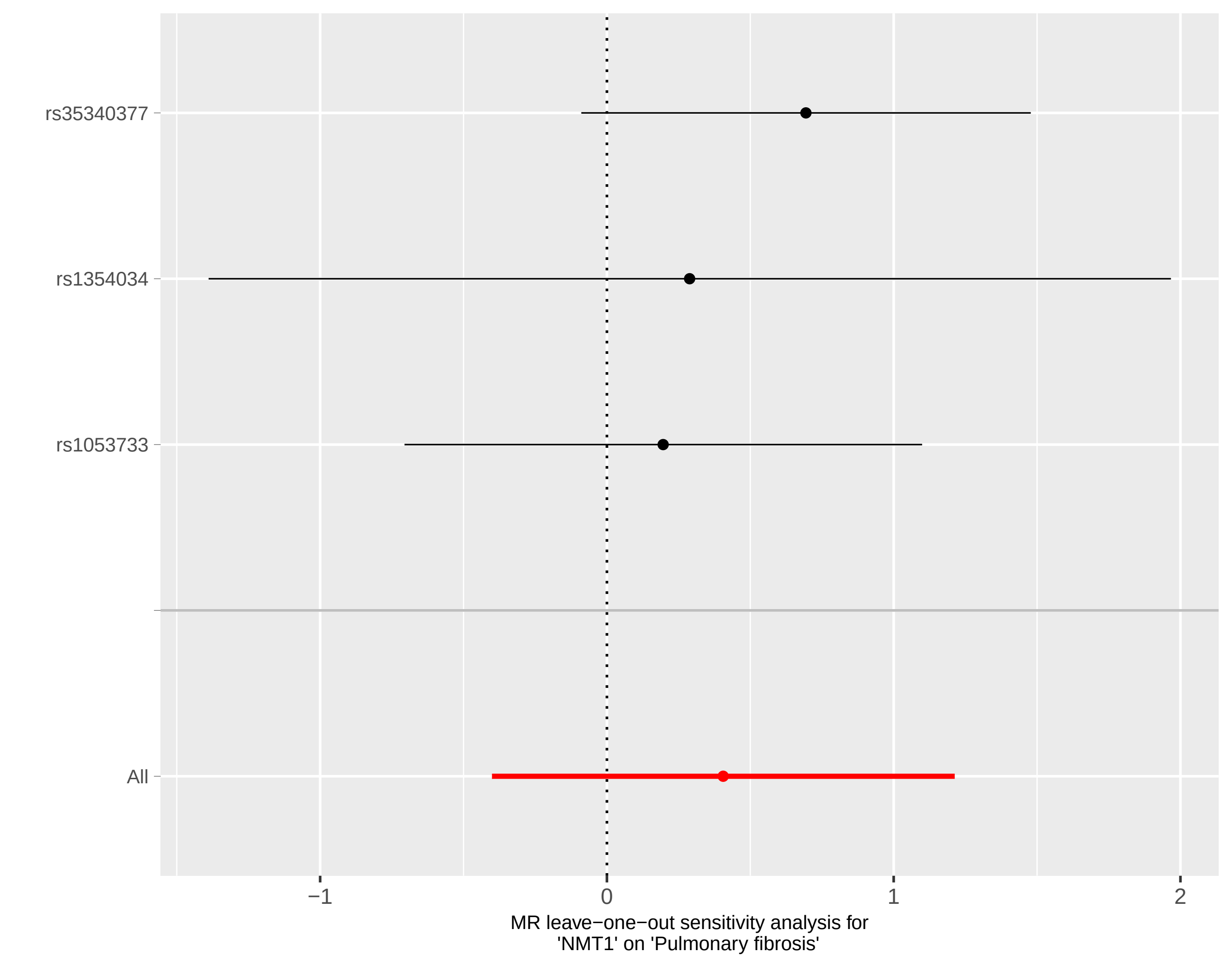
rs35340377
rs1354034
rs1053733
All
−1
0
1
2
MR leave−one−out sensitivity analysis for
'NMT1' on 'Pulmonary fibrosis'

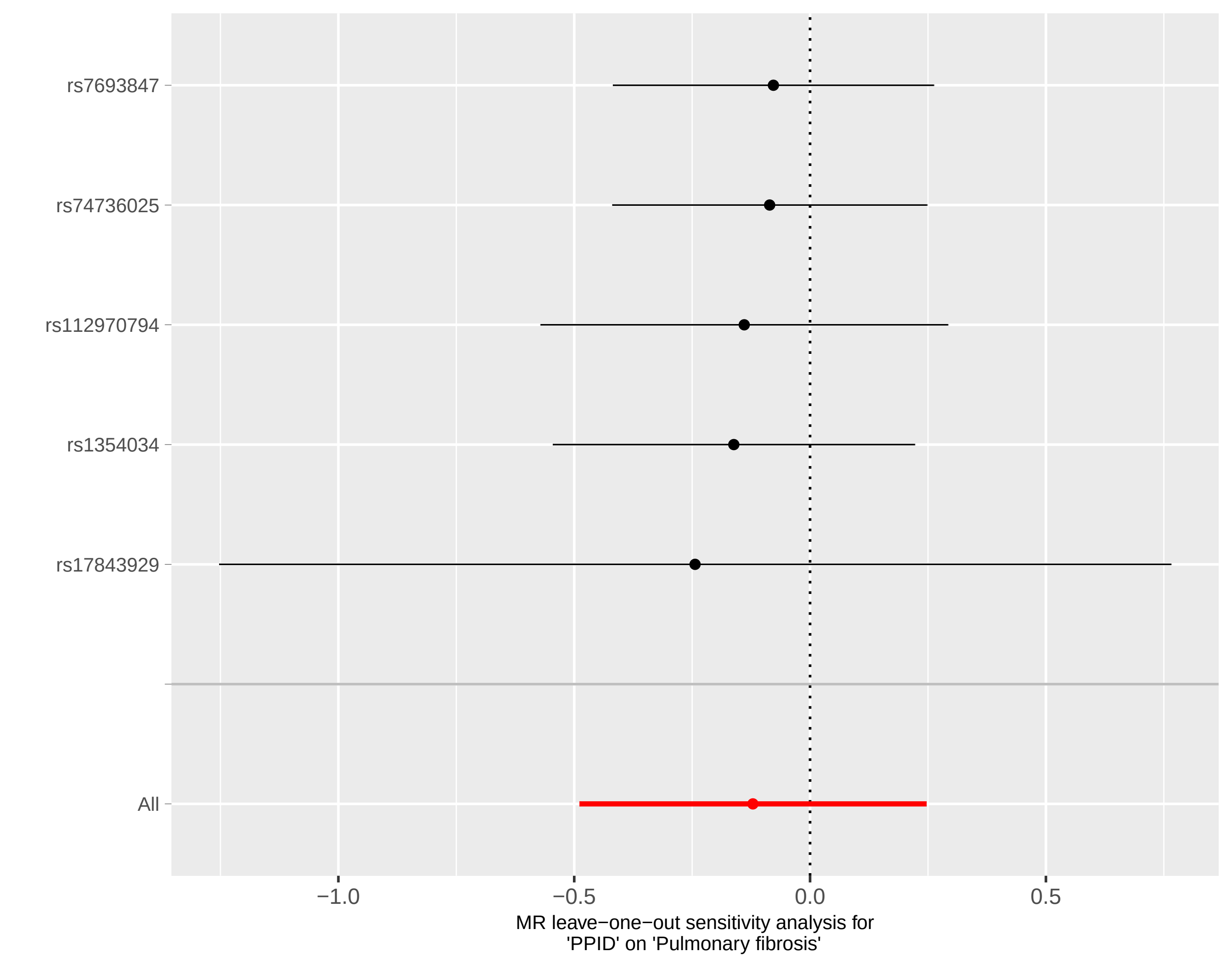
rs7693847
rs74736025
rs112970794
rs1354034
rs17843929
All
−1.0
−0.5
0.0
0.5
MR leave−one−out sensitivity analysis for
'PPID' on 'Pulmonary fibrosis'

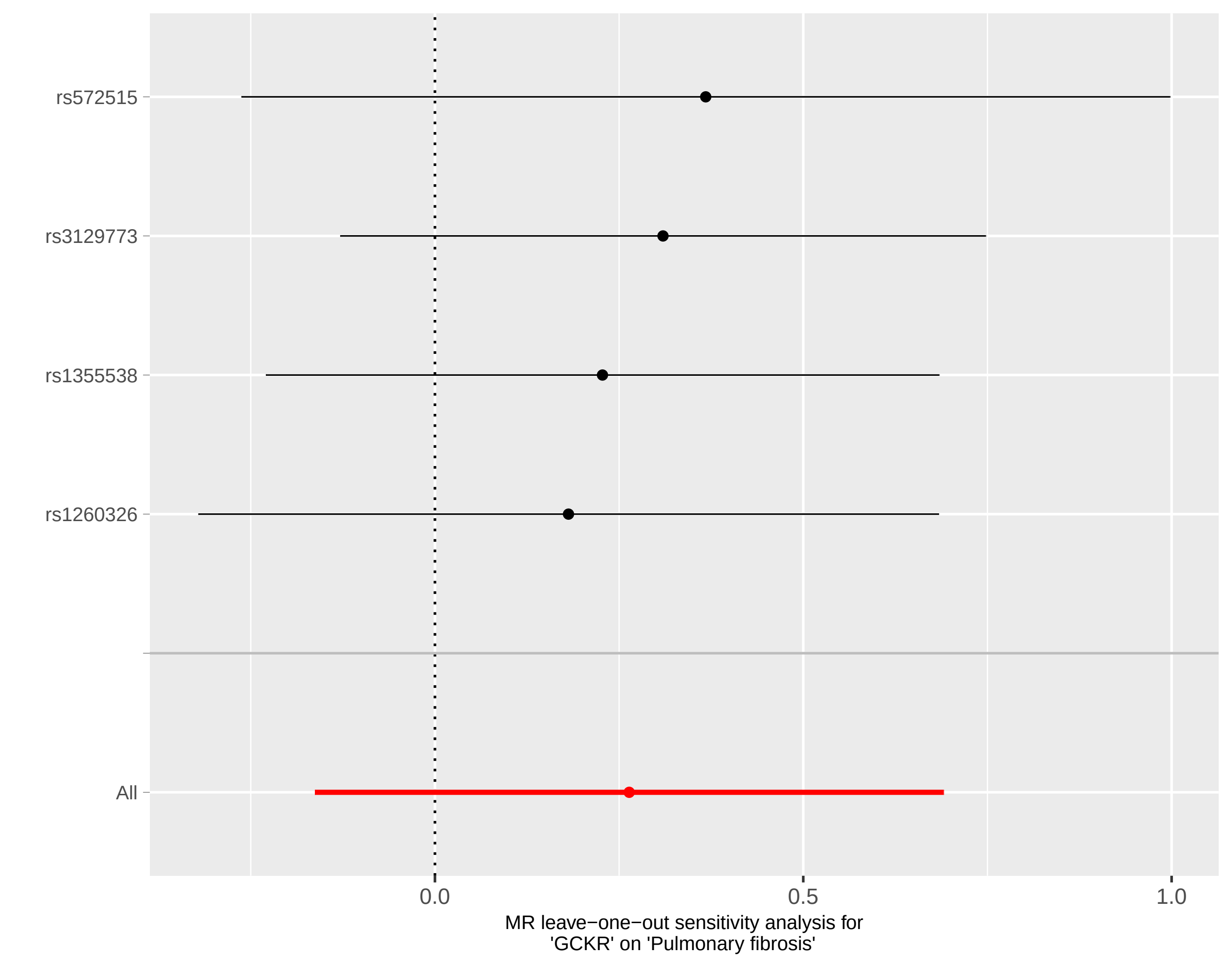
rs572515
rs3129773
rs1355538
rs1260326
All
0.0
0.5
1.0
MR leave−one−out sensitivity analysis for
'GCKR' on 'Pulmonary fibrosis'

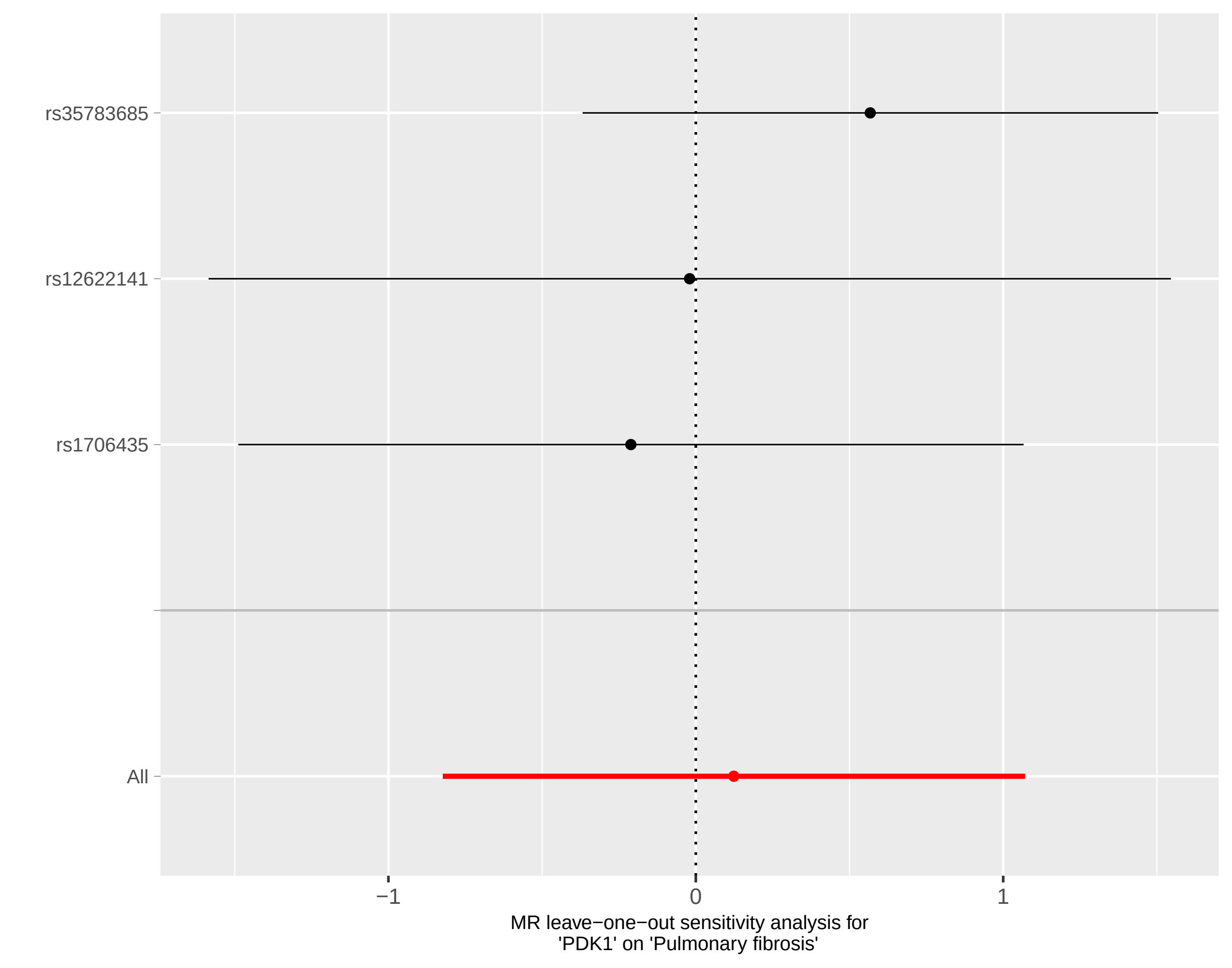
rs35783685
rs12622141
rs1706435
All
−1
0
1
MR leave−one−out sensitivity analysis for
'PDK1' on 'Pulmonary fibrosis'

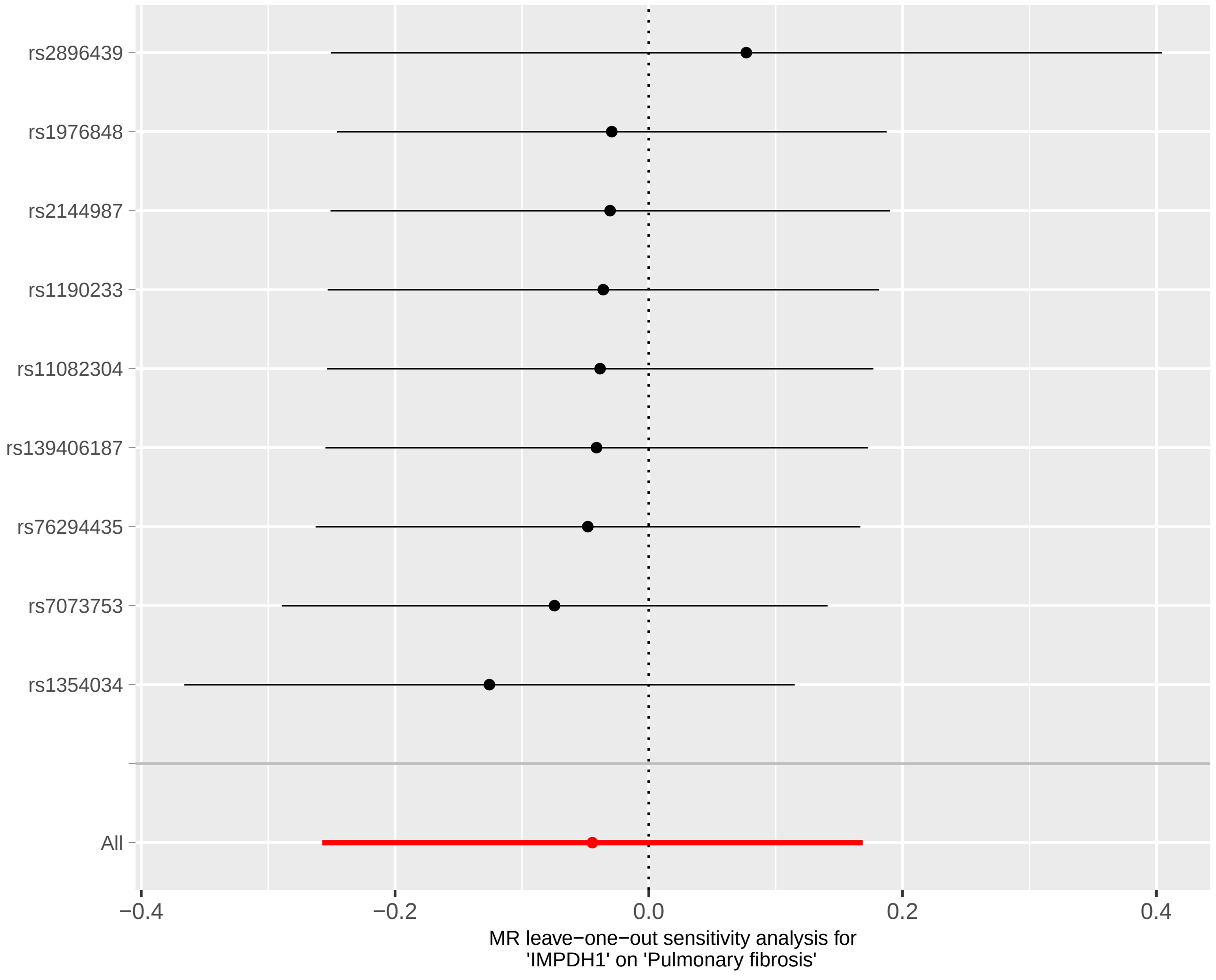

rs2896439
rs1976848
rs2144987
rs1190233
rs11082304
rs139406187
rs76294435
rs7073753
rs1354034
All
−0.4
−0.2
0.0
0.2
0.4
MR leave−one−out sensitivity analysis for
'IMPDH1' on 'Pulmonary fibrosis'

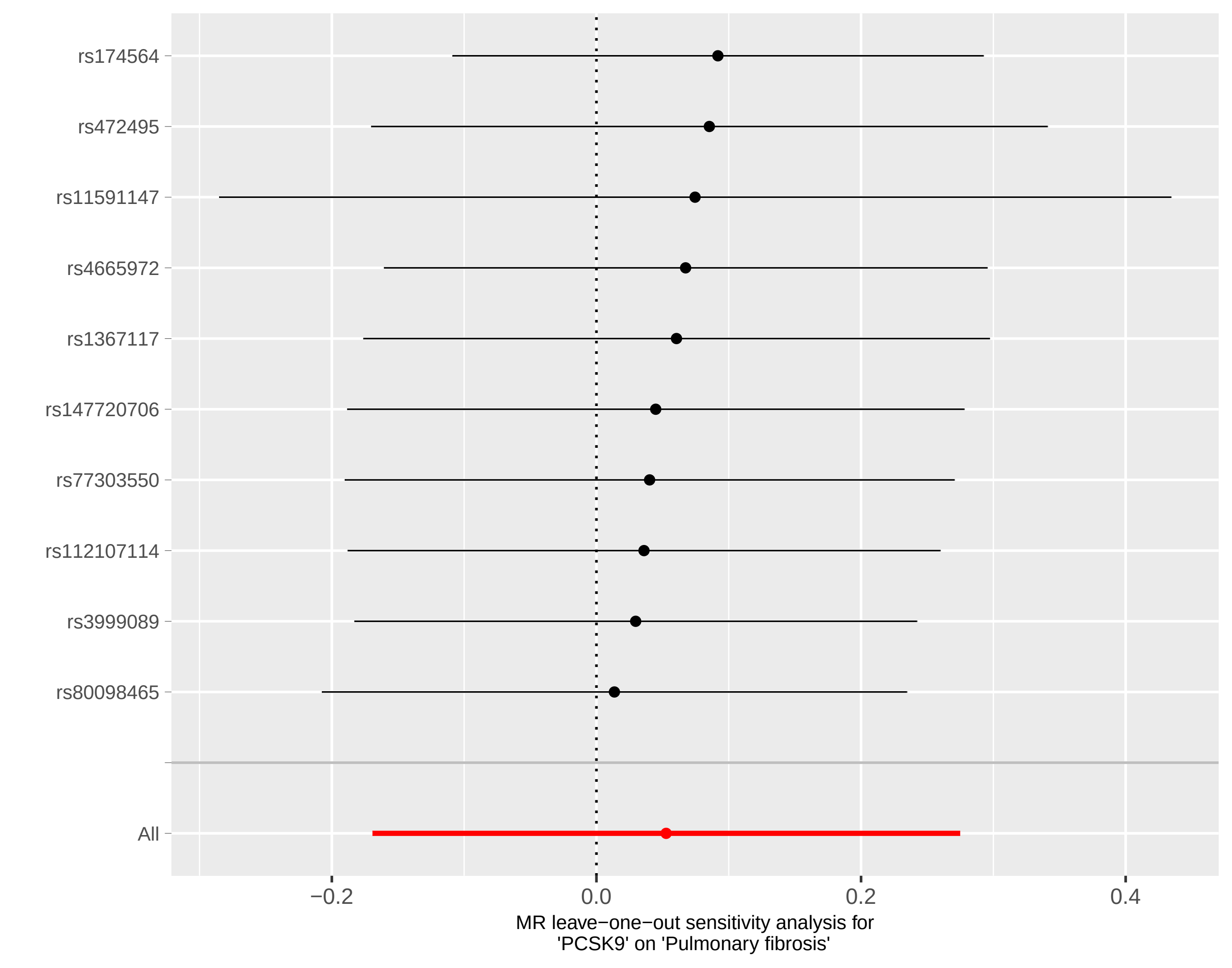
rs174564
rs472495
rs11591147
rs4665972
rs1367117
rs147720706
rs77303550
rs112107114
rs3999089
rs80098465
All
−0.2
0.0
0.2
0.4
MR leave−one−out sensitivity analysis for
'PCSK9' on 'Pulmonary fibrosis'

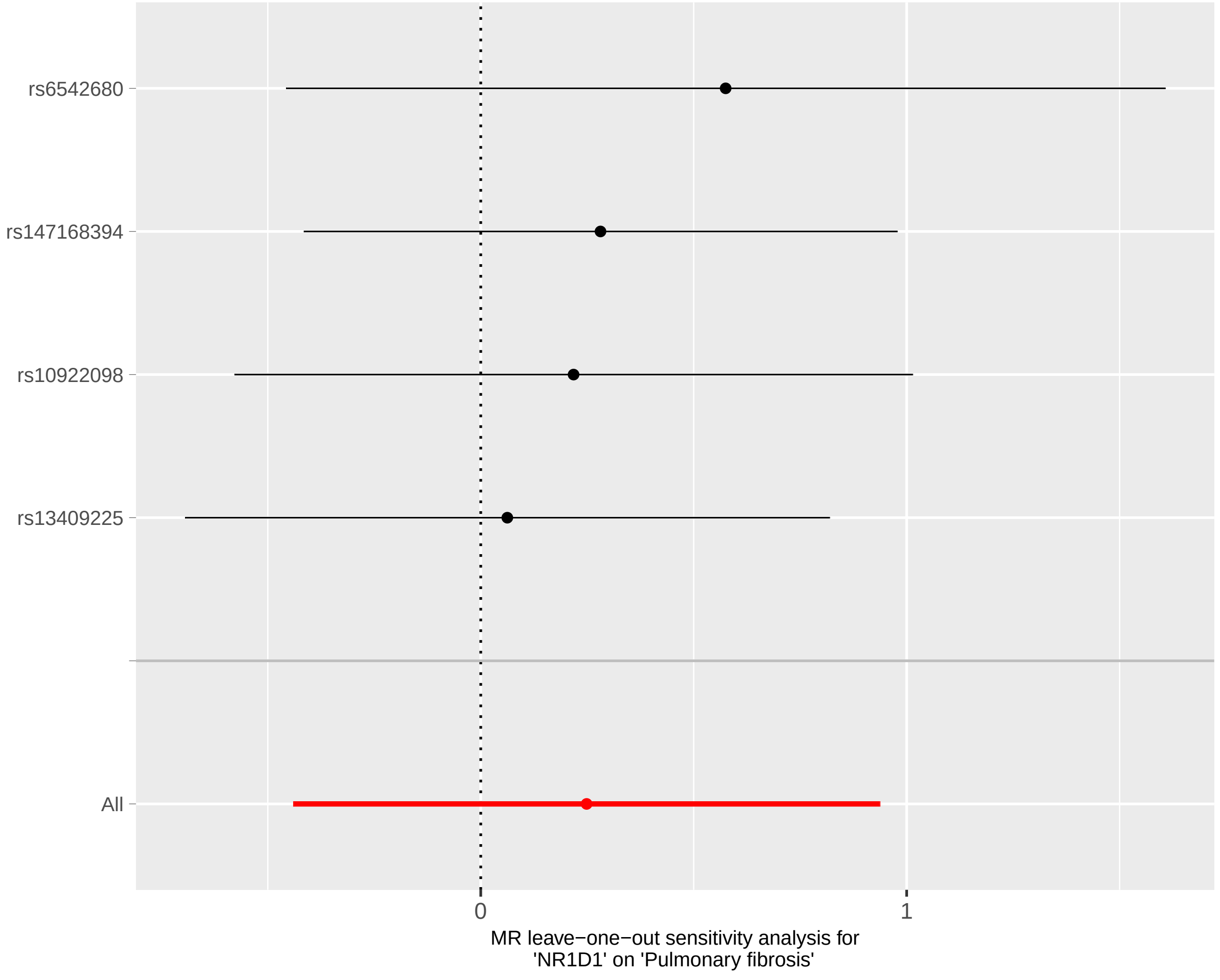
rs6542680
rs147168394
rs10922098
rs13409225
All
0
1
MR leave−one−out sensitivity analysis for
'NR1D1' on 'Pulmonary fibrosis'

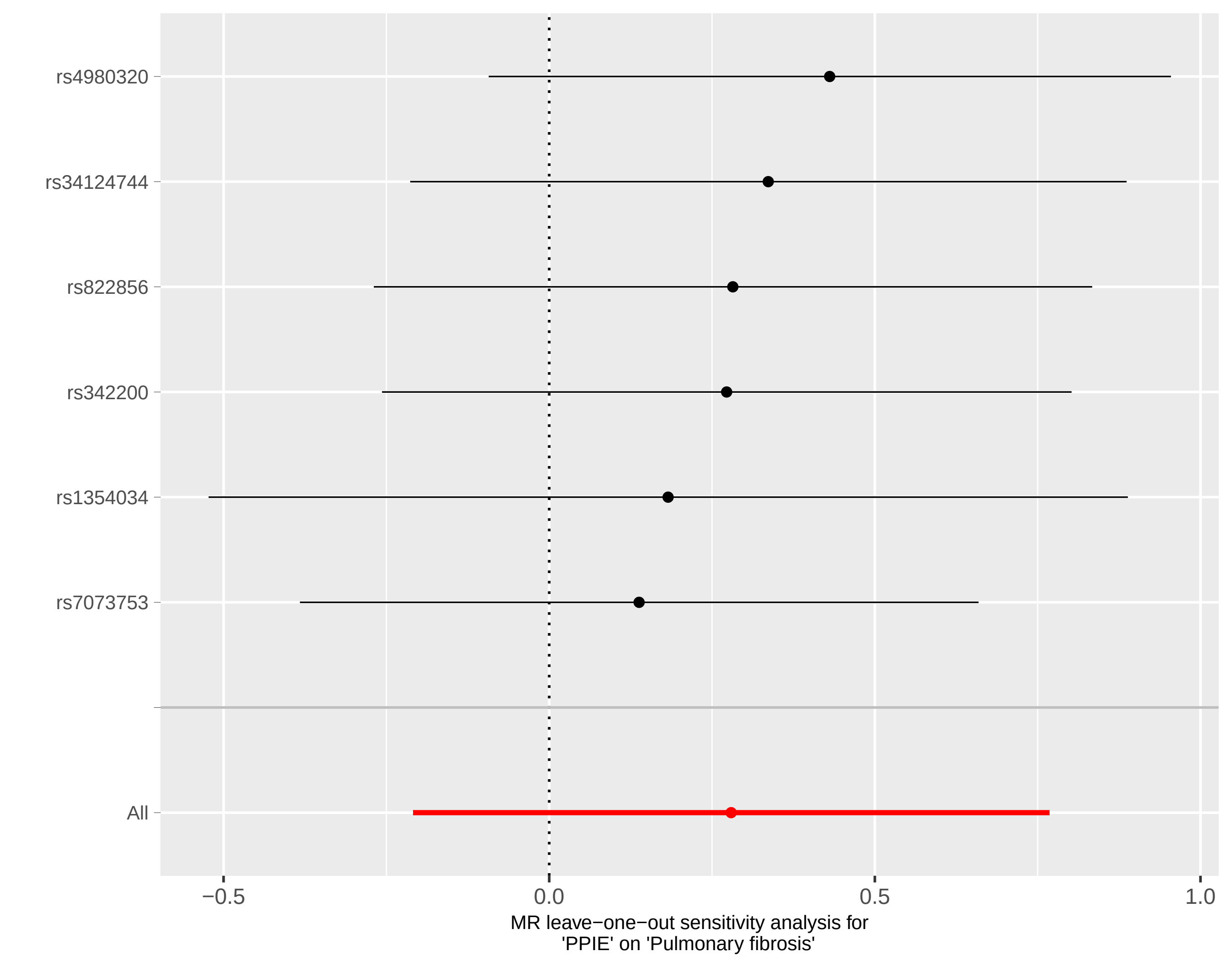
rs4980320
rs34124744
rs822856
rs342200
rs1354034
rs7073753
All
−0.5
0.0
0.5
1.0
MR leave−one−out sensitivity analysis for
'PPIE' on 'Pulmonary fibrosis'

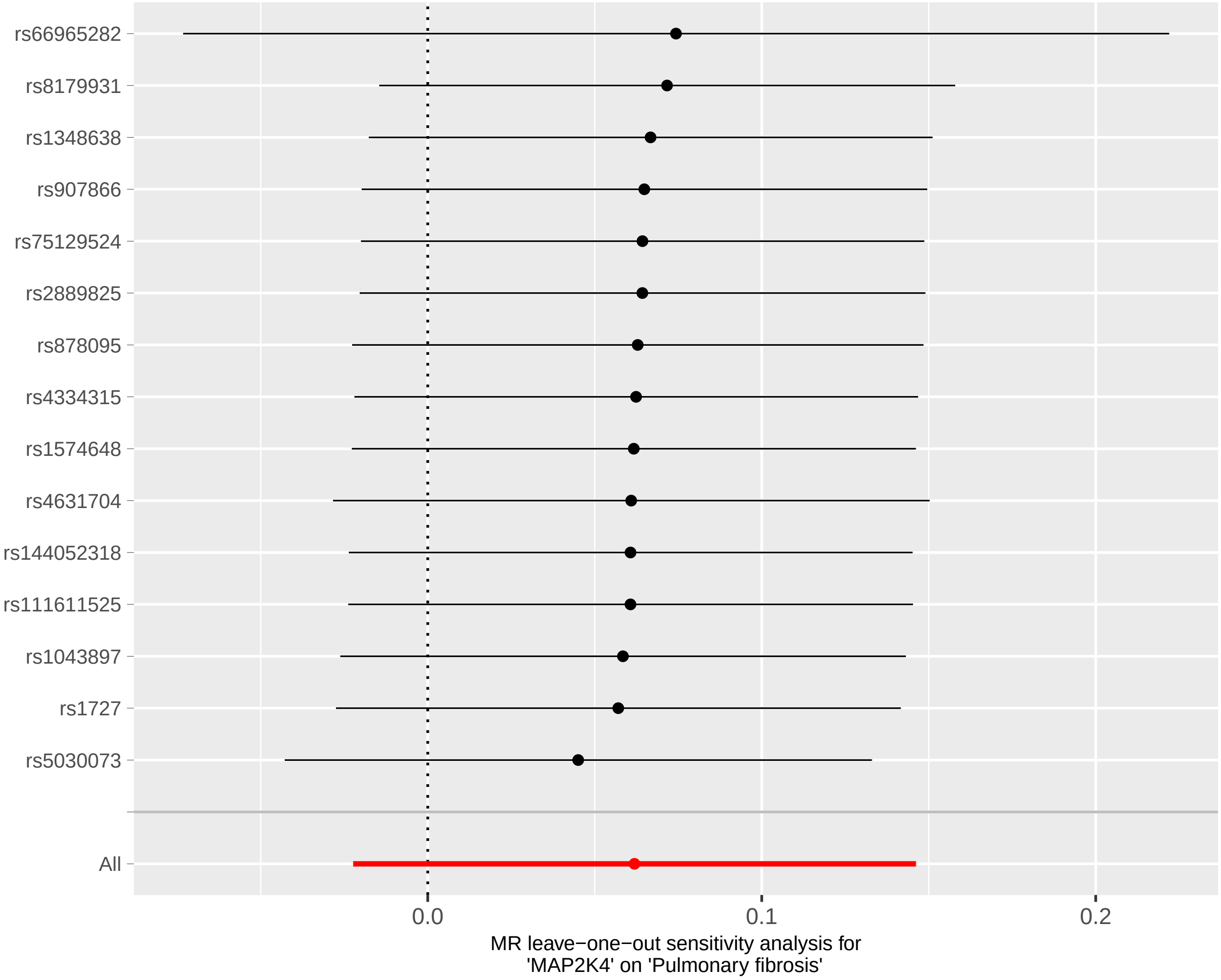
rs66965282
rs8179931
rs1348638
rs907866
rs75129524
rs2889825
rs878095
rs4334315
rs1574648
rs4631704
rs144052318
rs111611525
rs1043897
rs1727
rs5030073
All
0.0
0.1
0.2
MR leave−one−out sensitivity analysis for
'MAP2K4' on 'Pulmonary fibrosis'

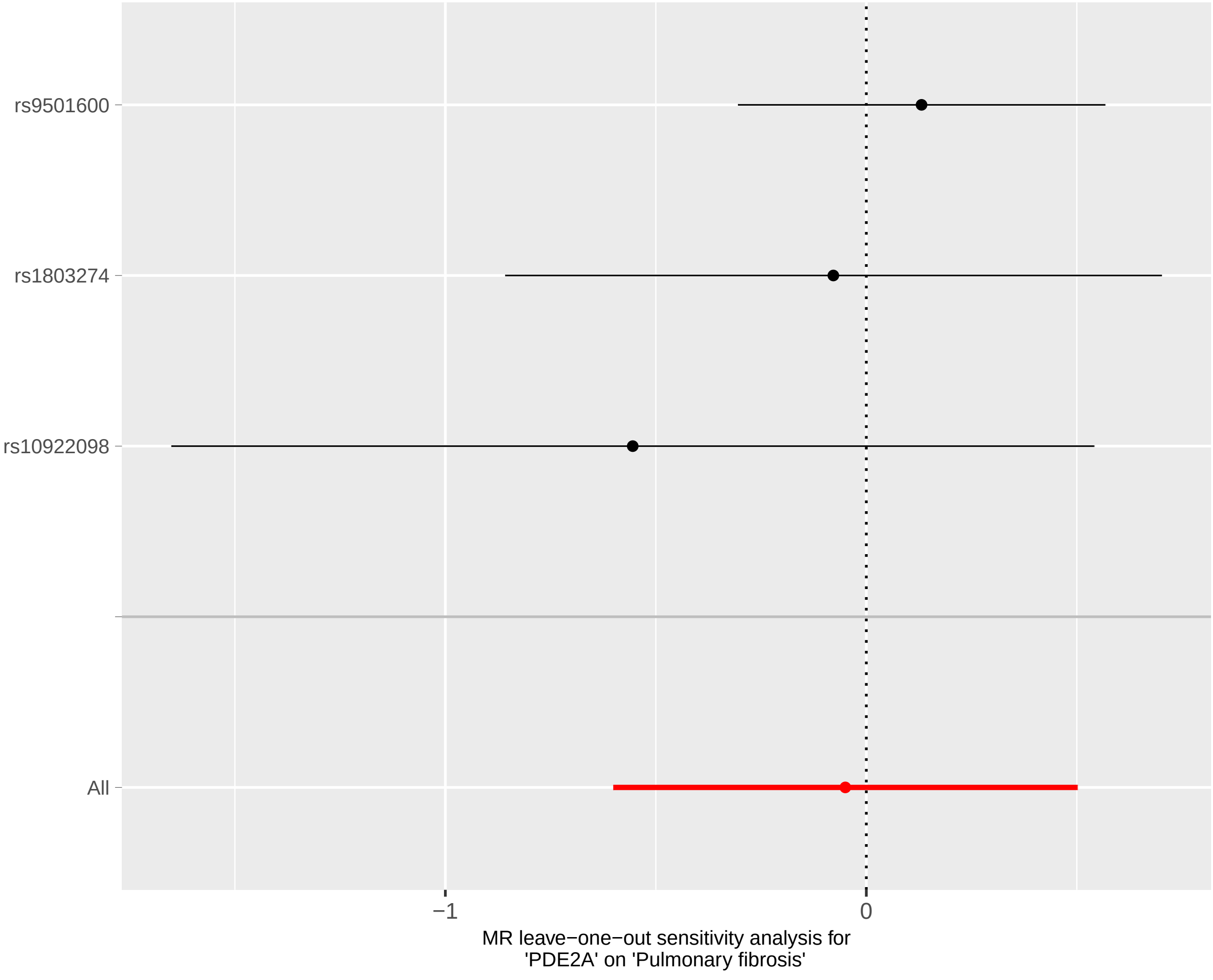
rs9501600
rs1803274
rs10922098
All
−1
0
MR leave−one−out sensitivity analysis for
'PDE2A' on 'Pulmonary fibrosis'

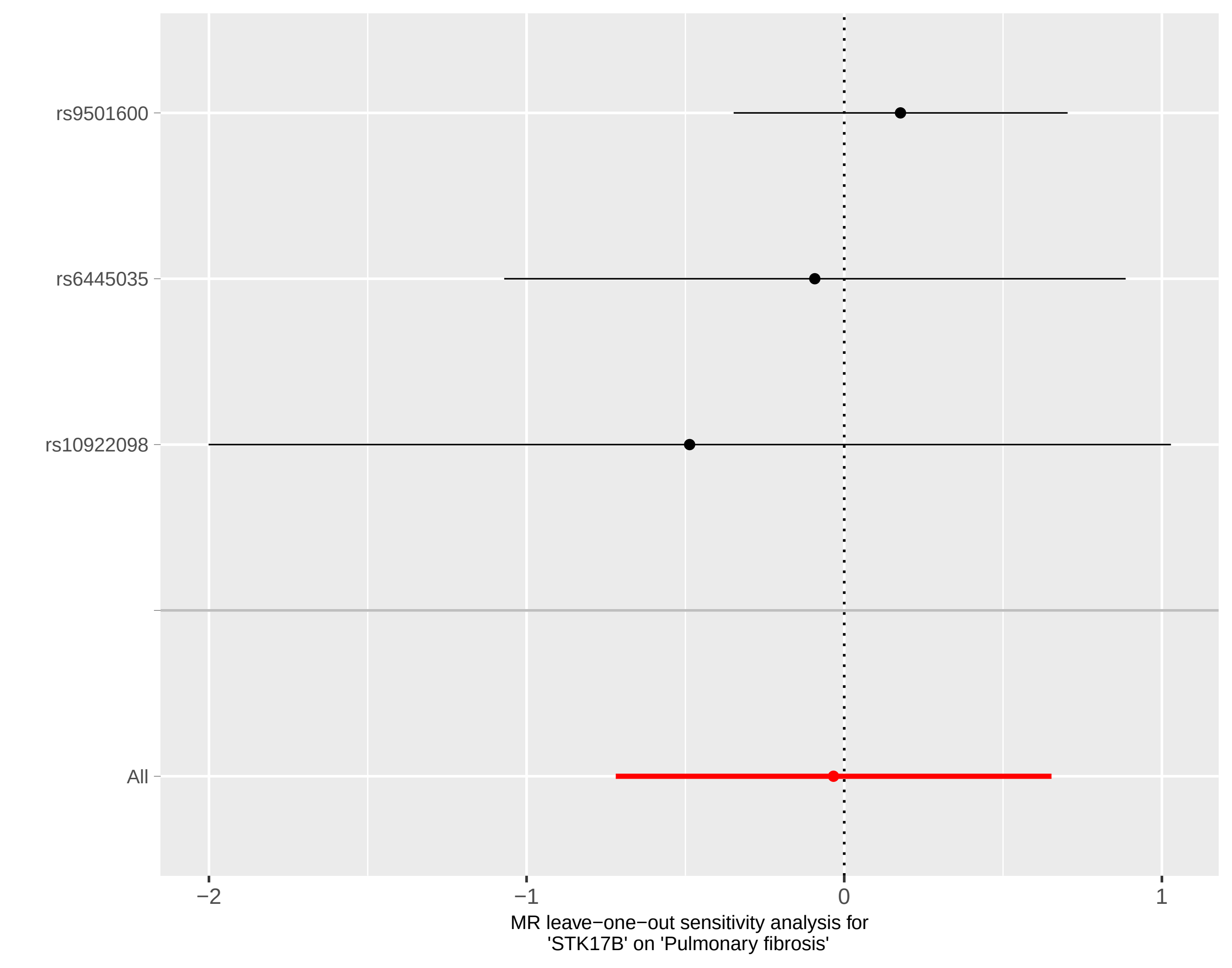
rs9501600
rs6445035
rs10922098
All
−2
−1
0
1
MR leave−one−out sensitivity analysis for
'STK17B' on 'Pulmonary fibrosis'

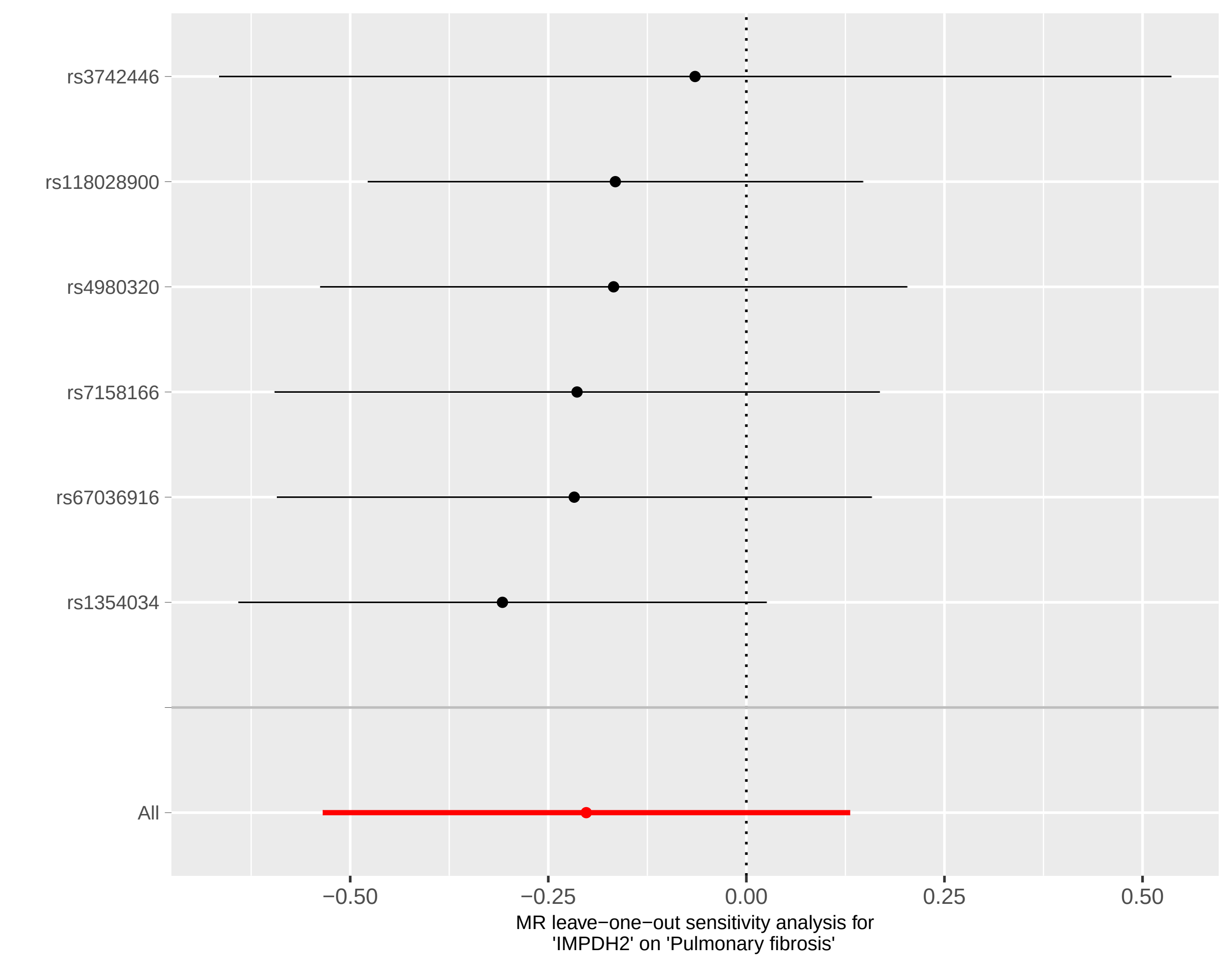
rs3742446
rs118028900
rs4980320
rs7158166
rs67036916
rs1354034
All
−0.50
−0.25
0.00
0.25
0.50
MR leave−one−out sensitivity analysis for
'IMPDH2' on 'Pulmonary fibrosis'

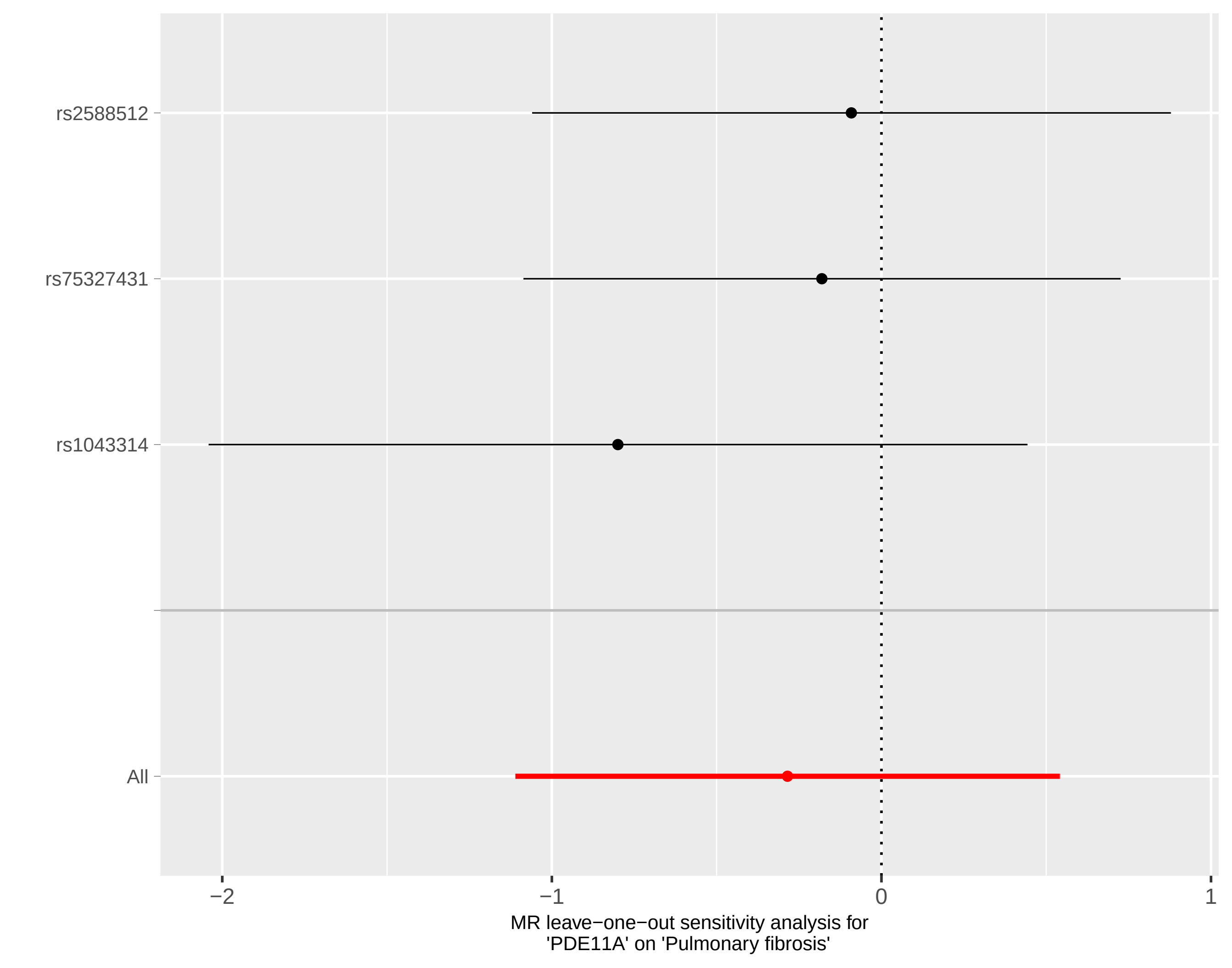
rs2588512
rs75327431
rs1043314
All
−2
−1
0
1
MR leave−one−out sensitivity analysis for
'PDE11A' on 'Pulmonary fibrosis'

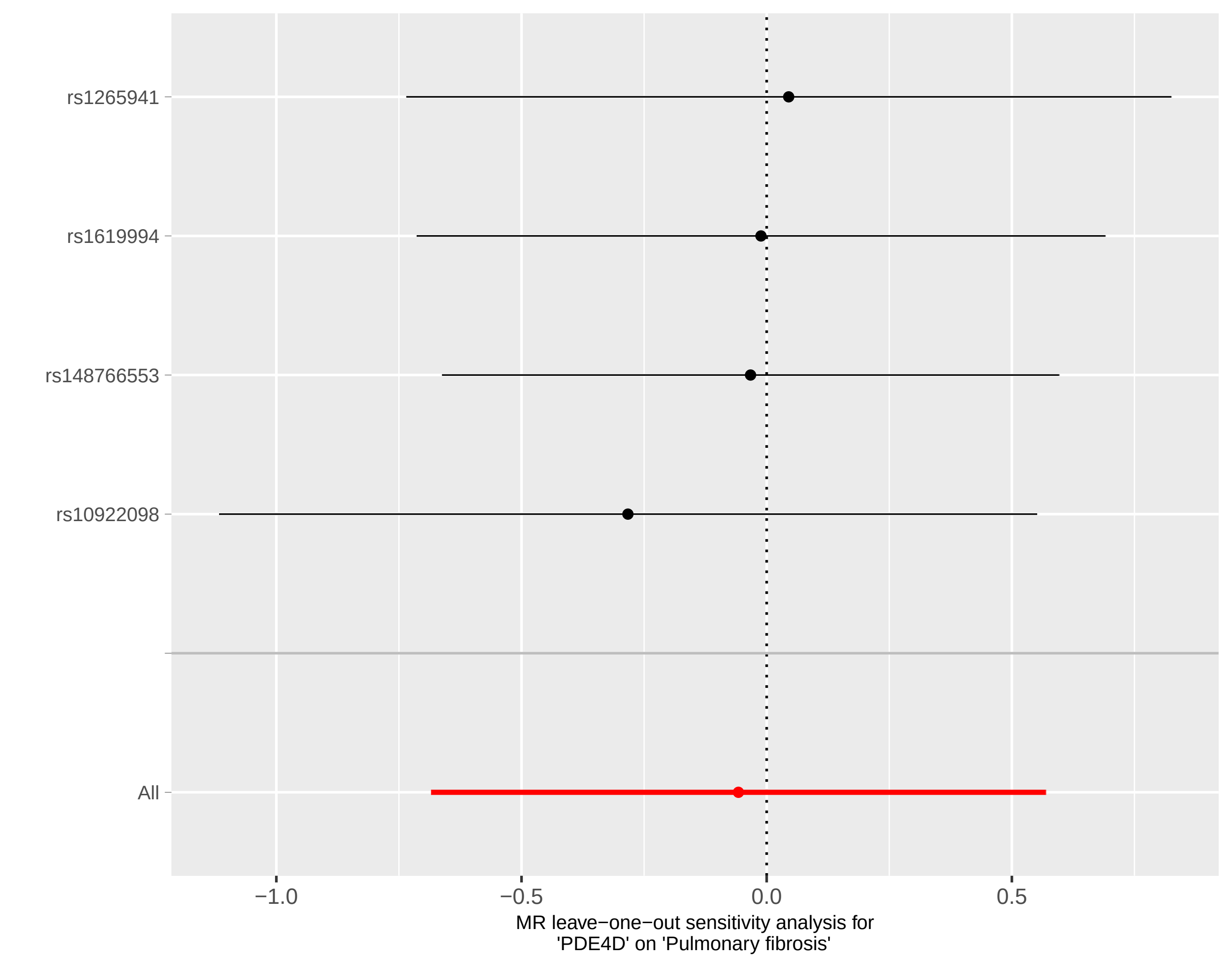
rs1265941
rs1619994
rs148766553
rs10922098
All
−1.0
−0.5
0.0
0.5
MR leave−one−out sensitivity analysis for
'PDE4D' on 'Pulmonary fibrosis'

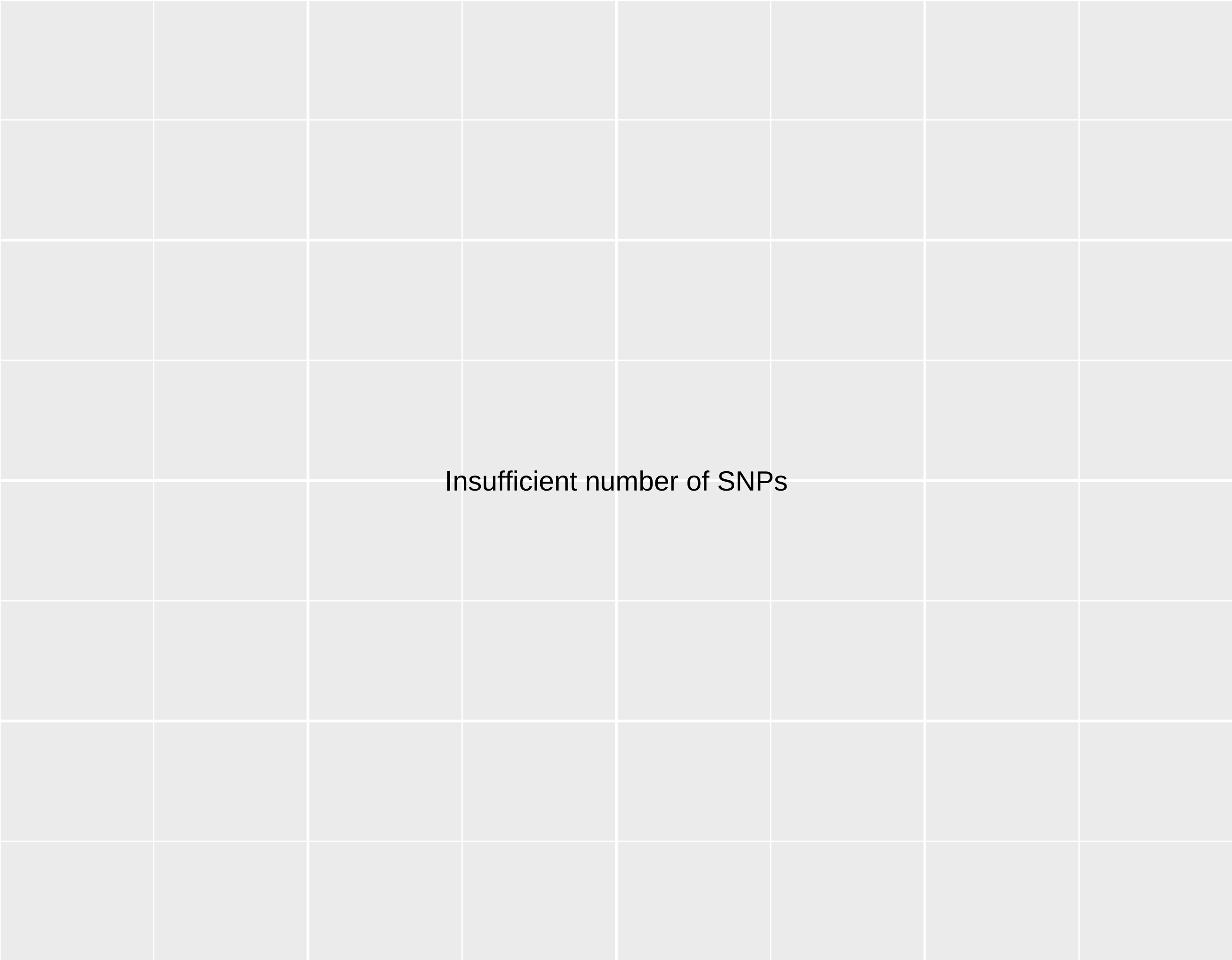
Insufficient number of SNPs

Insufficient number of SNPs

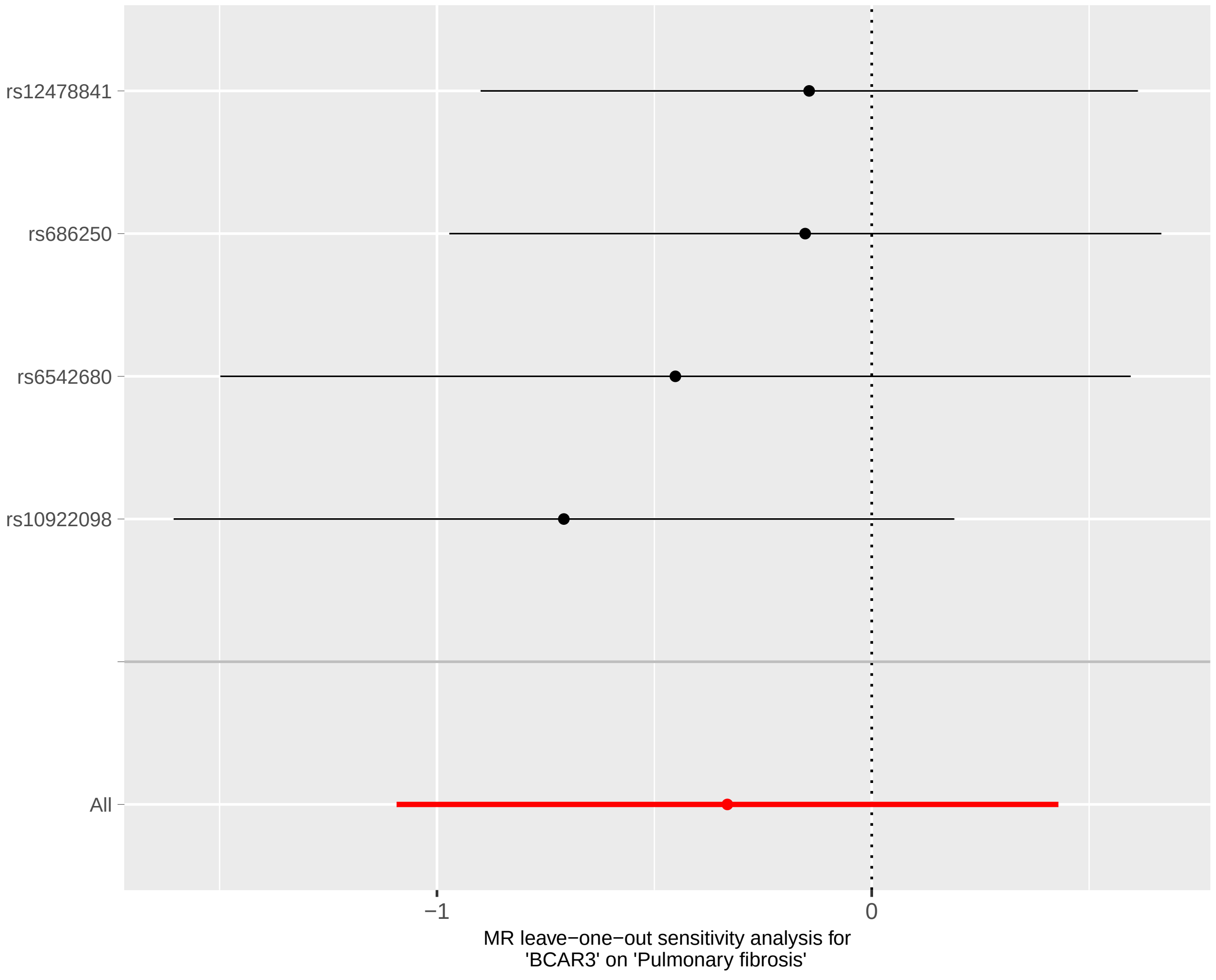
rs12478841
rs686250
rs6542680
rs10922098
All
−1
0
MR leave−one−out sensitivity analysis for
'BCAR3' on 'Pulmonary fibrosis'

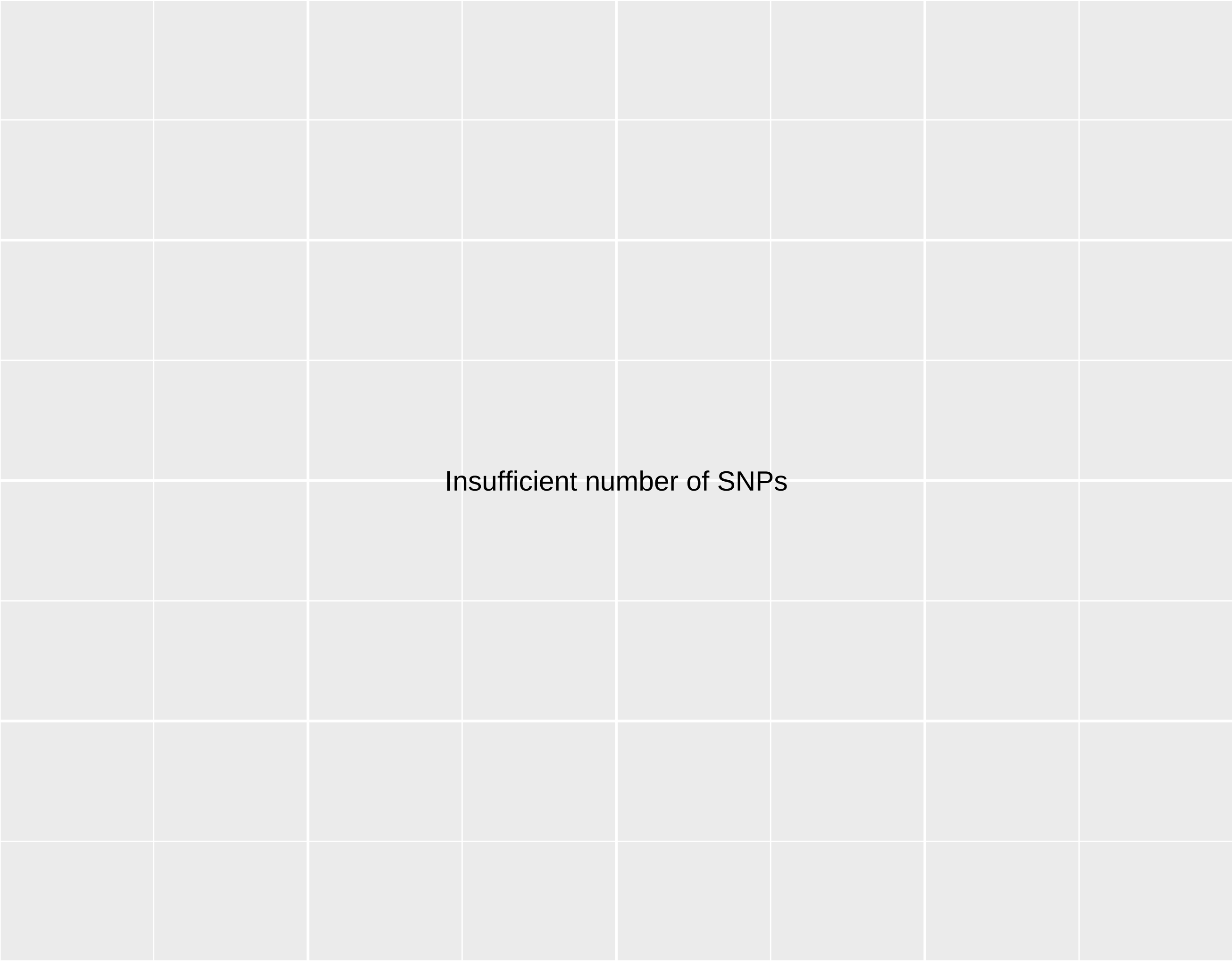
Insufficient number of SNPs

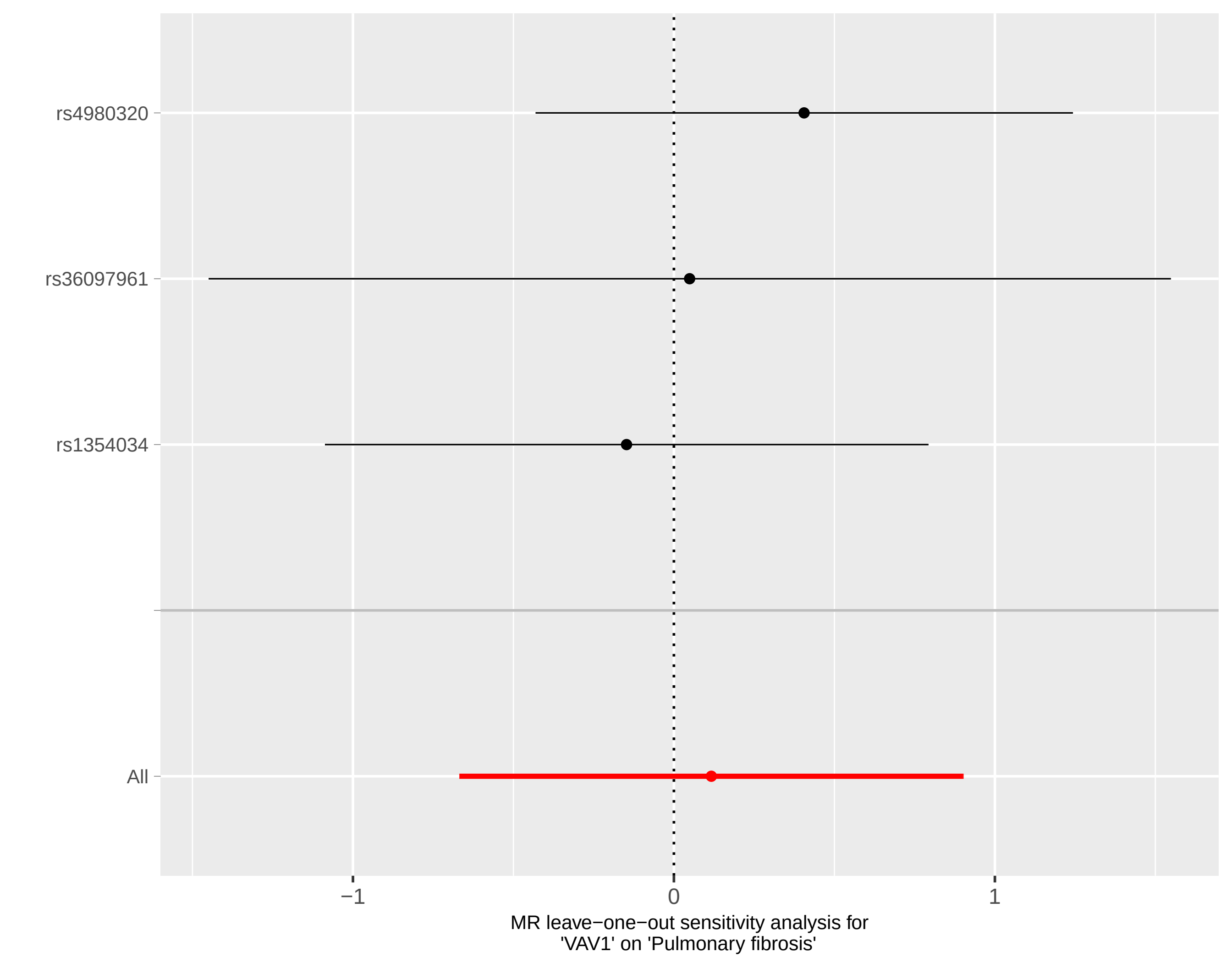
rs4980320
rs36097961
rs1354034
All
−1
0
1
MR leave−one−out sensitivity analysis for
'VAV1' on 'Pulmonary fibrosis'

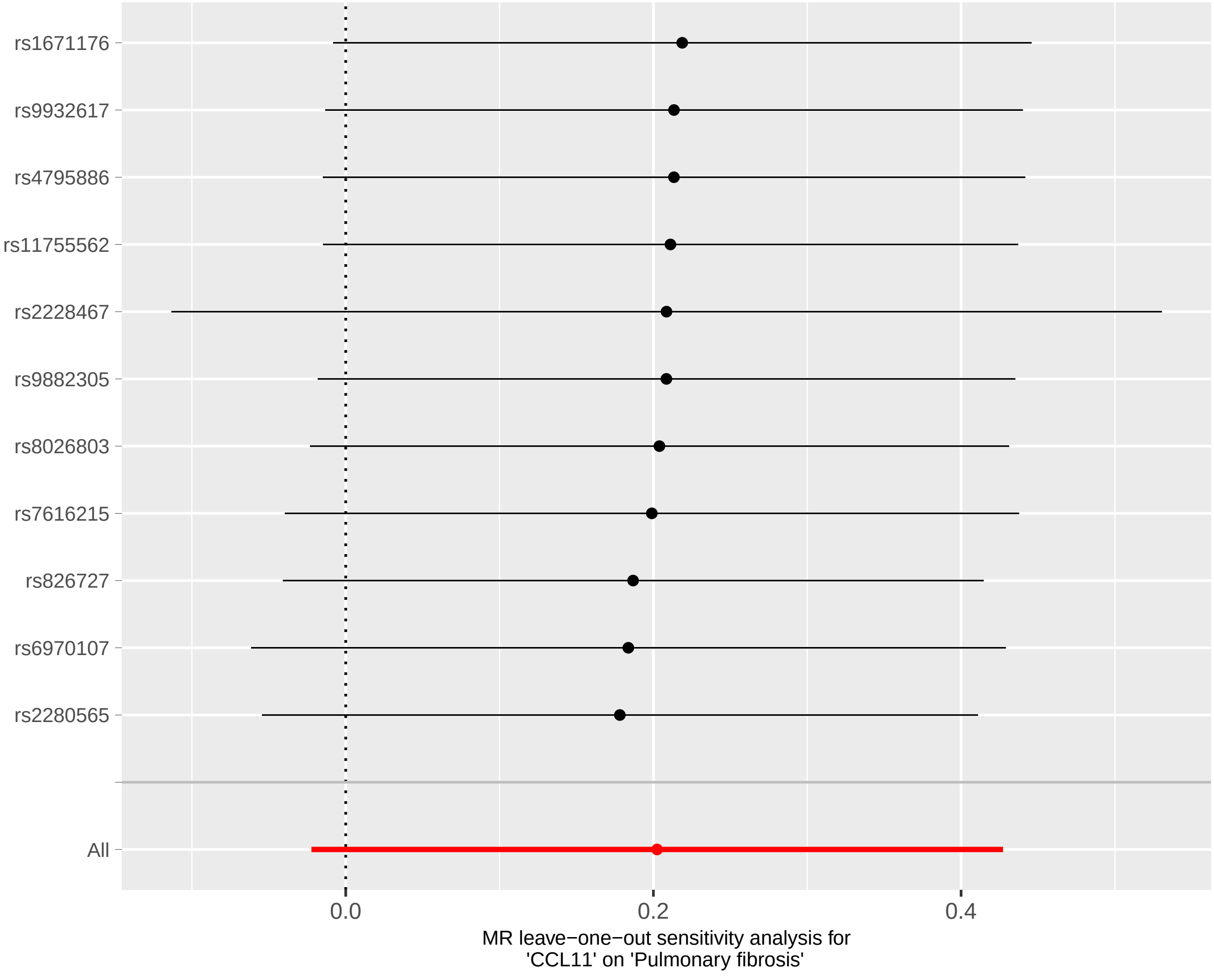
rs1671176
rs9932617
rs4795886
rs11755562
rs2228467
rs9882305
rs8026803
rs7616215
rs826727
rs6970107
rs2280565
All
0.0
0.2
0.4
MR leave−one−out sensitivity analysis for
'CCL11' on 'Pulmonary fibrosis'

Insufficient number of SNPs

Insufficient number of SNPs

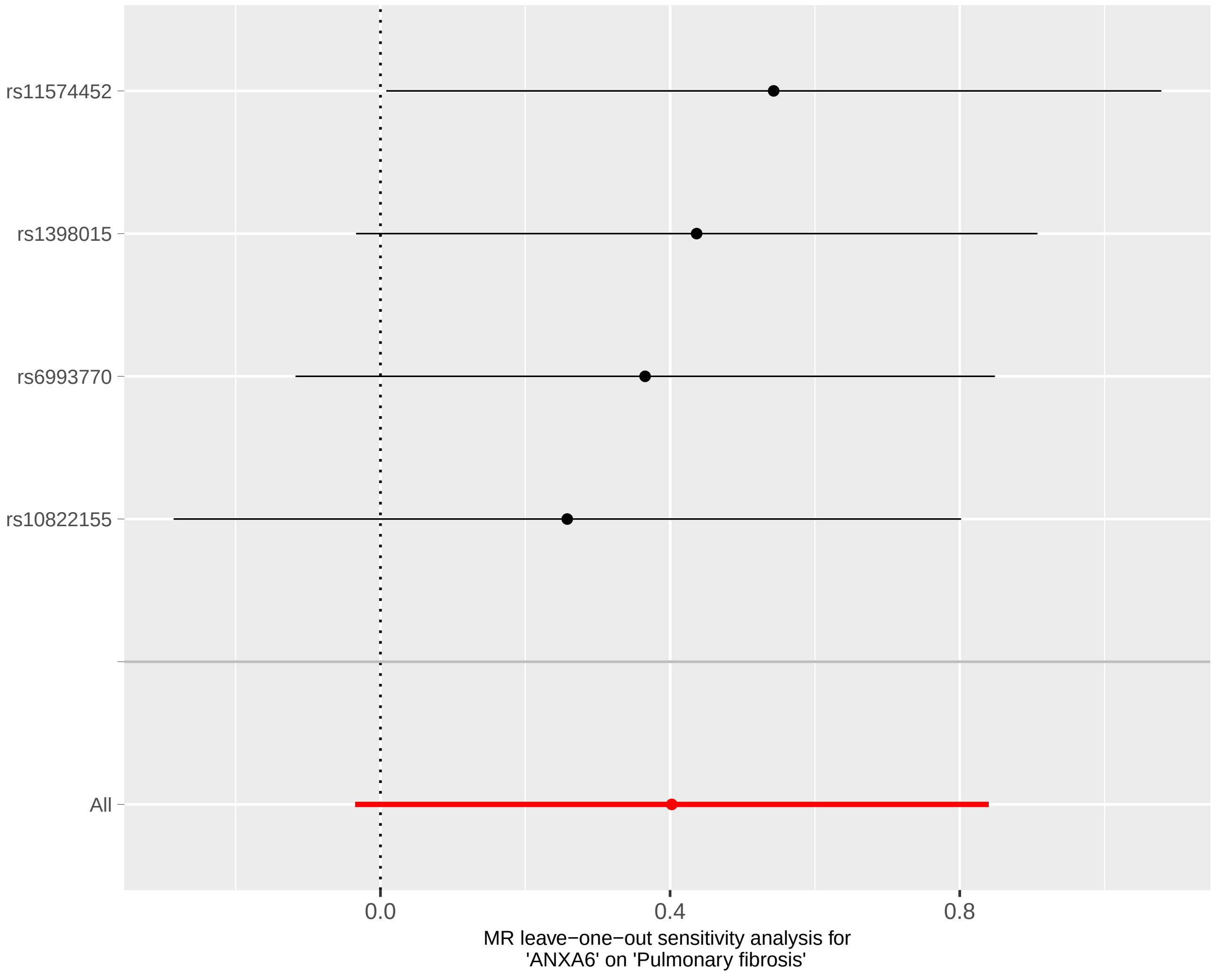

rs11574452
rs1398015
rs6993770
rs10822155
All
0.0
0.4
0.8
MR leave−one−out sensitivity analysis for
'ANXA6' on 'Pulmonary fibrosis'

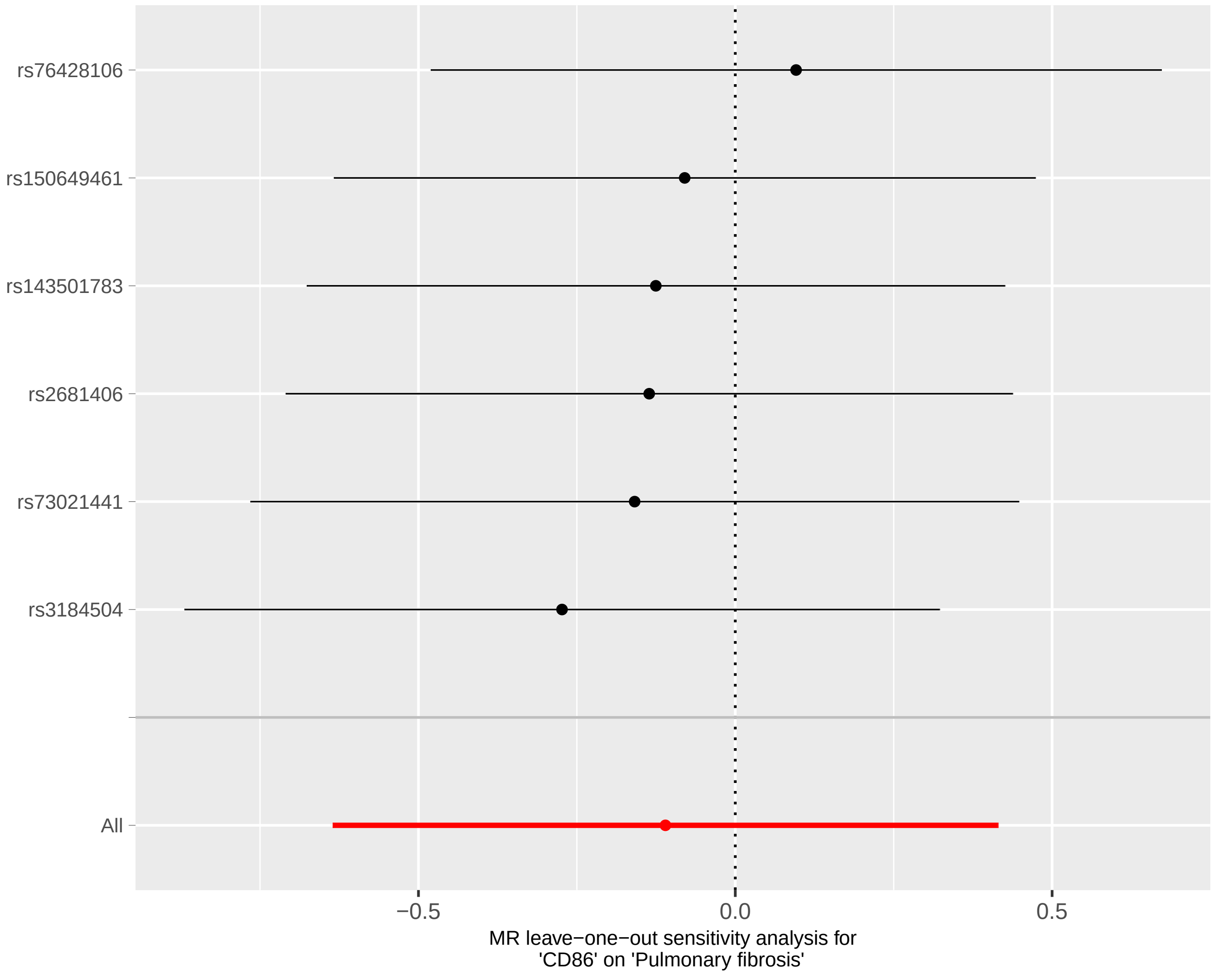
rs76428106
rs150649461
rs143501783
rs2681406
rs73021441
rs3184504
All
−0.5
0.0
0.5
MR leave−one−out sensitivity analysis for
'CD86' on 'Pulmonary fibrosis'

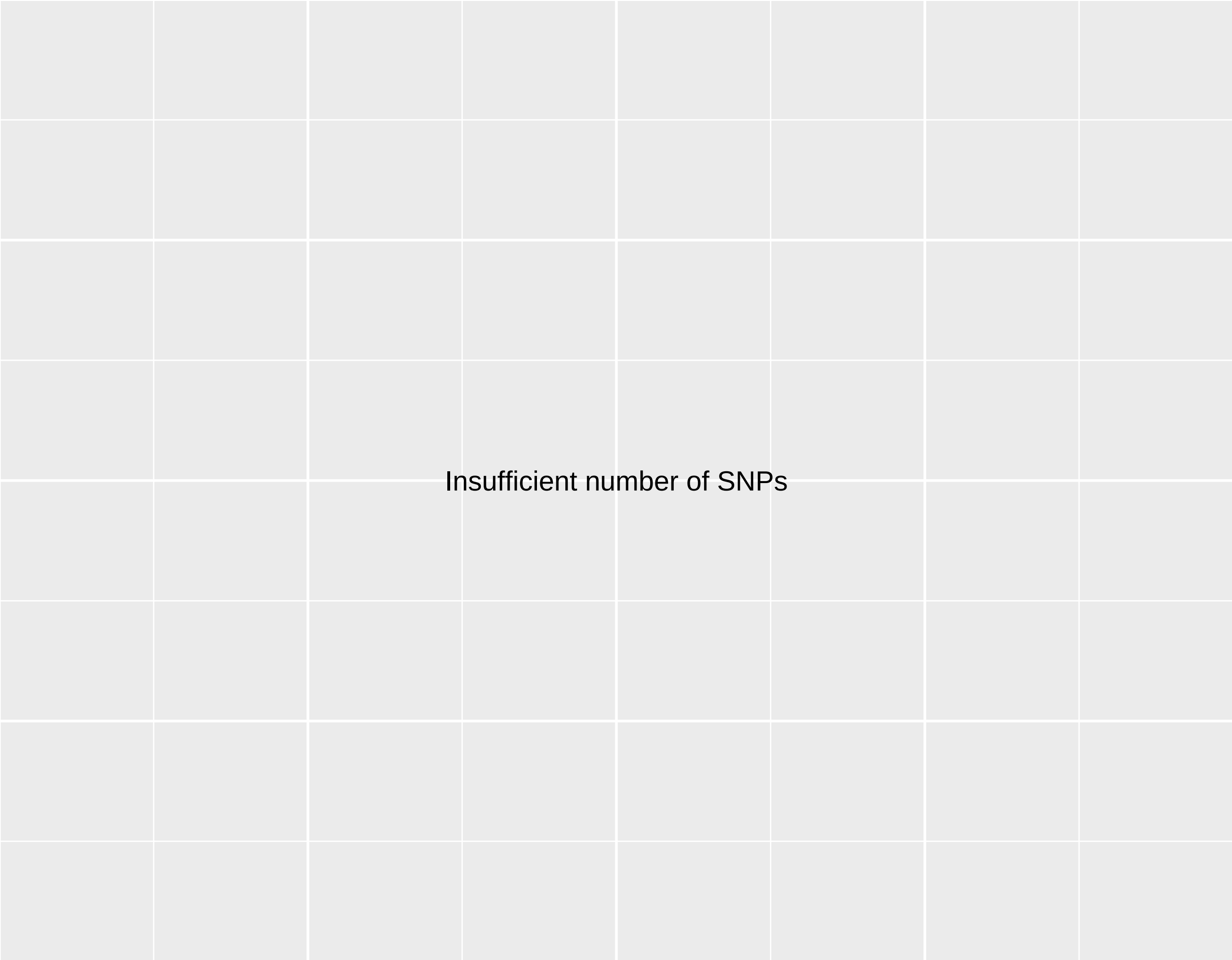
Insufficient number of SNPs

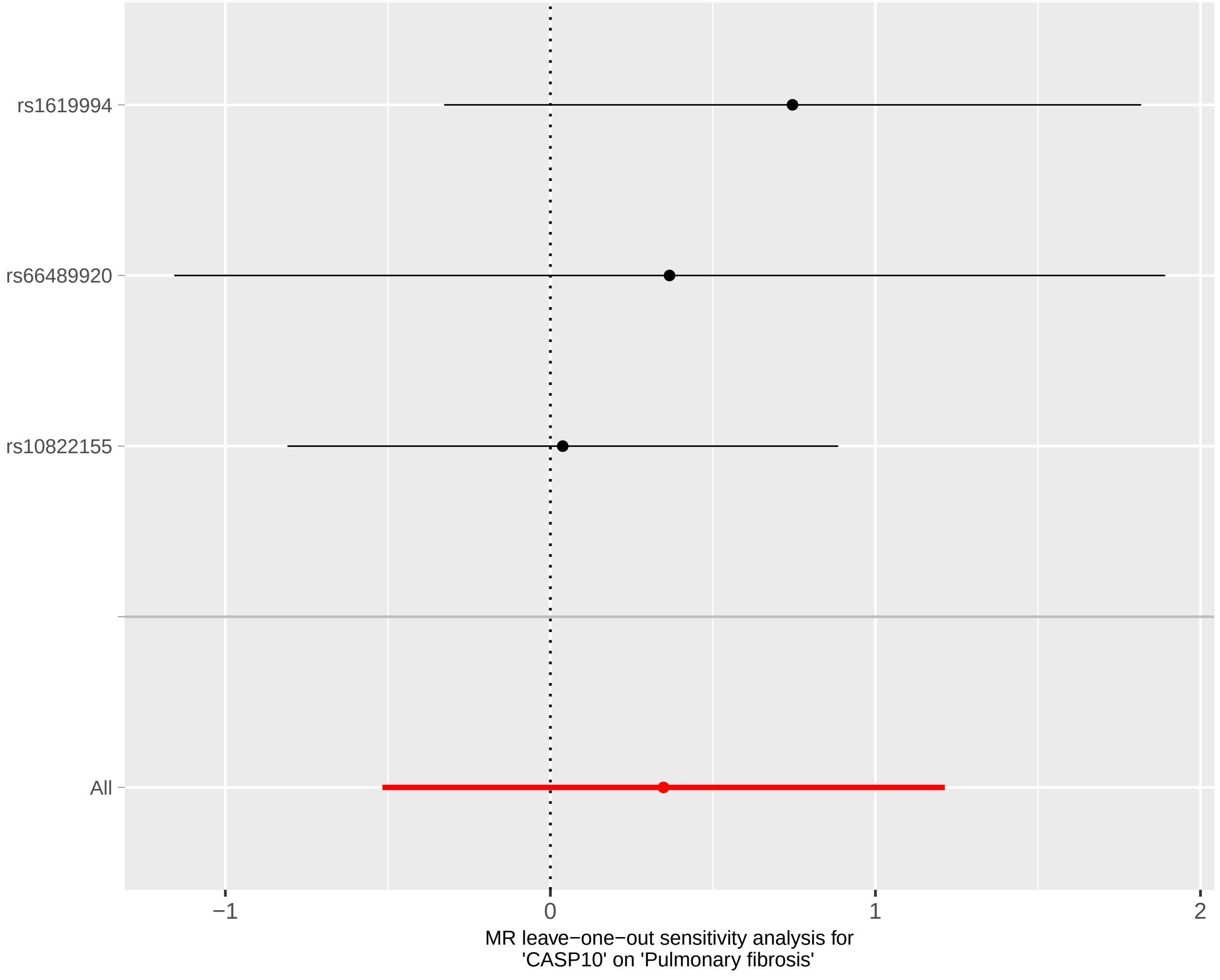

rs1619994
rs66489920
rs10822155
All
−1
0
1
2
MR leave−one−out sensitivity analysis for
'CASP10' on 'Pulmonary fibrosis'

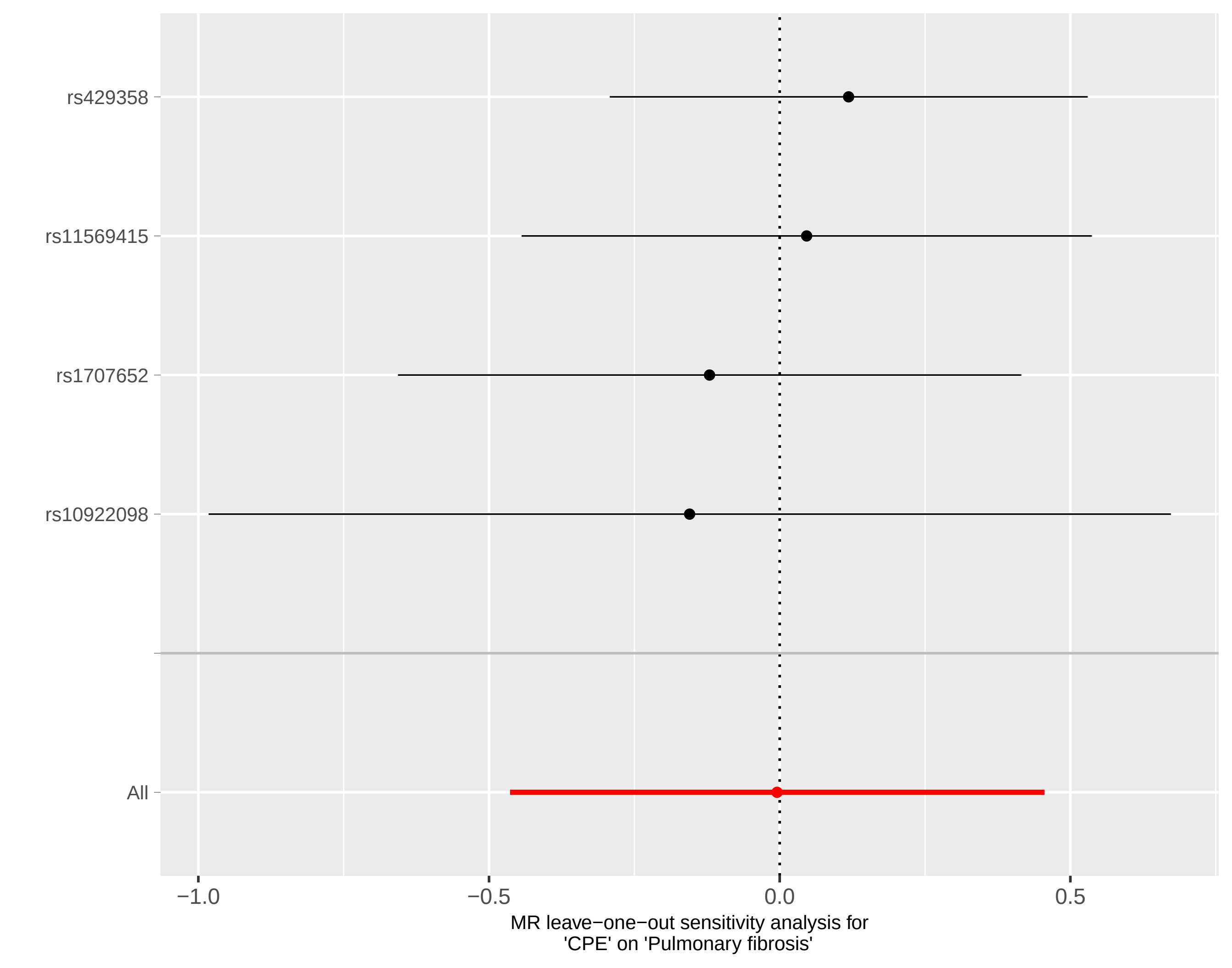
rs429358
rs11569415
rs1707652
rs10922098
All
−1.0
−0.5
0.0
0.5
MR leave−one−out sensitivity analysis for
'CPE' on 'Pulmonary fibrosis'

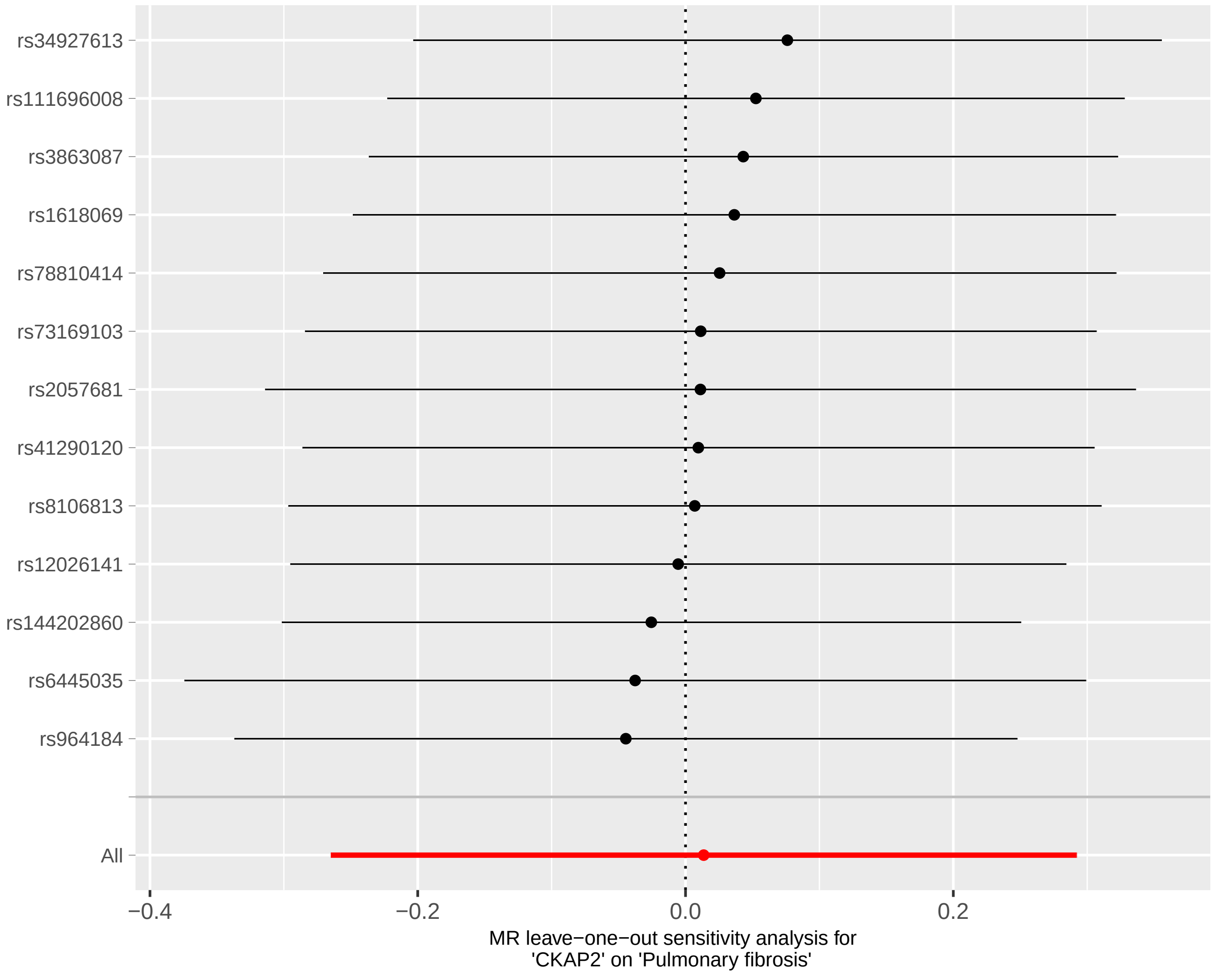

rs34927613
rs111696008
rs3863087
rs1618069
rs78810414
rs73169103
rs2057681
rs41290120
rs8106813
rs12026141
rs144202860
rs6445035
rs964184
All
−0.4
−0.2
0.0
0.2
MR leave−one−out sensitivity analysis for
'CKAP2' on 'Pulmonary fibrosis'

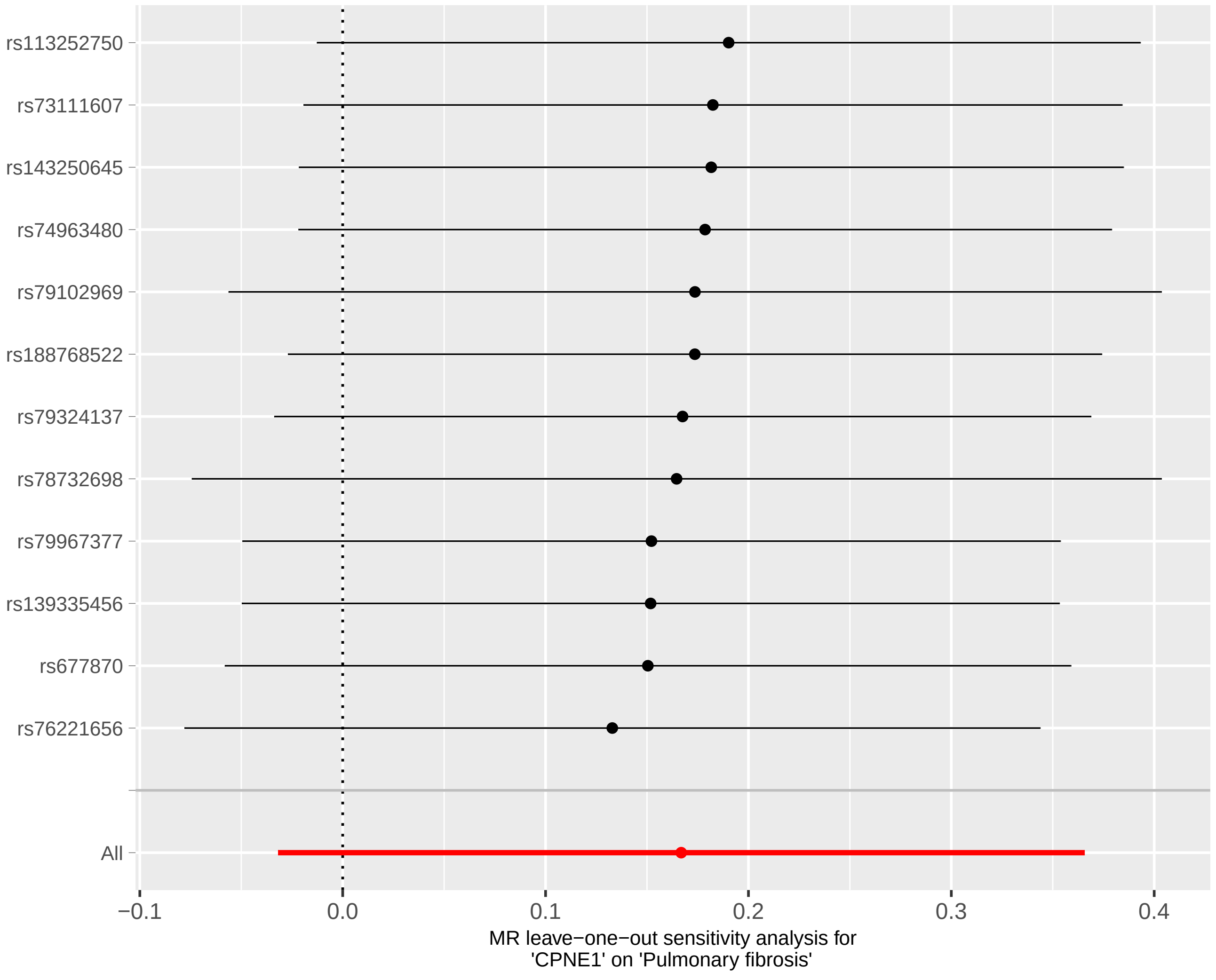
rs113252750
rs73111607
rs143250645
rs74963480
rs79102969
rs188768522
rs79324137
rs78732698
rs79967377
rs139335456
rs677870
rs76221656
All
−0.1
0.0
0.1
0.2
0.3
0.4
MR leave−one−out sensitivity analysis for
'CPNE1' on 'Pulmonary fibrosis'

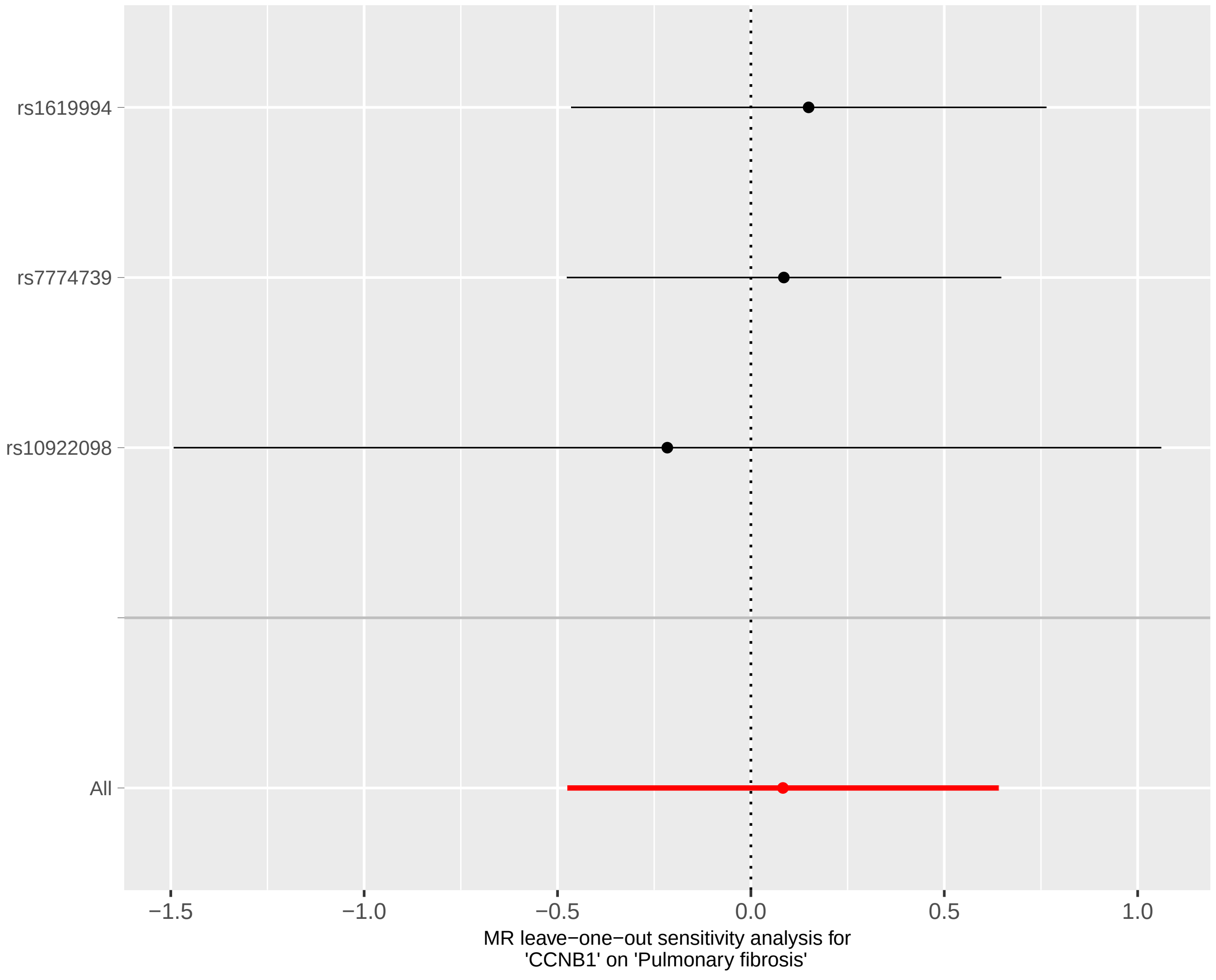
rs1619994
rs7774739
rs10922098
All
−1.5
−1.0
−0.5
0.0
0.5
1.0
MR leave−one−out sensitivity analysis for
'CCNB1' on 'Pulmonary fibrosis'

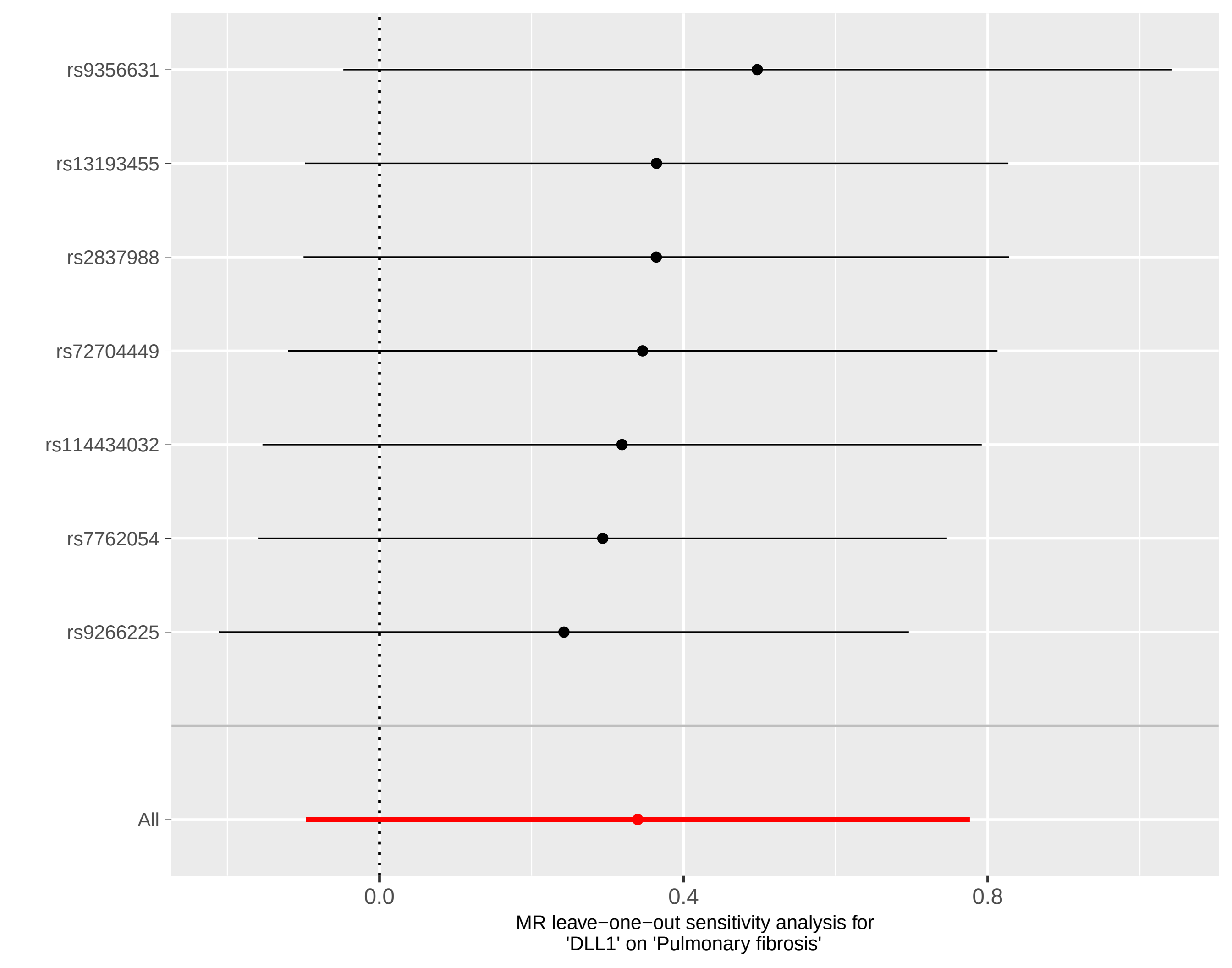
rs9356631
rs13193455
rs2837988
rs72704449
rs114434032
rs7762054
rs9266225
All
0.0
0.4
0.8
MR leave−one−out sensitivity analysis for
'DLL1' on 'Pulmonary fibrosis'

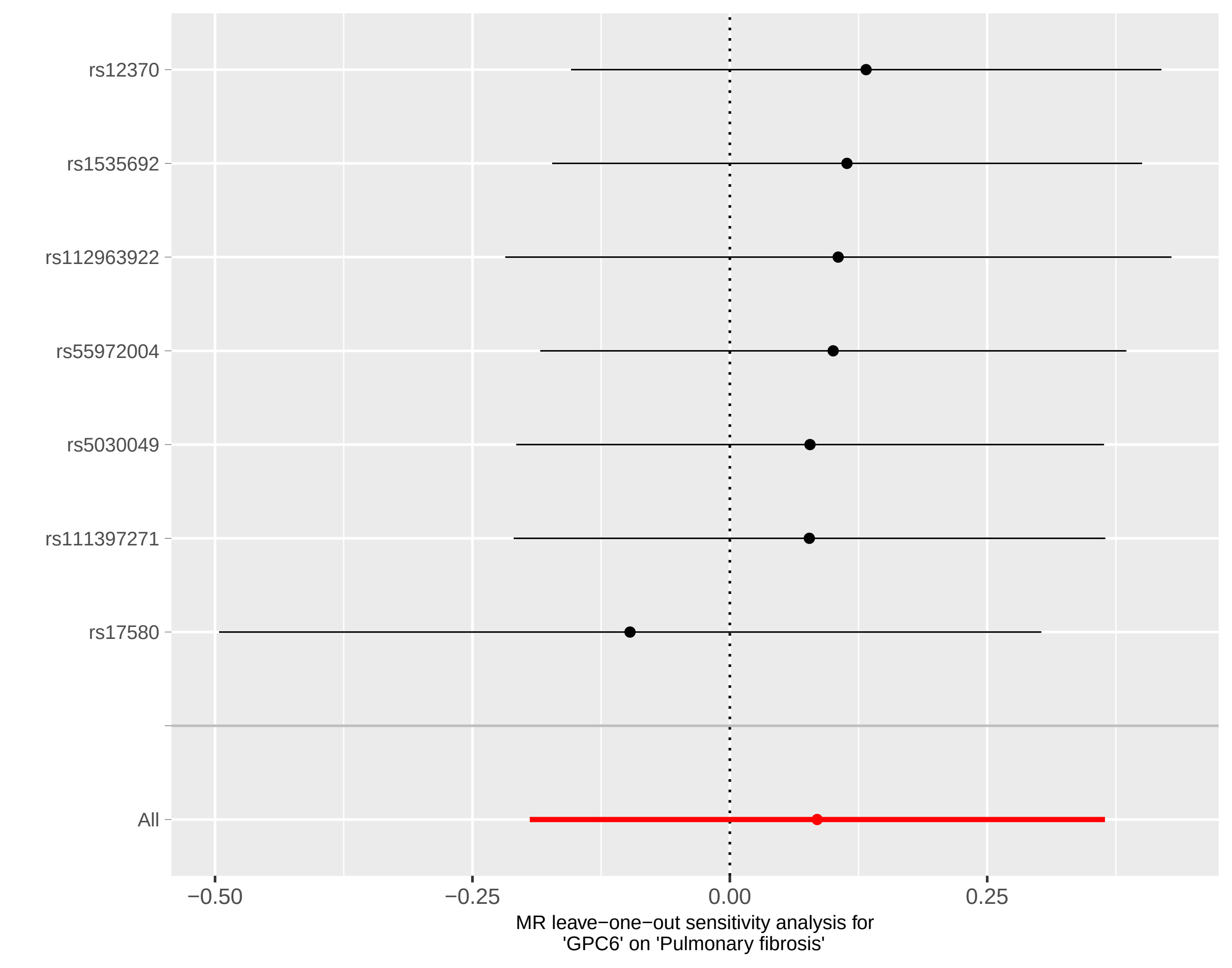

rs12370
rs1535692
rs112963922
rs55972004
rs5030049
rs111397271
rs17580
All
−0.50
−0.25
0.00
0.25
MR leave−one−out sensitivity analysis for
'GPC6' on 'Pulmonary fibrosis'

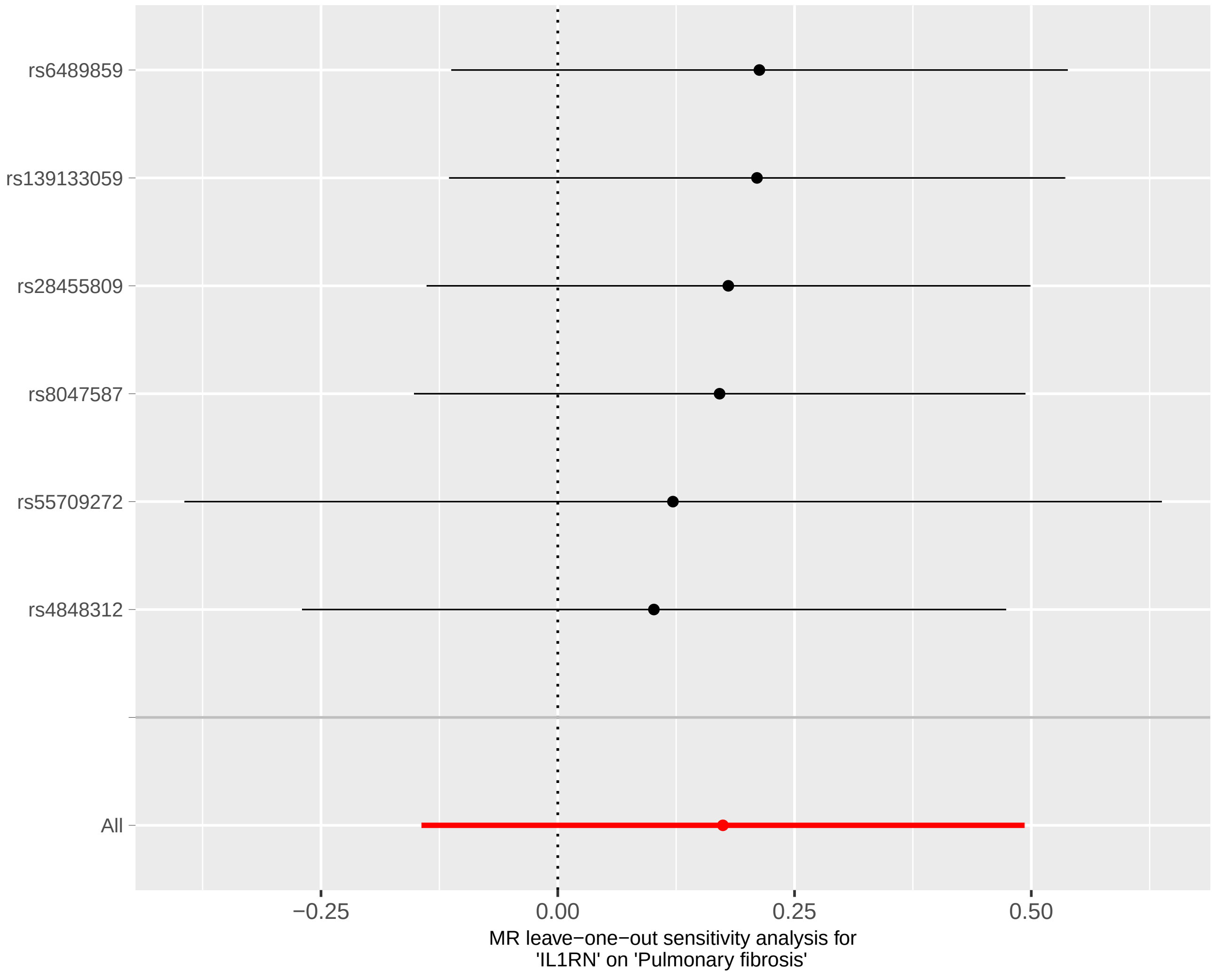
rs6489859
rs139133059
rs28455809
rs8047587
rs55709272
rs4848312
All
−0.25
0.00
0.25
0.50
MR leave−one−out sensitivity analysis for
'IL1RN' on 'Pulmonary fibrosis'

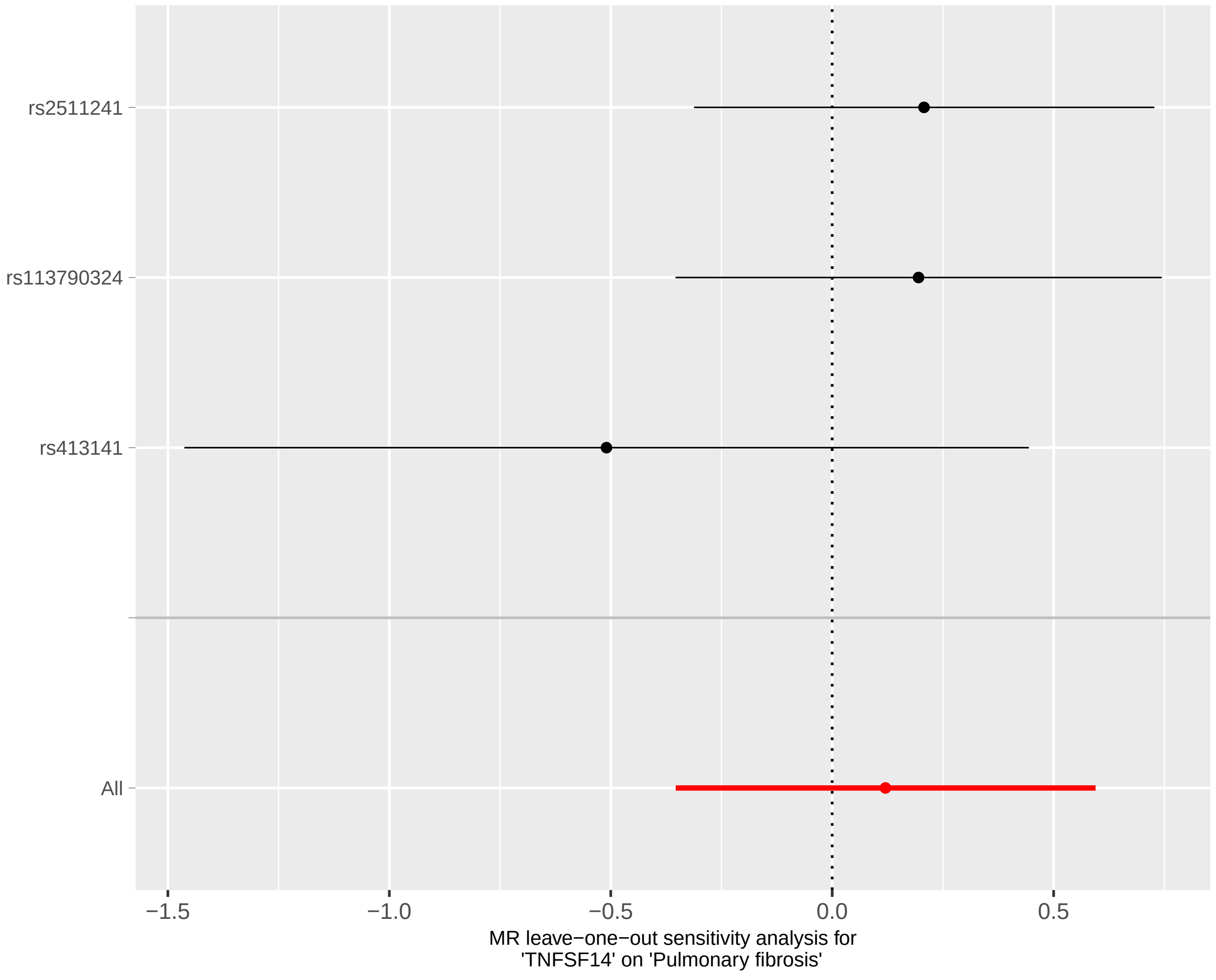
rs2511241
rs113790324
rs413141
All
−1.5
−1.0
−0.5
0.0
0.5
MR leave−one−out sensitivity analysis for
'TNFSF14' on 'Pulmonary fibrosis'

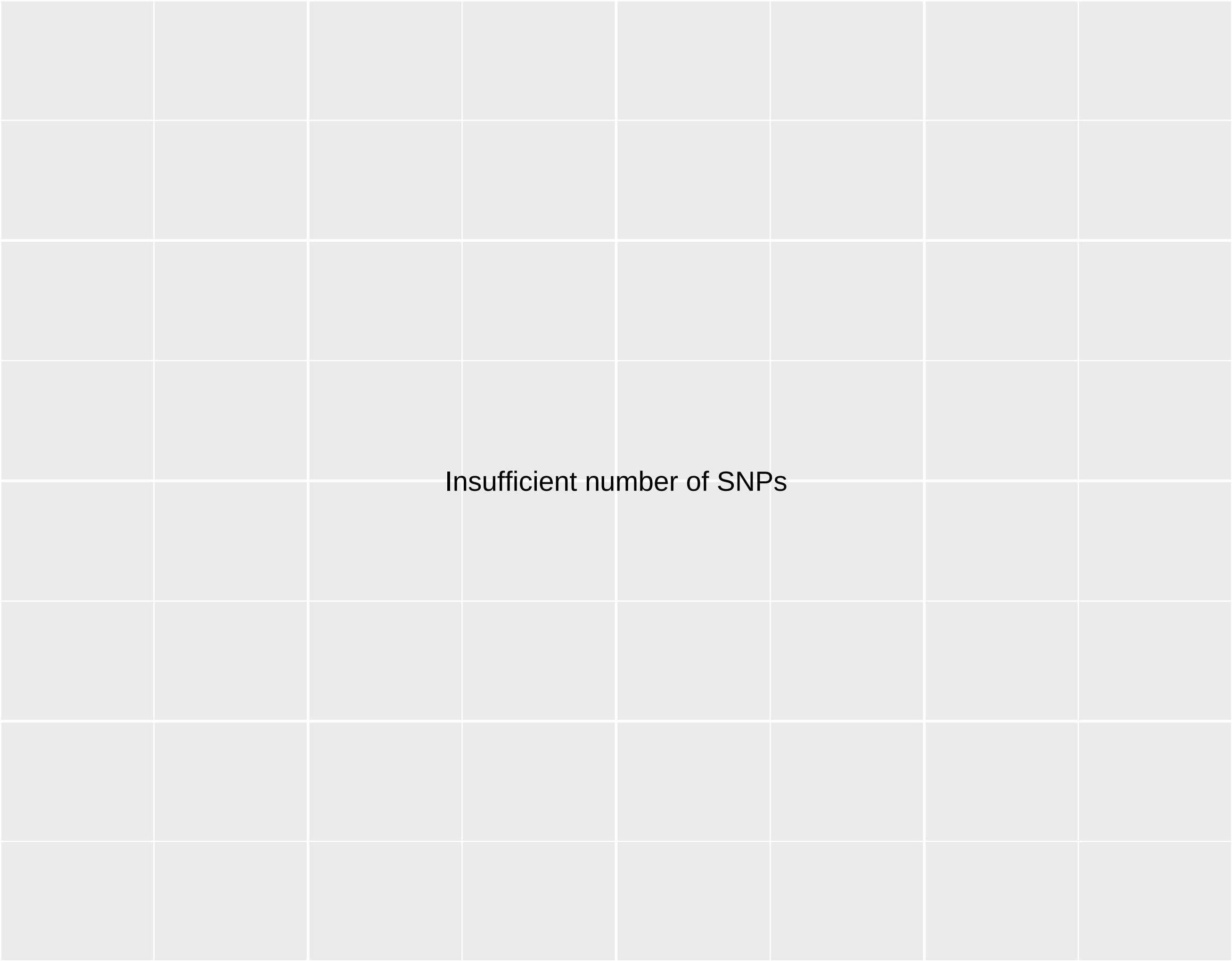
Insufficient number of SNPs

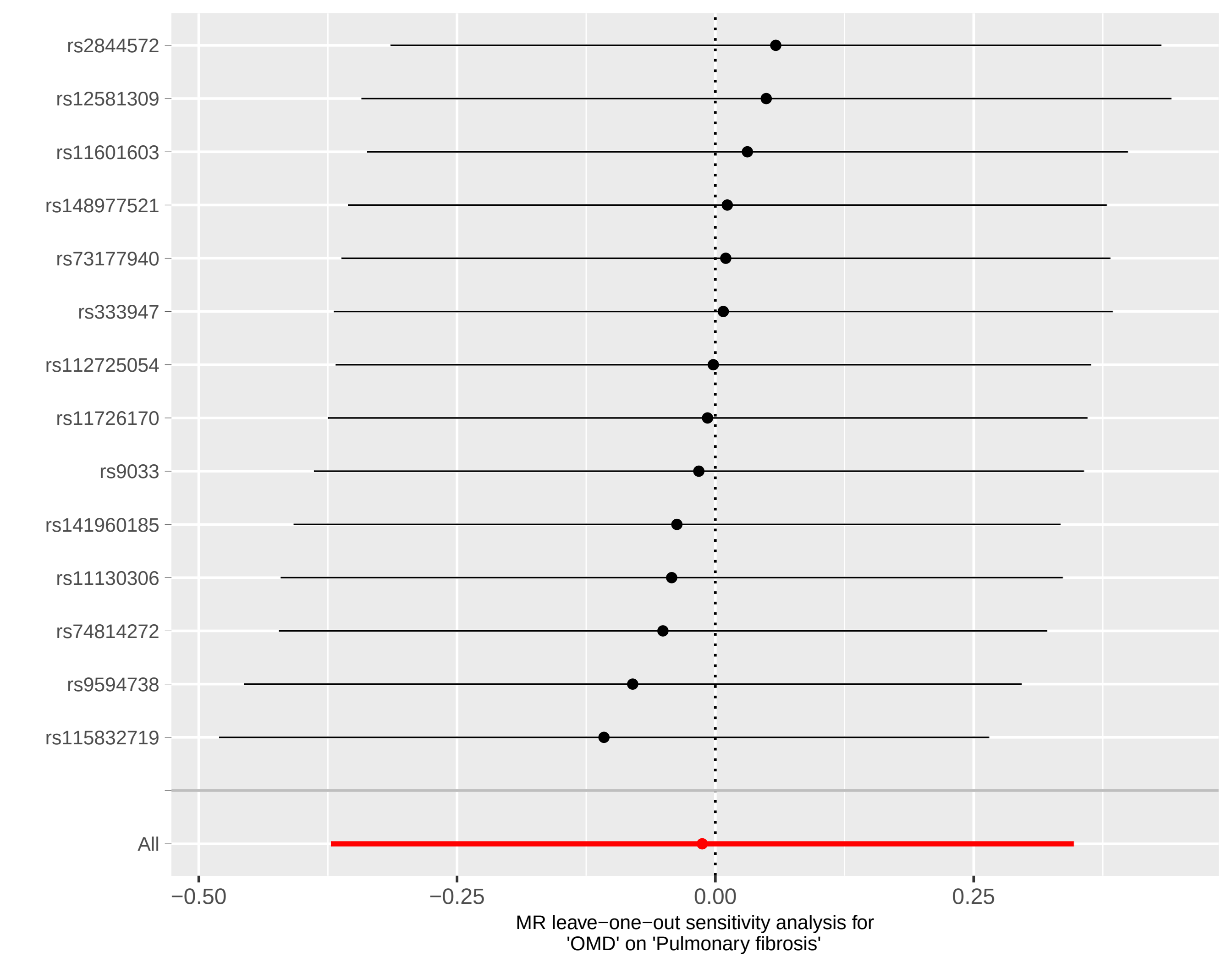

rs2844572
rs12581309
rs11601603
rs148977521
rs73177940
rs333947
rs112725054
rs11726170
rs9033
rs141960185
rs11130306
rs74814272
rs9594738
rs115832719
All
−0.50
−0.25
0.00
0.25
MR leave−one−out sensitivity analysis for
'OMD' on 'Pulmonary fibrosis'

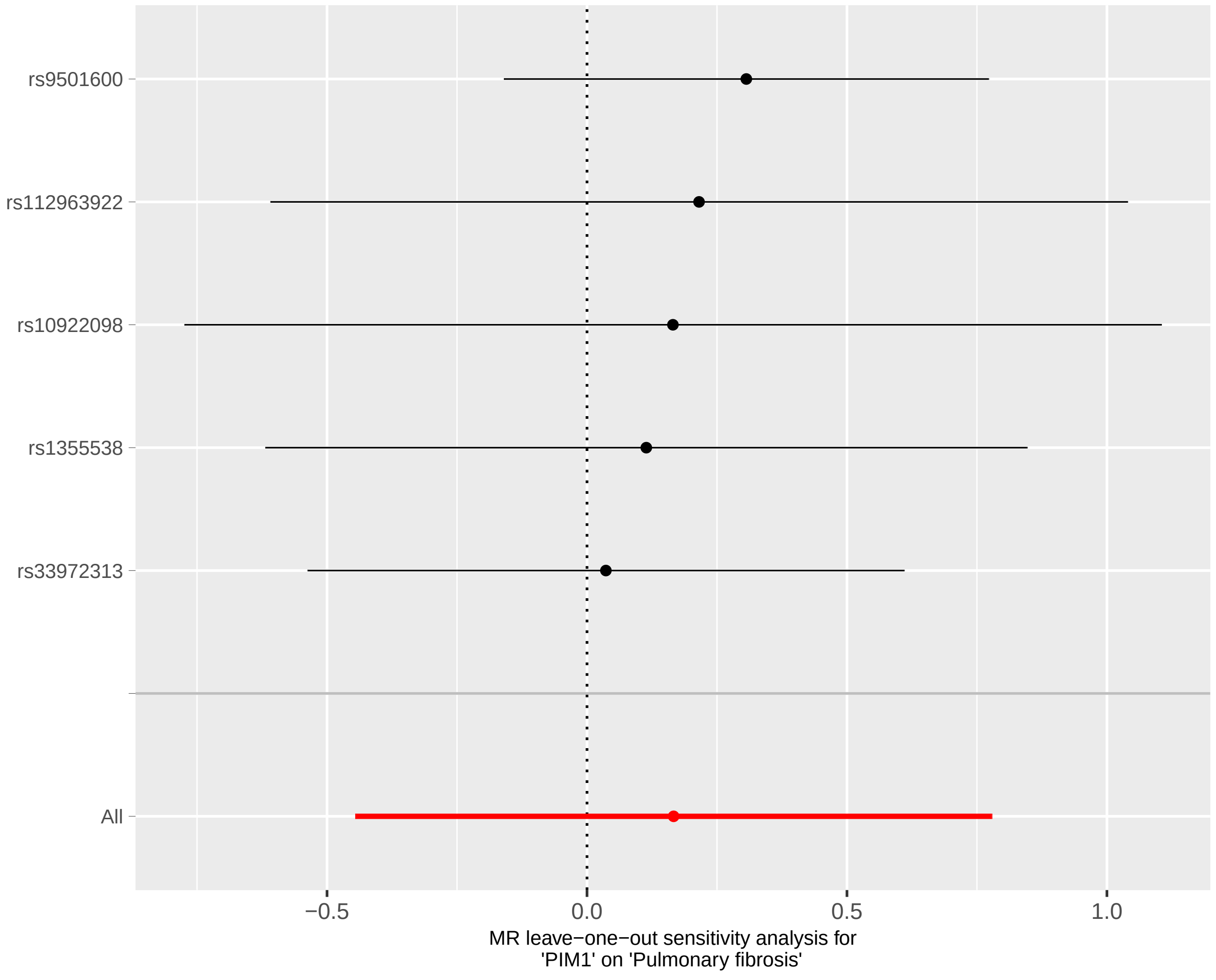
rs9501600
rs112963922
rs10922098
rs1355538
rs33972313
All
−0.5
0.0
0.5
1.0
MR leave−one−out sensitivity analysis for
'PIM1' on 'Pulmonary fibrosis'

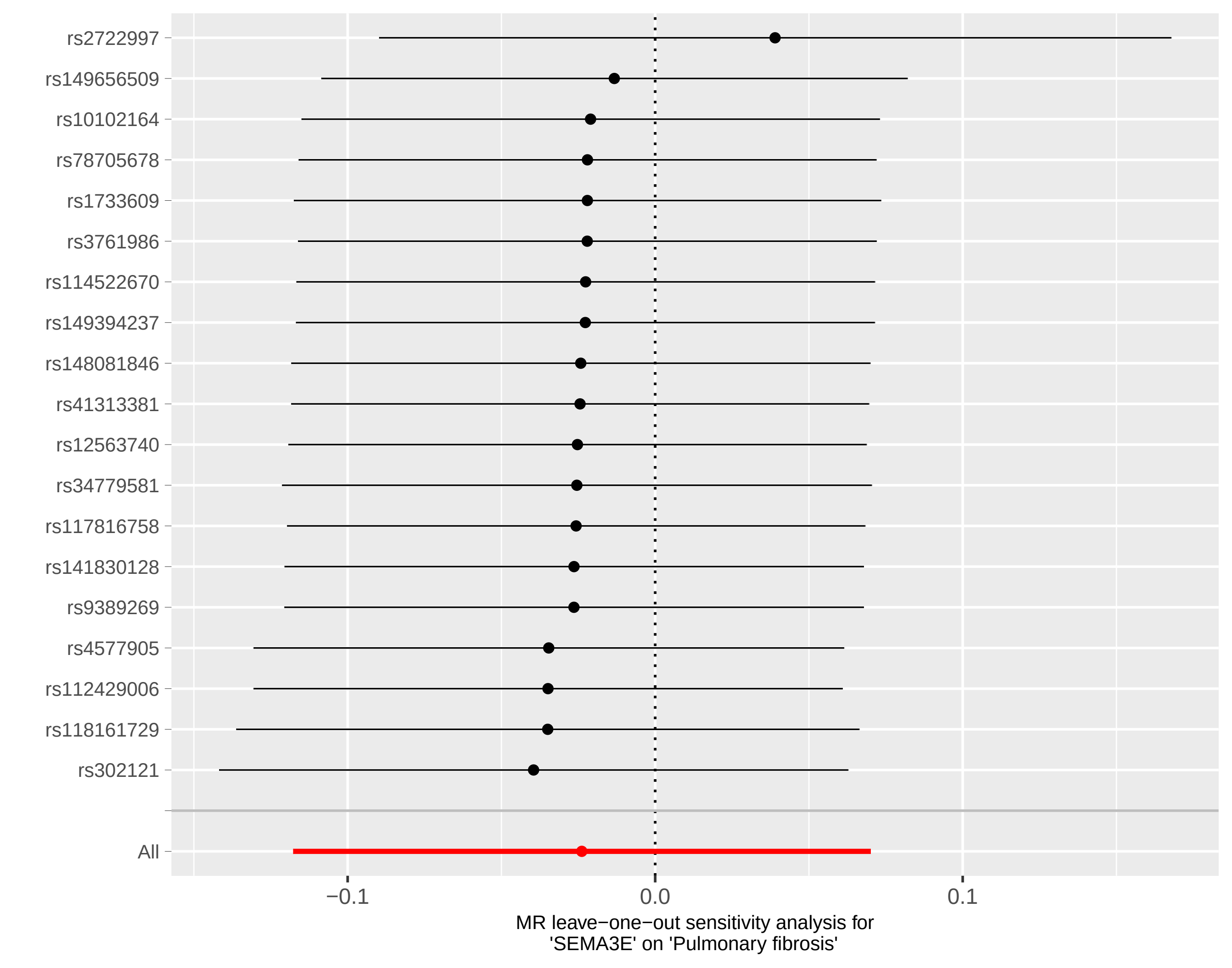

rs2722997
rs149656509
rs10102164
rs78705678
rs1733609
rs3761986
rs114522670
rs149394237
rs148081846
rs41313381
rs12563740
rs34779581
rs117816758
rs141830128
rs9389269
rs4577905
rs112429006
rs118161729
rs302121
All
−0.1
0.0
0.1
MR leave−one−out sensitivity analysis for
'SEMA3E' on 'Pulmonary fibrosis'

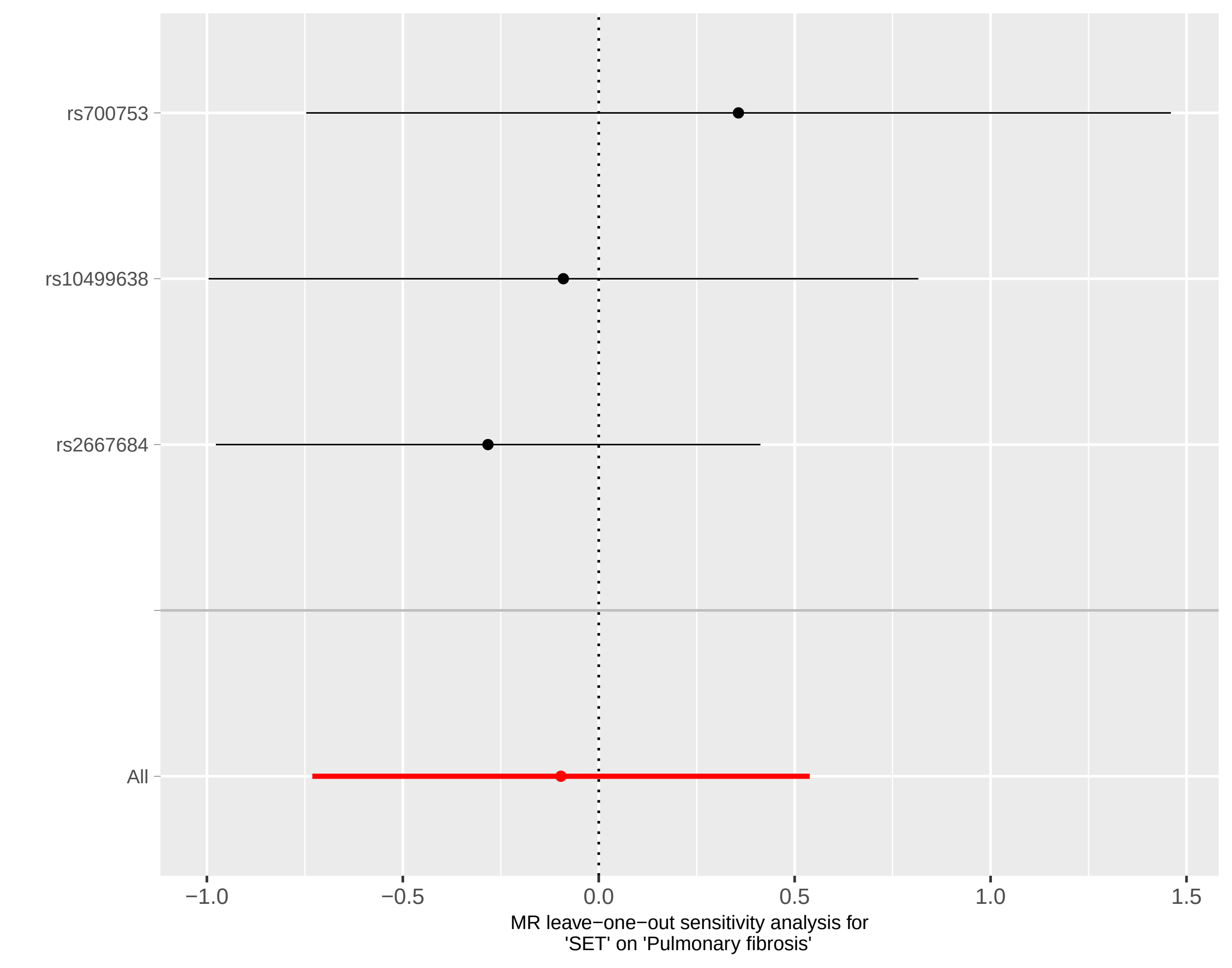
rs700753
rs10499638
rs2667684
All
−1.0
−0.5
0.0
0.5
1.0
1.5
MR leave−one−out sensitivity analysis for
'SET' on 'Pulmonary fibrosis'

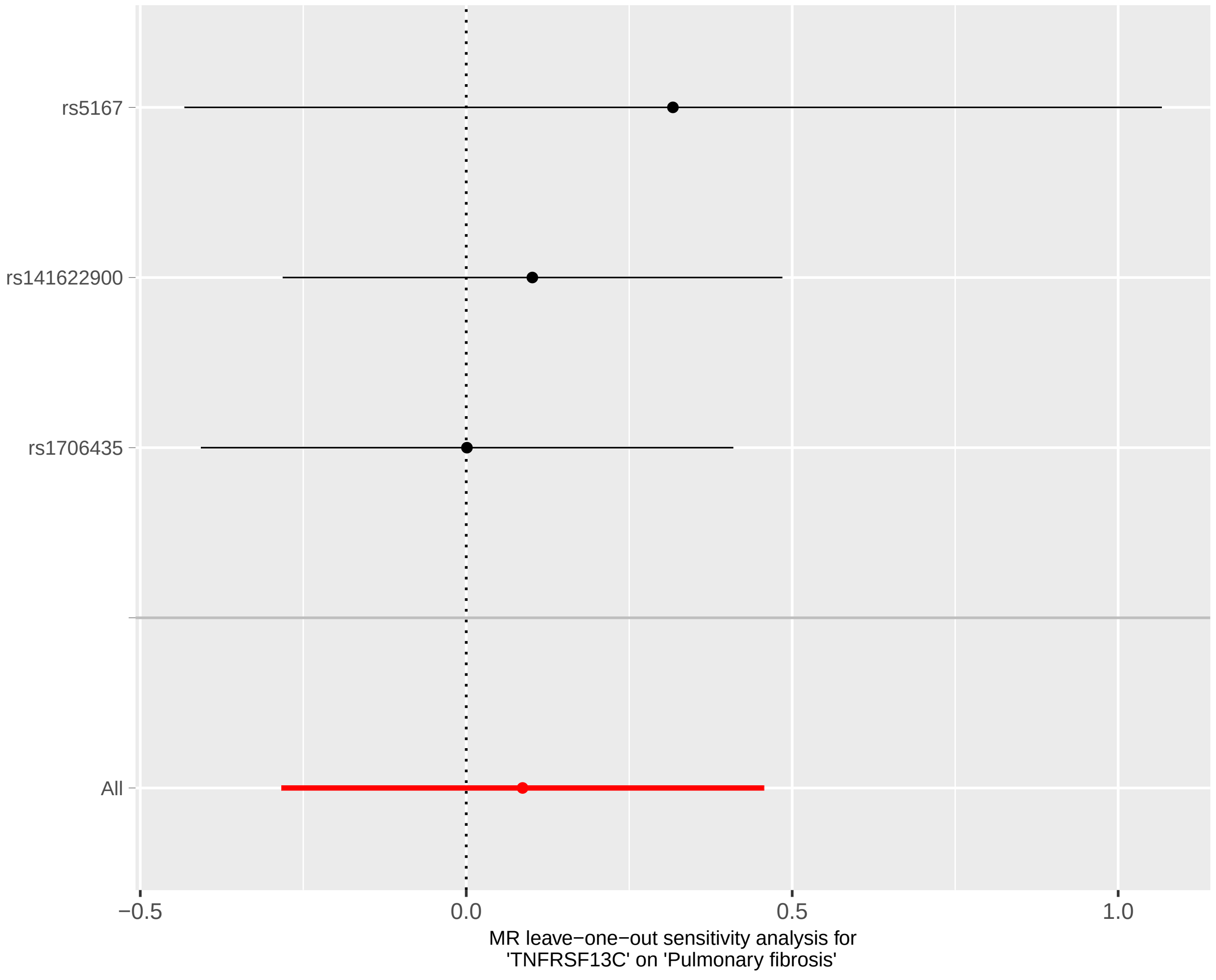
rs5167
rs141622900
rs1706435
All
−0.5
0.0
0.5
1.0
MR leave−one−out sensitivity analysis for
'TNFRSF13C' on 'Pulmonary fibrosis'

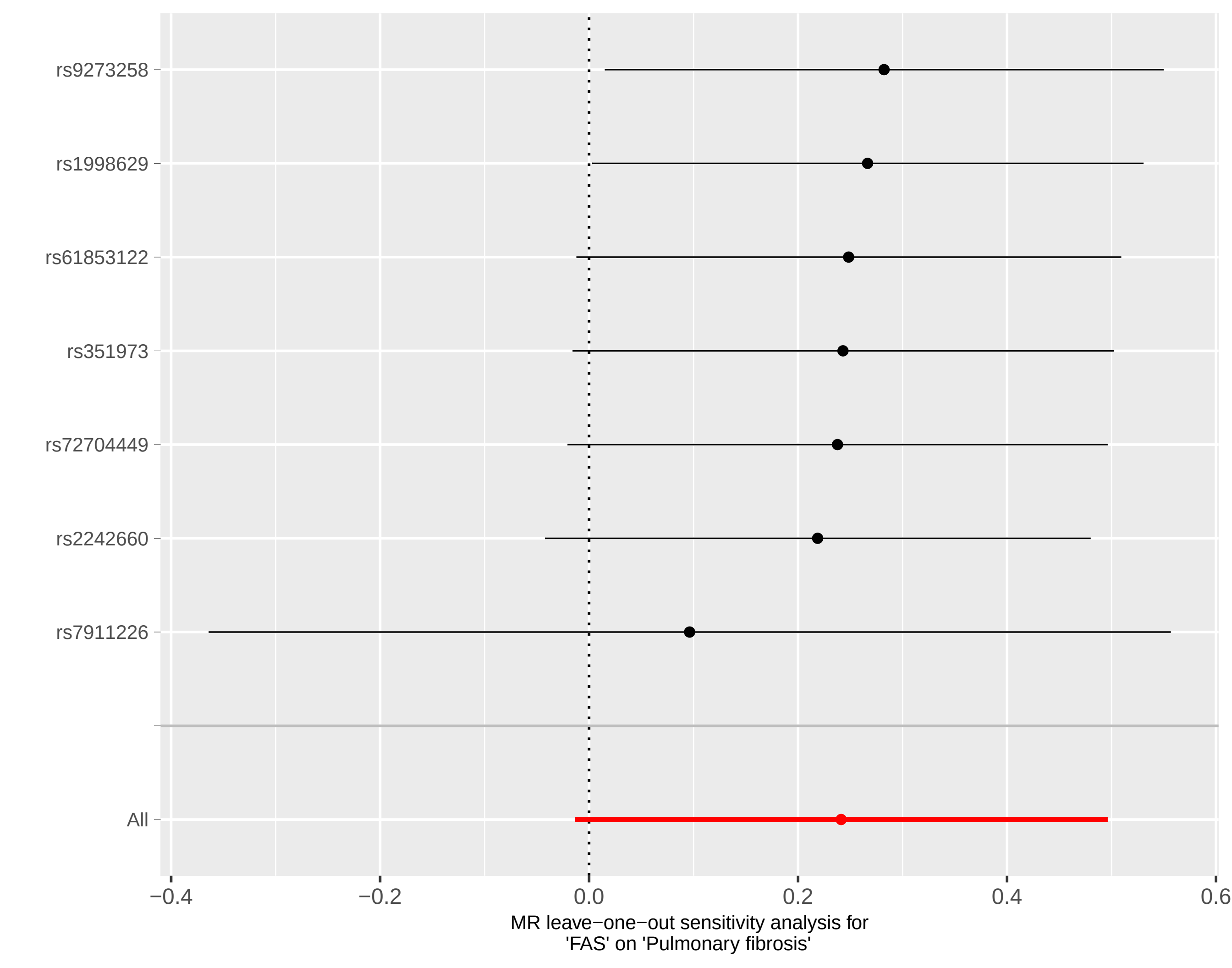
rs9273258
rs1998629
rs61853122
rs351973
rs72704449
rs2242660
rs7911226
All
−0.4
−0.2
0.0
0.2
0.4
0.6
MR leave−one−out sensitivity analysis for
'FAS' on 'Pulmonary fibrosis'

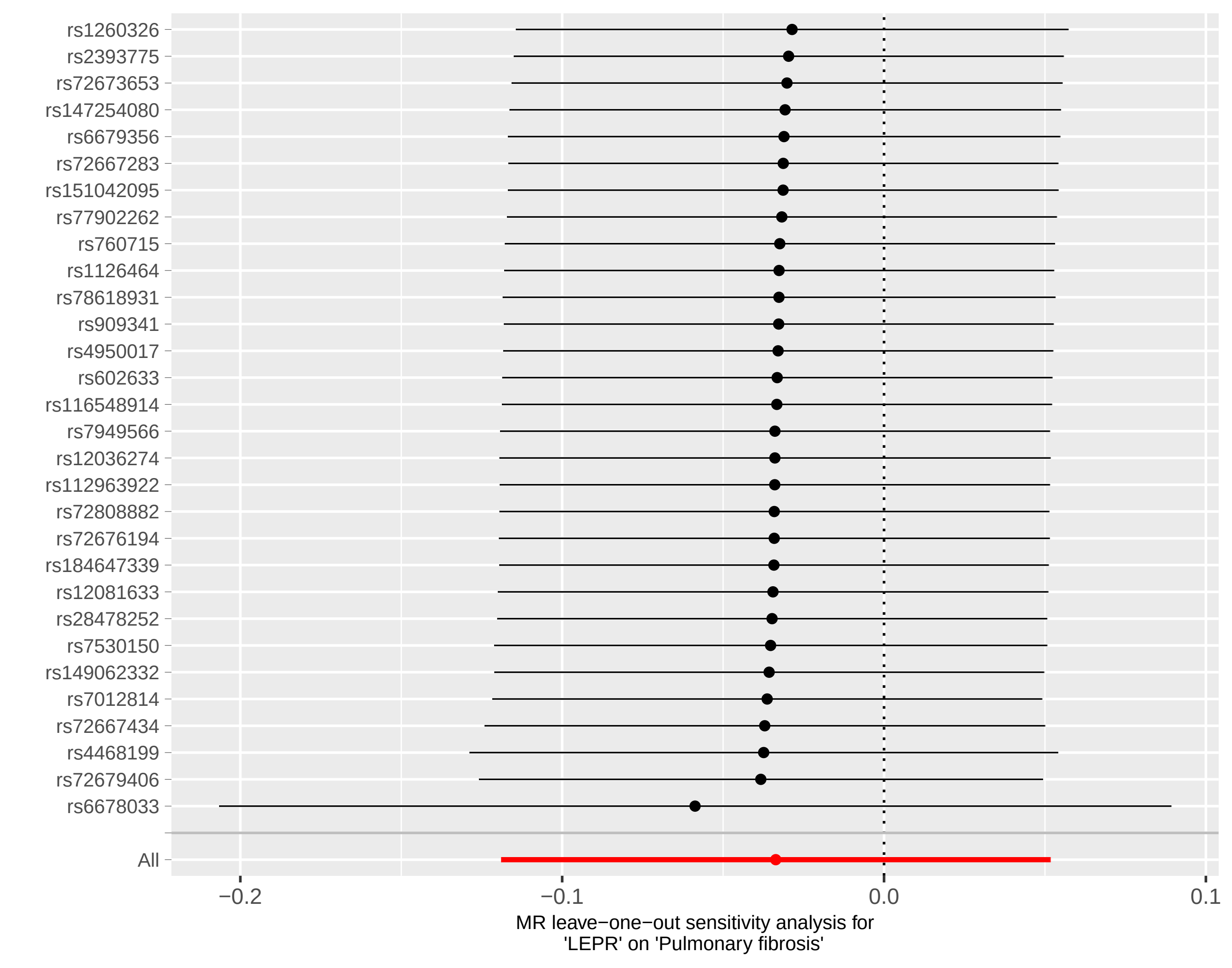
rs1260326
rs2393775
rs72673653
rs147254080
rs6679356
rs72667283
rs151042095
rs77902262
rs760715
rs1126464
rs78618931
rs909341
rs4950017
rs602633
rs116548914
rs7949566
rs12036274
rs112963922
rs72808882
rs72676194
rs184647339
rs12081633
rs28478252
rs7530150
rs149062332
rs7012814
rs72667434
rs4468199
rs72679406
rs6678033
All
−0.2
−0.1
0.0
0.1
MR leave−one−out sensitivity analysis for
'LEPR' on 'Pulmonary fibrosis'

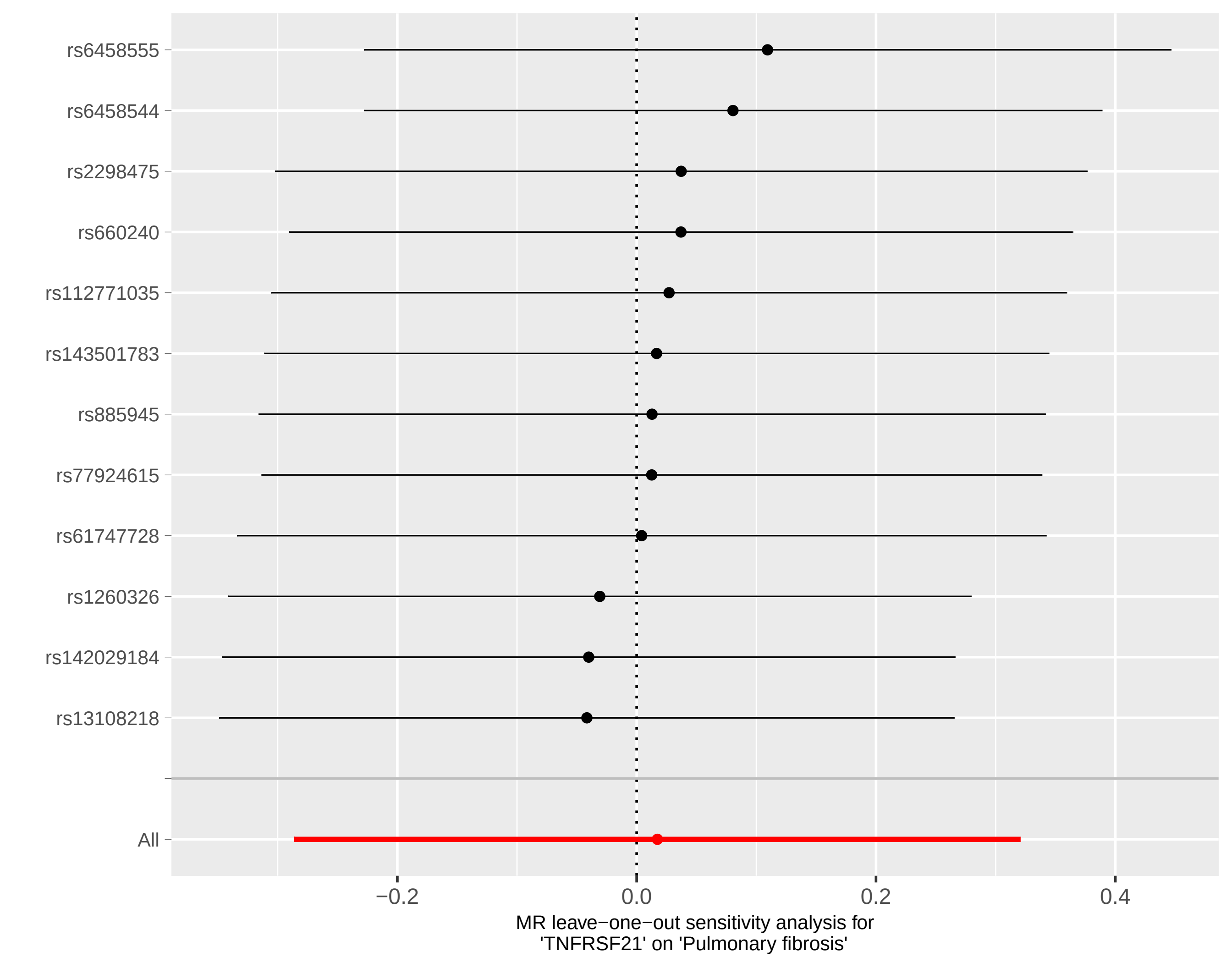
rs6458555
rs6458544
rs2298475
rs660240
rs112771035
rs143501783
rs885945
rs77924615
rs61747728
rs1260326
rs142029184
rs13108218
All
−0.2
0.0
0.2
0.4
MR leave−one−out sensitivity analysis for
'TNFRSF21' on 'Pulmonary fibrosis'

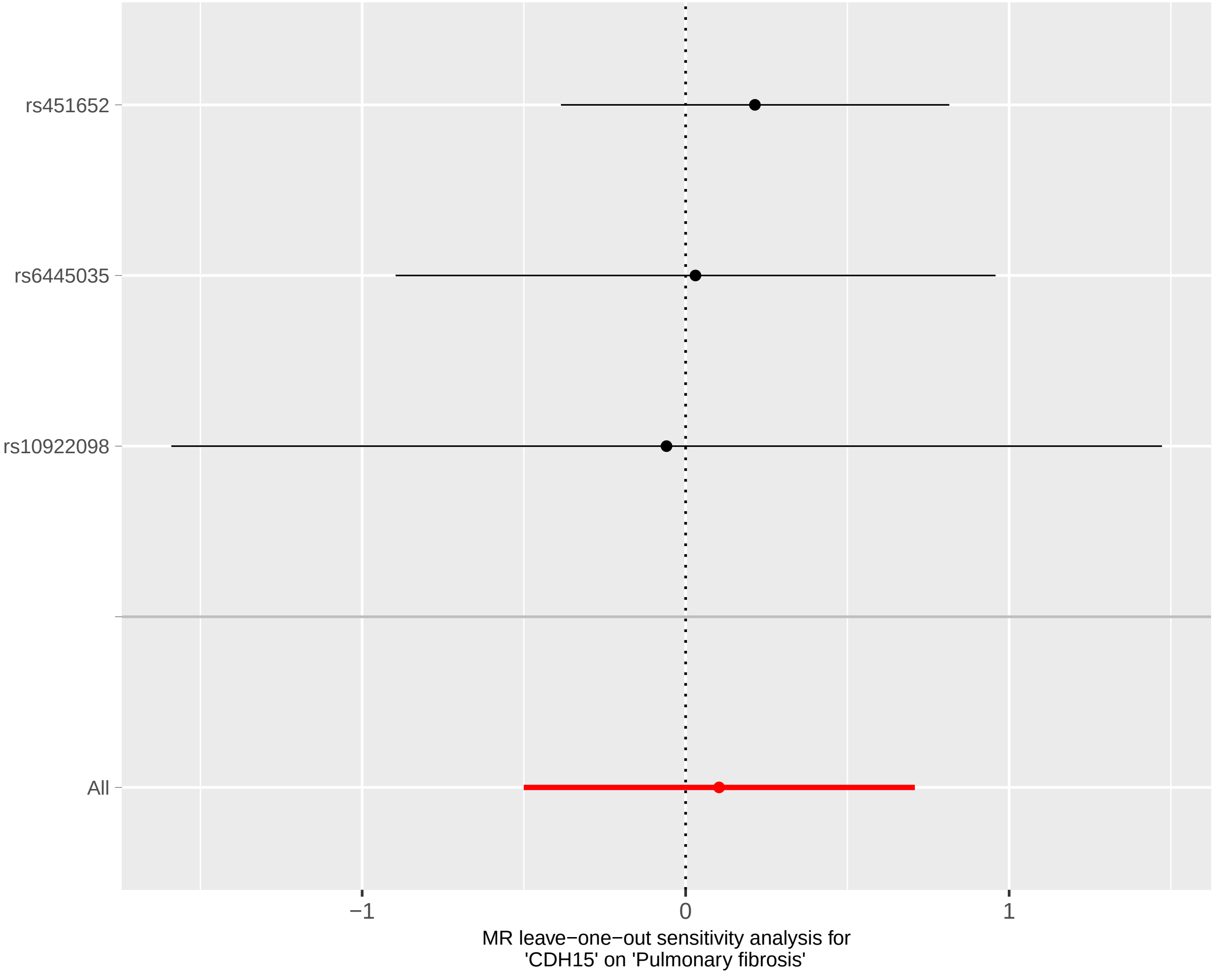
rs451652
rs6445035
rs10922098
All
−1
0
1
MR leave−one−out sensitivity analysis for
'CDH15' on 'Pulmonary fibrosis'

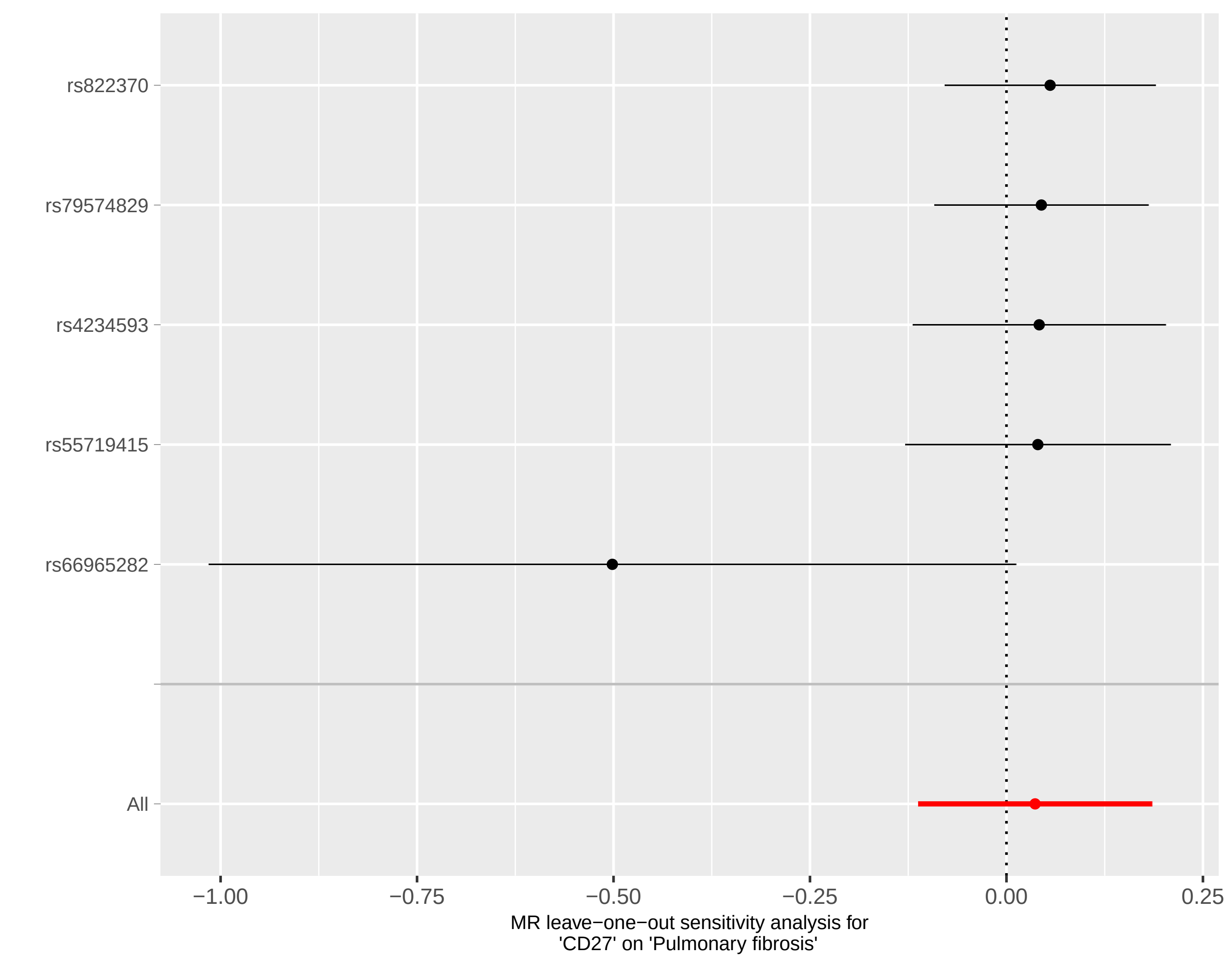

rs822370
rs79574829
rs4234593
rs55719415
rs66965282
All
−1.00
−0.75
−0.50
−0.25
0.00
0.25
MR leave−one−out sensitivity analysis for
'CD27' on 'Pulmonary fibrosis'

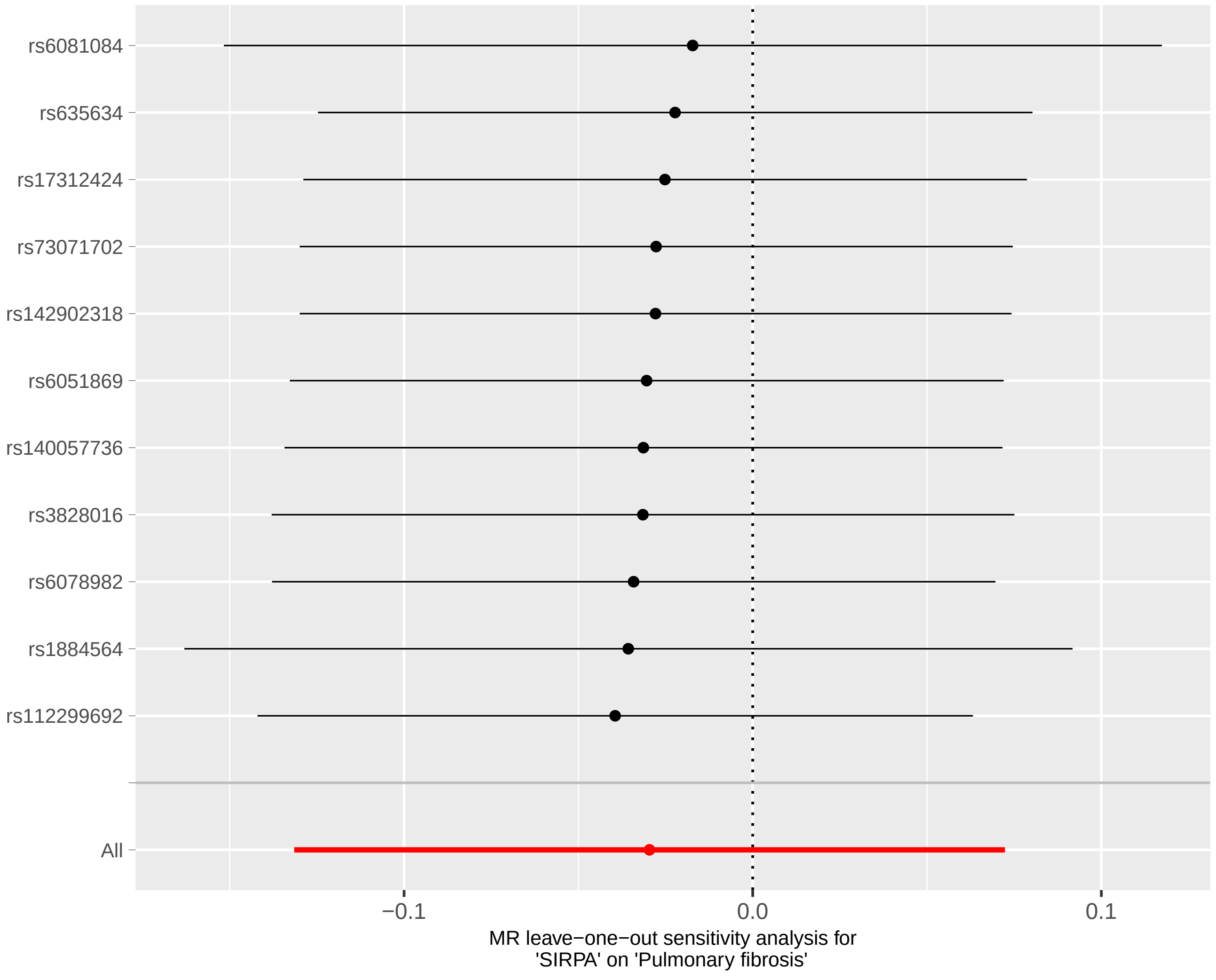
rs6081084
rs635634
rs17312424
rs73071702
rs142902318
rs6051869
rs140057736
rs3828016
rs6078982
rs1884564
rs112299692
All
−0.1
0.0
0.1
MR leave−one−out sensitivity analysis for
'SIRPA' on 'Pulmonary fibrosis'

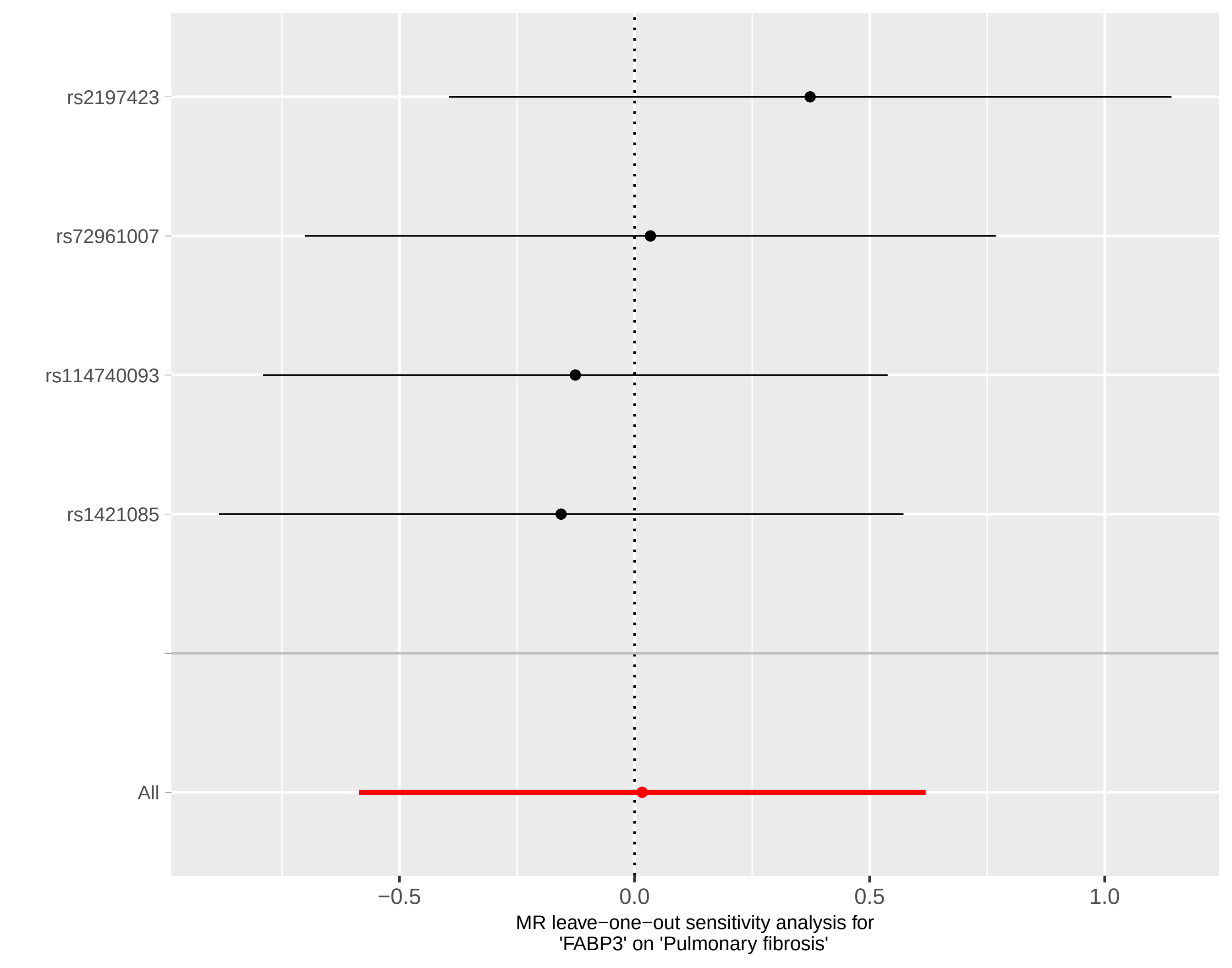
rs2197423
rs72961007
rs114740093
rs1421085
All
−0.5
0.0
0.5
1.0
MR leave−one−out sensitivity analysis for
'FABP3' on 'Pulmonary fibrosis'

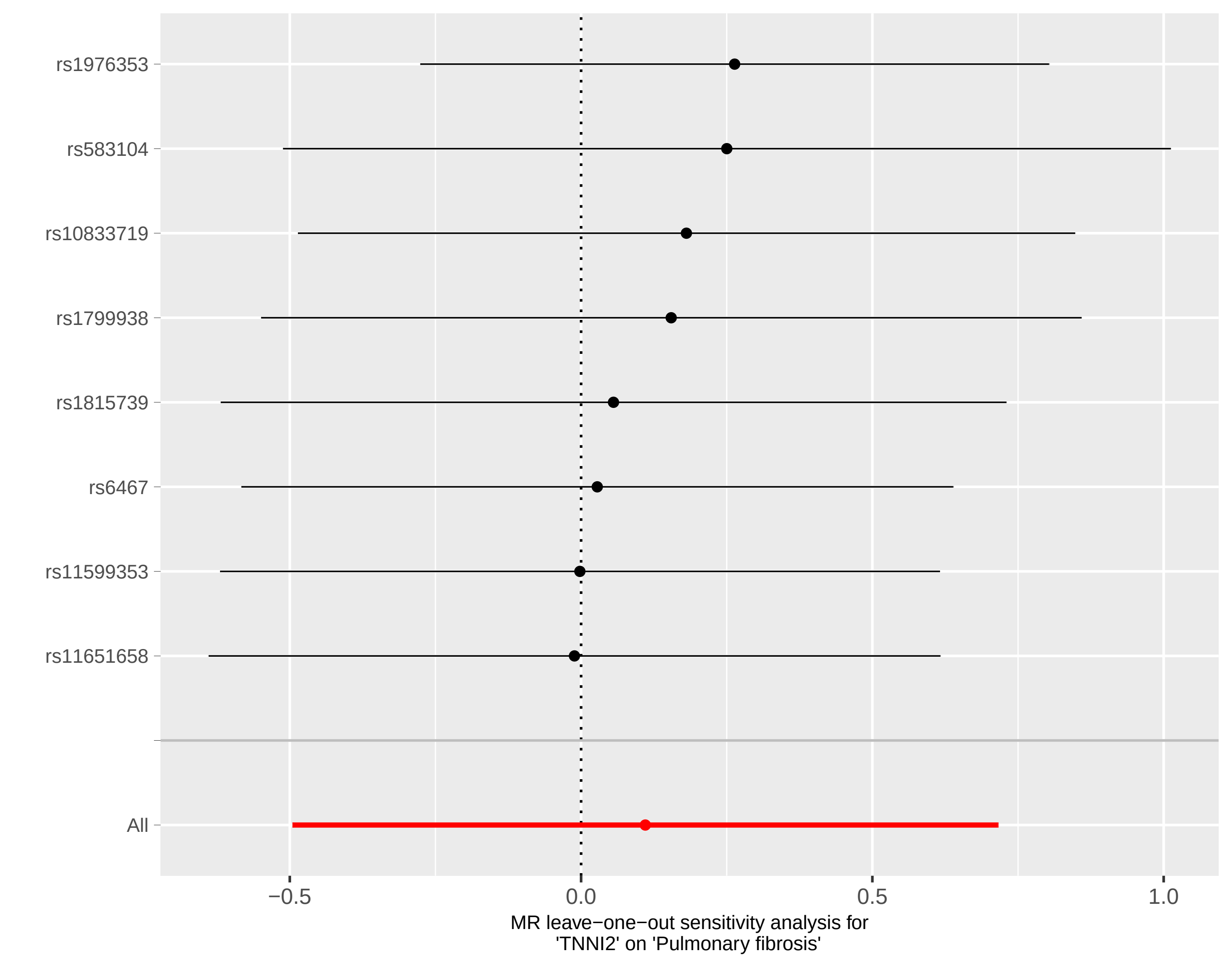
rs1976353
rs583104
rs10833719
rs1799938
rs1815739
rs6467
rs11599353
rs11651658
All
−0.5
0.0
0.5
1.0
MR leave−one−out sensitivity analysis for
'TNNI2' on 'Pulmonary fibrosis'

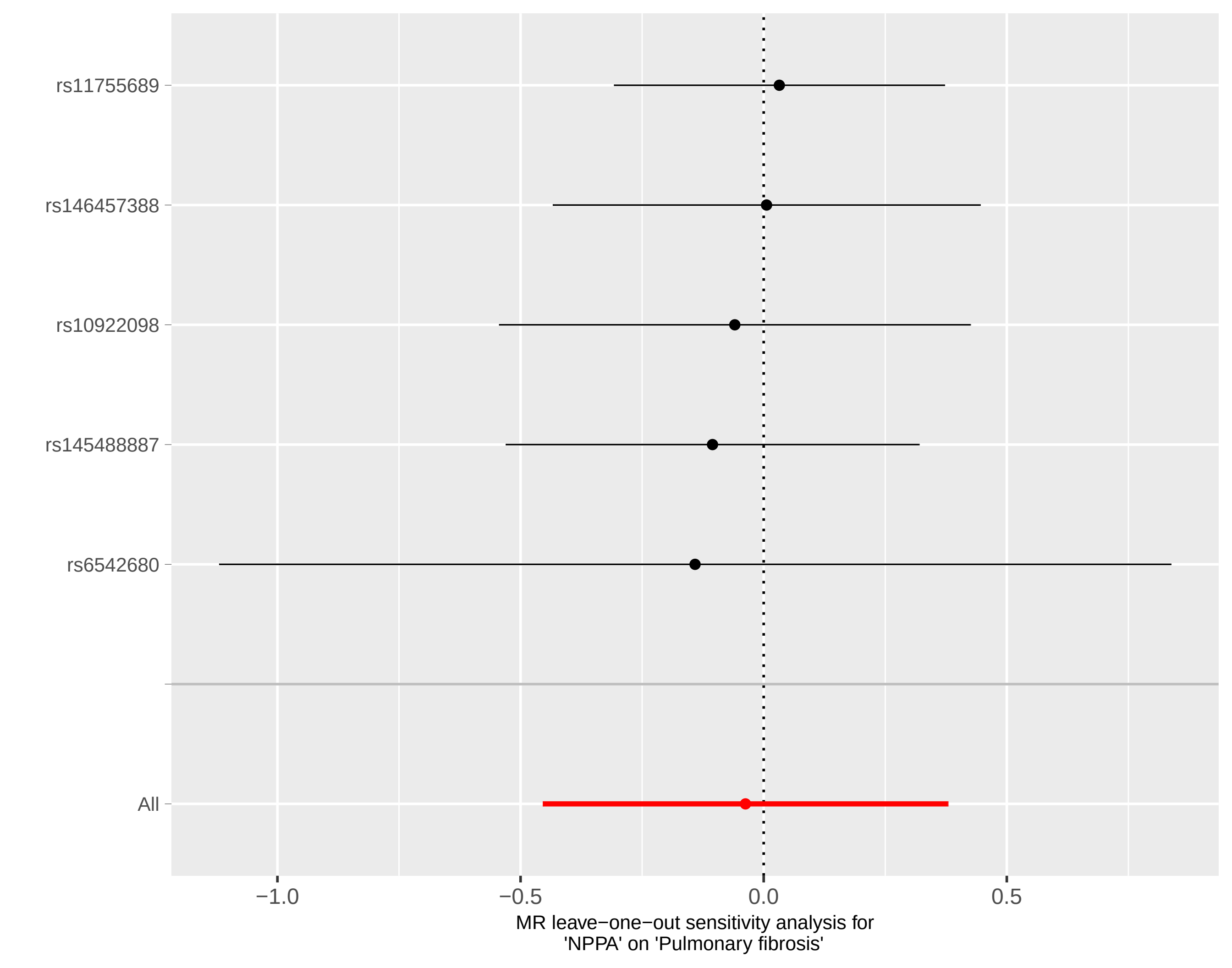
rs11755689
rs146457388
rs10922098
rs145488887
rs6542680
All
−1.0
−0.5
0.0
0.5
MR leave−one−out sensitivity analysis for
'NPPA' on 'Pulmonary fibrosis'

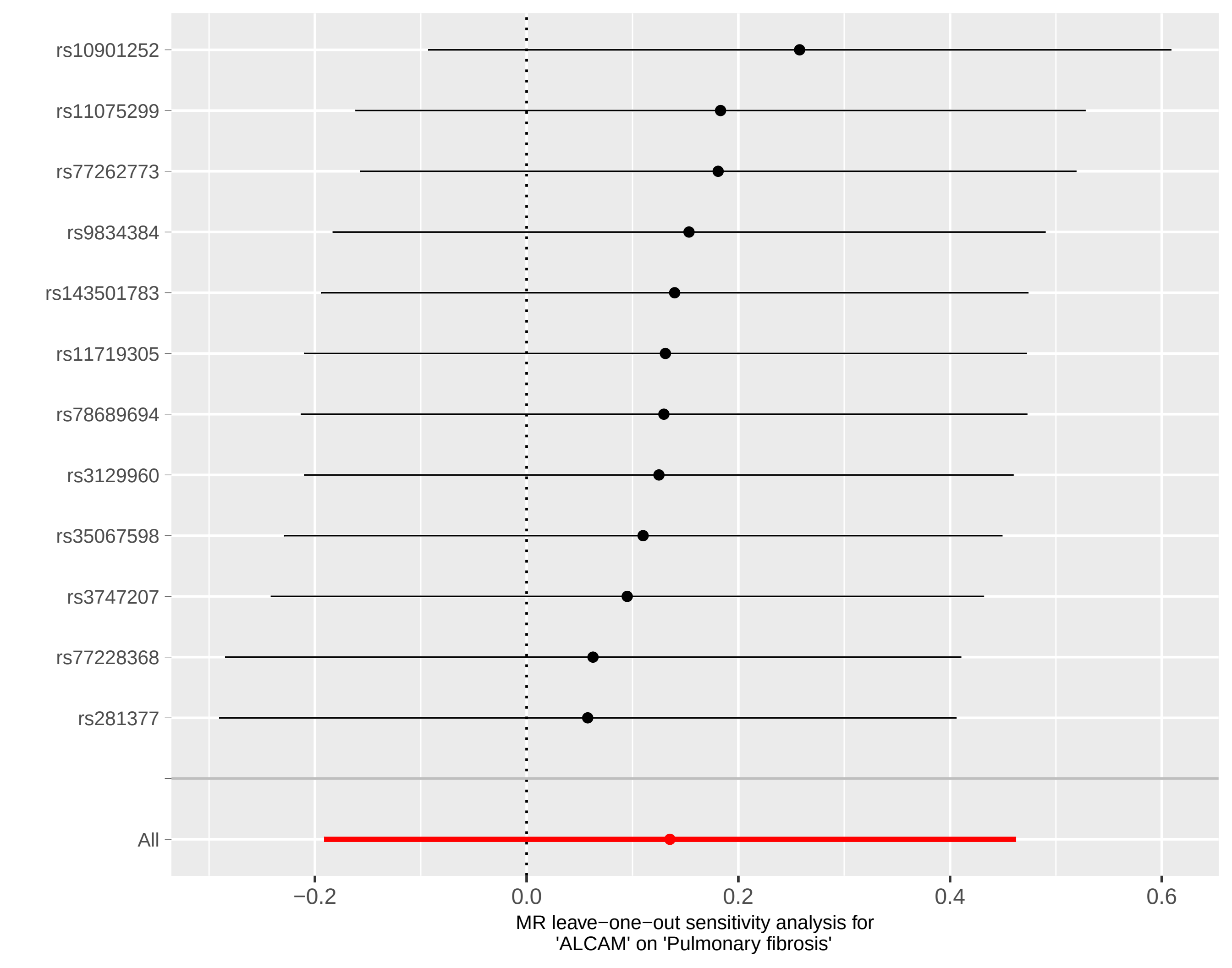

rs10901252
rs11075299
rs77262773
rs9834384
rs143501783
rs11719305
rs78689694
rs3129960
rs35067598
rs3747207
rs77228368
rs281377
All
−0.2
0.0
0.2
0.4
0.6
MR leave−one−out sensitivity analysis for
'ALCAM' on 'Pulmonary fibrosis'

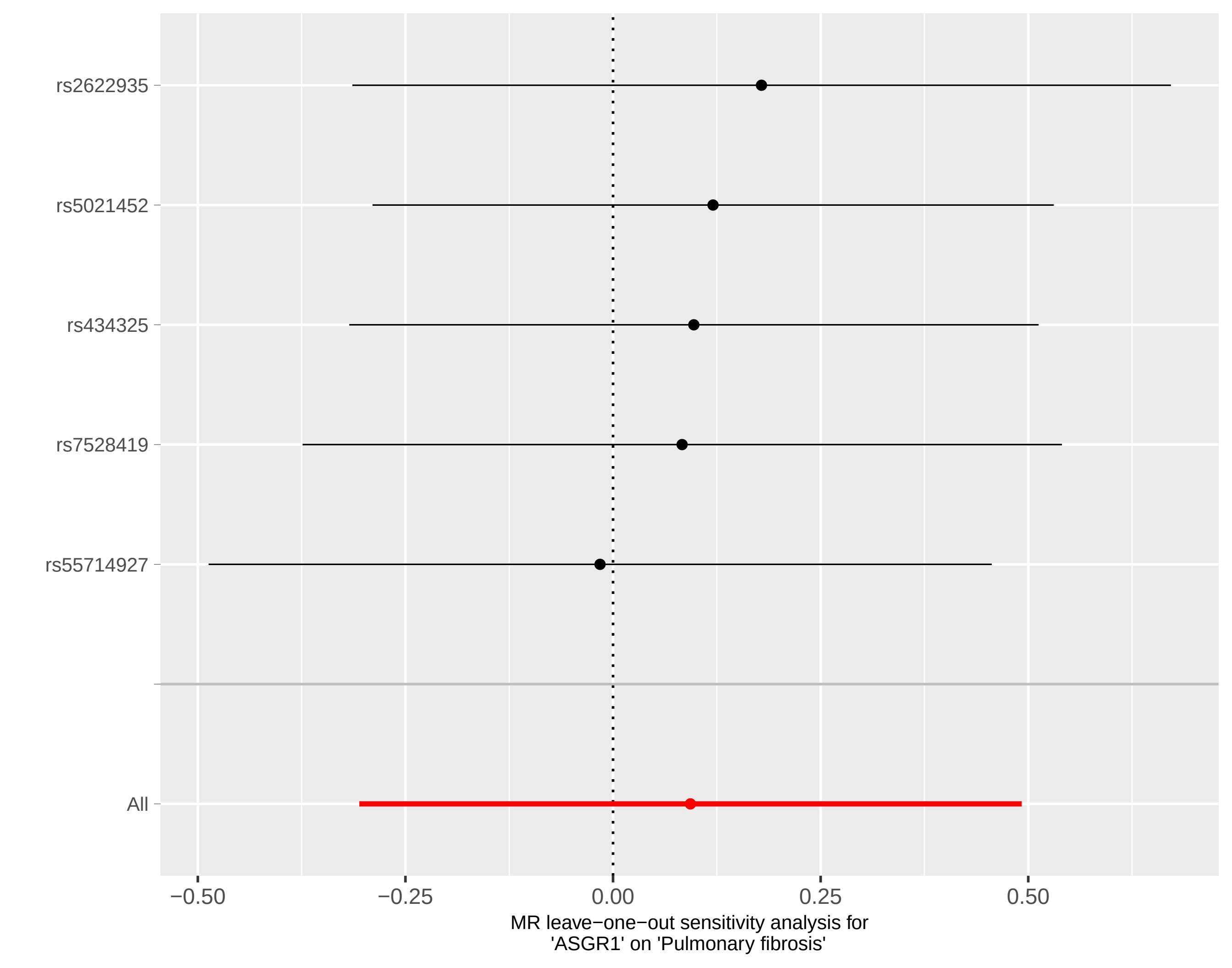

rs2622935
rs5021452
rs434325
rs7528419
rs55714927
All
−0.50
−0.25
0.00
0.25
0.50
MR leave−one−out sensitivity analysis for
'ASGR1' on 'Pulmonary fibrosis'

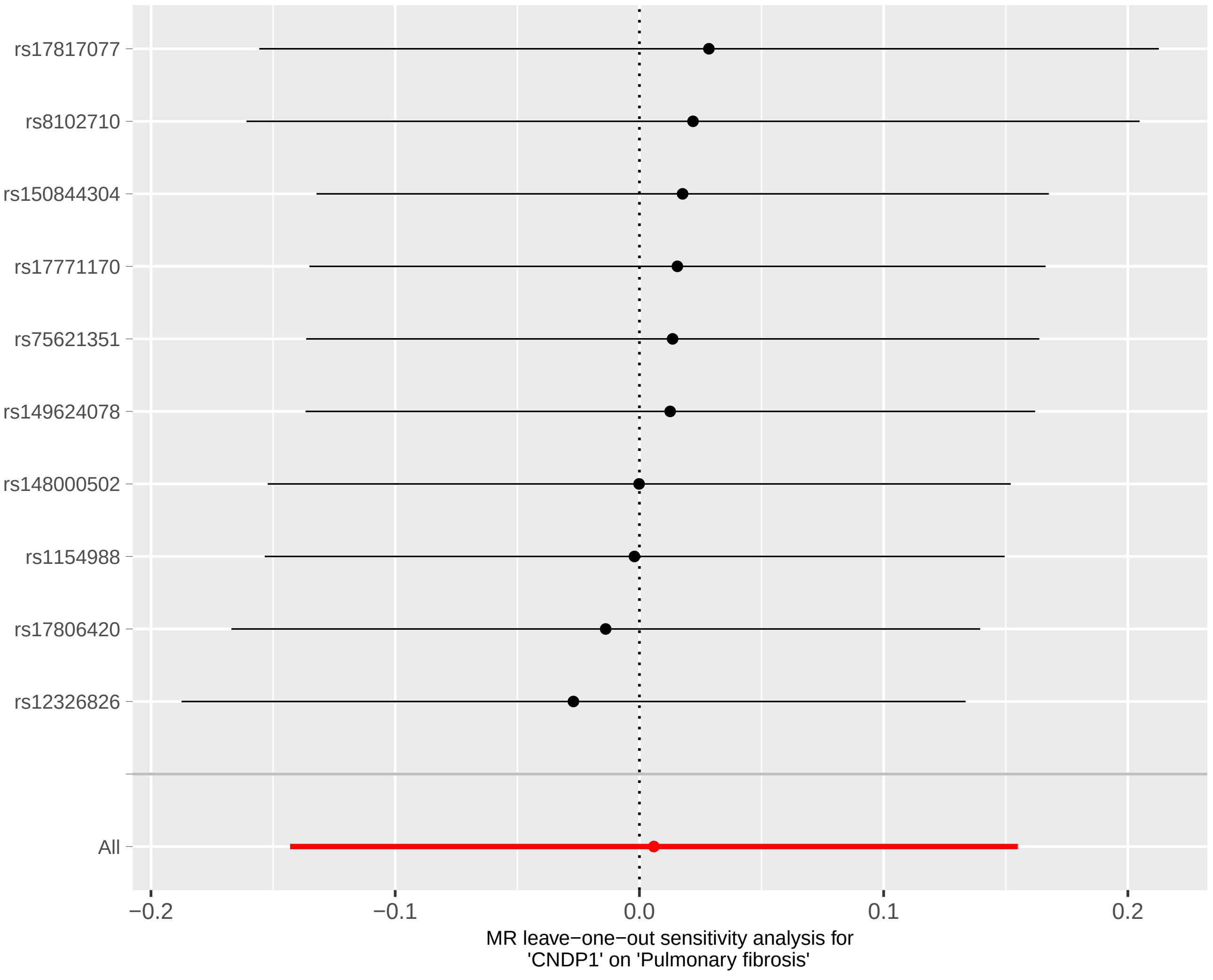
rs17817077
rs8102710
rs150844304
rs17771170
rs75621351
rs149624078
rs148000502
rs1154988
rs17806420
rs12326826
All
−0.2
−0.1
0.0
0.1
0.2
MR leave−one−out sensitivity analysis for
'CNDP1' on 'Pulmonary fibrosis'

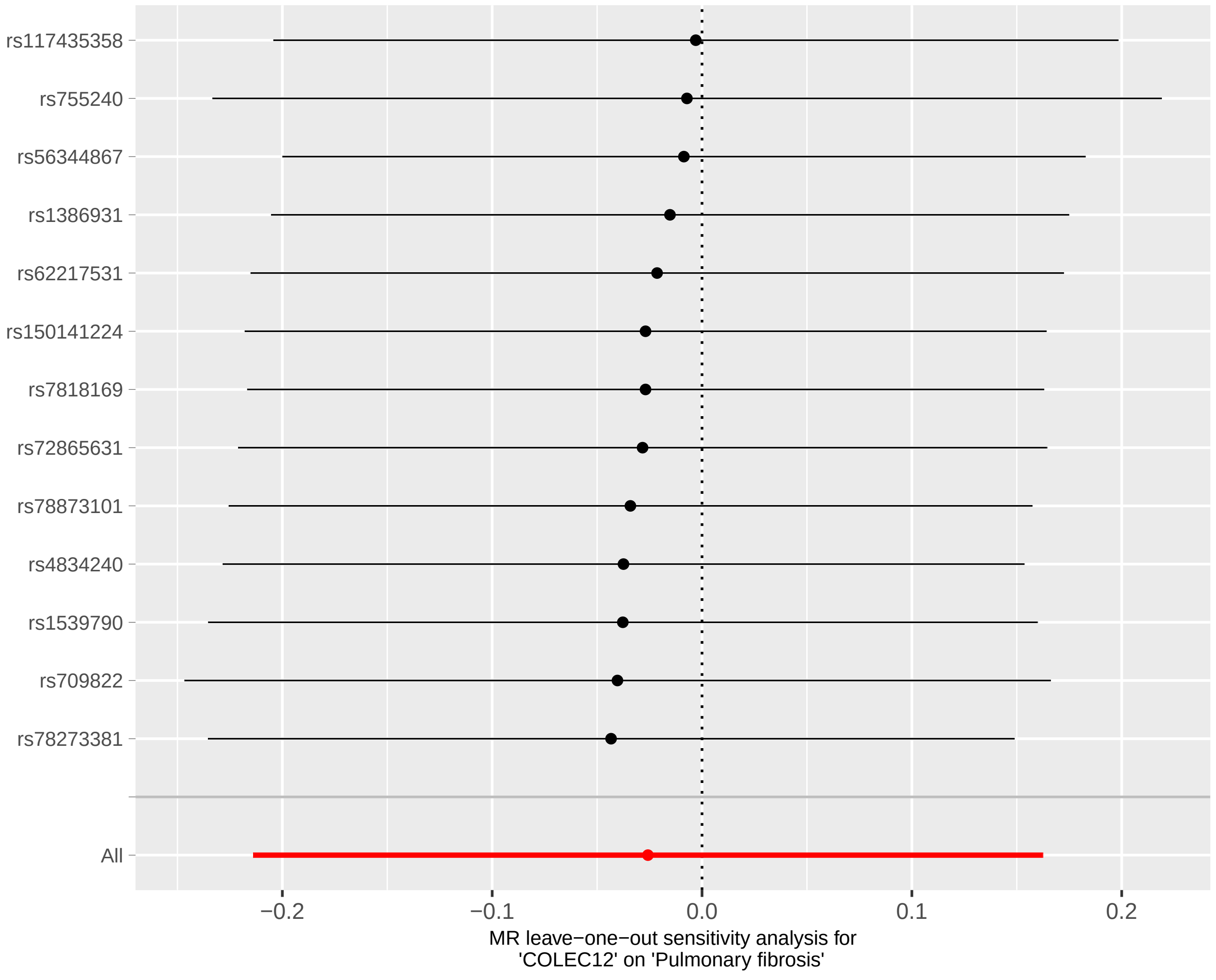
rs117435358
rs755240
rs56344867
rs1386931
rs62217531
rs150141224
rs7818169
rs72865631
rs78873101
rs4834240
rs1539790
rs709822
rs78273381
All
−0.2
−0.1
0.0
0.1
0.2
MR leave−one−out sensitivity analysis for
'COLEC12' on 'Pulmonary fibrosis'

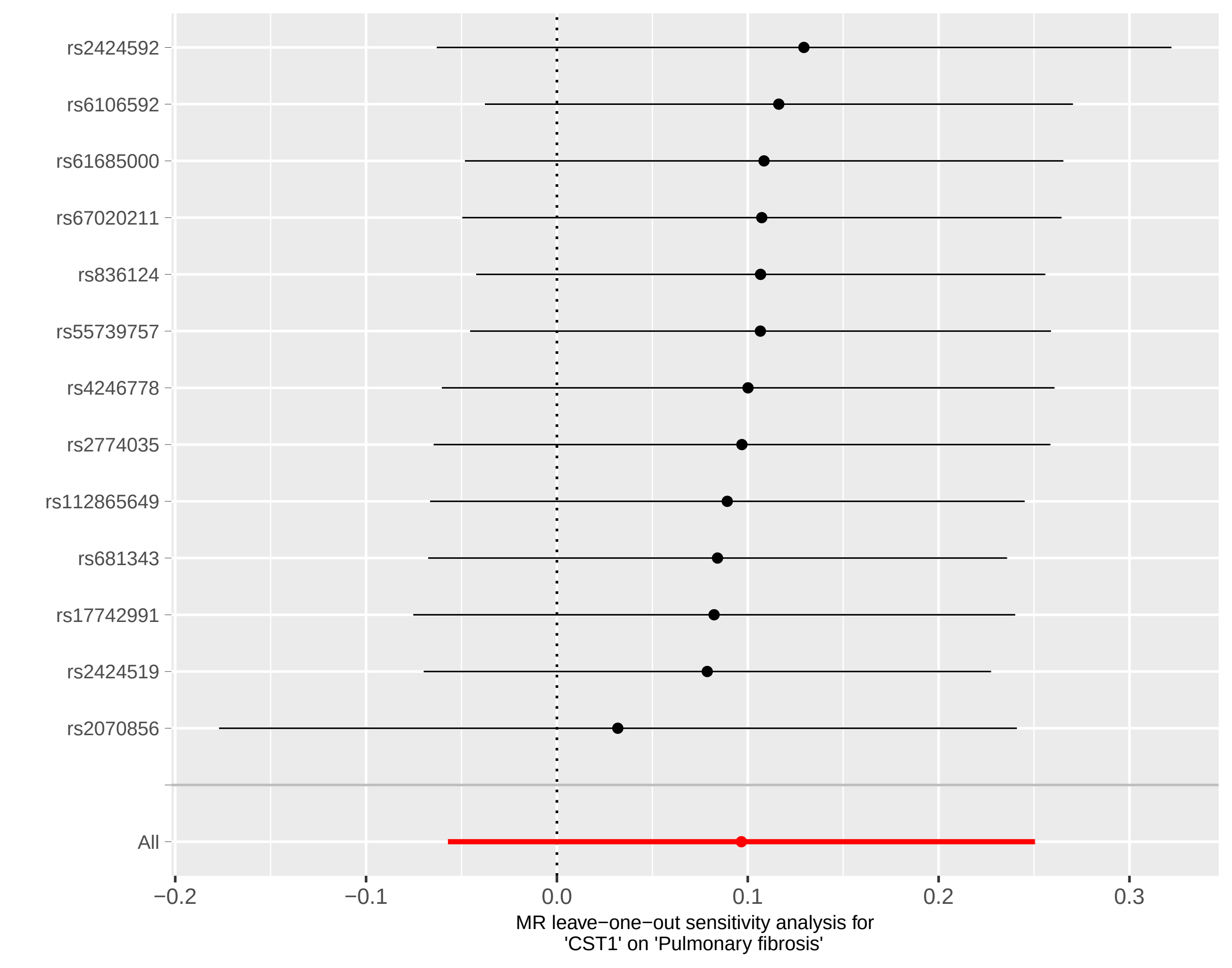
rs2424592
rs6106592
rs61685000
rs67020211
rs836124
rs55739757
rs4246778
rs2774035
rs112865649
rs681343
rs17742991
rs2424519
rs2070856
All
−0.2
−0.1
0.0
0.1
0.2
0.3
MR leave−one−out sensitivity analysis for
'CST1' on 'Pulmonary fibrosis'

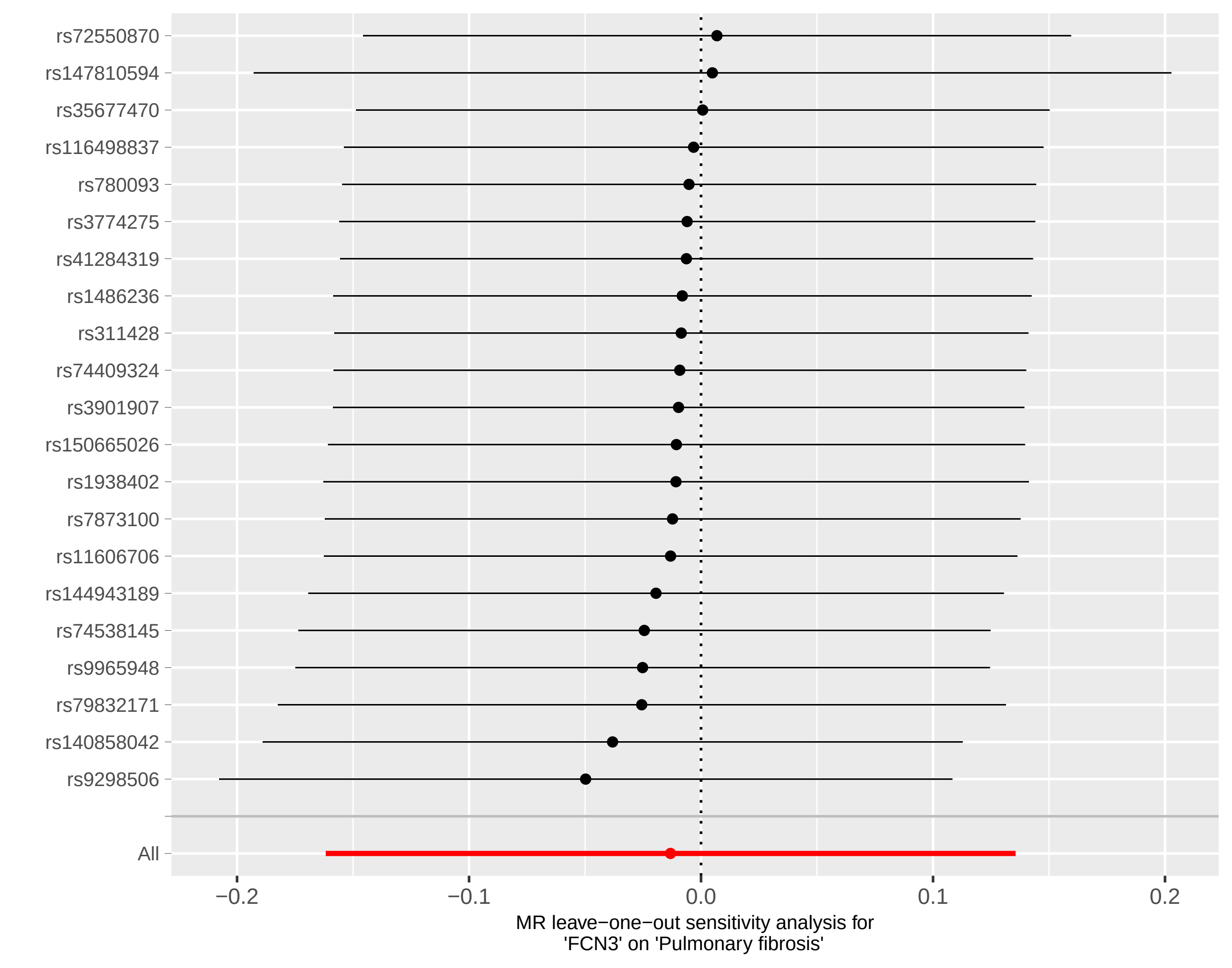
rs72550870
rs147810594
rs35677470
rs116498837
rs780093
rs3774275
rs41284319
rs1486236
rs311428
rs74409324
rs3901907
rs150665026
rs1938402
rs7873100
rs11606706
rs144943189
rs74538145
rs9965948
rs79832171
rs140858042
rs9298506
All
−0.2
−0.1
0.0
0.1
0.2
MR leave−one−out sensitivity analysis for
'FCN3' on 'Pulmonary fibrosis'

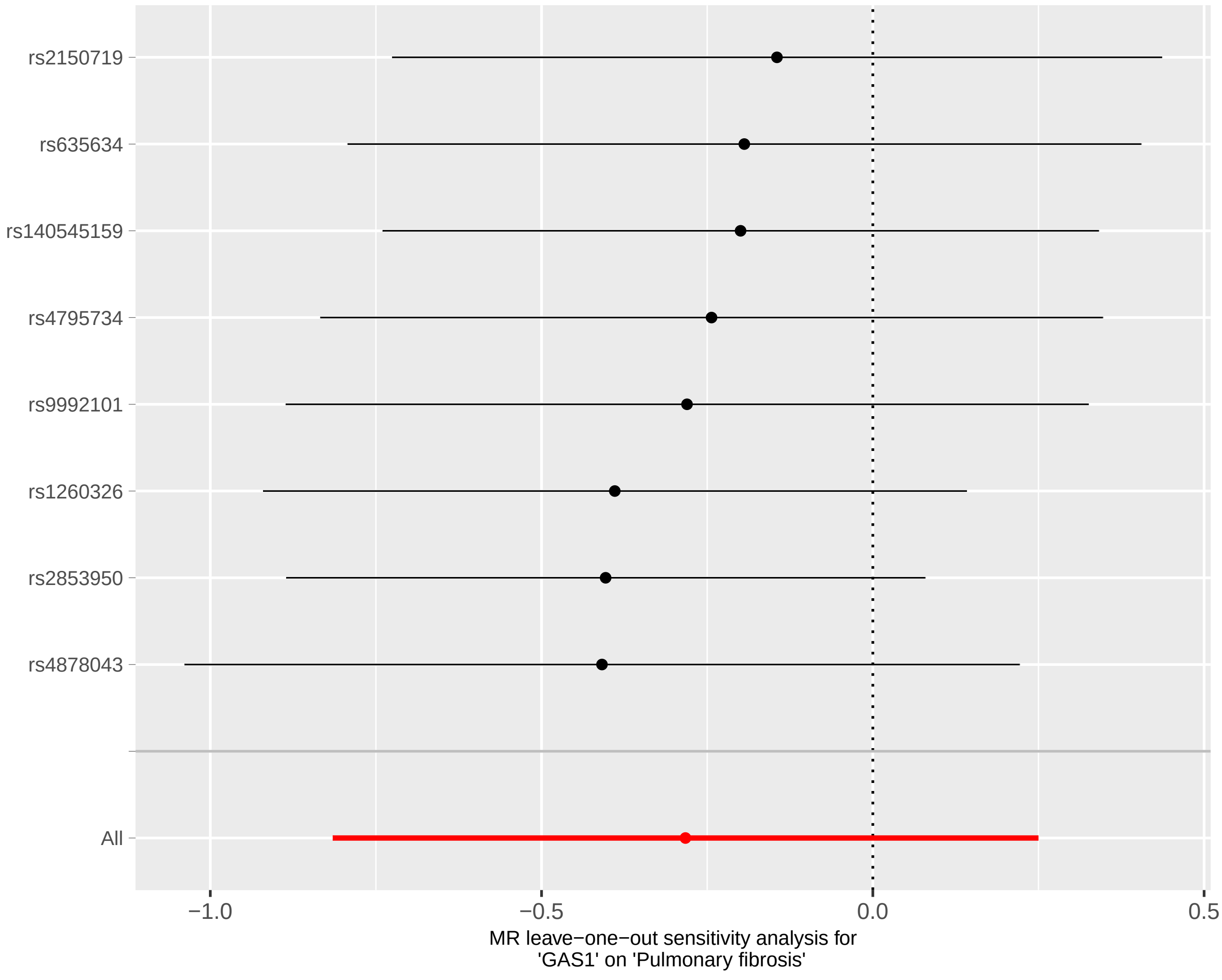
rs2150719
rs635634
rs140545159
rs4795734
rs9992101
rs1260326
rs2853950
rs4878043
All
−1.0
−0.5
0.0
0.5
MR leave−one−out sensitivity analysis for
'GAS1' on 'Pulmonary fibrosis'

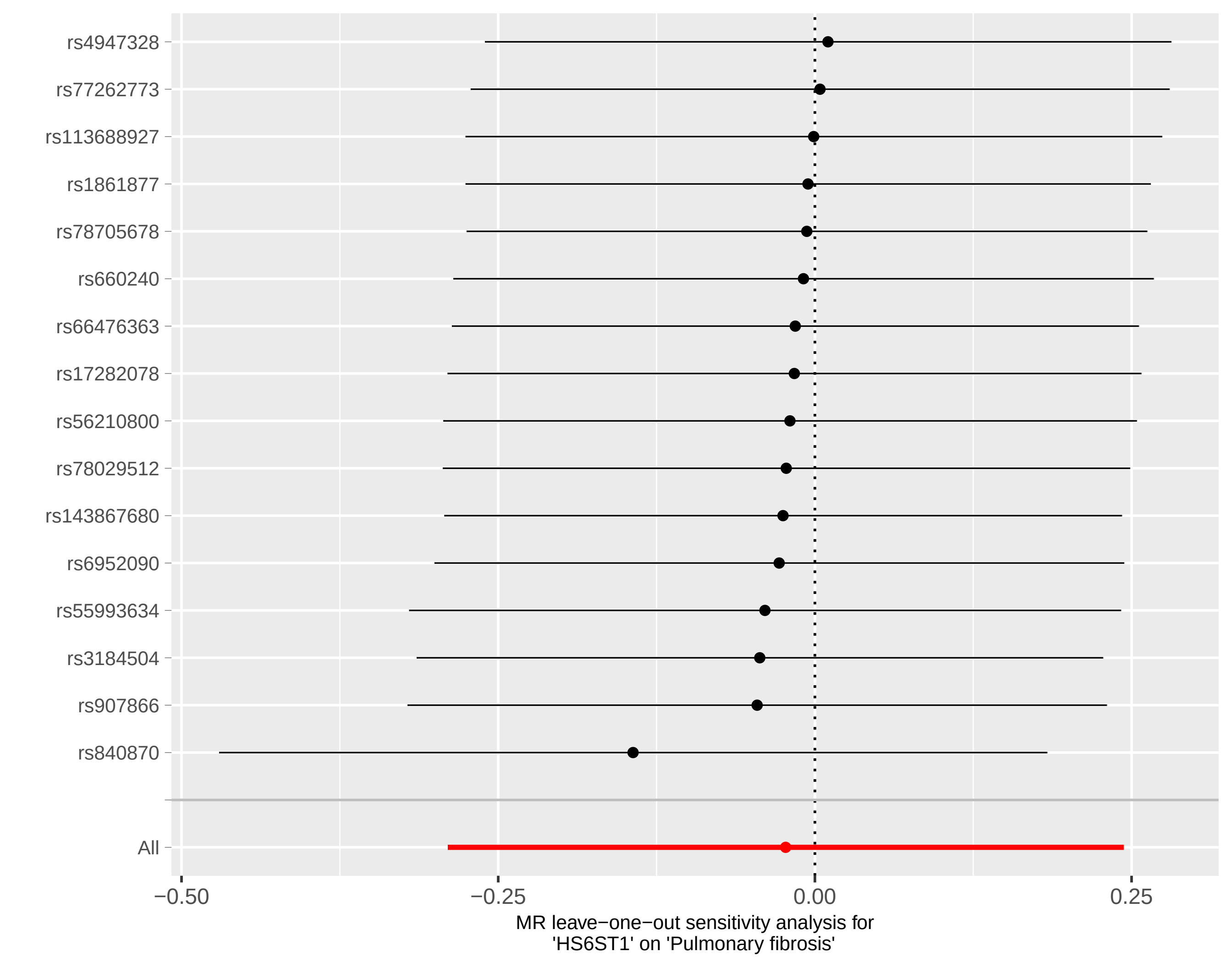
rs4947328
rs77262773
rs113688927
rs1861877
rs78705678
rs660240
rs66476363
rs17282078
rs56210800
rs78029512
rs143867680
rs6952090
rs55993634
rs3184504
rs907866
rs840870
All
−0.50
−0.25
0.00
0.25
MR leave−one−out sensitivity analysis for
'HS6ST1' on 'Pulmonary fibrosis'

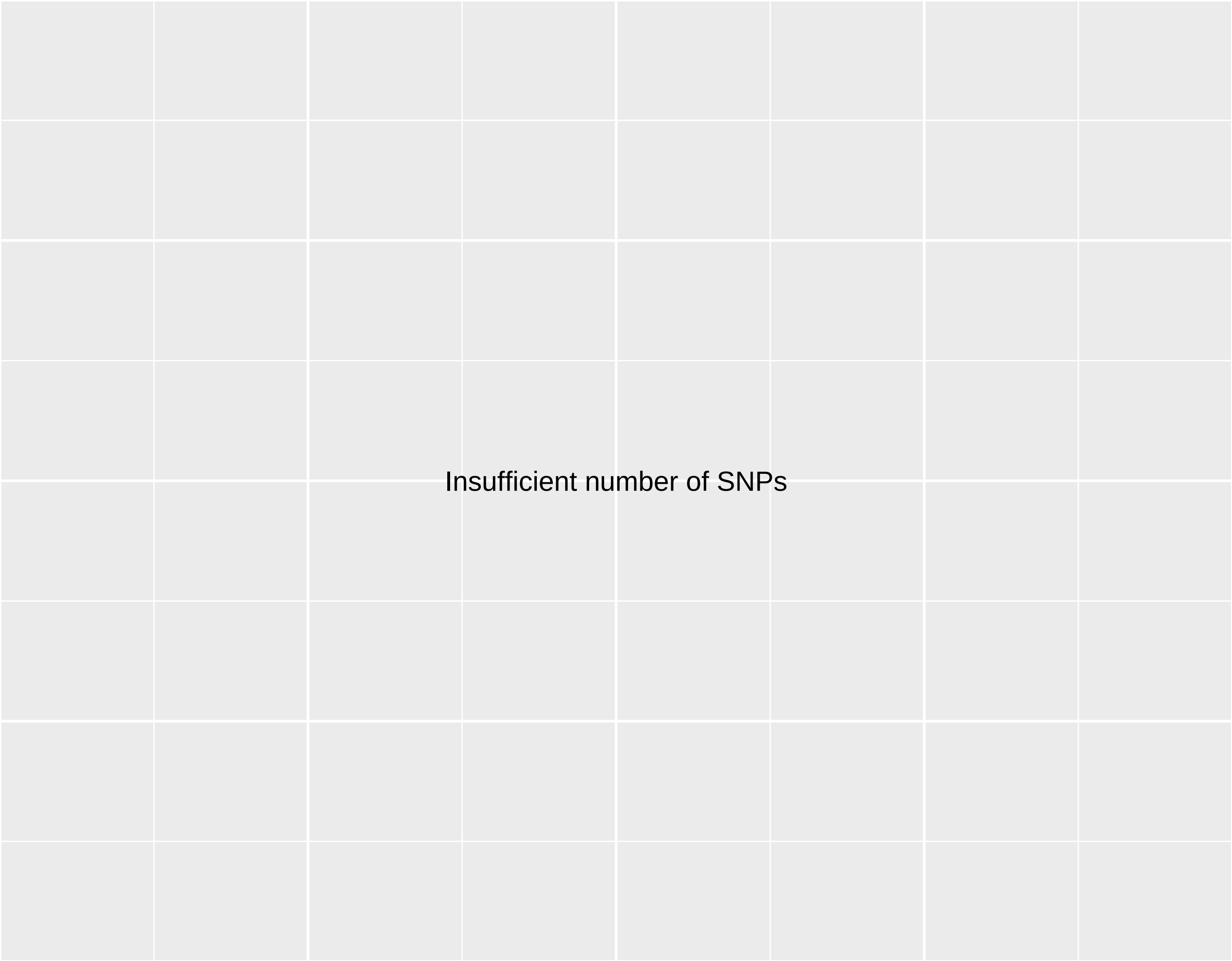
Insufficient number of SNPs

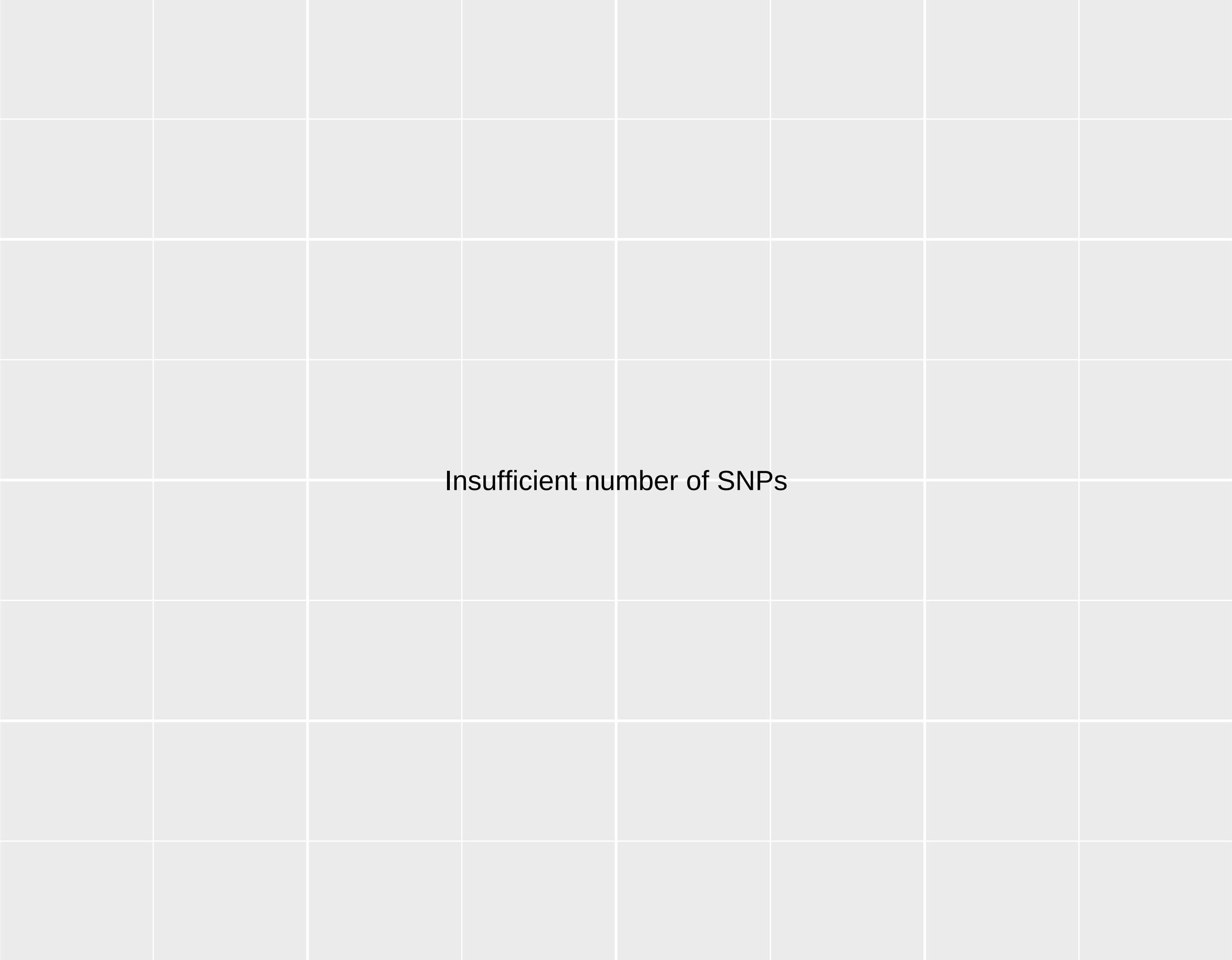
Insufficient number of SNPs

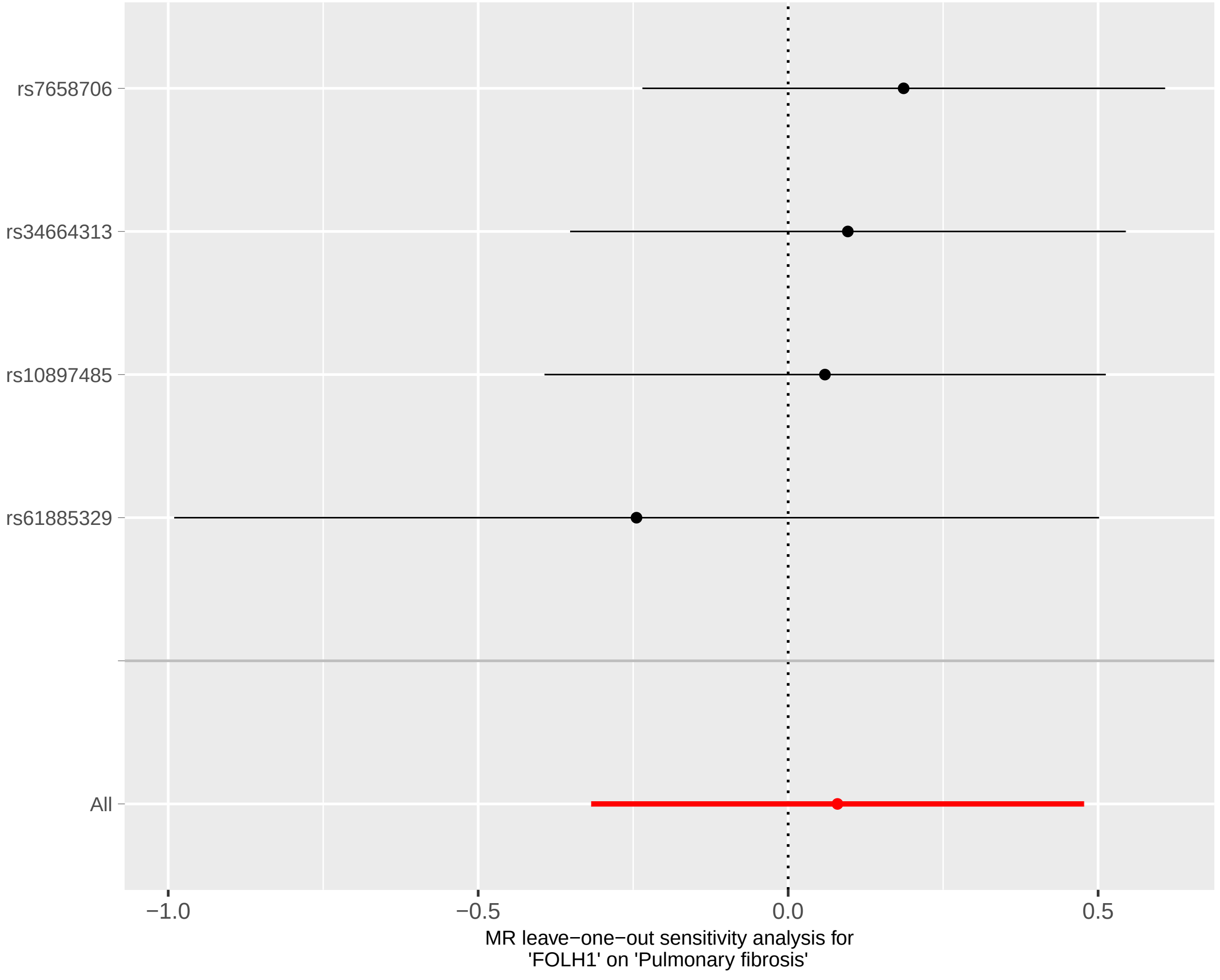
rs7658706
rs34664313
rs10897485
rs61885329
All
−1.0
−0.5
0.0
0.5
MR leave−one−out sensitivity analysis for
'FOLH1' on 'Pulmonary fibrosis'

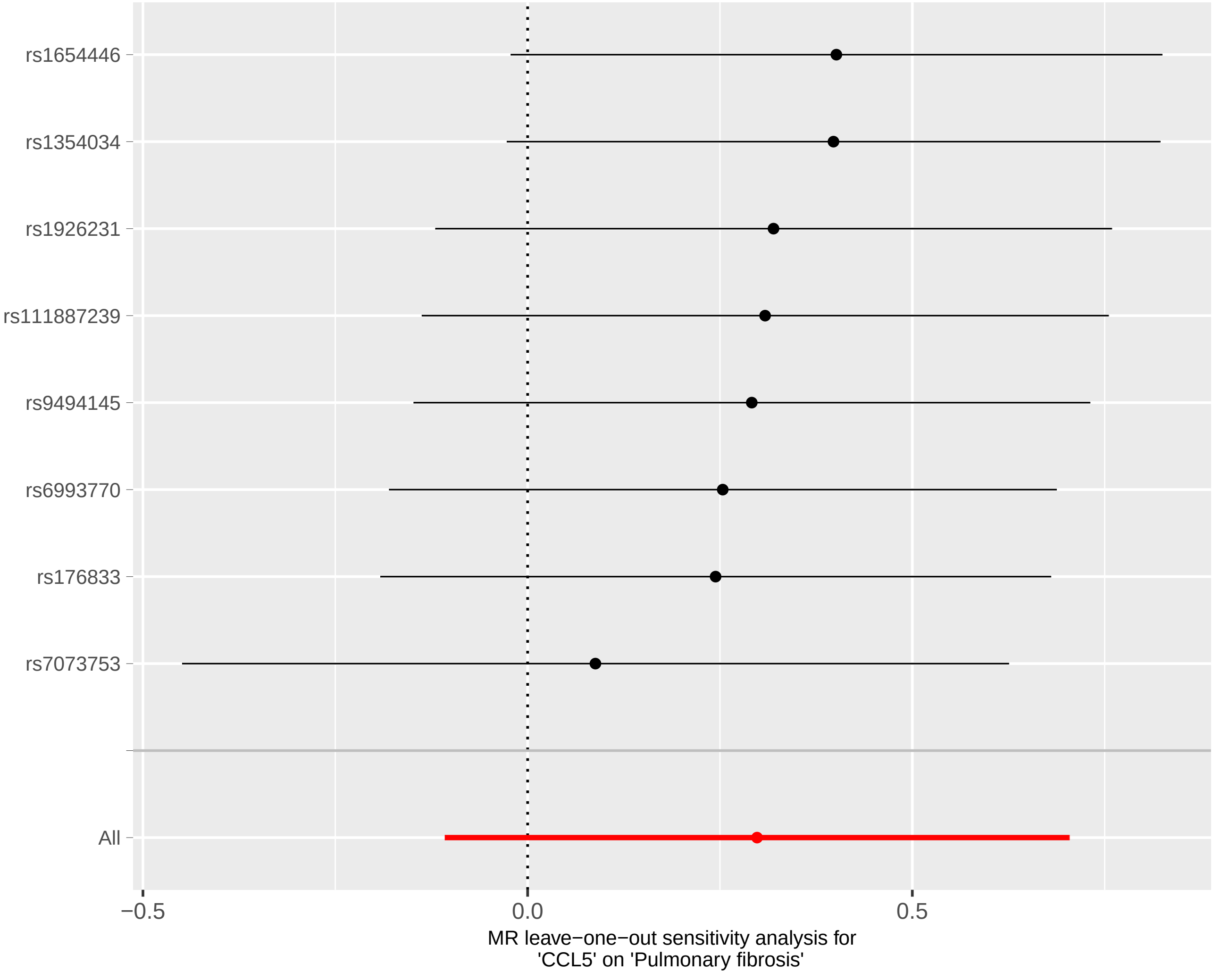
rs1654446
rs1354034
rs1926231
rs111887239
rs9494145
rs6993770
rs176833
rs7073753
All
−0.5
0.0
0.5
MR leave−one−out sensitivity analysis for
'CCL5' on 'Pulmonary fibrosis'

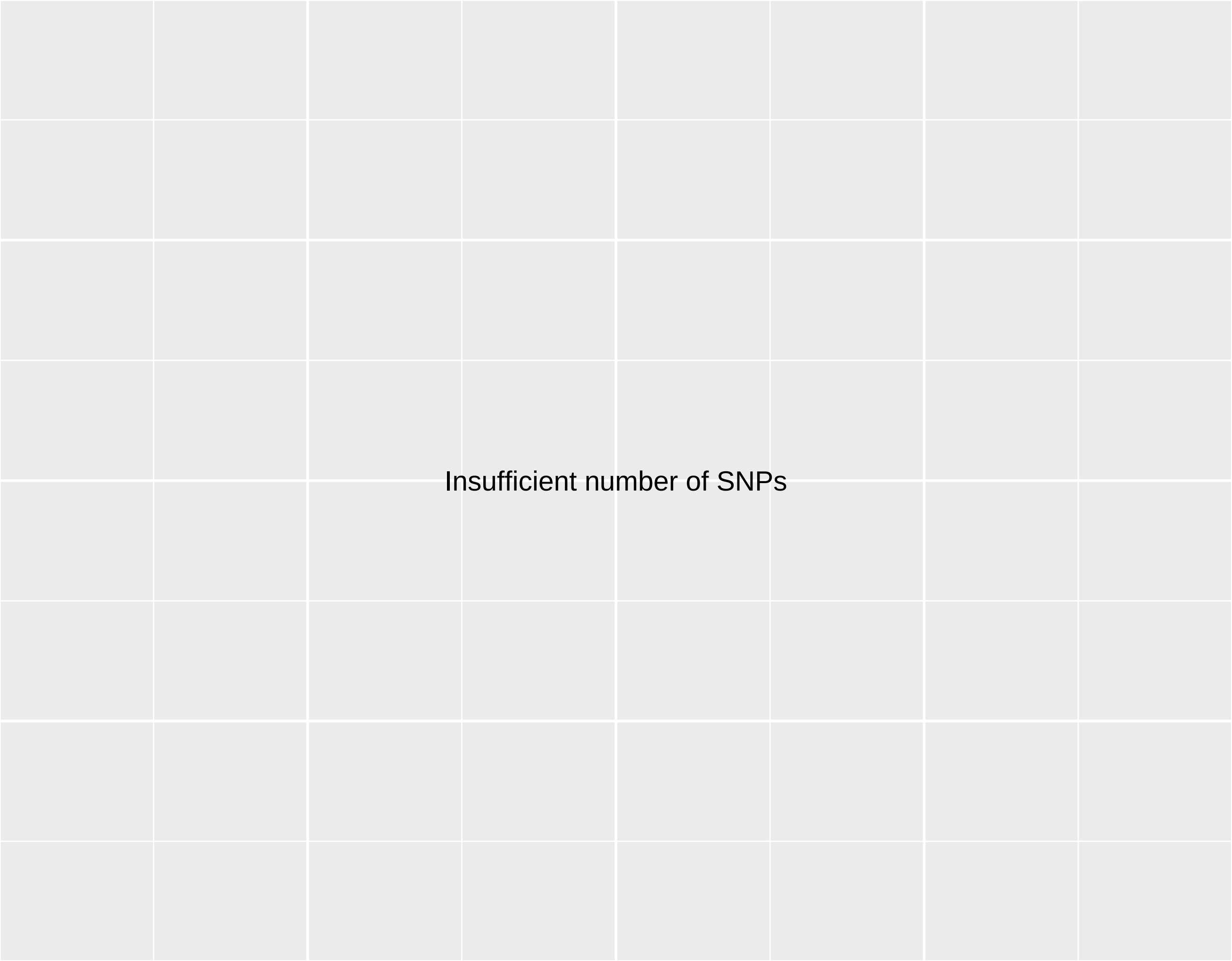
Insufficient number of SNPs

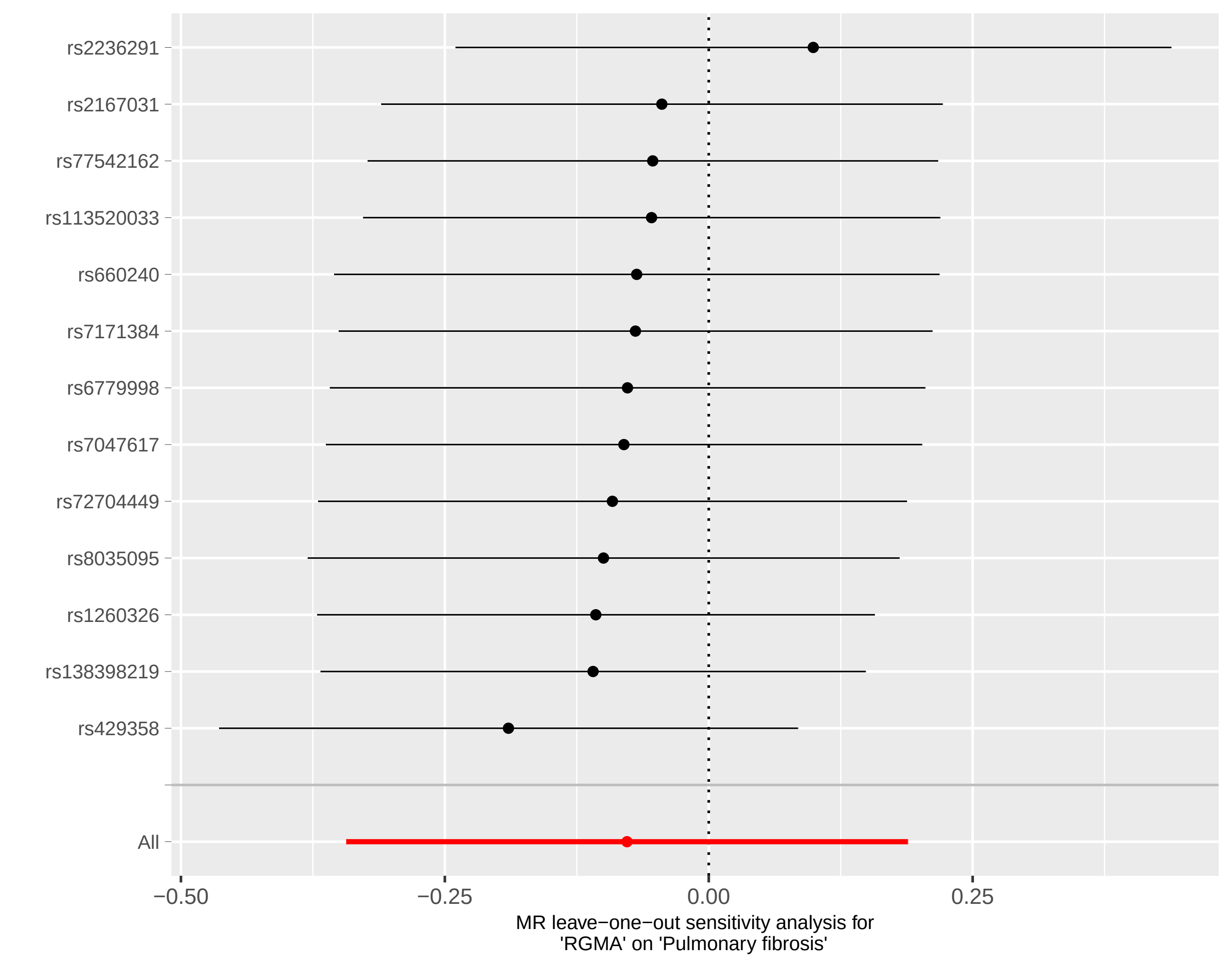
rs2236291
rs2167031
rs77542162
rs113520033
rs660240
rs7171384
rs6779998
rs7047617
rs72704449
rs8035095
rs1260326
rs138398219
rs429358
All
−0.50
−0.25
0.00
0.25
MR leave−one−out sensitivity analysis for
'RGMA' on 'Pulmonary fibrosis'

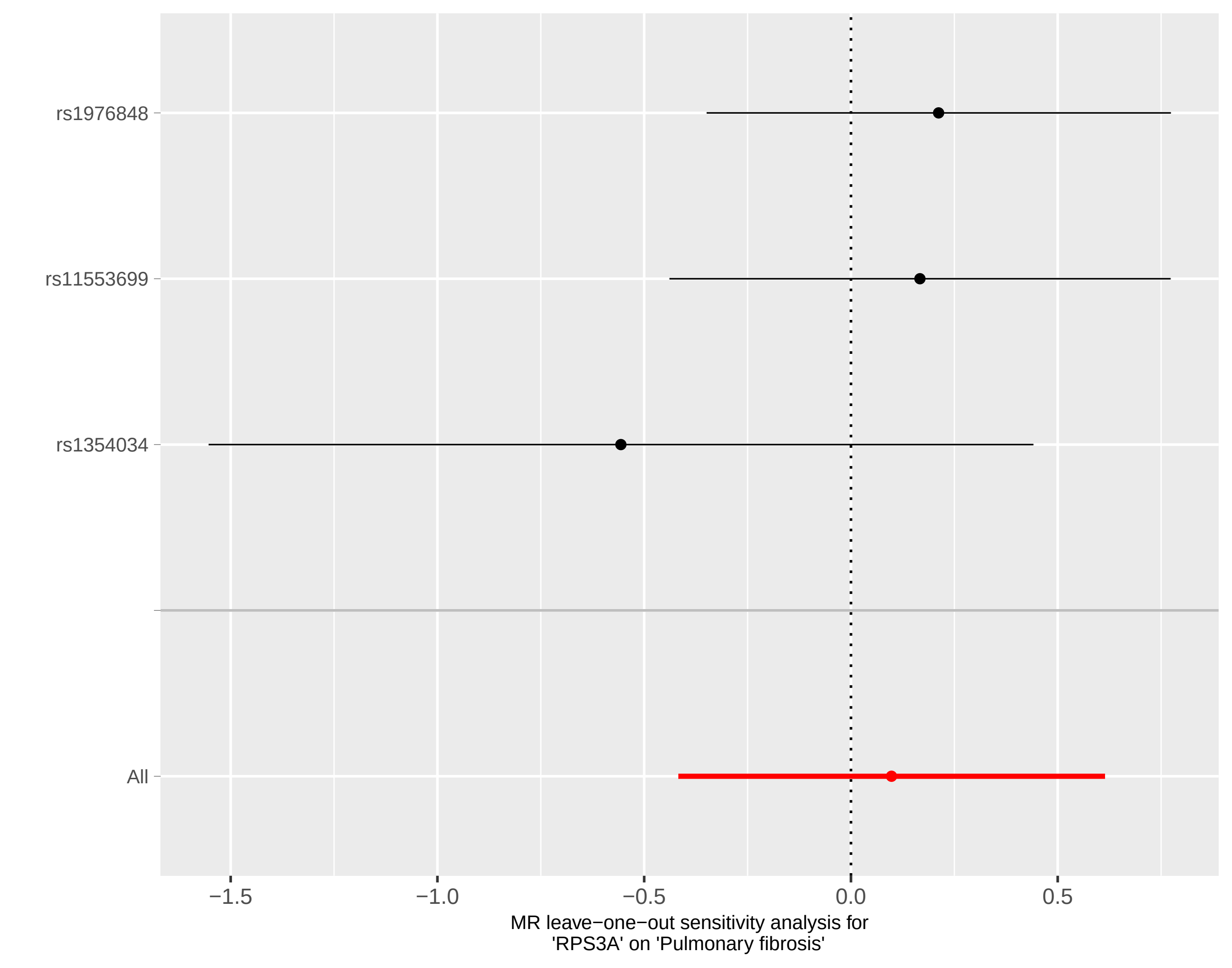
rs1976848
rs11553699
rs1354034
All
−1.5
−1.0
−0.5
0.0
0.5
MR leave−one−out sensitivity analysis for
'RPS3A' on 'Pulmonary fibrosis'

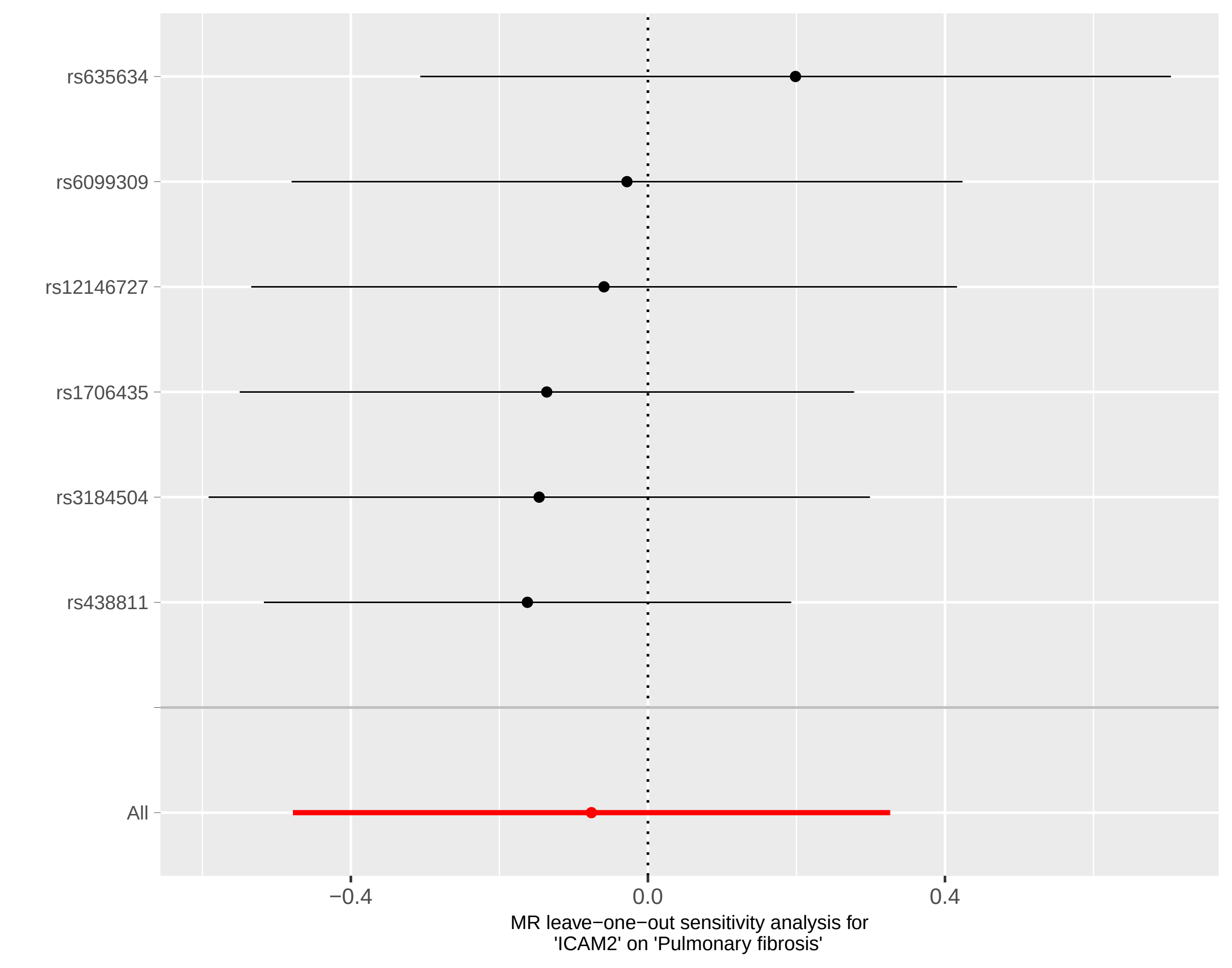
rs635634
rs6099309
rs12146727
rs1706435
rs3184504
rs438811
All
−0.4
0.0
0.4
MR leave−one−out sensitivity analysis for
'ICAM2' on 'Pulmonary fibrosis'

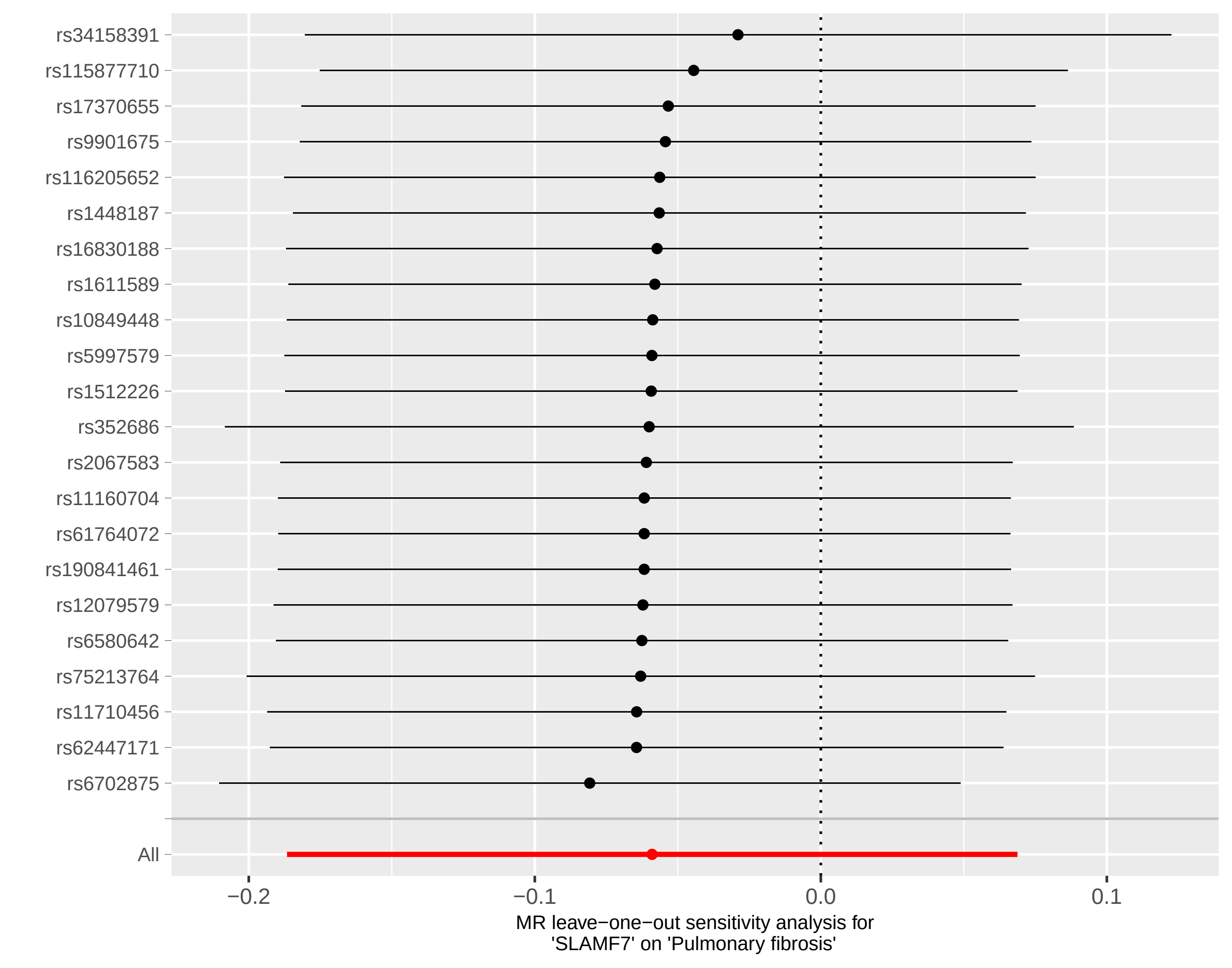
rs34158391
rs115877710
rs17370655
rs9901675
rs116205652
rs1448187
rs16830188
rs1611589
rs10849448
rs5997579
rs1512226
rs352686
rs2067583
rs11160704
rs61764072
rs190841461
rs12079579
rs6580642
rs75213764
rs11710456
rs62447171
rs6702875
All
−0.2
−0.1
0.0
0.1
MR leave−one−out sensitivity analysis for
'SLAMF7' on 'Pulmonary fibrosis'

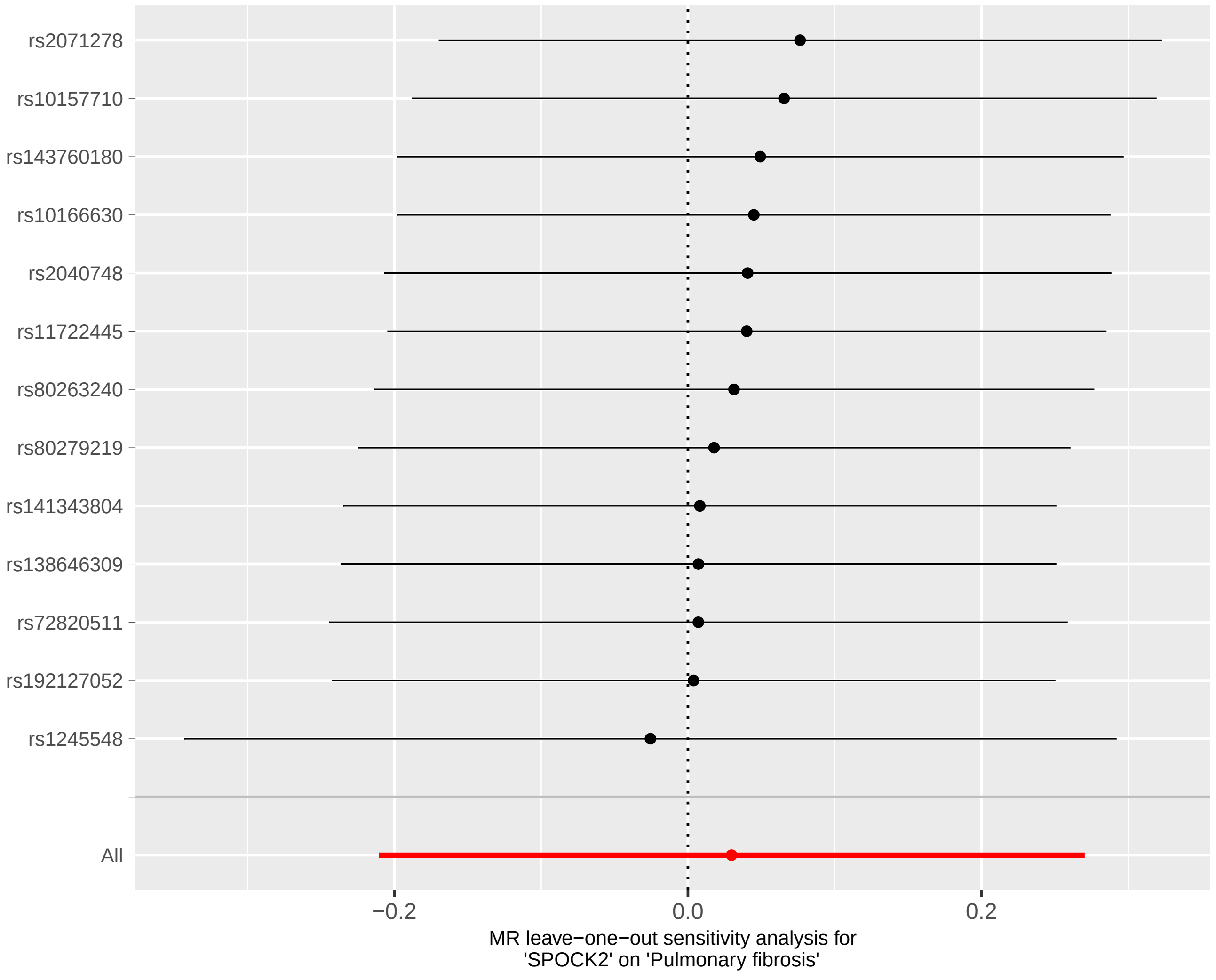
rs2071278
rs10157710
rs143760180
rs10166630
rs2040748
rs11722445
rs80263240
rs80279219
rs141343804
rs138646309
rs72820511
rs192127052
rs1245548
All
−0.2
0.0
0.2
MR leave−one−out sensitivity analysis for
'SPOCK2' on 'Pulmonary fibrosis'

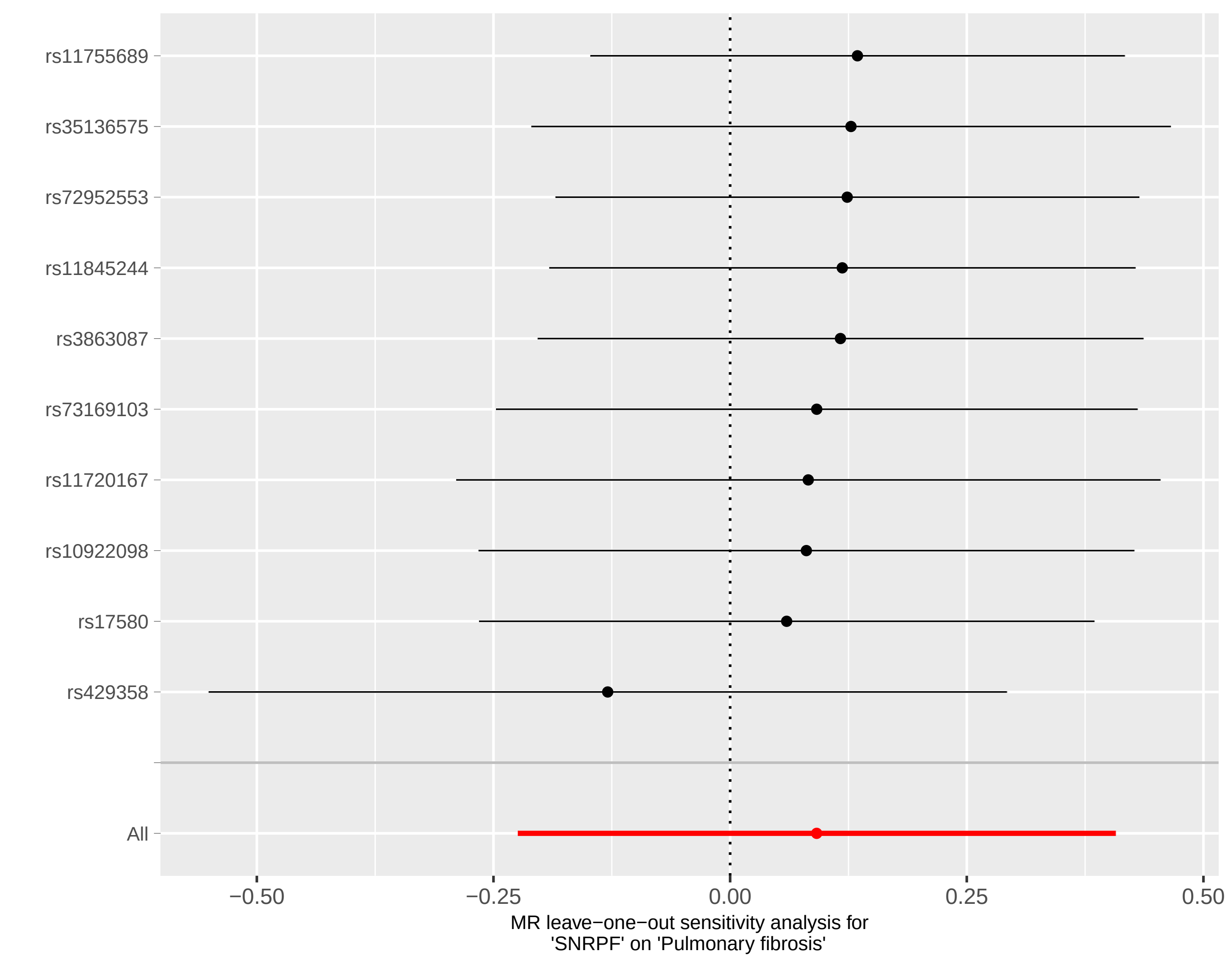
rs11755689
rs35136575
rs72952553
rs11845244
rs3863087
rs73169103
rs11720167
rs10922098
rs17580
rs429358
All
−0.50
−0.25
0.00
0.25
0.50
MR leave−one−out sensitivity analysis for
'SNRPF' on 'Pulmonary fibrosis'

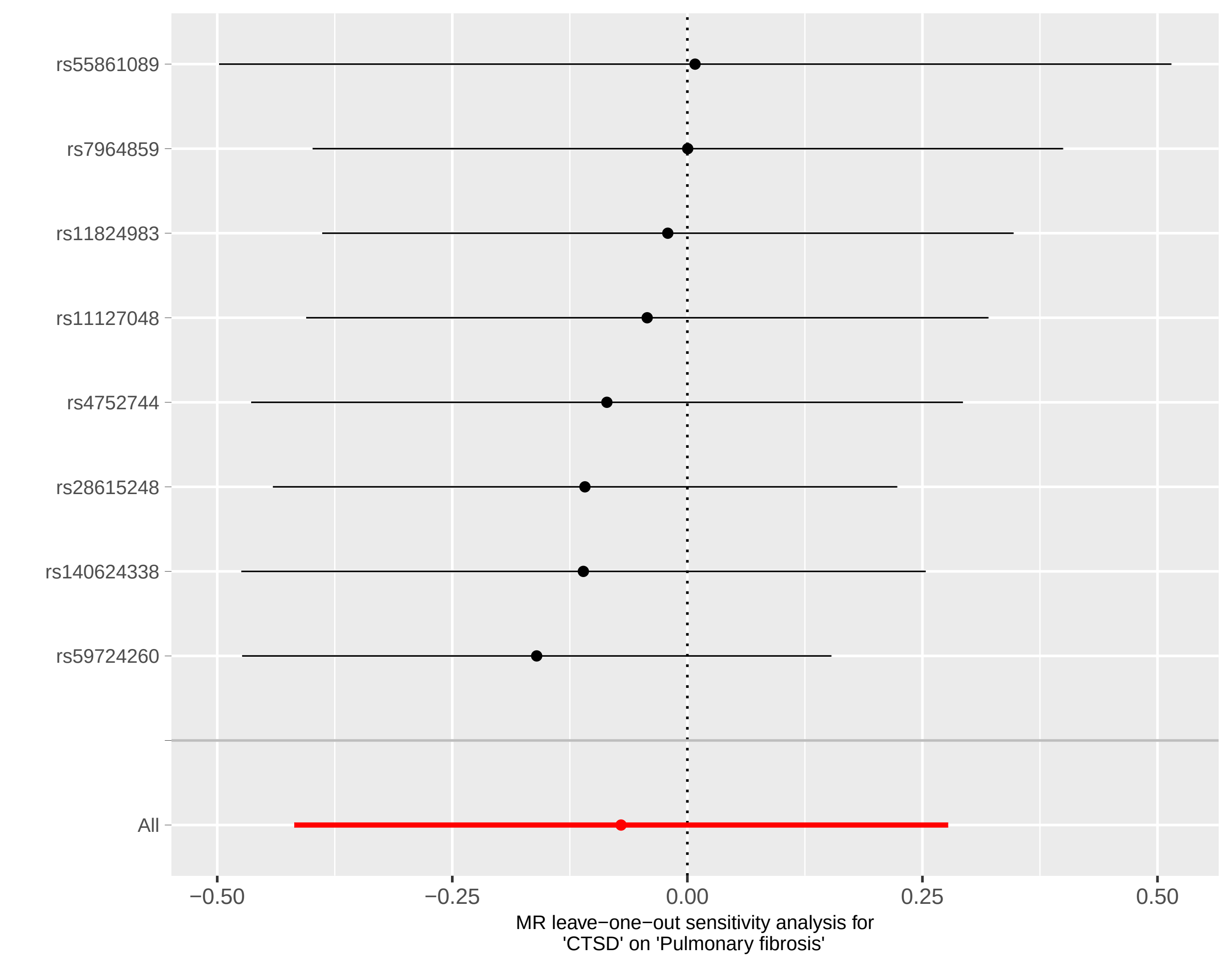
rs55861089
rs7964859
rs11824983
rs11127048
rs4752744
rs28615248
rs140624338
rs59724260
All
−0.50
−0.25
0.00
0.25
0.50
MR leave−one−out sensitivity analysis for
'CTSD' on 'Pulmonary fibrosis'

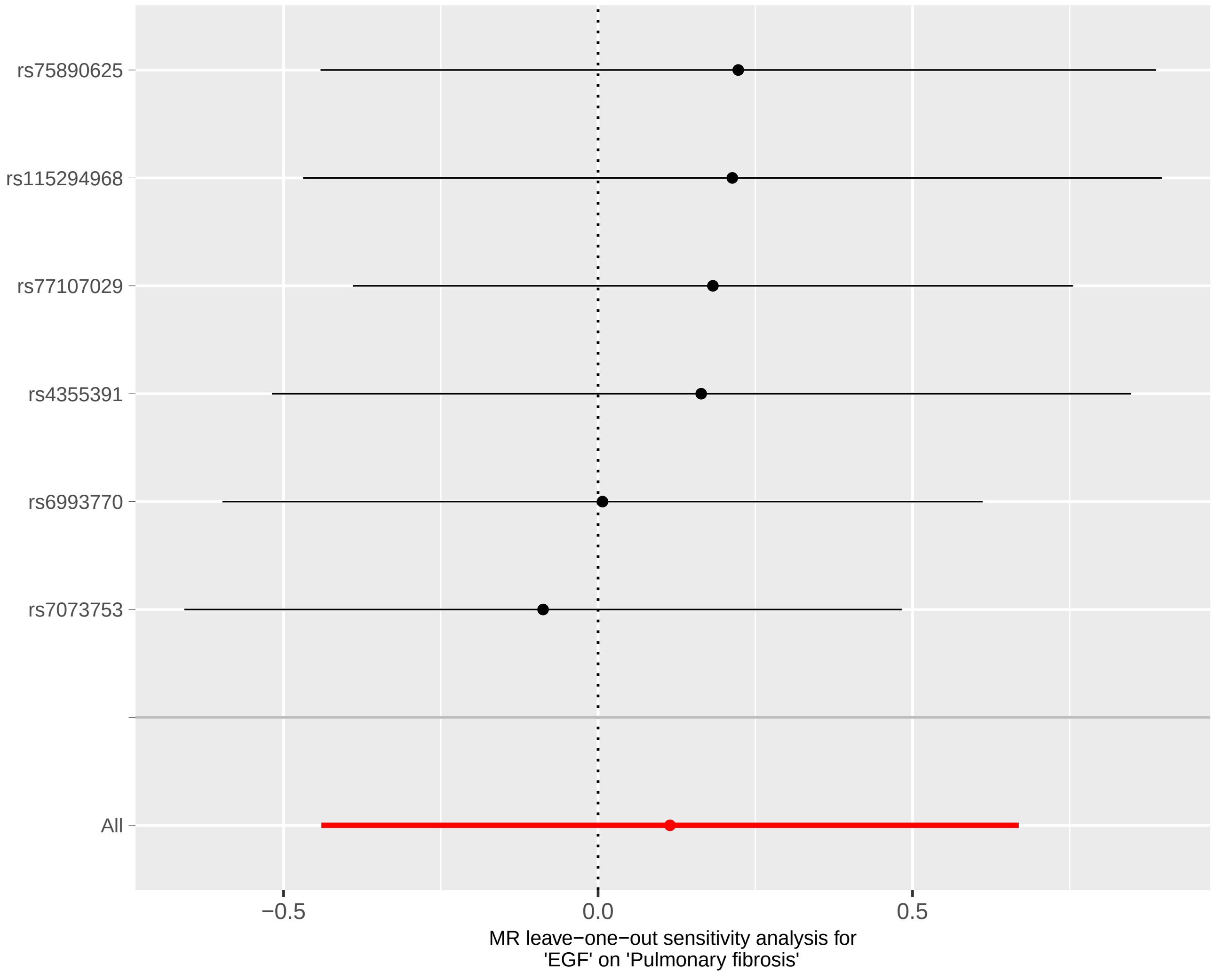
rs75890625
rs115294968
rs77107029
rs4355391
rs6993770
rs7073753
All
−0.5
0.0
0.5
MR leave−one−out sensitivity analysis for
'EGF' on 'Pulmonary fibrosis'

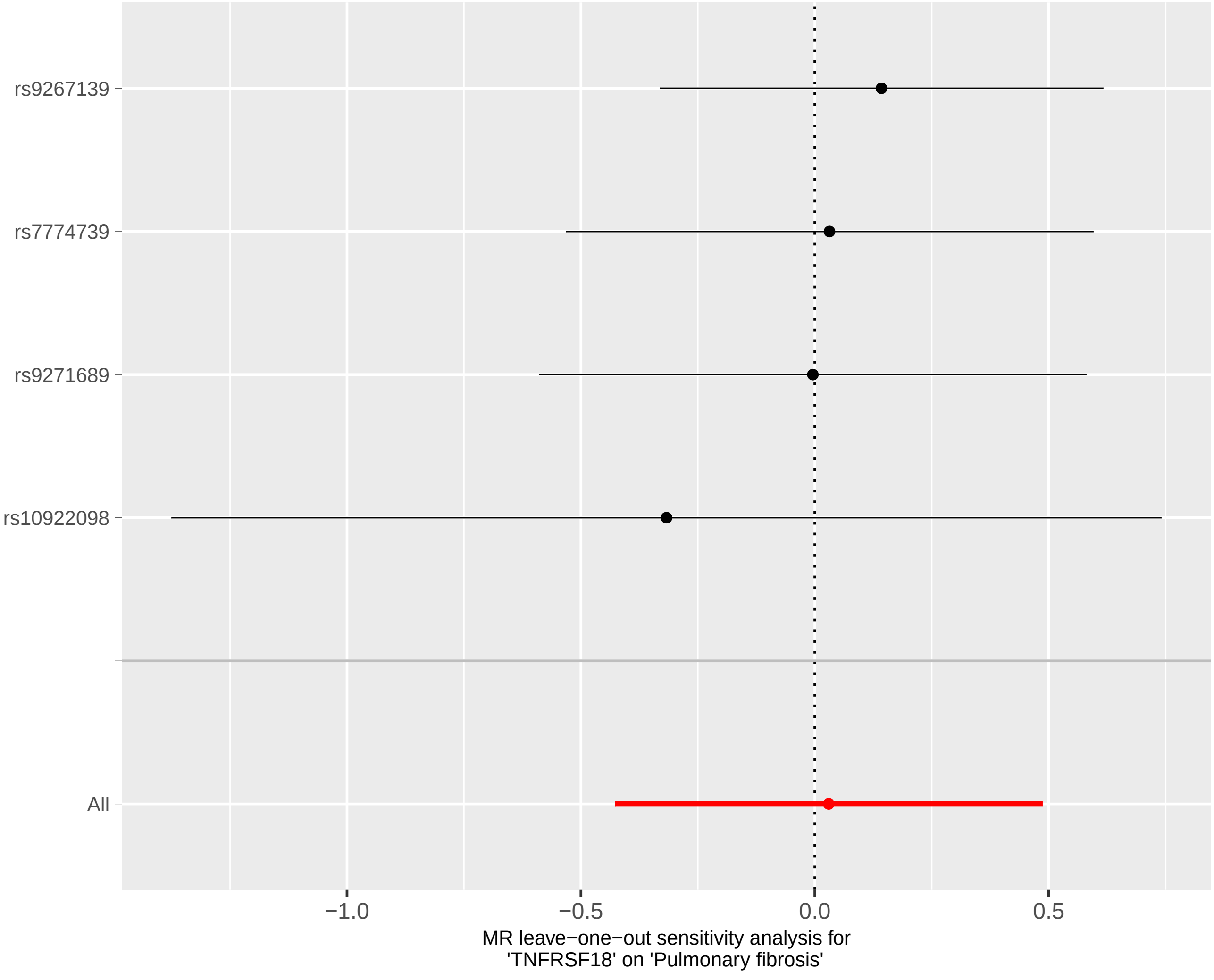
rs9267139
rs7774739
rs9271689
rs10922098
All
−1.0
−0.5
0.0
0.5
MR leave−one−out sensitivity analysis for
'TNFRSF18' on 'Pulmonary fibrosis'

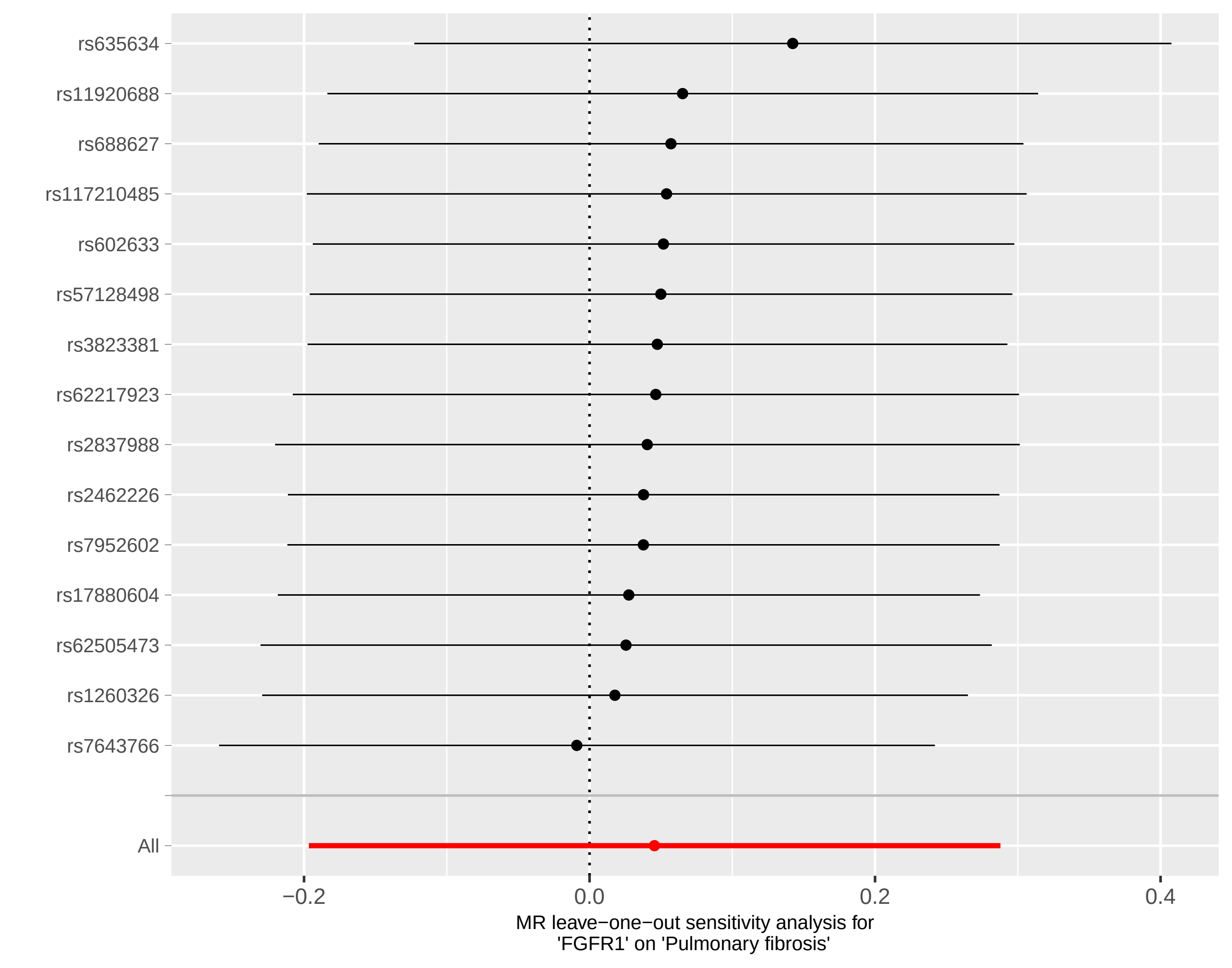
rs635634
rs11920688
rs688627
rs117210485
rs602633
rs57128498
rs3823381
rs62217923
rs2837988
rs2462226
rs7952602
rs17880604
rs62505473
rs1260326
rs7643766
All
−0.2
0.0
0.2
0.4
MR leave−one−out sensitivity analysis for
'FGFR1' on 'Pulmonary fibrosis'

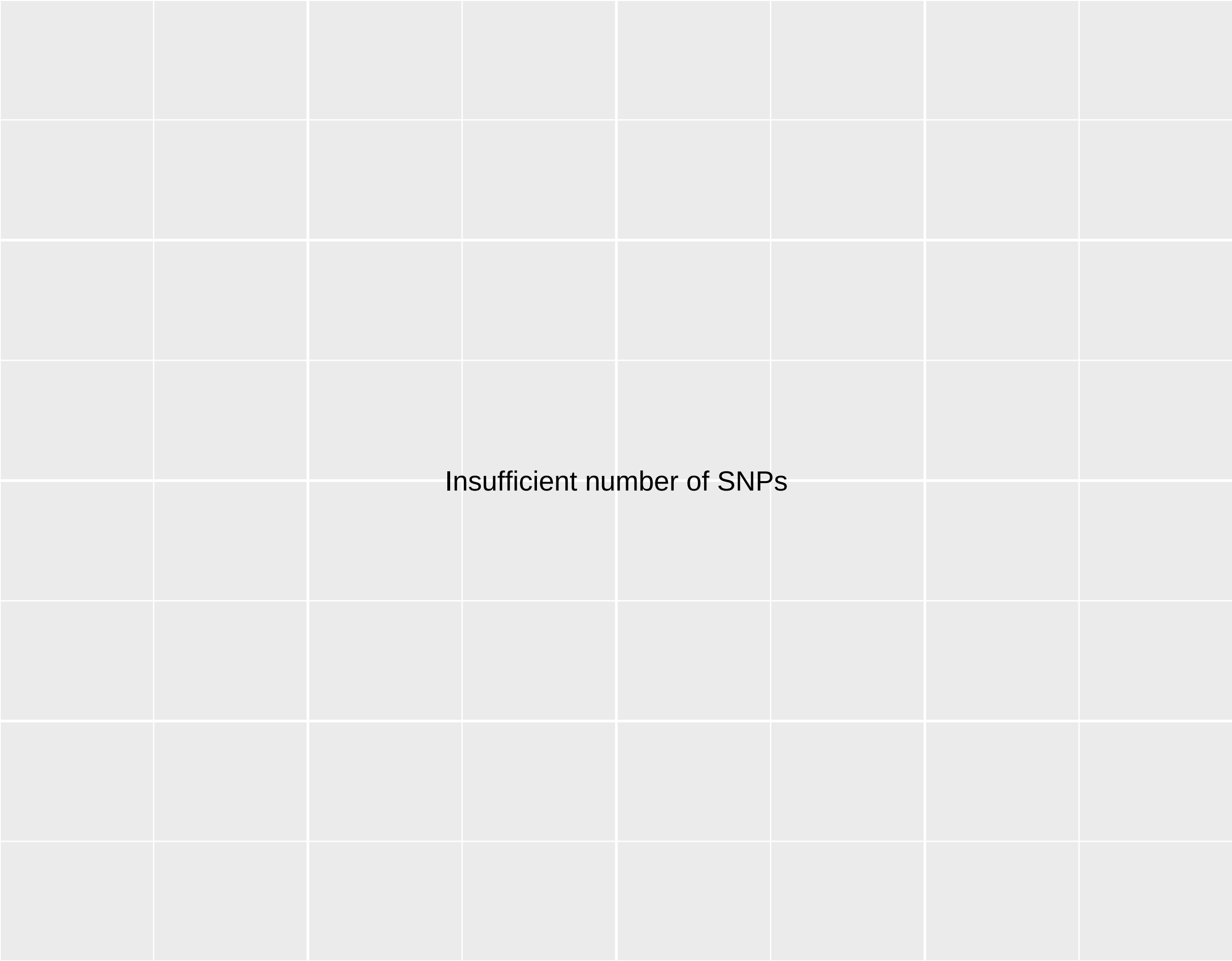
Insufficient number of SNPs

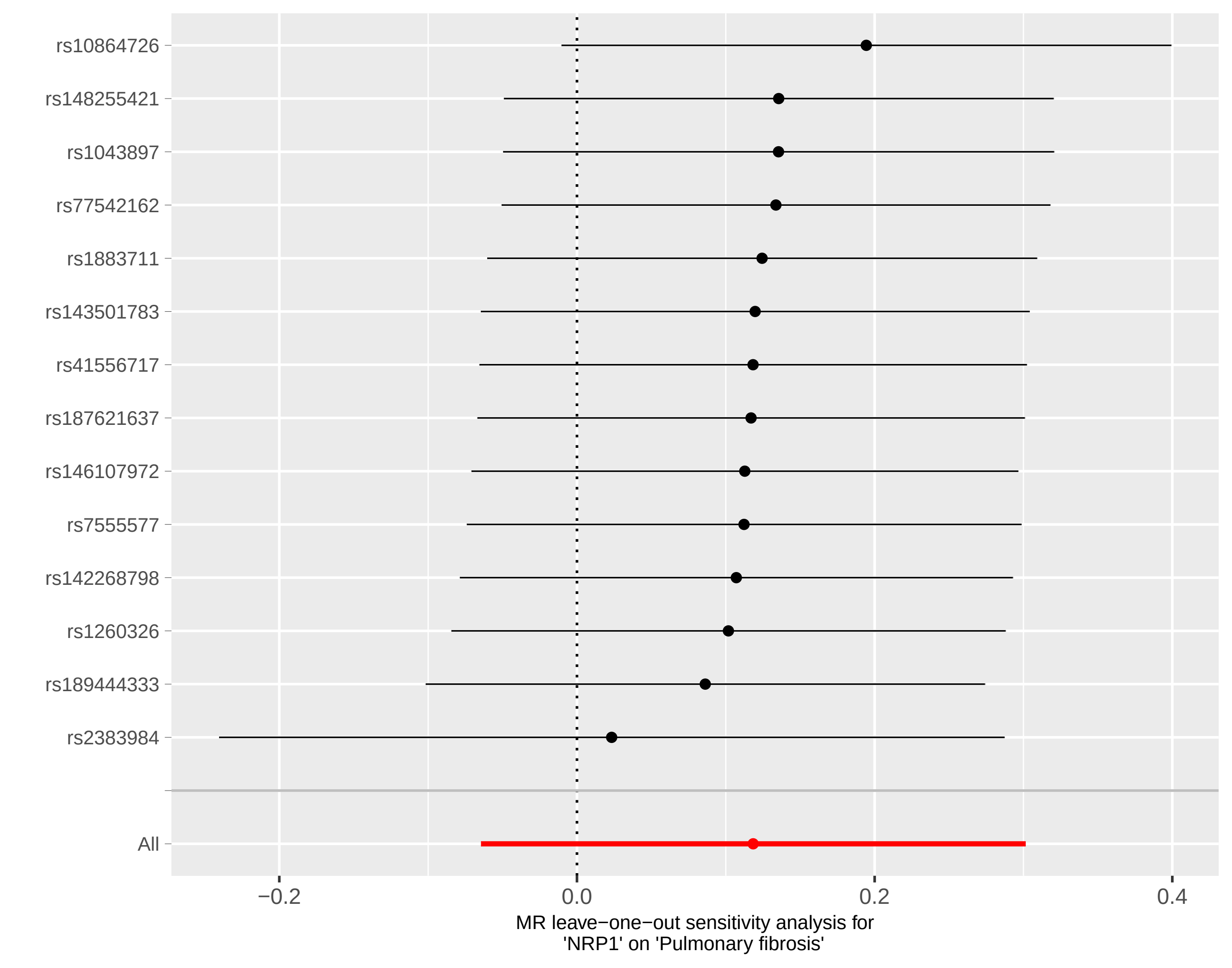
rs10864726
rs148255421
rs1043897
rs77542162
rs1883711
rs143501783
rs41556717
rs187621637
rs146107972
rs7555577
rs142268798
rs1260326
rs189444333
rs2383984
All
−0.2
0.0
0.2
0.4
MR leave−one−out sensitivity analysis for
'NRP1' on 'Pulmonary fibrosis'

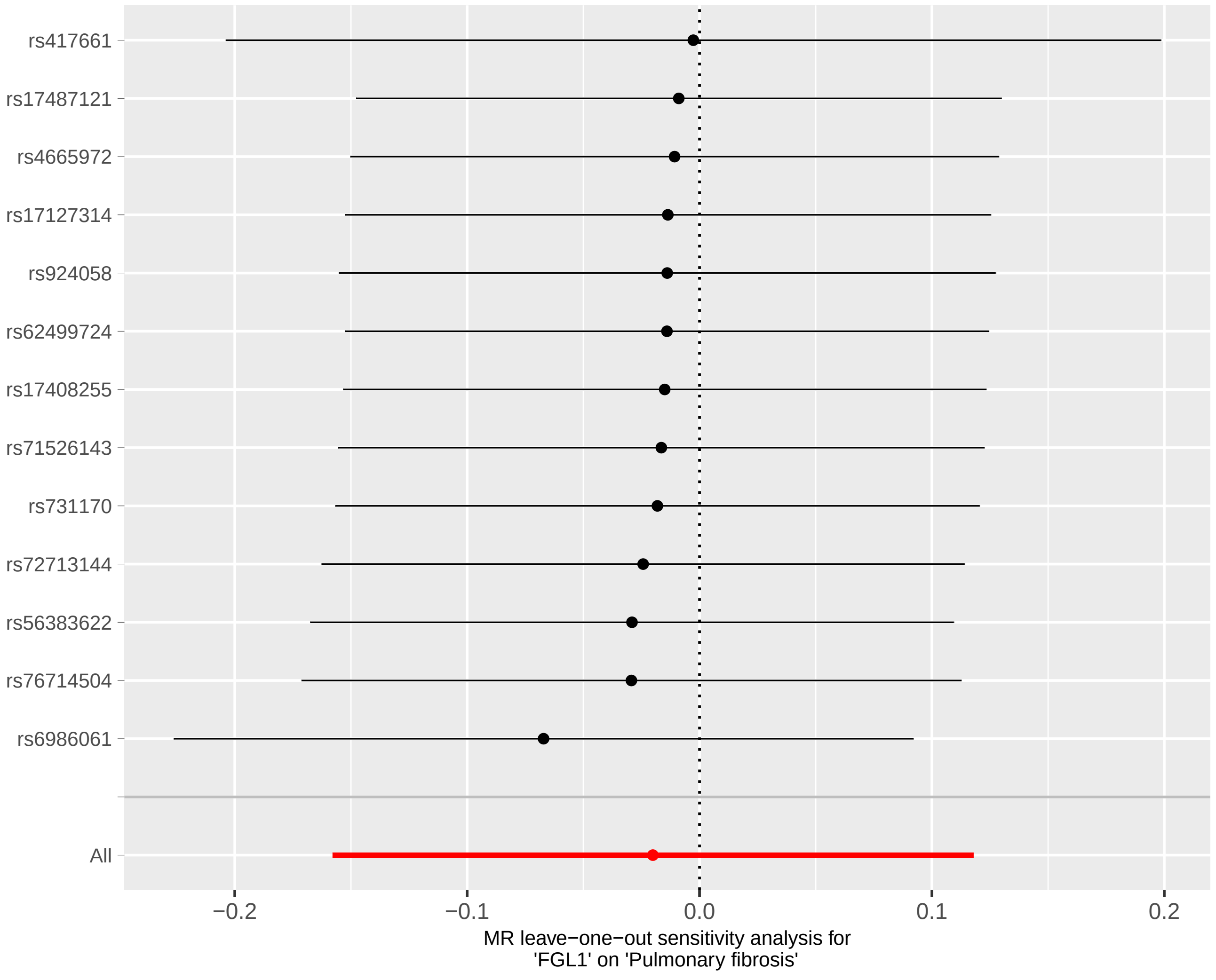
rs417661
rs17487121
rs4665972
rs17127314
rs924058
rs62499724
rs17408255
rs71526143
rs731170
rs72713144
rs56383622
rs76714504
rs6986061
All
−0.2
−0.1
0.0
0.1
0.2
MR leave−one−out sensitivity analysis for
'FGL1' on 'Pulmonary fibrosis'

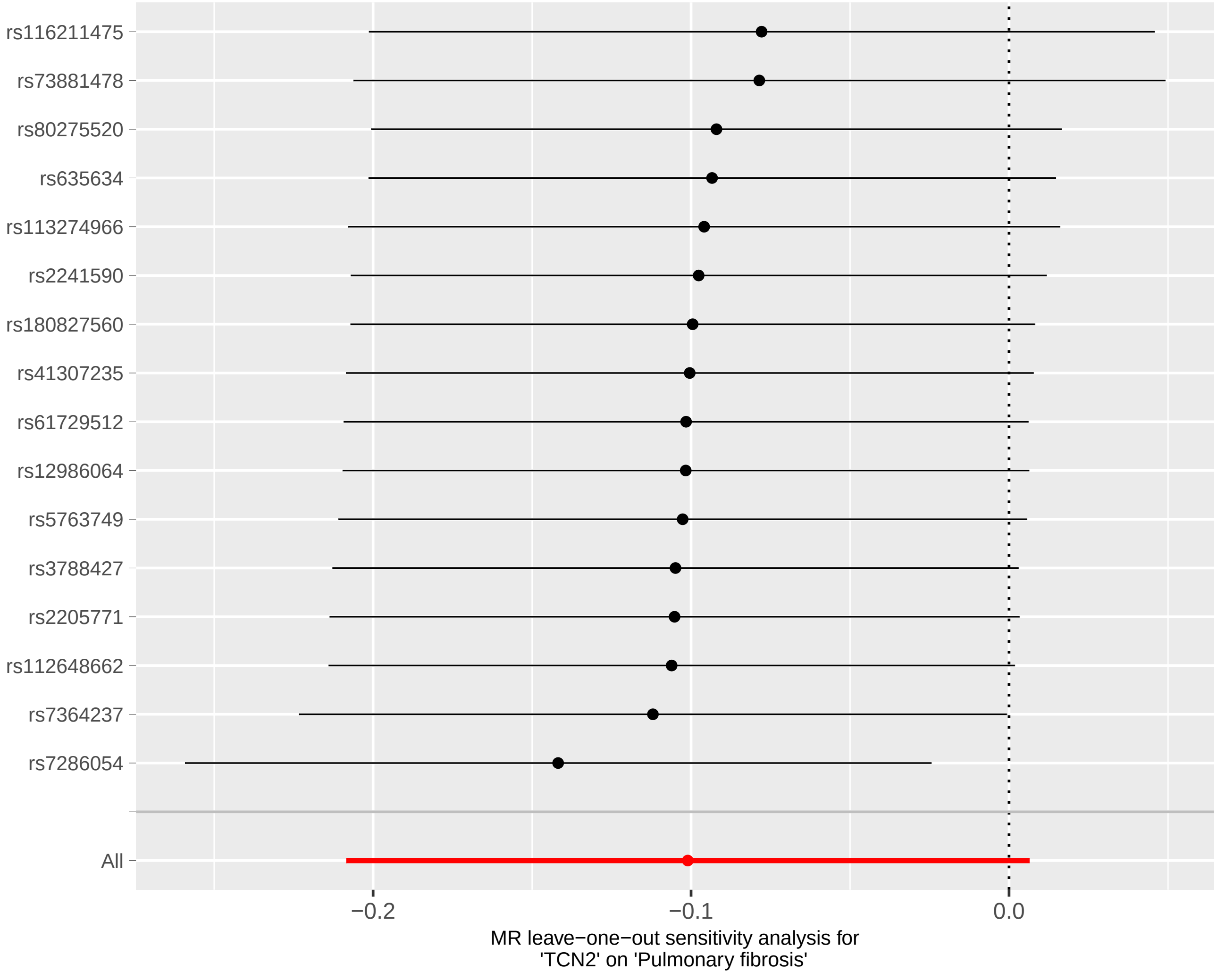
rs116211475
rs73881478
rs80275520
rs635634
rs113274966
rs2241590
rs180827560
rs41307235
rs61729512
rs12986064
rs5763749
rs3788427
rs2205771
rs112648662
rs7364237
rs7286054
All
−0.2
−0.1
0.0
MR leave−one−out sensitivity analysis for
'TCN2' on 'Pulmonary fibrosis'

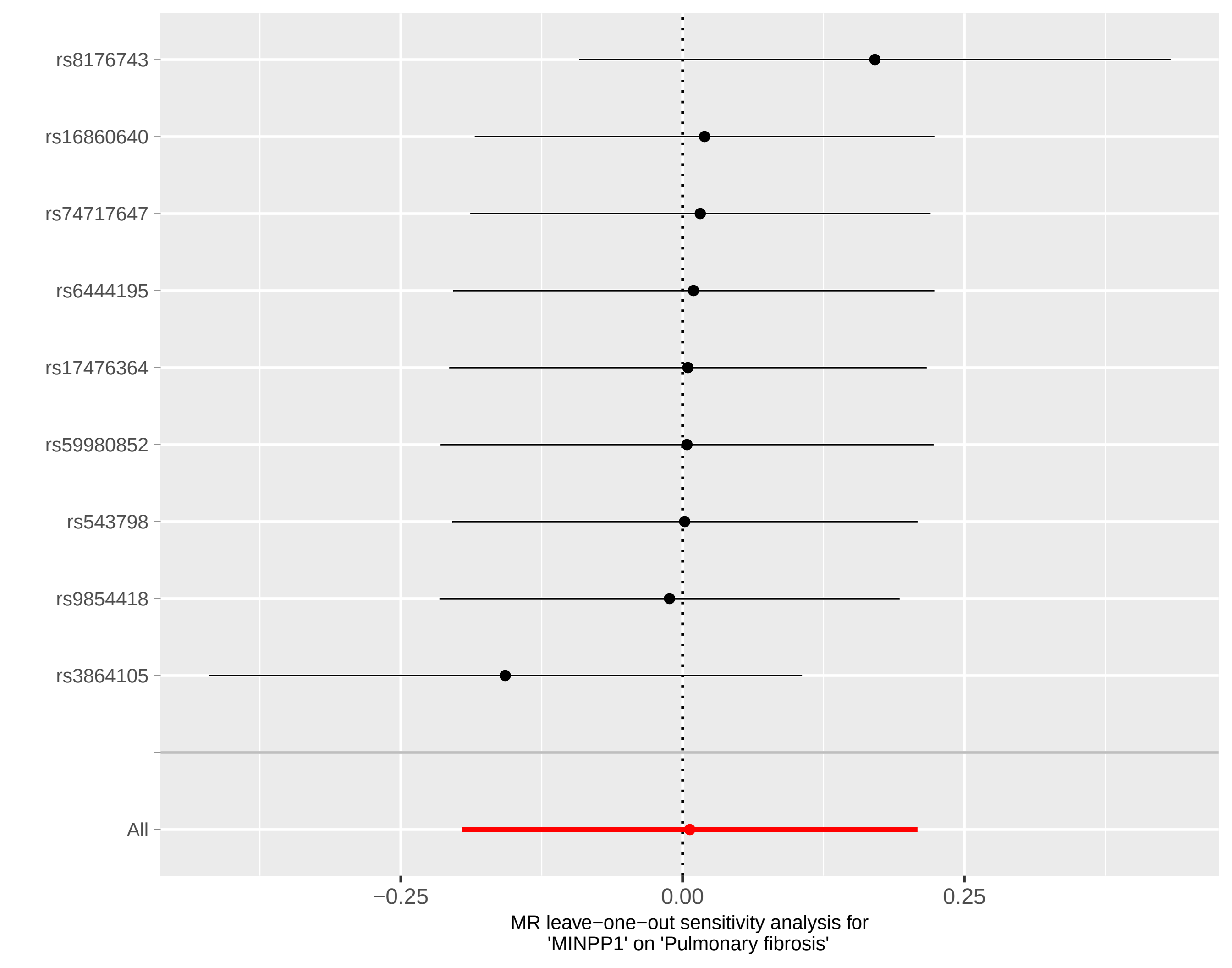
rs8176743
rs16860640
rs74717647
rs6444195
rs17476364
rs59980852
rs543798
rs9854418
rs3864105
All
−0.25
0.00
0.25
MR leave−one−out sensitivity analysis for
'MINPP1' on 'Pulmonary fibrosis'

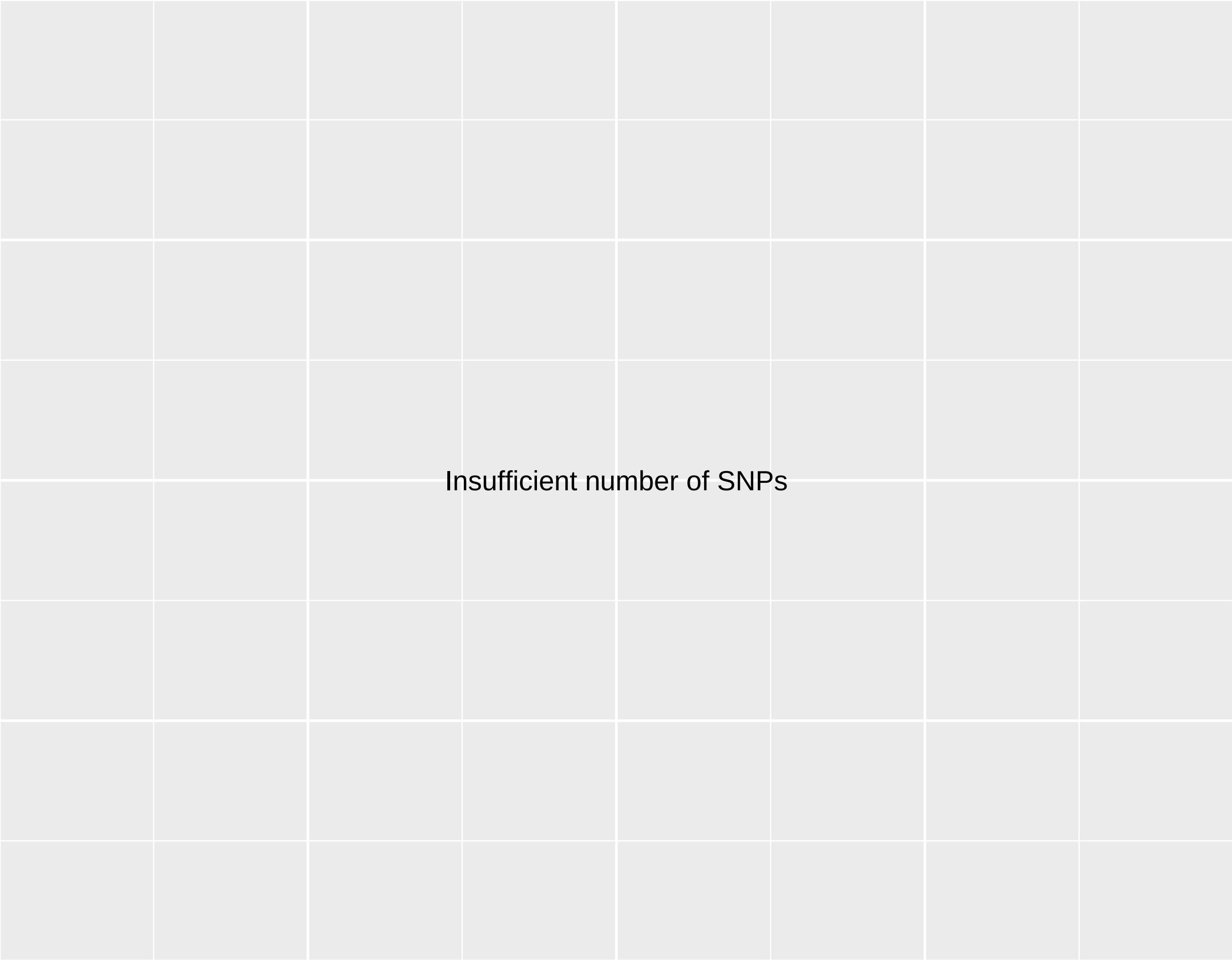
Insufficient number of SNPs

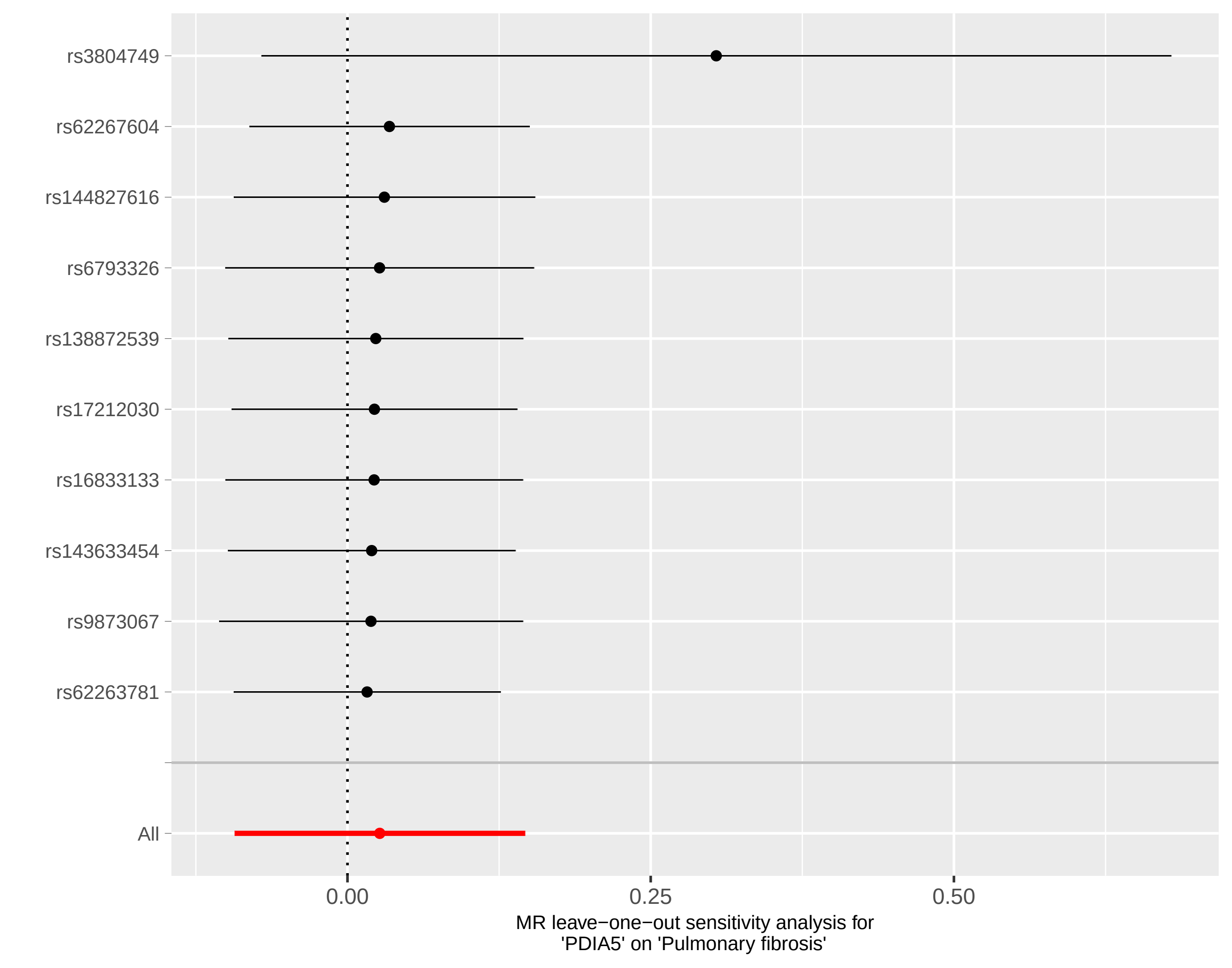

rs3804749
rs62267604
rs144827616
rs6793326
rs138872539
rs17212030
rs16833133
rs143633454
rs9873067
rs62263781
All
0.00
0.25
0.50
MR leave−one−out sensitivity analysis for
'PDIA5' on 'Pulmonary fibrosis'

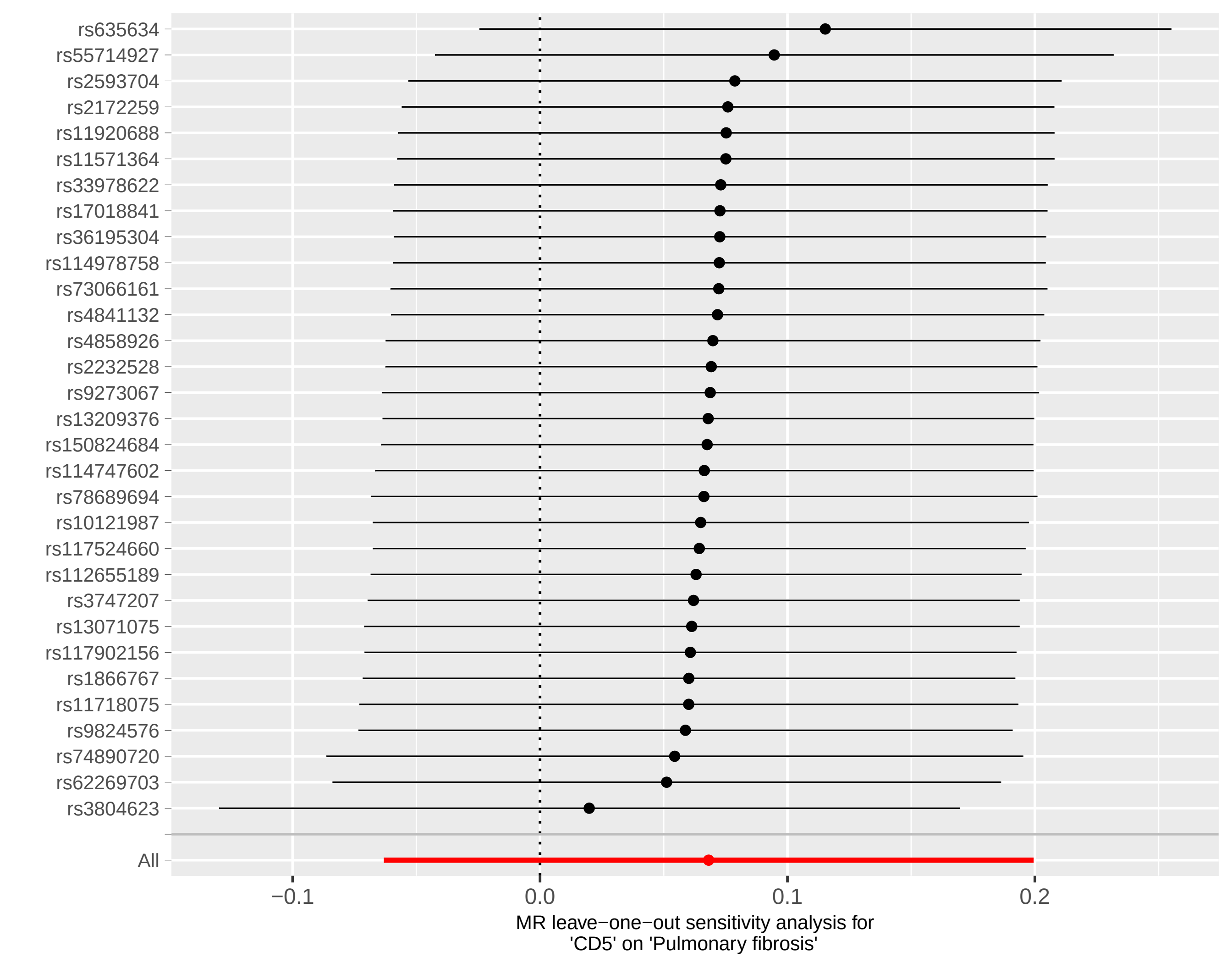

rs635634
rs55714927
rs2593704
rs2172259
rs11920688
rs11571364
rs33978622
rs17018841
rs36195304
rs114978758
rs73066161
rs4841132
rs4858926
rs2232528
rs9273067
rs13209376
rs150824684
rs114747602
rs78689694
rs10121987
rs117524660
rs112655189
rs3747207
rs13071075
rs117902156
rs1866767
rs11718075
rs9824576
rs74890720
rs62269703
rs3804623
All
−0.1
0.0
0.1
0.2
MR leave−one−out sensitivity analysis for
'CD5' on 'Pulmonary fibrosis'

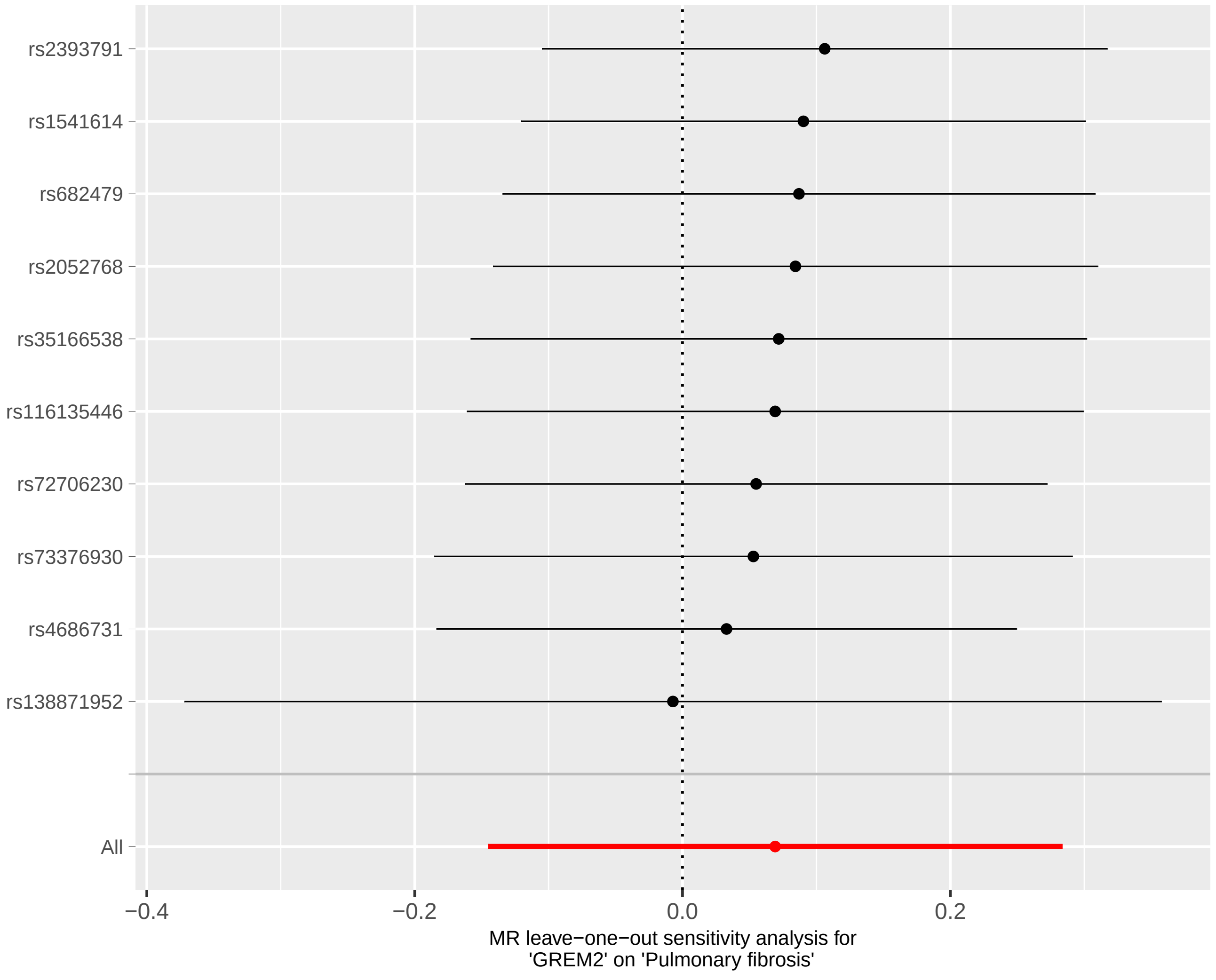

rs2393791
rs1541614
rs682479
rs2052768
rs35166538
rs116135446
rs72706230
rs73376930
rs4686731
rs138871952
All
−0.4
−0.2
0.0
0.2
MR leave−one−out sensitivity analysis for
'GREM2' on 'Pulmonary fibrosis'

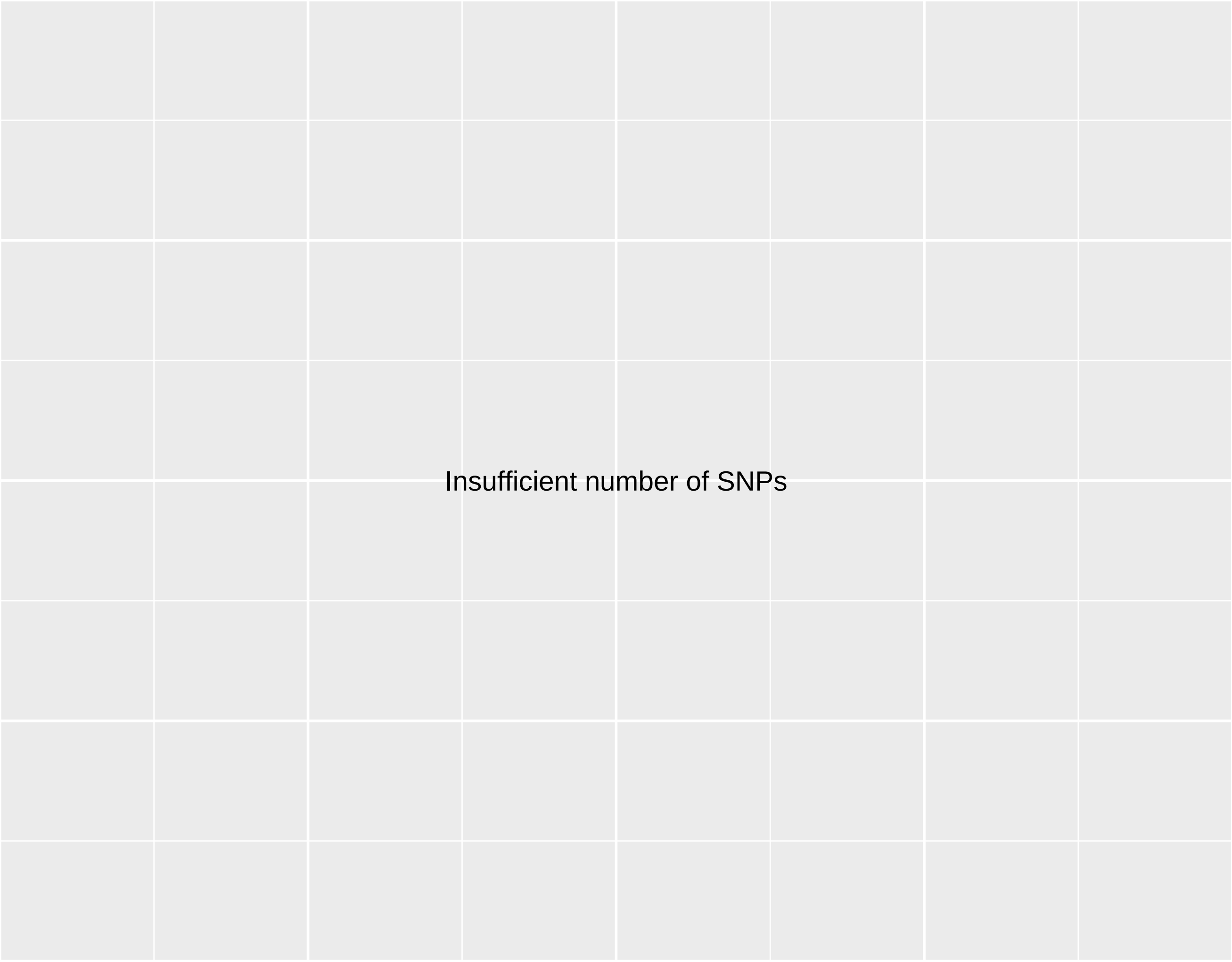
Insufficient number of SNPs

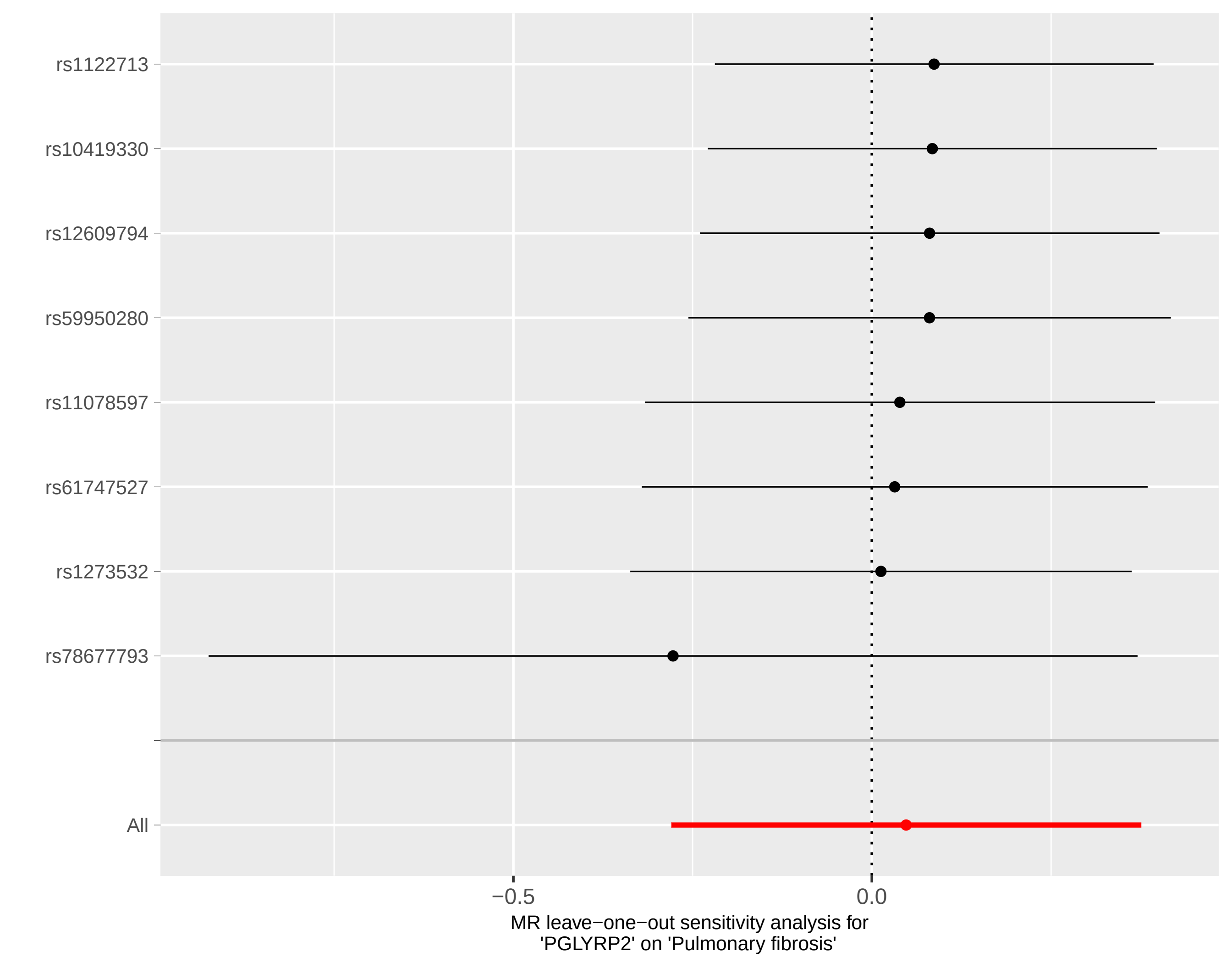
rs1122713
rs10419330
rs12609794
rs59950280
rs11078597
rs61747527
rs1273532
rs78677793
All
−0.5
0.0
MR leave−one−out sensitivity analysis for
'PGLYRP2' on 'Pulmonary fibrosis'

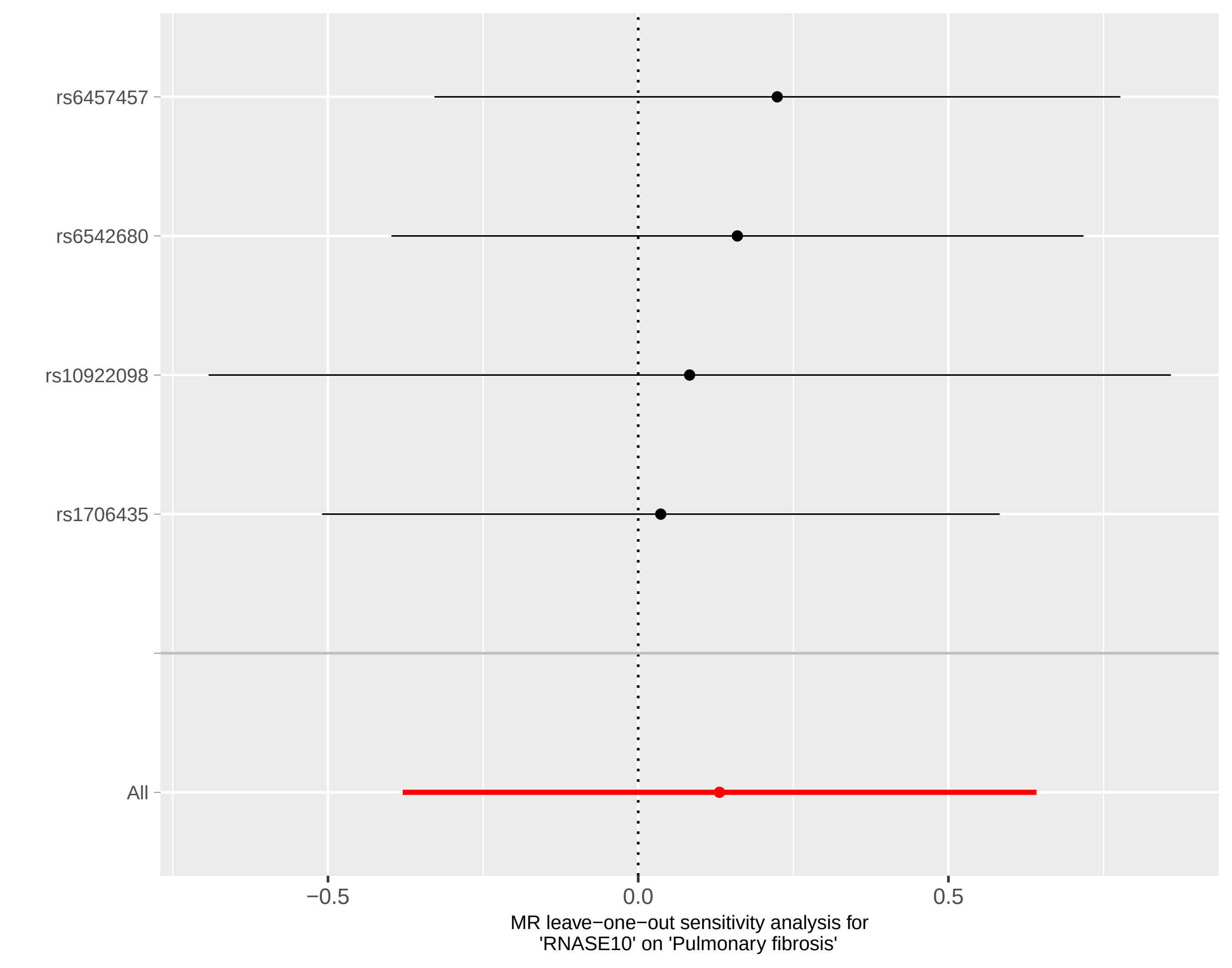
rs6457457
rs6542680
rs10922098
rs1706435
All
−0.5
0.0
0.5
MR leave−one−out sensitivity analysis for
'RNASE10' on 'Pulmonary fibrosis'

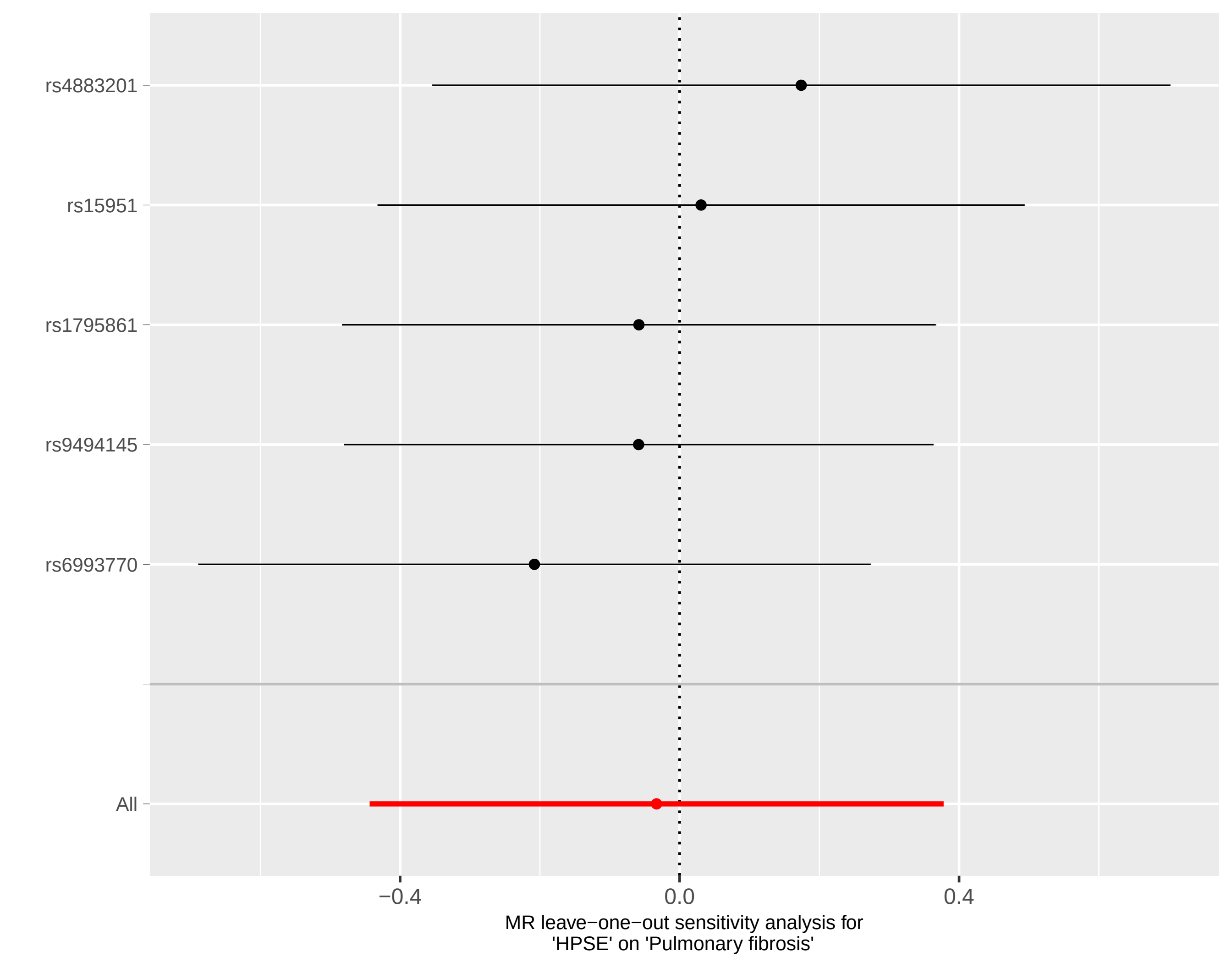
rs4883201
rs15951
rs1795861
rs9494145
rs6993770
All
−0.4
0.0
0.4
MR leave−one−out sensitivity analysis for
'HPSE' on 'Pulmonary fibrosis'

Insufficient number of SNPs

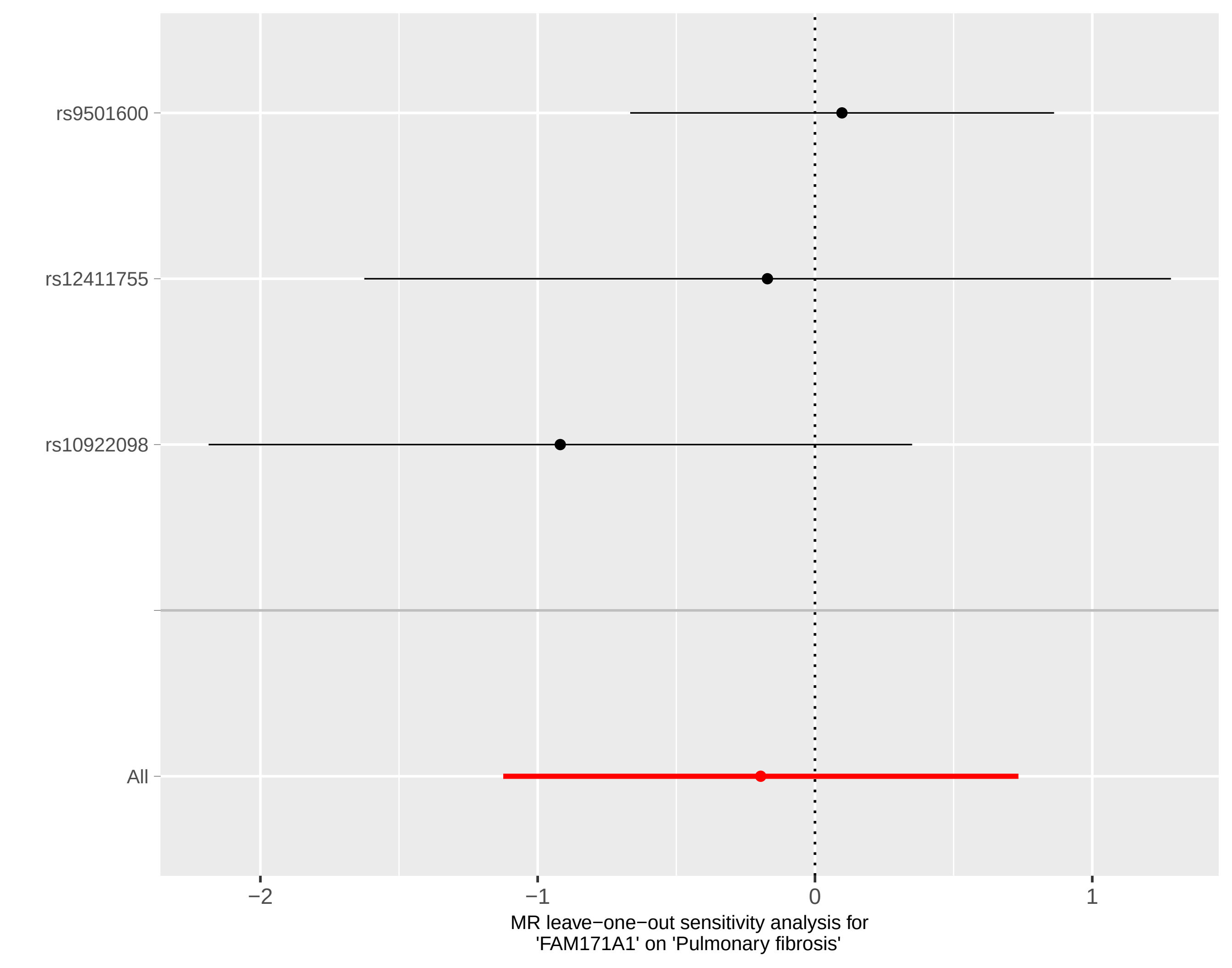
rs9501600
rs12411755
rs10922098
All
−2
−1
0
1
MR leave−one−out sensitivity analysis for
'FAM171A1' on 'Pulmonary fibrosis'

Insufficient number of SNPs

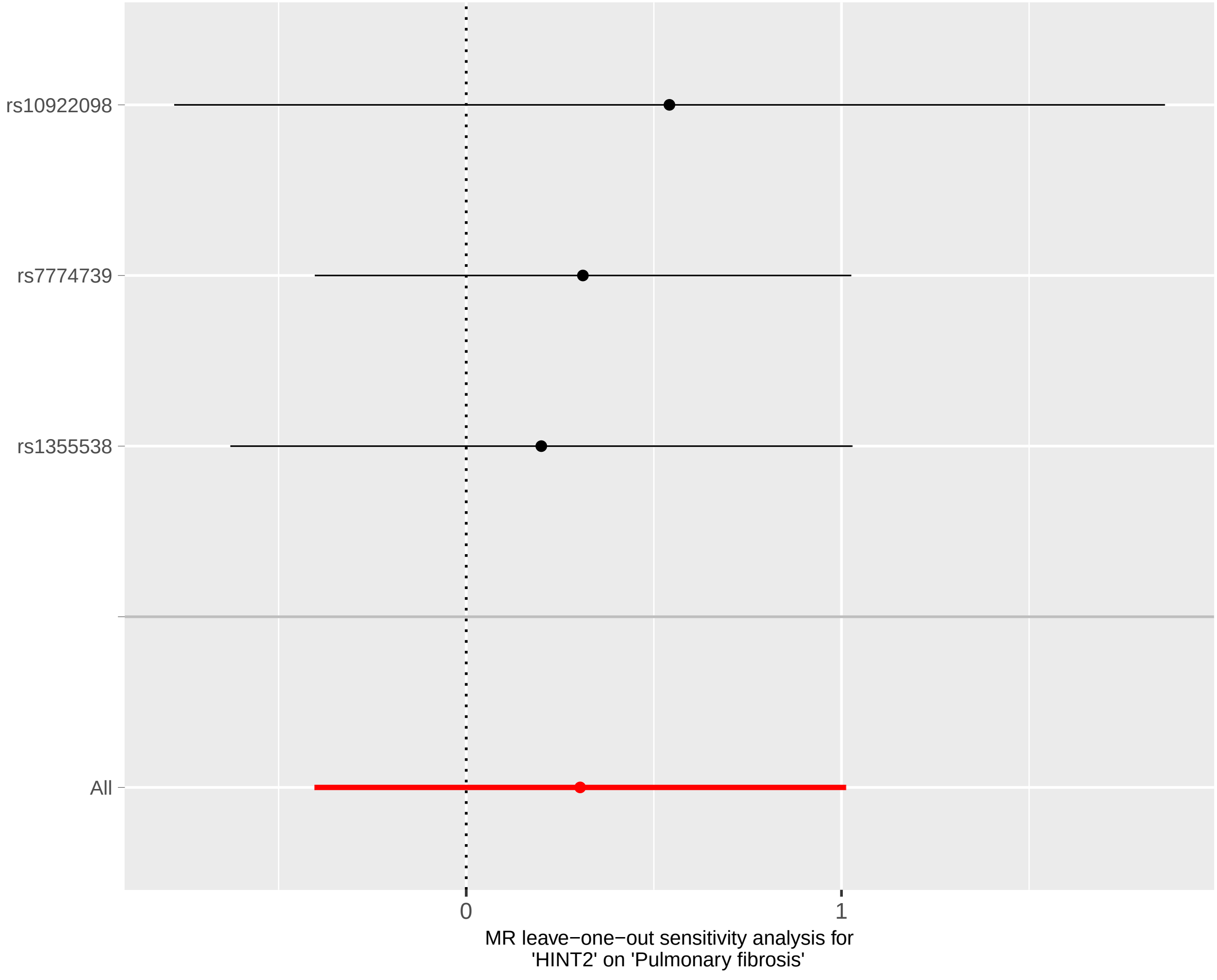
rs10922098
rs7774739
rs1355538
All
0
1
MR leave−one−out sensitivity analysis for
'HINT2' on 'Pulmonary fibrosis'

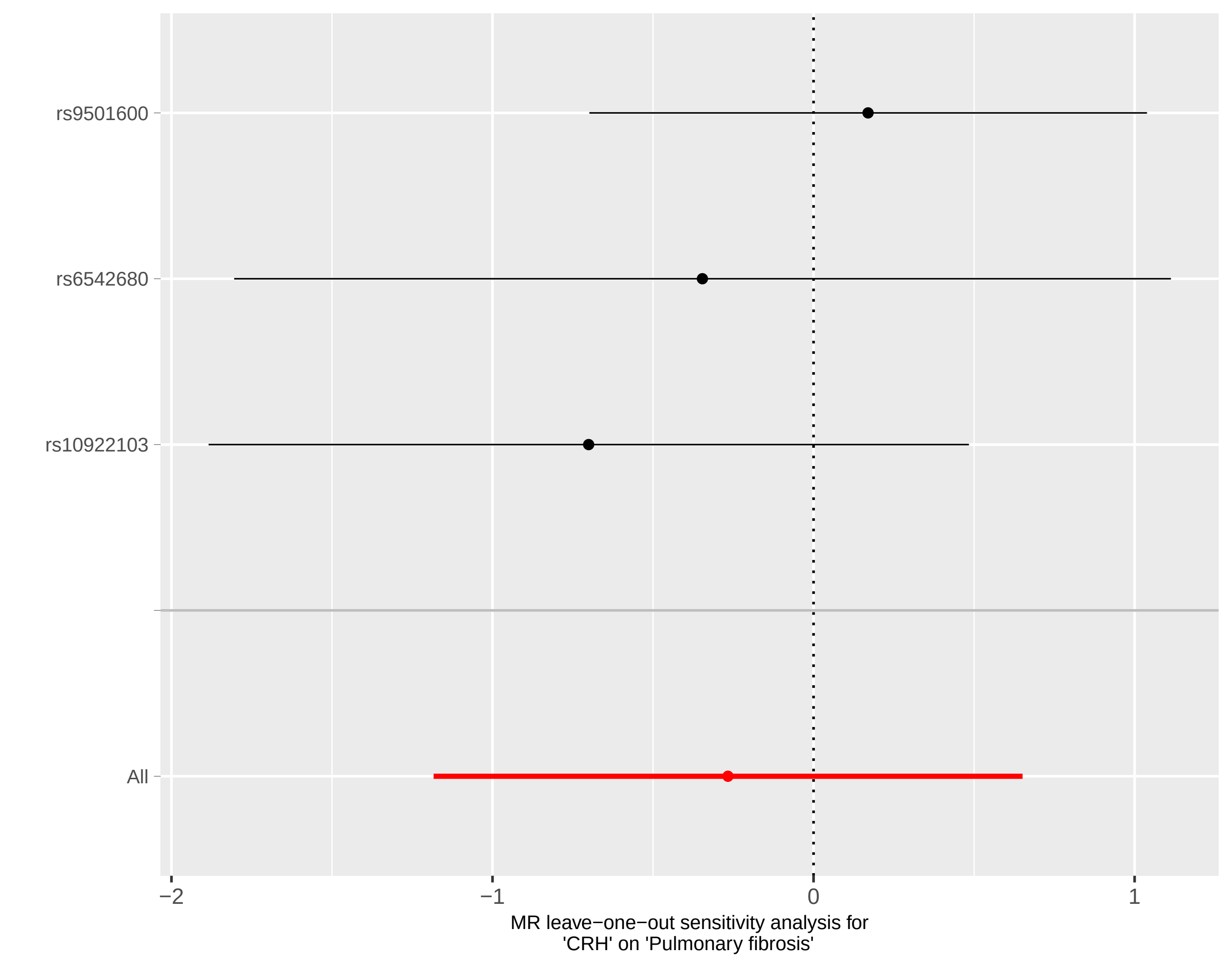
rs9501600
rs6542680
rs10922103
All
−2
−1
0
1
MR leave−one−out sensitivity analysis for
'CRH' on 'Pulmonary fibrosis'

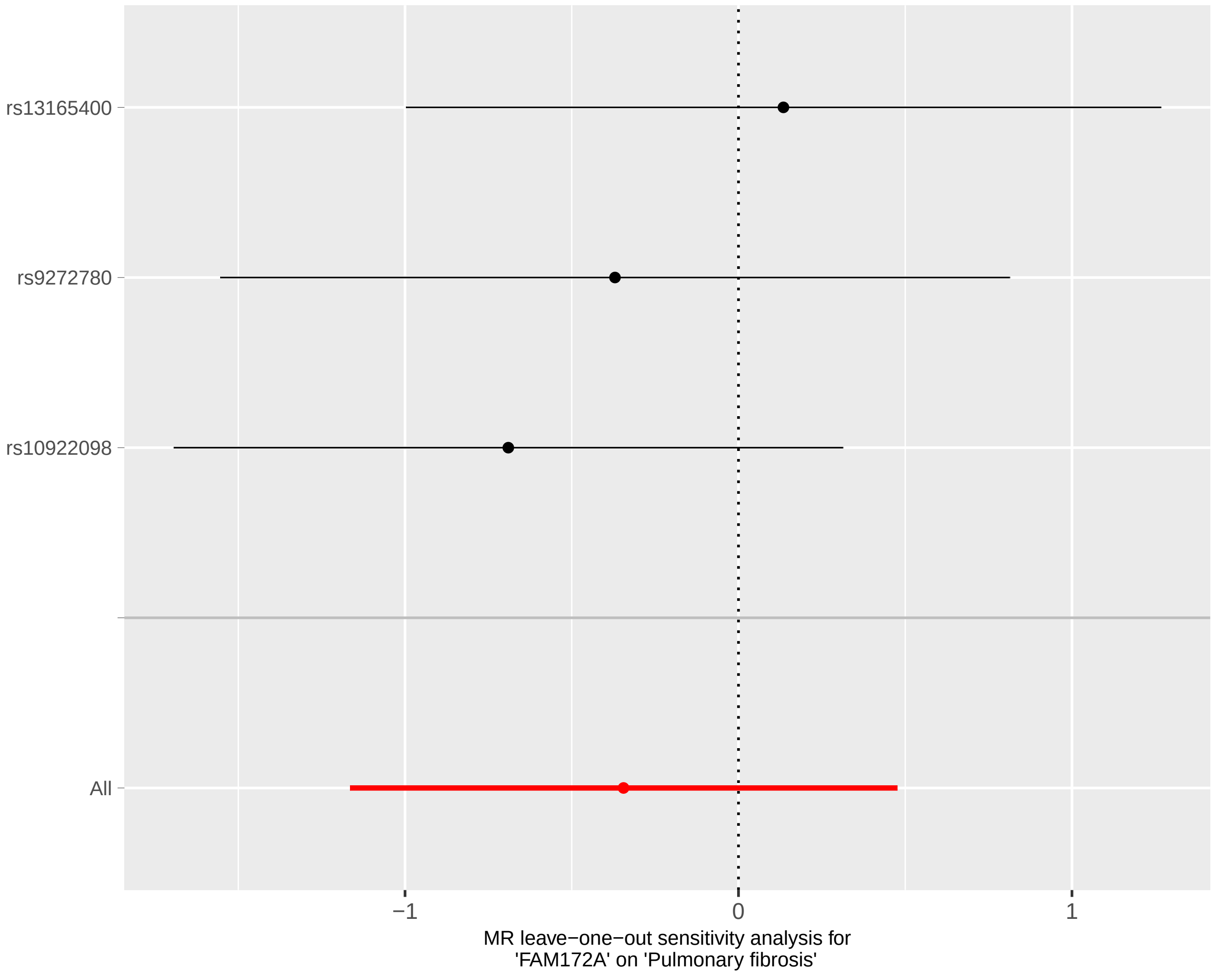
rs13165400
rs9272780
rs10922098
All
−1
0
1
MR leave−one−out sensitivity analysis for
'FAM172A' on 'Pulmonary fibrosis'

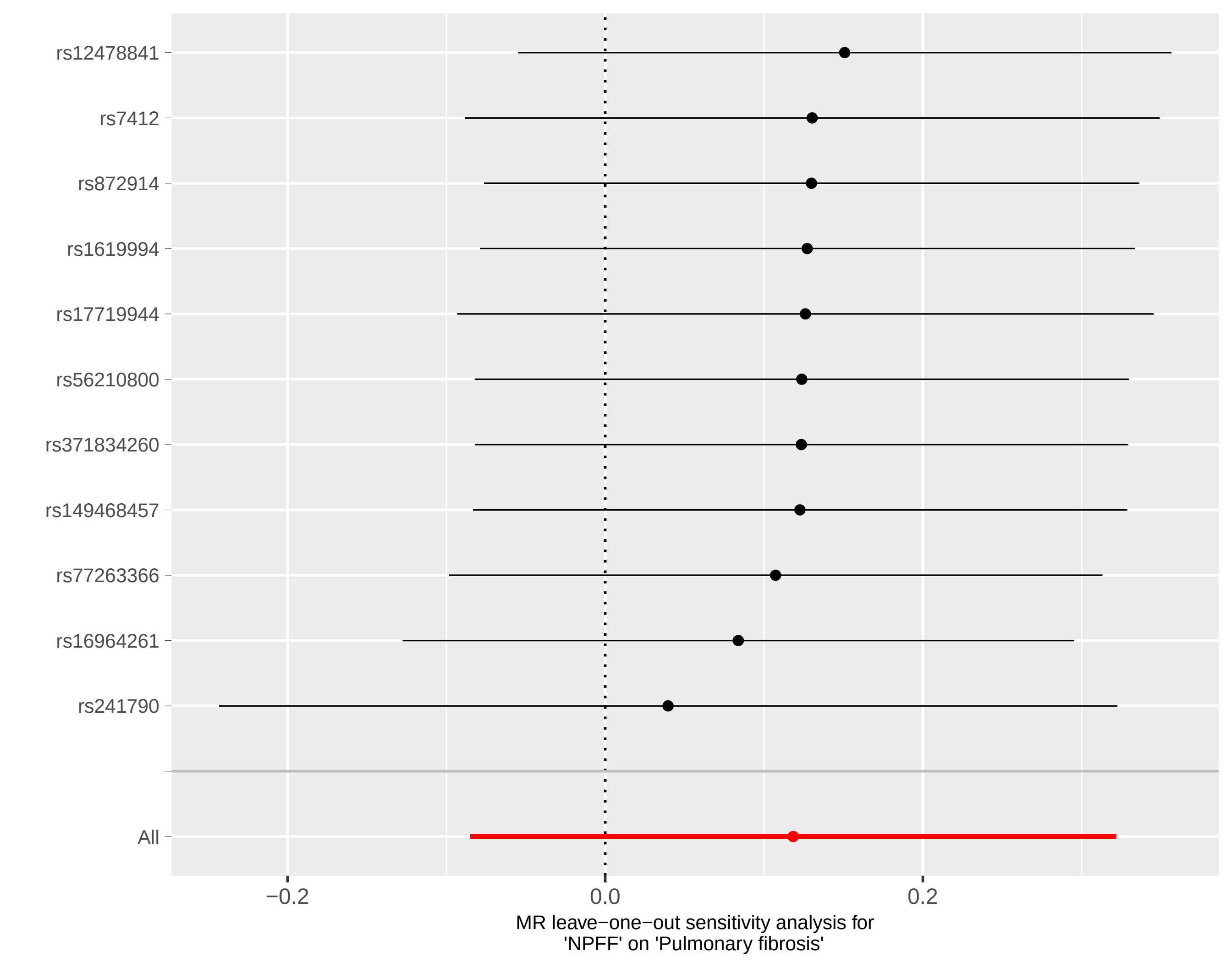
rs12478841
rs7412
rs872914
rs1619994
rs17719944
rs56210800
rs371834260
rs149468457
rs77263366
rs16964261
rs241790
All
−0.2
0.0
0.2
MR leave−one−out sensitivity analysis for
'NPFF' on 'Pulmonary fibrosis'

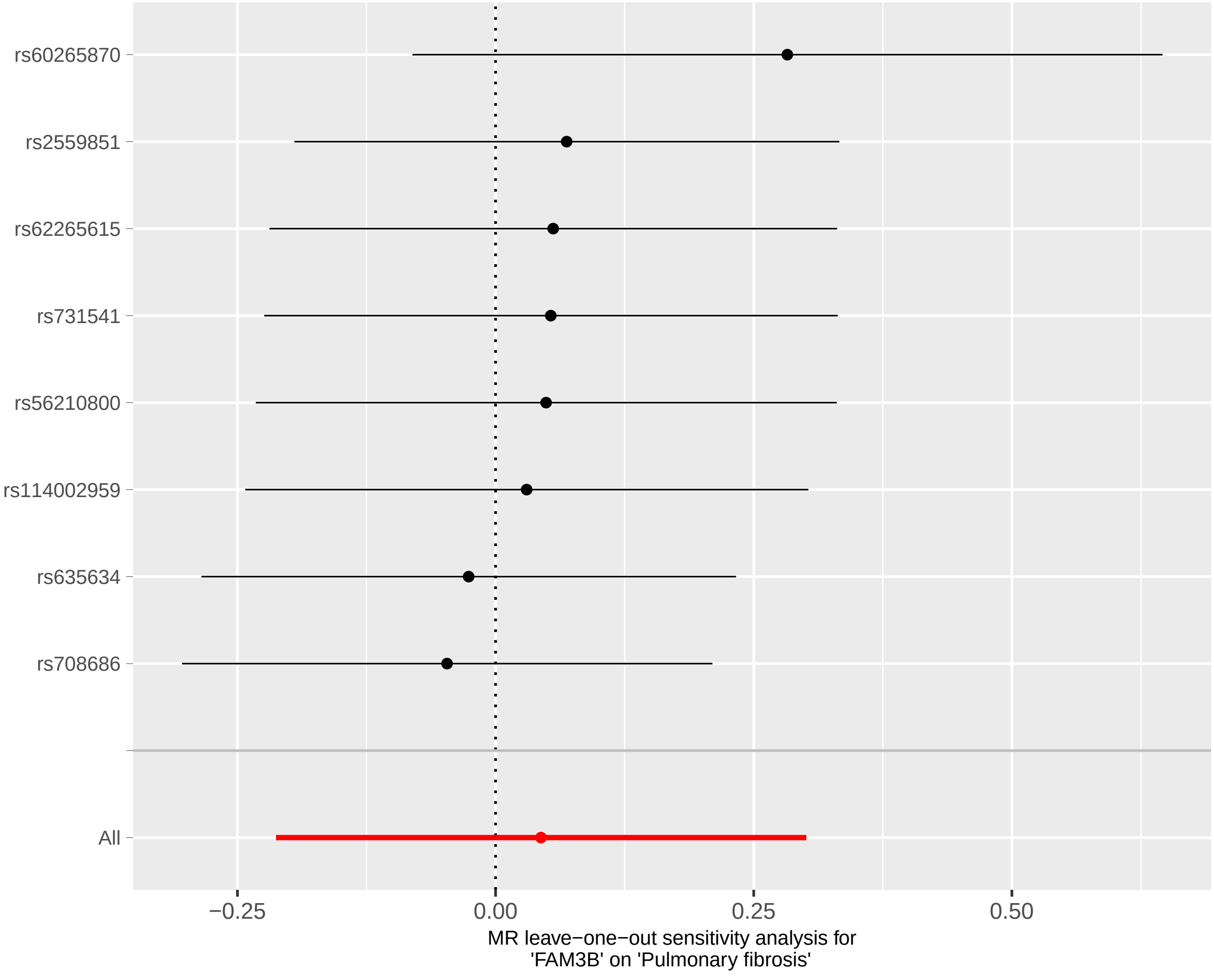
rs60265870
rs2559851
rs62265615
rs731541
rs56210800
rs114002959
rs635634
rs708686
All
−0.25
0.00
0.25
0.50
MR leave−one−out sensitivity analysis for
'FAM3B' on 'Pulmonary fibrosis'

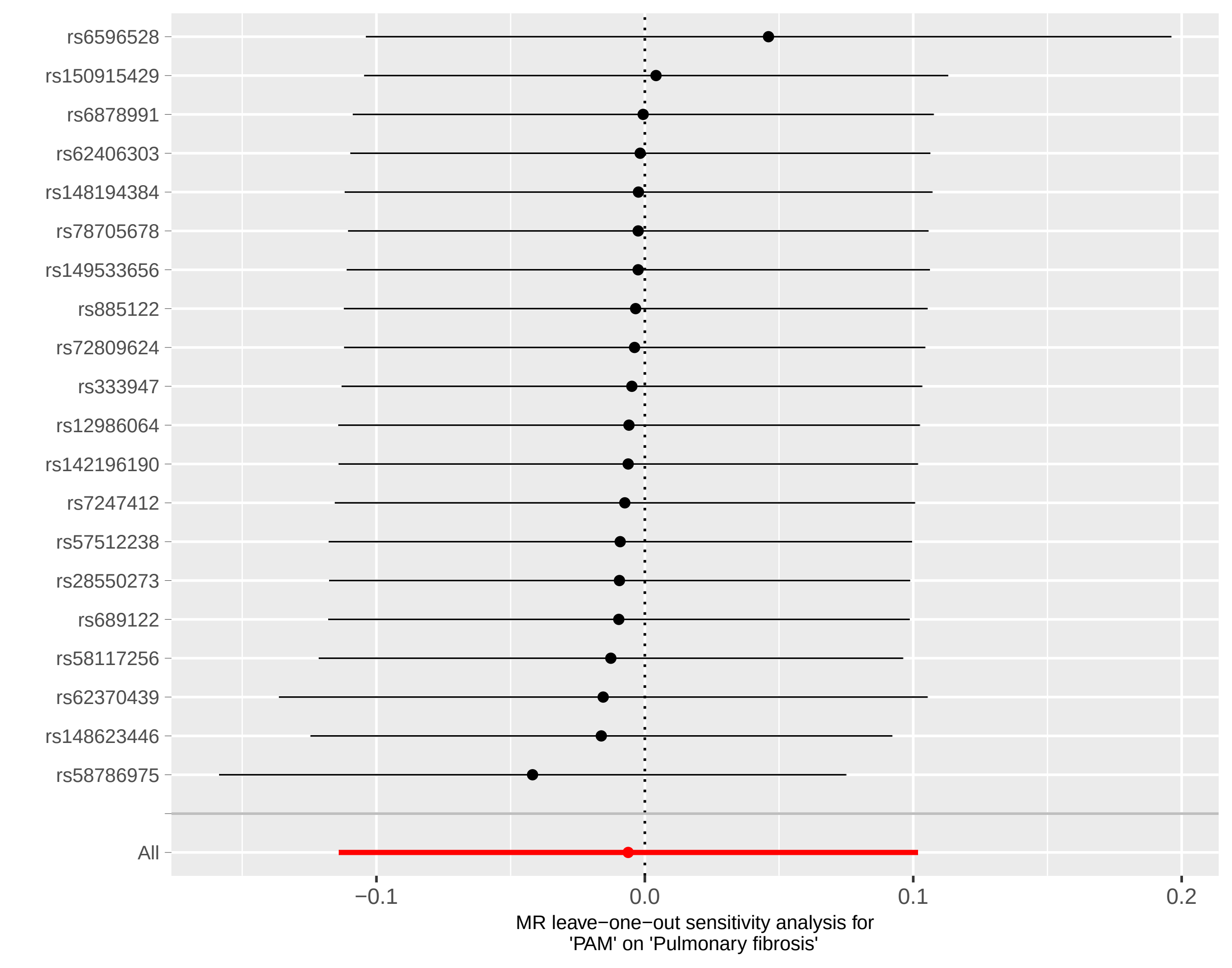

rs6596528
rs150915429
rs6878991
rs62406303
rs148194384
rs78705678
rs149533656
rs885122
rs72809624
rs333947
rs12986064
rs142196190
rs7247412
rs57512238
rs28550273
rs689122
rs58117256
rs62370439
rs148623446
rs58786975
All
−0.1
0.0
0.1
0.2
MR leave−one−out sensitivity analysis for
'PAM' on 'Pulmonary fibrosis'

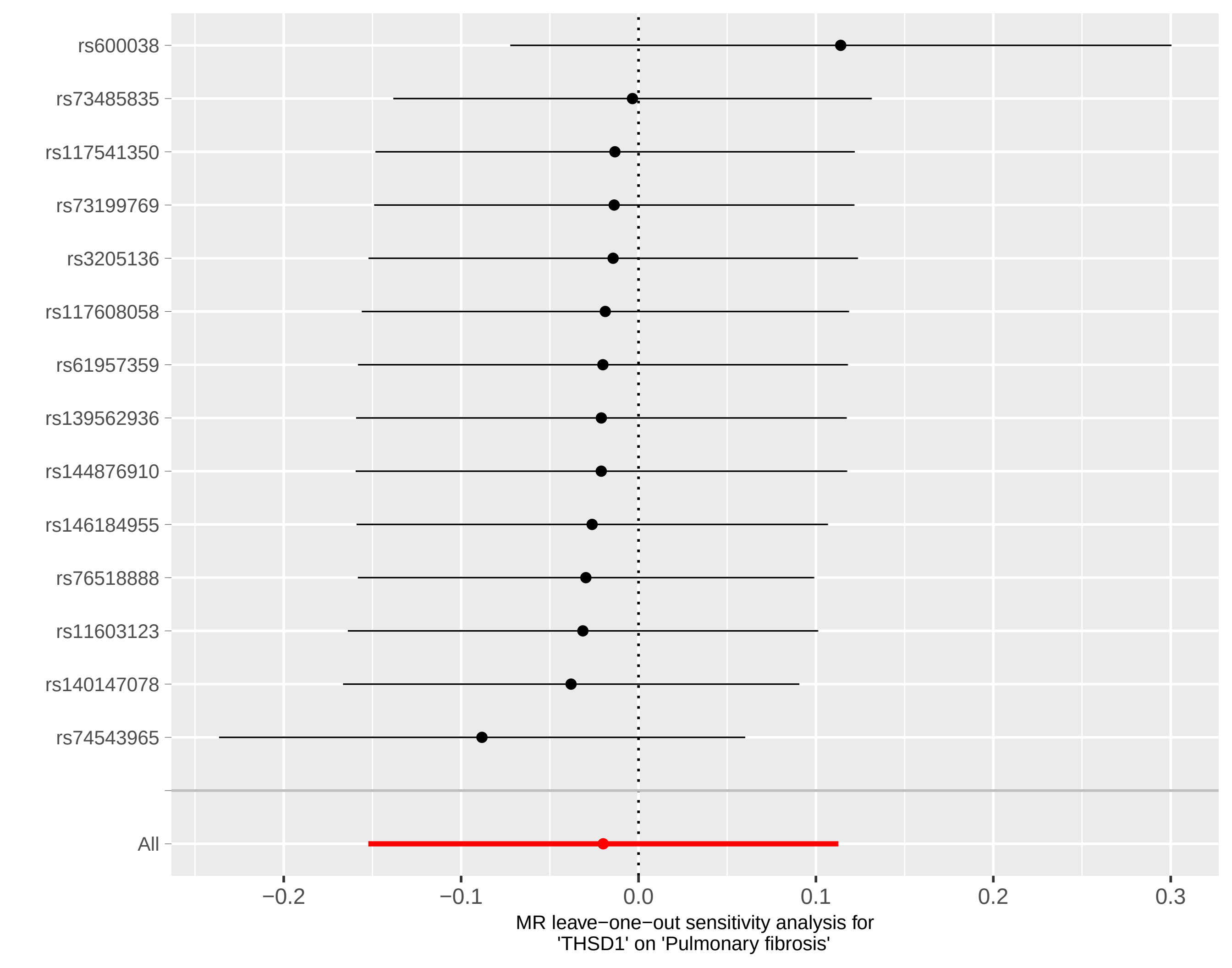

rs600038
rs73485835
rs117541350
rs73199769
rs3205136
rs117608058
rs61957359
rs139562936
rs144876910
rs146184955
rs76518888
rs11603123
rs140147078
rs74543965
All
−0.2
−0.1
0.0
0.1
0.2
0.3
MR leave−one−out sensitivity analysis for
'THSD1' on 'Pulmonary fibrosis'

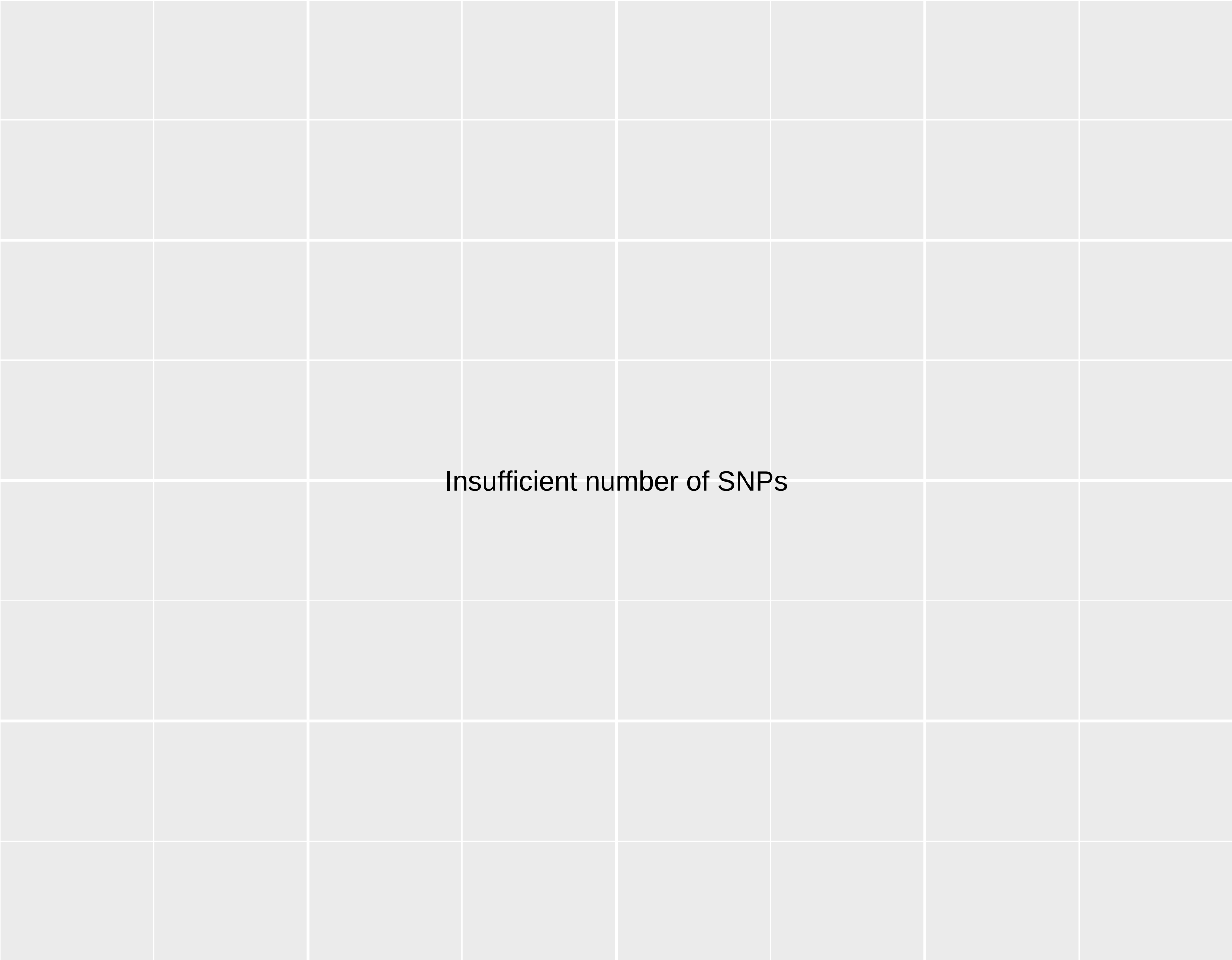
Insufficient number of SNPs

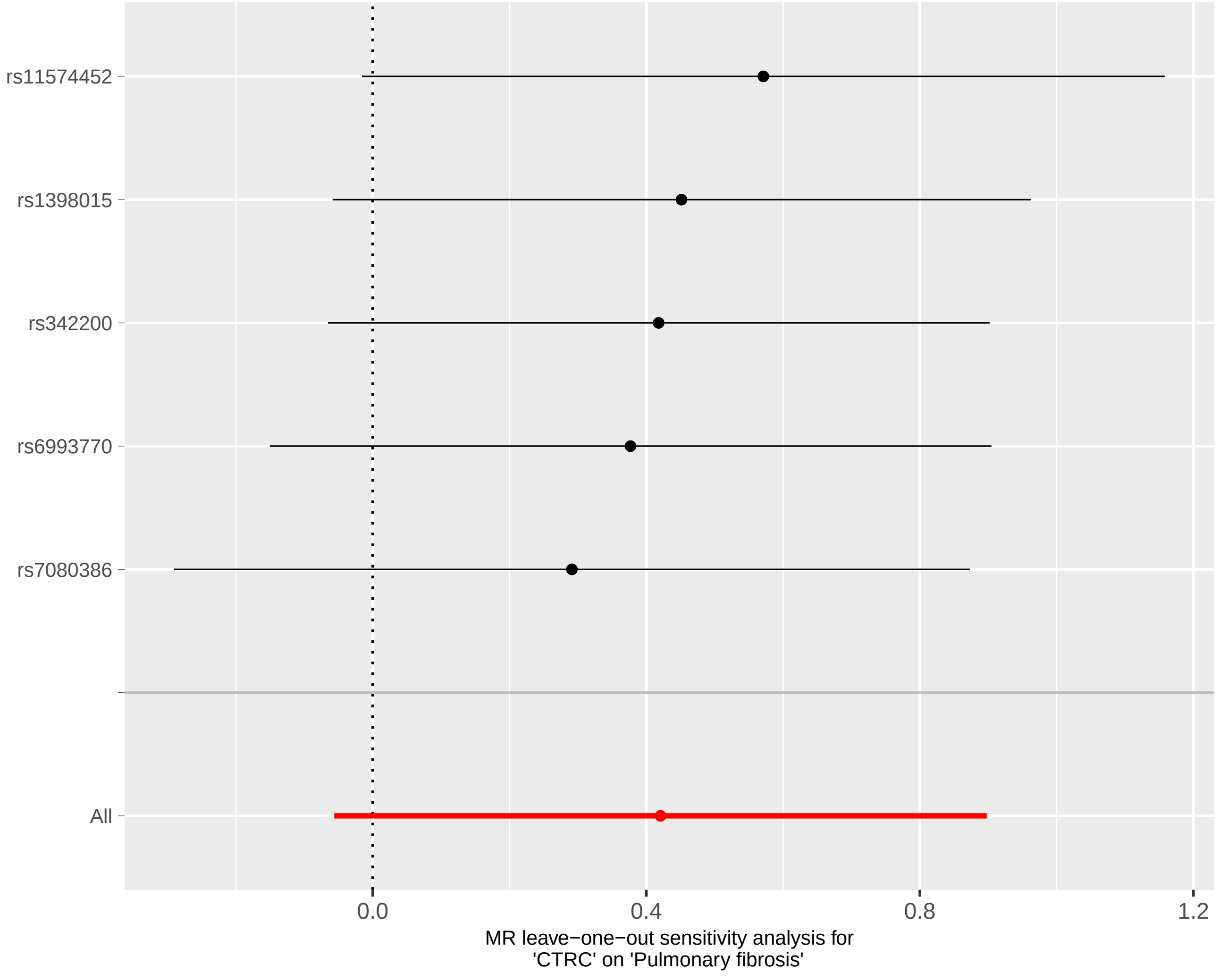
rs11574452
rs1398015
rs342200
rs6993770
rs7080386
All
0.0
0.4
0.8
1.2
MR leave−one−out sensitivity analysis for
'CTRC' on 'Pulmonary fibrosis'

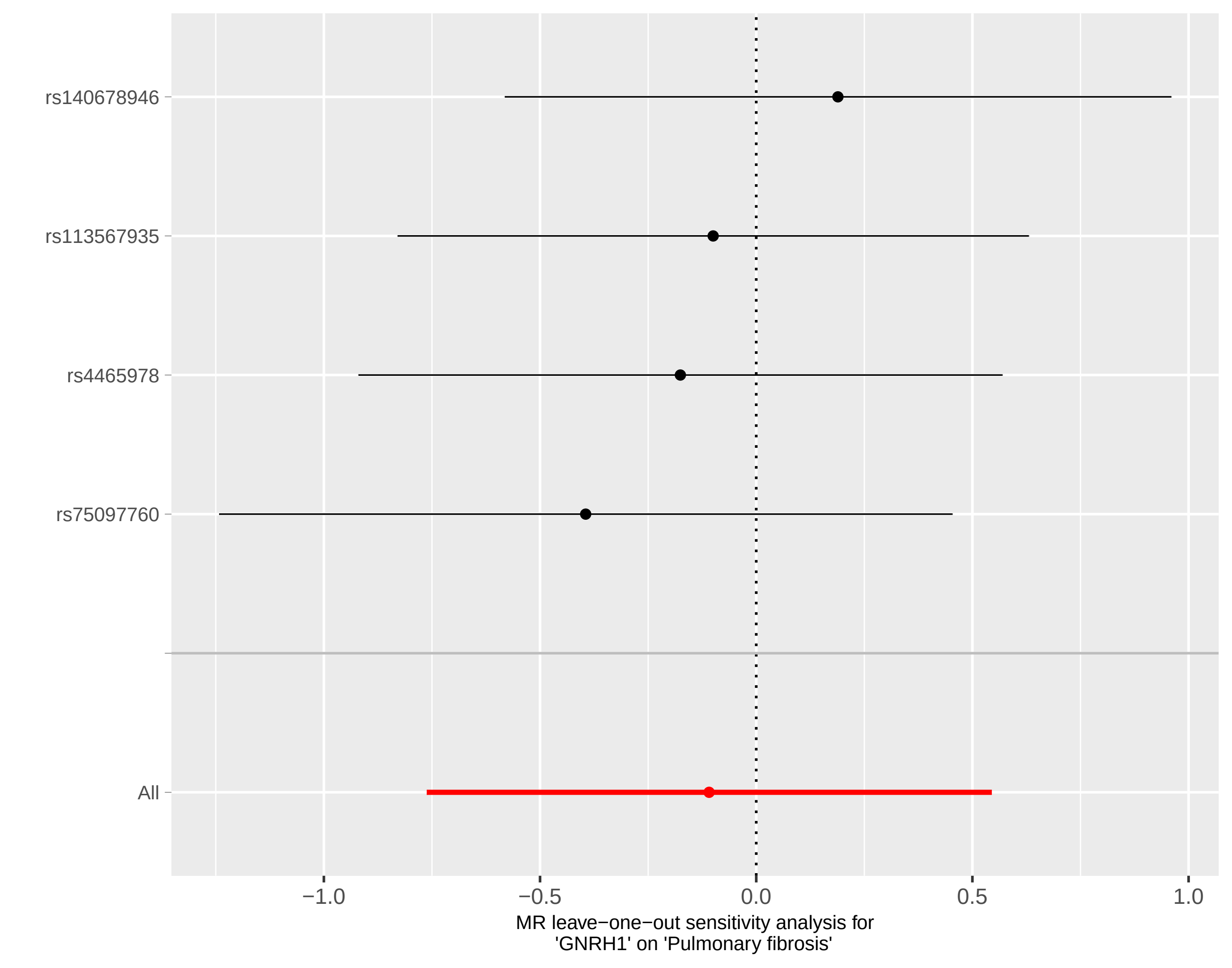
rs140678946
rs113567935
rs4465978
rs75097760
All
−1.0
−0.5
0.0
0.5
1.0
MR leave−one−out sensitivity analysis for
'GNRH1' on 'Pulmonary fibrosis'

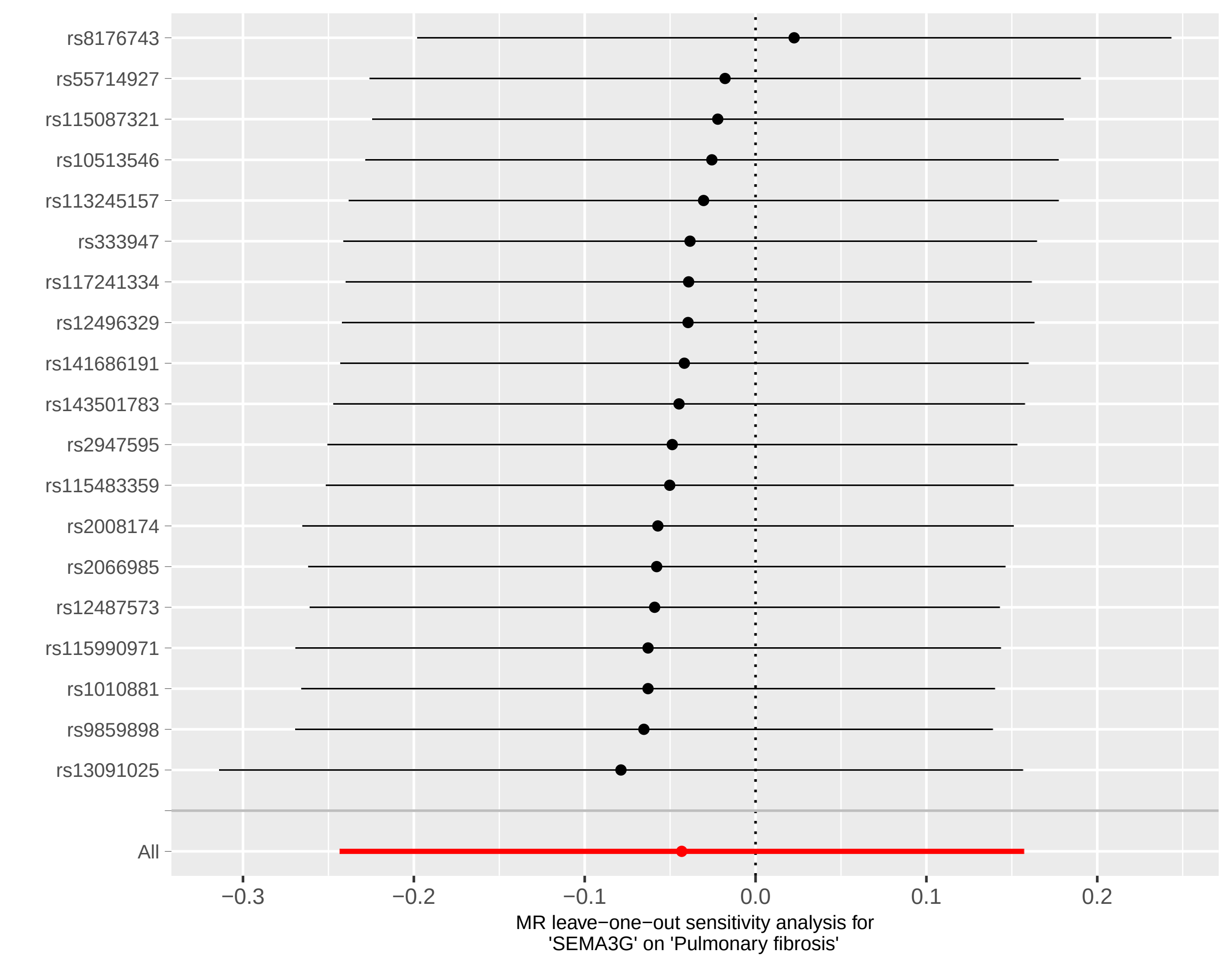
rs8176743
rs55714927
rs115087321
rs10513546
rs113245157
rs333947
rs117241334
rs12496329
rs141686191
rs143501783
rs2947595
rs115483359
rs2008174
rs2066985
rs12487573
rs115990971
rs1010881
rs9859898
rs13091025
All
−0.3
−0.2
−0.1
0.0
0.1
0.2
MR leave−one−out sensitivity analysis for
'SEMA3G' on 'Pulmonary fibrosis'

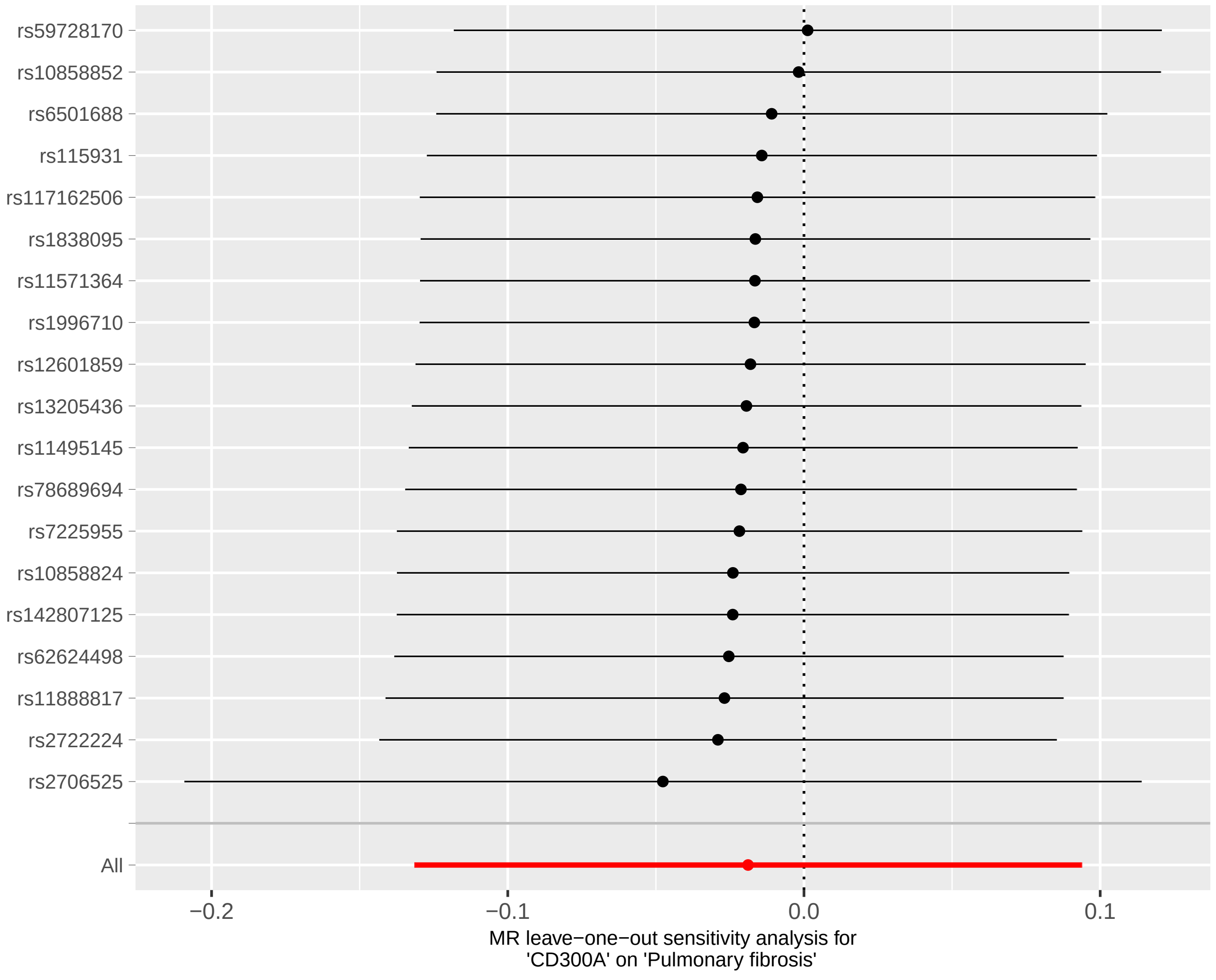
rs59728170
rs10858852
rs6501688
rs115931
rs117162506
rs1838095
rs11571364
rs1996710
rs12601859
rs13205436
rs11495145
rs78689694
rs7225955
rs10858824
rs142807125
rs62624498
rs11888817
rs2722224
rs2706525
All
−0.2
−0.1
0.0
0.1
MR leave−one−out sensitivity analysis for
'CD300A' on 'Pulmonary fibrosis'

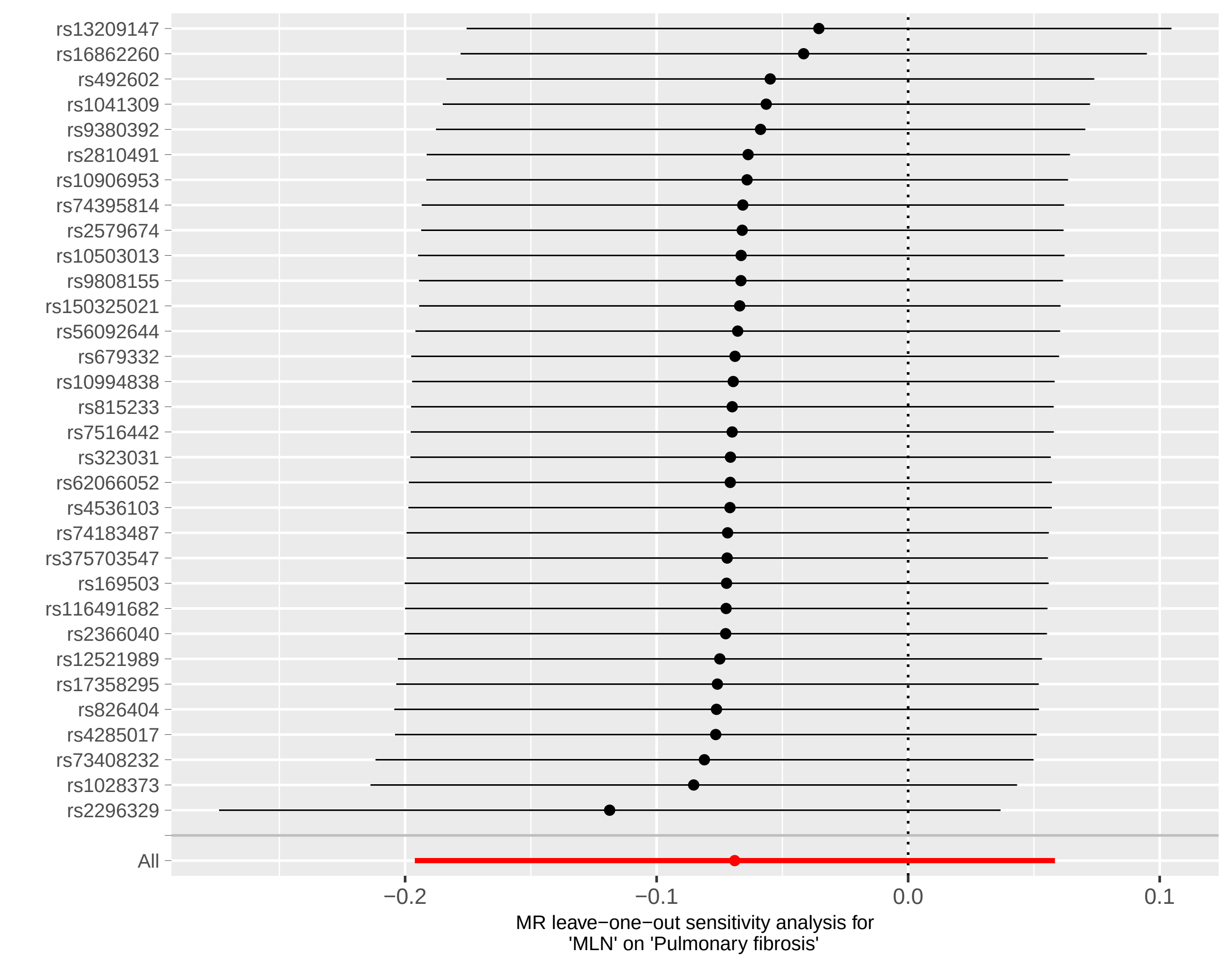
rs13209147
rs16862260
rs492602
rs1041309
rs9380392
rs2810491
rs10906953
rs74395814
rs2579674
rs10503013
rs9808155
rs150325021
rs56092644
rs679332
rs10994838
rs815233
rs7516442
rs323031
rs62066052
rs4536103
rs74183487
rs375703547
rs169503
rs116491682
rs2366040
rs12521989
rs17358295
rs826404
rs4285017
rs73408232
rs1028373
rs2296329
All
−0.2
−0.1
0.0
0.1
MR leave−one−out sensitivity analysis for
'MLN' on 'Pulmonary fibrosis'

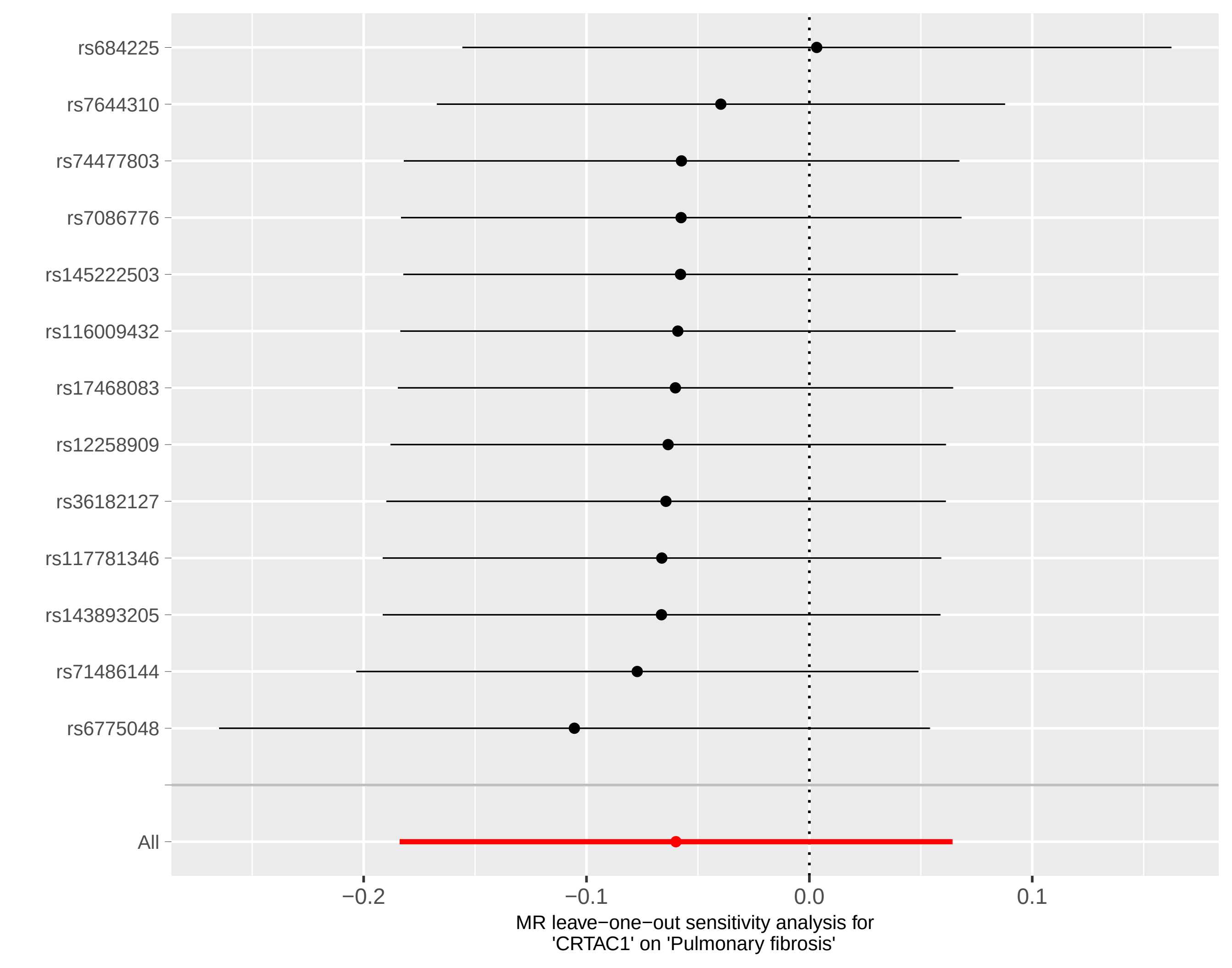
rs684225
rs7644310
rs74477803
rs7086776
rs145222503
rs116009432
rs17468083
rs12258909
rs36182127
rs117781346
rs143893205
rs71486144
rs6775048
All
−0.2
−0.1
0.0
0.1
MR leave−one−out sensitivity analysis for
'CRTAC1' on 'Pulmonary fibrosis'

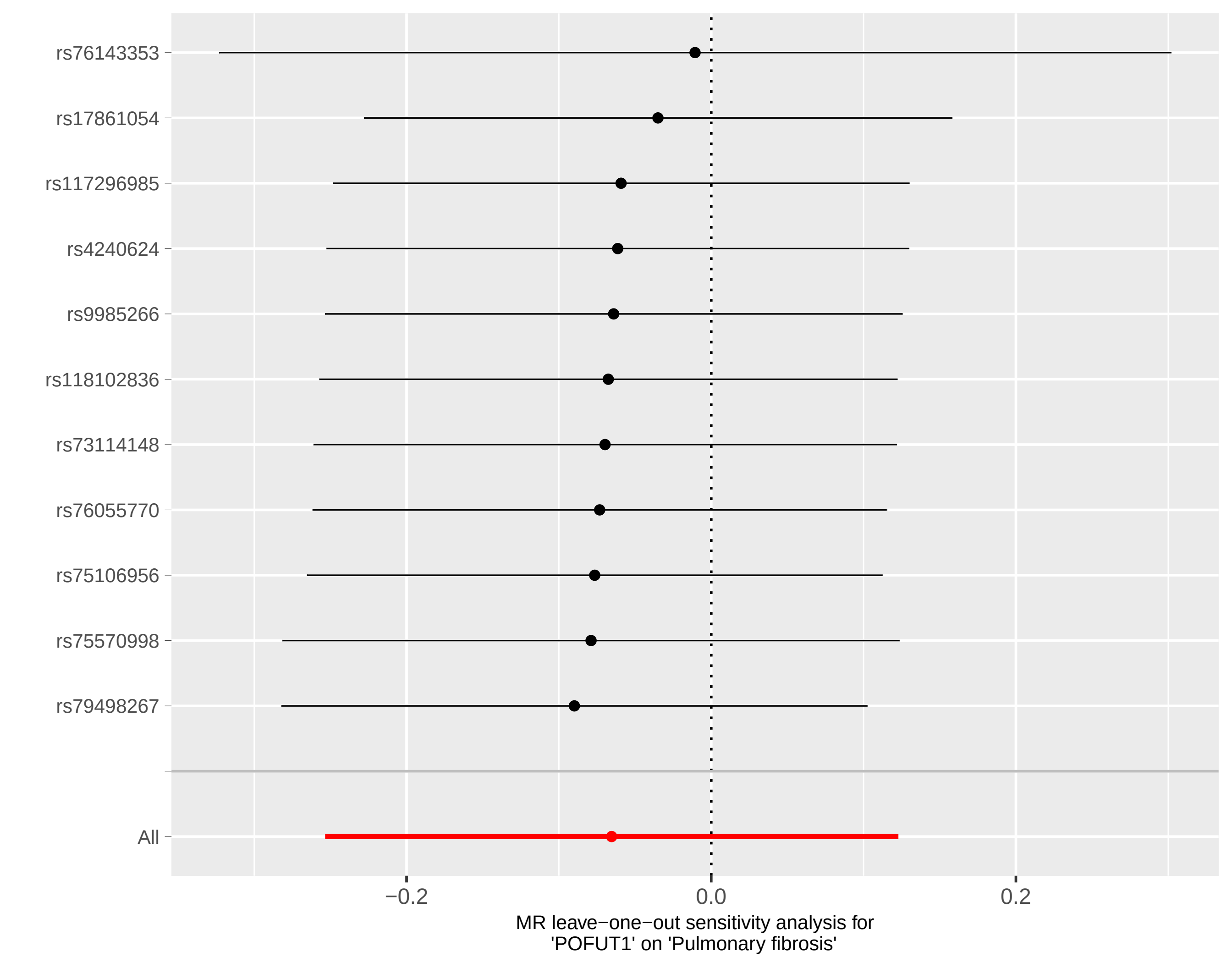
rs76143353
rs17861054
rs117296985
rs4240624
rs9985266
rs118102836
rs73114148
rs76055770
rs75106956
rs75570998
rs79498267
All
−0.2
0.0
0.2
MR leave−one−out sensitivity analysis for
'POFUT1' on 'Pulmonary fibrosis'

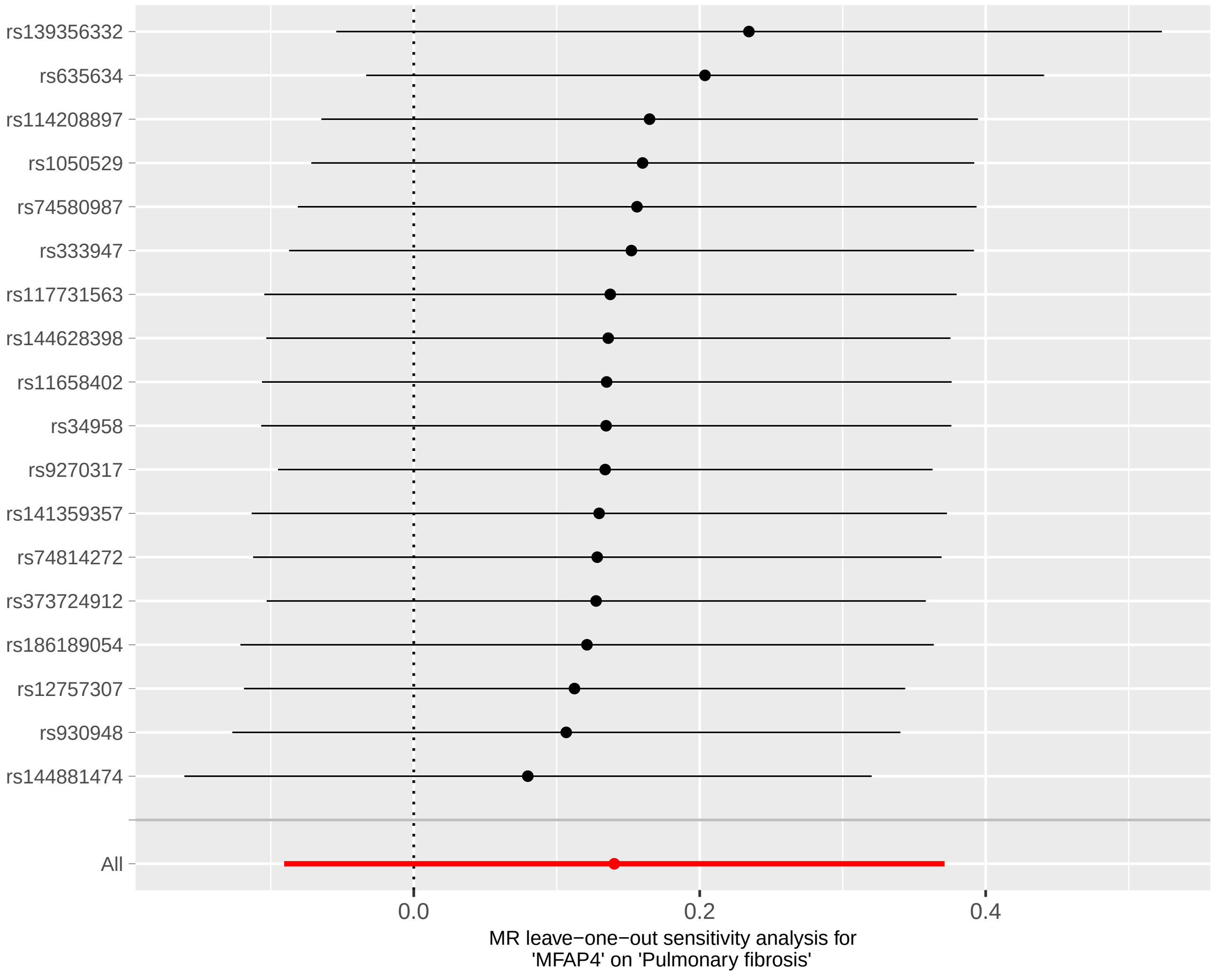
rs139356332
rs635634
rs114208897
rs1050529
rs74580987
rs333947
rs117731563
rs144628398
rs11658402
rs34958
rs9270317
rs141359357
rs74814272
rs373724912
rs186189054
rs12757307
rs930948
rs144881474
All
0.0
0.2
0.4
MR leave−one−out sensitivity analysis for
'MFAP4' on 'Pulmonary fibrosis'

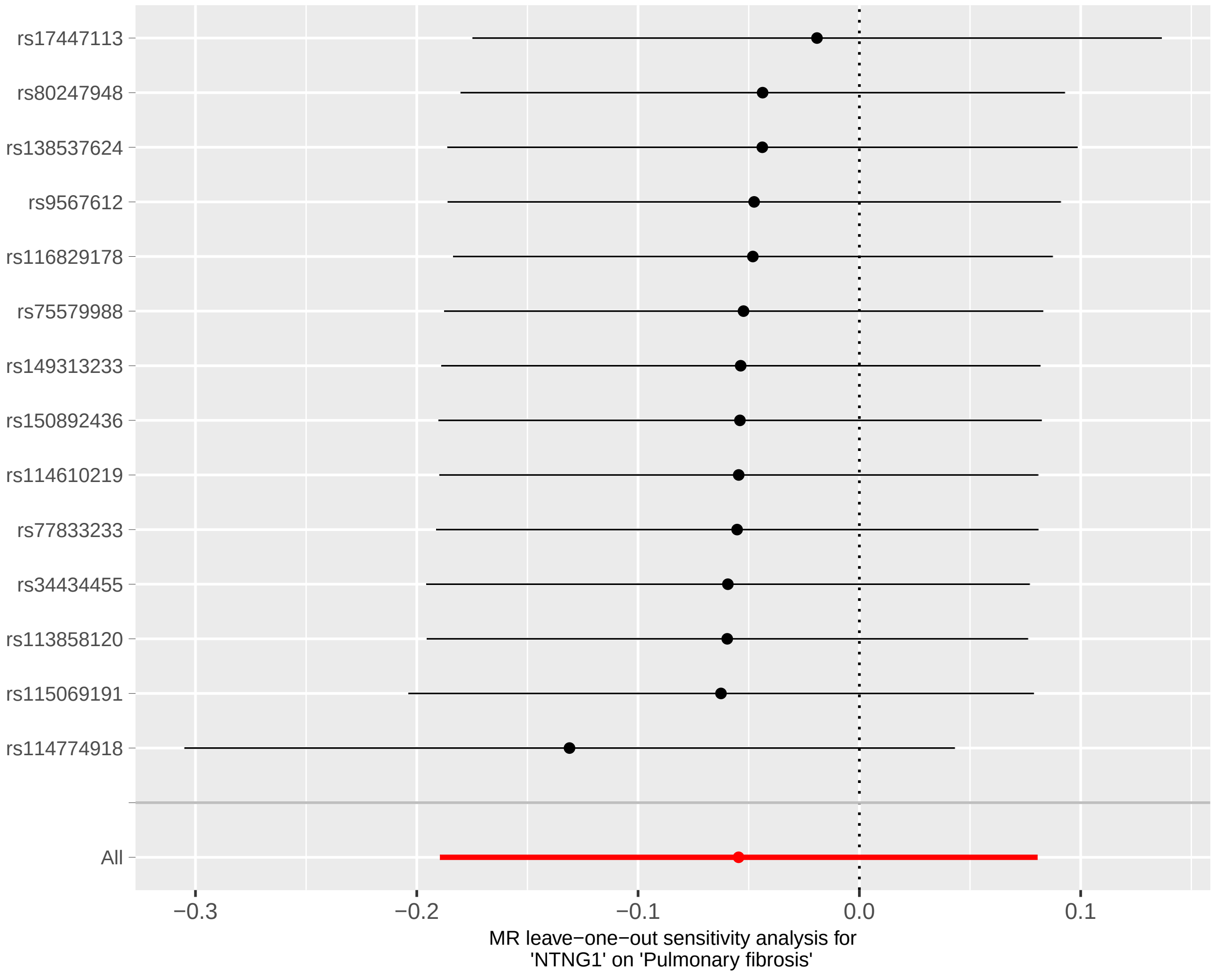

rs17447113
rs80247948
rs138537624
rs9567612
rs116829178
rs75579988
rs149313233
rs150892436
rs114610219
rs77833233
rs34434455
rs113858120
rs115069191
rs114774918
All
−0.3
−0.2
−0.1
0.0
0.1
MR leave−one−out sensitivity analysis for
'NTNG1' on 'Pulmonary fibrosis'

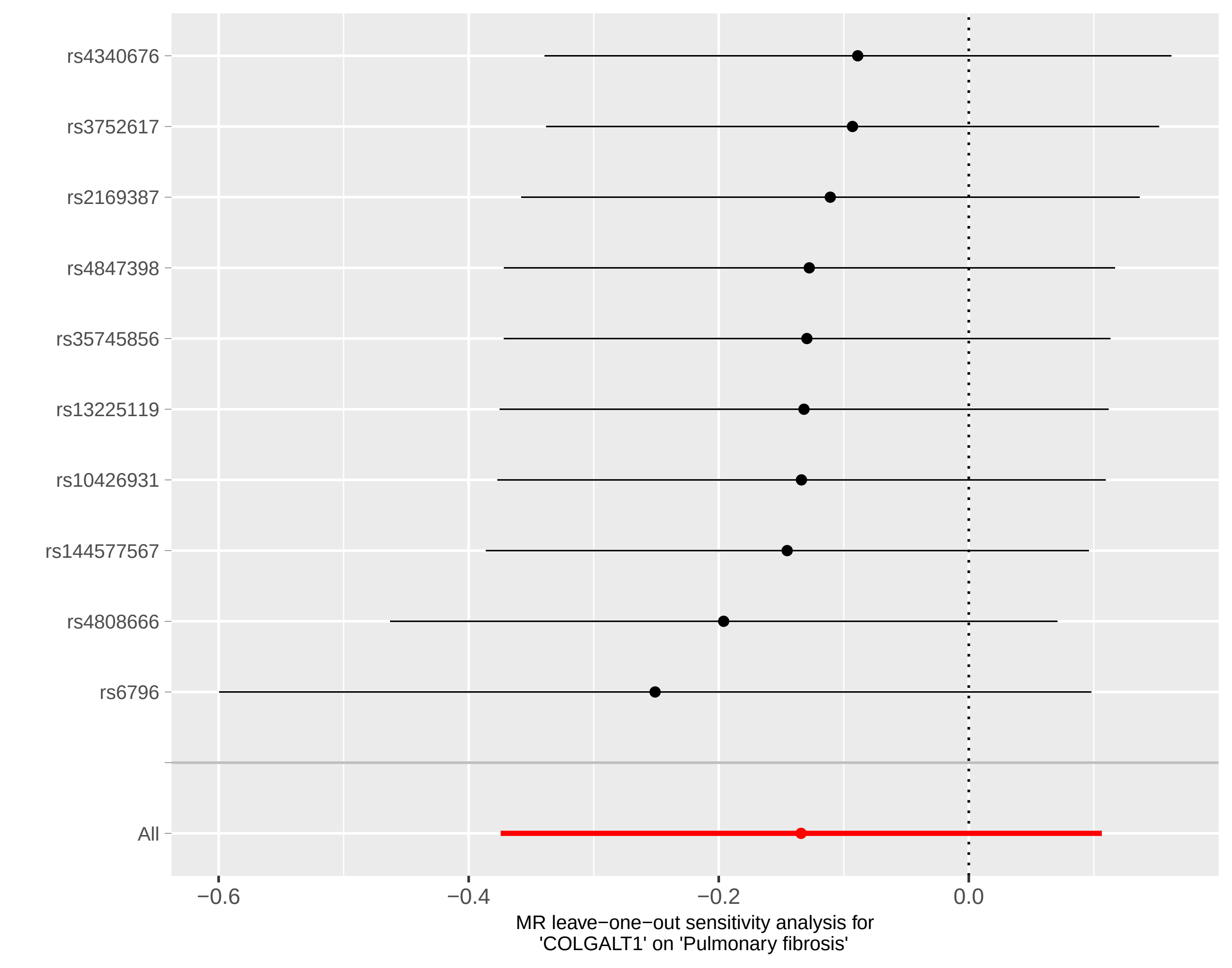
rs4340676
rs3752617
rs2169387
rs4847398
rs35745856
rs13225119
rs10426931
rs144577567
rs4808666
rs6796
All
−0.6
−0.4
−0.2
0.0
MR leave−one−out sensitivity analysis for
'COLGALT1' on 'Pulmonary fibrosis'

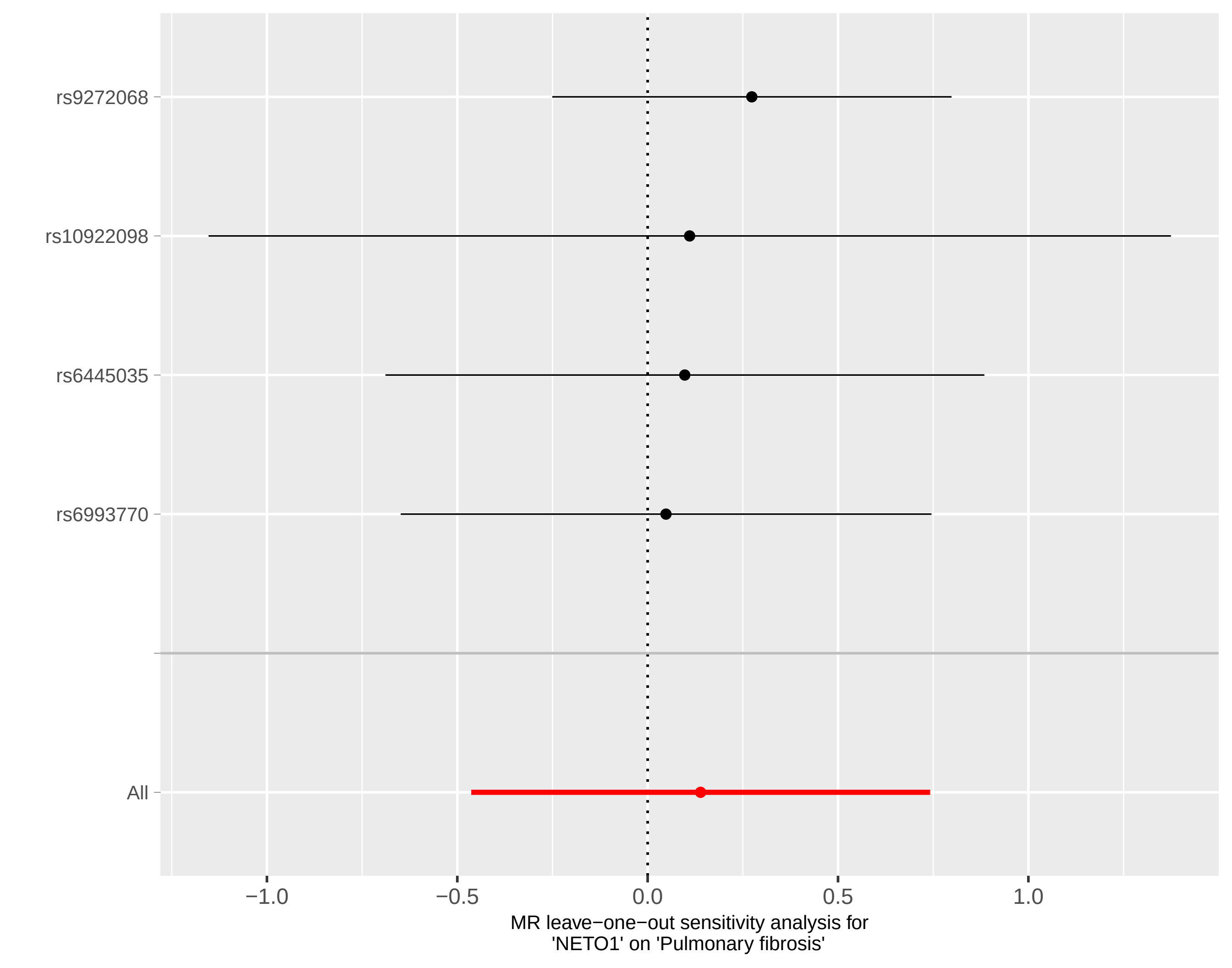
rs9272068
rs10922098
rs6445035
rs6993770
All
−1.0
−0.5
0.0
0.5
1.0
MR leave−one−out sensitivity analysis for
'NETO1' on 'Pulmonary fibrosis'

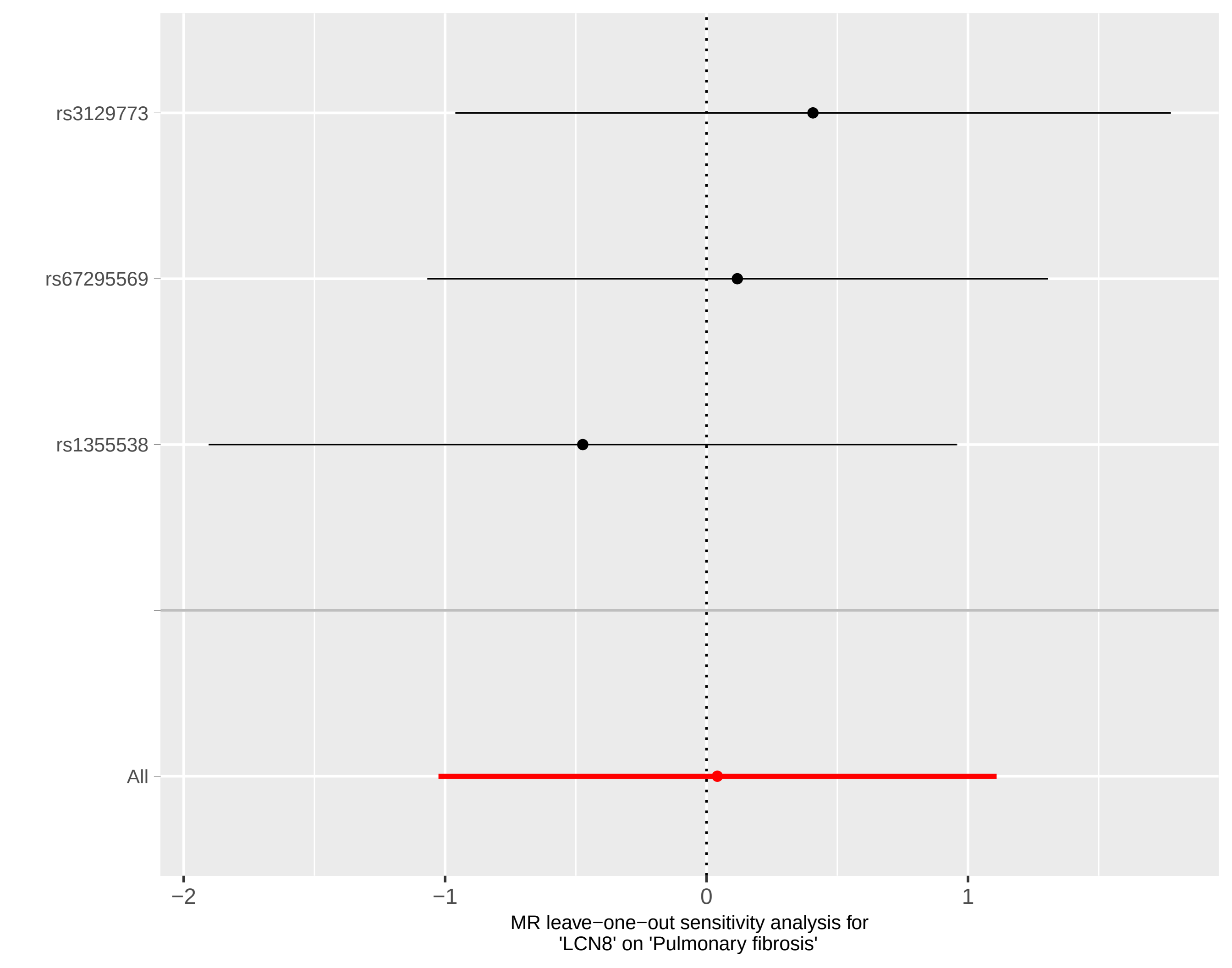
rs3129773
rs67295569
rs1355538
All
−2
−1
0
1
MR leave−one−out sensitivity analysis for
'LCN8' on 'Pulmonary fibrosis'

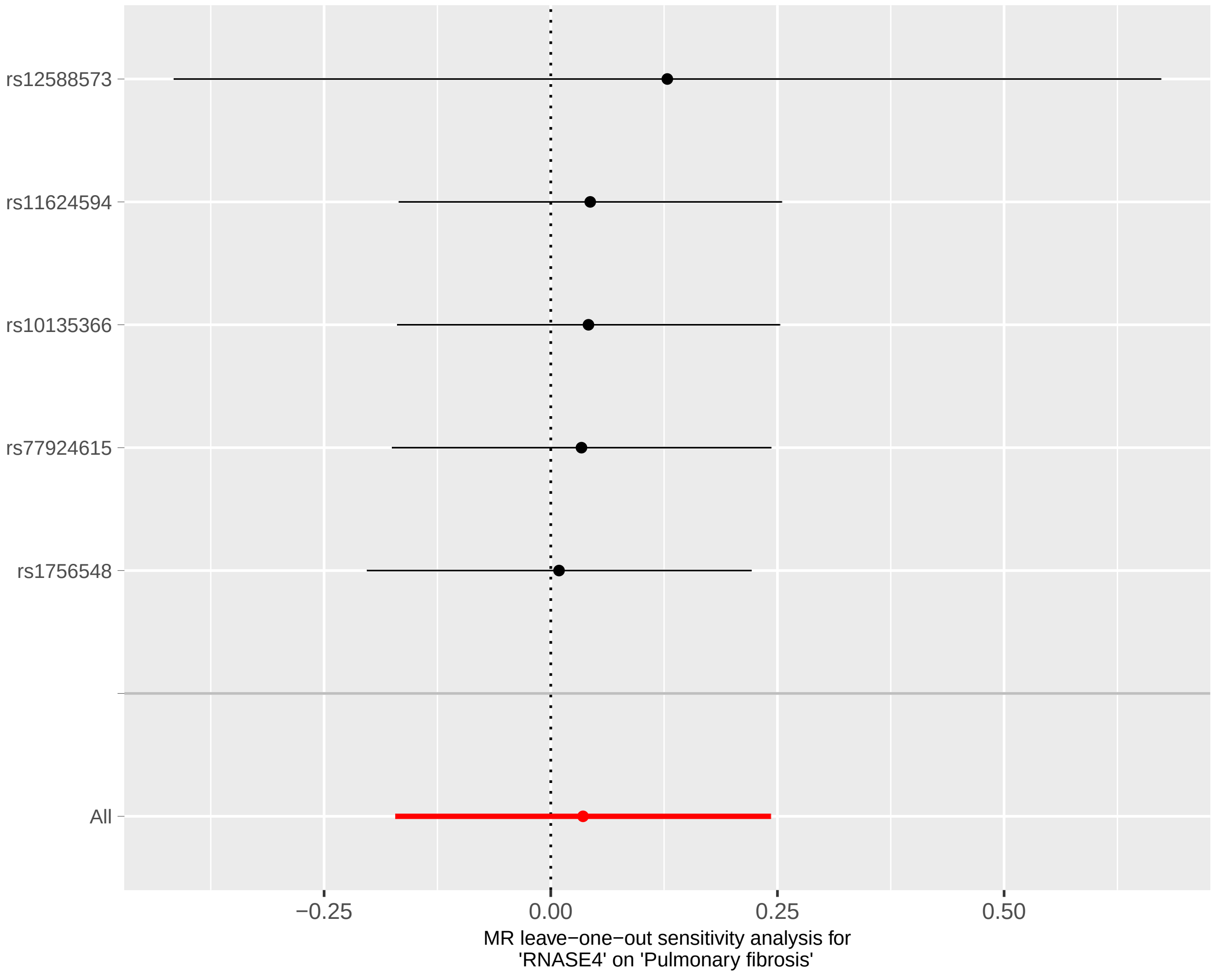

rs12588573
rs11624594
rs10135366
rs77924615
rs1756548
All
−0.25
0.00
0.25
0.50
MR leave−one−out sensitivity analysis for
'RNASE4' on 'Pulmonary fibrosis'

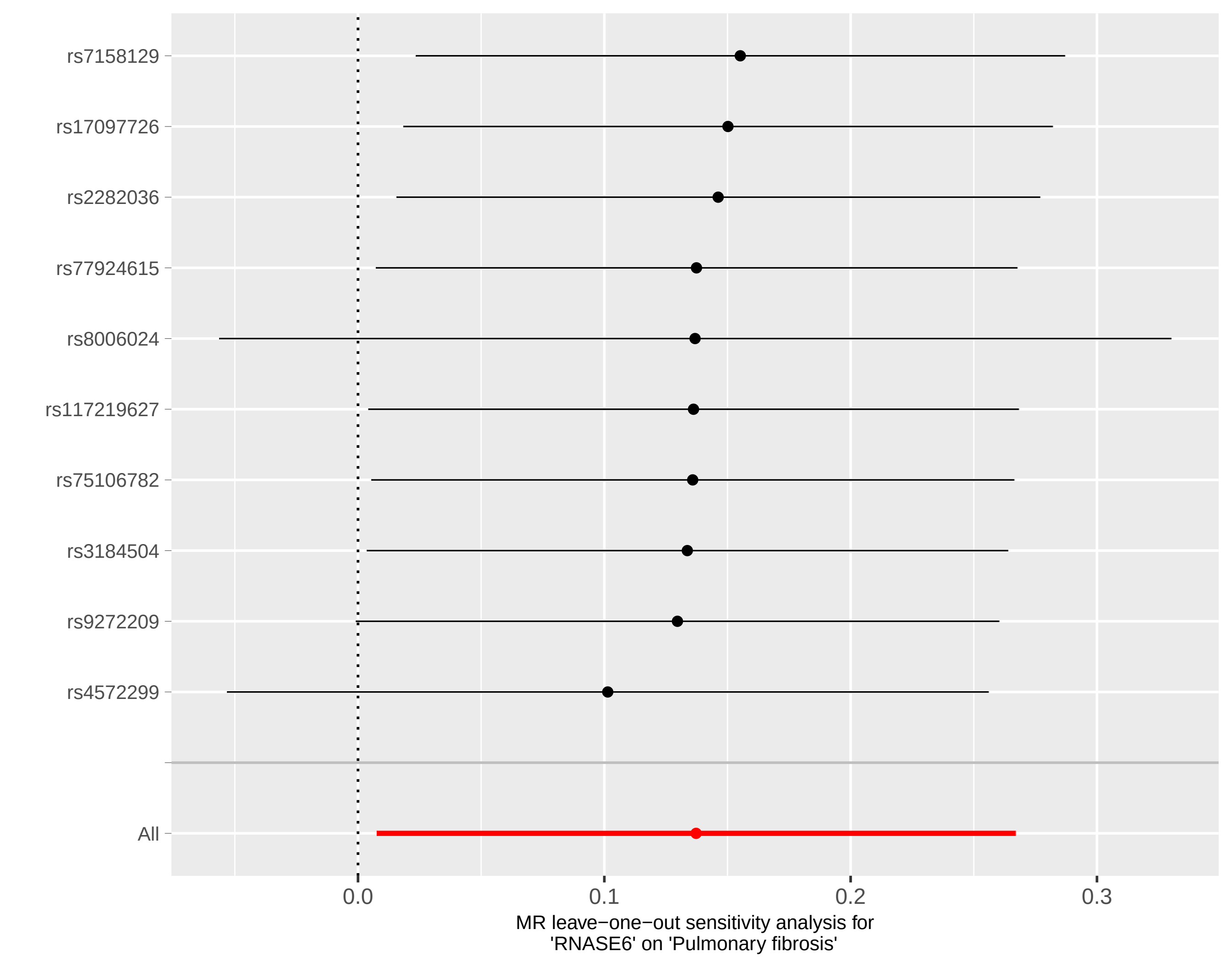
rs7158129
rs17097726
rs2282036
rs77924615
rs8006024
rs117219627
rs75106782
rs3184504
rs9272209
rs4572299
All
0.0
0.1
0.2
0.3
MR leave−one−out sensitivity analysis for
'RNASE6' on 'Pulmonary fibrosis'

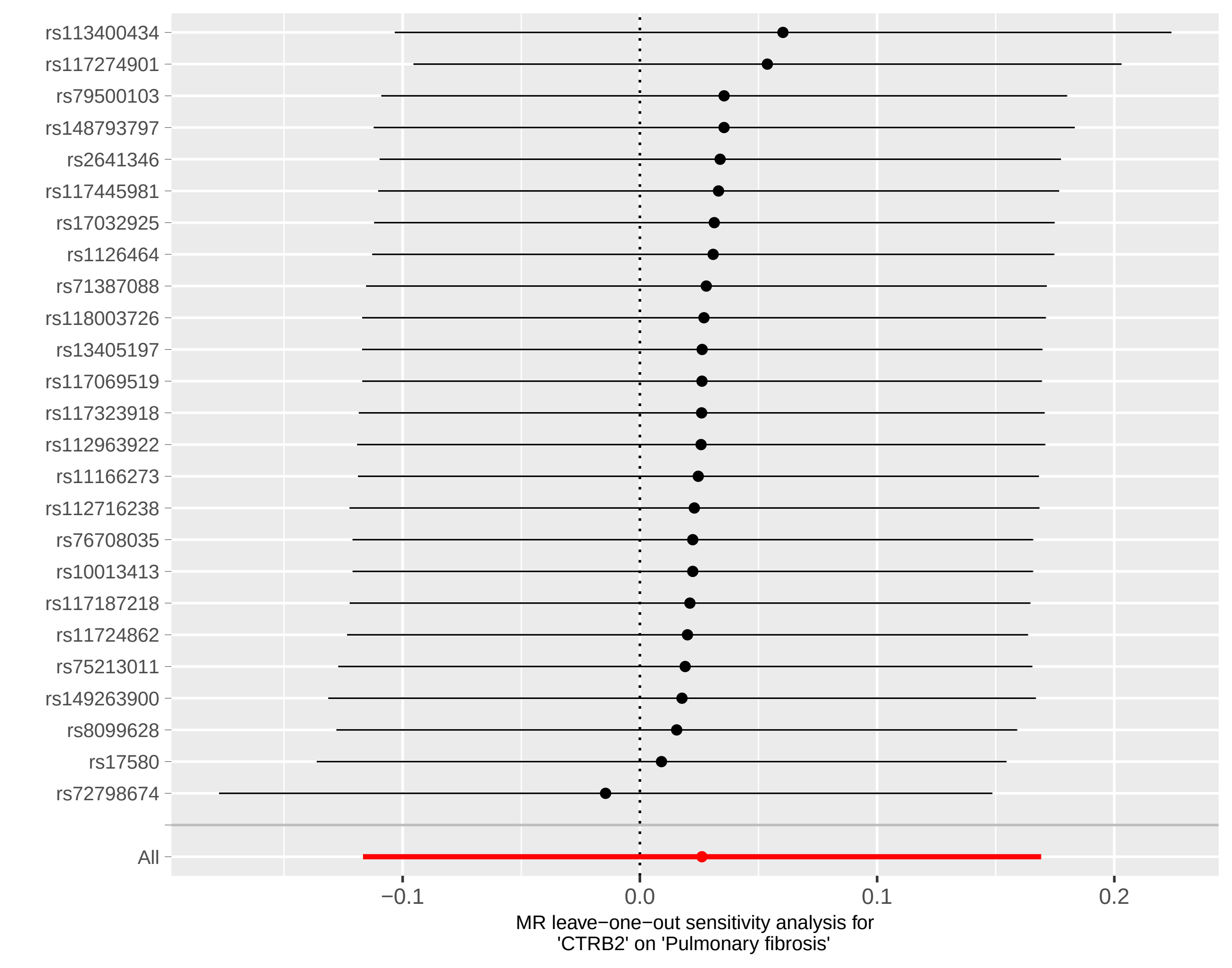
rs113400434
rs117274901
rs79500103
rs148793797
rs2641346
rs117445981
rs17032925
rs1126464
rs71387088
rs118003726
rs13405197
rs117069519
rs117323918
rs112963922
rs11166273
rs112716238
rs76708035
rs10013413
rs117187218
rs11724862
rs75213011
rs149263900
rs8099628
rs17580
rs72798674
All
−0.1
0.0
0.1
0.2
MR leave−one−out sensitivity analysis for
'CTRB2' on 'Pulmonary fibrosis'

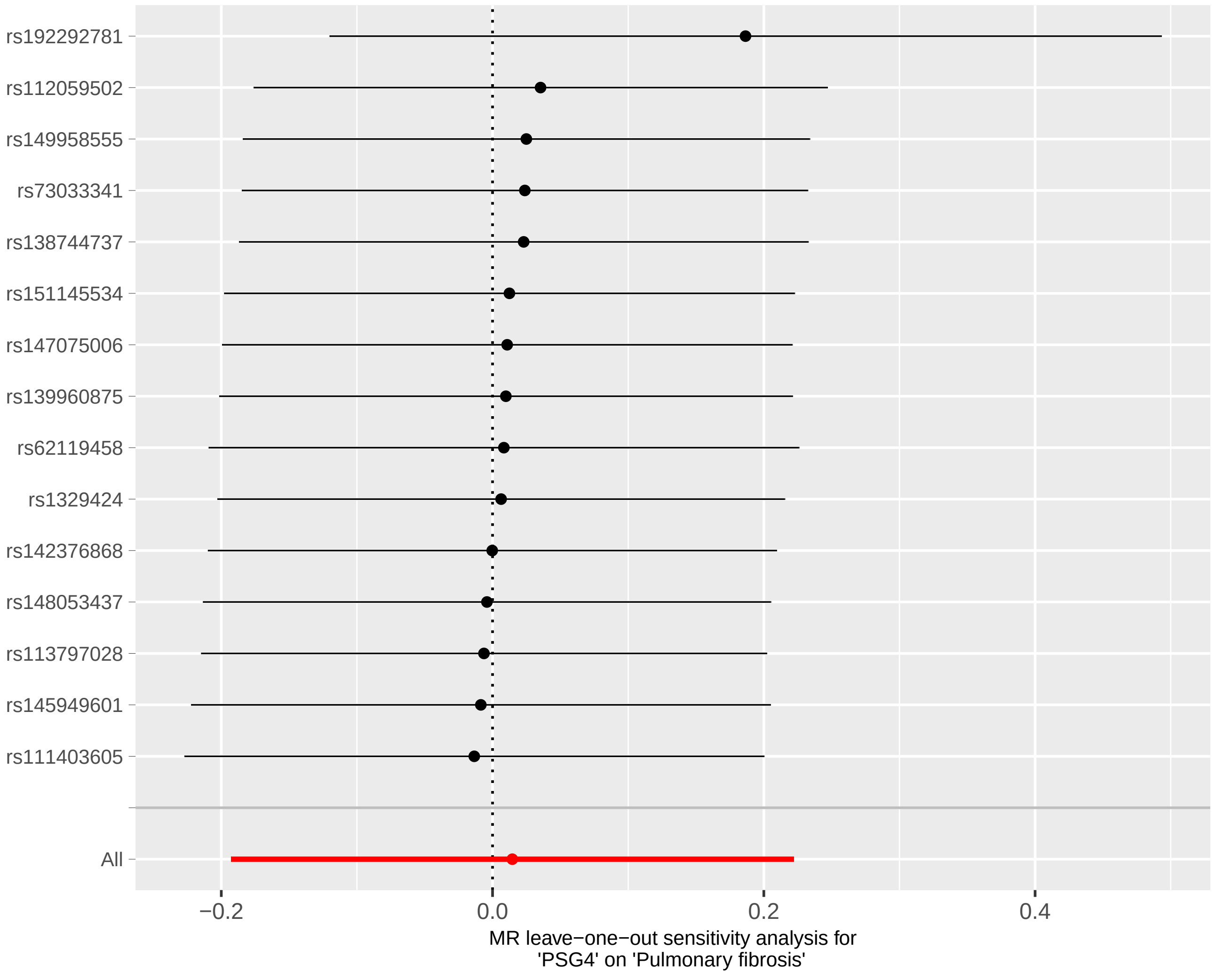

rs192292781
rs112059502
rs149958555
rs73033341
rs138744737
rs151145534
rs147075006
rs139960875
rs62119458
rs1329424
rs142376868
rs148053437
rs113797028
rs145949601
rs111403605
All
−0.2
0.0
0.2
0.4
MR leave−one−out sensitivity analysis for
'PSG4' on 'Pulmonary fibrosis'

Insufficient number of SNPs

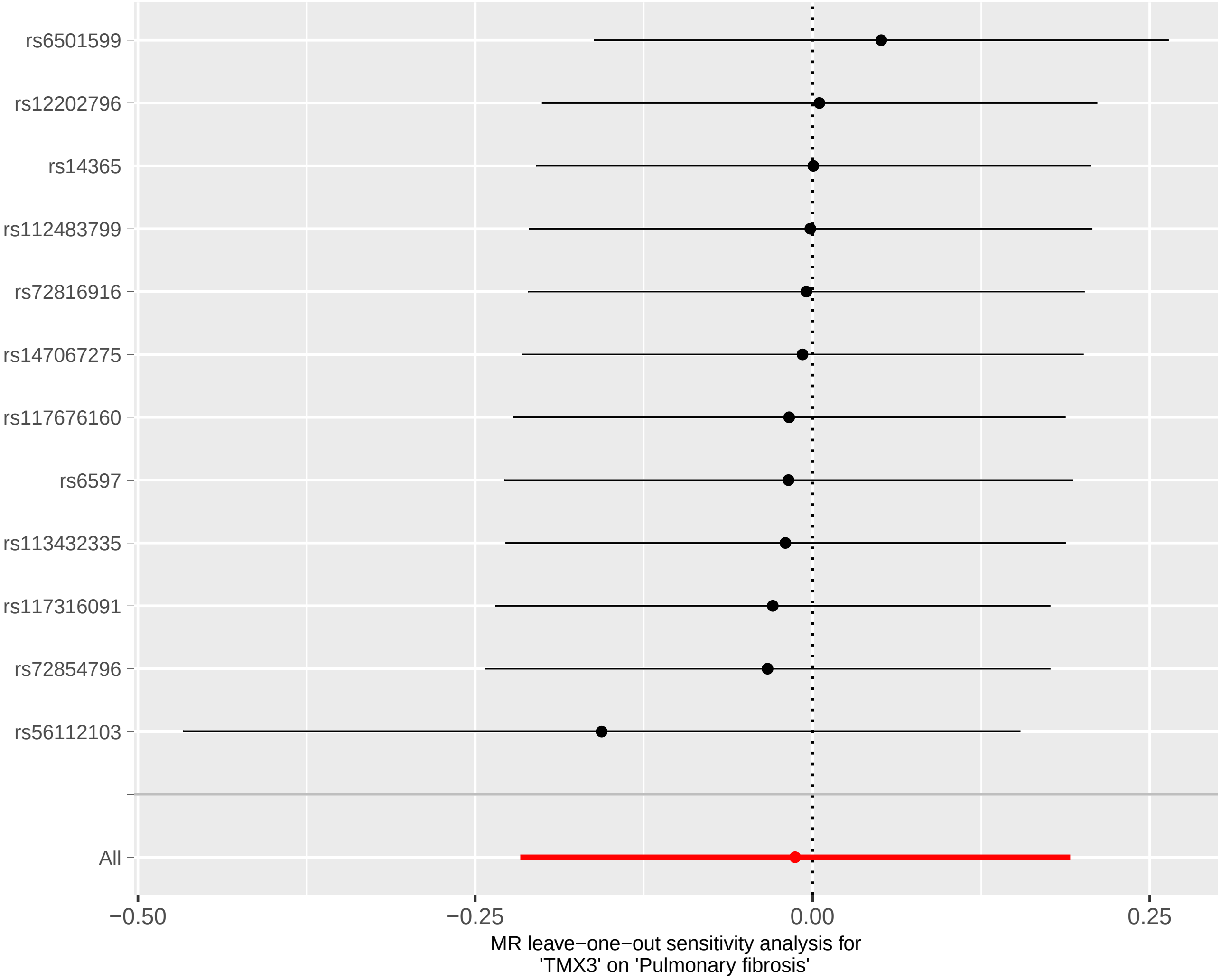
rs6501599
rs12202796
rs14365
rs112483799
rs72816916
rs147067275
rs117676160
rs6597
rs113432335
rs117316091
rs72854796
rs56112103
All
−0.50
−0.25
0.00
0.25
MR leave−one−out sensitivity analysis for
'TMX3' on 'Pulmonary fibrosis'

Insufficient number of SNPs

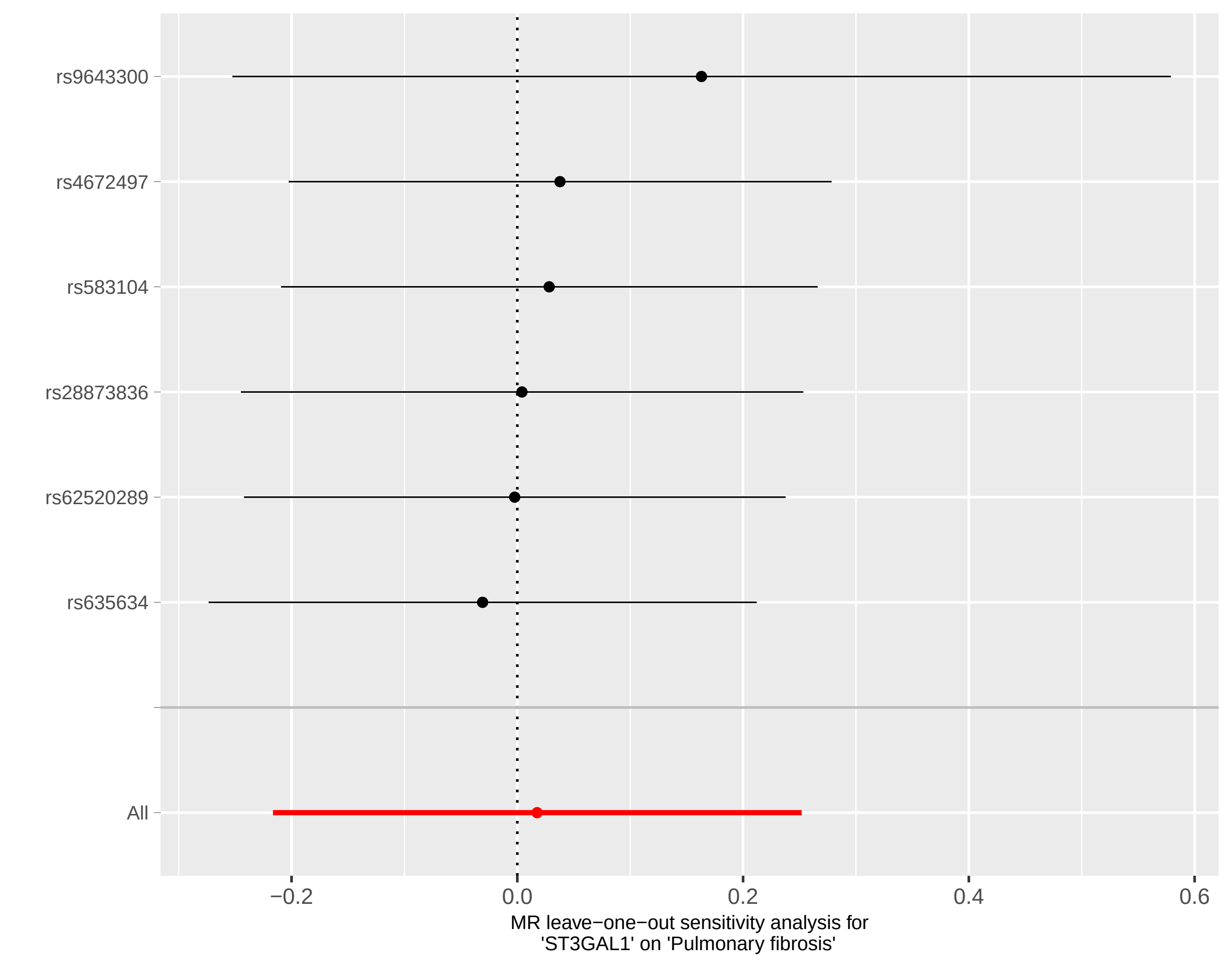
rs9643300
rs4672497
rs583104
rs28873836
rs62520289
rs635634
All
−0.2
0.0
0.2
0.4
0.6
MR leave−one−out sensitivity analysis for
'ST3GAL1' on 'Pulmonary fibrosis'

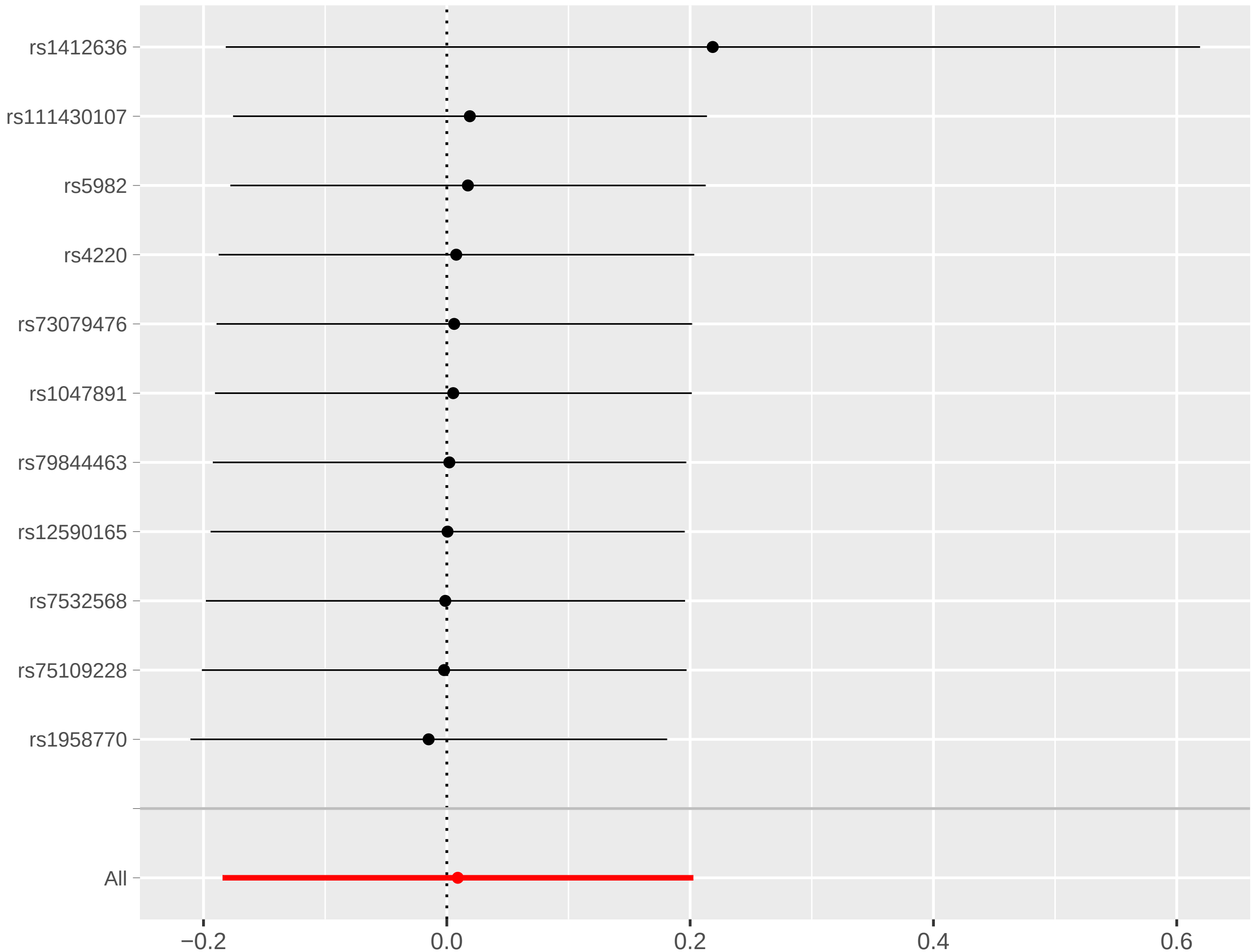

MR leave−one−out sensitivity analysis for
'F13B' on 'Pulmonary fibrosis'

Insufficient number of SNPs

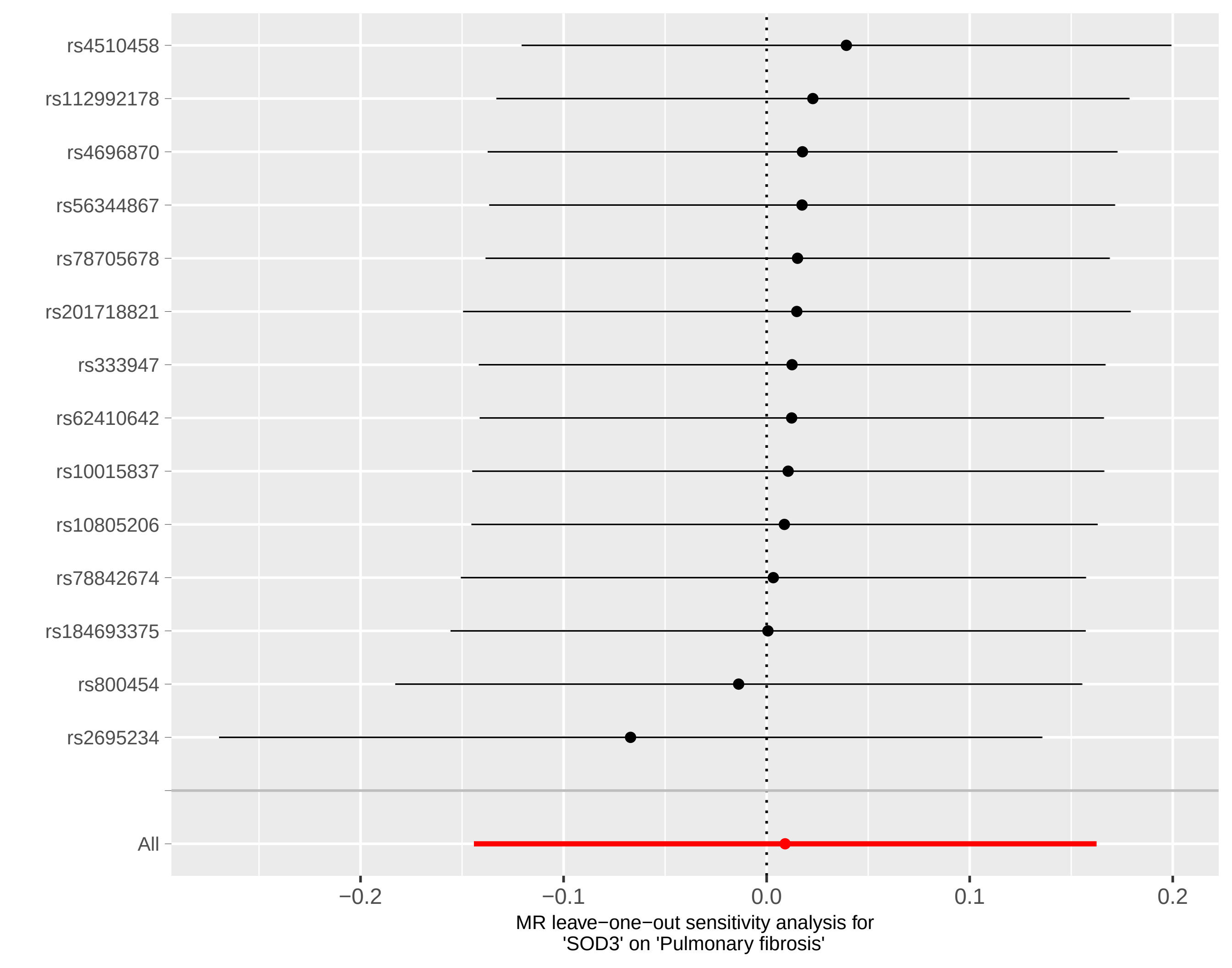
rs4510458
rs112992178
rs4696870
rs56344867
rs78705678
rs201718821
rs333947
rs62410642
rs10015837
rs10805206
rs78842674
rs184693375
rs800454
rs2695234
All
−0.2
−0.1
0.0
0.1
0.2
MR leave−one−out sensitivity analysis for
'SOD3' on 'Pulmonary fibrosis'

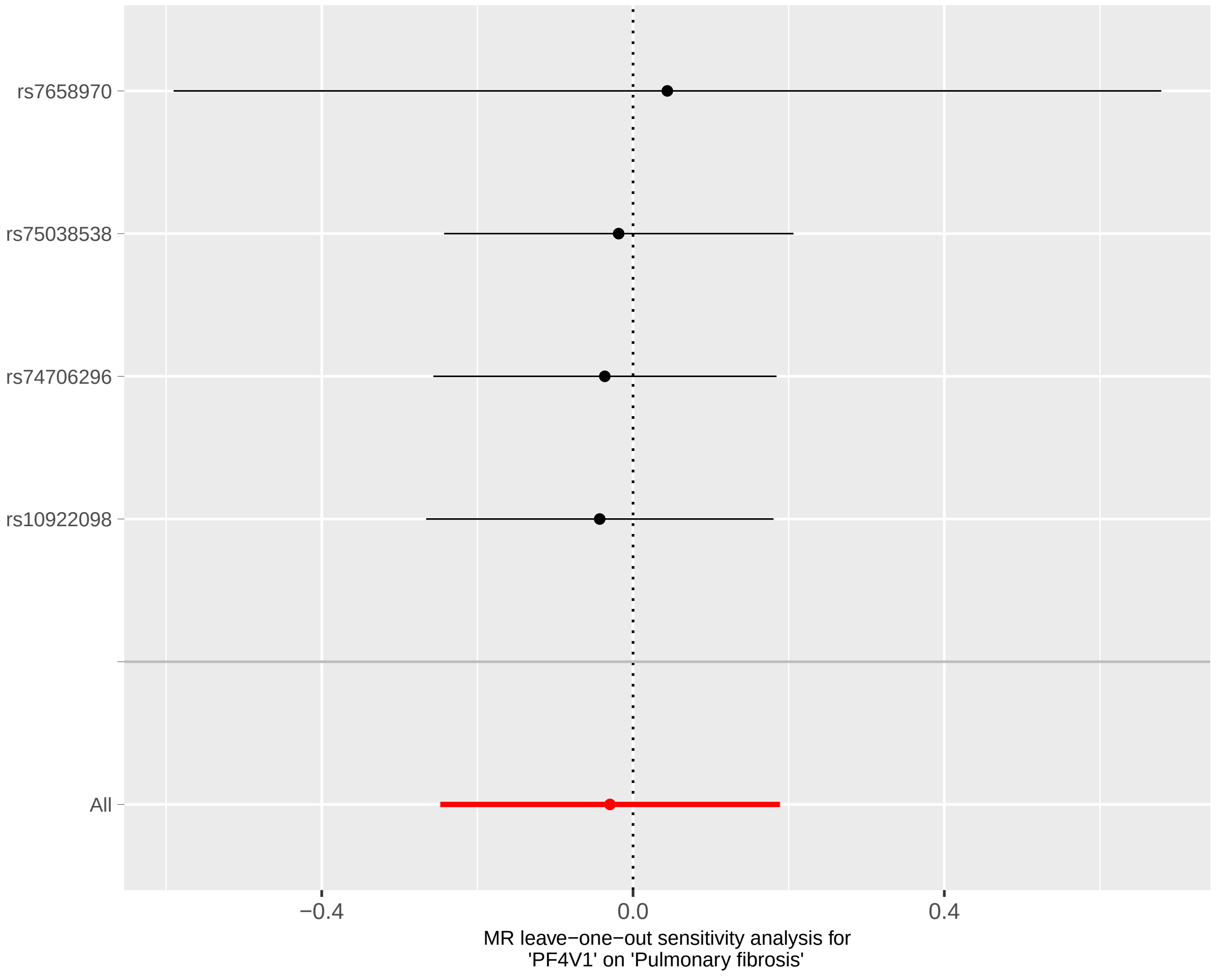

rs7658970
rs75038538
rs74706296
rs10922098
All
−0.4
0.0
0.4
MR leave−one−out sensitivity analysis for
'PF4V1' on 'Pulmonary fibrosis'

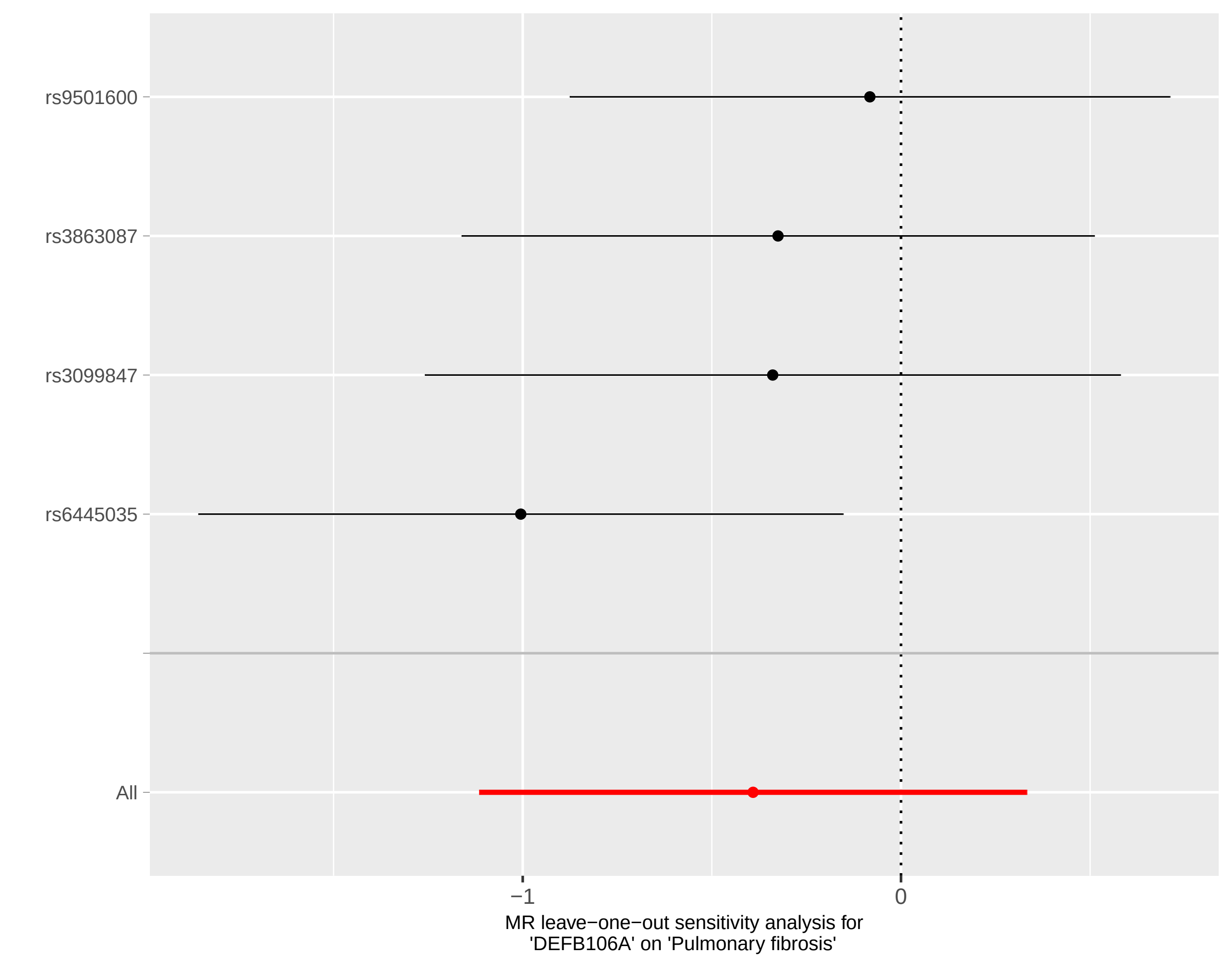
rs9501600
rs3863087
rs3099847
rs6445035
All
−1
0
MR leave−one−out sensitivity analysis for
'DEFB106A' on 'Pulmonary fibrosis'

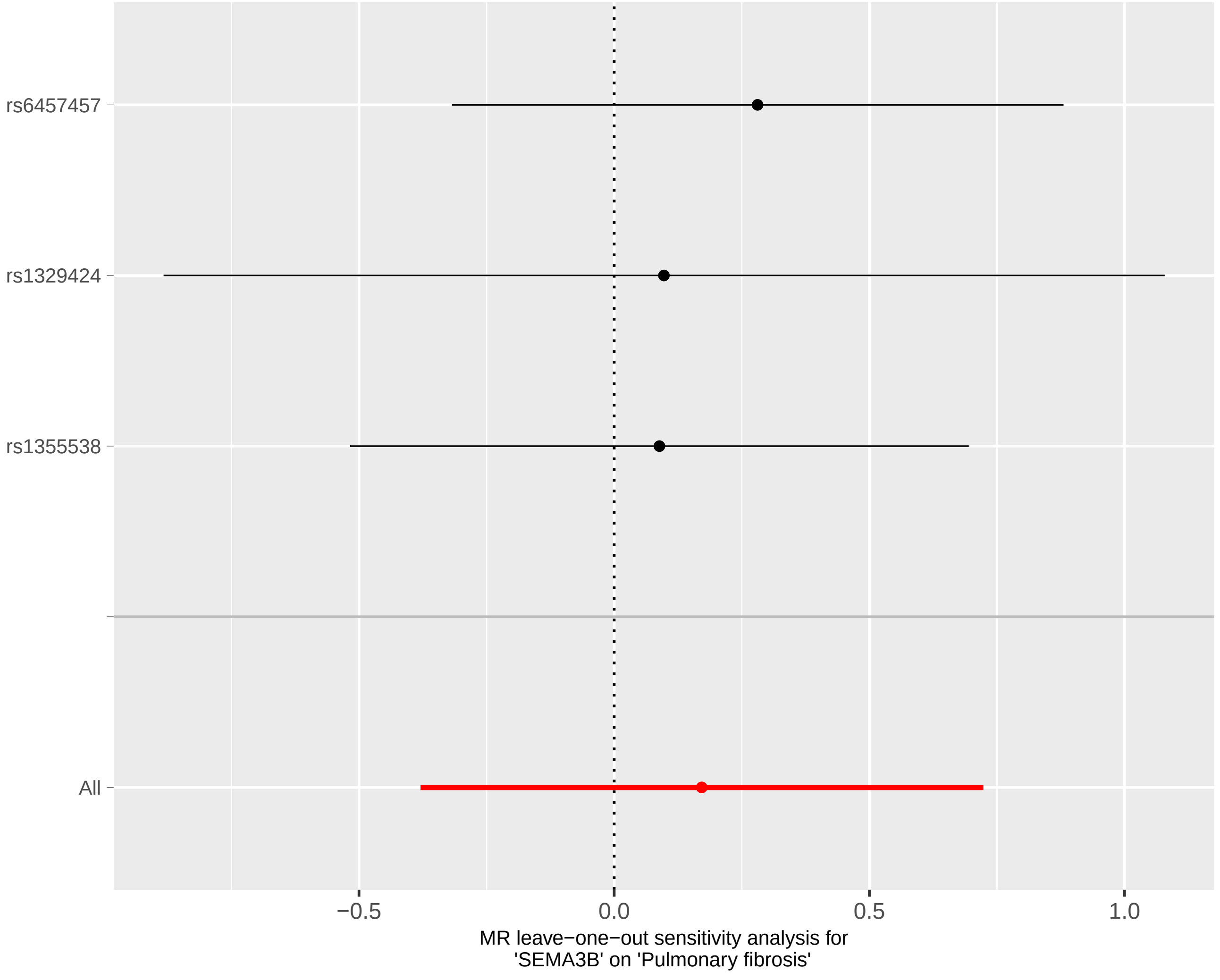
rs6457457
rs1329424
rs1355538
All
−0.5
0.0
0.5
1.0
MR leave−one−out sensitivity analysis for
'SEMA3B' on 'Pulmonary fibrosis'

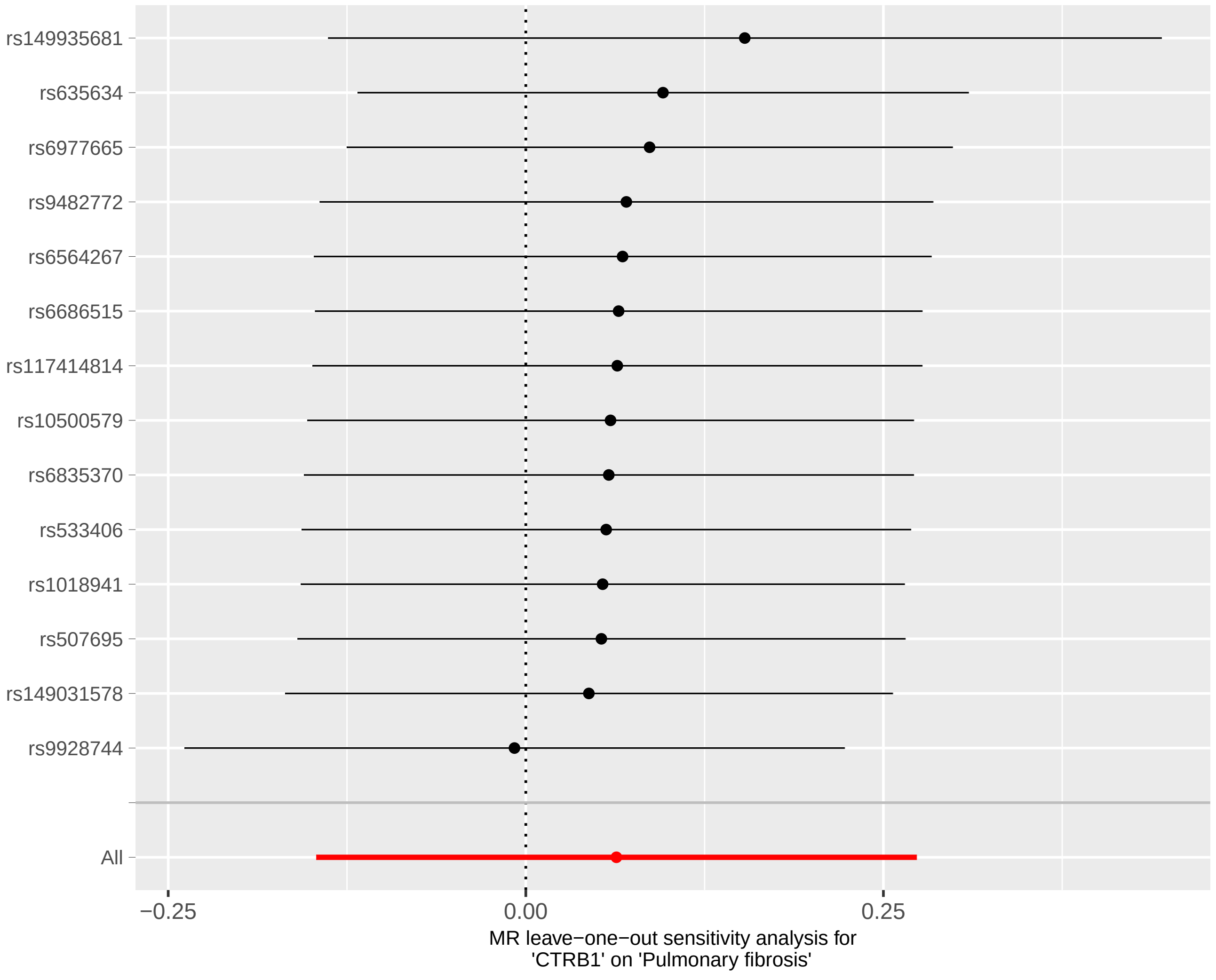
rs149935681
rs635634
rs6977665
rs9482772
rs6564267
rs6686515
rs117414814
rs10500579
rs6835370
rs533406
rs1018941
rs507695
rs149031578
rs9928744
All
−0.25
0.00
0.25
MR leave−one−out sensitivity analysis for
'CTRB1' on 'Pulmonary fibrosis'

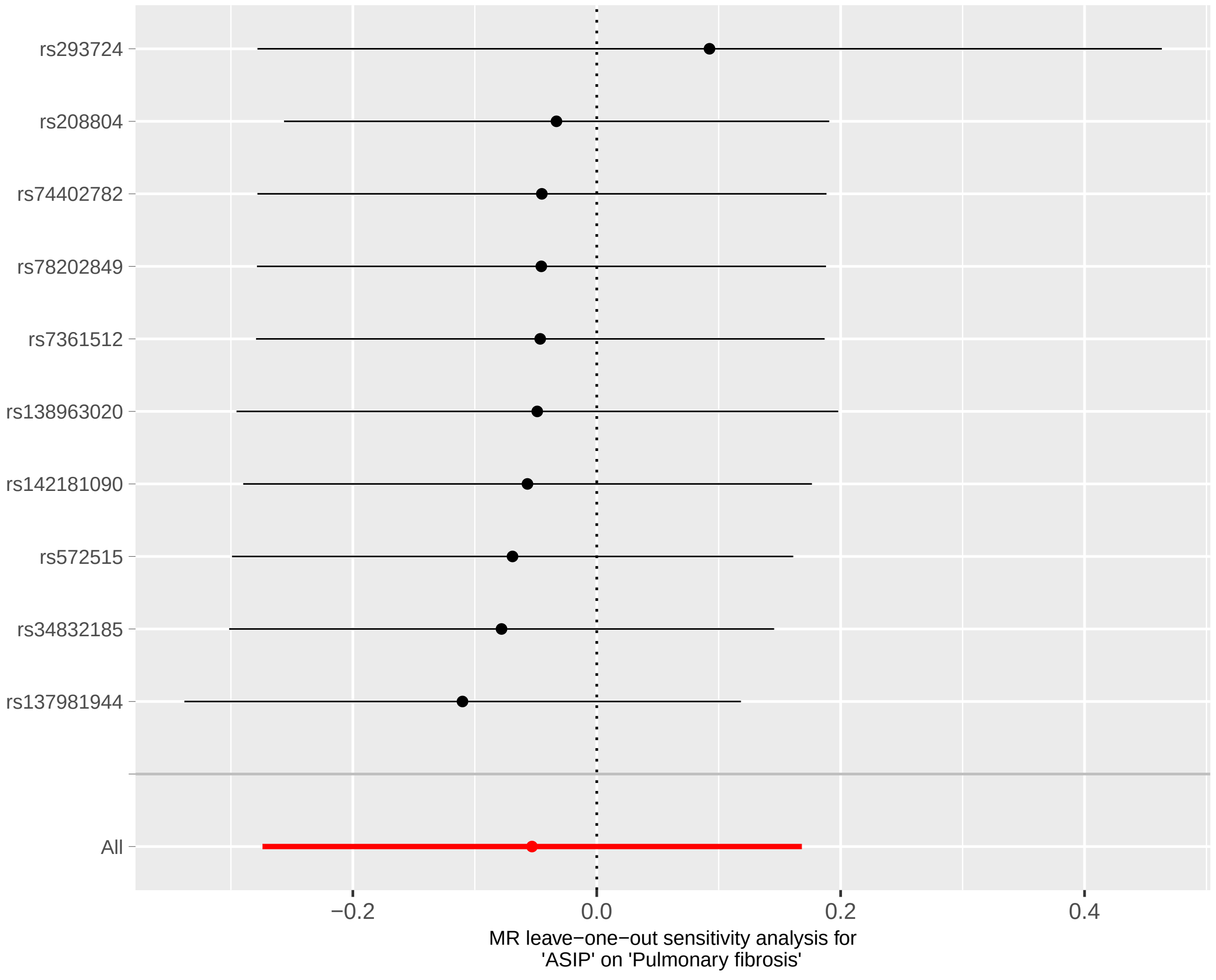
rs293724
rs208804
rs74402782
rs78202849
rs7361512
rs138963020
rs142181090
rs572515
rs34832185
rs137981944
All
−0.2
0.0
0.2
0.4
MR leave−one−out sensitivity analysis for
'ASIP' on 'Pulmonary fibrosis'

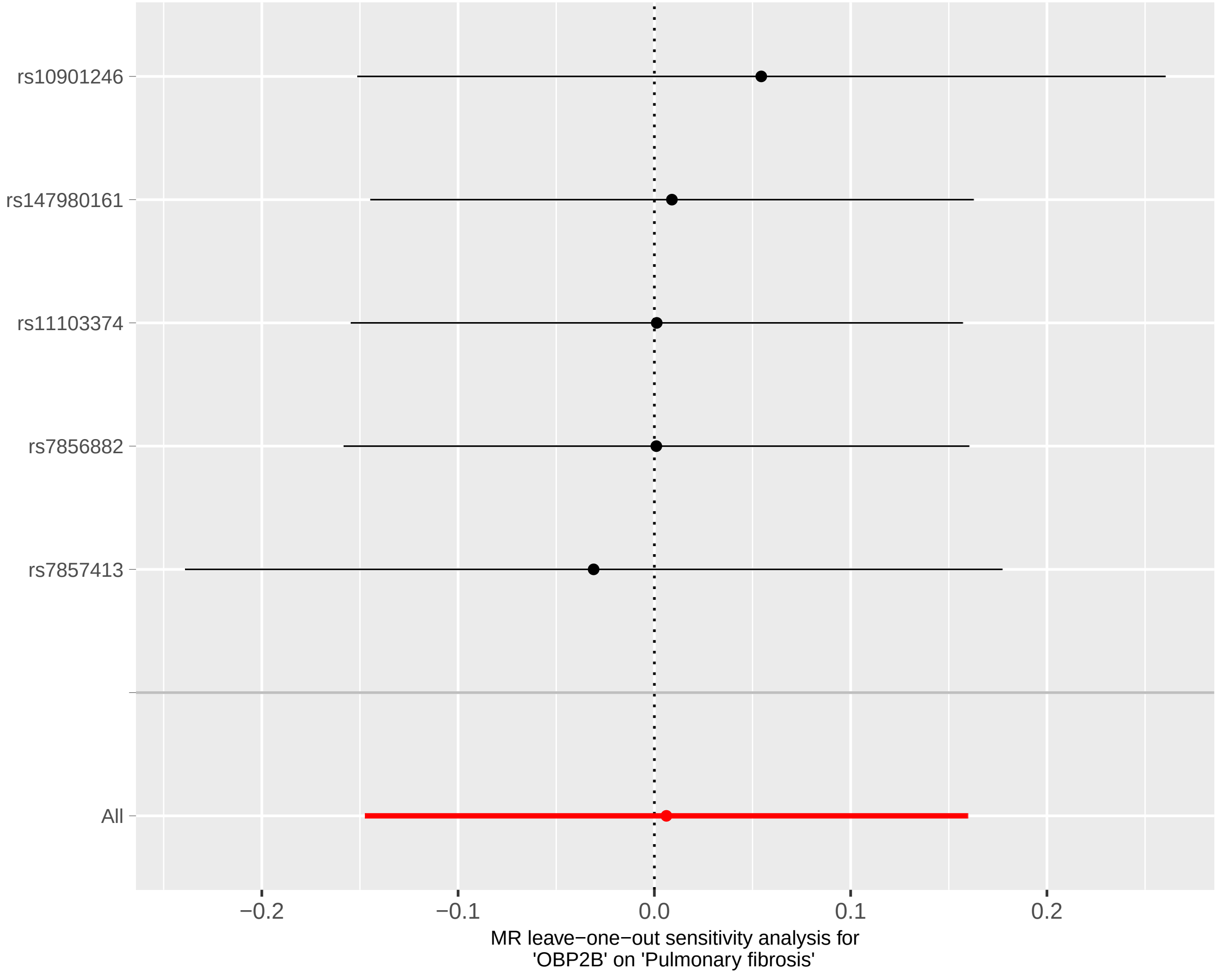

rs10901246
rs147980161
rs11103374
rs7856882
rs7857413
All
−0.2
−0.1
0.0
0.1
0.2
MR leave−one−out sensitivity analysis for
'OBP2B' on 'Pulmonary fibrosis'

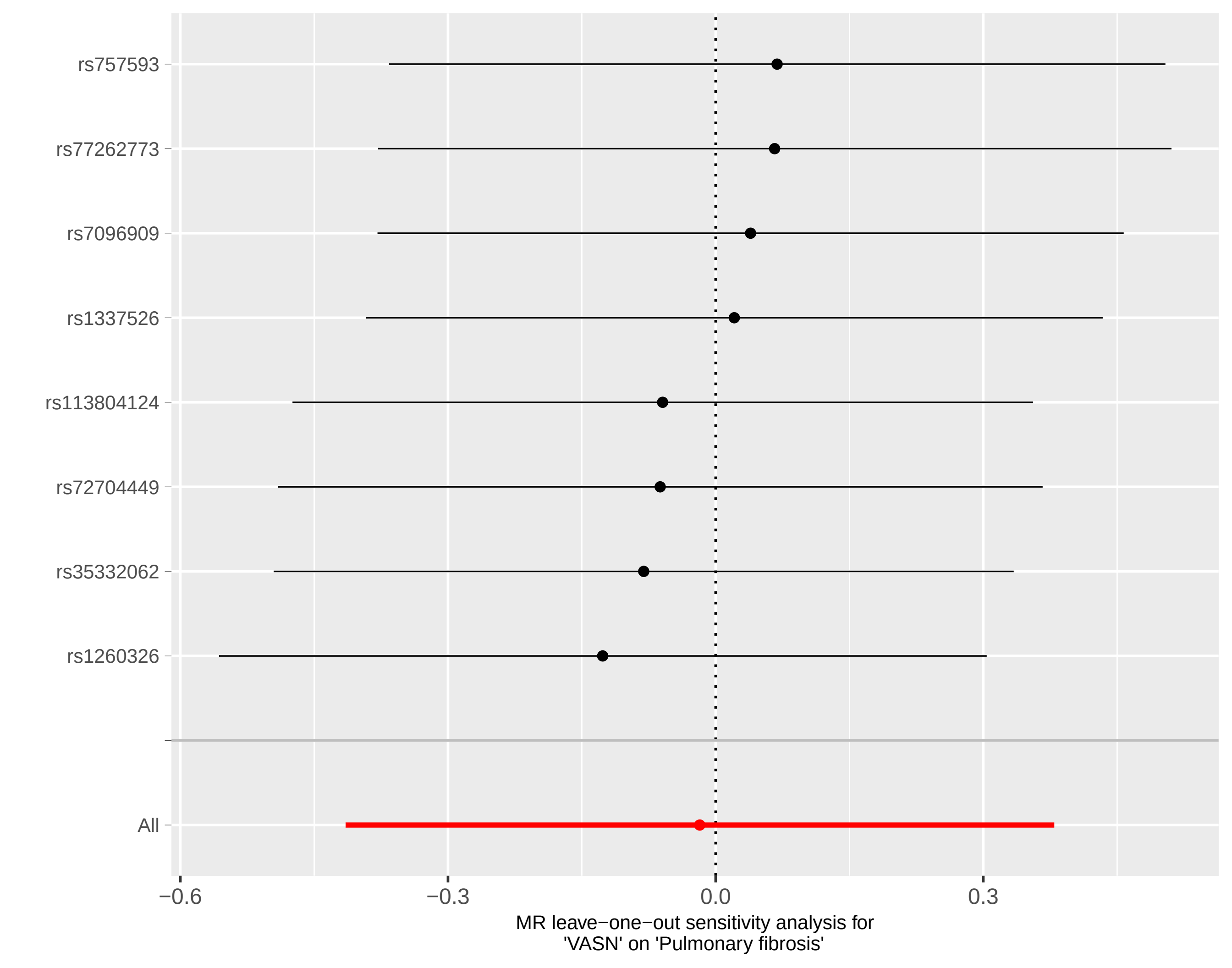
rs757593
rs77262773
rs7096909
rs1337526
rs113804124
rs72704449
rs35332062
rs1260326
All
−0.6
−0.3
0.0
0.3
MR leave−one−out sensitivity analysis for
'VASN' on 'Pulmonary fibrosis'

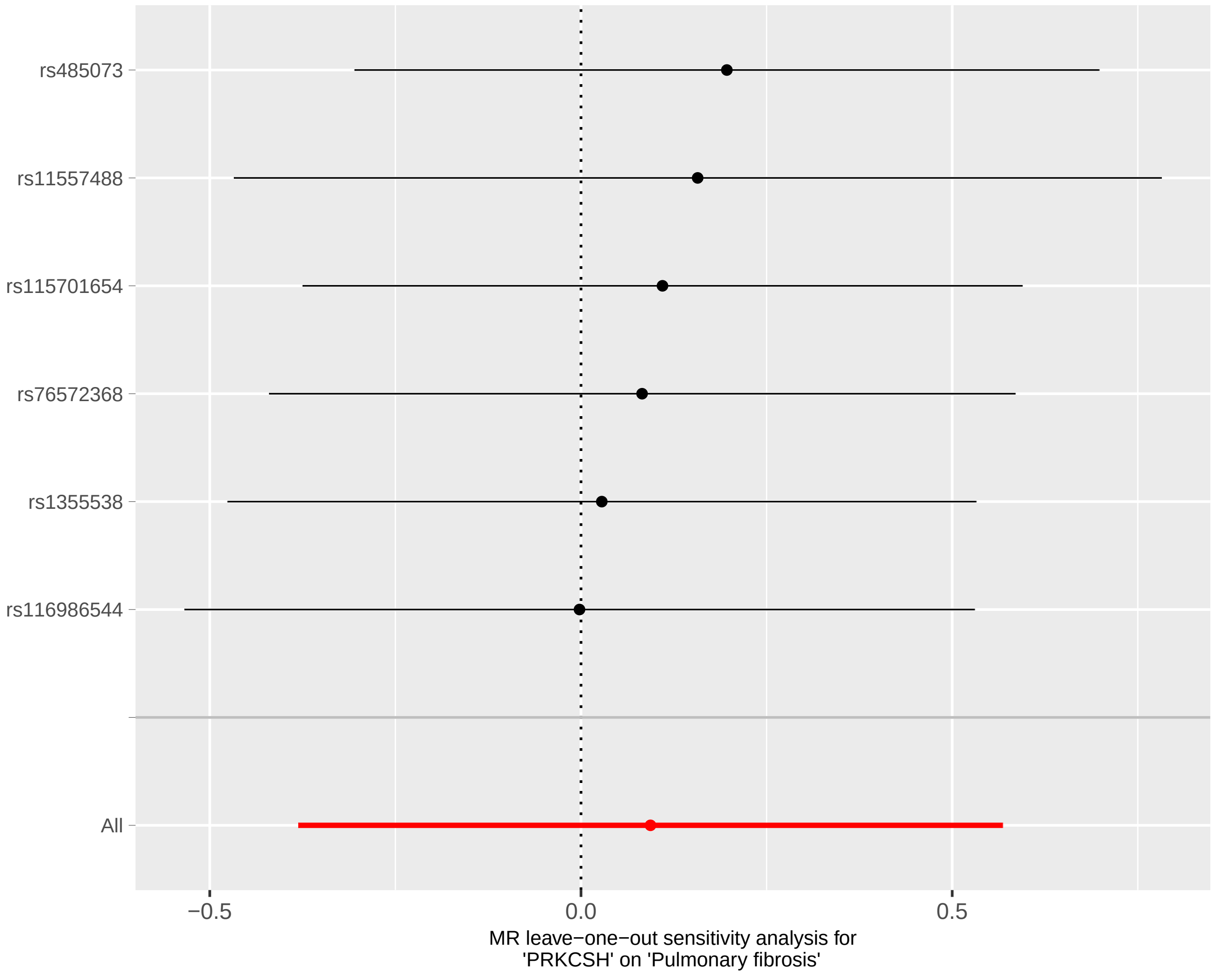

rs485073
rs11557488
rs115701654
rs76572368
rs1355538
rs116986544
All
−0.5
0.0
0.5
MR leave−one−out sensitivity analysis for
'PRKCSH' on 'Pulmonary fibrosis'

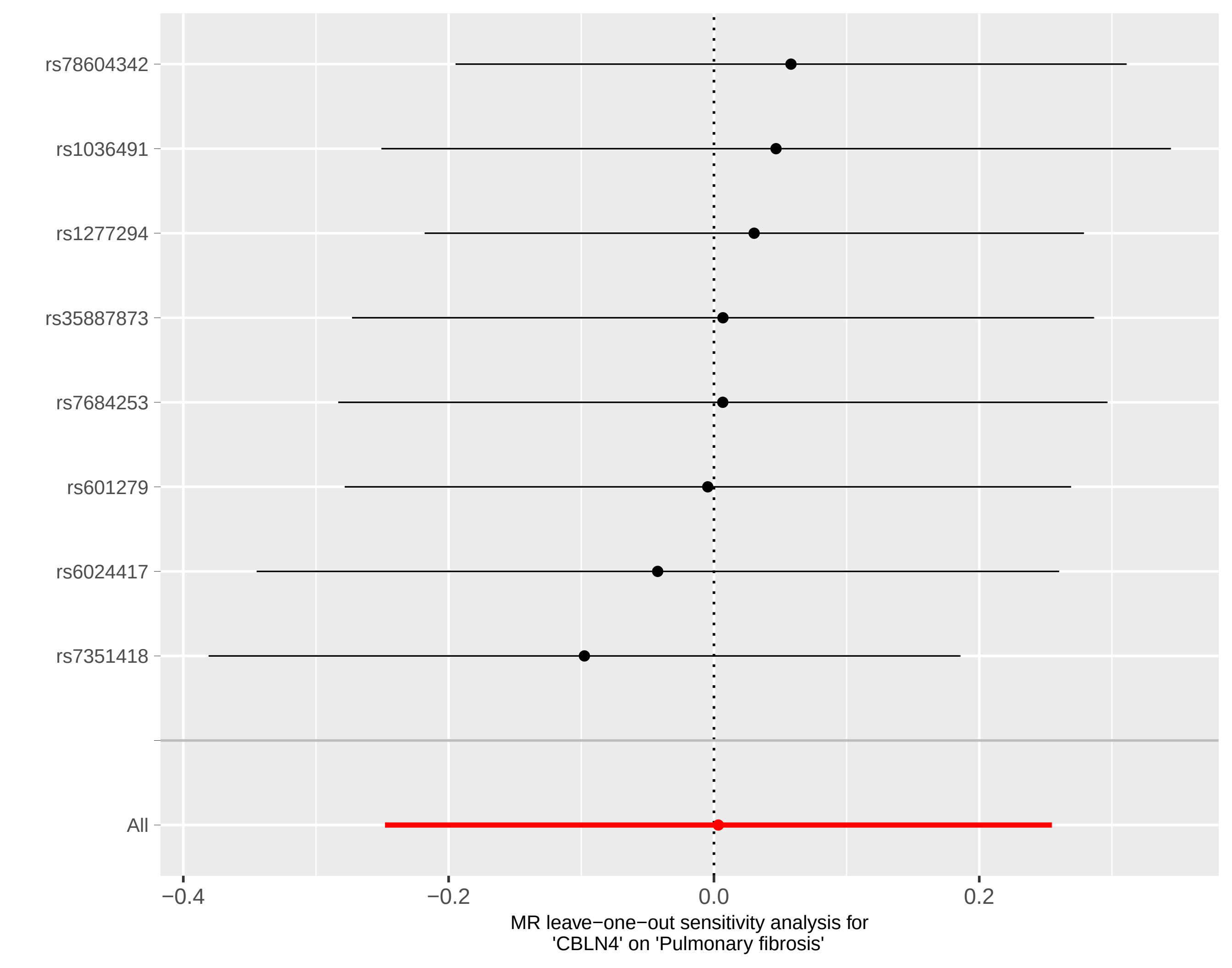

rs78604342
rs1036491
rs1277294
rs35887873
rs7684253
rs601279
rs6024417
rs7351418
All
−0.4
−0.2
0.0
0.2
MR leave−one−out sensitivity analysis for
'CBLN4' on 'Pulmonary fibrosis'

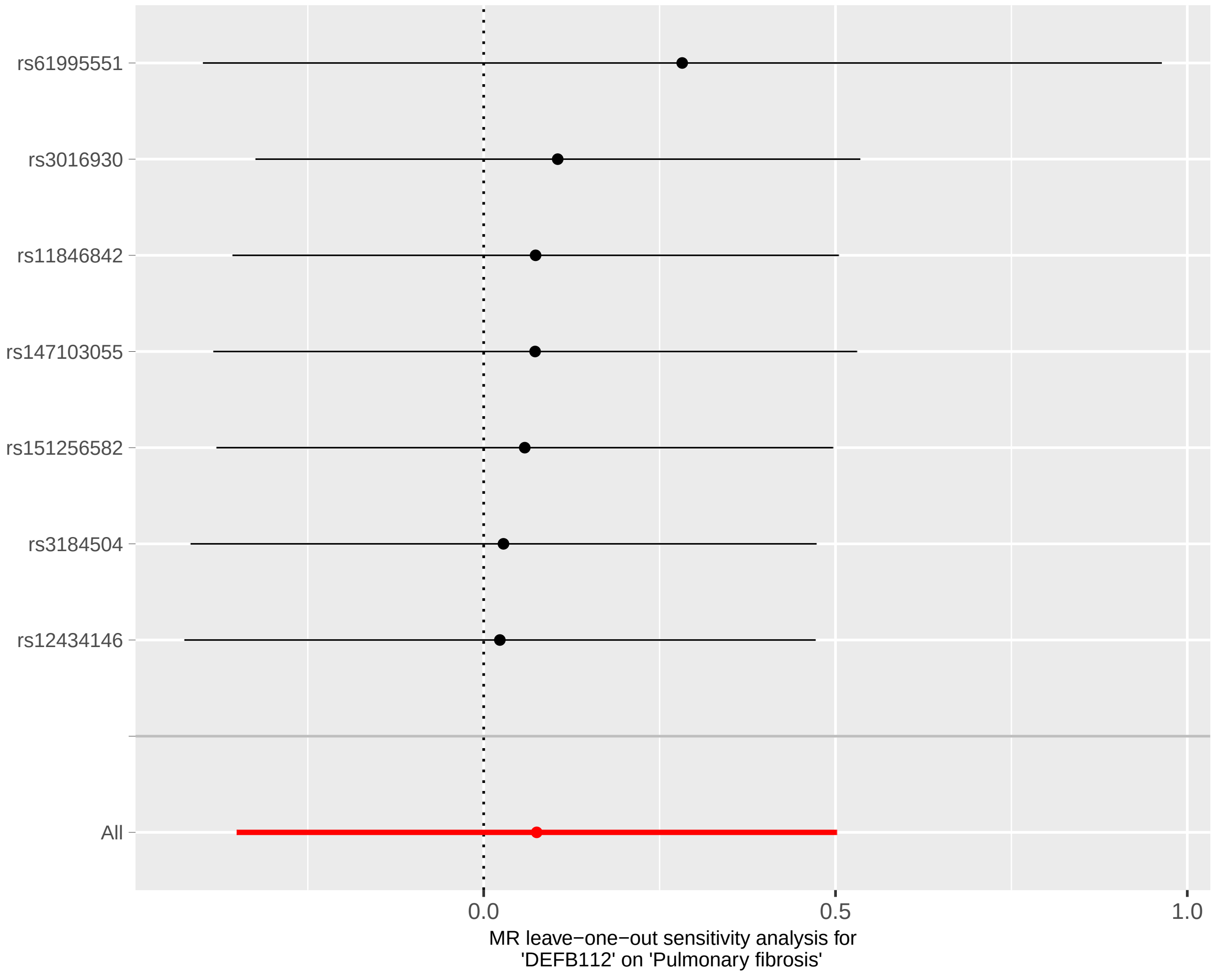
rs61995551
rs3016930
rs11846842
rs147103055
rs151256582
rs3184504
rs12434146
All
0.0
0.5
1.0
MR leave−one−out sensitivity analysis for
'DEFB112' on 'Pulmonary fibrosis'

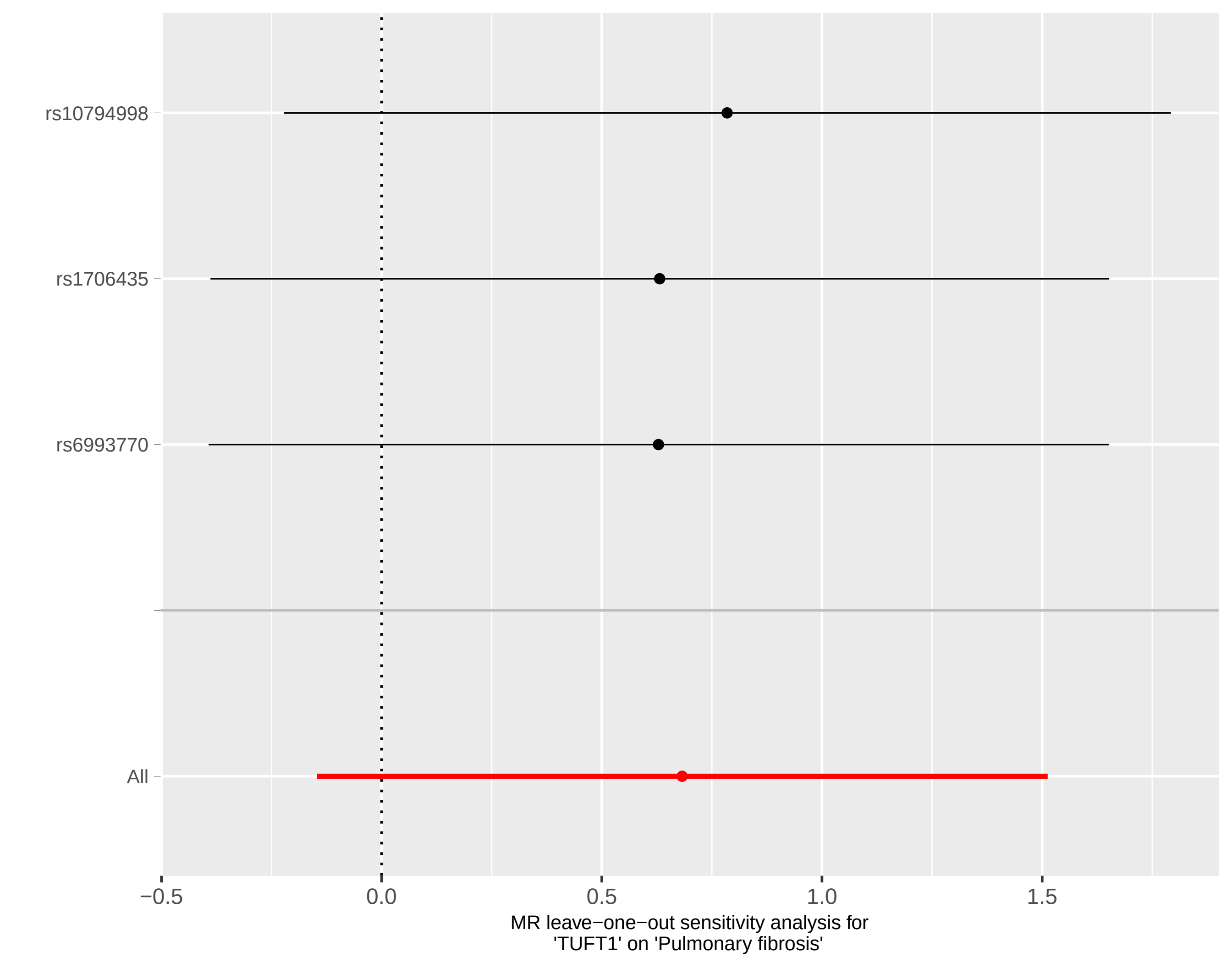

rs10794998
rs1706435
rs6993770
All
−0.5
0.0
0.5
1.0
1.5
MR leave−one−out sensitivity analysis for
'TUFT1' on 'Pulmonary fibrosis'

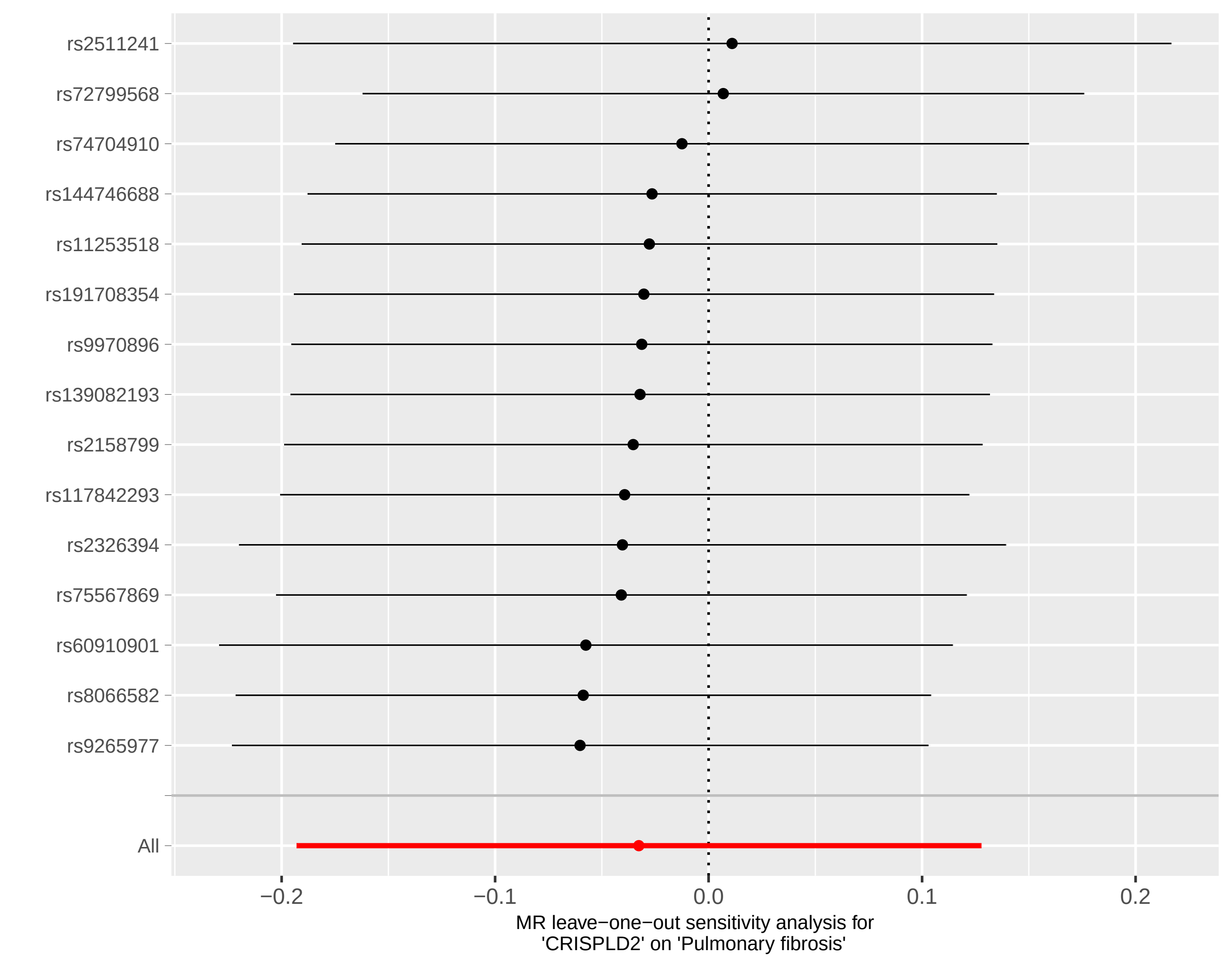
rs2511241
rs72799568
rs74704910
rs144746688
rs11253518
rs191708354
rs9970896
rs139082193
rs2158799
rs117842293
rs2326394
rs75567869
rs60910901
rs8066582
rs9265977
All
−0.2
−0.1
0.0
0.1
0.2
MR leave−one−out sensitivity analysis for
'CRISPLD2' on 'Pulmonary fibrosis'

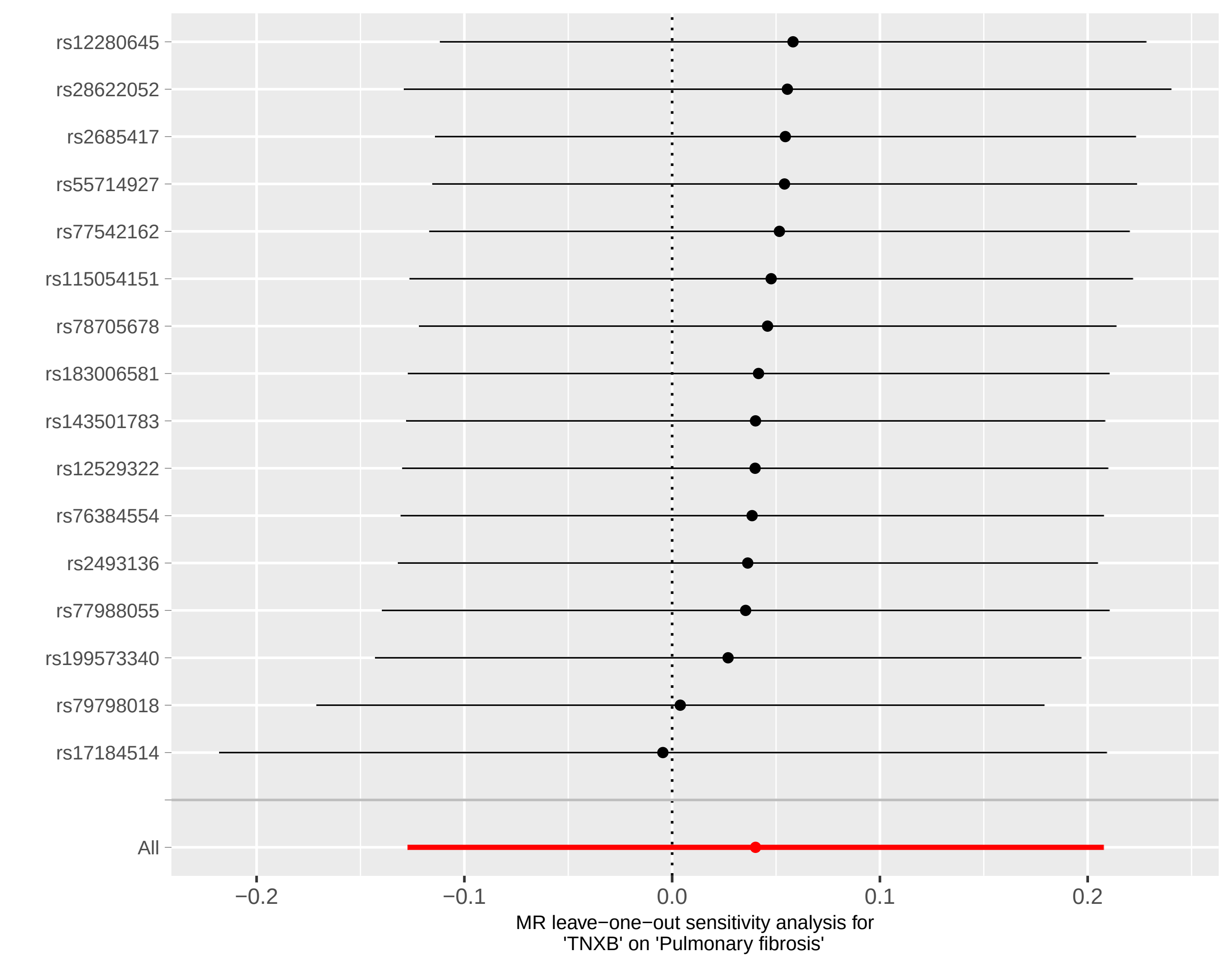
rs12280645
rs28622052
rs2685417
rs55714927
rs77542162
rs115054151
rs78705678
rs183006581
rs143501783
rs12529322
rs76384554
rs2493136
rs77988055
rs199573340
rs79798018
rs17184514
All
−0.2
−0.1
0.0
0.1
0.2
MR leave−one−out sensitivity analysis for
'TNXB' on 'Pulmonary fibrosis'

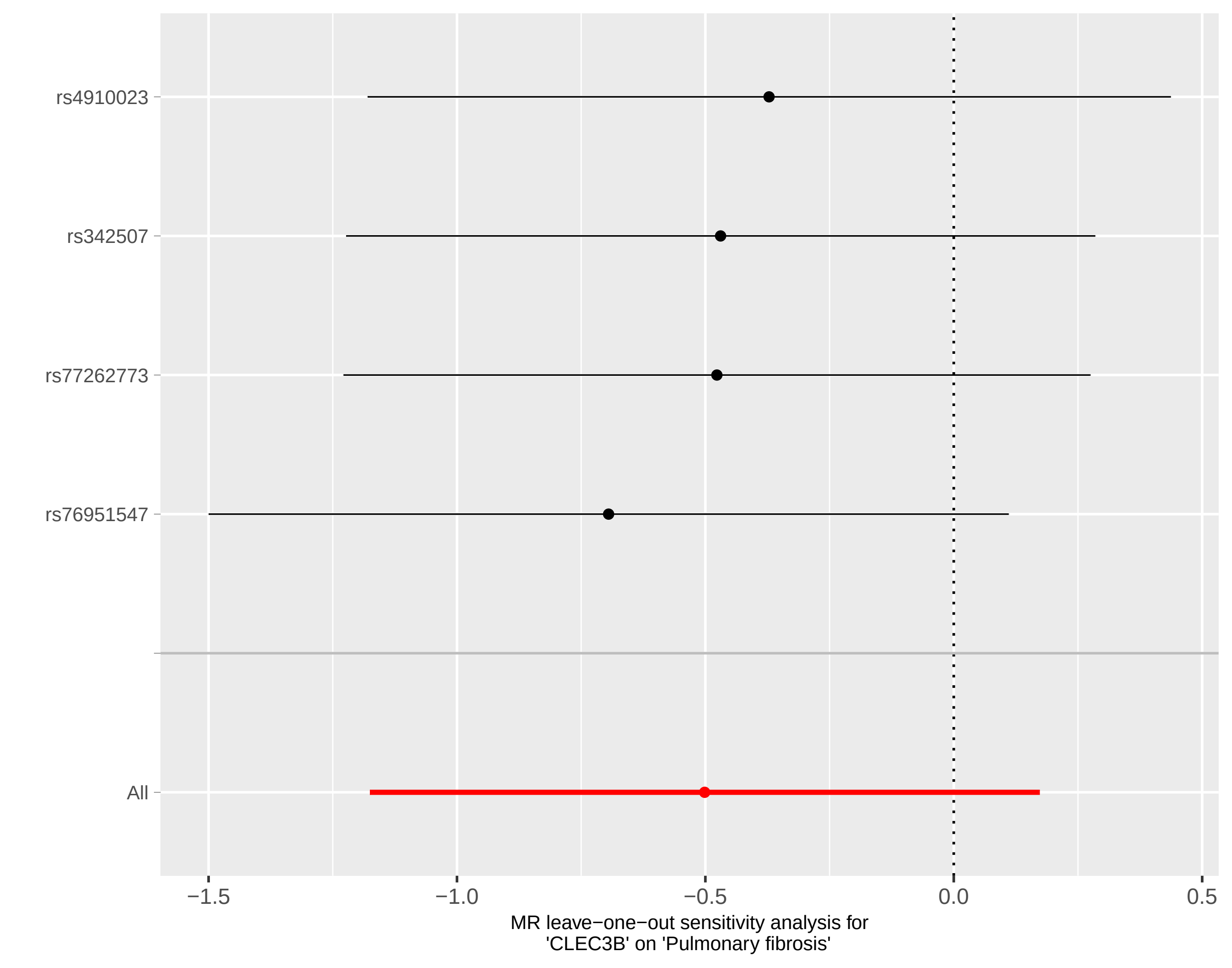

rs4910023
rs342507
rs77262773
rs76951547
All
−1.5
−1.0
−0.5
0.0
0.5
MR leave−one−out sensitivity analysis for
'CLEC3B' on 'Pulmonary fibrosis'

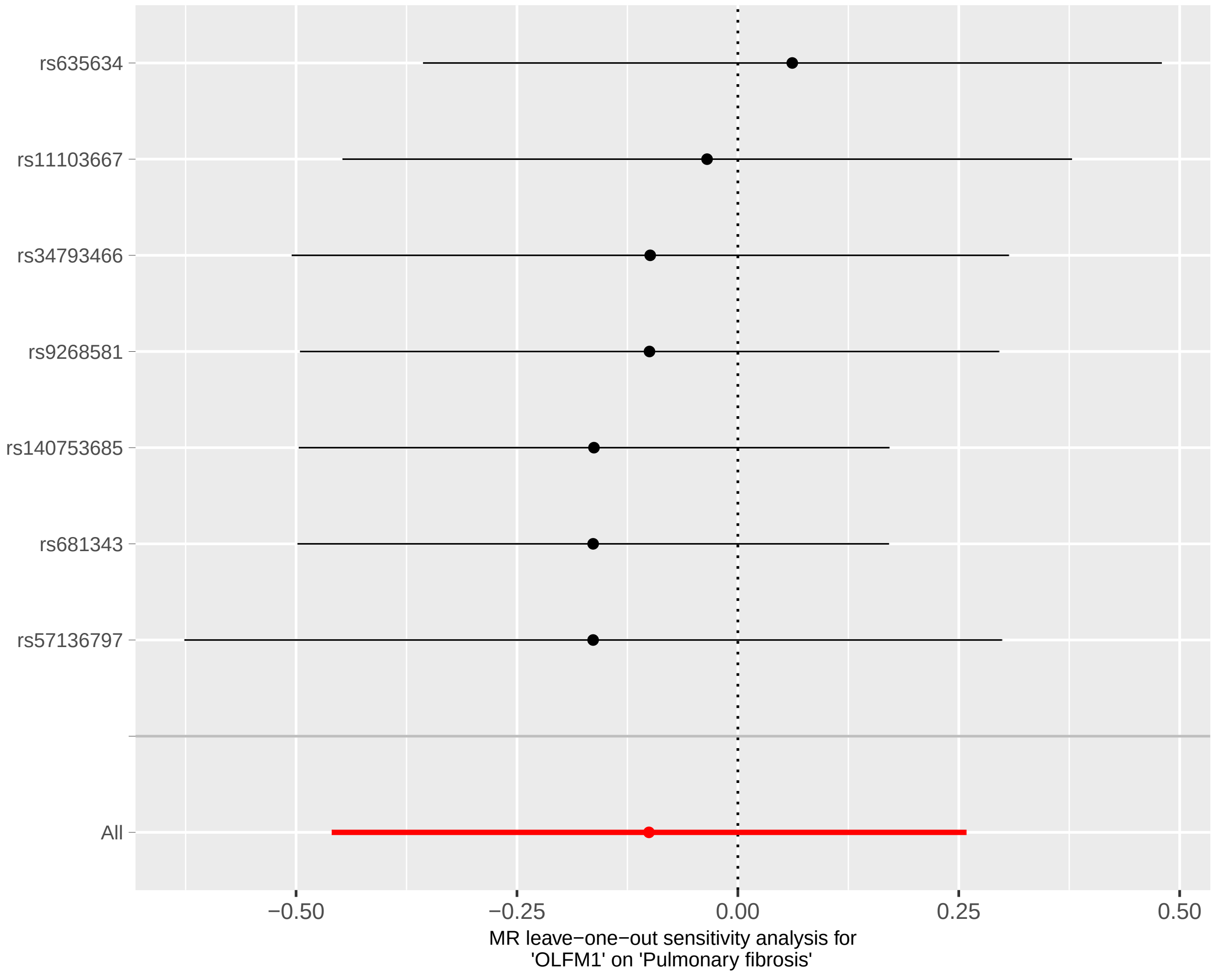
rs635634
rs11103667
rs34793466
rs9268581
rs140753685
rs681343
rs57136797
All
−0.50
−0.25
0.00
0.25
0.50
MR leave−one−out sensitivity analysis for
'OLFM1' on 'Pulmonary fibrosis'

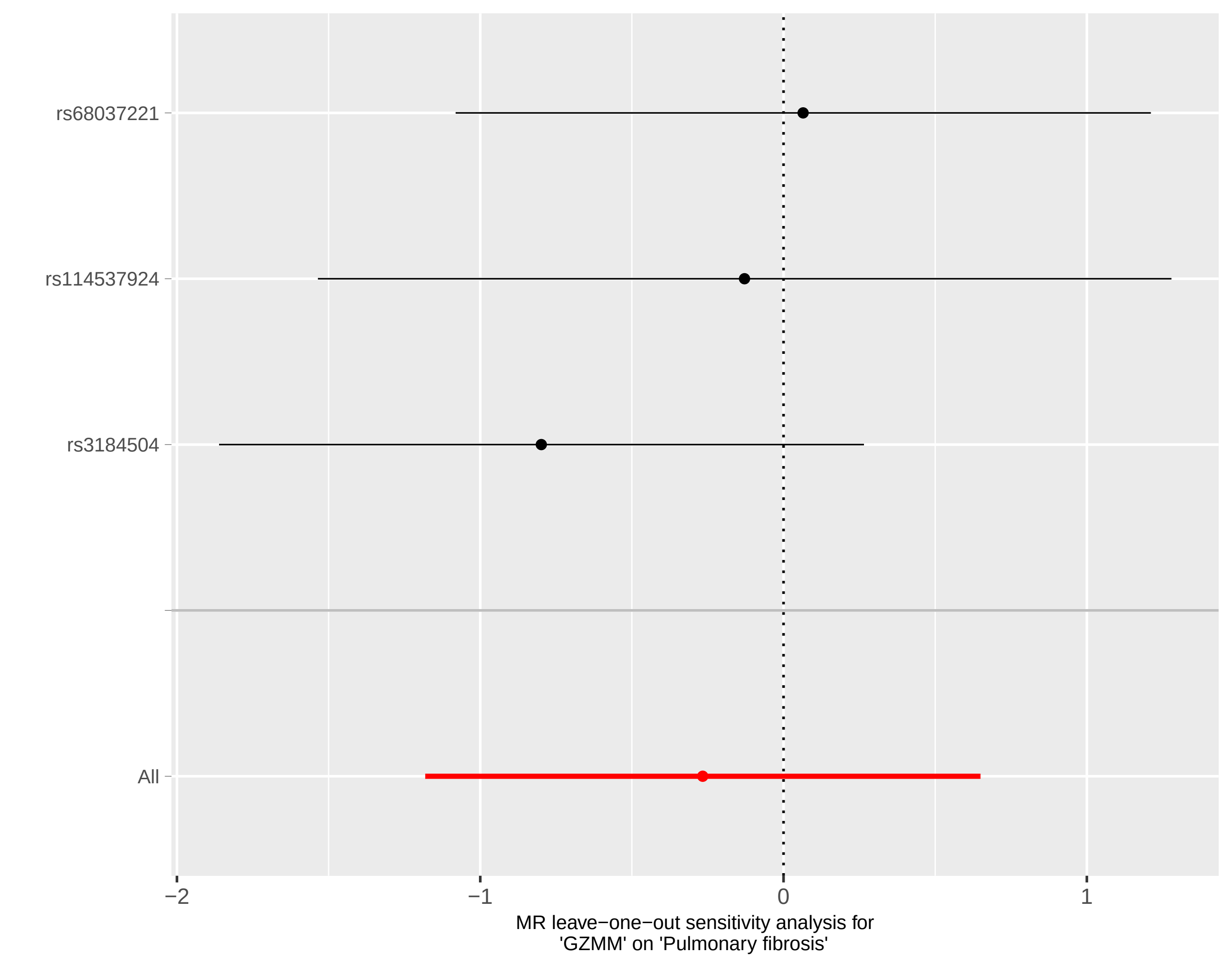
rs68037221
rs114537924
rs3184504
All
−2
−1
0
1
MR leave−one−out sensitivity analysis for
'GZMM' on 'Pulmonary fibrosis'

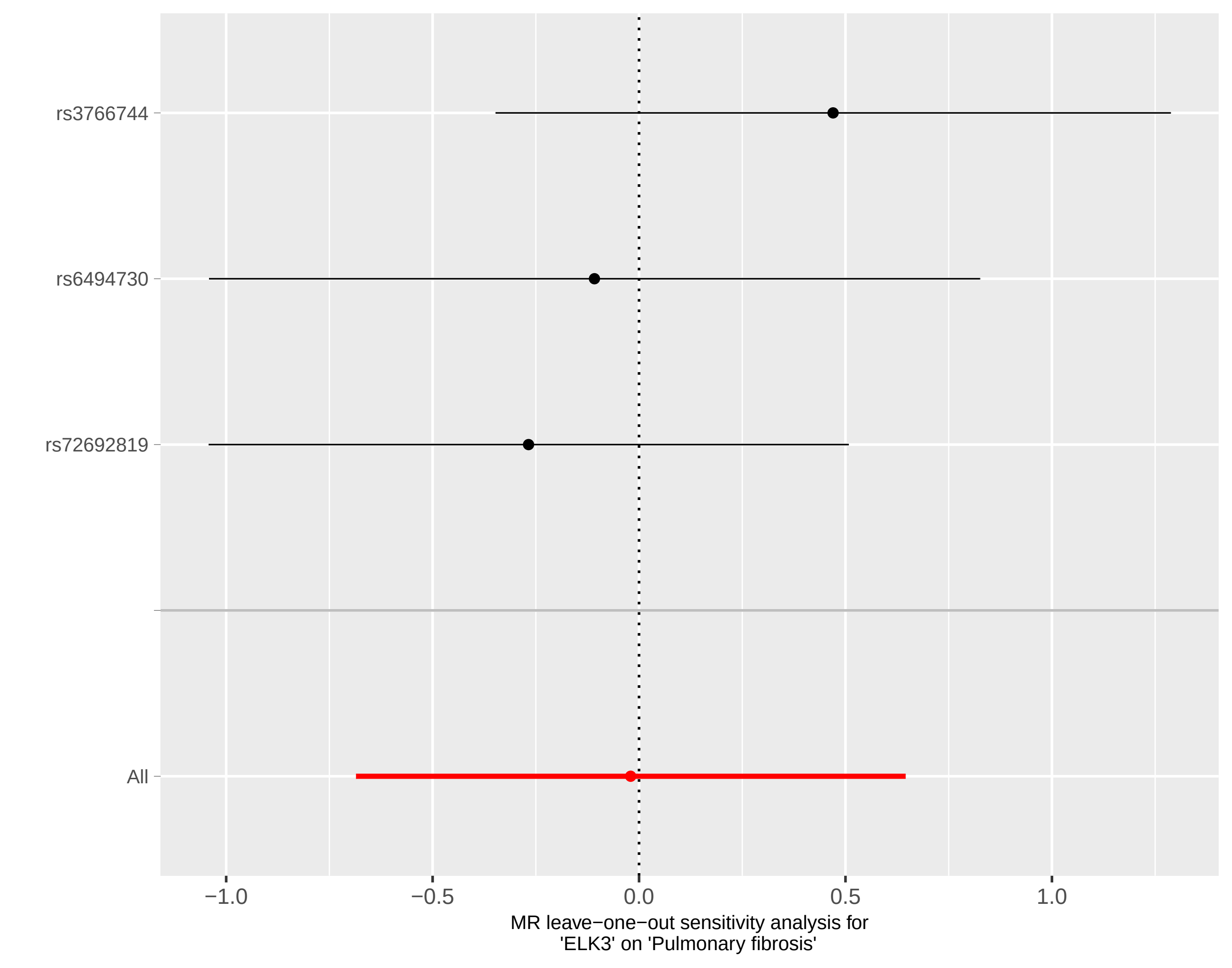
rs3766744
rs6494730
rs72692819
All
−1.0
−0.5
0.0
0.5
1.0
MR leave−one−out sensitivity analysis for
'ELK3' on 'Pulmonary fibrosis'

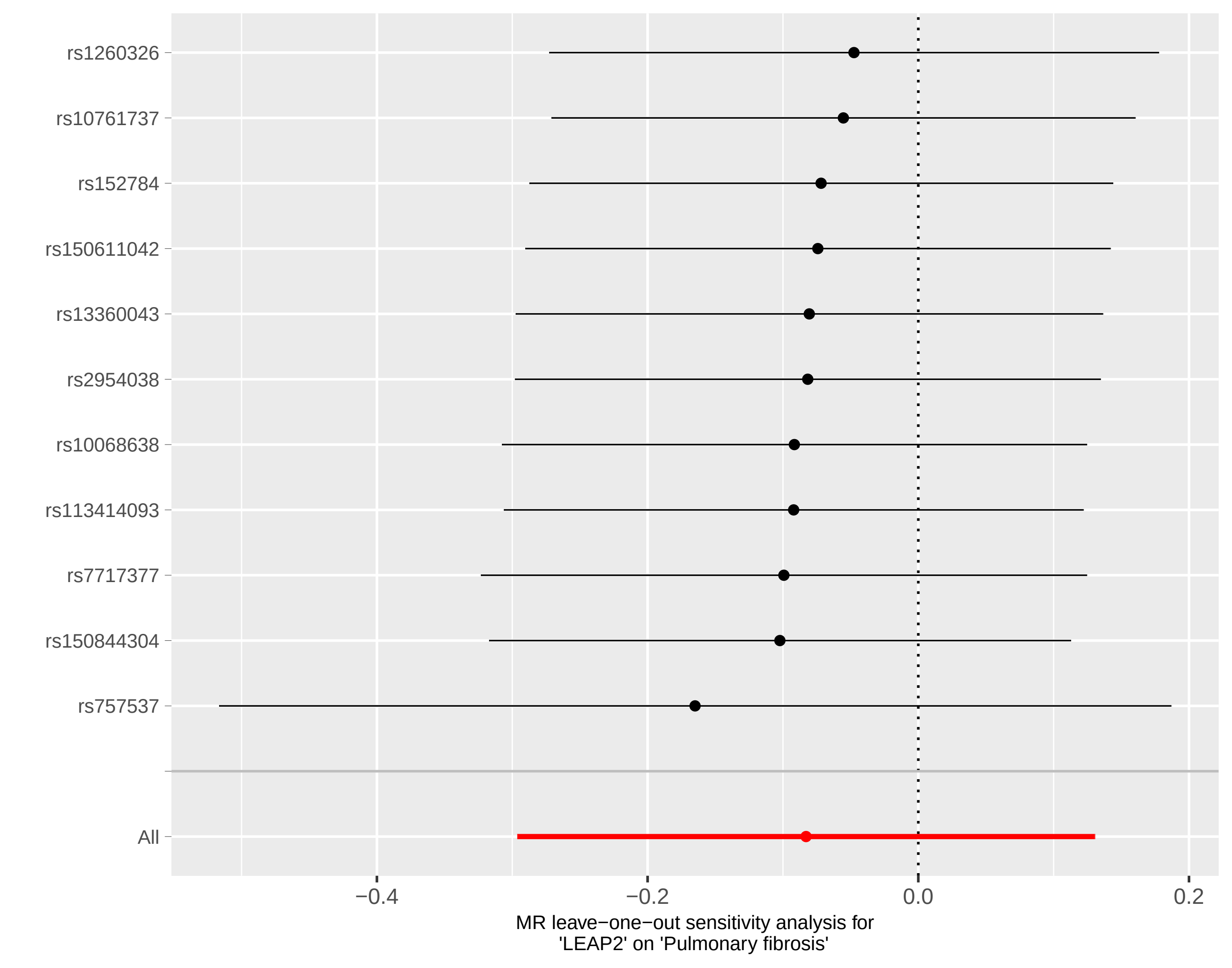

rs1260326
rs10761737
rs152784
rs150611042
rs13360043
rs2954038
rs10068638
rs113414093
rs7717377
rs150844304
rs757537
All
−0.4
−0.2
0.0
0.2
MR leave−one−out sensitivity analysis for
'LEAP2' on 'Pulmonary fibrosis'

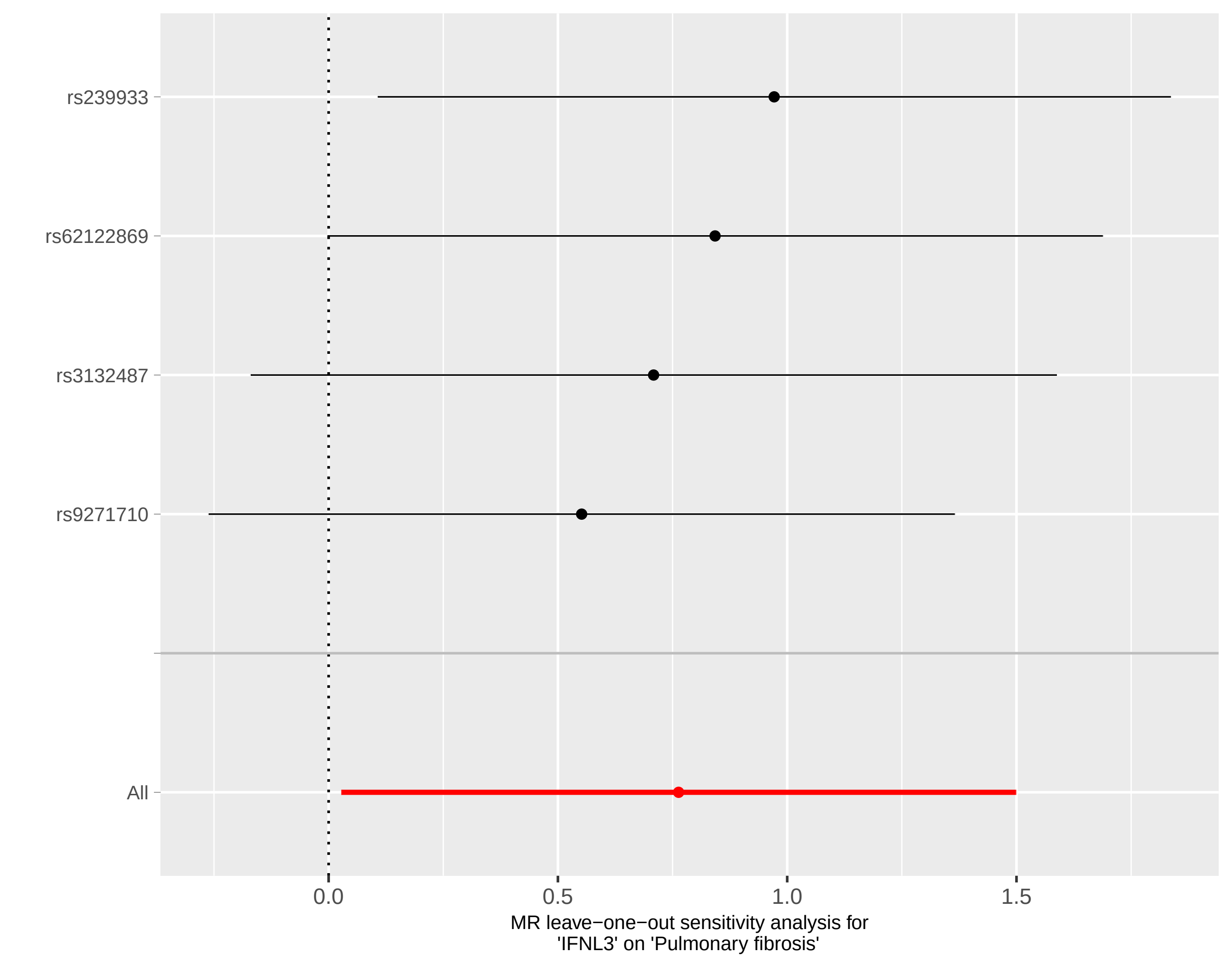
rs239933
rs62122869
rs3132487
rs9271710
All
0.0
0.5
1.0
1.5
MR leave−one−out sensitivity analysis for
'IFNL3' on 'Pulmonary fibrosis'

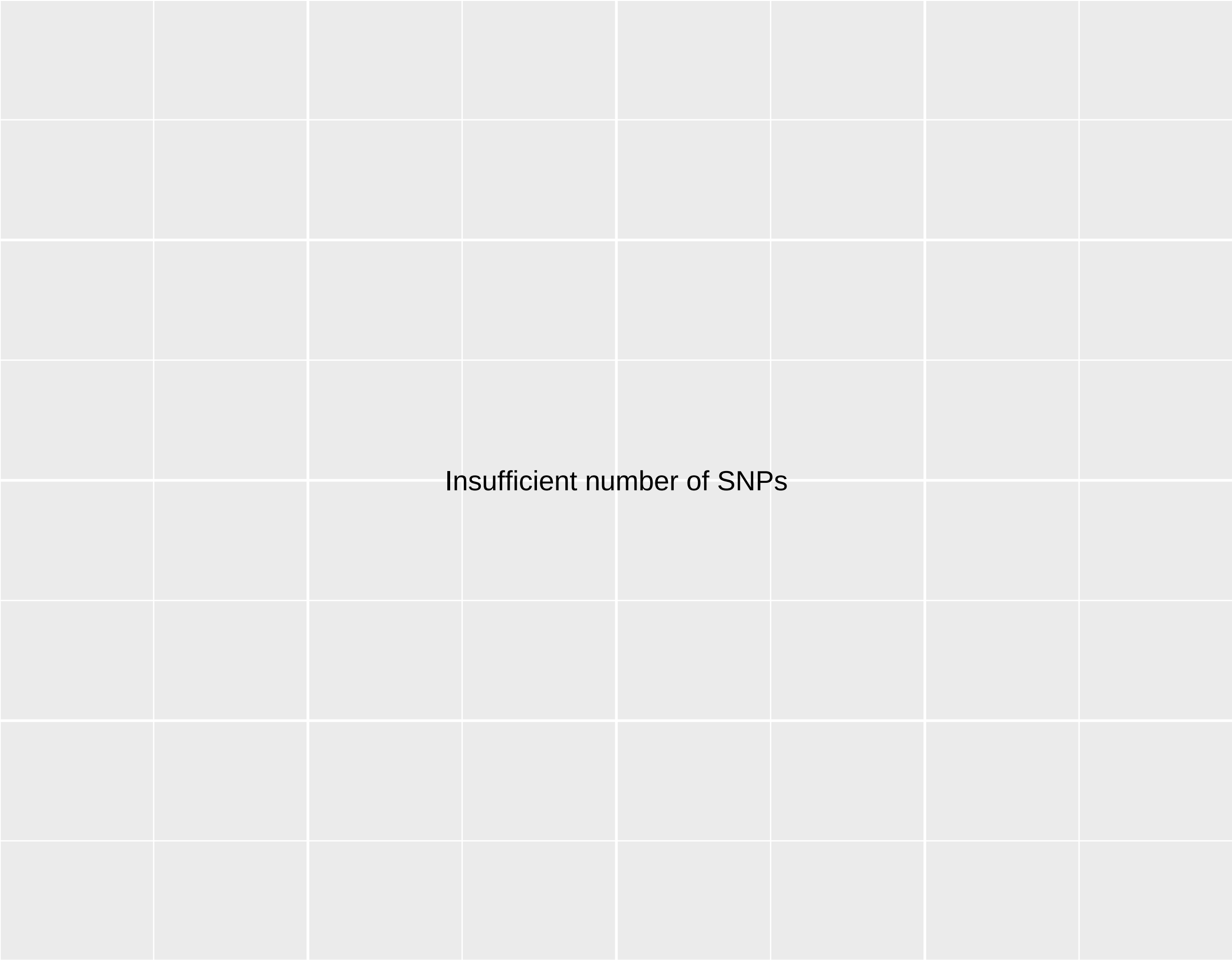
Insufficient number of SNPs

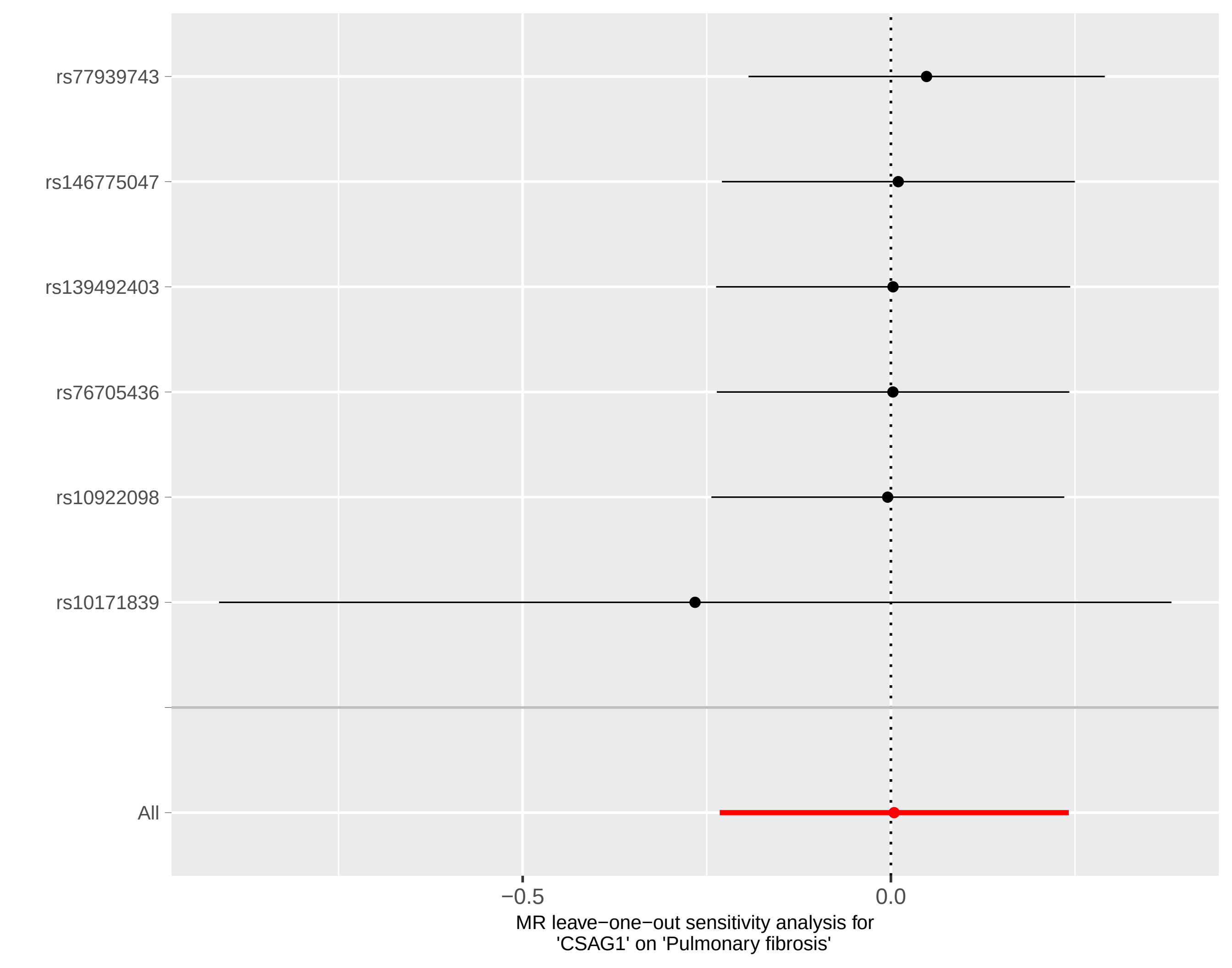
rs77939743
rs146775047
rs139492403
rs76705436
rs10922098
rs10171839
All
−0.5
0.0
MR leave−one−out sensitivity analysis for
'CSAG1' on 'Pulmonary fibrosis'

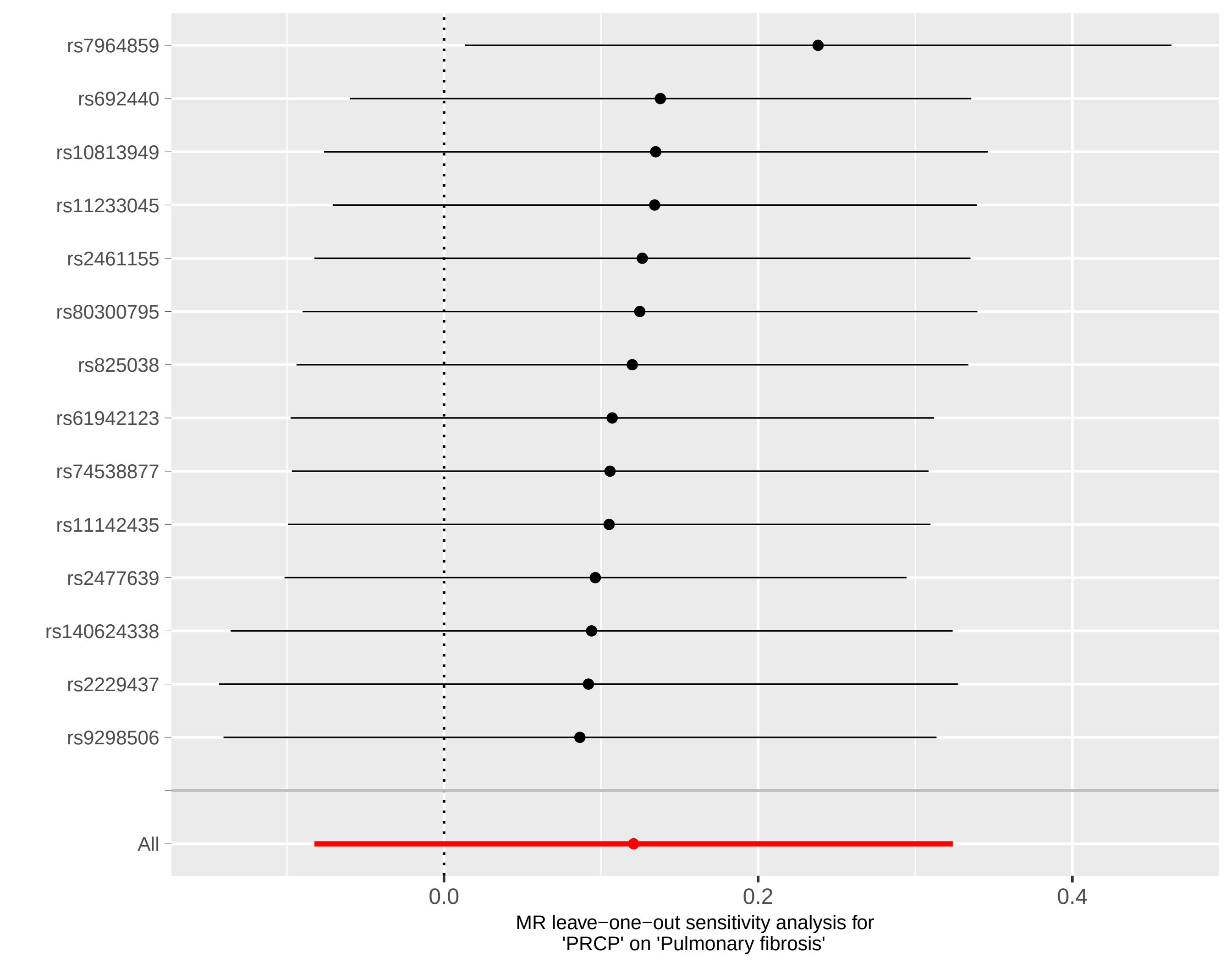

rs7964859
rs692440
rs10813949
rs11233045
rs2461155
rs80300795
rs825038
rs61942123
rs74538877
rs11142435
rs2477639
rs140624338
rs2229437
rs9298506
All
0.0
0.2
0.4
MR leave−one−out sensitivity analysis for
'PRCP' on 'Pulmonary fibrosis'

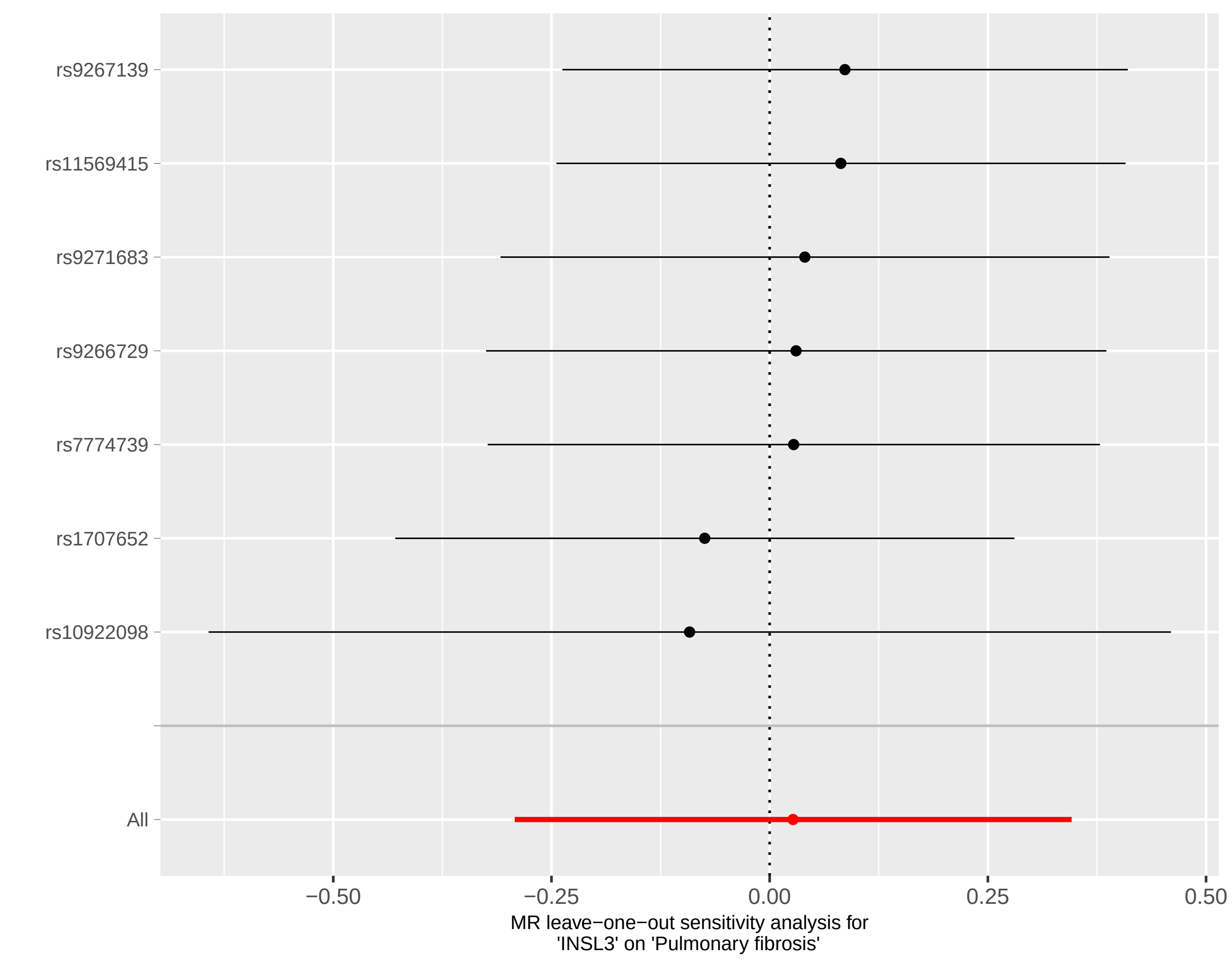
rs9267139
rs11569415
rs9271683
rs9266729
rs7774739
rs1707652
rs10922098
All
−0.50
−0.25
0.00
0.25
0.50
MR leave−one−out sensitivity analysis for
'INSL3' on 'Pulmonary fibrosis'

Insufficient number of SNPs

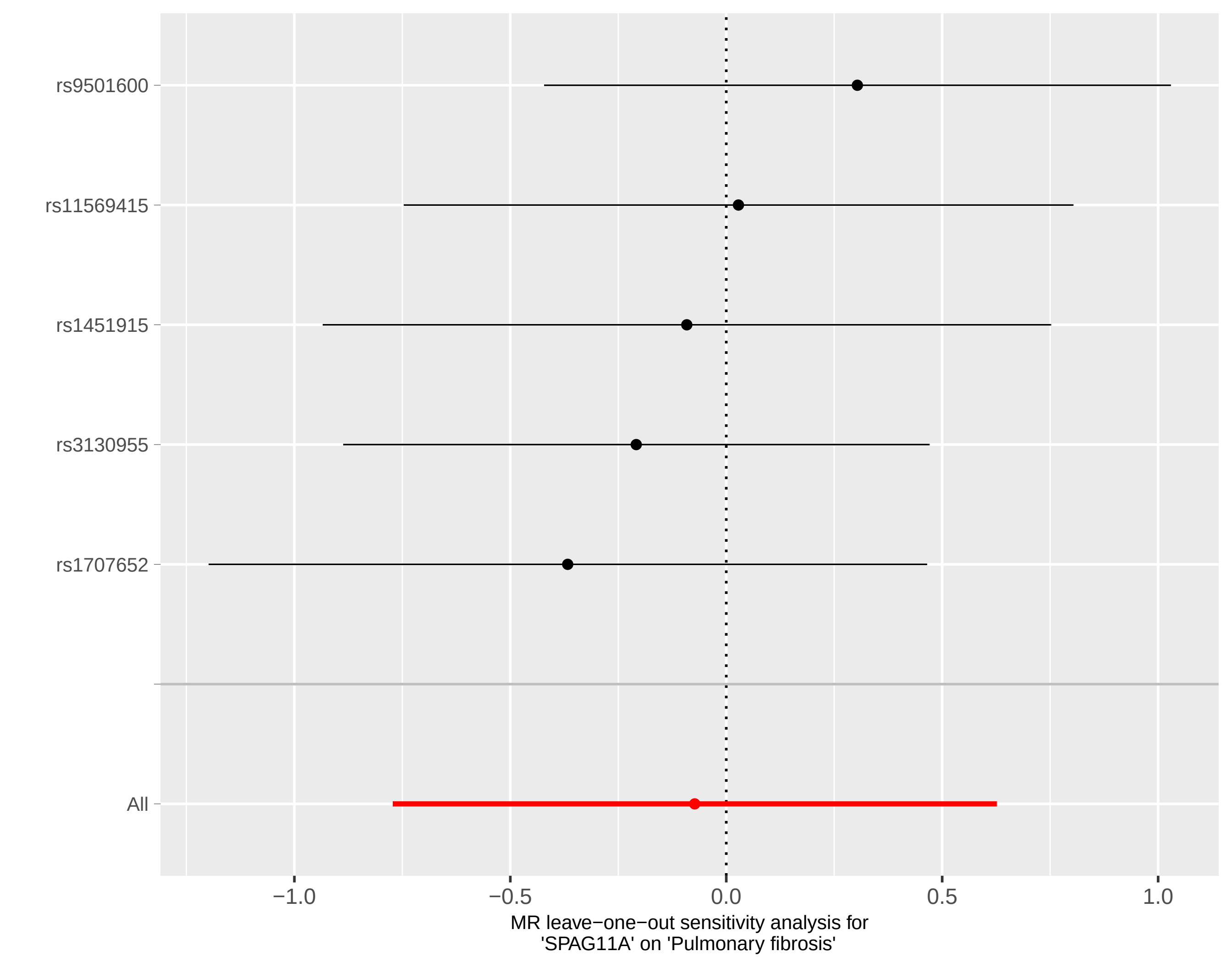
rs9501600
rs11569415
rs1451915
rs3130955
rs1707652
All
−1.0
−0.5
0.0
0.5
1.0
MR leave−one−out sensitivity analysis for
'SPAG11A' on 'Pulmonary fibrosis'

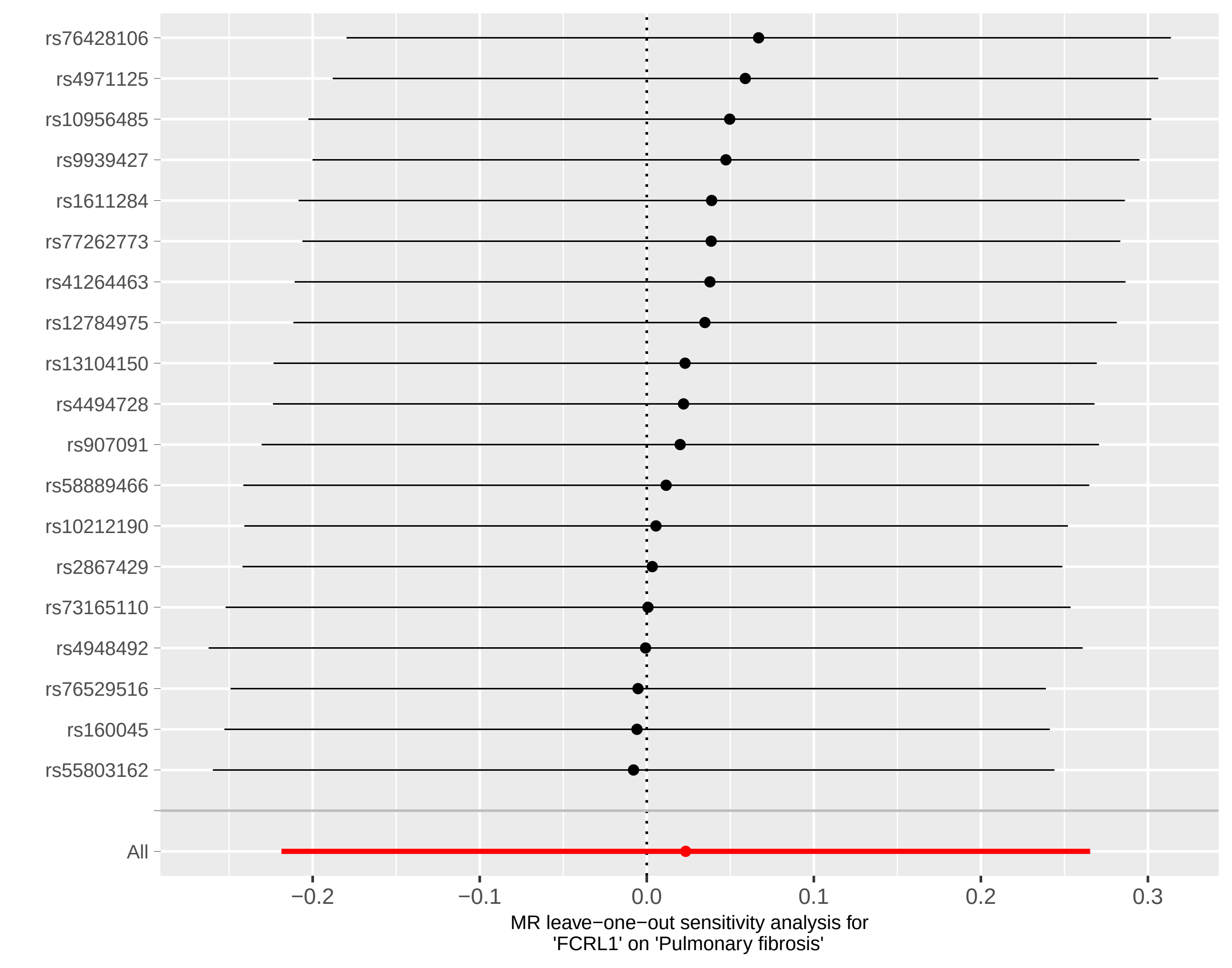
rs76428106
rs4971125
rs10956485
rs9939427
rs1611284
rs77262773
rs41264463
rs12784975
rs13104150
rs4494728
rs907091
rs58889466
rs10212190
rs2867429
rs73165110
rs4948492
rs76529516
rs160045
rs55803162
All
−0.2
−0.1
0.0
0.1
0.2
0.3
MR leave−one−out sensitivity analysis for
'FCRL1' on 'Pulmonary fibrosis'

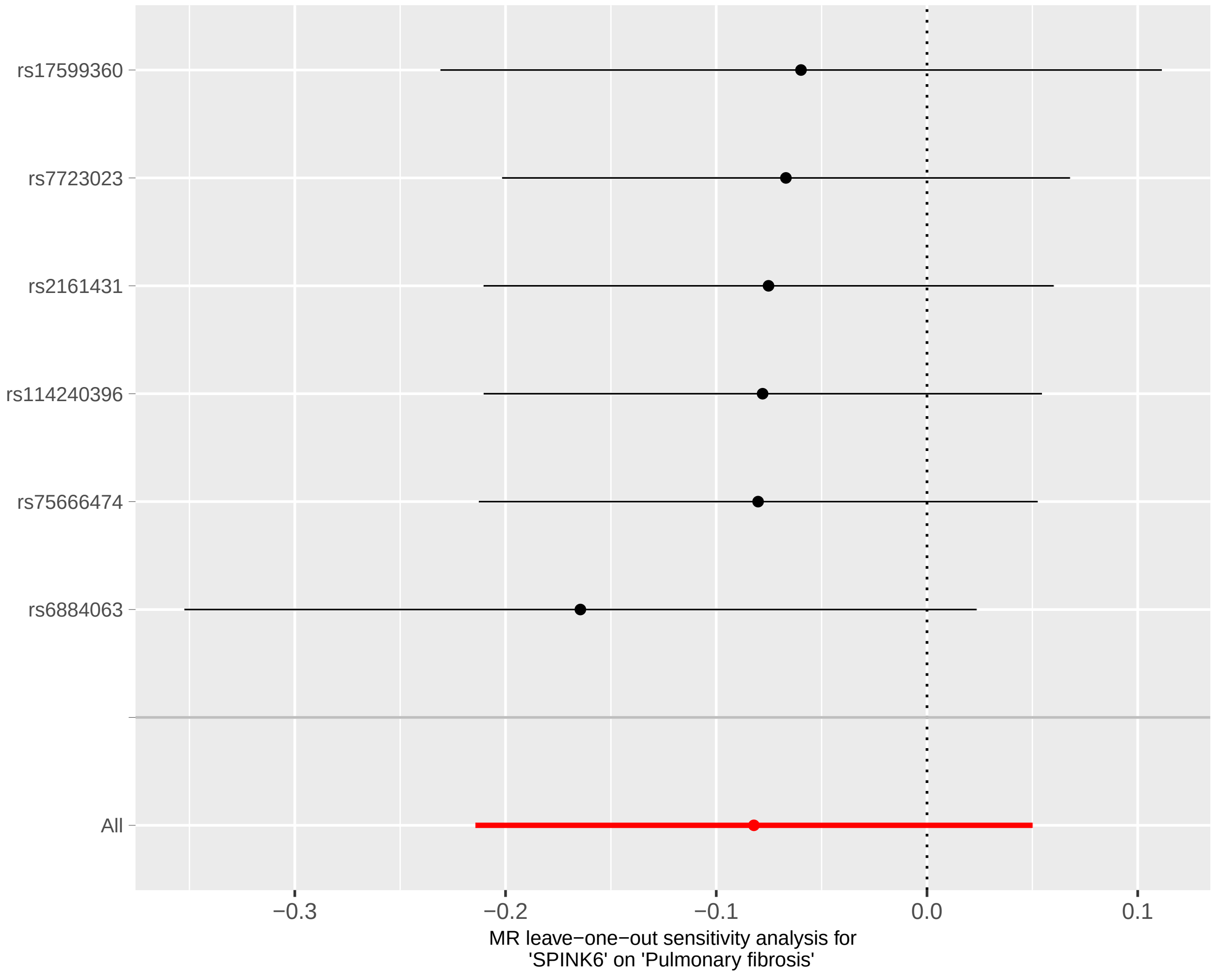
rs17599360
rs7723023
rs2161431
rs114240396
rs75666474
rs6884063
All
−0.3
−0.2
−0.1
0.0
0.1
MR leave−one−out sensitivity analysis for
'SPINK6' on 'Pulmonary fibrosis'

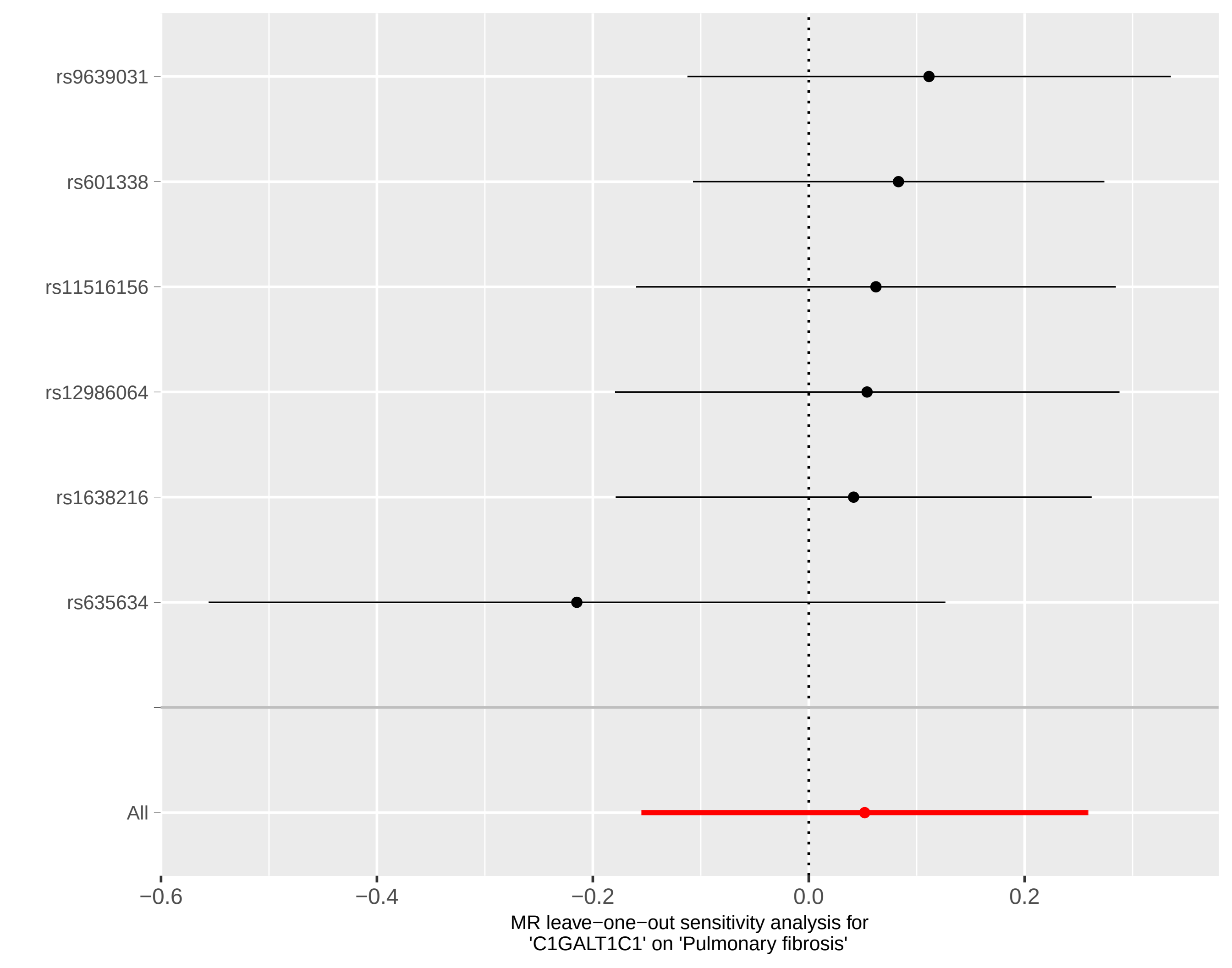
rs9639031
rs601338
rs11516156
rs12986064
rs1638216
rs635634
All
−0.6
−0.4
−0.2
0.0
0.2
MR leave−one−out sensitivity analysis for
'C1GALT1C1' on 'Pulmonary fibrosis'

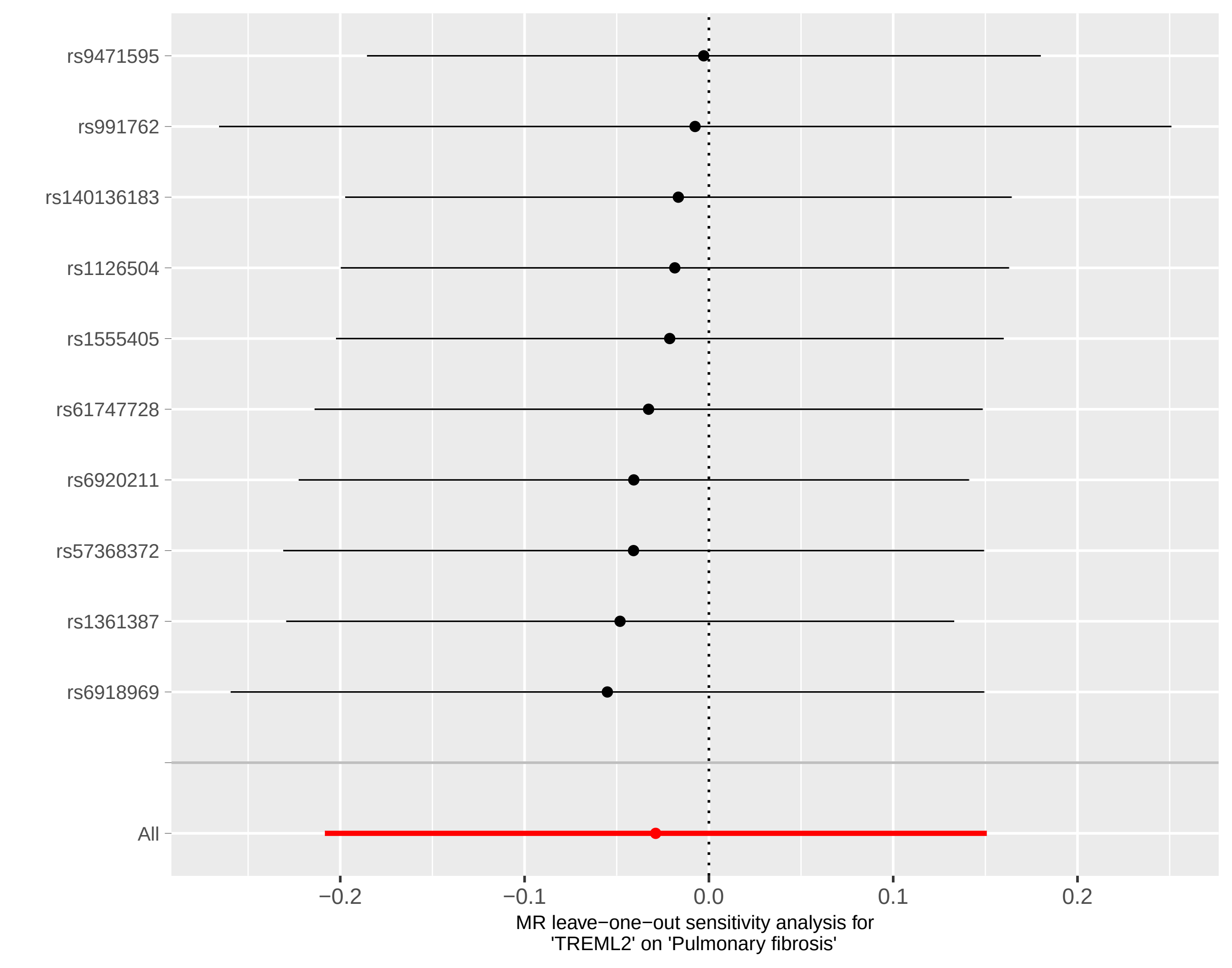
rs9471595
rs991762
rs140136183
rs1126504
rs1555405
rs61747728
rs6920211
rs57368372
rs1361387
rs6918969
All
−0.2
−0.1
0.0
0.1
0.2
MR leave−one−out sensitivity analysis for
'TREML2' on 'Pulmonary fibrosis'

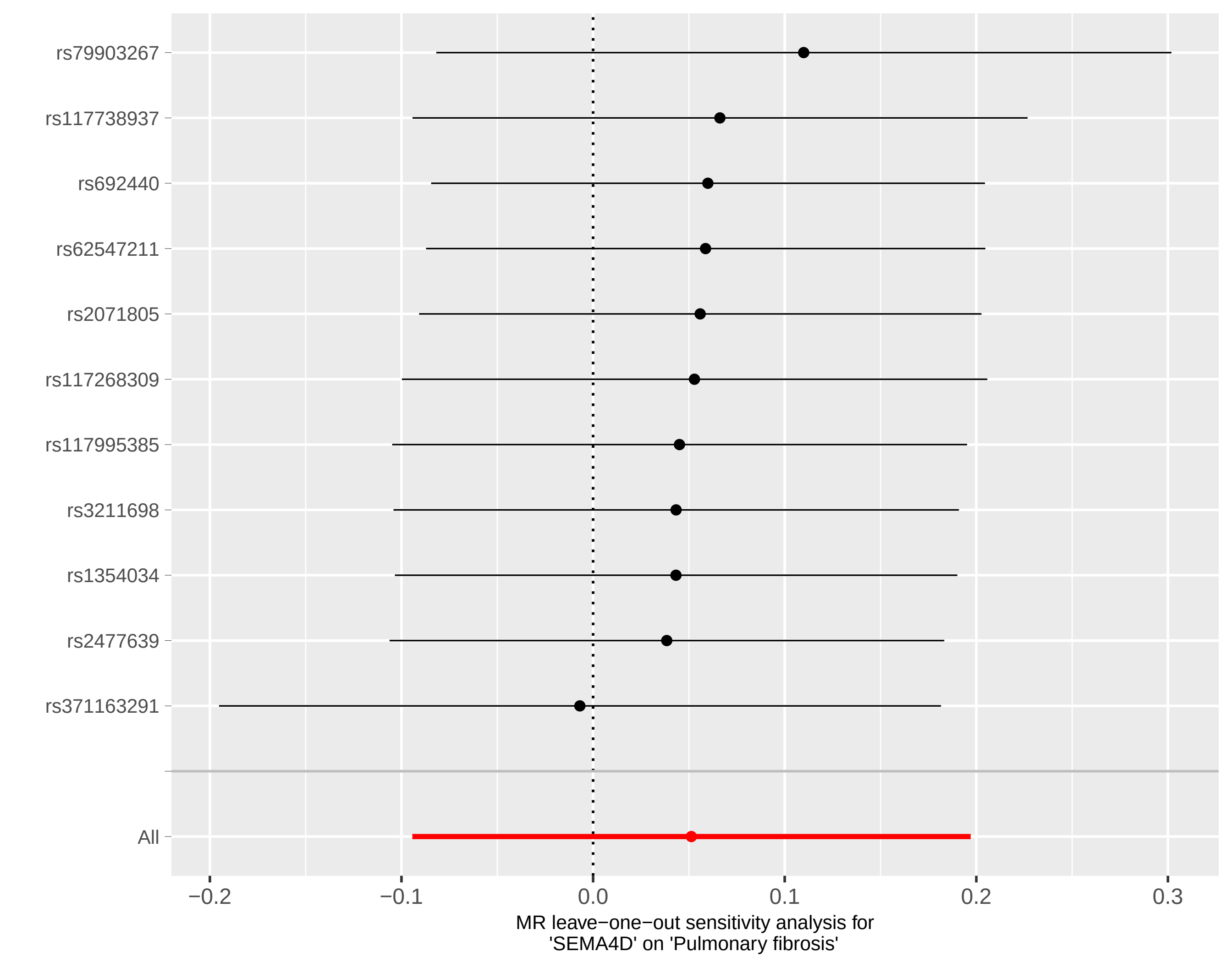

rs79903267
rs117738937
rs692440
rs62547211
rs2071805
rs117268309
rs117995385
rs3211698
rs1354034
rs2477639
rs371163291
All
−0.2
−0.1
0.0
0.1
0.2
0.3
MR leave−one−out sensitivity analysis for
'SEMA4D' on 'Pulmonary fibrosis'

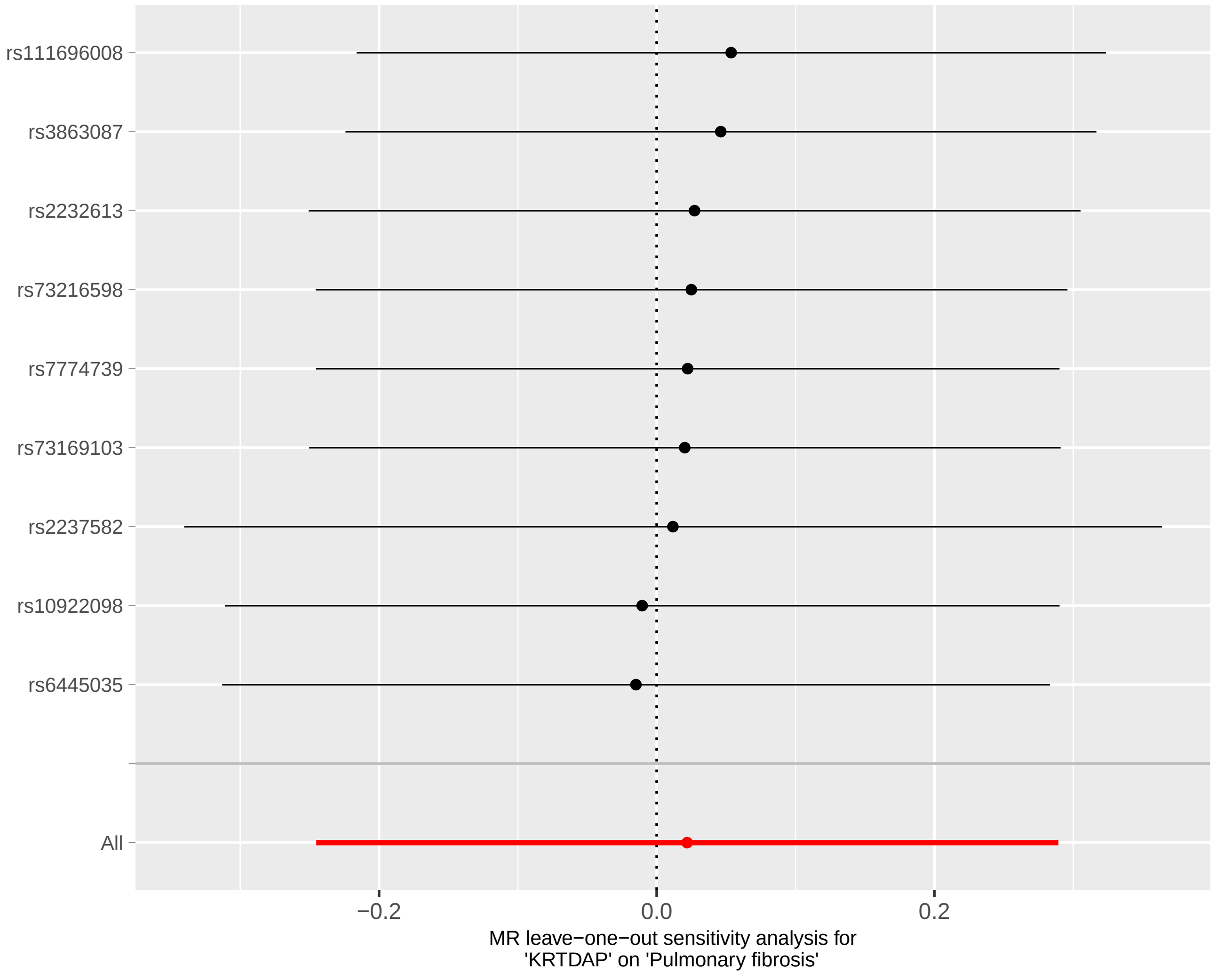
rs111696008
rs3863087
rs2232613
rs73216598
rs7774739
rs73169103
rs2237582
rs10922098
rs6445035
All
−0.2
0.0
0.2
MR leave−one−out sensitivity analysis for
'KRTDAP' on 'Pulmonary fibrosis'

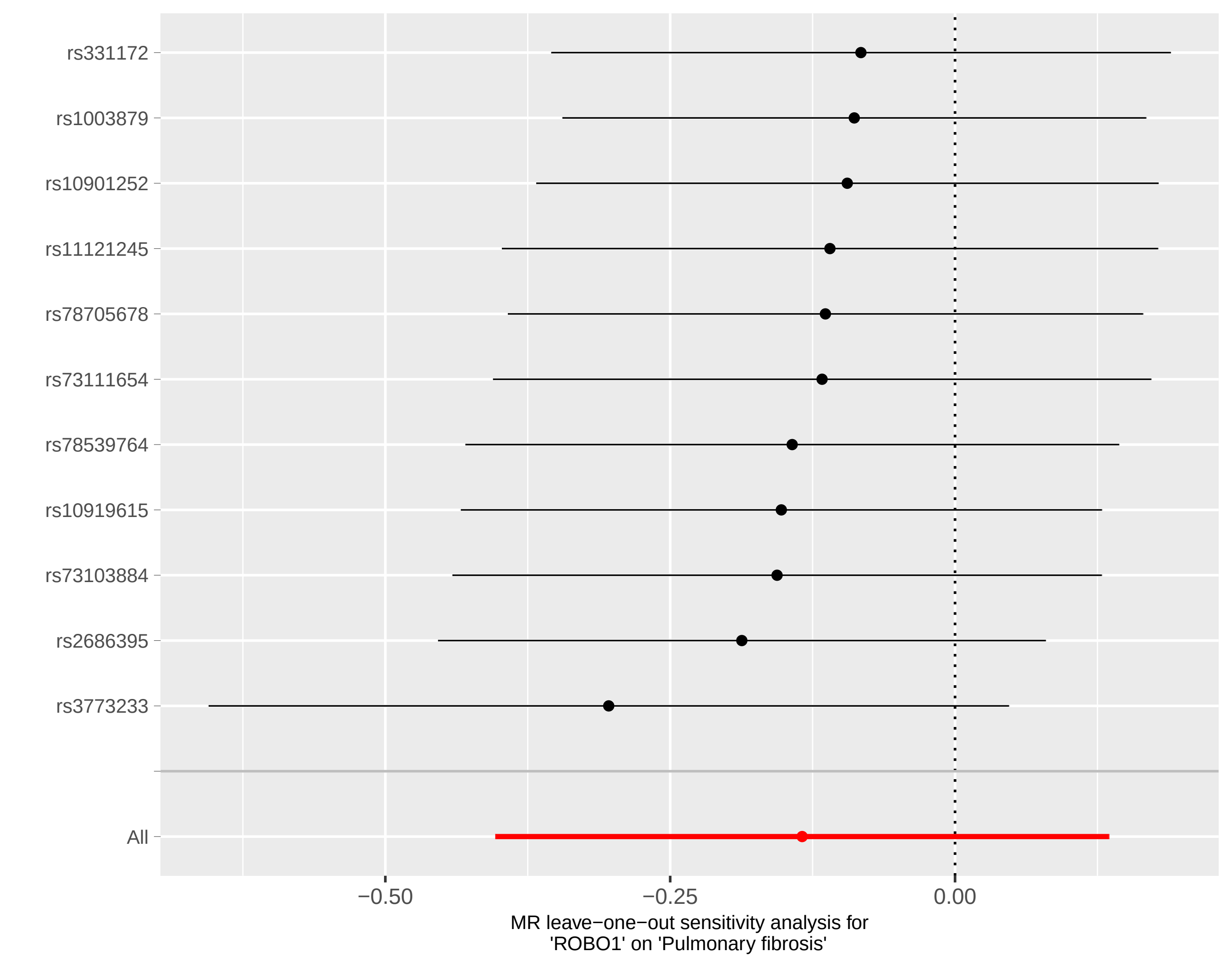
rs331172
rs1003879
rs10901252
rs11121245
rs78705678
rs73111654
rs78539764
rs10919615
rs73103884
rs2686395
rs3773233
All
−0.50
−0.25
0.00
MR leave−one−out sensitivity analysis for
'ROBO1' on 'Pulmonary fibrosis'

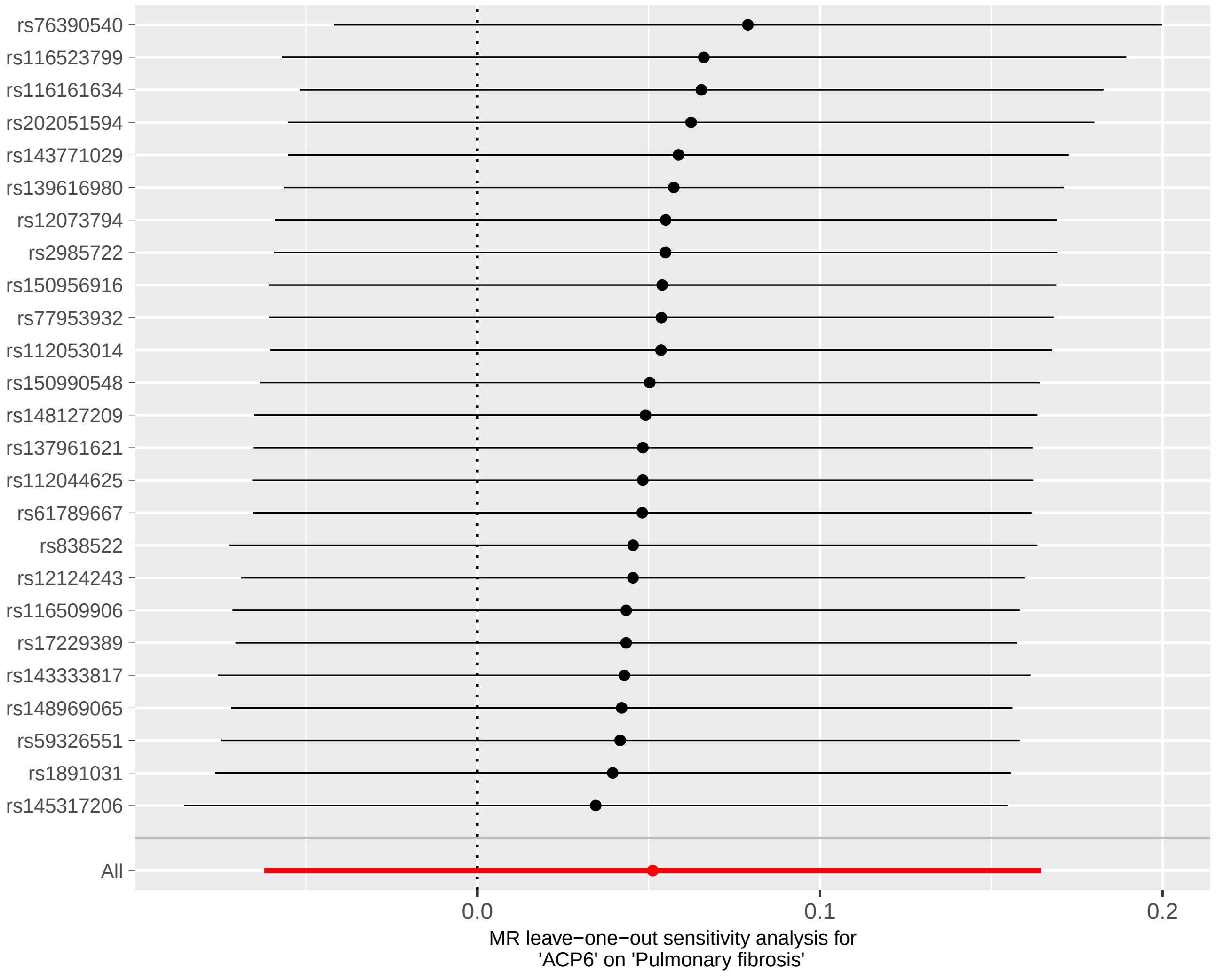
rs76390540
rs116523799
rs116161634
rs202051594
rs143771029
rs139616980
rs12073794
rs2985722
rs150956916
rs77953932
rs112053014
rs150990548
rs148127209
rs137961621
rs112044625
rs61789667
rs838522
rs12124243
rs116509906
rs17229389
rs143333817
rs148969065
rs59326551
rs1891031
rs145317206
All
0.0
0.1
0.2
MR leave−one−out sensitivity analysis for
'ACP6' on 'Pulmonary fibrosis'

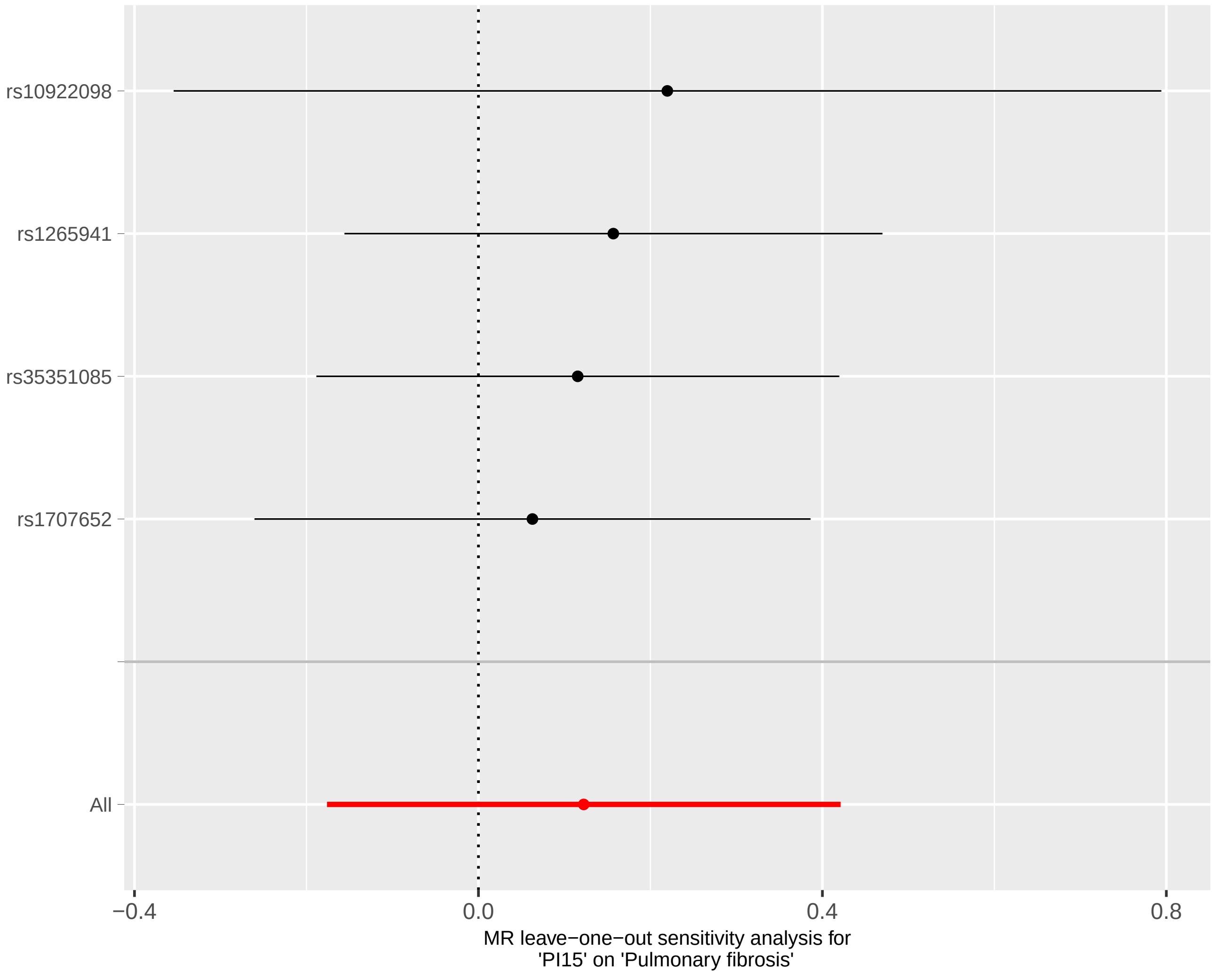
rs10922098
rs1265941
rs35351085
rs1707652
All
−0.4
0.0
0.4
0.8
MR leave−one−out sensitivity analysis for
'PI15' on 'Pulmonary fibrosis'

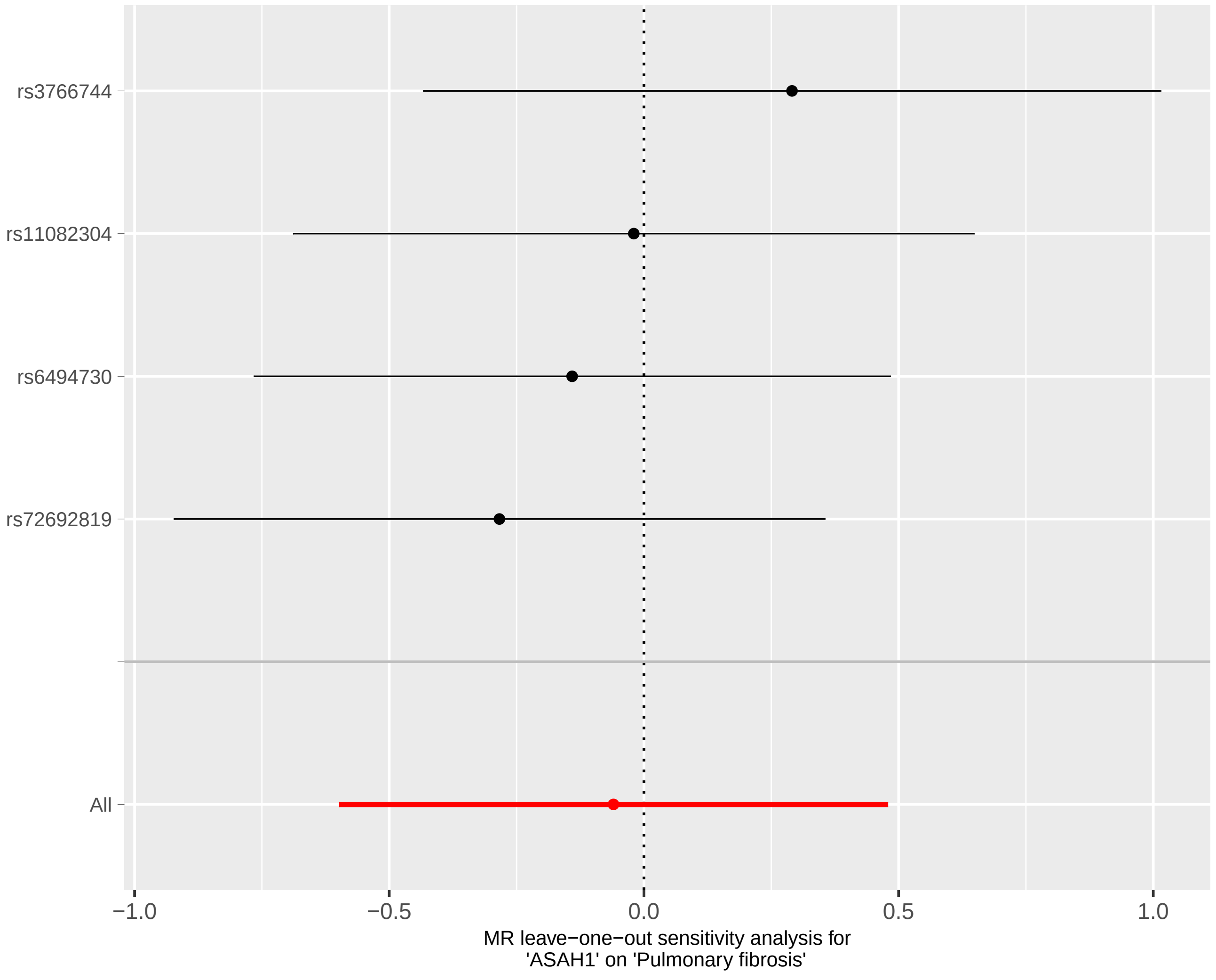
rs3766744
rs11082304
rs6494730
rs72692819
All
−1.0
−0.5
0.0
0.5
1.0
MR leave−one−out sensitivity analysis for
'ASAH1' on 'Pulmonary fibrosis'

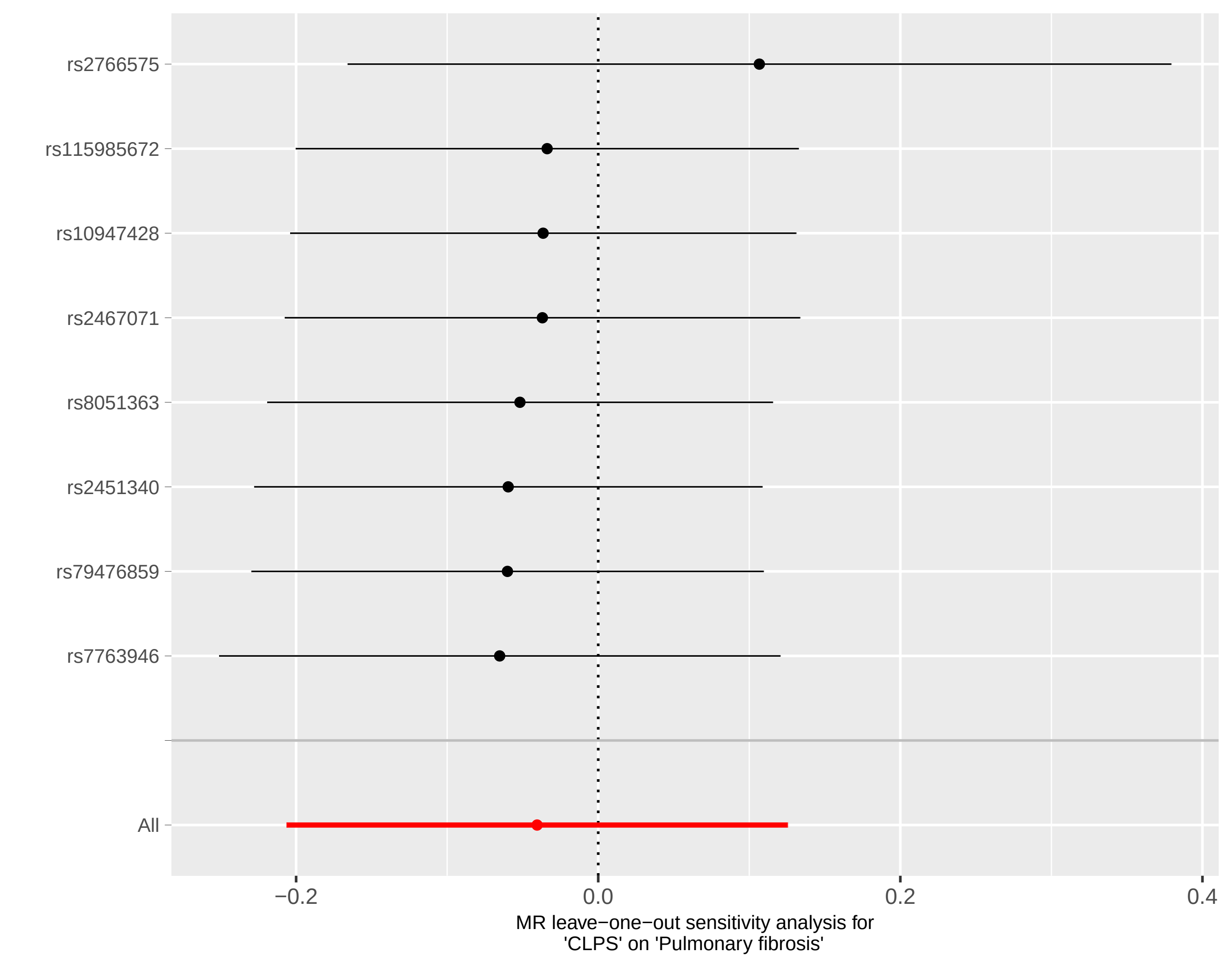
rs2766575
rs115985672
rs10947428
rs2467071
rs8051363
rs2451340
rs79476859
rs7763946
All
−0.2
0.0
0.2
0.4
MR leave−one−out sensitivity analysis for
'CLPS' on 'Pulmonary fibrosis'

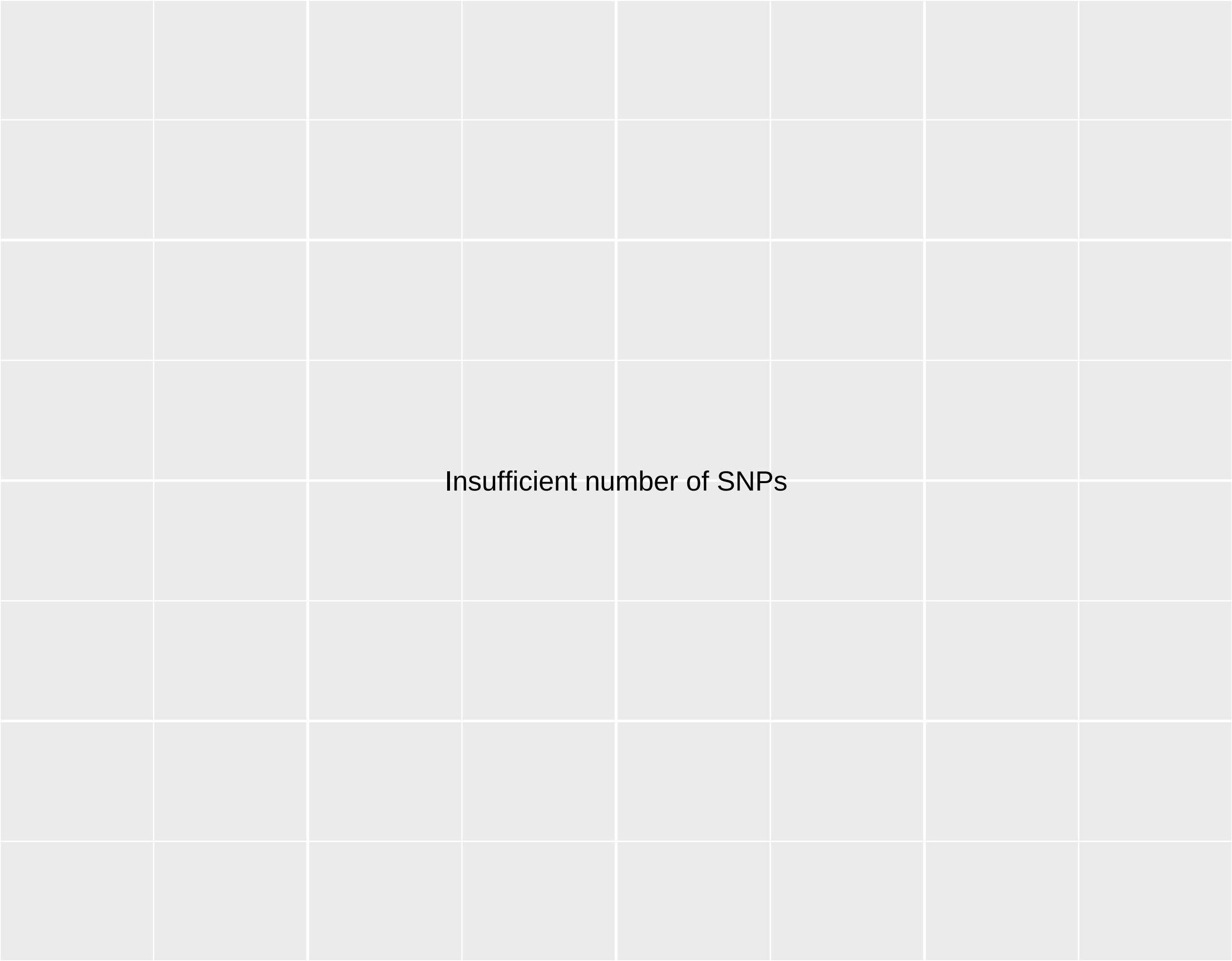
Insufficient number of SNPs

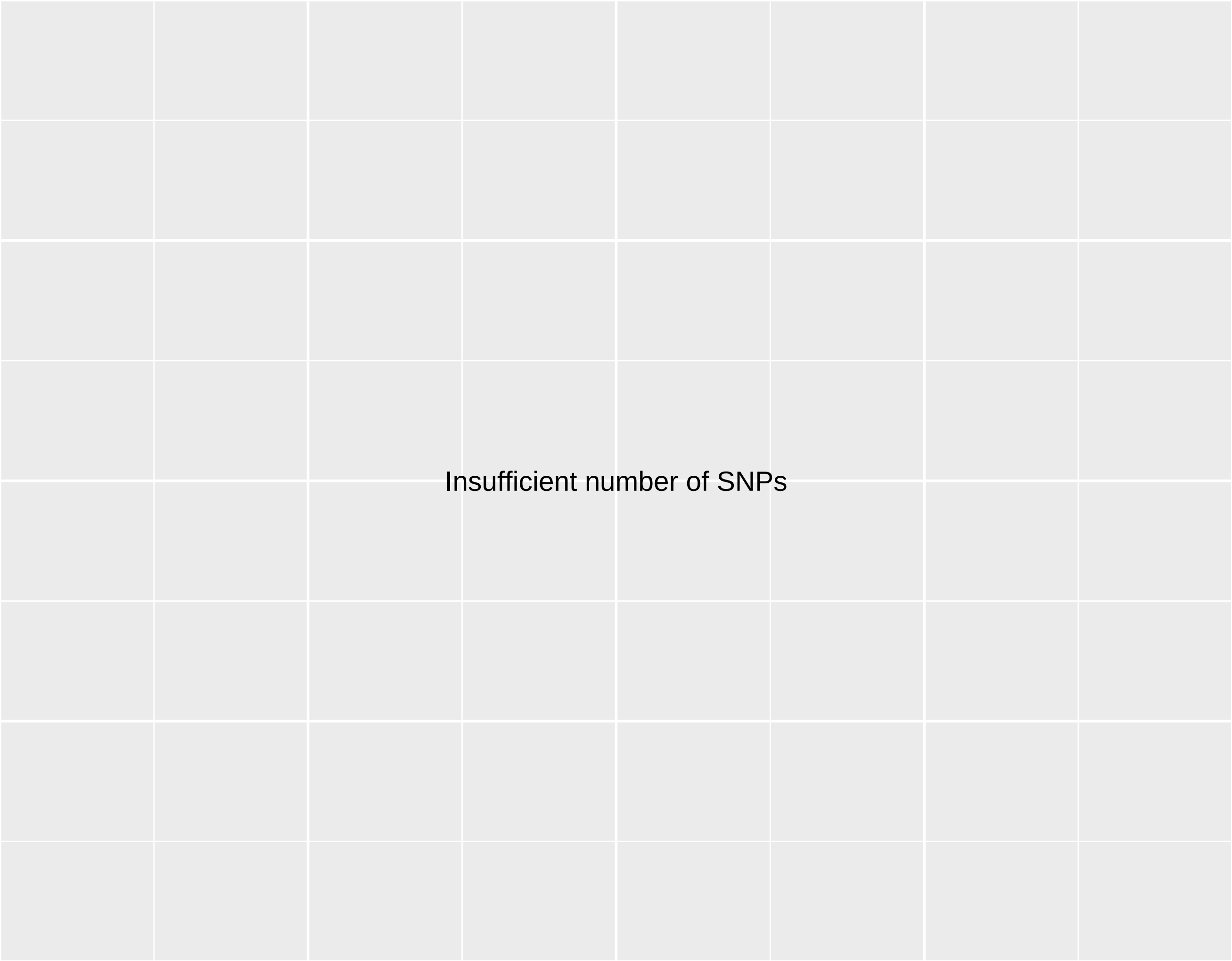
Insufficient number of SNPs

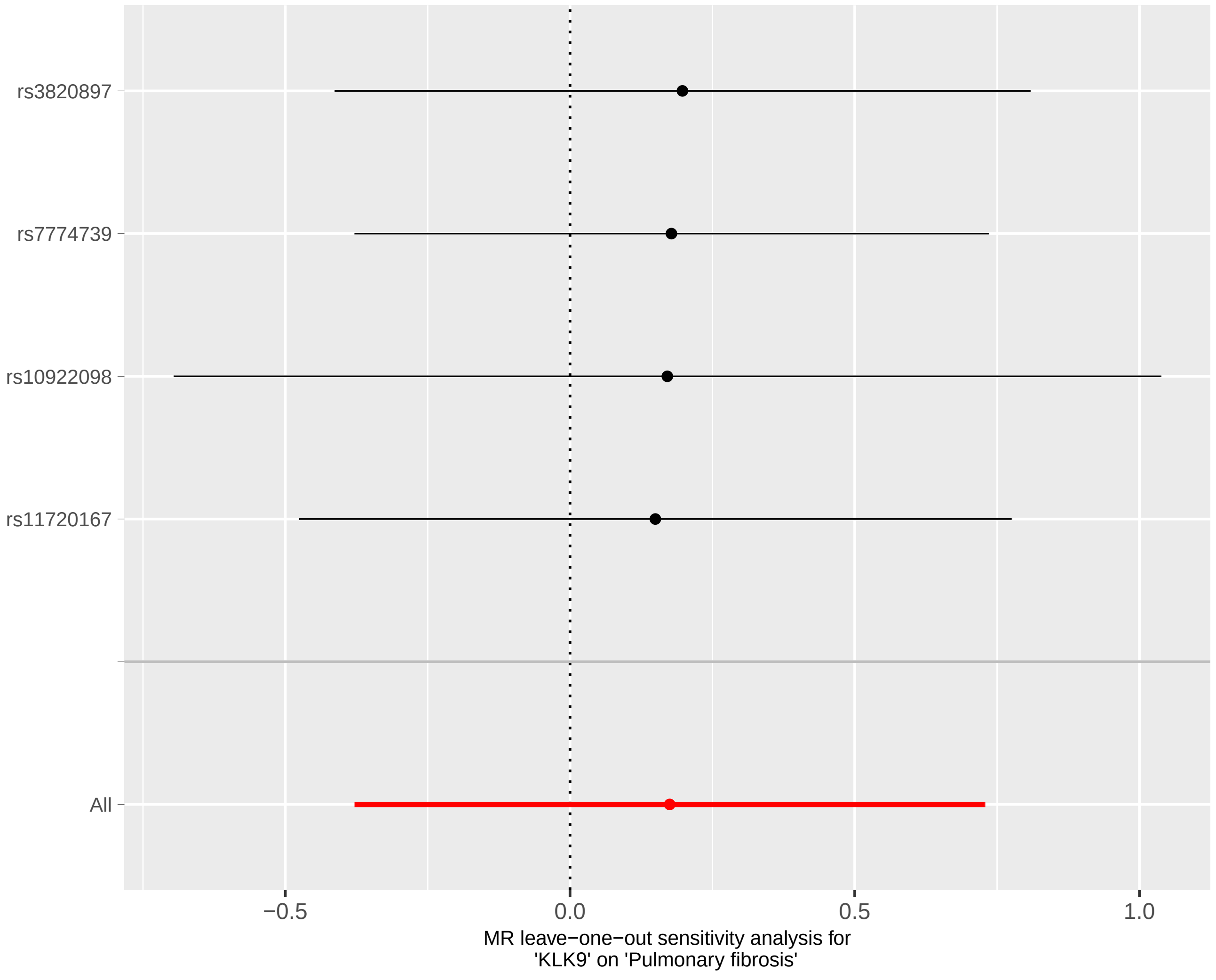
rs3820897
rs7774739
rs10922098
rs11720167
All
−0.5
0.0
0.5
1.0
MR leave−one−out sensitivity analysis for
'KLK9' on 'Pulmonary fibrosis'

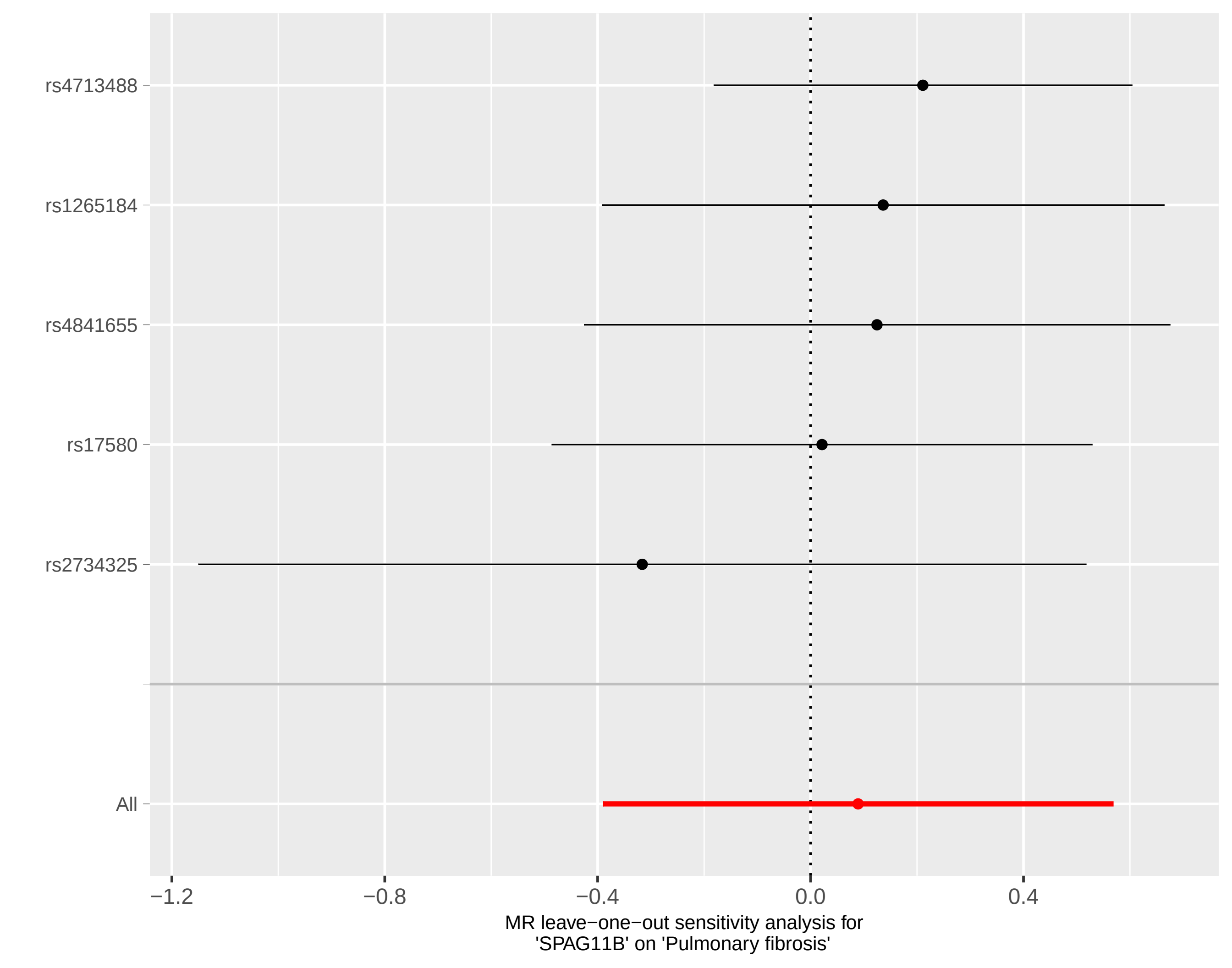

rs4713488
rs1265184
rs4841655
rs17580
rs2734325
All
−1.2
−0.8
−0.4
0.0
0.4
MR leave−one−out sensitivity analysis for
'SPAG11B' on 'Pulmonary fibrosis'

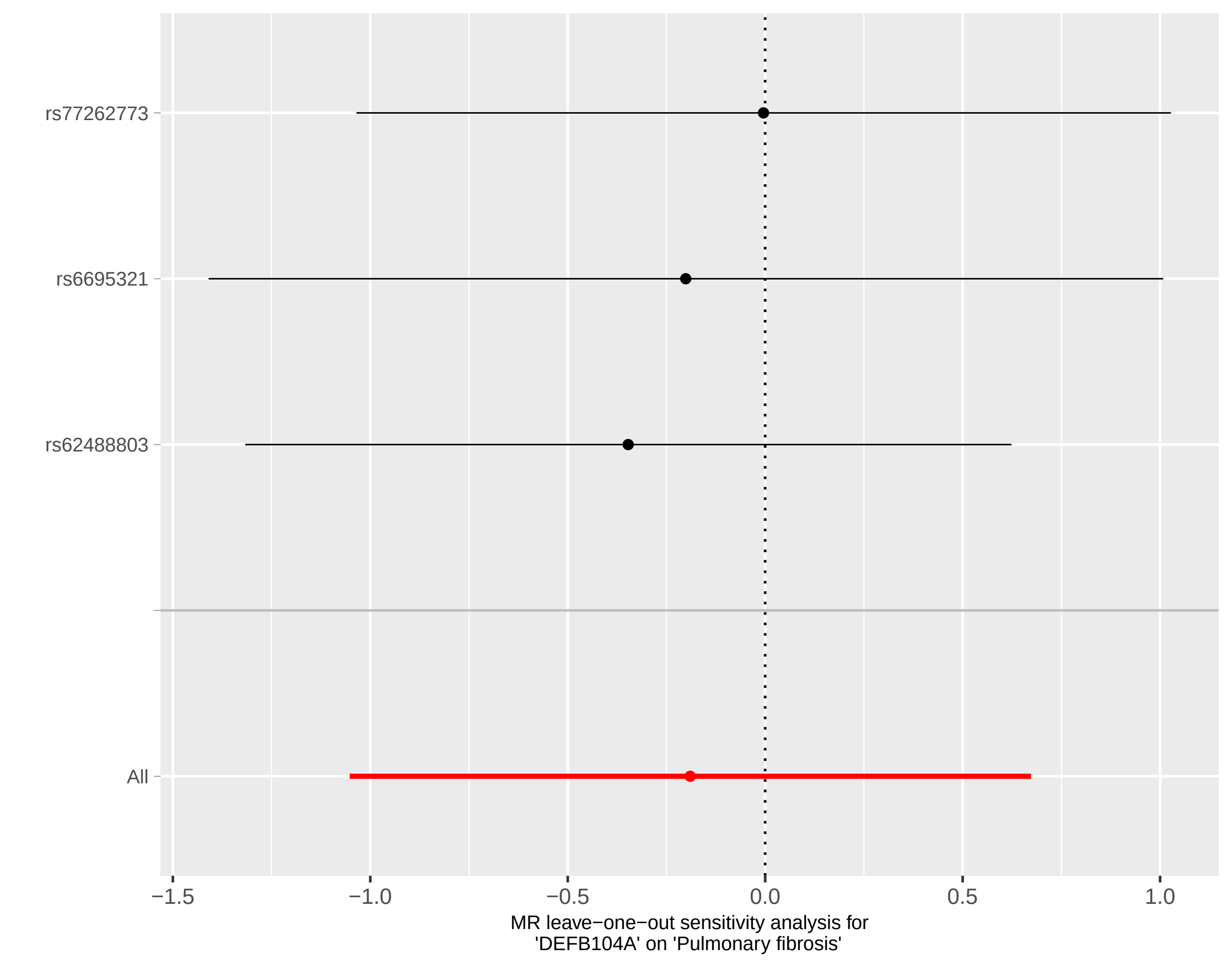
rs77262773
rs6695321
rs62488803
All
−1.5
−1.0
−0.5
0.0
0.5
1.0
MR leave−one−out sensitivity analysis for
'DEFB104A' on 'Pulmonary fibrosis'

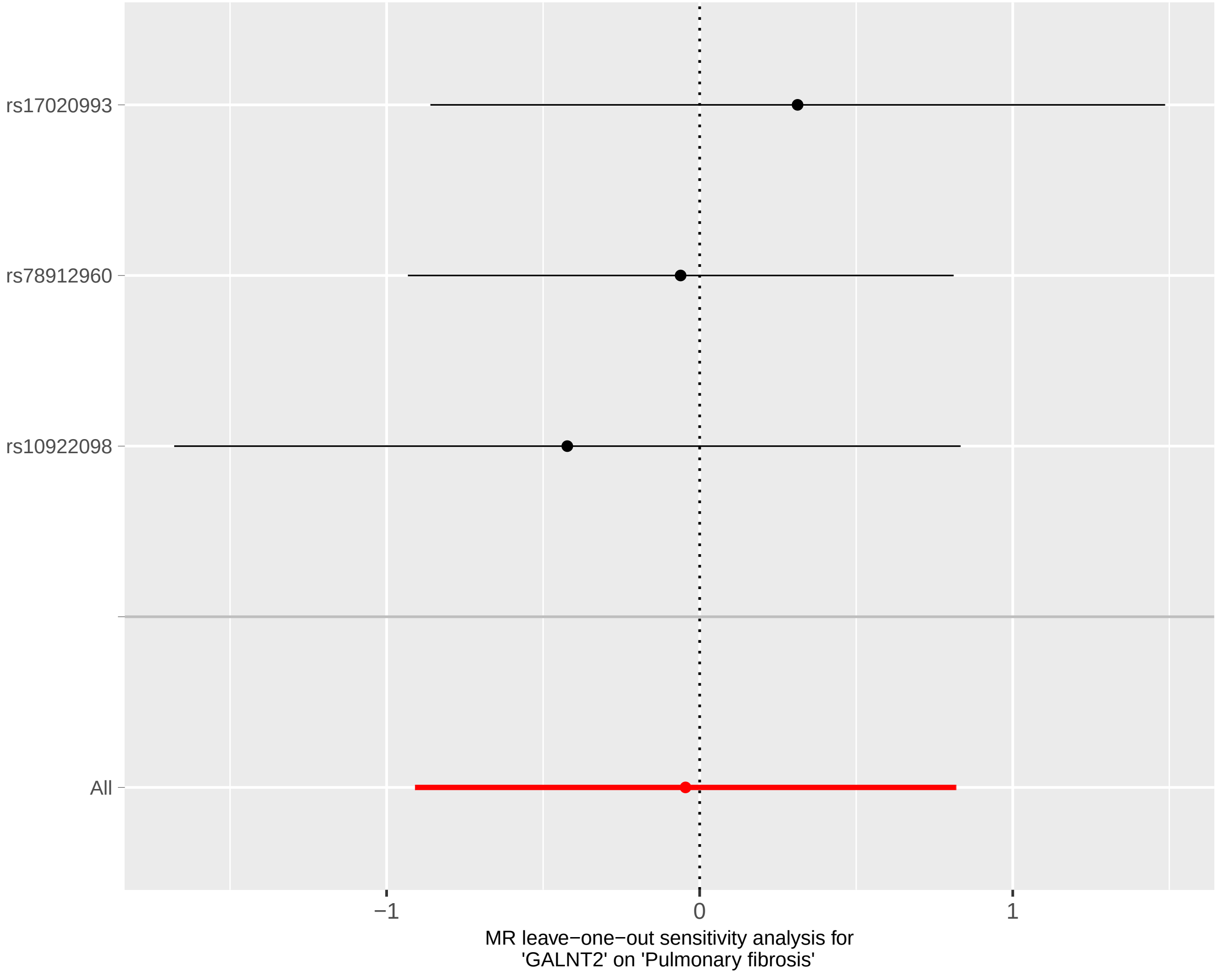
rs17020993
rs78912960
rs10922098
All
−1
0
1
MR leave−one−out sensitivity analysis for
'GALNT2' on 'Pulmonary fibrosis'

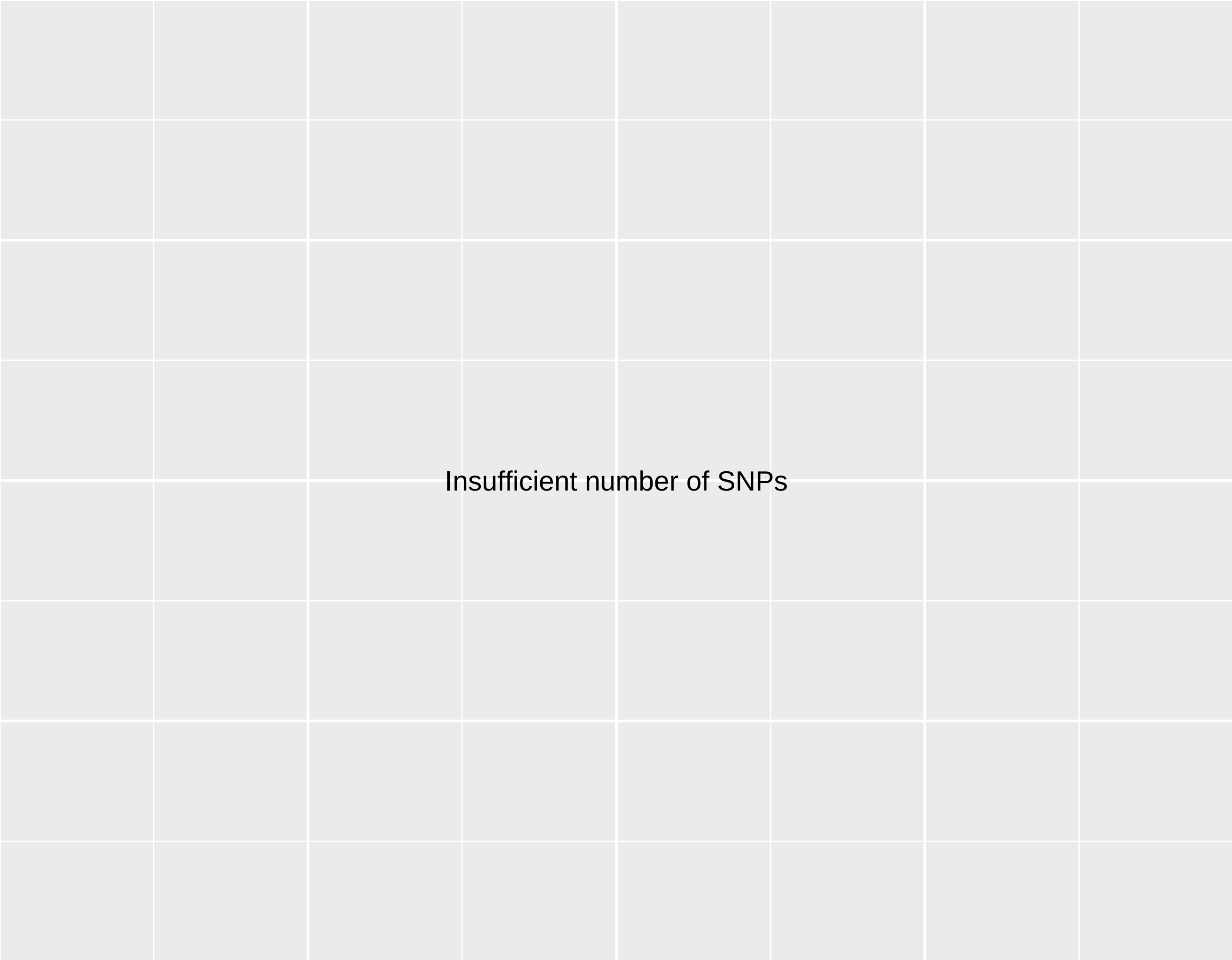
Insufficient number of SNPs

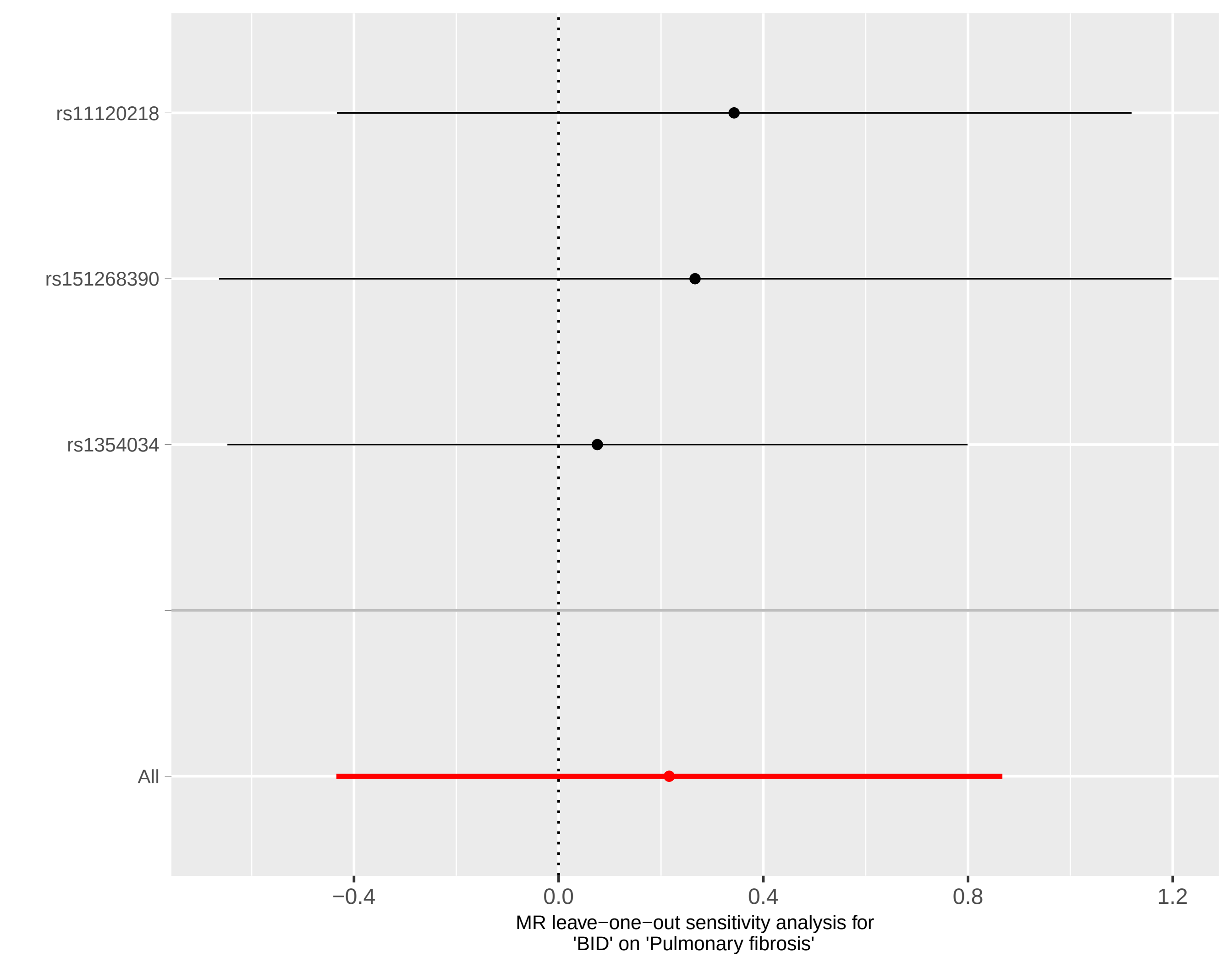
rs11120218
rs151268390
rs1354034
All
−0.4
0.0
0.4
0.8
1.2
MR leave−one−out sensitivity analysis for
'BID' on 'Pulmonary fibrosis'

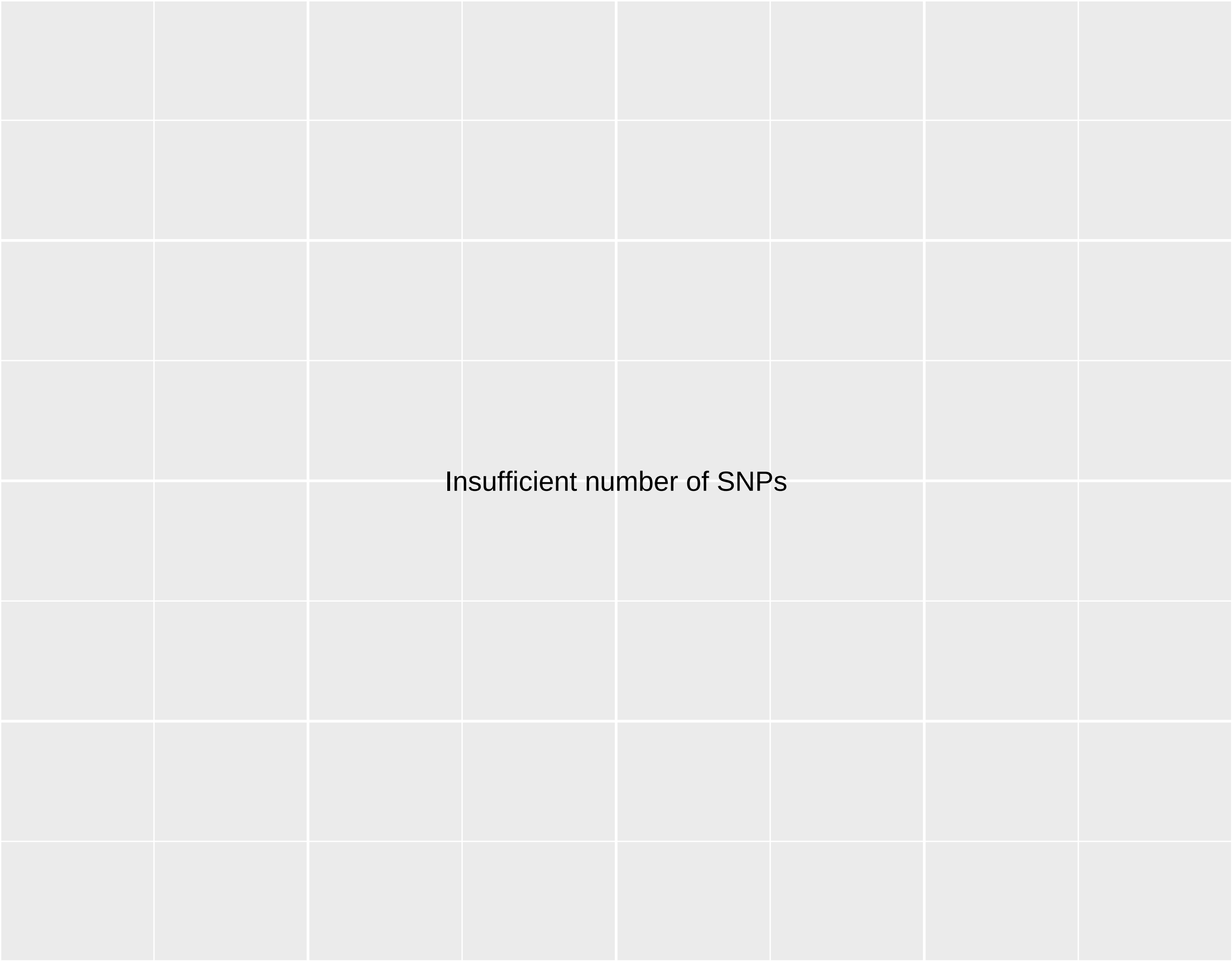
Insufficient number of SNPs

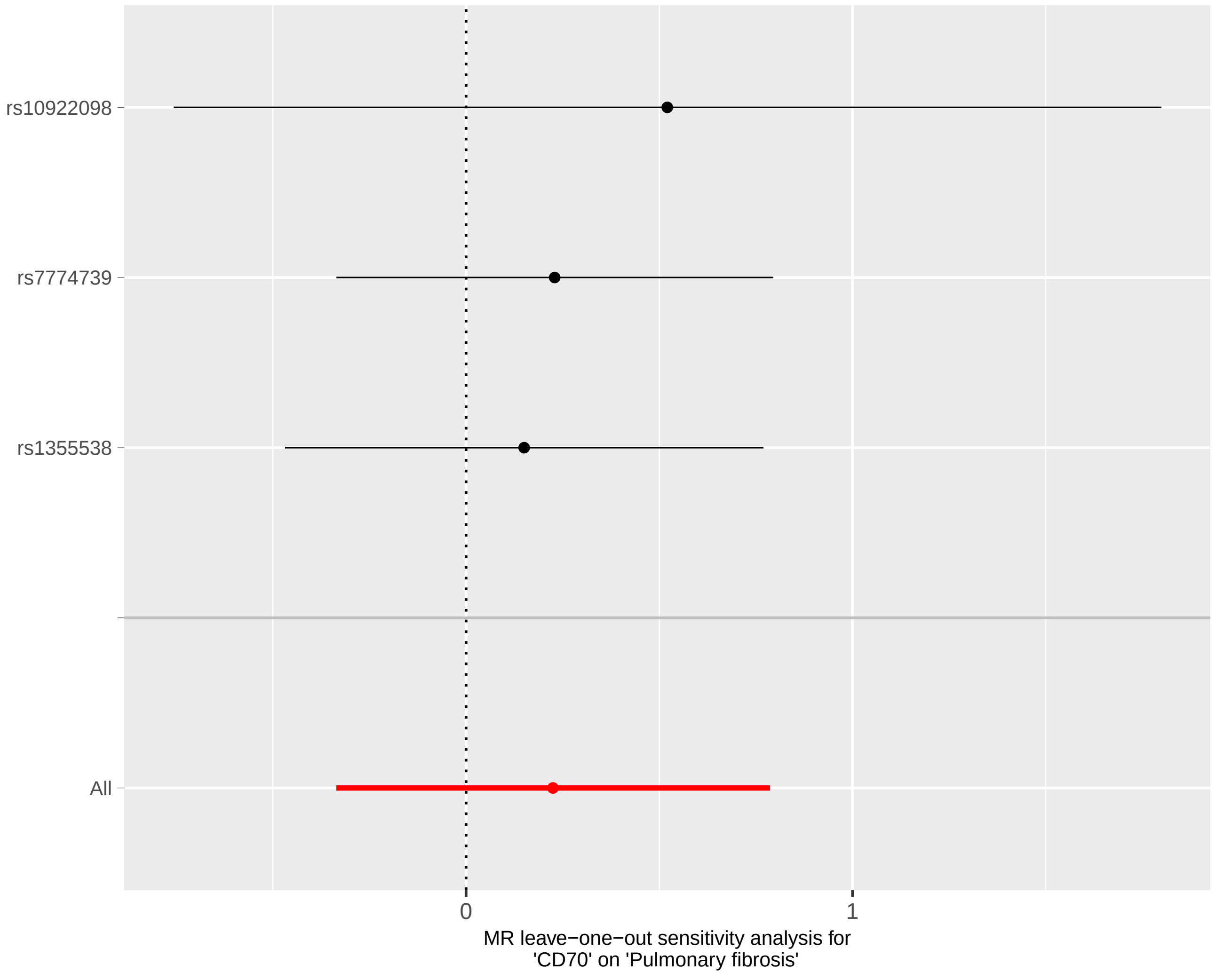
rs10922098
rs7774739
rs1355538
All
0
1
MR leave−one−out sensitivity analysis for
'CD70' on 'Pulmonary fibrosis'

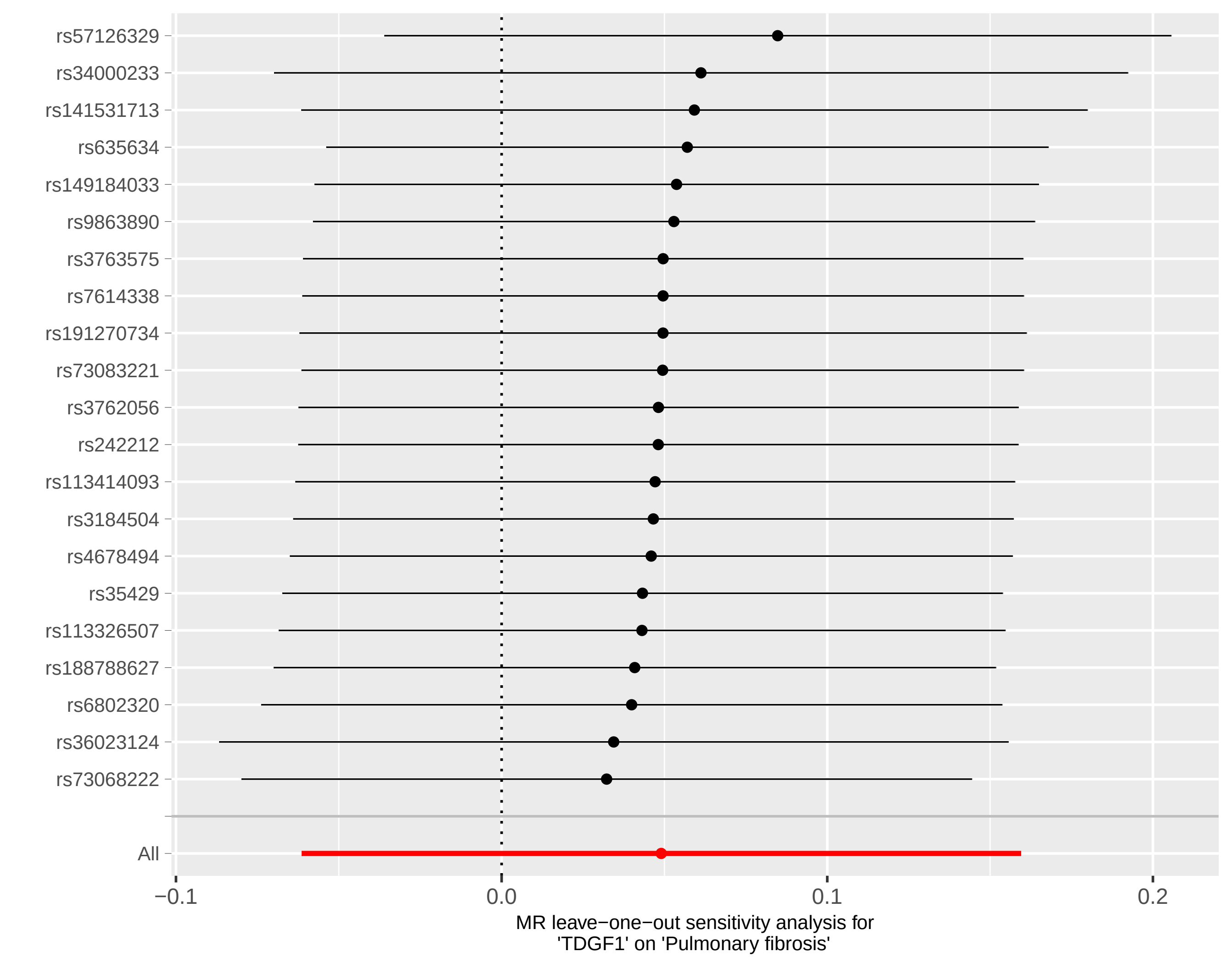
rs57126329
rs34000233
rs141531713
rs635634
rs149184033
rs9863890
rs3763575
rs7614338
rs191270734
rs73083221
rs3762056
rs242212
rs113414093
rs3184504
rs4678494
rs35429
rs113326507
rs188788627
rs6802320
rs36023124
rs73068222
All
−0.1
0.0
0.1
0.2
MR leave−one−out sensitivity analysis for
'TDGF1' on 'Pulmonary fibrosis'

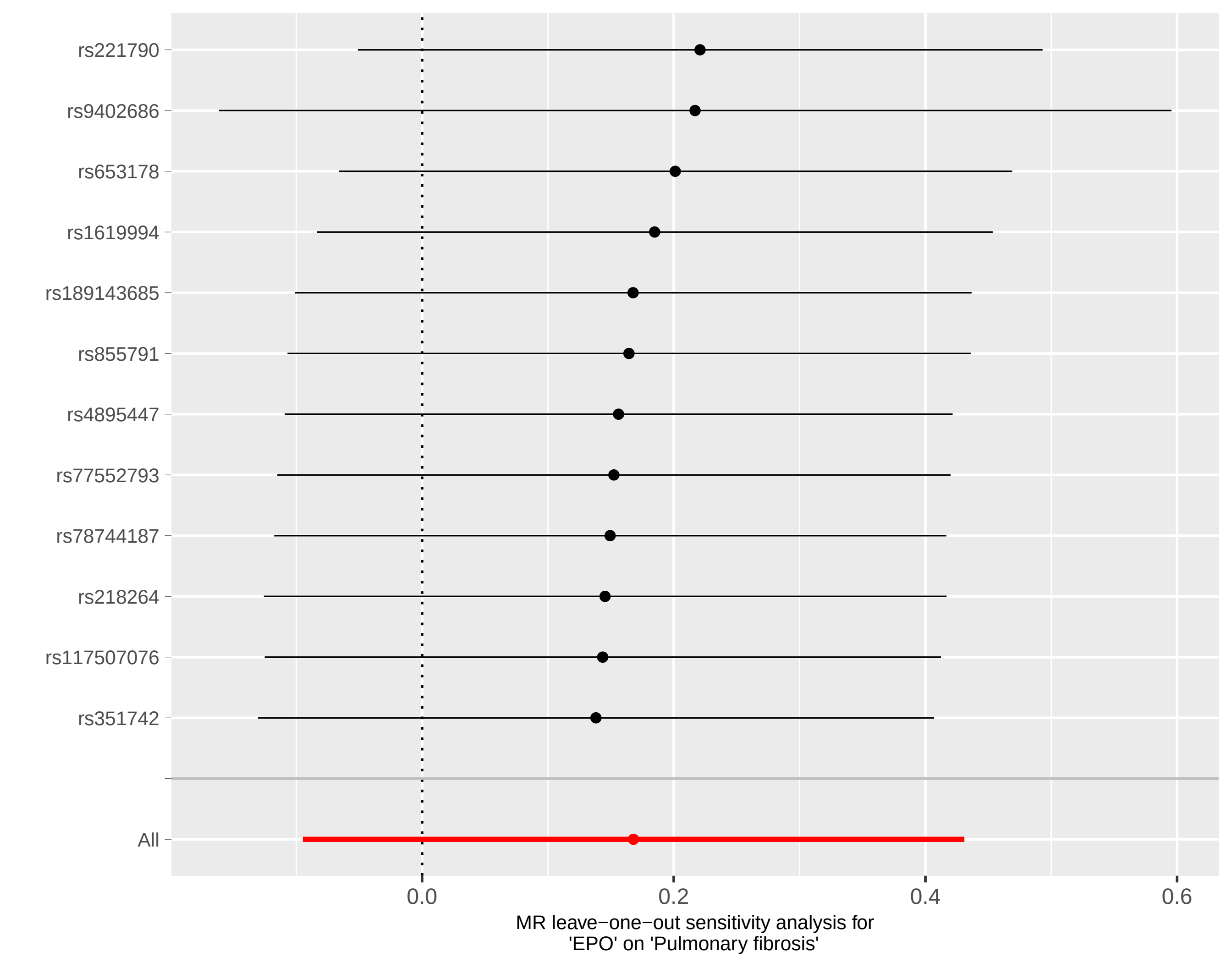
rs221790
rs9402686
rs653178
rs1619994
rs189143685
rs855791
rs4895447
rs77552793
rs78744187
rs218264
rs117507076
rs351742
All
0.0
0.2
0.4
0.6
MR leave−one−out sensitivity analysis for
'EPO' on 'Pulmonary fibrosis'

Insufficient number of SNPs

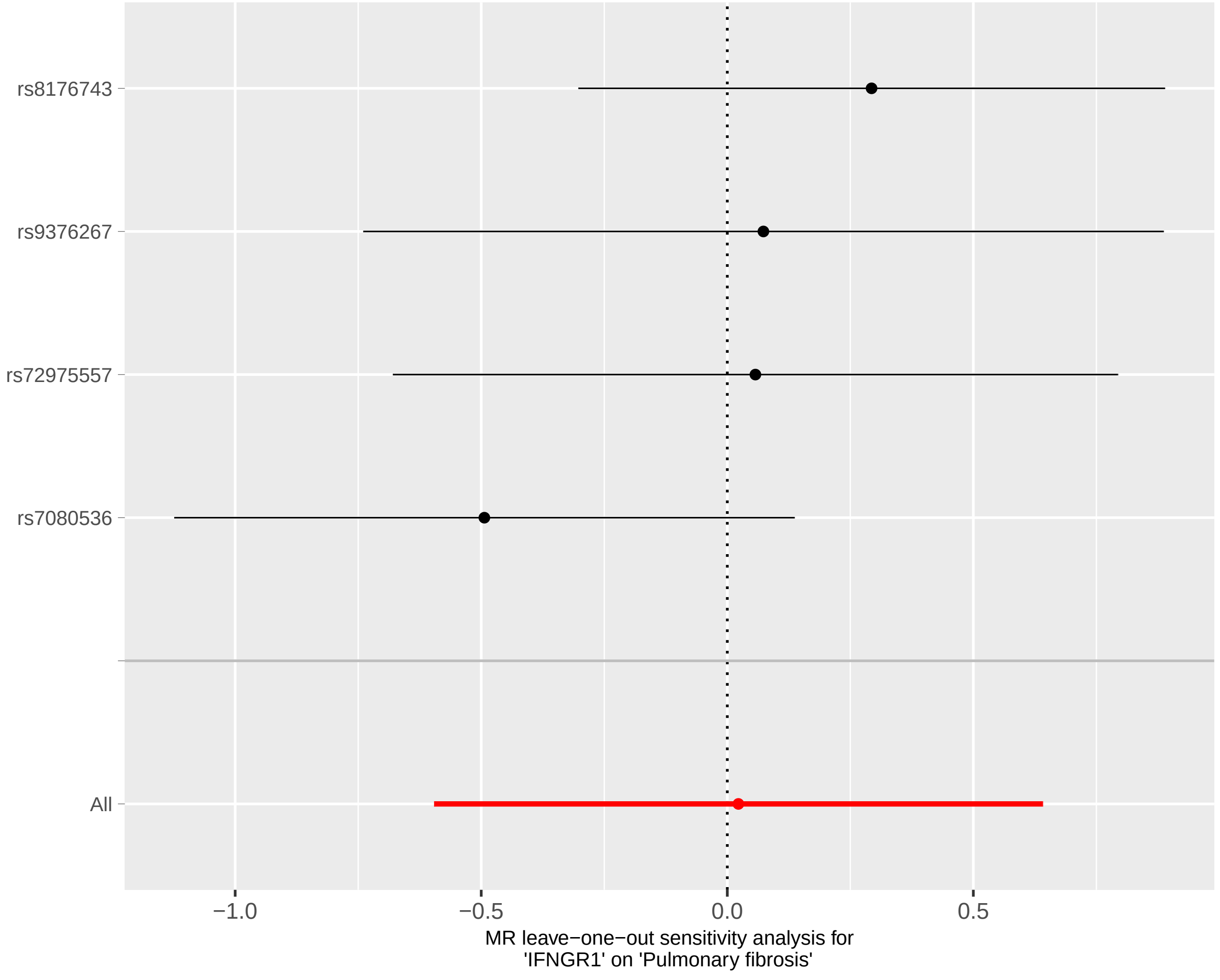
rs8176743
rs9376267
rs72975557
rs7080536
All
−1.0
−0.5
0.0
0.5
MR leave−one−out sensitivity analysis for
'IFNGR1' on 'Pulmonary fibrosis'

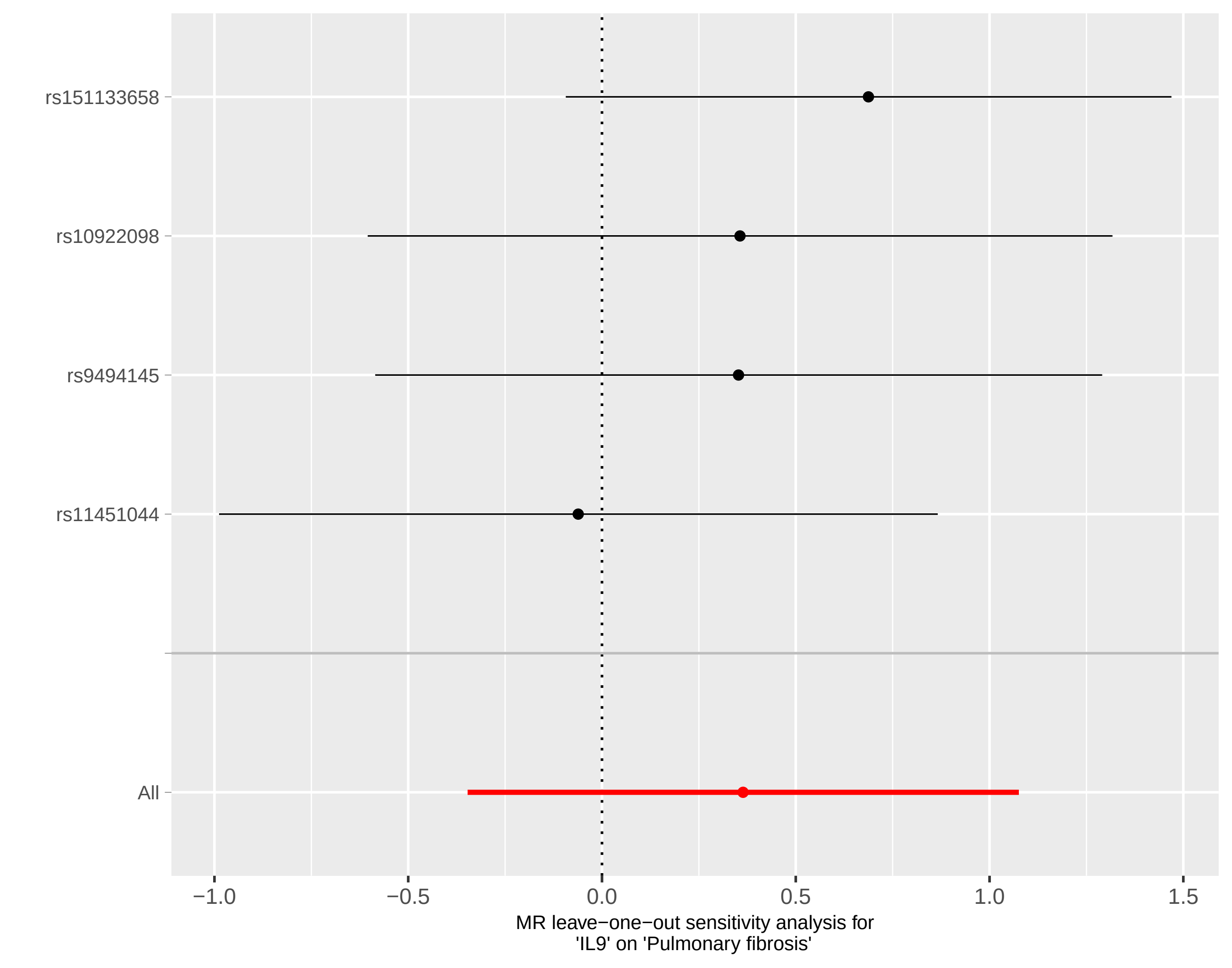

rs151133658
rs10922098
rs9494145
rs11451044
All
−1.0
−0.5
0.0
0.5
1.0
1.5
MR leave−one−out sensitivity analysis for
'IL9' on 'Pulmonary fibrosis'

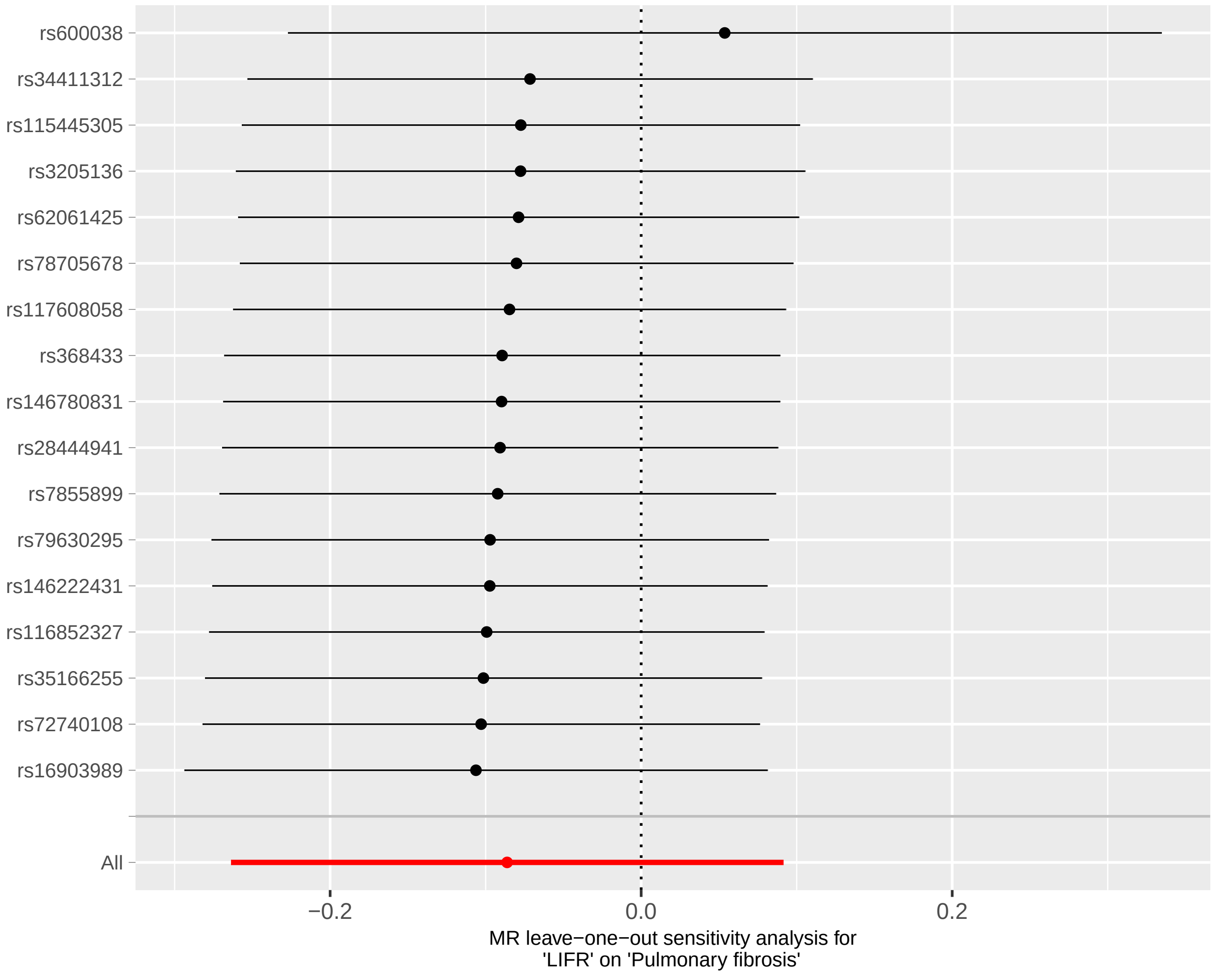
rs600038
rs34411312
rs115445305
rs3205136
rs62061425
rs78705678
rs117608058
rs368433
rs146780831
rs28444941
rs7855899
rs79630295
rs146222431
rs116852327
rs35166255
rs72740108
rs16903989
All
−0.2
0.0
0.2
MR leave−one−out sensitivity analysis for
'LIFR' on 'Pulmonary fibrosis'

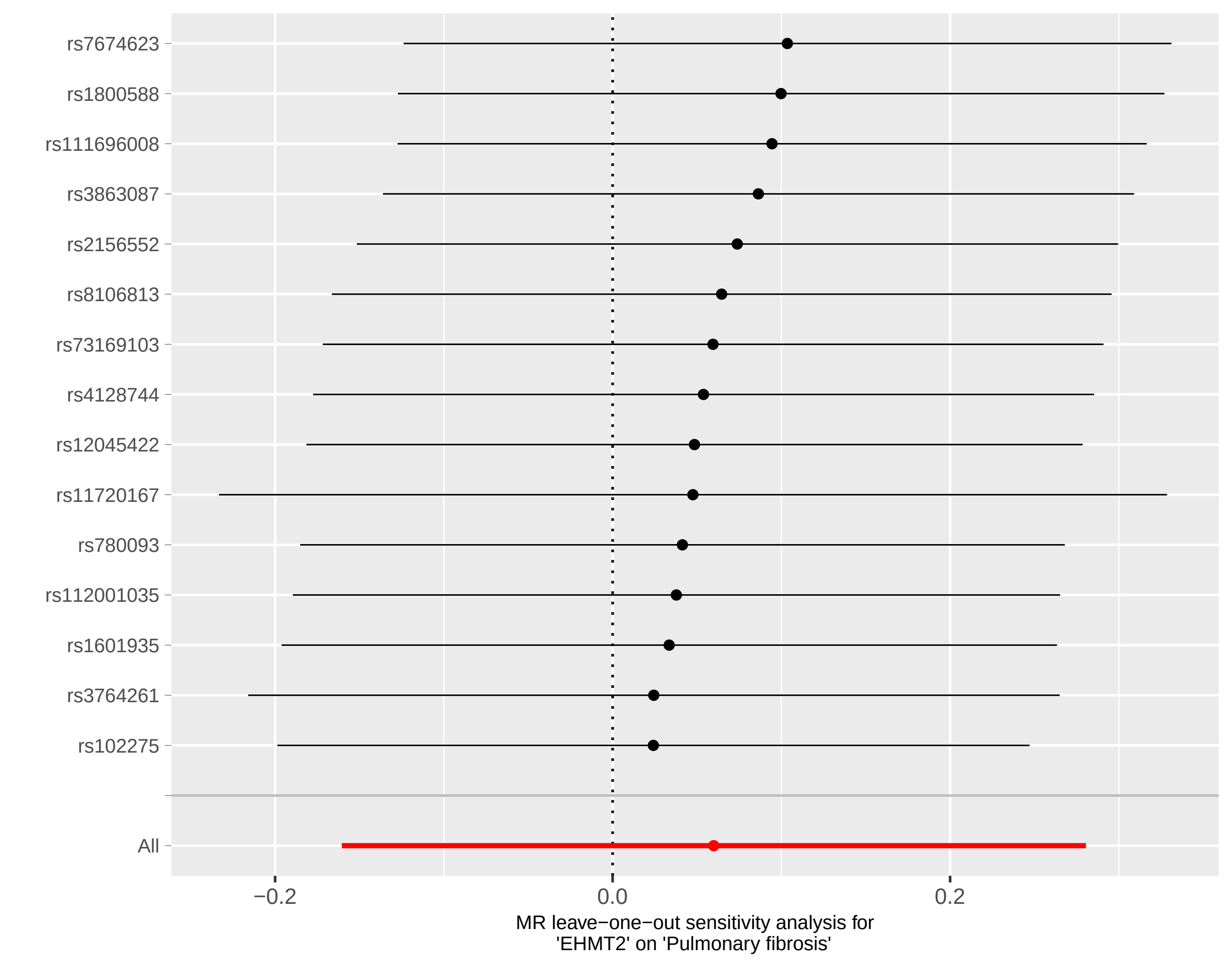
rs7674623
rs1800588
rs111696008
rs3863087
rs2156552
rs8106813
rs73169103
rs4128744
rs12045422
rs11720167
rs780093
rs112001035
rs1601935
rs3764261
rs102275
All
−0.2
0.0
0.2
MR leave−one−out sensitivity analysis for
'EHMT2' on 'Pulmonary fibrosis'

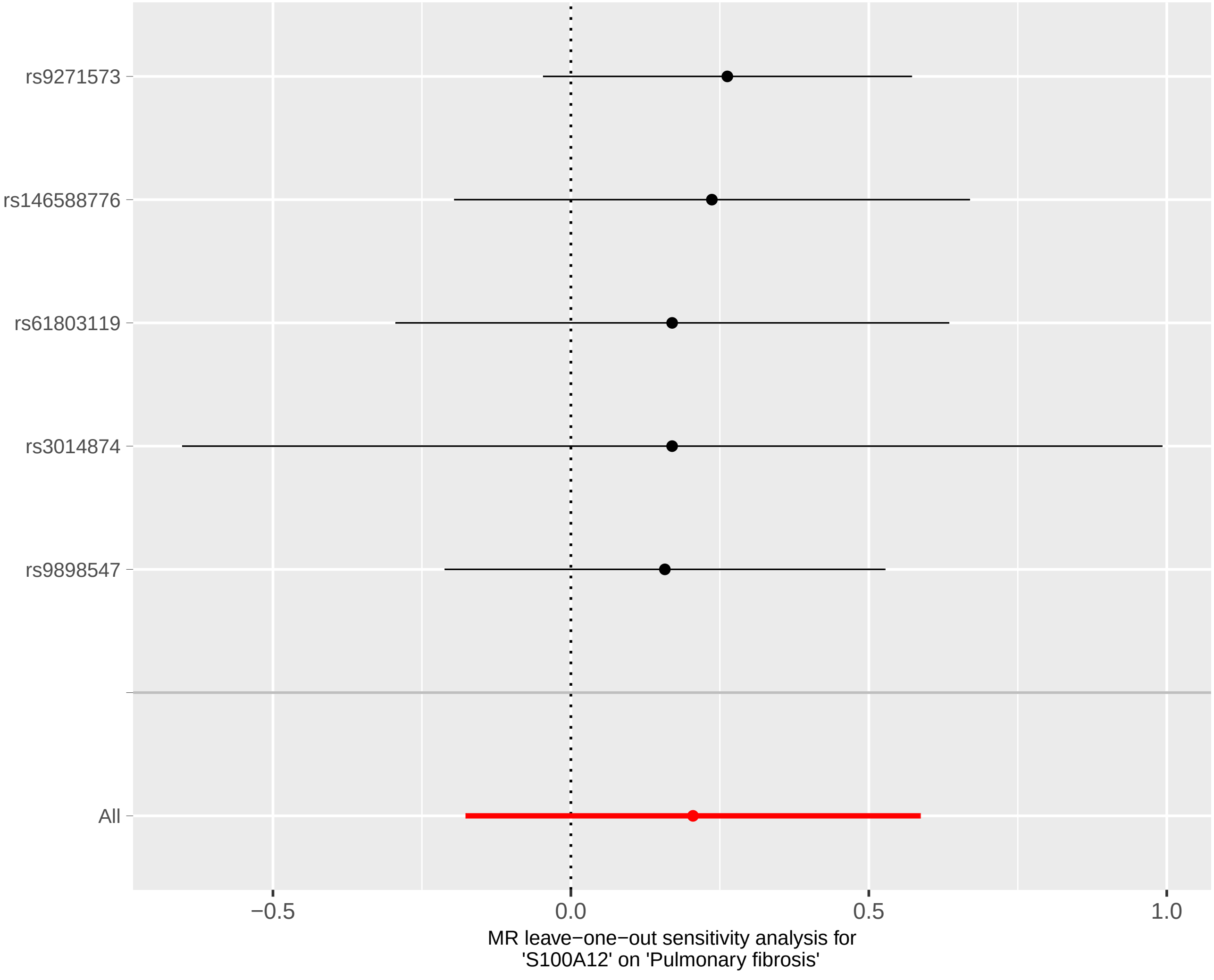
rs9271573
rs146588776
rs61803119
rs3014874
rs9898547
All
−0.5
0.0
0.5
1.0
MR leave−one−out sensitivity analysis for
'S100A12' on 'Pulmonary fibrosis'

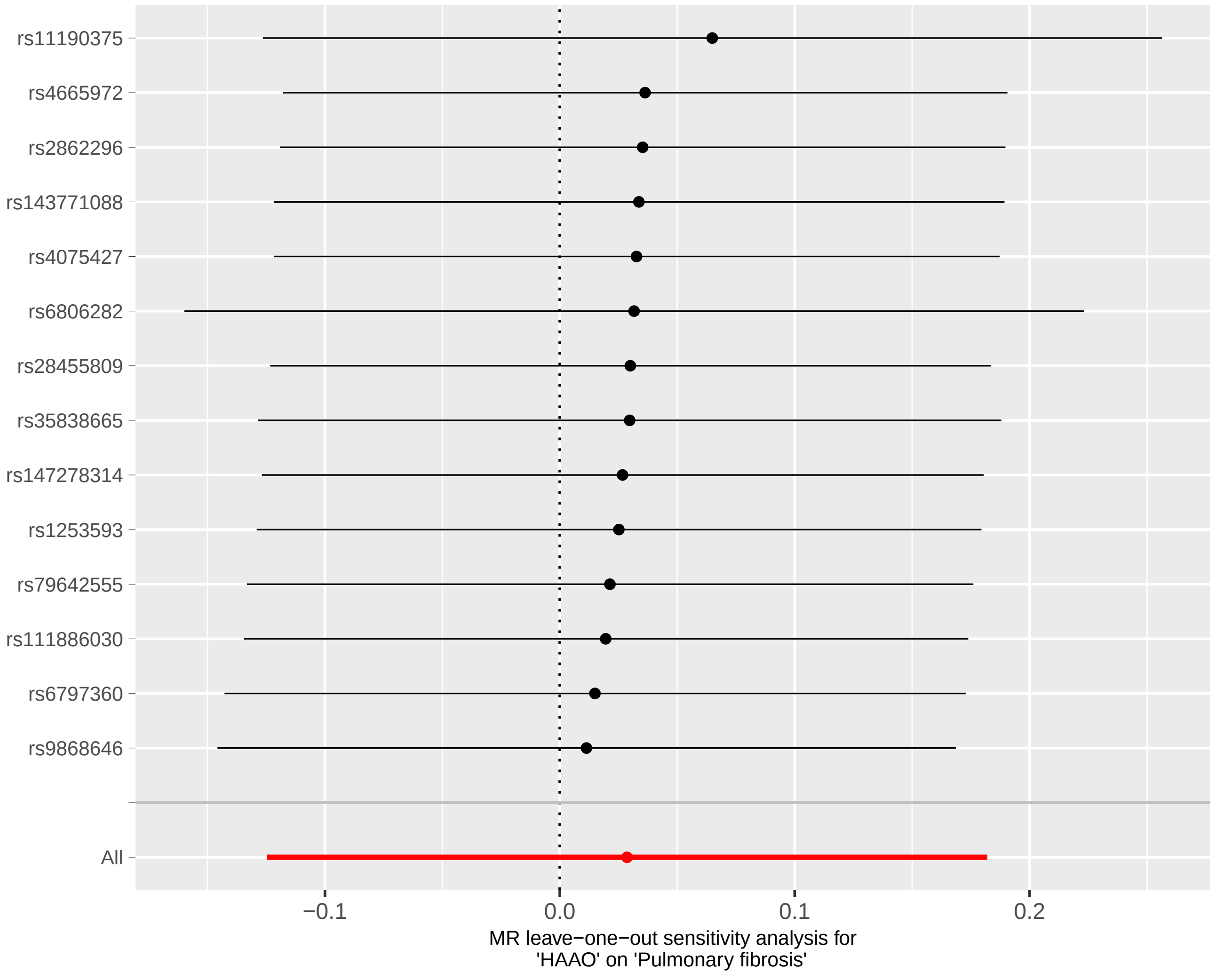
rs11190375
rs4665972
rs2862296
rs143771088
rs4075427
rs6806282
rs28455809
rs35838665
rs147278314
rs1253593
rs79642555
rs111886030
rs6797360
rs9868646
All
−0.1
0.0
0.1
0.2
MR leave−one−out sensitivity analysis for
'HAAO' on 'Pulmonary fibrosis'

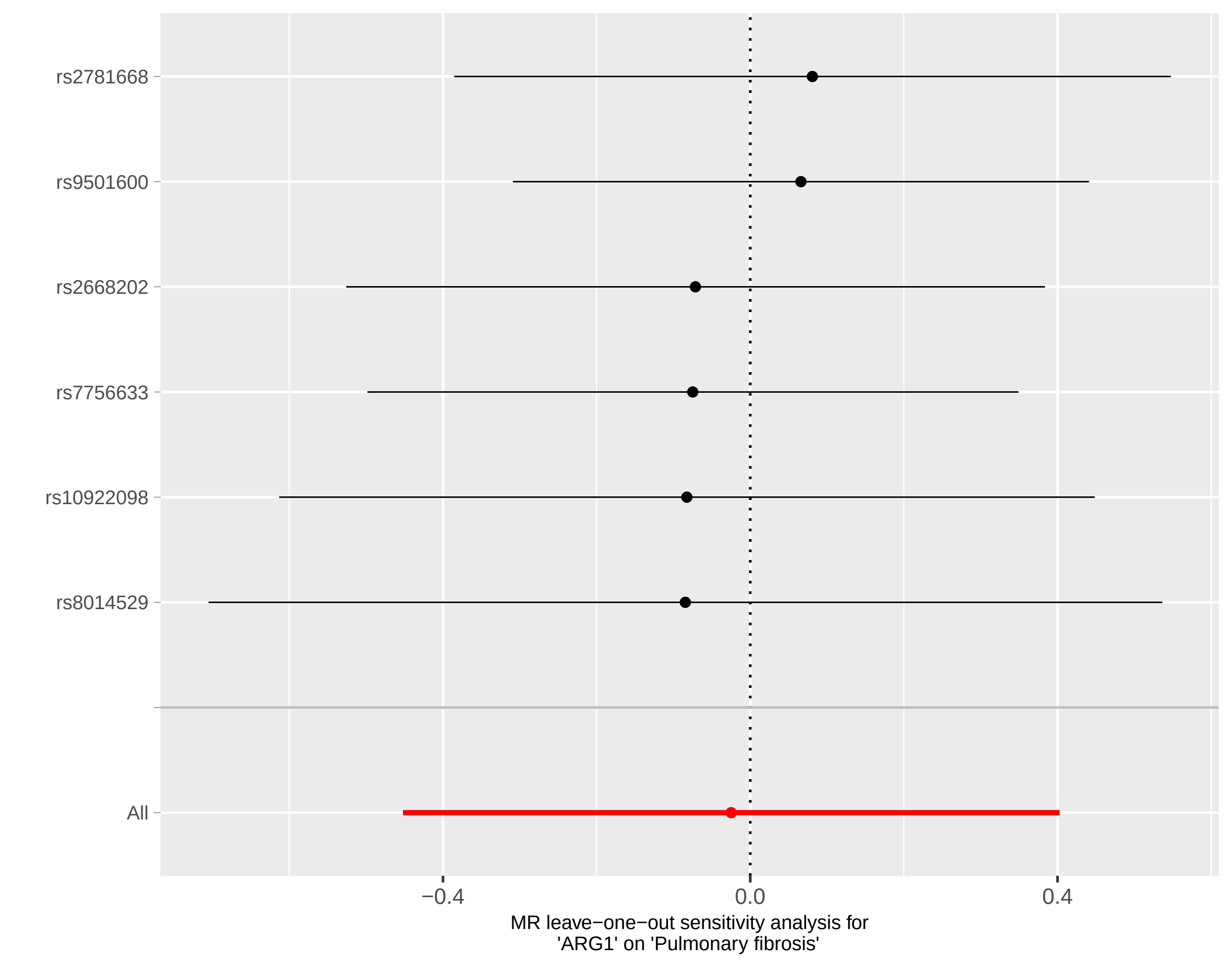
rs2781668
rs9501600
rs2668202
rs7756633
rs10922098
rs8014529
All
−0.4
0.0
0.4
MR leave−one−out sensitivity analysis for
'ARG1' on 'Pulmonary fibrosis'

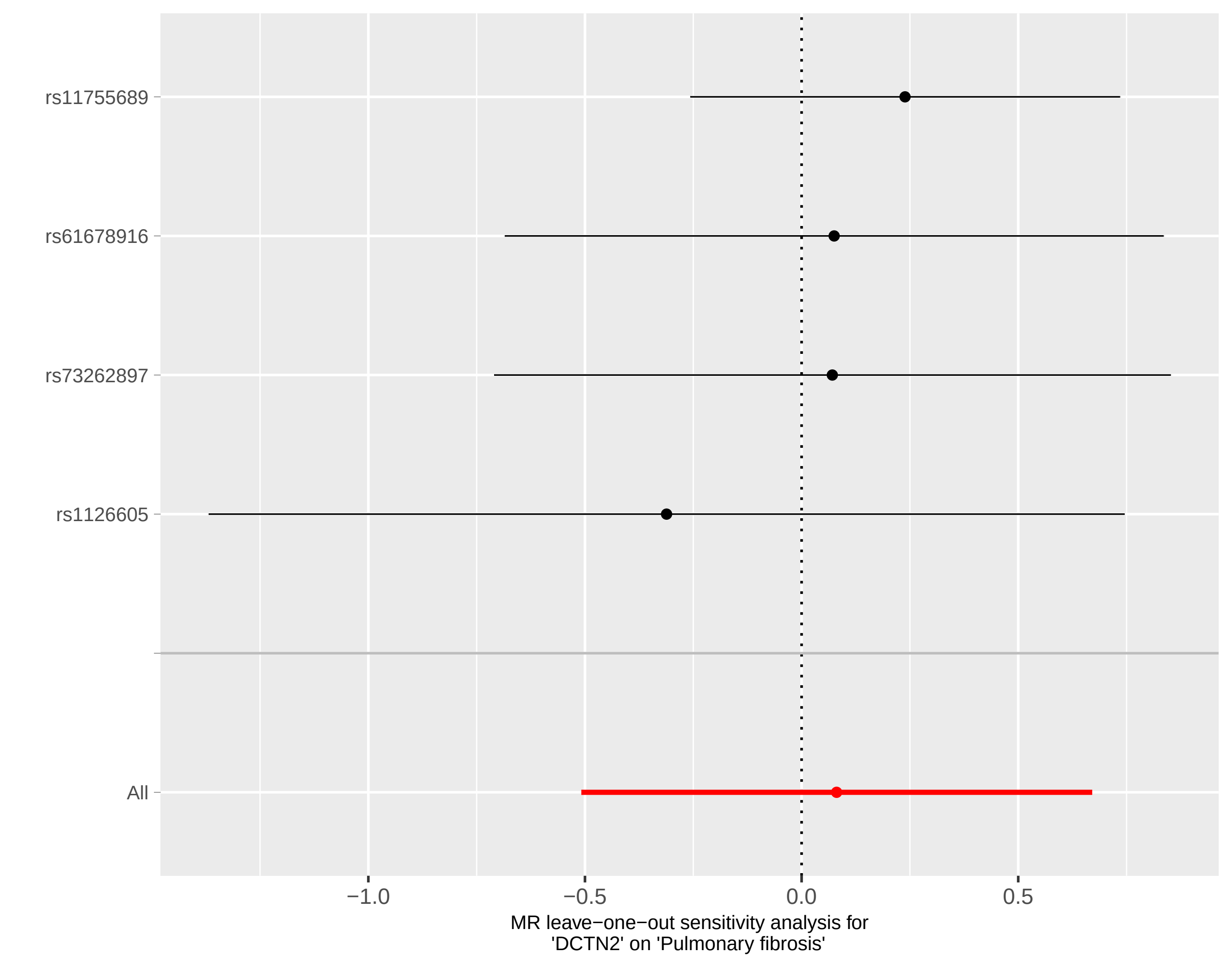
rs11755689
rs61678916
rs73262897
rs1126605
All
−1.0
−0.5
0.0
0.5
MR leave−one−out sensitivity analysis for
'DCTN2' on 'Pulmonary fibrosis'

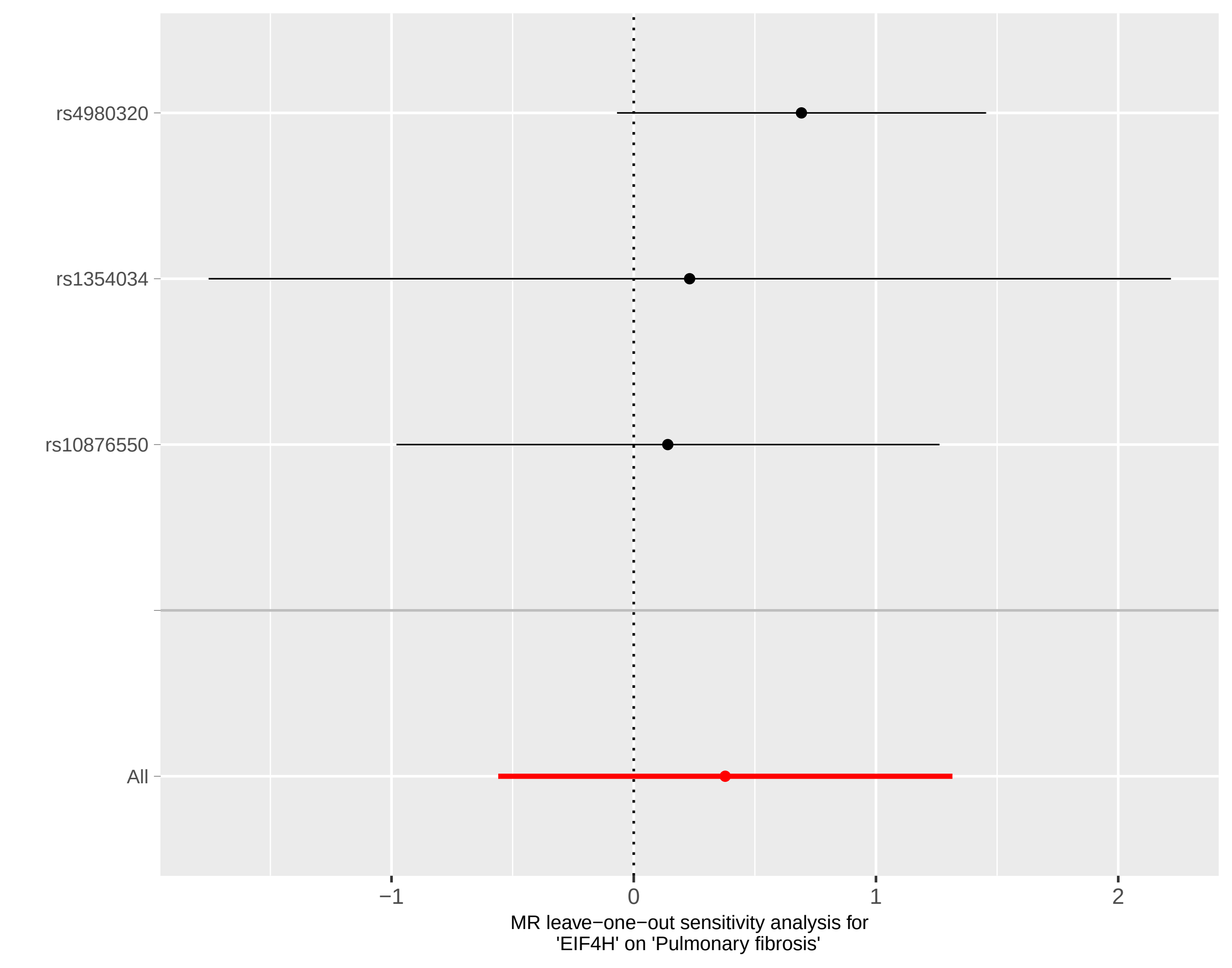

rs4980320
rs1354034
rs10876550
All
−1
0
1
2
MR leave−one−out sensitivity analysis for
'EIF4H' on 'Pulmonary fibrosis'

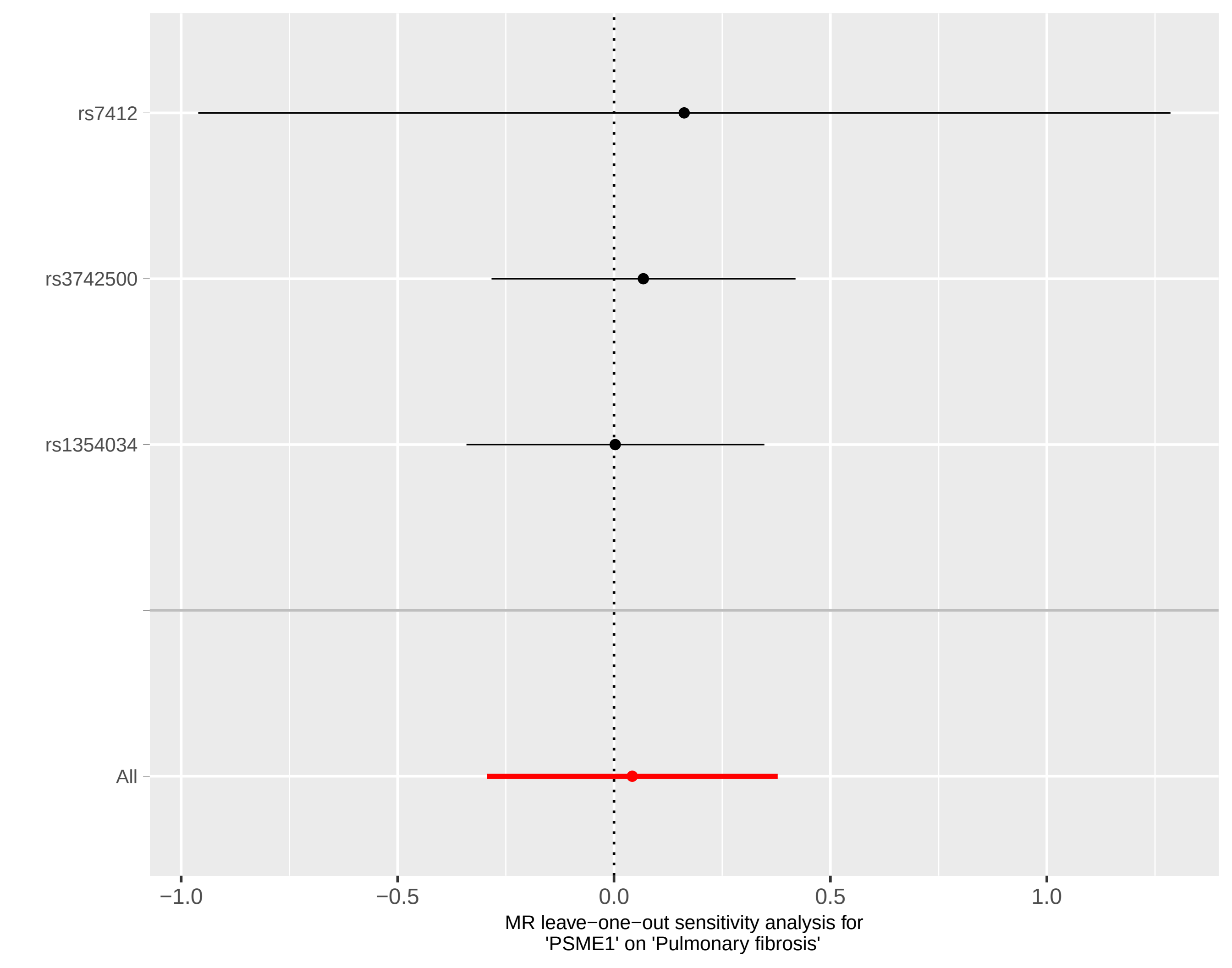
rs7412
rs3742500
rs1354034
All
−1.0
−0.5
0.0
0.5
1.0
MR leave−one−out sensitivity analysis for
'PSME1' on 'Pulmonary fibrosis'

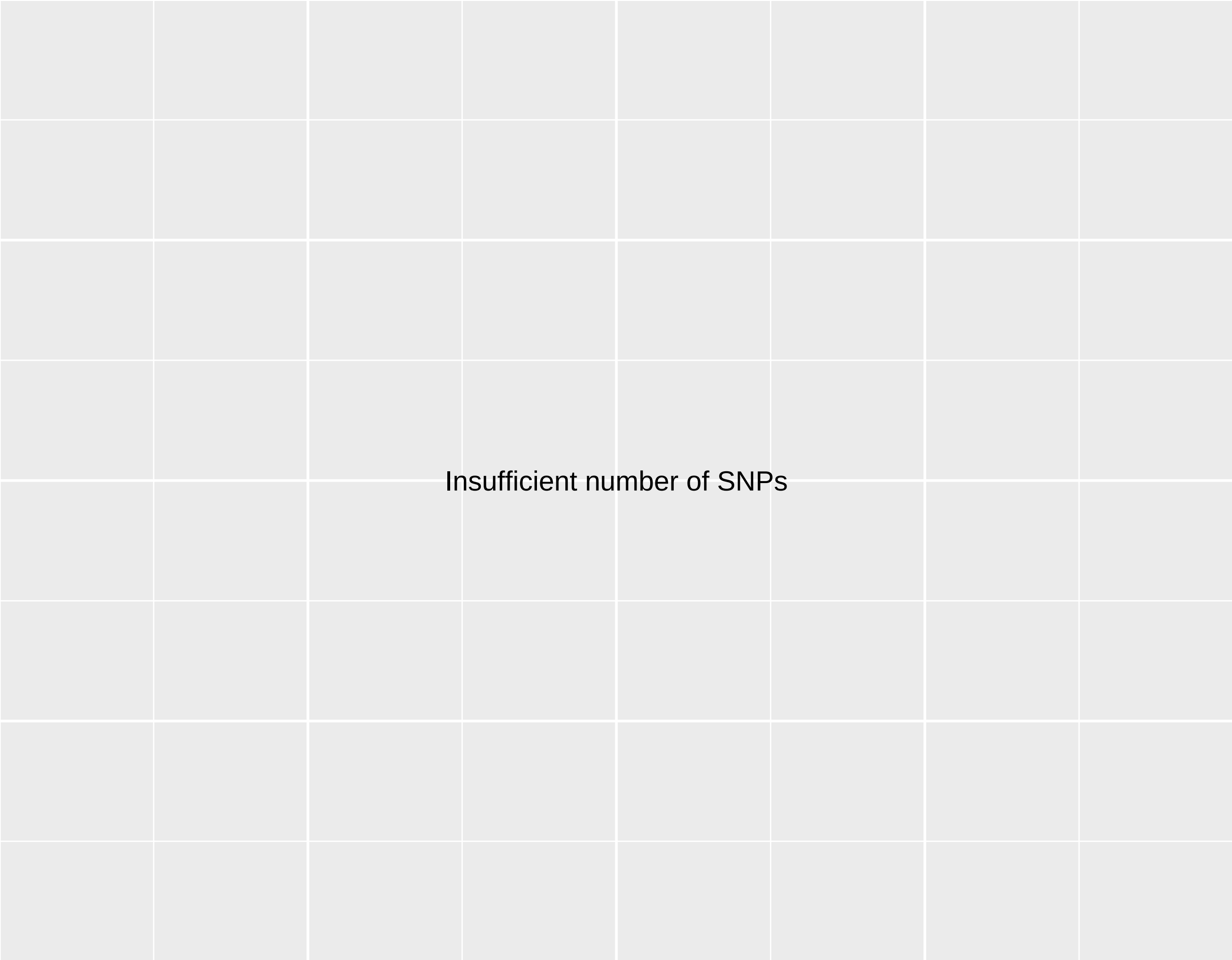
Insufficient number of SNPs

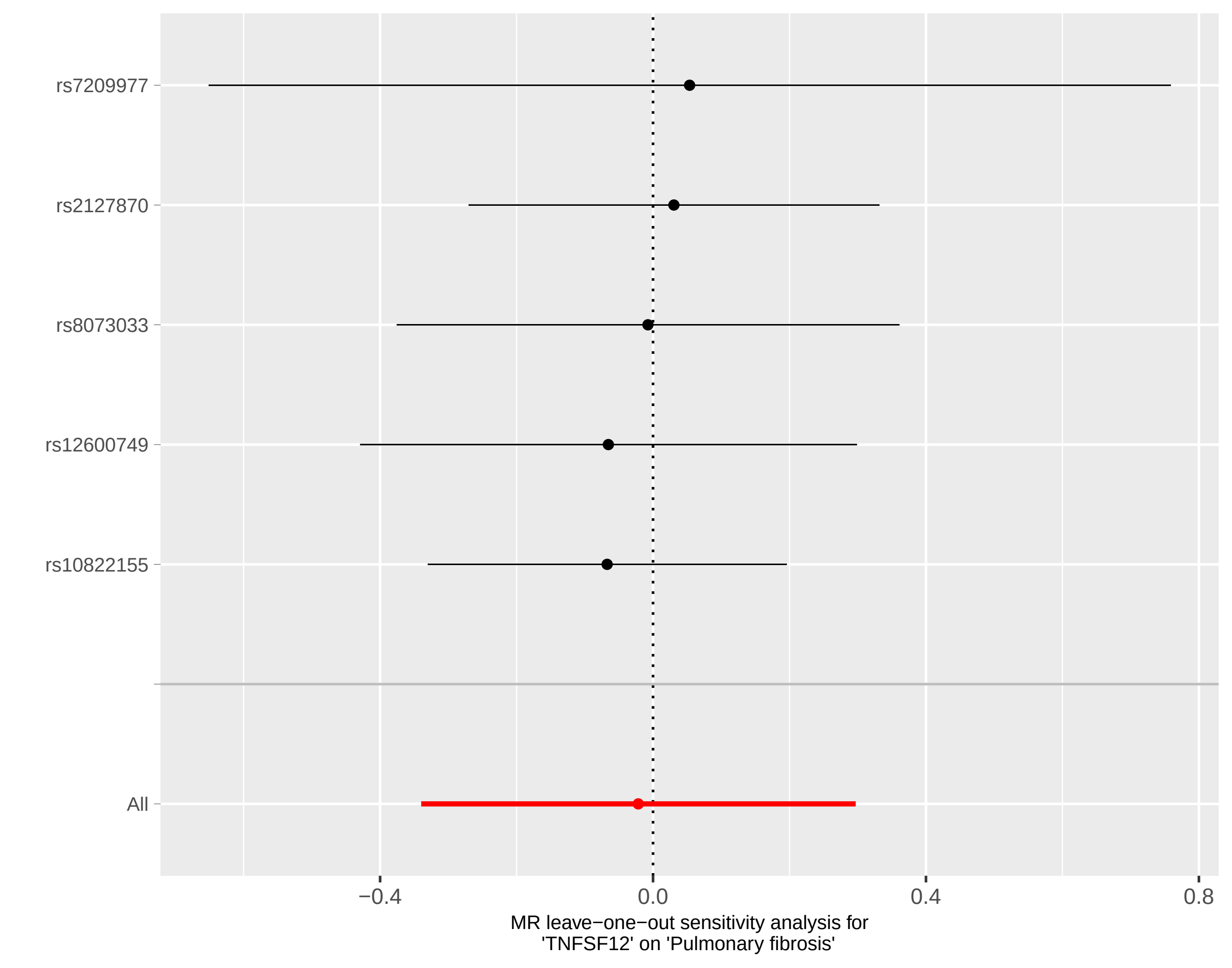
rs7209977
rs2127870
rs8073033
rs12600749
rs10822155
All
−0.4
0.0
0.4
0.8
MR leave−one−out sensitivity analysis for
'TNFSF12' on 'Pulmonary fibrosis'

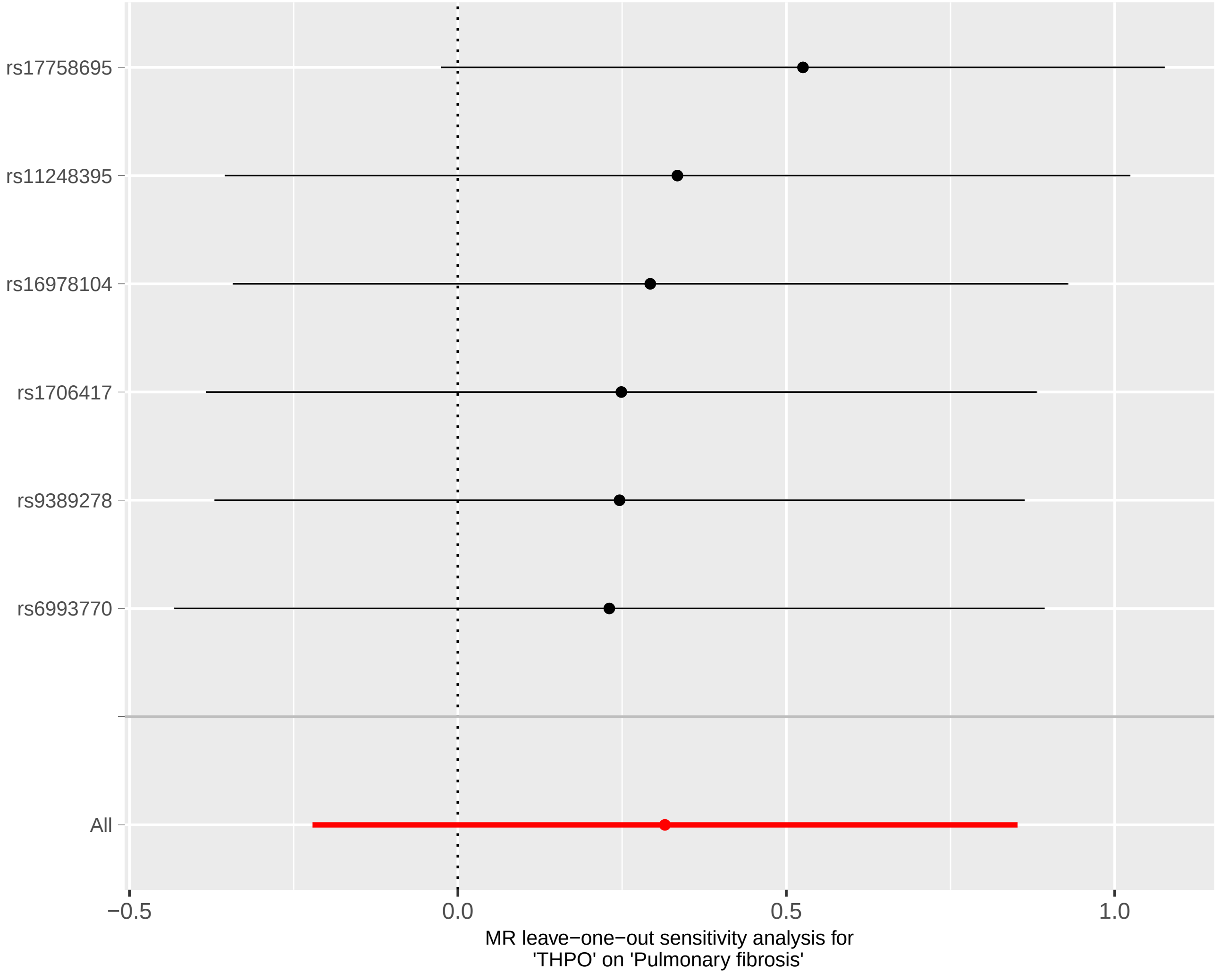
rs17758695
rs11248395
rs16978104
rs1706417
rs9389278
rs6993770
All
−0.5
0.0
0.5
1.0
MR leave−one−out sensitivity analysis for
'THPO' on 'Pulmonary fibrosis'

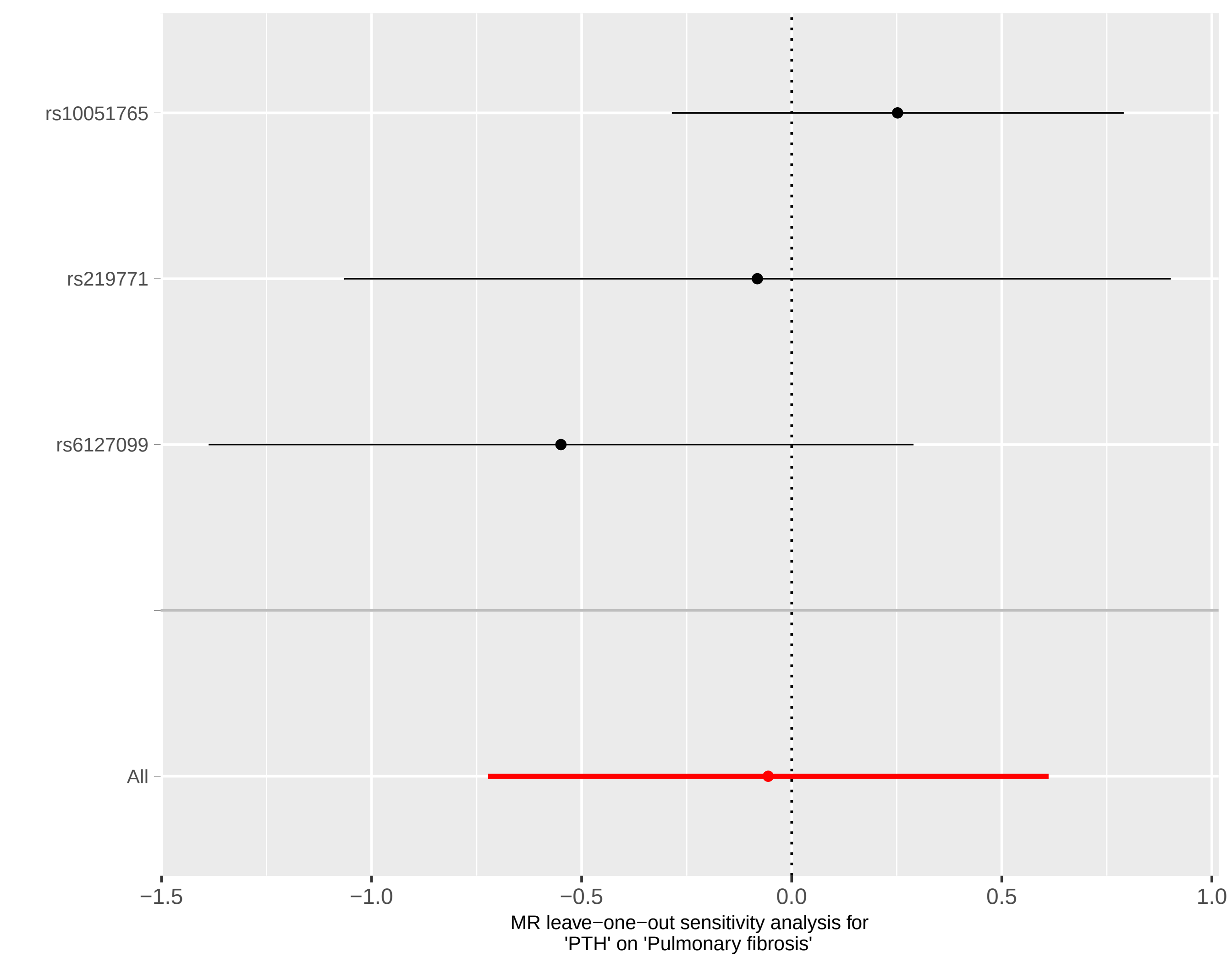
rs10051765
rs219771
rs6127099
All
−1.5
−1.0
−0.5
0.0
0.5
1.0
MR leave−one−out sensitivity analysis for
'PTH' on 'Pulmonary fibrosis'

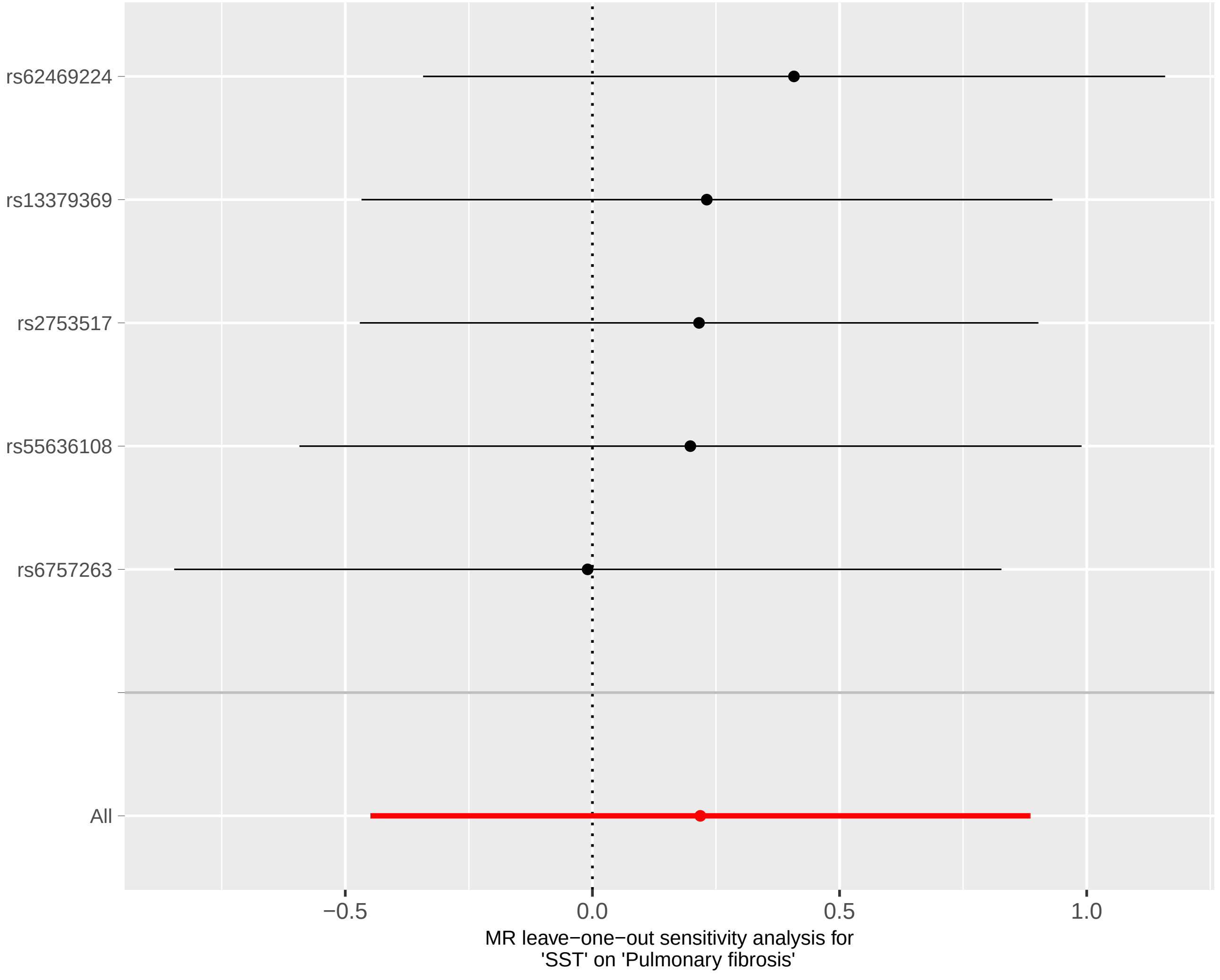
rs62469224
rs13379369
rs2753517
rs55636108
rs6757263
All
−0.5
0.0
0.5
1.0
MR leave−one−out sensitivity analysis for
'SST' on 'Pulmonary fibrosis'

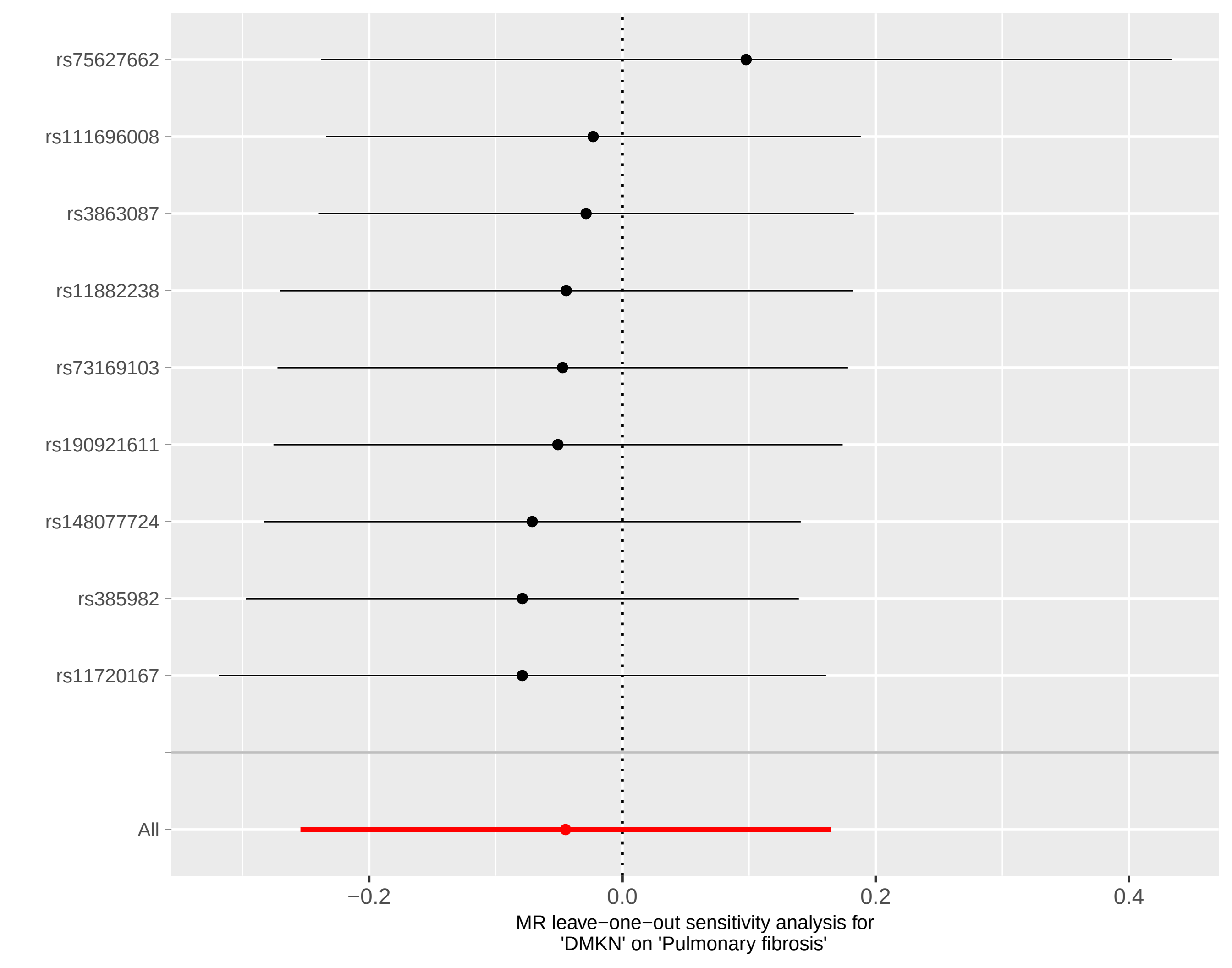
rs75627662
rs111696008
rs3863087
rs11882238
rs73169103
rs190921611
rs148077724
rs385982
rs11720167
All
−0.2
0.0
0.2
0.4
MR leave−one−out sensitivity analysis for
'DMKN' on 'Pulmonary fibrosis'

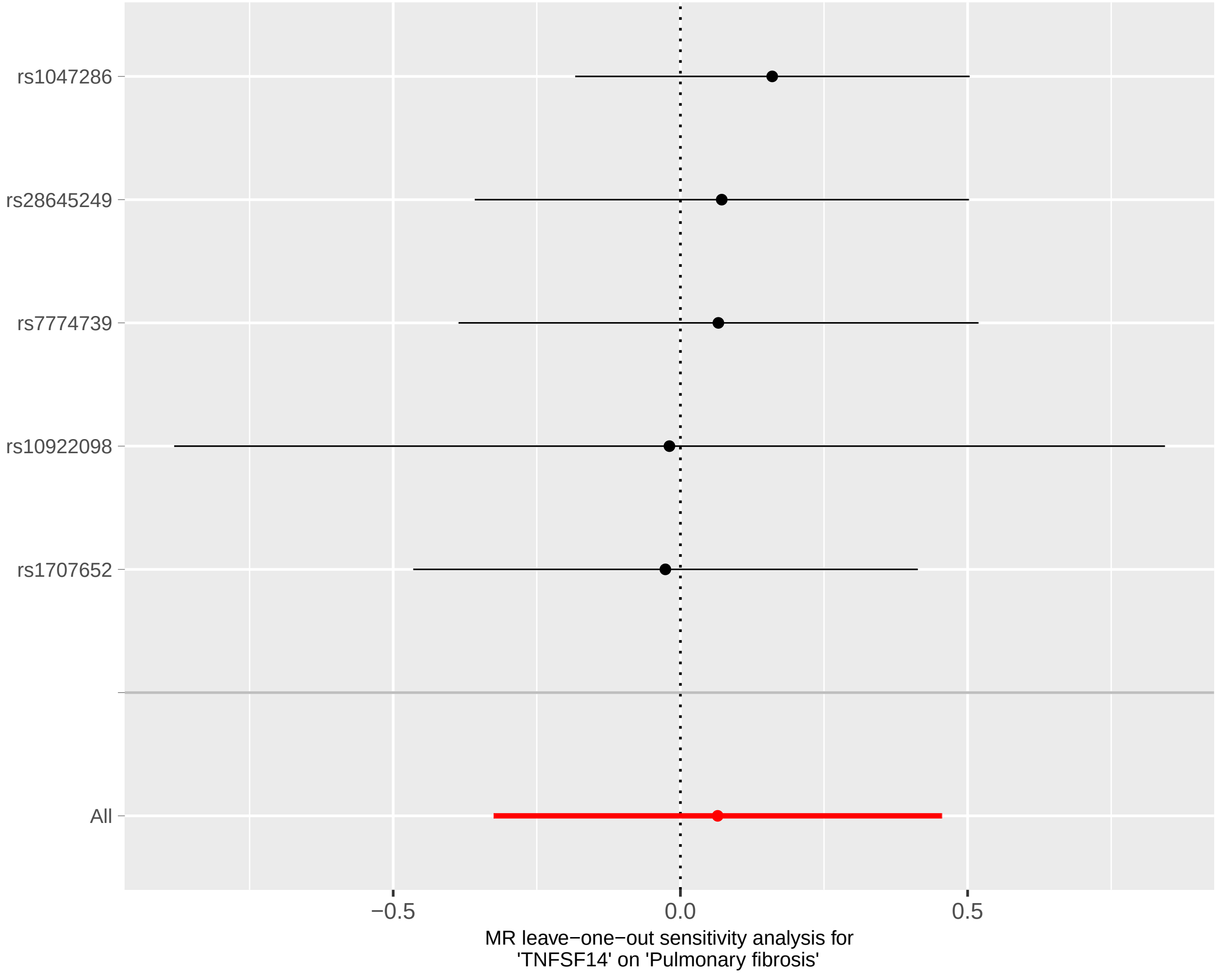
rs1047286
rs28645249
rs7774739
rs10922098
rs1707652
All
−0.5
0.0
0.5
MR leave−one−out sensitivity analysis for
'TNFSF14' on 'Pulmonary fibrosis'

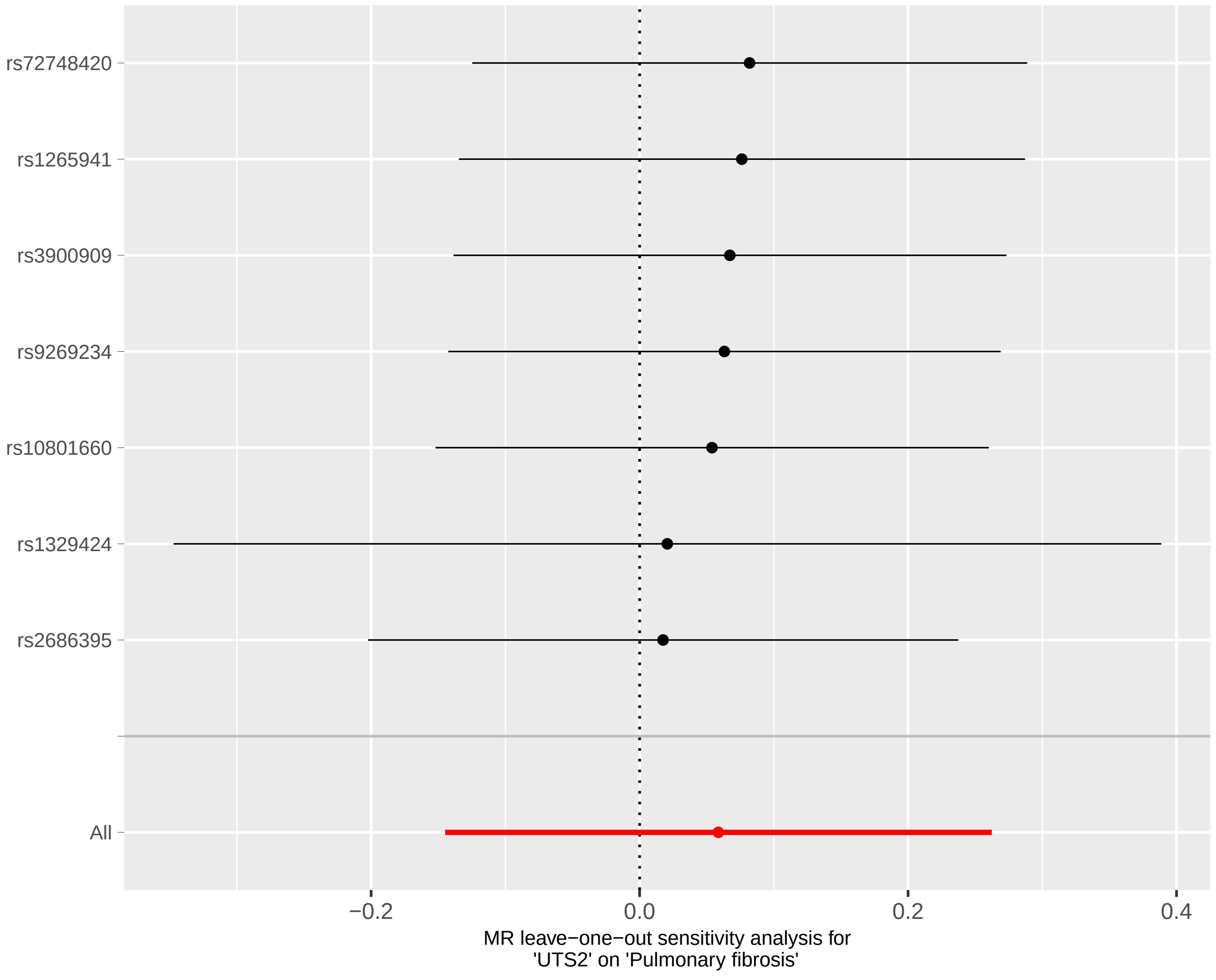
rs72748420
rs1265941
rs3900909
rs9269234
rs10801660
rs1329424
rs2686395
All
−0.2
0.0
0.2
0.4
MR leave−one−out sensitivity analysis for
'UTS2' on 'Pulmonary fibrosis'

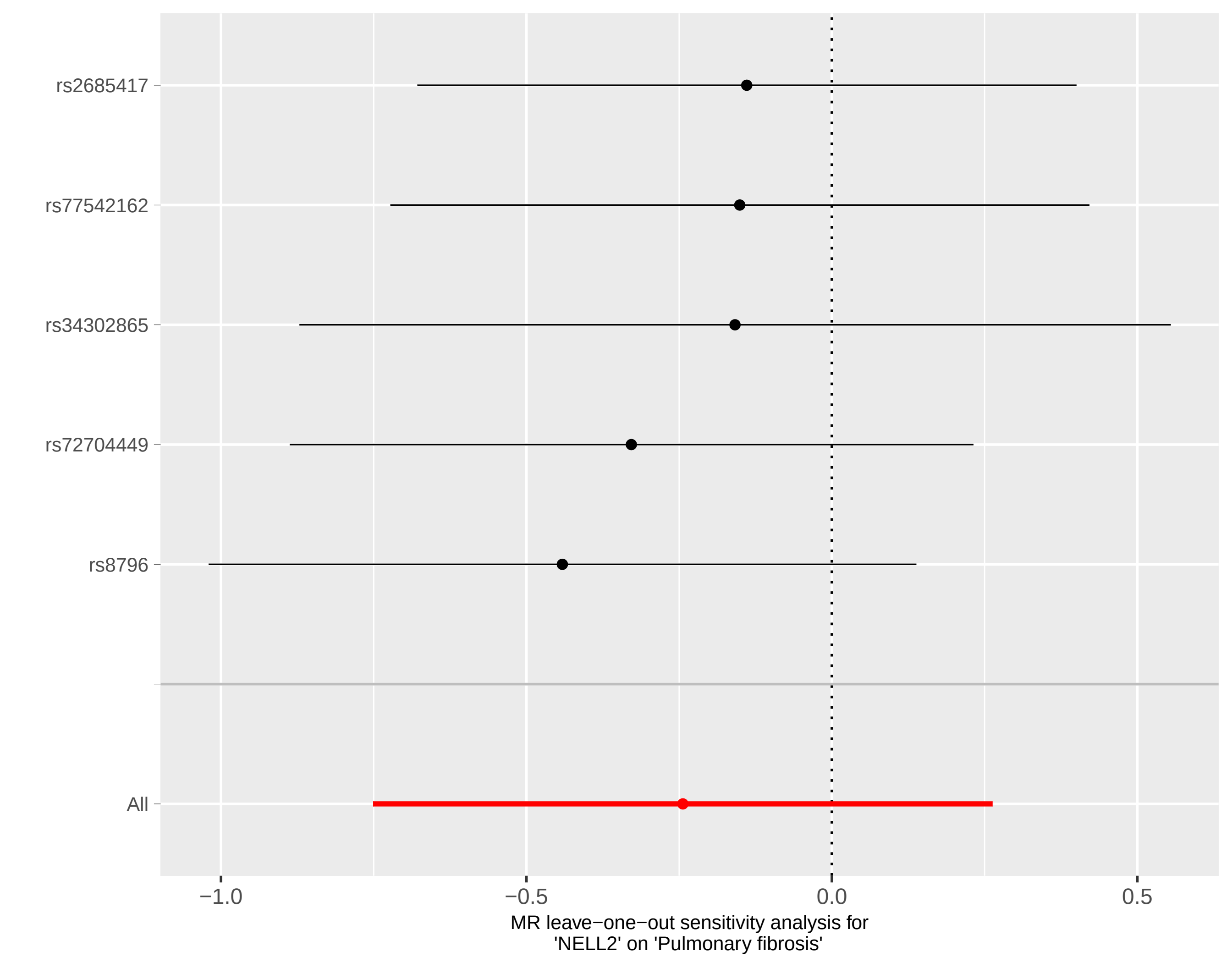
rs2685417
rs77542162
rs34302865
rs72704449
rs8796
All
−1.0
−0.5
0.0
0.5
MR leave−one−out sensitivity analysis for
'NELL2' on 'Pulmonary fibrosis'

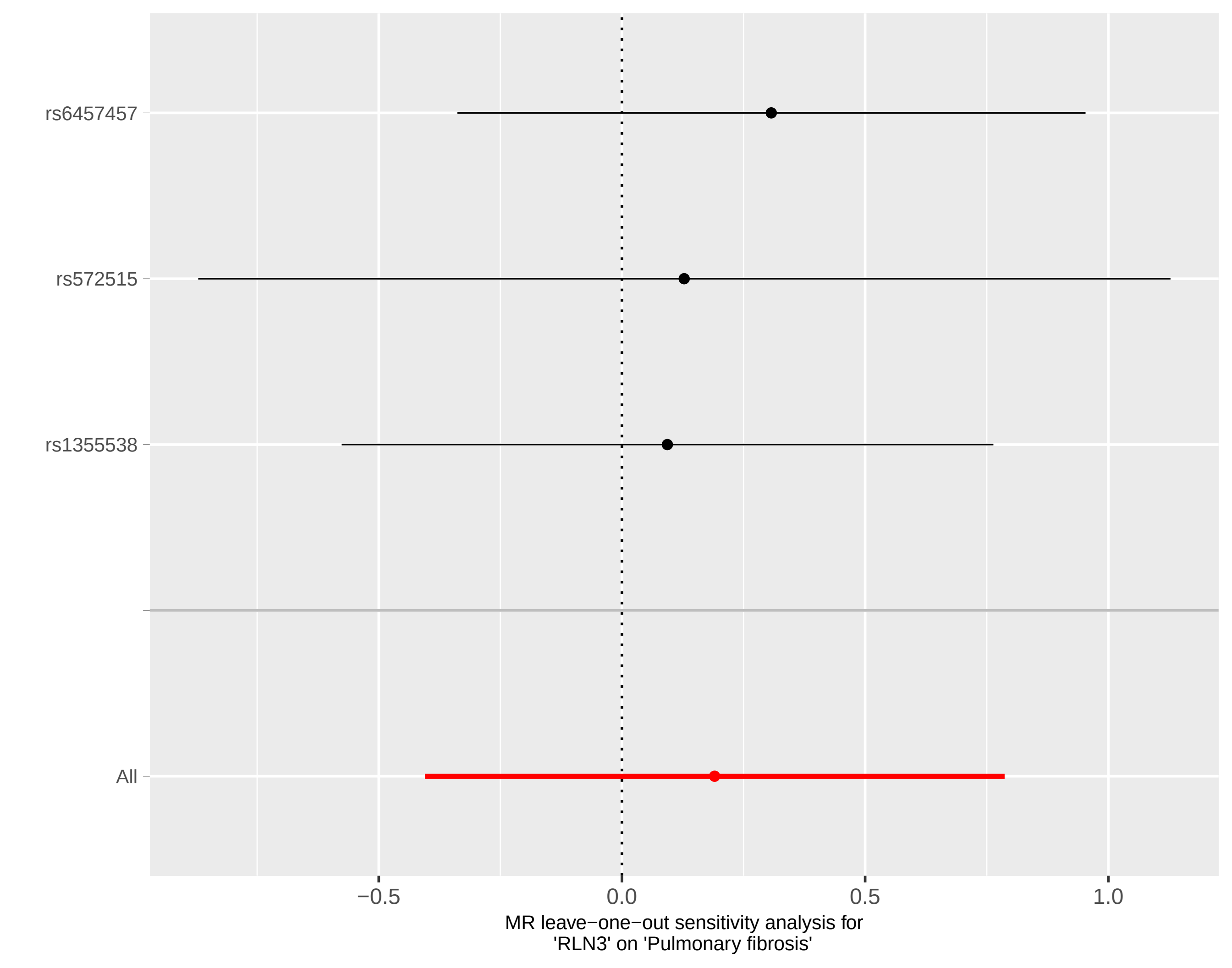
rs6457457
rs572515
rs1355538
All
−0.5
0.0
0.5
1.0
MR leave−one−out sensitivity analysis for
'RLN3' on 'Pulmonary fibrosis'

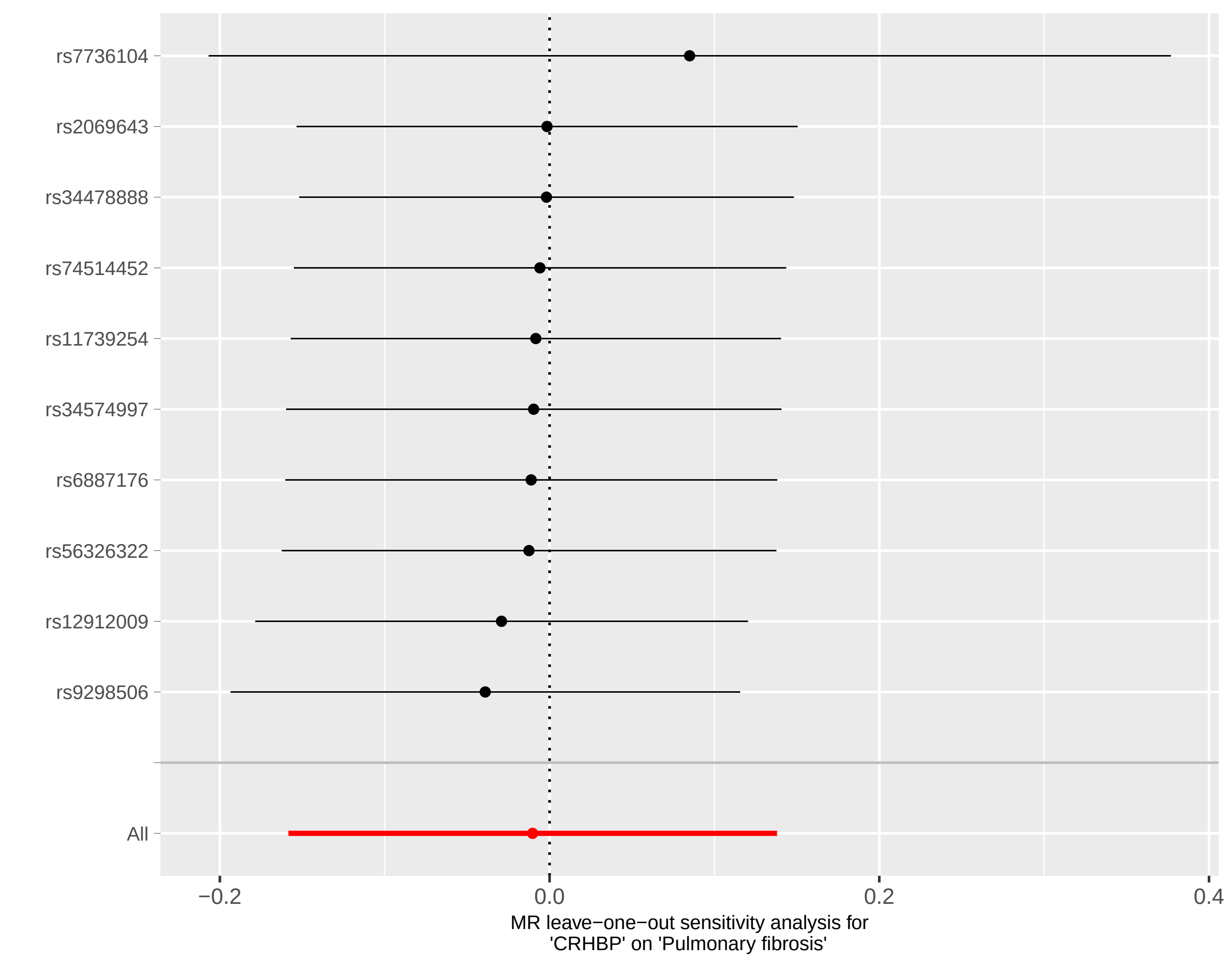
rs7736104
rs2069643
rs34478888
rs74514452
rs11739254
rs34574997
rs6887176
rs56326322
rs12912009
rs9298506
All
−0.2
0.0
0.2
0.4
MR leave−one−out sensitivity analysis for
'CRHBP' on 'Pulmonary fibrosis'

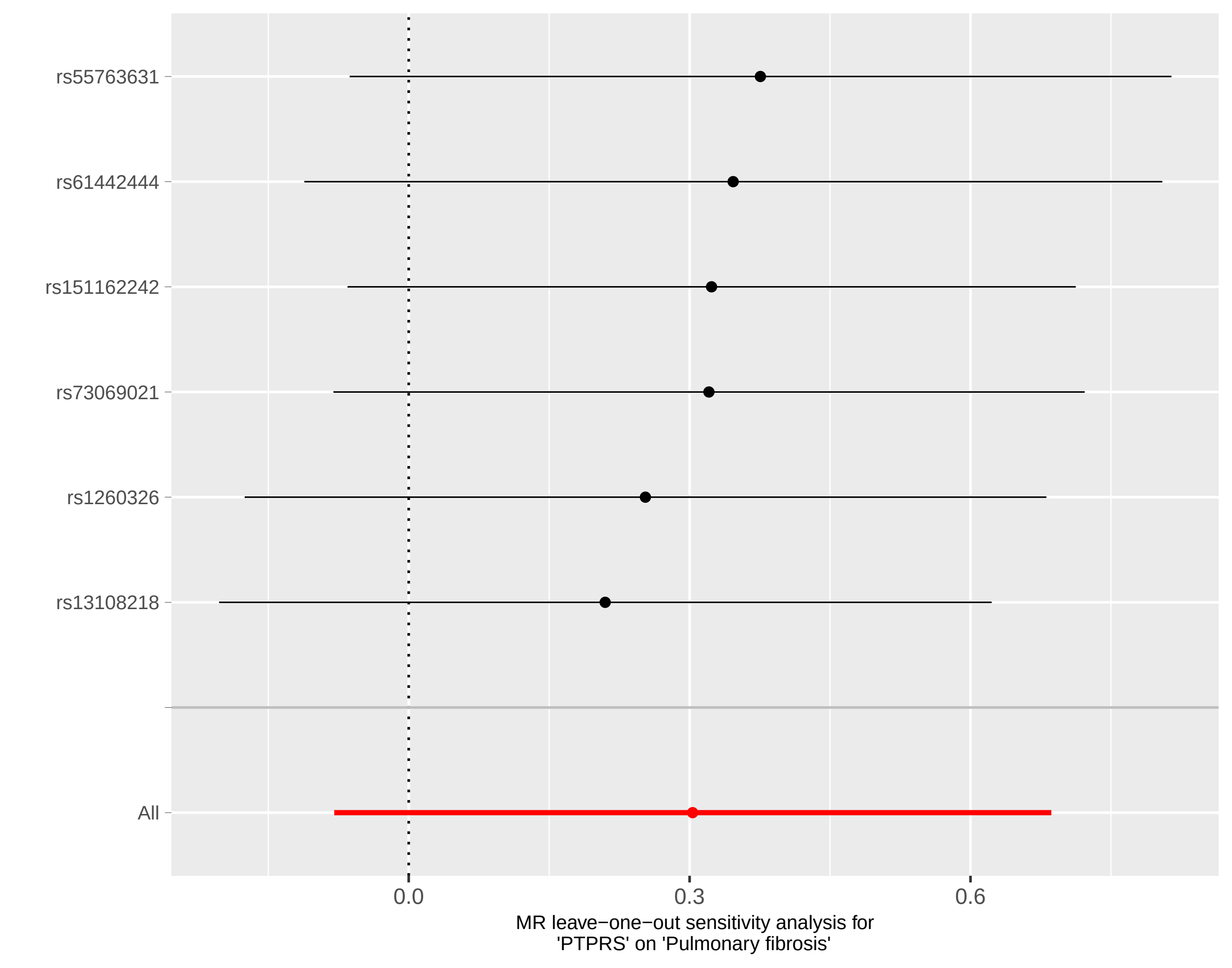
rs55763631
rs61442444
rs151162242
rs73069021
rs1260326
rs13108218
All
0.0
0.3
0.6
MR leave−one−out sensitivity analysis for
'PTPRS' on 'Pulmonary fibrosis'

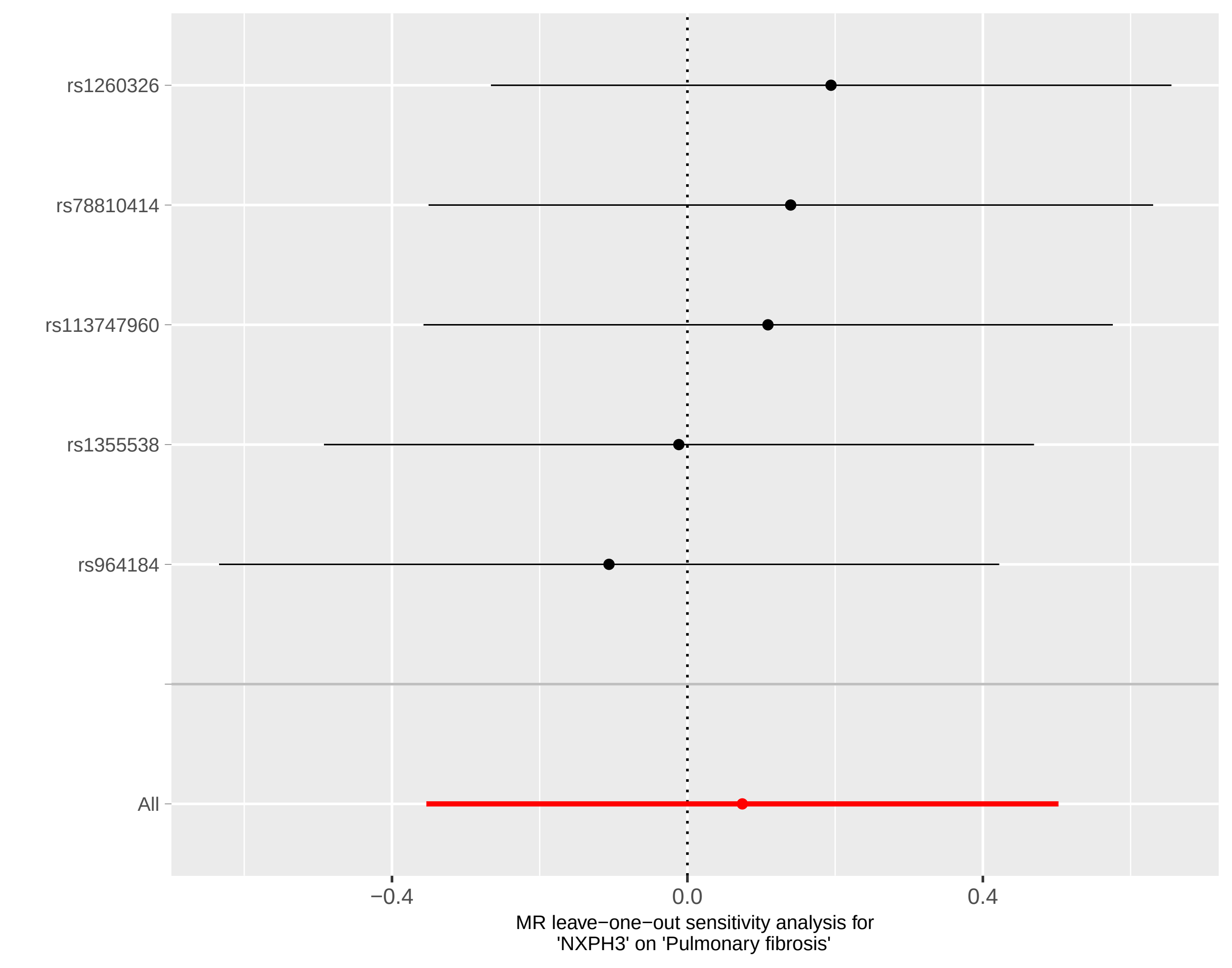
rs1260326
rs78810414
rs113747960
rs1355538
rs964184
All
−0.4
0.0
0.4
MR leave−one−out sensitivity analysis for
'NXPH3' on 'Pulmonary fibrosis'

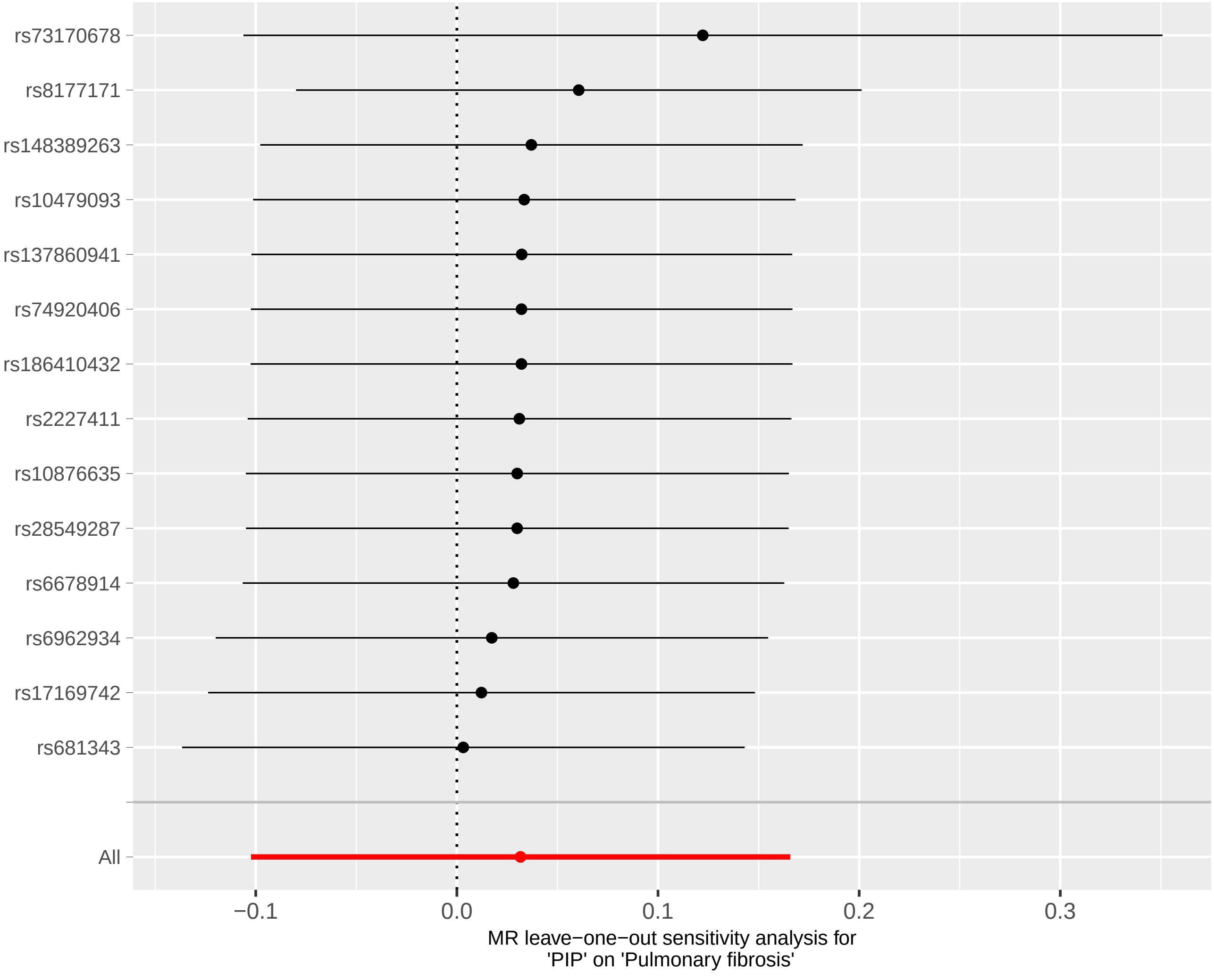
rs73170678
rs8177171
rs148389263
rs10479093
rs137860941
rs74920406
rs186410432
rs2227411
rs10876635
rs28549287
rs6678914
rs6962934
rs17169742
rs681343
All
−0.1
0.0
0.1
0.2
0.3
MR leave−one−out sensitivity analysis for
'PIP' on 'Pulmonary fibrosis'

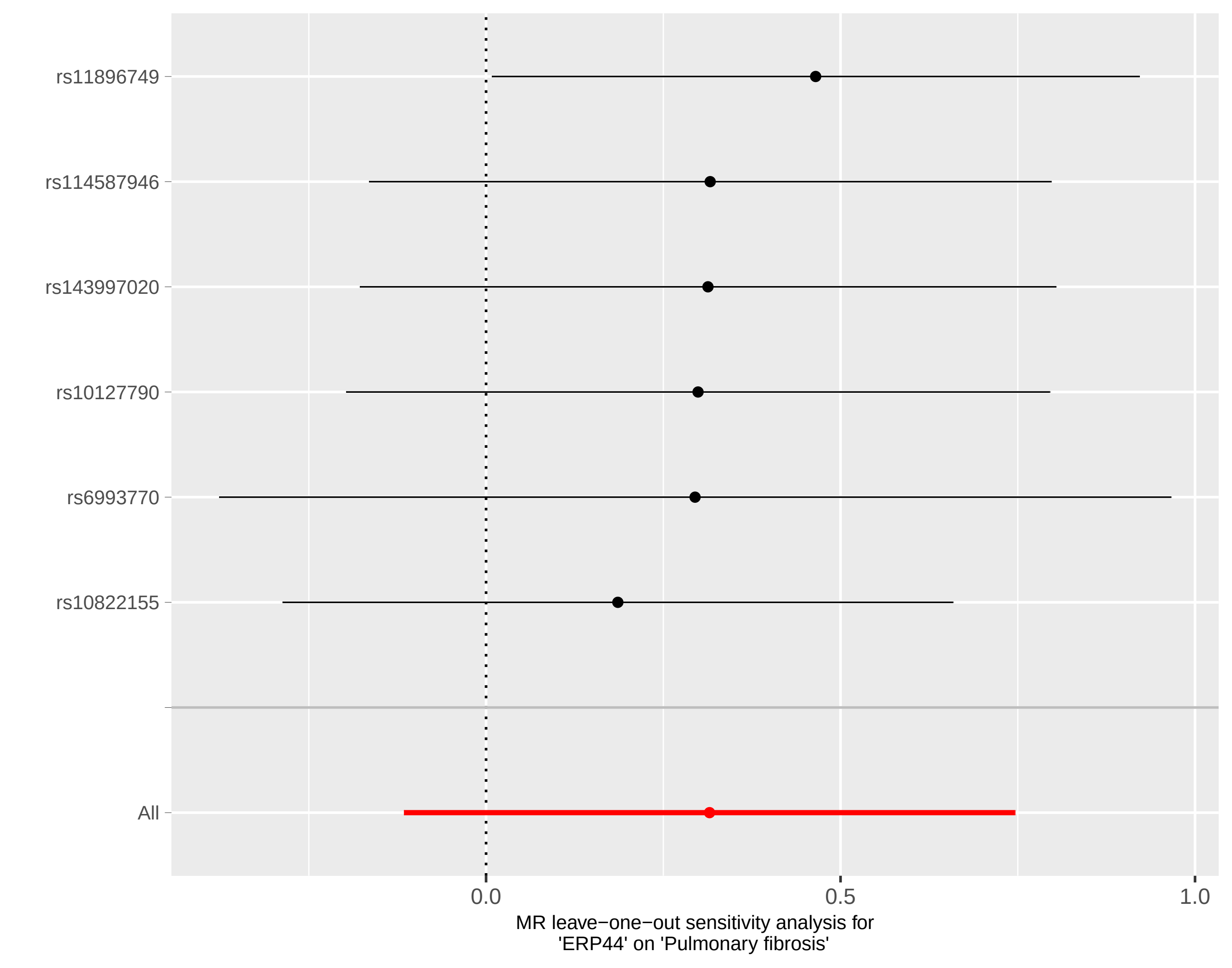
rs11896749
rs114587946
rs143997020
rs10127790
rs6993770
rs10822155
All
0.0
0.5
1.0
MR leave−one−out sensitivity analysis for
'ERP44' on 'Pulmonary fibrosis'

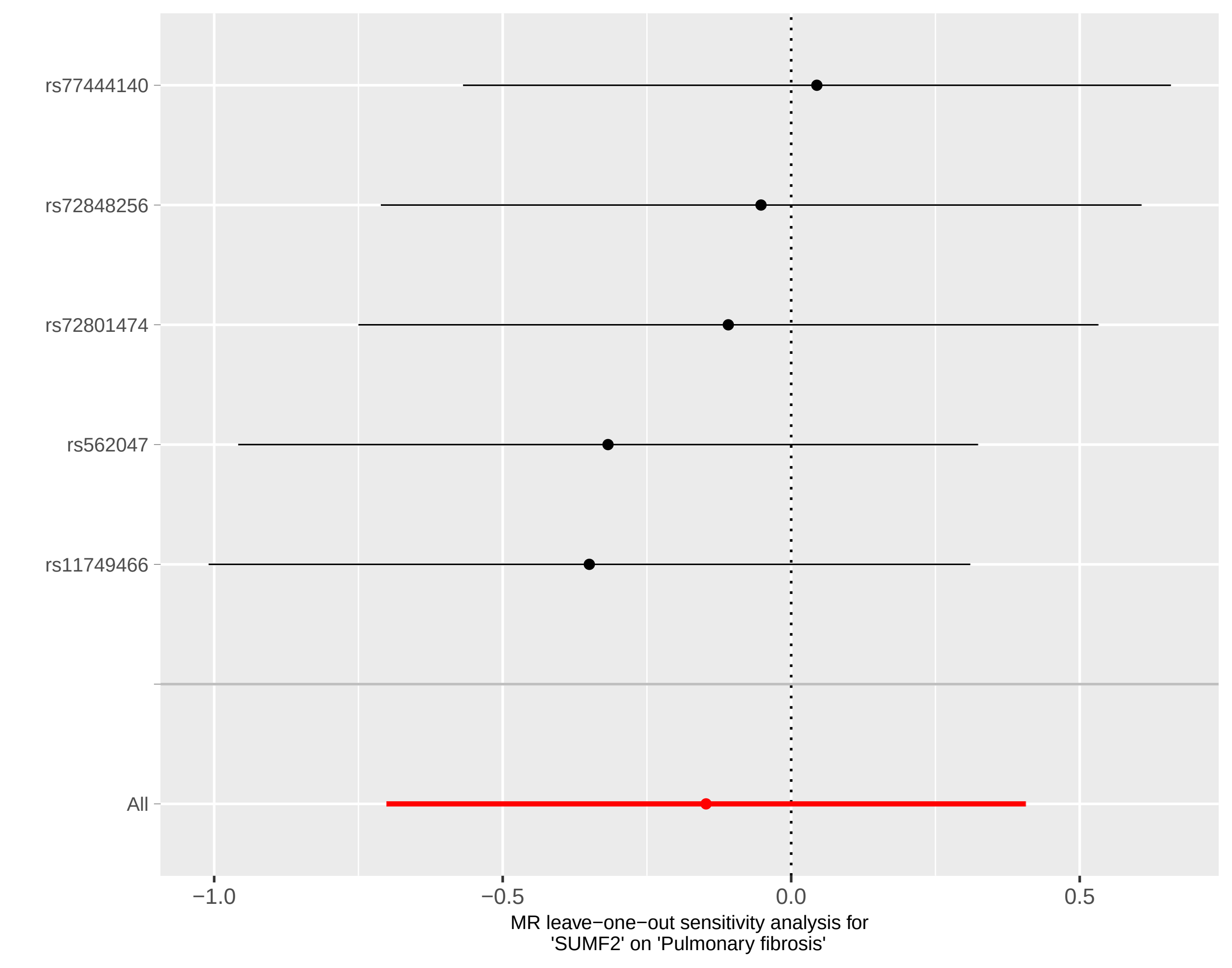
rs77444140
rs72848256
rs72801474
rs562047
rs11749466
All
−1.0
−0.5
0.0
0.5
MR leave−one−out sensitivity analysis for
'SUMF2' on 'Pulmonary fibrosis'

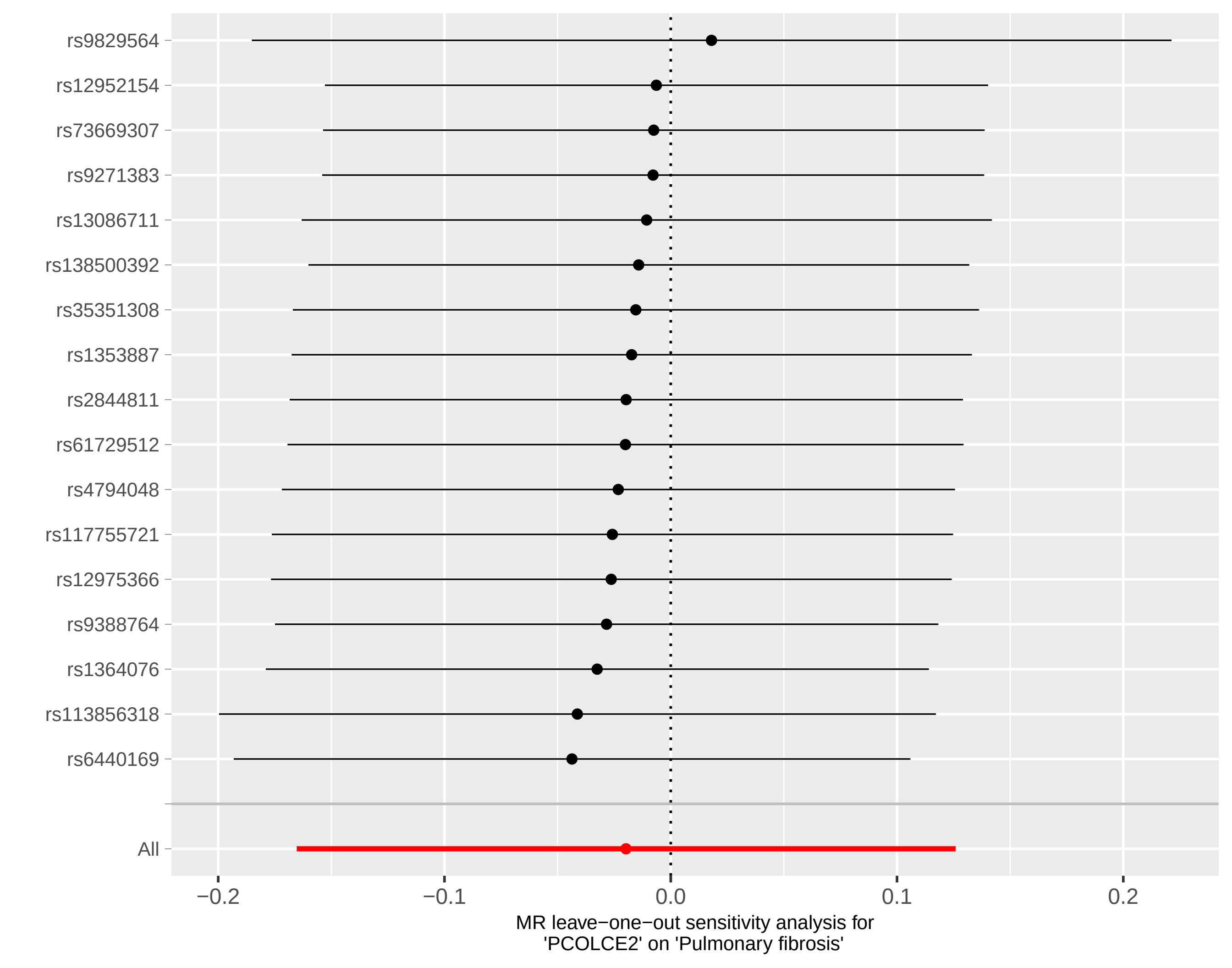
rs9829564
rs12952154
rs73669307
rs9271383
rs13086711
rs138500392
rs35351308
rs1353887
rs2844811
rs61729512
rs4794048
rs117755721
rs12975366
rs9388764
rs1364076
rs113856318
rs6440169
All
−0.2
−0.1
0.0
0.1
0.2
MR leave−one−out sensitivity analysis for
'PCOLCE2' on 'Pulmonary fibrosis'

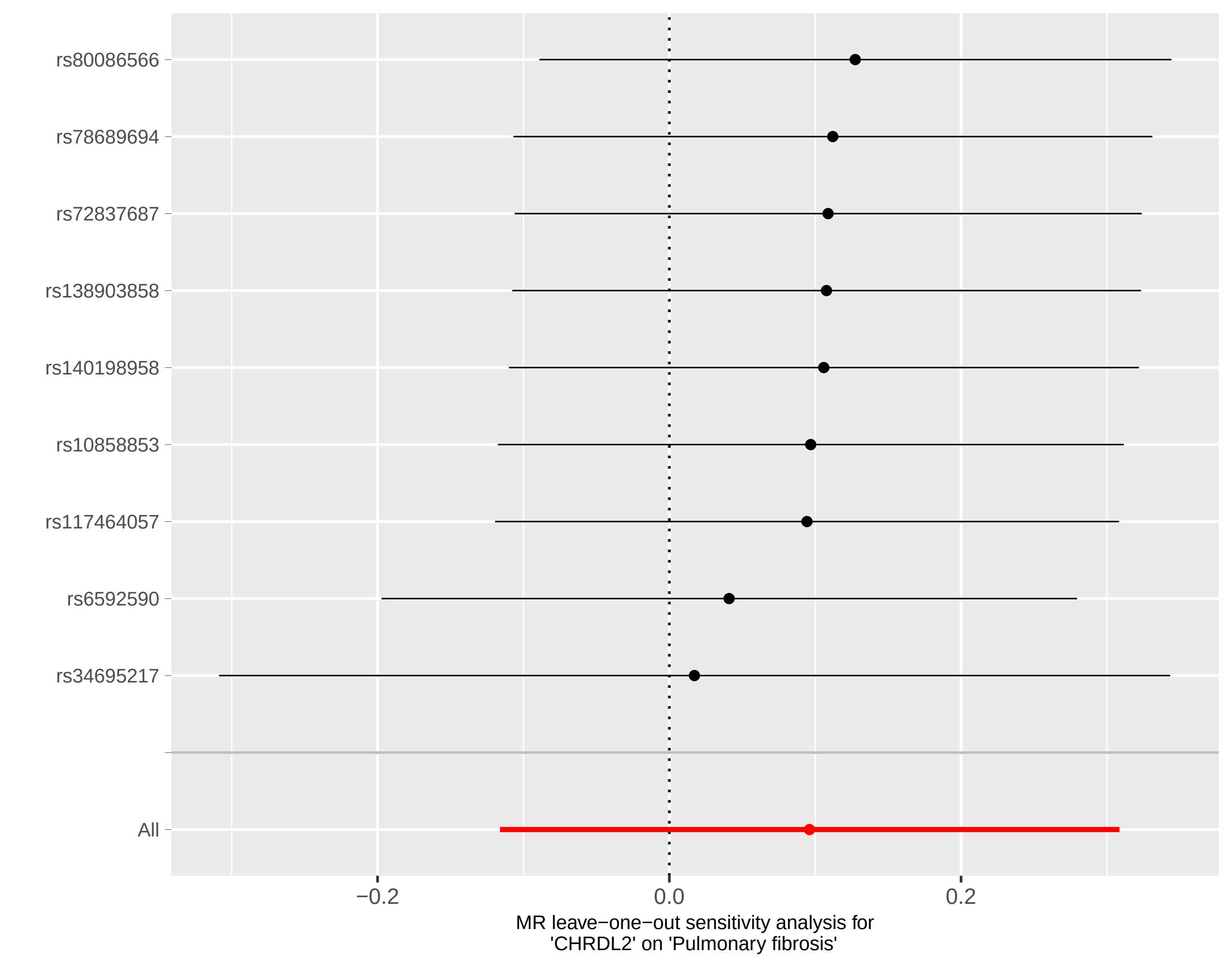
rs80086566
rs78689694
rs72837687
rs138903858
rs140198958
rs10858853
rs117464057
rs6592590
rs34695217
All
−0.2
0.0
0.2
MR leave−one−out sensitivity analysis for
'CHRDL2' on 'Pulmonary fibrosis'

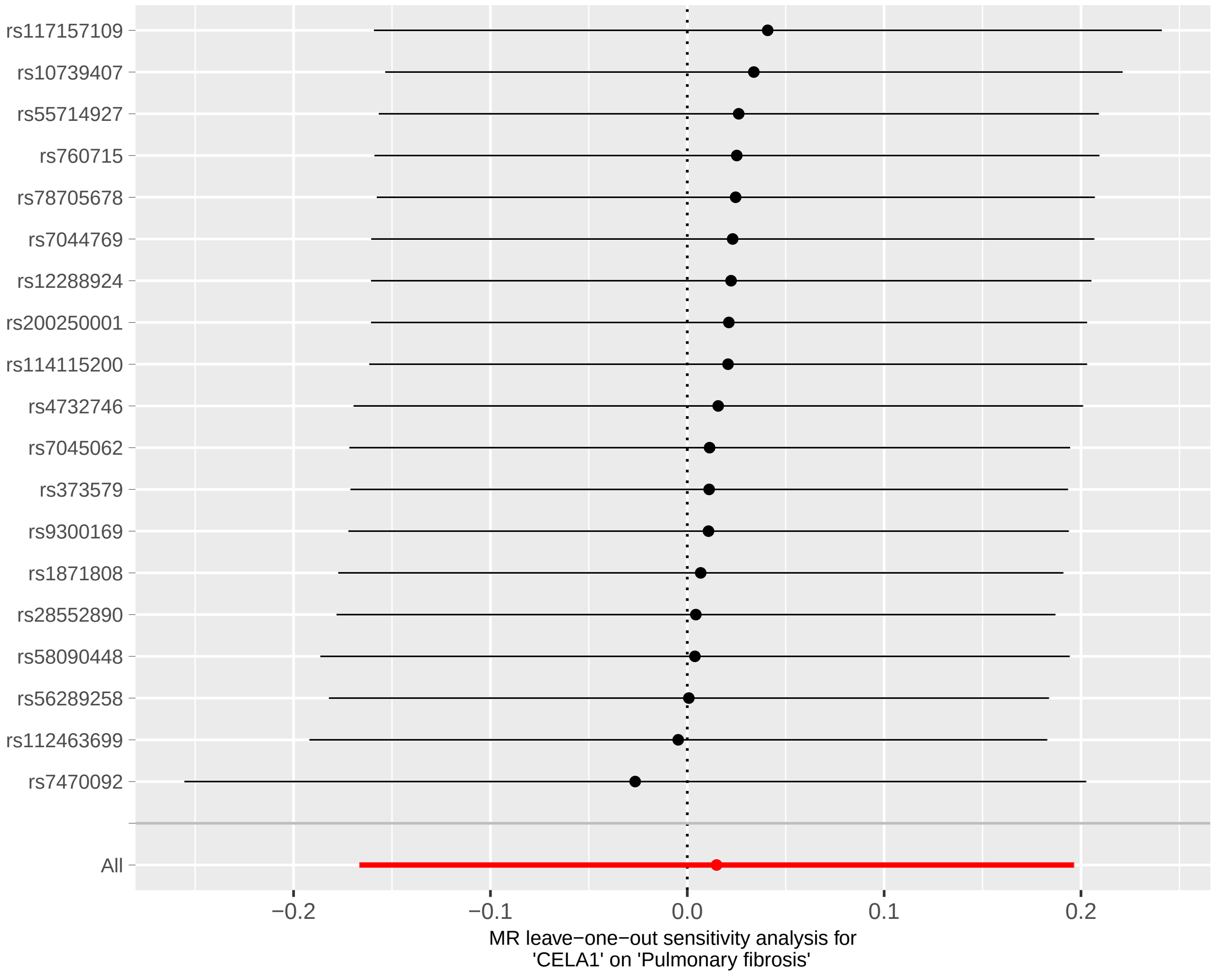
rs117157109
rs10739407
rs55714927
rs760715
rs78705678
rs7044769
rs12288924
rs200250001
rs114115200
rs4732746
rs7045062
rs373579
rs9300169
rs1871808
rs28552890
rs58090448
rs56289258
rs112463699
rs7470092
All
−0.2
−0.1
0.0
0.1
0.2
MR leave−one−out sensitivity analysis for
'CELA1' on 'Pulmonary fibrosis'

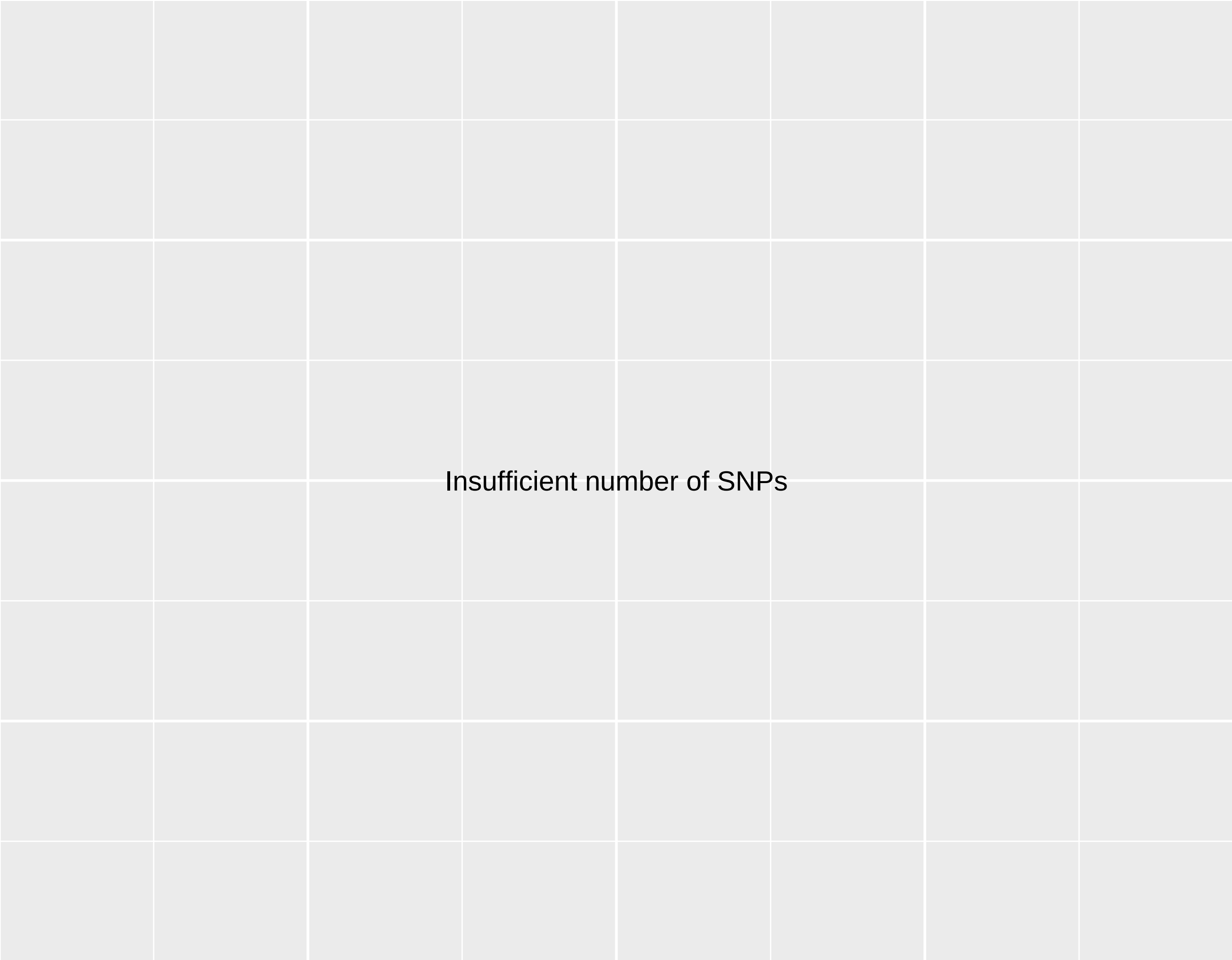
Insufficient number of SNPs

Insufficient number of SNPs

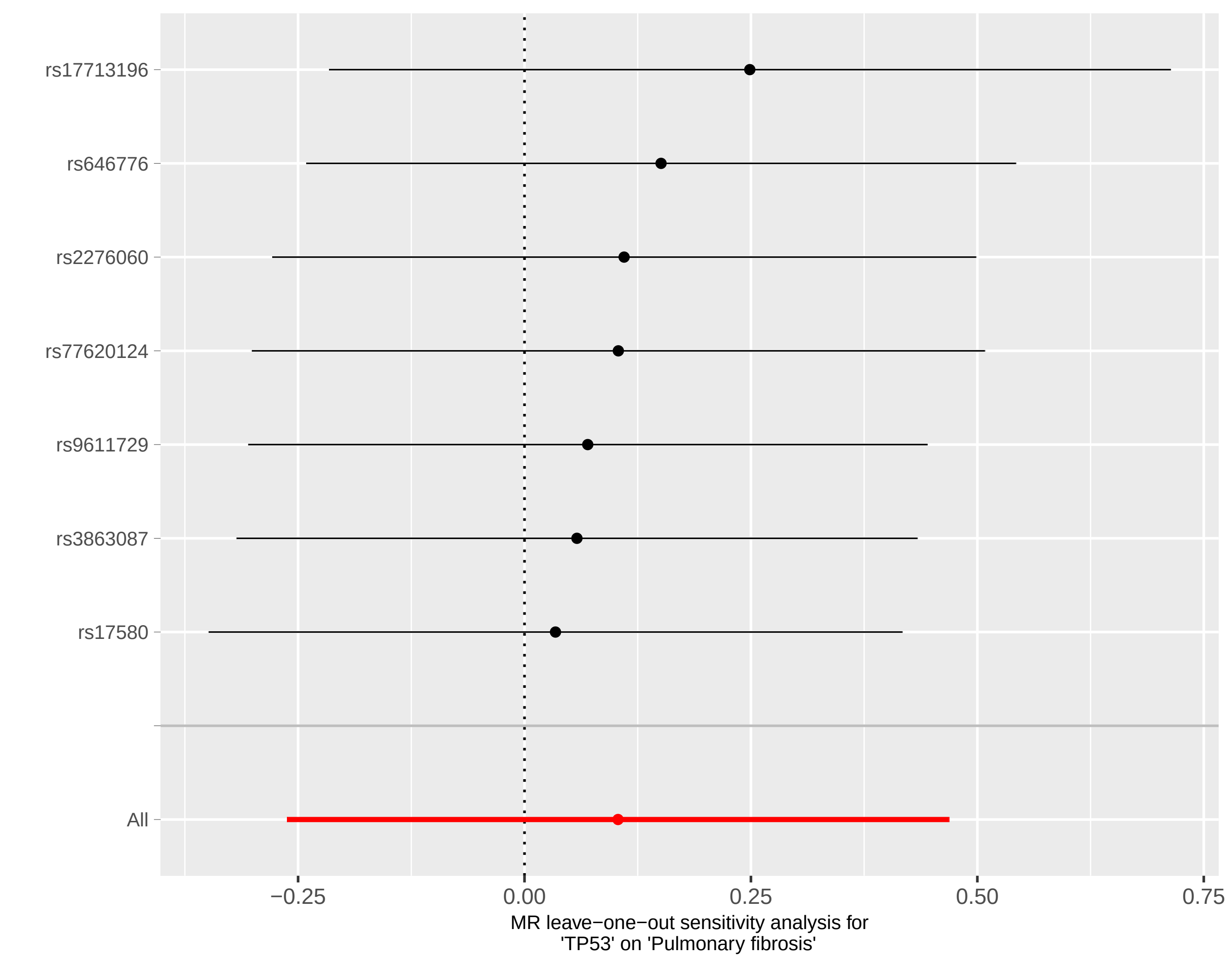
rs17713196
rs646776
rs2276060
rs77620124
rs9611729
rs3863087
rs17580
All
−0.25
0.00
0.25
0.50
0.75
MR leave−one−out sensitivity analysis for
'TP53' on 'Pulmonary fibrosis'

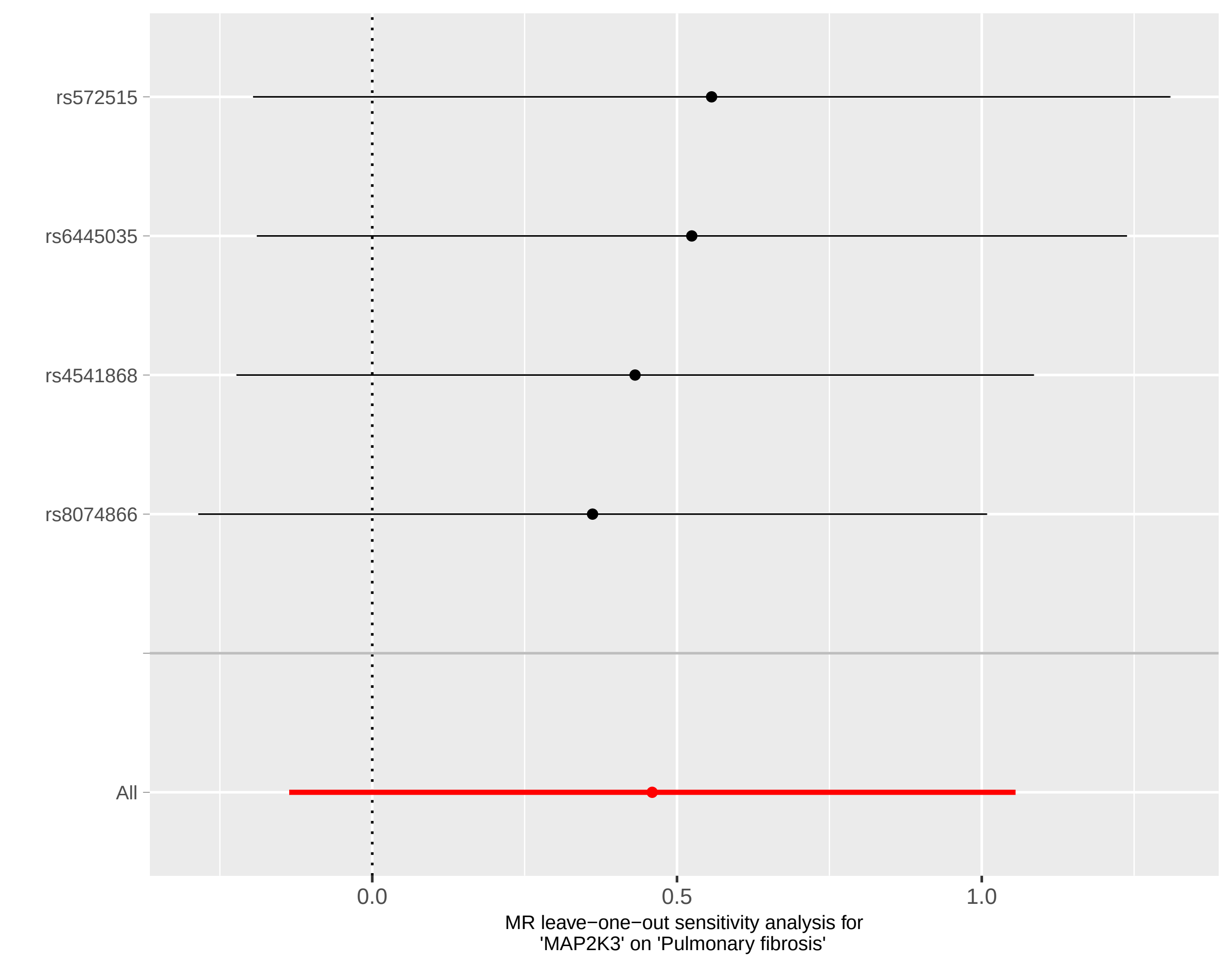
rs572515
rs6445035
rs4541868
rs8074866
All
0.0
0.5
1.0
MR leave−one−out sensitivity analysis for
'MAP2K3' on 'Pulmonary fibrosis'

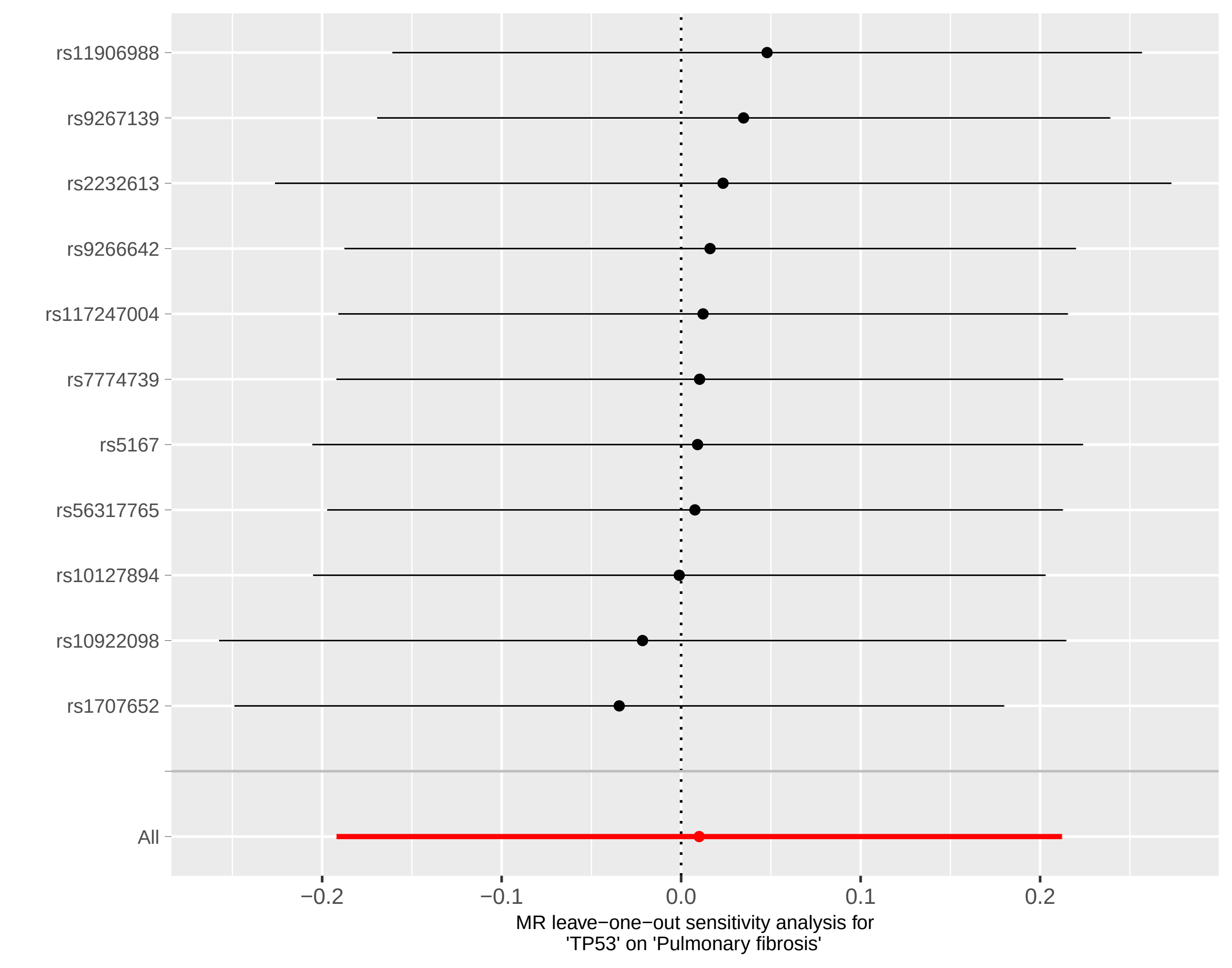
rs11906988
rs9267139
rs2232613
rs9266642
rs117247004
rs7774739
rs5167
rs56317765
rs10127894
rs10922098
rs1707652
All
−0.2
−0.1
0.0
0.1
0.2
MR leave−one−out sensitivity analysis for
'TP53' on 'Pulmonary fibrosis'

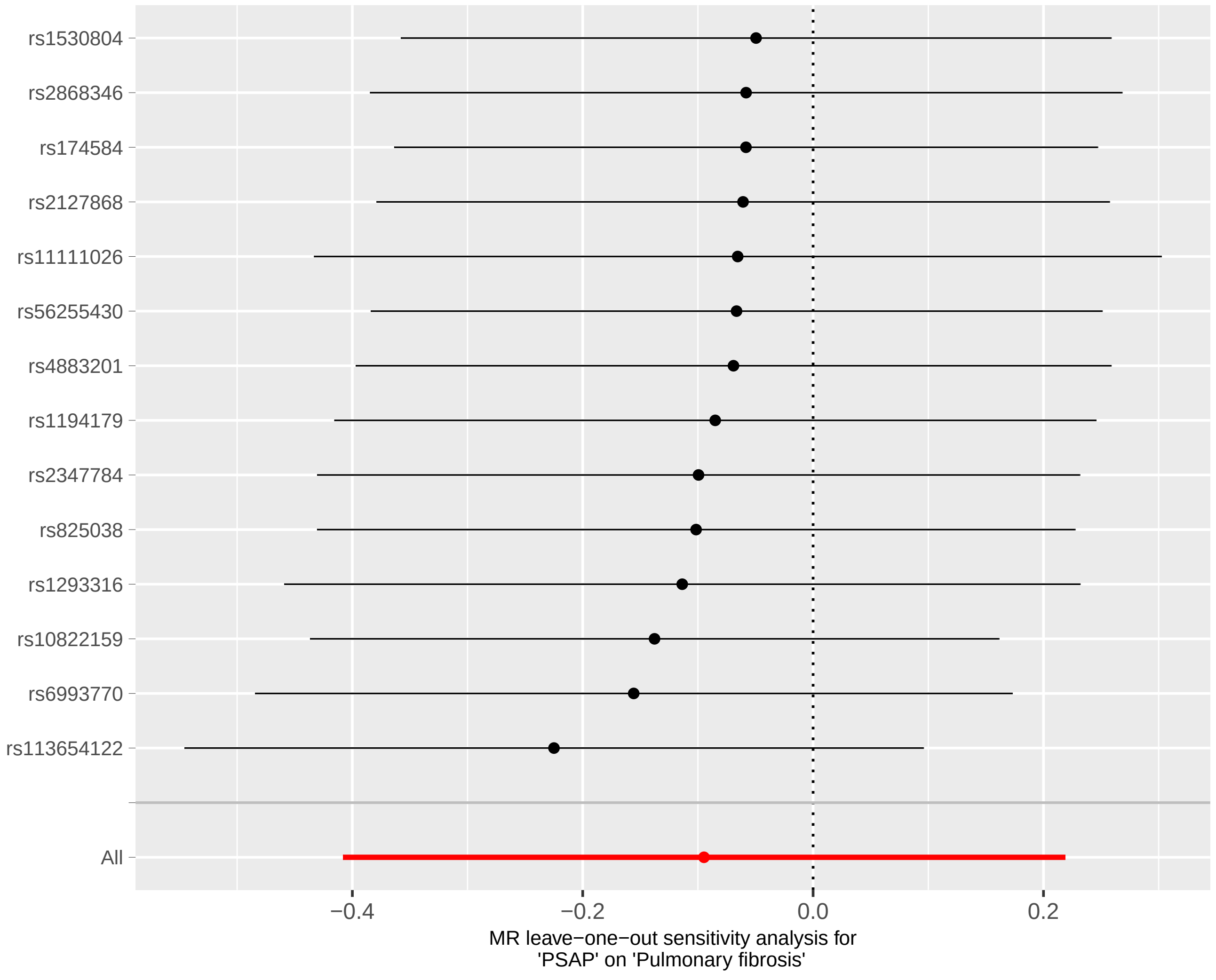

rs1530804
rs2868346
rs174584
rs2127868
rs11111026
rs56255430
rs4883201
rs1194179
rs2347784
rs825038
rs1293316
rs10822159
rs6993770
rs113654122
All
−0.4
−0.2
0.0
0.2
MR leave−one−out sensitivity analysis for
'PSAP' on 'Pulmonary fibrosis'

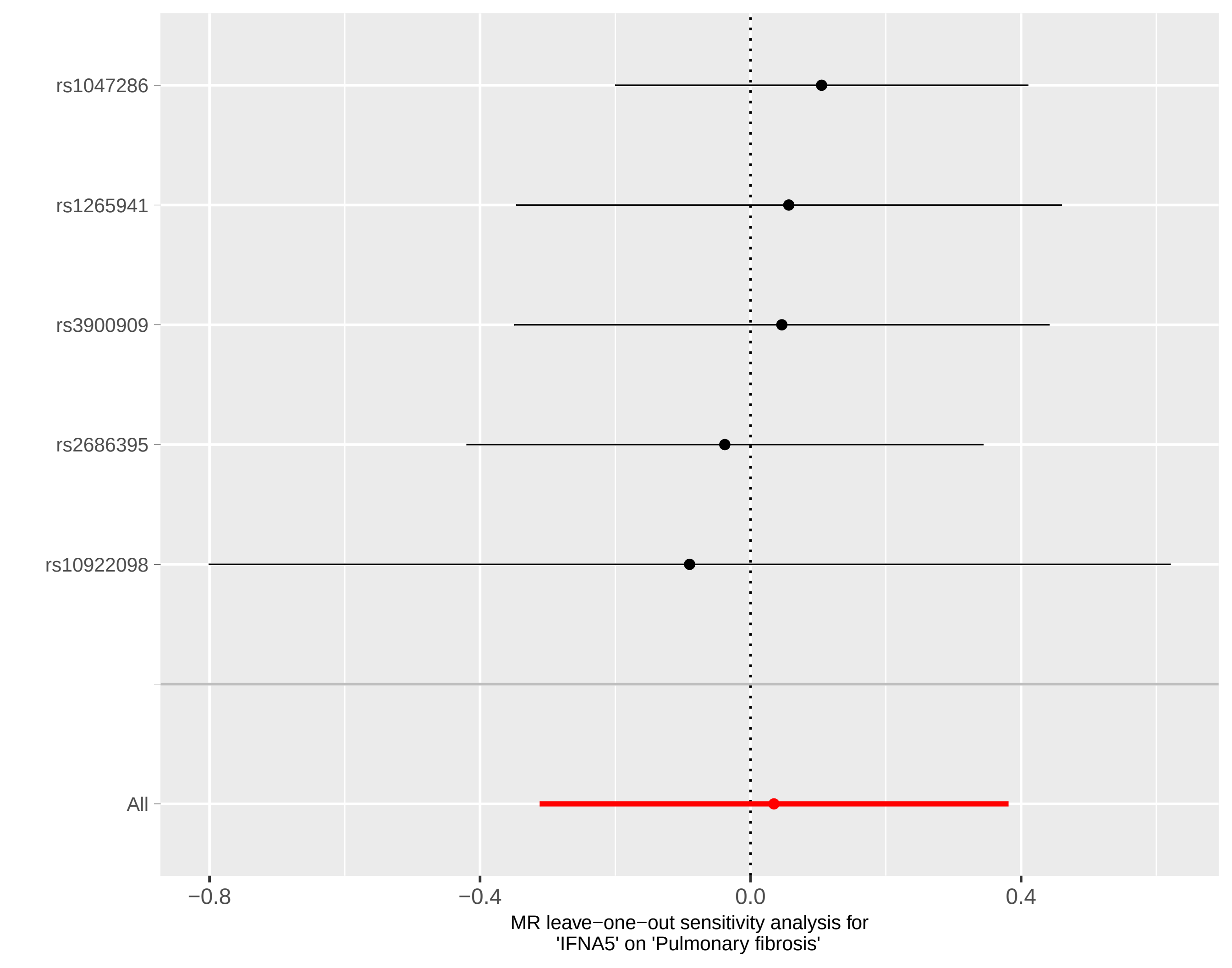

rs1047286
rs1265941
rs3900909
rs2686395
rs10922098
All
−0.8
−0.4
0.0
0.4
MR leave−one−out sensitivity analysis for
'IFNA5' on 'Pulmonary fibrosis'

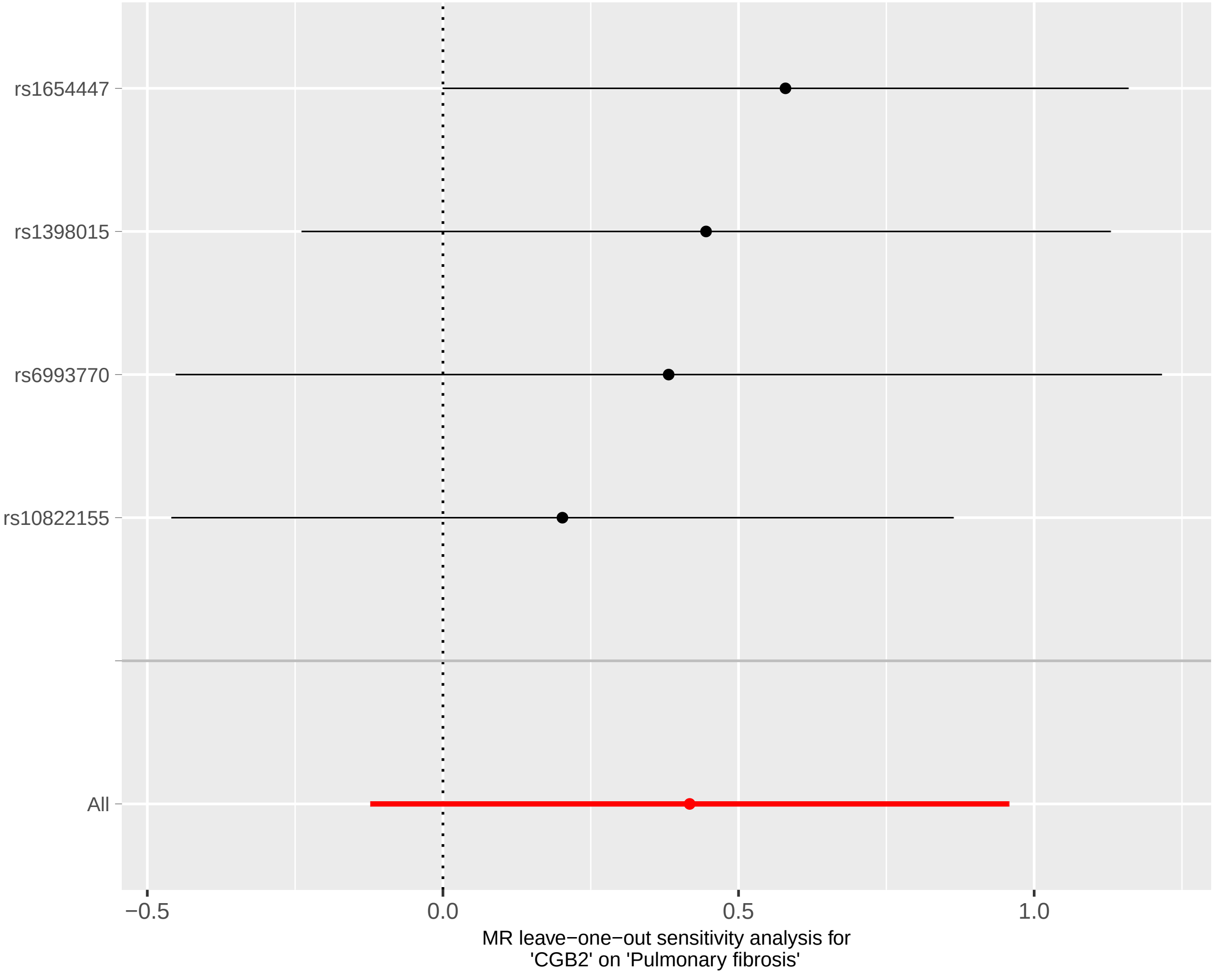
rs1654447
rs1398015
rs6993770
rs10822155
All
−0.5
0.0
0.5
1.0
MR leave−one−out sensitivity analysis for
'CGB2' on 'Pulmonary fibrosis'

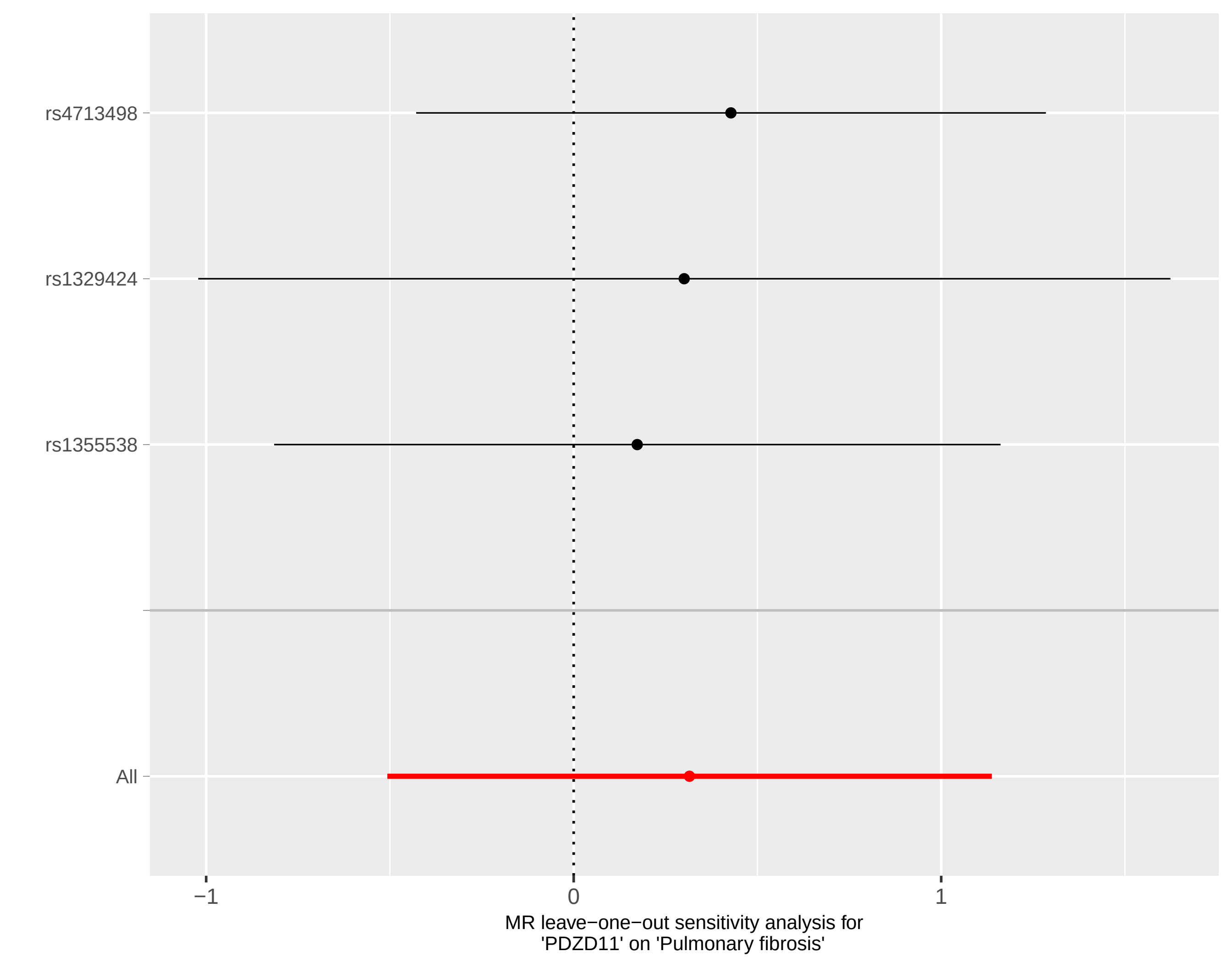
rs4713498
rs1329424
rs1355538
All
−1
0
1
MR leave−one−out sensitivity analysis for
'PDZD11' on 'Pulmonary fibrosis'

Insufficient number of SNPs

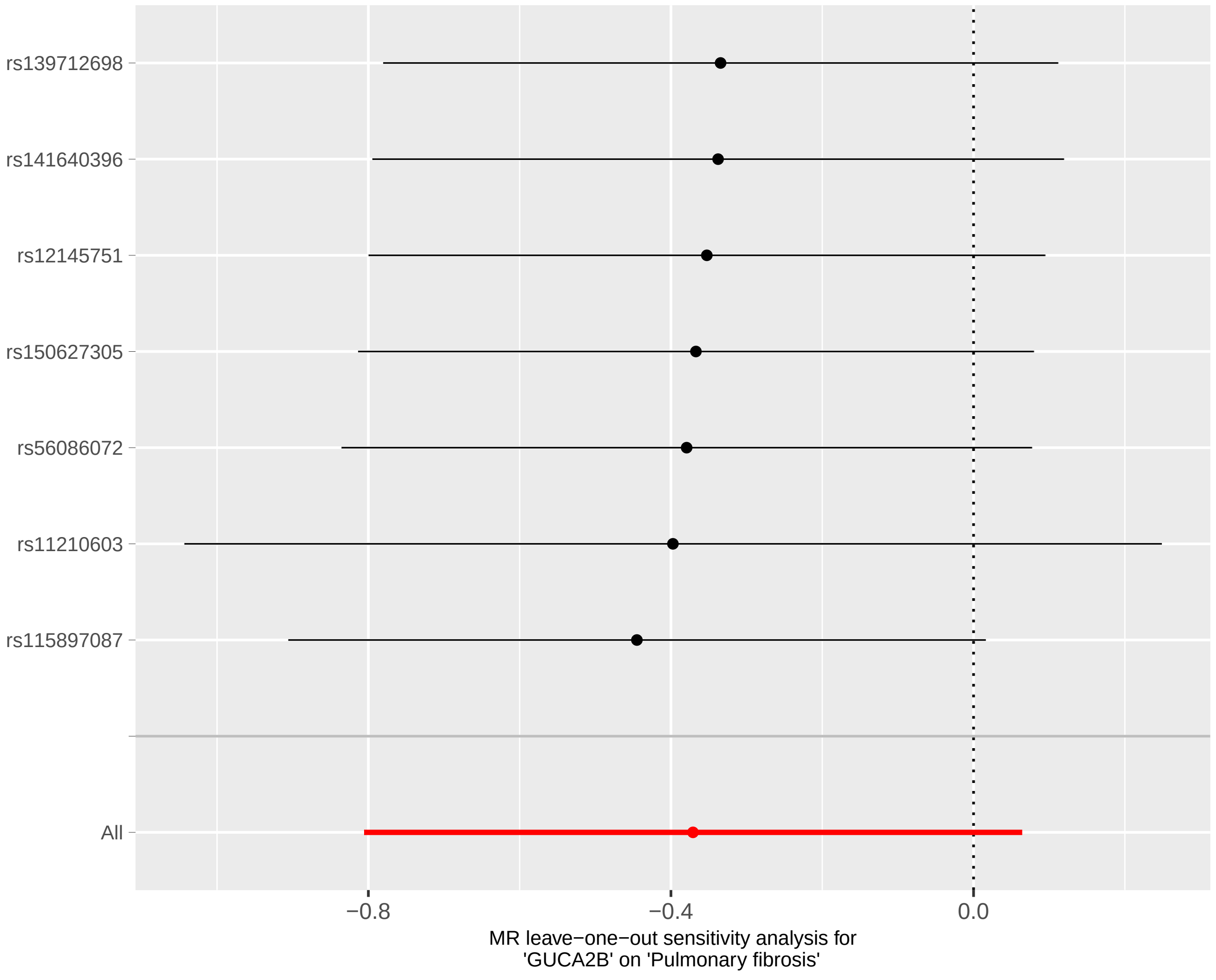
rs139712698
rs141640396
rs12145751
rs150627305
rs56086072
rs11210603
rs115897087
All
−0.8
−0.4
0.0
MR leave−one−out sensitivity analysis for
'GUCA2B' on 'Pulmonary fibrosis'

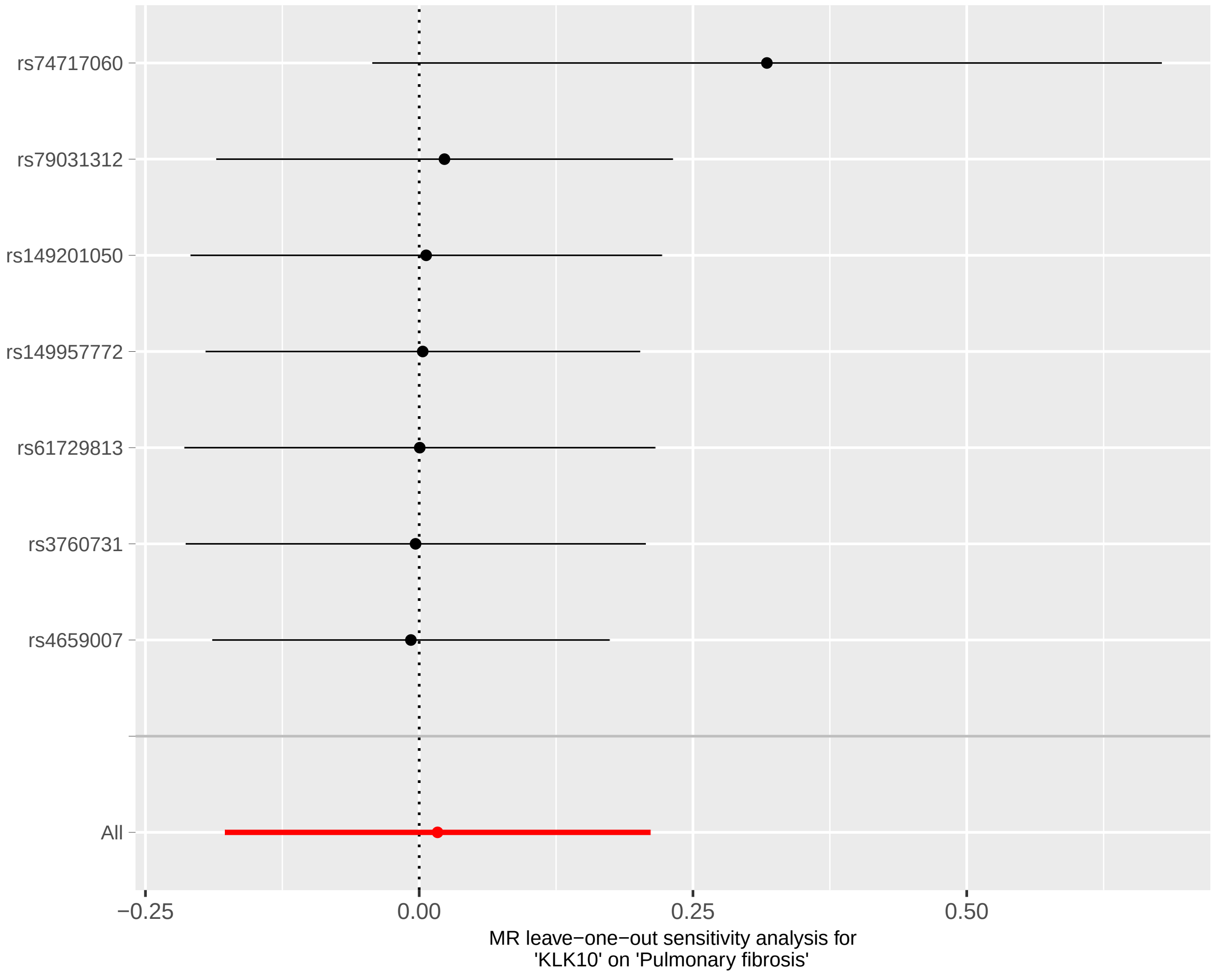

rs74717060
rs79031312
rs149201050
rs149957772
rs61729813
rs3760731
rs4659007
All
−0.25
0.00
0.25
0.50
MR leave−one−out sensitivity analysis for
'KLK10' on 'Pulmonary fibrosis'

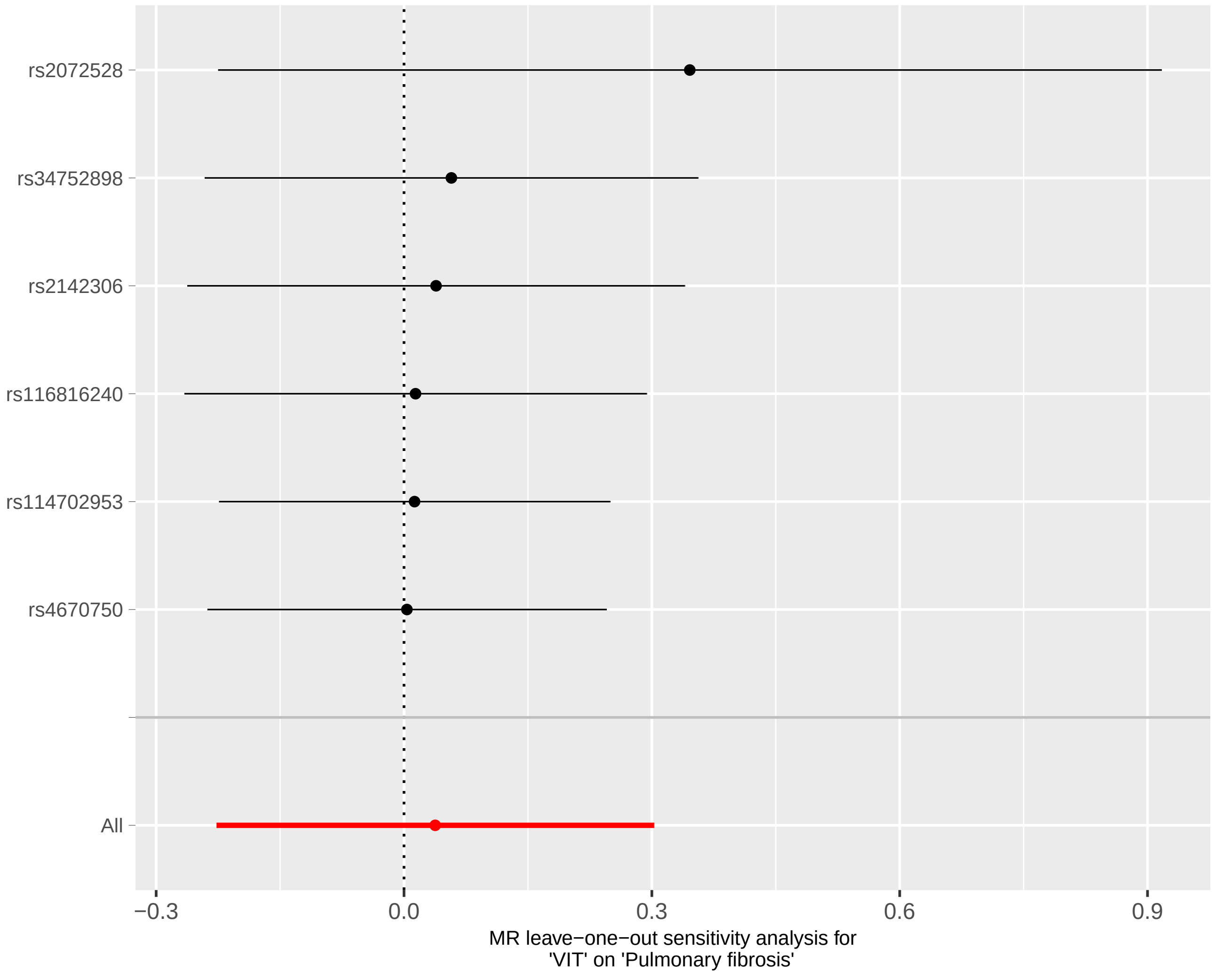
rs2072528
rs34752898
rs2142306
rs116816240
rs114702953
rs4670750
All
−0.3
0.0
0.3
0.6
0.9
MR leave−one−out sensitivity analysis for
'VIT' on 'Pulmonary fibrosis'

Insufficient number of SNPs

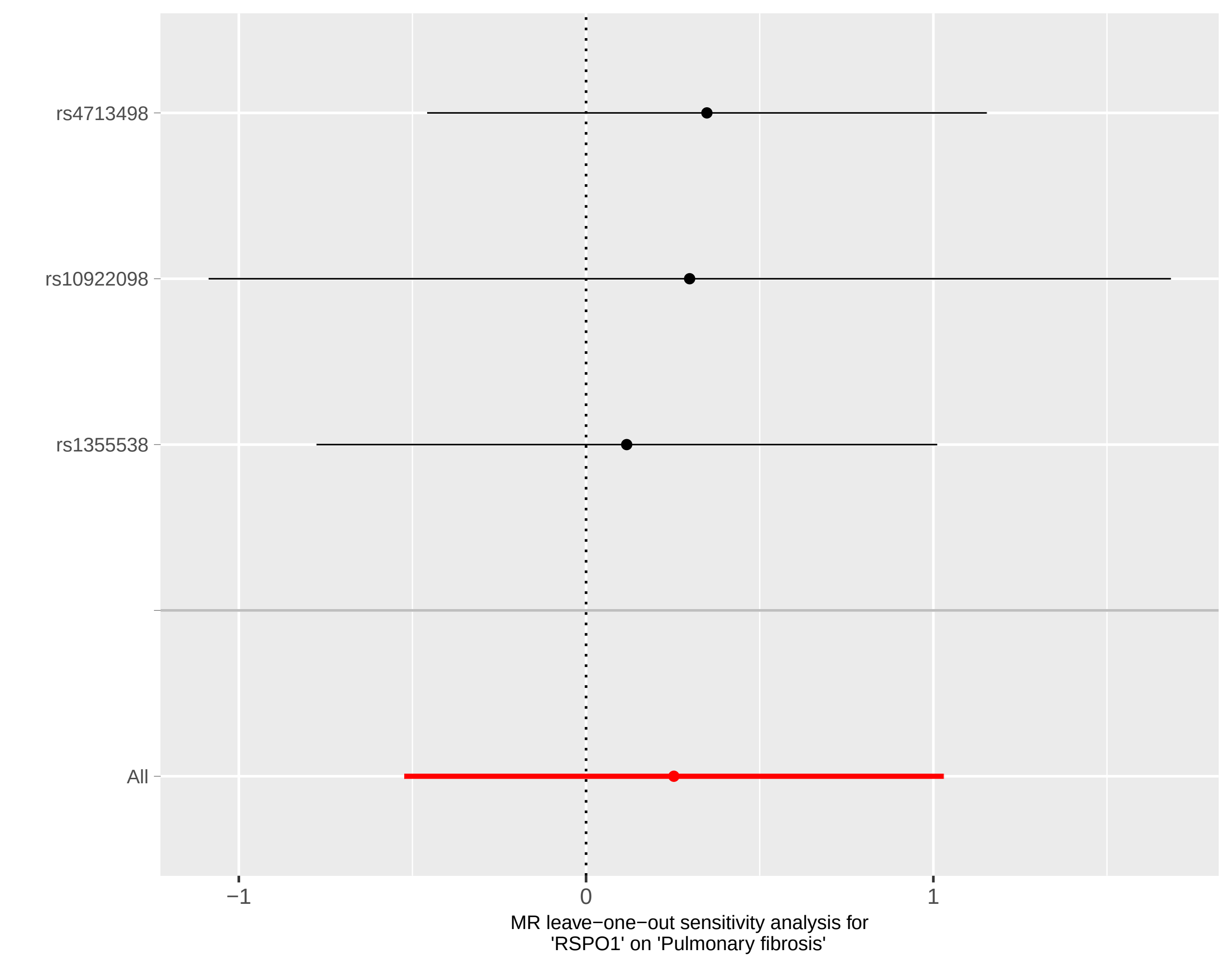
rs4713498
rs10922098
rs1355538
All
−1
0
1
MR leave−one−out sensitivity analysis for
'RSPO1' on 'Pulmonary fibrosis'

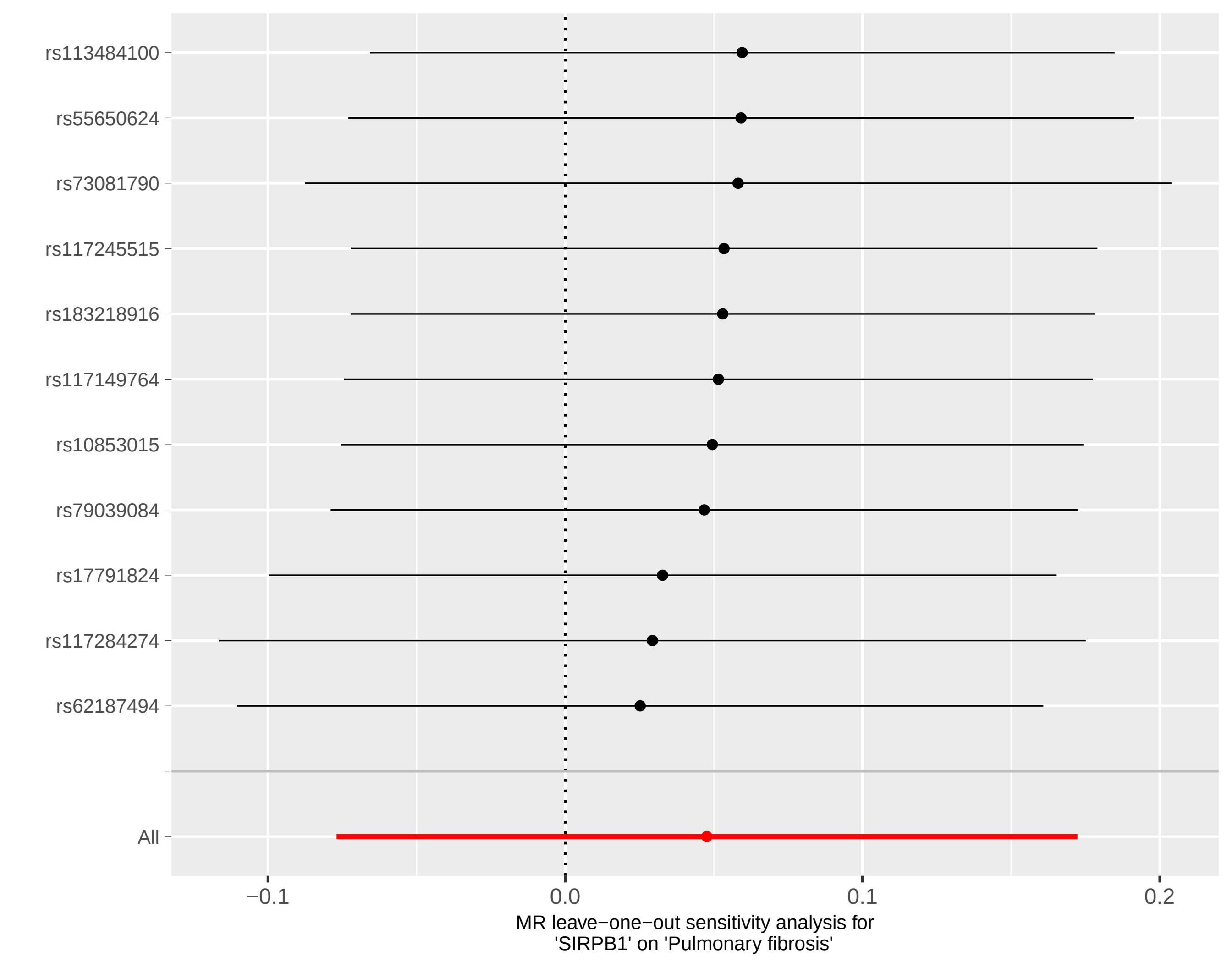

rs113484100
rs55650624
rs73081790
rs117245515
rs183218916
rs117149764
rs10853015
rs79039084
rs17791824
rs117284274
rs62187494
All
−0.1
0.0
0.1
0.2
MR leave−one−out sensitivity analysis for
'SIRPB1' on 'Pulmonary fibrosis'

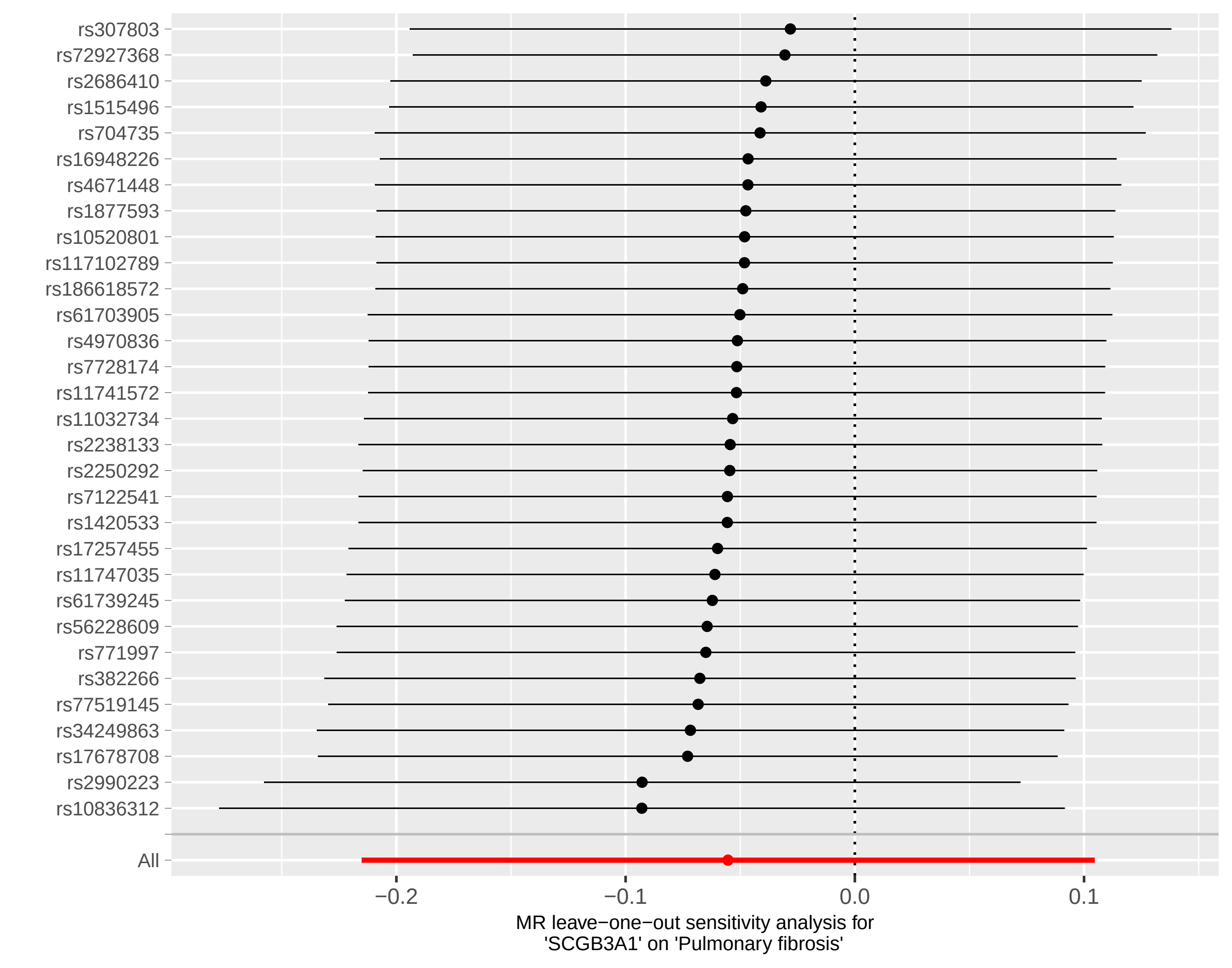
rs307803
rs72927368
rs2686410
rs1515496
rs704735
rs16948226
rs4671448
rs1877593
rs10520801
rs117102789
rs186618572
rs61703905
rs4970836
rs7728174
rs11741572
rs11032734
rs2238133
rs2250292
rs7122541
rs1420533
rs17257455
rs11747035
rs61739245
rs56228609
rs771997
rs382266
rs77519145
rs34249863
rs17678708
rs2990223
rs10836312
All
−0.2
−0.1
0.0
0.1
MR leave−one−out sensitivity analysis for
'SCGB3A1' on 'Pulmonary fibrosis'

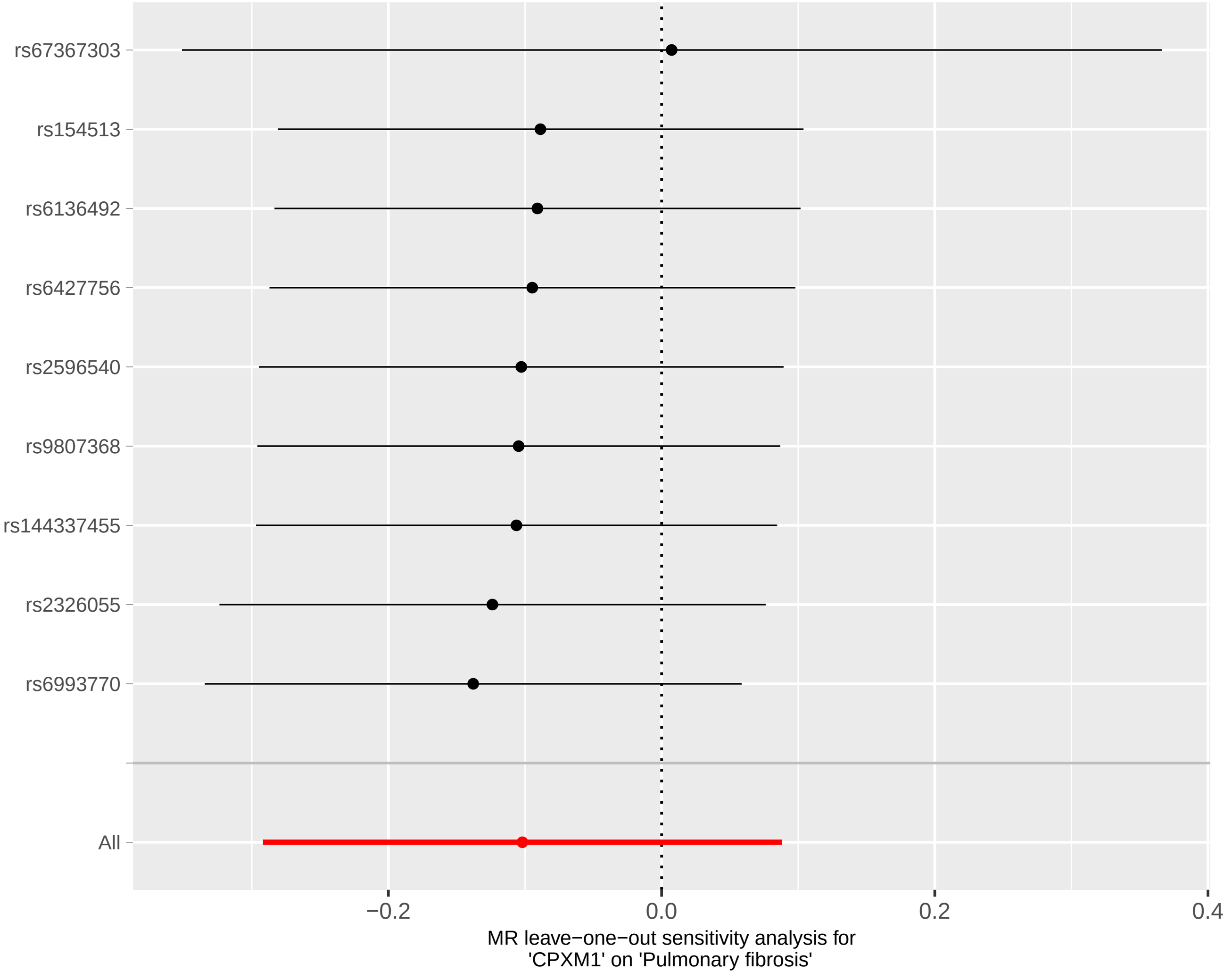
rs67367303
rs154513
rs6136492
rs6427756
rs2596540
rs9807368
rs144337455
rs2326055
rs6993770
All
−0.2
0.0
0.2
0.4
MR leave−one−out sensitivity analysis for
'CPXM1' on 'Pulmonary fibrosis'

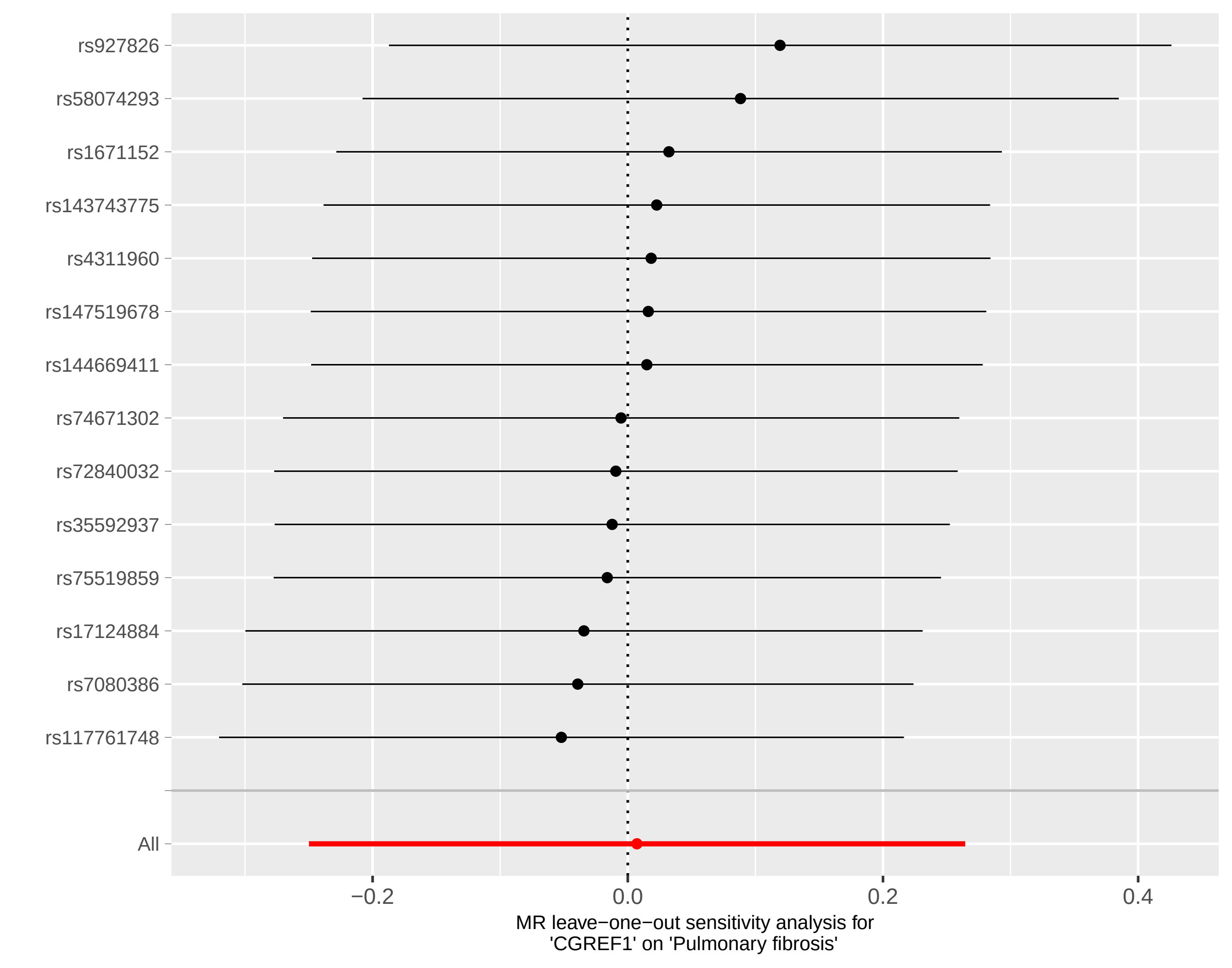
rs927826
rs58074293
rs1671152
rs143743775
rs4311960
rs147519678
rs144669411
rs74671302
rs72840032
rs35592937
rs75519859
rs17124884
rs7080386
rs117761748
All
−0.2
0.0
0.2
0.4
MR leave−one−out sensitivity analysis for
'CGREF1' on 'Pulmonary fibrosis'

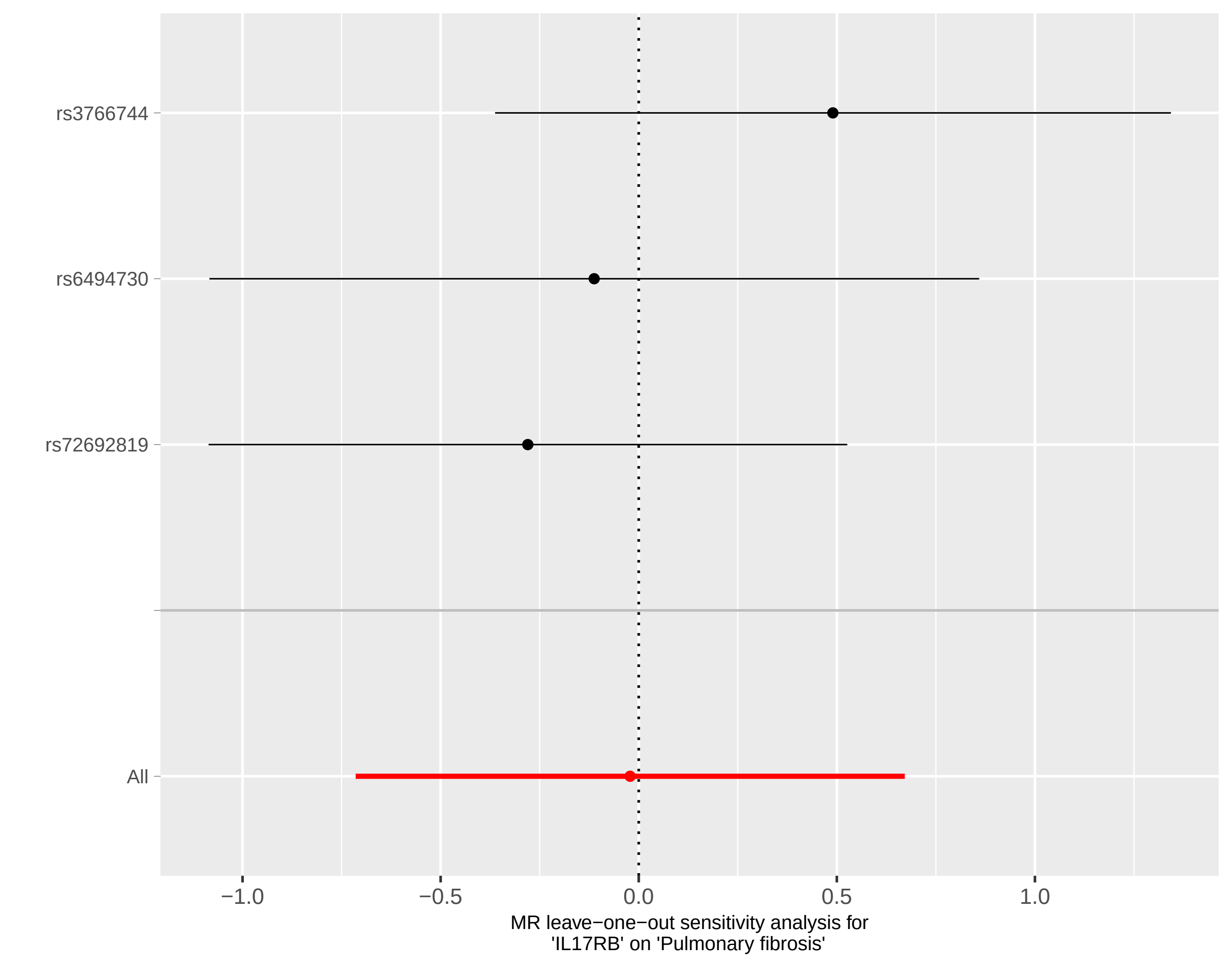
rs3766744
rs6494730
rs72692819
All
−1.0
−0.5
0.0
0.5
1.0
MR leave−one−out sensitivity analysis for
'IL17RB' on 'Pulmonary fibrosis'

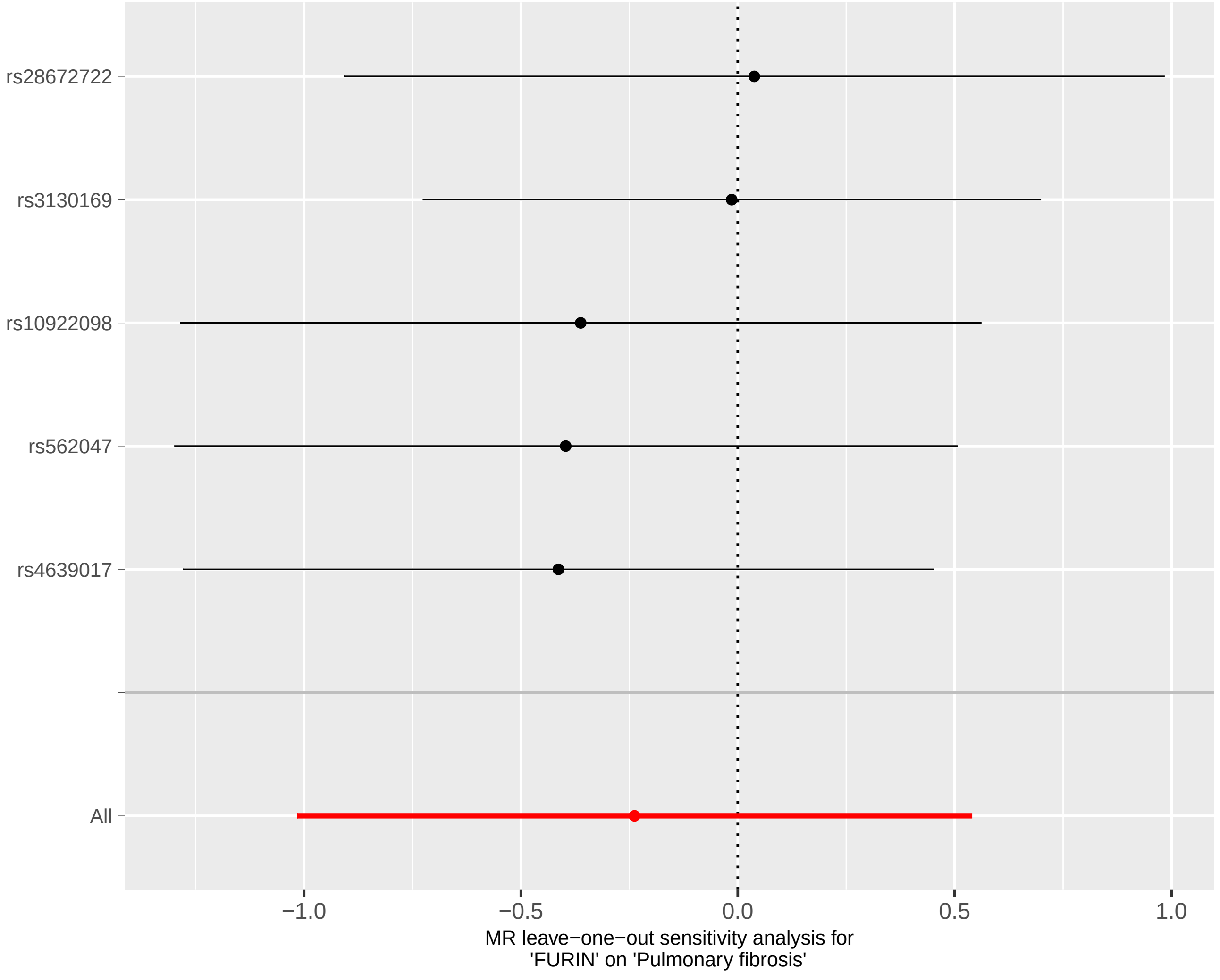
rs28672722
rs3130169
rs10922098
rs562047
rs4639017
All
−1.0
−0.5
0.0
0.5
1.0
MR leave−one−out sensitivity analysis for
'FURIN' on 'Pulmonary fibrosis'

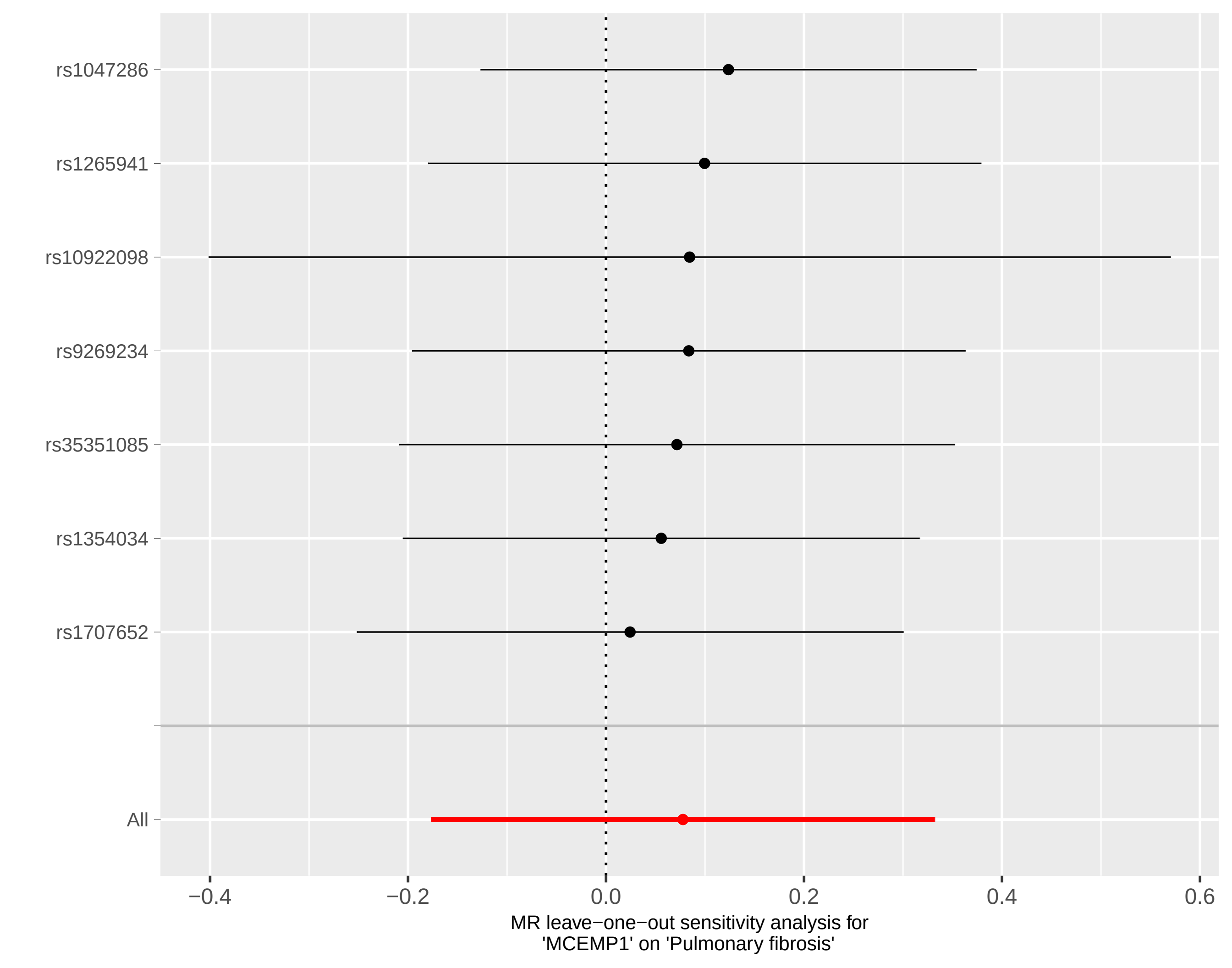
rs1047286
rs1265941
rs10922098
rs9269234
rs35351085
rs1354034
rs1707652
All
−0.4
−0.2
0.0
0.2
0.4
0.6
MR leave−one−out sensitivity analysis for
'MCEMP1' on 'Pulmonary fibrosis'

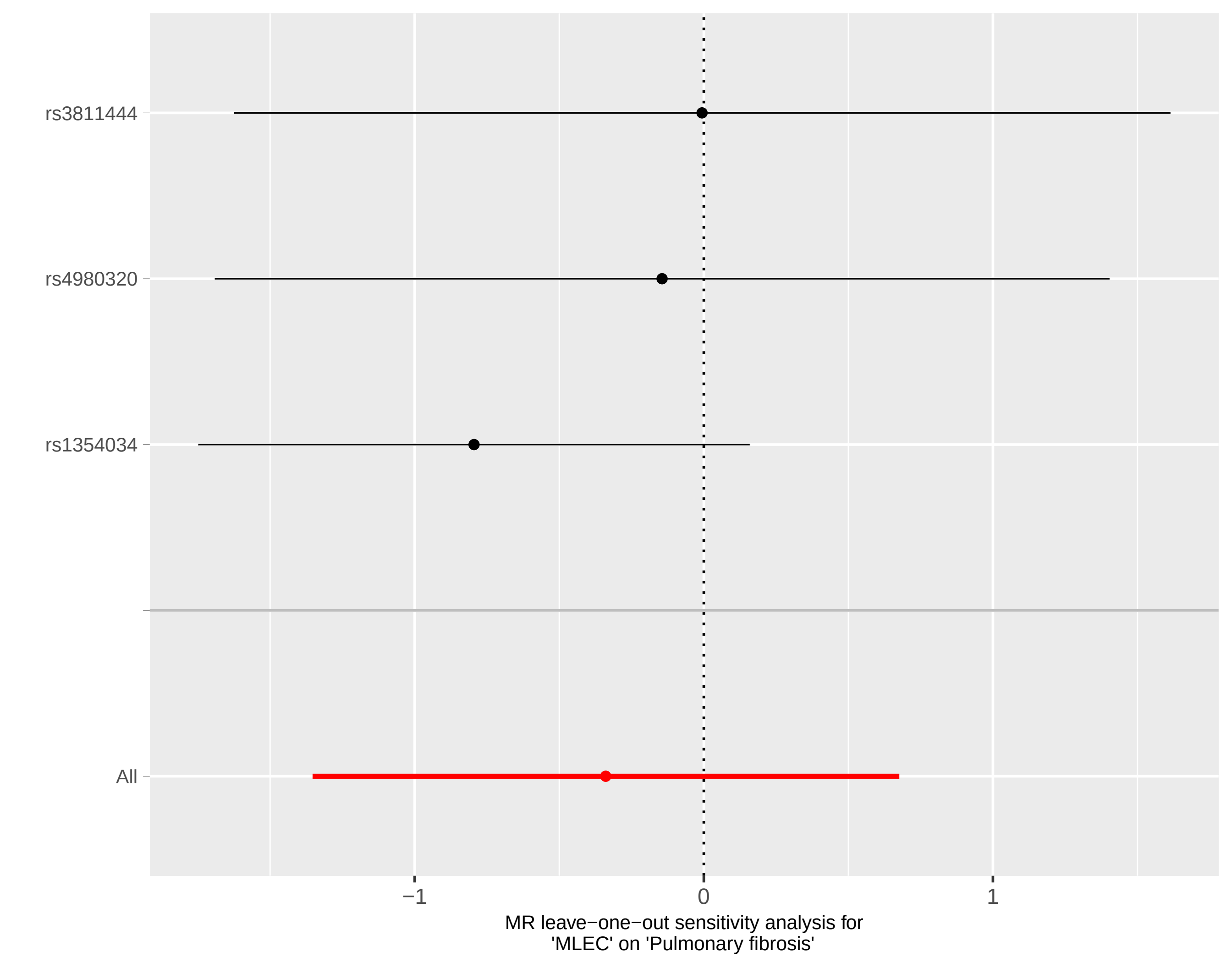

rs3811444
rs4980320
rs1354034
All
−1
0
1
MR leave−one−out sensitivity analysis for
'MLEC' on 'Pulmonary fibrosis'

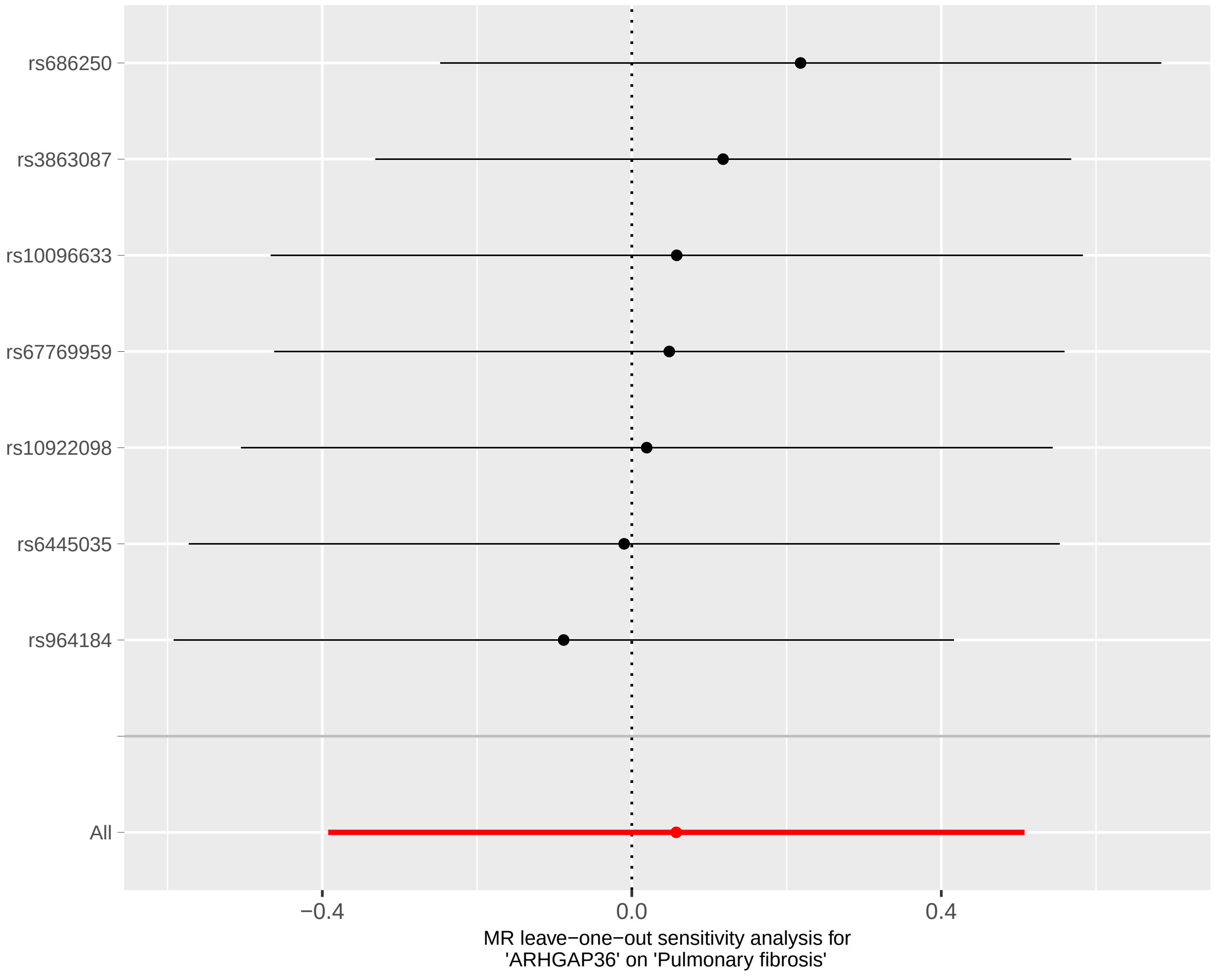
rs686250
rs3863087
rs10096633
rs67769959
rs10922098
rs6445035
rs964184
All
−0.4
0.0
0.4
MR leave−one−out sensitivity analysis for
'ARHGAP36' on 'Pulmonary fibrosis'

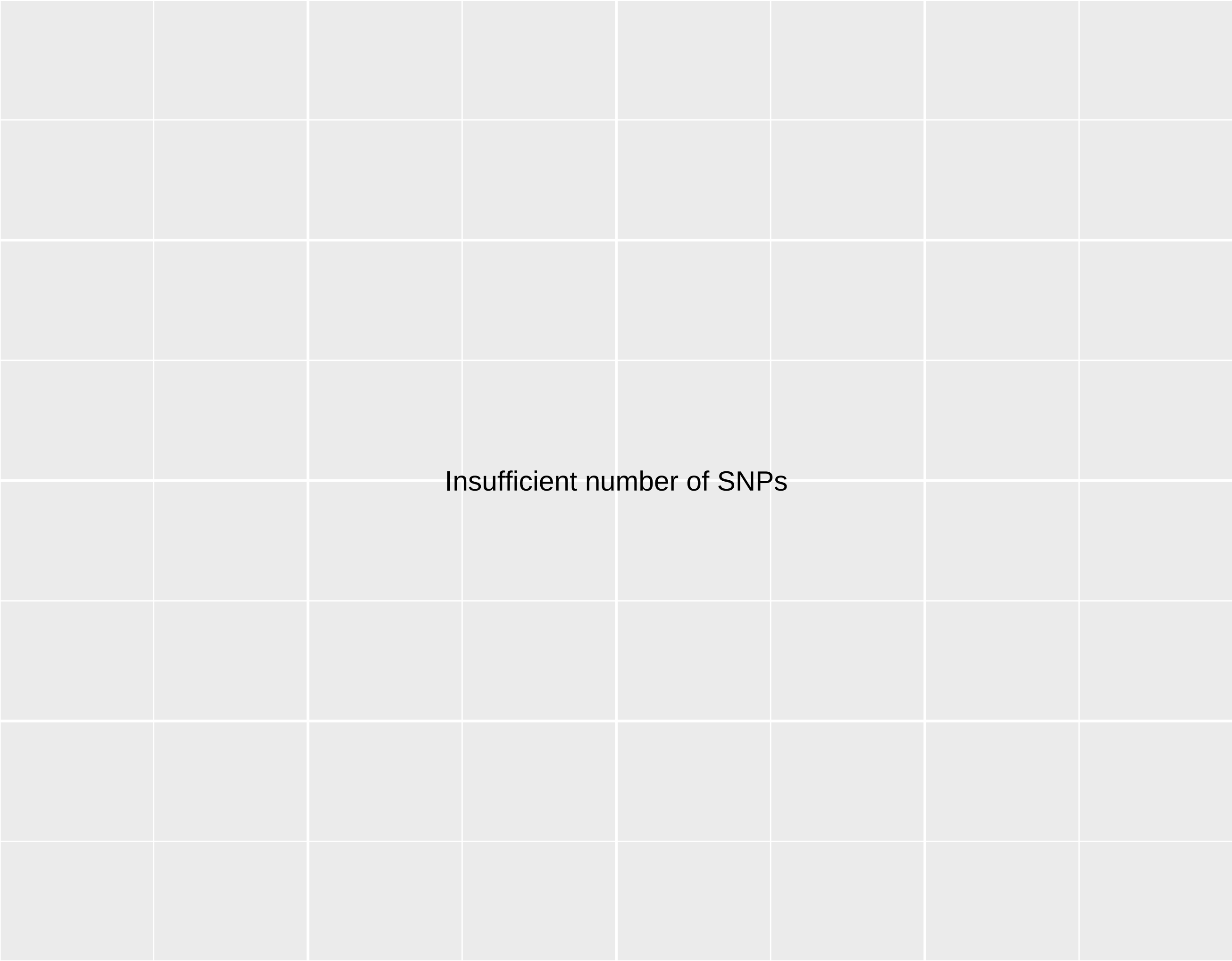
Insufficient number of SNPs

Insufficient number of SNPs

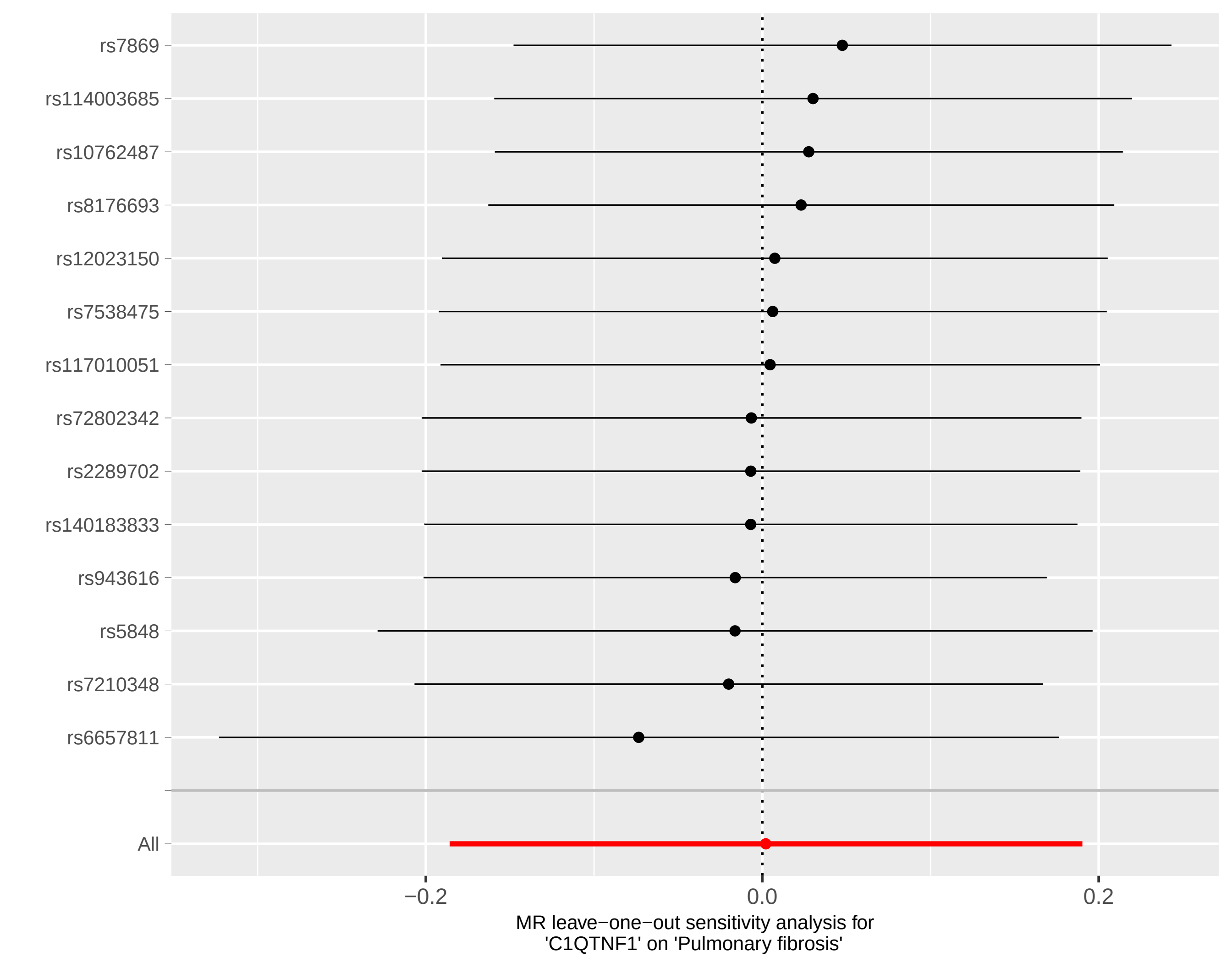

rs7869
rs114003685
rs10762487
rs8176693
rs12023150
rs7538475
rs117010051
rs72802342
rs2289702
rs140183833
rs943616
rs5848
rs7210348
rs6657811
All
−0.2
0.0
0.2
MR leave−one−out sensitivity analysis for
'C1QTNF1' on 'Pulmonary fibrosis'

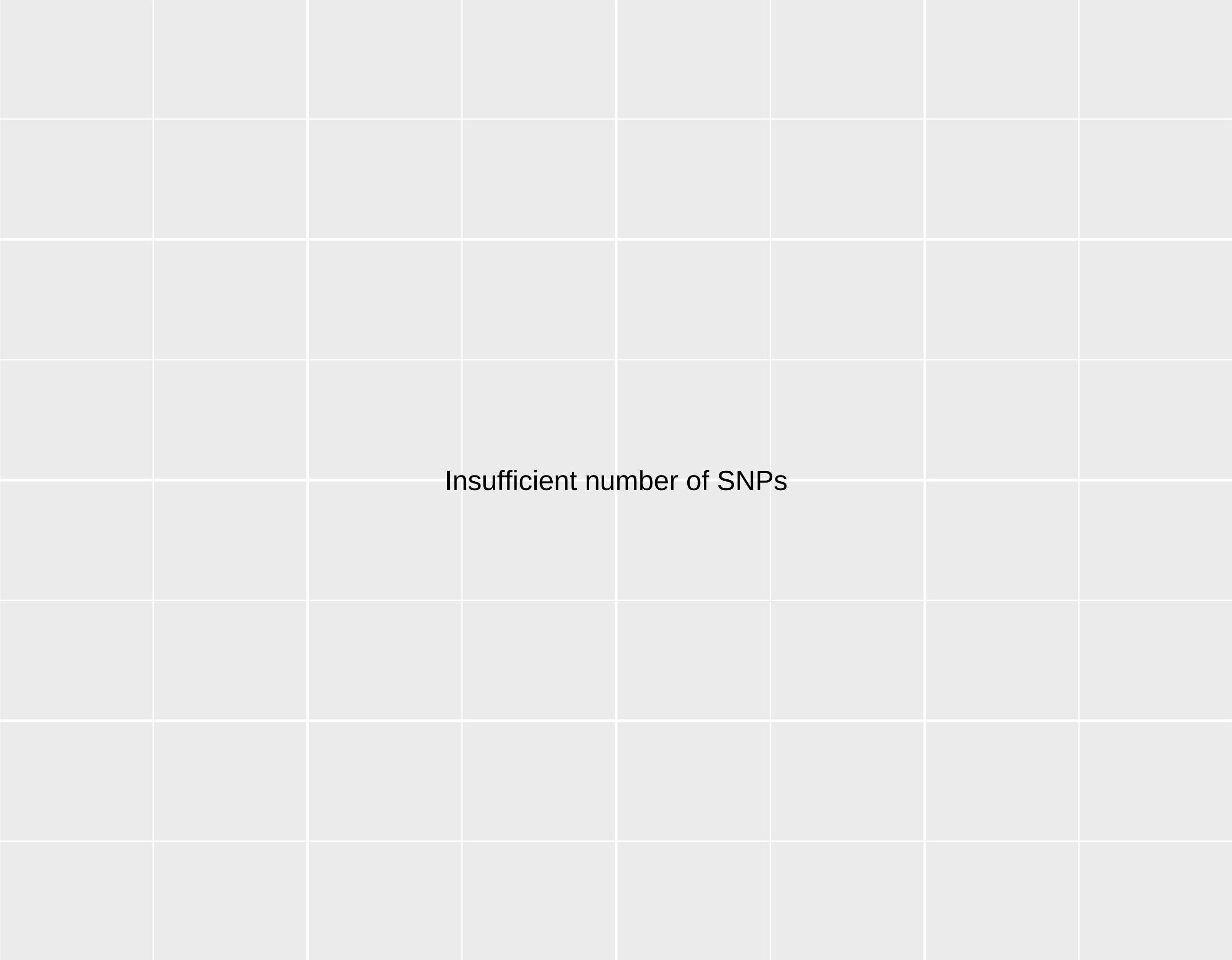
Insufficient number of SNPs

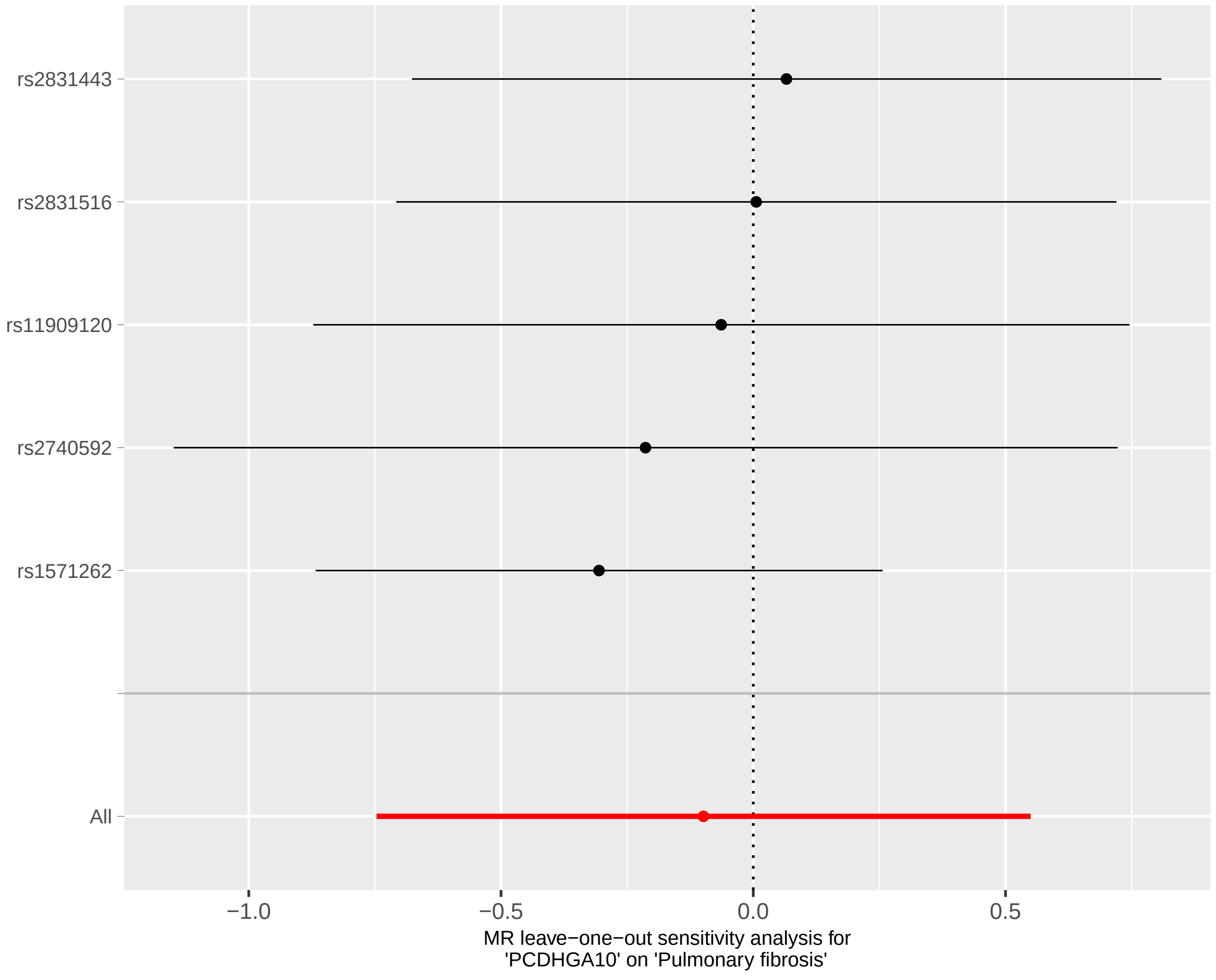
rs2831443
rs2831516
rs11909120
rs2740592
rs1571262
All
−1.0
−0.5
0.0
0.5
MR leave−one−out sensitivity analysis for
'PCDHGA10' on 'Pulmonary fibrosis'

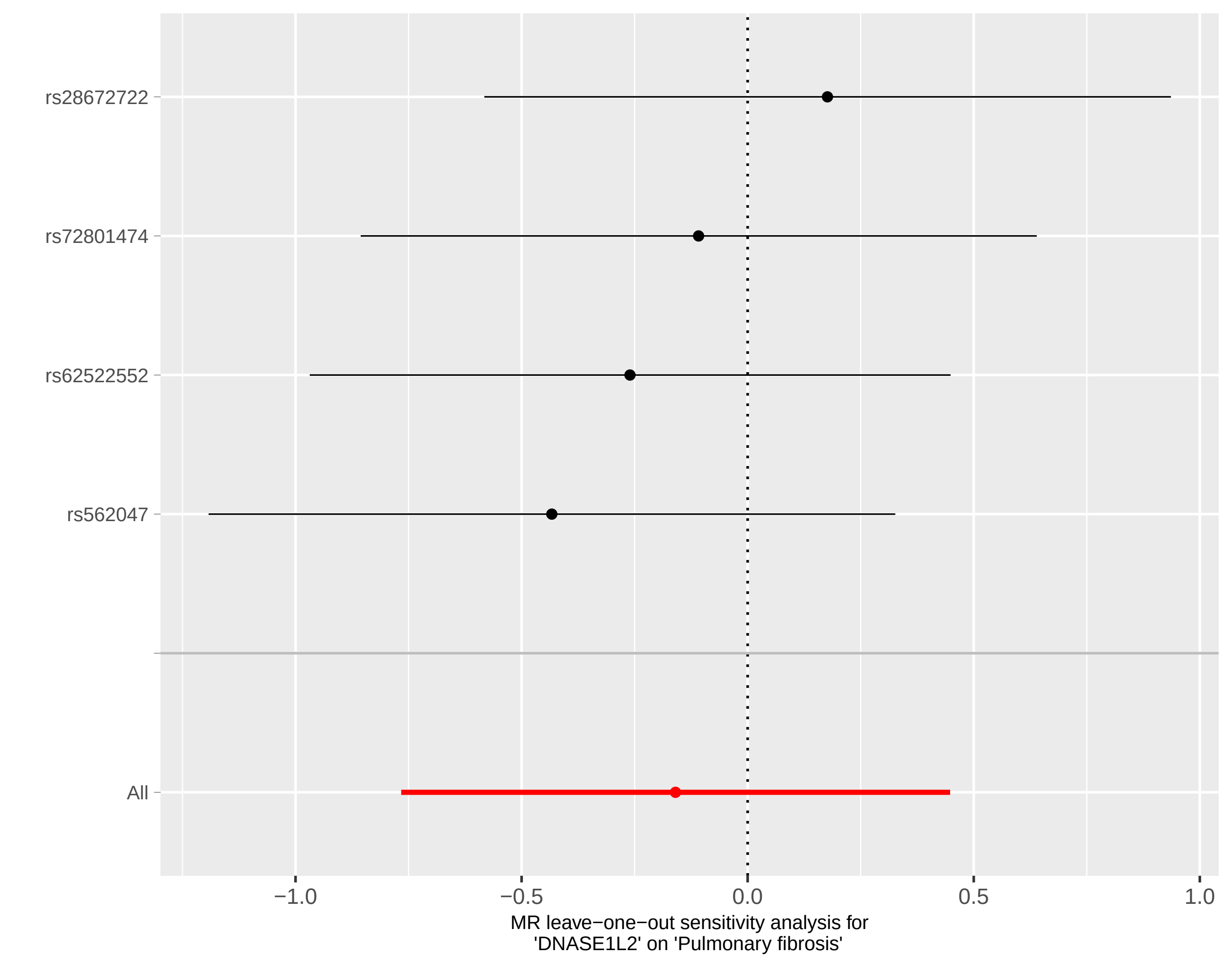
rs28672722
rs72801474
rs62522552
rs562047
All
−1.0
−0.5
0.0
0.5
1.0
MR leave−one−out sensitivity analysis for
'DNASE1L2' on 'Pulmonary fibrosis'

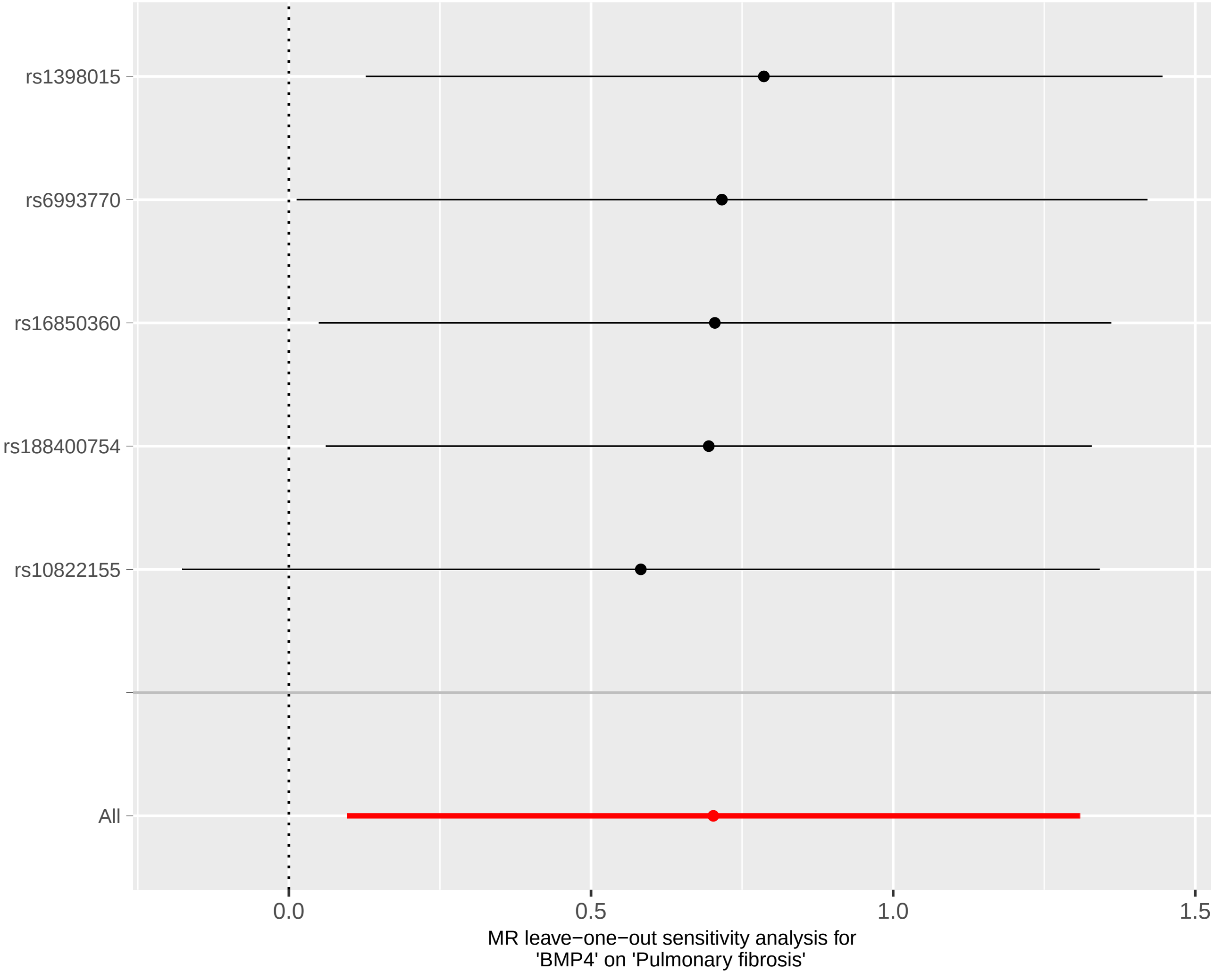
rs1398015
rs6993770
rs16850360
rs188400754
rs10822155
All
0.0
0.5
1.0
1.5
MR leave−one−out sensitivity analysis for
'BMP4' on 'Pulmonary fibrosis'

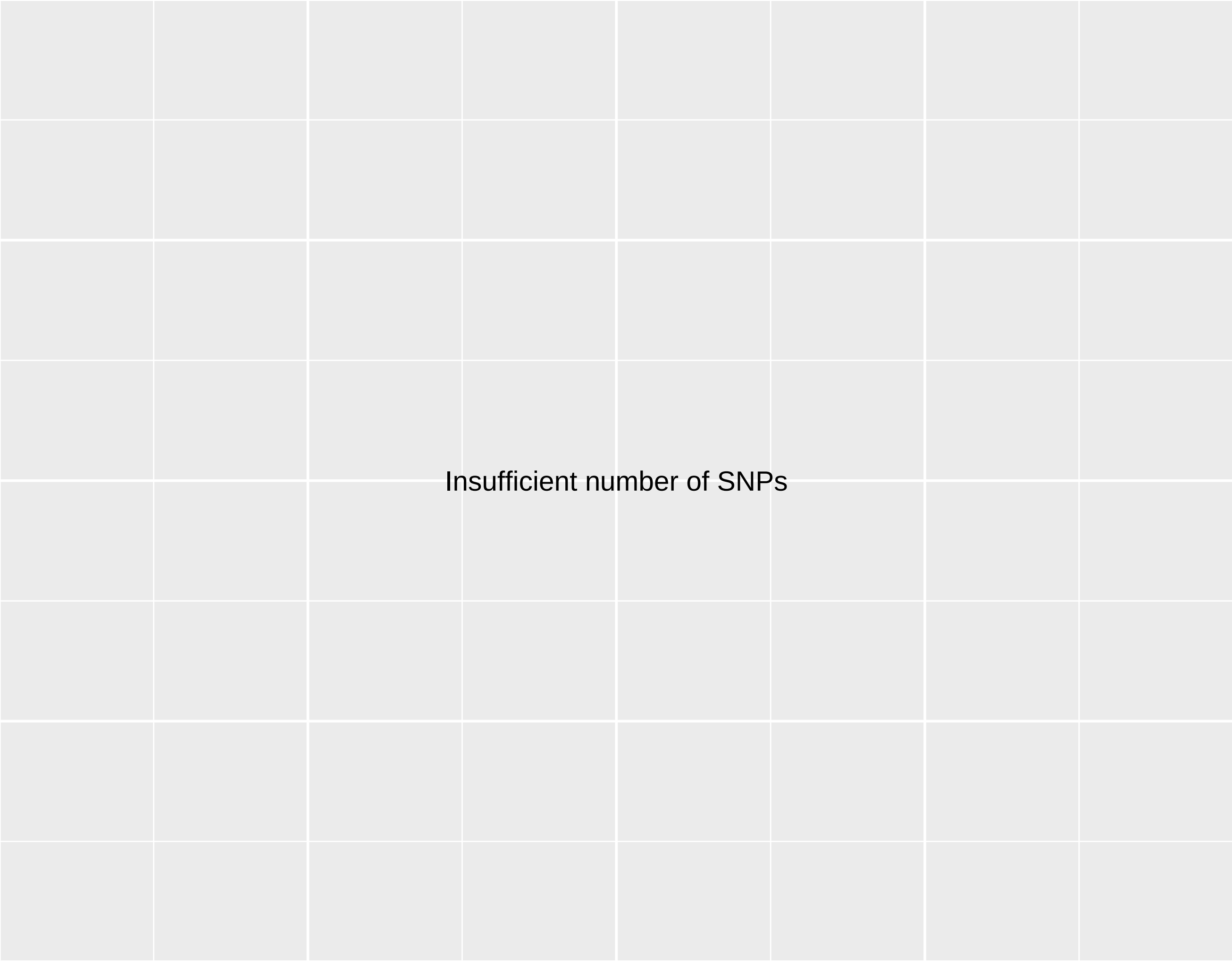
Insufficient number of SNPs

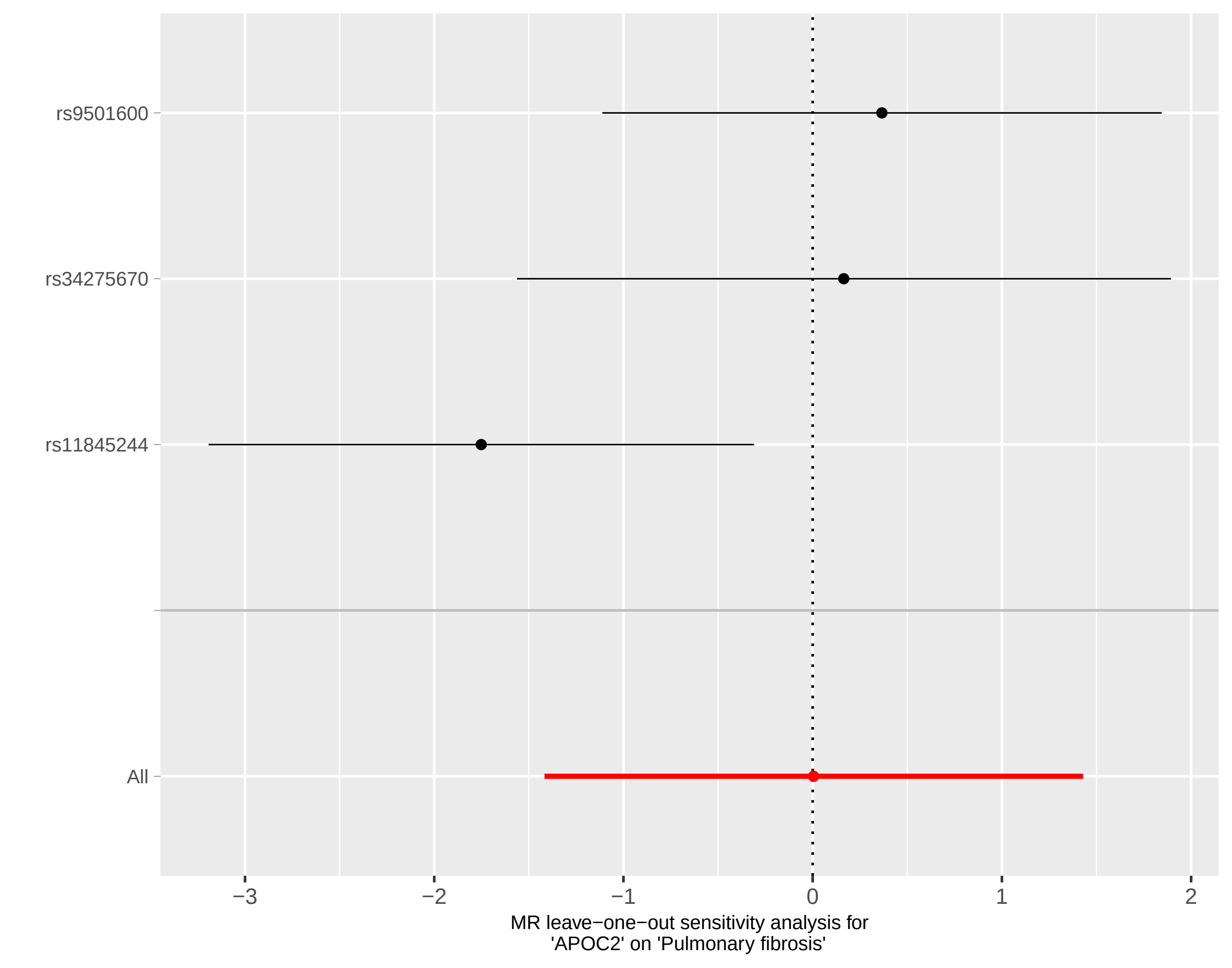
rs9501600
rs34275670
rs11845244
All
−3
−2
−1
0
1
2
MR leave−one−out sensitivity analysis for
'APOC2' on 'Pulmonary fibrosis'

Insufficient number of SNPs

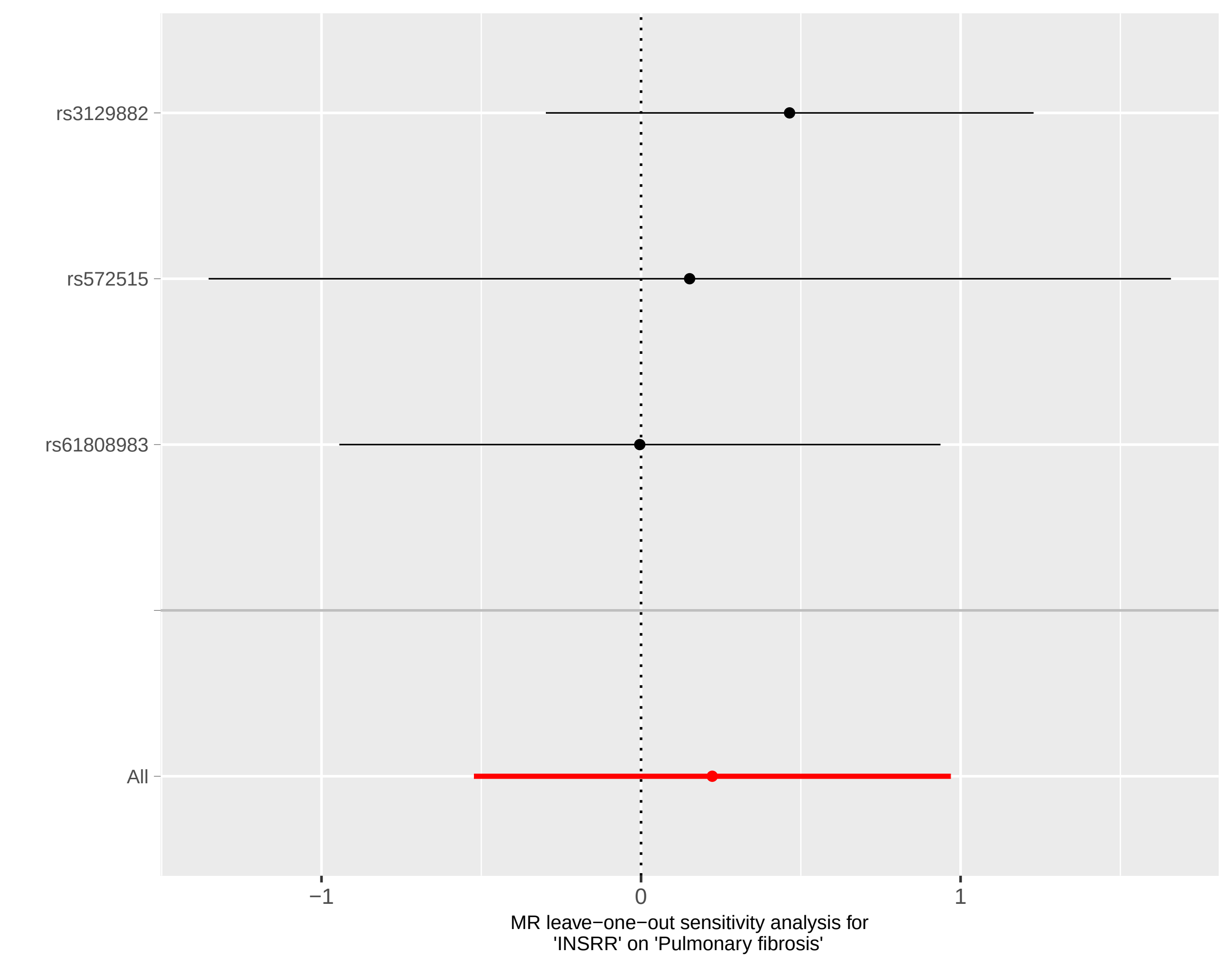
rs3129882
rs572515
rs61808983
All
−1
0
1
MR leave−one−out sensitivity analysis for
'INSRR' on 'Pulmonary fibrosis'

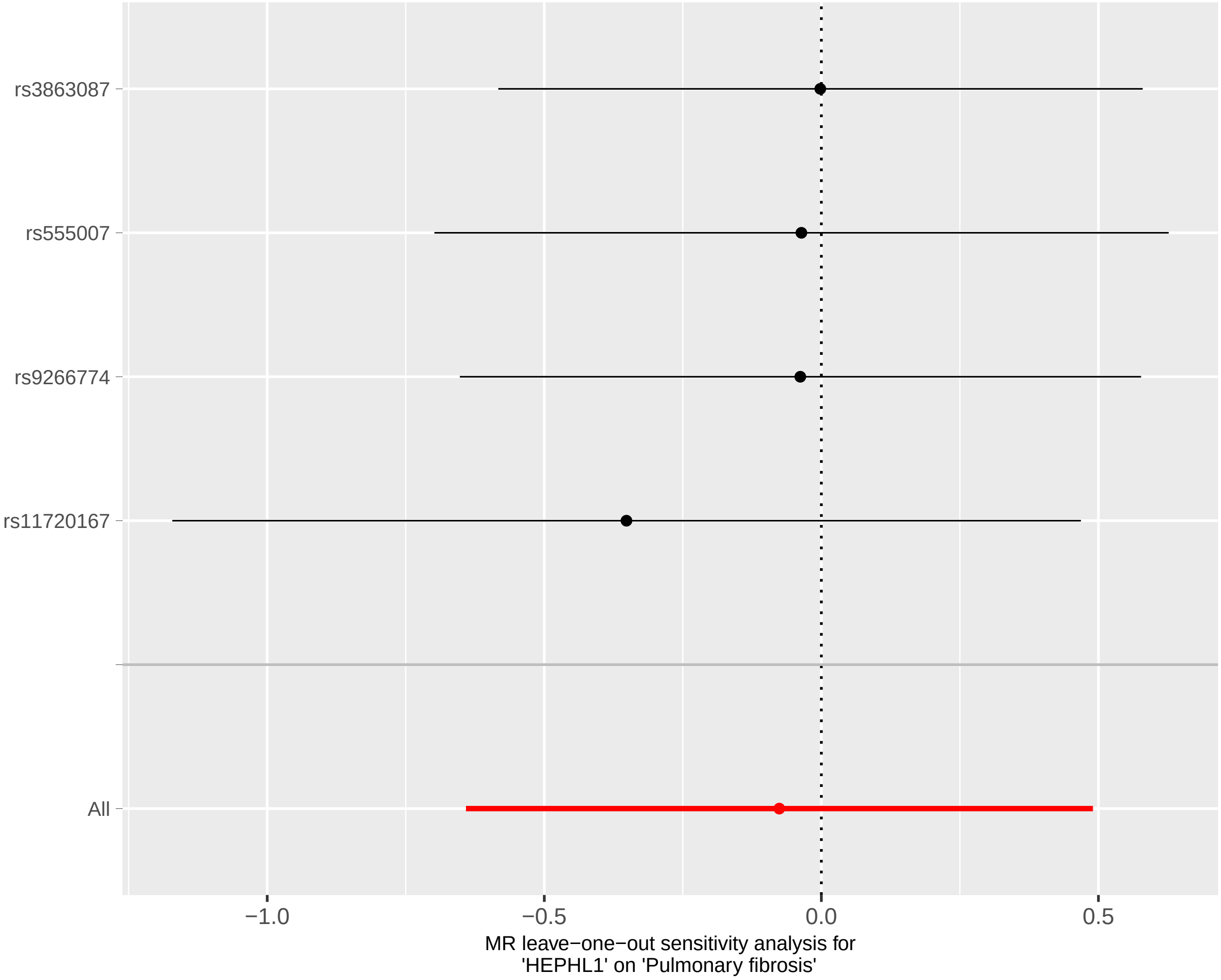
rs3863087
rs555007
rs9266774
rs11720167
All
−1.0
−0.5
0.0
0.5
MR leave−one−out sensitivity analysis for
'HEPHL1' on 'Pulmonary fibrosis'

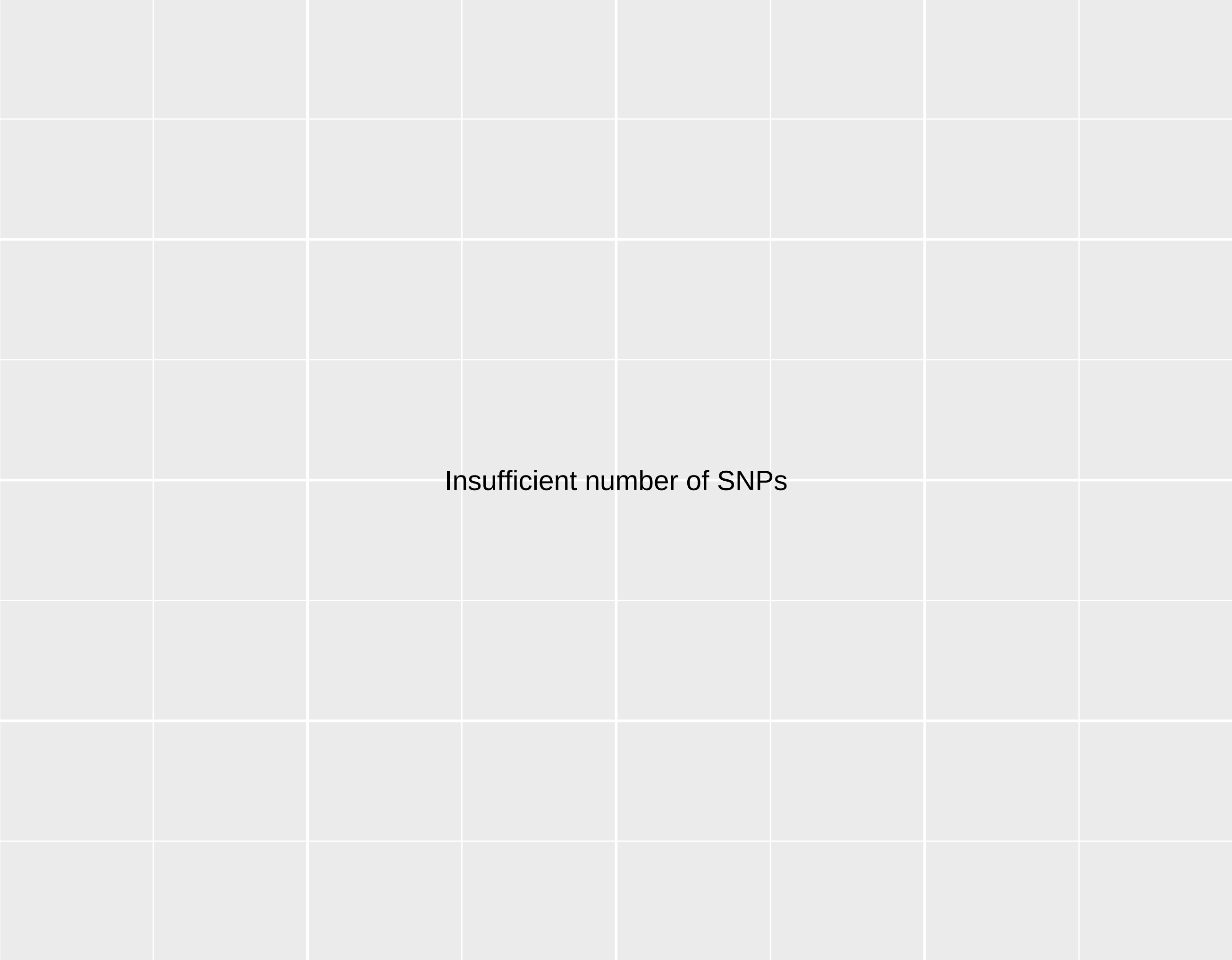
Insufficient number of SNPs

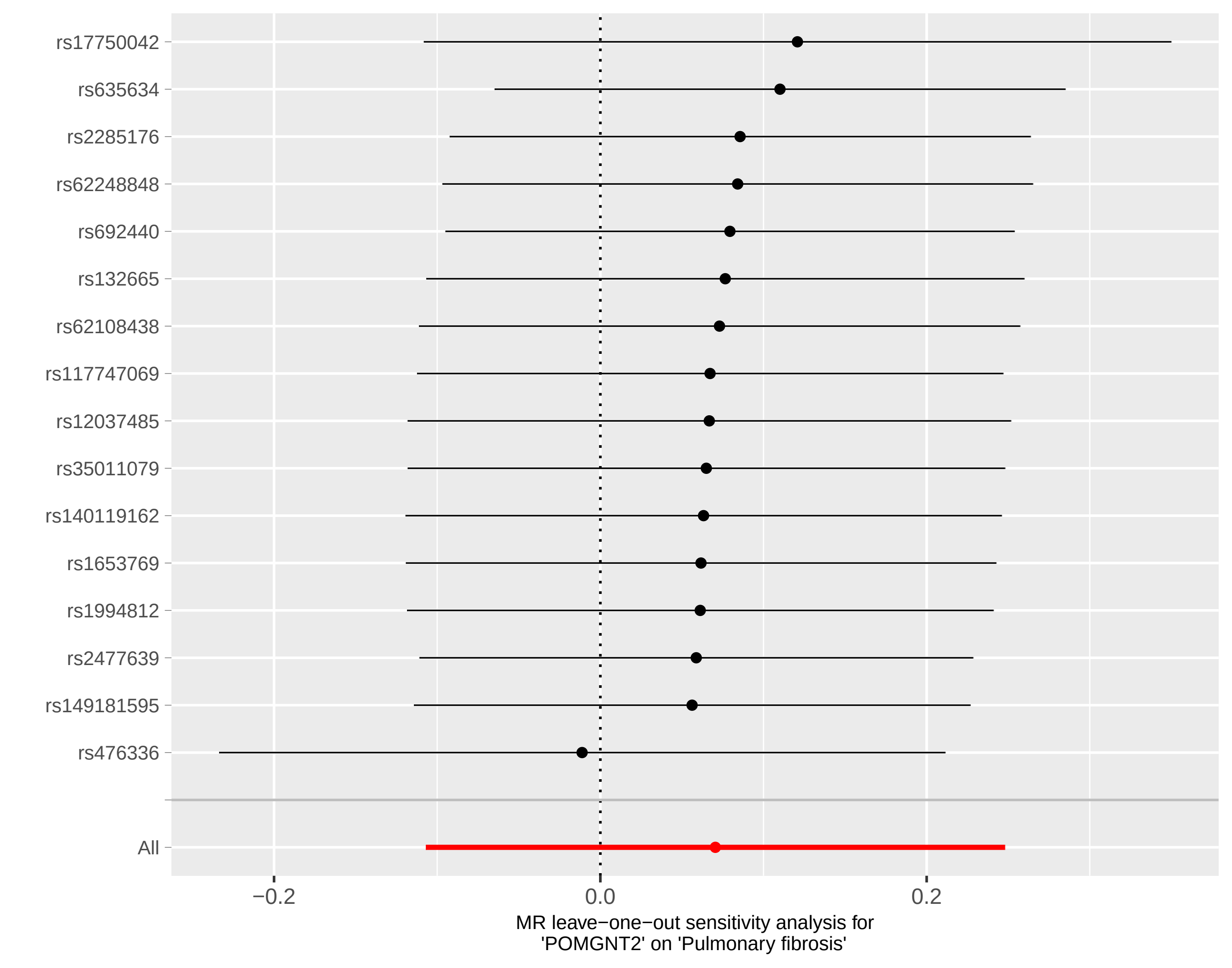
rs17750042
rs635634
rs2285176
rs62248848
rs692440
rs132665
rs62108438
rs117747069
rs12037485
rs35011079
rs140119162
rs1653769
rs1994812
rs2477639
rs149181595
rs476336
All
−0.2
0.0
0.2
MR leave−one−out sensitivity analysis for
'POMGNT2' on 'Pulmonary fibrosis'

Insufficient number of SNPs

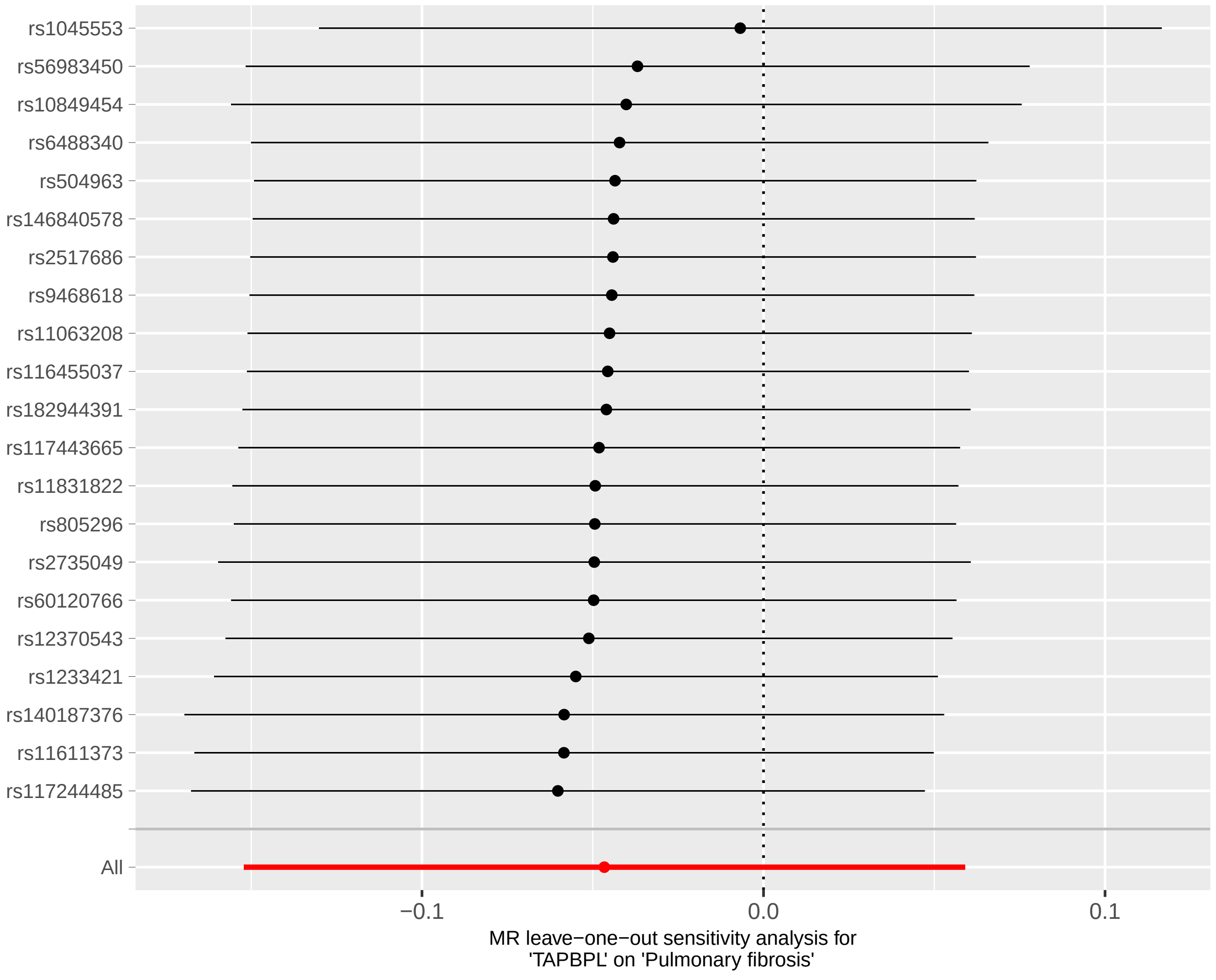
rs1045553
rs56983450
rs10849454
rs6488340
rs504963
rs146840578
rs2517686
rs9468618
rs11063208
rs116455037
rs182944391
rs117443665
rs11831822
rs805296
rs2735049
rs60120766
rs12370543
rs1233421
rs140187376
rs11611373
rs117244485
All
−0.1
0.0
0.1
MR leave−one−out sensitivity analysis for
'TAPBPL' on 'Pulmonary fibrosis'

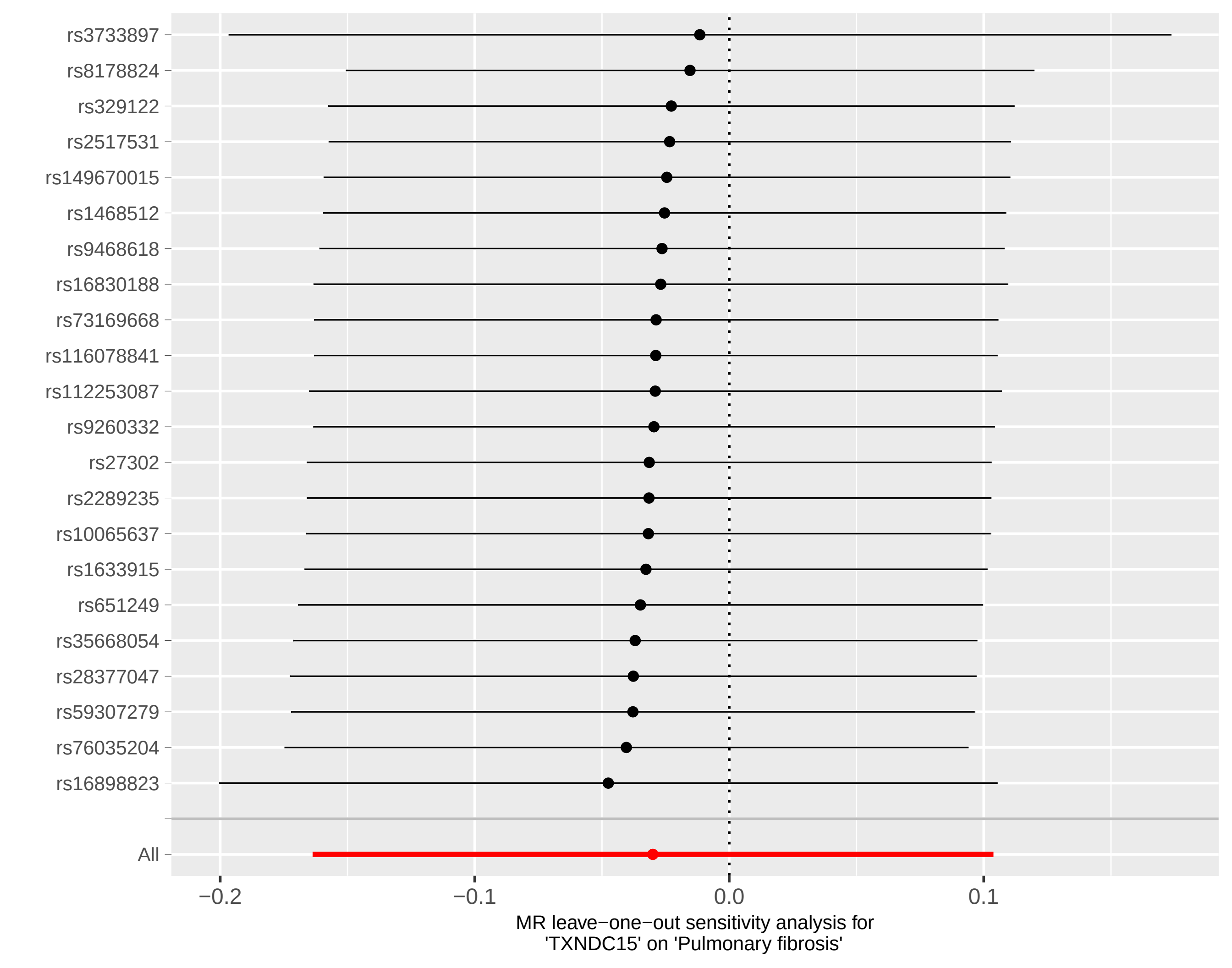
rs3733897
rs8178824
rs329122
rs2517531
rs149670015
rs1468512
rs9468618
rs16830188
rs73169668
rs116078841
rs112253087
rs9260332
rs27302
rs2289235
rs10065637
rs1633915
rs651249
rs35668054
rs28377047
rs59307279
rs76035204
rs16898823
All
−0.2
−0.1
0.0
0.1
MR leave−one−out sensitivity analysis for
'TXNDC15' on 'Pulmonary fibrosis'

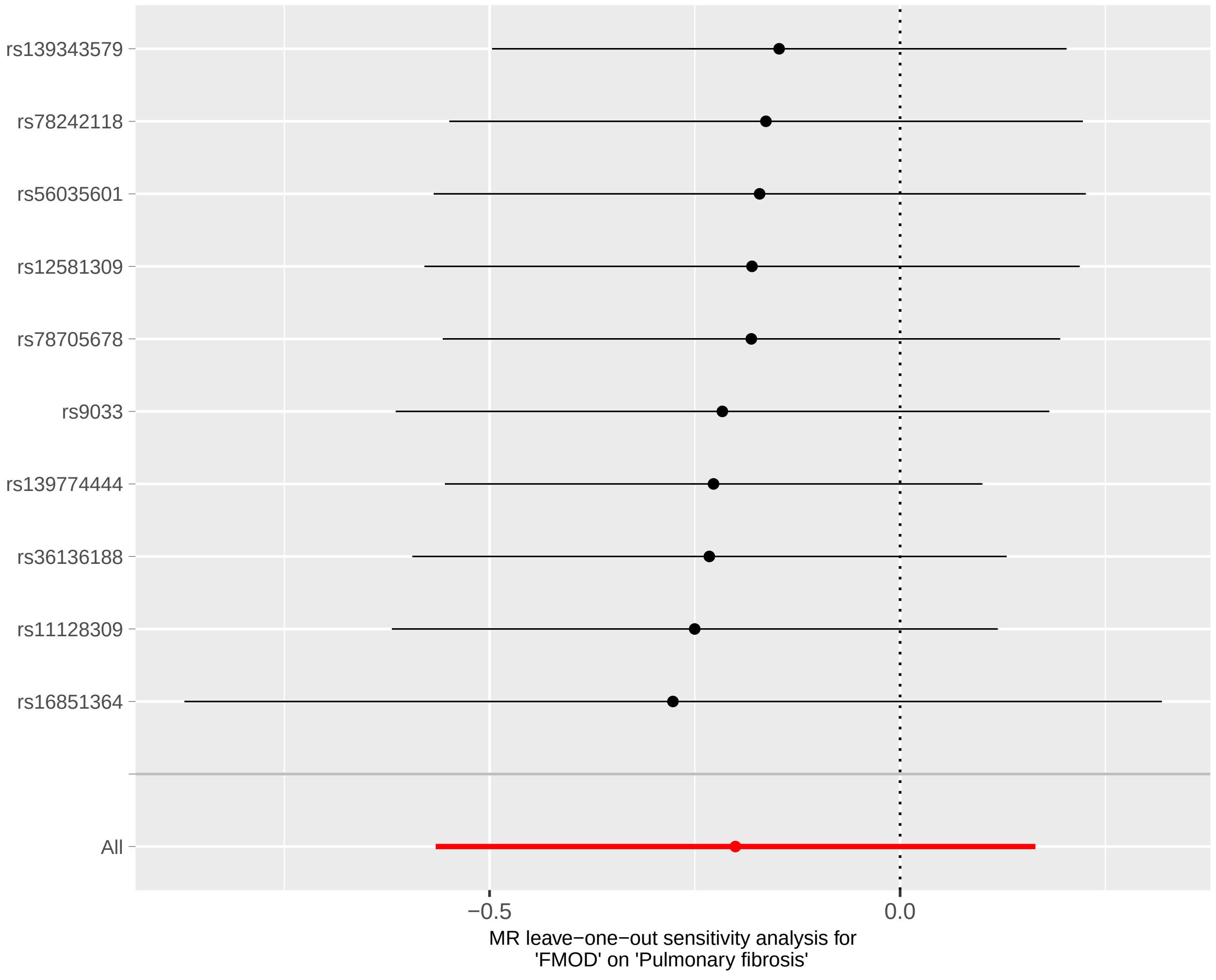

rs139343579
rs78242118
rs56035601
rs12581309
rs78705678
rs9033
rs139774444
rs36136188
rs11128309
rs16851364
All
−0.5
0.0
MR leave−one−out sensitivity analysis for
'FMOD' on 'Pulmonary fibrosis'

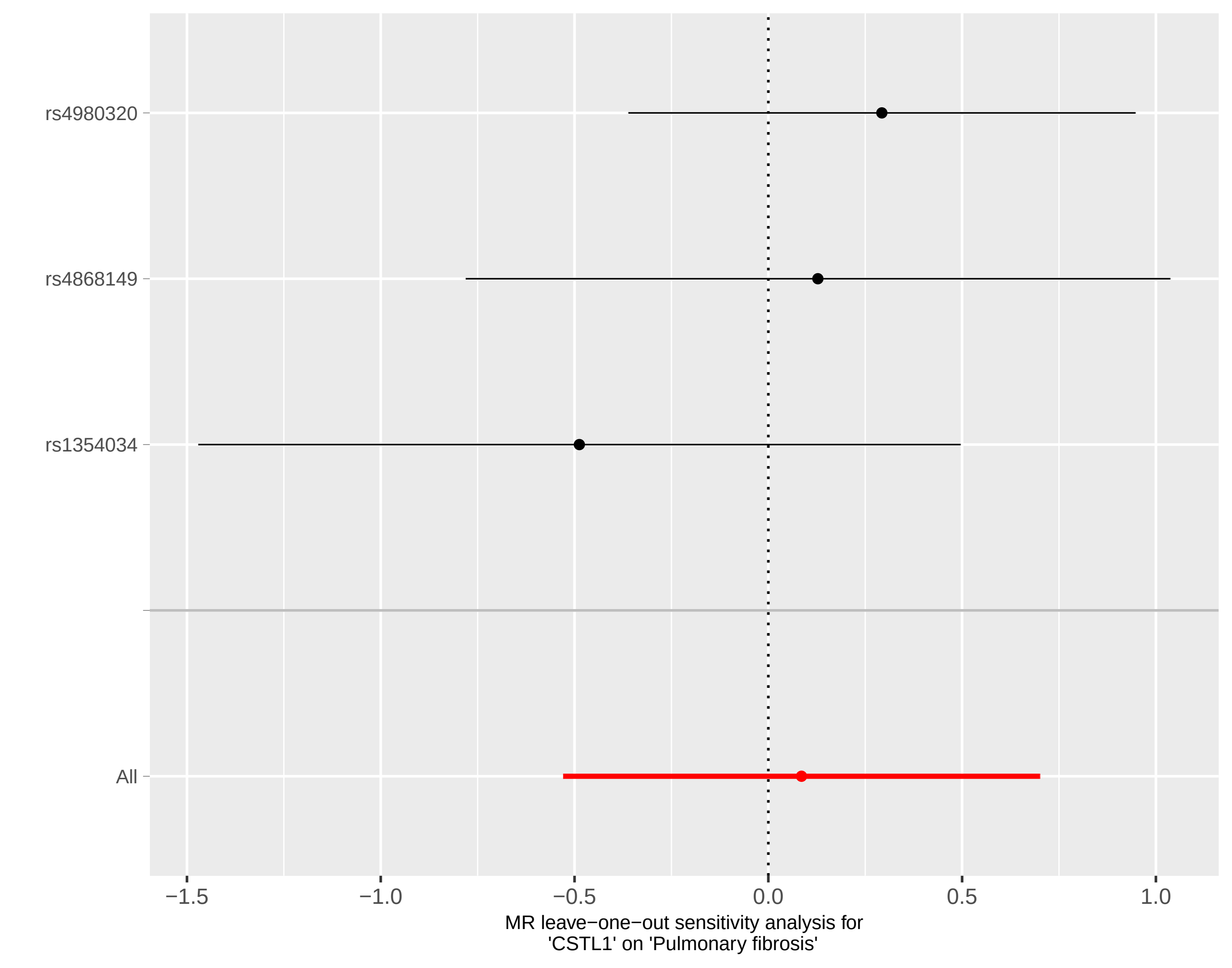
rs4980320
rs4868149
rs1354034
All
−1.5
−1.0
−0.5
0.0
0.5
1.0
MR leave−one−out sensitivity analysis for
'CSTL1' on 'Pulmonary fibrosis'

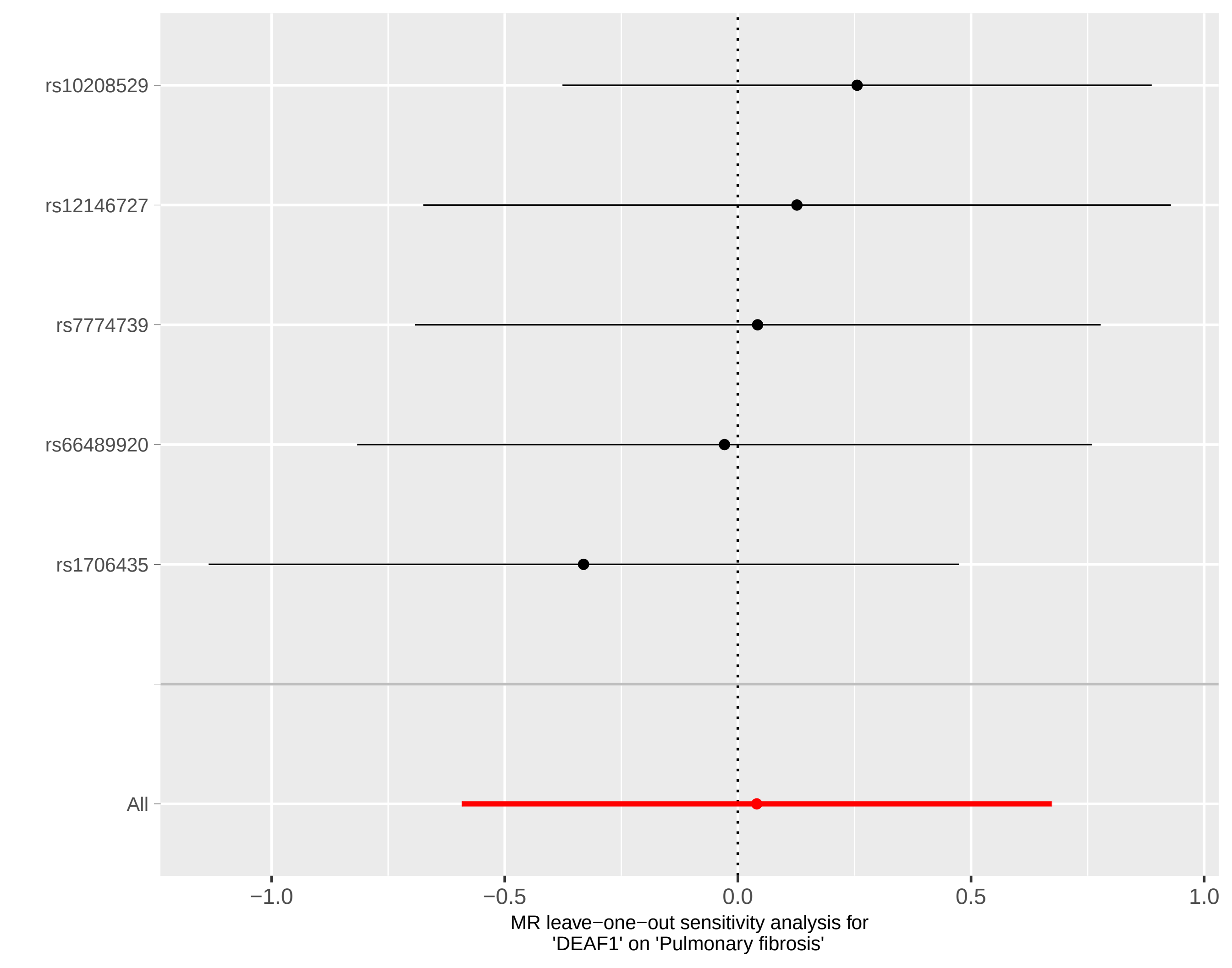
rs10208529
rs12146727
rs7774739
rs66489920
rs1706435
All
−1.0
−0.5
0.0
0.5
1.0
MR leave−one−out sensitivity analysis for
'DEAF1' on 'Pulmonary fibrosis'

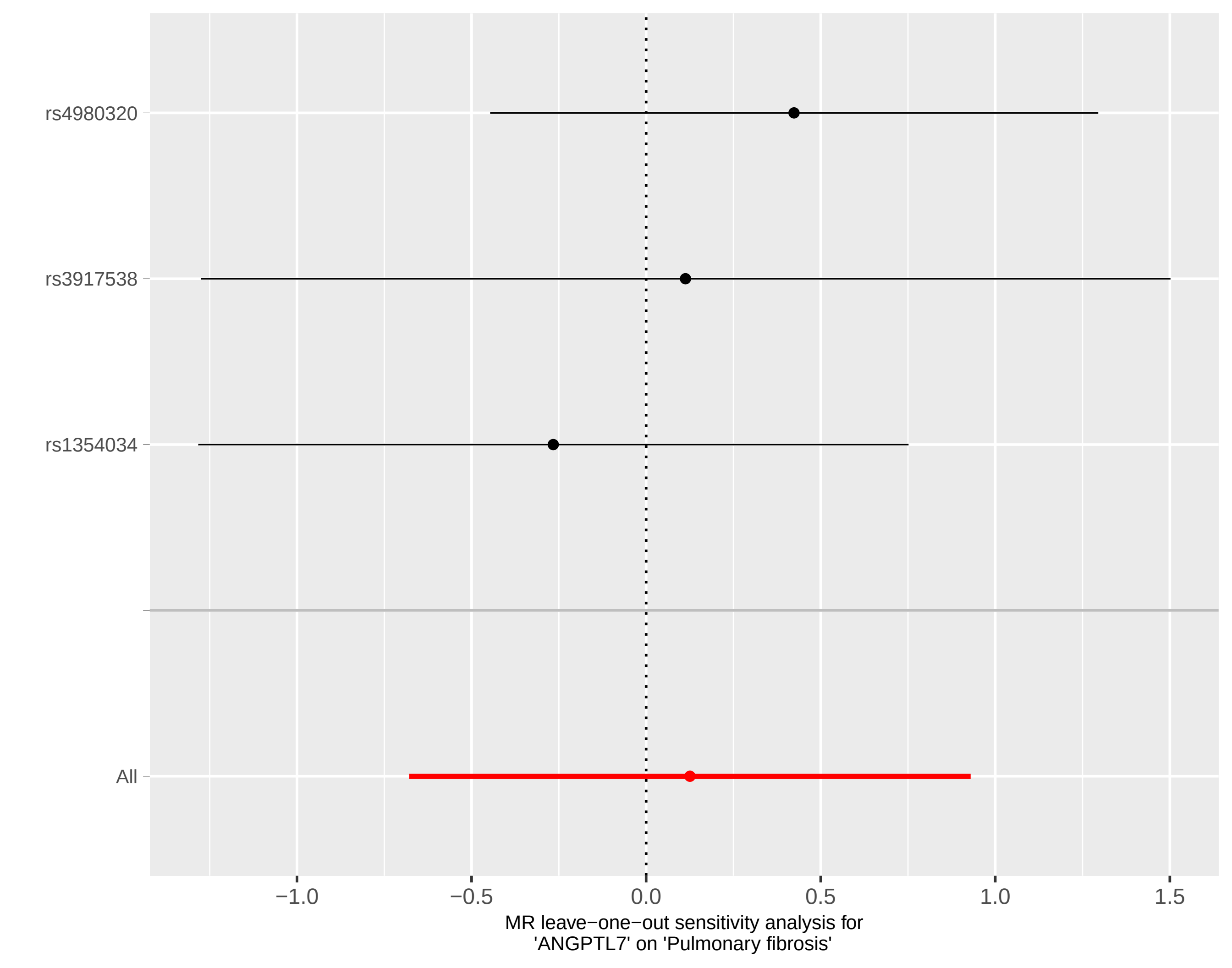
rs4980320
rs3917538
rs1354034
All
−1.0
−0.5
0.0
0.5
1.0
1.5
MR leave−one−out sensitivity analysis for
'ANGPTL7' on 'Pulmonary fibrosis'

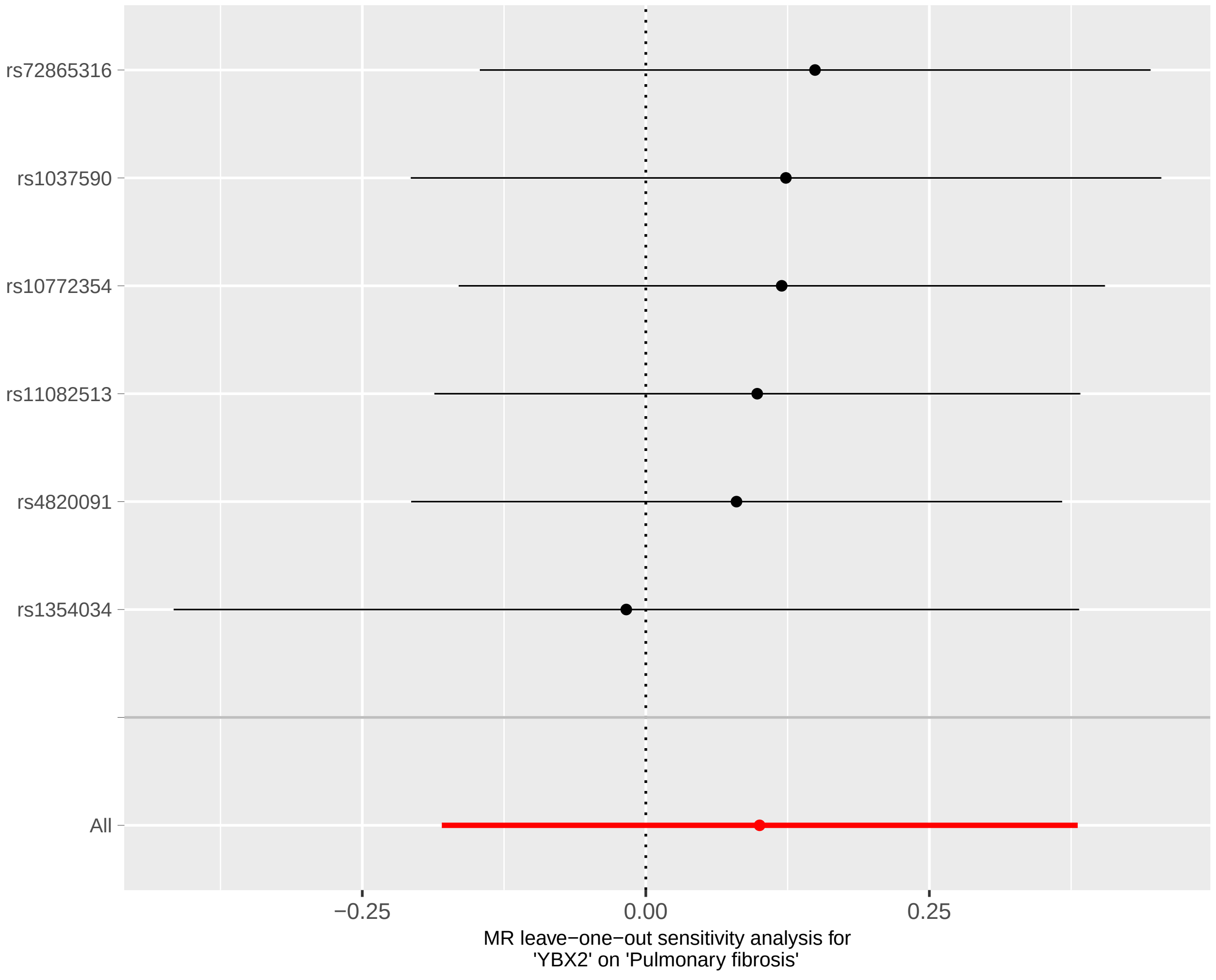
rs72865316
rs1037590
rs10772354
rs11082513
rs4820091
rs1354034
All
−0.25
0.00
0.25
MR leave−one−out sensitivity analysis for
'YBX2' on 'Pulmonary fibrosis'

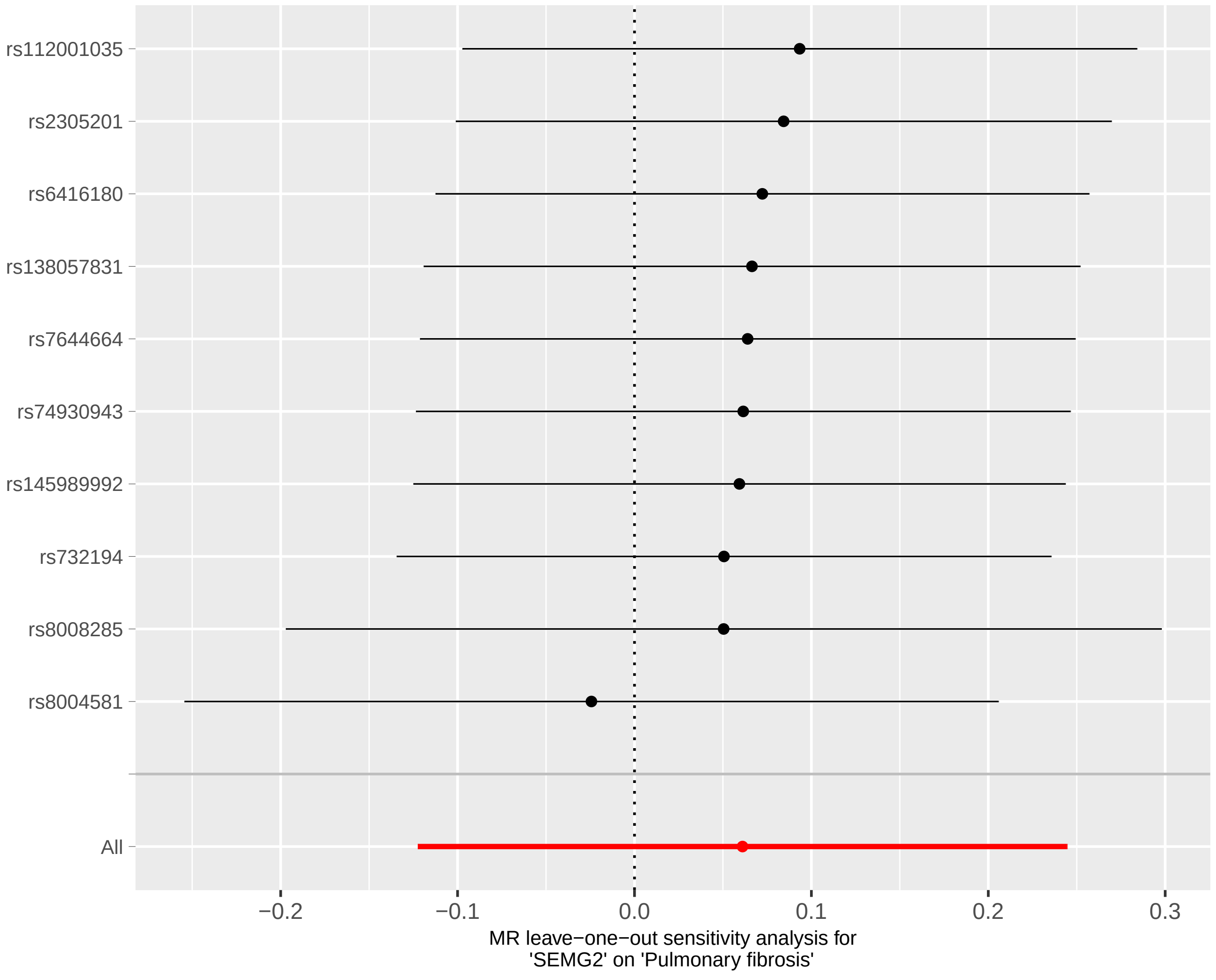
rs112001035
rs2305201
rs6416180
rs138057831
rs7644664
rs74930943
rs145989992
rs732194
rs8008285
rs8004581
All
−0.2
−0.1
0.0
0.1
0.2
0.3
MR leave−one−out sensitivity analysis for
'SEMG2' on 'Pulmonary fibrosis'

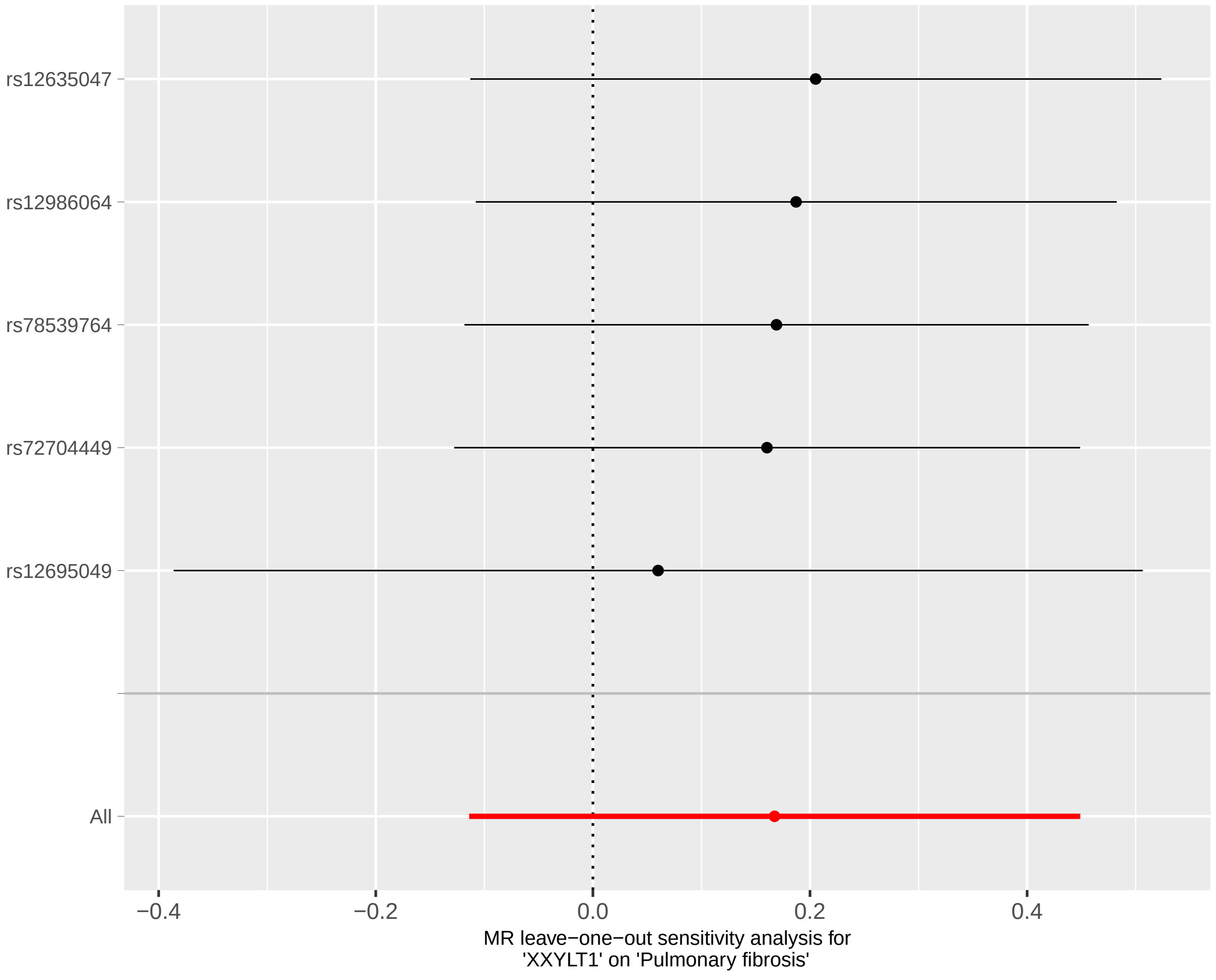
rs12635047
rs12986064
rs78539764
rs72704449
rs12695049
All
−0.4
−0.2
0.0
0.2
0.4
MR leave−one−out sensitivity analysis for
'XXYLT1' on 'Pulmonary fibrosis'

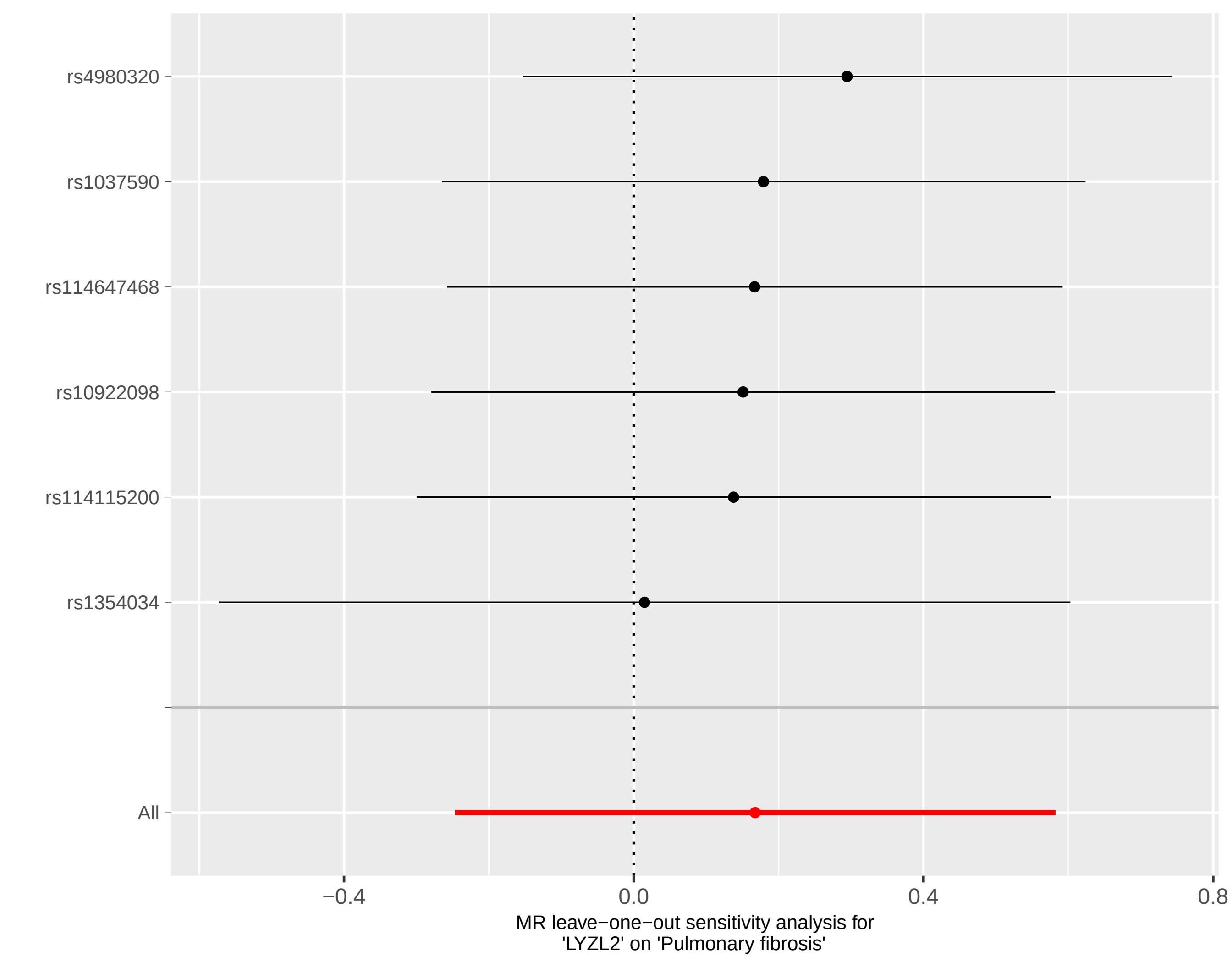
rs4980320
rs1037590
rs114647468
rs10922098
rs114115200
rs1354034
All
−0.4
0.0
0.4
0.8
MR leave−one−out sensitivity analysis for
'LYZL2' on 'Pulmonary fibrosis'

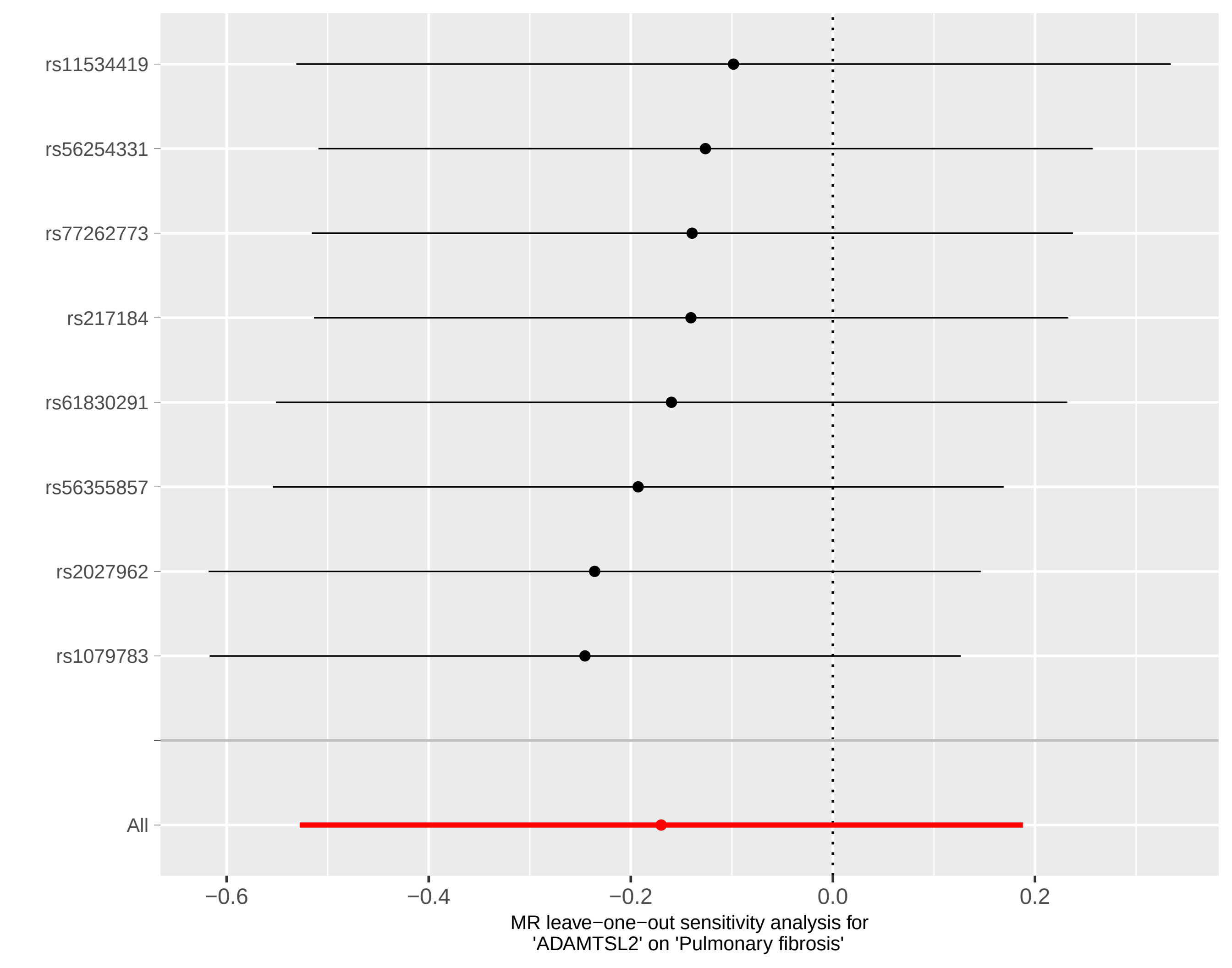
rs11534419
rs56254331
rs77262773
rs217184
rs61830291
rs56355857
rs2027962
rs1079783
All
−0.6
−0.4
−0.2
0.0
0.2
MR leave−one−out sensitivity analysis for
'ADAMTSL2' on 'Pulmonary fibrosis'

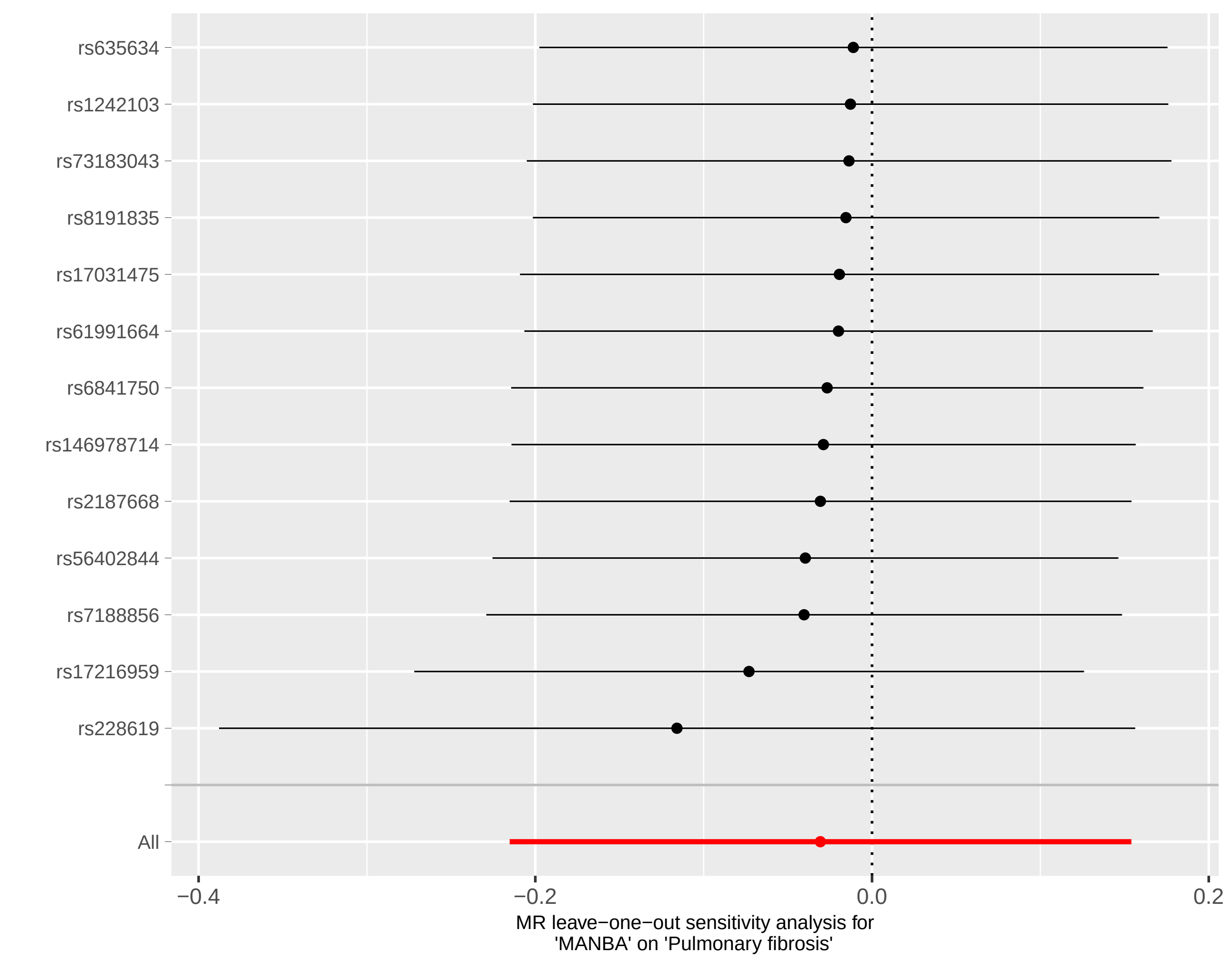

rs635634
rs1242103
rs73183043
rs8191835
rs17031475
rs61991664
rs6841750
rs146978714
rs2187668
rs56402844
rs7188856
rs17216959
rs228619
All
−0.4
−0.2
0.0
0.2
MR leave−one−out sensitivity analysis for
'MANBA' on 'Pulmonary fibrosis'

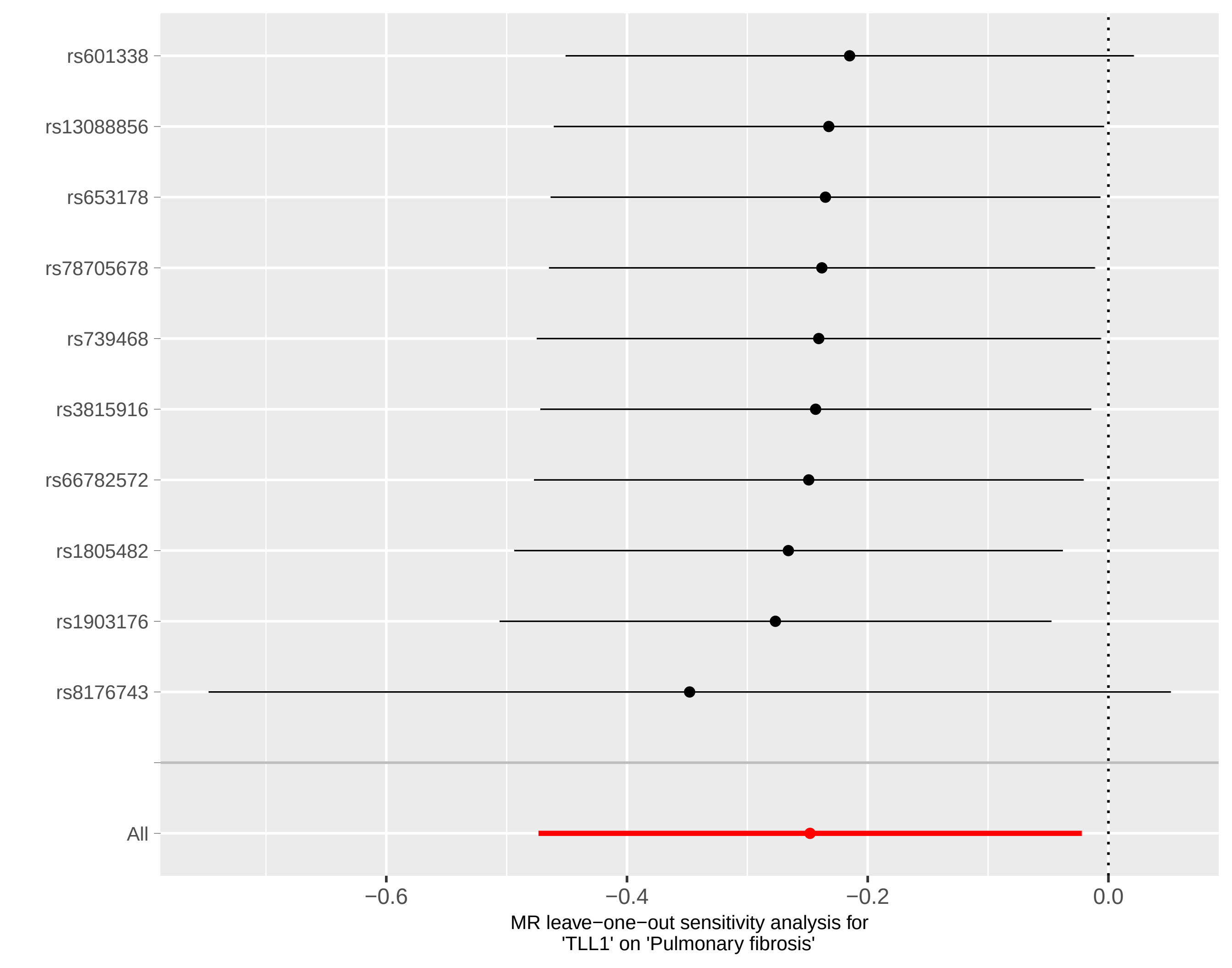
rs601338
rs13088856
rs653178
rs78705678
rs739468
rs3815916
rs66782572
rs1805482
rs1903176
rs8176743
All
−0.6
−0.4
−0.2
0.0
MR leave−one−out sensitivity analysis for
'TLL1' on 'Pulmonary fibrosis'

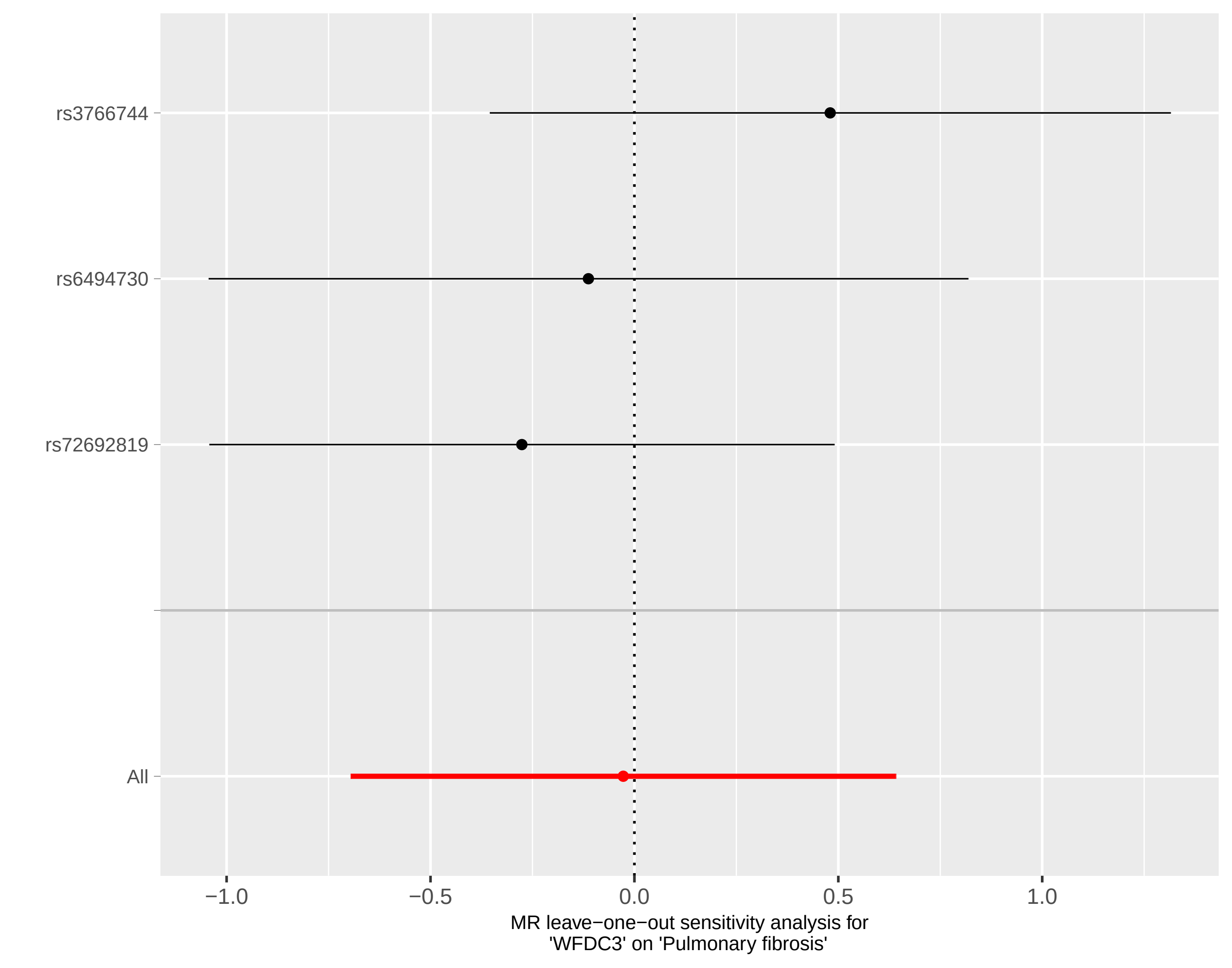
rs3766744
rs6494730
rs72692819
All
−1.0
−0.5
0.0
0.5
1.0
MR leave−one−out sensitivity analysis for
'WFDC3' on 'Pulmonary fibrosis'

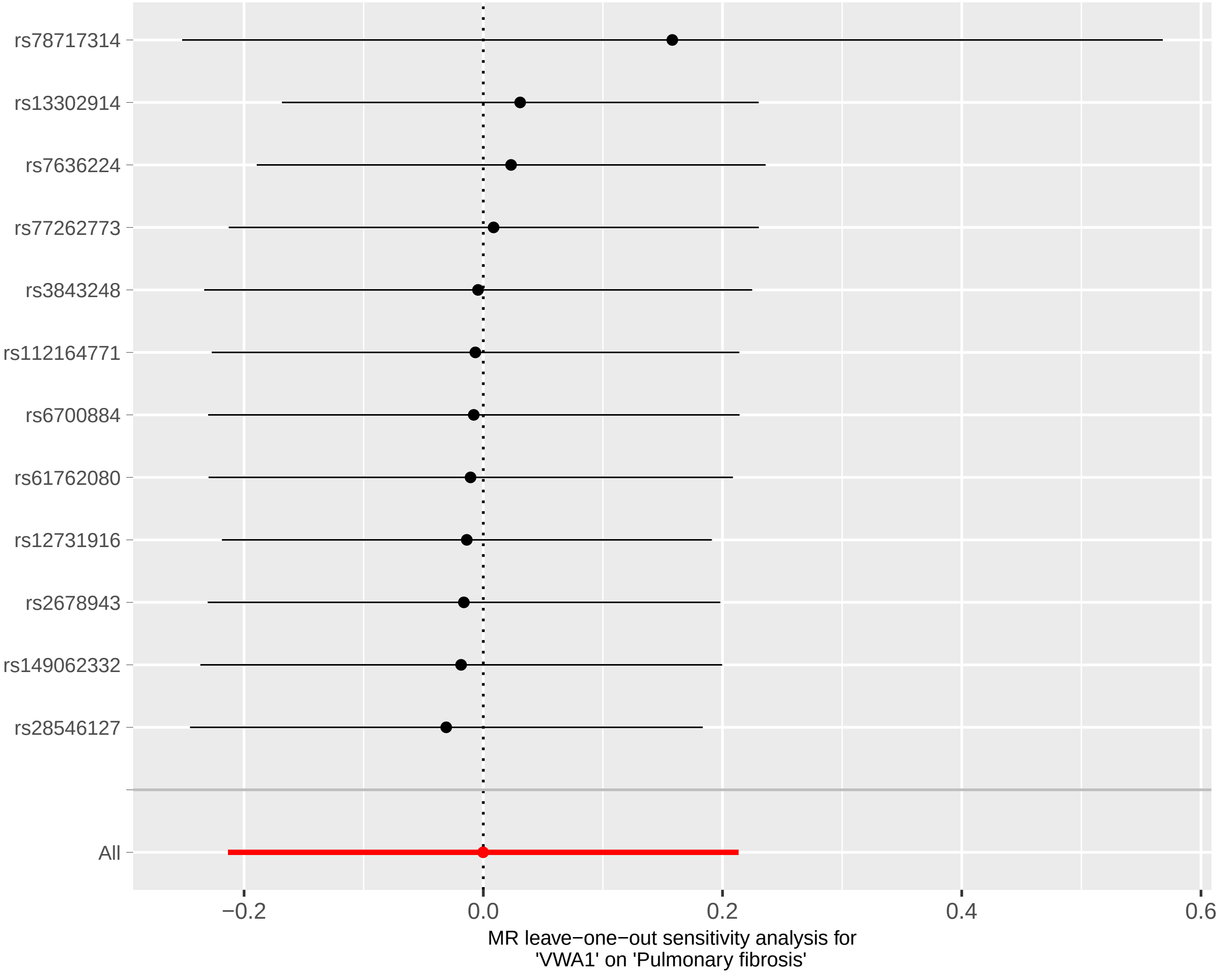
rs78717314
rs13302914
rs7636224
rs77262773
rs3843248
rs112164771
rs6700884
rs61762080
rs12731916
rs2678943
rs149062332
rs28546127
All
−0.2
0.0
0.2
0.4
0.6
MR leave−one−out sensitivity analysis for
'VWA1' on 'Pulmonary fibrosis'

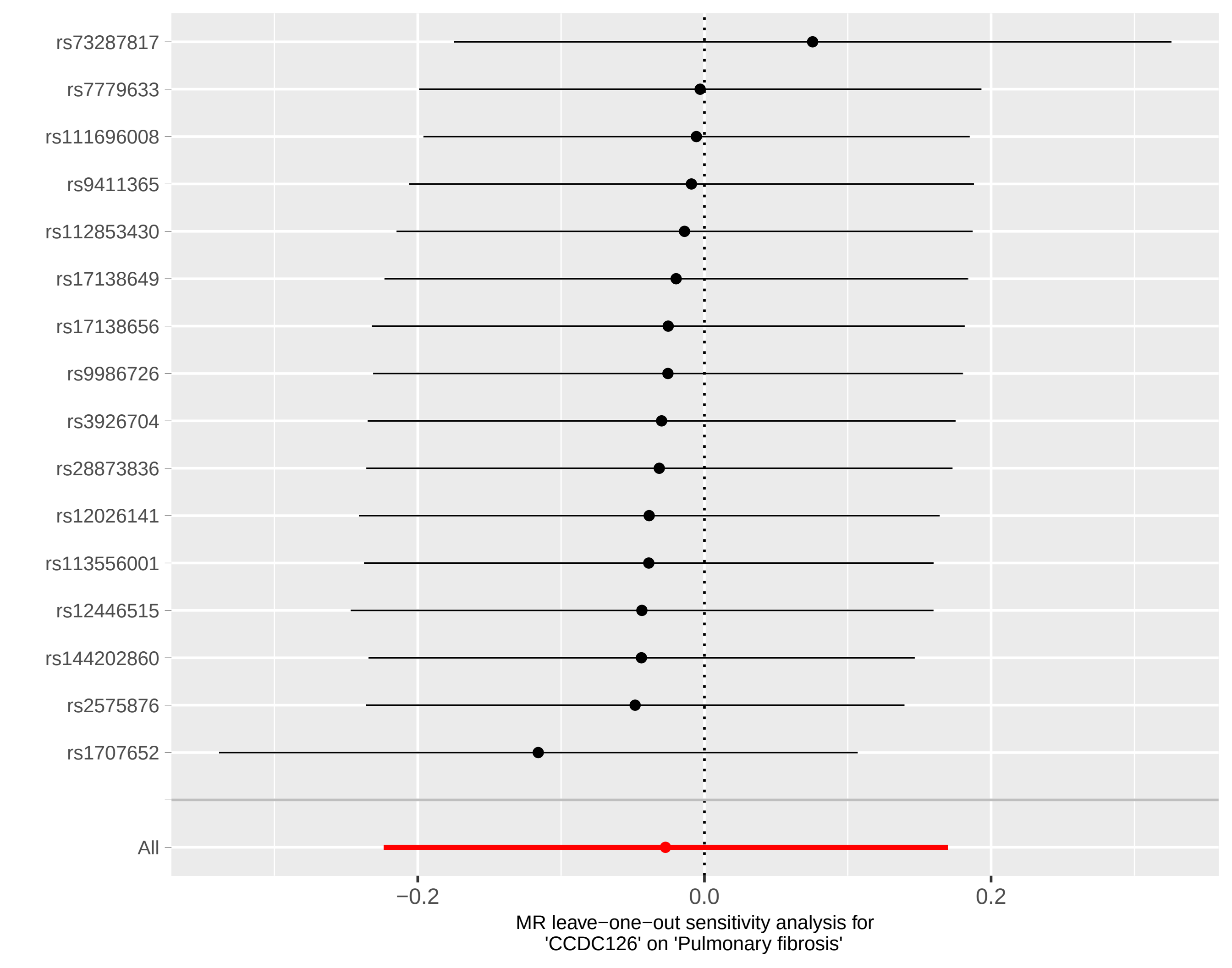
rs73287817
rs7779633
rs111696008
rs9411365
rs112853430
rs17138649
rs17138656
rs9986726
rs3926704
rs28873836
rs12026141
rs113556001
rs12446515
rs144202860
rs2575876
rs1707652
All
−0.2
0.0
0.2
MR leave−one−out sensitivity analysis for
'CCDC126' on 'Pulmonary fibrosis'

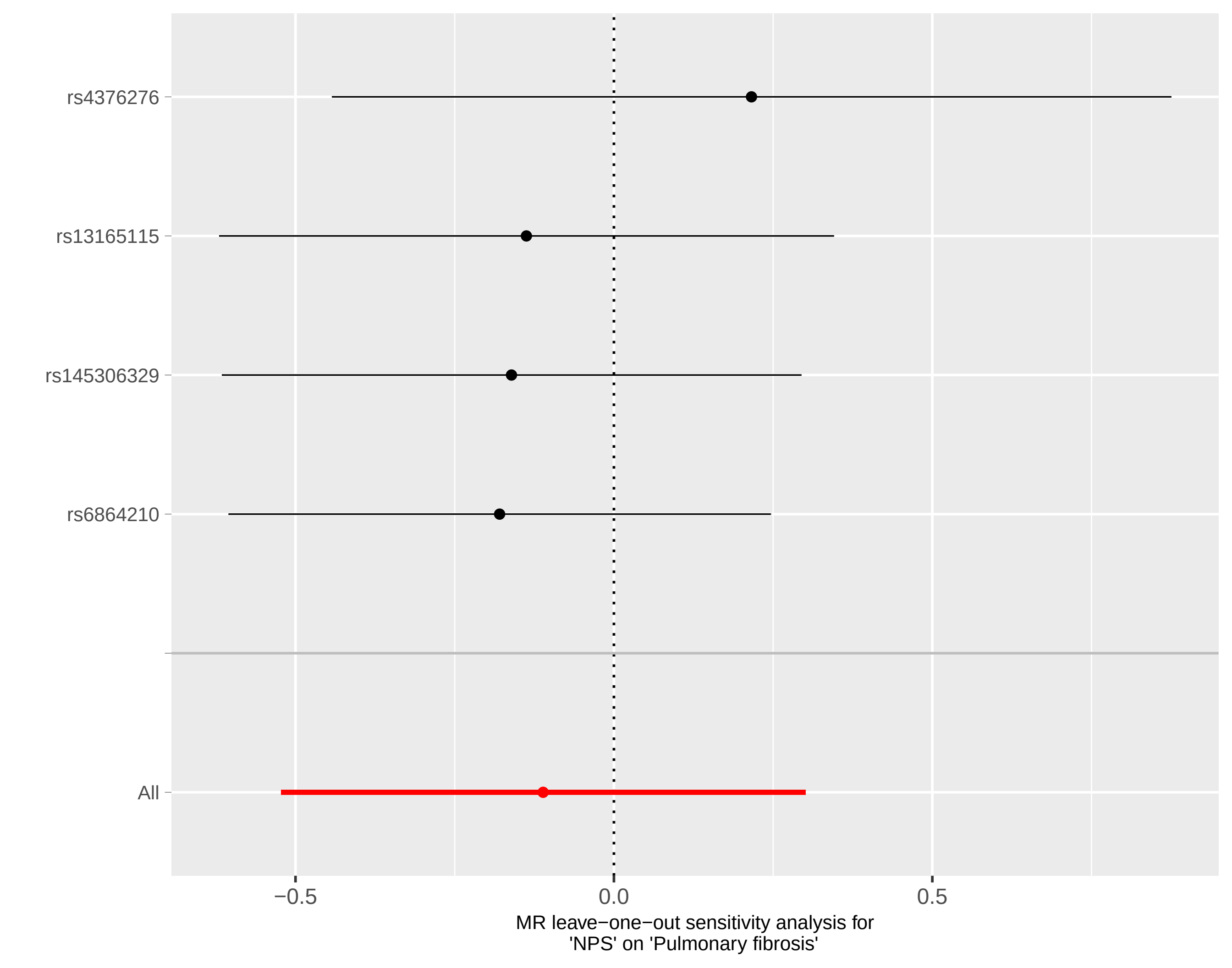
rs4376276
rs13165115
rs145306329
rs6864210
All
−0.5
0.0
0.5
MR leave−one−out sensitivity analysis for
'NPS' on 'Pulmonary fibrosis'

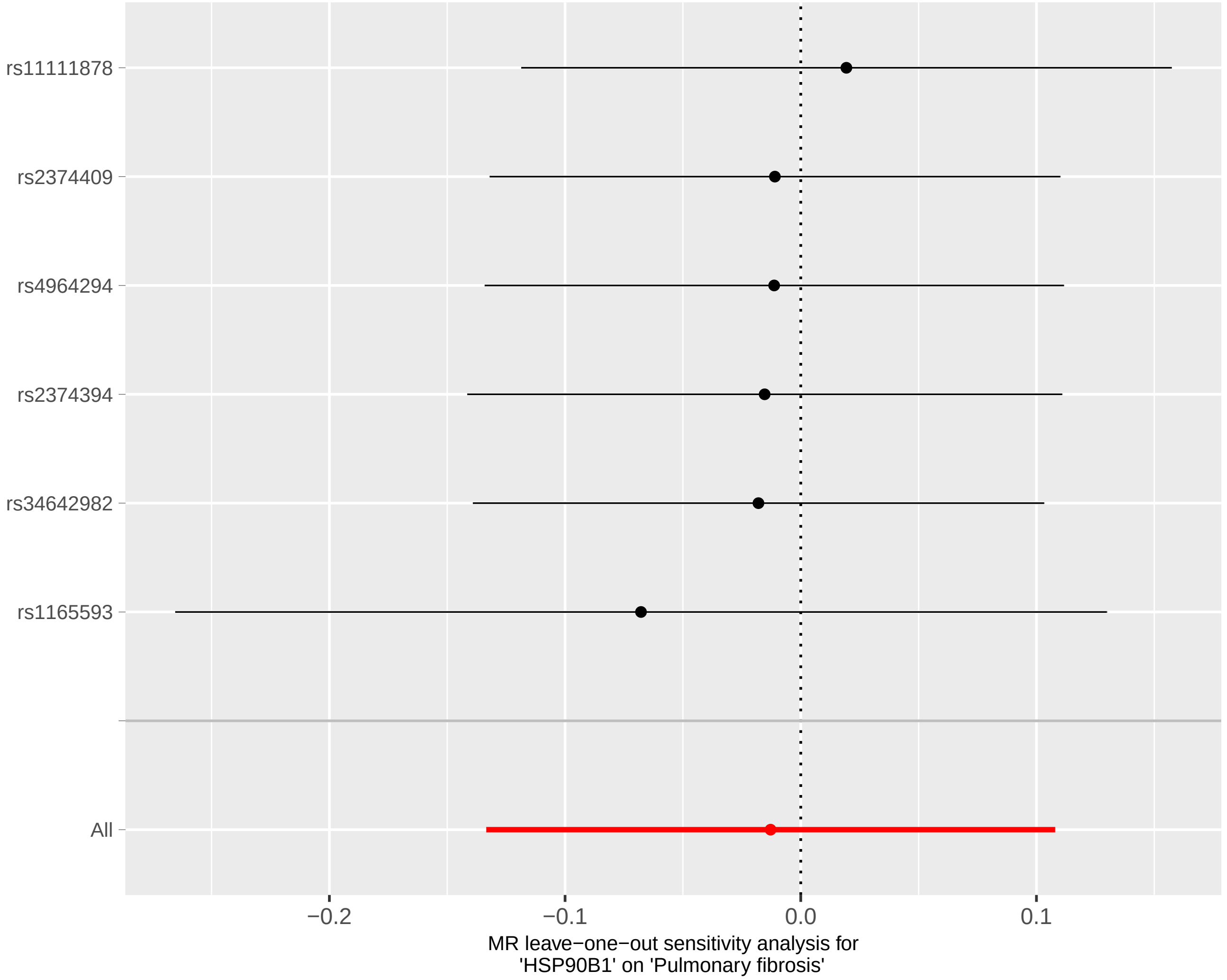
rs11111878
rs2374409
rs4964294
rs2374394
rs34642982
rs1165593
All
−0.2
−0.1
0.0
0.1
MR leave−one−out sensitivity analysis for
'HSP90B1' on 'Pulmonary fibrosis'

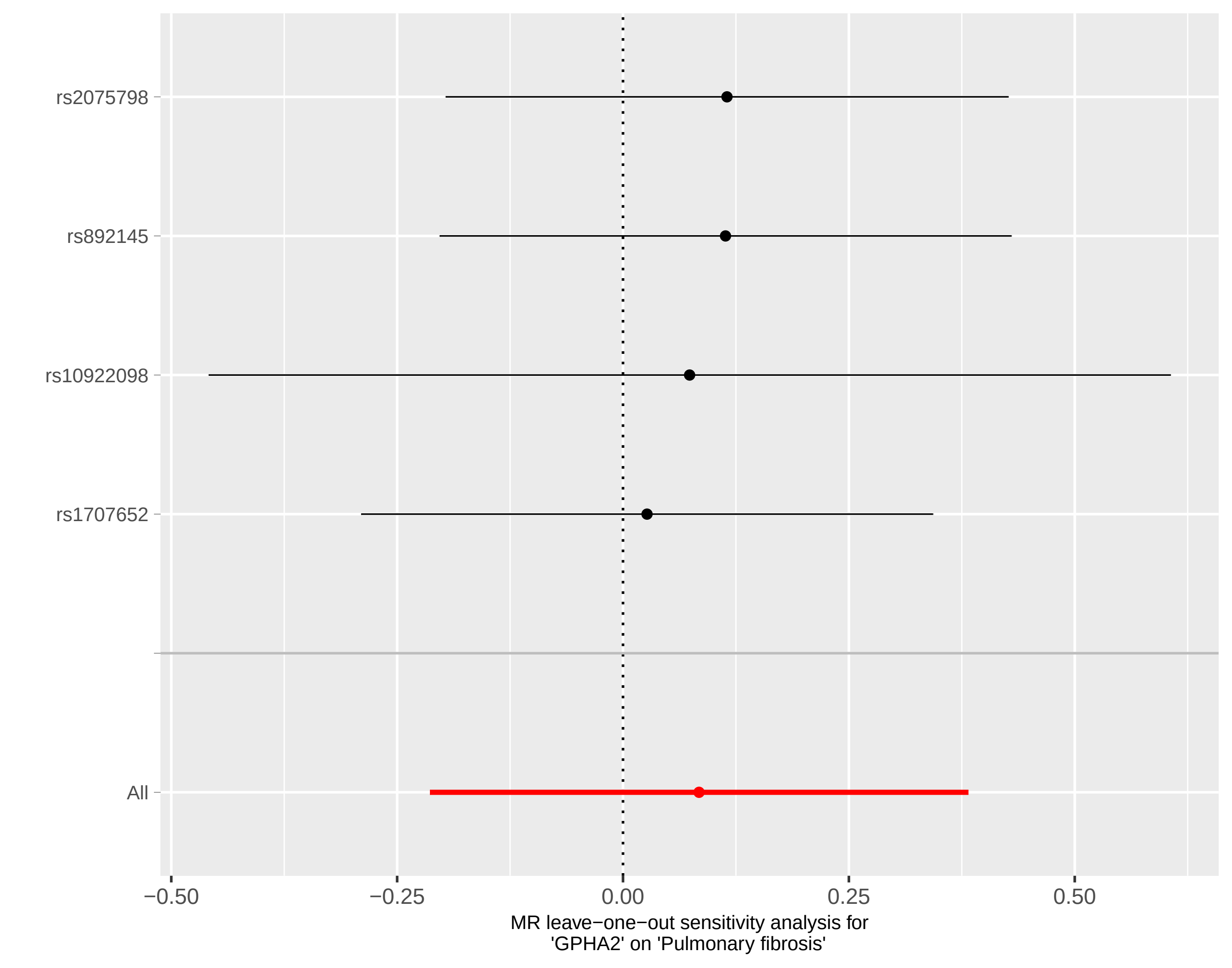

rs2075798
rs892145
rs10922098
rs1707652
All
−0.50
−0.25
0.00
0.25
0.50
MR leave−one−out sensitivity analysis for
'GPHA2' on 'Pulmonary fibrosis'

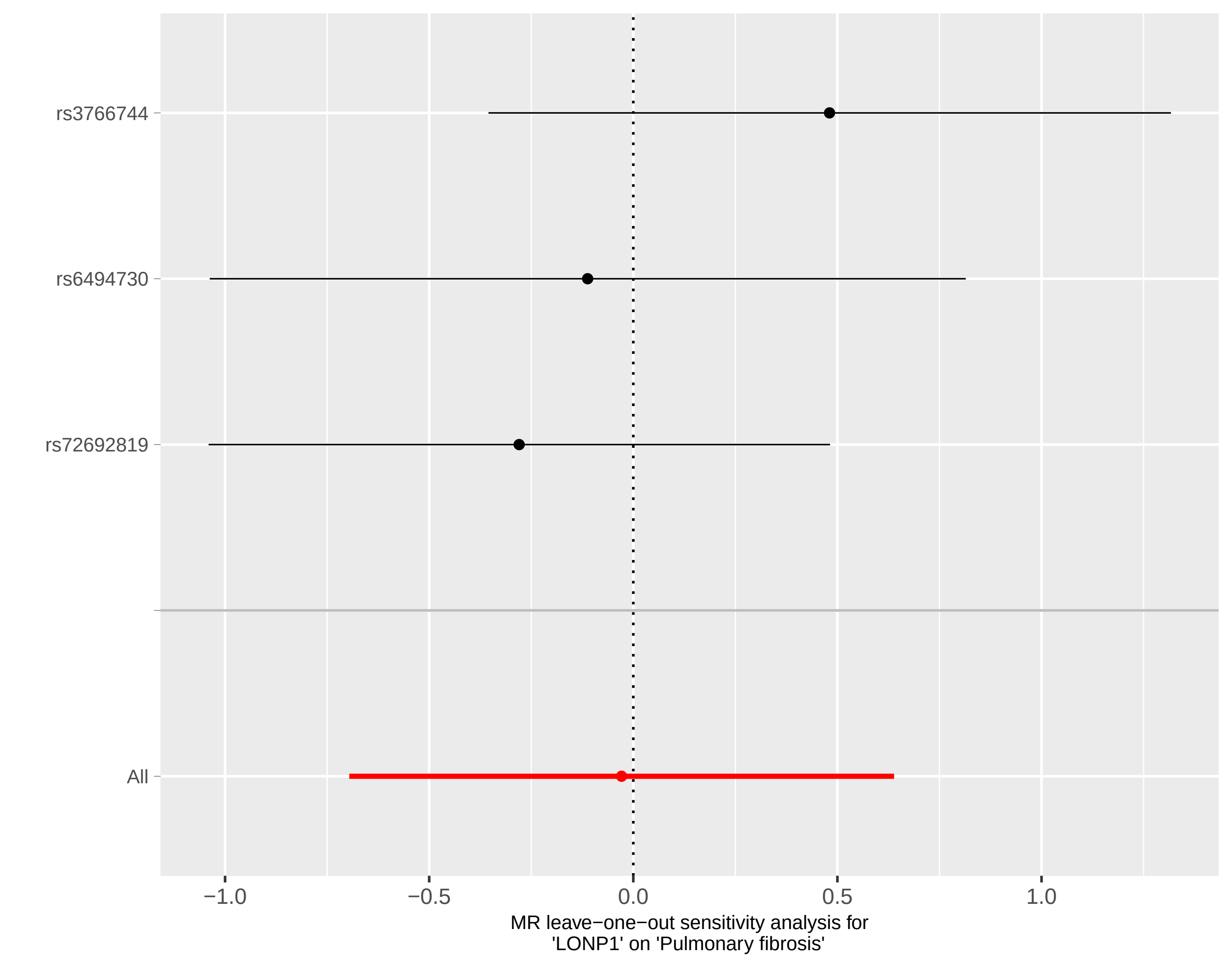

rs3766744
rs6494730
rs72692819
All
−1.0
−0.5
0.0
0.5
1.0
MR leave−one−out sensitivity analysis for
'LONP1' on 'Pulmonary fibrosis'

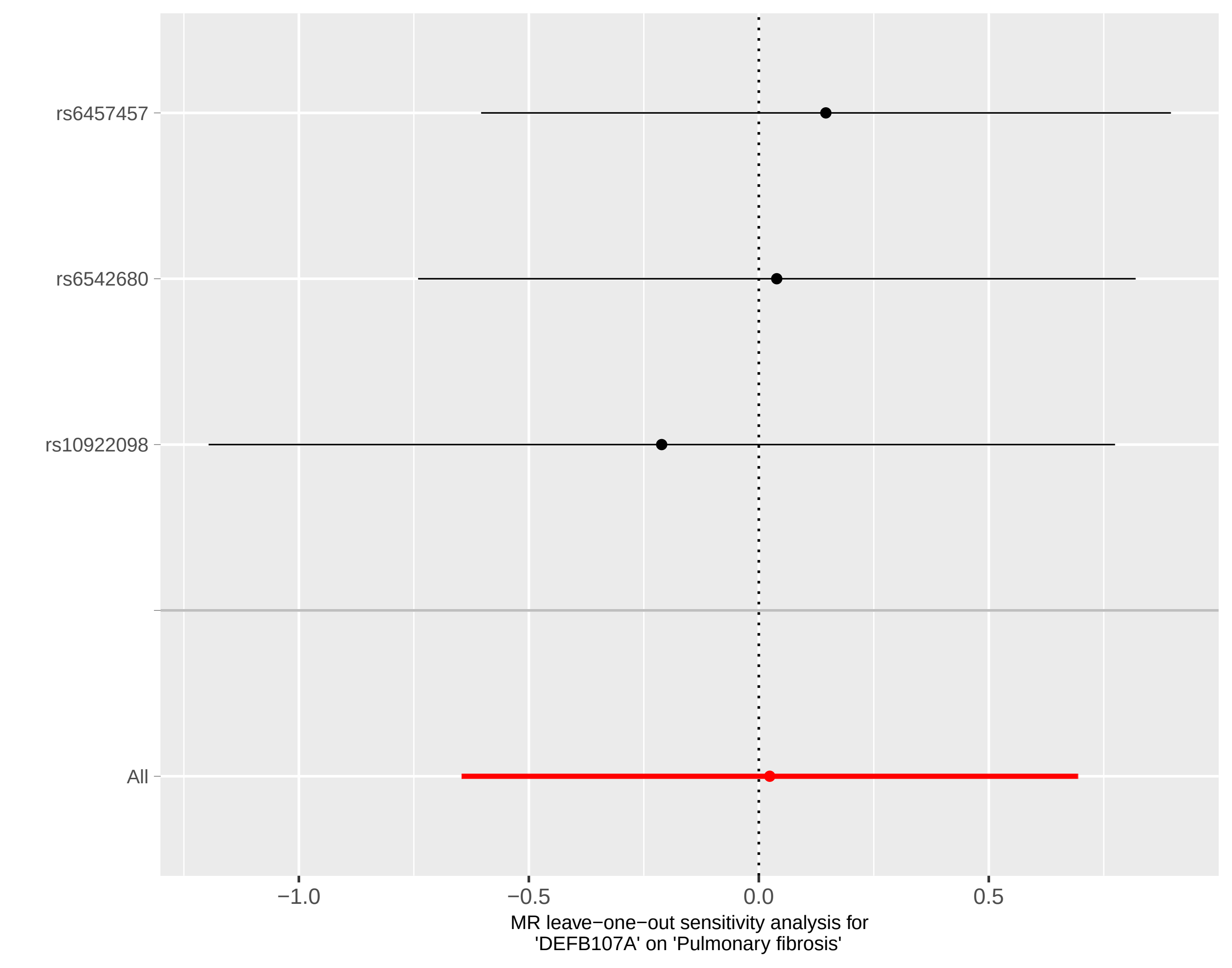
rs6457457
rs6542680
rs10922098
All
−1.0
−0.5
0.0
0.5
MR leave−one−out sensitivity analysis for
'DEFB107A' on 'Pulmonary fibrosis'

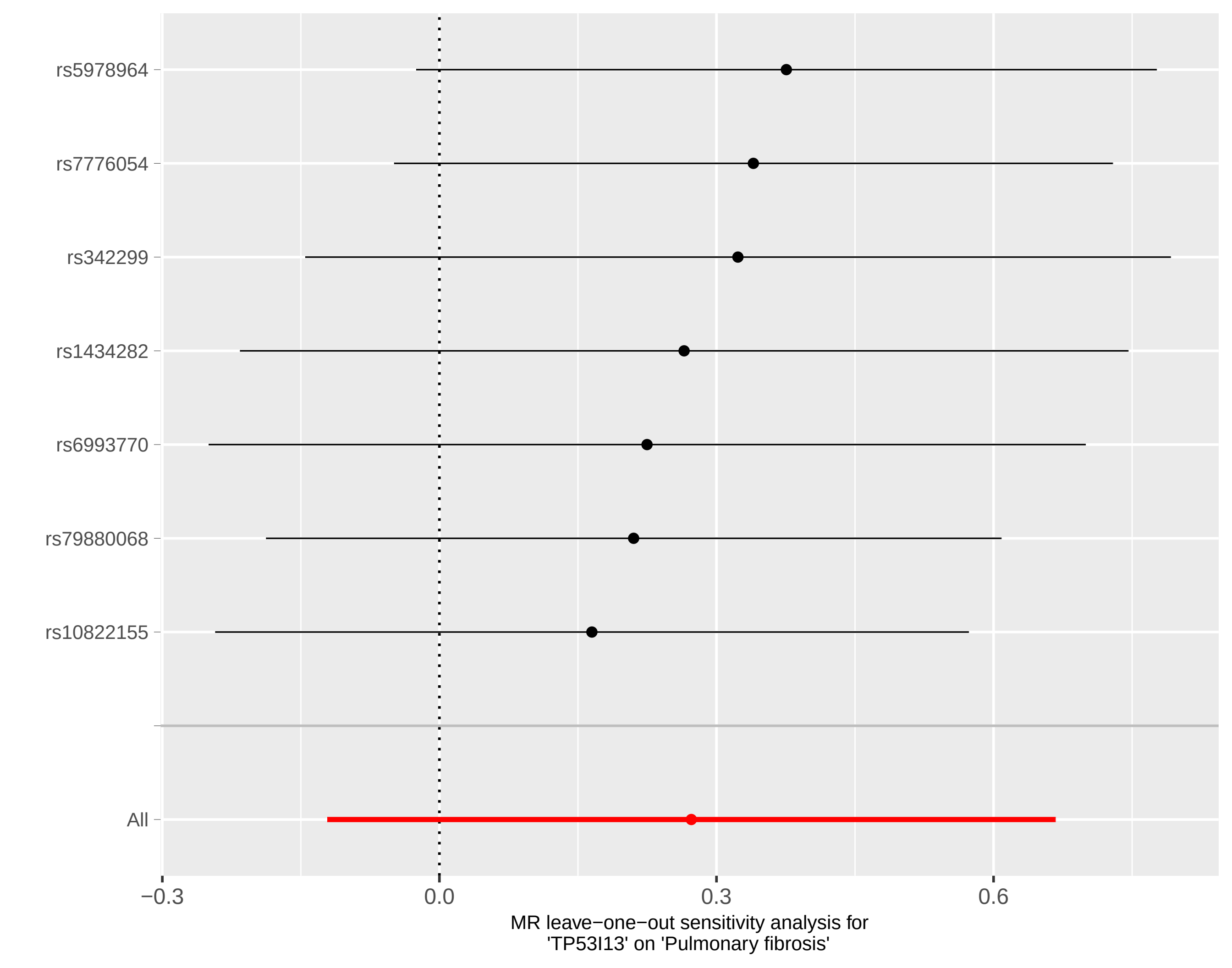
rs5978964
rs7776054
rs342299
rs1434282
rs6993770
rs79880068
rs10822155
All
−0.3
0.0
0.3
0.6
MR leave−one−out sensitivity analysis for
'TP53I13' on 'Pulmonary fibrosis'

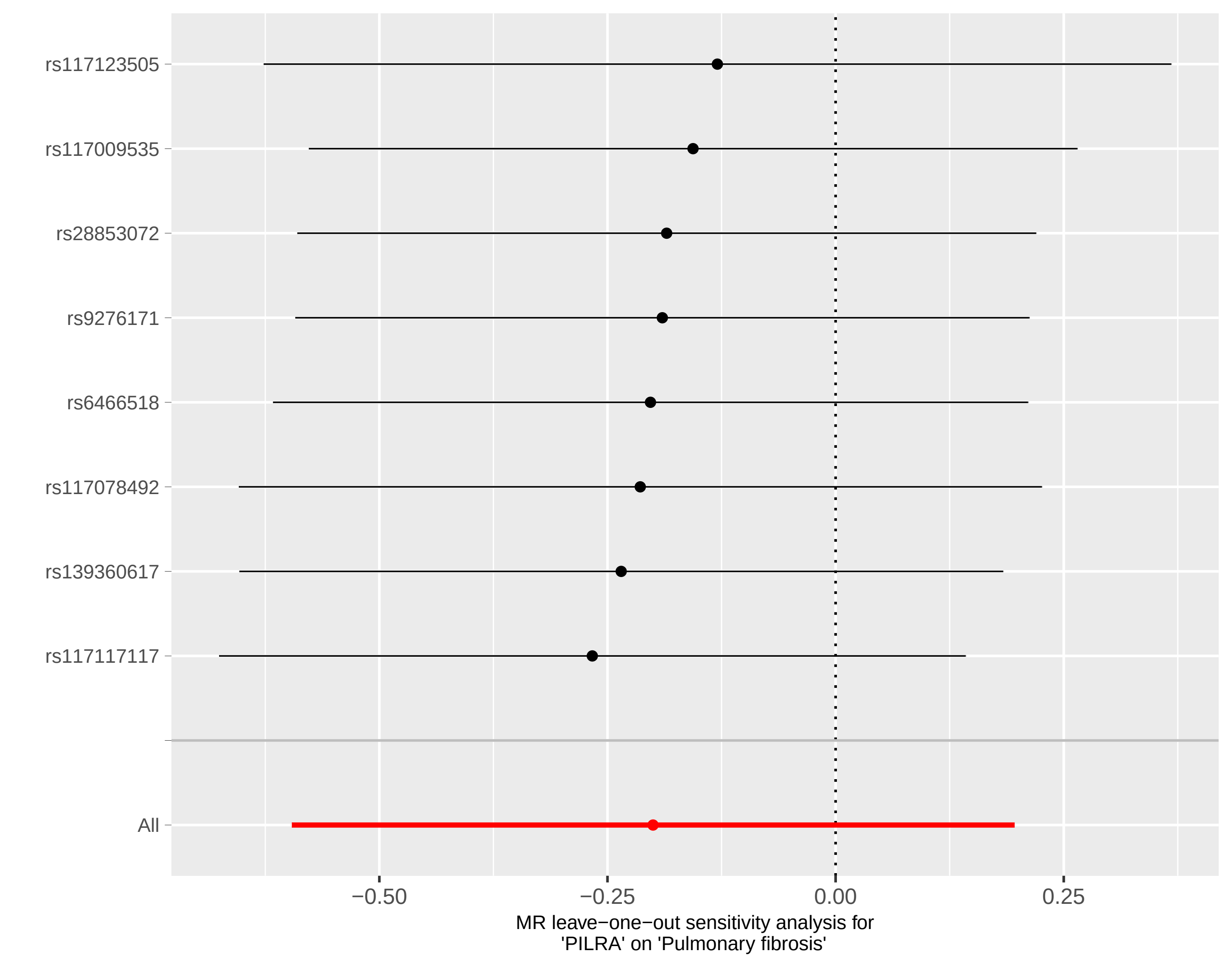

rs117123505
rs117009535
rs28853072
rs9276171
rs6466518
rs117078492
rs139360617
rs117117117
All
−0.50
−0.25
0.00
0.25
MR leave−one−out sensitivity analysis for
'PILRA' on 'Pulmonary fibrosis'

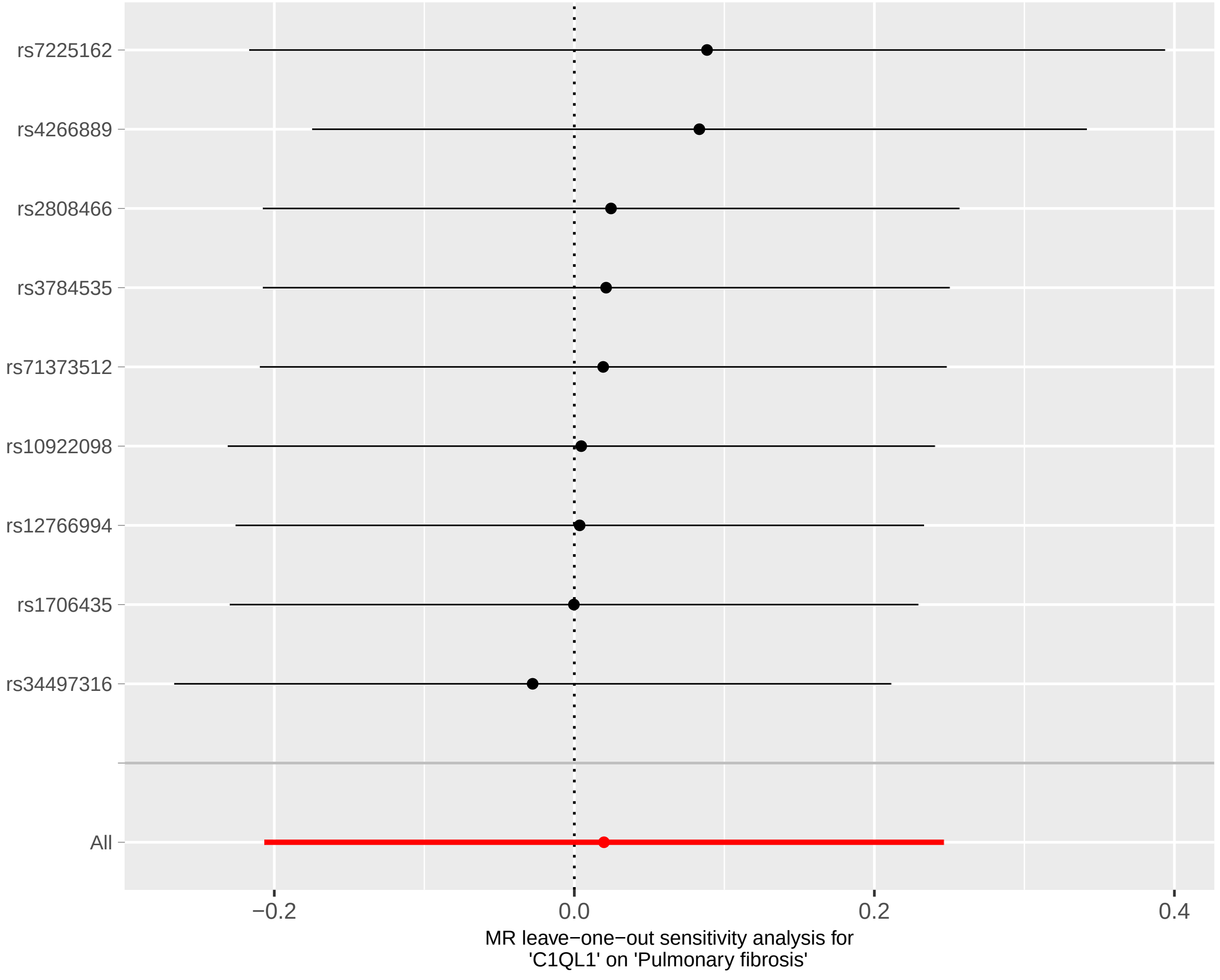
rs7225162
rs4266889
rs2808466
rs3784535
rs71373512
rs10922098
rs12766994
rs1706435
rs34497316
All
−0.2
0.0
0.2
0.4
MR leave−one−out sensitivity analysis for
'C1QL1' on 'Pulmonary fibrosis'

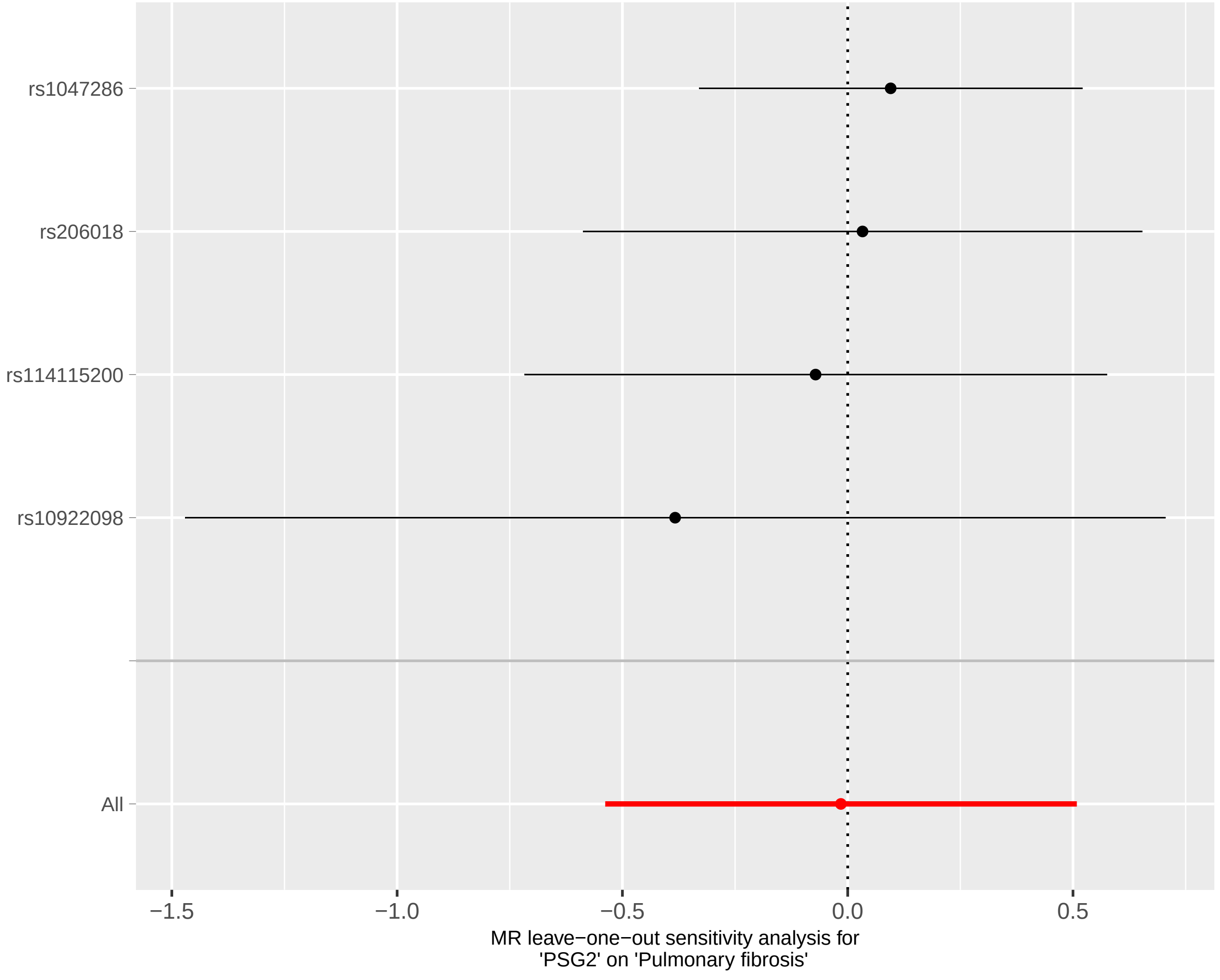
rs1047286
rs206018
rs114115200
rs10922098
All
−1.5
−1.0
−0.5
0.0
0.5
MR leave−one−out sensitivity analysis for
'PSG2' on 'Pulmonary fibrosis'

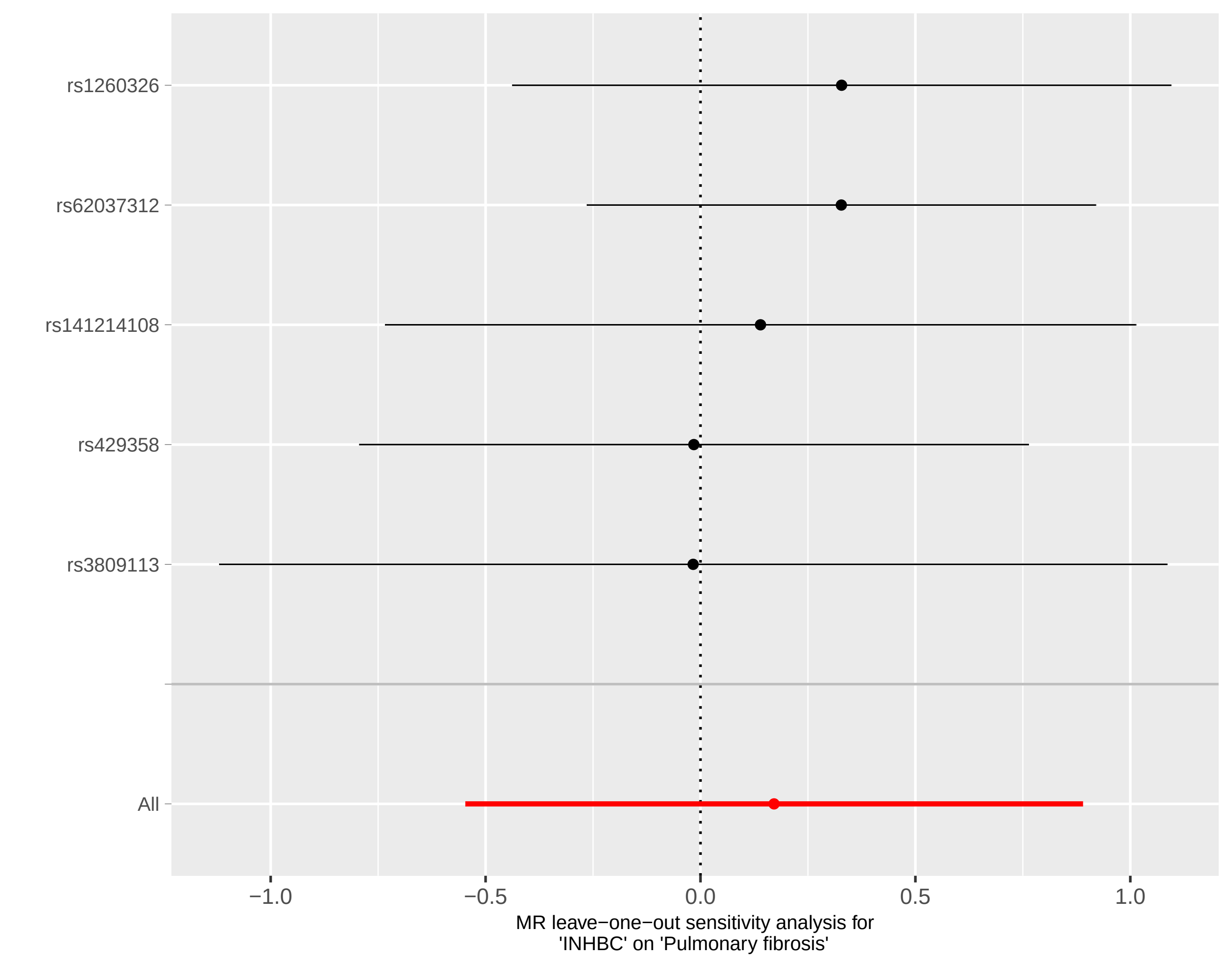
rs1260326
rs62037312
rs141214108
rs429358
rs3809113
All
−1.0
−0.5
0.0
0.5
1.0
MR leave−one−out sensitivity analysis for
'INHBC' on 'Pulmonary fibrosis'

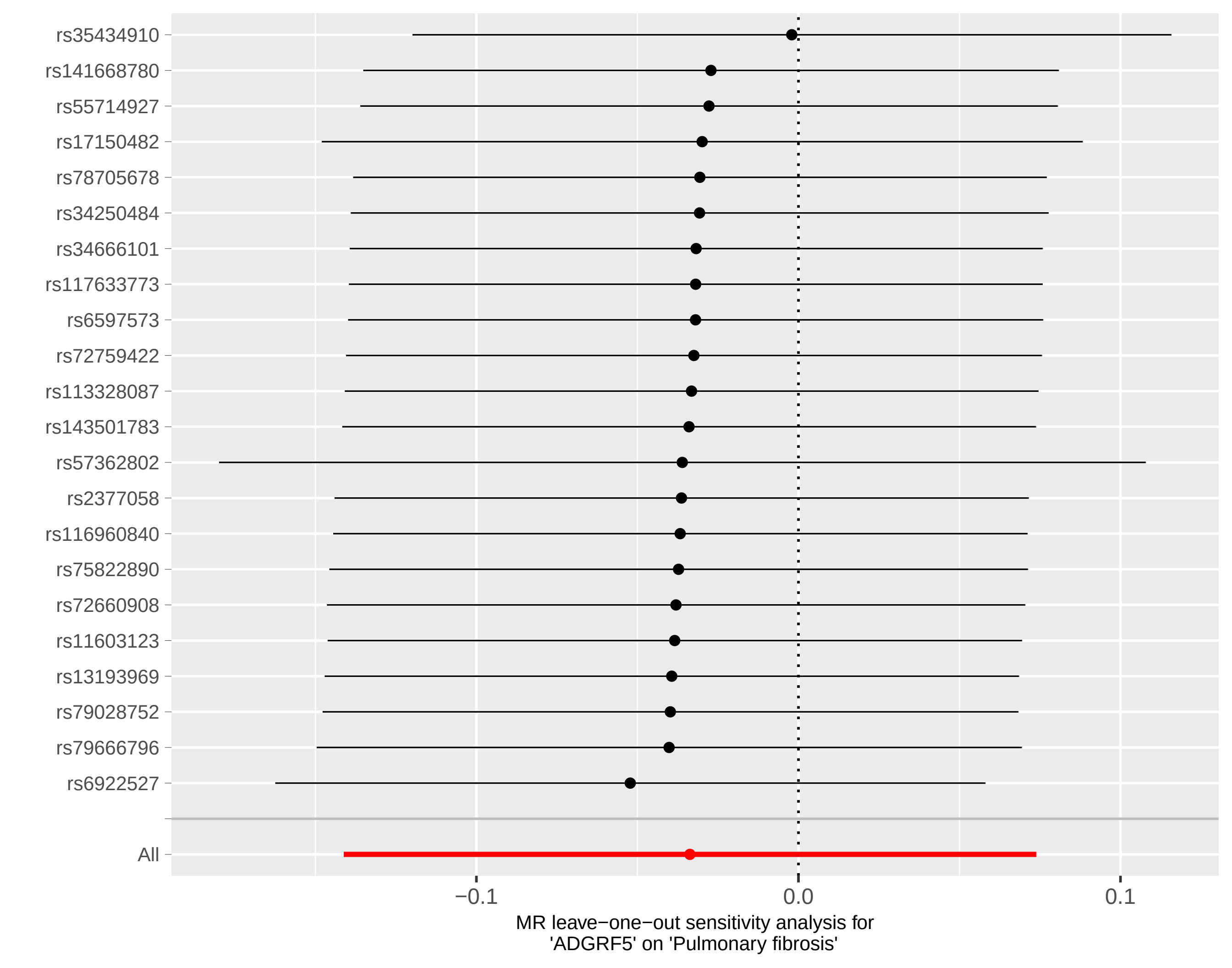
rs35434910
rs141668780
rs55714927
rs17150482
rs78705678
rs34250484
rs34666101
rs117633773
rs6597573
rs72759422
rs113328087
rs143501783
rs57362802
rs2377058
rs116960840
rs75822890
rs72660908
rs11603123
rs13193969
rs79028752
rs79666796
rs6922527
All
−0.1
0.0
0.1
MR leave−one−out sensitivity analysis for
'ADGRF5' on 'Pulmonary fibrosis'

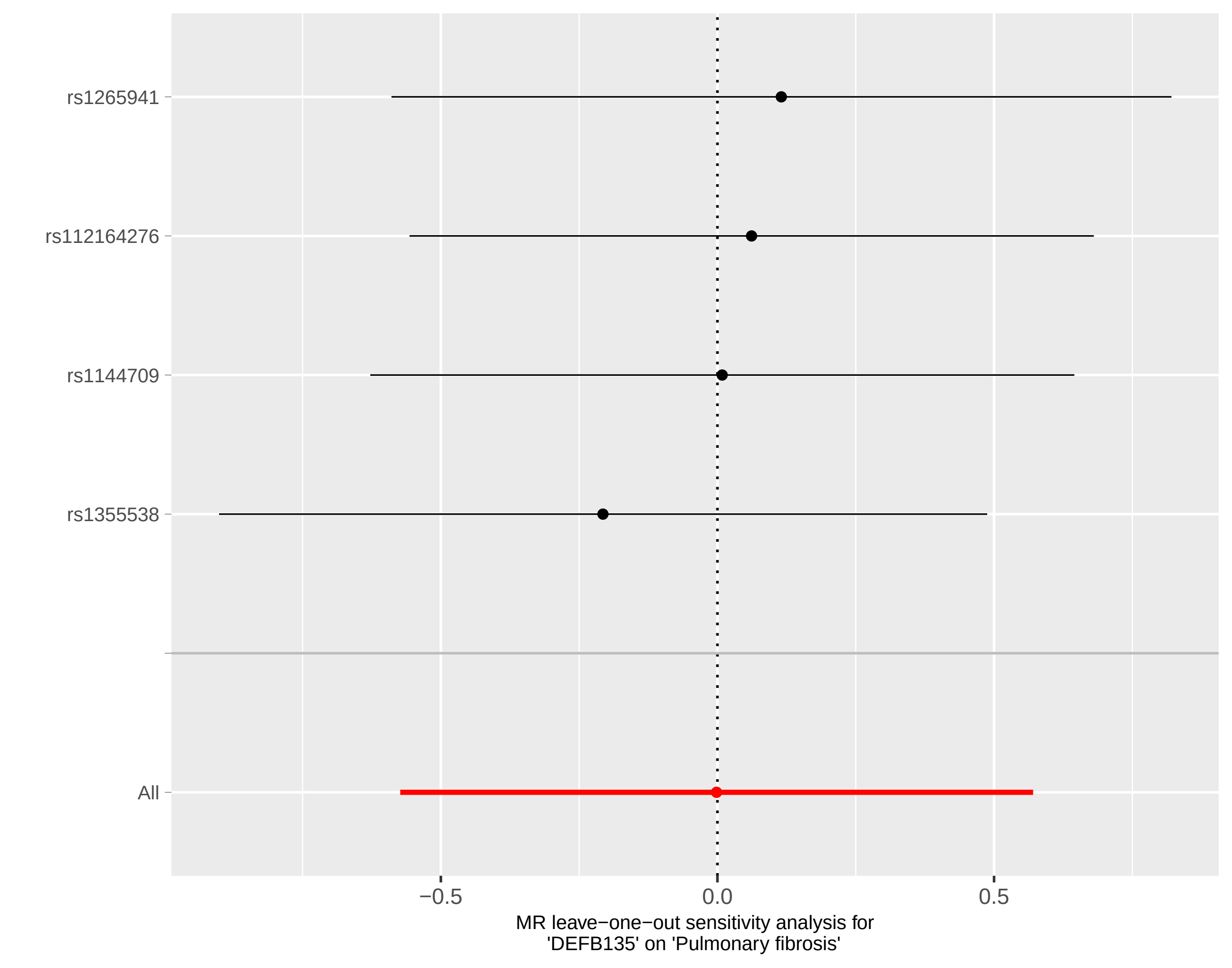
rs1265941
rs112164276
rs1144709
rs1355538
All
−0.5
0.0
0.5
MR leave−one−out sensitivity analysis for
'DEFB135' on 'Pulmonary fibrosis'

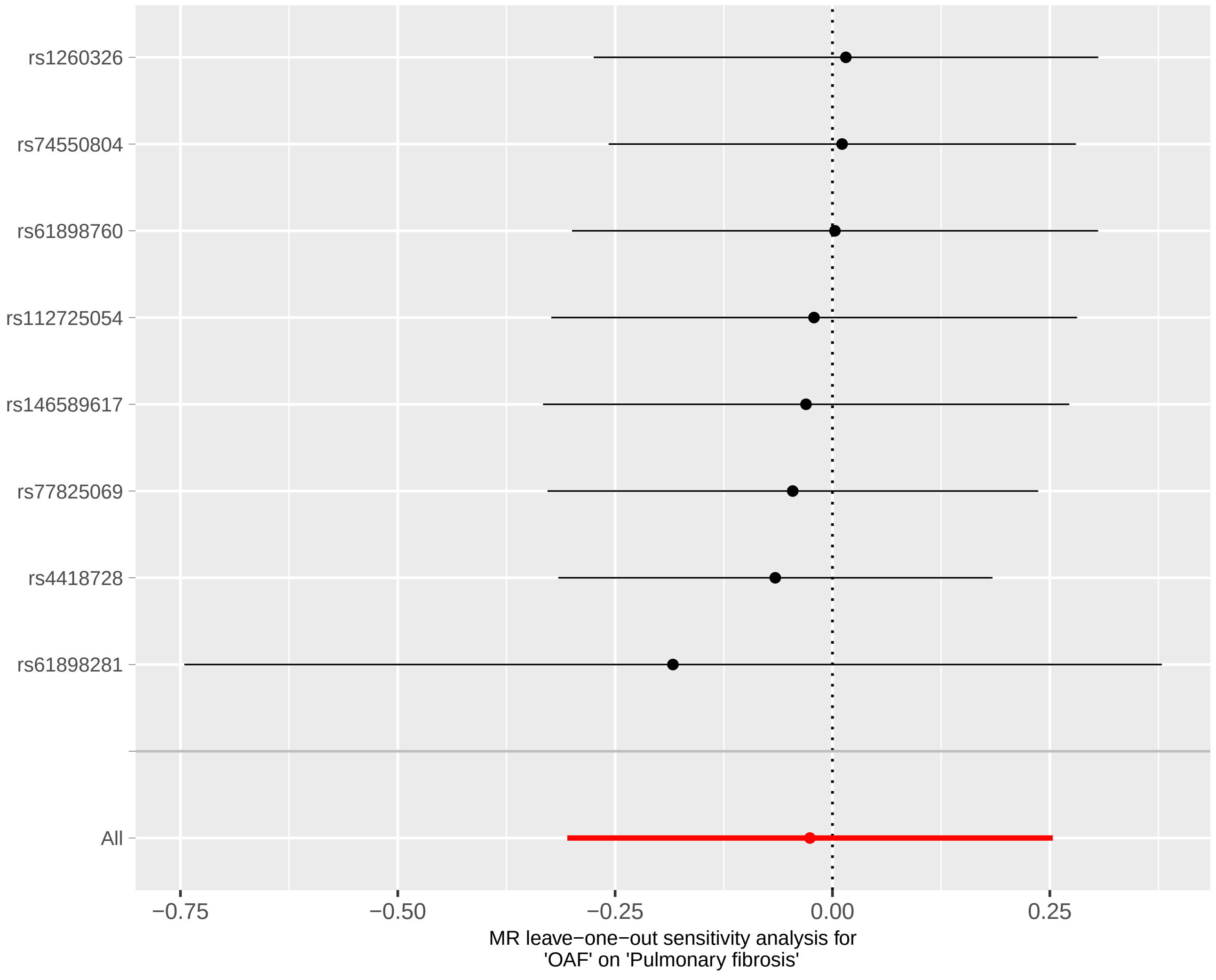

rs1260326
rs74550804
rs61898760
rs112725054
rs146589617
rs77825069
rs4418728
rs61898281
All
−0.75
−0.50
−0.25
0.00
0.25
MR leave−one−out sensitivity analysis for
'OAF' on 'Pulmonary fibrosis'

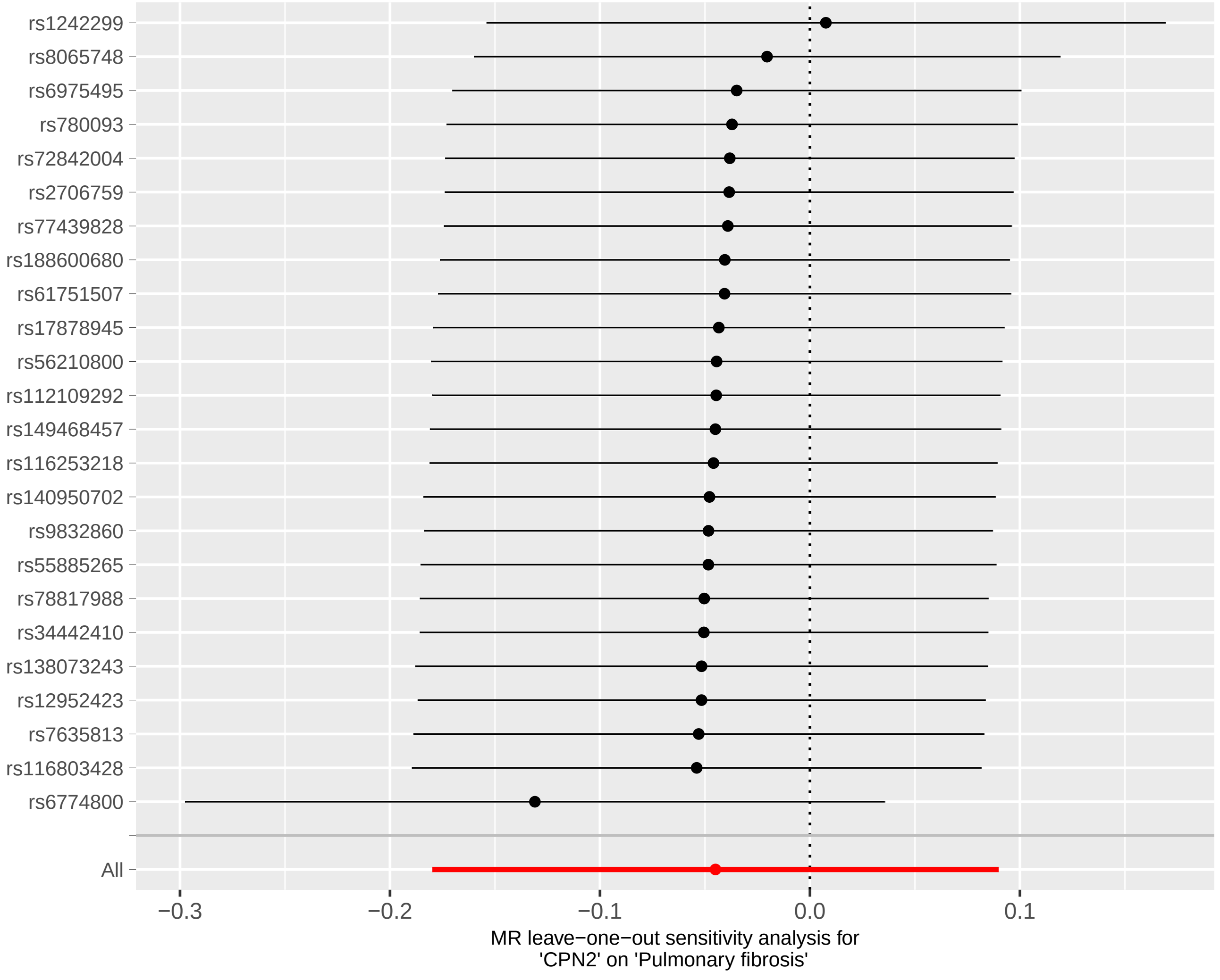
rs1242299
rs8065748
rs6975495
rs780093
rs72842004
rs2706759
rs77439828
rs188600680
rs61751507
rs17878945
rs56210800
rs112109292
rs149468457
rs116253218
rs140950702
rs9832860
rs55885265
rs78817988
rs34442410
rs138073243
rs12952423
rs7635813
rs116803428
rs6774800
All
−0.3
−0.2
−0.1
0.0
0.1
MR leave−one−out sensitivity analysis for
'CPN2' on 'Pulmonary fibrosis'

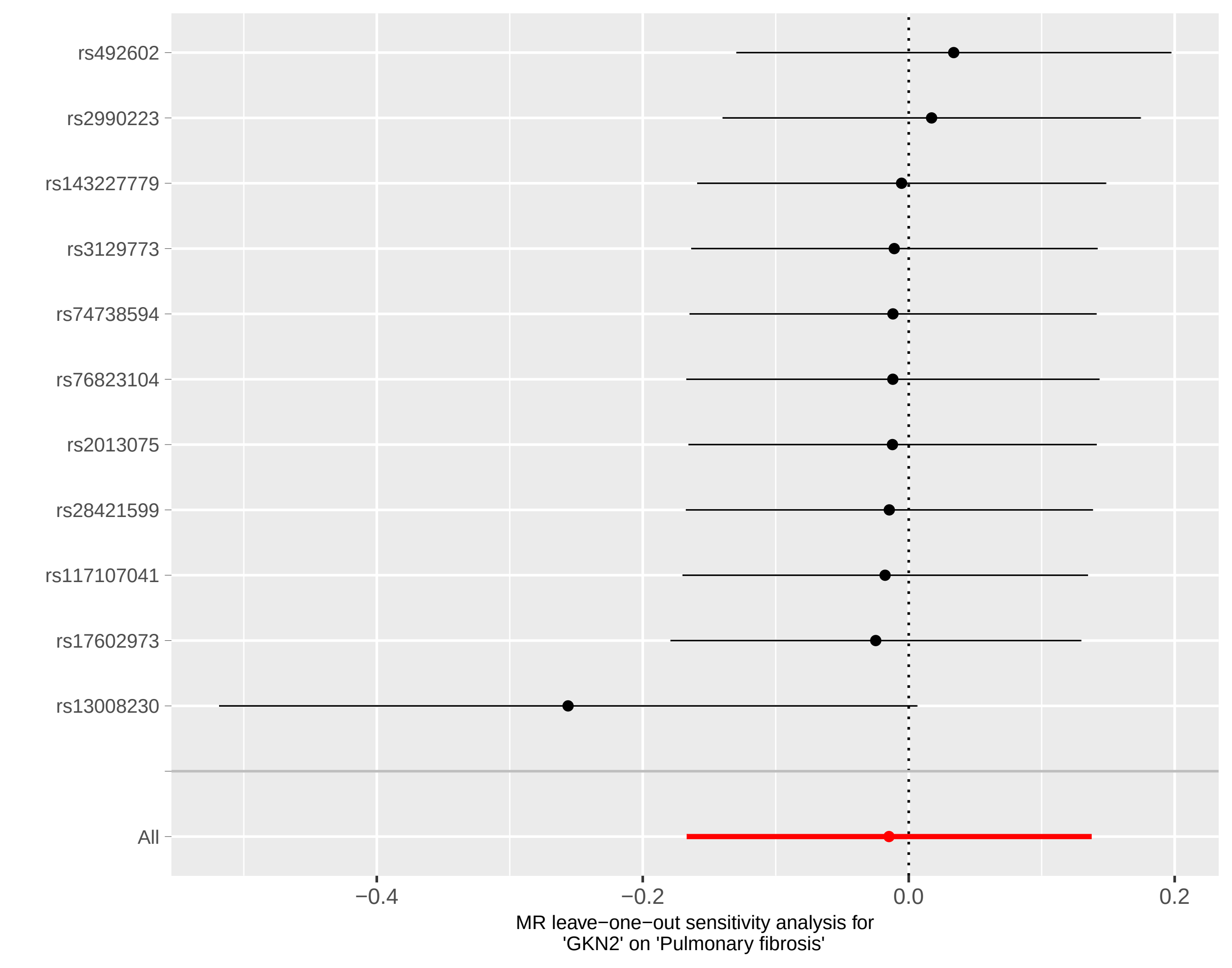

rs492602
rs2990223
rs143227779
rs3129773
rs74738594
rs76823104
rs2013075
rs28421599
rs117107041
rs17602973
rs13008230
All
−0.4
−0.2
0.0
0.2
MR leave−one−out sensitivity analysis for
'GKN2' on 'Pulmonary fibrosis'

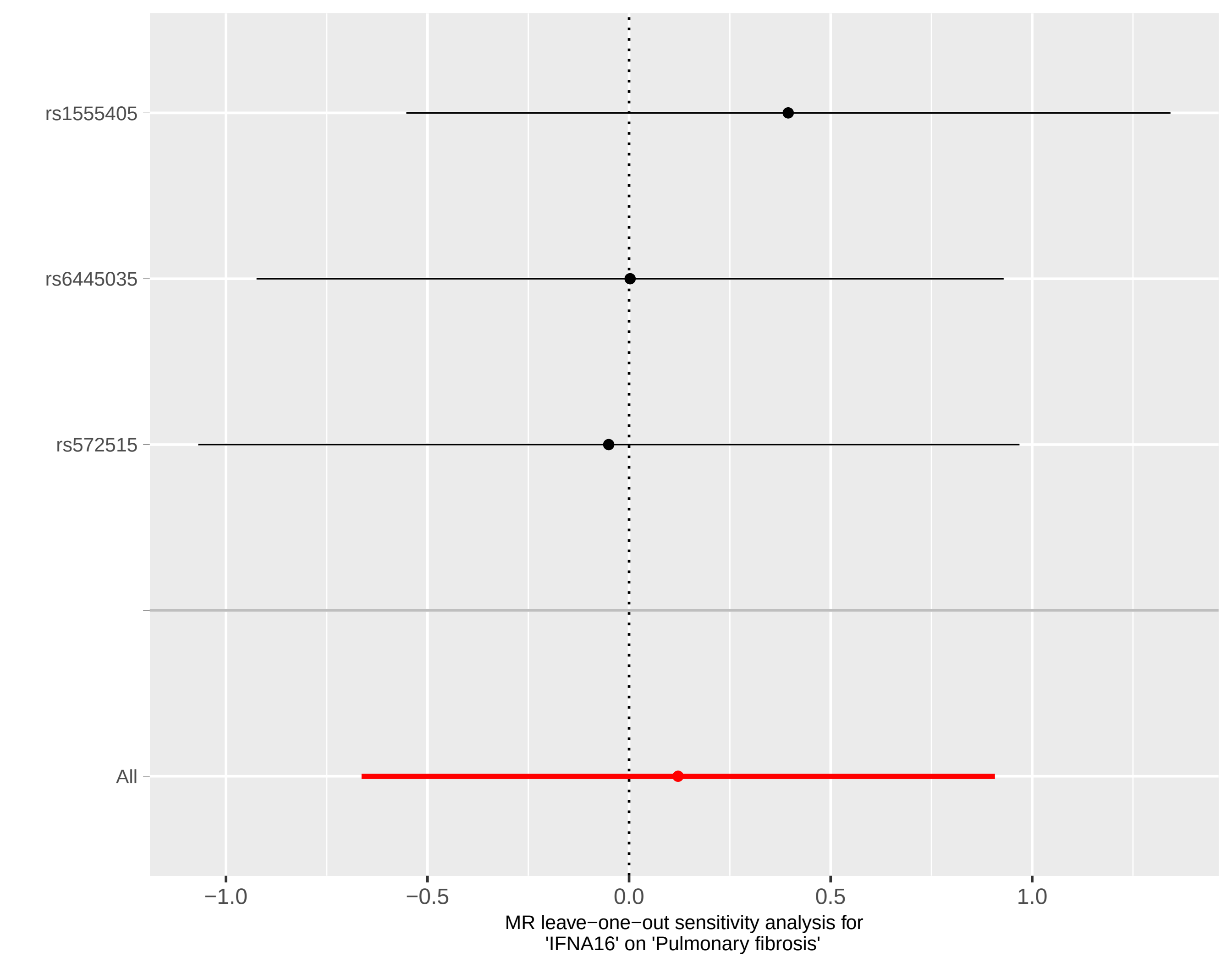
rs1555405
rs6445035
rs572515
All
−1.0
−0.5
0.0
0.5
1.0
MR leave−one−out sensitivity analysis for
'IFNA16' on 'Pulmonary fibrosis'

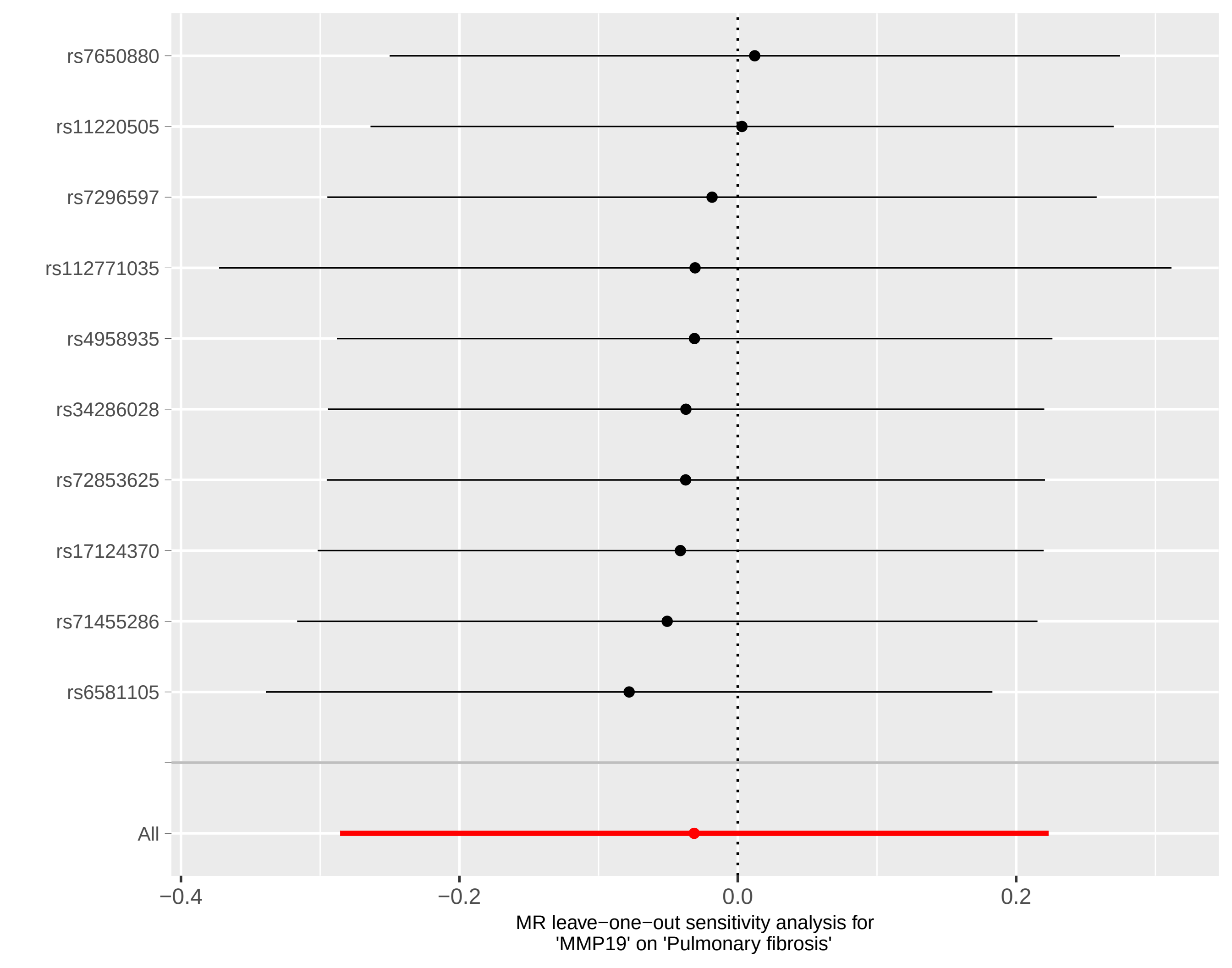

rs7650880
rs11220505
rs7296597
rs112771035
rs4958935
rs34286028
rs72853625
rs17124370
rs71455286
rs6581105
All
−0.4
−0.2
0.0
0.2
MR leave−one−out sensitivity analysis for
'MMP19' on 'Pulmonary fibrosis'

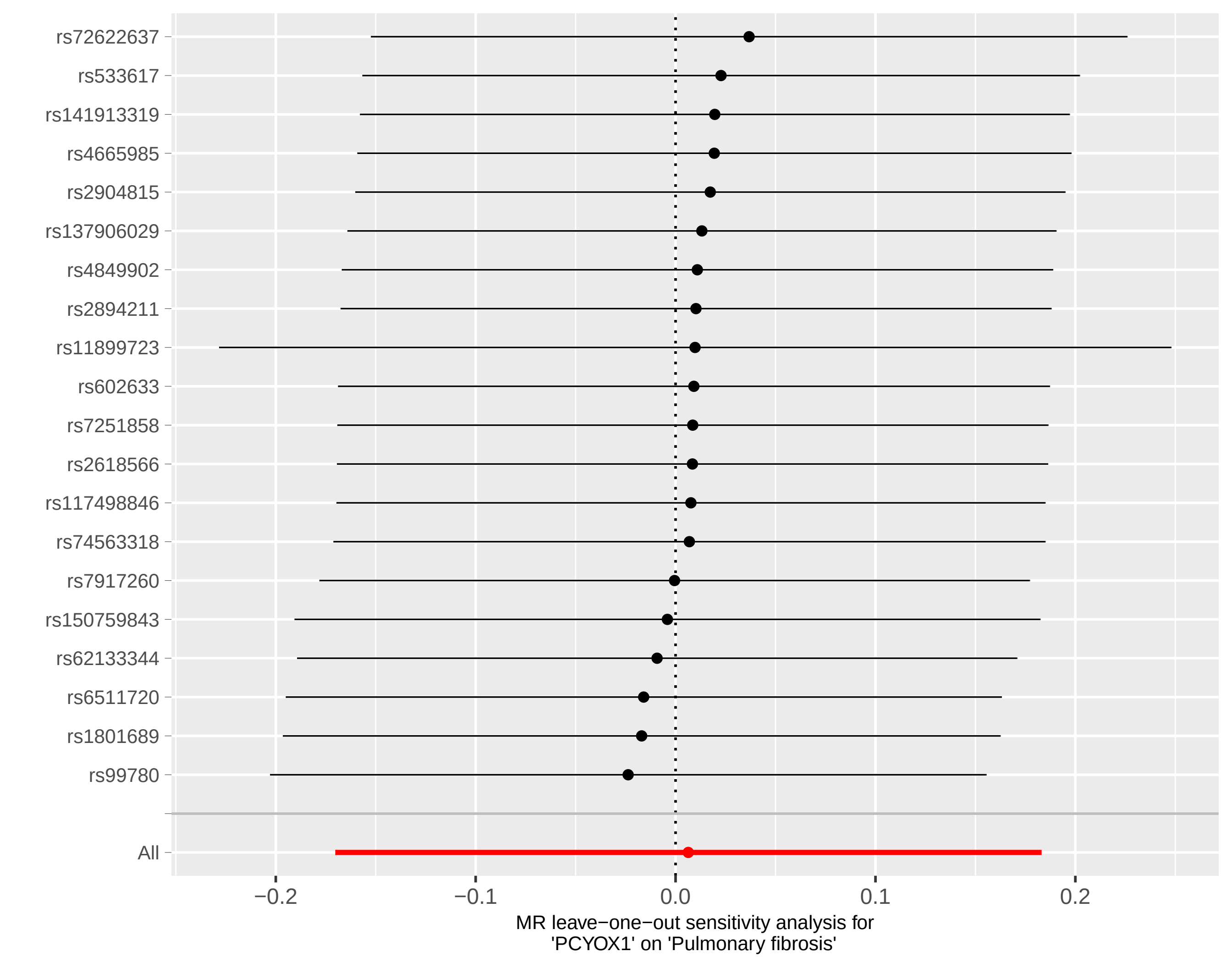
rs72622637
rs533617
rs141913319
rs4665985
rs2904815
rs137906029
rs4849902
rs2894211
rs11899723
rs602633
rs7251858
rs2618566
rs117498846
rs74563318
rs7917260
rs150759843
rs62133344
rs6511720
rs1801689
rs99780
All
−0.2
−0.1
0.0
0.1
0.2
MR leave−one−out sensitivity analysis for
'PCYOX1' on 'Pulmonary fibrosis'

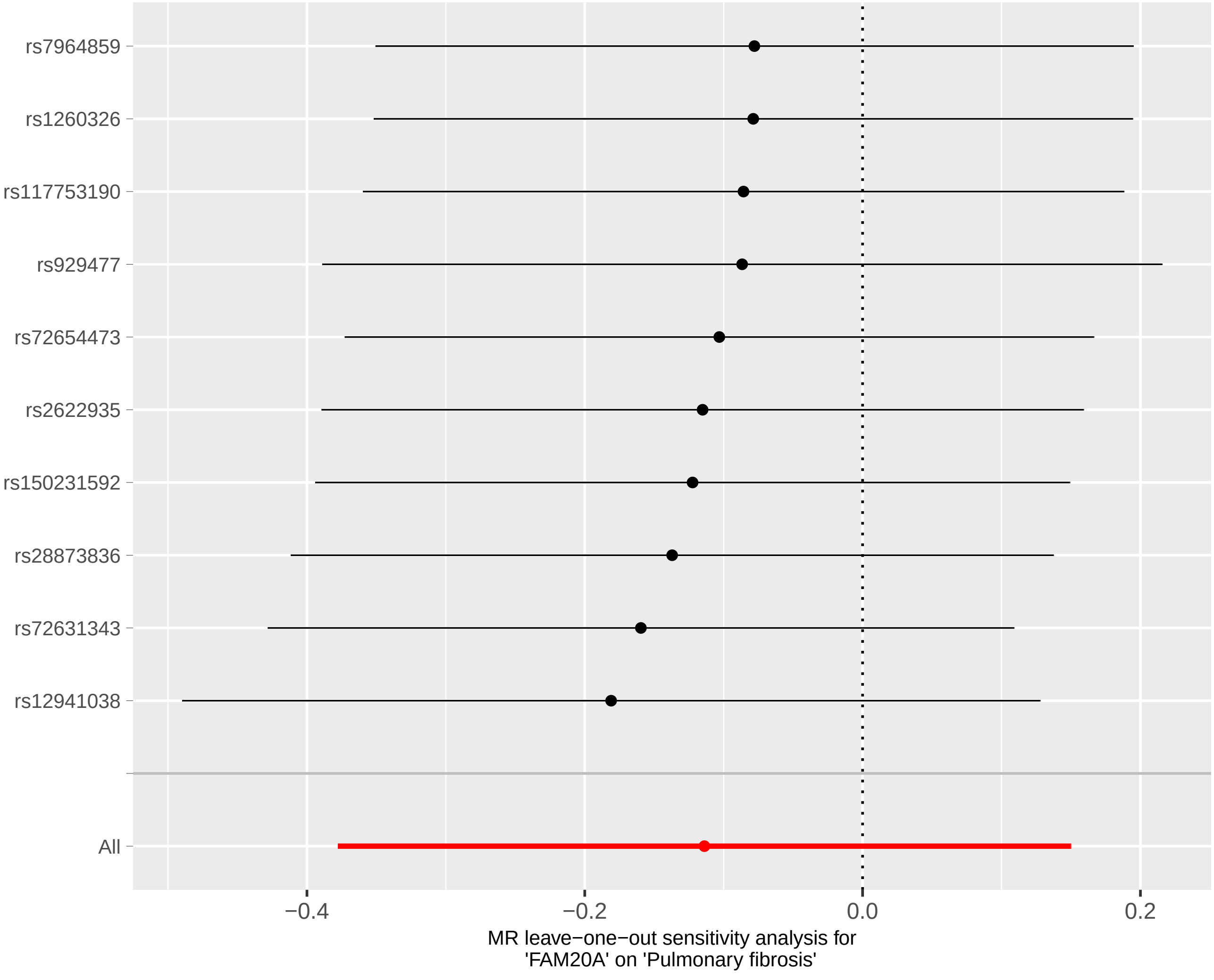
rs7964859
rs1260326
rs117753190
rs929477
rs72654473
rs2622935
rs150231592
rs28873836
rs72631343
rs12941038
All
−0.4
−0.2
0.0
0.2
MR leave−one−out sensitivity analysis for
'FAM20A' on 'Pulmonary fibrosis'

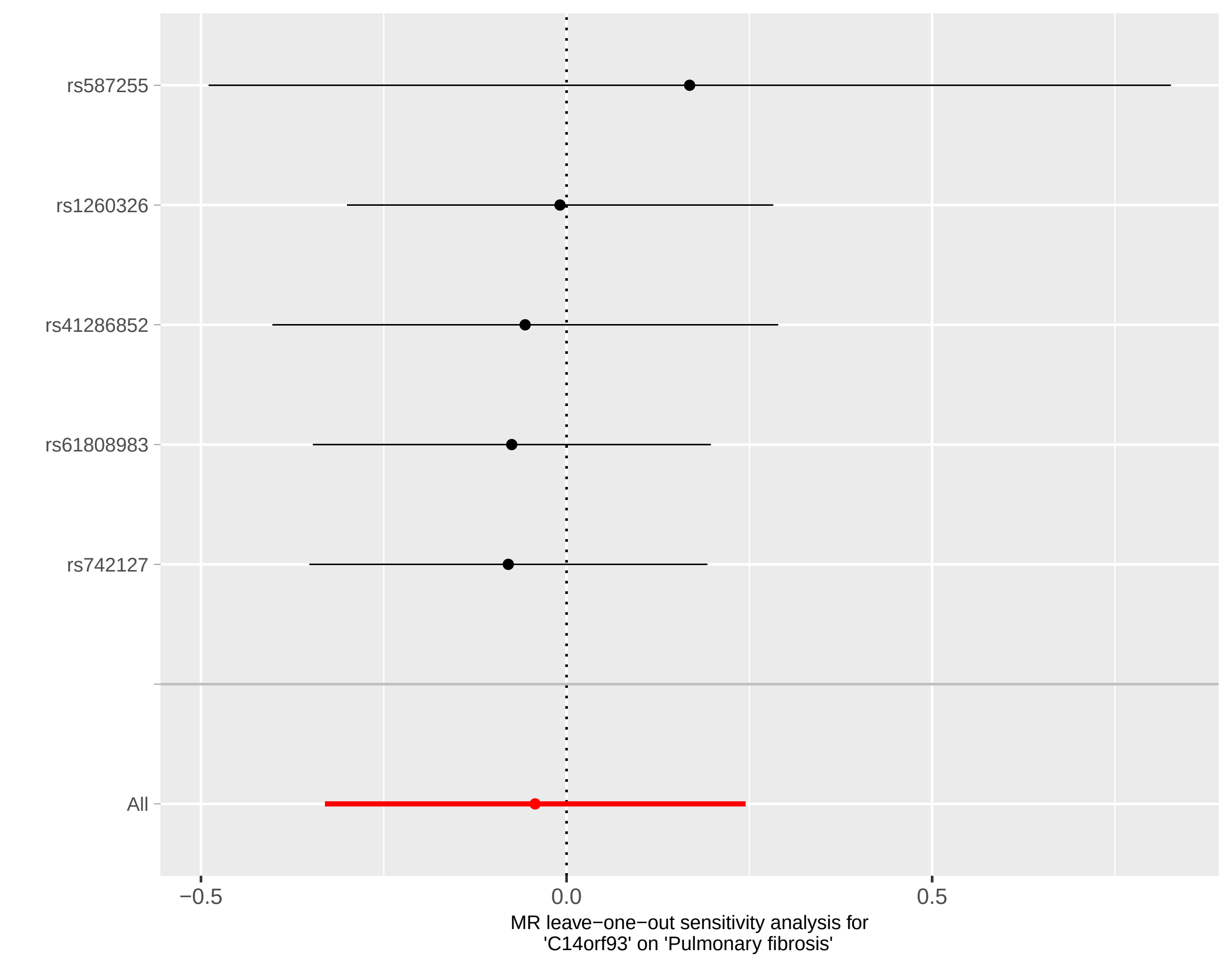
rs587255
rs1260326
rs41286852
rs61808983
rs742127
All
−0.5
0.0
0.5
MR leave−one−out sensitivity analysis for
'C14orf93' on 'Pulmonary fibrosis'

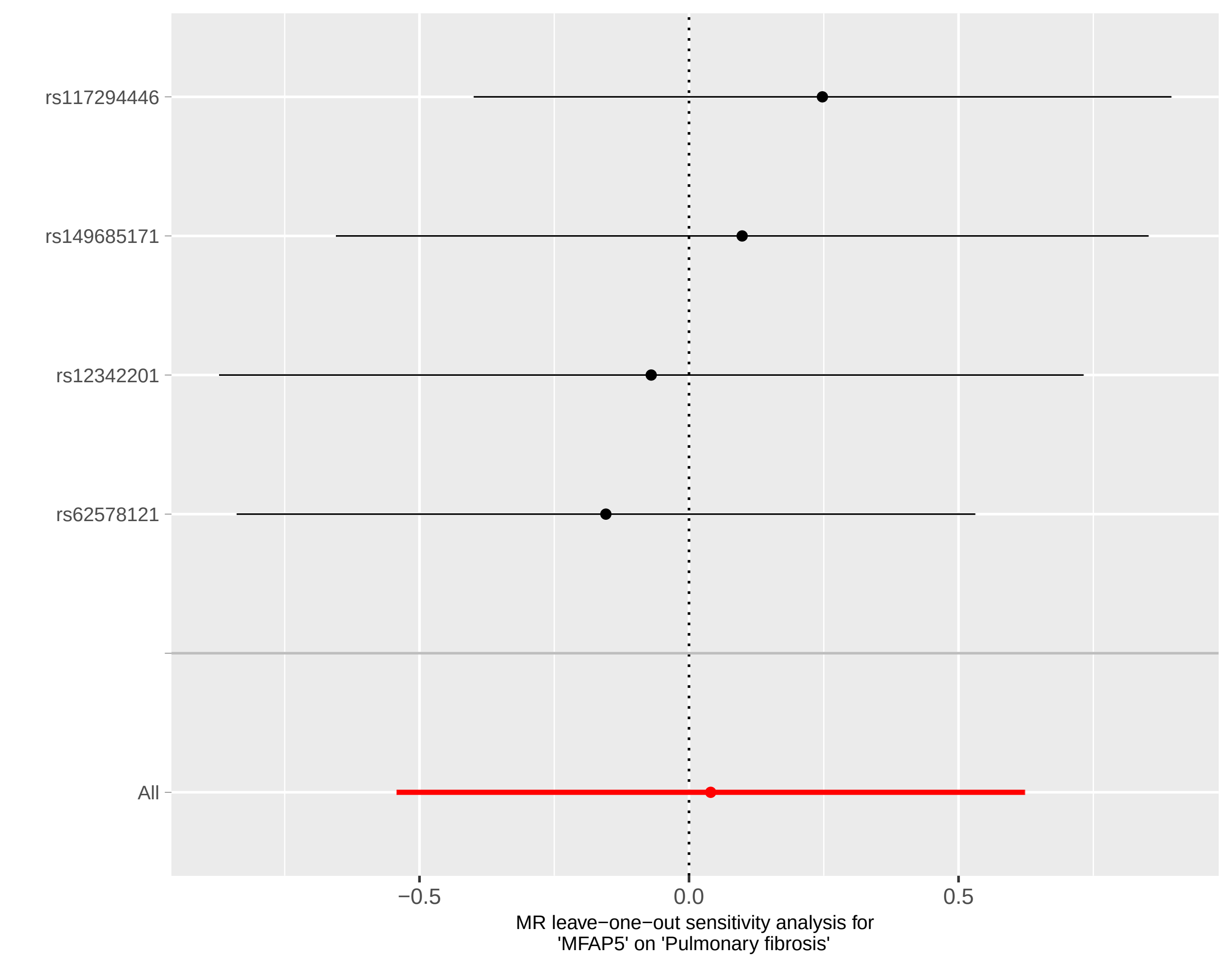

rs117294446
rs149685171
rs12342201
rs62578121
All
−0.5
0.0
0.5
MR leave−one−out sensitivity analysis for
'MFAP5' on 'Pulmonary fibrosis'

Insufficient number of SNPs

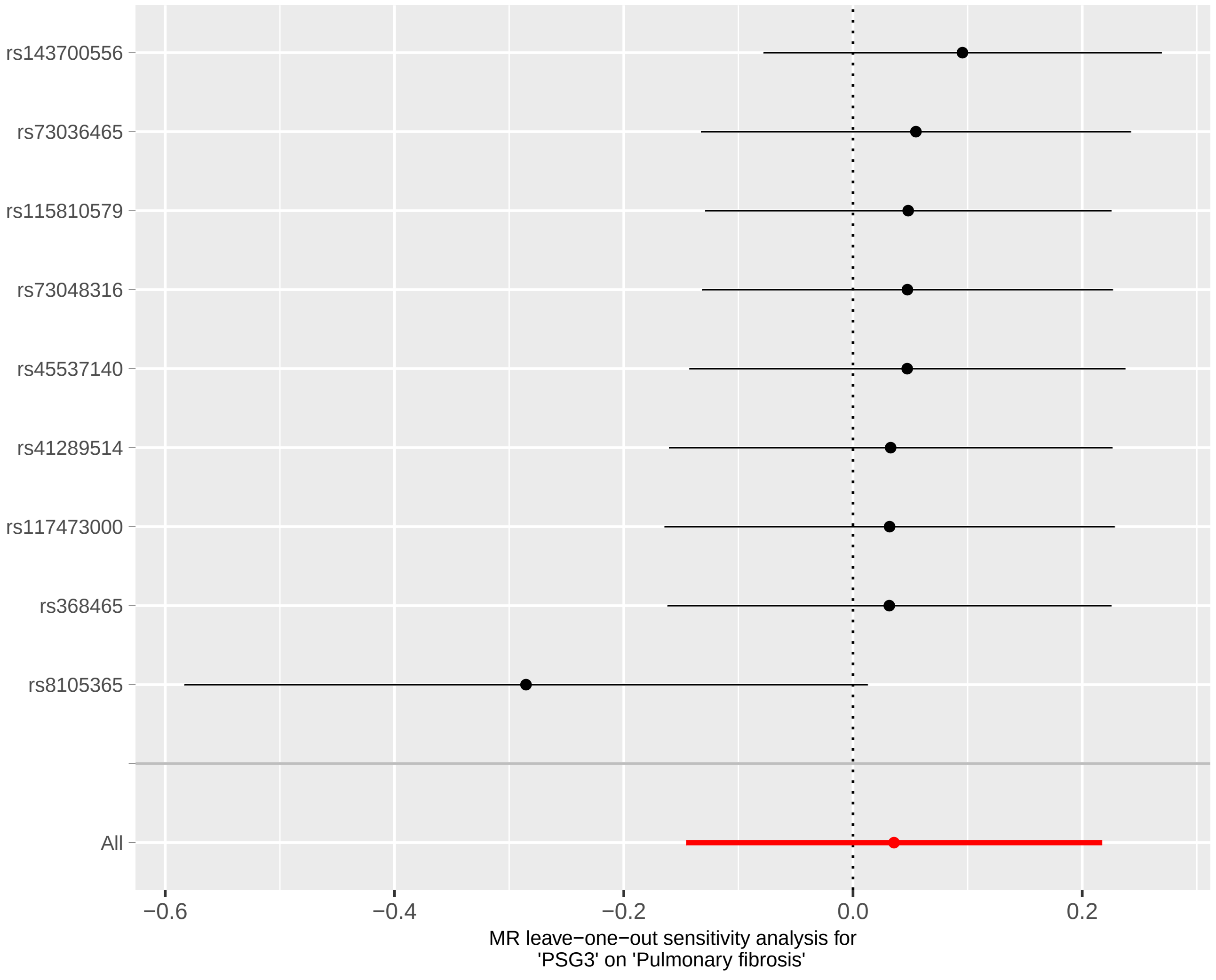
rs143700556
rs73036465
rs115810579
rs73048316
rs45537140
rs41289514
rs117473000
rs368465
rs8105365
All
−0.6
−0.4
−0.2
0.0
0.2
MR leave−one−out sensitivity analysis for
'PSG3' on 'Pulmonary fibrosis'

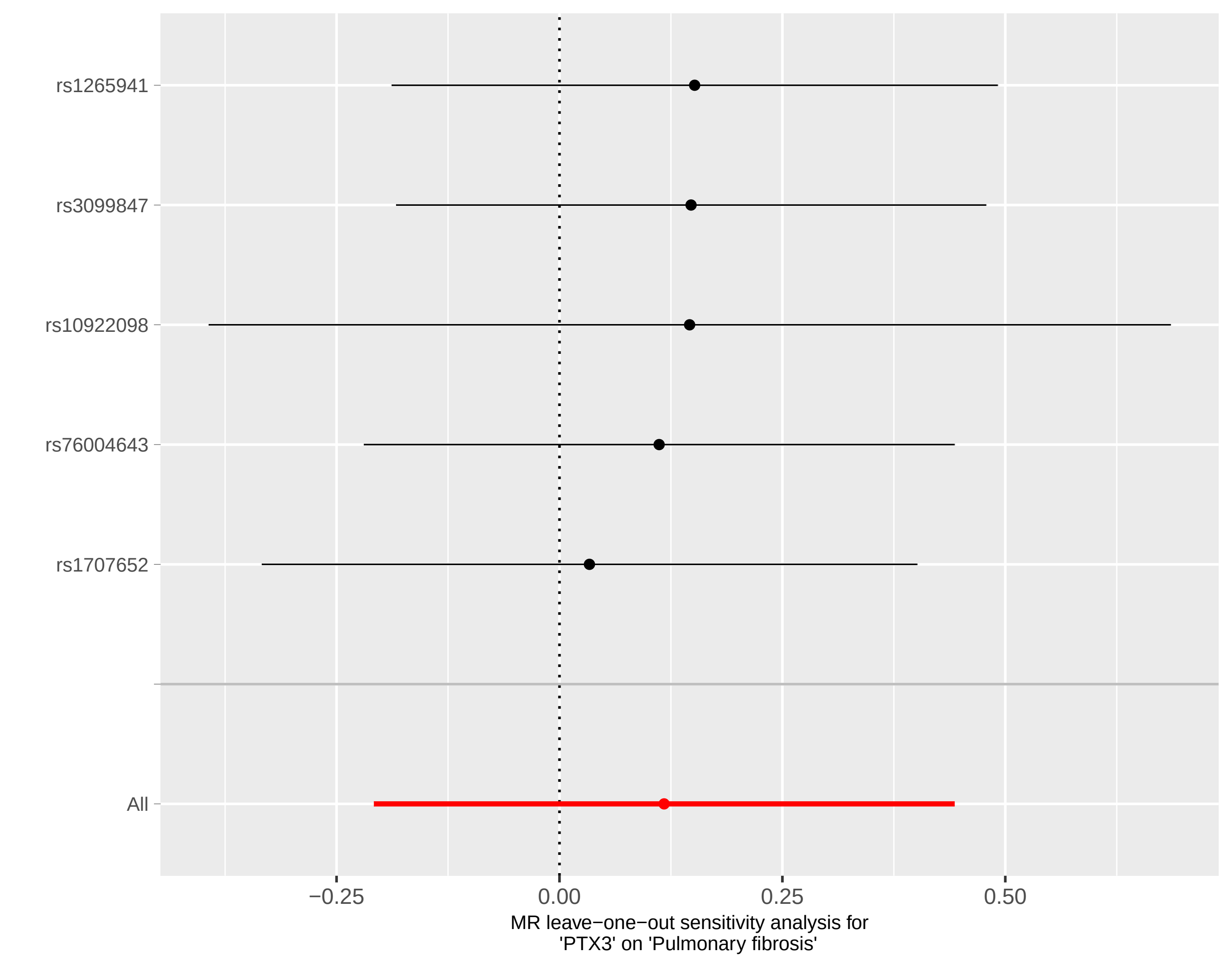
rs1265941
rs3099847
rs10922098
rs76004643
rs1707652
All
−0.25
0.00
0.25
0.50
MR leave−one−out sensitivity analysis for
'PTX3' on 'Pulmonary fibrosis'

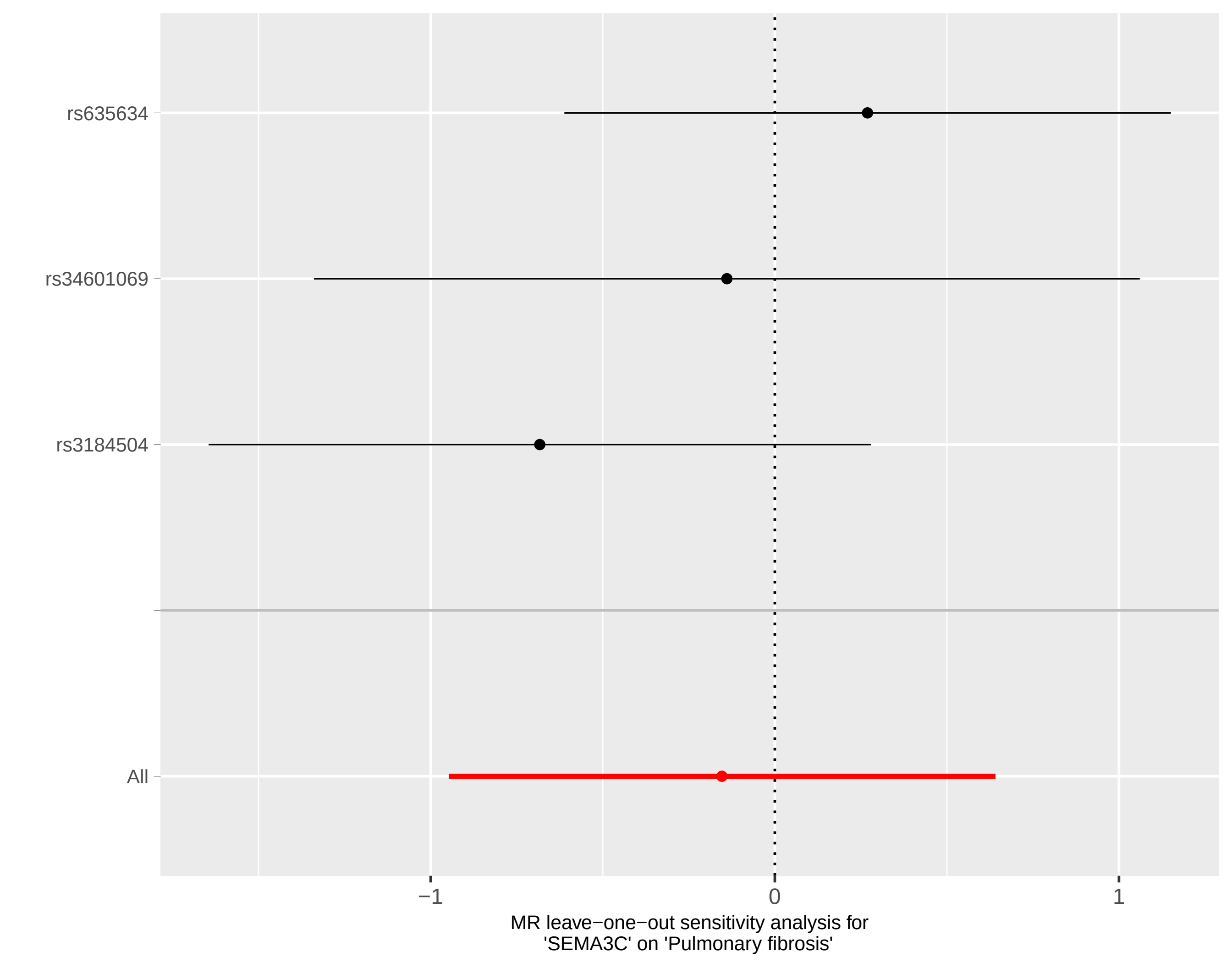
rs635634
rs34601069
rs3184504
All
−1
0
1
MR leave−one−out sensitivity analysis for
'SEMA3C' on 'Pulmonary fibrosis'

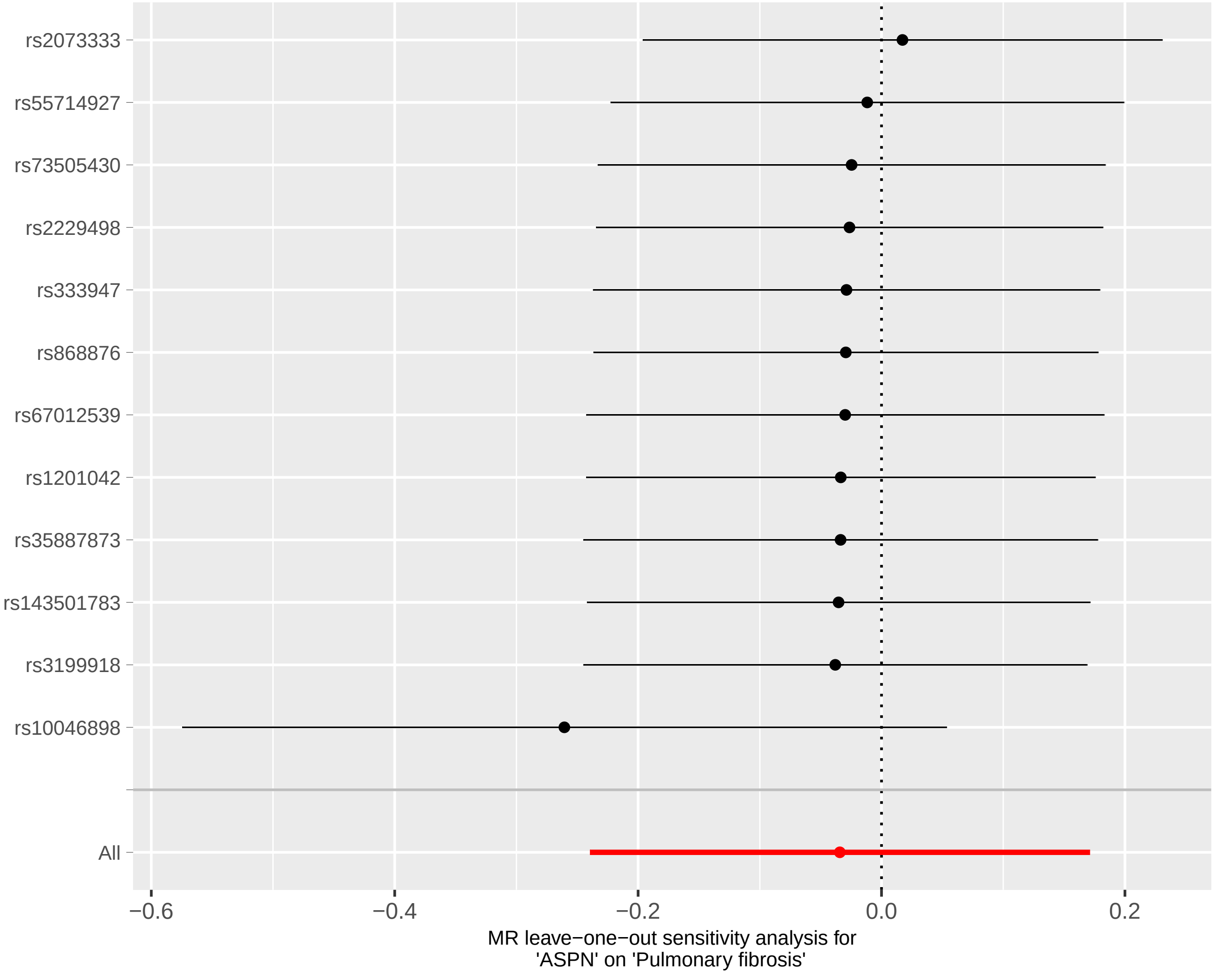
rs2073333
rs55714927
rs73505430
rs2229498
rs333947
rs868876
rs67012539
rs1201042
rs35887873
rs143501783
rs3199918
rs10046898
All
−0.6
−0.4
−0.2
0.0
0.2
MR leave−one−out sensitivity analysis for
'ASPN' on 'Pulmonary fibrosis'

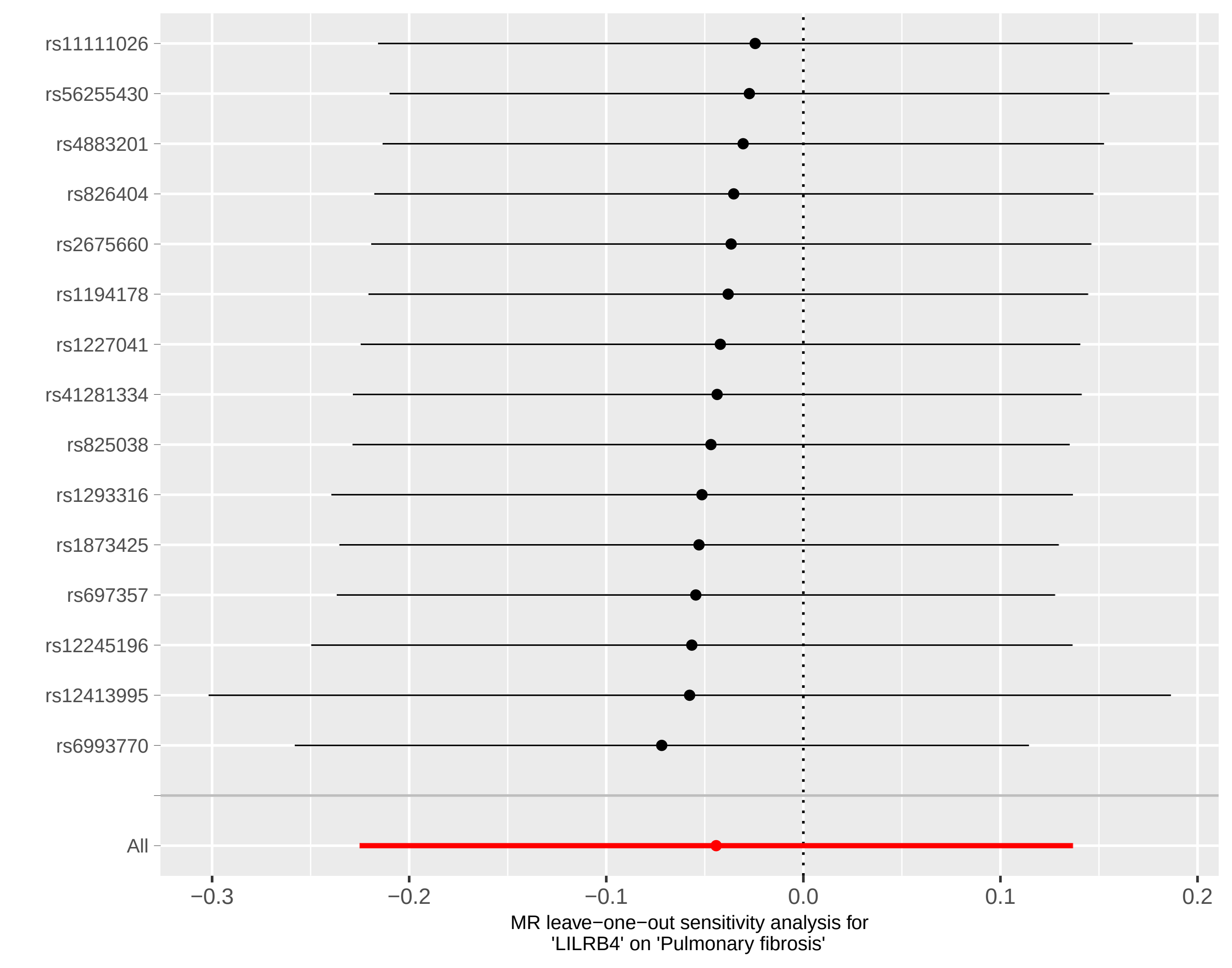
rs11111026
rs56255430
rs4883201
rs826404
rs2675660
rs1194178
rs1227041
rs41281334
rs825038
rs1293316
rs1873425
rs697357
rs12245196
rs12413995
rs6993770
All
−0.3
−0.2
−0.1
0.0
0.1
0.2
MR leave−one−out sensitivity analysis for
'LILRB4' on 'Pulmonary fibrosis'

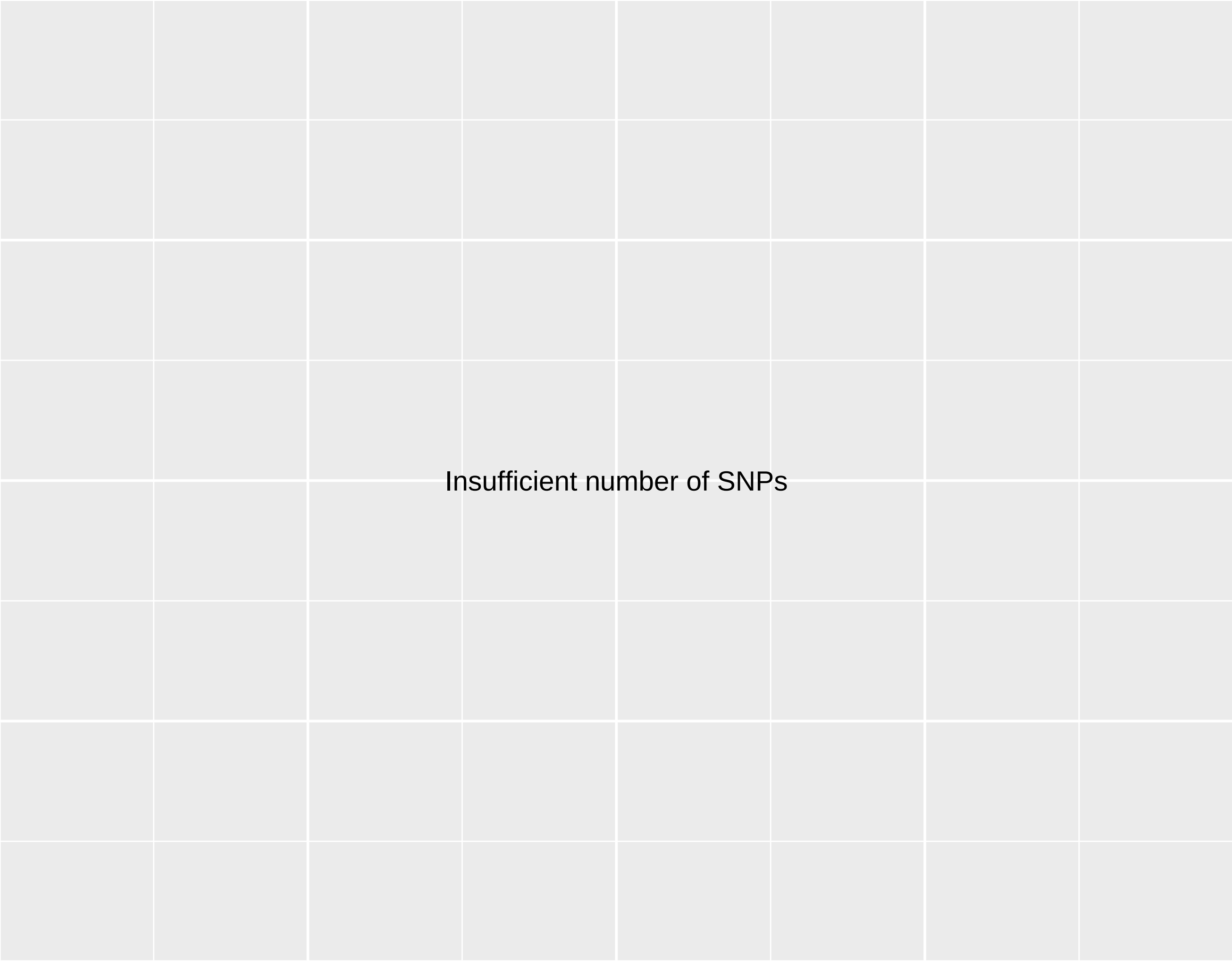
Insufficient number of SNPs

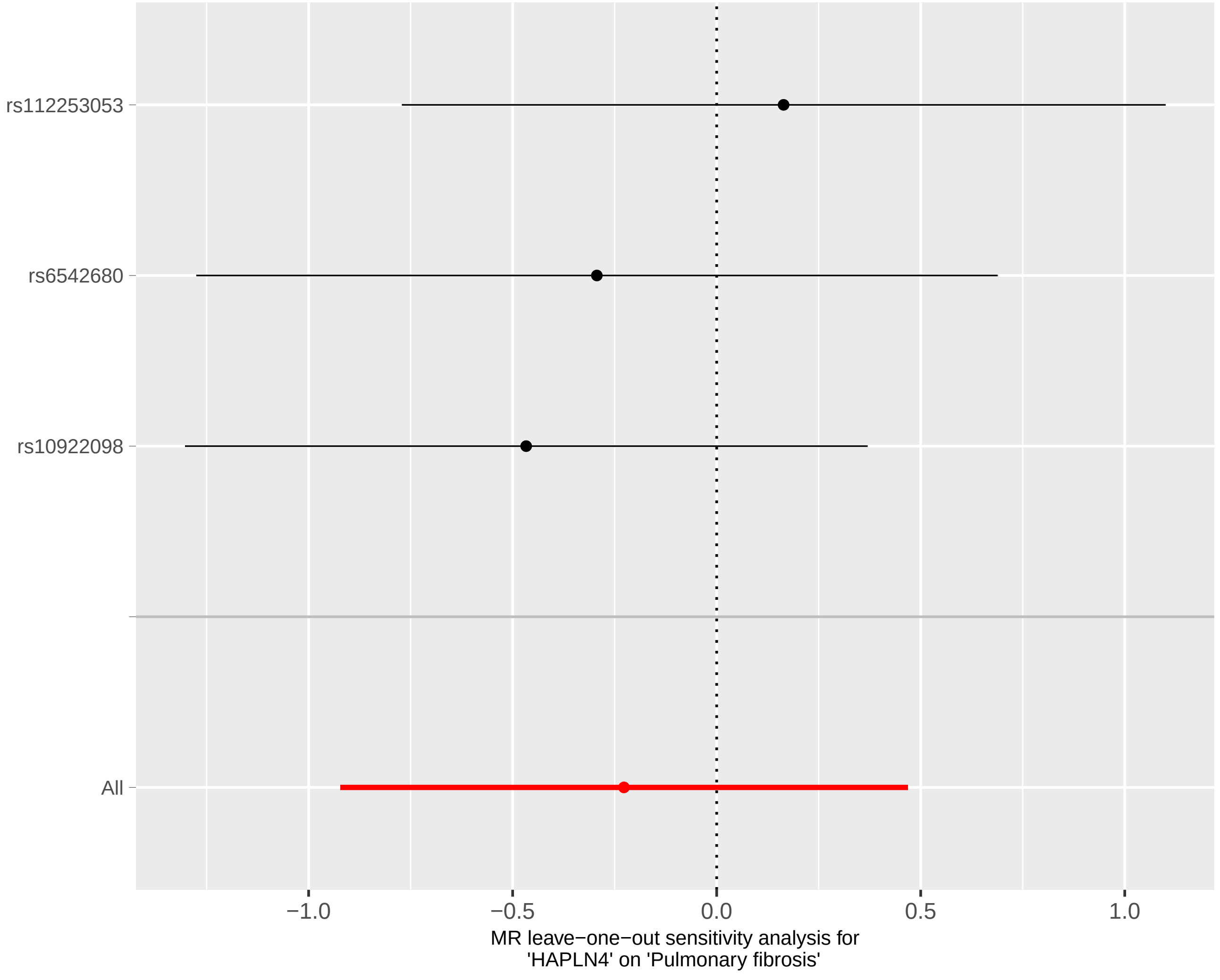
rs112253053
rs6542680
rs10922098
All
−1.0
−0.5
0.0
0.5
1.0
MR leave−one−out sensitivity analysis for
'HAPLN4' on 'Pulmonary fibrosis'

Insufficient number of SNPs

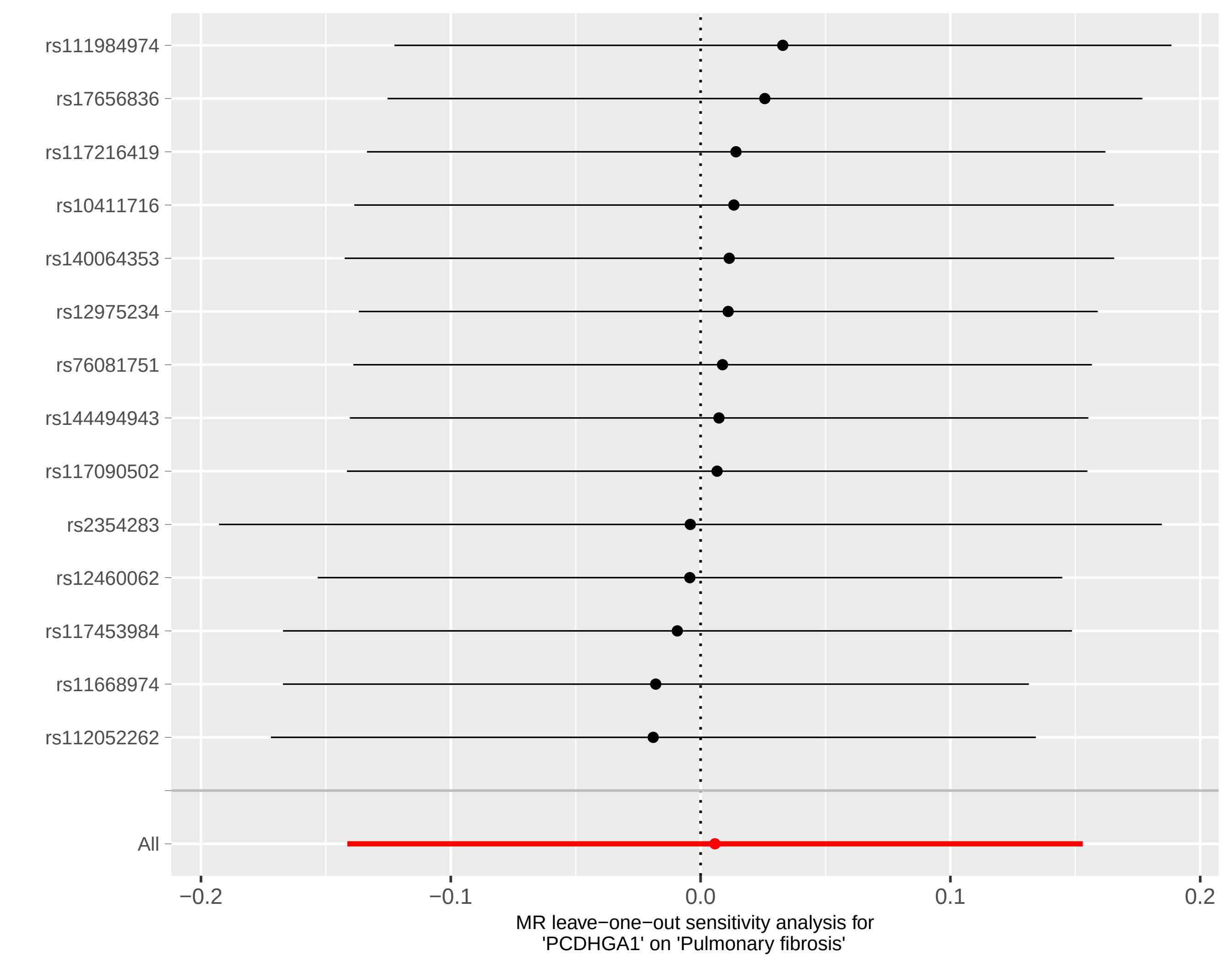
rs111984974
rs17656836
rs117216419
rs10411716
rs140064353
rs12975234
rs76081751
rs144494943
rs117090502
rs2354283
rs12460062
rs117453984
rs11668974
rs112052262
All
−0.2
−0.1
0.0
0.1
0.2
MR leave−one−out sensitivity analysis for
'PCDHGA1' on 'Pulmonary fibrosis'

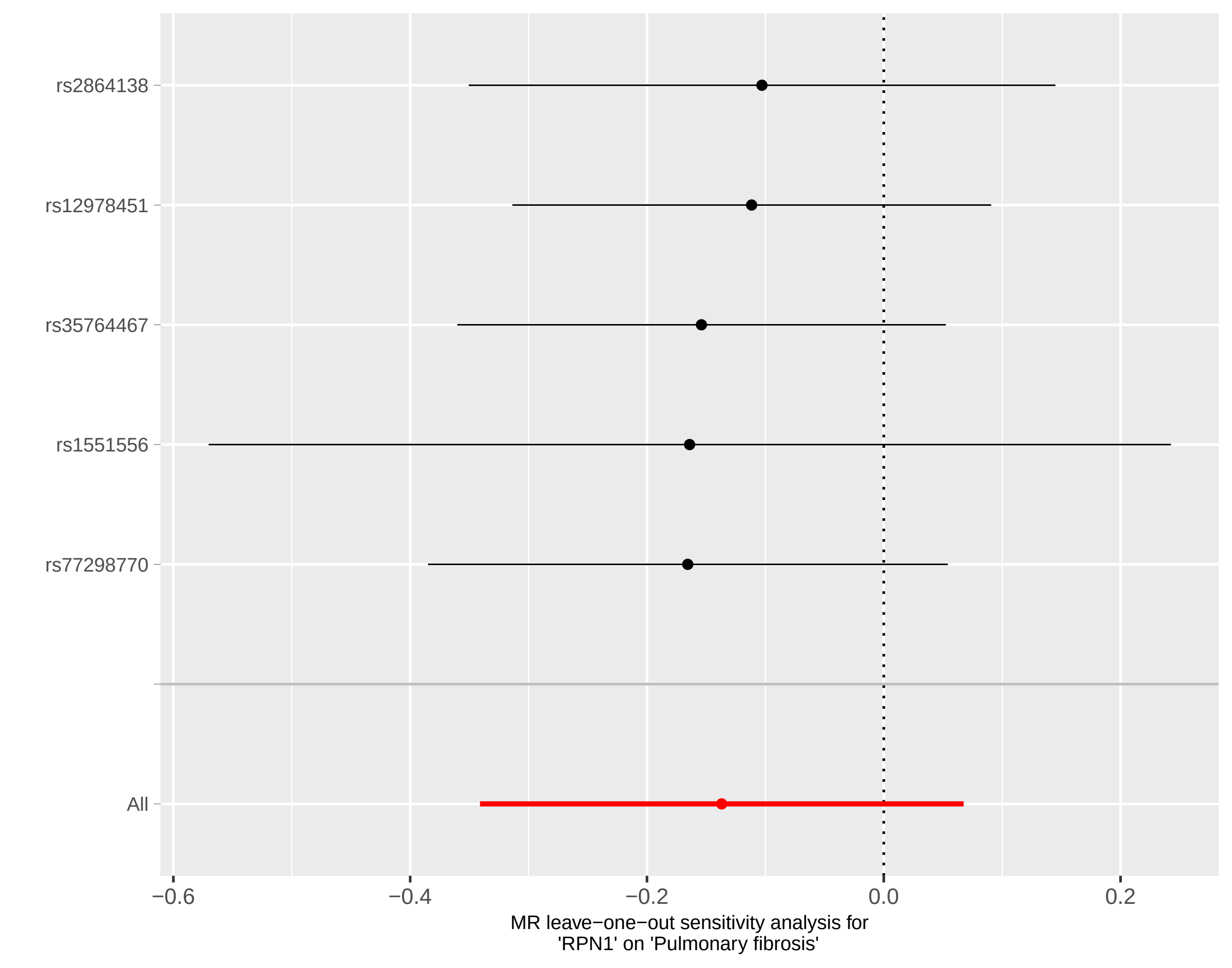
rs2864138
rs12978451
rs35764467
rs1551556
rs77298770
All
−0.6
−0.4
−0.2
0.0
0.2
MR leave−one−out sensitivity analysis for
'RPN1' on 'Pulmonary fibrosis'

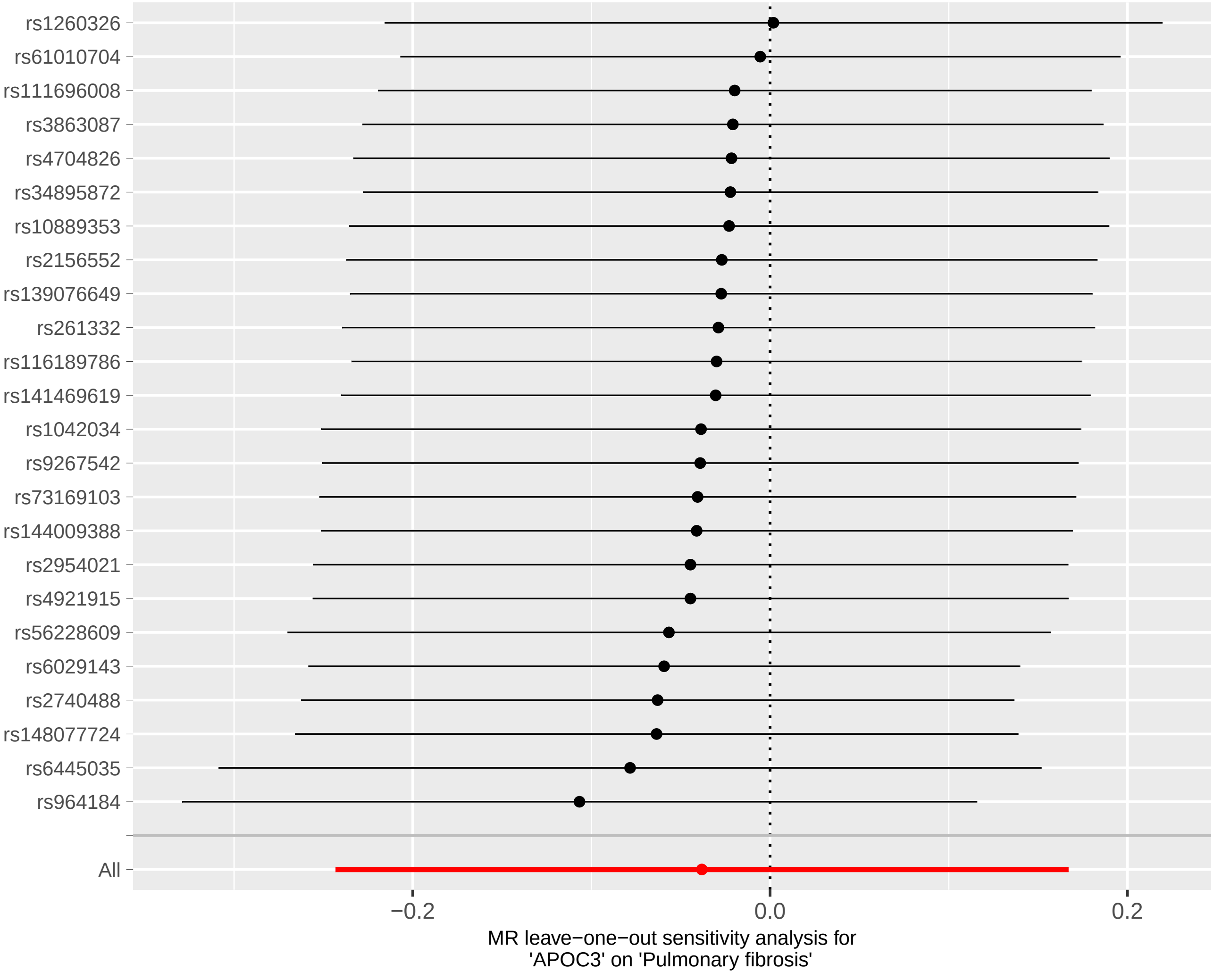
rs1260326
rs61010704
rs111696008
rs3863087
rs4704826
rs34895872
rs10889353
rs2156552
rs139076649
rs261332
rs116189786
rs141469619
rs1042034
rs9267542
rs73169103
rs144009388
rs2954021
rs4921915
rs56228609
rs6029143
rs2740488
rs148077724
rs6445035
rs964184
All
−0.2
0.0
0.2
MR leave−one−out sensitivity analysis for
'APOC3' on 'Pulmonary fibrosis'

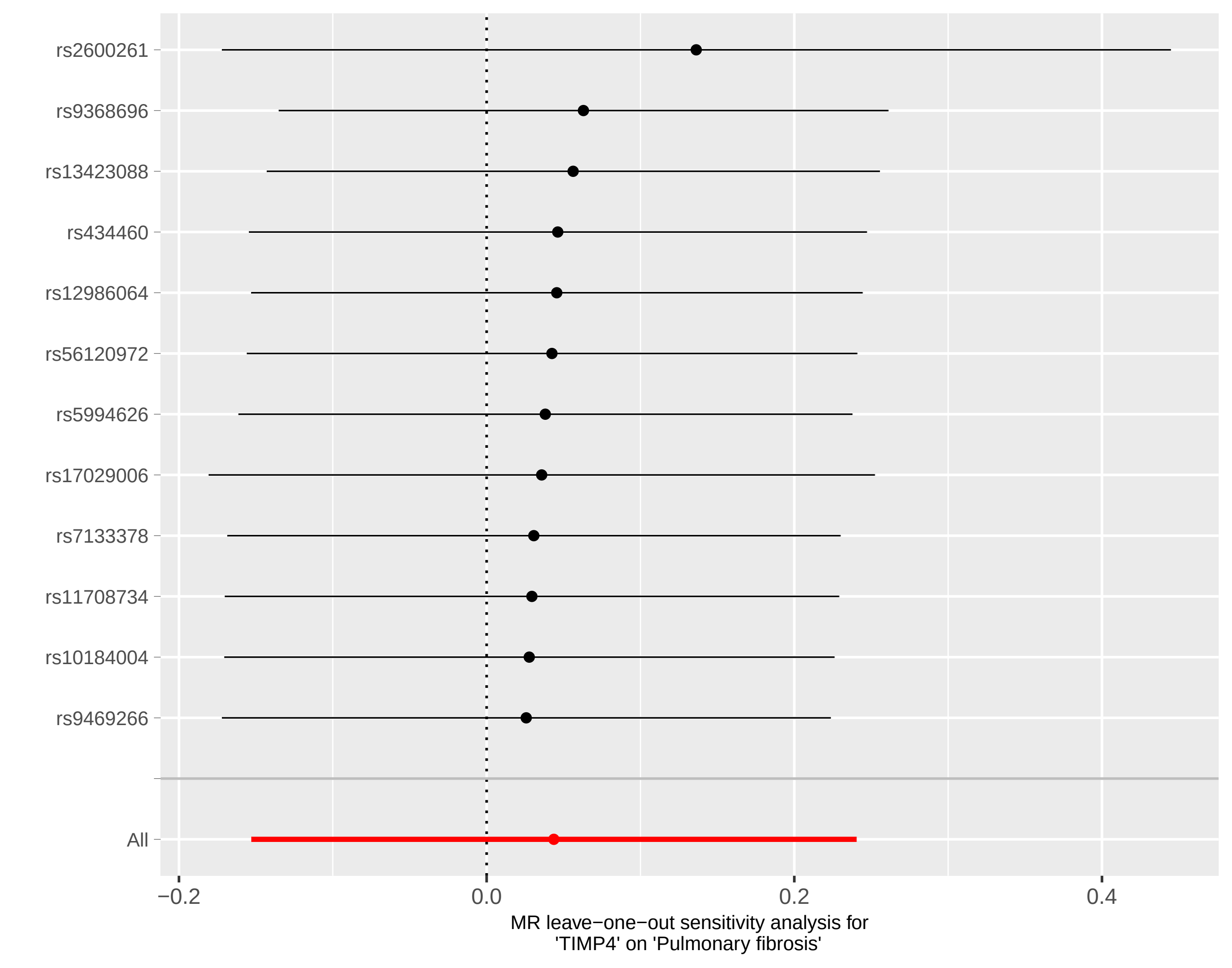
rs2600261
rs9368696
rs13423088
rs434460
rs12986064
rs56120972
rs5994626
rs17029006
rs7133378
rs11708734
rs10184004
rs9469266
All
−0.2
0.0
0.2
0.4
MR leave−one−out sensitivity analysis for
'TIMP4' on 'Pulmonary fibrosis'

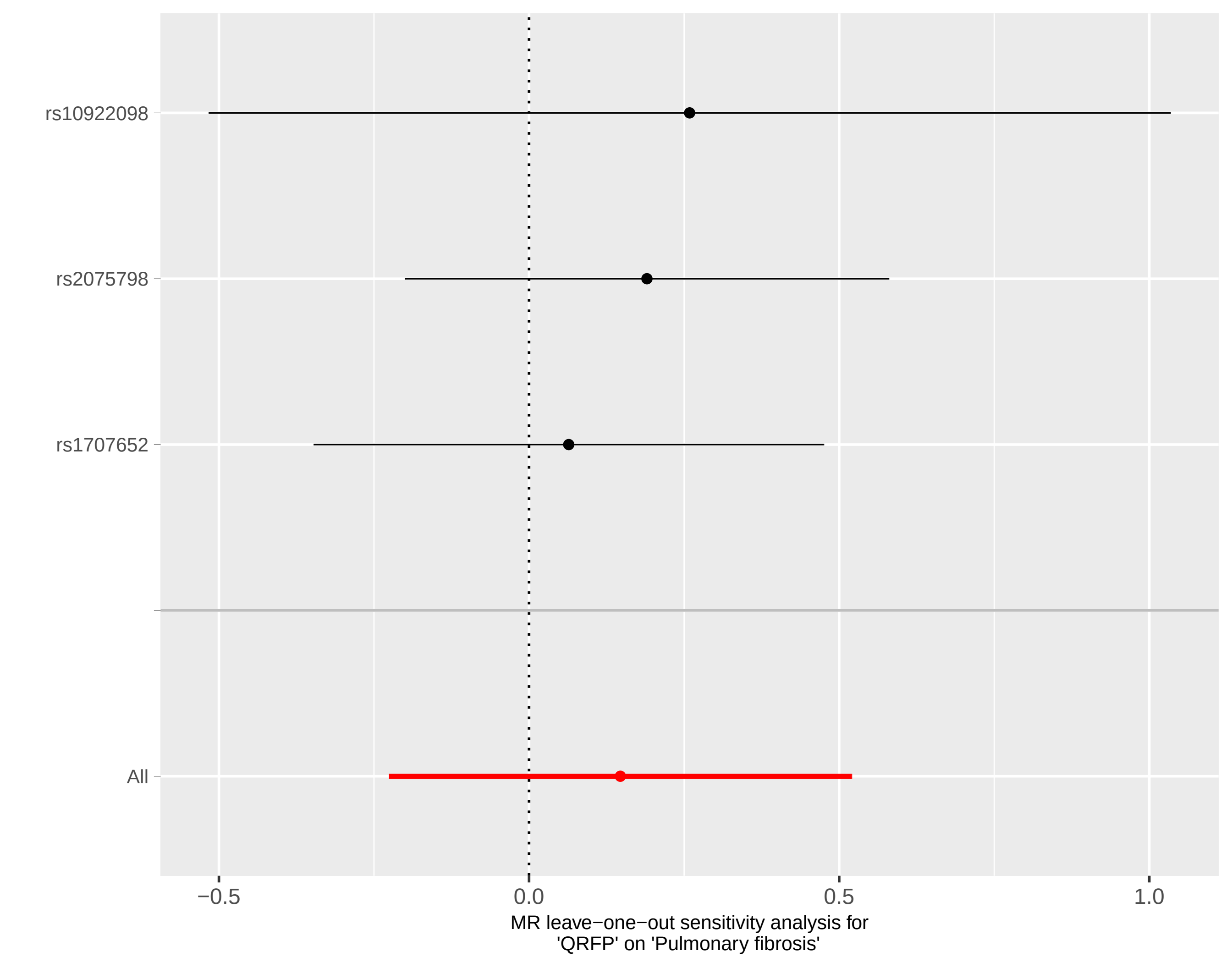
rs10922098
rs2075798
rs1707652
All
−0.5
0.0
0.5
1.0
MR leave−one−out sensitivity analysis for
'QRFP' on 'Pulmonary fibrosis'

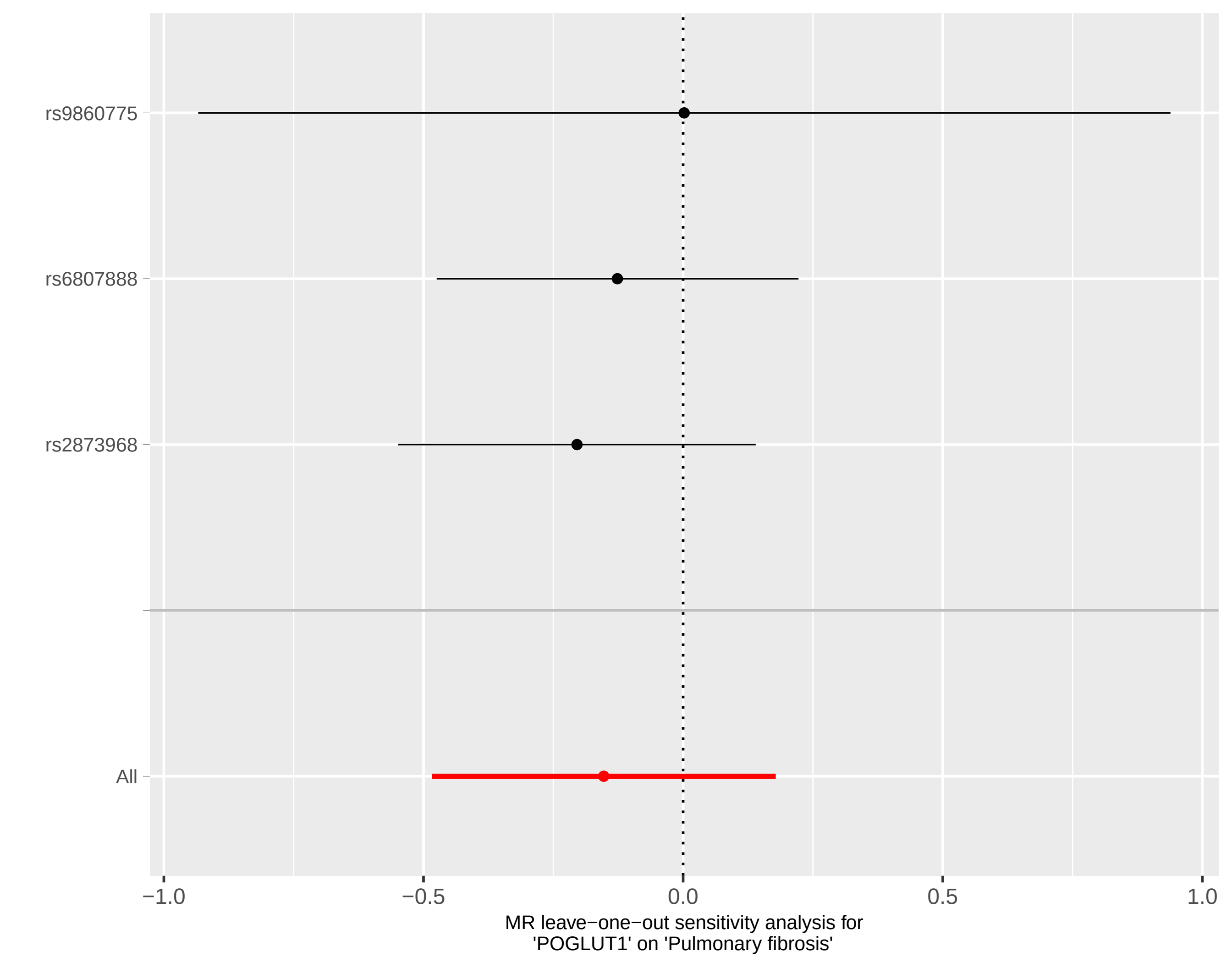
rs9860775
rs6807888
rs2873968
All
−1.0
−0.5
0.0
0.5
1.0
MR leave−one−out sensitivity analysis for
'POGLUT1' on 'Pulmonary fibrosis'

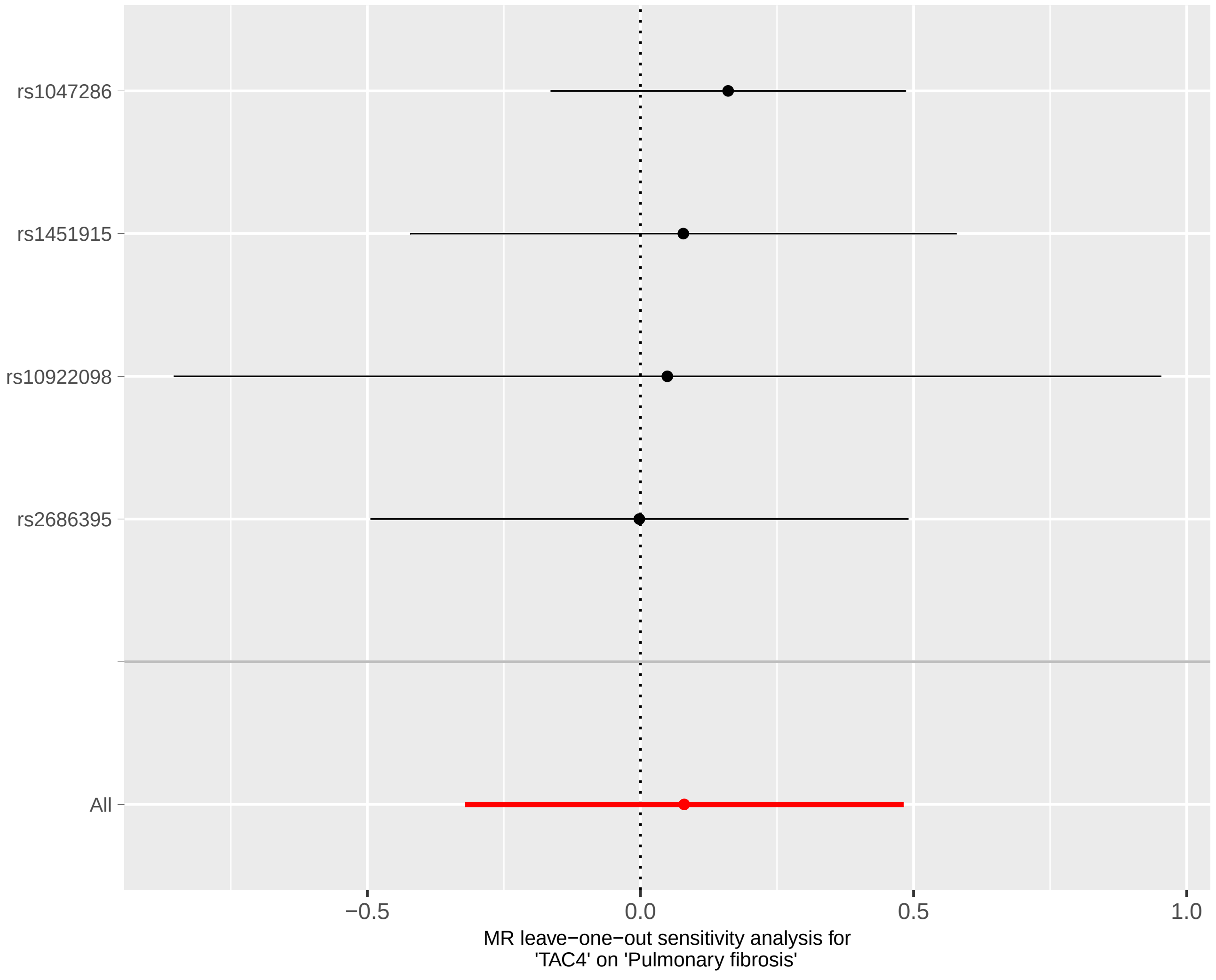
rs1047286
rs1451915
rs10922098
rs2686395
All
−0.5
0.0
0.5
1.0
MR leave−one−out sensitivity analysis for
'TAC4' on 'Pulmonary fibrosis'

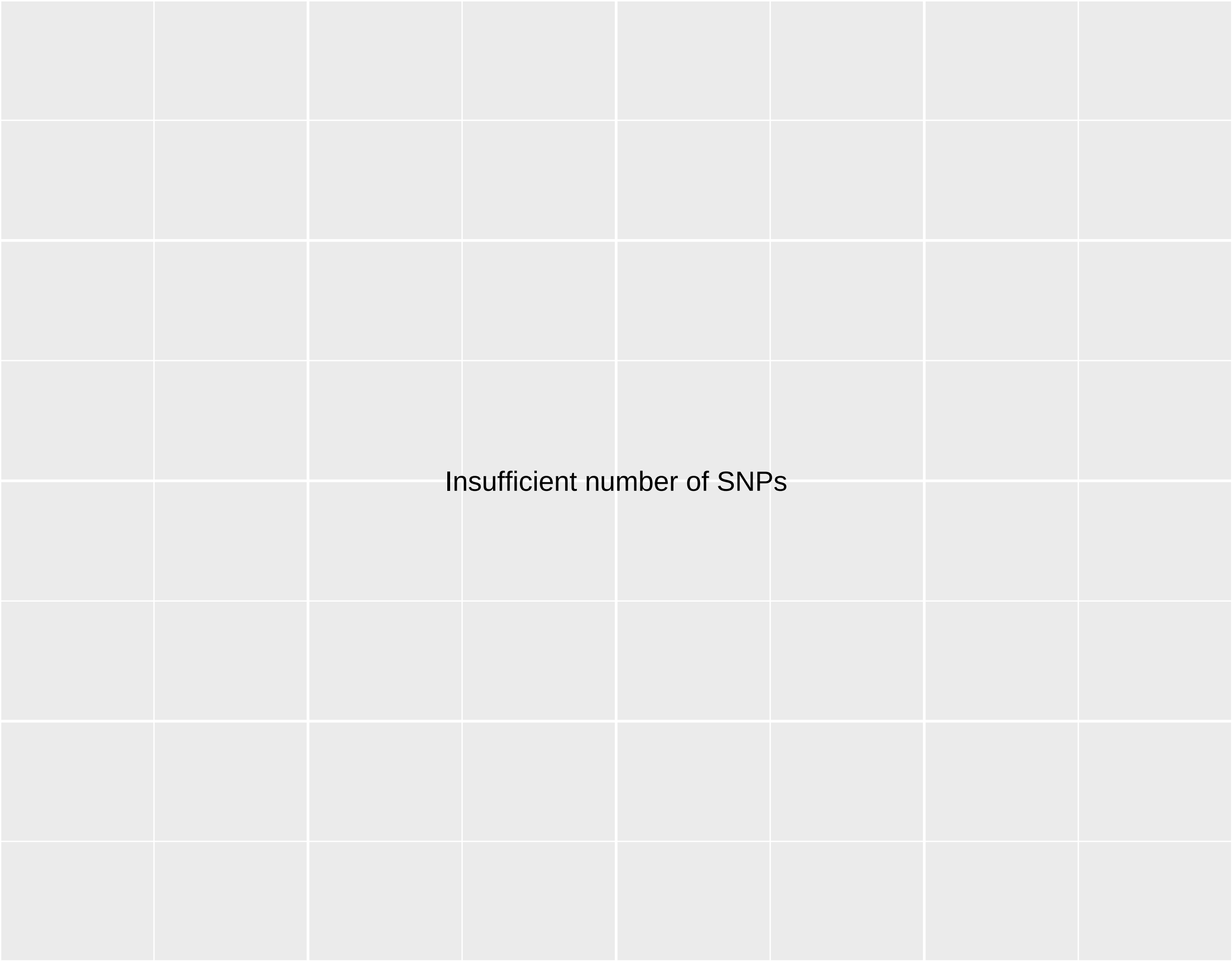
Insufficient number of SNPs

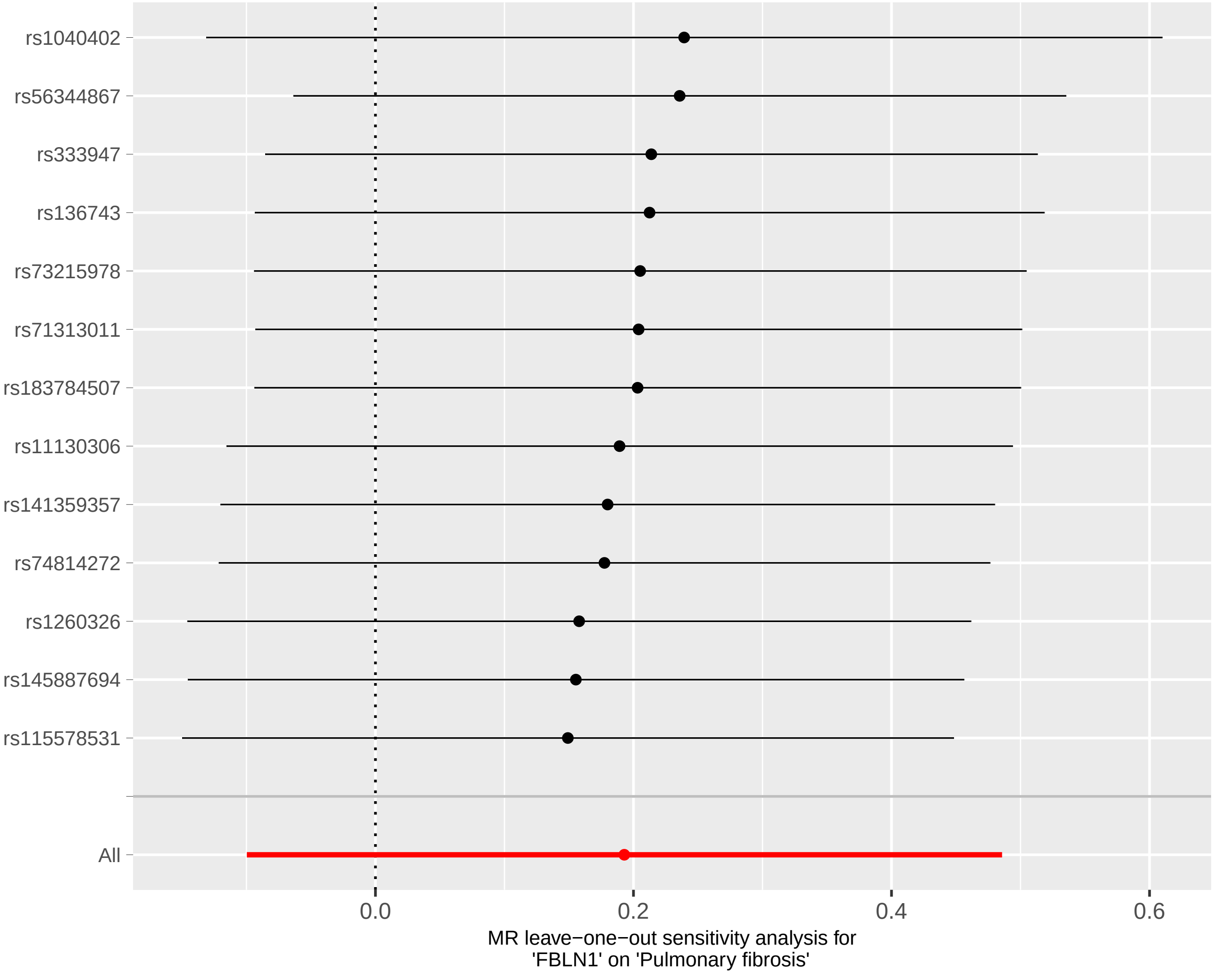

rs1040402
rs56344867
rs333947
rs136743
rs73215978
rs71313011
rs183784507
rs11130306
rs141359357
rs74814272
rs1260326
rs145887694
rs115578531
All
0.0
0.2
0.4
0.6
MR leave−one−out sensitivity analysis for
'FBLN1' on 'Pulmonary fibrosis'

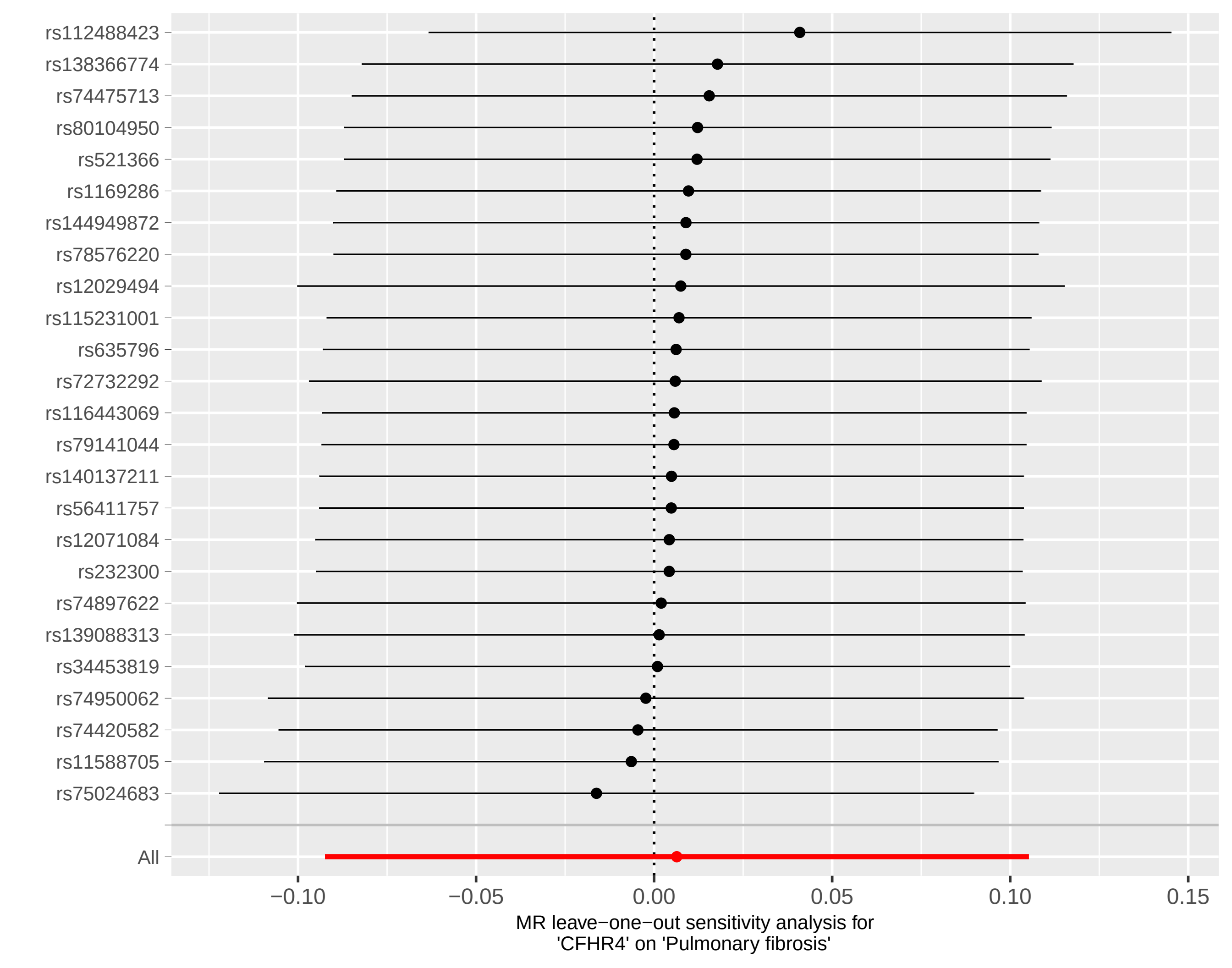
rs112488423
rs138366774
rs74475713
rs80104950
rs521366
rs1169286
rs144949872
rs78576220
rs12029494
rs115231001
rs635796
rs72732292
rs116443069
rs79141044
rs140137211
rs56411757
rs12071084
rs232300
rs74897622
rs139088313
rs34453819
rs74950062
rs74420582
rs11588705
rs75024683
All
−0.10
−0.05
0.00
0.05
0.10
0.15
MR leave−one−out sensitivity analysis for
'CFHR4' on 'Pulmonary fibrosis'

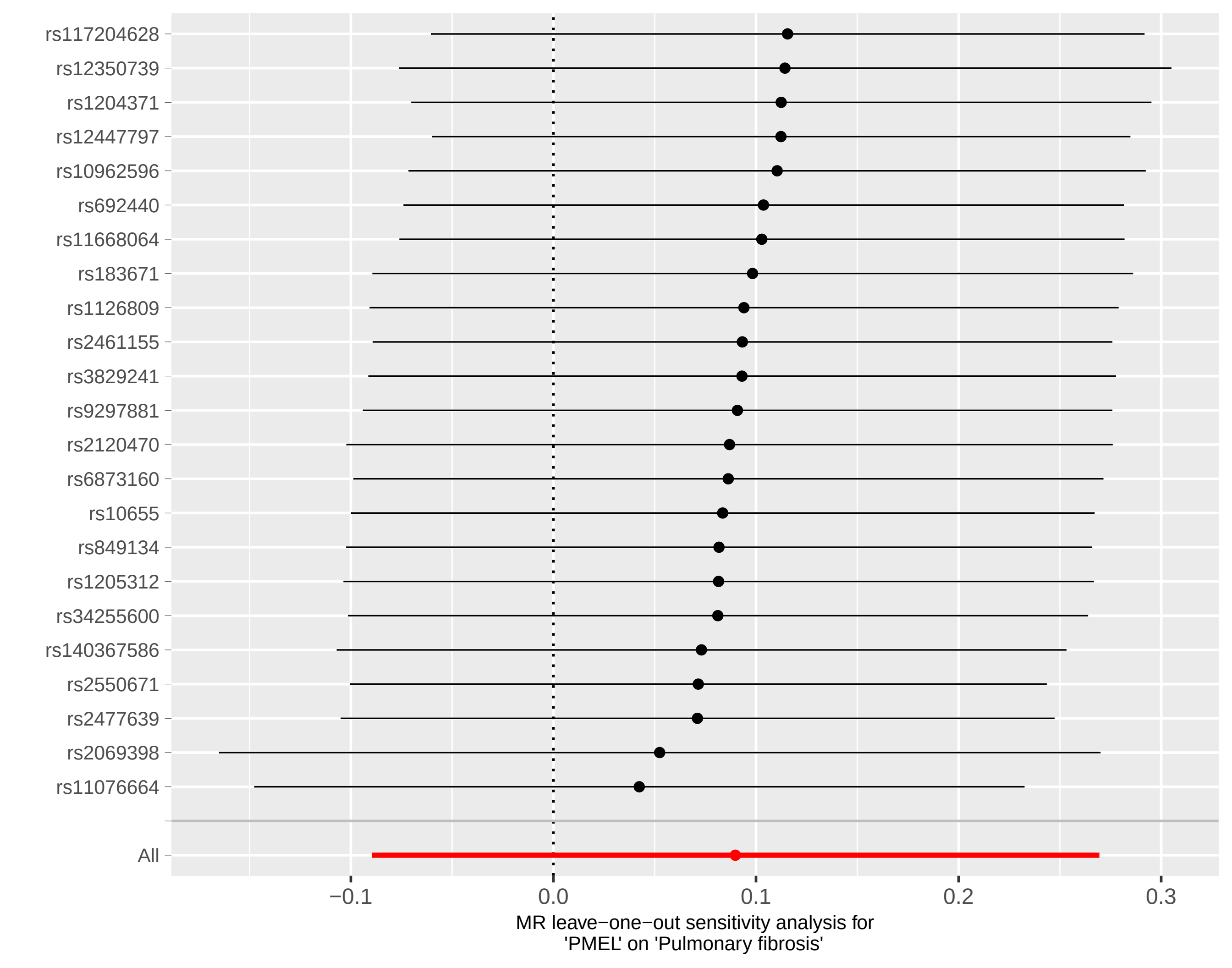

rs117204628
rs12350739
rs1204371
rs12447797
rs10962596
rs692440
rs11668064
rs183671
rs1126809
rs2461155
rs3829241
rs9297881
rs2120470
rs6873160
rs10655
rs849134
rs1205312
rs34255600
rs140367586
rs2550671
rs2477639
rs2069398
rs11076664
All
−0.1
0.0
0.1
0.2
0.3
MR leave−one−out sensitivity analysis for
'PMEL' on 'Pulmonary fibrosis'

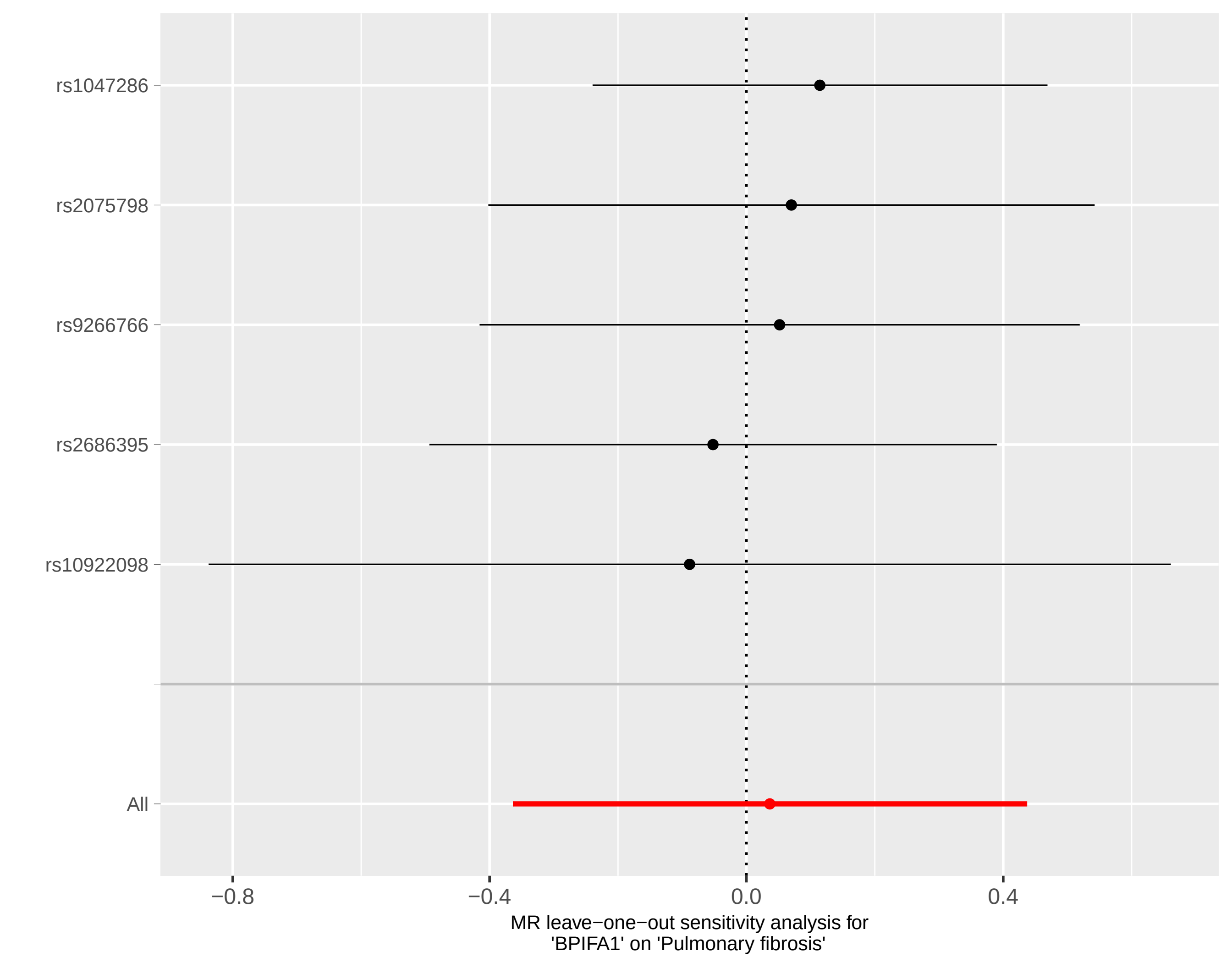

rs1047286
rs2075798
rs9266766
rs2686395
rs10922098
All
−0.8
−0.4
0.0
0.4
MR leave−one−out sensitivity analysis for
'BPIFA1' on 'Pulmonary fibrosis'

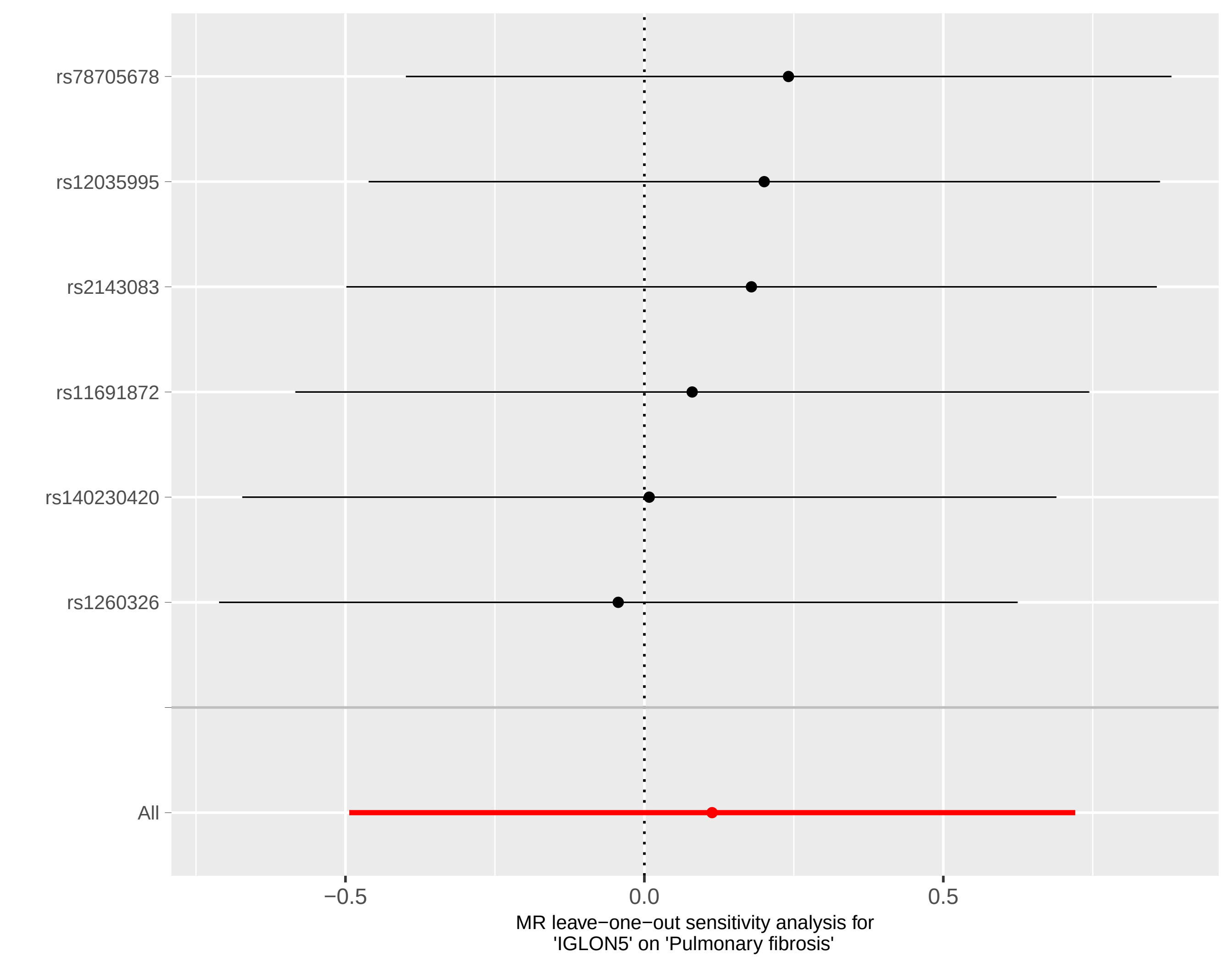
rs78705678
rs12035995
rs2143083
rs11691872
rs140230420
rs1260326
All
−0.5
0.0
0.5
MR leave−one−out sensitivity analysis for
'IGLON5' on 'Pulmonary fibrosis'

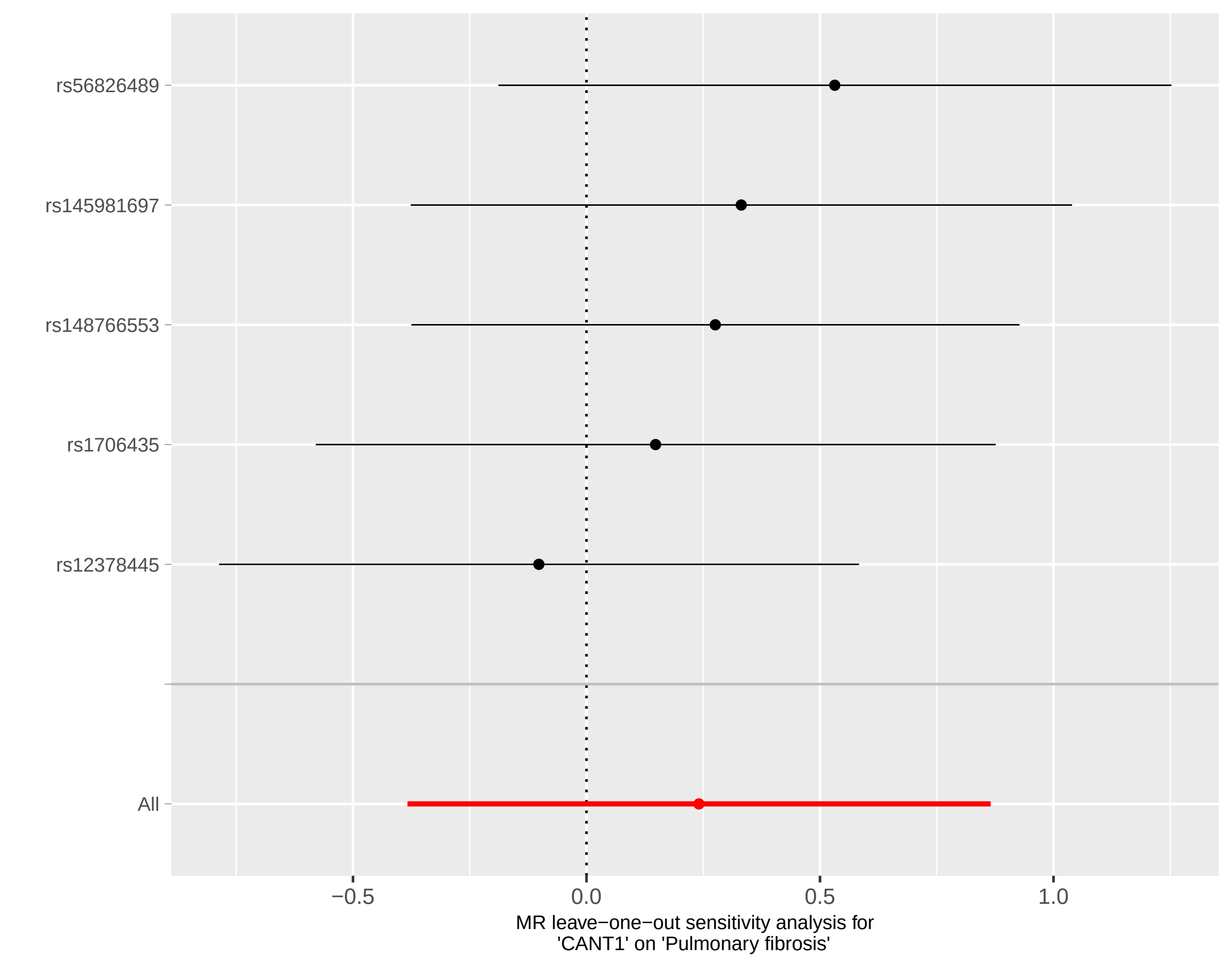
rs56826489
rs145981697
rs148766553
rs1706435
rs12378445
All
−0.5
0.0
0.5
1.0
MR leave−one−out sensitivity analysis for
'CANT1' on 'Pulmonary fibrosis'

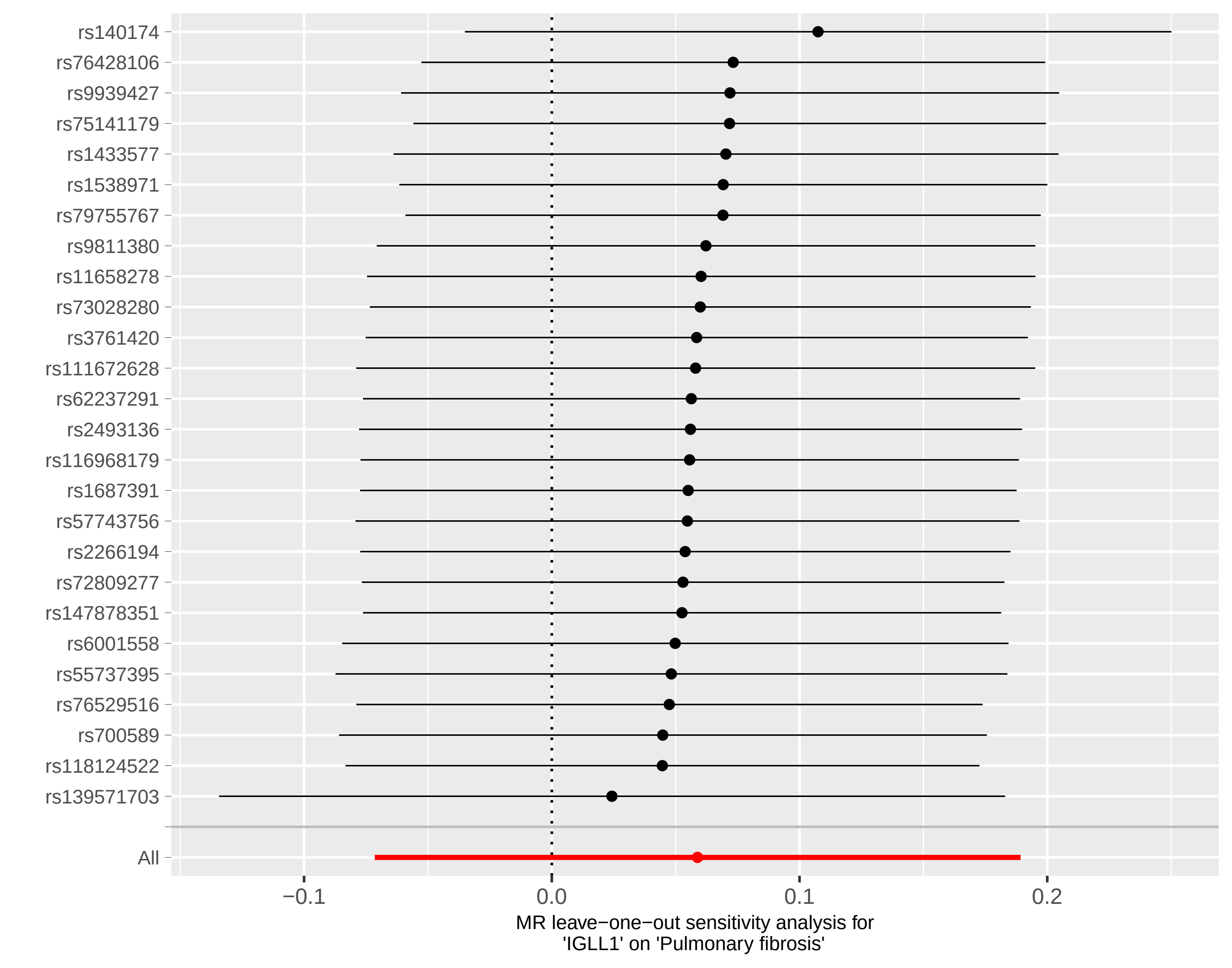
rs140174
rs76428106
rs9939427
rs75141179
rs1433577
rs1538971
rs79755767
rs9811380
rs11658278
rs73028280
rs3761420
rs111672628
rs62237291
rs2493136
rs116968179
rs1687391
rs57743756
rs2266194
rs72809277
rs147878351
rs6001558
rs55737395
rs76529516
rs700589
rs118124522
rs139571703
All
−0.1
0.0
0.1
0.2
MR leave−one−out sensitivity analysis for
'IGLL1' on 'Pulmonary fibrosis'

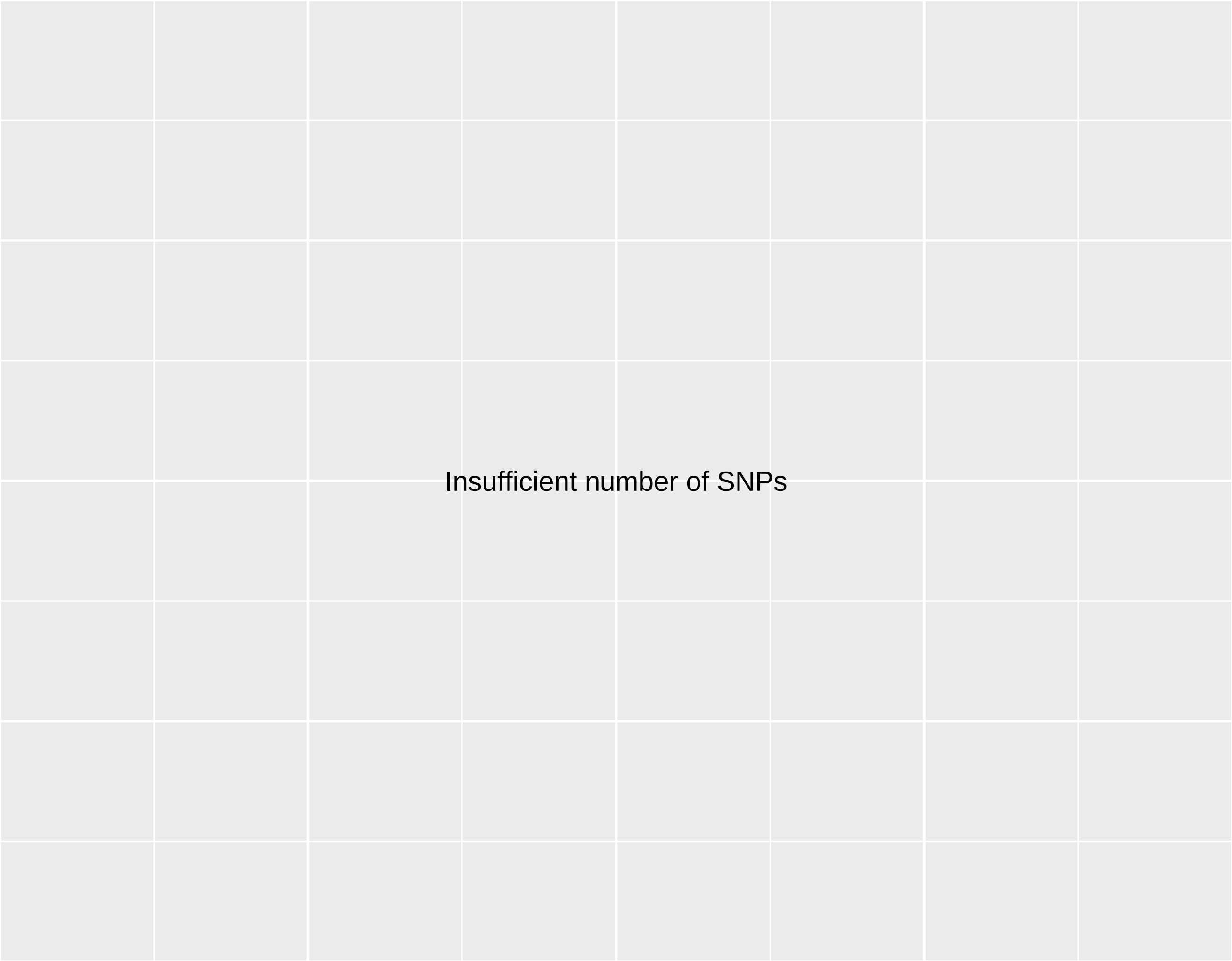
Insufficient number of SNPs

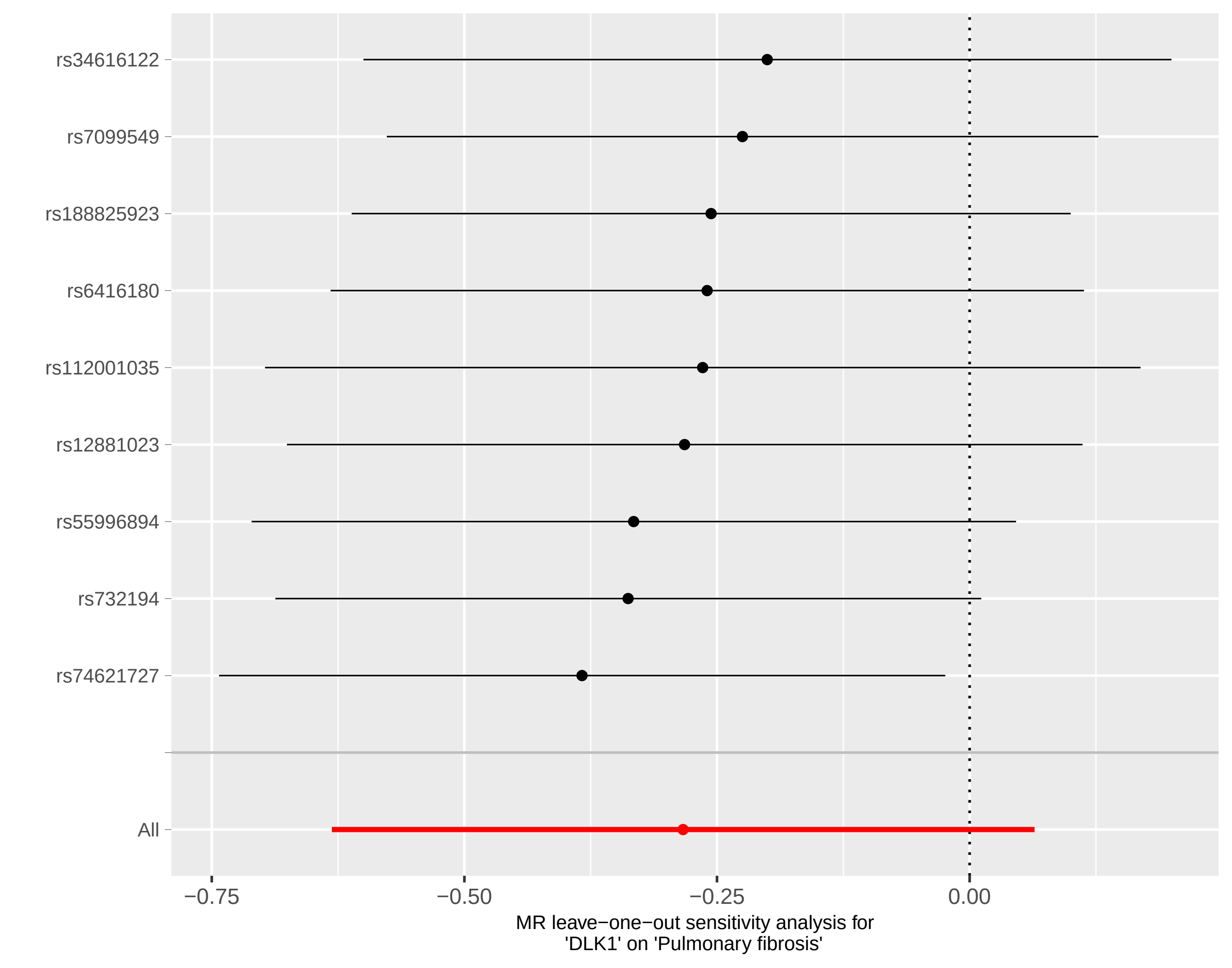

rs34616122
rs7099549
rs188825923
rs6416180
rs112001035
rs12881023
rs55996894
rs732194
rs74621727
All
−0.75
−0.50
−0.25
0.00
MR leave−one−out sensitivity analysis for
'DLK1' on 'Pulmonary fibrosis'

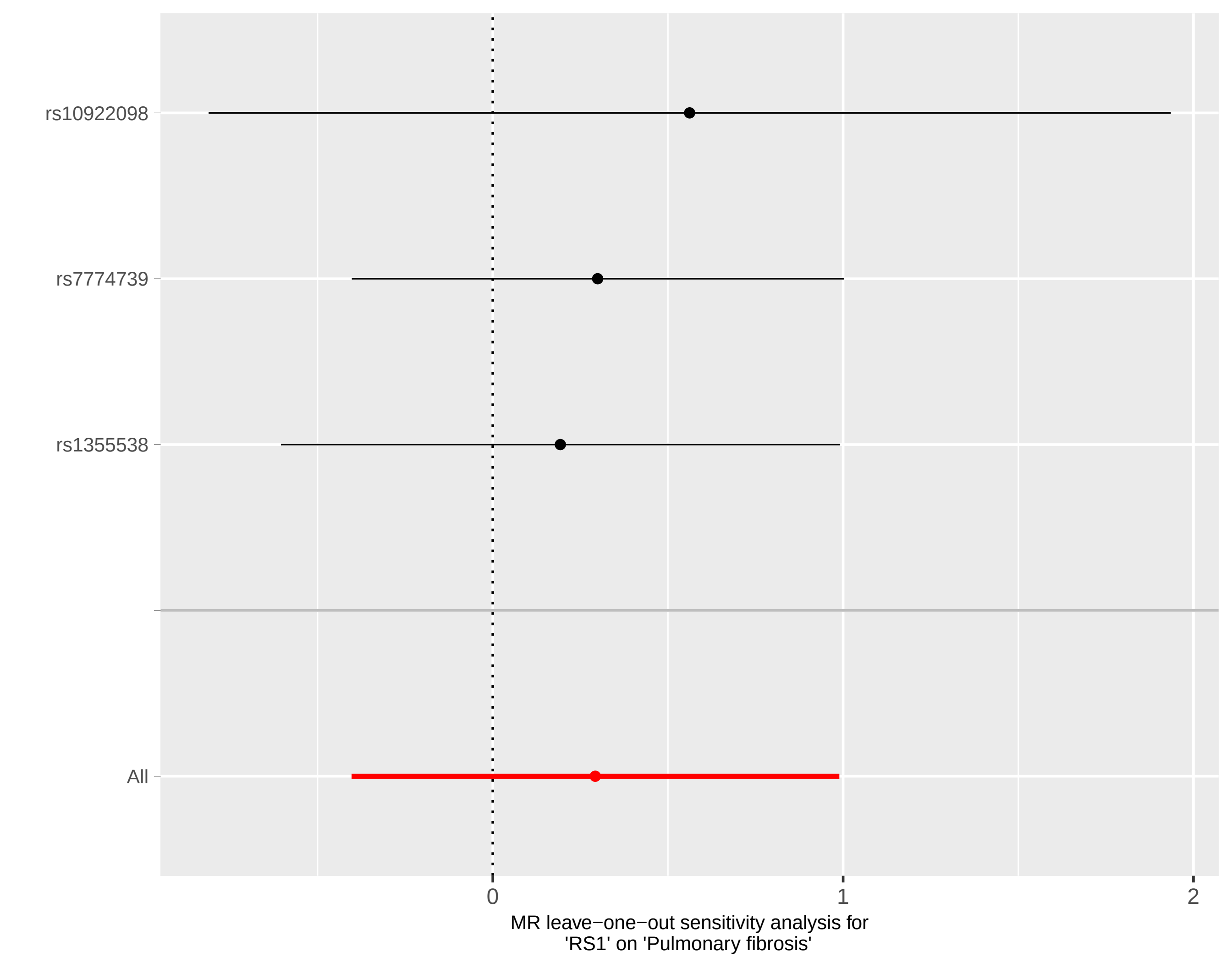
rs10922098
rs7774739
rs1355538
All
0
1
2
MR leave−one−out sensitivity analysis for
'RS1' on 'Pulmonary fibrosis'

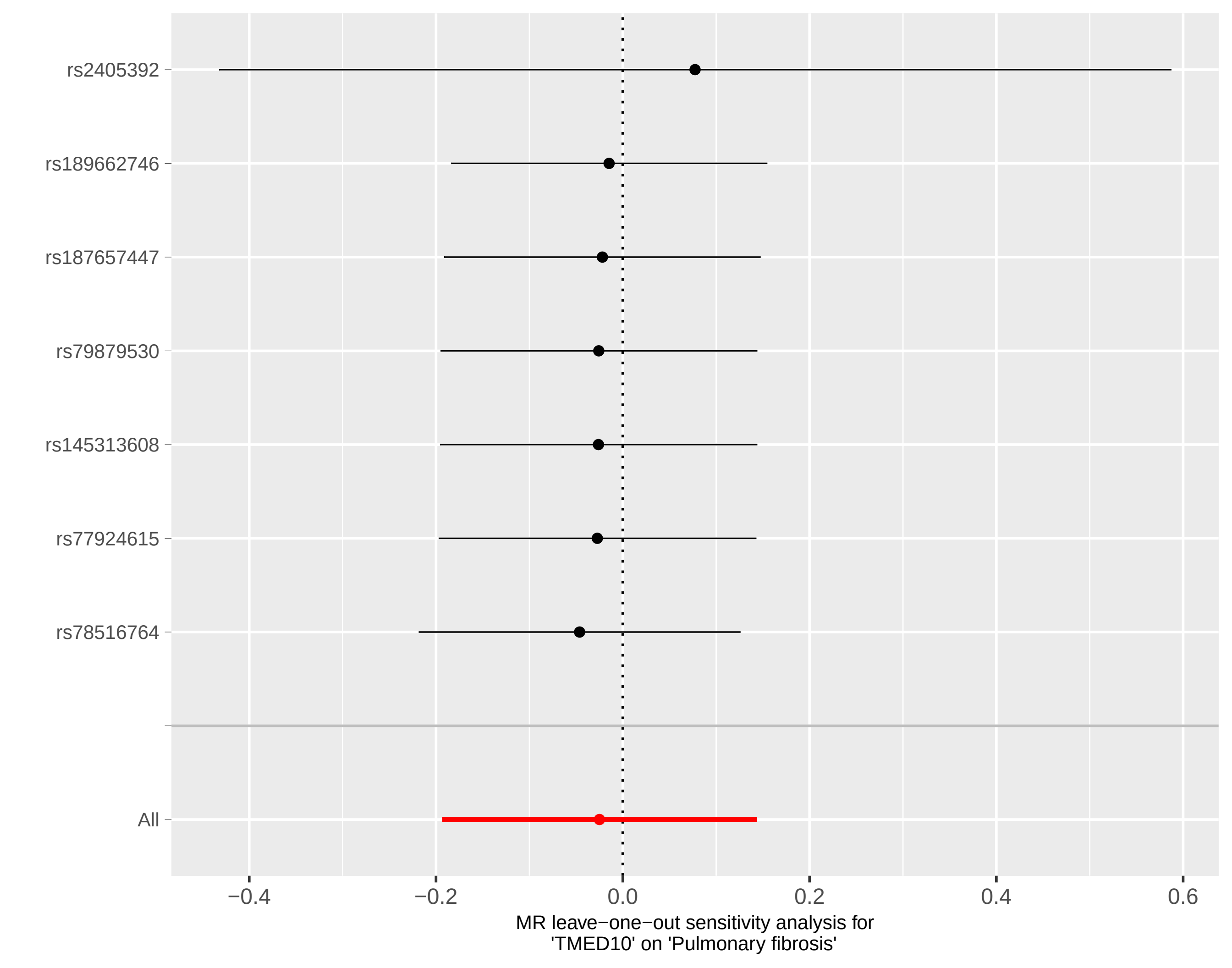

rs2405392
rs189662746
rs187657447
rs79879530
rs145313608
rs77924615
rs78516764
All
−0.4
−0.2
0.0
0.2
0.4
0.6
MR leave−one−out sensitivity analysis for
'TMED10' on 'Pulmonary fibrosis'

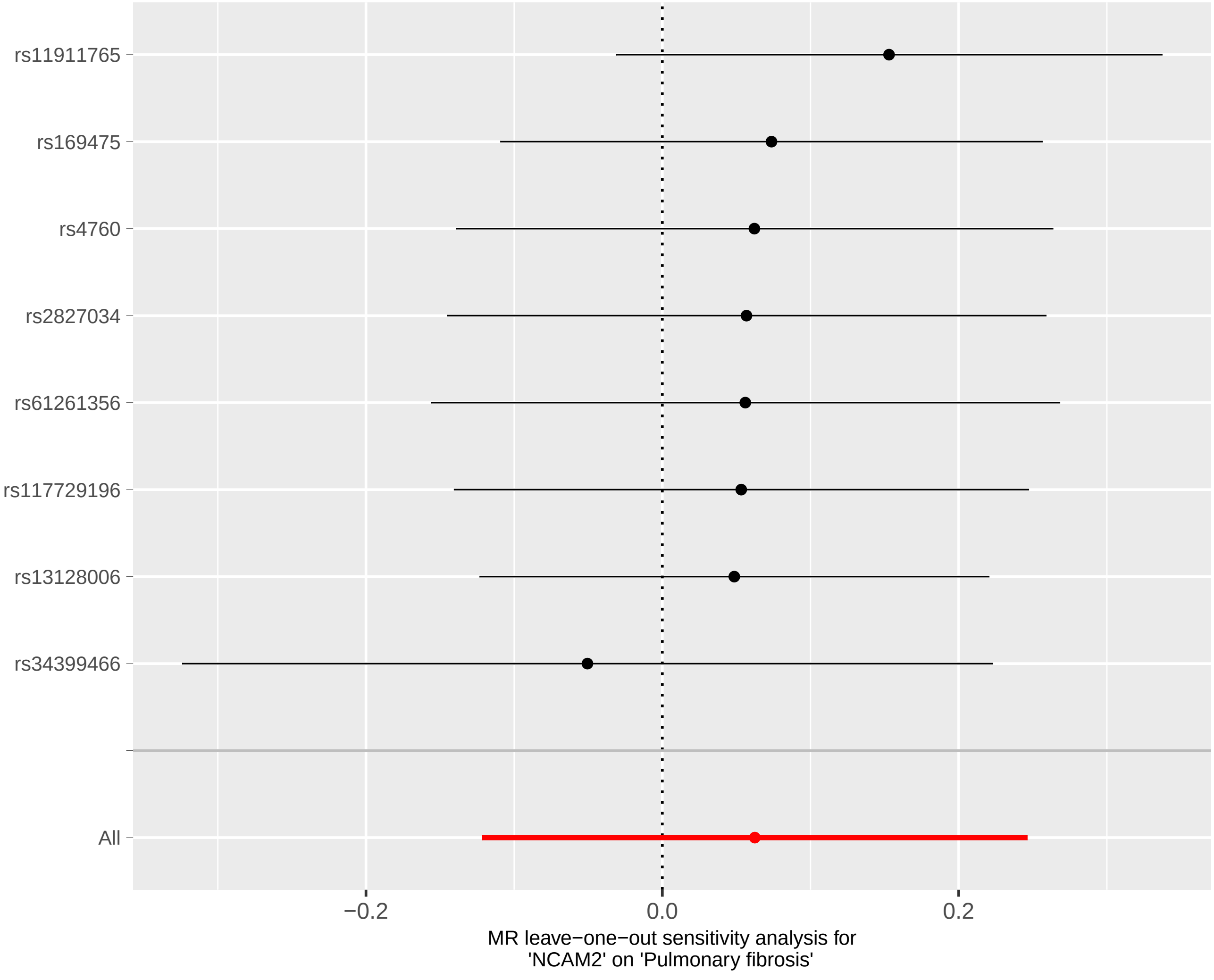
rs11911765
rs169475
rs4760
rs2827034
rs61261356
rs117729196
rs13128006
rs34399466
All
−0.2
0.0
0.2
MR leave−one−out sensitivity analysis for
'NCAM2' on 'Pulmonary fibrosis'

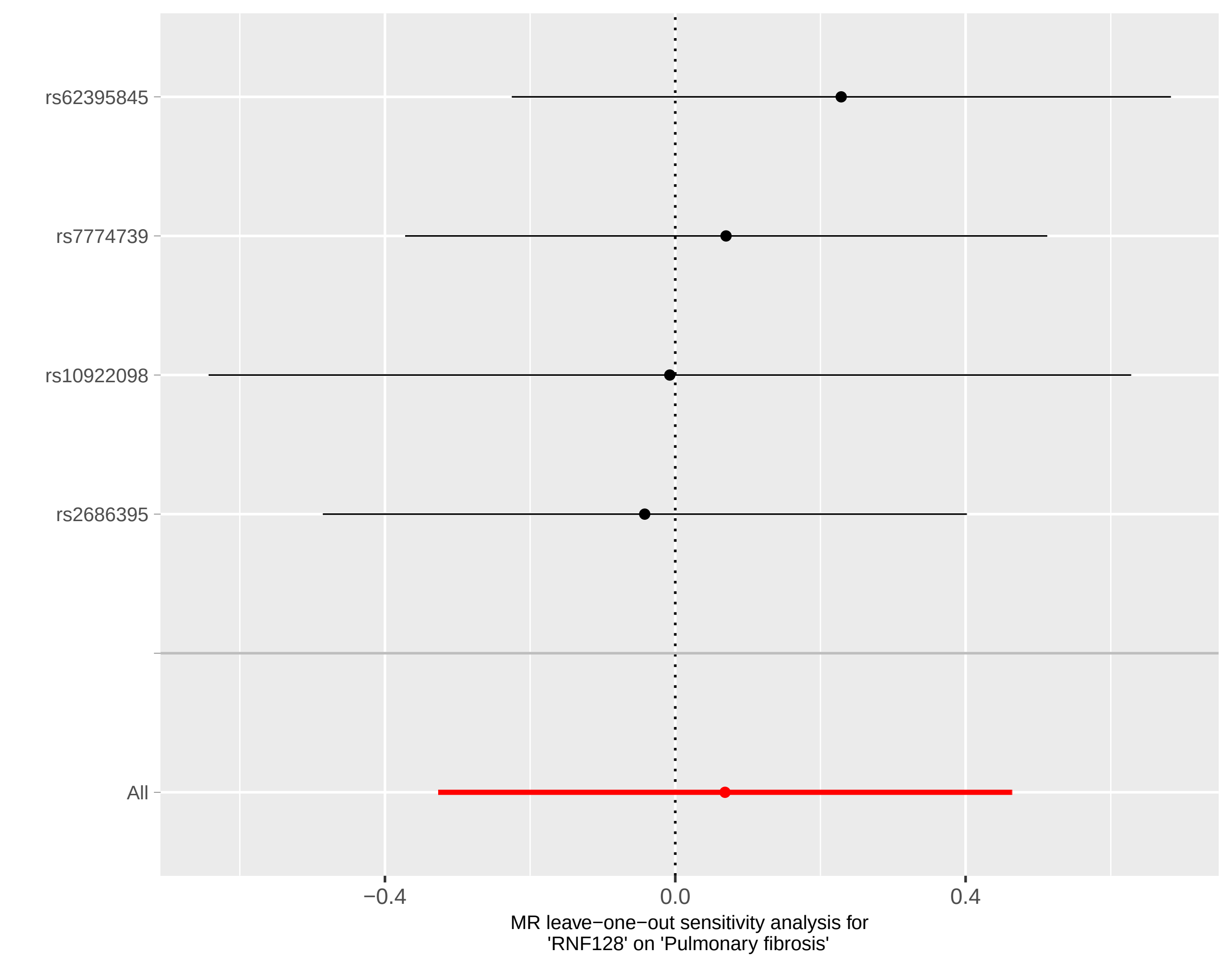
rs62395845
rs7774739
rs10922098
rs2686395
All
−0.4
0.0
0.4
MR leave−one−out sensitivity analysis for
'RNF128' on 'Pulmonary fibrosis'

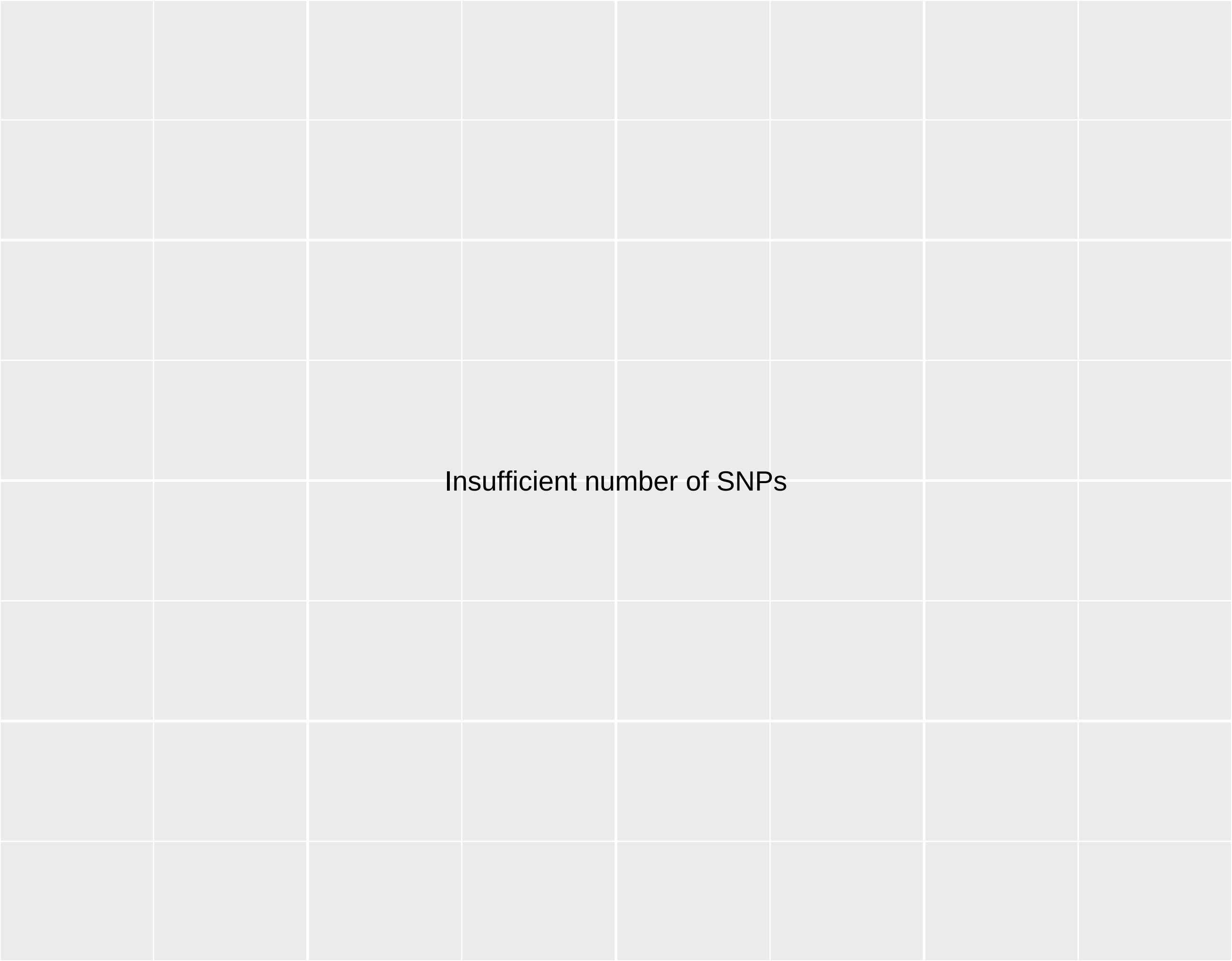
Insufficient number of SNPs

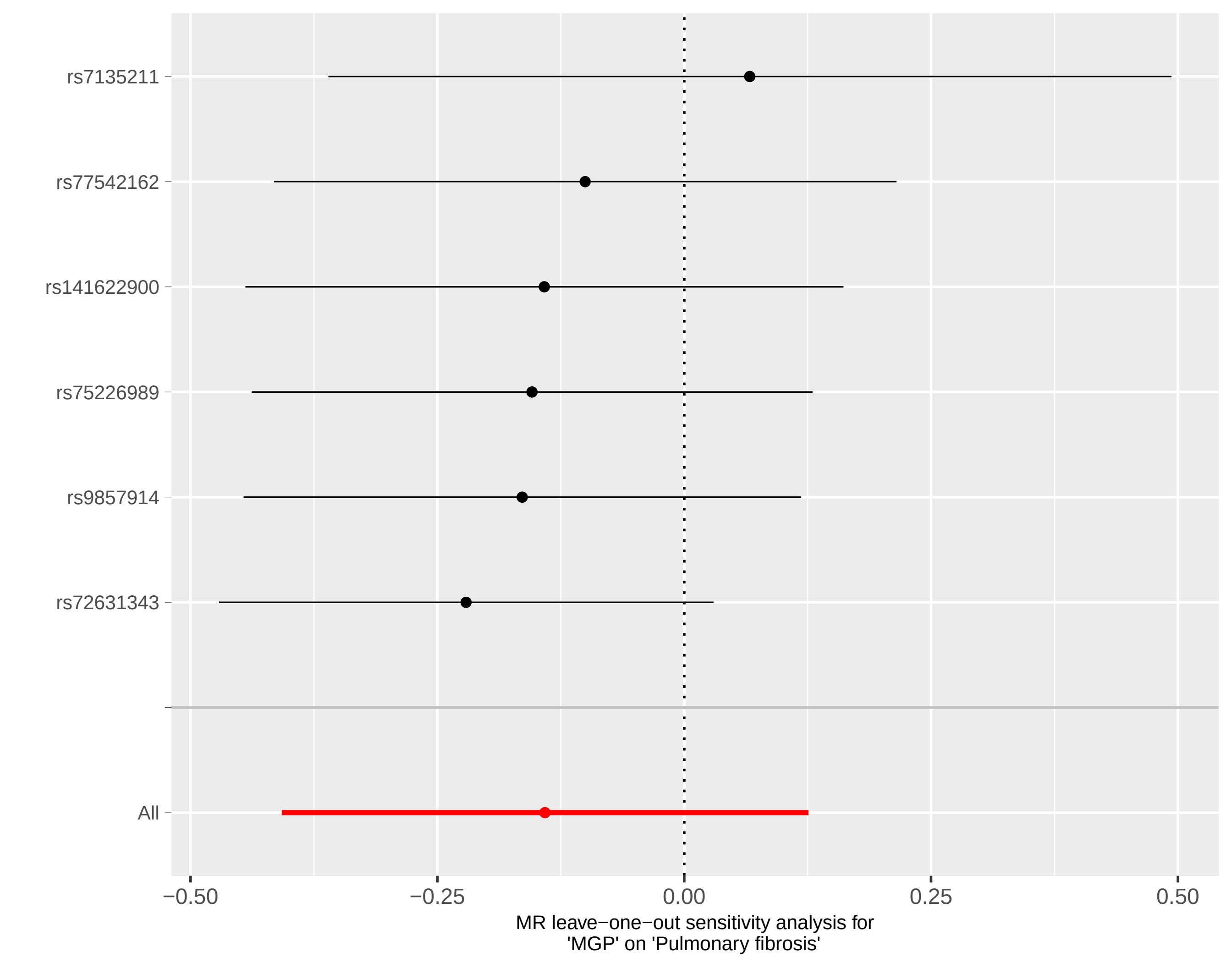
rs7135211
rs77542162
rs141622900
rs75226989
rs9857914
rs72631343
All
−0.50
−0.25
0.00
0.25
0.50
MR leave−one−out sensitivity analysis for
'MGP' on 'Pulmonary fibrosis'

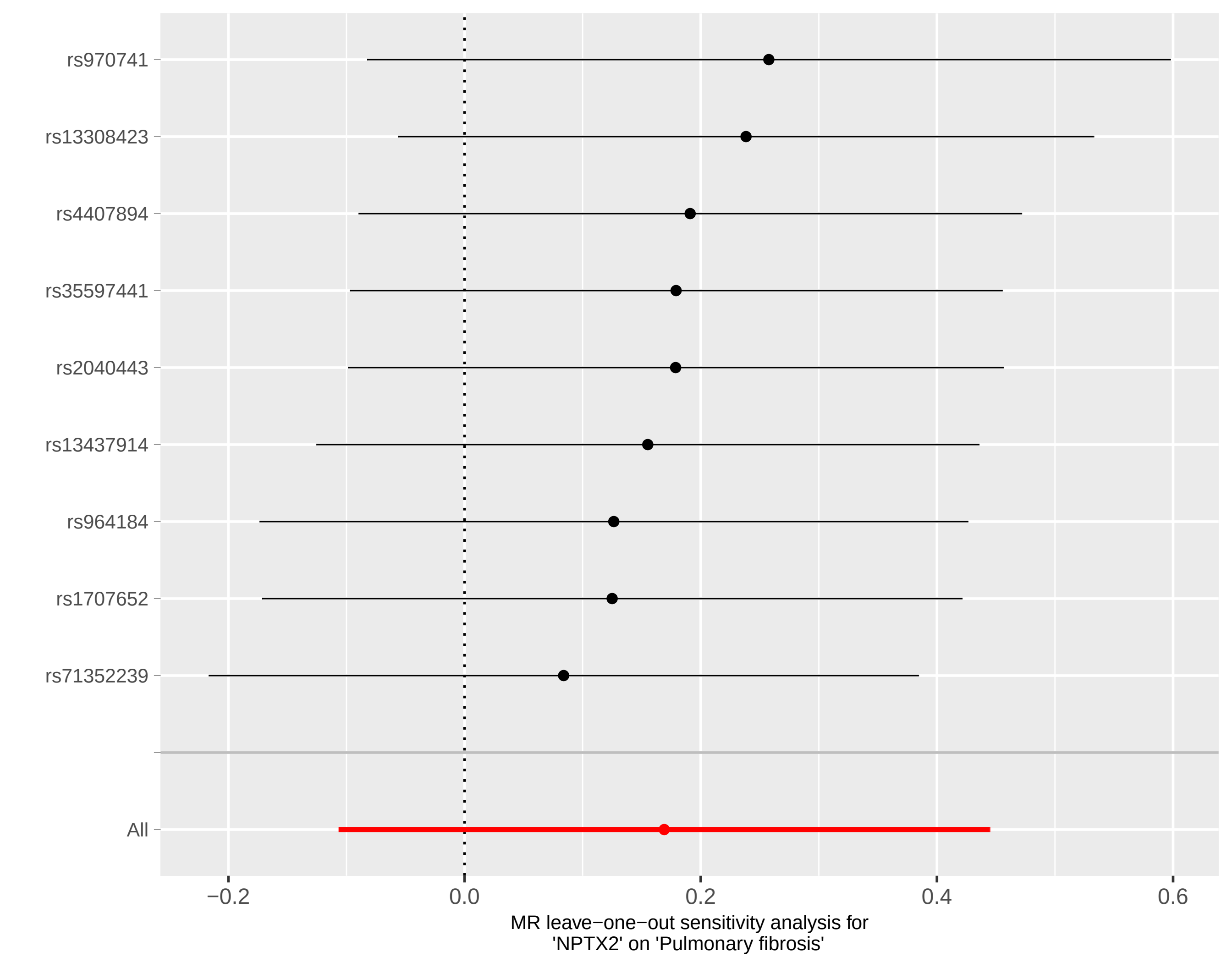
rs970741
rs13308423
rs4407894
rs35597441
rs2040443
rs13437914
rs964184
rs1707652
rs71352239
All
−0.2
0.0
0.2
0.4
0.6
MR leave−one−out sensitivity analysis for
'NPTX2' on 'Pulmonary fibrosis'

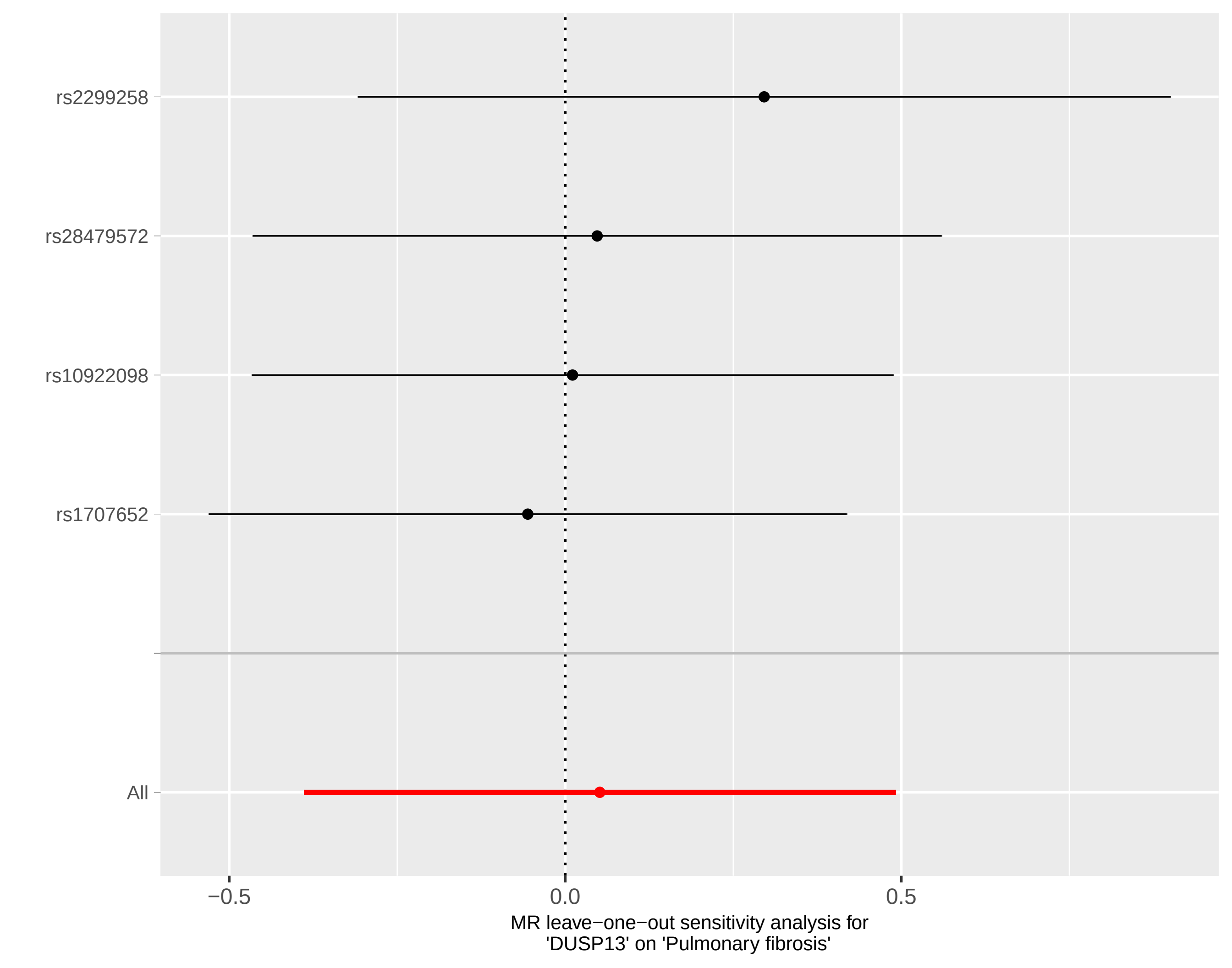
rs2299258
rs28479572
rs10922098
rs1707652
All
−0.5
0.0
0.5
MR leave−one−out sensitivity analysis for
'DUSP13' on 'Pulmonary fibrosis'

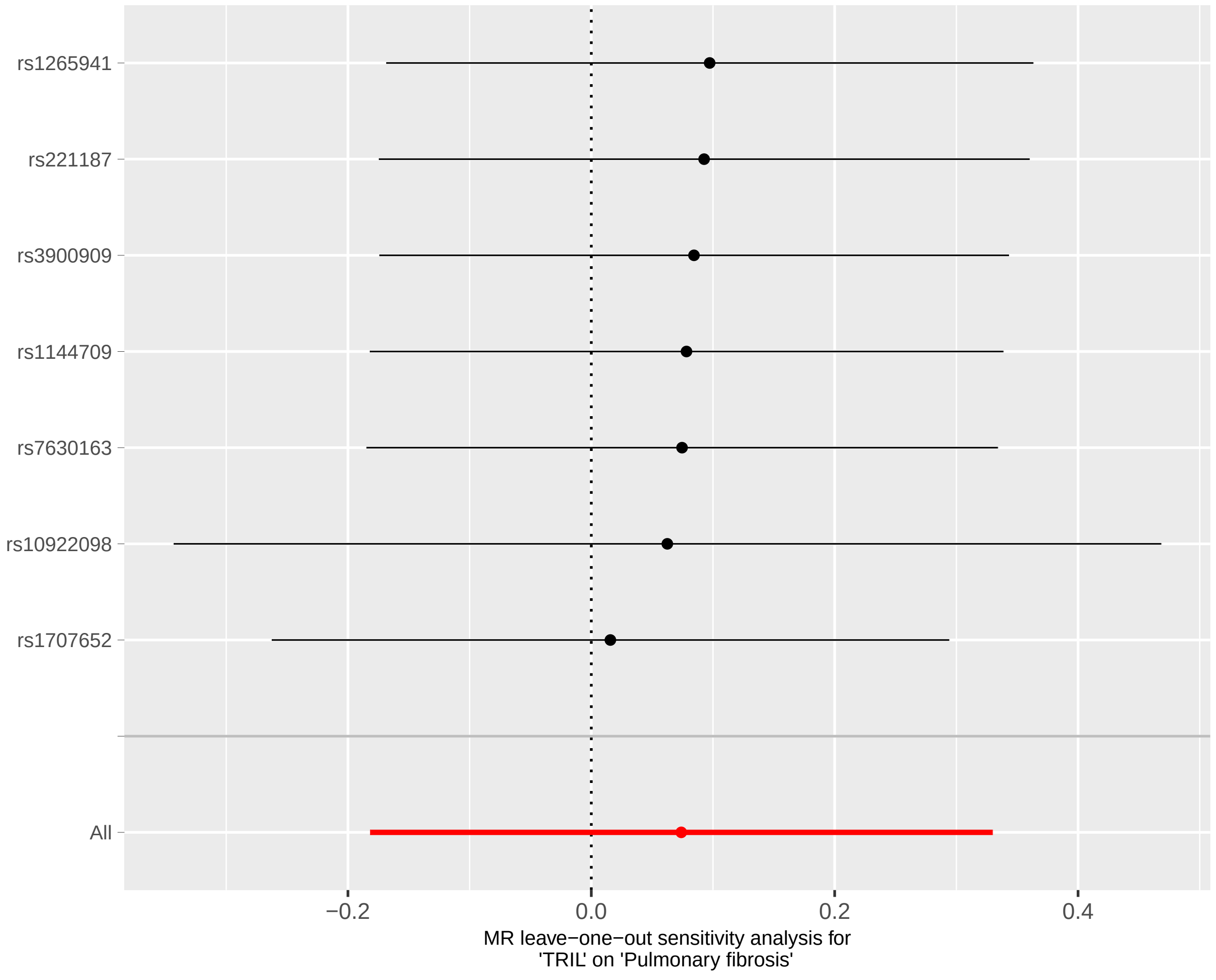
rs1265941
rs221187
rs3900909
rs1144709
rs7630163
rs10922098
rs1707652
All
−0.2
0.0
0.2
0.4
MR leave−one−out sensitivity analysis for
'TRIL' on 'Pulmonary fibrosis'

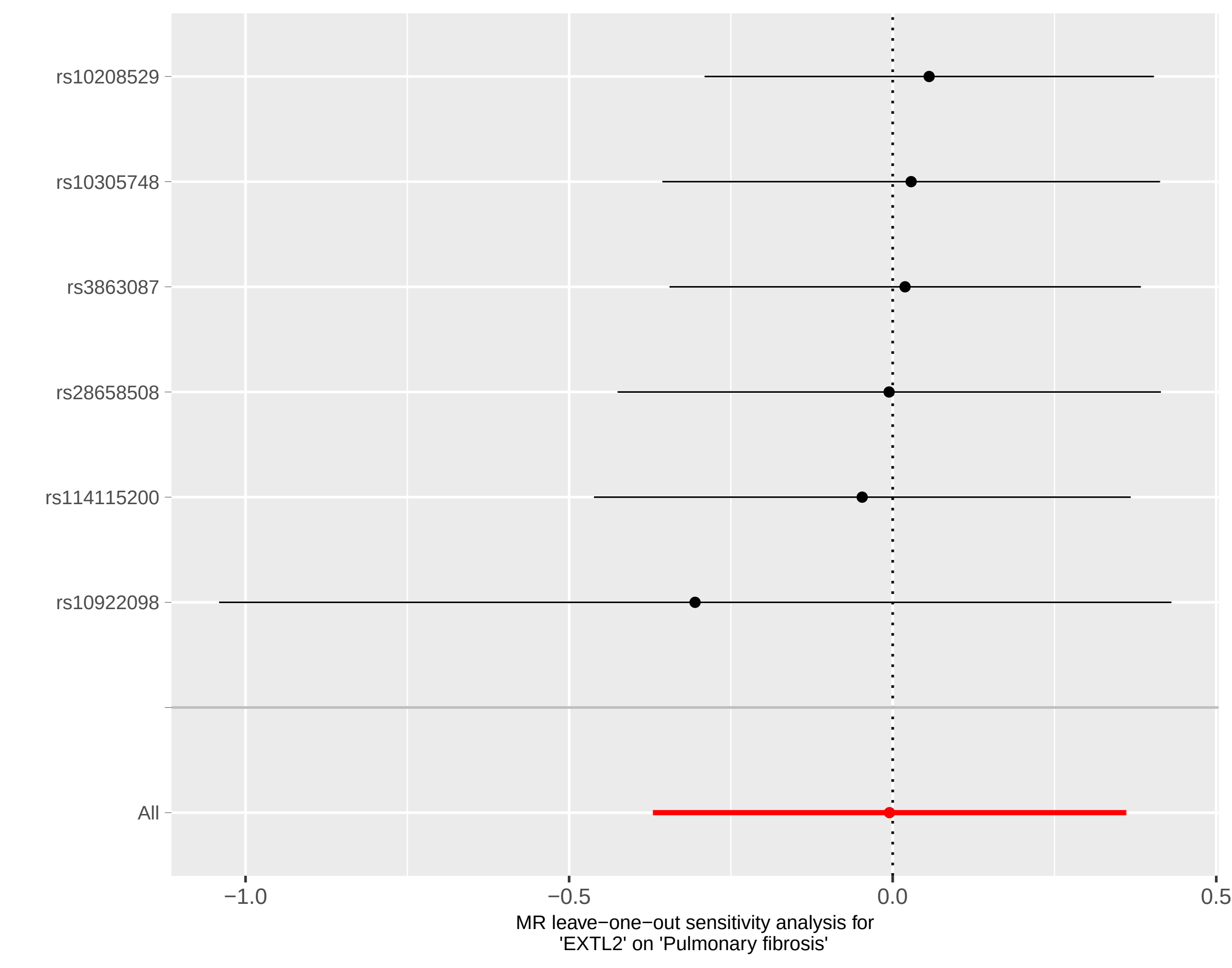
rs10208529
rs10305748
rs3863087
rs28658508
rs114115200
rs10922098
All
−1.0
−0.5
0.0
0.5
MR leave−one−out sensitivity analysis for
'EXTL2' on 'Pulmonary fibrosis'

Insufficient number of SNPs

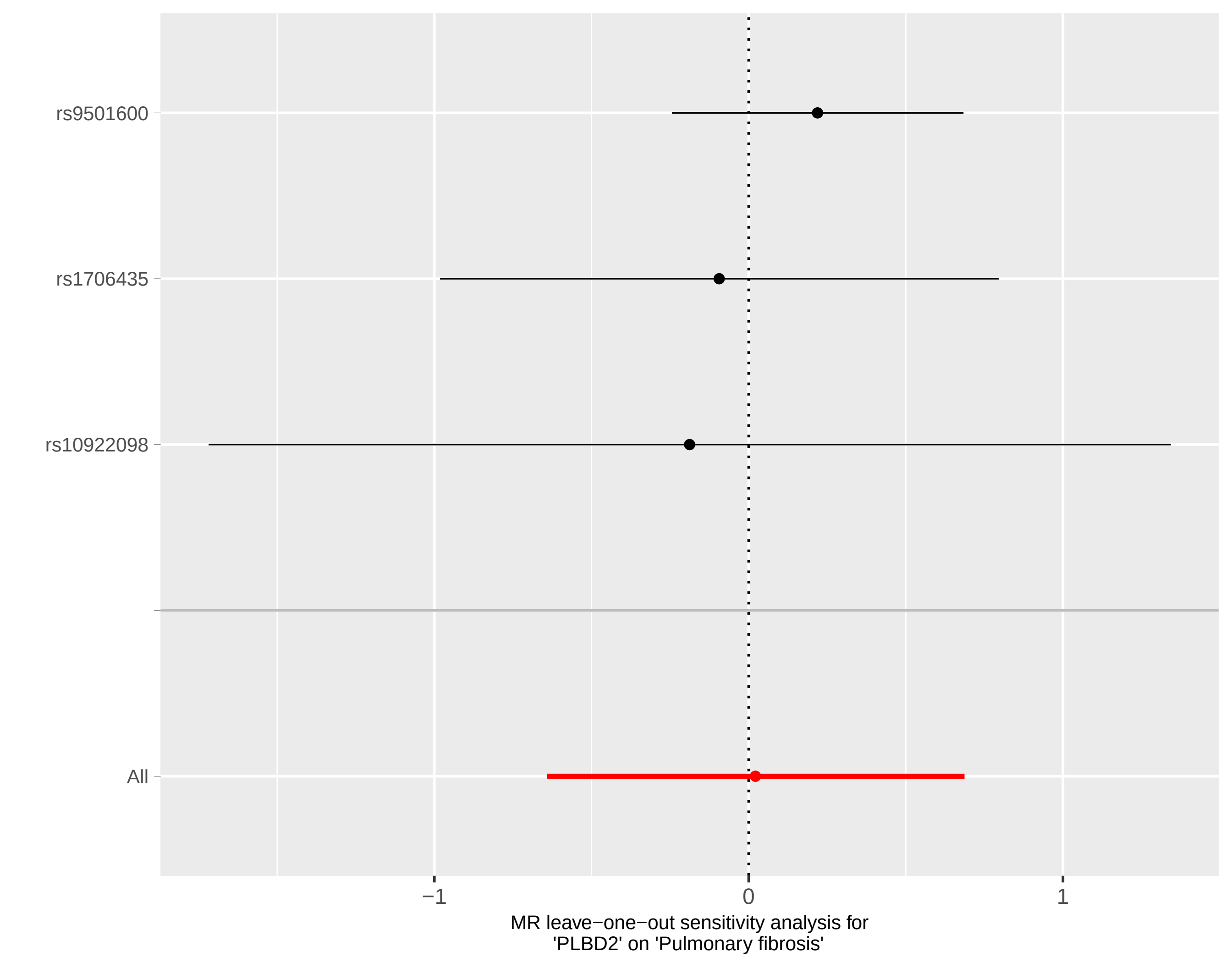

rs9501600
rs1706435
rs10922098
All
−1
0
1
MR leave−one−out sensitivity analysis for
'PLBD2' on 'Pulmonary fibrosis'

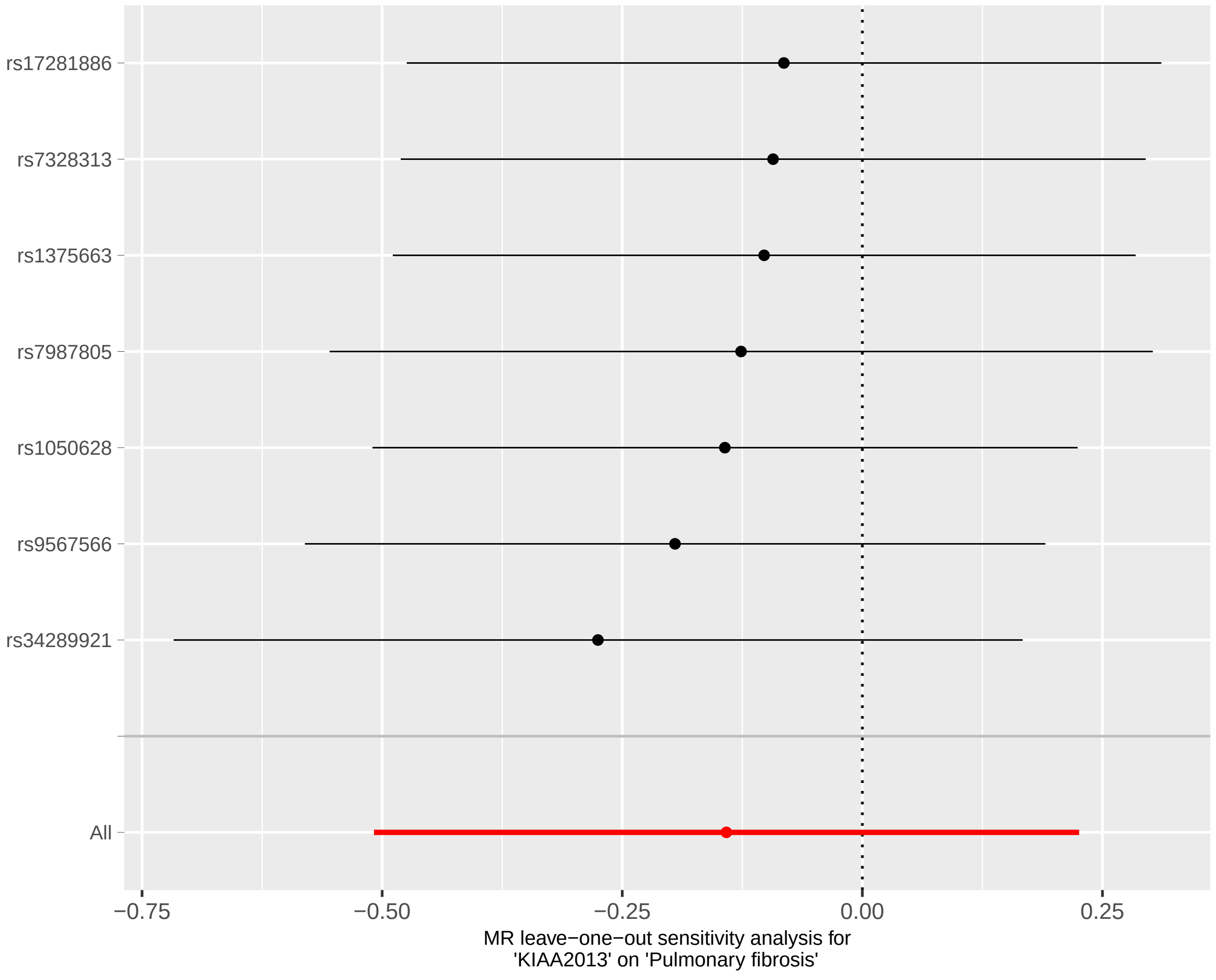

rs17281886
rs7328313
rs1375663
rs7987805
rs1050628
rs9567566
rs34289921
All
−0.75
−0.50
−0.25
0.00
0.25
MR leave−one−out sensitivity analysis for
'KIAA2013' on 'Pulmonary fibrosis'

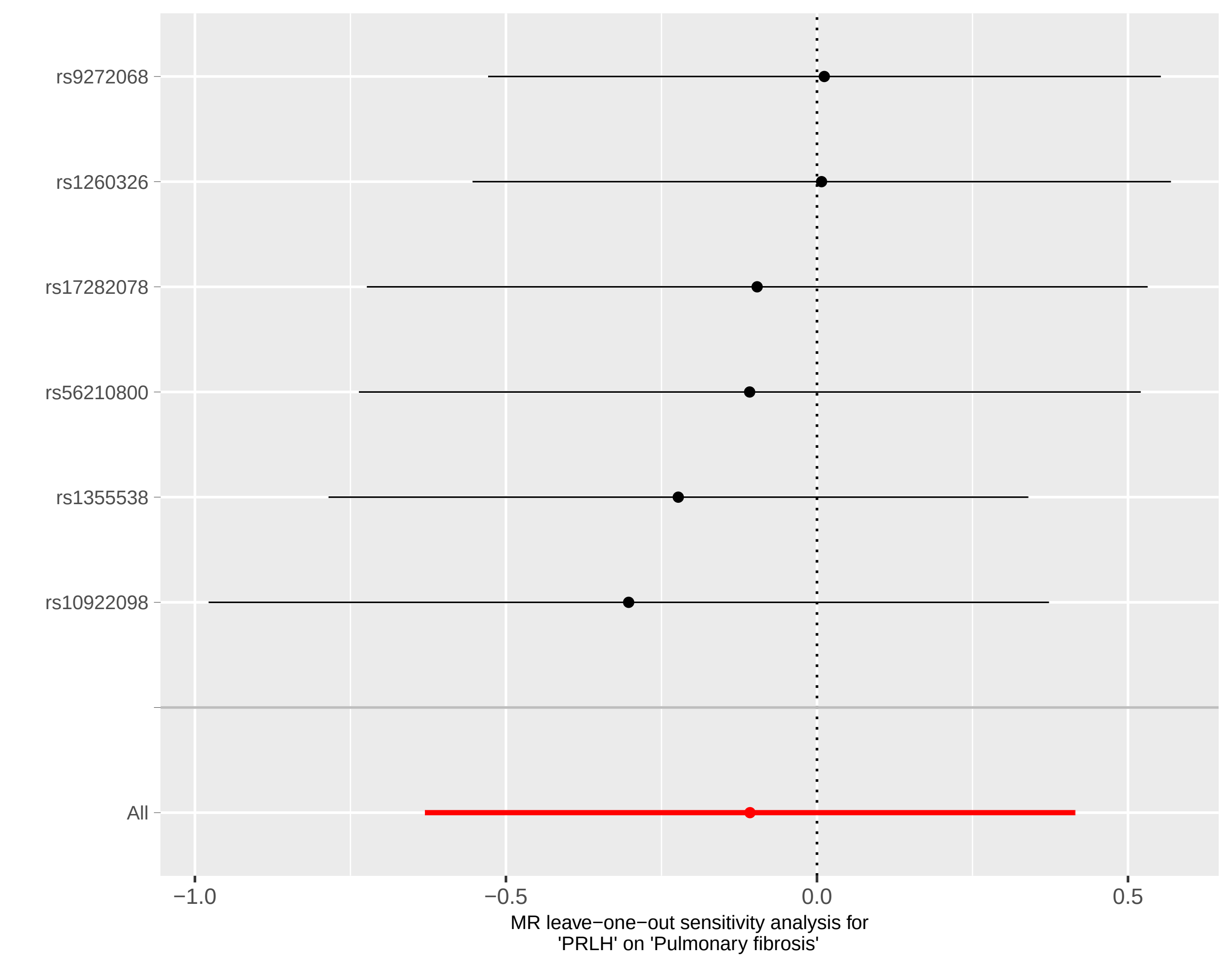
rs9272068
rs1260326
rs17282078
rs56210800
rs1355538
rs10922098
All
−1.0
−0.5
0.0
0.5
MR leave−one−out sensitivity analysis for
'PRLH' on 'Pulmonary fibrosis'

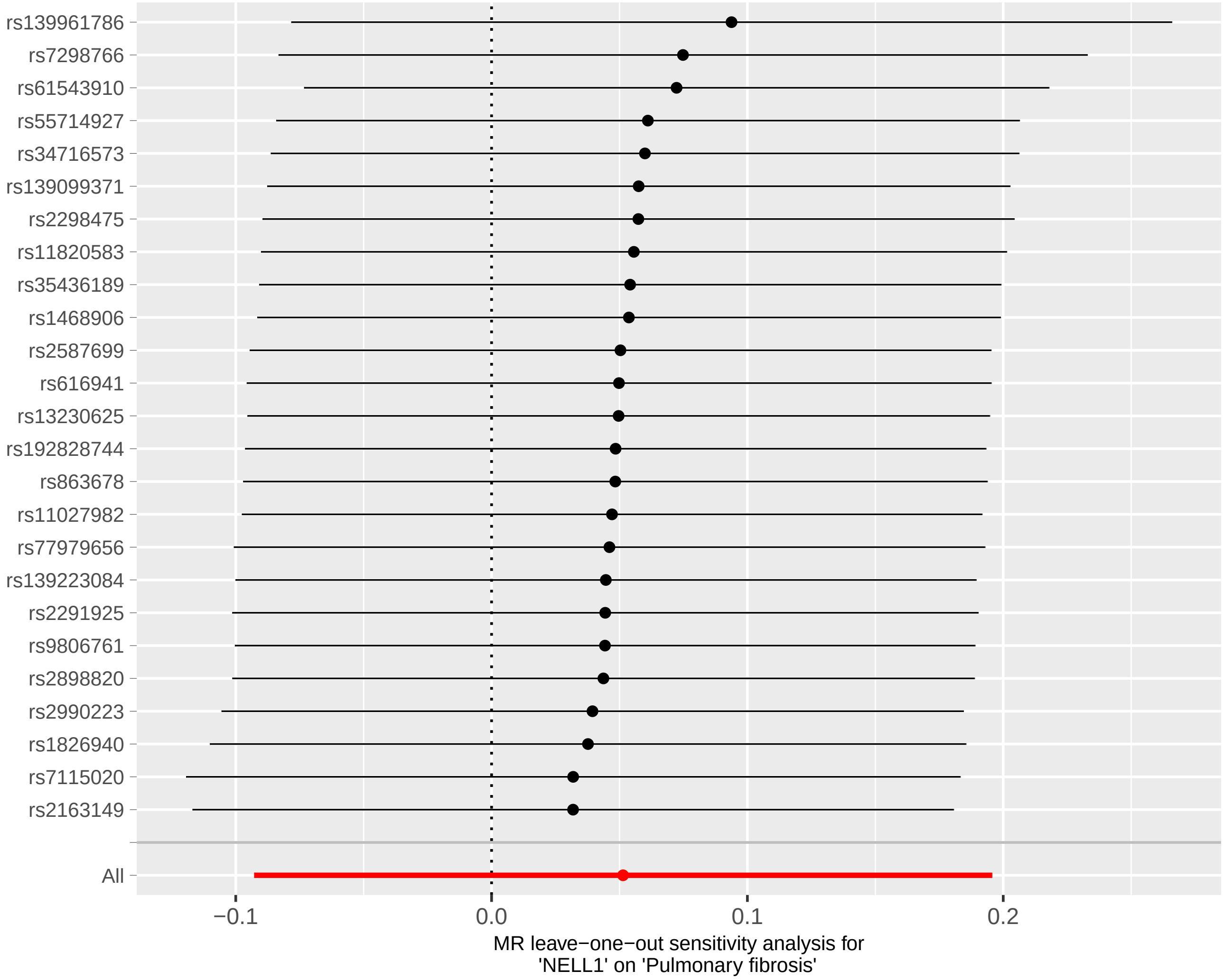
rs139961786
rs7298766
rs61543910
rs55714927
rs34716573
rs139099371
rs2298475
rs11820583
rs35436189
rs1468906
rs2587699
rs616941
rs13230625
rs192828744
rs863678
rs11027982
rs77979656
rs139223084
rs2291925
rs9806761
rs2898820
rs2990223
rs1826940
rs7115020
rs2163149
All
−0.1
0.0
0.1
0.2
MR leave−one−out sensitivity analysis for
'NELL1' on 'Pulmonary fibrosis'

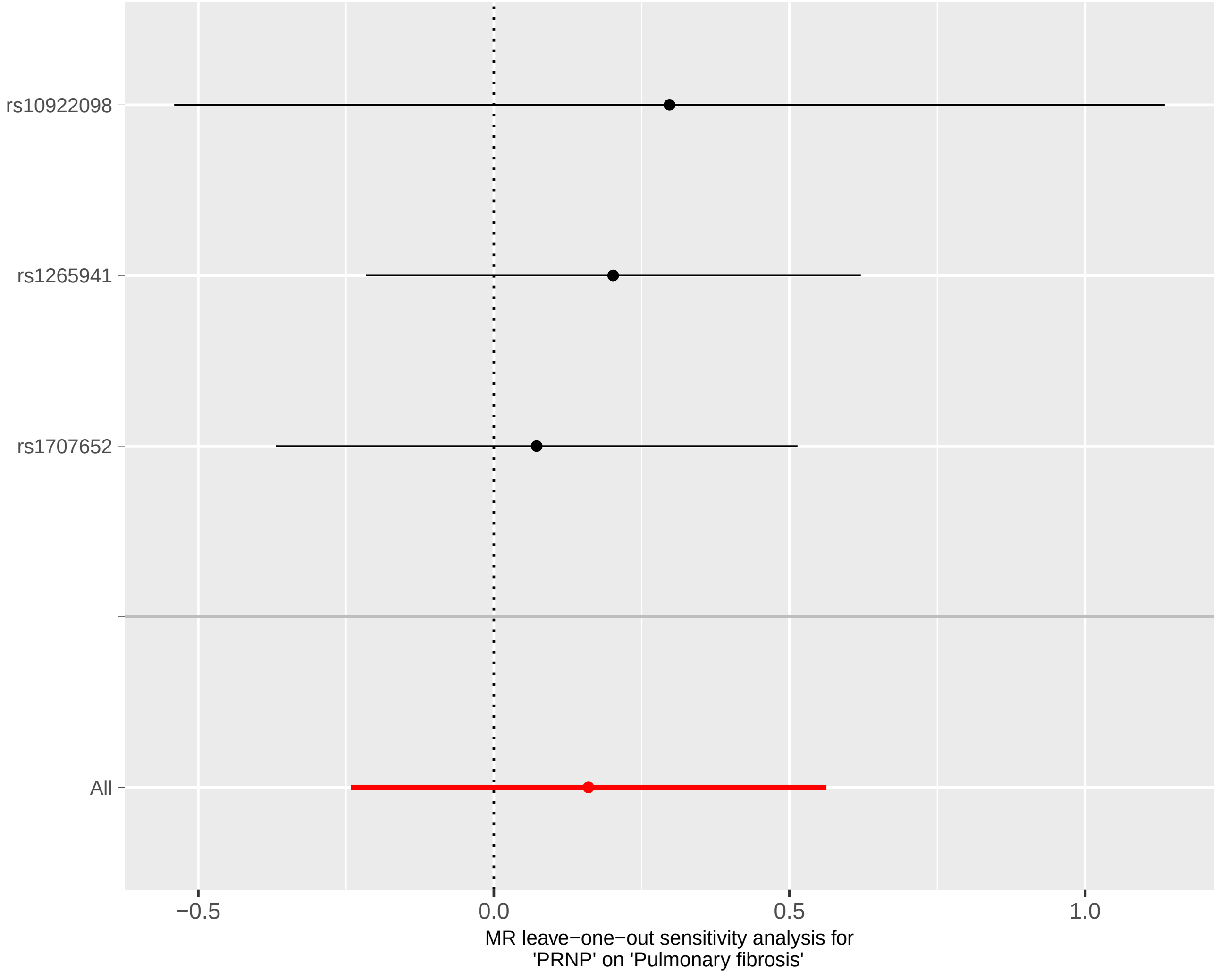
rs10922098
rs1265941
rs1707652
All
−0.5
0.0
0.5
1.0
MR leave−one−out sensitivity analysis for
'PRNP' on 'Pulmonary fibrosis'

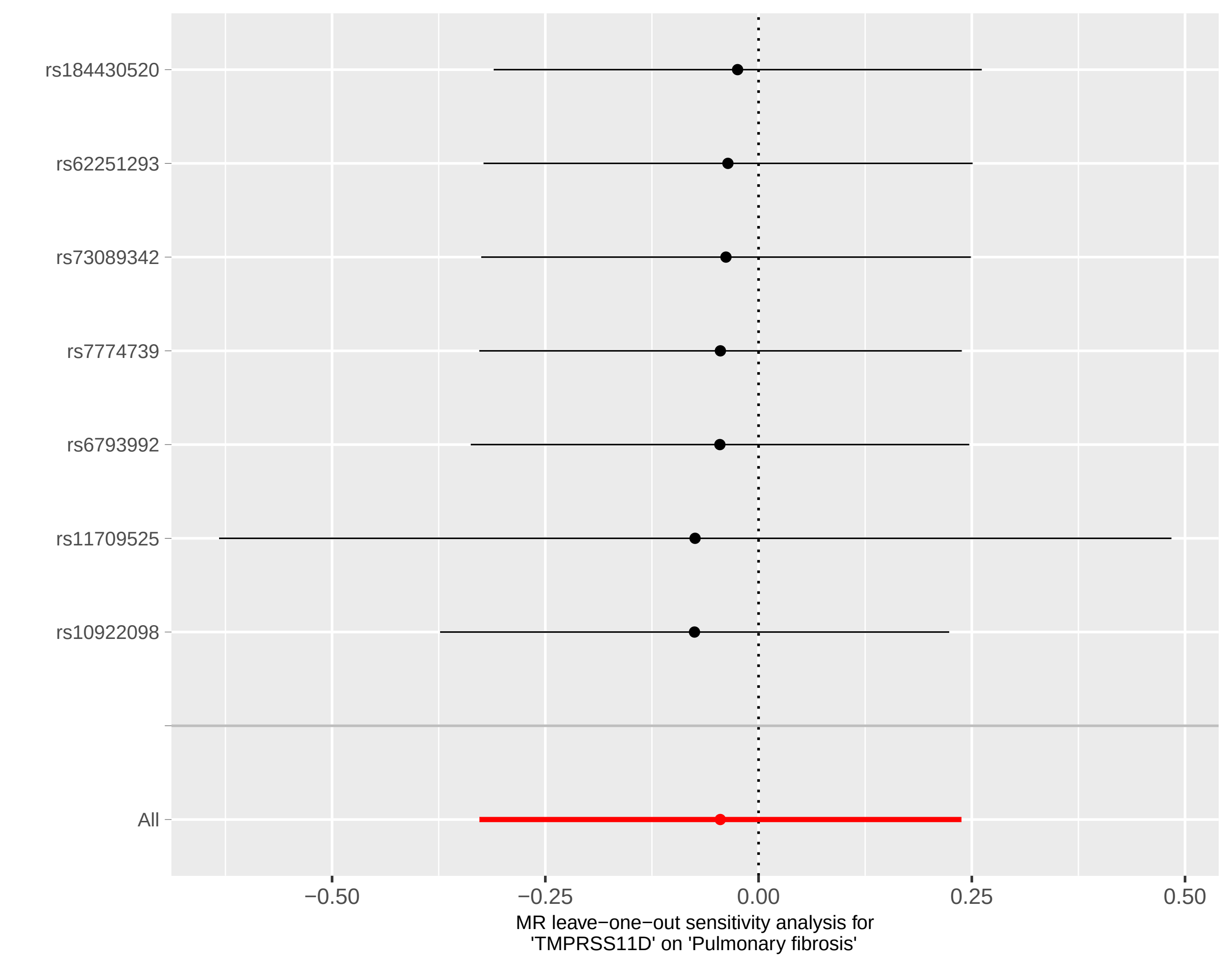
rs184430520
rs62251293
rs73089342
rs7774739
rs6793992
rs11709525
rs10922098
All
−0.50
−0.25
0.00
0.25
0.50
MR leave−one−out sensitivity analysis for
'TMPRSS11D' on 'Pulmonary fibrosis'

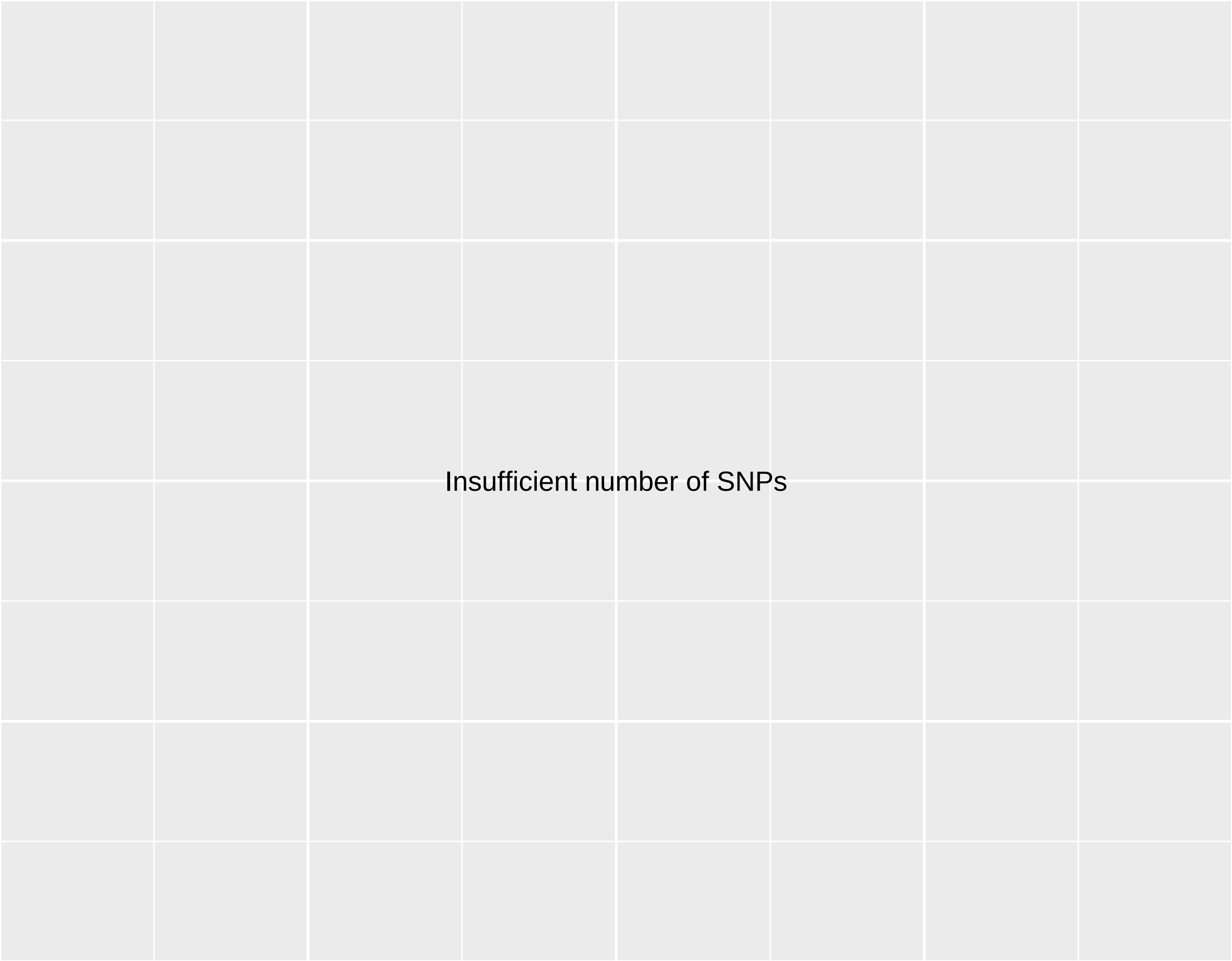
Insufficient number of SNPs

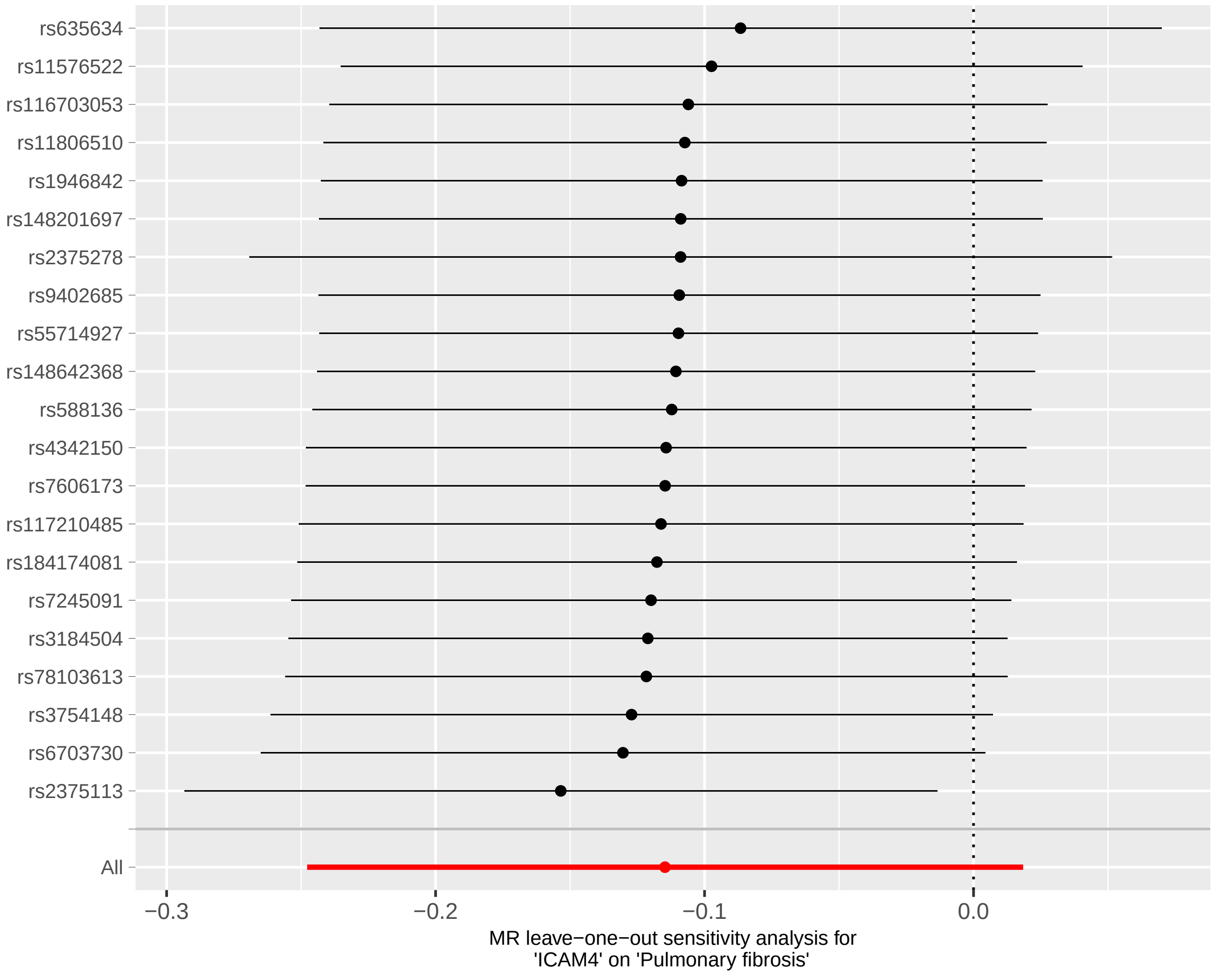
rs635634
rs11576522
rs116703053
rs11806510
rs1946842
rs148201697
rs2375278
rs9402685
rs55714927
rs148642368
rs588136
rs4342150
rs7606173
rs117210485
rs184174081
rs7245091
rs3184504
rs78103613
rs3754148
rs6703730
rs2375113
All
−0.3
−0.2
−0.1
0.0
MR leave−one−out sensitivity analysis for
'ICAM4' on 'Pulmonary fibrosis'

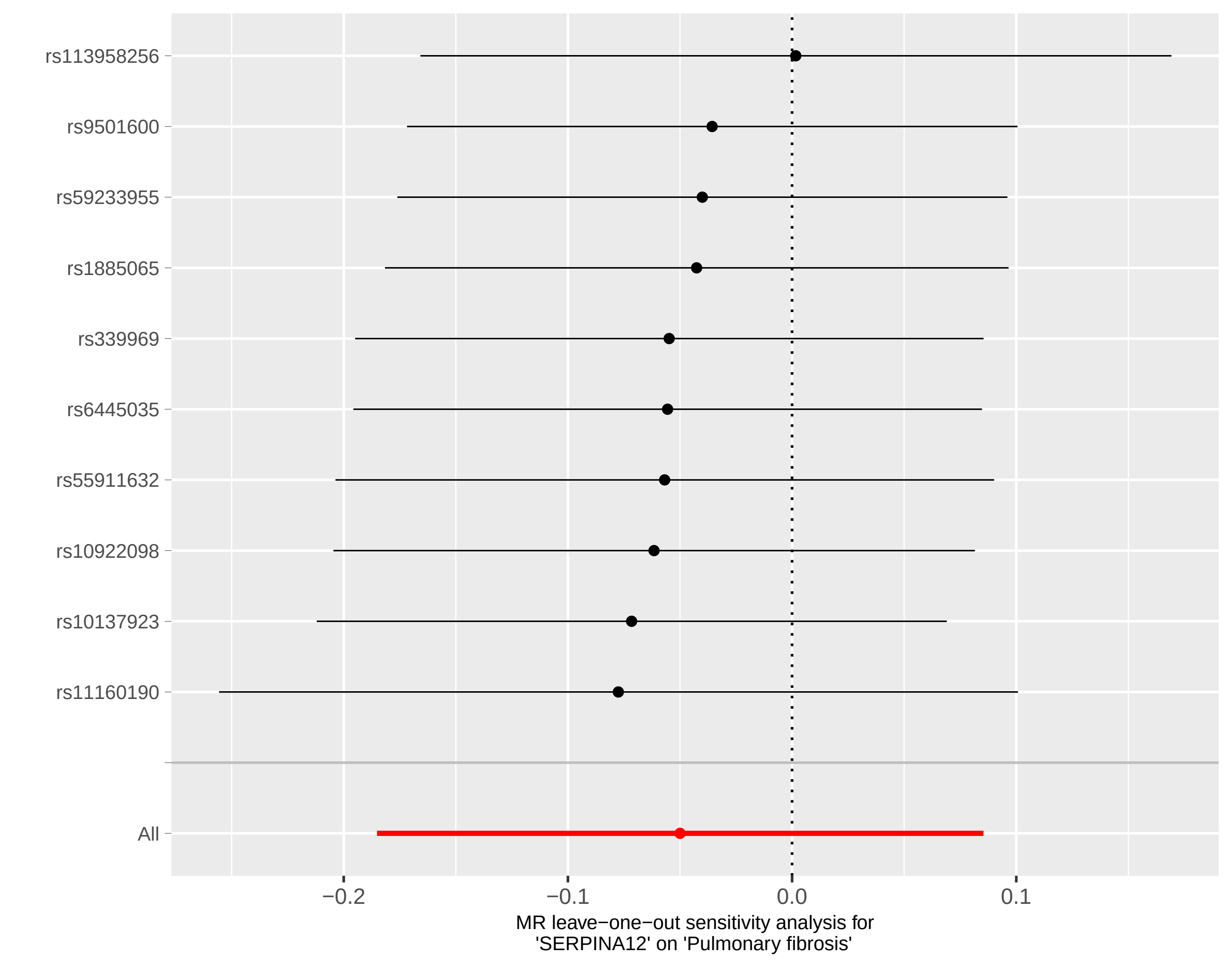
rs113958256
rs9501600
rs59233955
rs1885065
rs339969
rs6445035
rs55911632
rs10922098
rs10137923
rs11160190
All
−0.2
−0.1
0.0
0.1
MR leave−one−out sensitivity analysis for
'SERPINA12' on 'Pulmonary fibrosis'

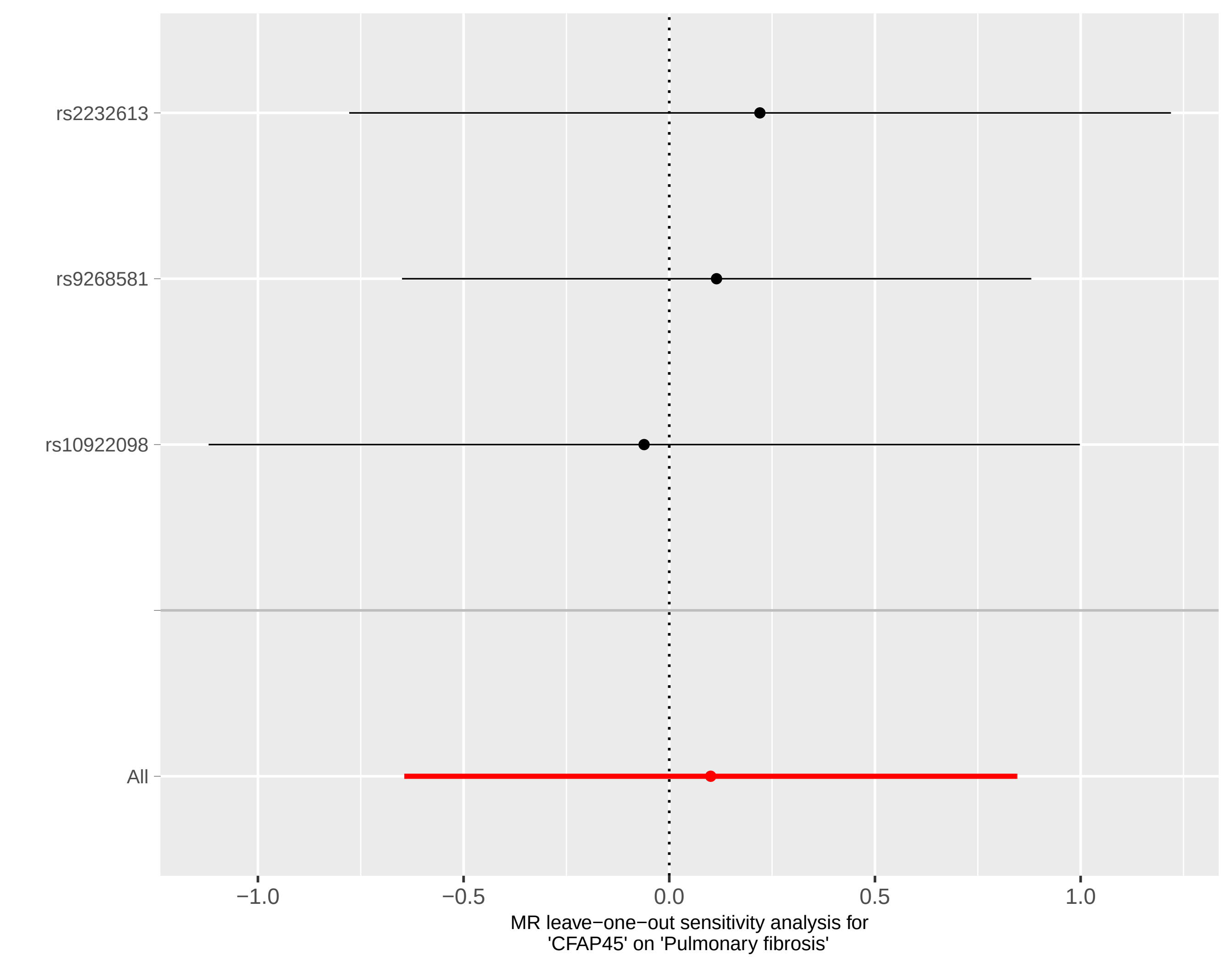
rs2232613
rs9268581
rs10922098
All
−1.0
−0.5
0.0
0.5
1.0
MR leave−one−out sensitivity analysis for
'CFAP45' on 'Pulmonary fibrosis'

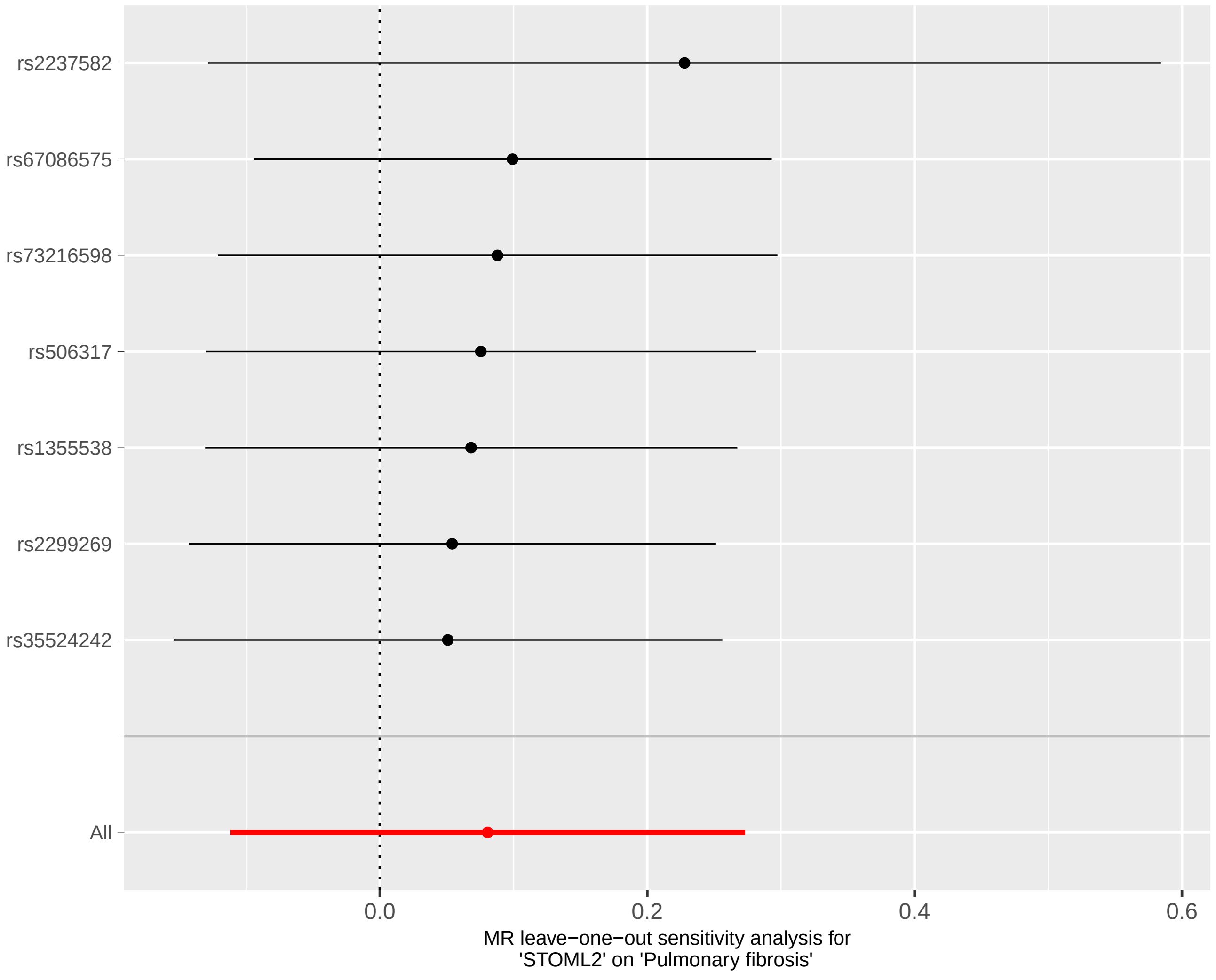

rs2237582
rs67086575
rs73216598
rs506317
rs1355538
rs2299269
rs35524242
All
0.0
0.2
0.4
0.6
MR leave−one−out sensitivity analysis for
'STOML2' on 'Pulmonary fibrosis'

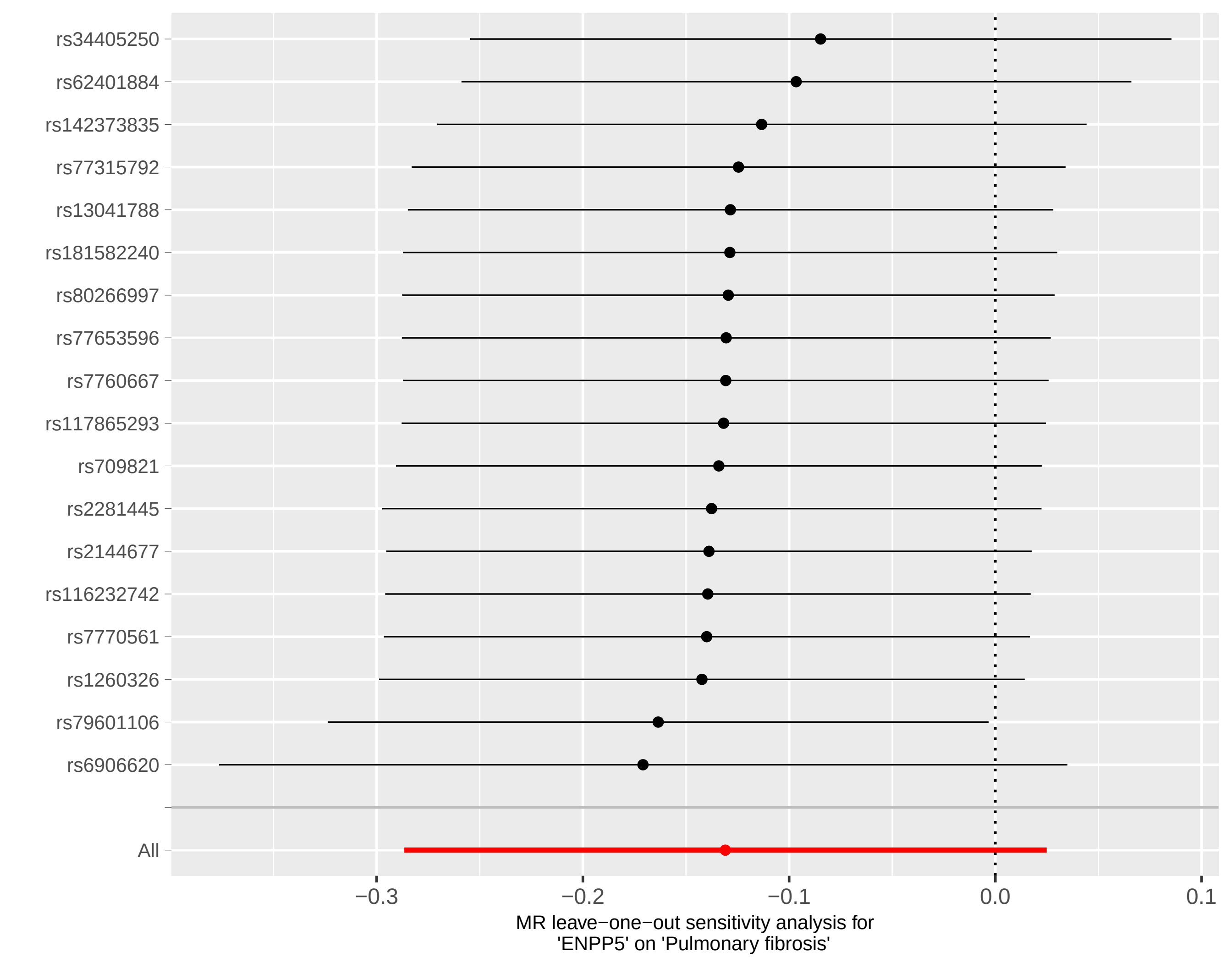
rs34405250
rs62401884
rs142373835
rs77315792
rs13041788
rs181582240
rs80266997
rs77653596
rs7760667
rs117865293
rs709821
rs2281445
rs2144677
rs116232742
rs7770561
rs1260326
rs79601106
rs6906620
All
−0.3
−0.2
−0.1
0.0
0.1
MR leave−one−out sensitivity analysis for
'ENPP5' on 'Pulmonary fibrosis'

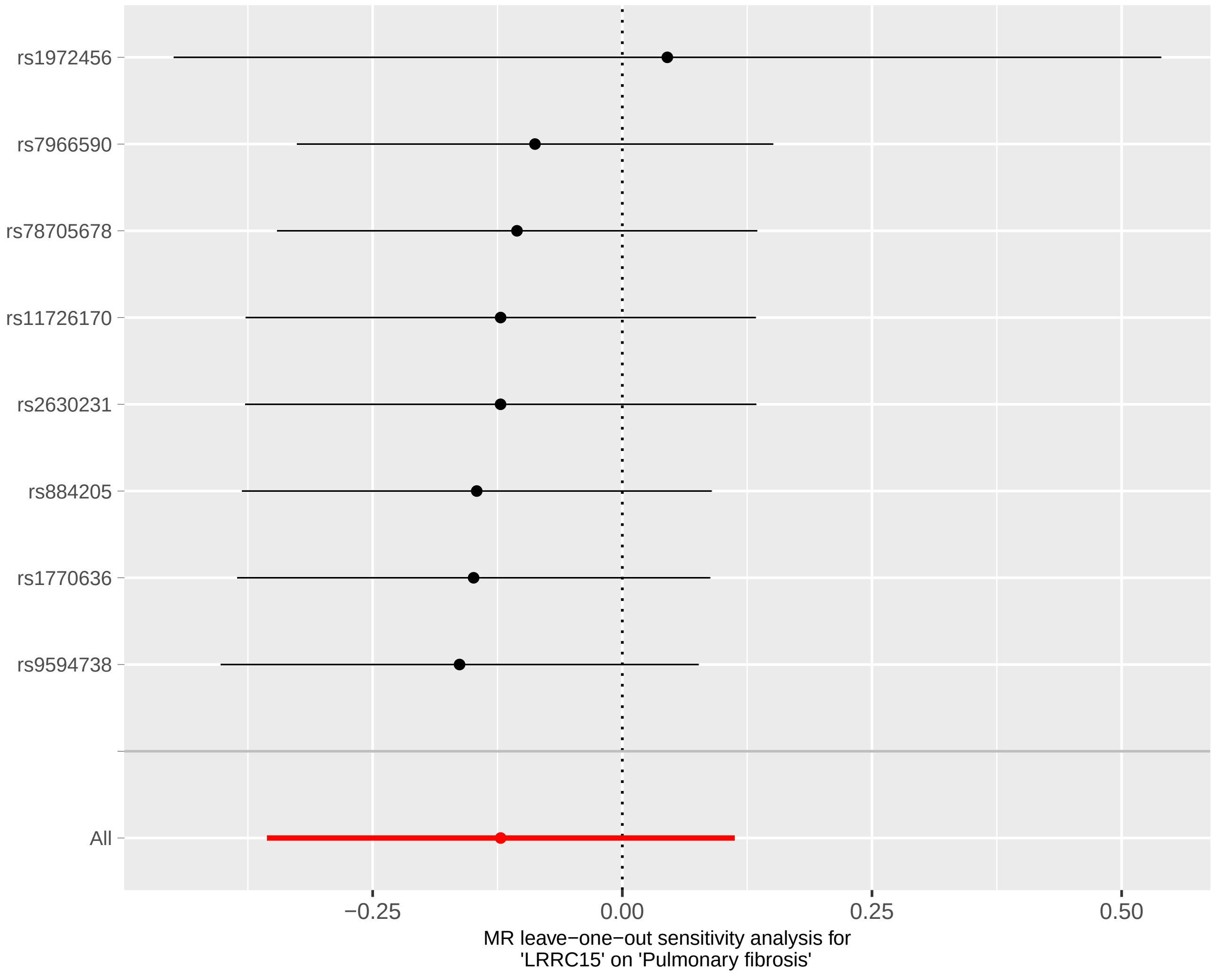
rs1972456
rs7966590
rs78705678
rs11726170
rs2630231
rs884205
rs1770636
rs9594738
All
−0.25
0.00
0.25
0.50
MR leave−one−out sensitivity analysis for
'LRRC15' on 'Pulmonary fibrosis'

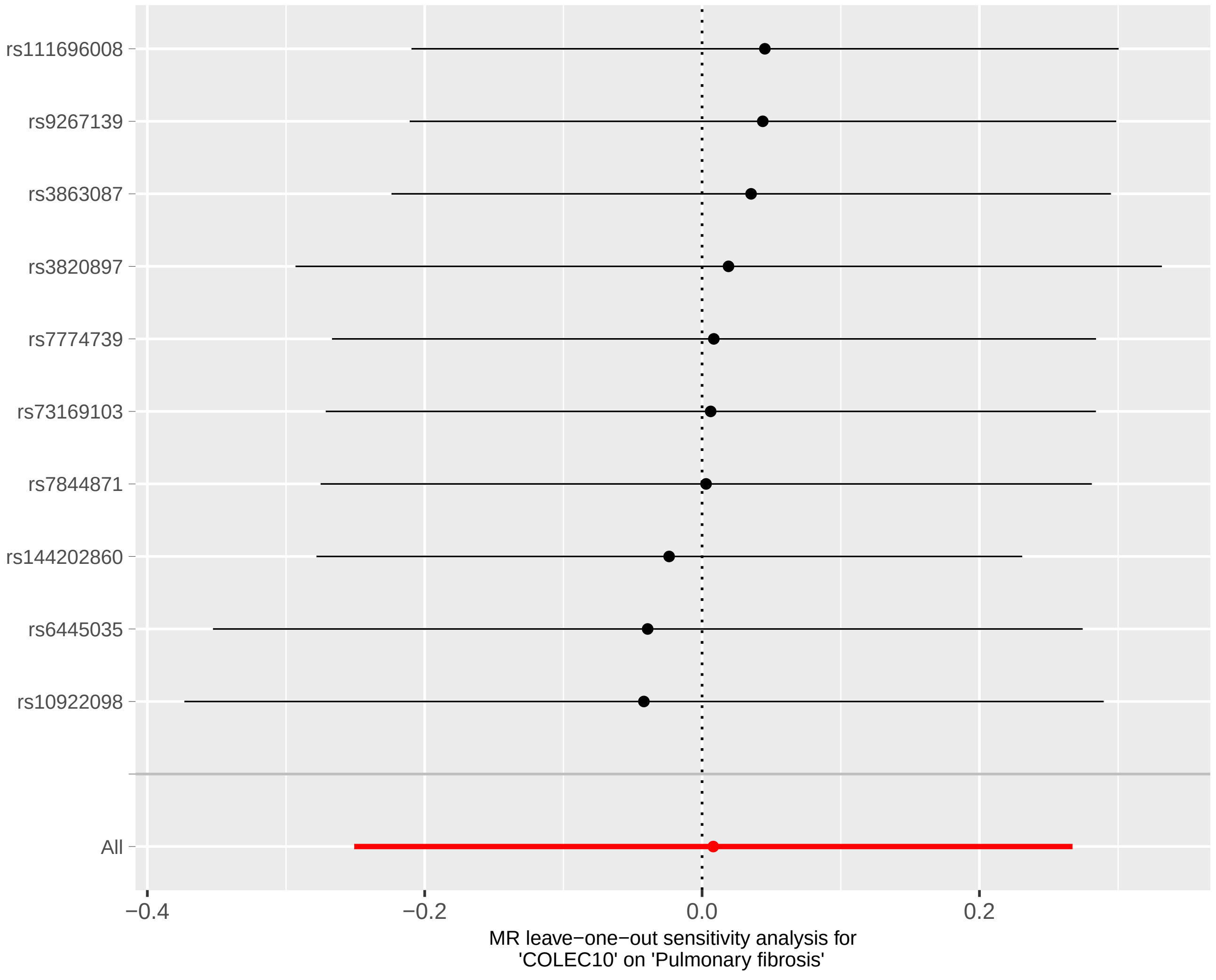
rs111696008
rs9267139
rs3863087
rs3820897
rs7774739
rs73169103
rs7844871
rs144202860
rs6445035
rs10922098
All
−0.4
−0.2
0.0
0.2
MR leave−one−out sensitivity analysis for
'COLEC10' on 'Pulmonary fibrosis'

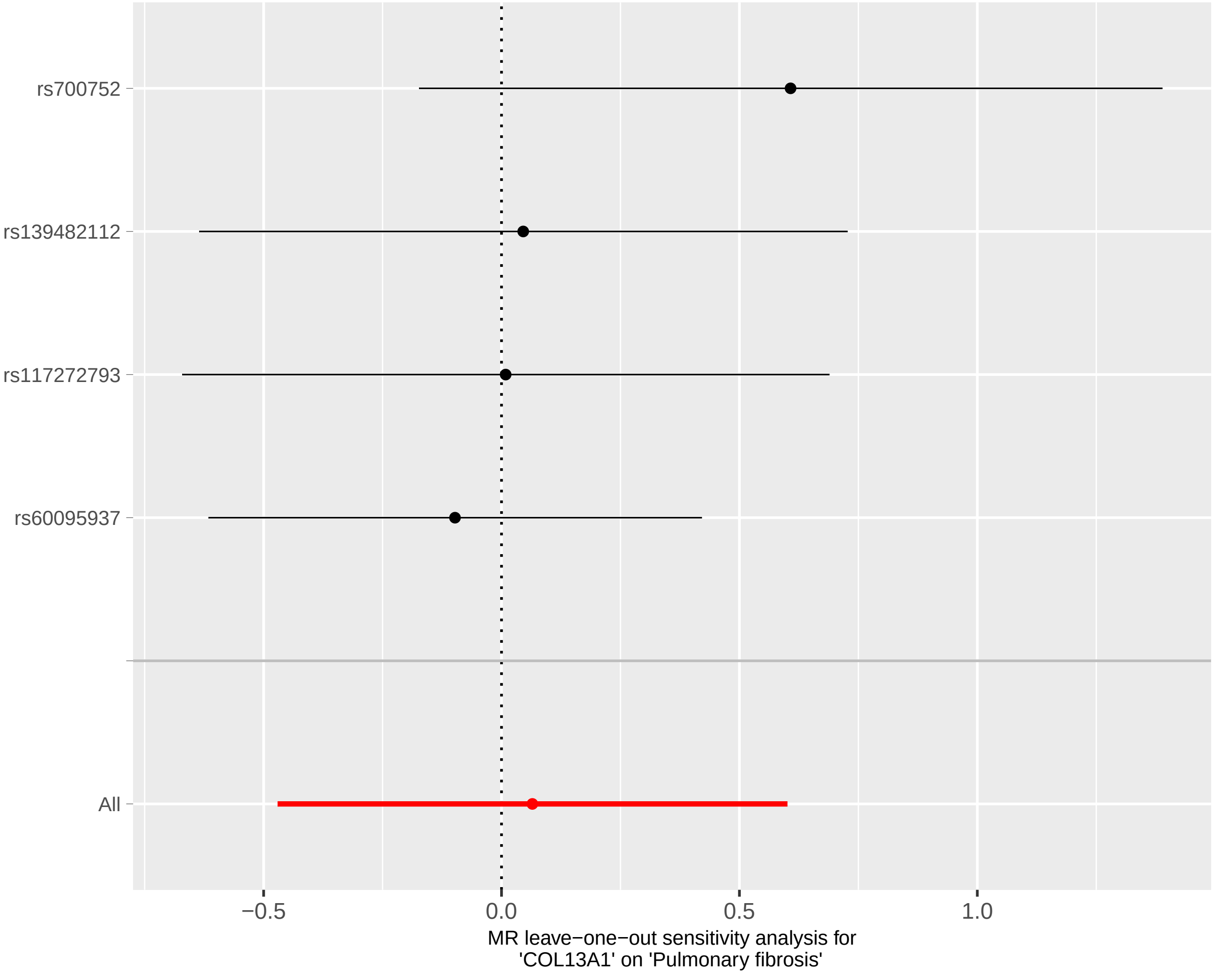
rs700752
rs139482112
rs117272793
rs60095937
All
−0.5
0.0
0.5
1.0
MR leave−one−out sensitivity analysis for
'COL13A1' on 'Pulmonary fibrosis'

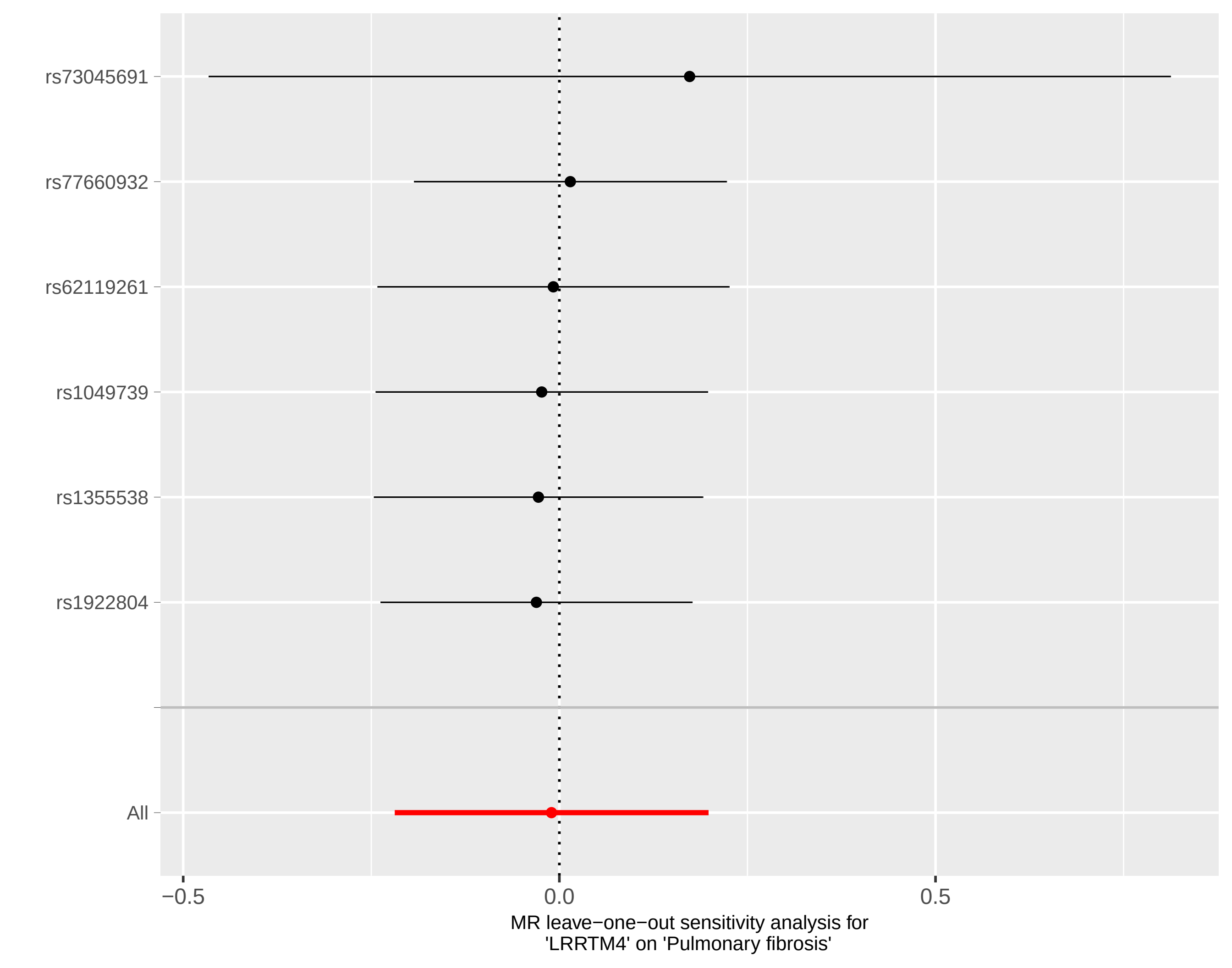
rs73045691
rs77660932
rs62119261
rs1049739
rs1355538
rs1922804
All
−0.5
0.0
0.5
MR leave−one−out sensitivity analysis for
'LRRTM4' on 'Pulmonary fibrosis'

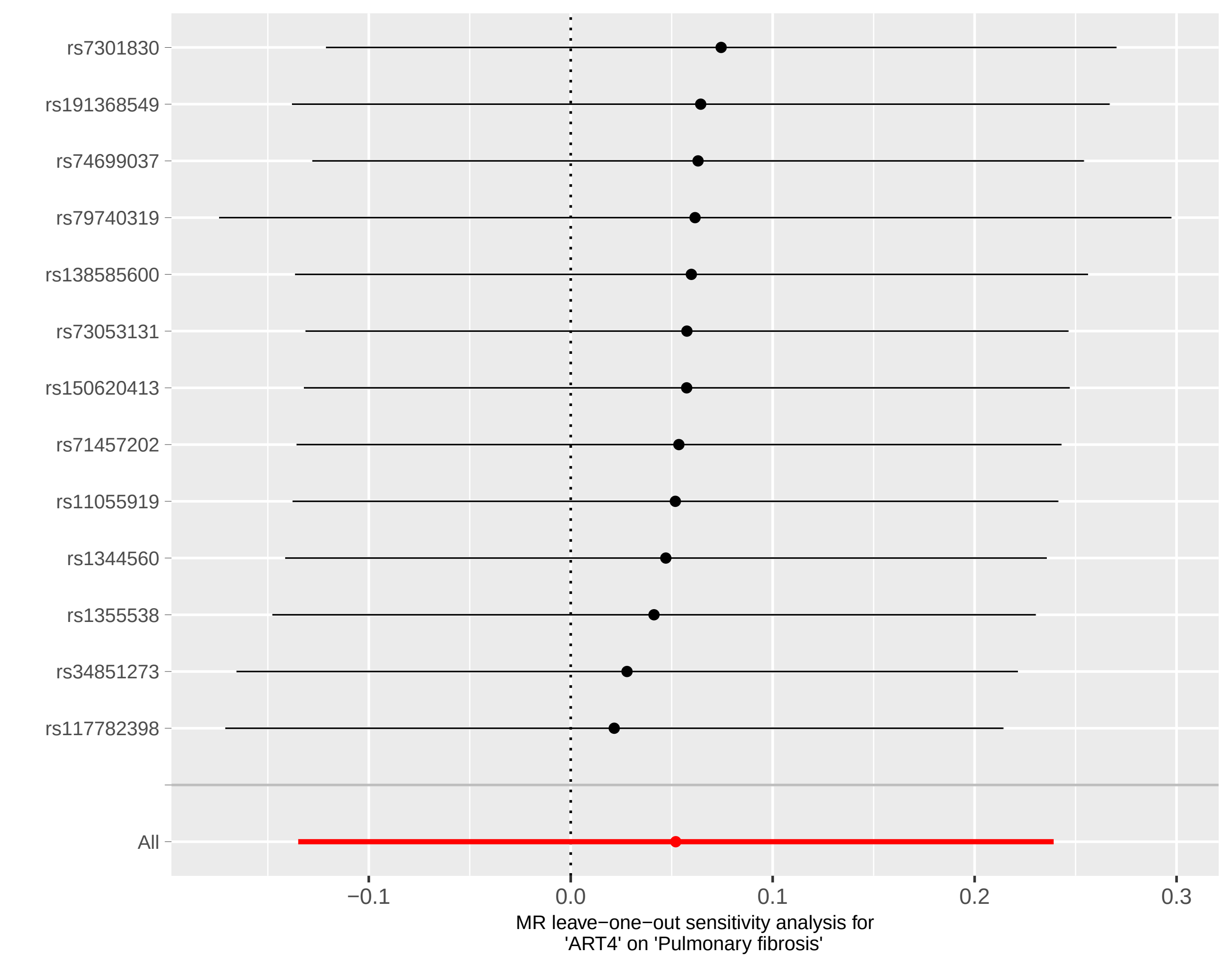
rs7301830
rs191368549
rs74699037
rs79740319
rs138585600
rs73053131
rs150620413
rs71457202
rs11055919
rs1344560
rs1355538
rs34851273
rs117782398
All
−0.1
0.0
0.1
0.2
0.3
MR leave−one−out sensitivity analysis for
'ART4' on 'Pulmonary fibrosis'

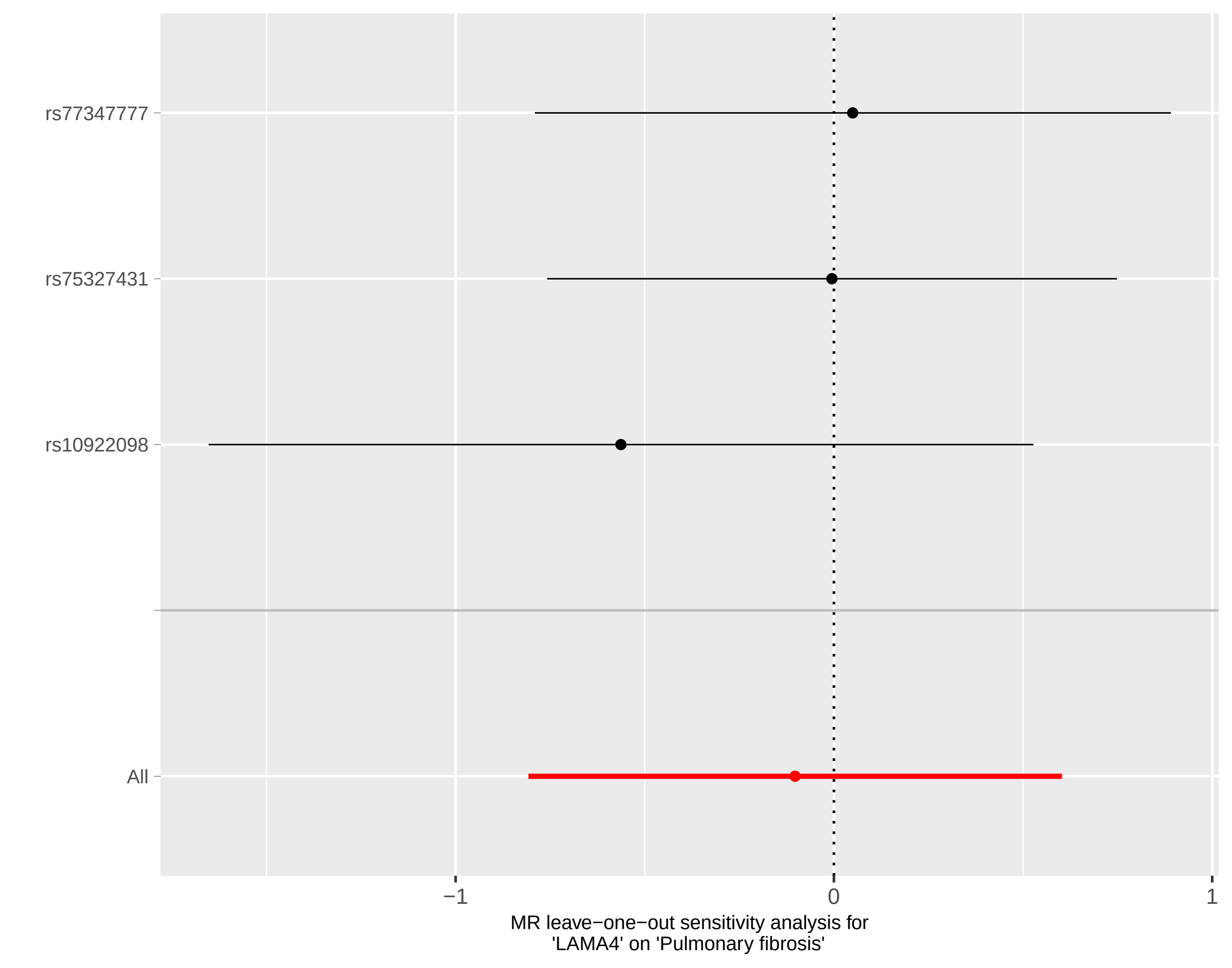
rs77347777
rs75327431
rs10922098
All
−1
0
1
MR leave−one−out sensitivity analysis for
'LAMA4' on 'Pulmonary fibrosis'

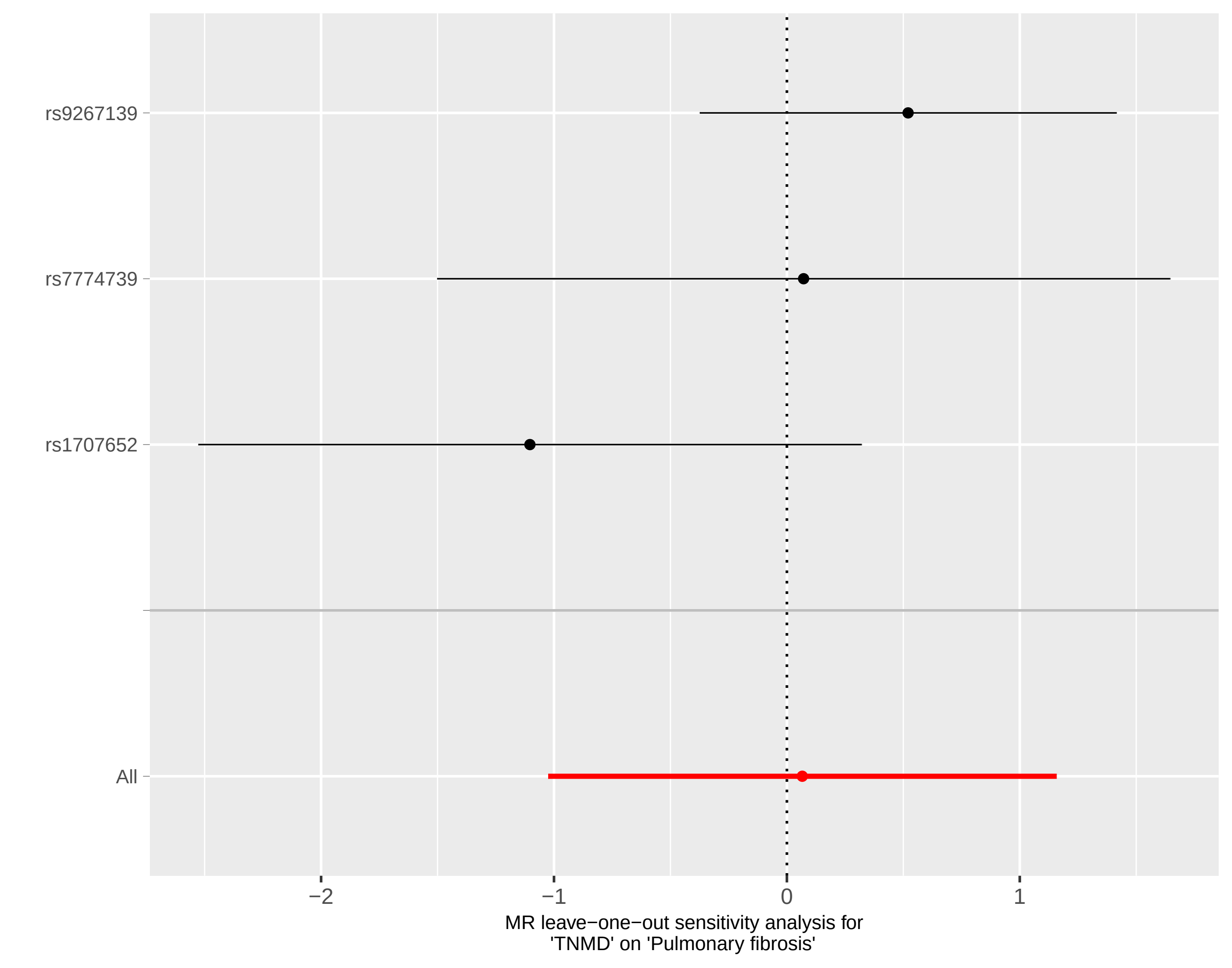
rs9267139
rs7774739
rs1707652
All
−2
−1
0
1
MR leave−one−out sensitivity analysis for
'TNMD' on 'Pulmonary fibrosis'

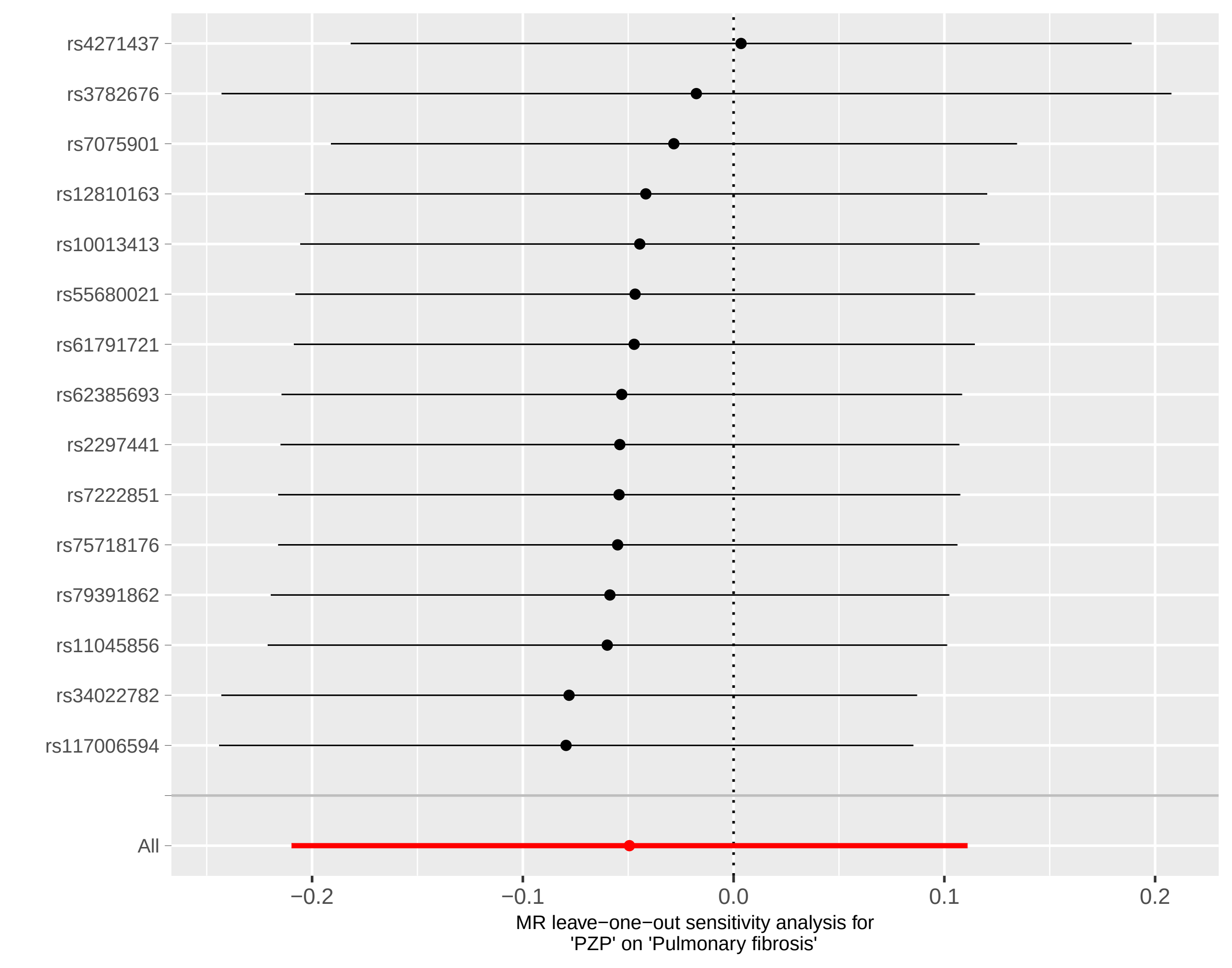

rs4271437
rs3782676
rs7075901
rs12810163
rs10013413
rs55680021
rs61791721
rs62385693
rs2297441
rs7222851
rs75718176
rs79391862
rs11045856
rs34022782
rs117006594
All
−0.2
−0.1
0.0
0.1
0.2
MR leave−one−out sensitivity analysis for
'PZP' on 'Pulmonary fibrosis'

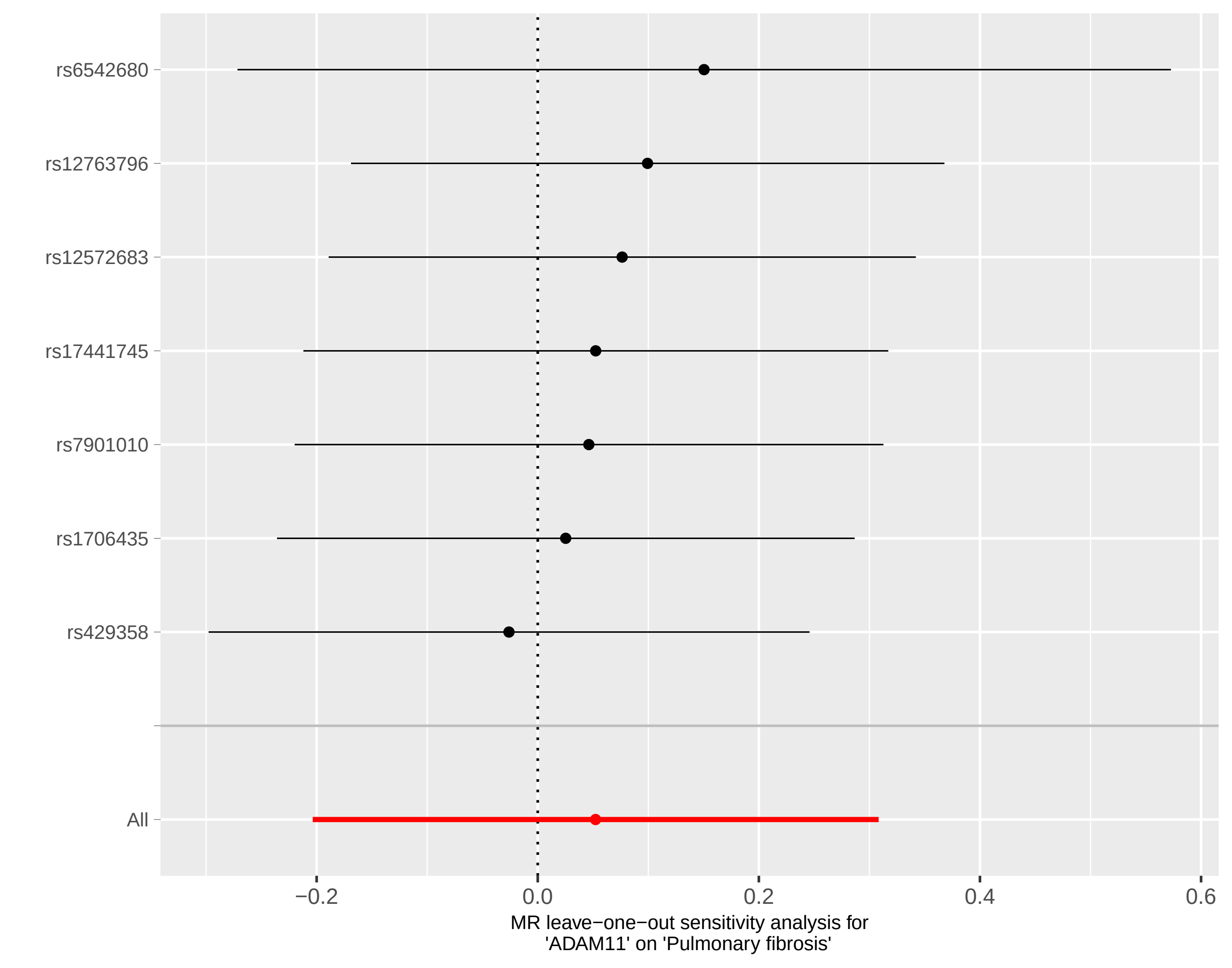
rs6542680
rs12763796
rs12572683
rs17441745
rs7901010
rs1706435
rs429358
All
−0.2
0.0
0.2
0.4
0.6
MR leave−one−out sensitivity analysis for
'ADAM11' on 'Pulmonary fibrosis'

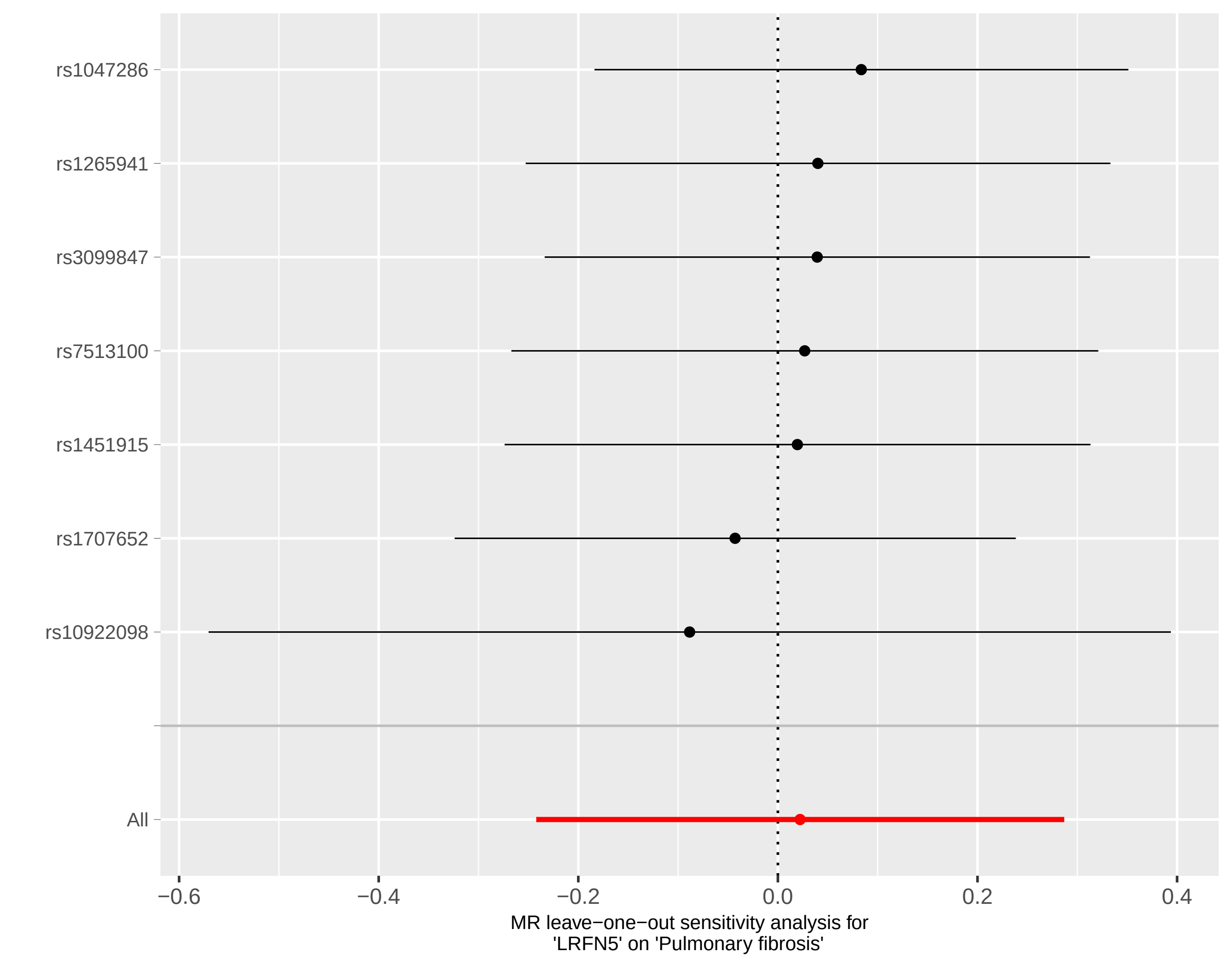
rs1047286
rs1265941
rs3099847
rs7513100
rs1451915
rs1707652
rs10922098
All
−0.6
−0.4
−0.2
0.0
0.2
0.4
MR leave−one−out sensitivity analysis for
'LRFN5' on 'Pulmonary fibrosis'

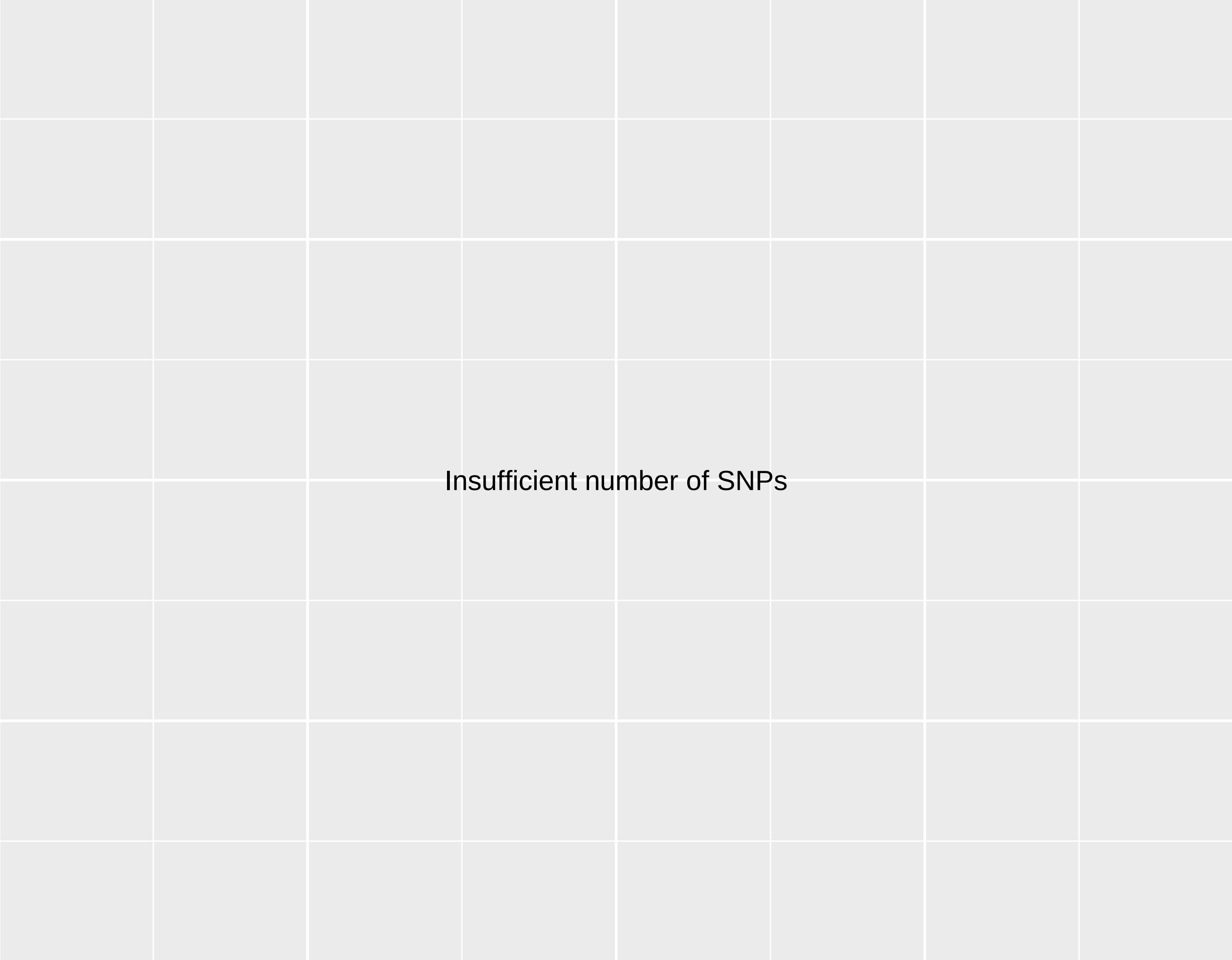
Insufficient number of SNPs

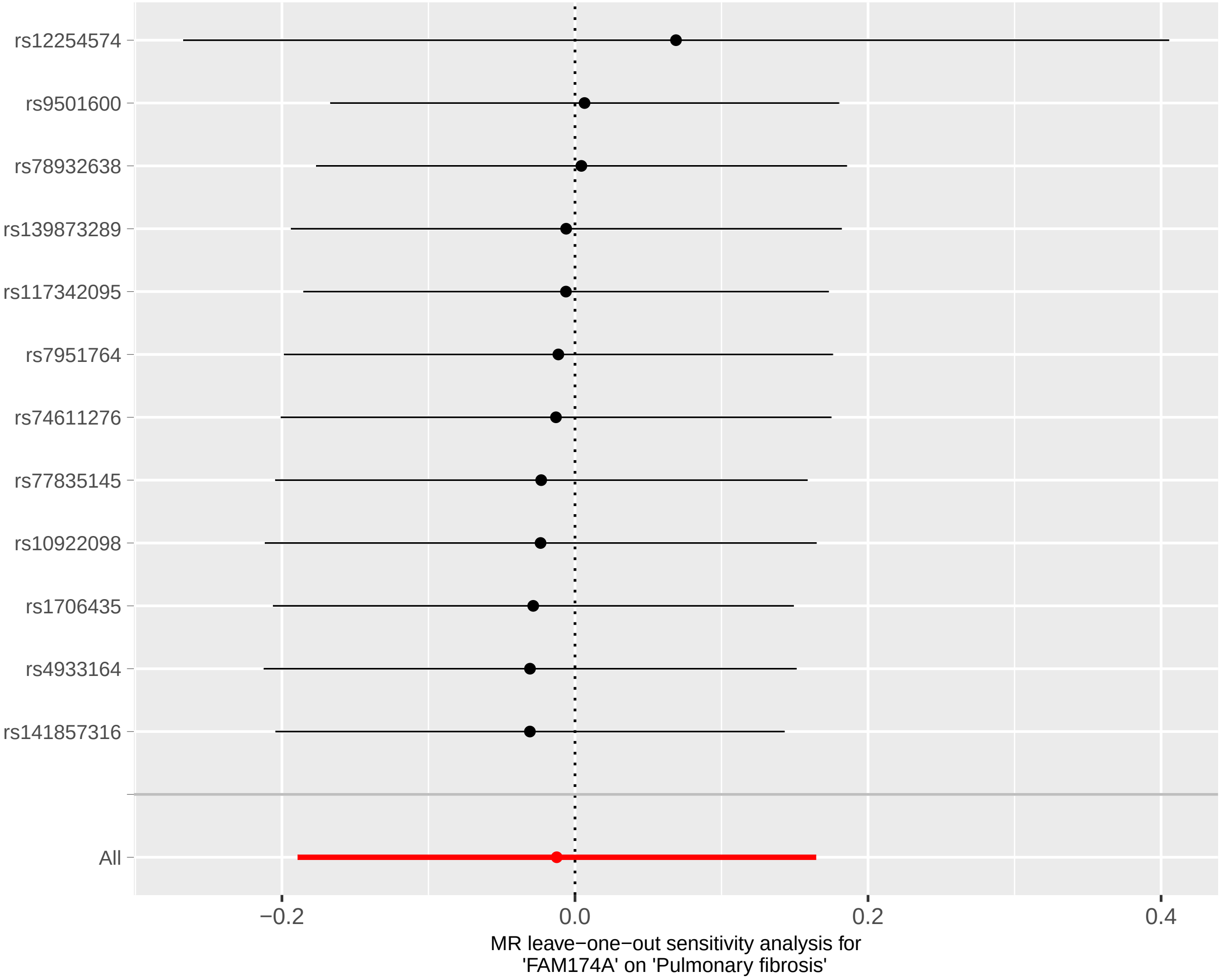

rs12254574
rs9501600
rs78932638
rs139873289
rs117342095
rs7951764
rs74611276
rs77835145
rs10922098
rs1706435
rs4933164
rs141857316
All
−0.2
0.0
0.2
0.4
MR leave−one−out sensitivity analysis for
'FAM174A' on 'Pulmonary fibrosis'

Insufficient number of SNPs

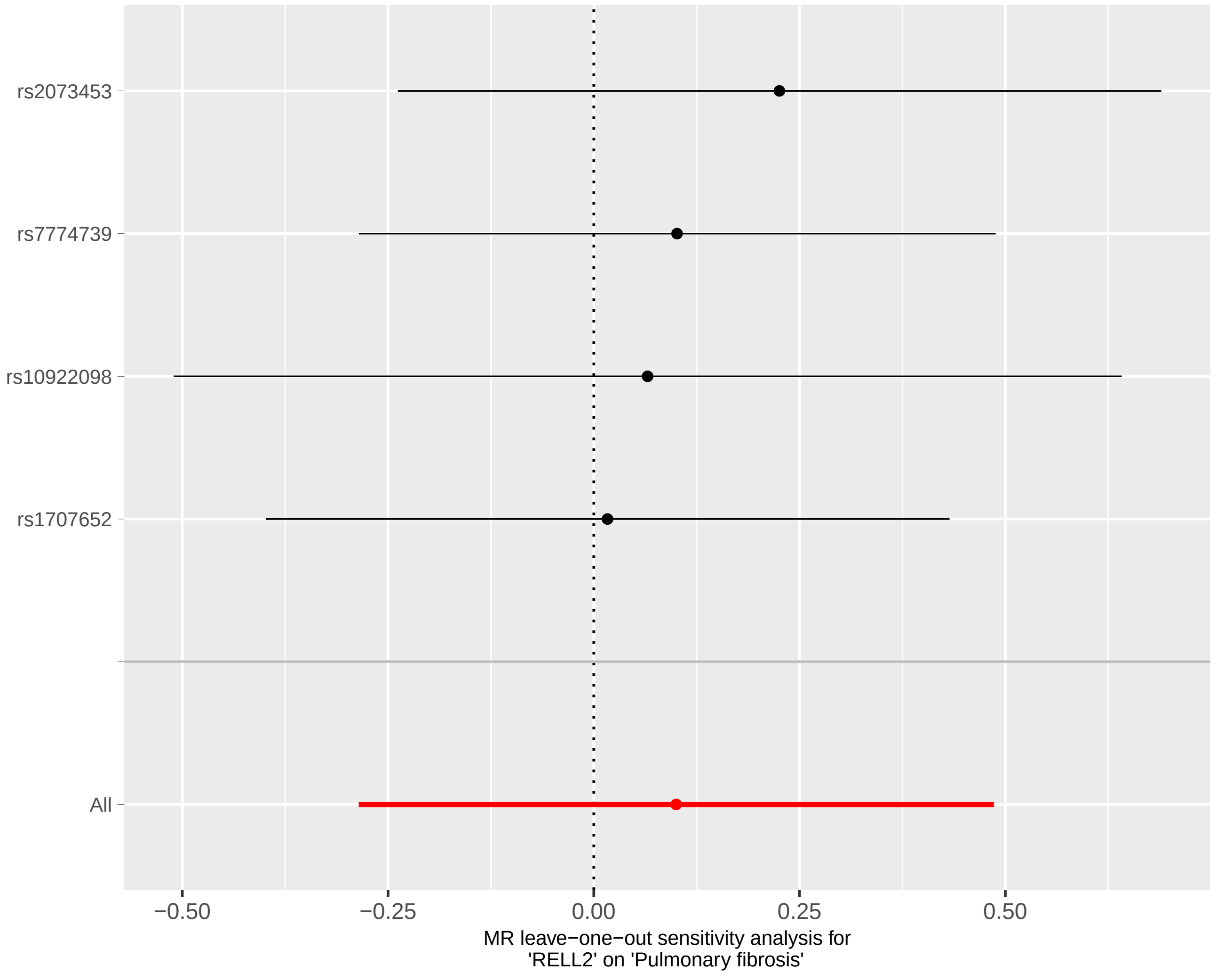
rs2073453
rs7774739
rs10922098
rs1707652
All
−0.50
−0.25
0.00
0.25
0.50
MR leave−one−out sensitivity analysis for
'RELL2' on 'Pulmonary fibrosis'

Insufficient number of SNPs

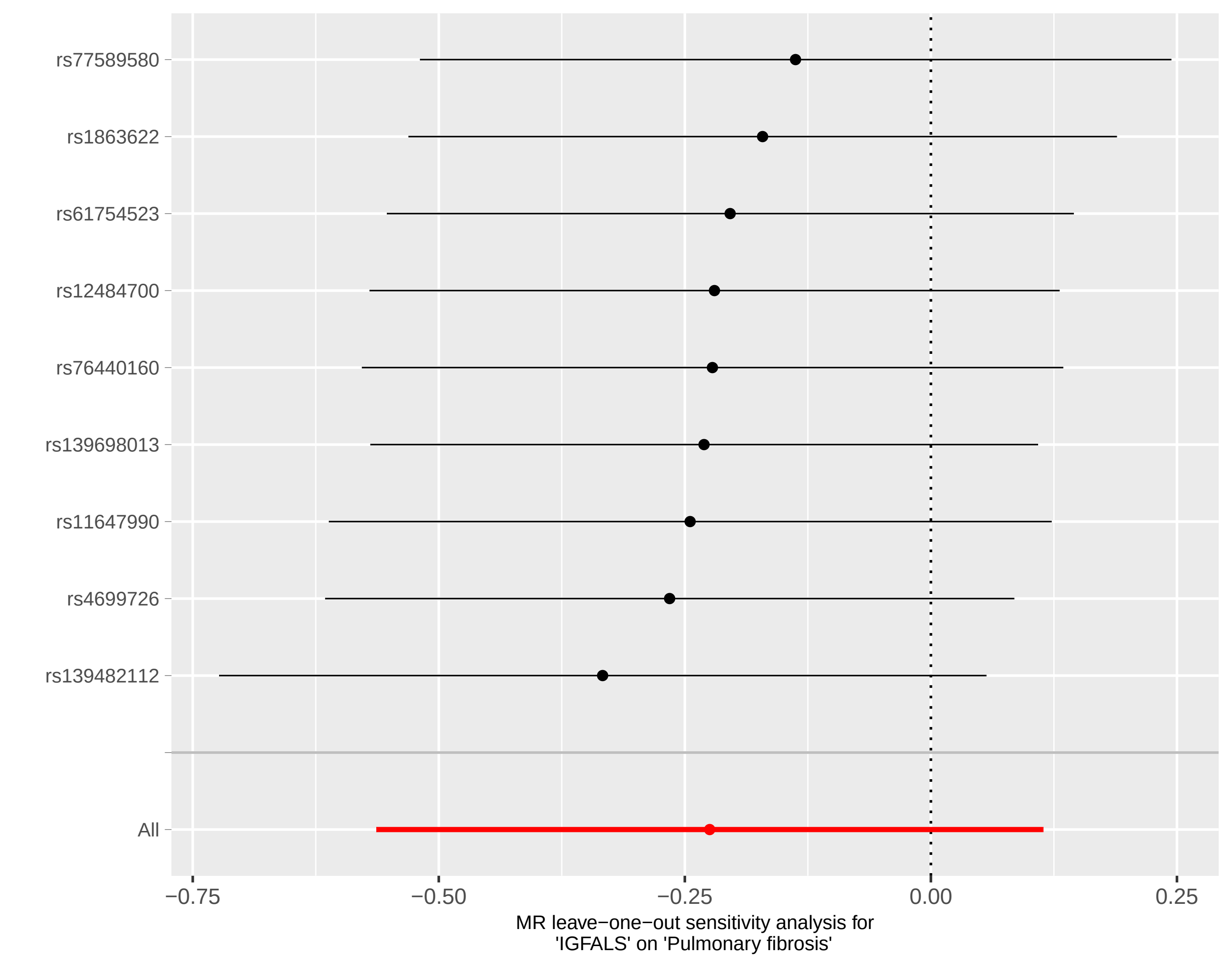
rs77589580
rs1863622
rs61754523
rs12484700
rs76440160
rs139698013
rs11647990
rs4699726
rs139482112
All
−0.75
−0.50
−0.25
0.00
0.25
MR leave−one−out sensitivity analysis for
'IGFALS' on 'Pulmonary fibrosis'

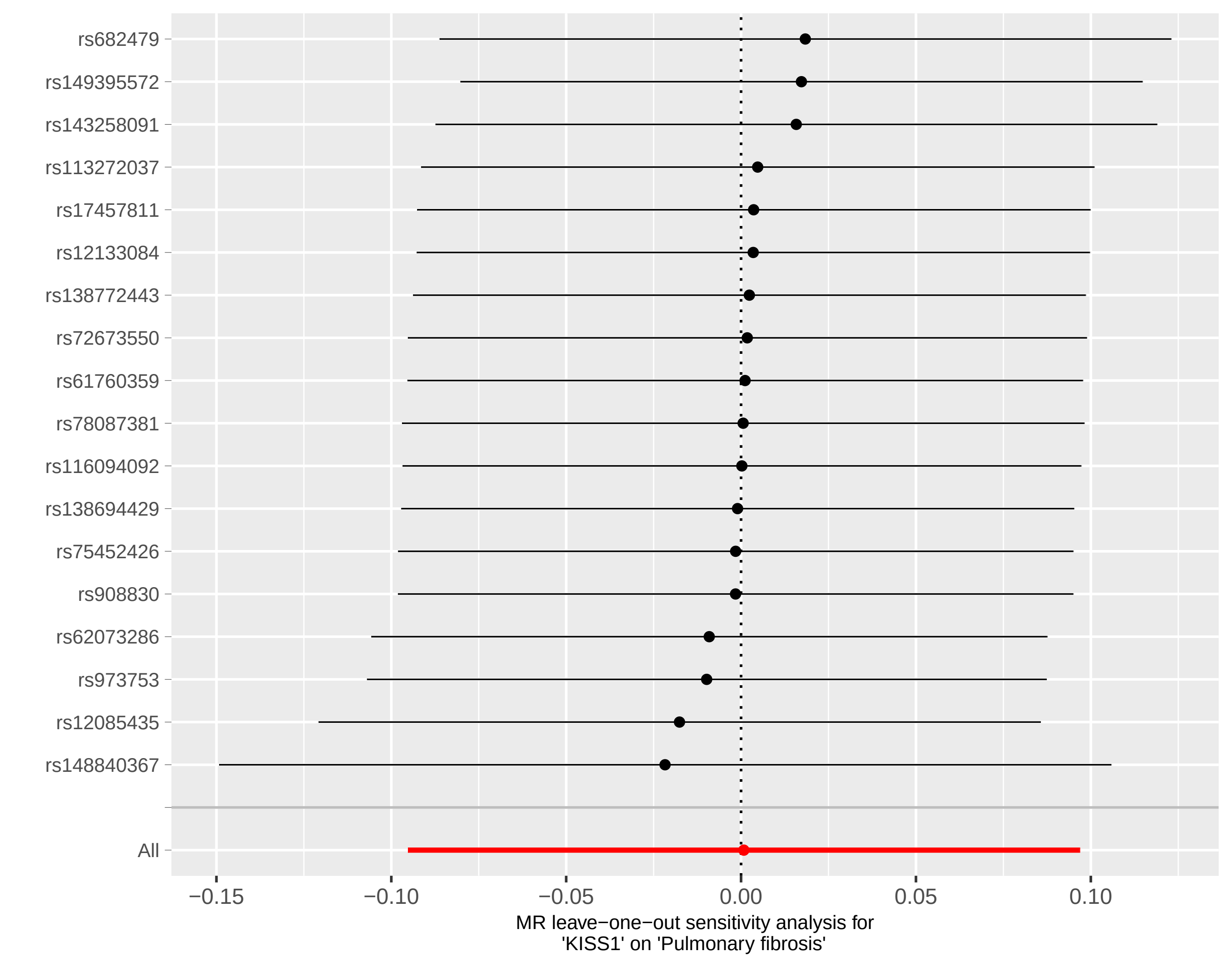
rs682479
rs149395572
rs143258091
rs113272037
rs17457811
rs12133084
rs138772443
rs72673550
rs61760359
rs78087381
rs116094092
rs138694429
rs75452426
rs908830
rs62073286
rs973753
rs12085435
rs148840367
All
−0.15
−0.10
−0.05
0.00
0.05
0.10
MR leave−one−out sensitivity analysis for
'KISS1' on 'Pulmonary fibrosis'

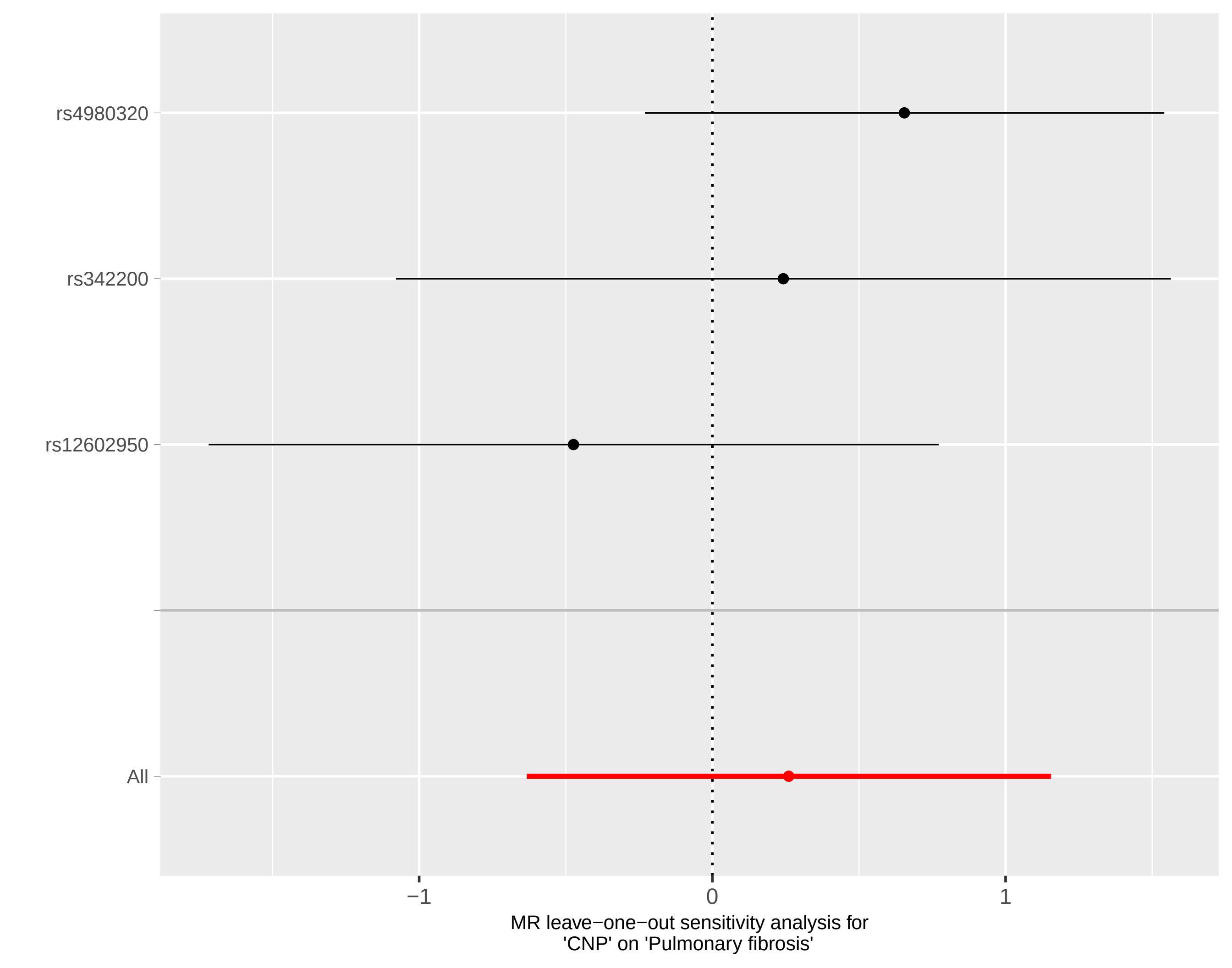
rs4980320
rs342200
rs12602950
All
−1
0
1
MR leave−one−out sensitivity analysis for
'CNP' on 'Pulmonary fibrosis'

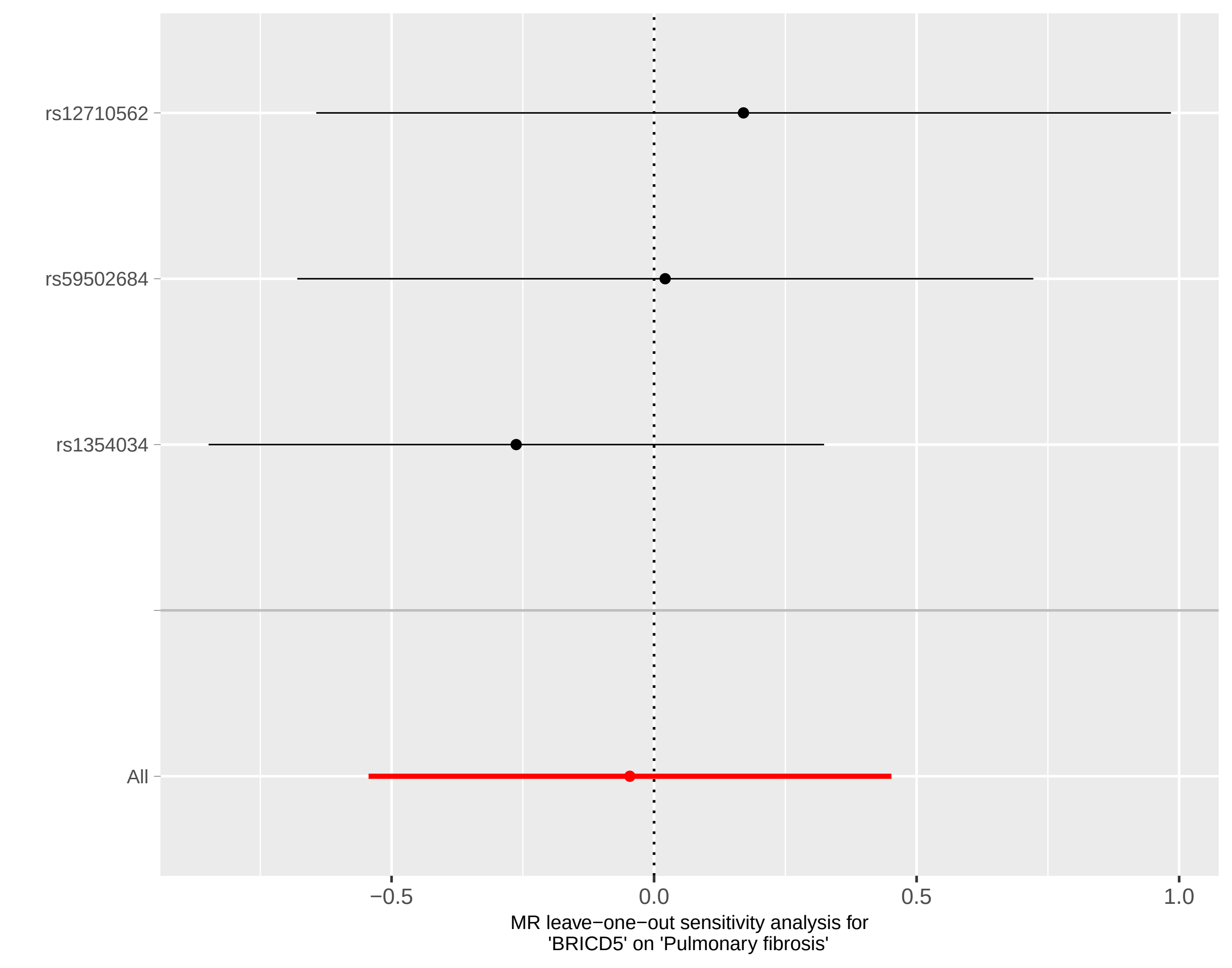

rs12710562
rs59502684
rs1354034
All
−0.5
0.0
0.5
1.0
MR leave−one−out sensitivity analysis for
'BRICD5' on 'Pulmonary fibrosis'

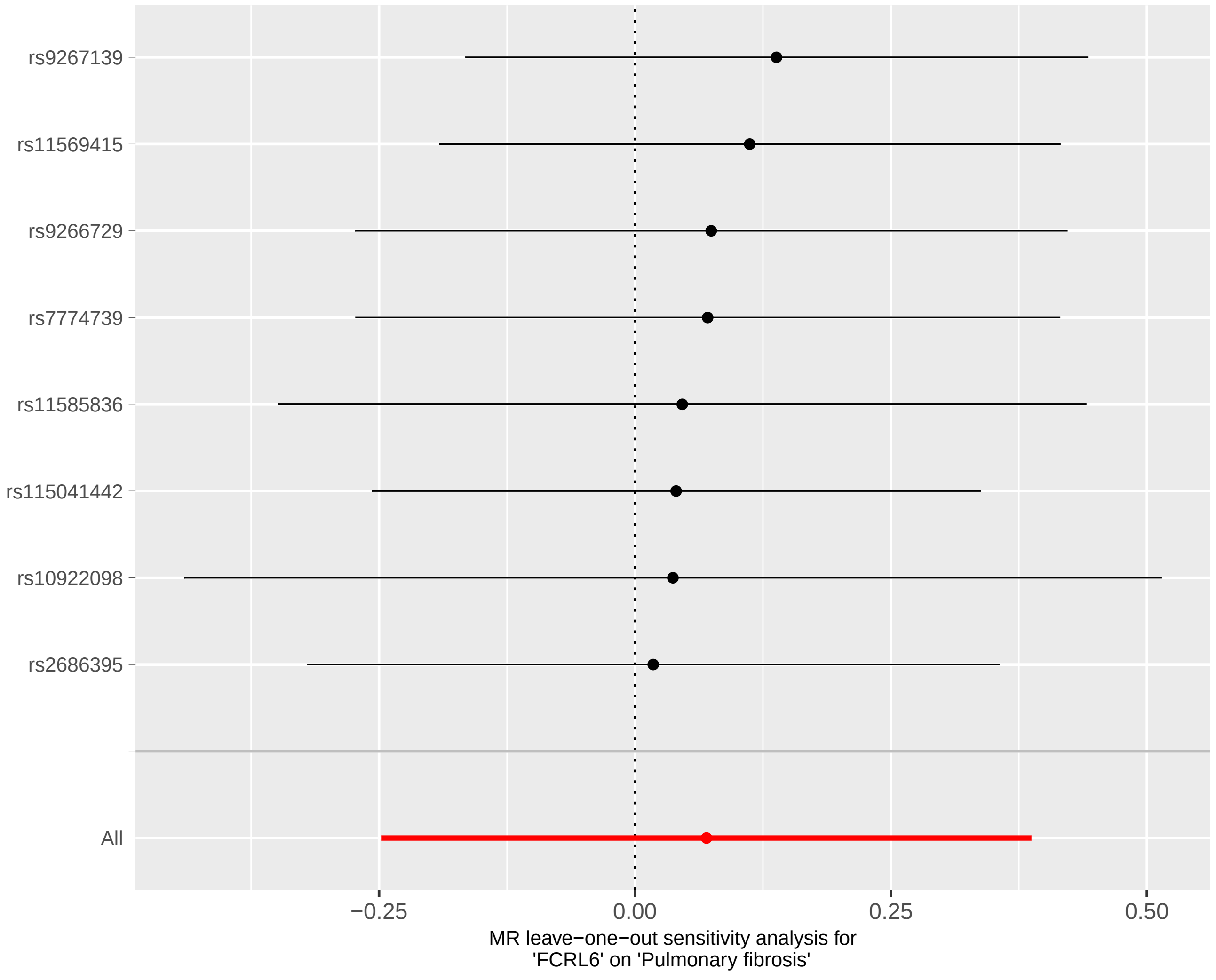
rs9267139
rs11569415
rs9266729
rs7774739
rs11585836
rs115041442
rs10922098
rs2686395
All
−0.25
0.00
0.25
0.50
MR leave−one−out sensitivity analysis for
'FCRL6' on 'Pulmonary fibrosis'

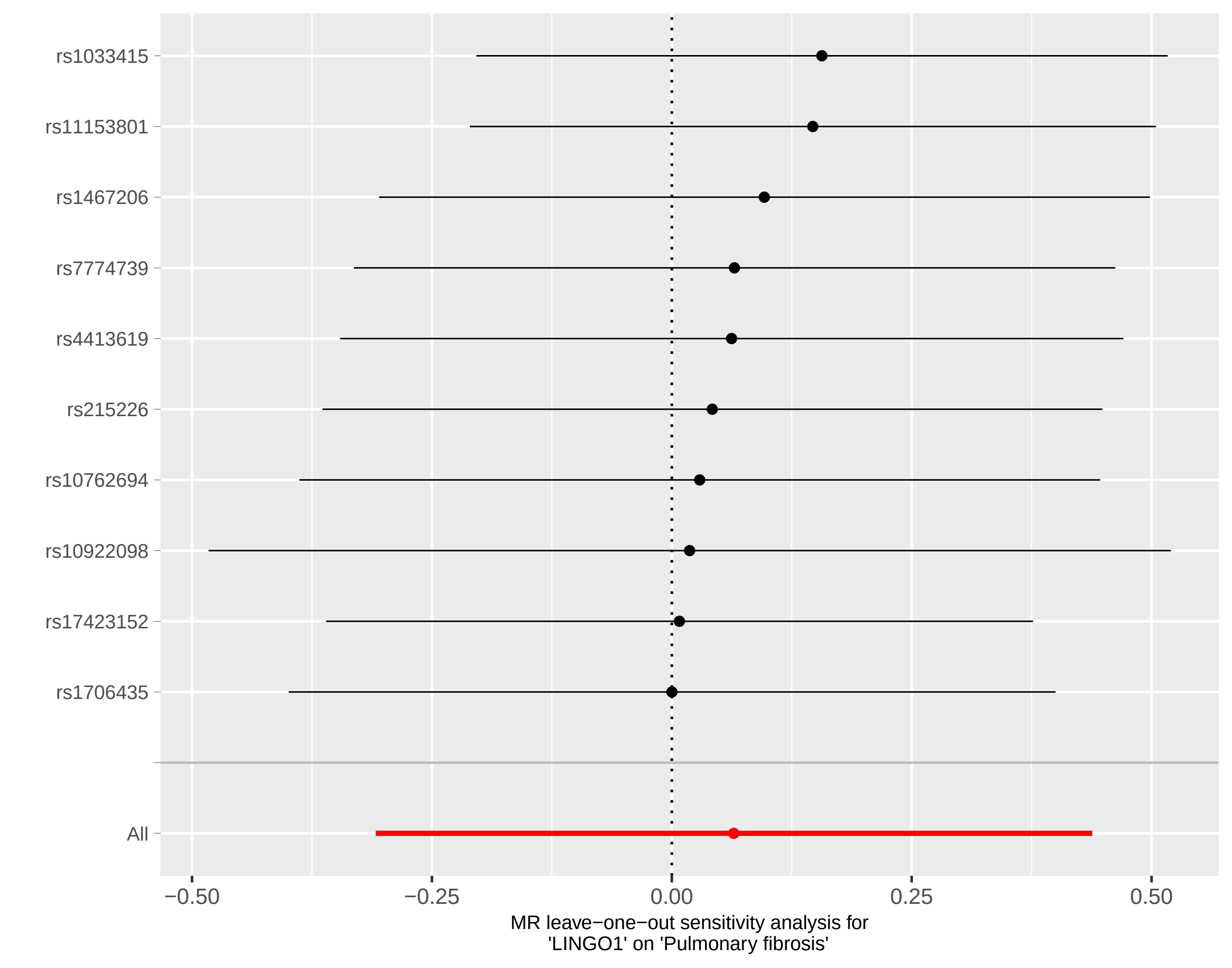

rs1033415
rs11153801
rs1467206
rs7774739
rs4413619
rs215226
rs10762694
rs10922098
rs17423152
rs1706435
All
−0.50
−0.25
0.00
0.25
0.50
MR leave−one−out sensitivity analysis for
'LINGO1' on 'Pulmonary fibrosis'

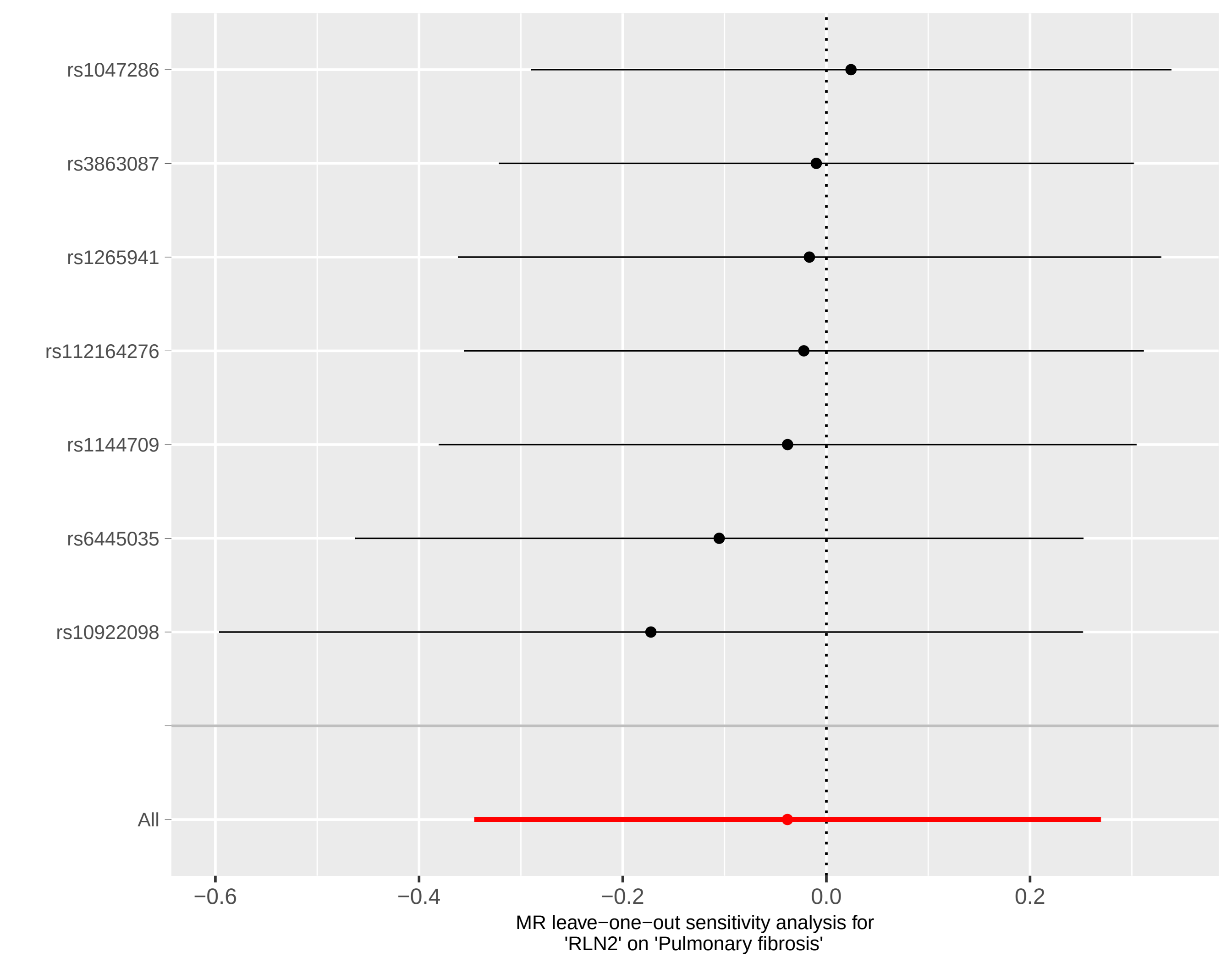
rs1047286
rs3863087
rs1265941
rs112164276
rs1144709
rs6445035
rs10922098
All
−0.6
−0.4
−0.2
0.0
0.2
MR leave−one−out sensitivity analysis for
'RLN2' on 'Pulmonary fibrosis'

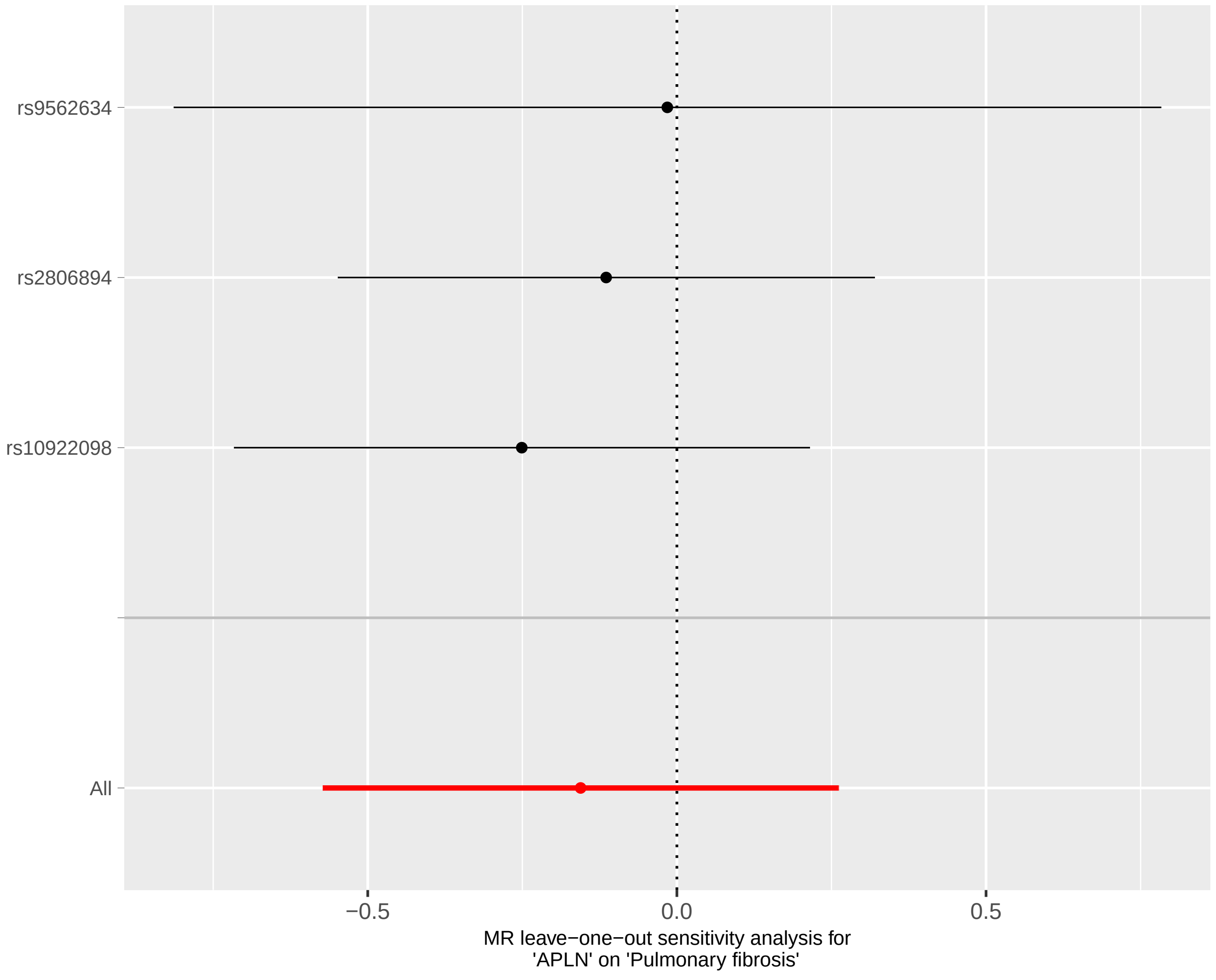
rs9562634
rs2806894
rs10922098
All
−0.5
0.0
0.5
MR leave−one−out sensitivity analysis for
'APLN' on 'Pulmonary fibrosis'

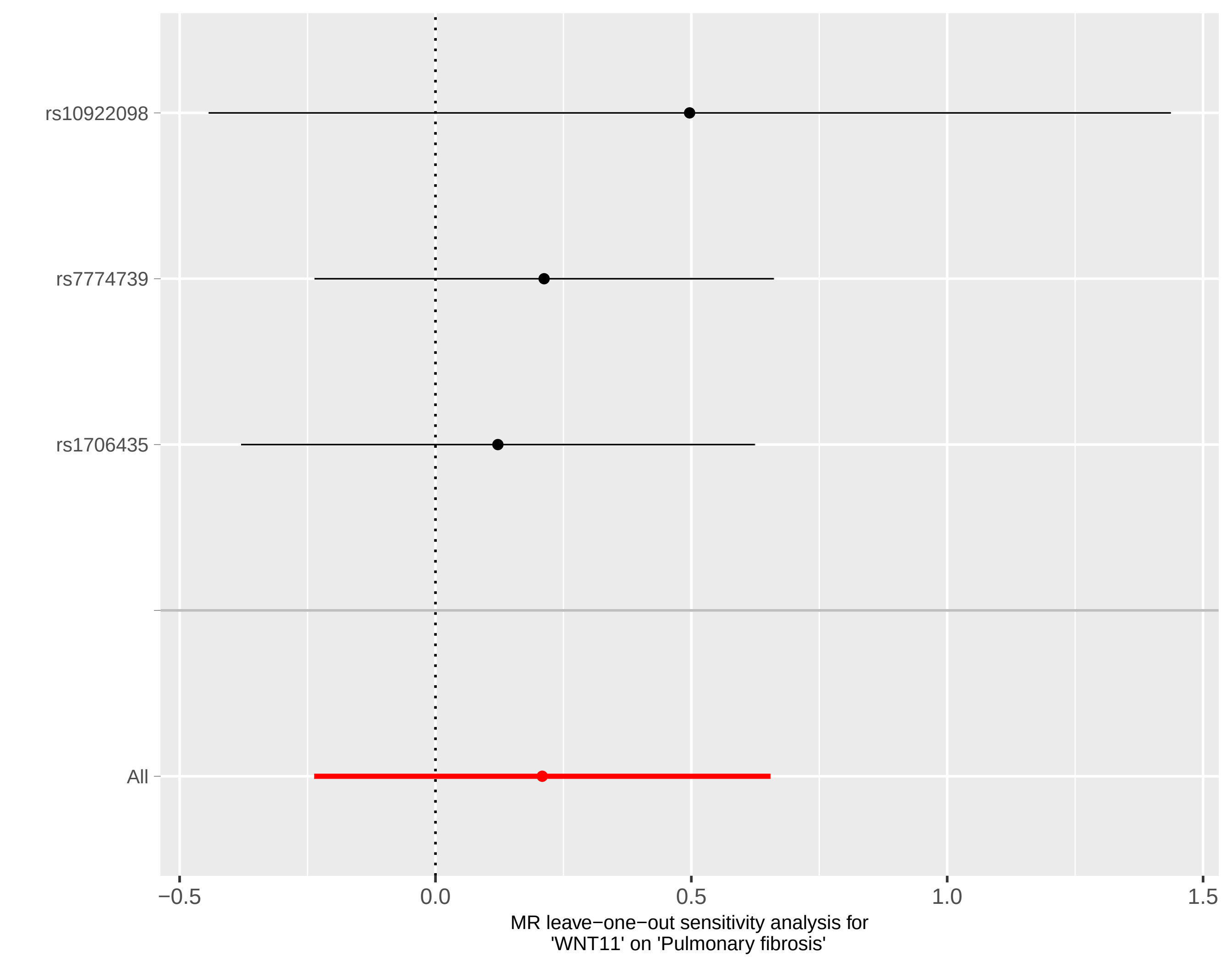
rs10922098
rs7774739
rs1706435
All
−0.5
0.0
0.5
1.0
1.5
MR leave−one−out sensitivity analysis for
'WNT11' on 'Pulmonary fibrosis'

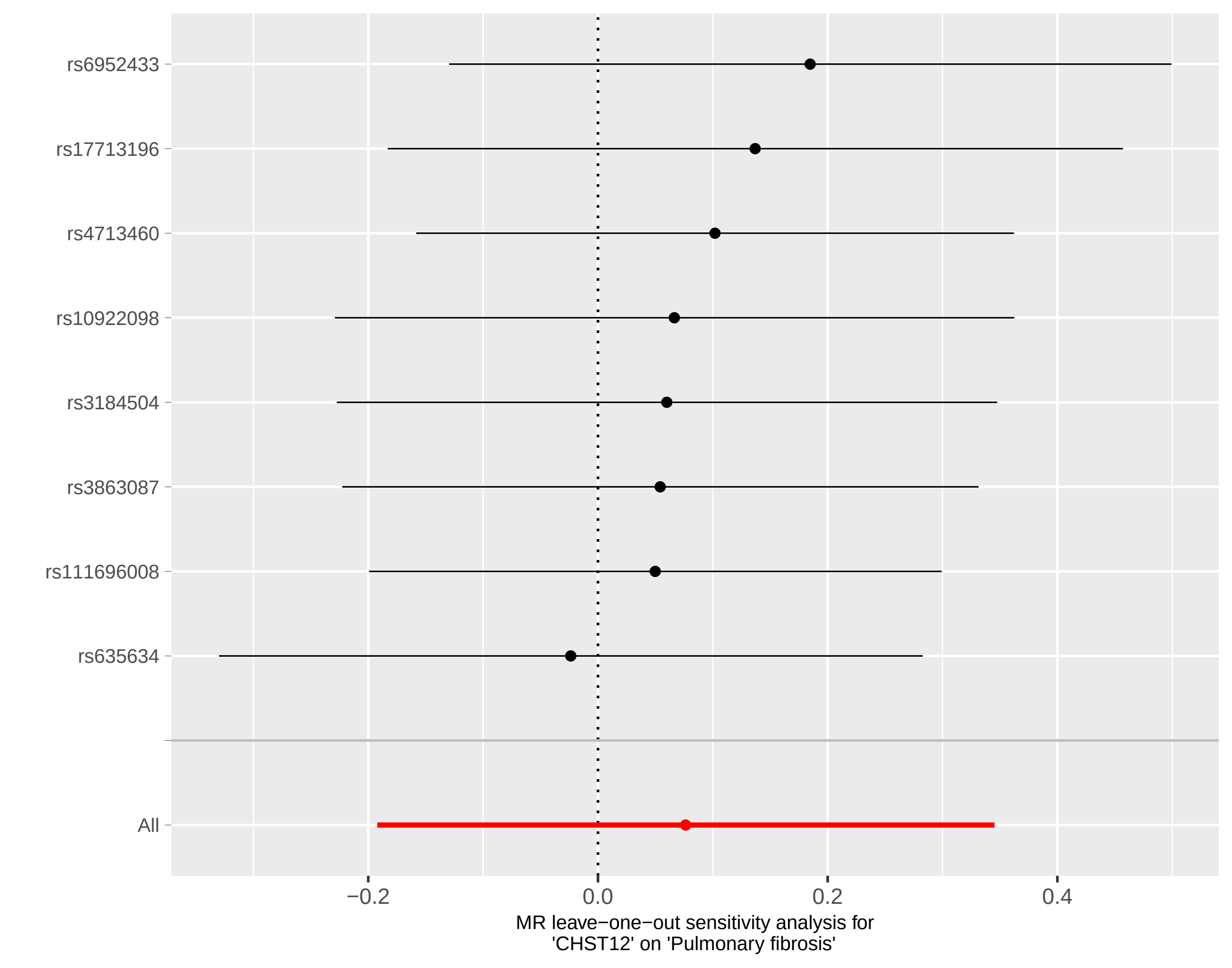
rs6952433
rs17713196
rs4713460
rs10922098
rs3184504
rs3863087
rs111696008
rs635634
All
−0.2
0.0
0.2
0.4
MR leave−one−out sensitivity analysis for
'CHST12' on 'Pulmonary fibrosis'

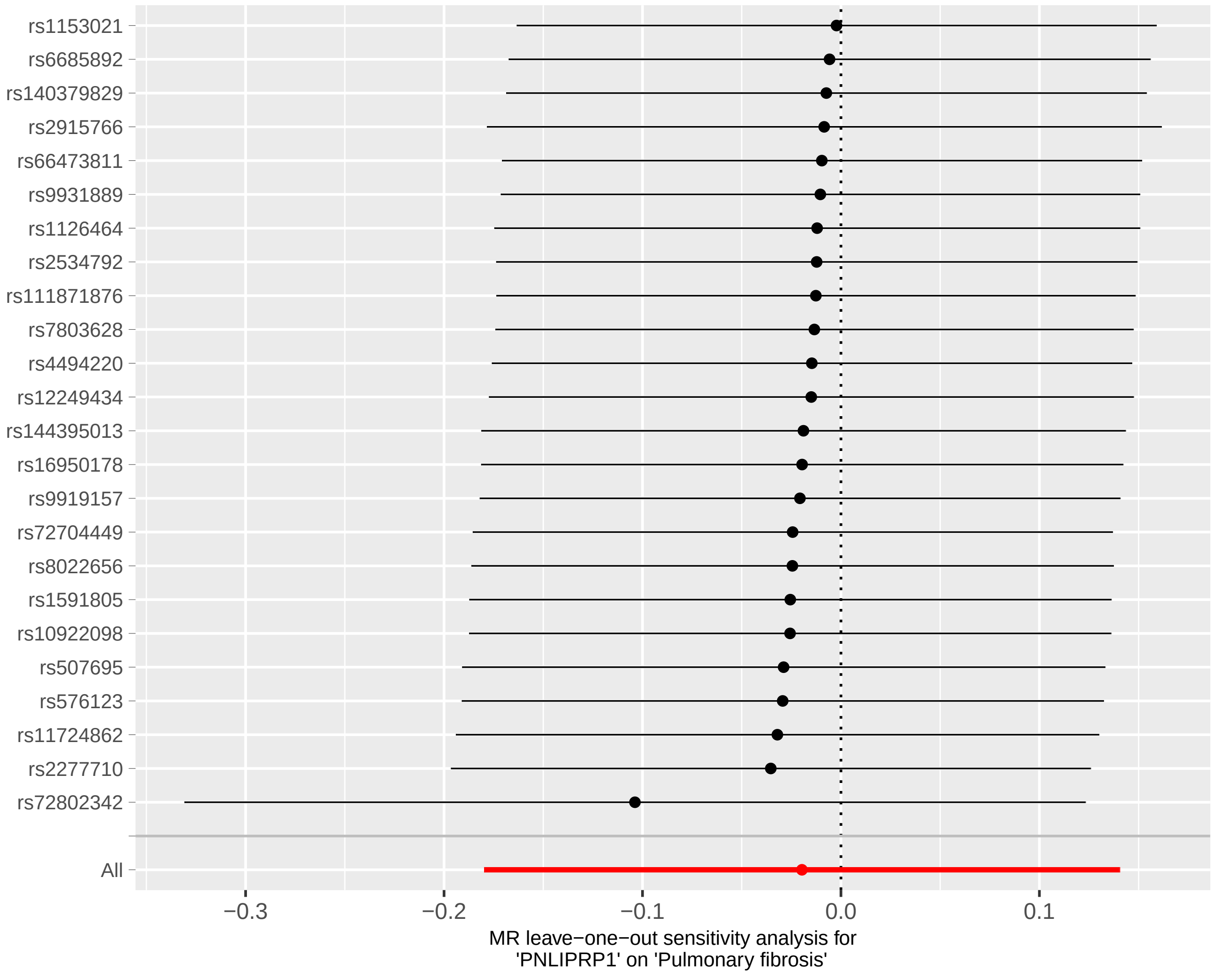

rs1153021
rs6685892
rs140379829
rs2915766
rs66473811
rs9931889
rs1126464
rs2534792
rs111871876
rs7803628
rs4494220
rs12249434
rs144395013
rs16950178
rs9919157
rs72704449
rs8022656
rs1591805
rs10922098
rs507695
rs576123
rs11724862
rs2277710
rs72802342
All
−0.3
−0.2
−0.1
0.0
0.1
MR leave−one−out sensitivity analysis for
'PNLIPRP1' on 'Pulmonary fibrosis'

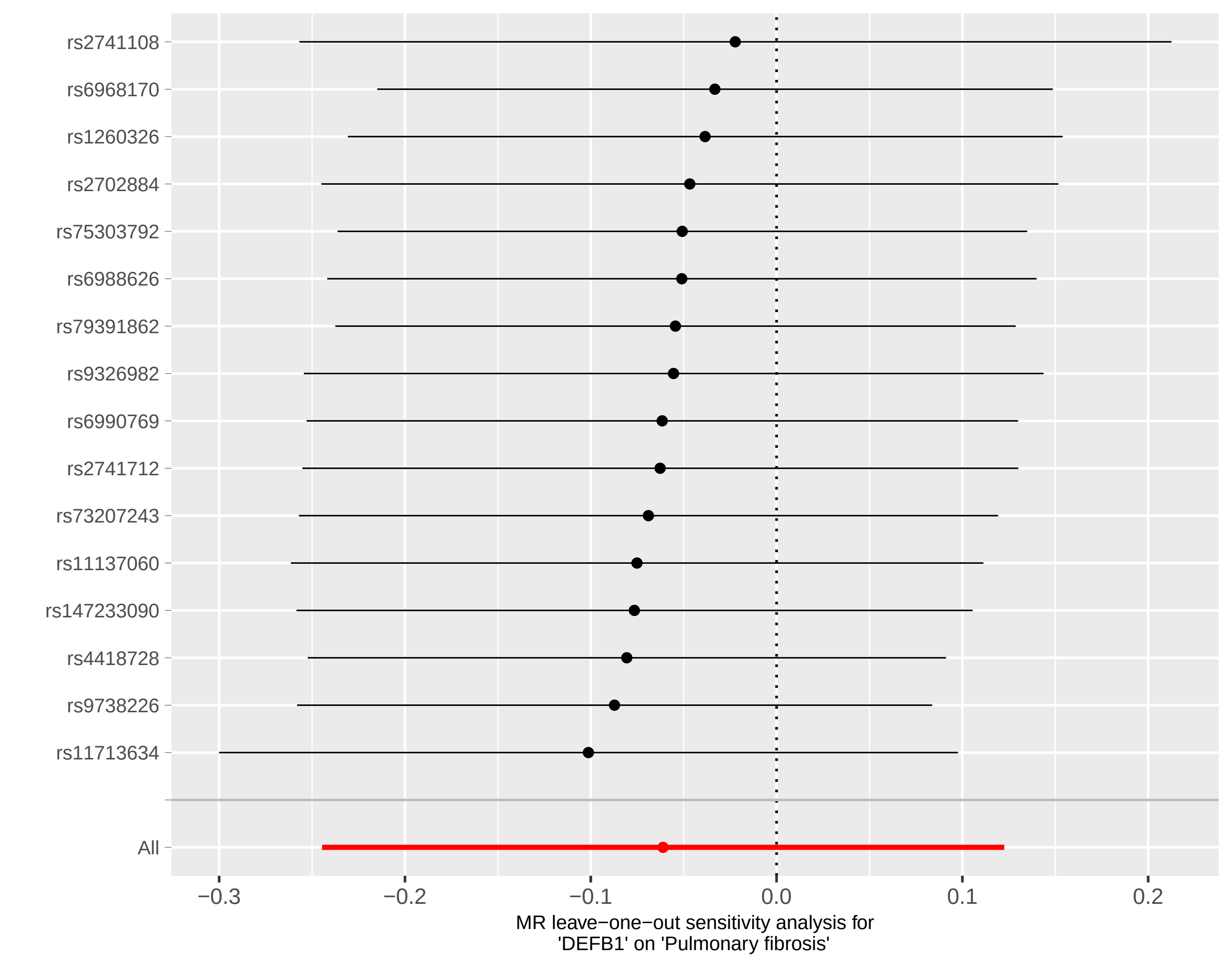

rs2741108
rs6968170
rs1260326
rs2702884
rs75303792
rs6988626
rs79391862
rs9326982
rs6990769
rs2741712
rs73207243
rs11137060
rs147233090
rs4418728
rs9738226
rs11713634
All
−0.3
−0.2
−0.1
0.0
0.1
0.2
MR leave−one−out sensitivity analysis for
'DEFB1' on 'Pulmonary fibrosis'

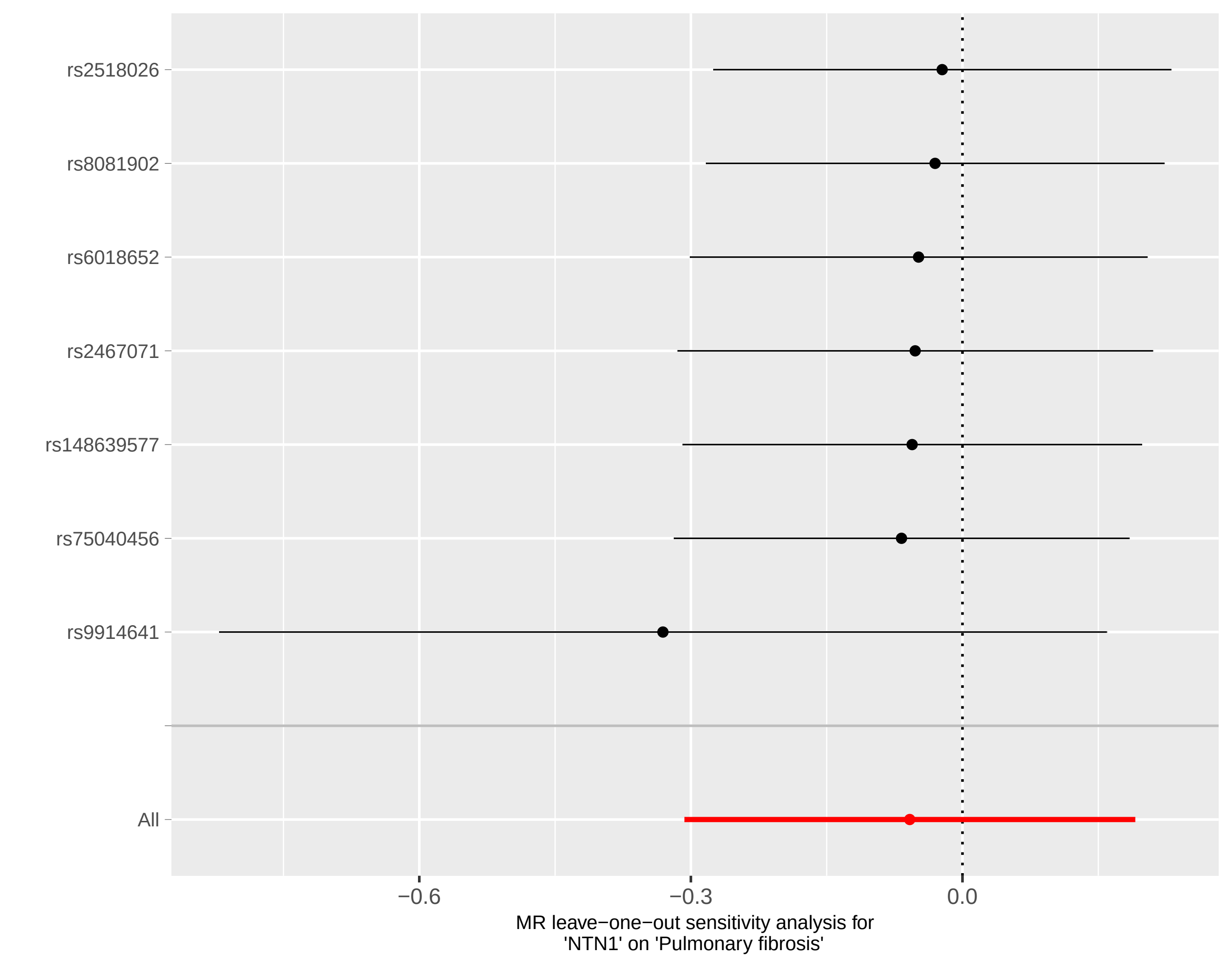
rs2518026
rs8081902
rs6018652
rs2467071
rs148639577
rs75040456
rs9914641
All
−0.6
−0.3
0.0
MR leave−one−out sensitivity analysis for
'NTN1' on 'Pulmonary fibrosis'

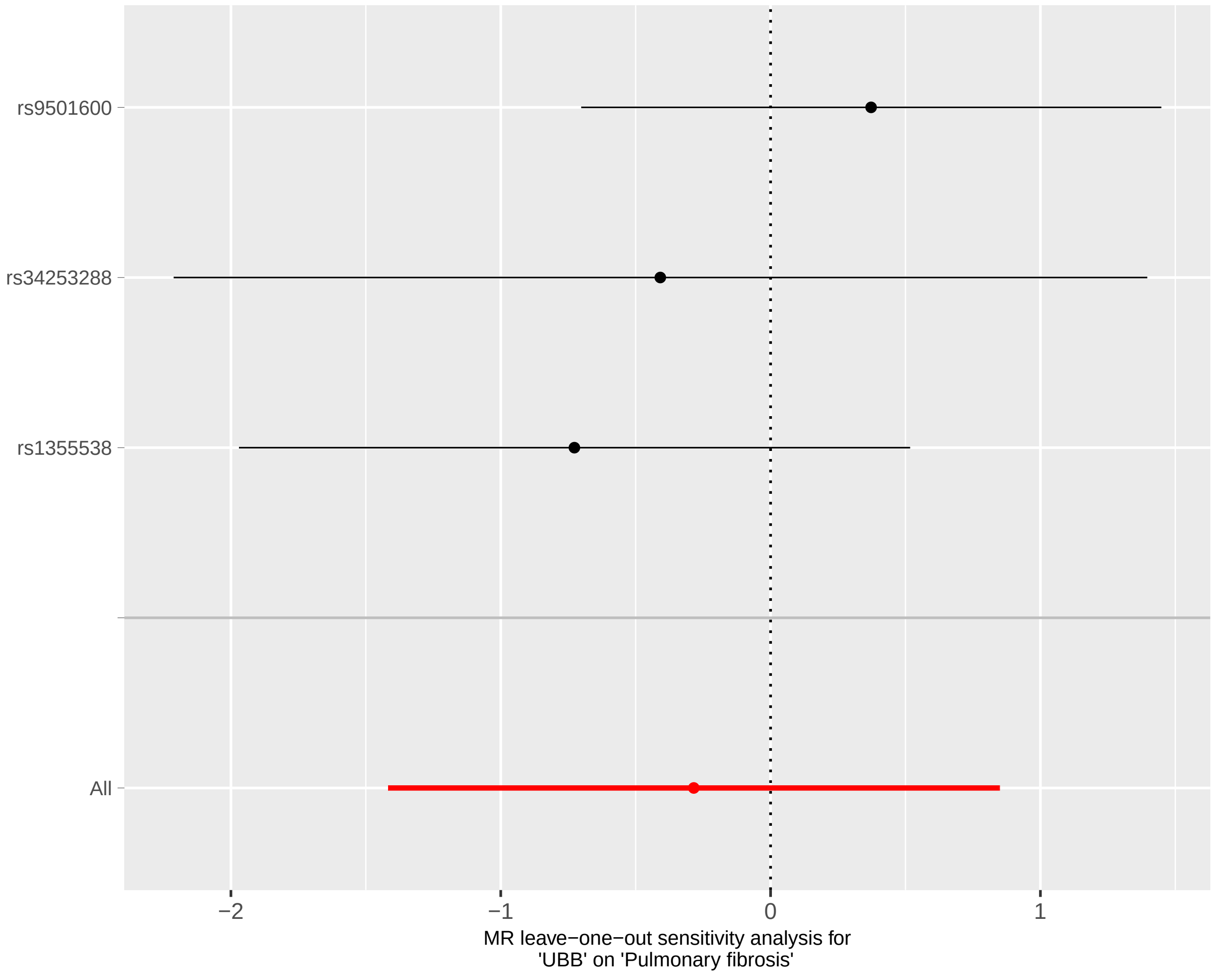
rs9501600
rs34253288
rs1355538
All
−2
−1
0
1
MR leave−one−out sensitivity analysis for
'UBB' on 'Pulmonary fibrosis'

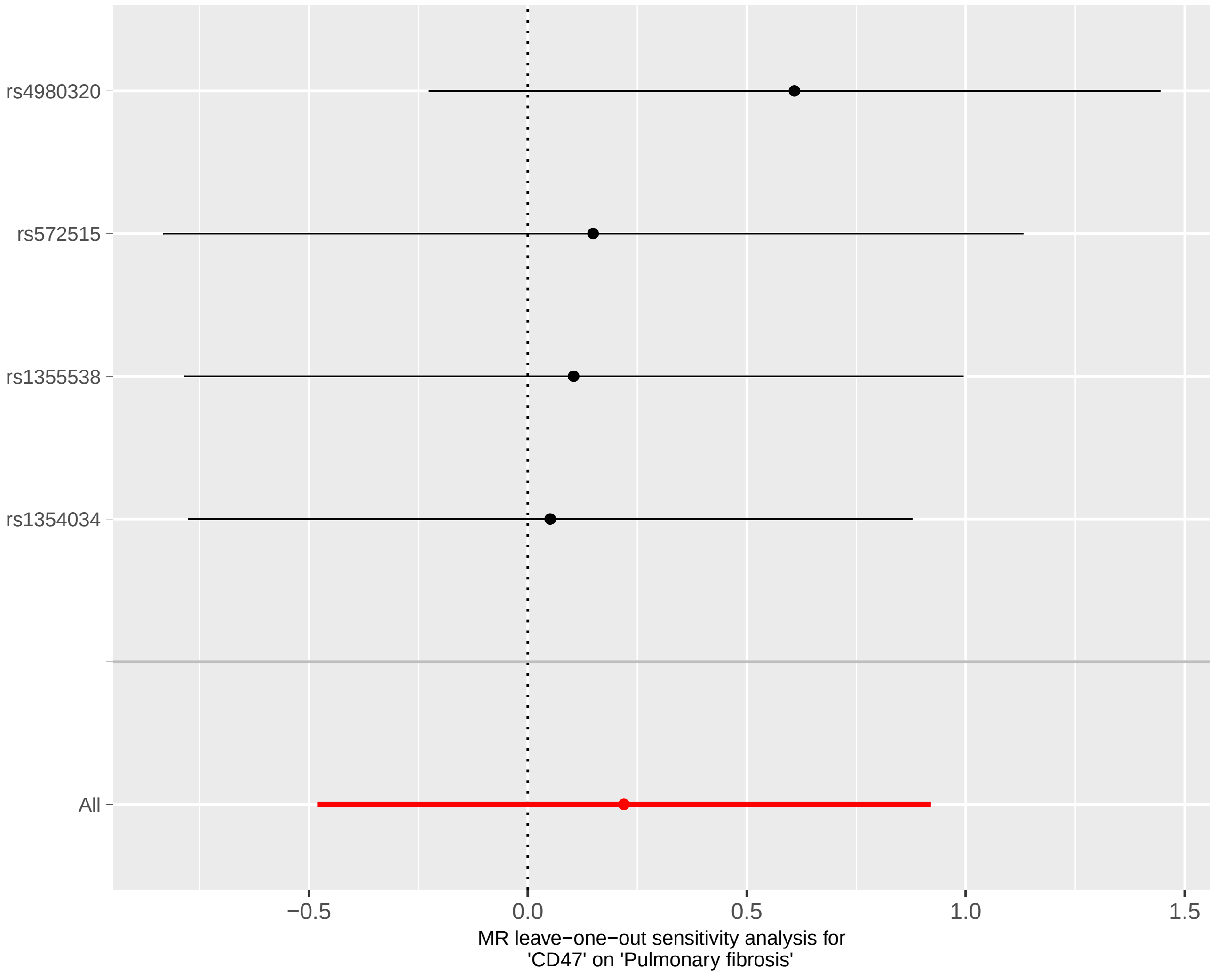
rs4980320
rs572515
rs1355538
rs1354034
All
−0.5
0.0
0.5
1.0
1.5
MR leave−one−out sensitivity analysis for
'CD47' on 'Pulmonary fibrosis'

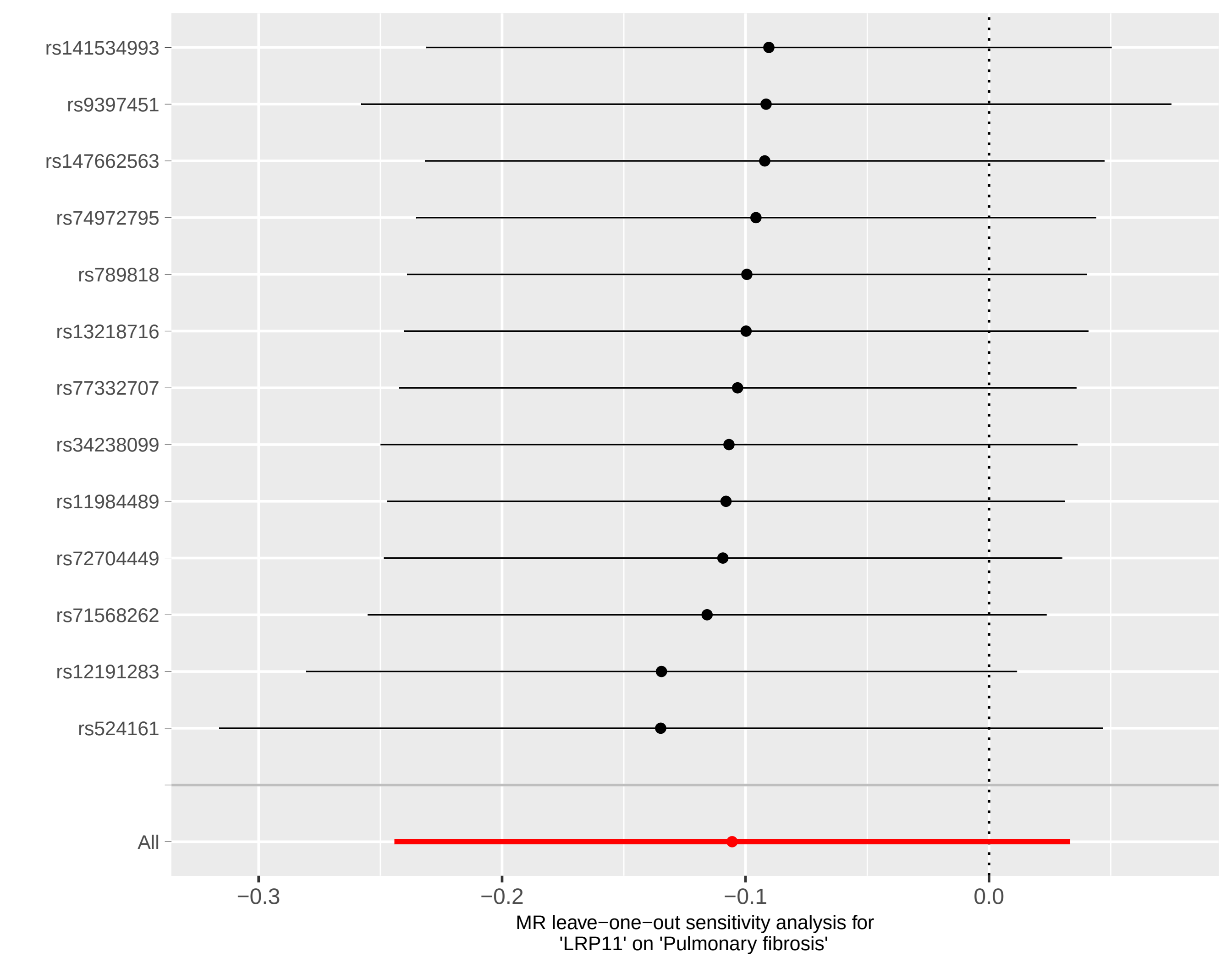

rs141534993
rs9397451
rs147662563
rs74972795
rs789818
rs13218716
rs77332707
rs34238099
rs11984489
rs72704449
rs71568262
rs12191283
rs524161
All
−0.3
−0.2
−0.1
0.0
MR leave−one−out sensitivity analysis for
'LRP11' on 'Pulmonary fibrosis'

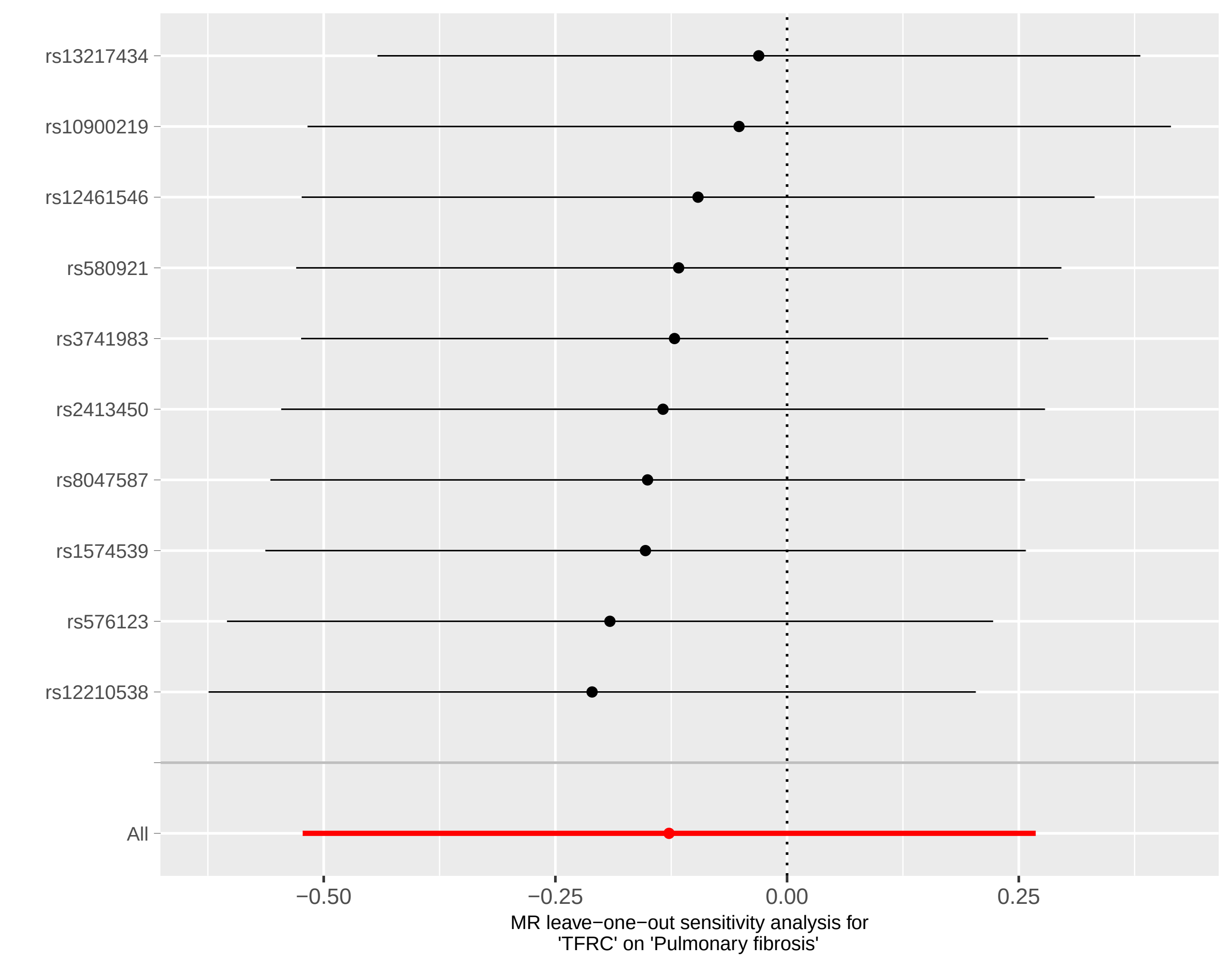

rs13217434
rs10900219
rs12461546
rs580921
rs3741983
rs2413450
rs8047587
rs1574539
rs576123
rs12210538
All
−0.50
−0.25
0.00
0.25
MR leave−one−out sensitivity analysis for
'TFRC' on 'Pulmonary fibrosis'

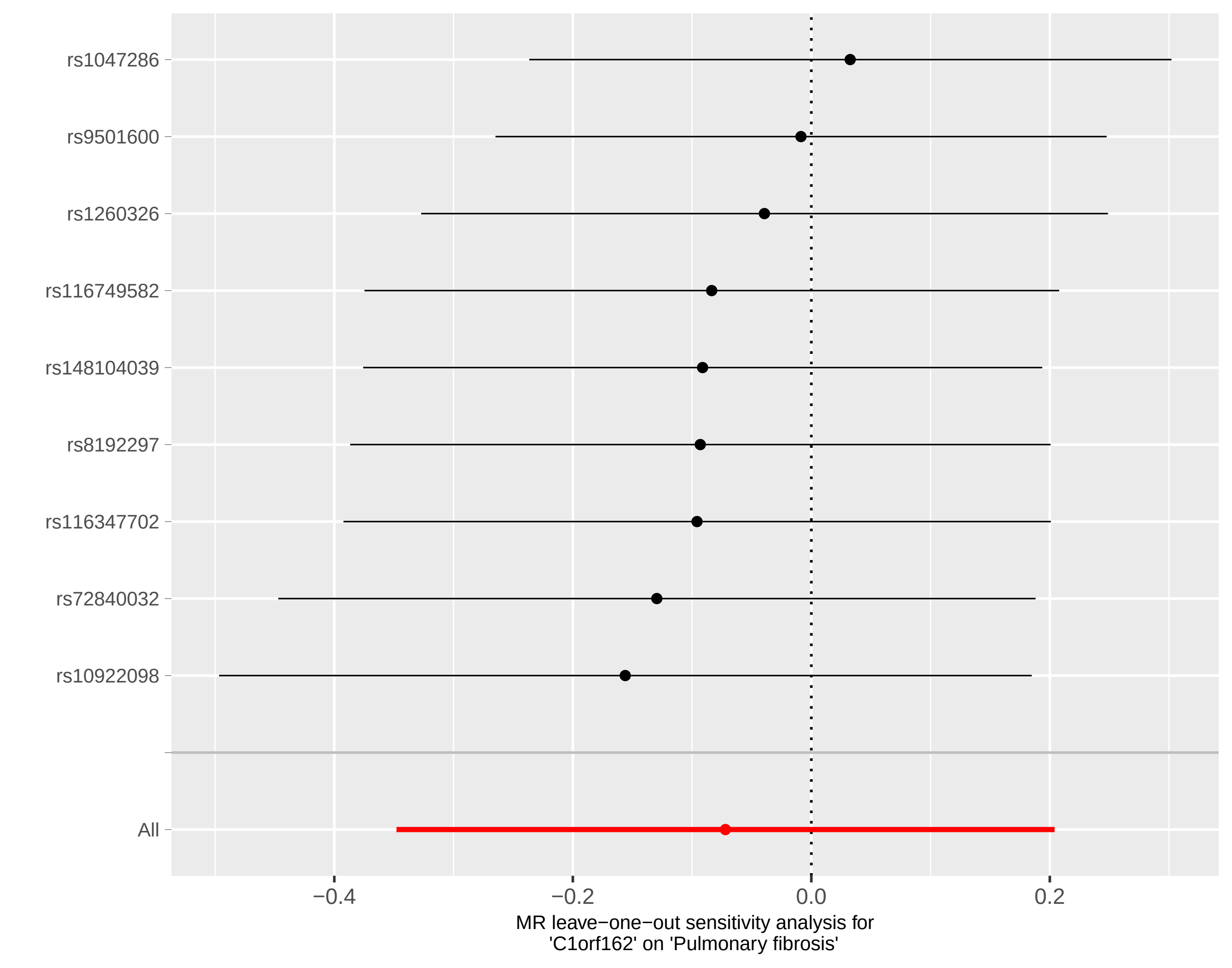
rs1047286
rs9501600
rs1260326
rs116749582
rs148104039
rs8192297
rs116347702
rs72840032
rs10922098
All
−0.4
−0.2
0.0
0.2
MR leave−one−out sensitivity analysis for
'C1orf162' on 'Pulmonary fibrosis'

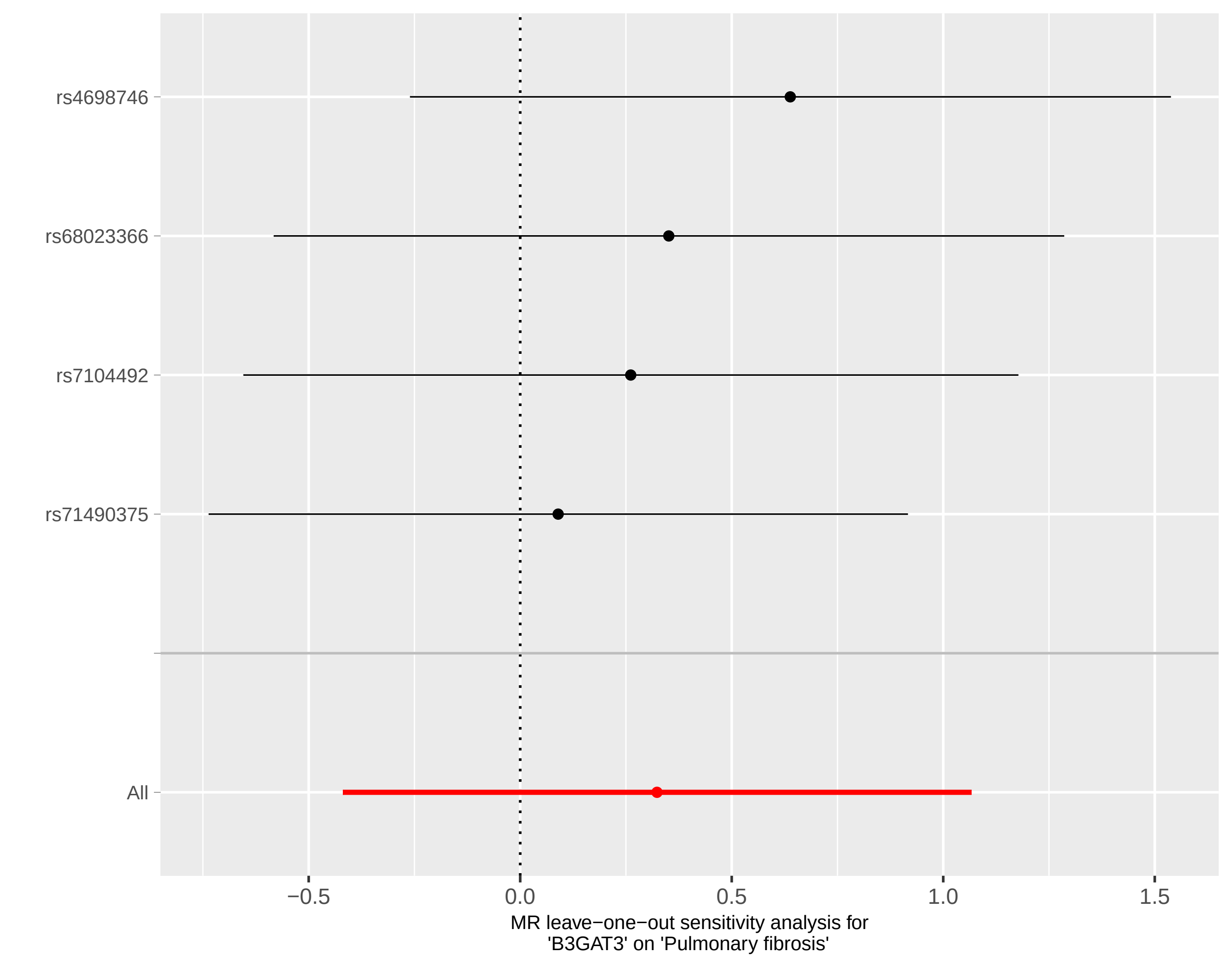
rs4698746
rs68023366
rs7104492
rs71490375
All
−0.5
0.0
0.5
1.0
1.5
MR leave−one−out sensitivity analysis for
'B3GAT3' on 'Pulmonary fibrosis'

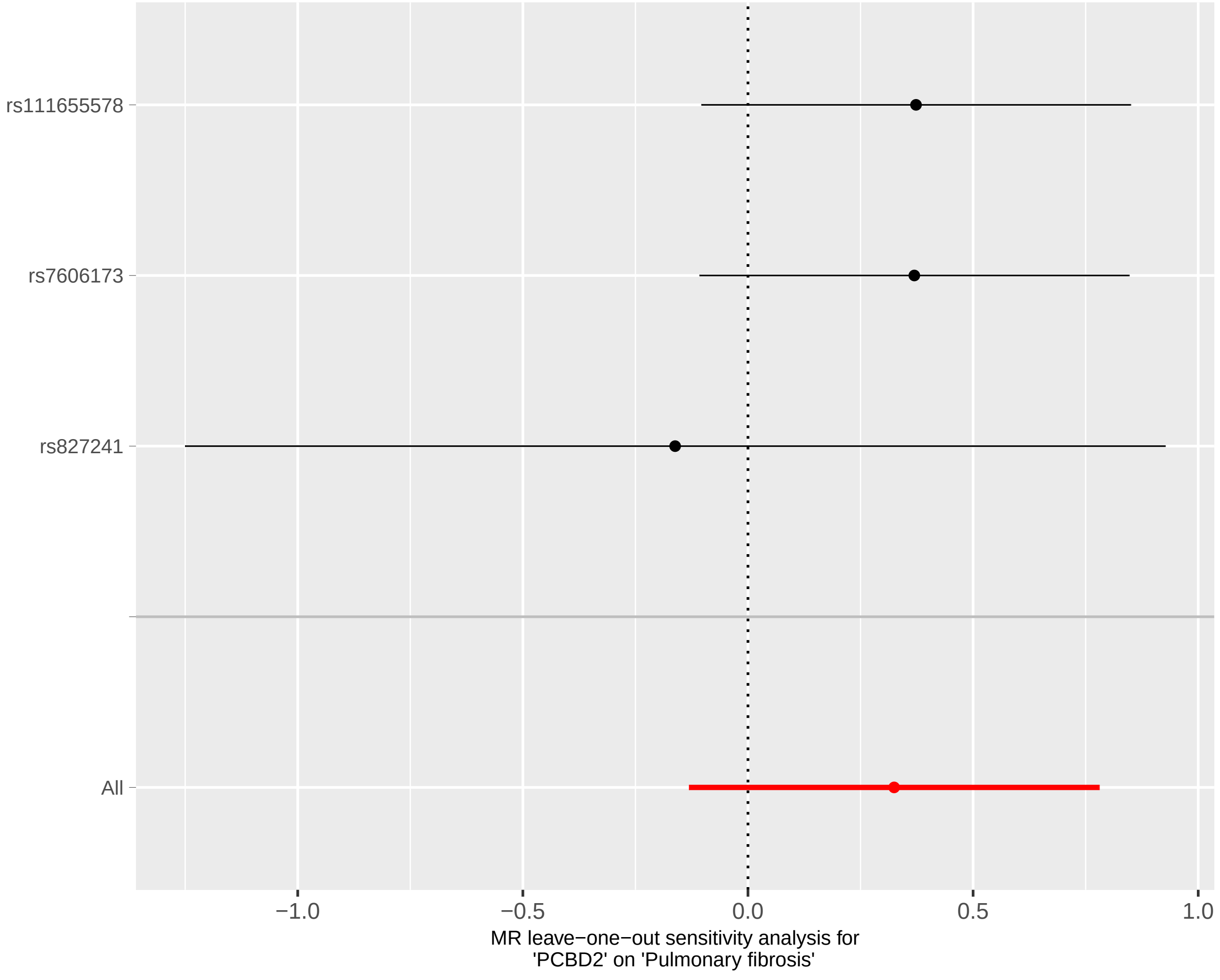
rs111655578
rs7606173
rs827241
All
−1.0
−0.5
0.0
0.5
1.0
MR leave−one−out sensitivity analysis for
'PCBD2' on 'Pulmonary fibrosis'

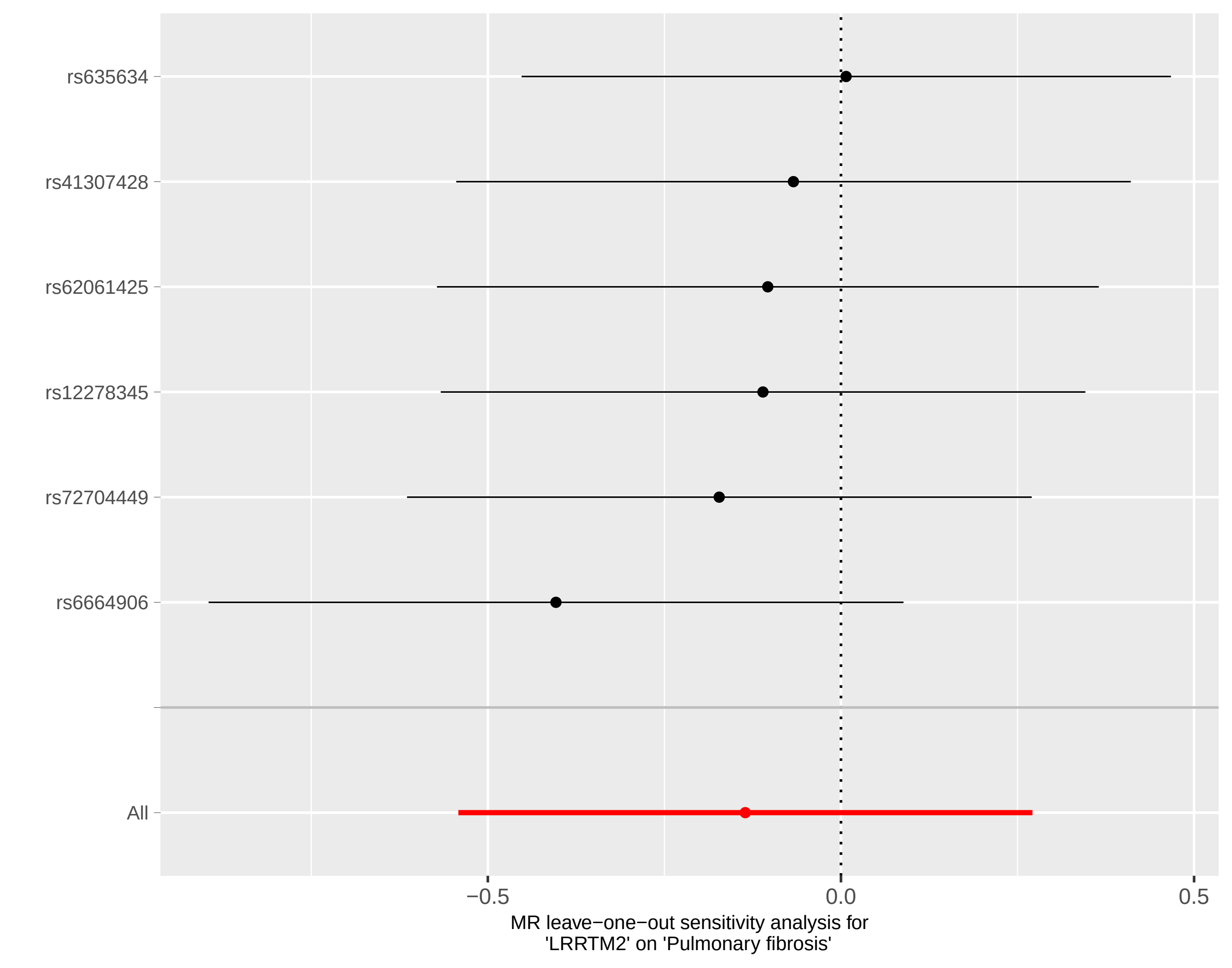
rs635634
rs41307428
rs62061425
rs12278345
rs72704449
rs6664906
All
−0.5
0.0
0.5
MR leave−one−out sensitivity analysis for
'LRRTM2' on 'Pulmonary fibrosis'

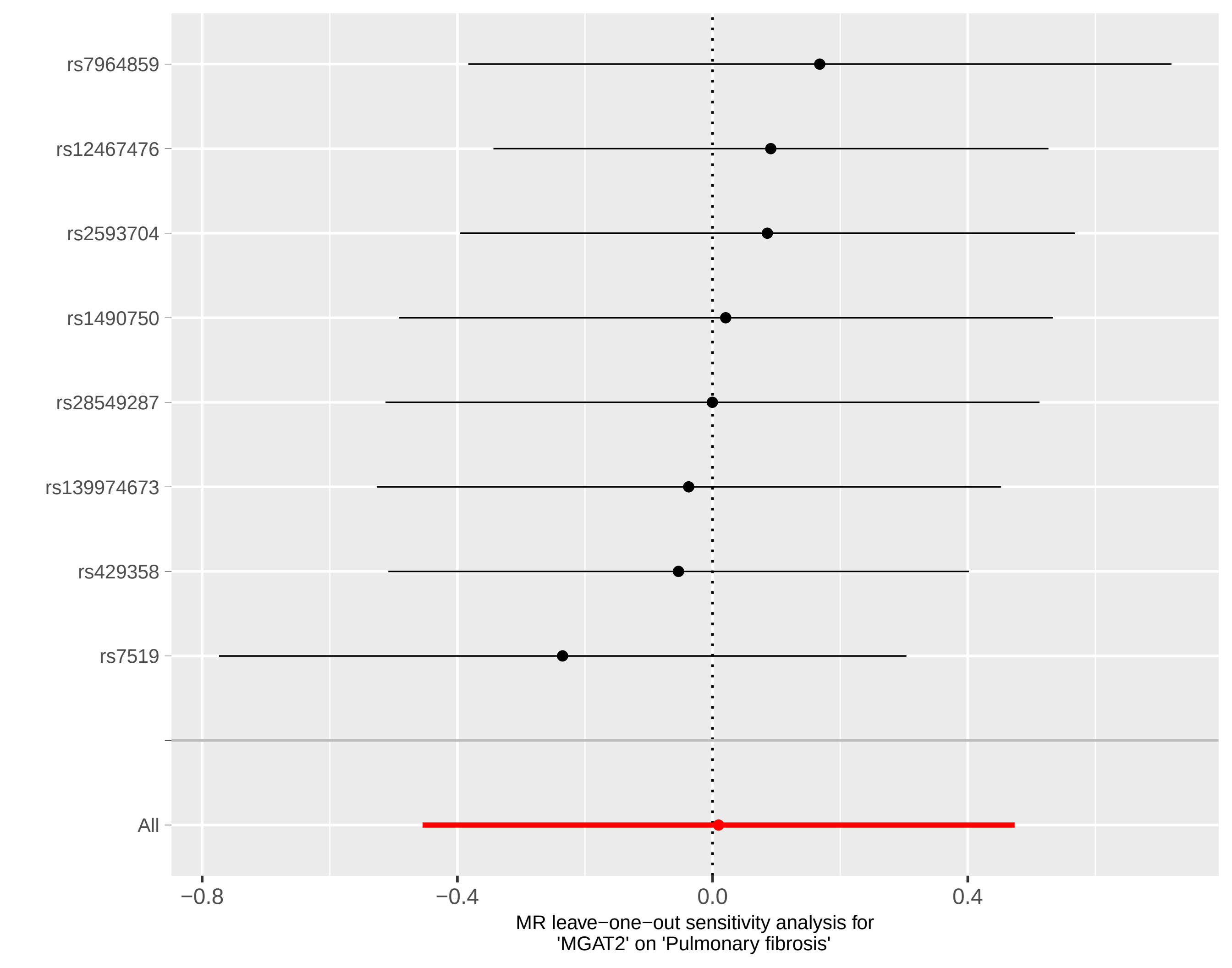

rs7964859
rs12467476
rs2593704
rs1490750
rs28549287
rs139974673
rs429358
rs7519
All
−0.8
−0.4
0.0
0.4
MR leave−one−out sensitivity analysis for
'MGAT2' on 'Pulmonary fibrosis'

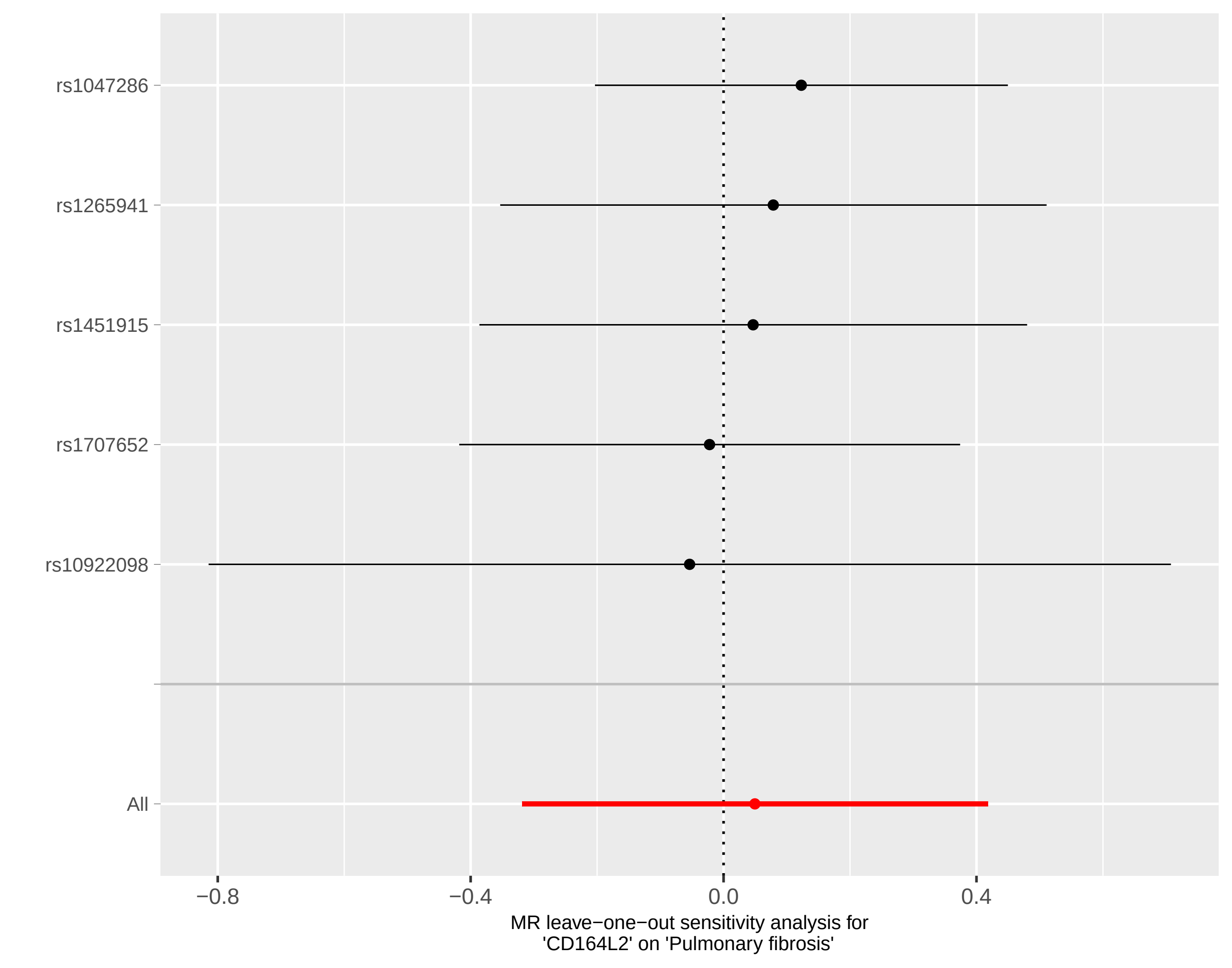
rs1047286
rs1265941
rs1451915
rs1707652
rs10922098
All
−0.8
−0.4
0.0
0.4
MR leave−one−out sensitivity analysis for
'CD164L2' on 'Pulmonary fibrosis'

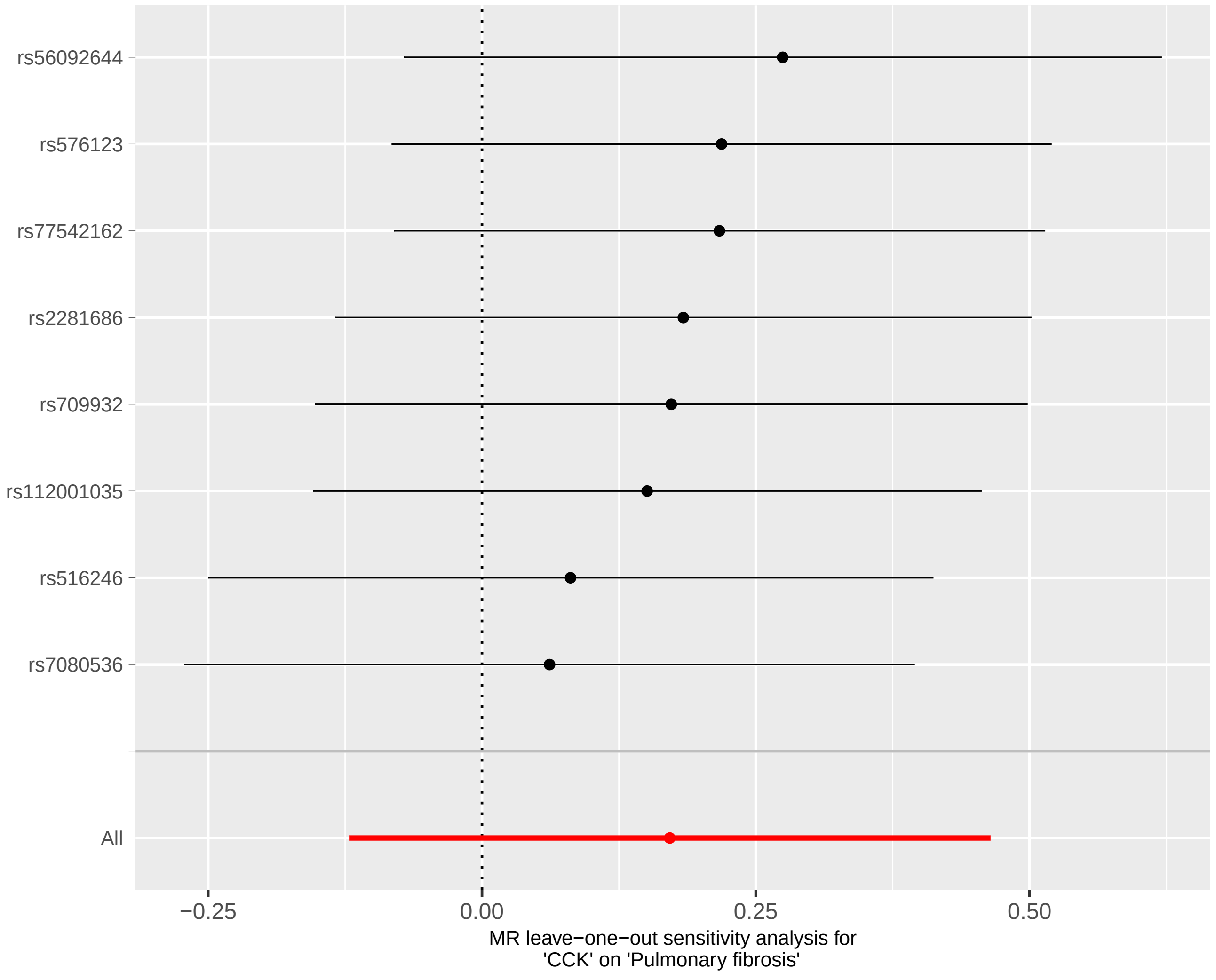

rs56092644
rs576123
rs77542162
rs2281686
rs709932
rs112001035
rs516246
rs7080536
All
−0.25
0.00
0.25
0.50
MR leave−one−out sensitivity analysis for
'CCK' on 'Pulmonary fibrosis'

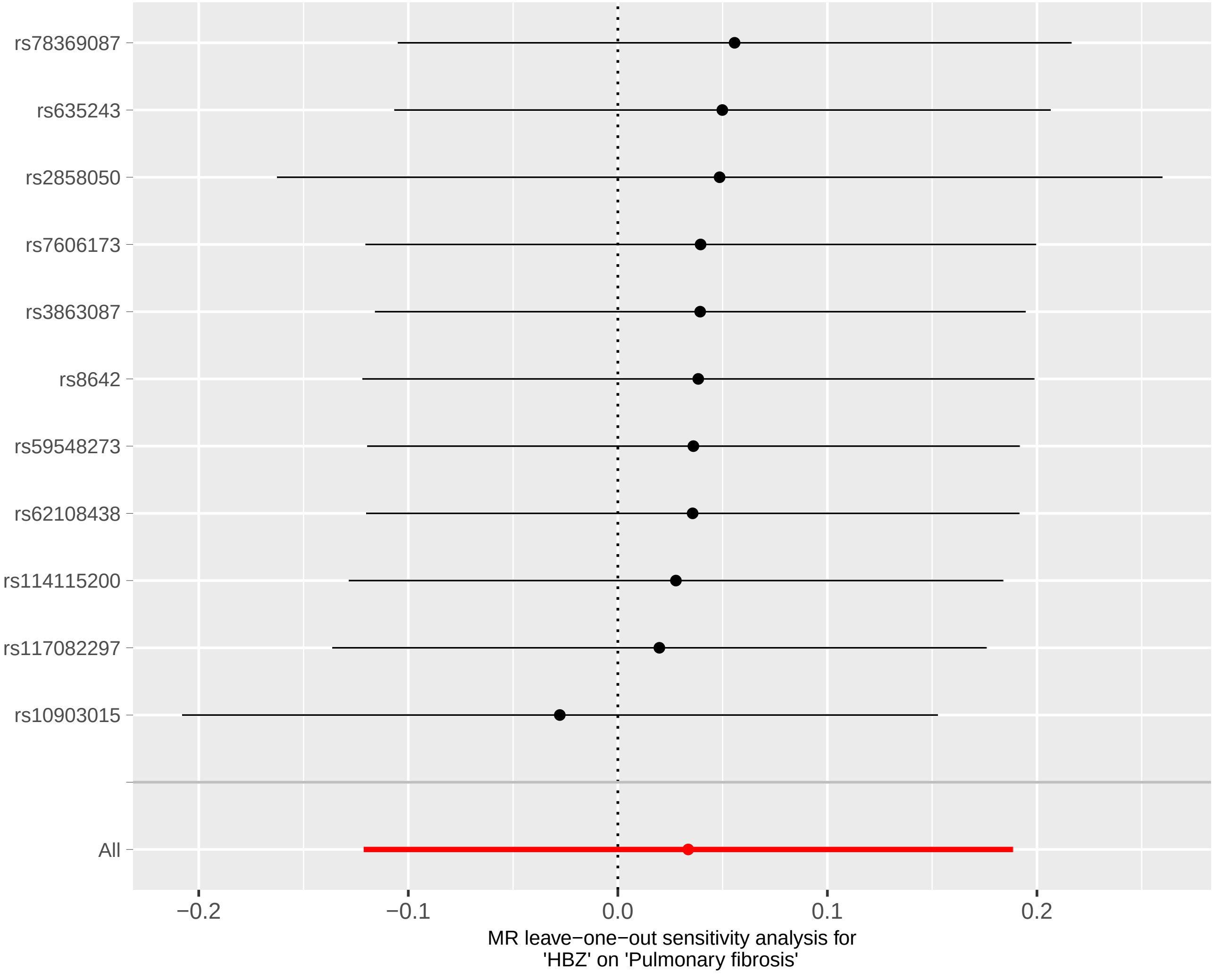
rs78369087
rs635243
rs2858050
rs7606173
rs3863087
rs8642
rs59548273
rs62108438
rs114115200
rs117082297
rs10903015
All
−0.2
−0.1
0.0
0.1
0.2
MR leave−one−out sensitivity analysis for
'HBZ' on 'Pulmonary fibrosis'

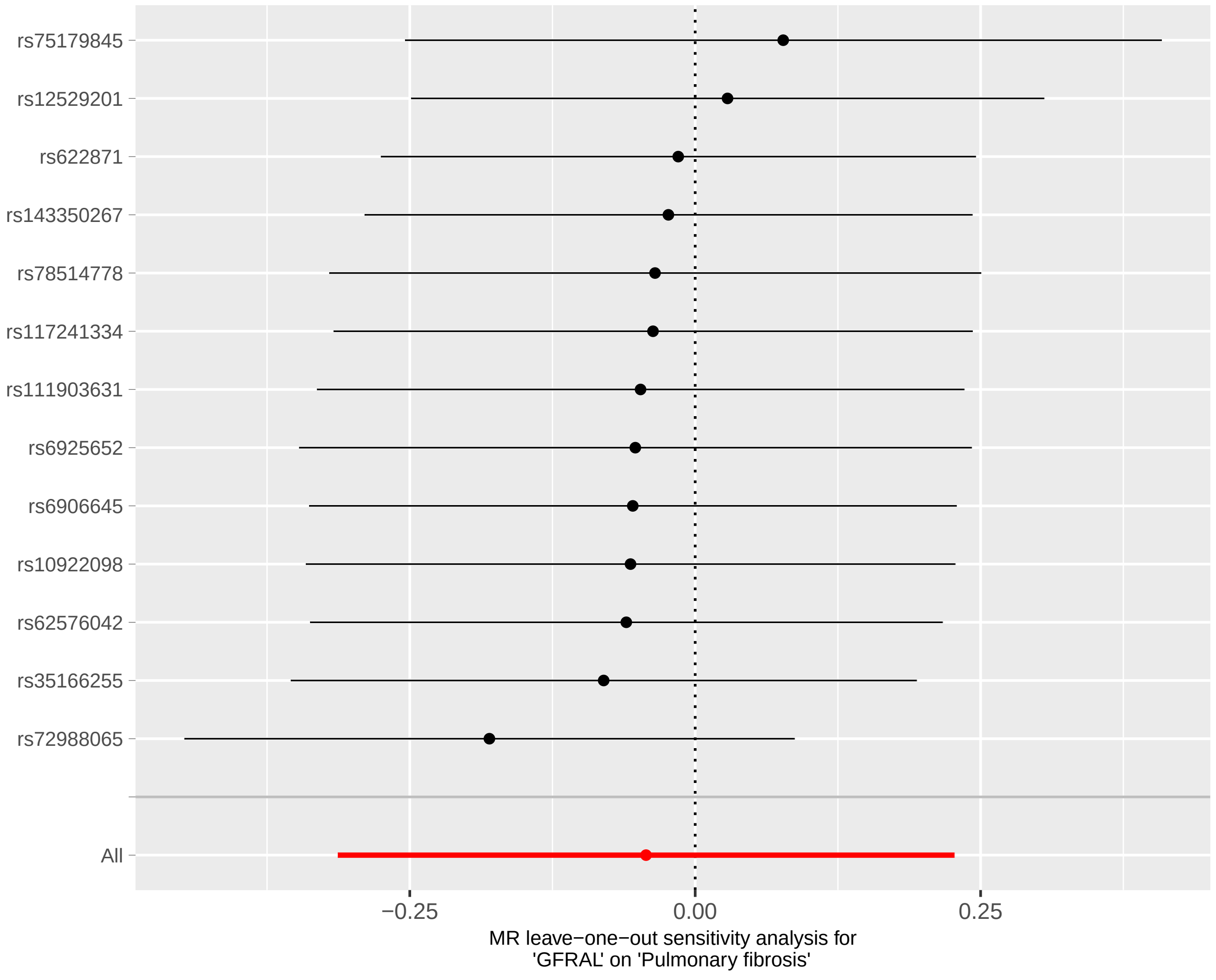
rs75179845
rs12529201
rs622871
rs143350267
rs78514778
rs117241334
rs111903631
rs6925652
rs6906645
rs10922098
rs62576042
rs35166255
rs72988065
All
−0.25
0.00
0.25
MR leave−one−out sensitivity analysis for
'GFRAL' on 'Pulmonary fibrosis'

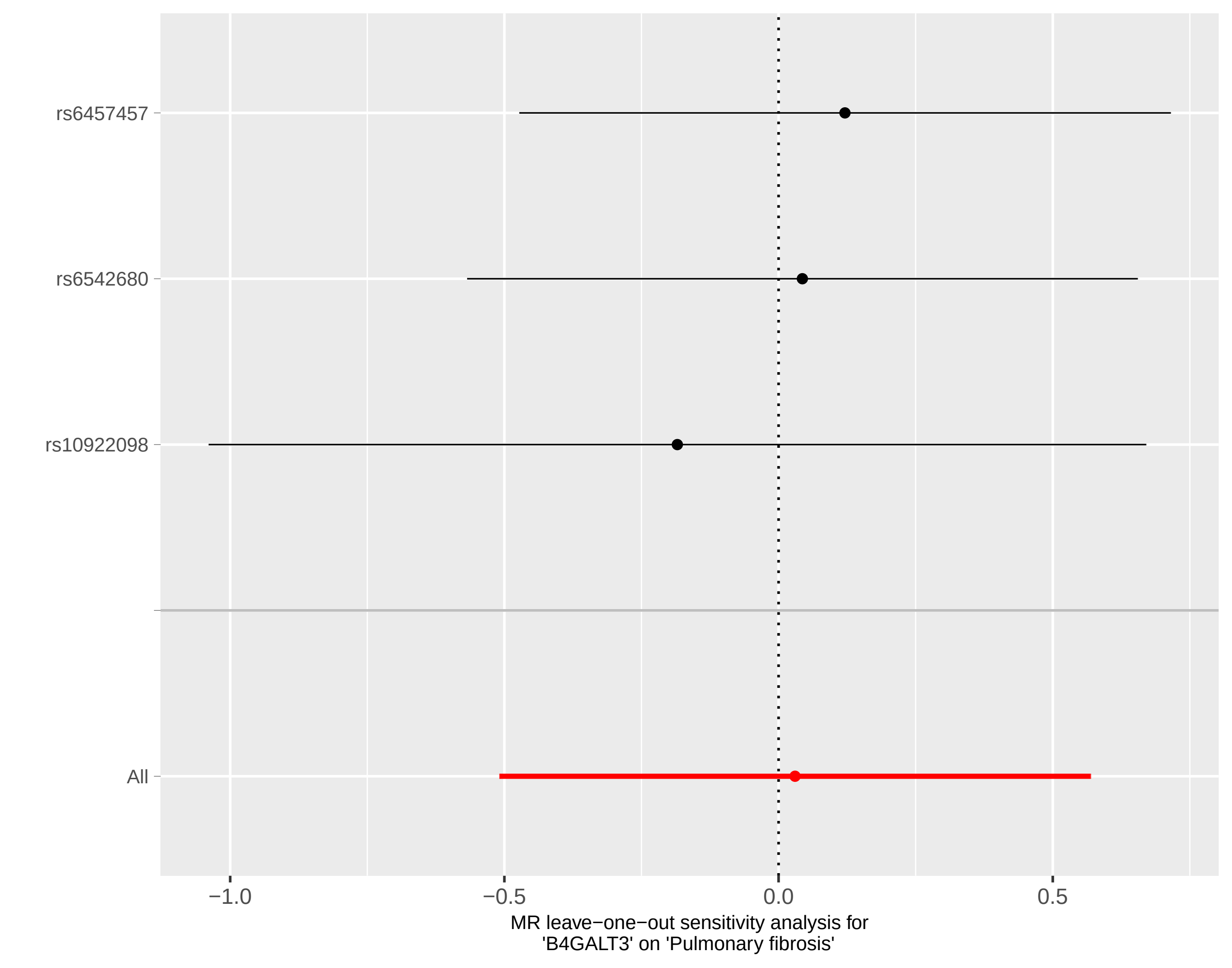
rs6457457
rs6542680
rs10922098
All
−1.0
−0.5
0.0
0.5
MR leave−one−out sensitivity analysis for
'B4GALT3' on 'Pulmonary fibrosis'

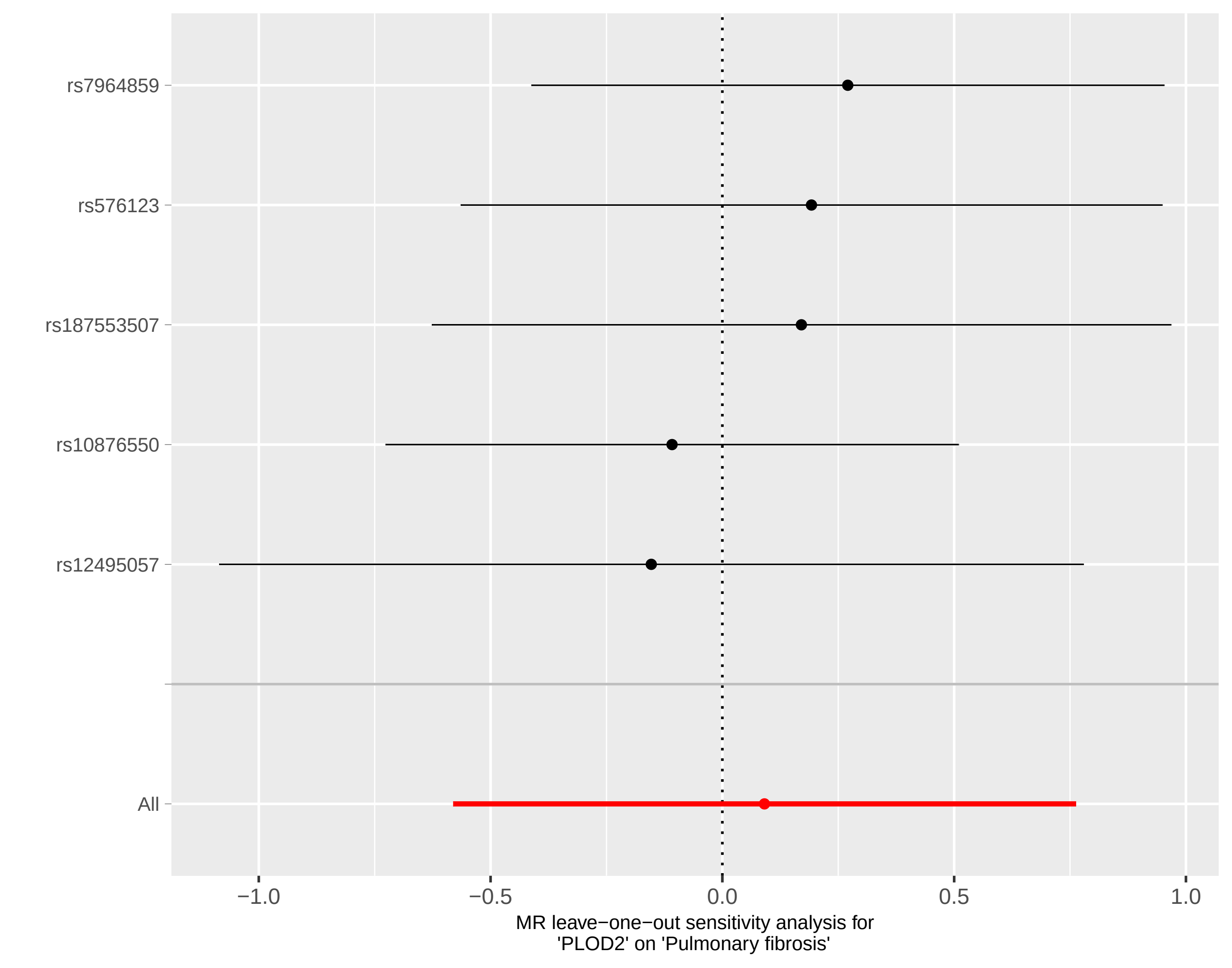
rs7964859
rs576123
rs187553507
rs10876550
rs12495057
All
−1.0
−0.5
0.0
0.5
1.0
MR leave−one−out sensitivity analysis for
'PLOD2' on 'Pulmonary fibrosis'

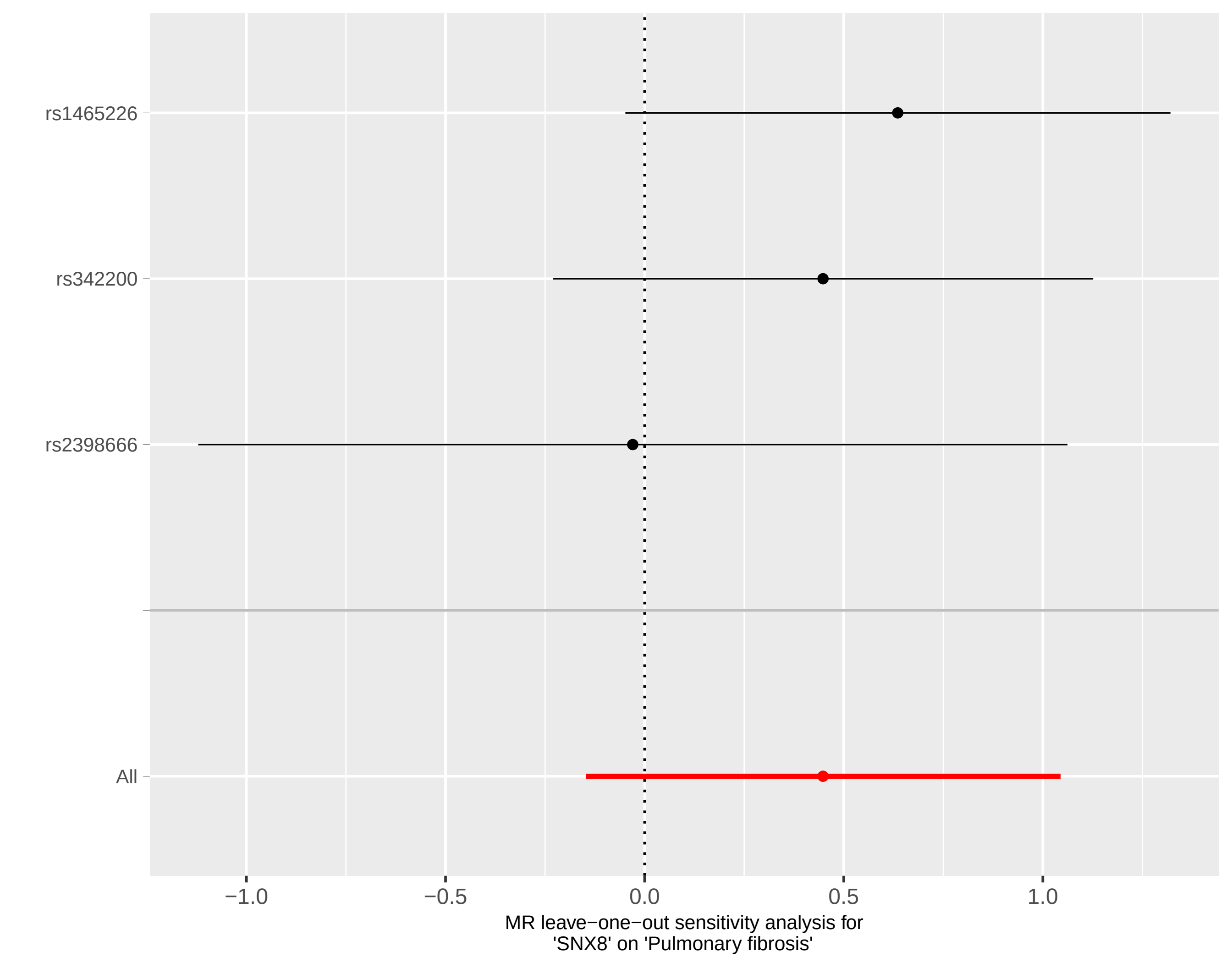

rs1465226
rs342200
rs2398666
All
−1.0
−0.5
0.0
0.5
1.0
MR leave−one−out sensitivity analysis for
'SNX8' on 'Pulmonary fibrosis'

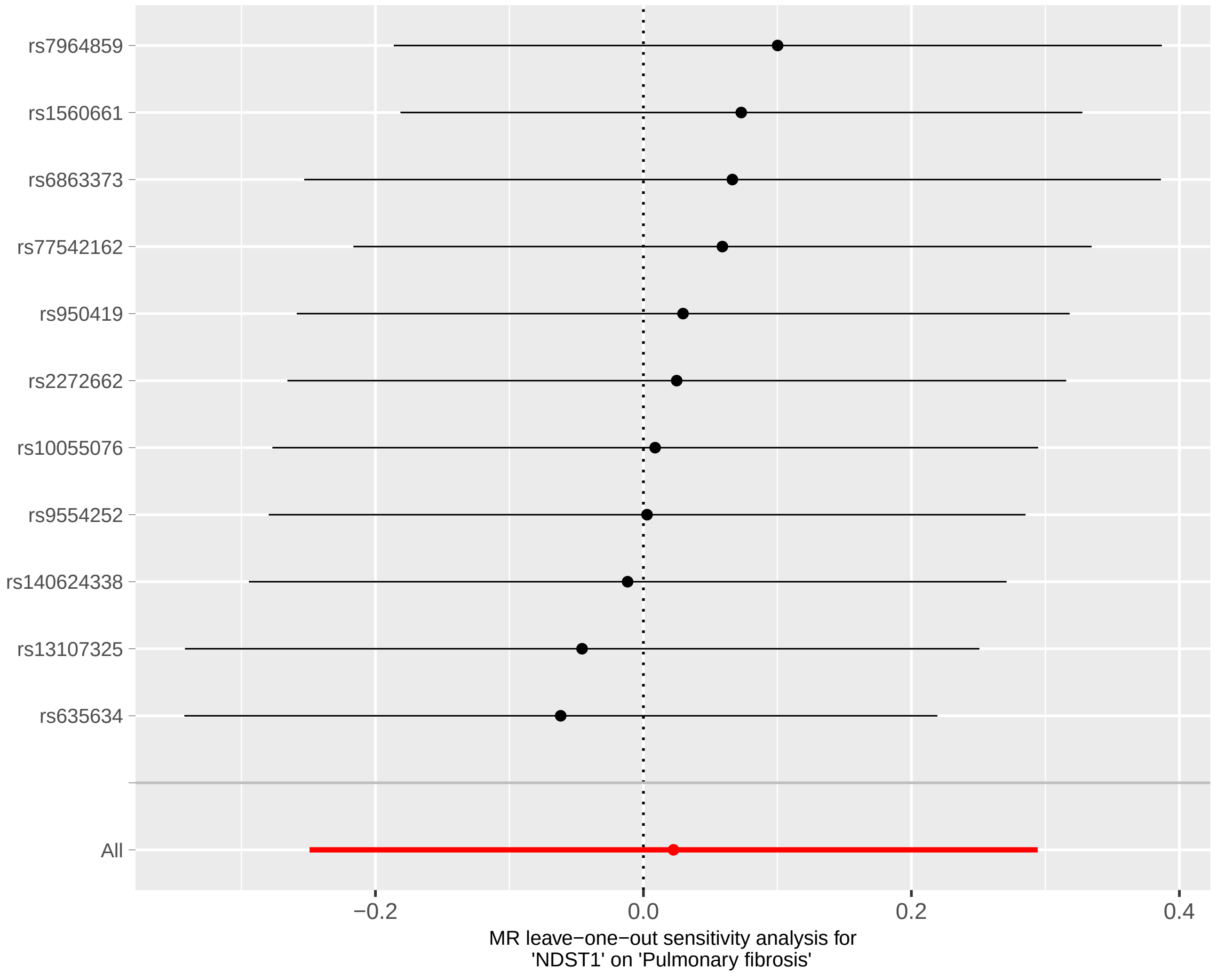
rs7964859
rs1560661
rs6863373
rs77542162
rs950419
rs2272662
rs10055076
rs9554252
rs140624338
rs13107325
rs635634
All
−0.2
0.0
0.2
0.4
MR leave−one−out sensitivity analysis for
'NDST1' on 'Pulmonary fibrosis'

Insufficient number of SNPs

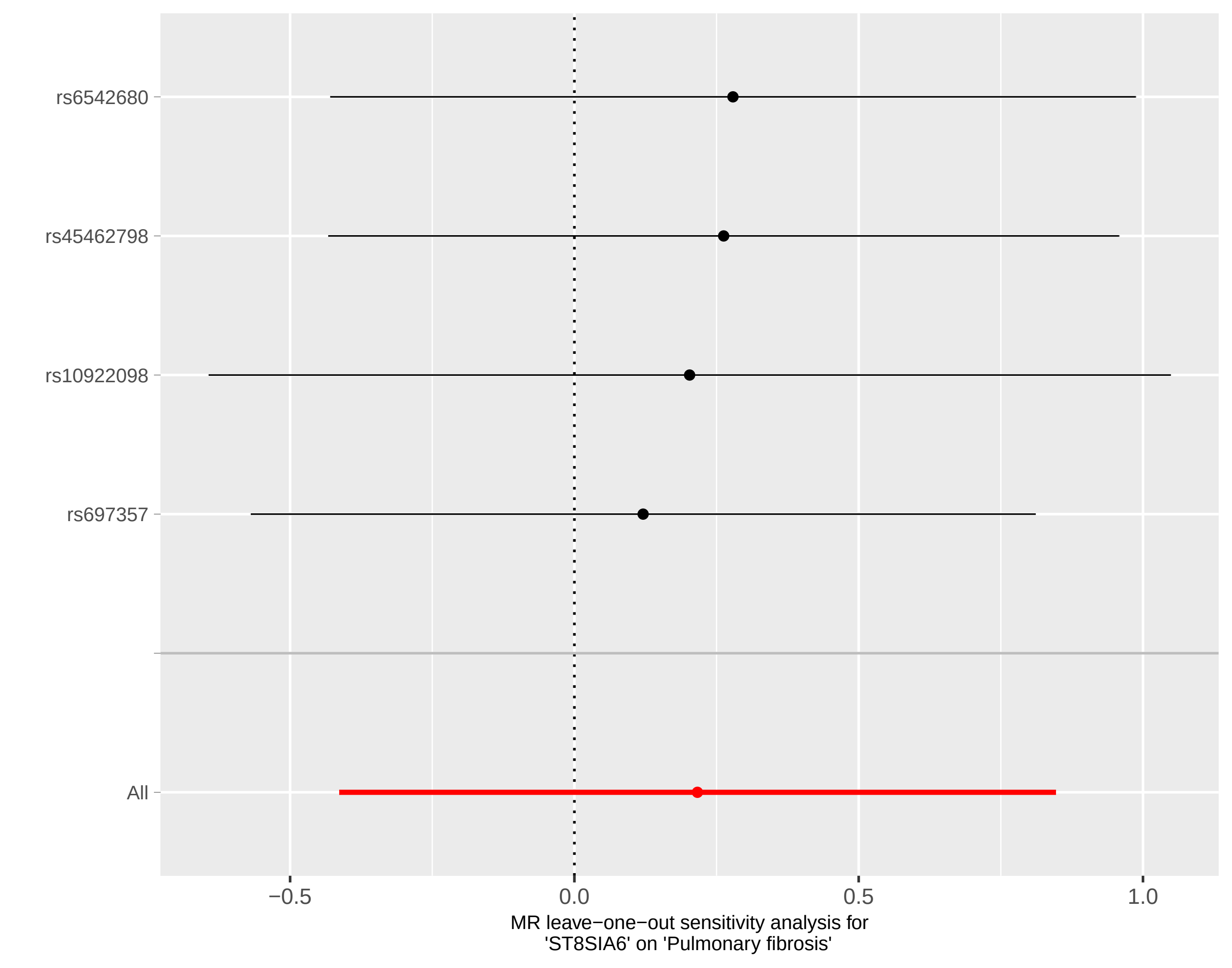
rs6542680
rs45462798
rs10922098
rs697357
All
−0.5
0.0
0.5
1.0
MR leave−one−out sensitivity analysis for
'ST8SIA6' on 'Pulmonary fibrosis'

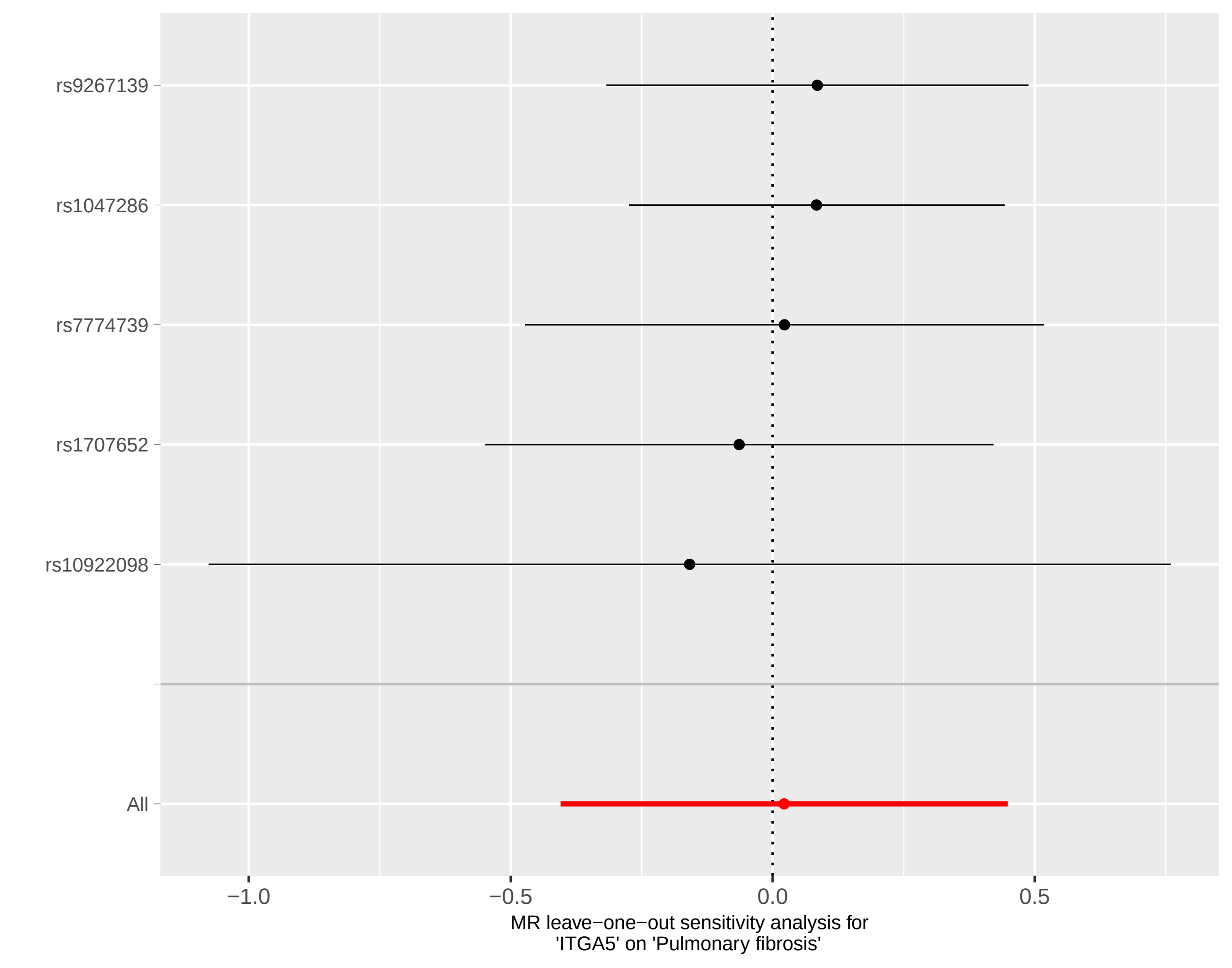
rs9267139
rs1047286
rs7774739
rs1707652
rs10922098
All
−1.0
−0.5
0.0
0.5
MR leave−one−out sensitivity analysis for
'ITGA5' on 'Pulmonary fibrosis'

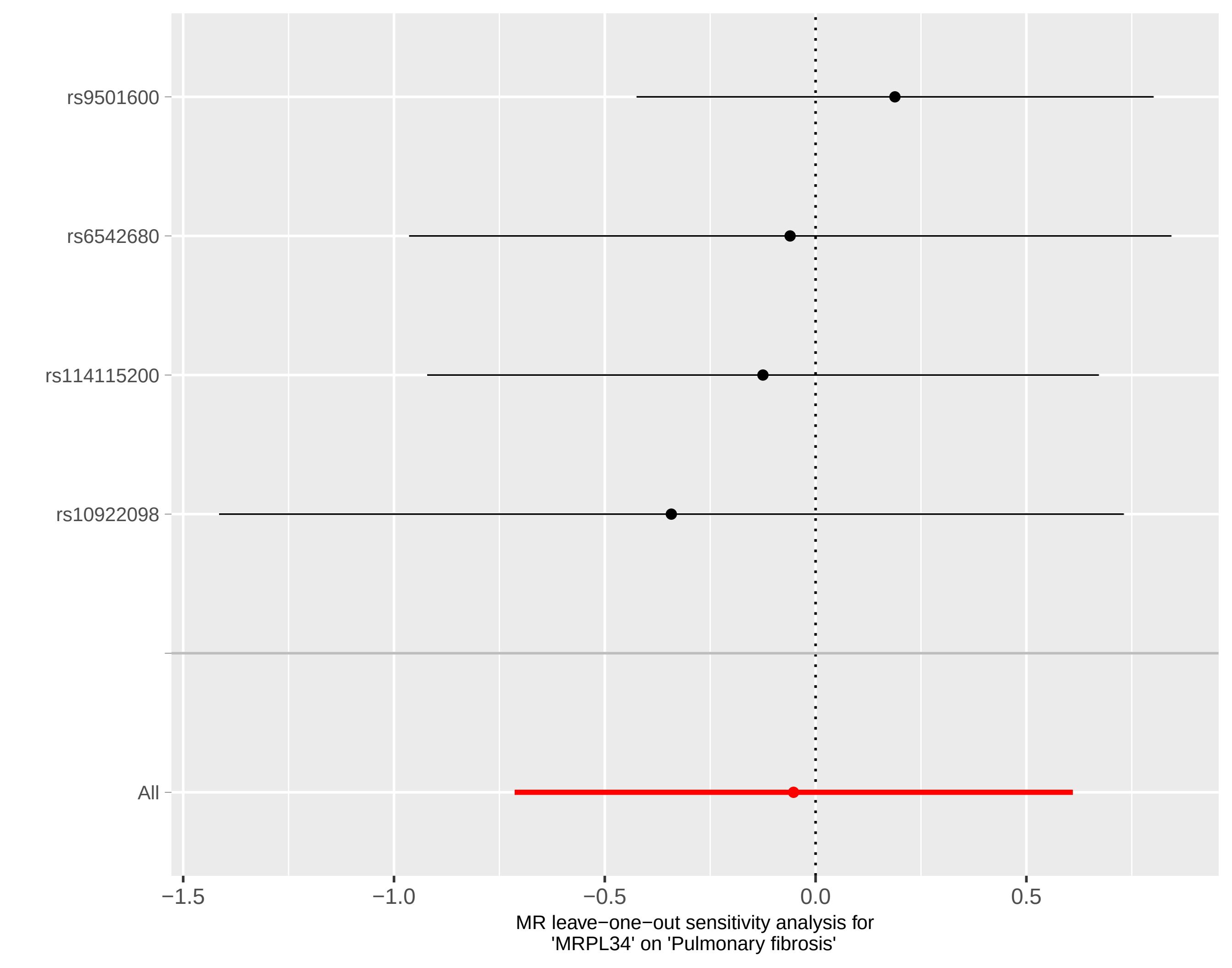
rs9501600
rs6542680
rs114115200
rs10922098
All
−1.5
−1.0
−0.5
0.0
0.5
MR leave−one−out sensitivity analysis for
'MRPL34' on 'Pulmonary fibrosis'

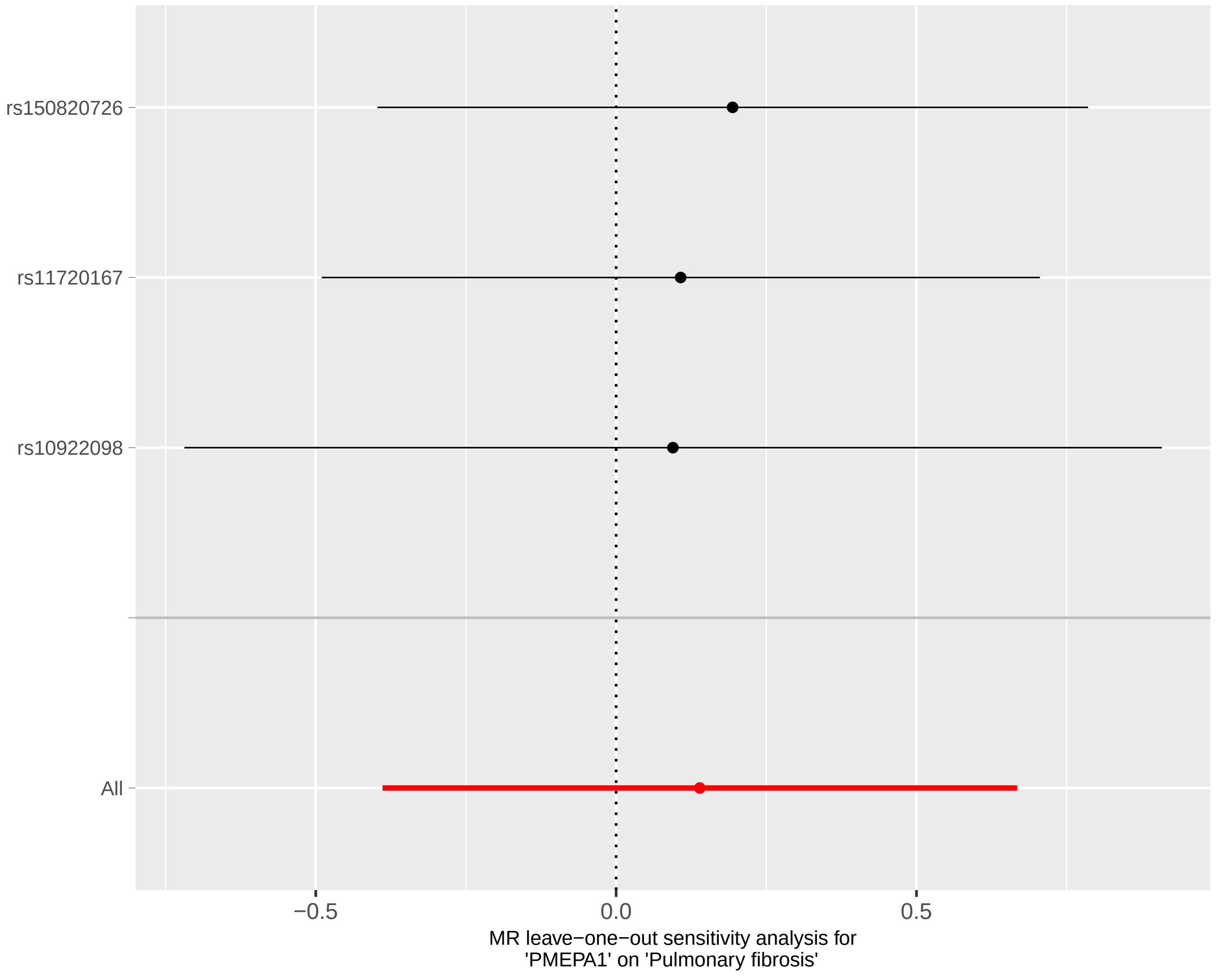
rs150820726
rs11720167
rs10922098
All
−0.5
0.0
0.5
MR leave−one−out sensitivity analysis for
'PMEPA1' on 'Pulmonary fibrosis'

Insufficient number of SNPs

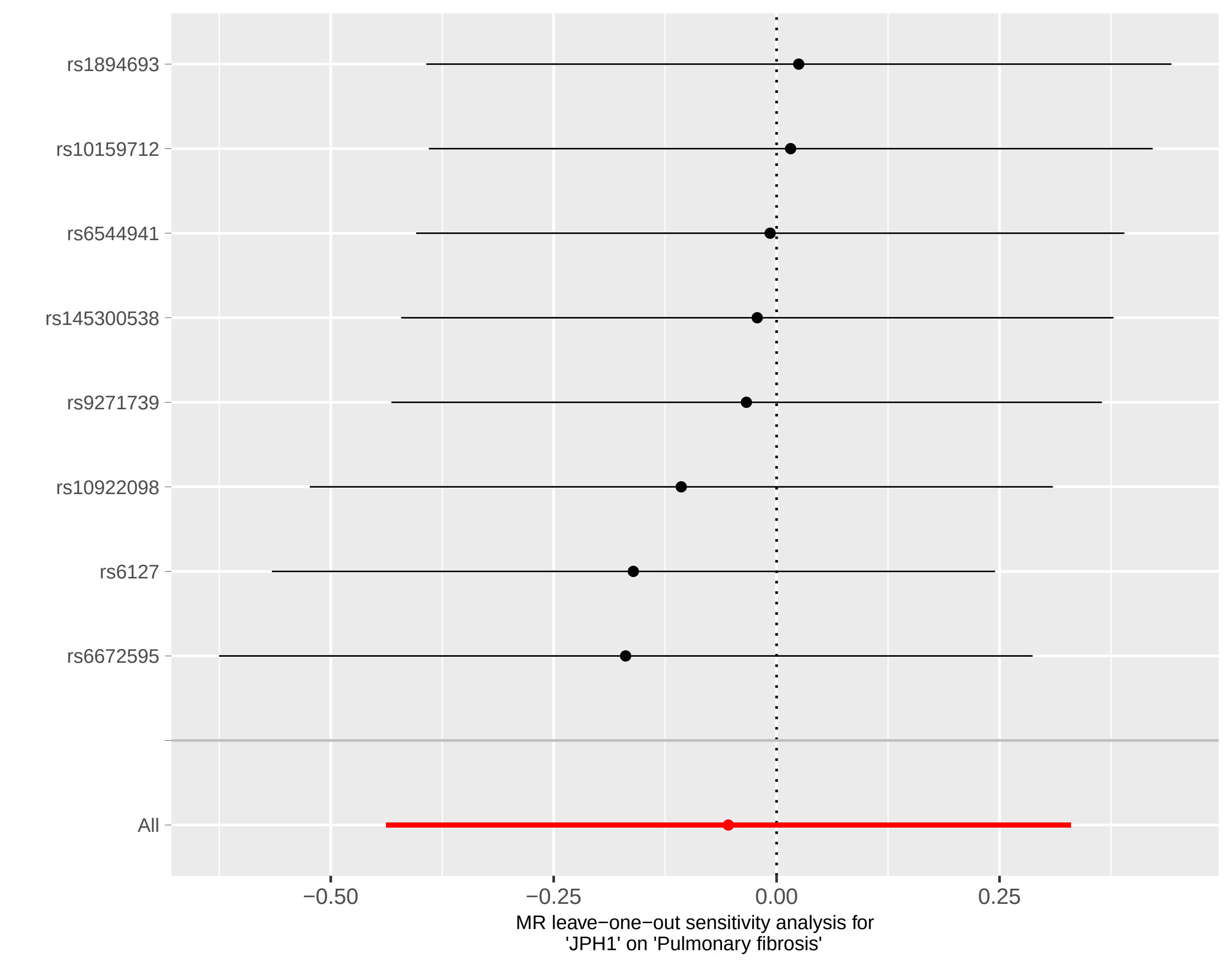
rs1894693
rs10159712
rs6544941
rs145300538
rs9271739
rs10922098
rs6127
rs6672595
All
−0.50
−0.25
0.00
0.25
MR leave−one−out sensitivity analysis for
'JPH1' on 'Pulmonary fibrosis'

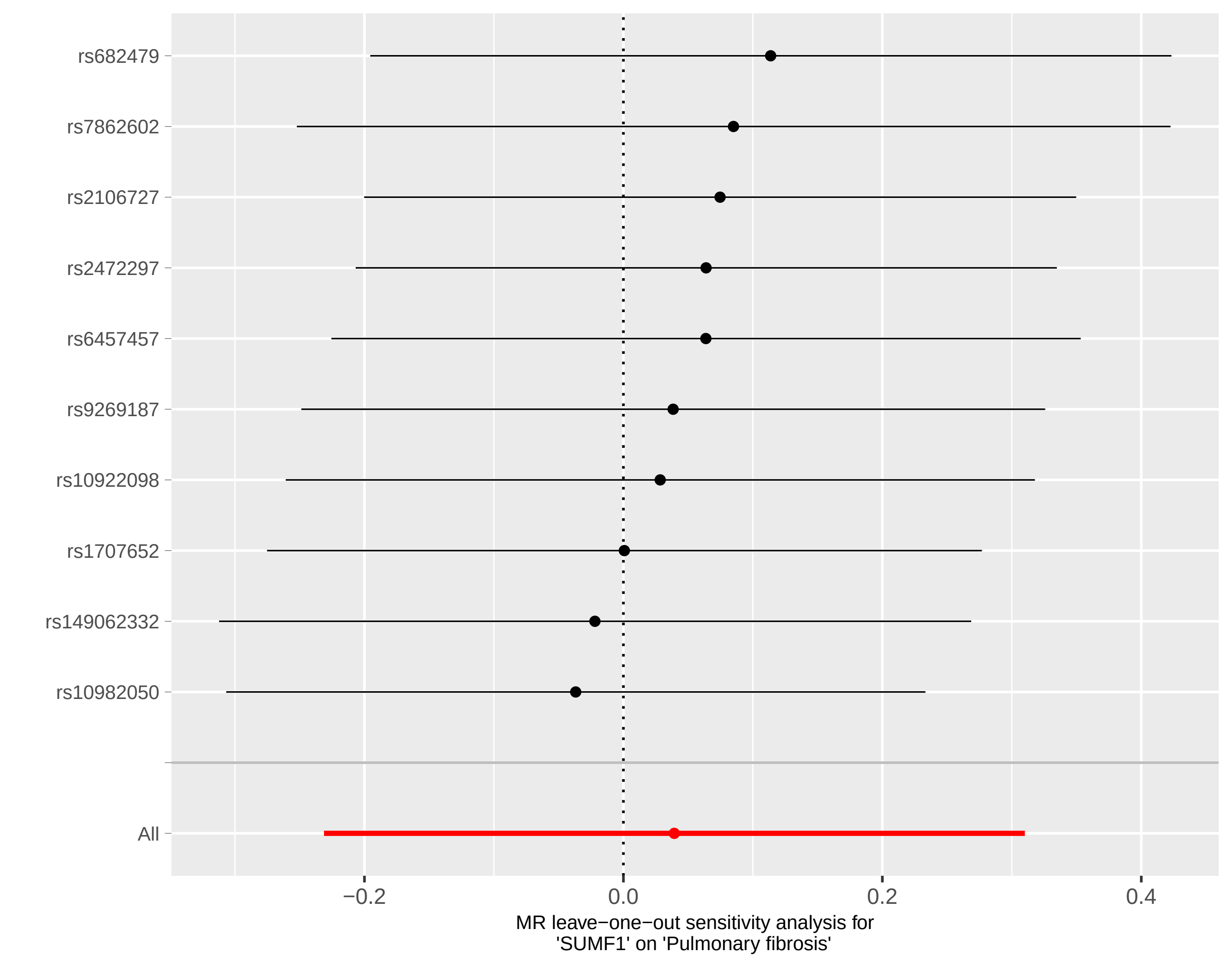
rs682479
rs7862602
rs2106727
rs2472297
rs6457457
rs9269187
rs10922098
rs1707652
rs149062332
rs10982050
All
−0.2
0.0
0.2
0.4
MR leave−one−out sensitivity analysis for
'SUMF1' on 'Pulmonary fibrosis'

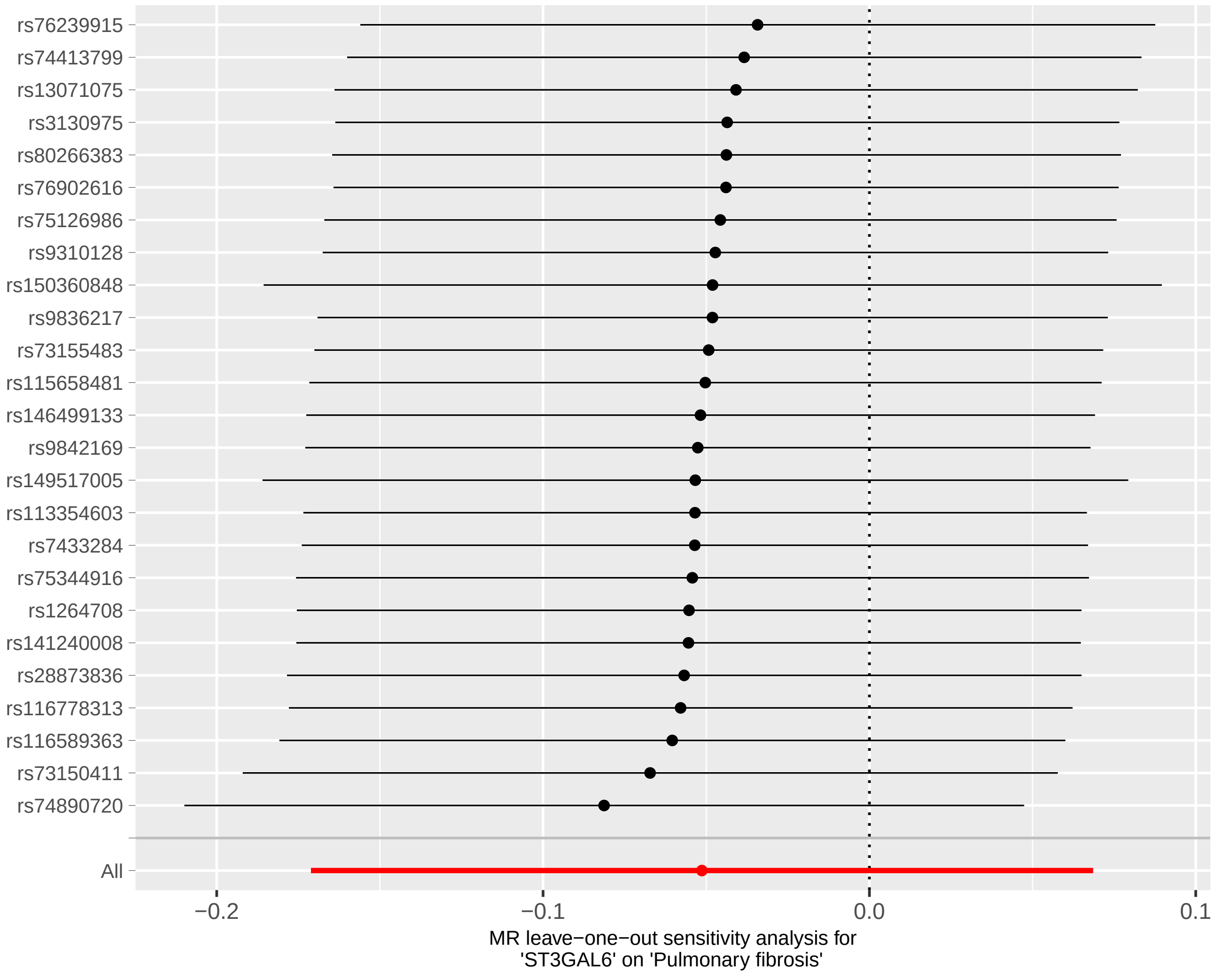
rs76239915
rs74413799
rs13071075
rs3130975
rs80266383
rs76902616
rs75126986
rs9310128
rs150360848
rs9836217
rs73155483
rs115658481
rs146499133
rs9842169
rs149517005
rs113354603
rs7433284
rs75344916
rs1264708
rs141240008
rs28873836
rs116778313
rs116589363
rs73150411
rs74890720
All
−0.2
−0.1
0.0
0.1
MR leave−one−out sensitivity analysis for
'ST3GAL6' on 'Pulmonary fibrosis'

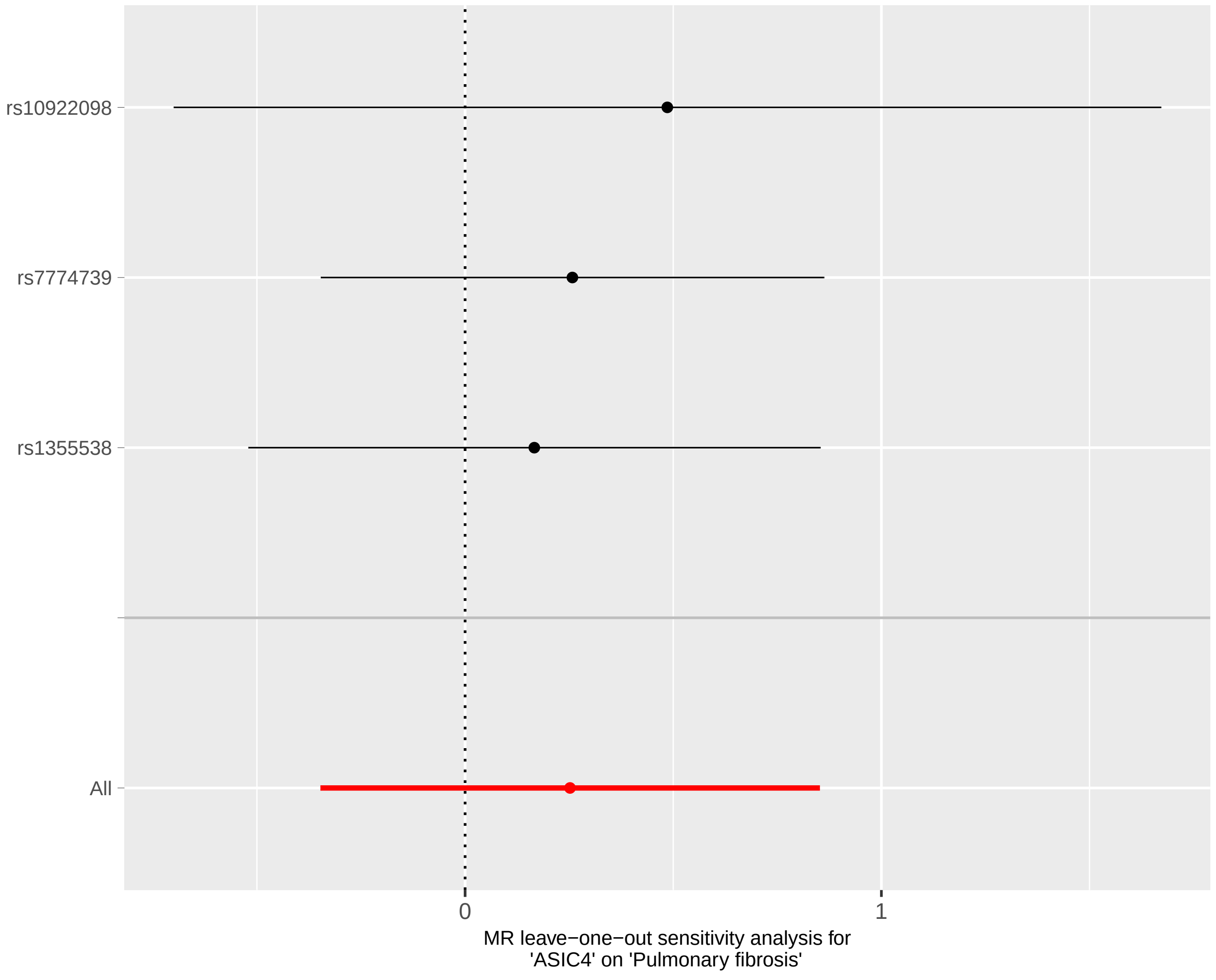
rs10922098
rs7774739
rs1355538
All
0
1
MR leave−one−out sensitivity analysis for
'ASIC4' on 'Pulmonary fibrosis'

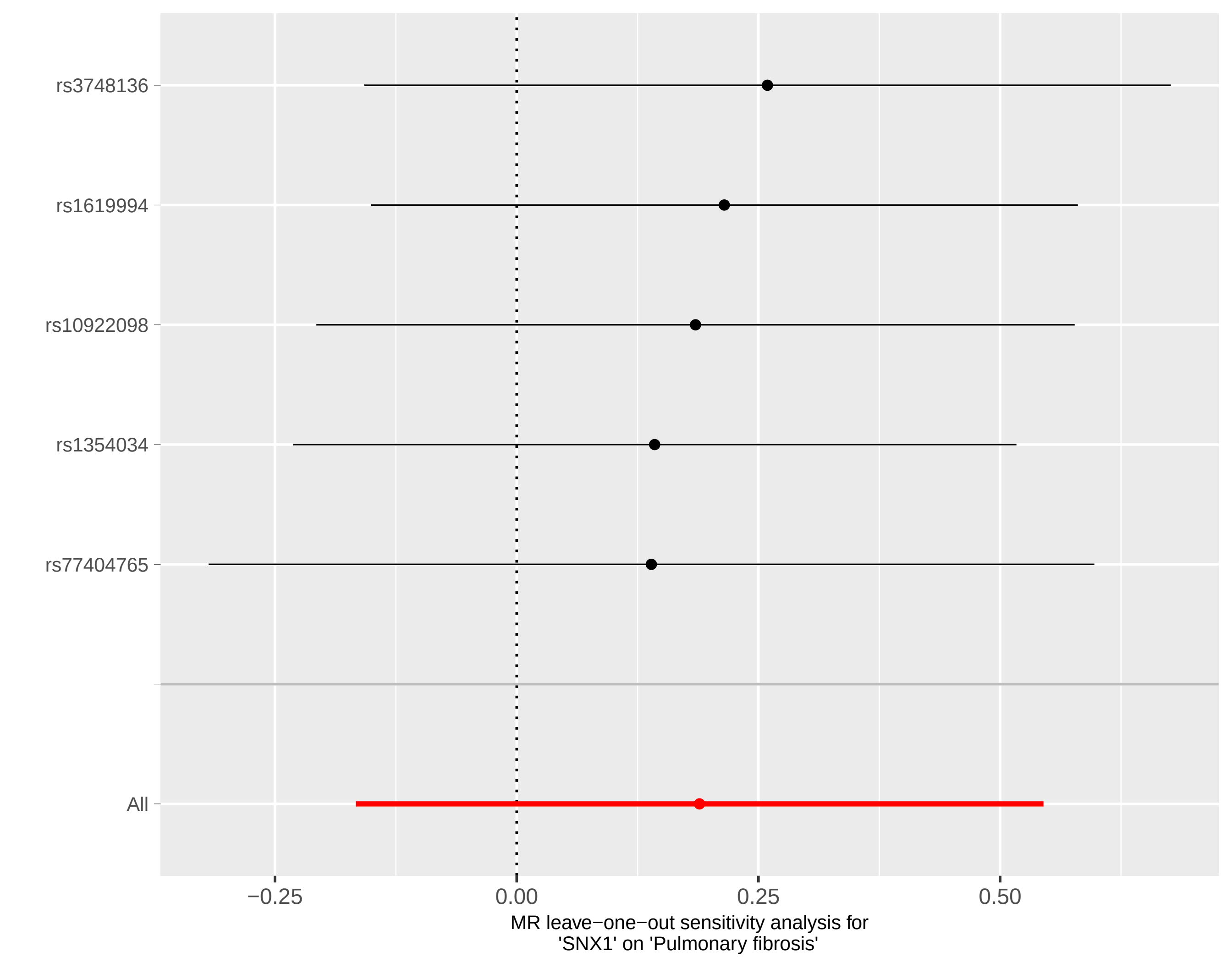
rs3748136
rs1619994
rs10922098
rs1354034
rs77404765
All
−0.25
0.00
0.25
0.50
MR leave−one−out sensitivity analysis for
'SNX1' on 'Pulmonary fibrosis'

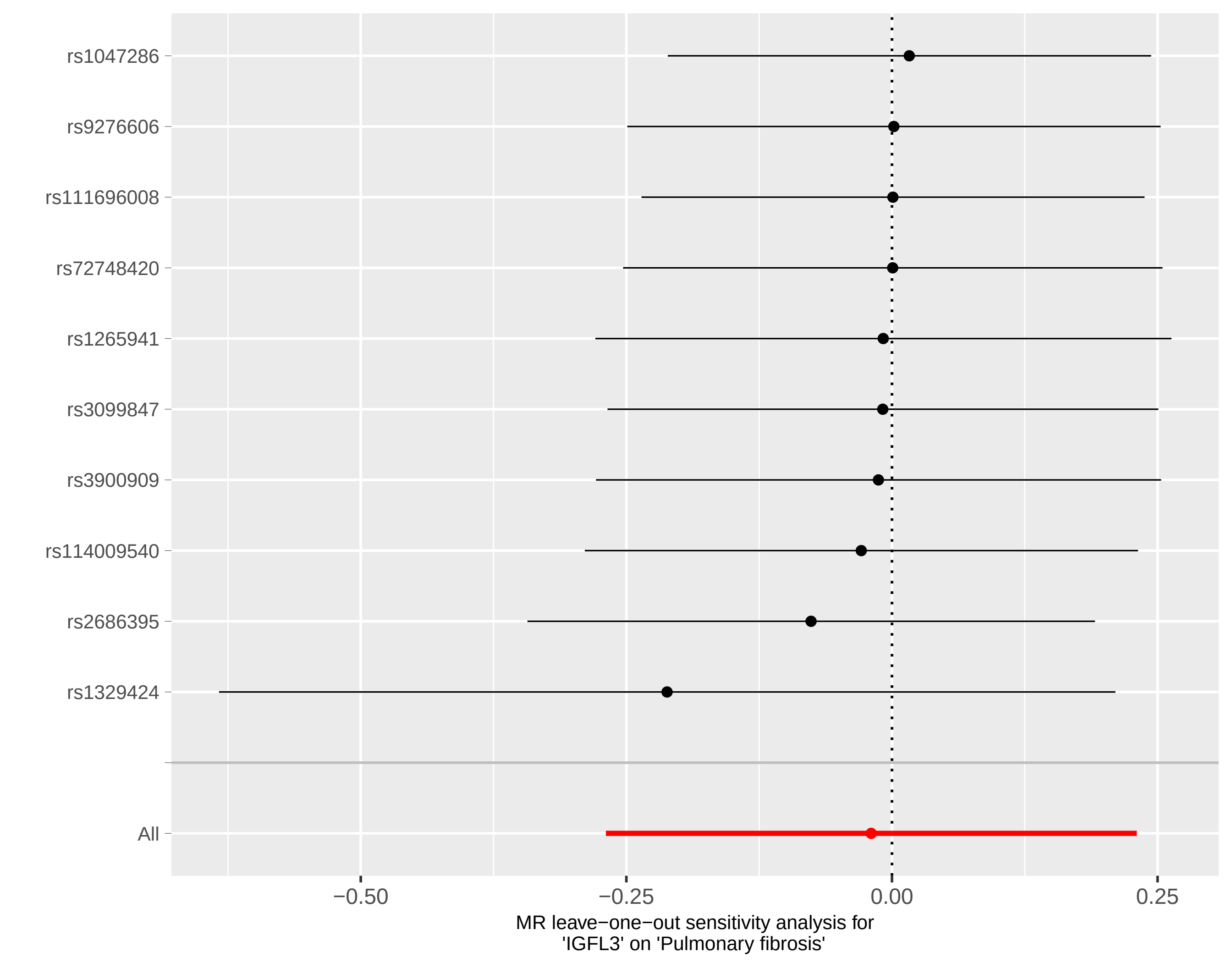

rs1047286
rs9276606
rs111696008
rs72748420
rs1265941
rs3099847
rs3900909
rs114009540
rs2686395
rs1329424
All
−0.50
−0.25
0.00
0.25
MR leave−one−out sensitivity analysis for
'IGFL3' on 'Pulmonary fibrosis'

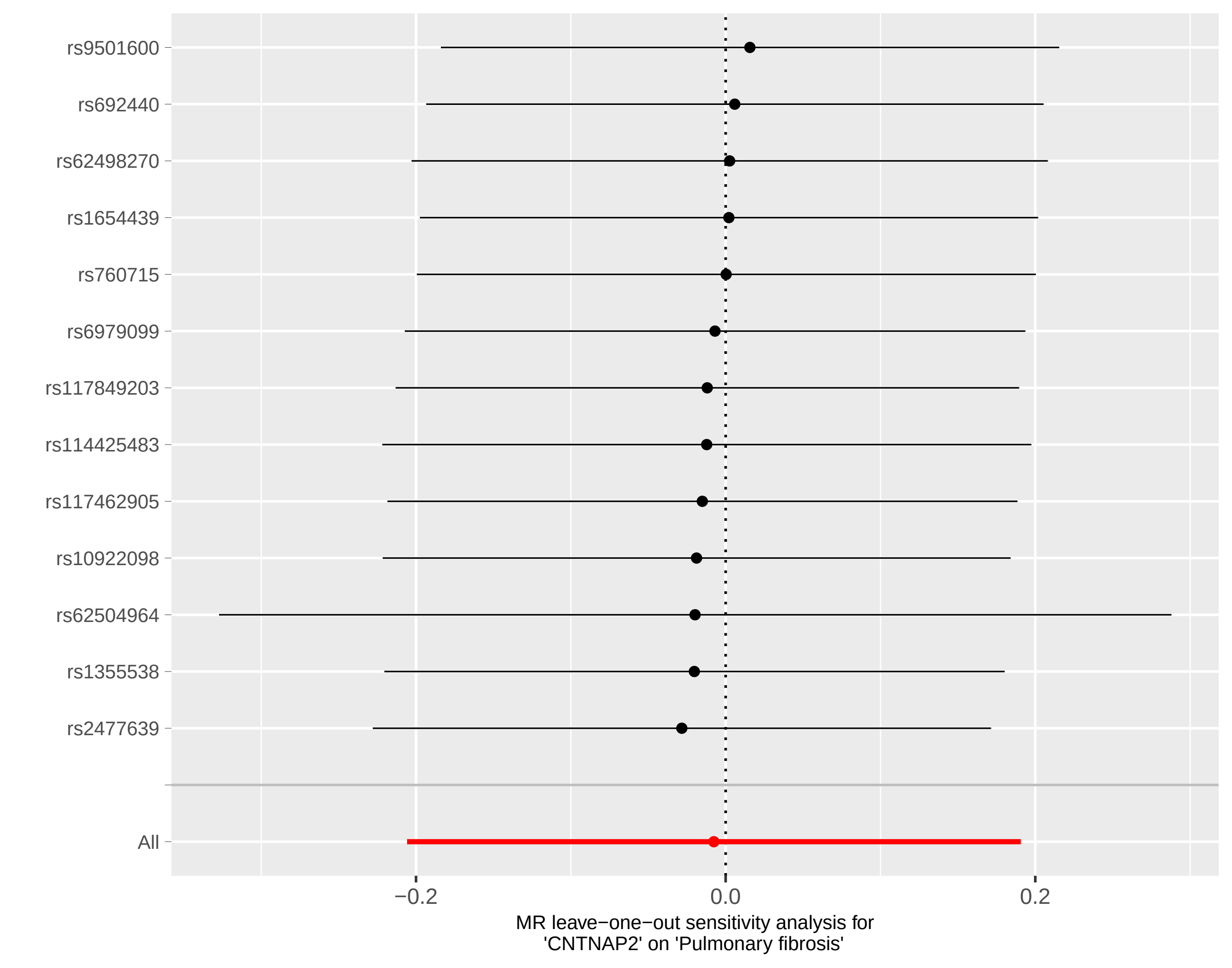
rs9501600
rs692440
rs62498270
rs1654439
rs760715
rs6979099
rs117849203
rs114425483
rs117462905
rs10922098
rs62504964
rs1355538
rs2477639
All
−0.2
0.0
0.2
MR leave−one−out sensitivity analysis for
'CNTNAP2' on 'Pulmonary fibrosis'

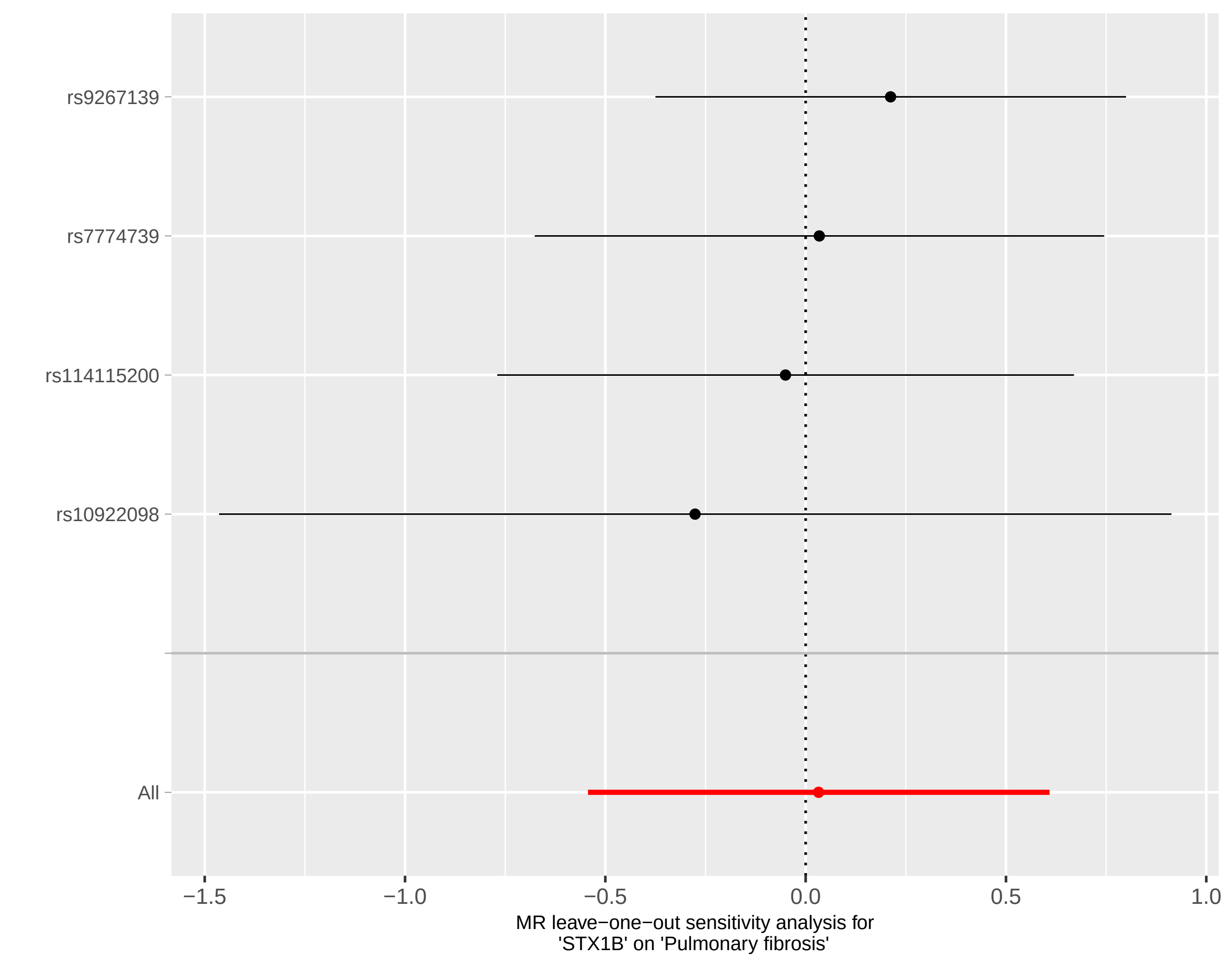
rs9267139
rs7774739
rs114115200
rs10922098
All
−1.5
−1.0
−0.5
0.0
0.5
1.0
MR leave−one−out sensitivity analysis for
'STX1B' on 'Pulmonary fibrosis'

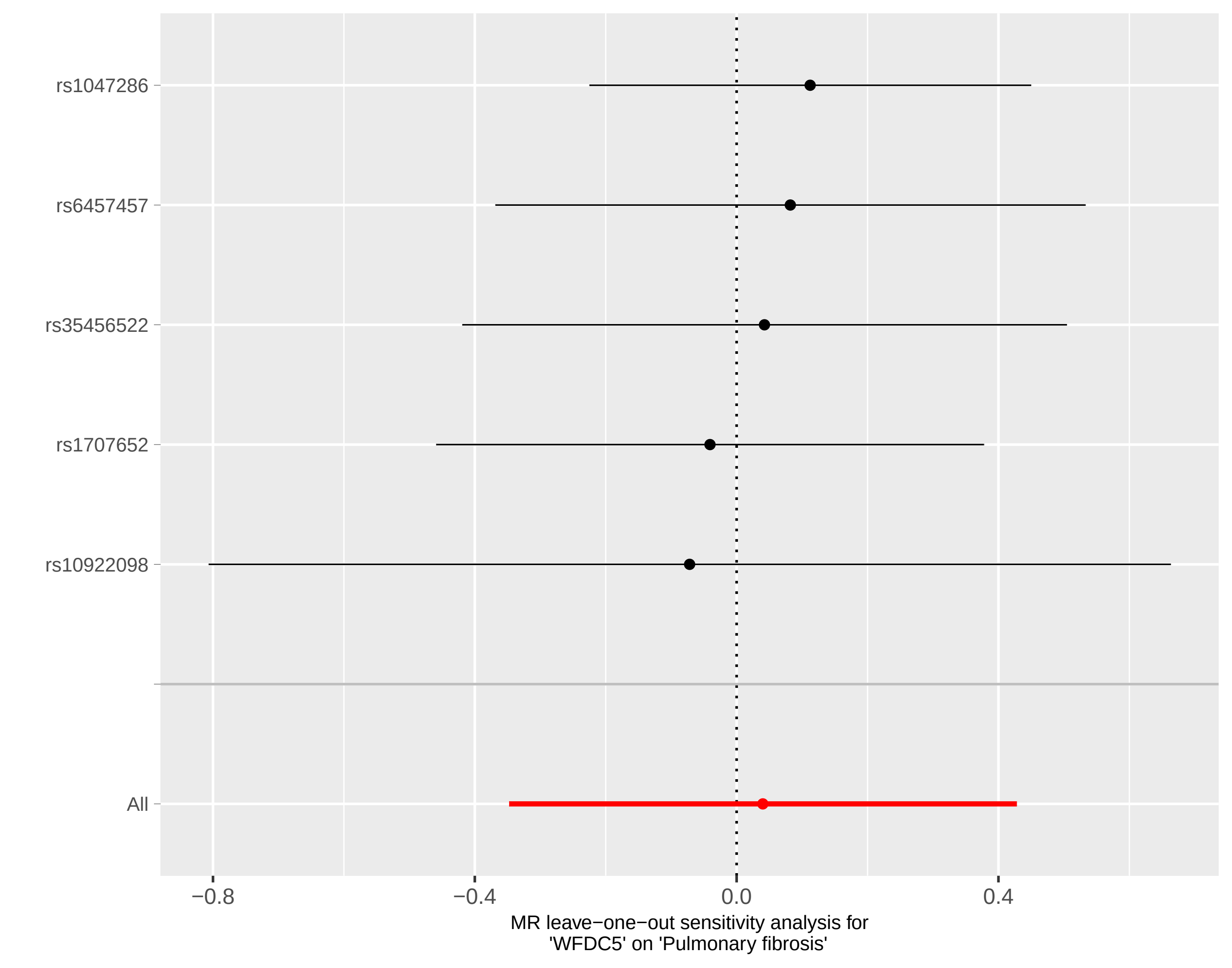

rs1047286
rs6457457
rs35456522
rs1707652
rs10922098
All
−0.8
−0.4
0.0
0.4
MR leave−one−out sensitivity analysis for
'WFDC5' on 'Pulmonary fibrosis'

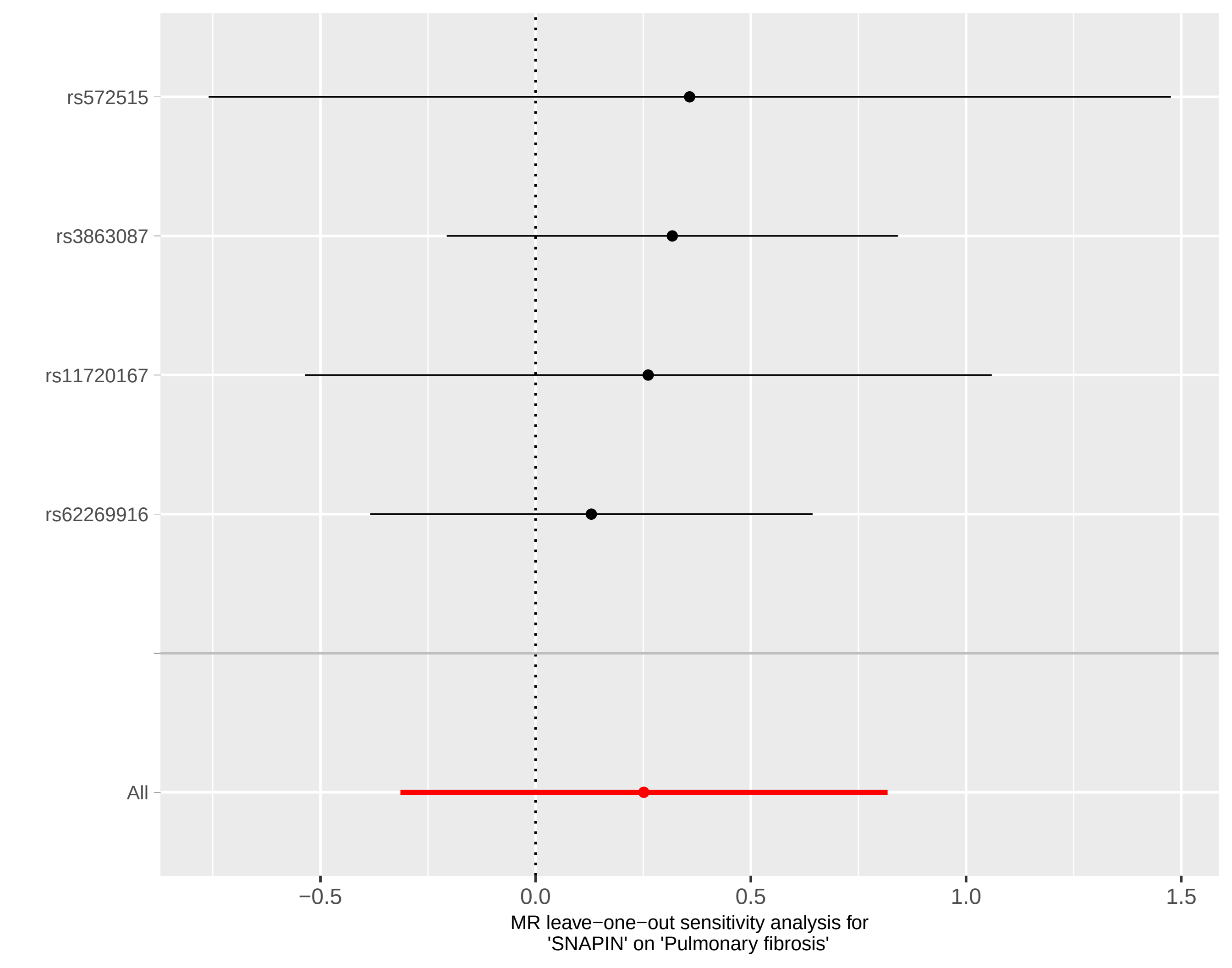
rs572515
rs3863087
rs11720167
rs62269916
All
−0.5
0.0
0.5
1.0
1.5
MR leave−one−out sensitivity analysis for
'SNAPIN' on 'Pulmonary fibrosis'

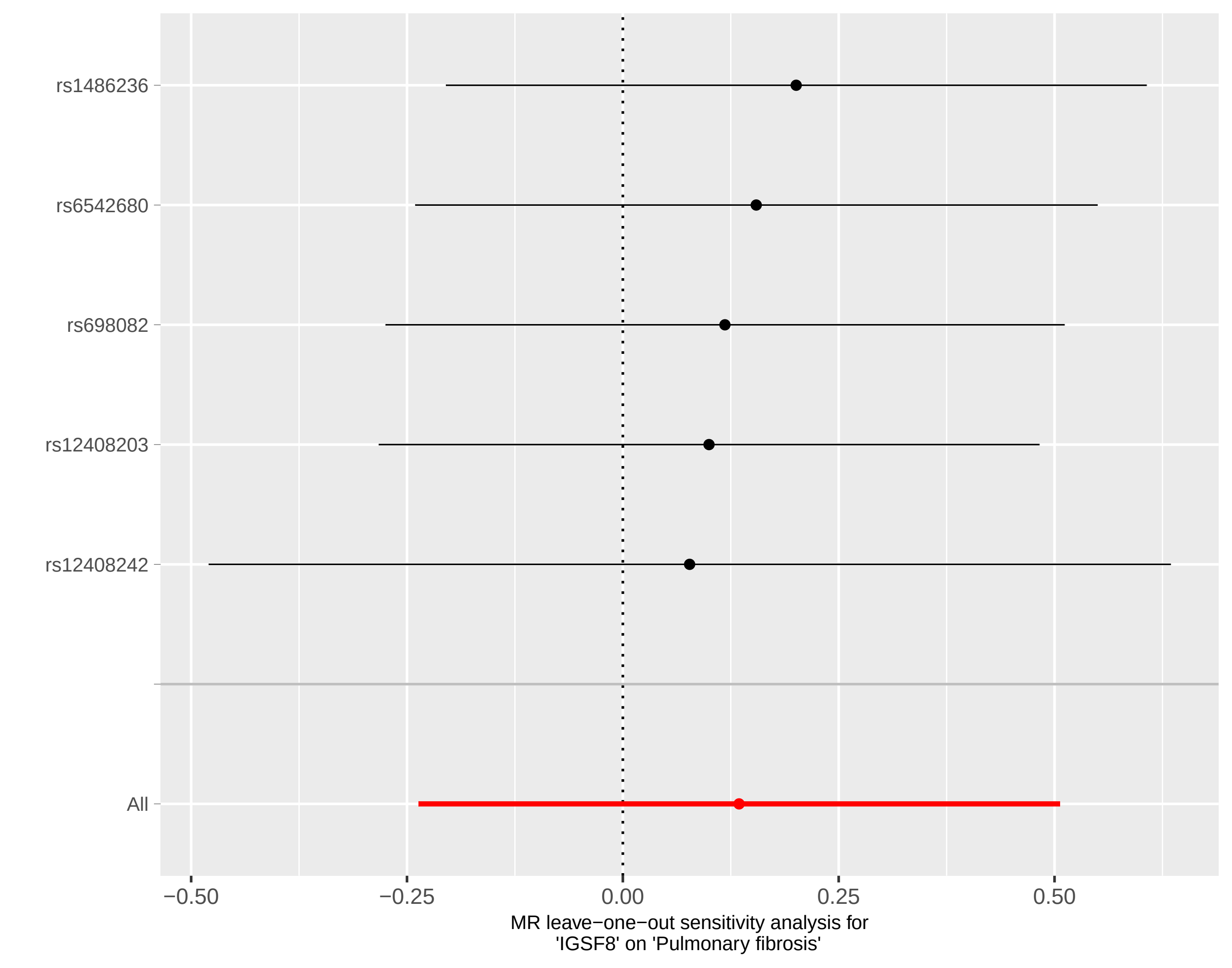
rs1486236
rs6542680
rs698082
rs12408203
rs12408242
All
−0.50
−0.25
0.00
0.25
0.50
MR leave−one−out sensitivity analysis for
'IGSF8' on 'Pulmonary fibrosis'

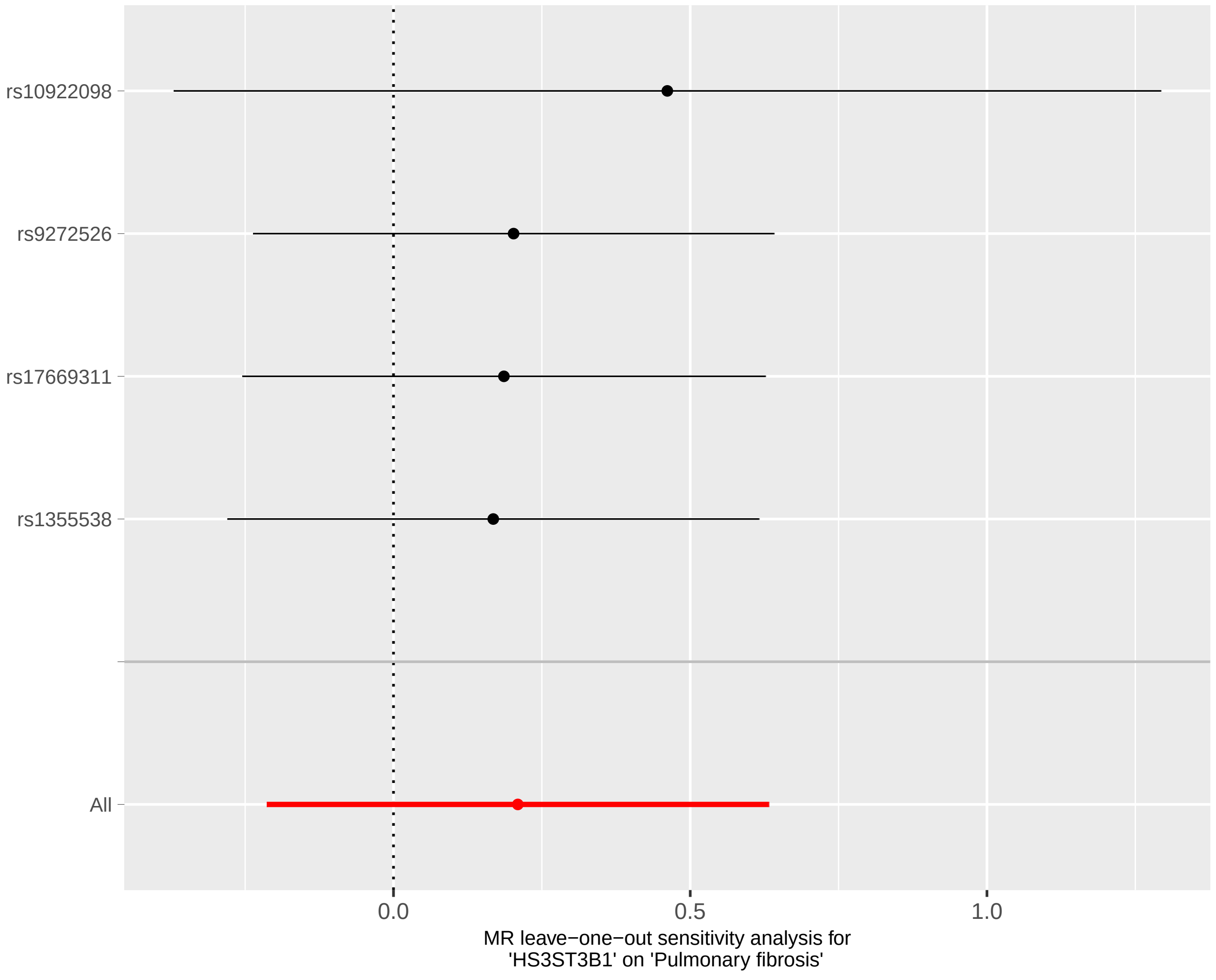
rs10922098
rs9272526
rs17669311
rs1355538
All
0.0
0.5
1.0
MR leave−one−out sensitivity analysis for
'HS3ST3B1' on 'Pulmonary fibrosis'

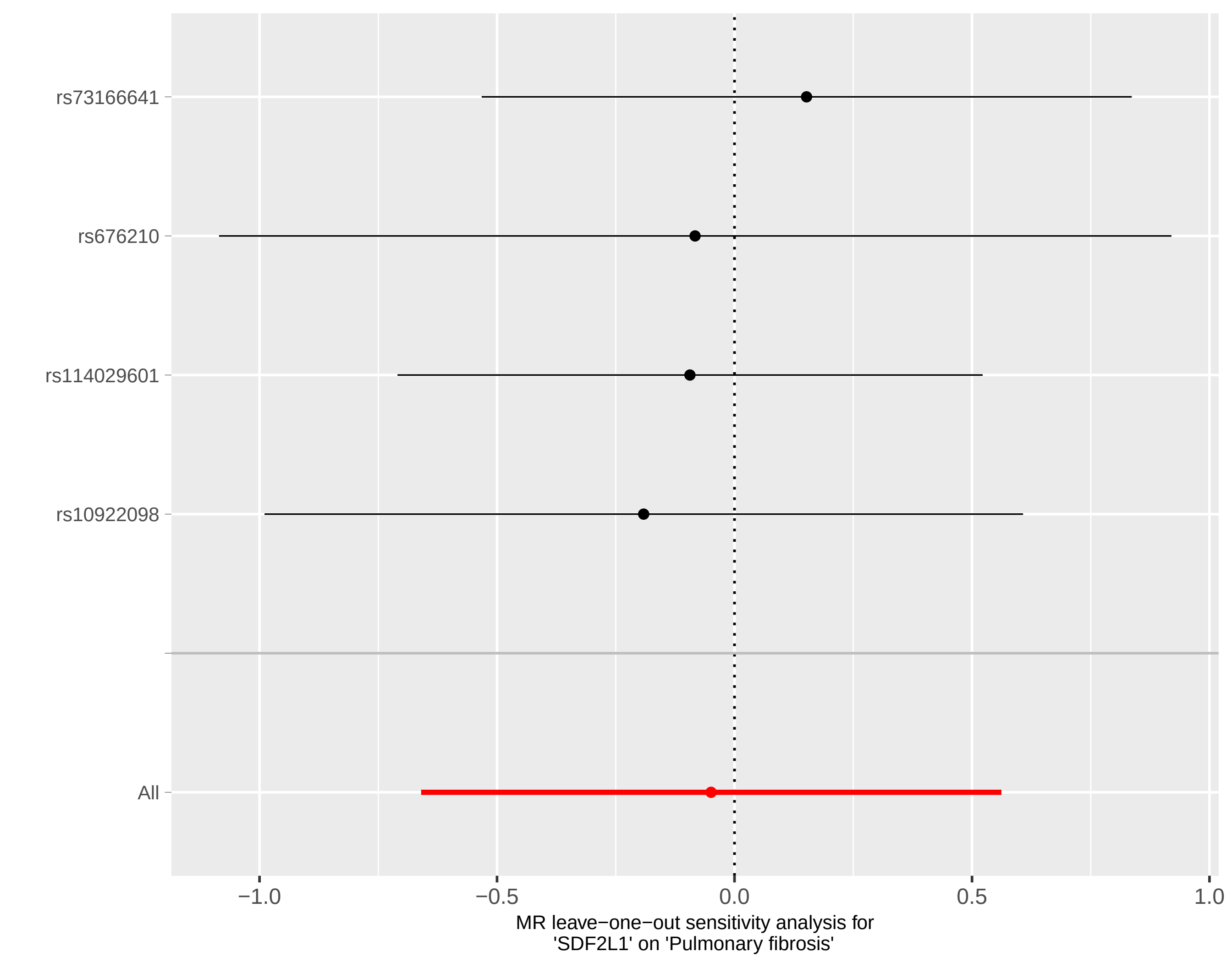
rs73166641
rs676210
rs114029601
rs10922098
All
−1.0
−0.5
0.0
0.5
1.0
MR leave−one−out sensitivity analysis for
'SDF2L1' on 'Pulmonary fibrosis'

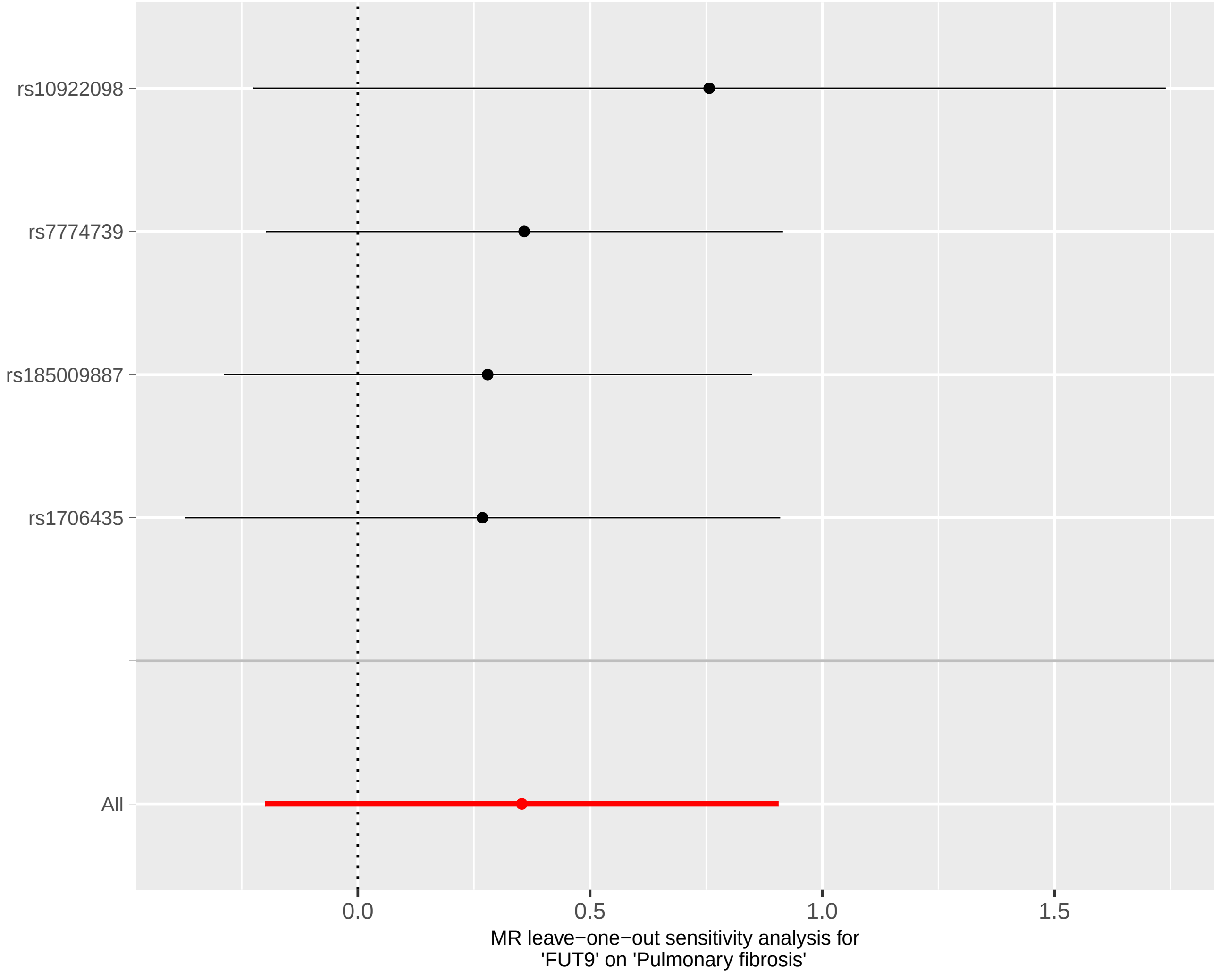
rs10922098
rs7774739
rs185009887
rs1706435
All
0.0
0.5
1.0
1.5
MR leave−one−out sensitivity analysis for
'FUT9' on 'Pulmonary fibrosis'

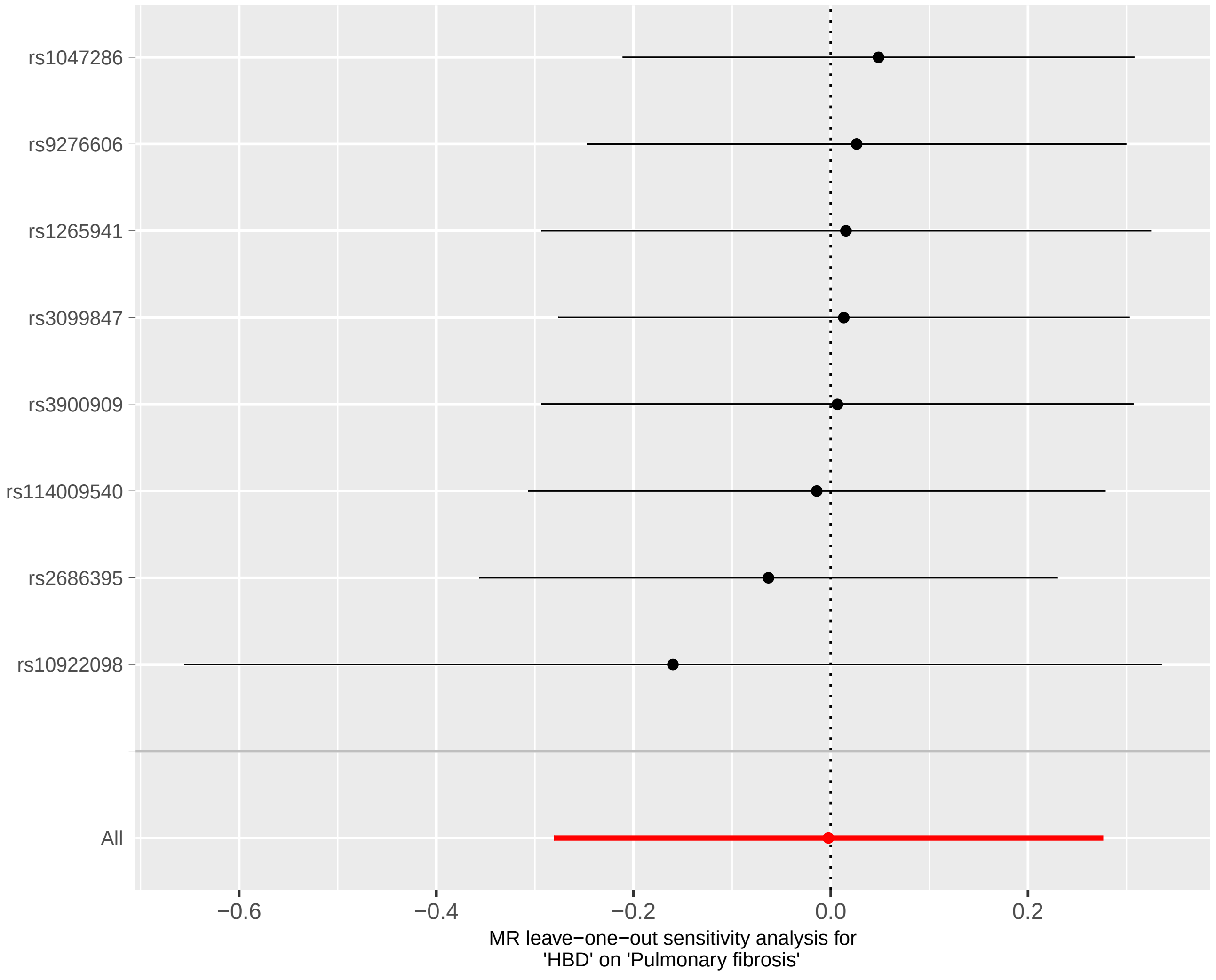

rs1047286
rs9276606
rs1265941
rs3099847
rs3900909
rs114009540
rs2686395
rs10922098
All
−0.6
−0.4
−0.2
0.0
0.2
MR leave−one−out sensitivity analysis for
'HBD' on 'Pulmonary fibrosis'

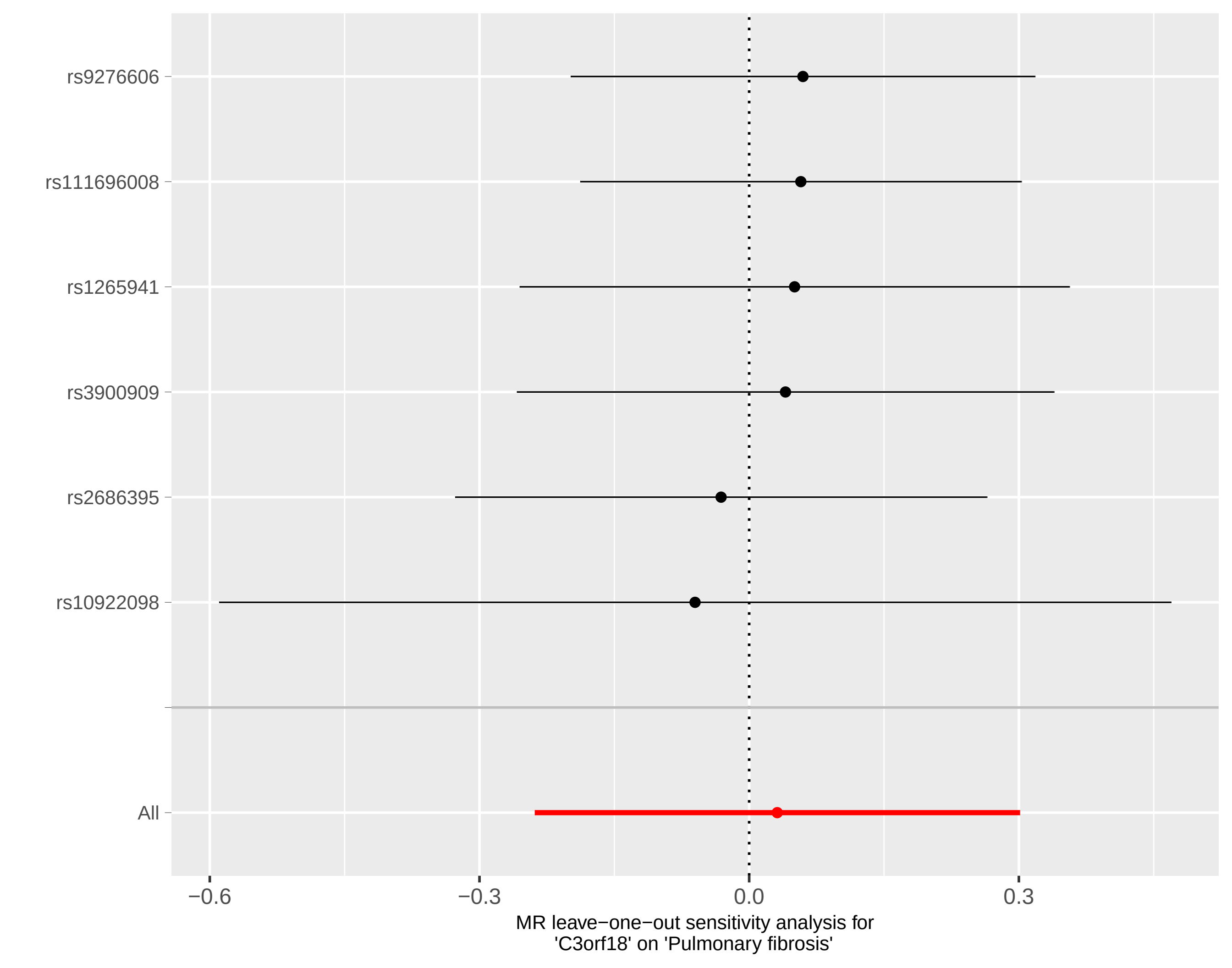
rs9276606
rs111696008
rs1265941
rs3900909
rs2686395
rs10922098
All
−0.6
−0.3
0.0
0.3
MR leave−one−out sensitivity analysis for
'C3orf18' on 'Pulmonary fibrosis'

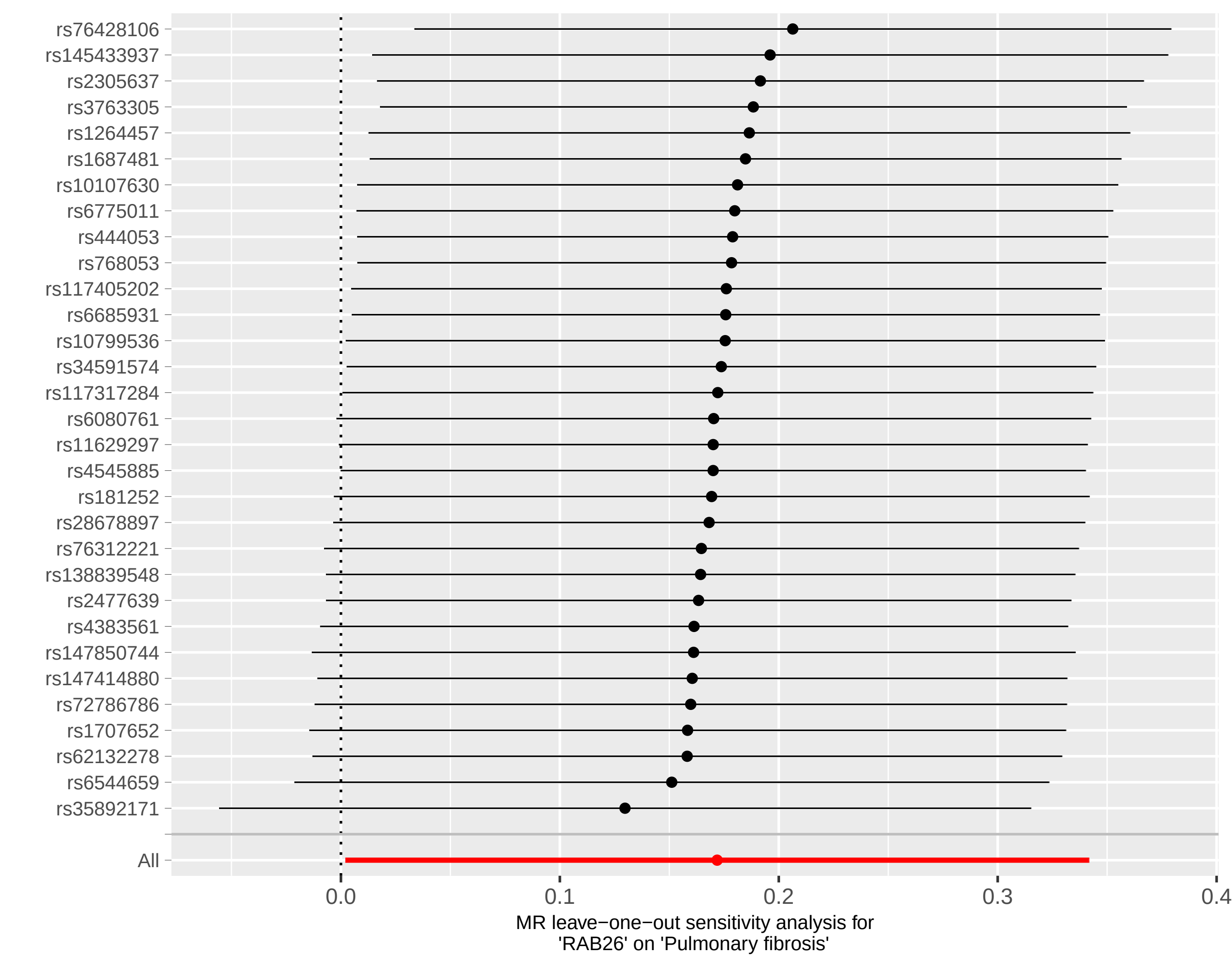
rs76428106
rs145433937
rs2305637
rs3763305
rs1264457
rs1687481
rs10107630
rs6775011
rs444053
rs768053
rs117405202
rs6685931
rs10799536
rs34591574
rs117317284
rs6080761
rs11629297
rs4545885
rs181252
rs28678897
rs76312221
rs138839548
rs2477639
rs4383561
rs147850744
rs147414880
rs72786786
rs1707652
rs62132278
rs6544659
rs35892171
All
0.0
0.1
0.2
0.3
0.4
MR leave−one−out sensitivity analysis for
'RAB26' on 'Pulmonary fibrosis'

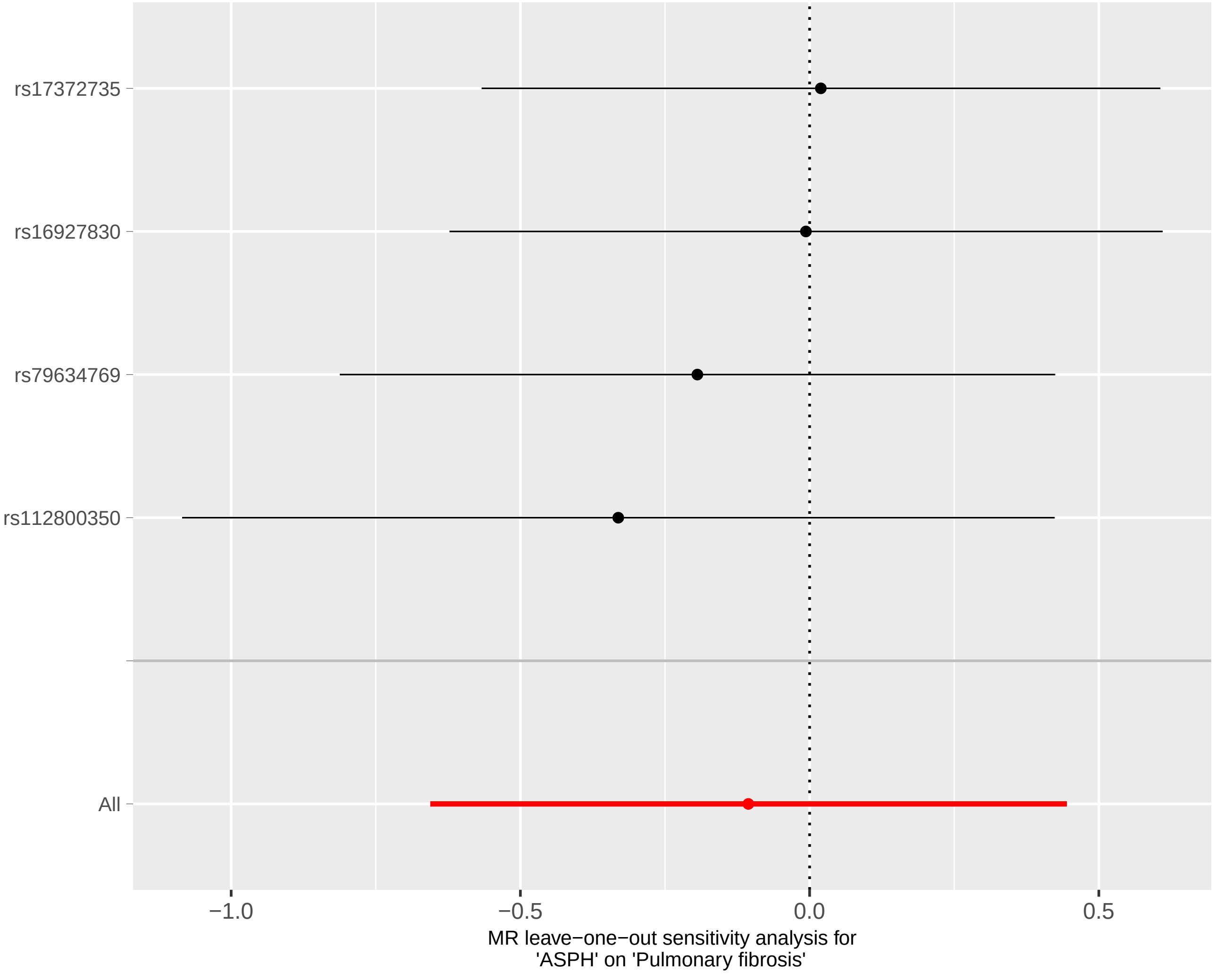

rs17372735
rs16927830
rs79634769
rs112800350
All
−1.0
−0.5
0.0
0.5
MR leave−one−out sensitivity analysis for
'ASPH' on 'Pulmonary fibrosis'

Insufficient number of SNPs

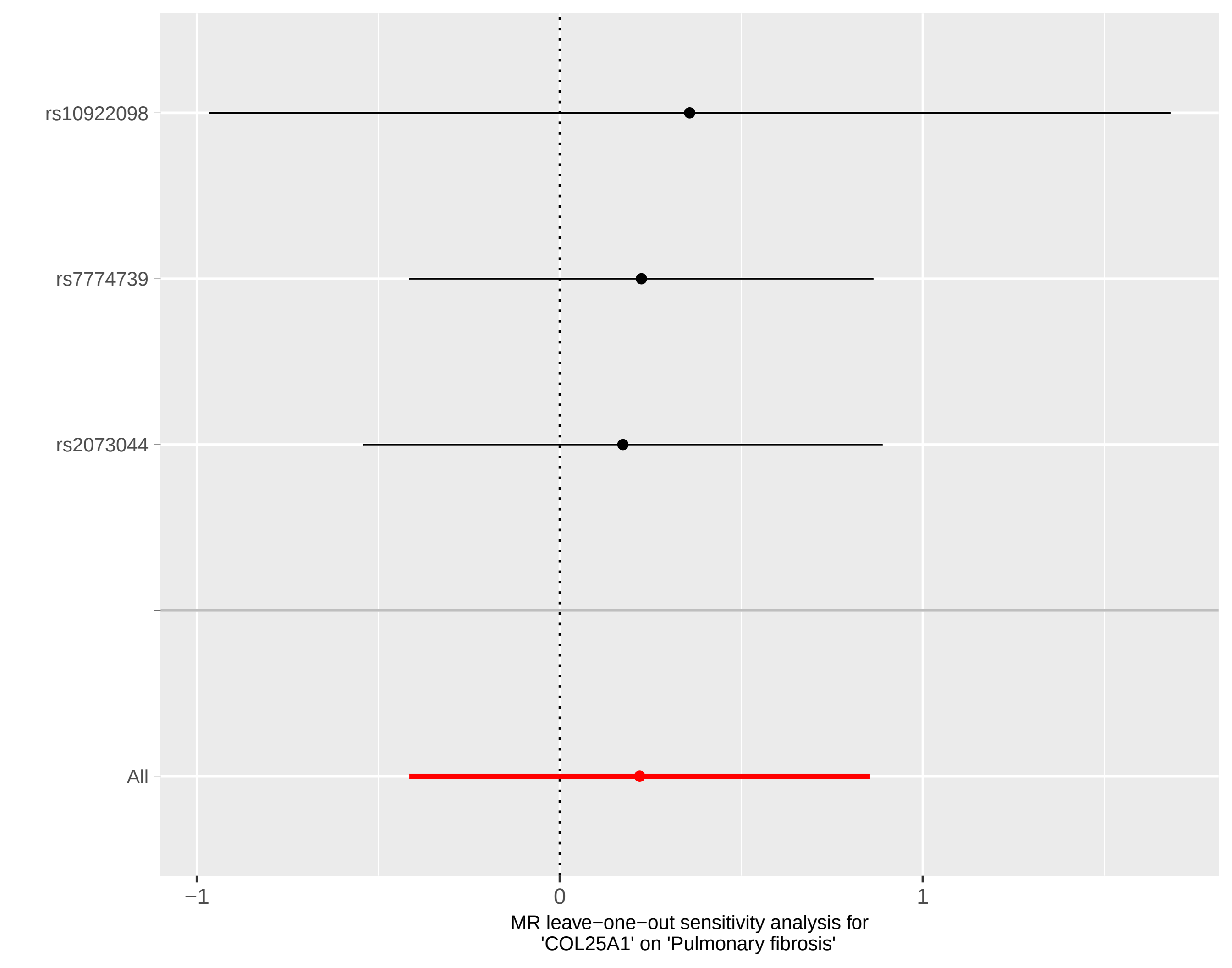
rs10922098
rs7774739
rs2073044
All
−1
0
1
MR leave−one−out sensitivity analysis for
'COL25A1' on 'Pulmonary fibrosis'

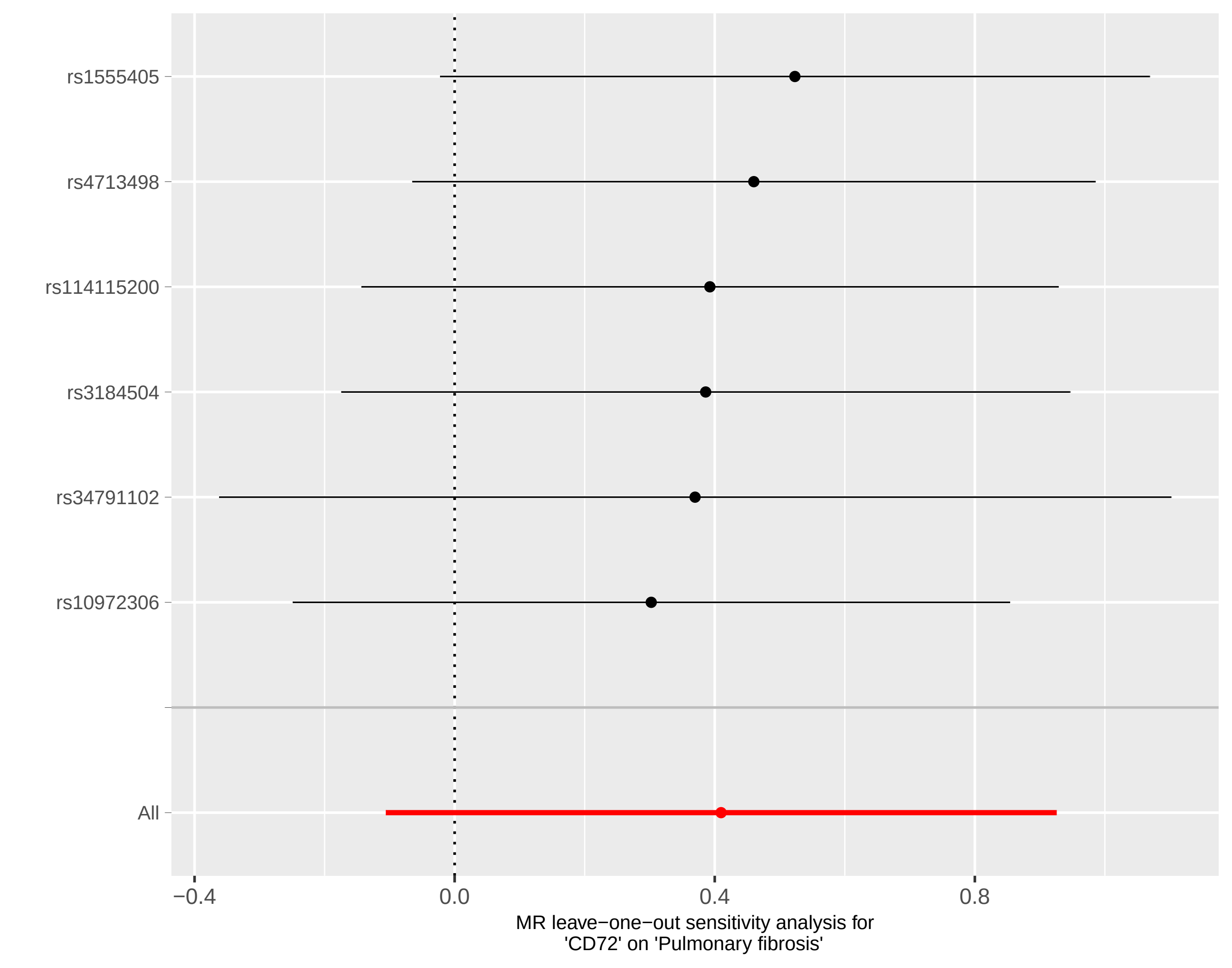
rs1555405
rs4713498
rs114115200
rs3184504
rs34791102
rs10972306
All
−0.4
0.0
0.4
0.8
MR leave−one−out sensitivity analysis for
'CD72' on 'Pulmonary fibrosis'

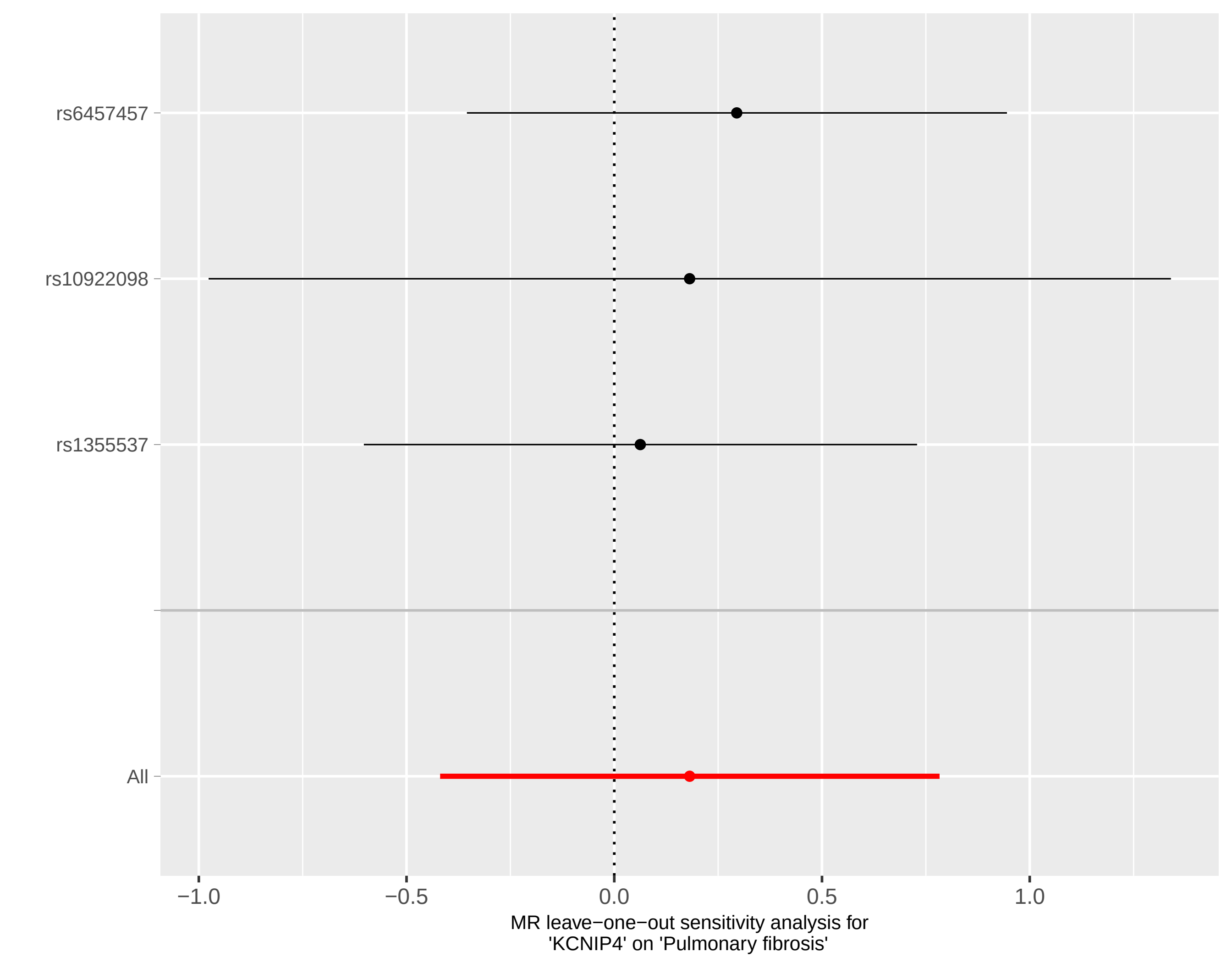
rs6457457
rs10922098
rs1355537
All
−1.0
−0.5
0.0
0.5
1.0
MR leave−one−out sensitivity analysis for
'KCNIP4' on 'Pulmonary fibrosis'

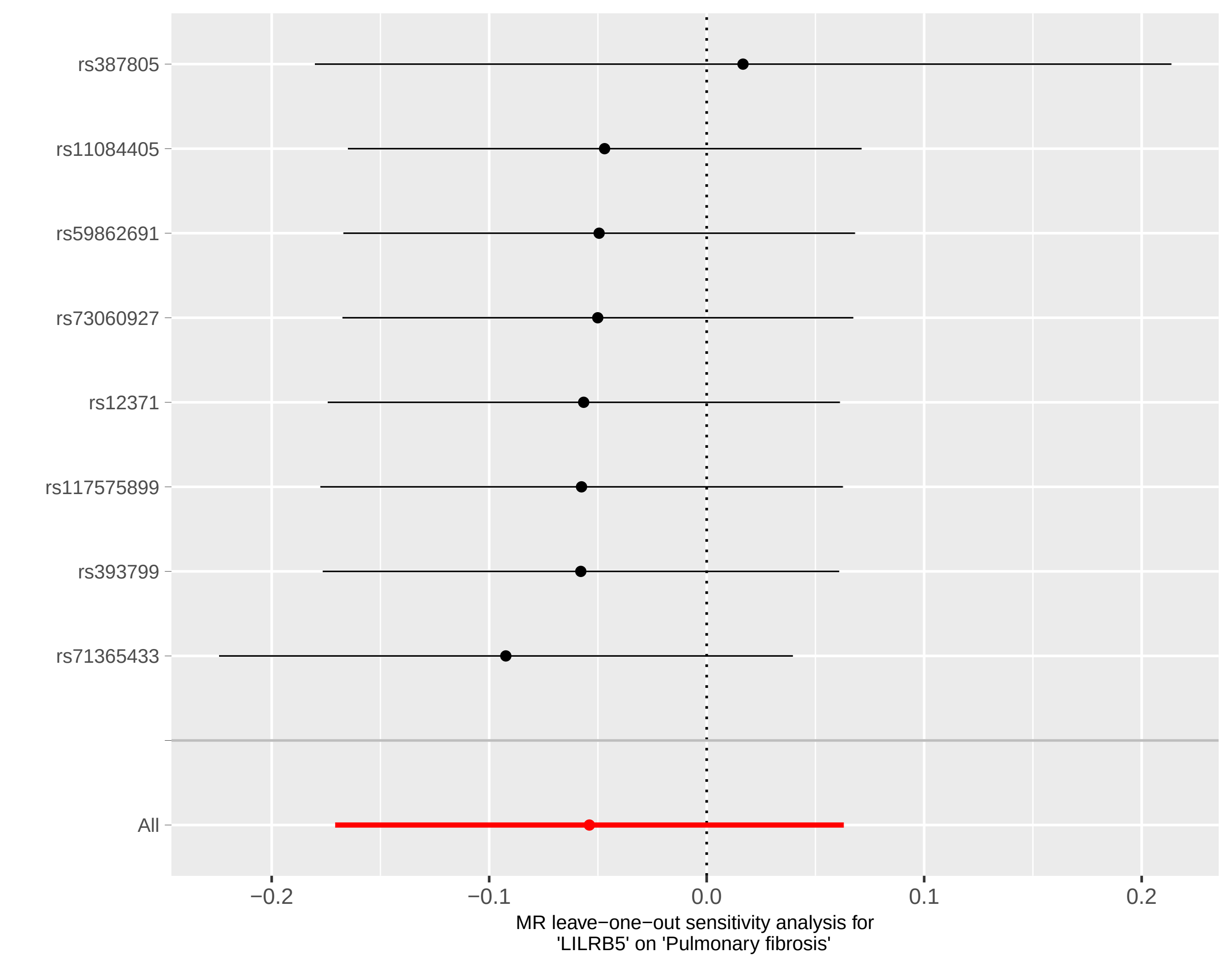
rs387805
rs11084405
rs59862691
rs73060927
rs12371
rs117575899
rs393799
rs71365433
All
−0.2
−0.1
0.0
0.1
0.2
MR leave−one−out sensitivity analysis for
'LILRB5' on 'Pulmonary fibrosis'

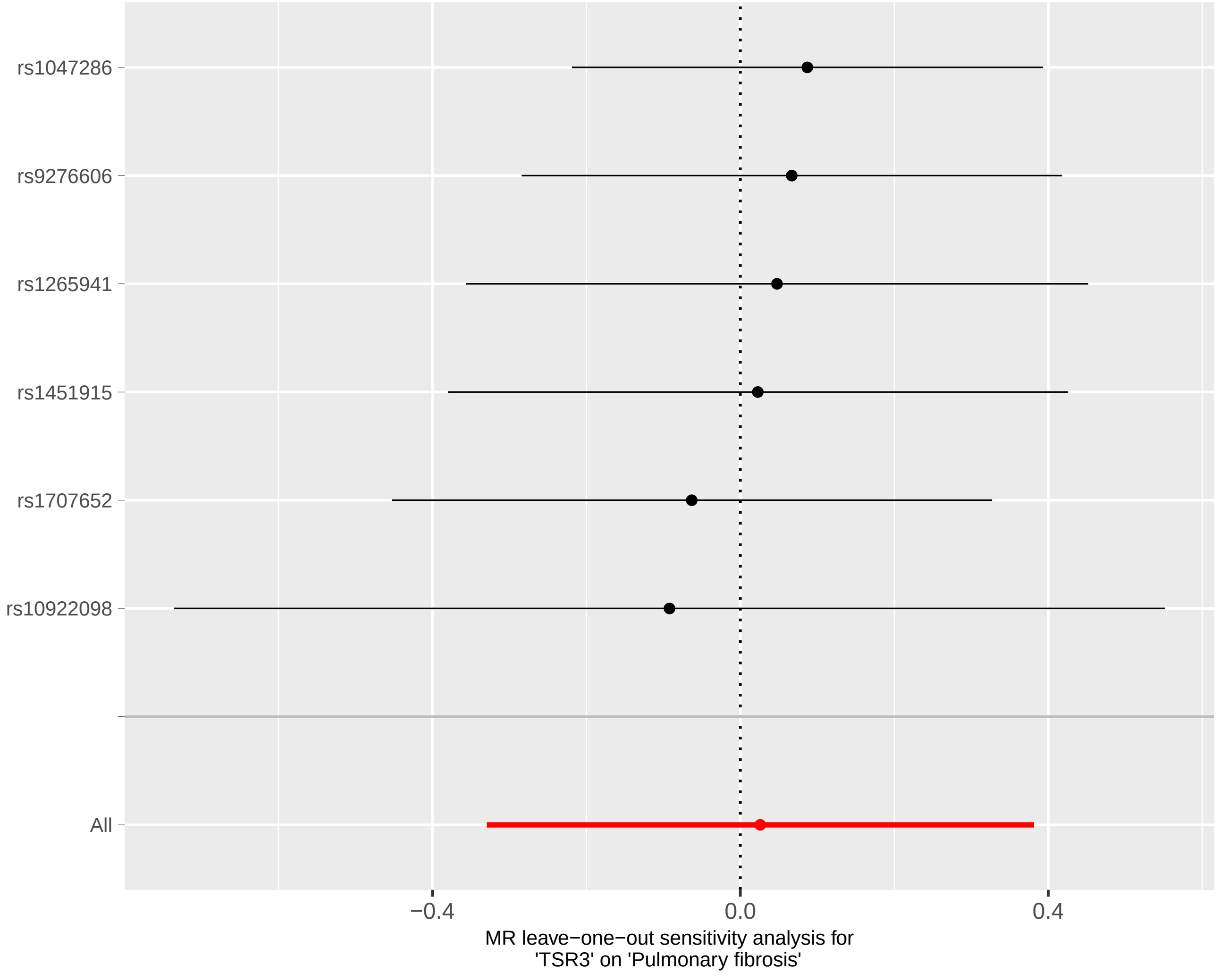
rs1047286
rs9276606
rs1265941
rs1451915
rs1707652
rs10922098
All
−0.4
0.0
0.4
MR leave−one−out sensitivity analysis for
'TSR3' on 'Pulmonary fibrosis'

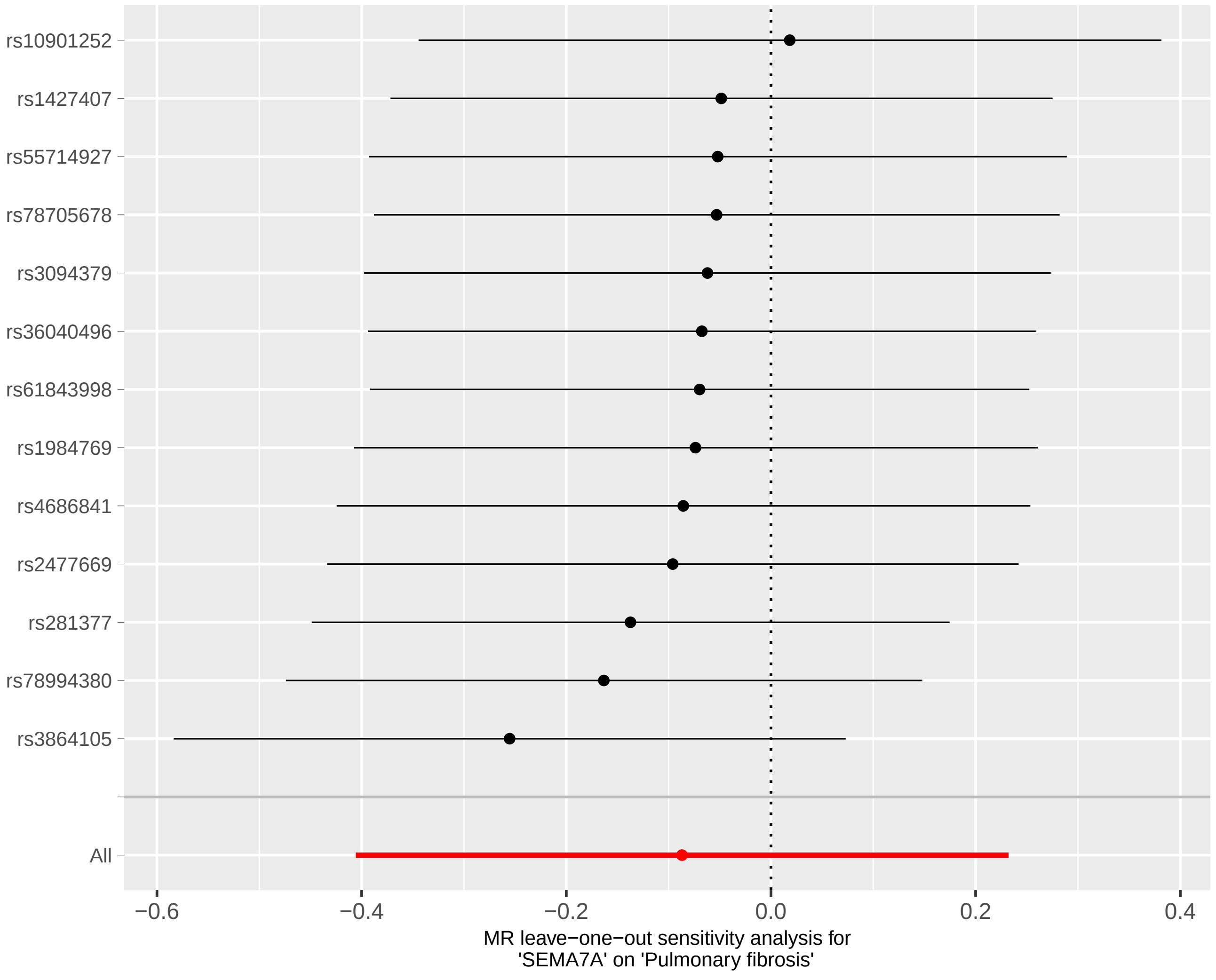
rs10901252
rs1427407
rs55714927
rs78705678
rs3094379
rs36040496
rs61843998
rs1984769
rs4686841
rs2477669
rs281377
rs78994380
rs3864105
All
−0.6
−0.4
−0.2
0.0
0.2
0.4
MR leave−one−out sensitivity analysis for
'SEMA7A' on 'Pulmonary fibrosis'

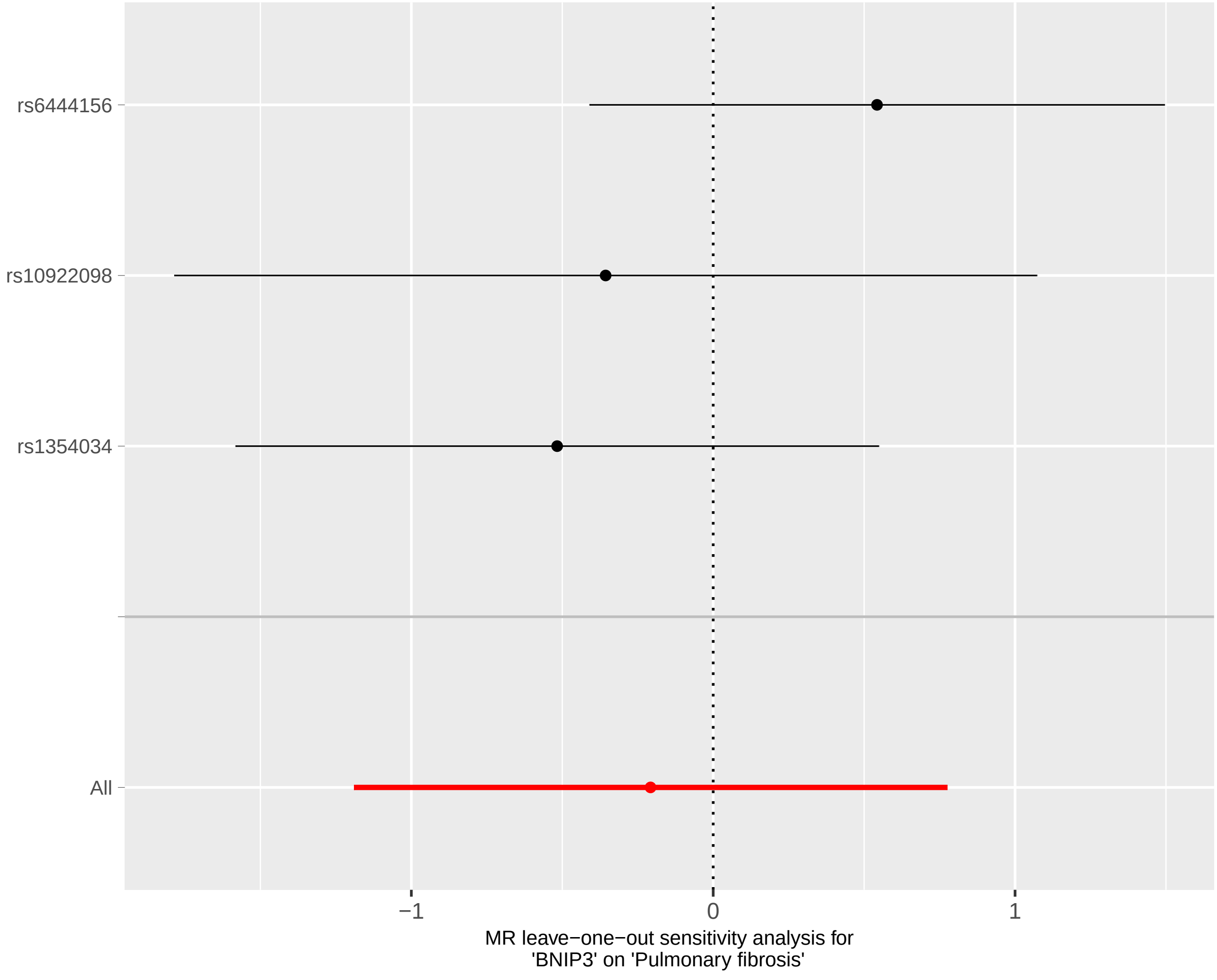
rs6444156
rs10922098
rs1354034
All
−1
0
1
MR leave−one−out sensitivity analysis for
'BNIP3' on 'Pulmonary fibrosis'

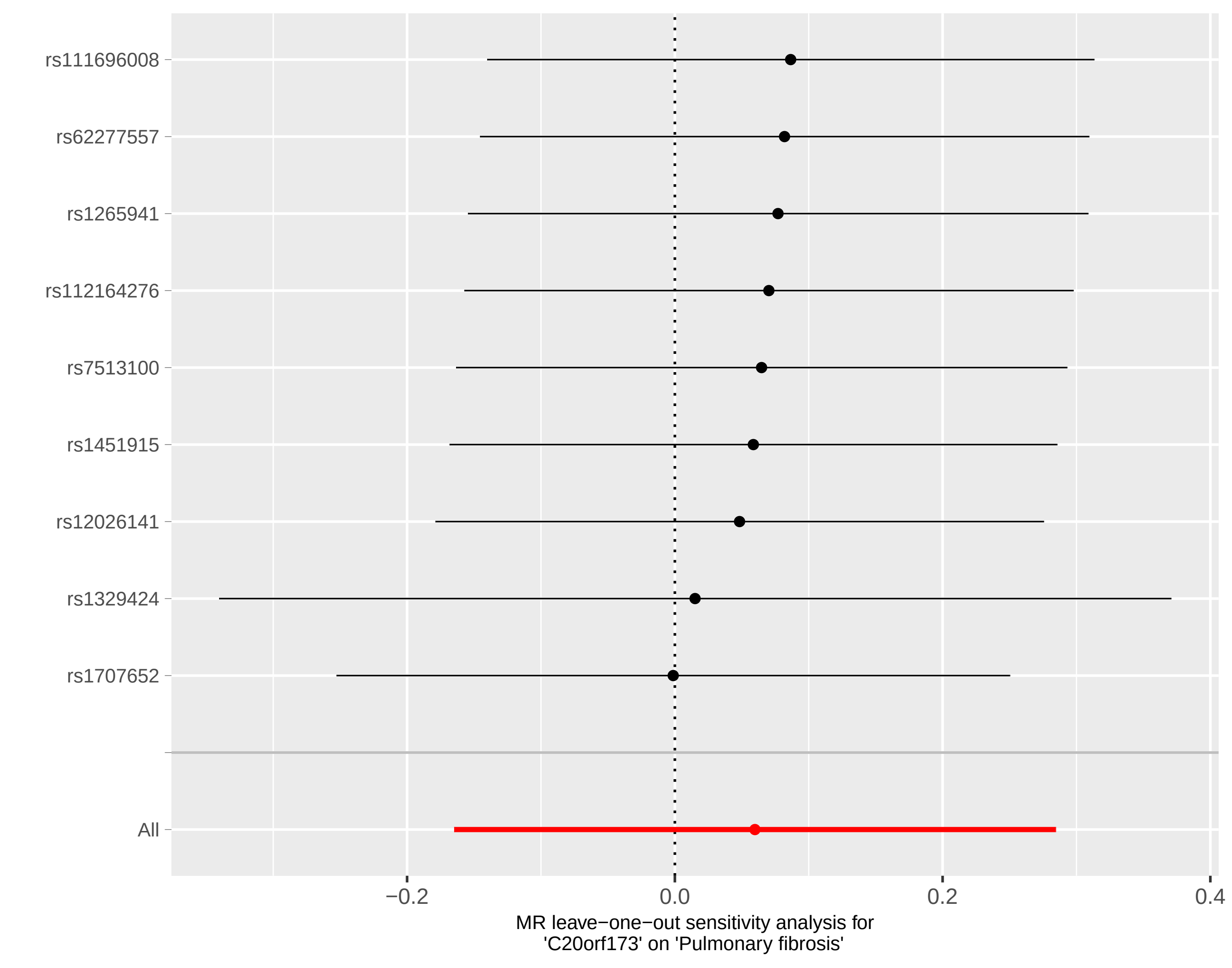
rs111696008
rs62277557
rs1265941
rs112164276
rs7513100
rs1451915
rs12026141
rs1329424
rs1707652
All
−0.2
0.0
0.2
0.4
MR leave−one−out sensitivity analysis for
'C20orf173' on 'Pulmonary fibrosis'

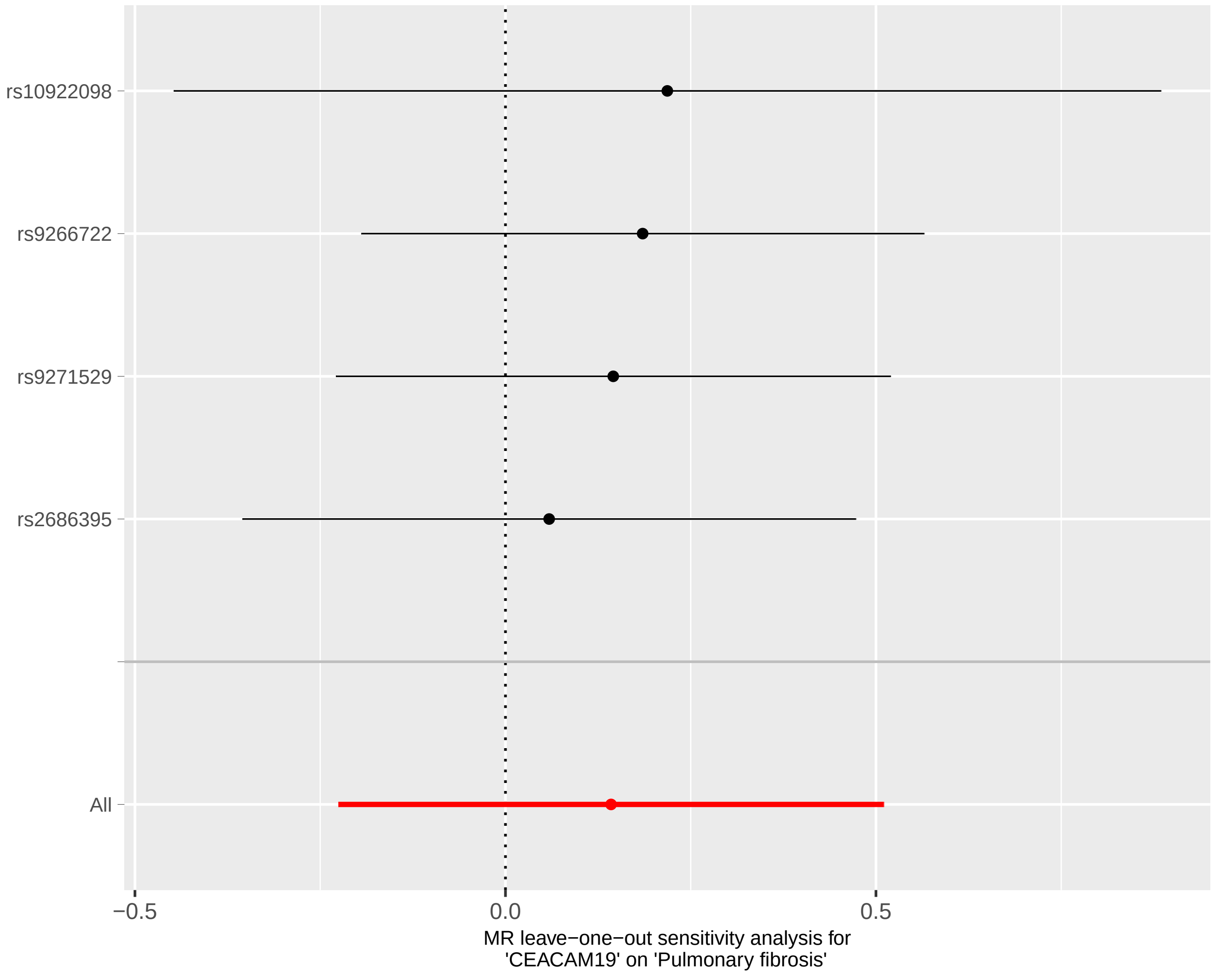
rs10922098
rs9266722
rs9271529
rs2686395
All
−0.5
0.0
0.5
MR leave−one−out sensitivity analysis for
'CEACAM19' on 'Pulmonary fibrosis'

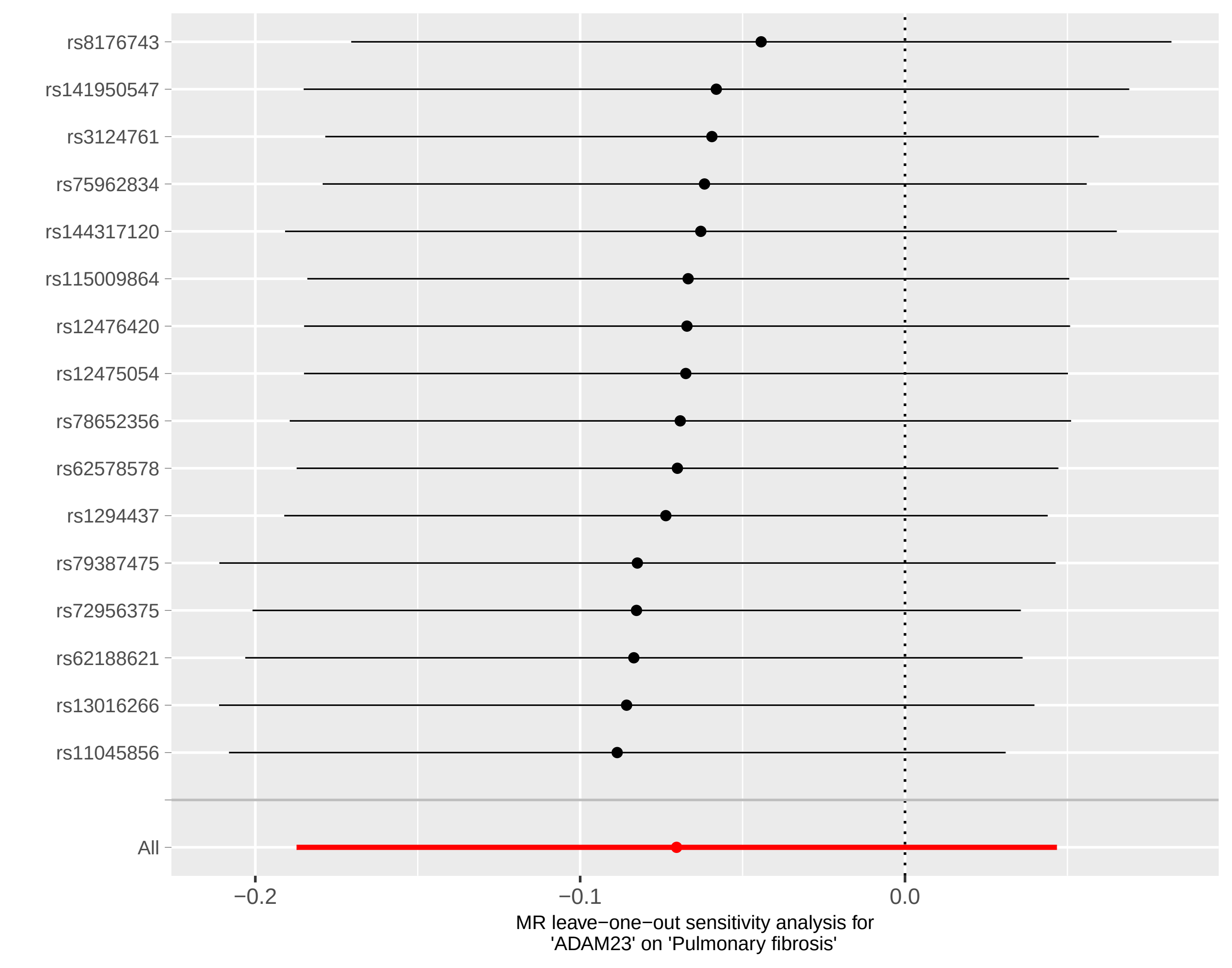
rs8176743
rs141950547
rs3124761
rs75962834
rs144317120
rs115009864
rs12476420
rs12475054
rs78652356
rs62578578
rs1294437
rs79387475
rs72956375
rs62188621
rs13016266
rs11045856
All
−0.2
−0.1
0.0
MR leave−one−out sensitivity analysis for
'ADAM23' on 'Pulmonary fibrosis'

Insufficient number of SNPs

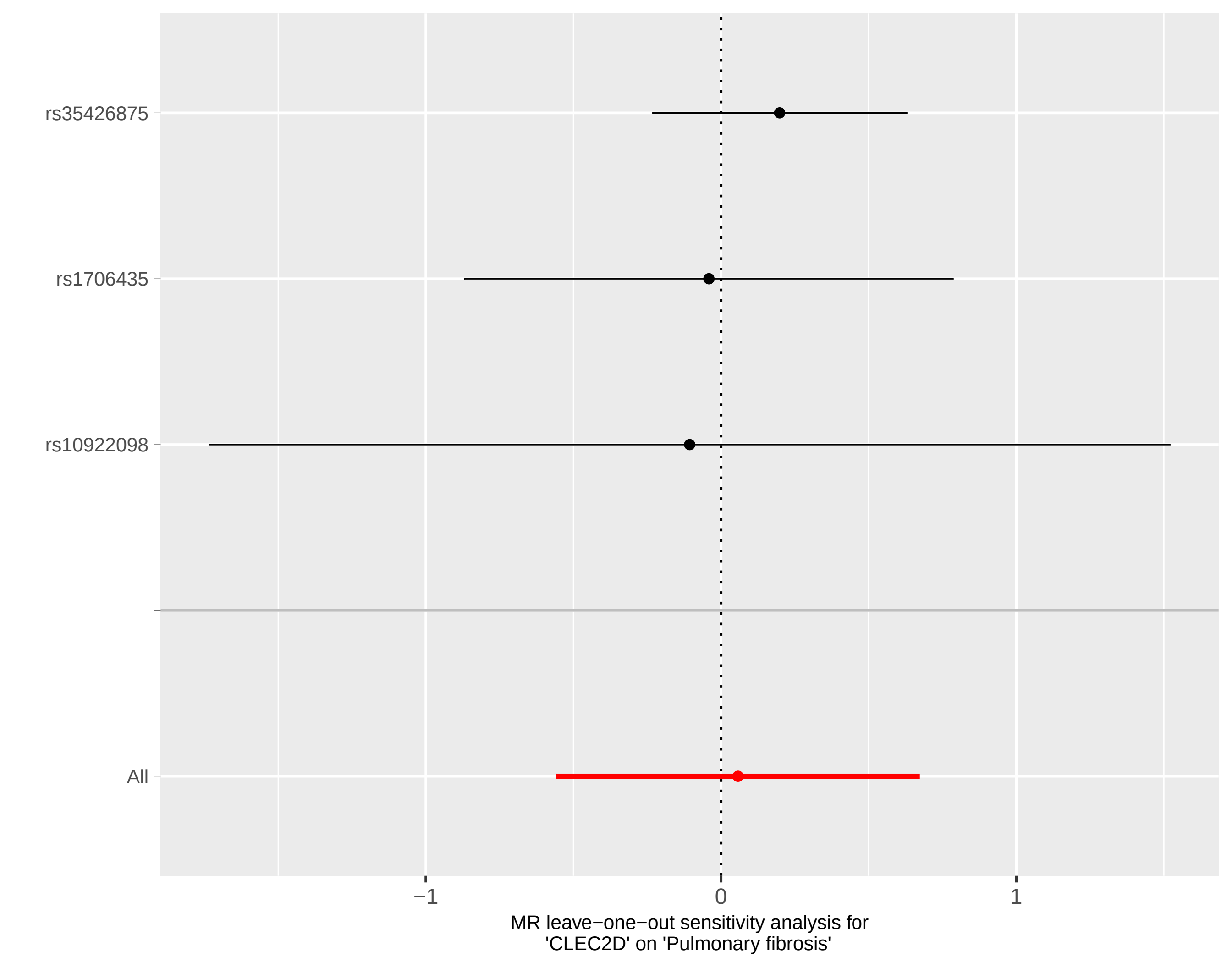
rs35426875
rs1706435
rs10922098
All
−1
0
1
MR leave−one−out sensitivity analysis for
'CLEC2D' on 'Pulmonary fibrosis'

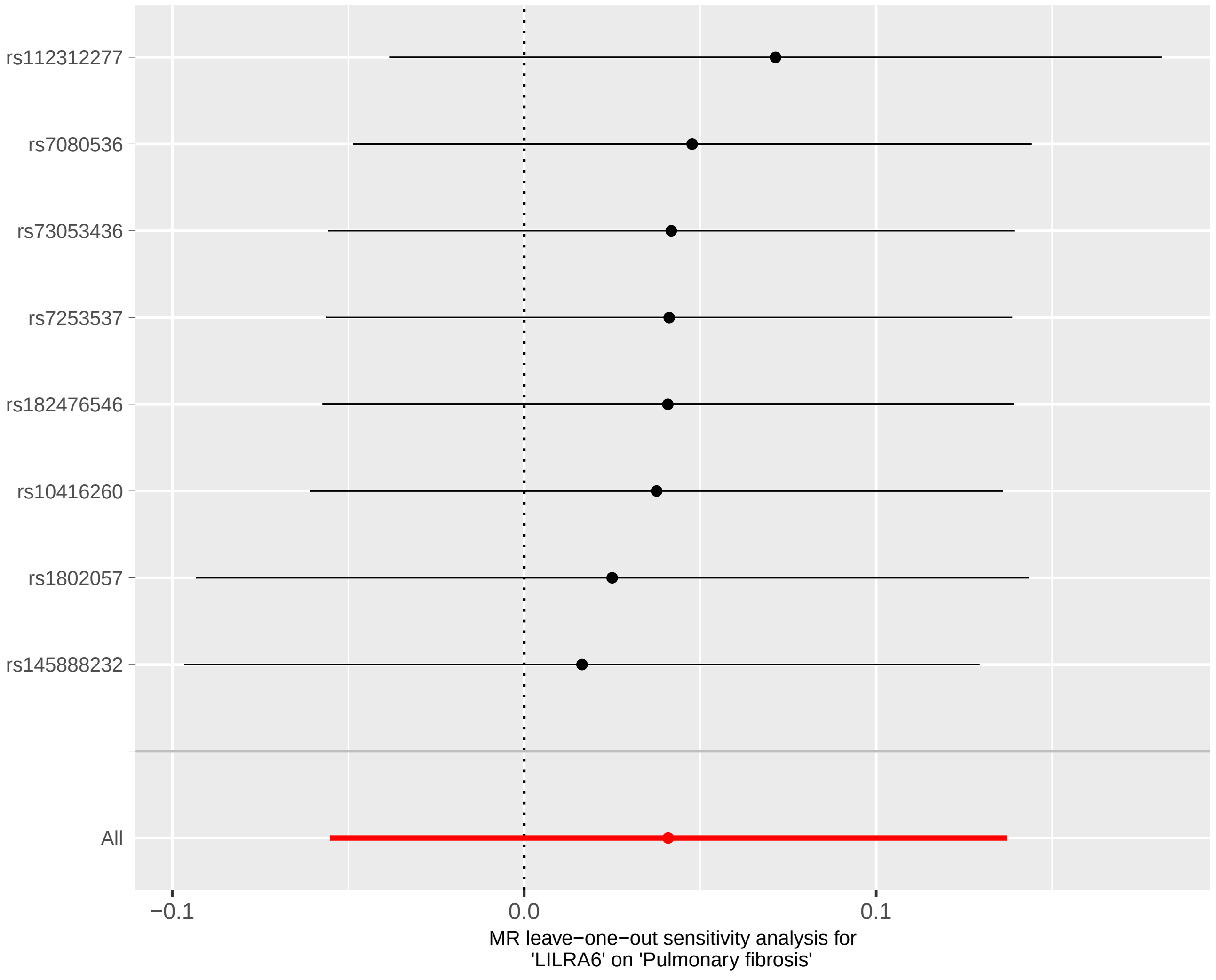
rs112312277
rs7080536
rs73053436
rs7253537
rs182476546
rs10416260
rs1802057
rs145888232
All
−0.1
0.0
0.1
MR leave−one−out sensitivity analysis for
'LILRA6' on 'Pulmonary fibrosis'

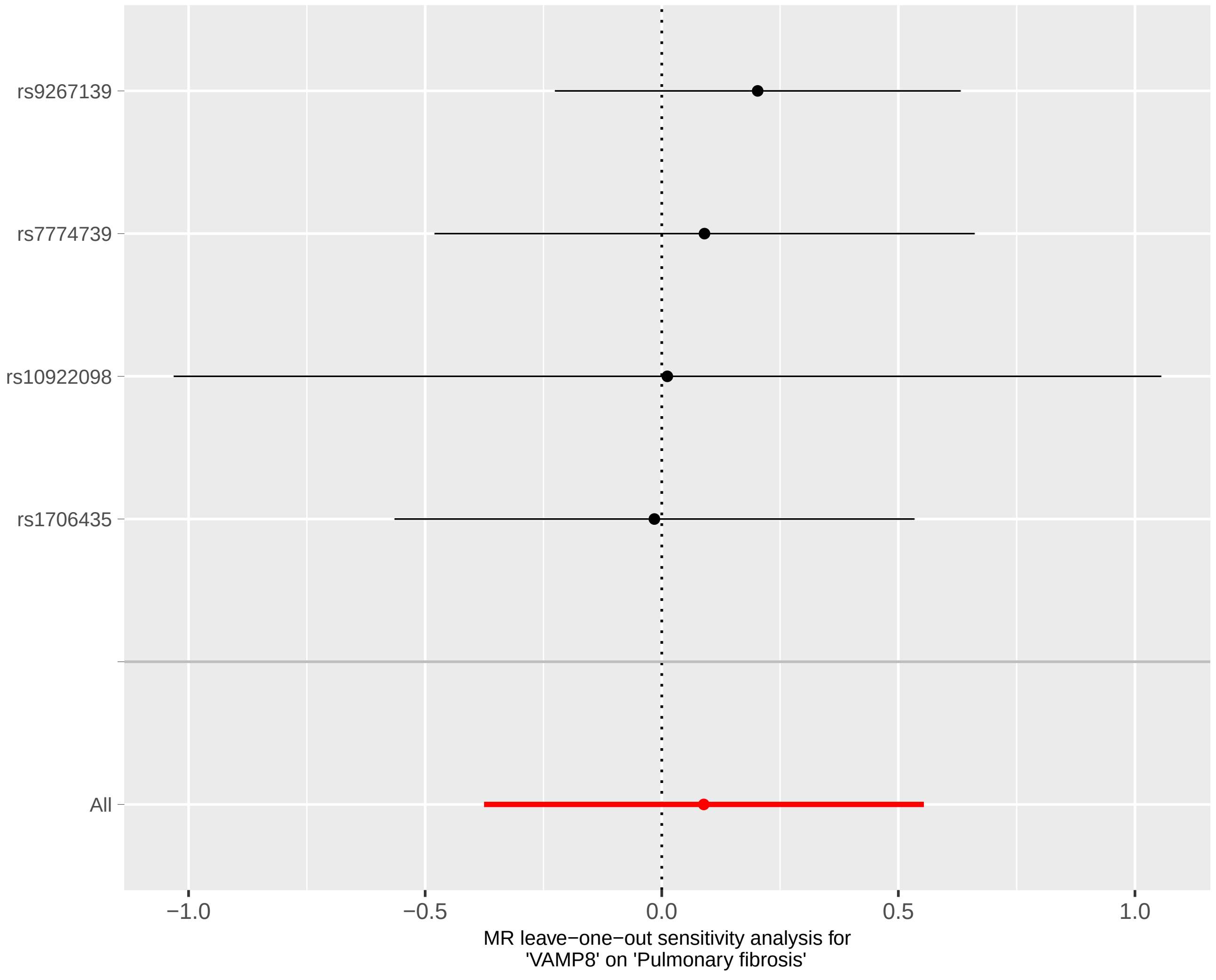

rs9267139
rs7774739
rs10922098
rs1706435
All
−1.0
−0.5
0.0
0.5
1.0
MR leave−one−out sensitivity analysis for
'VAMP8' on 'Pulmonary fibrosis'

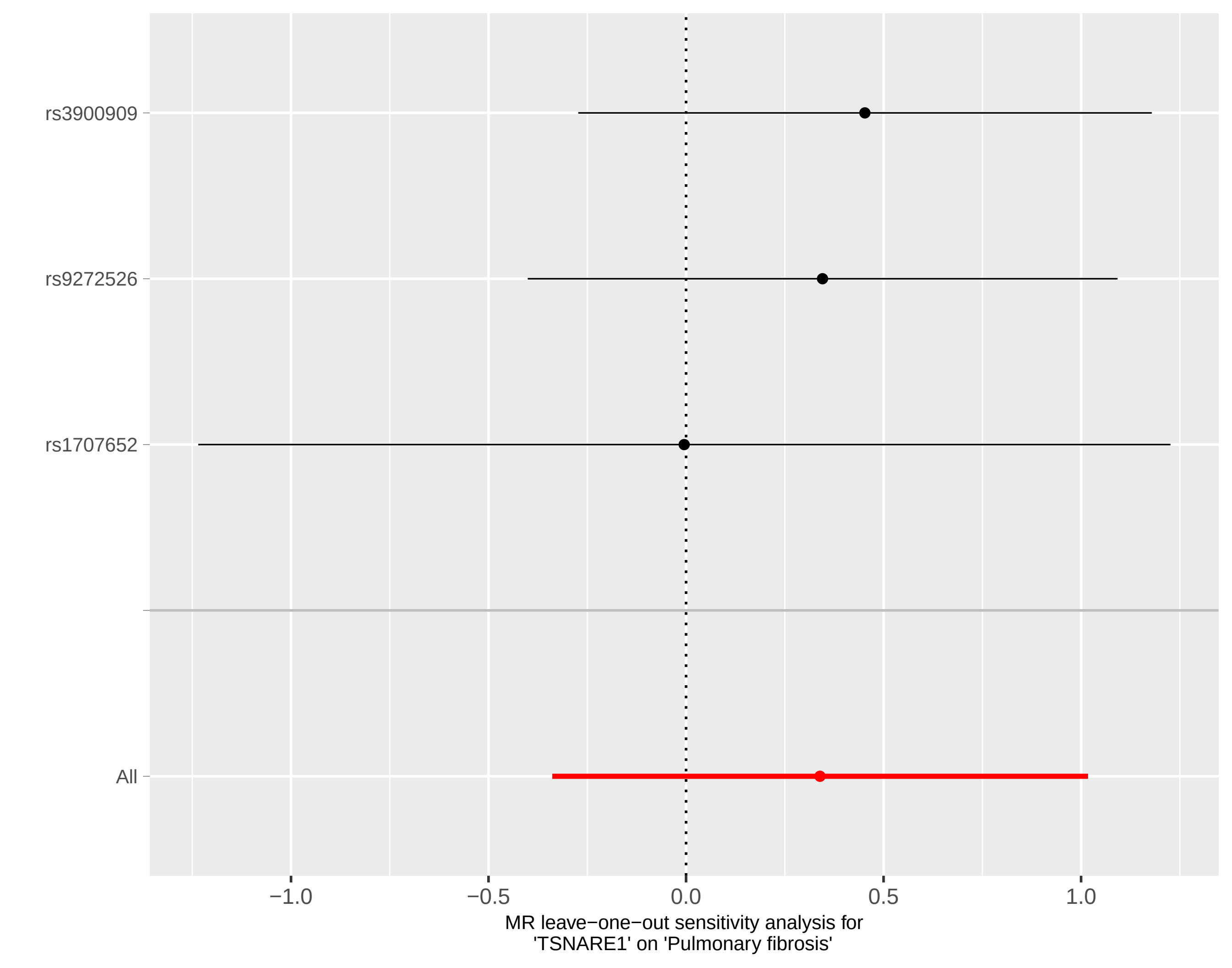
rs3900909
rs9272526
rs1707652
All
−1.0
−0.5
0.0
0.5
1.0
MR leave−one−out sensitivity analysis for
'TSNARE1' on 'Pulmonary fibrosis'

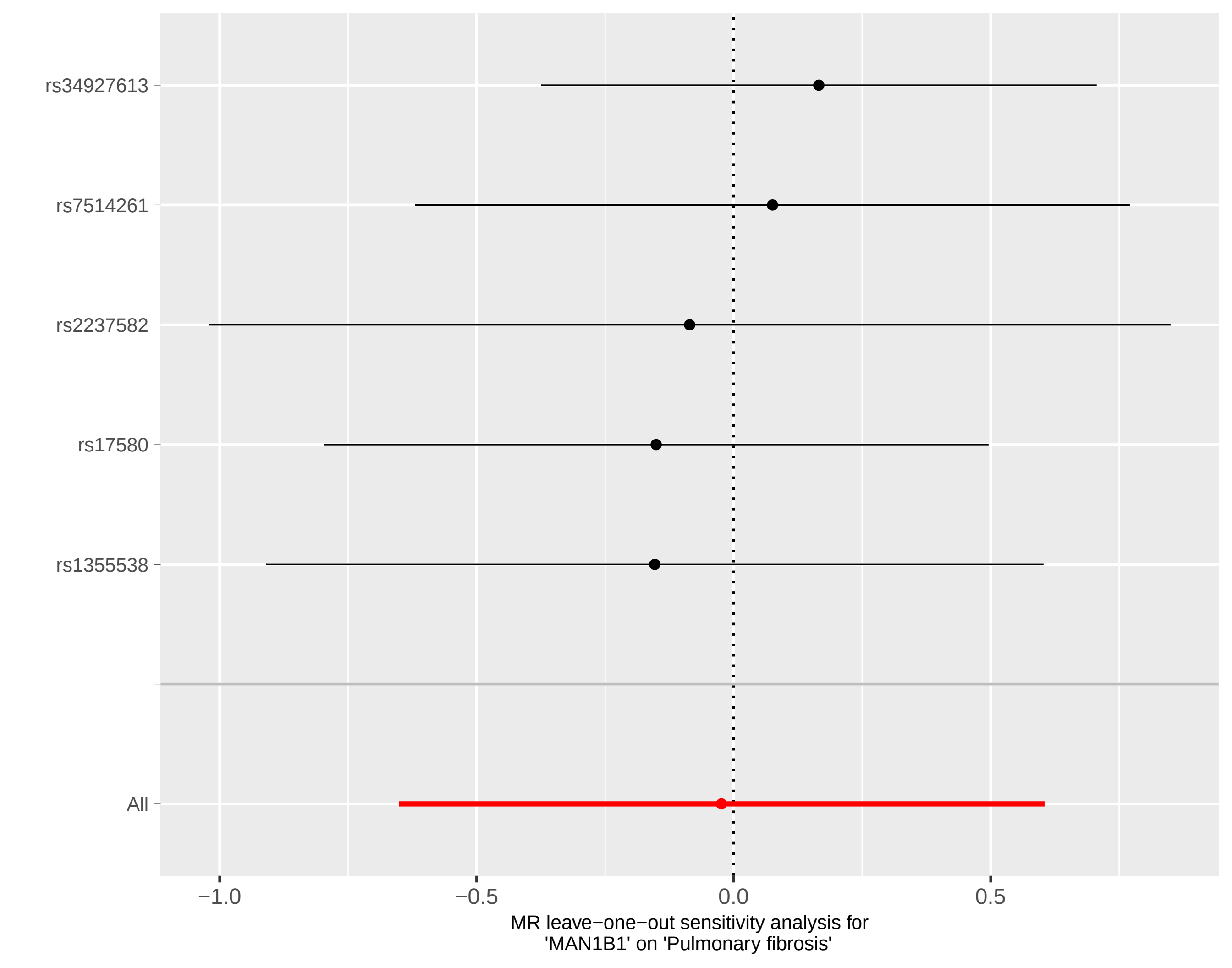

rs34927613
rs7514261
rs2237582
rs17580
rs1355538
All
−1.0
−0.5
0.0
0.5
MR leave−one−out sensitivity analysis for
'MAN1B1' on 'Pulmonary fibrosis'

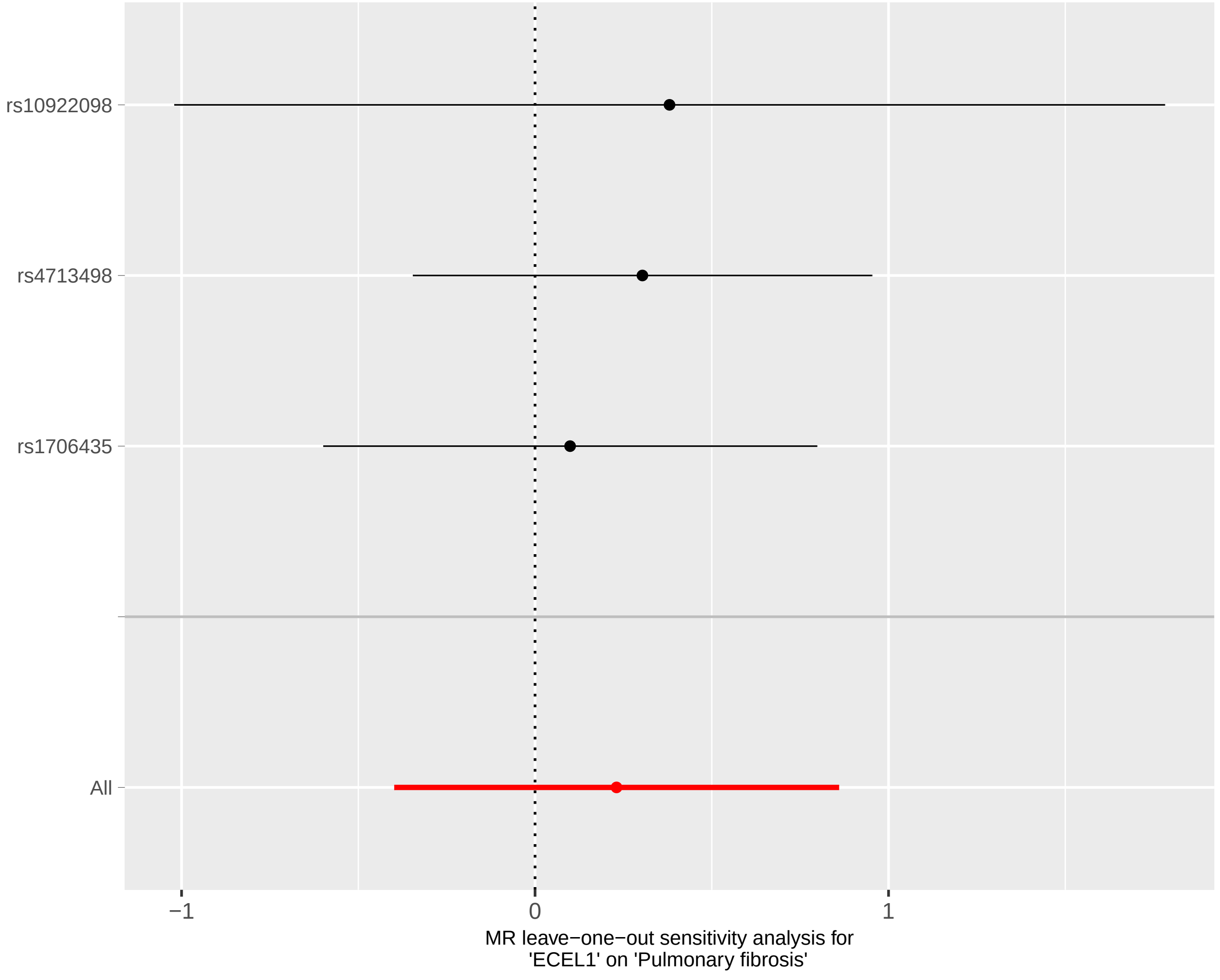

rs10922098
rs4713498
rs1706435
All
−1
0
1
MR leave−one−out sensitivity analysis for
'ECEL1' on 'Pulmonary fibrosis'

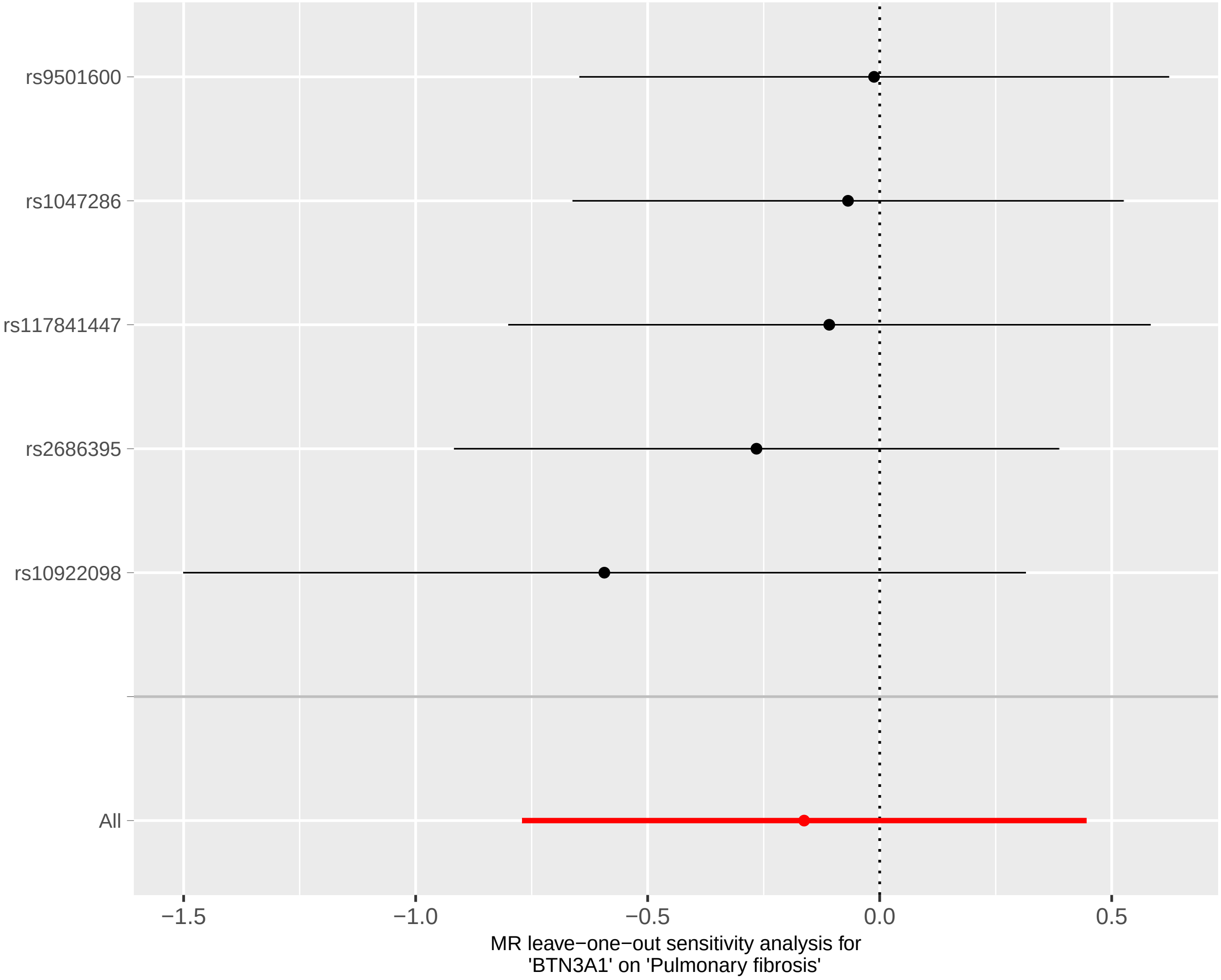
rs9501600
rs1047286
rs117841447
rs2686395
rs10922098
All
−1.5
−1.0
−0.5
0.0
0.5
MR leave−one−out sensitivity analysis for
'BTN3A1' on 'Pulmonary fibrosis'

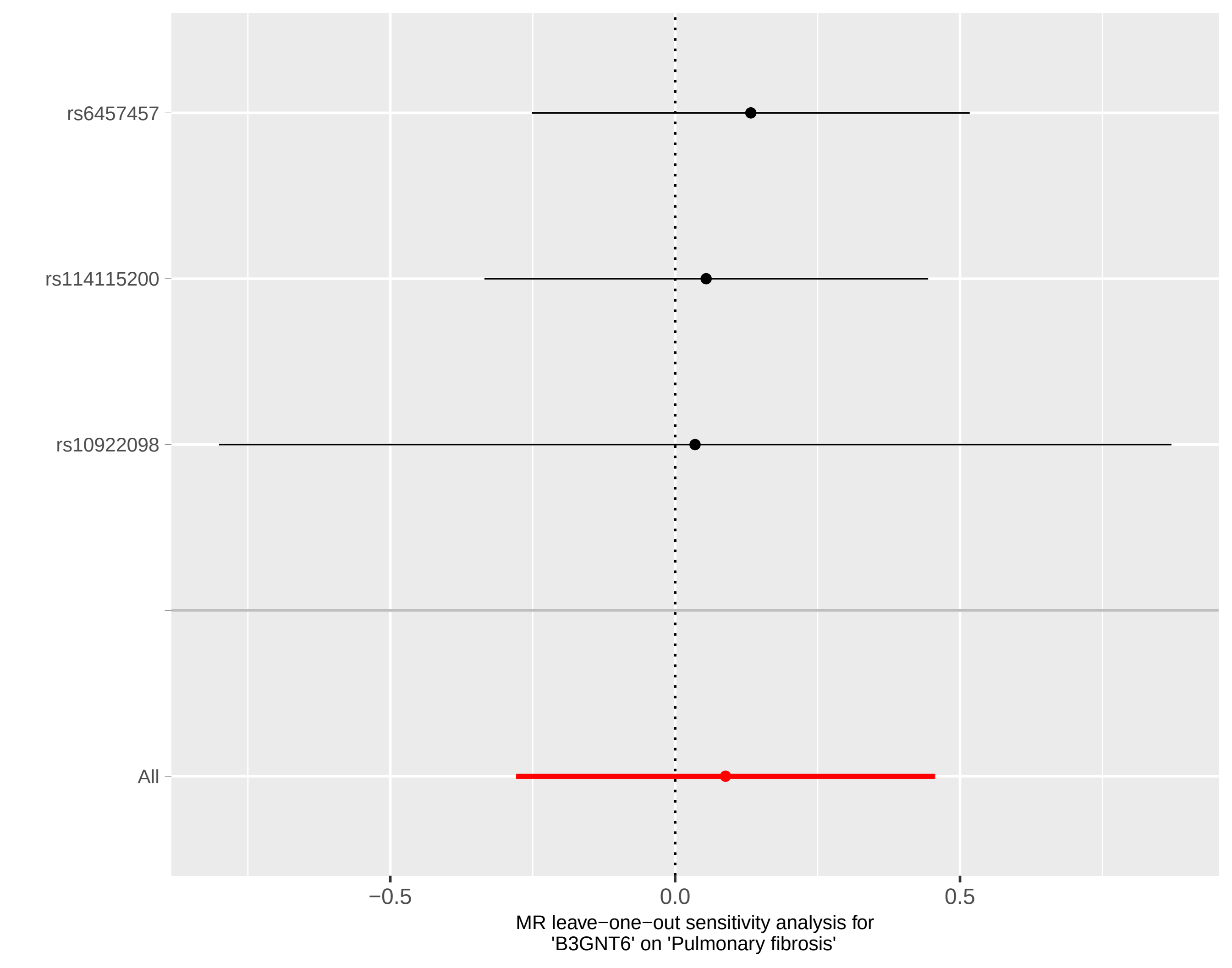
rs6457457
rs114115200
rs10922098
All
−0.5
0.0
0.5
MR leave−one−out sensitivity analysis for
'B3GNT6' on 'Pulmonary fibrosis'

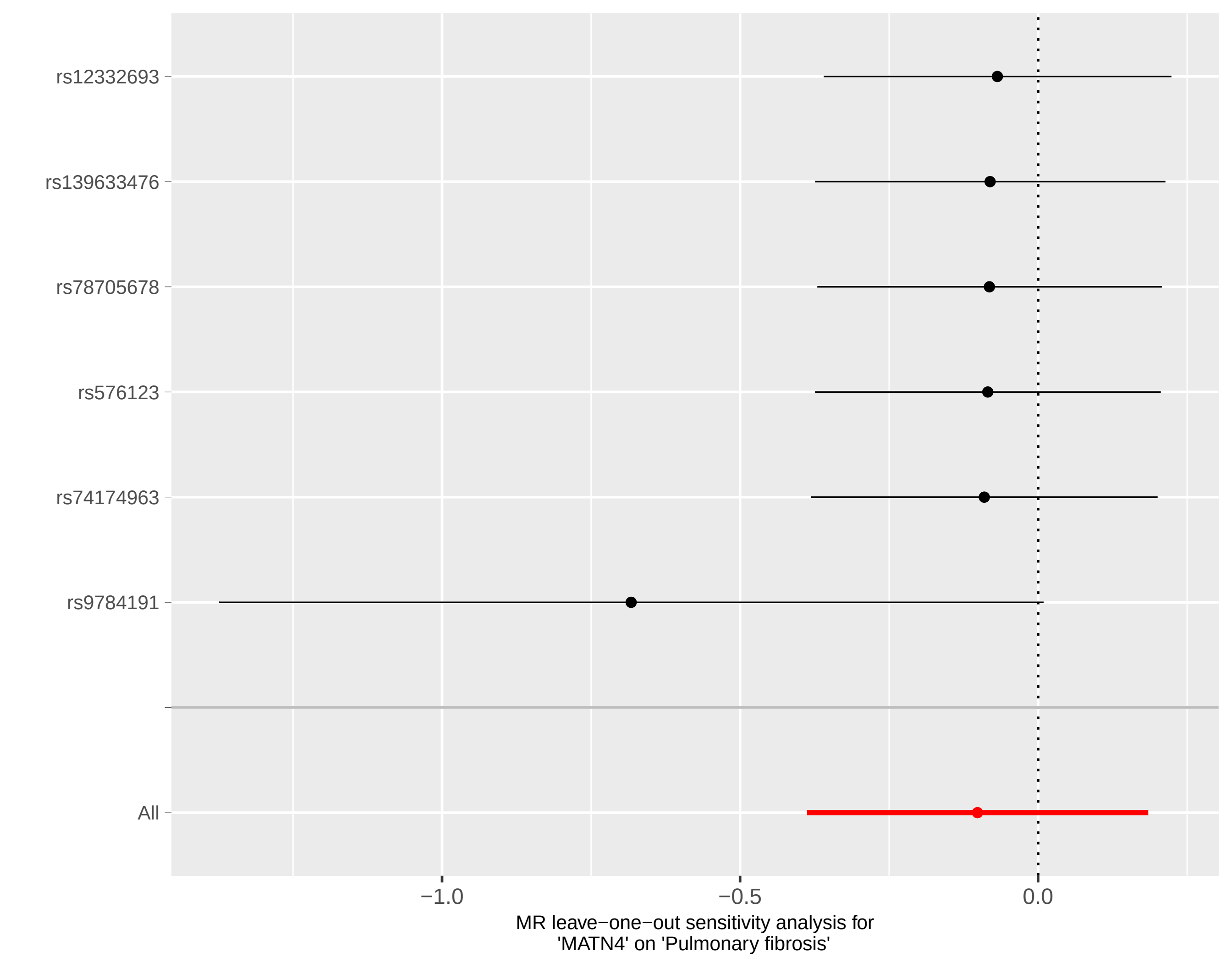
rs12332693
rs139633476
rs78705678
rs576123
rs74174963
rs9784191
All
−1.0
−0.5
0.0
MR leave−one−out sensitivity analysis for
'MATN4' on 'Pulmonary fibrosis'

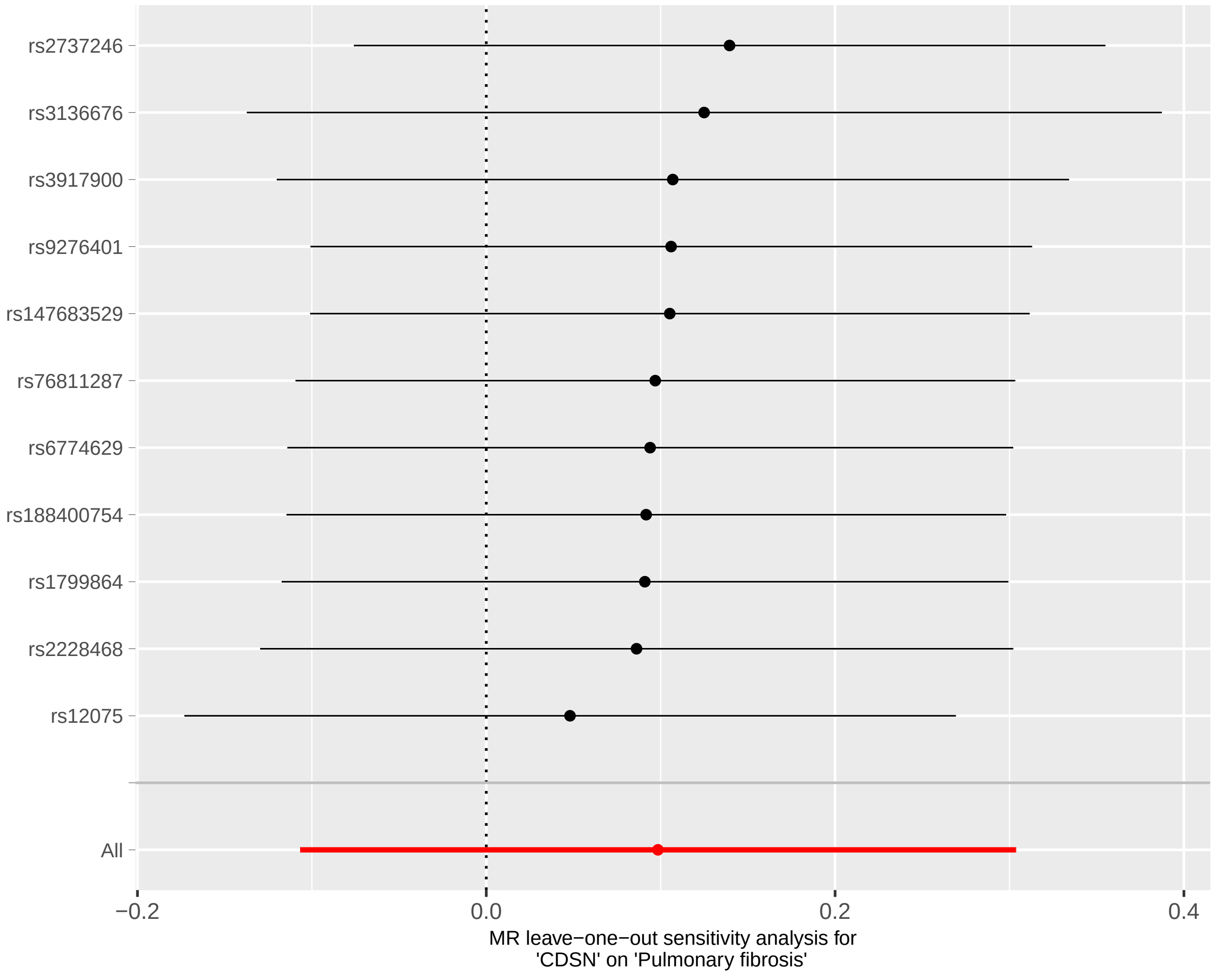
rs2737246
rs3136676
rs3917900
rs9276401
rs147683529
rs76811287
rs6774629
rs188400754
rs1799864
rs2228468
rs12075
All
−0.2
0.0
0.2
0.4
MR leave−one−out sensitivity analysis for
'CDSN' on 'Pulmonary fibrosis'

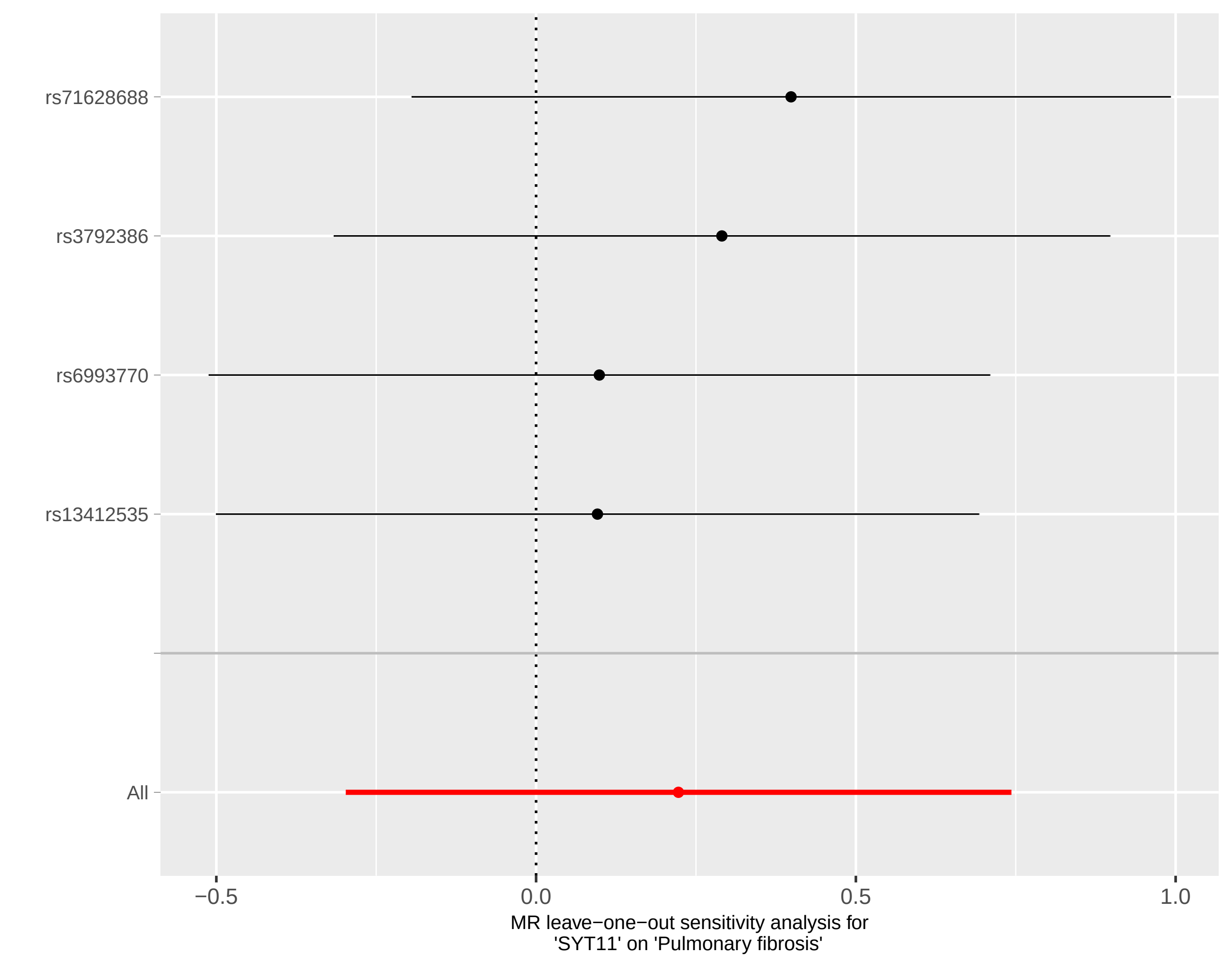
rs71628688
rs3792386
rs6993770
rs13412535
All
−0.5
0.0
0.5
1.0
MR leave−one−out sensitivity analysis for
'SYT11' on 'Pulmonary fibrosis'

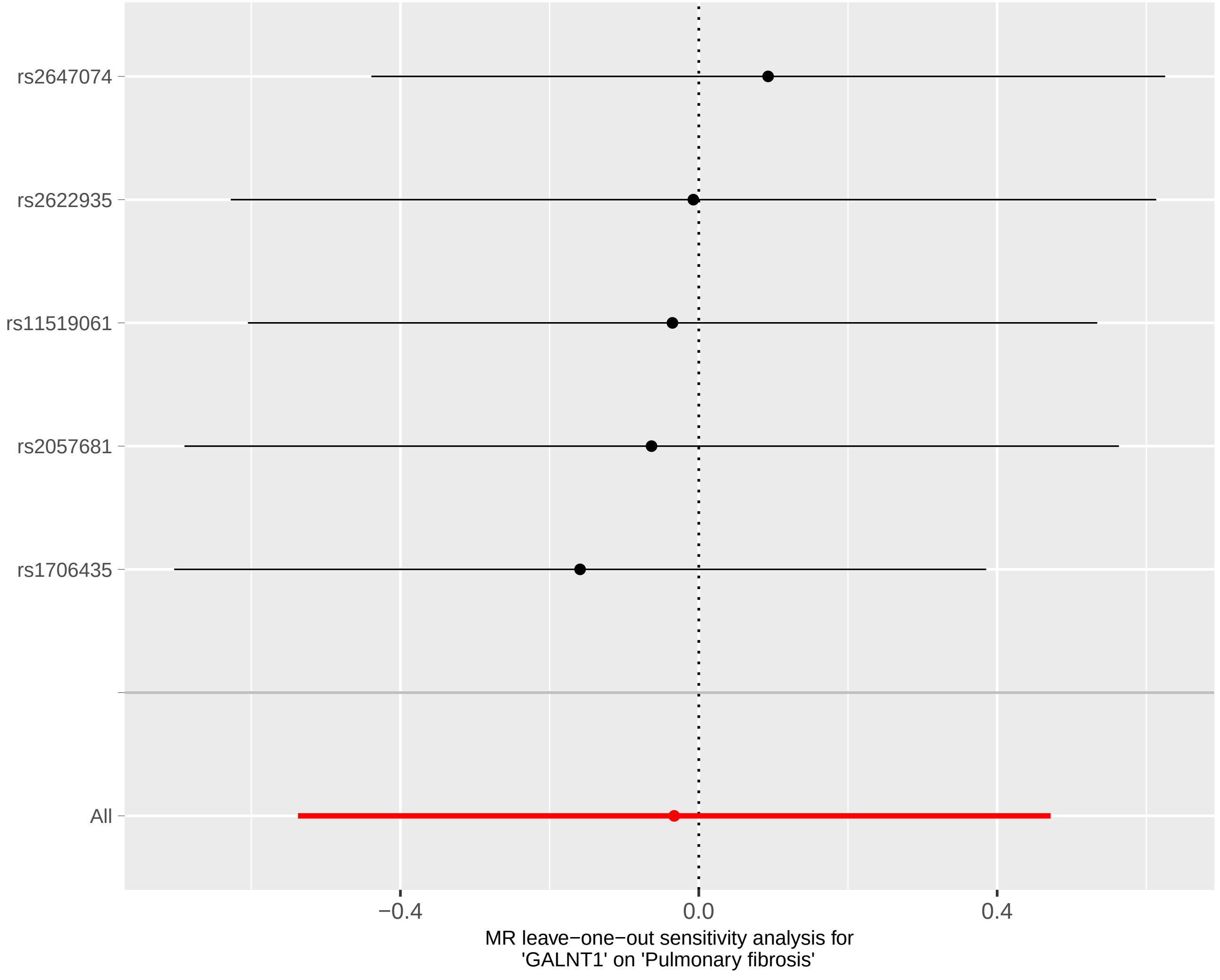
rs2647074
rs2622935
rs11519061
rs2057681
rs1706435
All
−0.4
0.0
0.4
MR leave−one−out sensitivity analysis for
'GALNT1' on 'Pulmonary fibrosis'

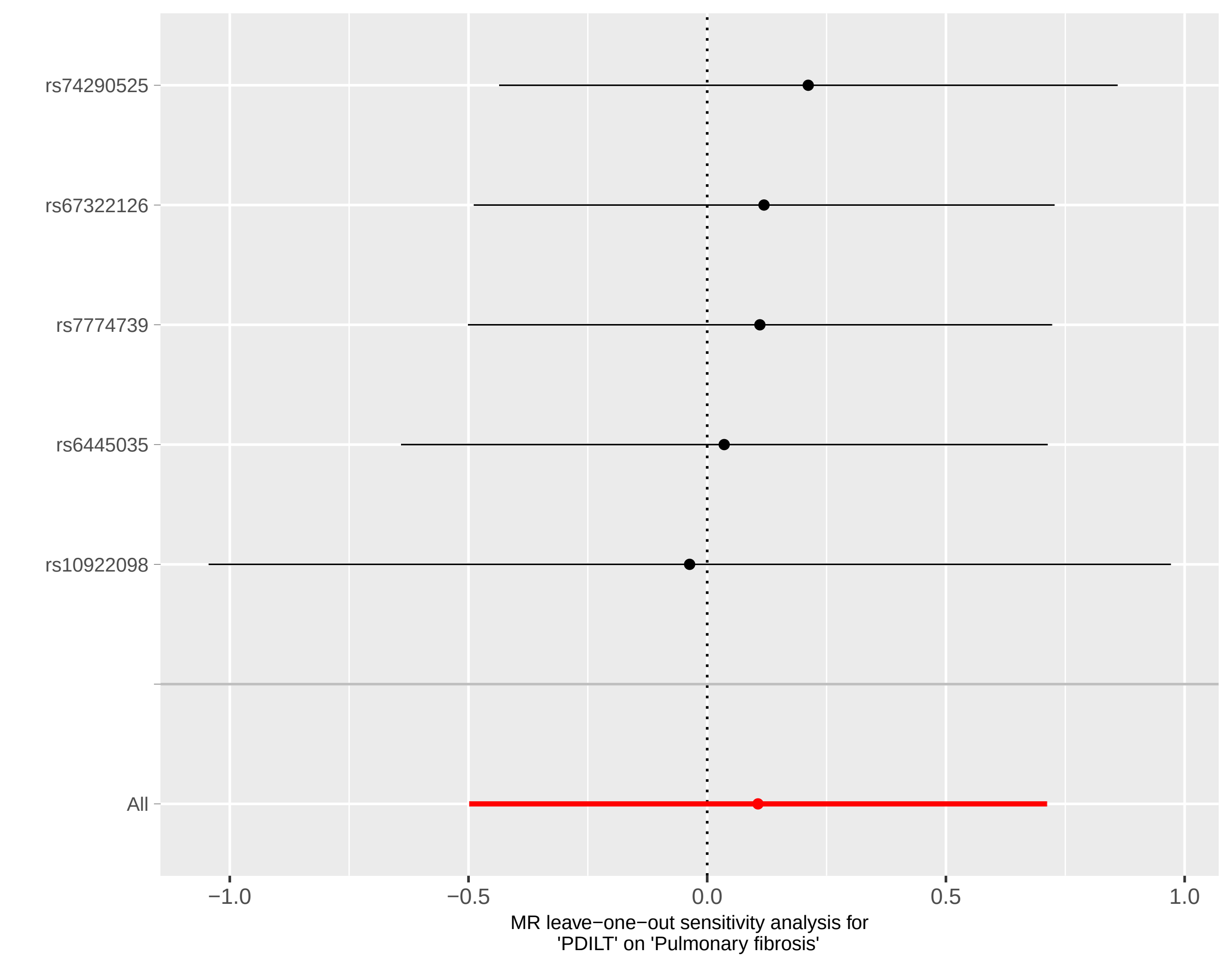
rs74290525
rs67322126
rs7774739
rs6445035
rs10922098
All
−1.0
−0.5
0.0
0.5
1.0
MR leave−one−out sensitivity analysis for
'PDILT' on 'Pulmonary fibrosis'

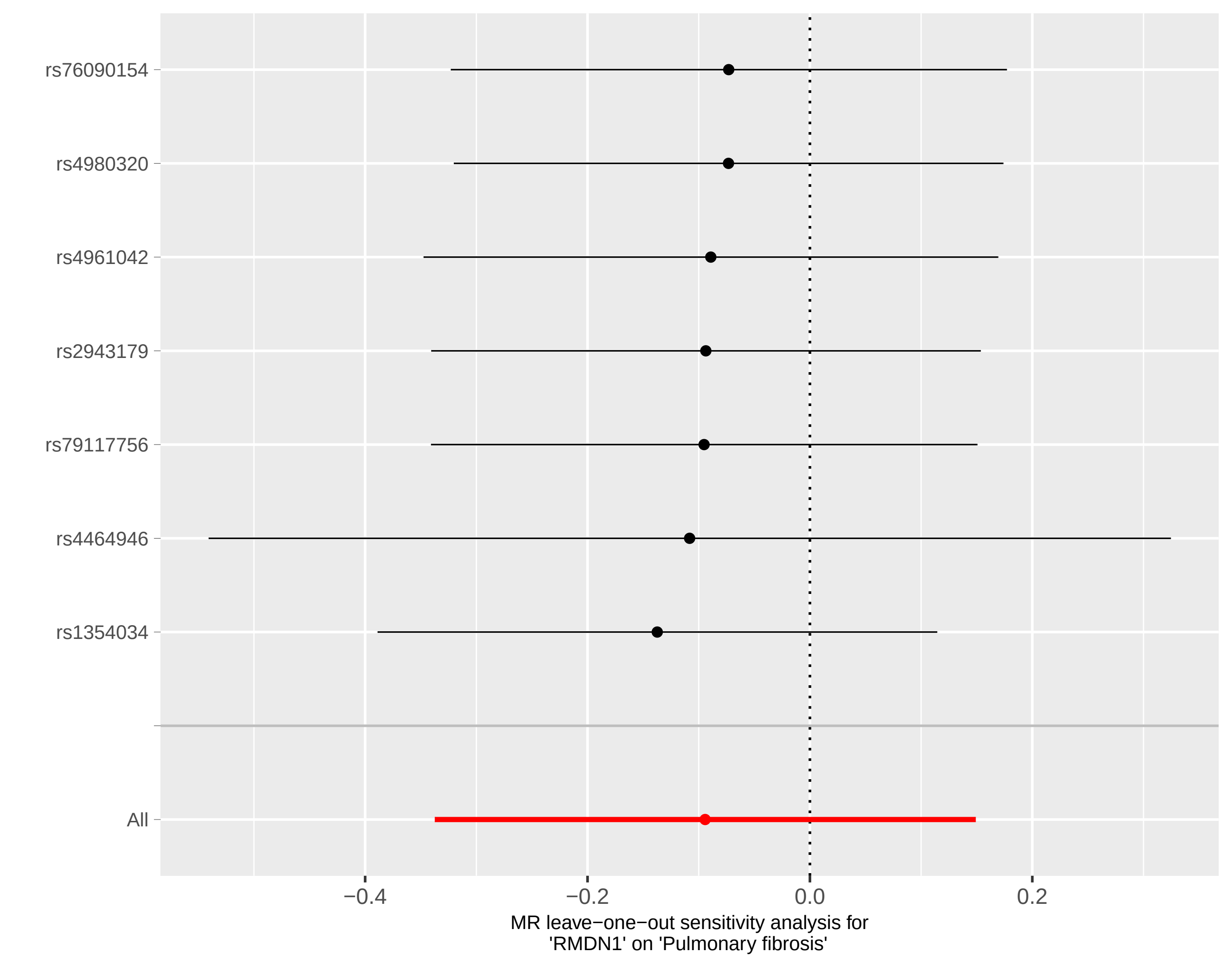
rs76090154
rs4980320
rs4961042
rs2943179
rs79117756
rs4464946
rs1354034
All
−0.4
−0.2
0.0
0.2
MR leave−one−out sensitivity analysis for
'RMDN1' on 'Pulmonary fibrosis'

Insufficient number of SNPs

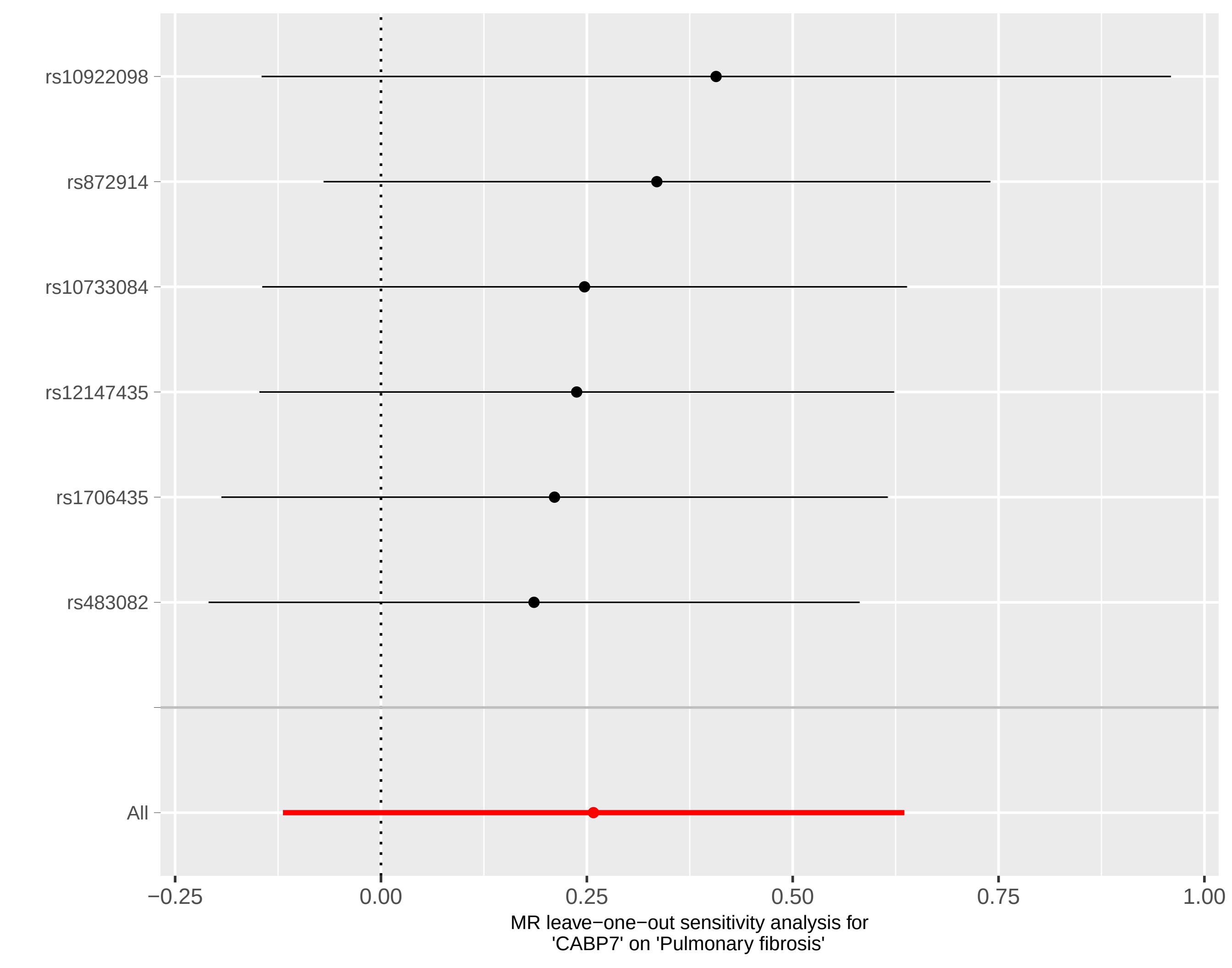

rs10922098
rs872914
rs10733084
rs12147435
rs1706435
rs483082
All
−0.25
0.00
0.25
0.50
0.75
1.00
MR leave−one−out sensitivity analysis for
'CABP7' on 'Pulmonary fibrosis'

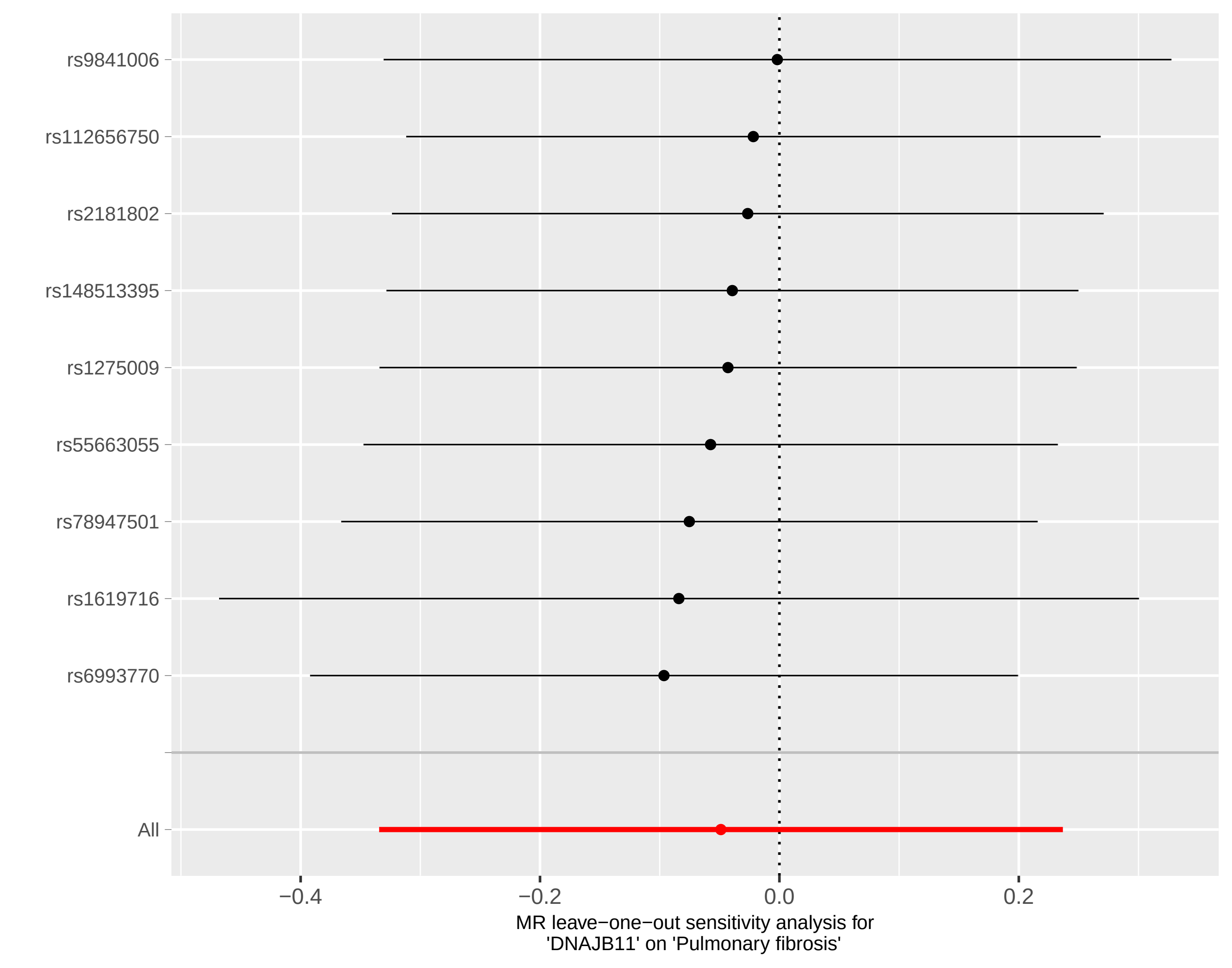

rs9841006
rs112656750
rs2181802
rs148513395
rs1275009
rs55663055
rs78947501
rs1619716
rs6993770
All
−0.4
−0.2
0.0
0.2
MR leave−one−out sensitivity analysis for
'DNAJB11' on 'Pulmonary fibrosis'

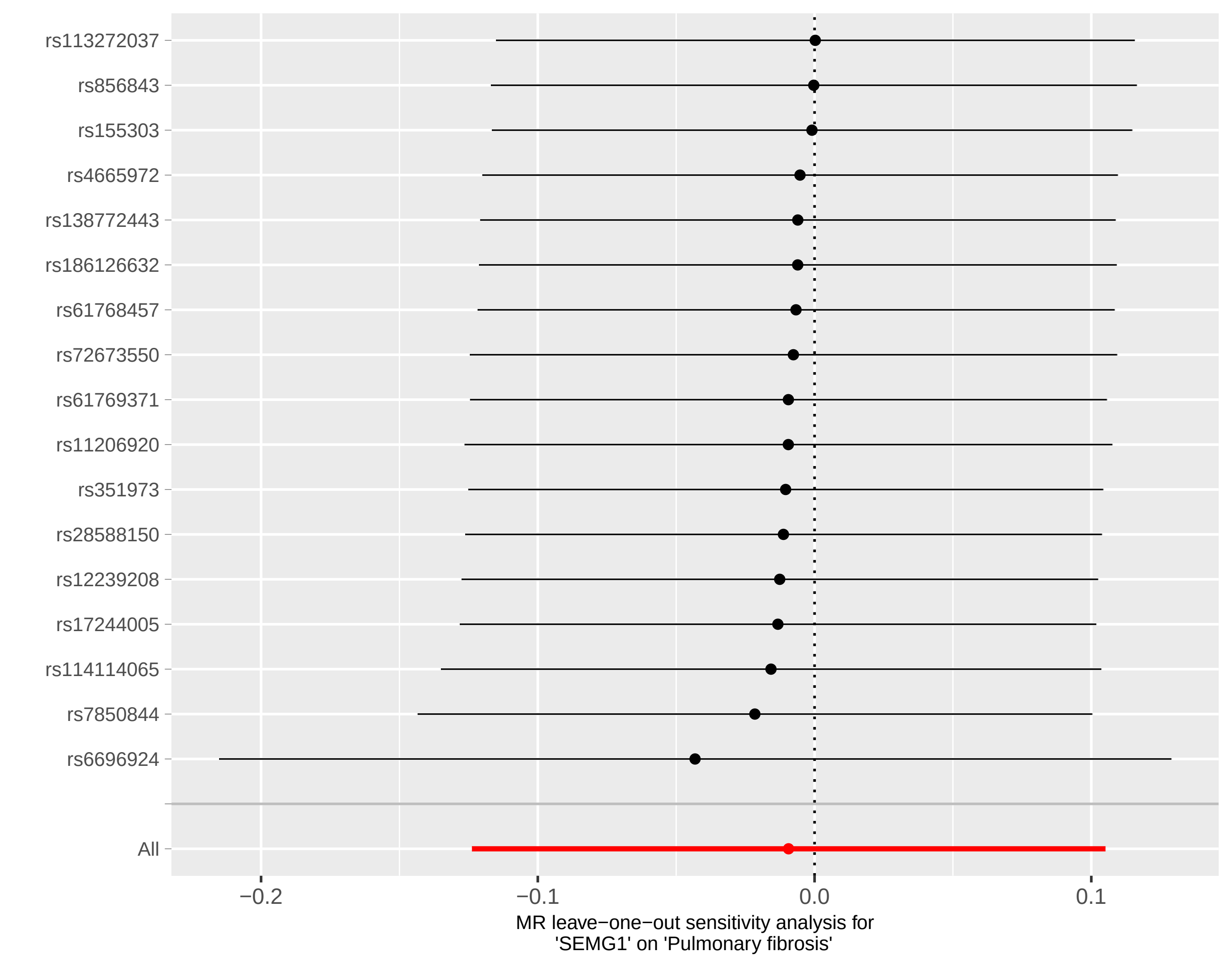
rs113272037
rs856843
rs155303
rs4665972
rs138772443
rs186126632
rs61768457
rs72673550
rs61769371
rs11206920
rs351973
rs28588150
rs12239208
rs17244005
rs114114065
rs7850844
rs6696924
All
−0.2
−0.1
0.0
0.1
MR leave−one−out sensitivity analysis for
'SEMG1' on 'Pulmonary fibrosis'

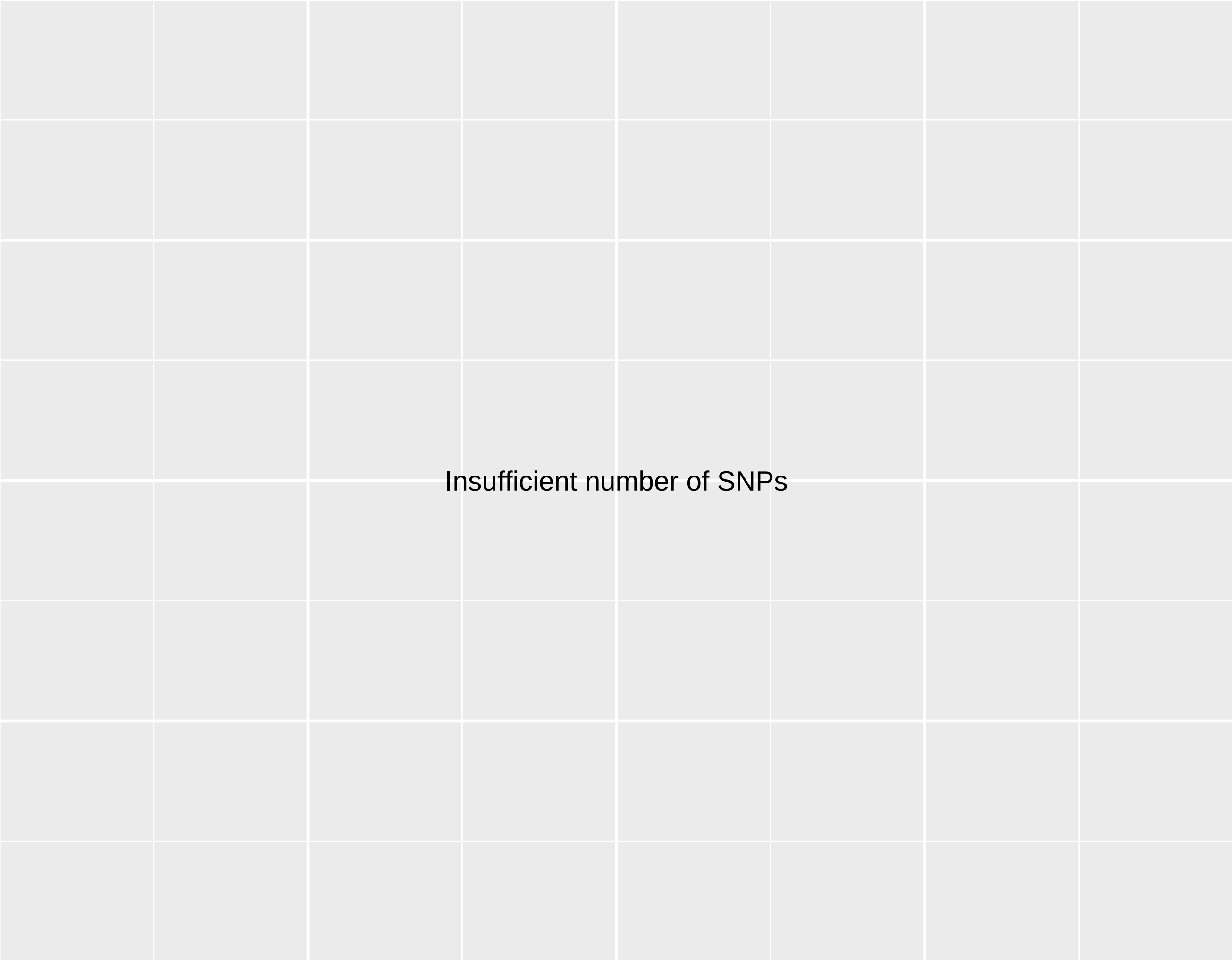
Insufficient number of SNPs

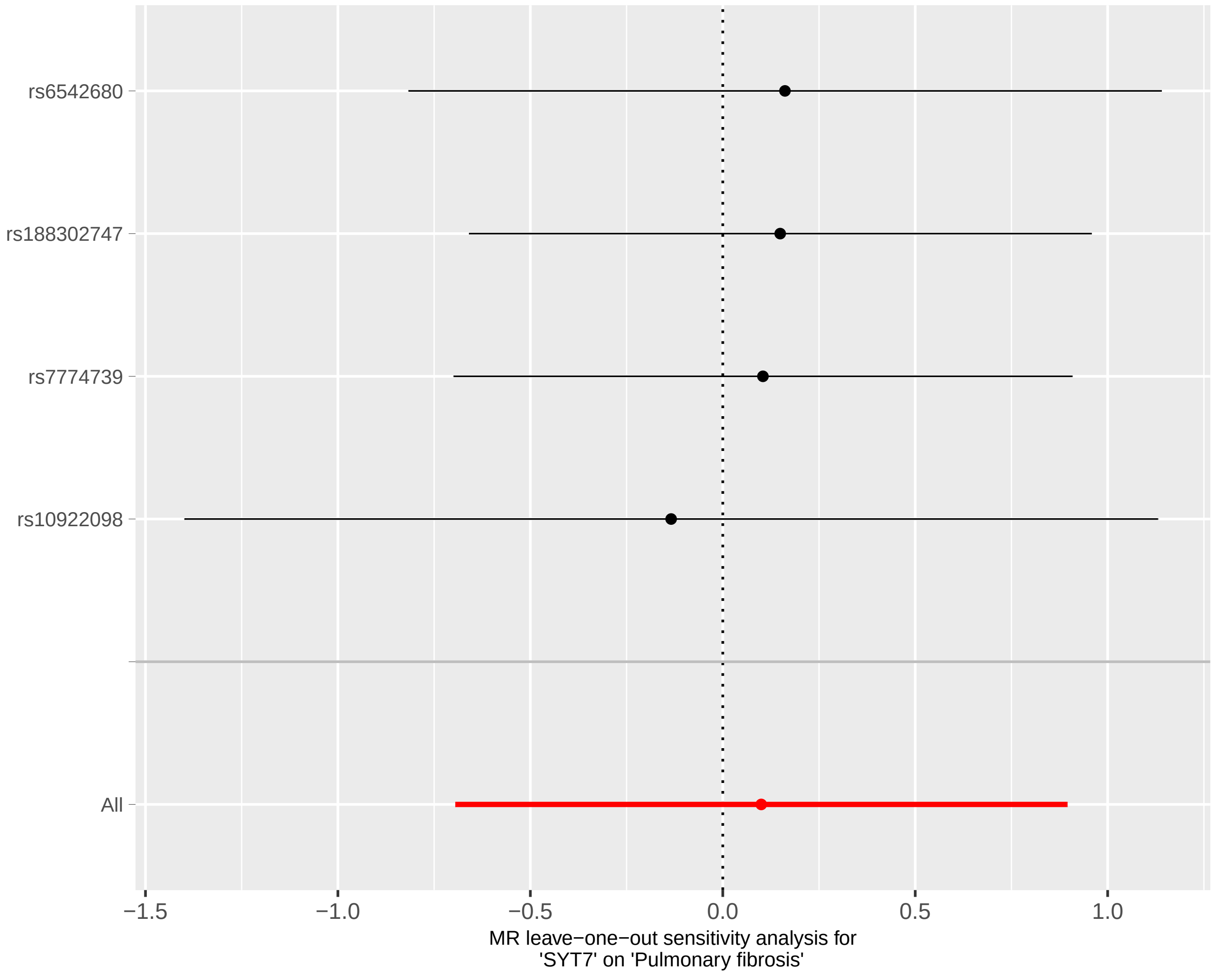
rs6542680
rs188302747
rs7774739
rs10922098
All
−1.5
−1.0
−0.5
0.0
0.5
1.0
MR leave−one−out sensitivity analysis for
'SYT7' on 'Pulmonary fibrosis'

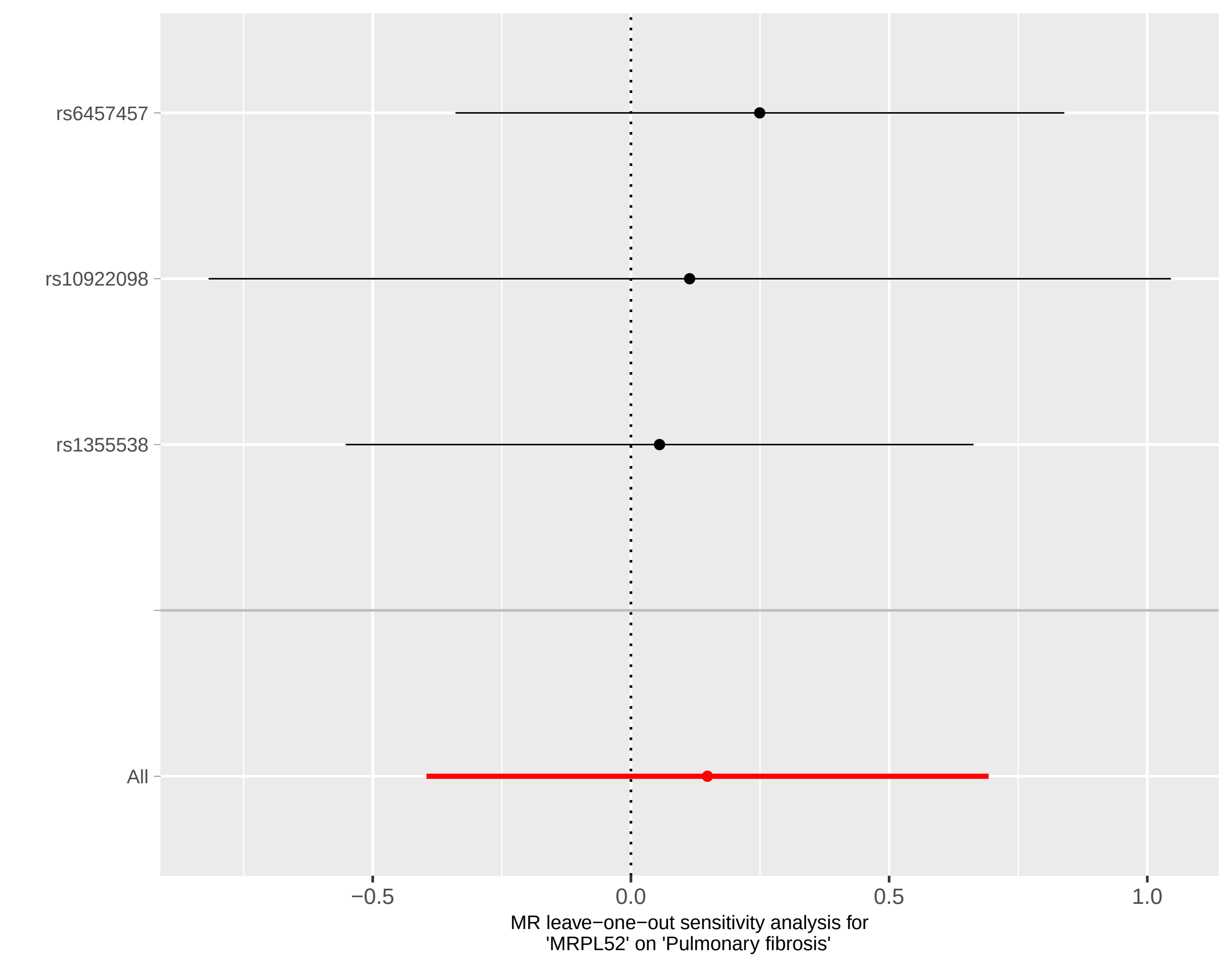
rs6457457
rs10922098
rs1355538
All
−0.5
0.0
0.5
1.0
MR leave−one−out sensitivity analysis for
'MRPL52' on 'Pulmonary fibrosis'

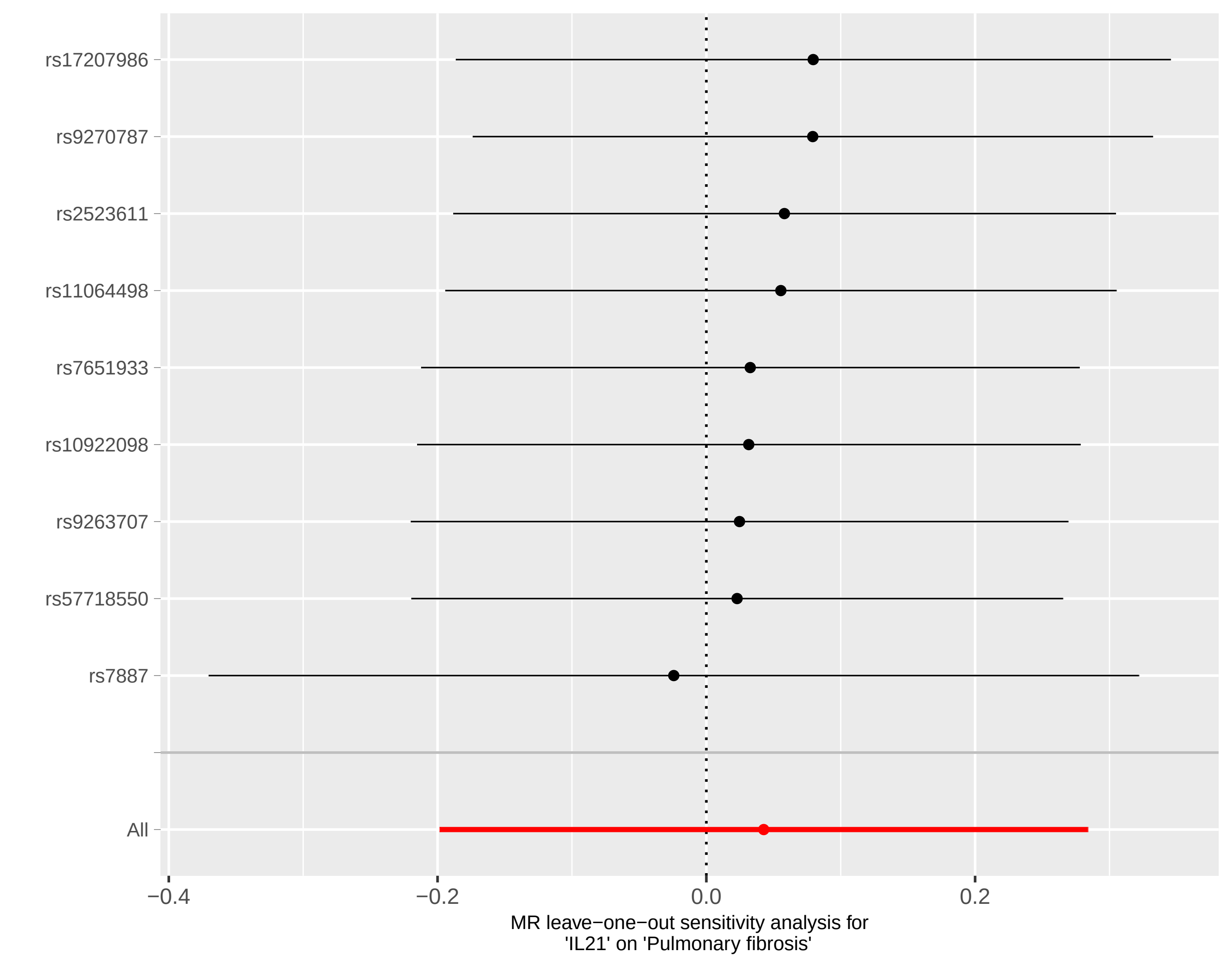
rs17207986
rs9270787
rs2523611
rs11064498
rs7651933
rs10922098
rs9263707
rs57718550
rs7887
All
−0.4
−0.2
0.0
0.2
MR leave−one−out sensitivity analysis for
'IL21' on 'Pulmonary fibrosis'

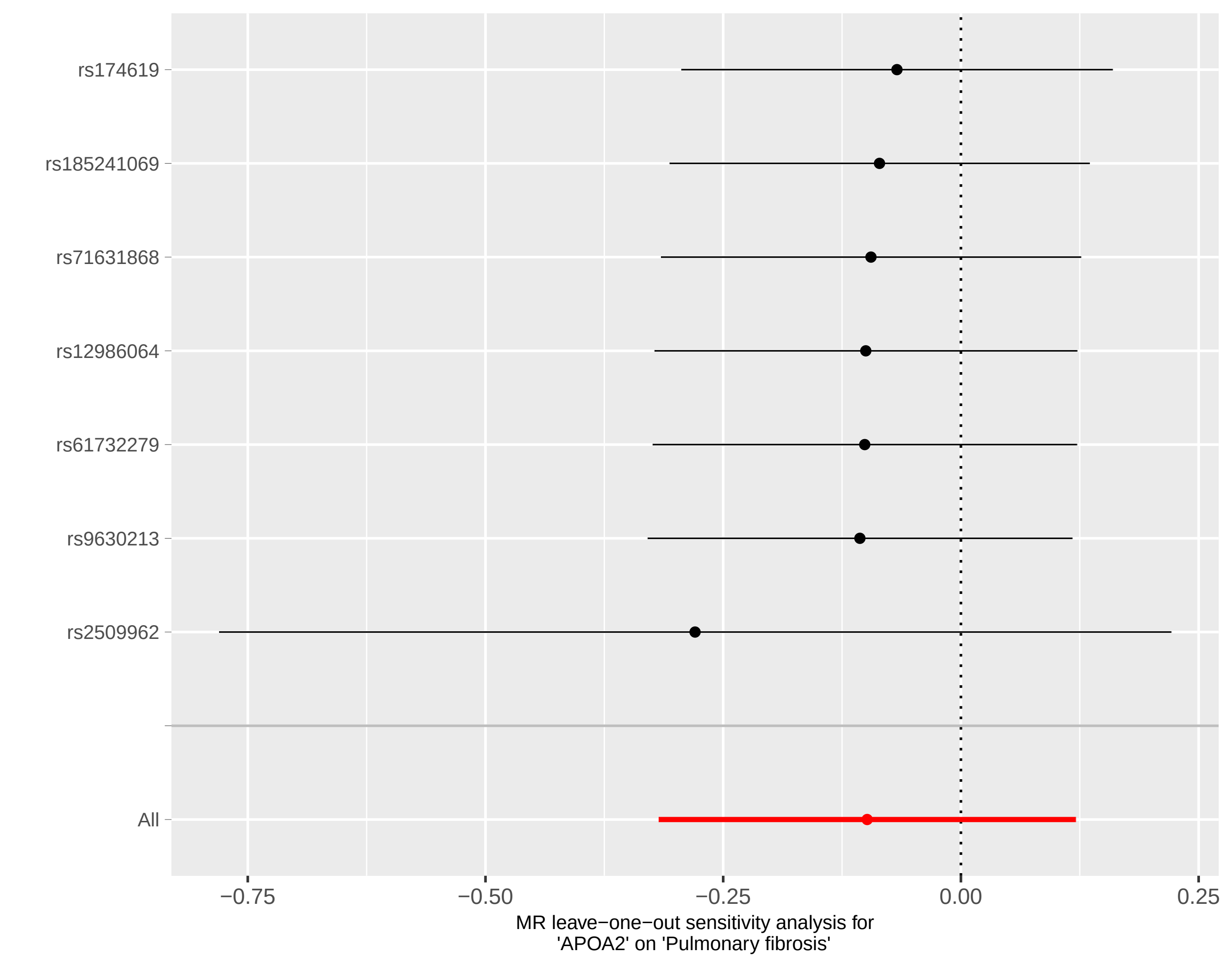
rs174619
rs185241069
rs71631868
rs12986064
rs61732279
rs9630213
rs2509962
All
−0.75
−0.50
−0.25
0.00
0.25
MR leave−one−out sensitivity analysis for
'APOA2' on 'Pulmonary fibrosis'

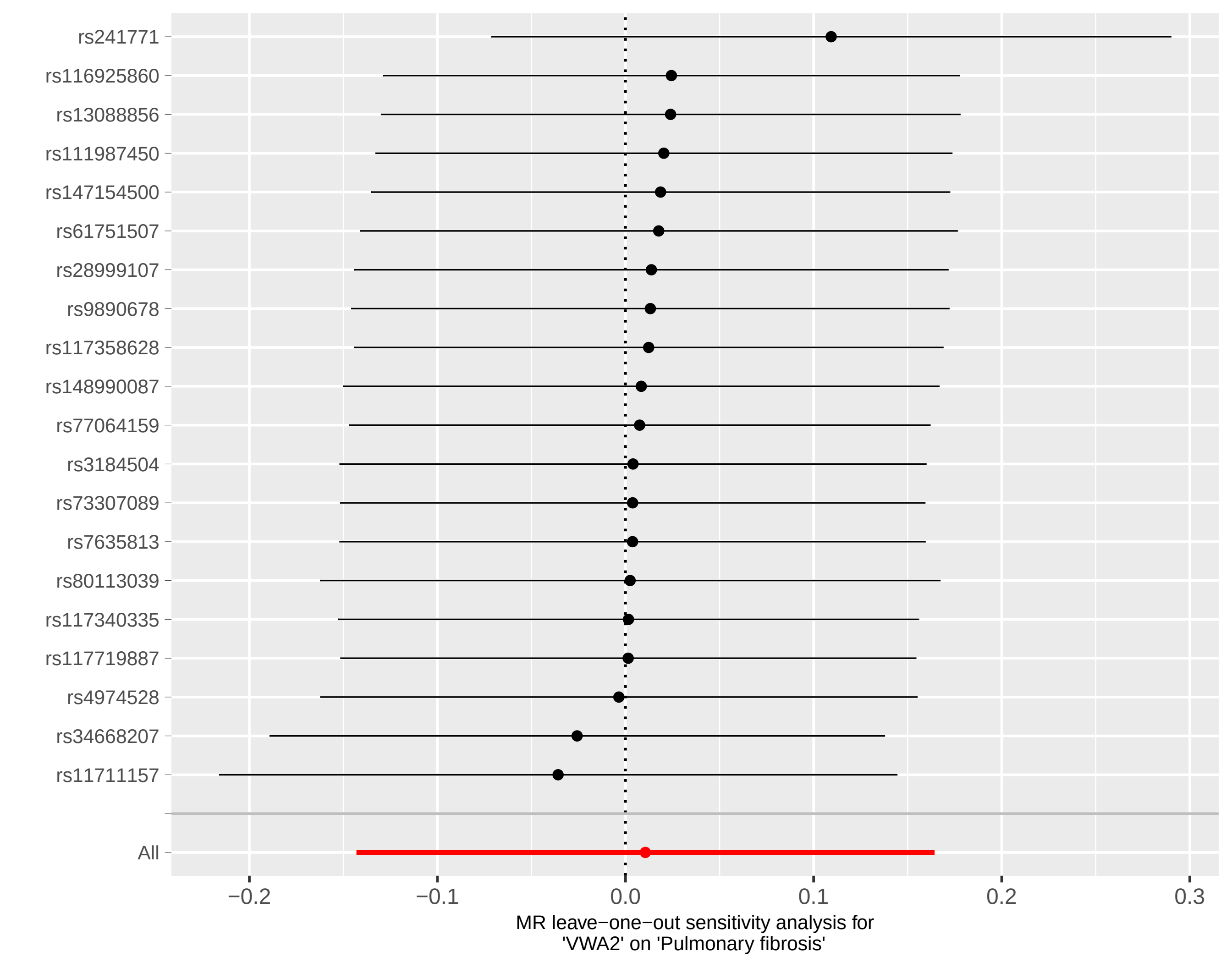

rs241771
rs116925860
rs13088856
rs111987450
rs147154500
rs61751507
rs28999107
rs9890678
rs117358628
rs148990087
rs77064159
rs3184504
rs73307089
rs7635813
rs80113039
rs117340335
rs117719887
rs4974528
rs34668207
rs11711157
All
−0.2
−0.1
0.0
0.1
0.2
0.3
MR leave−one−out sensitivity analysis for
'VWA2' on 'Pulmonary fibrosis'

Insufficient number of SNPs

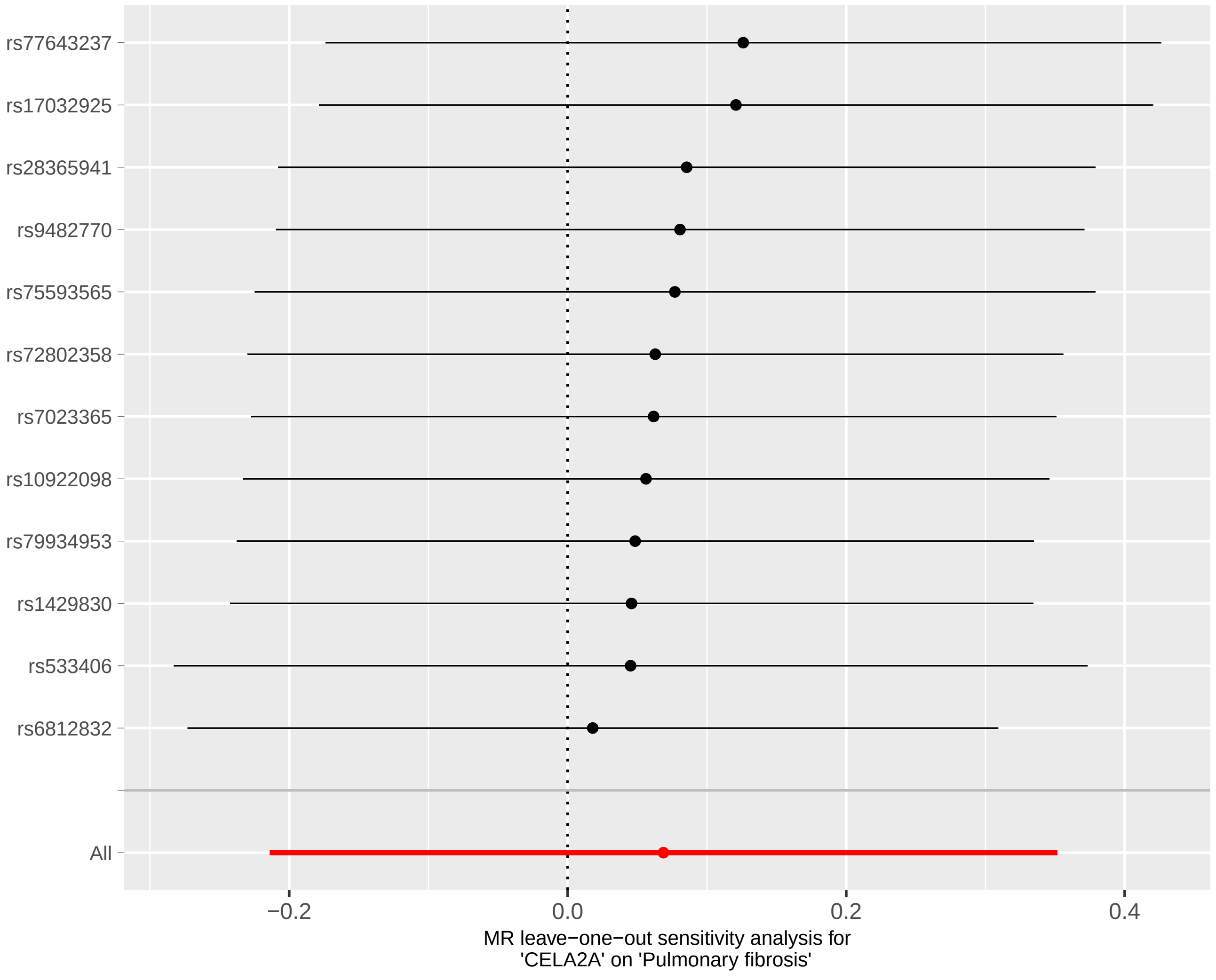

rs77643237
rs17032925
rs28365941
rs9482770
rs75593565
rs72802358
rs7023365
rs10922098
rs79934953
rs1429830
rs533406
rs6812832
All
−0.2
0.0
0.2
0.4
MR leave−one−out sensitivity analysis for
'CELA2A' on 'Pulmonary fibrosis'

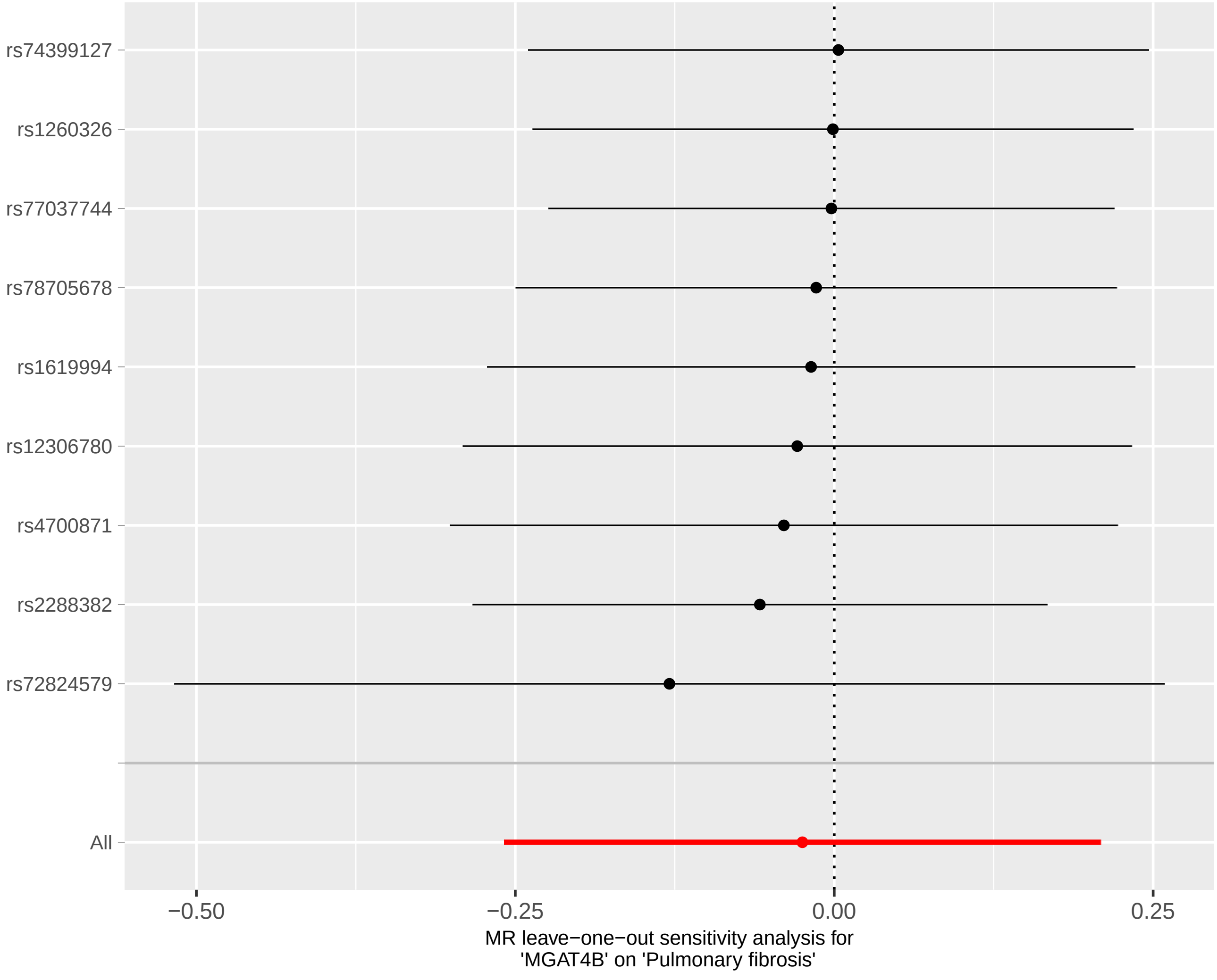
rs74399127
rs1260326
rs77037744
rs78705678
rs1619994
rs12306780
rs4700871
rs2288382
rs72824579
All
−0.50
−0.25
0.00
0.25
MR leave−one−out sensitivity analysis for
'MGAT4B' on 'Pulmonary fibrosis'

Insufficient number of SNPs

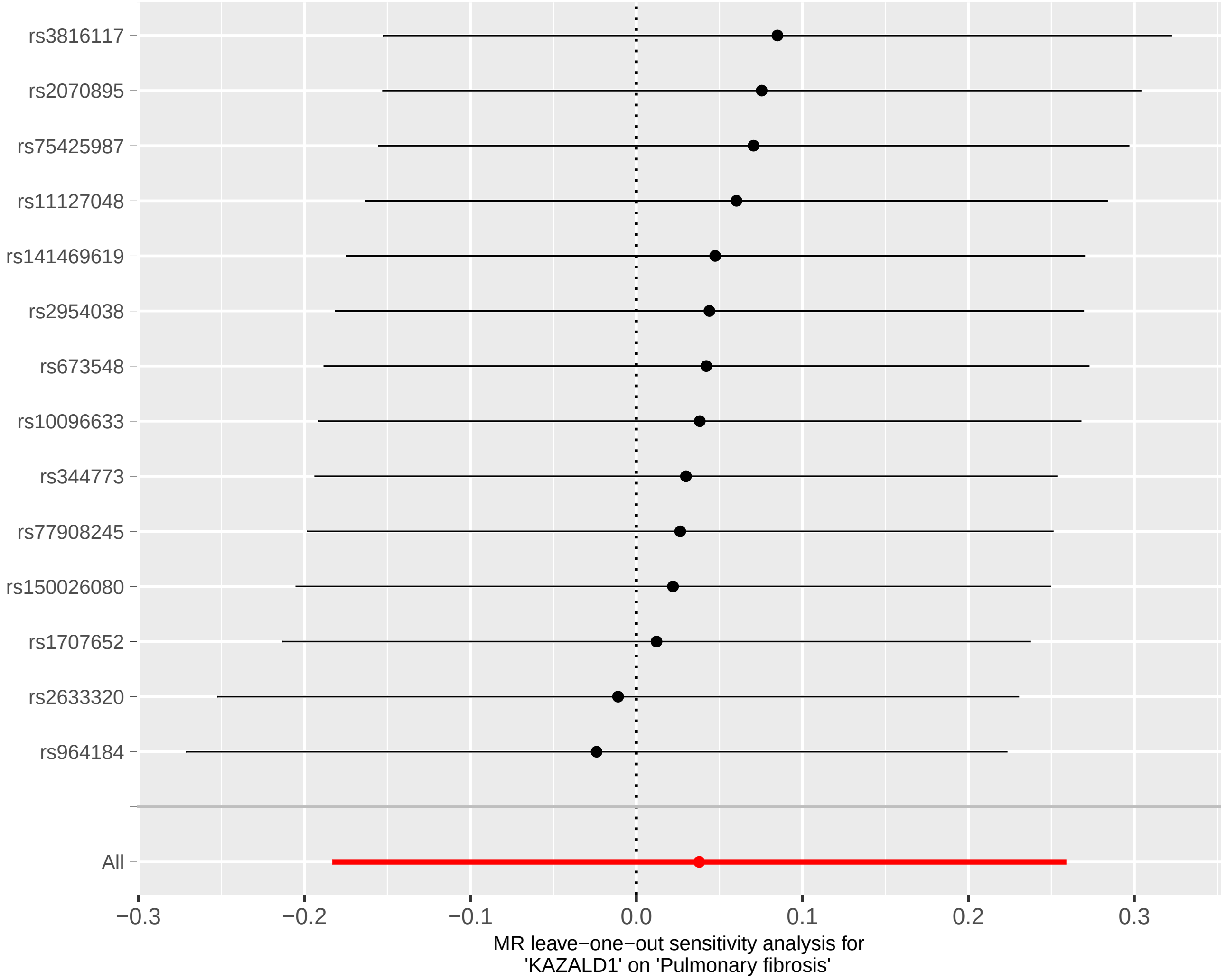
rs3816117
rs2070895
rs75425987
rs11127048
rs141469619
rs2954038
rs673548
rs10096633
rs344773
rs77908245
rs150026080
rs1707652
rs2633320
rs964184
All
−0.3
−0.2
−0.1
0.0
0.1
0.2
0.3
MR leave−one−out sensitivity analysis for
'KAZALD1' on 'Pulmonary fibrosis'

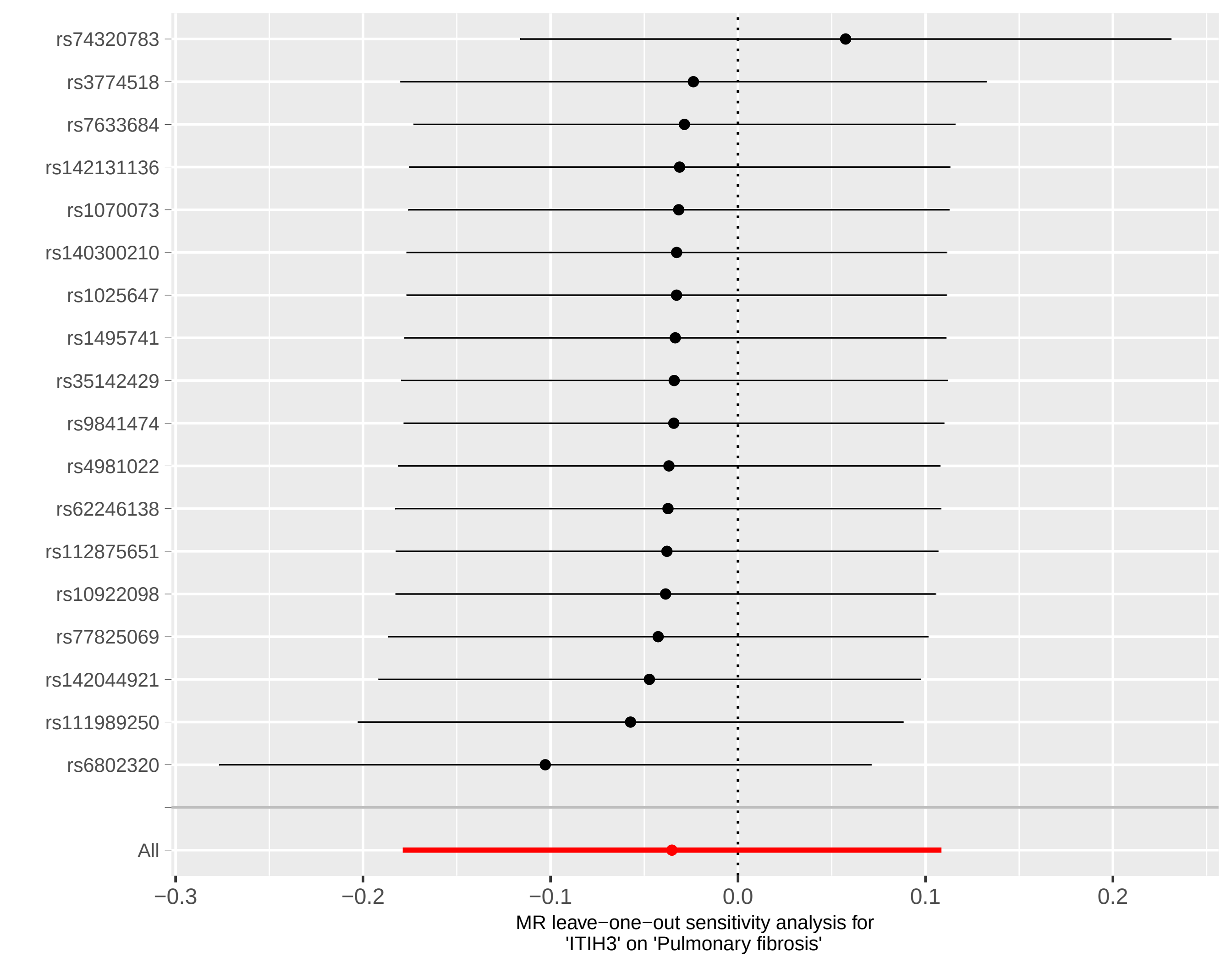
rs74320783
rs3774518
rs7633684
rs142131136
rs1070073
rs140300210
rs1025647
rs1495741
rs35142429
rs9841474
rs4981022
rs62246138
rs112875651
rs10922098
rs77825069
rs142044921
rs111989250
rs6802320
All
−0.3
−0.2
−0.1
0.0
0.1
0.2
MR leave−one−out sensitivity analysis for
'ITIH3' on 'Pulmonary fibrosis'

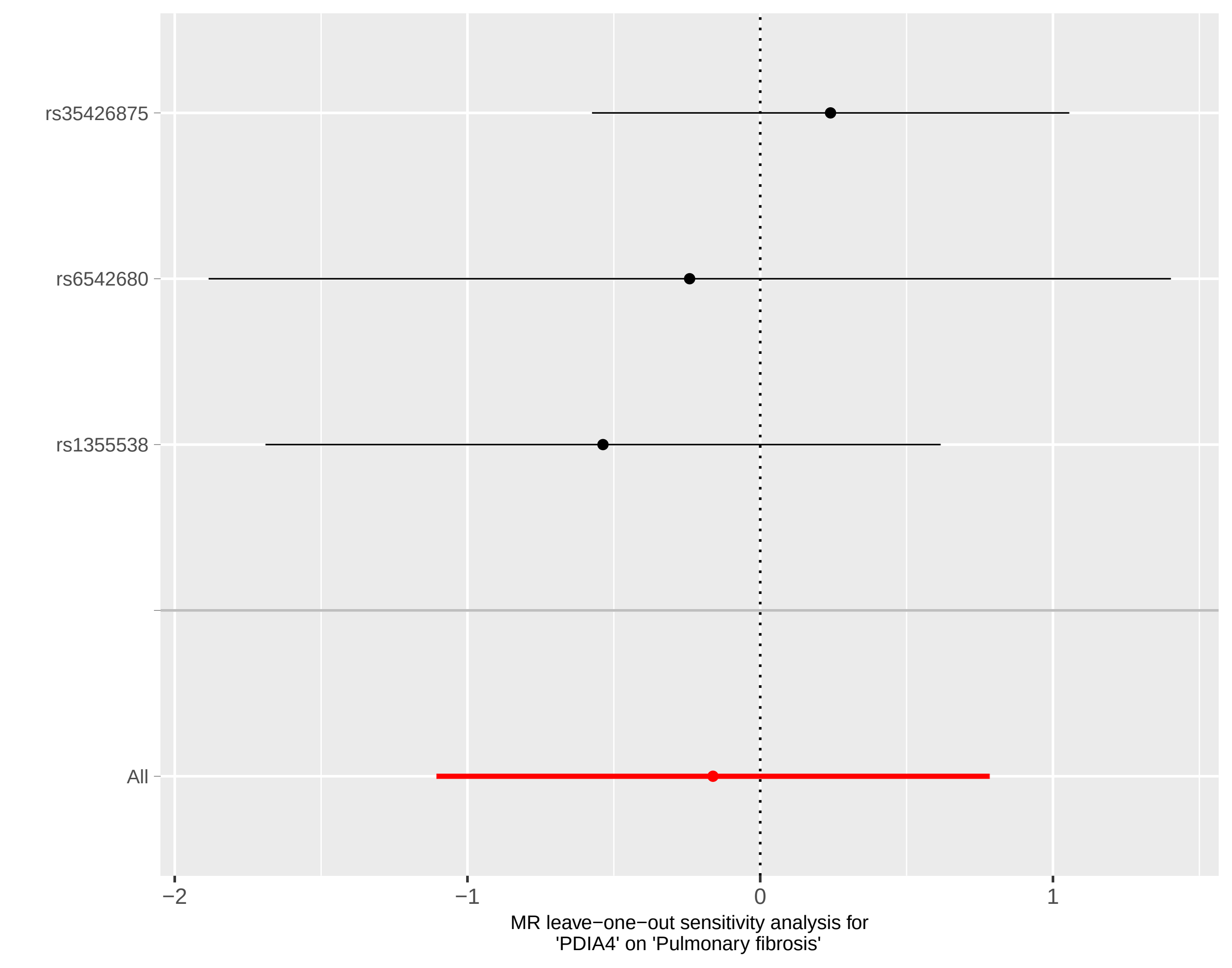
rs35426875
rs6542680
rs1355538
All
−2
−1
0
1
MR leave−one−out sensitivity analysis for
'PDIA4' on 'Pulmonary fibrosis'

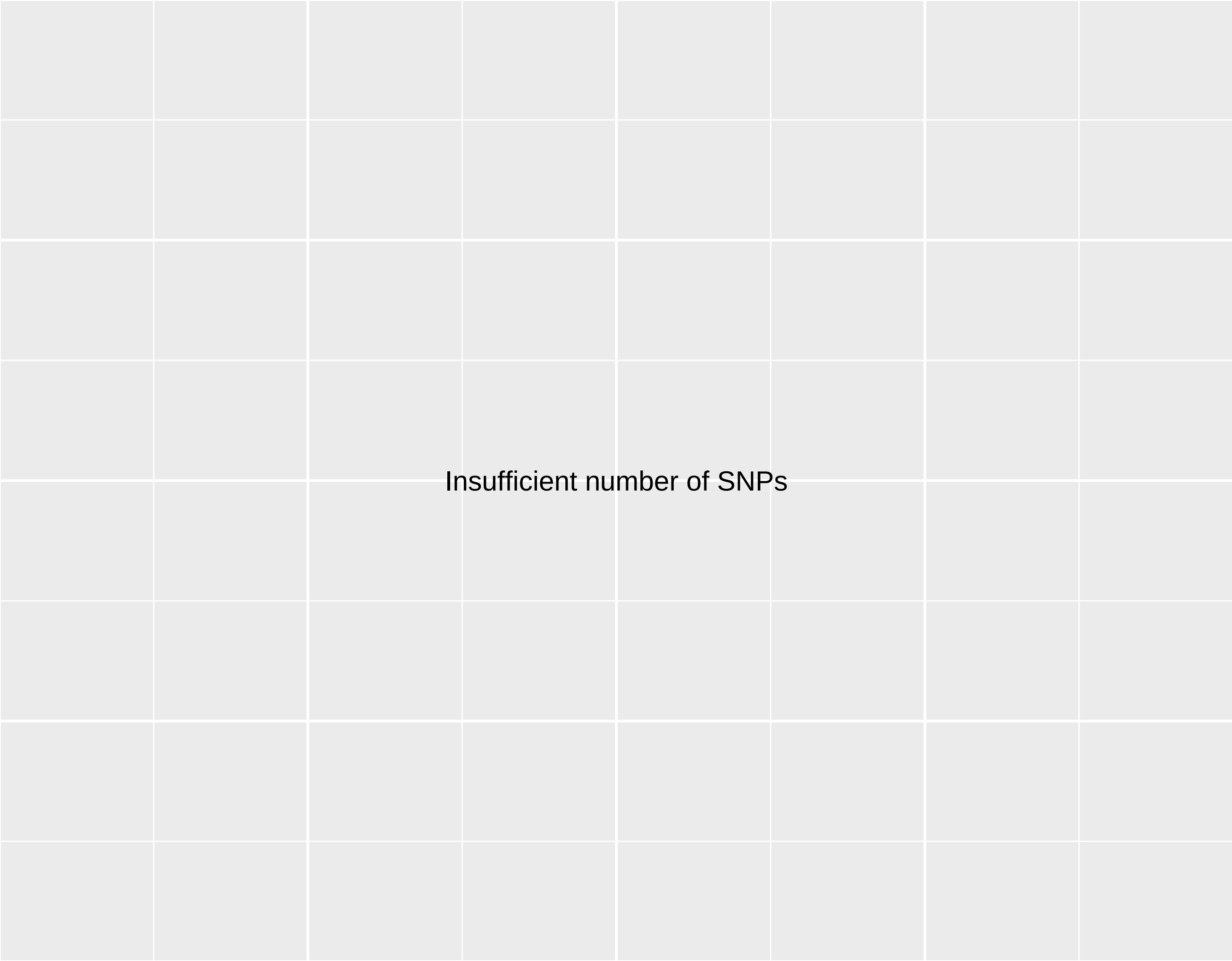
Insufficient number of SNPs

Insufficient number of SNPs

Insufficient number of SNPs

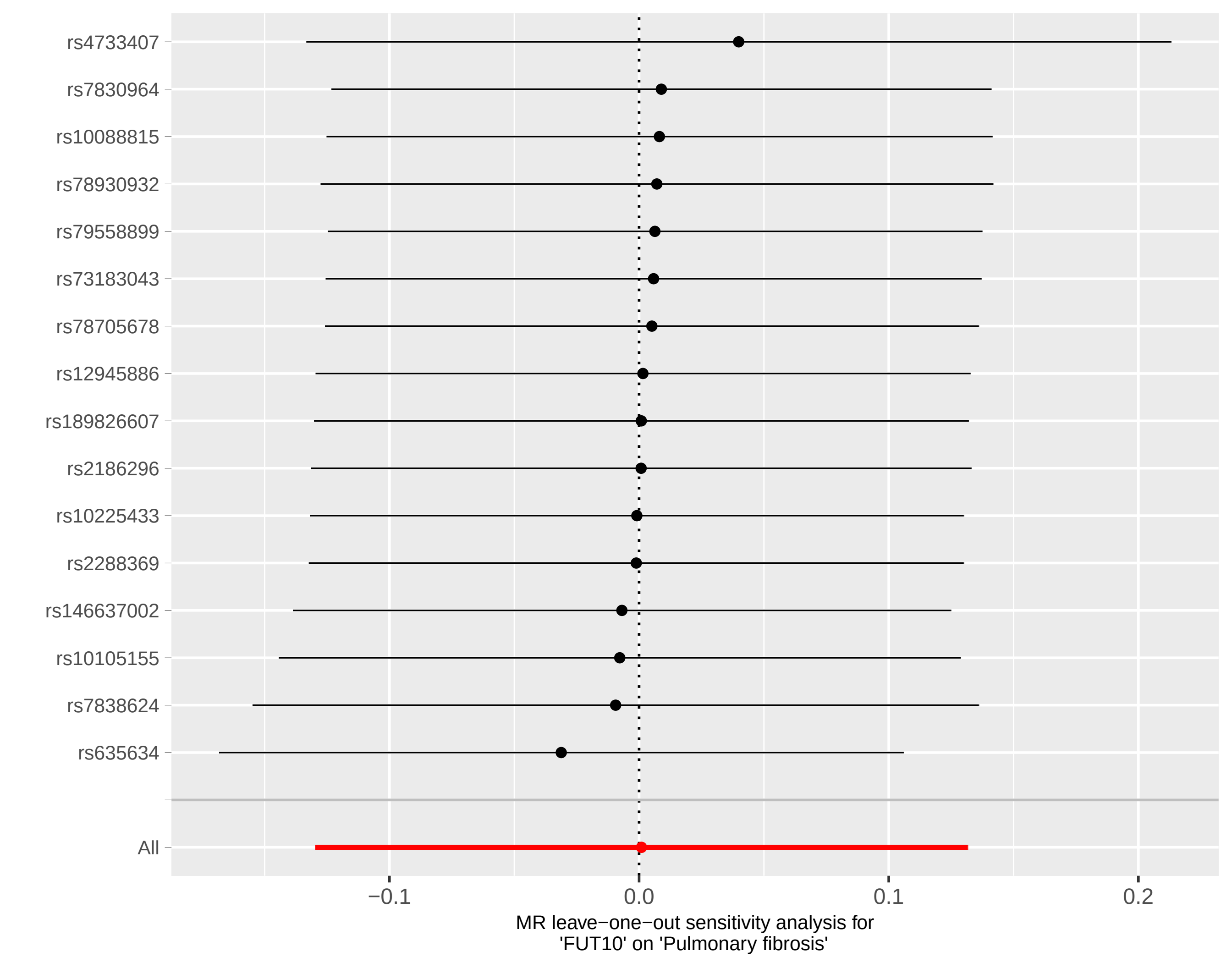
rs4733407
rs7830964
rs10088815
rs78930932
rs79558899
rs73183043
rs78705678
rs12945886
rs189826607
rs2186296
rs10225433
rs2288369
rs146637002
rs10105155
rs7838624
rs635634
All
−0.1
0.0
0.1
0.2
MR leave−one−out sensitivity analysis for
'FUT10' on 'Pulmonary fibrosis'

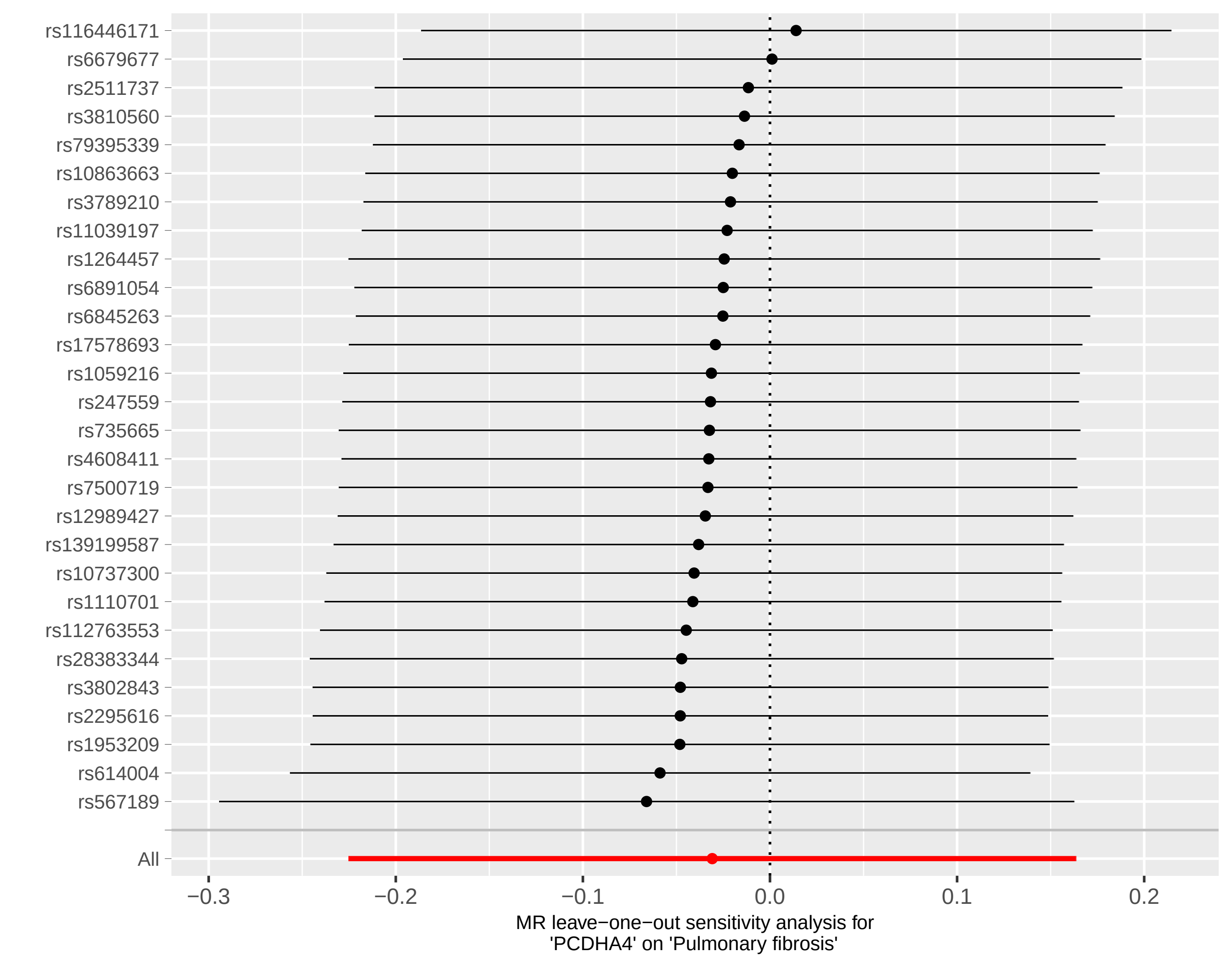
rs116446171
rs6679677
rs2511737
rs3810560
rs79395339
rs10863663
rs3789210
rs11039197
rs1264457
rs6891054
rs6845263
rs17578693
rs1059216
rs247559
rs735665
rs4608411
rs7500719
rs12989427
rs139199587
rs10737300
rs1110701
rs112763553
rs28383344
rs3802843
rs2295616
rs1953209
rs614004
rs567189
All
−0.3
−0.2
−0.1
0.0
0.1
0.2
MR leave−one−out sensitivity analysis for
'PCDHA4' on 'Pulmonary fibrosis'

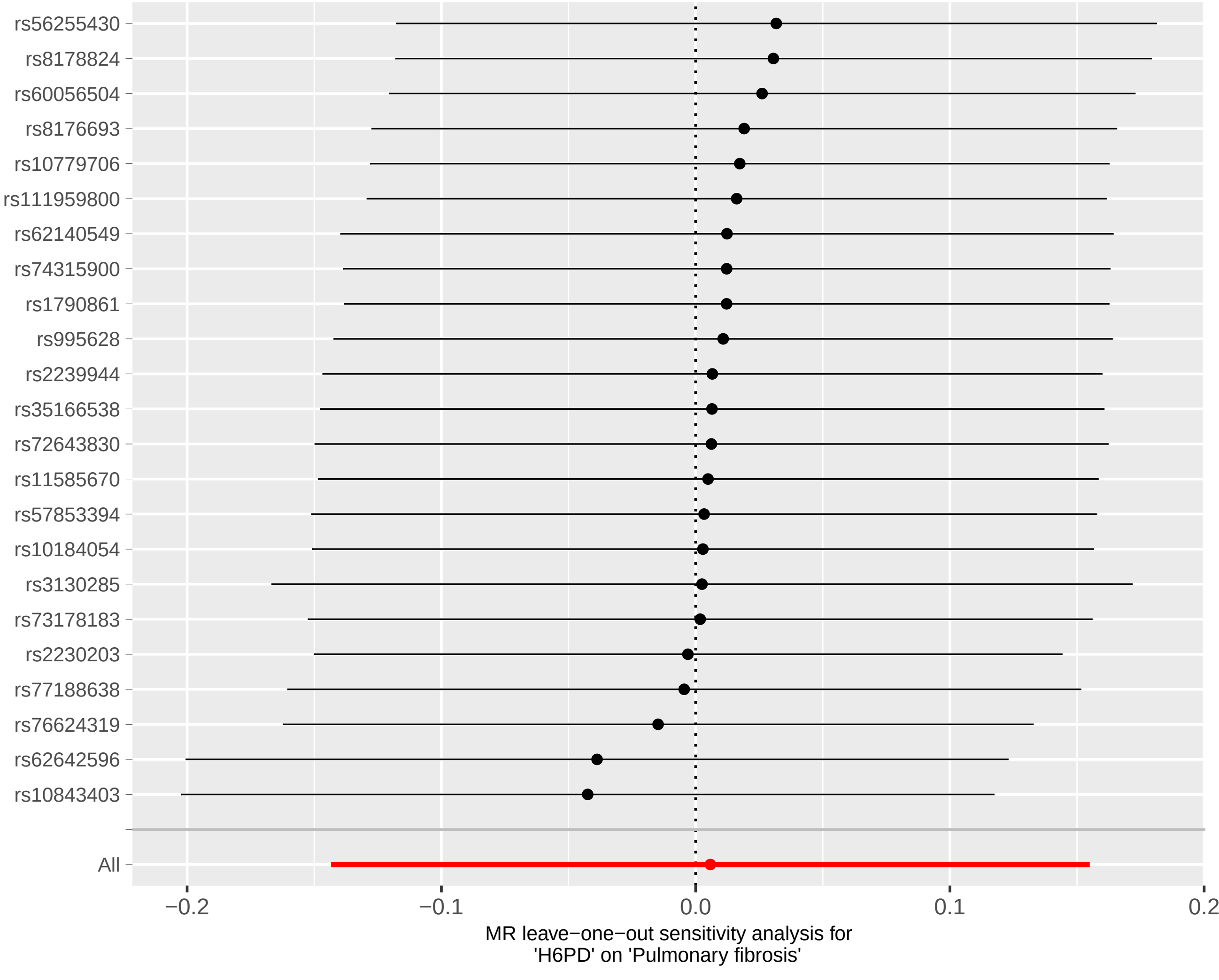
rs56255430
rs8178824
rs60056504
rs8176693
rs10779706
rs111959800
rs62140549
rs74315900
rs1790861
rs995628
rs2239944
rs35166538
rs72643830
rs11585670
rs57853394
rs10184054
rs3130285
rs73178183
rs2230203
rs77188638
rs76624319
rs62642596
rs10843403
All
−0.2
−0.1
0.0
0.1
0.2
MR leave−one−out sensitivity analysis for
'H6PD' on 'Pulmonary fibrosis'

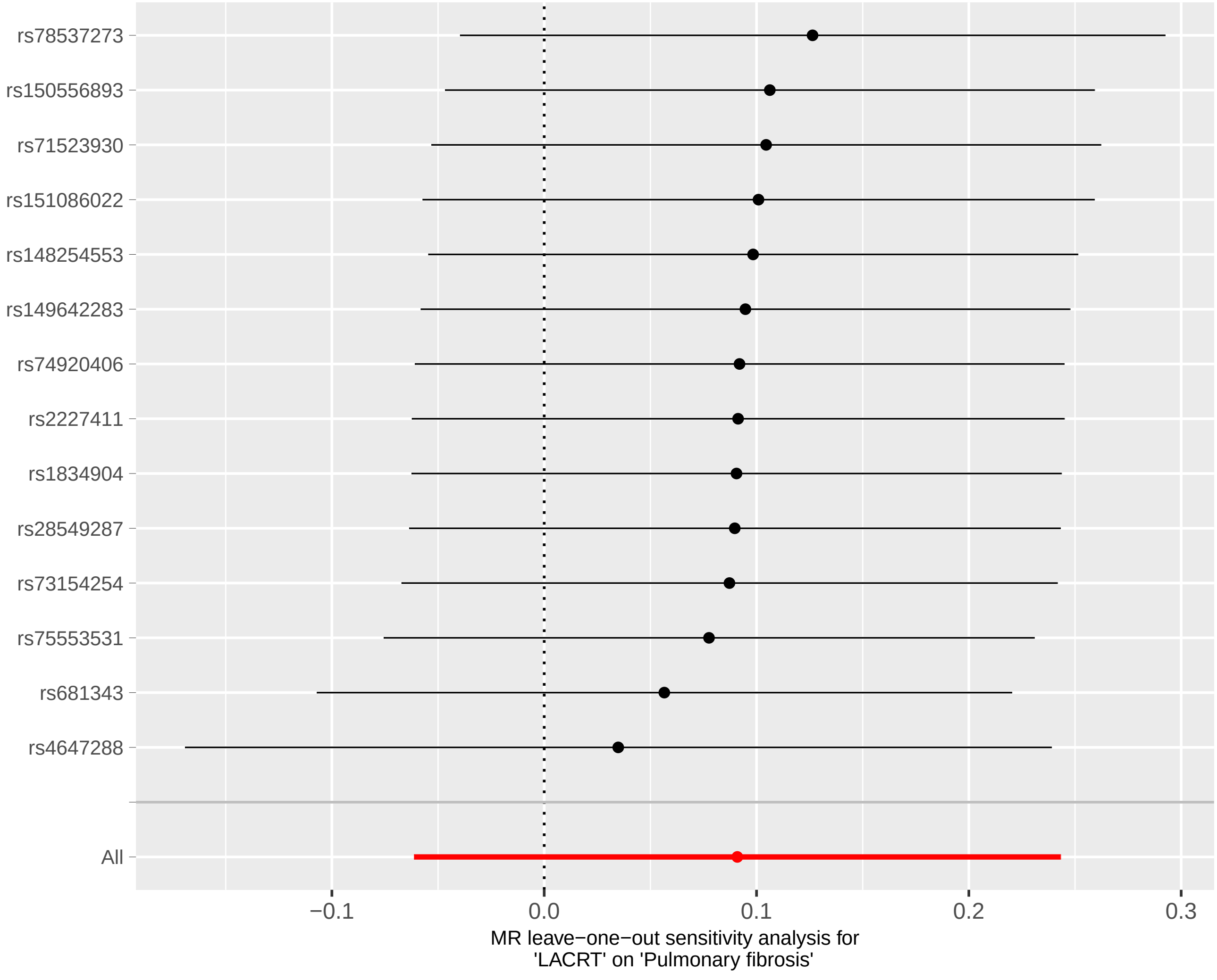
rs78537273
rs150556893
rs71523930
rs151086022
rs148254553
rs149642283
rs74920406
rs2227411
rs1834904
rs28549287
rs73154254
rs75553531
rs681343
rs4647288
All
−0.1
0.0
0.1
0.2
0.3
MR leave−one−out sensitivity analysis for
'LACRT' on 'Pulmonary fibrosis'

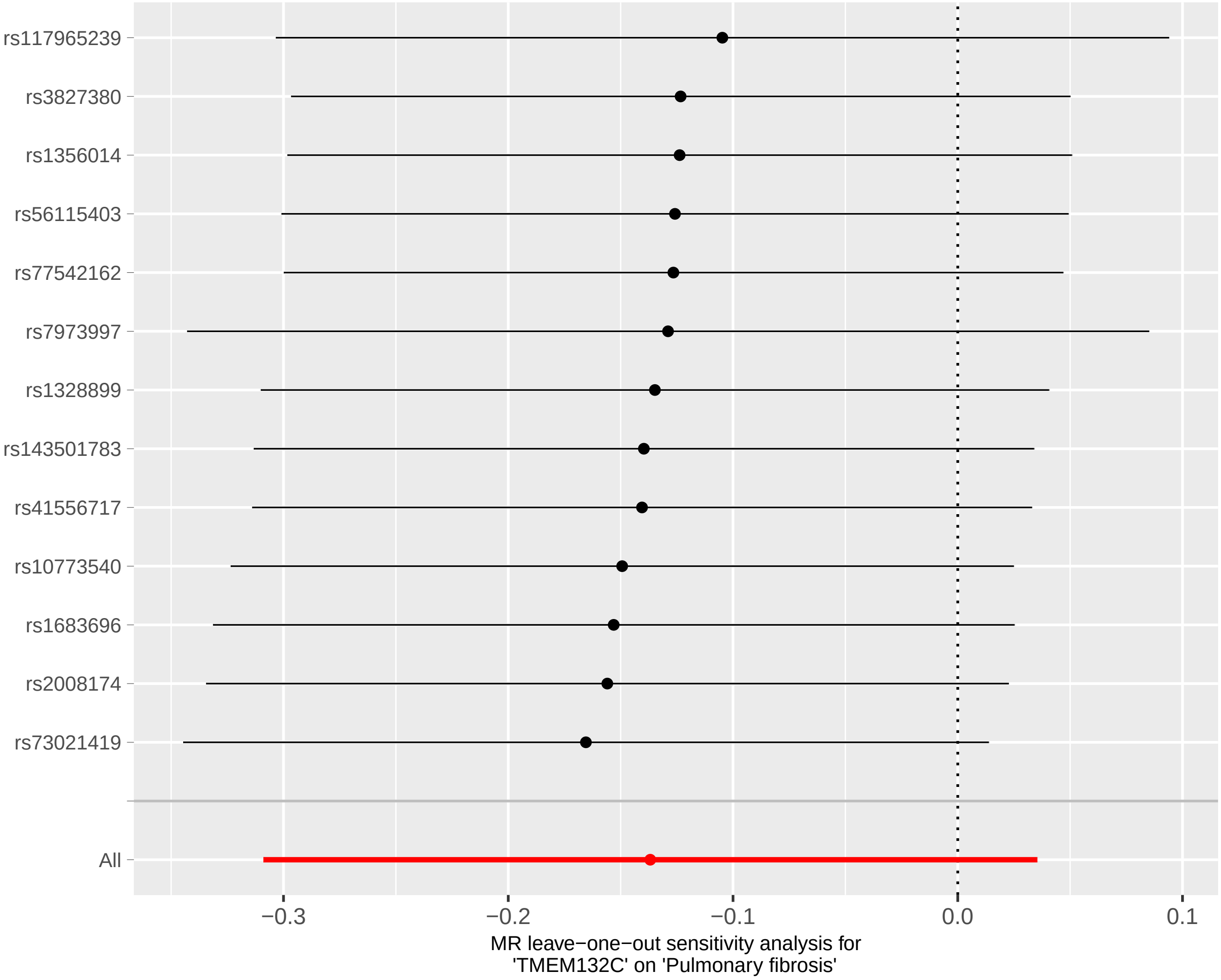
rs117965239
rs3827380
rs1356014
rs56115403
rs77542162
rs7973997
rs1328899
rs143501783
rs41556717
rs10773540
rs1683696
rs2008174
rs73021419
All
−0.3
−0.2
−0.1
0.0
0.1
MR leave−one−out sensitivity analysis for
'TMEM132C' on 'Pulmonary fibrosis'

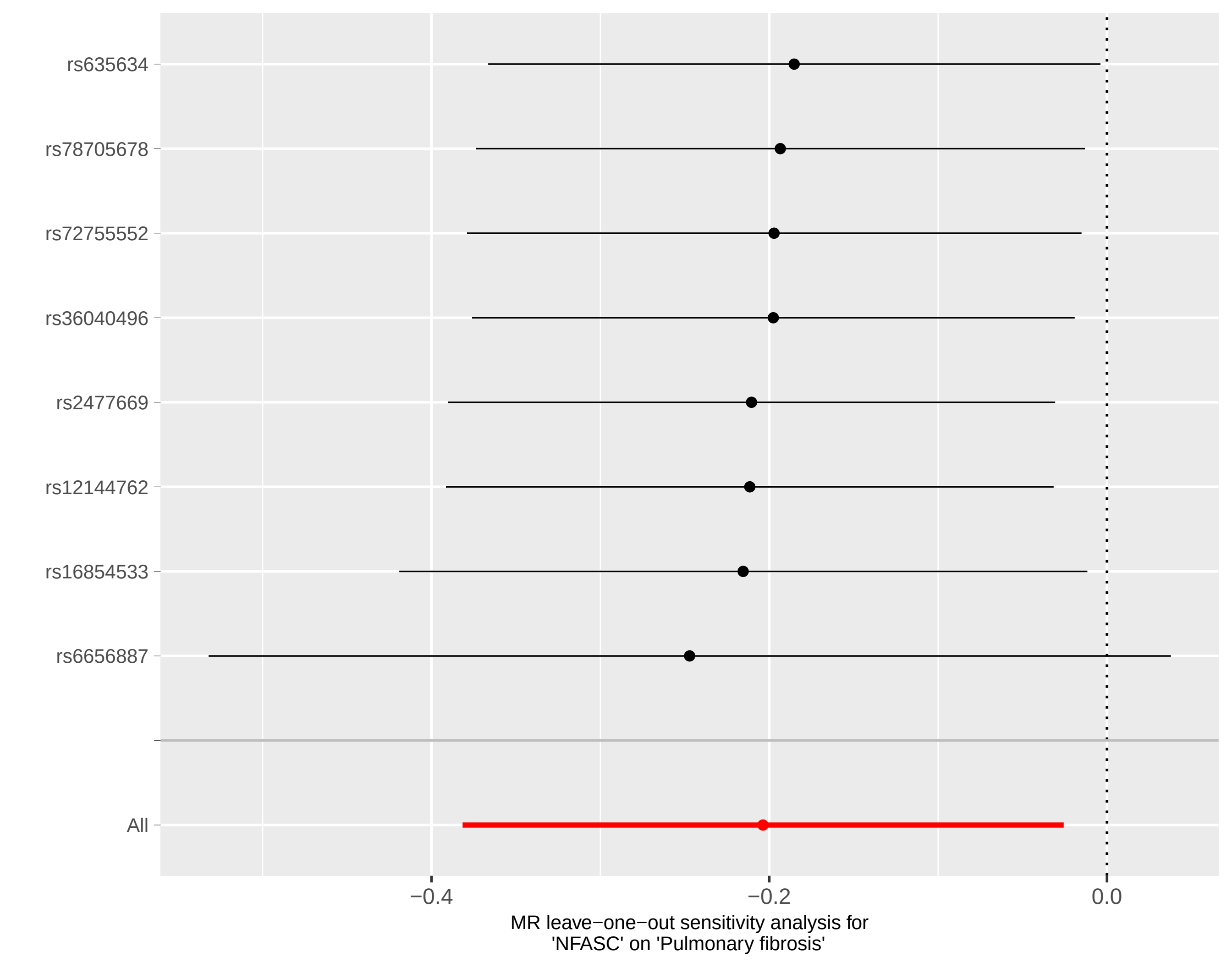
rs635634
rs78705678
rs72755552
rs36040496
rs2477669
rs12144762
rs16854533
rs6656887
All
−0.4
−0.2
0.0
MR leave−one−out sensitivity analysis for
'NFASC' on 'Pulmonary fibrosis'

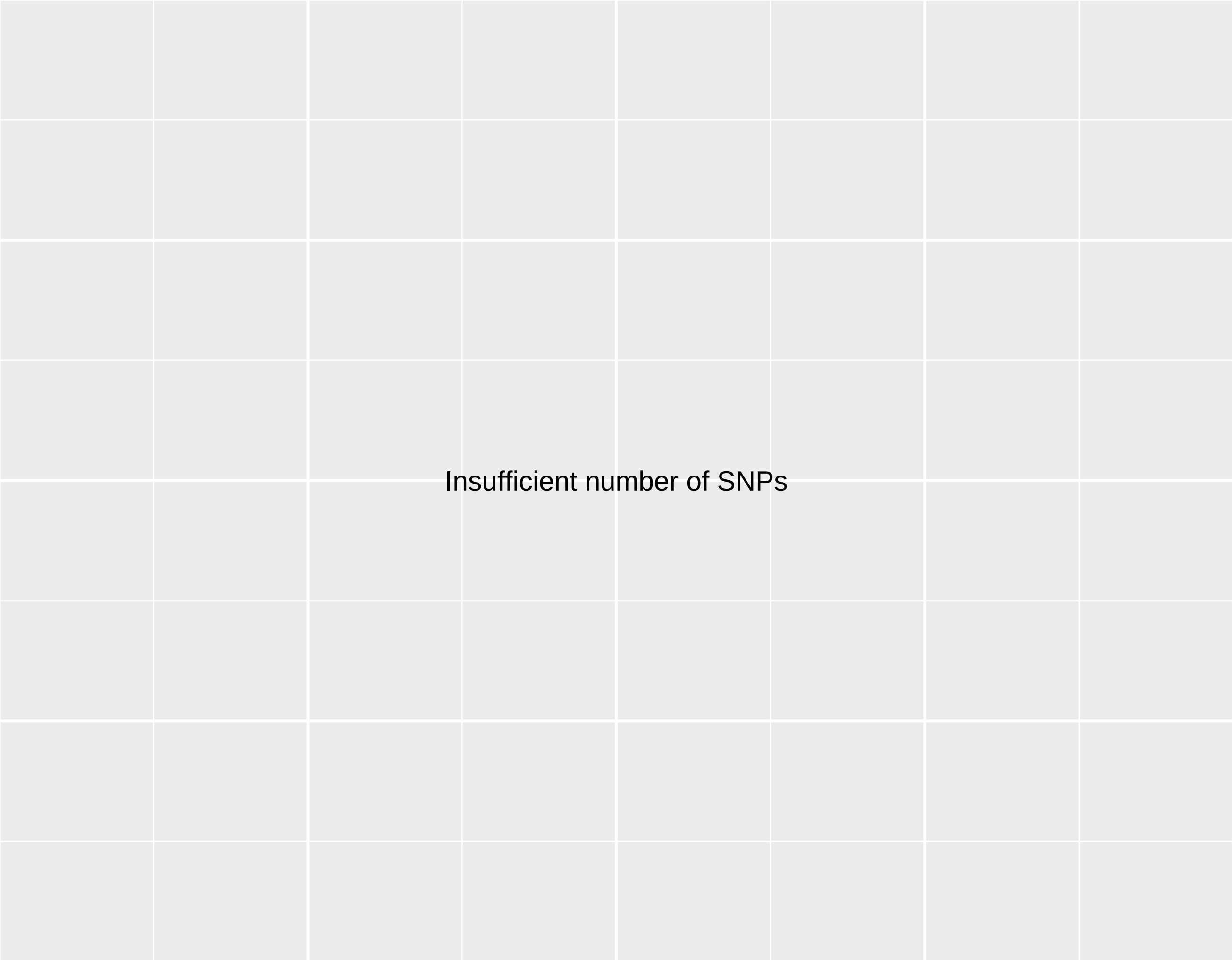
Insufficient number of SNPs

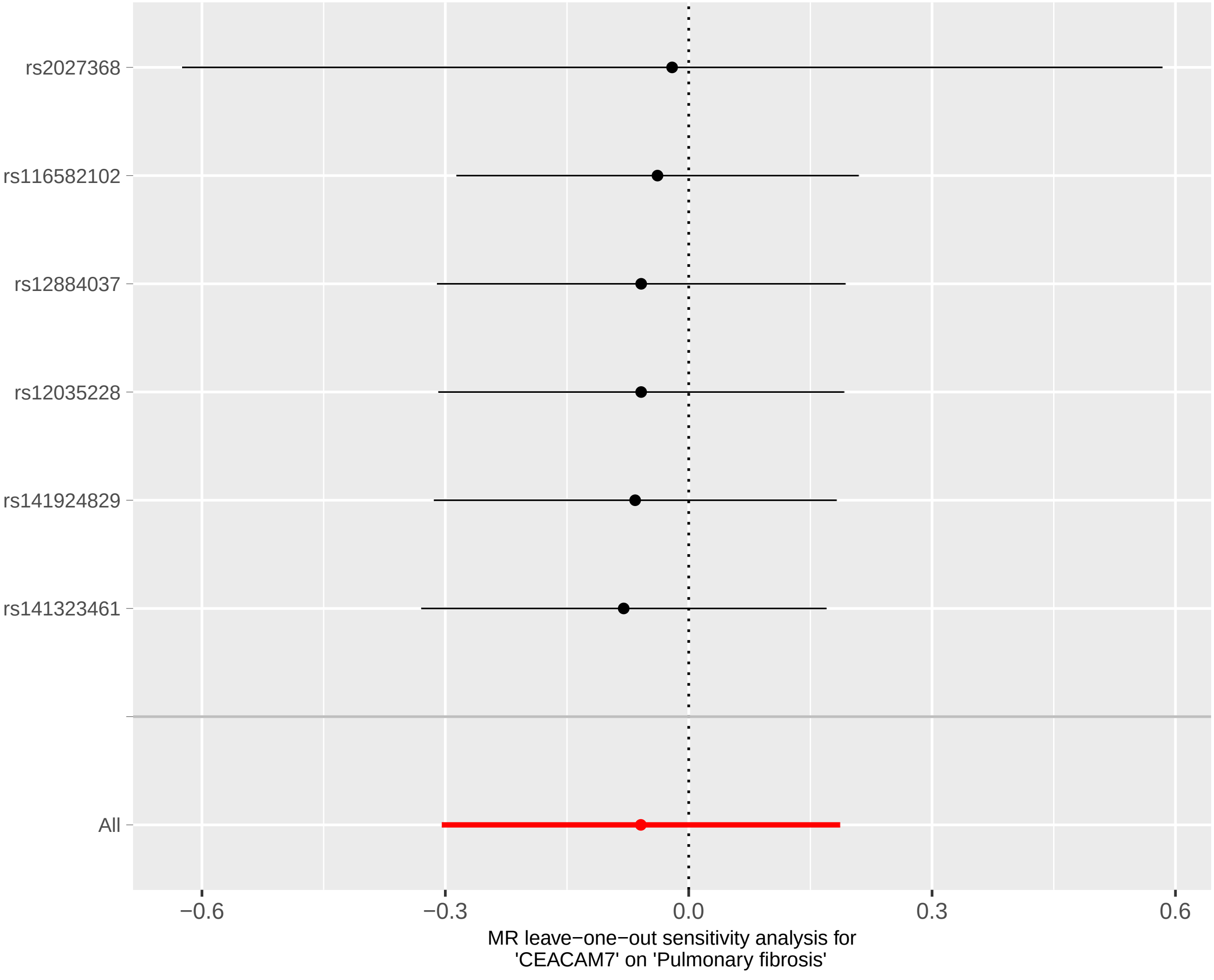
rs2027368
rs116582102
rs12884037
rs12035228
rs141924829
rs141323461
All
−0.6
−0.3
0.0
0.3
0.6
MR leave−one−out sensitivity analysis for
'CEACAM7' on 'Pulmonary fibrosis'

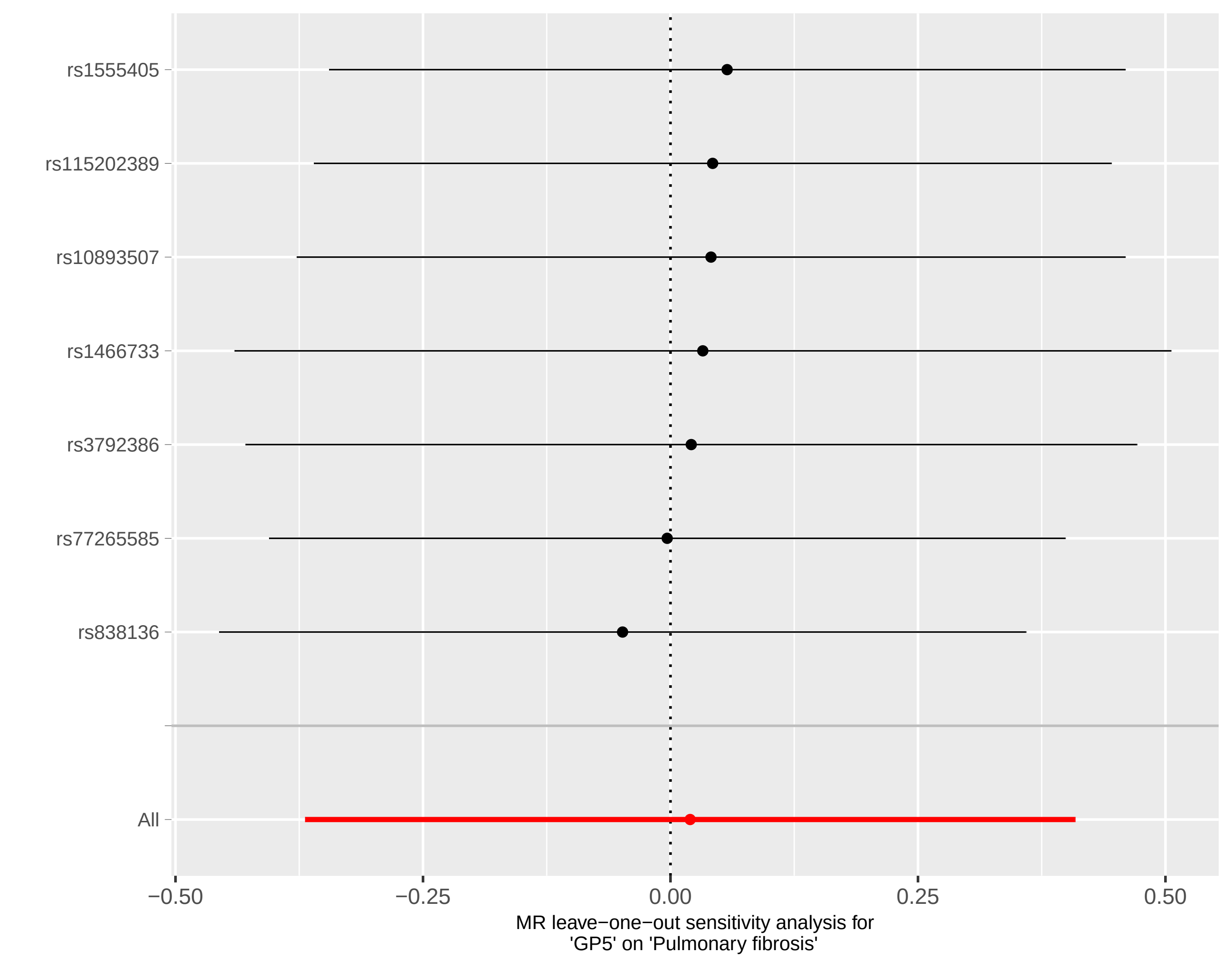
rs1555405
rs115202389
rs10893507
rs1466733
rs3792386
rs77265585
rs838136
All
−0.50
−0.25
0.00
0.25
0.50
MR leave−one−out sensitivity analysis for
'GP5' on 'Pulmonary fibrosis'

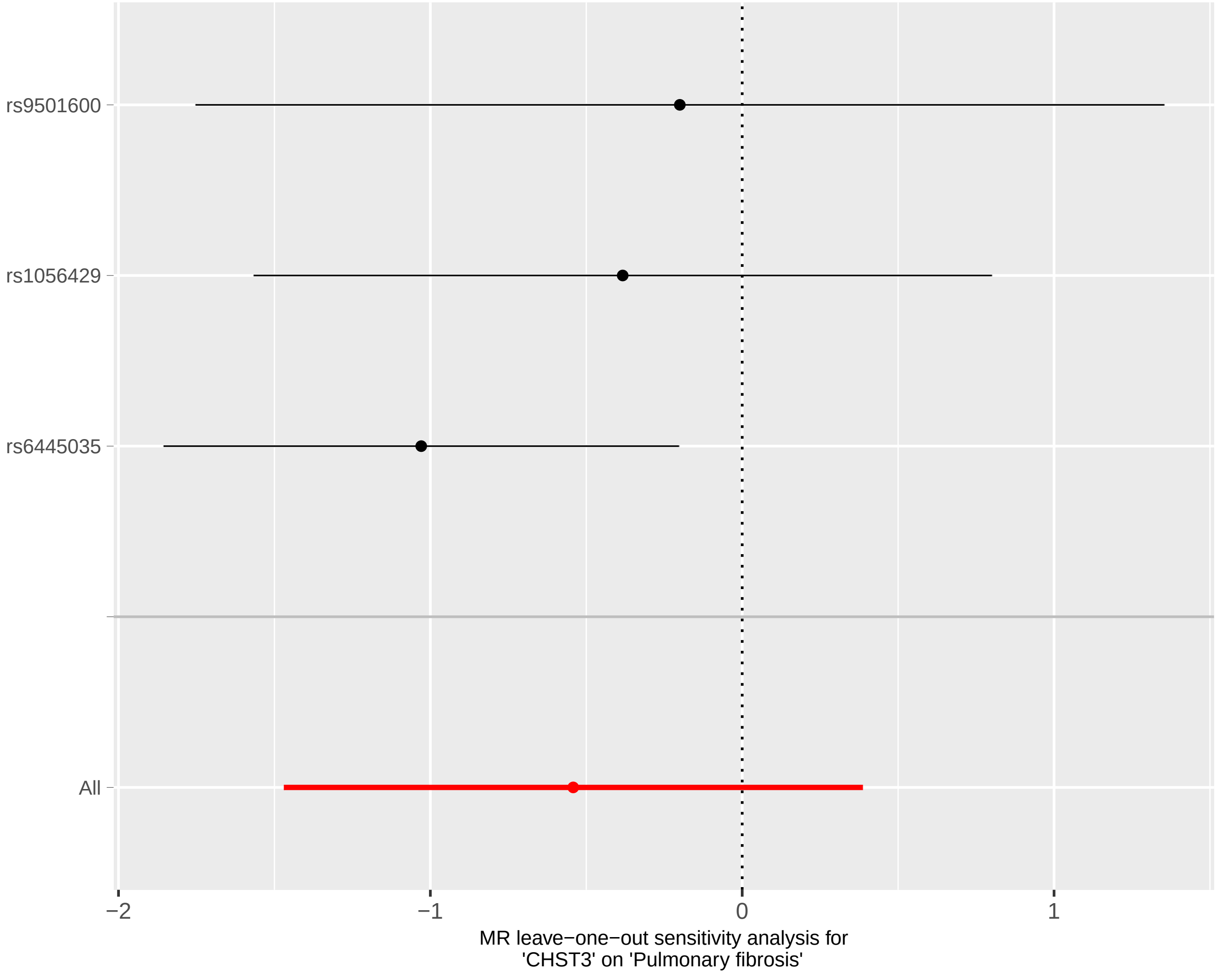
rs9501600
rs1056429
rs6445035
All
−2
−1
0
1
MR leave−one−out sensitivity analysis for
'CHST3' on 'Pulmonary fibrosis'

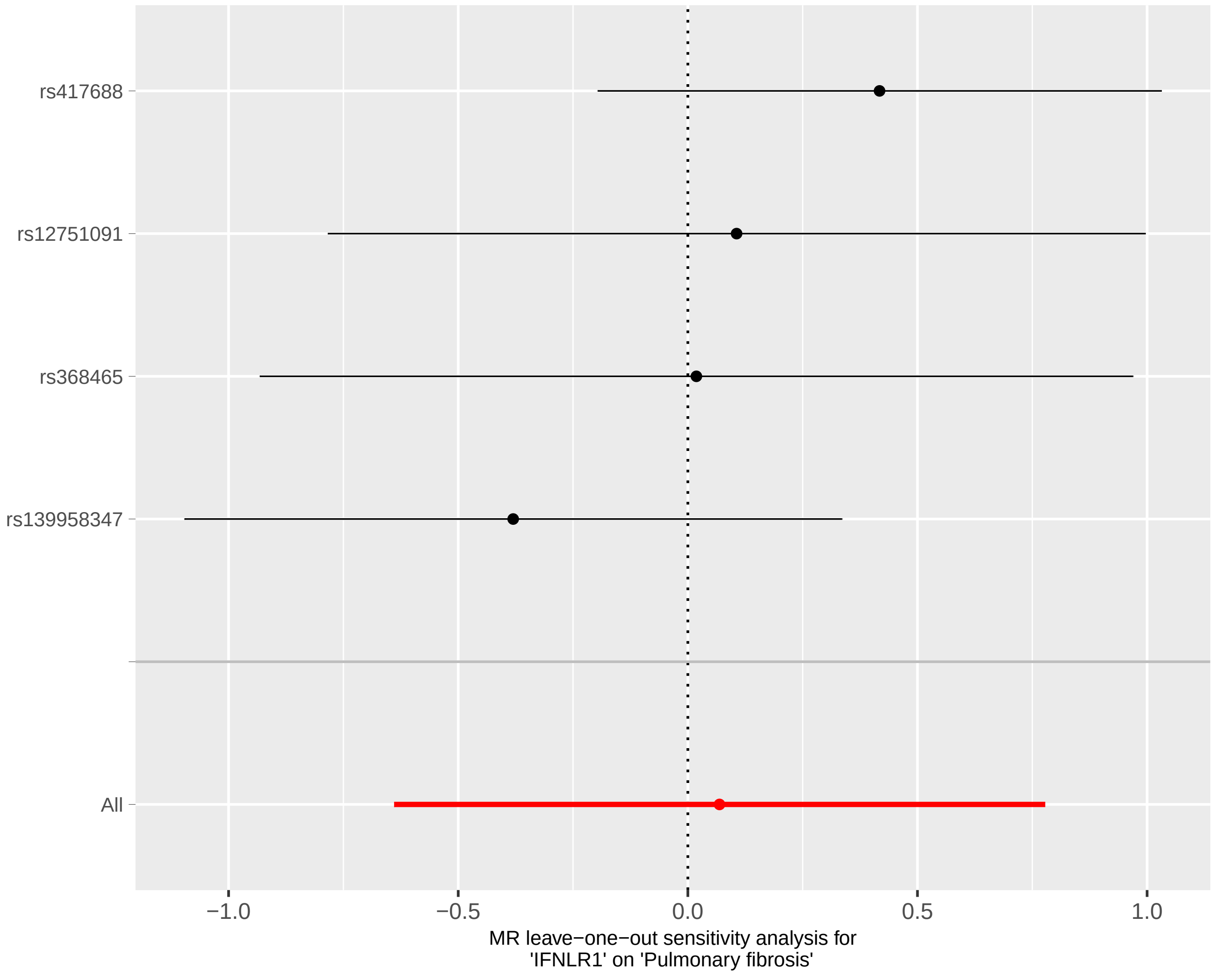
rs417688
rs12751091
rs368465
rs139958347
All
−1.0
−0.5
0.0
0.5
1.0
MR leave−one−out sensitivity analysis for
'IFNLR1' on 'Pulmonary fibrosis'

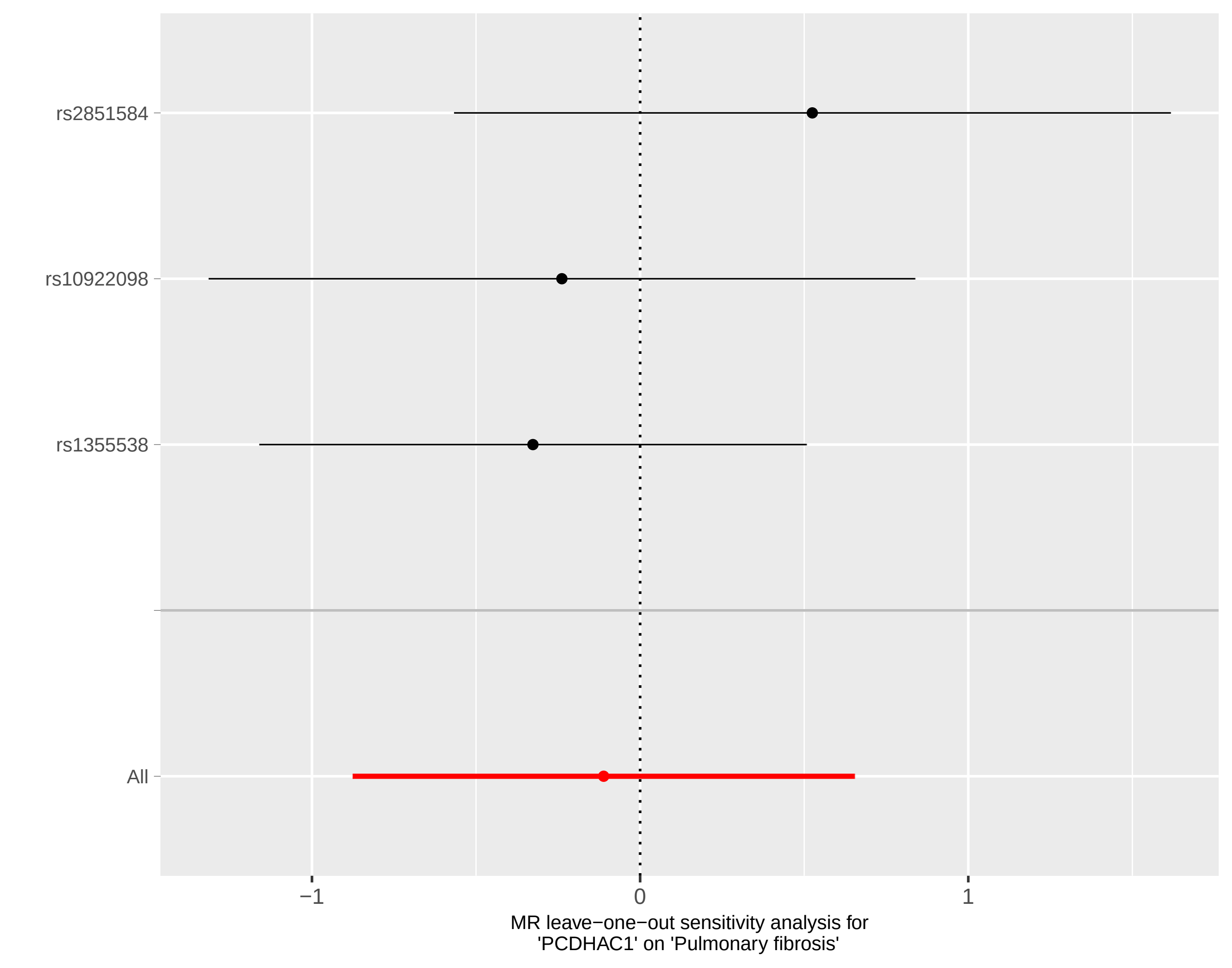
rs2851584
rs10922098
rs1355538
All
−1
0
1
MR leave−one−out sensitivity analysis for
'PCDHAC1' on 'Pulmonary fibrosis'

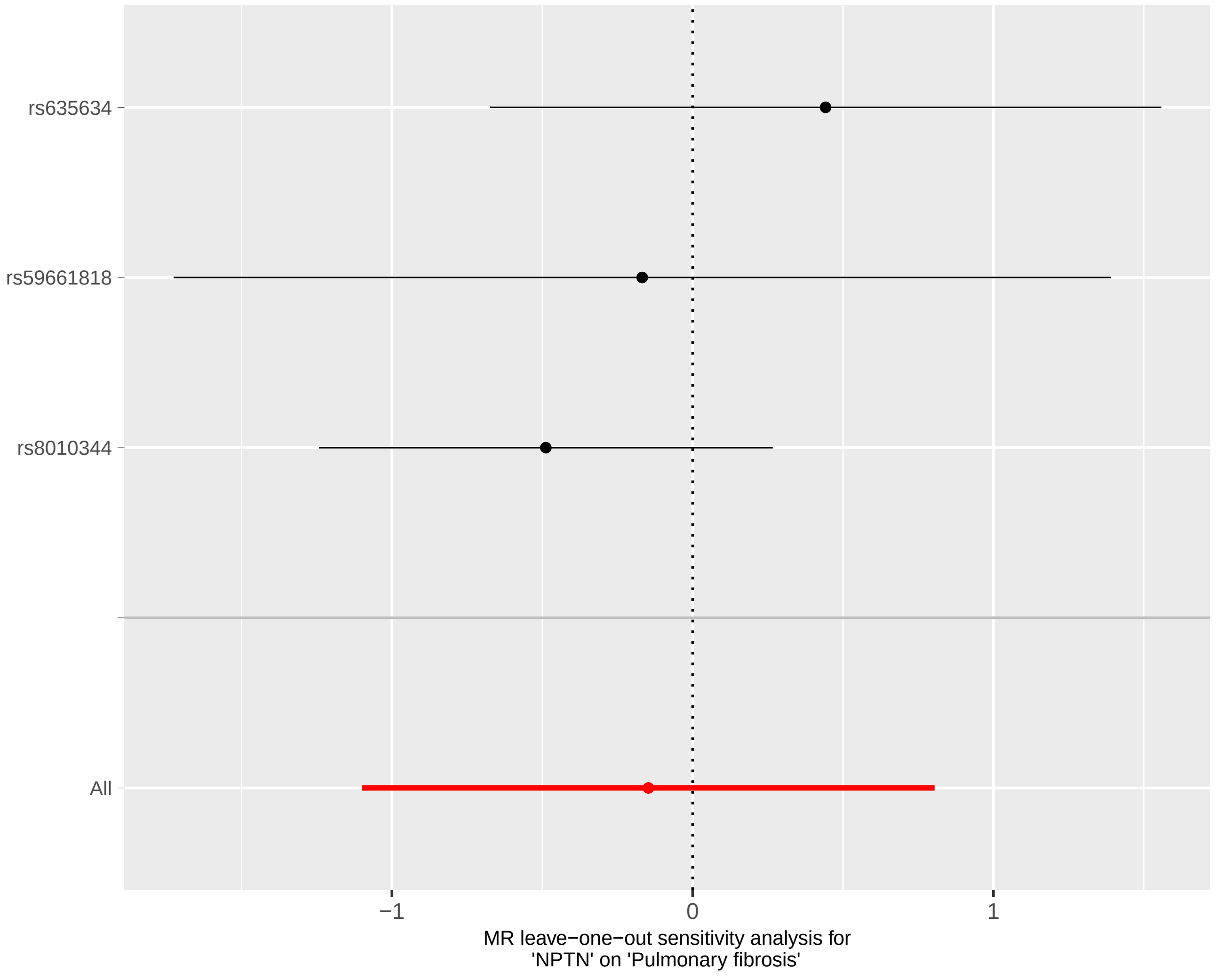
rs635634
rs59661818
rs8010344
All
−1
0
1
MR leave−one−out sensitivity analysis for
'NPTN' on 'Pulmonary fibrosis'

Insufficient number of SNPs

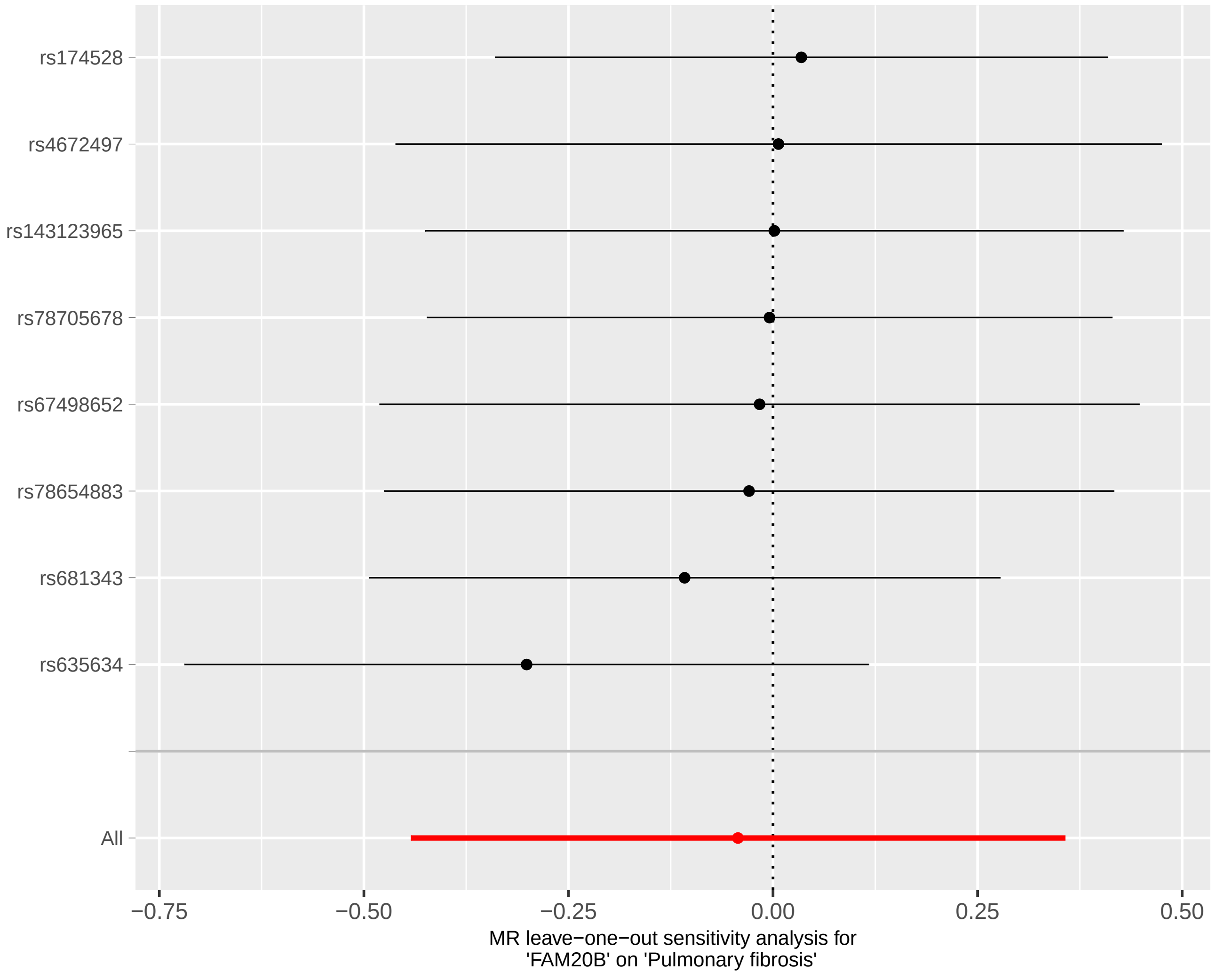

rs174528
rs4672497
rs143123965
rs78705678
rs67498652
rs78654883
rs681343
rs635634
All
−0.75
−0.50
−0.25
0.00
0.25
0.50
MR leave−one−out sensitivity analysis for
'FAM20B' on 'Pulmonary fibrosis'

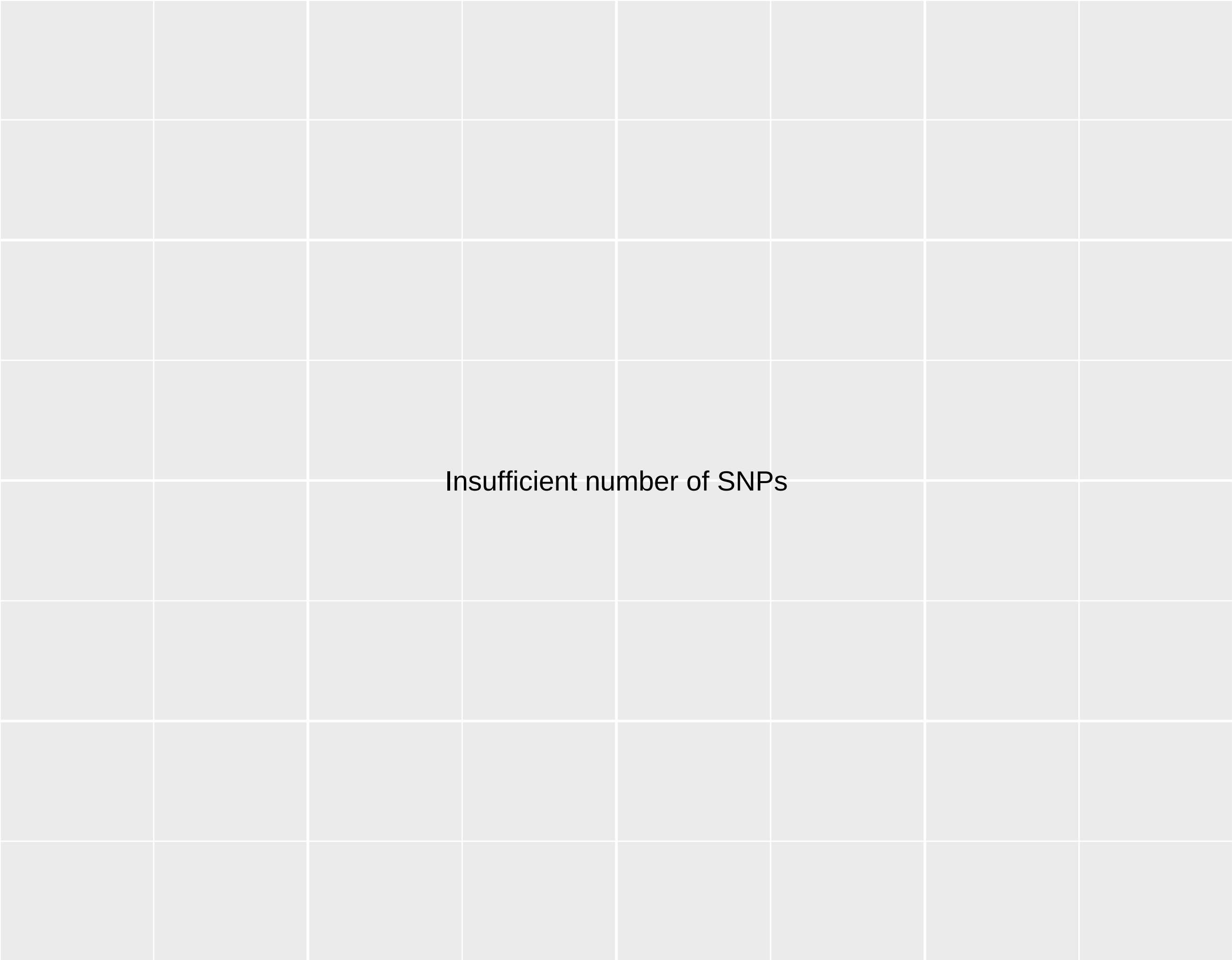
Insufficient number of SNPs

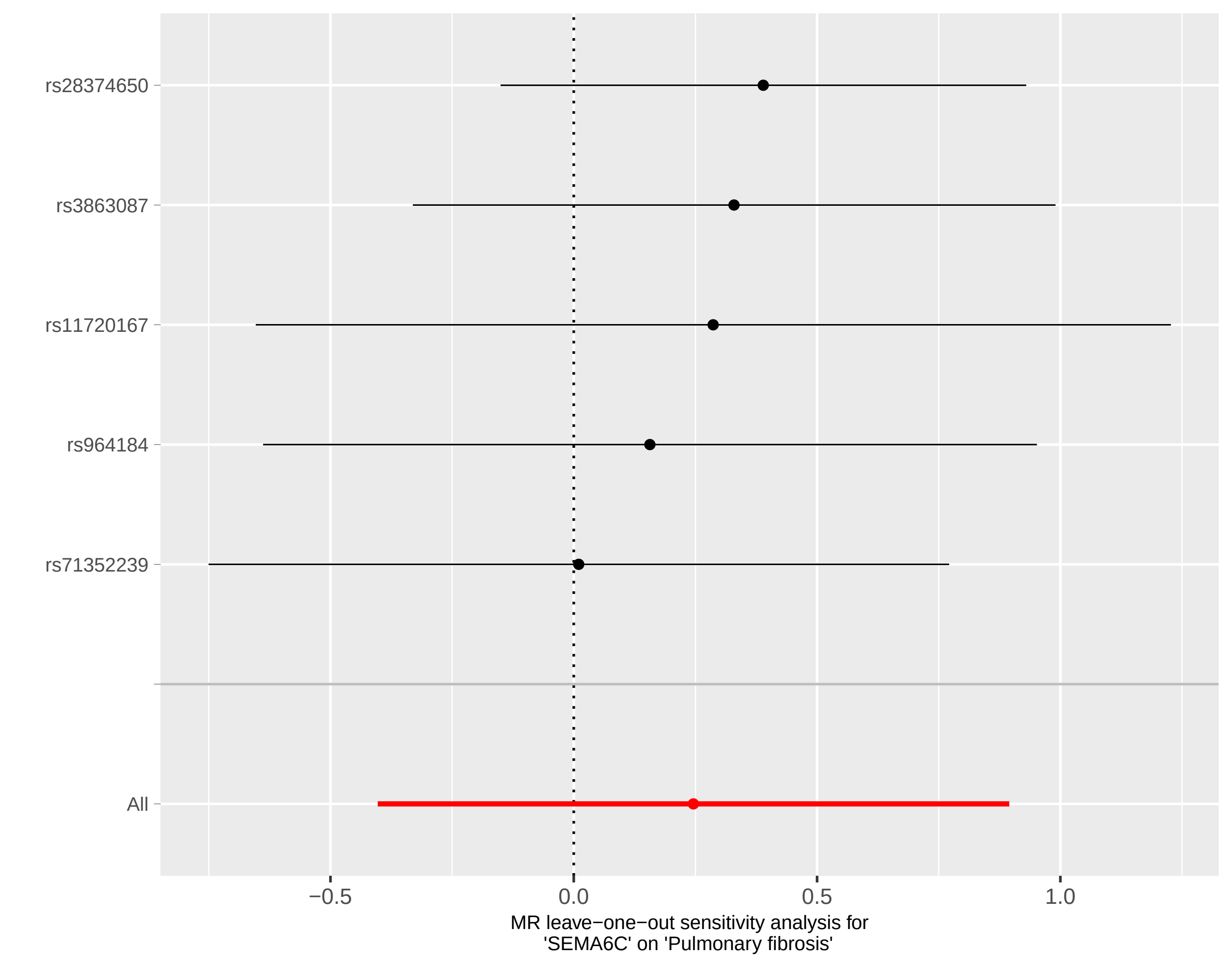
rs28374650
rs3863087
rs11720167
rs964184
rs71352239
All
−0.5
0.0
0.5
1.0
MR leave−one−out sensitivity analysis for
'SEMA6C' on 'Pulmonary fibrosis'

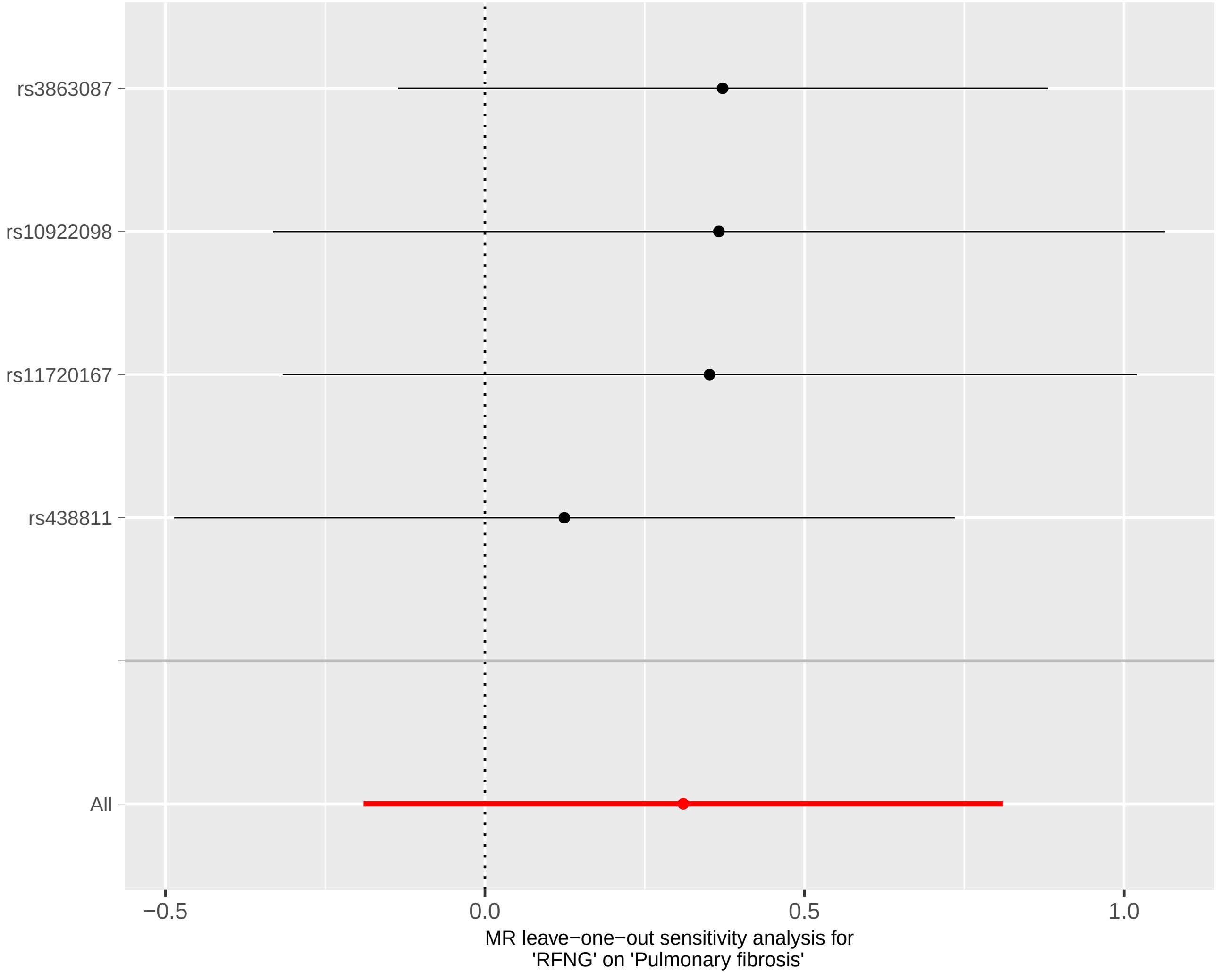

rs3863087
rs10922098
rs11720167
rs438811
All
−0.5
0.0
0.5
1.0
MR leave−one−out sensitivity analysis for
'RFNG' on 'Pulmonary fibrosis'

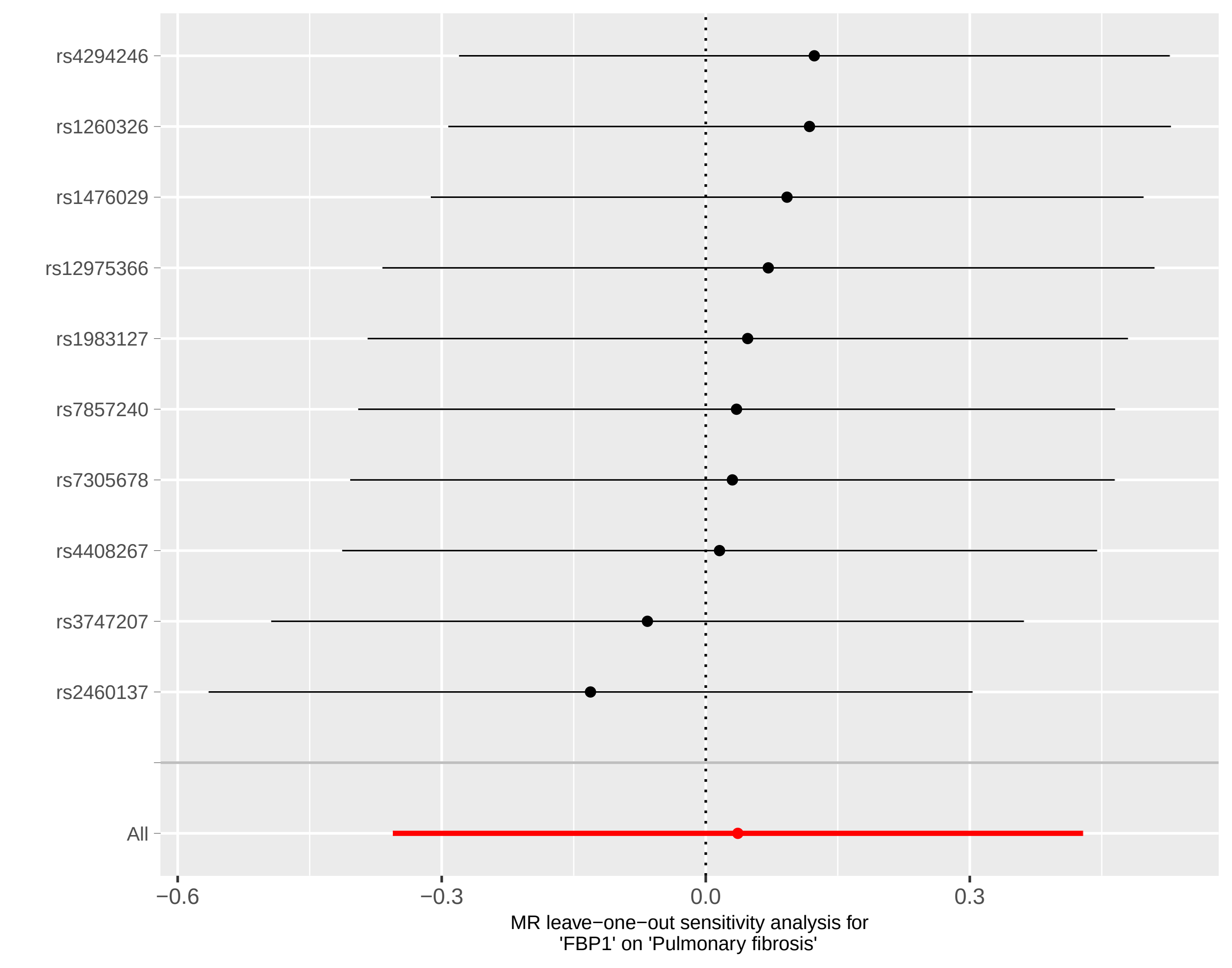
rs4294246
rs1260326
rs1476029
rs12975366
rs1983127
rs7857240
rs7305678
rs4408267
rs3747207
rs2460137
All
−0.6
−0.3
0.0
0.3
MR leave−one−out sensitivity analysis for
'FBP1' on 'Pulmonary fibrosis'

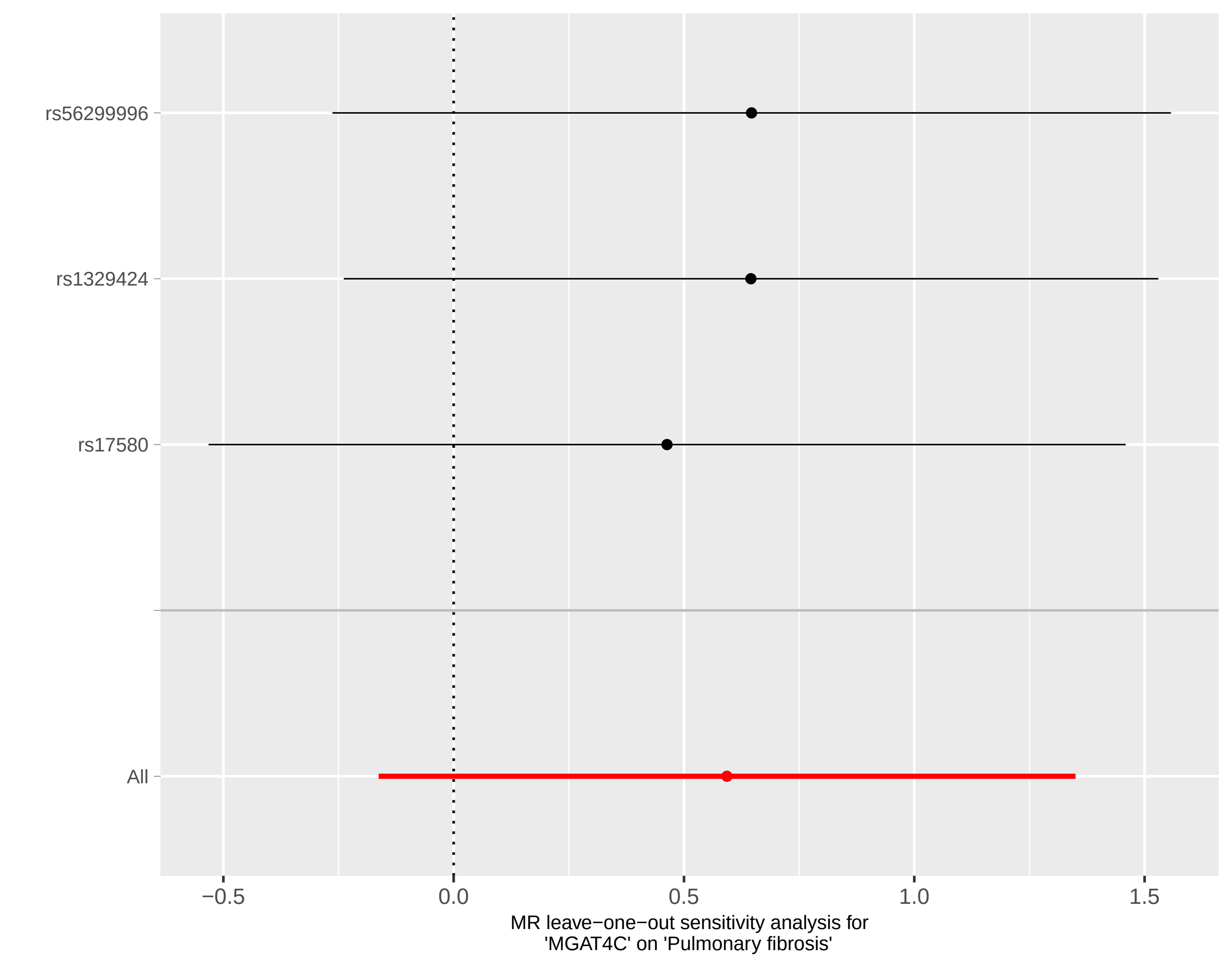
rs56299996
rs1329424
rs17580
All
−0.5
0.0
0.5
1.0
1.5
MR leave−one−out sensitivity analysis for
'MGAT4C' on 'Pulmonary fibrosis'

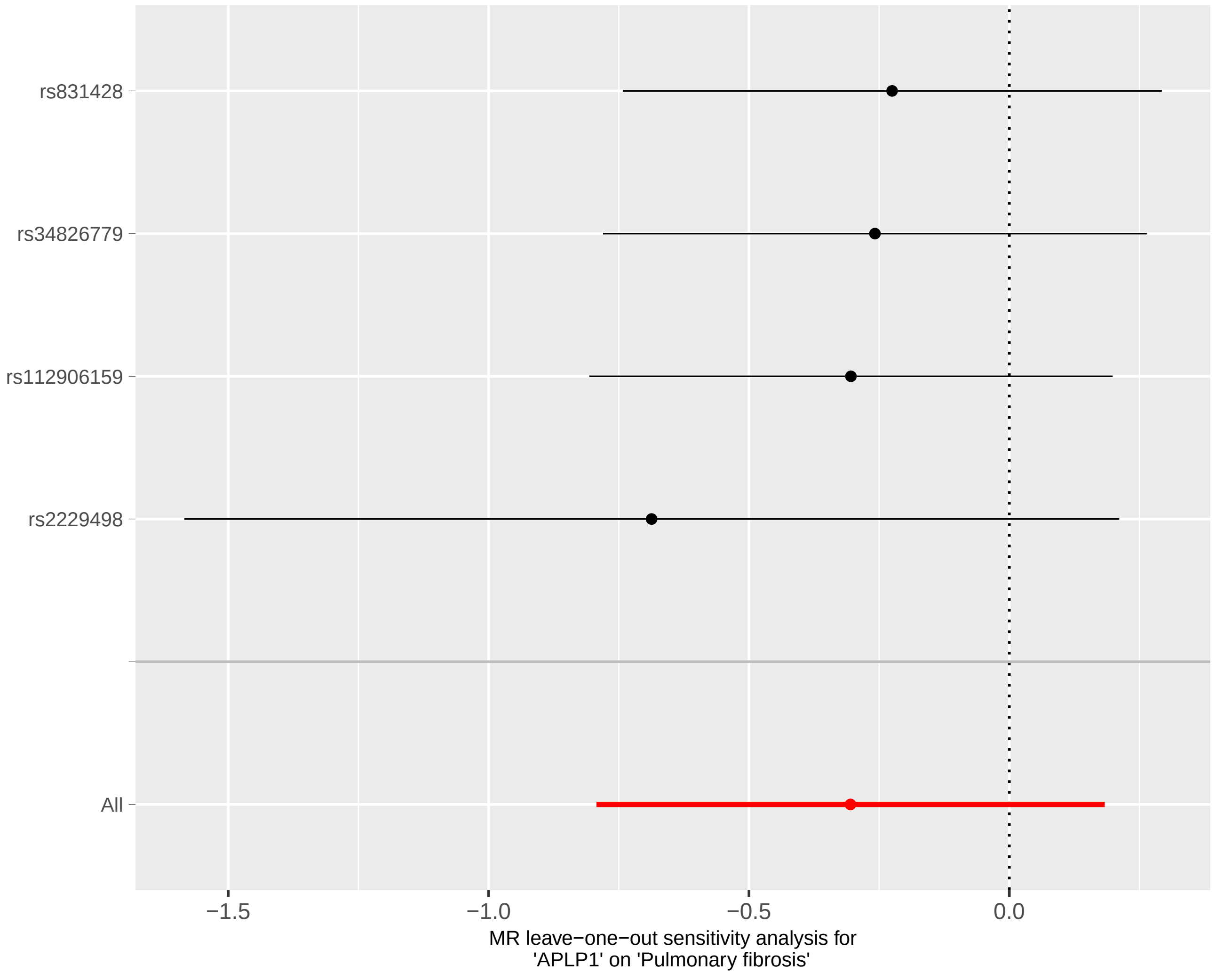
rs831428
rs34826779
rs112906159
rs2229498
All
−1.5
−1.0
−0.5
0.0
MR leave−one−out sensitivity analysis for
'APLP1' on 'Pulmonary fibrosis'

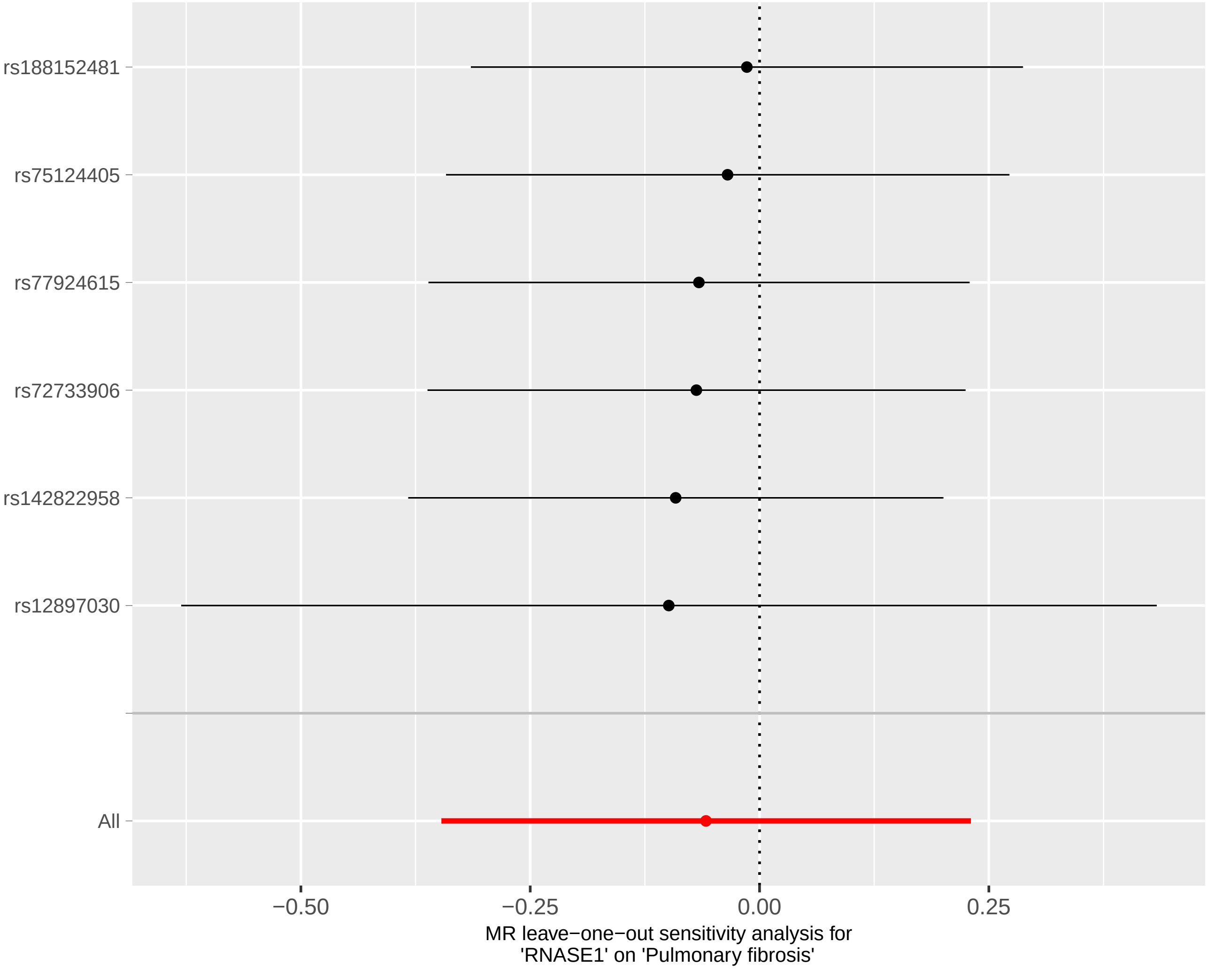
rs188152481
rs75124405
rs77924615
rs72733906
rs142822958
rs12897030
All
−0.50
−0.25
0.00
0.25
MR leave−one−out sensitivity analysis for
'RNASE1' on 'Pulmonary fibrosis'

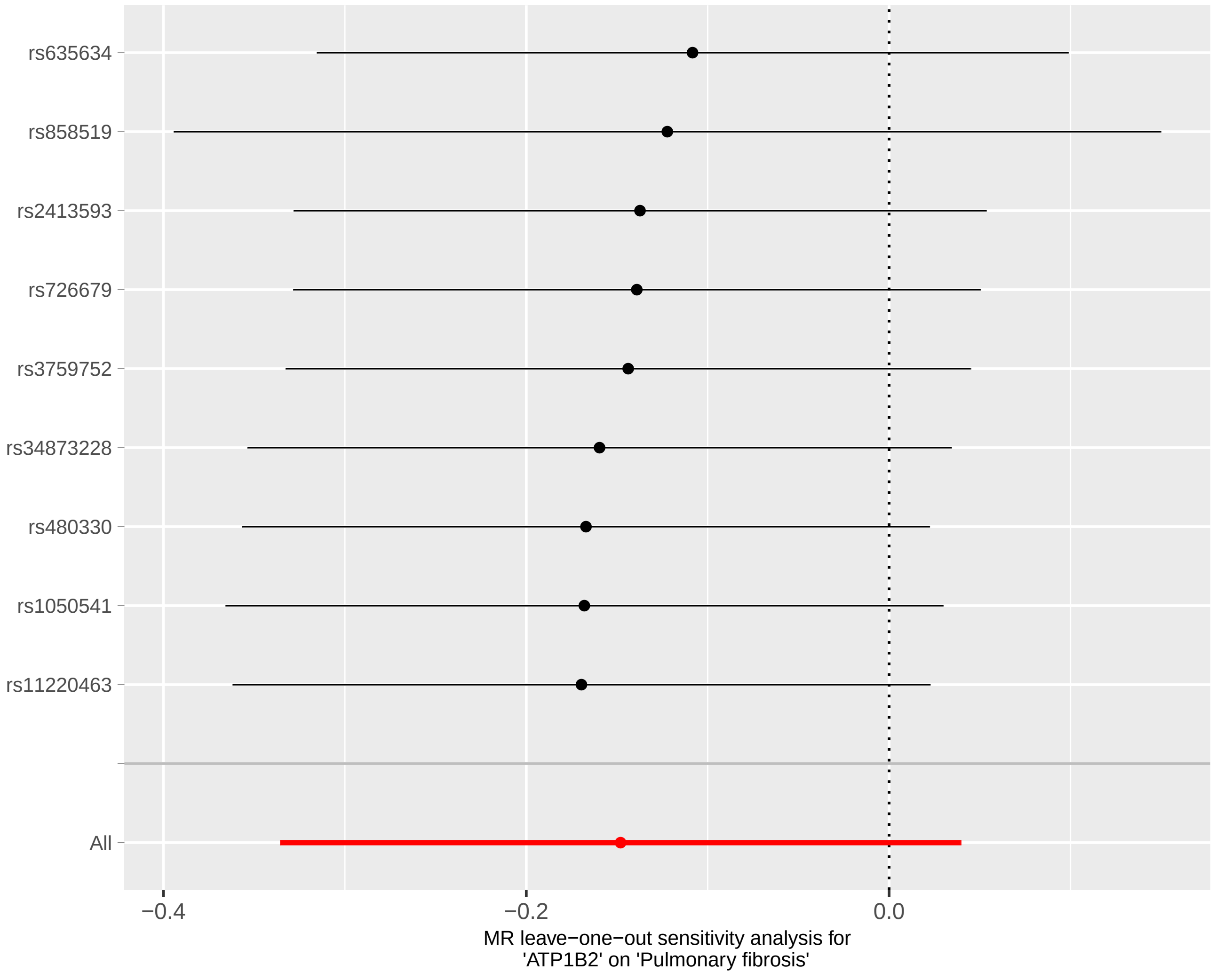

rs635634
rs858519
rs2413593
rs726679
rs3759752
rs34873228
rs480330
rs1050541
rs11220463
All
−0.4
−0.2
0.0
MR leave−one−out sensitivity analysis for
'ATP1B2' on 'Pulmonary fibrosis'

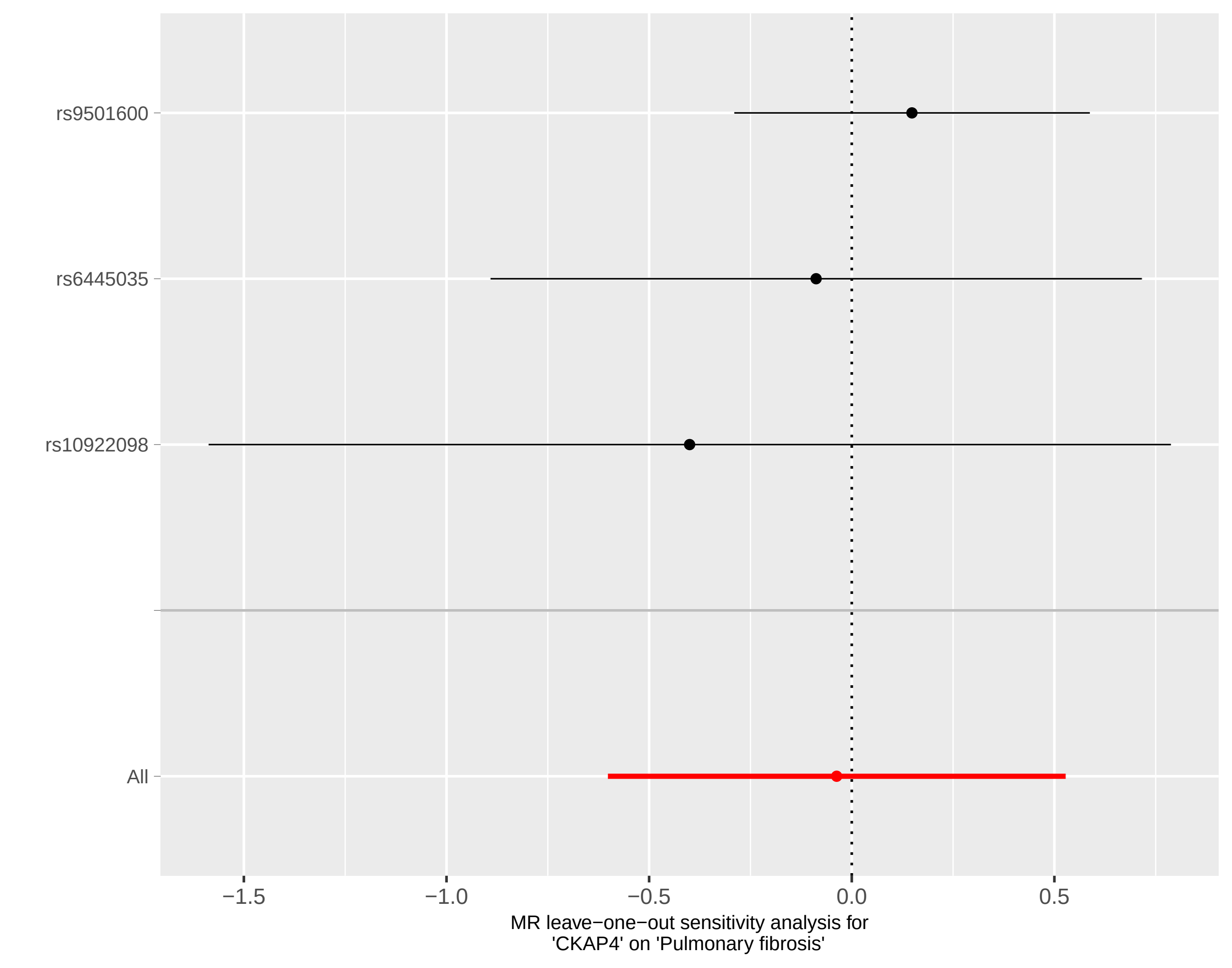

rs9501600
rs6445035
rs10922098
All
−1.5
−1.0
−0.5
0.0
0.5
MR leave−one−out sensitivity analysis for
'CKAP4' on 'Pulmonary fibrosis'

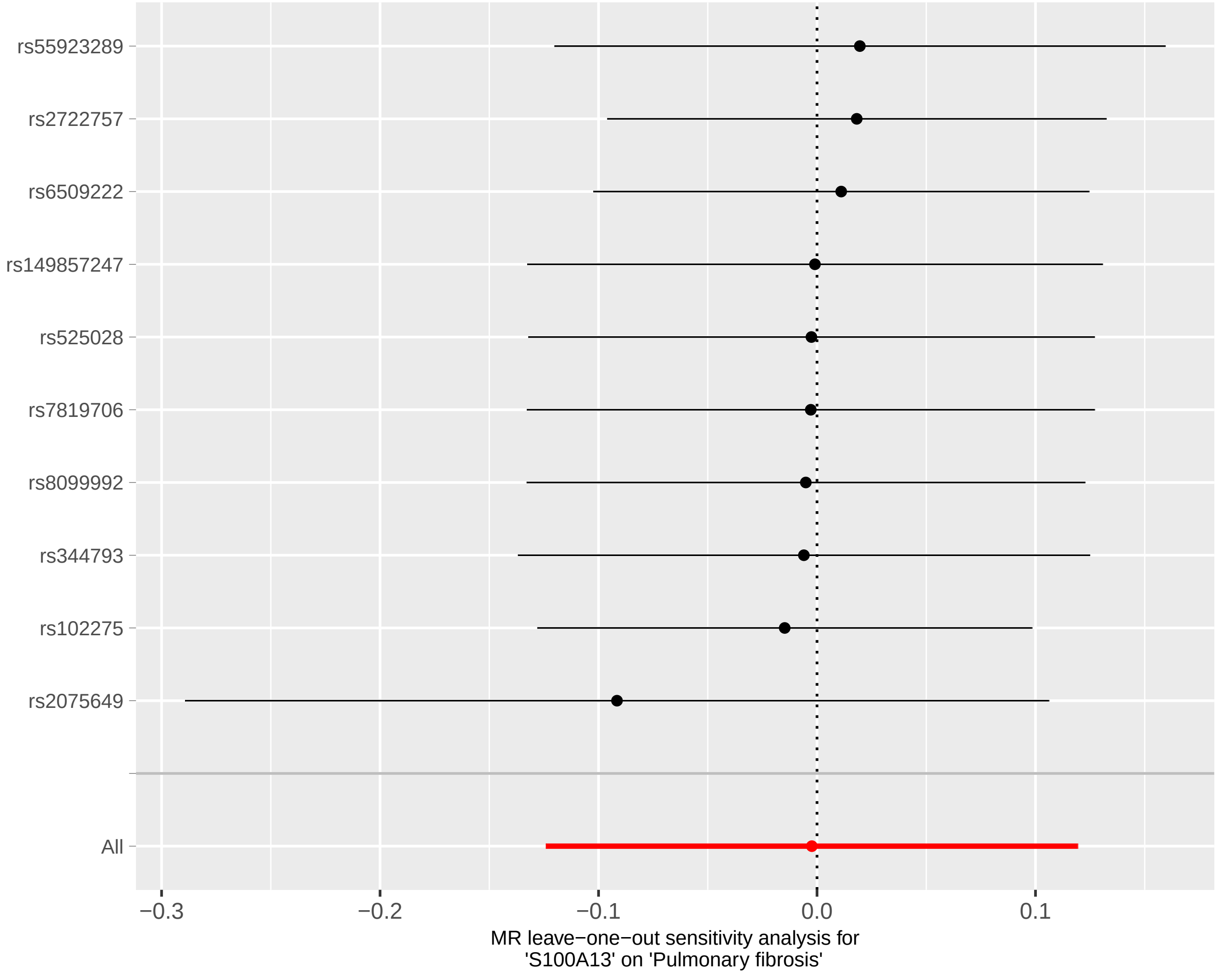

rs55923289
rs2722757
rs6509222
rs149857247
rs525028
rs7819706
rs8099992
rs344793
rs102275
rs2075649
All
−0.3
−0.2
−0.1
0.0
0.1
MR leave−one−out sensitivity analysis for
'S100A13' on 'Pulmonary fibrosis'

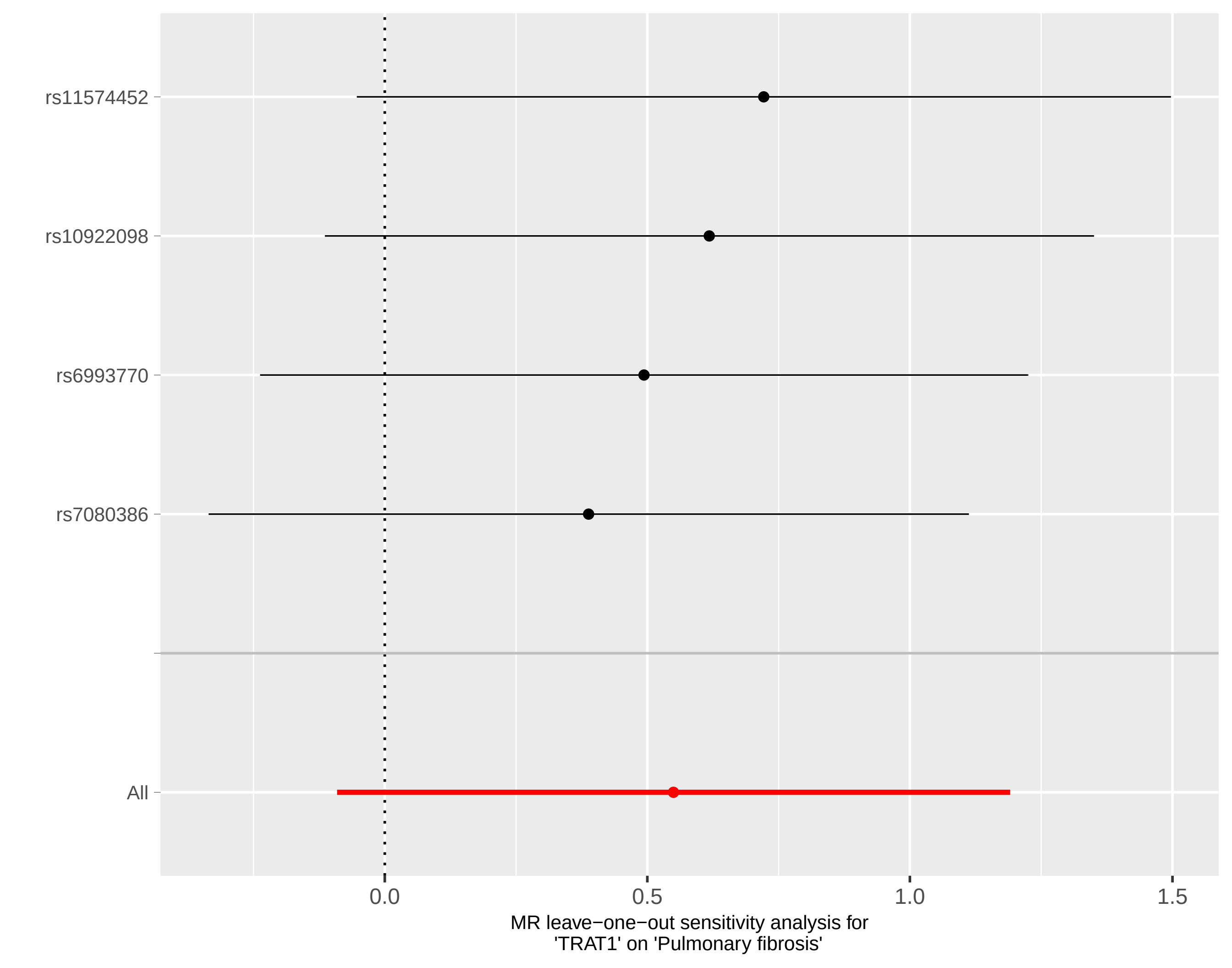
rs11574452
rs10922098
rs6993770
rs7080386
All
0.0
0.5
1.0
1.5
MR leave−one−out sensitivity analysis for
'TRAT1' on 'Pulmonary fibrosis'

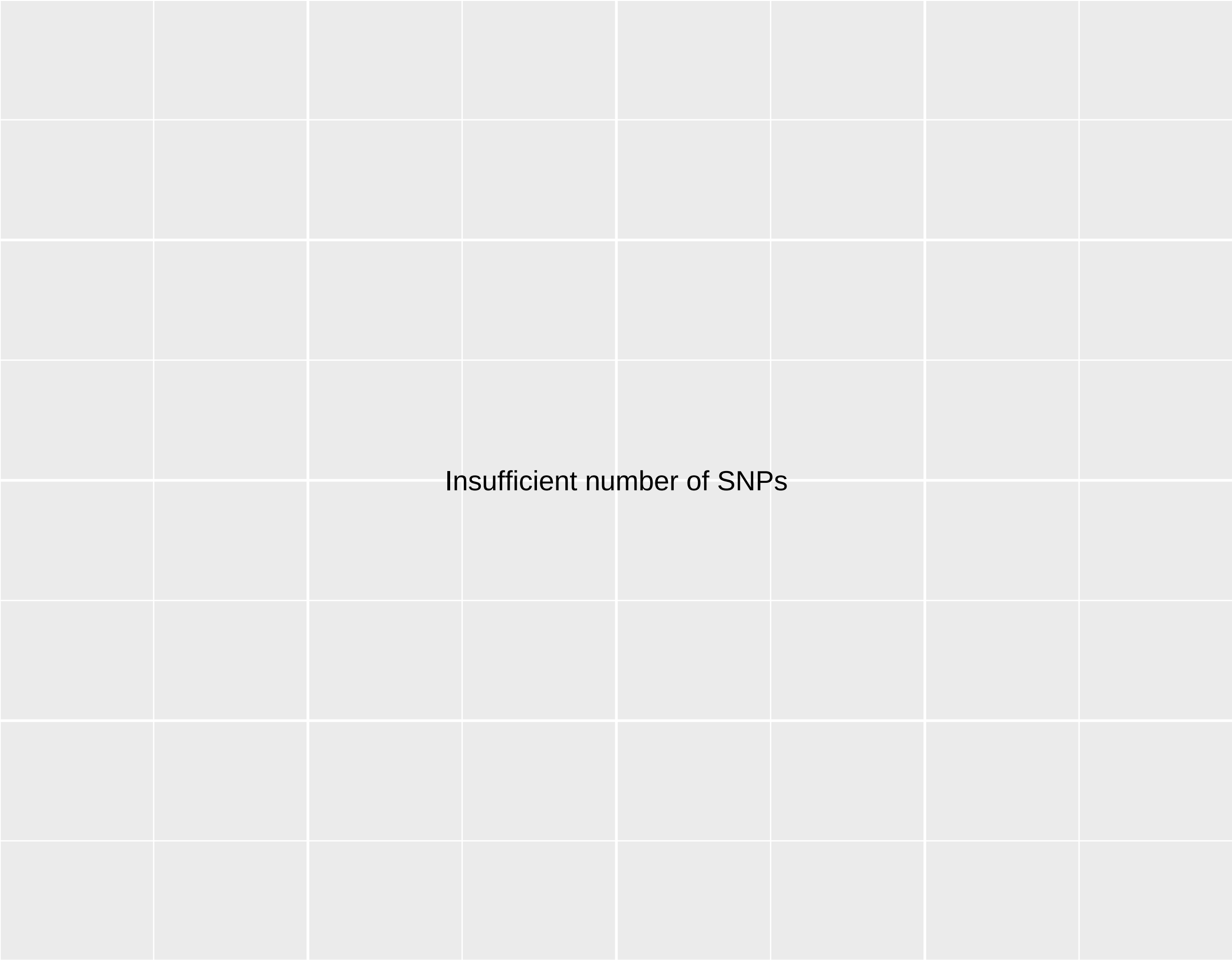
Insufficient number of SNPs

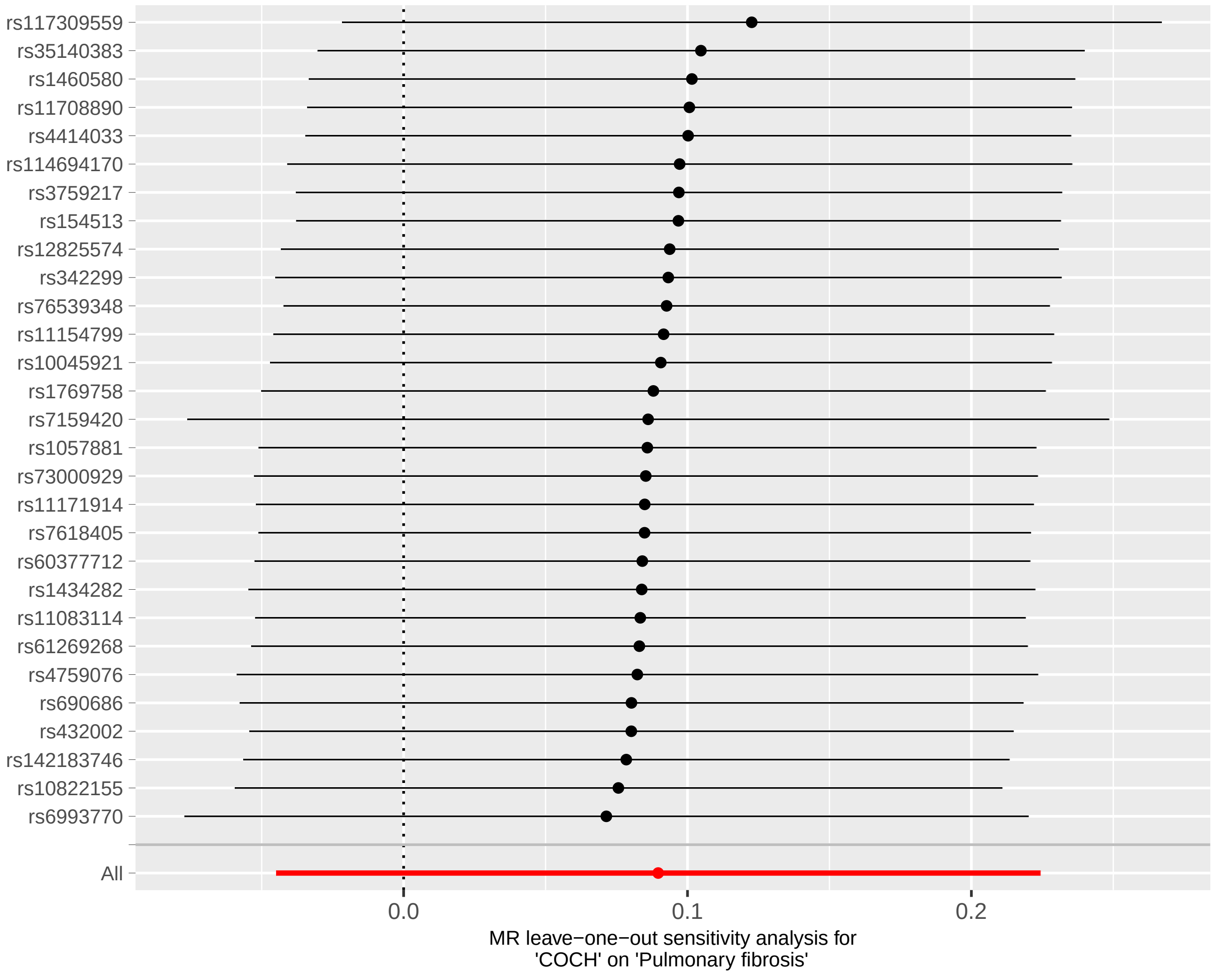
rs117309559
rs35140383
rs1460580
rs11708890
rs4414033
rs114694170
rs3759217
rs154513
rs12825574
rs342299
rs76539348
rs11154799
rs10045921
rs1769758
rs7159420
rs1057881
rs73000929
rs11171914
rs7618405
rs60377712
rs1434282
rs11083114
rs61269268
rs4759076
rs690686
rs432002
rs142183746
rs10822155
rs6993770
All
0.0
0.1
0.2
MR leave−one−out sensitivity analysis for
'COCH' on 'Pulmonary fibrosis'

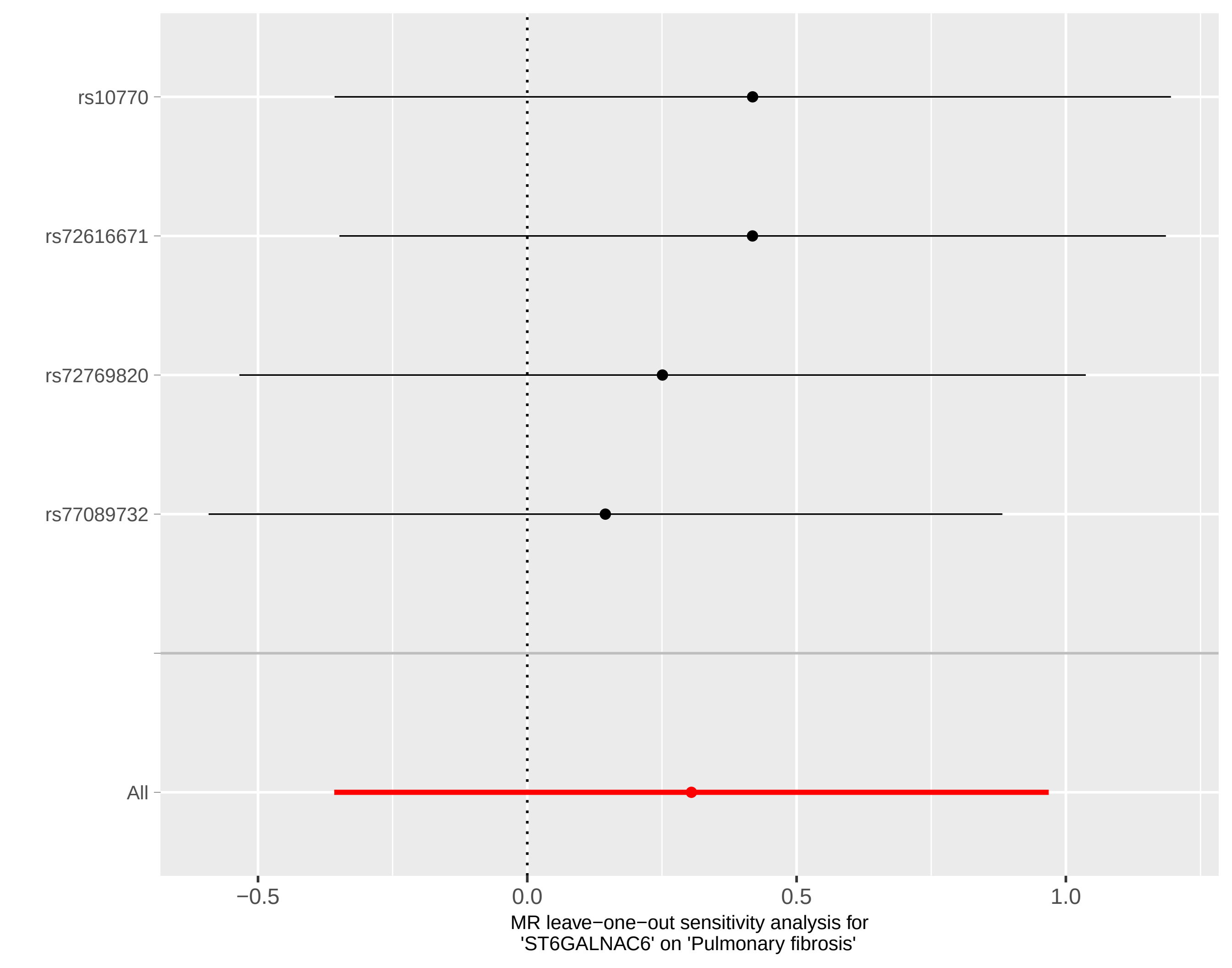
rs10770
rs72616671
rs72769820
rs77089732
All
−0.5
0.0
0.5
1.0
MR leave−one−out sensitivity analysis for
'ST6GALNAC6' on 'Pulmonary fibrosis'

Insufficient number of SNPs

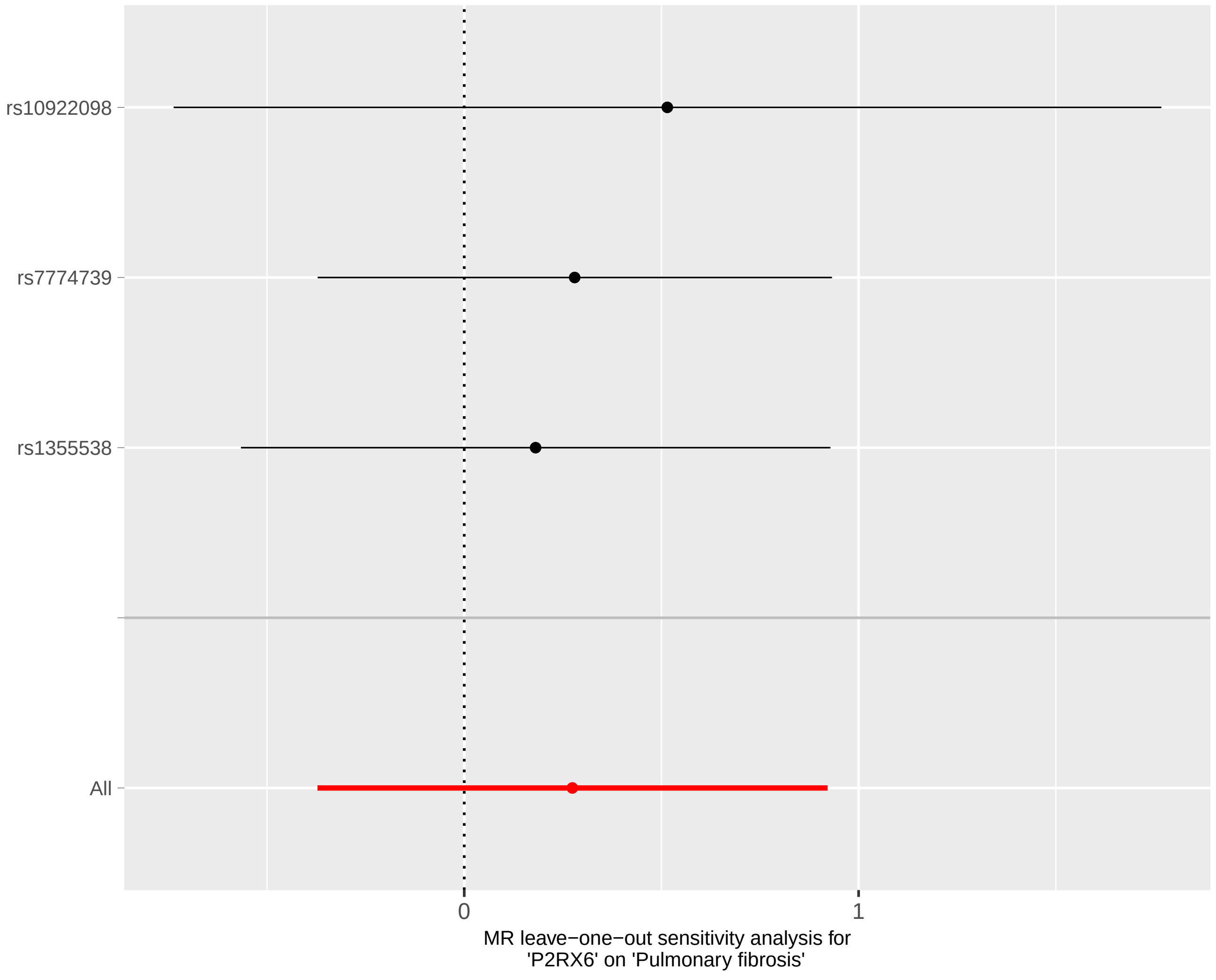
rs10922098
rs7774739
rs1355538
All
0
1
MR leave−one−out sensitivity analysis for
'P2RX6' on 'Pulmonary fibrosis'

Insufficient number of SNPs

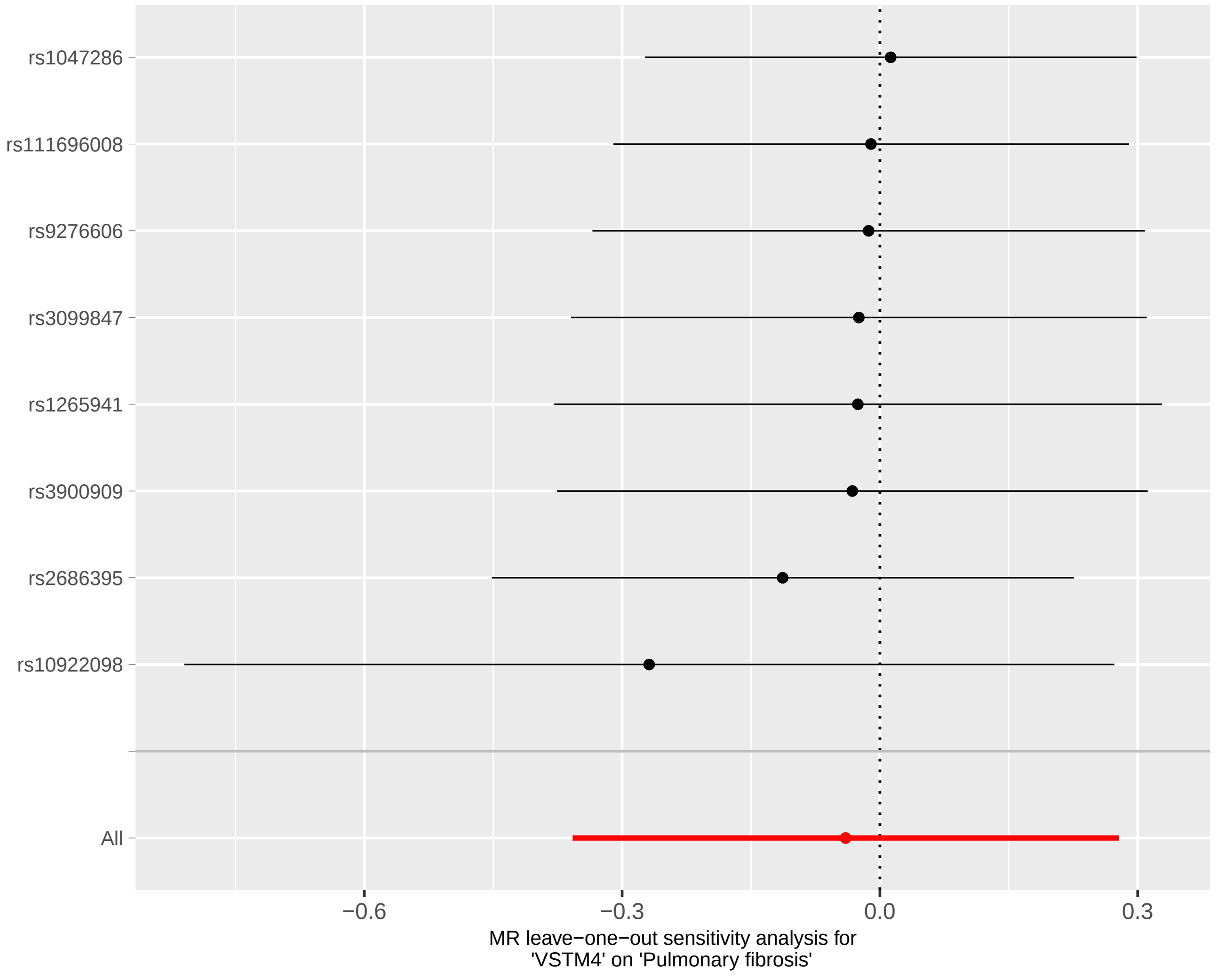
rs1047286
rs111696008
rs9276606
rs3099847
rs1265941
rs3900909
rs2686395
rs10922098
All
−0.6
−0.3
0.0
0.3
MR leave−one−out sensitivity analysis for
'VSTM4' on 'Pulmonary fibrosis'

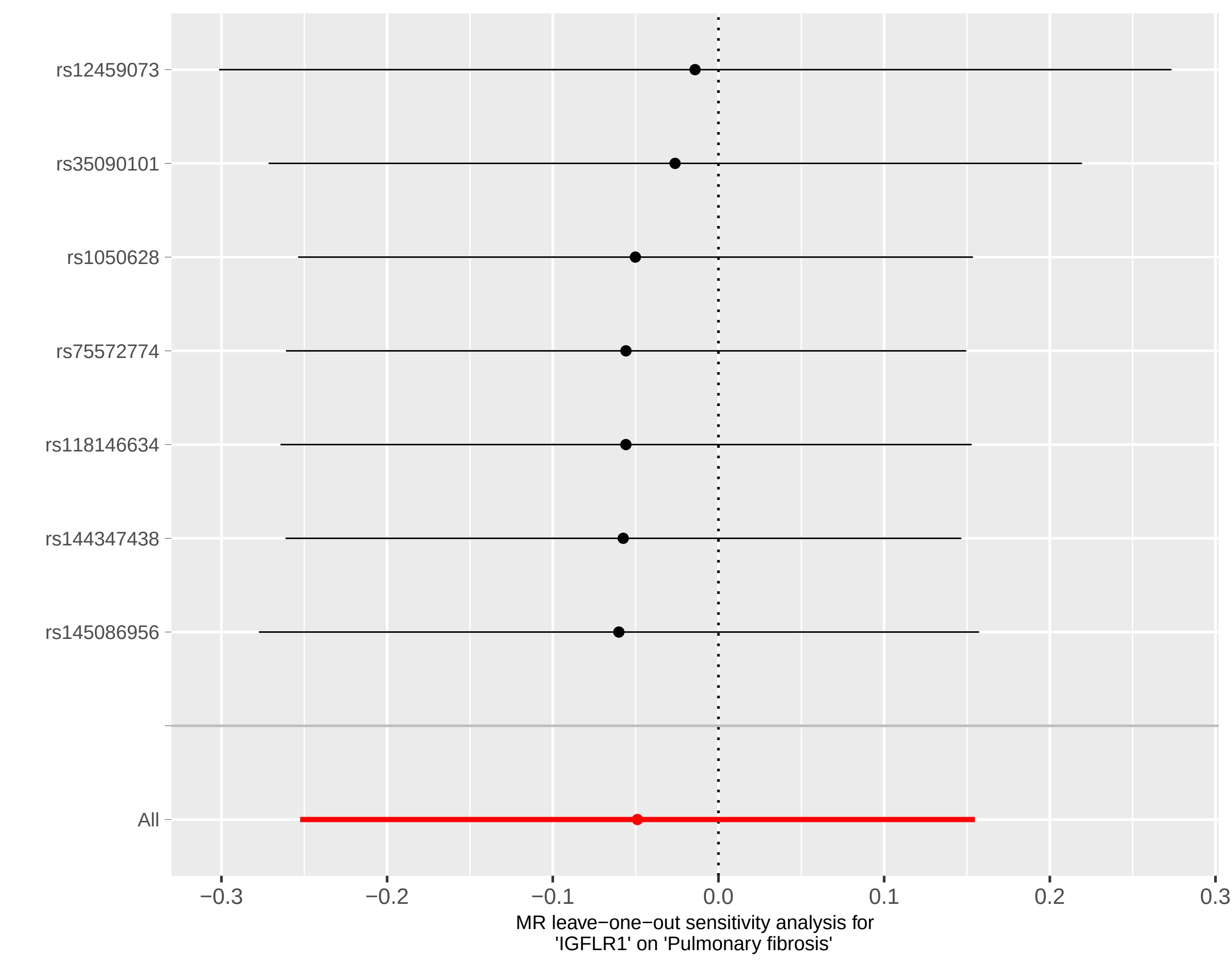
rs12459073
rs35090101
rs1050628
rs75572774
rs118146634
rs144347438
rs145086956
All
−0.3
−0.2
−0.1
0.0
0.1
0.2
0.3
MR leave−one−out sensitivity analysis for
'IGFLR1' on 'Pulmonary fibrosis'

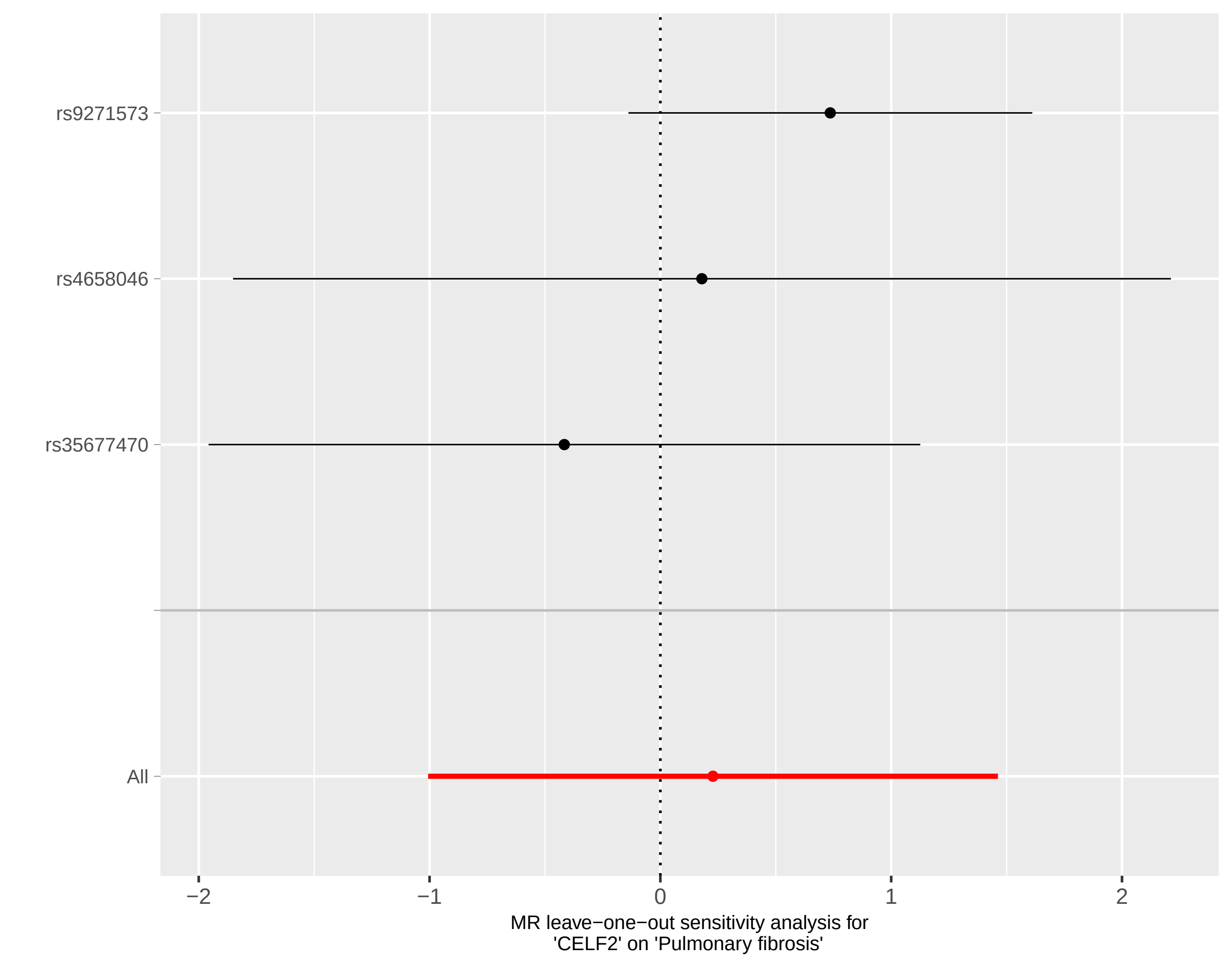
rs9271573
rs4658046
rs35677470
All
−2
−1
0
1
2
MR leave−one−out sensitivity analysis for
'CELF2' on 'Pulmonary fibrosis'

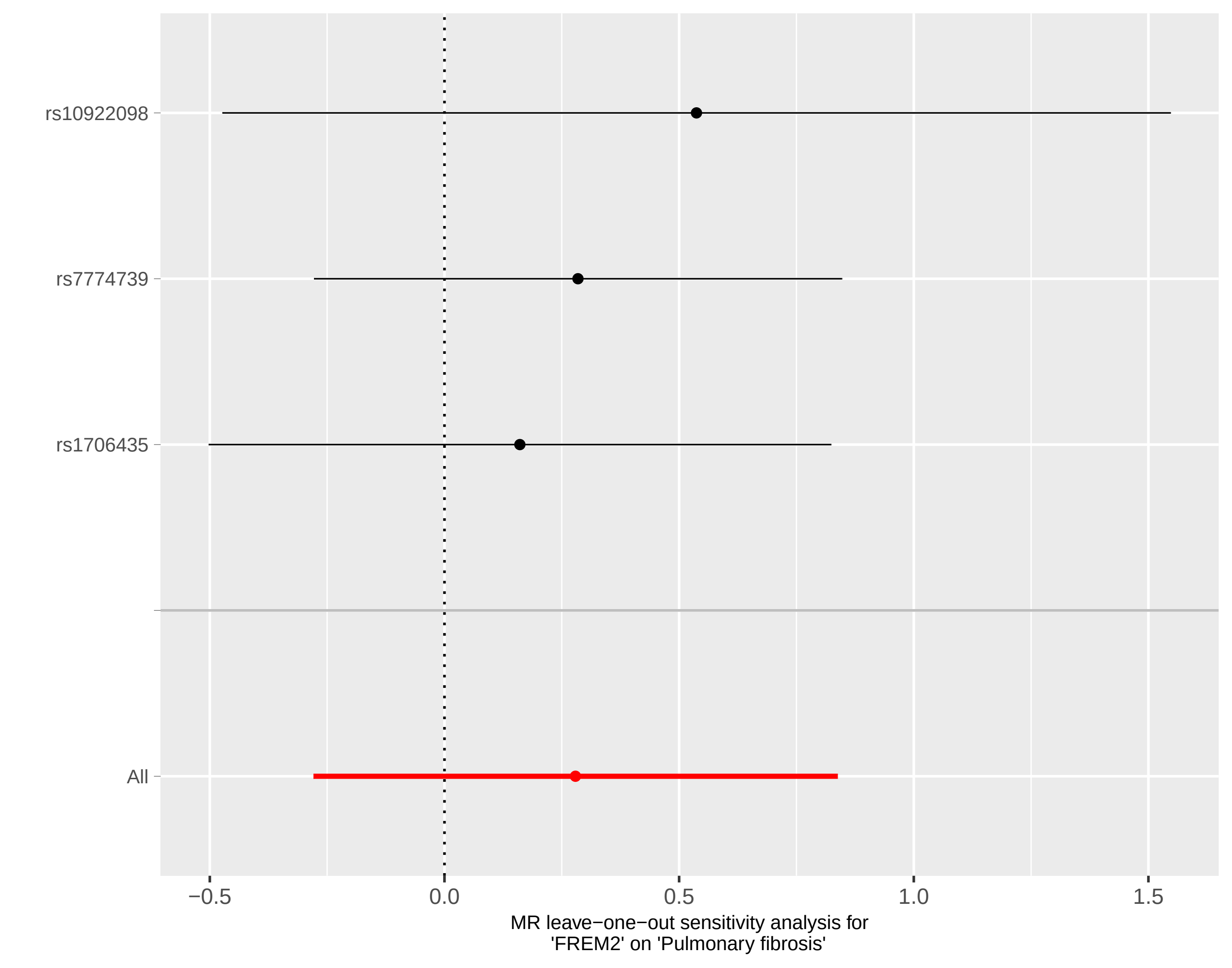
rs10922098
rs7774739
rs1706435
All
−0.5
0.0
0.5
1.0
1.5
MR leave−one−out sensitivity analysis for
'FREM2' on 'Pulmonary fibrosis'

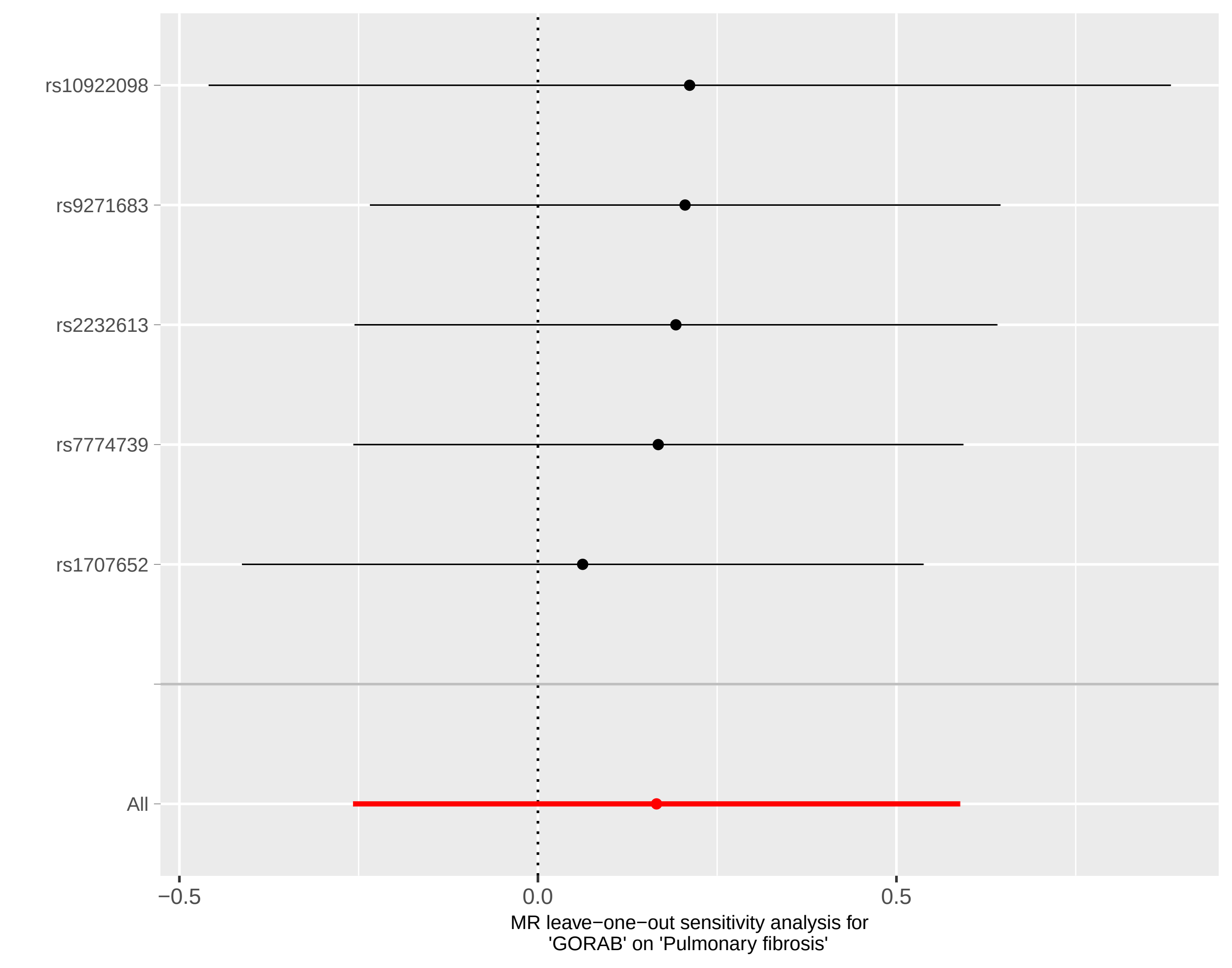
rs10922098
rs9271683
rs2232613
rs7774739
rs1707652
All
−0.5
0.0
0.5
MR leave−one−out sensitivity analysis for
'GORAB' on 'Pulmonary fibrosis'

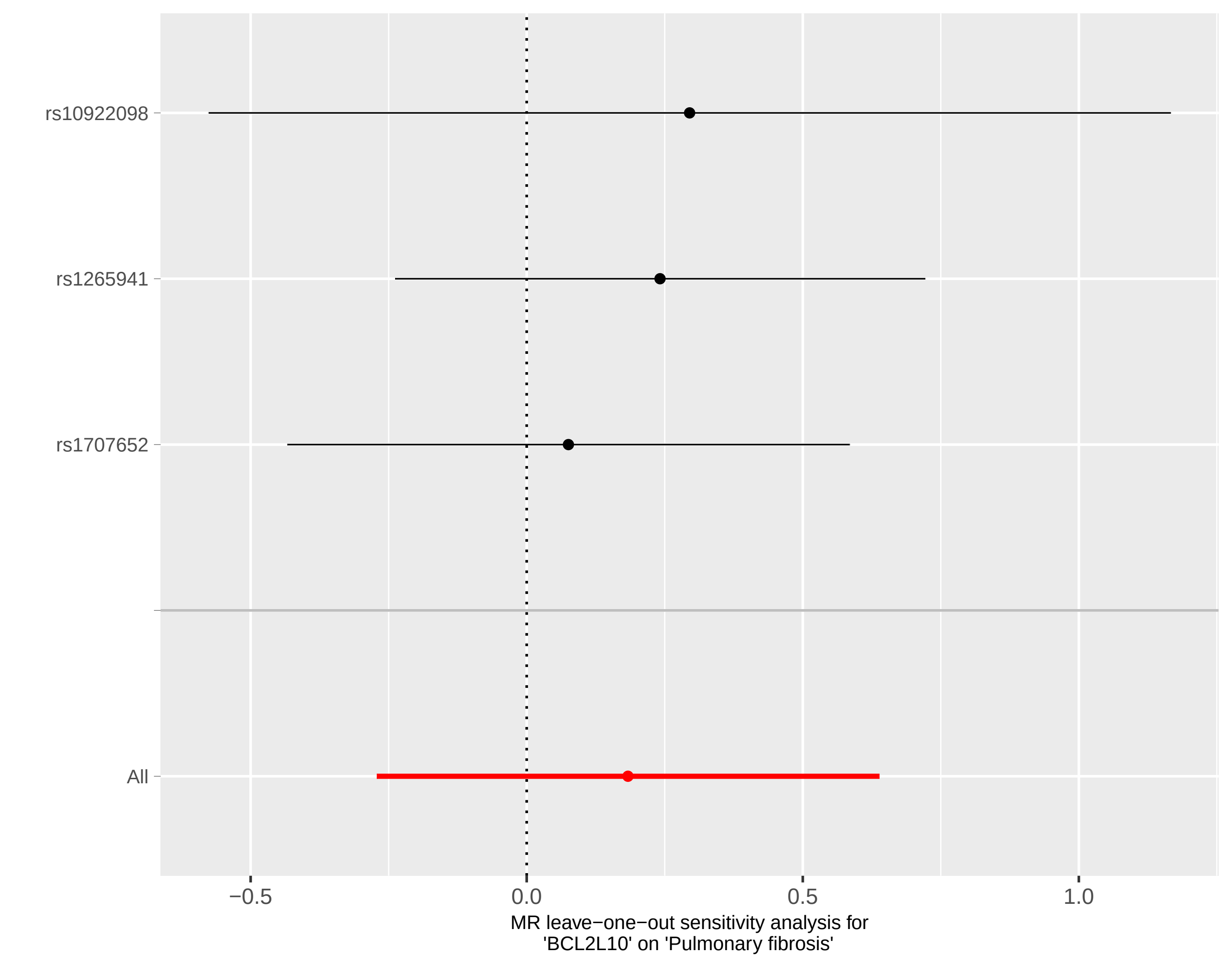
rs10922098
rs1265941
rs1707652
All
−0.5
0.0
0.5
1.0
MR leave−one−out sensitivity analysis for
'BCL2L10' on 'Pulmonary fibrosis'

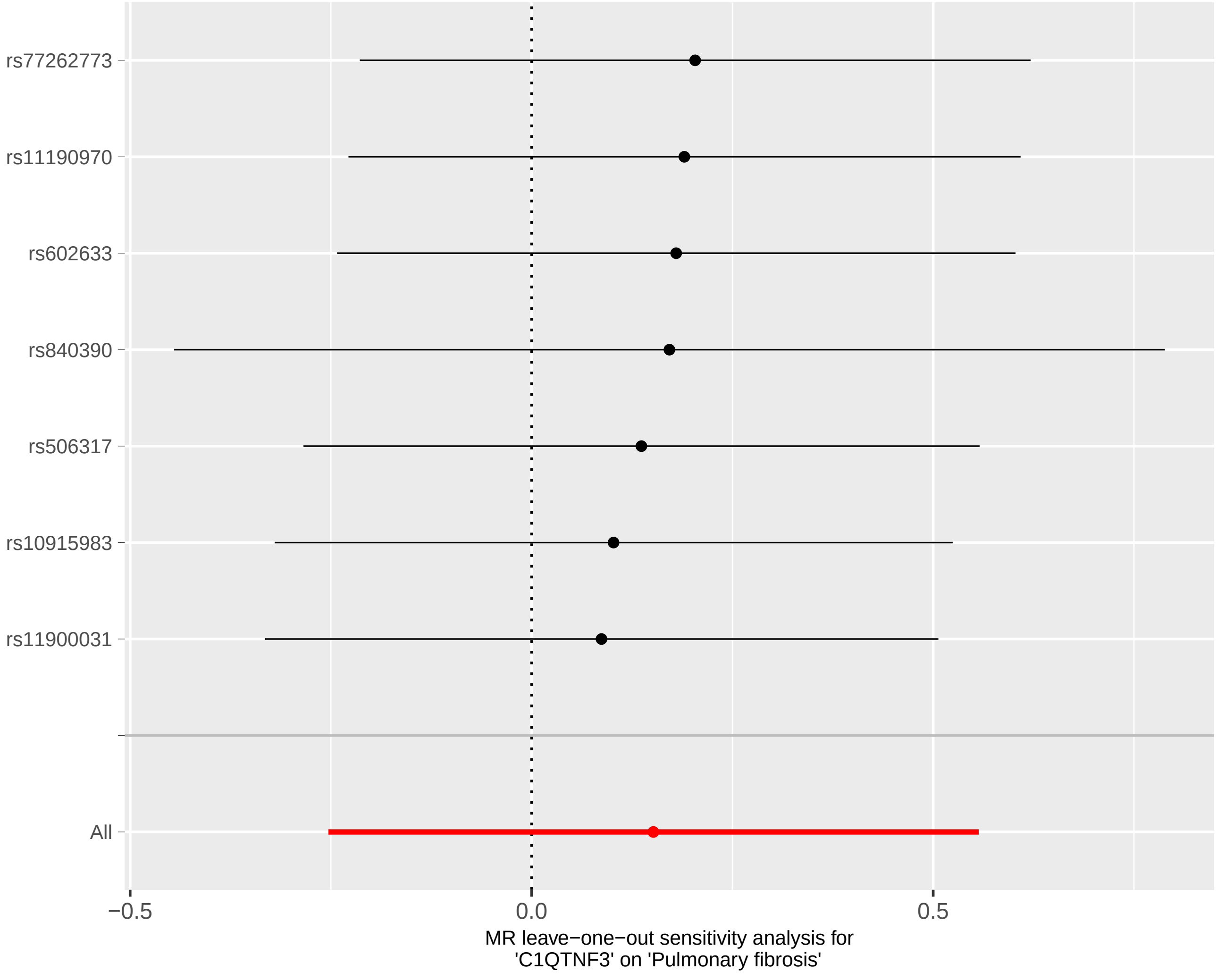
rs77262773
rs11190970
rs602633
rs840390
rs506317
rs10915983
rs11900031
All
−0.5
0.0
0.5
MR leave−one−out sensitivity analysis for
'C1QTNF3' on 'Pulmonary fibrosis'

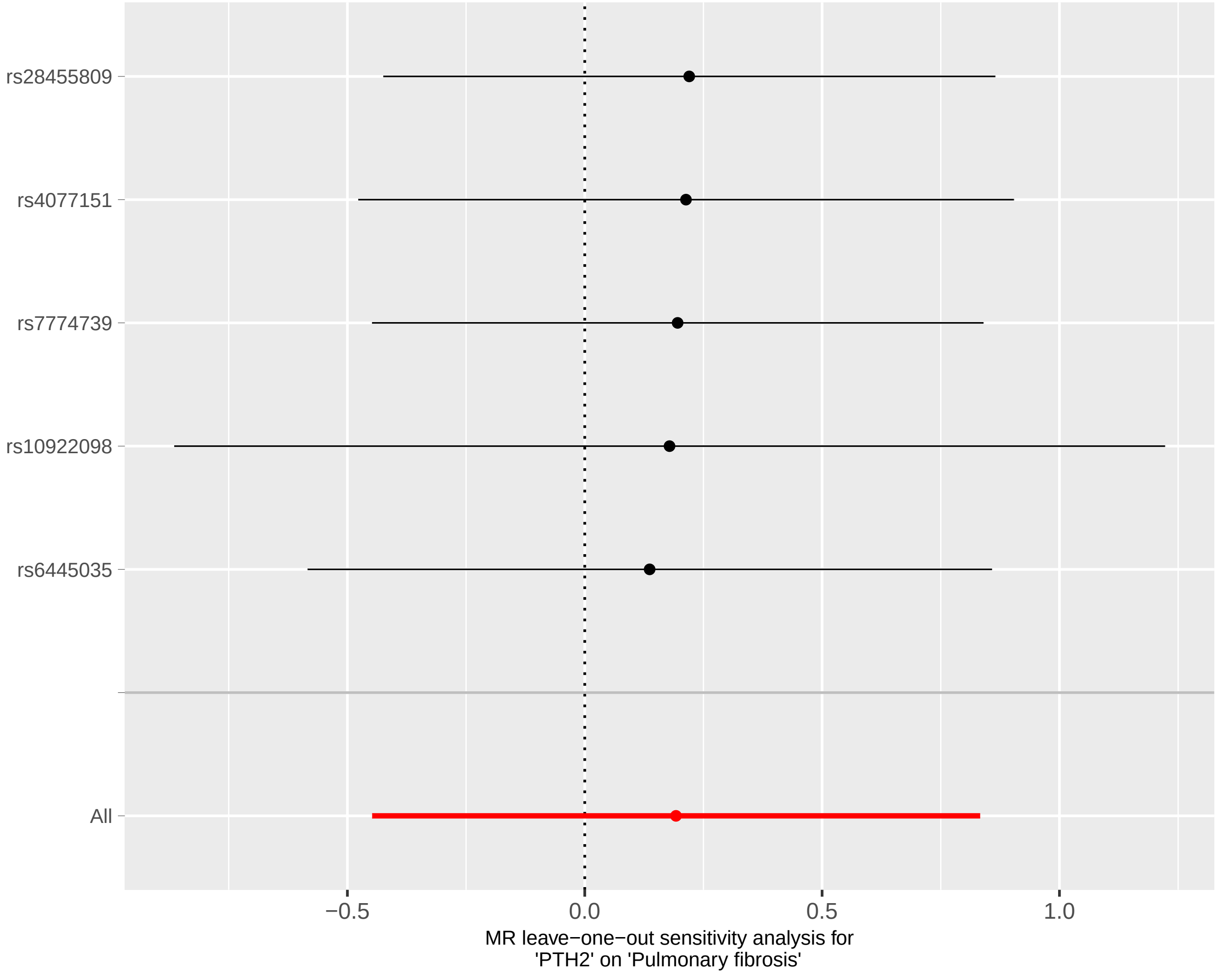
rs28455809
rs4077151
rs7774739
rs10922098
rs6445035
All
−0.5
0.0
0.5
1.0
MR leave−one−out sensitivity analysis for
'PTH2' on 'Pulmonary fibrosis'

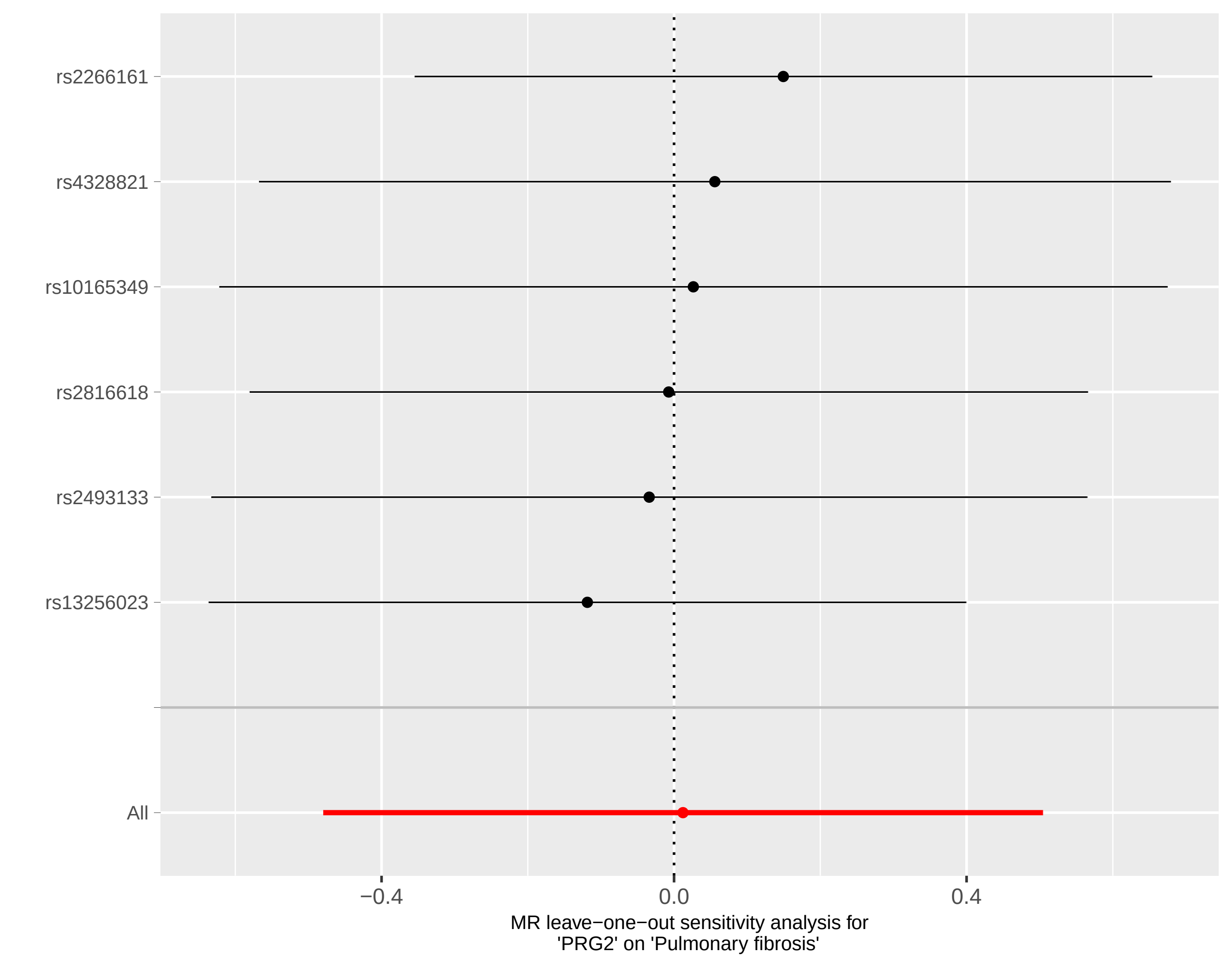

rs2266161
rs4328821
rs10165349
rs2816618
rs2493133
rs13256023
All
−0.4
0.0
0.4
MR leave−one−out sensitivity analysis for
'PRG2' on 'Pulmonary fibrosis'

Insufficient number of SNPs

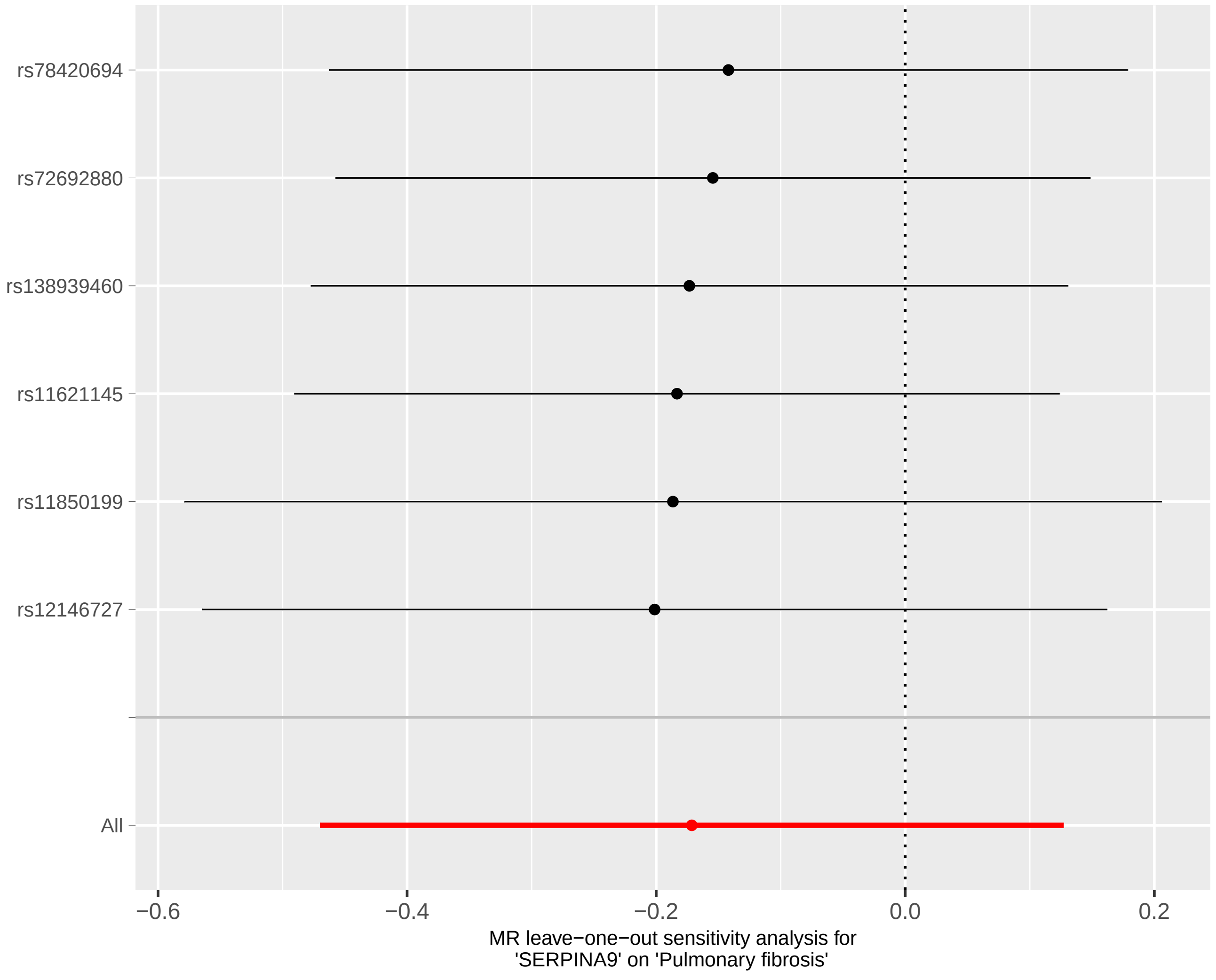

rs78420694
rs72692880
rs138939460
rs11621145
rs11850199
rs12146727
All
−0.6
−0.4
−0.2
0.0
0.2
MR leave−one−out sensitivity analysis for
'SERPINA9' on 'Pulmonary fibrosis'

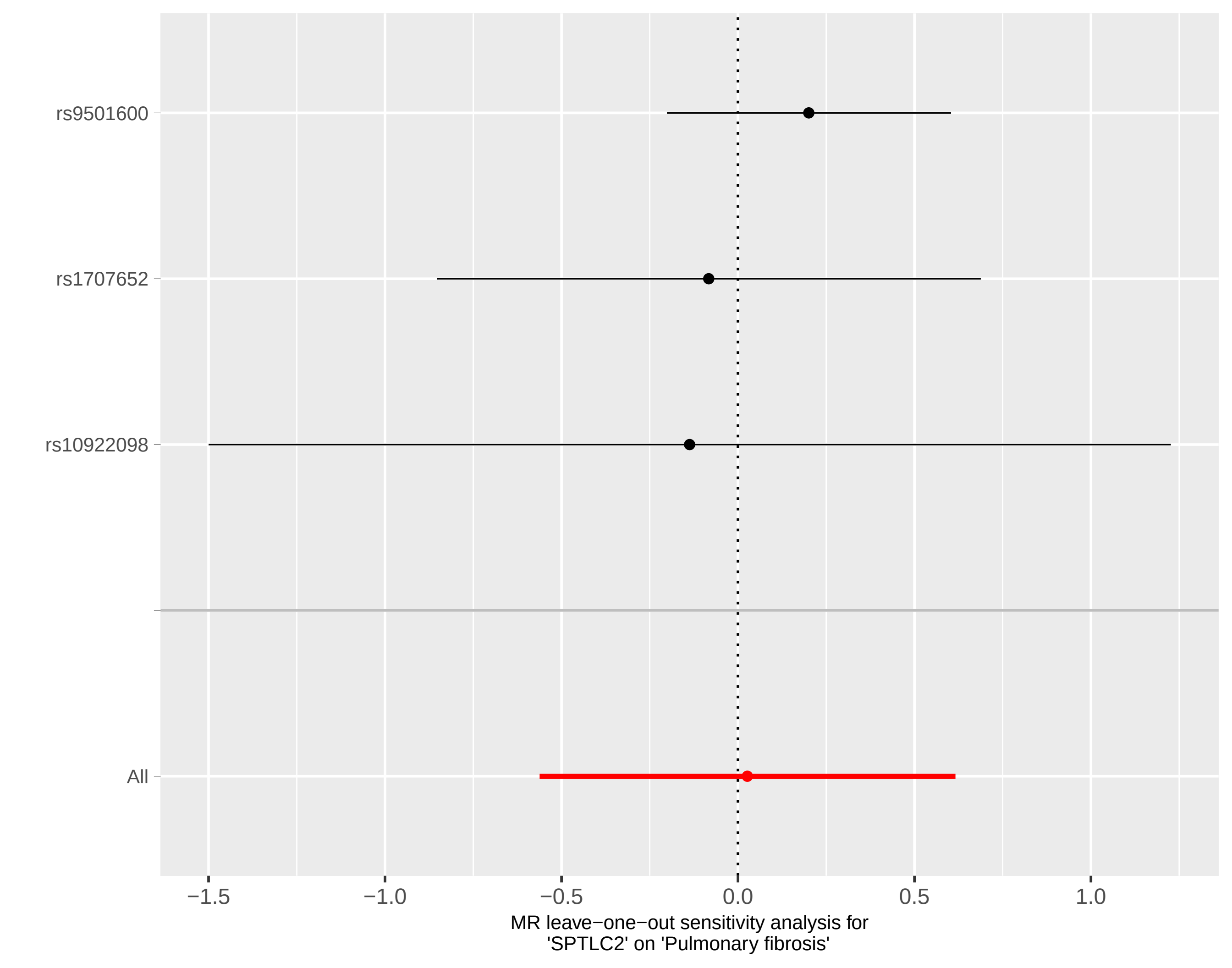
rs9501600
rs1707652
rs10922098
All
−1.5
−1.0
−0.5
0.0
0.5
1.0
MR leave−one−out sensitivity analysis for
'SPTLC2' on 'Pulmonary fibrosis'

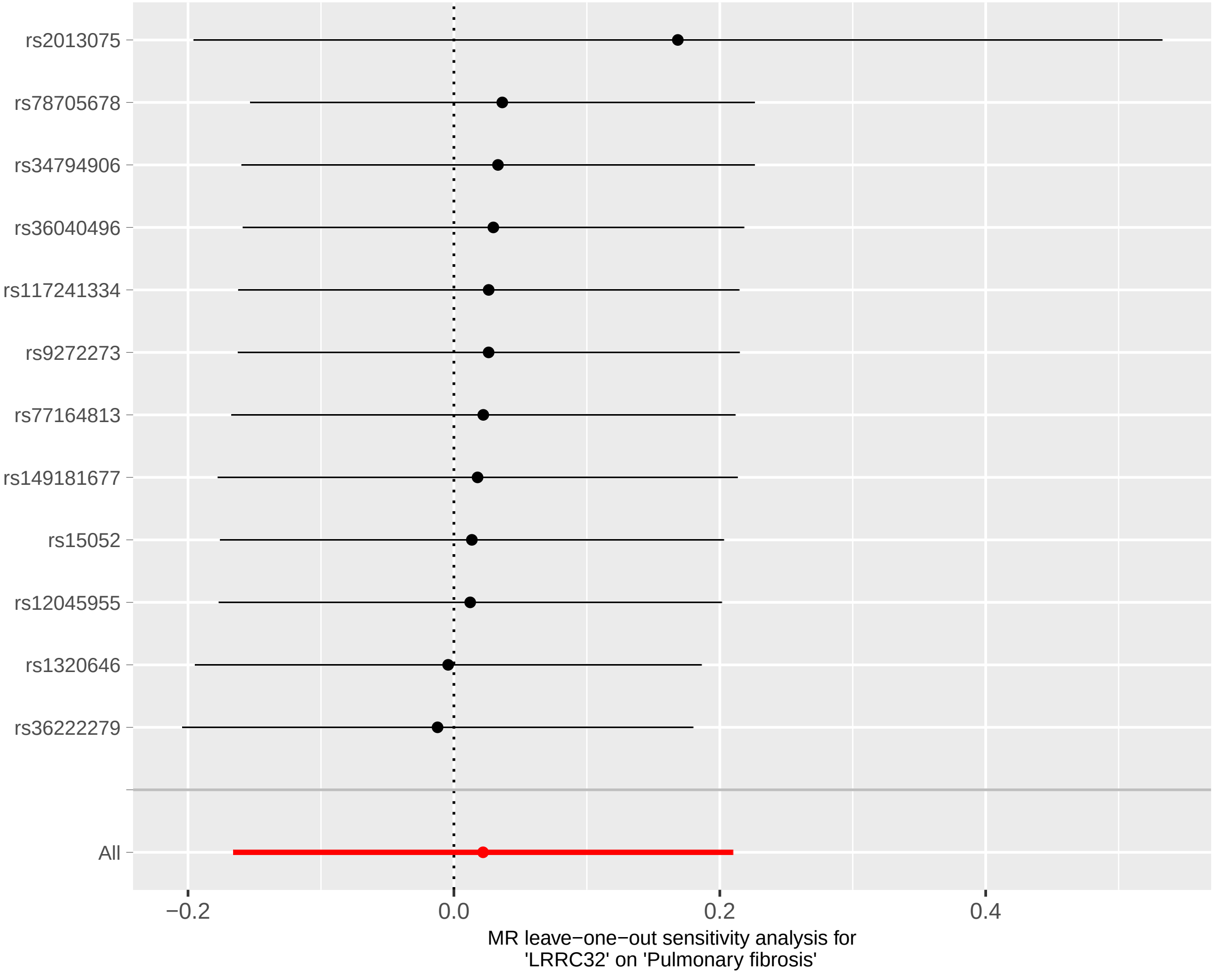
rs2013075
rs78705678
rs34794906
rs36040496
rs117241334
rs9272273
rs77164813
rs149181677
rs15052
rs12045955
rs1320646
rs36222279
All
−0.2
0.0
0.2
0.4
MR leave−one−out sensitivity analysis for
'LRRC32' on 'Pulmonary fibrosis'

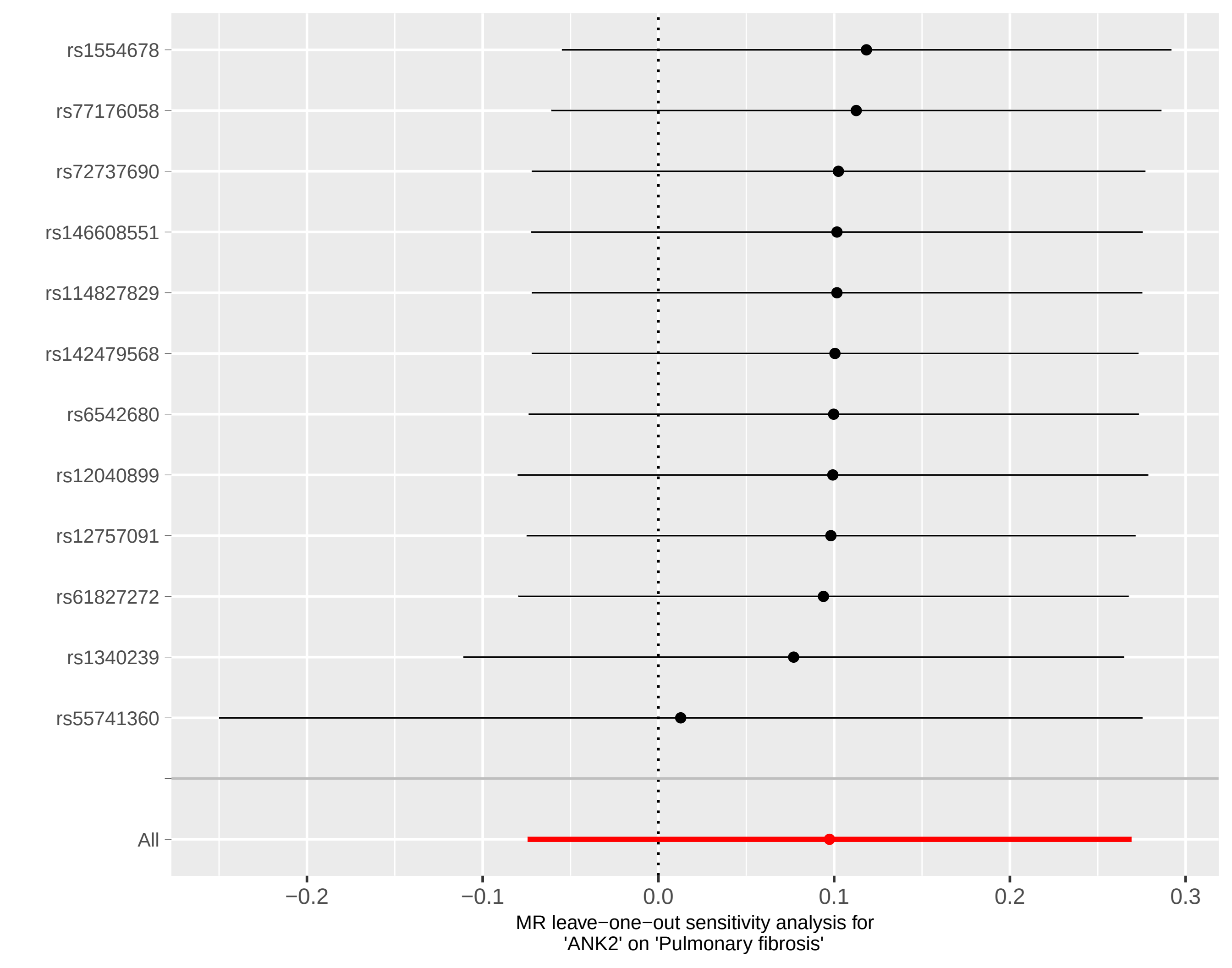
rs1554678
rs77176058
rs72737690
rs146608551
rs114827829
rs142479568
rs6542680
rs12040899
rs12757091
rs61827272
rs1340239
rs55741360
All
−0.2
−0.1
0.0
0.1
0.2
0.3
MR leave−one−out sensitivity analysis for
'ANK2' on 'Pulmonary fibrosis'

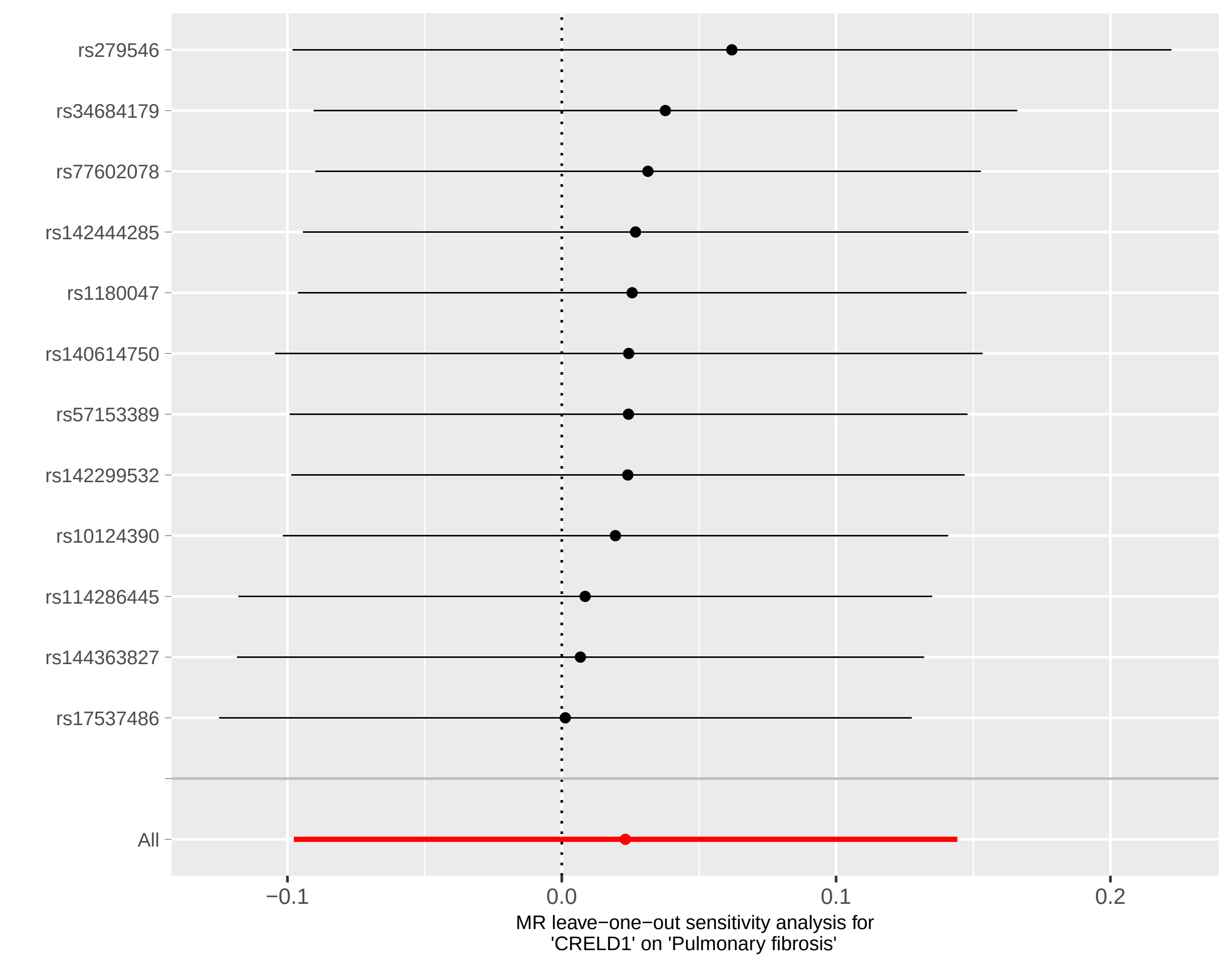
rs279546
rs34684179
rs77602078
rs142444285
rs1180047
rs140614750
rs57153389
rs142299532
rs10124390
rs114286445
rs144363827
rs17537486
All
−0.1
0.0
0.1
0.2
MR leave−one−out sensitivity analysis for
'CRELD1' on 'Pulmonary fibrosis'

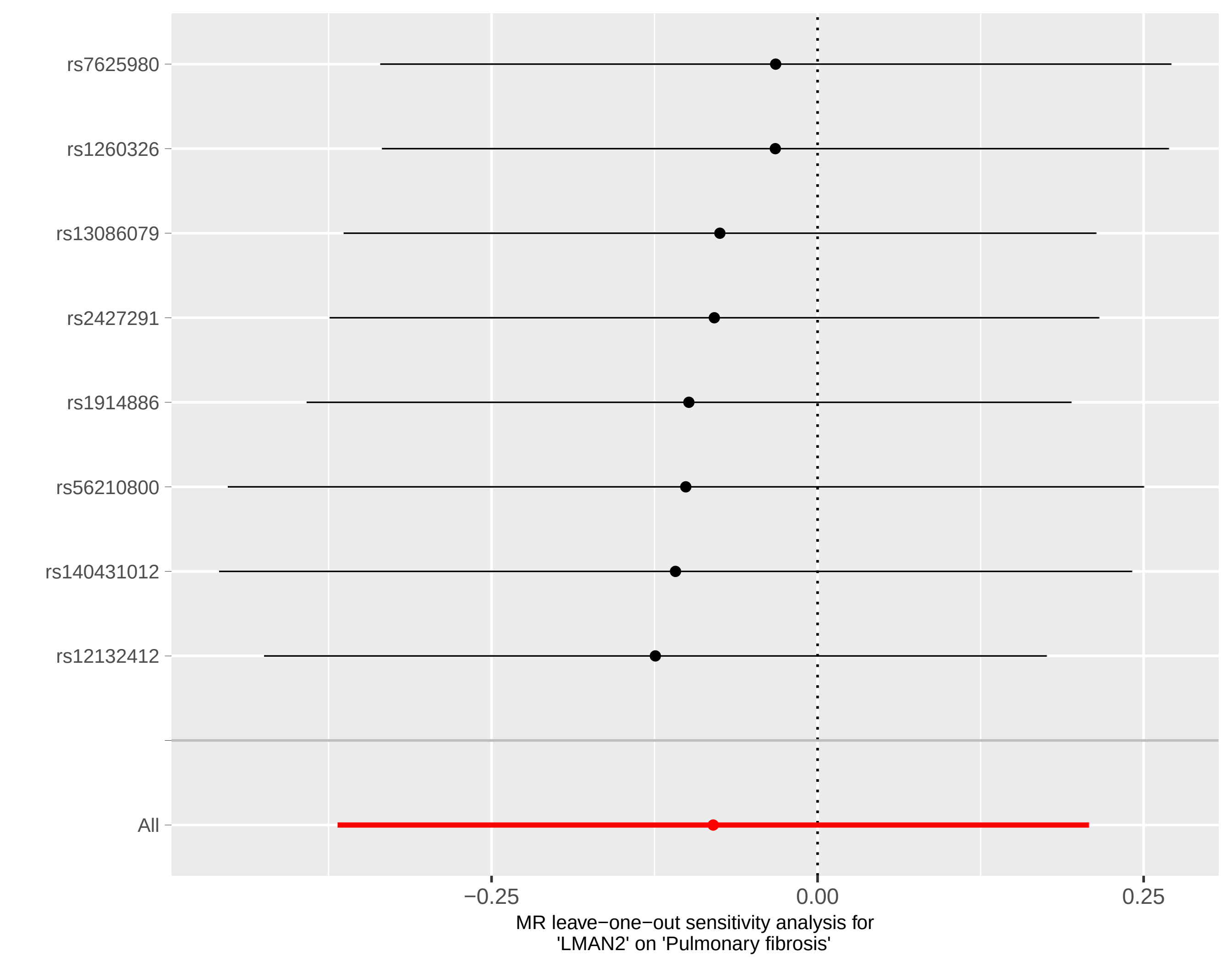

rs7625980
rs1260326
rs13086079
rs2427291
rs1914886
rs56210800
rs140431012
rs12132412
All
−0.25
0.00
0.25
MR leave−one−out sensitivity analysis for
'LMAN2' on 'Pulmonary fibrosis'

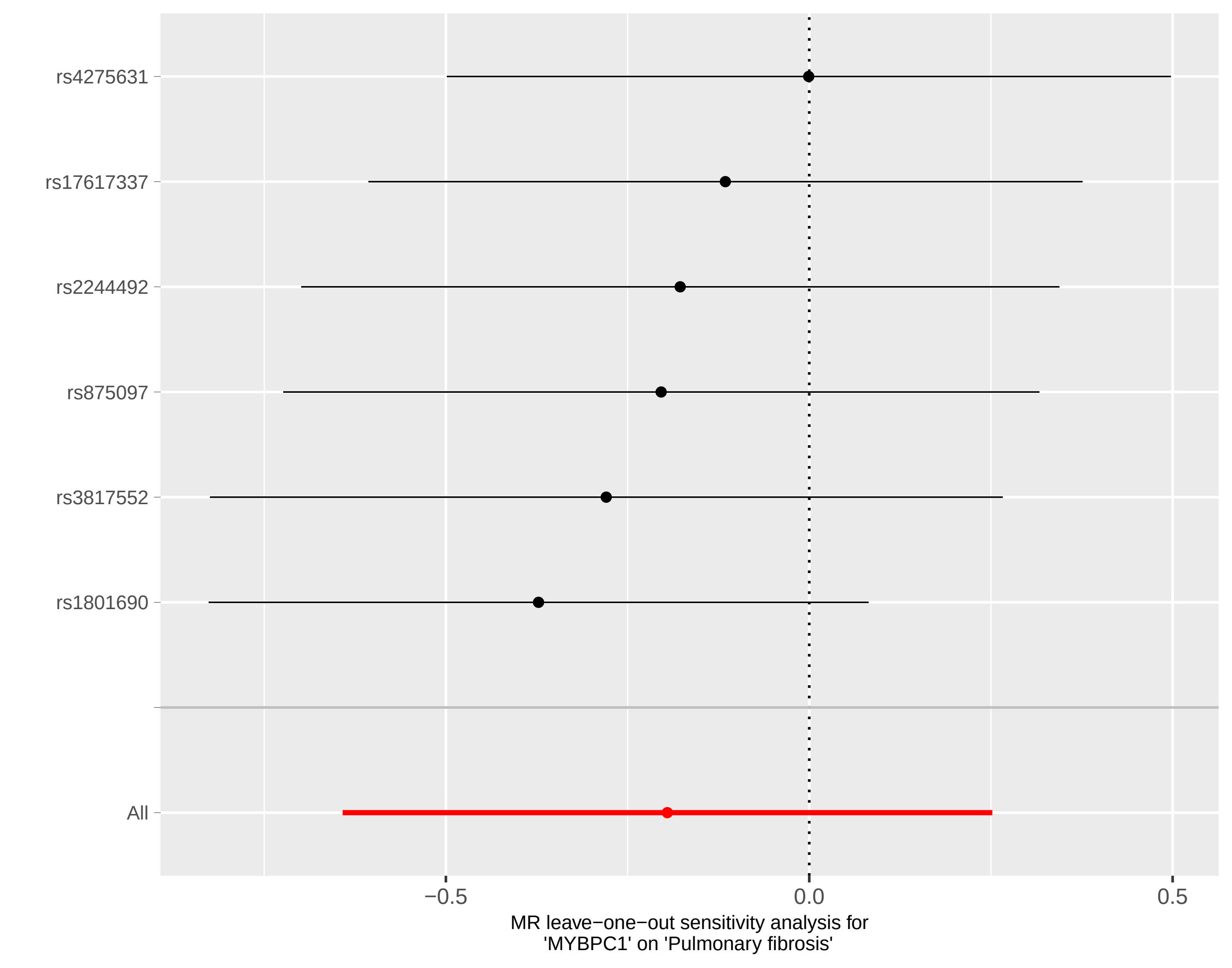
rs4275631
rs17617337
rs2244492
rs875097
rs3817552
rs1801690
All
−0.5
0.0
0.5
MR leave−one−out sensitivity analysis for
'MYBPC1' on 'Pulmonary fibrosis'

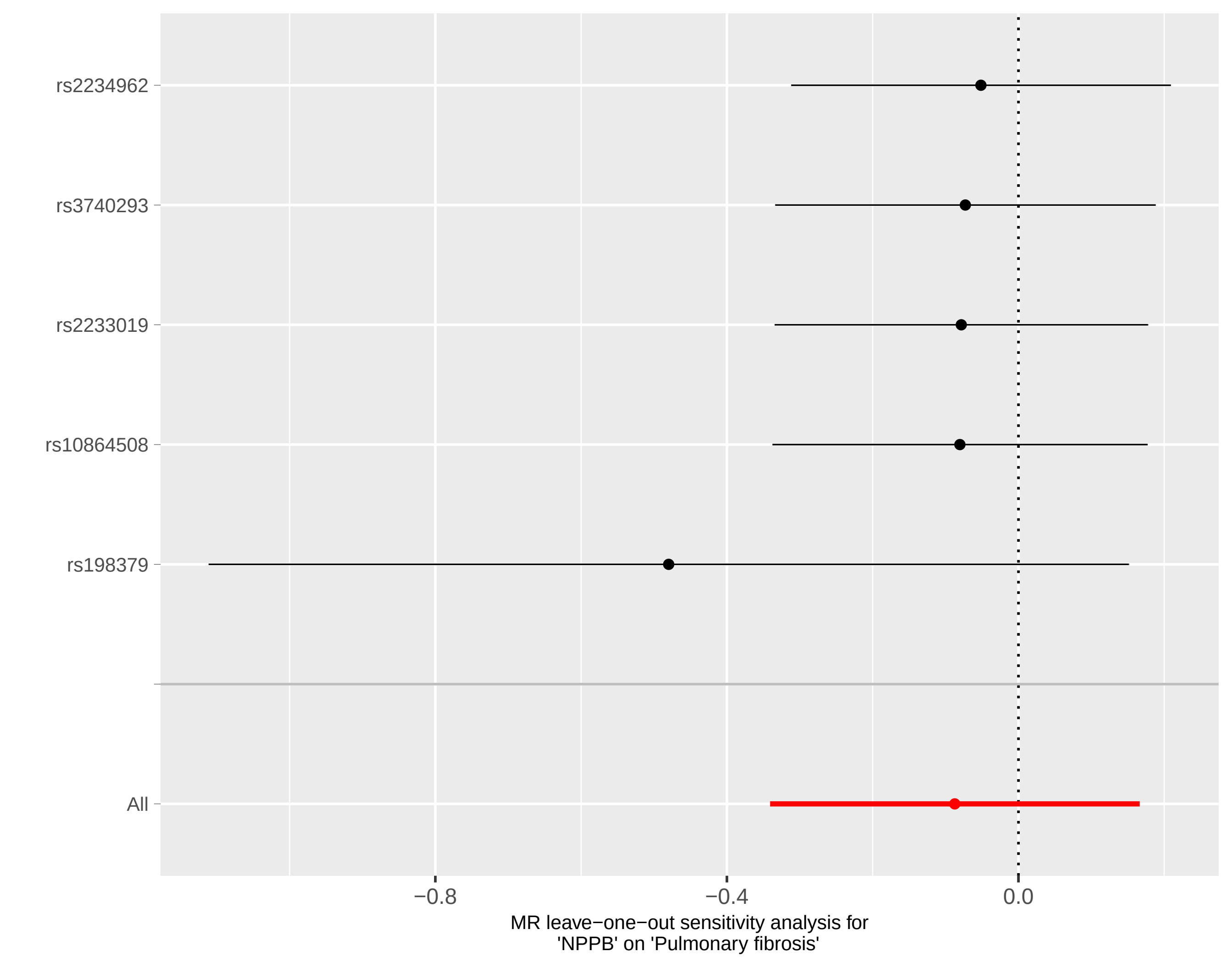

rs2234962
rs3740293
rs2233019
rs10864508
rs198379
All
−0.8
−0.4
0.0
MR leave−one−out sensitivity analysis for
'NPPB' on 'Pulmonary fibrosis'

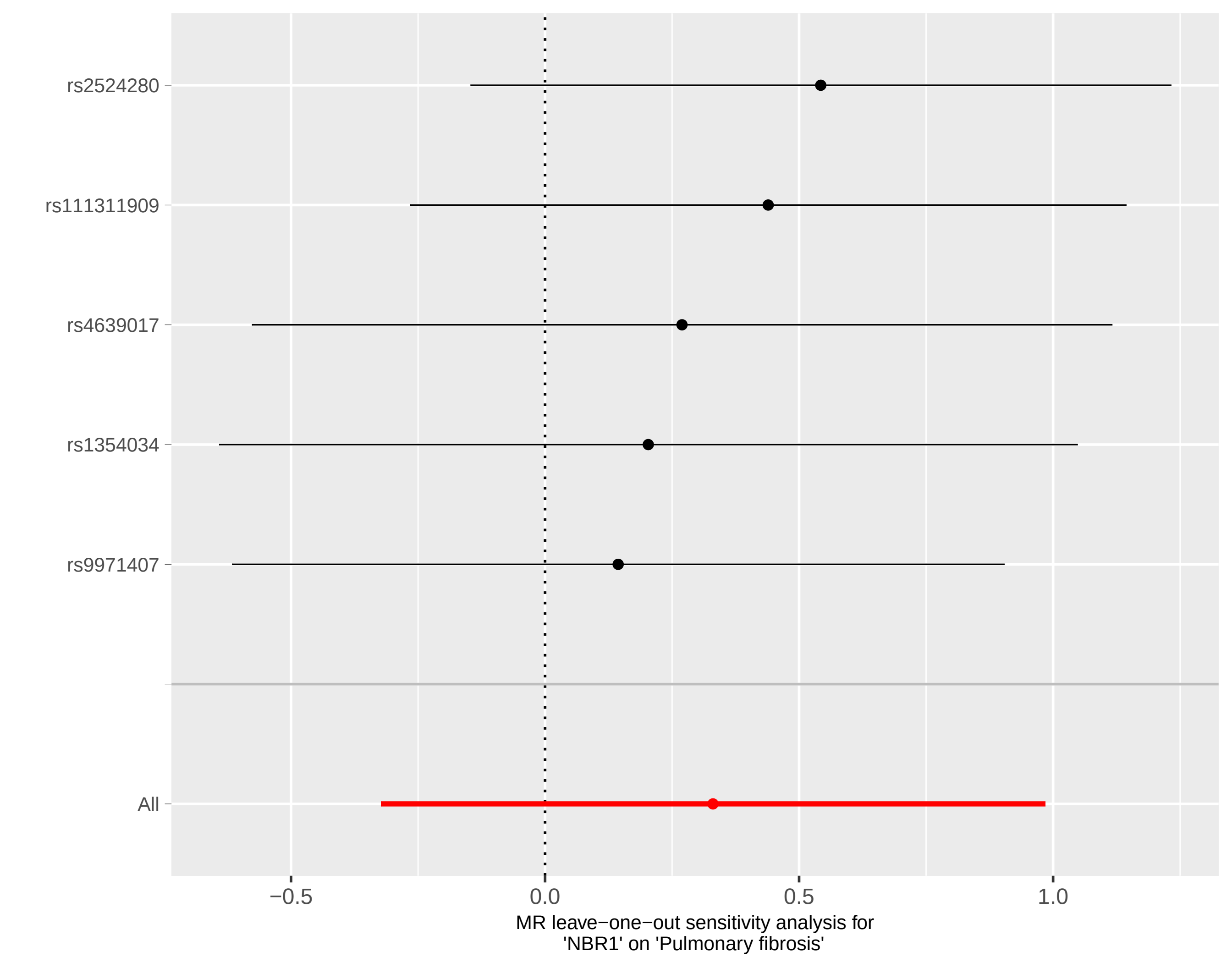

rs2524280
rs111311909
rs4639017
rs1354034
rs9971407
All
−0.5
0.0
0.5
1.0
MR leave−one−out sensitivity analysis for
'NBR1' on 'Pulmonary fibrosis'

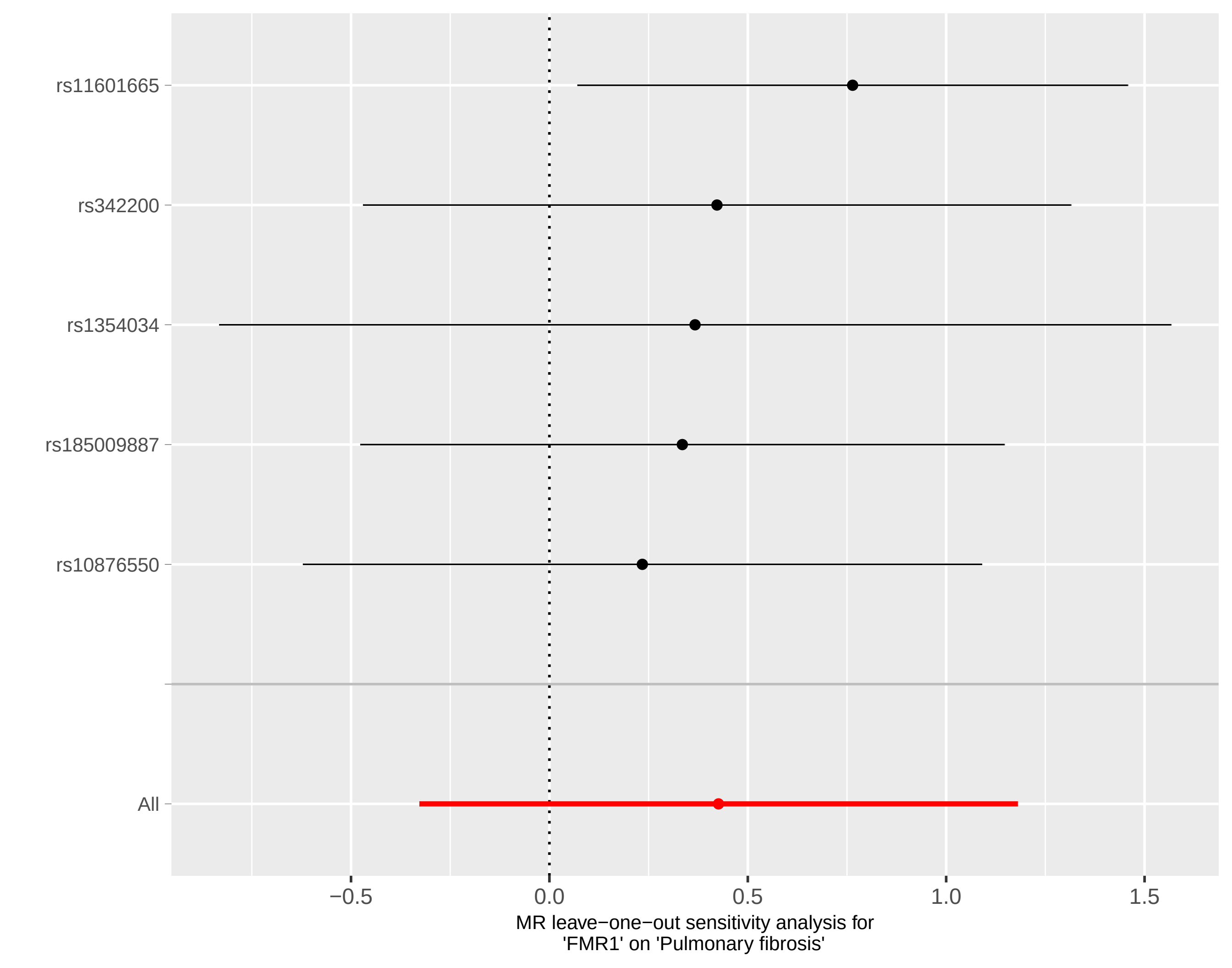
rs11601665
rs342200
rs1354034
rs185009887
rs10876550
All
−0.5
0.0
0.5
1.0
1.5
MR leave−one−out sensitivity analysis for
'FMR1' on 'Pulmonary fibrosis'

Insufficient number of SNPs

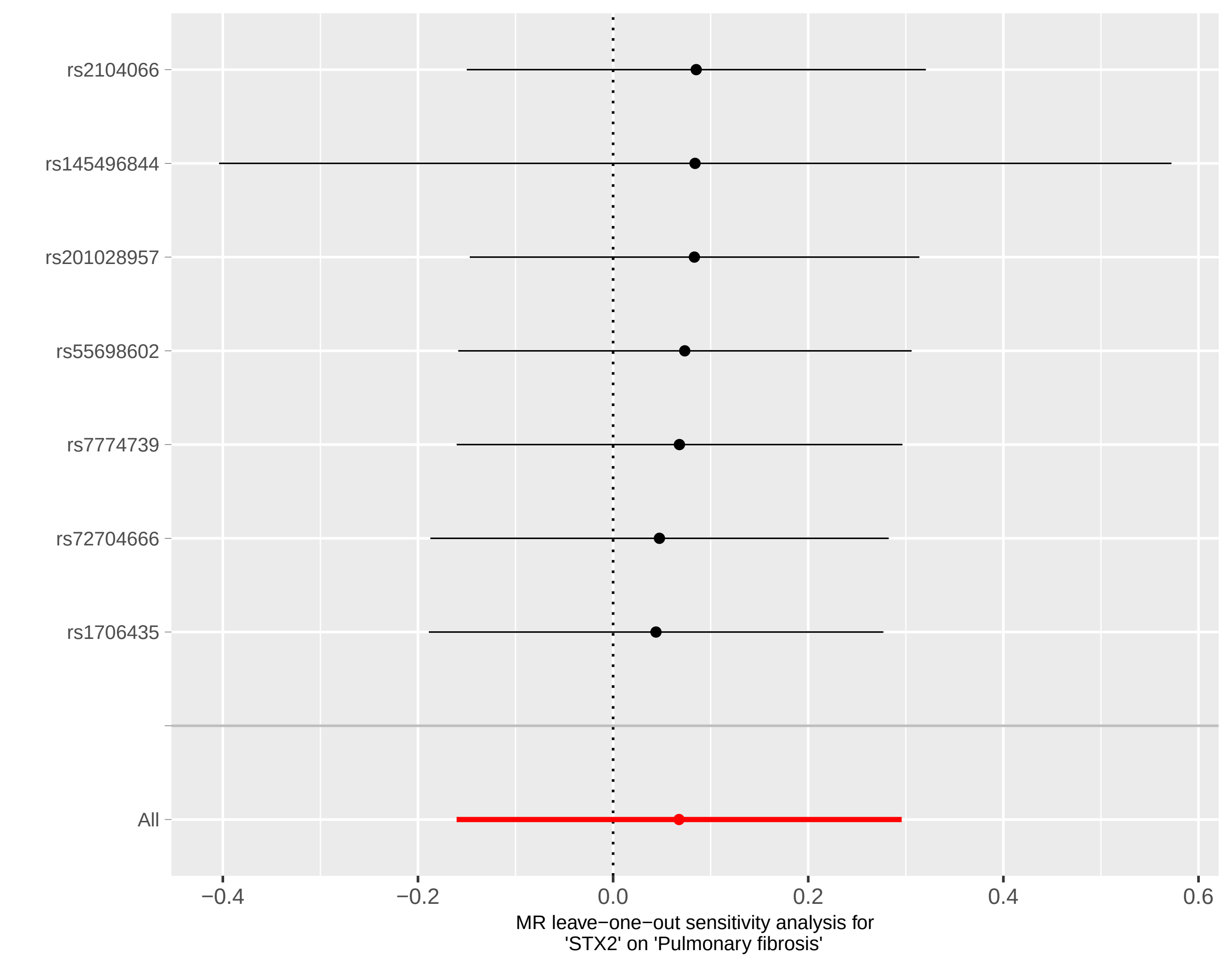
rs2104066
rs145496844
rs201028957
rs55698602
rs7774739
rs72704666
rs1706435
All
−0.4
−0.2
0.0
0.2
0.4
0.6
MR leave−one−out sensitivity analysis for
'STX2' on 'Pulmonary fibrosis'

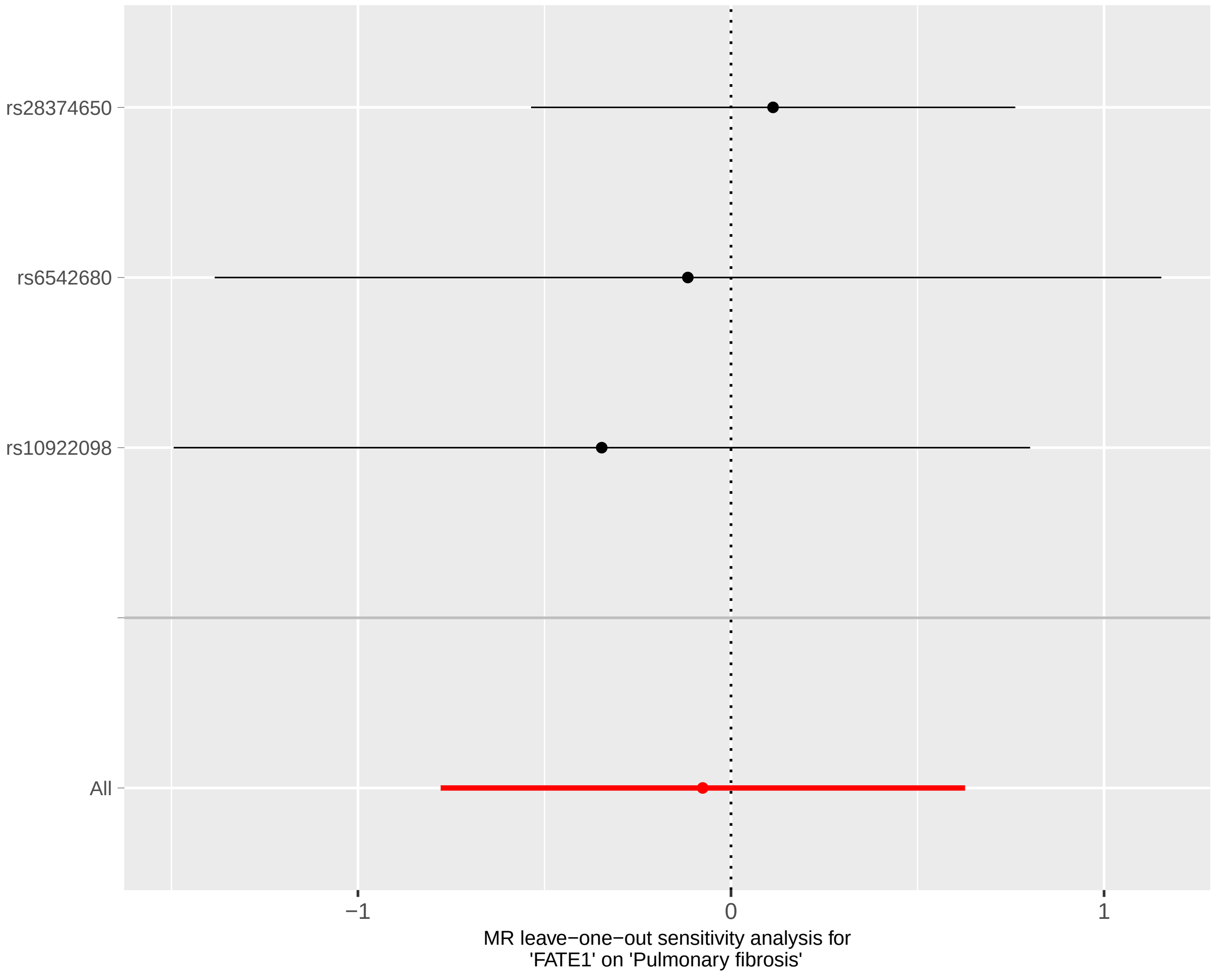
rs28374650
rs6542680
rs10922098
All
−1
0
1
MR leave−one−out sensitivity analysis for
'FATE1' on 'Pulmonary fibrosis'

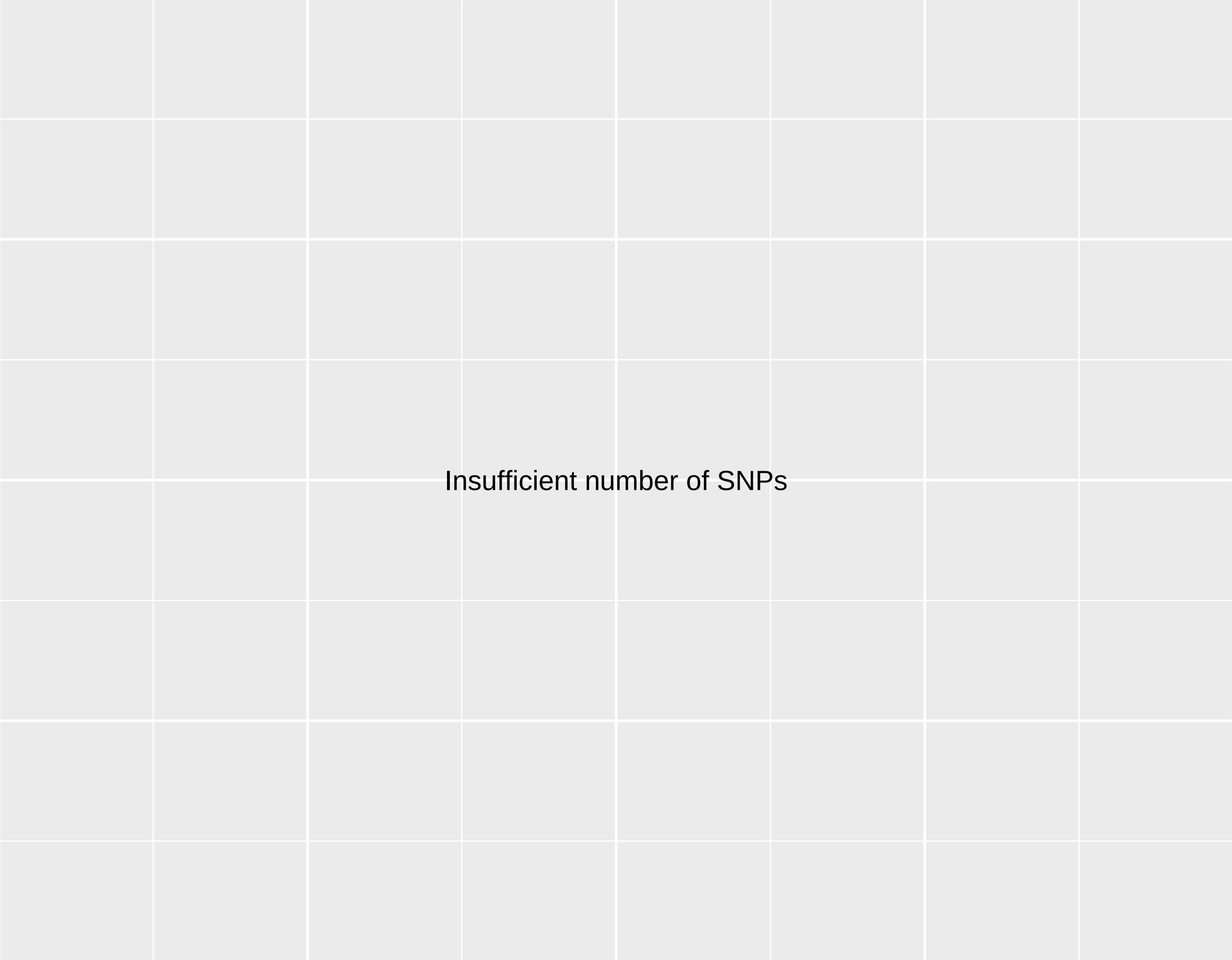
Insufficient number of SNPs

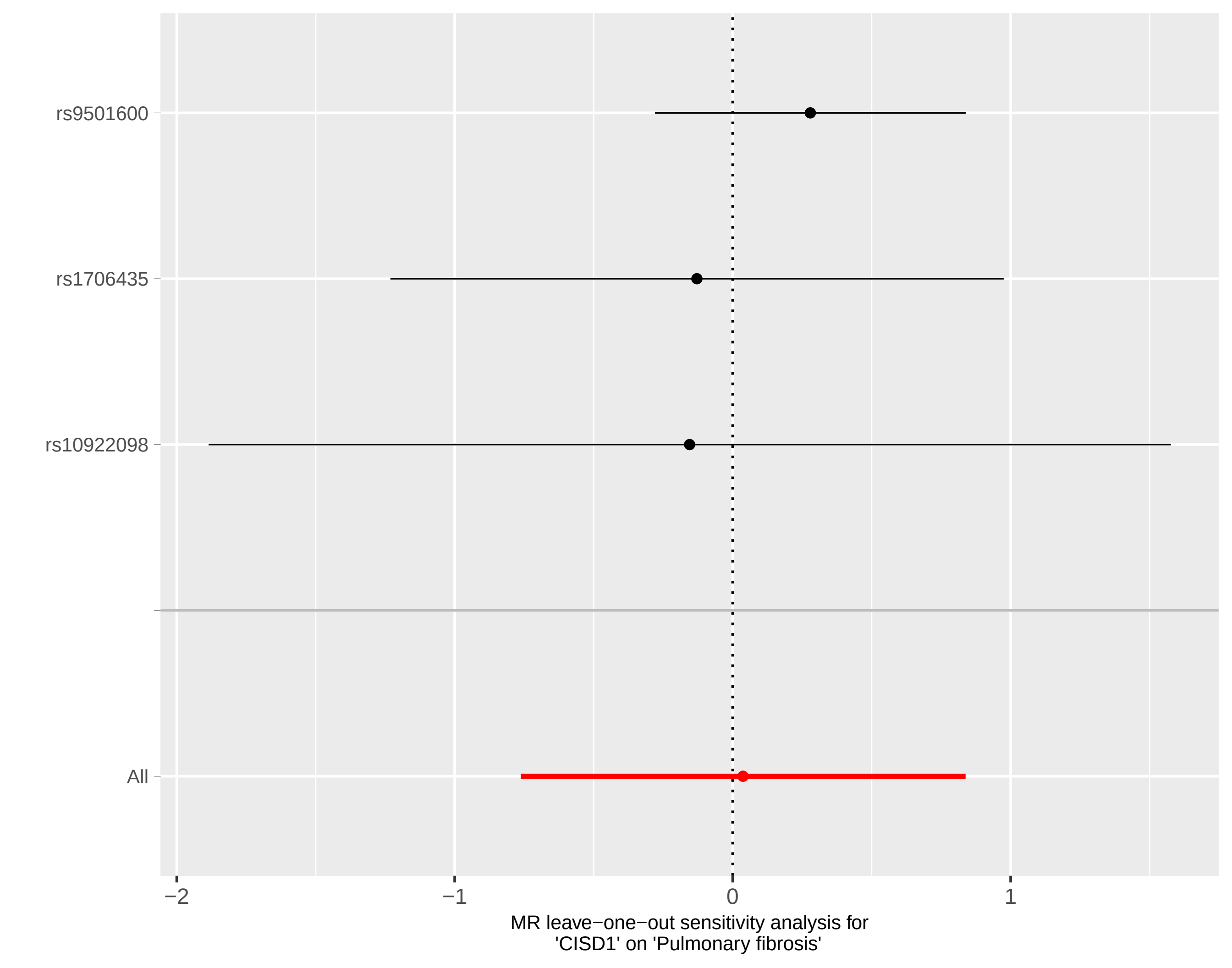
rs9501600
rs1706435
rs10922098
All
−2
−1
0
1
MR leave−one−out sensitivity analysis for
'CISD1' on 'Pulmonary fibrosis'

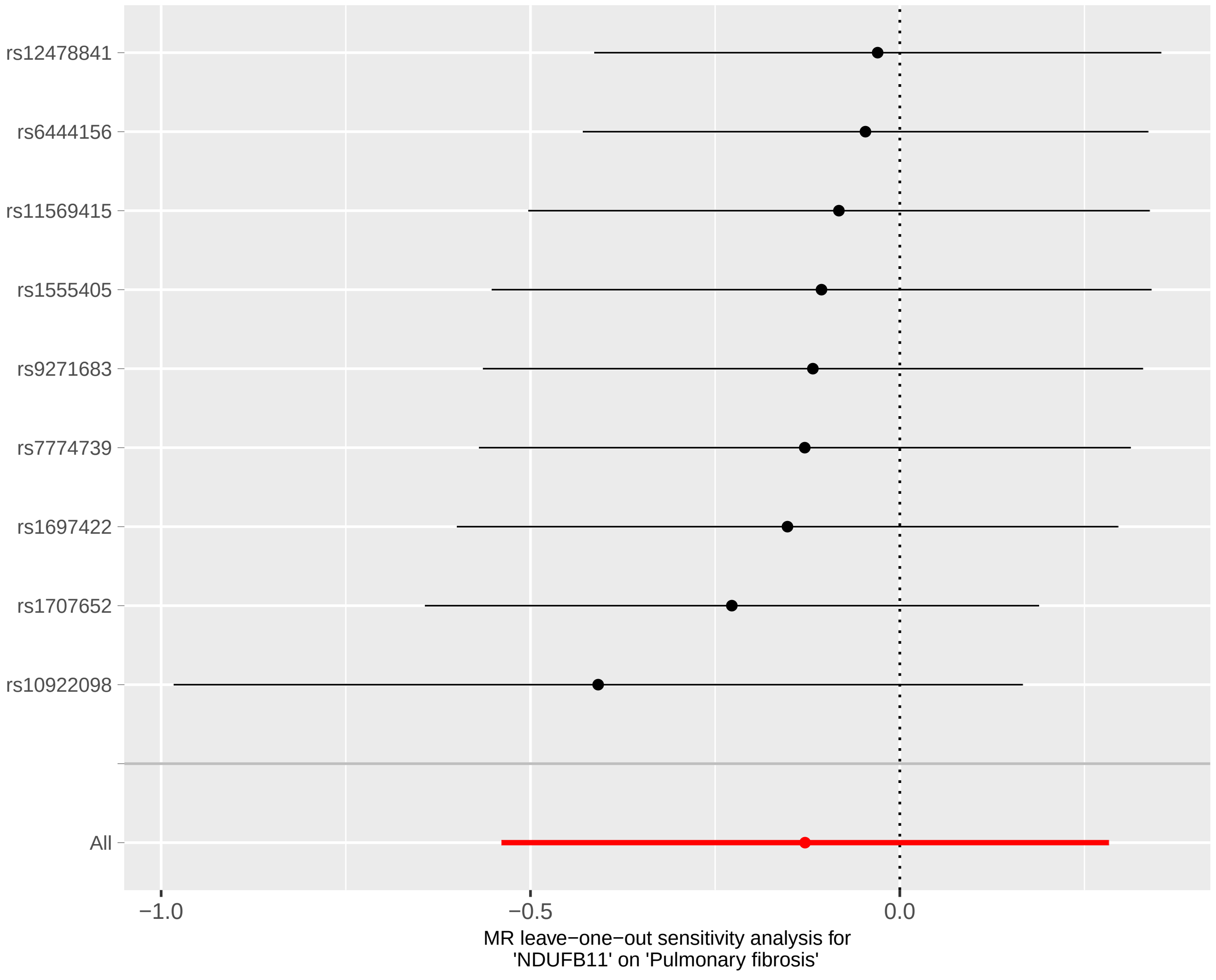

rs12478841
rs6444156
rs11569415
rs1555405
rs9271683
rs7774739
rs1697422
rs1707652
rs10922098
All
−1.0
−0.5
0.0
MR leave−one−out sensitivity analysis for
'NDUFB11' on 'Pulmonary fibrosis'

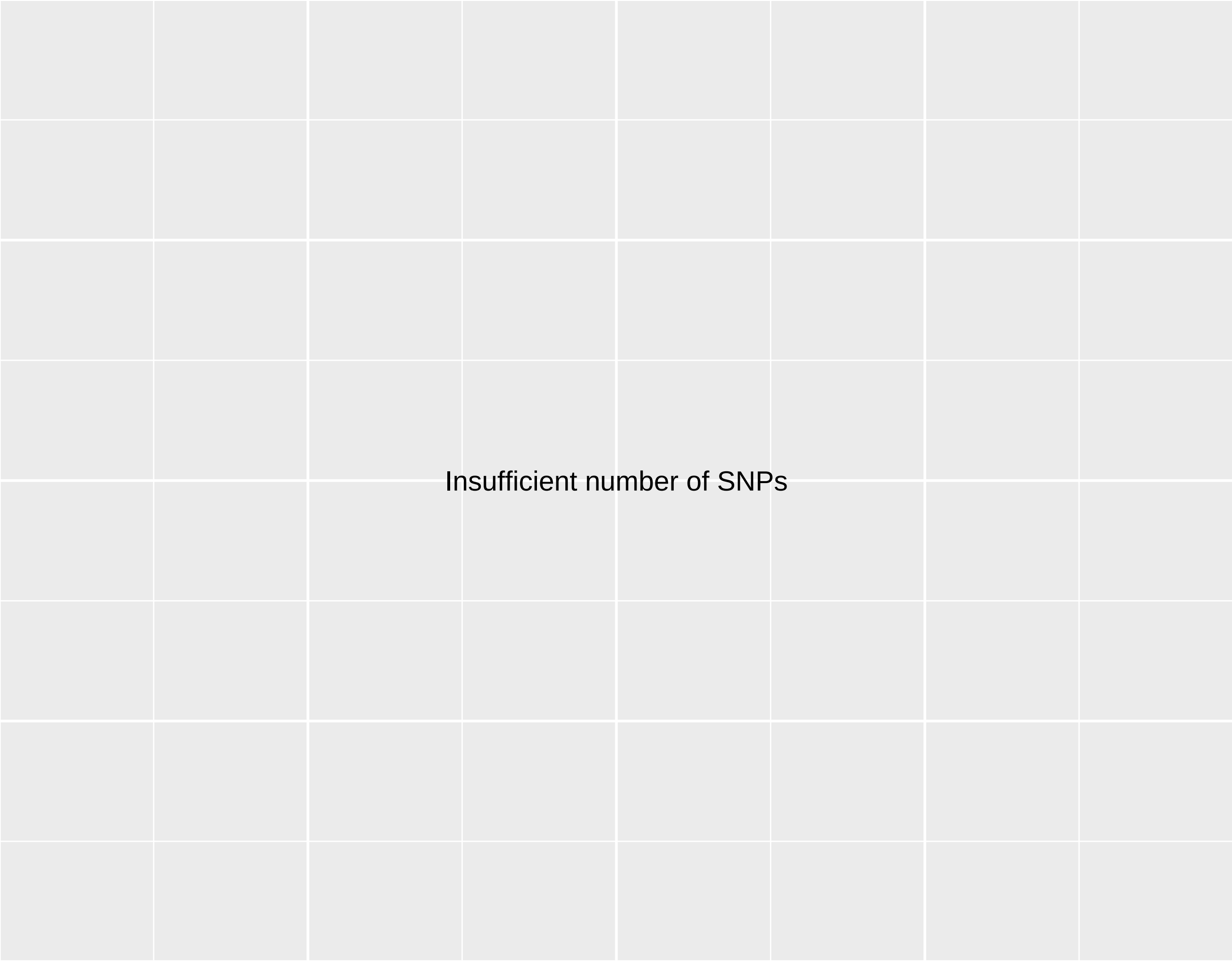
Insufficient number of SNPs

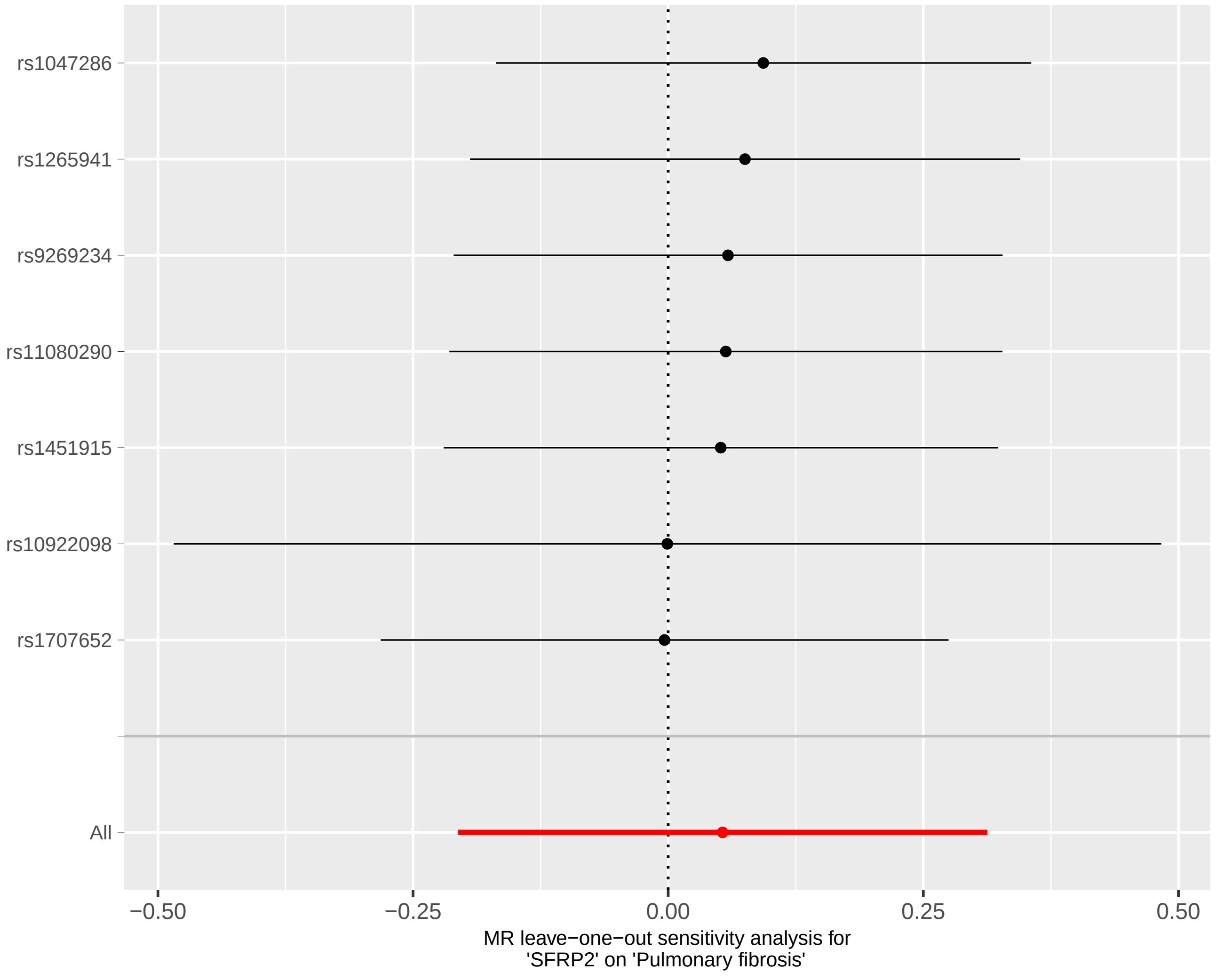
rs1047286
rs1265941
rs9269234
rs11080290
rs1451915
rs10922098
rs1707652
All
−0.50
−0.25
0.00
0.25
0.50
MR leave−one−out sensitivity analysis for
'SFRP2' on 'Pulmonary fibrosis'

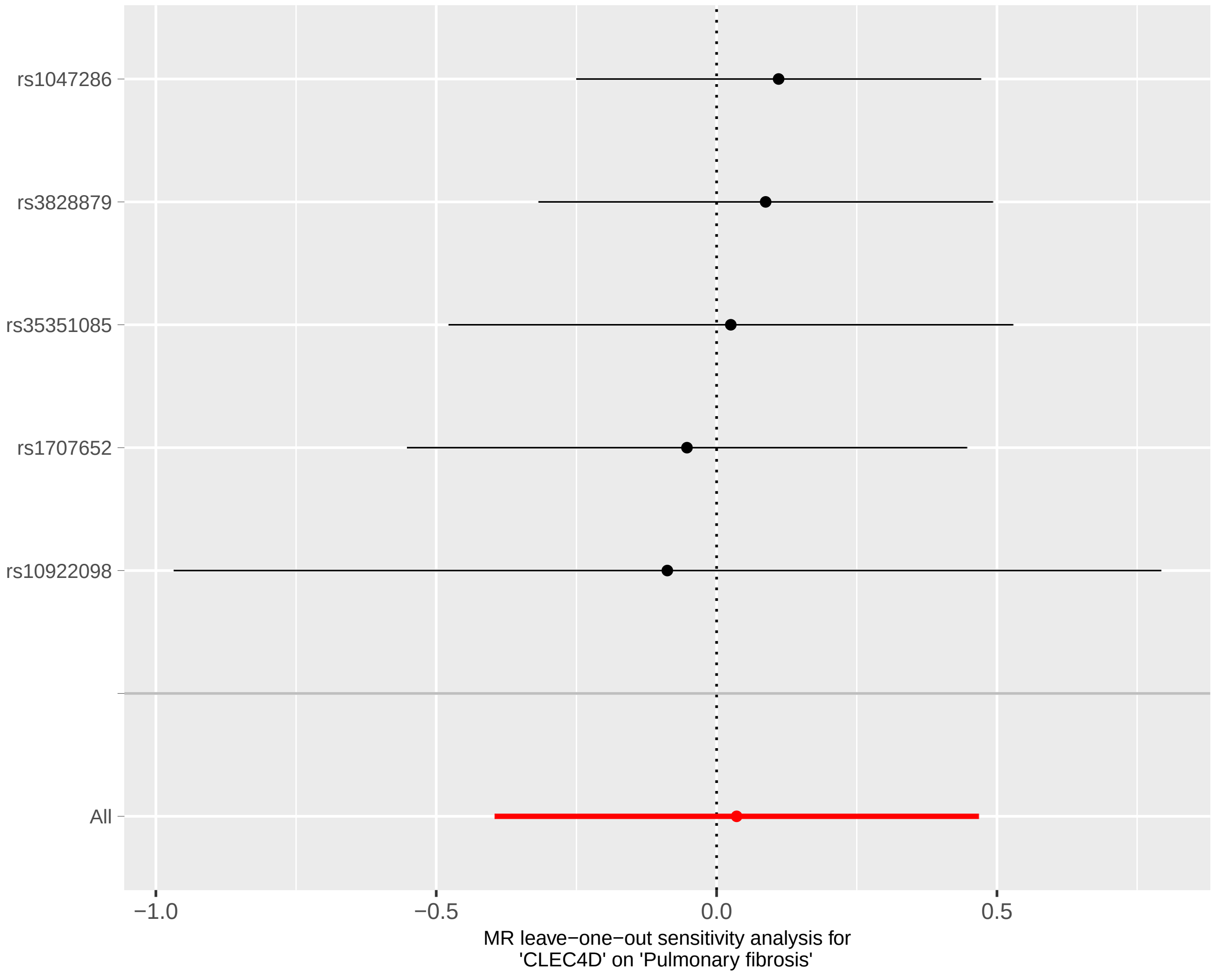

rs1047286
rs3828879
rs35351085
rs1707652
rs10922098
All
−1.0
−0.5
0.0
0.5
MR leave−one−out sensitivity analysis for
'CLEC4D' on 'Pulmonary fibrosis'

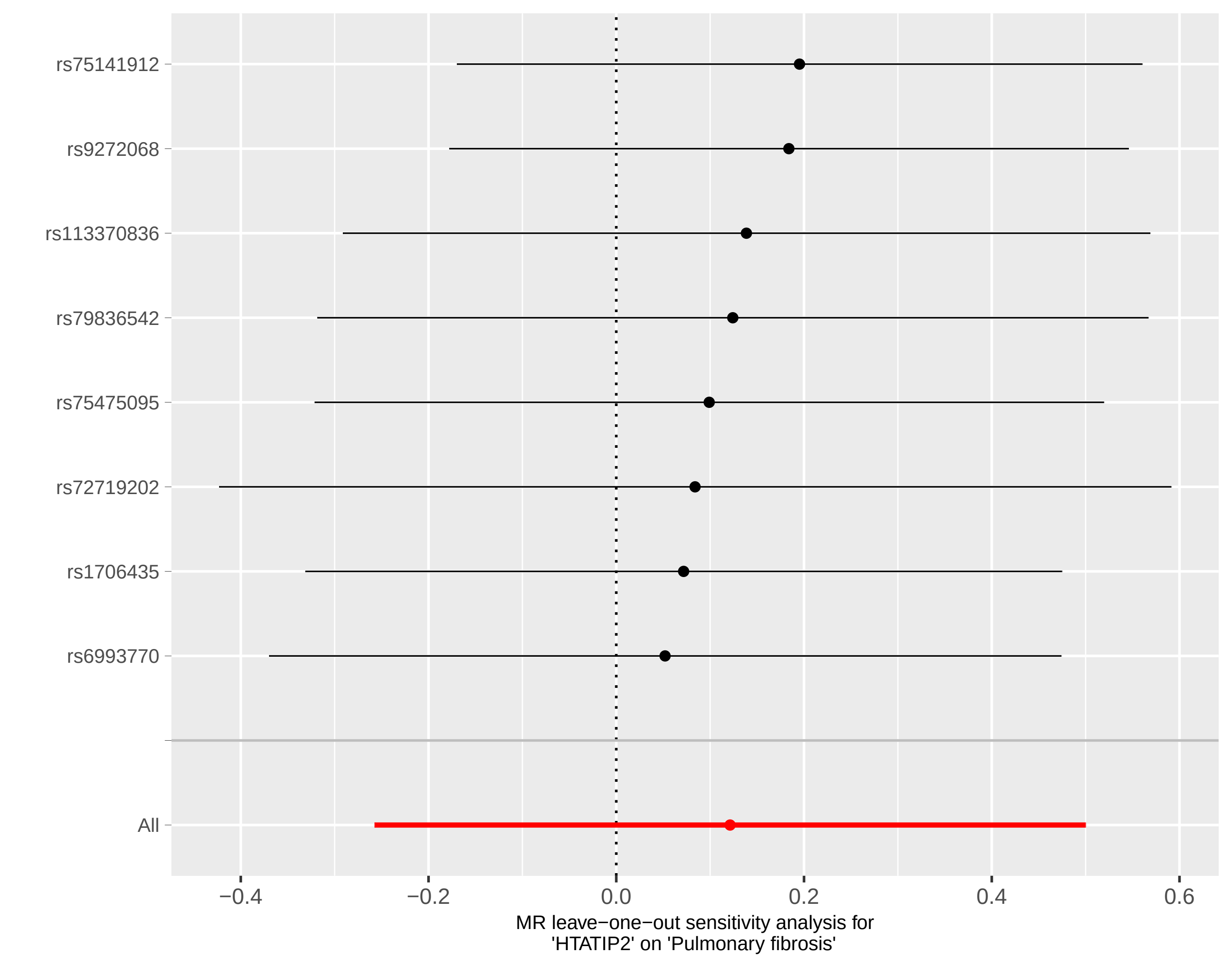
rs75141912
rs9272068
rs113370836
rs79836542
rs75475095
rs72719202
rs1706435
rs6993770
All
−0.4
−0.2
0.0
0.2
0.4
0.6
MR leave−one−out sensitivity analysis for
'HTATIP2' on 'Pulmonary fibrosis'

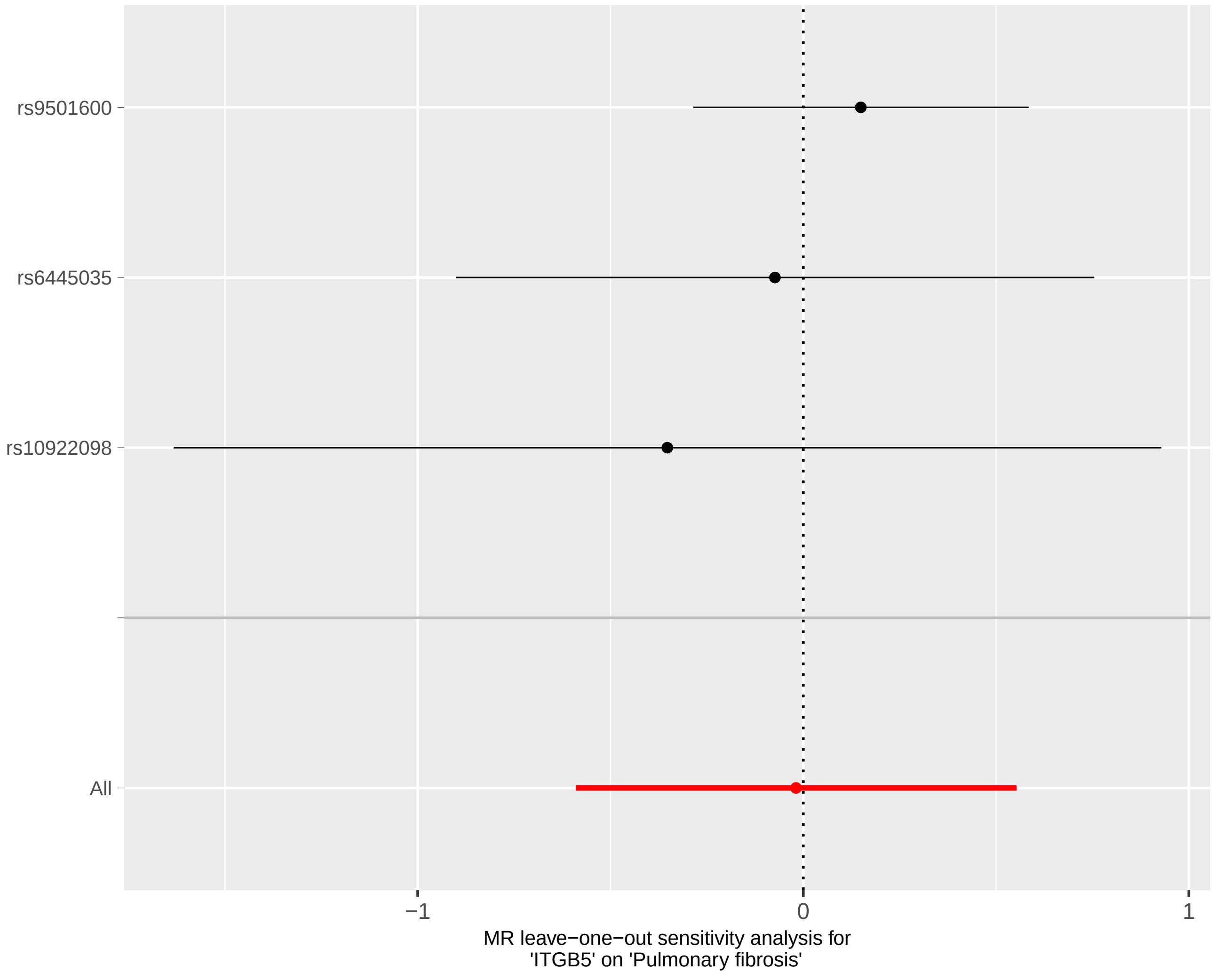
rs9501600
rs6445035
rs10922098
All
−1
0
1
MR leave−one−out sensitivity analysis for
'ITGB5' on 'Pulmonary fibrosis'

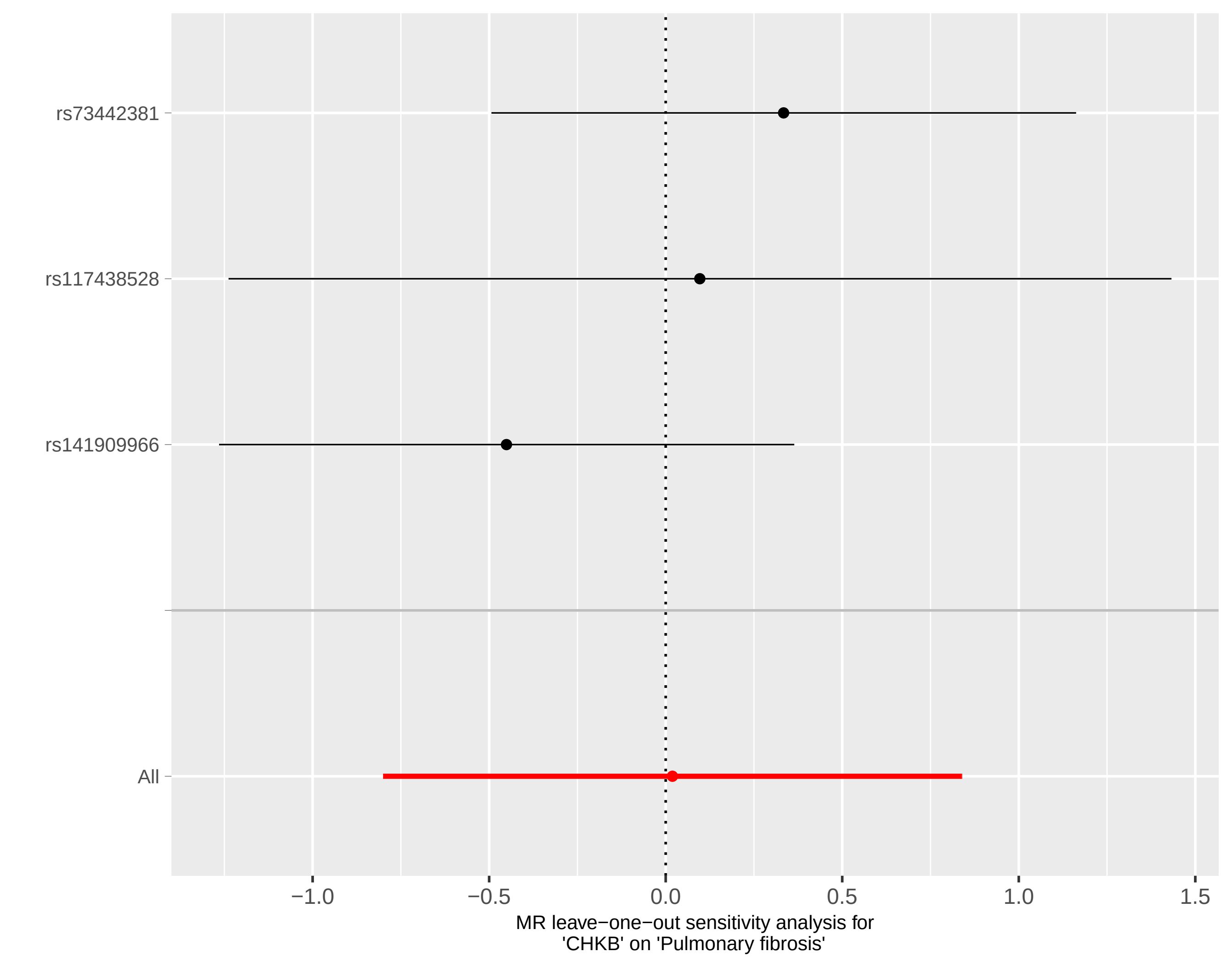
rs73442381
rs117438528
rs141909966
All
−1.0
−0.5
0.0
0.5
1.0
1.5
MR leave−one−out sensitivity analysis for
'CHKB' on 'Pulmonary fibrosis'

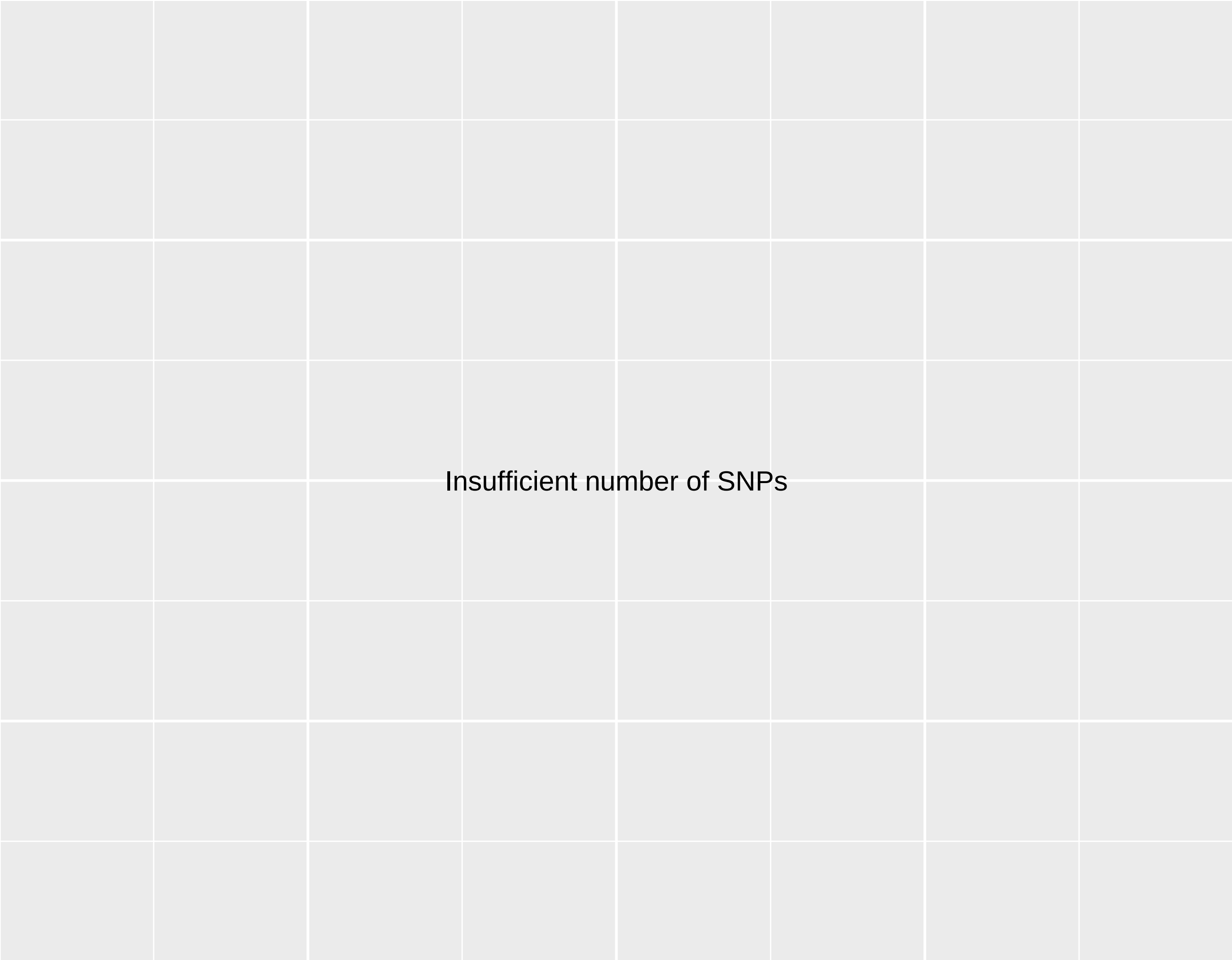
Insufficient number of SNPs

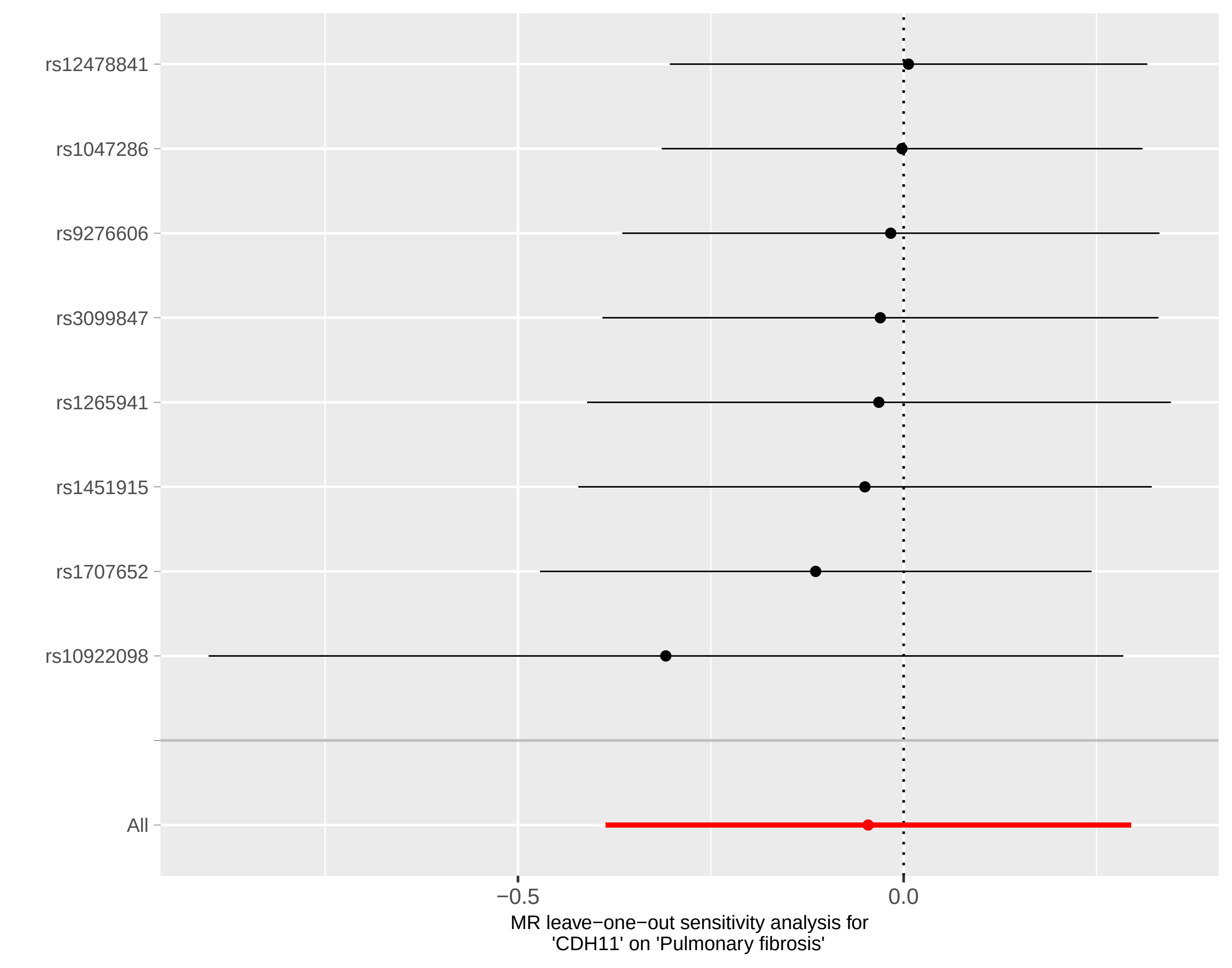
rs12478841
rs1047286
rs9276606
rs3099847
rs1265941
rs1451915
rs1707652
rs10922098
All
−0.5
0.0
MR leave−one−out sensitivity analysis for
'CDH11' on 'Pulmonary fibrosis'

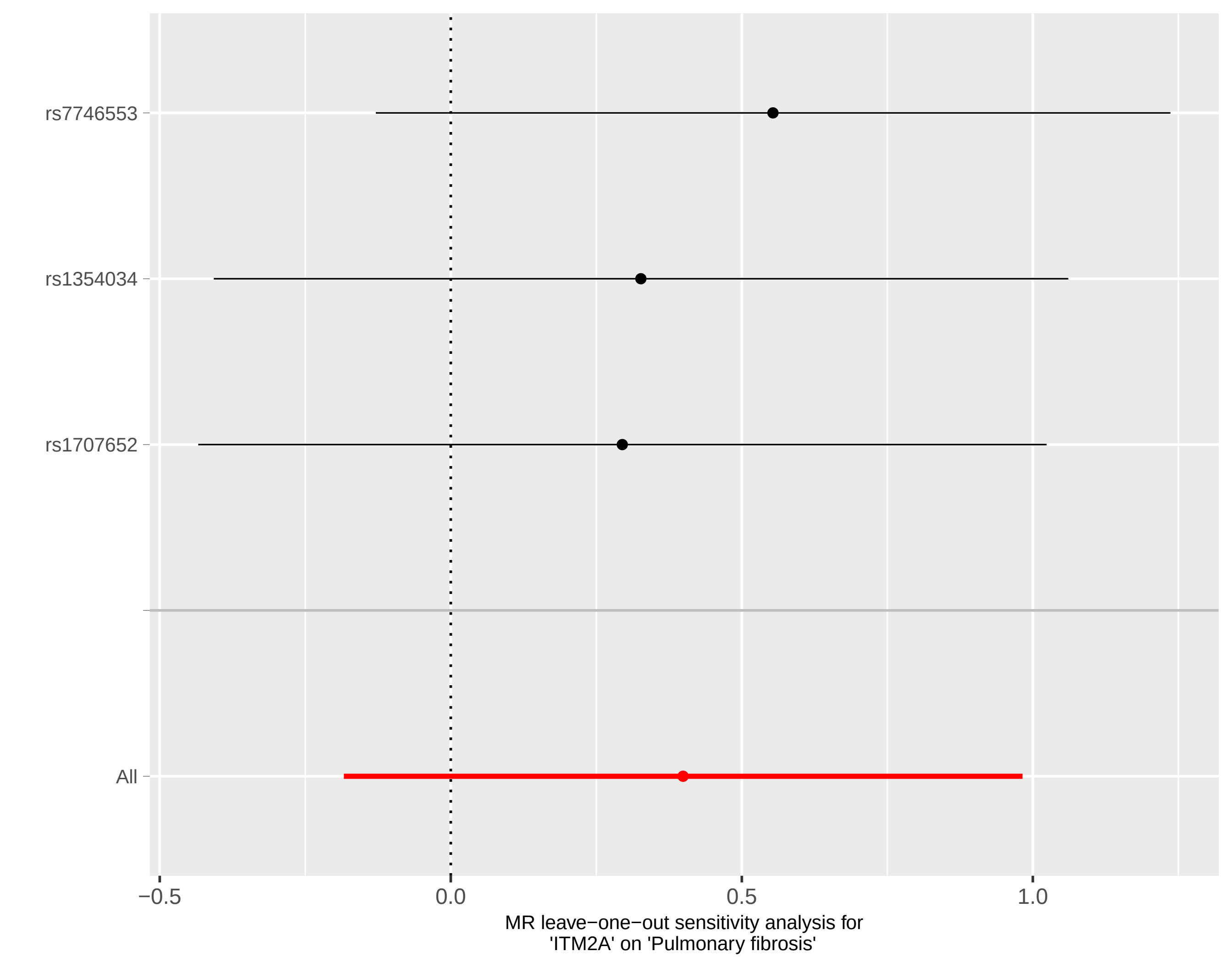
rs7746553
rs1354034
rs1707652
All
−0.5
0.0
0.5
1.0
MR leave−one−out sensitivity analysis for
'ITM2A' on 'Pulmonary fibrosis'

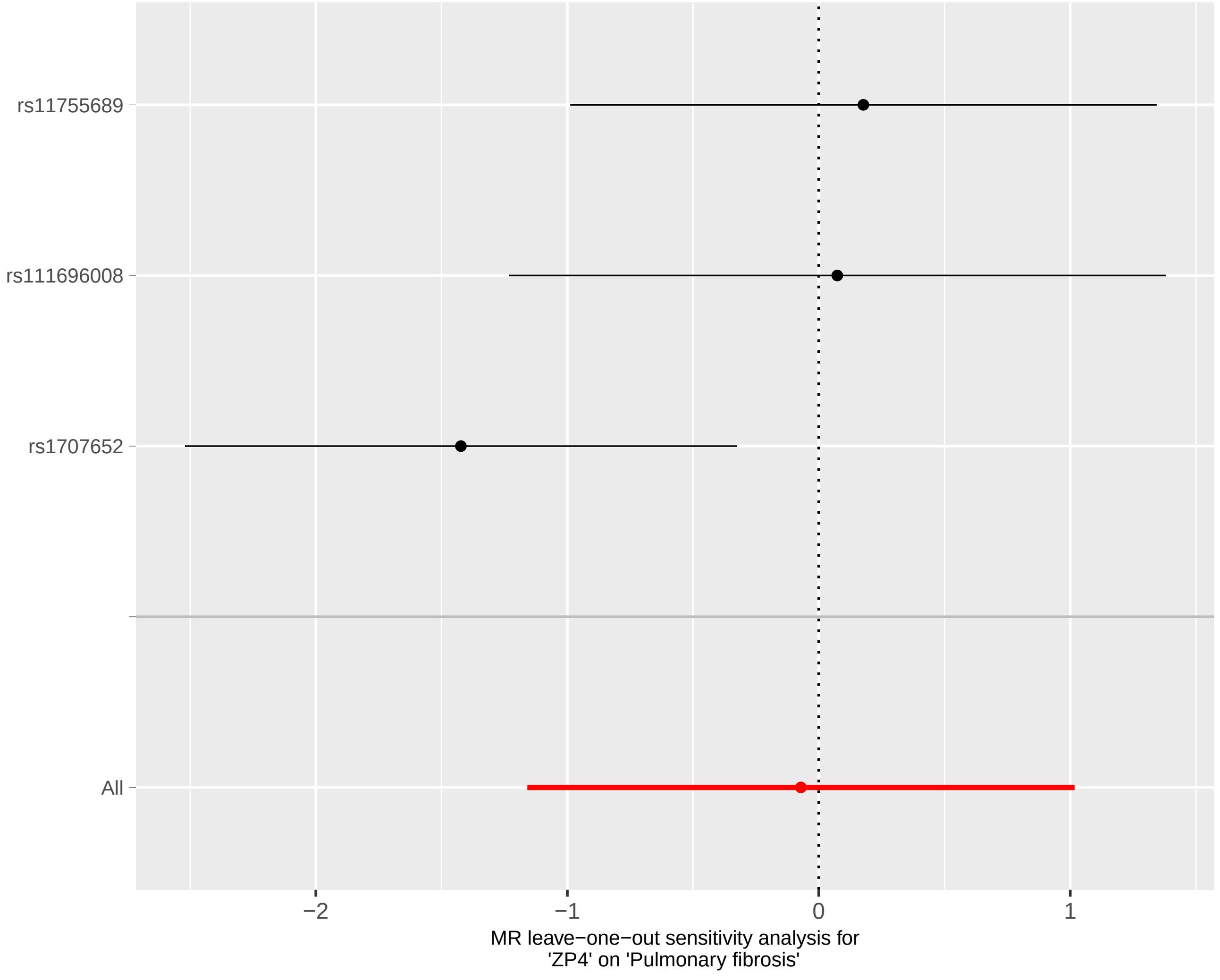
rs11755689
rs111696008
rs1707652
All
−2
−1
0
1
MR leave−one−out sensitivity analysis for
'ZP4' on 'Pulmonary fibrosis'

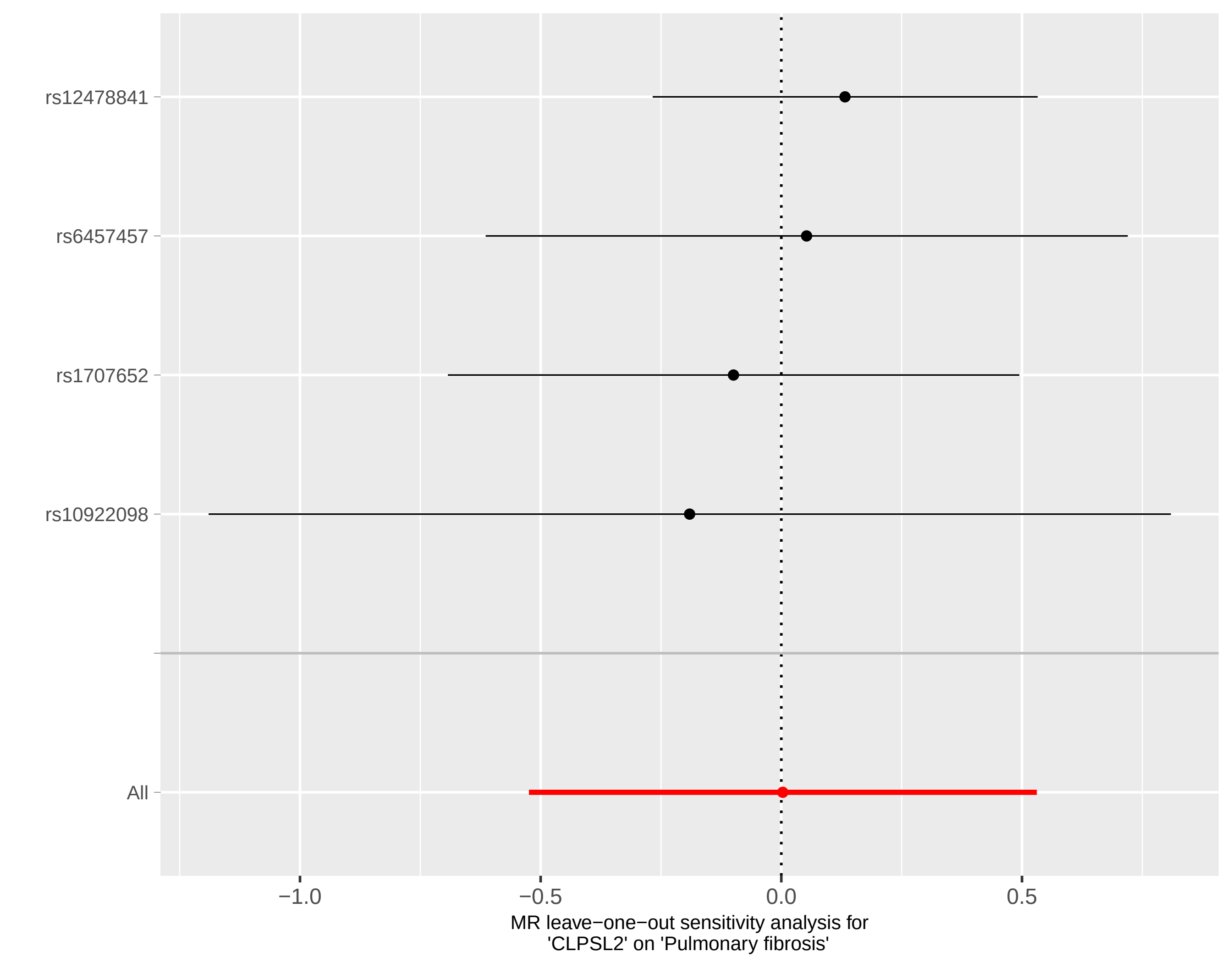
rs12478841
rs6457457
rs1707652
rs10922098
All
−1.0
−0.5
0.0
0.5
MR leave−one−out sensitivity analysis for
'CLPSL2' on 'Pulmonary fibrosis'

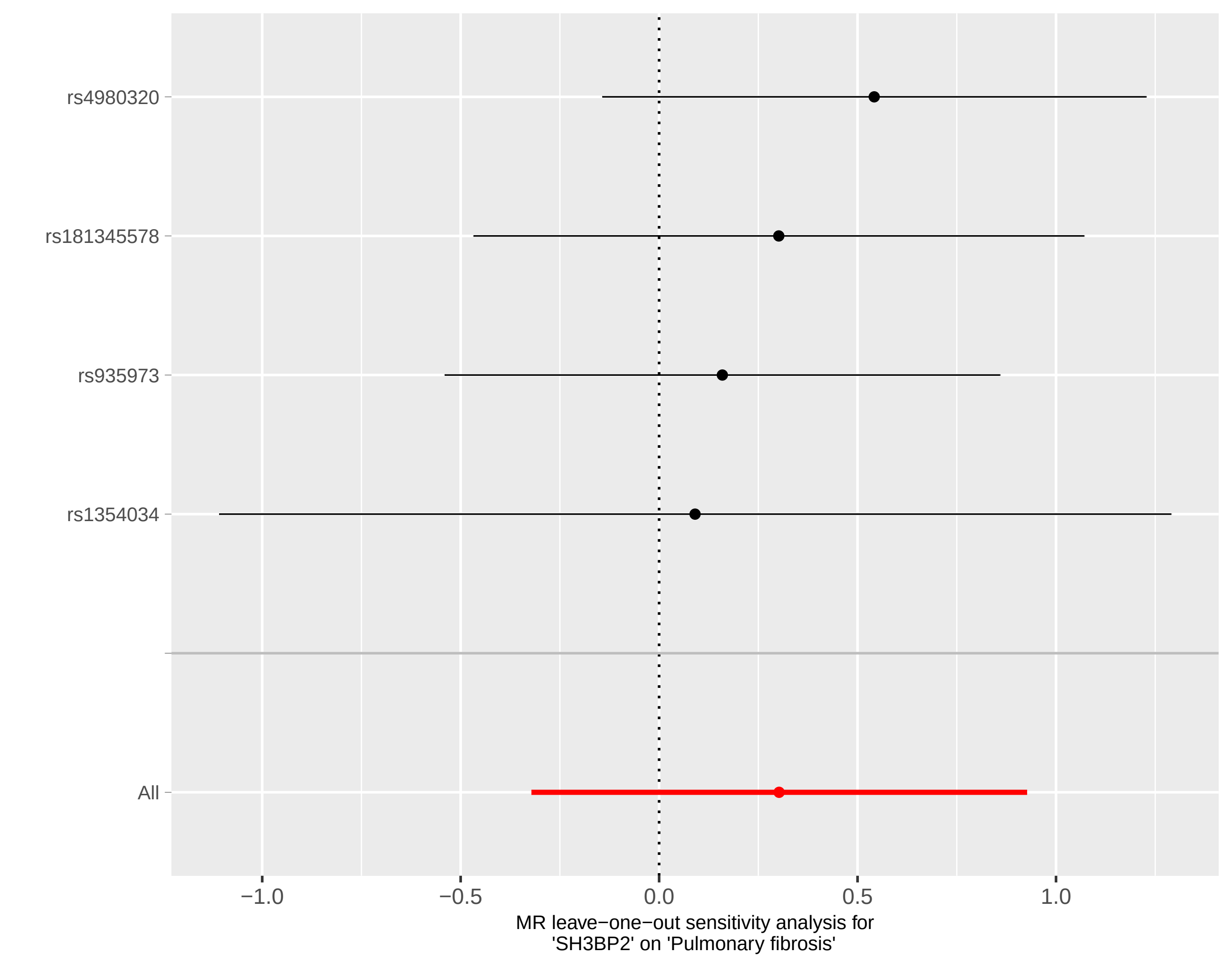
rs4980320
rs181345578
rs935973
rs1354034
All
−1.0
−0.5
0.0
0.5
1.0
MR leave−one−out sensitivity analysis for
'SH3BP2' on 'Pulmonary fibrosis'

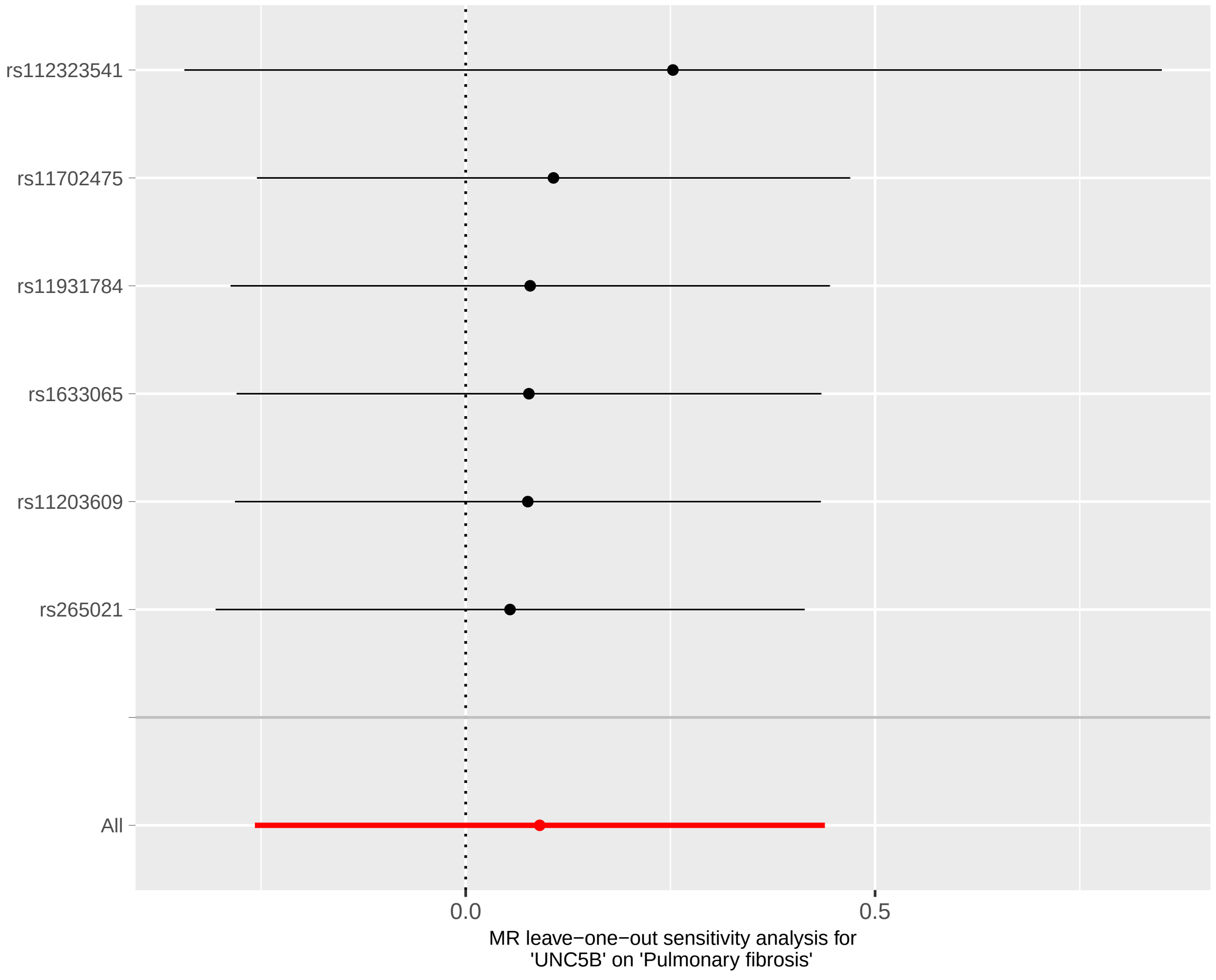

rs112323541
rs11702475
rs11931784
rs1633065
rs11203609
rs265021
All
0.0
0.5
MR leave−one−out sensitivity analysis for
'UNC5B' on 'Pulmonary fibrosis'

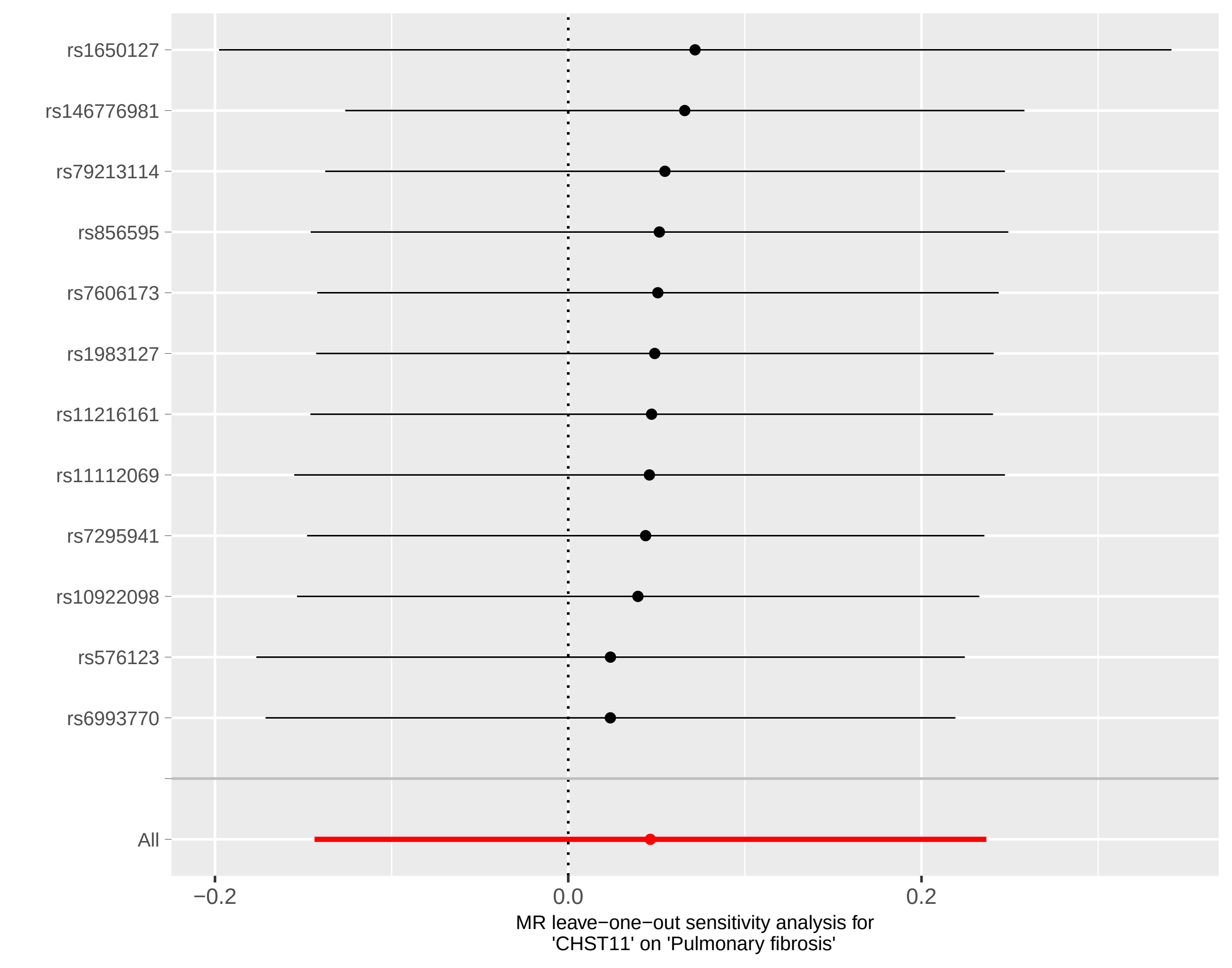
rs1650127
rs146776981
rs79213114
rs856595
rs7606173
rs1983127
rs11216161
rs11112069
rs7295941
rs10922098
rs576123
rs6993770
All
−0.2
0.0
0.2
MR leave−one−out sensitivity analysis for
'CHST11' on 'Pulmonary fibrosis'

Insufficient number of SNPs

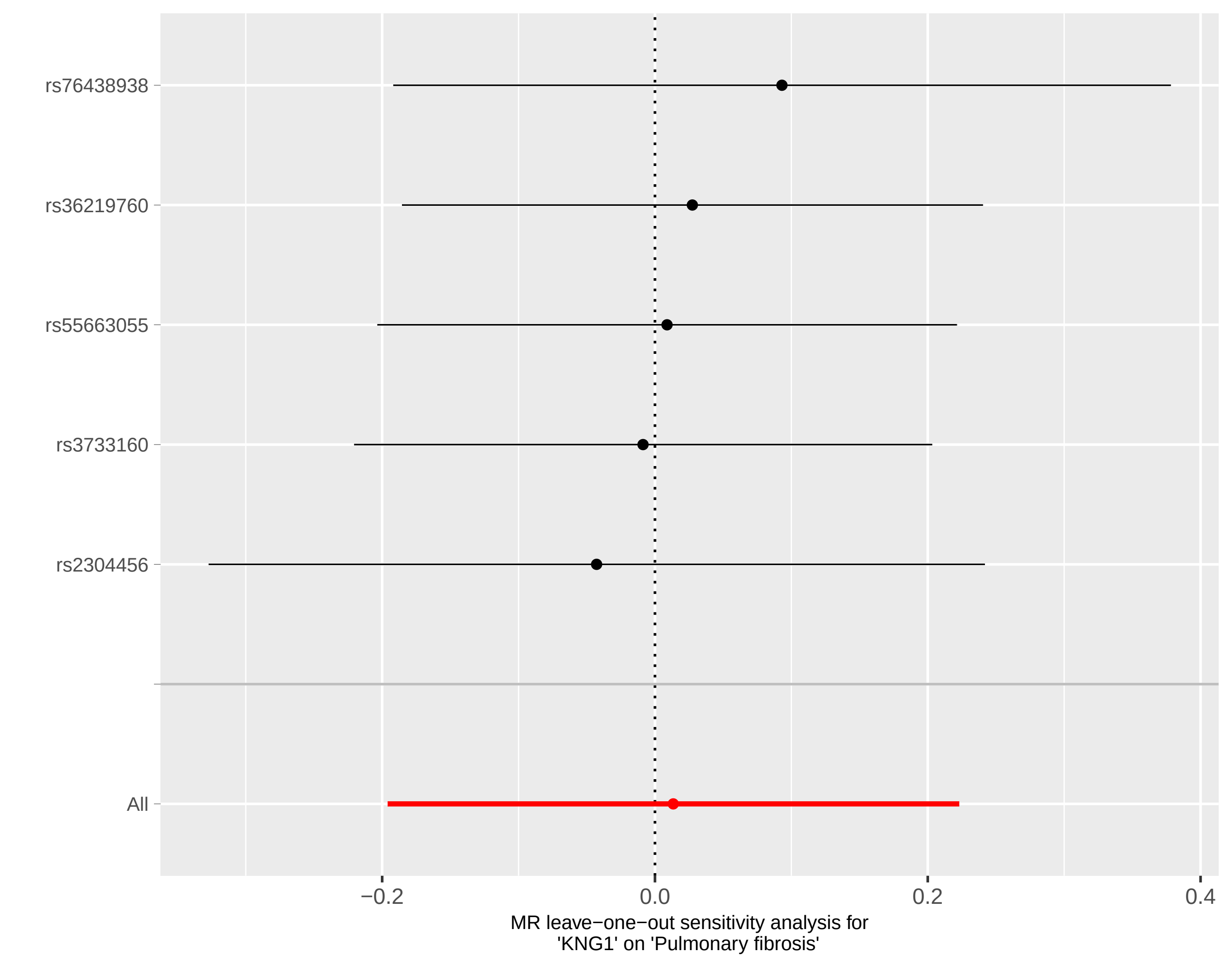
rs76438938
rs36219760
rs55663055
rs3733160
rs2304456
All
−0.2
0.0
0.2
0.4
MR leave−one−out sensitivity analysis for
'KNG1' on 'Pulmonary fibrosis'

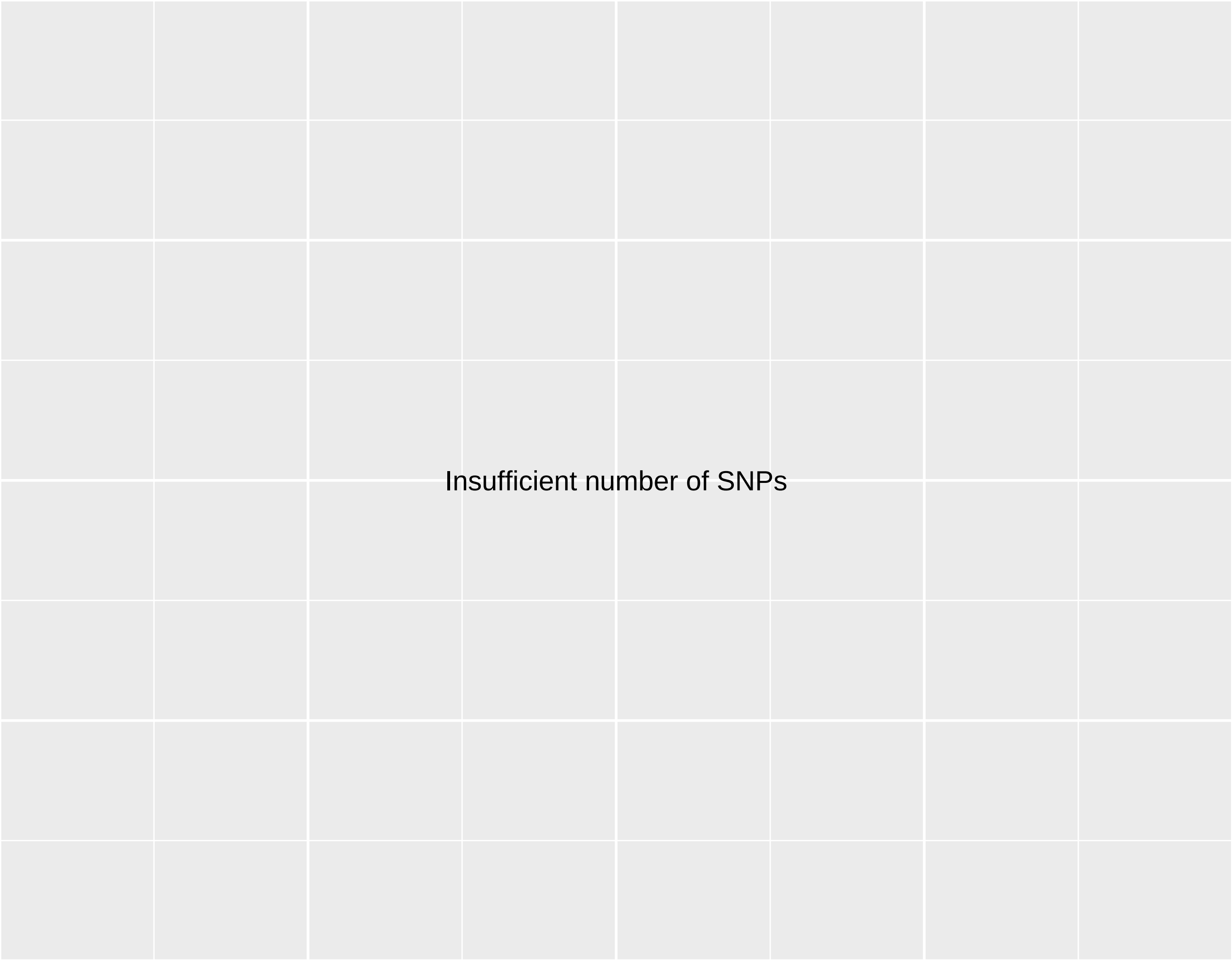
Insufficient number of SNPs

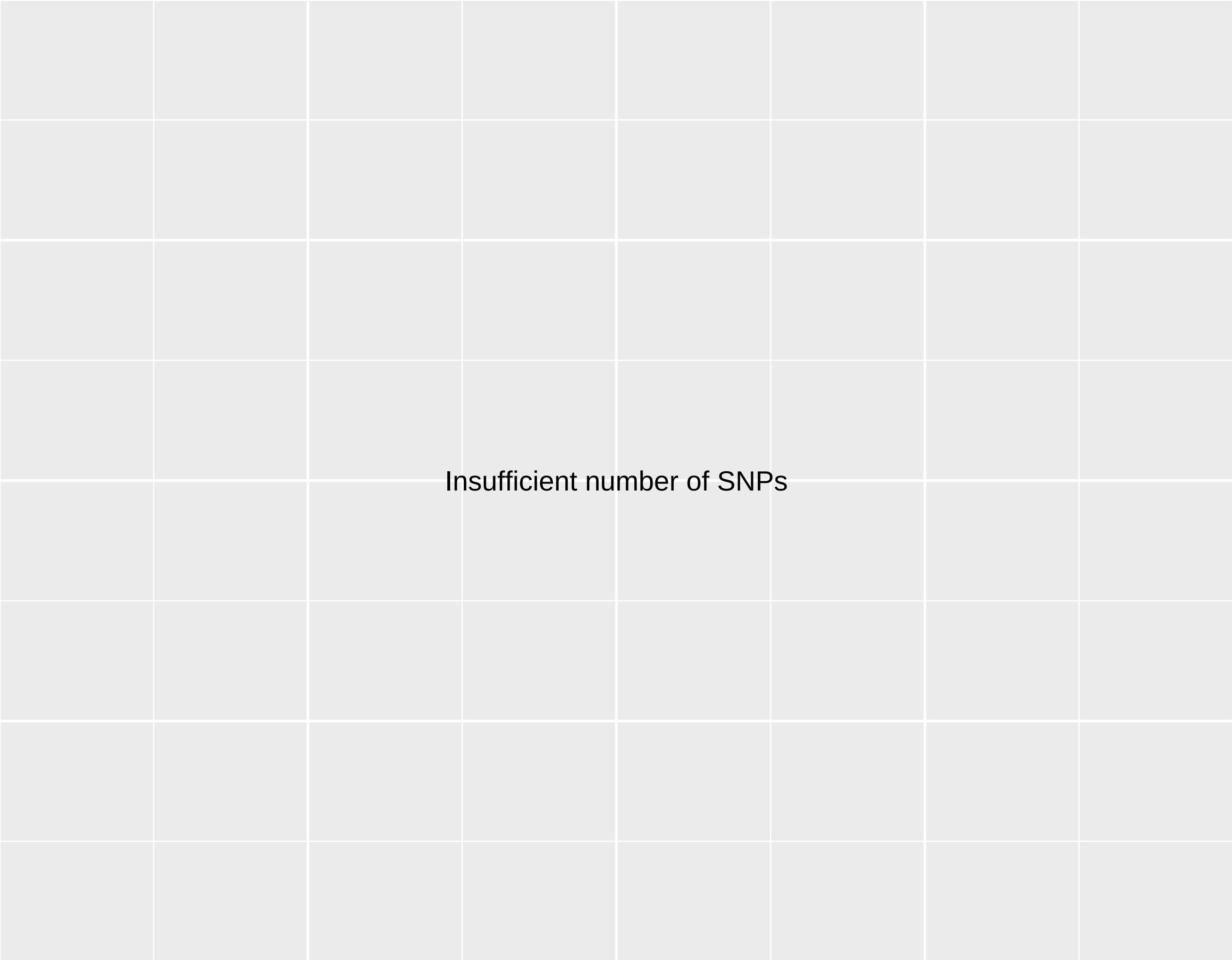
Insufficient number of SNPs

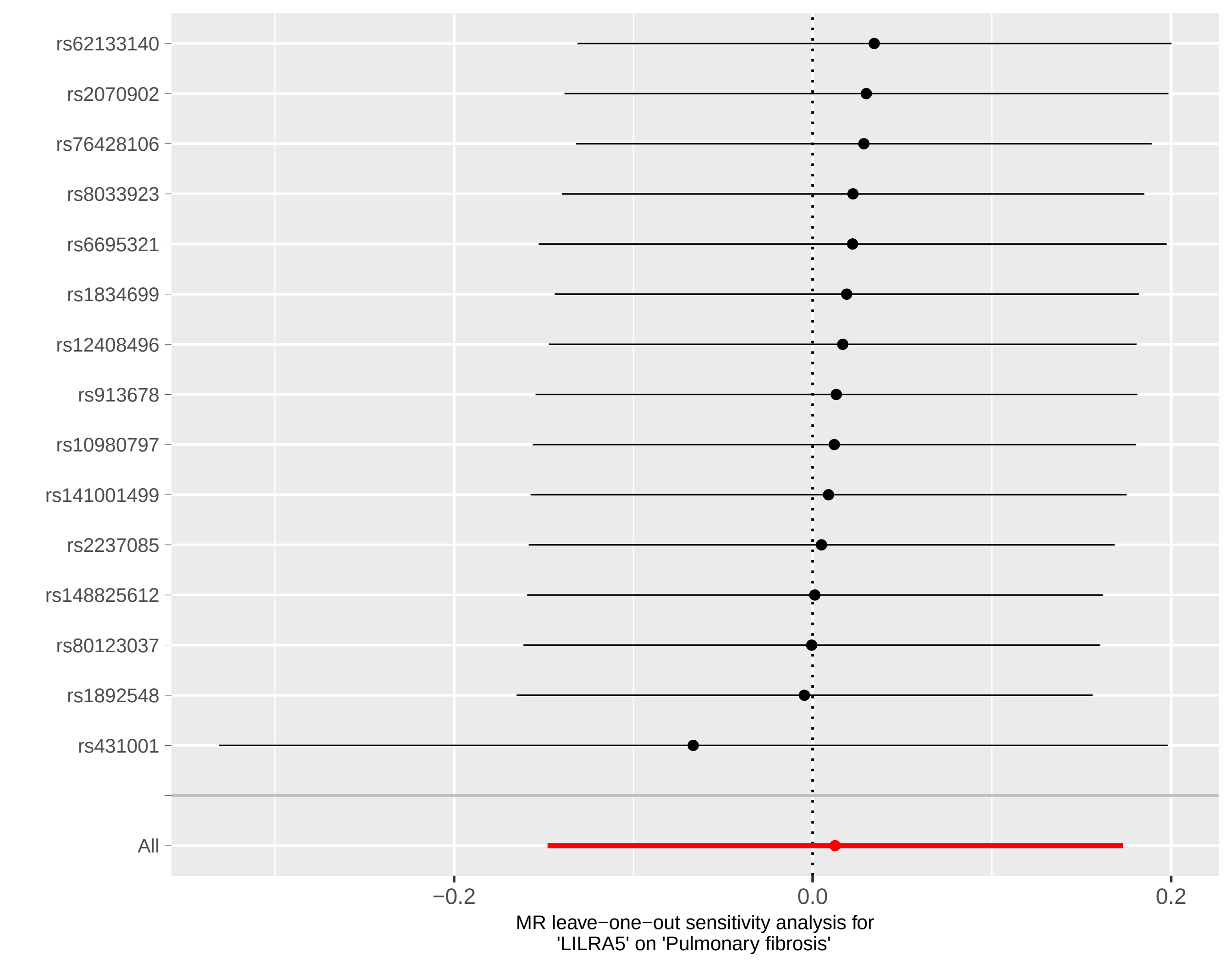
rs62133140
rs2070902
rs76428106
rs8033923
rs6695321
rs1834699
rs12408496
rs913678
rs10980797
rs141001499
rs2237085
rs148825612
rs80123037
rs1892548
rs431001
All
−0.2
0.0
0.2
MR leave−one−out sensitivity analysis for
'LILRA5' on 'Pulmonary fibrosis'

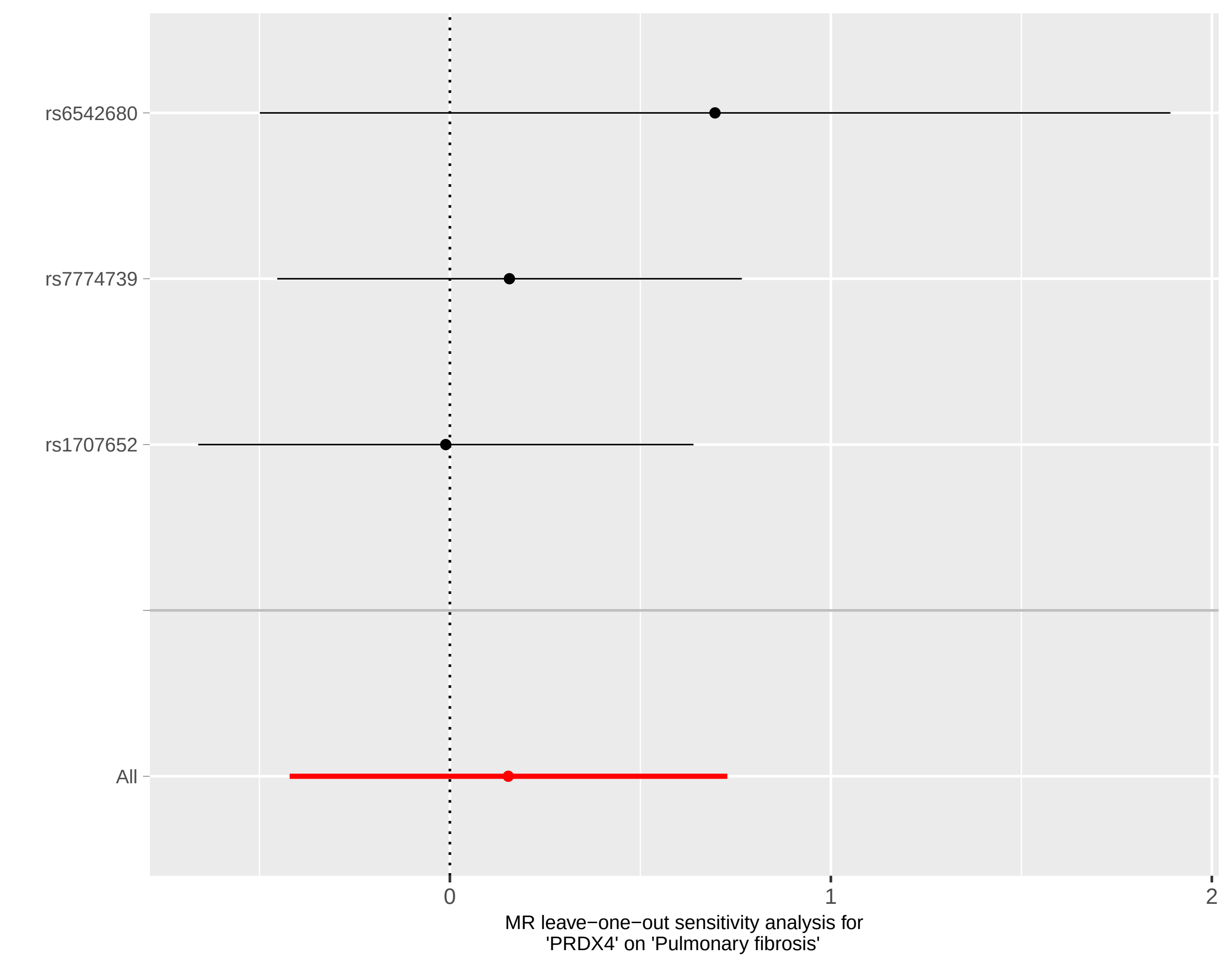
rs6542680
rs7774739
rs1707652
All
0
1
2
MR leave−one−out sensitivity analysis for
'PRDX4' on 'Pulmonary fibrosis'

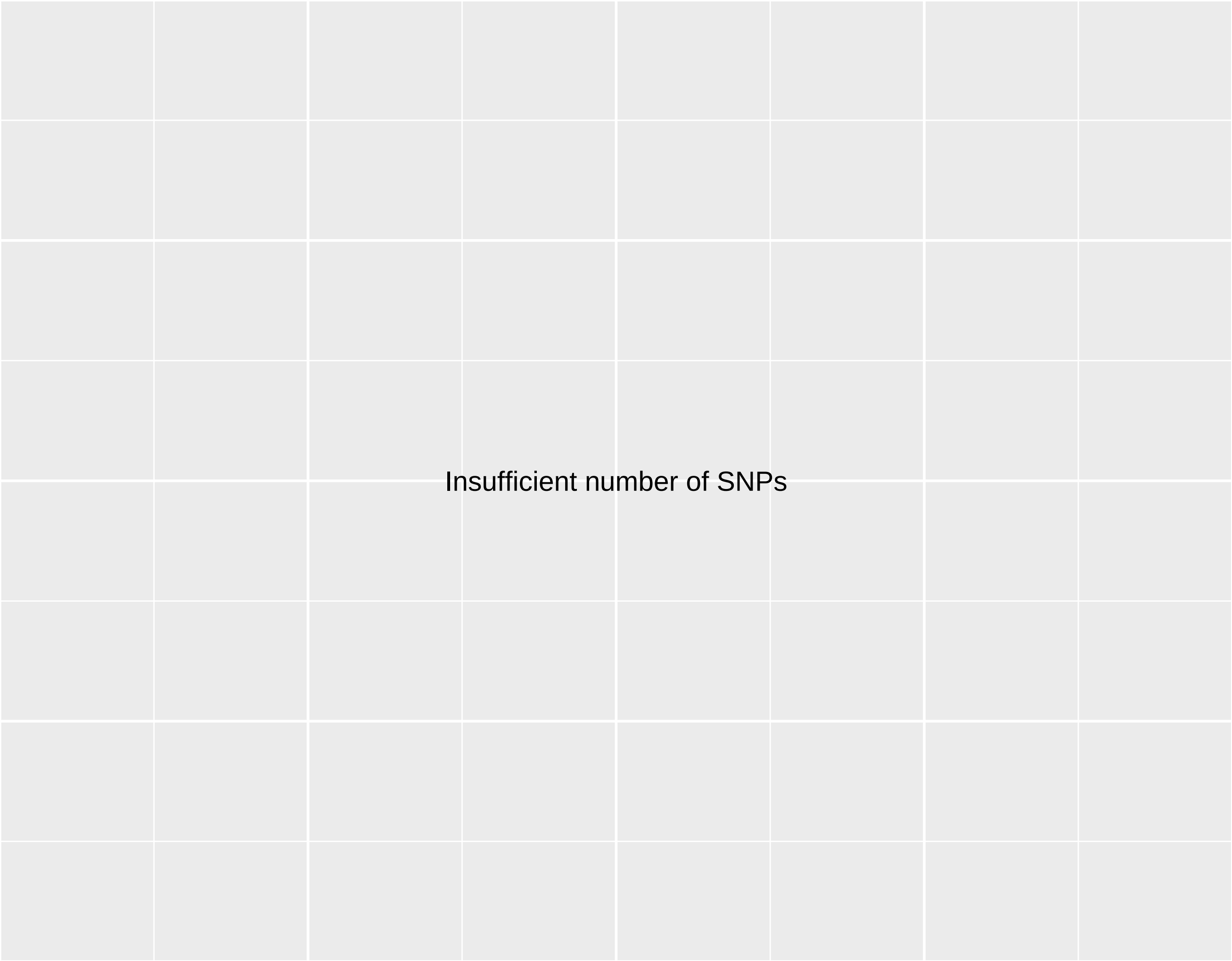
Insufficient number of SNPs

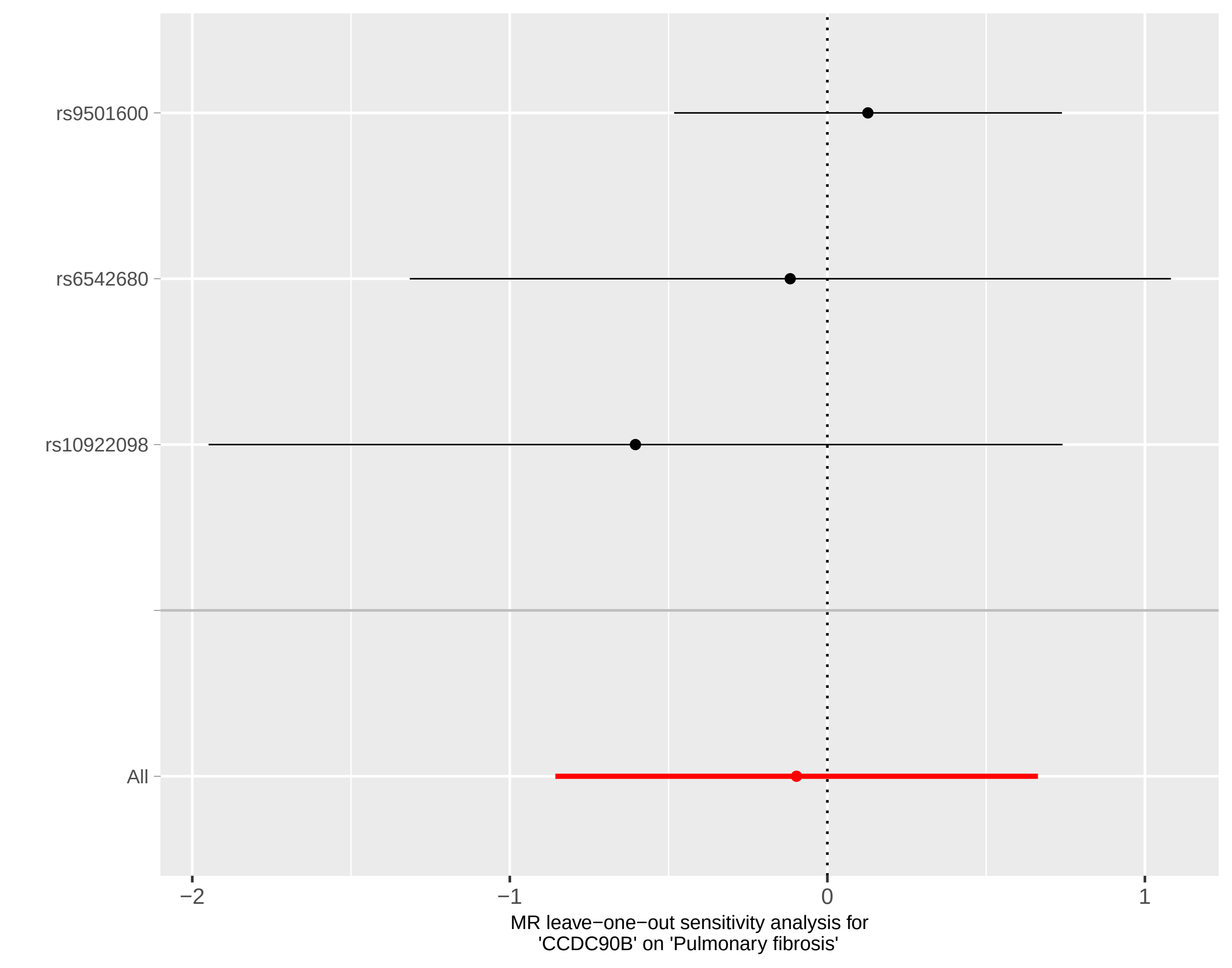
rs9501600
rs6542680
rs10922098
All
−2
−1
0
1
MR leave−one−out sensitivity analysis for
'CCDC90B' on 'Pulmonary fibrosis'

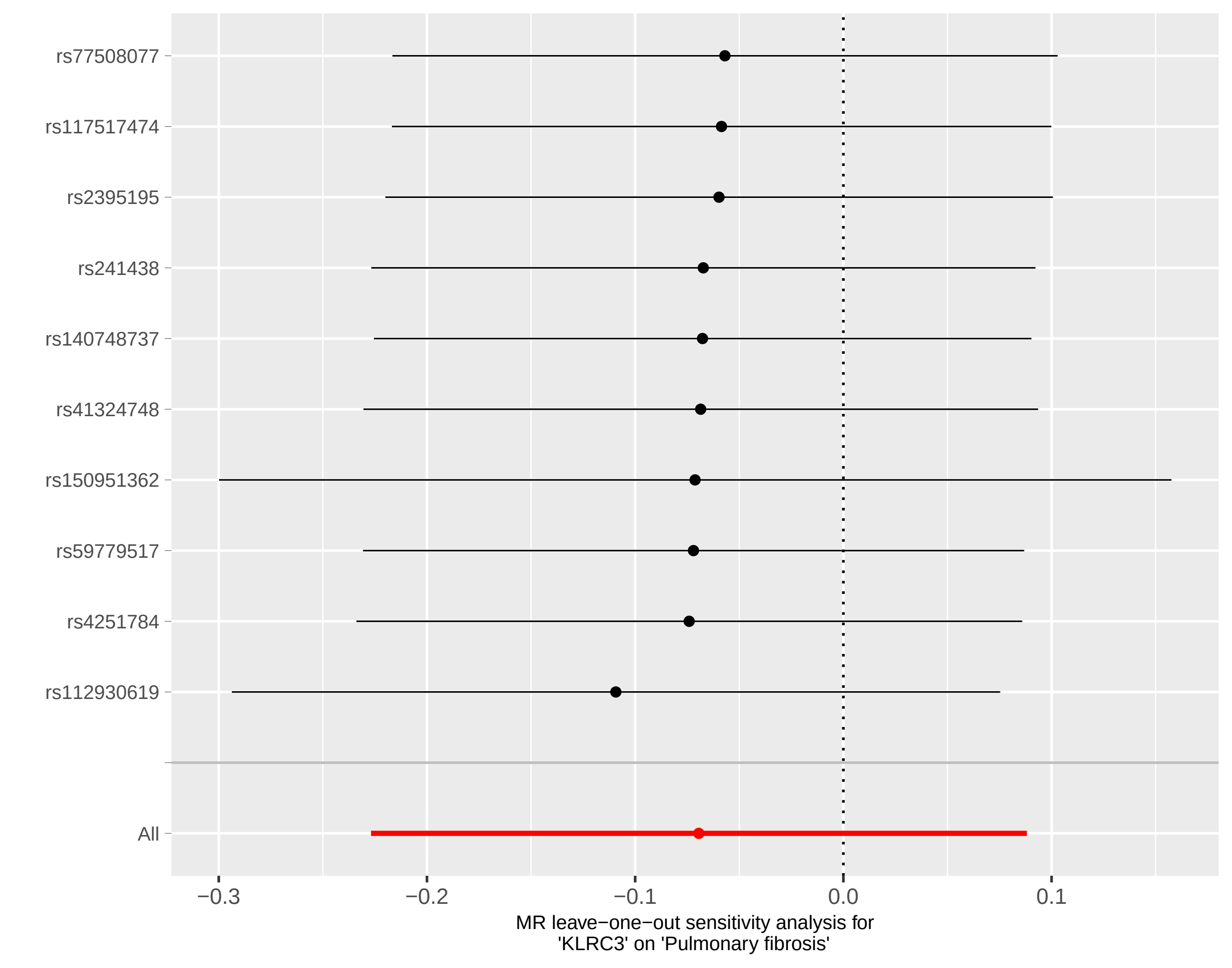
rs77508077
rs117517474
rs2395195
rs241438
rs140748737
rs41324748
rs150951362
rs59779517
rs4251784
rs112930619
All
−0.3
−0.2
−0.1
0.0
0.1
MR leave−one−out sensitivity analysis for
'KLRC3' on 'Pulmonary fibrosis'

Insufficient number of SNPs

Insufficient number of SNPs

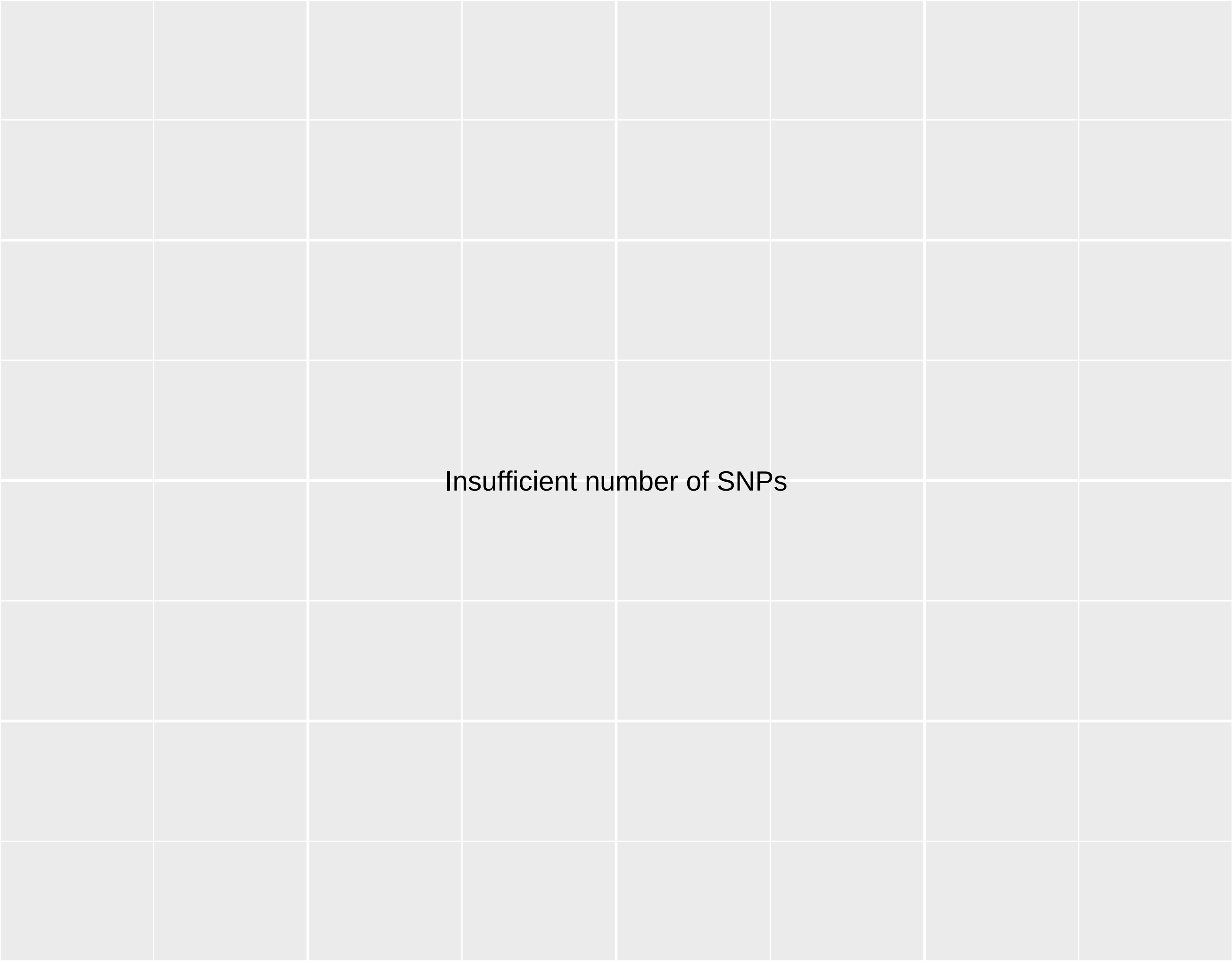
Insufficient number of SNPs

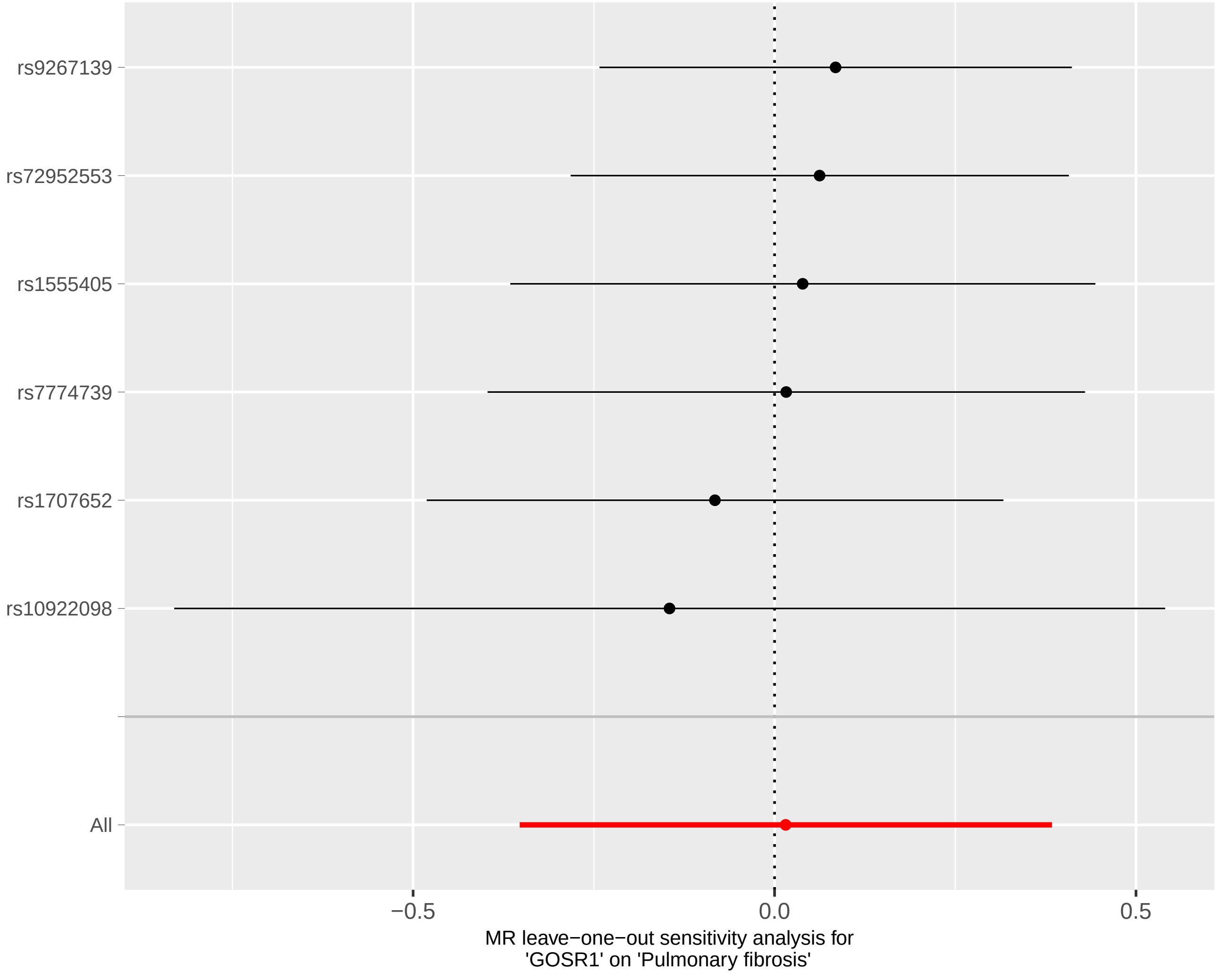
rs9267139
rs72952553
rs1555405
rs7774739
rs1707652
rs10922098
All
−0.5
0.0
0.5
MR leave−one−out sensitivity analysis for
'GOSR1' on 'Pulmonary fibrosis'

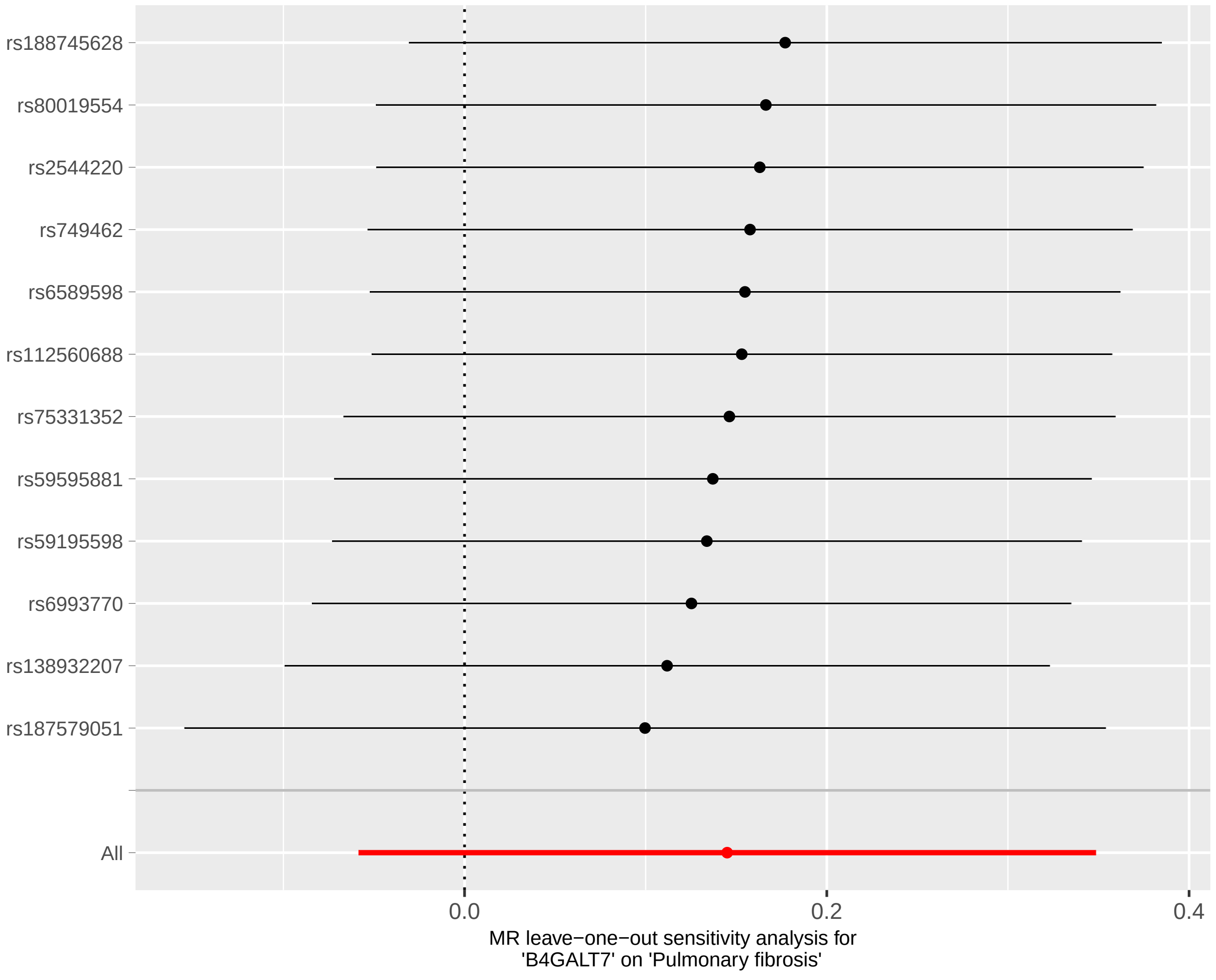
rs188745628
rs80019554
rs2544220
rs749462
rs6589598
rs112560688
rs75331352
rs59595881
rs59195598
rs6993770
rs138932207
rs187579051
All
0.0
0.2
0.4
MR leave−one−out sensitivity analysis for
'B4GALT7' on 'Pulmonary fibrosis'

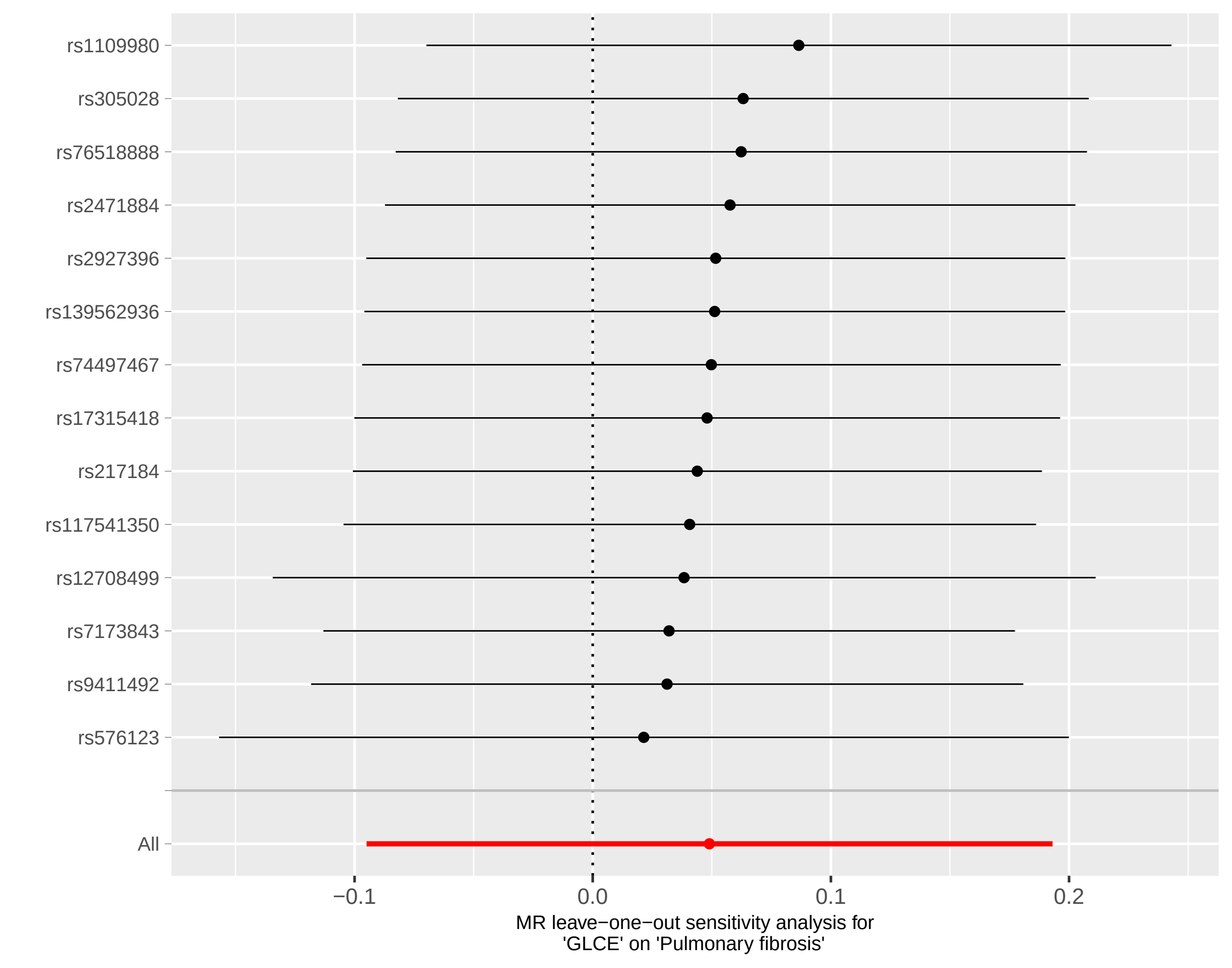

rs1109980
rs305028
rs76518888
rs2471884
rs2927396
rs139562936
rs74497467
rs17315418
rs217184
rs117541350
rs12708499
rs7173843
rs9411492
rs576123
All
−0.1
0.0
0.1
0.2
MR leave−one−out sensitivity analysis for
'GLCE' on 'Pulmonary fibrosis'

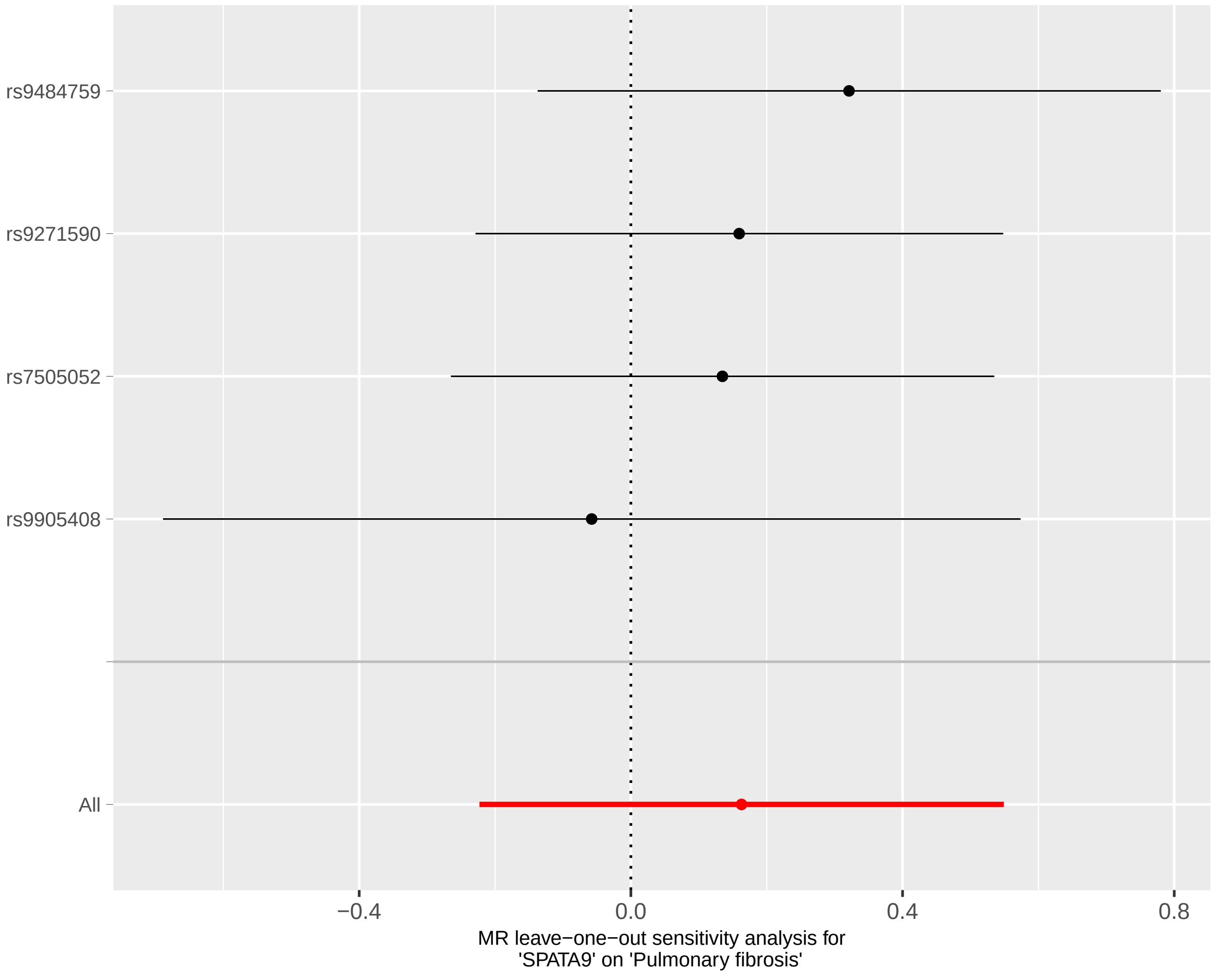
rs9484759
rs9271590
rs7505052
rs9905408
All
−0.4
0.0
0.4
0.8
MR leave−one−out sensitivity analysis for
'SPATA9' on 'Pulmonary fibrosis'

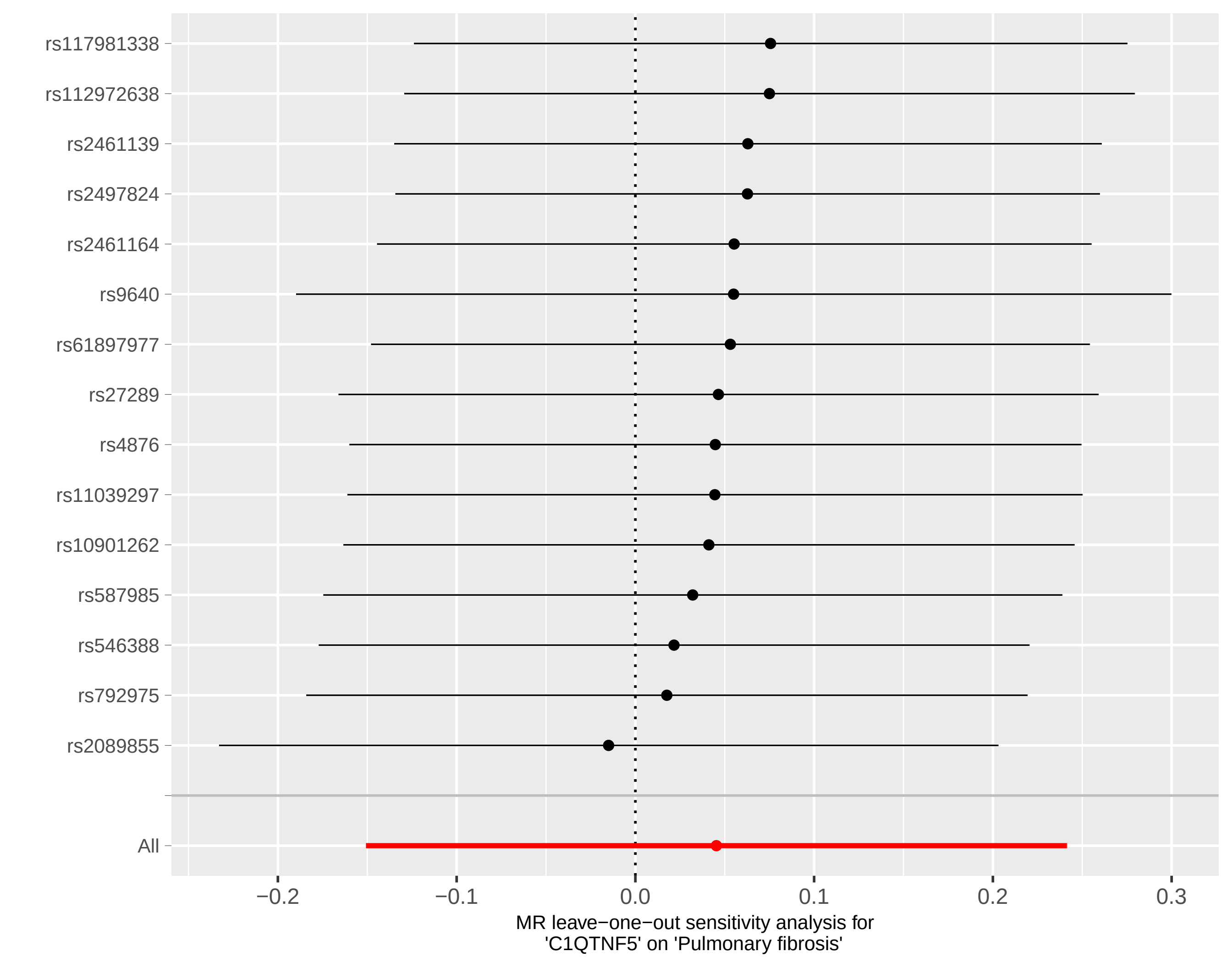
rs117981338
rs112972638
rs2461139
rs2497824
rs2461164
rs9640
rs61897977
rs27289
rs4876
rs11039297
rs10901262
rs587985
rs546388
rs792975
rs2089855
All
−0.2
−0.1
0.0
0.1
0.2
0.3
MR leave−one−out sensitivity analysis for
'C1QTNF5' on 'Pulmonary fibrosis'

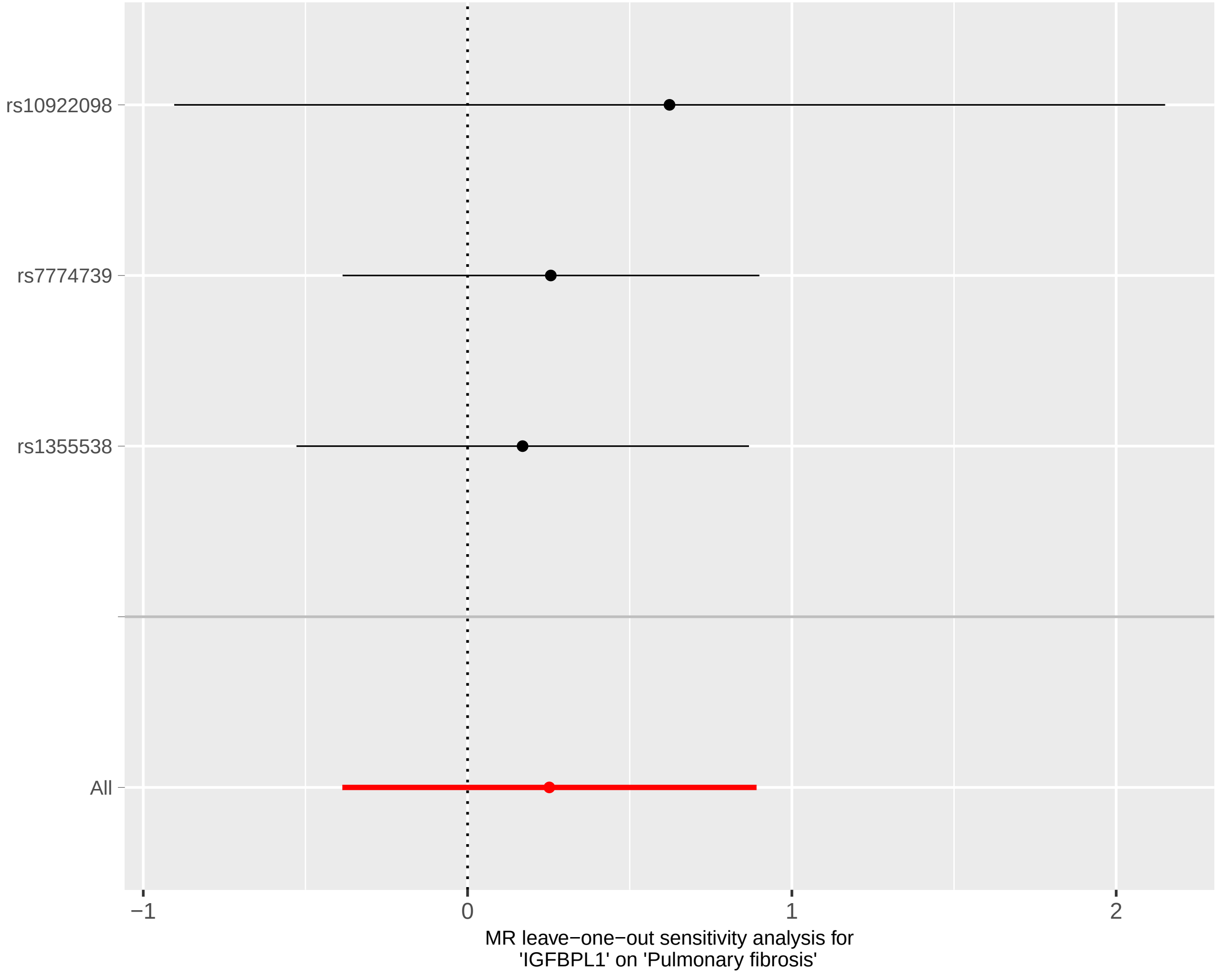

rs10922098
rs7774739
rs1355538
All
−1
0
1
2
MR leave−one−out sensitivity analysis for
'IGFBPL1' on 'Pulmonary fibrosis'

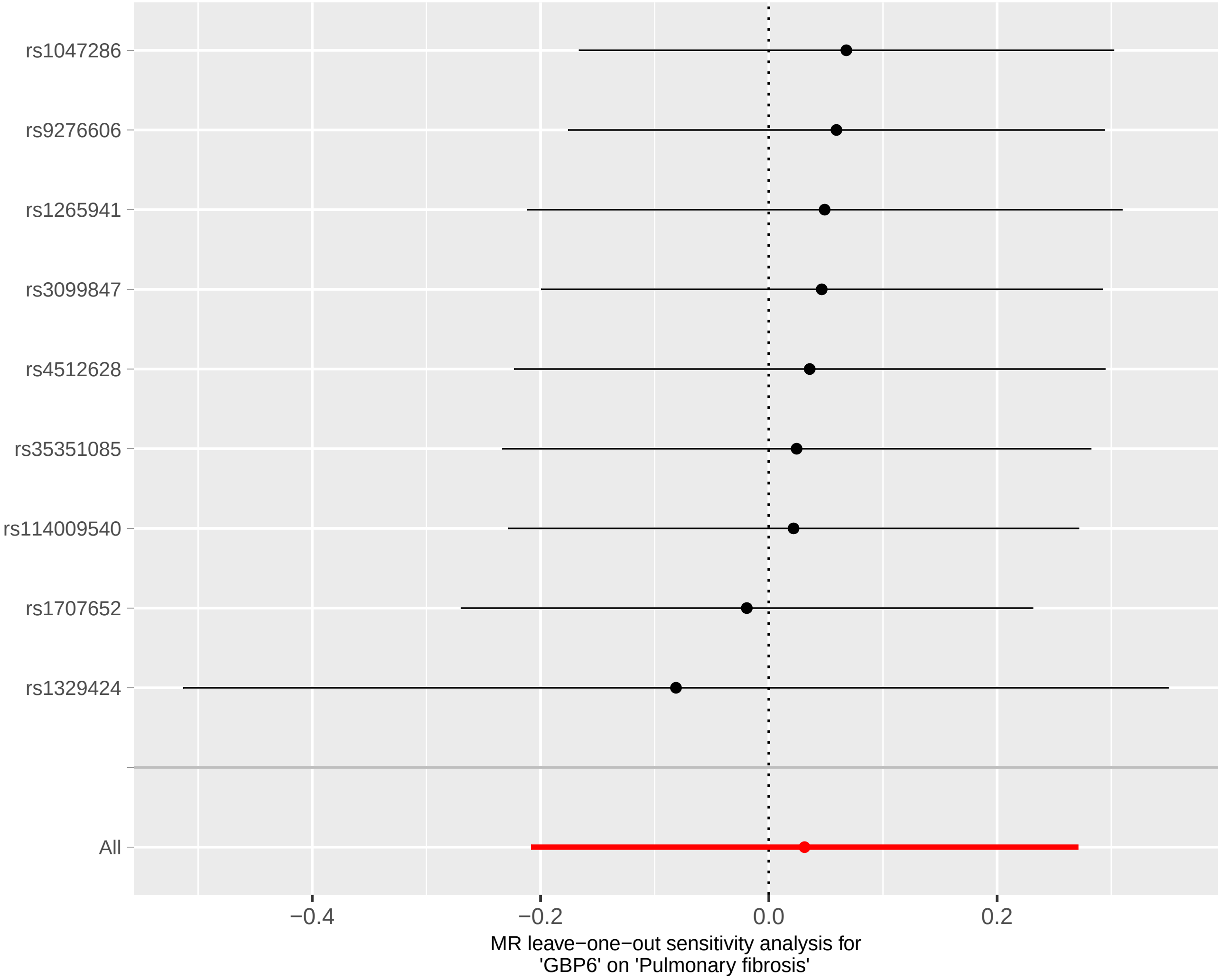
rs1047286
rs9276606
rs1265941
rs3099847
rs4512628
rs35351085
rs114009540
rs1707652
rs1329424
All
−0.4
−0.2
0.0
0.2
MR leave−one−out sensitivity analysis for
'GBP6' on 'Pulmonary fibrosis'

Insufficient number of SNPs

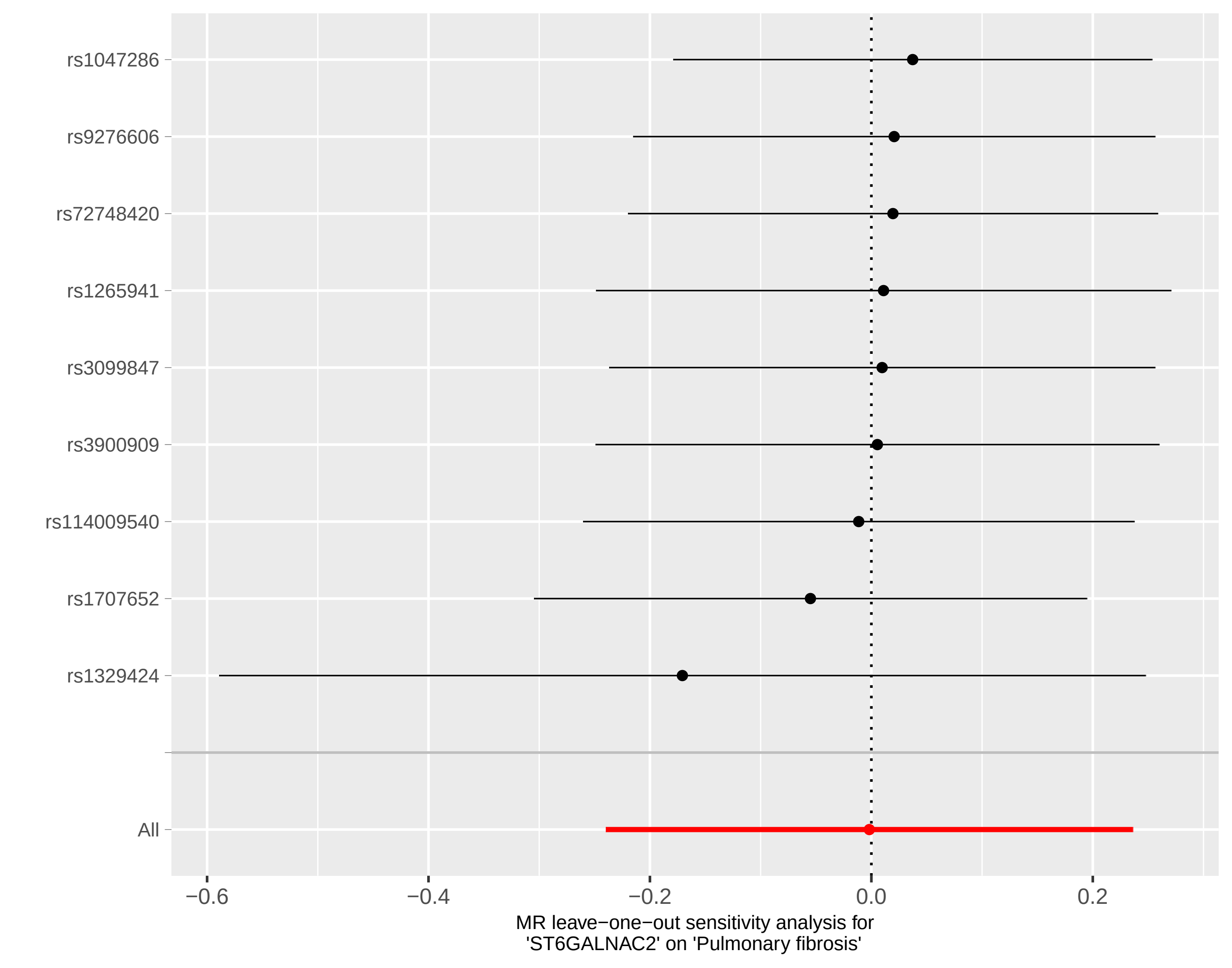
rs1047286
rs9276606
rs72748420
rs1265941
rs3099847
rs3900909
rs114009540
rs1707652
rs1329424
All
−0.6
−0.4
−0.2
0.0
0.2
MR leave−one−out sensitivity analysis for
'ST6GALNAC2' on 'Pulmonary fibrosis'

Insufficient number of SNPs

Insufficient number of SNPs

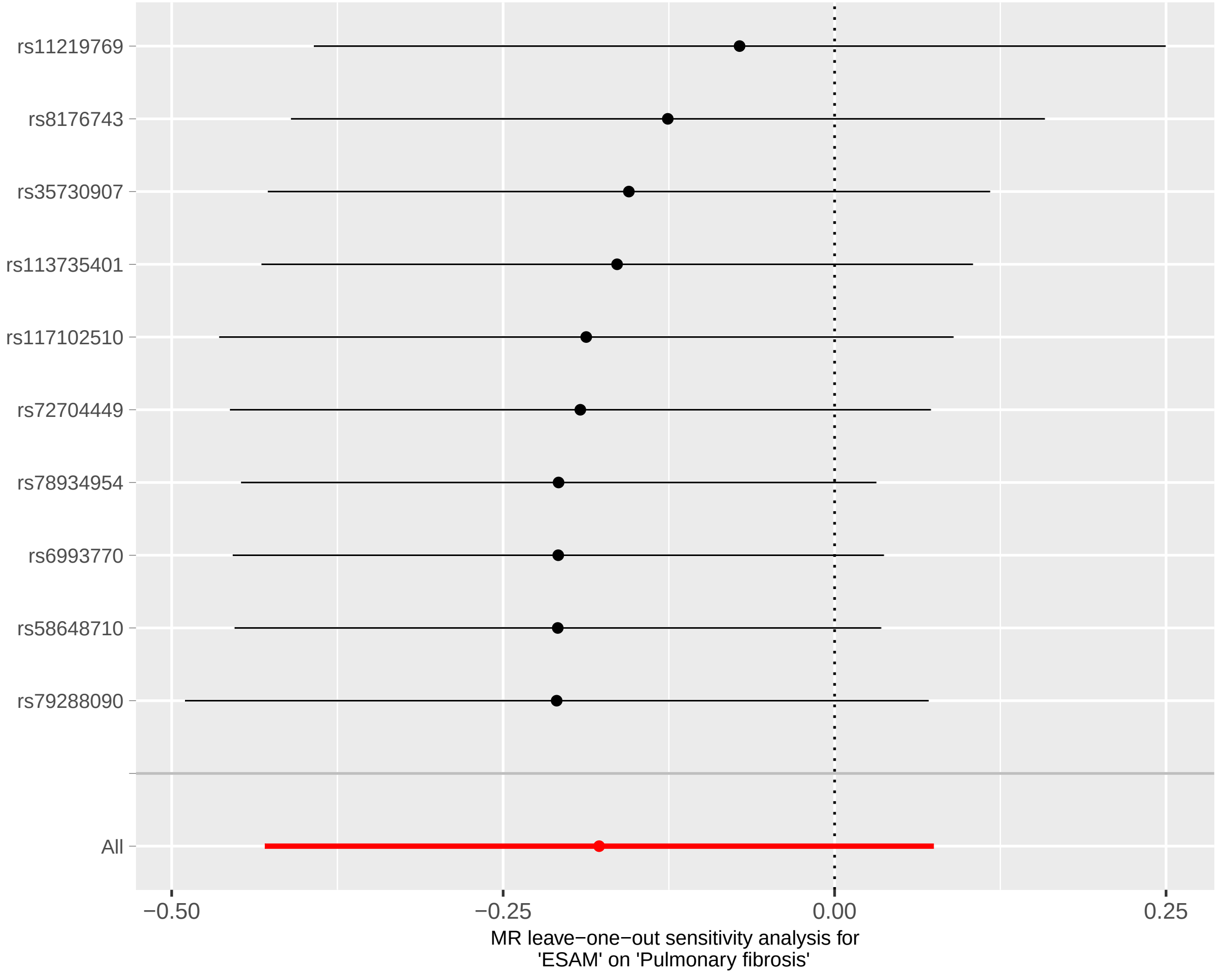
rs11219769
rs8176743
rs35730907
rs113735401
rs117102510
rs72704449
rs78934954
rs6993770
rs58648710
rs79288090
All
−0.50
−0.25
0.00
0.25
MR leave−one−out sensitivity analysis for
'ESAM' on 'Pulmonary fibrosis'

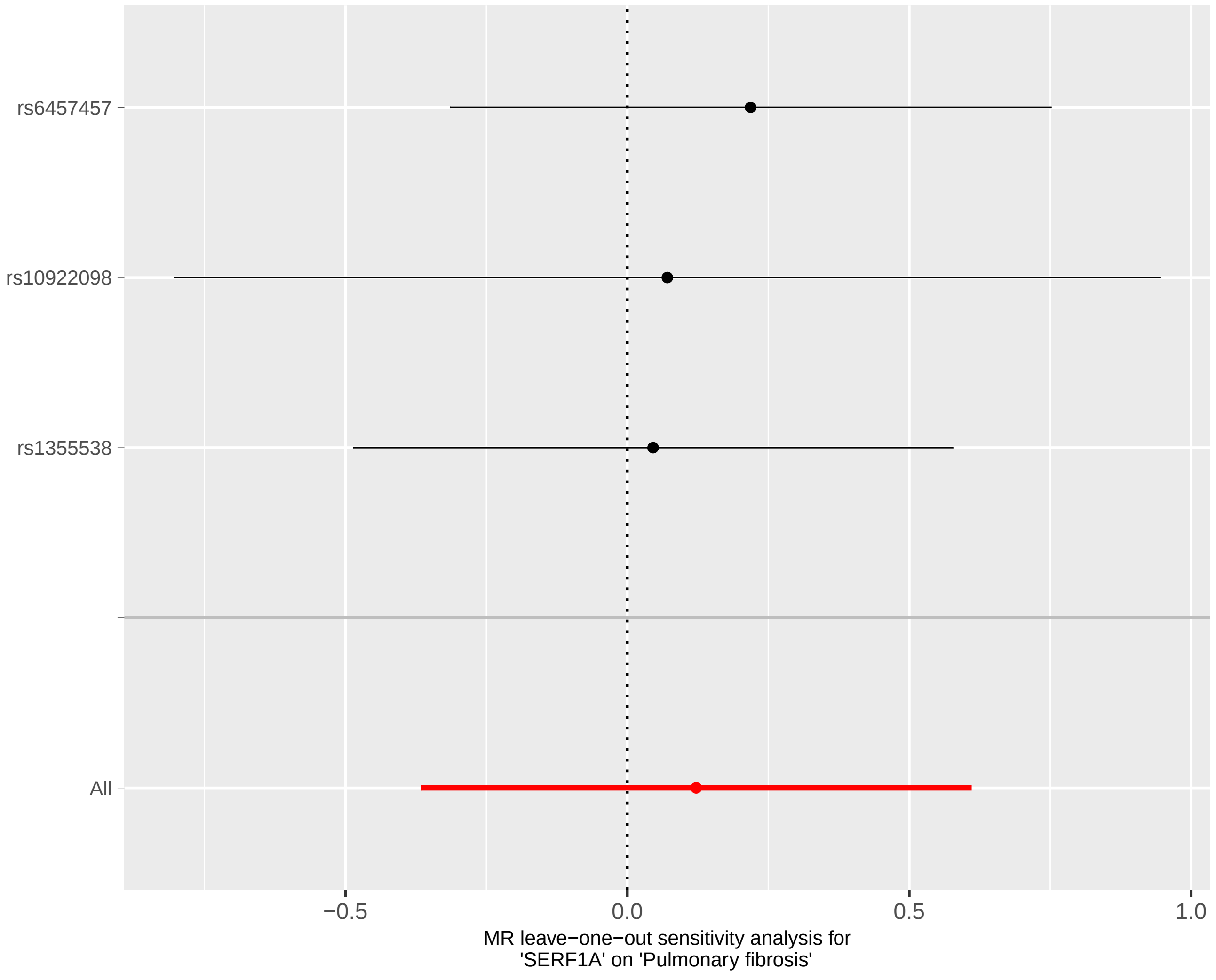
rs6457457
rs10922098
rs1355538
All
−0.5
0.0
0.5
1.0
MR leave−one−out sensitivity analysis for
'SERF1A' on 'Pulmonary fibrosis'

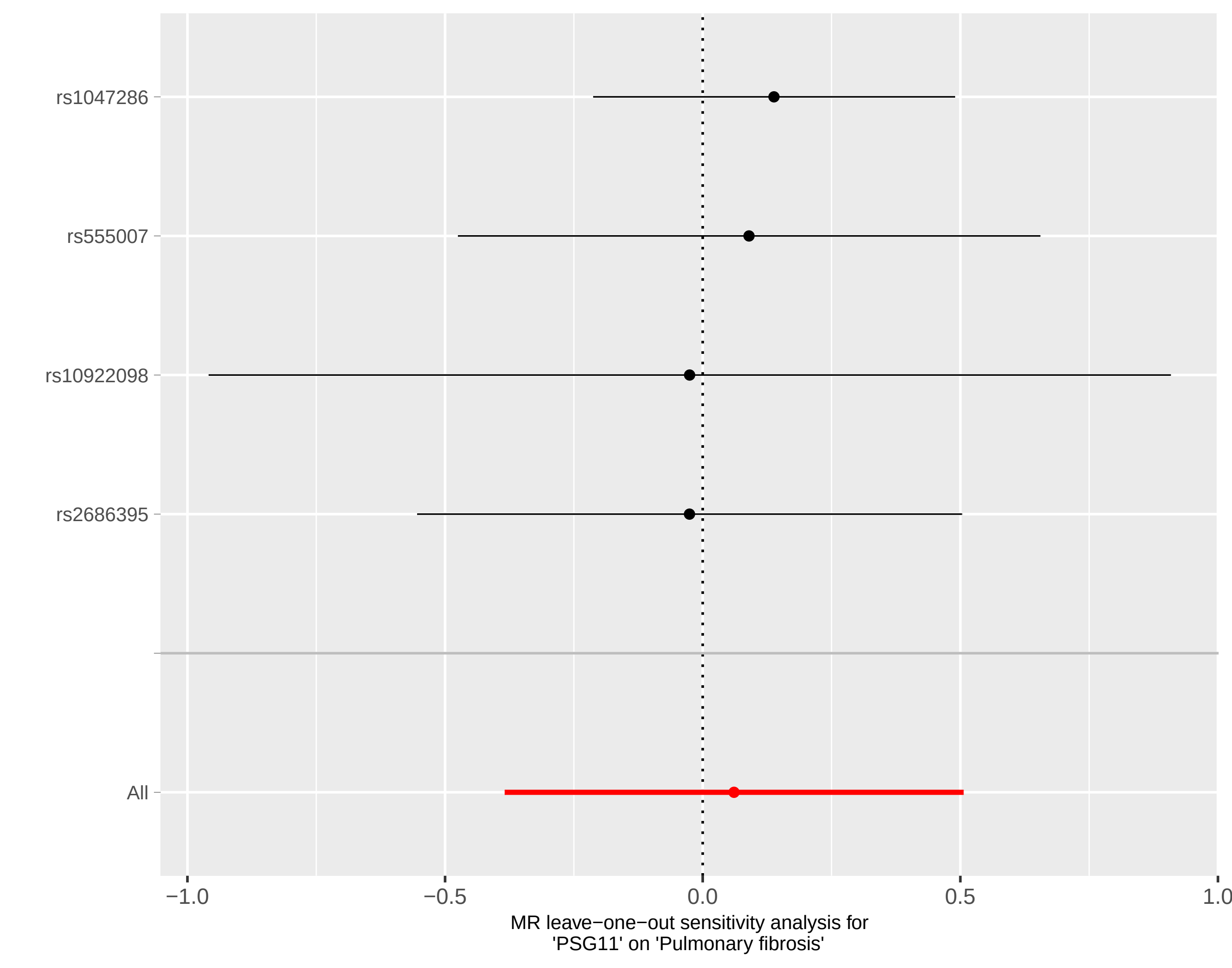
rs1047286
rs555007
rs10922098
rs2686395
All
−1.0
−0.5
0.0
0.5
1.0
MR leave−one−out sensitivity analysis for
'PSG11' on 'Pulmonary fibrosis'

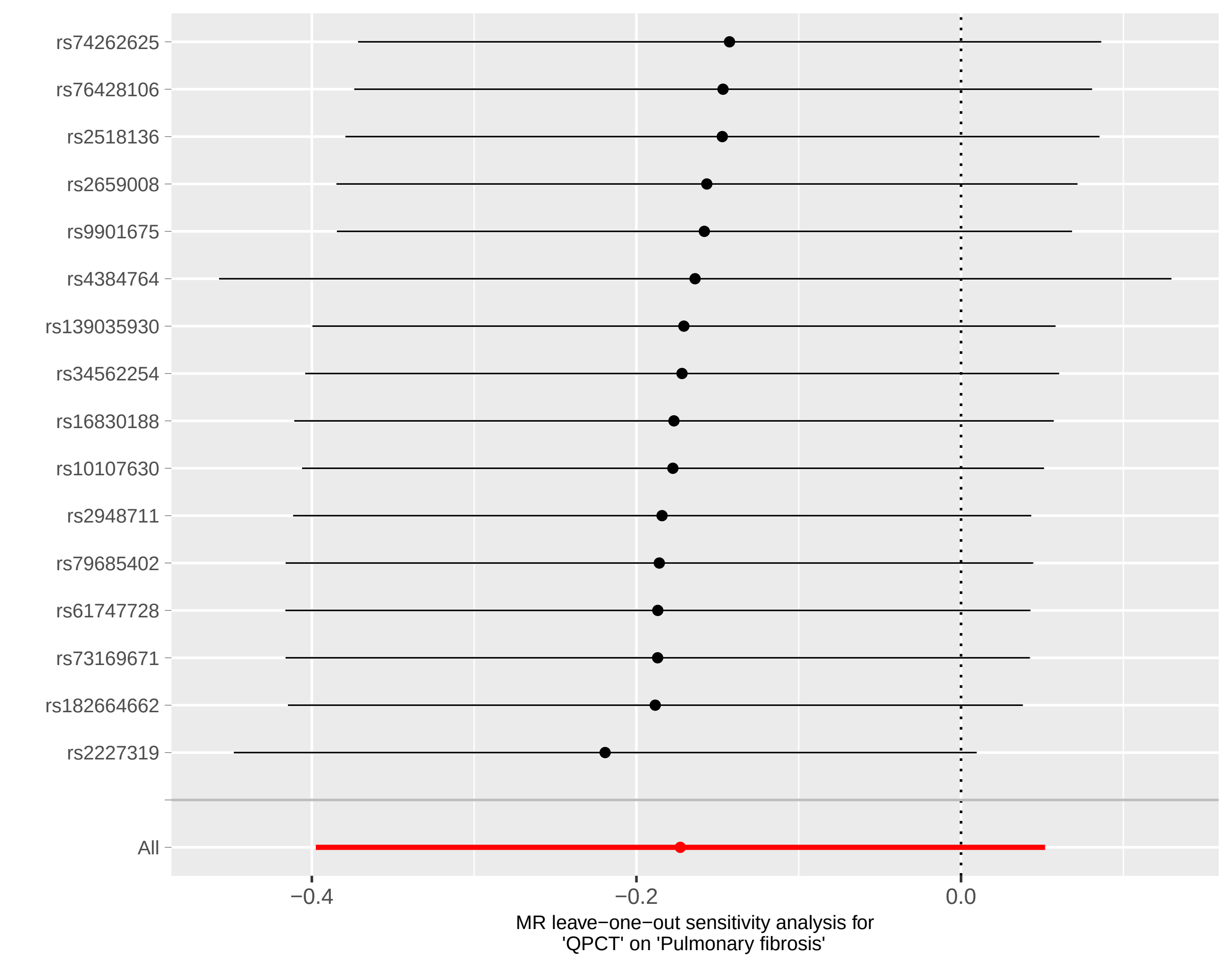

rs74262625
rs76428106
rs2518136
rs2659008
rs9901675
rs4384764
rs139035930
rs34562254
rs16830188
rs10107630
rs2948711
rs79685402
rs61747728
rs73169671
rs182664662
rs2227319
All
−0.4
−0.2
0.0
MR leave−one−out sensitivity analysis for
'QPCT' on 'Pulmonary fibrosis'

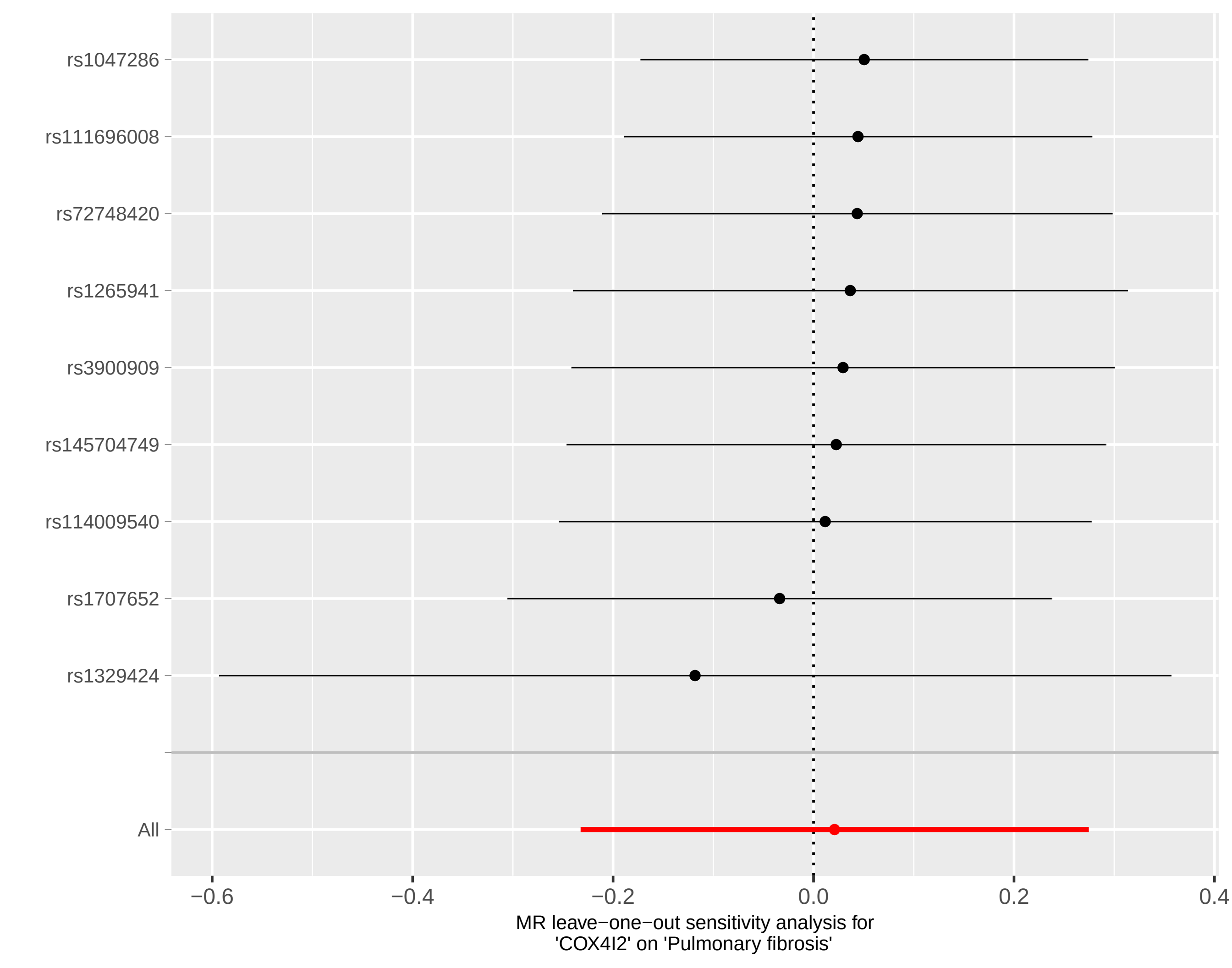

rs1047286
rs111696008
rs72748420
rs1265941
rs3900909
rs145704749
rs114009540
rs1707652
rs1329424
All
−0.6
−0.4
−0.2
0.0
0.2
0.4
MR leave−one−out sensitivity analysis for
'COX4I2' on 'Pulmonary fibrosis'

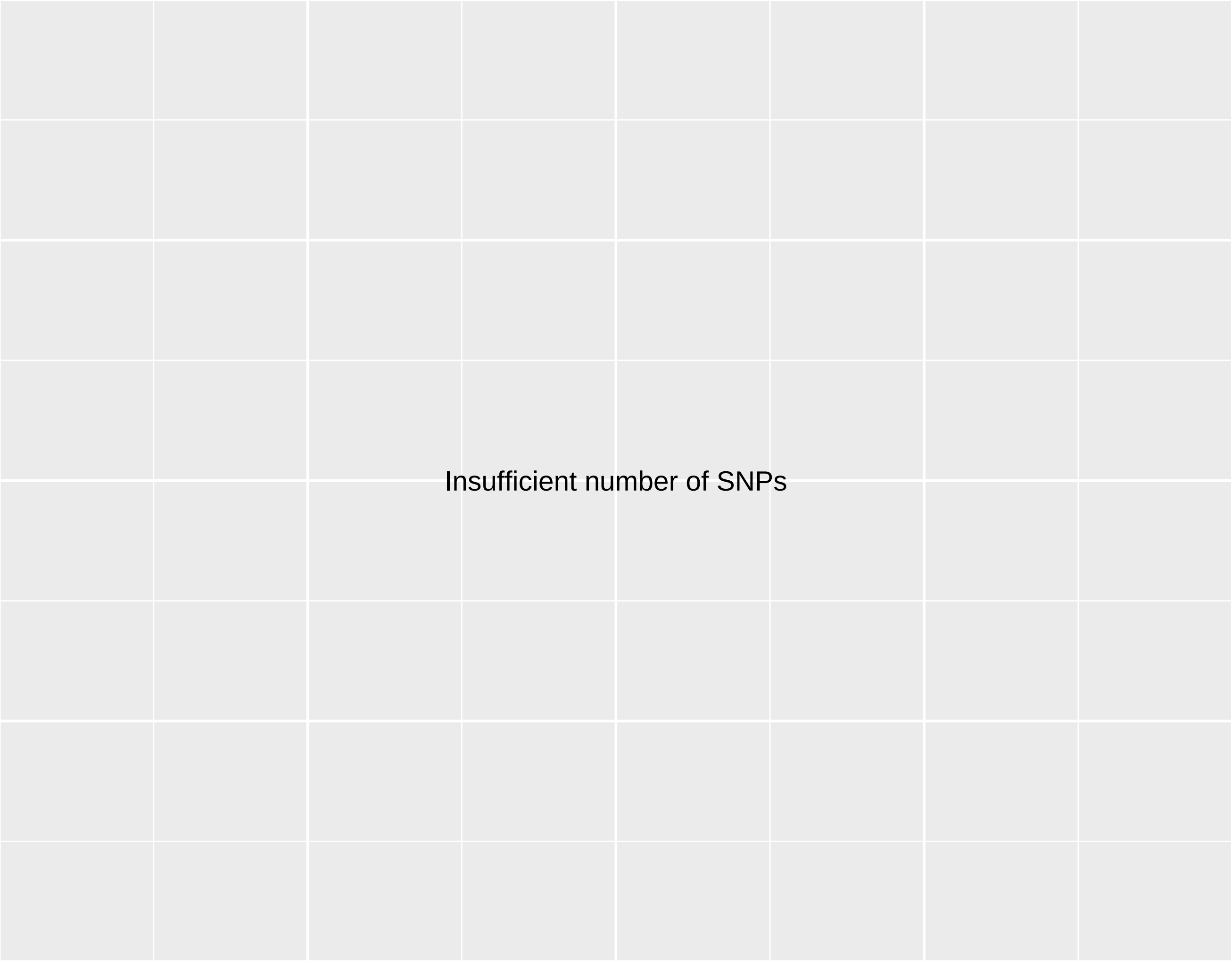
Insufficient number of SNPs

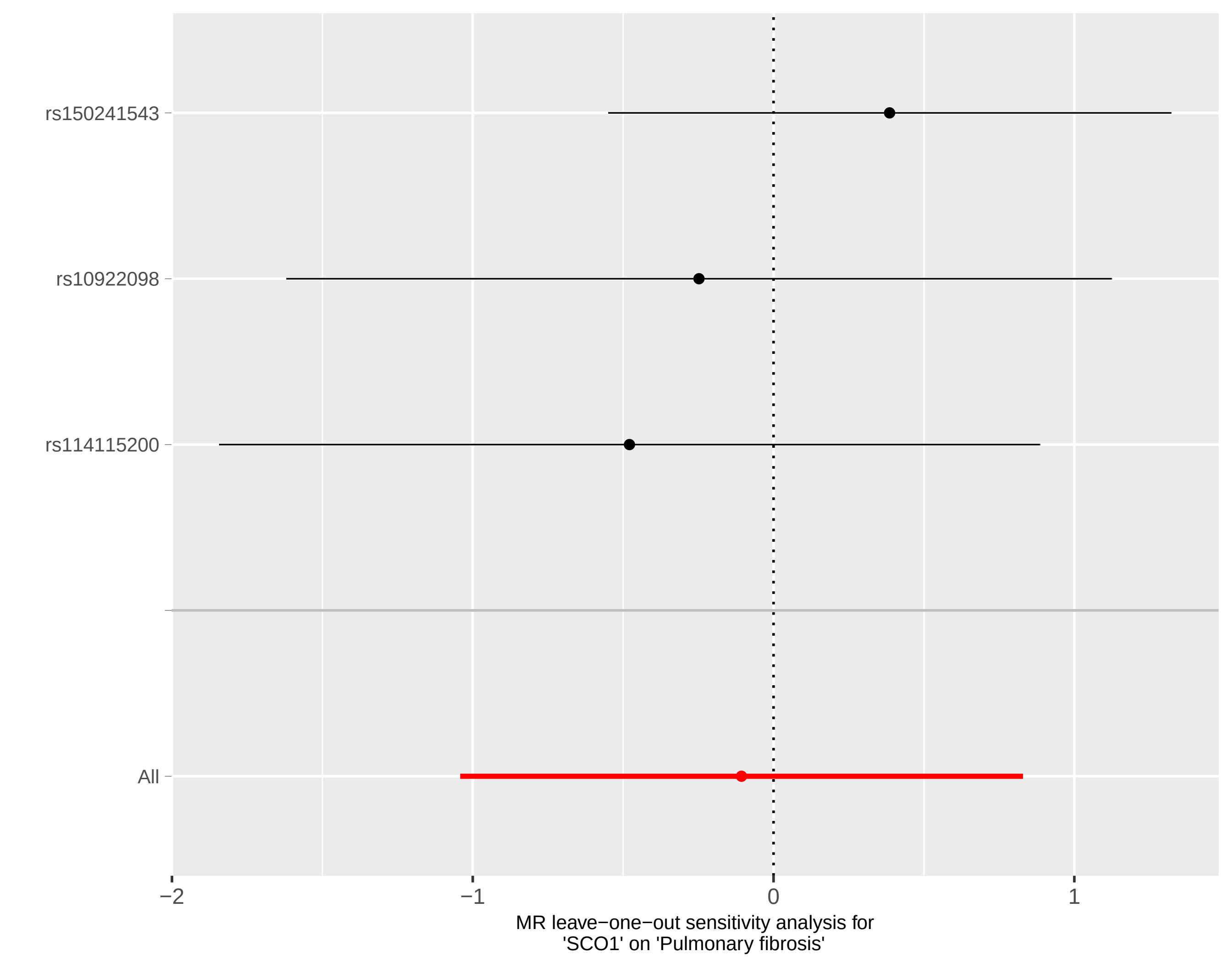
rs150241543
rs10922098
rs114115200
All
−2
−1
0
1
MR leave−one−out sensitivity analysis for
'SCO1' on 'Pulmonary fibrosis'

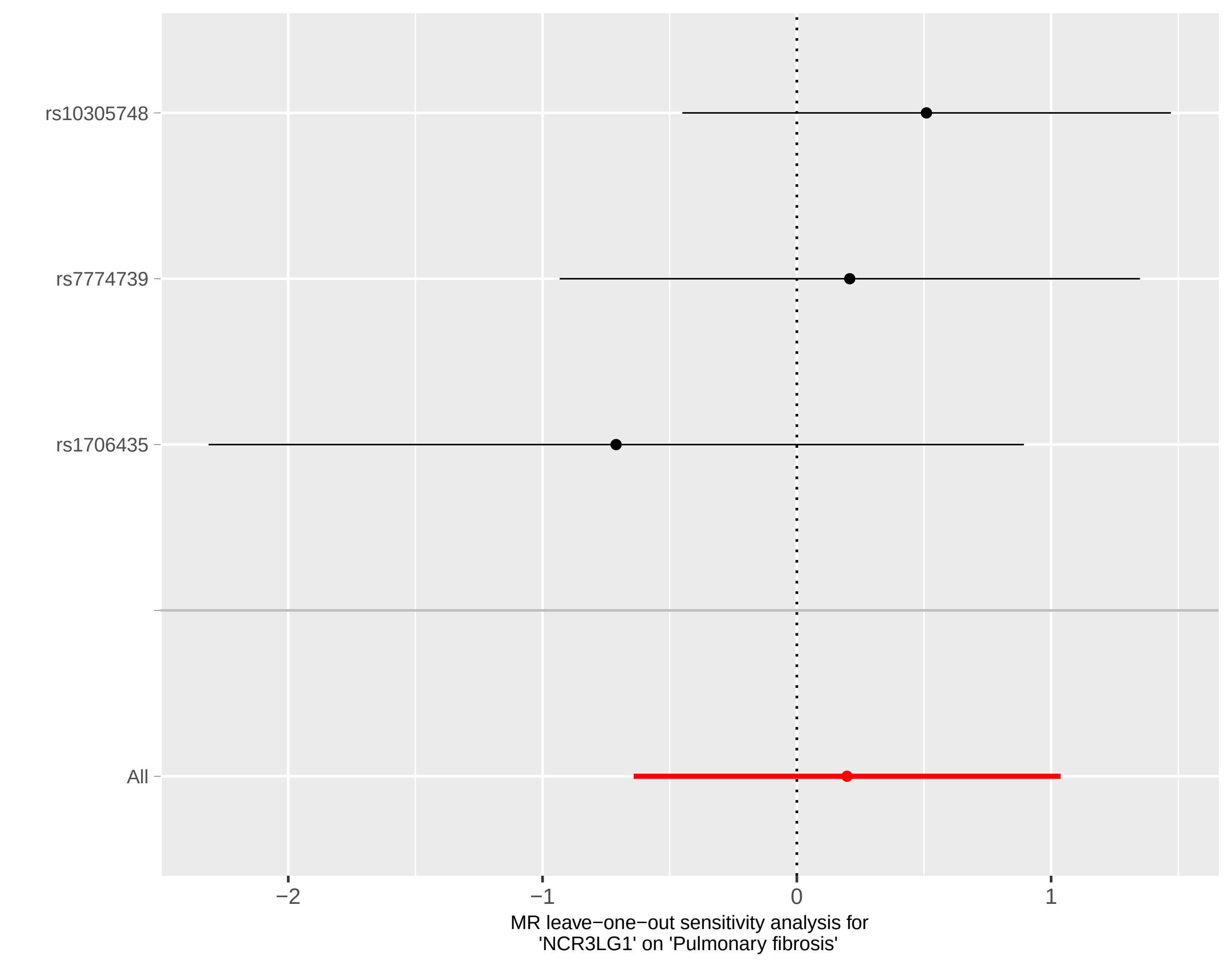
rs10305748
rs7774739
rs1706435
All
−2
−1
0
1
MR leave−one−out sensitivity analysis for
'NCR3LG1' on 'Pulmonary fibrosis'

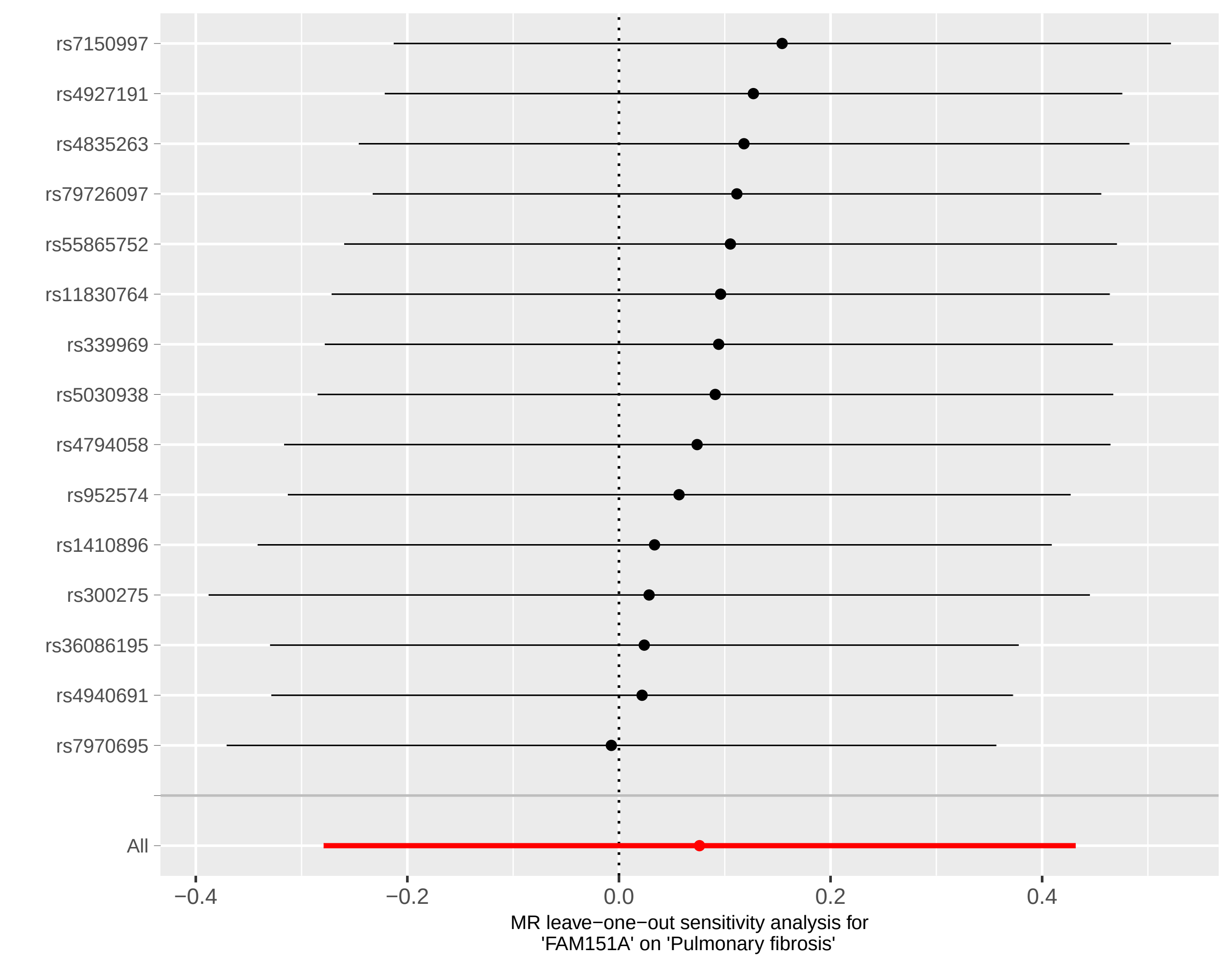

rs7150997
rs4927191
rs4835263
rs79726097
rs55865752
rs11830764
rs339969
rs5030938
rs4794058
rs952574
rs1410896
rs300275
rs36086195
rs4940691
rs7970695
All
−0.4
−0.2
0.0
0.2
0.4
MR leave−one−out sensitivity analysis for
'FAM151A' on 'Pulmonary fibrosis'

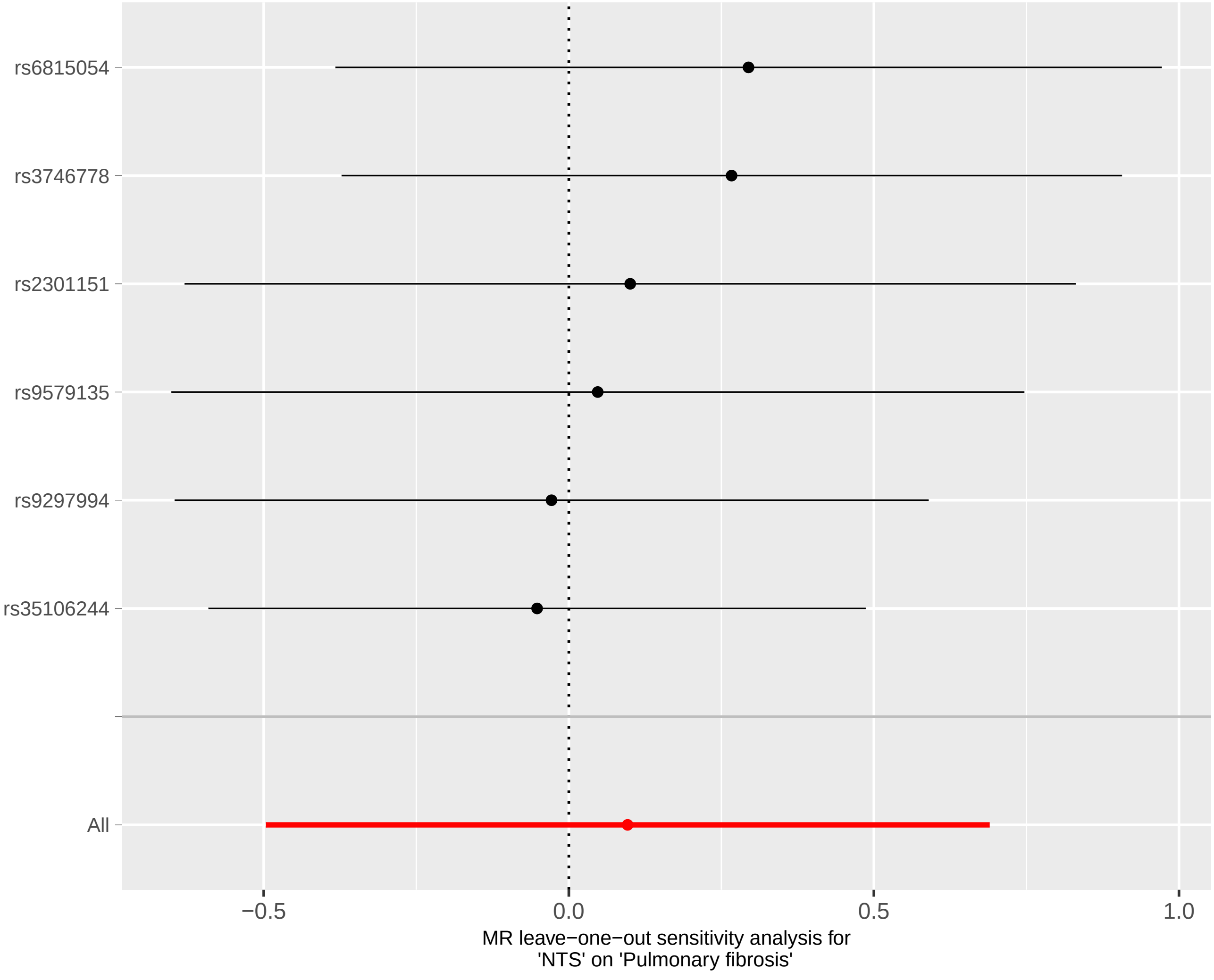
rs6815054
rs3746778
rs2301151
rs9579135
rs9297994
rs35106244
All
−0.5
0.0
0.5
1.0
MR leave−one−out sensitivity analysis for
'NTS' on 'Pulmonary fibrosis'

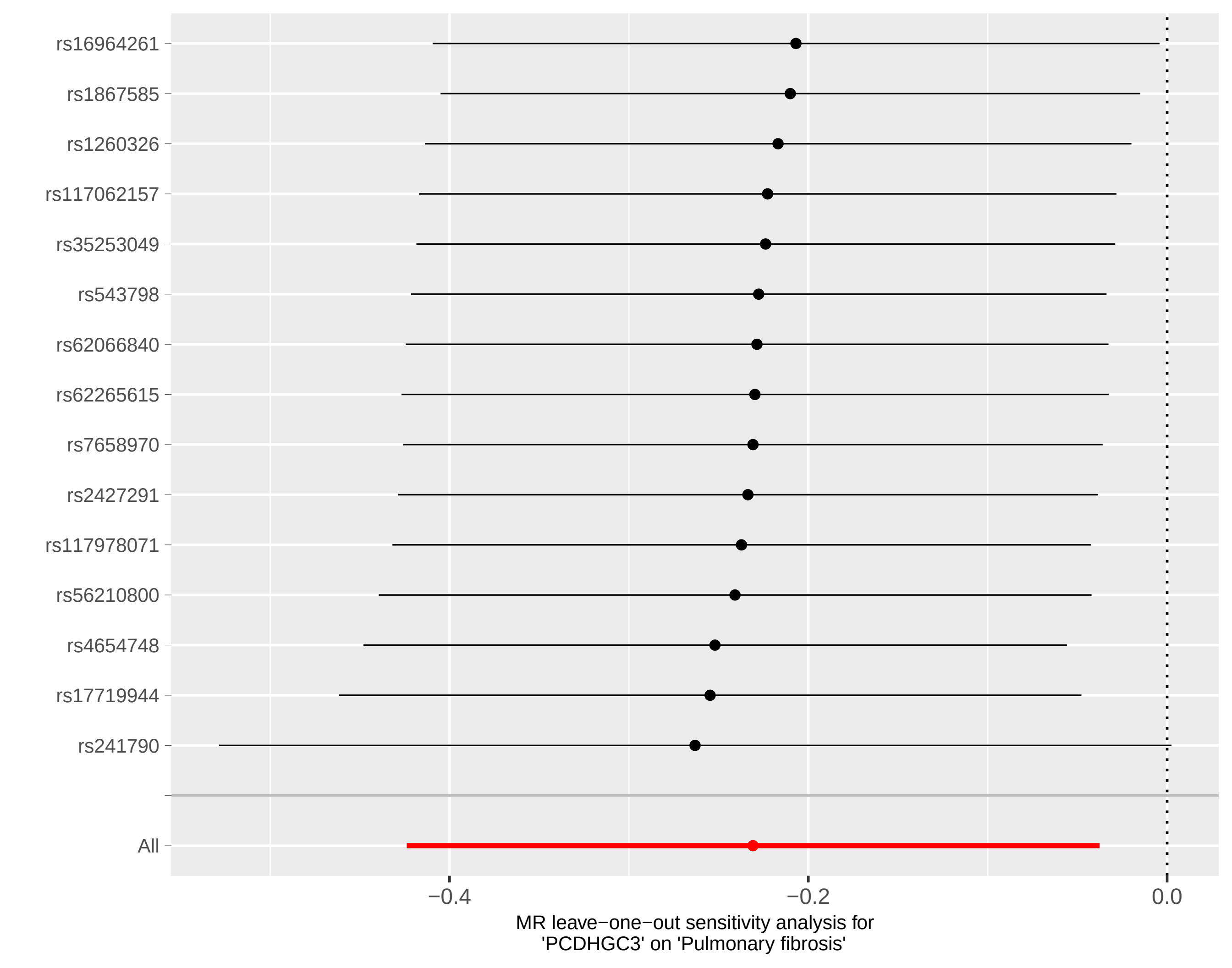

rs16964261
rs1867585
rs1260326
rs117062157
rs35253049
rs543798
rs62066840
rs62265615
rs7658970
rs2427291
rs117978071
rs56210800
rs4654748
rs17719944
rs241790
All
−0.4
−0.2
0.0
MR leave−one−out sensitivity analysis for
'PCDHGC3' on 'Pulmonary fibrosis'

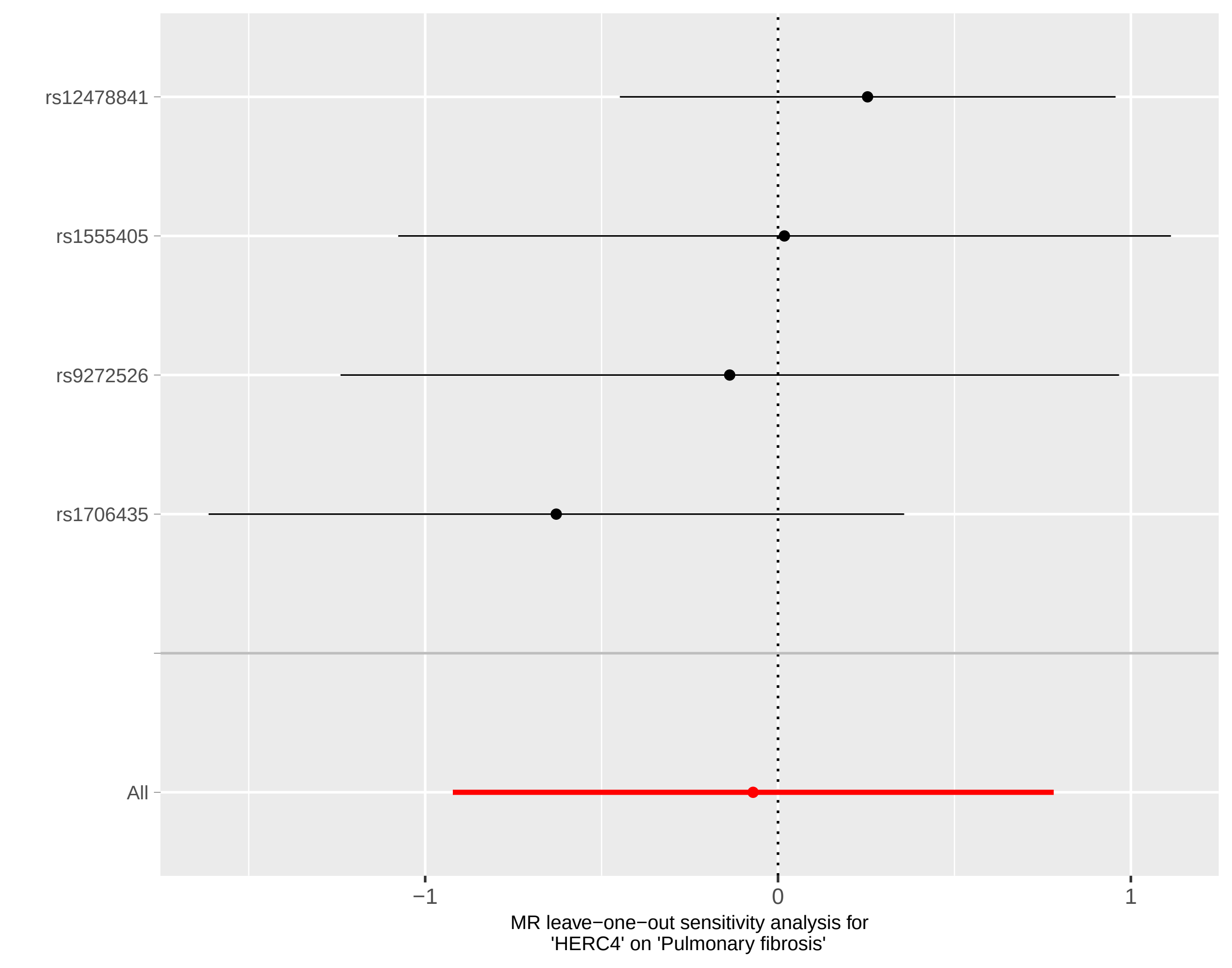
rs12478841
rs1555405
rs9272526
rs1706435
All
−1
0
1
MR leave−one−out sensitivity analysis for
'HERC4' on 'Pulmonary fibrosis'

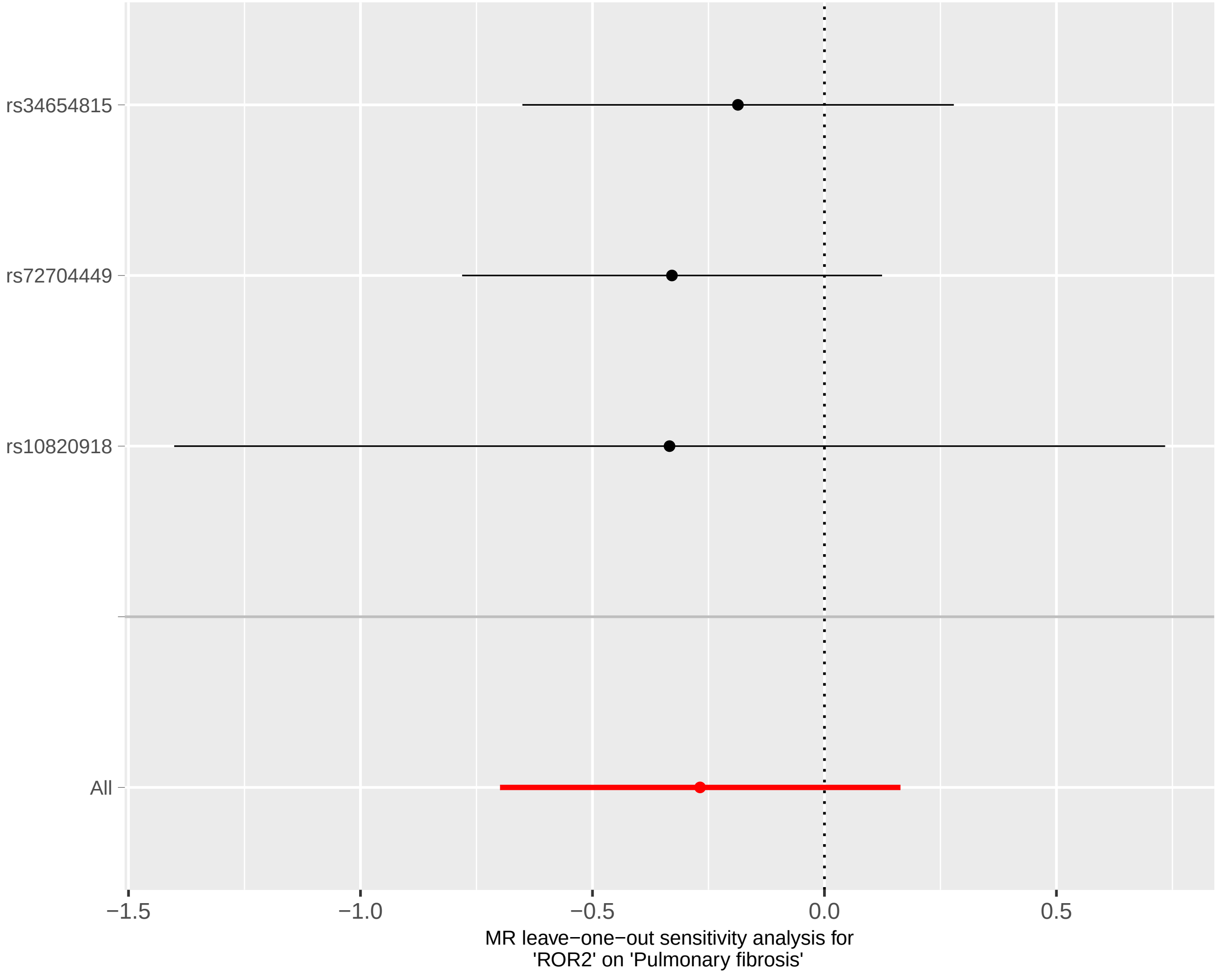
rs34654815
rs72704449
rs10820918
All
−1.5
−1.0
−0.5
0.0
0.5
MR leave−one−out sensitivity analysis for
'ROR2' on 'Pulmonary fibrosis'

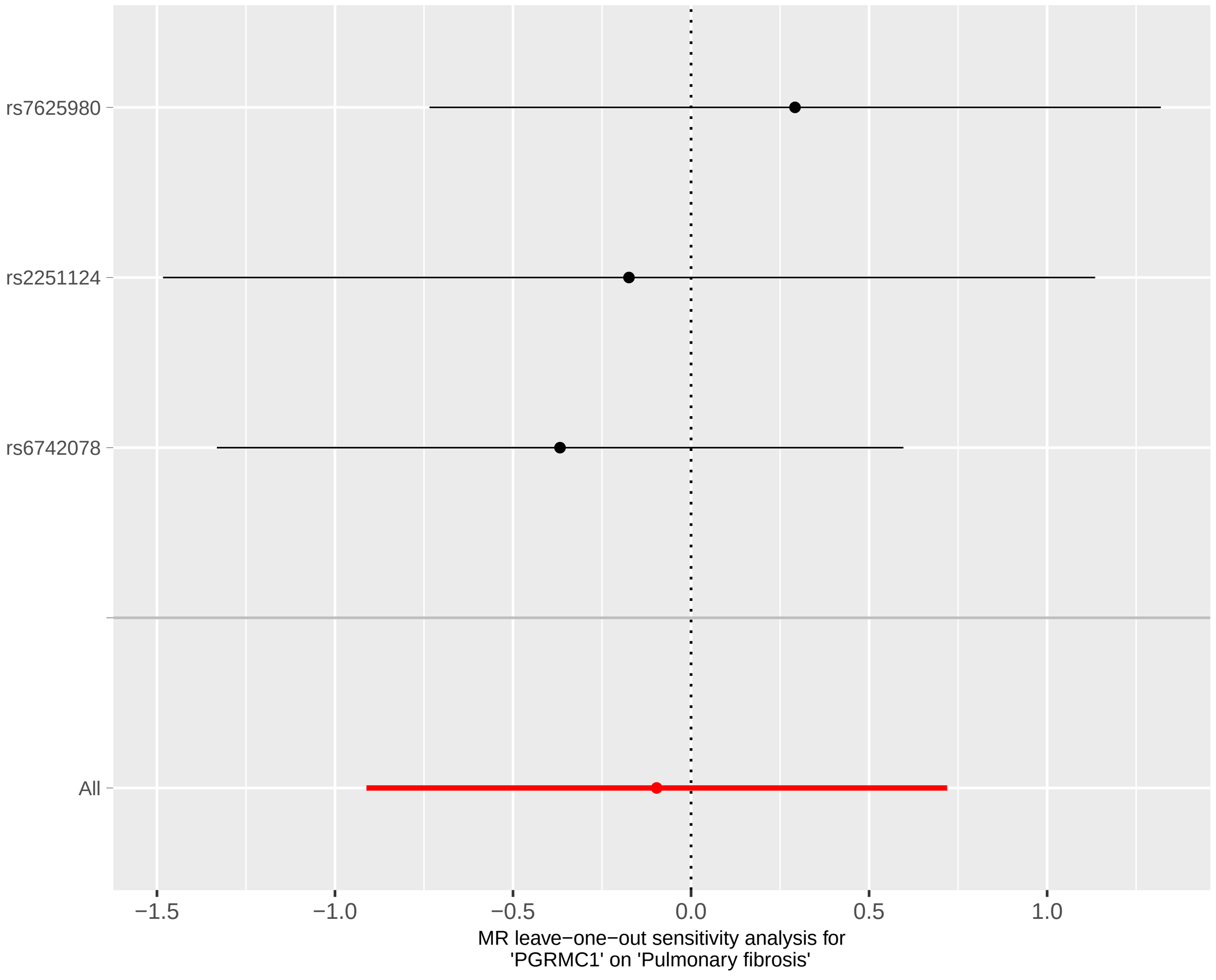
rs7625980
rs2251124
rs6742078
All
−1.5
−1.0
−0.5
0.0
0.5
1.0
MR leave−one−out sensitivity analysis for
'PGRMC1' on 'Pulmonary fibrosis'

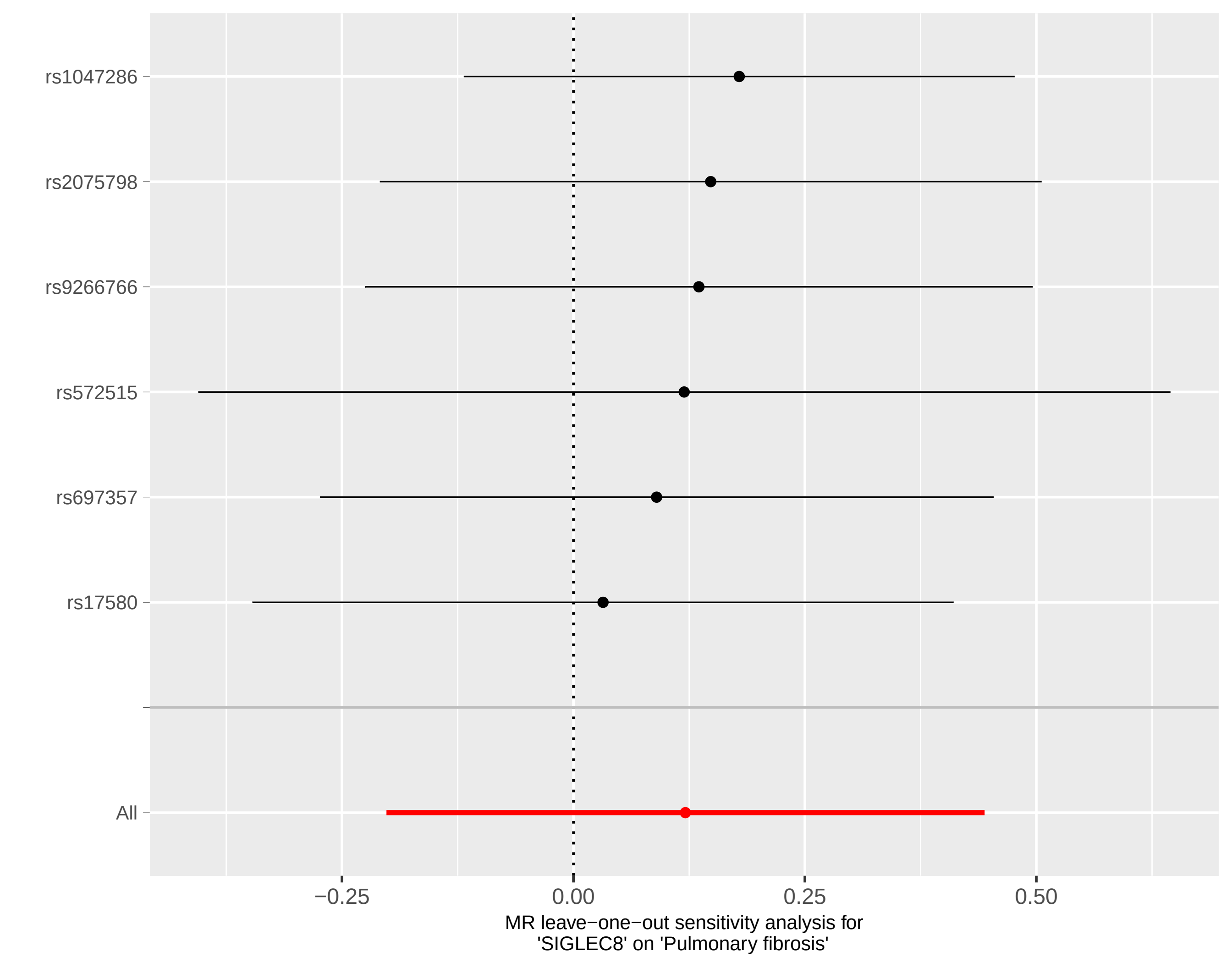

rs1047286
rs2075798
rs9266766
rs572515
rs697357
rs17580
All
−0.25
0.00
0.25
0.50
MR leave−one−out sensitivity analysis for
'SIGLEC8' on 'Pulmonary fibrosis'

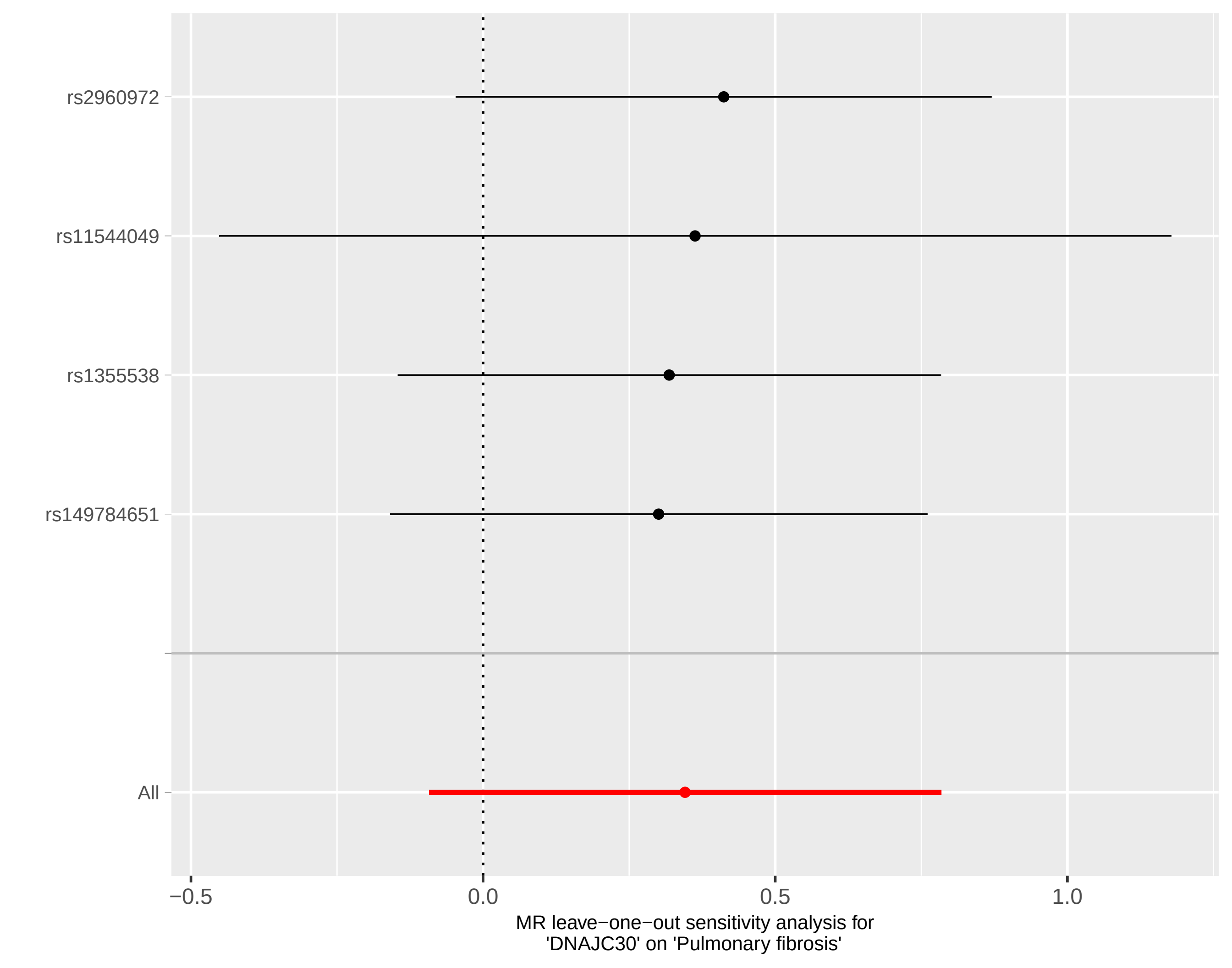
rs2960972
rs11544049
rs1355538
rs149784651
All
−0.5
0.0
0.5
1.0
MR leave−one−out sensitivity analysis for
'DNAJC30' on 'Pulmonary fibrosis'

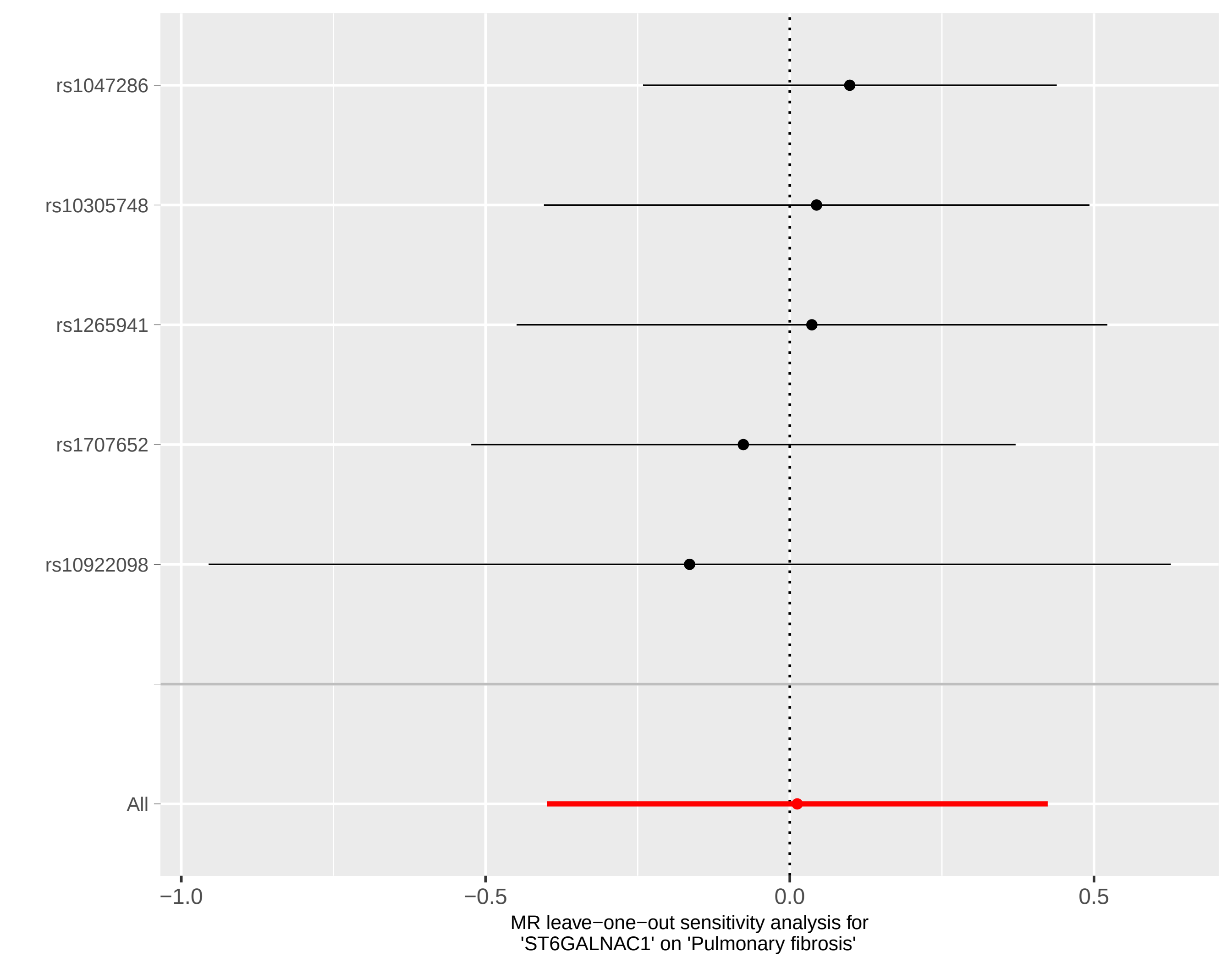

rs1047286
rs10305748
rs1265941
rs1707652
rs10922098
All
−1.0
−0.5
0.0
0.5
MR leave−one−out sensitivity analysis for
'ST6GALNAC1' on 'Pulmonary fibrosis'

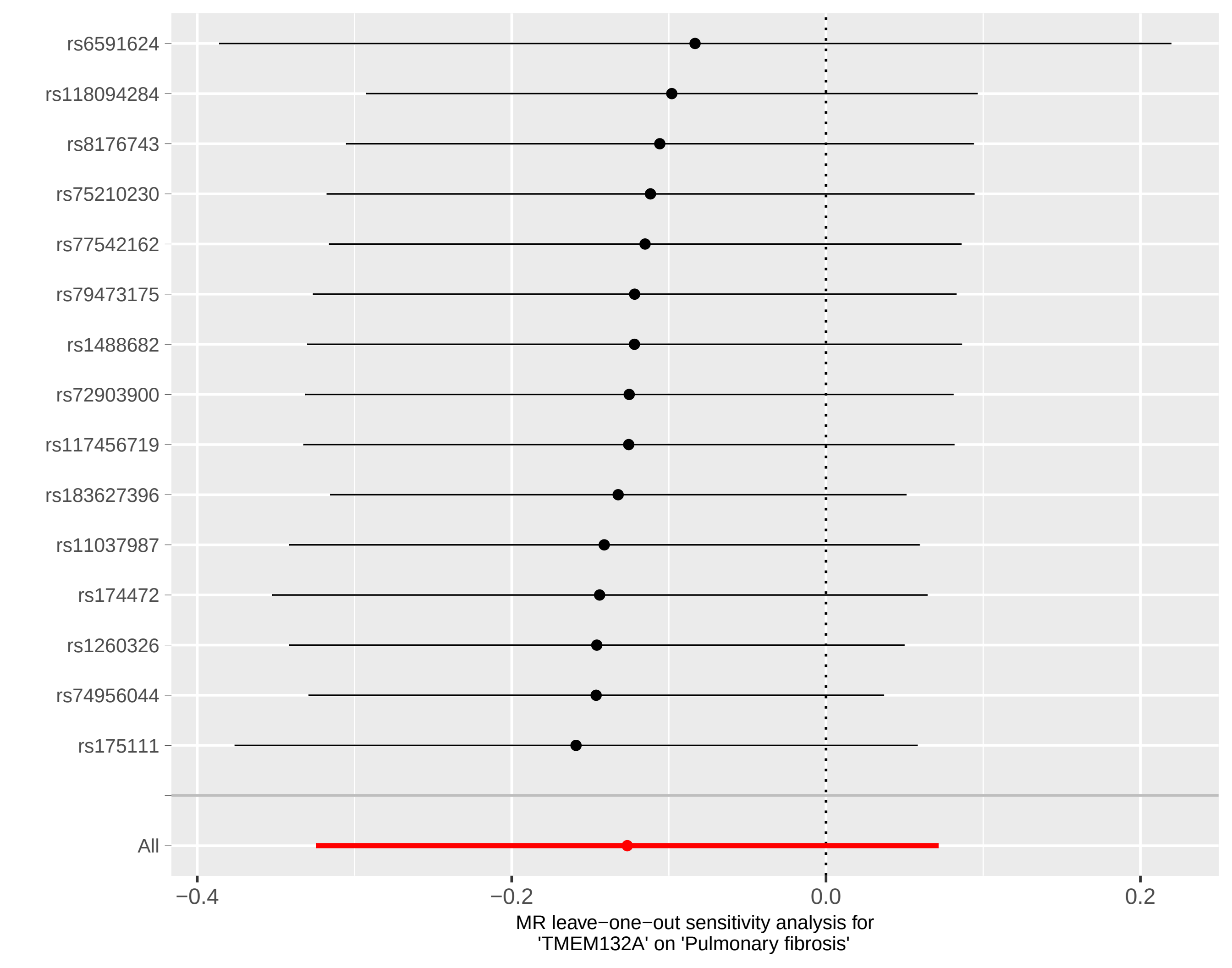

rs6591624
rs118094284
rs8176743
rs75210230
rs77542162
rs79473175
rs1488682
rs72903900
rs117456719
rs183627396
rs11037987
rs174472
rs1260326
rs74956044
rs175111
All
−0.4
−0.2
0.0
0.2
MR leave−one−out sensitivity analysis for
'TMEM132A' on 'Pulmonary fibrosis'

Insufficient number of SNPs

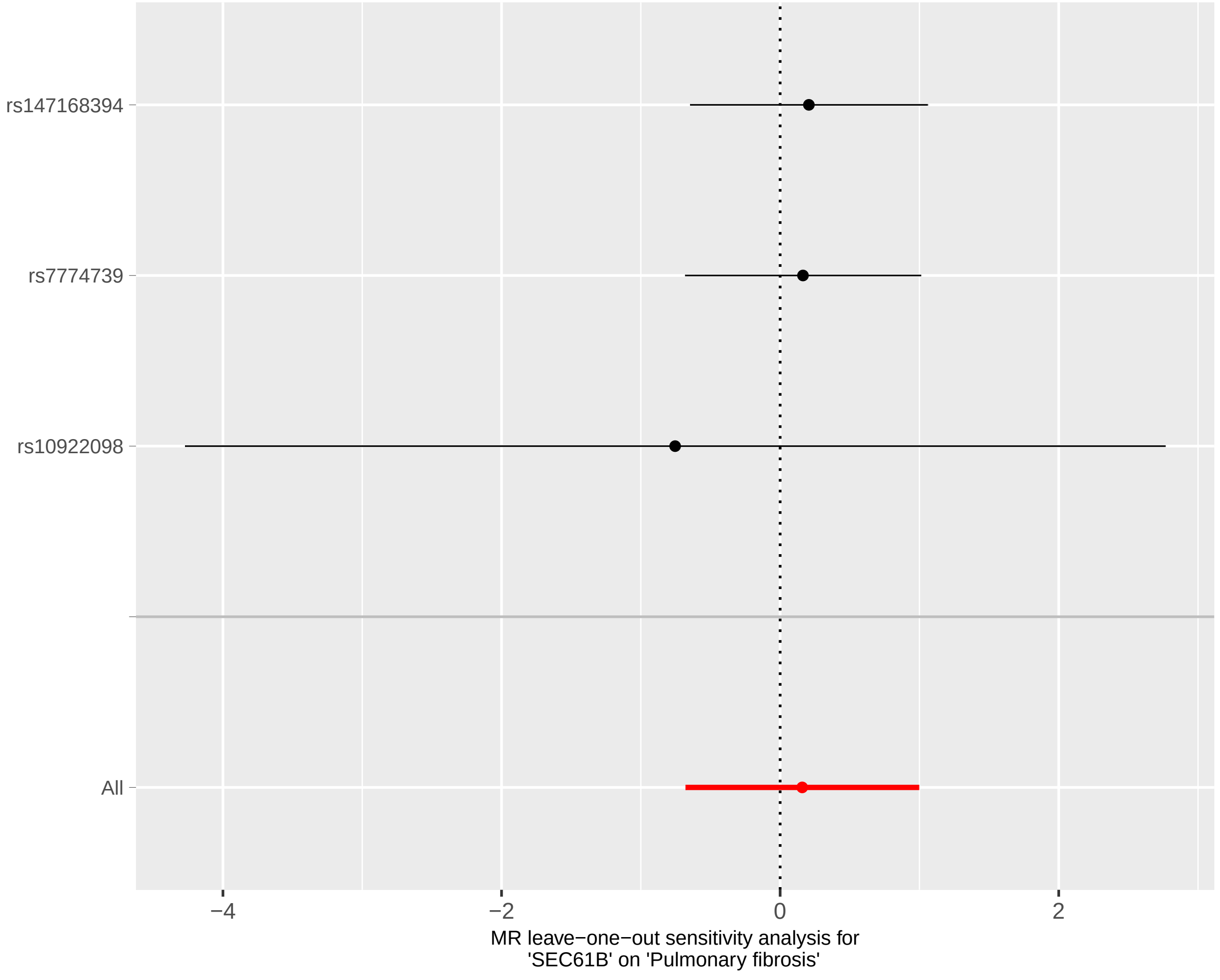
rs147168394
rs7774739
rs10922098
All
−4
−2
0
2
MR leave−one−out sensitivity analysis for
'SEC61B' on 'Pulmonary fibrosis'

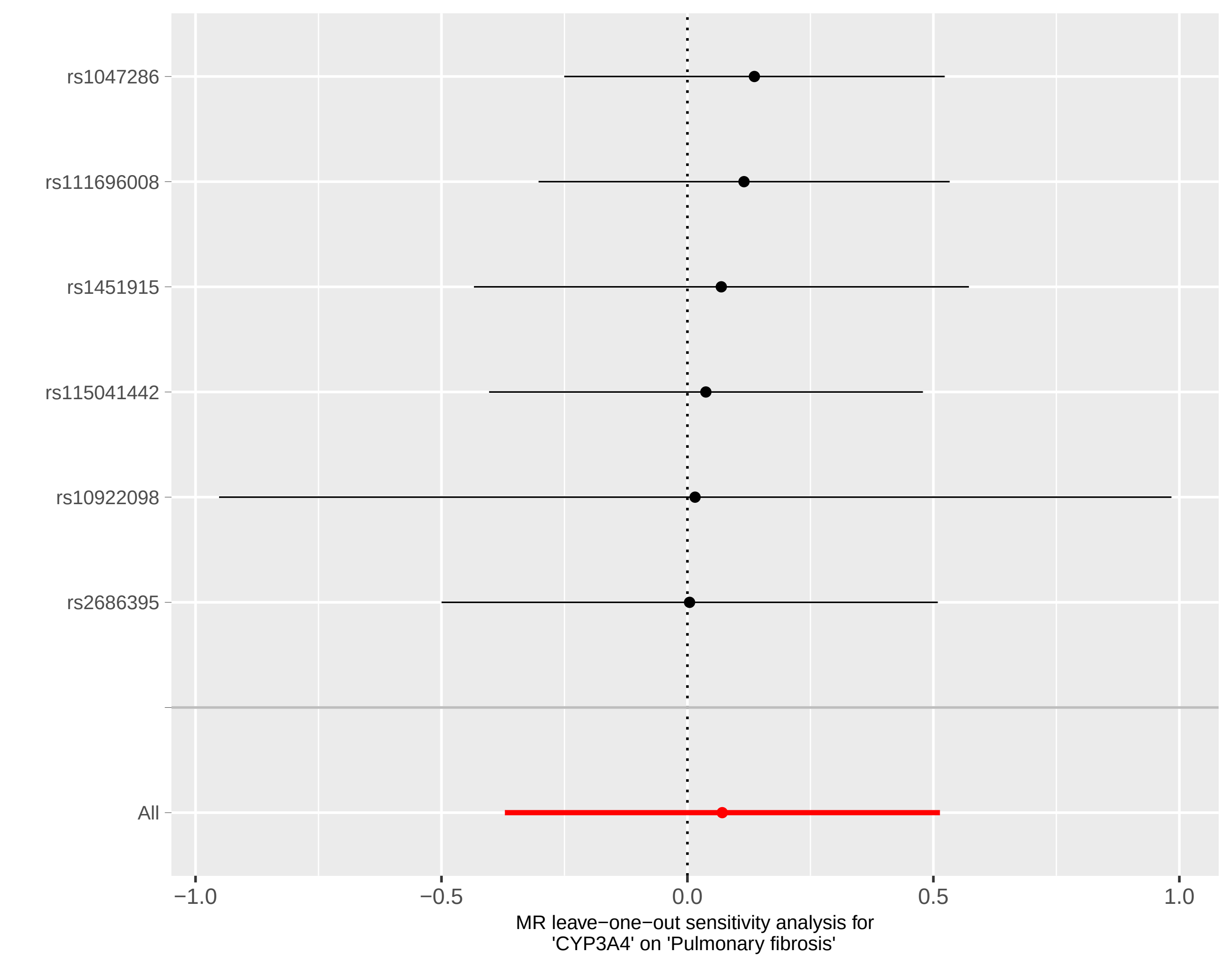
rs1047286
rs111696008
rs1451915
rs115041442
rs10922098
rs2686395
All
−1.0
−0.5
0.0
0.5
1.0
MR leave−one−out sensitivity analysis for
'CYP3A4' on 'Pulmonary fibrosis'

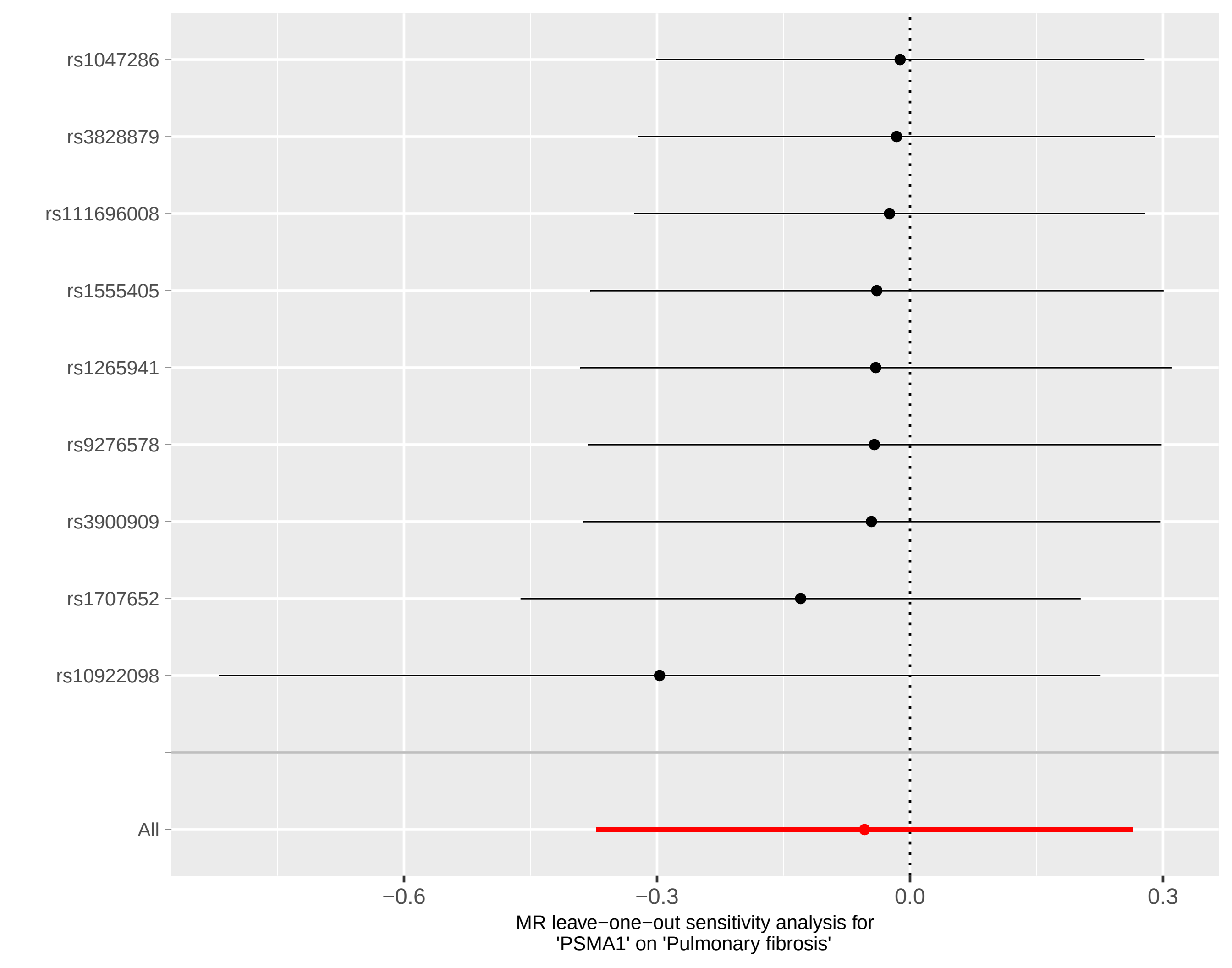
rs1047286
rs3828879
rs111696008
rs1555405
rs1265941
rs9276578
rs3900909
rs1707652
rs10922098
All
−0.6
−0.3
0.0
0.3
MR leave−one−out sensitivity analysis for
'PSMA1' on 'Pulmonary fibrosis'

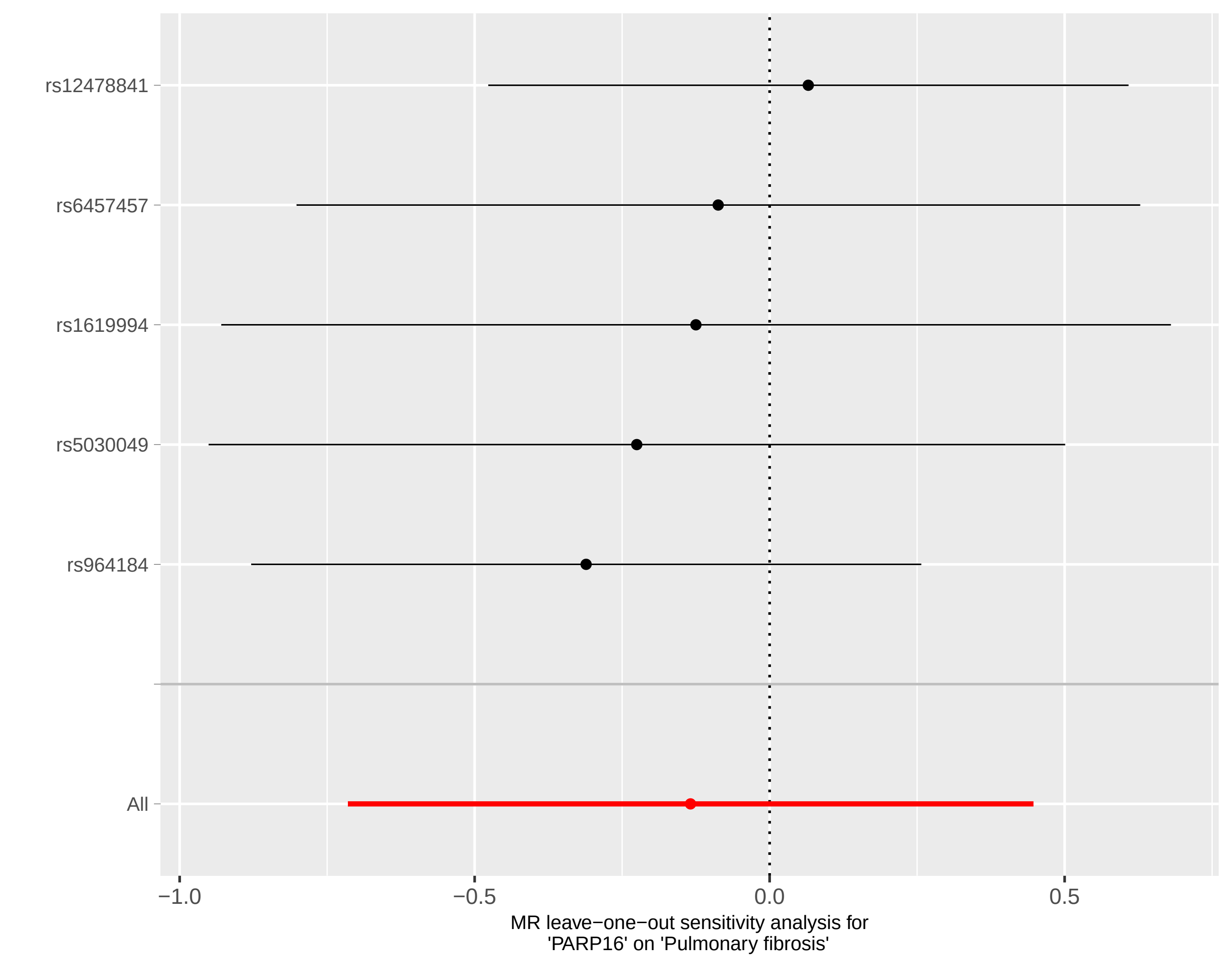
rs12478841
rs6457457
rs1619994
rs5030049
rs964184
All
−1.0
−0.5
0.0
0.5
MR leave−one−out sensitivity analysis for
'PARP16' on 'Pulmonary fibrosis'

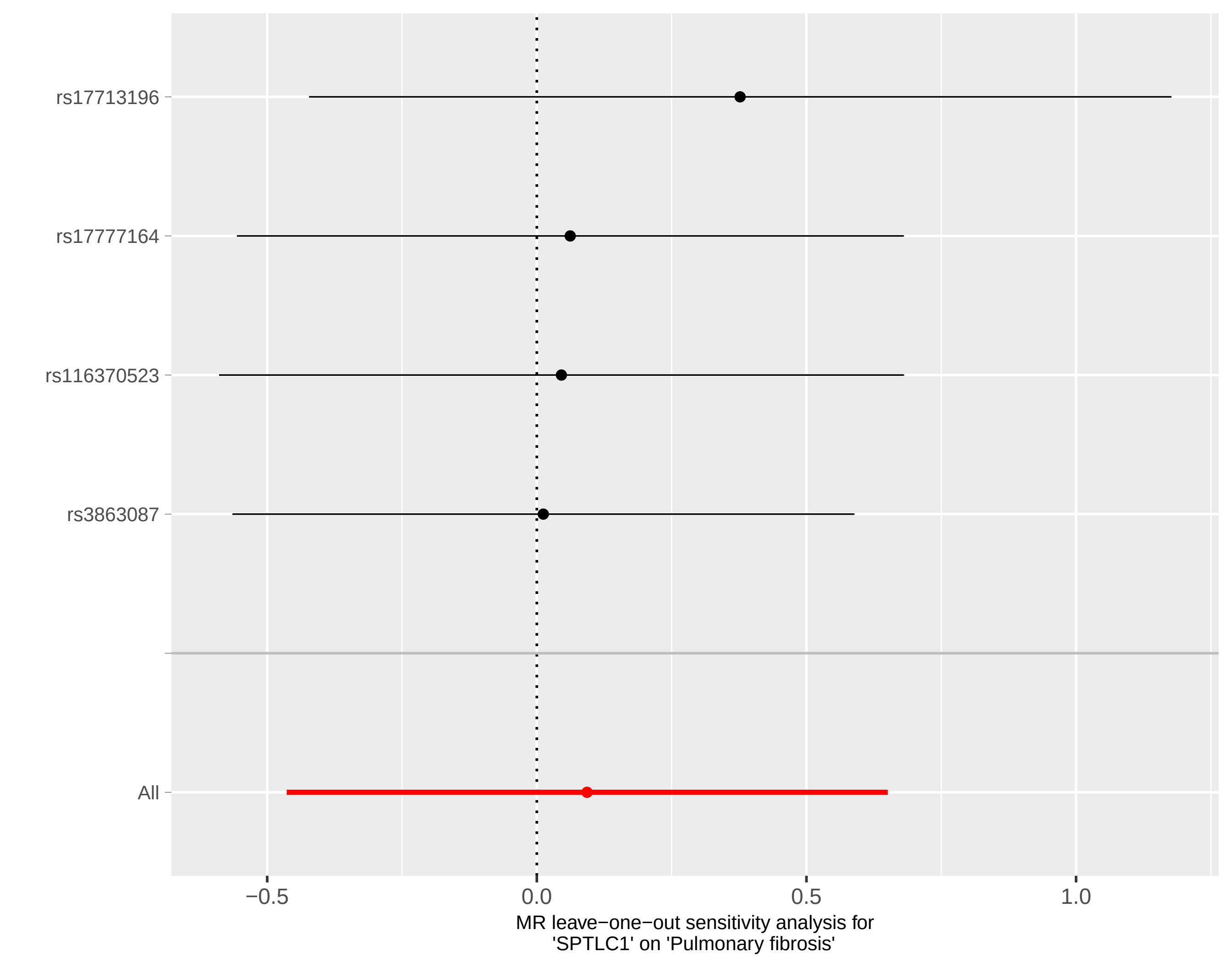
rs17713196
rs17777164
rs116370523
rs3863087
All
−0.5
0.0
0.5
1.0
MR leave−one−out sensitivity analysis for
'SPTLC1' on 'Pulmonary fibrosis'

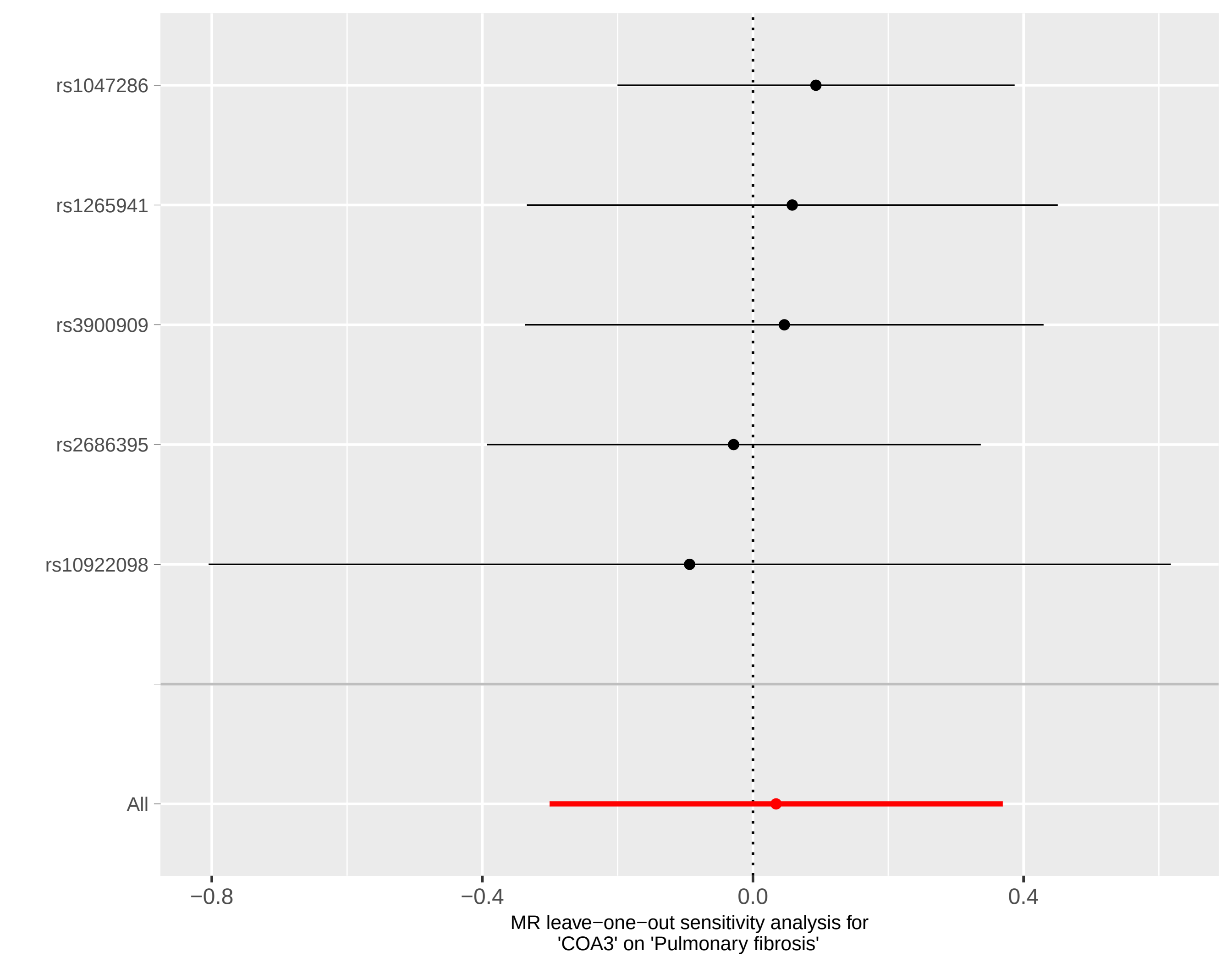
rs1047286
rs1265941
rs3900909
rs2686395
rs10922098
All
−0.8
−0.4
0.0
0.4
MR leave−one−out sensitivity analysis for
'COA3' on 'Pulmonary fibrosis'

Insufficient number of SNPs

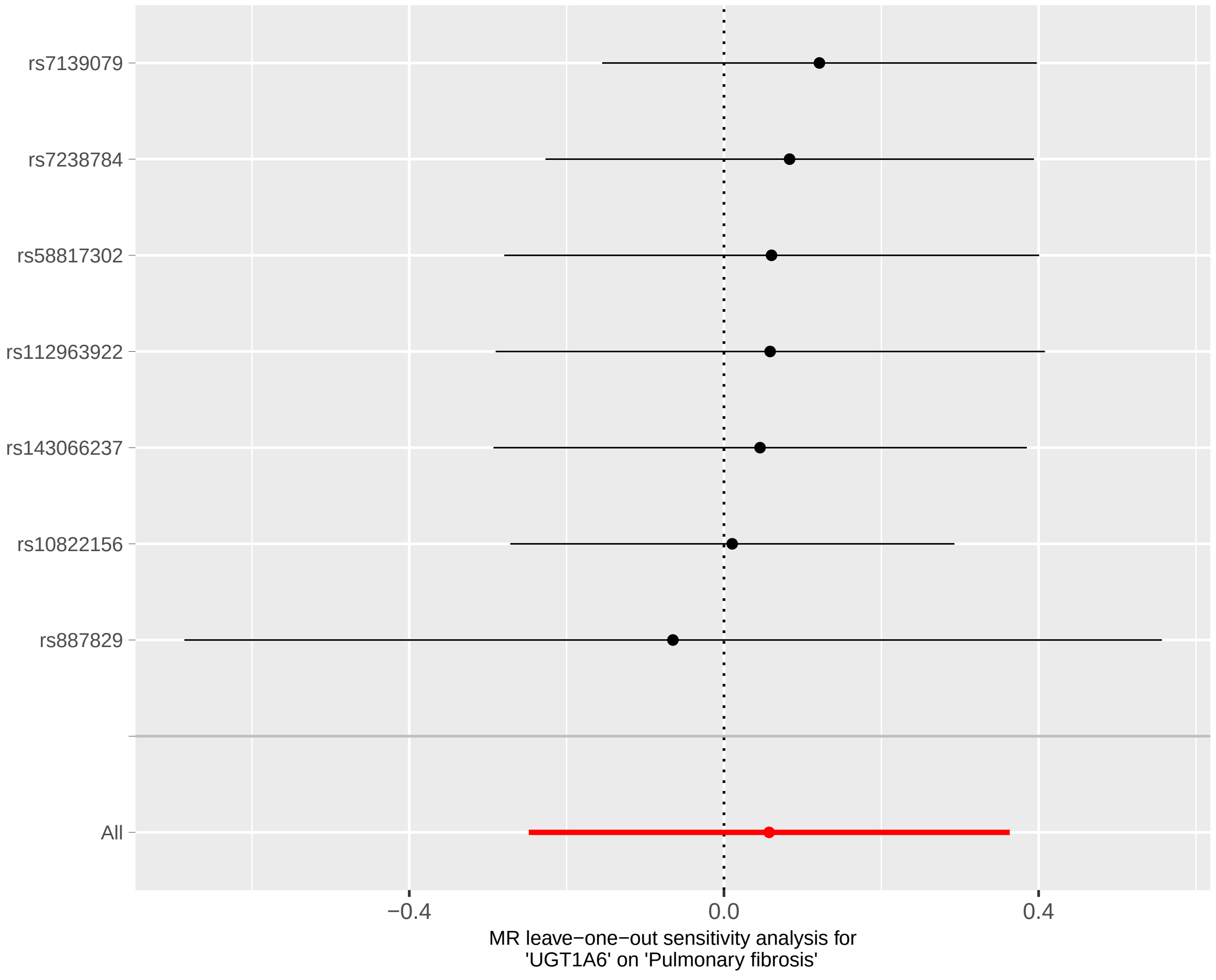

rs7139079
rs7238784
rs58817302
rs112963922
rs143066237
rs10822156
rs887829
All
−0.4
0.0
0.4
MR leave−one−out sensitivity analysis for
'UGT1A6' on 'Pulmonary fibrosis'

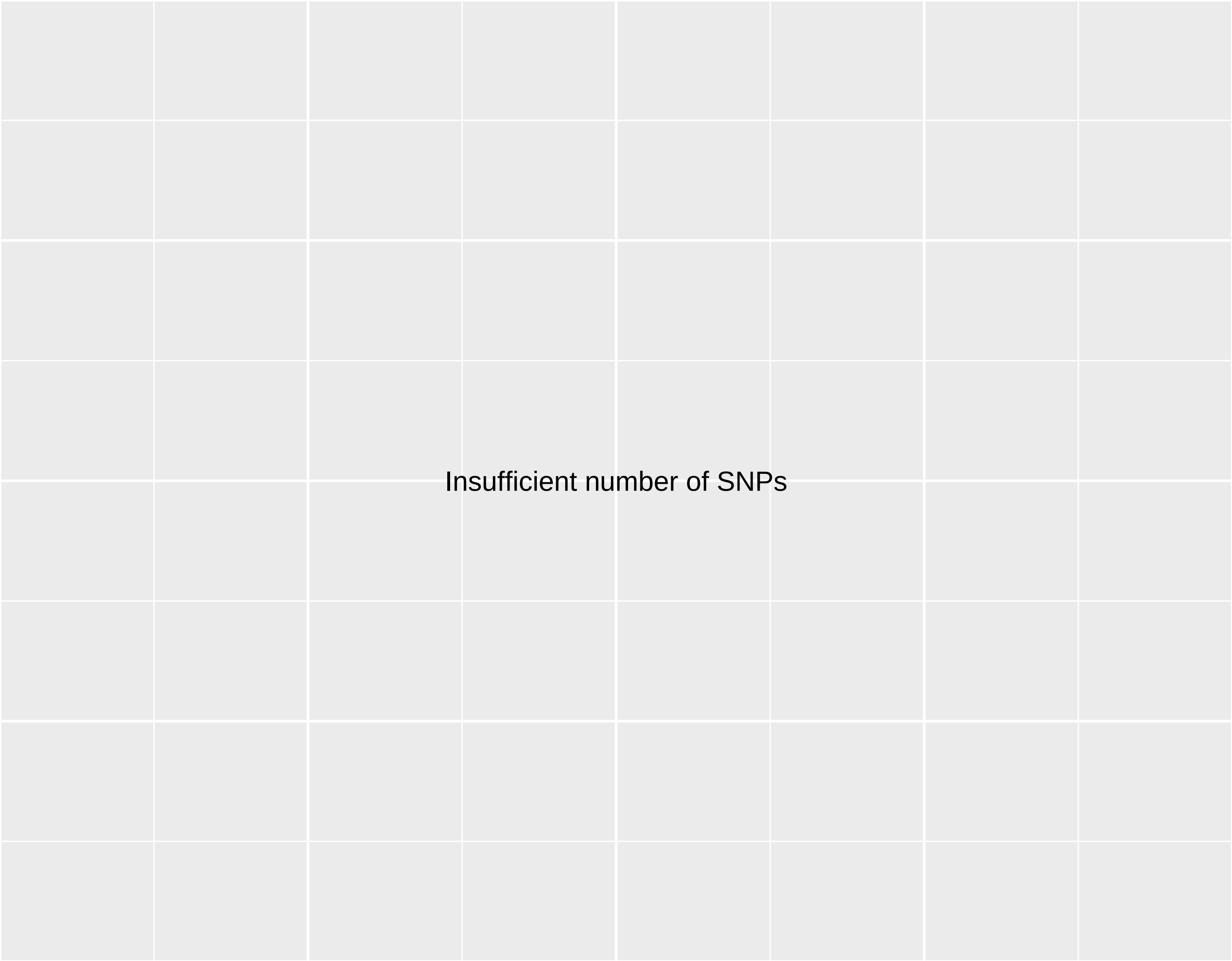
Insufficient number of SNPs

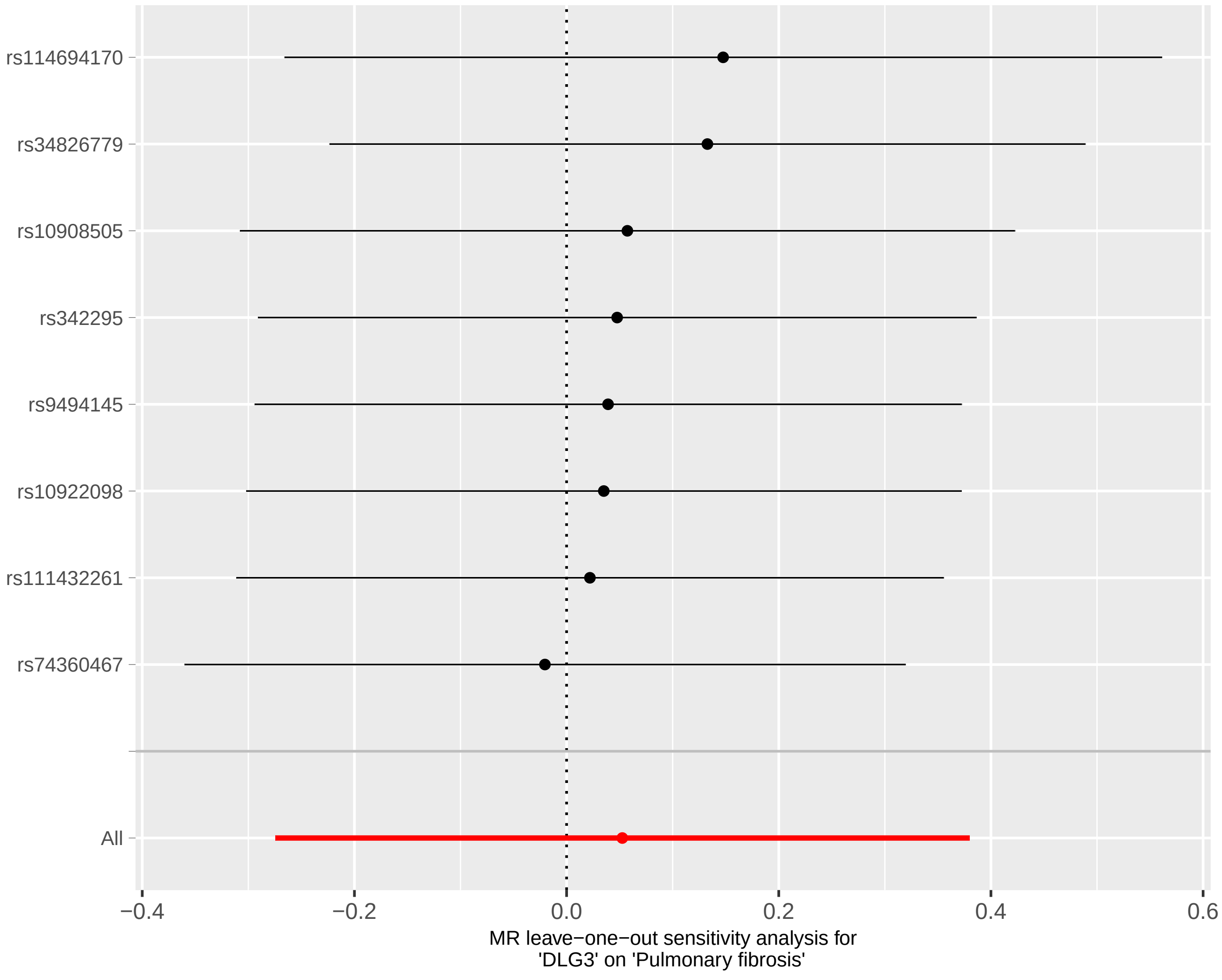
rs114694170
rs34826779
rs10908505
rs342295
rs9494145
rs10922098
rs111432261
rs74360467
All
−0.4
−0.2
0.0
0.2
0.4
0.6
MR leave−one−out sensitivity analysis for
'DLG3' on 'Pulmonary fibrosis'

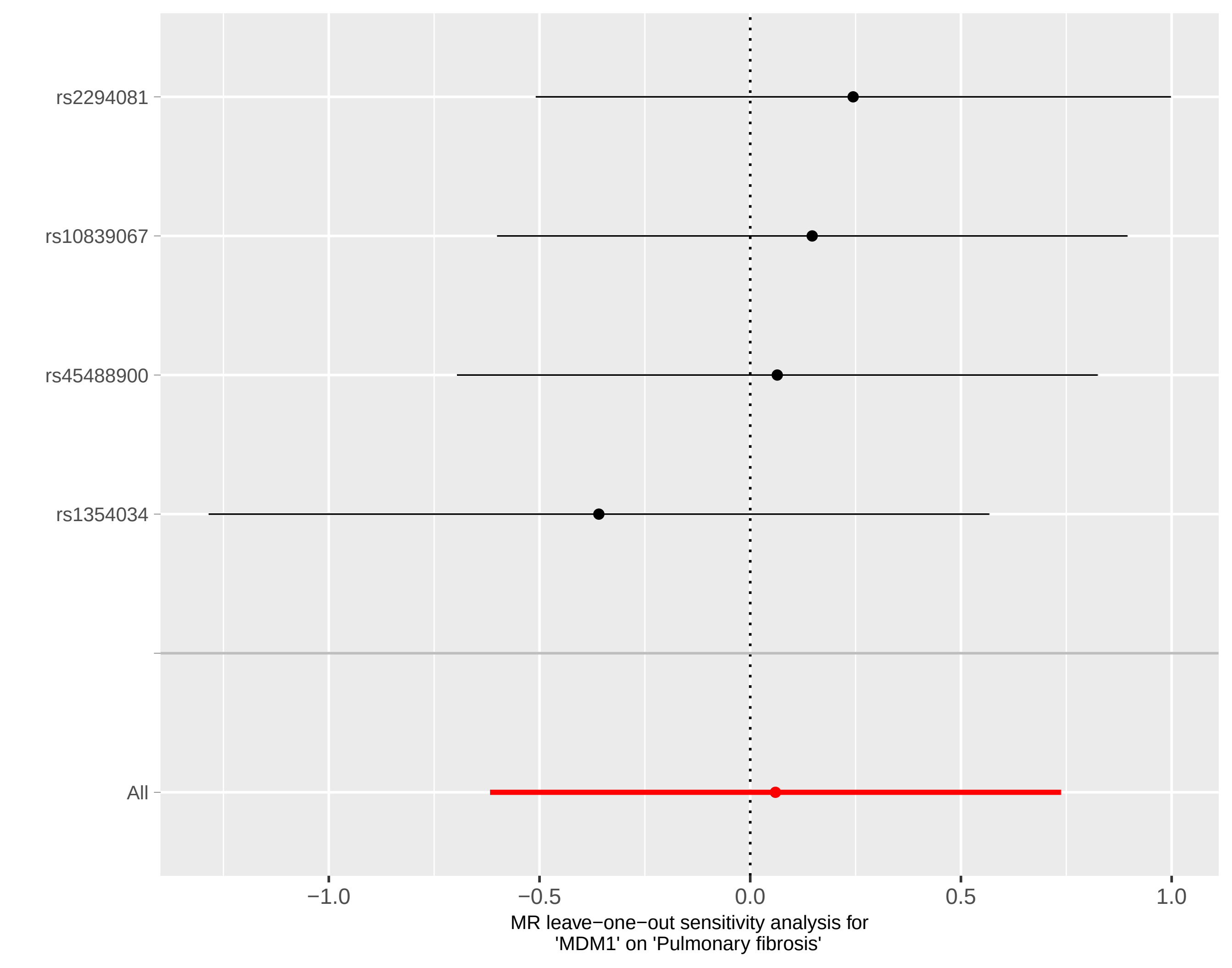
rs2294081
rs10839067
rs45488900
rs1354034
All
−1.0
−0.5
0.0
0.5
1.0
MR leave−one−out sensitivity analysis for
'MDM1' on 'Pulmonary fibrosis'

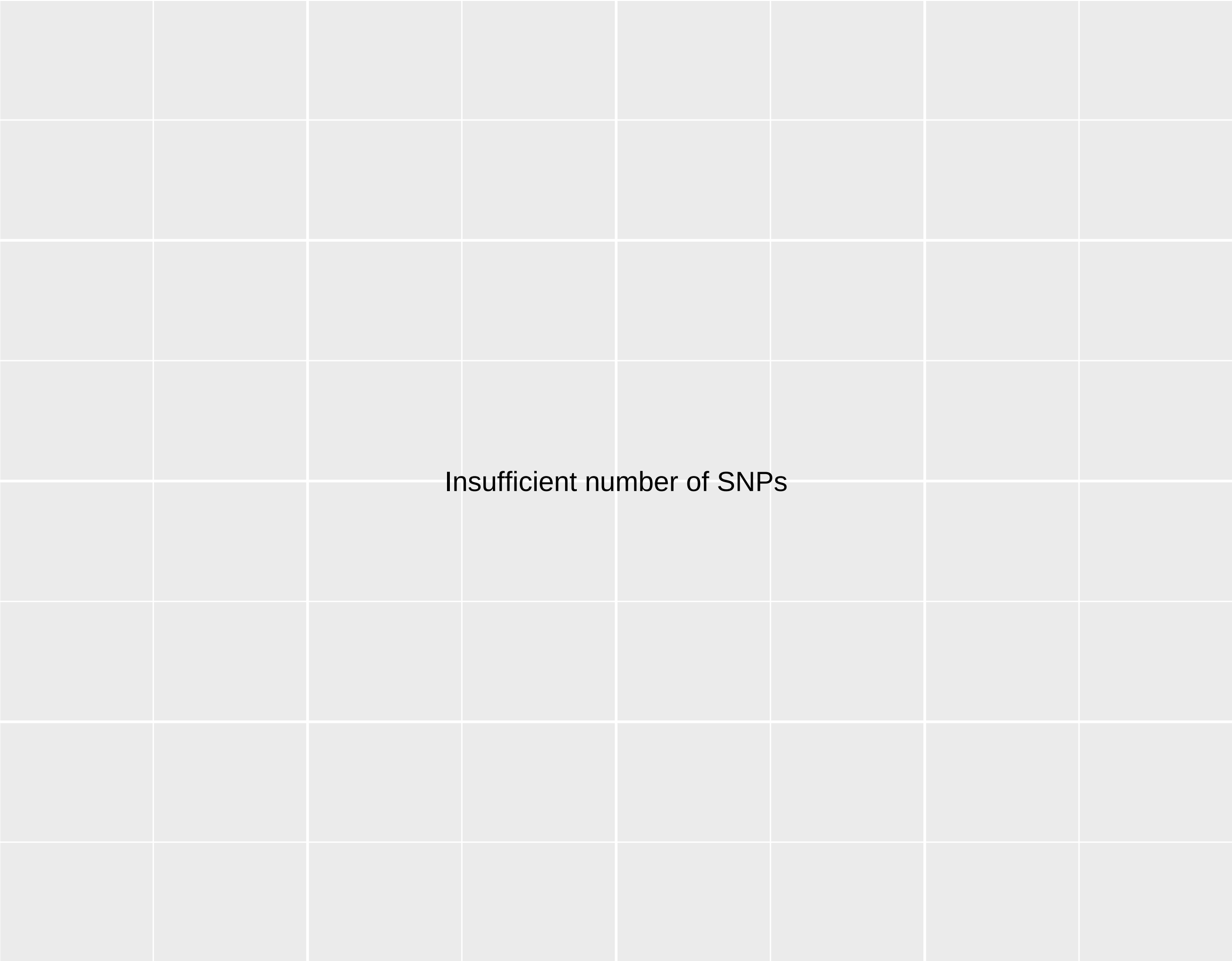
Insufficient number of SNPs

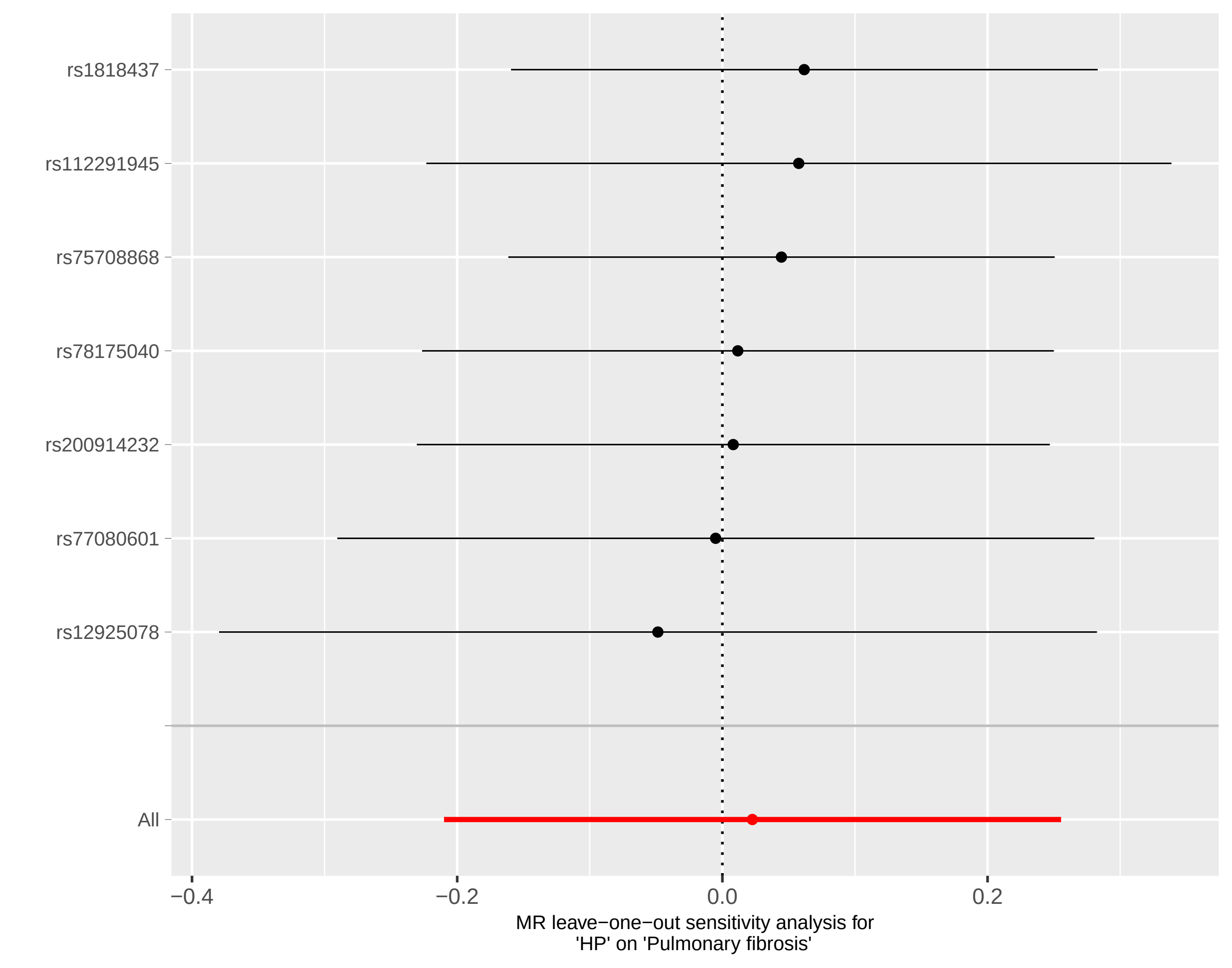
rs1818437
rs112291945
rs75708868
rs78175040
rs200914232
rs77080601
rs12925078
All
−0.4
−0.2
0.0
0.2
MR leave−one−out sensitivity analysis for
'HP' on 'Pulmonary fibrosis'

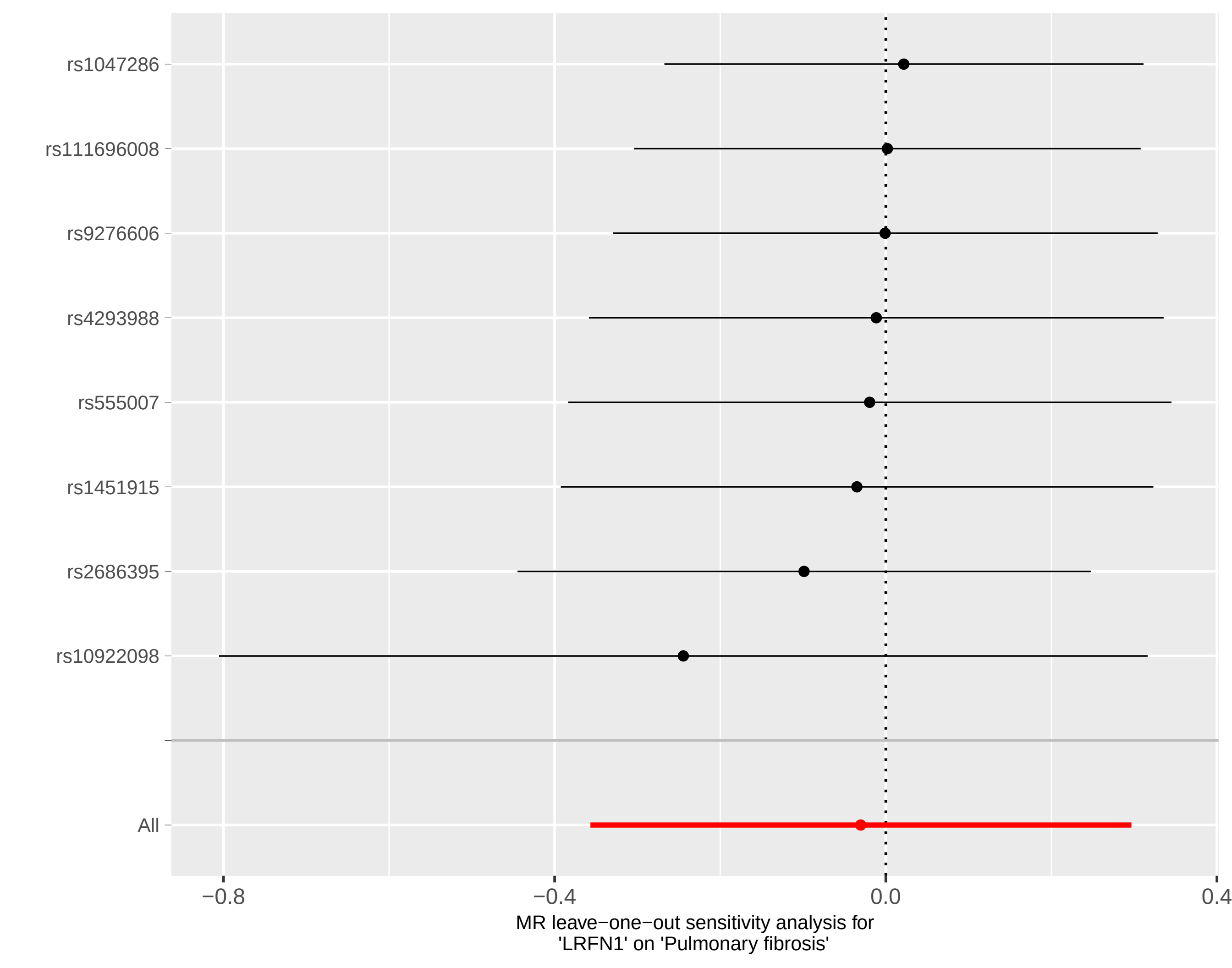

rs1047286
rs111696008
rs9276606
rs4293988
rs555007
rs1451915
rs2686395
rs10922098
All
−0.8
−0.4
0.0
0.4
MR leave−one−out sensitivity analysis for
'LRFN1' on 'Pulmonary fibrosis'

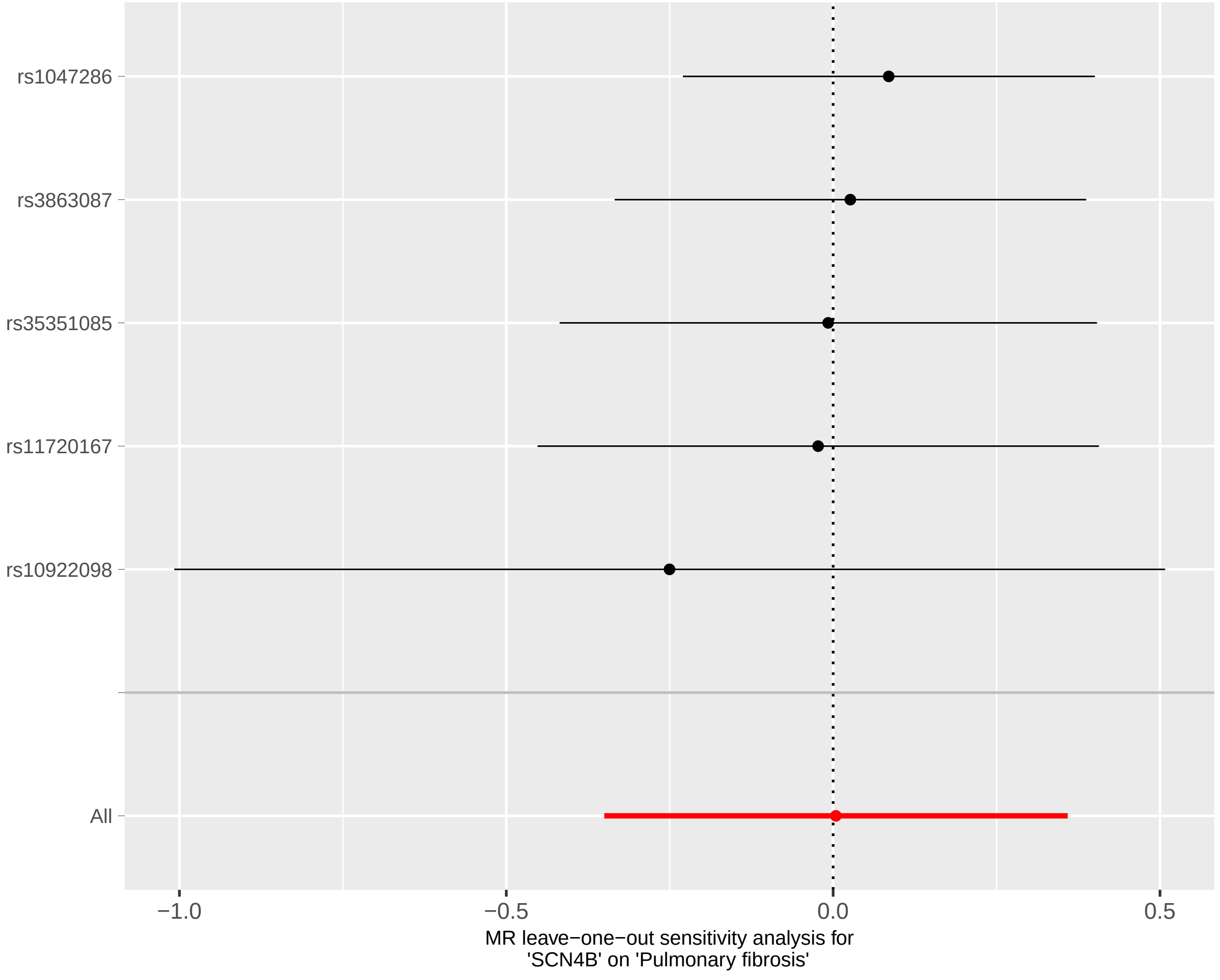
rs1047286
rs3863087
rs35351085
rs11720167
rs10922098
All
−1.0
−0.5
0.0
0.5
MR leave−one−out sensitivity analysis for
'SCN4B' on 'Pulmonary fibrosis'

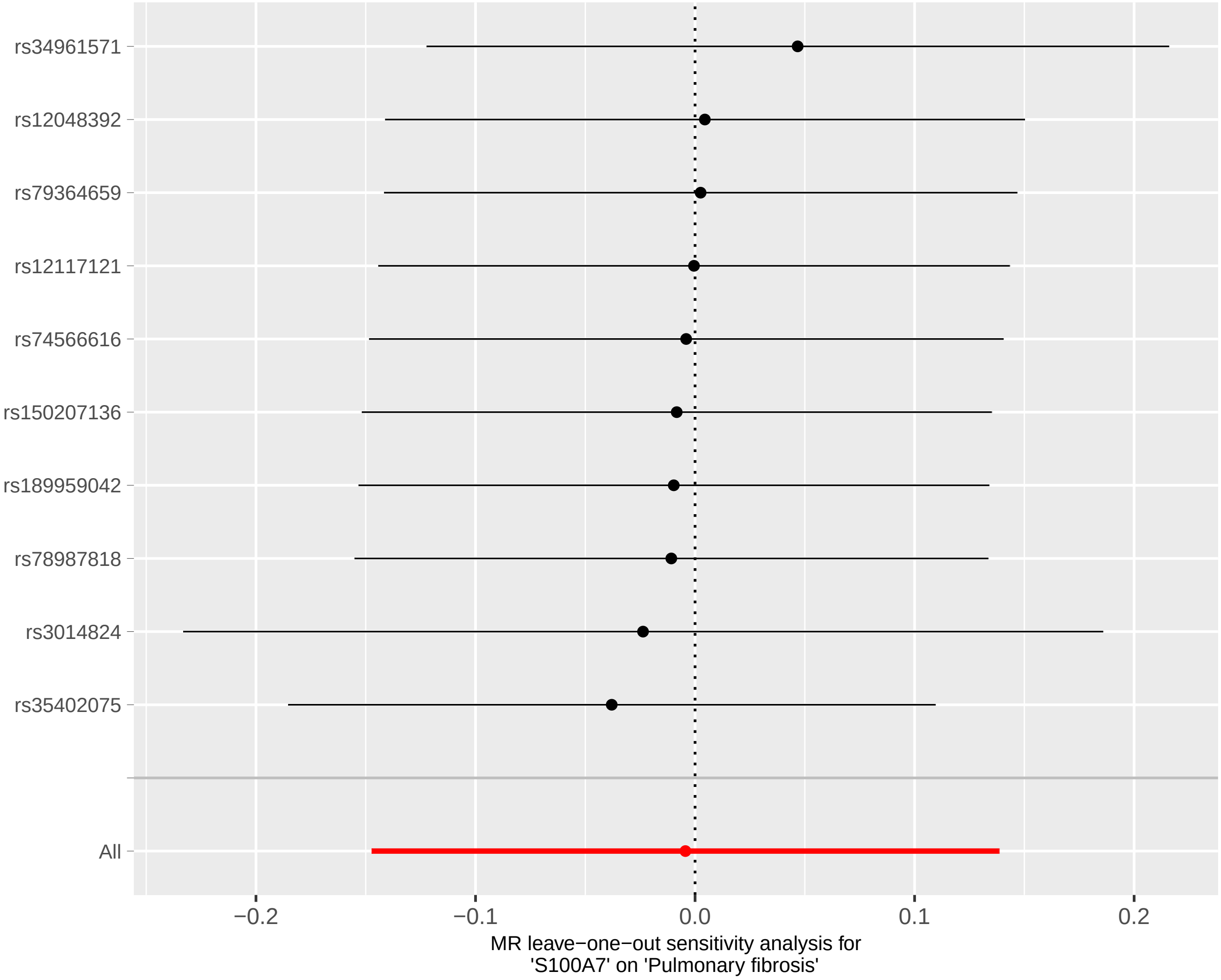
rs34961571
rs12048392
rs79364659
rs12117121
rs74566616
rs150207136
rs189959042
rs78987818
rs3014824
rs35402075
All
−0.2
−0.1
0.0
0.1
0.2
MR leave−one−out sensitivity analysis for
'S100A7' on 'Pulmonary fibrosis'

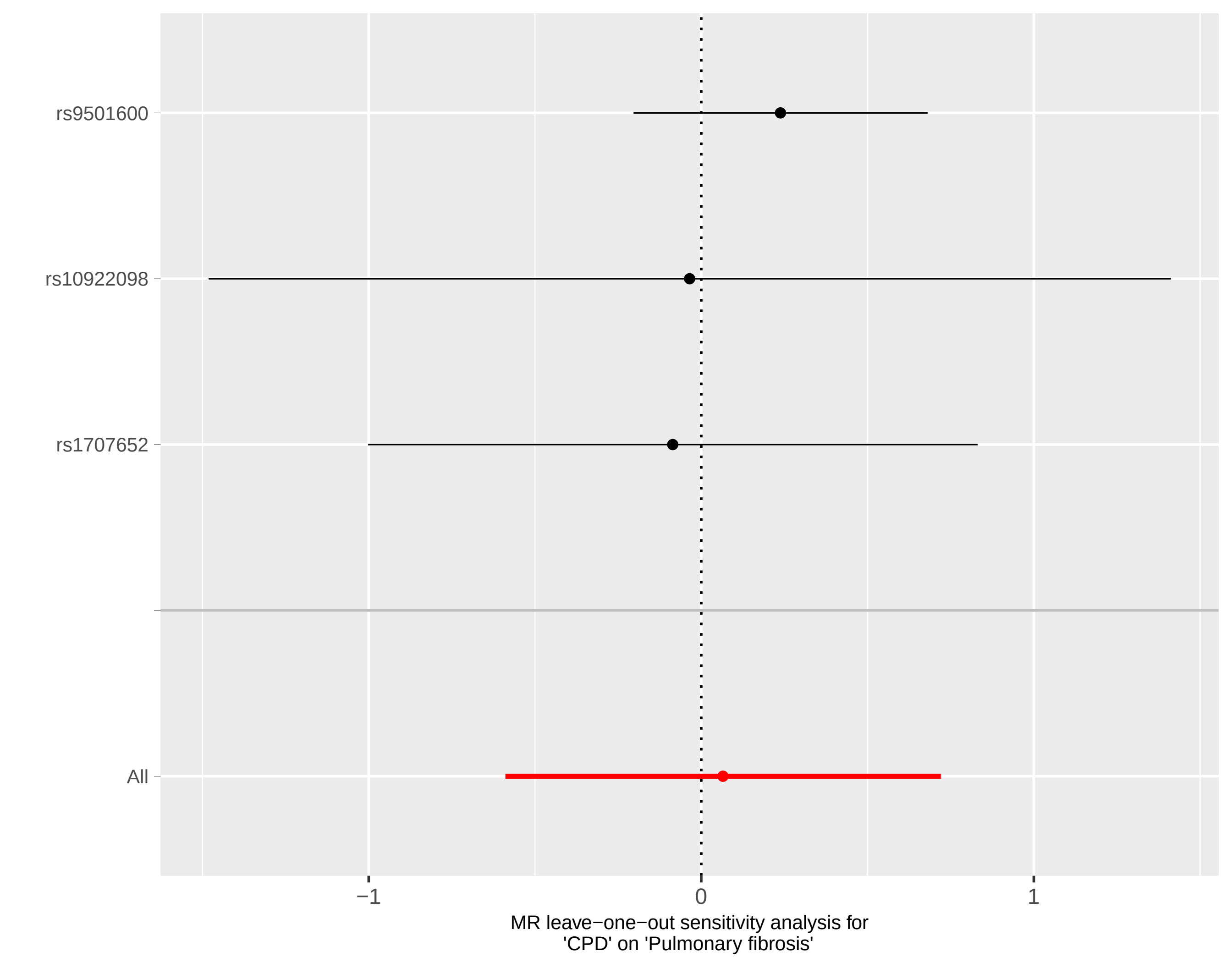
rs9501600
rs10922098
rs1707652
All
−1
0
1
MR leave−one−out sensitivity analysis for
'CPD' on 'Pulmonary fibrosis'

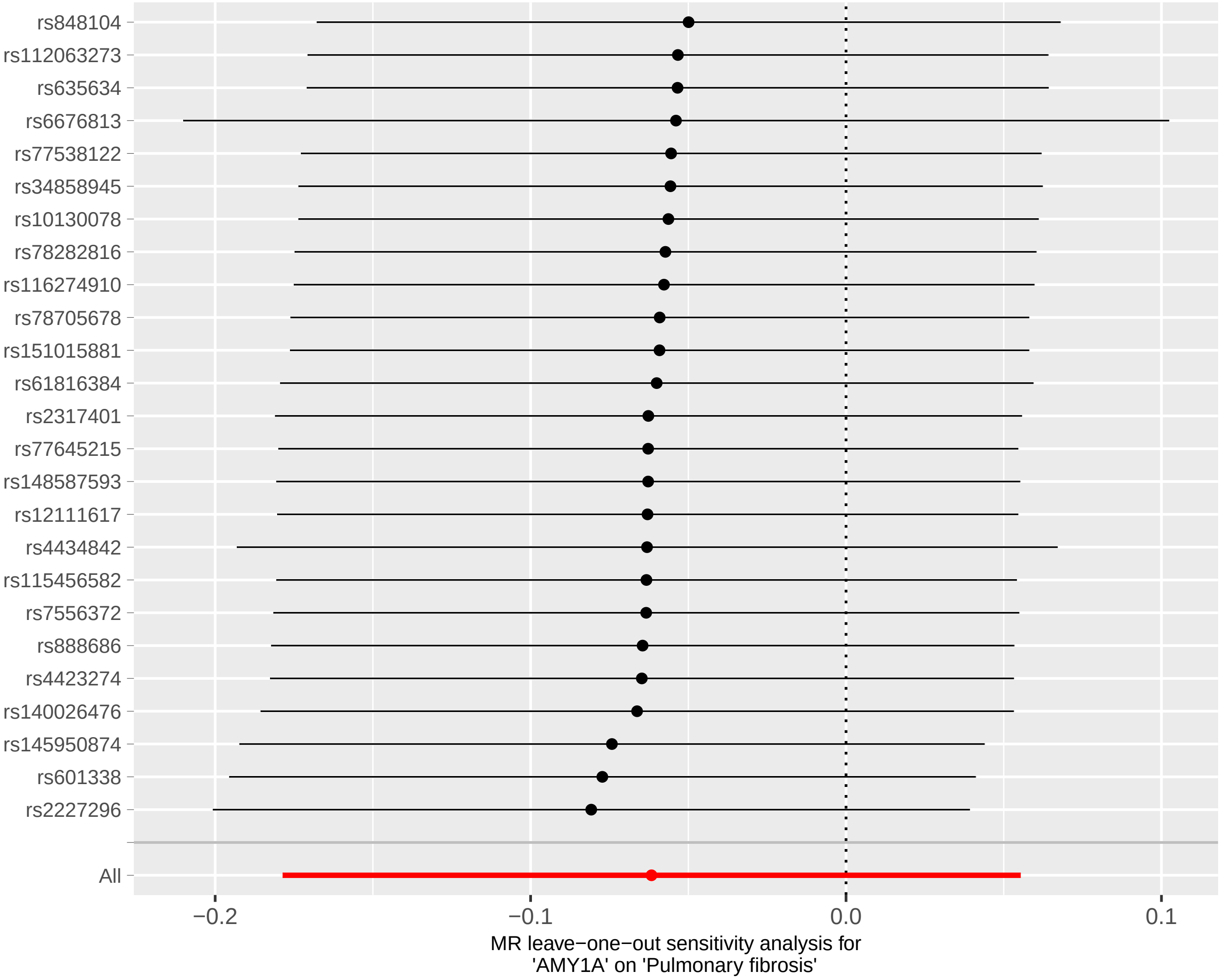
rs848104
rs112063273
rs635634
rs6676813
rs77538122
rs34858945
rs10130078
rs78282816
rs116274910
rs78705678
rs151015881
rs61816384
rs2317401
rs77645215
rs148587593
rs12111617
rs4434842
rs115456582
rs7556372
rs888686
rs4423274
rs140026476
rs145950874
rs601338
rs2227296
All
−0.2
−0.1
0.0
0.1
MR leave−one−out sensitivity analysis for
'AMY1A' on 'Pulmonary fibrosis'

Insufficient number of SNPs

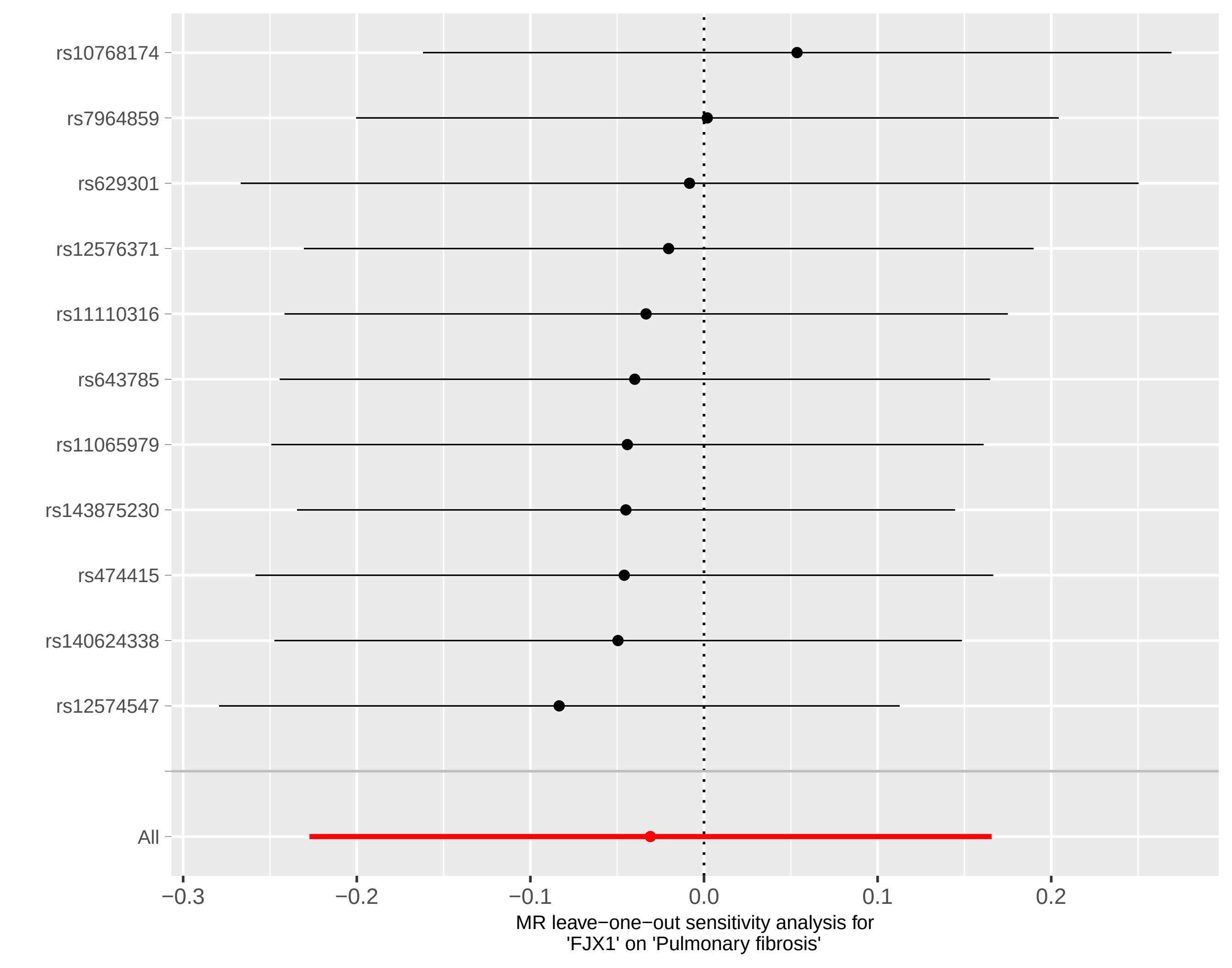
rs10768174
rs7964859
rs629301
rs12576371
rs11110316
rs643785
rs11065979
rs143875230
rs474415
rs140624338
rs12574547
All
−0.3
−0.2
−0.1
0.0
0.1
0.2
MR leave−one−out sensitivity analysis for
'FJX1' on 'Pulmonary fibrosis'

Insufficient number of SNPs

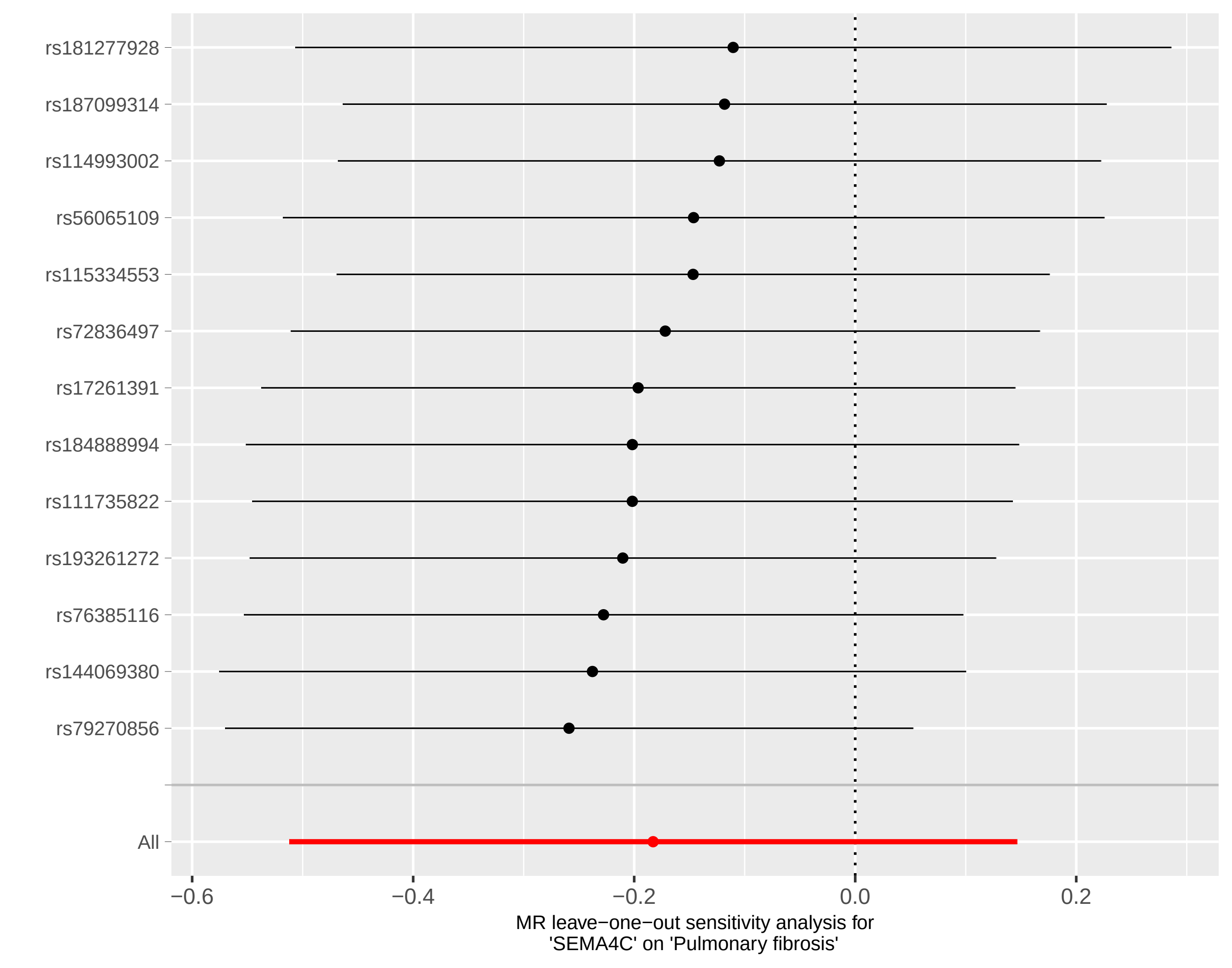
rs181277928
rs187099314
rs114993002
rs56065109
rs115334553
rs72836497
rs17261391
rs184888994
rs111735822
rs193261272
rs76385116
rs144069380
rs79270856
All
−0.6
−0.4
−0.2
0.0
0.2
MR leave−one−out sensitivity analysis for
'SEMA4C' on 'Pulmonary fibrosis'

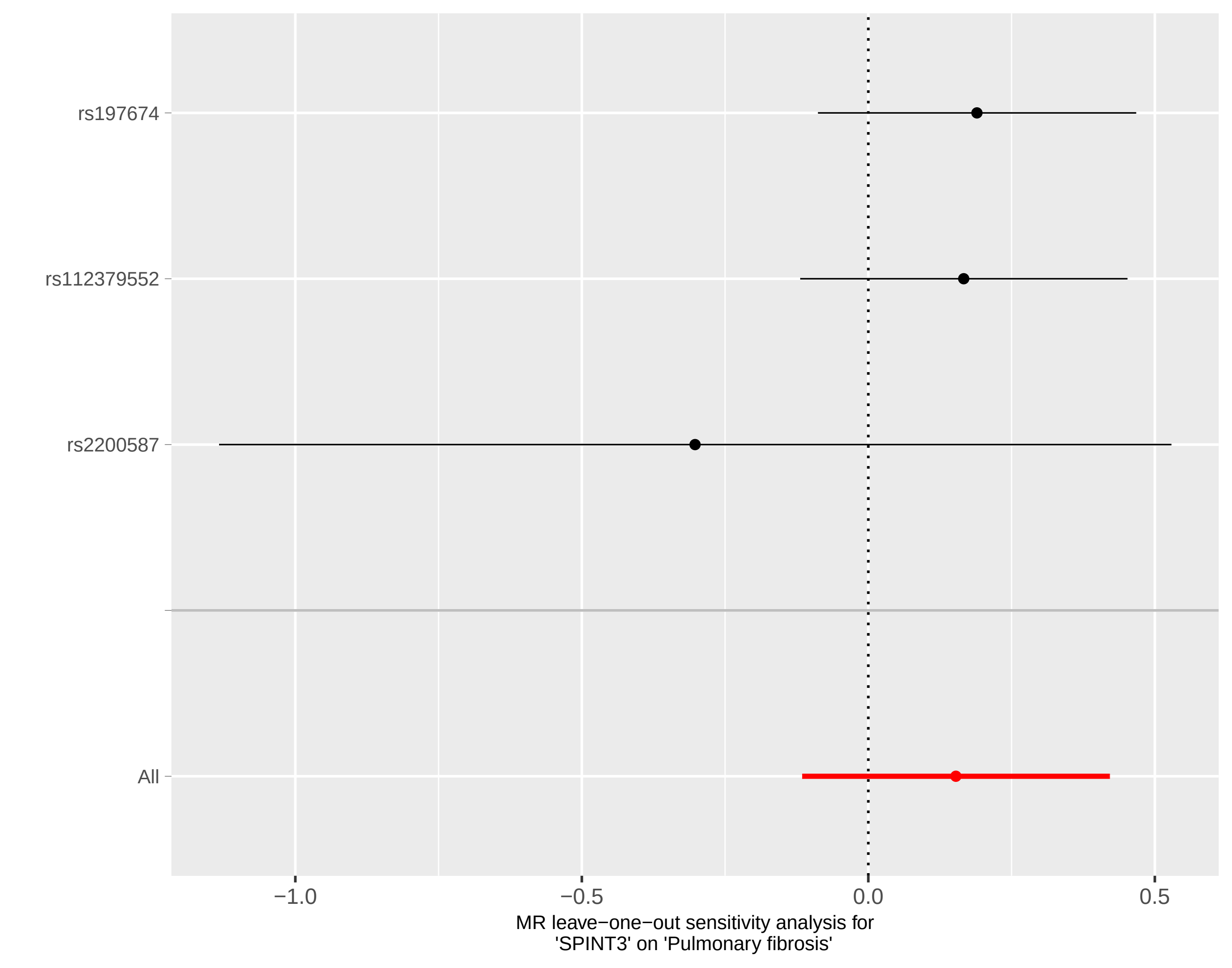
rs197674
rs112379552
rs2200587
All
−1.0
−0.5
0.0
0.5
MR leave−one−out sensitivity analysis for
'SPINT3' on 'Pulmonary fibrosis'

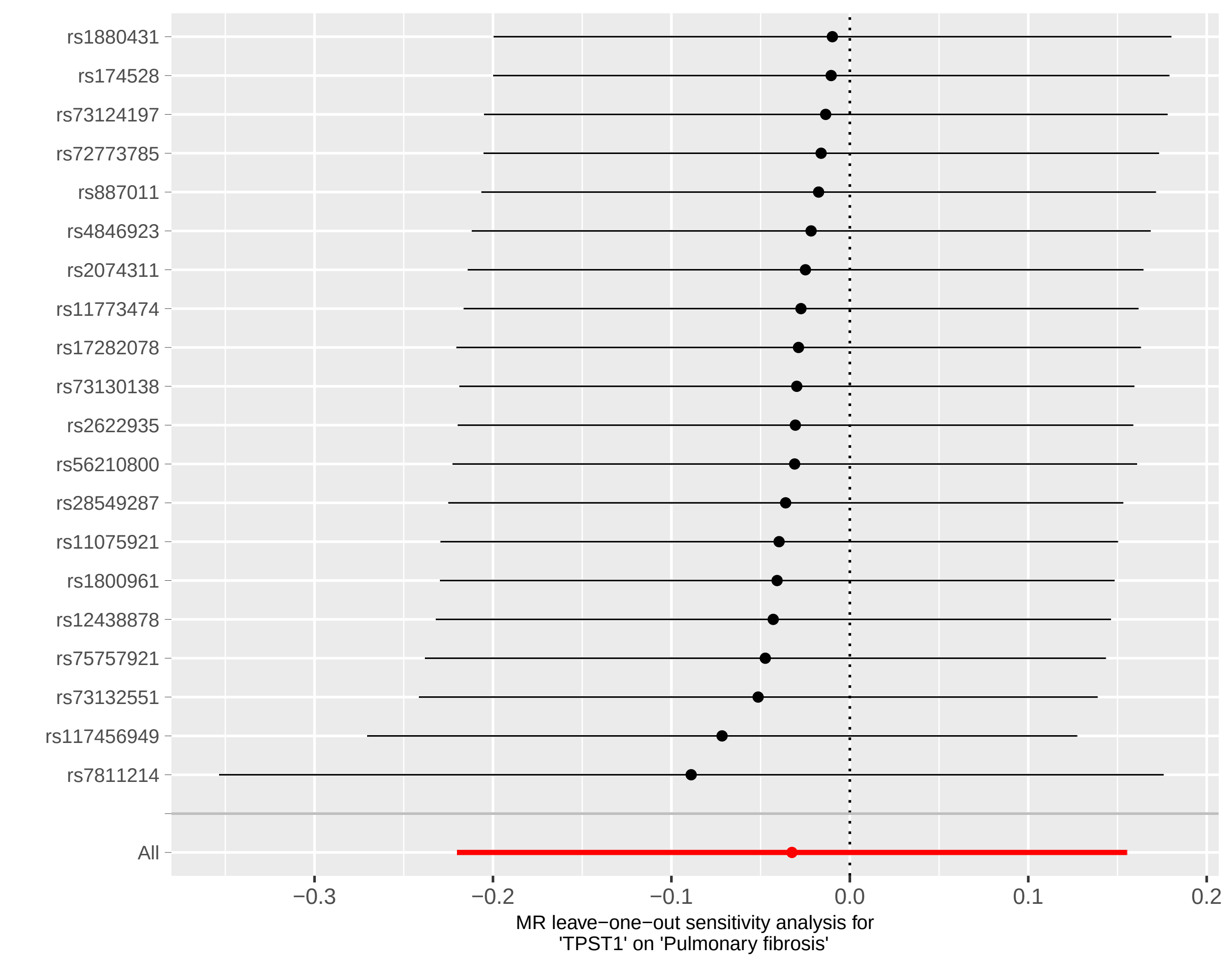
rs1880431
rs174528
rs73124197
rs72773785
rs887011
rs4846923
rs2074311
rs11773474
rs17282078
rs73130138
rs2622935
rs56210800
rs28549287
rs11075921
rs1800961
rs12438878
rs75757921
rs73132551
rs117456949
rs7811214
All
−0.3
−0.2
−0.1
0.0
0.1
0.2
MR leave−one−out sensitivity analysis for
'TPST1' on 'Pulmonary fibrosis'

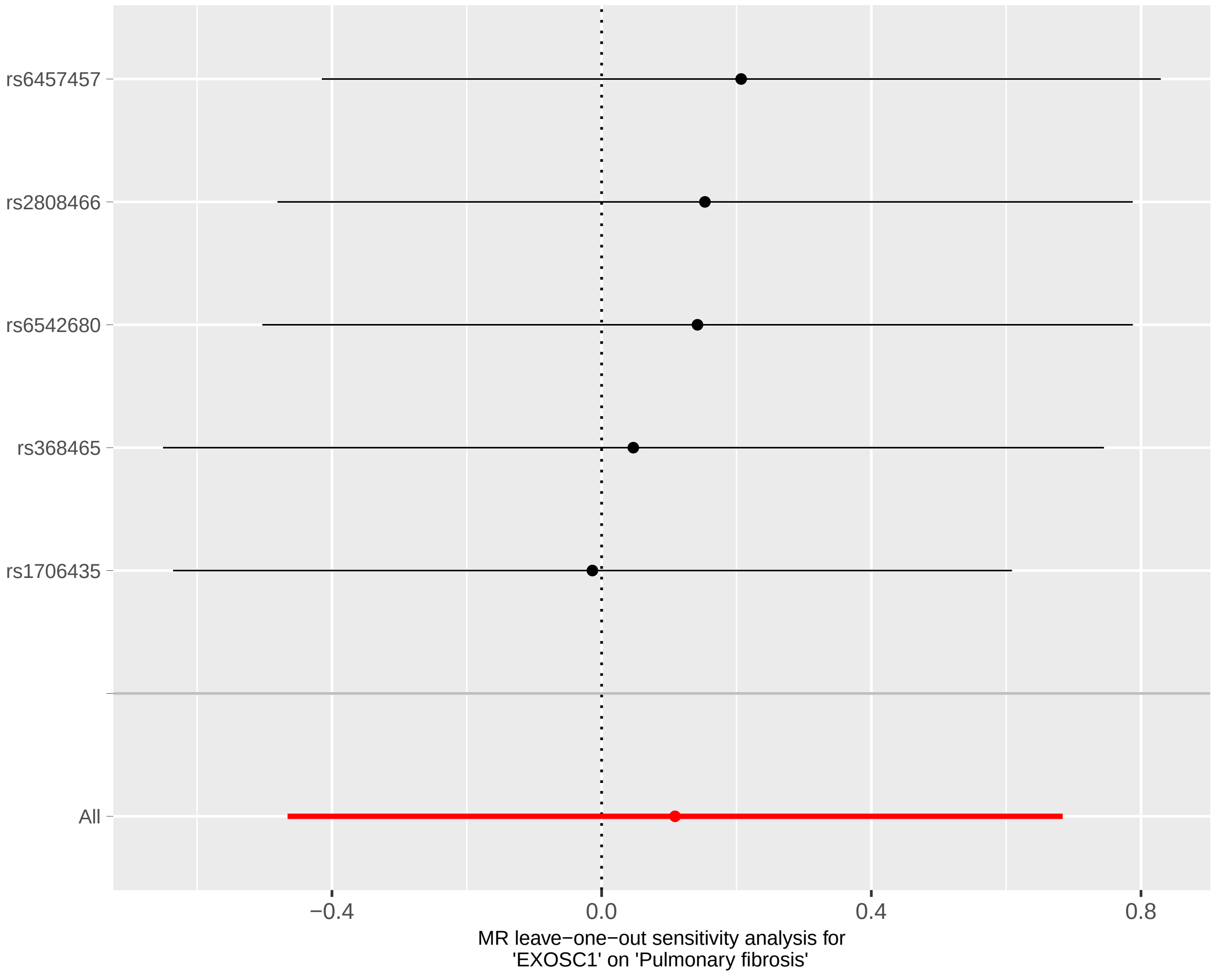
rs6457457
rs2808466
rs6542680
rs368465
rs1706435
All
−0.4
0.0
0.4
0.8
MR leave−one−out sensitivity analysis for
'EXOSC1' on 'Pulmonary fibrosis'

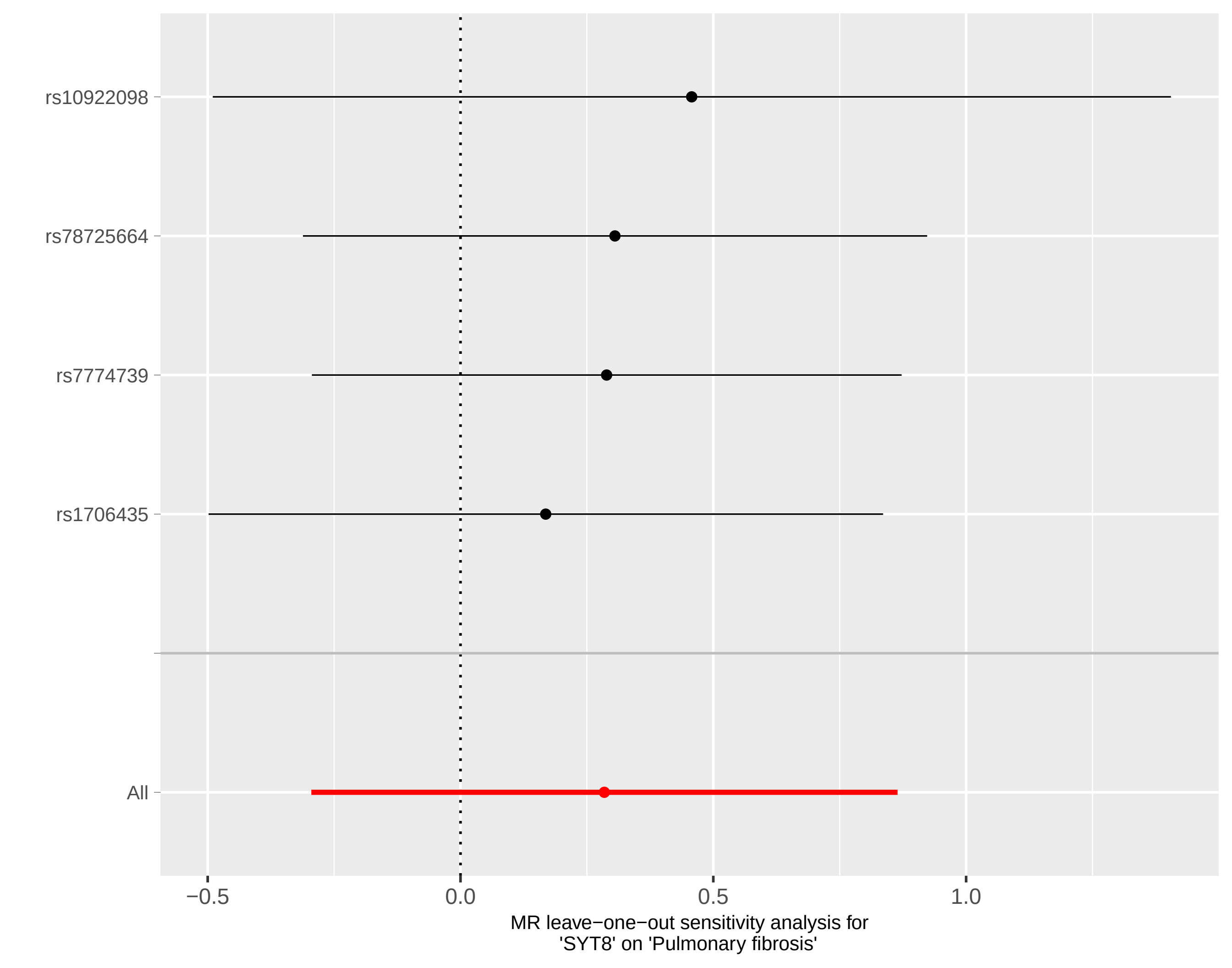
rs10922098
rs78725664
rs7774739
rs1706435
All
−0.5
0.0
0.5
1.0
MR leave−one−out sensitivity analysis for
'SYT8' on 'Pulmonary fibrosis'

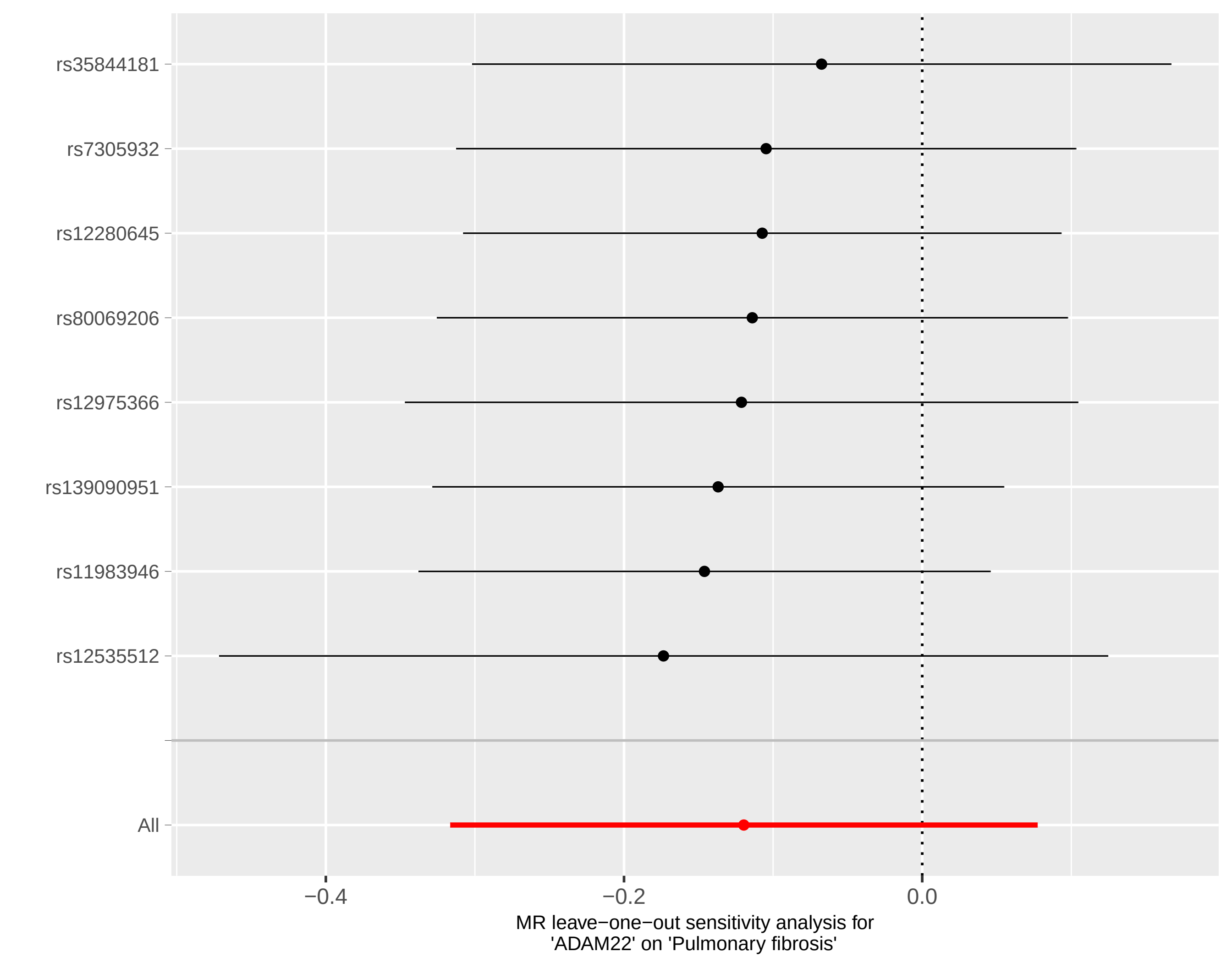
rs35844181
rs7305932
rs12280645
rs80069206
rs12975366
rs139090951
rs11983946
rs12535512
All
−0.4
−0.2
0.0
MR leave−one−out sensitivity analysis for
'ADAM22' on 'Pulmonary fibrosis'

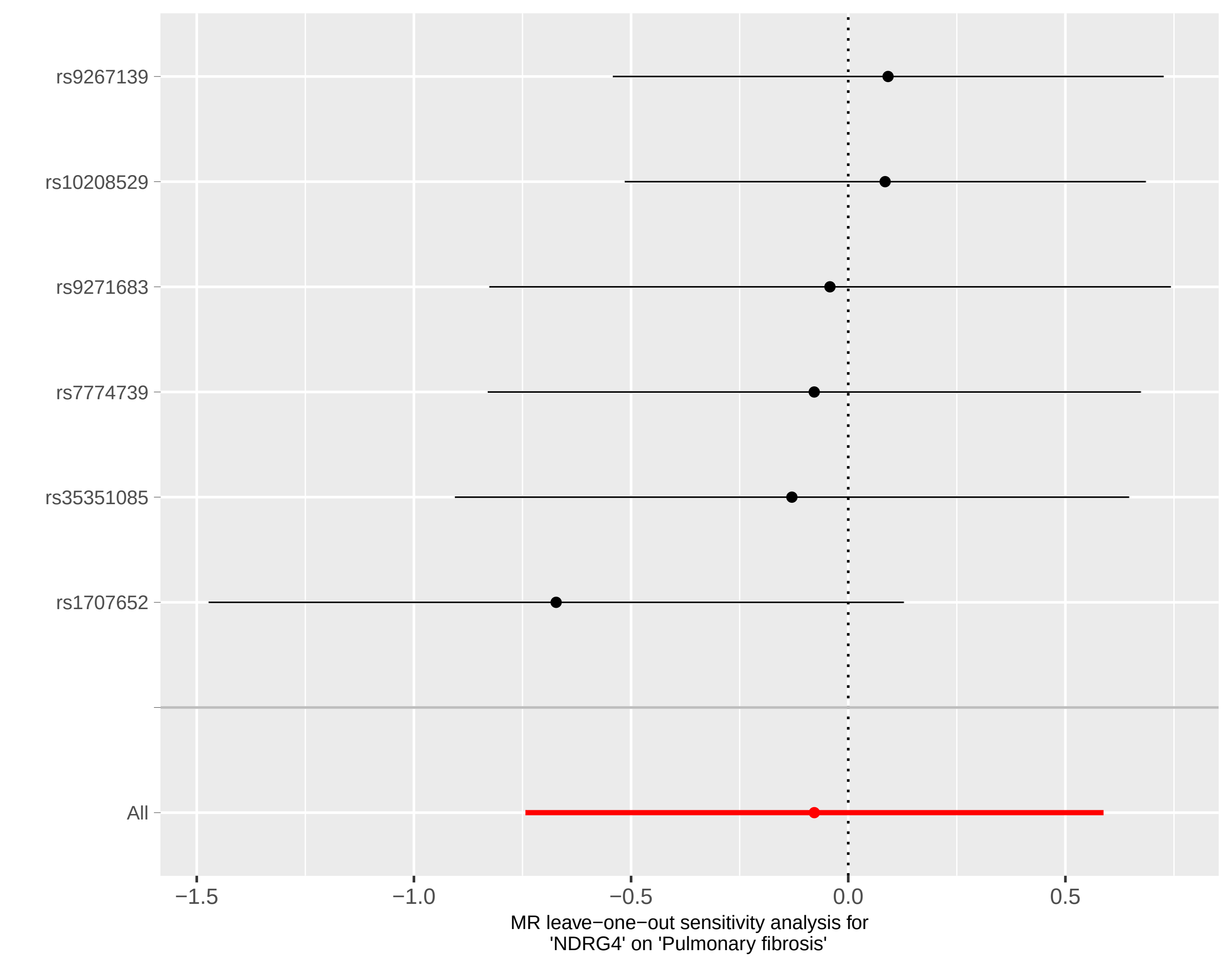
rs9267139
rs10208529
rs9271683
rs7774739
rs35351085
rs1707652
All
−1.5
−1.0
−0.5
0.0
0.5
MR leave−one−out sensitivity analysis for
'NDRG4' on 'Pulmonary fibrosis'

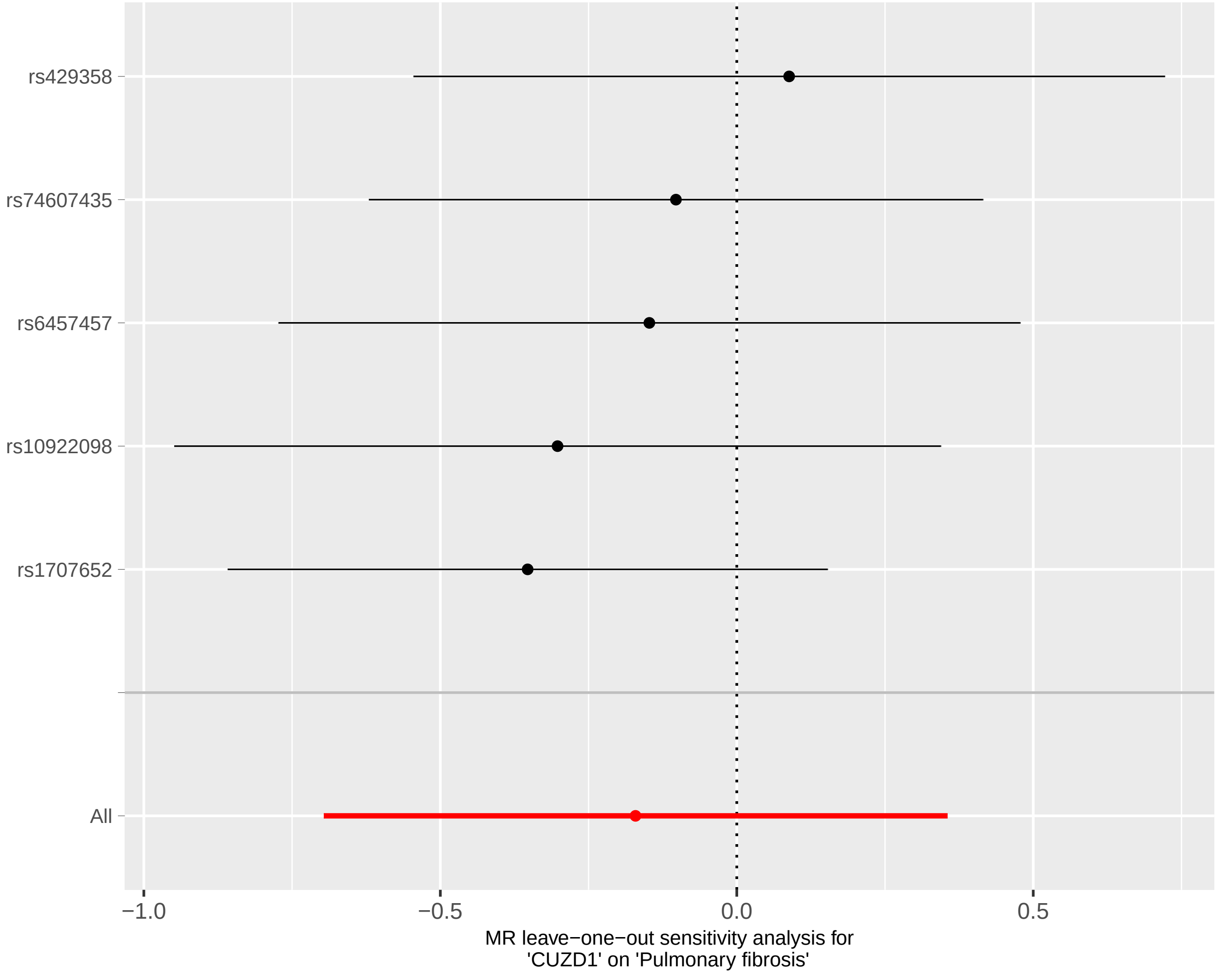
rs429358
rs74607435
rs6457457
rs10922098
rs1707652
All
−1.0
−0.5
0.0
0.5
MR leave−one−out sensitivity analysis for
'CUZD1' on 'Pulmonary fibrosis'

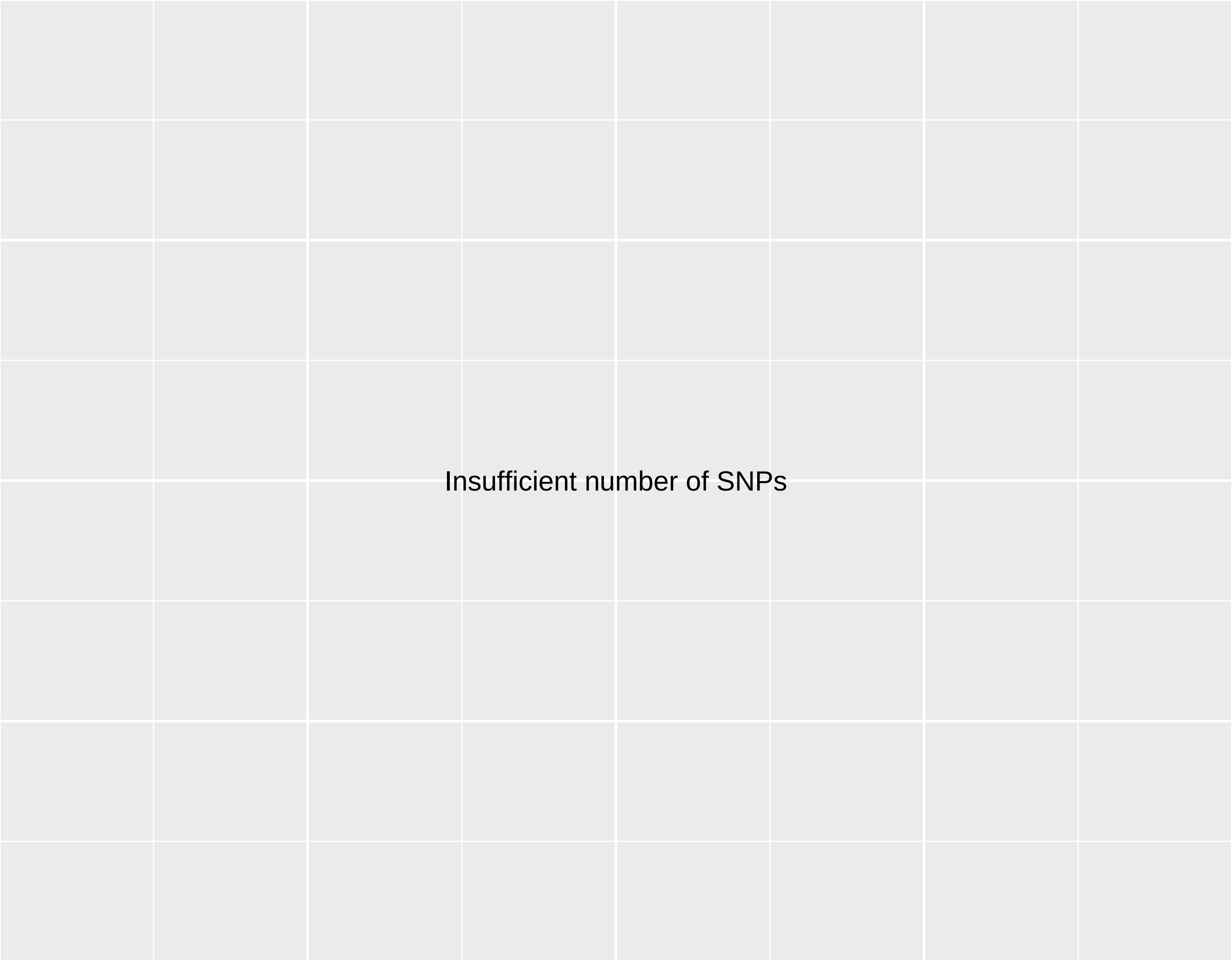
Insufficient number of SNPs

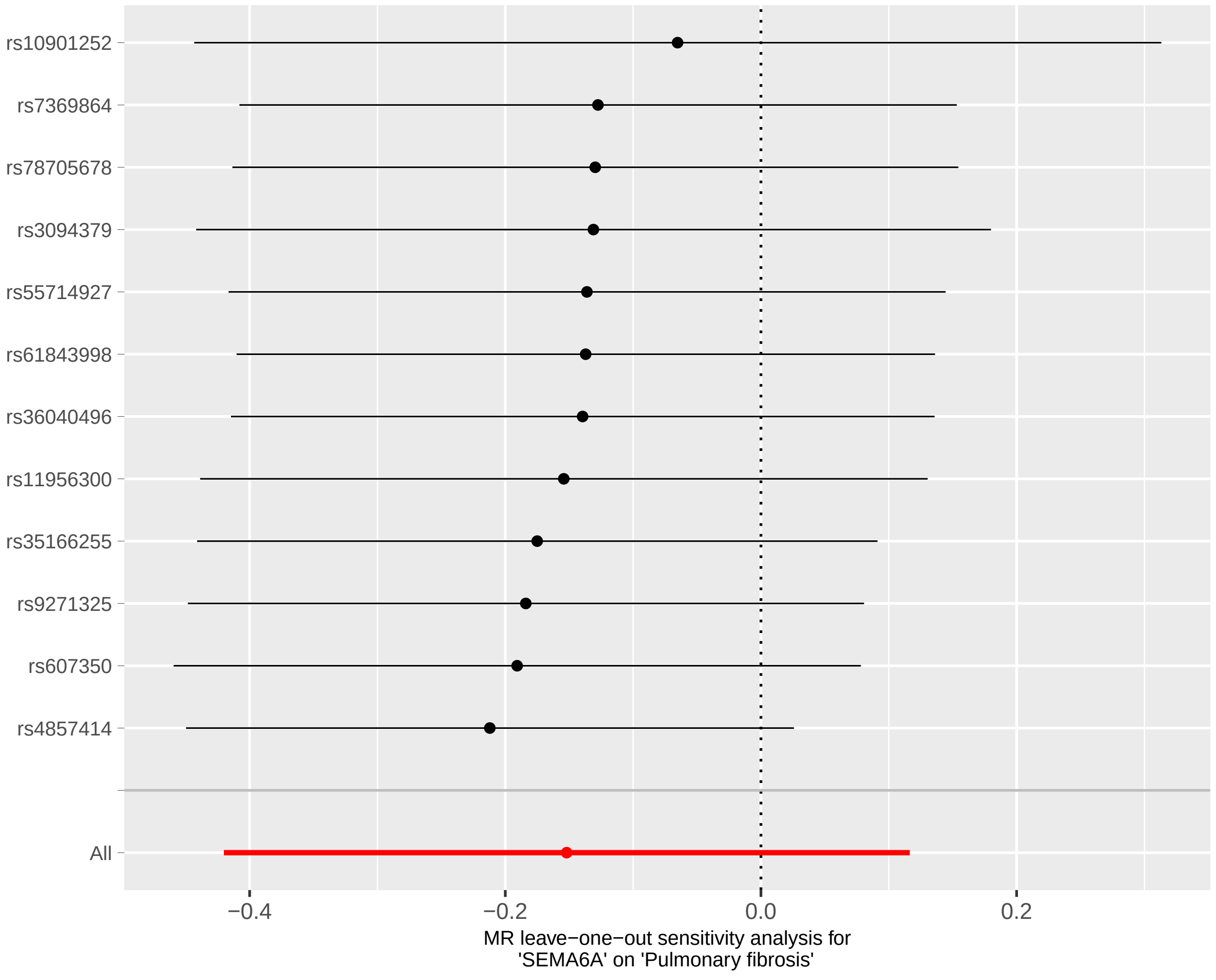
rs10901252
rs7369864
rs78705678
rs3094379
rs55714927
rs61843998
rs36040496
rs11956300
rs35166255
rs9271325
rs607350
rs4857414
All
−0.4
−0.2
0.0
0.2
MR leave−one−out sensitivity analysis for
'SEMA6A' on 'Pulmonary fibrosis'

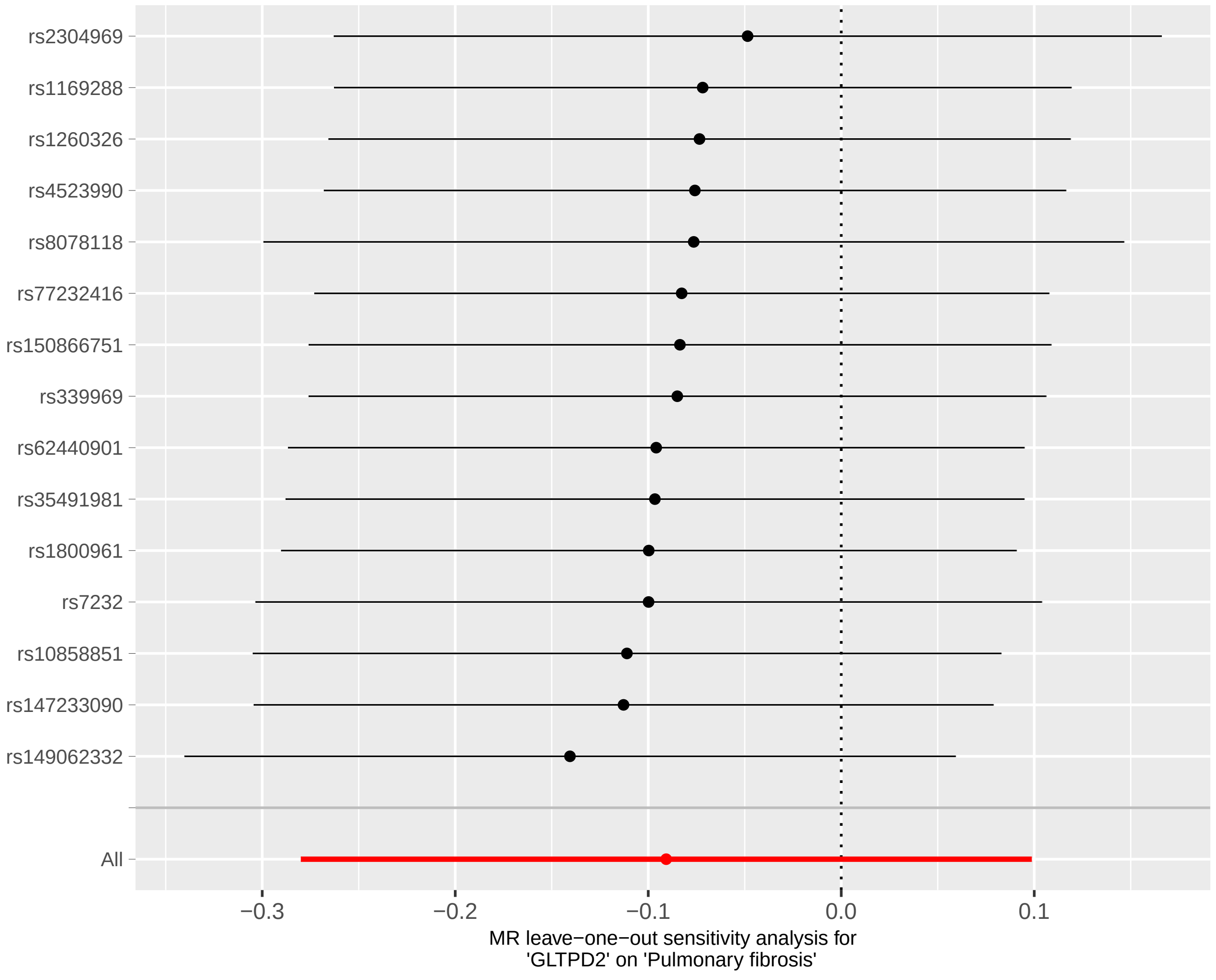
rs2304969
rs1169288
rs1260326
rs4523990
rs8078118
rs77232416
rs150866751
rs339969
rs62440901
rs35491981
rs1800961
rs7232
rs10858851
rs147233090
rs149062332
All
−0.3
−0.2
−0.1
0.0
0.1
MR leave−one−out sensitivity analysis for
'GLTPD2' on 'Pulmonary fibrosis'

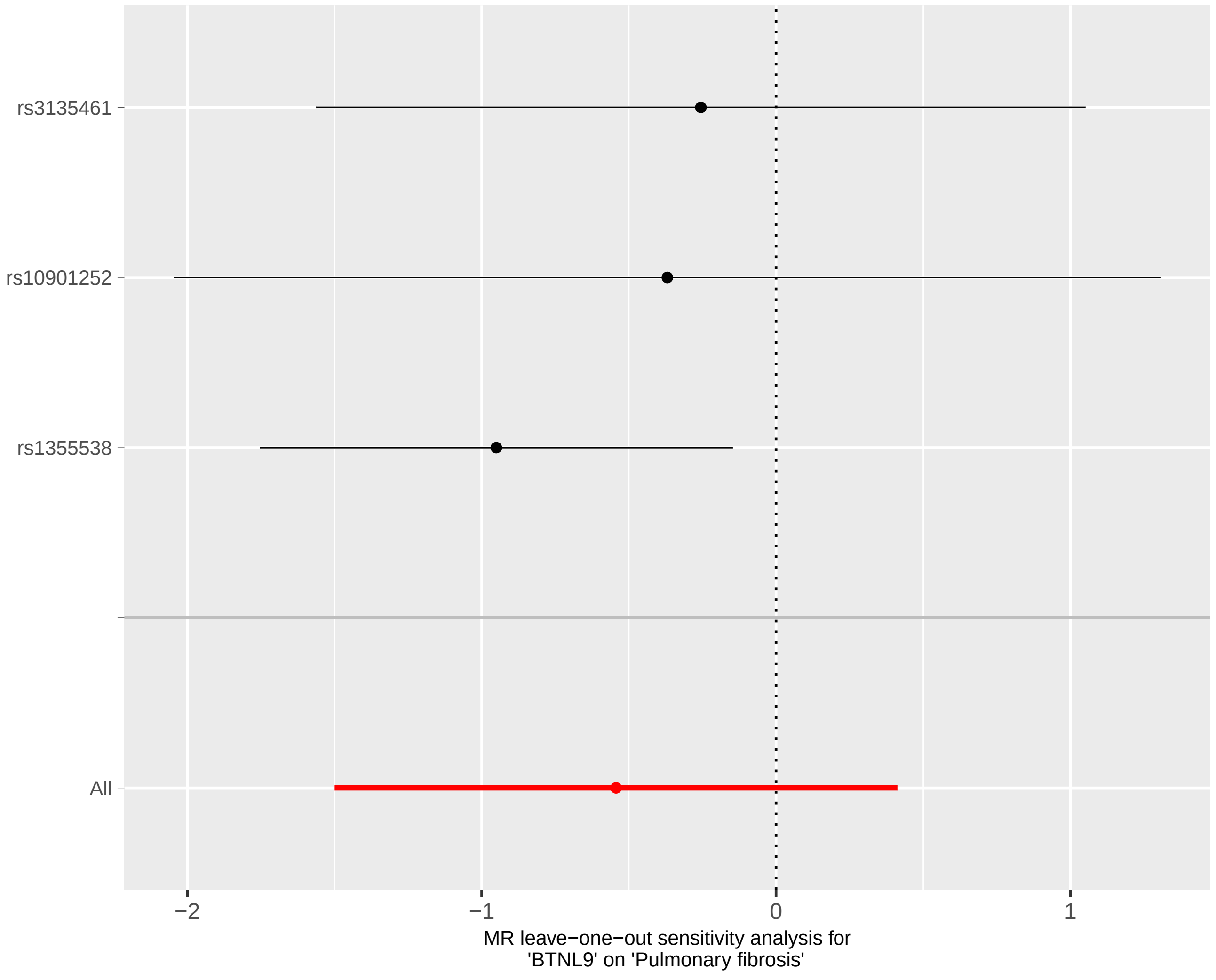
rs3135461
rs10901252
rs1355538
All
−2
−1
0
1
MR leave−one−out sensitivity analysis for
'BTNL9' on 'Pulmonary fibrosis'

Insufficient number of SNPs

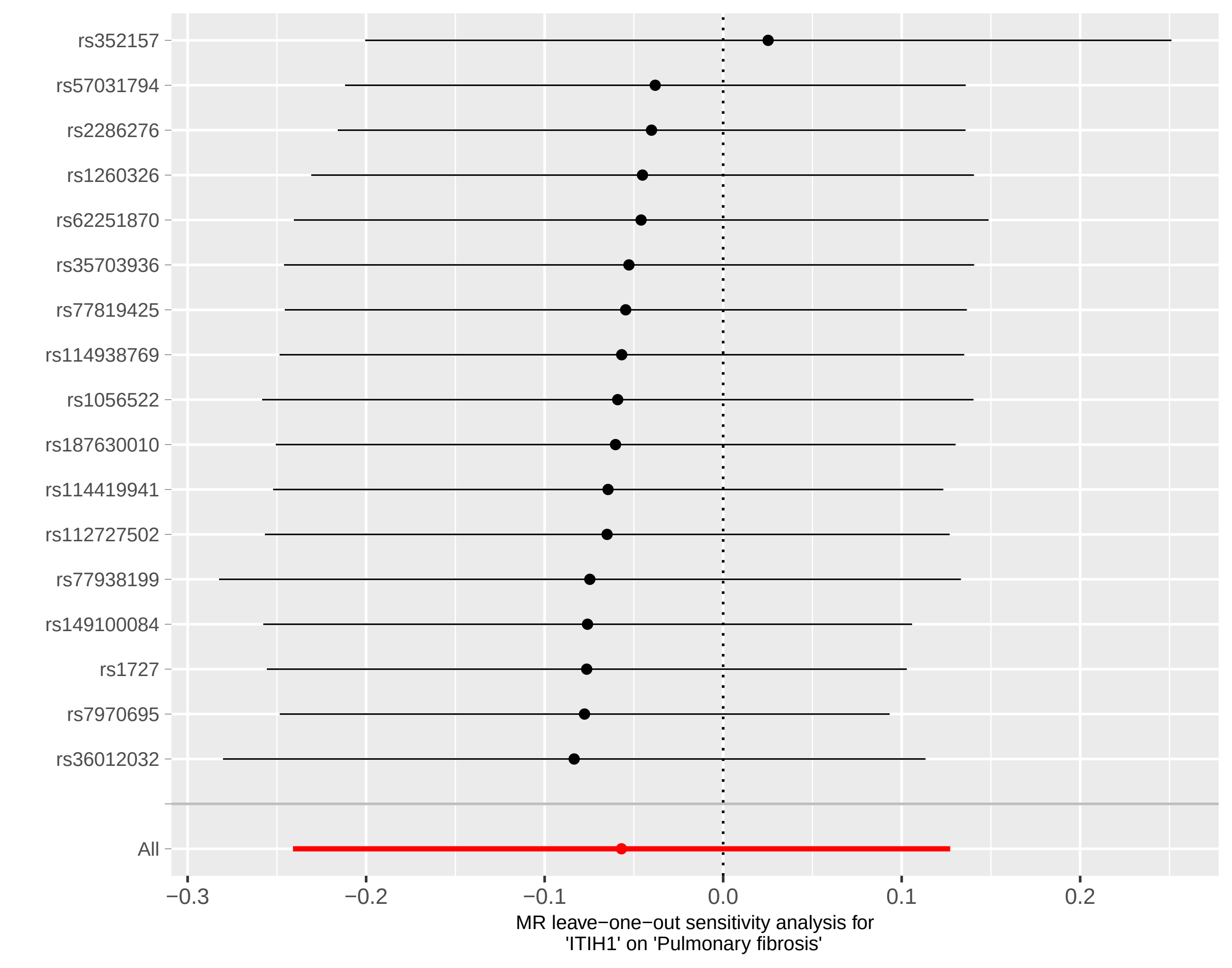
rs352157
rs57031794
rs2286276
rs1260326
rs62251870
rs35703936
rs77819425
rs114938769
rs1056522
rs187630010
rs114419941
rs112727502
rs77938199
rs149100084
rs1727
rs7970695
rs36012032
All
−0.3
−0.2
−0.1
0.0
0.1
0.2
MR leave−one−out sensitivity analysis for
'ITIH1' on 'Pulmonary fibrosis'

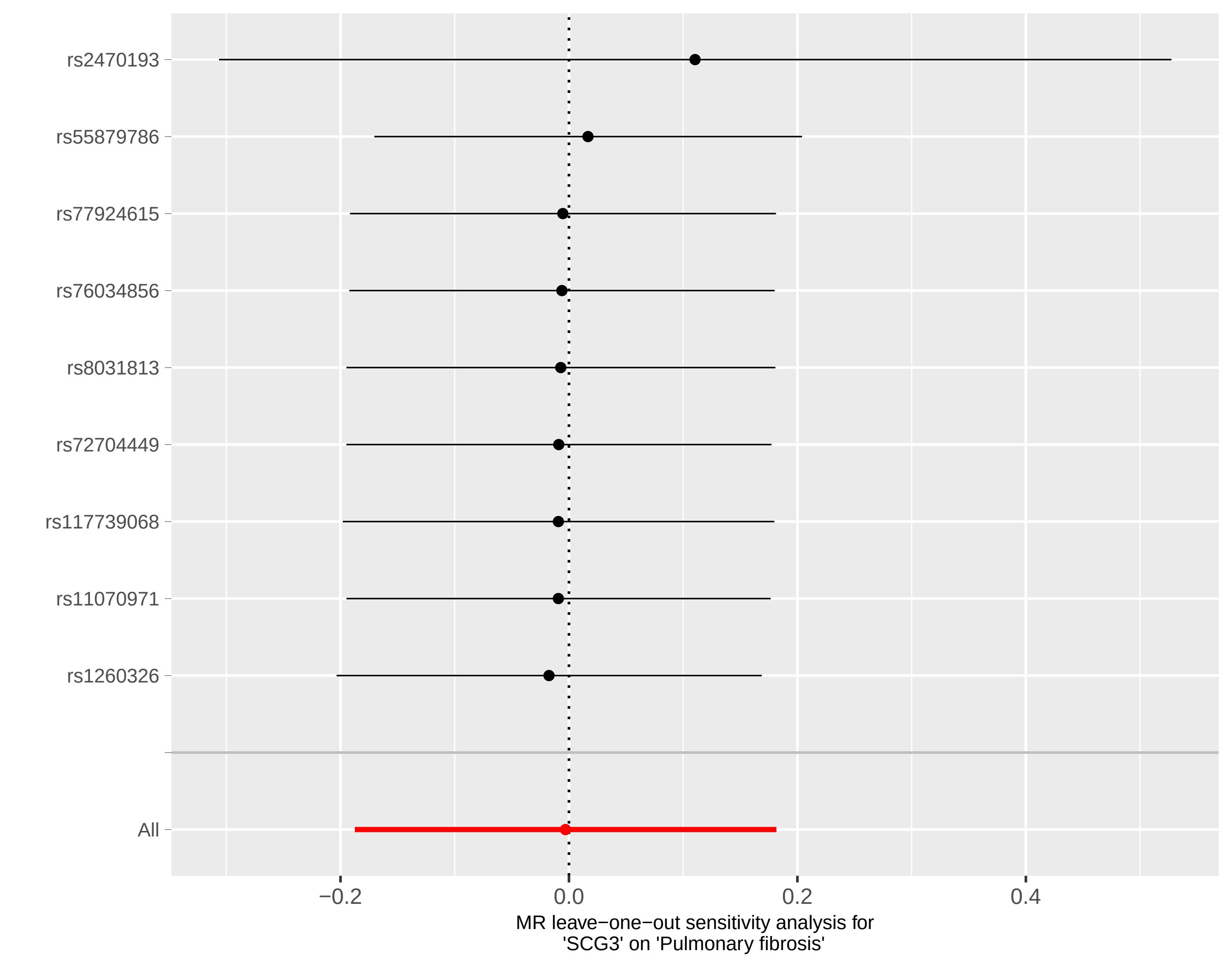
rs2470193
rs55879786
rs77924615
rs76034856
rs8031813
rs72704449
rs117739068
rs11070971
rs1260326
All
−0.2
0.0
0.2
0.4
MR leave−one−out sensitivity analysis for
'SCG3' on 'Pulmonary fibrosis'

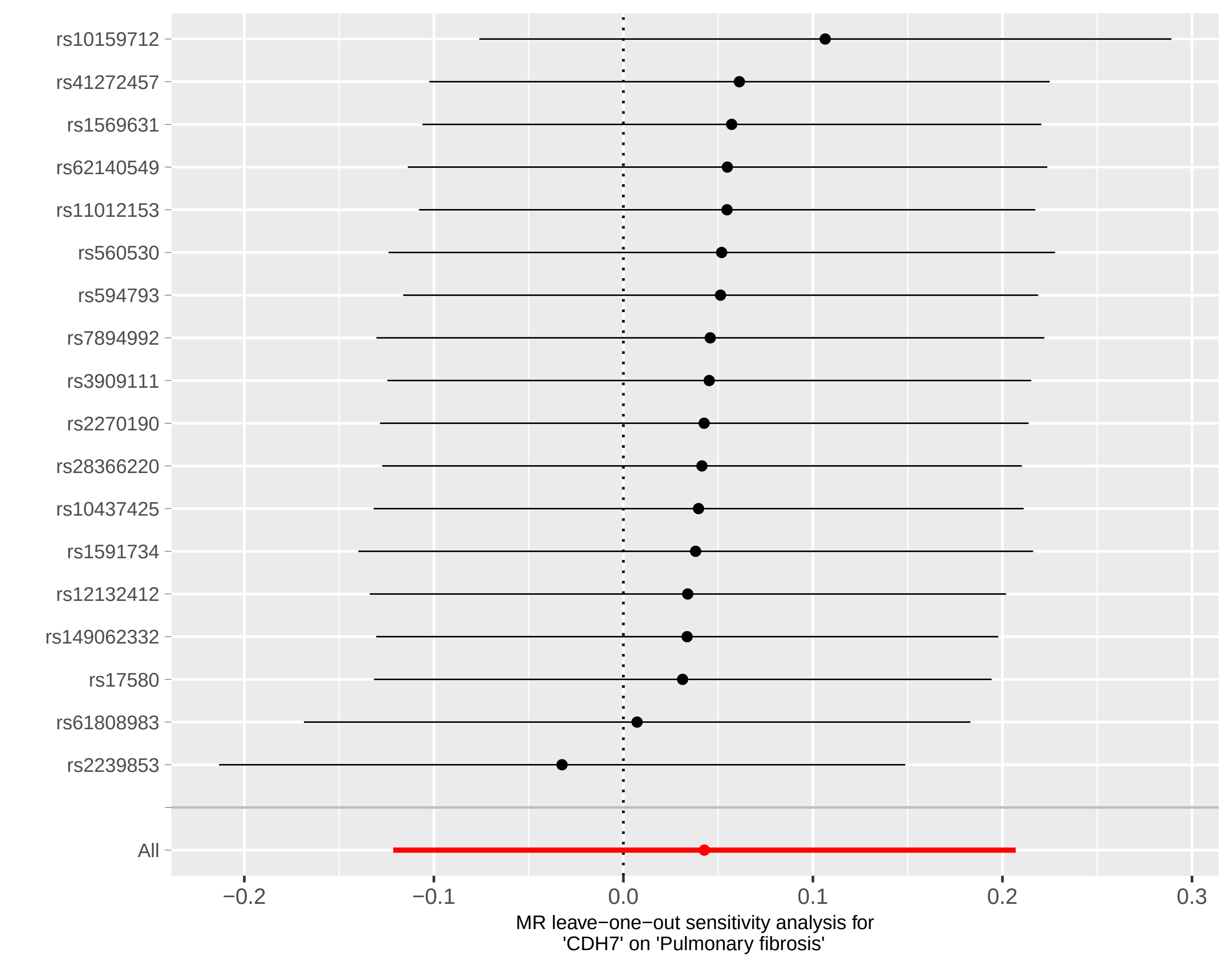
rs10159712
rs41272457
rs1569631
rs62140549
rs11012153
rs560530
rs594793
rs7894992
rs3909111
rs2270190
rs28366220
rs10437425
rs1591734
rs12132412
rs149062332
rs17580
rs61808983
rs2239853
All
−0.2
−0.1
0.0
0.1
0.2
0.3
MR leave−one−out sensitivity analysis for
'CDH7' on 'Pulmonary fibrosis'

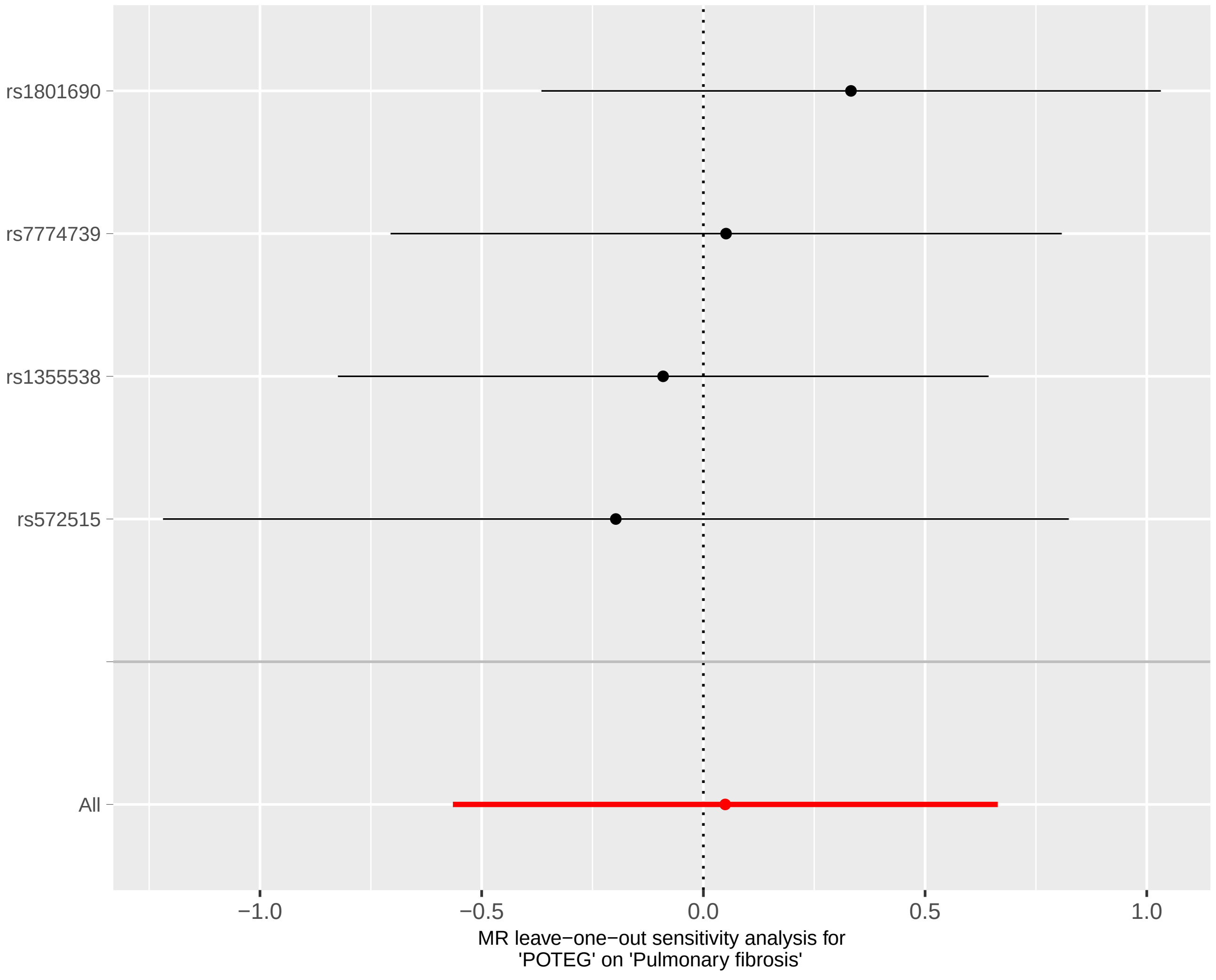
rs1801690
rs7774739
rs1355538
rs572515
All
−1.0
−0.5
0.0
0.5
1.0
MR leave−one−out sensitivity analysis for
'POTEG' on 'Pulmonary fibrosis'

Insufficient number of SNPs

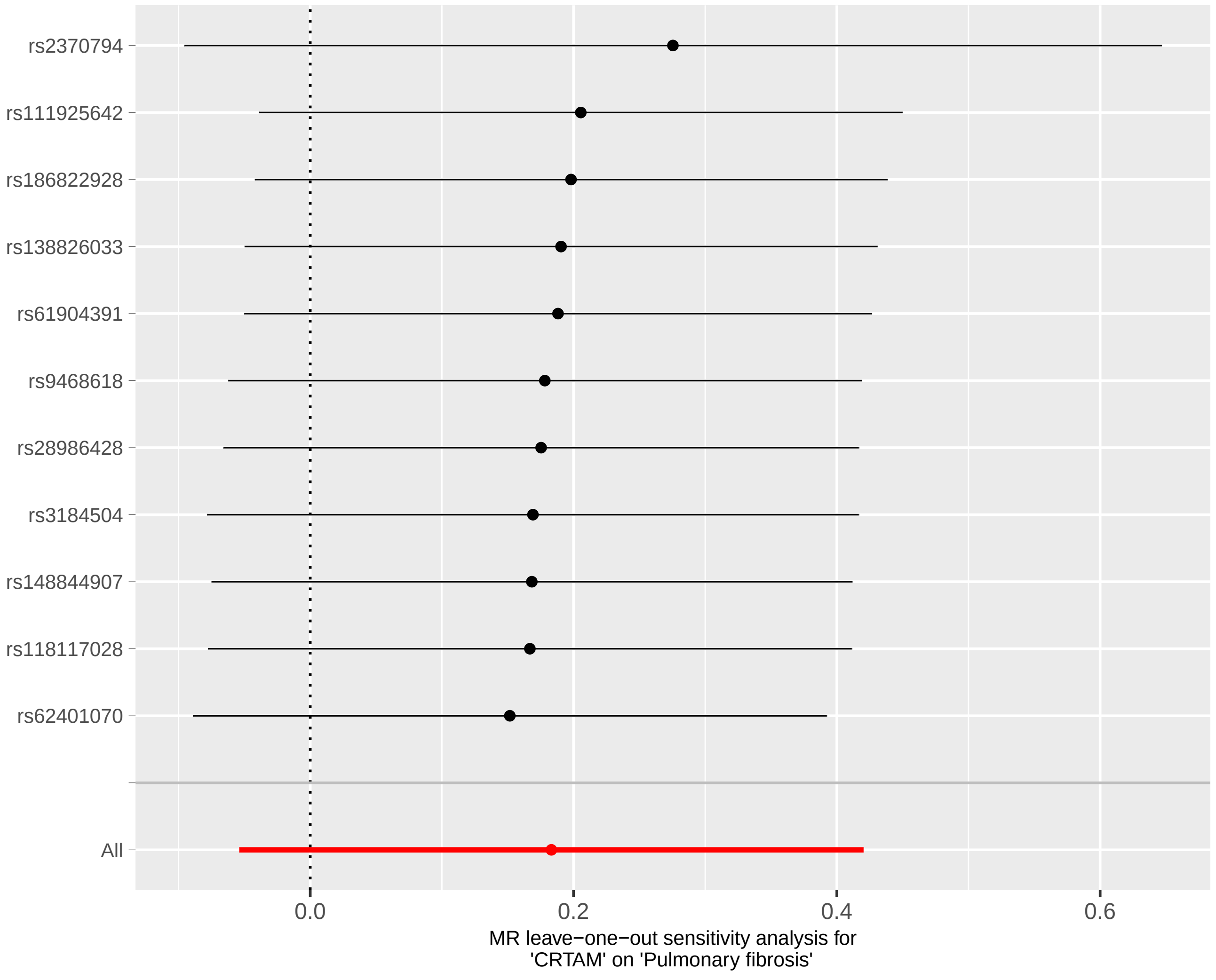
rs2370794
rs111925642
rs186822928
rs138826033
rs61904391
rs9468618
rs28986428
rs3184504
rs148844907
rs118117028
rs62401070
All
0.0
0.2
0.4
0.6
MR leave−one−out sensitivity analysis for
'CRTAM' on 'Pulmonary fibrosis'

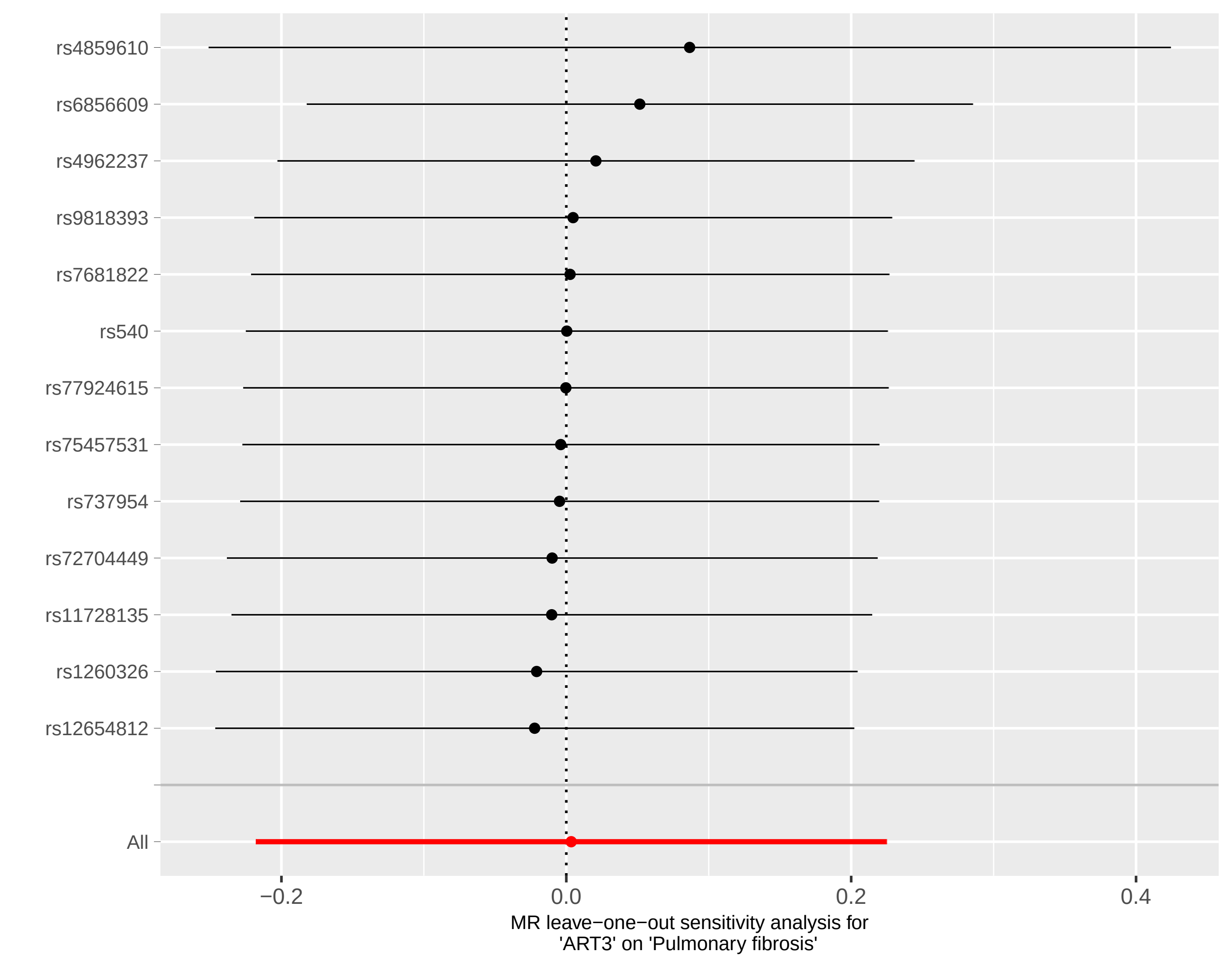
rs4859610
rs6856609
rs4962237
rs9818393
rs7681822
rs540
rs77924615
rs75457531
rs737954
rs72704449
rs11728135
rs1260326
rs12654812
All
−0.2
0.0
0.2
0.4
MR leave−one−out sensitivity analysis for
'ART3' on 'Pulmonary fibrosis'

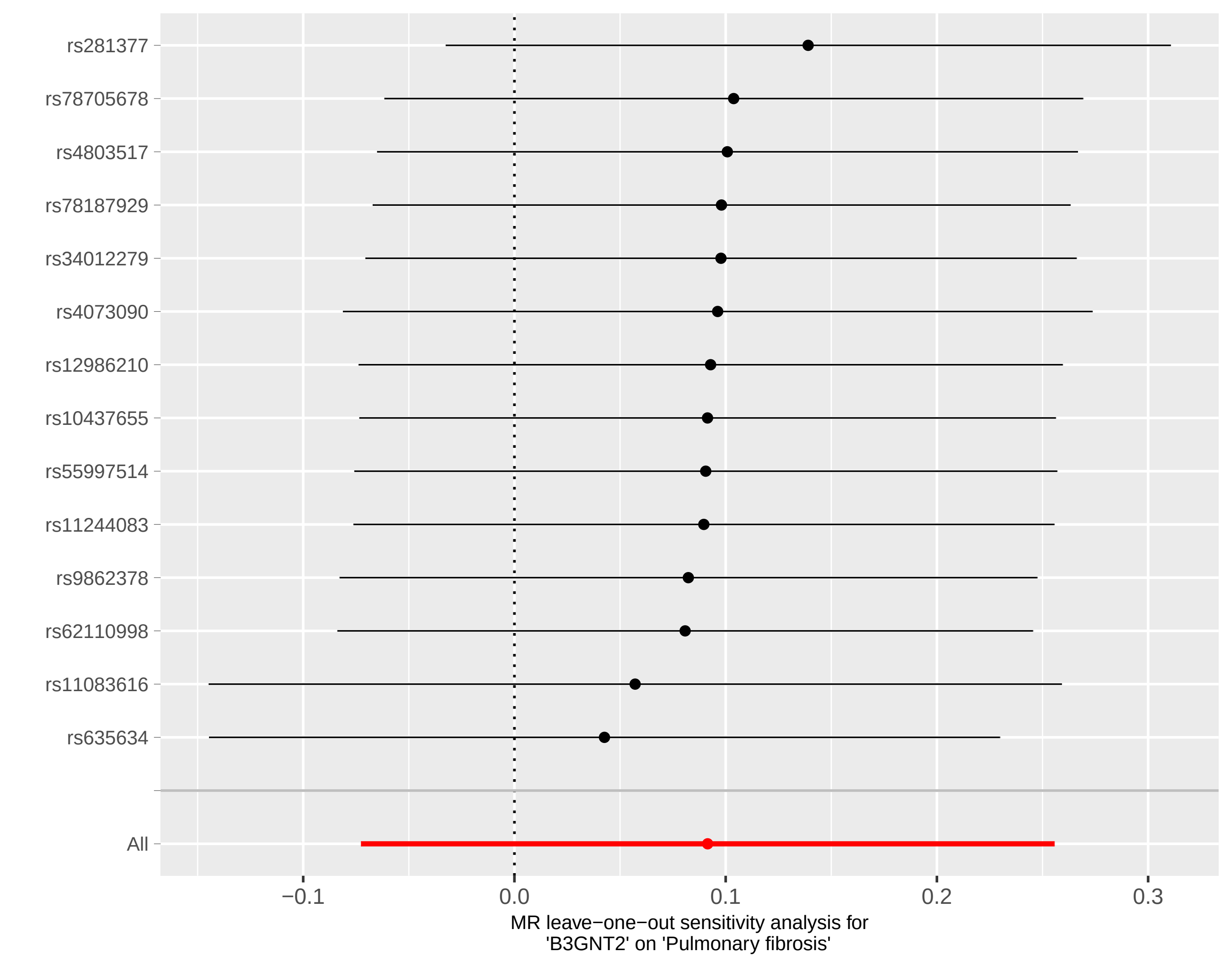
rs281377
rs78705678
rs4803517
rs78187929
rs34012279
rs4073090
rs12986210
rs10437655
rs55997514
rs11244083
rs9862378
rs62110998
rs11083616
rs635634
All
−0.1
0.0
0.1
0.2
0.3
MR leave−one−out sensitivity analysis for
'B3GNT2' on 'Pulmonary fibrosis'

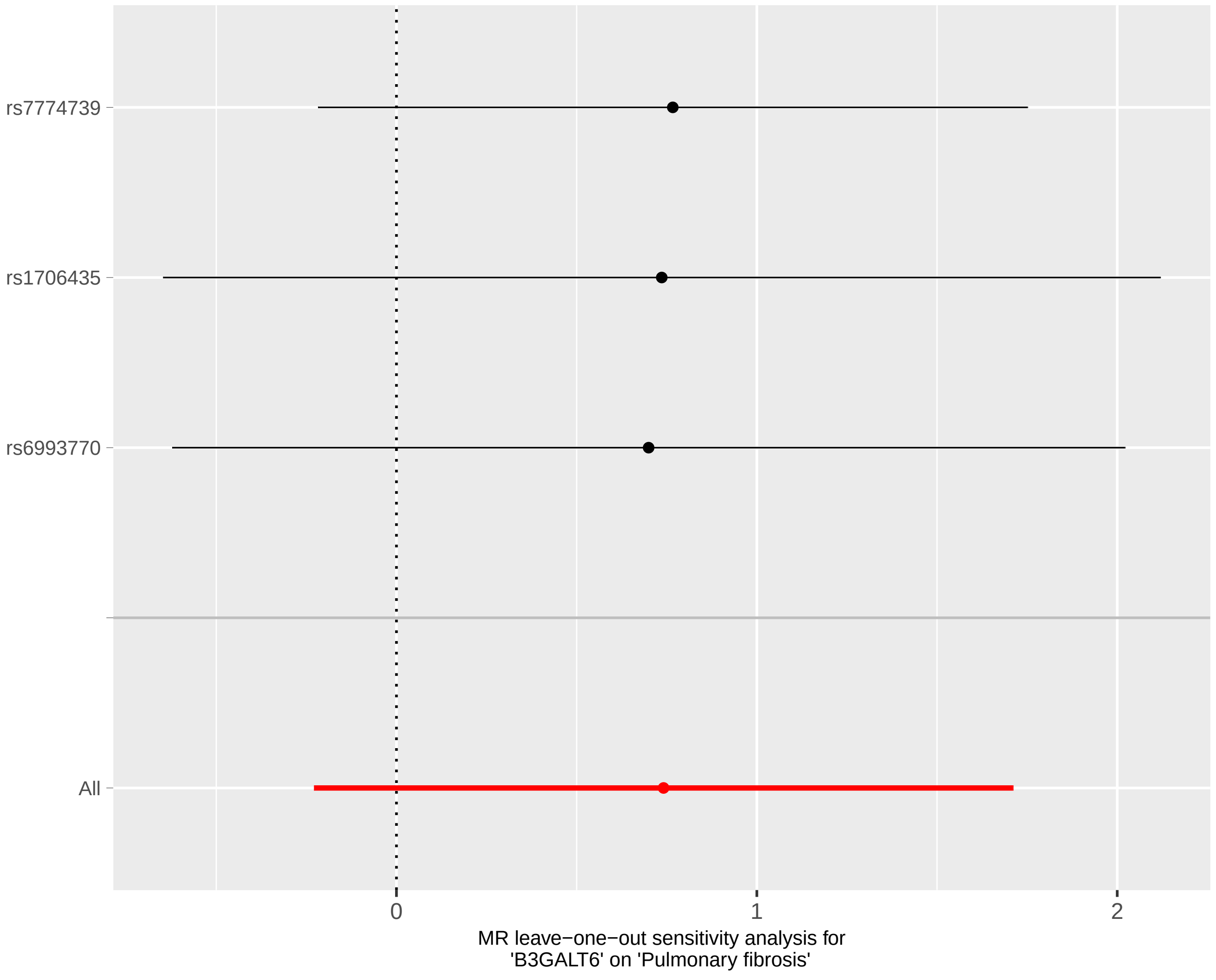
rs7774739
rs1706435
rs6993770
All
0
1
2
MR leave−one−out sensitivity analysis for
'B3GALT6' on 'Pulmonary fibrosis'

Insufficient number of SNPs

Insufficient number of SNPs

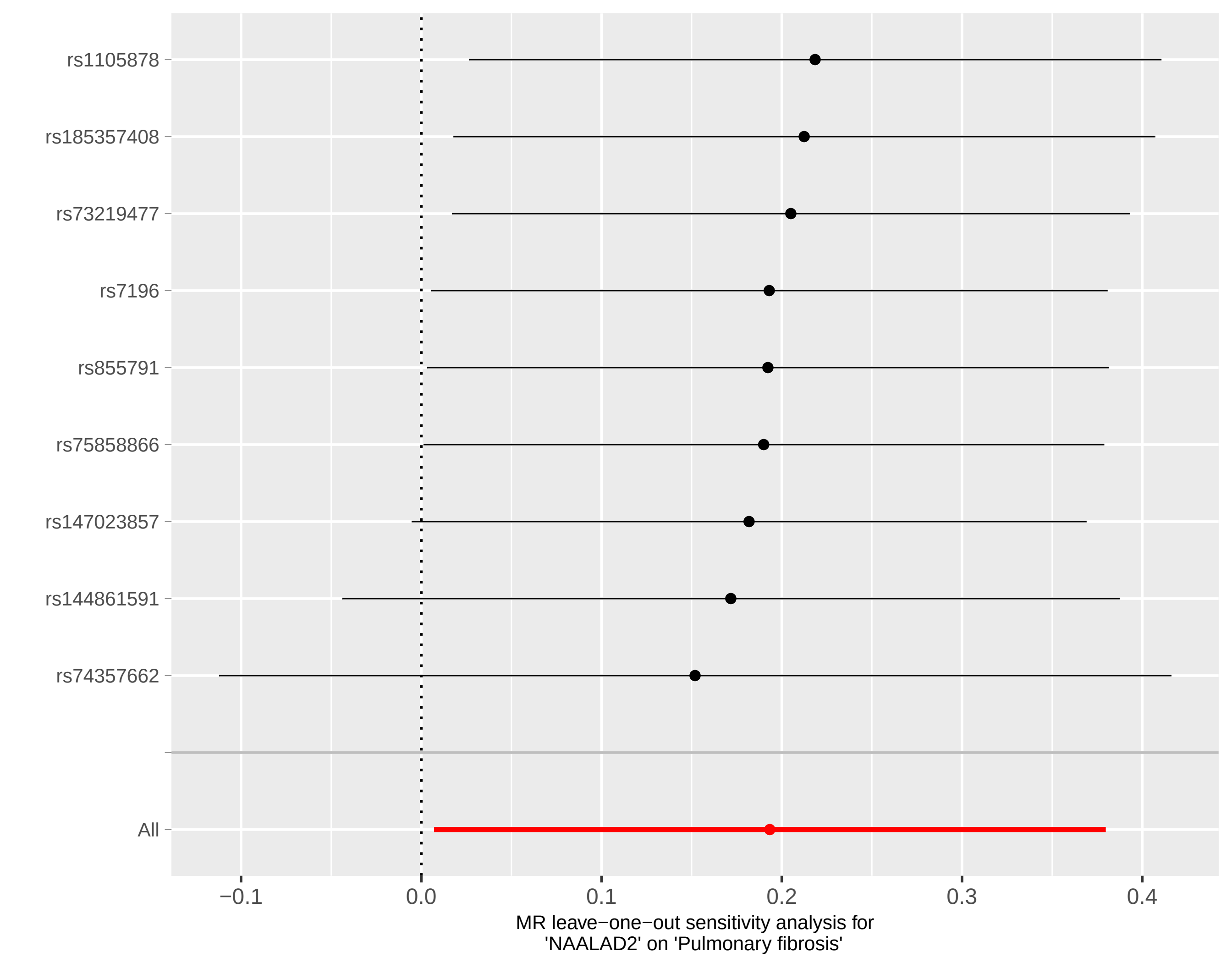
rs1105878
rs185357408
rs73219477
rs7196
rs855791
rs75858866
rs147023857
rs144861591
rs74357662
All
−0.1
0.0
0.1
0.2
0.3
0.4
MR leave−one−out sensitivity analysis for
'NAALAD2' on 'Pulmonary fibrosis'

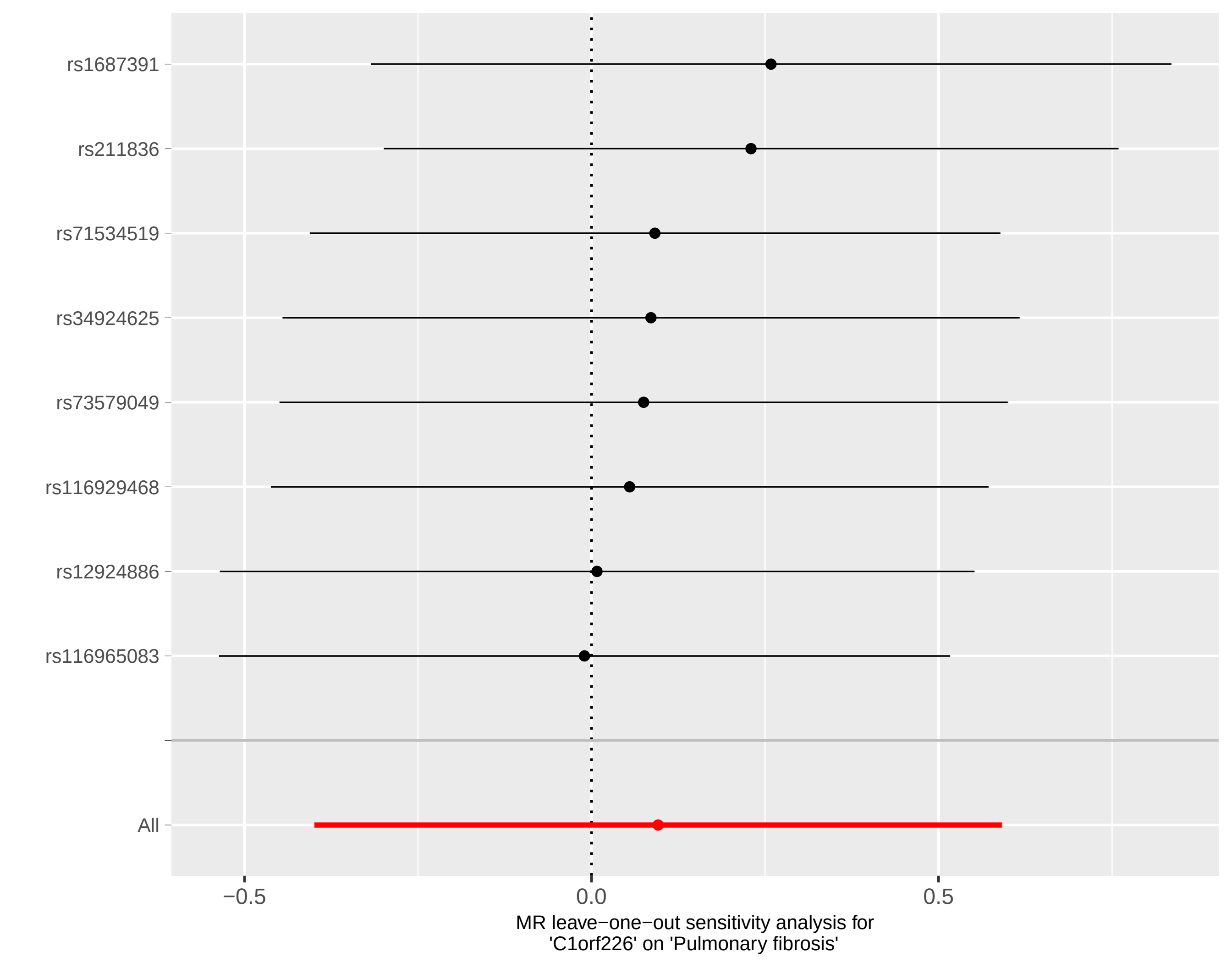

rs1687391
rs211836
rs71534519
rs34924625
rs73579049
rs116929468
rs12924886
rs116965083
All
−0.5
0.0
0.5
MR leave−one−out sensitivity analysis for
'C1orf226' on 'Pulmonary fibrosis'

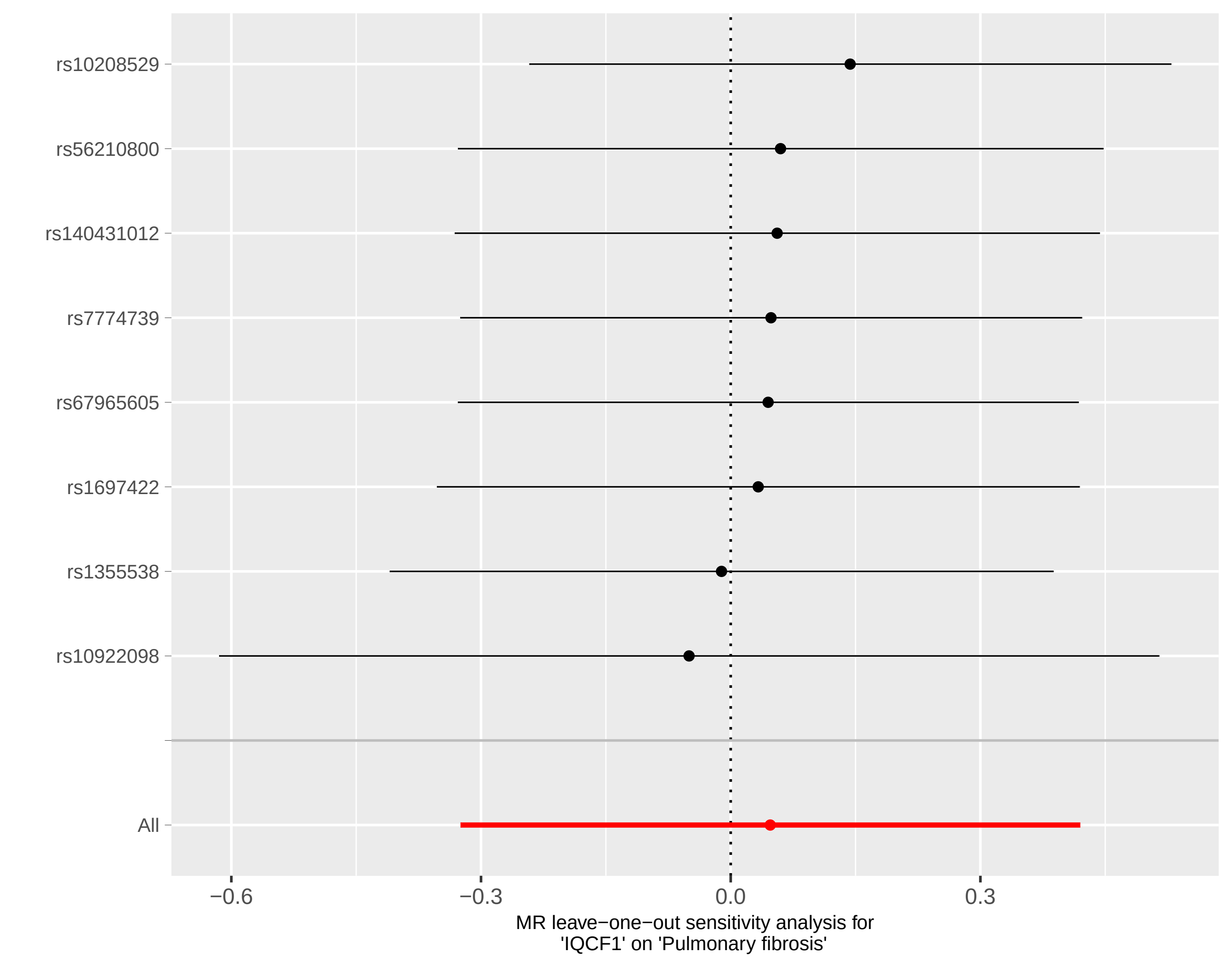
rs10208529
rs56210800
rs140431012
rs7774739
rs67965605
rs1697422
rs1355538
rs10922098
All
−0.6
−0.3
0.0
0.3
MR leave−one−out sensitivity analysis for
'IQCF1' on 'Pulmonary fibrosis'

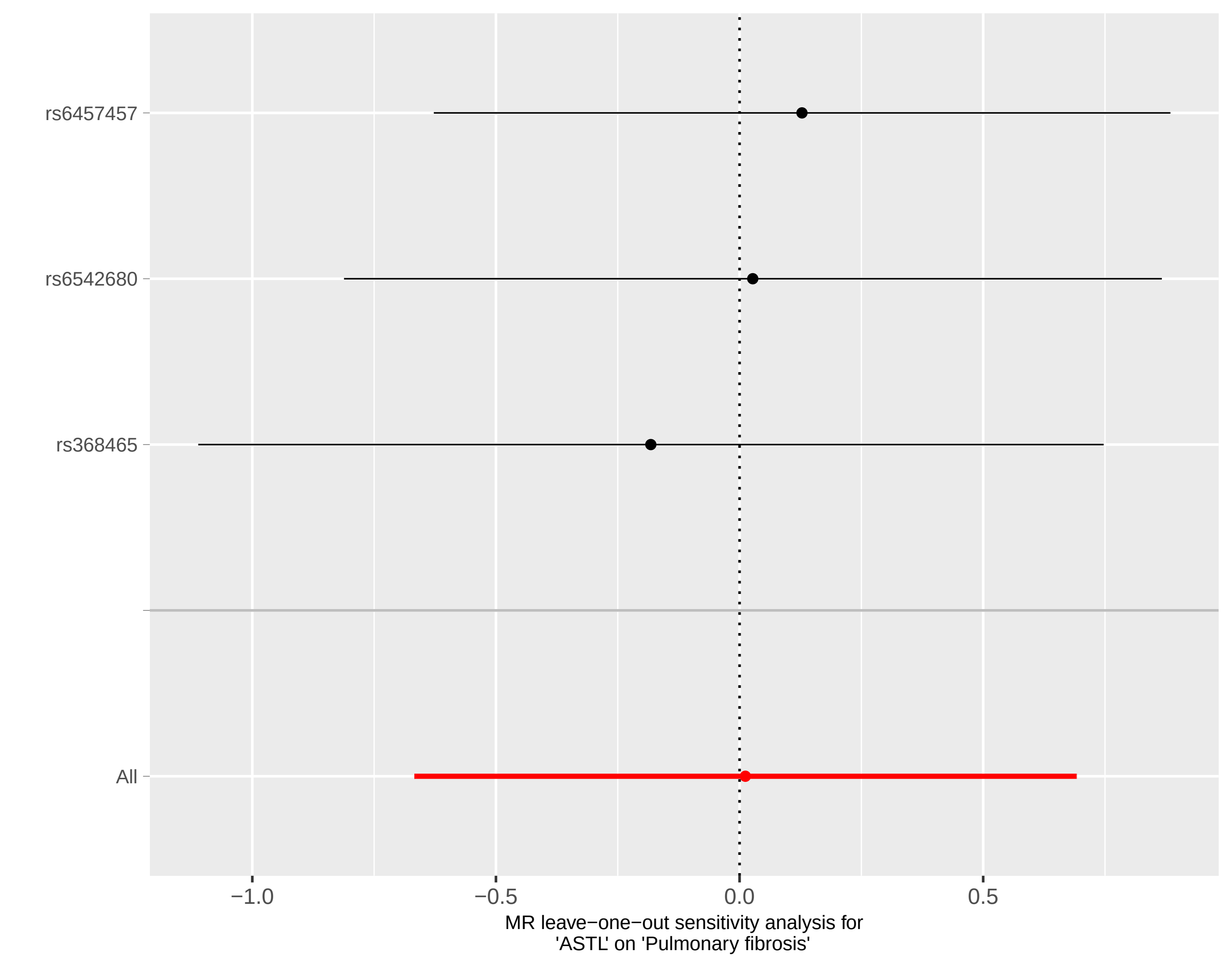
rs6457457
rs6542680
rs368465
All
−1.0
−0.5
0.0
0.5
MR leave−one−out sensitivity analysis for
'ASTL' on 'Pulmonary fibrosis'

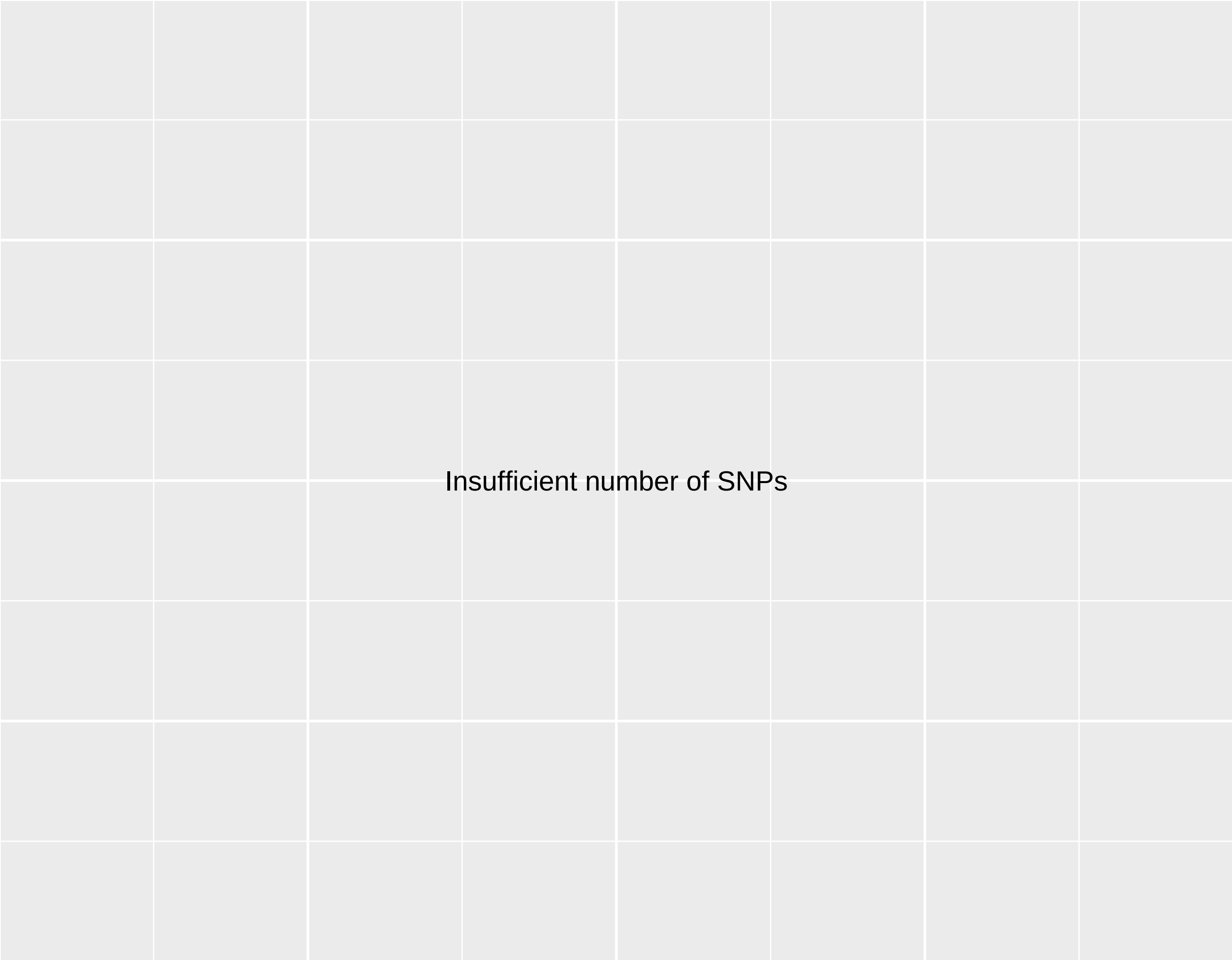
Insufficient number of SNPs

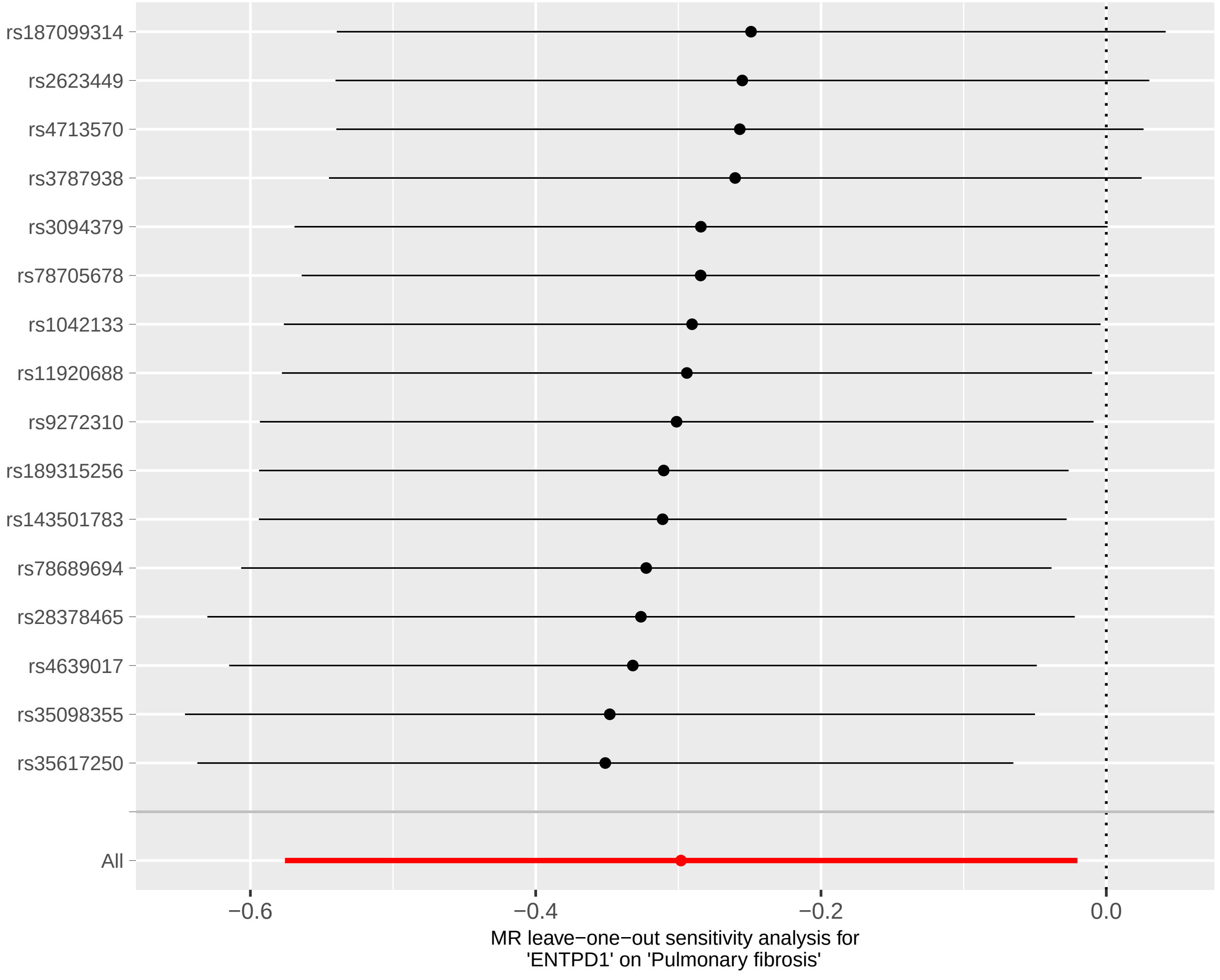
rs187099314
rs2623449
rs4713570
rs3787938
rs3094379
rs78705678
rs1042133
rs11920688
rs9272310
rs189315256
rs143501783
rs78689694
rs28378465
rs4639017
rs35098355
rs35617250
All
−0.6
−0.4
−0.2
0.0
MR leave−one−out sensitivity analysis for
'ENTPD1' on 'Pulmonary fibrosis'

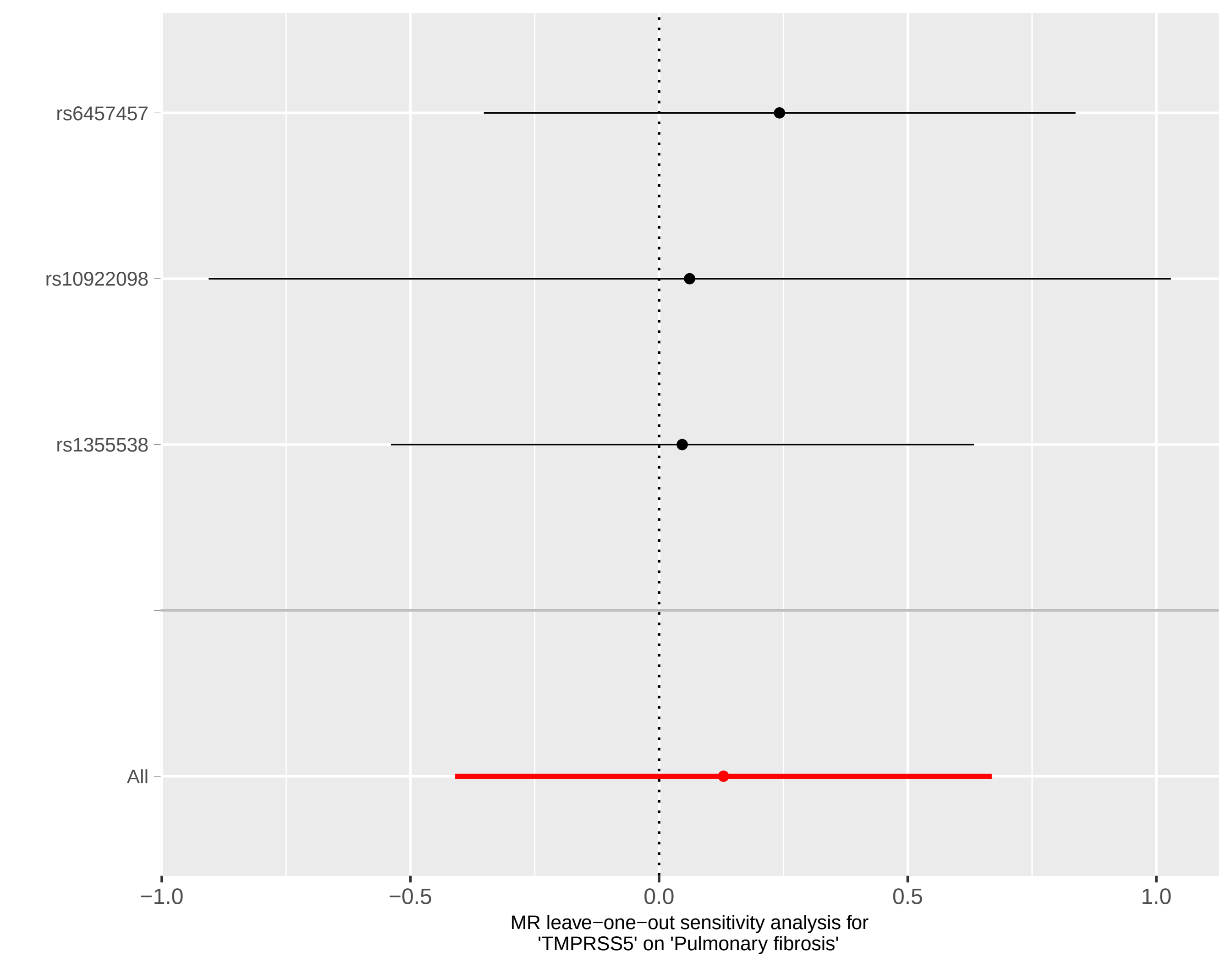
rs6457457
rs10922098
rs1355538
All
−1.0
−0.5
0.0
0.5
1.0
MR leave−one−out sensitivity analysis for
'TMPRSS5' on 'Pulmonary fibrosis'

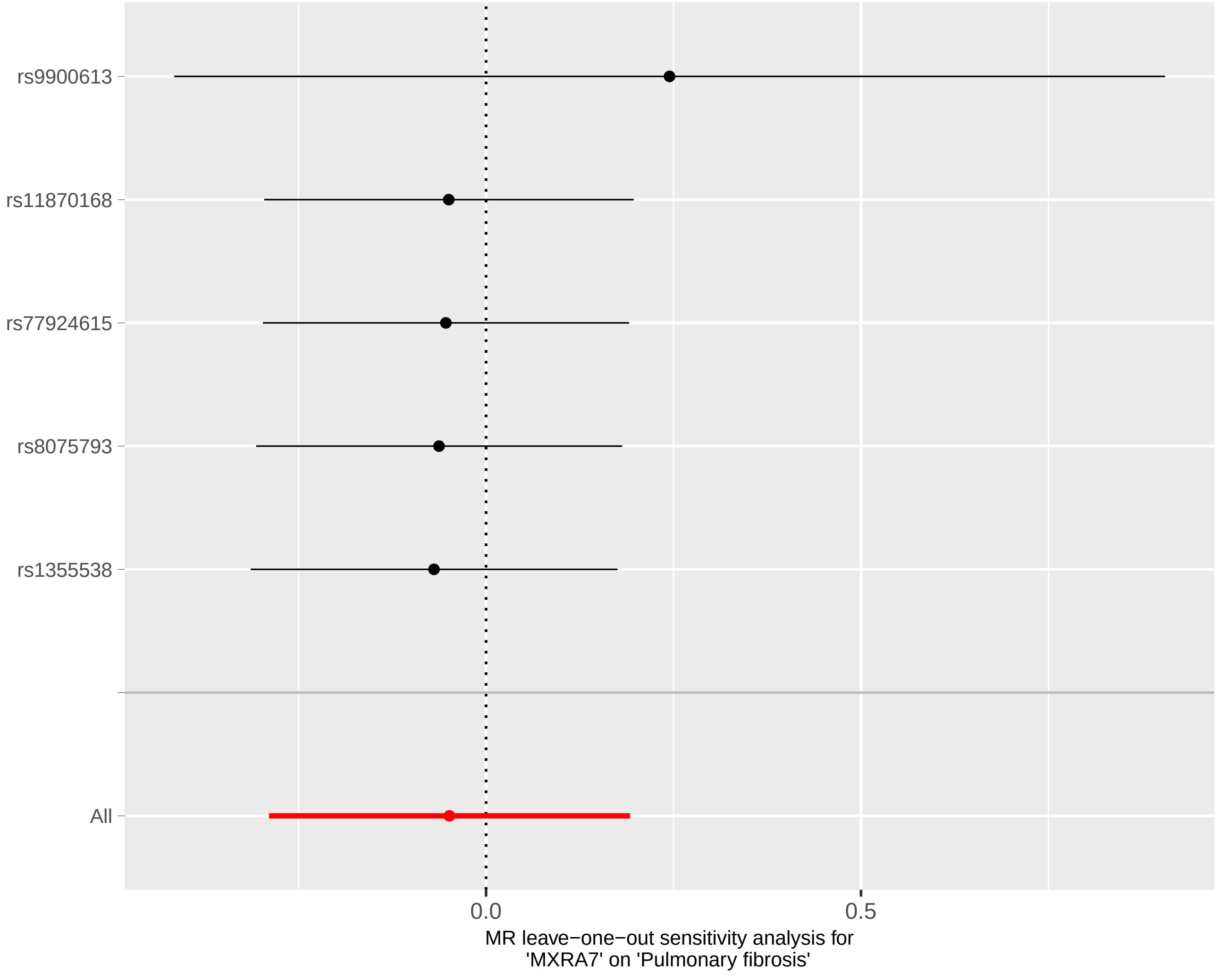
rs9900613
rs11870168
rs77924615
rs8075793
rs1355538
All
0.0
0.5
MR leave−one−out sensitivity analysis for
'MXRA7' on 'Pulmonary fibrosis'

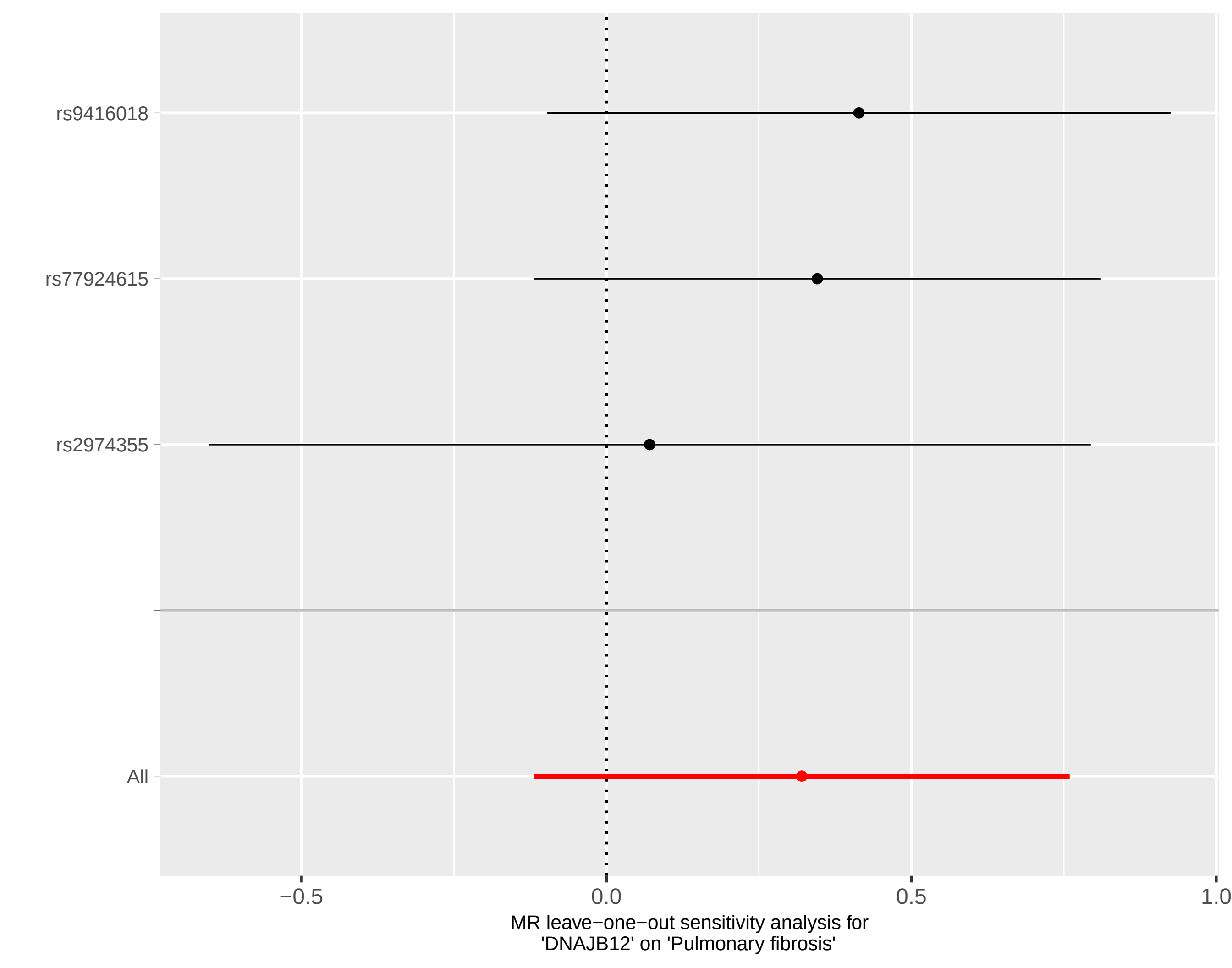
rs9416018
rs77924615
rs2974355
All
−0.5
0.0
0.5
1.0
MR leave−one−out sensitivity analysis for
'DNAJB12' on 'Pulmonary fibrosis'

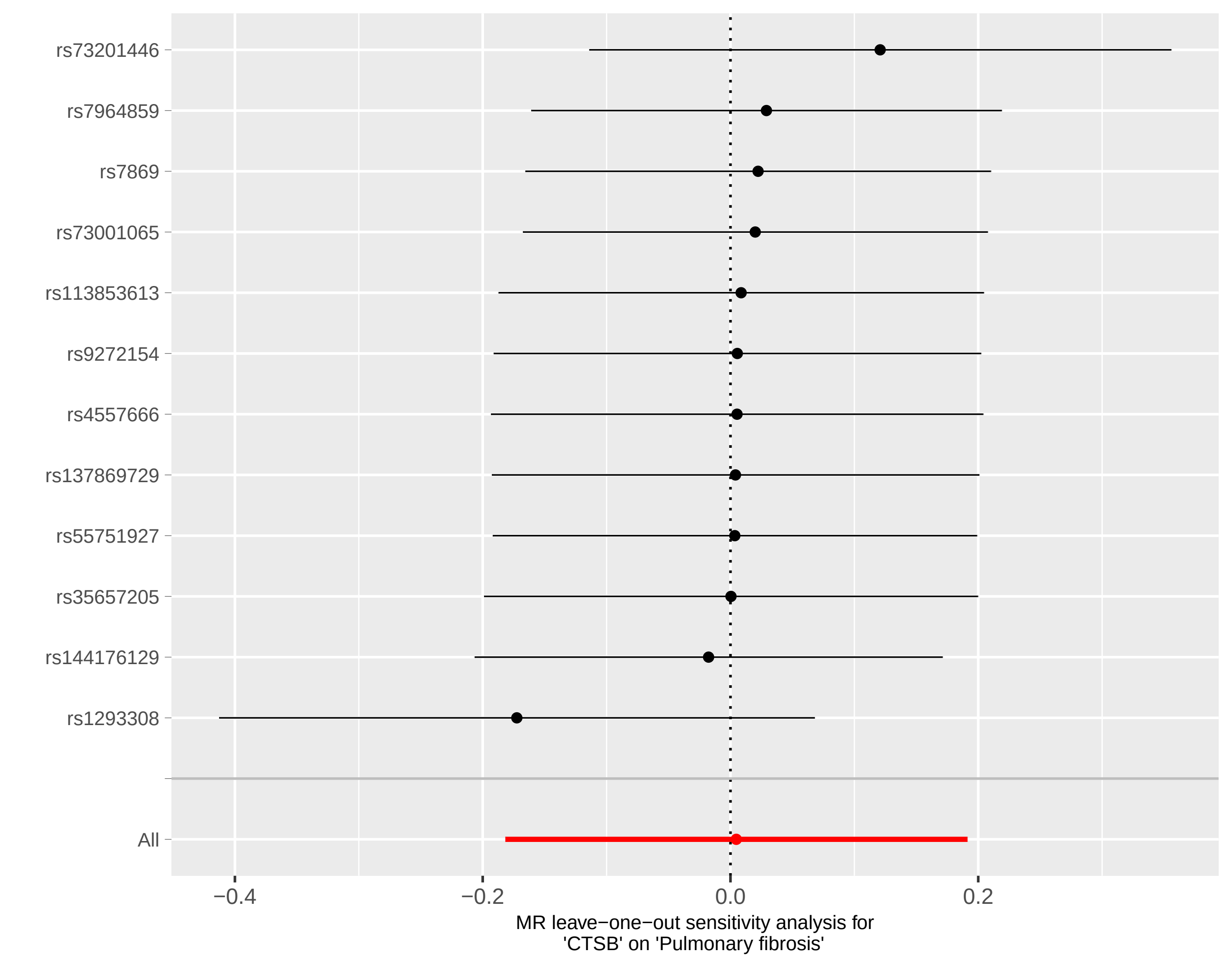
rs73201446
rs7964859
rs7869
rs73001065
rs113853613
rs9272154
rs4557666
rs137869729
rs55751927
rs35657205
rs144176129
rs1293308
All
−0.4
−0.2
0.0
0.2
MR leave−one−out sensitivity analysis for
'CTSB' on 'Pulmonary fibrosis'

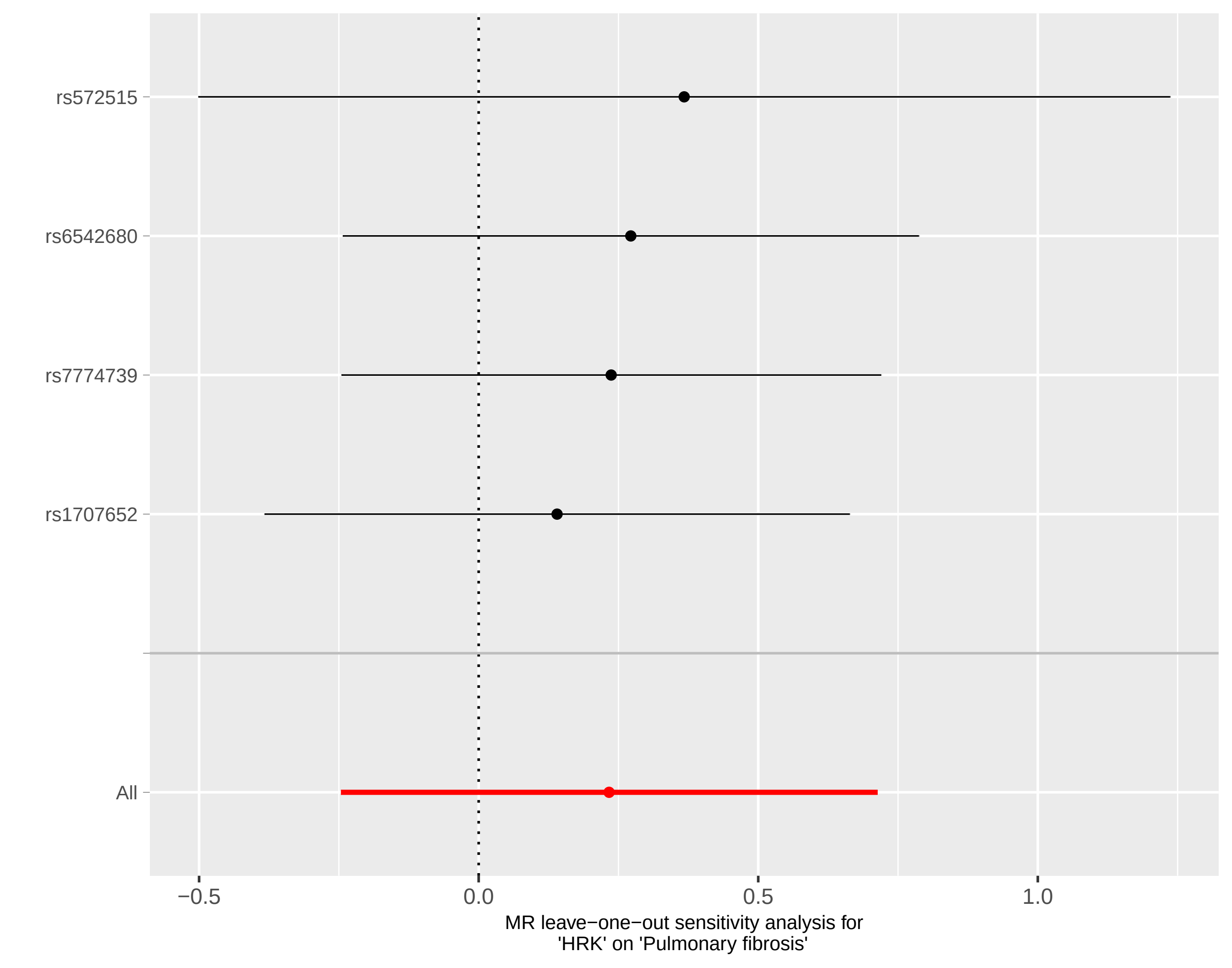
rs572515
rs6542680
rs7774739
rs1707652
All
−0.5
0.0
0.5
1.0
MR leave−one−out sensitivity analysis for
'HRK' on 'Pulmonary fibrosis'

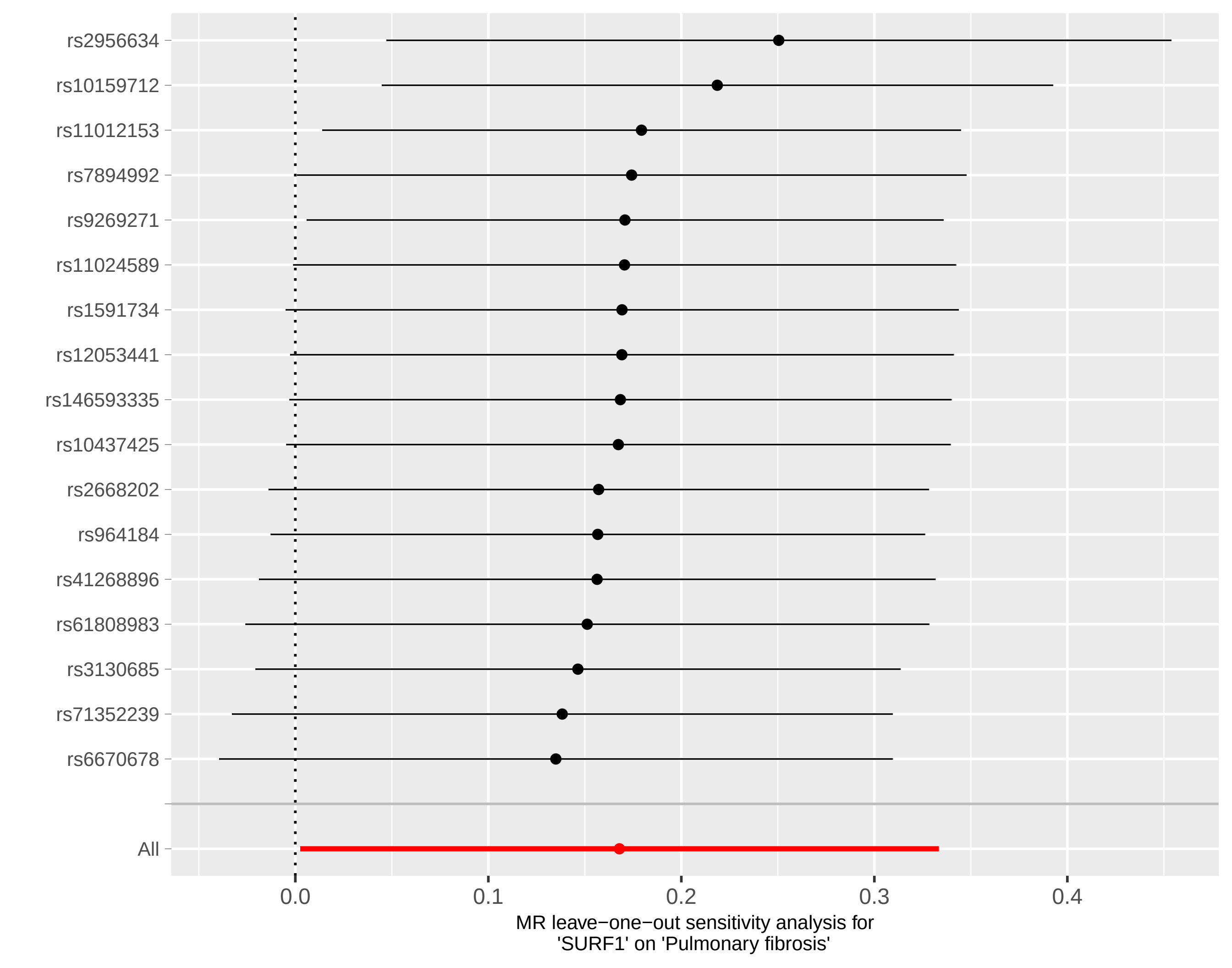

rs2956634
rs10159712
rs11012153
rs7894992
rs9269271
rs11024589
rs1591734
rs12053441
rs146593335
rs10437425
rs2668202
rs964184
rs41268896
rs61808983
rs3130685
rs71352239
rs6670678
All
0.0
0.1
0.2
0.3
0.4
MR leave−one−out sensitivity analysis for
'SURF1' on 'Pulmonary fibrosis'

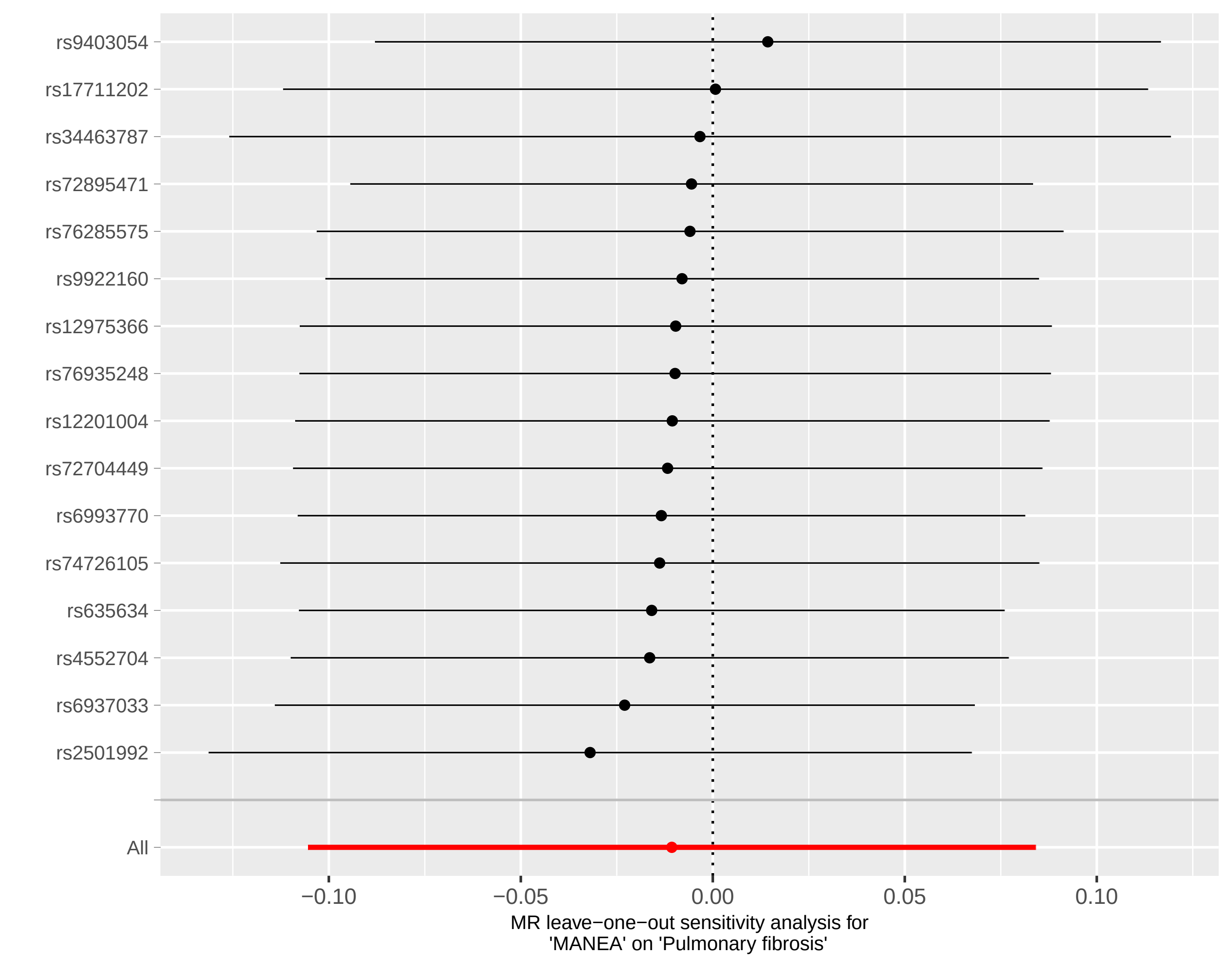
rs9403054
rs17711202
rs34463787
rs72895471
rs76285575
rs9922160
rs12975366
rs76935248
rs12201004
rs72704449
rs6993770
rs74726105
rs635634
rs4552704
rs6937033
rs2501992
All
−0.10
−0.05
0.00
0.05
0.10
MR leave−one−out sensitivity analysis for
'MANEA' on 'Pulmonary fibrosis'

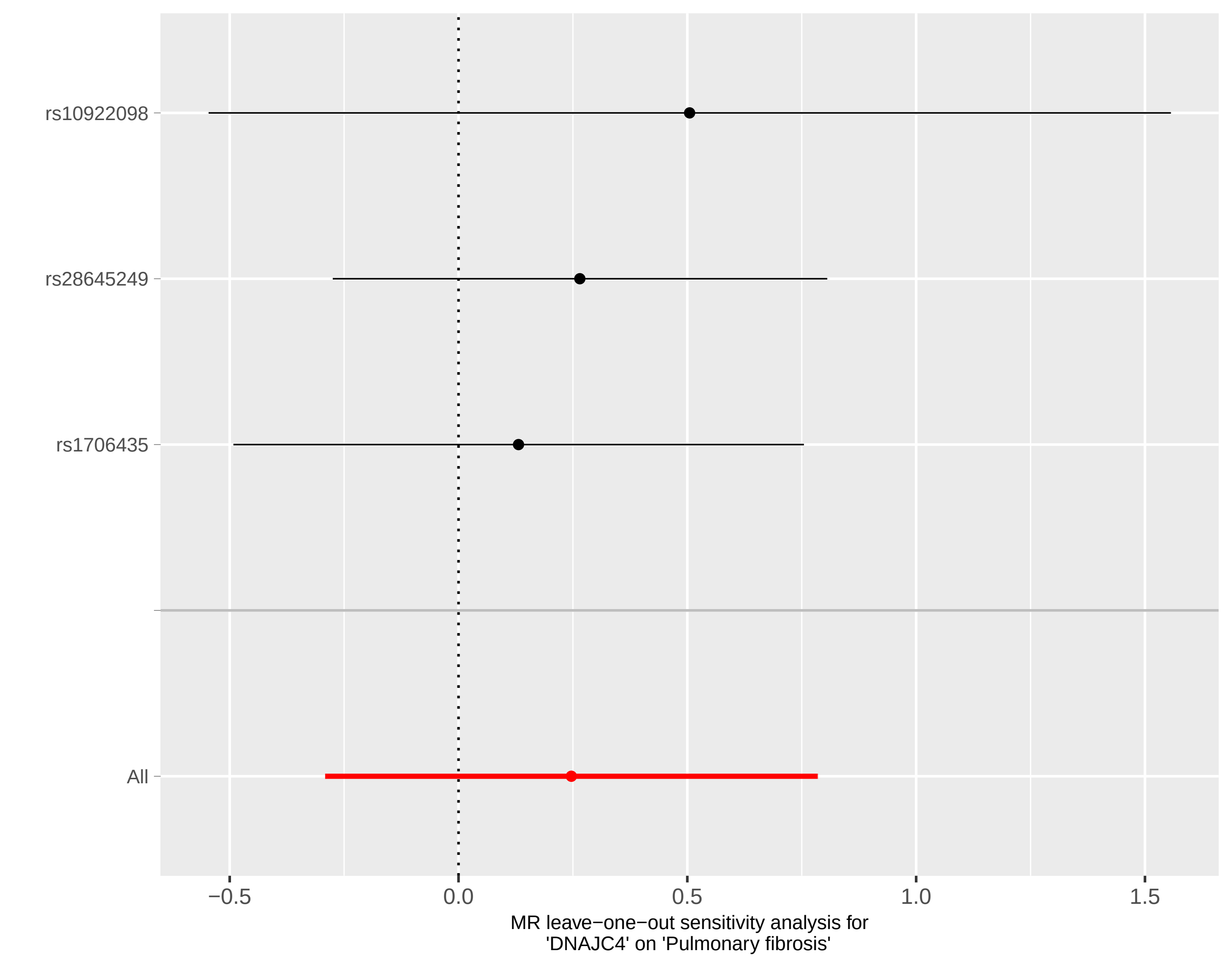
rs10922098
rs28645249
rs1706435
All
−0.5
0.0
0.5
1.0
1.5
MR leave−one−out sensitivity analysis for
'DNAJC4' on 'Pulmonary fibrosis'

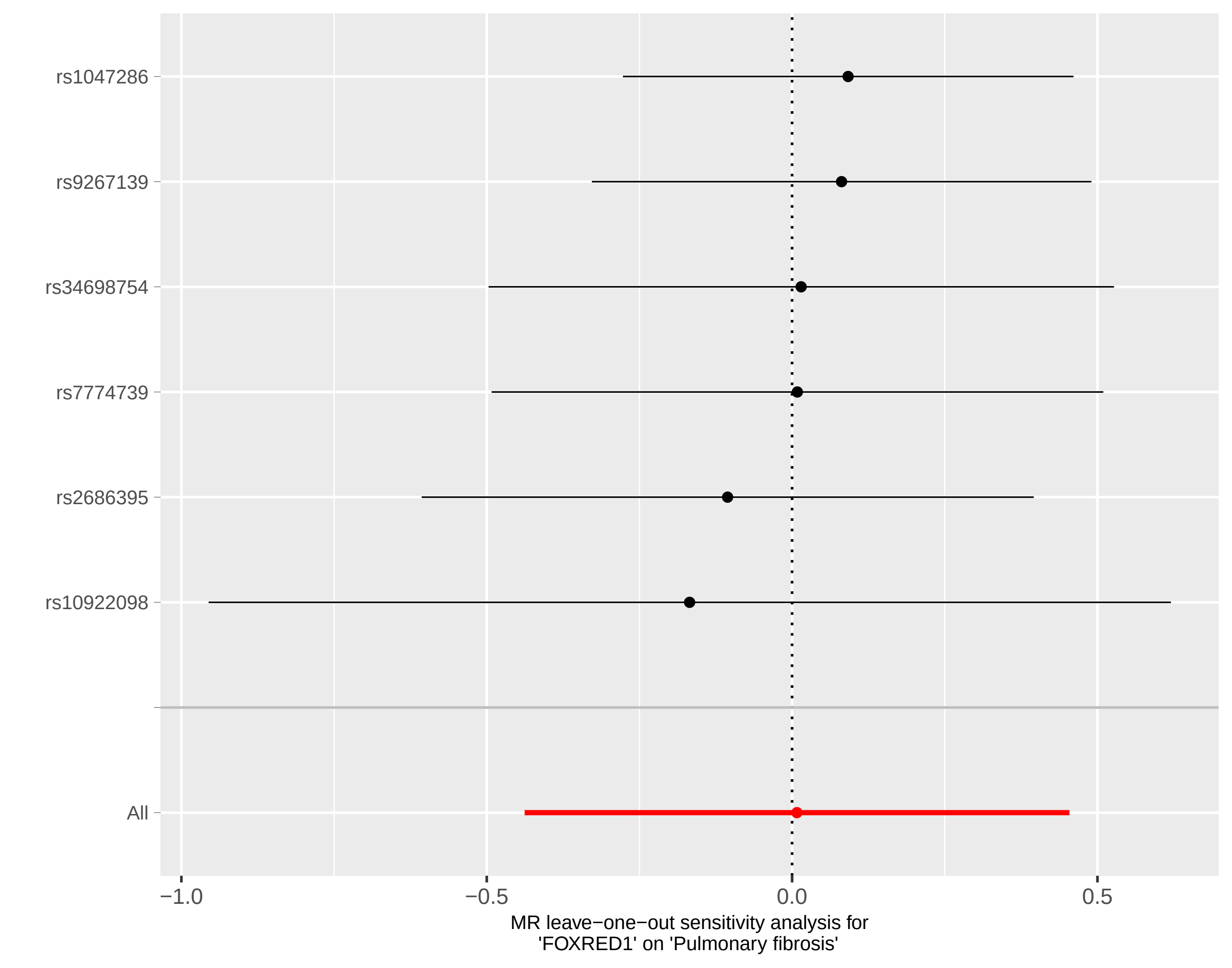
rs1047286
rs9267139
rs34698754
rs7774739
rs2686395
rs10922098
All
−1.0
−0.5
0.0
0.5
MR leave−one−out sensitivity analysis for
'FOXRED1' on 'Pulmonary fibrosis'

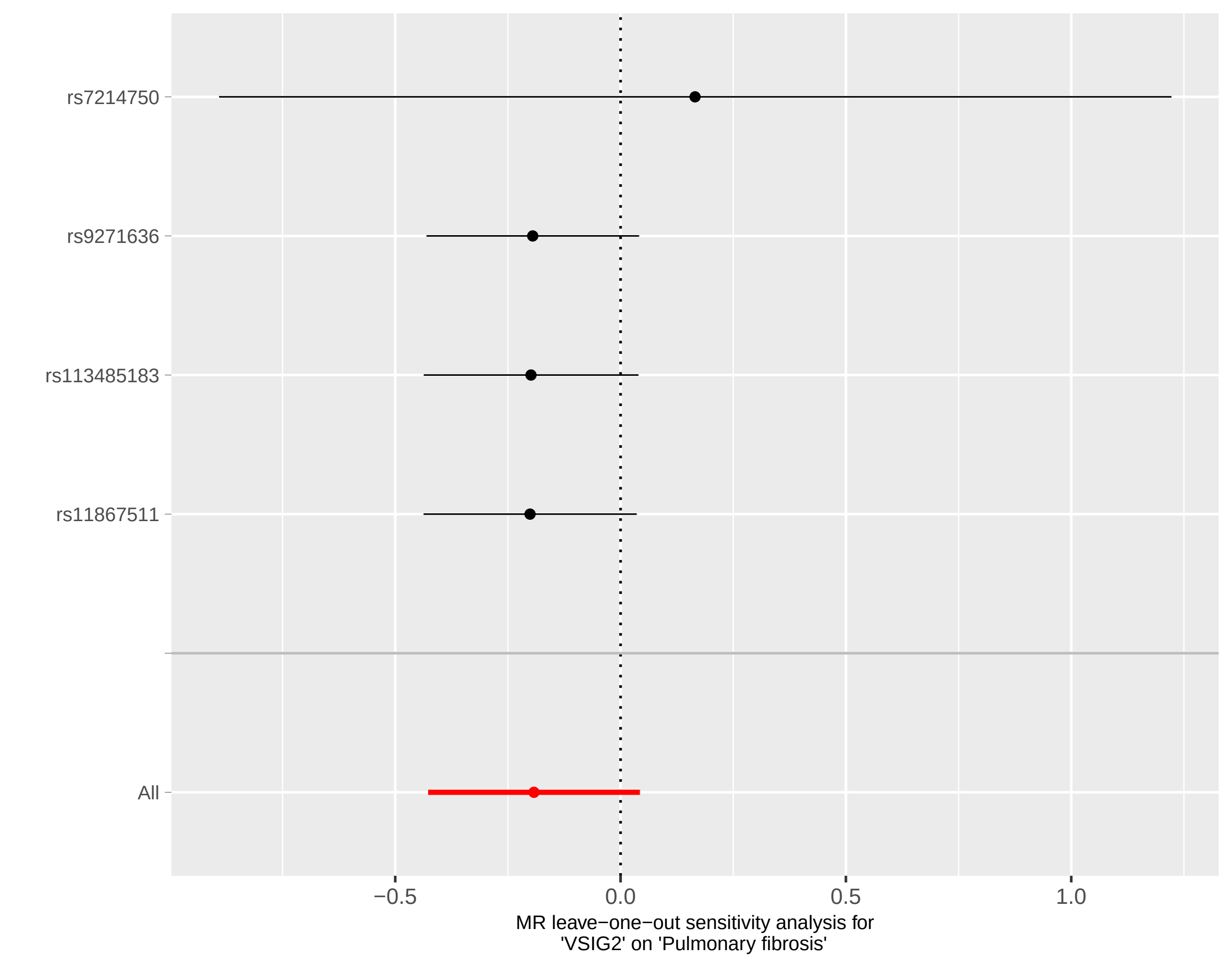

rs7214750
rs9271636
rs113485183
rs11867511
All
−0.5
0.0
0.5
1.0
MR leave−one−out sensitivity analysis for
'VSIG2' on 'Pulmonary fibrosis'

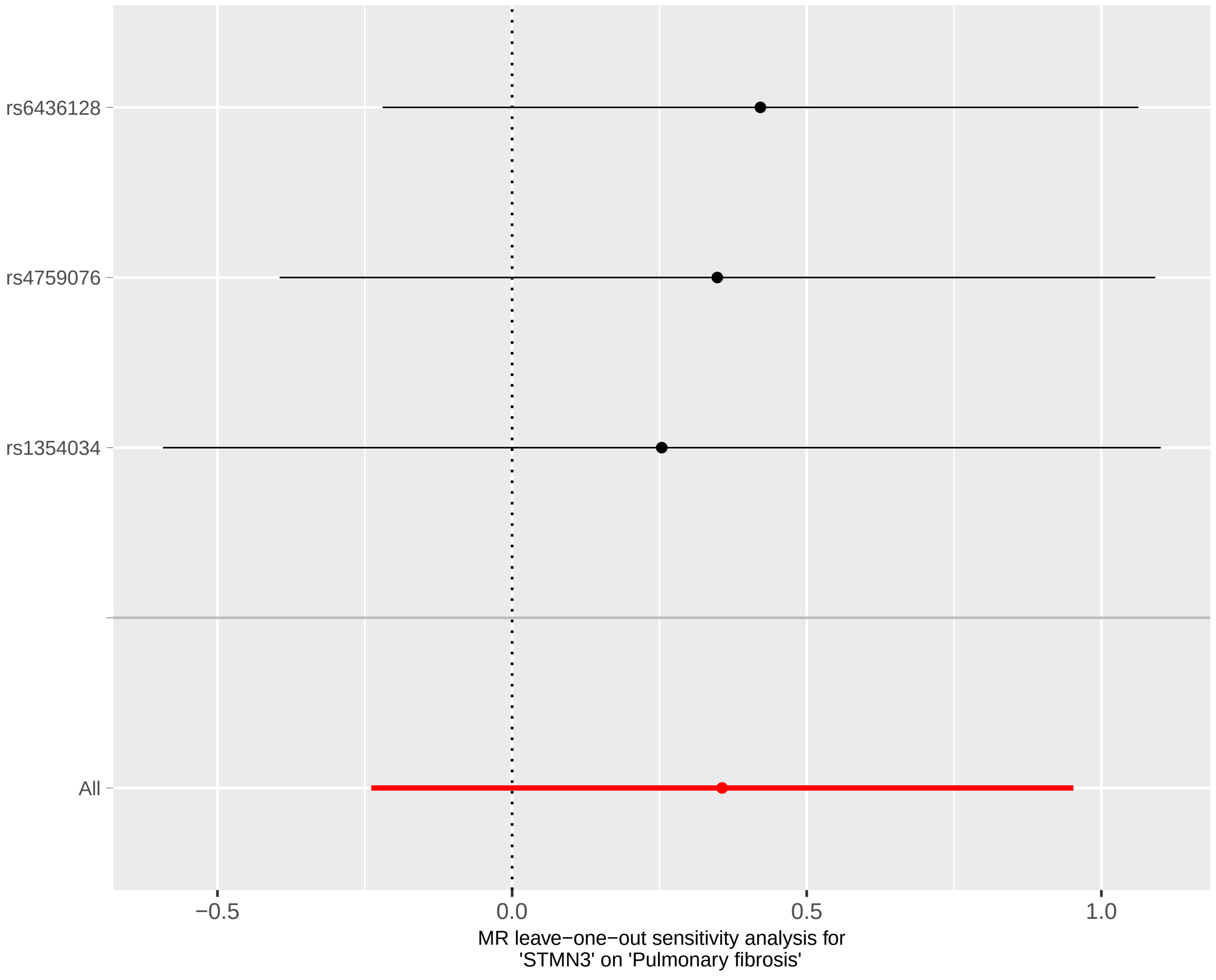
rs6436128
rs4759076
rs1354034
All
−0.5
0.0
0.5
1.0
MR leave−one−out sensitivity analysis for
'STMN3' on 'Pulmonary fibrosis'

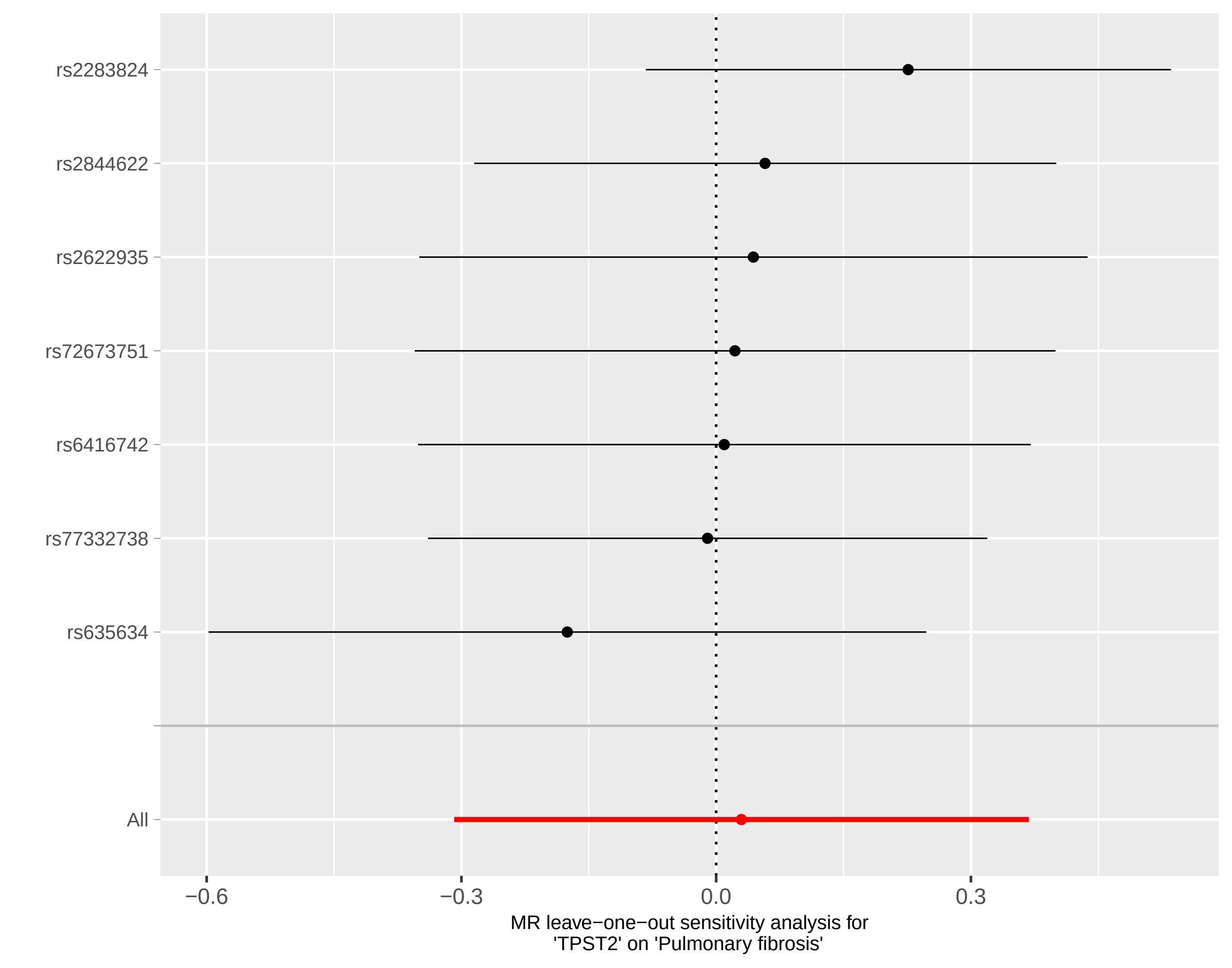
rs2283824
rs2844622
rs2622935
rs72673751
rs6416742
rs77332738
rs635634
All
−0.6
−0.3
0.0
0.3
MR leave−one−out sensitivity analysis for
'TPST2' on 'Pulmonary fibrosis'

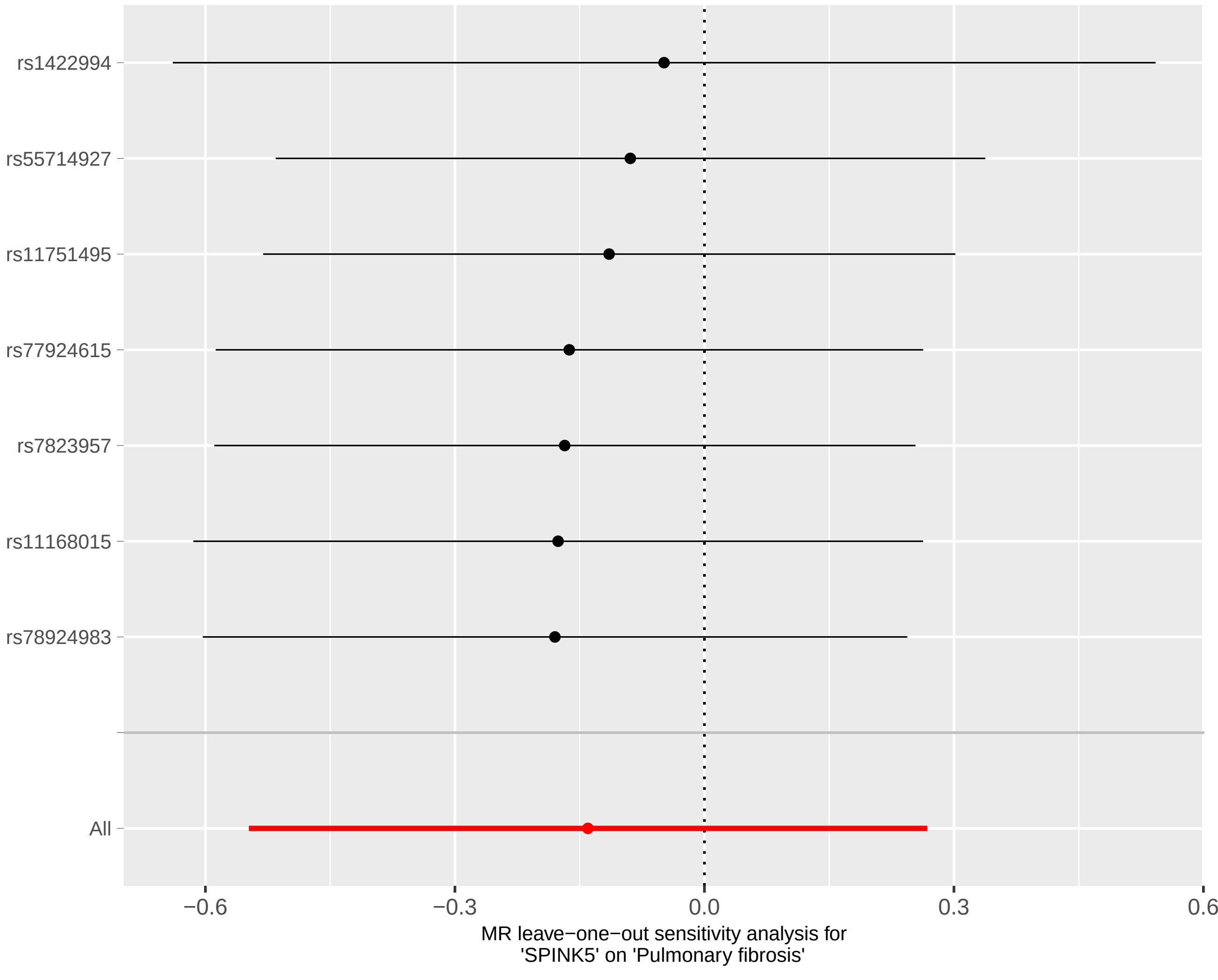

rs1422994
rs55714927
rs11751495
rs77924615
rs7823957
rs11168015
rs78924983
All
−0.6
−0.3
0.0
0.3
0.6
MR leave−one−out sensitivity analysis for
'SPINK5' on 'Pulmonary fibrosis'

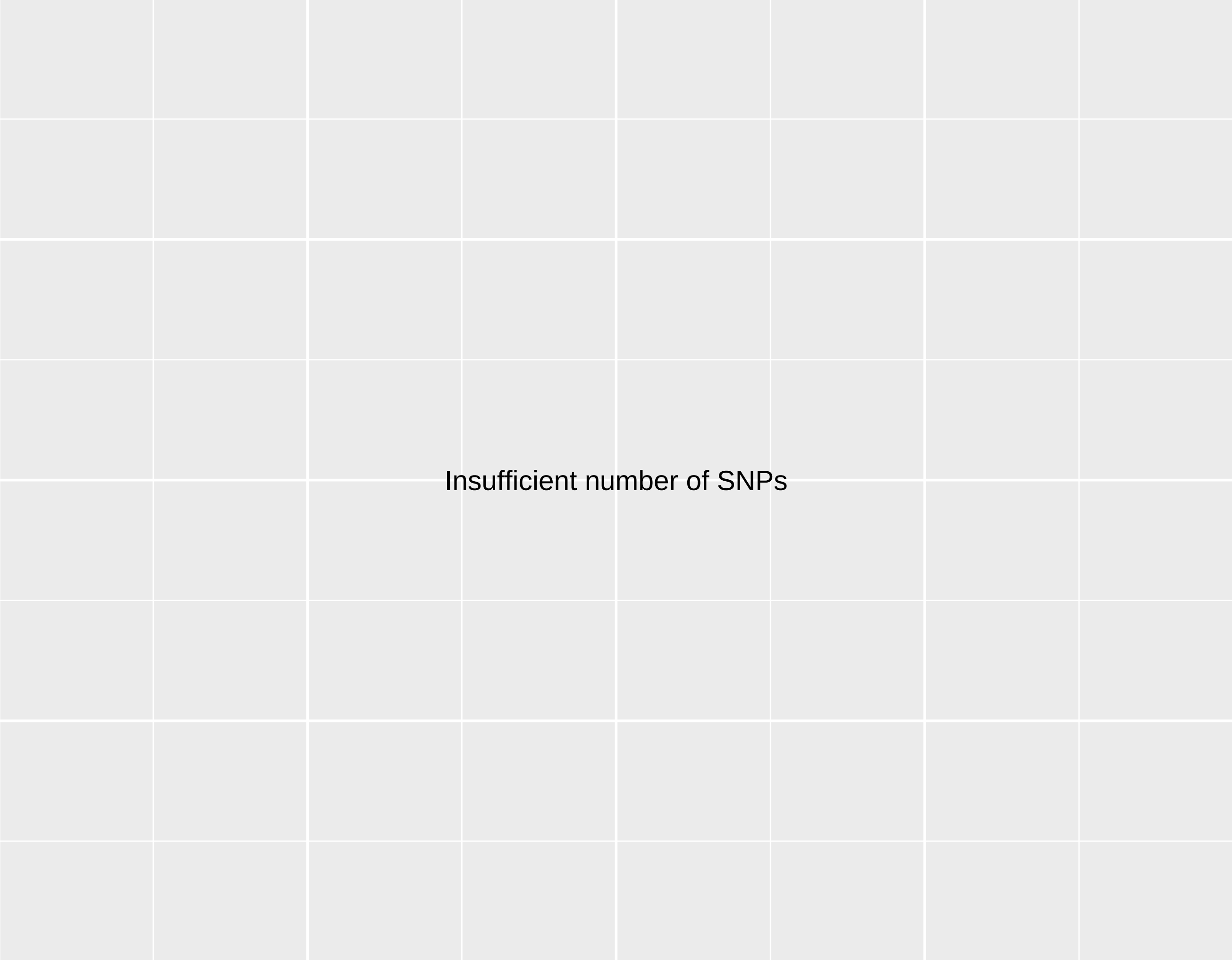
Insufficient number of SNPs

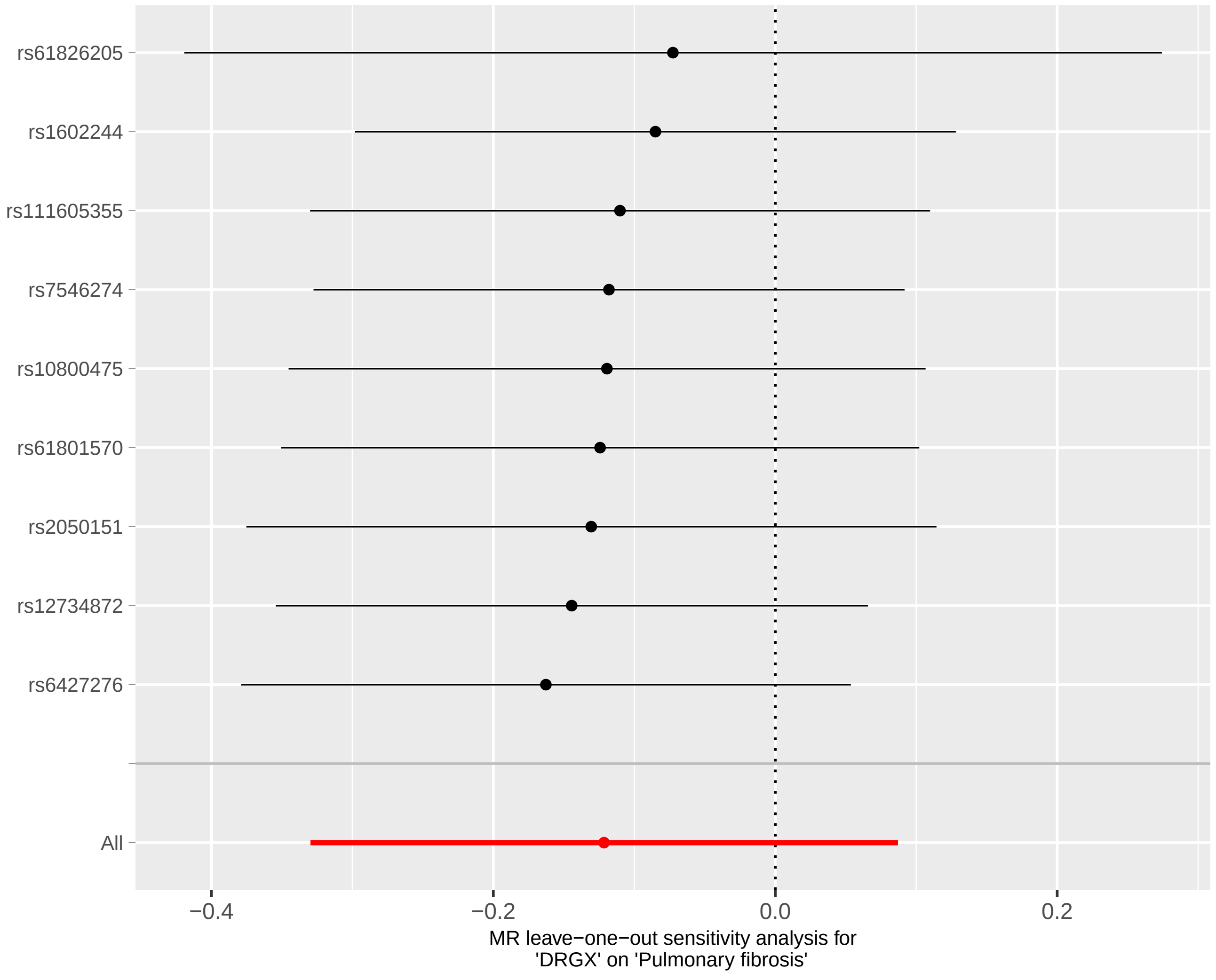
rs61826205
rs1602244
rs111605355
rs7546274
rs10800475
rs61801570
rs2050151
rs12734872
rs6427276
All
−0.4
−0.2
0.0
0.2
MR leave−one−out sensitivity analysis for
'DRGX' on 'Pulmonary fibrosis'

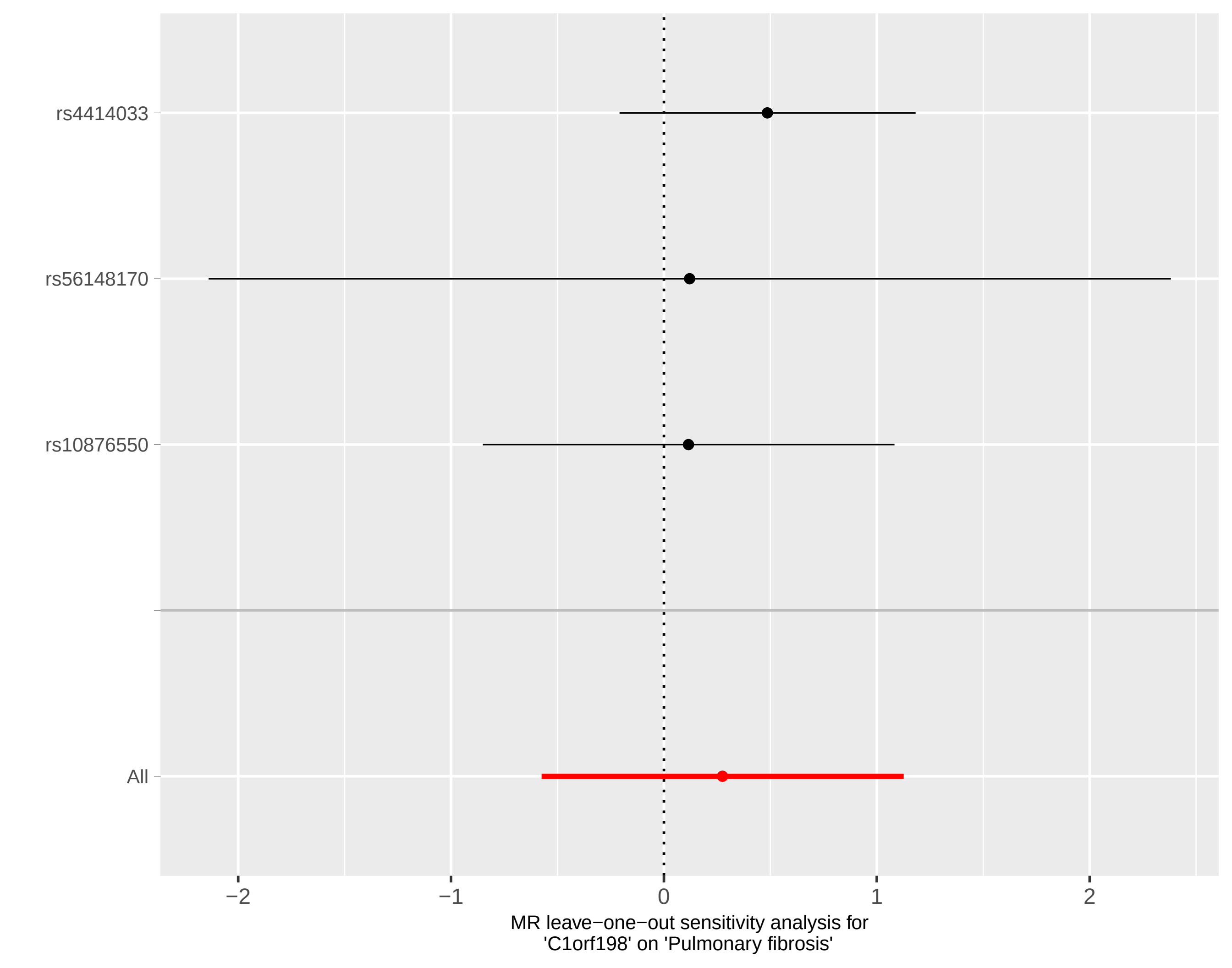

rs4414033
rs56148170
rs10876550
All
−2
−1
0
1
2
MR leave−one−out sensitivity analysis for
'C1orf198' on 'Pulmonary fibrosis'

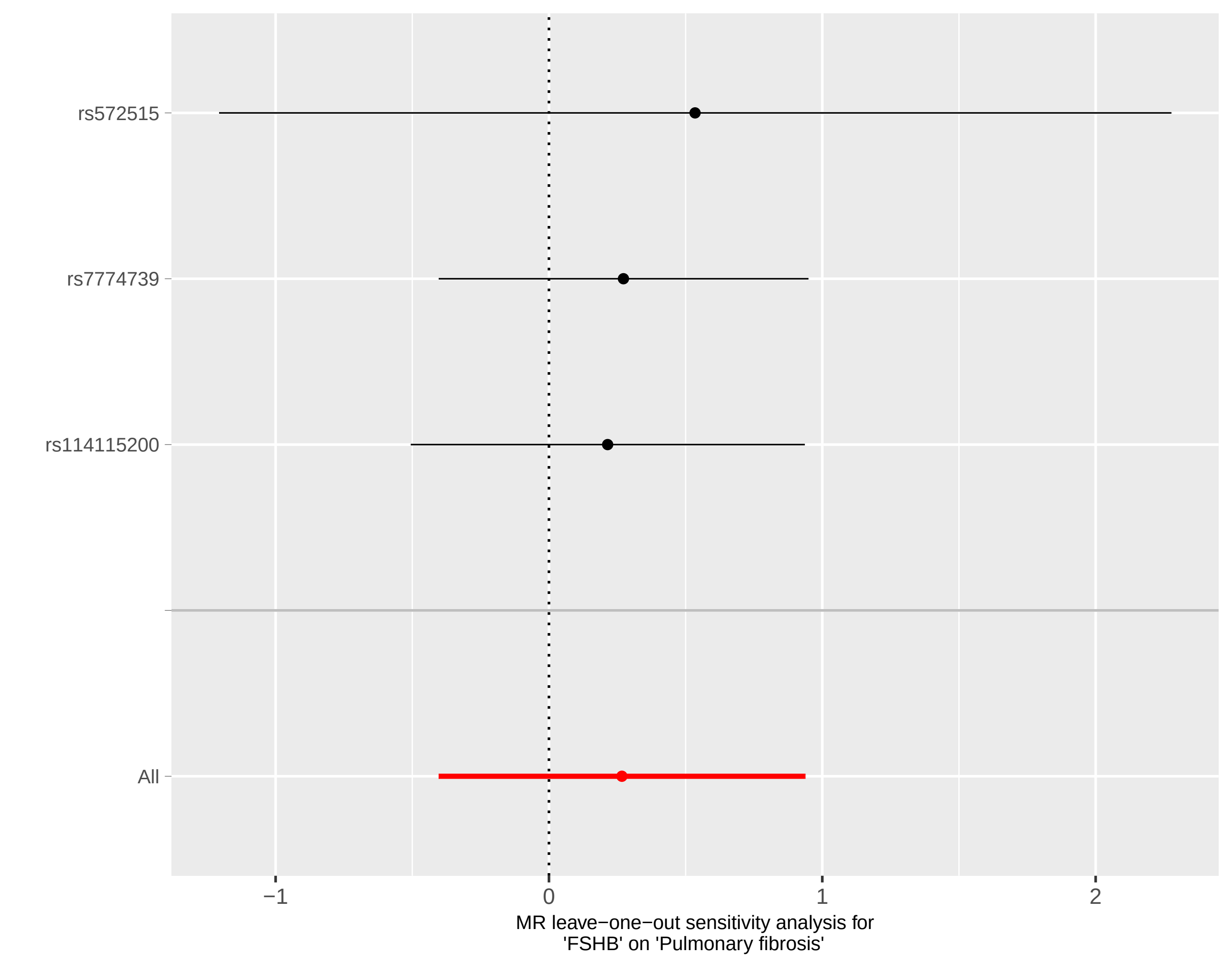
rs572515
rs7774739
rs114115200
All
−1
0
1
2
MR leave−one−out sensitivity analysis for
'FSHB' on 'Pulmonary fibrosis'

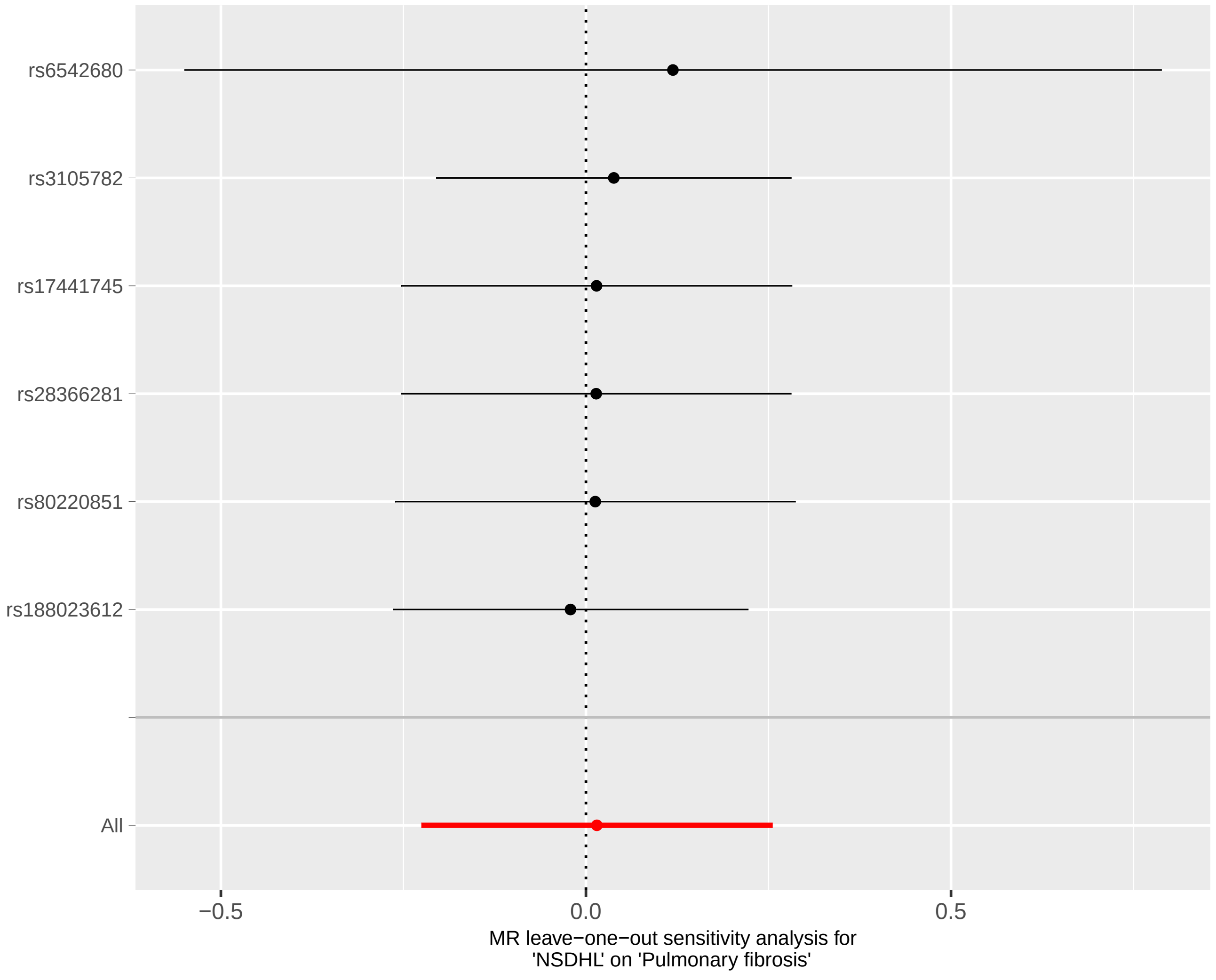
rs6542680
rs3105782
rs17441745
rs28366281
rs80220851
rs188023612
All
−0.5
0.0
0.5
MR leave−one−out sensitivity analysis for
'NSDHL' on 'Pulmonary fibrosis'

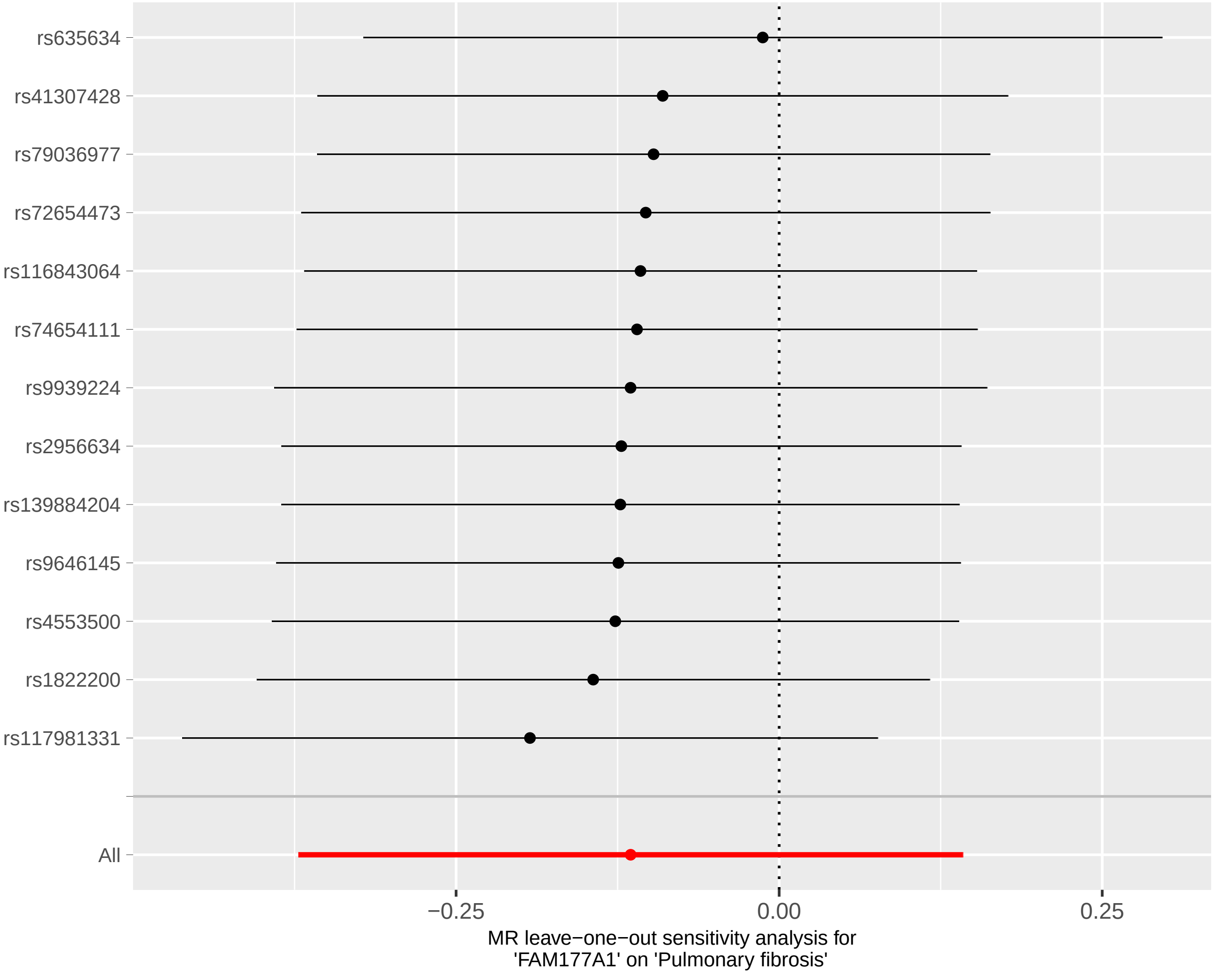
rs635634
rs41307428
rs79036977
rs72654473
rs116843064
rs74654111
rs9939224
rs2956634
rs139884204
rs9646145
rs4553500
rs1822200
rs117981331
All
−0.25
0.00
0.25
MR leave−one−out sensitivity analysis for
'FAM177A1' on 'Pulmonary fibrosis'

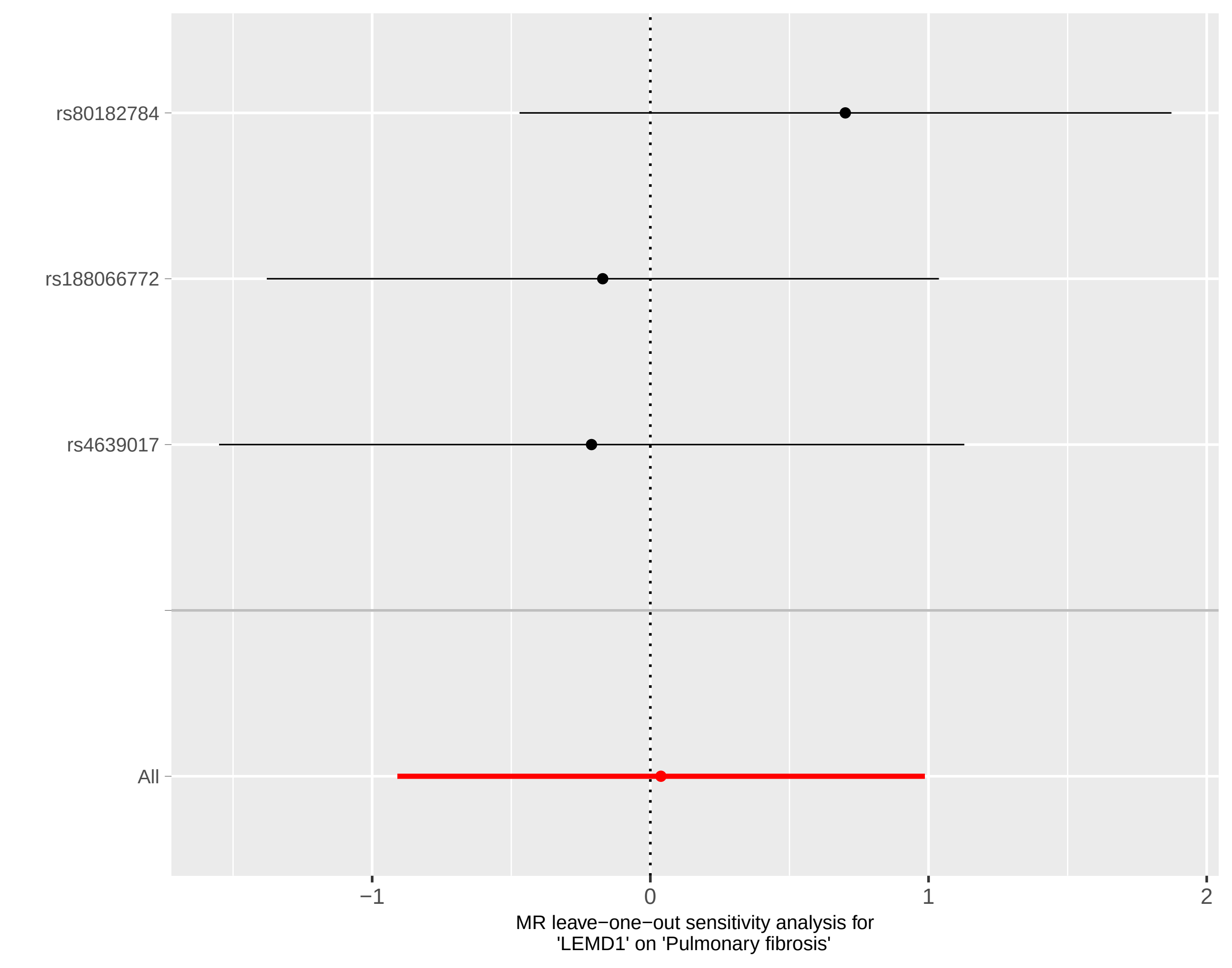
rs80182784
rs188066772
rs4639017
All
−1
0
1
2
MR leave−one−out sensitivity analysis for
'LEMD1' on 'Pulmonary fibrosis'

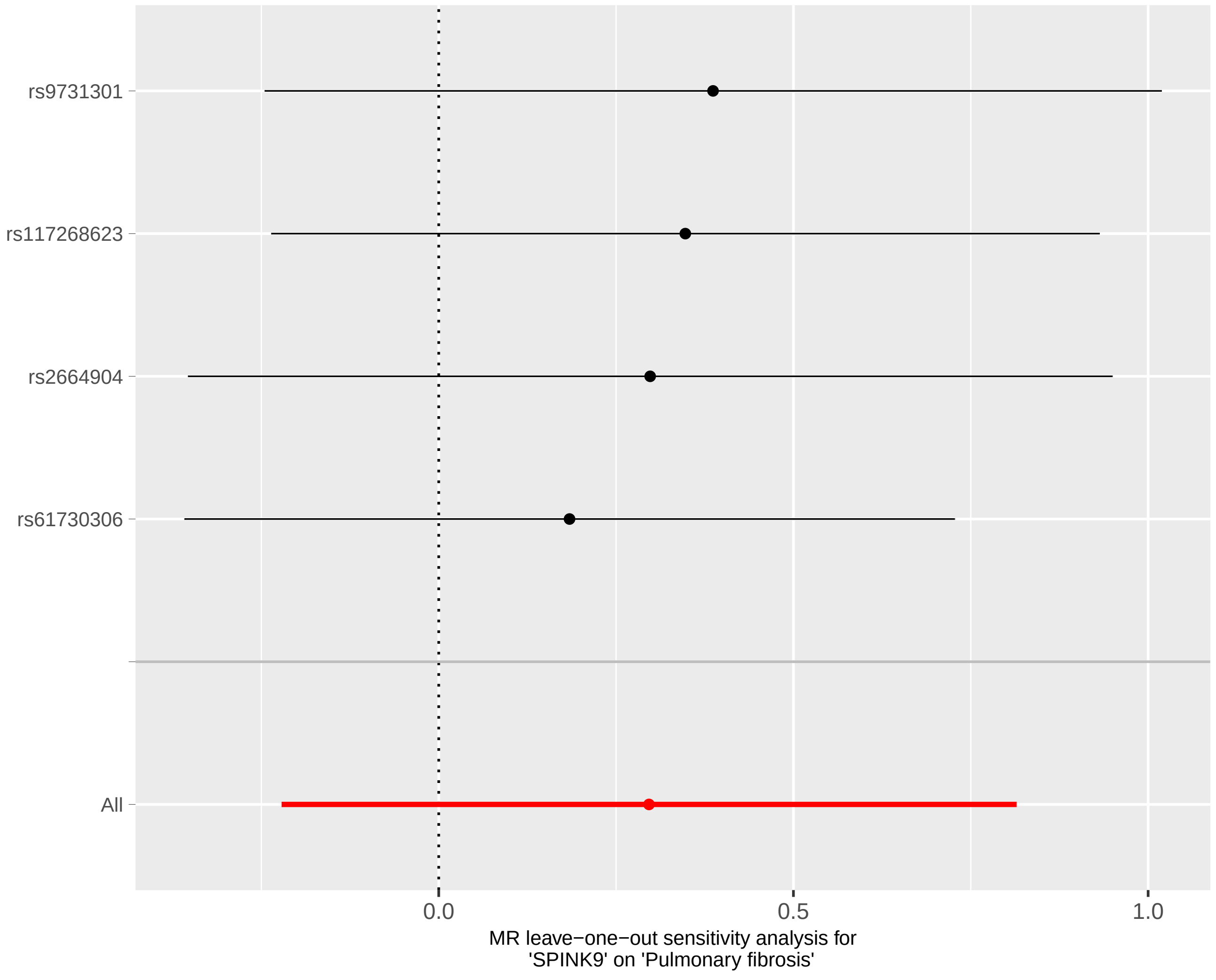

rs9731301
rs117268623
rs2664904
rs61730306
All
0.0
0.5
1.0
MR leave−one−out sensitivity analysis for
'SPINK9' on 'Pulmonary fibrosis'

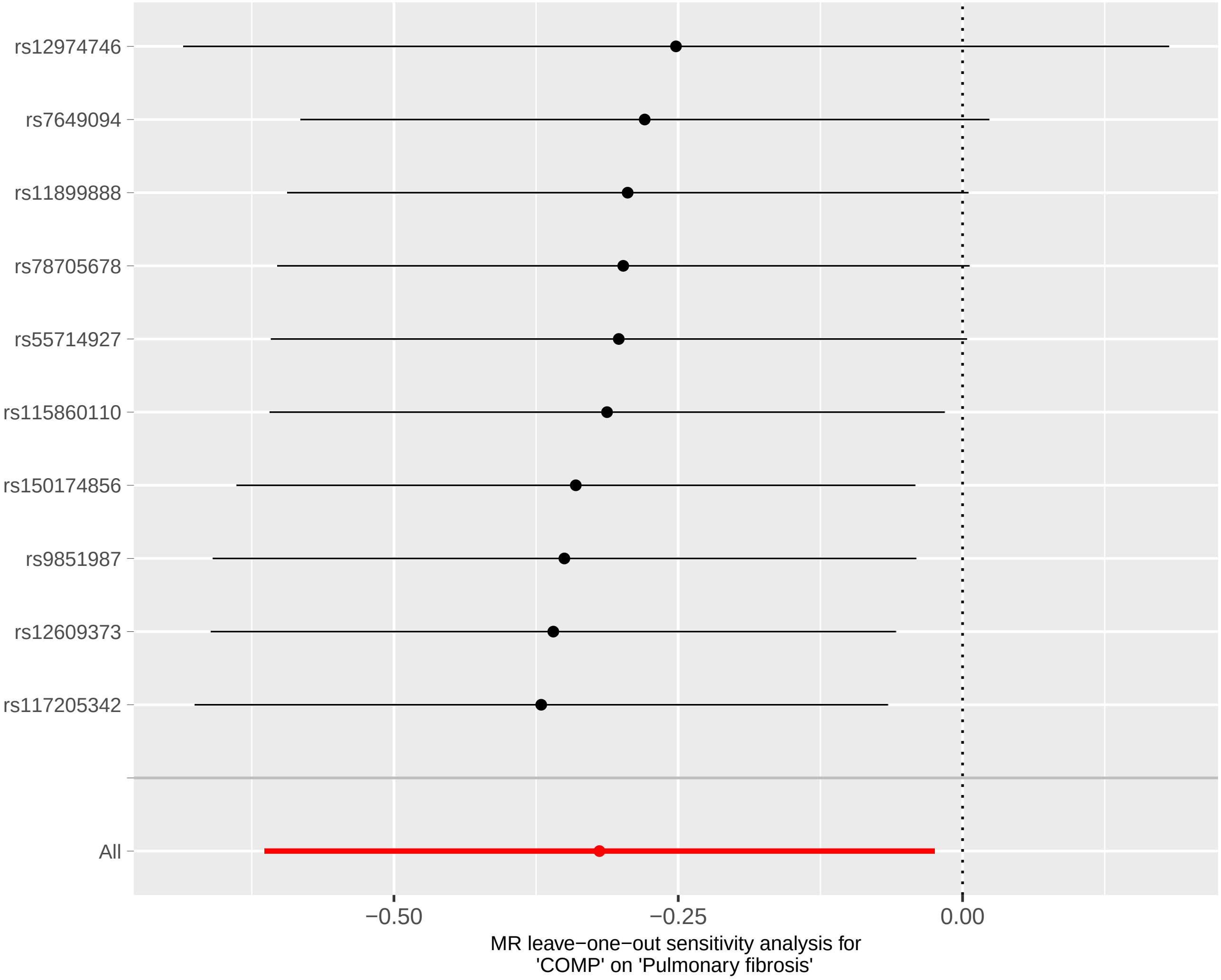
rs12974746
rs7649094
rs11899888
rs78705678
rs55714927
rs115860110
rs150174856
rs9851987
rs12609373
rs117205342
All
−0.50
−0.25
0.00
MR leave−one−out sensitivity analysis for
'COMP' on 'Pulmonary fibrosis'

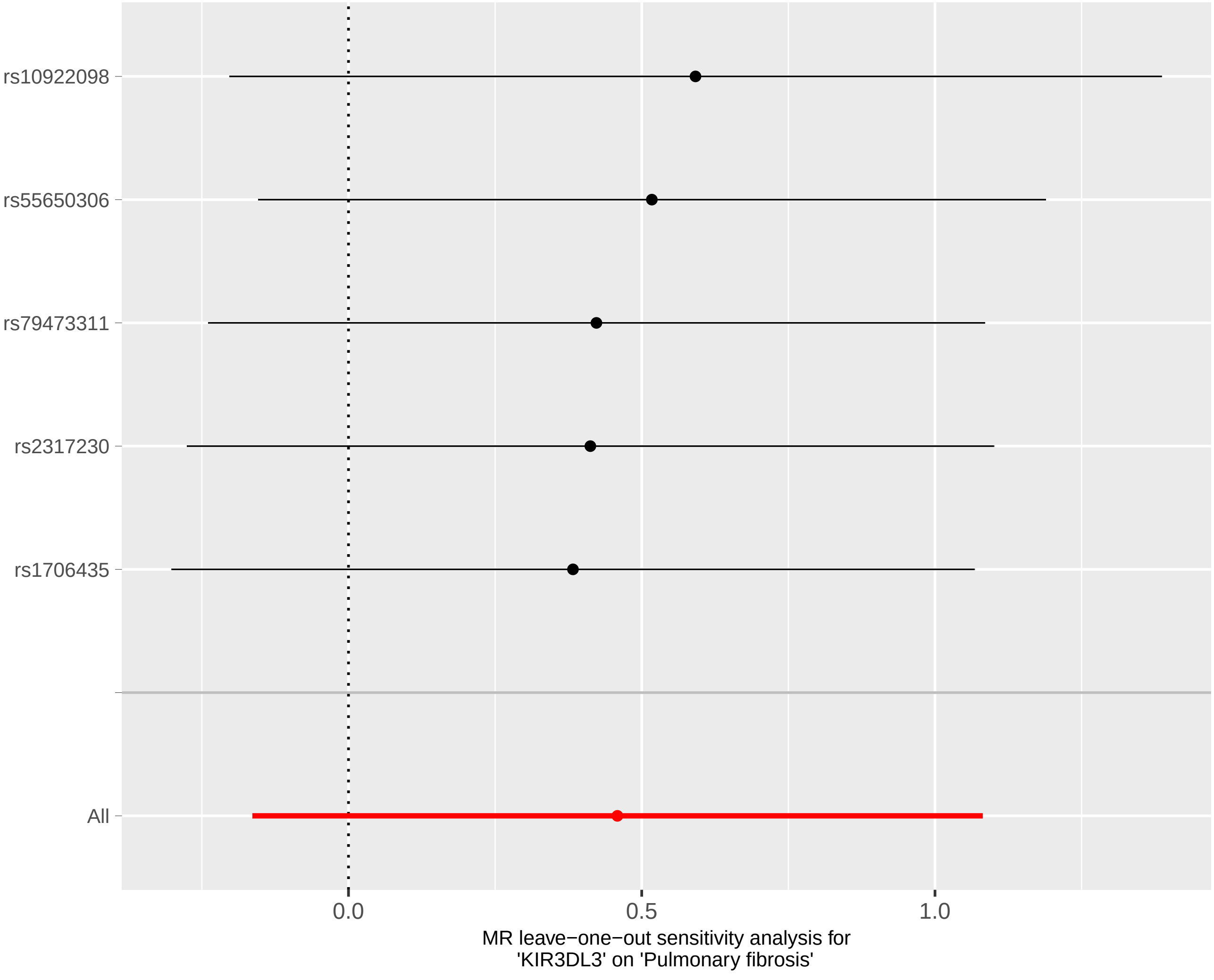

rs10922098
rs55650306
rs79473311
rs2317230
rs1706435
All
0.0
0.5
1.0
MR leave−one−out sensitivity analysis for
'KIR3DL3' on 'Pulmonary fibrosis'

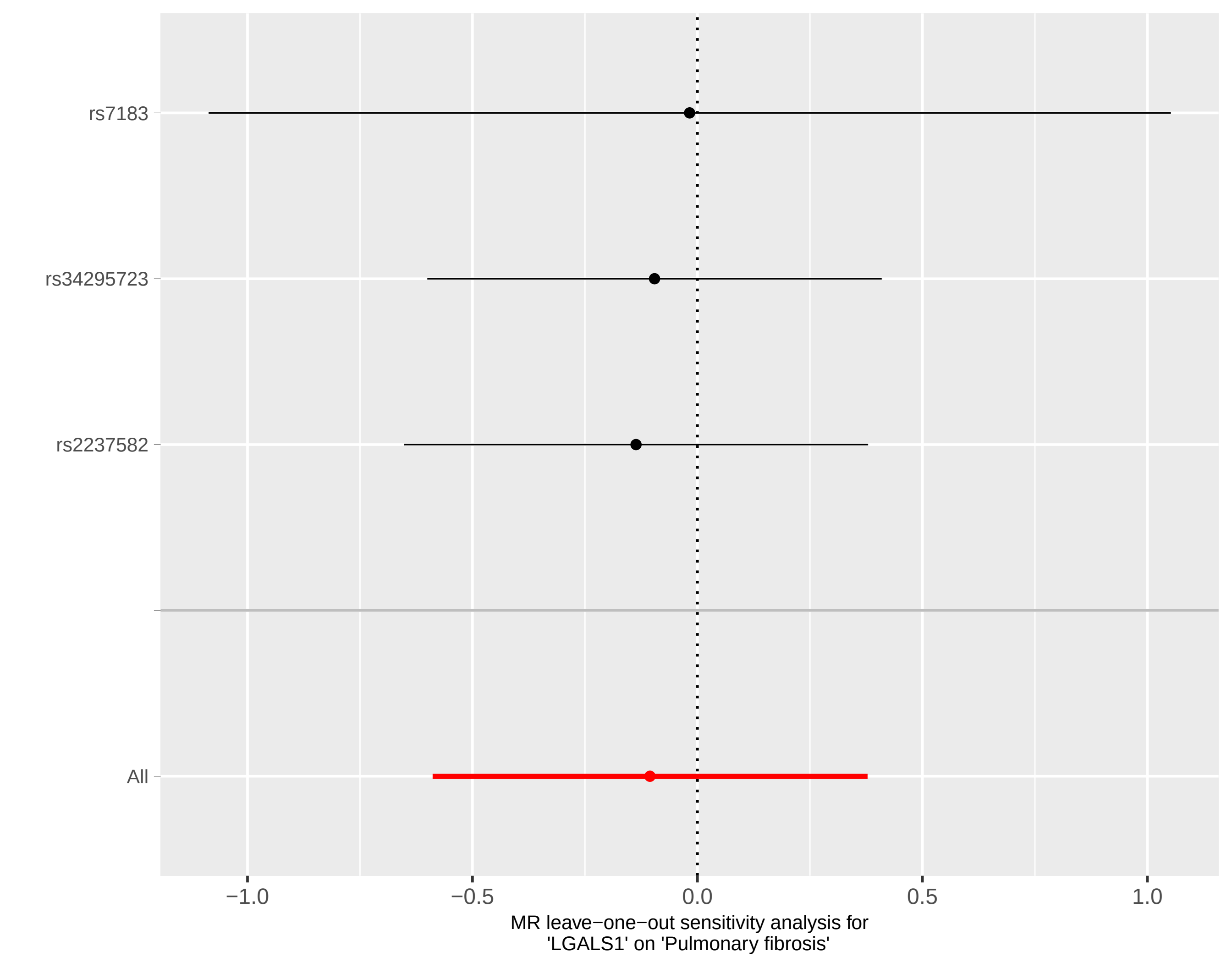
rs7183
rs34295723
rs2237582
All
−1.0
−0.5
0.0
0.5
1.0
MR leave−one−out sensitivity analysis for
'LGALS1' on 'Pulmonary fibrosis'

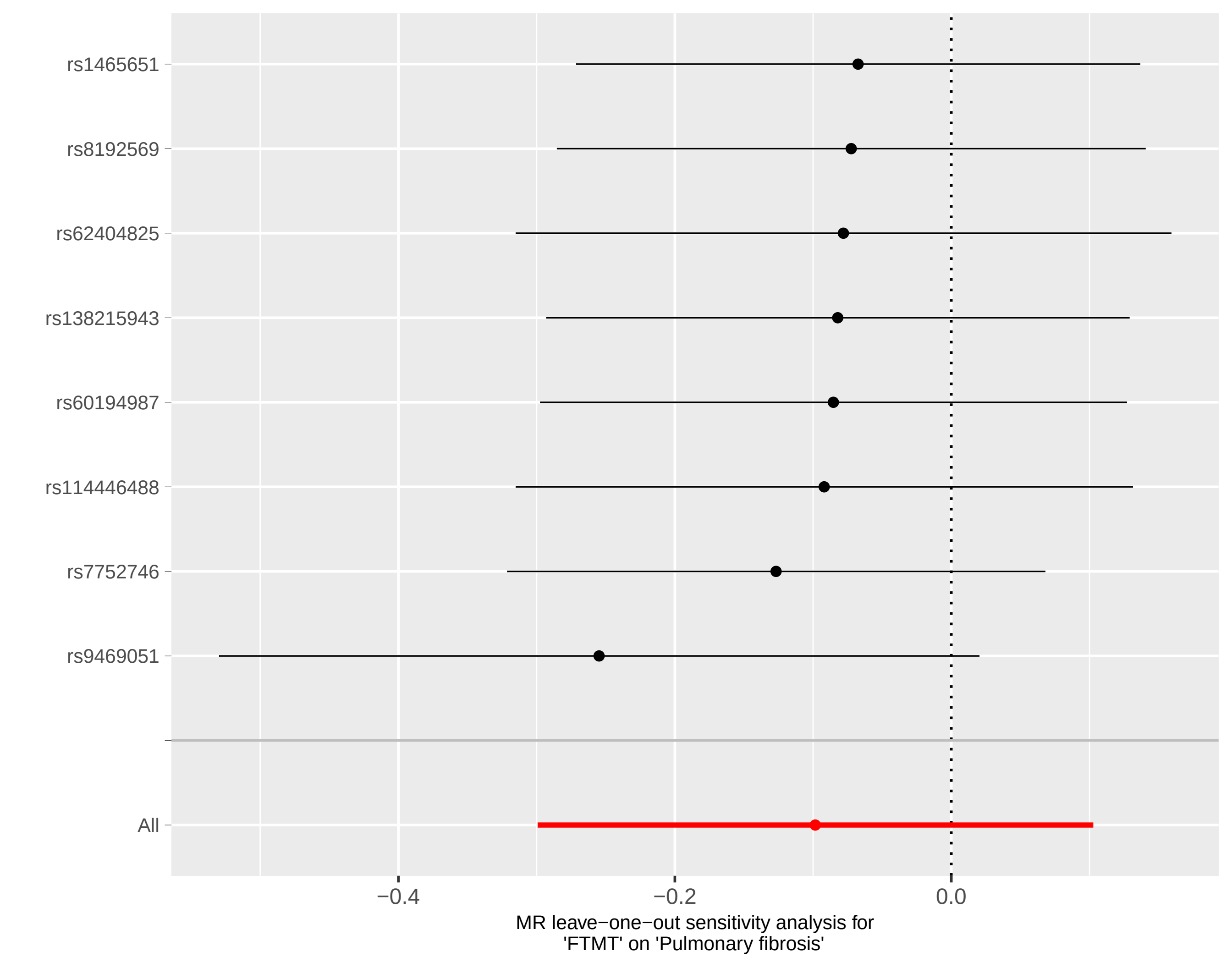
rs1465651
rs8192569
rs62404825
rs138215943
rs60194987
rs114446488
rs7752746
rs9469051
All
−0.4
−0.2
0.0
MR leave−one−out sensitivity analysis for
'FTMT' on 'Pulmonary fibrosis'

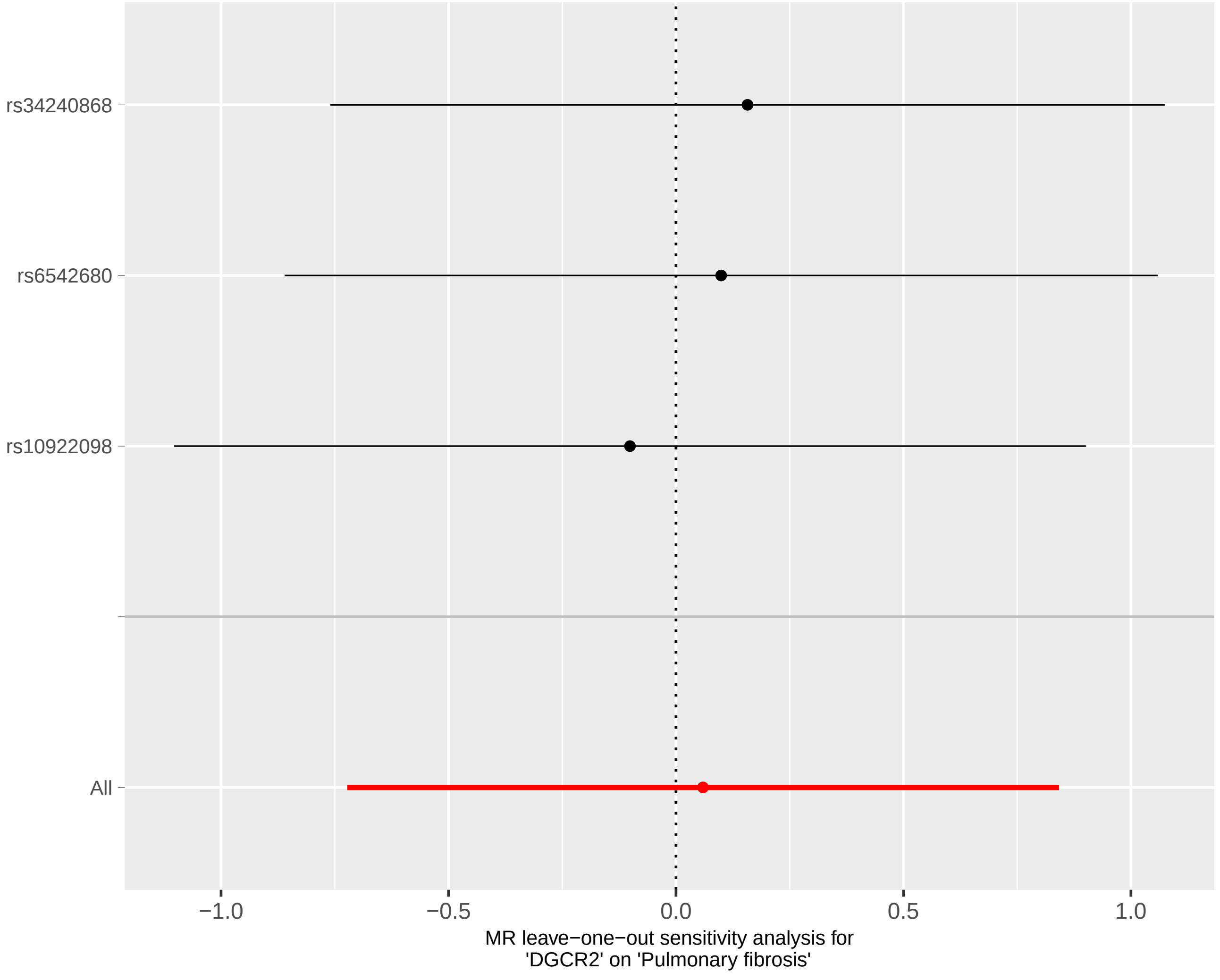
rs34240868
rs6542680
rs10922098
All
−1.0
−0.5
0.0
0.5
1.0
MR leave−one−out sensitivity analysis for
'DGCR2' on 'Pulmonary fibrosis'

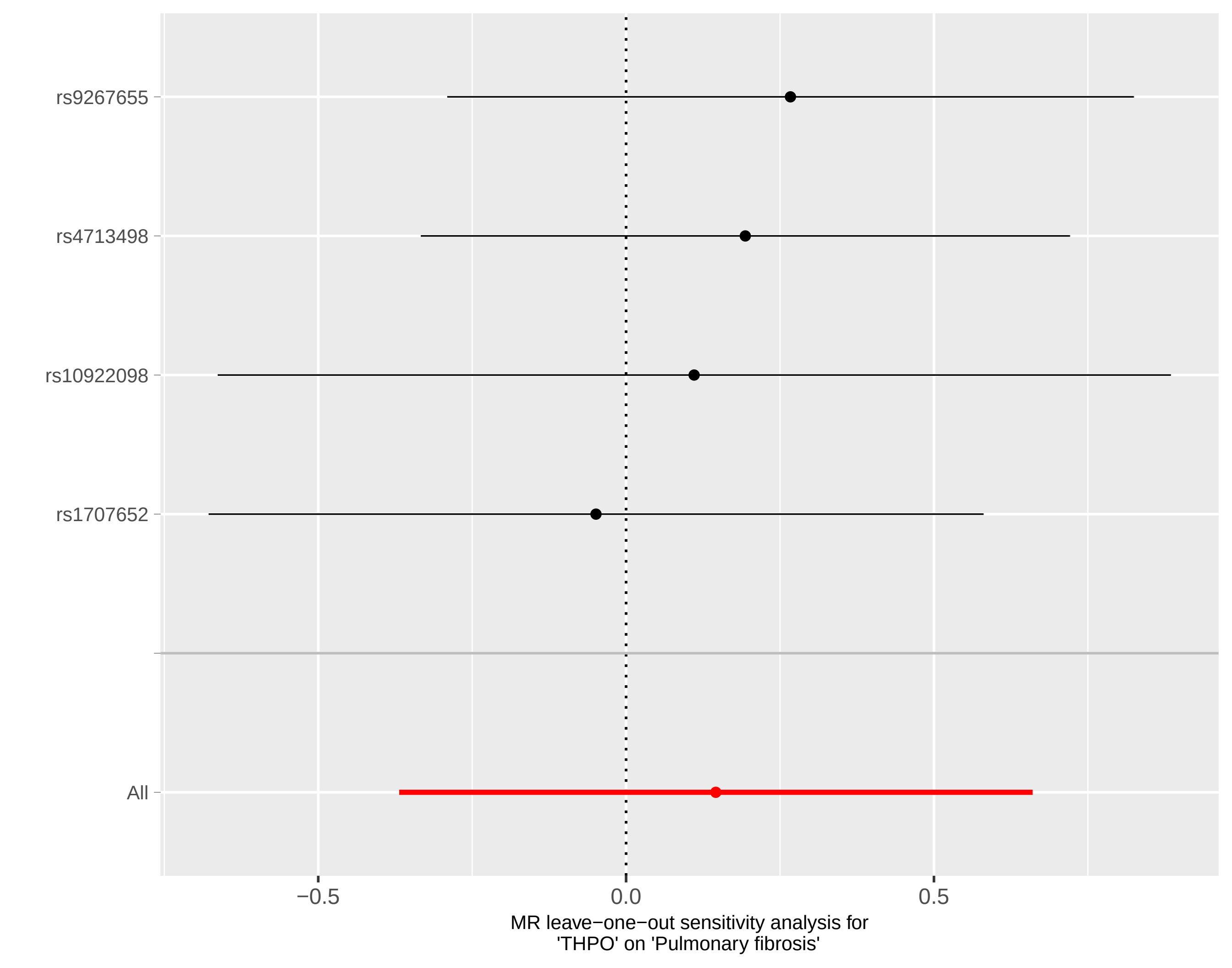
rs9267655
rs4713498
rs10922098
rs1707652
All
−0.5
0.0
0.5
MR leave−one−out sensitivity analysis for
'THPO' on 'Pulmonary fibrosis'

Insufficient number of SNPs

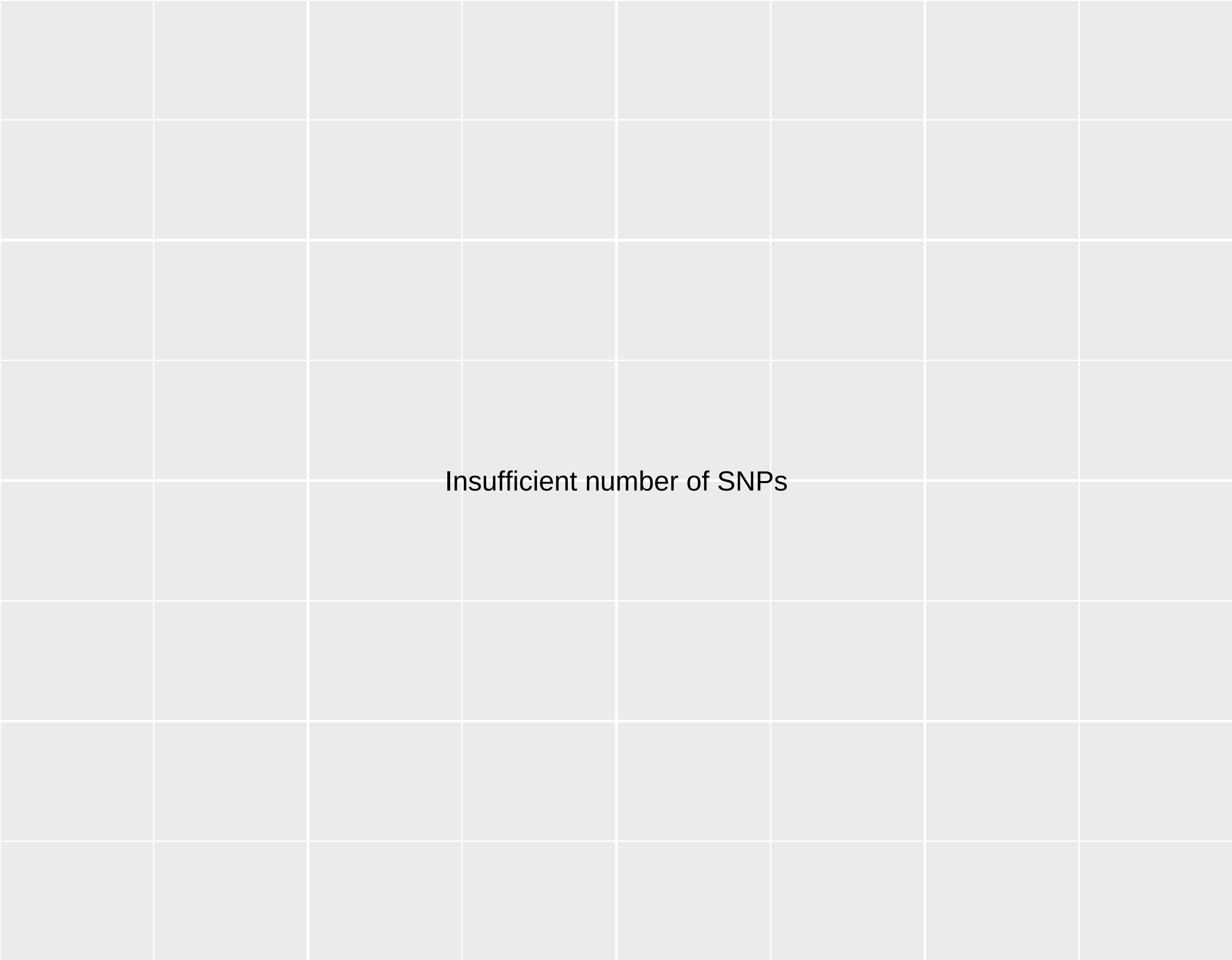
Insufficient number of SNPs

Insufficient number of SNPs

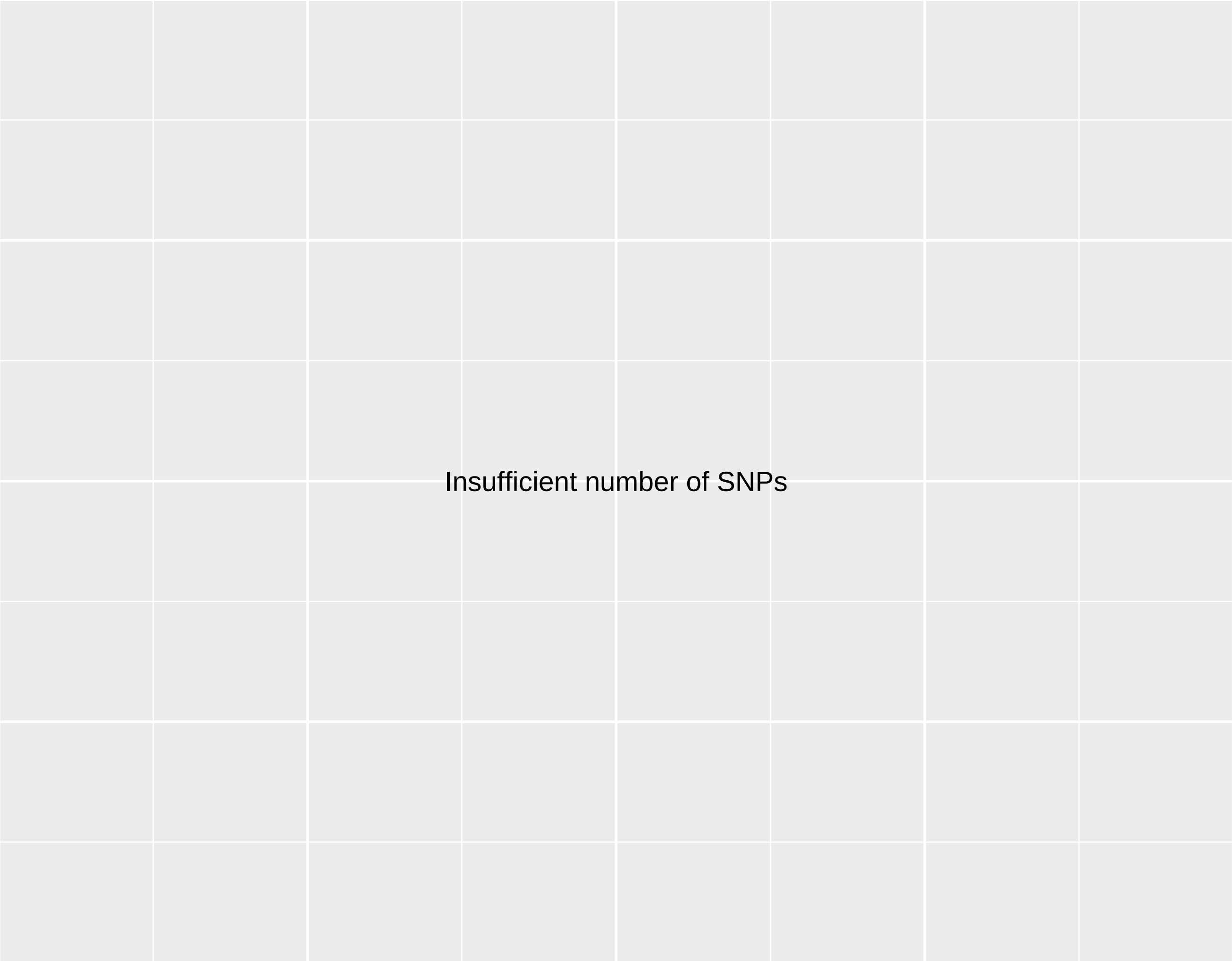
Insufficient number of SNPs

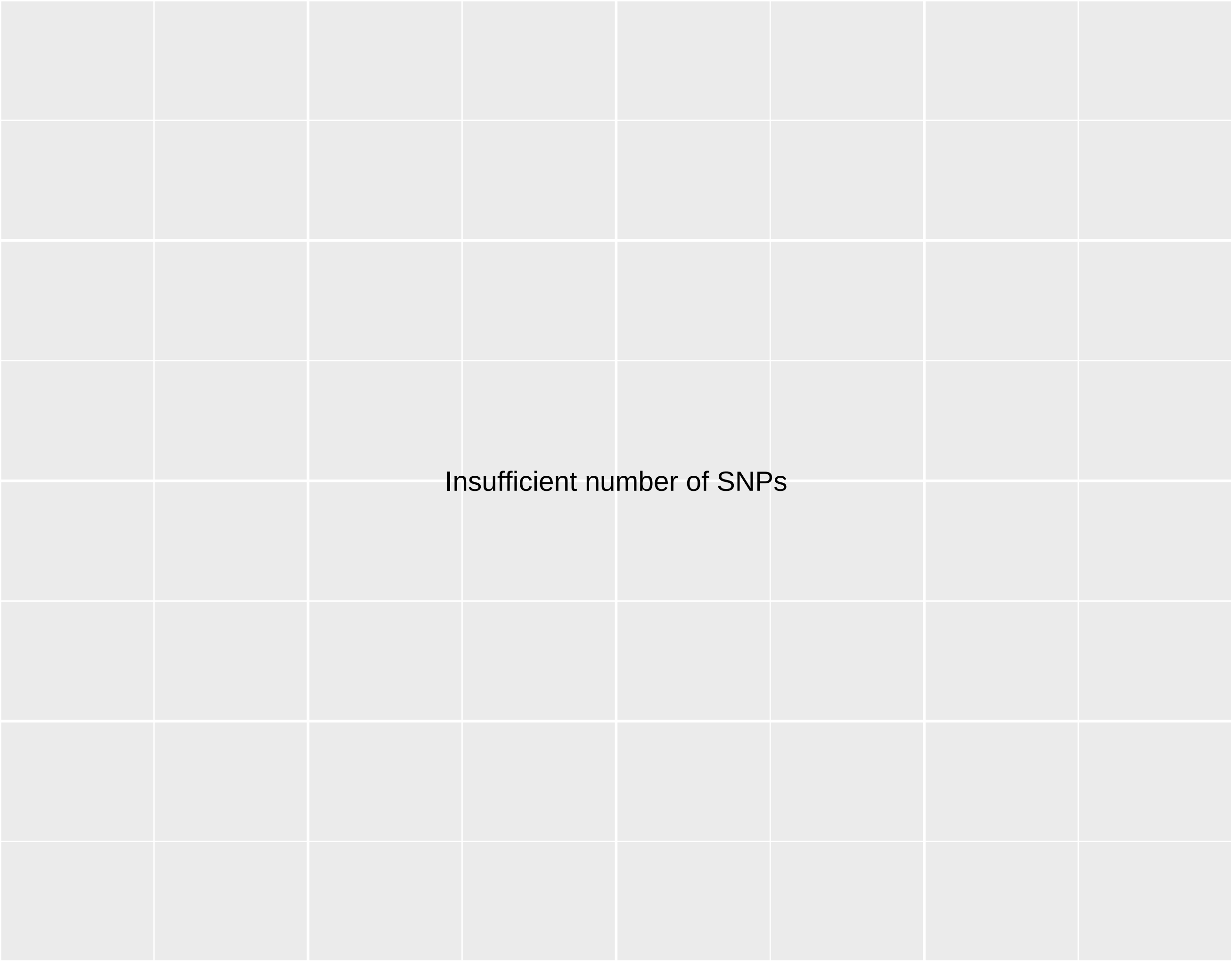
Insufficient number of SNPs

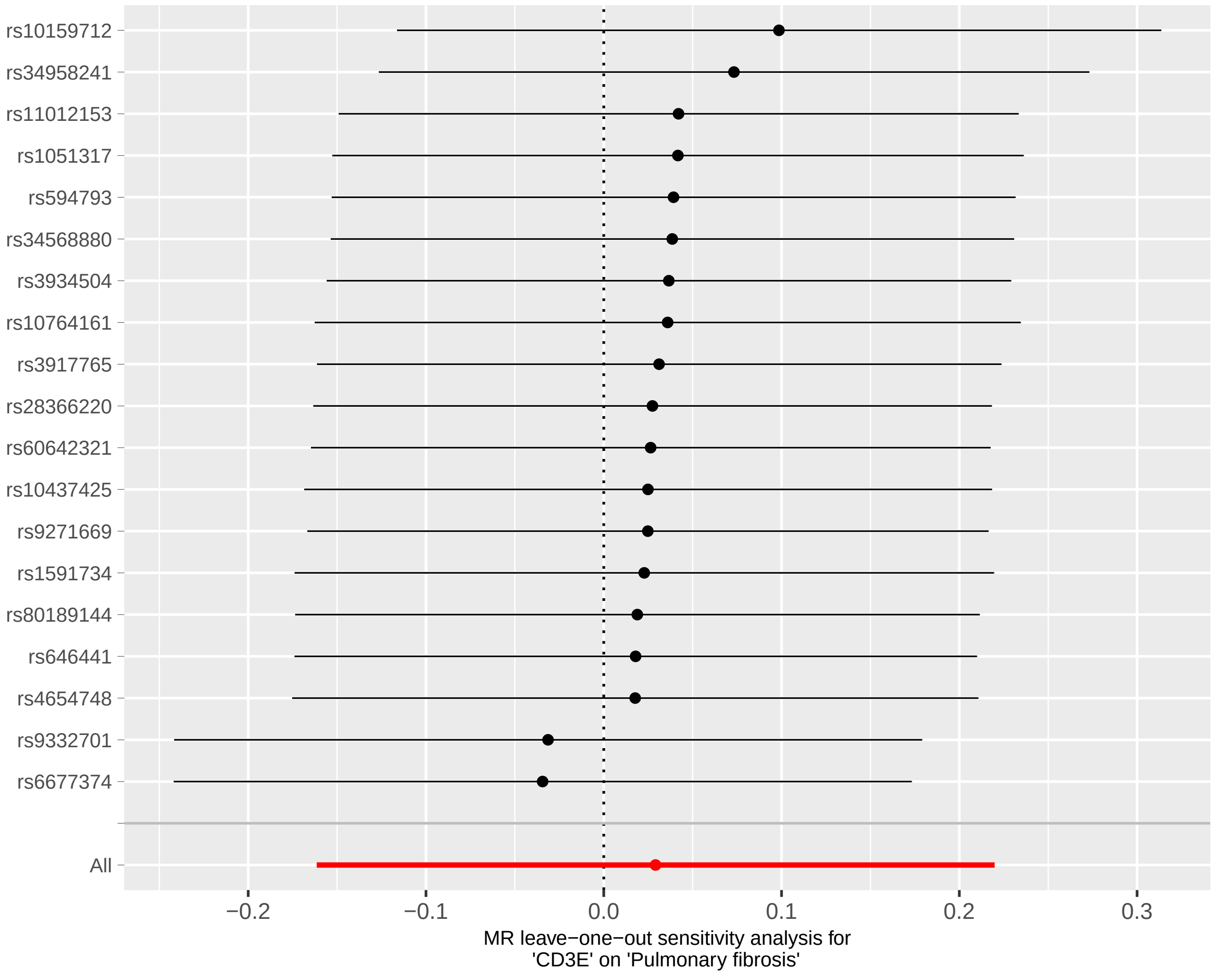
rs10159712
rs34958241
rs11012153
rs1051317
rs594793
rs34568880
rs3934504
rs10764161
rs3917765
rs28366220
rs60642321
rs10437425
rs9271669
rs1591734
rs80189144
rs646441
rs4654748
rs9332701
rs6677374
All
−0.2
−0.1
0.0
0.1
0.2
0.3
MR leave−one−out sensitivity analysis for
'CD3E' on 'Pulmonary fibrosis'

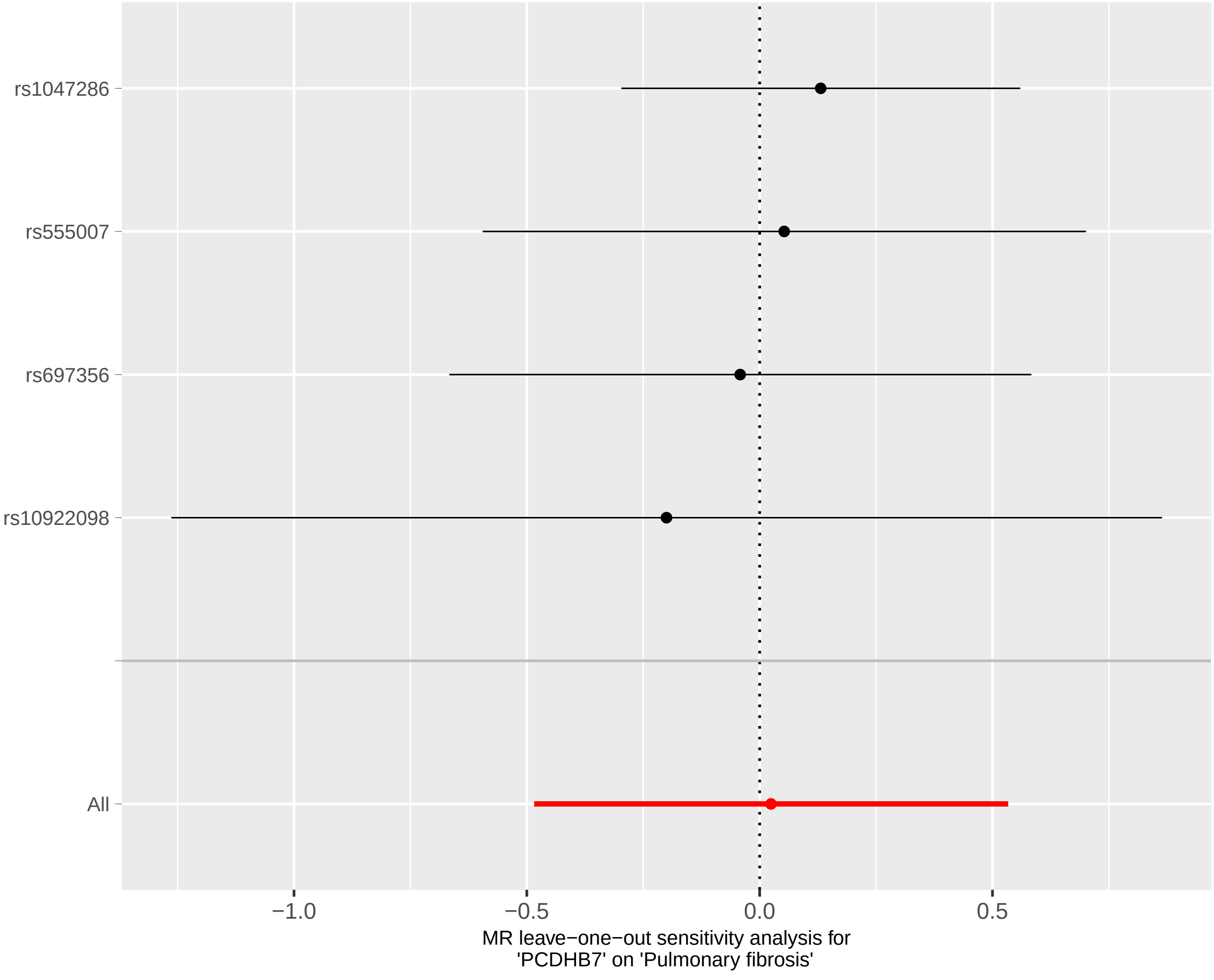
rs1047286
rs555007
rs697356
rs10922098
All
−1.0
−0.5
0.0
0.5
MR leave−one−out sensitivity analysis for
'PCDHB7' on 'Pulmonary fibrosis'

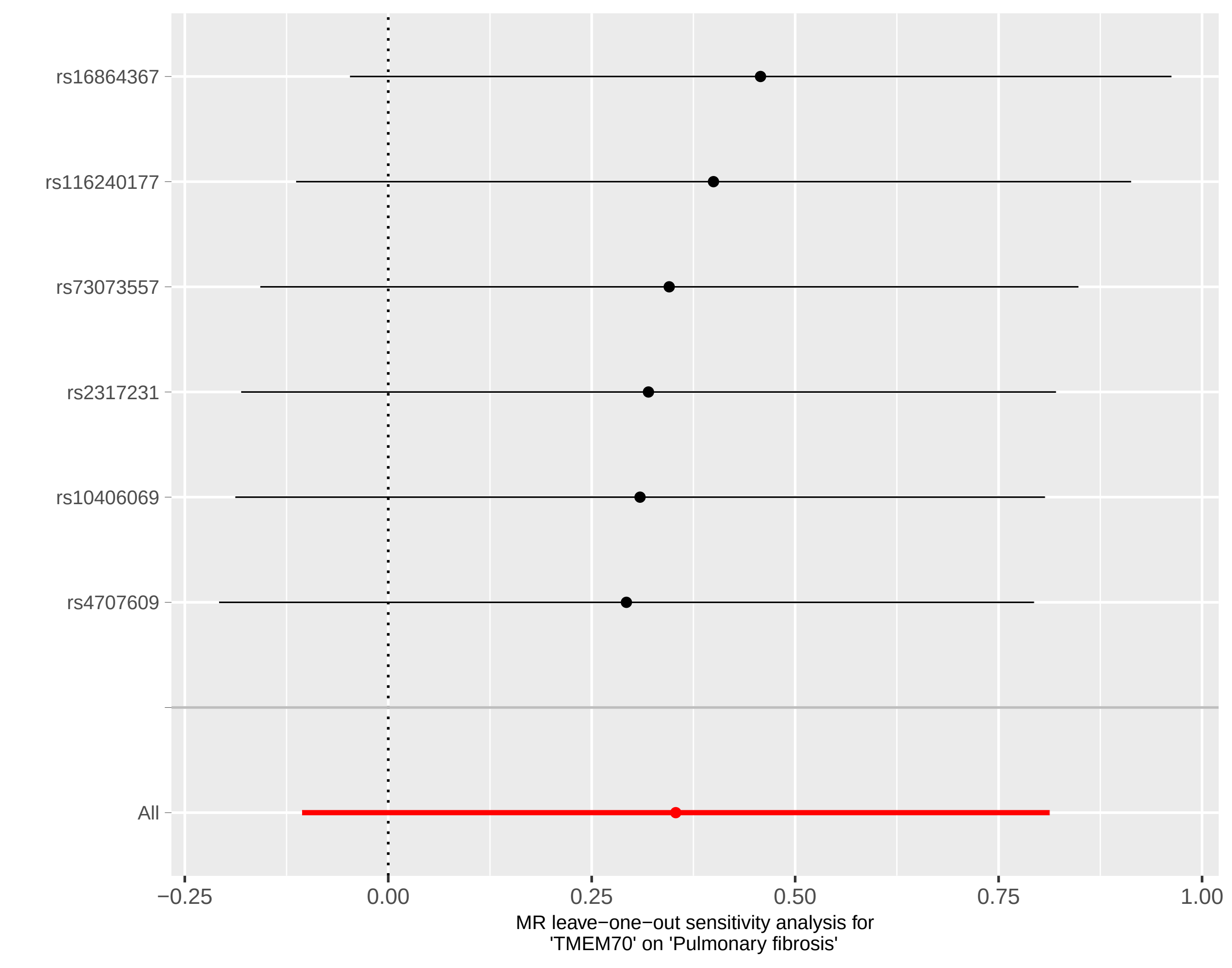
rs16864367
rs116240177
rs73073557
rs2317231
rs10406069
rs4707609
All
−0.25
0.00
0.25
0.50
0.75
1.00
MR leave−one−out sensitivity analysis for
'TMEM70' on 'Pulmonary fibrosis'

Insufficient number of SNPs

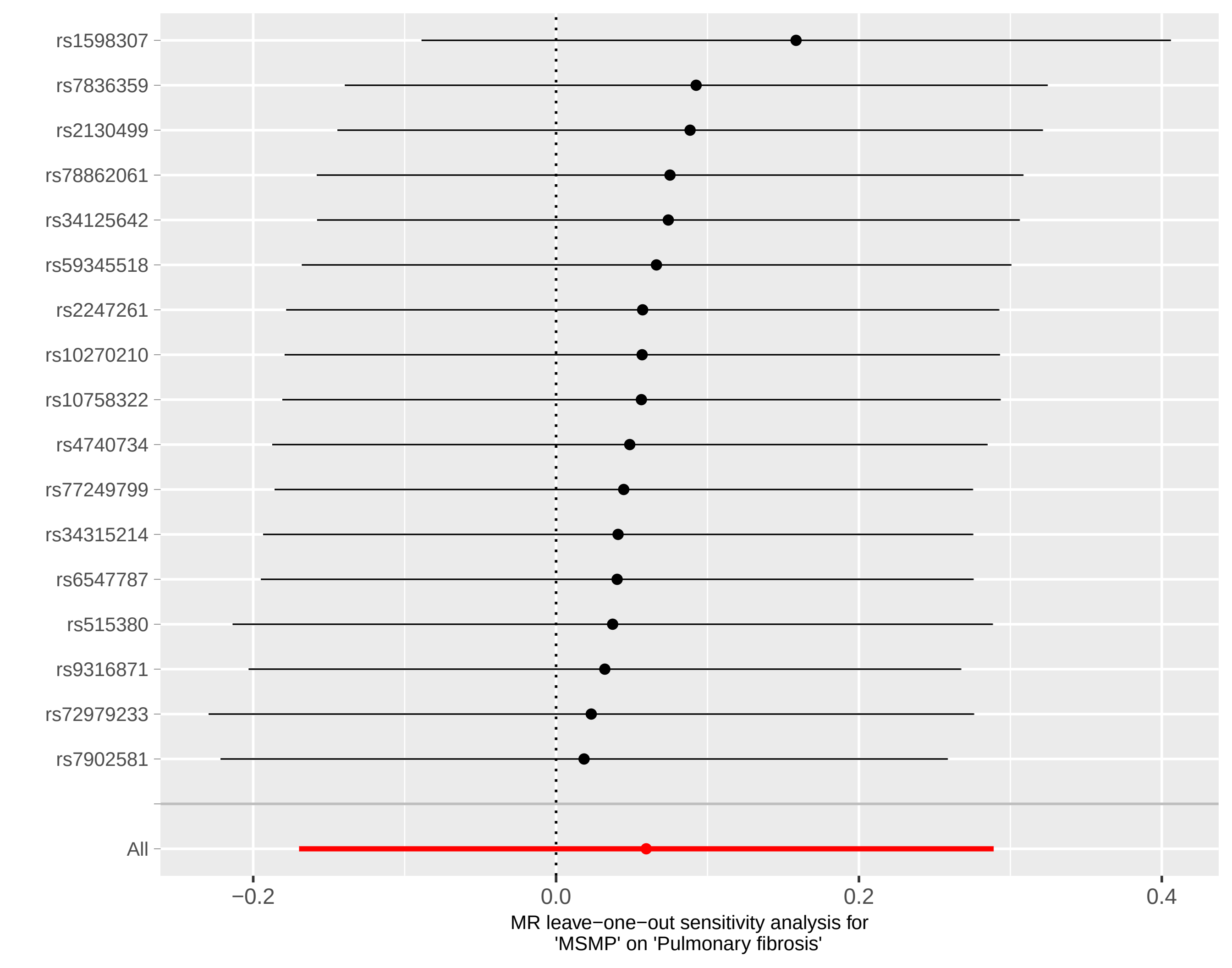

rs1598307
rs7836359
rs2130499
rs78862061
rs34125642
rs59345518
rs2247261
rs10270210
rs10758322
rs4740734
rs77249799
rs34315214
rs6547787
rs515380
rs9316871
rs72979233
rs7902581
All
−0.2
0.0
0.2
0.4
MR leave−one−out sensitivity analysis for
'MSMP' on 'Pulmonary fibrosis'

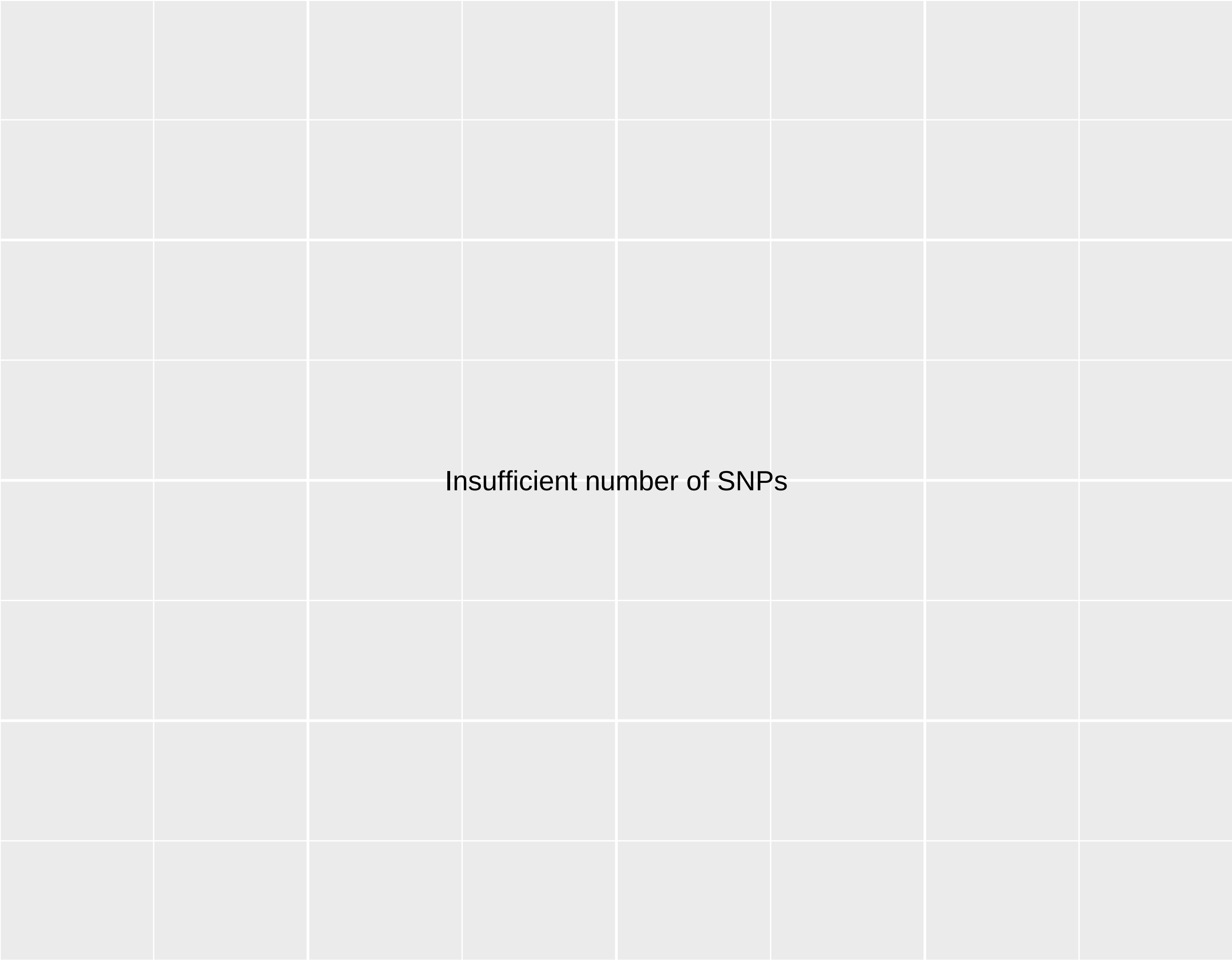
Insufficient number of SNPs

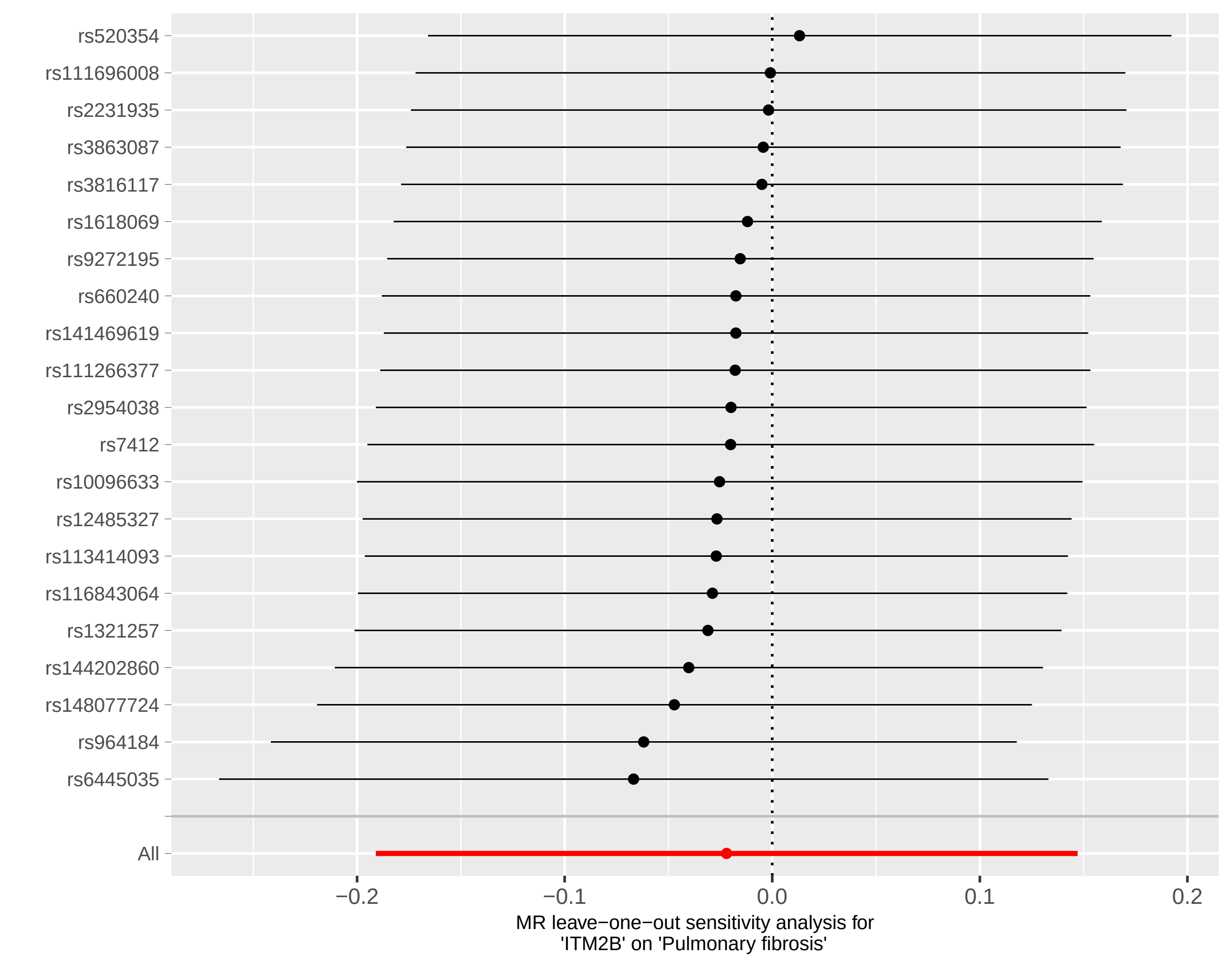
rs520354
rs111696008
rs2231935
rs3863087
rs3816117
rs1618069
rs9272195
rs660240
rs141469619
rs111266377
rs2954038
rs7412
rs10096633
rs12485327
rs113414093
rs116843064
rs1321257
rs144202860
rs148077724
rs964184
rs6445035
All
−0.2
−0.1
0.0
0.1
0.2
MR leave−one−out sensitivity analysis for
'ITM2B' on 'Pulmonary fibrosis'

Insufficient number of SNPs

Insufficient number of SNPs

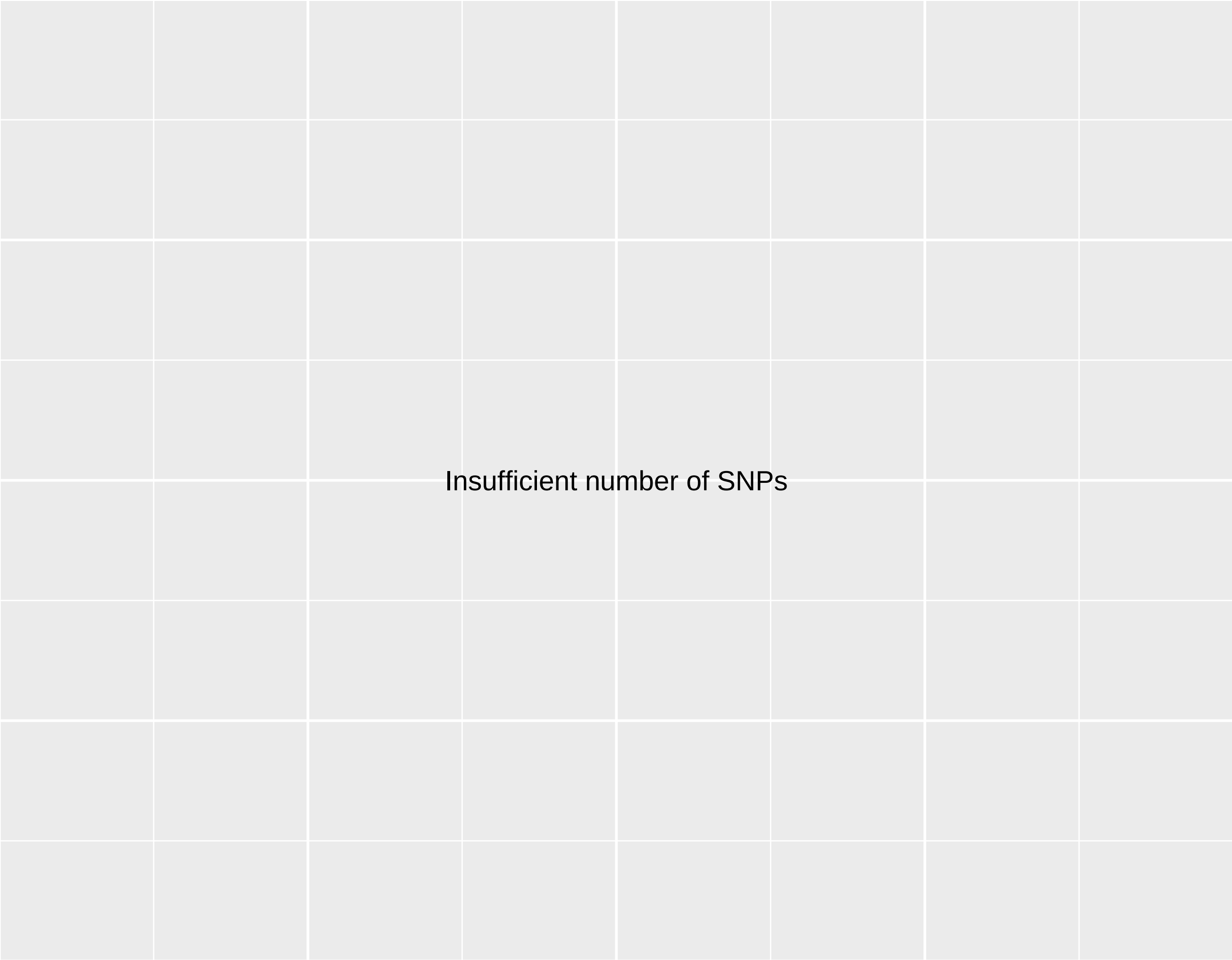
Insufficient number of SNPs

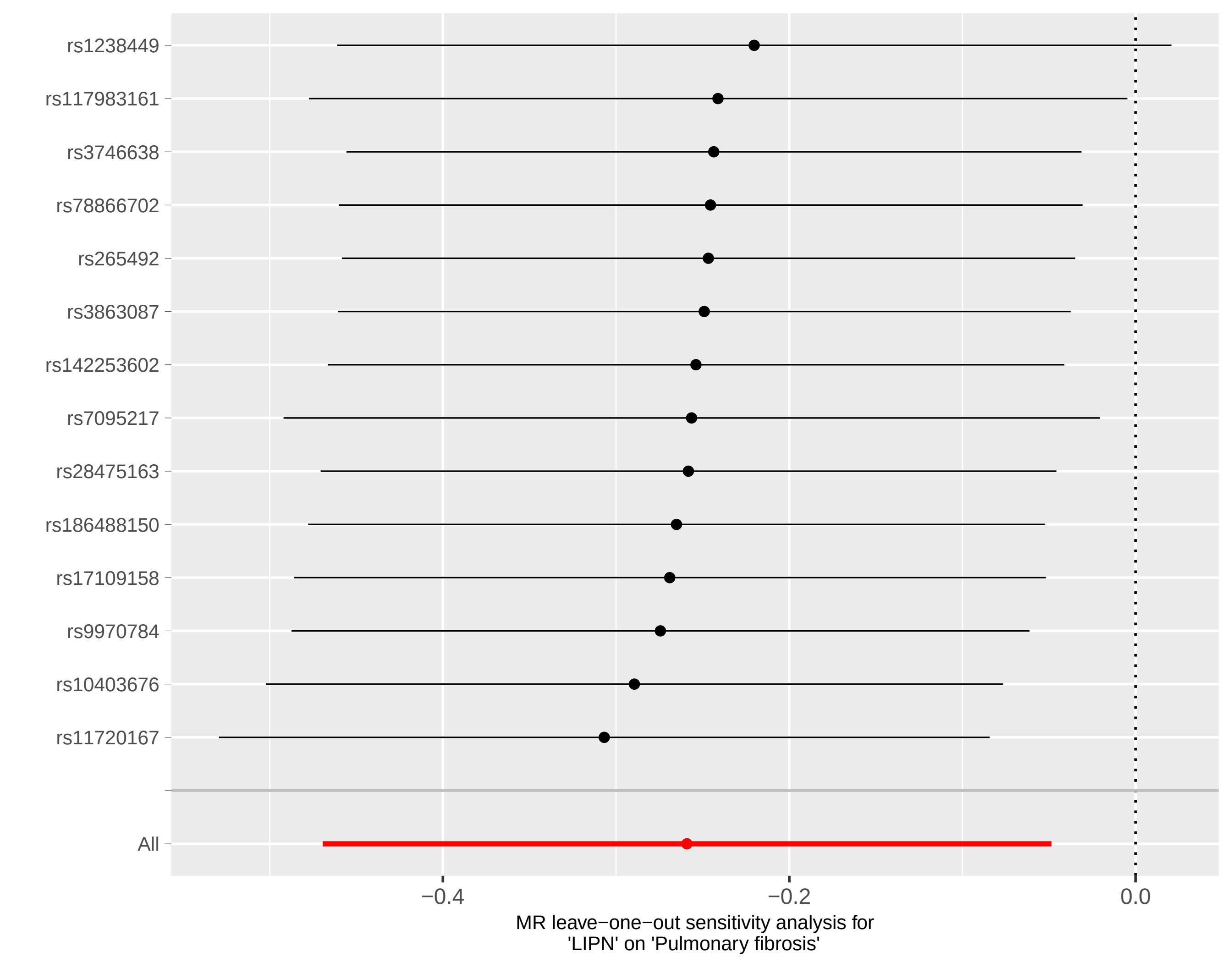
rs1238449
rs117983161
rs3746638
rs78866702
rs265492
rs3863087
rs142253602
rs7095217
rs28475163
rs186488150
rs17109158
rs9970784
rs10403676
rs11720167
All
−0.4
−0.2
0.0
MR leave−one−out sensitivity analysis for
'LIPN' on 'Pulmonary fibrosis'

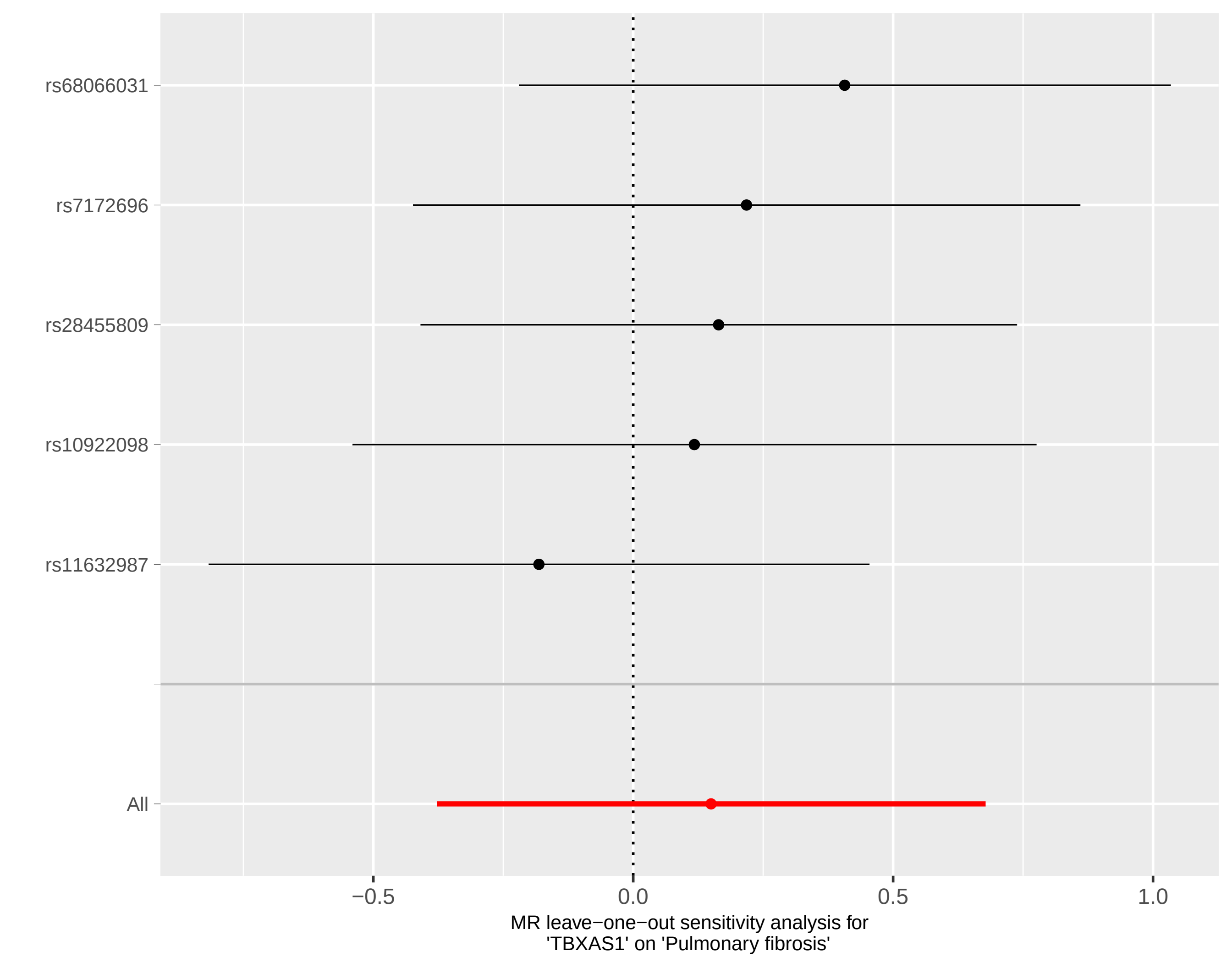
rs68066031
rs7172696
rs28455809
rs10922098
rs11632987
All
−0.5
0.0
0.5
1.0
MR leave−one−out sensitivity analysis for
'TBXAS1' on 'Pulmonary fibrosis'

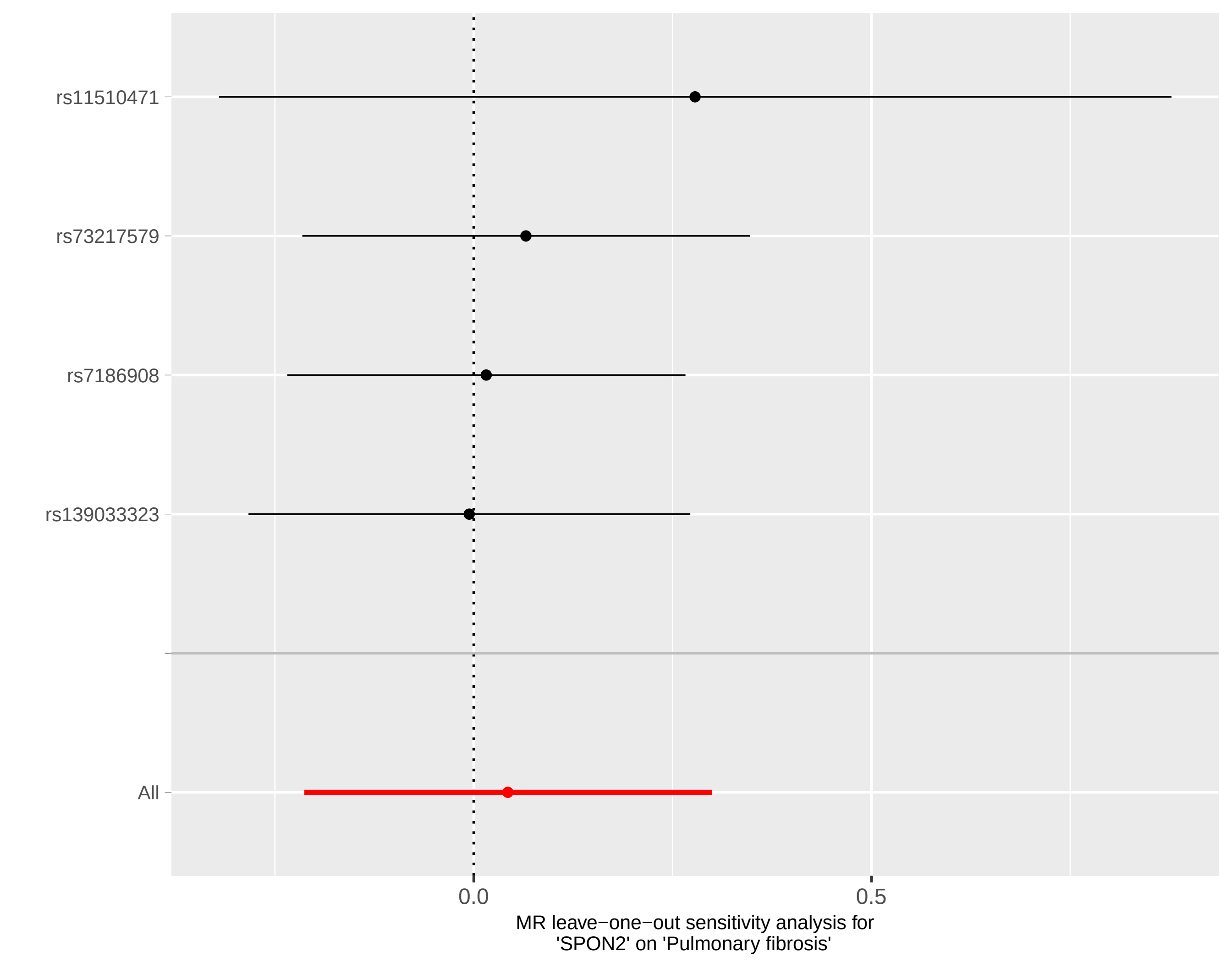
rs11510471
rs73217579
rs7186908
rs139033323
All
0.0
0.5
MR leave−one−out sensitivity analysis for
'SPON2' on 'Pulmonary fibrosis'

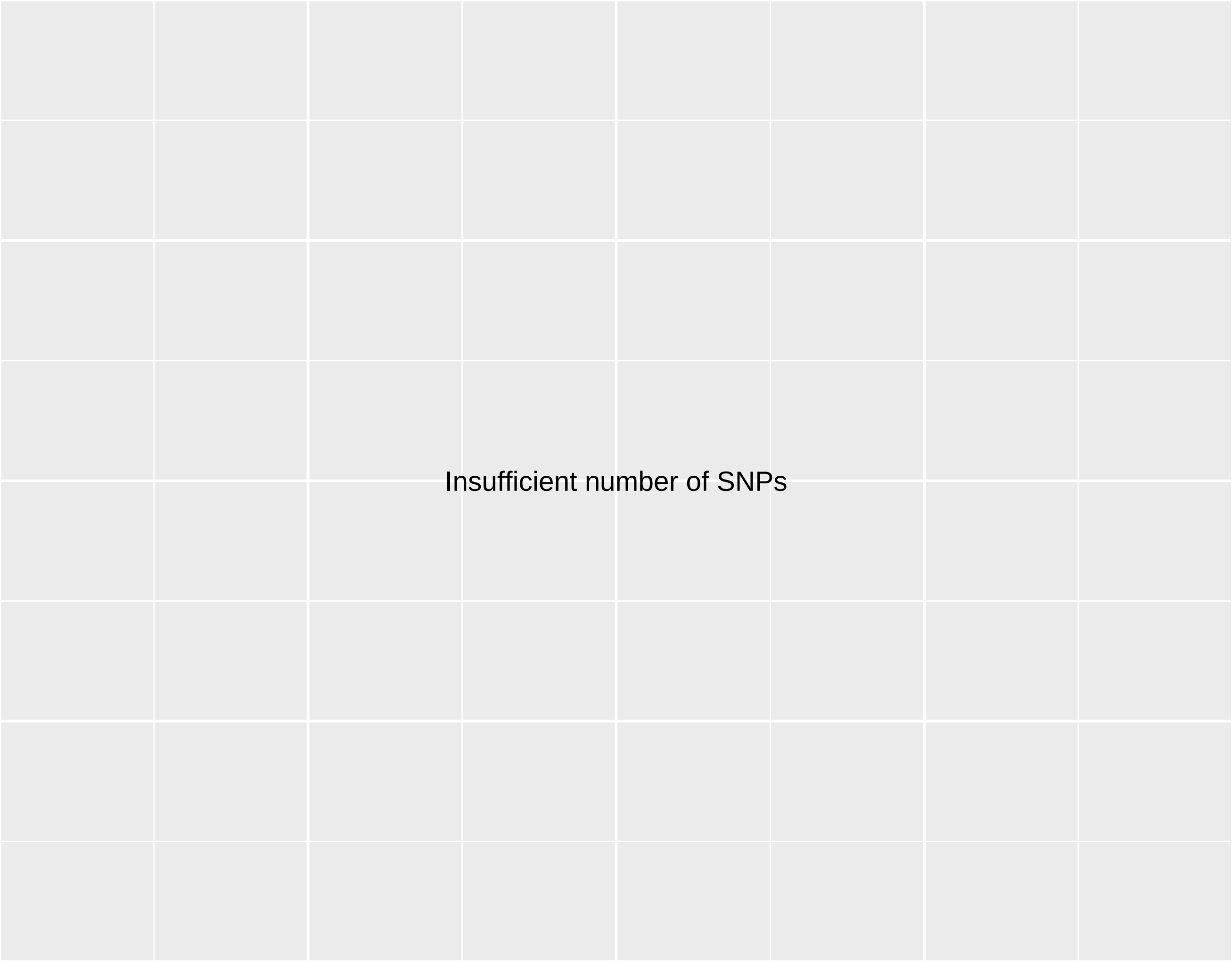
Insufficient number of SNPs

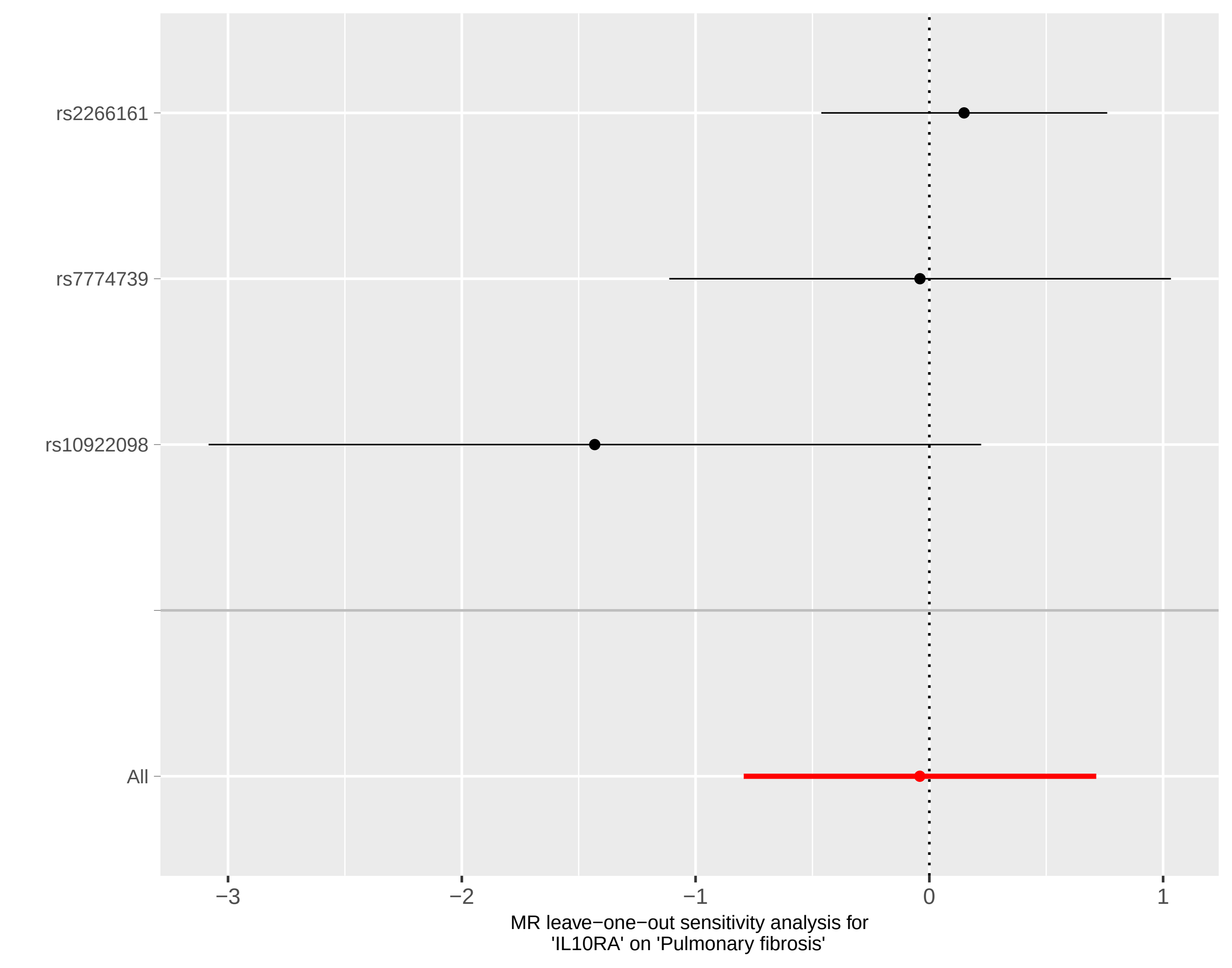
rs2266161
rs7774739
rs10922098
All
−3
−2
−1
0
1
MR leave−one−out sensitivity analysis for
'IL10RA' on 'Pulmonary fibrosis'

Insufficient number of SNPs

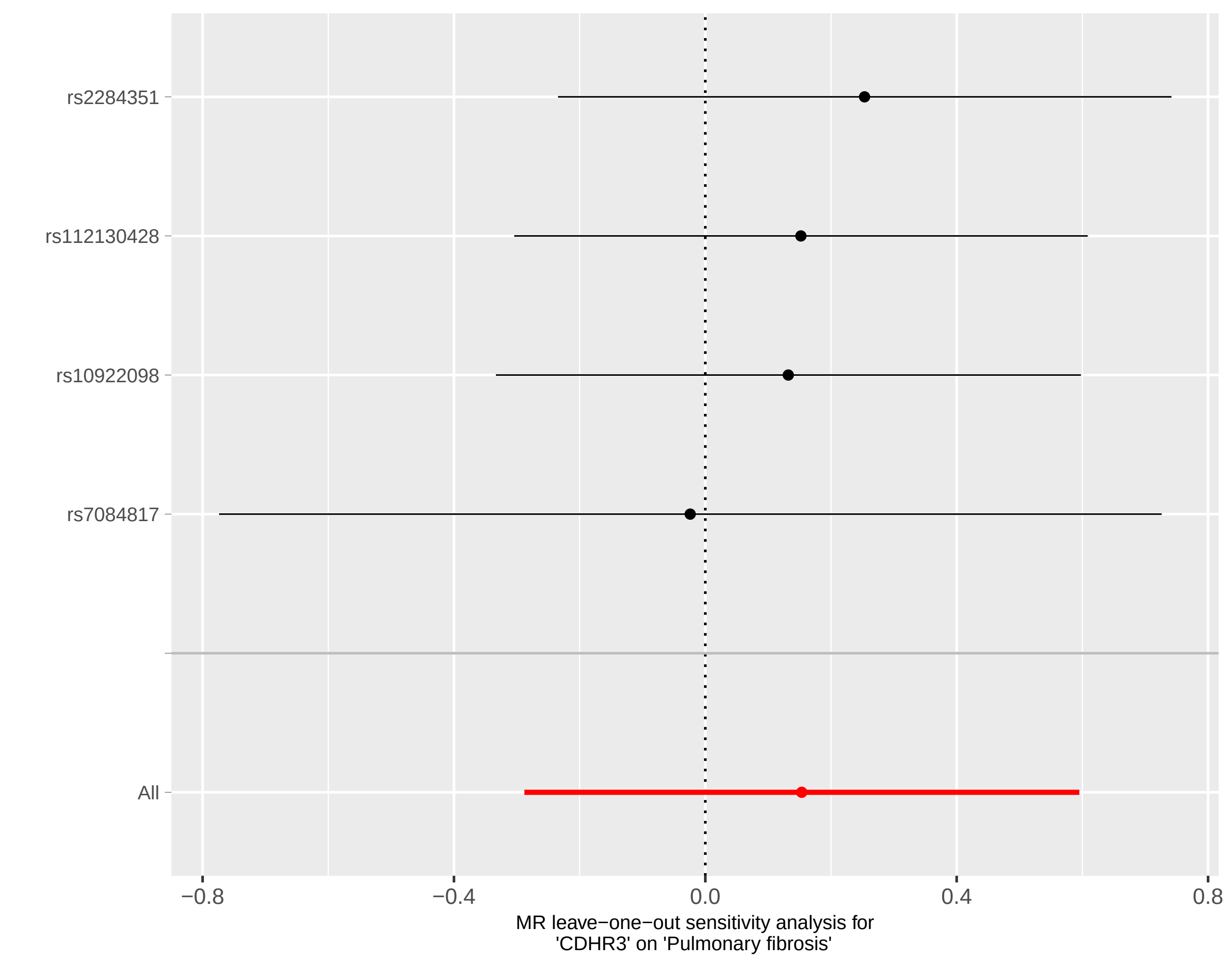
rs2284351
rs112130428
rs10922098
rs7084817
All
−0.8
−0.4
0.0
0.4
0.8
MR leave−one−out sensitivity analysis for
'CDHR3' on 'Pulmonary fibrosis'

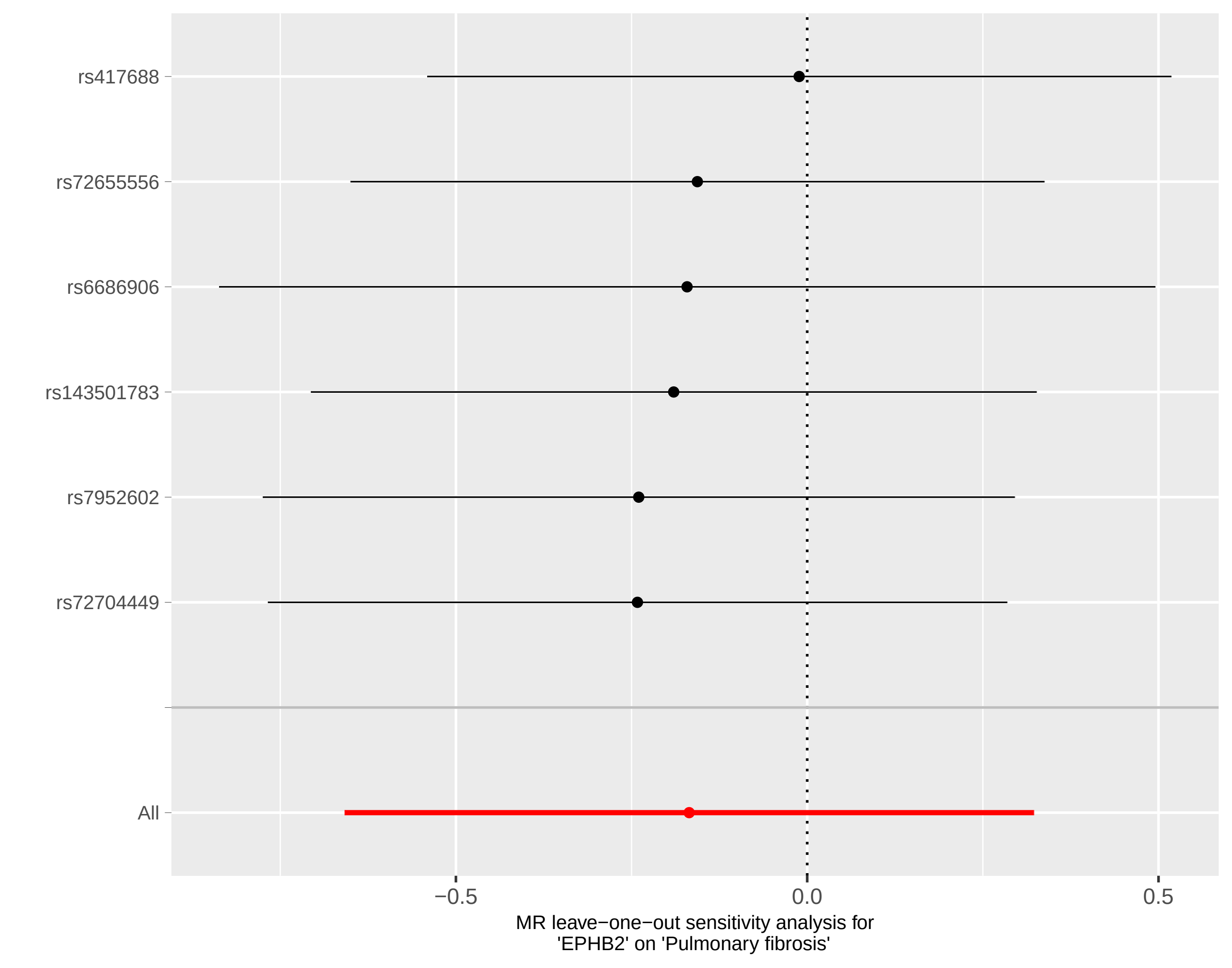
rs417688
rs72655556
rs6686906
rs143501783
rs7952602
rs72704449
All
−0.5
0.0
0.5
MR leave−one−out sensitivity analysis for
'EPHB2' on 'Pulmonary fibrosis'

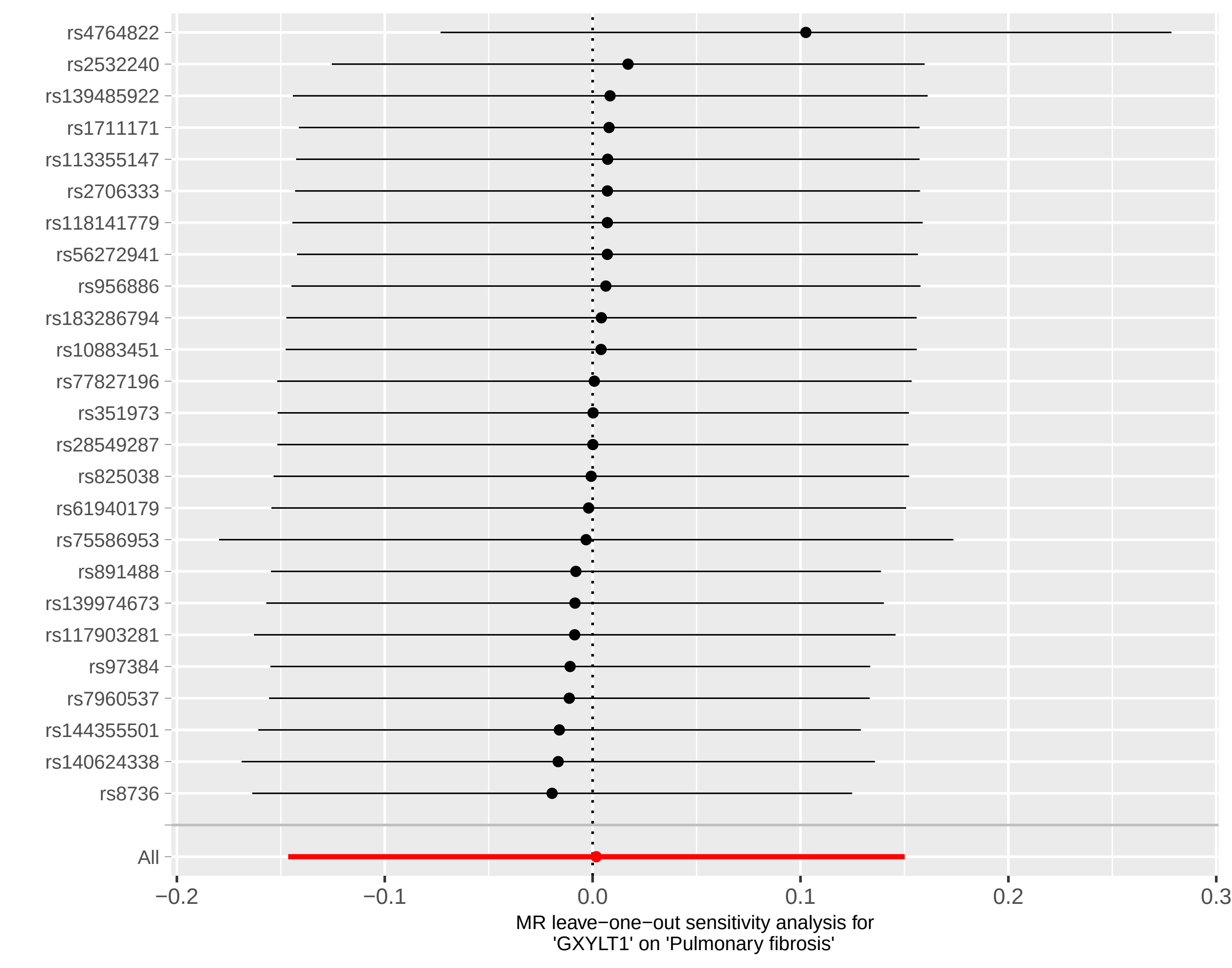
rs4764822
rs2532240
rs139485922
rs1711171
rs113355147
rs2706333
rs118141779
rs56272941
rs956886
rs183286794
rs10883451
rs77827196
rs351973
rs28549287
rs825038
rs61940179
rs75586953
rs891488
rs139974673
rs117903281
rs97384
rs7960537
rs144355501
rs140624338
rs8736
All
−0.2
−0.1
0.0
0.1
0.2
0.3
MR leave−one−out sensitivity analysis for
'GXYLT1' on 'Pulmonary fibrosis'

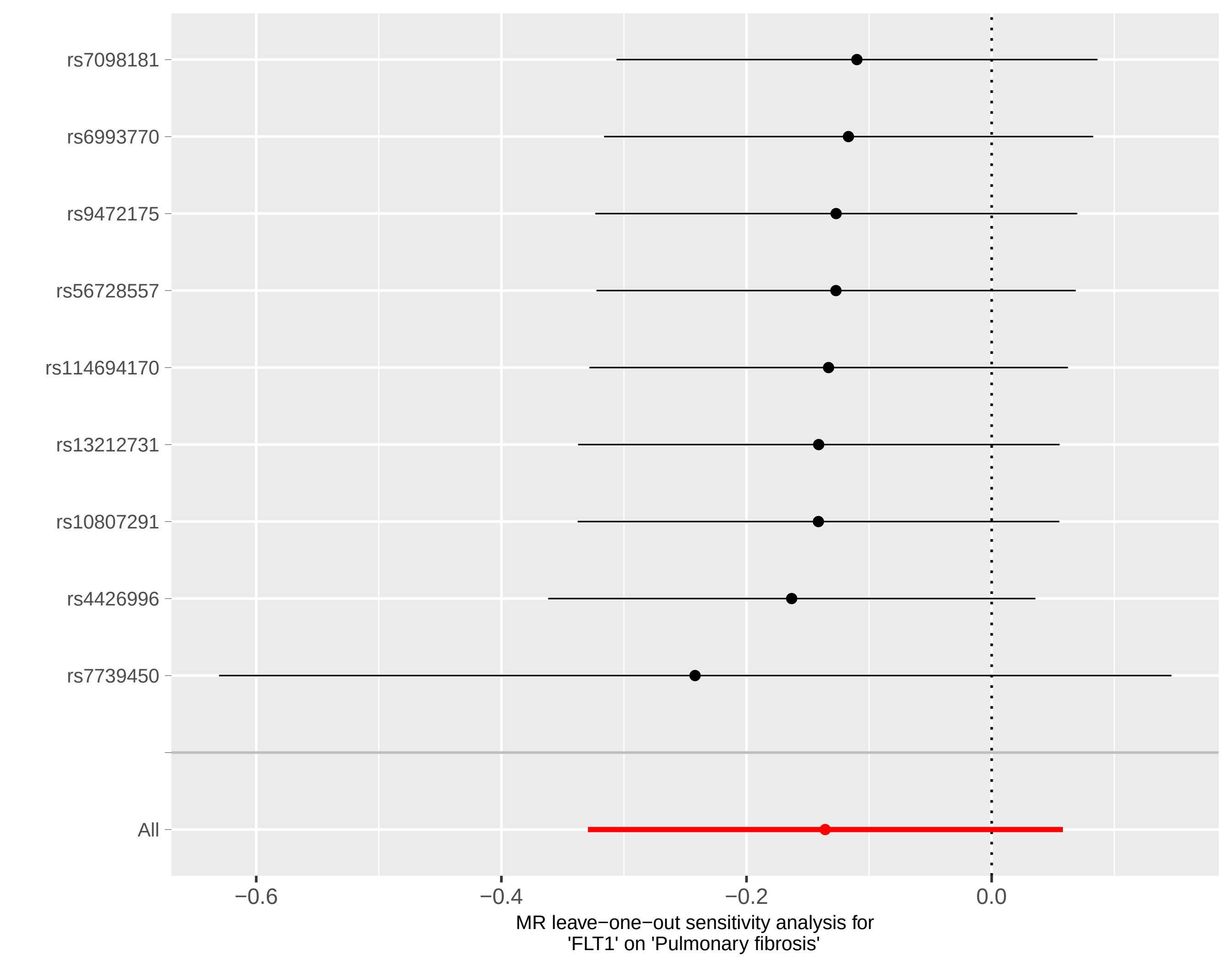
rs7098181
rs6993770
rs9472175
rs56728557
rs114694170
rs13212731
rs10807291
rs4426996
rs7739450
All
−0.6
−0.4
−0.2
0.0
MR leave−one−out sensitivity analysis for
'FLT1' on 'Pulmonary fibrosis'

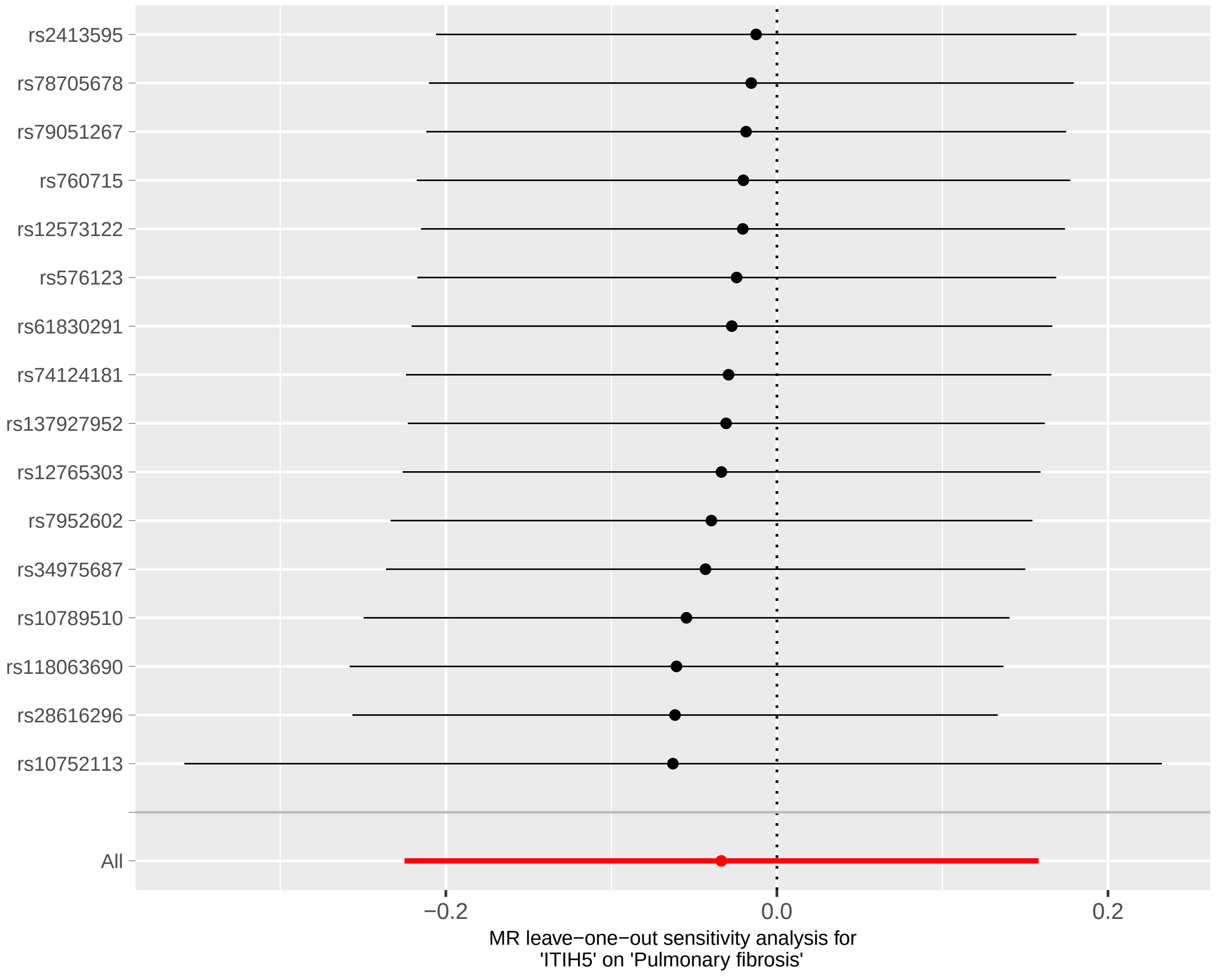
rs2413595
rs78705678
rs79051267
rs760715
rs12573122
rs576123
rs61830291
rs74124181
rs137927952
rs12765303
rs7952602
rs34975687
rs10789510
rs118063690
rs28616296
rs10752113
All
−0.2
0.0
0.2
MR leave−one−out sensitivity analysis for
'ITIH5' on 'Pulmonary fibrosis'

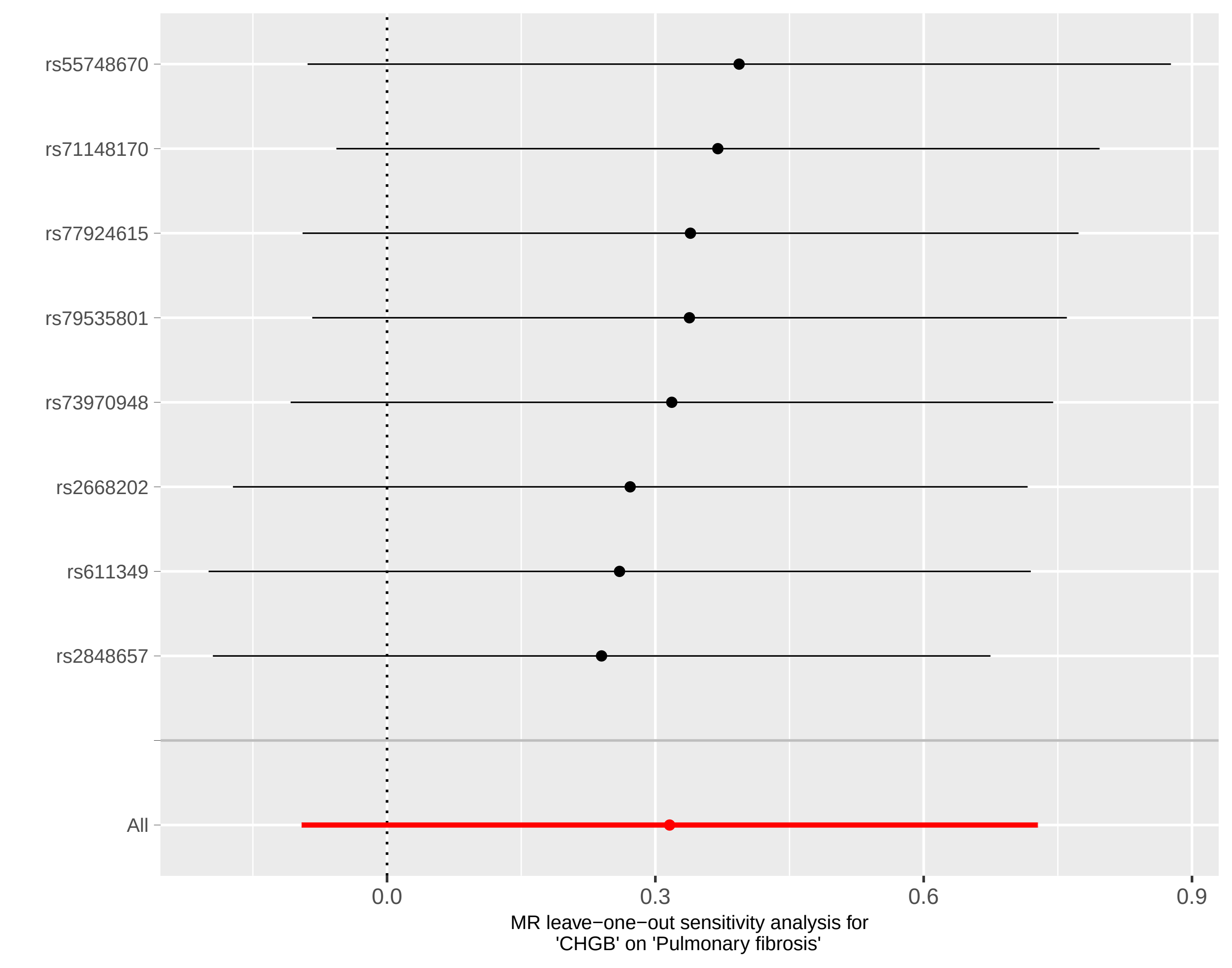
rs55748670
rs71148170
rs77924615
rs79535801
rs73970948
rs2668202
rs611349
rs2848657
All
0.0
0.3
0.6
0.9
MR leave−one−out sensitivity analysis for
'CHGB' on 'Pulmonary fibrosis'

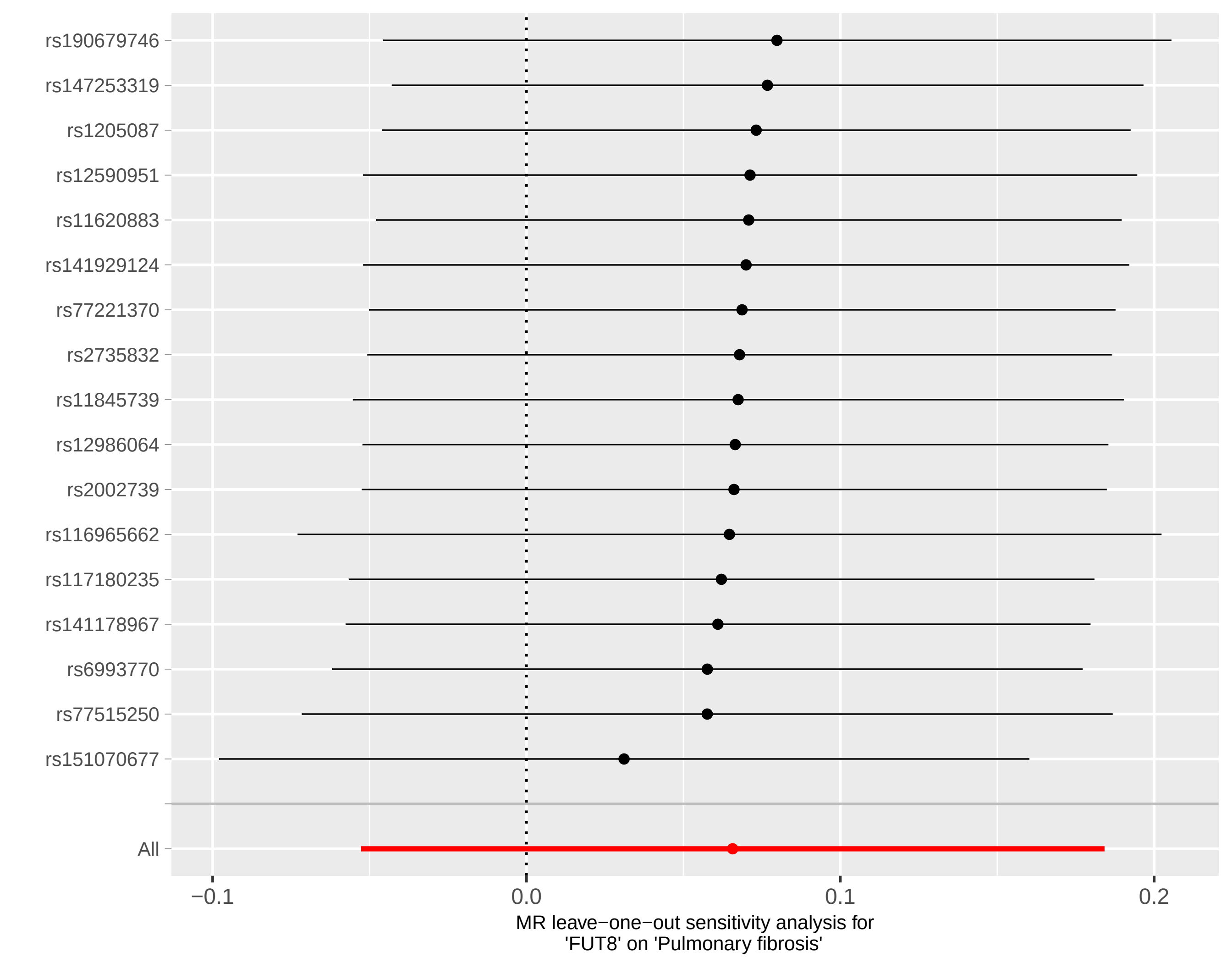

rs190679746
rs147253319
rs1205087
rs12590951
rs11620883
rs141929124
rs77221370
rs2735832
rs11845739
rs12986064
rs2002739
rs116965662
rs117180235
rs141178967
rs6993770
rs77515250
rs151070677
All
−0.1
0.0
0.1
0.2
MR leave−one−out sensitivity analysis for
'FUT8' on 'Pulmonary fibrosis'

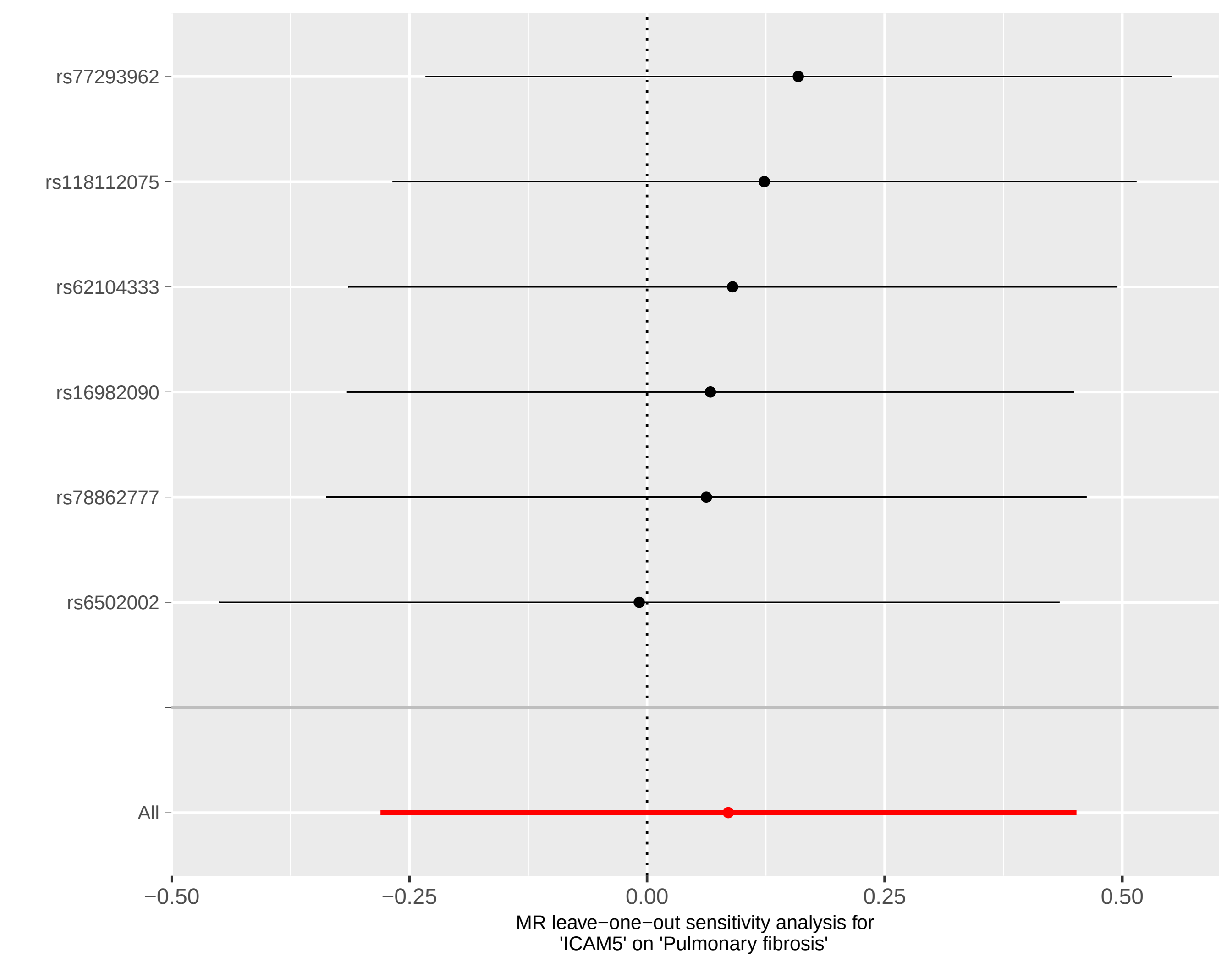

rs77293962
rs118112075
rs62104333
rs16982090
rs78862777
rs6502002
All
−0.50
−0.25
0.00
0.25
0.50
MR leave−one−out sensitivity analysis for
'ICAM5' on 'Pulmonary fibrosis'

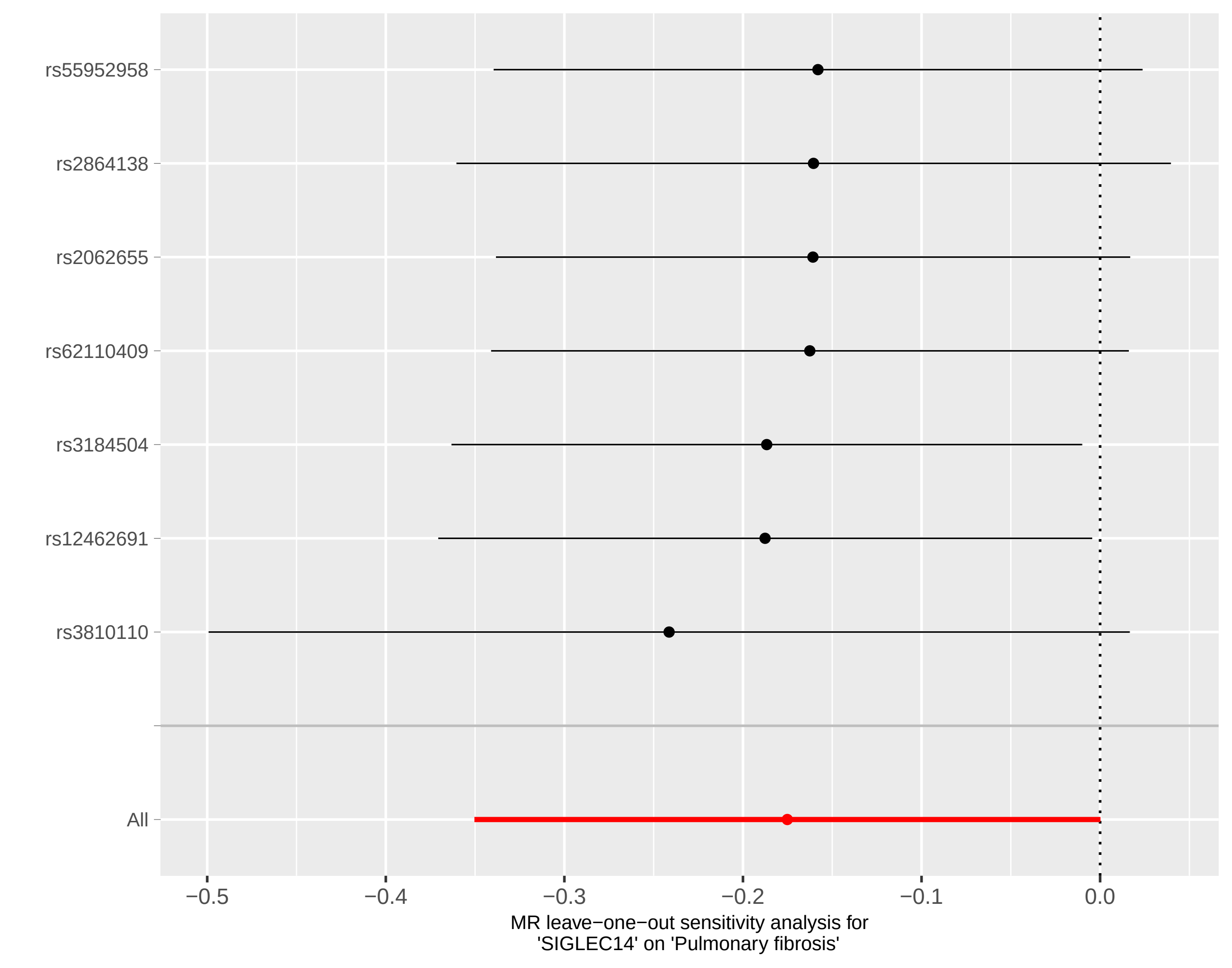
rs55952958
rs2864138
rs2062655
rs62110409
rs3184504
rs12462691
rs3810110
All
−0.5
−0.4
−0.3
−0.2
−0.1
0.0
MR leave−one−out sensitivity analysis for
'SIGLEC14' on 'Pulmonary fibrosis'

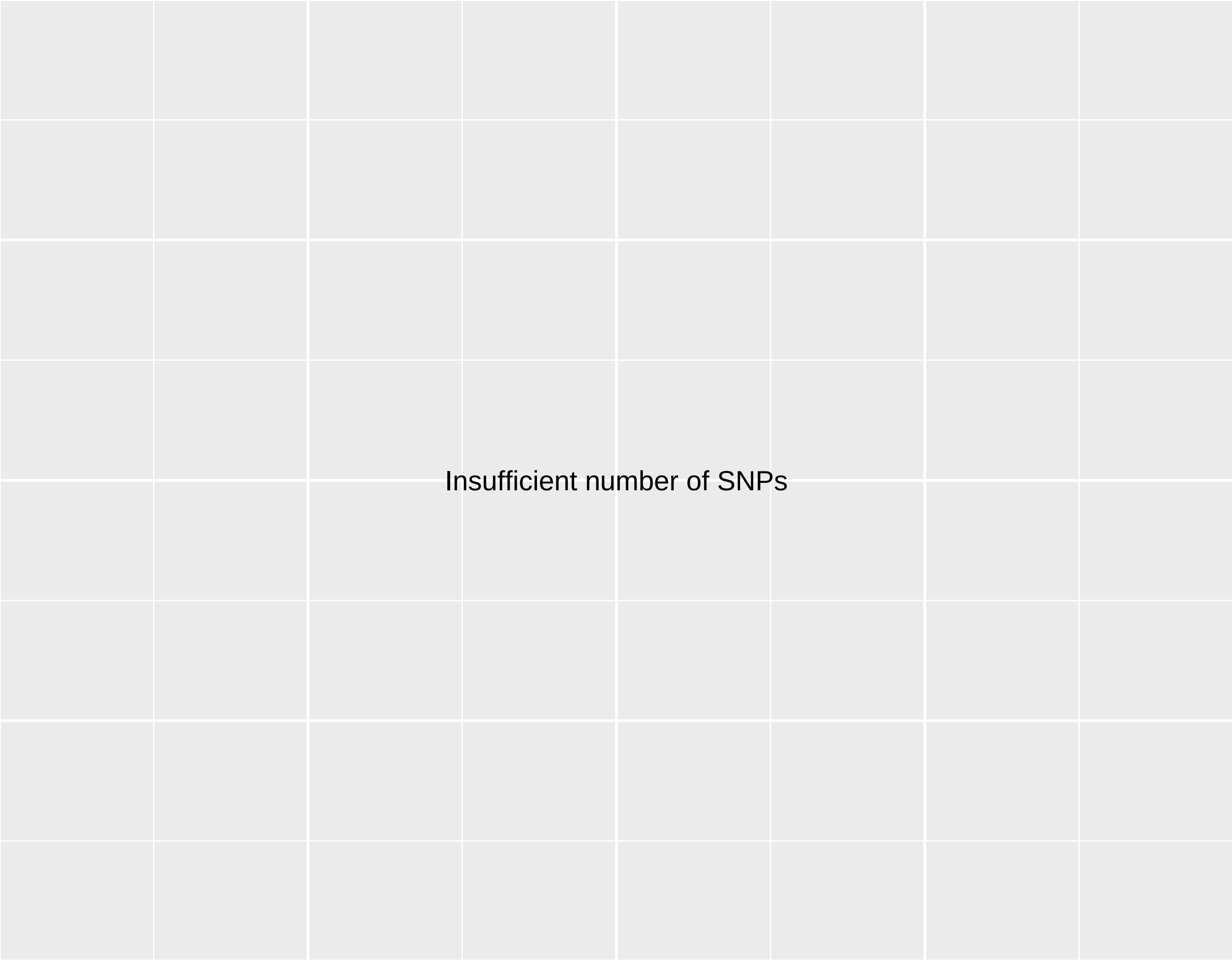
Insufficient number of SNPs

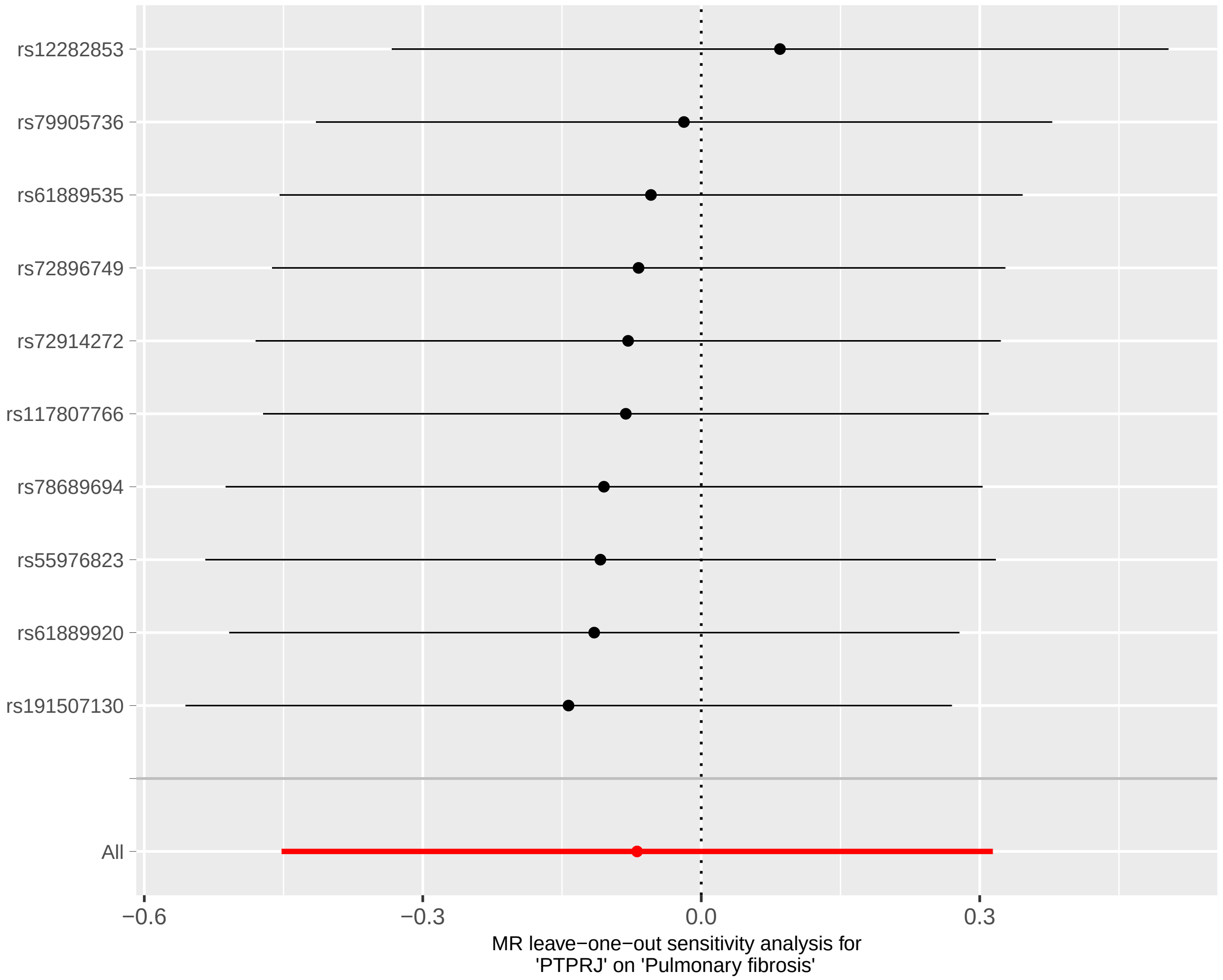

rs12282853
rs79905736
rs61889535
rs72896749
rs72914272
rs117807766
rs78689694
rs55976823
rs61889920
rs191507130
All
−0.6
−0.3
0.0
0.3
MR leave−one−out sensitivity analysis for
'PTPRJ' on 'Pulmonary fibrosis'

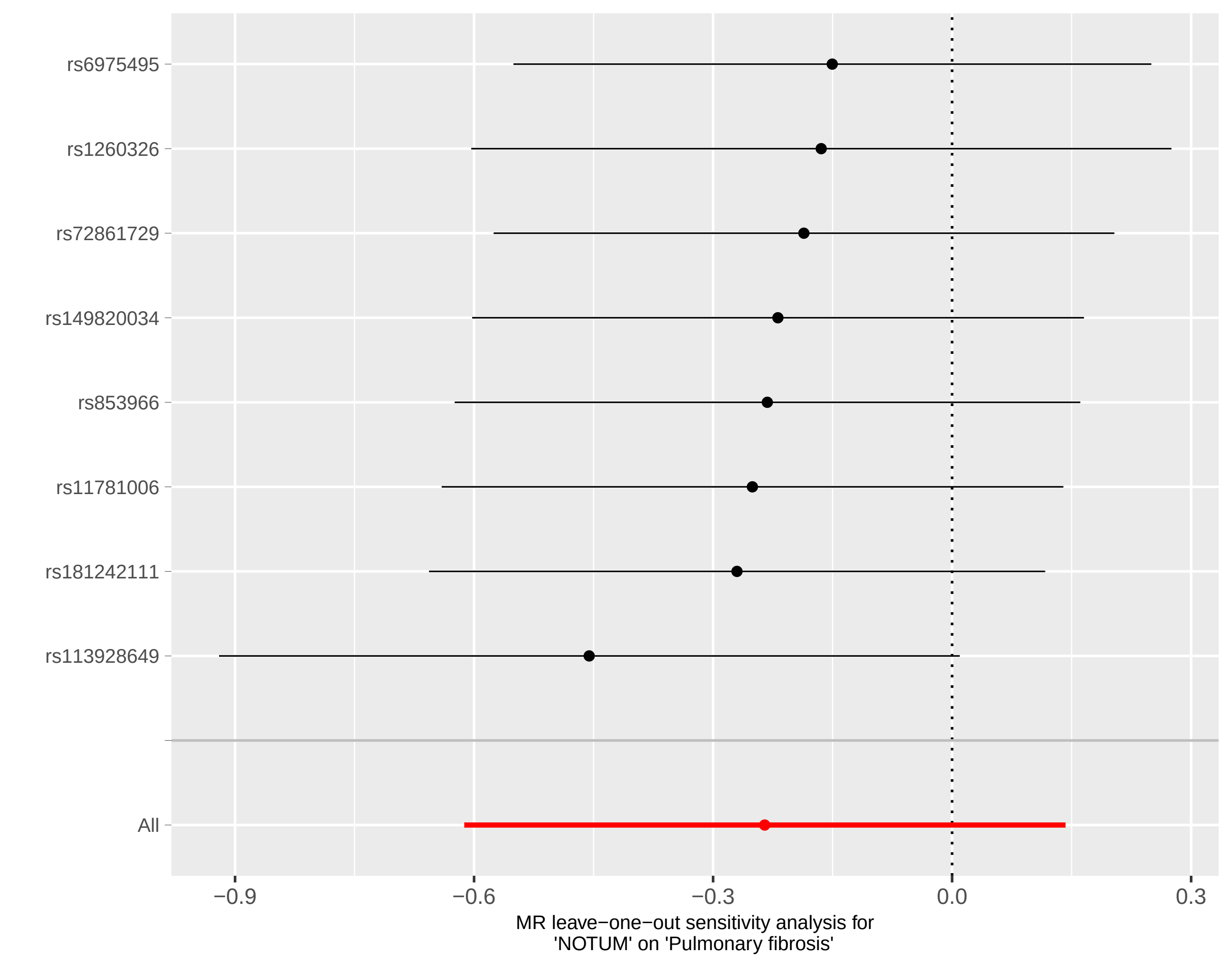
rs6975495
rs1260326
rs72861729
rs149820034
rs853966
rs11781006
rs181242111
rs113928649
All
−0.9
−0.6
−0.3
0.0
0.3
MR leave−one−out sensitivity analysis for
'NOTUM' on 'Pulmonary fibrosis'

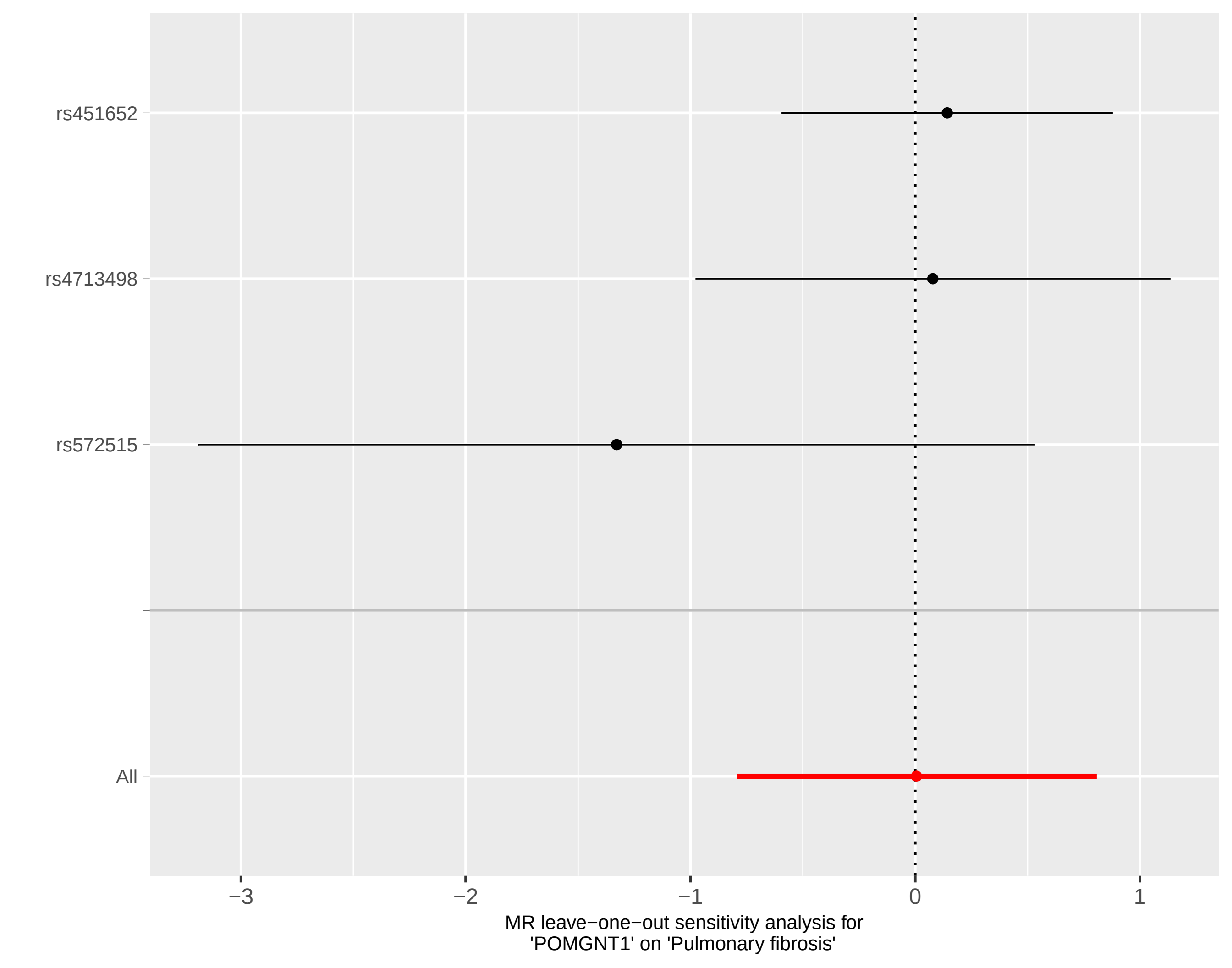
rs451652
rs4713498
rs572515
All
−3
−2
−1
0
1
MR leave−one−out sensitivity analysis for
'POMGNT1' on 'Pulmonary fibrosis'

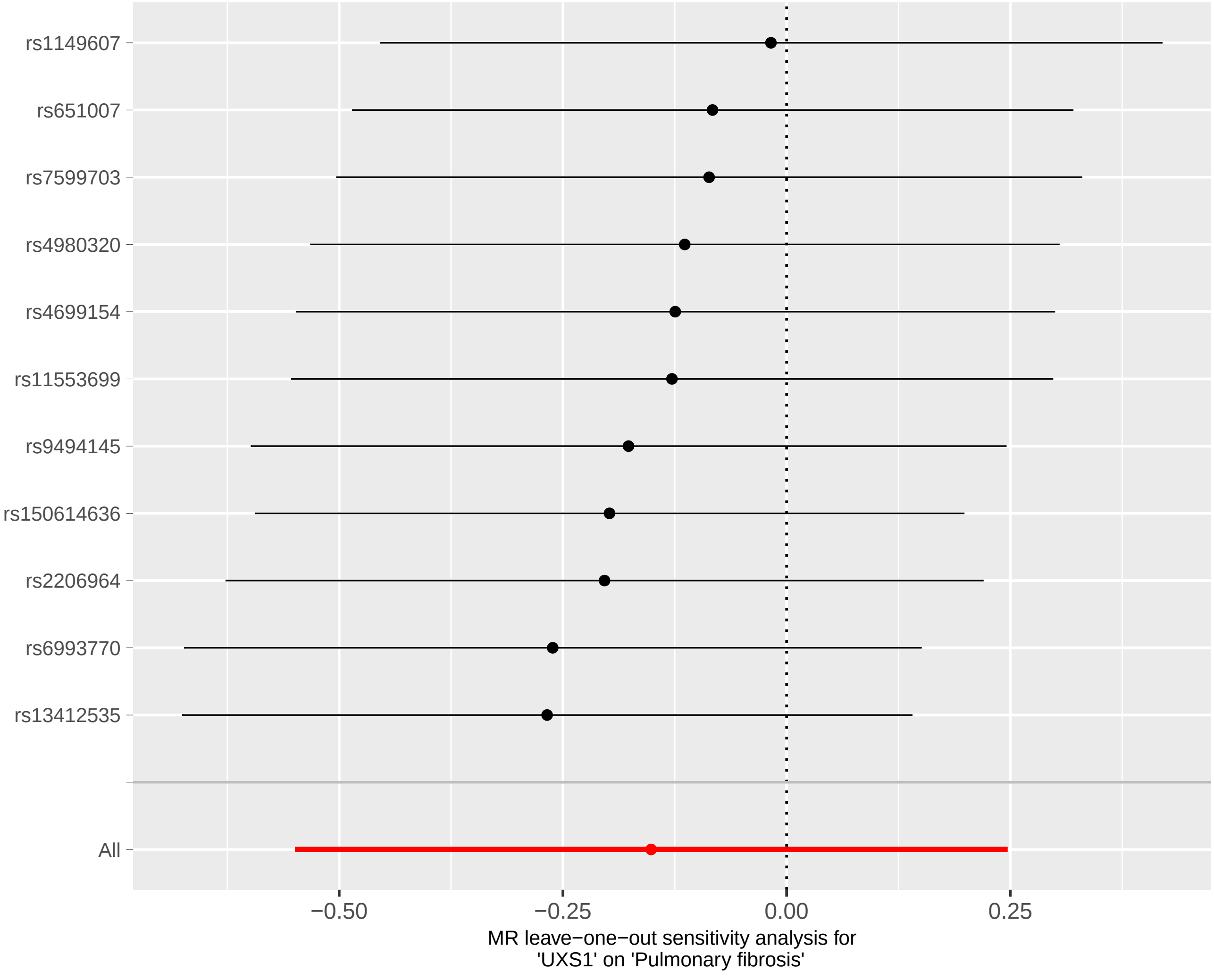
rs1149607
rs651007
rs7599703
rs4980320
rs4699154
rs11553699
rs9494145
rs150614636
rs2206964
rs6993770
rs13412535
All
−0.50
−0.25
0.00
0.25
MR leave−one−out sensitivity analysis for
'UXS1' on 'Pulmonary fibrosis'

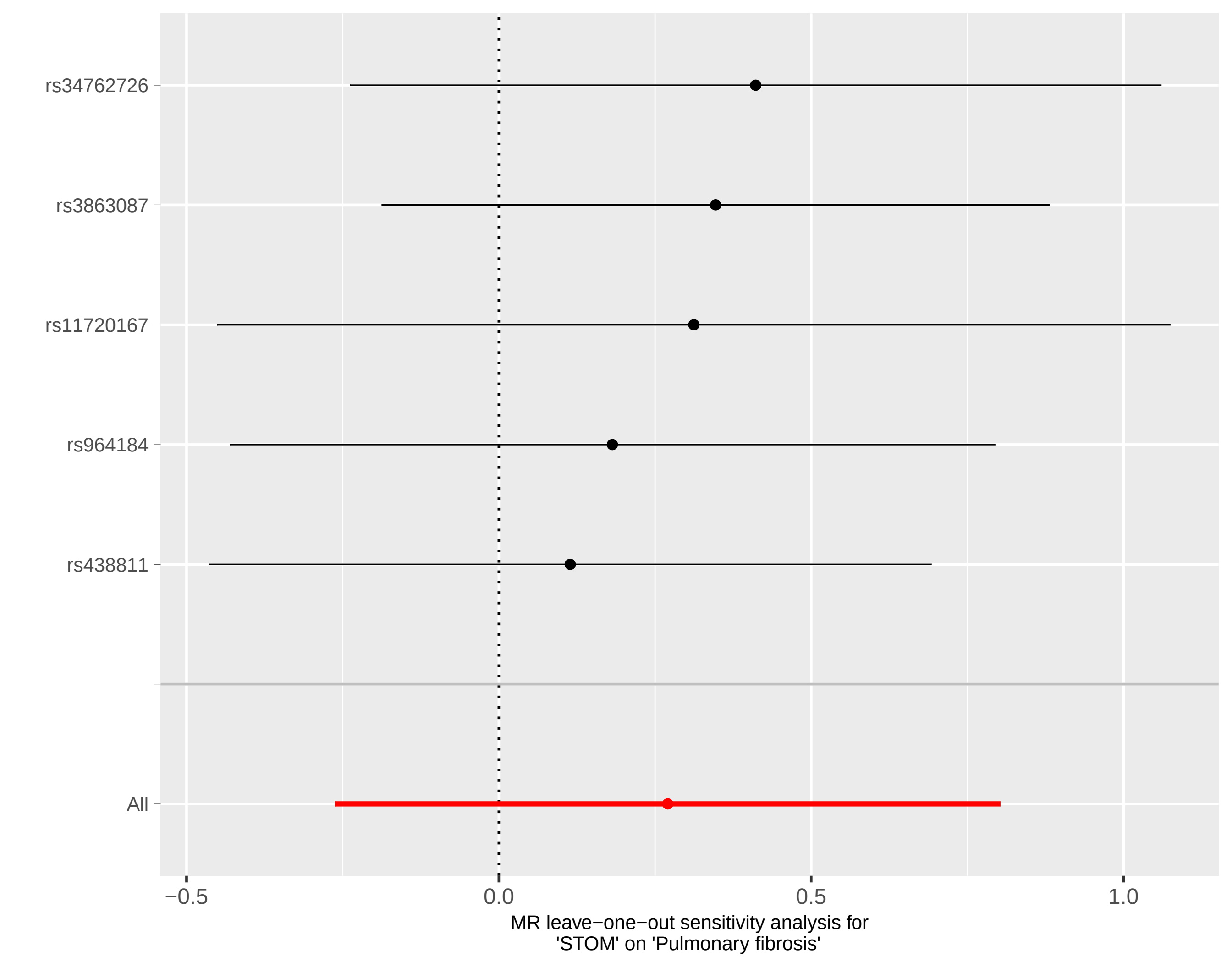
rs34762726
rs3863087
rs11720167
rs964184
rs438811
All
−0.5
0.0
0.5
1.0
MR leave−one−out sensitivity analysis for
'STOM' on 'Pulmonary fibrosis'

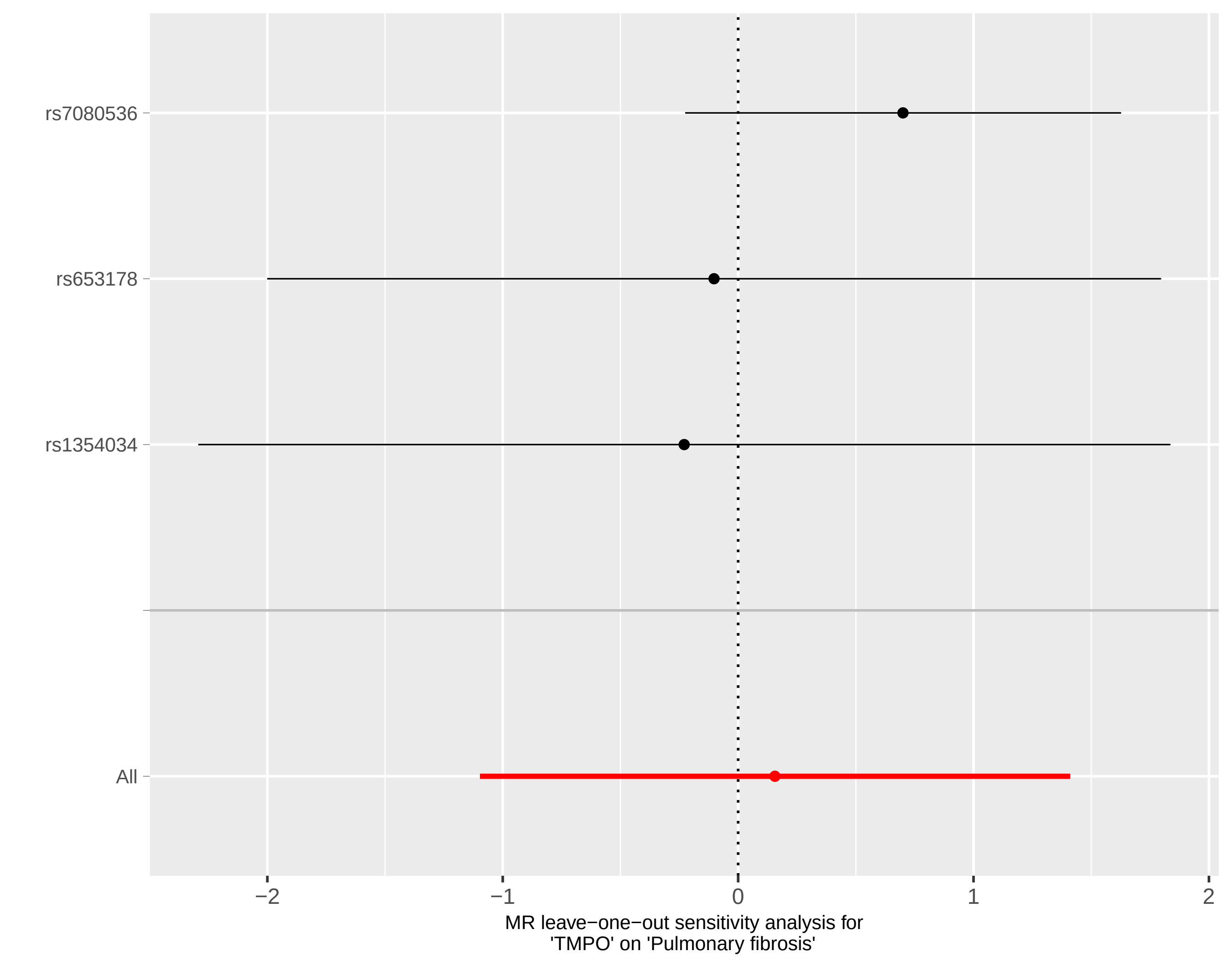
rs7080536
rs653178
rs1354034
All
−2
−1
0
1
2
MR leave−one−out sensitivity analysis for
'TMPO' on 'Pulmonary fibrosis'

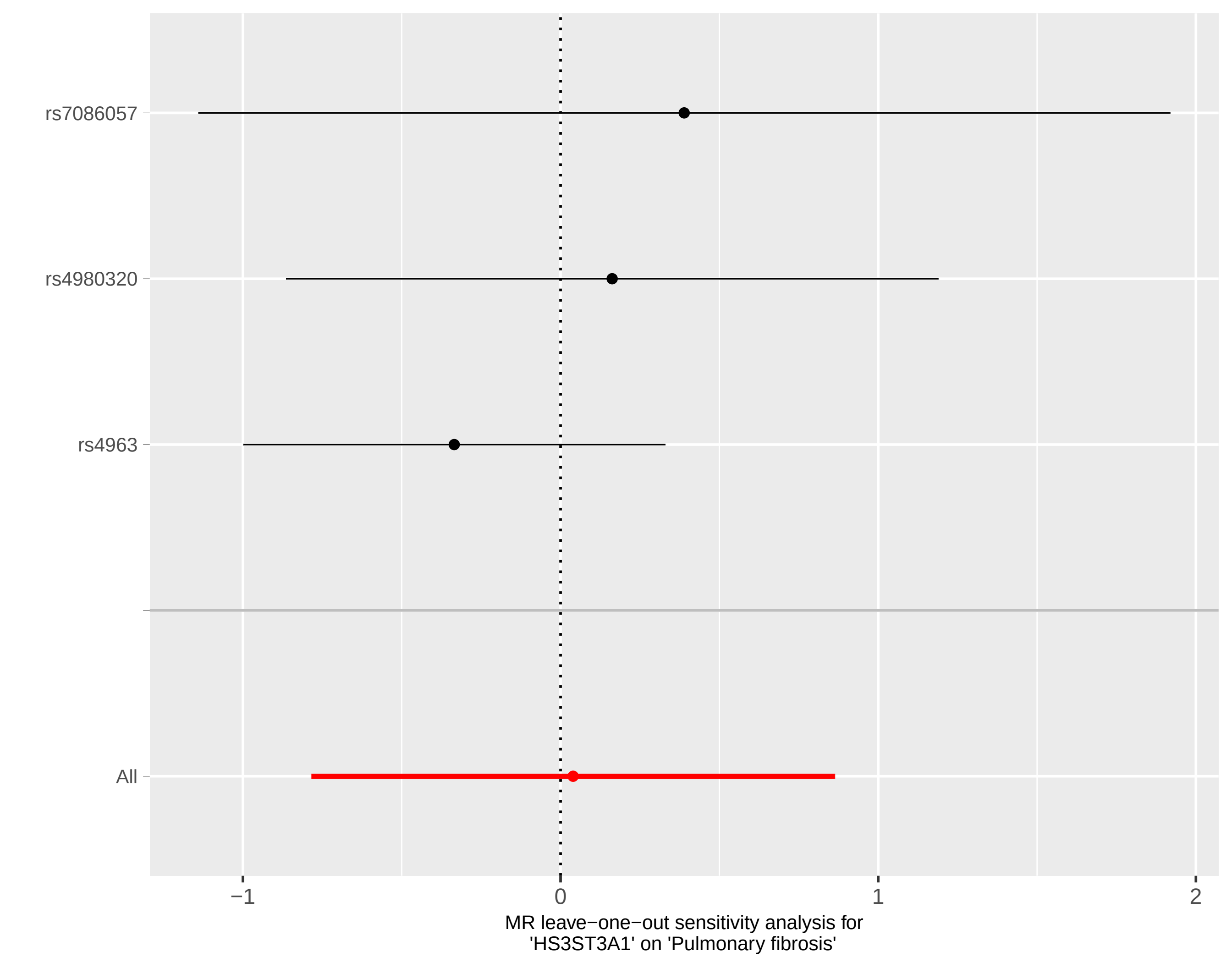
rs7086057
rs4980320
rs4963
All
−1
0
1
2
MR leave−one−out sensitivity analysis for
'HS3ST3A1' on 'Pulmonary fibrosis'

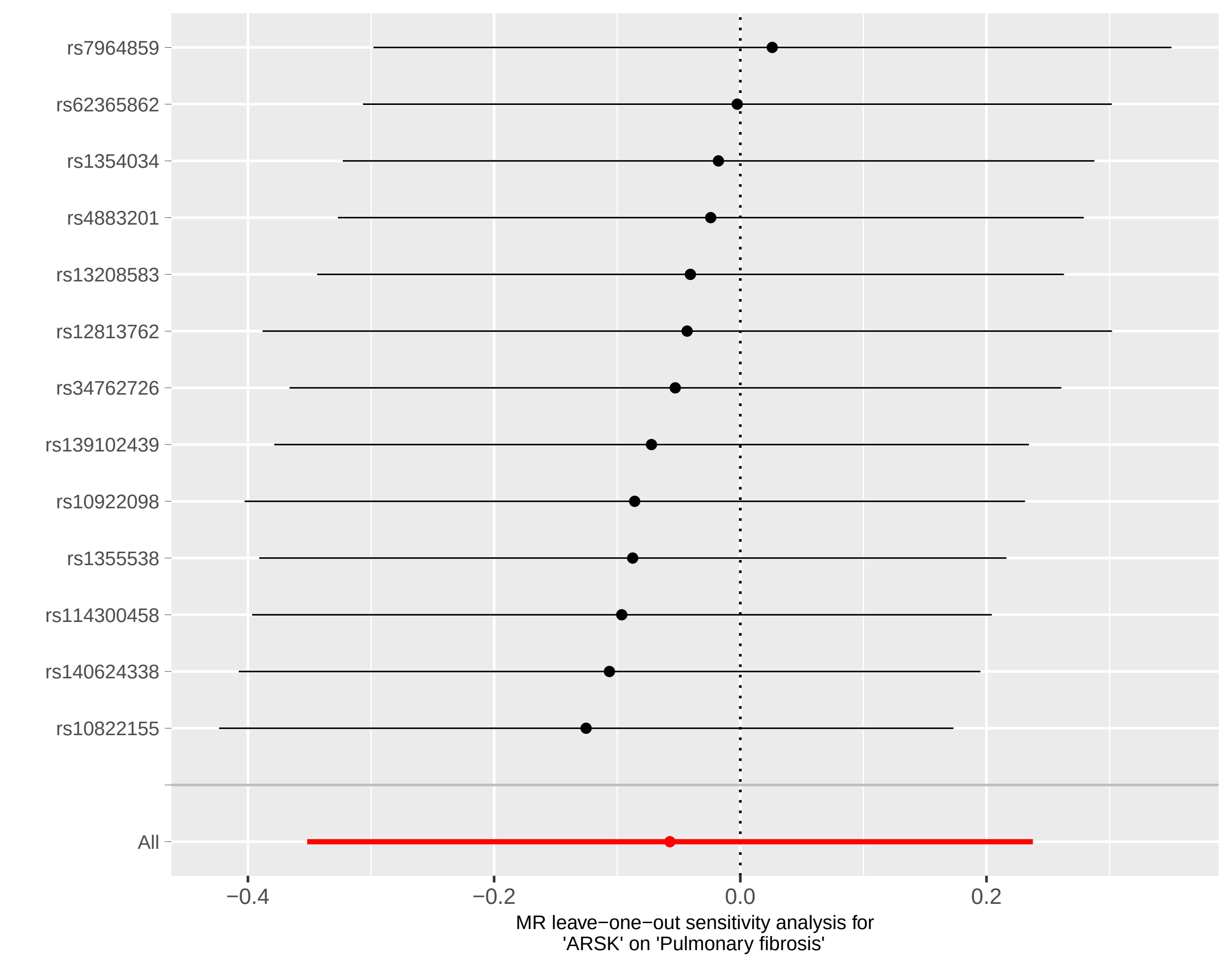
rs7964859
rs62365862
rs1354034
rs4883201
rs13208583
rs12813762
rs34762726
rs139102439
rs10922098
rs1355538
rs114300458
rs140624338
rs10822155
All
−0.4
−0.2
0.0
0.2
MR leave−one−out sensitivity analysis for
'ARSK' on 'Pulmonary fibrosis'

Insufficient number of SNPs

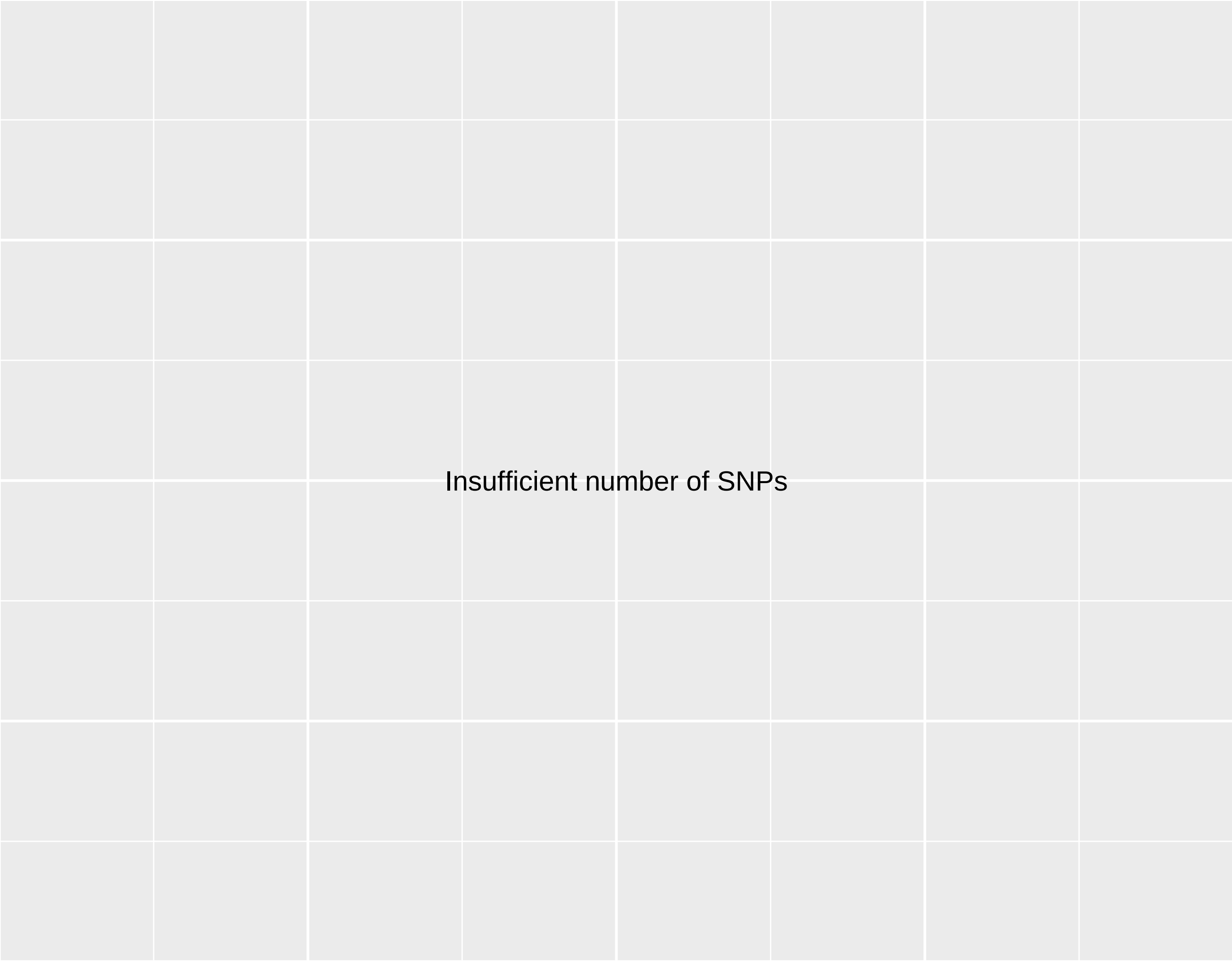
Insufficient number of SNPs

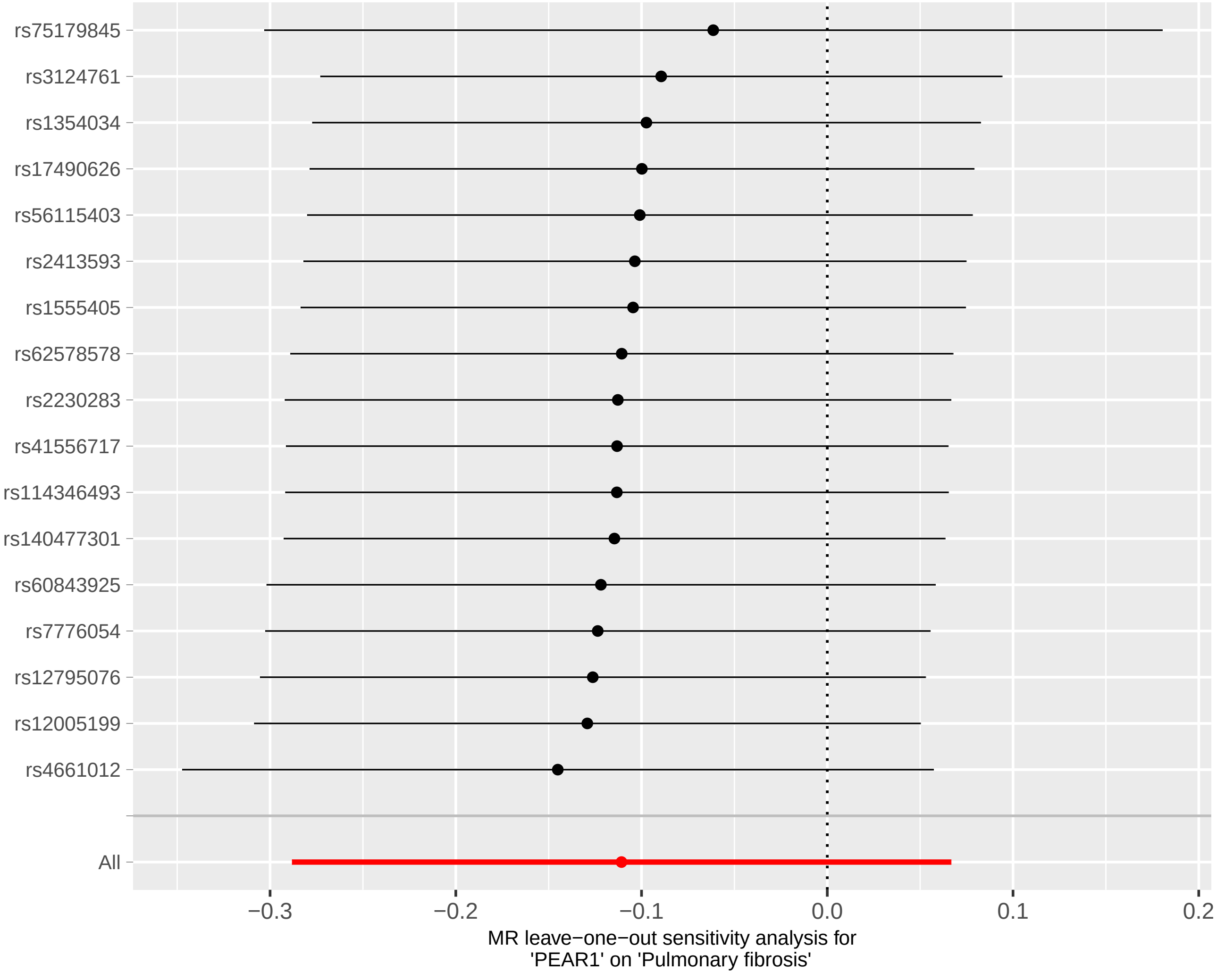
rs75179845
rs3124761
rs1354034
rs17490626
rs56115403
rs2413593
rs1555405
rs62578578
rs2230283
rs41556717
rs114346493
rs140477301
rs60843925
rs7776054
rs12795076
rs12005199
rs4661012
All
−0.3
−0.2
−0.1
0.0
0.1
0.2
MR leave−one−out sensitivity analysis for
'PEAR1' on 'Pulmonary fibrosis'

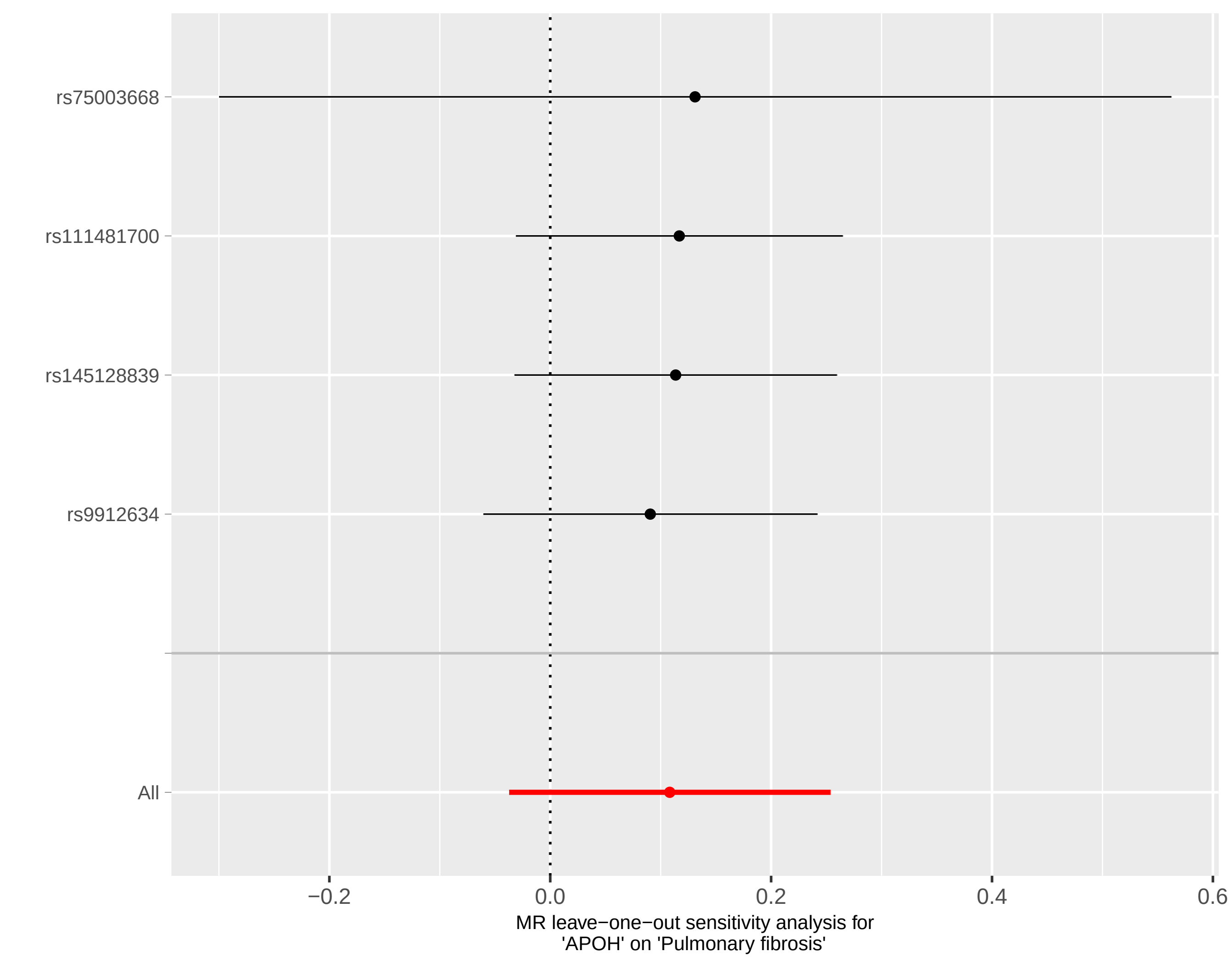
rs75003668
rs111481700
rs145128839
rs9912634
All
−0.2
0.0
0.2
0.4
0.6
MR leave−one−out sensitivity analysis for
'APOH' on 'Pulmonary fibrosis'

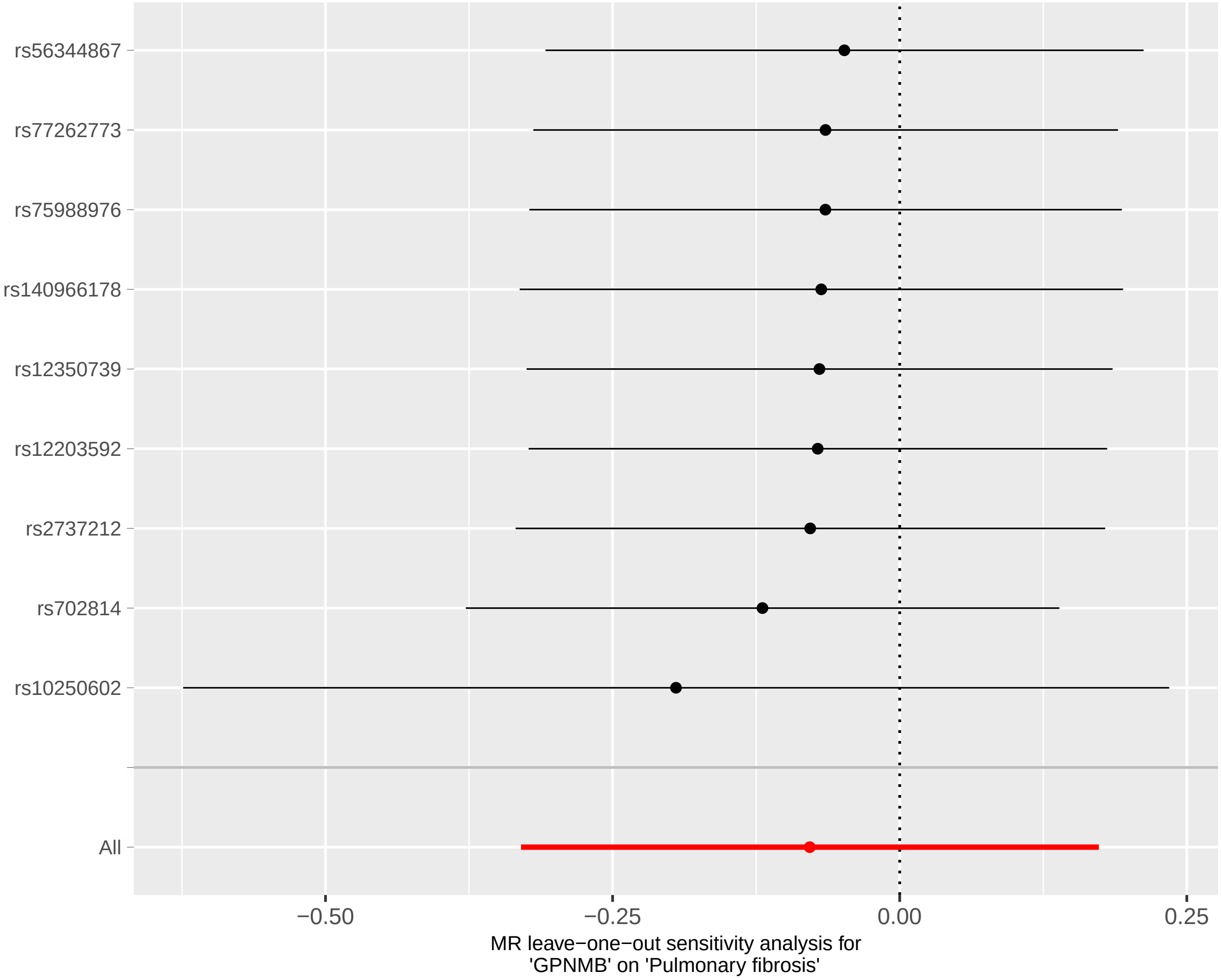
rs56344867
rs77262773
rs75988976
rs140966178
rs12350739
rs12203592
rs2737212
rs702814
rs10250602
All
−0.50
−0.25
0.00
0.25
MR leave−one−out sensitivity analysis for
'GPNMB' on 'Pulmonary fibrosis'

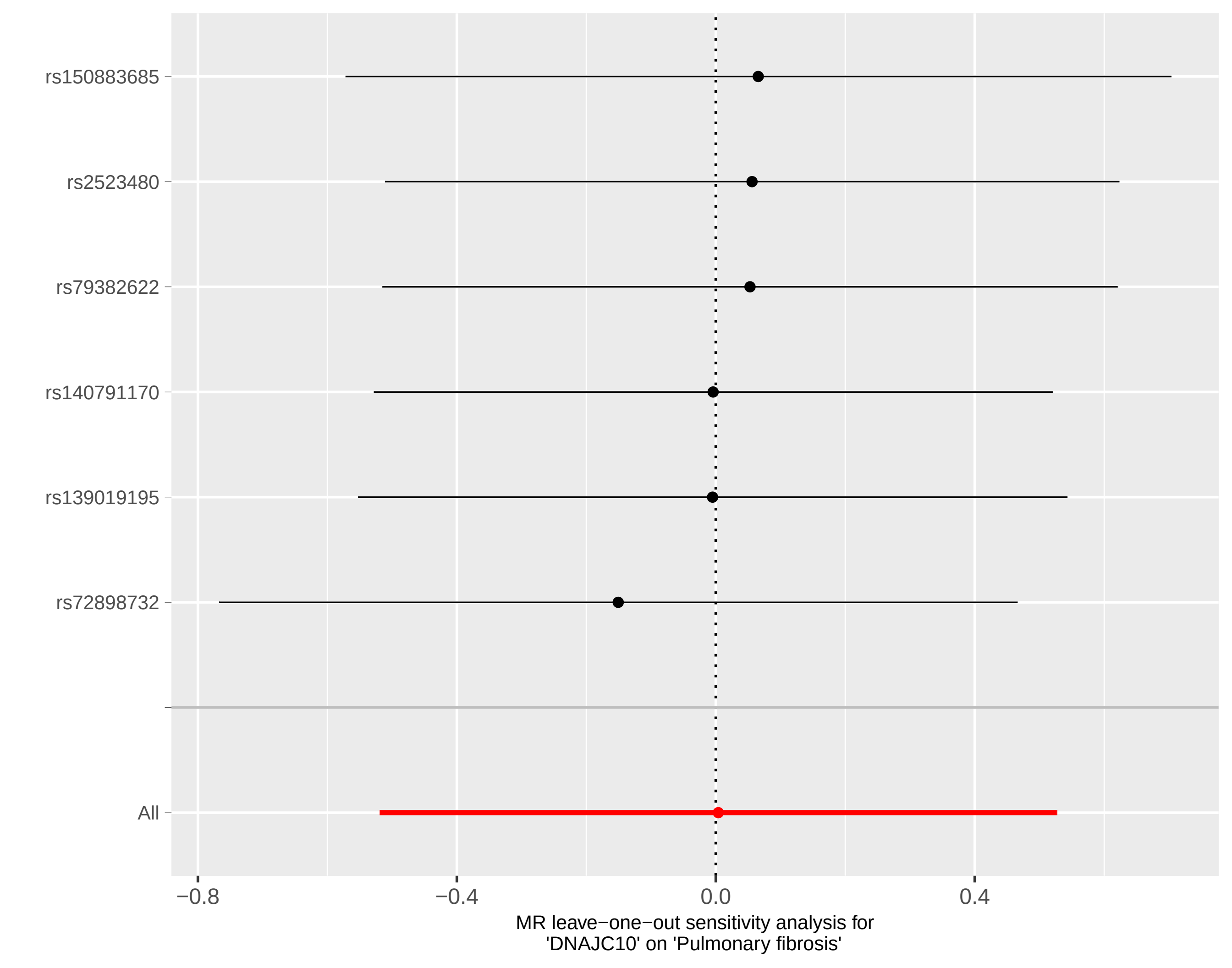
rs150883685
rs2523480
rs79382622
rs140791170
rs139019195
rs72898732
All
−0.8
−0.4
0.0
0.4
MR leave−one−out sensitivity analysis for
'DNAJC10' on 'Pulmonary fibrosis'

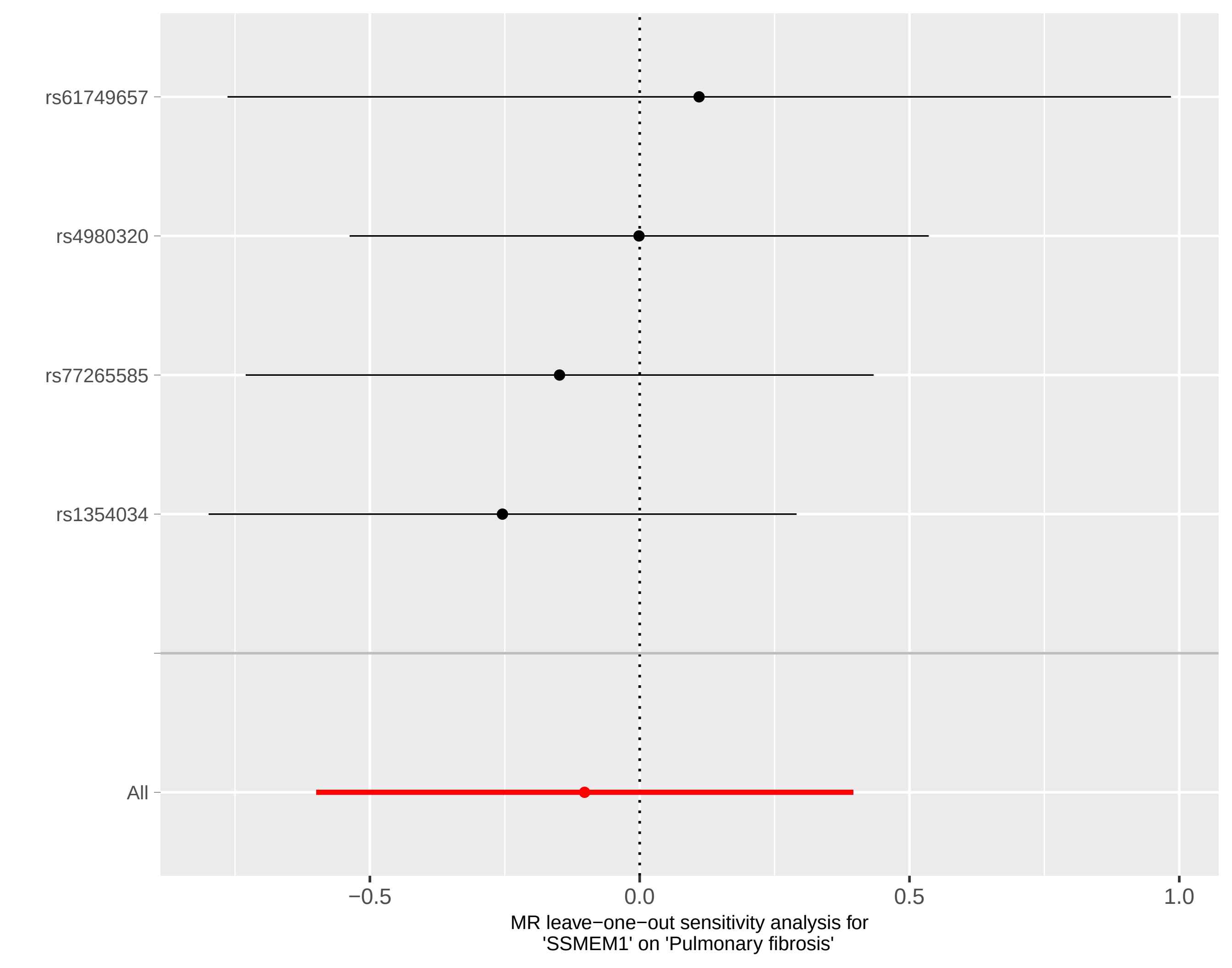
rs61749657
rs4980320
rs77265585
rs1354034
All
−0.5
0.0
0.5
1.0
MR leave−one−out sensitivity analysis for
'SSMEM1' on 'Pulmonary fibrosis'

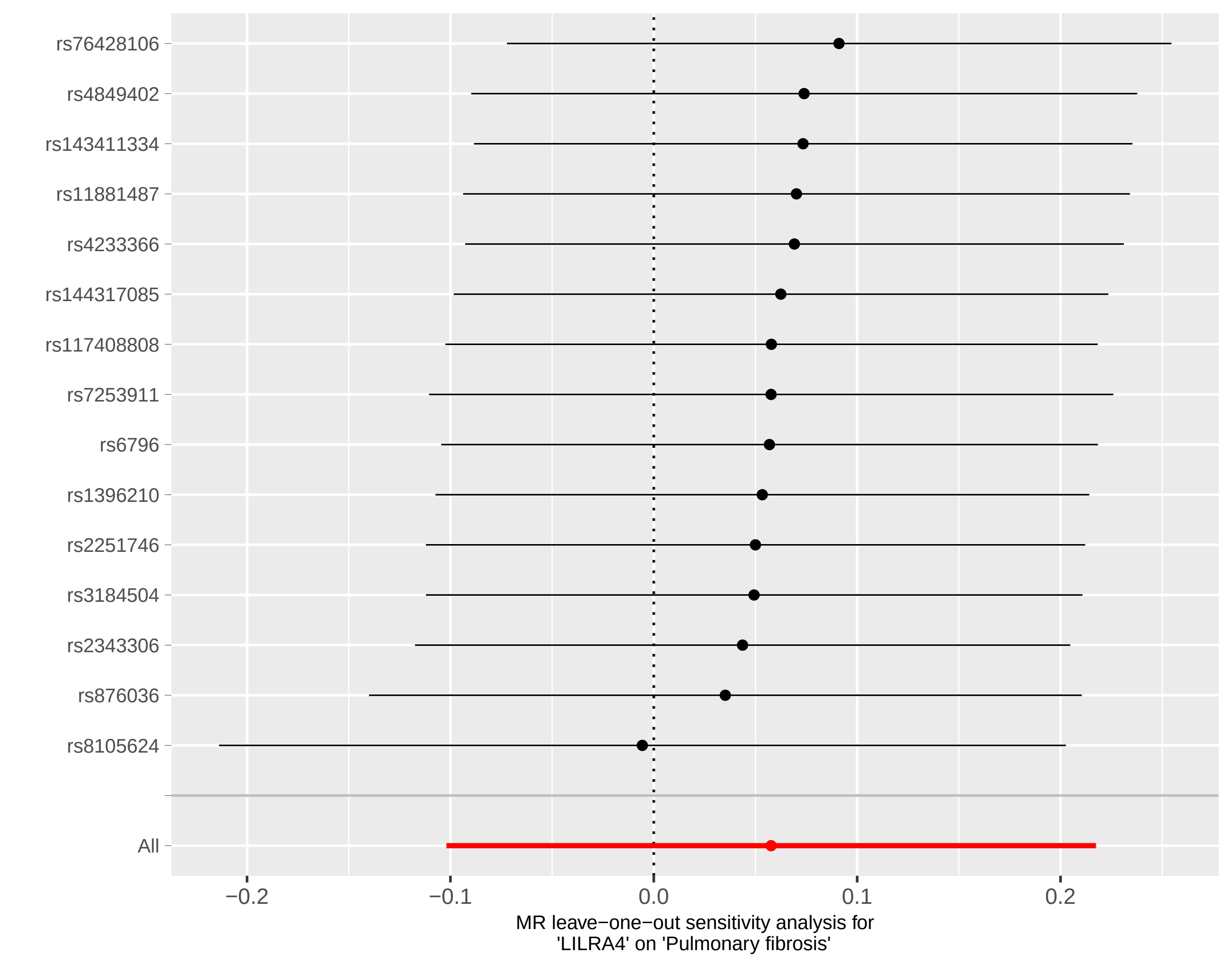
rs76428106
rs4849402
rs143411334
rs11881487
rs4233366
rs144317085
rs117408808
rs7253911
rs6796
rs1396210
rs2251746
rs3184504
rs2343306
rs876036
rs8105624
All
−0.2
−0.1
0.0
0.1
0.2
MR leave−one−out sensitivity analysis for
'LILRA4' on 'Pulmonary fibrosis'

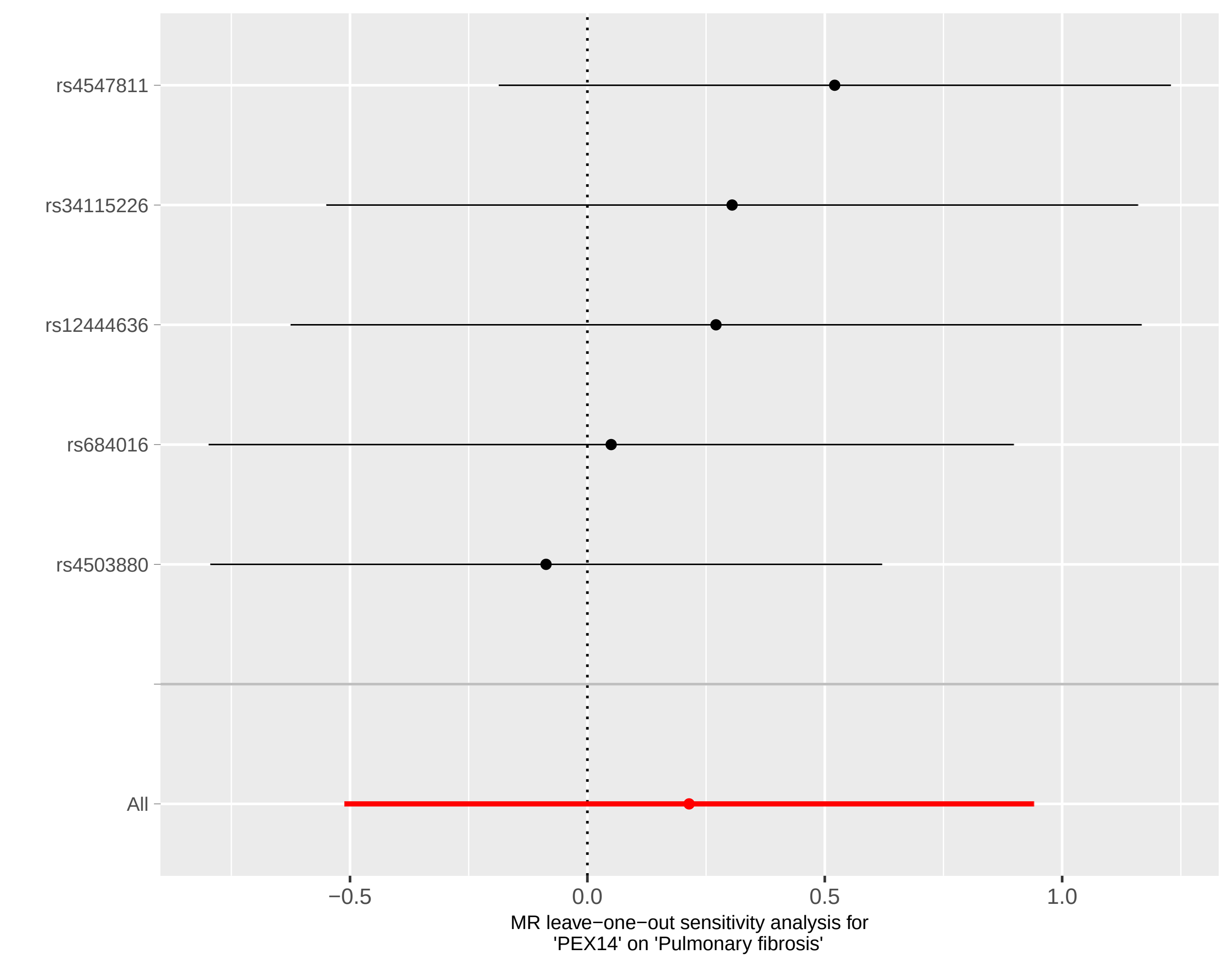
rs4547811
rs34115226
rs12444636
rs684016
rs4503880
All
−0.5
0.0
0.5
1.0
MR leave−one−out sensitivity analysis for
'PEX14' on 'Pulmonary fibrosis'

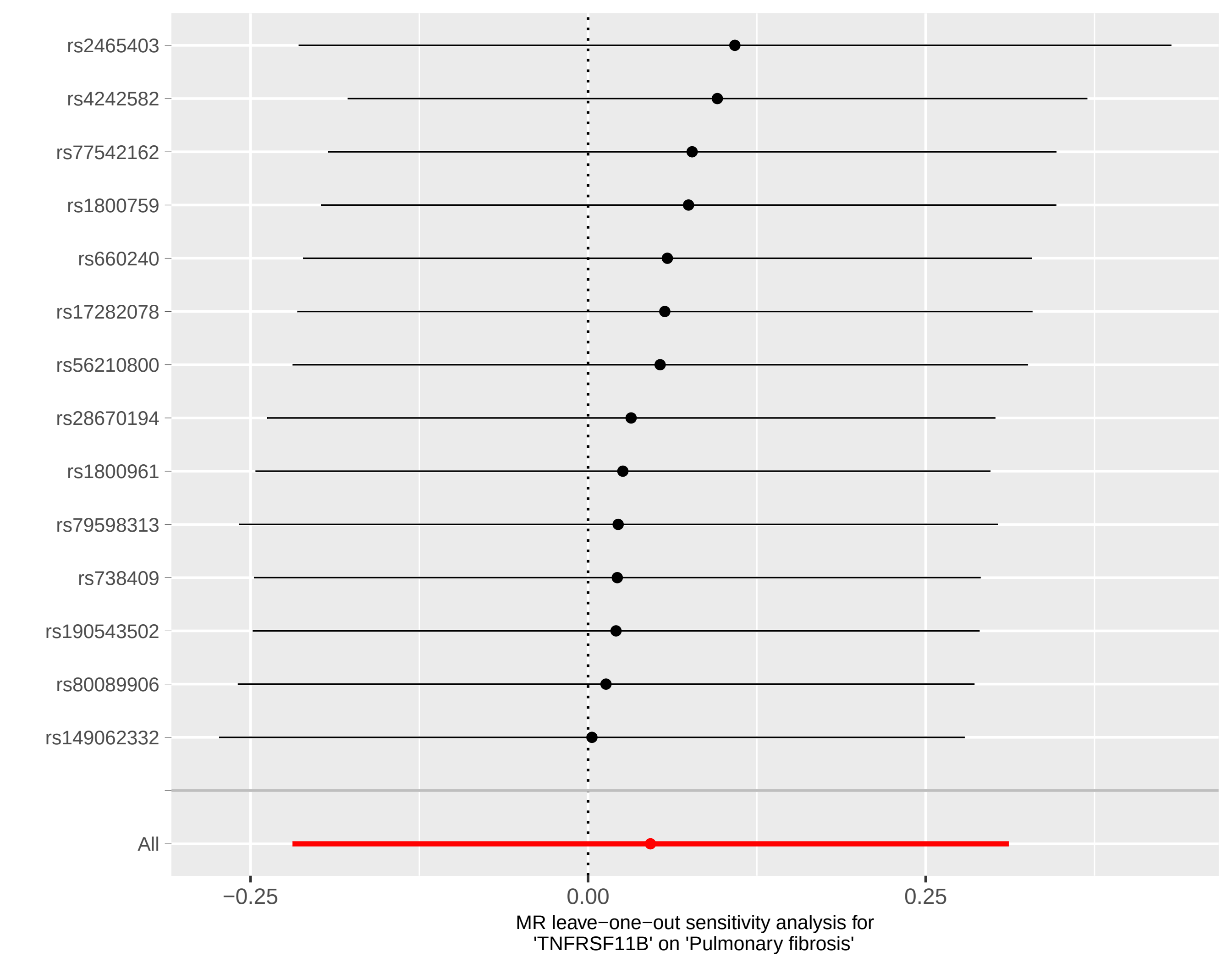
rs2465403
rs4242582
rs77542162
rs1800759
rs660240
rs17282078
rs56210800
rs28670194
rs1800961
rs79598313
rs738409
rs190543502
rs80089906
rs149062332
All
−0.25
0.00
0.25
MR leave−one−out sensitivity analysis for
'TNFRSF11B' on 'Pulmonary fibrosis'

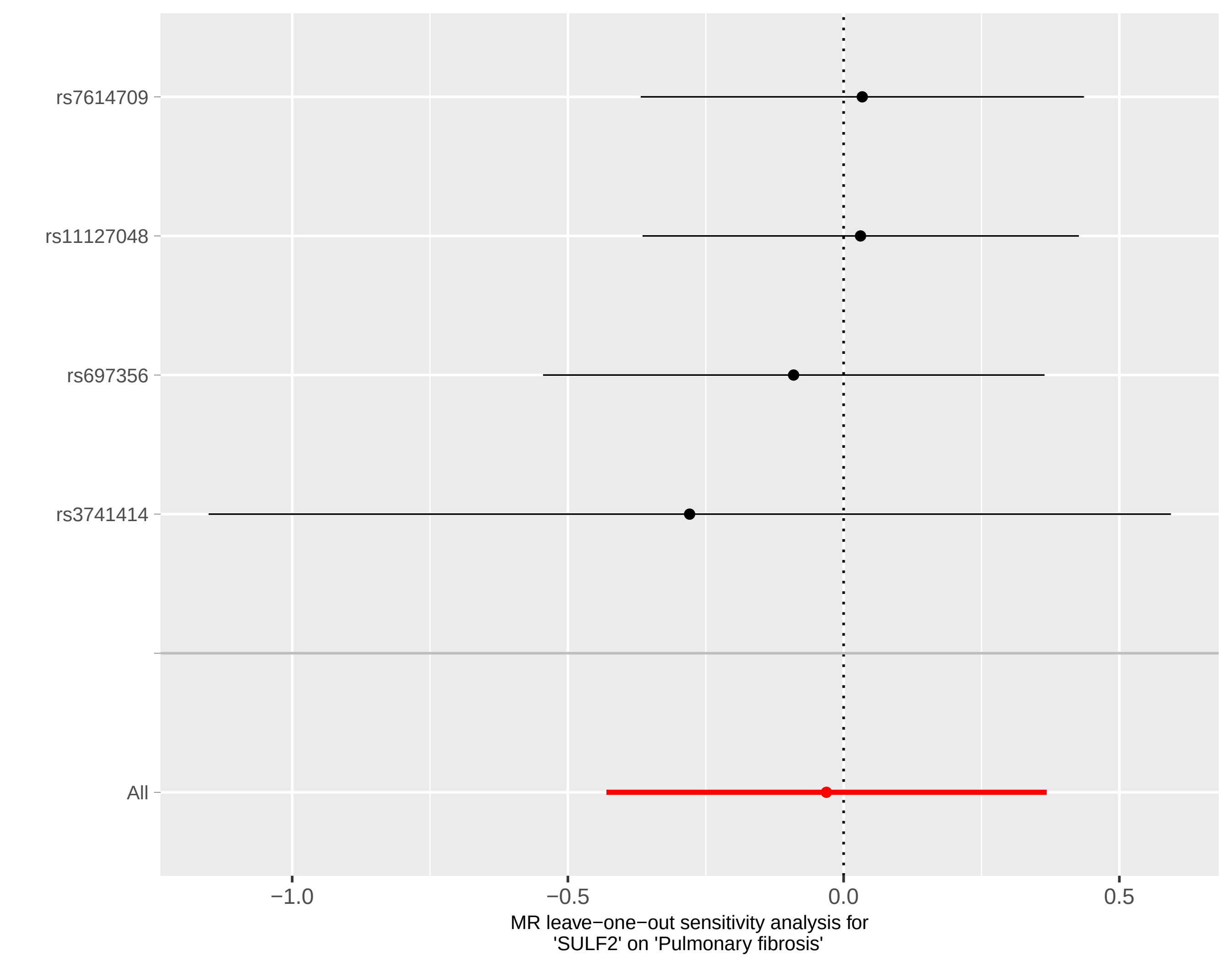
rs7614709
rs11127048
rs697356
rs3741414
All
−1.0
−0.5
0.0
0.5
MR leave−one−out sensitivity analysis for
'SULF2' on 'Pulmonary fibrosis'

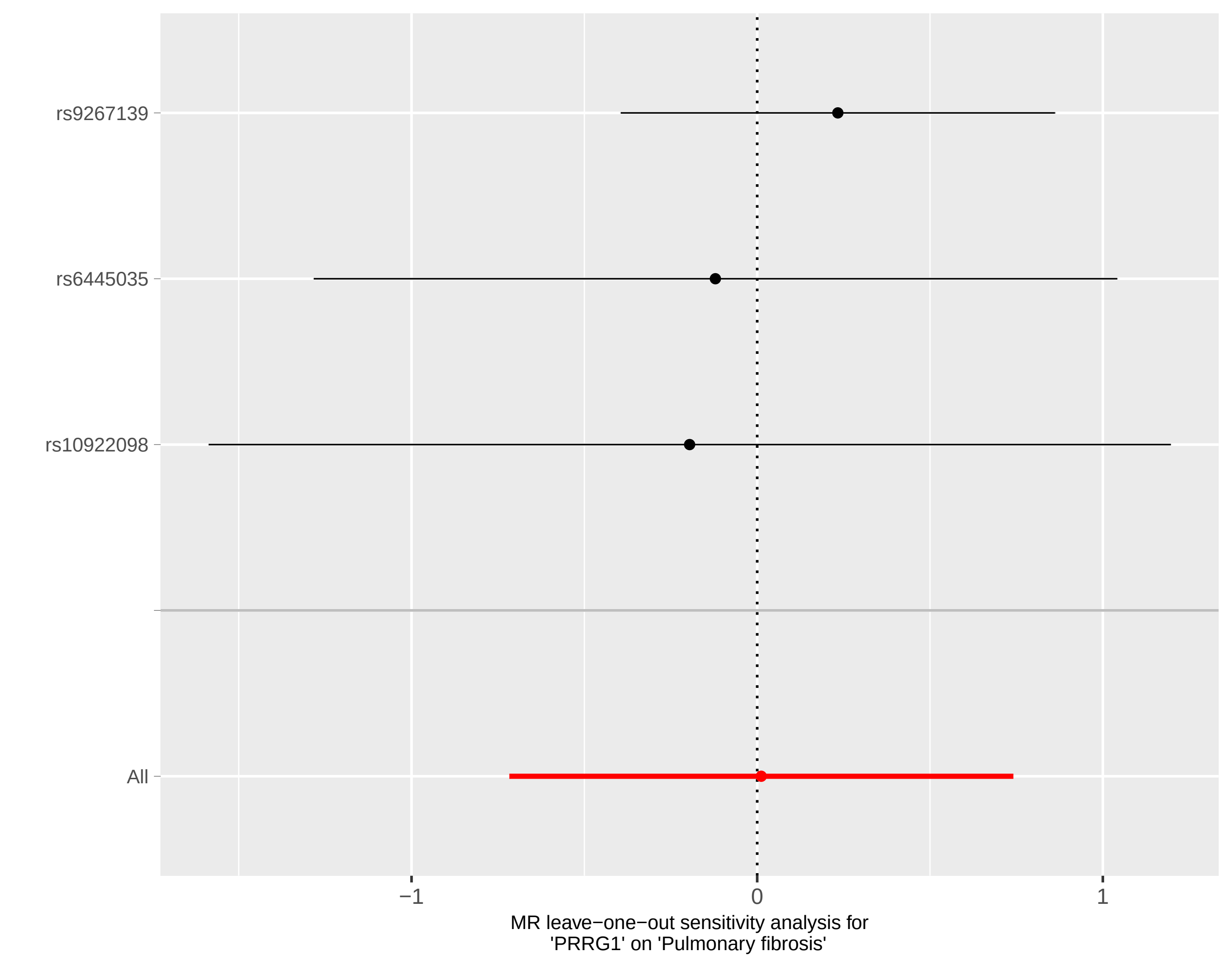
rs9267139
rs6445035
rs10922098
All
−1
0
1
MR leave−one−out sensitivity analysis for
'PRRG1' on 'Pulmonary fibrosis'

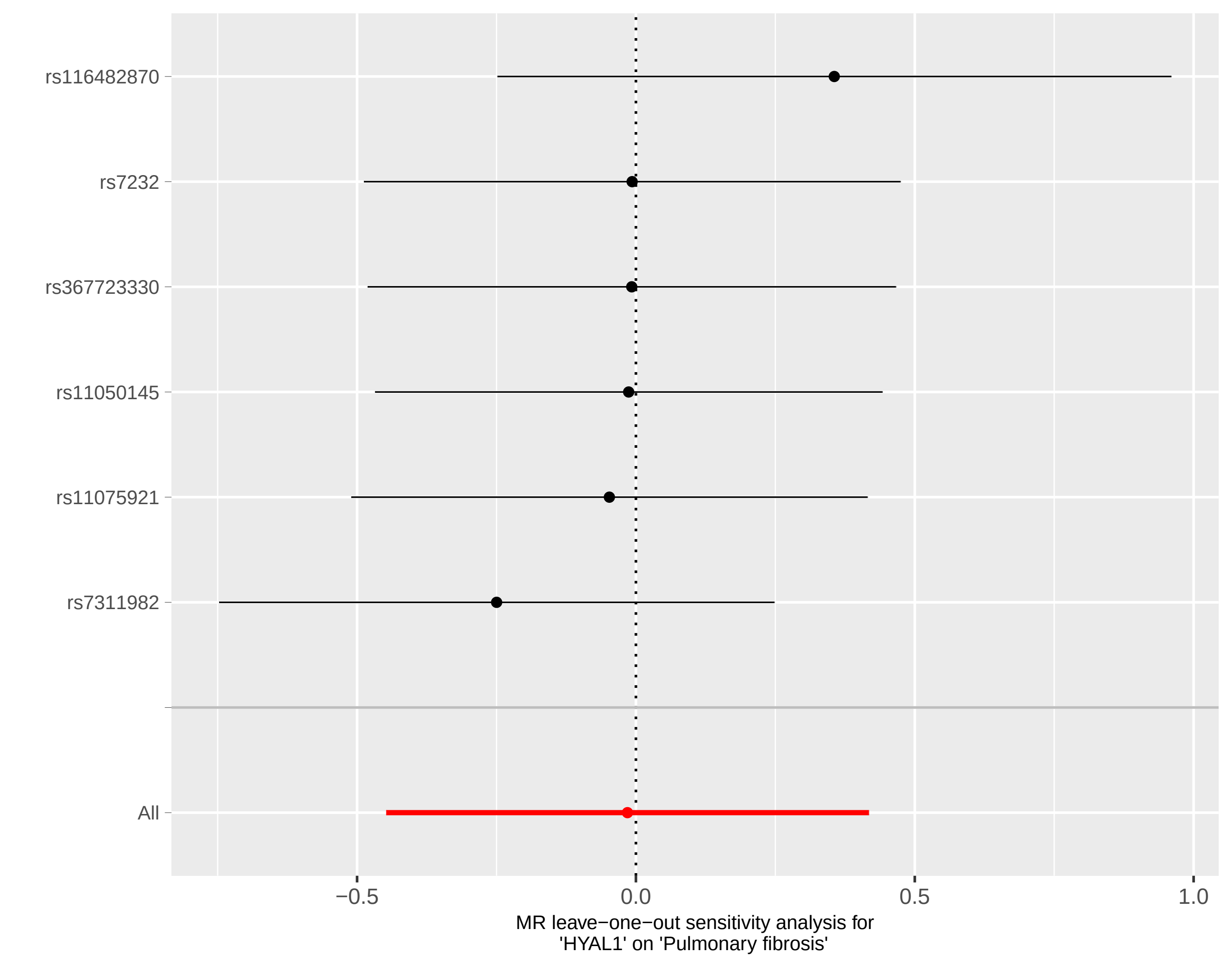

rs116482870
rs7232
rs367723330
rs11050145
rs11075921
rs7311982
All
−0.5
0.0
0.5
1.0
MR leave−one−out sensitivity analysis for
'HYAL1' on 'Pulmonary fibrosis'

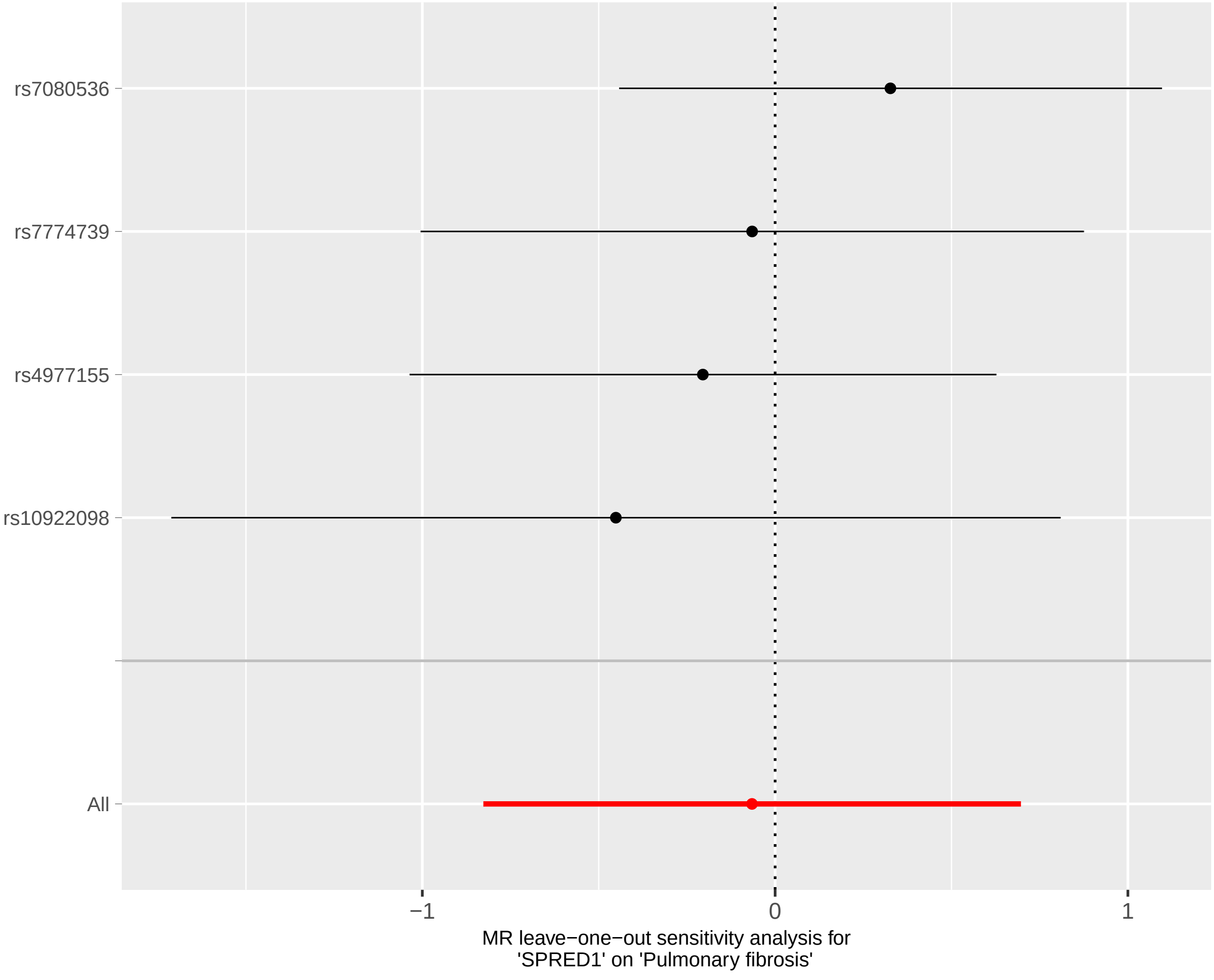

rs7080536
rs7774739
rs4977155
rs10922098
All
−1
0
1
MR leave−one−out sensitivity analysis for
'SPRED1' on 'Pulmonary fibrosis'

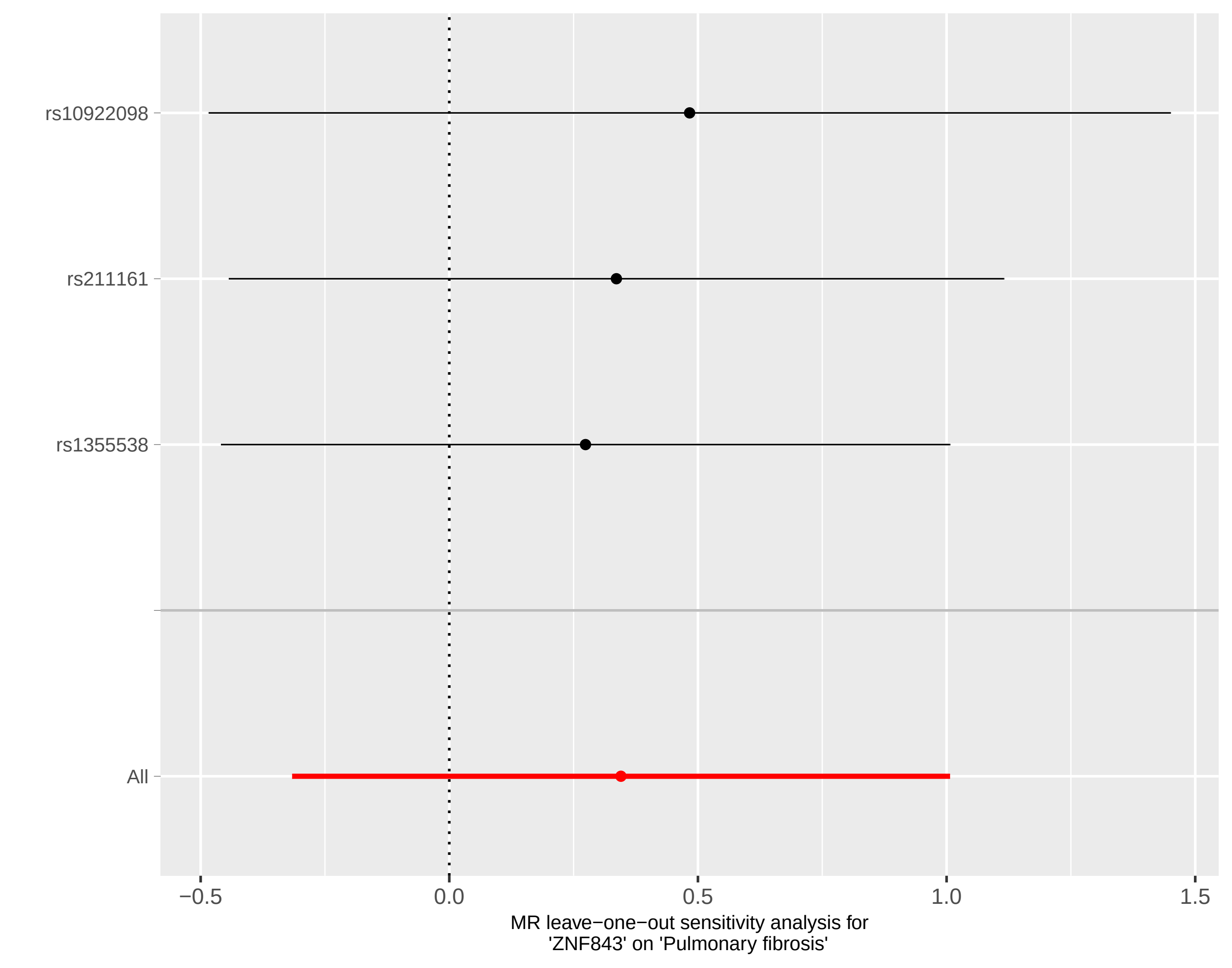
rs10922098
rs211161
rs1355538
All
−0.5
0.0
0.5
1.0
1.5
MR leave−one−out sensitivity analysis for
'ZNF843' on 'Pulmonary fibrosis'

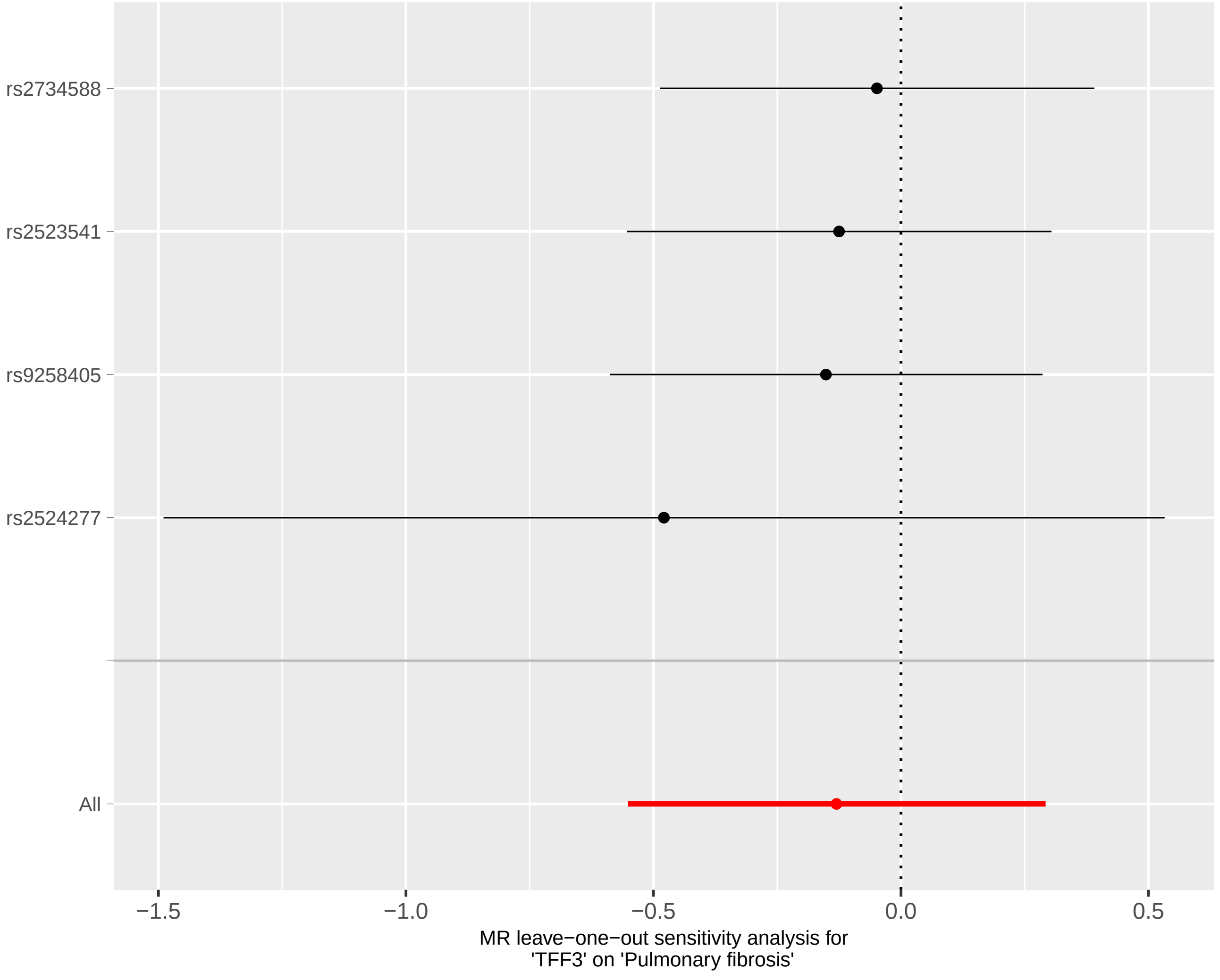
rs2734588
rs2523541
rs9258405
rs2524277
All
−1.5
−1.0
−0.5
0.0
0.5
MR leave−one−out sensitivity analysis for
'TFF3' on 'Pulmonary fibrosis'

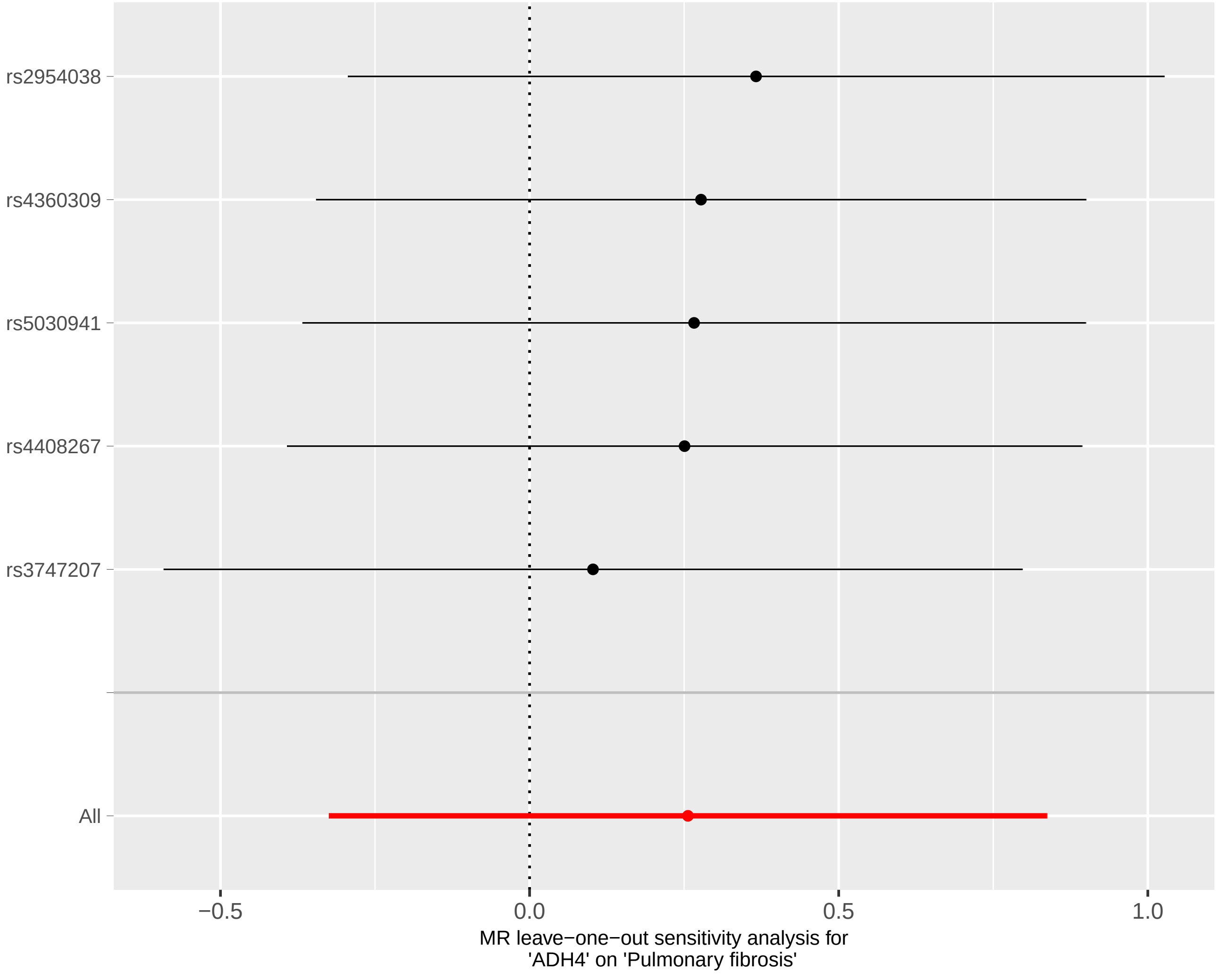
rs2954038
rs4360309
rs5030941
rs4408267
rs3747207
All
−0.5
0.0
0.5
1.0
MR leave−one−out sensitivity analysis for
'ADH4' on 'Pulmonary fibrosis'

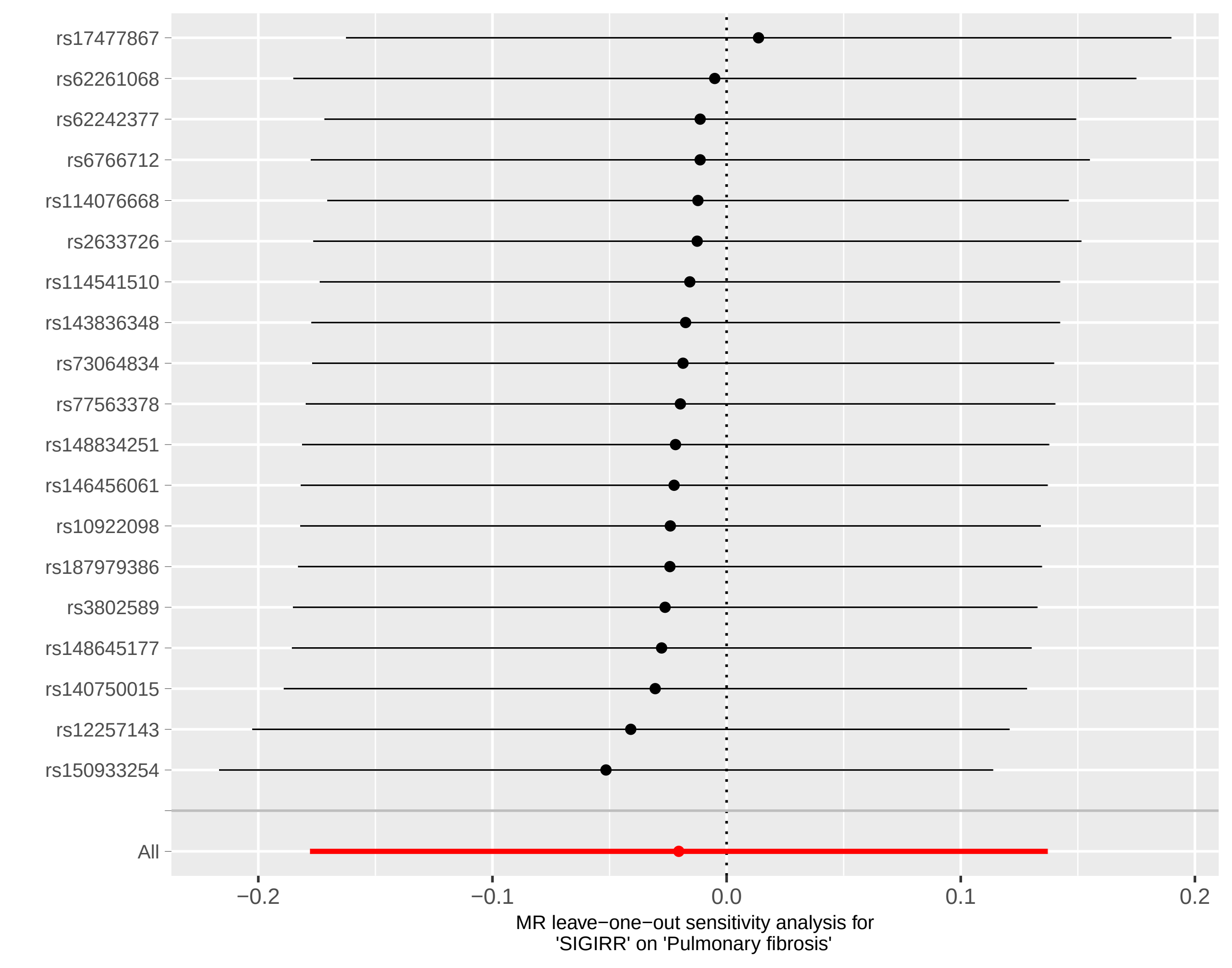

rs17477867
rs62261068
rs62242377
rs6766712
rs114076668
rs2633726
rs114541510
rs143836348
rs73064834
rs77563378
rs148834251
rs146456061
rs10922098
rs187979386
rs3802589
rs148645177
rs140750015
rs12257143
rs150933254
All
−0.2
−0.1
0.0
0.1
0.2
MR leave−one−out sensitivity analysis for
'SIGIRR' on 'Pulmonary fibrosis'

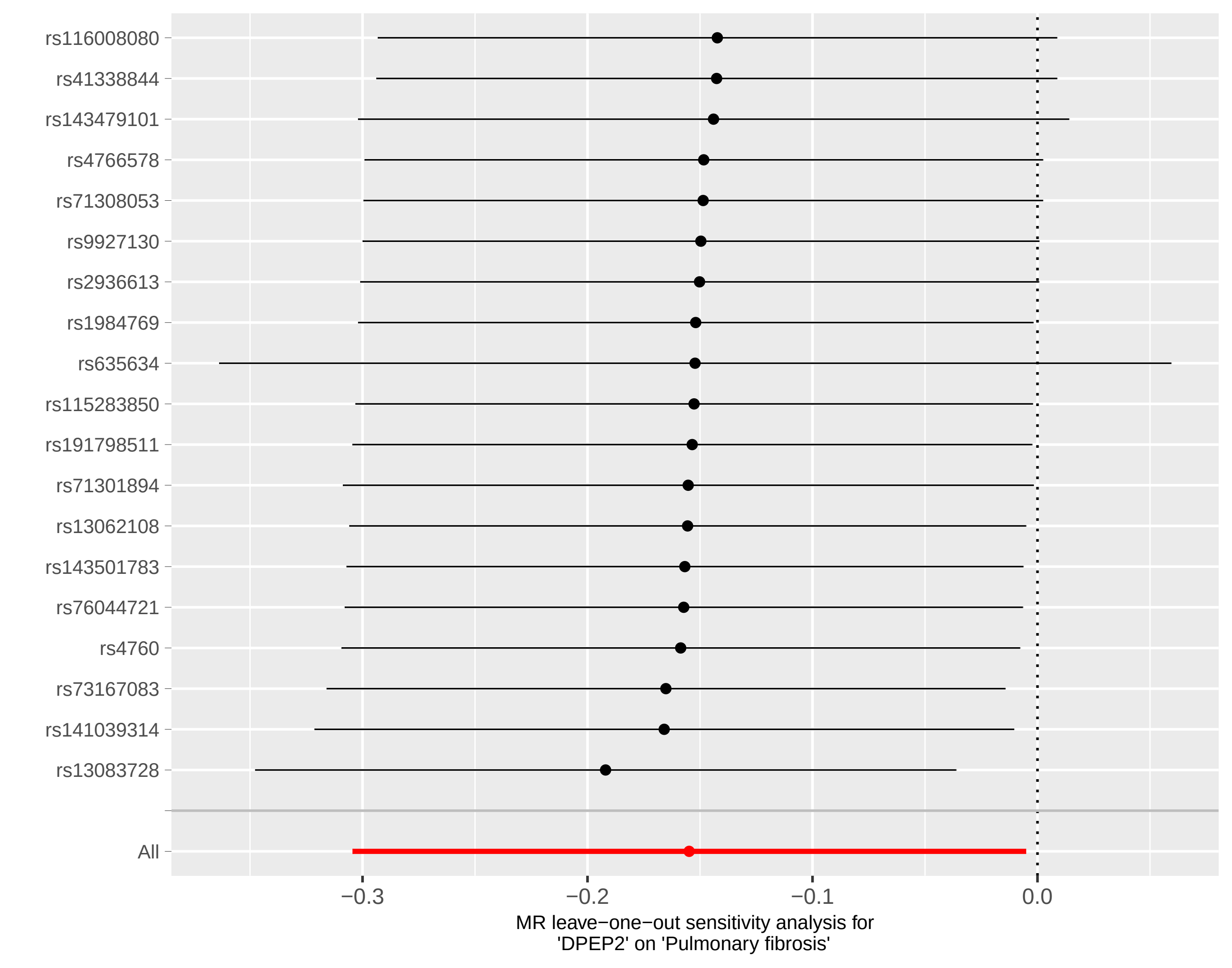
rs116008080
rs41338844
rs143479101
rs4766578
rs71308053
rs9927130
rs2936613
rs1984769
rs635634
rs115283850
rs191798511
rs71301894
rs13062108
rs143501783
rs76044721
rs4760
rs73167083
rs141039314
rs13083728
All
−0.3
−0.2
−0.1
0.0
MR leave−one−out sensitivity analysis for
'DPEP2' on 'Pulmonary fibrosis'

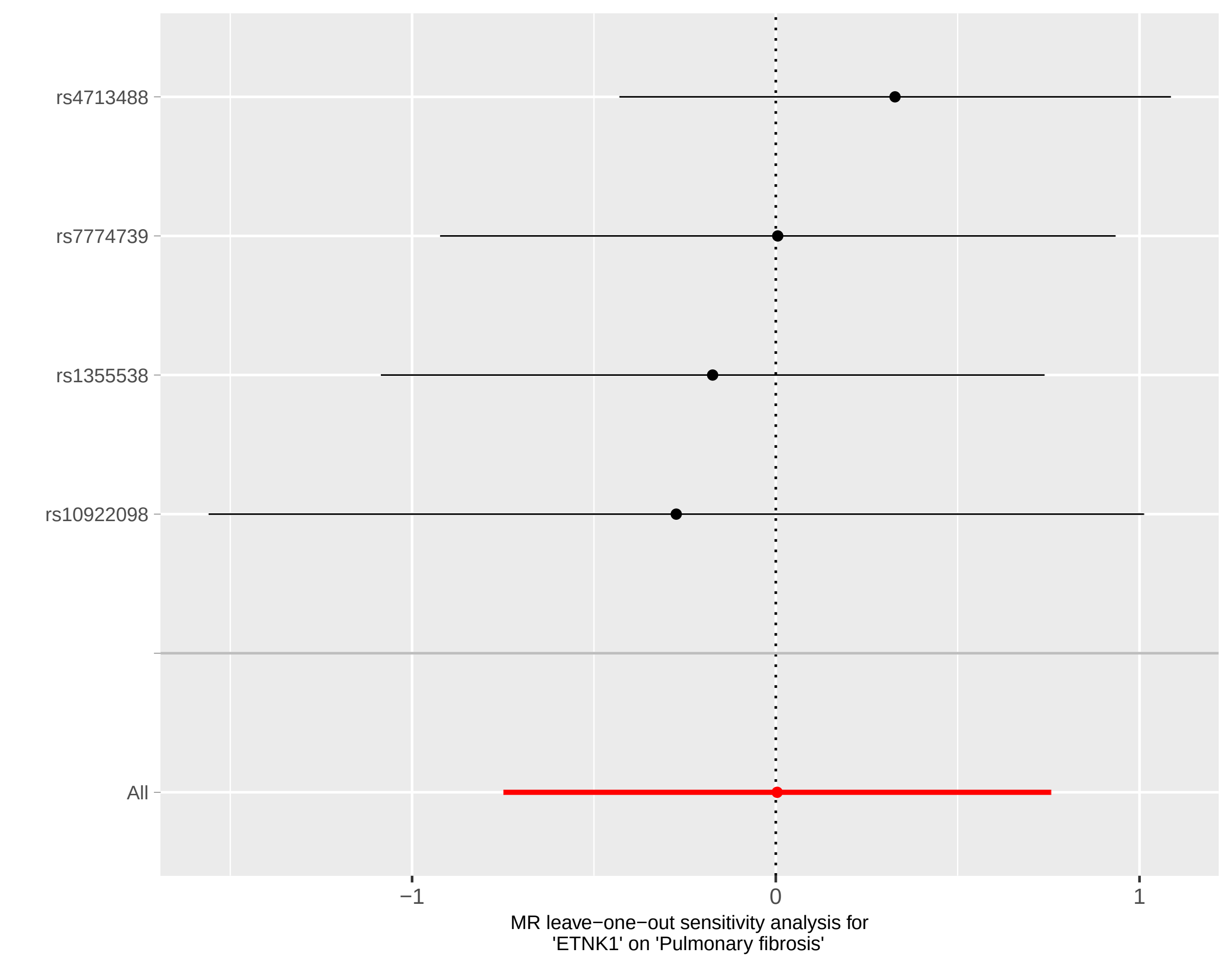
rs4713488
rs7774739
rs1355538
rs10922098
All
−1
0
1
MR leave−one−out sensitivity analysis for
'ETNK1' on 'Pulmonary fibrosis'

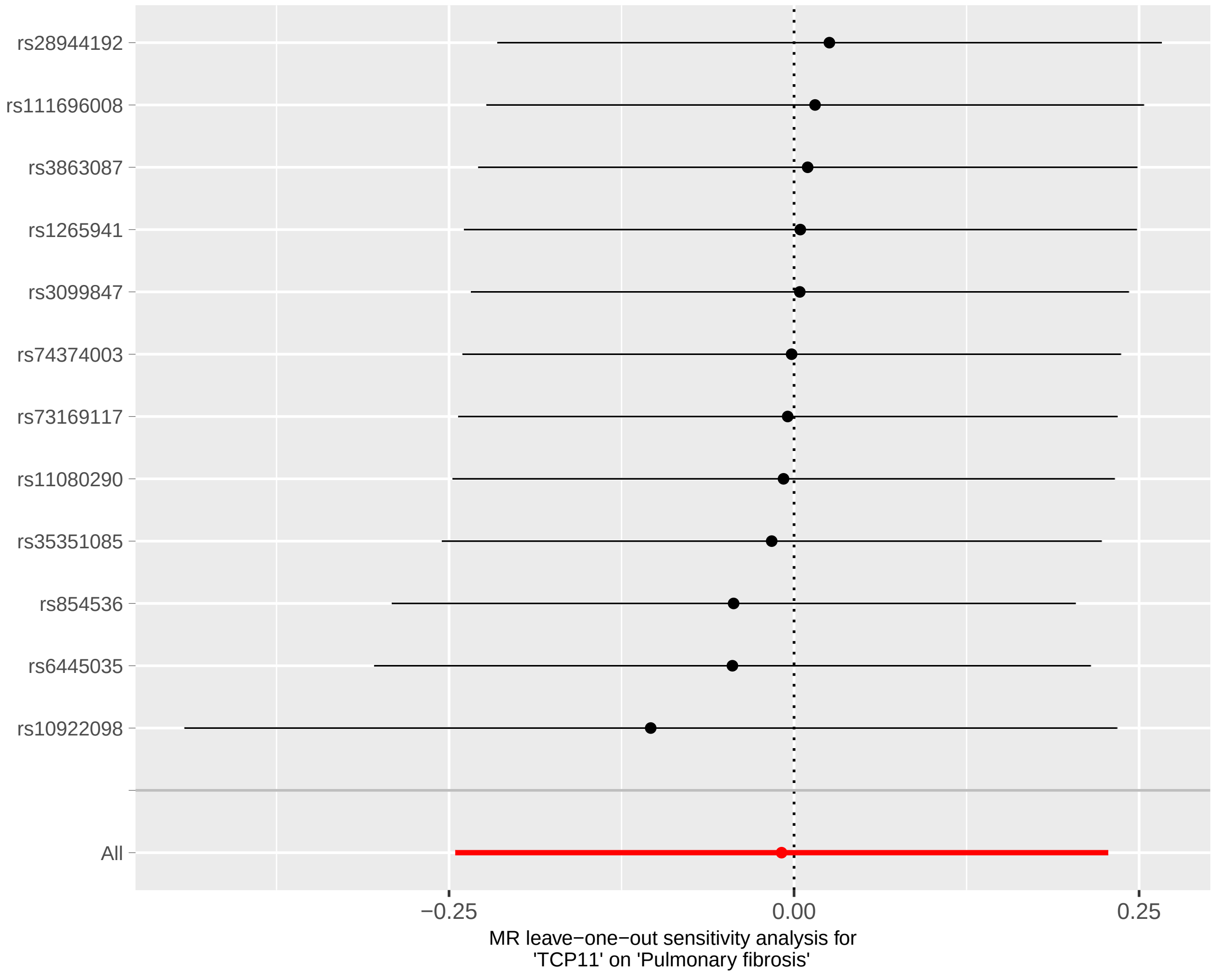
rs28944192
rs111696008
rs3863087
rs1265941
rs3099847
rs74374003
rs73169117
rs11080290
rs35351085
rs854536
rs6445035
rs10922098
All
−0.25
0.00
0.25
MR leave−one−out sensitivity analysis for
'TCP11' on 'Pulmonary fibrosis'

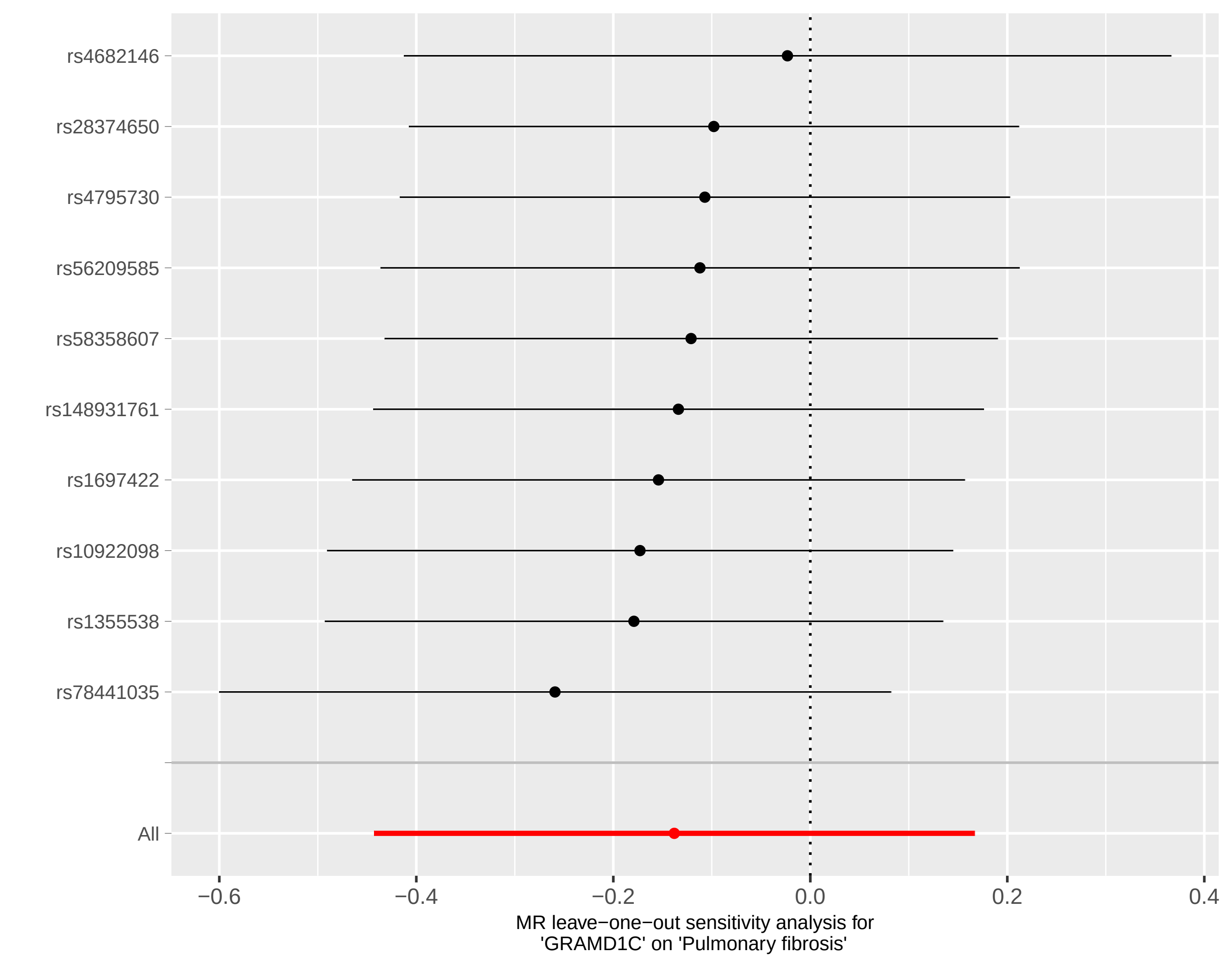
rs4682146
rs28374650
rs4795730
rs56209585
rs58358607
rs148931761
rs1697422
rs10922098
rs1355538
rs78441035
All
−0.6
−0.4
−0.2
0.0
0.2
0.4
MR leave−one−out sensitivity analysis for
'GRAMD1C' on 'Pulmonary fibrosis'

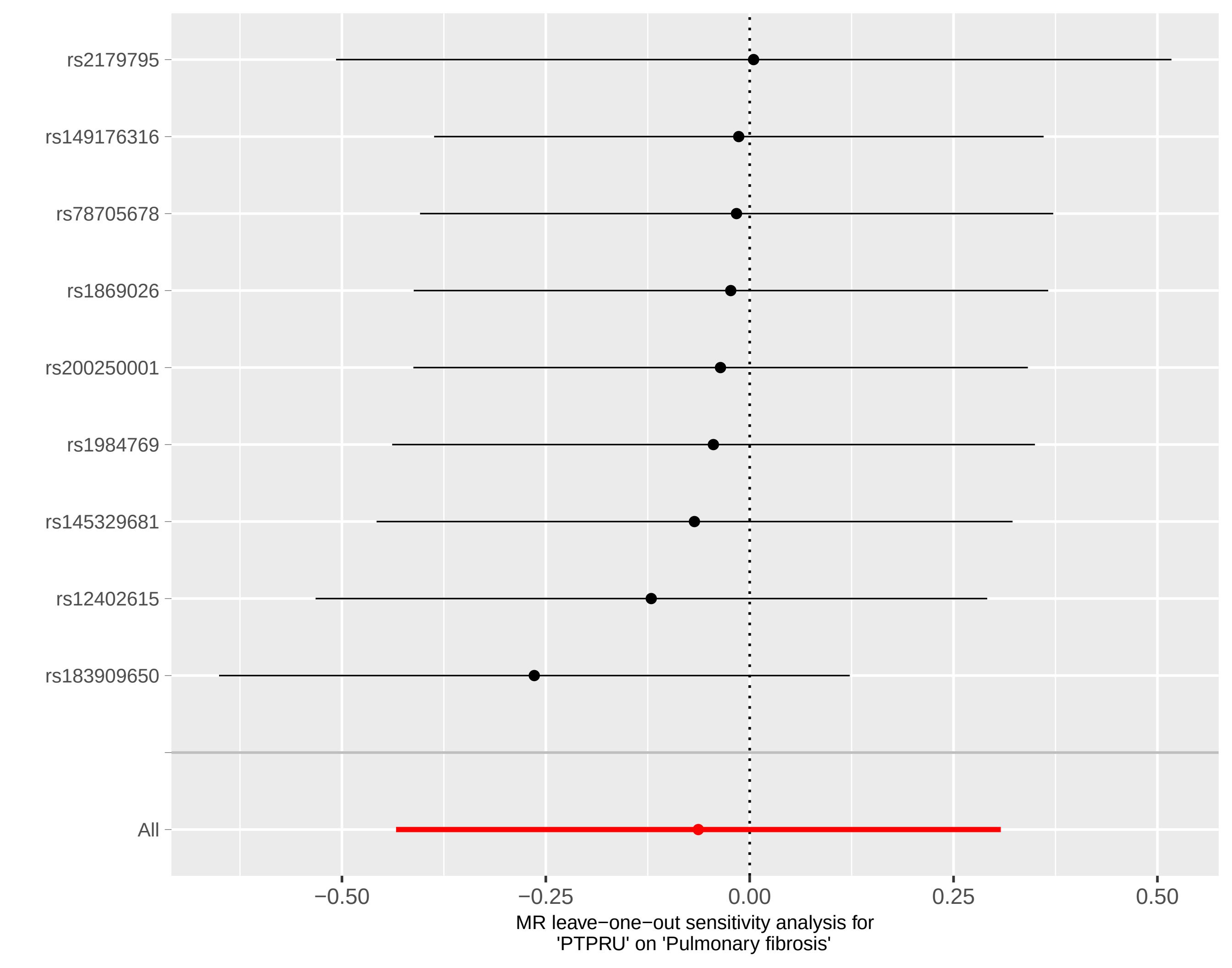
rs2179795
rs149176316
rs78705678
rs1869026
rs200250001
rs1984769
rs145329681
rs12402615
rs183909650
All
−0.50
−0.25
0.00
0.25
0.50
MR leave−one−out sensitivity analysis for
'PTPRU' on 'Pulmonary fibrosis'

Insufficient number of SNPs

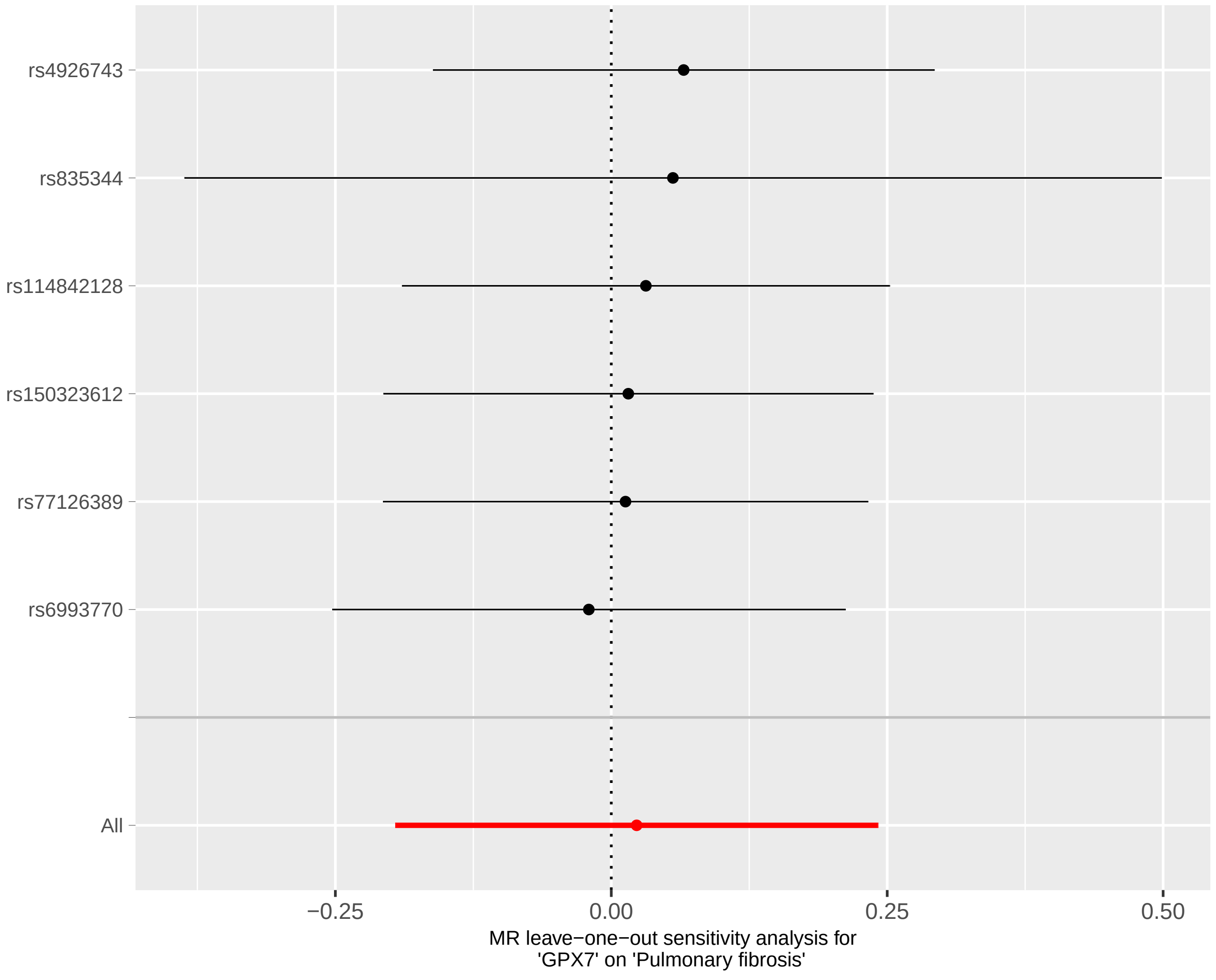
rs4926743
rs835344
rs114842128
rs150323612
rs77126389
rs6993770
All
−0.25
0.00
0.25
0.50
MR leave−one−out sensitivity analysis for
'GPX7' on 'Pulmonary fibrosis'

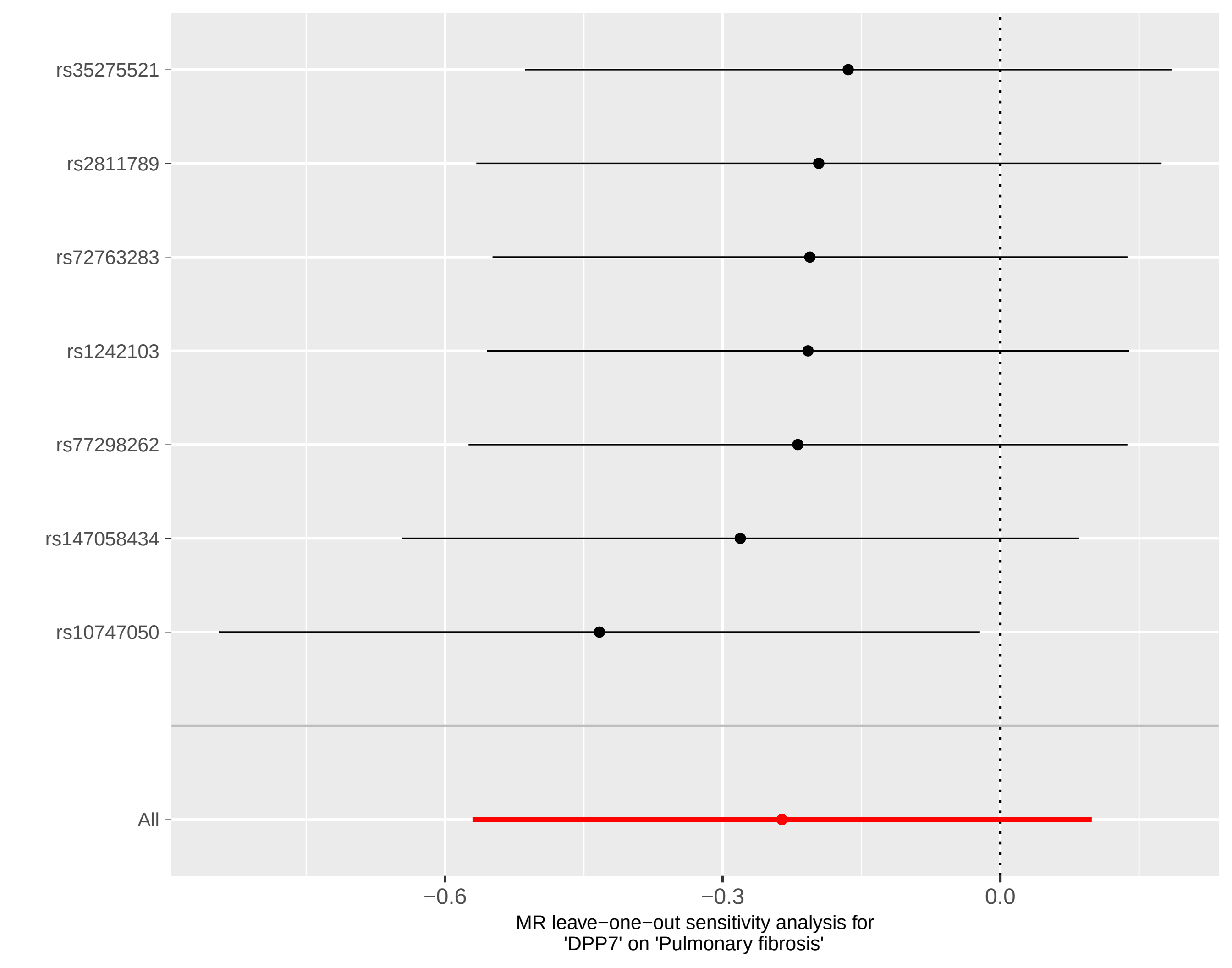

rs35275521
rs2811789
rs72763283
rs1242103
rs77298262
rs147058434
rs10747050
All
−0.6
−0.3
0.0
MR leave−one−out sensitivity analysis for
'DPP7' on 'Pulmonary fibrosis'

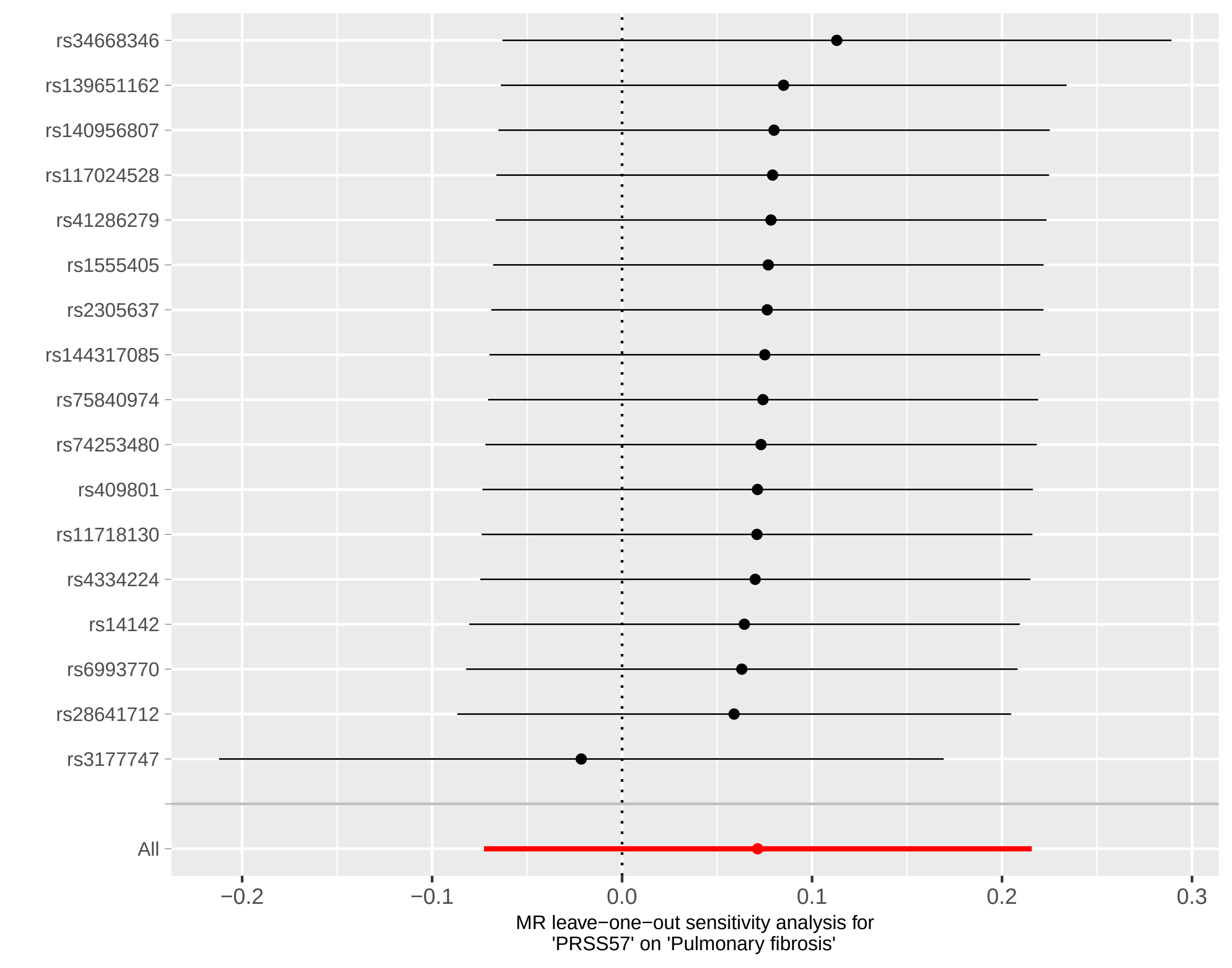
rs34668346
rs139651162
rs140956807
rs117024528
rs41286279
rs1555405
rs2305637
rs144317085
rs75840974
rs74253480
rs409801
rs11718130
rs4334224
rs14142
rs6993770
rs28641712
rs3177747
All
−0.2
−0.1
0.0
0.1
0.2
0.3
MR leave−one−out sensitivity analysis for
'PRSS57' on 'Pulmonary fibrosis'

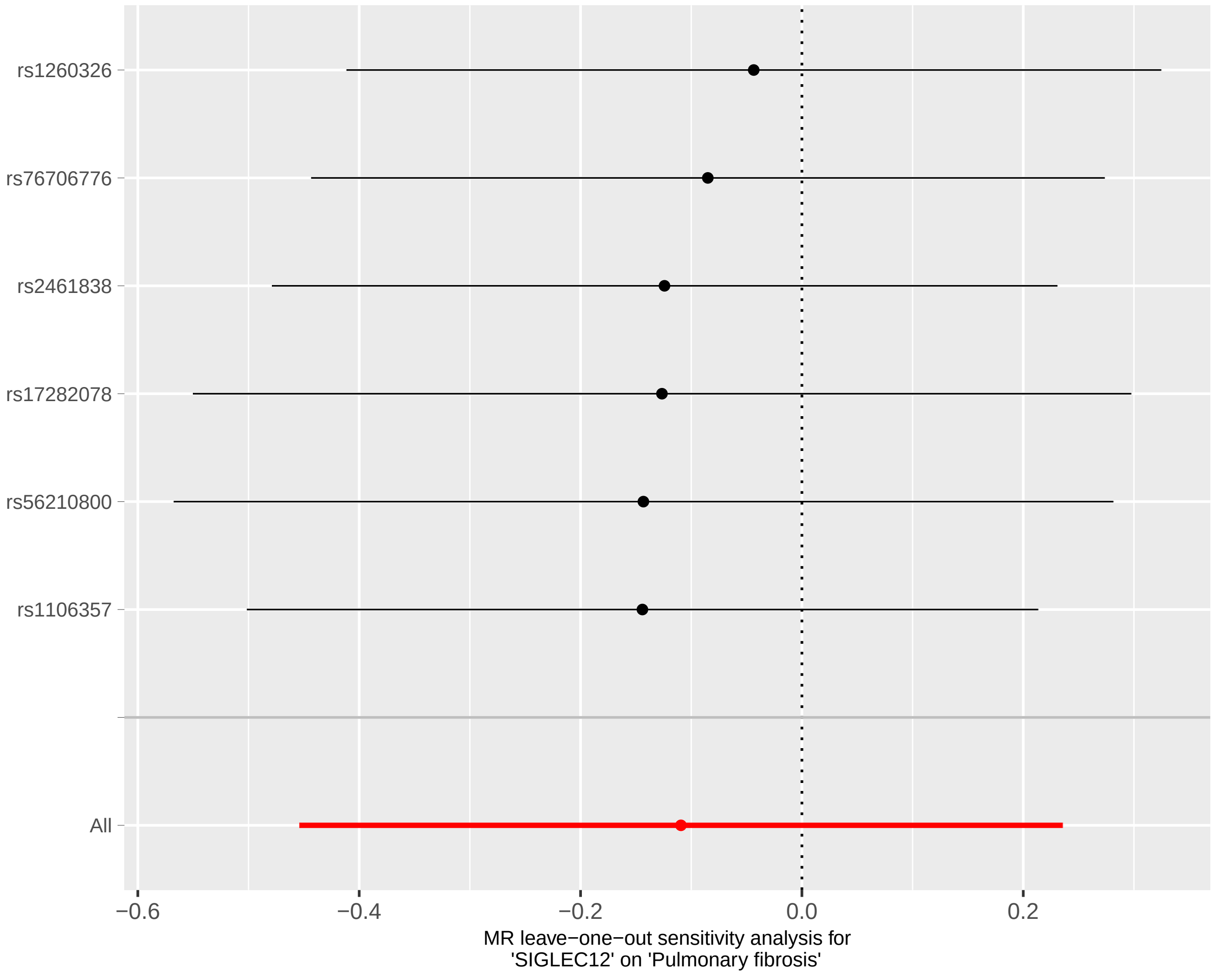

rs1260326
rs76706776
rs2461838
rs17282078
rs56210800
rs1106357
All
−0.6
−0.4
−0.2
0.0
0.2
MR leave−one−out sensitivity analysis for
'SIGLEC12' on 'Pulmonary fibrosis'

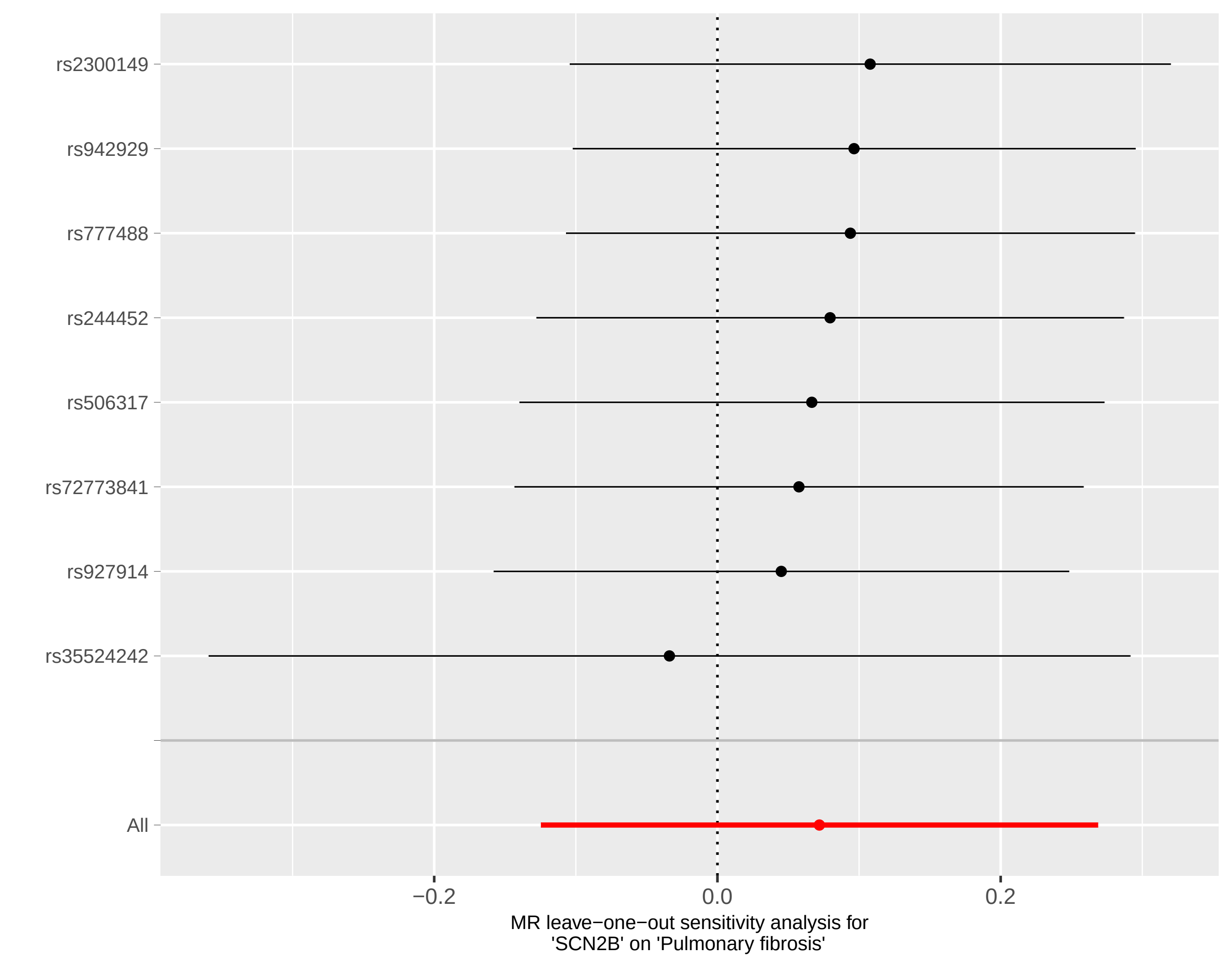
rs2300149
rs942929
rs777488
rs244452
rs506317
rs72773841
rs927914
rs35524242
All
−0.2
0.0
0.2
MR leave−one−out sensitivity analysis for
'SCN2B' on 'Pulmonary fibrosis'

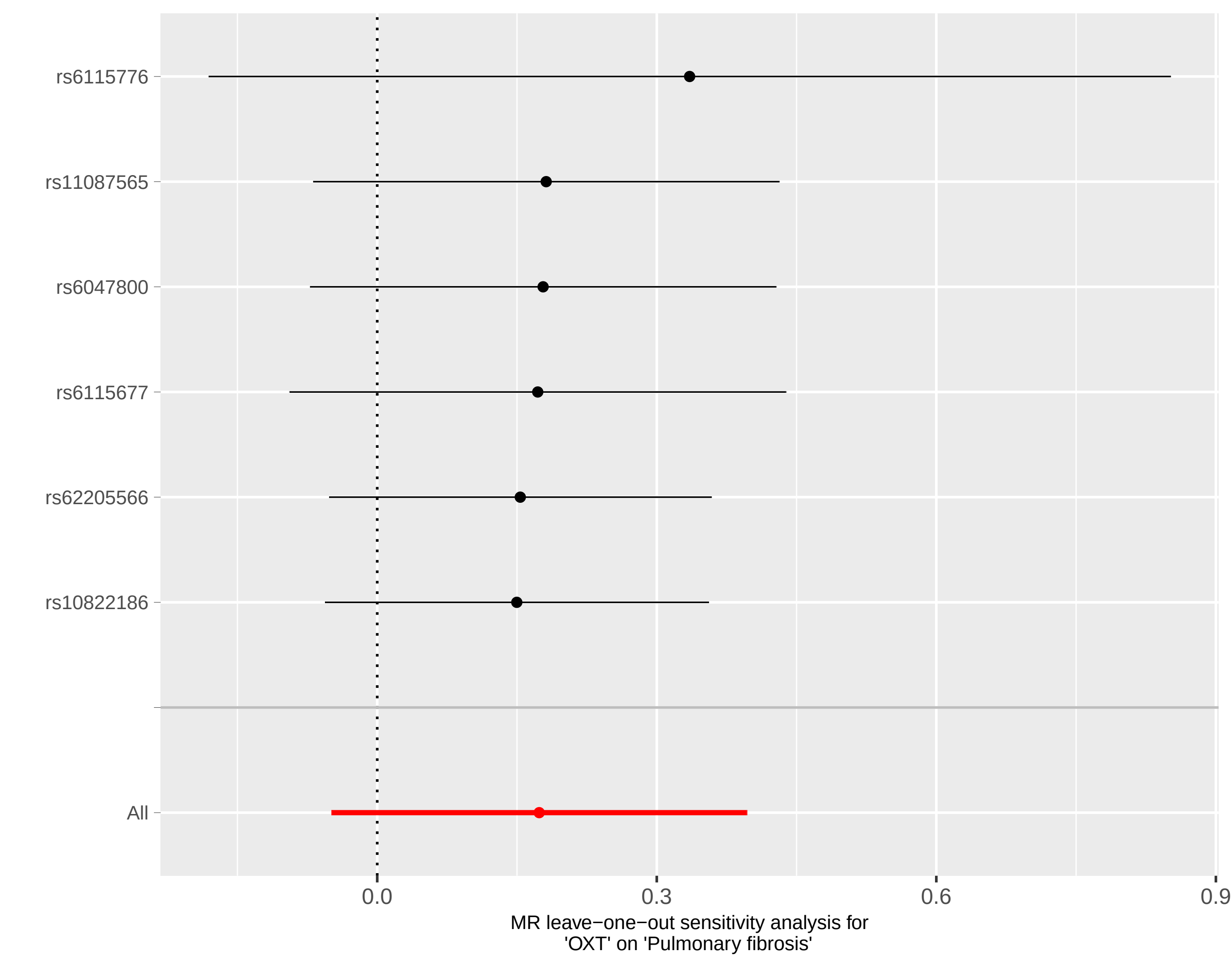
rs6115776
rs11087565
rs6047800
rs6115677
rs62205566
rs10822186
All
0.0
0.3
0.6
0.9
MR leave−one−out sensitivity analysis for
'OXT' on 'Pulmonary fibrosis'

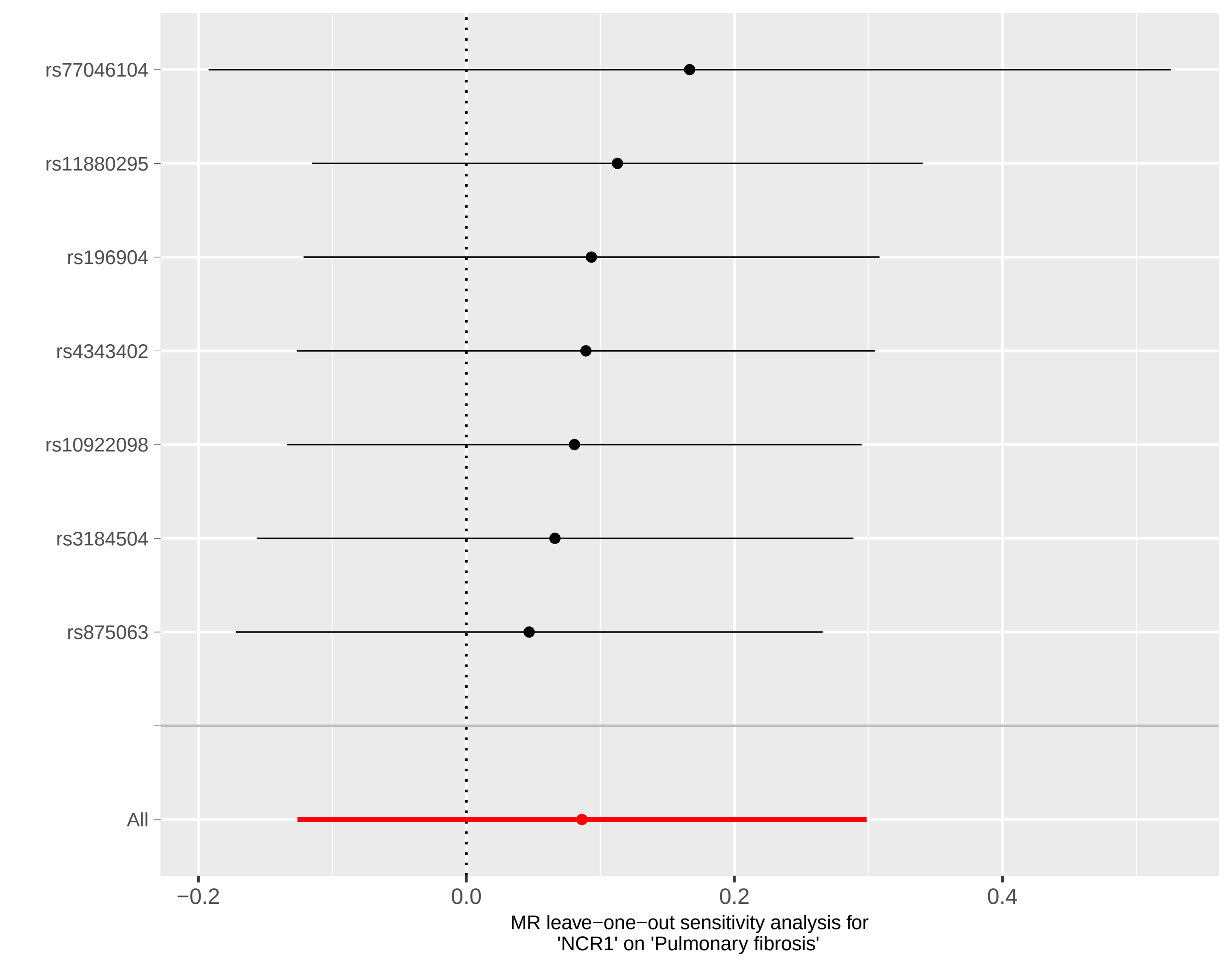
rs77046104
rs11880295
rs196904
rs4343402
rs10922098
rs3184504
rs875063
All
−0.2
0.0
0.2
0.4
MR leave−one−out sensitivity analysis for
'NCR1' on 'Pulmonary fibrosis'

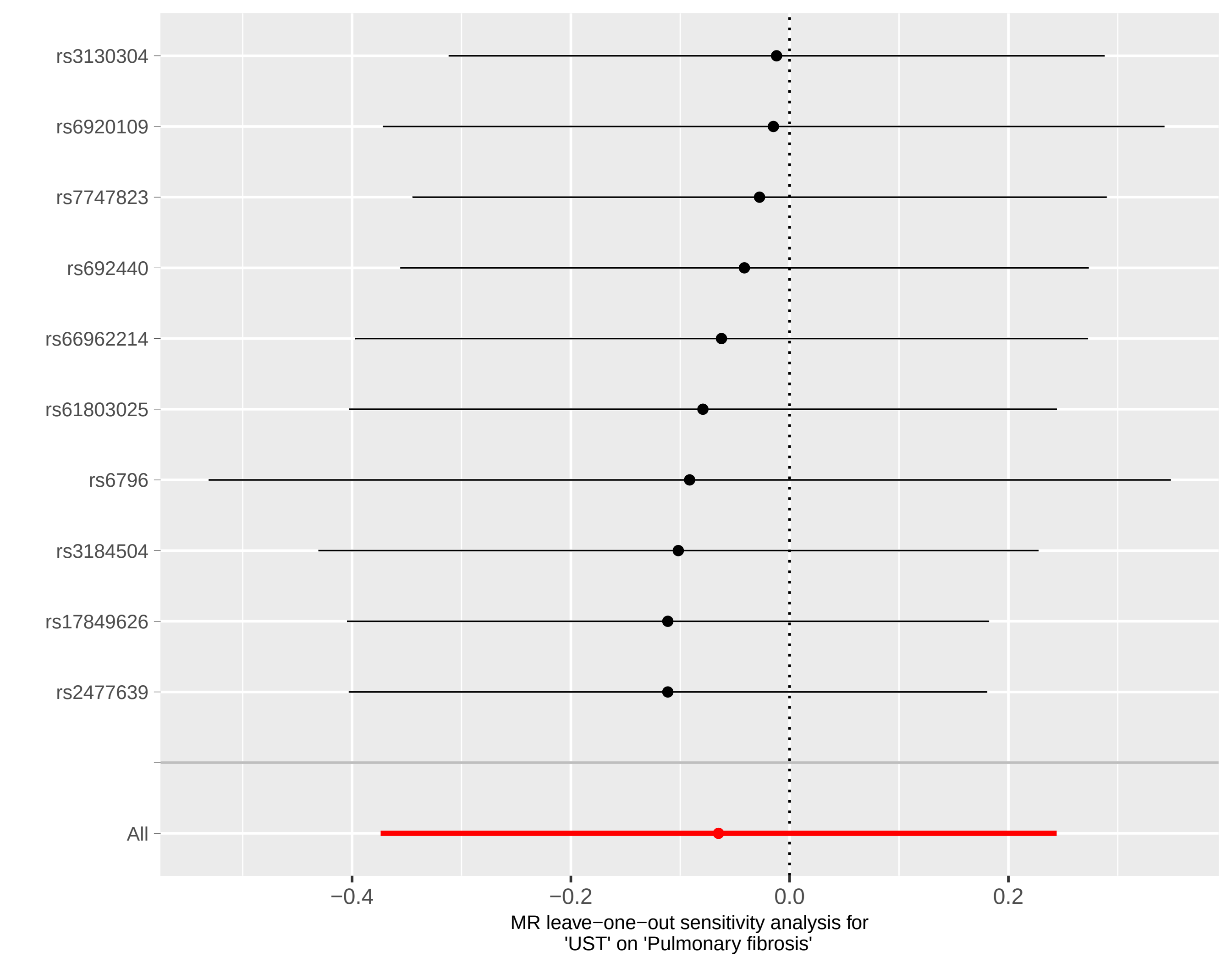
rs3130304
rs6920109
rs7747823
rs692440
rs66962214
rs61803025
rs6796
rs3184504
rs17849626
rs2477639
All
−0.4
−0.2
0.0
0.2
MR leave−one−out sensitivity analysis for
'UST' on 'Pulmonary fibrosis'

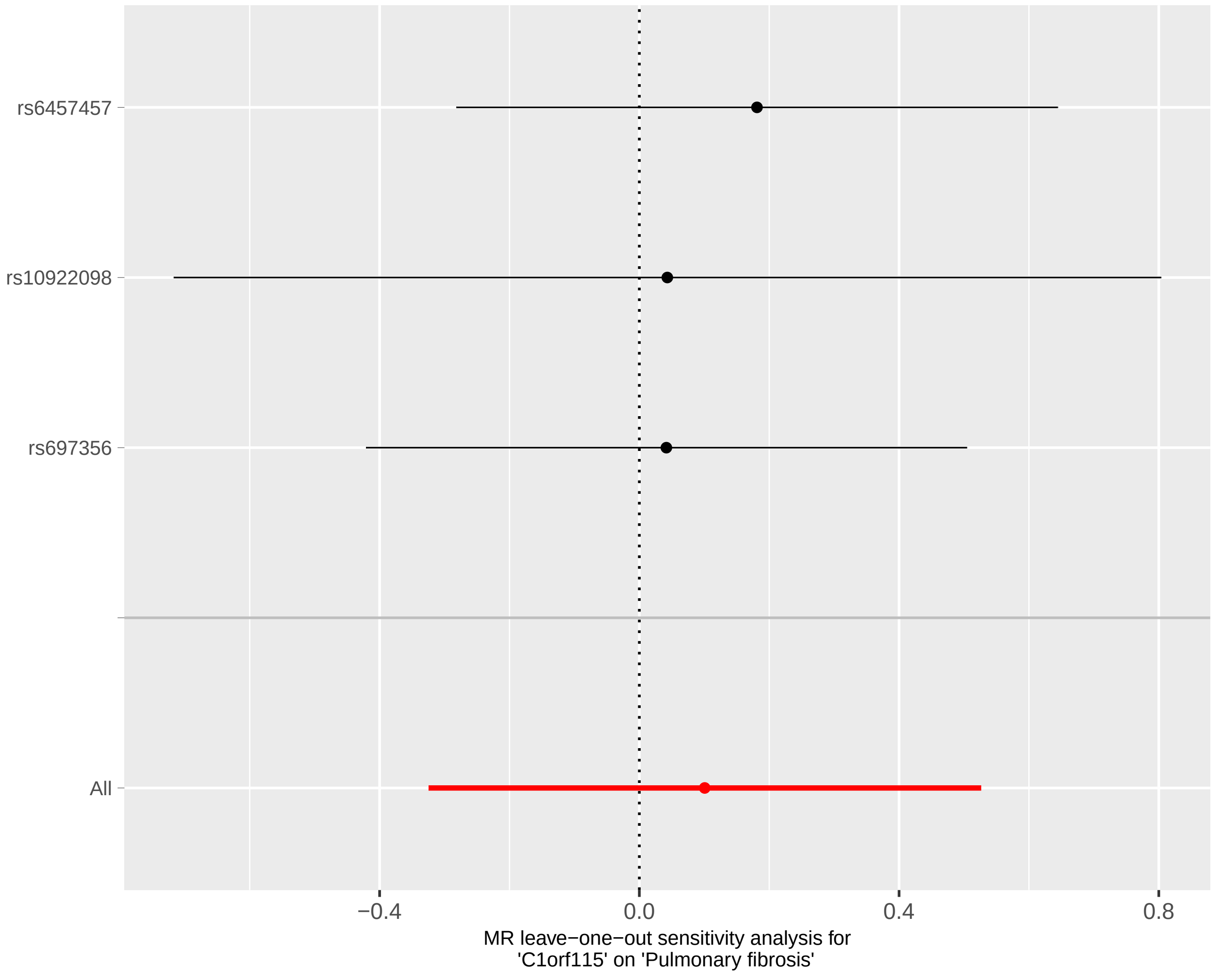

rs6457457
rs10922098
rs697356
All
−0.4
0.0
0.4
0.8
MR leave−one−out sensitivity analysis for
'C1orf115' on 'Pulmonary fibrosis'

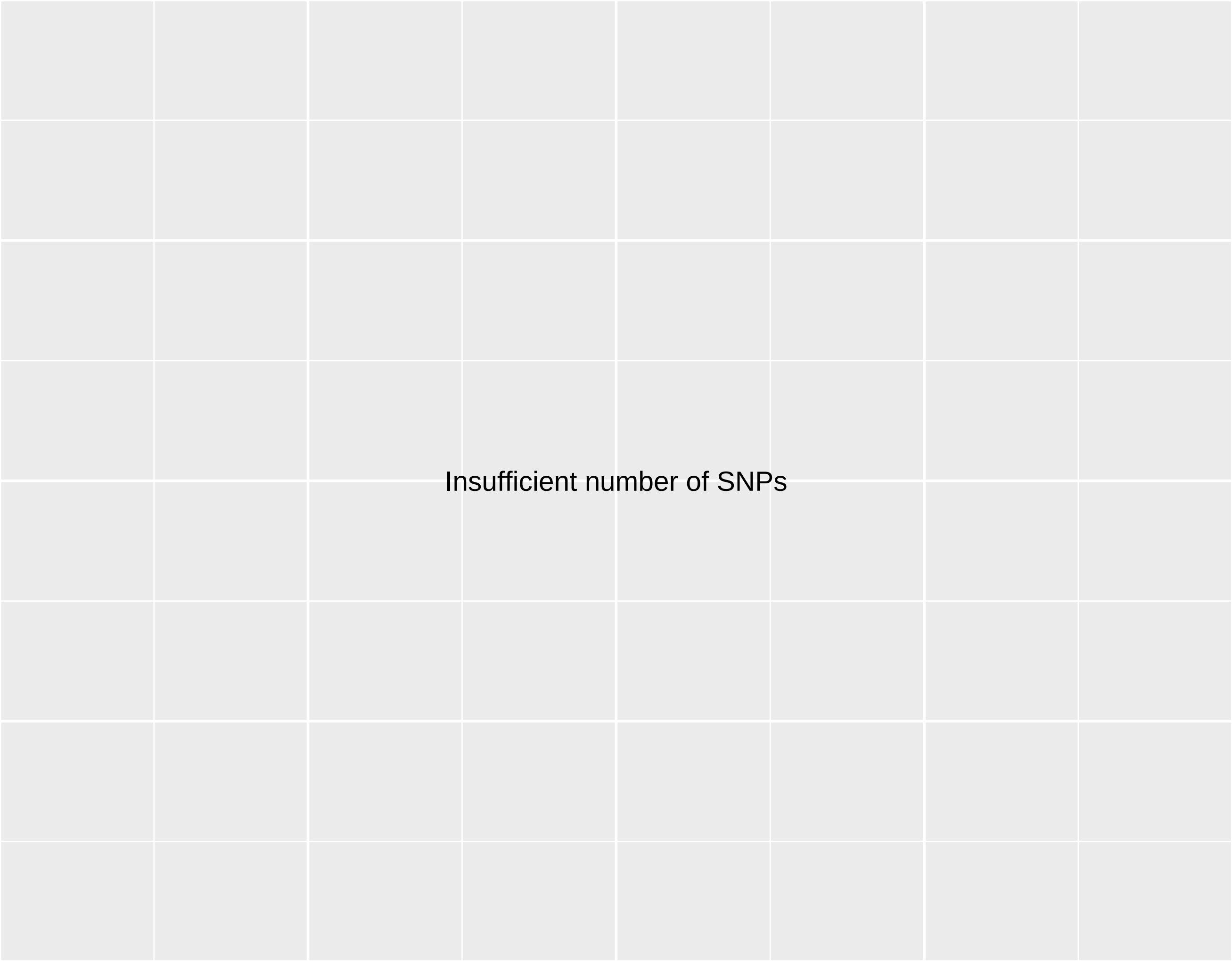
Insufficient number of SNPs

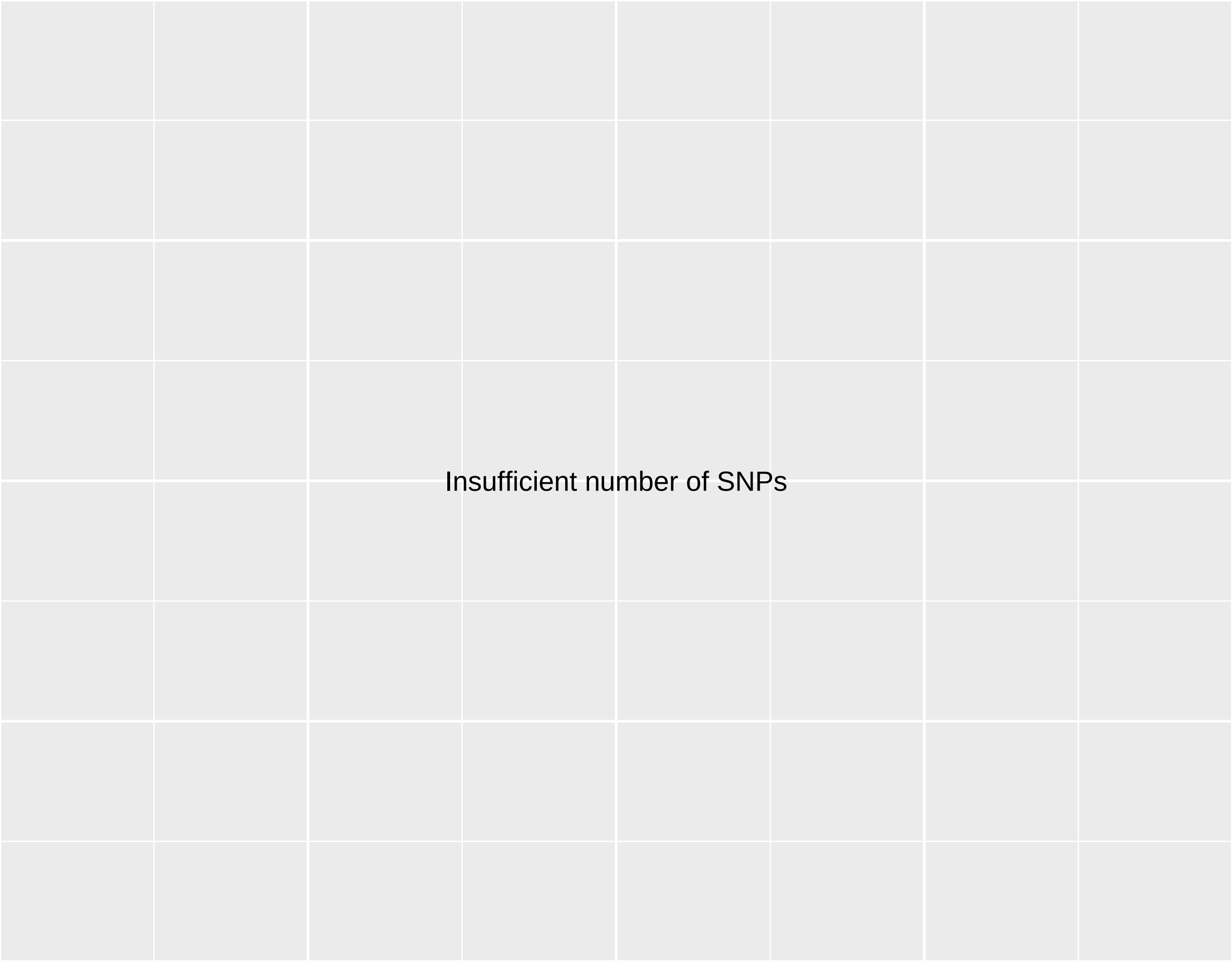
Insufficient number of SNPs

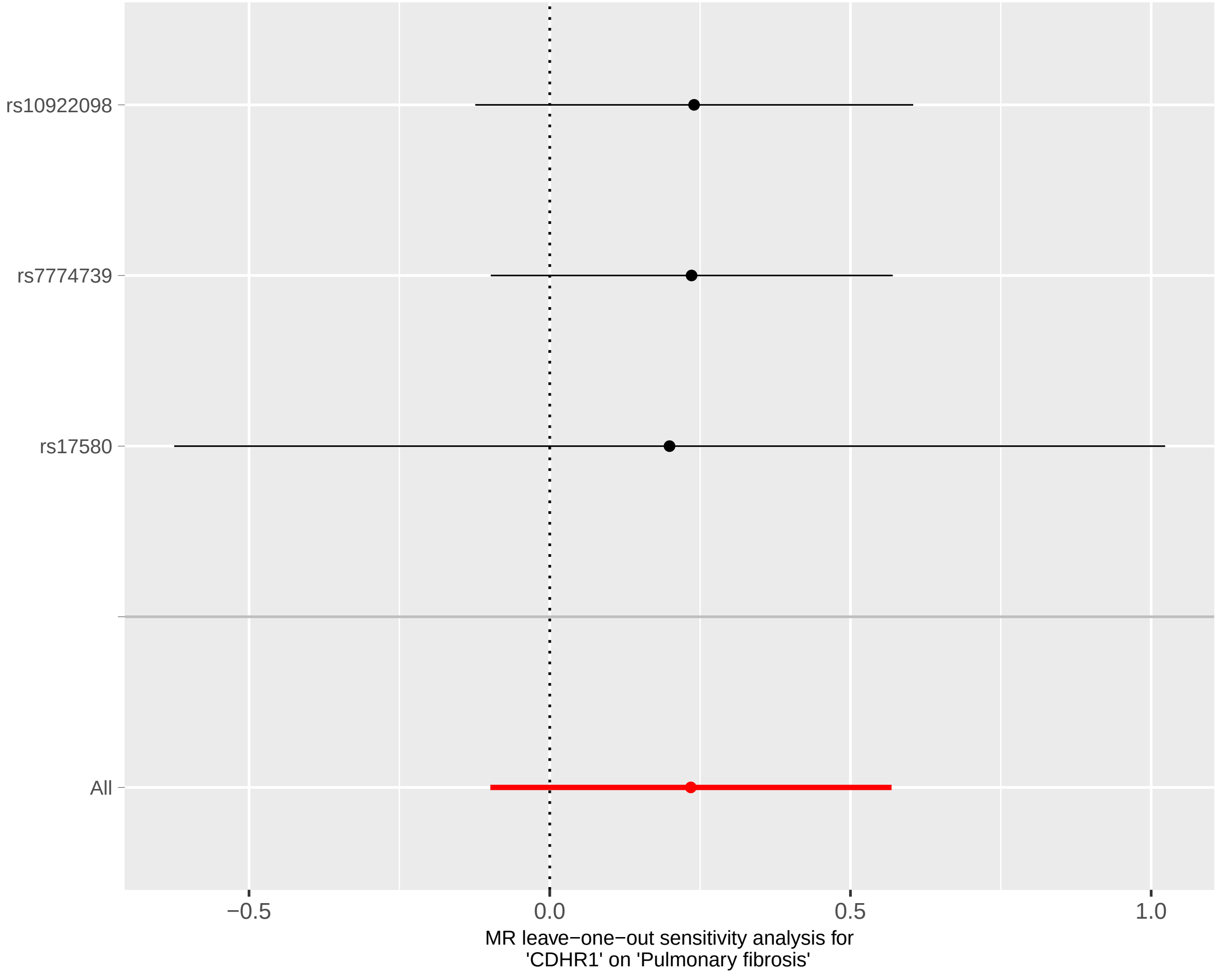
rs10922098
rs7774739
rs17580
All
−0.5
0.0
0.5
1.0
MR leave−one−out sensitivity analysis for
'CDHR1' on 'Pulmonary fibrosis'

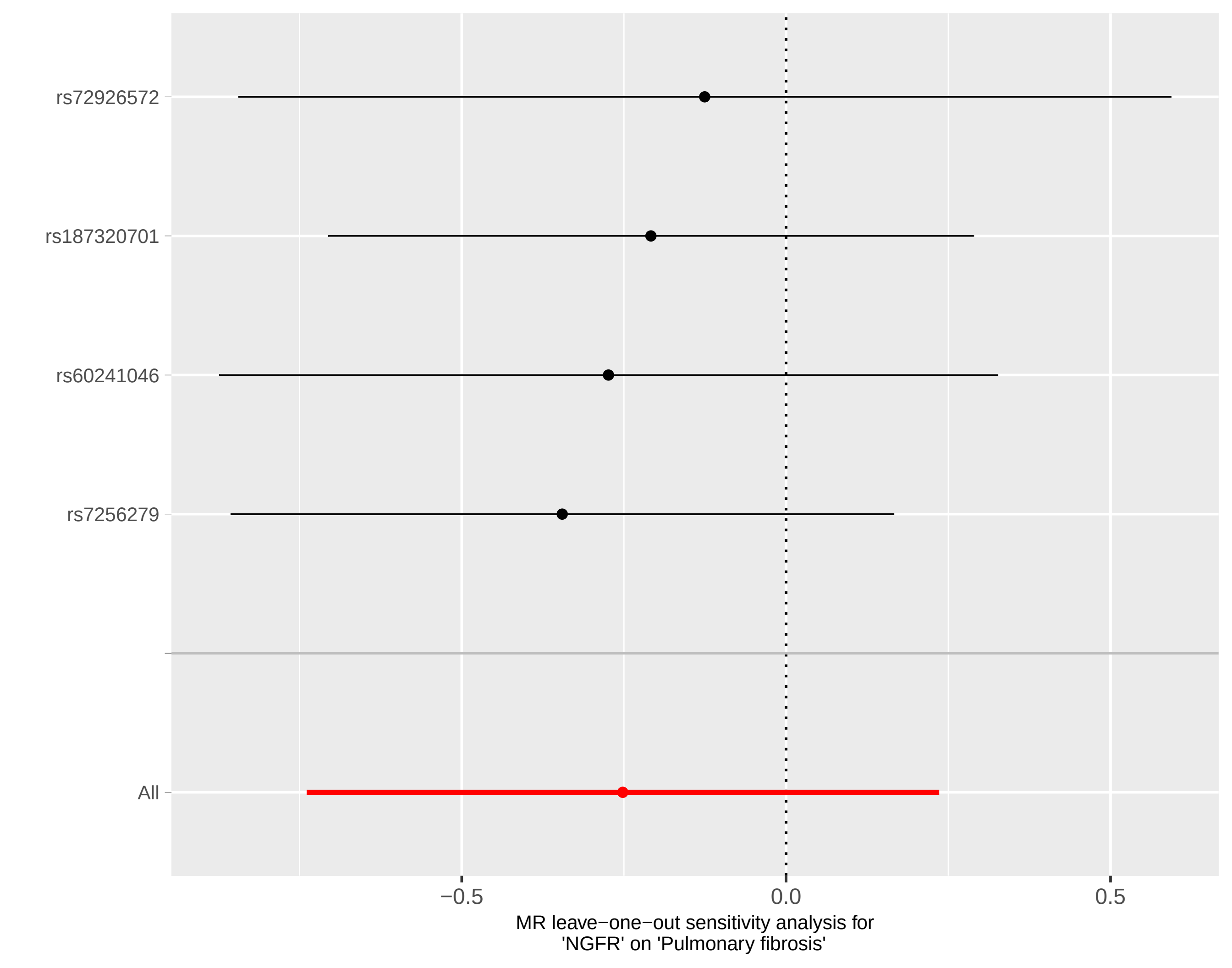
rs72926572
rs187320701
rs60241046
rs7256279
All
−0.5
0.0
0.5
MR leave−one−out sensitivity analysis for
'NGFR' on 'Pulmonary fibrosis'

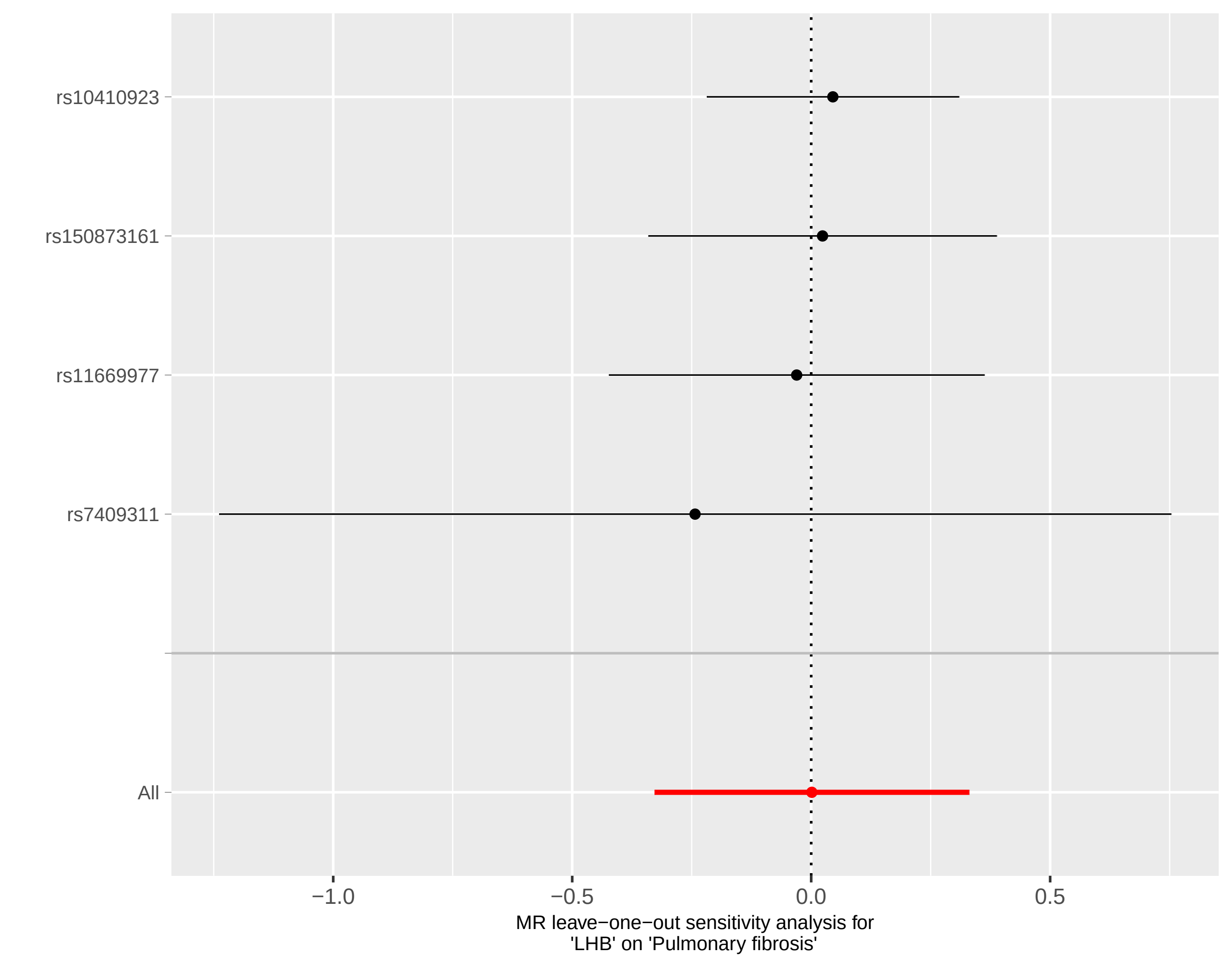
rs10410923
rs150873161
rs11669977
rs7409311
All
−1.0
−0.5
0.0
0.5
MR leave−one−out sensitivity analysis for
'LHB' on 'Pulmonary fibrosis'

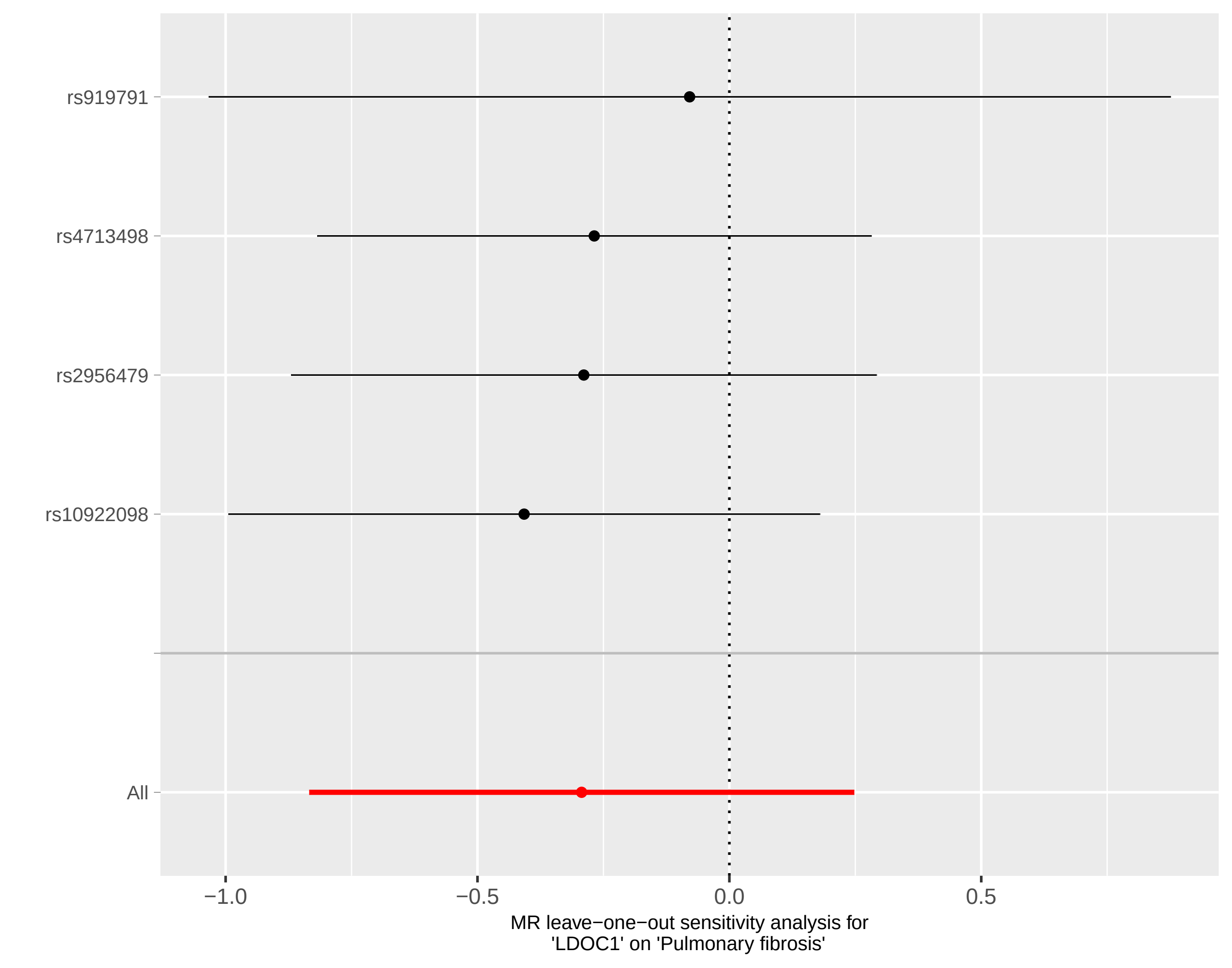
rs919791
rs4713498
rs2956479
rs10922098
All
−1.0
−0.5
0.0
0.5
MR leave−one−out sensitivity analysis for
'LDOC1' on 'Pulmonary fibrosis'

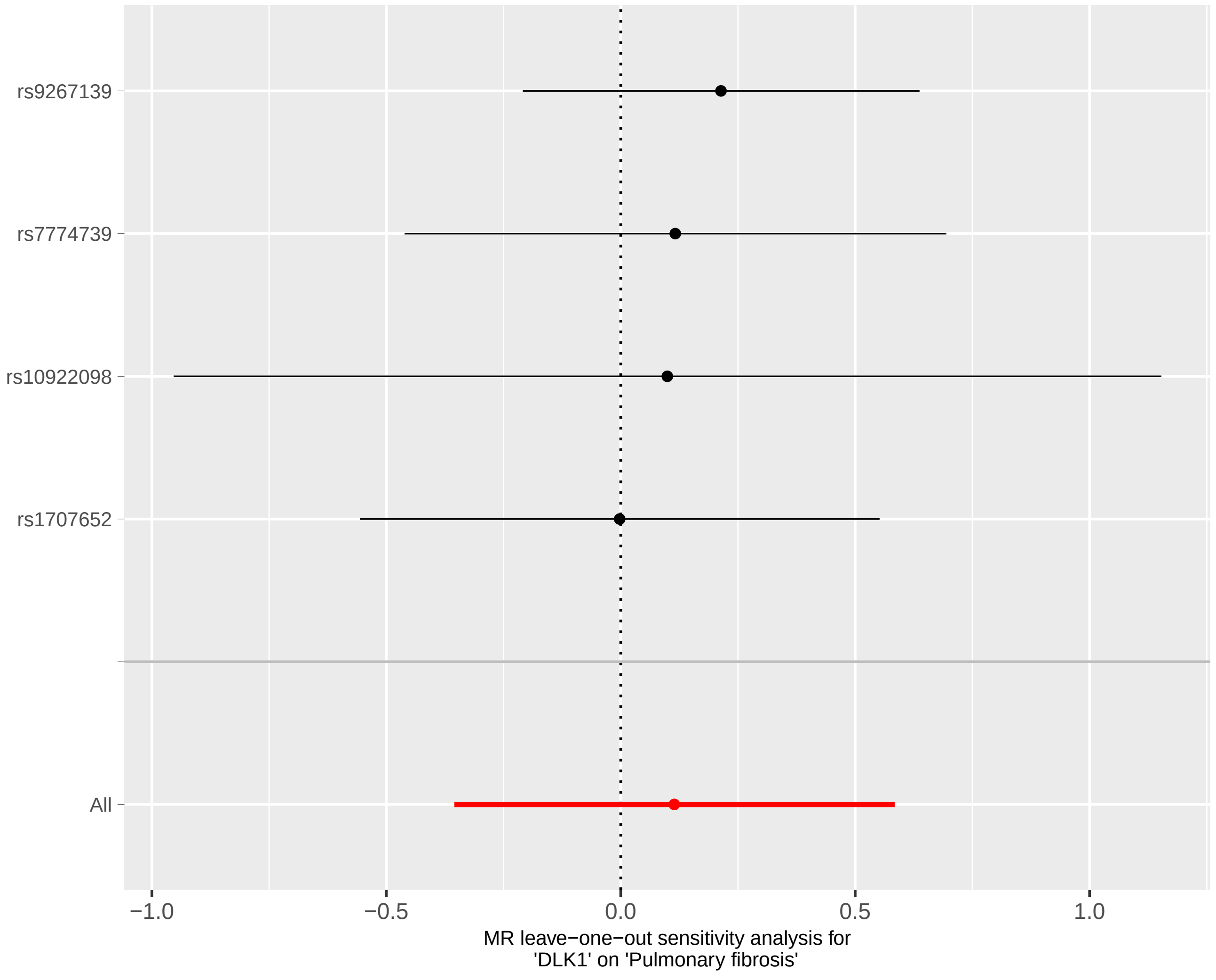

rs9267139
rs7774739
rs10922098
rs1707652
All
−1.0
−0.5
0.0
0.5
1.0
MR leave−one−out sensitivity analysis for
'DLK1' on 'Pulmonary fibrosis'

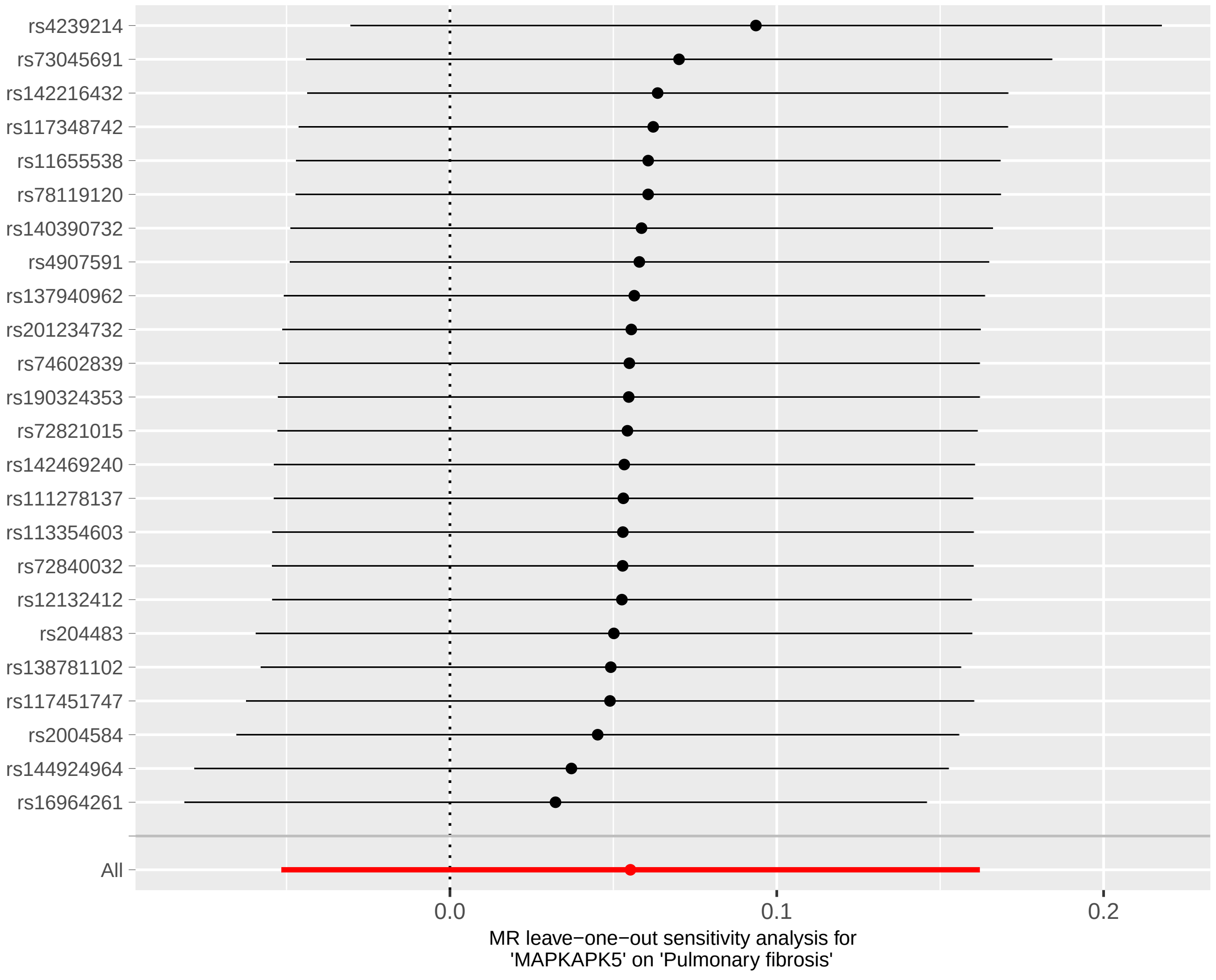
rs4239214
rs73045691
rs142216432
rs117348742
rs11655538
rs78119120
rs140390732
rs4907591
rs137940962
rs201234732
rs74602839
rs190324353
rs72821015
rs142469240
rs111278137
rs113354603
rs72840032
rs12132412
rs204483
rs138781102
rs117451747
rs2004584
rs144924964
rs16964261
All
0.0
0.1
0.2
MR leave−one−out sensitivity analysis for
'MAPKAPK5' on 'Pulmonary fibrosis'

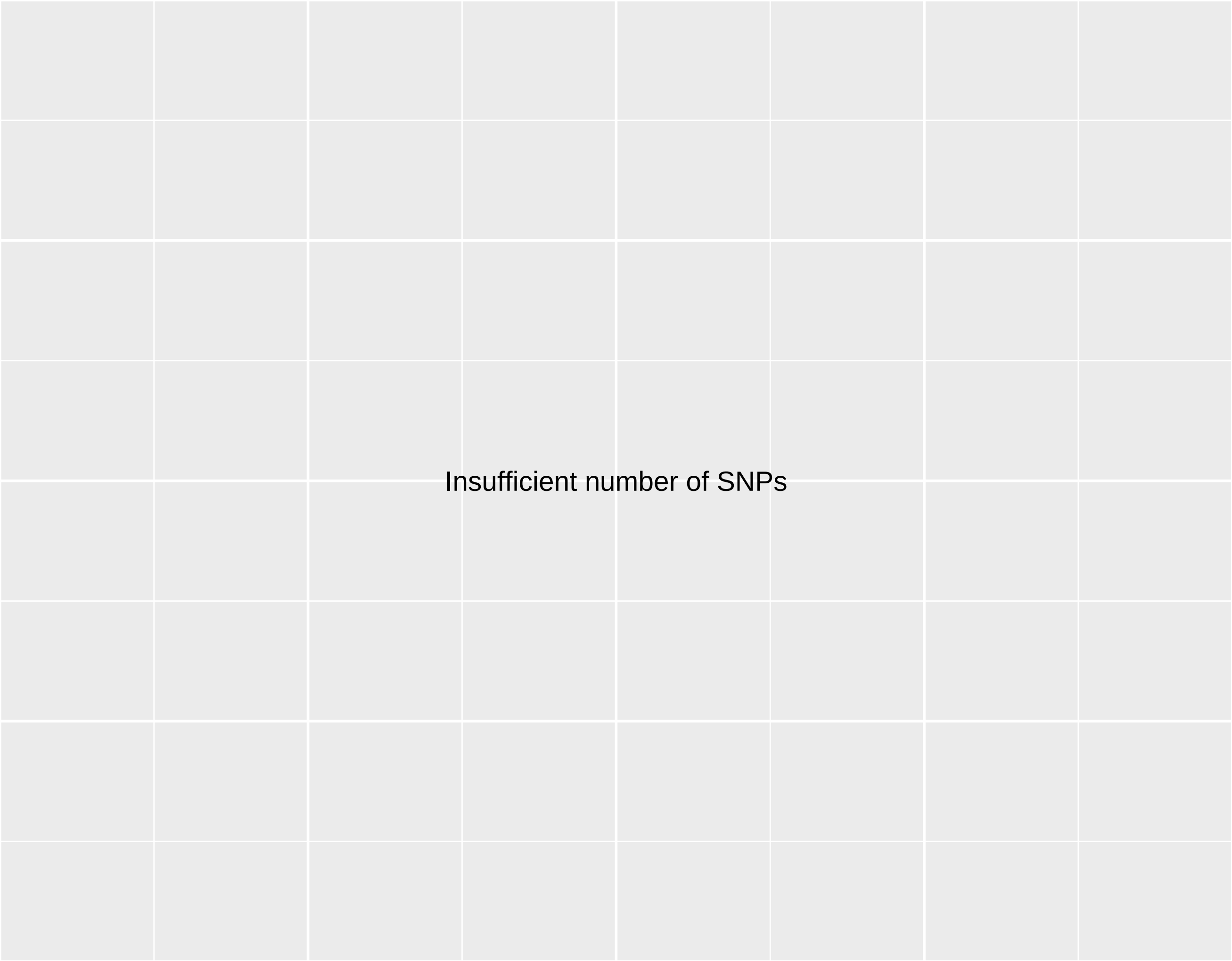
Insufficient number of SNPs

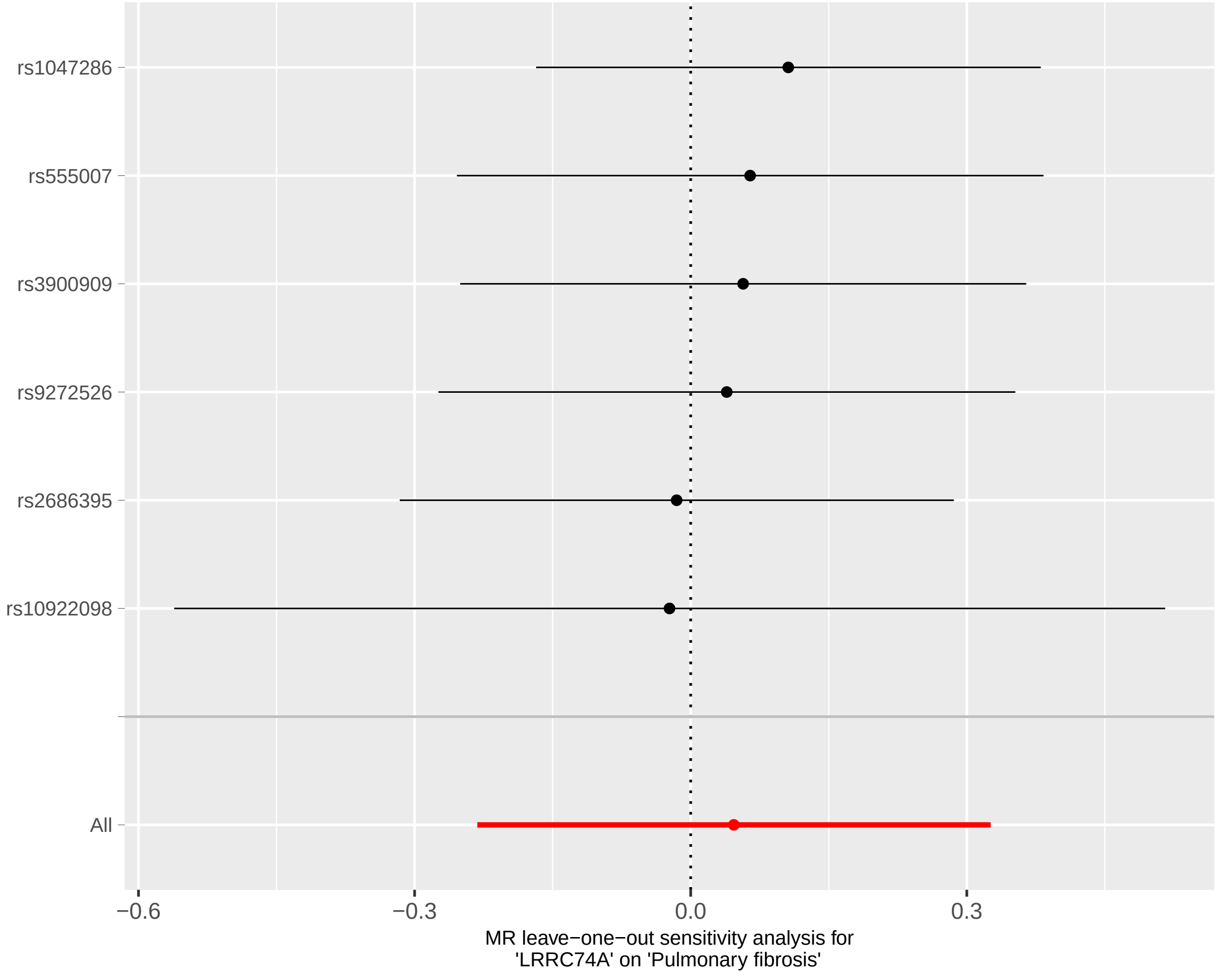
rs1047286
rs555007
rs3900909
rs9272526
rs2686395
rs10922098
All
−0.6
−0.3
0.0
0.3
MR leave−one−out sensitivity analysis for
'LRRC74A' on 'Pulmonary fibrosis'

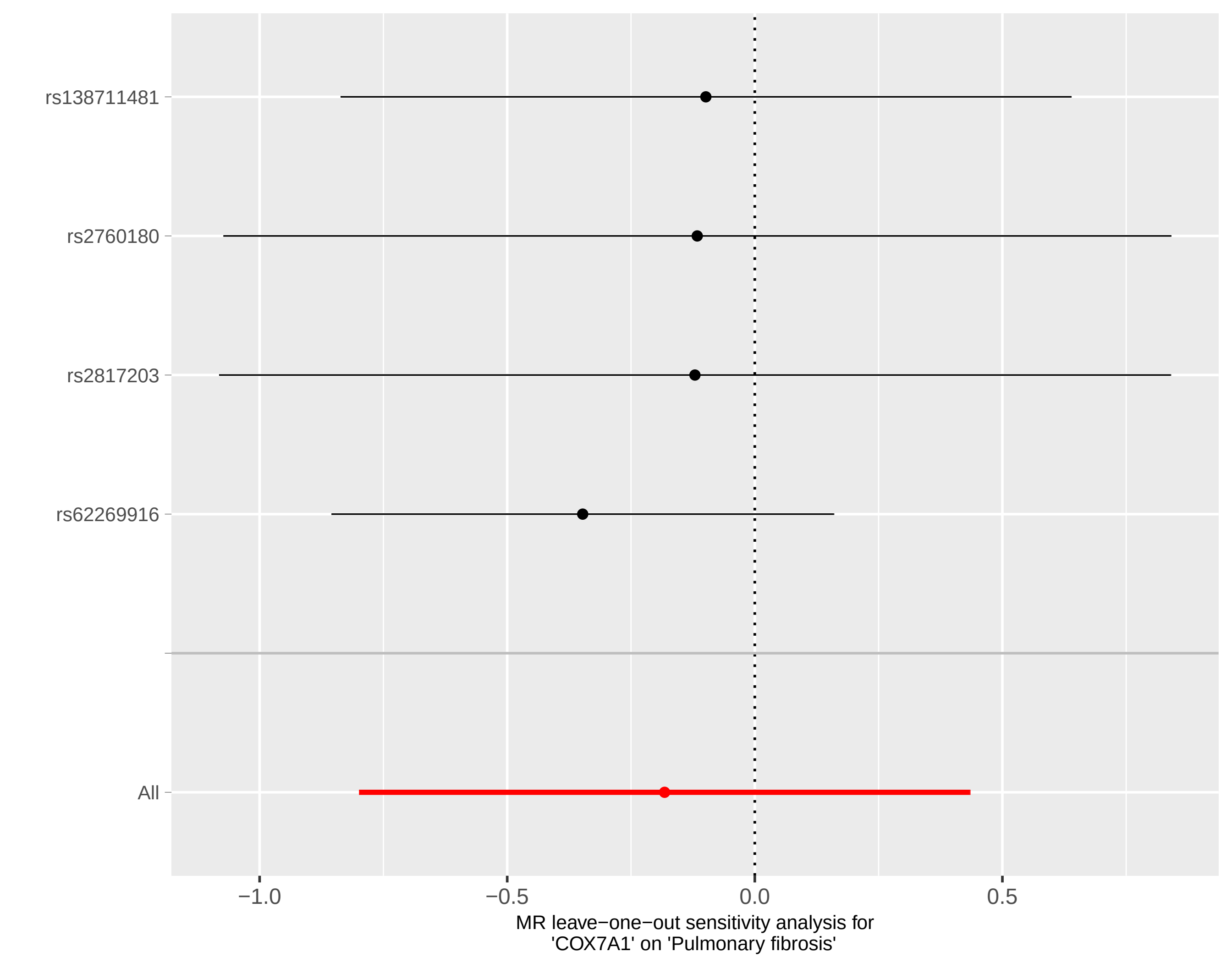
rs138711481
rs2760180
rs2817203
rs62269916
All
−1.0
−0.5
0.0
0.5
MR leave−one−out sensitivity analysis for
'COX7A1' on 'Pulmonary fibrosis'

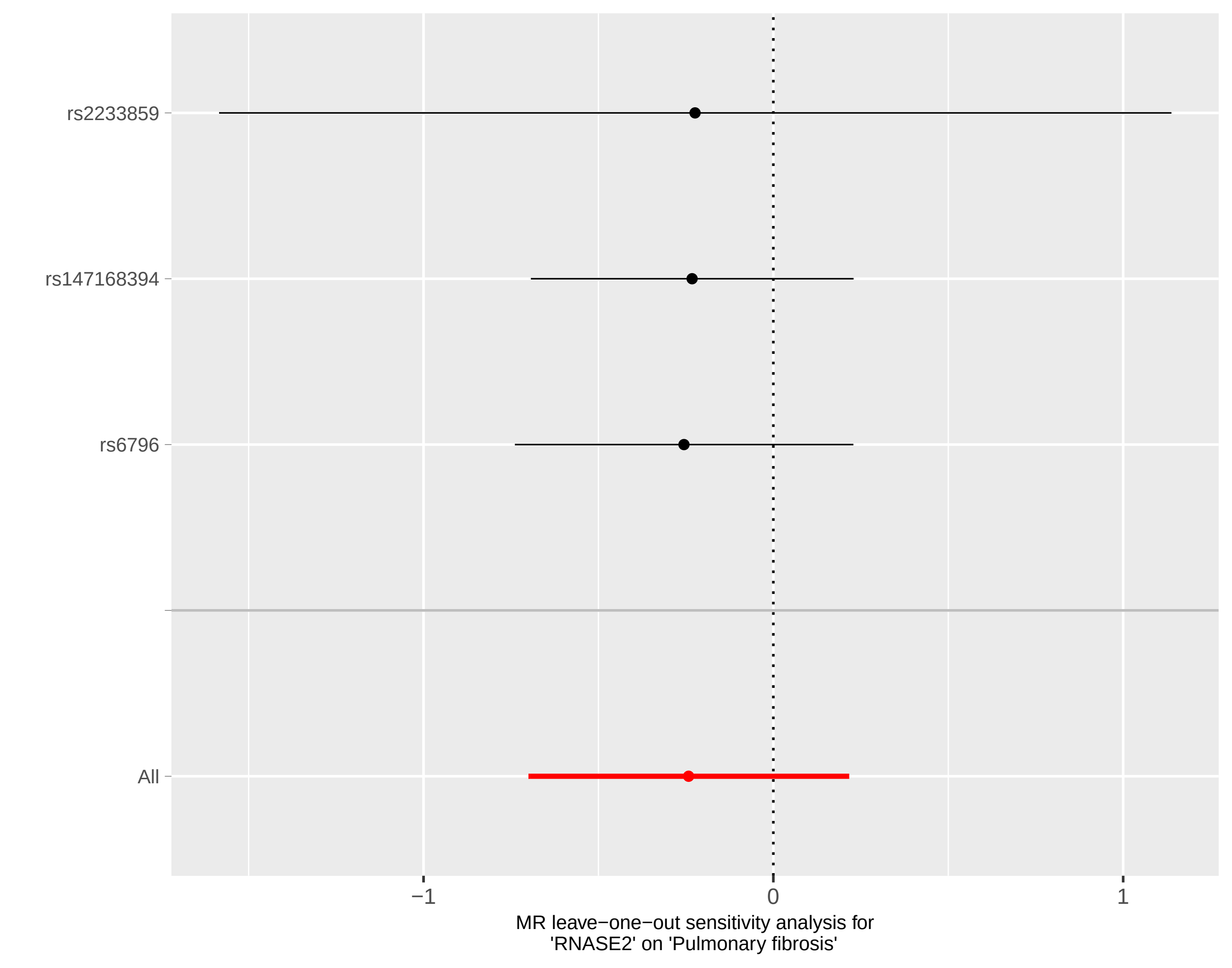
rs2233859
rs147168394
rs6796
All
−1
0
1
MR leave−one−out sensitivity analysis for
'RNASE2' on 'Pulmonary fibrosis'

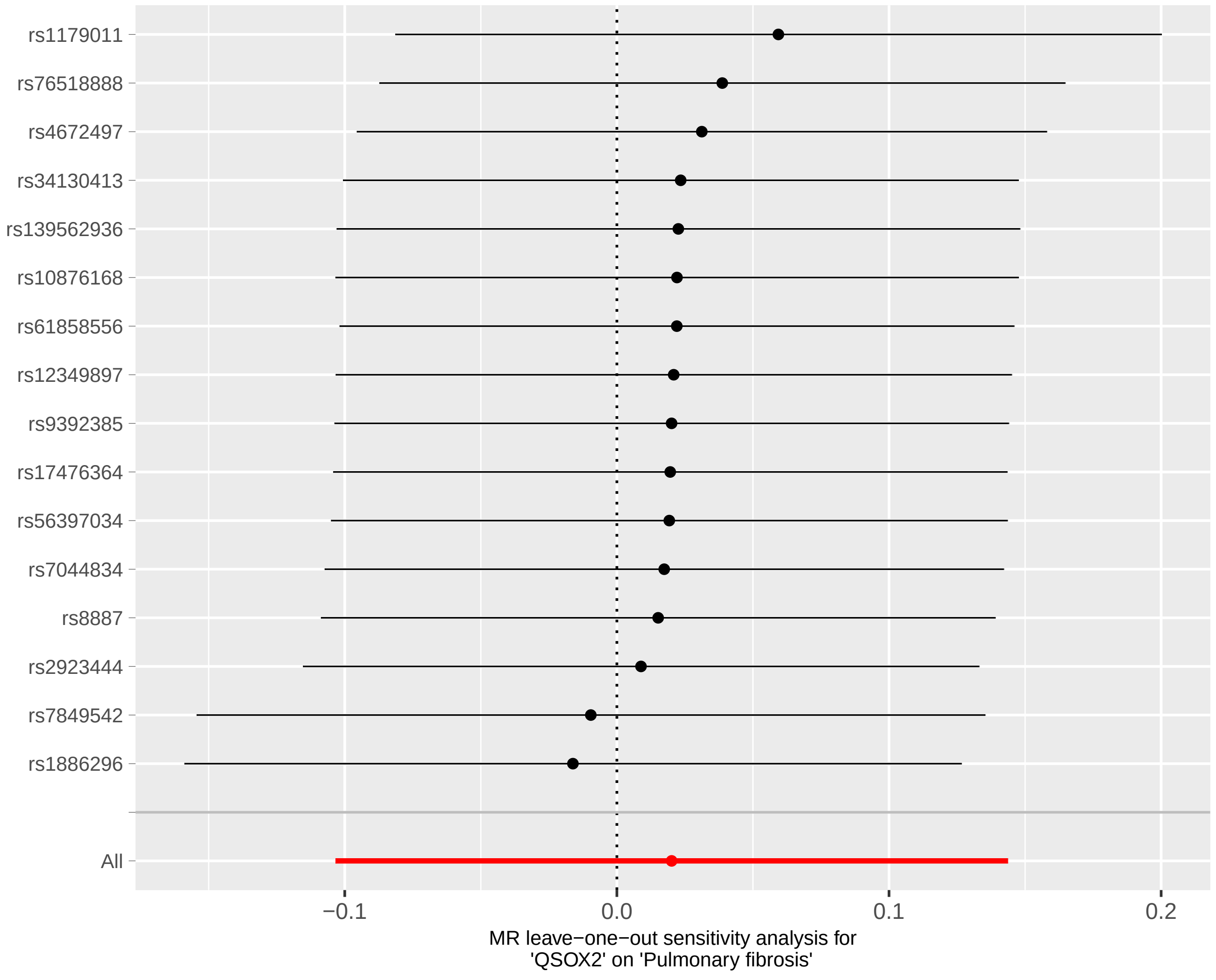
rs1179011
rs76518888
rs4672497
rs34130413
rs139562936
rs10876168
rs61858556
rs12349897
rs9392385
rs17476364
rs56397034
rs7044834
rs8887
rs2923444
rs7849542
rs1886296
All
−0.1
0.0
0.1
0.2
MR leave−one−out sensitivity analysis for
'QSOX2' on 'Pulmonary fibrosis'

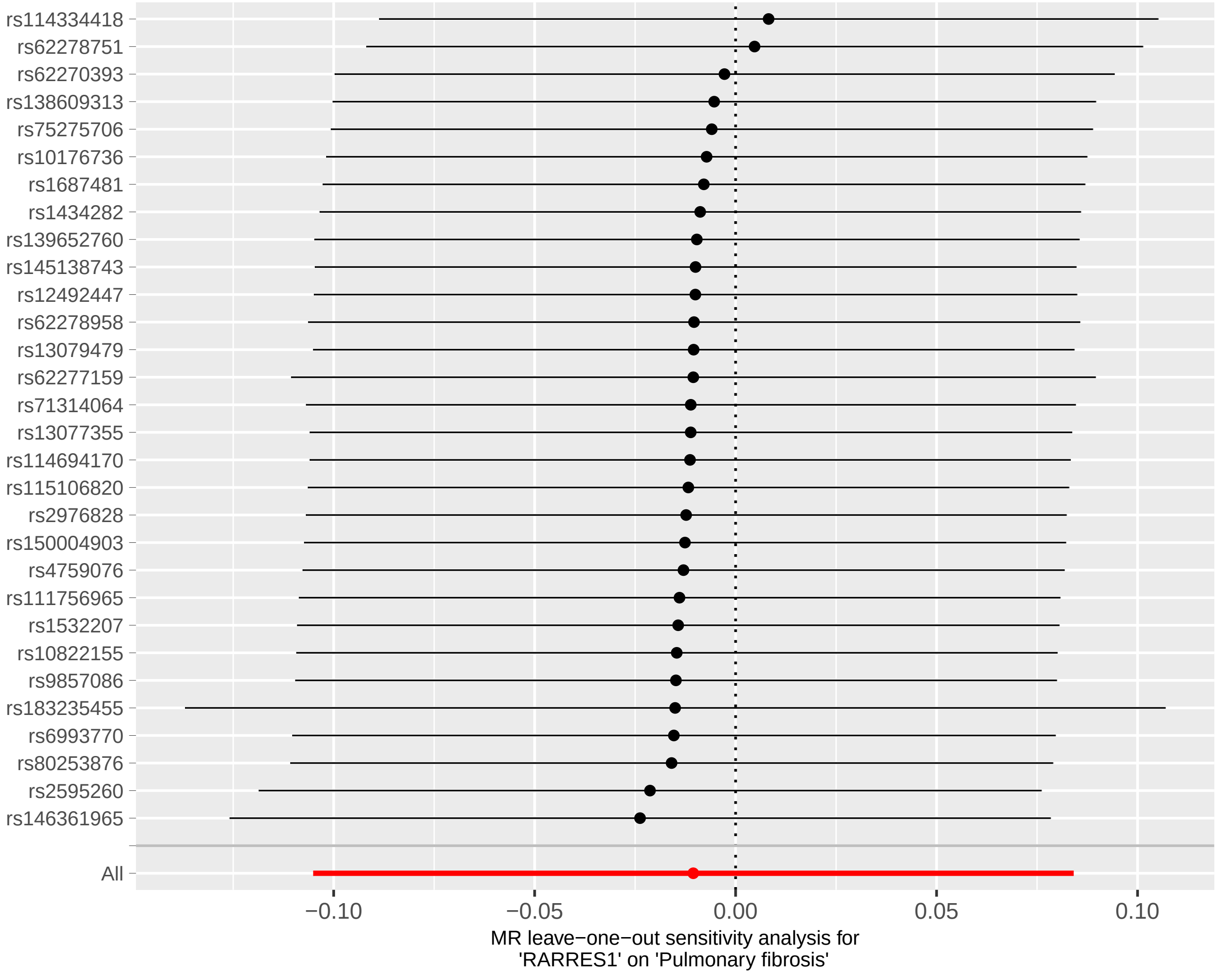
rs114334418
rs62278751
rs62270393
rs138609313
rs75275706
rs10176736
rs1687481
rs1434282
rs139652760
rs145138743
rs12492447
rs62278958
rs13079479
rs62277159
rs71314064
rs13077355
rs114694170
rs115106820
rs2976828
rs150004903
rs4759076
rs111756965
rs1532207
rs10822155
rs9857086
rs183235455
rs6993770
rs80253876
rs2595260
rs146361965
All
−0.10
−0.05
0.00
0.05
0.10
MR leave−one−out sensitivity analysis for
'RARRES1' on 'Pulmonary fibrosis'

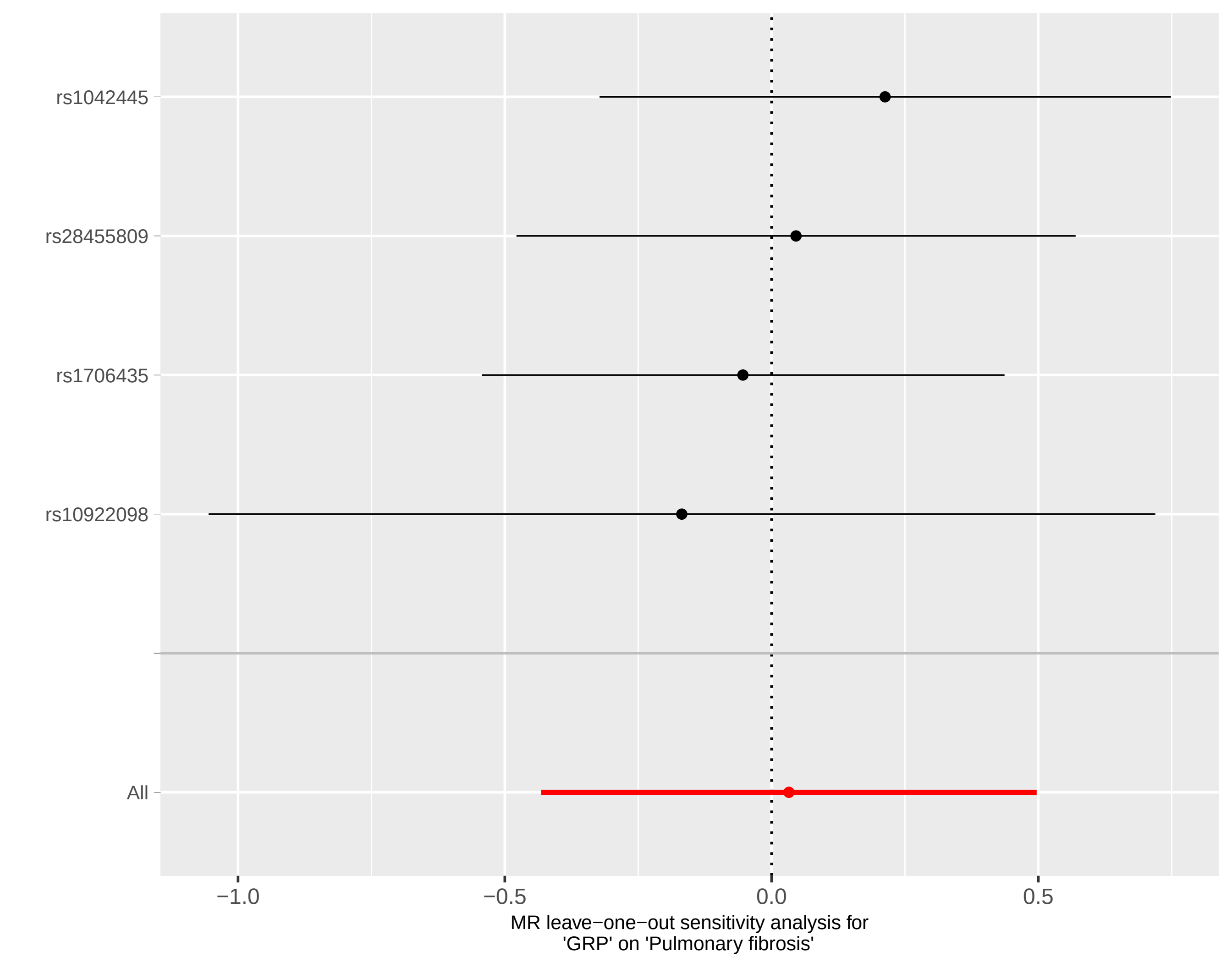
rs1042445
rs28455809
rs1706435
rs10922098
All
−1.0
−0.5
0.0
0.5
MR leave−one−out sensitivity analysis for
'GRP' on 'Pulmonary fibrosis'

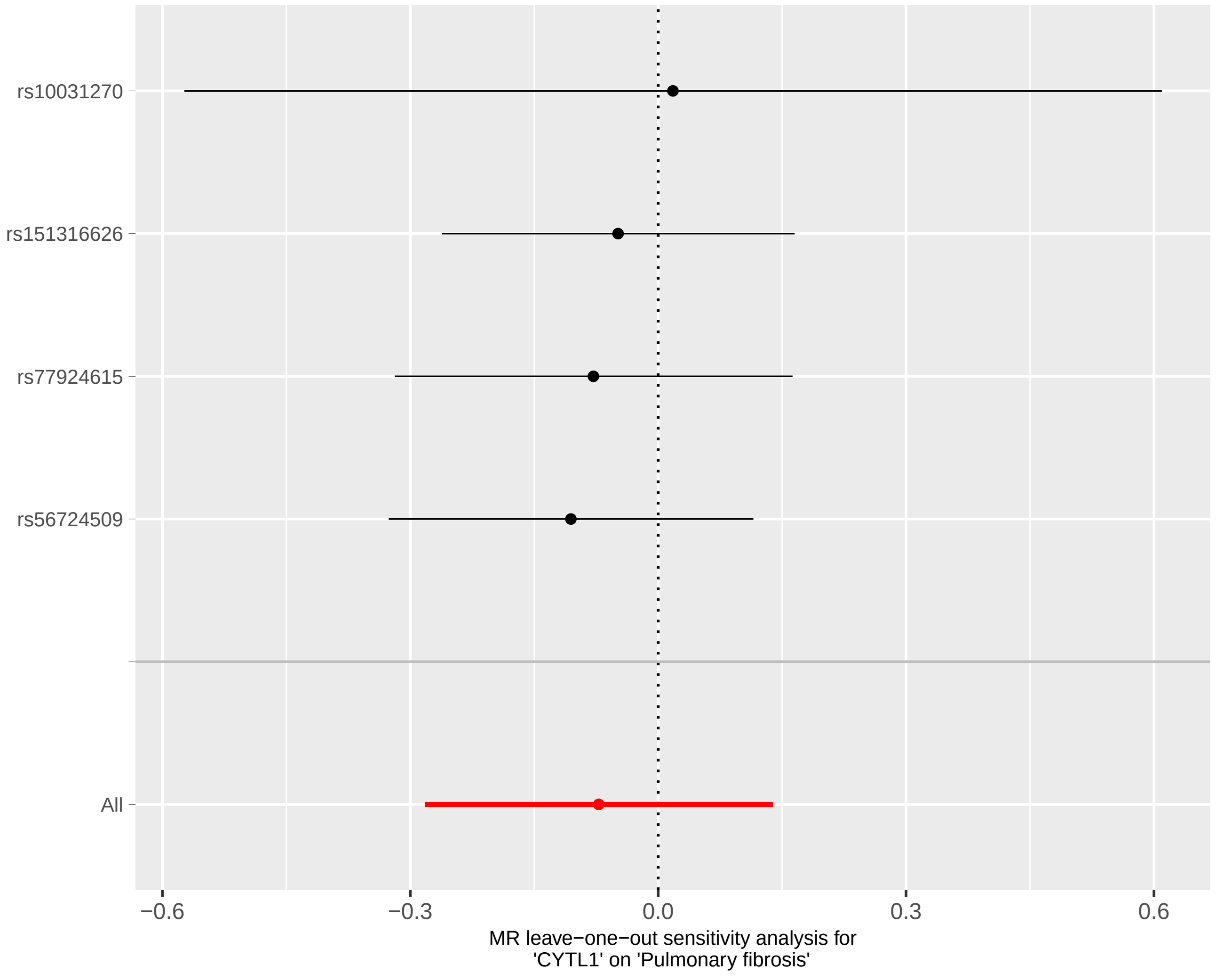
rs10031270
rs151316626
rs77924615
rs56724509
All
−0.6
−0.3
0.0
0.3
0.6
MR leave−one−out sensitivity analysis for
'CYTL1' on 'Pulmonary fibrosis'

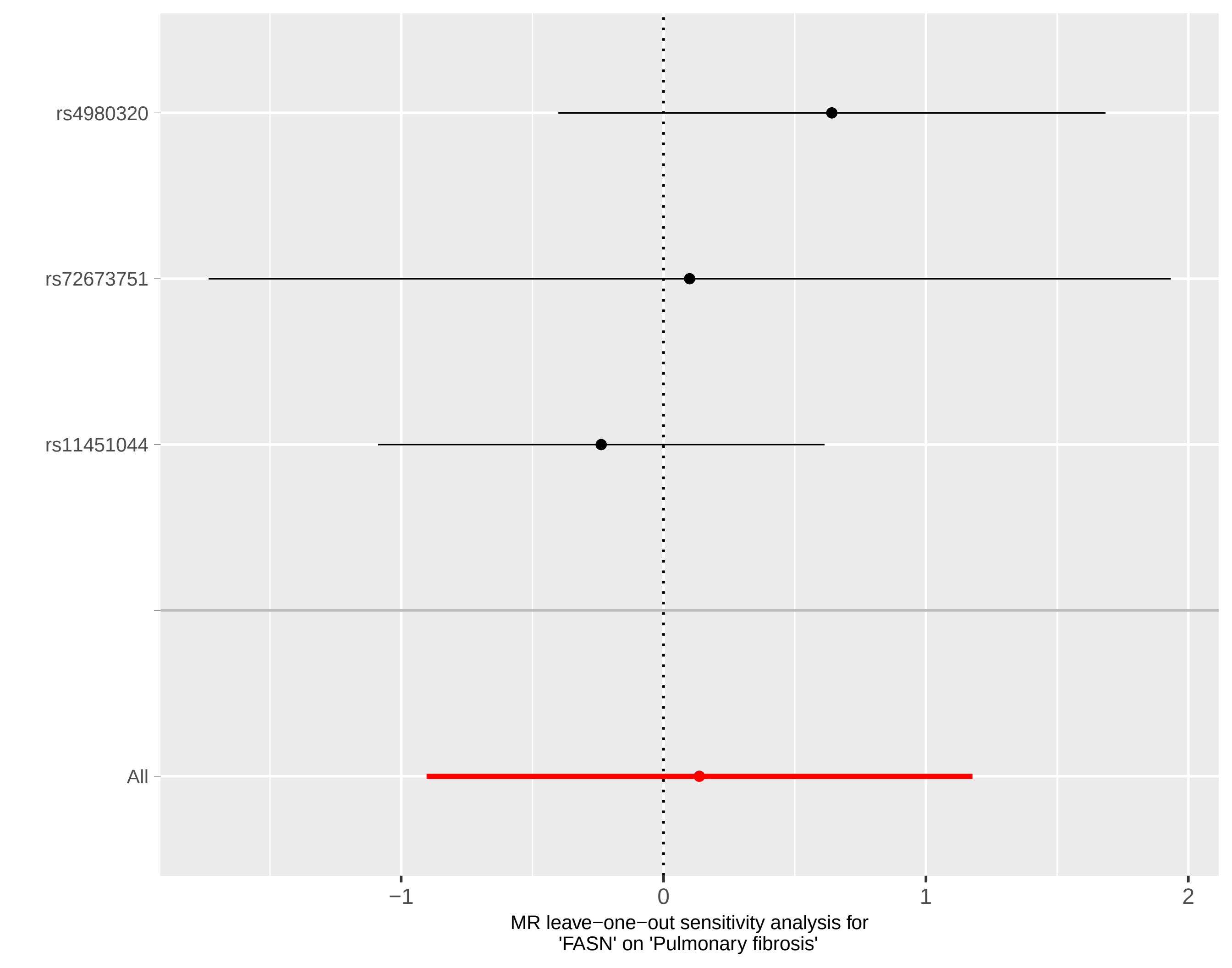
rs4980320
rs72673751
rs11451044
All
−1
0
1
2
MR leave−one−out sensitivity analysis for
'FASN' on 'Pulmonary fibrosis'

Insufficient number of SNPs

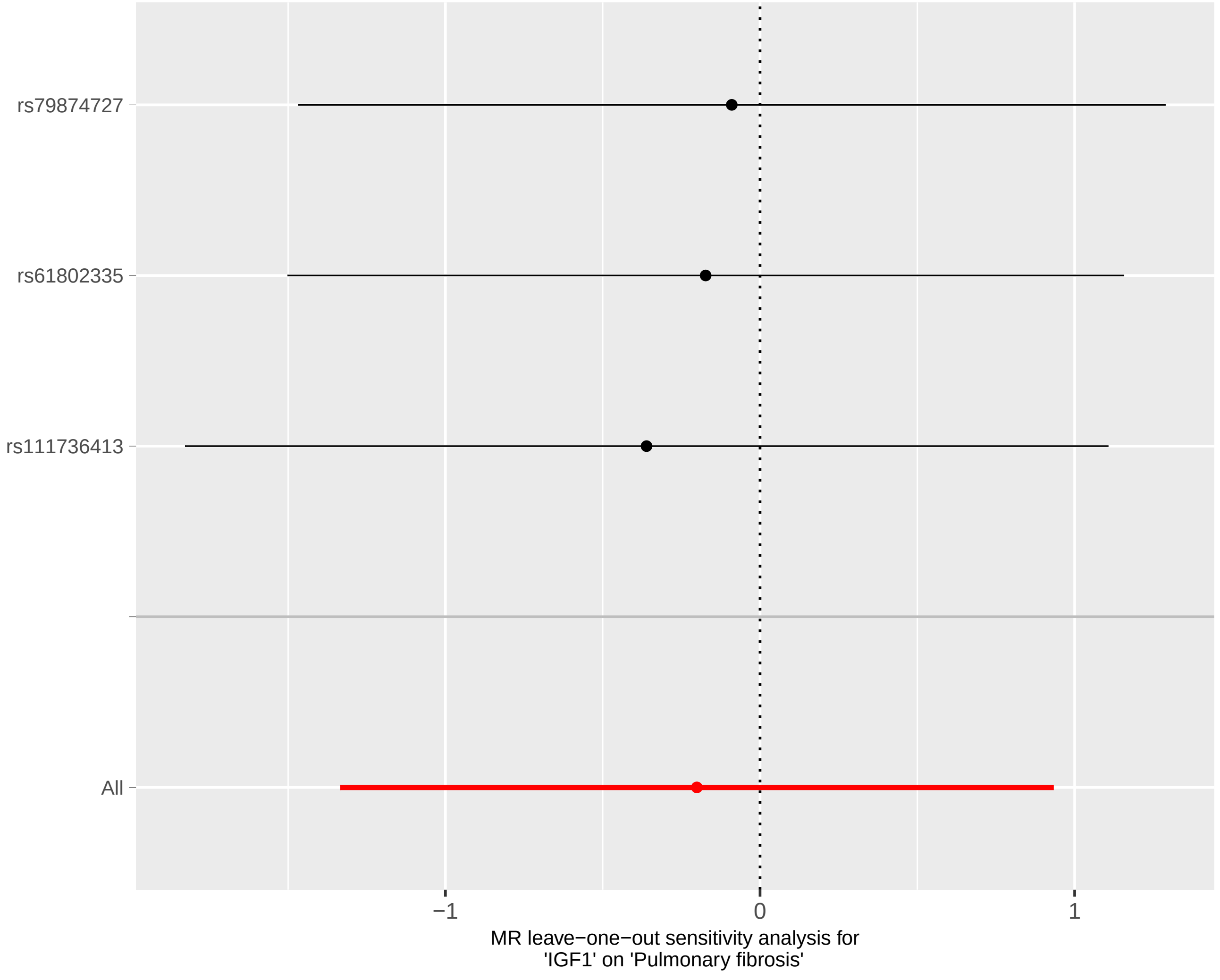
rs79874727
rs61802335
rs111736413
All
−1
0
1
MR leave−one−out sensitivity analysis for
'IGF1' on 'Pulmonary fibrosis'

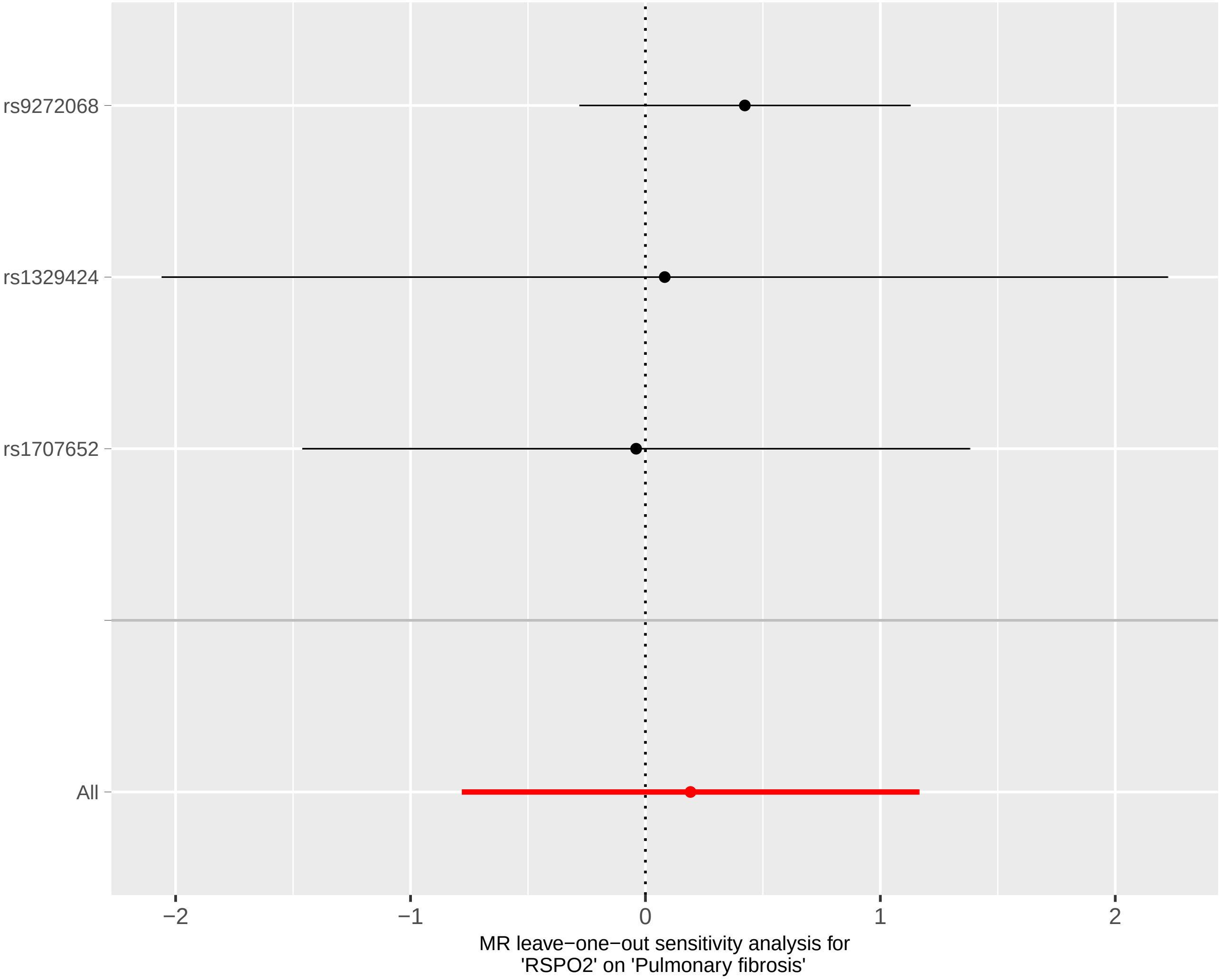

rs9272068
rs1329424
rs1707652
All
−2
−1
0
1
2
MR leave−one−out sensitivity analysis for
'RSPO2' on 'Pulmonary fibrosis'

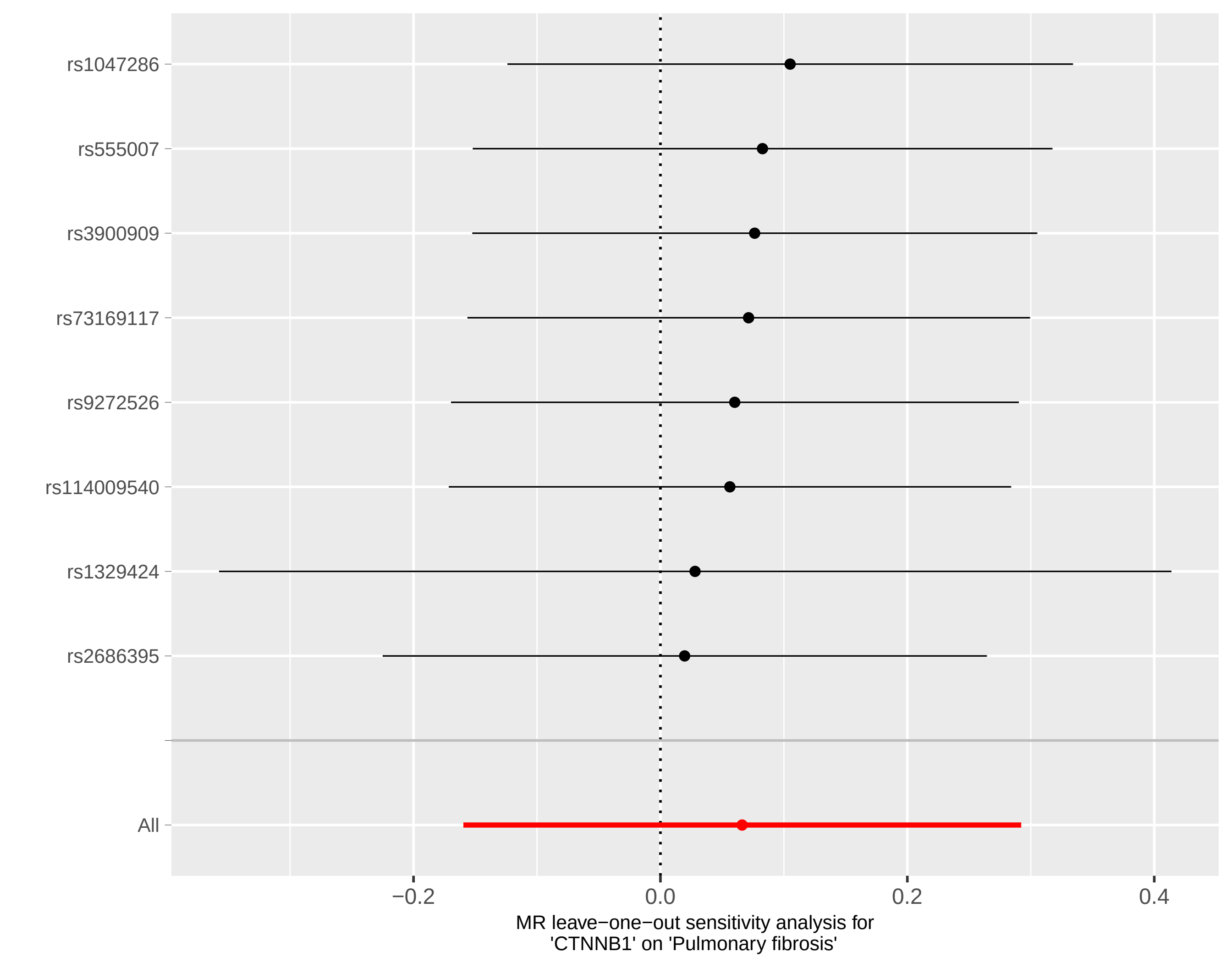
rs1047286
rs555007
rs3900909
rs73169117
rs9272526
rs114009540
rs1329424
rs2686395
All
−0.2
0.0
0.2
0.4
MR leave−one−out sensitivity analysis for
'CTNNB1' on 'Pulmonary fibrosis'

Insufficient number of SNPs

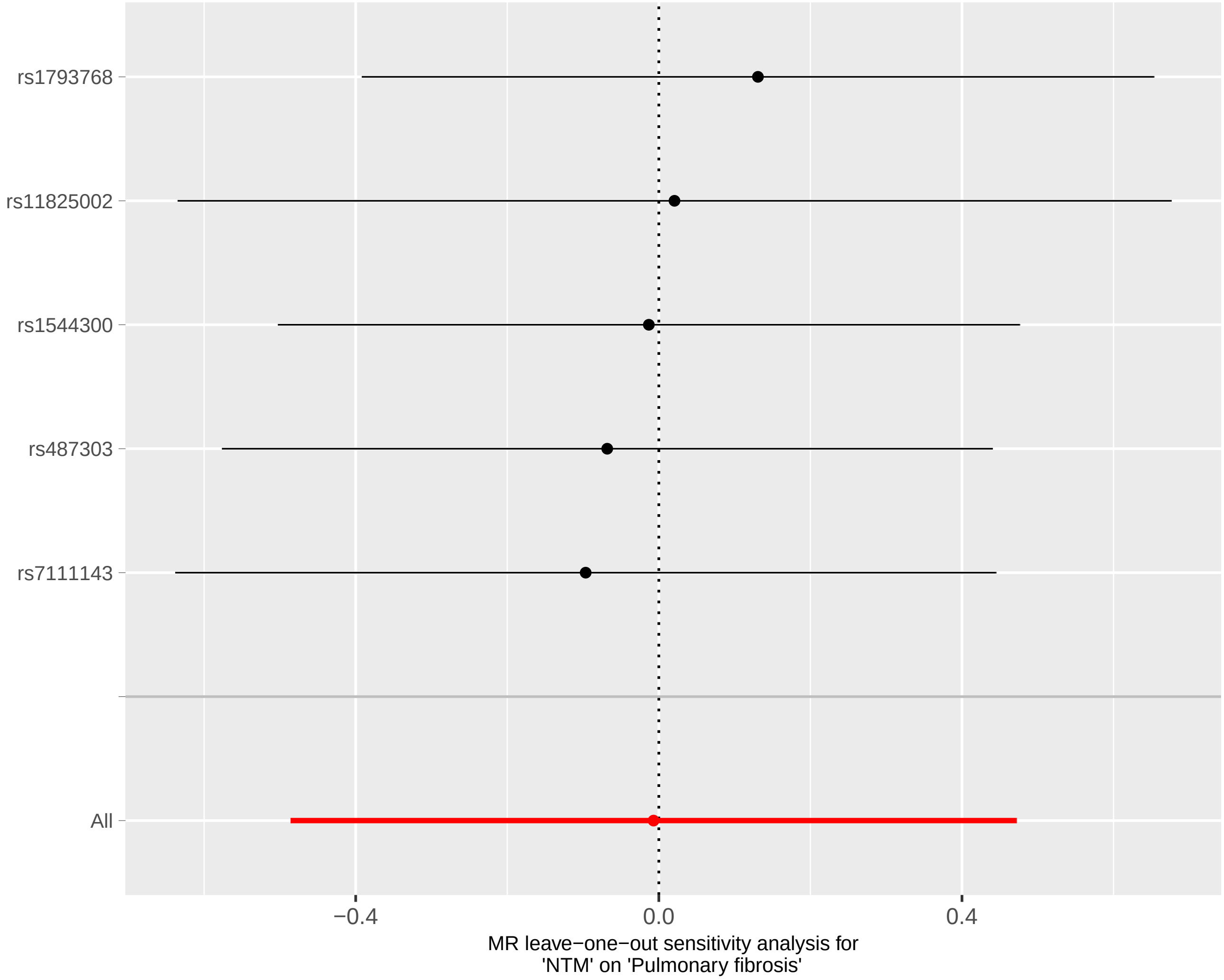
rs1793768
rs11825002
rs1544300
rs487303
rs7111143
All
−0.4
0.0
0.4
MR leave−one−out sensitivity analysis for
'NTM' on 'Pulmonary fibrosis'

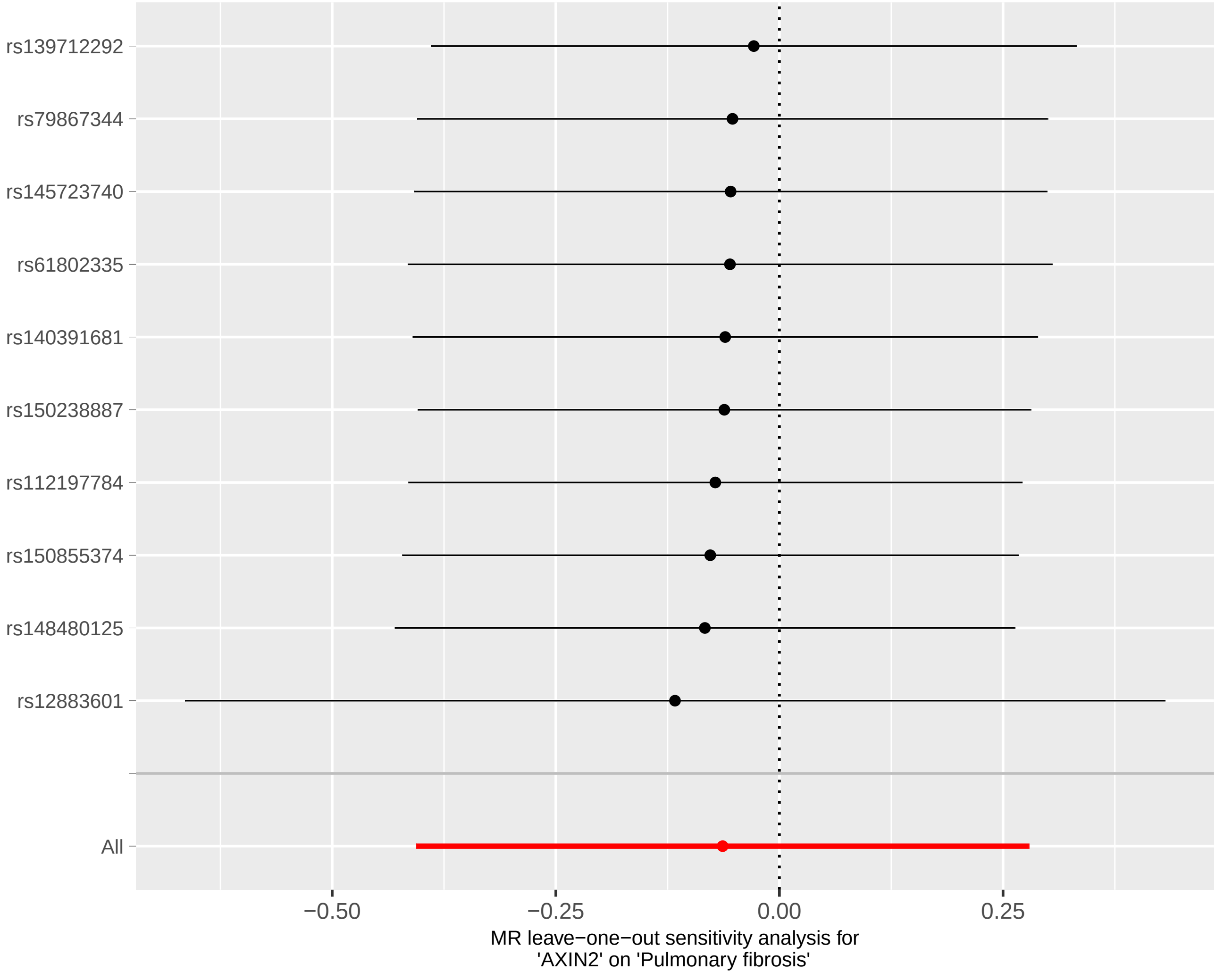

rs139712292
rs79867344
rs145723740
rs61802335
rs140391681
rs150238887
rs112197784
rs150855374
rs148480125
rs12883601
All
−0.50
−0.25
0.00
0.25
MR leave−one−out sensitivity analysis for
'AXIN2' on 'Pulmonary fibrosis'

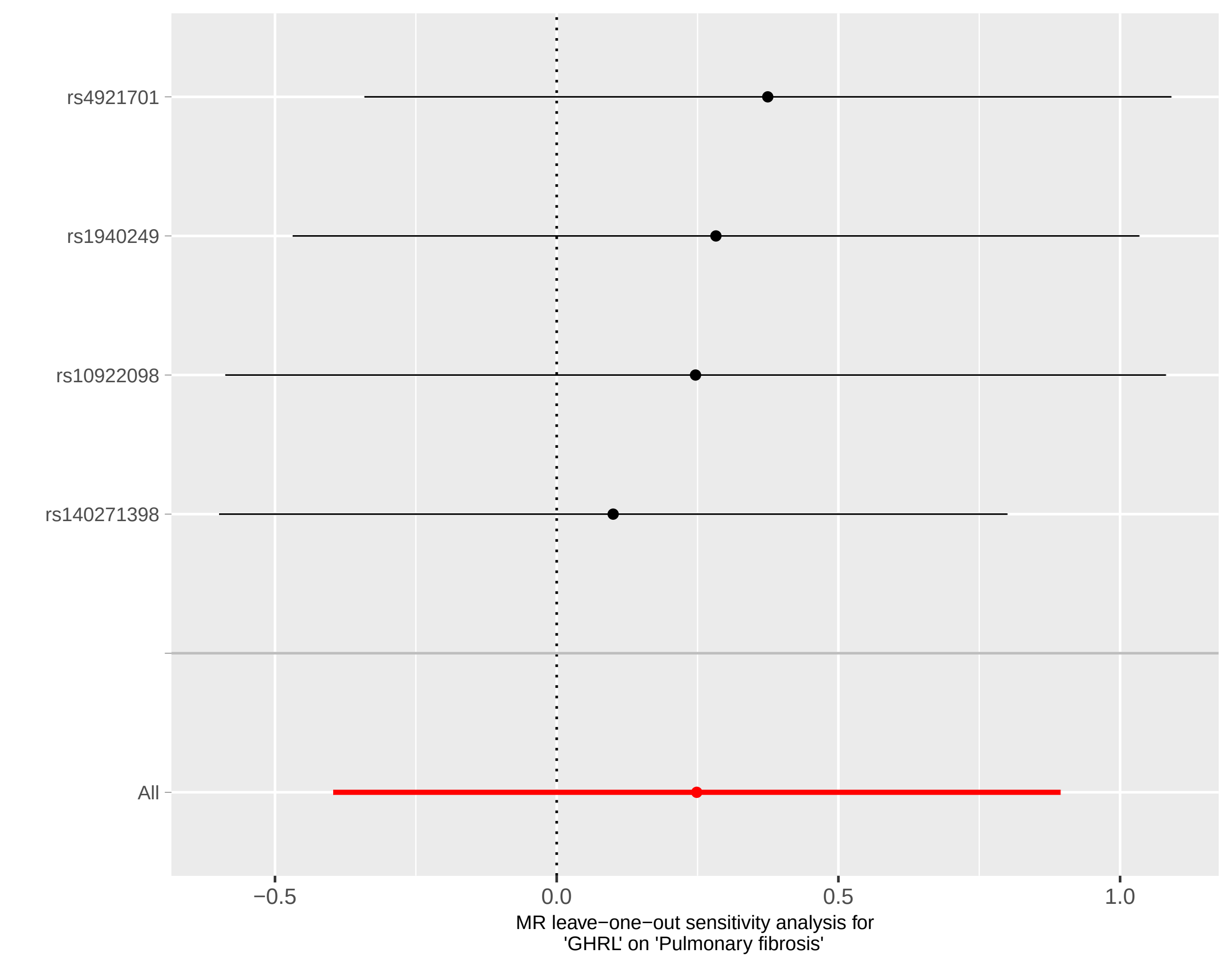

rs4921701
rs1940249
rs10922098
rs140271398
All
−0.5
0.0
0.5
1.0
MR leave−one−out sensitivity analysis for
'GHRL' on 'Pulmonary fibrosis'

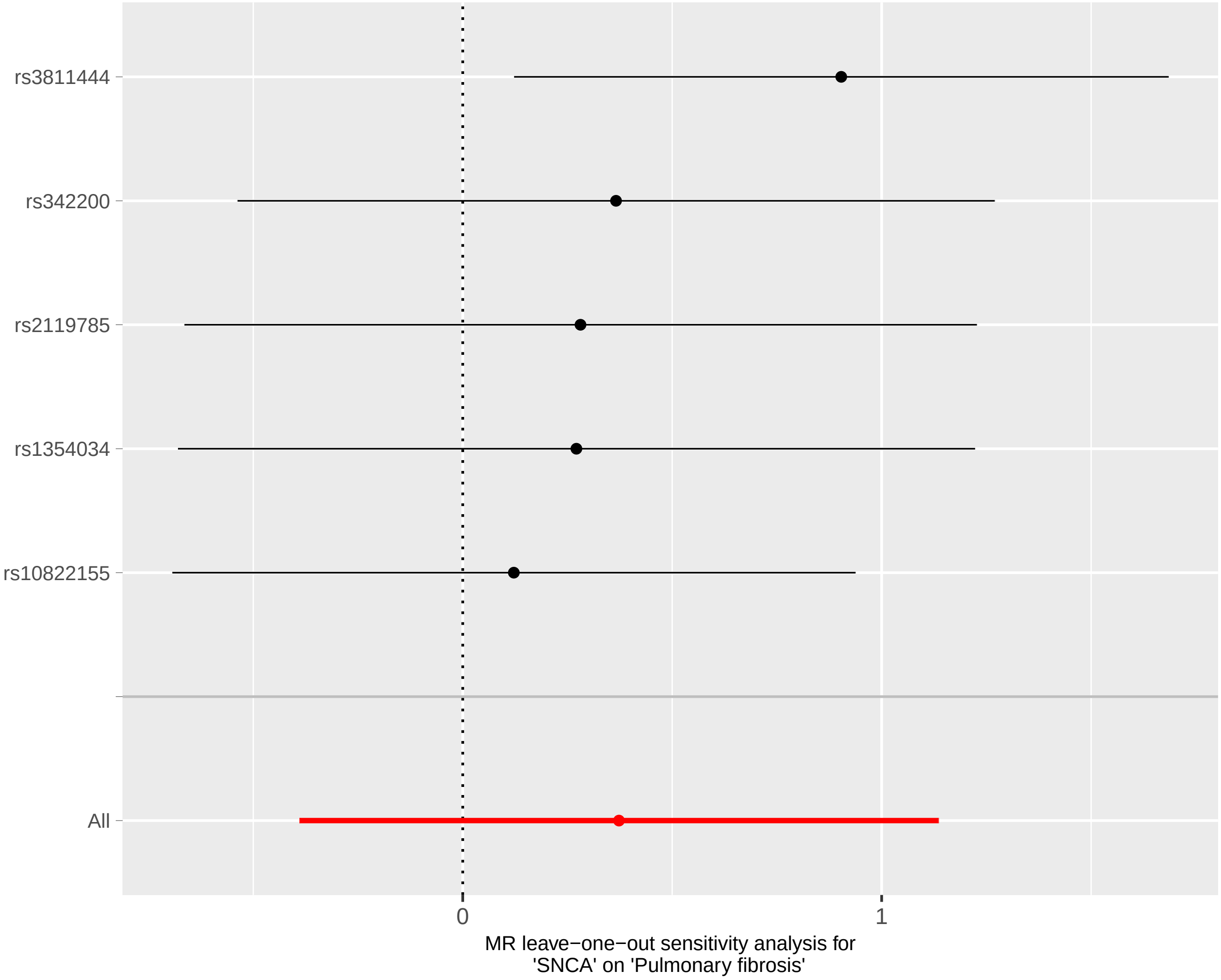
rs3811444
rs342200
rs2119785
rs1354034
rs10822155
All
0
1
MR leave−one−out sensitivity analysis for
'SNCA' on 'Pulmonary fibrosis'

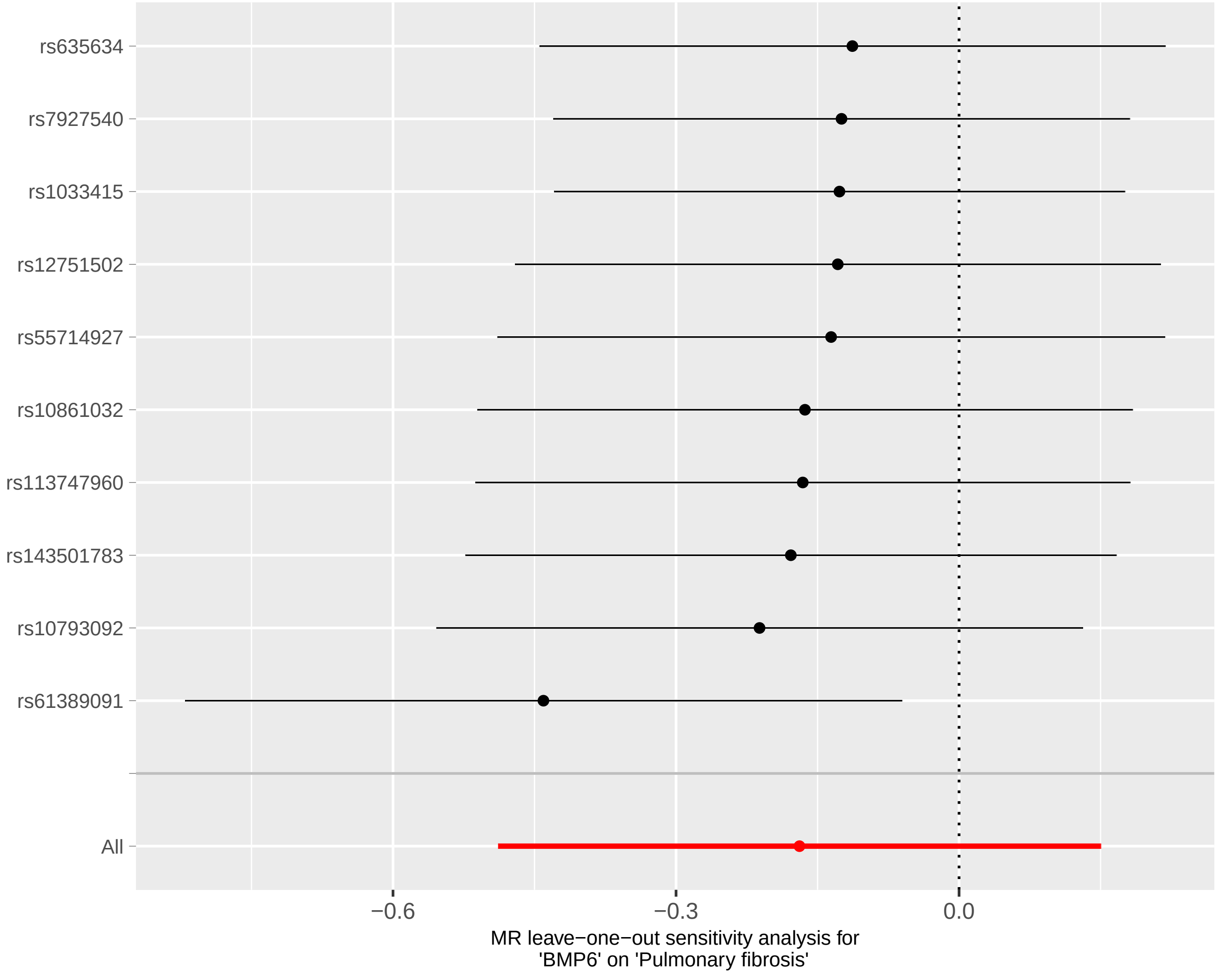
rs635634
rs7927540
rs1033415
rs12751502
rs55714927
rs10861032
rs113747960
rs143501783
rs10793092
rs61389091
All
−0.6
−0.3
0.0
MR leave−one−out sensitivity analysis for
'BMP6' on 'Pulmonary fibrosis'

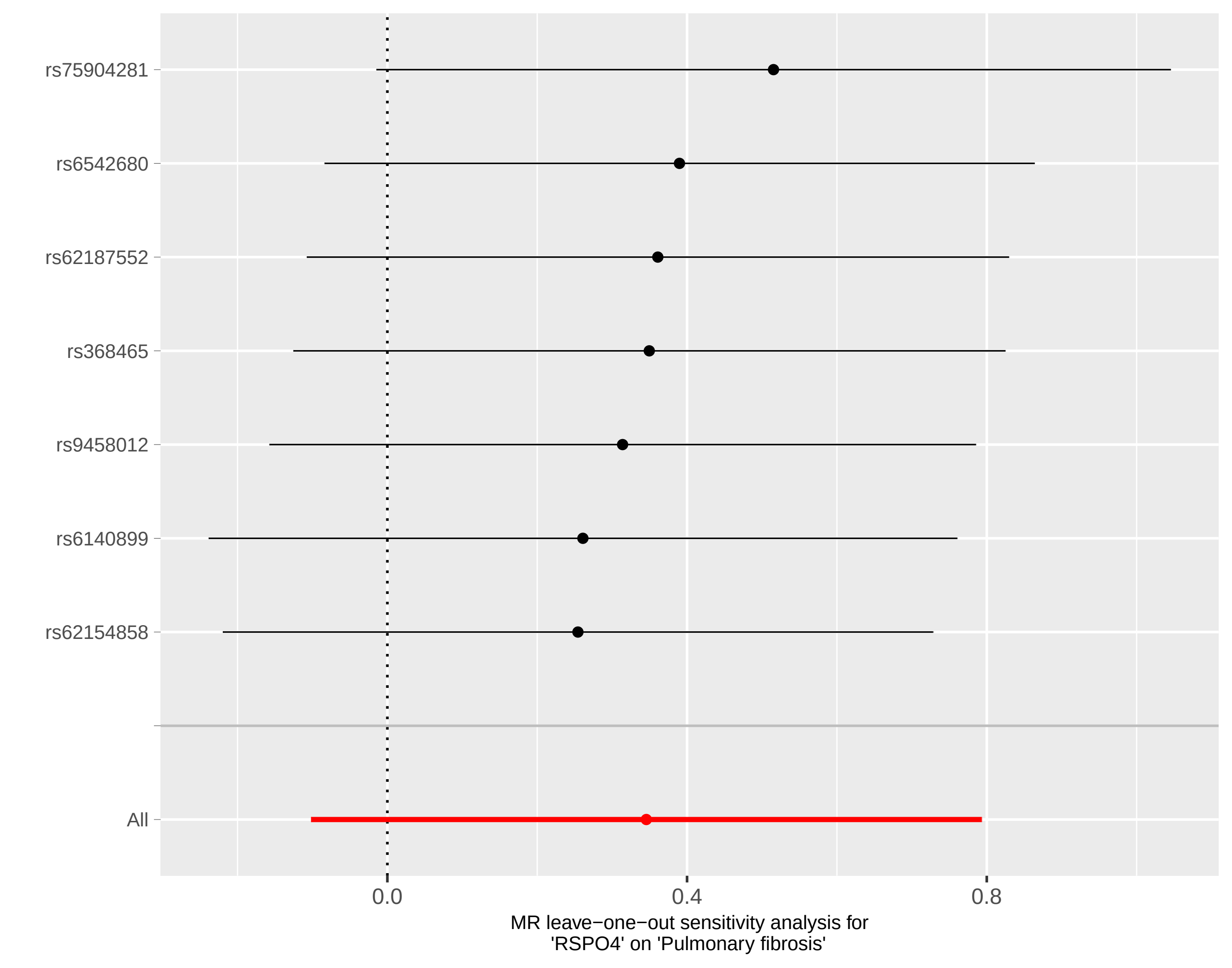
rs75904281
rs6542680
rs62187552
rs368465
rs9458012
rs6140899
rs62154858
All
0.0
0.4
0.8
MR leave−one−out sensitivity analysis for
'RSPO4' on 'Pulmonary fibrosis'

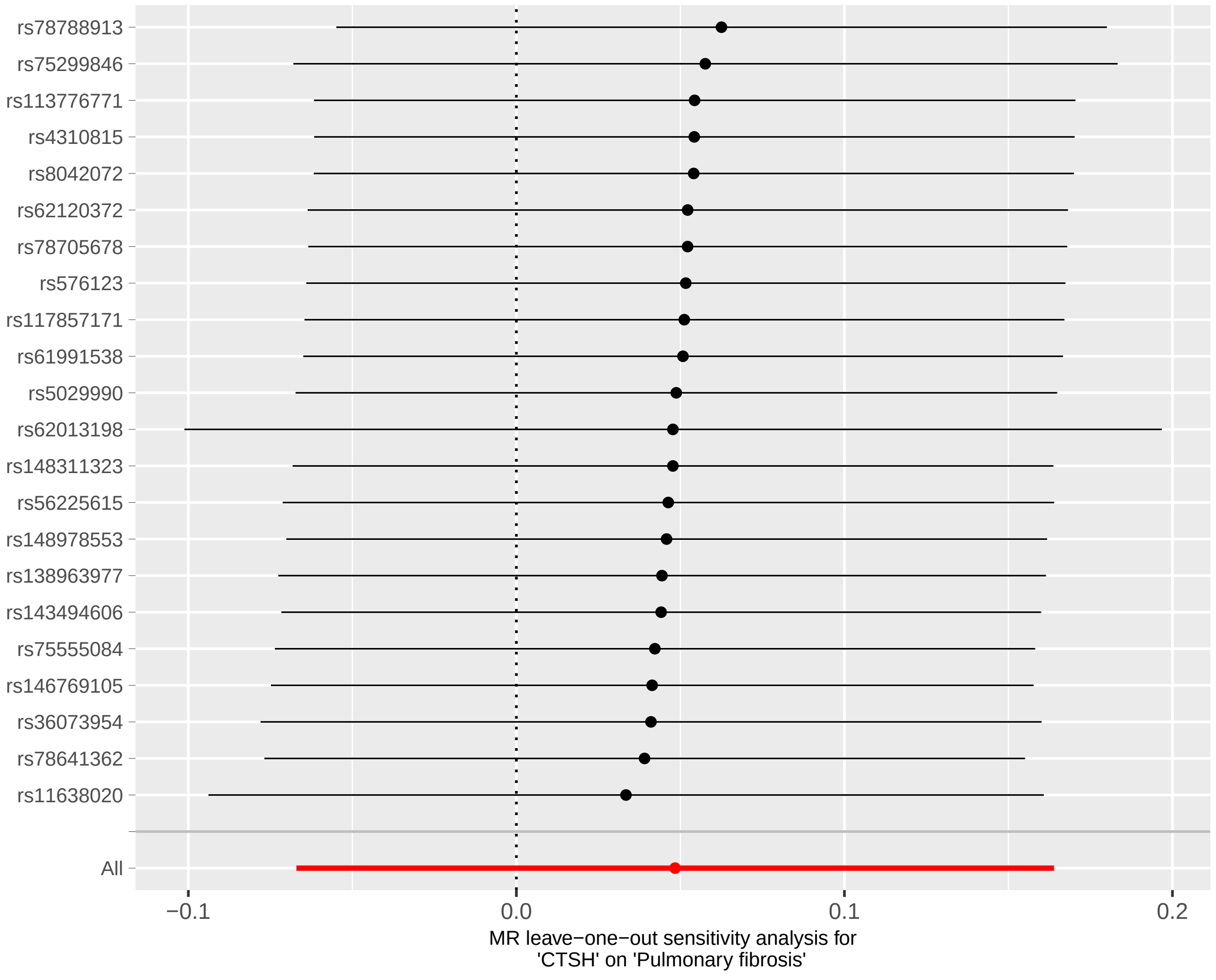
rs78788913
rs75299846
rs113776771
rs4310815
rs8042072
rs62120372
rs78705678
rs576123
rs117857171
rs61991538
rs5029990
rs62013198
rs148311323
rs56225615
rs148978553
rs138963977
rs143494606
rs75555084
rs146769105
rs36073954
rs78641362
rs11638020
All
−0.1
0.0
0.1
0.2
MR leave−one−out sensitivity analysis for
'CTSH' on 'Pulmonary fibrosis'

Insufficient number of SNPs

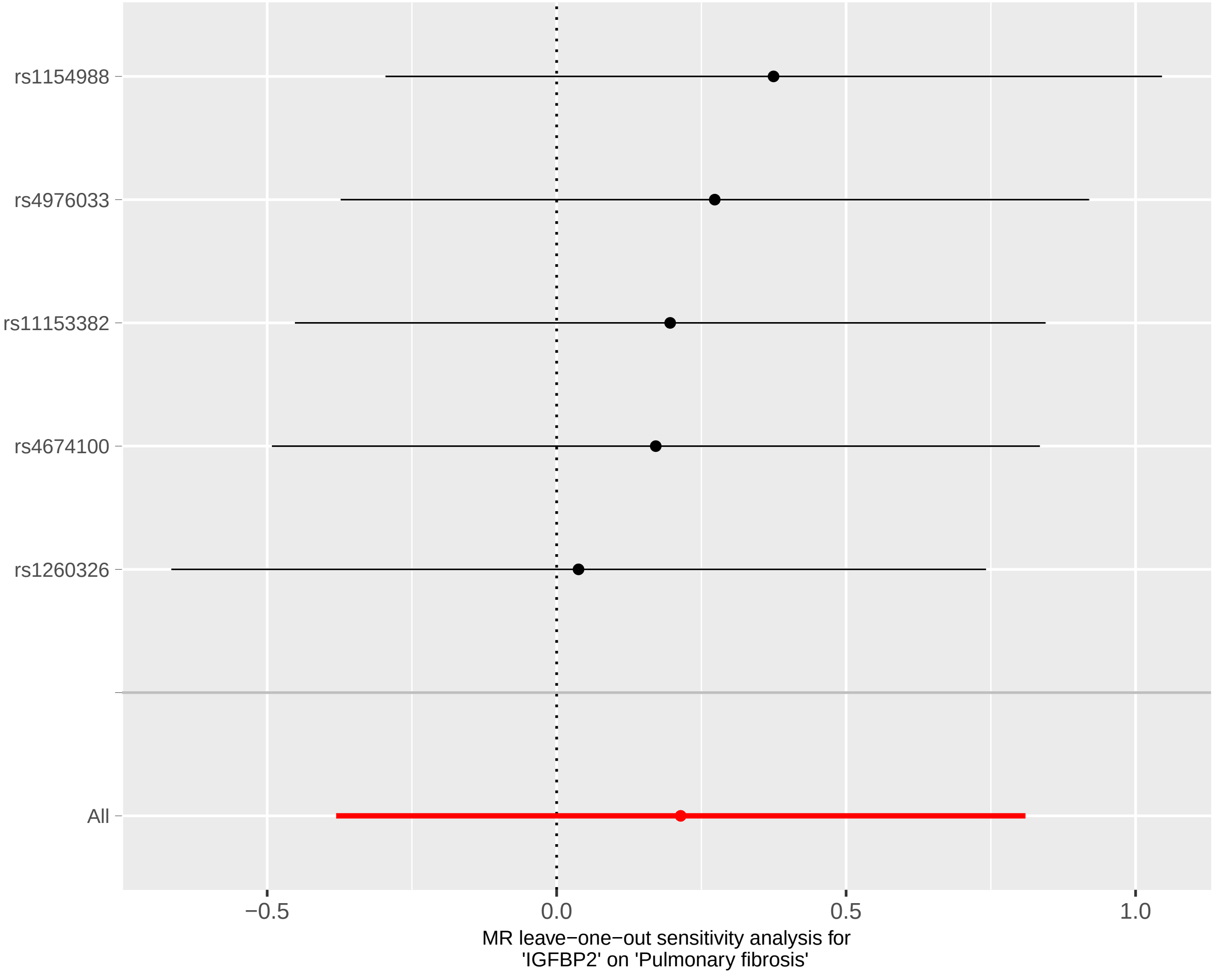
rs1154988
rs4976033
rs11153382
rs4674100
rs1260326
All
−0.5
0.0
0.5
1.0
MR leave−one−out sensitivity analysis for
'IGFBP2' on 'Pulmonary fibrosis'

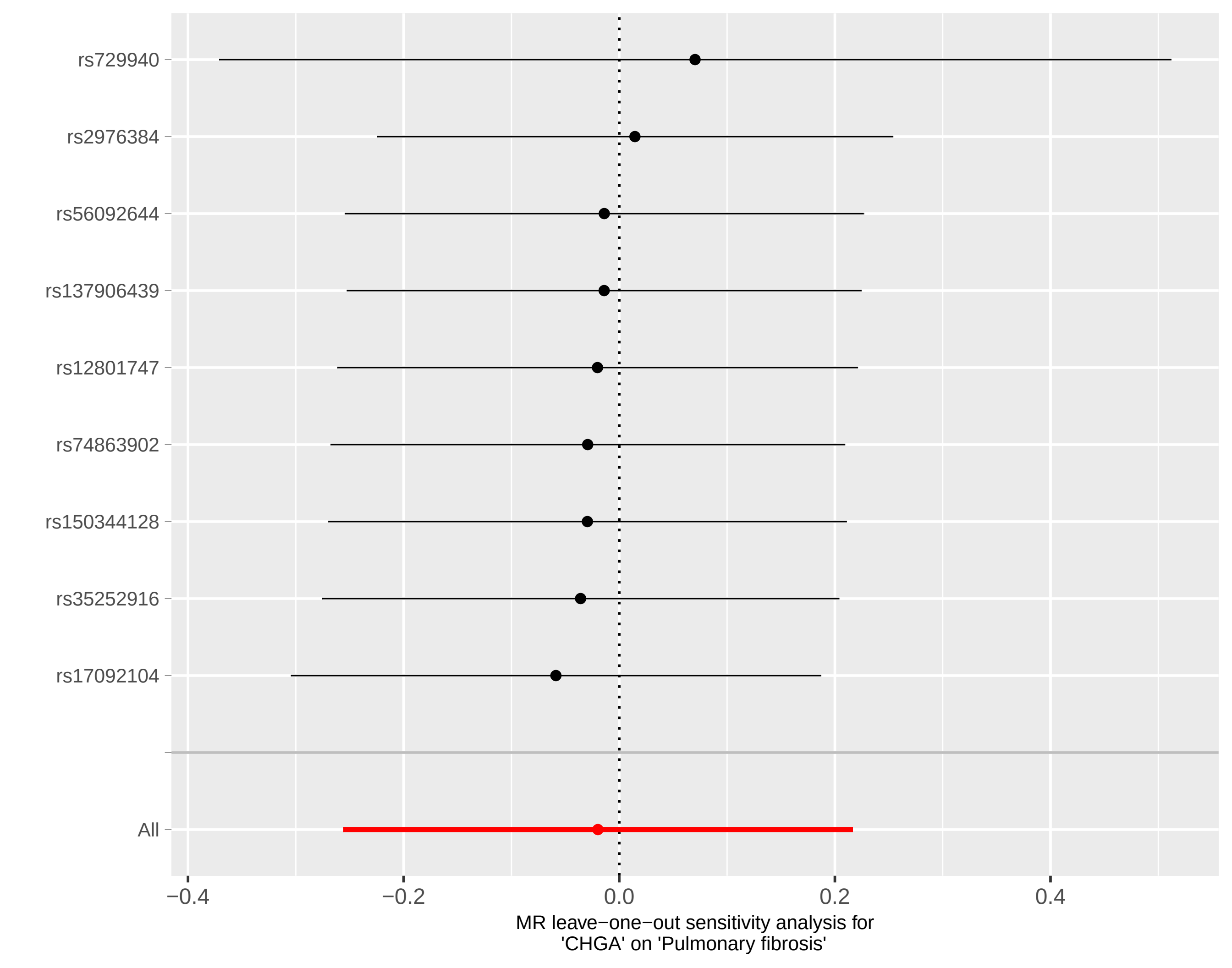
rs729940
rs2976384
rs56092644
rs137906439
rs12801747
rs74863902
rs150344128
rs35252916
rs17092104
All
−0.4
−0.2
0.0
0.2
0.4
MR leave−one−out sensitivity analysis for
'CHGA' on 'Pulmonary fibrosis'

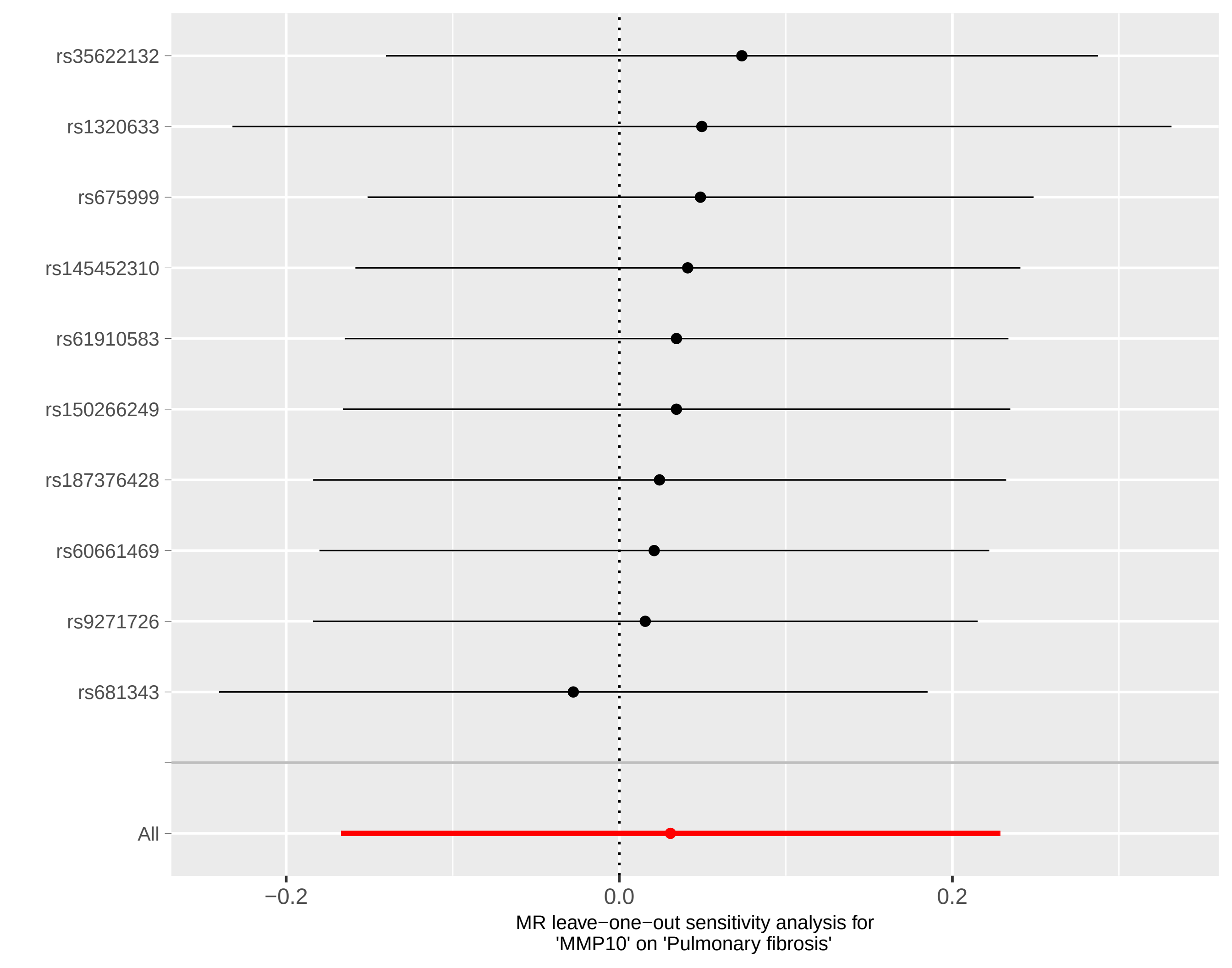
rs35622132
rs1320633
rs675999
rs145452310
rs61910583
rs150266249
rs187376428
rs60661469
rs9271726
rs681343
All
−0.2
0.0
0.2
MR leave−one−out sensitivity analysis for
'MMP10' on 'Pulmonary fibrosis'

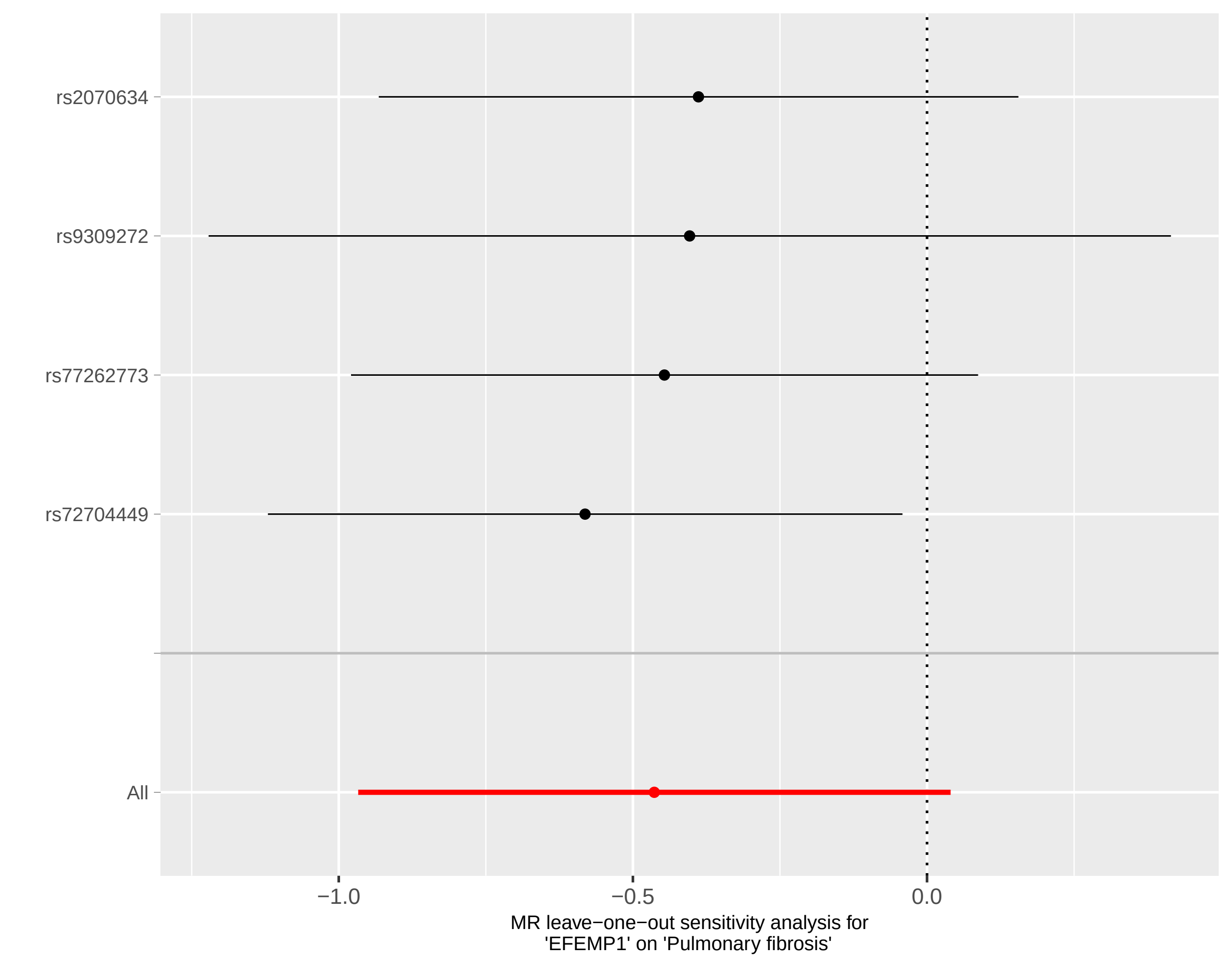
rs2070634
rs9309272
rs77262773
rs72704449
All
−1.0
−0.5
0.0
MR leave−one−out sensitivity analysis for
'EFEMP1' on 'Pulmonary fibrosis'

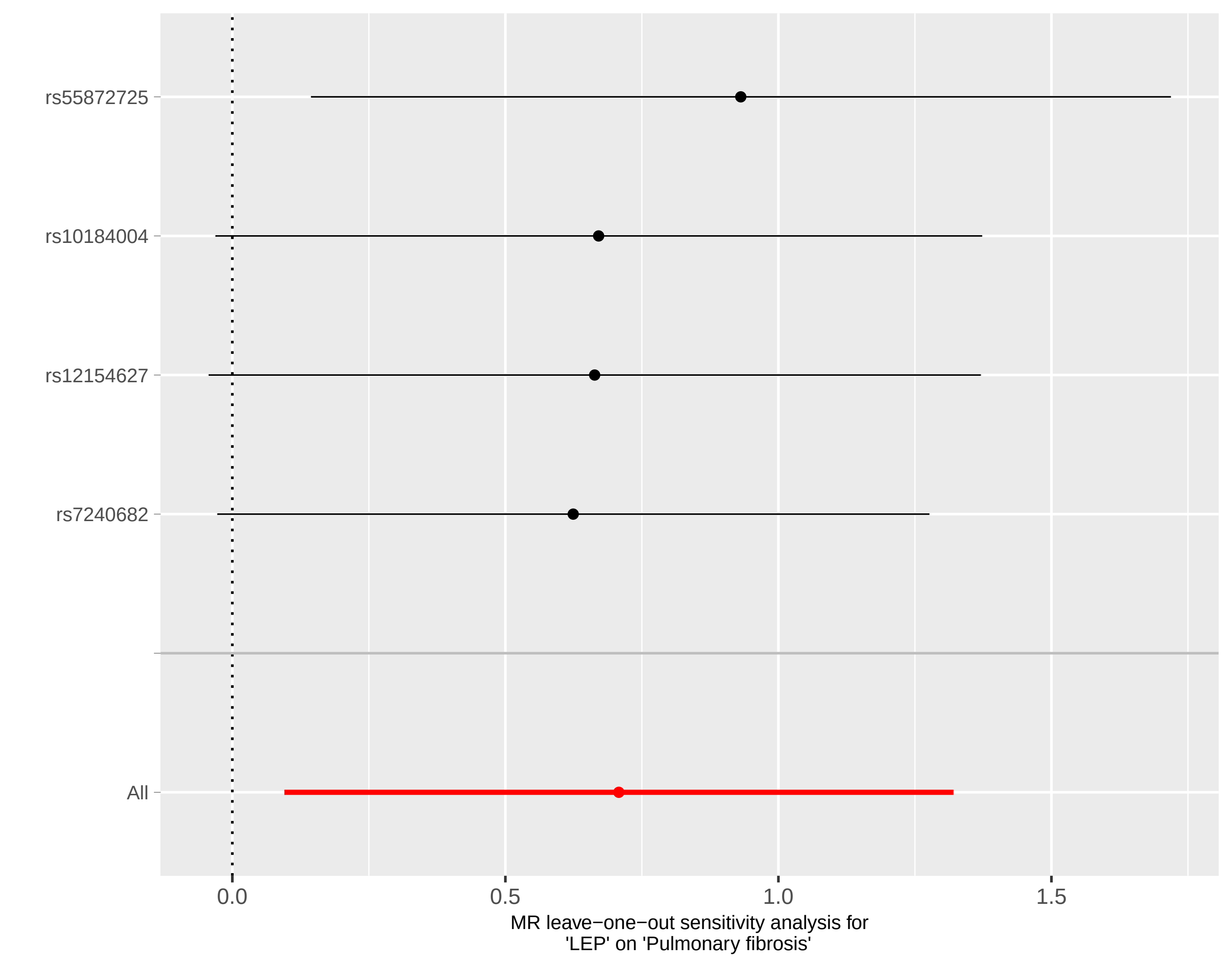
rs55872725
rs10184004
rs12154627
rs7240682
All
0.0
0.5
1.0
1.5
MR leave−one−out sensitivity analysis for
'LEP' on 'Pulmonary fibrosis'

Insufficient number of SNPs

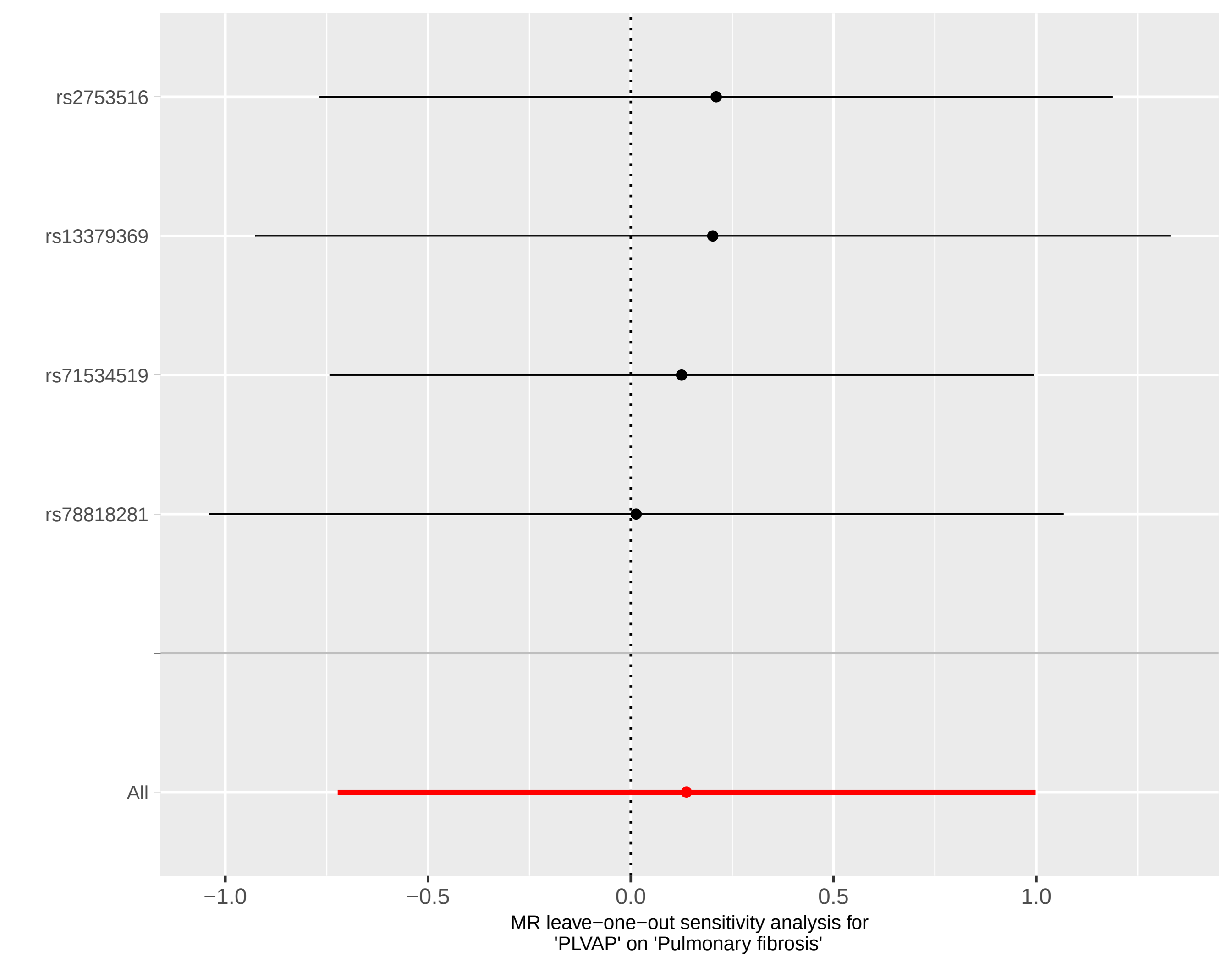
rs2753516
rs13379369
rs71534519
rs78818281
All
−1.0
−0.5
0.0
0.5
1.0
MR leave−one−out sensitivity analysis for
'PLVAP' on 'Pulmonary fibrosis'

Insufficient number of SNPs

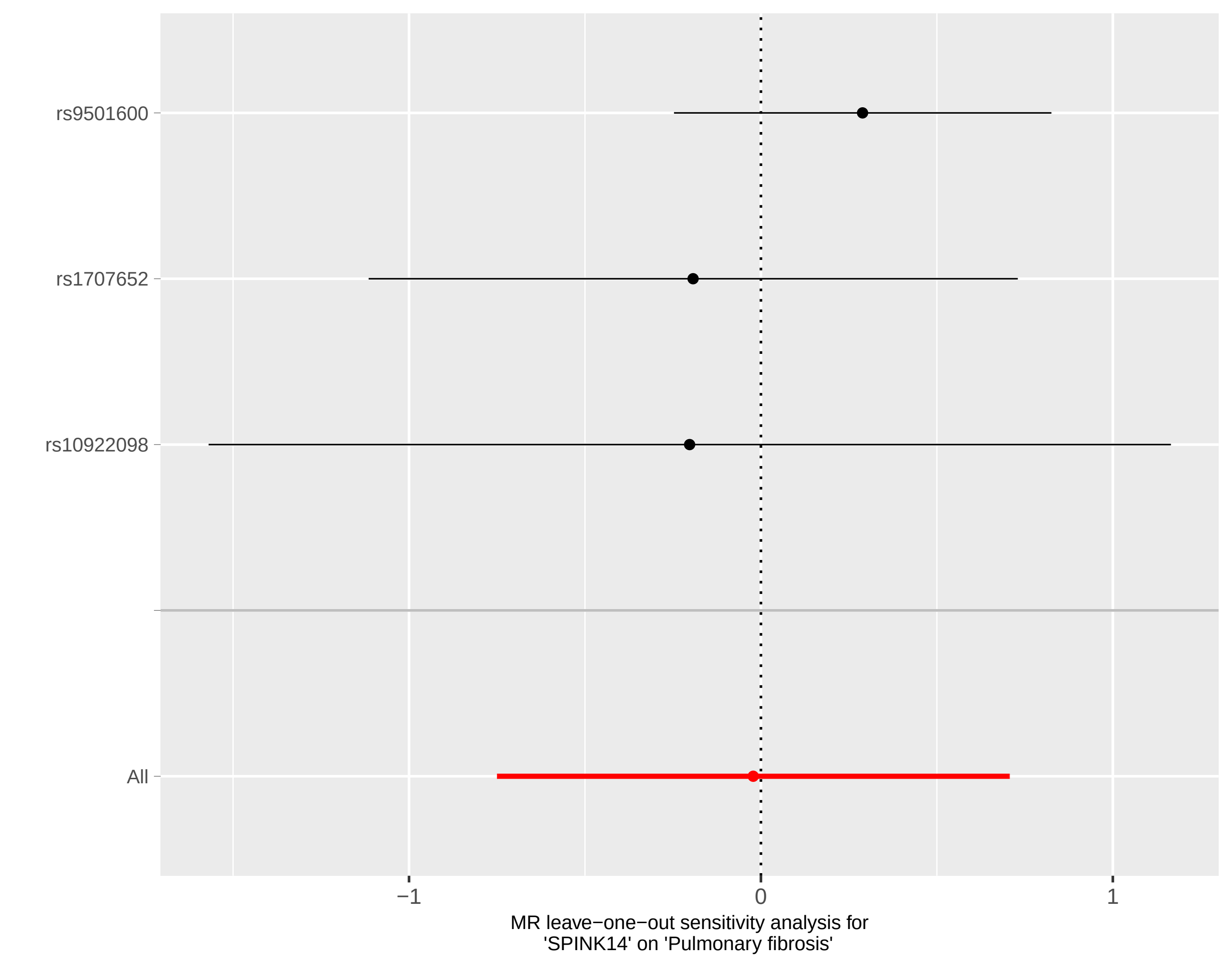
rs9501600
rs1707652
rs10922098
All
−1
0
1
MR leave−one−out sensitivity analysis for
'SPINK14' on 'Pulmonary fibrosis'

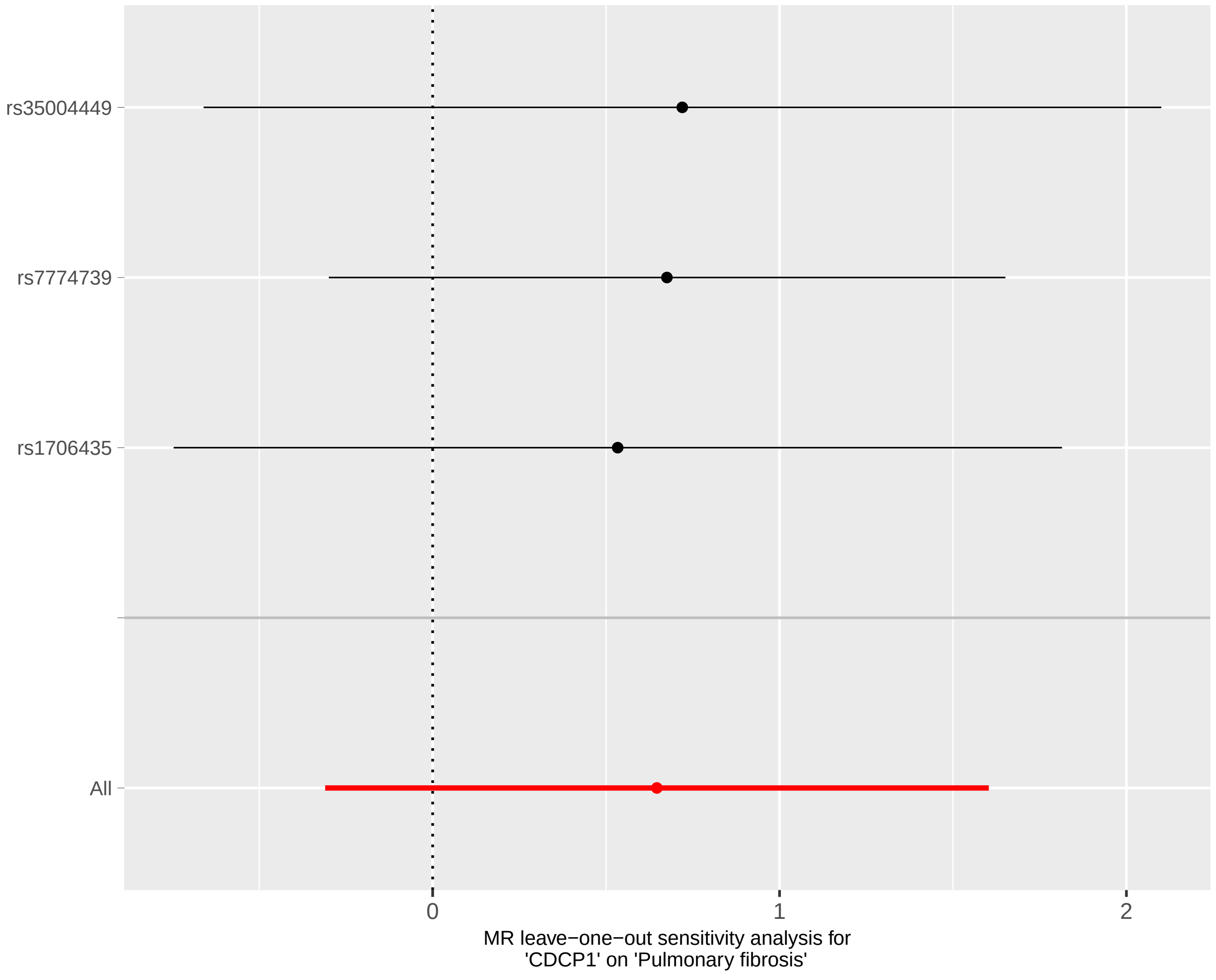

rs35004449
rs7774739
rs1706435
All
0
1
2
MR leave−one−out sensitivity analysis for
'CDCP1' on 'Pulmonary fibrosis'

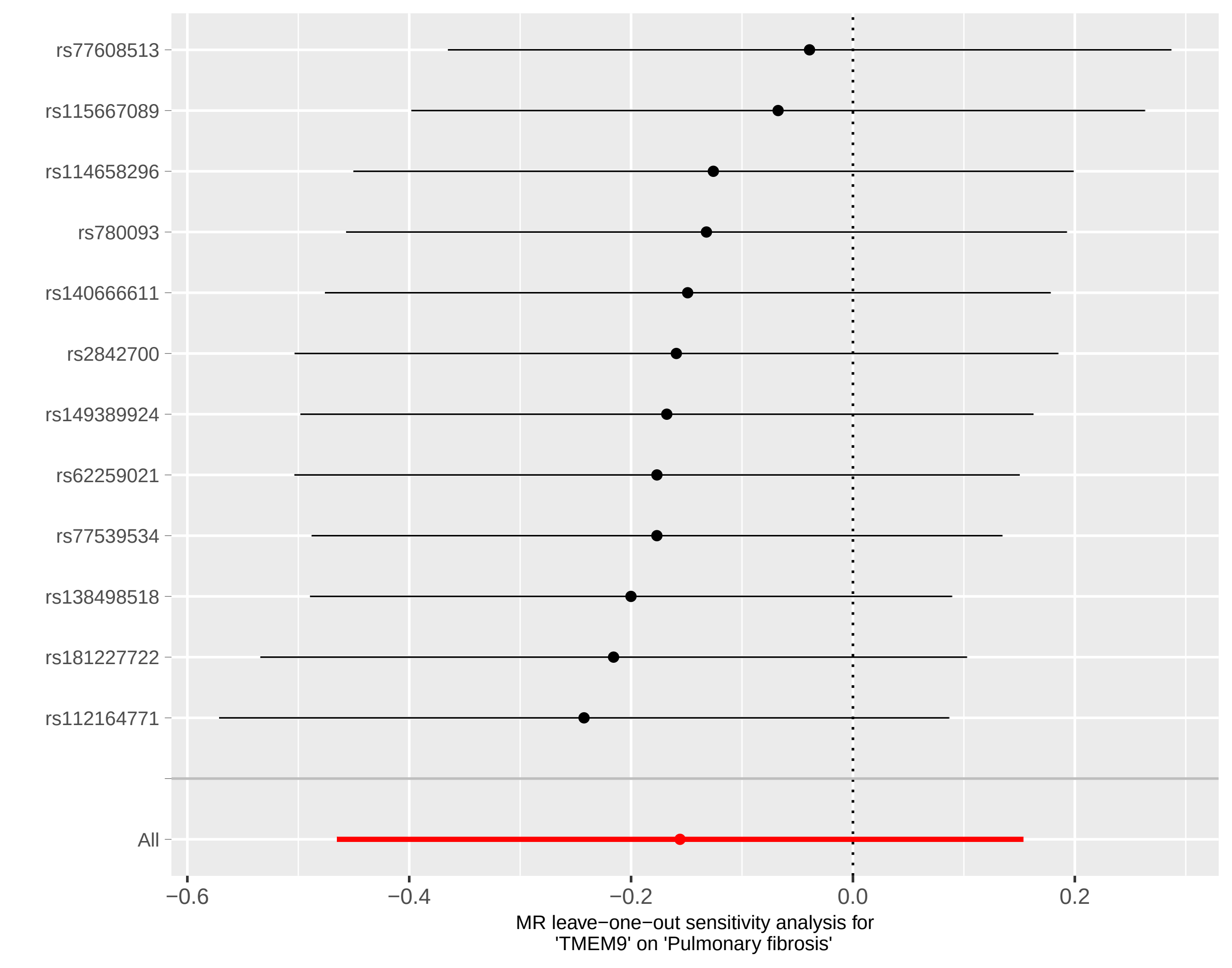

rs77608513
rs115667089
rs114658296
rs780093
rs140666611
rs2842700
rs149389924
rs62259021
rs77539534
rs138498518
rs181227722
rs112164771
All
−0.6
−0.4
−0.2
0.0
0.2
MR leave−one−out sensitivity analysis for
'TMEM9' on 'Pulmonary fibrosis'

Insufficient number of SNPs

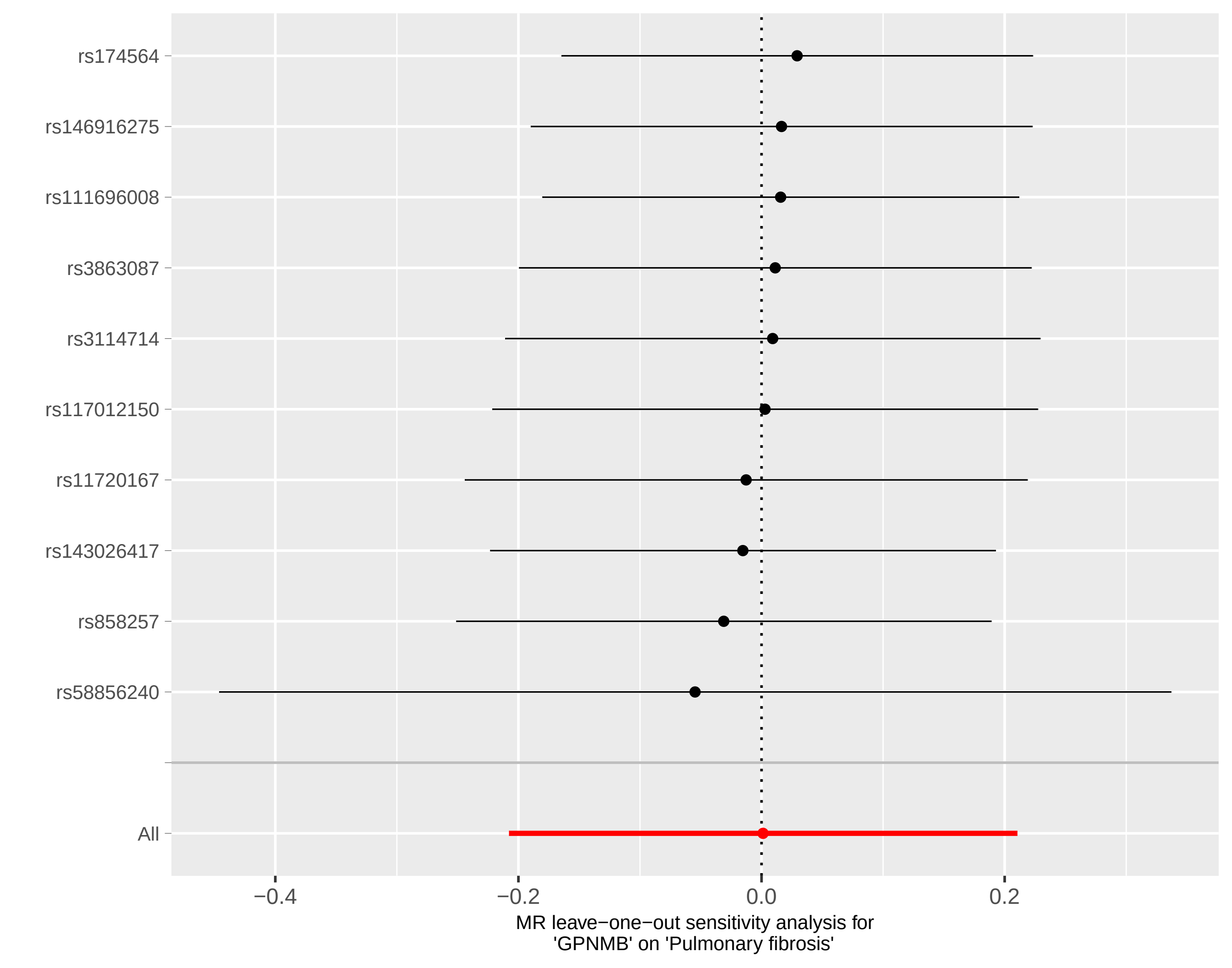
rs174564
rs146916275
rs111696008
rs3863087
rs3114714
rs117012150
rs11720167
rs143026417
rs858257
rs58856240
All
−0.4
−0.2
0.0
0.2
MR leave−one−out sensitivity analysis for
'GPNMB' on 'Pulmonary fibrosis'

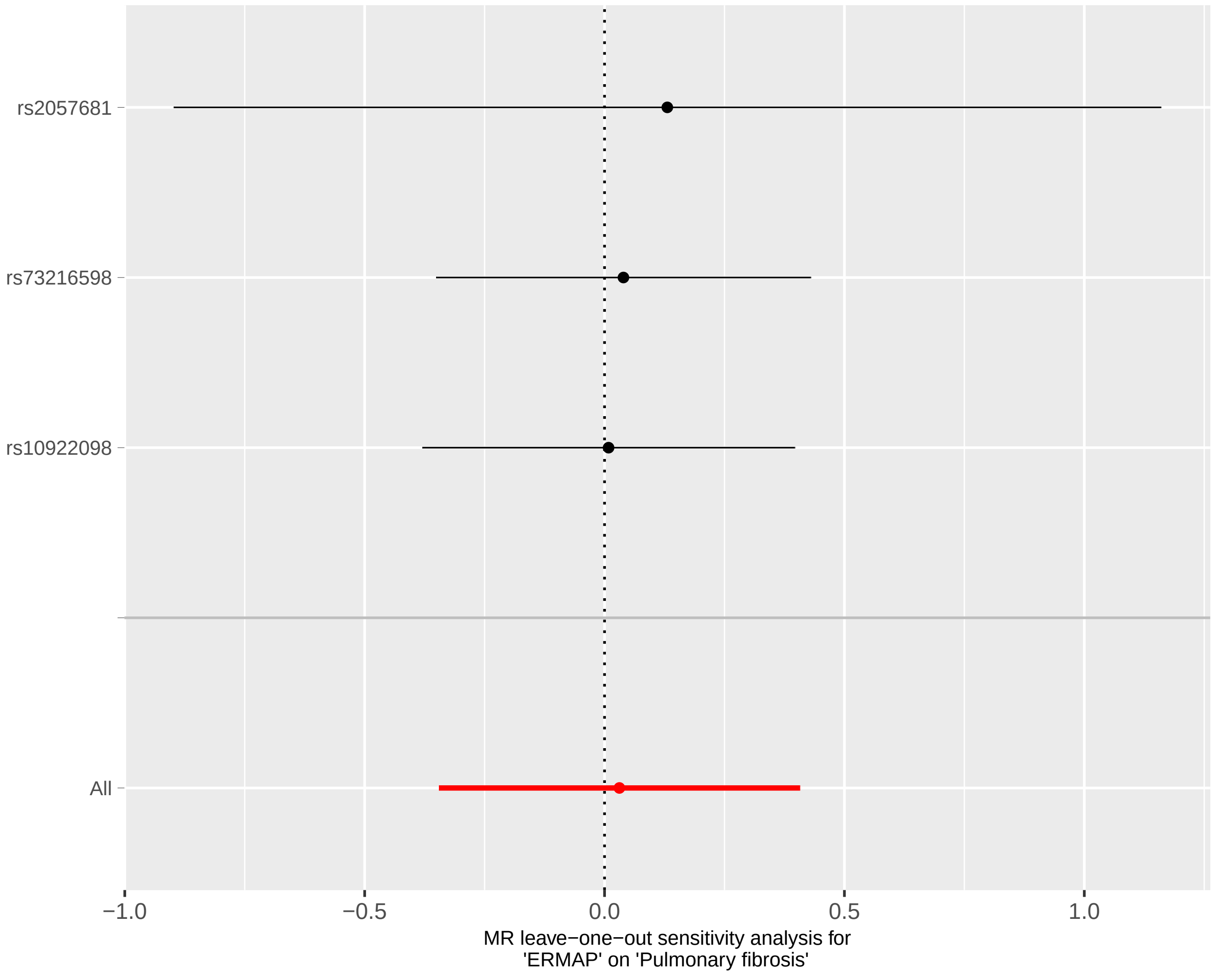
rs2057681
rs73216598
rs10922098
All
−1.0
−0.5
0.0
0.5
1.0
MR leave−one−out sensitivity analysis for
'ERMAP' on 'Pulmonary fibrosis'

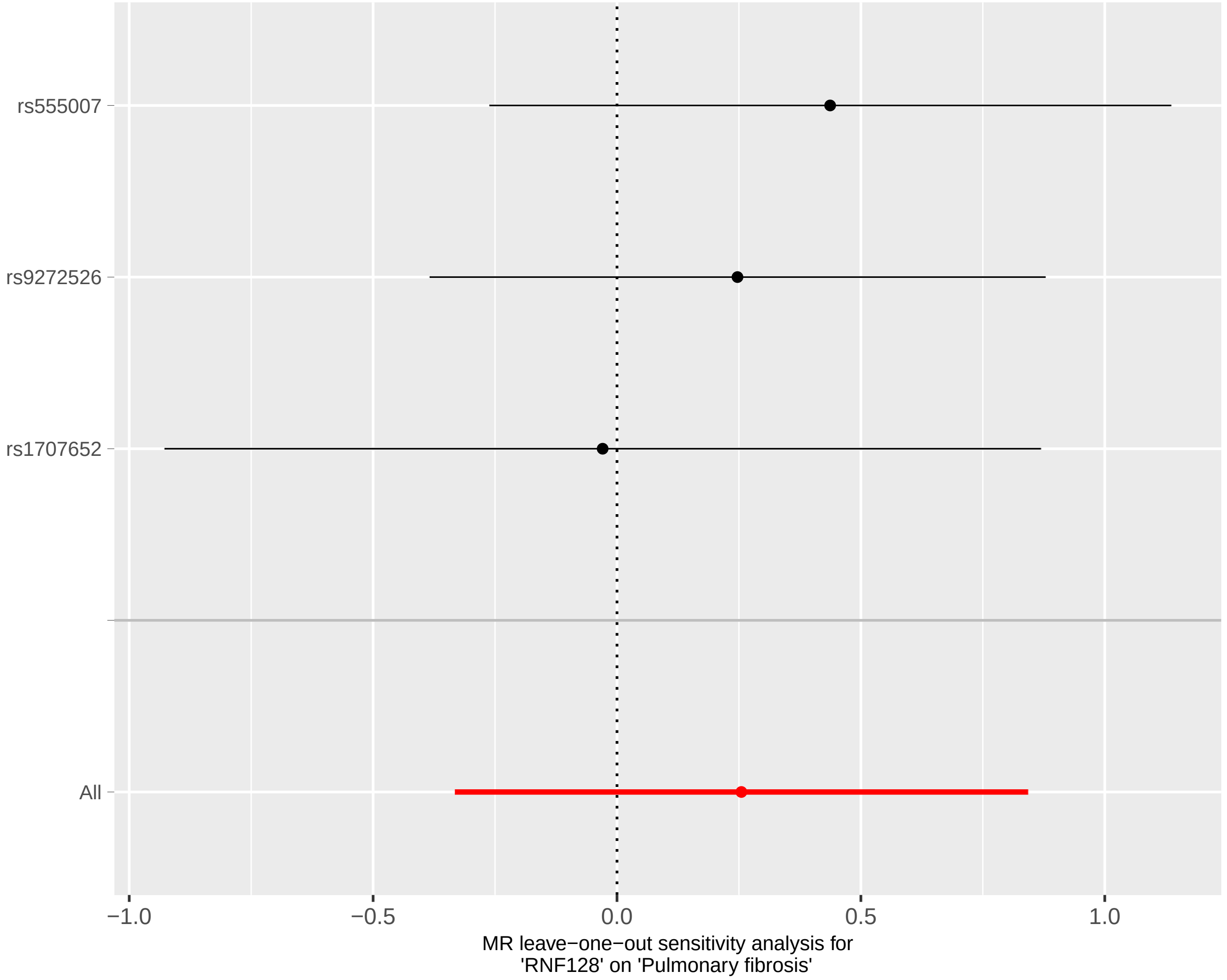
rs555007
rs9272526
rs1707652
All
−1.0
−0.5
0.0
0.5
1.0
MR leave−one−out sensitivity analysis for
'RNF128' on 'Pulmonary fibrosis'

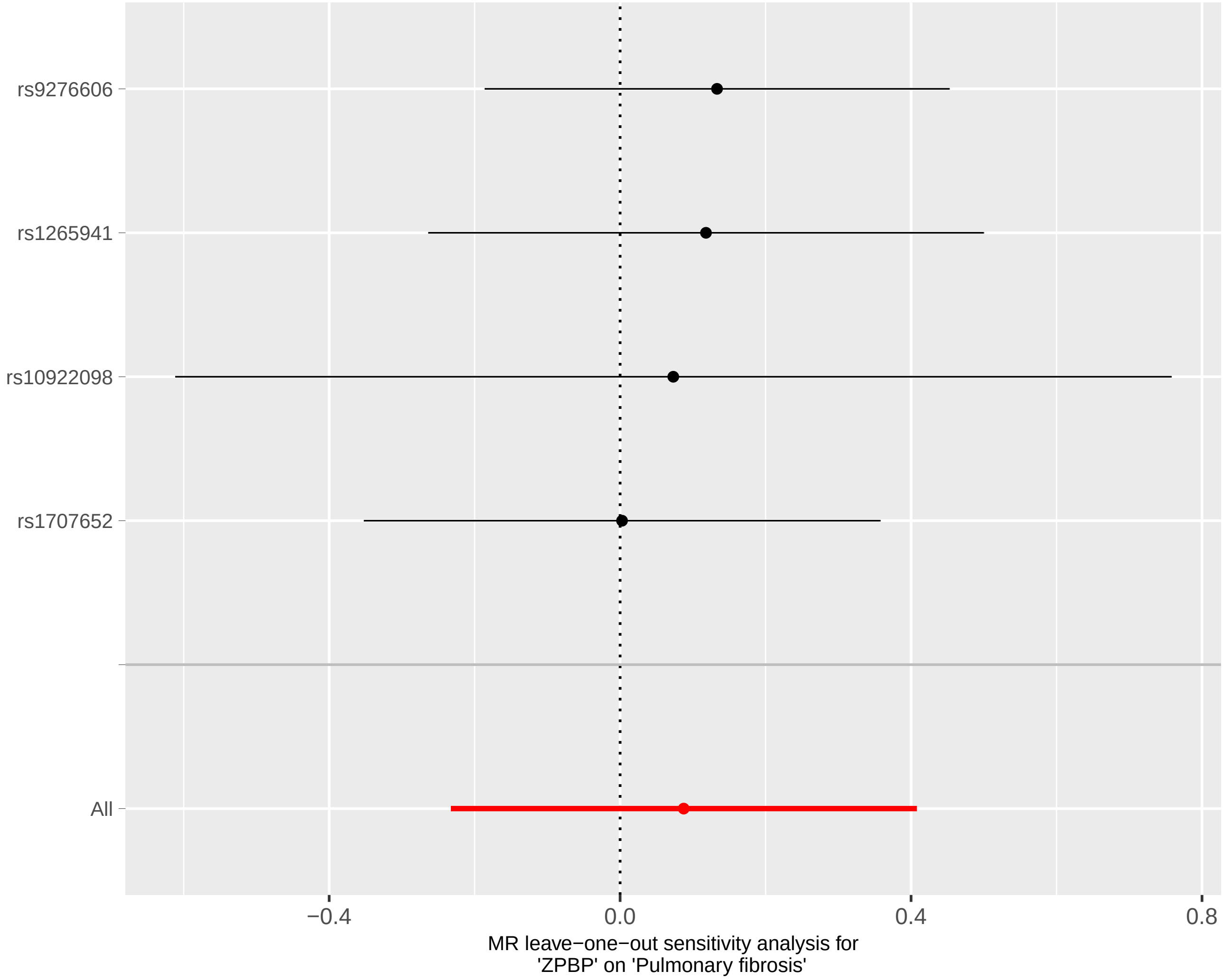
rs9276606
rs1265941
rs10922098
rs1707652
All
−0.4
0.0
0.4
0.8
MR leave−one−out sensitivity analysis for
'ZPBP' on 'Pulmonary fibrosis'

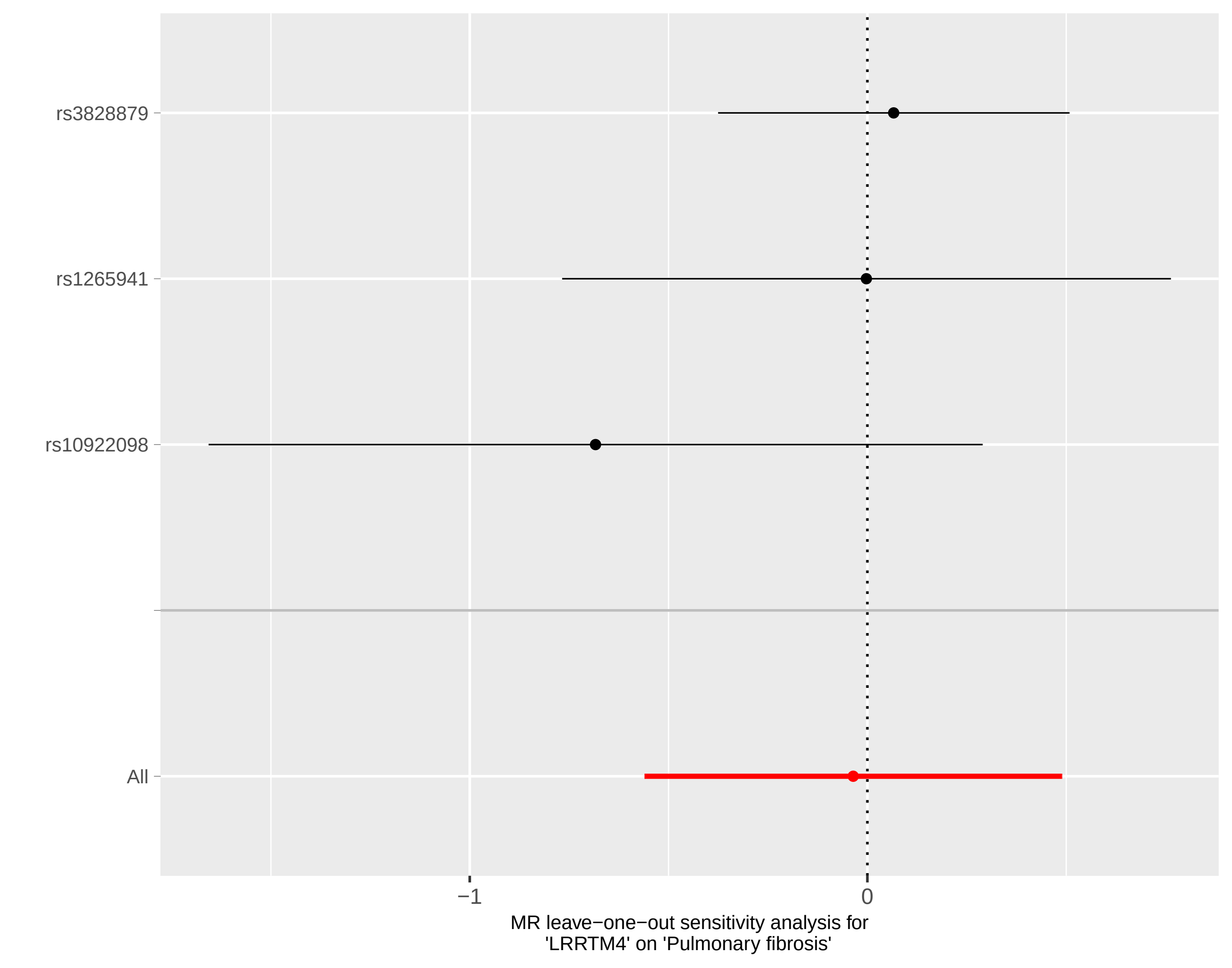
rs3828879
rs1265941
rs10922098
All
−1
0
MR leave−one−out sensitivity analysis for
'LRRTM4' on 'Pulmonary fibrosis'

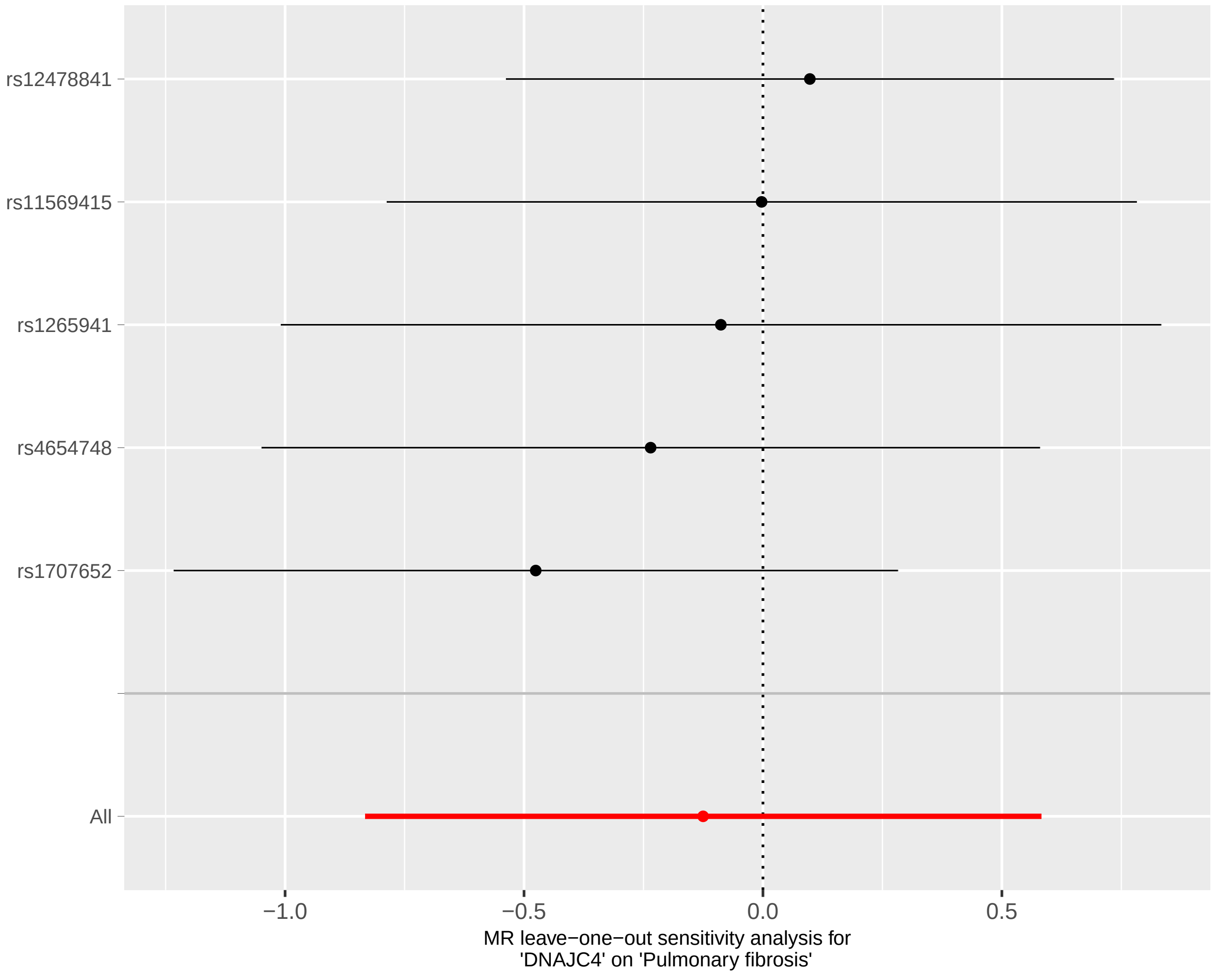
rs12478841
rs11569415
rs1265941
rs4654748
rs1707652
All
−1.0
−0.5
0.0
0.5
MR leave−one−out sensitivity analysis for
'DNAJC4' on 'Pulmonary fibrosis'

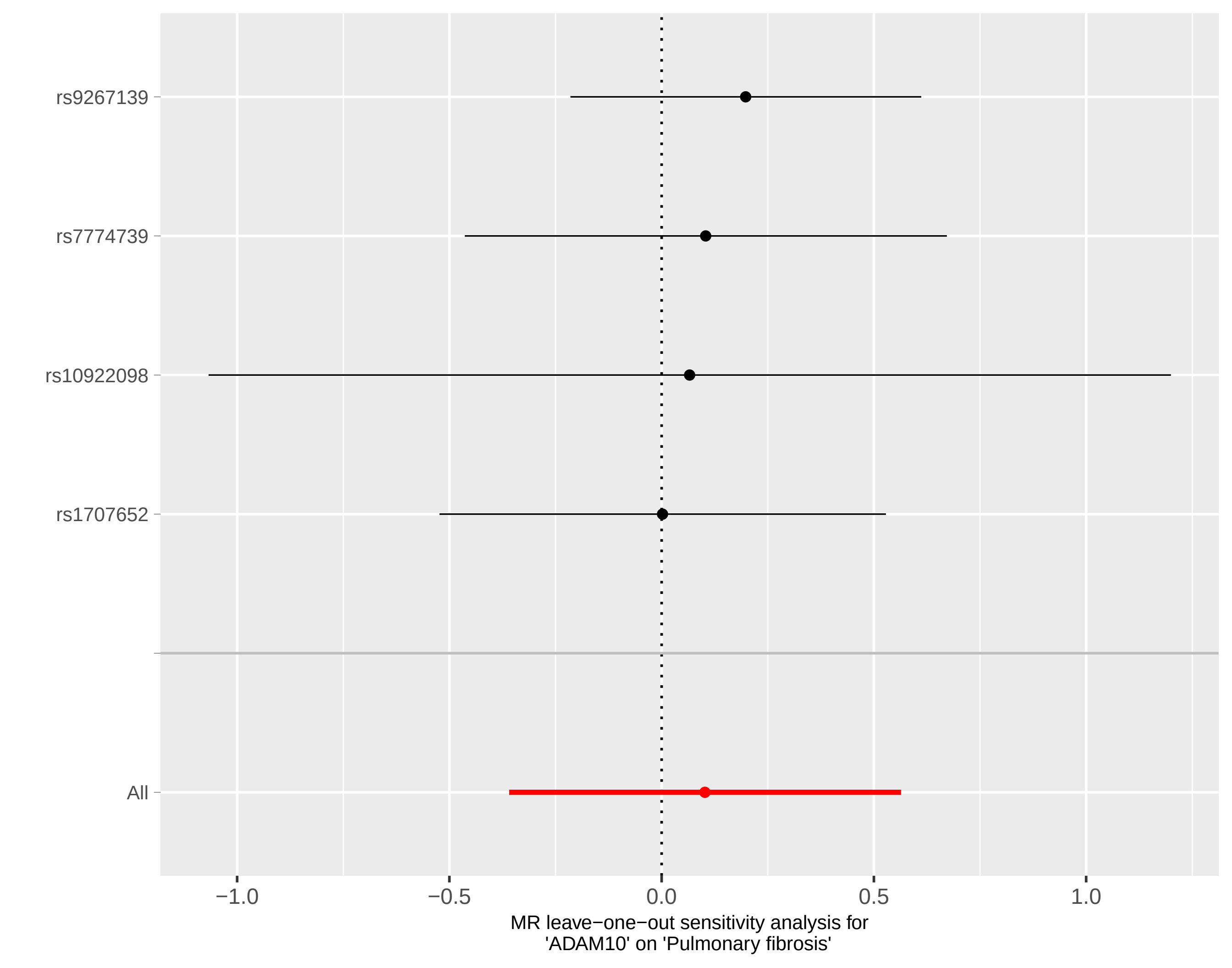
rs9267139
rs7774739
rs10922098
rs1707652
All
−1.0
−0.5
0.0
0.5
1.0
MR leave−one−out sensitivity analysis for
'ADAM10' on 'Pulmonary fibrosis'

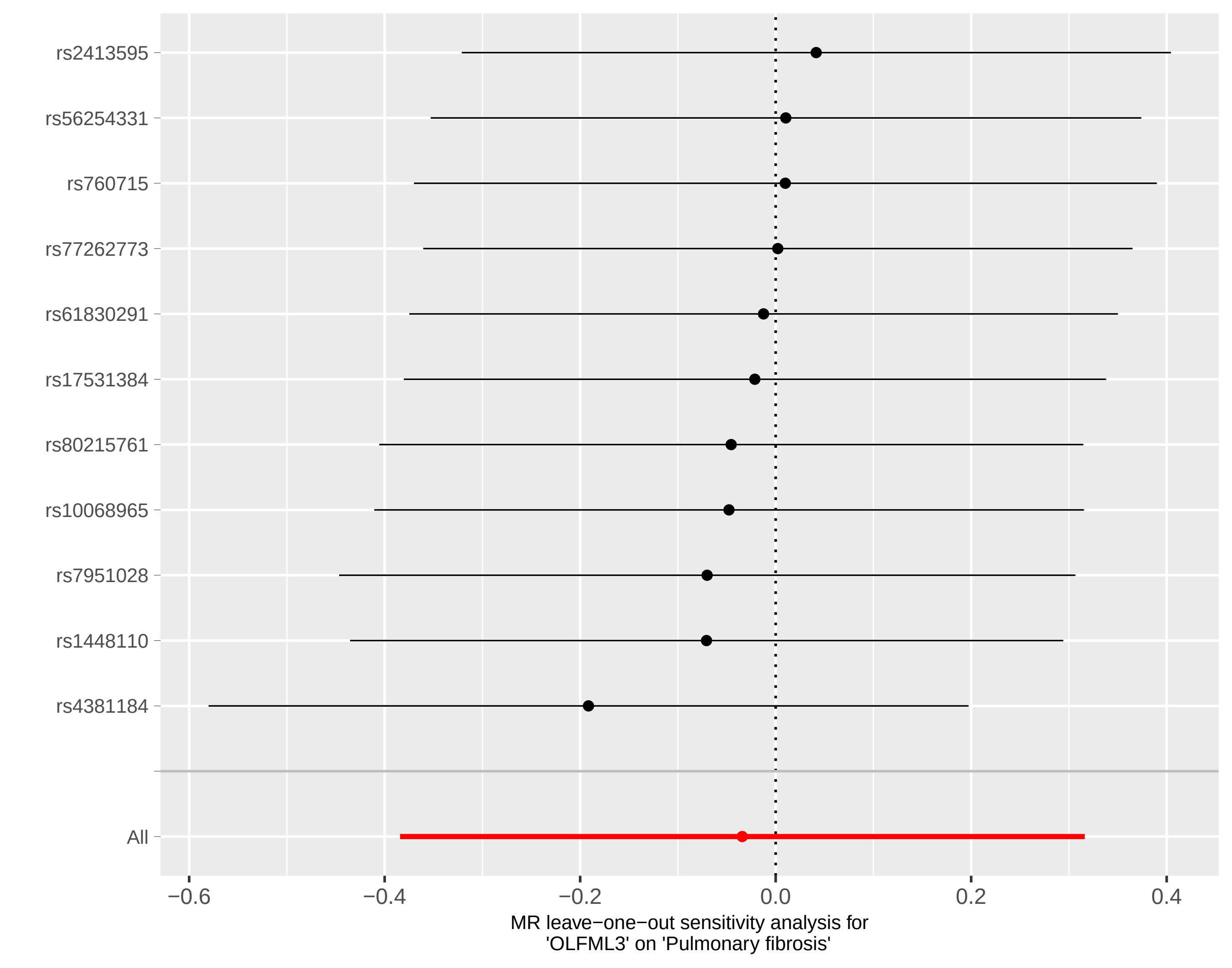
rs2413595
rs56254331
rs760715
rs77262773
rs61830291
rs17531384
rs80215761
rs10068965
rs7951028
rs1448110
rs4381184
All
−0.6
−0.4
−0.2
0.0
0.2
0.4
MR leave−one−out sensitivity analysis for
'OLFML3' on 'Pulmonary fibrosis'

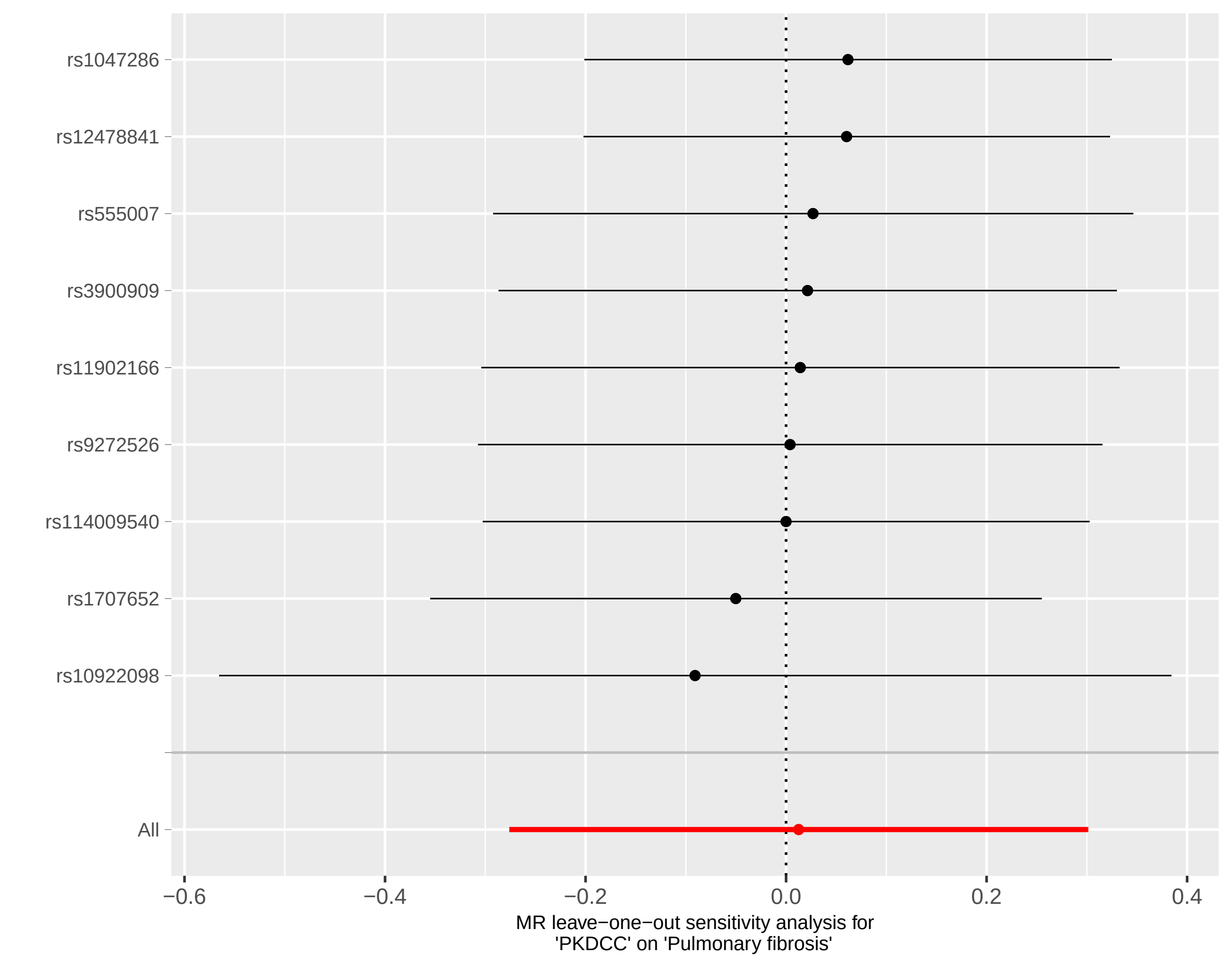
rs1047286
rs12478841
rs555007
rs3900909
rs11902166
rs9272526
rs114009540
rs1707652
rs10922098
All
−0.6
−0.4
−0.2
0.0
0.2
0.4
MR leave−one−out sensitivity analysis for
'PKDCC' on 'Pulmonary fibrosis'

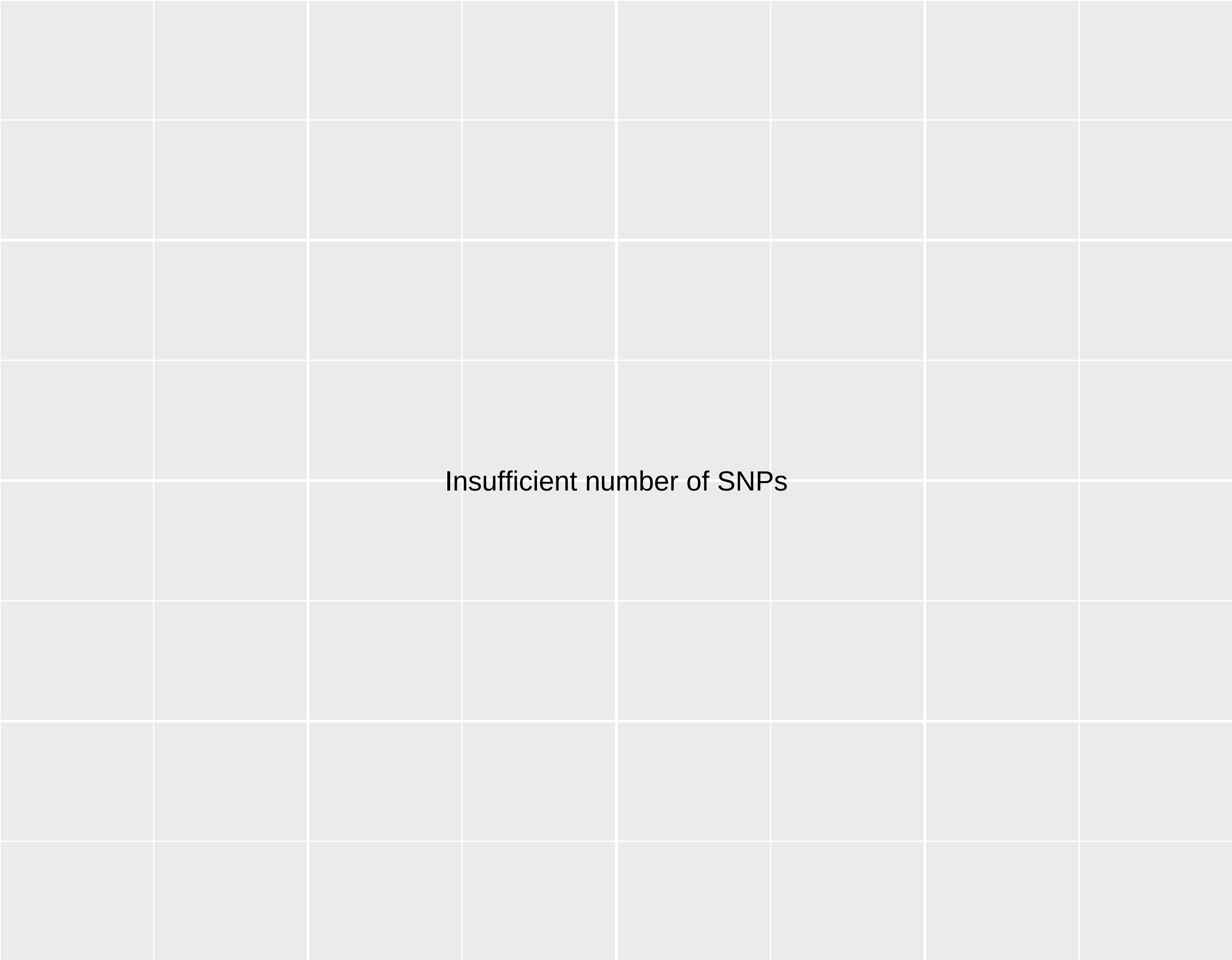
Insufficient number of SNPs

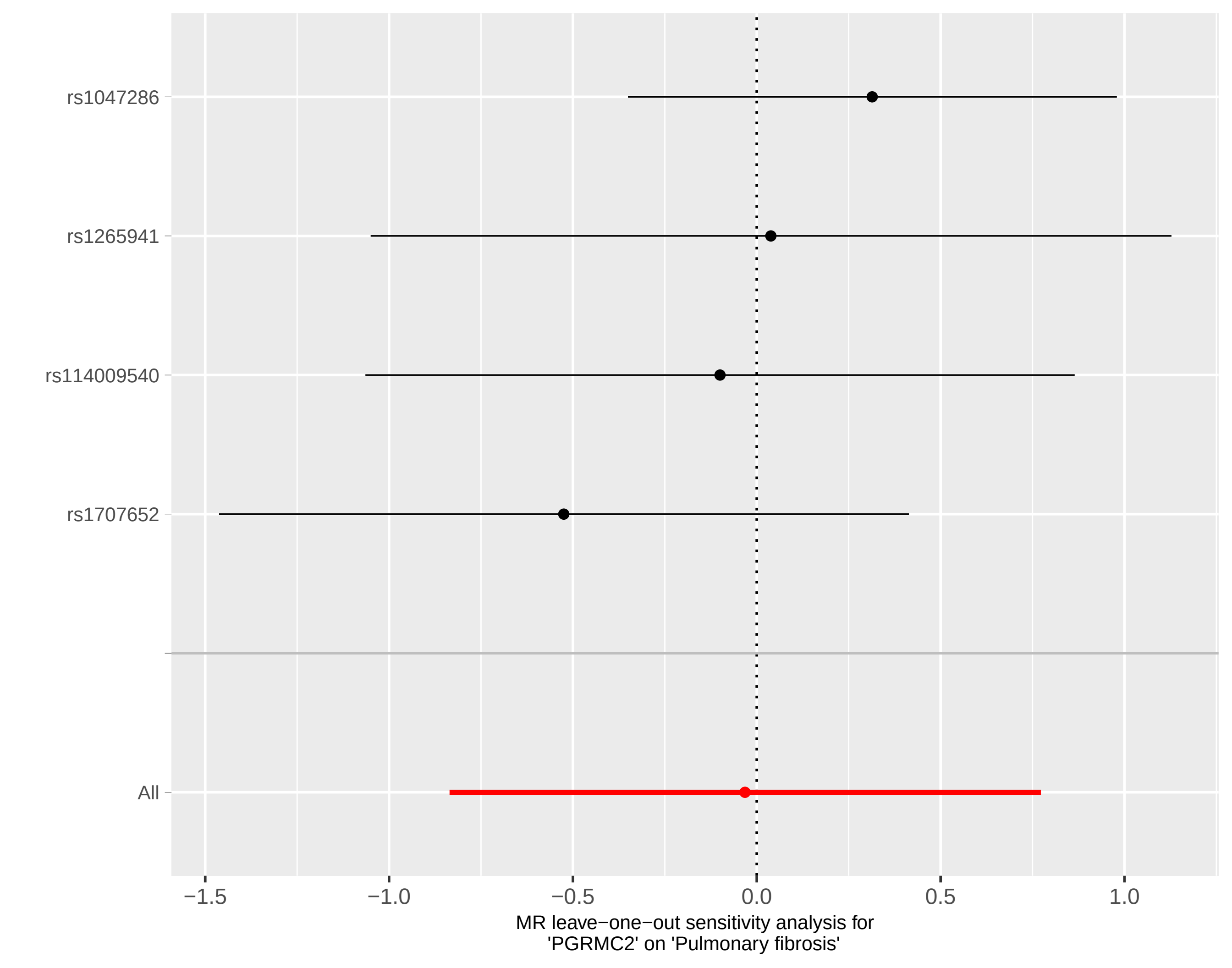
rs1047286
rs1265941
rs114009540
rs1707652
All
−1.5
−1.0
−0.5
0.0
0.5
1.0
MR leave−one−out sensitivity analysis for
'PGRMC2' on 'Pulmonary fibrosis'

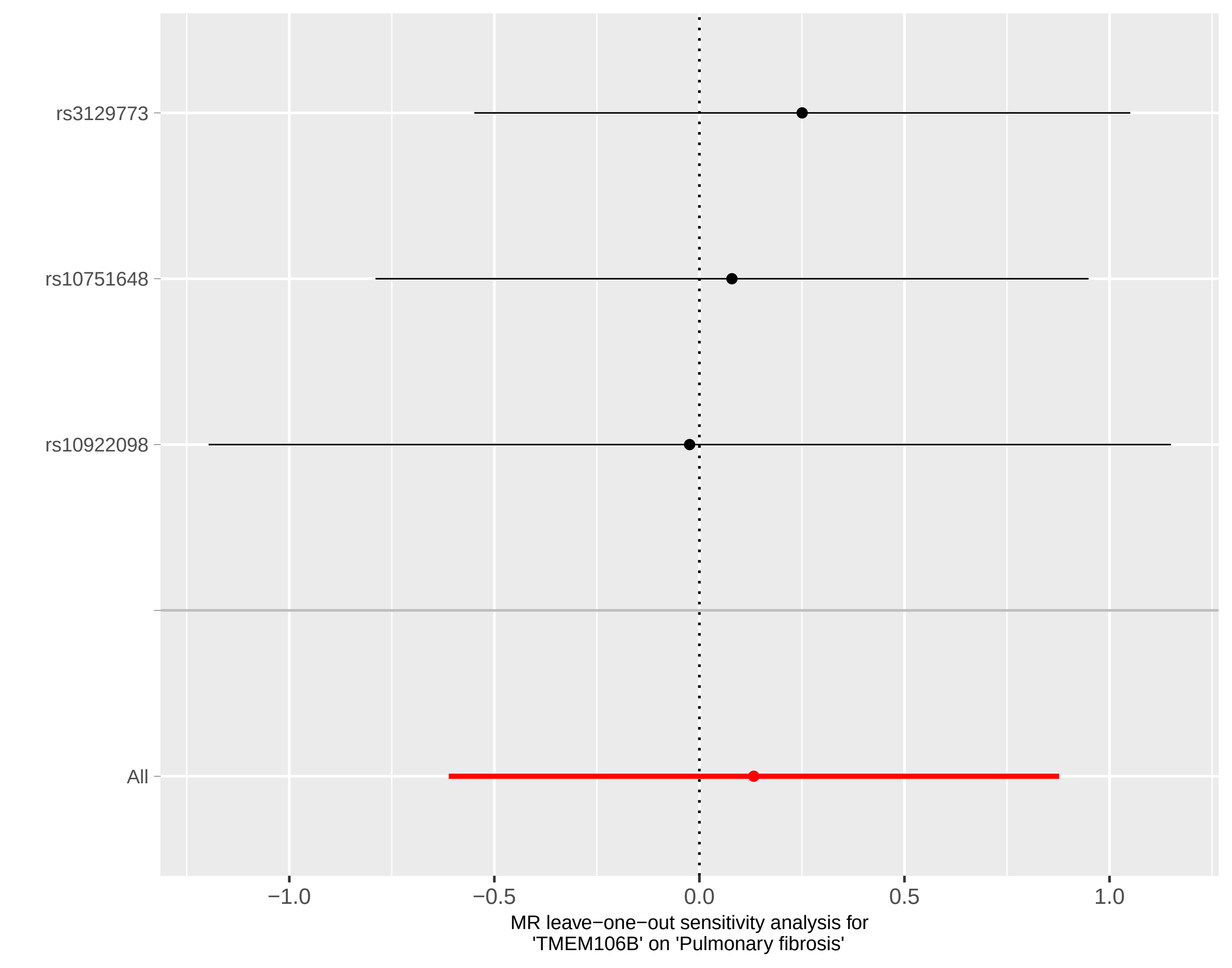

rs3129773
rs10751648
rs10922098
All
−1.0
−0.5
0.0
0.5
1.0
MR leave−one−out sensitivity analysis for
'TMEM106B' on 'Pulmonary fibrosis'

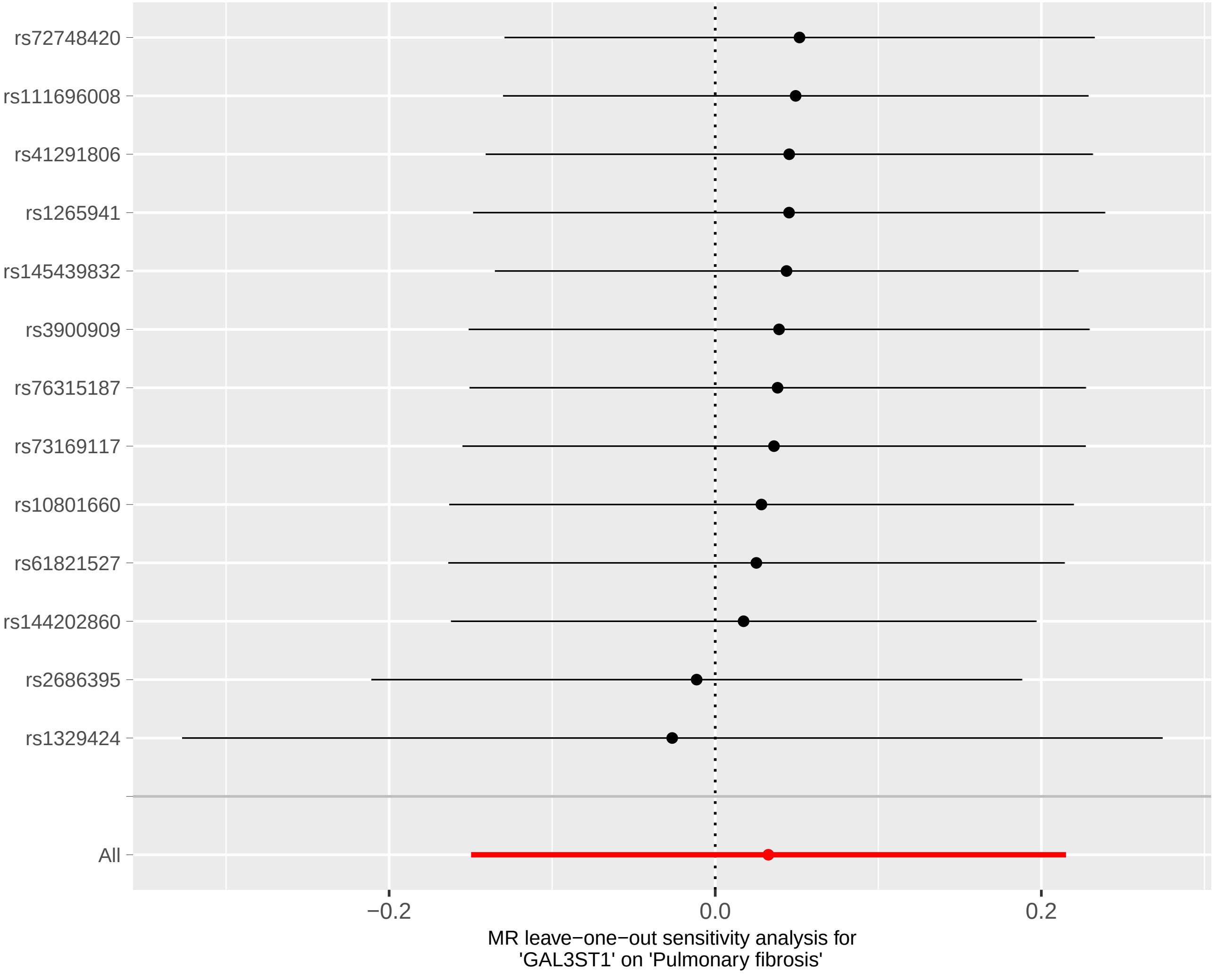
rs72748420
rs111696008
rs41291806
rs1265941
rs145439832
rs3900909
rs76315187
rs73169117
rs10801660
rs61821527
rs144202860
rs2686395
rs1329424
All
−0.2
0.0
0.2
MR leave−one−out sensitivity analysis for
'GAL3ST1' on 'Pulmonary fibrosis'

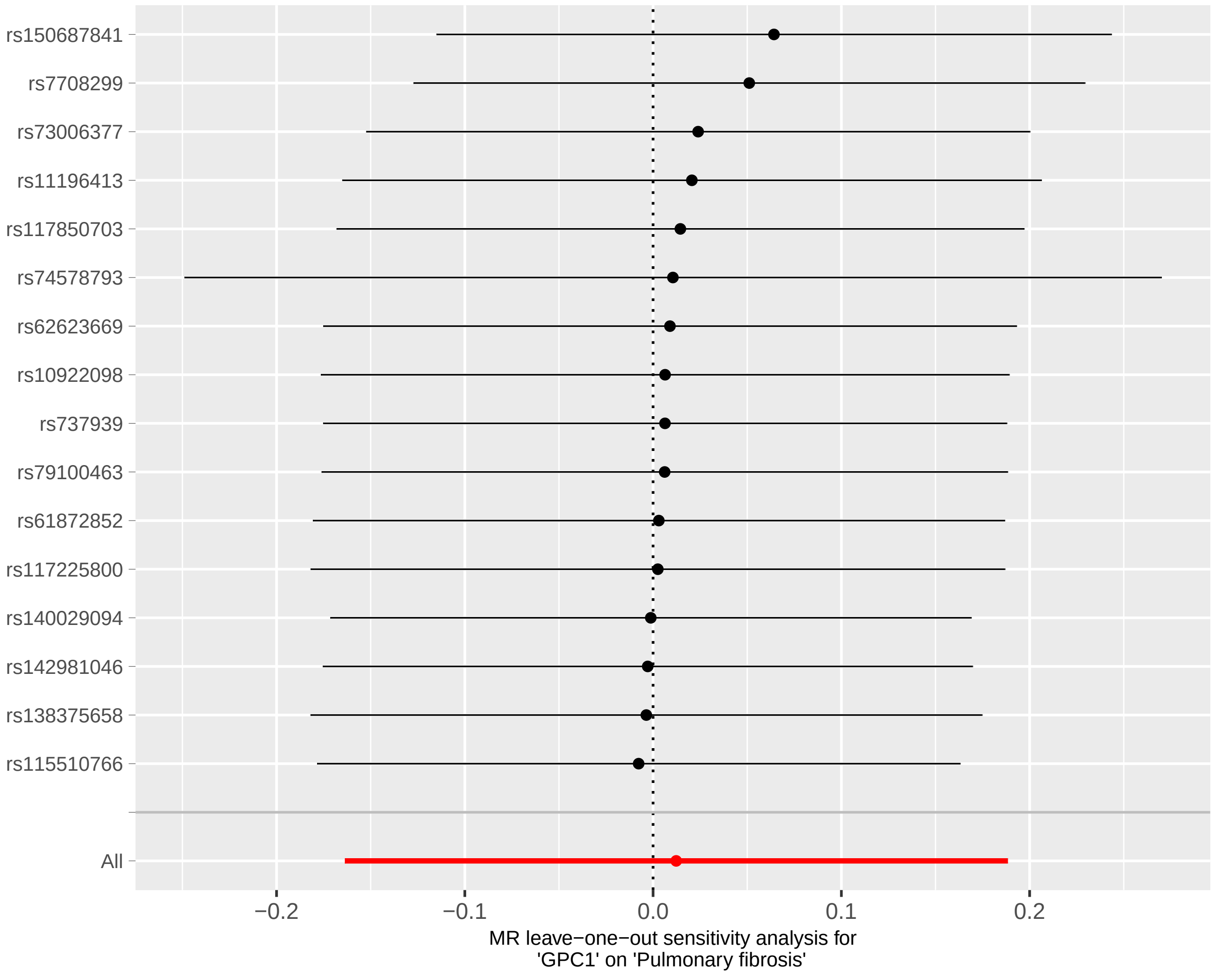
rs150687841
rs7708299
rs73006377
rs11196413
rs117850703
rs74578793
rs62623669
rs10922098
rs737939
rs79100463
rs61872852
rs117225800
rs140029094
rs142981046
rs138375658
rs115510766
All
−0.2
−0.1
0.0
0.1
0.2
MR leave−one−out sensitivity analysis for
'GPC1' on 'Pulmonary fibrosis'

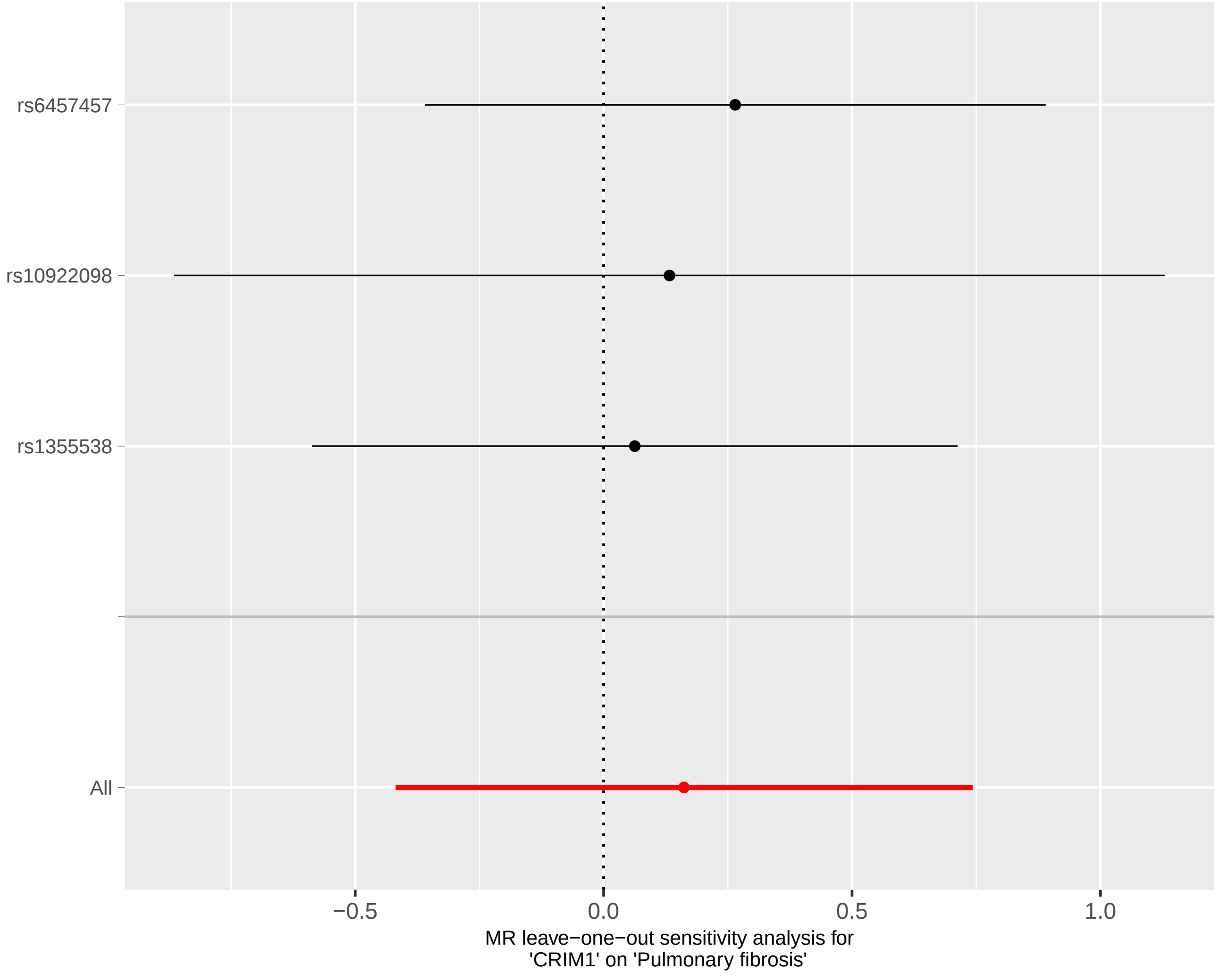
rs6457457
rs10922098
rs1355538
All
−0.5
0.0
0.5
1.0
MR leave−one−out sensitivity analysis for
'CRIM1' on 'Pulmonary fibrosis'

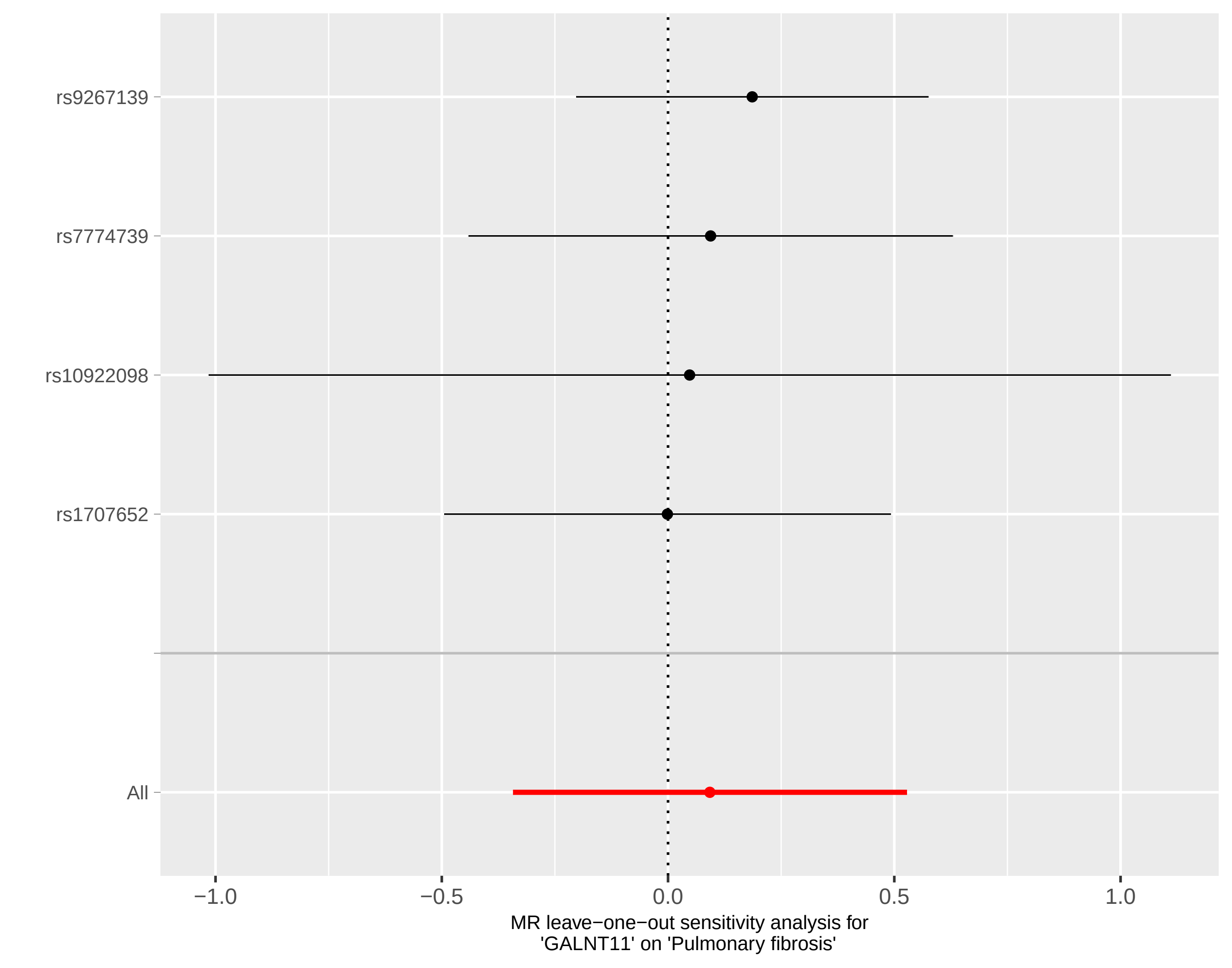
rs9267139
rs7774739
rs10922098
rs1707652
All
−1.0
−0.5
0.0
0.5
1.0
MR leave−one−out sensitivity analysis for
'GALNT11' on 'Pulmonary fibrosis'

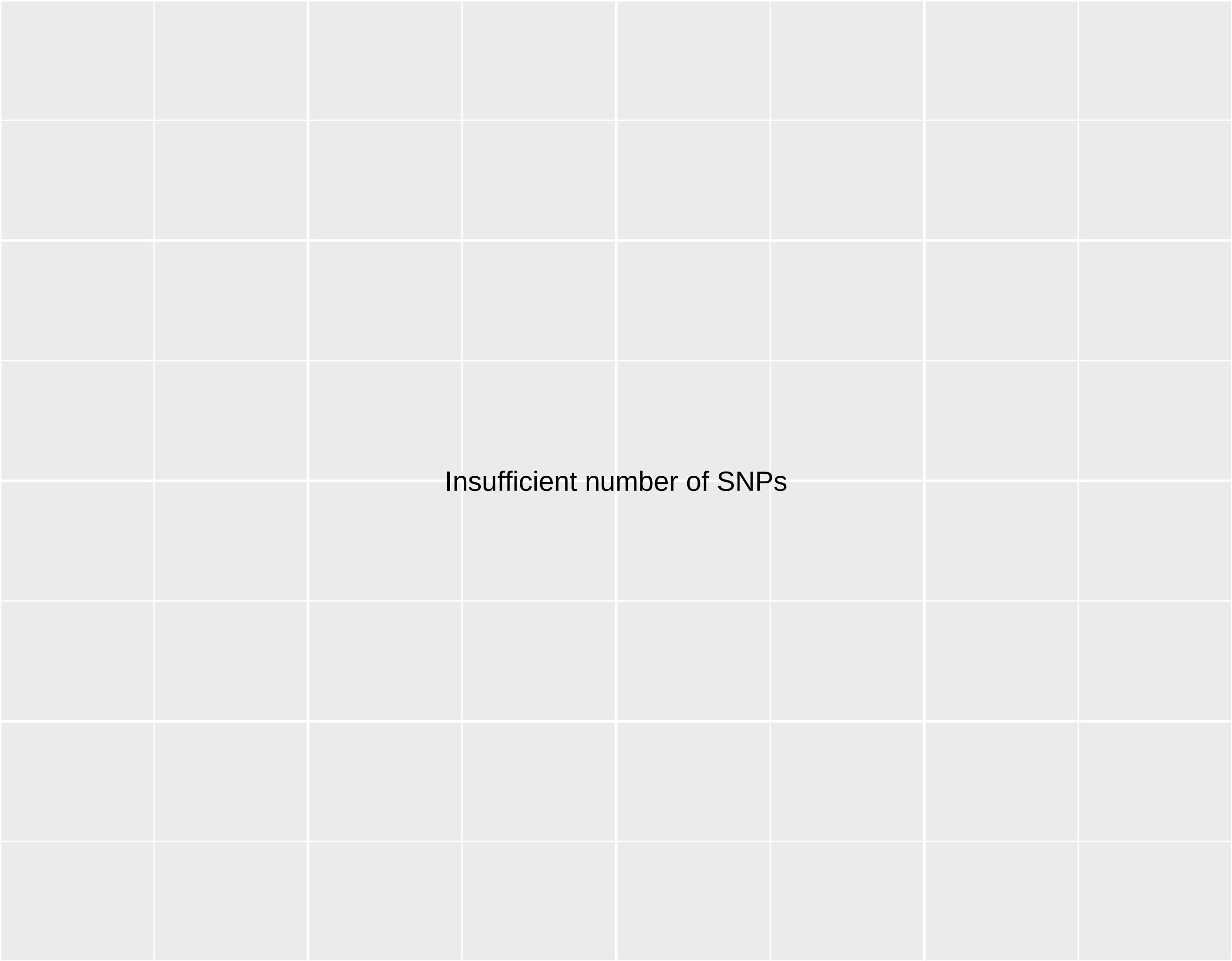
Insufficient number of SNPs

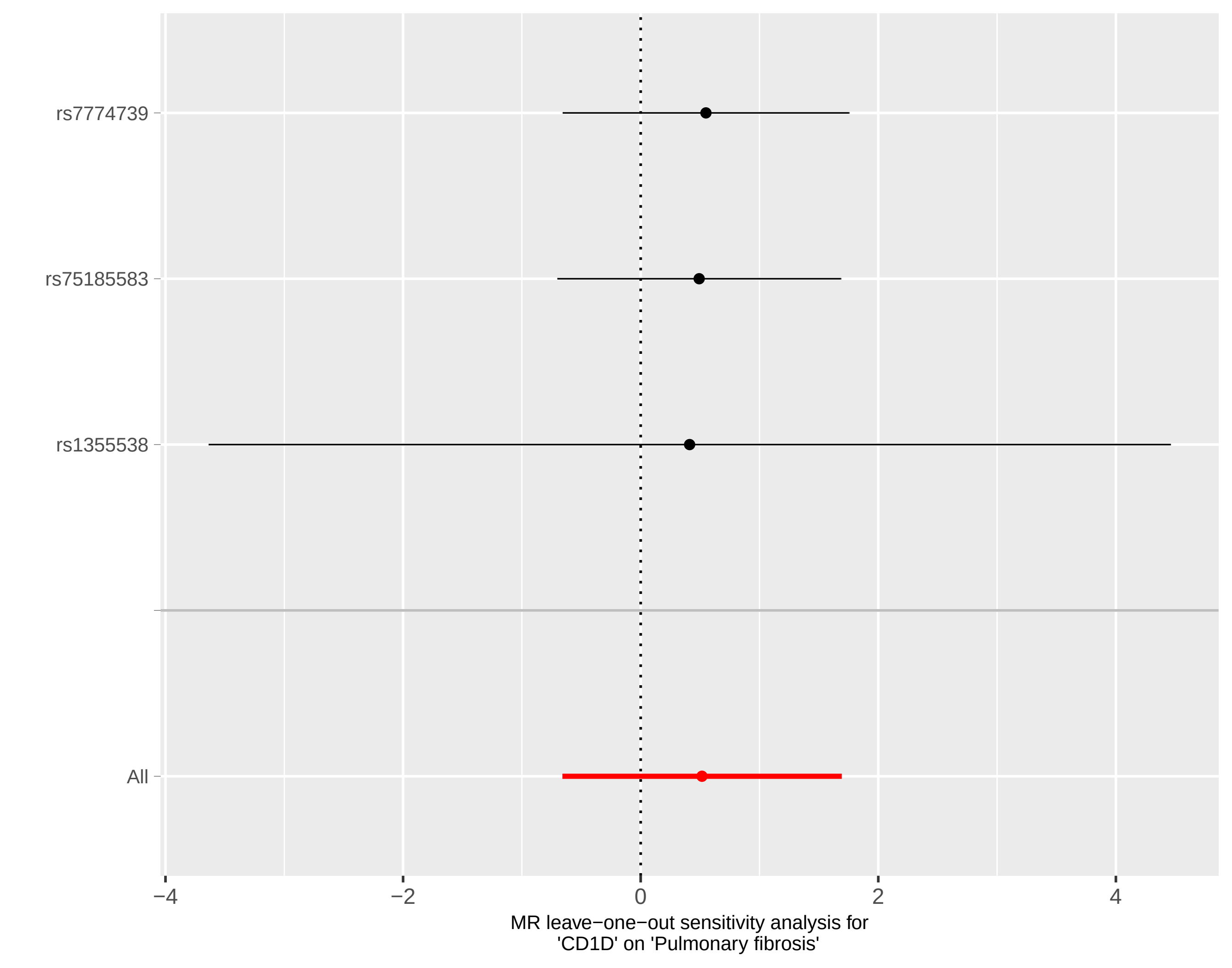
rs7774739
rs75185583
rs1355538
All
−4
−2
0
2
4
MR leave−one−out sensitivity analysis for
'CD1D' on 'Pulmonary fibrosis'

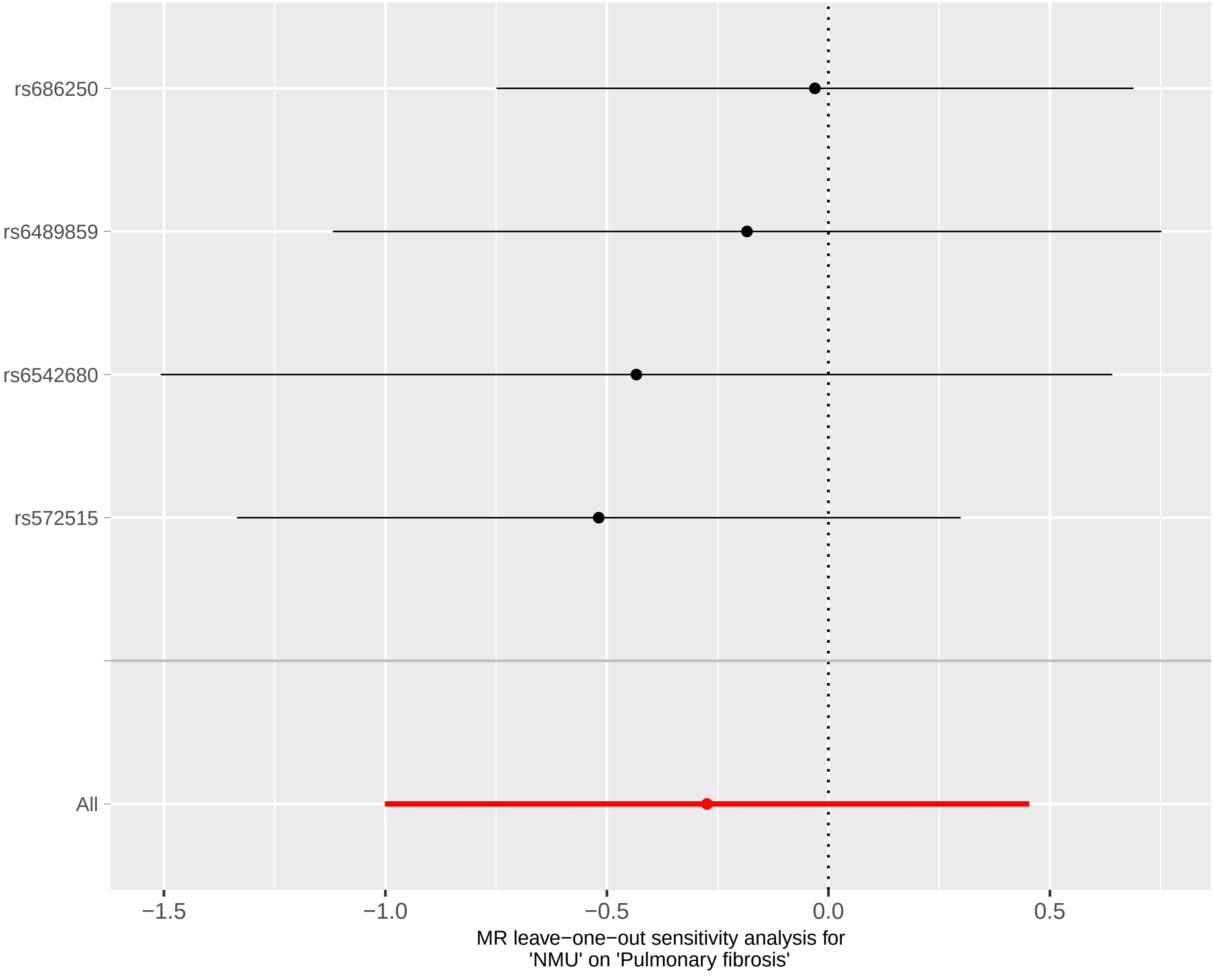
rs686250
rs6489859
rs6542680
rs572515
All
−1.5
−1.0
−0.5
0.0
0.5
MR leave−one−out sensitivity analysis for
'NMU' on 'Pulmonary fibrosis'

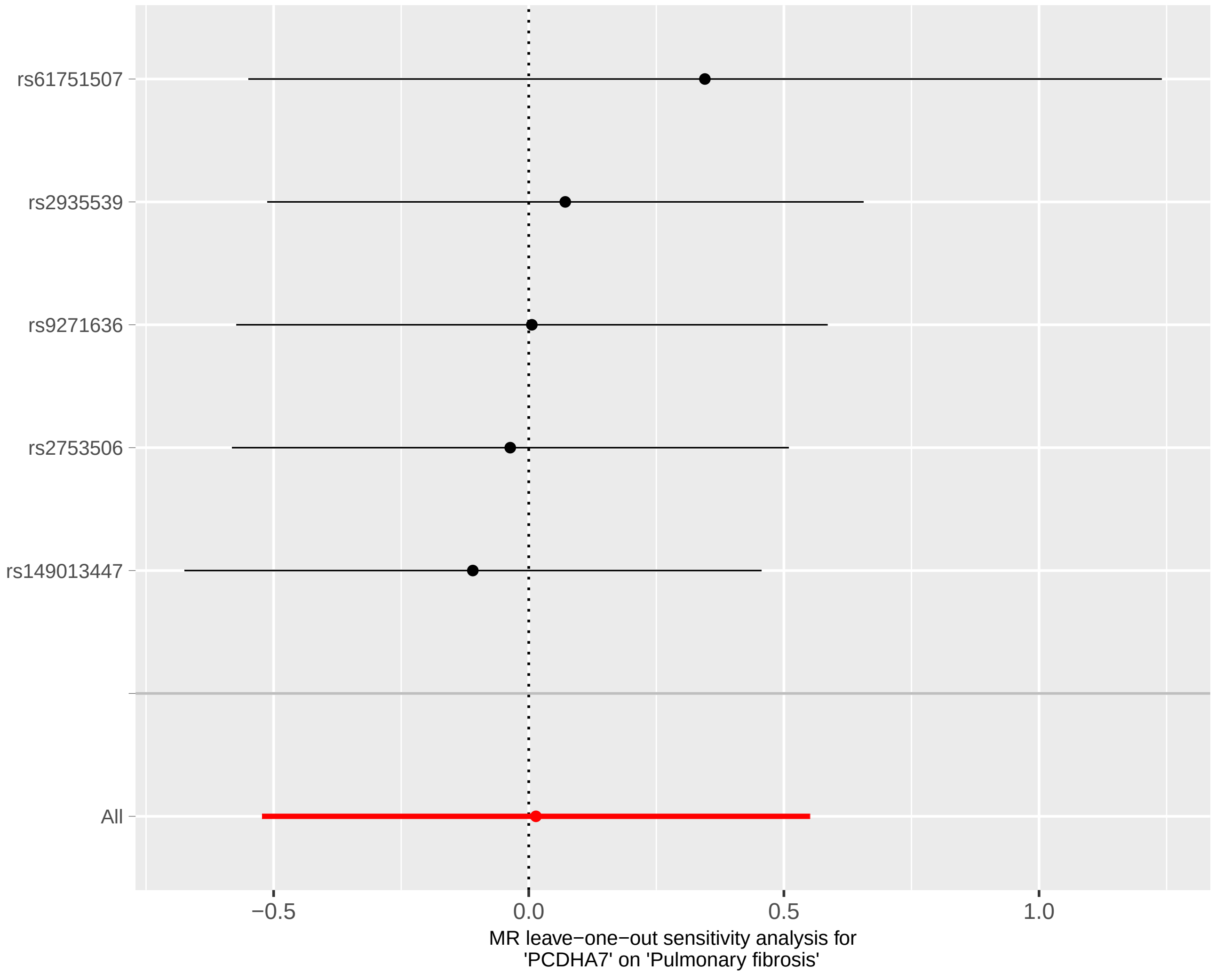
rs61751507
rs2935539
rs9271636
rs2753506
rs149013447
All
−0.5
0.0
0.5
1.0
MR leave−one−out sensitivity analysis for
'PCDHA7' on 'Pulmonary fibrosis'

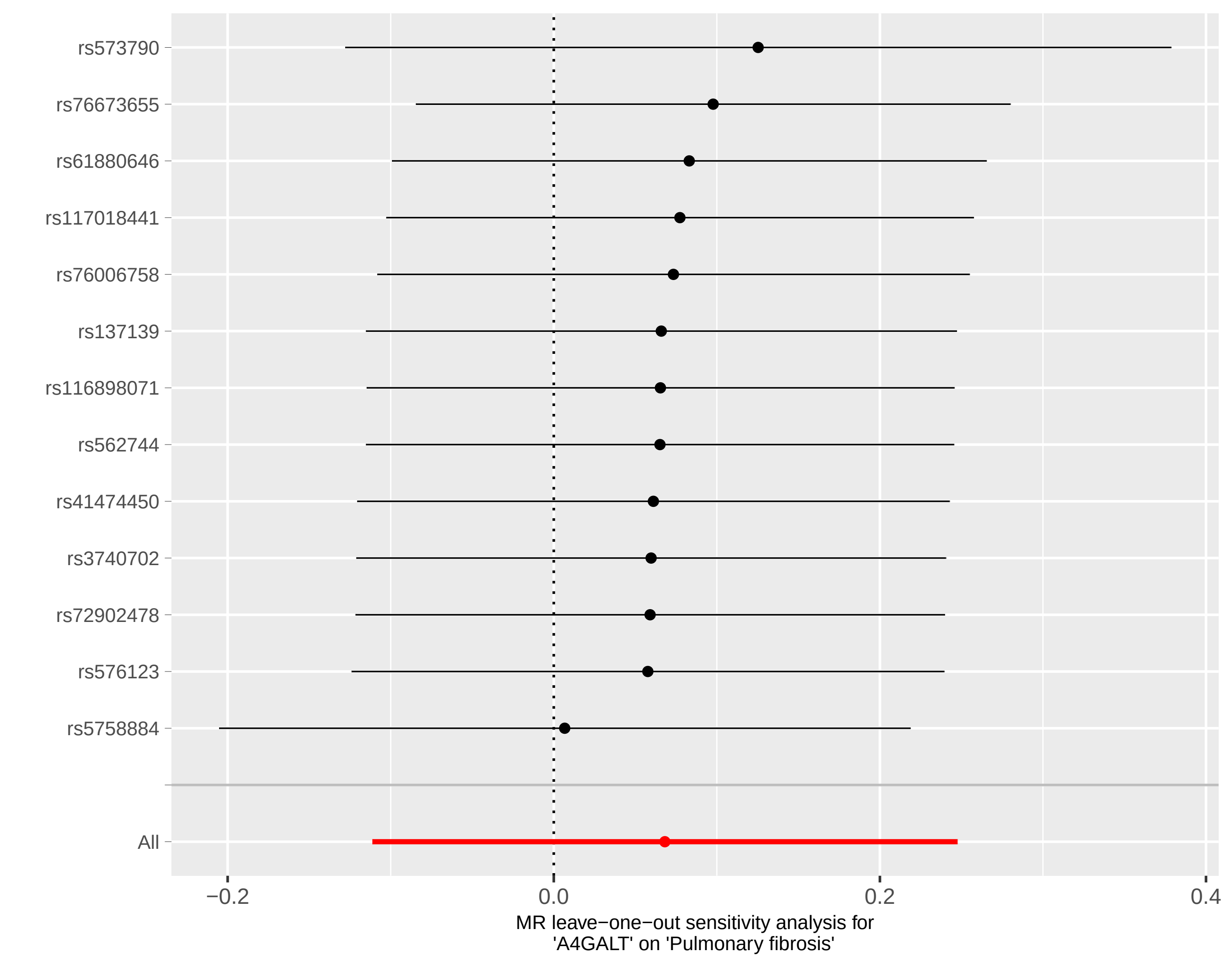

rs573790
rs76673655
rs61880646
rs117018441
rs76006758
rs137139
rs116898071
rs562744
rs41474450
rs3740702
rs72902478
rs576123
rs5758884
All
−0.2
0.0
0.2
0.4
MR leave−one−out sensitivity analysis for
'A4GALT' on 'Pulmonary fibrosis'

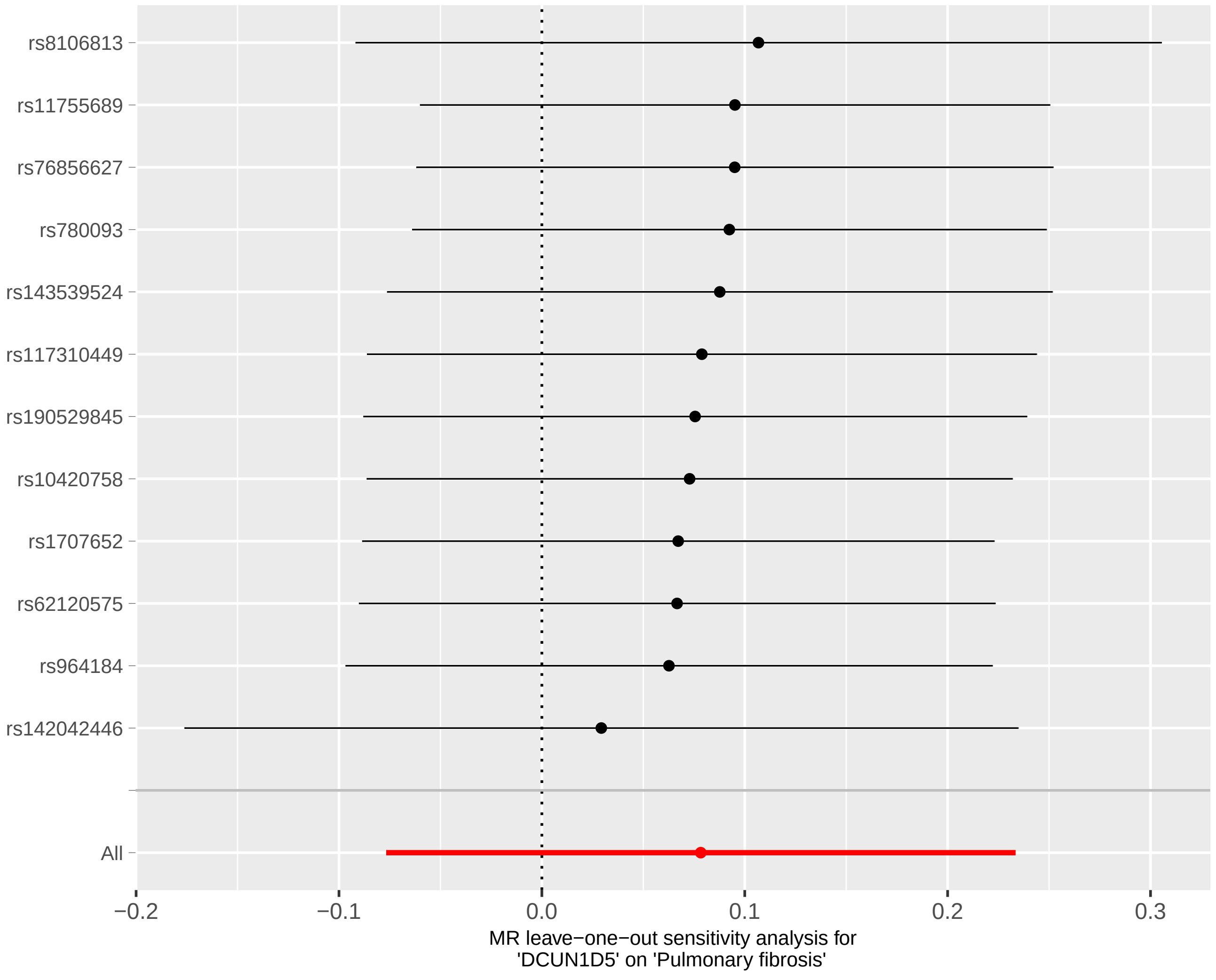
rs8106813
rs11755689
rs76856627
rs780093
rs143539524
rs117310449
rs190529845
rs10420758
rs1707652
rs62120575
rs964184
rs142042446
All
−0.2
−0.1
0.0
0.1
0.2
0.3
MR leave−one−out sensitivity analysis for
'DCUN1D5' on 'Pulmonary fibrosis'

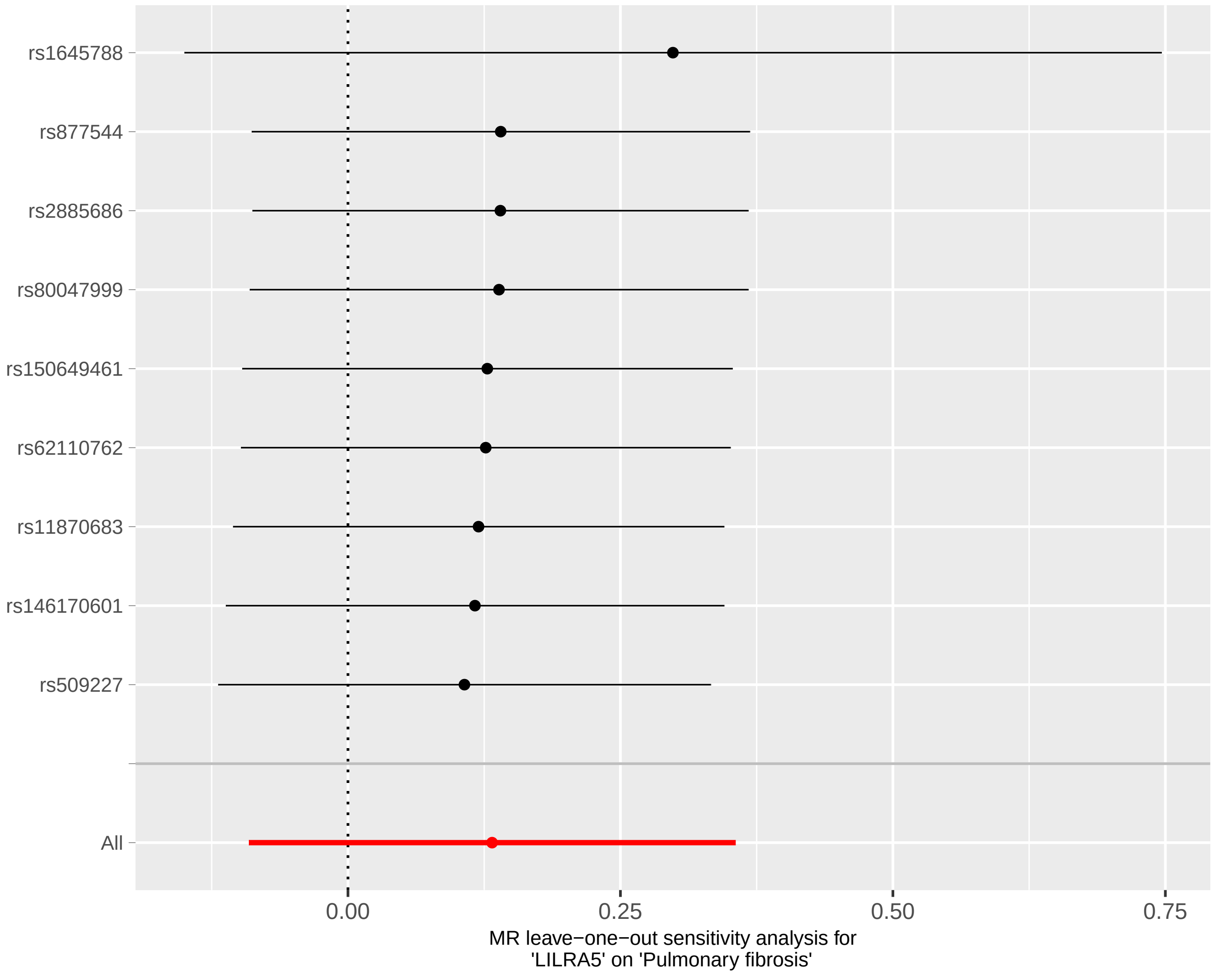

rs1645788
rs877544
rs2885686
rs80047999
rs150649461
rs62110762
rs11870683
rs146170601
rs509227
All
0.00
0.25
0.50
0.75
MR leave−one−out sensitivity analysis for
'LILRA5' on 'Pulmonary fibrosis'

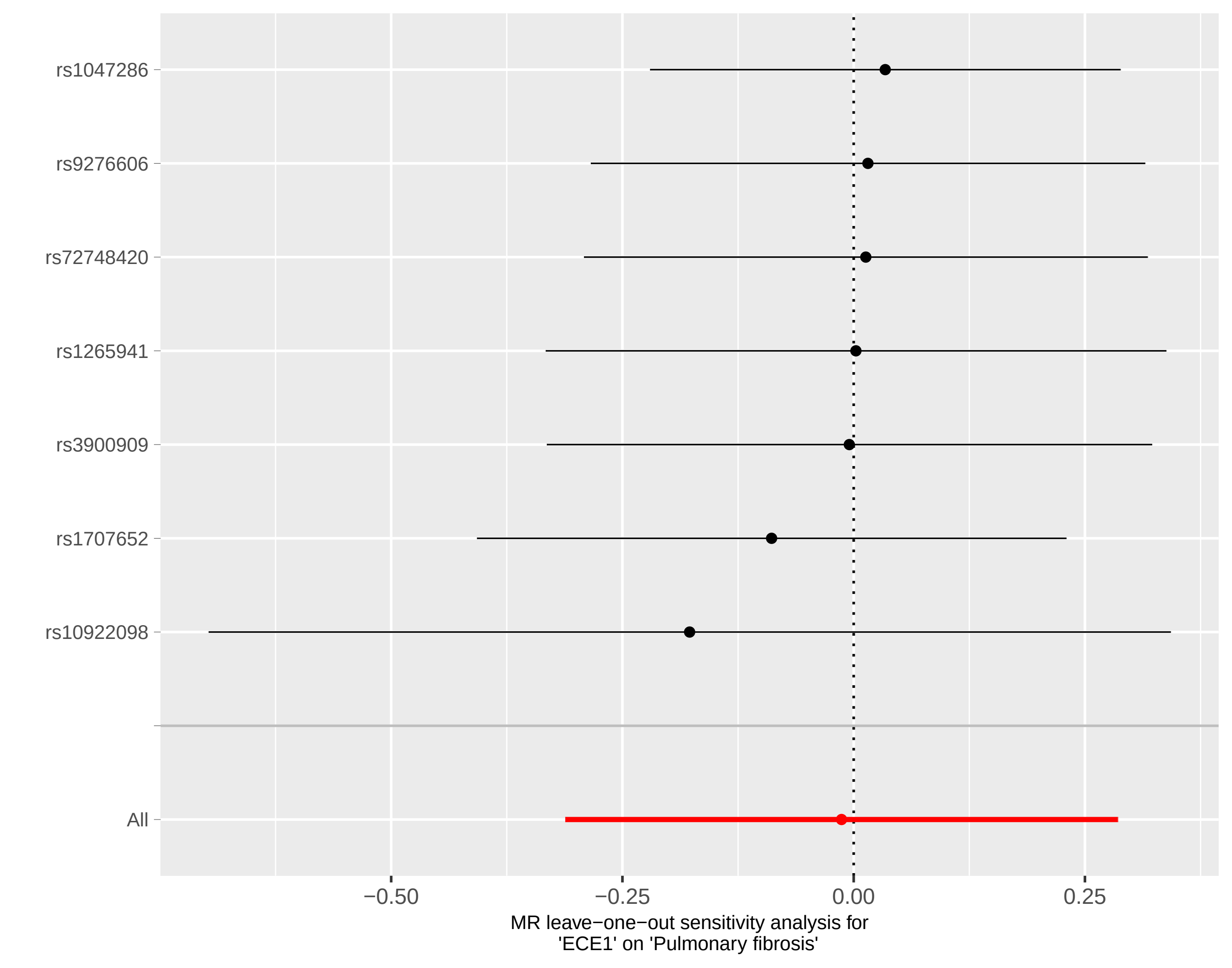
rs1047286
rs9276606
rs72748420
rs1265941
rs3900909
rs1707652
rs10922098
All
−0.50
−0.25
0.00
0.25
MR leave−one−out sensitivity analysis for
'ECE1' on 'Pulmonary fibrosis'

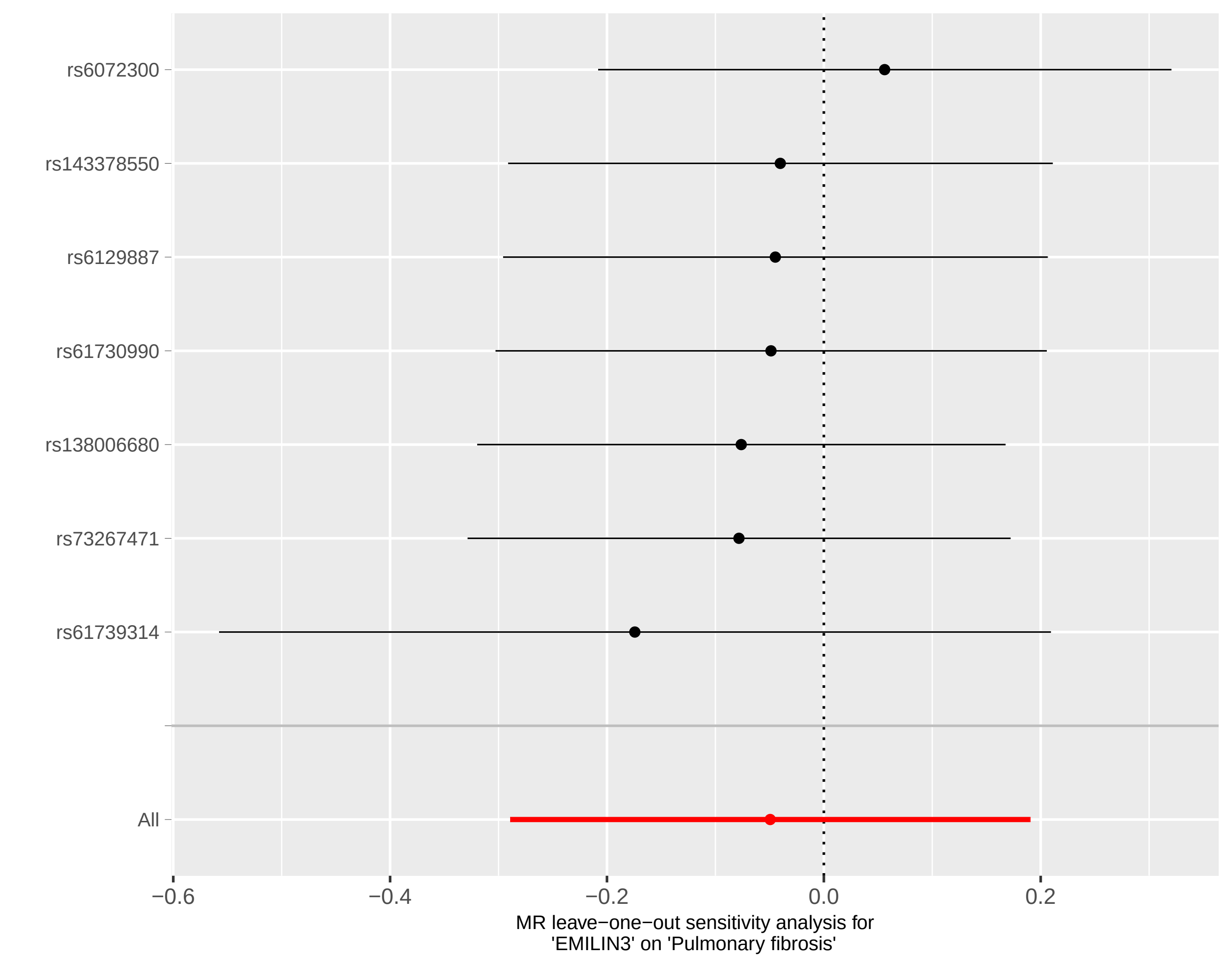
rs6072300
rs143378550
rs6129887
rs61730990
rs138006680
rs73267471
rs61739314
All
−0.6
−0.4
−0.2
0.0
0.2
MR leave−one−out sensitivity analysis for
'EMILIN3' on 'Pulmonary fibrosis'

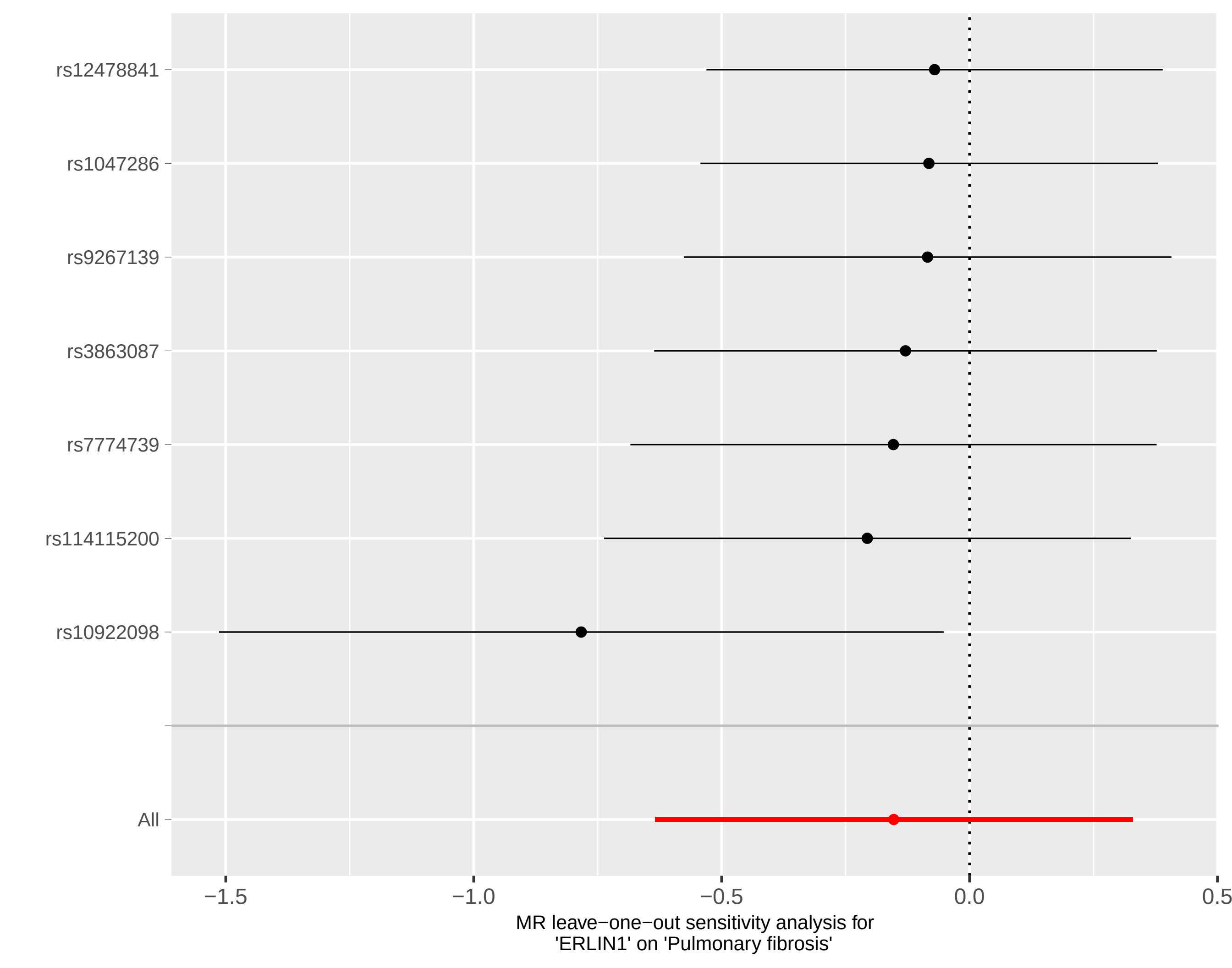
rs12478841
rs1047286
rs9267139
rs3863087
rs7774739
rs114115200
rs10922098
All
−1.5
−1.0
−0.5
0.0
0.5
MR leave−one−out sensitivity analysis for
'ERLIN1' on 'Pulmonary fibrosis'

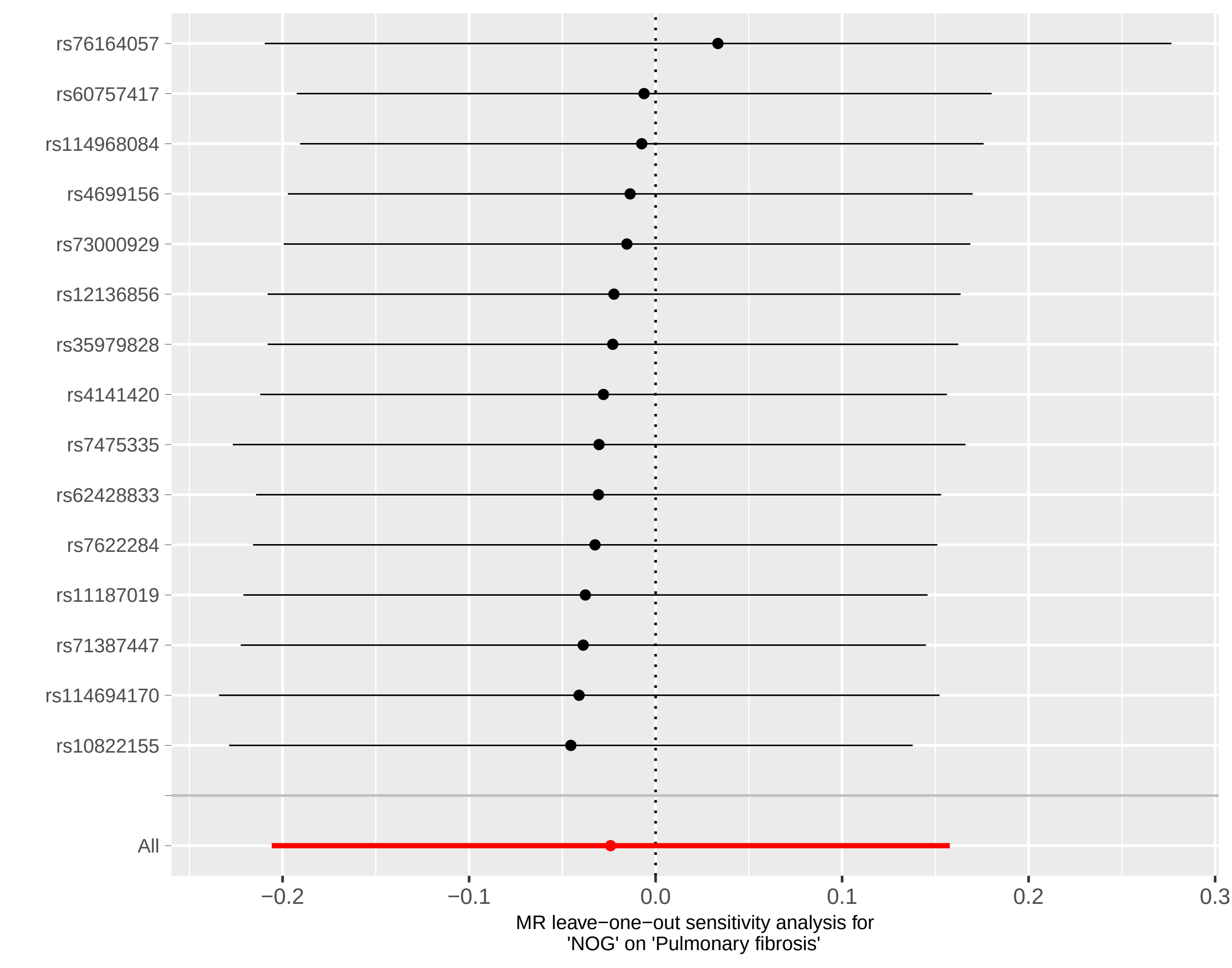
rs76164057
rs60757417
rs114968084
rs4699156
rs73000929
rs12136856
rs35979828
rs4141420
rs7475335
rs62428833
rs7622284
rs11187019
rs71387447
rs114694170
rs10822155
All
−0.2
−0.1
0.0
0.1
0.2
0.3
MR leave−one−out sensitivity analysis for
'NOG' on 'Pulmonary fibrosis'

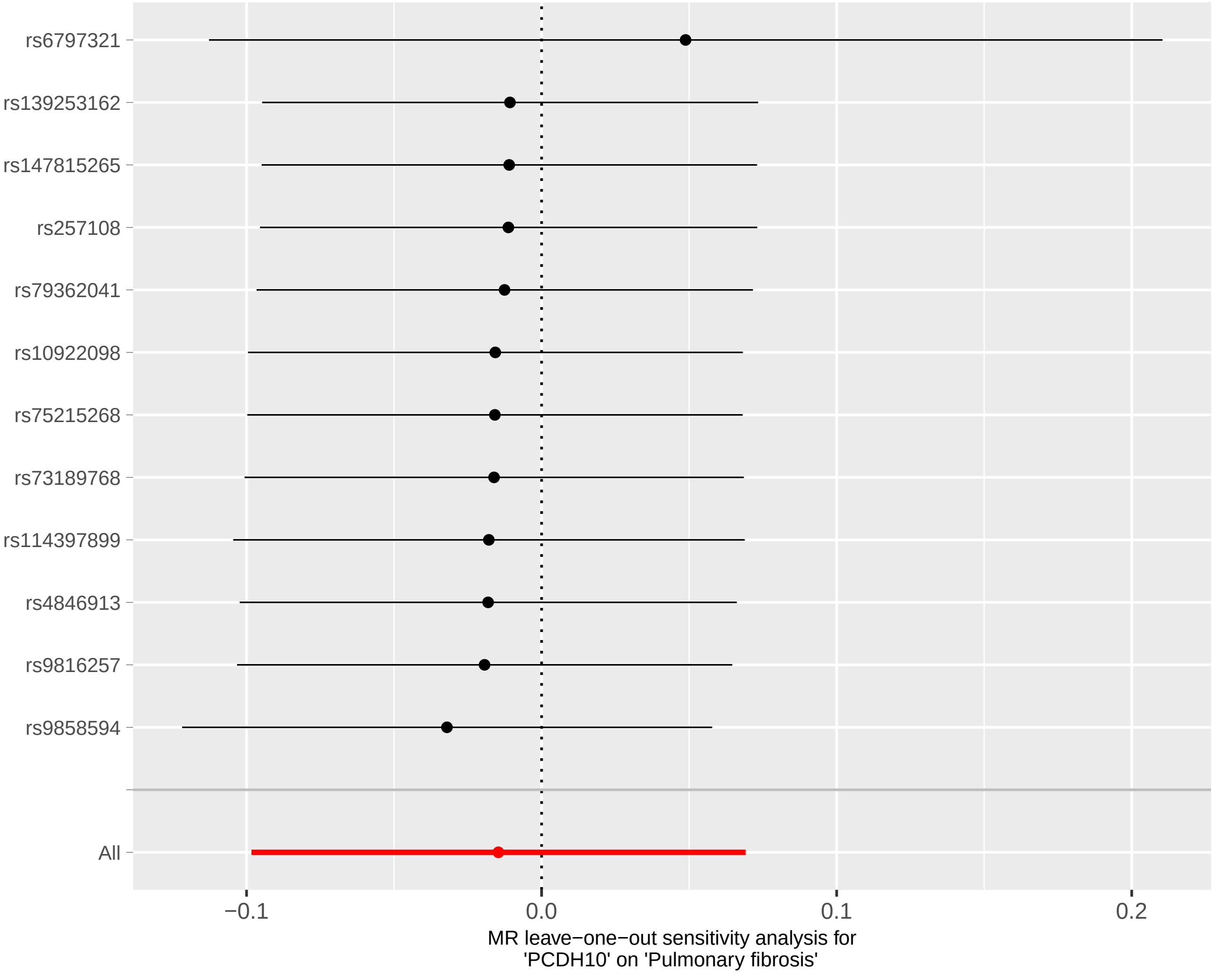
rs6797321
rs139253162
rs147815265
rs257108
rs79362041
rs10922098
rs75215268
rs73189768
rs114397899
rs4846913
rs9816257
rs9858594
All
−0.1
0.0
0.1
0.2
MR leave−one−out sensitivity analysis for
'PCDH10' on 'Pulmonary fibrosis'

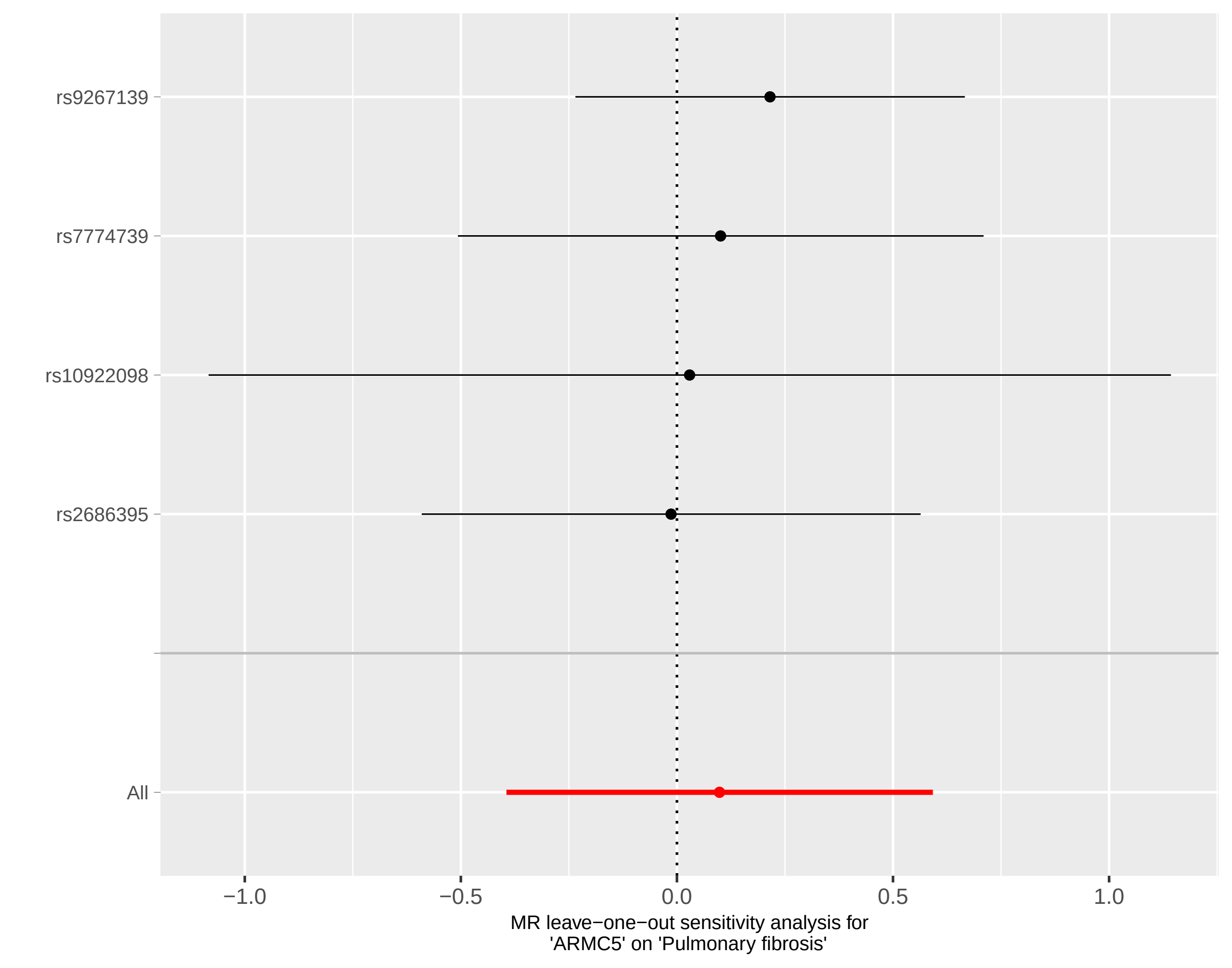
rs9267139
rs7774739
rs10922098
rs2686395
All
−1.0
−0.5
0.0
0.5
1.0
MR leave−one−out sensitivity analysis for
'ARMC5' on 'Pulmonary fibrosis'

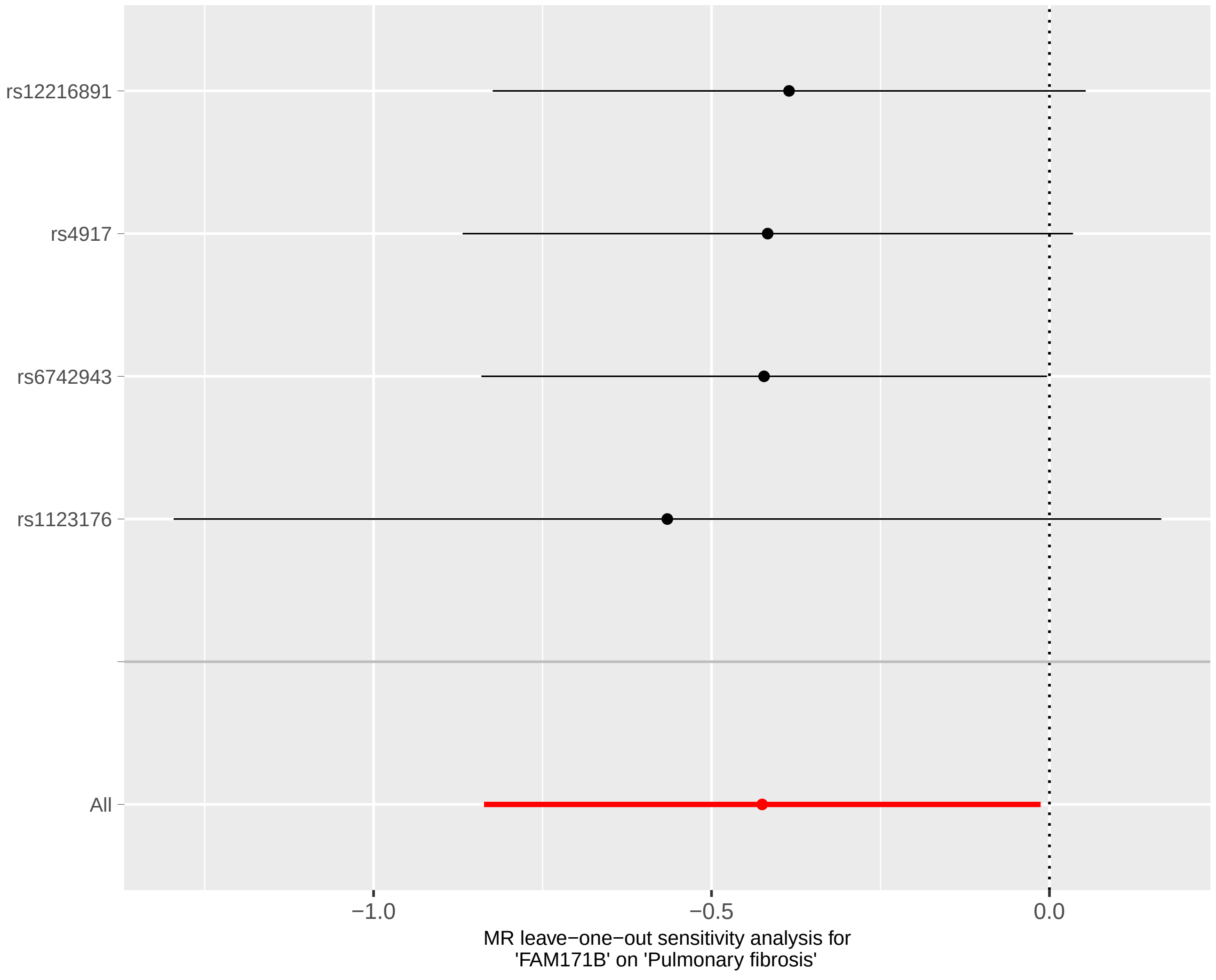
rs12216891
rs4917
rs6742943
rs1123176
All
−1.0
−0.5
0.0
MR leave−one−out sensitivity analysis for
'FAM171B' on 'Pulmonary fibrosis'

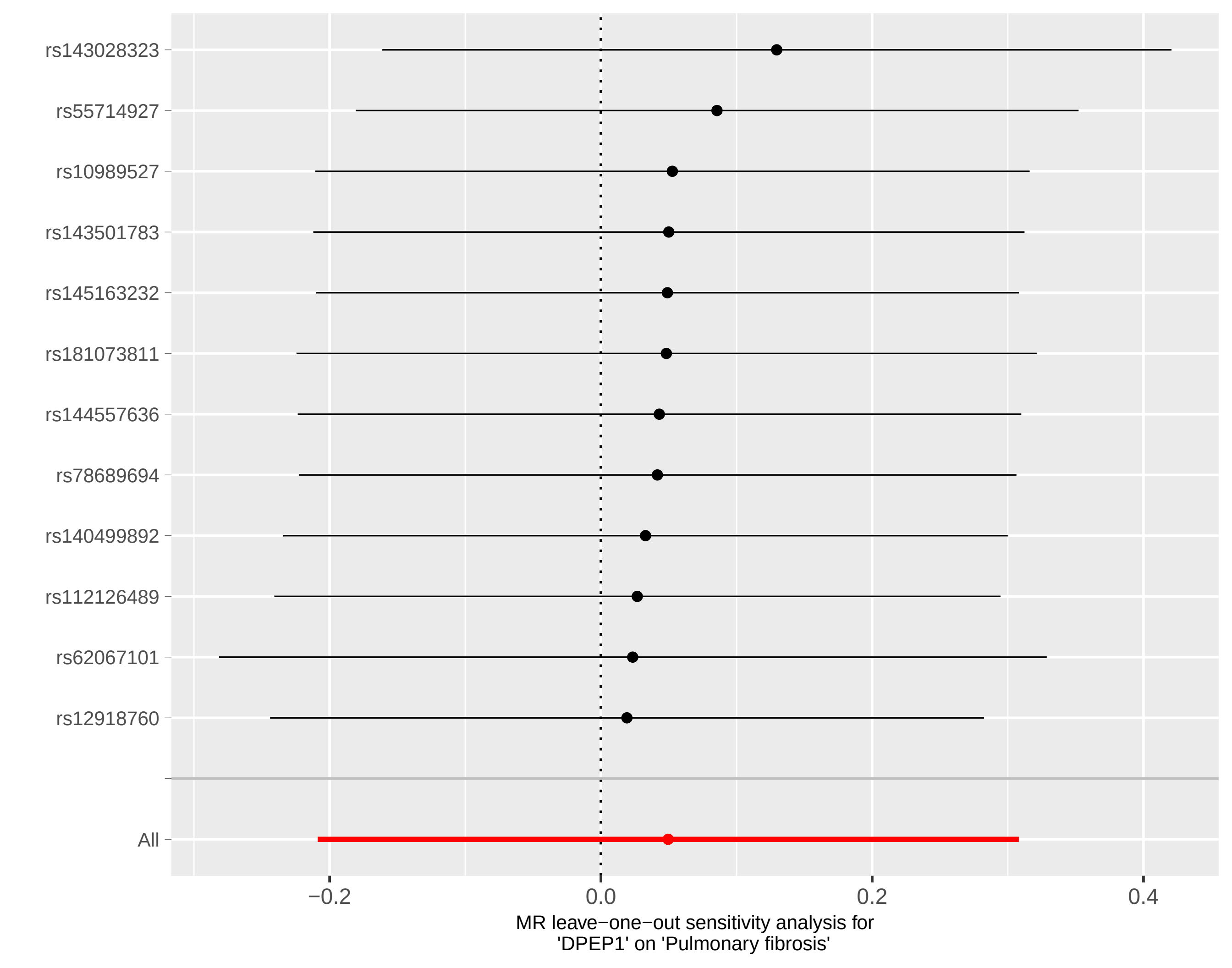
rs143028323
rs55714927
rs10989527
rs143501783
rs145163232
rs181073811
rs144557636
rs78689694
rs140499892
rs112126489
rs62067101
rs12918760
All
−0.2
0.0
0.2
0.4
MR leave−one−out sensitivity analysis for
'DPEP1' on 'Pulmonary fibrosis'

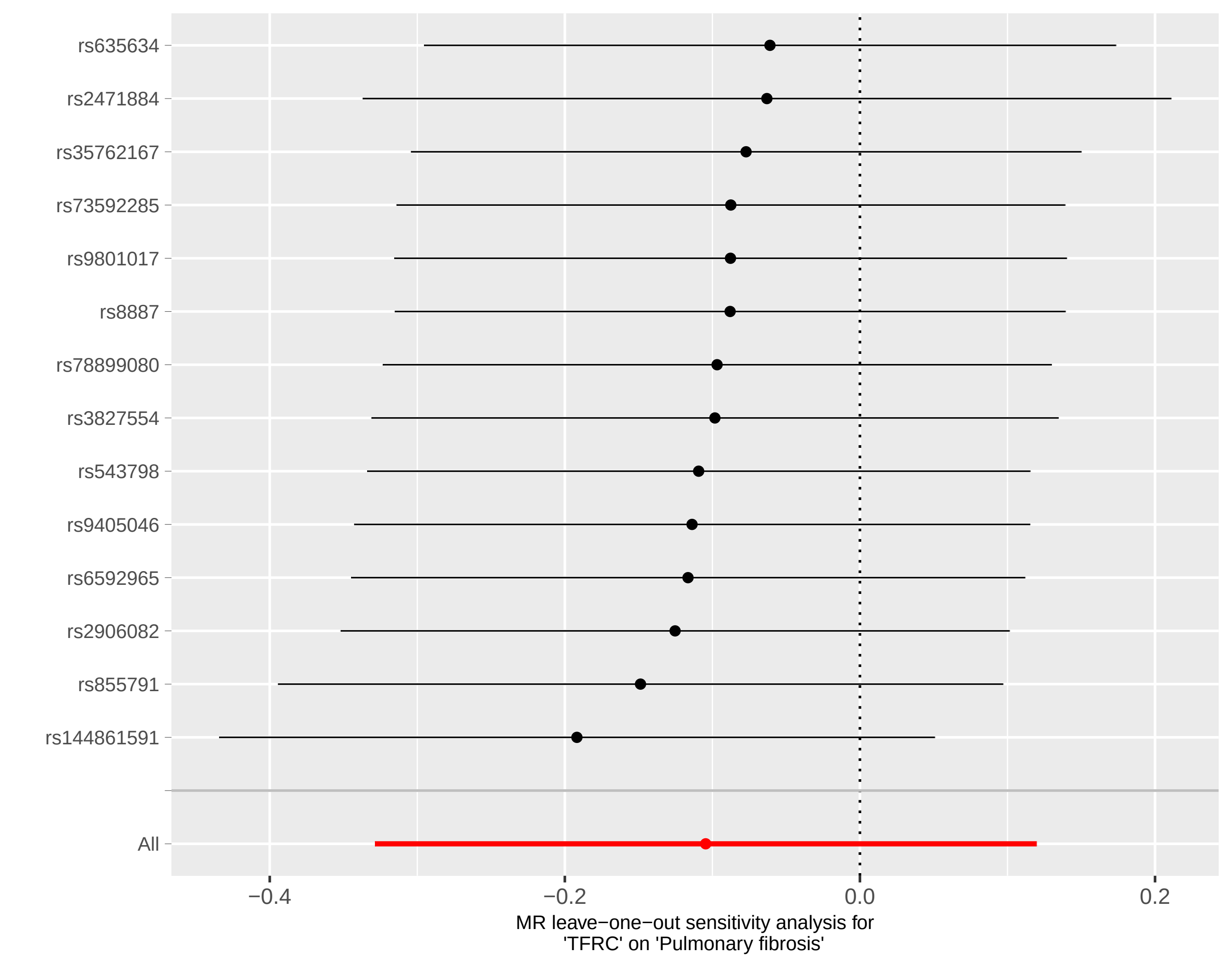
rs635634
rs2471884
rs35762167
rs73592285
rs9801017
rs8887
rs78899080
rs3827554
rs543798
rs9405046
rs6592965
rs2906082
rs855791
rs144861591
All
−0.4
−0.2
0.0
0.2
MR leave−one−out sensitivity analysis for
'TFRC' on 'Pulmonary fibrosis'

Insufficient number of SNPs

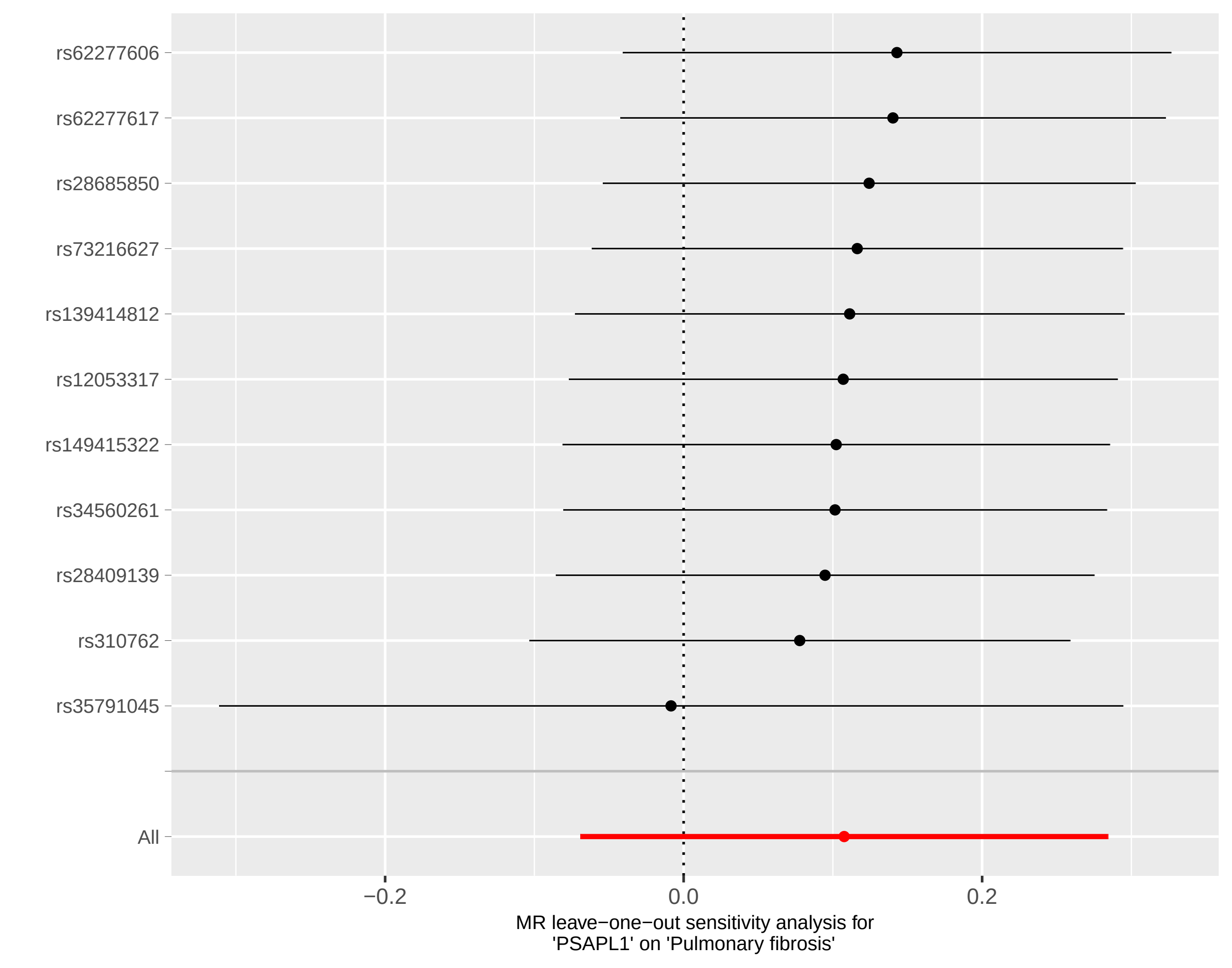
rs62277606
rs62277617
rs28685850
rs73216627
rs139414812
rs12053317
rs149415322
rs34560261
rs28409139
rs310762
rs35791045
All
−0.2
0.0
0.2
MR leave−one−out sensitivity analysis for
'PSAPL1' on 'Pulmonary fibrosis'

Insufficient number of SNPs

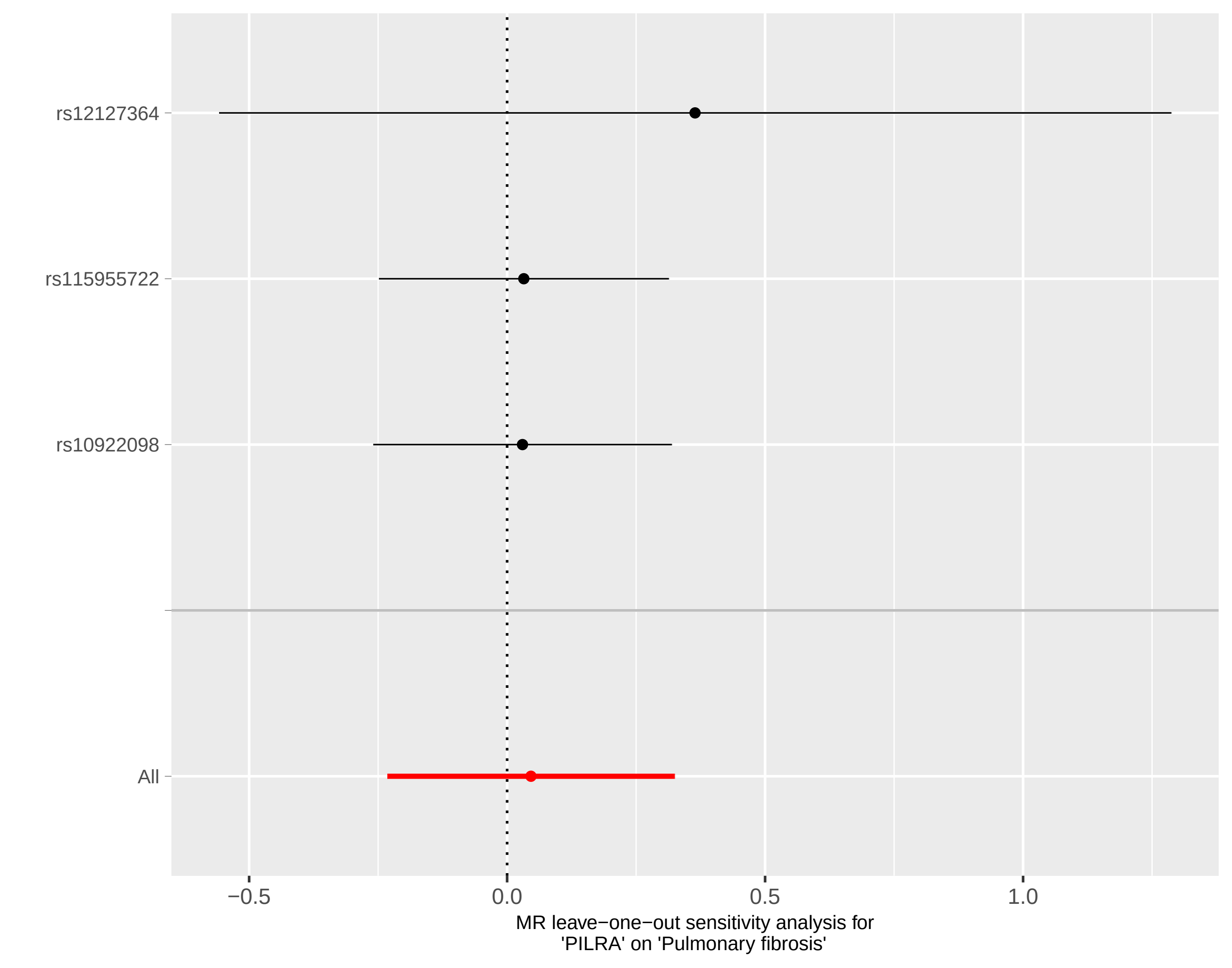

rs12127364
rs115955722
rs10922098
All
−0.5
0.0
0.5
1.0
MR leave−one−out sensitivity analysis for
'PILRA' on 'Pulmonary fibrosis'

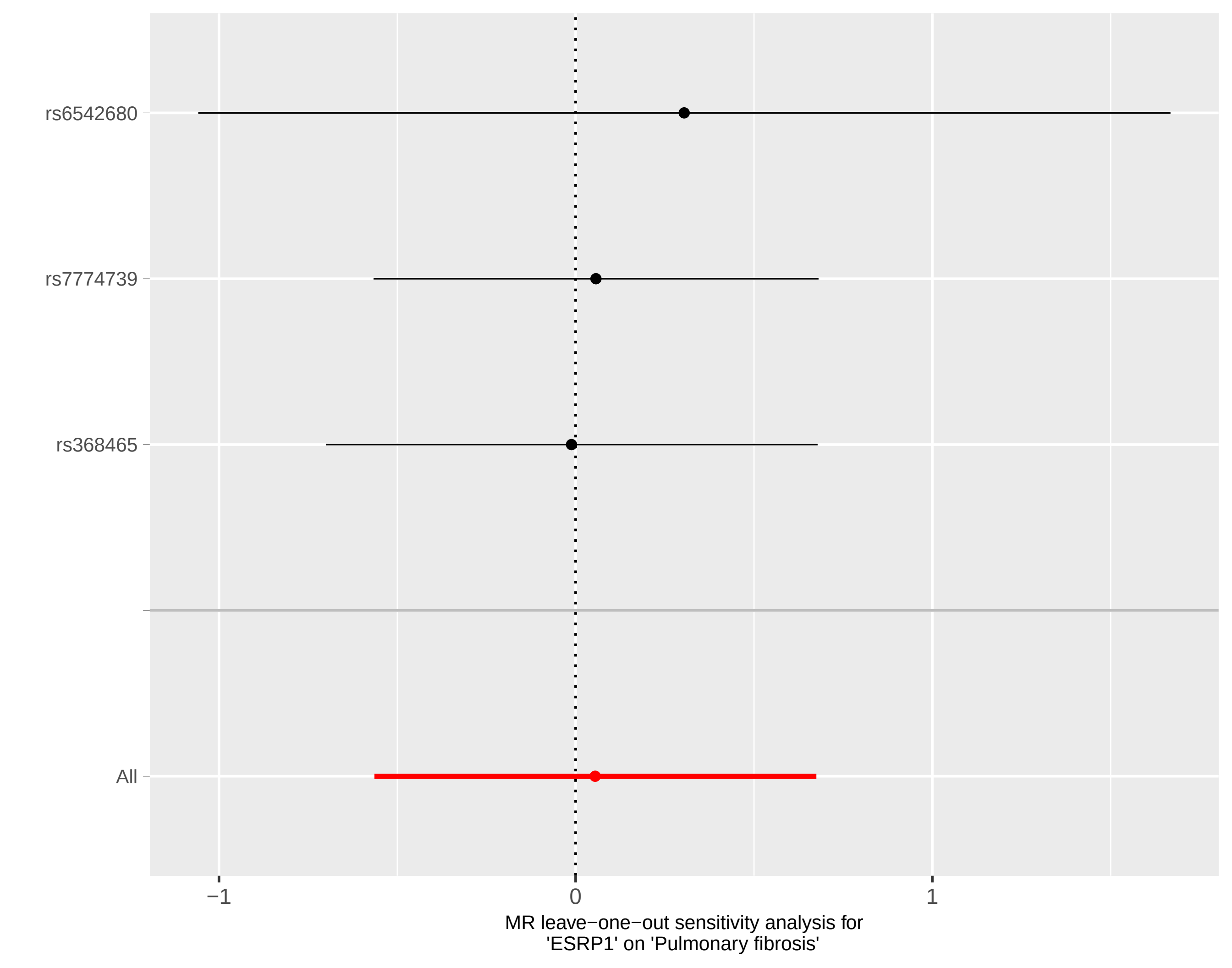

rs6542680
rs7774739
rs368465
All
−1
0
1
MR leave−one−out sensitivity analysis for
'ESRP1' on 'Pulmonary fibrosis'

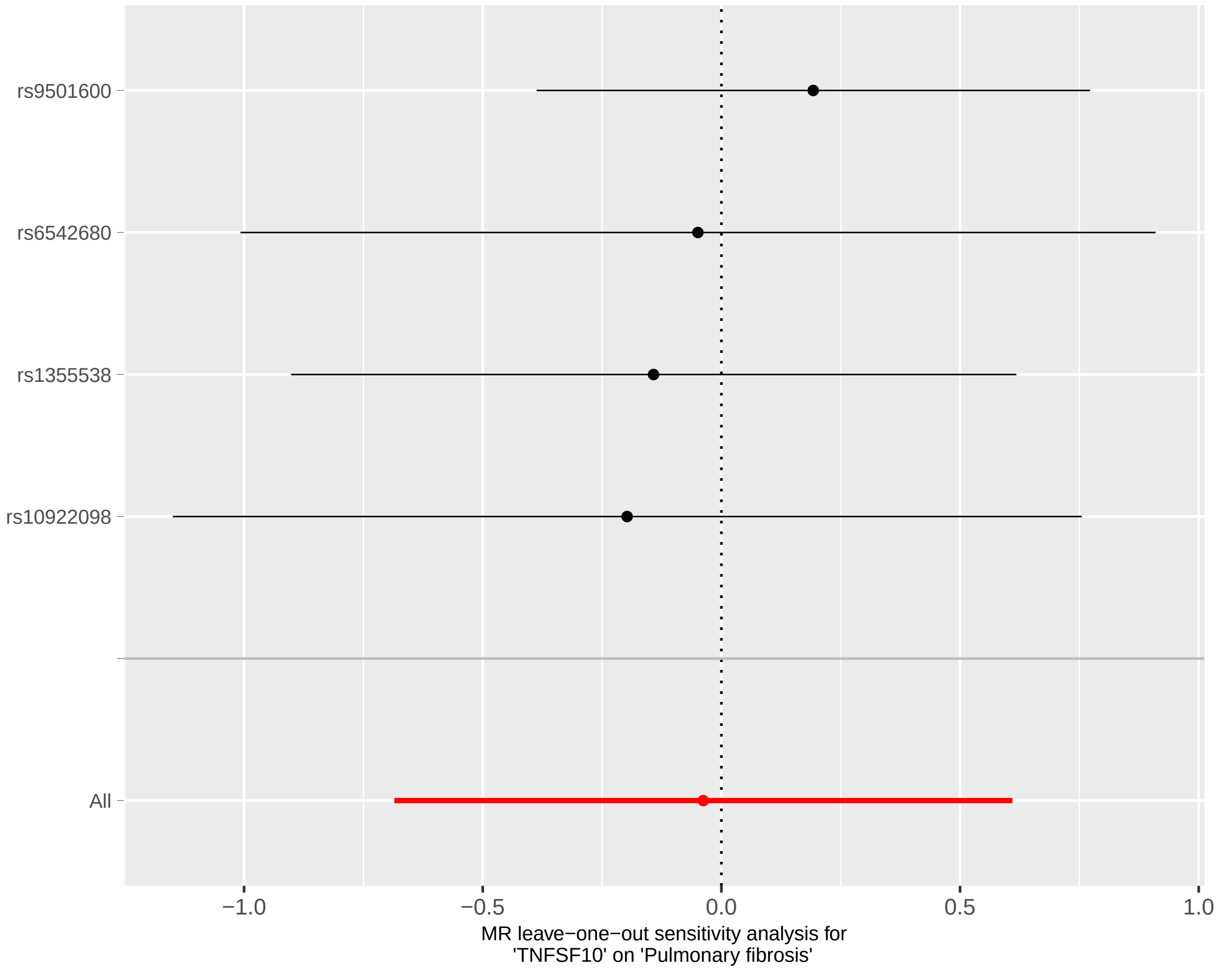
rs9501600
rs6542680
rs1355538
rs10922098
All
−1.0
−0.5
0.0
0.5
1.0
MR leave−one−out sensitivity analysis for
'TNFSF10' on 'Pulmonary fibrosis'

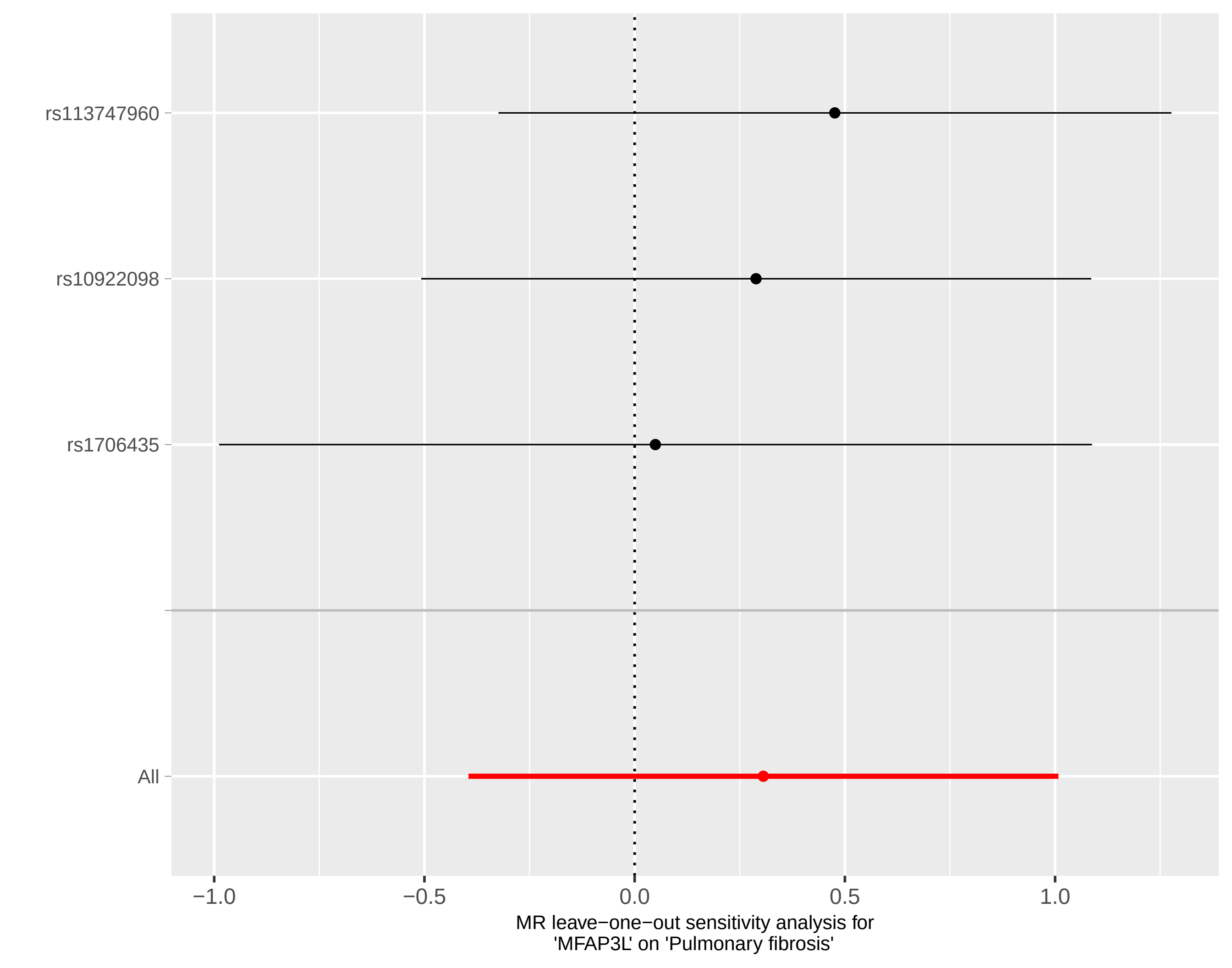

rs113747960
rs10922098
rs1706435
All
−1.0
−0.5
0.0
0.5
1.0
MR leave−one−out sensitivity analysis for
'MFAP3L' on 'Pulmonary fibrosis'

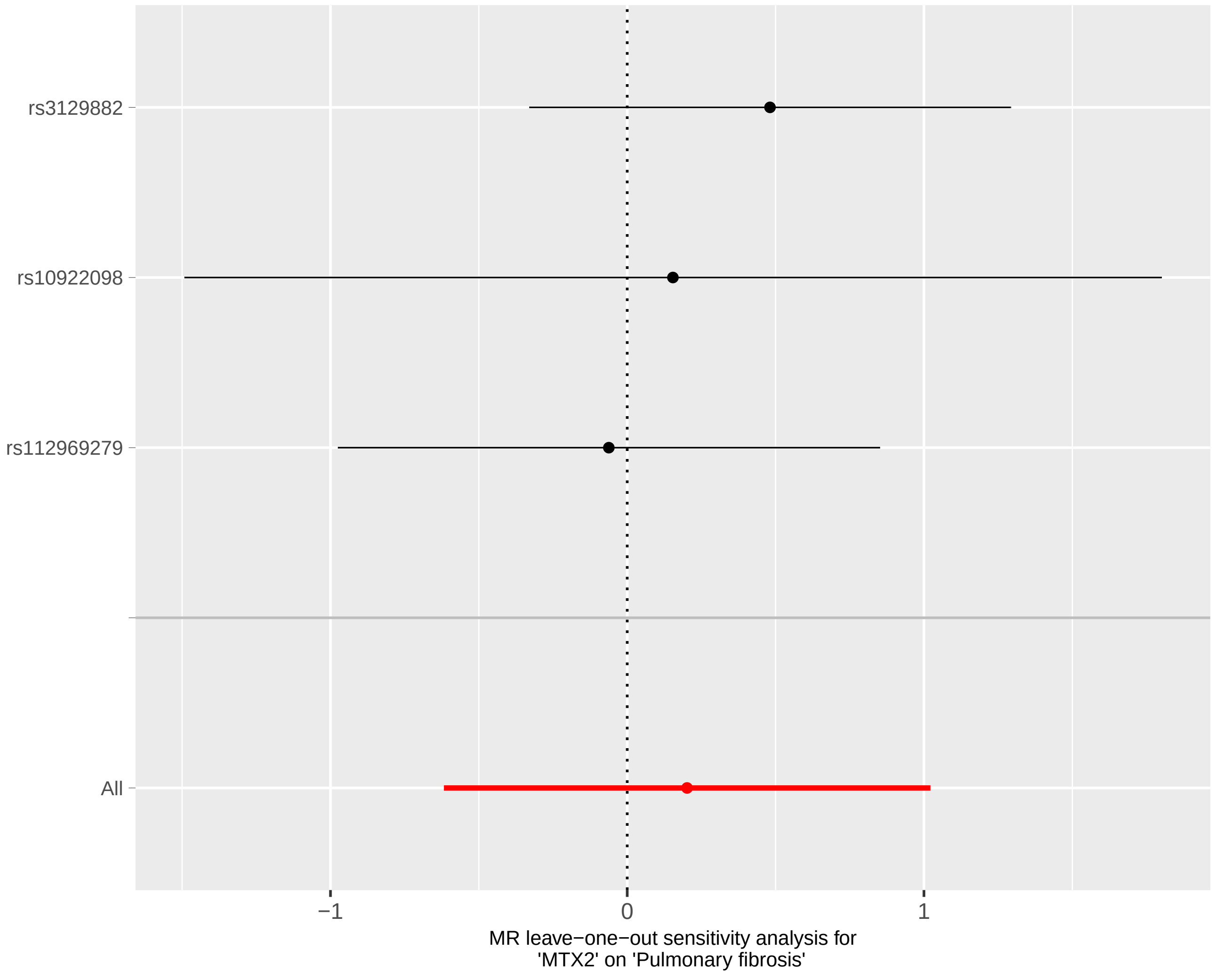
rs3129882
rs10922098
rs112969279
All
−1
0
1
MR leave−one−out sensitivity analysis for
'MTX2' on 'Pulmonary fibrosis'

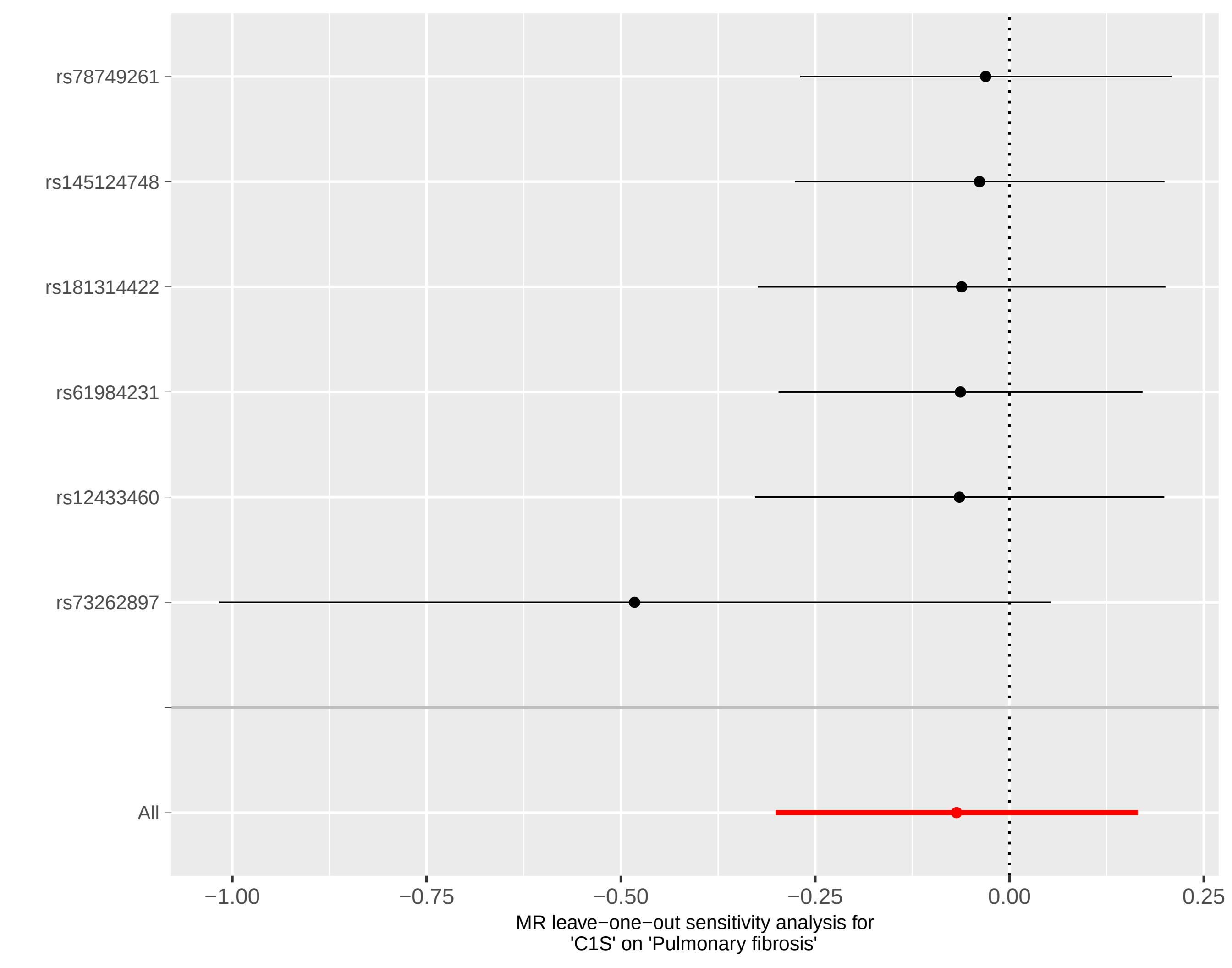
rs78749261
rs145124748
rs181314422
rs61984231
rs12433460
rs73262897
All
−1.00
−0.75
−0.50
−0.25
0.00
0.25
MR leave−one−out sensitivity analysis for
'C1S' on 'Pulmonary fibrosis'

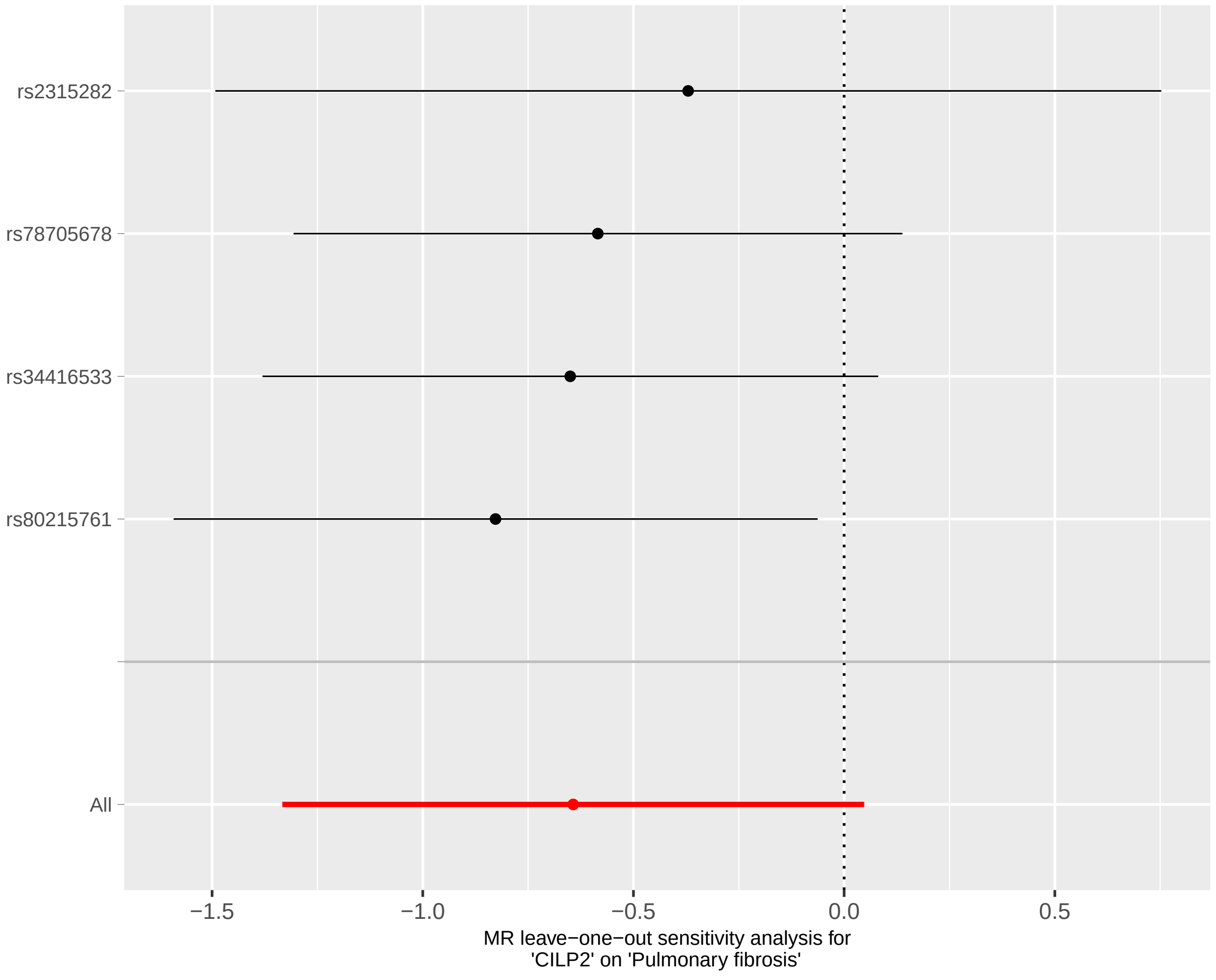
rs2315282
rs78705678
rs34416533
rs80215761
All
−1.5
−1.0
−0.5
0.0
0.5
MR leave−one−out sensitivity analysis for
'CILP2' on 'Pulmonary fibrosis'

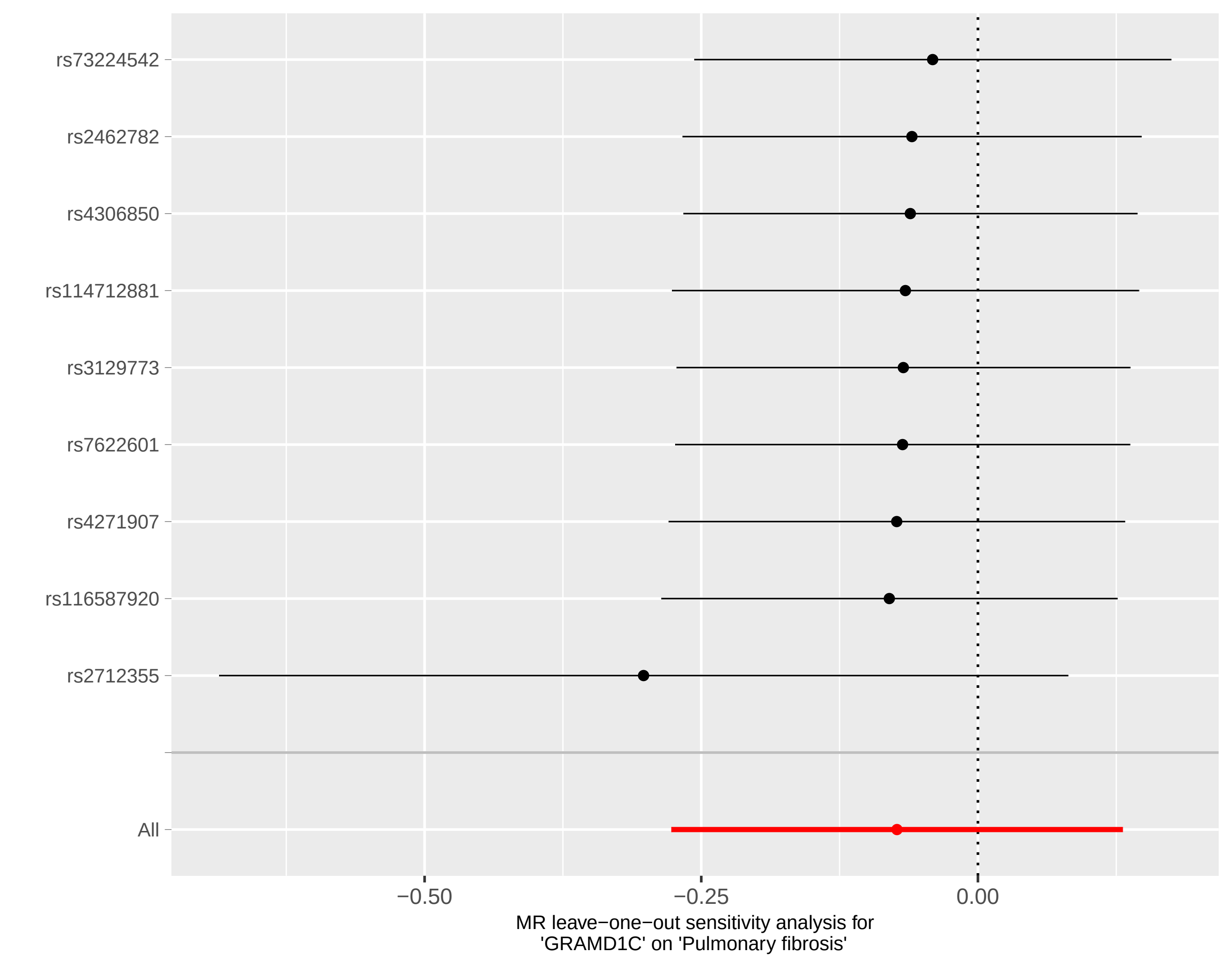
rs73224542
rs2462782
rs4306850
rs114712881
rs3129773
rs7622601
rs4271907
rs116587920
rs2712355
All
−0.50
−0.25
0.00
MR leave−one−out sensitivity analysis for
'GRAMD1C' on 'Pulmonary fibrosis'

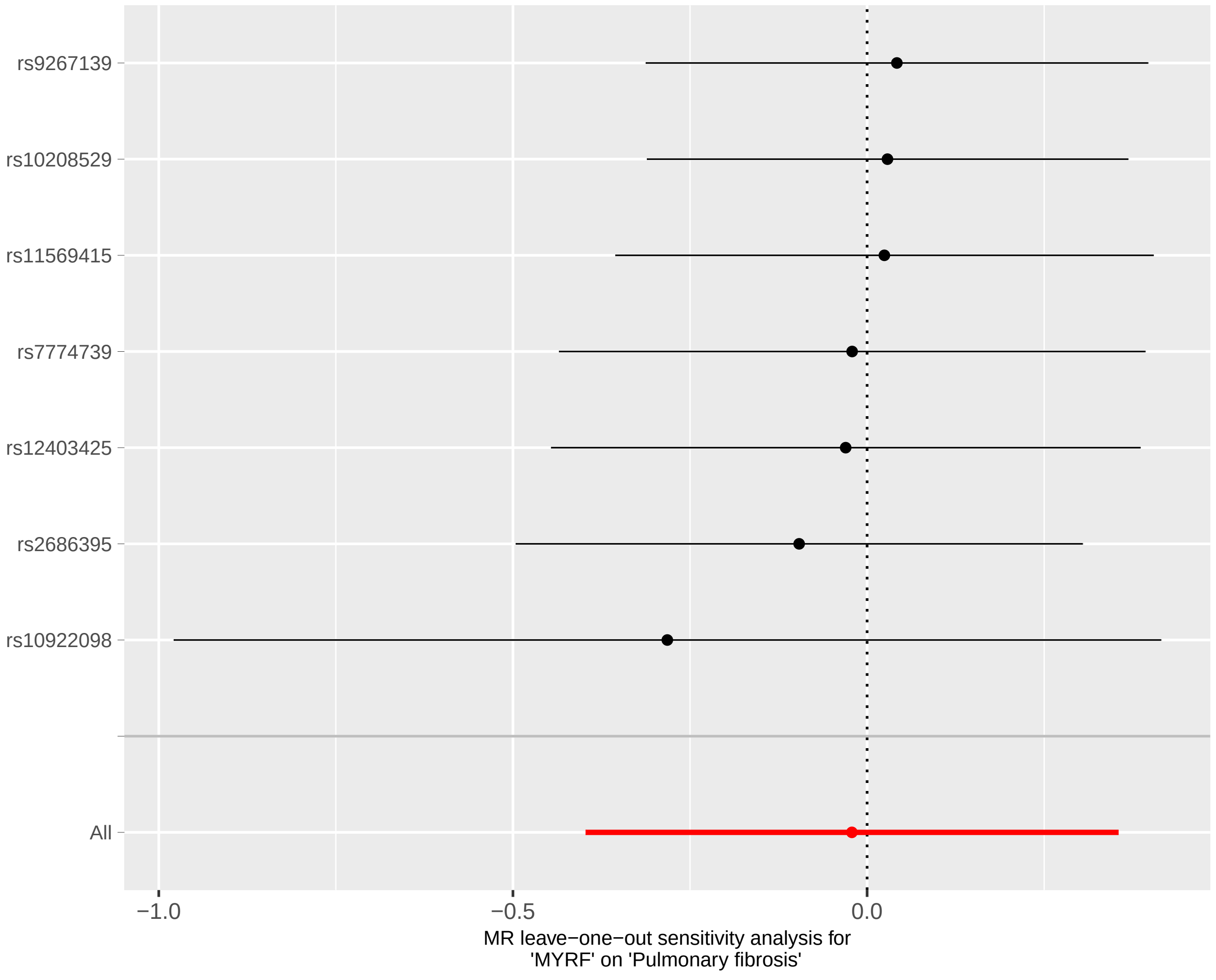

rs9267139
rs10208529
rs11569415
rs7774739
rs12403425
rs2686395
rs10922098
All
−1.0
−0.5
0.0
MR leave−one−out sensitivity analysis for
'MYRF' on 'Pulmonary fibrosis'

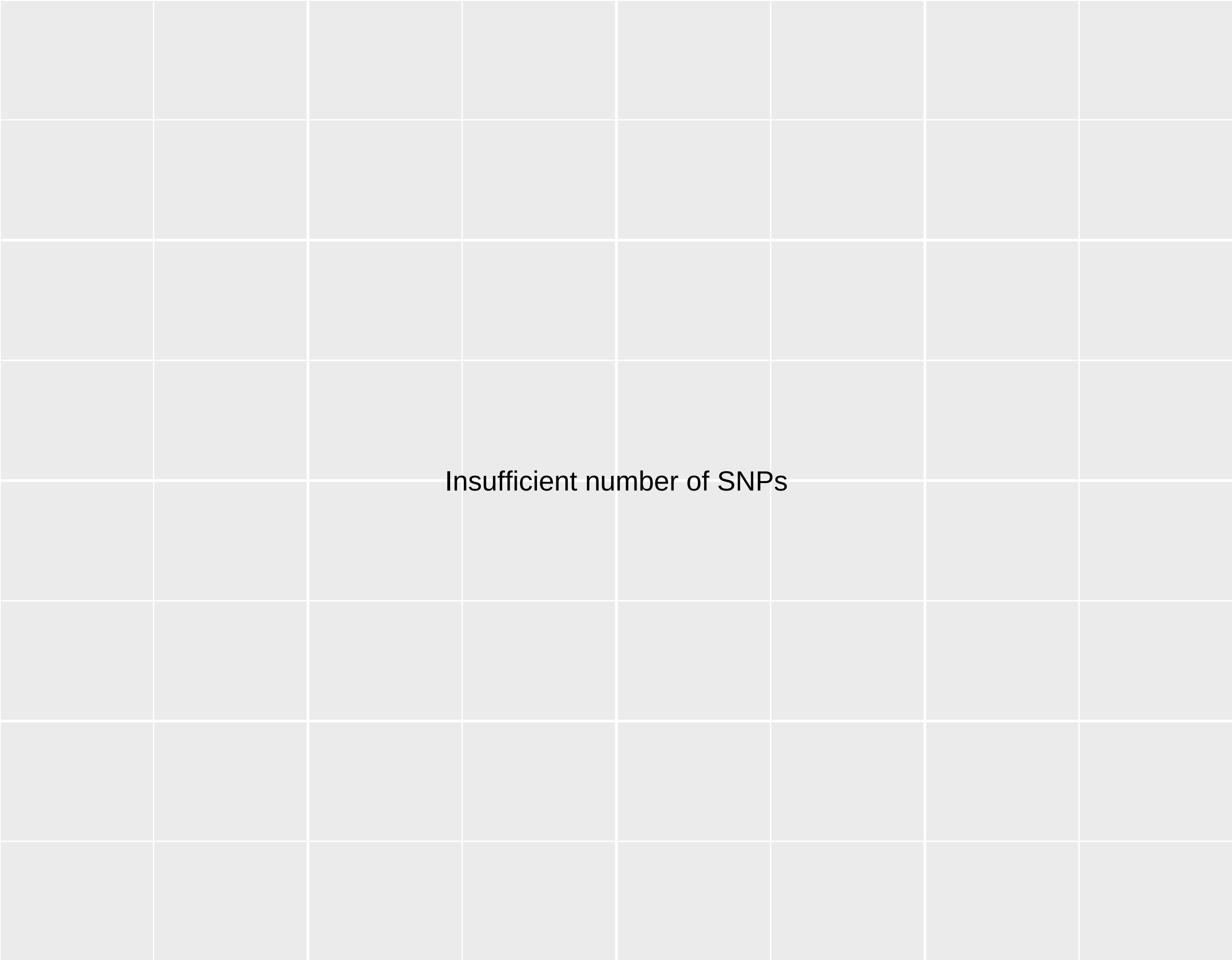
Insufficient number of SNPs

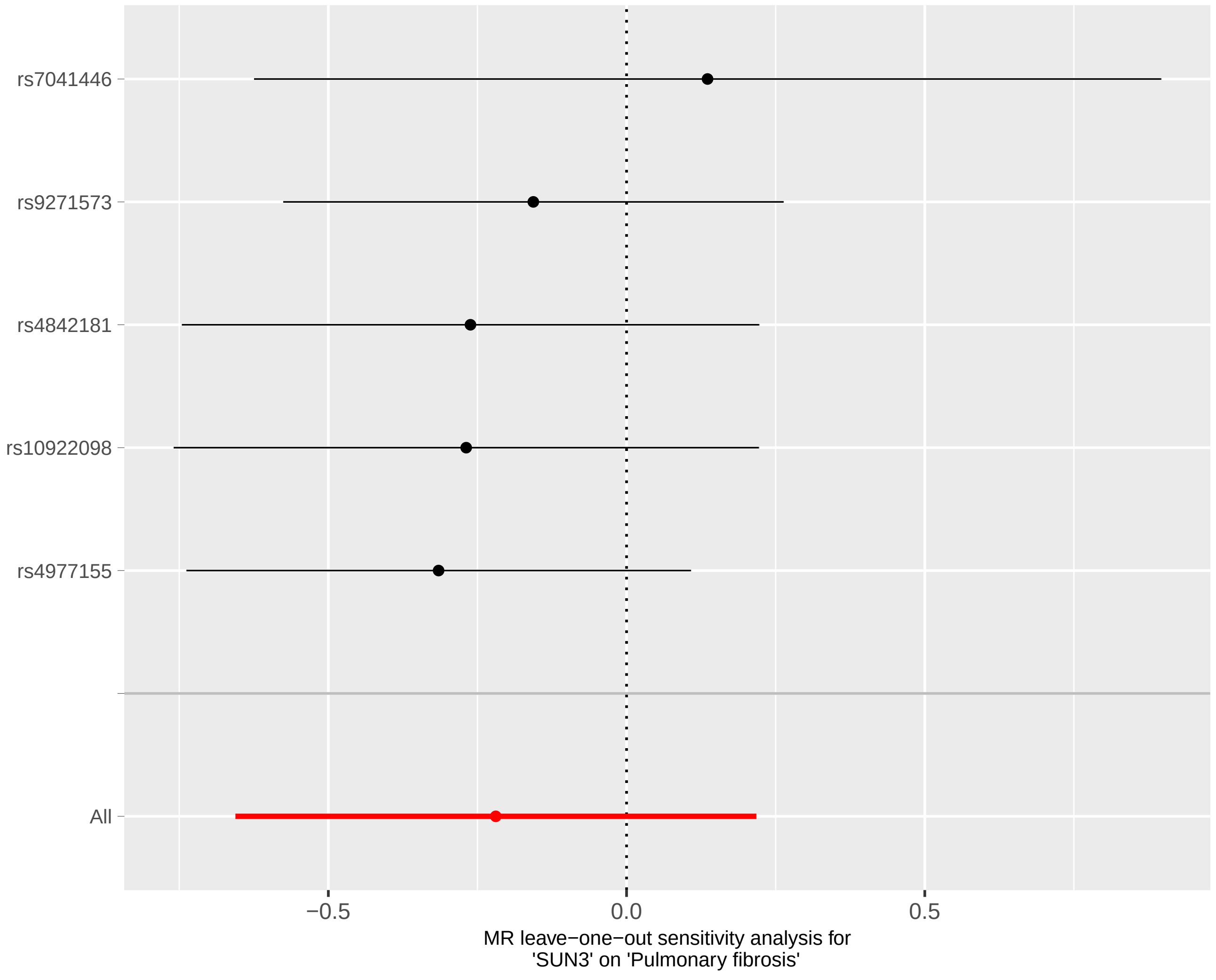
rs7041446
rs9271573
rs4842181
rs10922098
rs4977155
All
−0.5
0.0
0.5
MR leave−one−out sensitivity analysis for
'SUN3' on 'Pulmonary fibrosis'

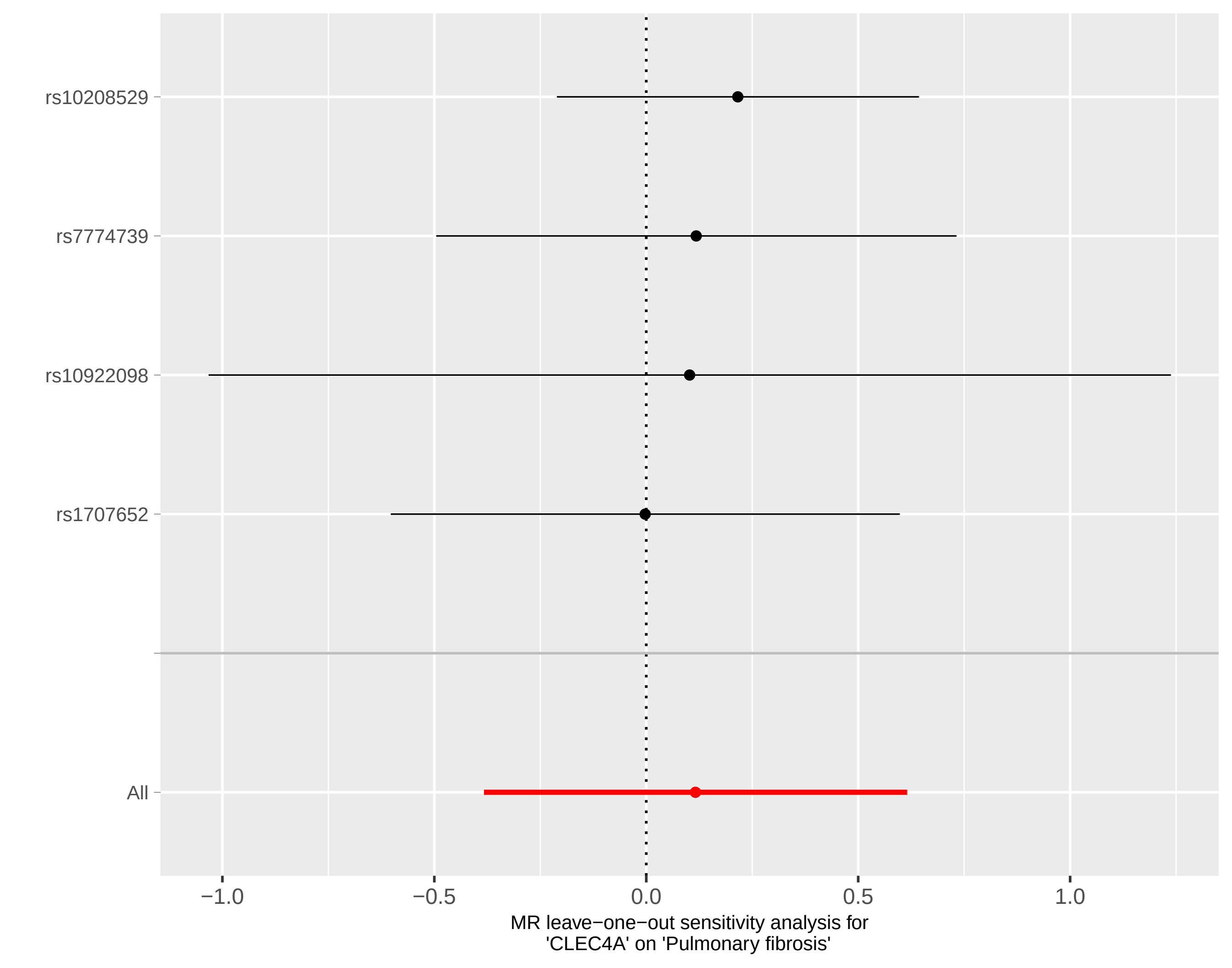
rs10208529
rs7774739
rs10922098
rs1707652
All
−1.0
−0.5
0.0
0.5
1.0
MR leave−one−out sensitivity analysis for
'CLEC4A' on 'Pulmonary fibrosis'

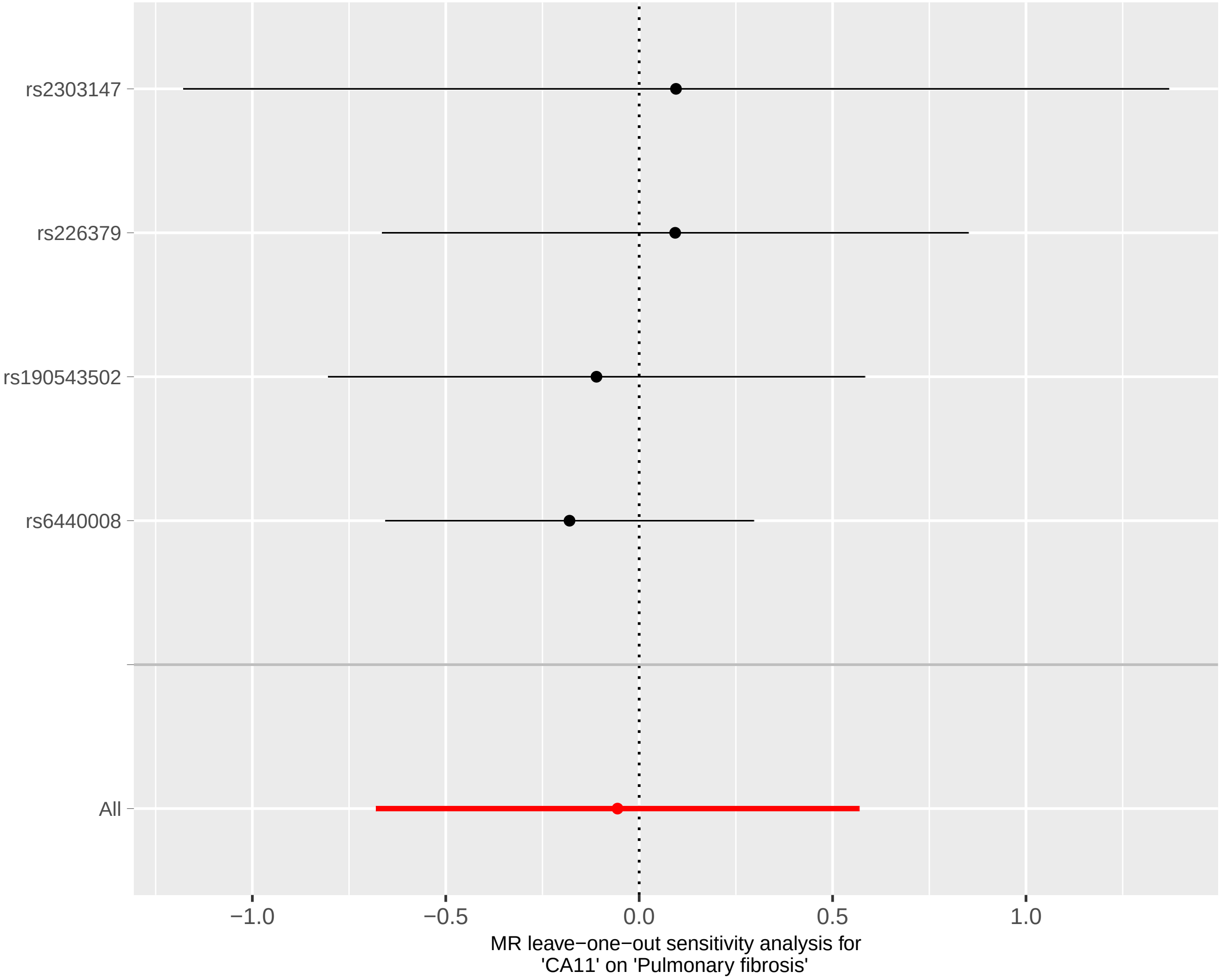
rs2303147
rs226379
rs190543502
rs6440008
All
−1.0
−0.5
0.0
0.5
1.0
MR leave−one−out sensitivity analysis for
'CA11' on 'Pulmonary fibrosis'

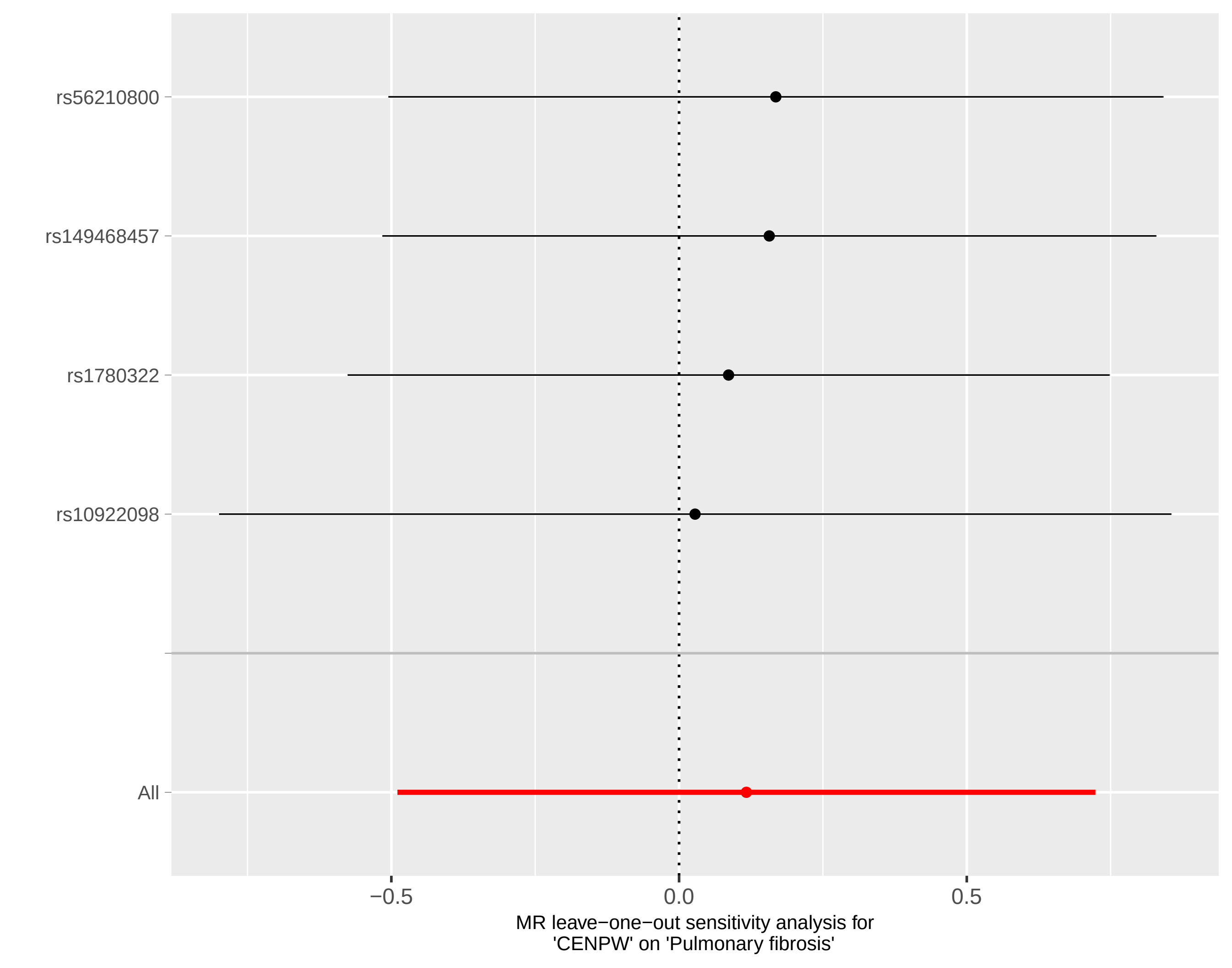
rs56210800
rs149468457
rs1780322
rs10922098
All
−0.5
0.0
0.5
MR leave−one−out sensitivity analysis for
'CENPW' on 'Pulmonary fibrosis'

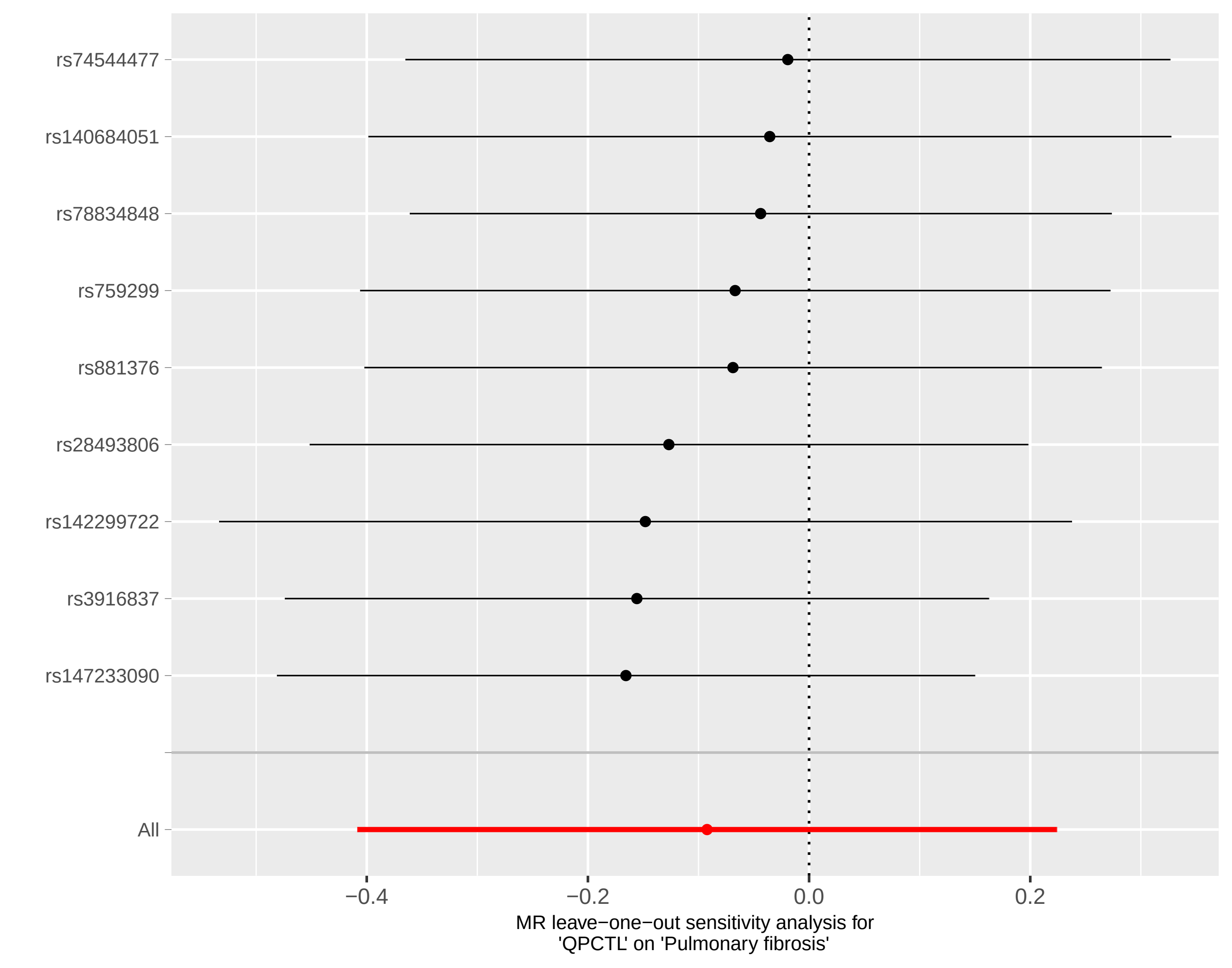
rs74544477
rs140684051
rs78834848
rs759299
rs881376
rs28493806
rs142299722
rs3916837
rs147233090
All
−0.4
−0.2
0.0
0.2
MR leave−one−out sensitivity analysis for
'QPCTL' on 'Pulmonary fibrosis'

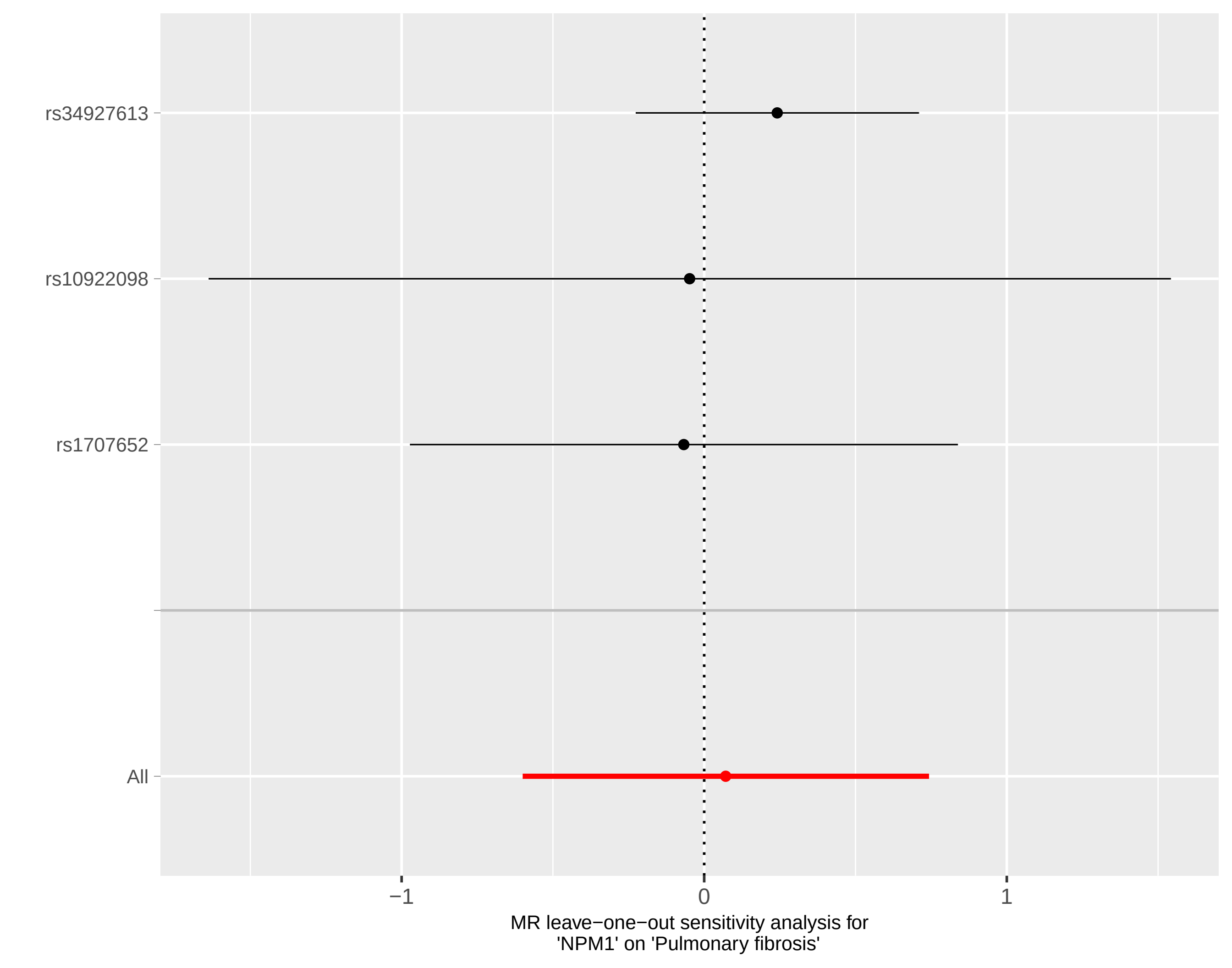
rs34927613
rs10922098
rs1707652
All
−1
0
1
MR leave−one−out sensitivity analysis for
'NPM1' on 'Pulmonary fibrosis'

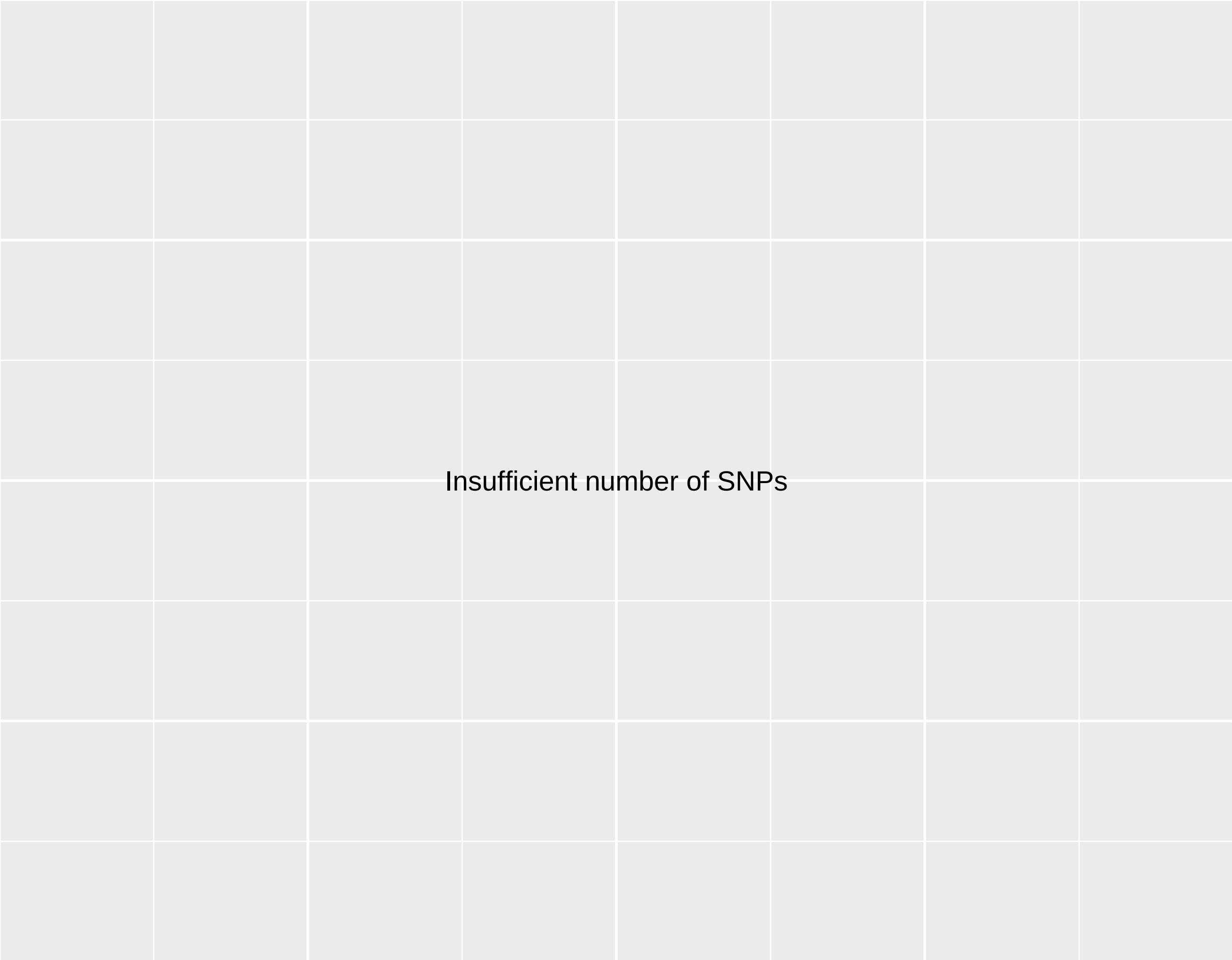
Insufficient number of SNPs

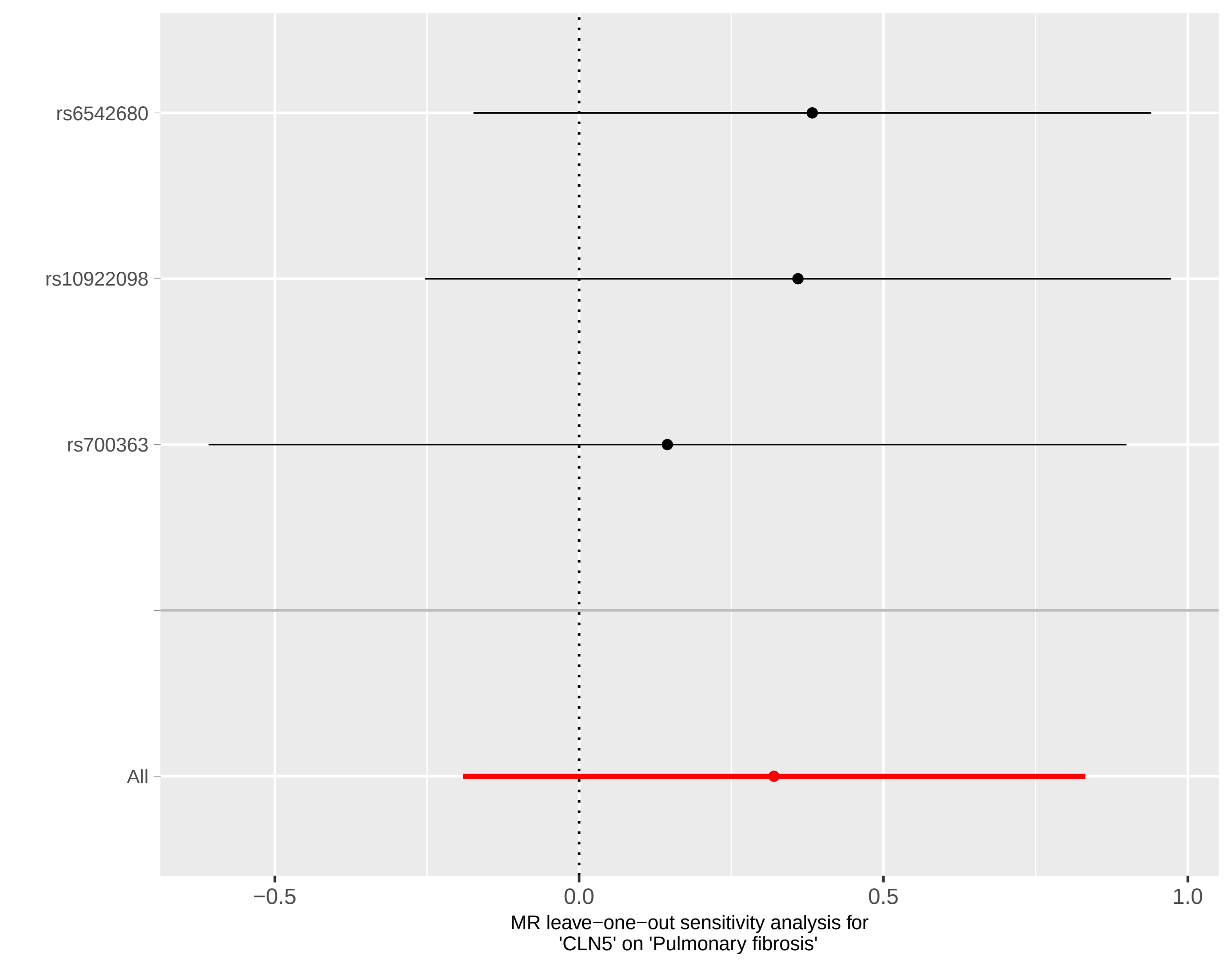

rs6542680
rs10922098
rs700363
All
−0.5
0.0
0.5
1.0
MR leave−one−out sensitivity analysis for
'CLN5' on 'Pulmonary fibrosis'

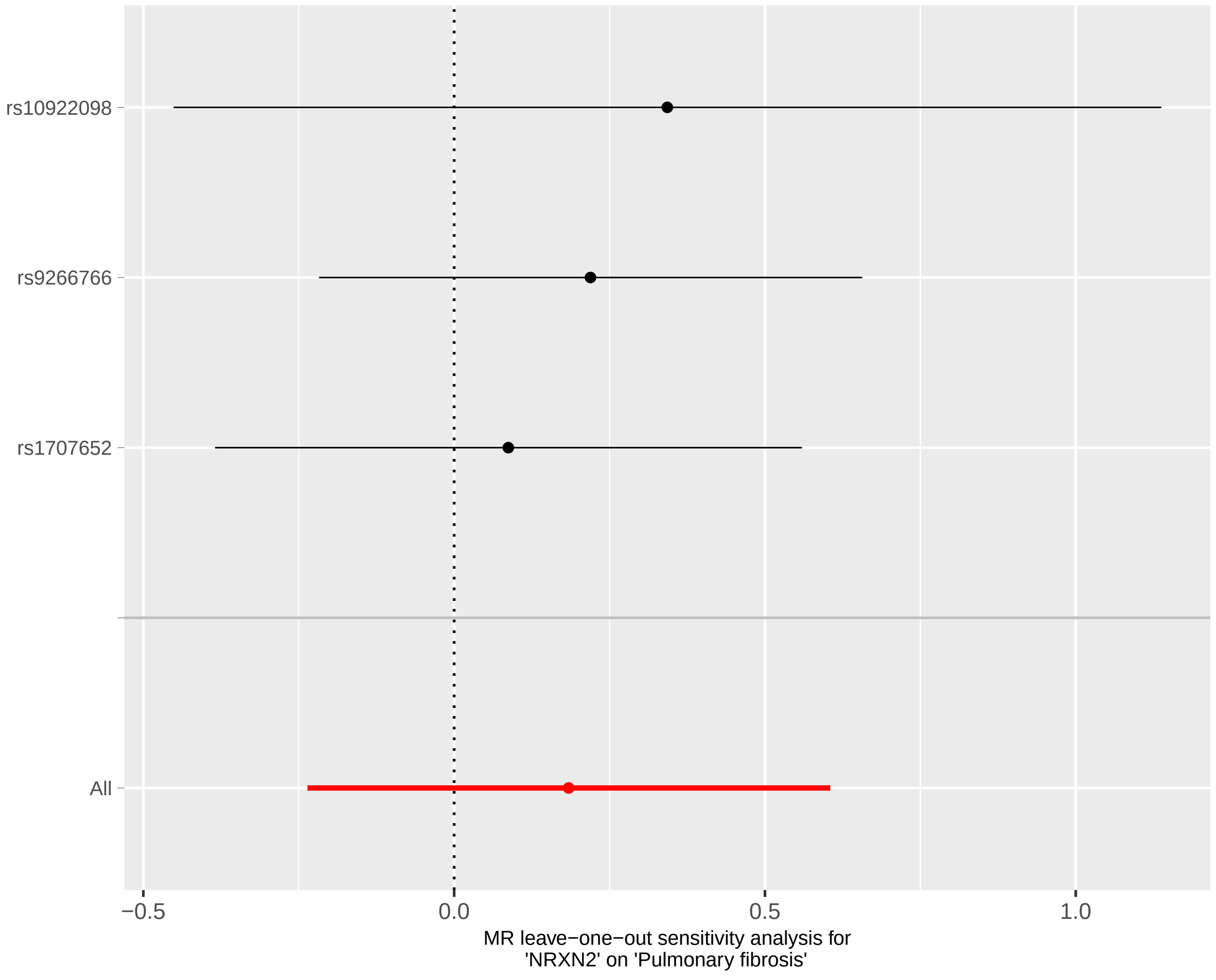
rs10922098
rs9266766
rs1707652
All
−0.5
0.0
0.5
1.0
MR leave−one−out sensitivity analysis for
'NRXN2' on 'Pulmonary fibrosis'

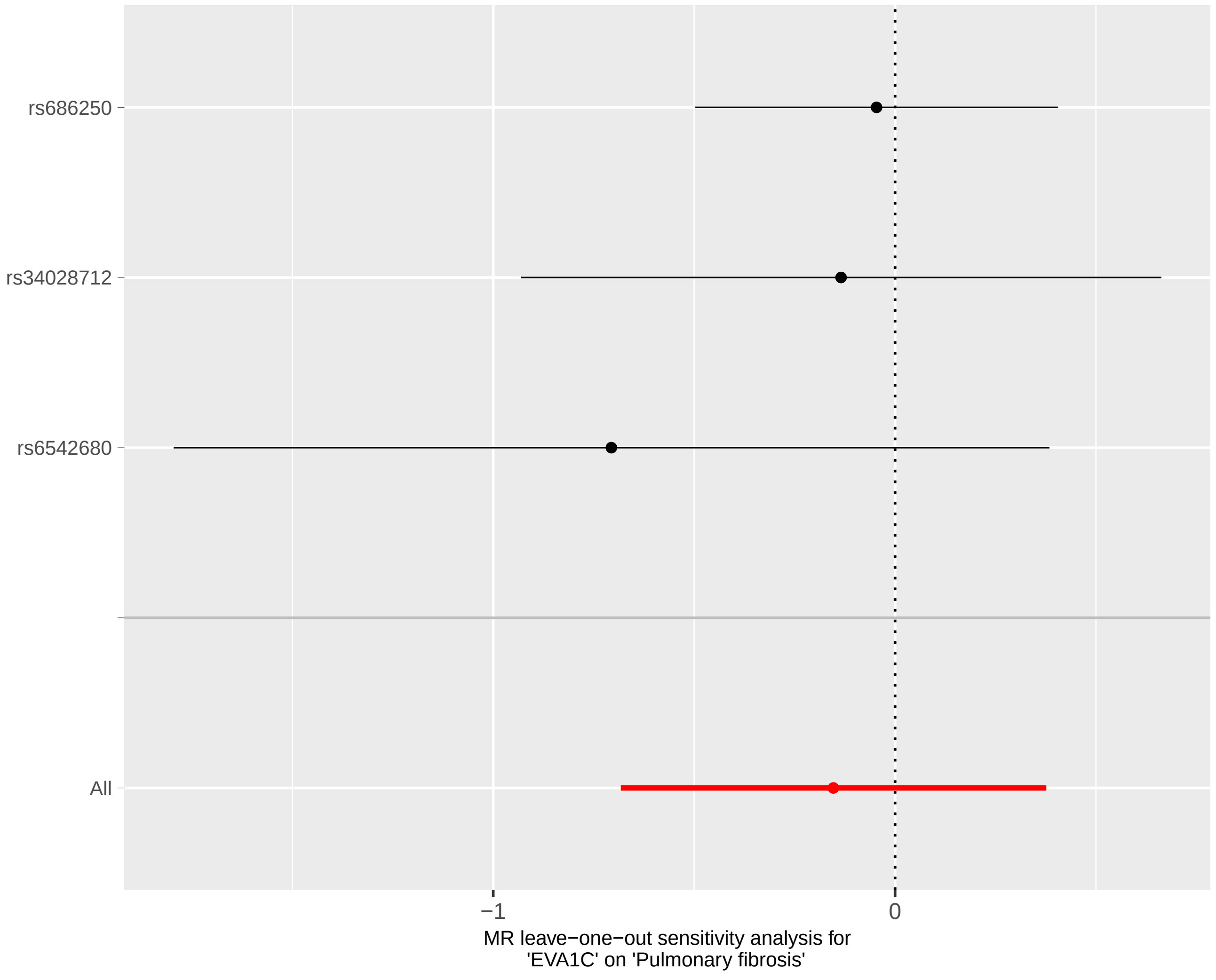
rs686250
rs34028712
rs6542680
All
−1
0
MR leave−one−out sensitivity analysis for
'EVA1C' on 'Pulmonary fibrosis'

Insufficient number of SNPs

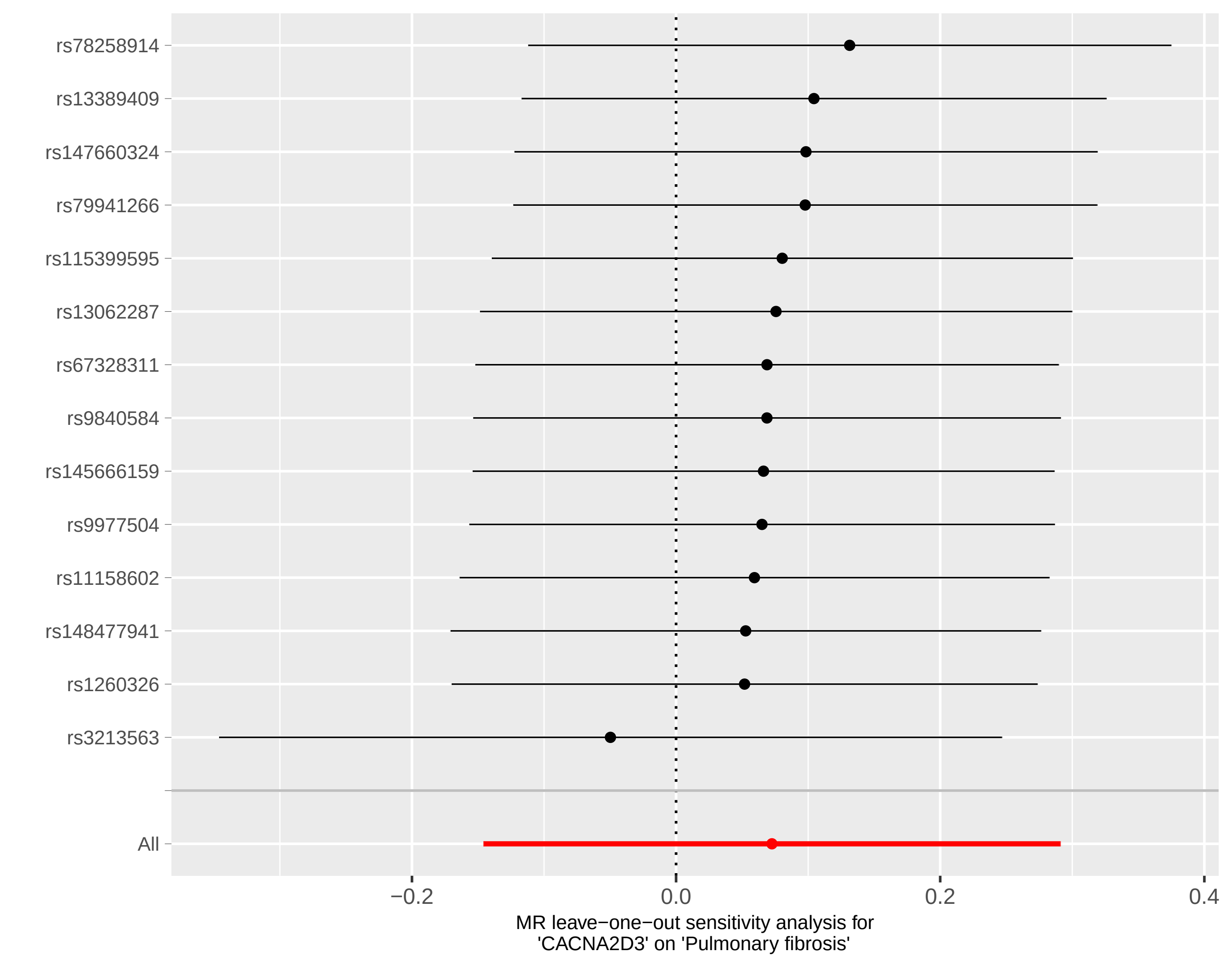

rs78258914
rs13389409
rs147660324
rs79941266
rs115399595
rs13062287
rs67328311
rs9840584
rs145666159
rs9977504
rs11158602
rs148477941
rs1260326
rs3213563
All
−0.2
0.0
0.2
0.4
MR leave−one−out sensitivity analysis for
'CACNA2D3' on 'Pulmonary fibrosis'

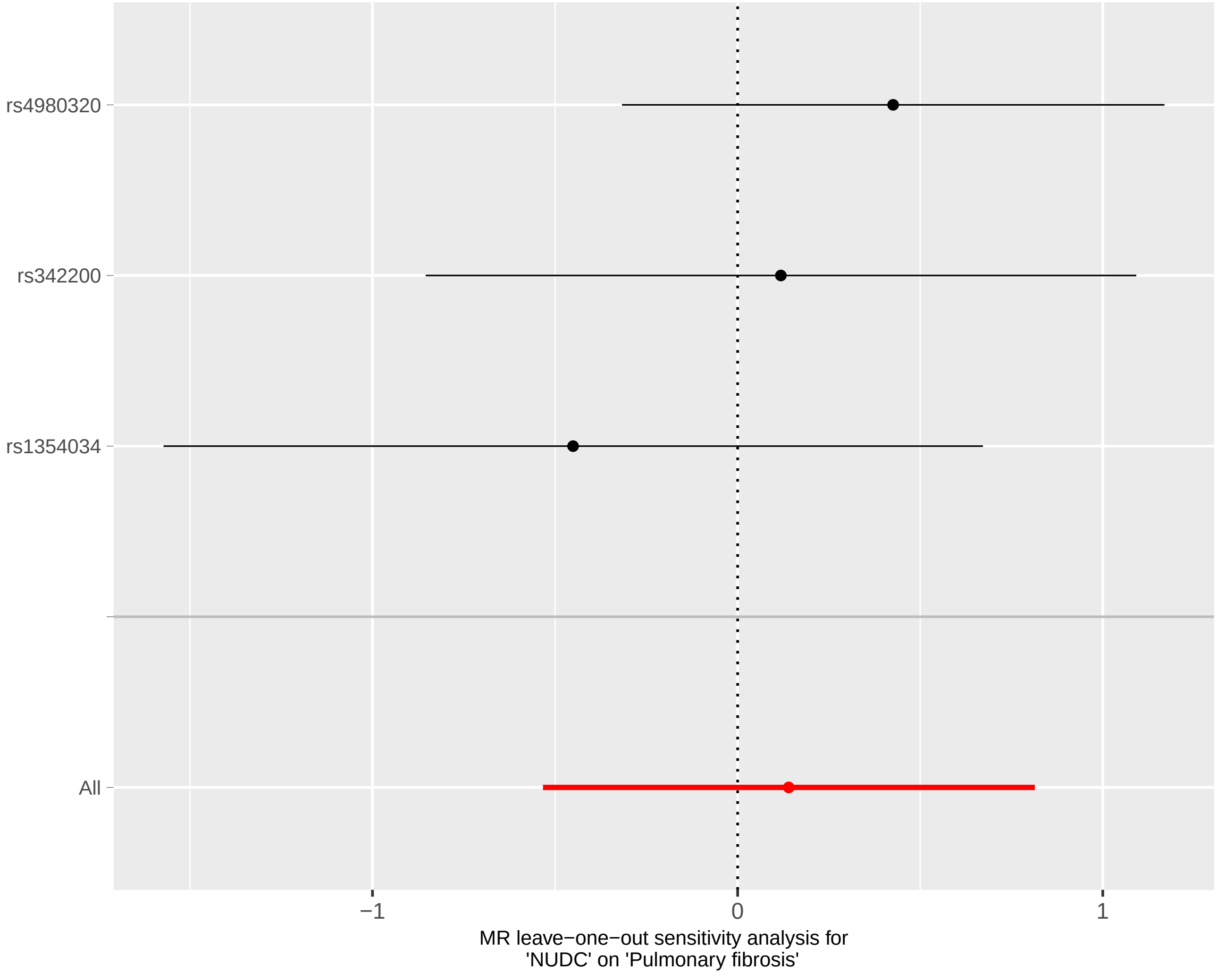
rs4980320
rs342200
rs1354034
All
−1
0
1
MR leave−one−out sensitivity analysis for
'NUDC' on 'Pulmonary fibrosis'

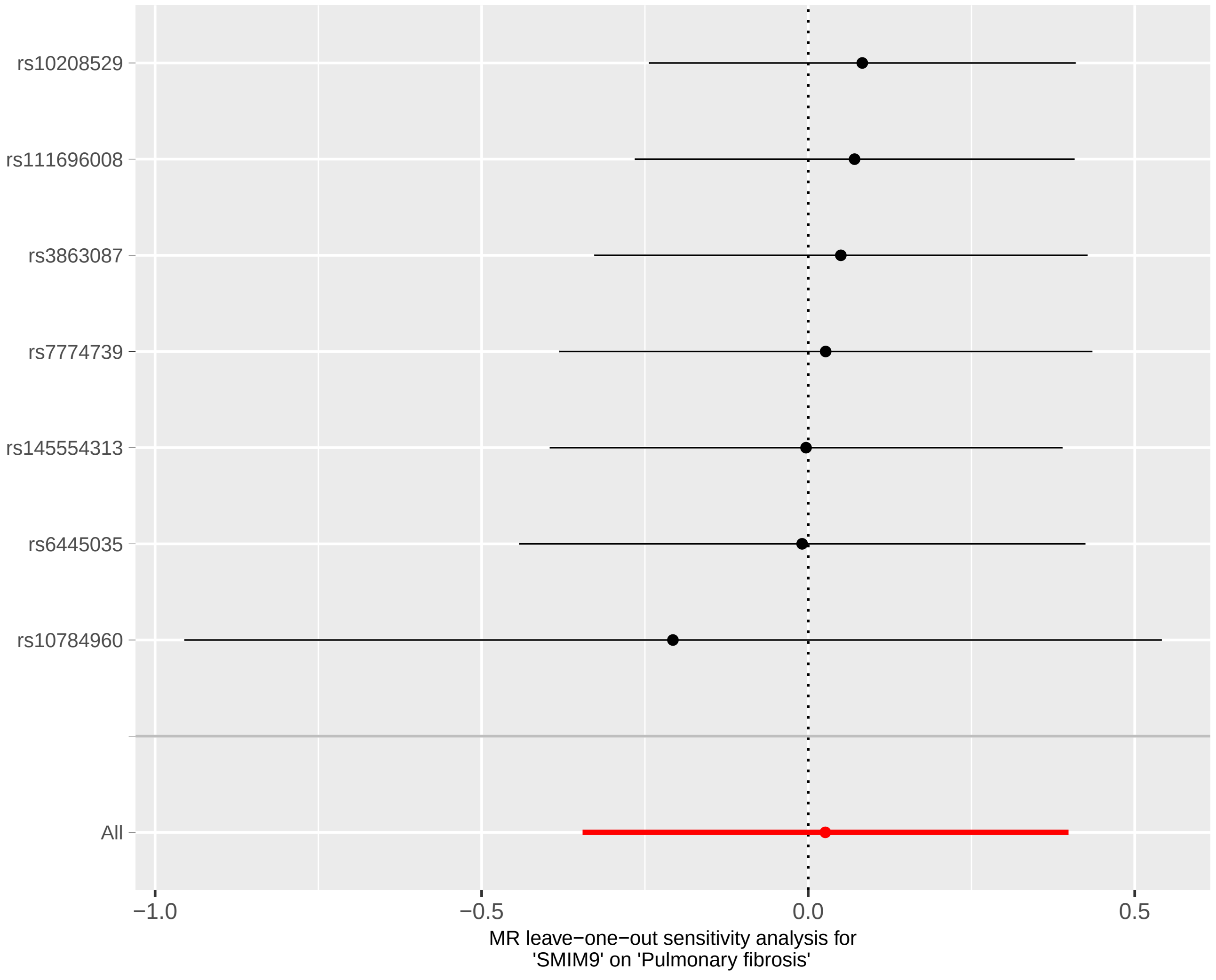
rs10208529
rs111696008
rs3863087
rs7774739
rs145554313
rs6445035
rs10784960
All
−1.0
−0.5
0.0
0.5
MR leave−one−out sensitivity analysis for
'SMIM9' on 'Pulmonary fibrosis'

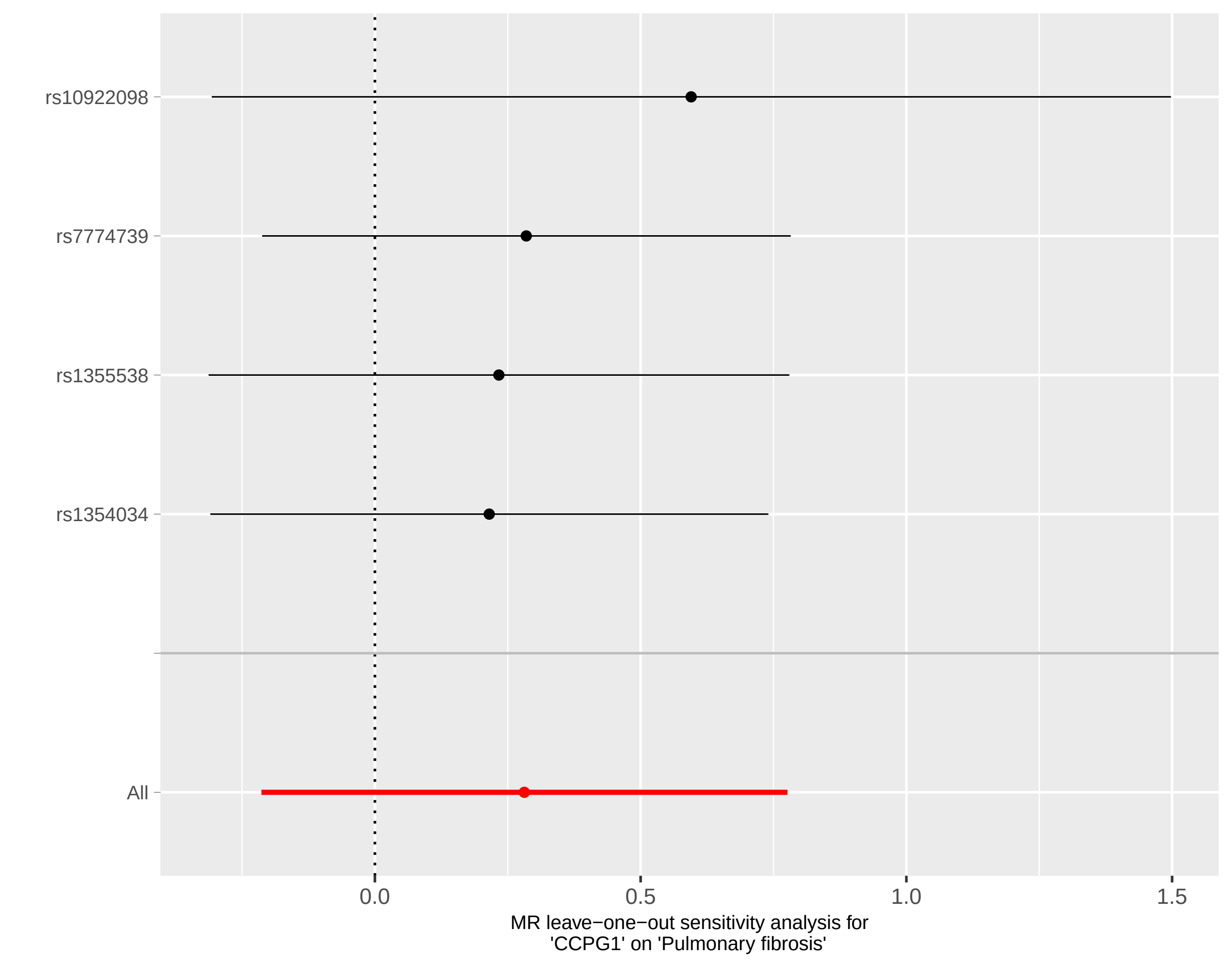
rs10922098
rs7774739
rs1355538
rs1354034
All
0.0
0.5
1.0
1.5
MR leave−one−out sensitivity analysis for
'CCPG1' on 'Pulmonary fibrosis'

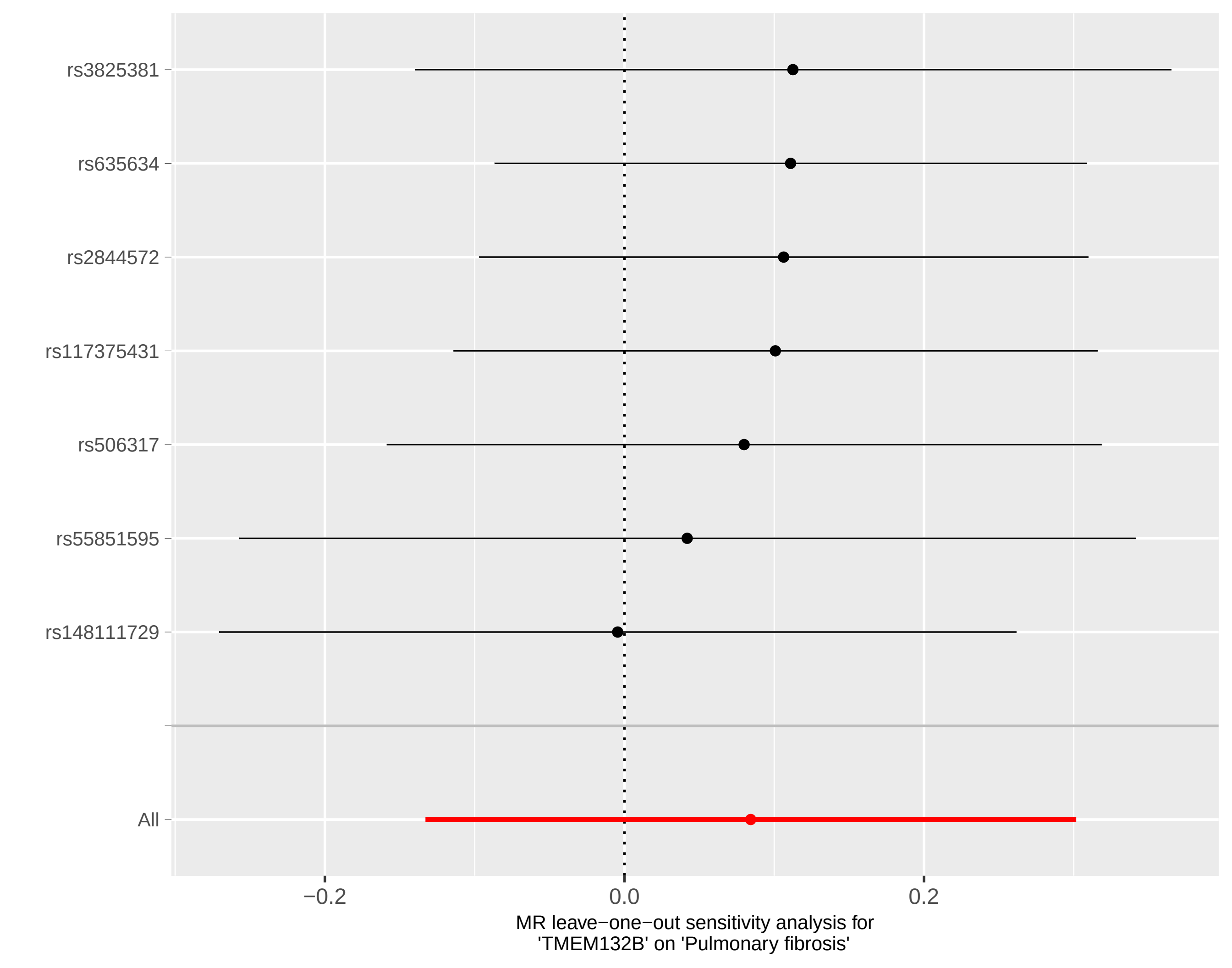

rs3825381
rs635634
rs2844572
rs117375431
rs506317
rs55851595
rs148111729
All
−0.2
0.0
0.2
MR leave−one−out sensitivity analysis for
'TMEM132B' on 'Pulmonary fibrosis'

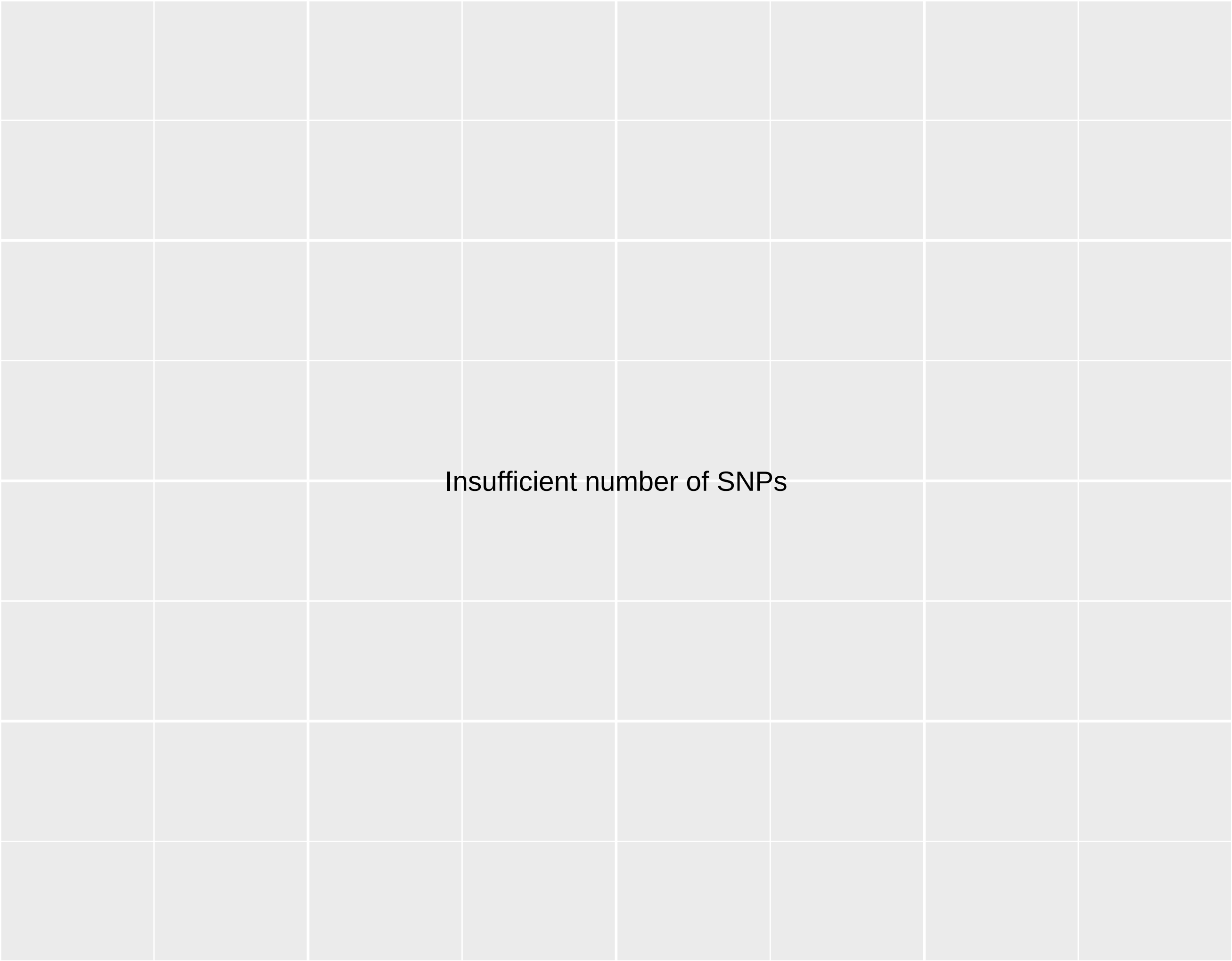
Insufficient number of SNPs

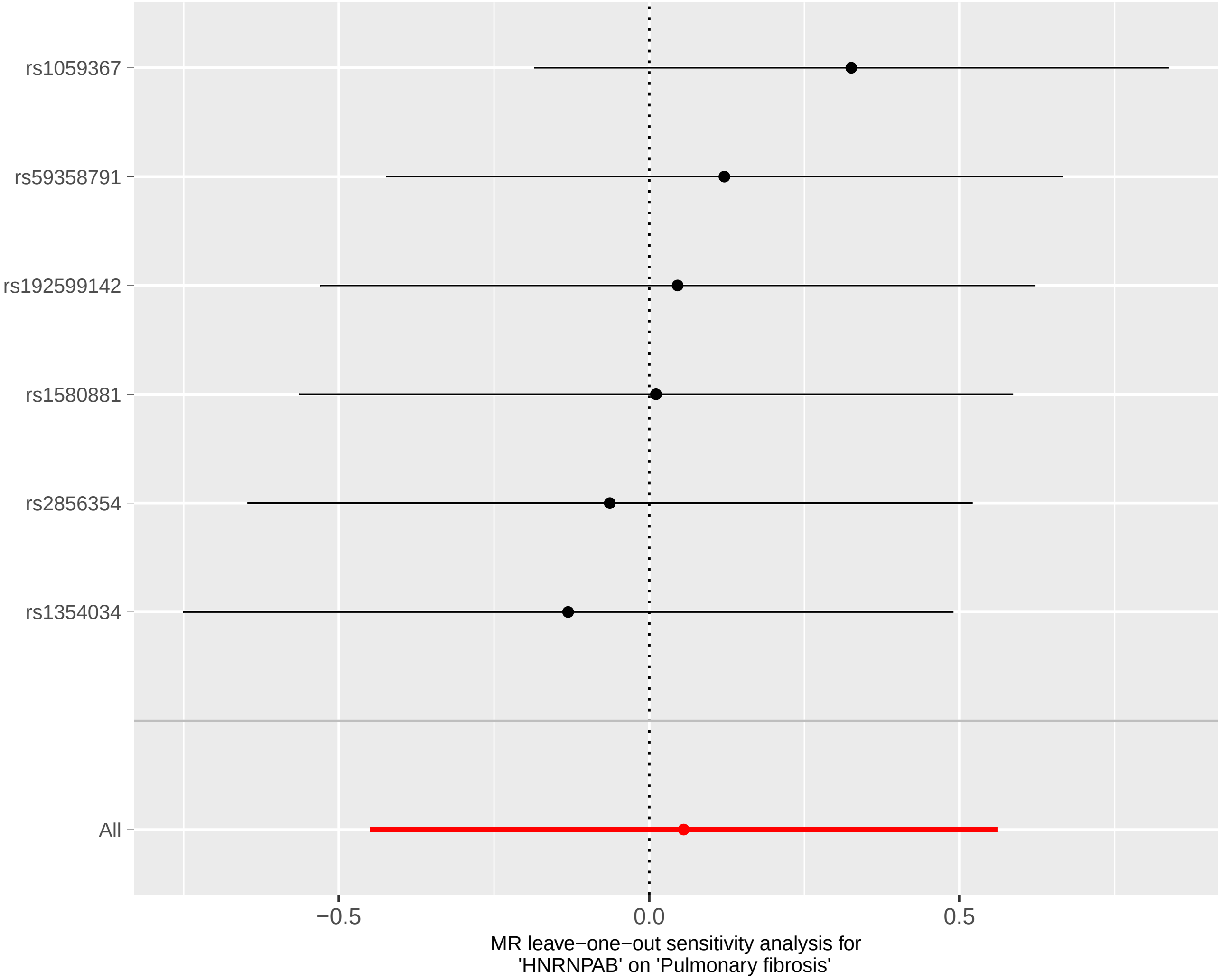
rs1059367
rs59358791
rs192599142
rs1580881
rs2856354
rs1354034
All
−0.5
0.0
0.5
MR leave−one−out sensitivity analysis for
'HNRNPAB' on 'Pulmonary fibrosis'

Insufficient number of SNPs

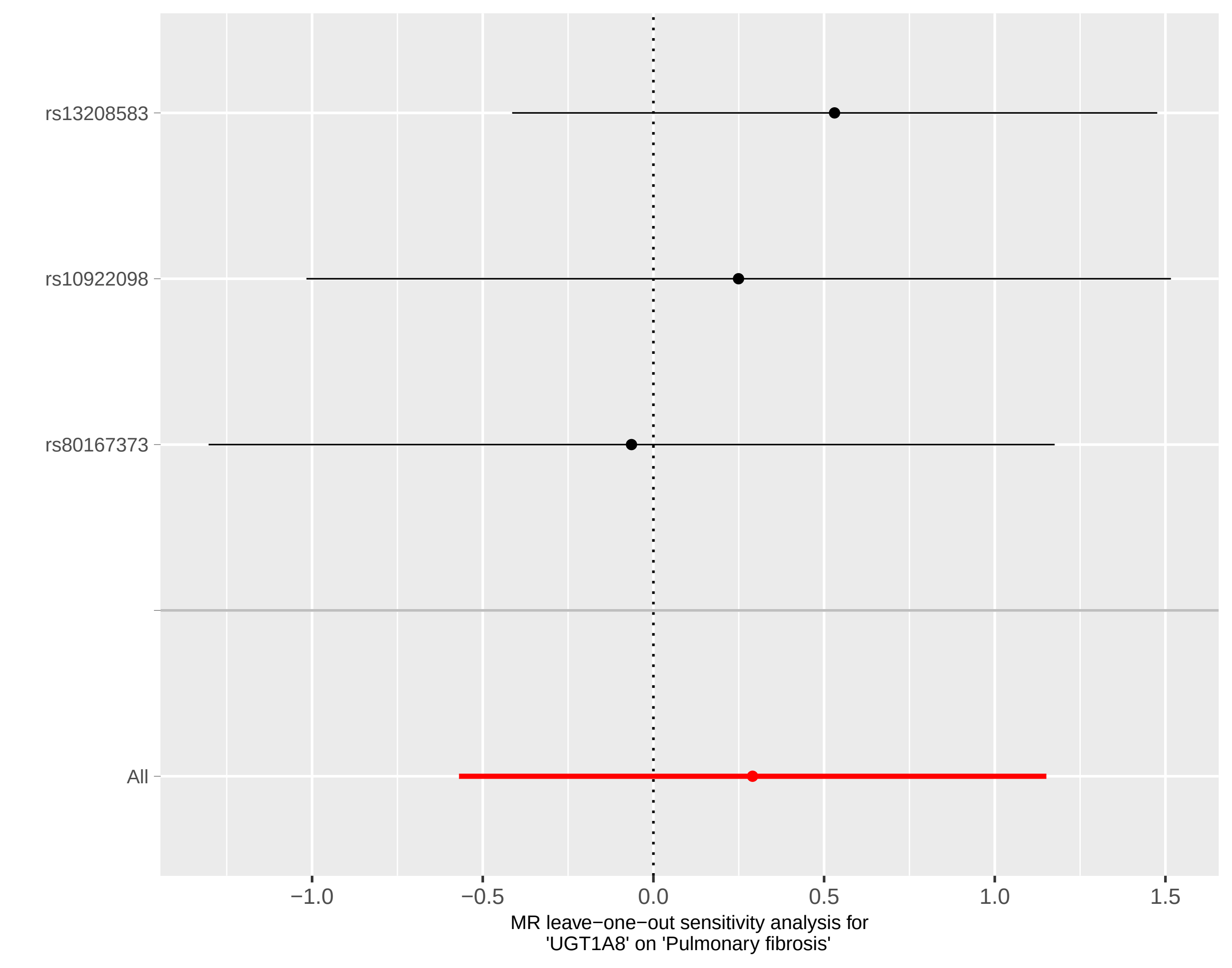
rs13208583
rs10922098
rs80167373
All
−1.0
−0.5
0.0
0.5
1.0
1.5
MR leave−one−out sensitivity analysis for
'UGT1A8' on 'Pulmonary fibrosis'

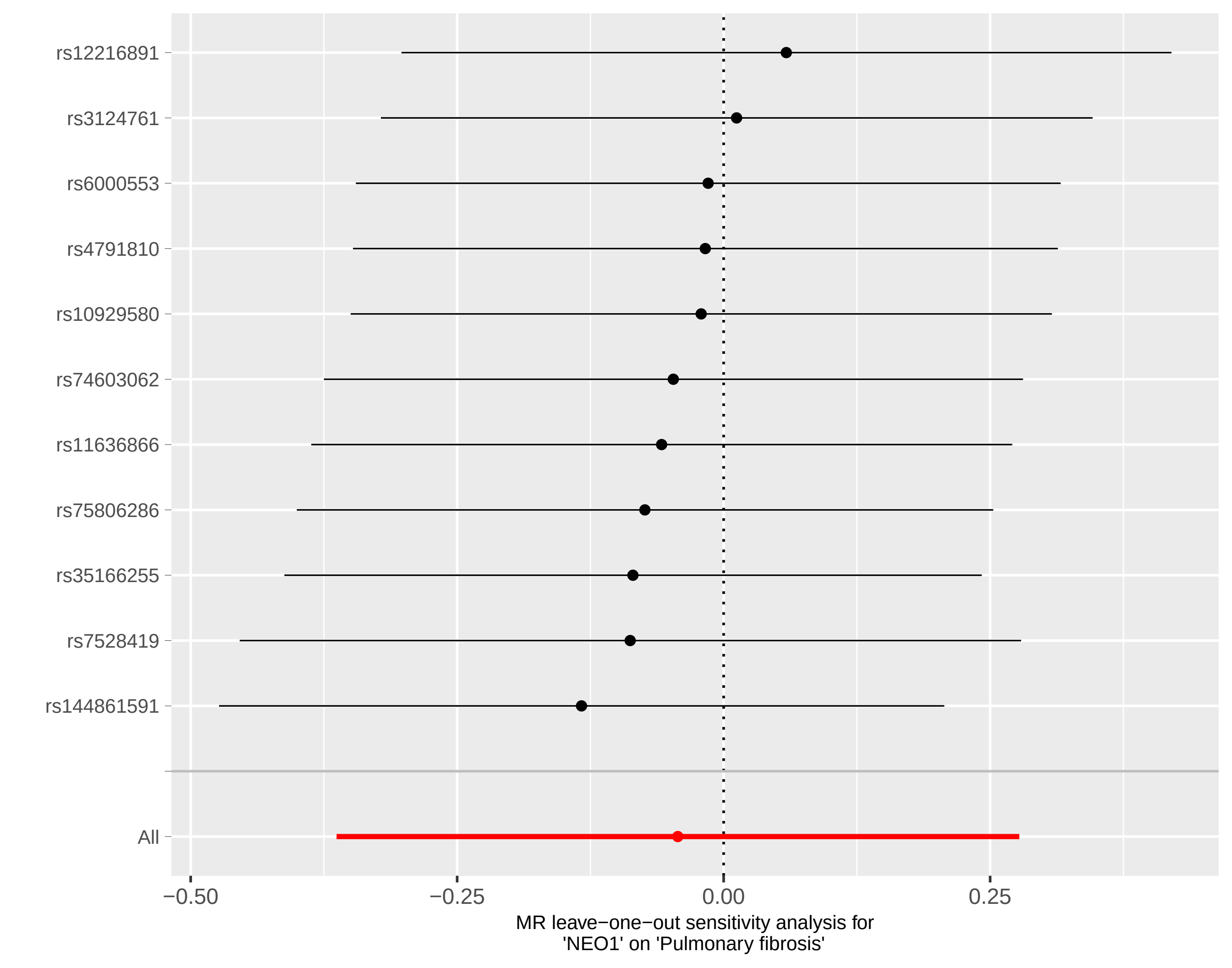

rs12216891
rs3124761
rs6000553
rs4791810
rs10929580
rs74603062
rs11636866
rs75806286
rs35166255
rs7528419
rs144861591
All
−0.50
−0.25
0.00
0.25
MR leave−one−out sensitivity analysis for
'NEO1' on 'Pulmonary fibrosis'

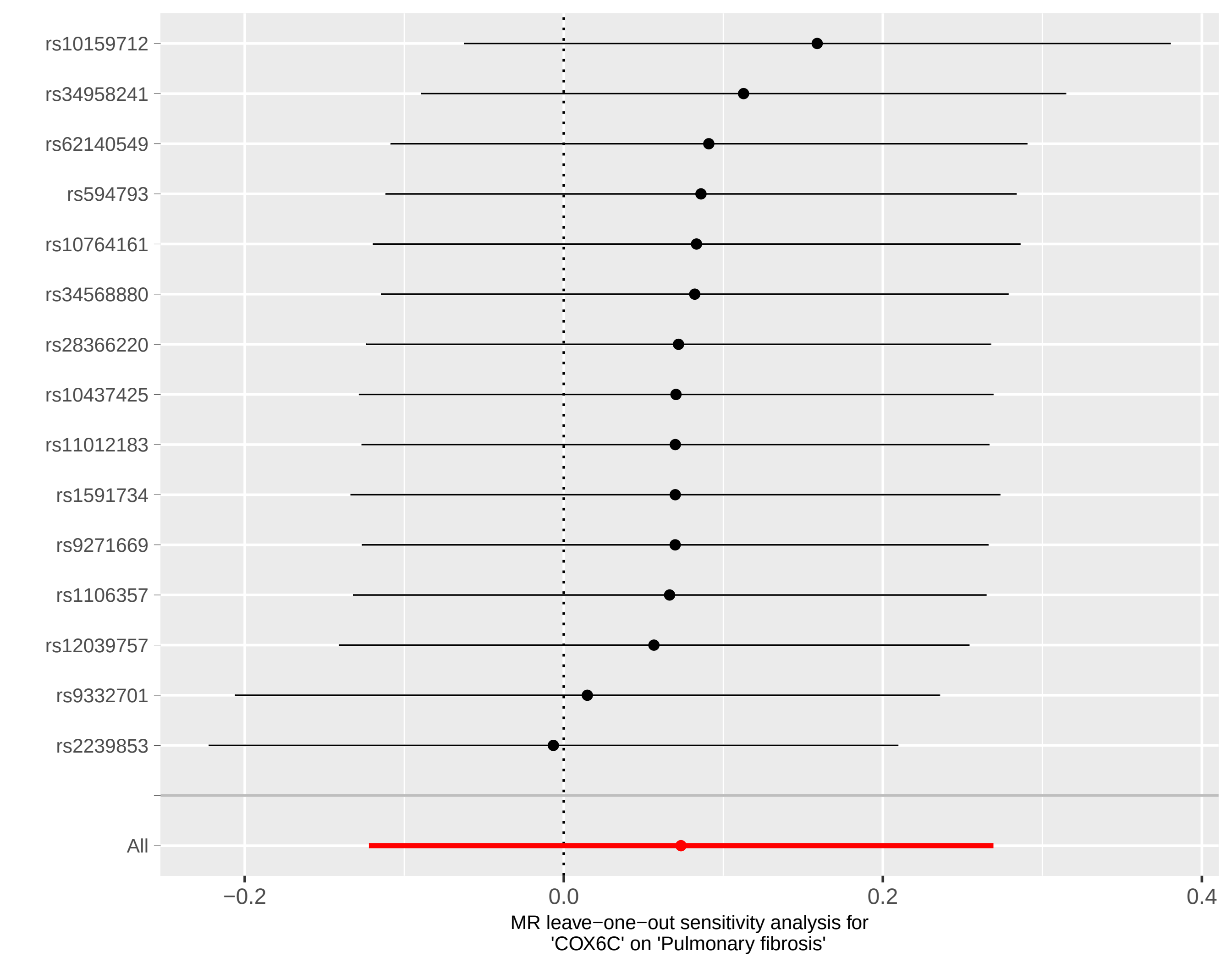
rs10159712
rs34958241
rs62140549
rs594793
rs10764161
rs34568880
rs28366220
rs10437425
rs11012183
rs1591734
rs9271669
rs1106357
rs12039757
rs9332701
rs2239853
All
−0.2
0.0
0.2
0.4
MR leave−one−out sensitivity analysis for
'COX6C' on 'Pulmonary fibrosis'

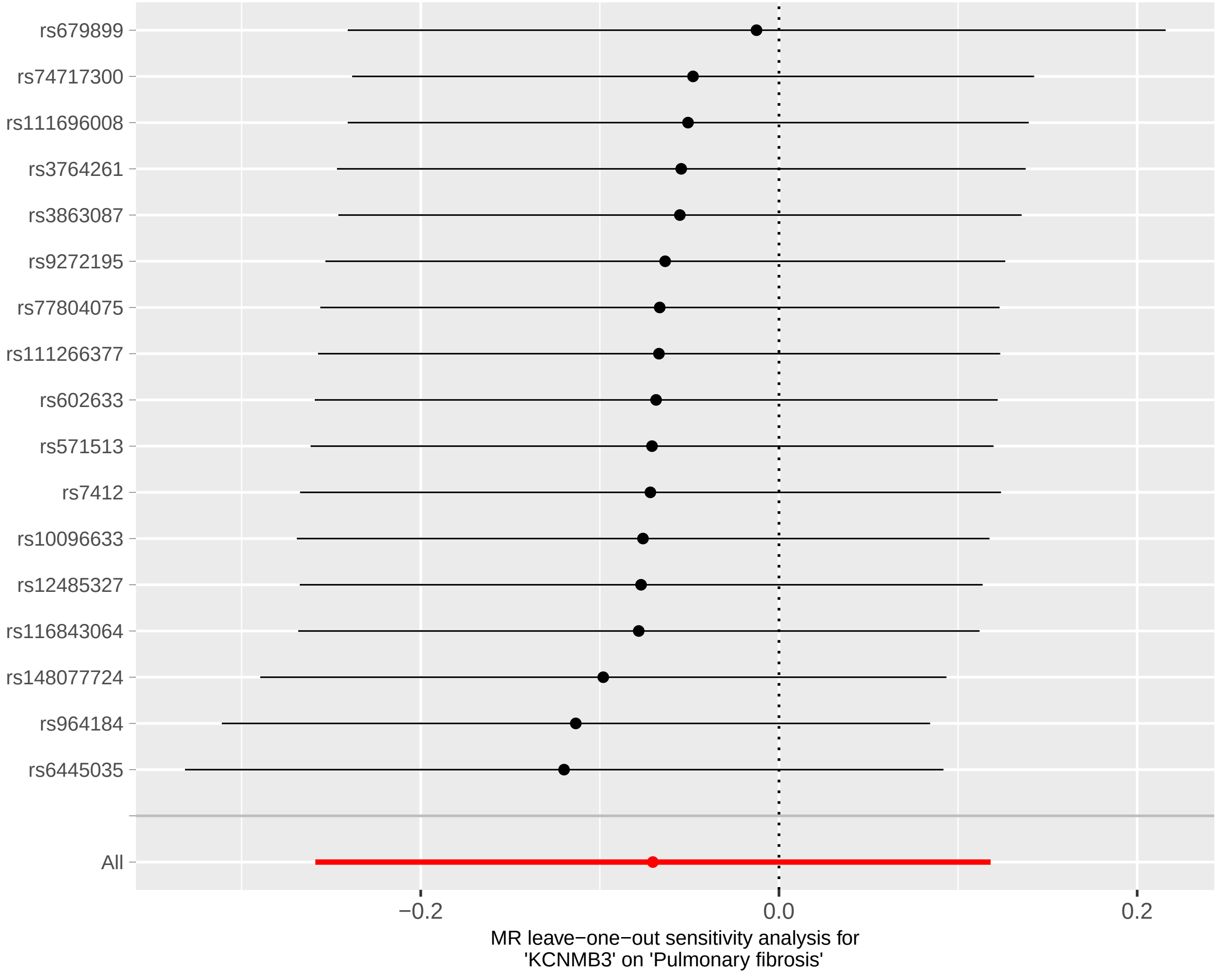
rs679899
rs74717300
rs111696008
rs3764261
rs3863087
rs9272195
rs77804075
rs111266377
rs602633
rs571513
rs7412
rs10096633
rs12485327
rs116843064
rs148077724
rs964184
rs6445035
All
−0.2
0.0
0.2
MR leave−one−out sensitivity analysis for
'KCNMB3' on 'Pulmonary fibrosis'

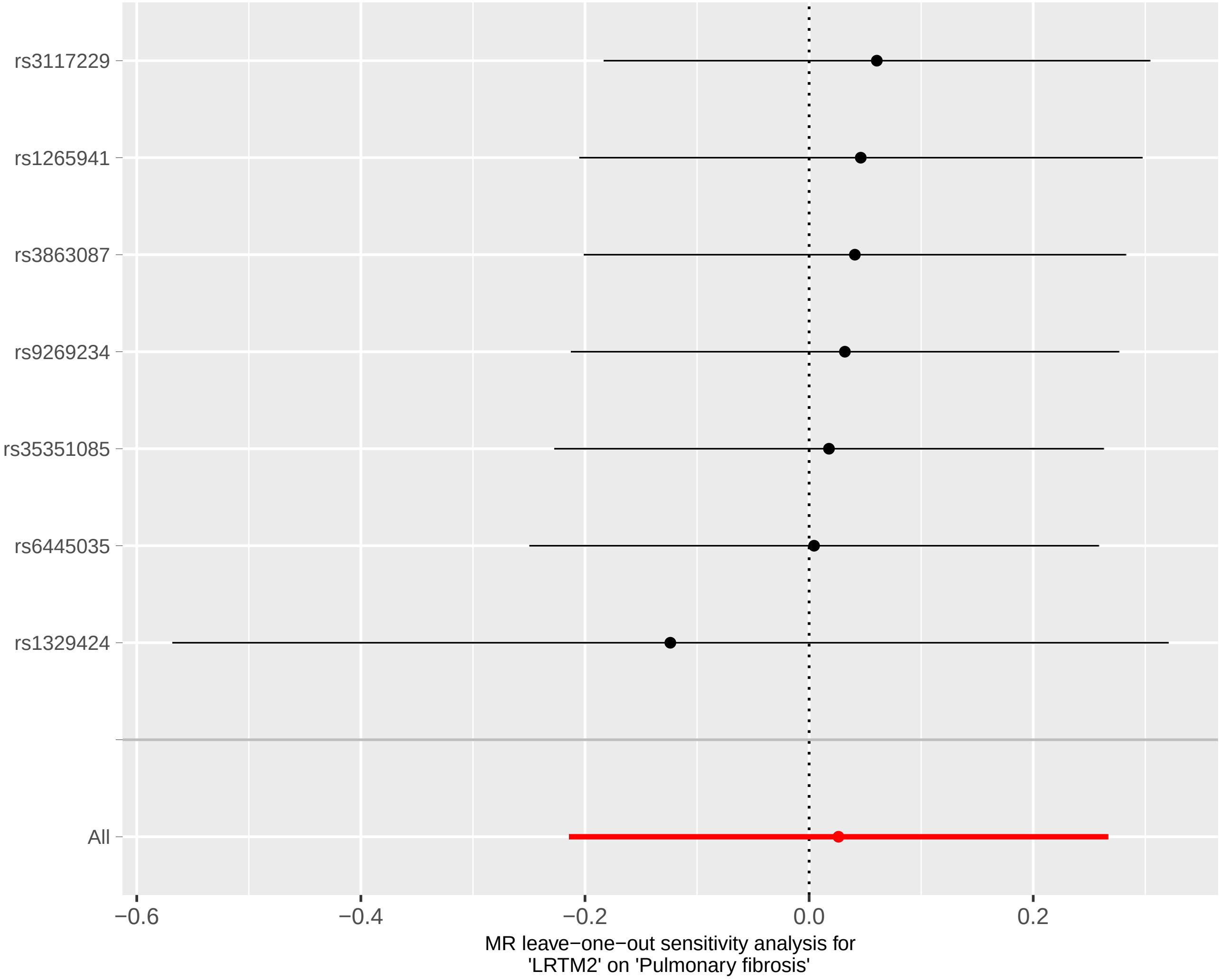

rs3117229
rs1265941
rs3863087
rs9269234
rs35351085
rs6445035
rs1329424
All
−0.6
−0.4
−0.2
0.0
0.2
MR leave−one−out sensitivity analysis for
'LRTM2' on 'Pulmonary fibrosis'

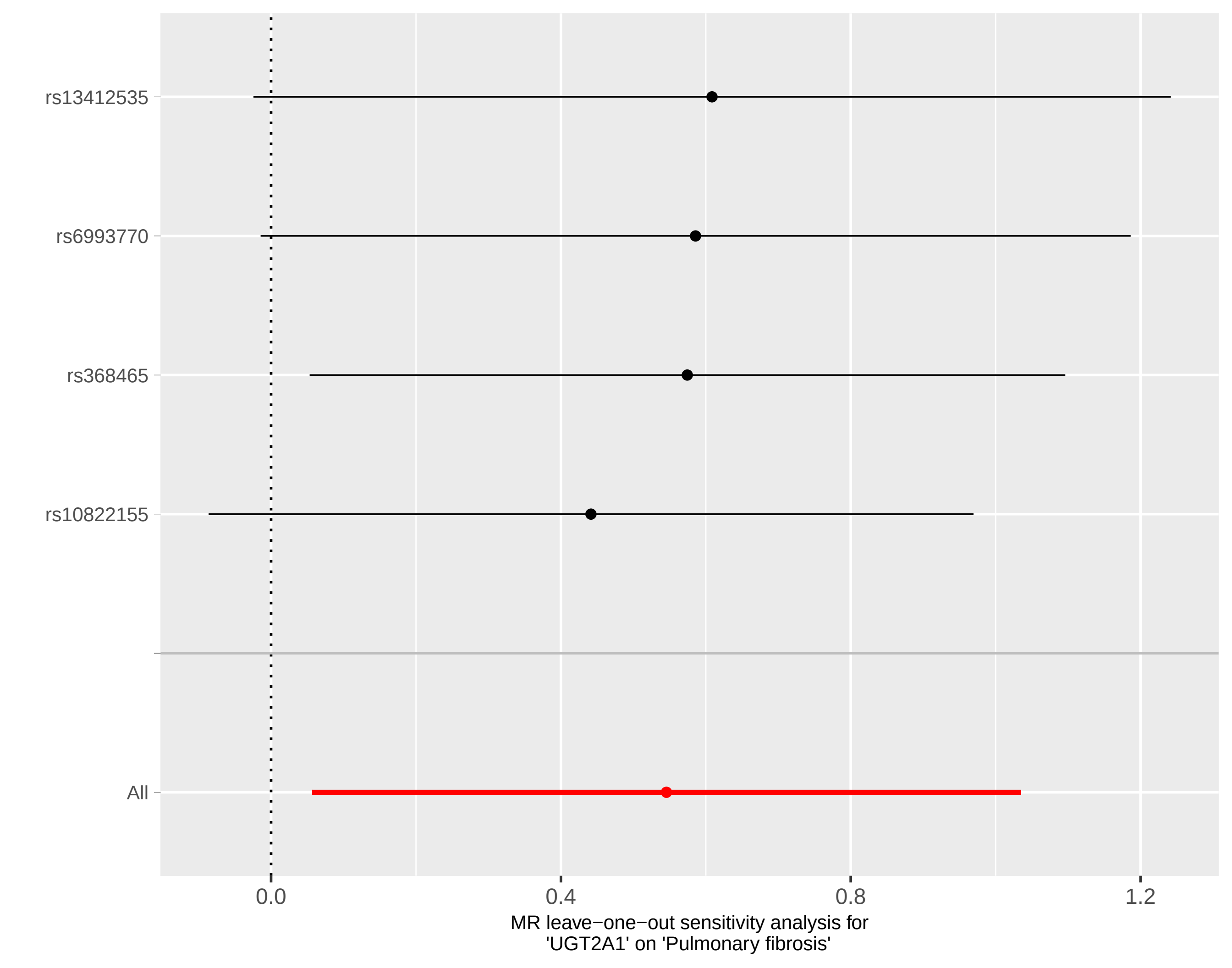
rs13412535
rs6993770
rs368465
rs10822155
All
0.0
0.4
0.8
1.2
MR leave−one−out sensitivity analysis for
'UGT2A1' on 'Pulmonary fibrosis'

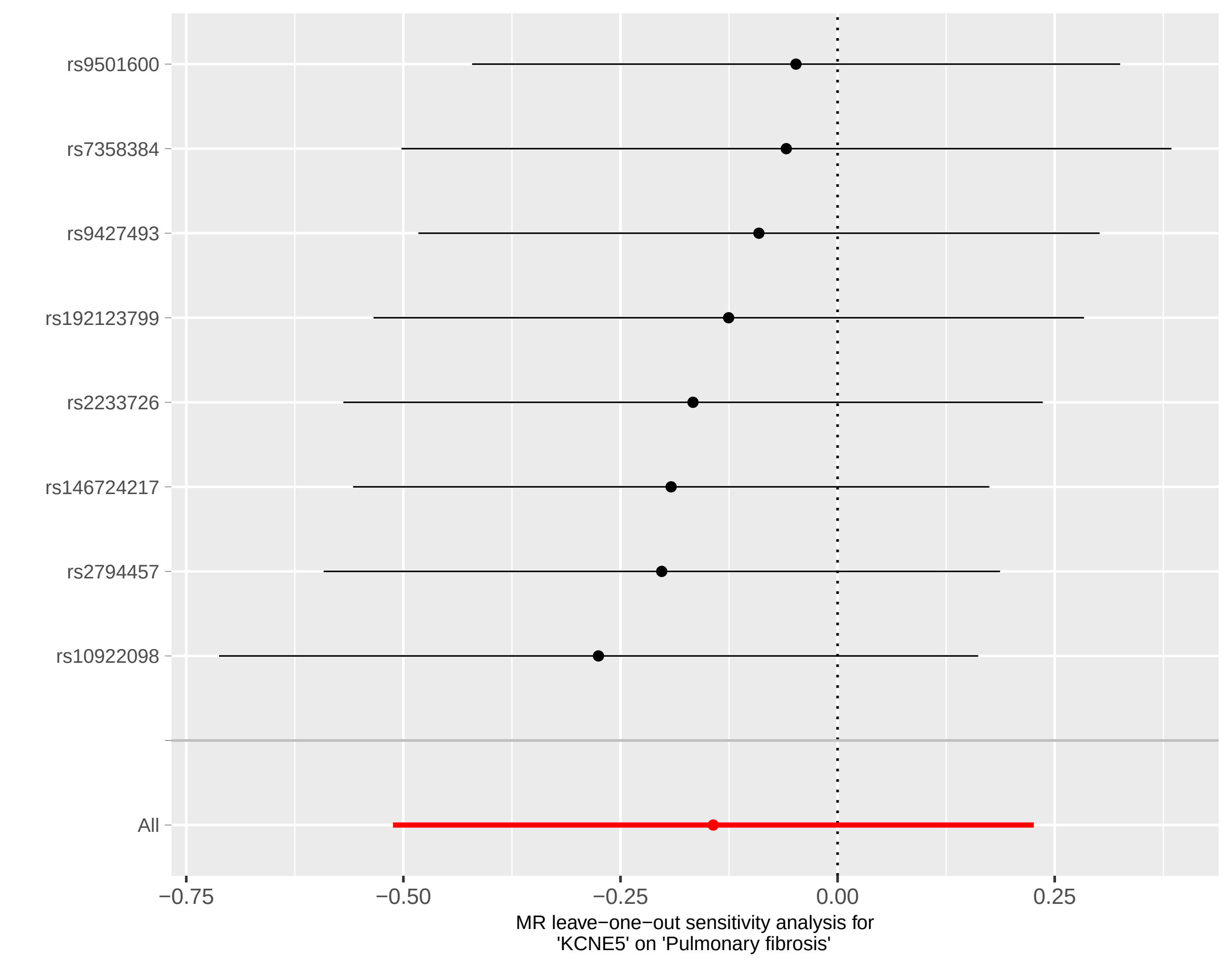
rs9501600
rs7358384
rs9427493
rs192123799
rs2233726
rs146724217
rs2794457
rs10922098
All
−0.75
−0.50
−0.25
0.00
0.25
MR leave−one−out sensitivity analysis for
'KCNE5' on 'Pulmonary fibrosis'

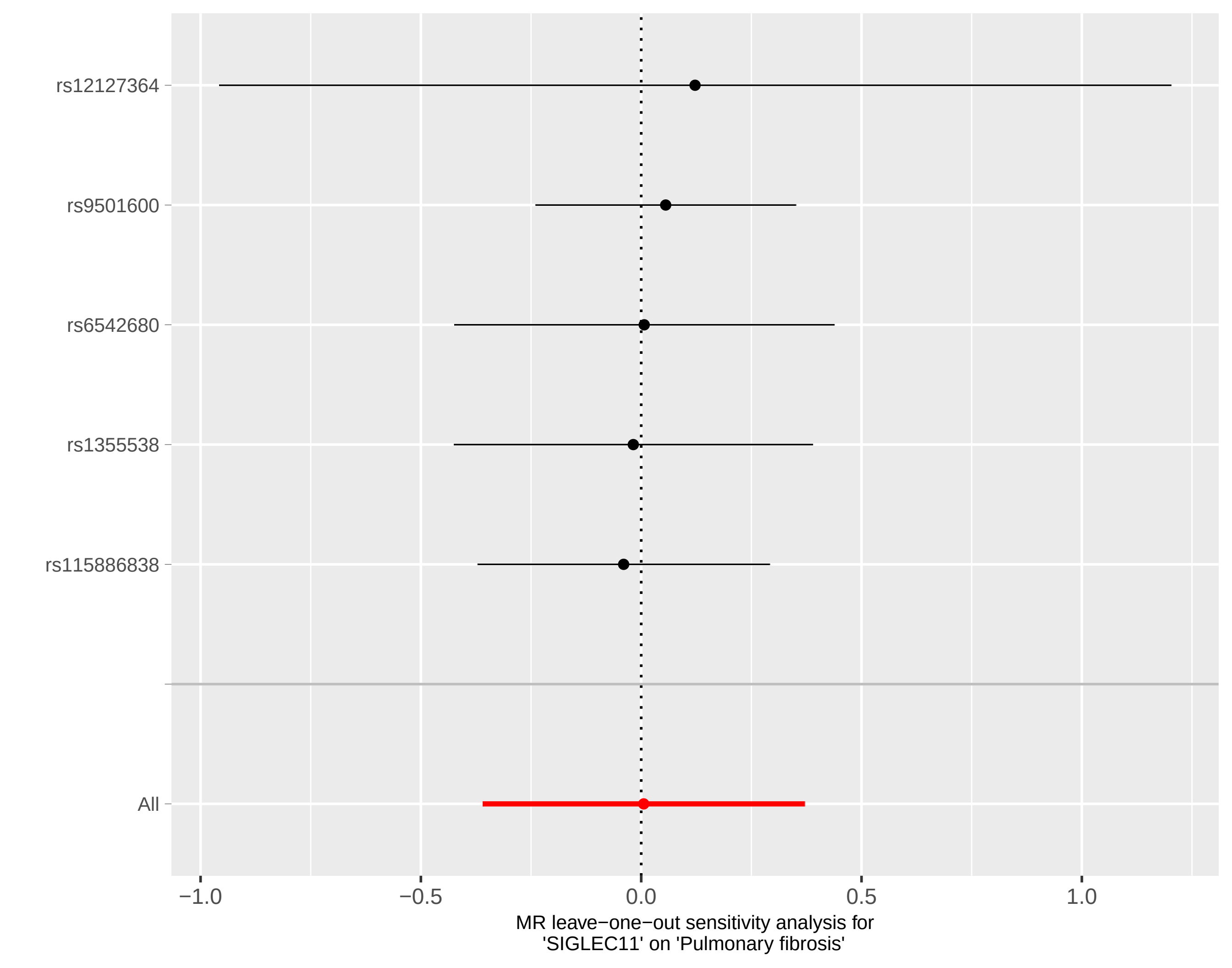

rs12127364
rs9501600
rs6542680
rs1355538
rs115886838
All
−1.0
−0.5
0.0
0.5
1.0
MR leave−one−out sensitivity analysis for
'SIGLEC11' on 'Pulmonary fibrosis'

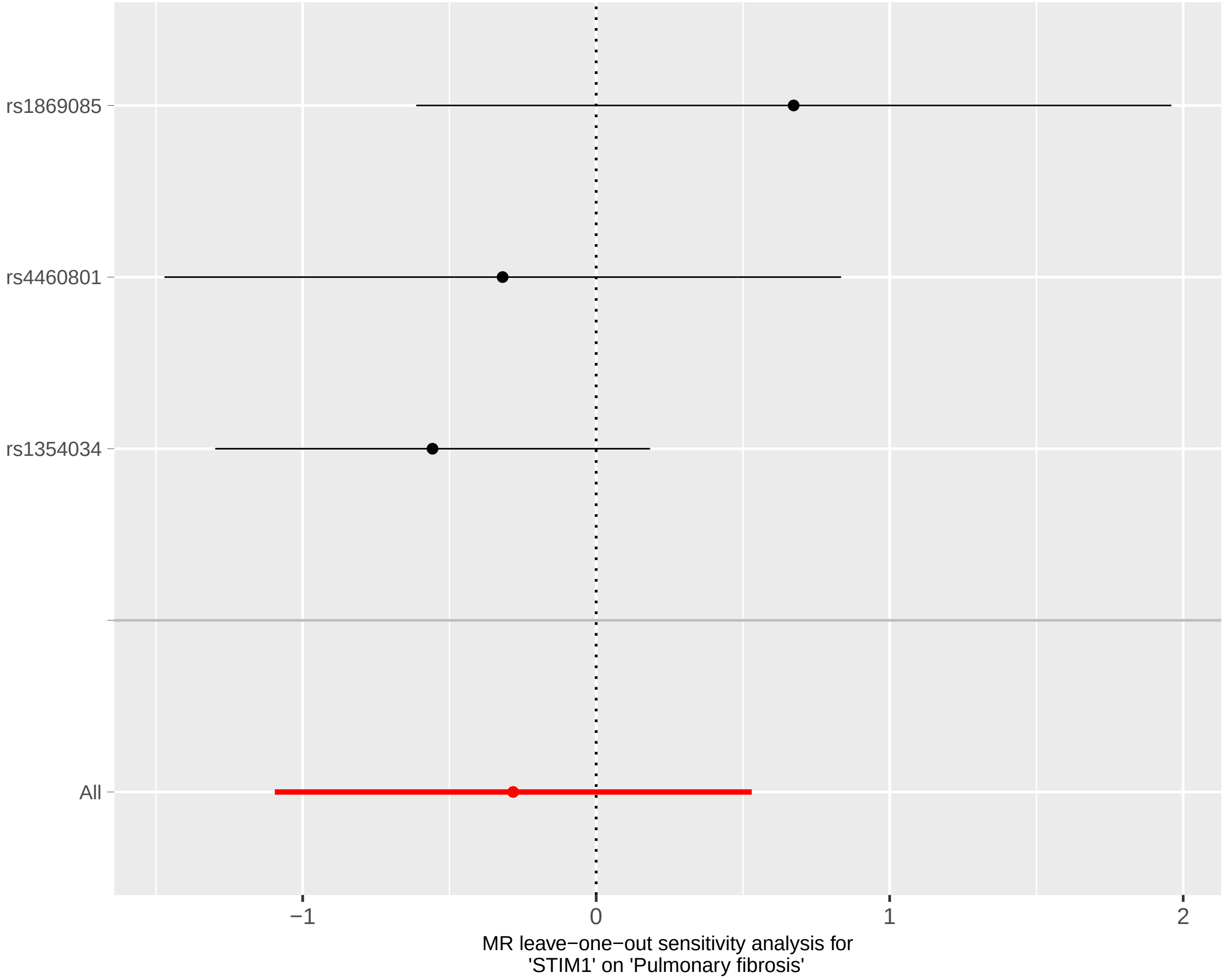

rs1869085
rs4460801
rs1354034
All
−1
0
1
2
MR leave−one−out sensitivity analysis for
'STIM1' on 'Pulmonary fibrosis'

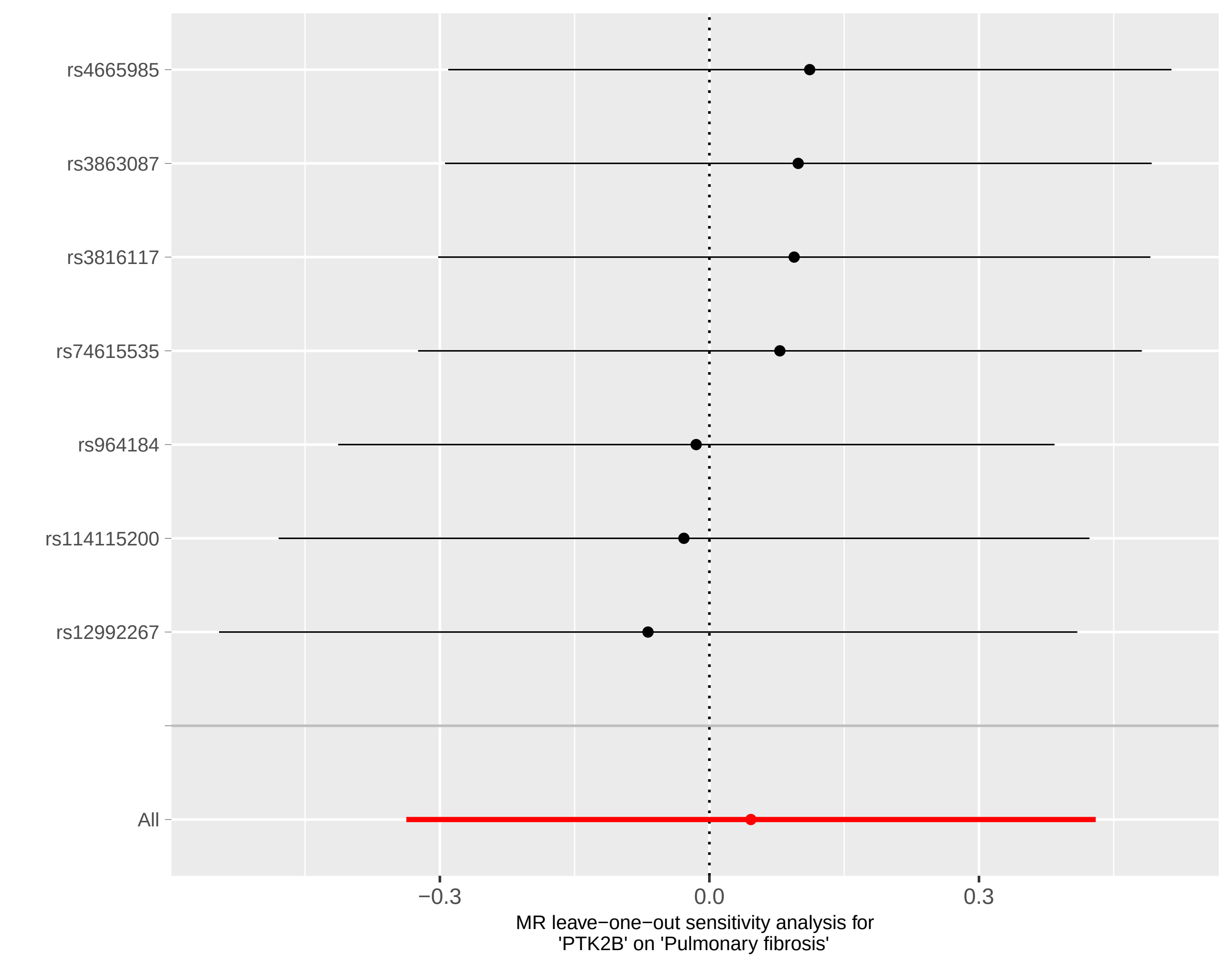
rs4665985
rs3863087
rs3816117
rs74615535
rs964184
rs114115200
rs12992267
All
−0.3
0.0
0.3
MR leave−one−out sensitivity analysis for
'PTK2B' on 'Pulmonary fibrosis'

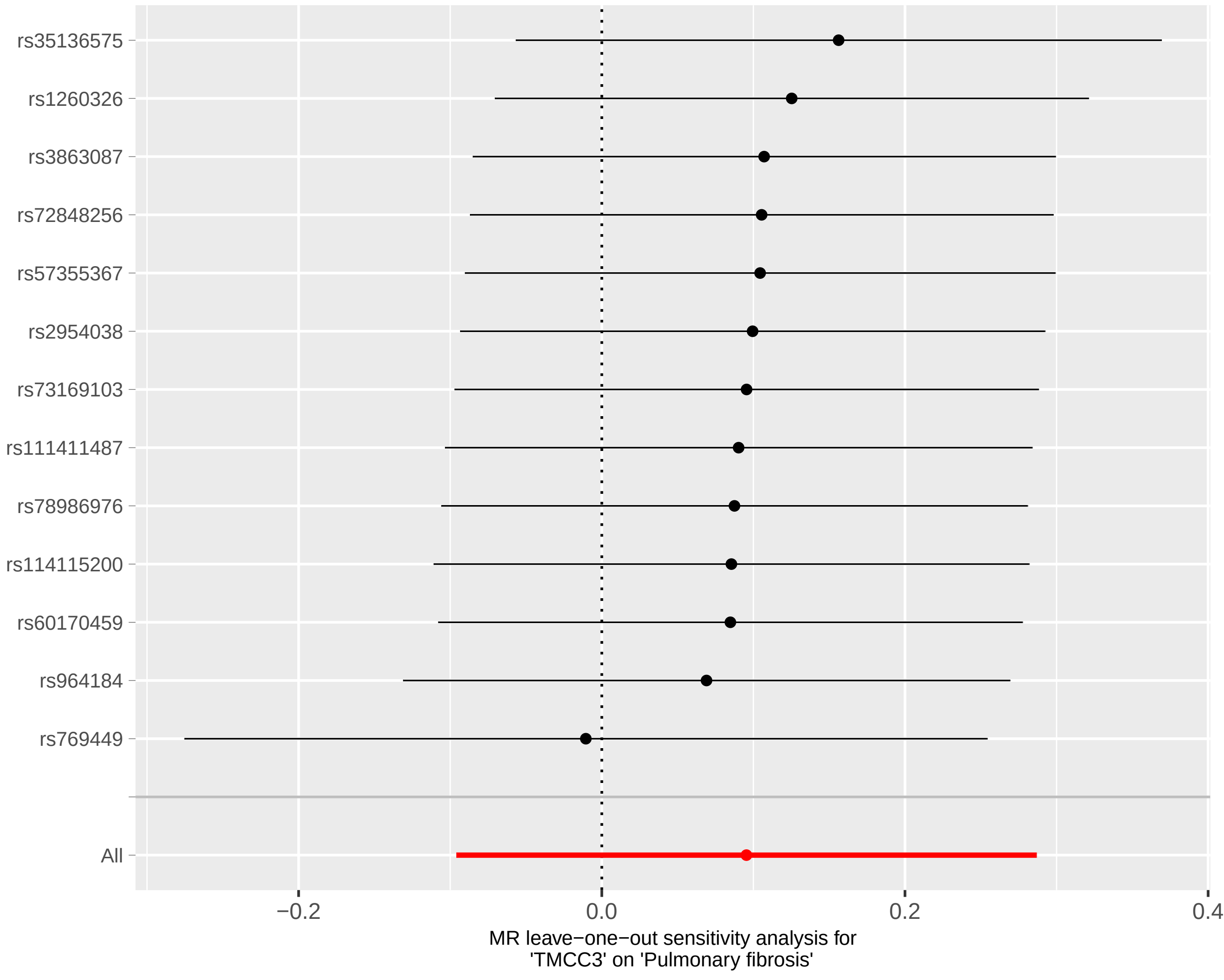
rs35136575
rs1260326
rs3863087
rs72848256
rs57355367
rs2954038
rs73169103
rs111411487
rs78986976
rs114115200
rs60170459
rs964184
rs769449
All
−0.2
0.0
0.2
0.4
MR leave−one−out sensitivity analysis for
'TMCC3' on 'Pulmonary fibrosis'

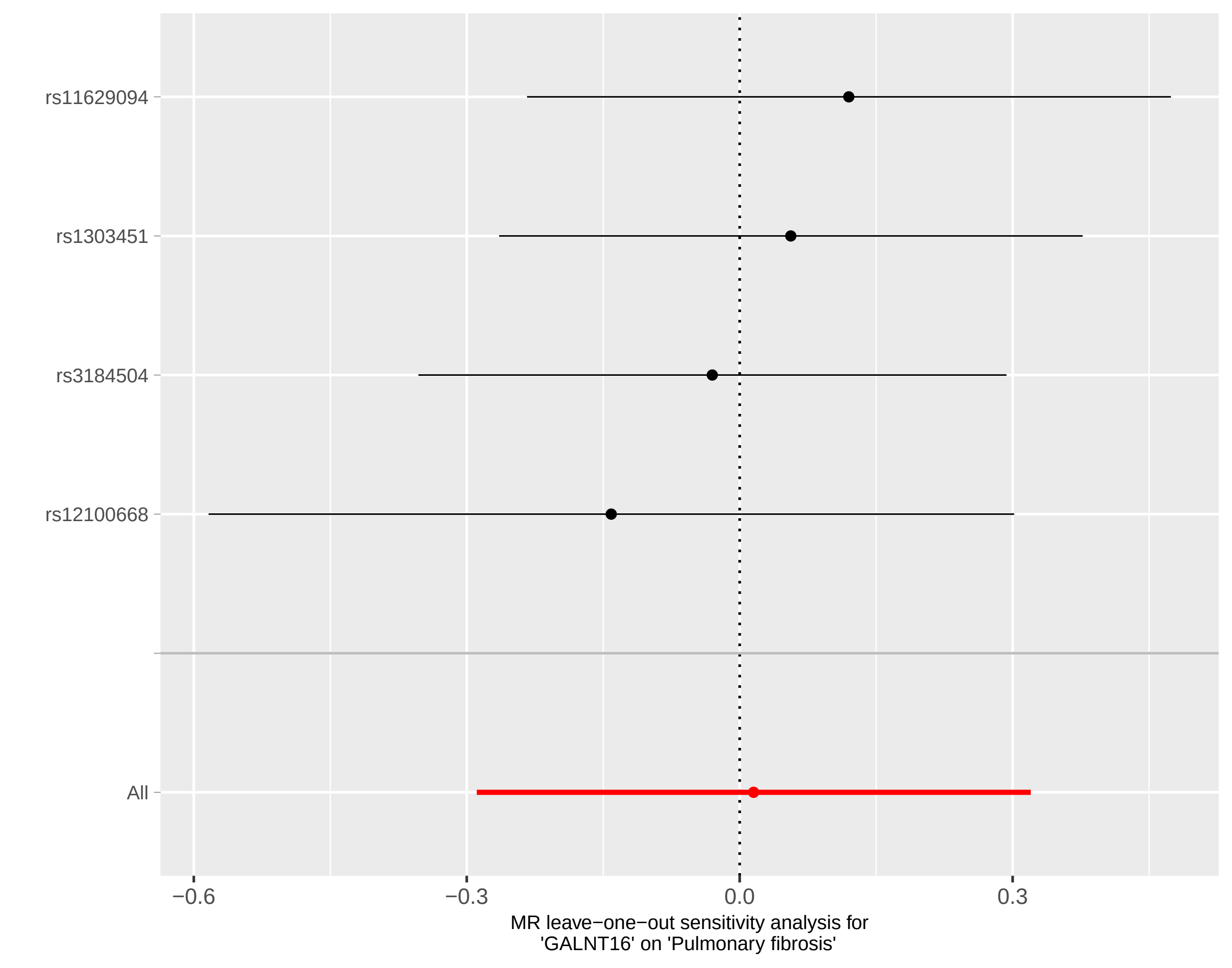
rs11629094
rs1303451
rs3184504
rs12100668
All
−0.6
−0.3
0.0
0.3
MR leave−one−out sensitivity analysis for
'GALNT16' on 'Pulmonary fibrosis'

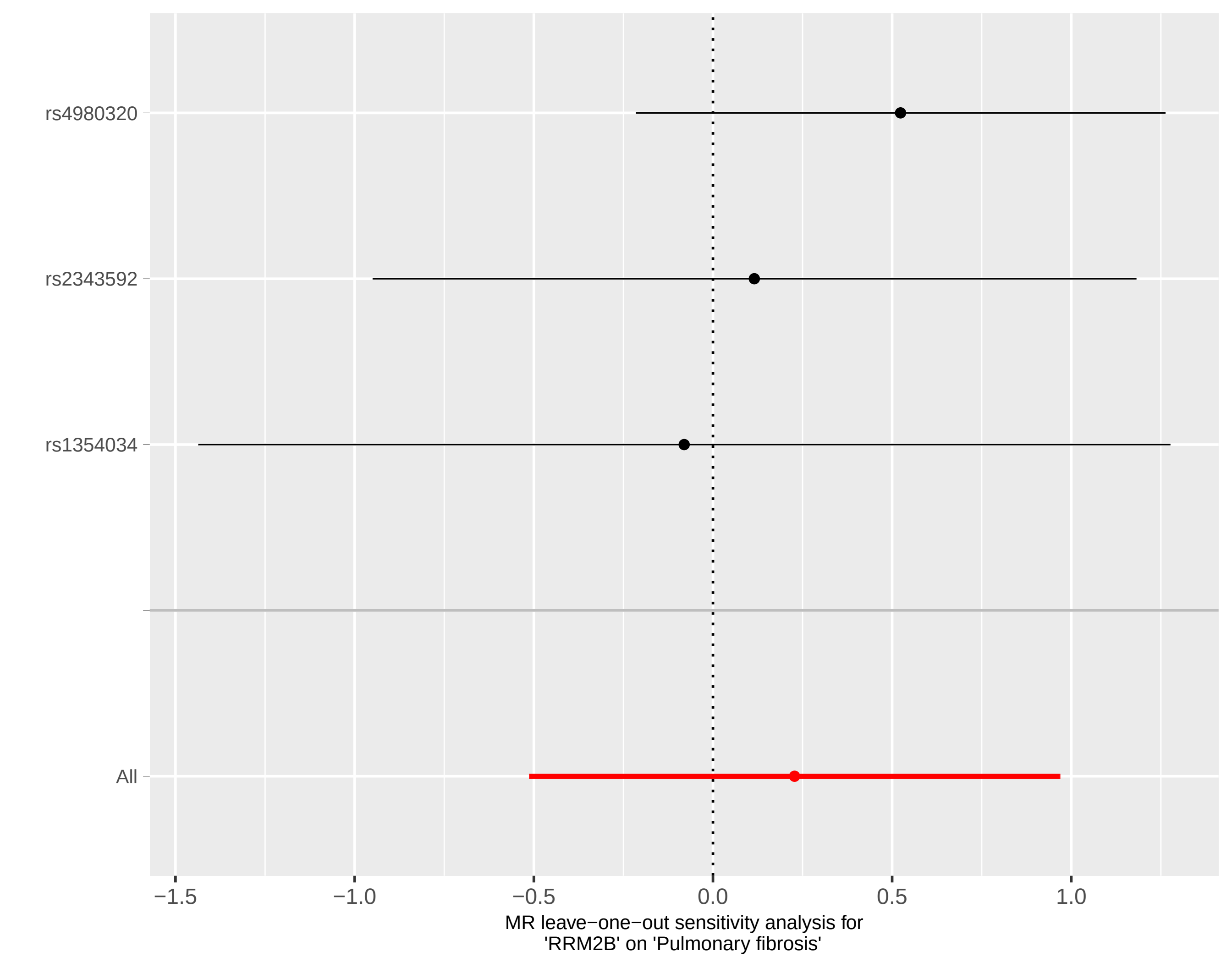

rs4980320
rs2343592
rs1354034
All
−1.5
−1.0
−0.5
0.0
0.5
1.0
MR leave−one−out sensitivity analysis for
'RRM2B' on 'Pulmonary fibrosis'

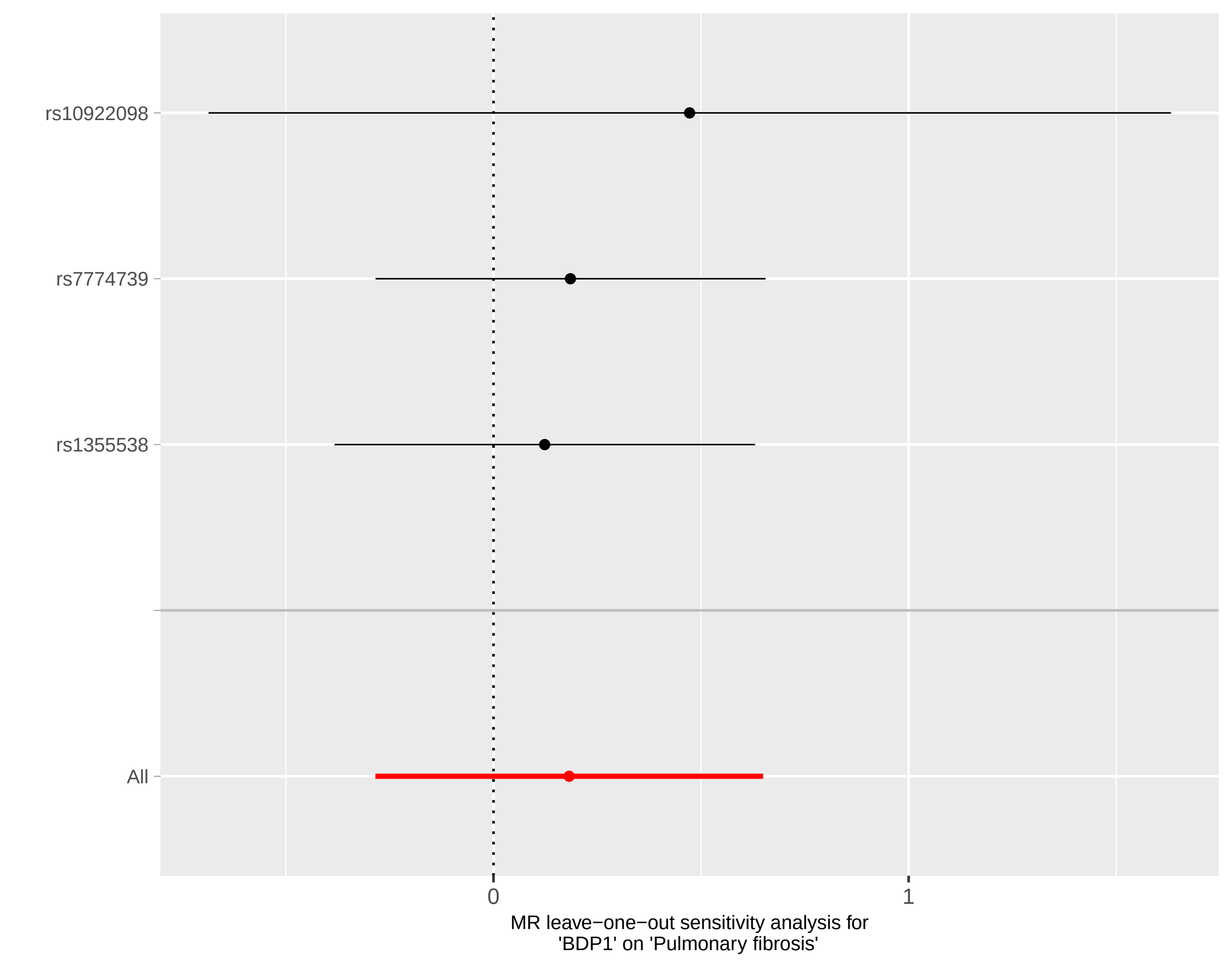
rs10922098
rs7774739
rs1355538
All
0
1
MR leave−one−out sensitivity analysis for
'BDP1' on 'Pulmonary fibrosis'

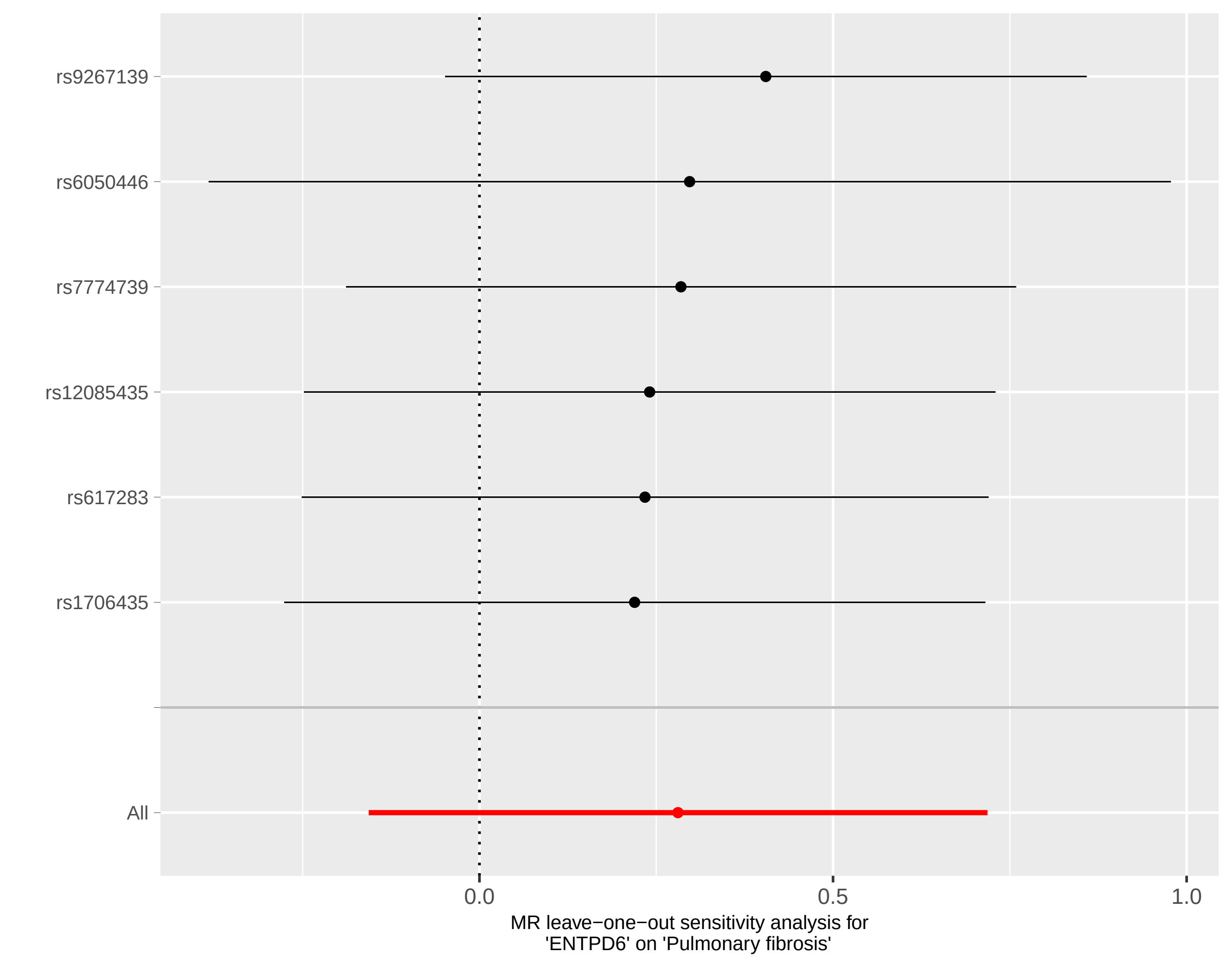
rs9267139
rs6050446
rs7774739
rs12085435
rs617283
rs1706435
All
0.0
0.5
1.0
MR leave−one−out sensitivity analysis for
'ENTPD6' on 'Pulmonary fibrosis'

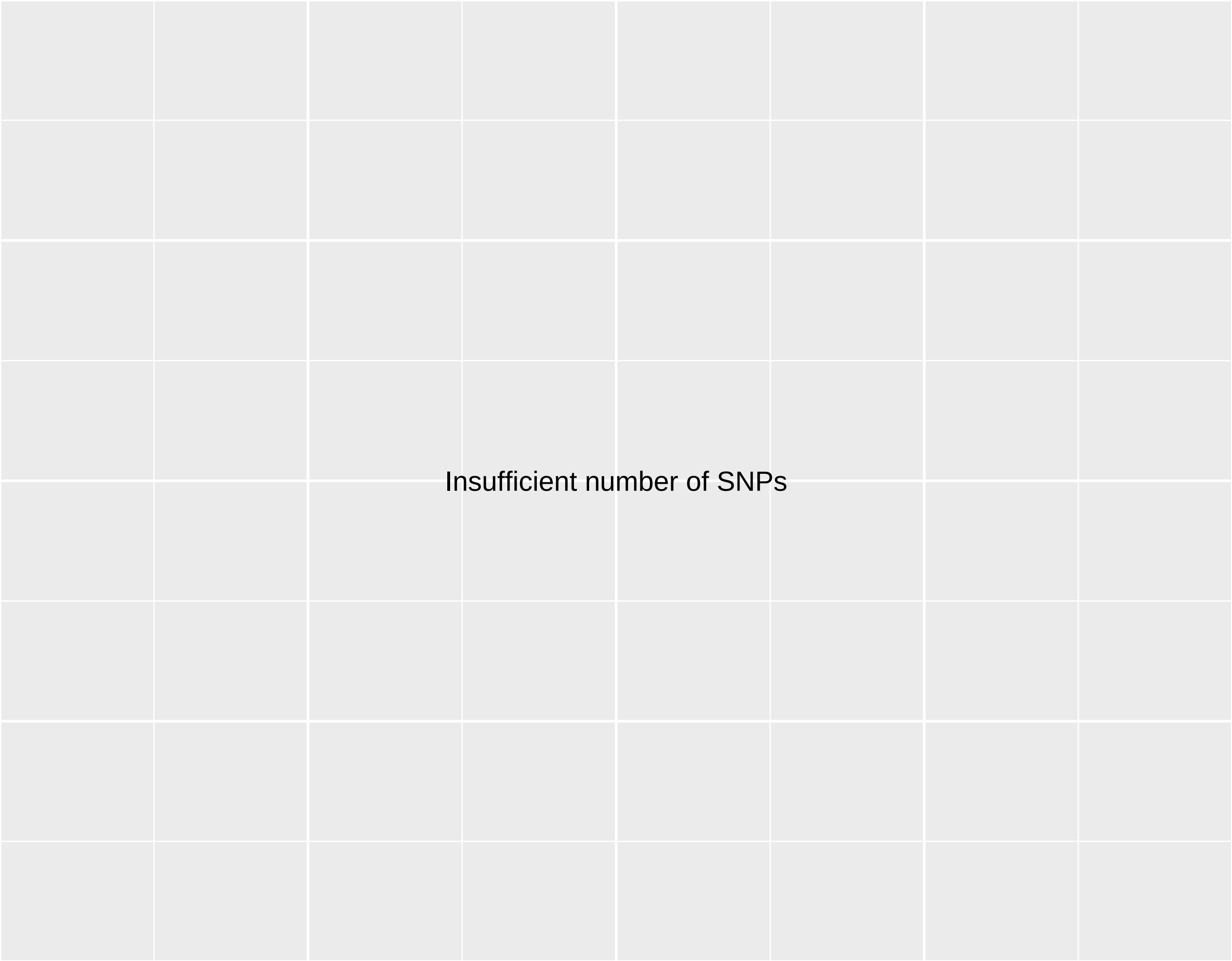
Insufficient number of SNPs

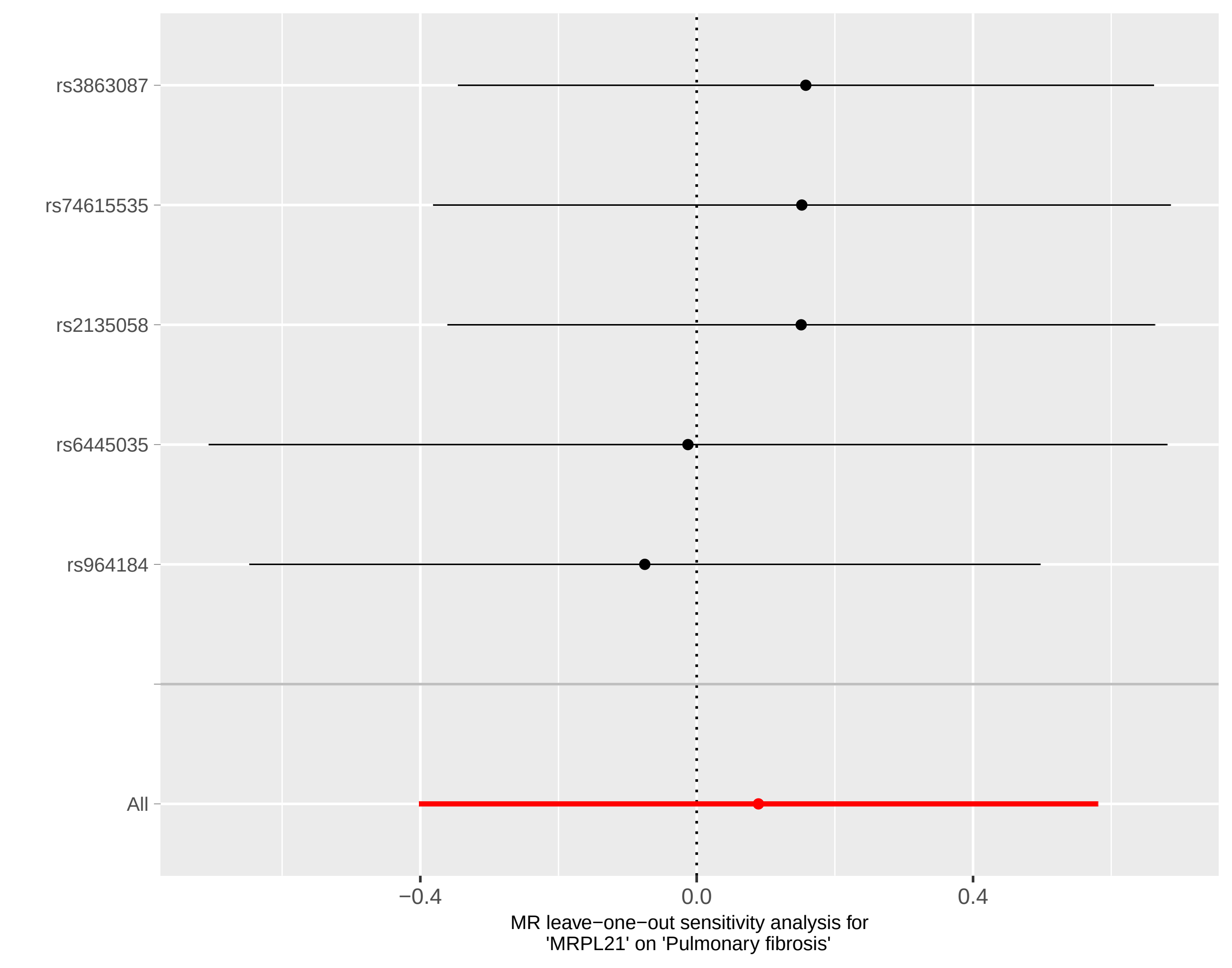
rs3863087
rs74615535
rs2135058
rs6445035
rs964184
All
−0.4
0.0
0.4
MR leave−one−out sensitivity analysis for
'MRPL21' on 'Pulmonary fibrosis'

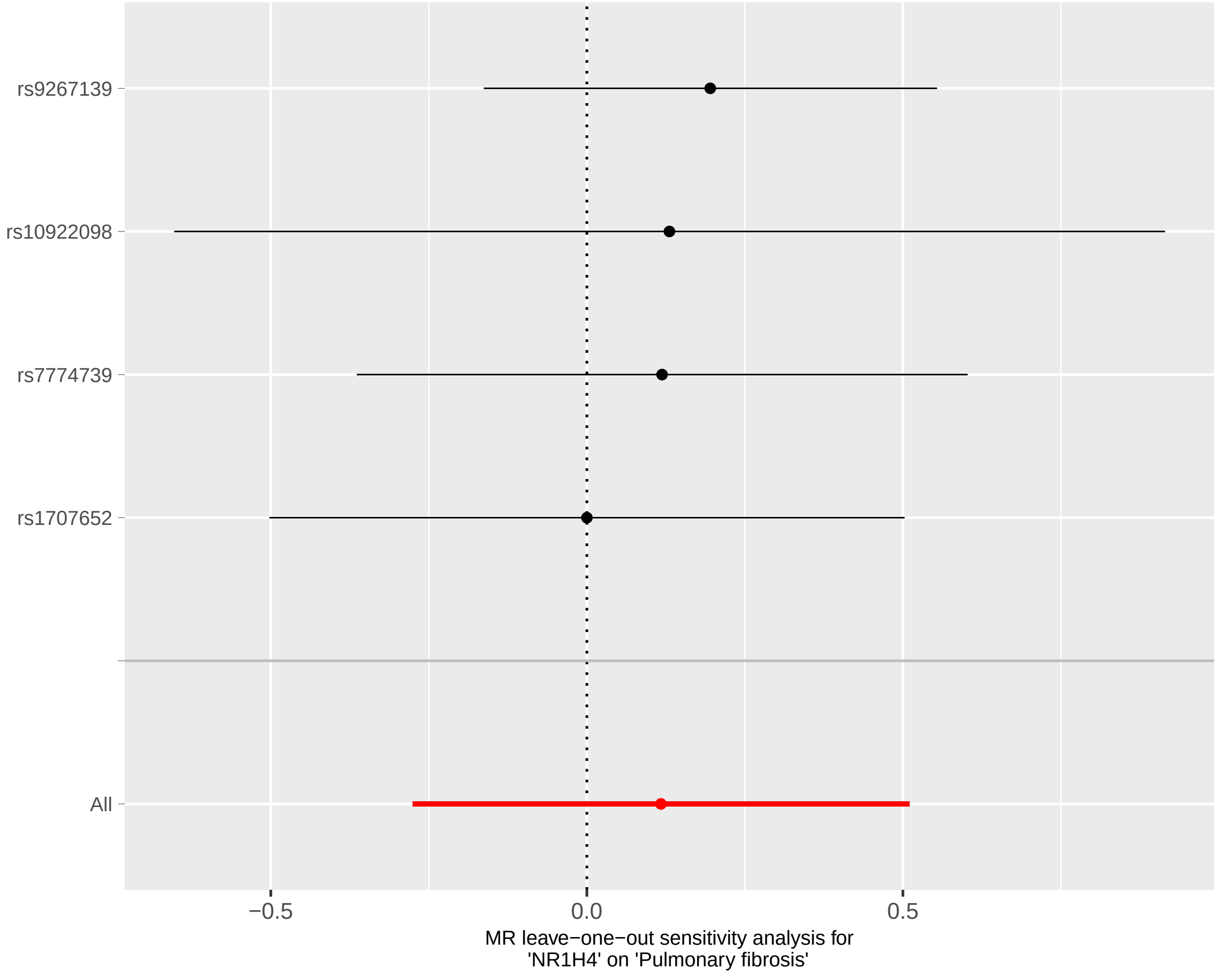
rs9267139
rs10922098
rs7774739
rs1707652
All
−0.5
0.0
0.5
MR leave−one−out sensitivity analysis for
'NR1H4' on 'Pulmonary fibrosis'

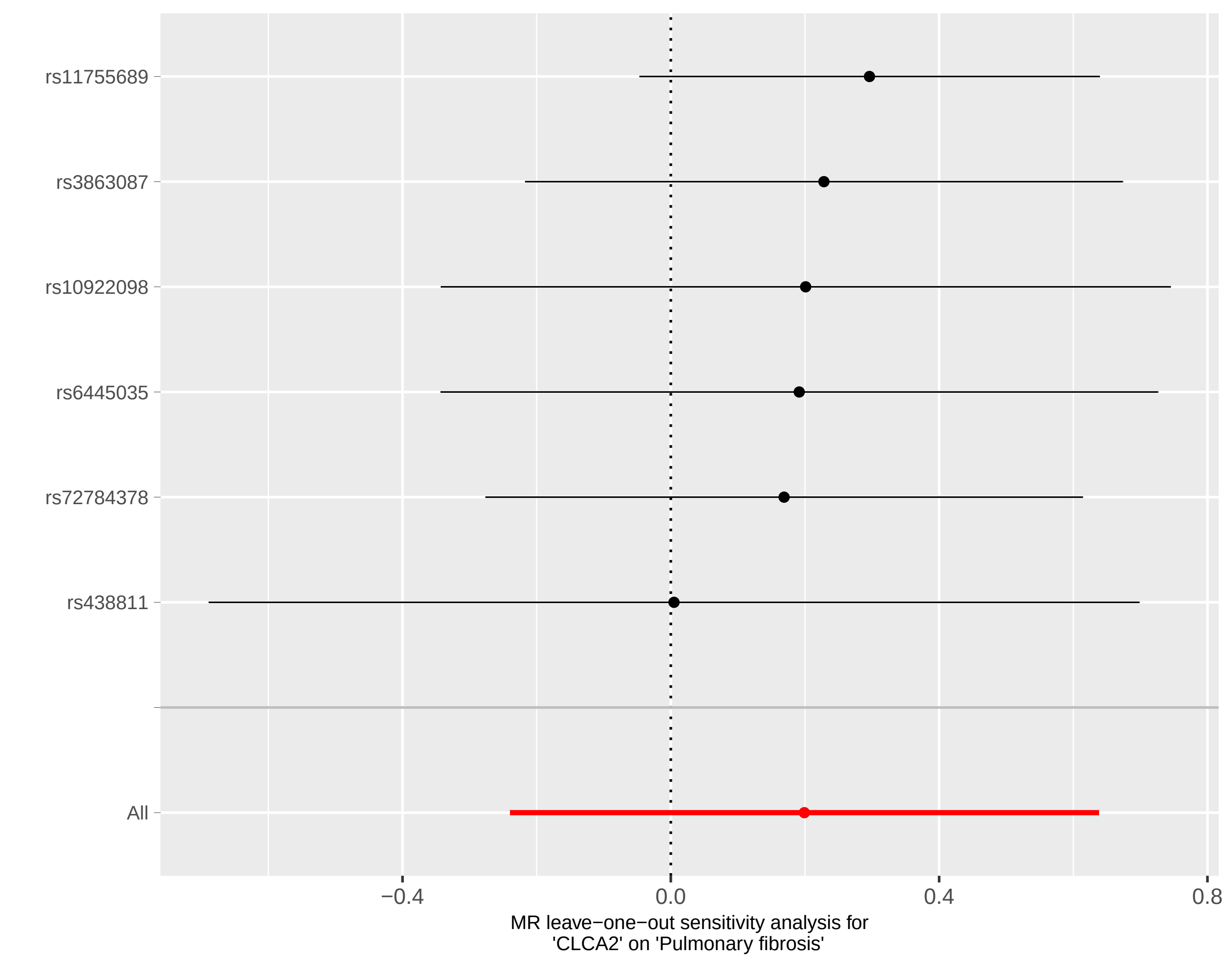

rs11755689
rs3863087
rs10922098
rs6445035
rs72784378
rs438811
All
−0.4
0.0
0.4
0.8
MR leave−one−out sensitivity analysis for
'CLCA2' on 'Pulmonary fibrosis'

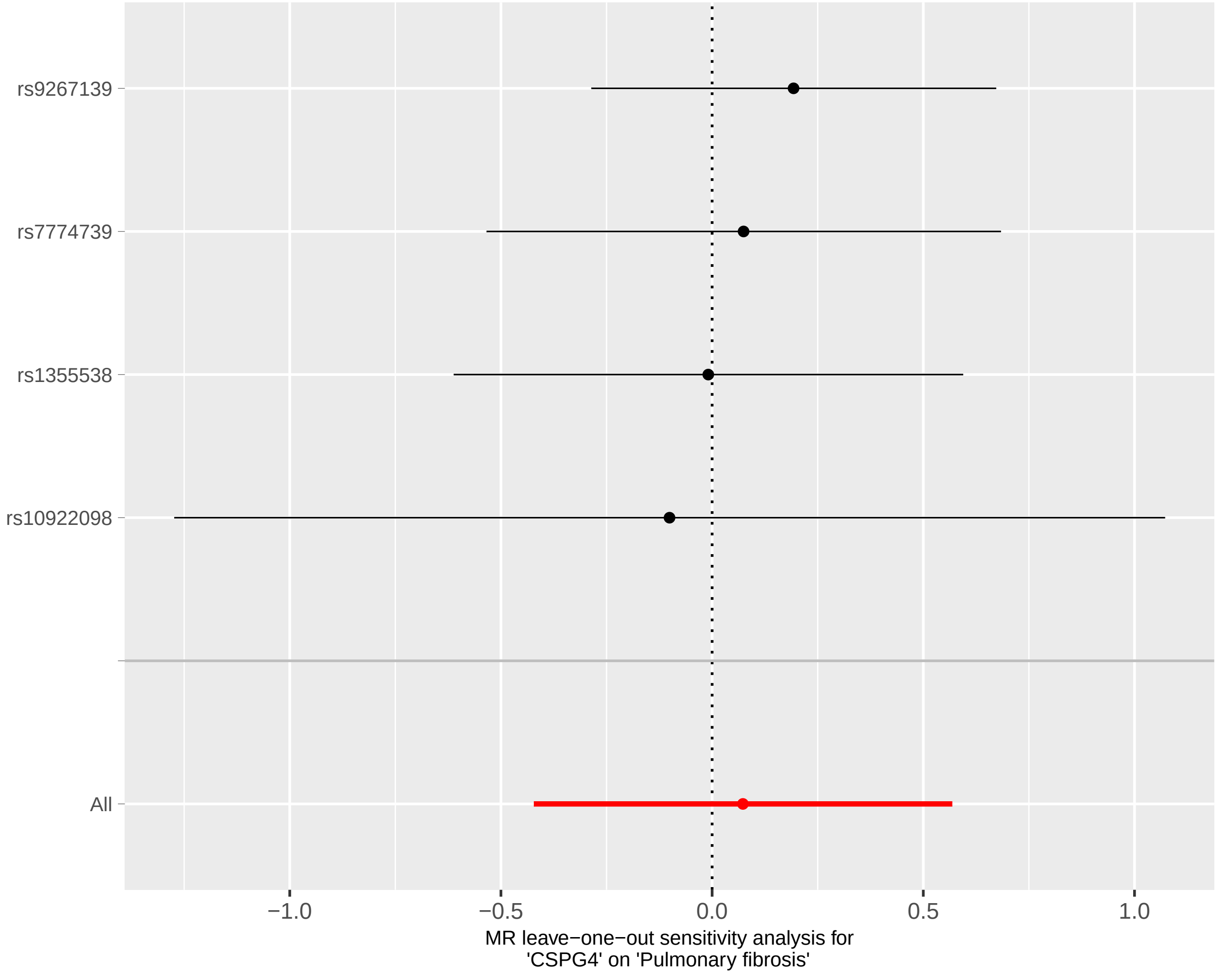
rs9267139
rs7774739
rs1355538
rs10922098
All
−1.0
−0.5
0.0
0.5
1.0
MR leave−one−out sensitivity analysis for
'CSPG4' on 'Pulmonary fibrosis'

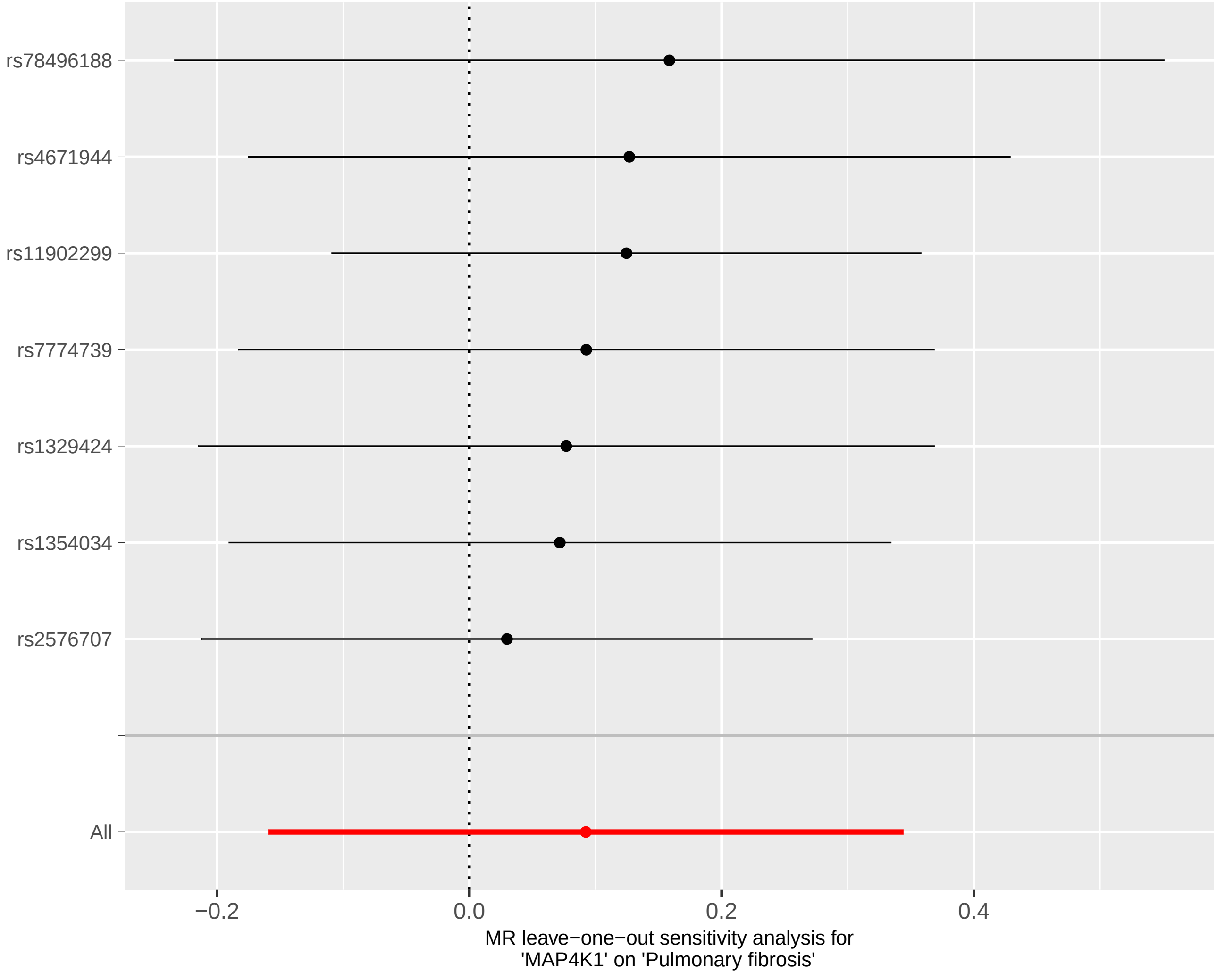

rs78496188
rs4671944
rs11902299
rs7774739
rs1329424
rs1354034
rs2576707
All
−0.2
0.0
0.2
0.4
MR leave−one−out sensitivity analysis for
'MAP4K1' on 'Pulmonary fibrosis'

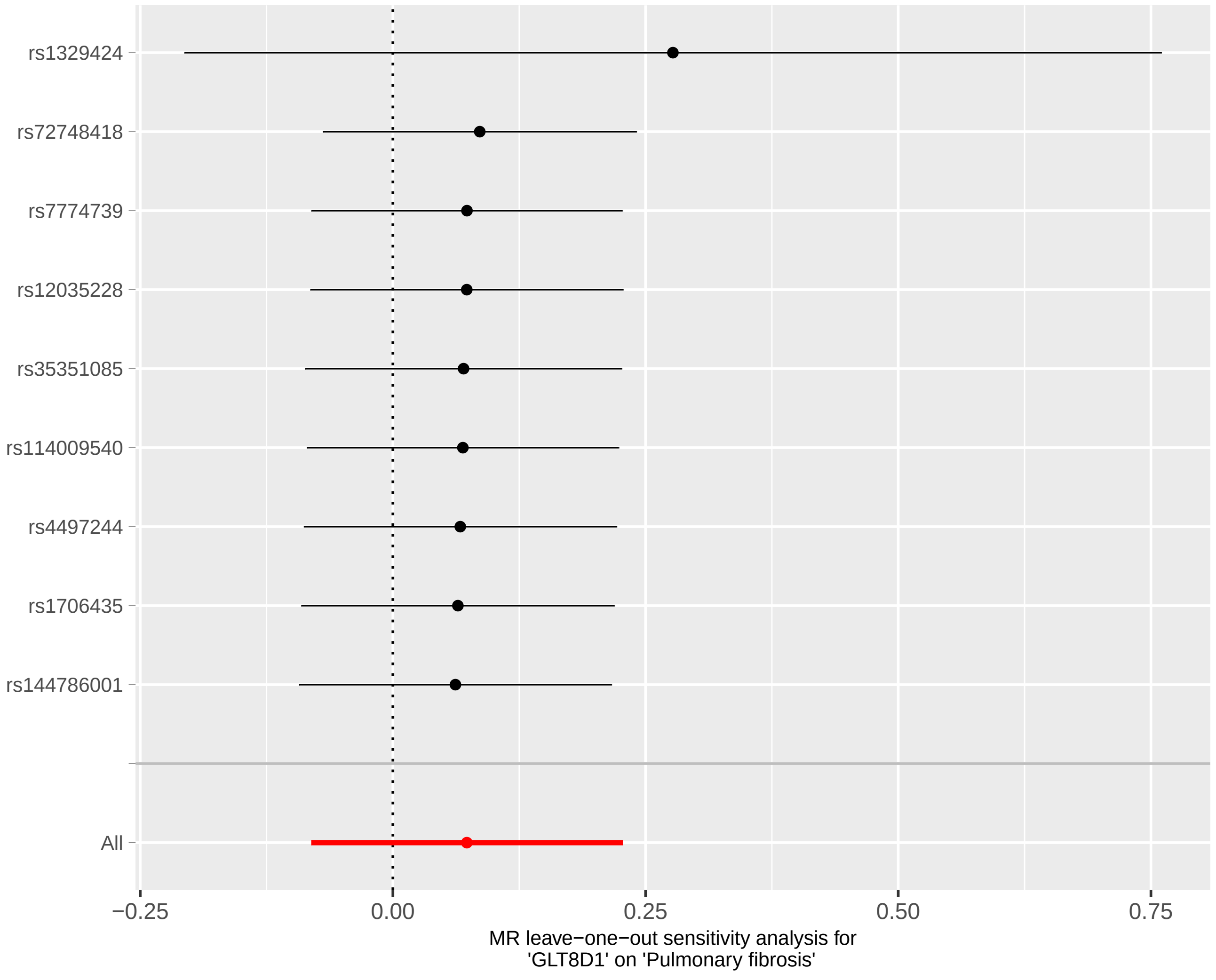
rs1329424
rs72748418
rs7774739
rs12035228
rs35351085
rs114009540
rs4497244
rs1706435
rs144786001
All
−0.25
0.00
0.25
0.50
0.75
MR leave−one−out sensitivity analysis for
'GLT8D1' on 'Pulmonary fibrosis'

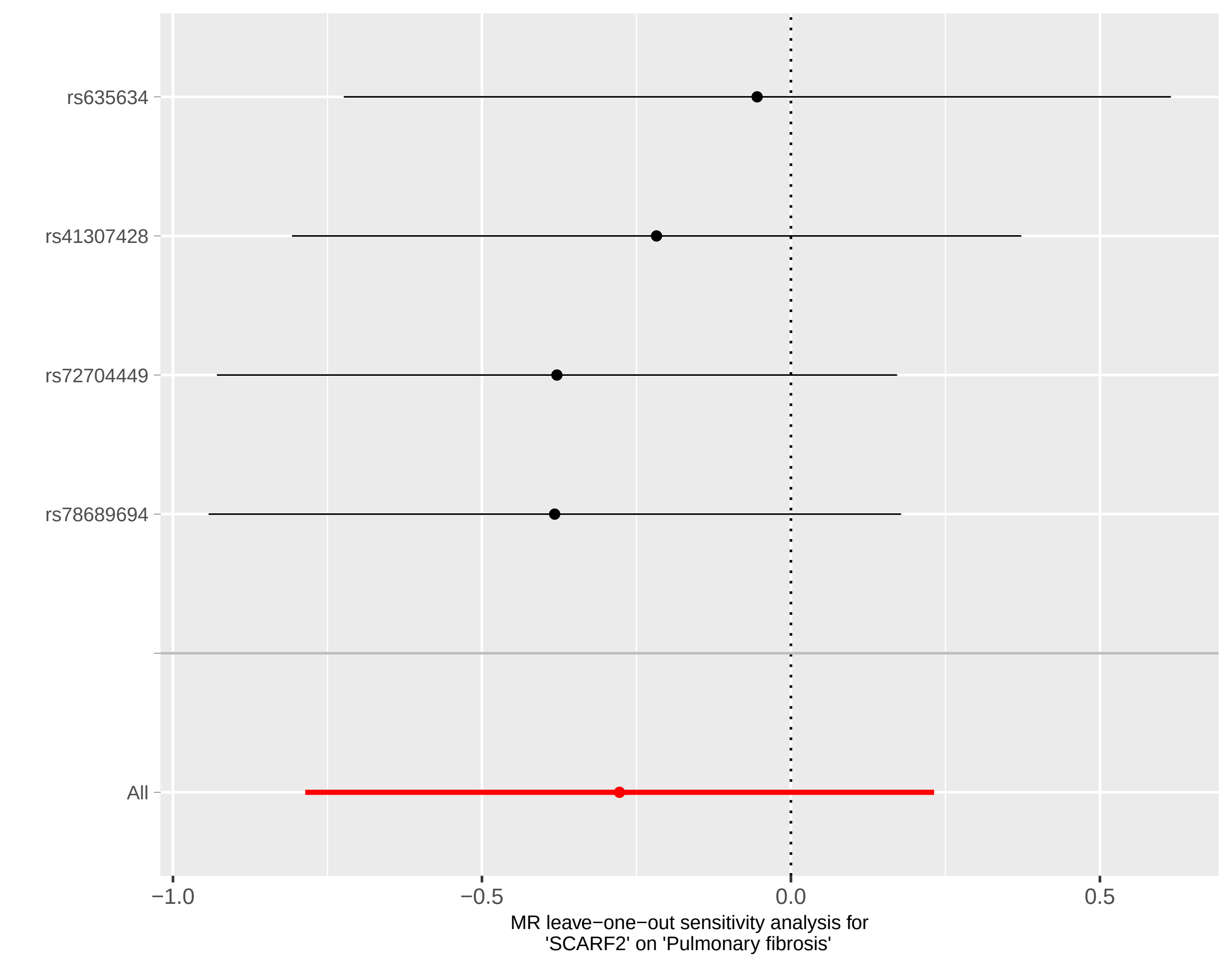
rs635634
rs41307428
rs72704449
rs78689694
All
−1.0
−0.5
0.0
0.5
MR leave−one−out sensitivity analysis for
'SCARF2' on 'Pulmonary fibrosis'

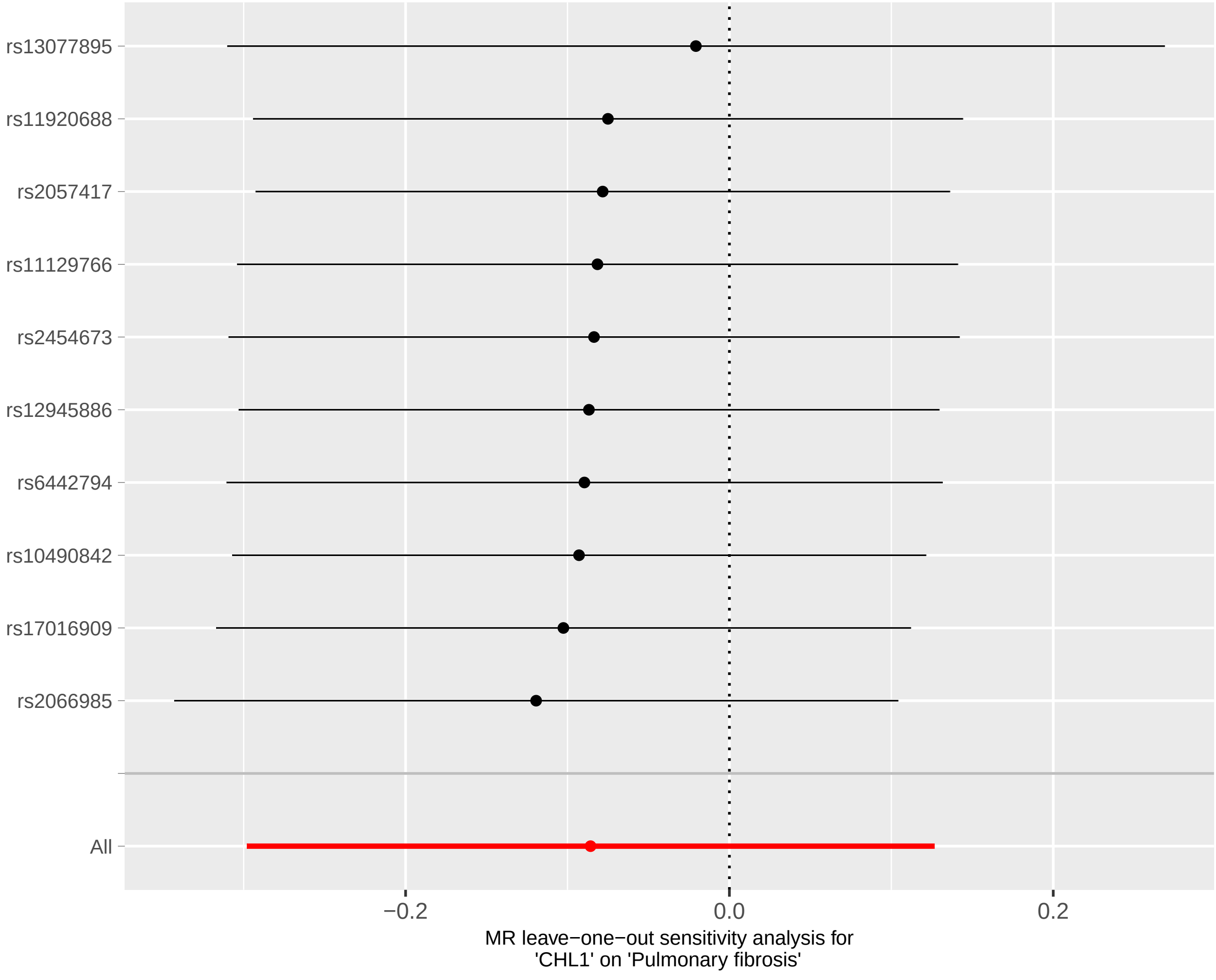
rs13077895
rs11920688
rs2057417
rs11129766
rs2454673
rs12945886
rs6442794
rs10490842
rs17016909
rs2066985
All
−0.2
0.0
0.2
MR leave−one−out sensitivity analysis for
'CHL1' on 'Pulmonary fibrosis'

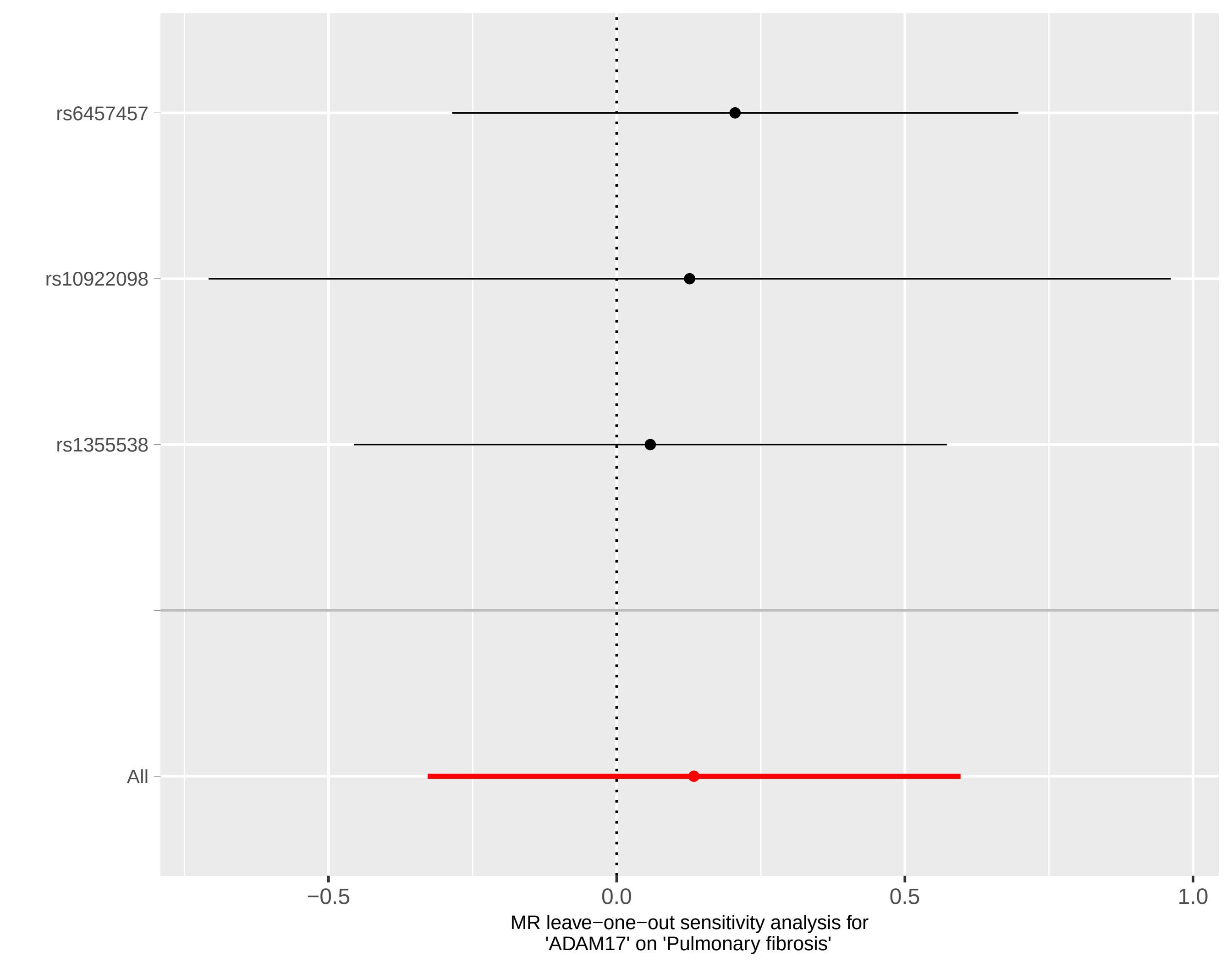
rs6457457
rs10922098
rs1355538
All
−0.5
0.0
0.5
1.0
MR leave−one−out sensitivity analysis for
'ADAM17' on 'Pulmonary fibrosis'

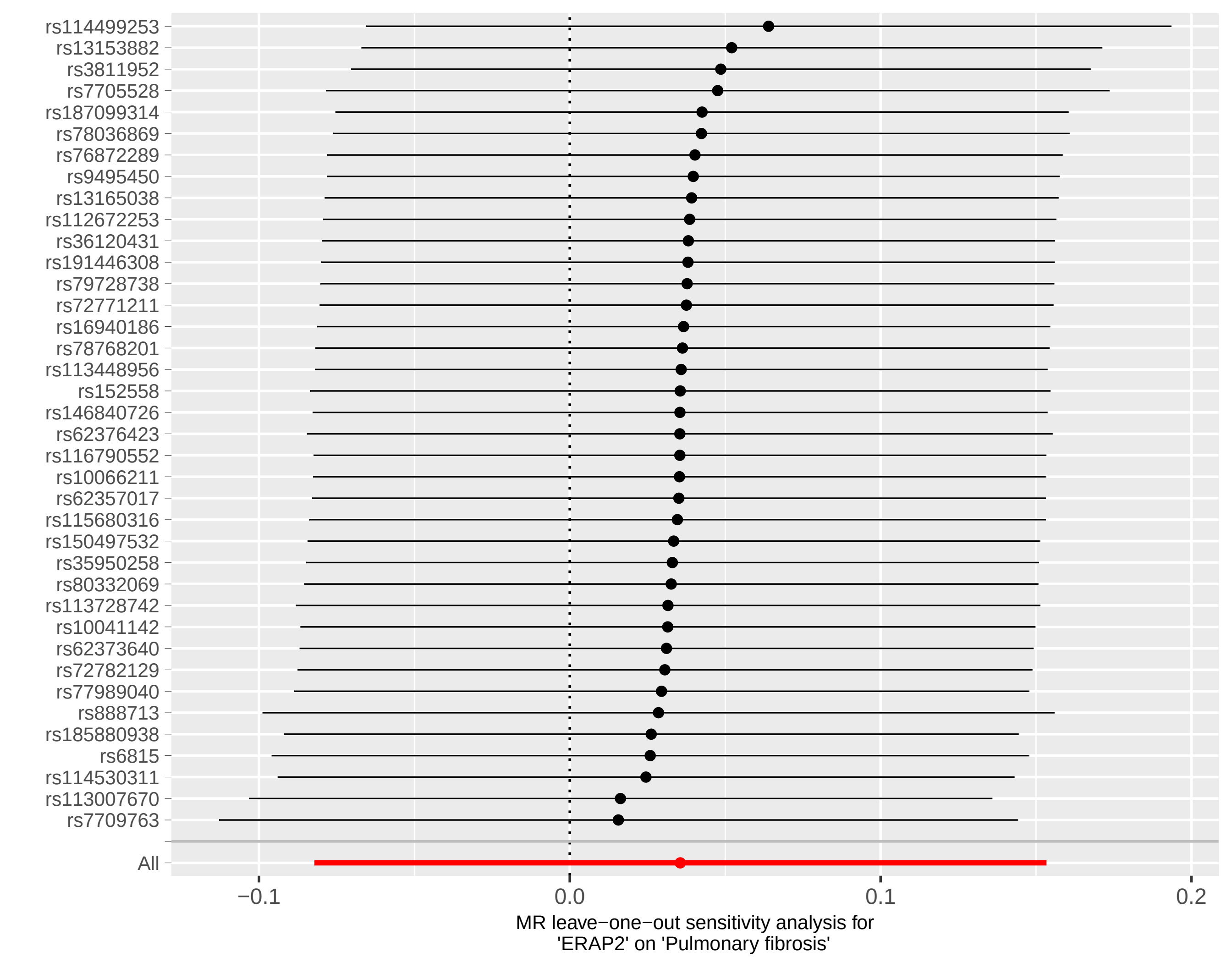

rs114499253
rs13153882
rs3811952
rs7705528
rs187099314
rs78036869
rs76872289
rs9495450
rs13165038
rs112672253
rs36120431
rs191446308
rs79728738
rs72771211
rs16940186
rs78768201
rs113448956
rs152558
rs146840726
rs62376423
rs116790552
rs10066211
rs62357017
rs115680316
rs150497532
rs35950258
rs80332069
rs113728742
rs10041142
rs62373640
rs72782129
rs77989040
rs888713
rs185880938
rs6815
rs114530311
rs113007670
rs7709763
All
−0.1
0.0
0.1
0.2
MR leave−one−out sensitivity analysis for
'ERAP2' on 'Pulmonary fibrosis'

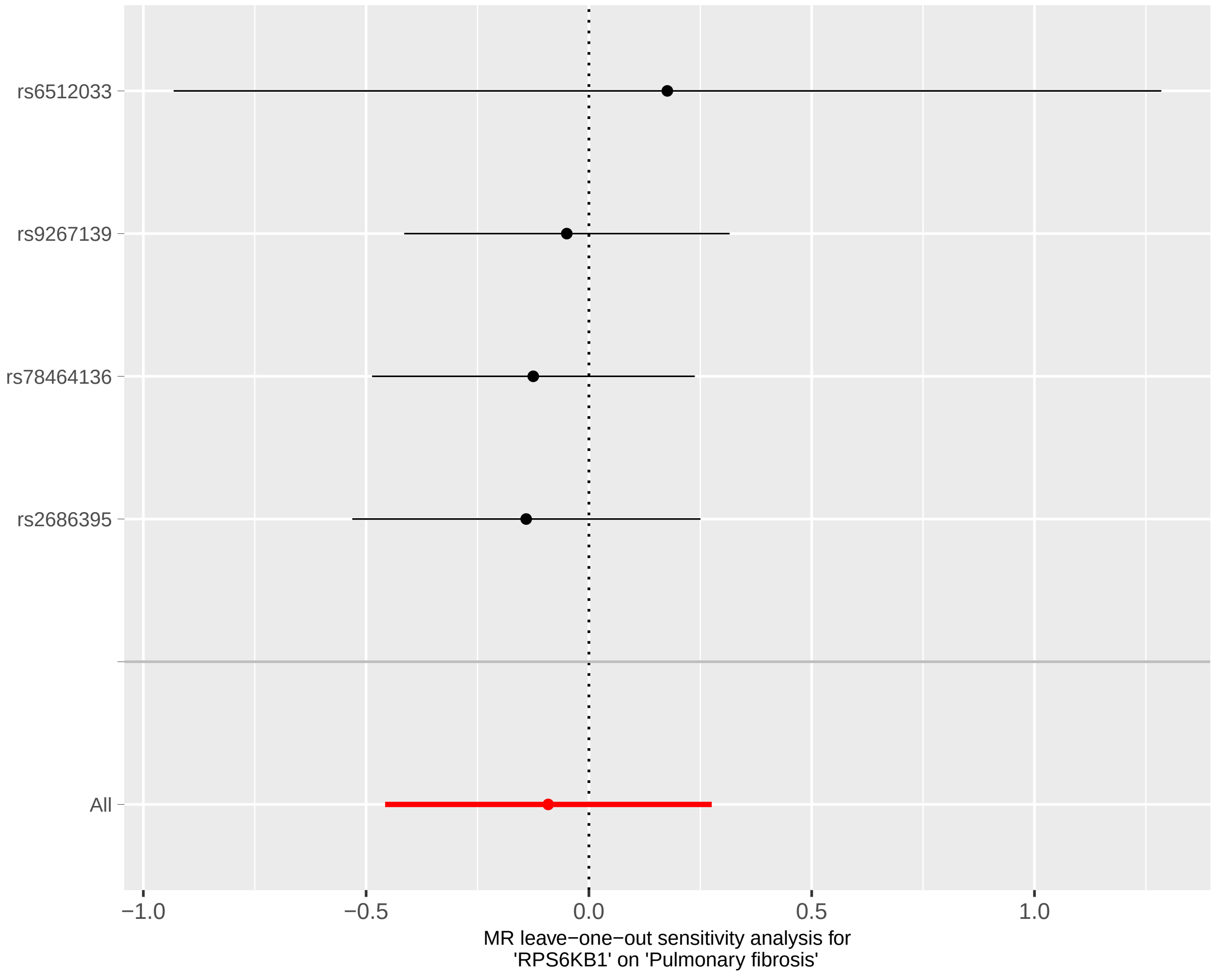
rs6512033
rs9267139
rs78464136
rs2686395
All
−1.0
−0.5
0.0
0.5
1.0
MR leave−one−out sensitivity analysis for
'RPS6KB1' on 'Pulmonary fibrosis'

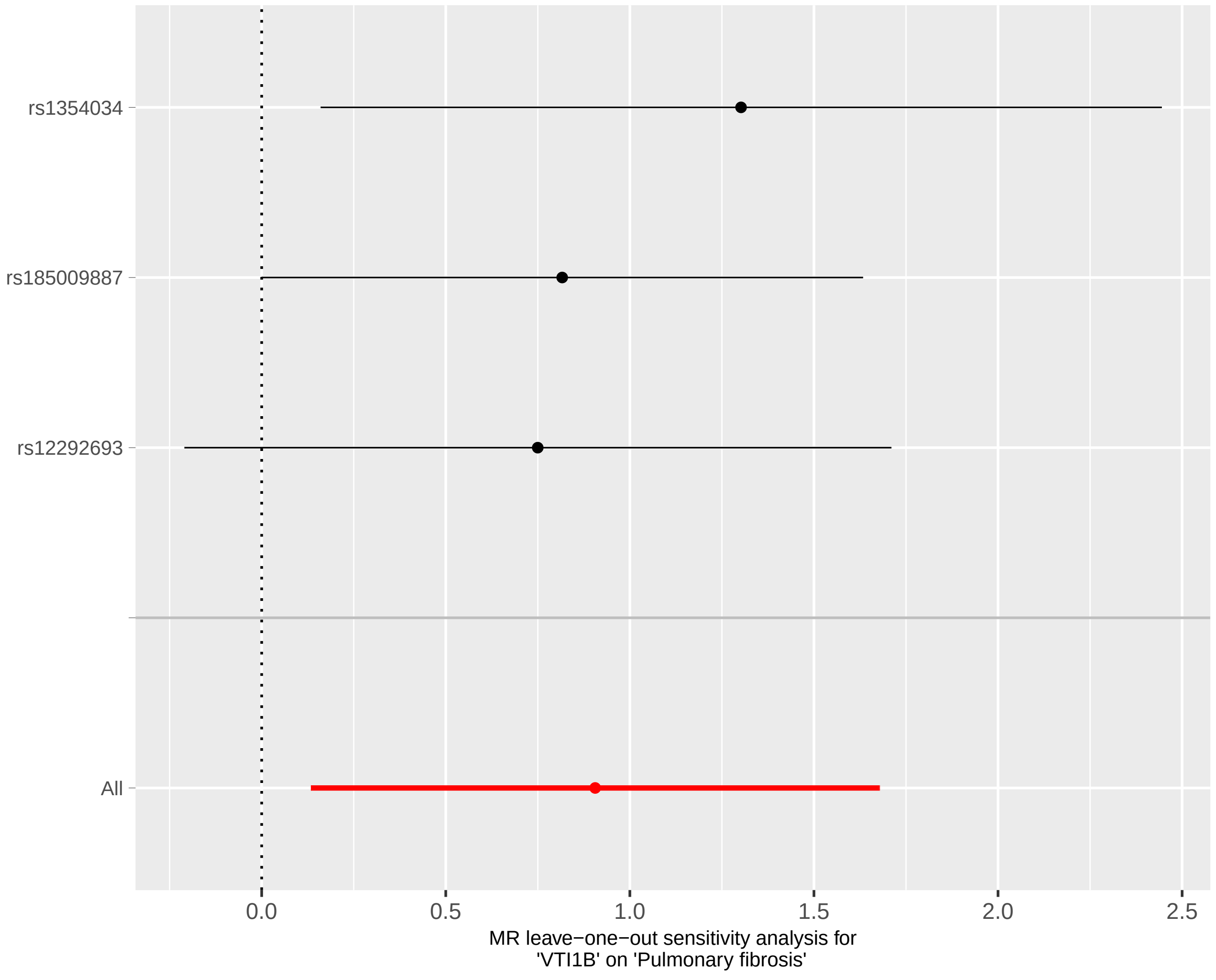
rs1354034
rs185009887
rs12292693
All
0.0
0.5
1.0
1.5
2.0
2.5
MR leave−one−out sensitivity analysis for
'VTI1B' on 'Pulmonary fibrosis'

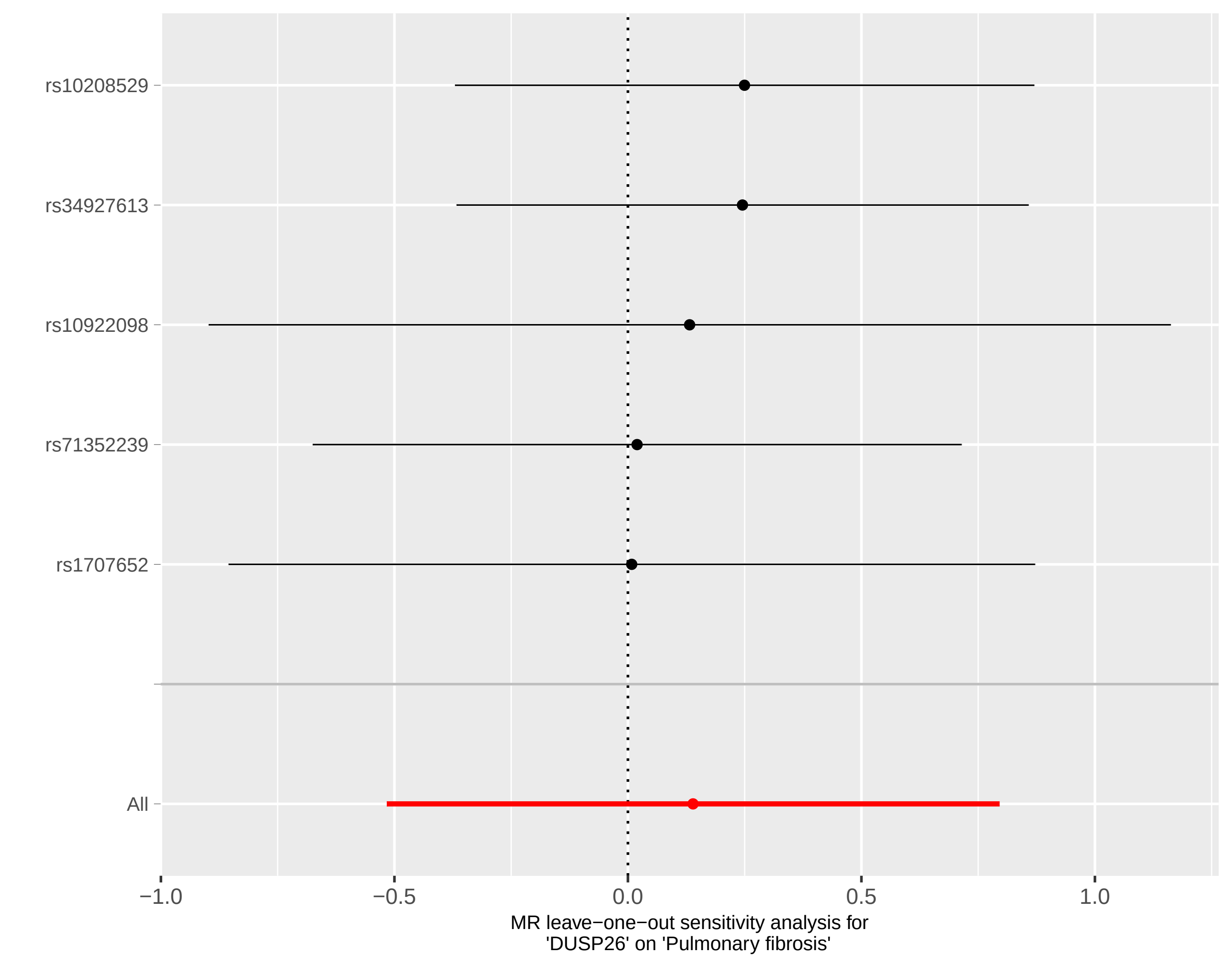
rs10208529
rs34927613
rs10922098
rs71352239
rs1707652
All
−1.0
−0.5
0.0
0.5
1.0
MR leave−one−out sensitivity analysis for
'DUSP26' on 'Pulmonary fibrosis'

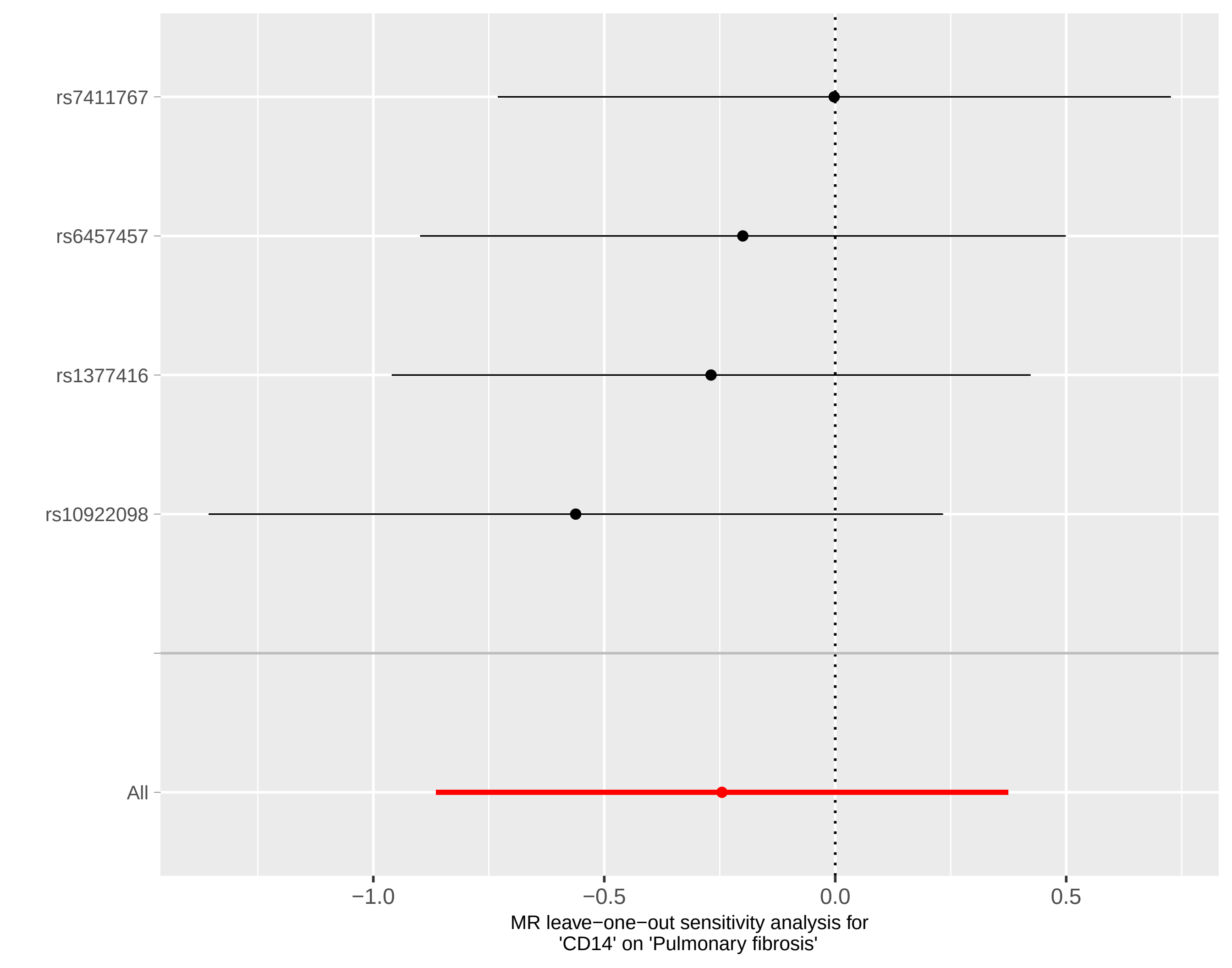
rs7411767
rs6457457
rs1377416
rs10922098
All
−1.0
−0.5
0.0
0.5
MR leave−one−out sensitivity analysis for
'CD14' on 'Pulmonary fibrosis'

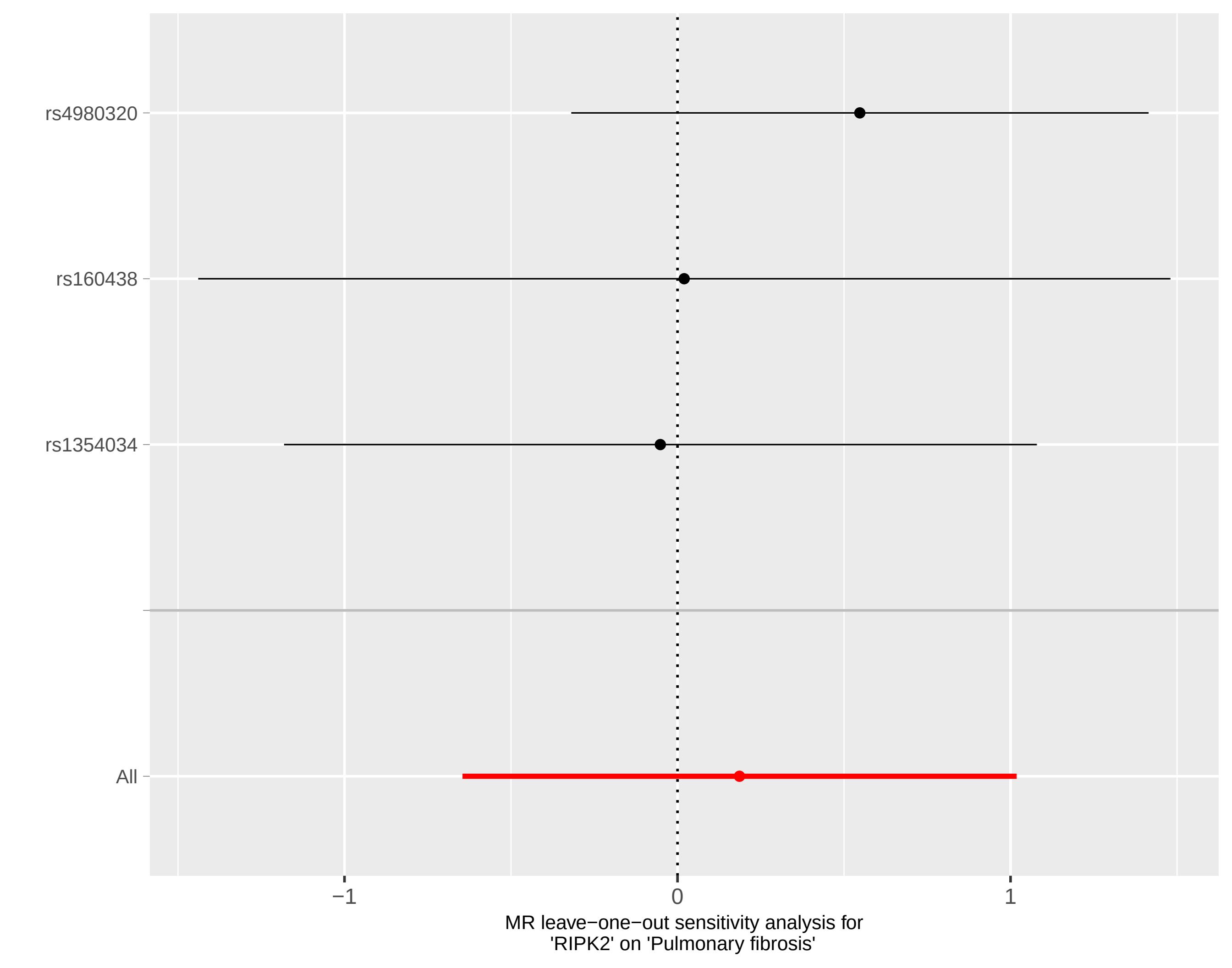
rs4980320
rs160438
rs1354034
All
−1
0
1
MR leave−one−out sensitivity analysis for
'RIPK2' on 'Pulmonary fibrosis'

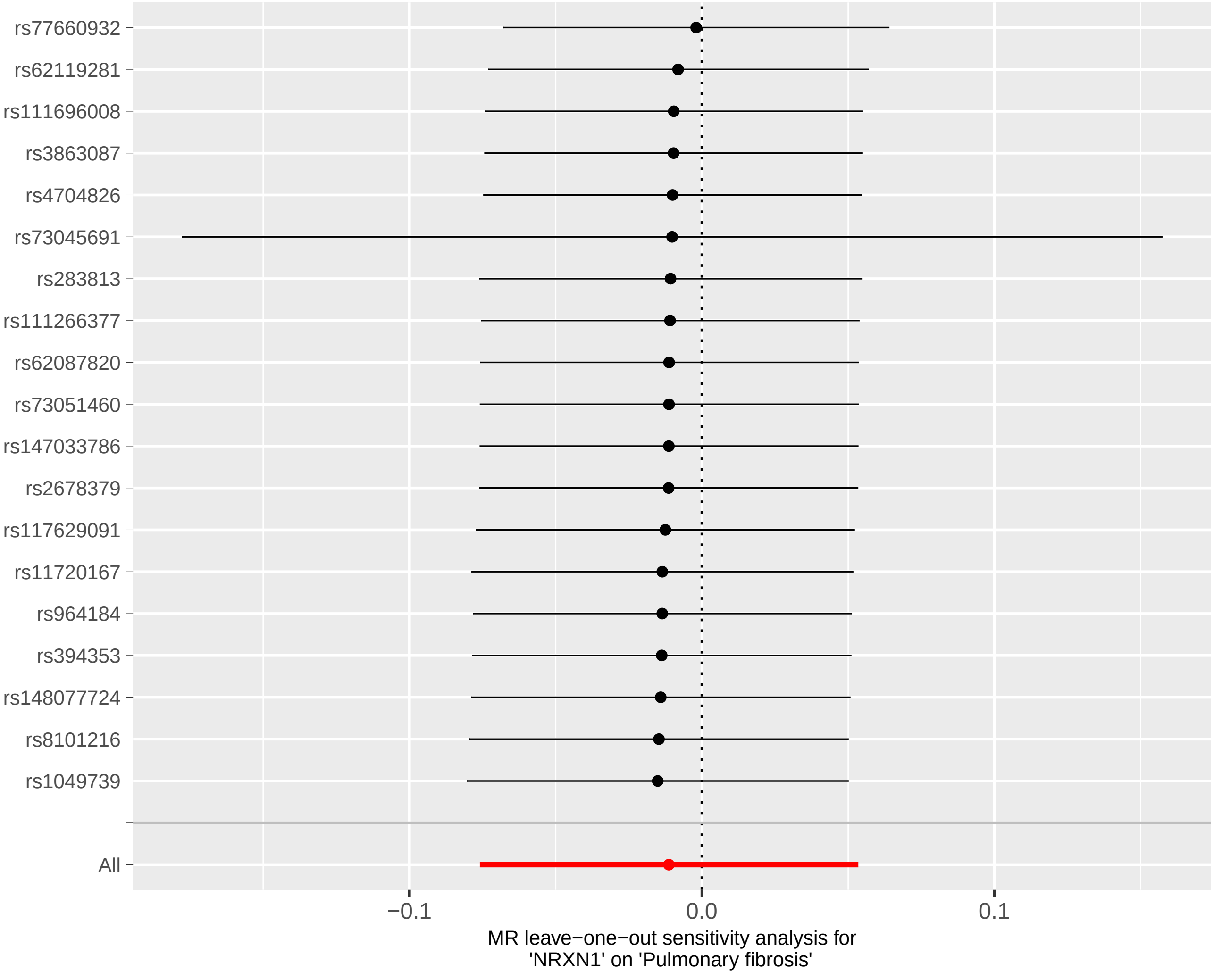
rs77660932
rs62119281
rs111696008
rs3863087
rs4704826
rs73045691
rs283813
rs111266377
rs62087820
rs73051460
rs147033786
rs2678379
rs117629091
rs11720167
rs964184
rs394353
rs148077724
rs8101216
rs1049739
All
−0.1
0.0
0.1
MR leave−one−out sensitivity analysis for
'NRXN1' on 'Pulmonary fibrosis'

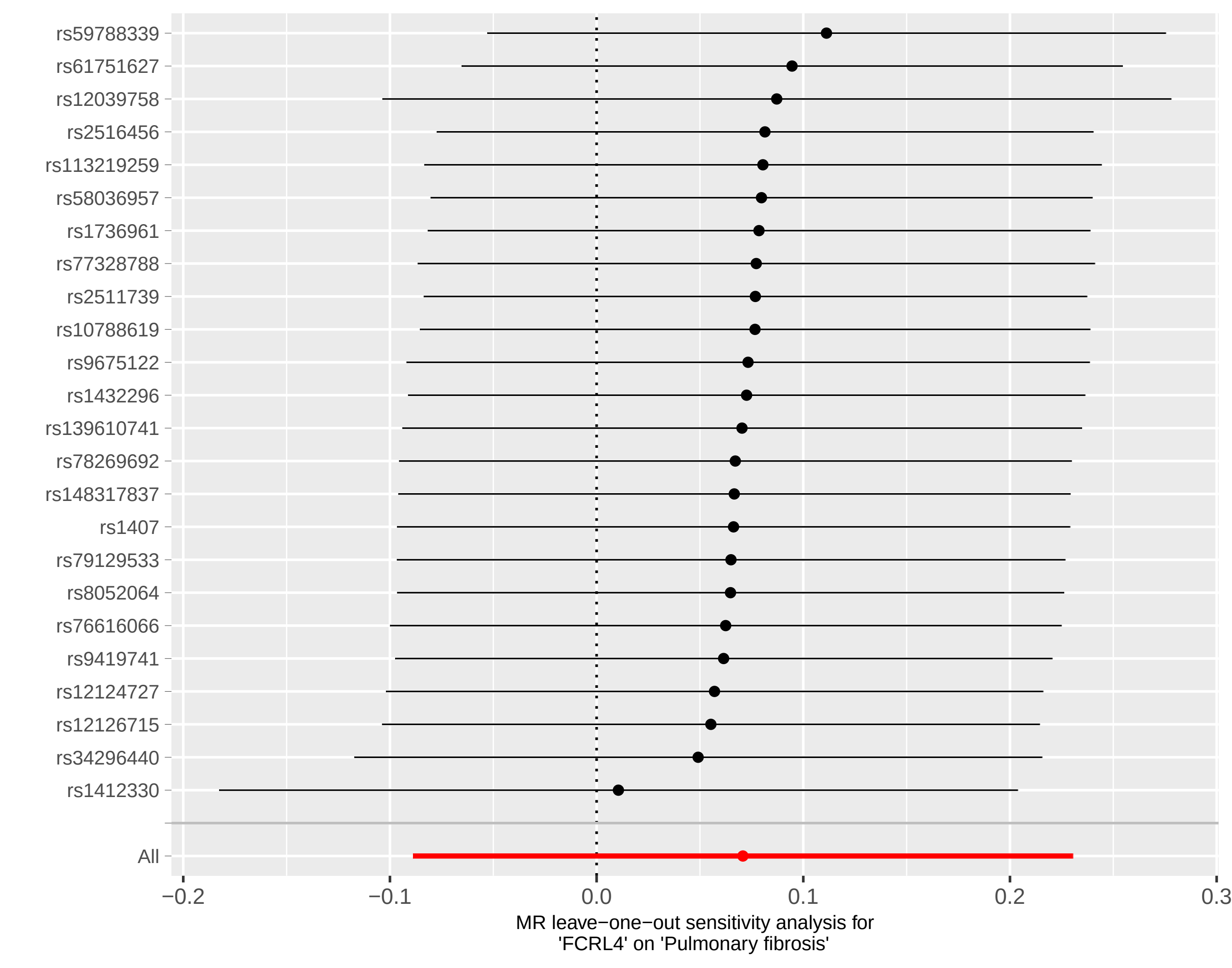
rs59788339
rs61751627
rs12039758
rs2516456
rs113219259
rs58036957
rs1736961
rs77328788
rs2511739
rs10788619
rs9675122
rs1432296
rs139610741
rs78269692
rs148317837
rs1407
rs79129533
rs8052064
rs76616066
rs9419741
rs12124727
rs12126715
rs34296440
rs1412330
All
−0.2
−0.1
0.0
0.1
0.2
0.3
MR leave−one−out sensitivity analysis for
'FCRL4' on 'Pulmonary fibrosis'

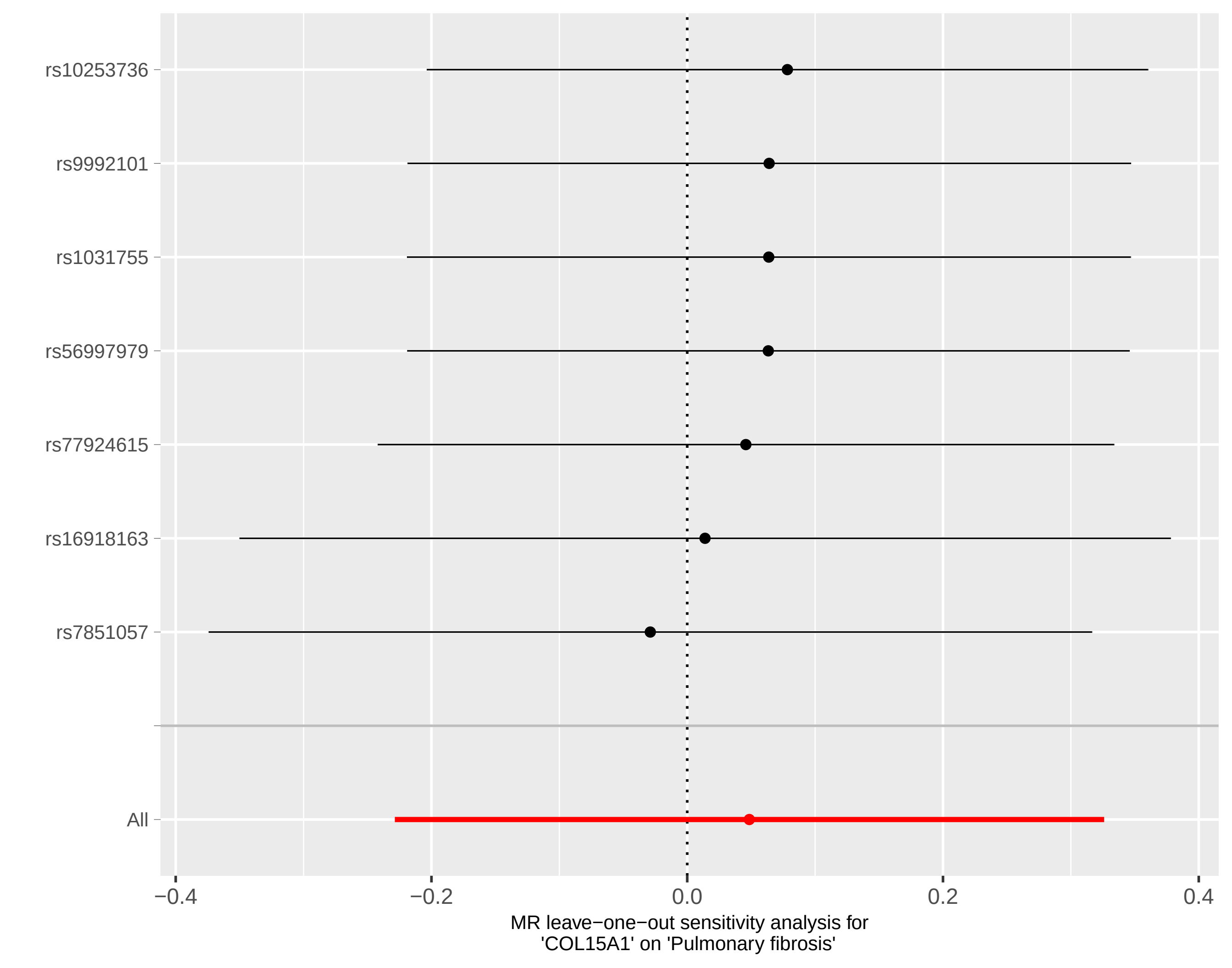
rs10253736
rs9992101
rs1031755
rs56997979
rs77924615
rs16918163
rs7851057
All
−0.4
−0.2
0.0
0.2
0.4
MR leave−one−out sensitivity analysis for
'COL15A1' on 'Pulmonary fibrosis'

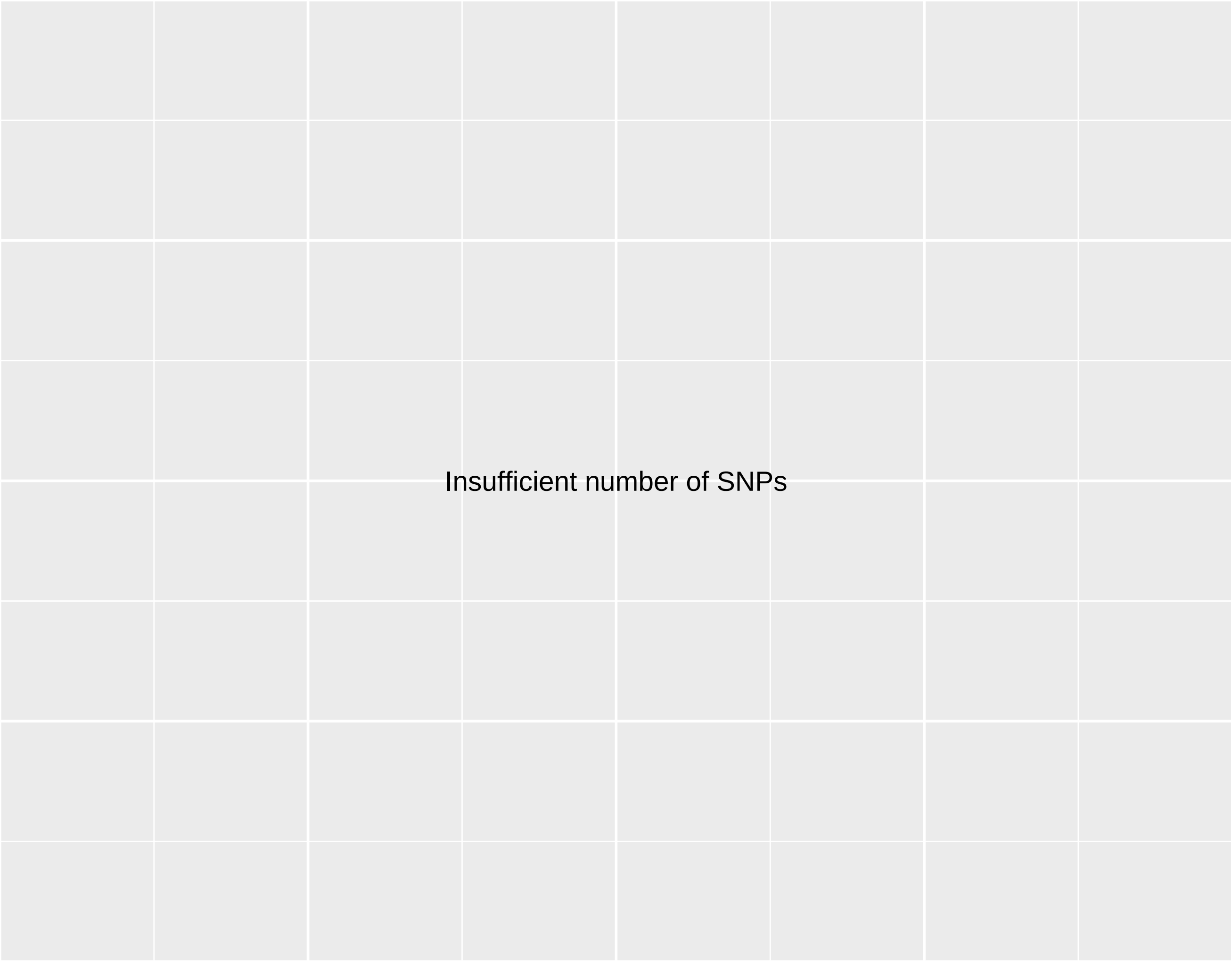
Insufficient number of SNPs

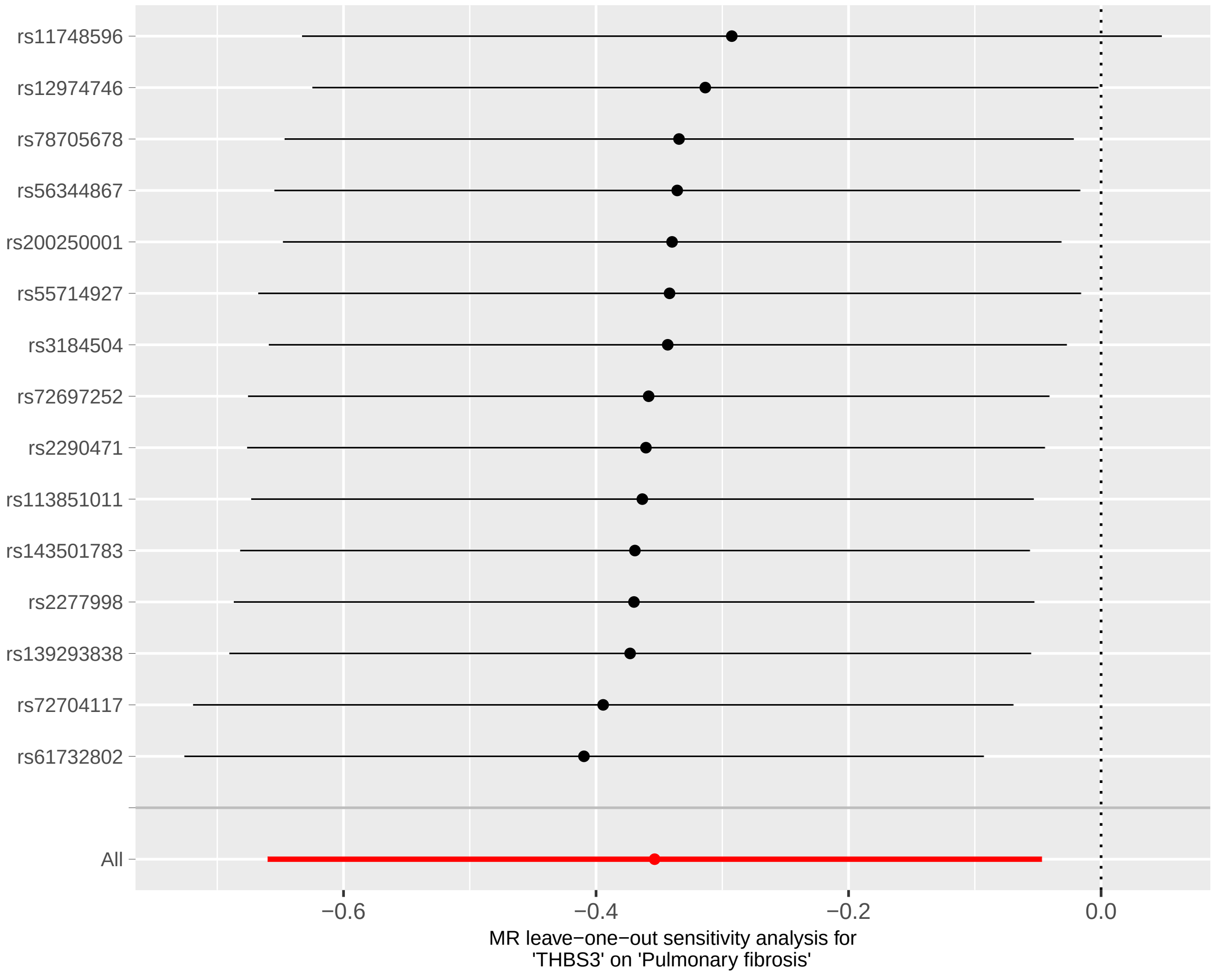
rs11748596
rs12974746
rs78705678
rs56344867
rs200250001
rs55714927
rs3184504
rs72697252
rs2290471
rs113851011
rs143501783
rs2277998
rs139293838
rs72704117
rs61732802
All
−0.6
−0.4
−0.2
0.0
MR leave−one−out sensitivity analysis for
'THBS3' on 'Pulmonary fibrosis'

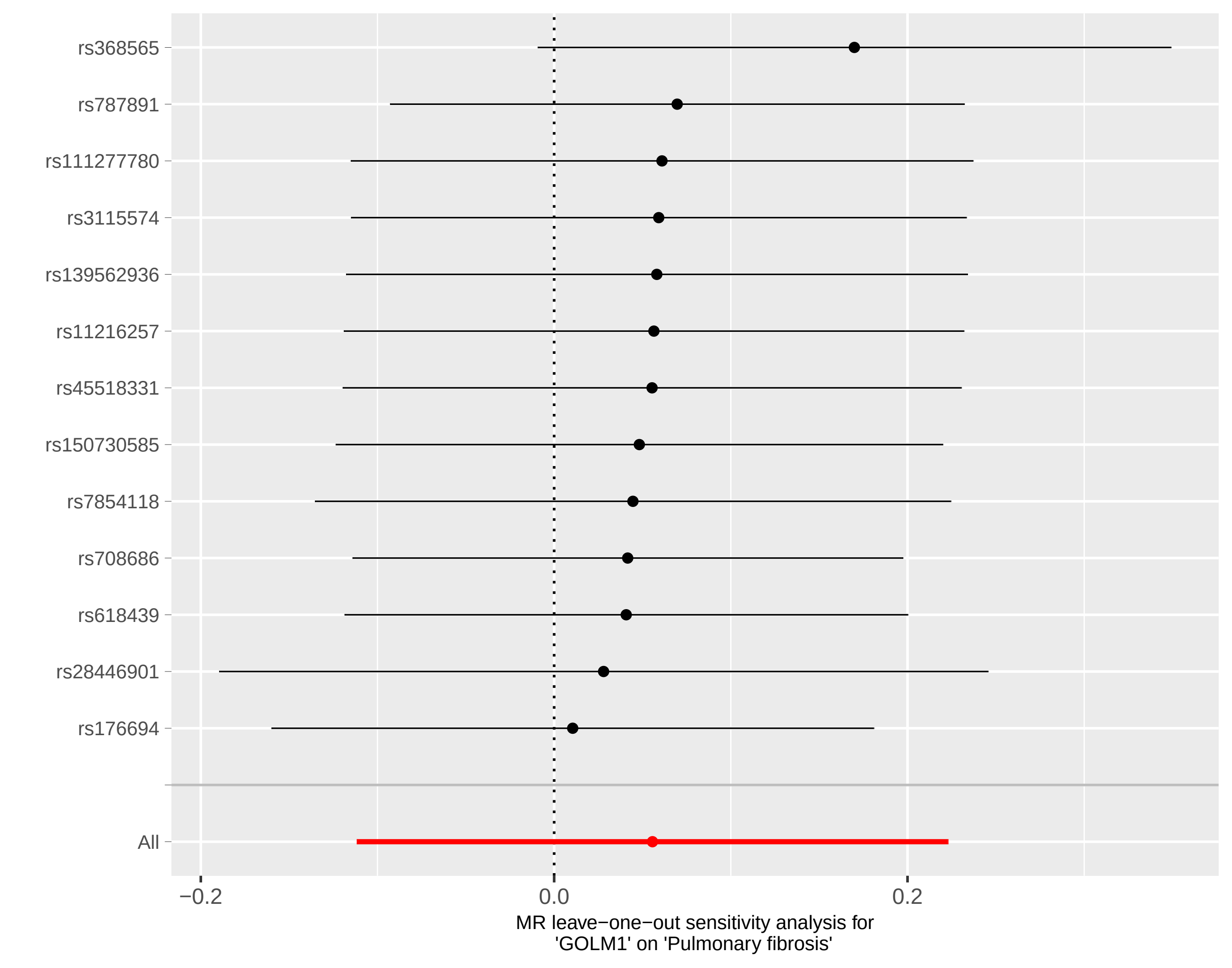
rs368565
rs787891
rs111277780
rs3115574
rs139562936
rs11216257
rs45518331
rs150730585
rs7854118
rs708686
rs618439
rs28446901
rs176694
All
−0.2
0.0
0.2
MR leave−one−out sensitivity analysis for
'GOLM1' on 'Pulmonary fibrosis'

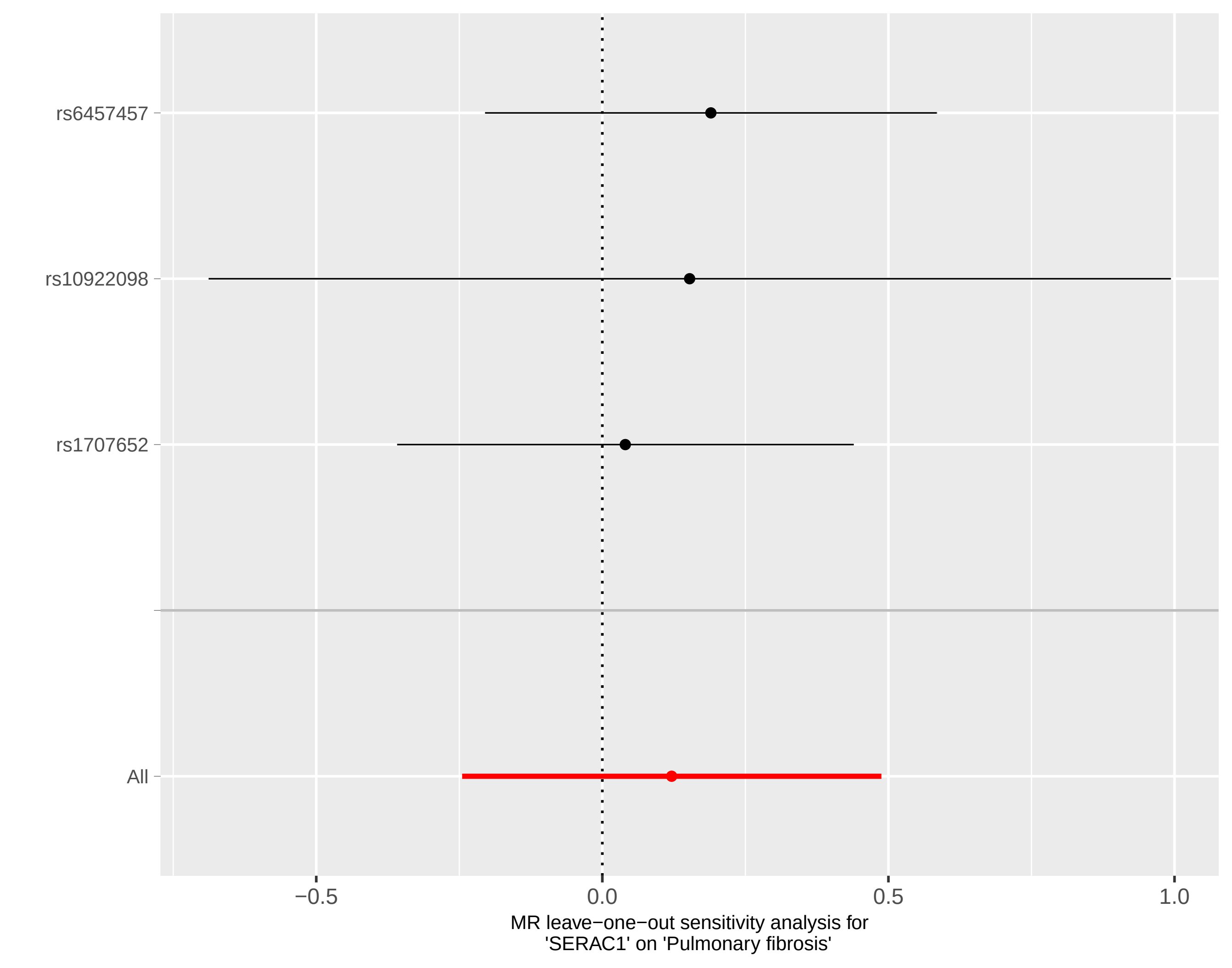

rs6457457
rs10922098
rs1707652
All
−0.5
0.0
0.5
1.0
MR leave−one−out sensitivity analysis for
'SERAC1' on 'Pulmonary fibrosis'

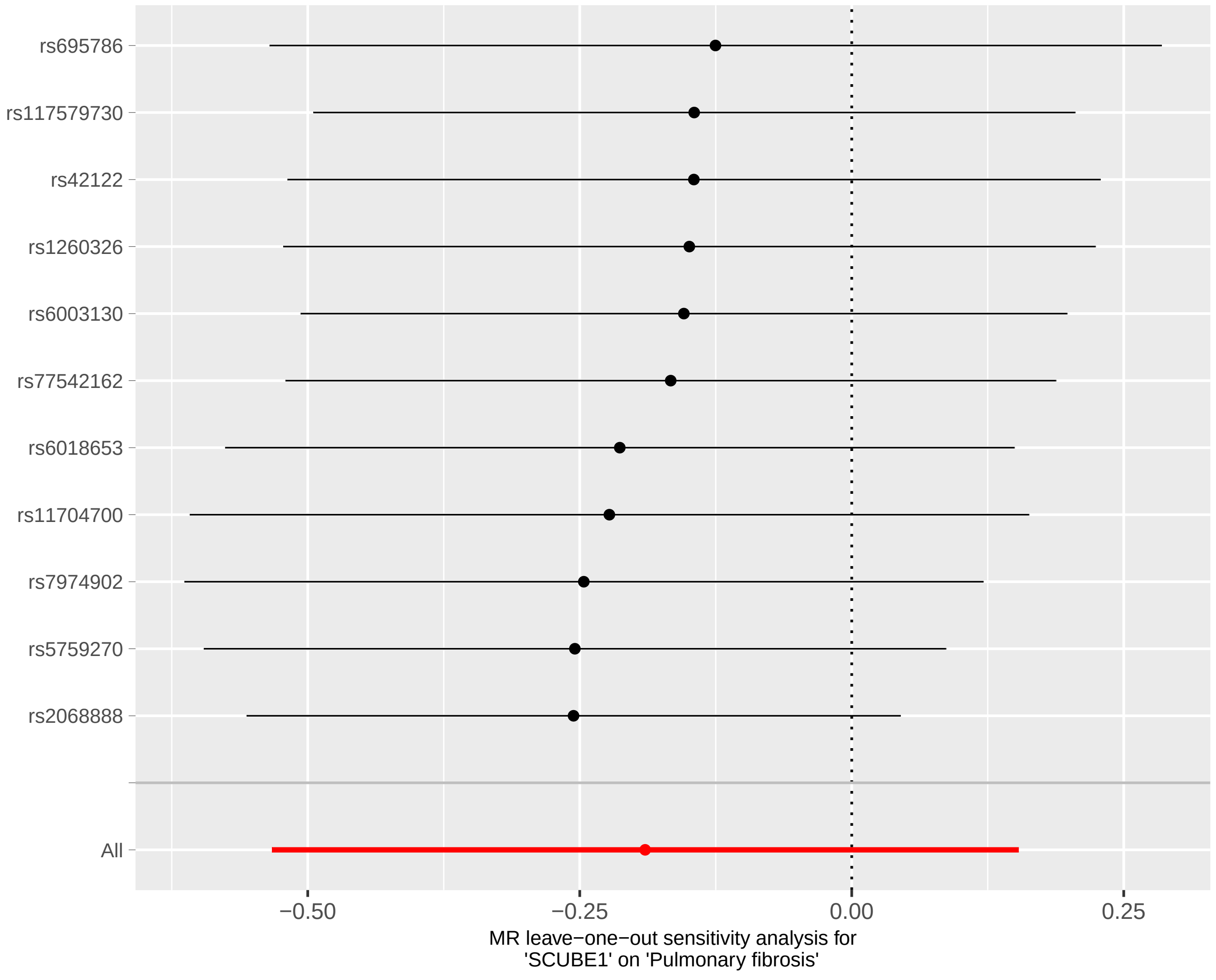
rs695786
rs117579730
rs42122
rs1260326
rs6003130
rs77542162
rs6018653
rs11704700
rs7974902
rs5759270
rs2068888
All
−0.50
−0.25
0.00
0.25
MR leave−one−out sensitivity analysis for
'SCUBE1' on 'Pulmonary fibrosis'

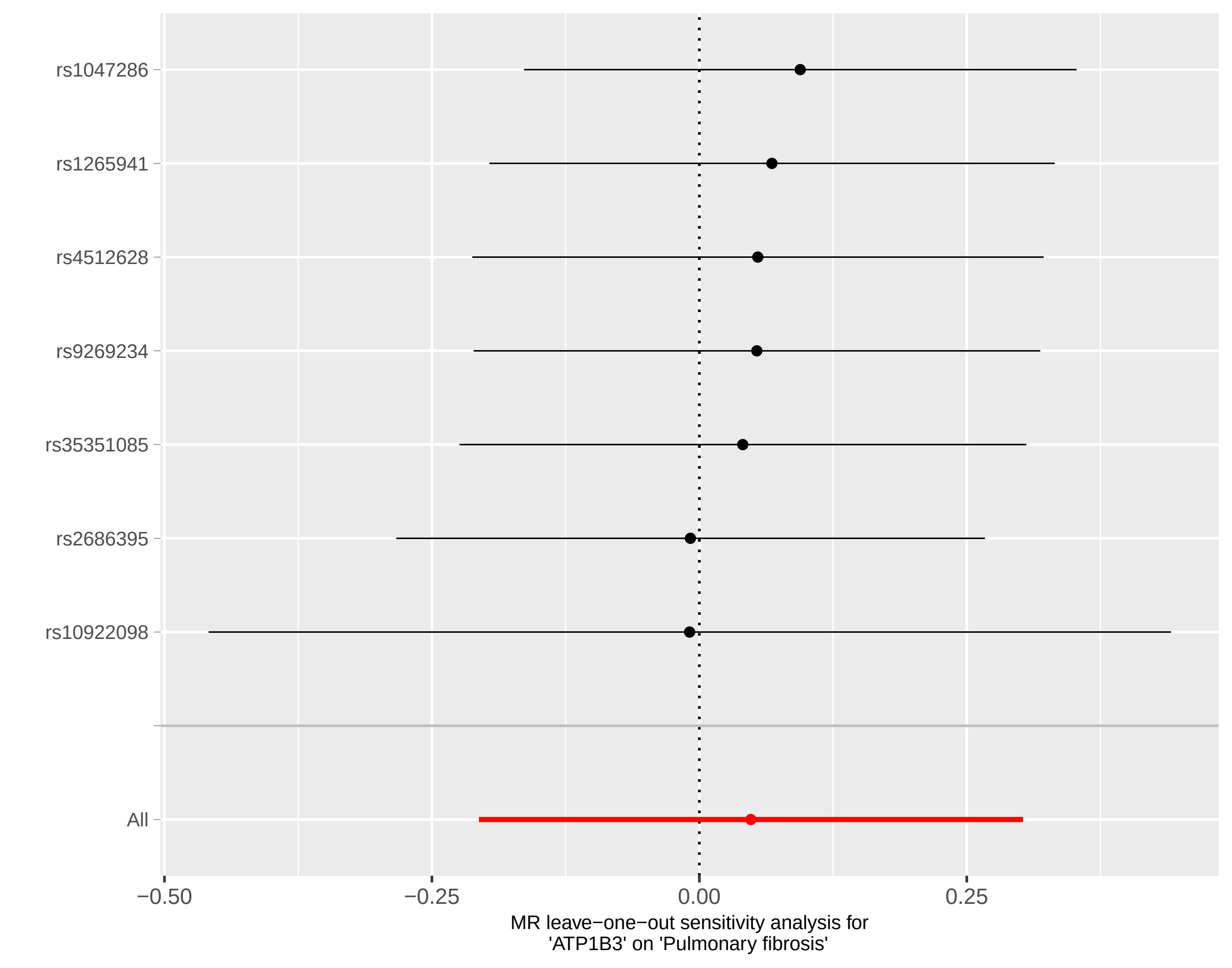
rs1047286
rs1265941
rs4512628
rs9269234
rs35351085
rs2686395
rs10922098
All
−0.50
−0.25
0.00
0.25
MR leave−one−out sensitivity analysis for
'ATP1B3' on 'Pulmonary fibrosis'

Insufficient number of SNPs

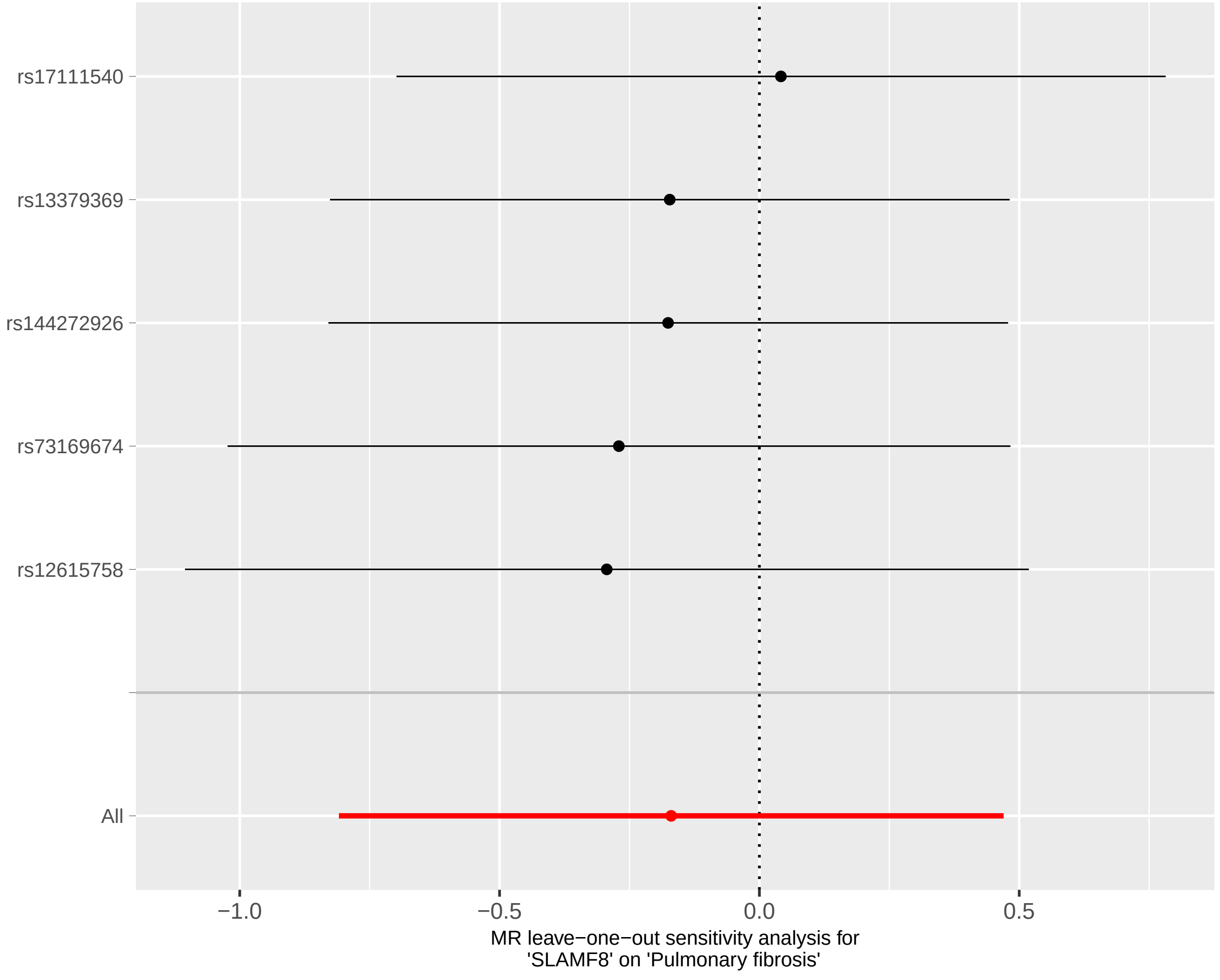
rs17111540
rs13379369
rs144272926
rs73169674
rs12615758
All
−1.0
−0.5
0.0
0.5
MR leave−one−out sensitivity analysis for
'SLAMF8' on 'Pulmonary fibrosis'

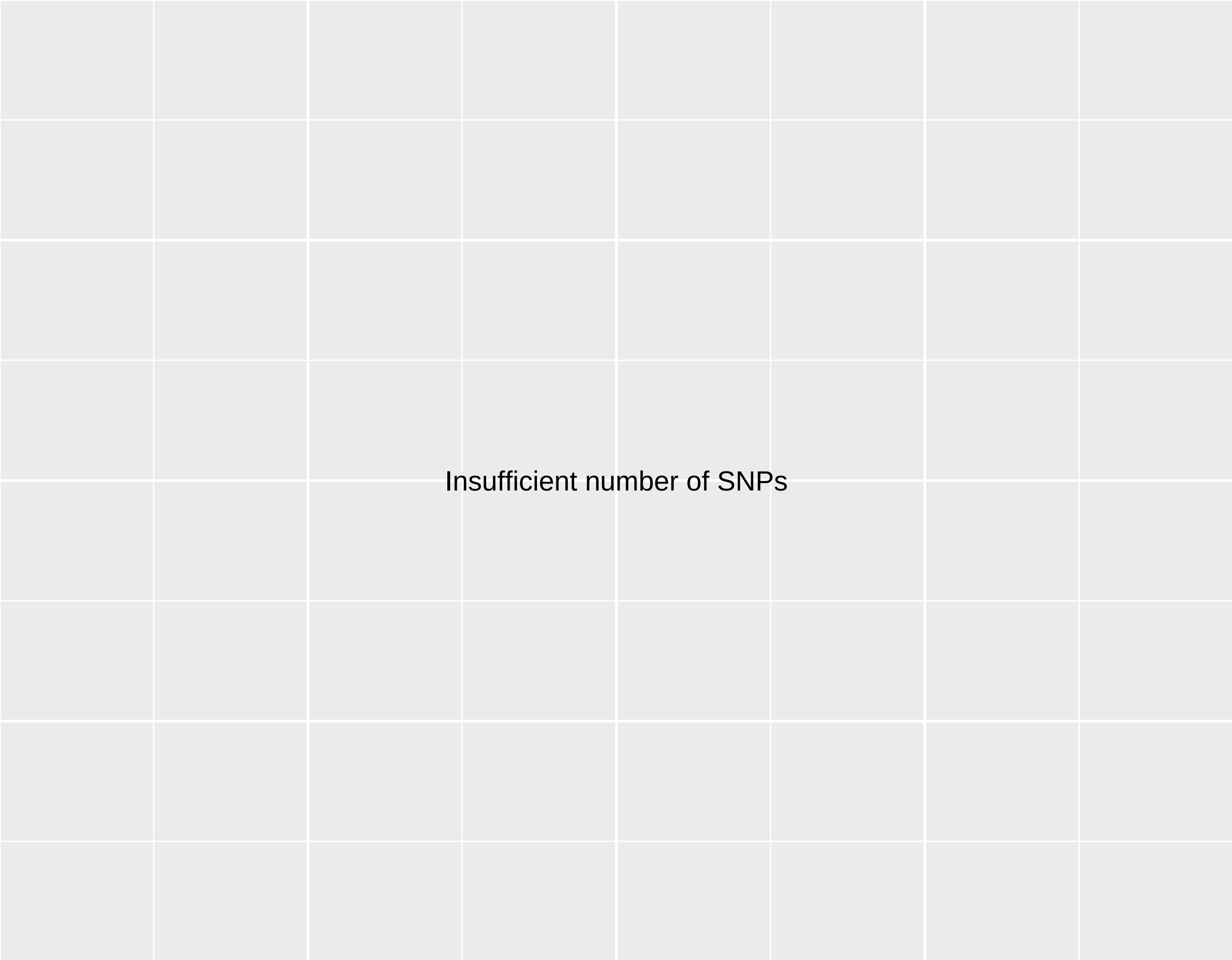
Insufficient number of SNPs

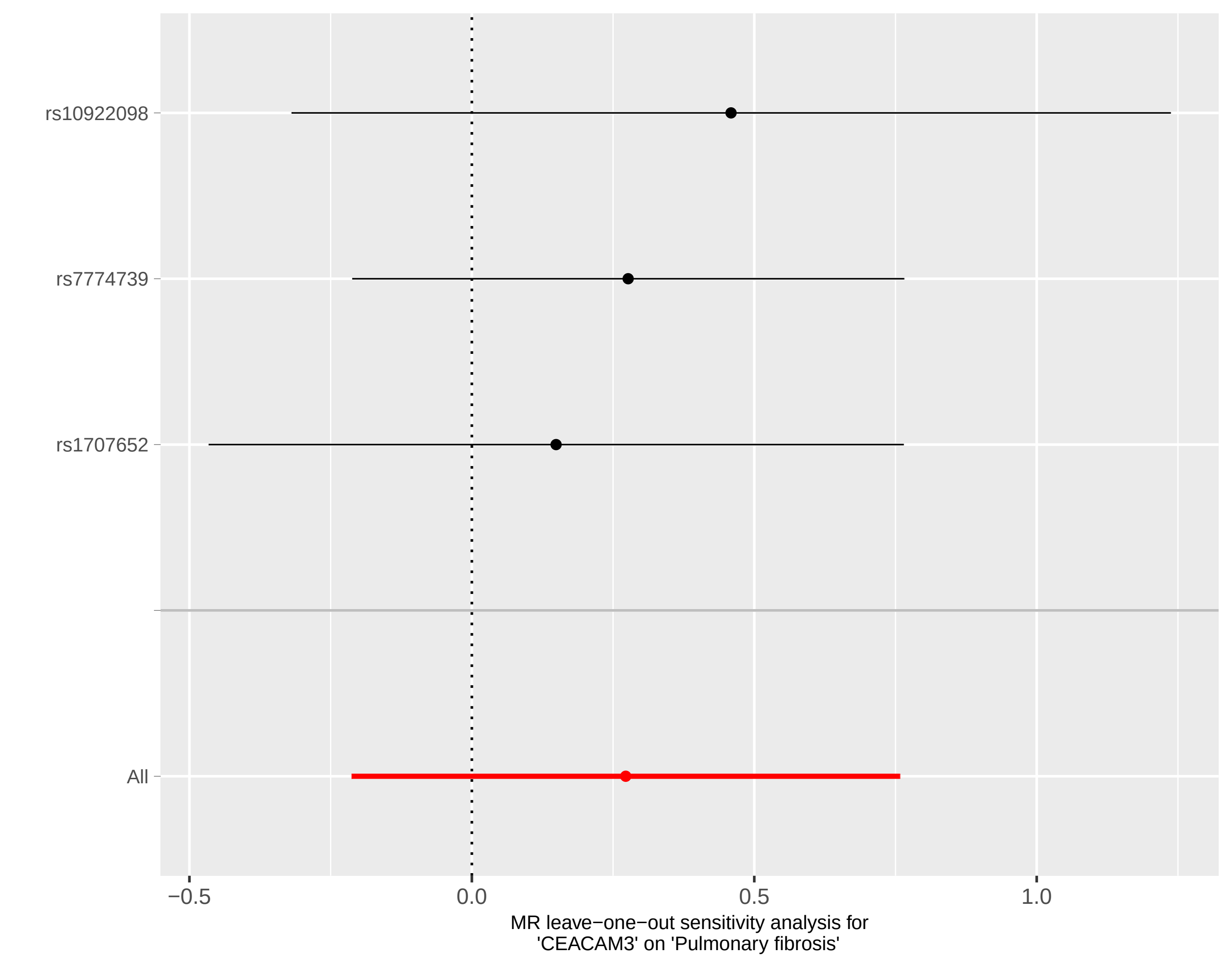

rs10922098
rs7774739
rs1707652
All
−0.5
0.0
0.5
1.0
MR leave−one−out sensitivity analysis for
'CEACAM3' on 'Pulmonary fibrosis'

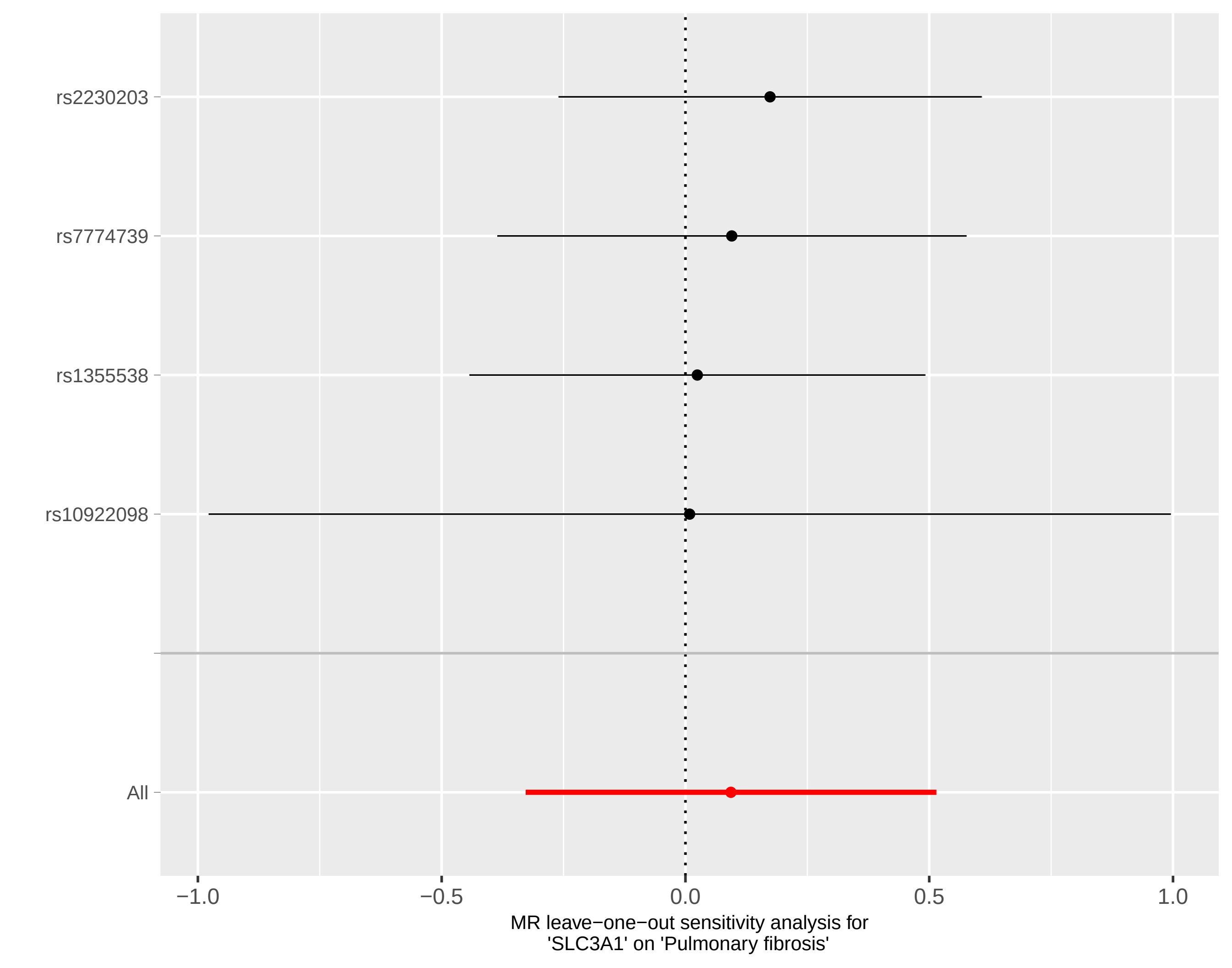
rs2230203
rs7774739
rs1355538
rs10922098
All
−1.0
−0.5
0.0
0.5
1.0
MR leave−one−out sensitivity analysis for
'SLC3A1' on 'Pulmonary fibrosis'

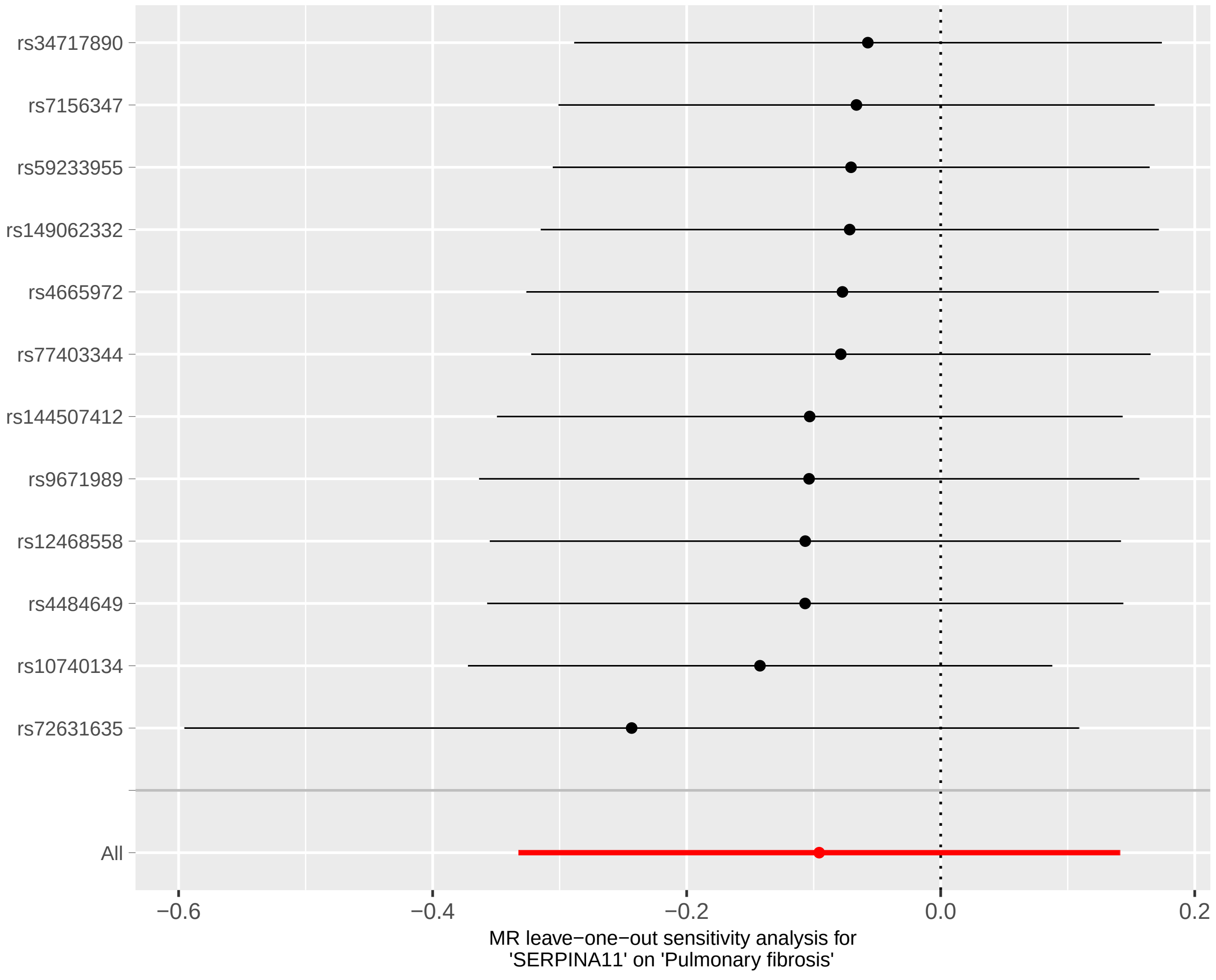
rs34717890
rs7156347
rs59233955
rs149062332
rs4665972
rs77403344
rs144507412
rs9671989
rs12468558
rs4484649
rs10740134
rs72631635
All
−0.6
−0.4
−0.2
0.0
0.2
MR leave−one−out sensitivity analysis for
'SERPINA11' on 'Pulmonary fibrosis'

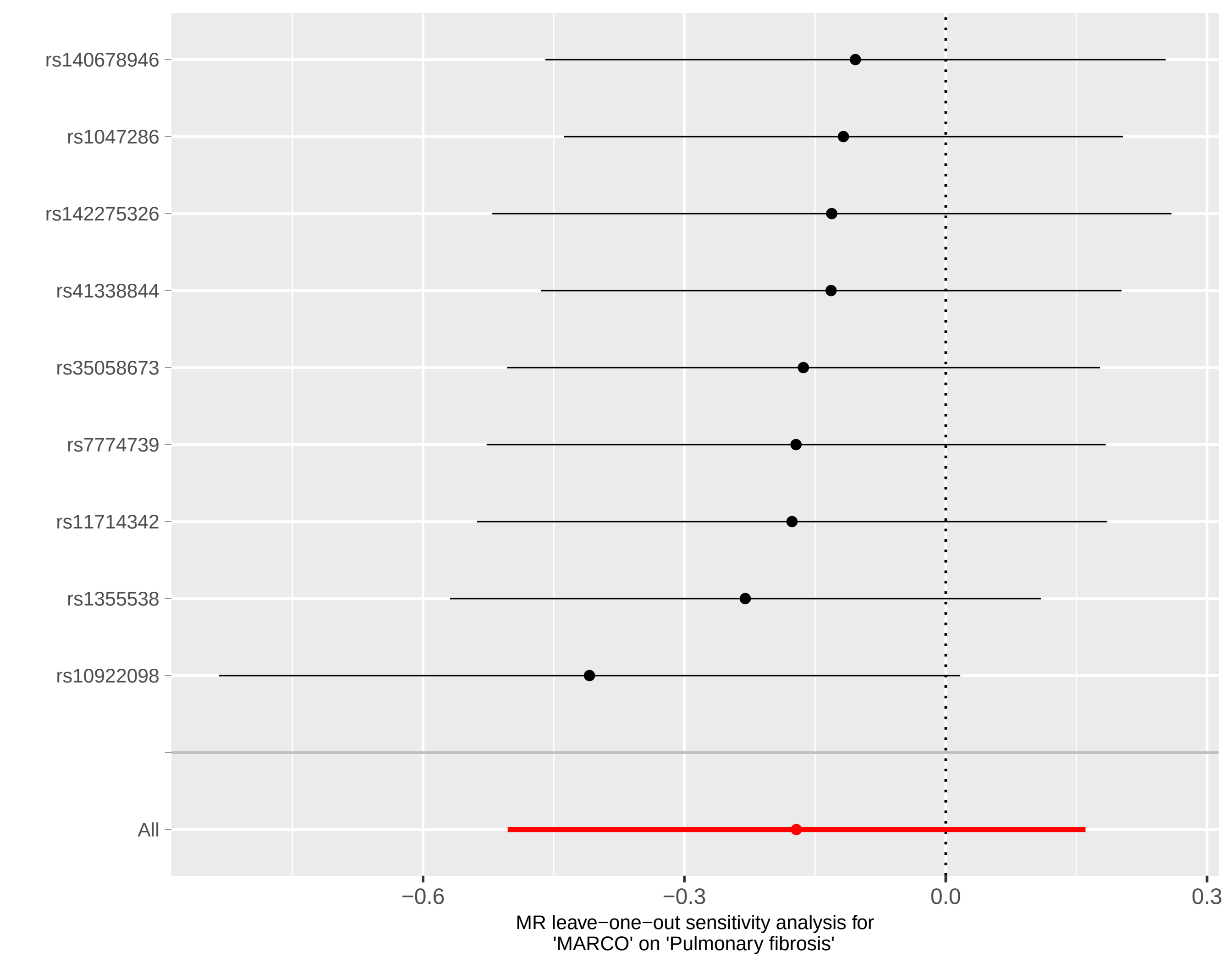
rs140678946
rs1047286
rs142275326
rs41338844
rs35058673
rs7774739
rs11714342
rs1355538
rs10922098
All
−0.6
−0.3
0.0
0.3
MR leave−one−out sensitivity analysis for
'MARCO' on 'Pulmonary fibrosis'

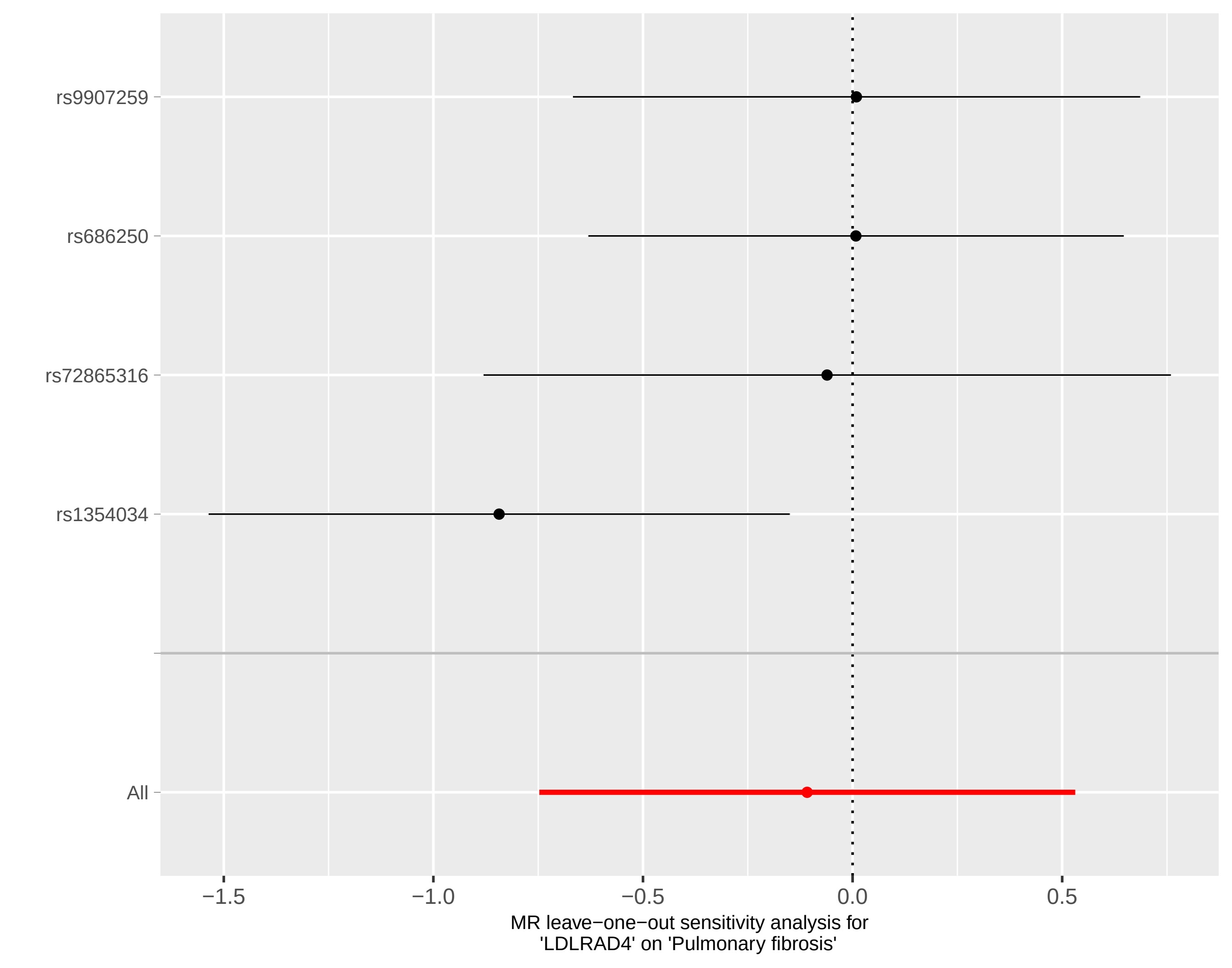
rs9907259
rs686250
rs72865316
rs1354034
All
−1.5
−1.0
−0.5
0.0
0.5
MR leave−one−out sensitivity analysis for
'LDLRAD4' on 'Pulmonary fibrosis'

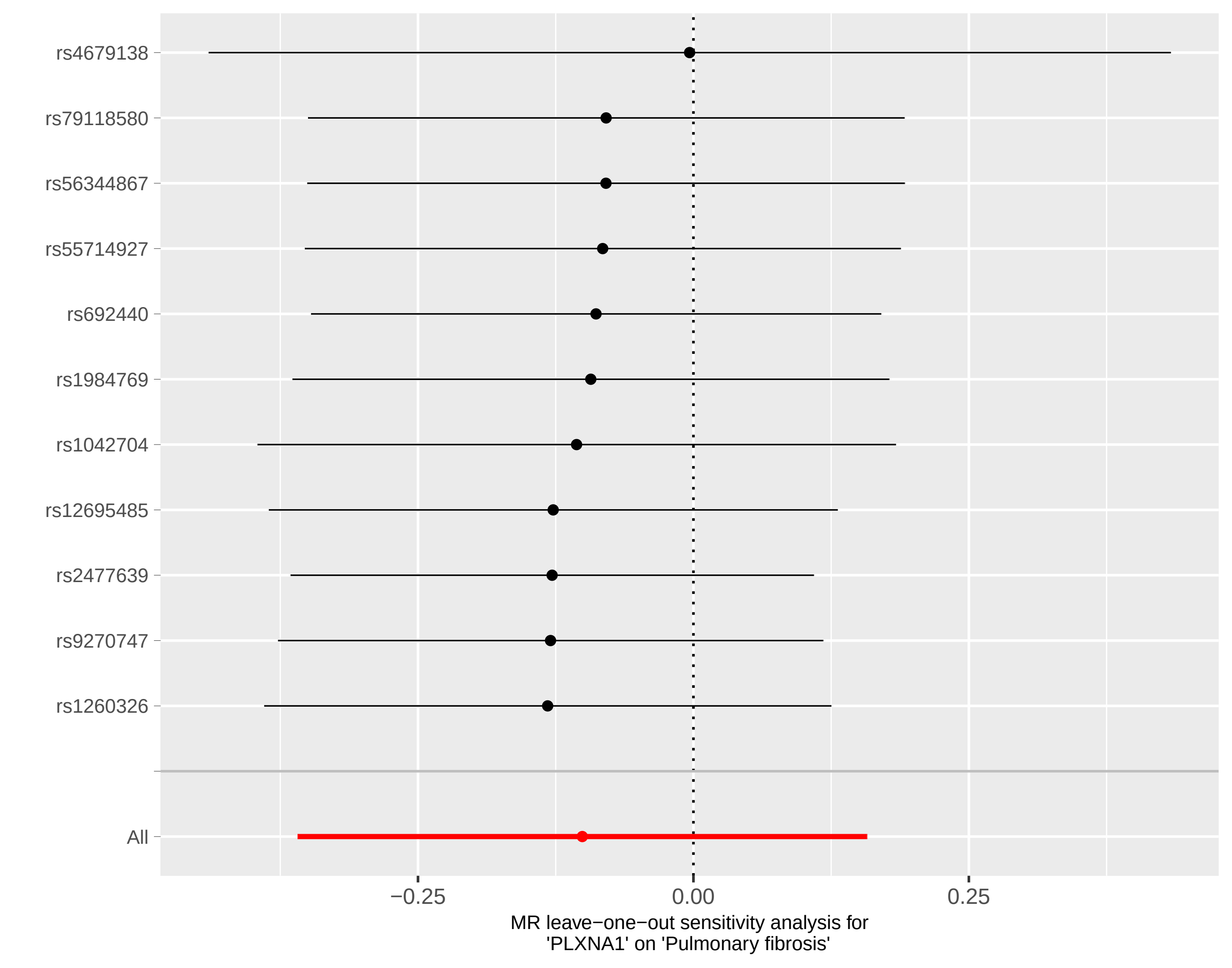
rs4679138
rs79118580
rs56344867
rs55714927
rs692440
rs1984769
rs1042704
rs12695485
rs2477639
rs9270747
rs1260326
All
−0.25
0.00
0.25
MR leave−one−out sensitivity analysis for
'PLXNA1' on 'Pulmonary fibrosis'

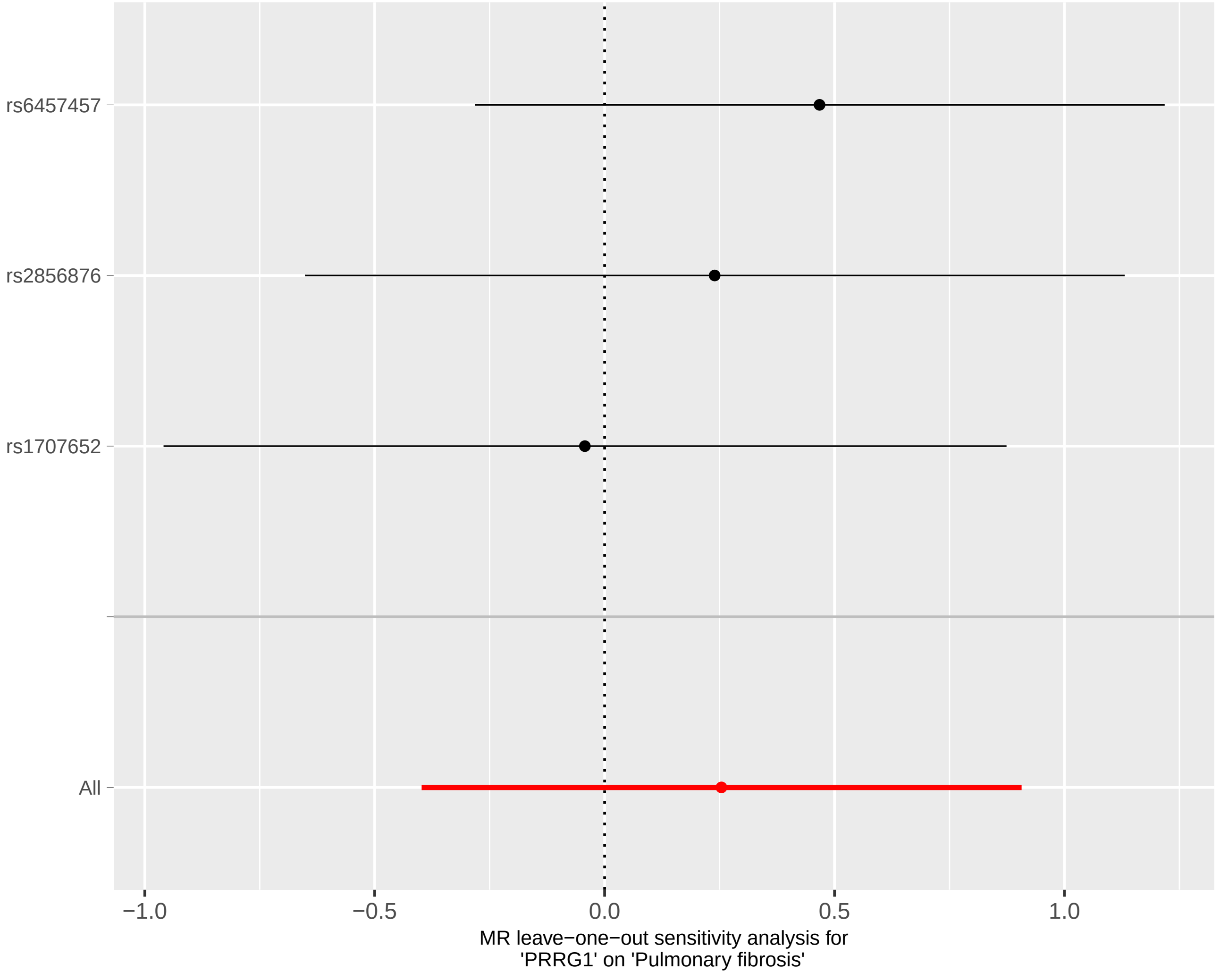

rs6457457
rs2856876
rs1707652
All
−1.0
−0.5
0.0
0.5
1.0
MR leave−one−out sensitivity analysis for
'PRRG1' on 'Pulmonary fibrosis'

Insufficient number of SNPs

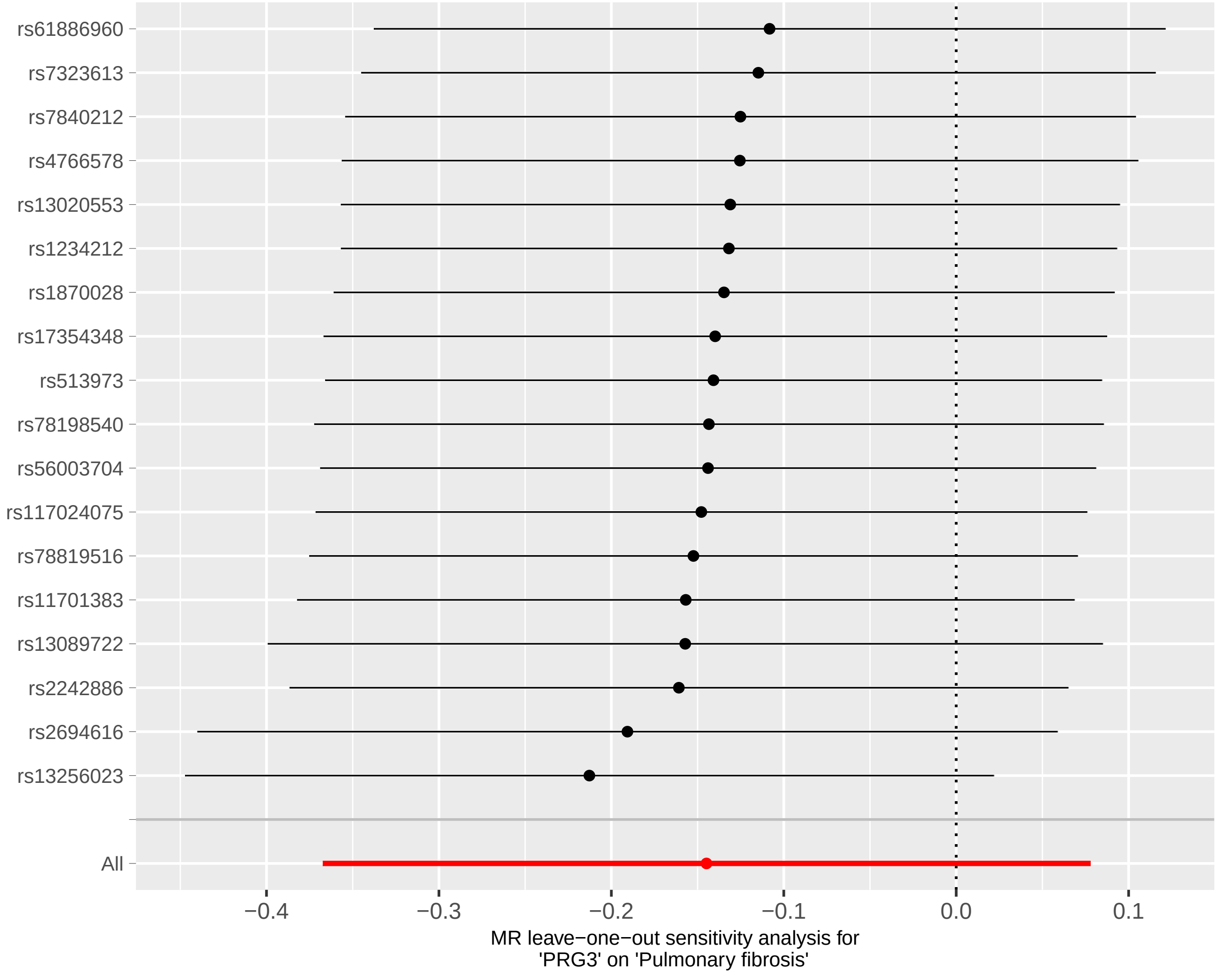
rs61886960
rs7323613
rs7840212
rs4766578
rs13020553
rs1234212
rs1870028
rs17354348
rs513973
rs78198540
rs56003704
rs117024075
rs78819516
rs11701383
rs13089722
rs2242886
rs2694616
rs13256023
All
−0.4
−0.3
−0.2
−0.1
0.0
0.1
MR leave−one−out sensitivity analysis for
'PRG3' on 'Pulmonary fibrosis'

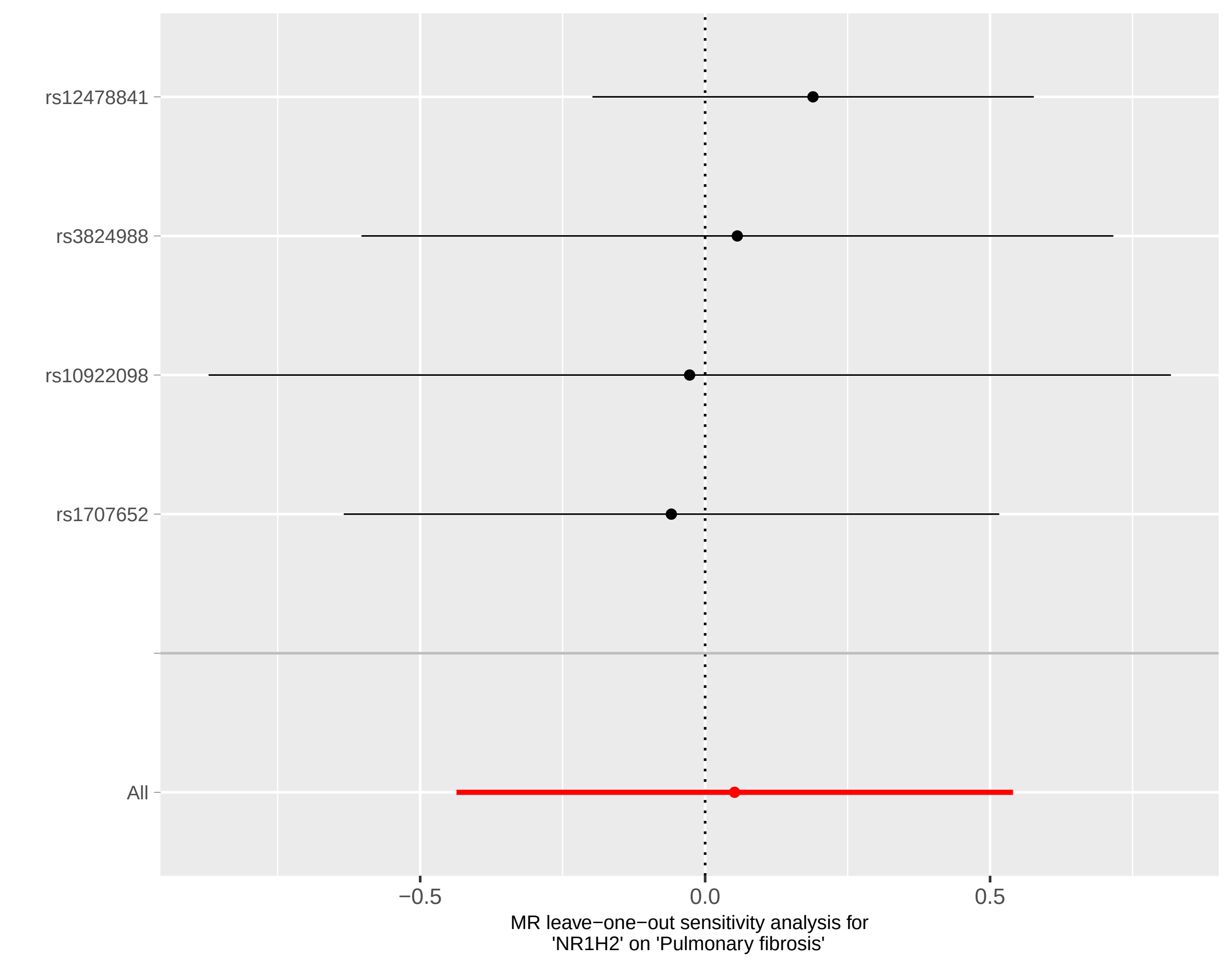
rs12478841
rs3824988
rs10922098
rs1707652
All
−0.5
0.0
0.5
MR leave−one−out sensitivity analysis for
'NR1H2' on 'Pulmonary fibrosis'

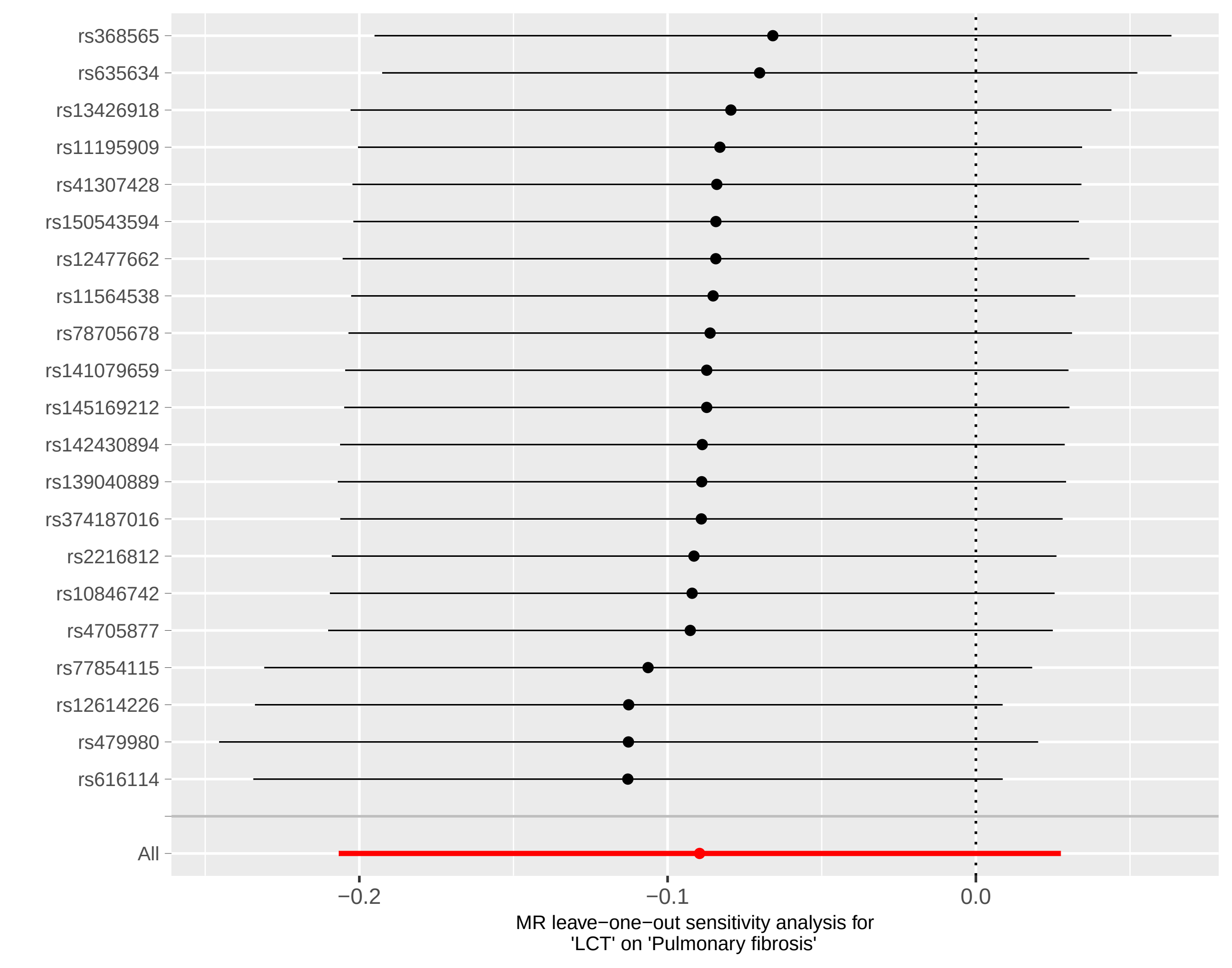

rs368565
rs635634
rs13426918
rs11195909
rs41307428
rs150543594
rs12477662
rs11564538
rs78705678
rs141079659
rs145169212
rs142430894
rs139040889
rs374187016
rs2216812
rs10846742
rs4705877
rs77854115
rs12614226
rs479980
rs616114
All
−0.2
−0.1
0.0
MR leave−one−out sensitivity analysis for
'LCT' on 'Pulmonary fibrosis'

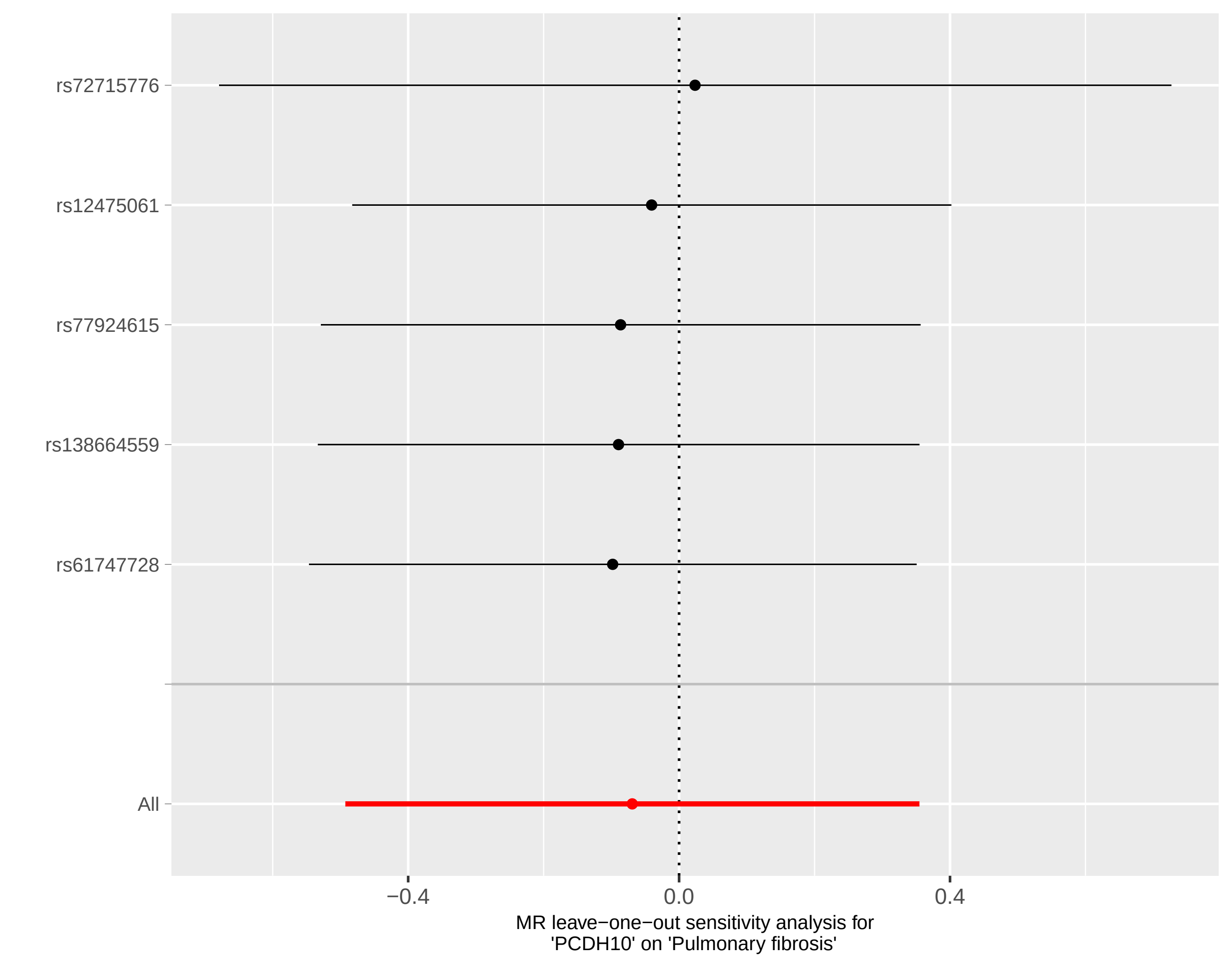

rs72715776
rs12475061
rs77924615
rs138664559
rs61747728
All
−0.4
0.0
0.4
MR leave−one−out sensitivity analysis for
'PCDH10' on 'Pulmonary fibrosis'

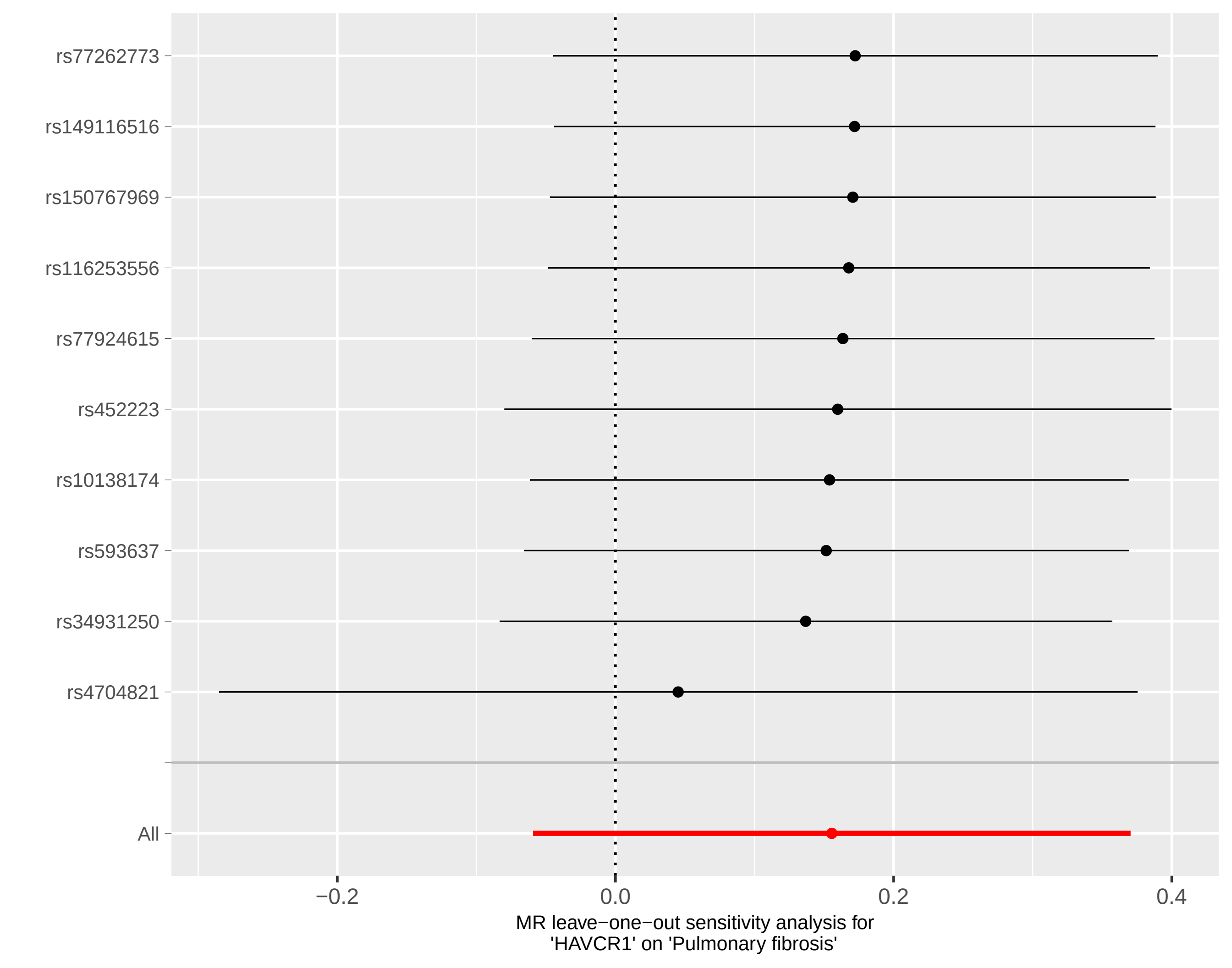
rs77262773
rs149116516
rs150767969
rs116253556
rs77924615
rs452223
rs10138174
rs593637
rs34931250
rs4704821
All
−0.2
0.0
0.2
0.4
MR leave−one−out sensitivity analysis for
'HAVCR1' on 'Pulmonary fibrosis'

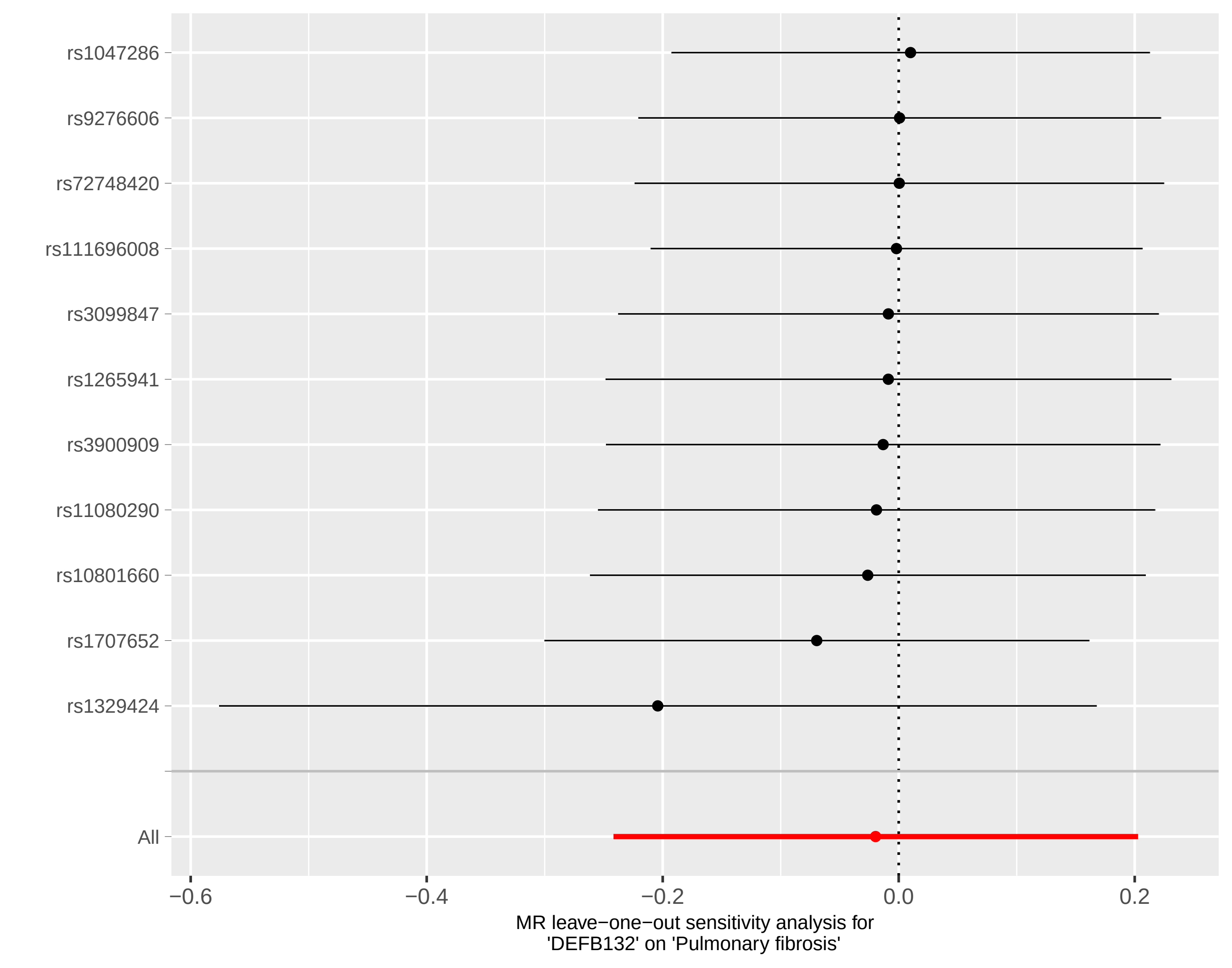
rs1047286
rs9276606
rs72748420
rs111696008
rs3099847
rs1265941
rs3900909
rs11080290
rs10801660
rs1707652
rs1329424
All
−0.6
−0.4
−0.2
0.0
0.2
MR leave−one−out sensitivity analysis for
'DEFB132' on 'Pulmonary fibrosis'

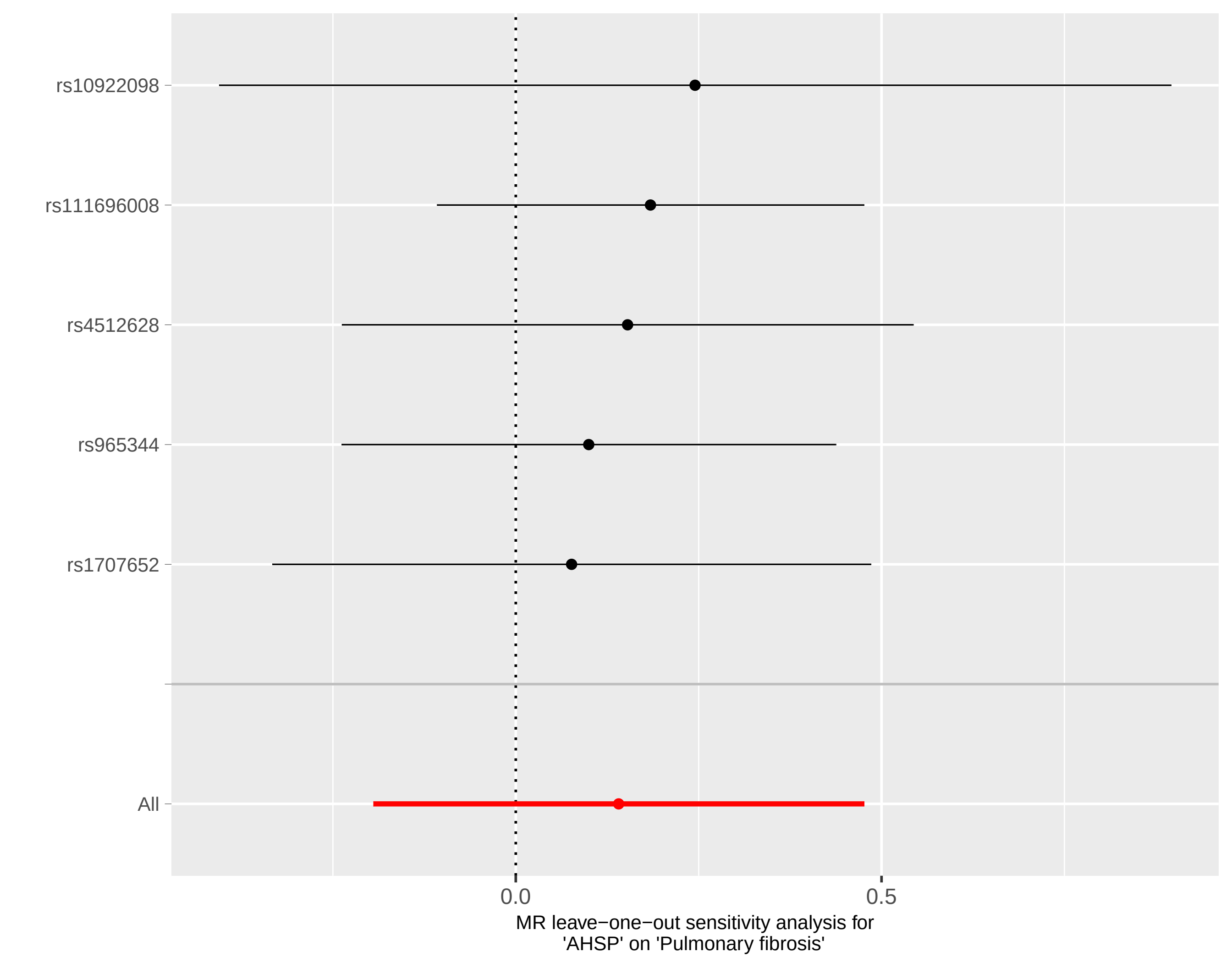
rs10922098
rs111696008
rs4512628
rs965344
rs1707652
All
0.0
0.5
MR leave−one−out sensitivity analysis for
'AHSP' on 'Pulmonary fibrosis'

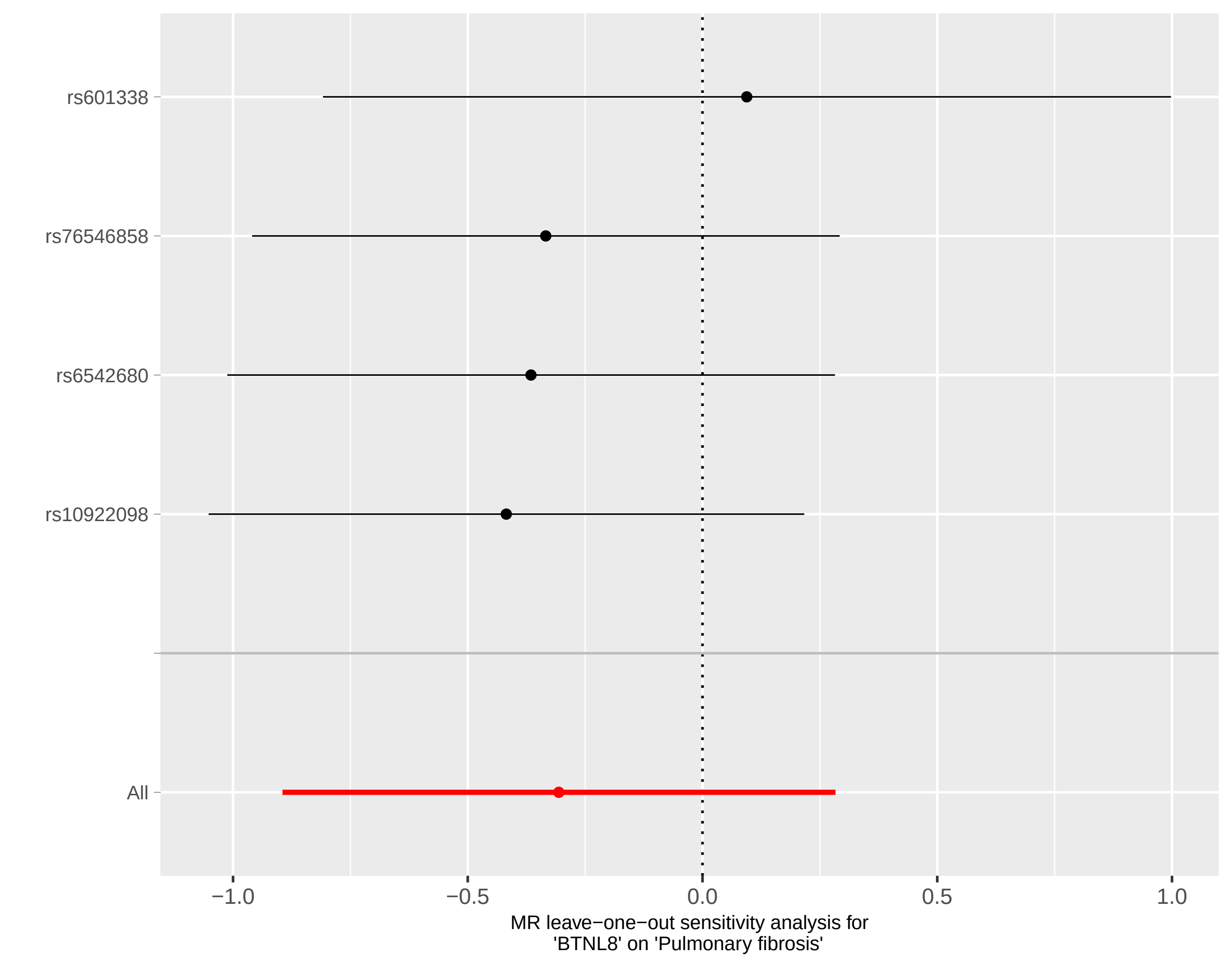
rs601338
rs76546858
rs6542680
rs10922098
All
−1.0
−0.5
0.0
0.5
1.0
MR leave−one−out sensitivity analysis for
'BTNL8' on 'Pulmonary fibrosis'

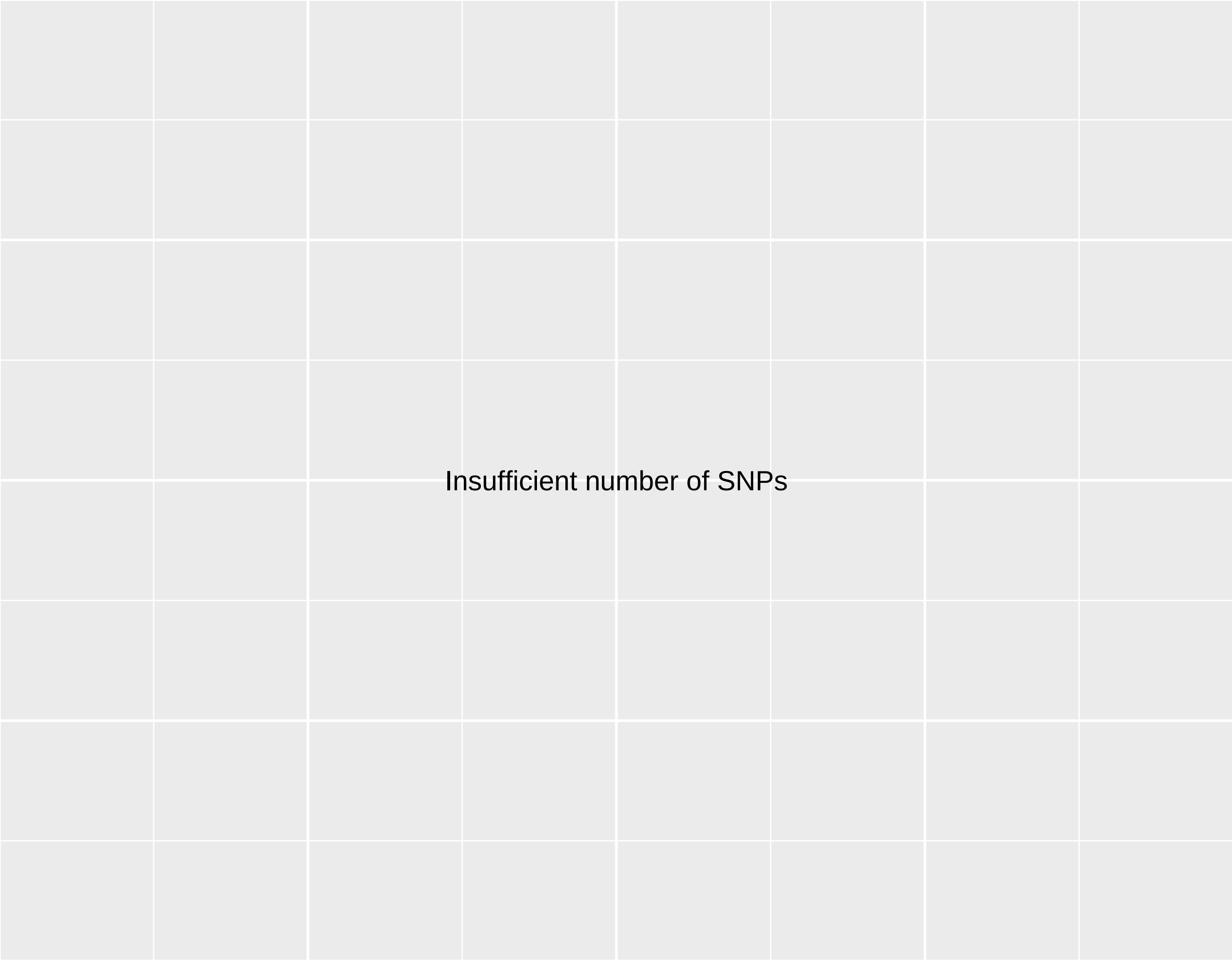
Insufficient number of SNPs

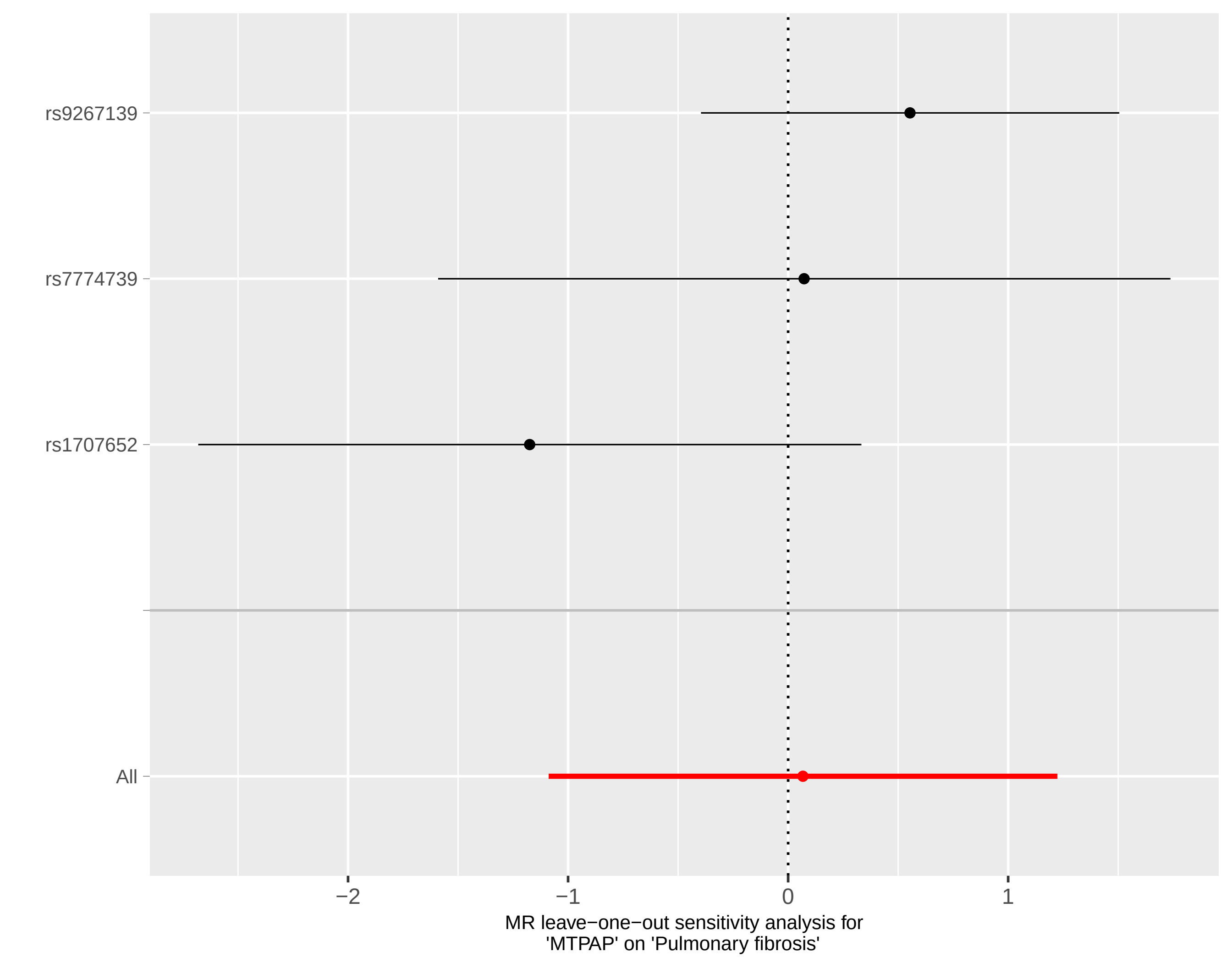
rs9267139
rs7774739
rs1707652
All
−2
−1
0
1
MR leave−one−out sensitivity analysis for
'MTPAP' on 'Pulmonary fibrosis'

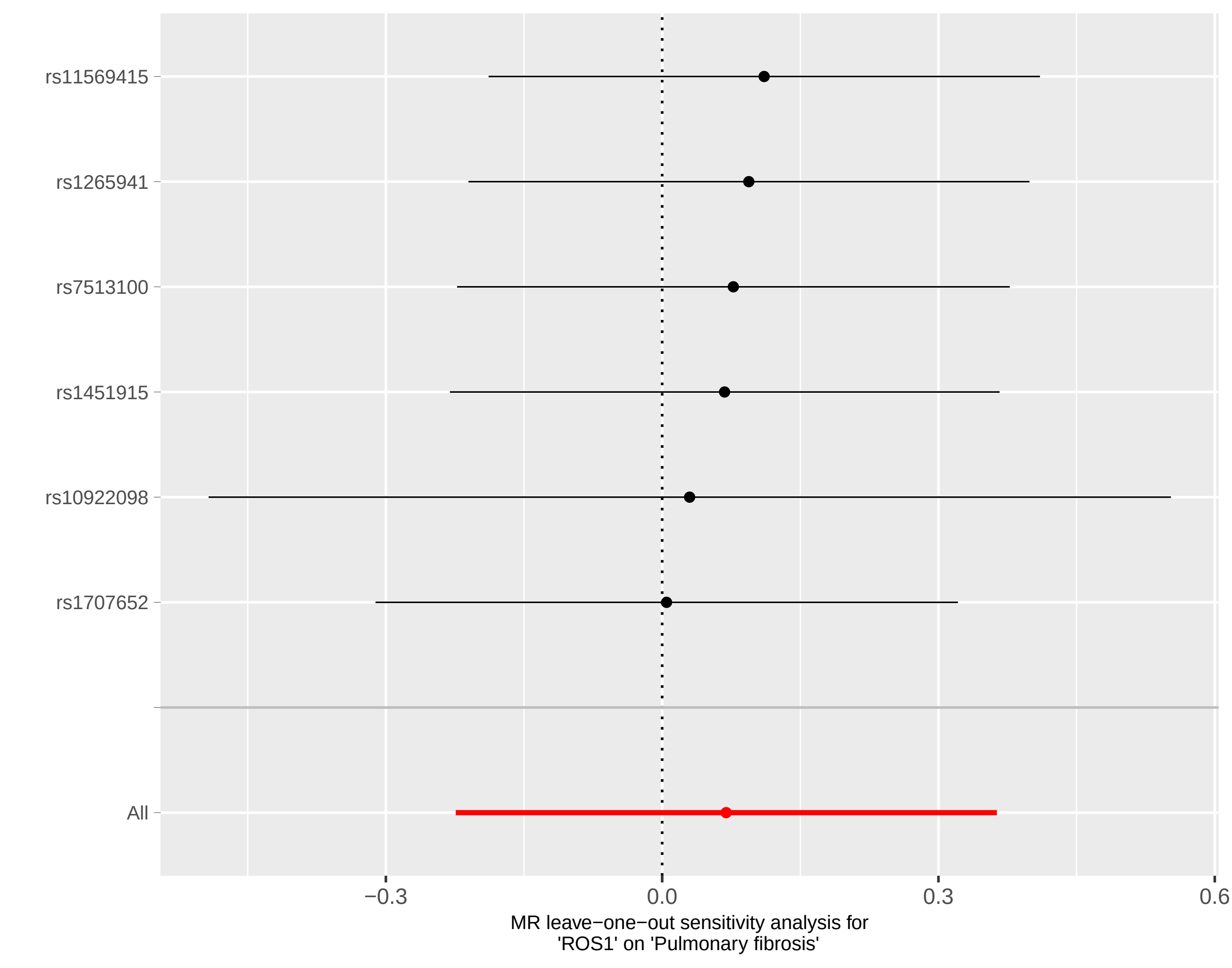
rs11569415
rs1265941
rs7513100
rs1451915
rs10922098
rs1707652
All
−0.3
0.0
0.3
0.6
MR leave−one−out sensitivity analysis for
'ROS1' on 'Pulmonary fibrosis'

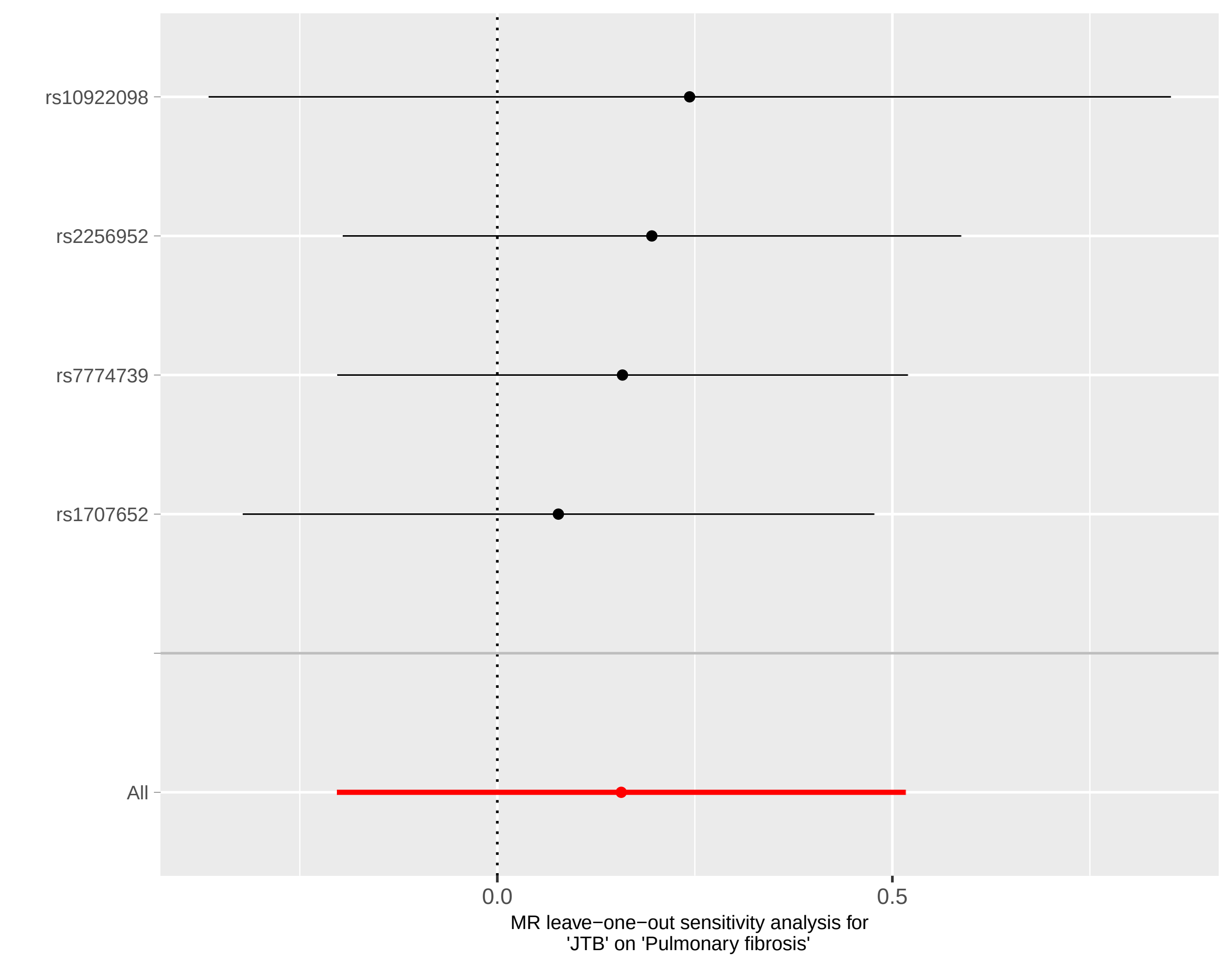
rs10922098
rs2256952
rs7774739
rs1707652
All
0.0
0.5
MR leave−one−out sensitivity analysis for
'JTB' on 'Pulmonary fibrosis'

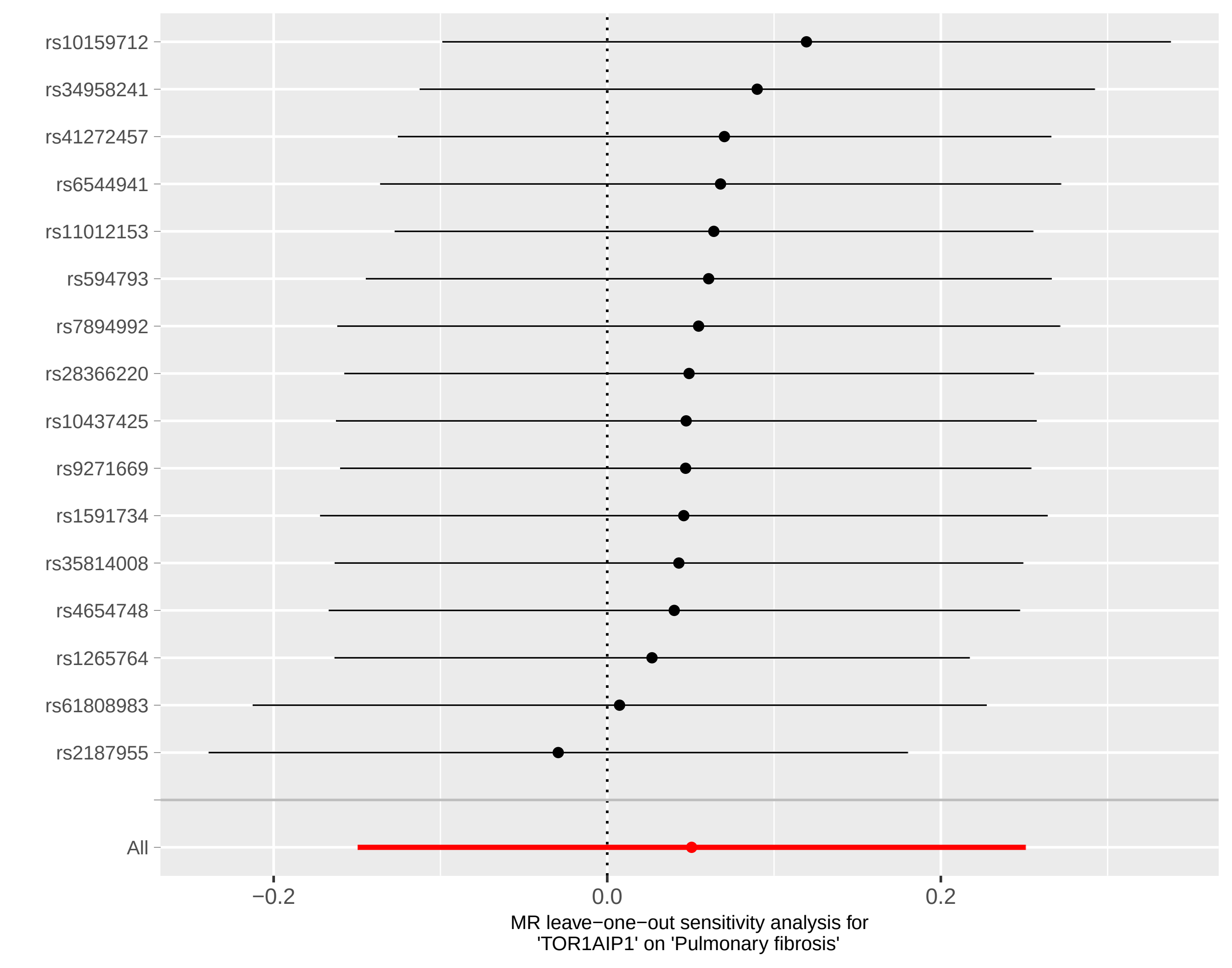
rs10159712
rs34958241
rs41272457
rs6544941
rs11012153
rs594793
rs7894992
rs28366220
rs10437425
rs9271669
rs1591734
rs35814008
rs4654748
rs1265764
rs61808983
rs2187955
All
−0.2
0.0
0.2
MR leave−one−out sensitivity analysis for
'TOR1AIP1' on 'Pulmonary fibrosis'

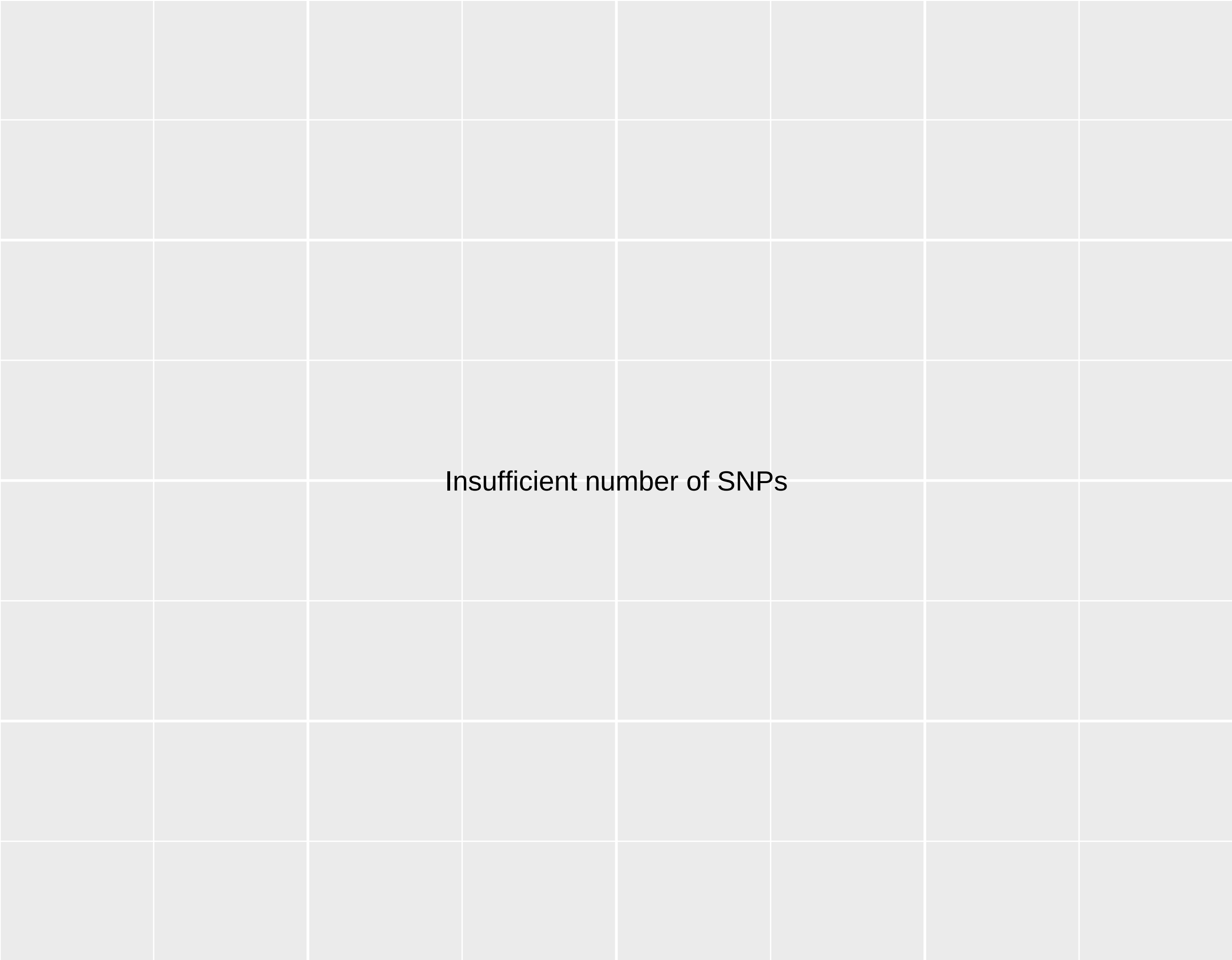
Insufficient number of SNPs

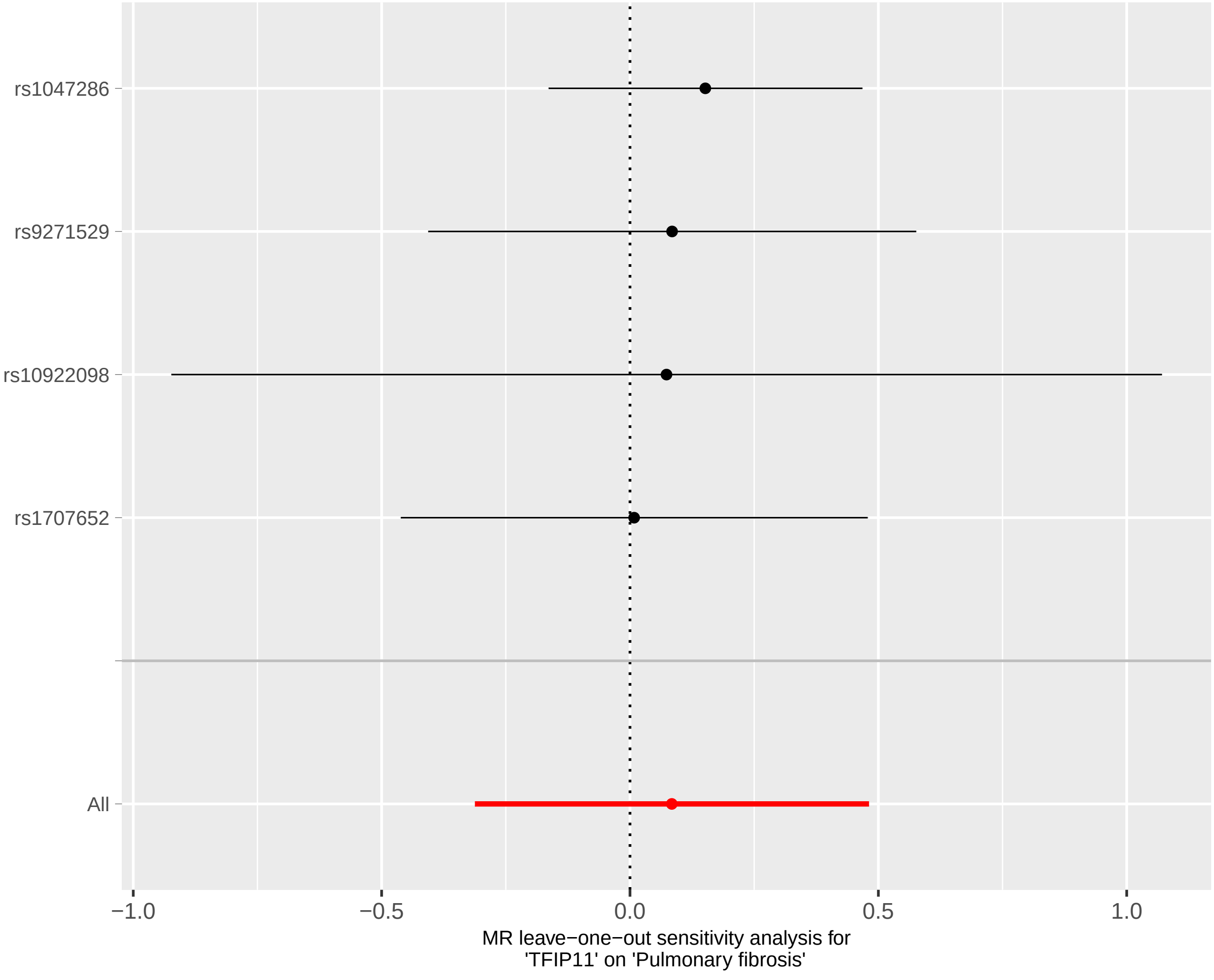
rs1047286
rs9271529
rs10922098
rs1707652
All
−1.0
−0.5
0.0
0.5
1.0
MR leave−one−out sensitivity analysis for
'TFIP11' on 'Pulmonary fibrosis'

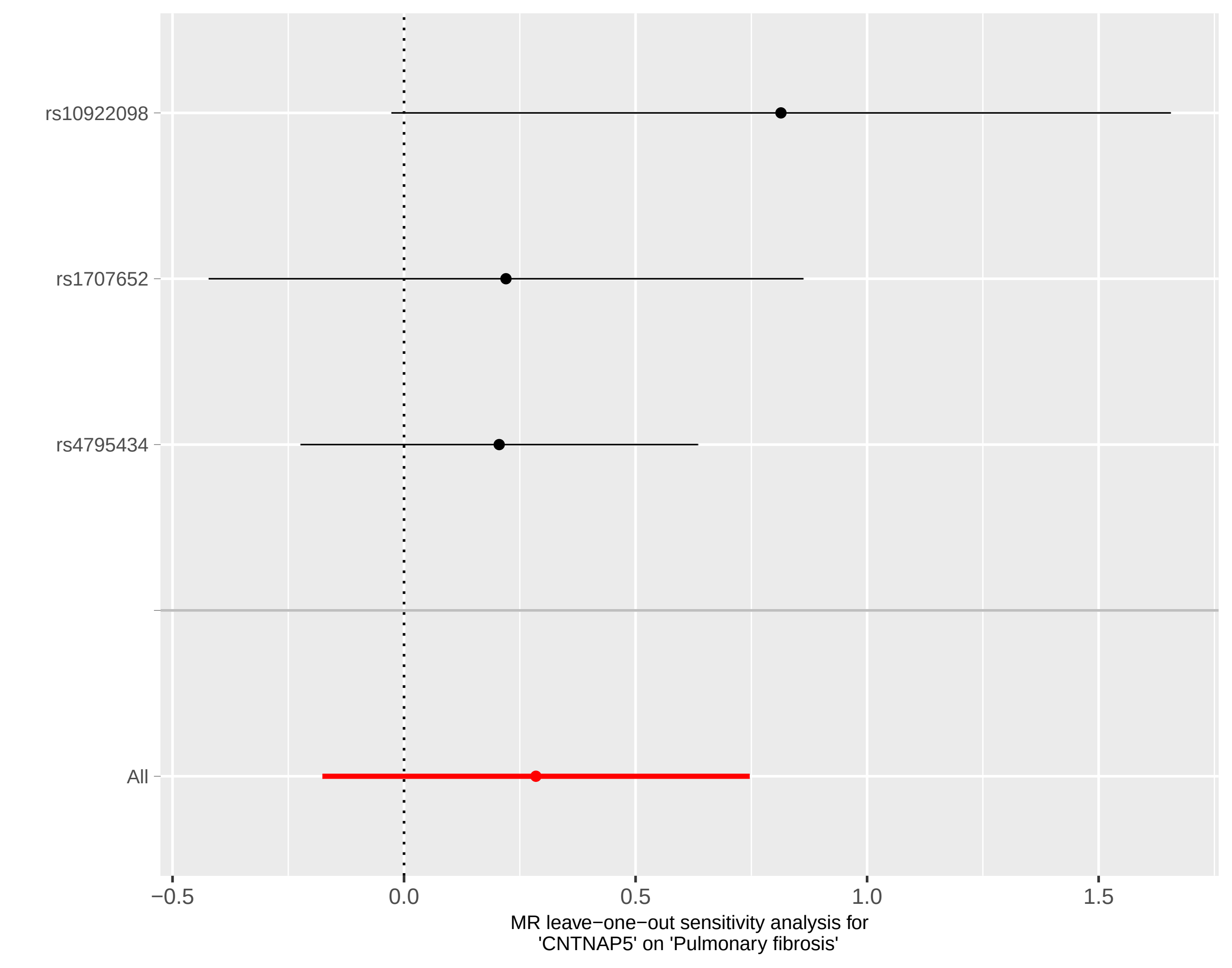
rs10922098
rs1707652
rs4795434
All
−0.5
0.0
0.5
1.0
1.5
MR leave−one−out sensitivity analysis for
'CNTNAP5' on 'Pulmonary fibrosis'

Insufficient number of SNPs

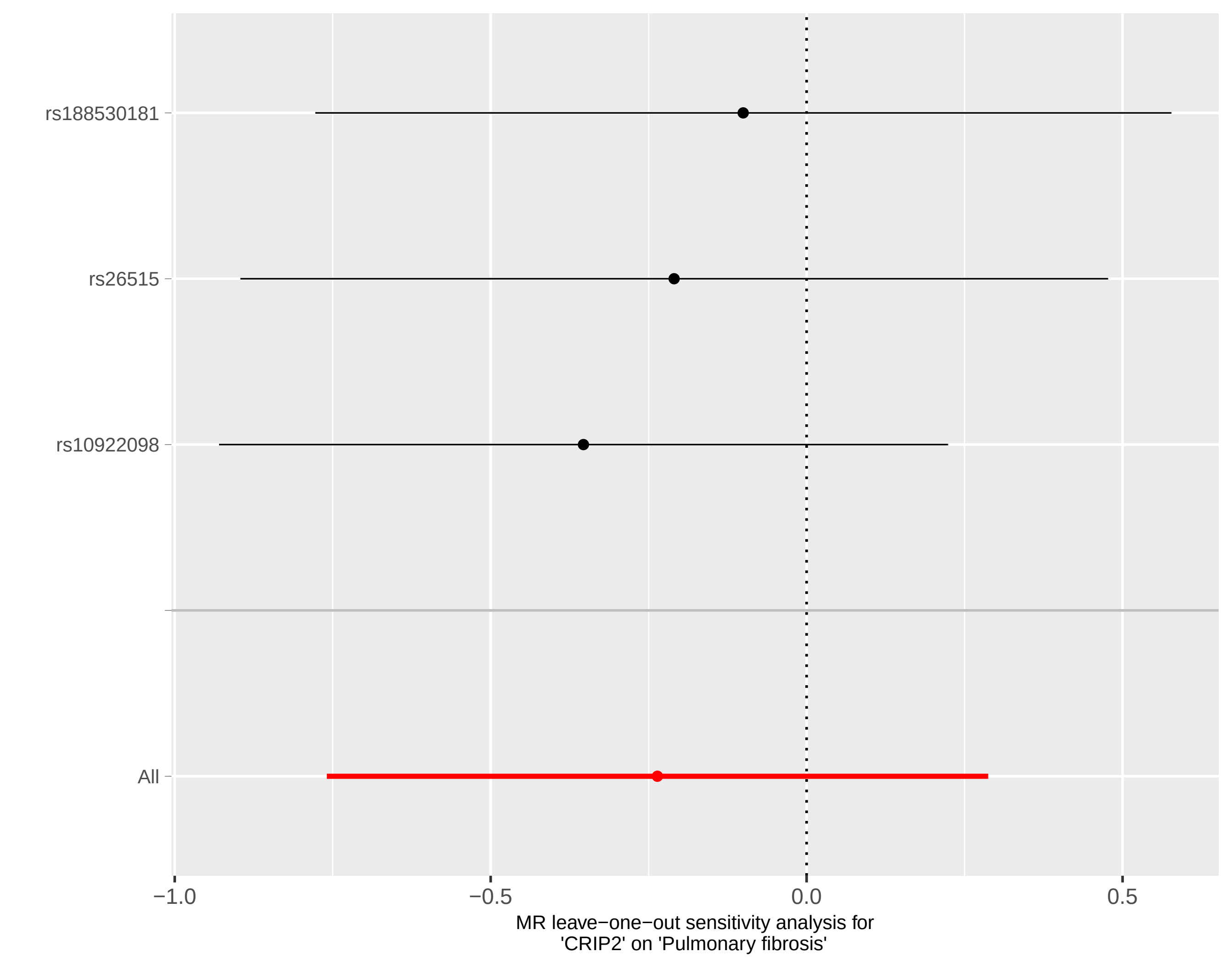
rs188530181
rs26515
rs10922098
All
−1.0
−0.5
0.0
0.5
MR leave−one−out sensitivity analysis for
'CRIP2' on 'Pulmonary fibrosis'

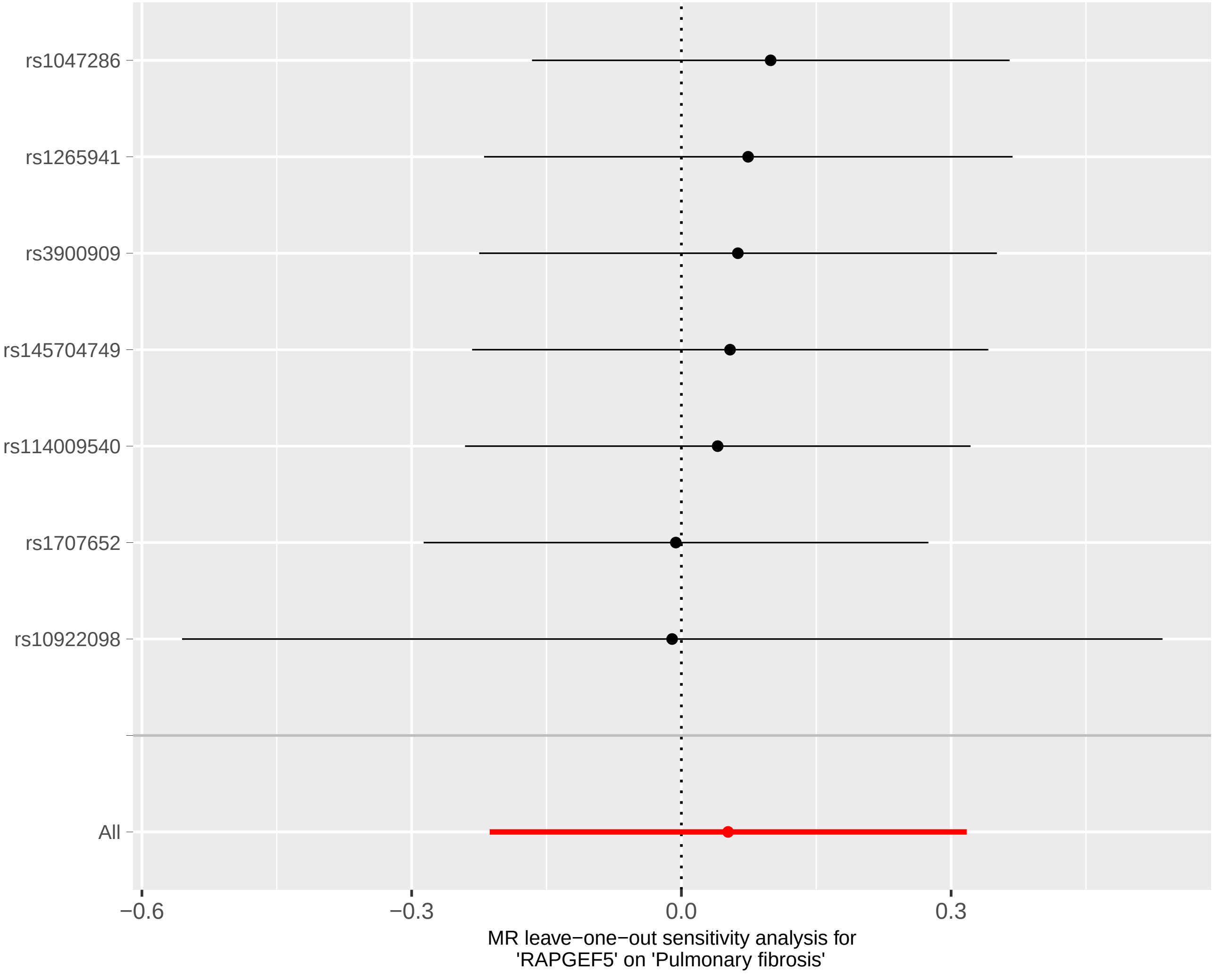
rs1047286
rs1265941
rs3900909
rs145704749
rs114009540
rs1707652
rs10922098
All
−0.6
−0.3
0.0
0.3
MR leave−one−out sensitivity analysis for
'RAPGEF5' on 'Pulmonary fibrosis'

Insufficient number of SNPs

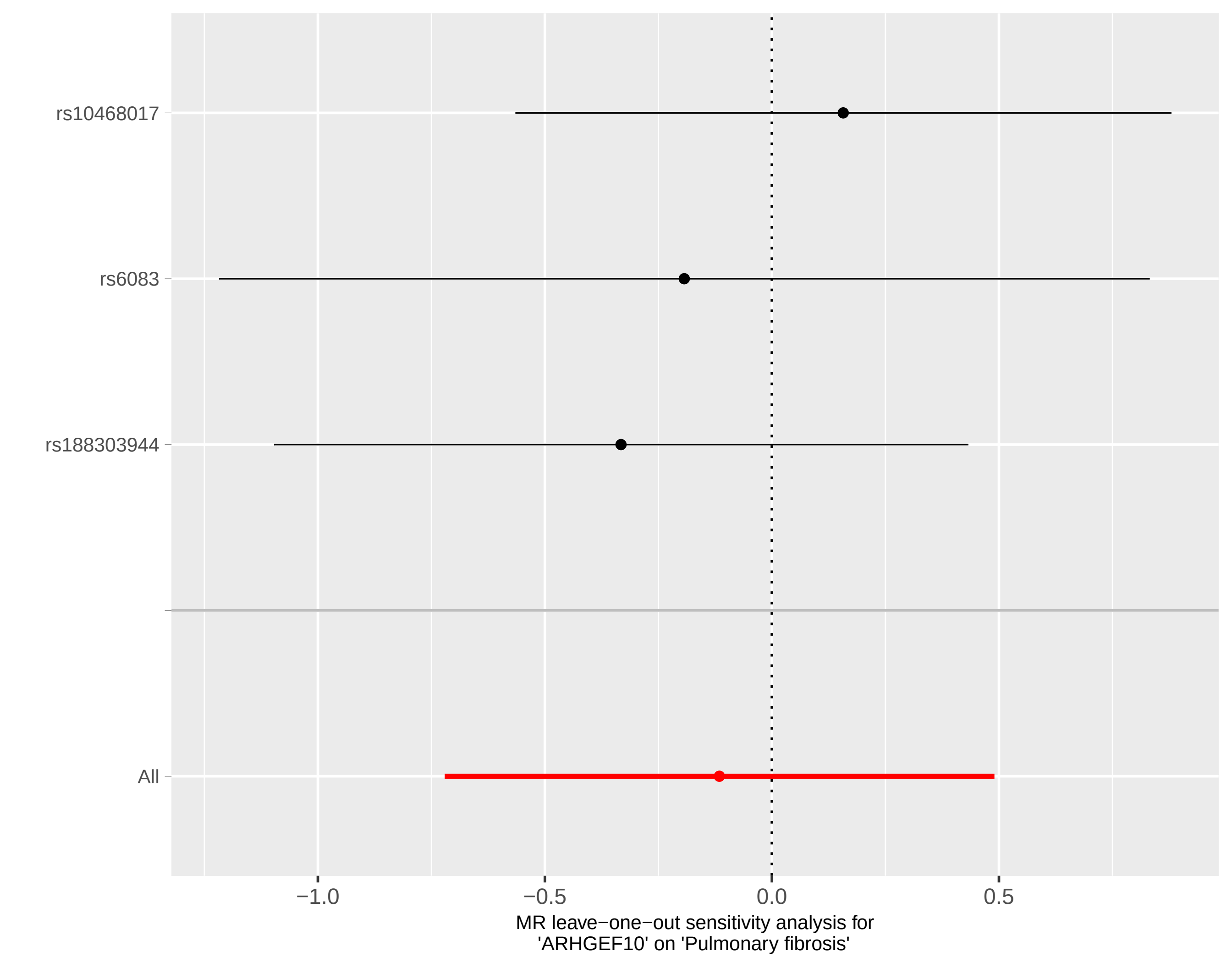
rs10468017
rs6083
rs188303944
All
−1.0
−0.5
0.0
0.5
MR leave−one−out sensitivity analysis for
'ARHGEF10' on 'Pulmonary fibrosis'

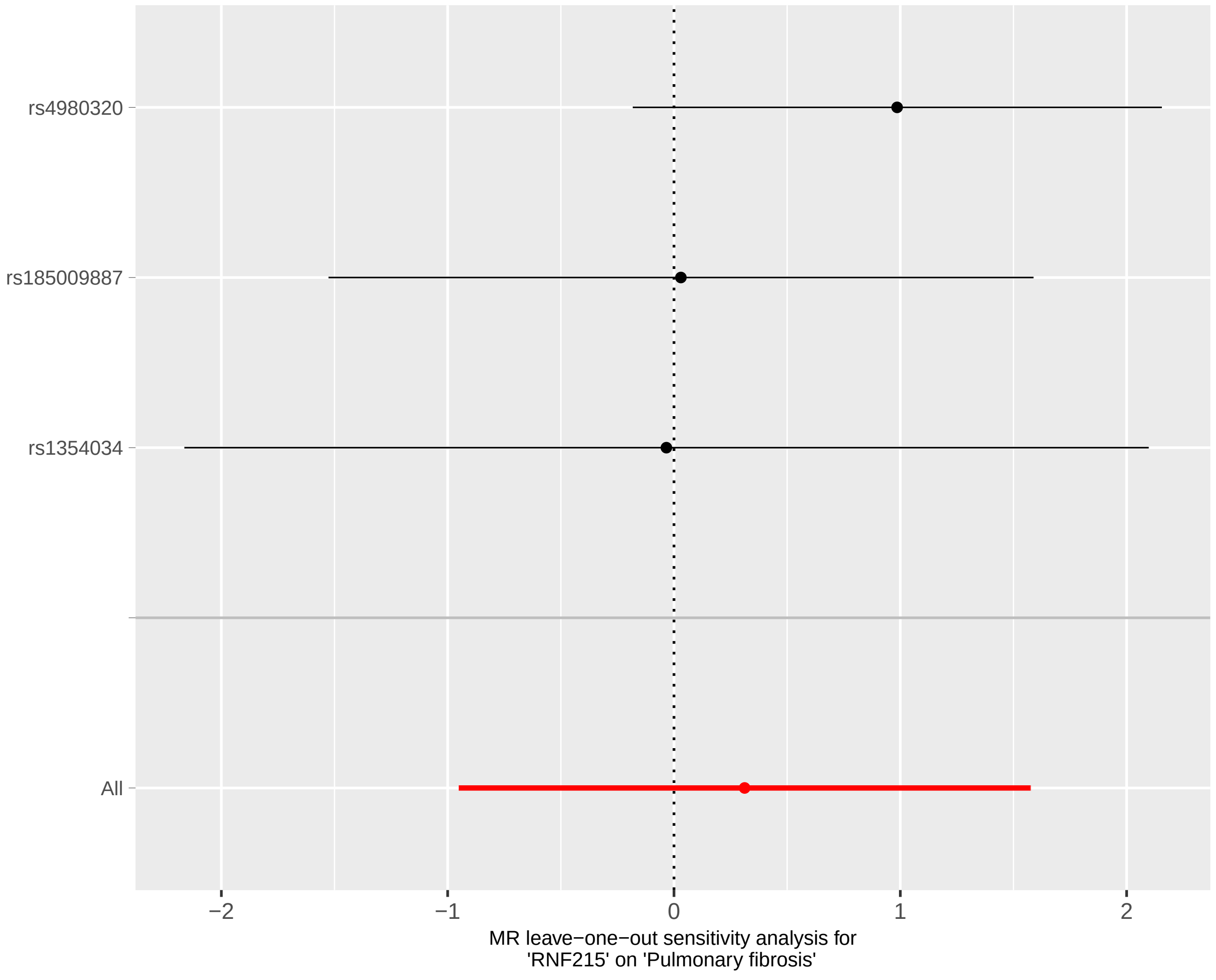
rs4980320
rs185009887
rs1354034
All
−2
−1
0
1
2
MR leave−one−out sensitivity analysis for
'RNF215' on 'Pulmonary fibrosis'

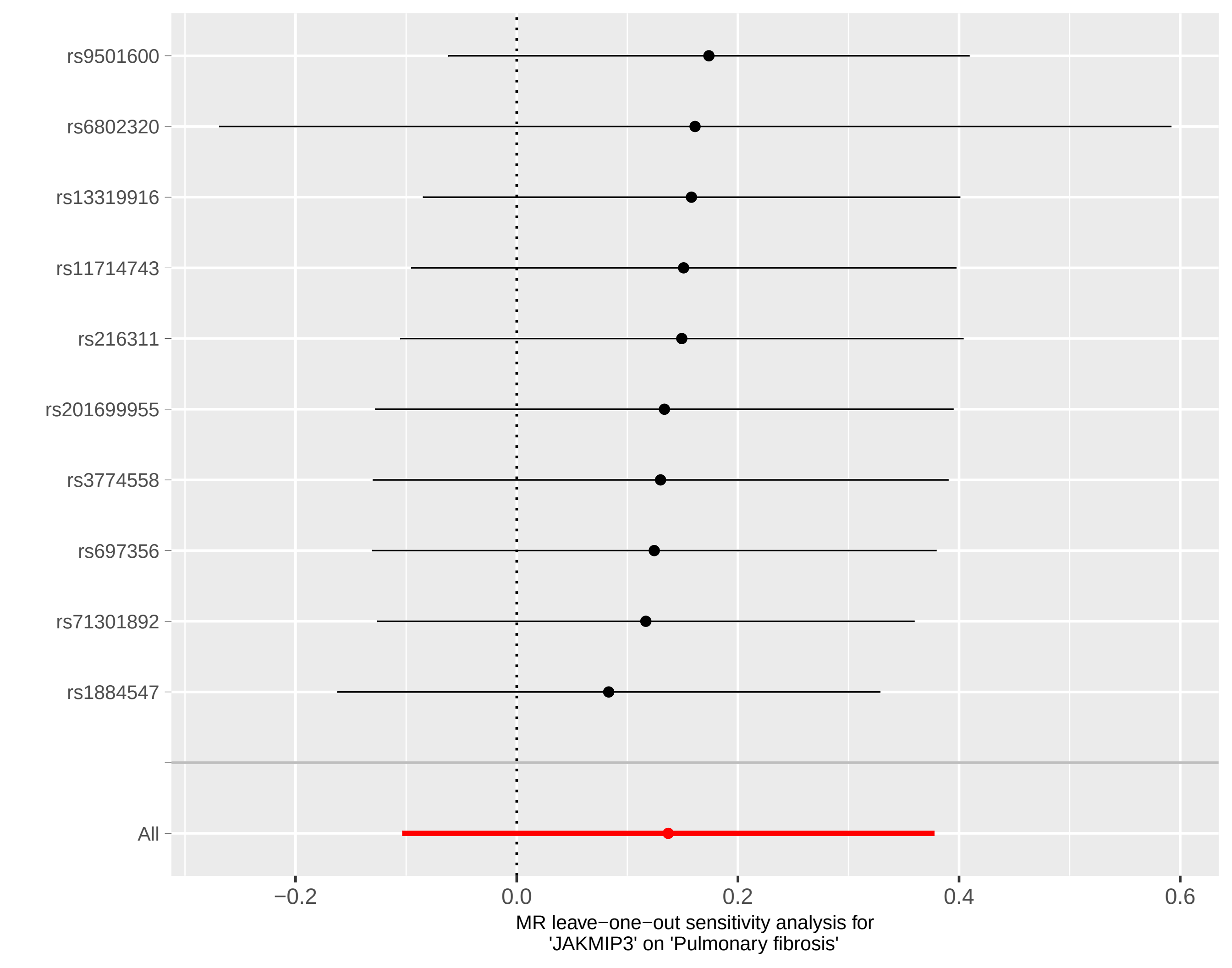

rs9501600
rs6802320
rs13319916
rs11714743
rs216311
rs201699955
rs3774558
rs697356
rs71301892
rs1884547
All
−0.2
0.0
0.2
0.4
0.6
MR leave−one−out sensitivity analysis for
'JAKMIP3' on 'Pulmonary fibrosis'

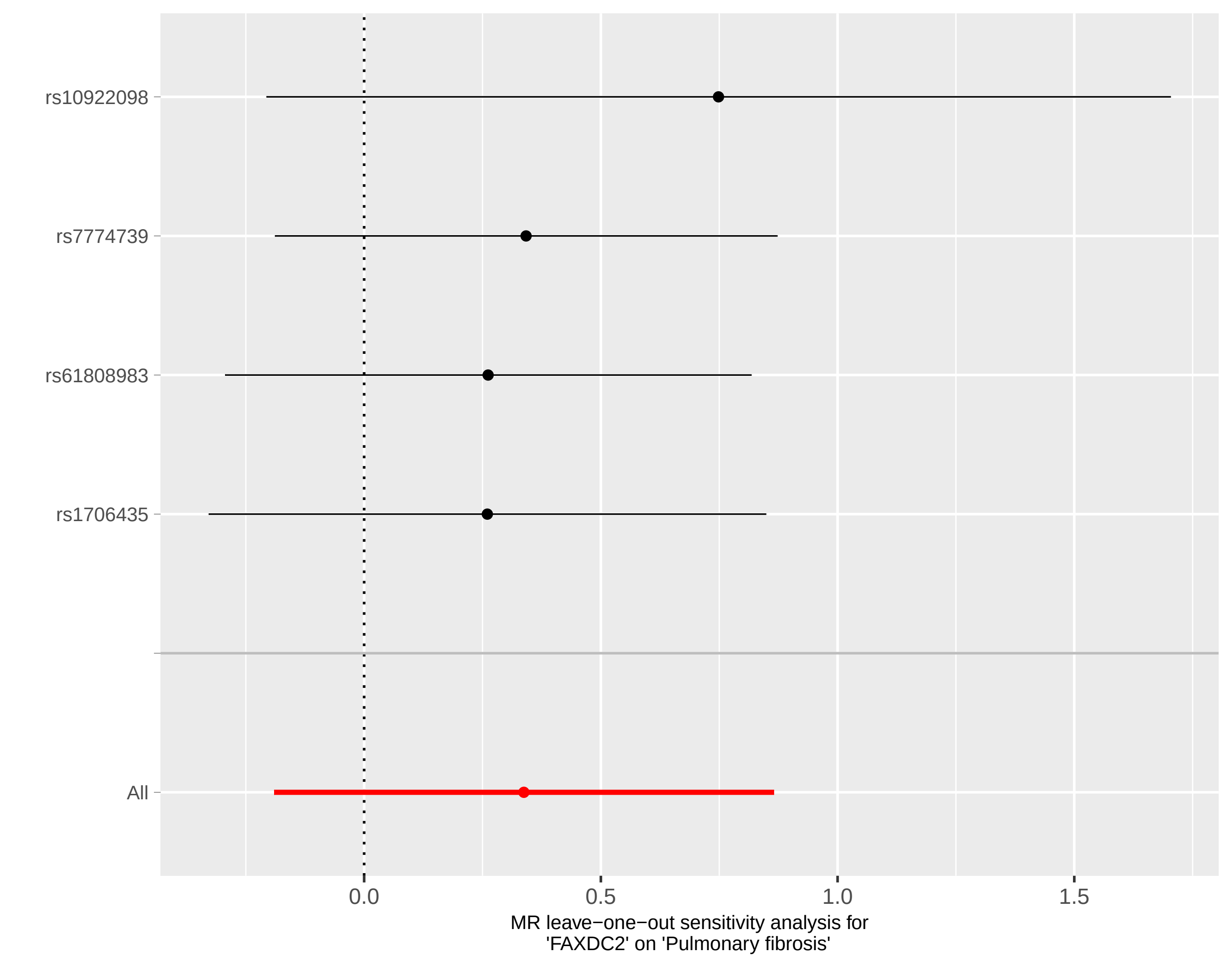
rs10922098
rs7774739
rs61808983
rs1706435
All
0.0
0.5
1.0
1.5
MR leave−one−out sensitivity analysis for
'FAXDC2' on 'Pulmonary fibrosis'

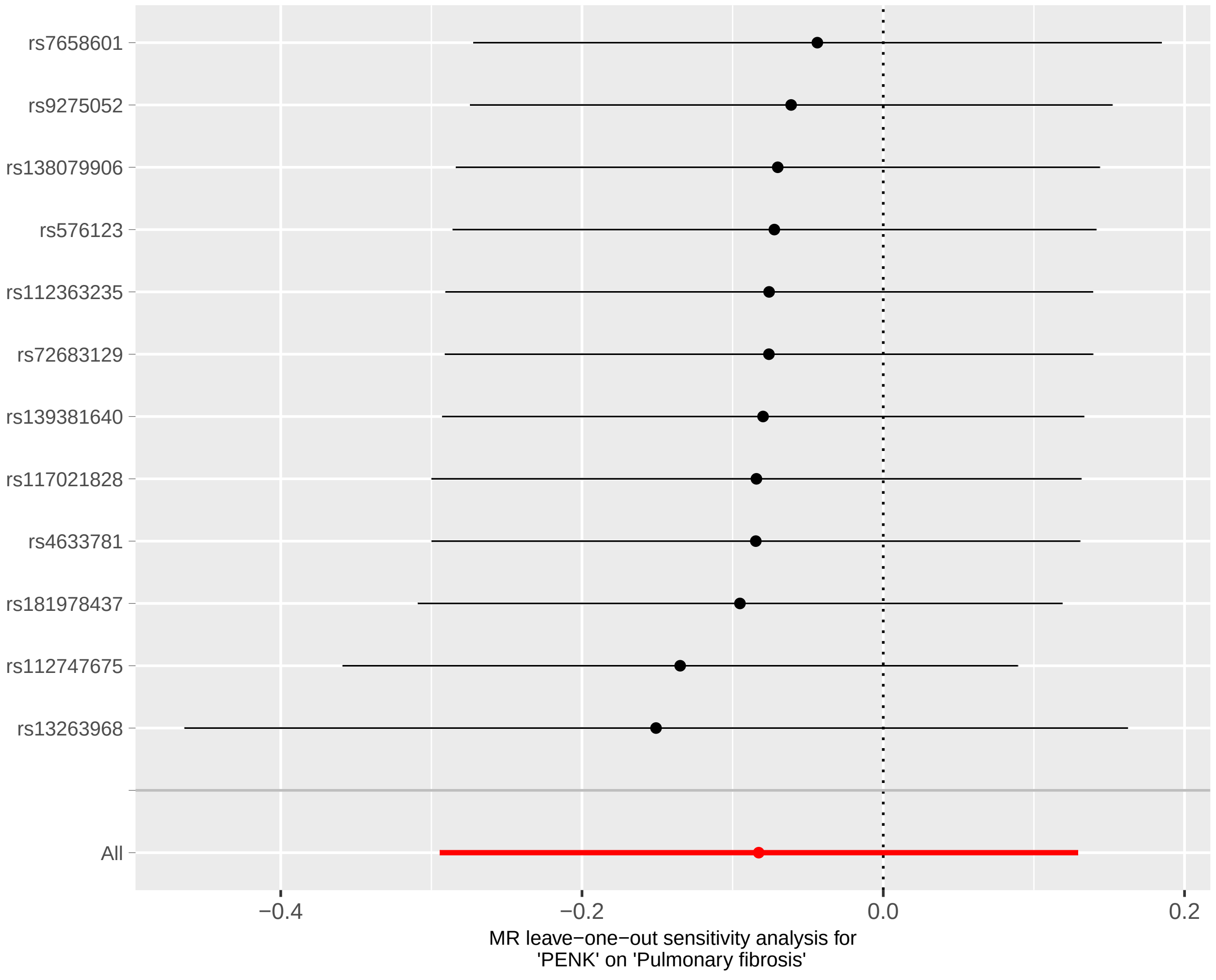
rs7658601
rs9275052
rs138079906
rs576123
rs112363235
rs72683129
rs139381640
rs117021828
rs4633781
rs181978437
rs112747675
rs13263968
All
−0.4
−0.2
0.0
0.2
MR leave−one−out sensitivity analysis for
'PENK' on 'Pulmonary fibrosis'

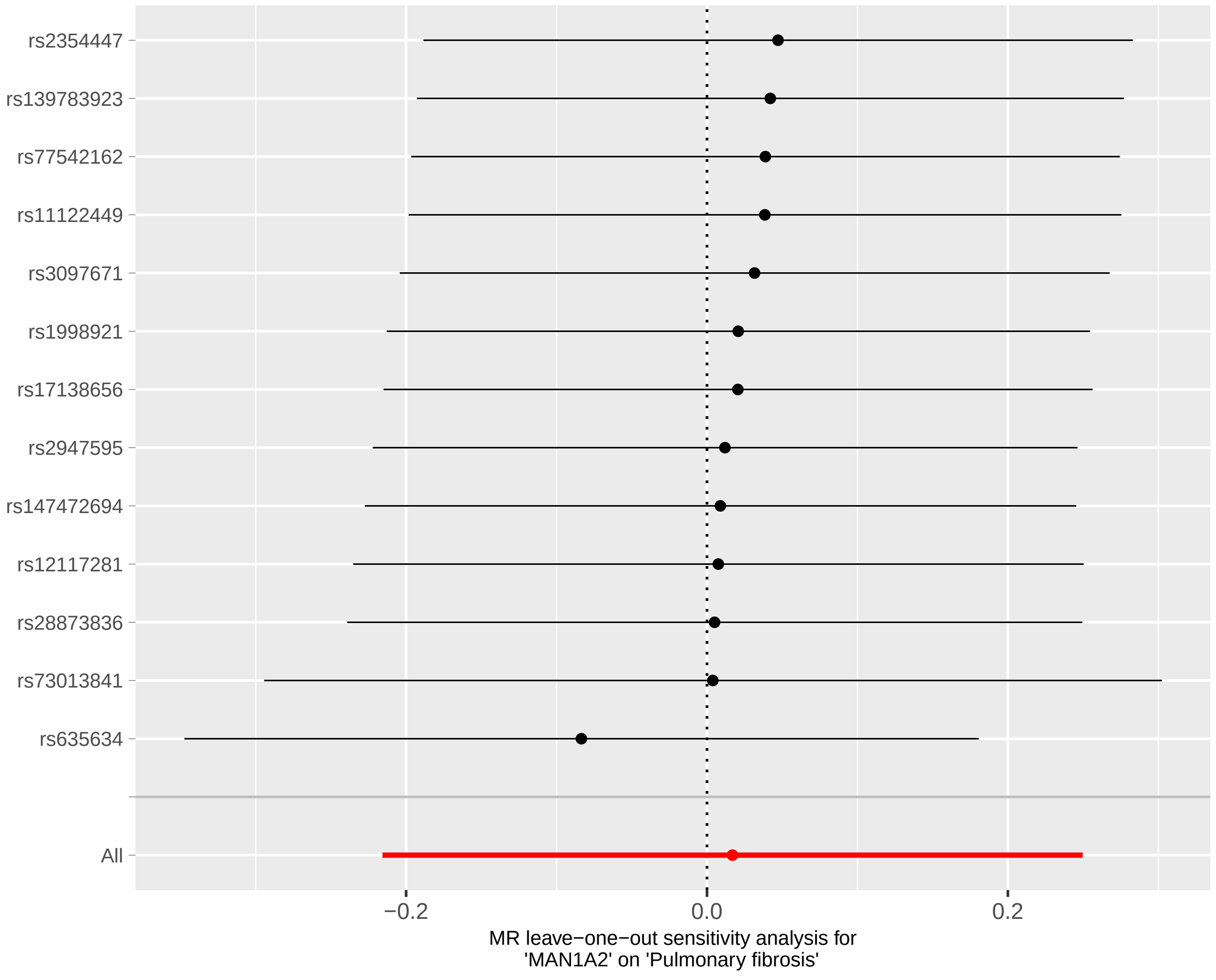
rs2354447
rs139783923
rs77542162
rs11122449
rs3097671
rs1998921
rs17138656
rs2947595
rs147472694
rs12117281
rs28873836
rs73013841
rs635634
All
−0.2
0.0
0.2
MR leave−one−out sensitivity analysis for
'MAN1A2' on 'Pulmonary fibrosis'

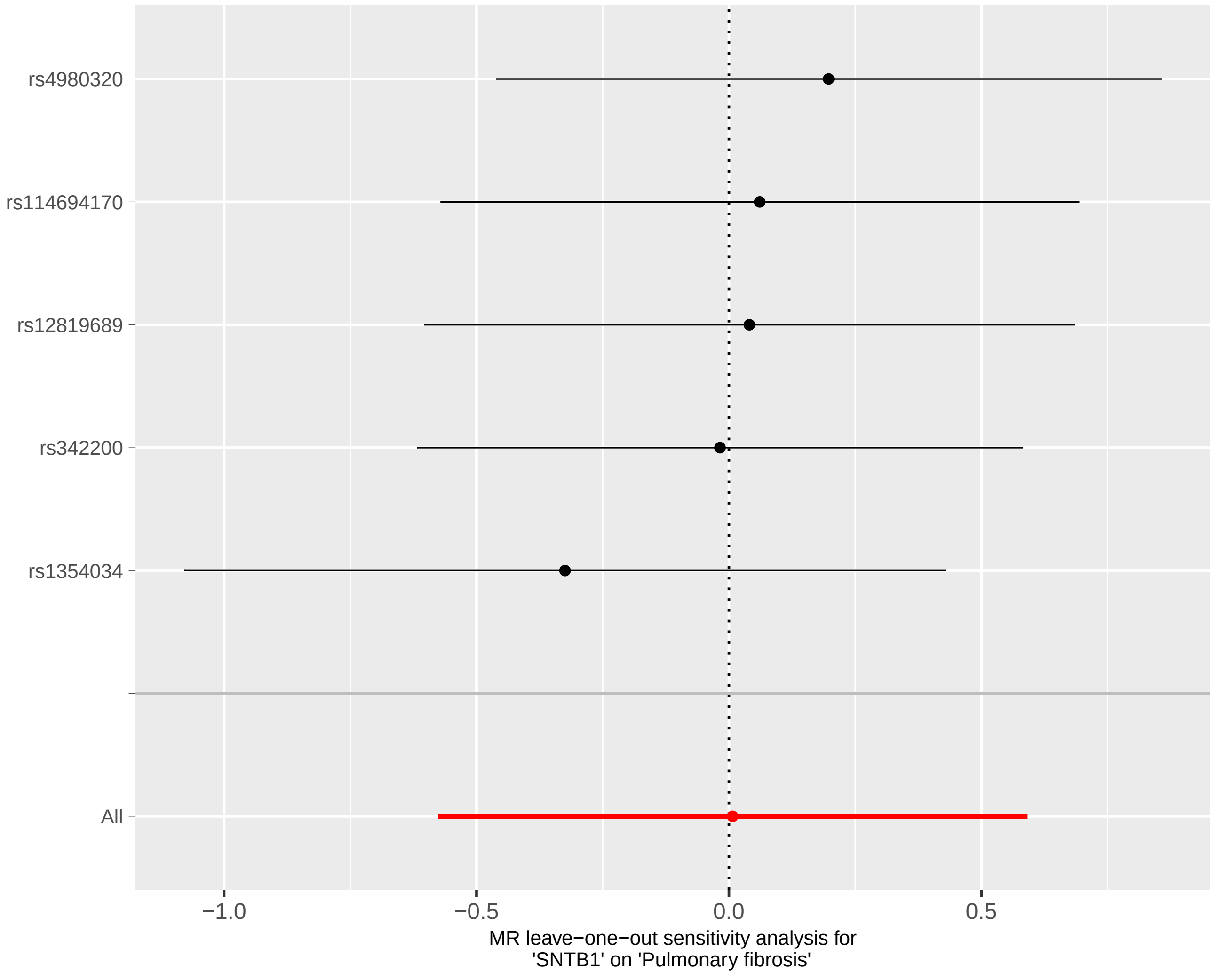
rs4980320
rs114694170
rs12819689
rs342200
rs1354034
All
−1.0
−0.5
0.0
0.5
MR leave−one−out sensitivity analysis for
'SNTB1' on 'Pulmonary fibrosis'

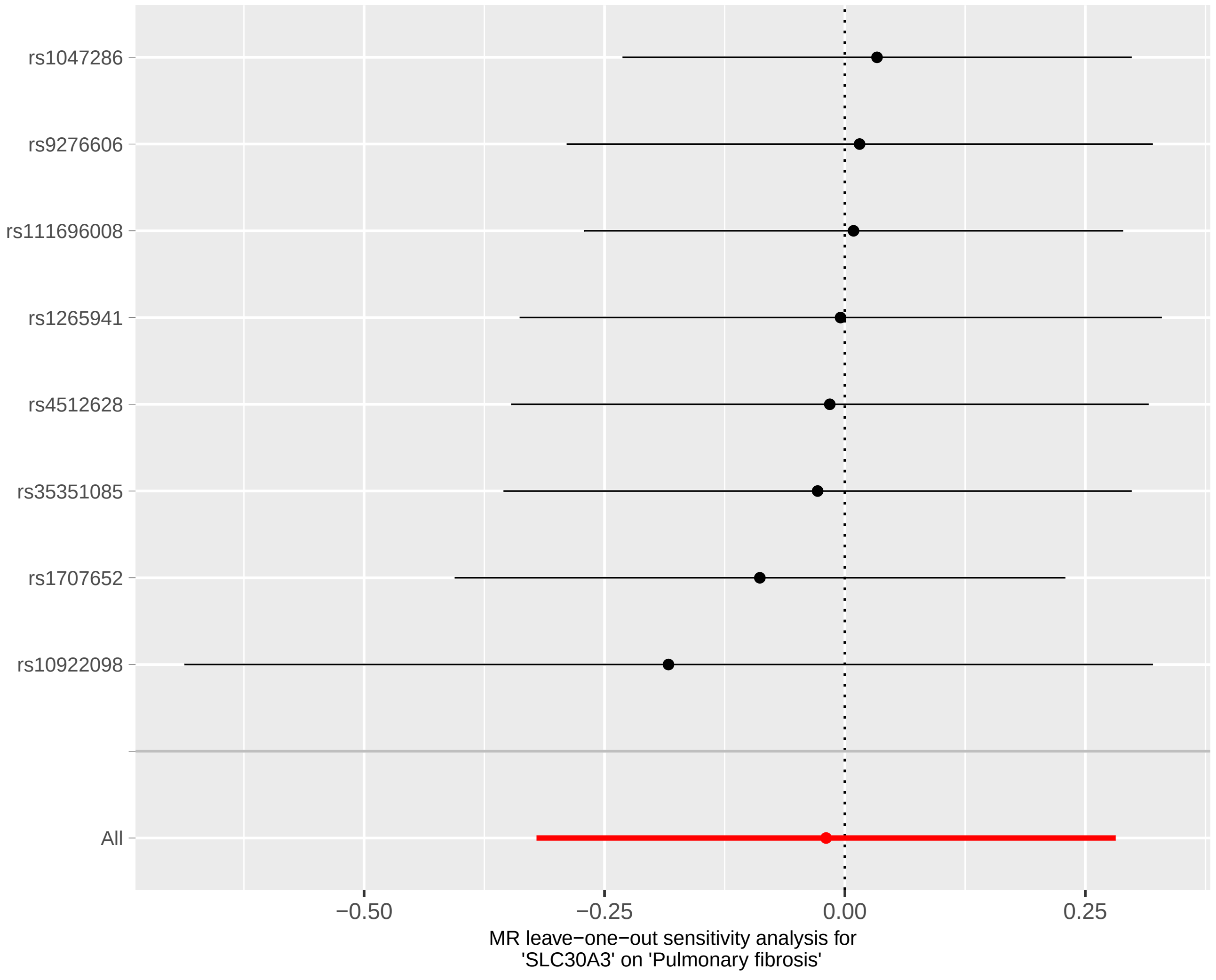
rs1047286
rs9276606
rs111696008
rs1265941
rs4512628
rs35351085
rs1707652
rs10922098
All
−0.50
−0.25
0.00
0.25
MR leave−one−out sensitivity analysis for
'SLC30A3' on 'Pulmonary fibrosis'

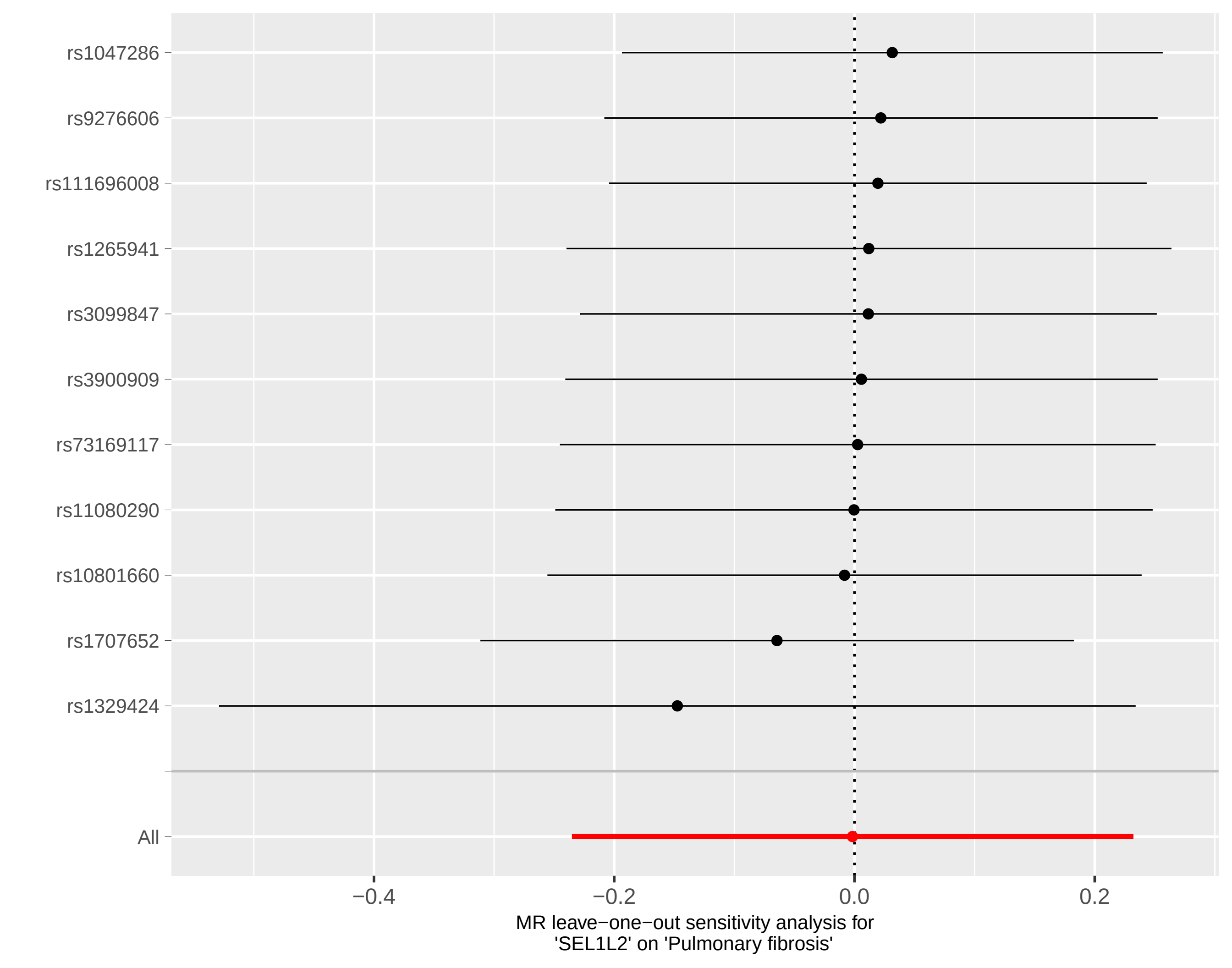
rs1047286
rs9276606
rs111696008
rs1265941
rs3099847
rs3900909
rs73169117
rs11080290
rs10801660
rs1707652
rs1329424
All
−0.4
−0.2
0.0
0.2
MR leave−one−out sensitivity analysis for
'SEL1L2' on 'Pulmonary fibrosis'

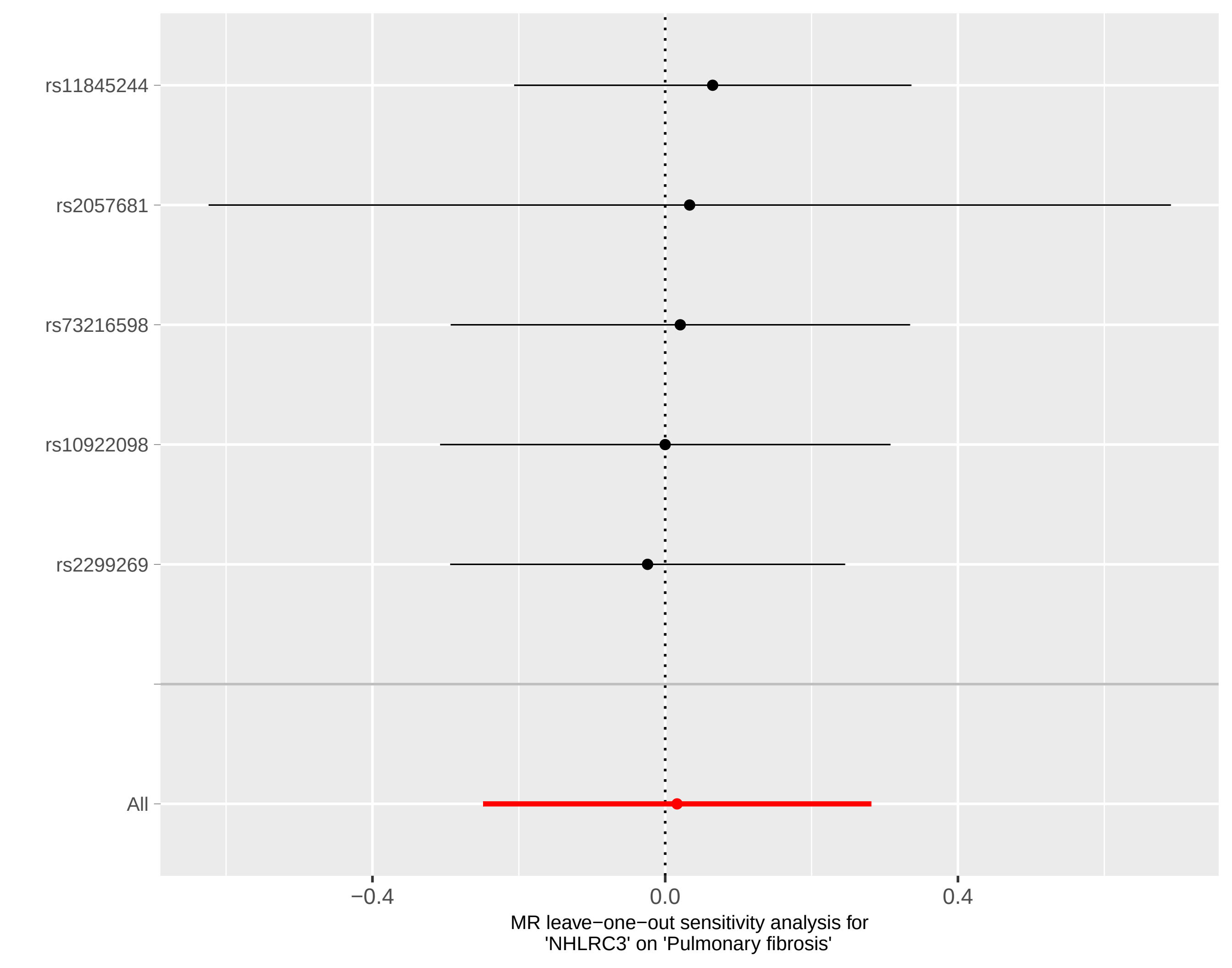
rs11845244
rs2057681
rs73216598
rs10922098
rs2299269
All
−0.4
0.0
0.4
MR leave−one−out sensitivity analysis for
'NHLRC3' on 'Pulmonary fibrosis'

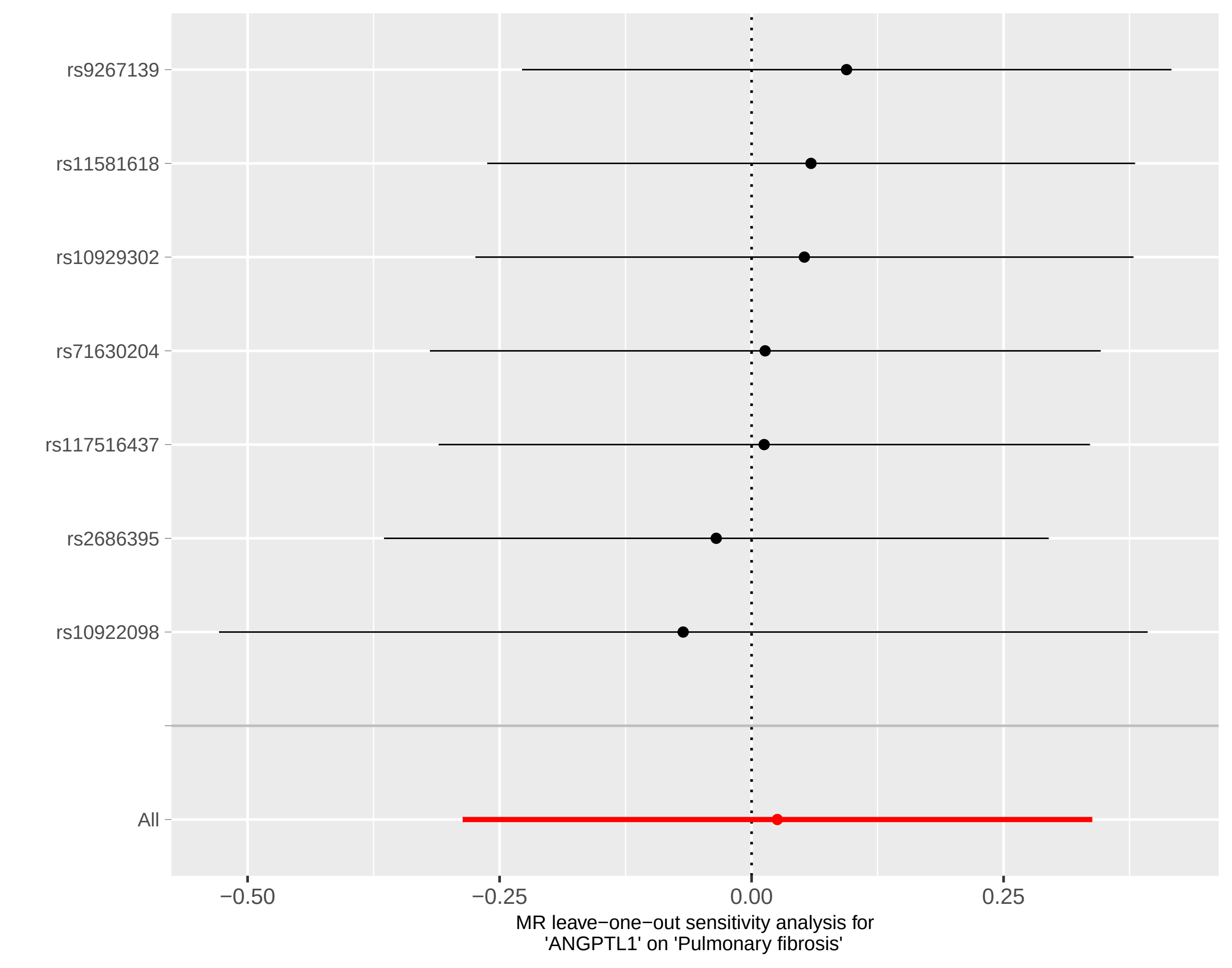
rs9267139
rs11581618
rs10929302
rs71630204
rs117516437
rs2686395
rs10922098
All
−0.50
−0.25
0.00
0.25
MR leave−one−out sensitivity analysis for
'ANGPTL1' on 'Pulmonary fibrosis'

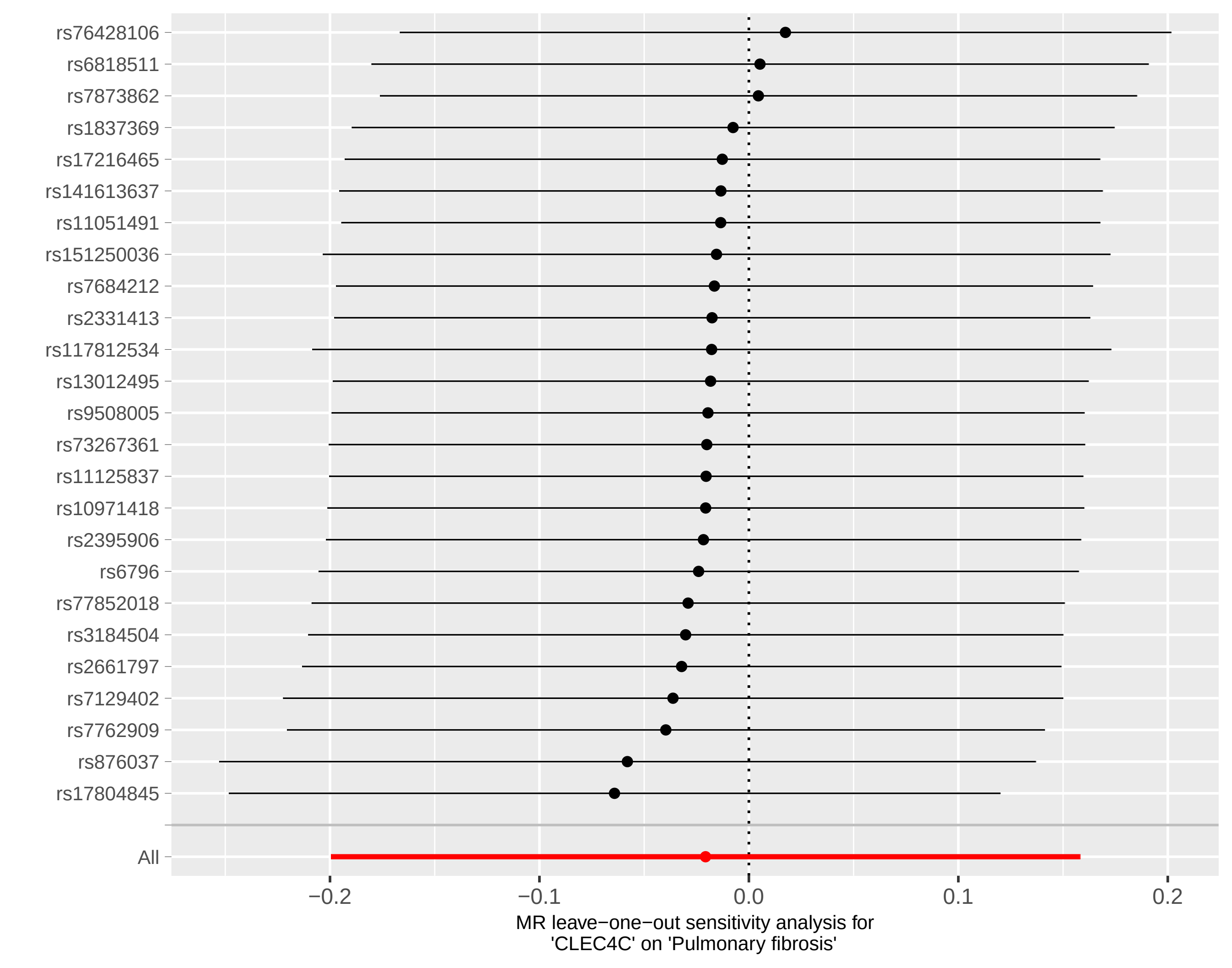

rs76428106
rs6818511
rs7873862
rs1837369
rs17216465
rs141613637
rs11051491
rs151250036
rs7684212
rs2331413
rs117812534
rs13012495
rs9508005
rs73267361
rs11125837
rs10971418
rs2395906
rs6796
rs77852018
rs3184504
rs2661797
rs7129402
rs7762909
rs876037
rs17804845
All
−0.2
−0.1
0.0
0.1
0.2
MR leave−one−out sensitivity analysis for
'CLEC4C' on 'Pulmonary fibrosis'

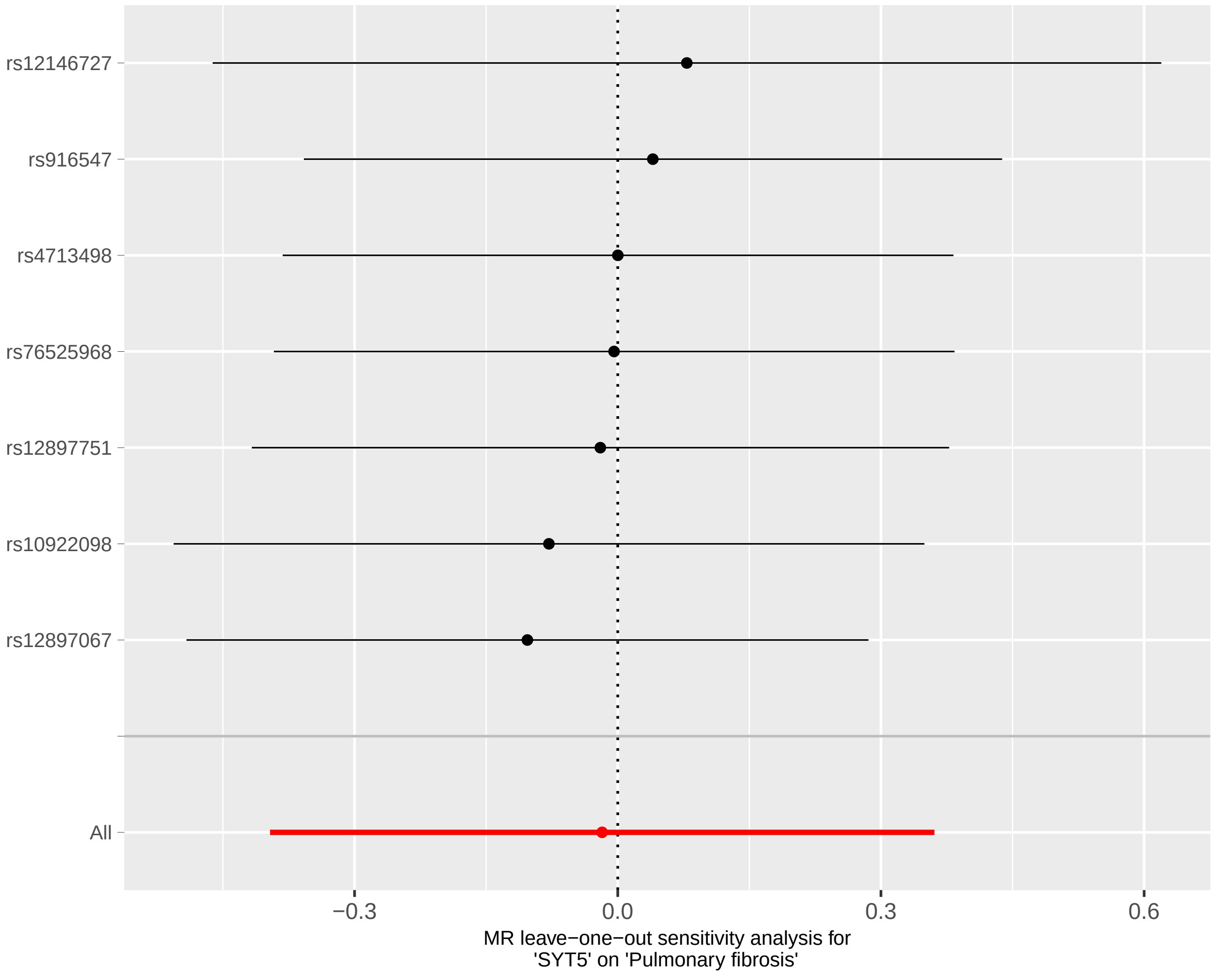

rs12146727
rs916547
rs4713498
rs76525968
rs12897751
rs10922098
rs12897067
All
−0.3
0.0
0.3
0.6
MR leave−one−out sensitivity analysis for
'SYT5' on 'Pulmonary fibrosis'

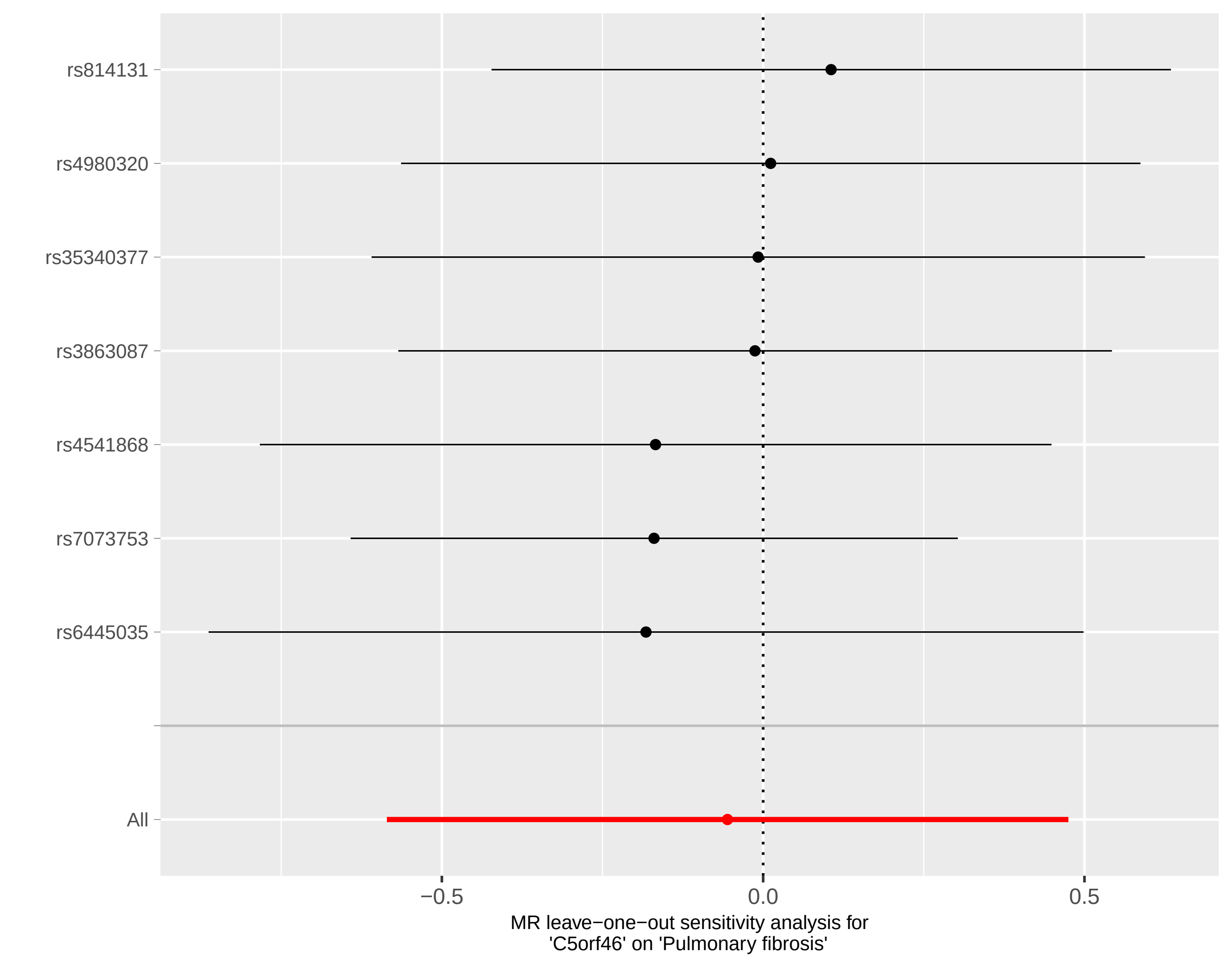
rs814131
rs4980320
rs35340377
rs3863087
rs4541868
rs7073753
rs6445035
All
−0.5
0.0
0.5
MR leave−one−out sensitivity analysis for
'C5orf46' on 'Pulmonary fibrosis'

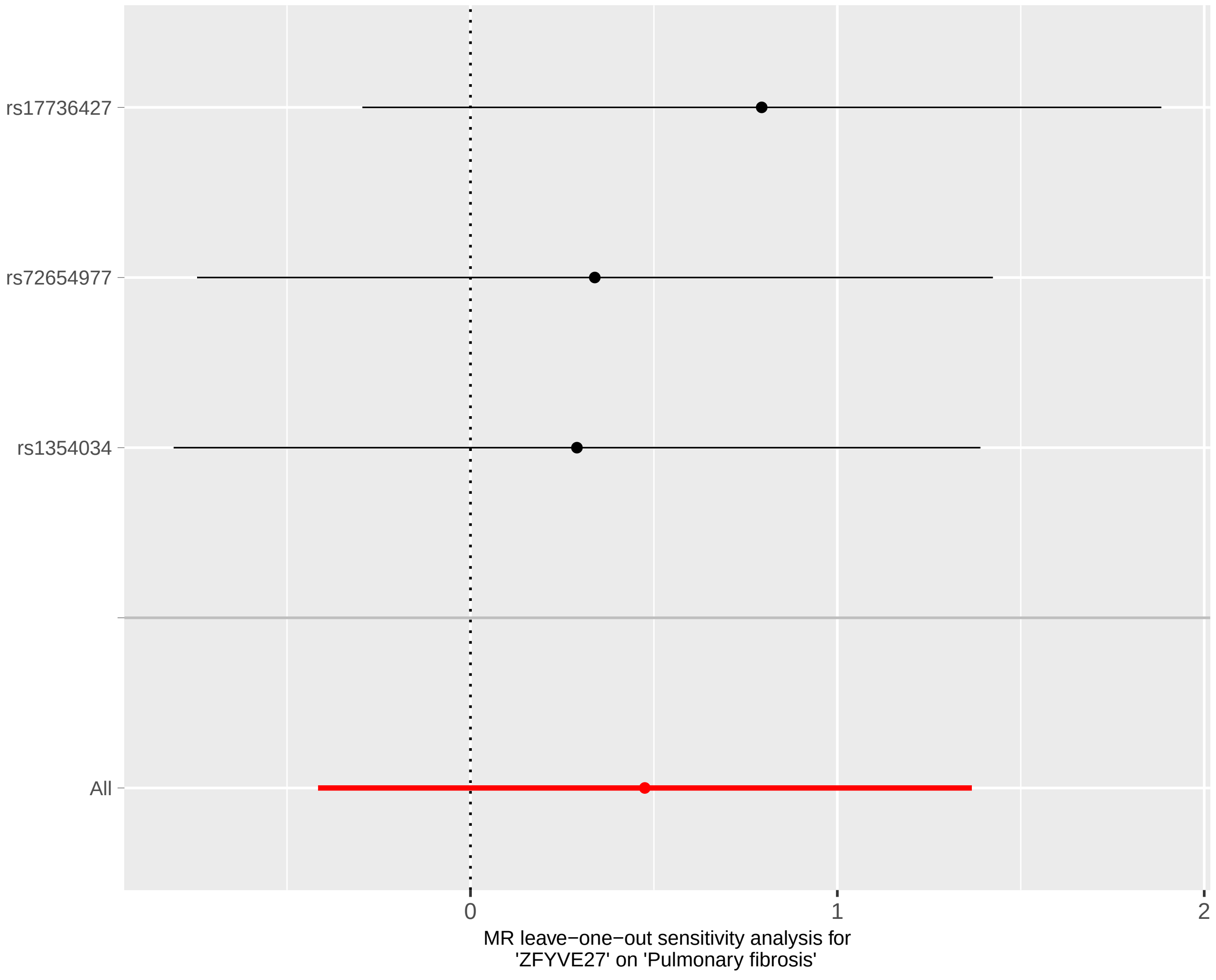
rs17736427
rs72654977
rs1354034
All
0
1
2
MR leave−one−out sensitivity analysis for
'ZFYVE27' on 'Pulmonary fibrosis'

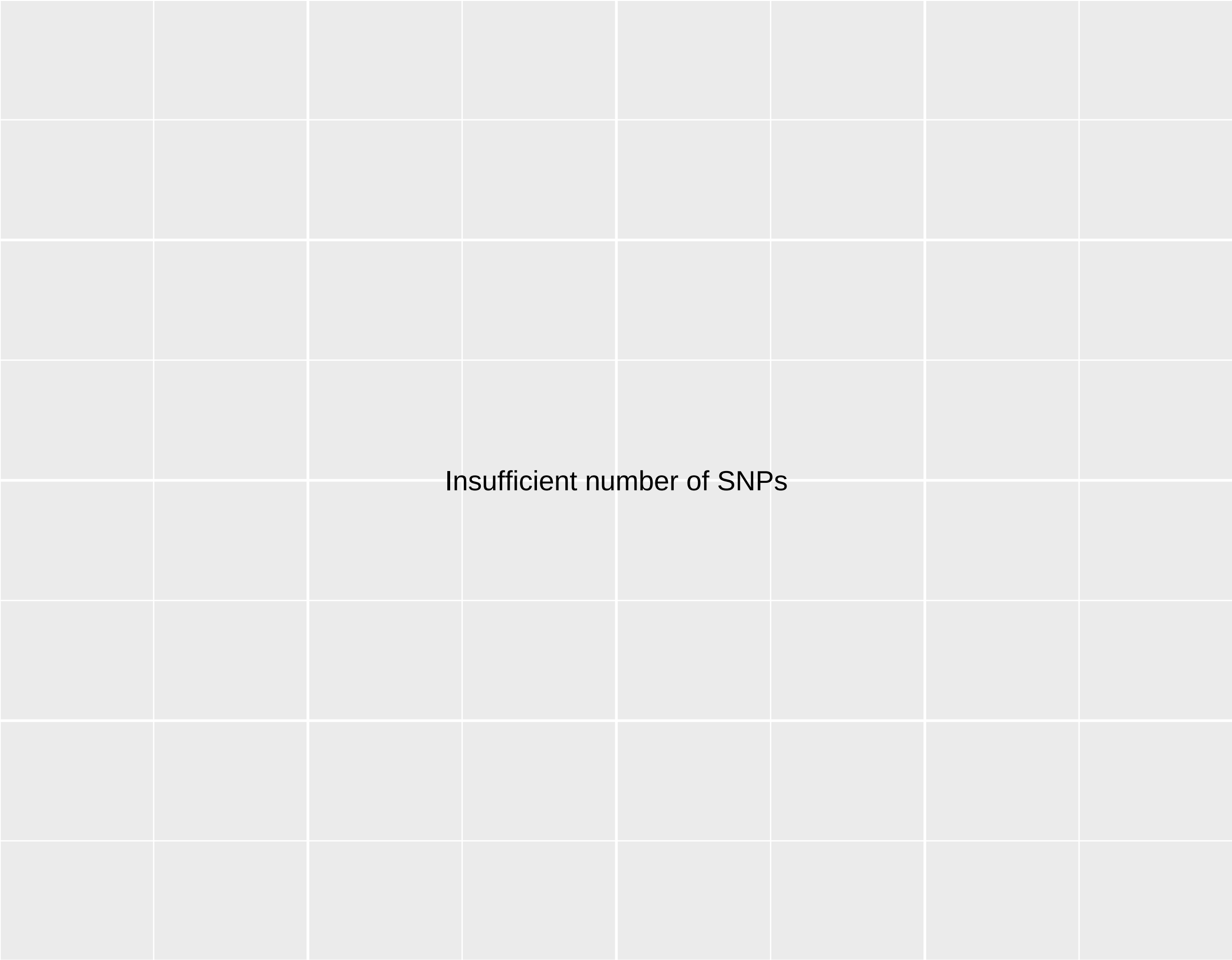
Insufficient number of SNPs

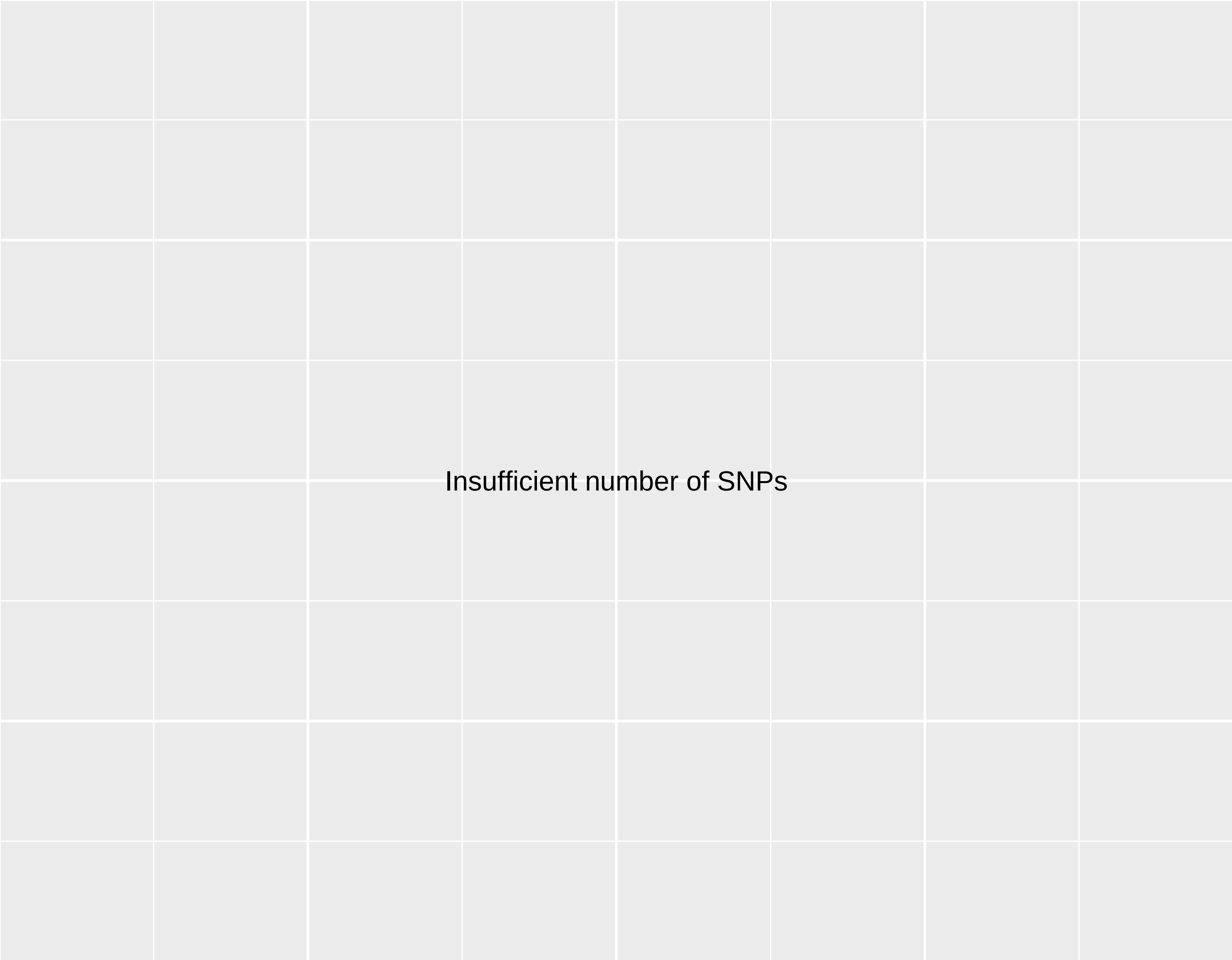
Insufficient number of SNPs

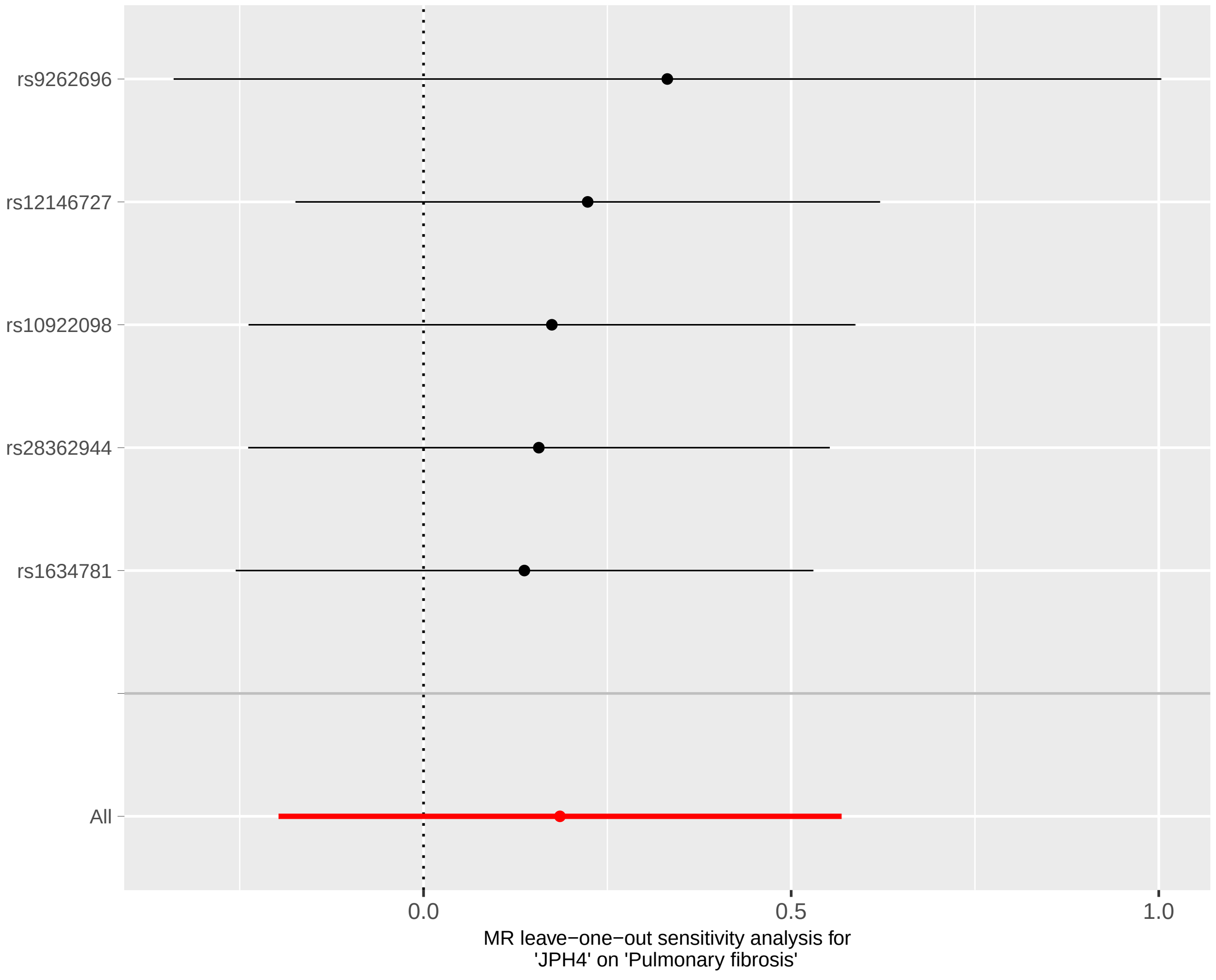
rs9262696
rs12146727
rs10922098
rs28362944
rs1634781
All
0.0
0.5
1.0
MR leave−one−out sensitivity analysis for
'JPH4' on 'Pulmonary fibrosis'

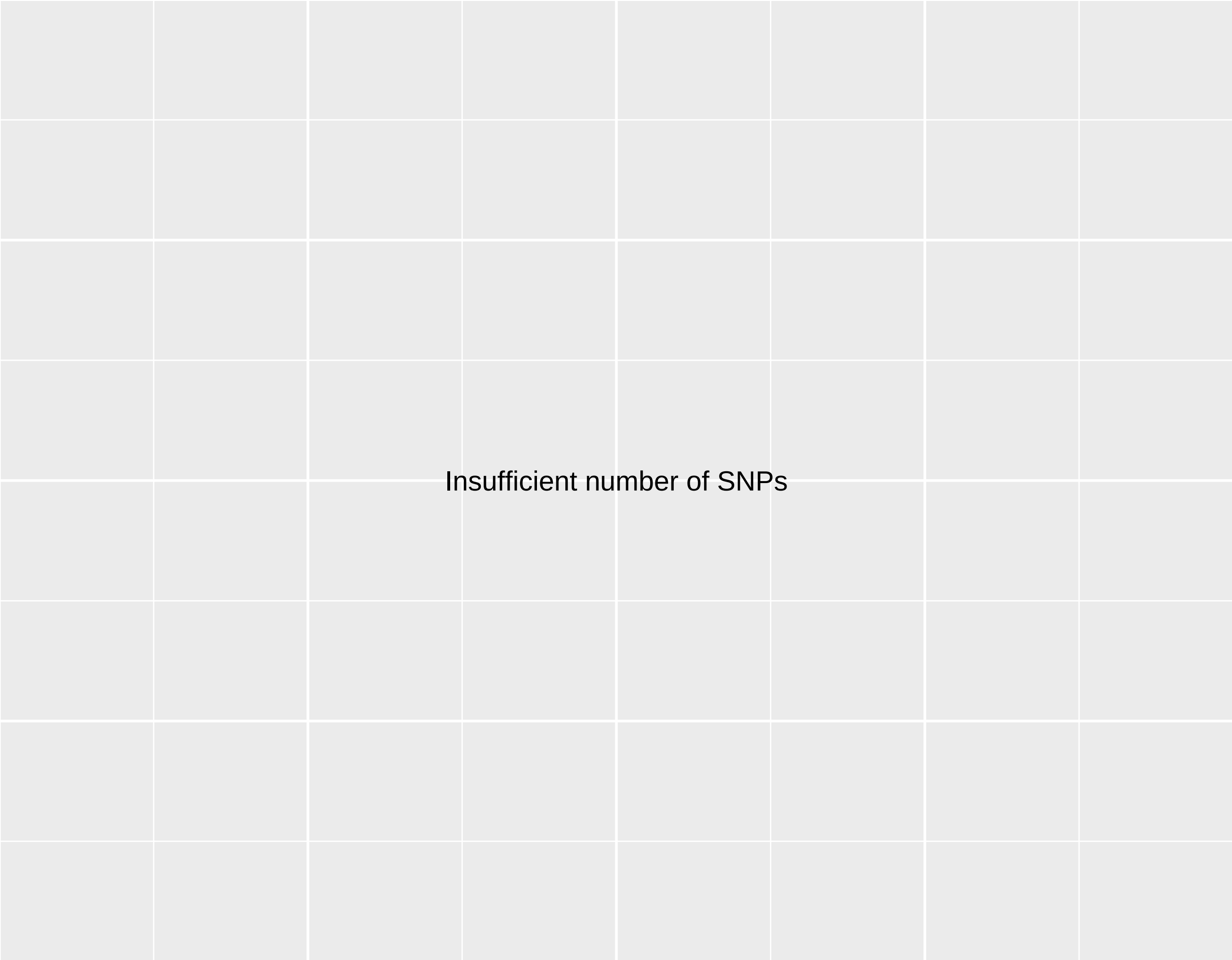
Insufficient number of SNPs

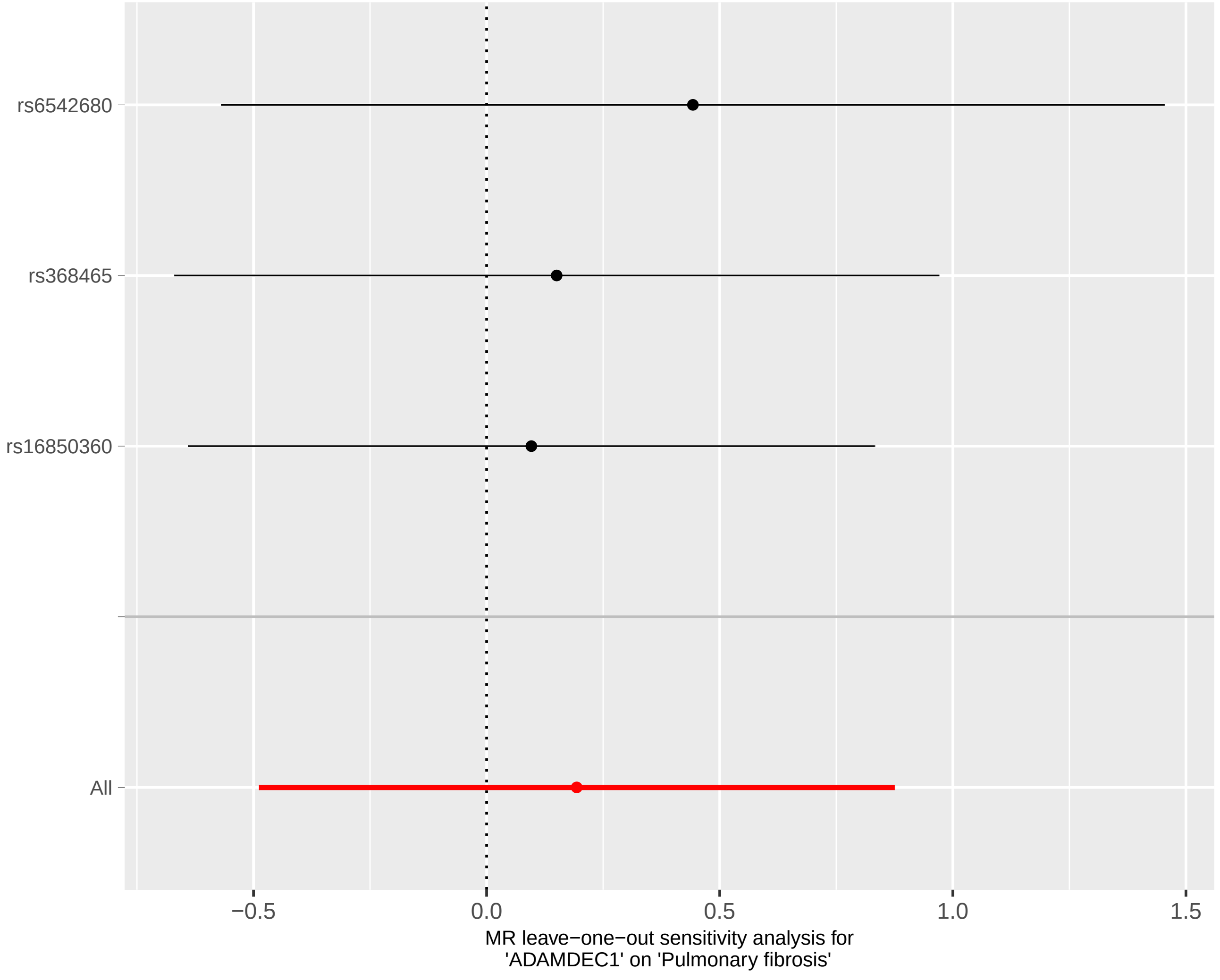
rs6542680
rs368465
rs16850360
All
−0.5
0.0
0.5
1.0
1.5
MR leave−one−out sensitivity analysis for
'ADAMDEC1' on 'Pulmonary fibrosis'

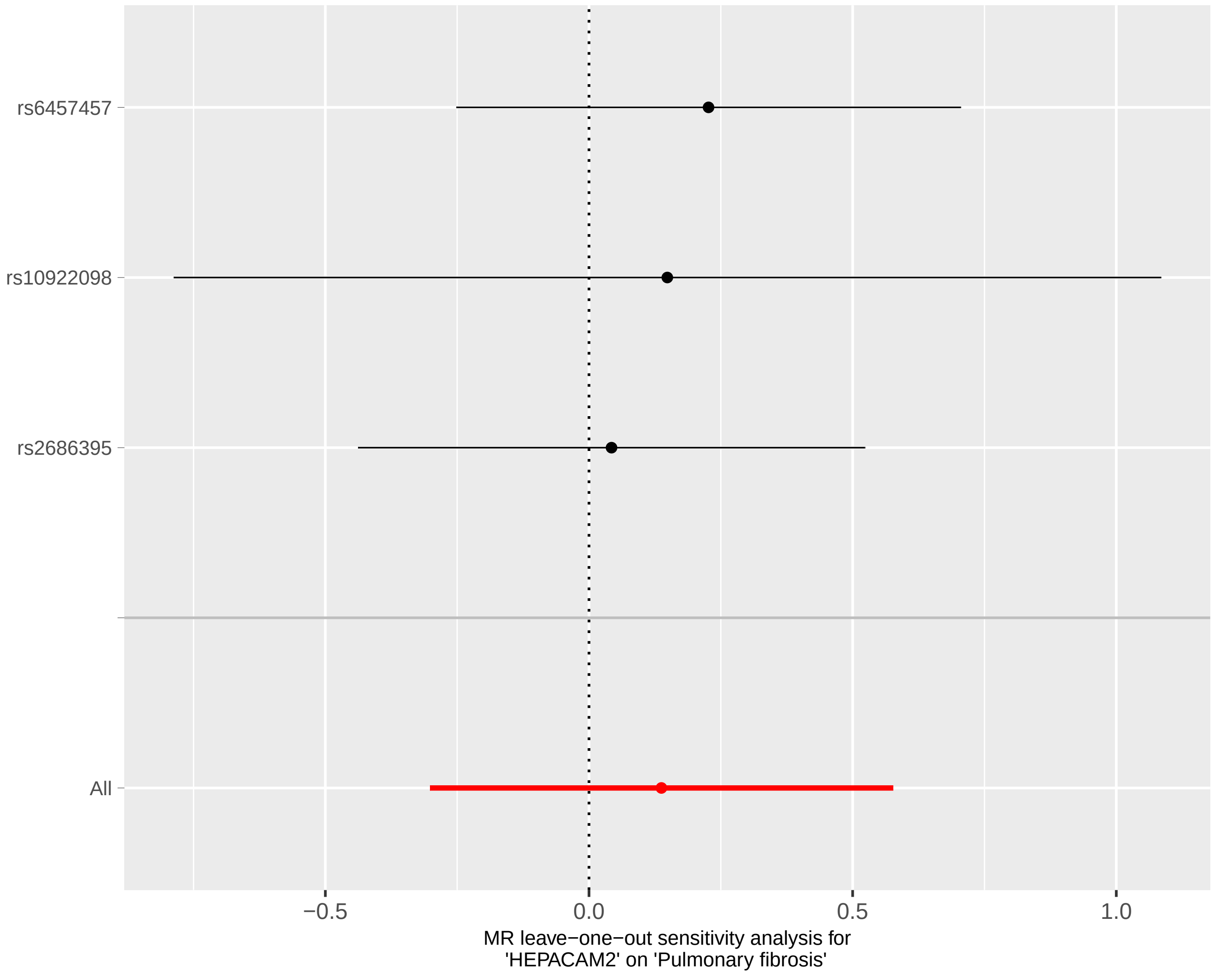
rs6457457
rs10922098
rs2686395
All
−0.5
0.0
0.5
1.0
MR leave−one−out sensitivity analysis for
'HEPACAM2' on 'Pulmonary fibrosis'

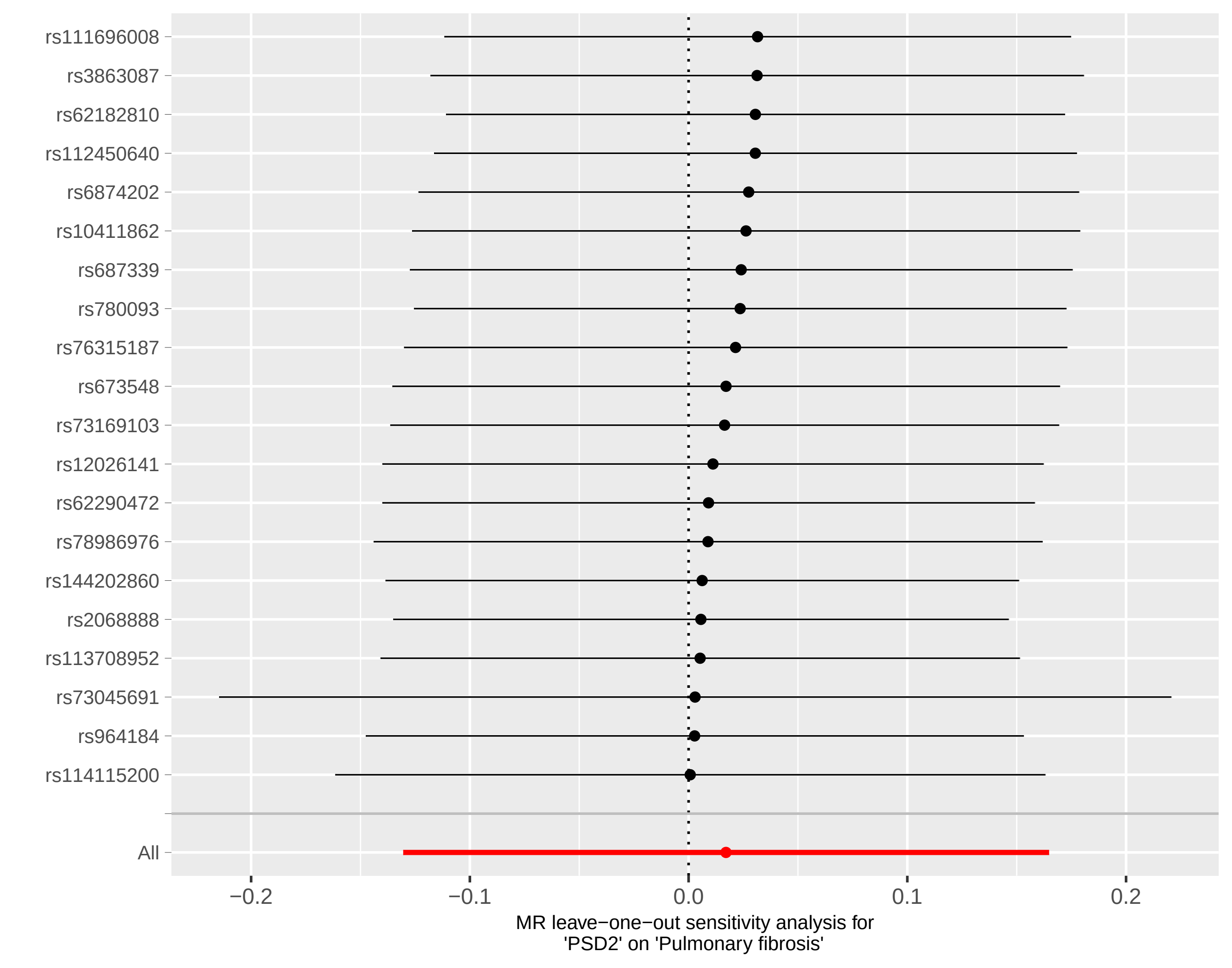

rs111696008
rs3863087
rs62182810
rs112450640
rs6874202
rs10411862
rs687339
rs780093
rs76315187
rs673548
rs73169103
rs12026141
rs62290472
rs78986976
rs144202860
rs2068888
rs113708952
rs73045691
rs964184
rs114115200
All
−0.2
−0.1
0.0
0.1
0.2
MR leave−one−out sensitivity analysis for
'PSD2' on 'Pulmonary fibrosis'

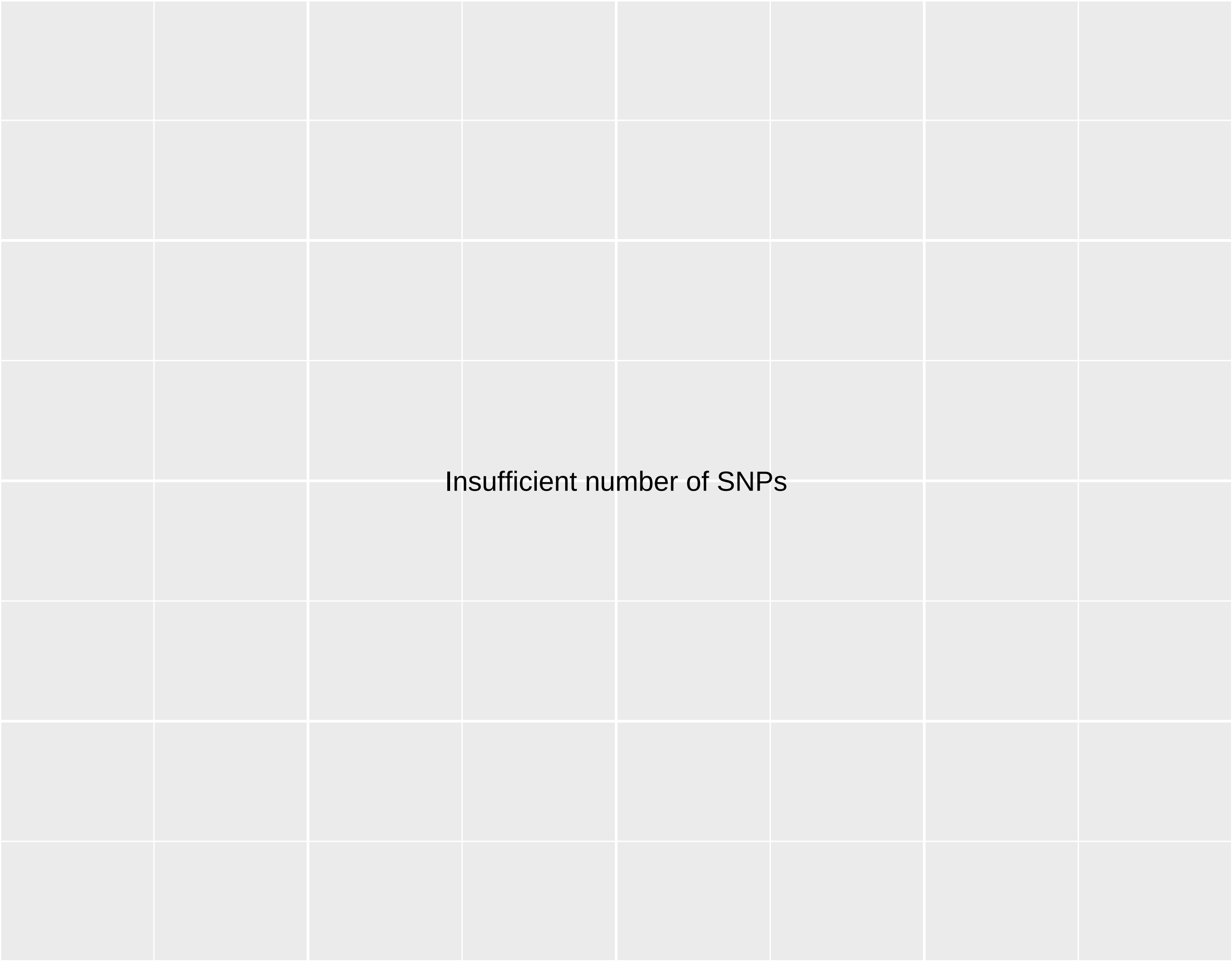
Insufficient number of SNPs

Insufficient number of SNPs

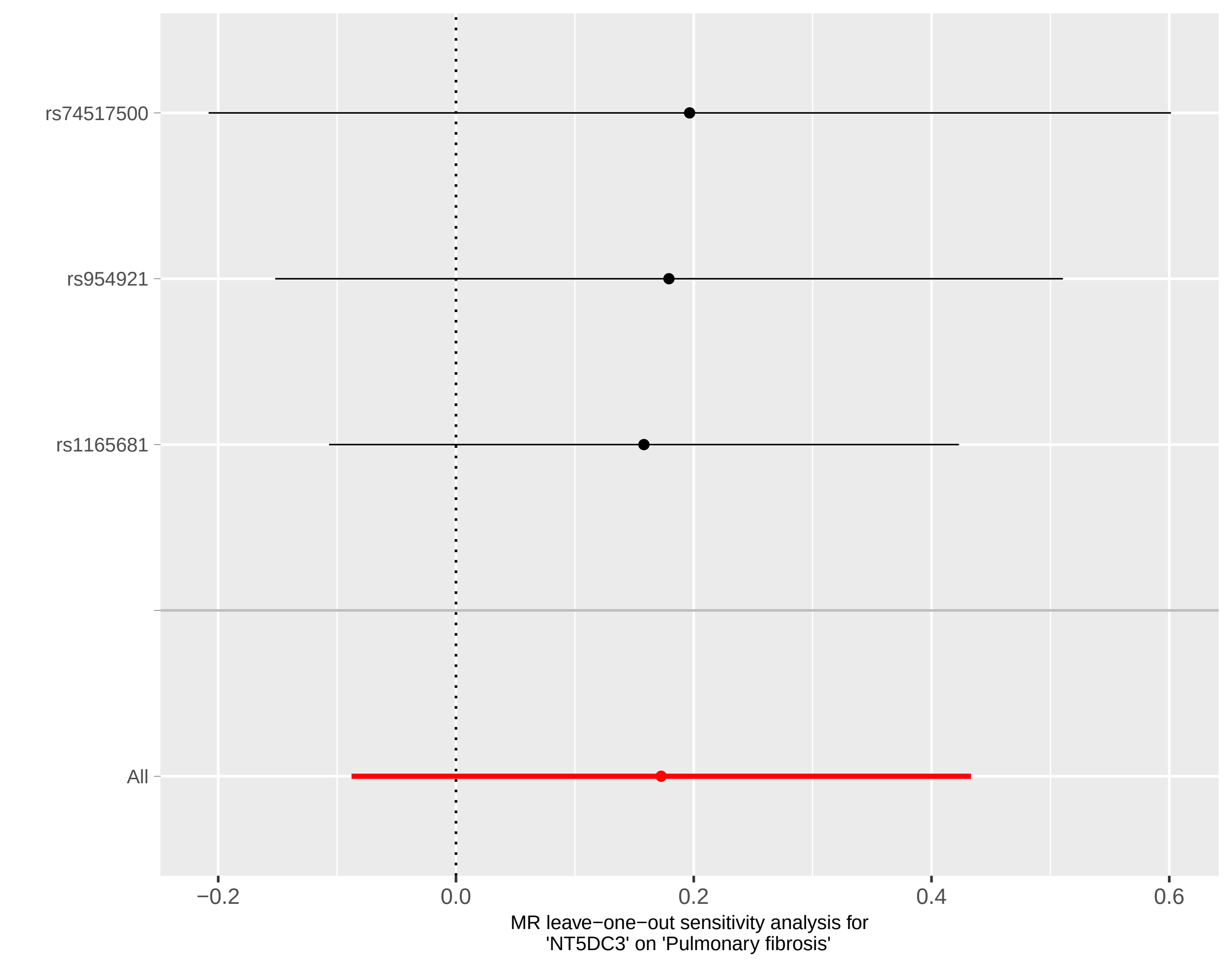
rs74517500
rs954921
rs1165681
All
−0.2
0.0
0.2
0.4
0.6
MR leave−one−out sensitivity analysis for
'NT5DC3' on 'Pulmonary fibrosis'

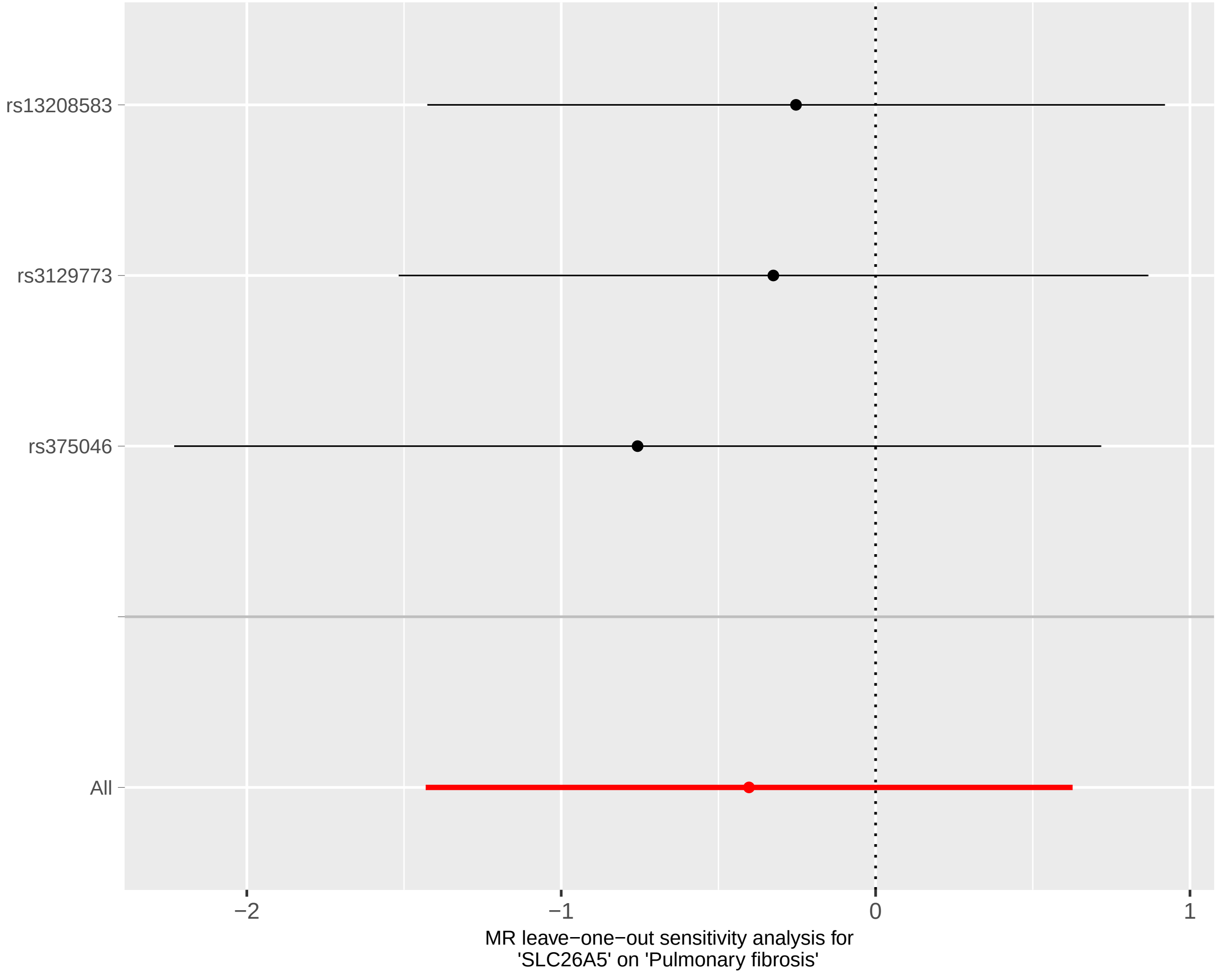
rs13208583
rs3129773
rs375046
All
−2
−1
0
1
MR leave−one−out sensitivity analysis for
'SLC26A5' on 'Pulmonary fibrosis'

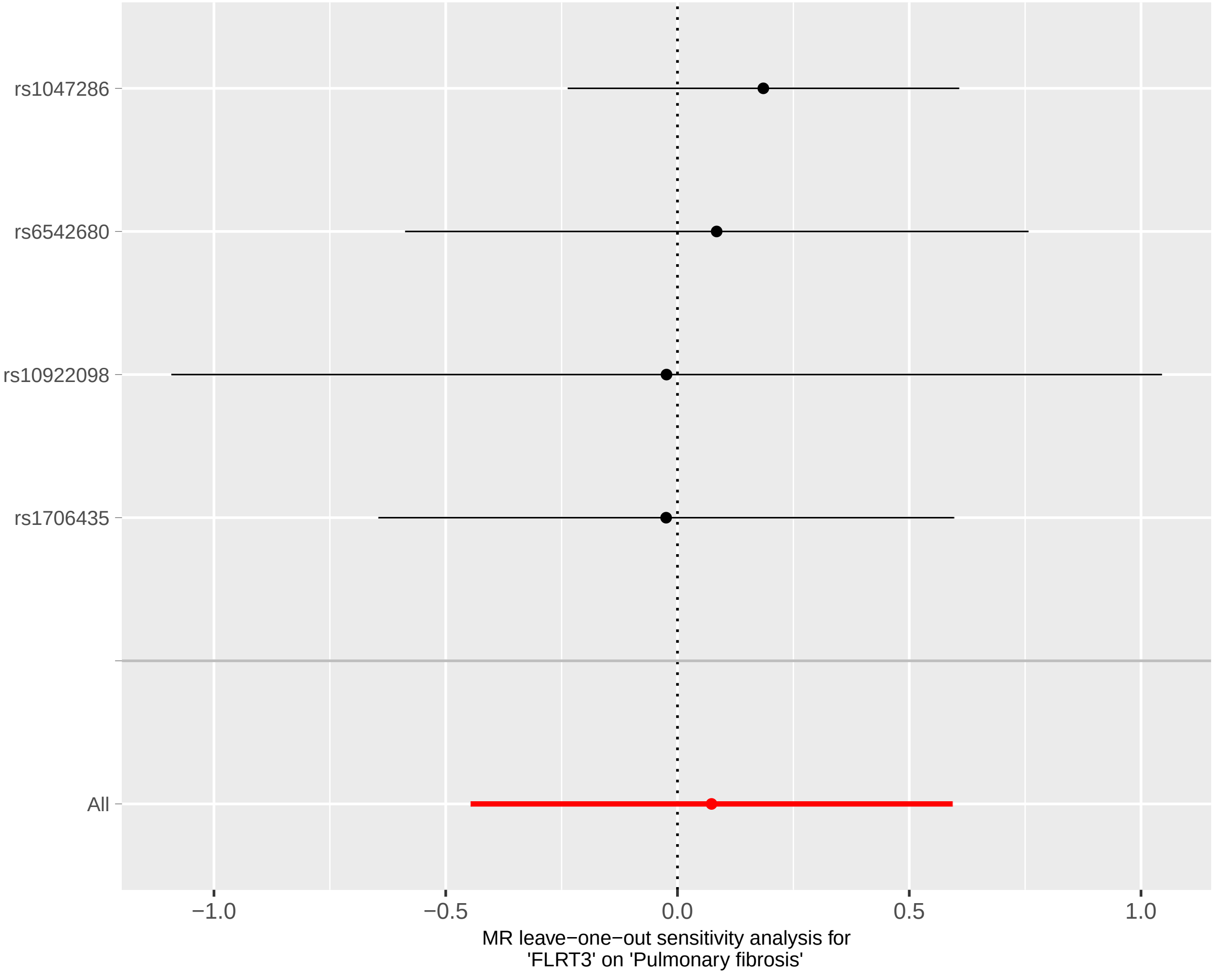
rs1047286
rs6542680
rs10922098
rs1706435
All
−1.0
−0.5
0.0
0.5
1.0
MR leave−one−out sensitivity analysis for
'FLRT3' on 'Pulmonary fibrosis'

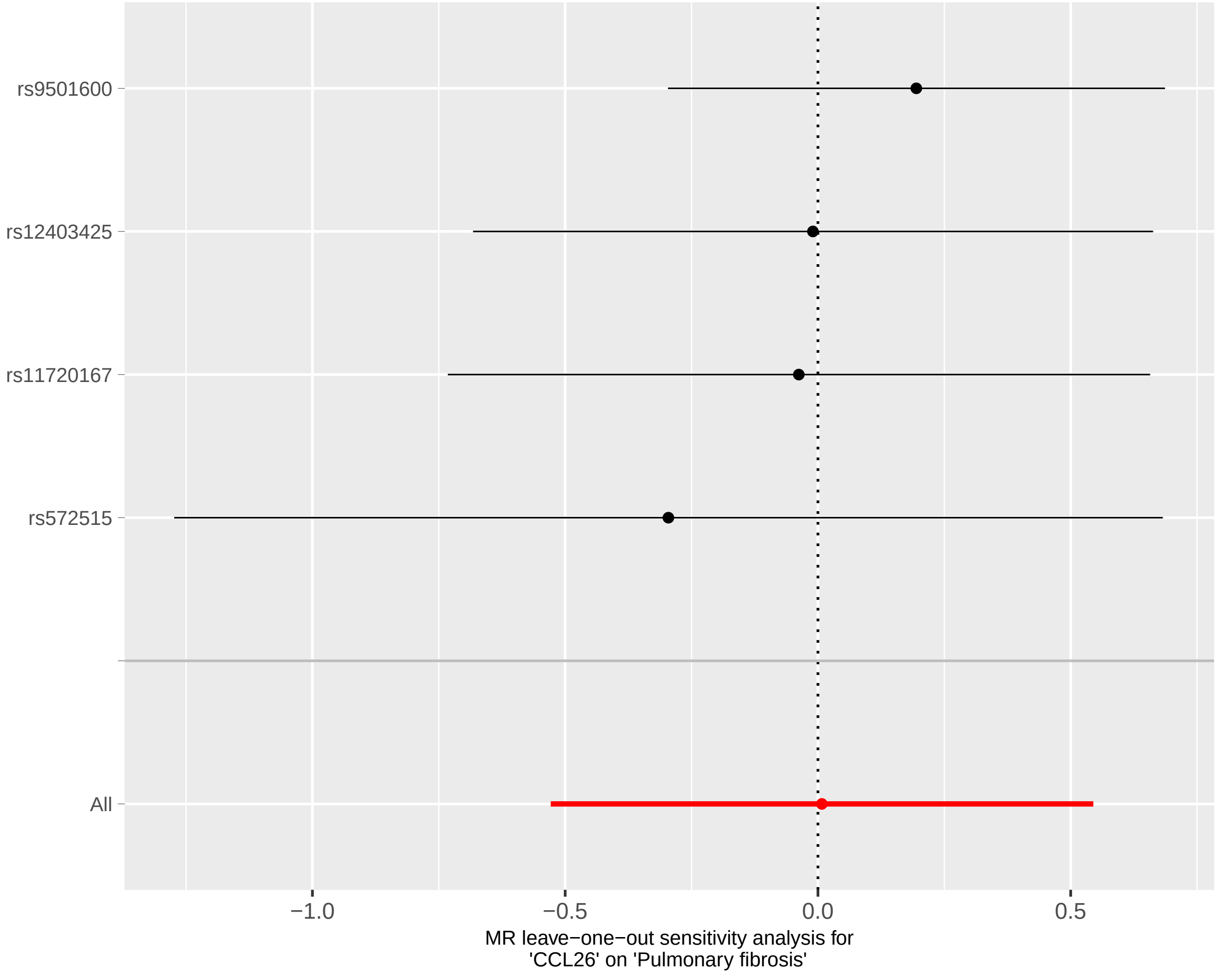
rs9501600
rs12403425
rs11720167
rs572515
All
−1.0
−0.5
0.0
0.5
MR leave−one−out sensitivity analysis for
'CCL26' on 'Pulmonary fibrosis'

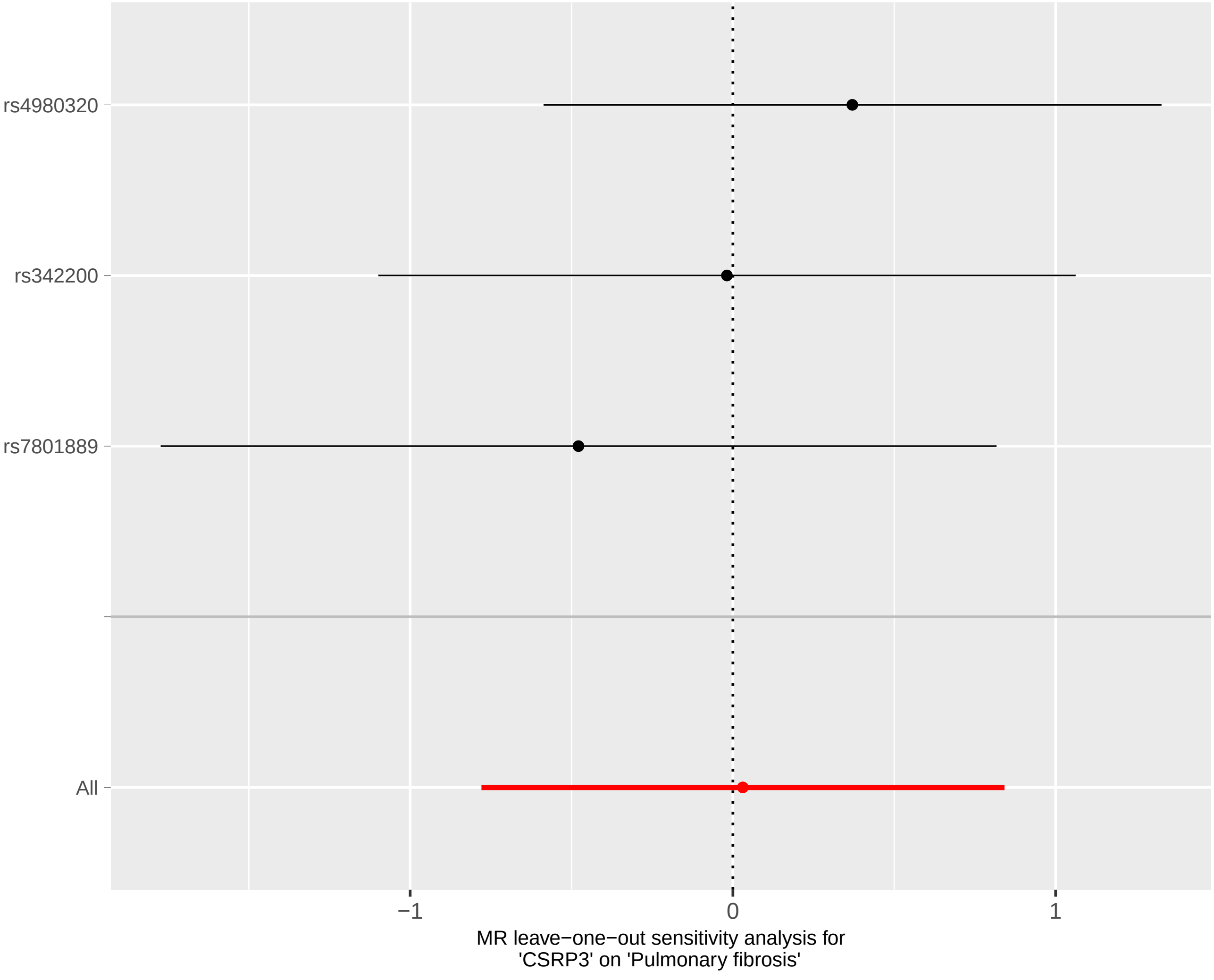
rs4980320
rs342200
rs7801889
All
−1
0
1
MR leave−one−out sensitivity analysis for
'CSRP3' on 'Pulmonary fibrosis'

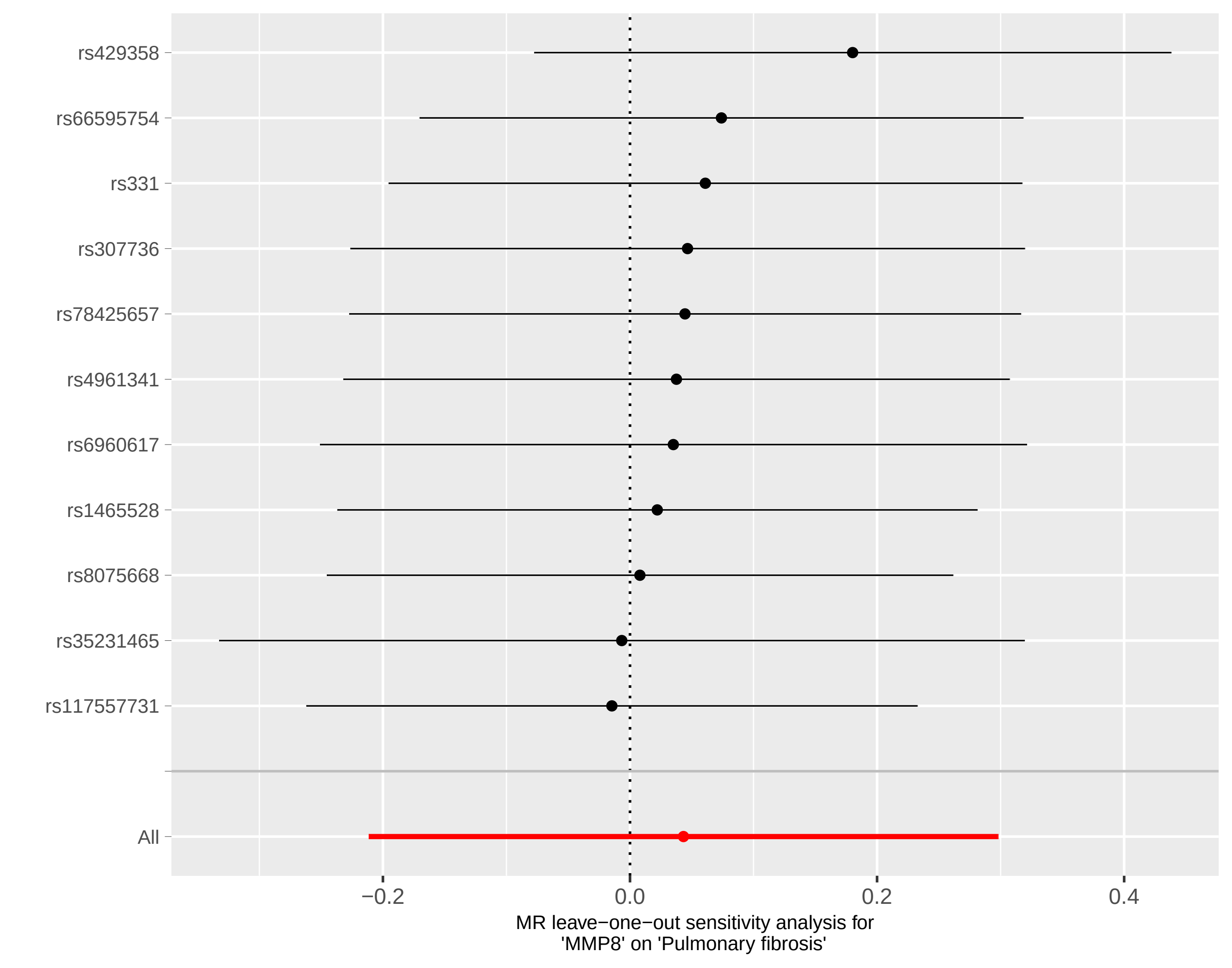
rs429358
rs66595754
rs331
rs307736
rs78425657
rs4961341
rs6960617
rs1465528
rs8075668
rs35231465
rs117557731
All
−0.2
0.0
0.2
0.4
MR leave−one−out sensitivity analysis for
'MMP8' on 'Pulmonary fibrosis'

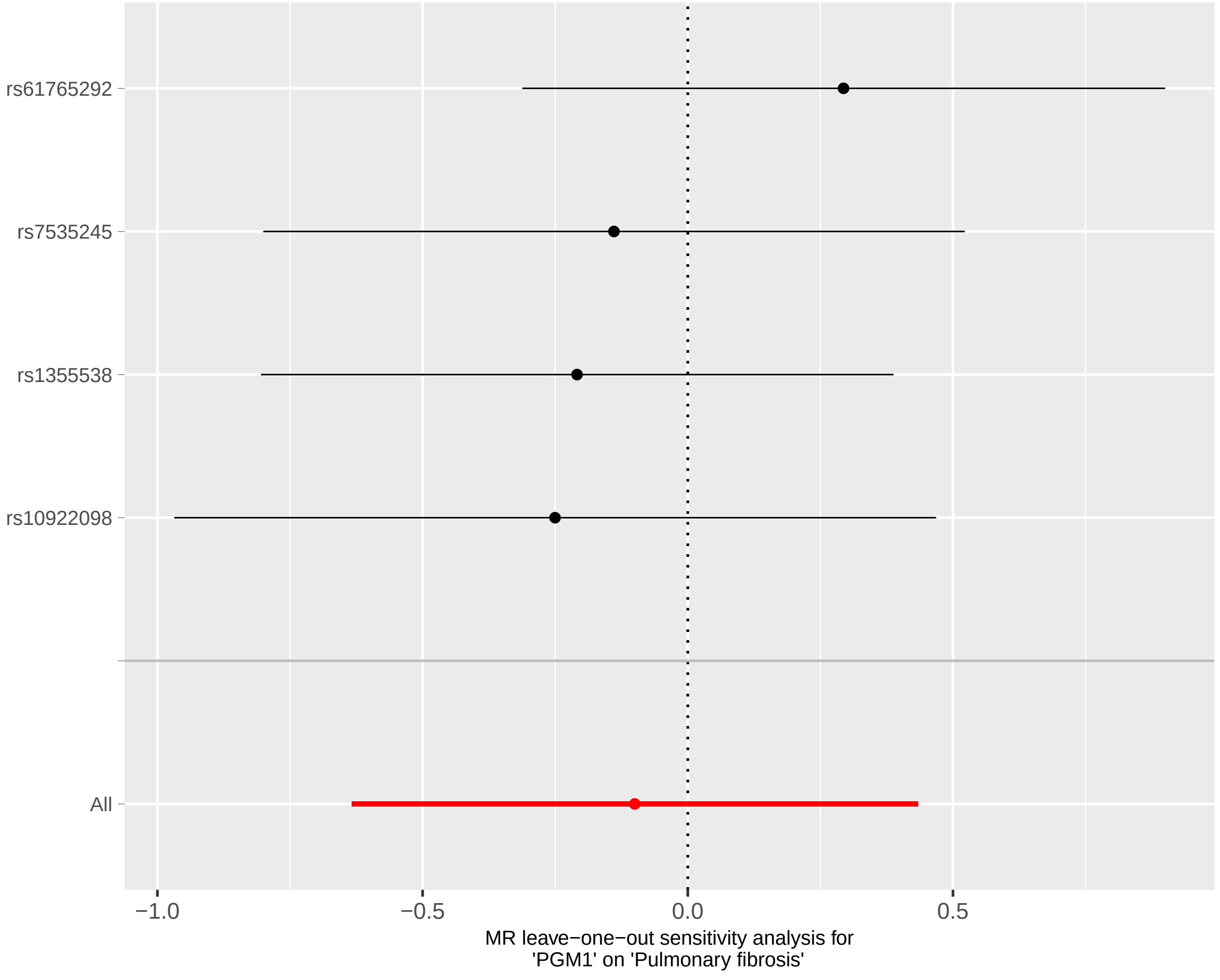
rs61765292
rs7535245
rs1355538
rs10922098
All
−1.0
−0.5
0.0
0.5
MR leave−one−out sensitivity analysis for
'PGM1' on 'Pulmonary fibrosis'

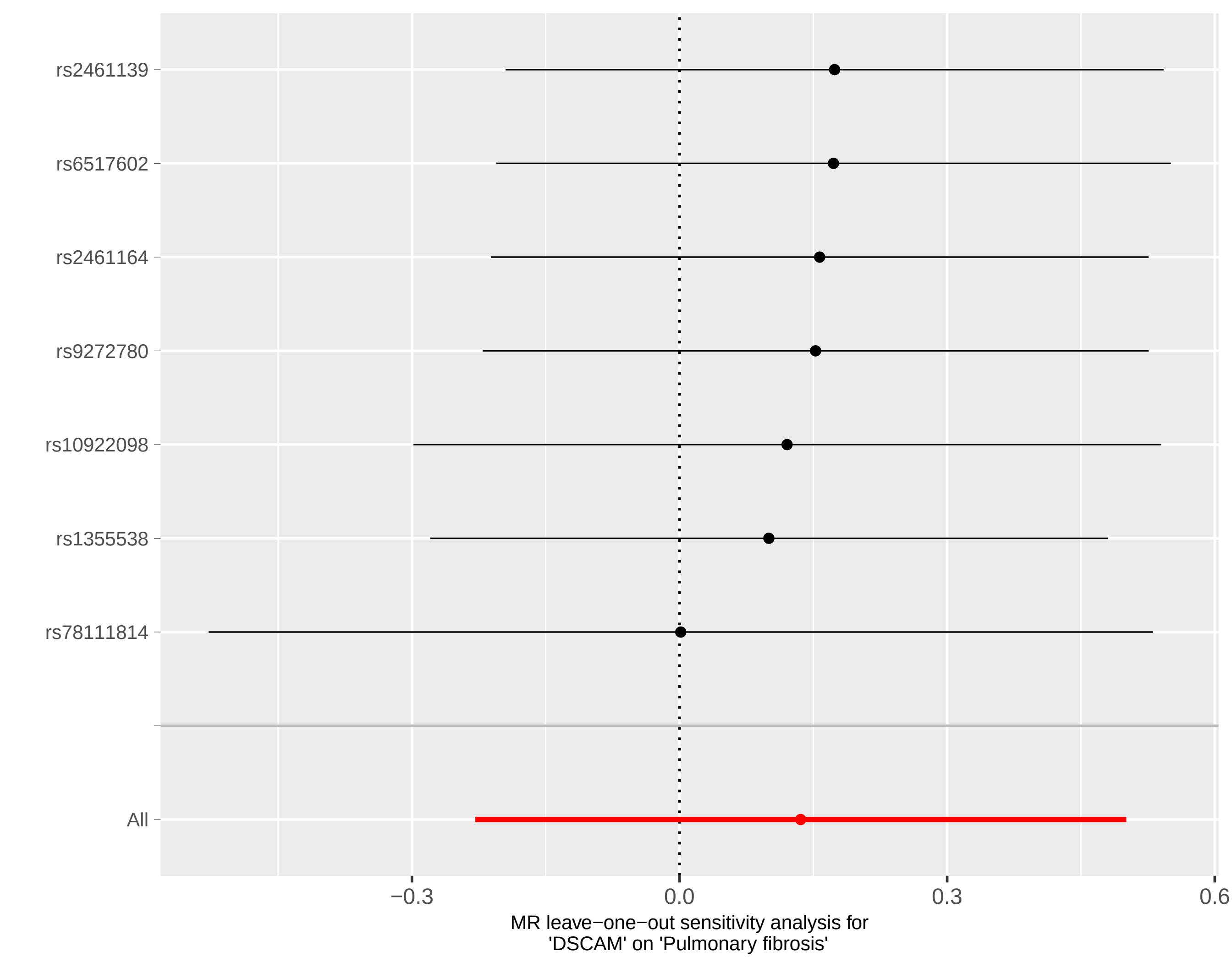

rs2461139
rs6517602
rs2461164
rs9272780
rs10922098
rs1355538
rs78111814
All
−0.3
0.0
0.3
0.6
MR leave−one−out sensitivity analysis for
'DSCAM' on 'Pulmonary fibrosis'

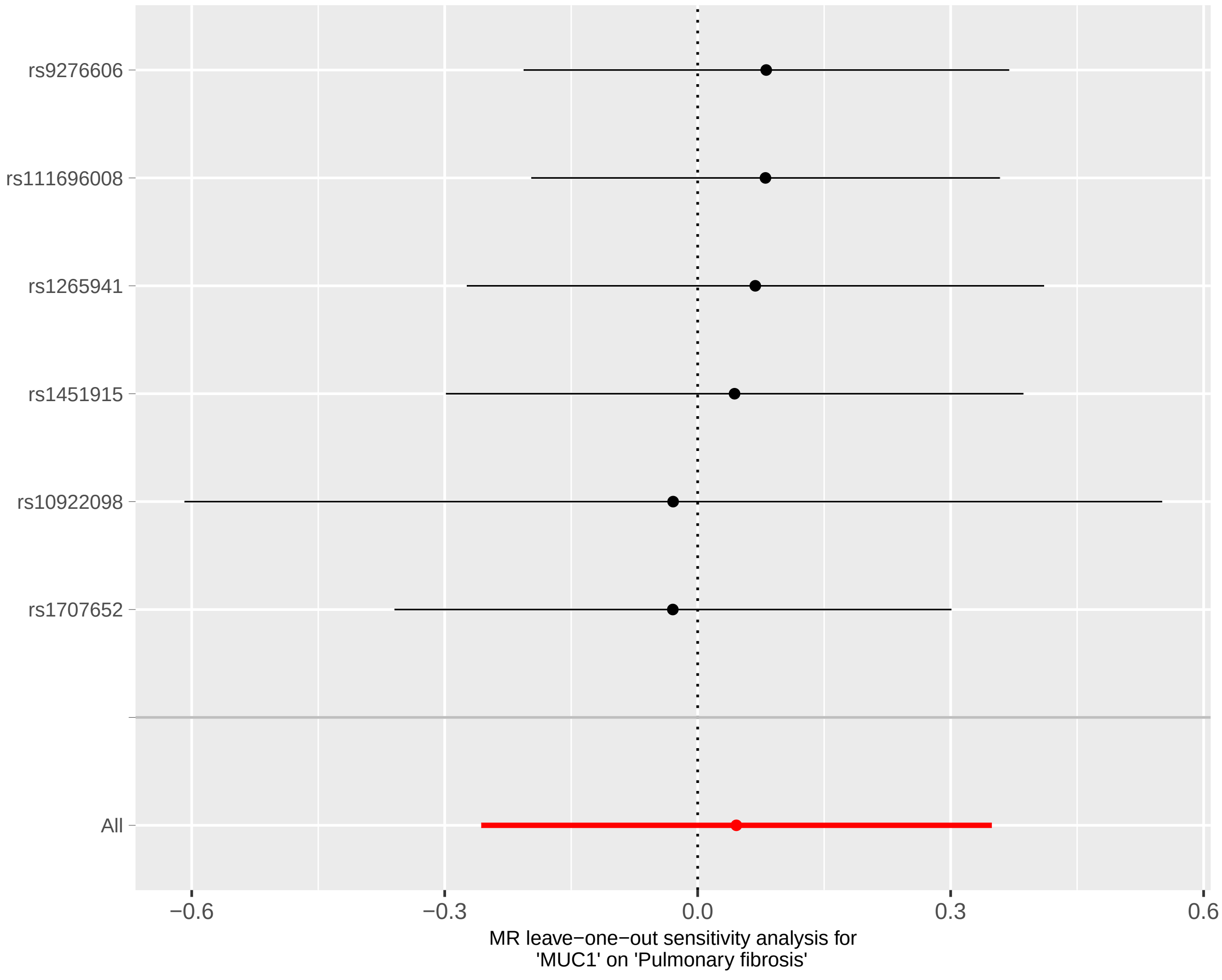
rs9276606
rs111696008
rs1265941
rs1451915
rs10922098
rs1707652
All
−0.6
−0.3
0.0
0.3
0.6
MR leave−one−out sensitivity analysis for
'MUC1' on 'Pulmonary fibrosis'

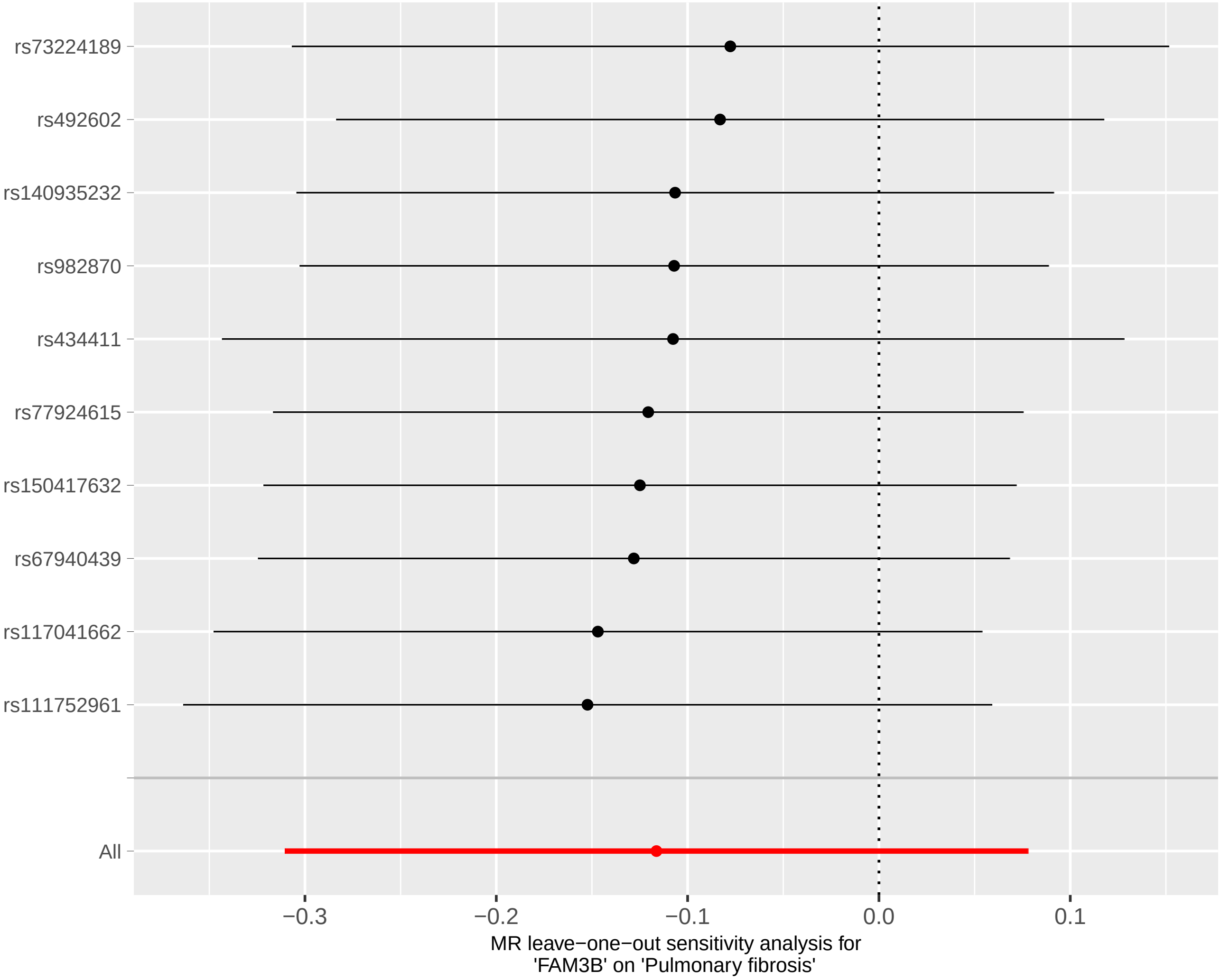
rs73224189
rs492602
rs140935232
rs982870
rs434411
rs77924615
rs150417632
rs67940439
rs117041662
rs111752961
All
−0.3
−0.2
−0.1
0.0
0.1
MR leave−one−out sensitivity analysis for
'FAM3B' on 'Pulmonary fibrosis'

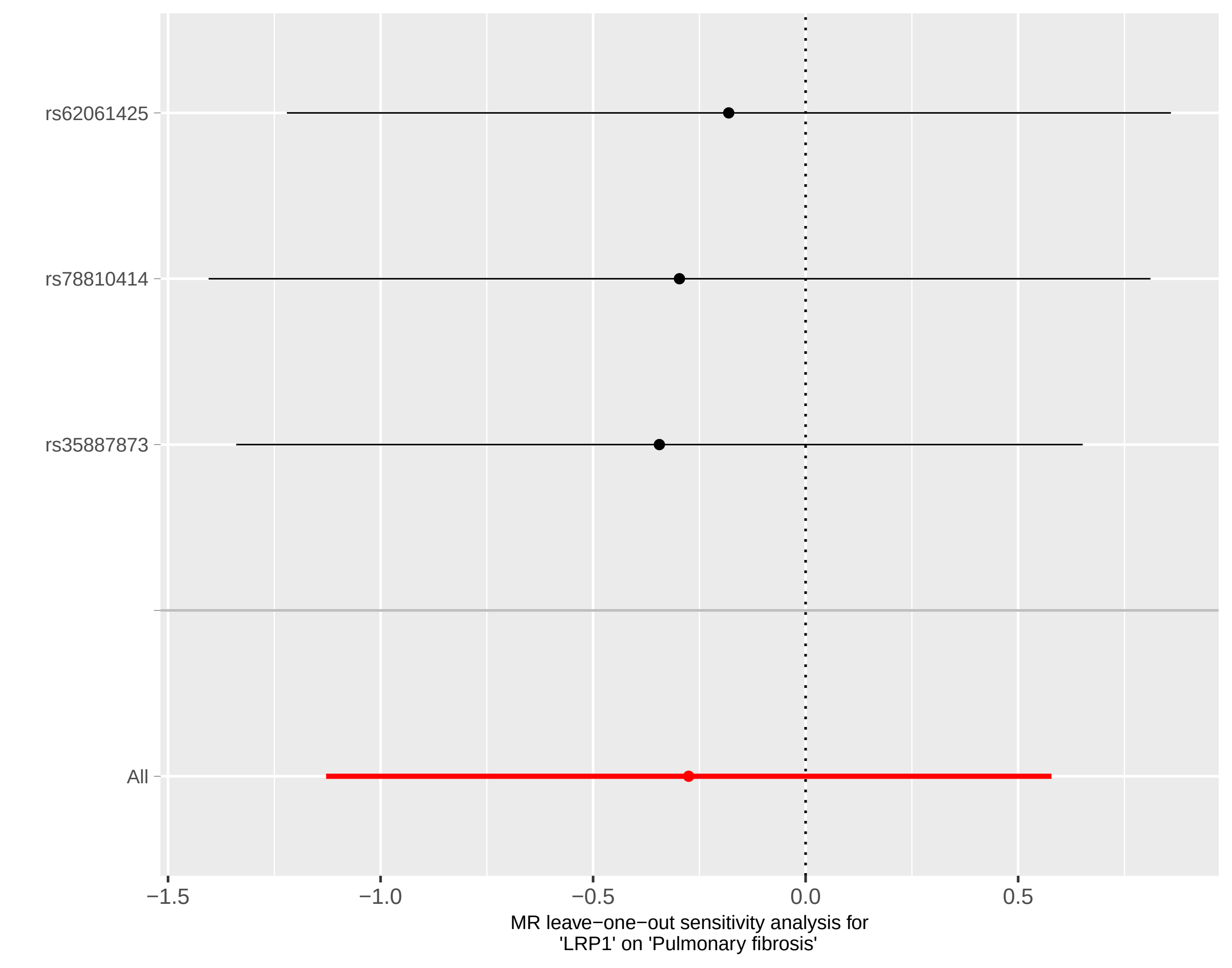

rs62061425
rs78810414
rs35887873
All
−1.5
−1.0
−0.5
0.0
0.5
MR leave−one−out sensitivity analysis for
'LRP1' on 'Pulmonary fibrosis'

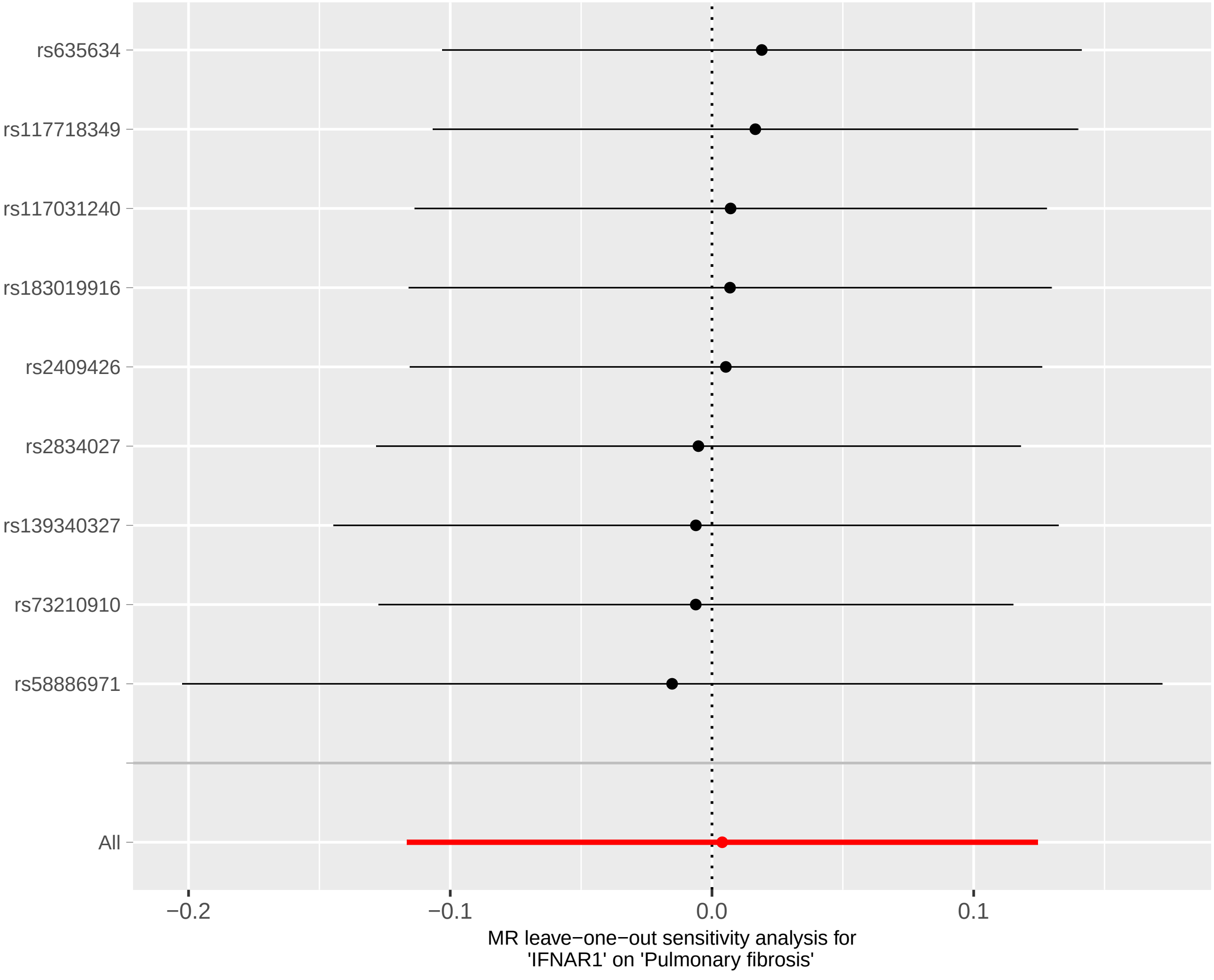

rs635634
rs117718349
rs117031240
rs183019916
rs2409426
rs2834027
rs139340327
rs73210910
rs58886971
All
−0.2
−0.1
0.0
0.1
MR leave−one−out sensitivity analysis for
'IFNAR1' on 'Pulmonary fibrosis'

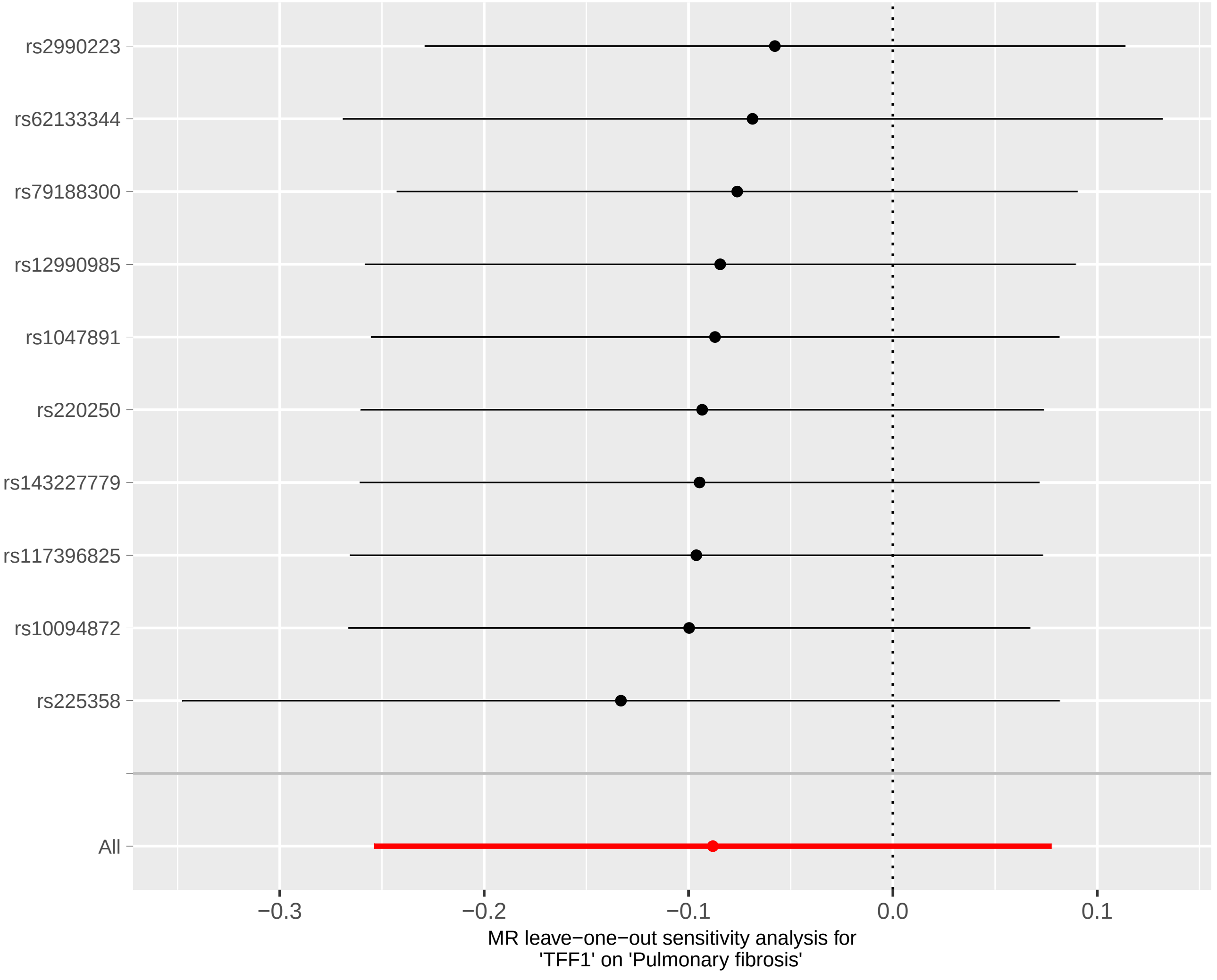
rs2990223
rs62133344
rs79188300
rs12990985
rs1047891
rs220250
rs143227779
rs117396825
rs10094872
rs225358
All
−0.3
−0.2
−0.1
0.0
0.1
MR leave−one−out sensitivity analysis for
'TFF1' on 'Pulmonary fibrosis'

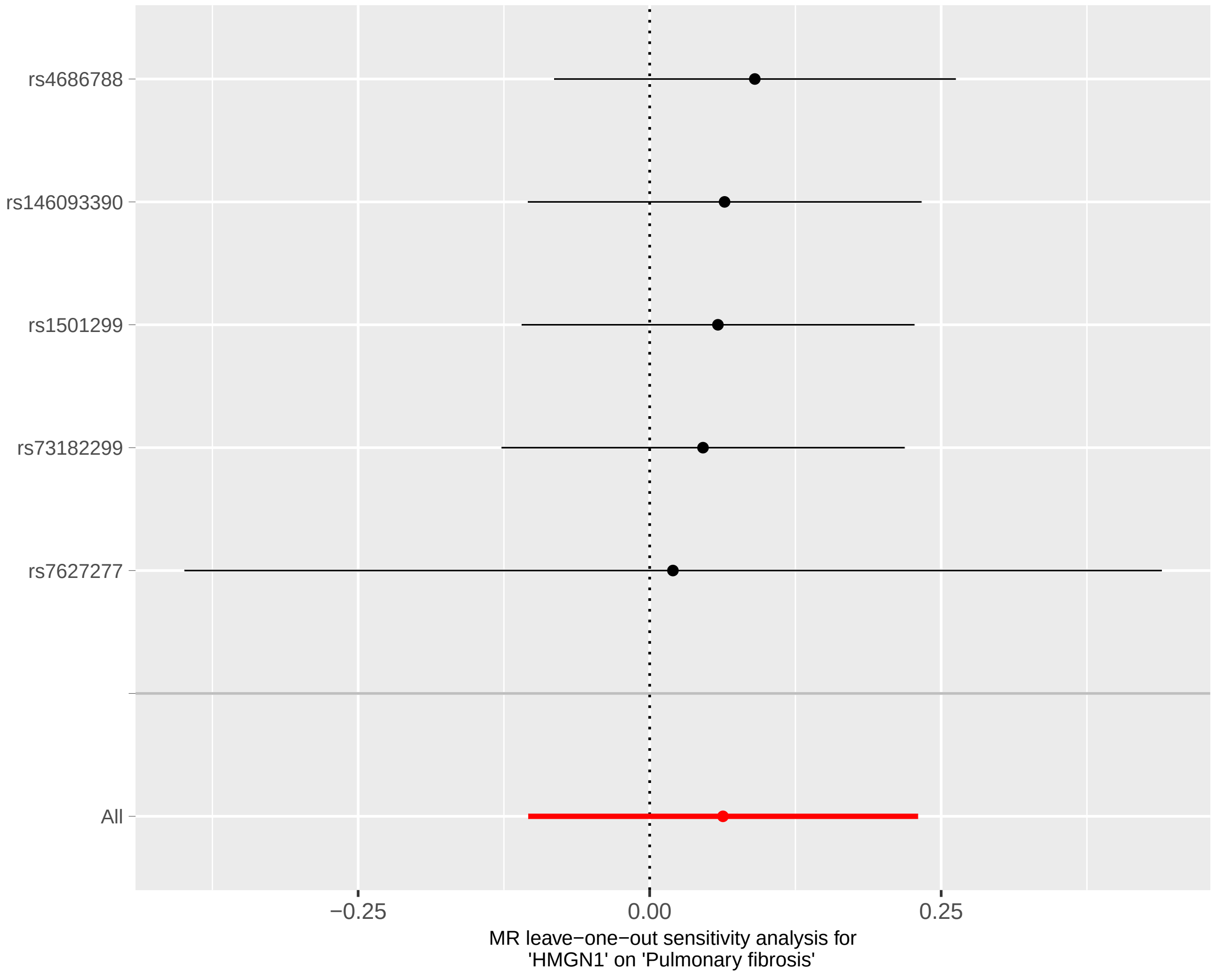
rs4686788
rs146093390
rs1501299
rs73182299
rs7627277
All
−0.25
0.00
0.25
MR leave−one−out sensitivity analysis for
'HMGN1' on 'Pulmonary fibrosis'

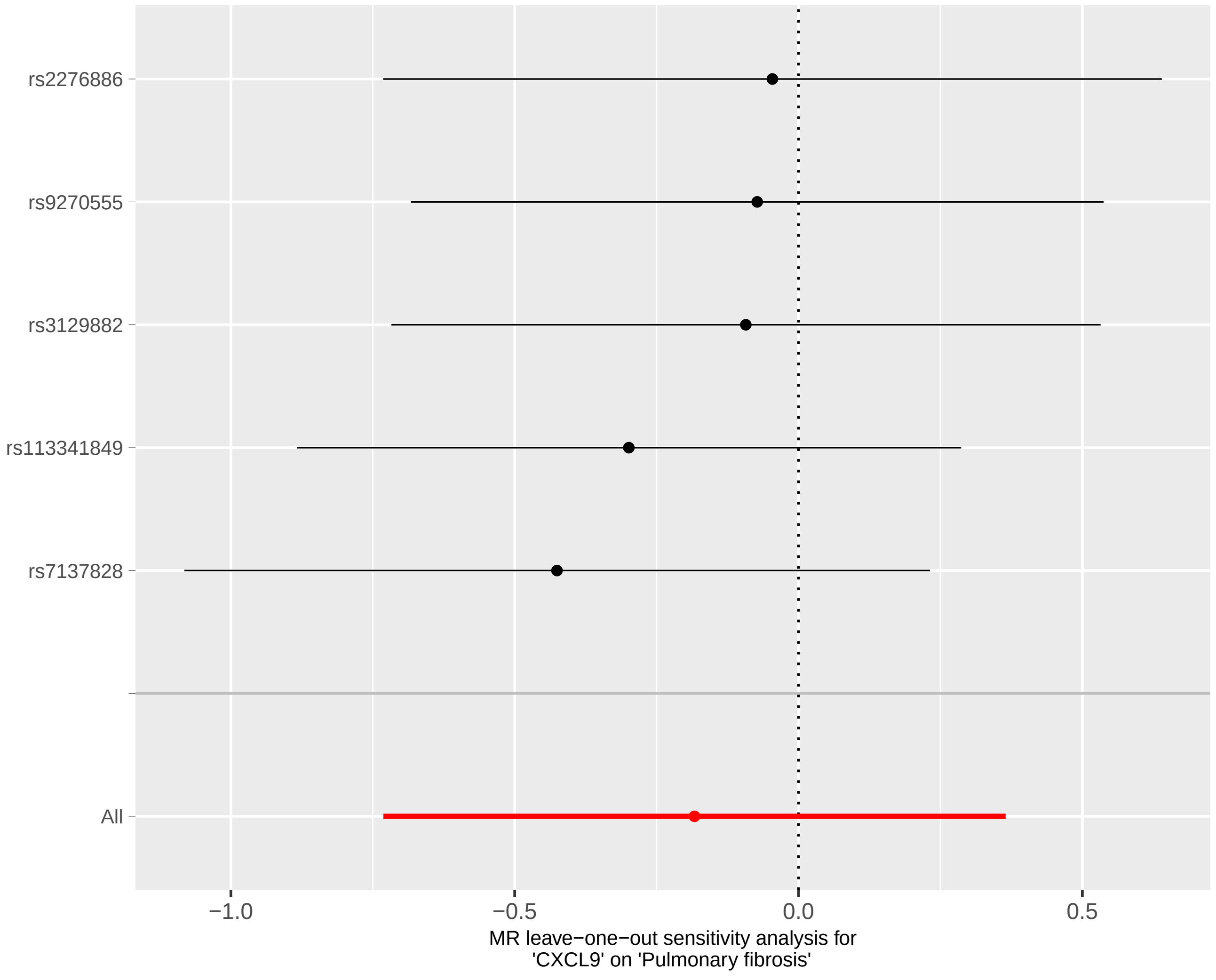
rs2276886
rs9270555
rs3129882
rs113341849
rs7137828
All
−1.0
−0.5
0.0
0.5
MR leave−one−out sensitivity analysis for
'CXCL9' on 'Pulmonary fibrosis'

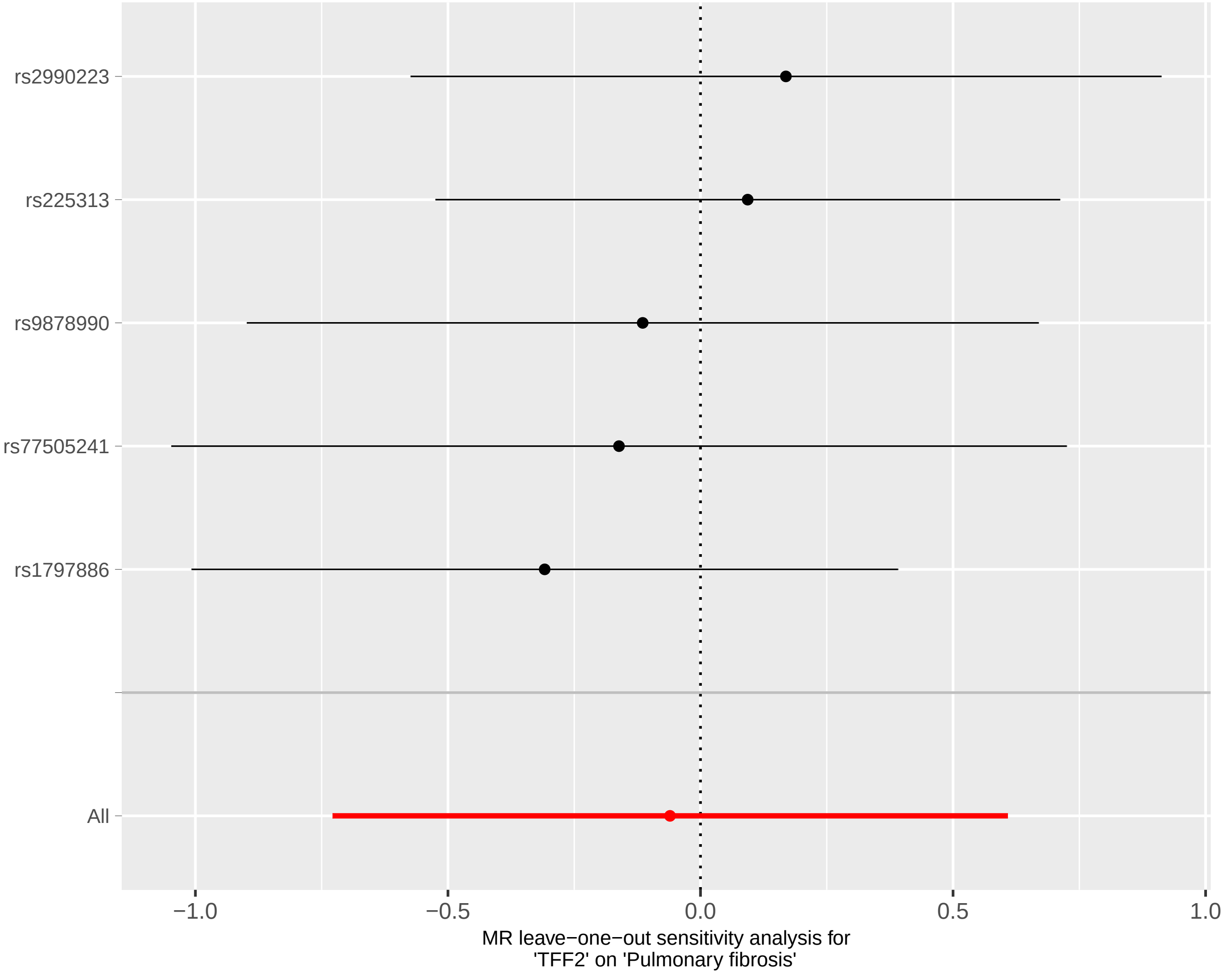
rs2990223
rs225313
rs9878990
rs77505241
rs1797886
All
−1.0
−0.5
0.0
0.5
1.0
MR leave−one−out sensitivity analysis for
'TFF2' on 'Pulmonary fibrosis'

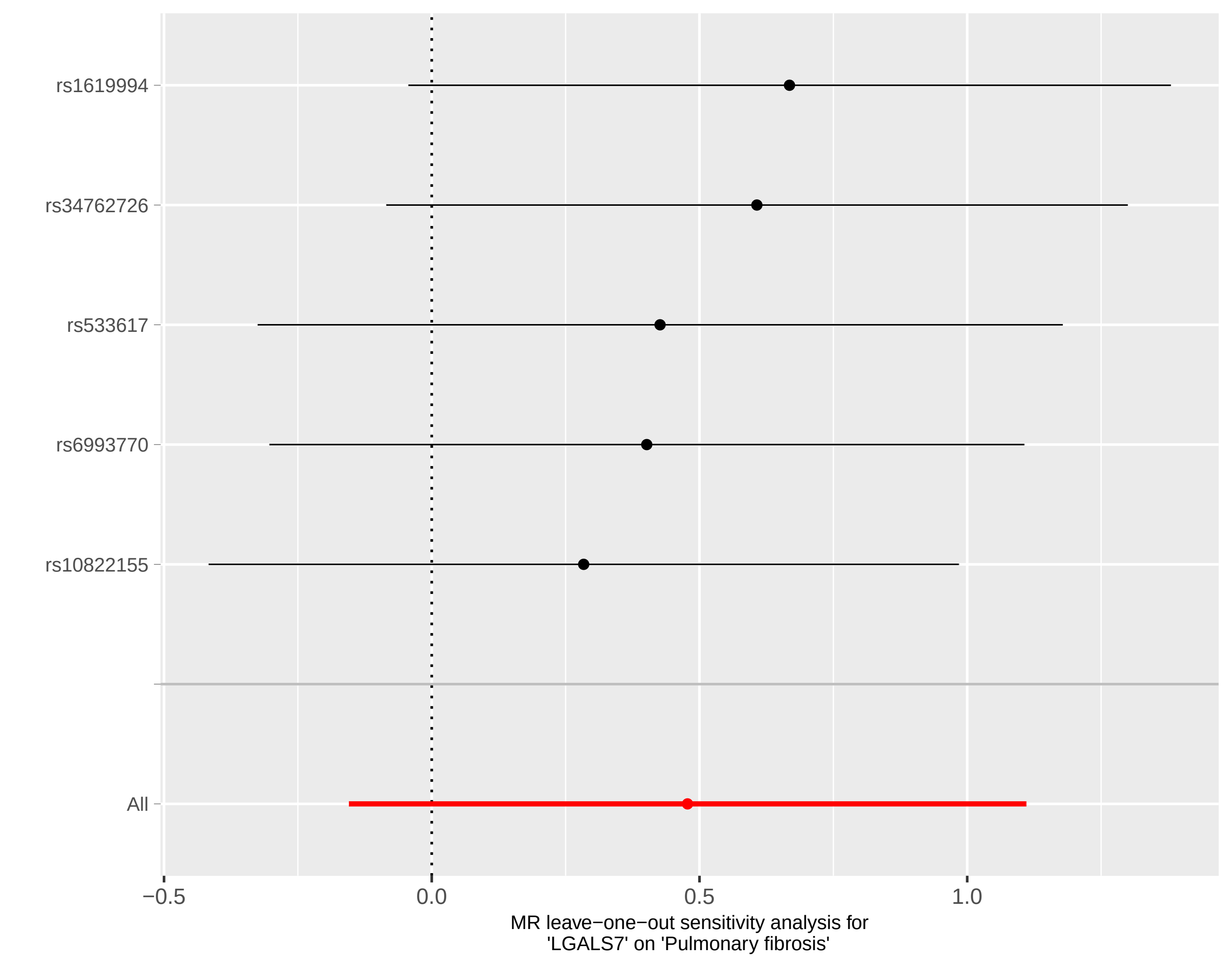
rs1619994
rs34762726
rs533617
rs6993770
rs10822155
All
−0.5
0.0
0.5
1.0
MR leave−one−out sensitivity analysis for
'LGALS7' on 'Pulmonary fibrosis'

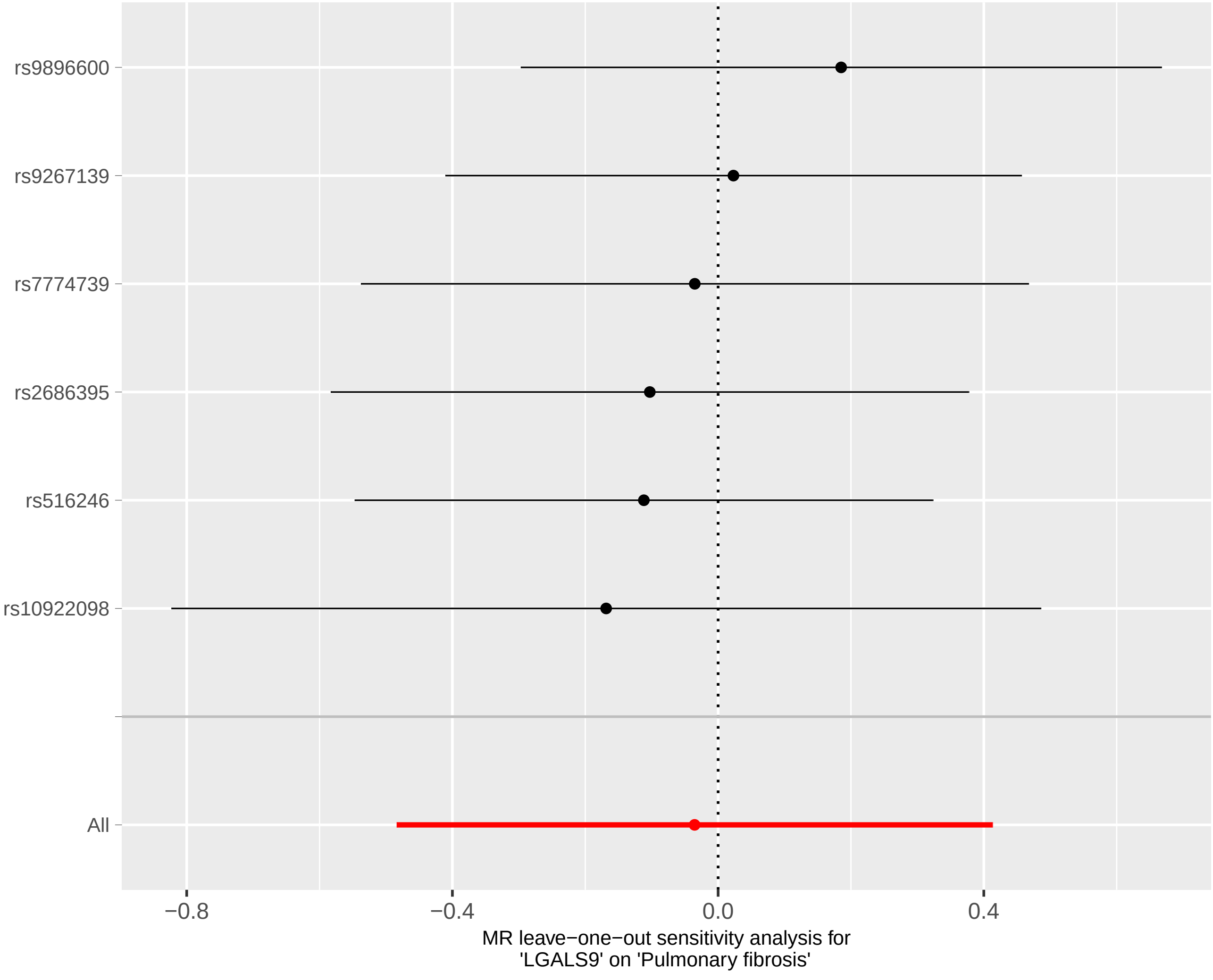
rs9896600
rs9267139
rs7774739
rs2686395
rs516246
rs10922098
All
−0.8
−0.4
0.0
0.4
MR leave−one−out sensitivity analysis for
'LGALS9' on 'Pulmonary fibrosis'

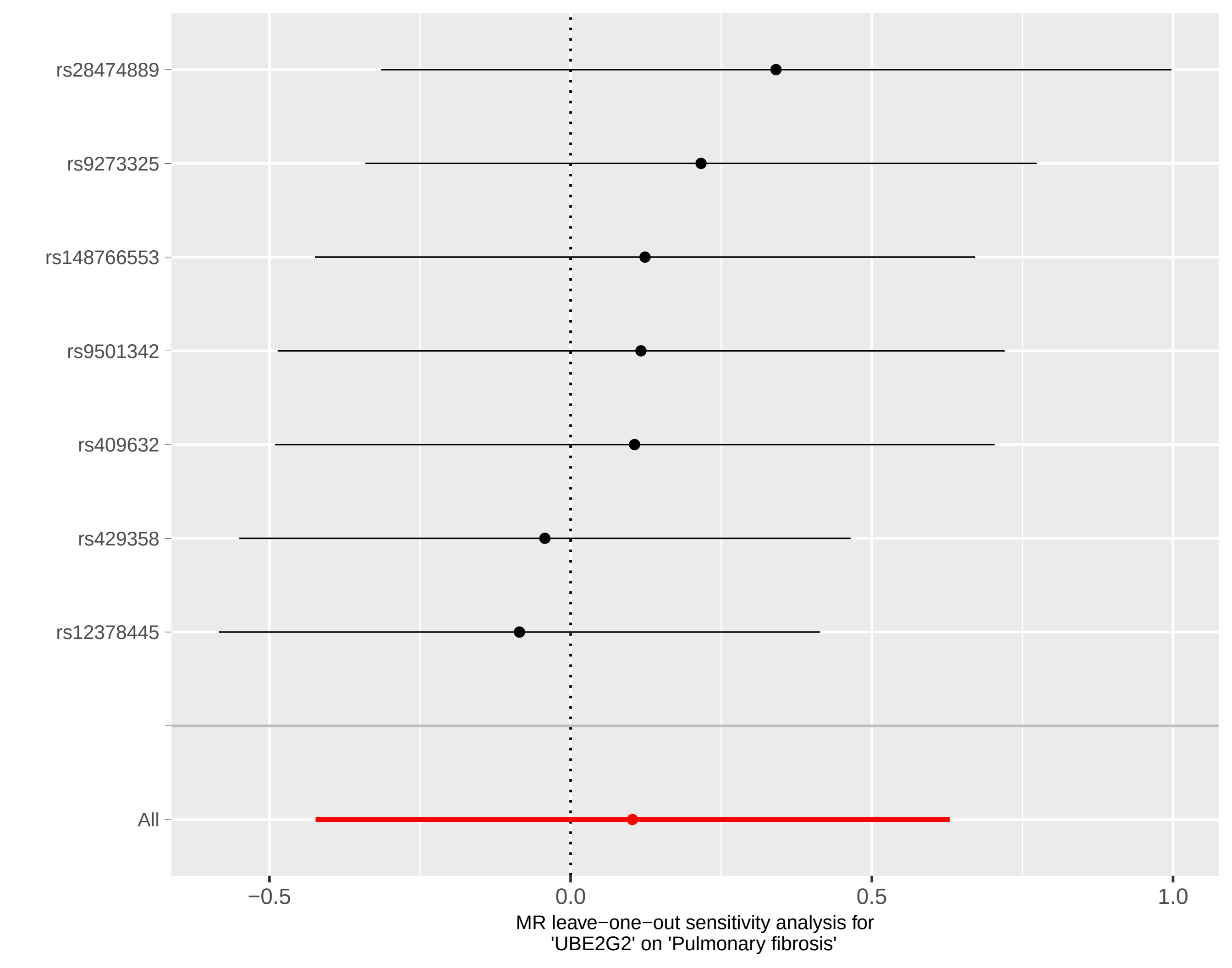
rs28474889
rs9273325
rs148766553
rs9501342
rs409632
rs429358
rs12378445
All
−0.5
0.0
0.5
1.0
MR leave−one−out sensitivity analysis for
'UBE2G2' on 'Pulmonary fibrosis'

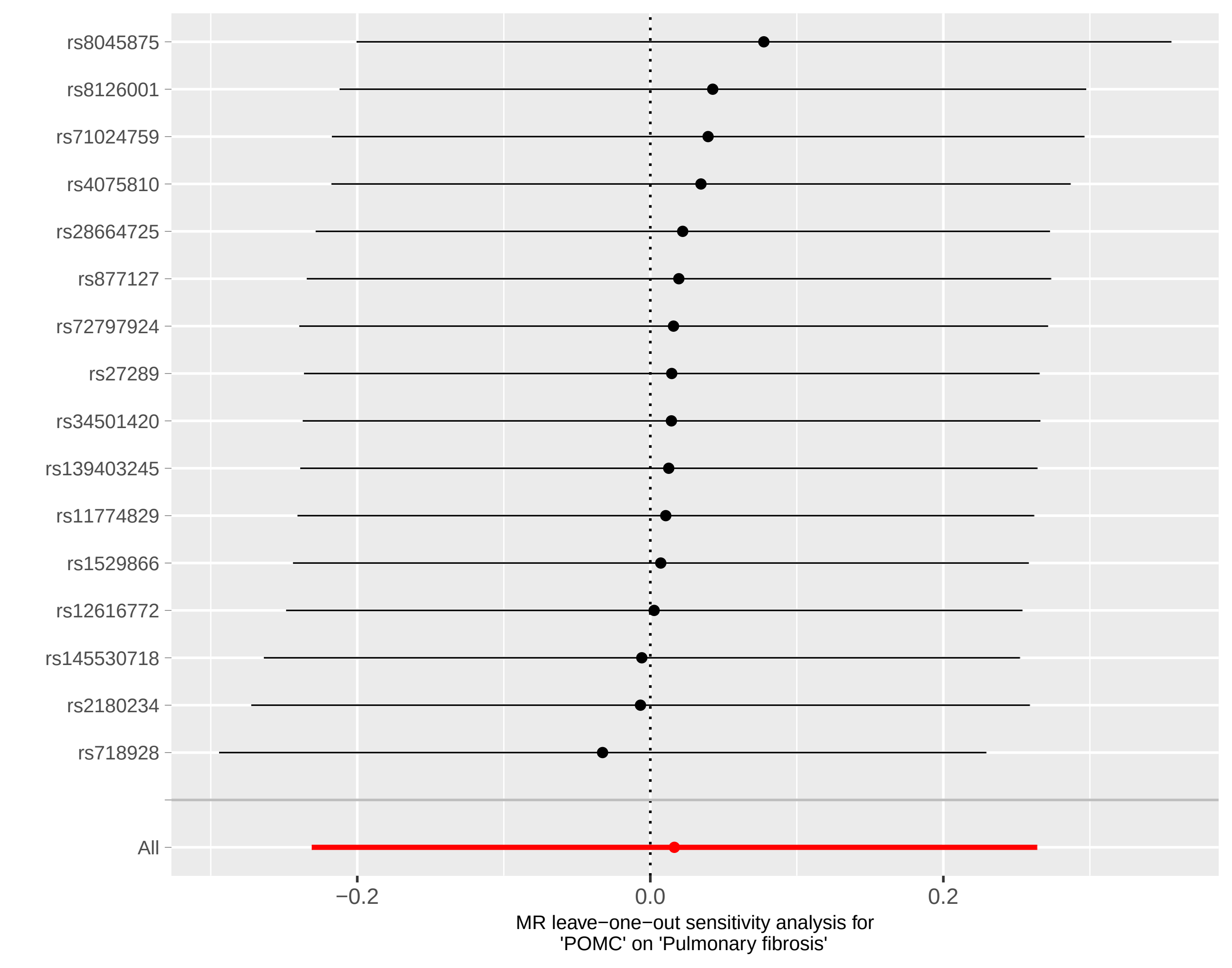
rs8045875
rs8126001
rs71024759
rs4075810
rs28664725
rs877127
rs72797924
rs27289
rs34501420
rs139403245
rs11774829
rs1529866
rs12616772
rs145530718
rs2180234
rs718928
All
−0.2
0.0
0.2
MR leave−one−out sensitivity analysis for
'POMC' on 'Pulmonary fibrosis'

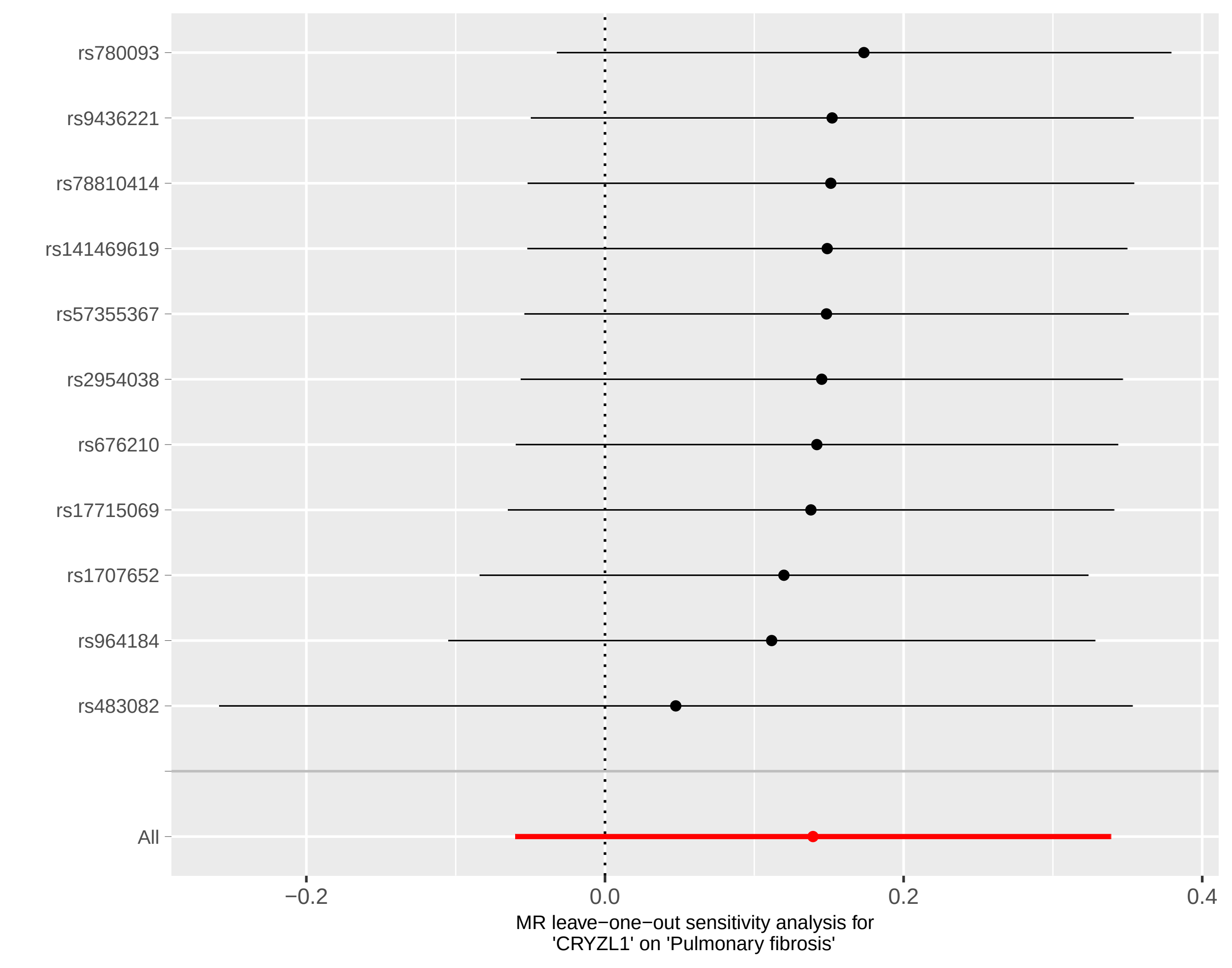
rs780093
rs9436221
rs78810414
rs141469619
rs57355367
rs2954038
rs676210
rs17715069
rs1707652
rs964184
rs483082
All
−0.2
0.0
0.2
0.4
MR leave−one−out sensitivity analysis for
'CRYZL1' on 'Pulmonary fibrosis'

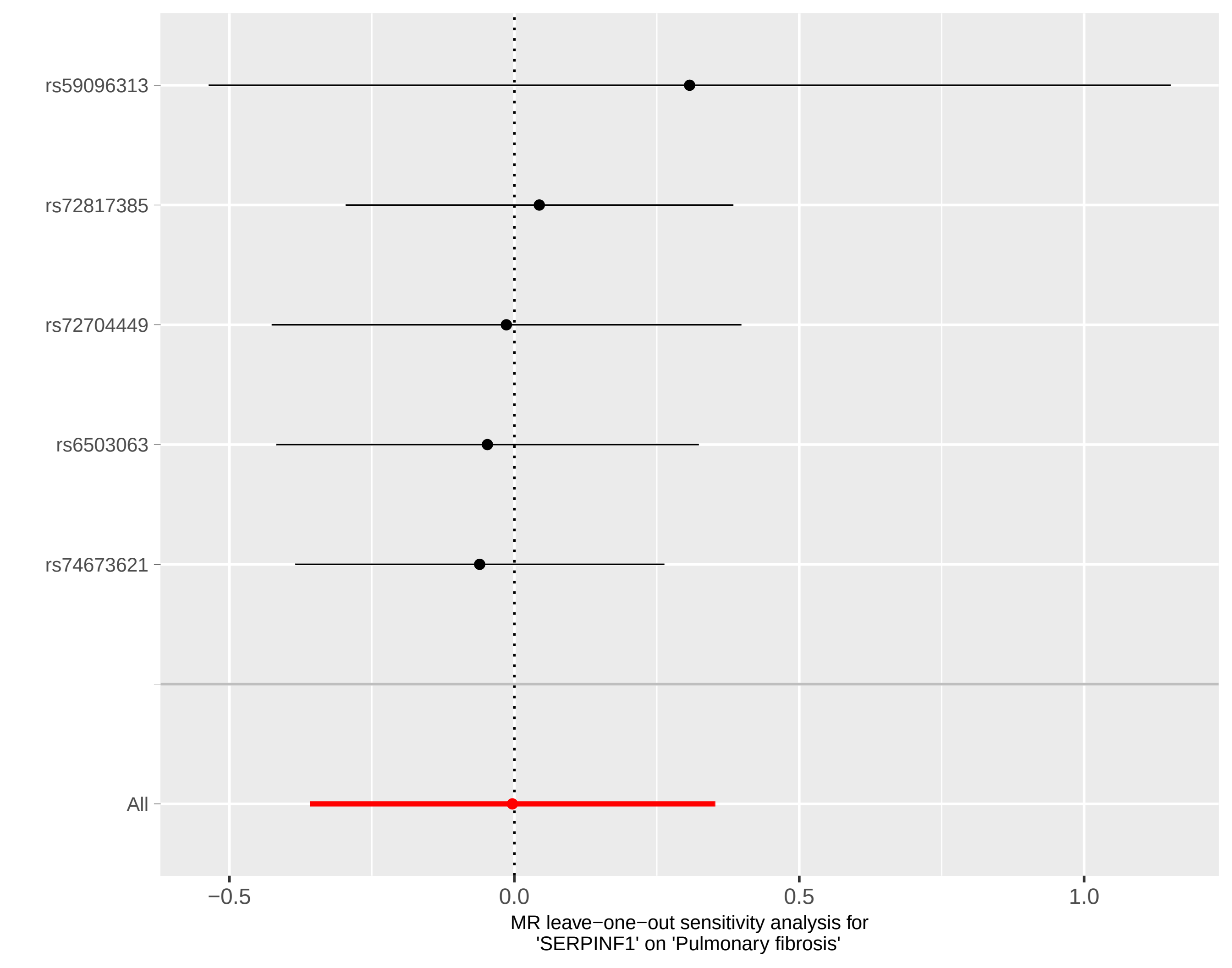
rs59096313
rs72817385
rs72704449
rs6503063
rs74673621
All
−0.5
0.0
0.5
1.0
MR leave−one−out sensitivity analysis for
'SERPINF1' on 'Pulmonary fibrosis'

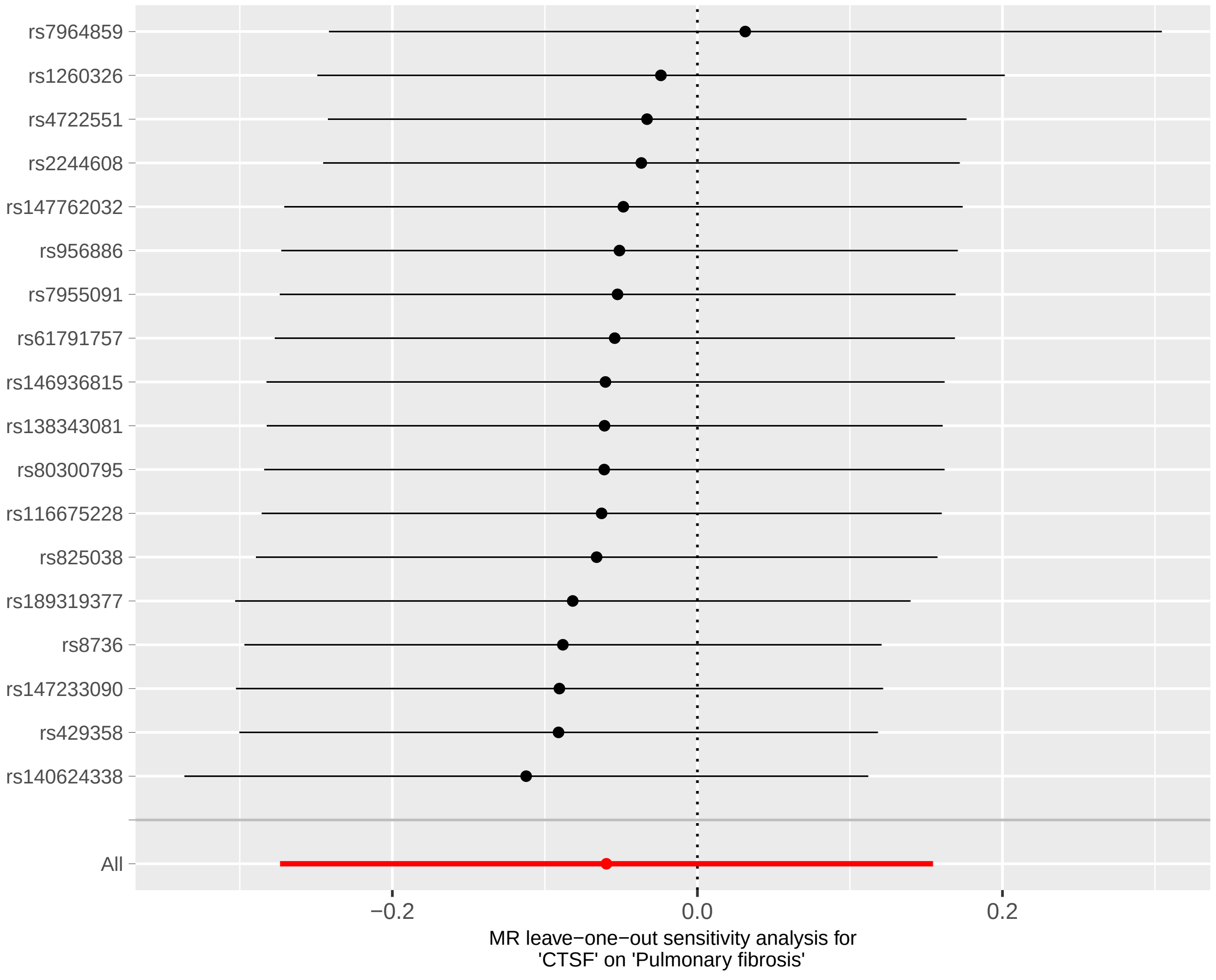

rs7964859
rs1260326
rs4722551
rs2244608
rs147762032
rs956886
rs7955091
rs61791757
rs146936815
rs138343081
rs80300795
rs116675228
rs825038
rs189319377
rs8736
rs147233090
rs429358
rs140624338
All
−0.2
0.0
0.2
MR leave−one−out sensitivity analysis for
'CTSF' on 'Pulmonary fibrosis'

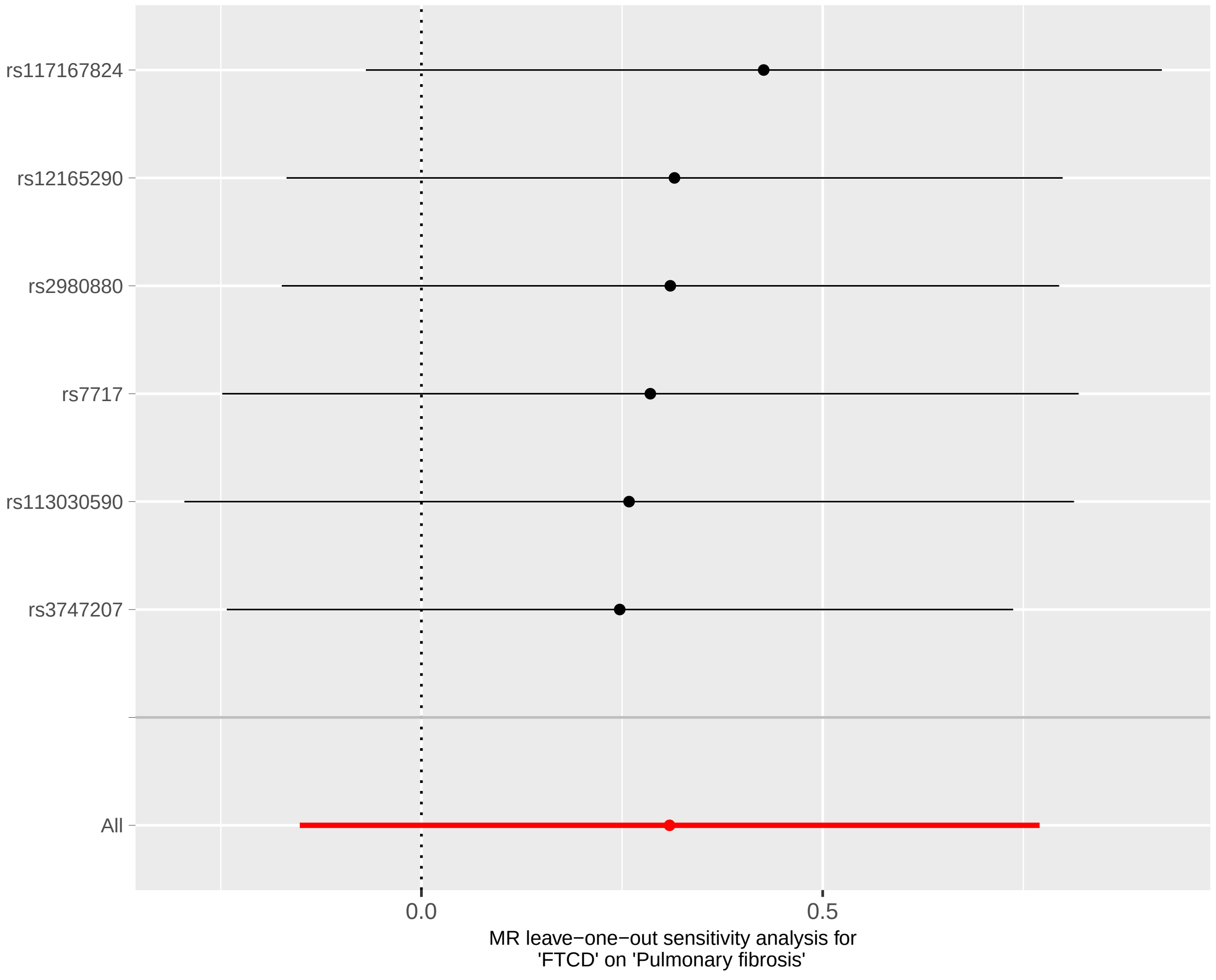
rs117167824
rs12165290
rs2980880
rs7717
rs113030590
rs3747207
All
0.0
0.5
MR leave−one−out sensitivity analysis for
'FTCD' on 'Pulmonary fibrosis'

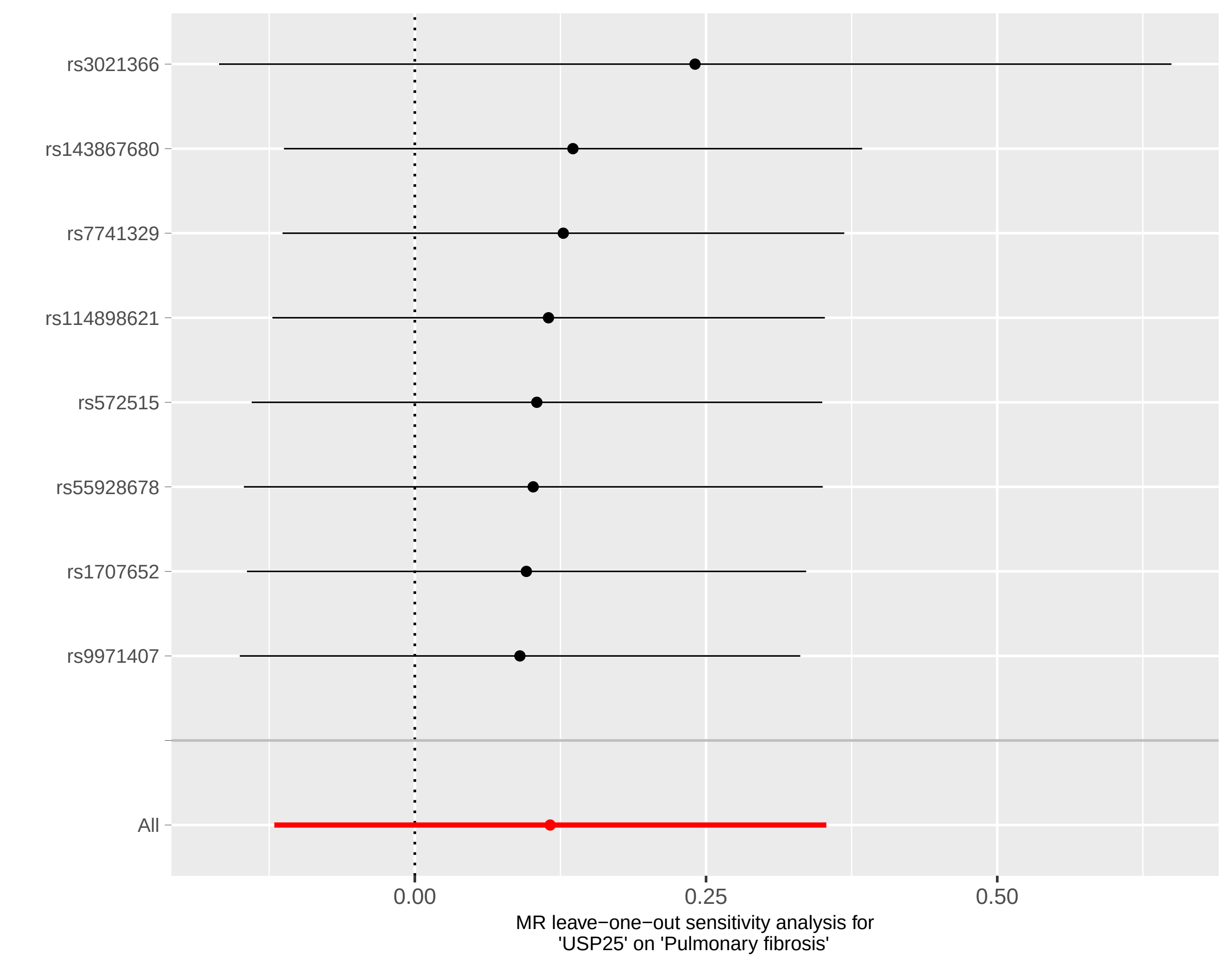
rs3021366
rs143867680
rs7741329
rs114898621
rs572515
rs55928678
rs1707652
rs9971407
All
0.00
0.25
0.50
MR leave−one−out sensitivity analysis for
'USP25' on 'Pulmonary fibrosis'

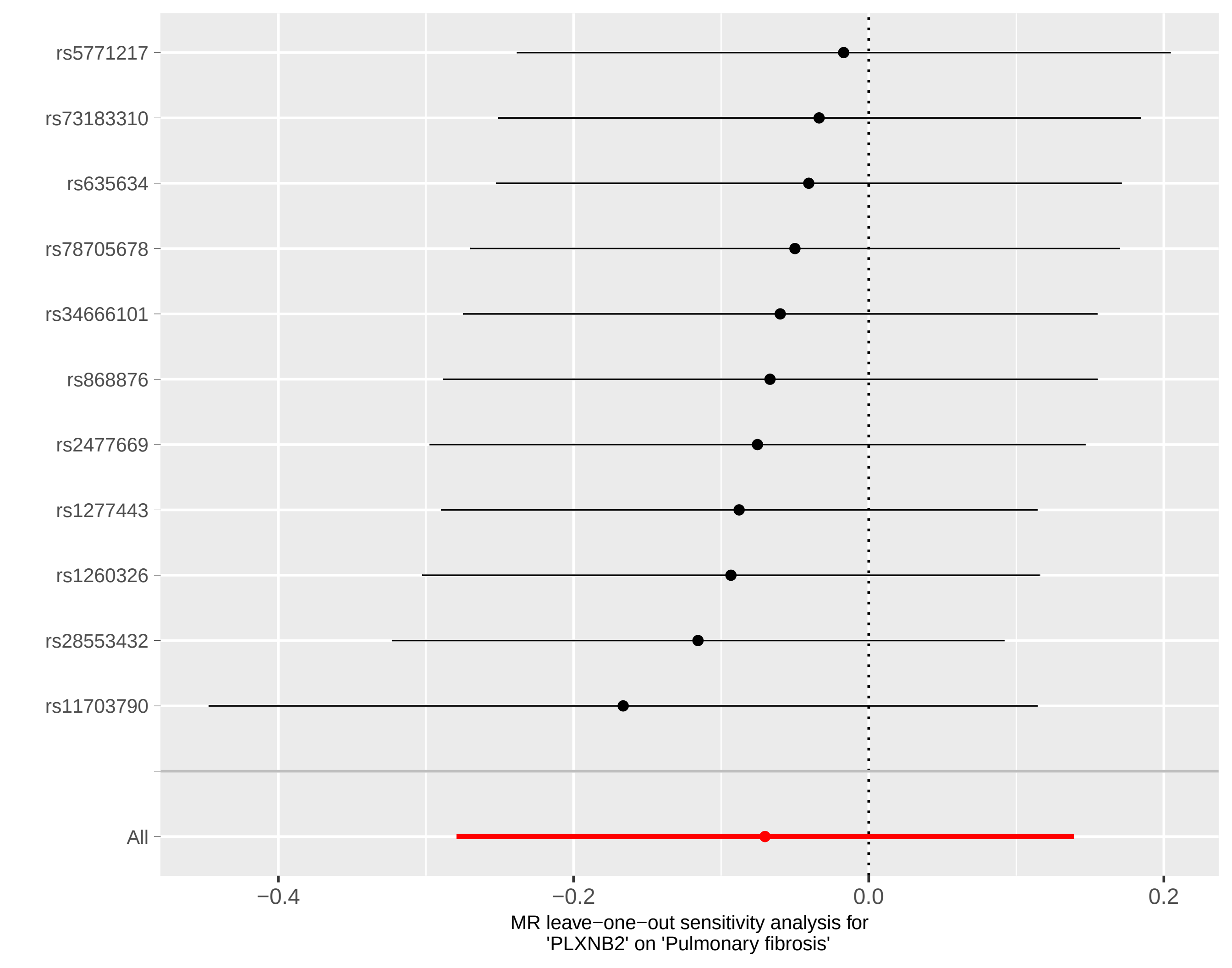
rs5771217
rs73183310
rs635634
rs78705678
rs34666101
rs868876
rs2477669
rs1277443
rs1260326
rs28553432
rs11703790
All
−0.4
−0.2
0.0
0.2
MR leave−one−out sensitivity analysis for
'PLXNB2' on 'Pulmonary fibrosis'

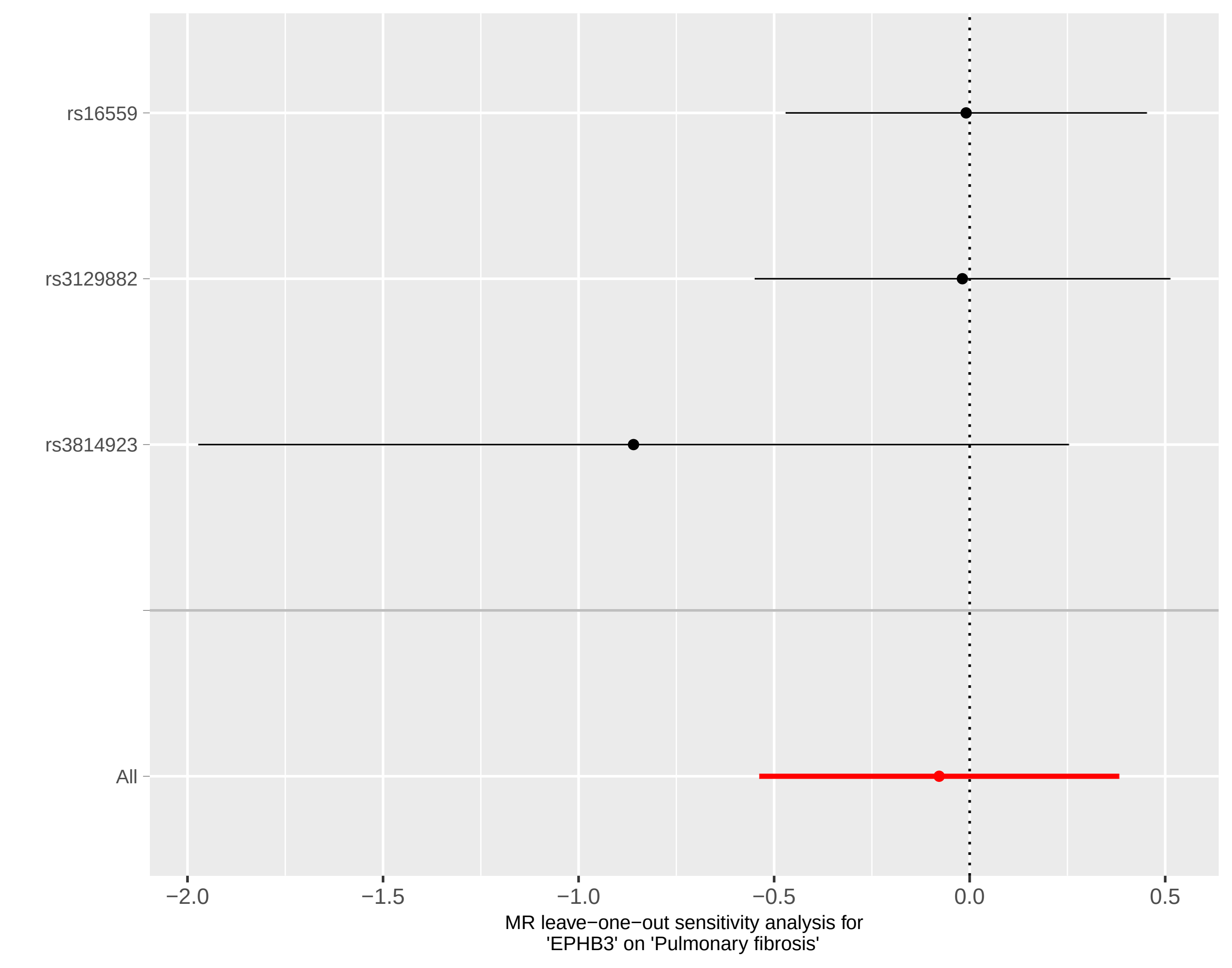

rs16559
rs3129882
rs3814923
All
−2.0
−1.5
−1.0
−0.5
0.0
0.5
MR leave−one−out sensitivity analysis for
'EPHB3' on 'Pulmonary fibrosis'

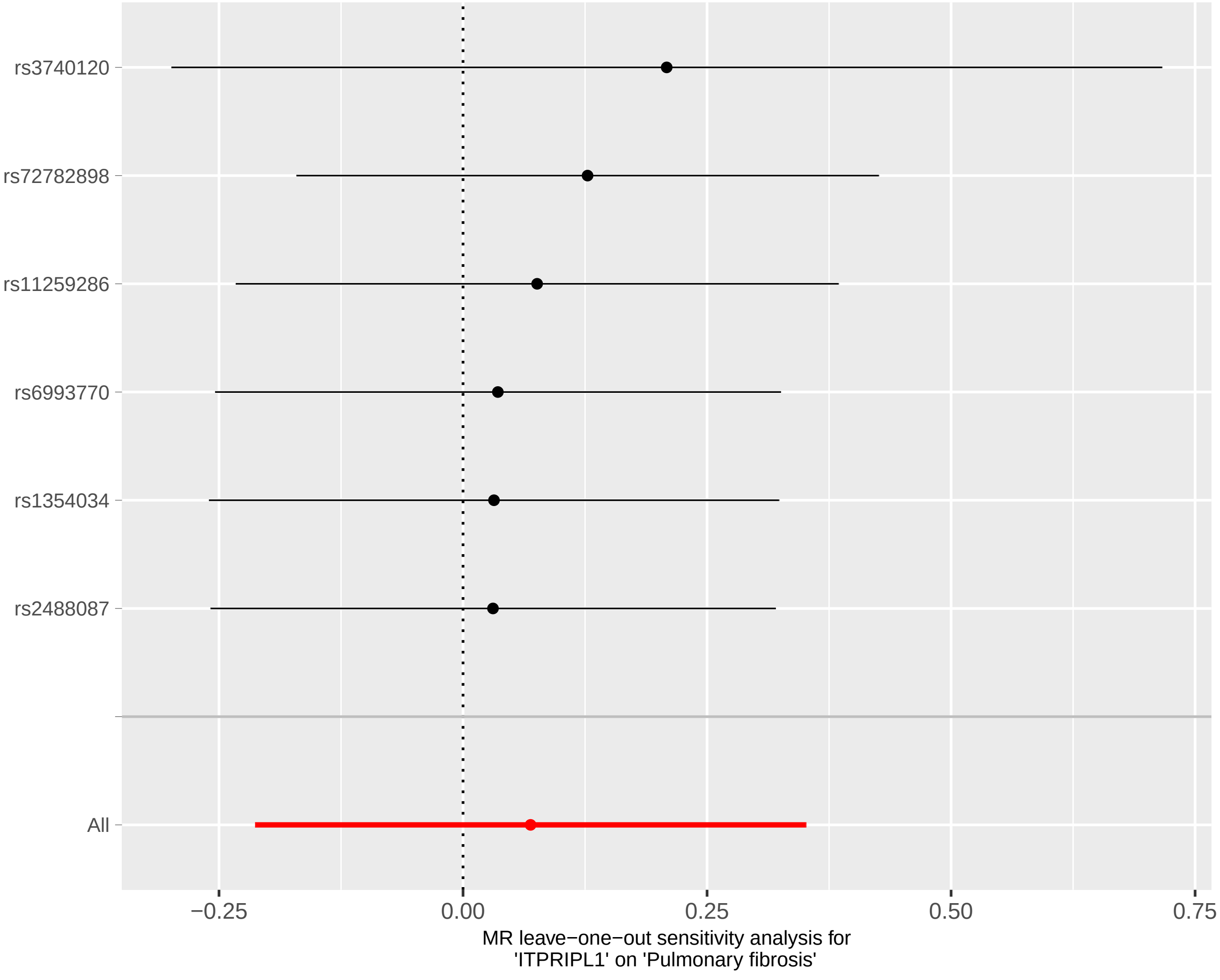
rs3740120
rs72782898
rs11259286
rs6993770
rs1354034
rs2488087
All
−0.25
0.00
0.25
0.50
0.75
MR leave−one−out sensitivity analysis for
'ITPRIPL1' on 'Pulmonary fibrosis'

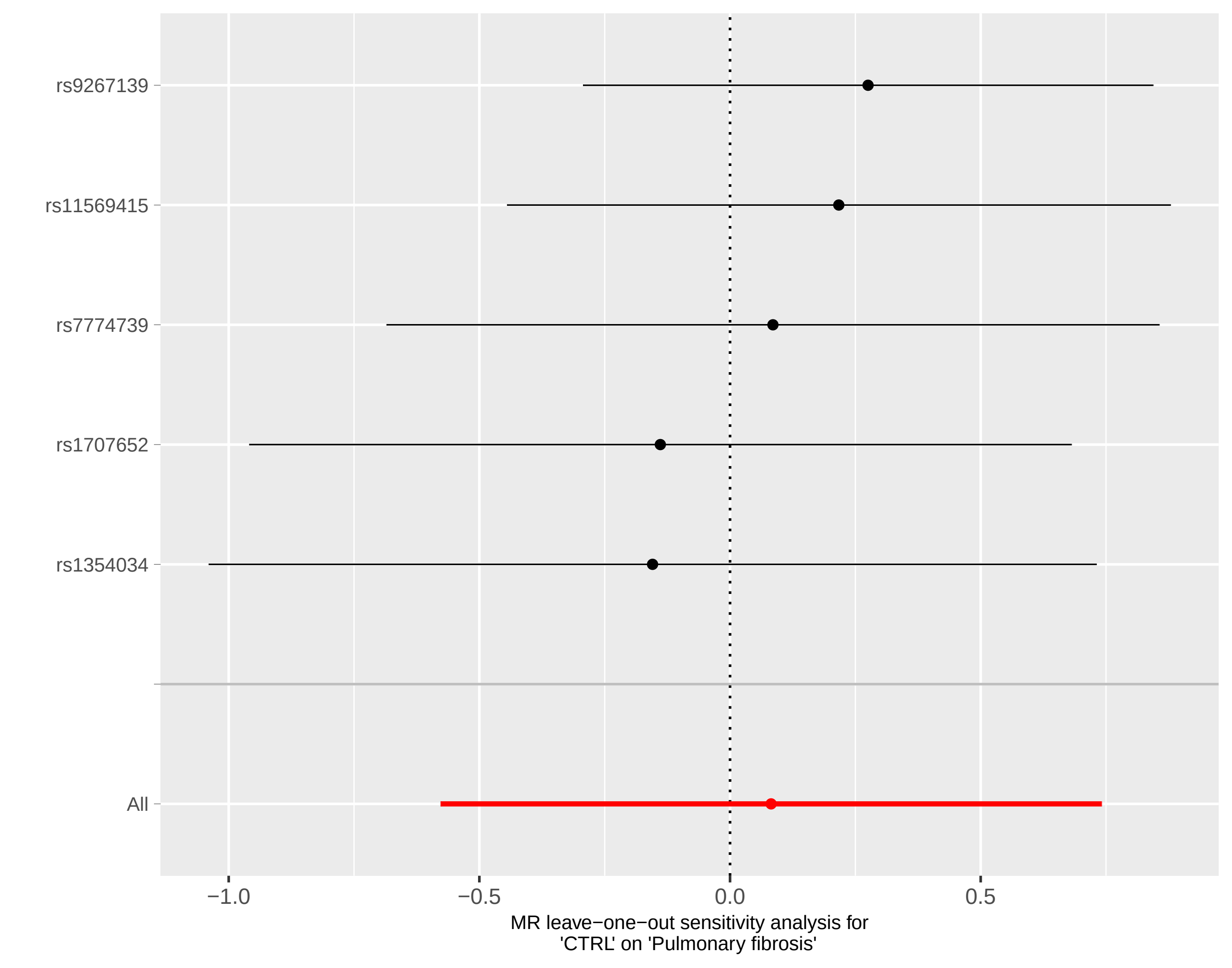

rs9267139
rs11569415
rs7774739
rs1707652
rs1354034
All
−1.0
−0.5
0.0
0.5
MR leave−one−out sensitivity analysis for
'CTRL' on 'Pulmonary fibrosis'

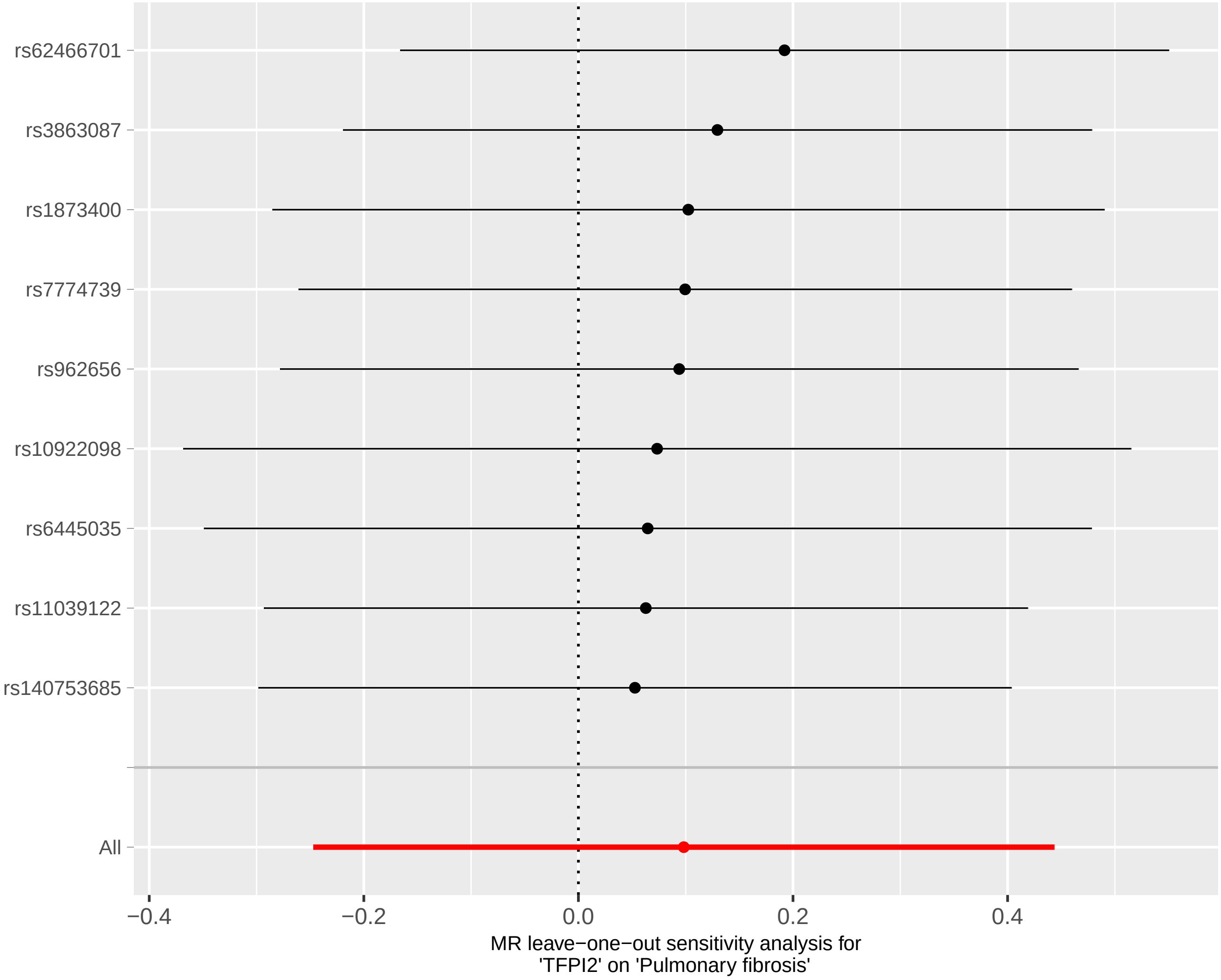
rs62466701
rs3863087
rs1873400
rs7774739
rs962656
rs10922098
rs6445035
rs11039122
rs140753685
All
−0.4
−0.2
0.0
0.2
0.4
MR leave−one−out sensitivity analysis for
'TFPI2' on 'Pulmonary fibrosis'

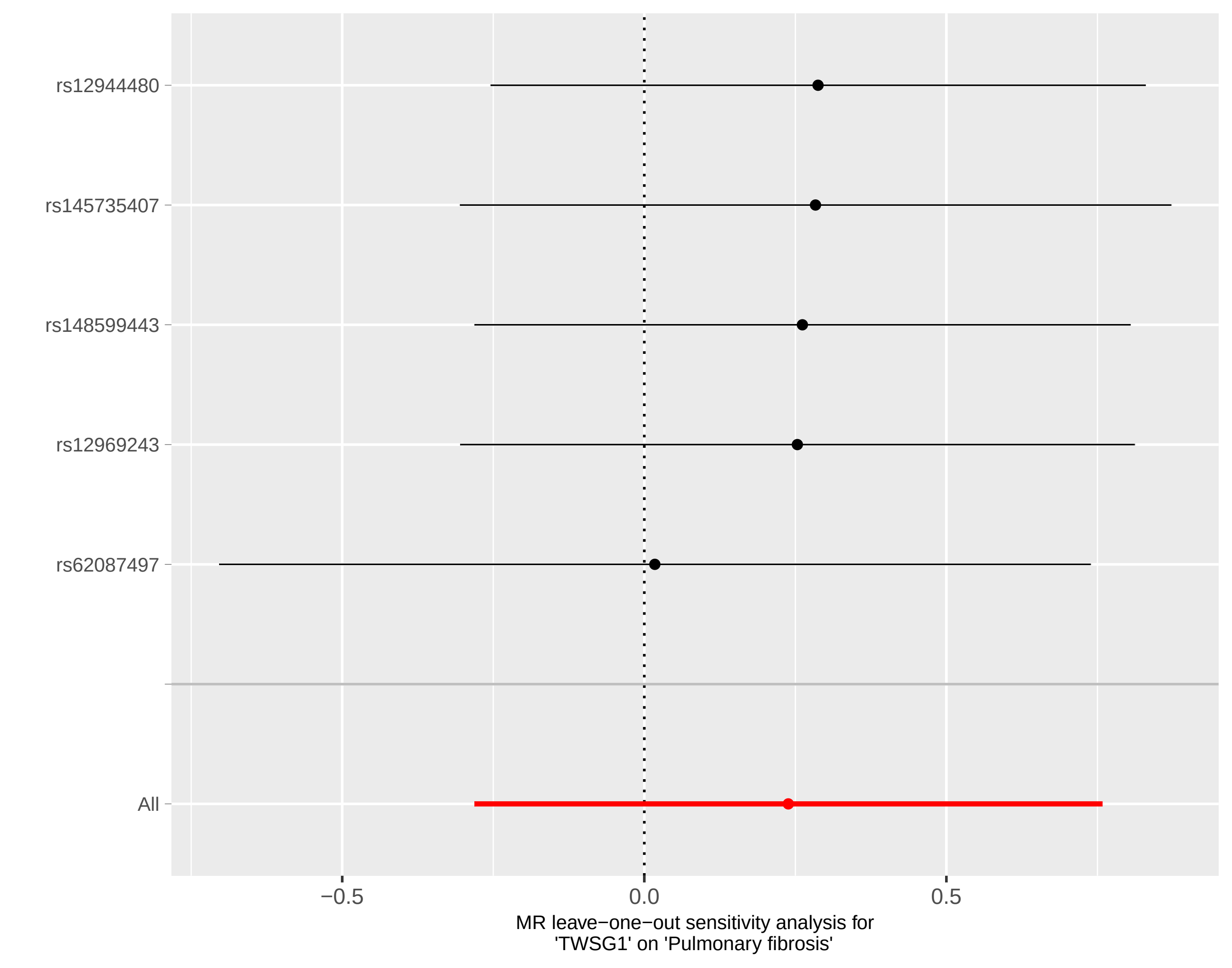
rs12944480
rs145735407
rs148599443
rs12969243
rs62087497
All
−0.5
0.0
0.5
MR leave−one−out sensitivity analysis for
'TWSG1' on 'Pulmonary fibrosis'

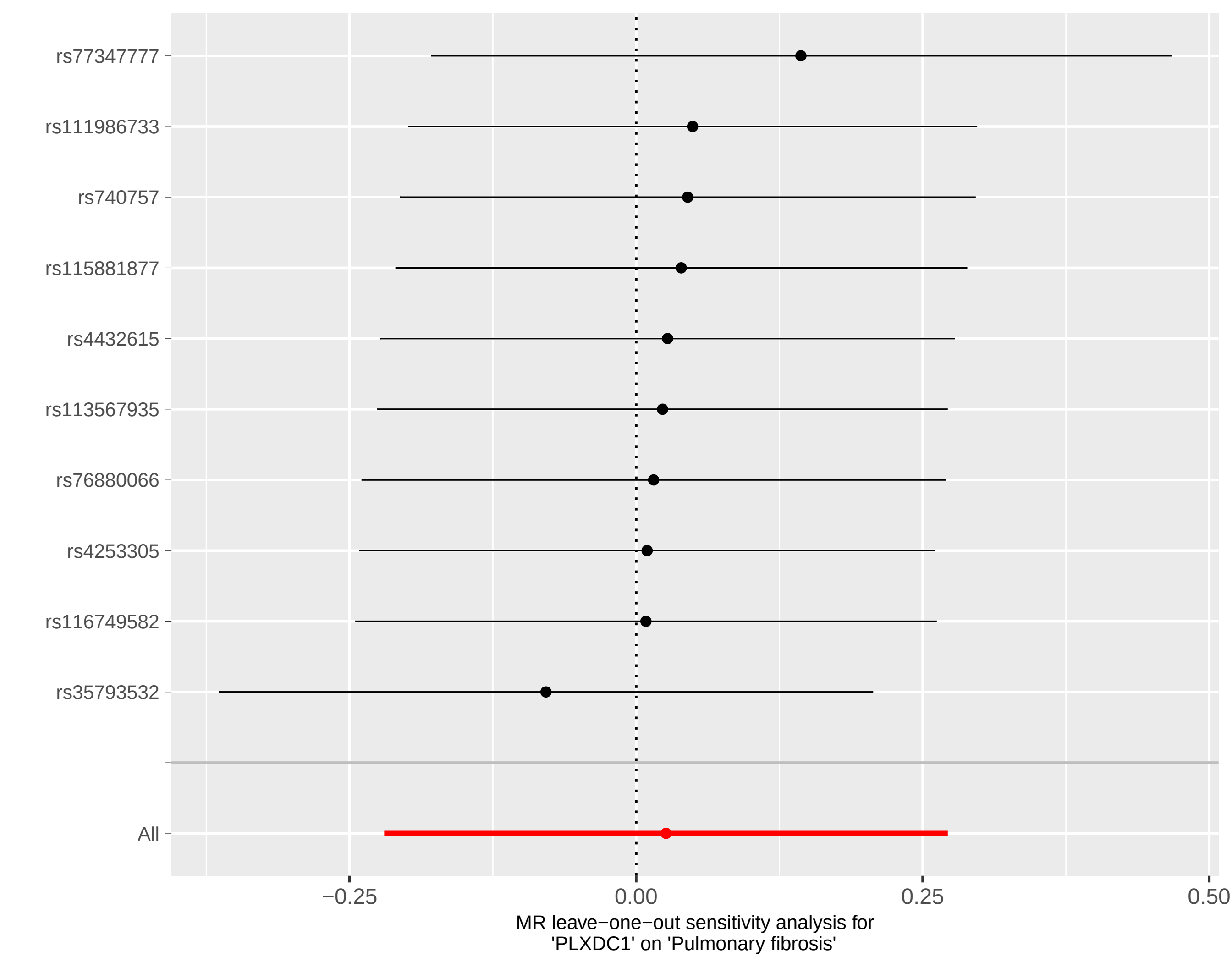
rs77347777
rs111986733
rs740757
rs115881877
rs4432615
rs113567935
rs76880066
rs4253305
rs116749582
rs35793532
All
−0.25
0.00
0.25
0.50
MR leave−one−out sensitivity analysis for
'PLXDC1' on 'Pulmonary fibrosis'

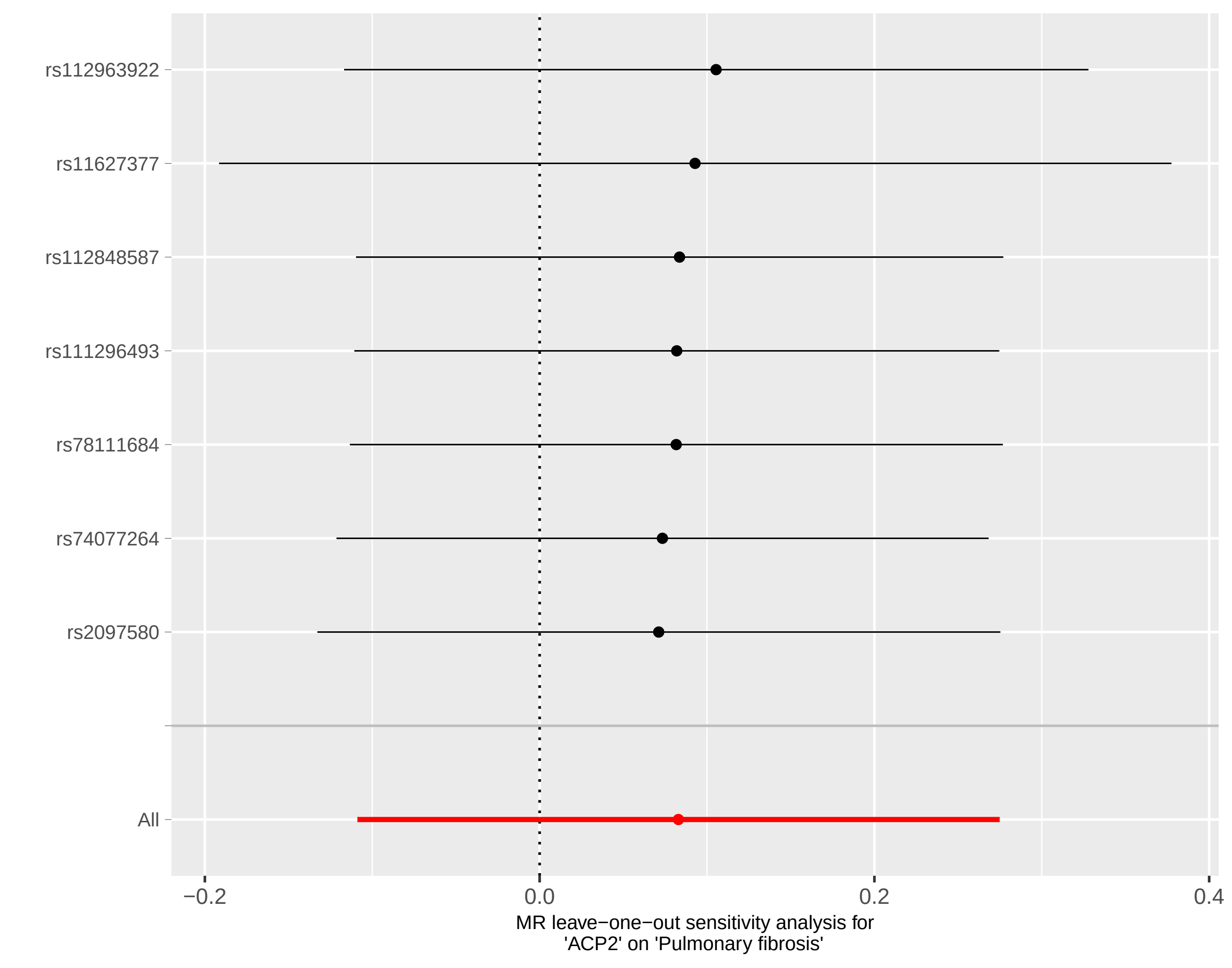

rs112963922
rs11627377
rs112848587
rs111296493
rs78111684
rs74077264
rs2097580
All
−0.2
0.0
0.2
0.4
MR leave−one−out sensitivity analysis for
'ACP2' on 'Pulmonary fibrosis'

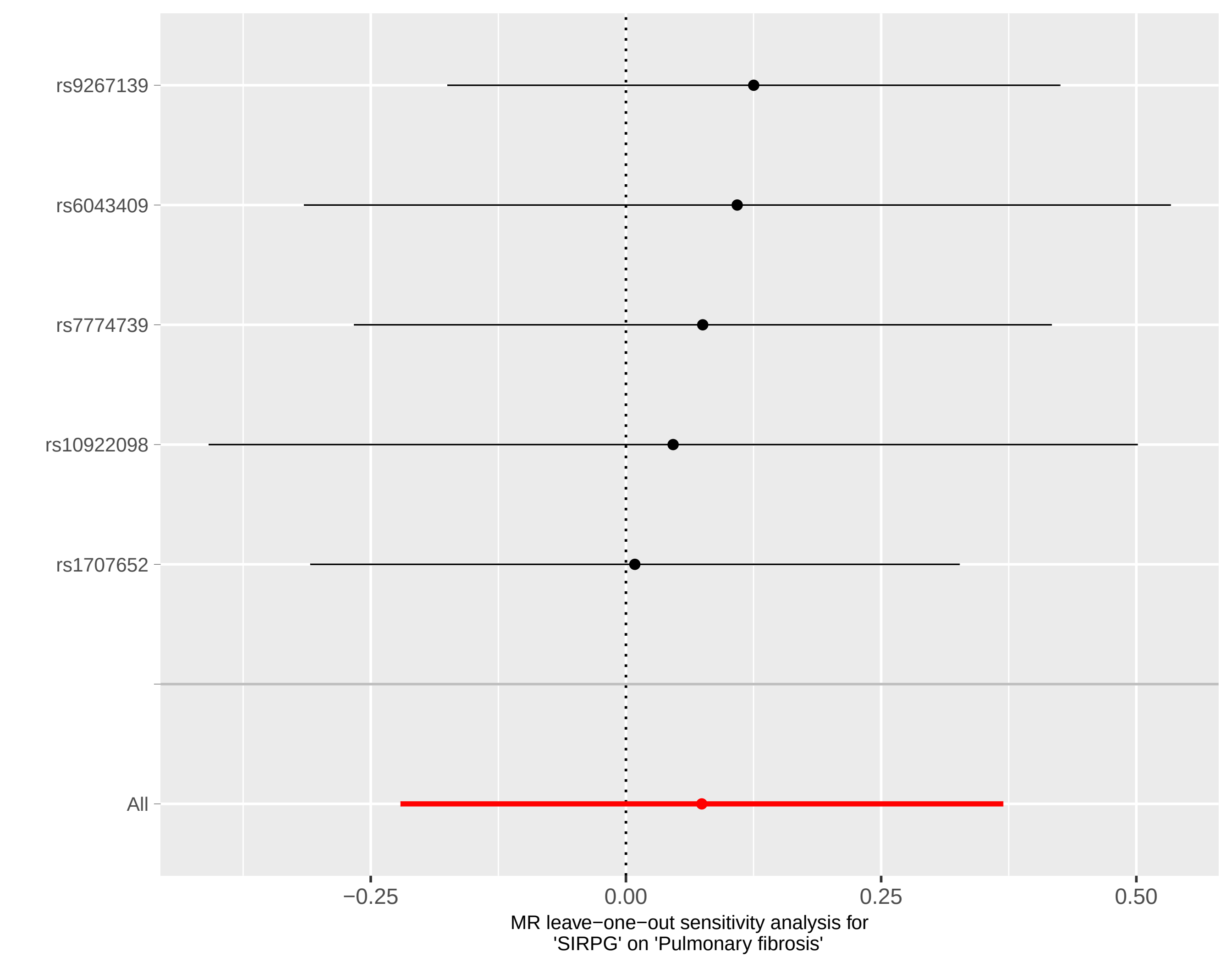
rs9267139
rs6043409
rs7774739
rs10922098
rs1707652
All
−0.25
0.00
0.25
0.50
MR leave−one−out sensitivity analysis for
'SIRPG' on 'Pulmonary fibrosis'

Insufficient number of SNPs

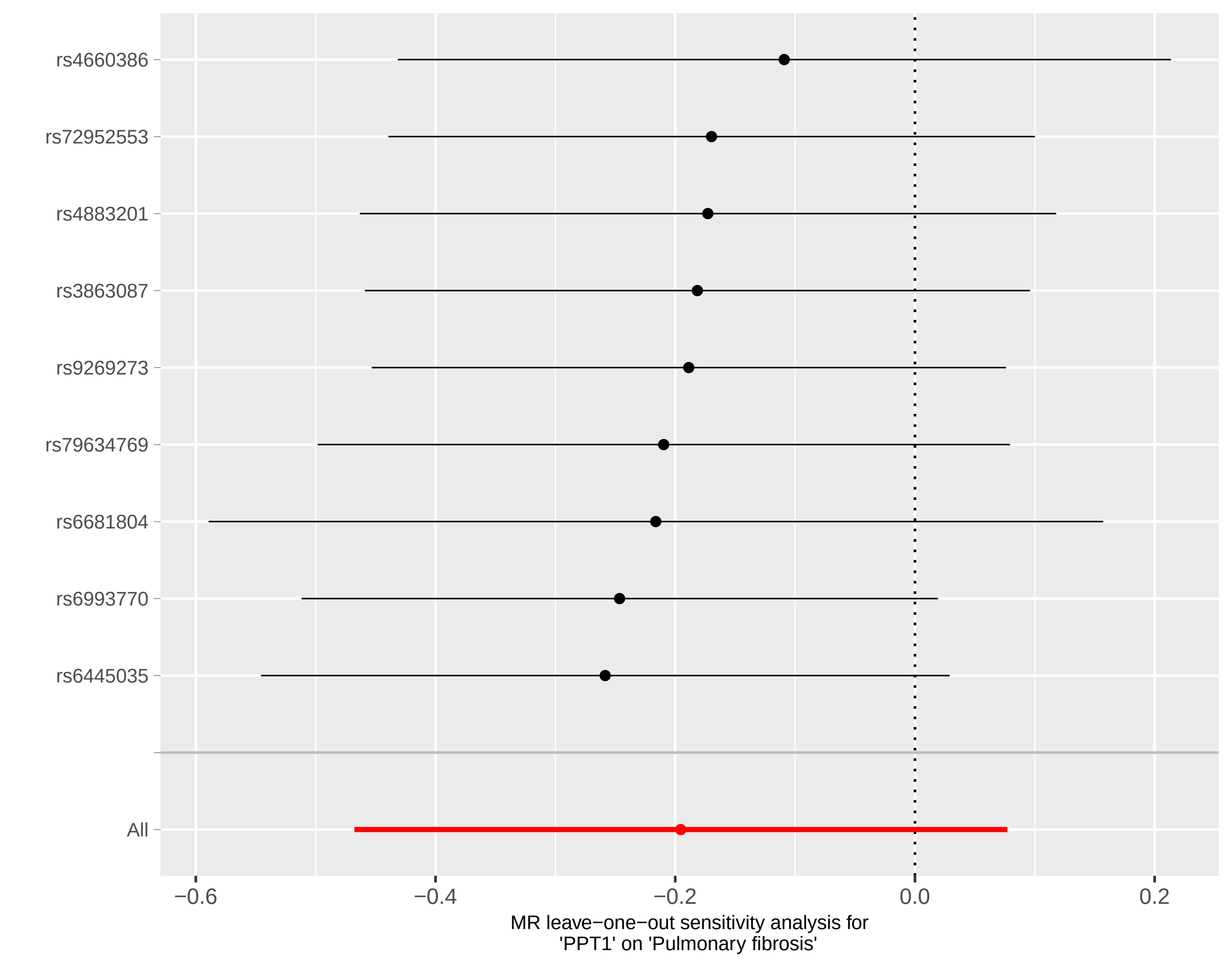
rs4660386
rs72952553
rs4883201
rs3863087
rs9269273
rs79634769
rs6681804
rs6993770
rs6445035
All
−0.6
−0.4
−0.2
0.0
0.2
MR leave−one−out sensitivity analysis for
'PPT1' on 'Pulmonary fibrosis'

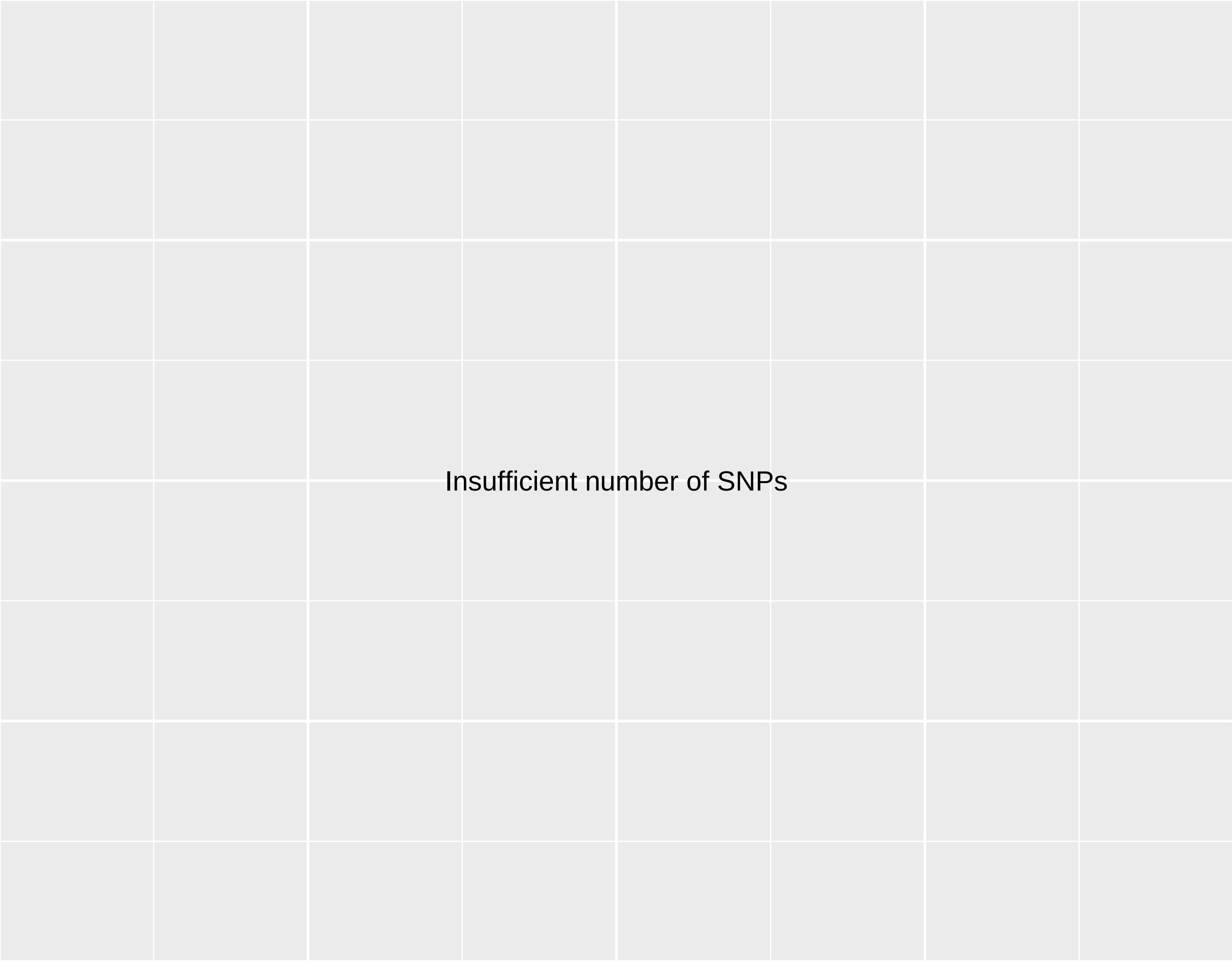
Insufficient number of SNPs

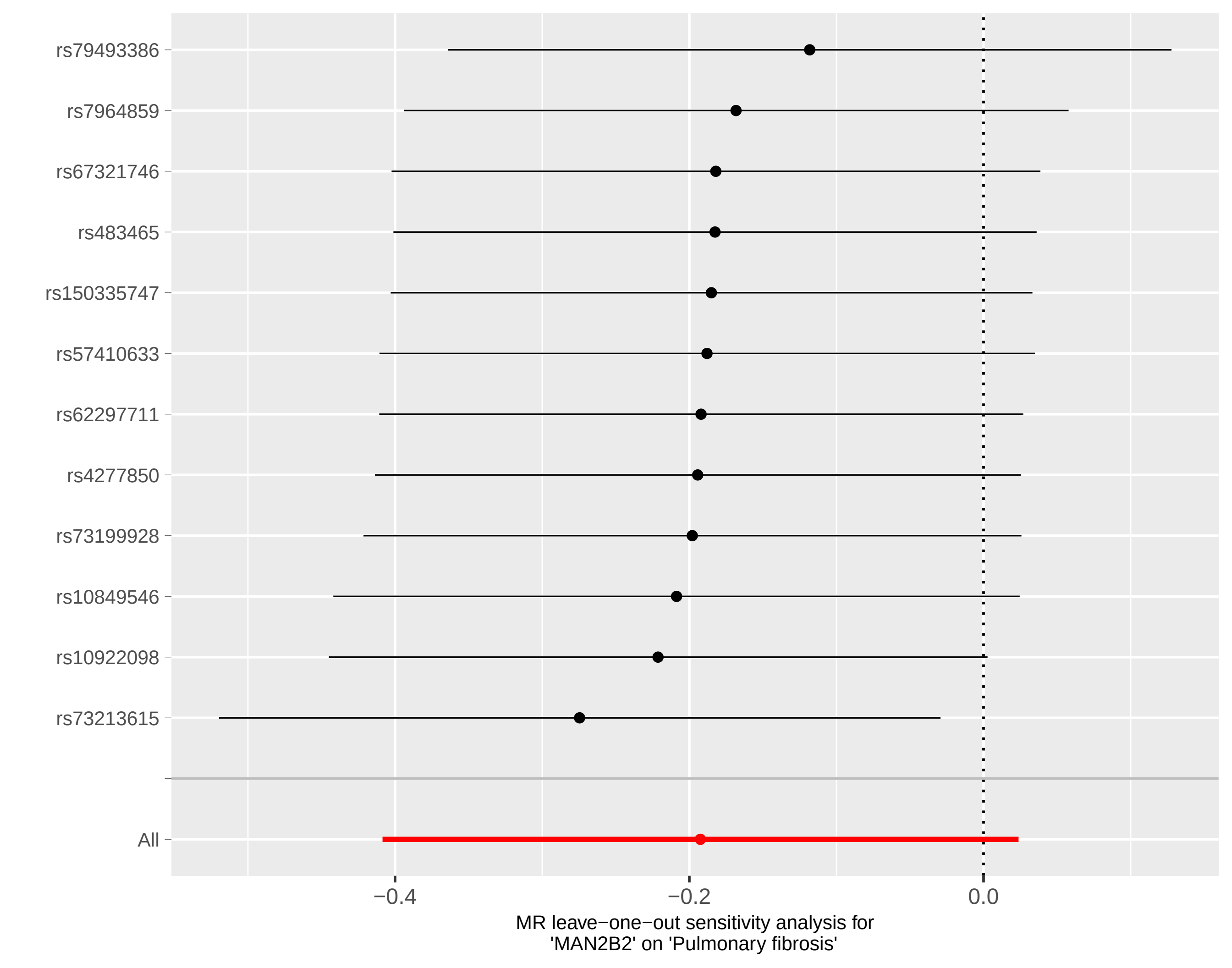
rs79493386
rs7964859
rs67321746
rs483465
rs150335747
rs57410633
rs62297711
rs4277850
rs73199928
rs10849546
rs10922098
rs73213615
All
−0.4
−0.2
0.0
MR leave−one−out sensitivity analysis for
'MAN2B2' on 'Pulmonary fibrosis'

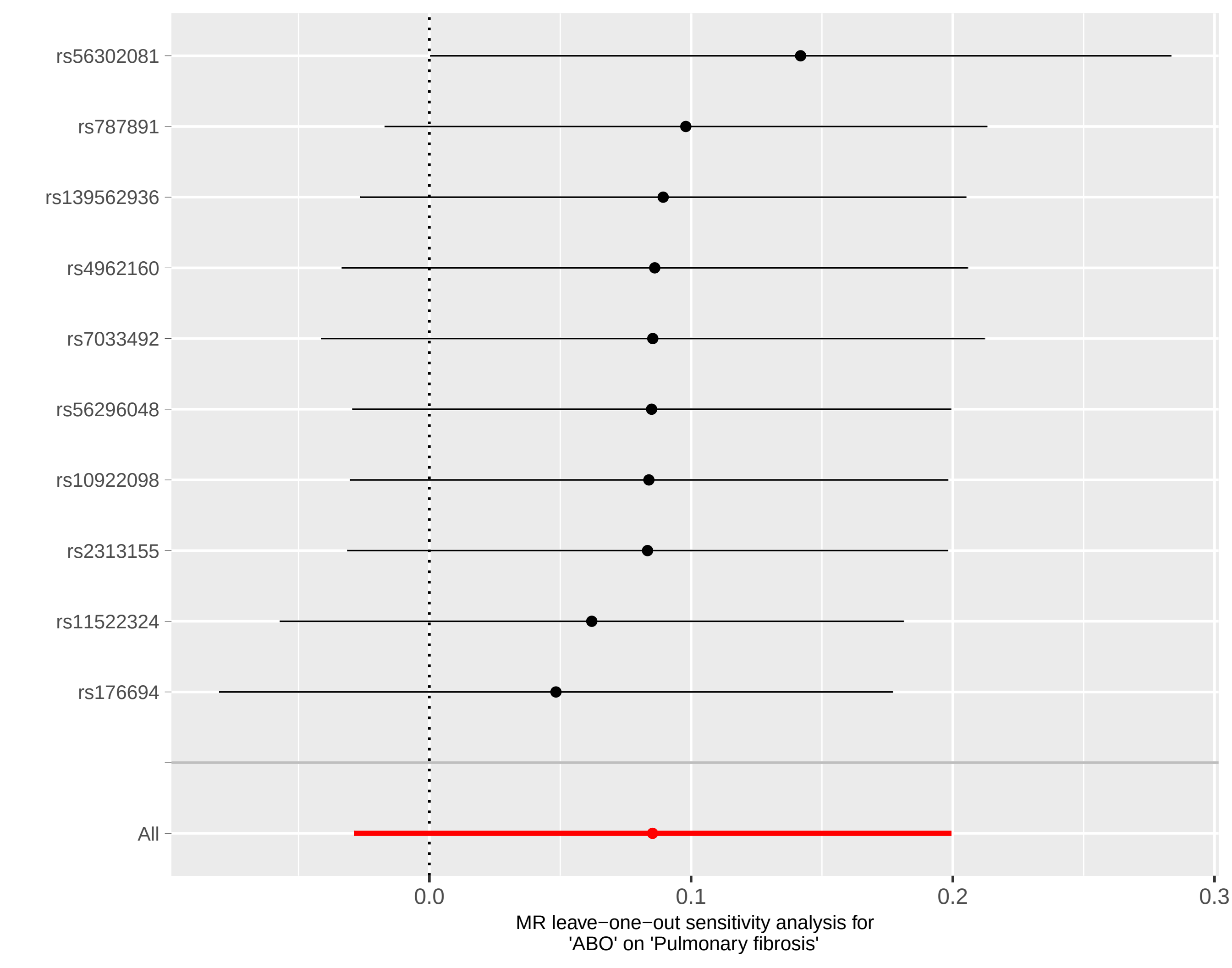
rs56302081
rs787891
rs139562936
rs4962160
rs7033492
rs56296048
rs10922098
rs2313155
rs11522324
rs176694
All
0.0
0.1
0.2
0.3
MR leave−one−out sensitivity analysis for
'ABO' on 'Pulmonary fibrosis'

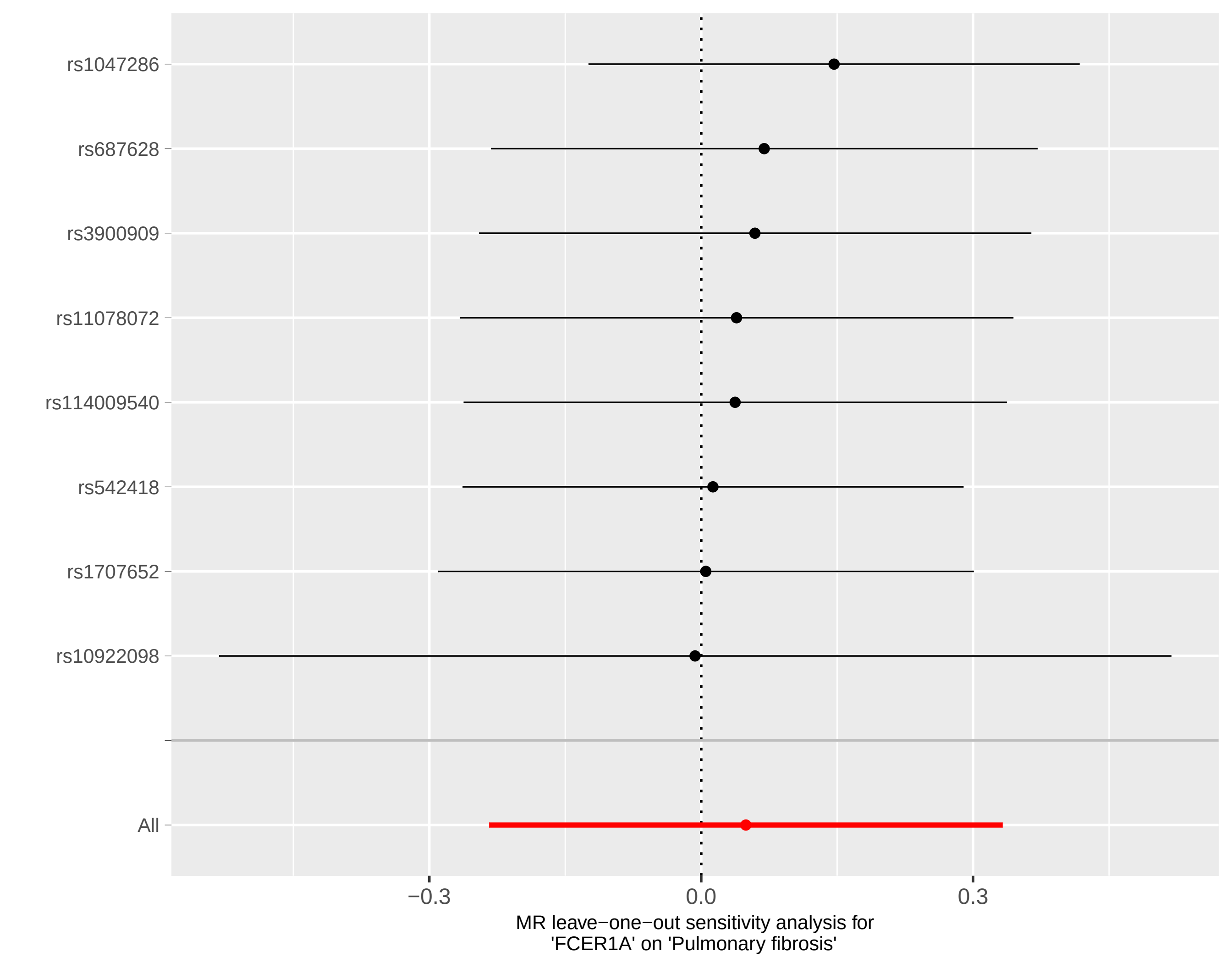
rs1047286
rs687628
rs3900909
rs11078072
rs114009540
rs542418
rs1707652
rs10922098
All
−0.3
0.0
0.3
MR leave−one−out sensitivity analysis for
'FCER1A' on 'Pulmonary fibrosis'

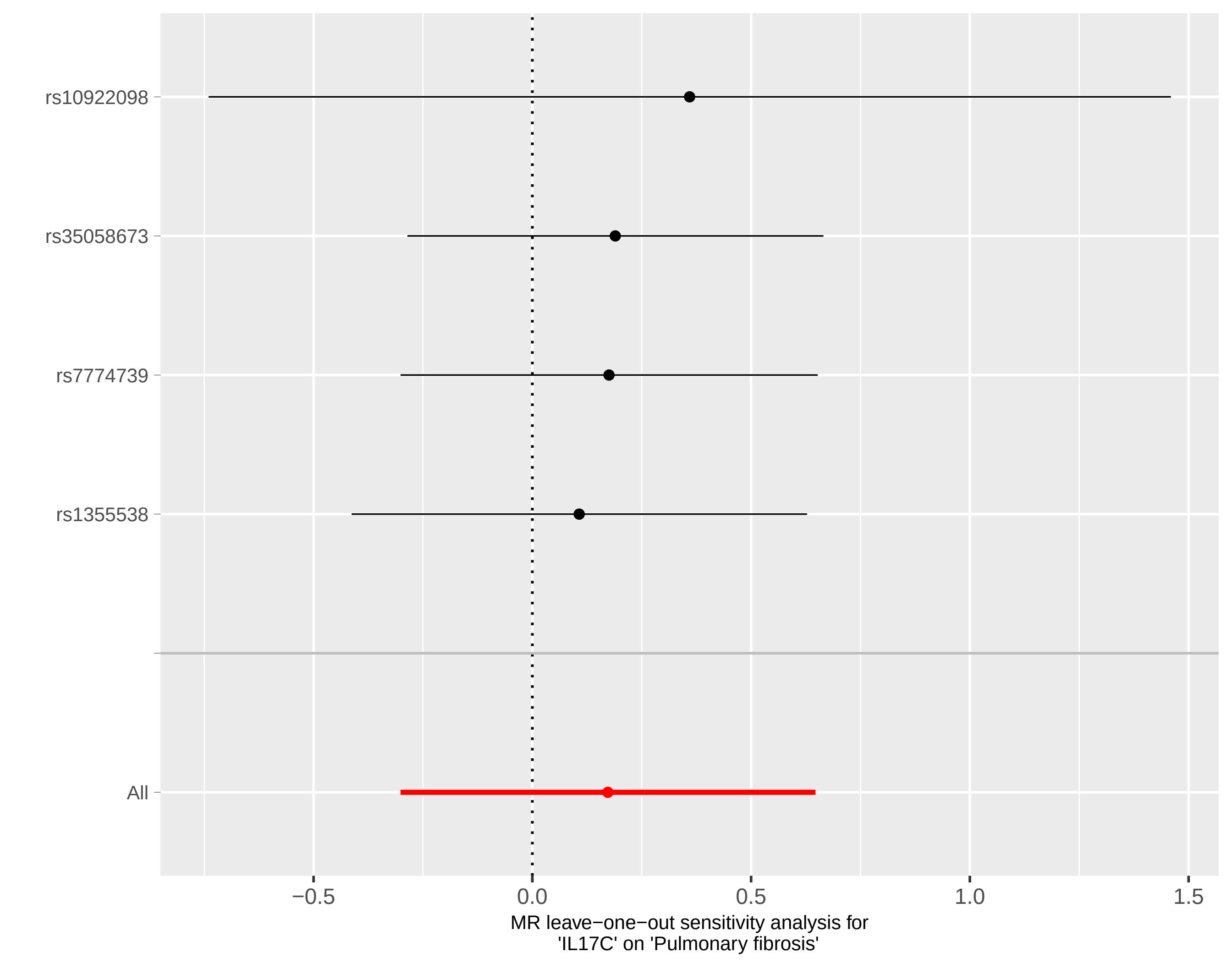
rs10922098
rs35058673
rs7774739
rs1355538
All
−0.5
0.0
0.5
1.0
1.5
MR leave−one−out sensitivity analysis for
'IL17C' on 'Pulmonary fibrosis'

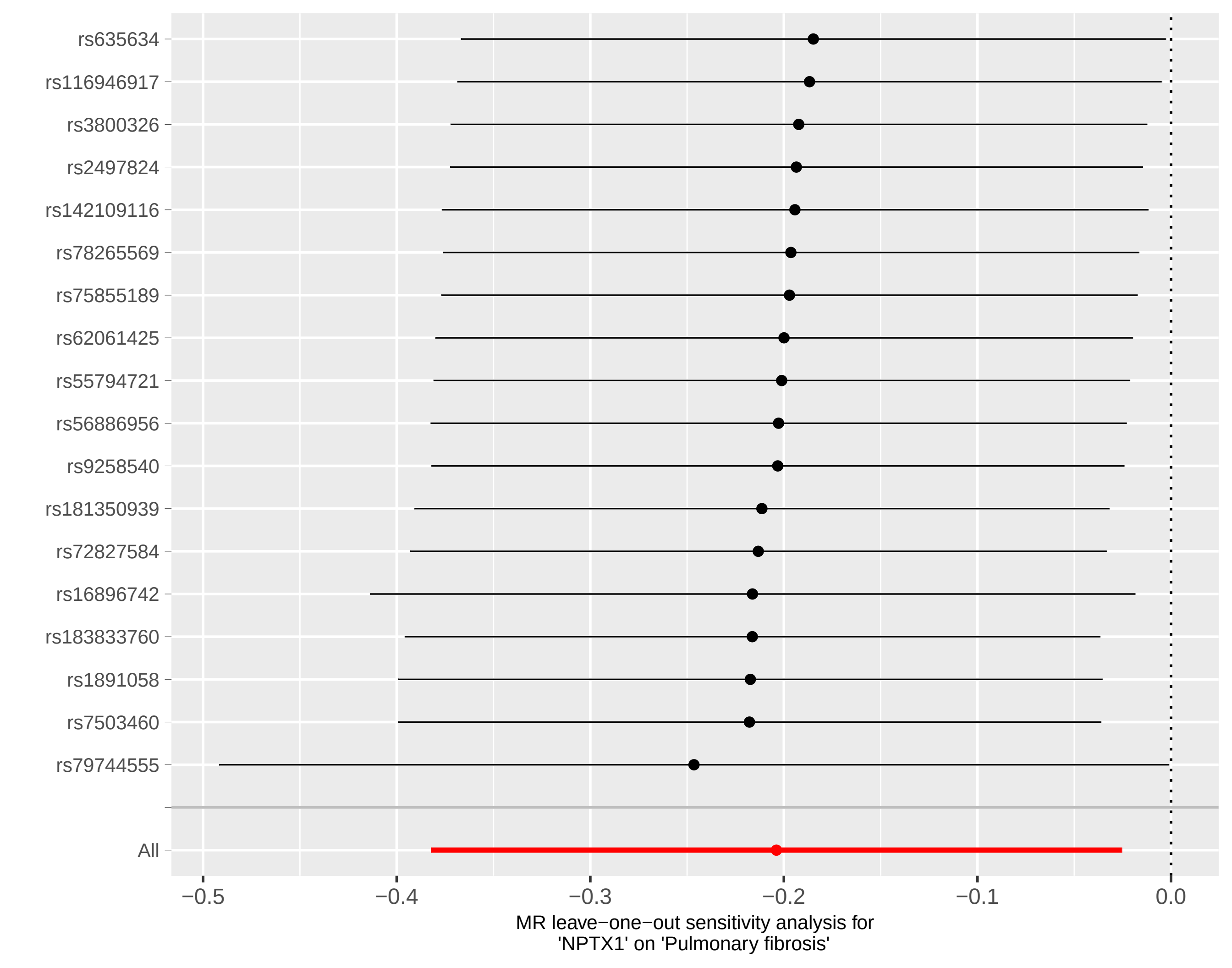
rs635634
rs116946917
rs3800326
rs2497824
rs142109116
rs78265569
rs75855189
rs62061425
rs55794721
rs56886956
rs9258540
rs181350939
rs72827584
rs16896742
rs183833760
rs1891058
rs7503460
rs79744555
All
−0.5
−0.4
−0.3
−0.2
−0.1
0.0
MR leave−one−out sensitivity analysis for
'NPTX1' on 'Pulmonary fibrosis'

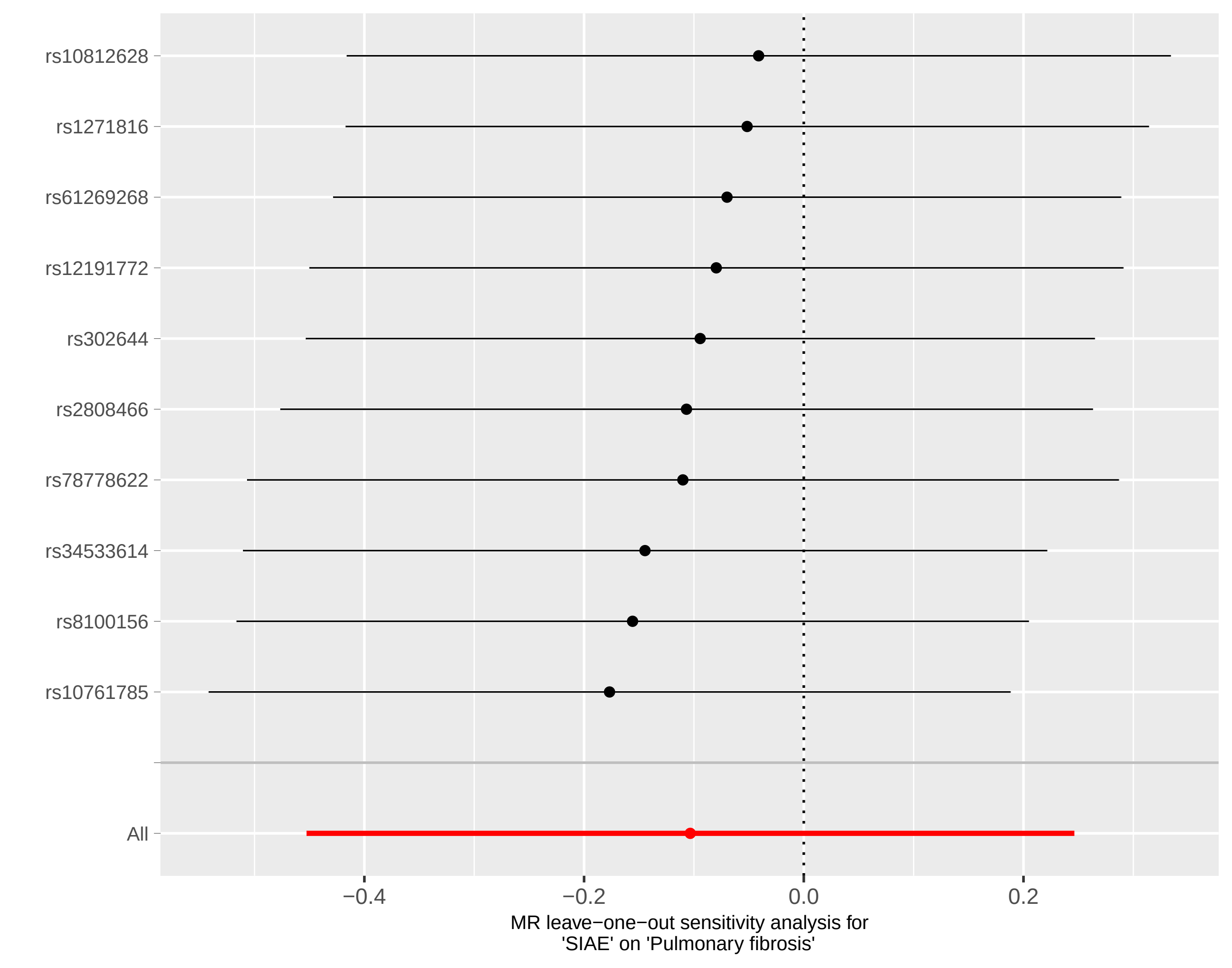
rs10812628
rs1271816
rs61269268
rs12191772
rs302644
rs2808466
rs78778622
rs34533614
rs8100156
rs10761785
All
−0.4
−0.2
0.0
0.2
MR leave−one−out sensitivity analysis for
'SIAE' on 'Pulmonary fibrosis'

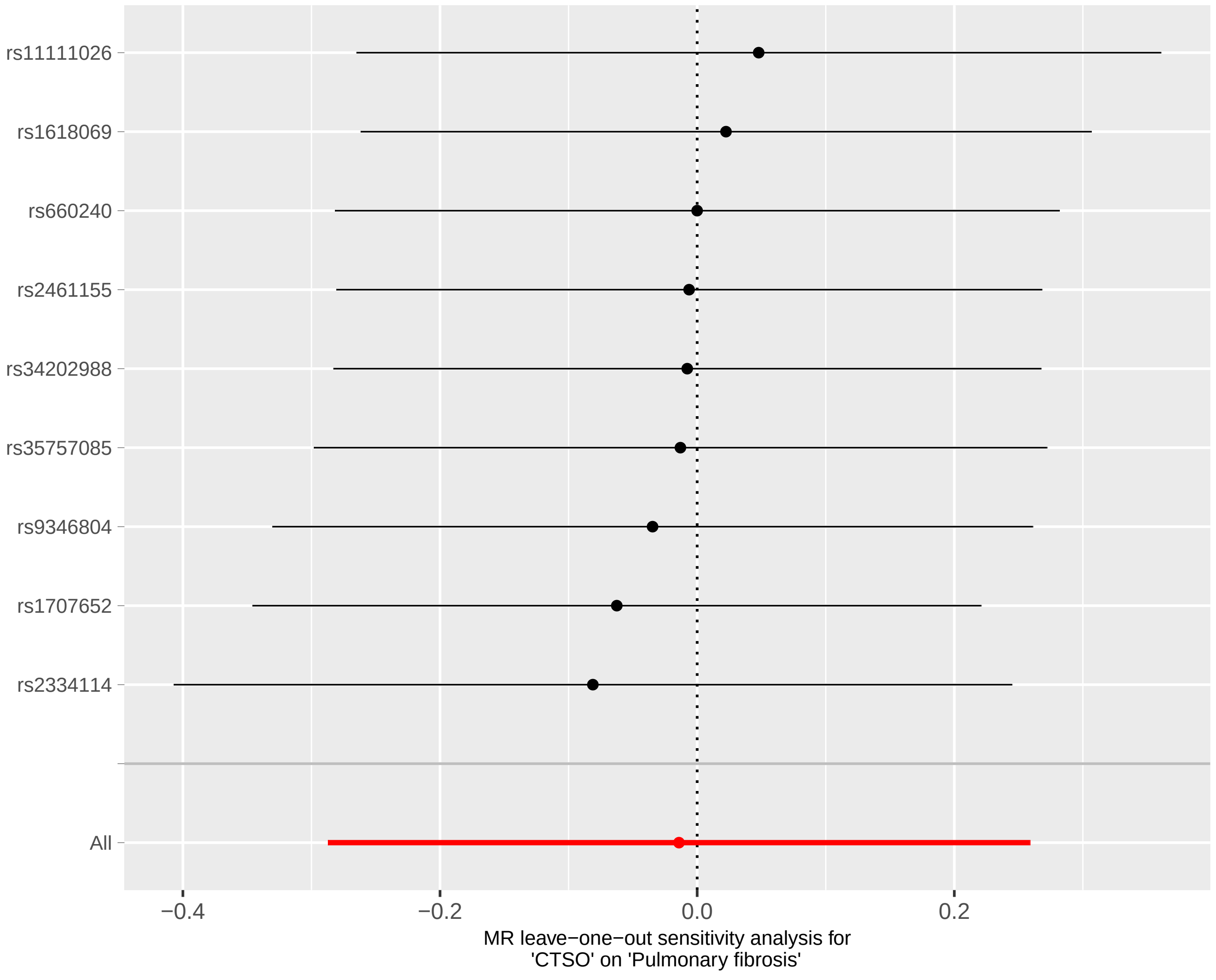
rs11111026
rs1618069
rs660240
rs2461155
rs34202988
rs35757085
rs9346804
rs1707652
rs2334114
All
−0.4
−0.2
0.0
0.2
MR leave−one−out sensitivity analysis for
'CTSO' on 'Pulmonary fibrosis'

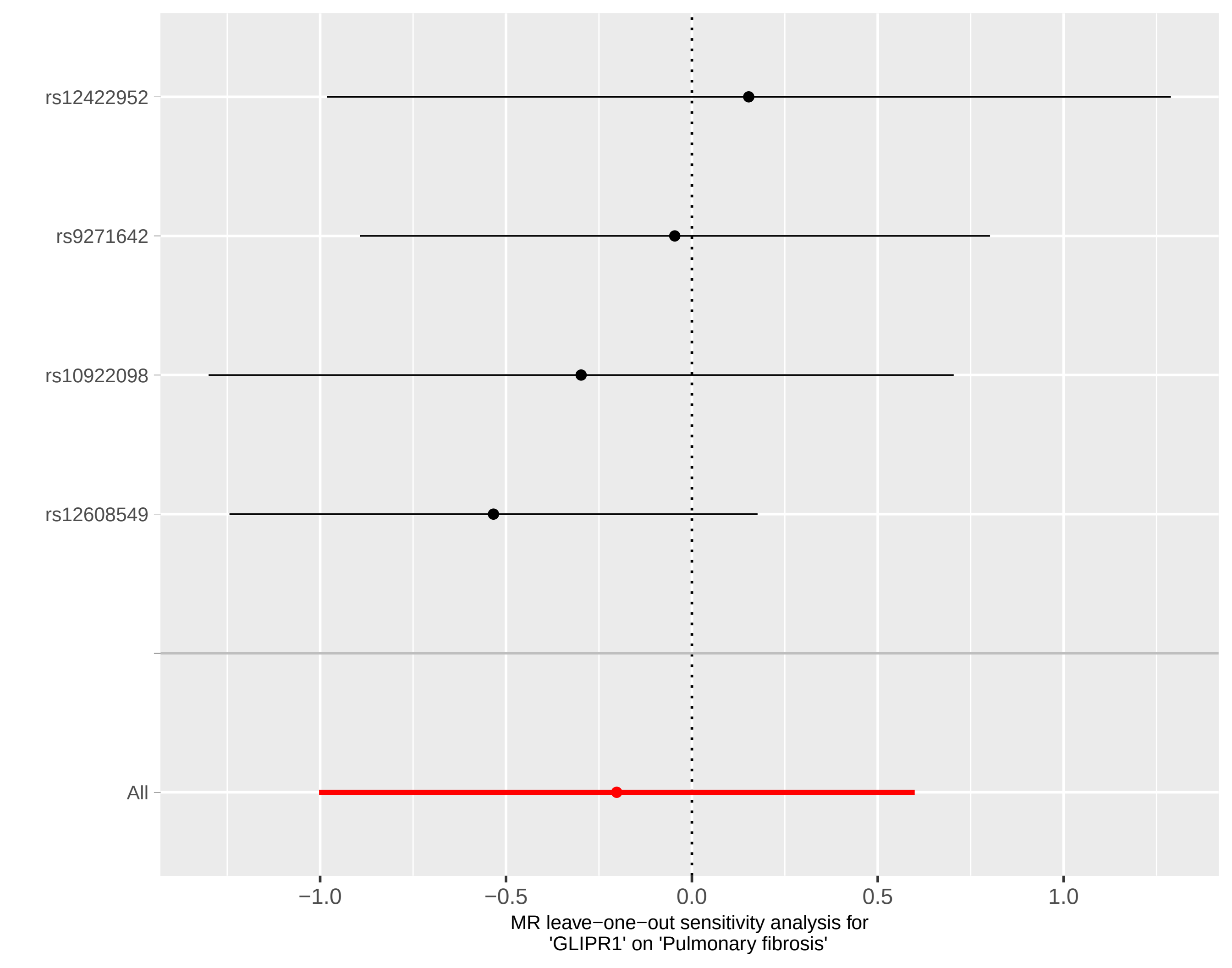
rs12422952
rs9271642
rs10922098
rs12608549
All
−1.0
−0.5
0.0
0.5
1.0
MR leave−one−out sensitivity analysis for
'GLIPR1' on 'Pulmonary fibrosis'

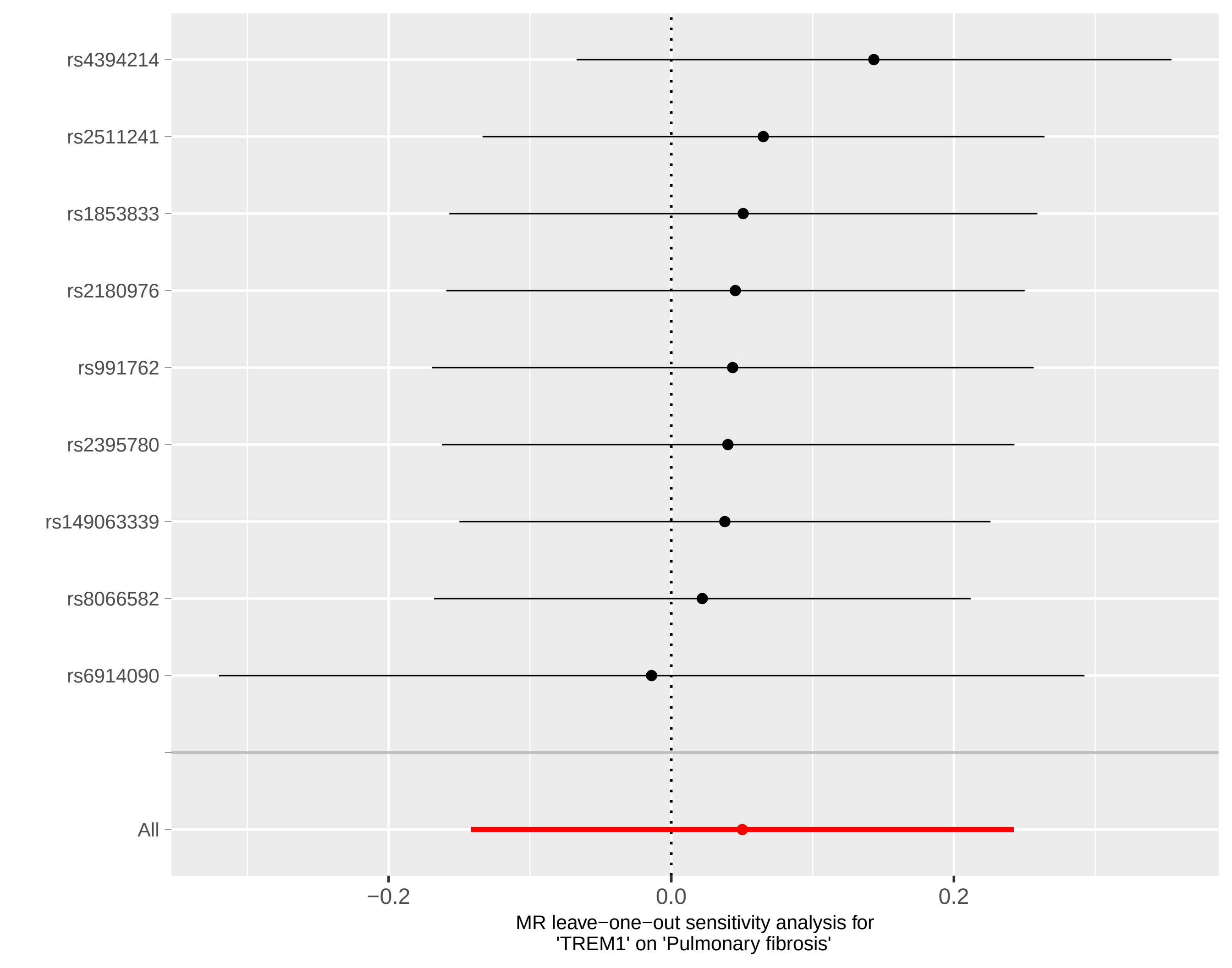
rs4394214
rs2511241
rs1853833
rs2180976
rs991762
rs2395780
rs149063339
rs8066582
rs6914090
All
−0.2
0.0
0.2
MR leave−one−out sensitivity analysis for
'TREM1' on 'Pulmonary fibrosis'

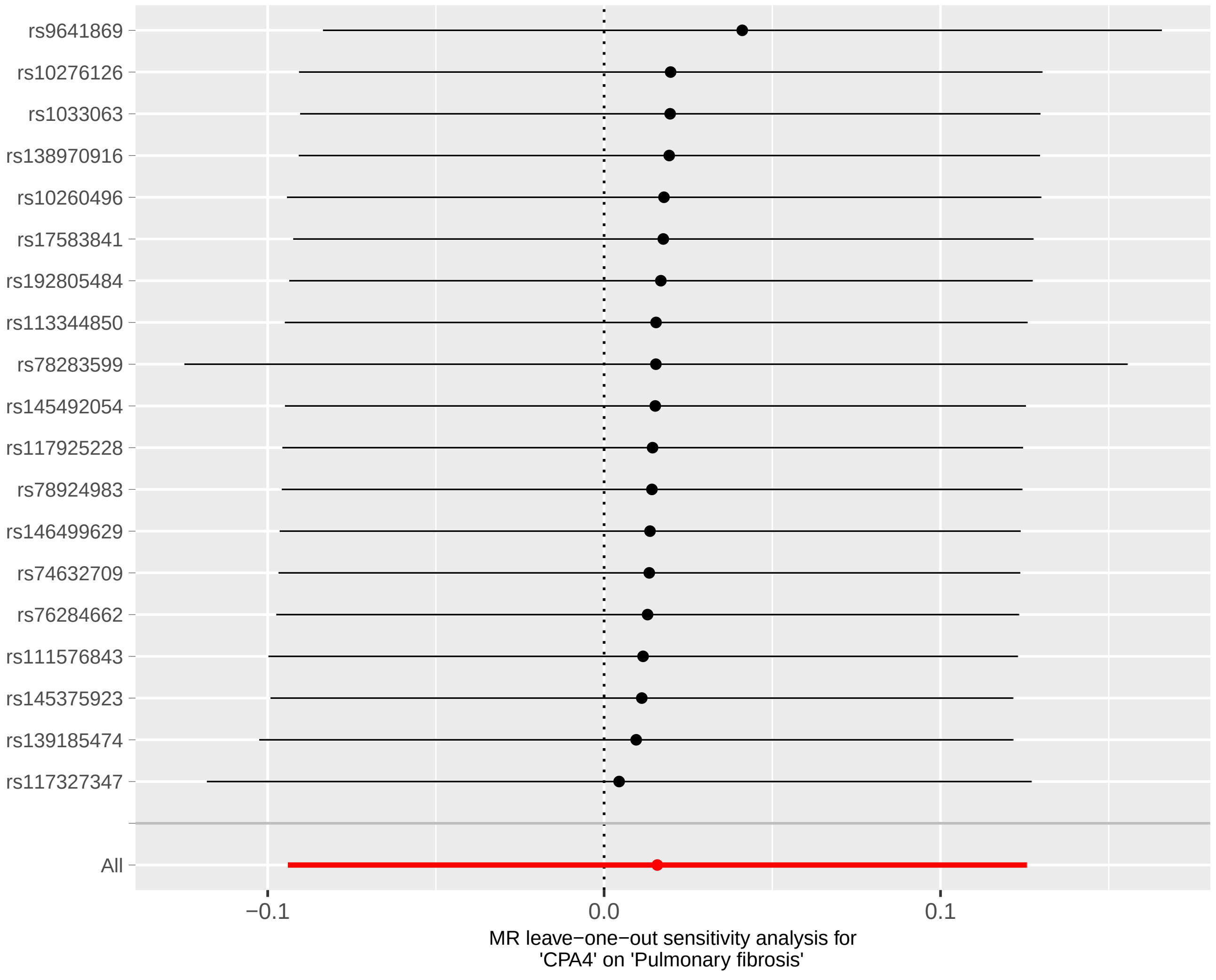

rs9641869
rs10276126
rs1033063
rs138970916
rs10260496
rs17583841
rs192805484
rs113344850
rs78283599
rs145492054
rs117925228
rs78924983
rs146499629
rs74632709
rs76284662
rs111576843
rs145375923
rs139185474
rs117327347
All
−0.1
0.0
0.1
MR leave−one−out sensitivity analysis for
'CPA4' on 'Pulmonary fibrosis'

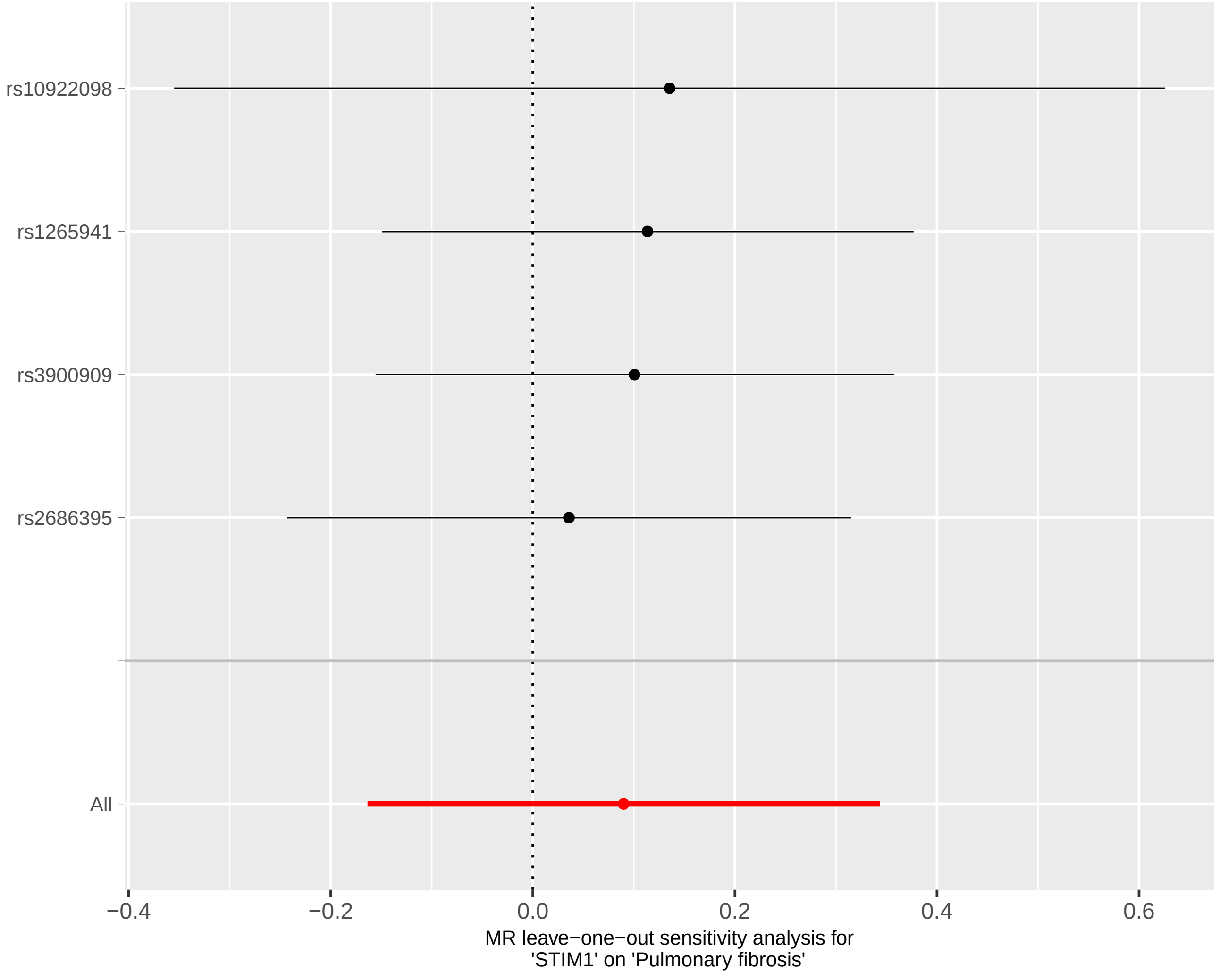
rs10922098
rs1265941
rs3900909
rs2686395
All
−0.4
−0.2
0.0
0.2
0.4
0.6
MR leave−one−out sensitivity analysis for
'STIM1' on 'Pulmonary fibrosis'

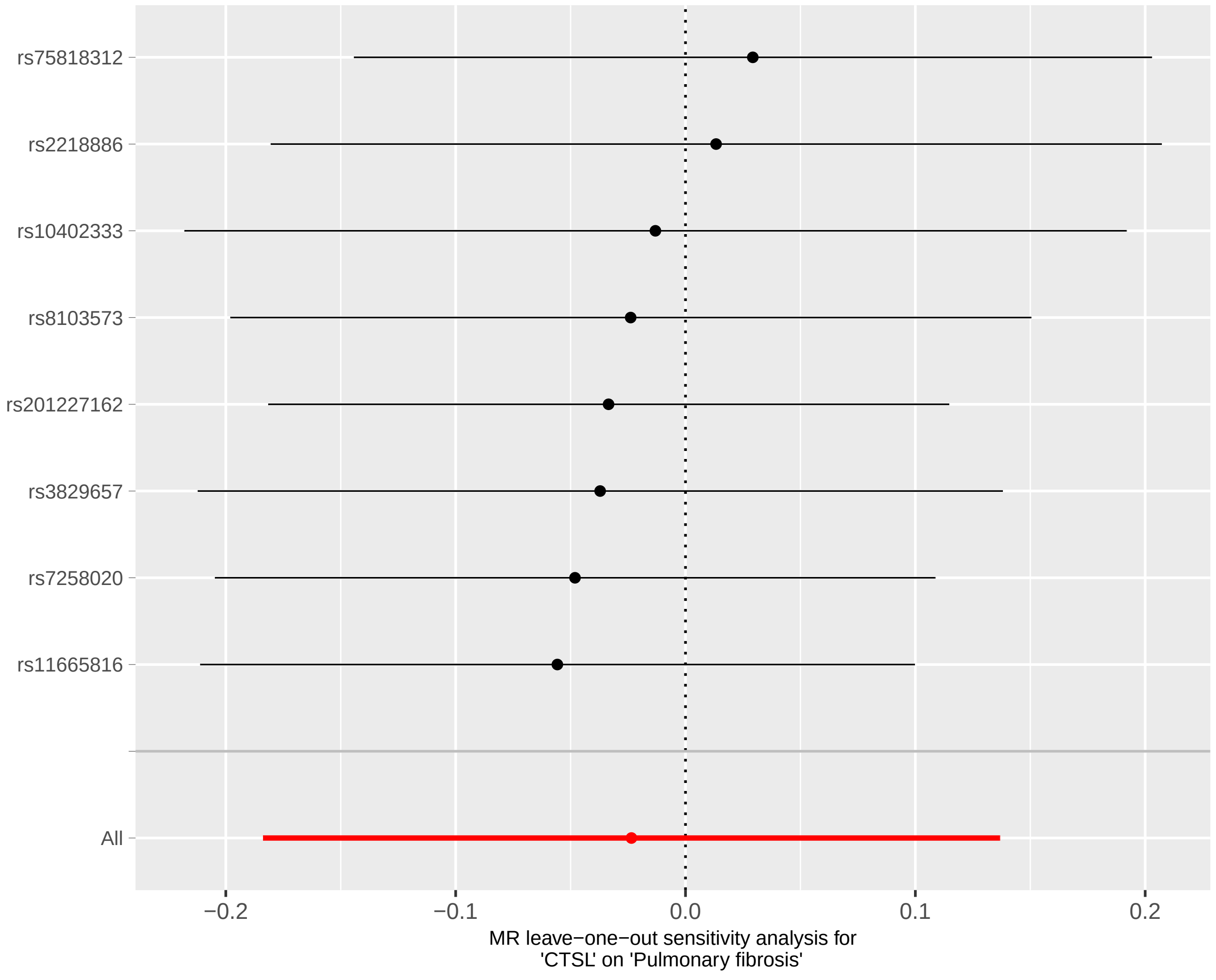
rs75818312
rs2218886
rs10402333
rs8103573
rs201227162
rs3829657
rs7258020
rs11665816
All
−0.2
−0.1
0.0
0.1
0.2
MR leave−one−out sensitivity analysis for
'CTSL' on 'Pulmonary fibrosis'

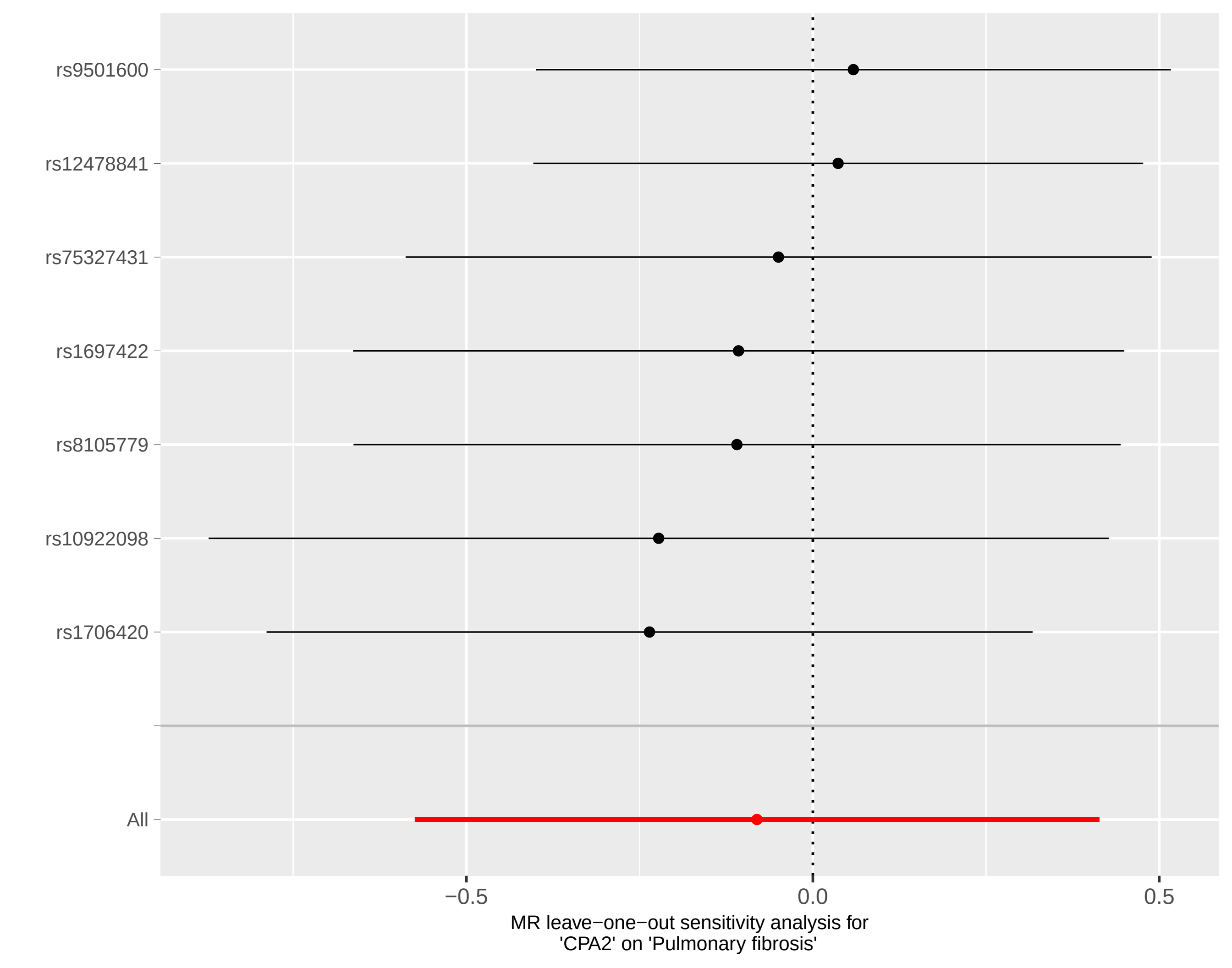

rs9501600
rs12478841
rs75327431
rs1697422
rs8105779
rs10922098
rs1706420
All
−0.5
0.0
0.5
MR leave−one−out sensitivity analysis for
'CPA2' on 'Pulmonary fibrosis'

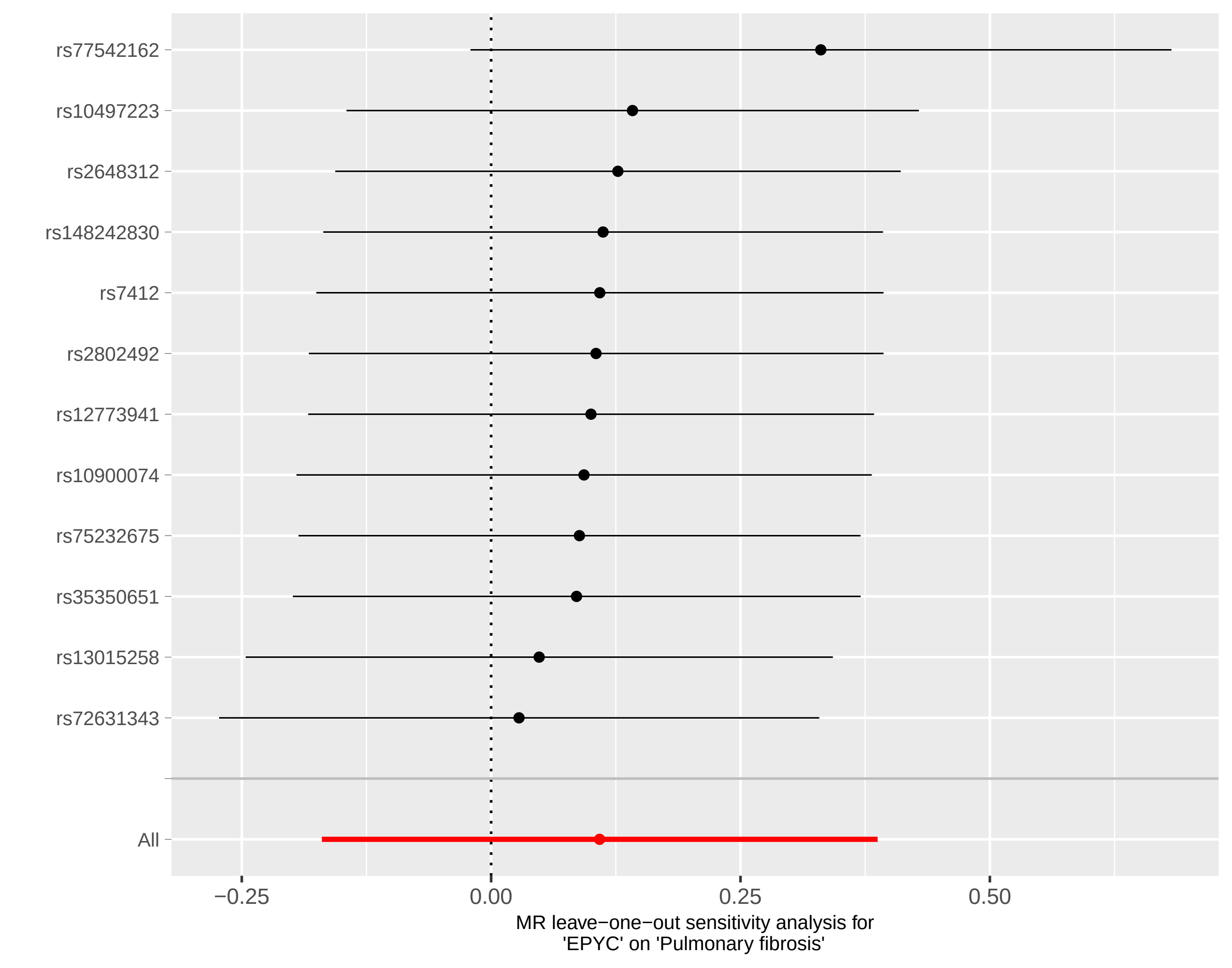
rs77542162
rs10497223
rs2648312
rs148242830
rs7412
rs2802492
rs12773941
rs10900074
rs75232675
rs35350651
rs13015258
rs72631343
All
−0.25
0.00
0.25
0.50
MR leave−one−out sensitivity analysis for
'EPYC' on 'Pulmonary fibrosis'

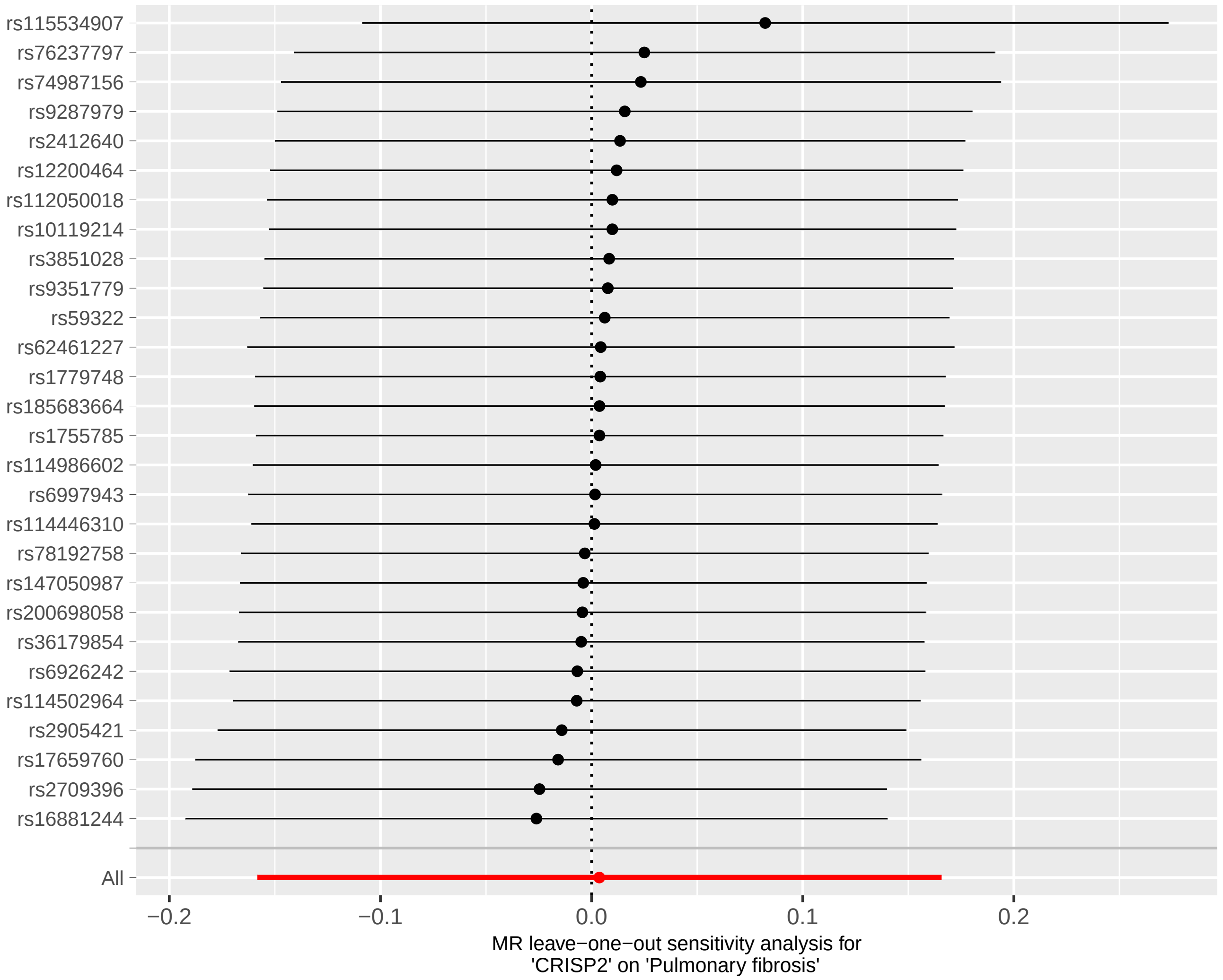
rs115534907
rs76237797
rs74987156
rs9287979
rs2412640
rs12200464
rs112050018
rs10119214
rs3851028
rs9351779
rs59322
rs62461227
rs1779748
rs185683664
rs1755785
rs114986602
rs6997943
rs114446310
rs78192758
rs147050987
rs200698058
rs36179854
rs6926242
rs114502964
rs2905421
rs17659760
rs2709396
rs16881244
All
−0.2
−0.1
0.0
0.1
0.2
MR leave−one−out sensitivity analysis for
'CRISP2' on 'Pulmonary fibrosis'

Insufficient number of SNPs

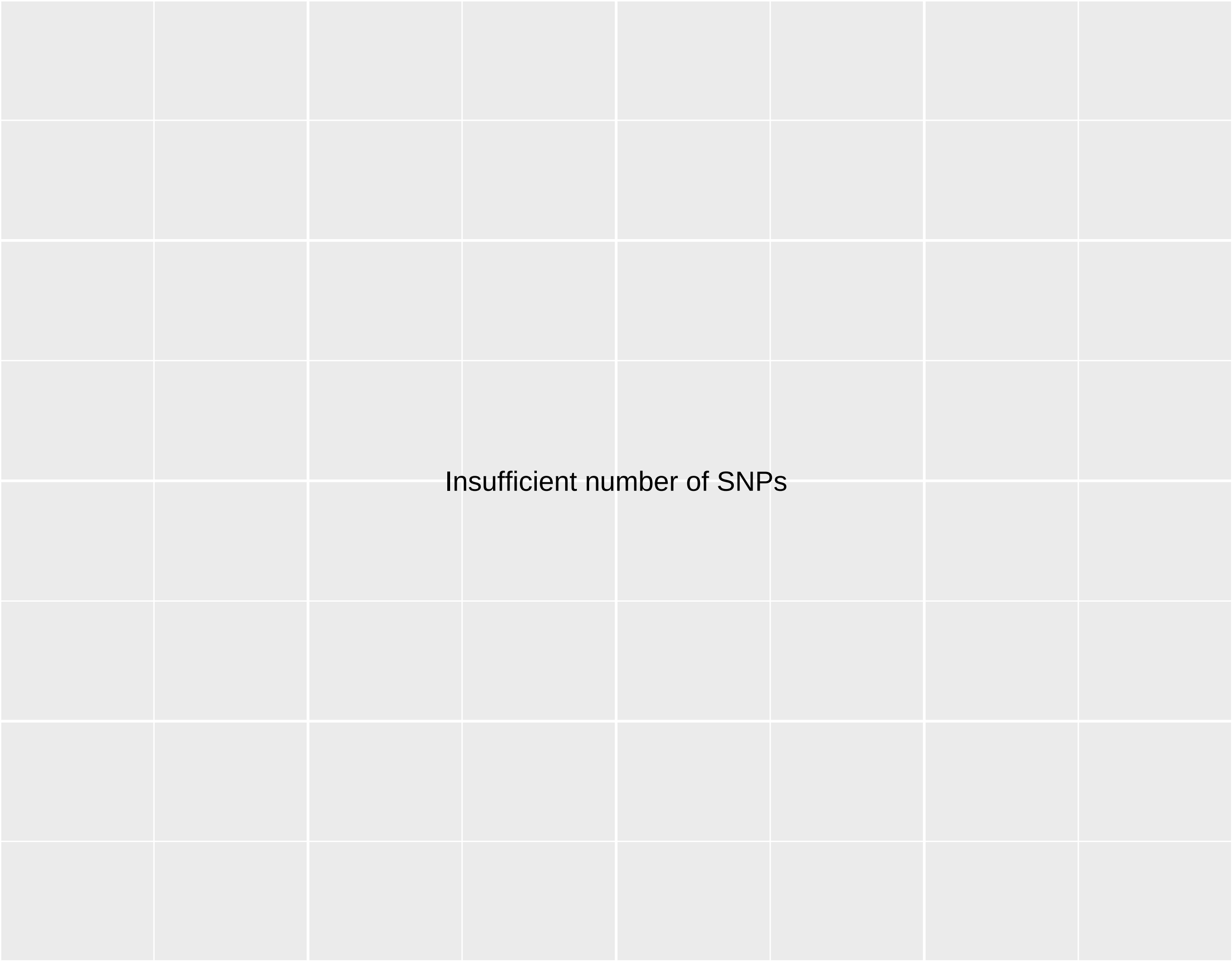
Insufficient number of SNPs

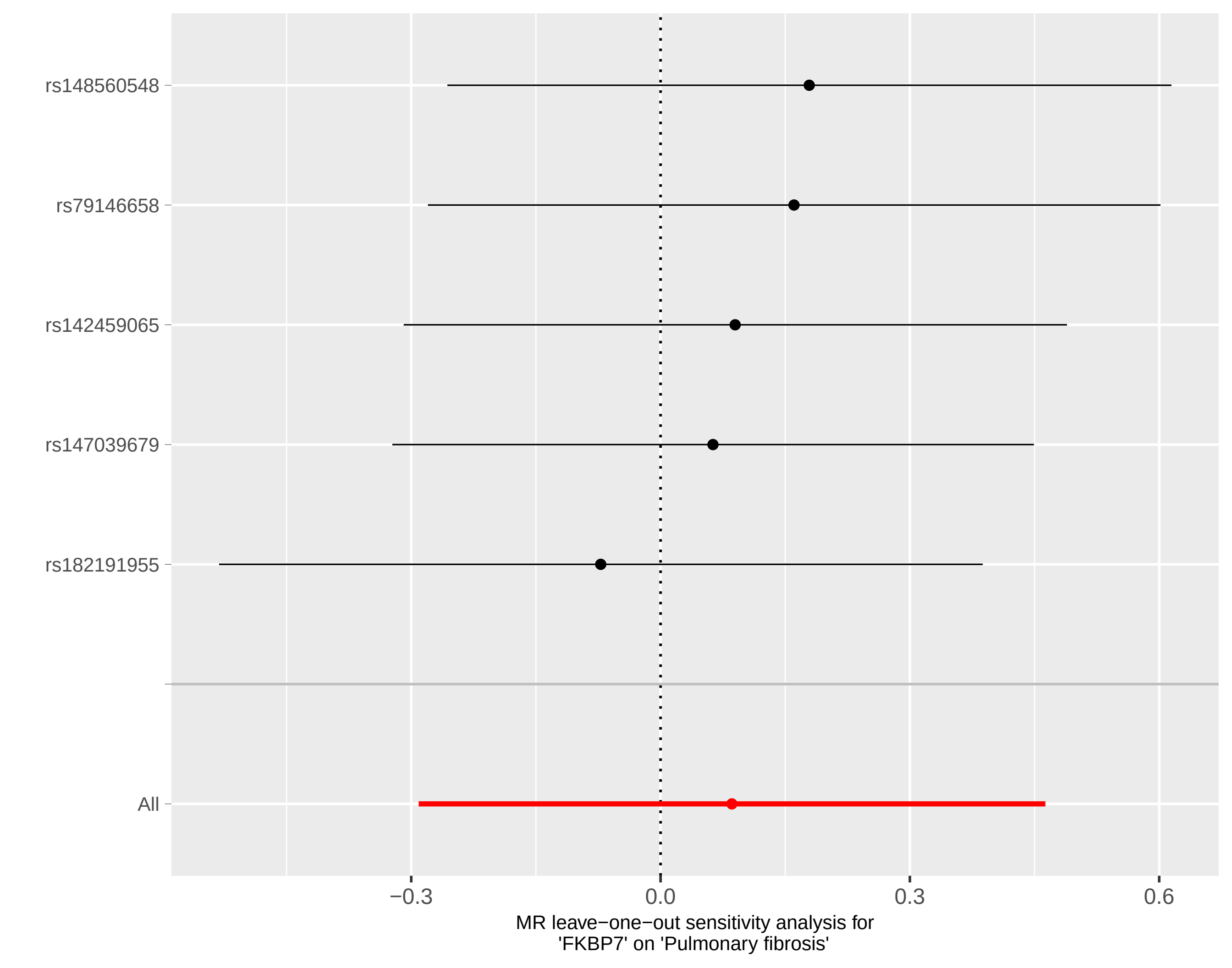

rs148560548
rs79146658
rs142459065
rs147039679
rs182191955
All
−0.3
0.0
0.3
0.6
MR leave−one−out sensitivity analysis for
'FKBP7' on 'Pulmonary fibrosis'

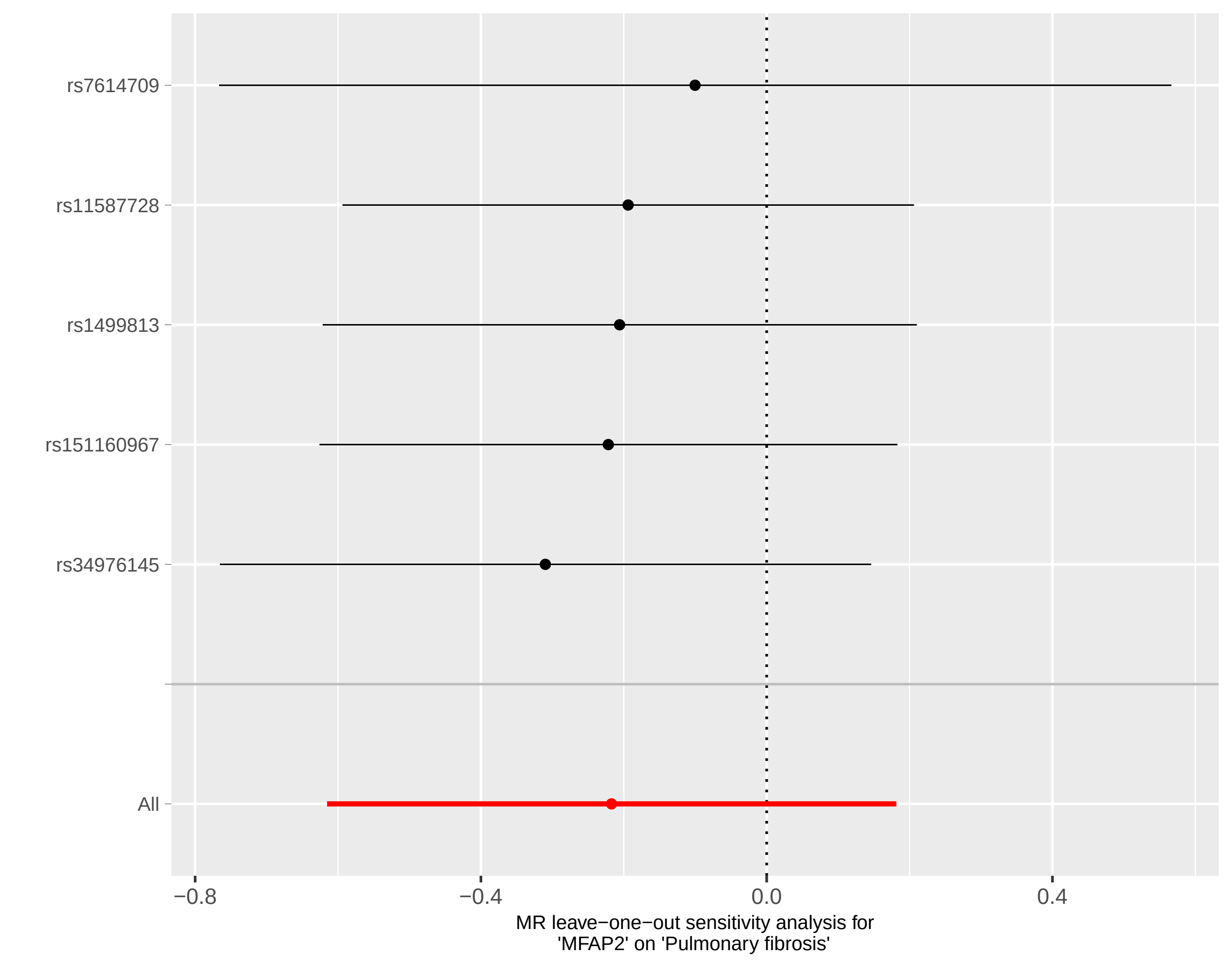

rs7614709
rs11587728
rs1499813
rs151160967
rs34976145
All
−0.8
−0.4
0.0
0.4
MR leave−one−out sensitivity analysis for
'MFAP2' on 'Pulmonary fibrosis'

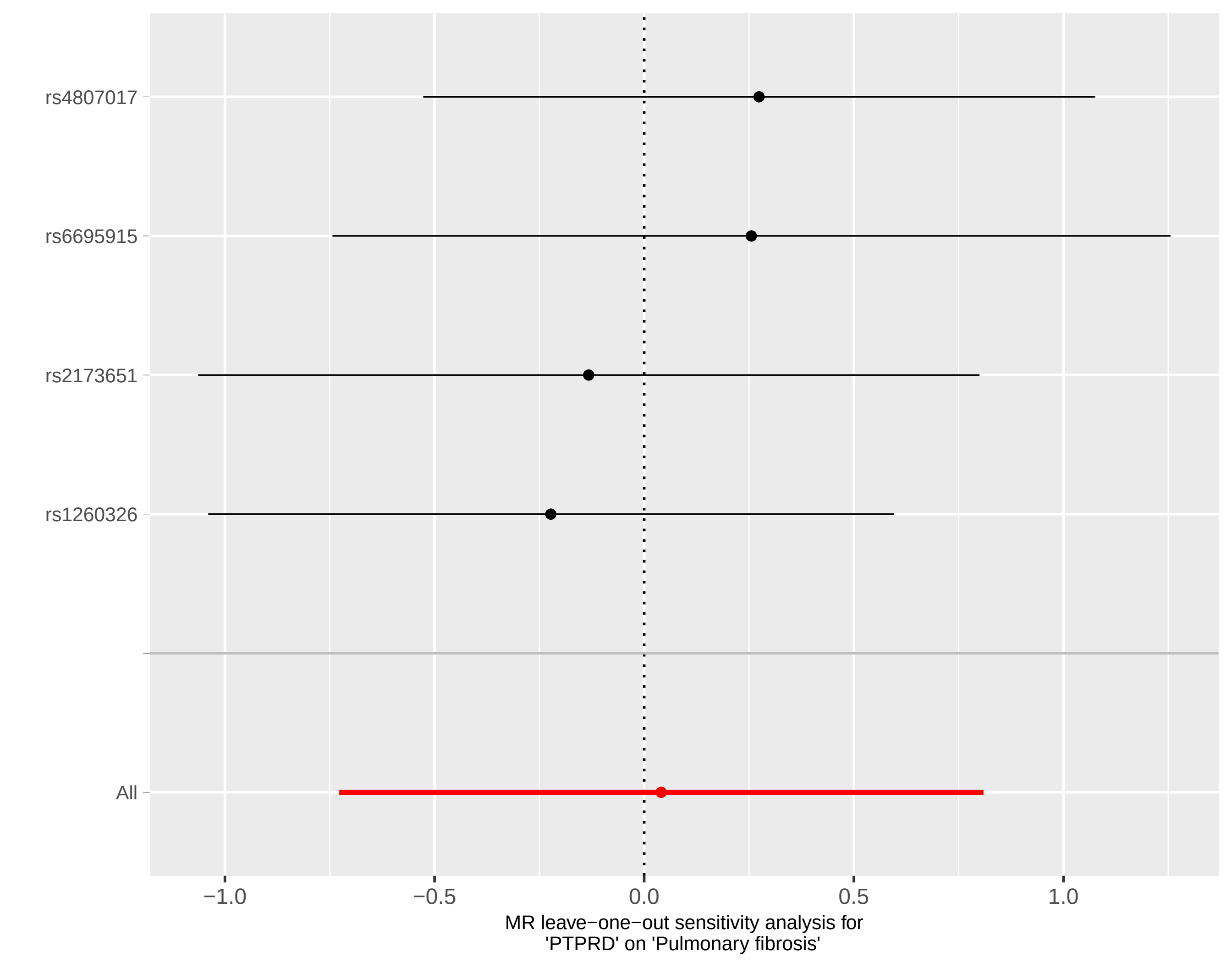
rs4807017
rs6695915
rs2173651
rs1260326
All
−1.0
−0.5
0.0
0.5
1.0
MR leave−one−out sensitivity analysis for
'PTPRD' on 'Pulmonary fibrosis'

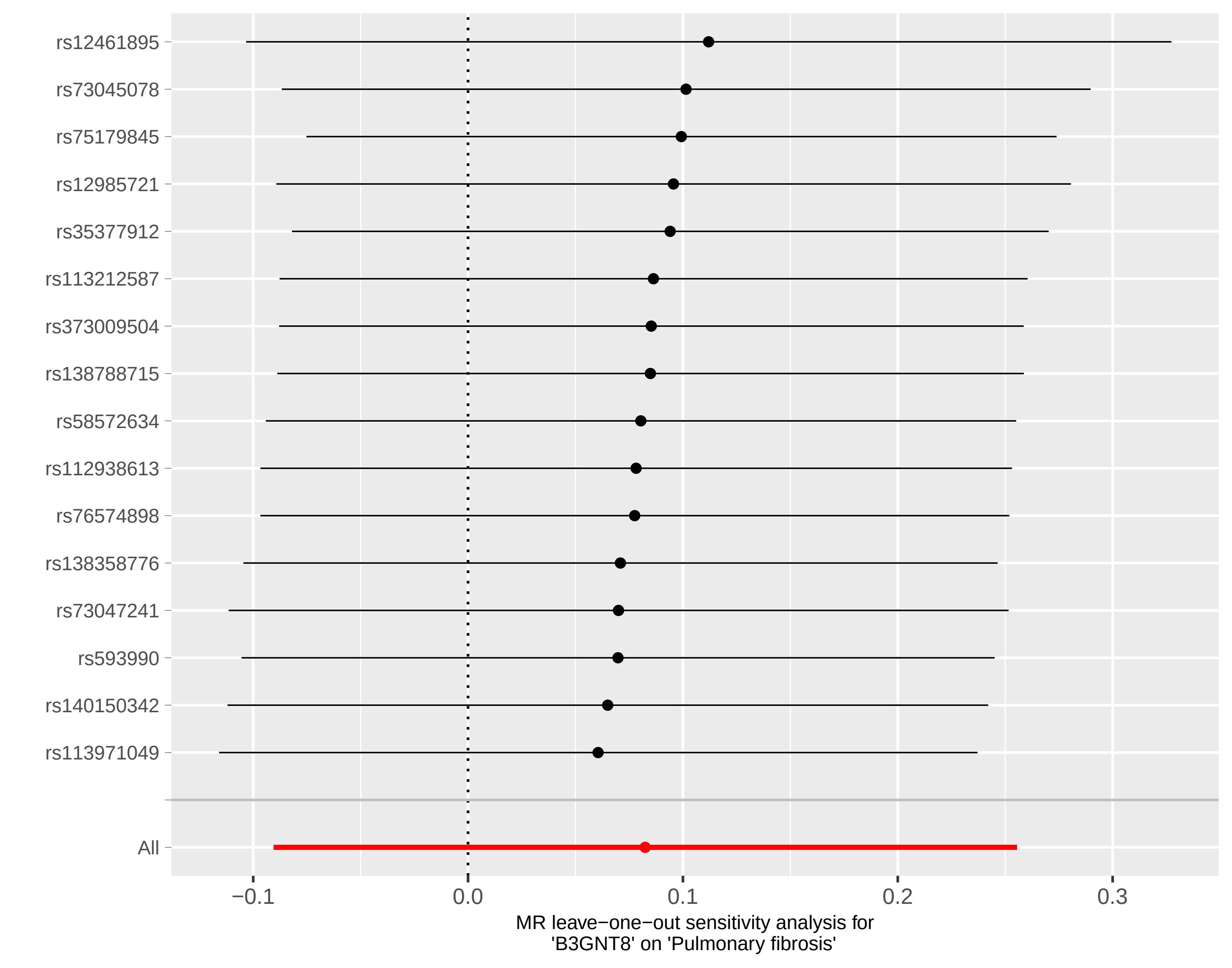
rs12461895
rs73045078
rs75179845
rs12985721
rs35377912
rs113212587
rs373009504
rs138788715
rs58572634
rs112938613
rs76574898
rs138358776
rs73047241
rs593990
rs140150342
rs113971049
All
−0.1
0.0
0.1
0.2
0.3
MR leave−one−out sensitivity analysis for
'B3GNT8' on 'Pulmonary fibrosis'

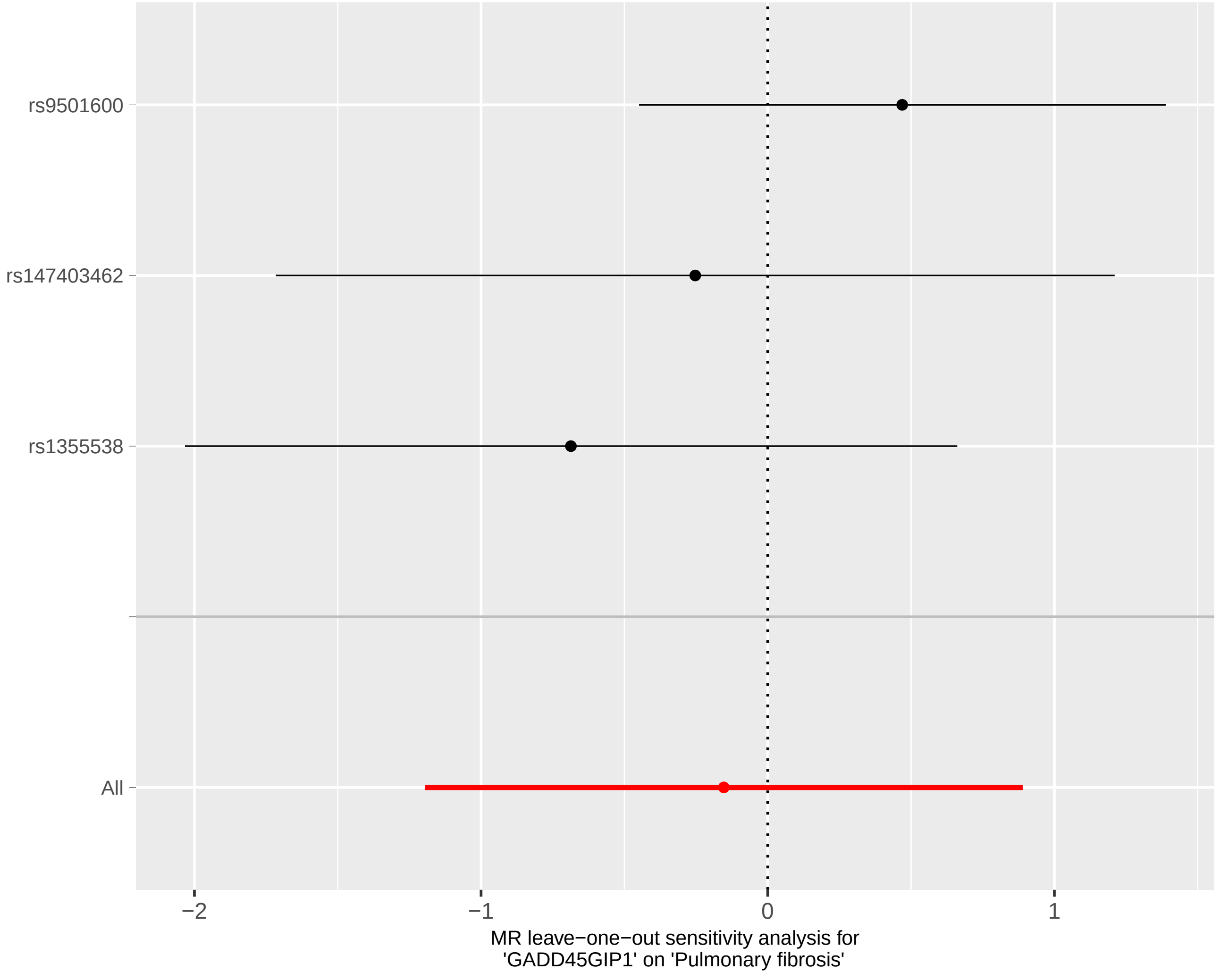
rs9501600
rs147403462
rs1355538
All
−2
−1
0
1
MR leave−one−out sensitivity analysis for
'GADD45GIP1' on 'Pulmonary fibrosis'

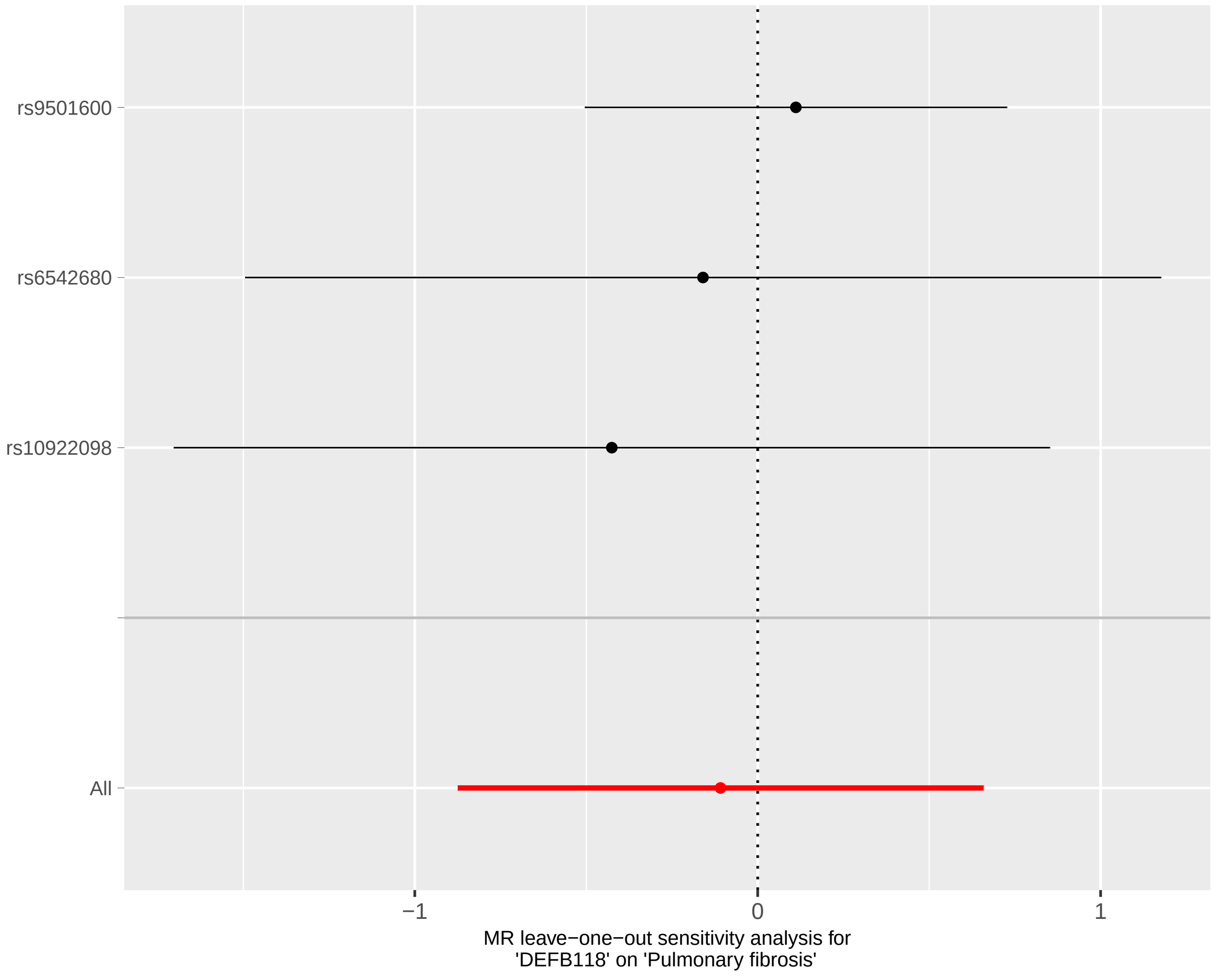
rs9501600
rs6542680
rs10922098
All
−1
0
1
MR leave−one−out sensitivity analysis for
'DEFB118' on 'Pulmonary fibrosis'

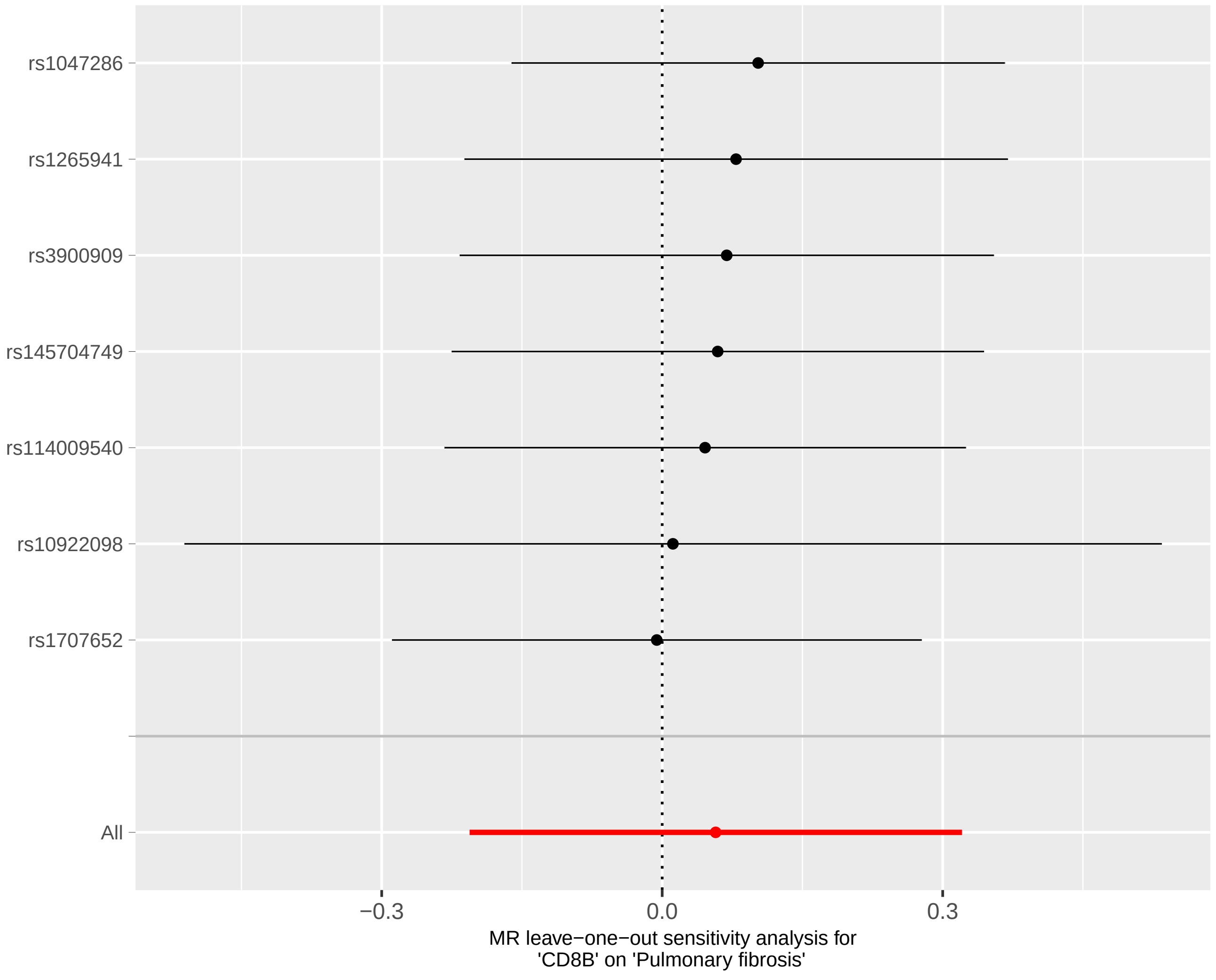
rs1047286
rs1265941
rs3900909
rs145704749
rs114009540
rs10922098
rs1707652
All
−0.3
0.0
0.3
MR leave−one−out sensitivity analysis for
'CD8B' on 'Pulmonary fibrosis'

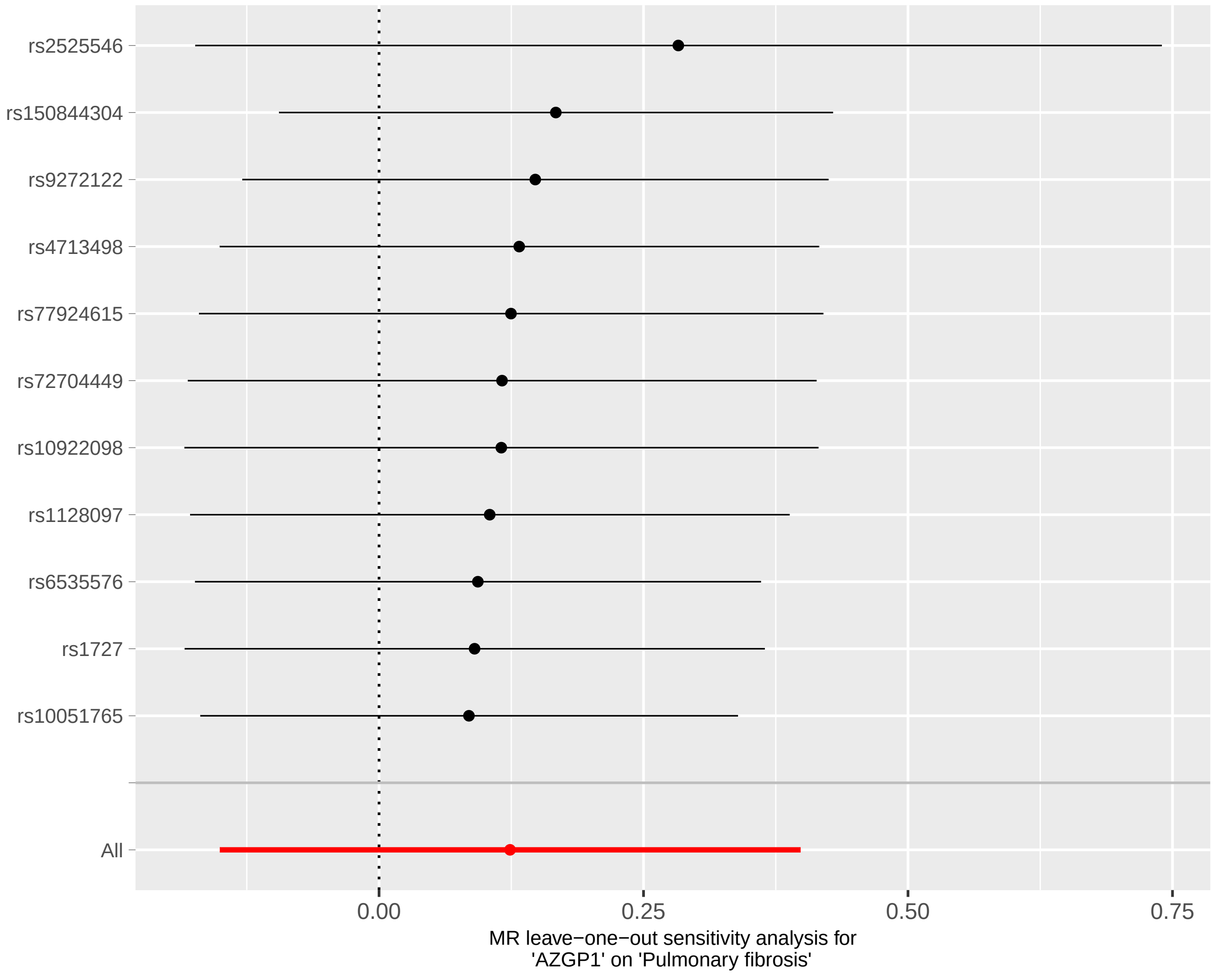
rs2525546
rs150844304
rs9272122
rs4713498
rs77924615
rs72704449
rs10922098
rs1128097
rs6535576
rs1727
rs10051765
All
0.00
0.25
0.50
0.75
MR leave−one−out sensitivity analysis for
'AZGP1' on 'Pulmonary fibrosis'

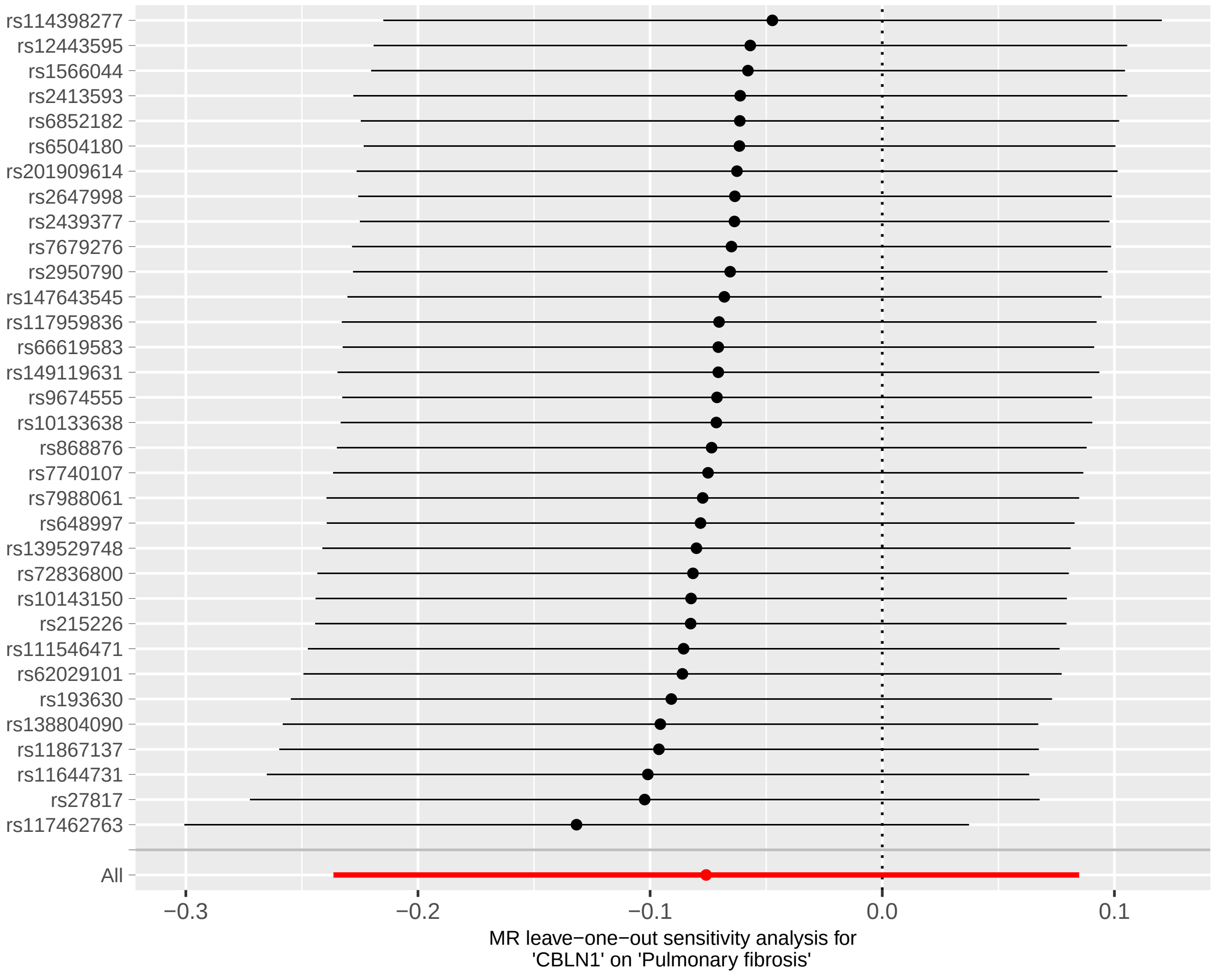

rs114398277
rs12443595
rs1566044
rs2413593
rs6852182
rs6504180
rs201909614
rs2647998
rs2439377
rs7679276
rs2950790
rs147643545
rs117959836
rs66619583
rs149119631
rs9674555
rs10133638
rs868876
rs7740107
rs7988061
rs648997
rs139529748
rs72836800
rs10143150
rs215226
rs111546471
rs62029101
rs193630
rs138804090
rs11867137
rs11644731
rs27817
rs117462763
All
−0.3
−0.2
−0.1
0.0
0.1
MR leave−one−out sensitivity analysis for
'CBLN1' on 'Pulmonary fibrosis'

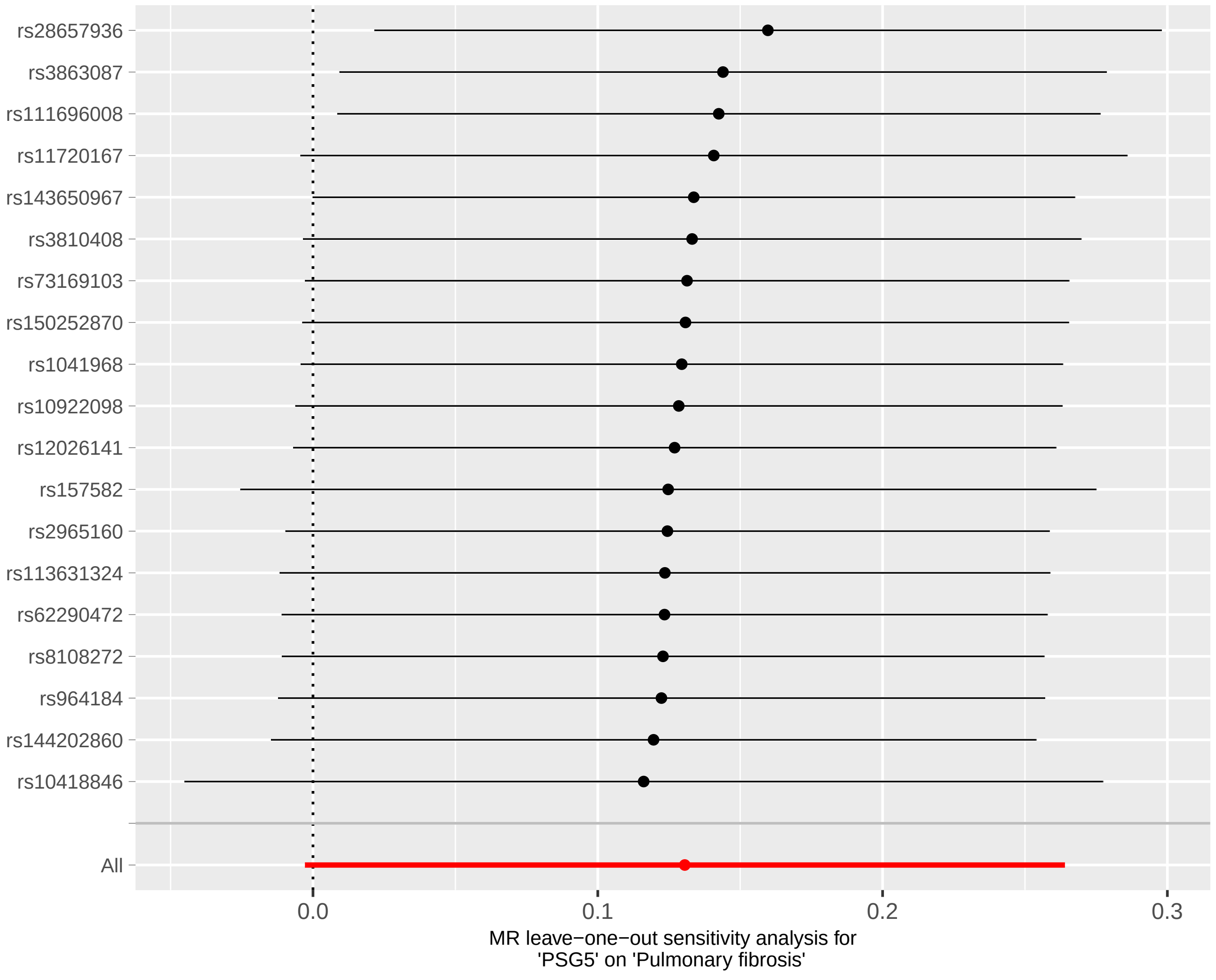
rs28657936
rs3863087
rs111696008
rs11720167
rs143650967
rs3810408
rs73169103
rs150252870
rs1041968
rs10922098
rs12026141
rs157582
rs2965160
rs113631324
rs62290472
rs8108272
rs964184
rs144202860
rs10418846
All
0.0
0.1
0.2
0.3
MR leave−one−out sensitivity analysis for
'PSG5' on 'Pulmonary fibrosis'

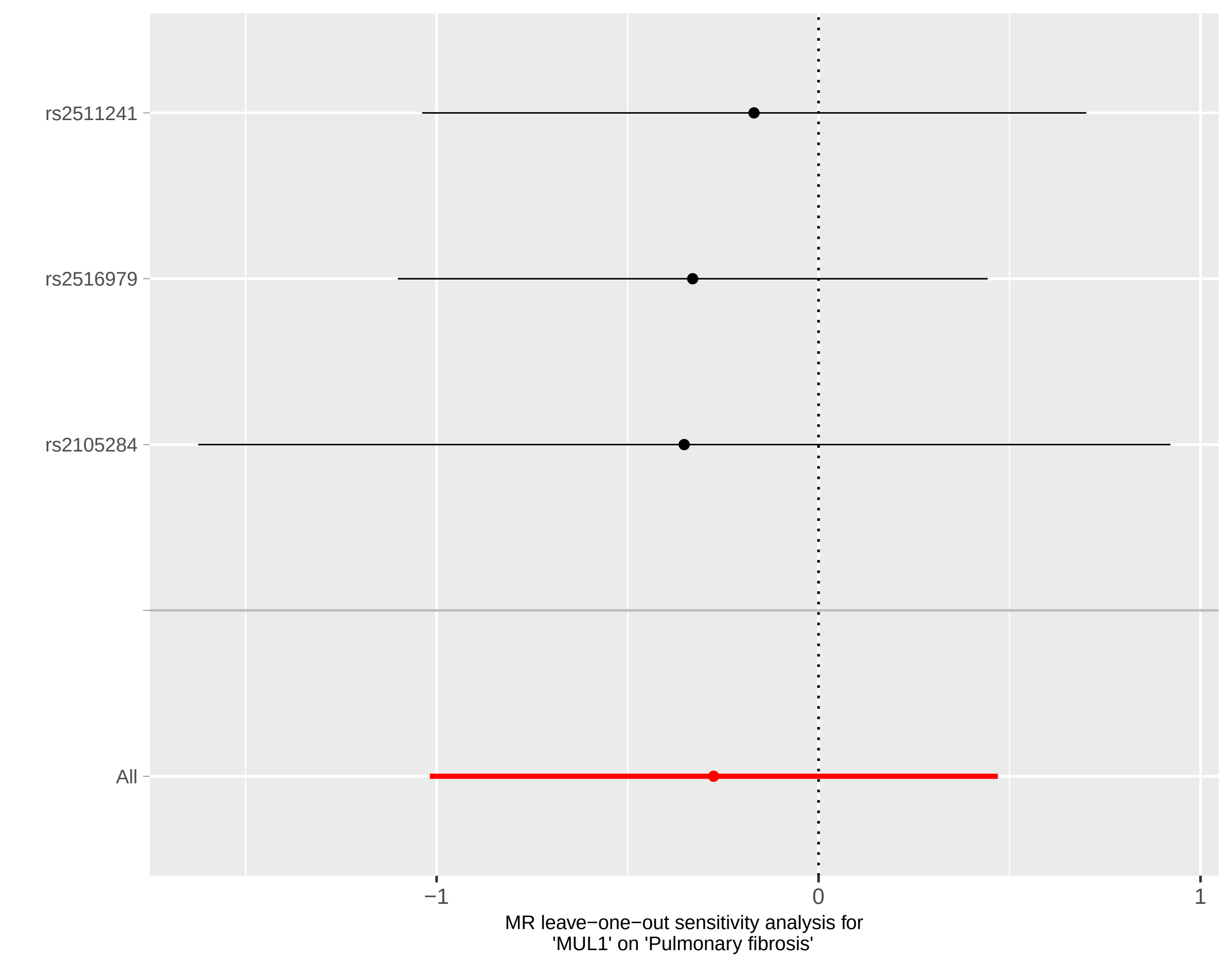
rs2511241
rs2516979
rs2105284
All
−1
0
1
MR leave−one−out sensitivity analysis for
'MUL1' on 'Pulmonary fibrosis'

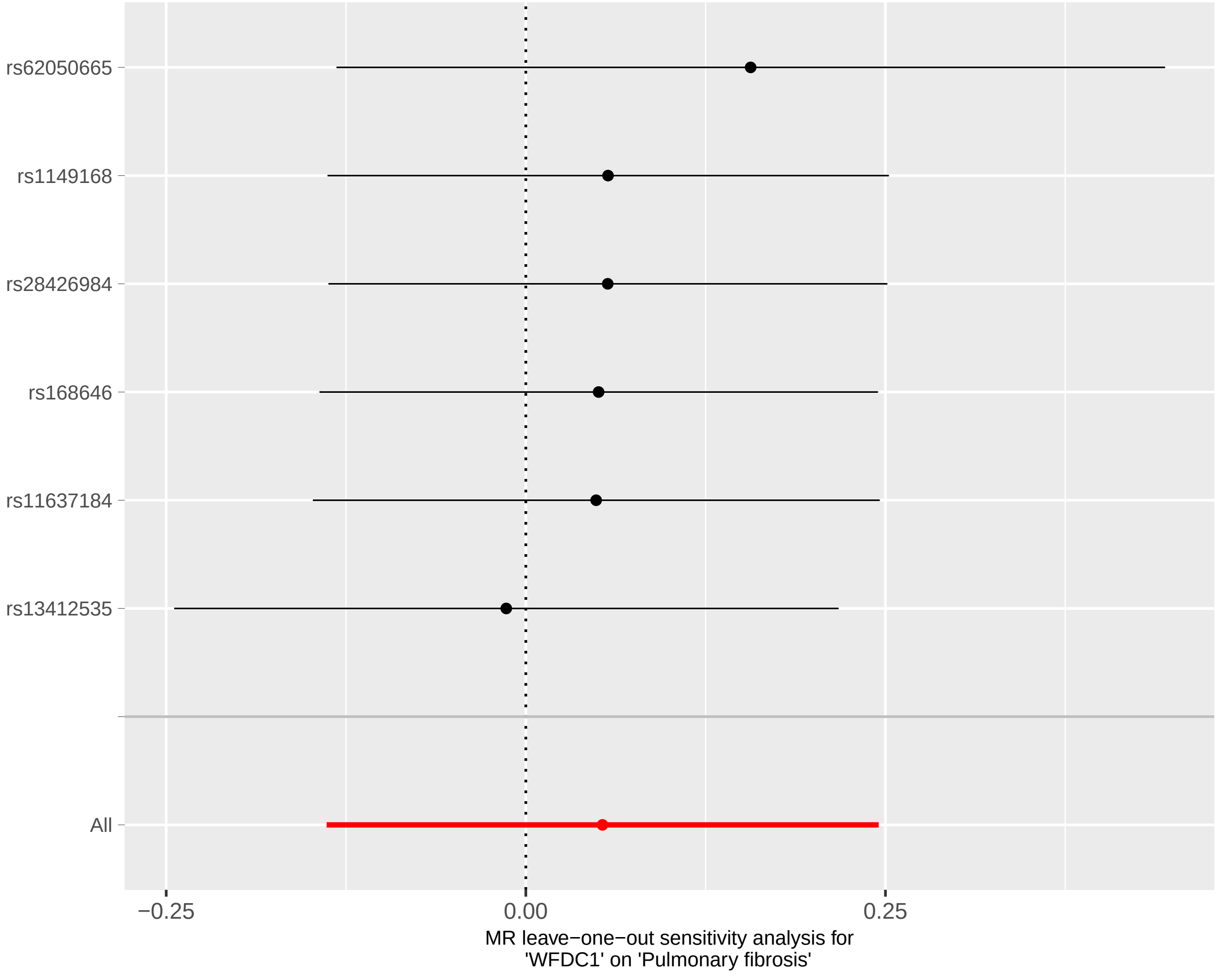
rs62050665
rs1149168
rs28426984
rs168646
rs11637184
rs13412535
All
−0.25
0.00
0.25
MR leave−one−out sensitivity analysis for
'WFDC1' on 'Pulmonary fibrosis'

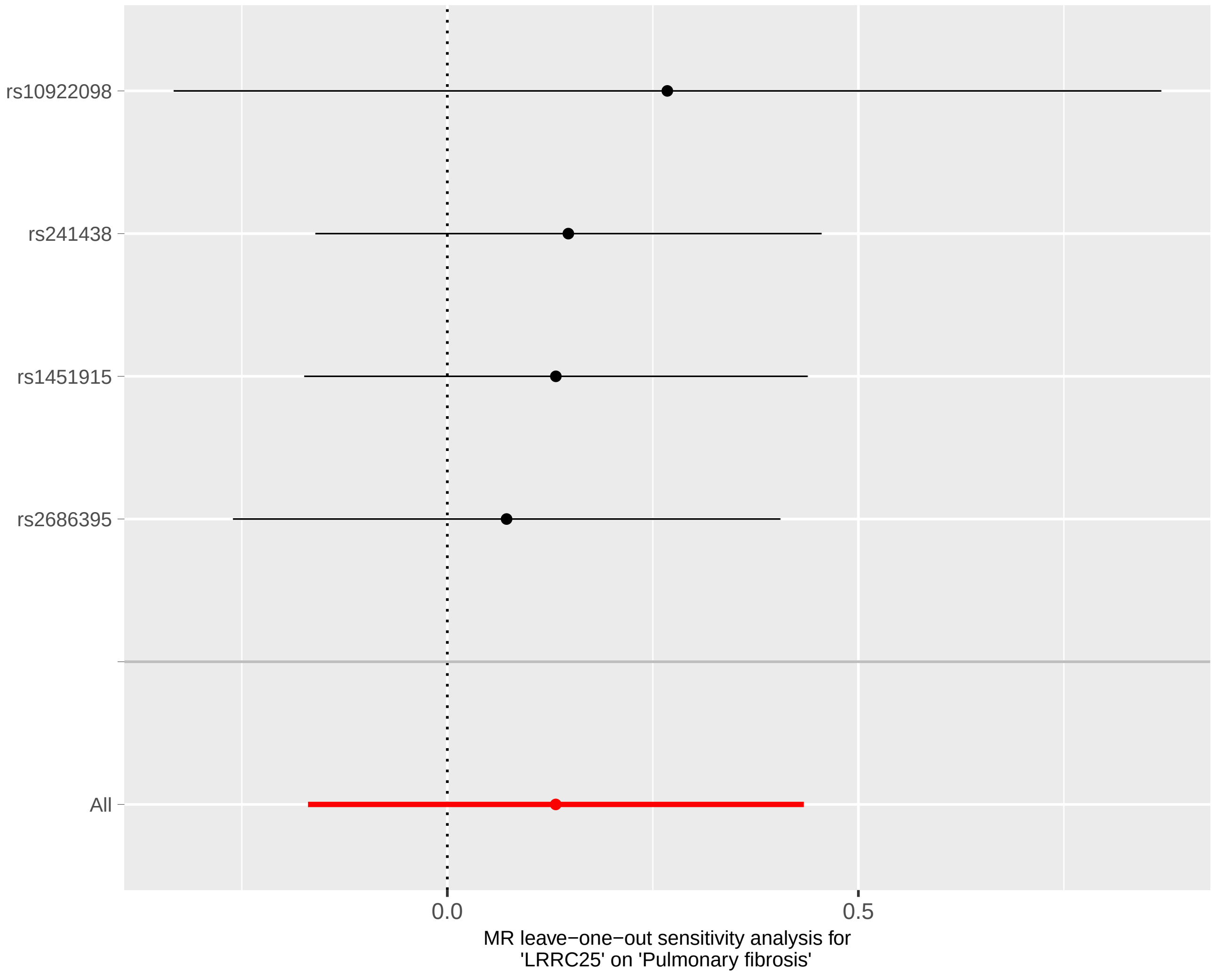
rs10922098
rs241438
rs1451915
rs2686395
All
0.0
0.5
MR leave−one−out sensitivity analysis for
'LRRC25' on 'Pulmonary fibrosis'

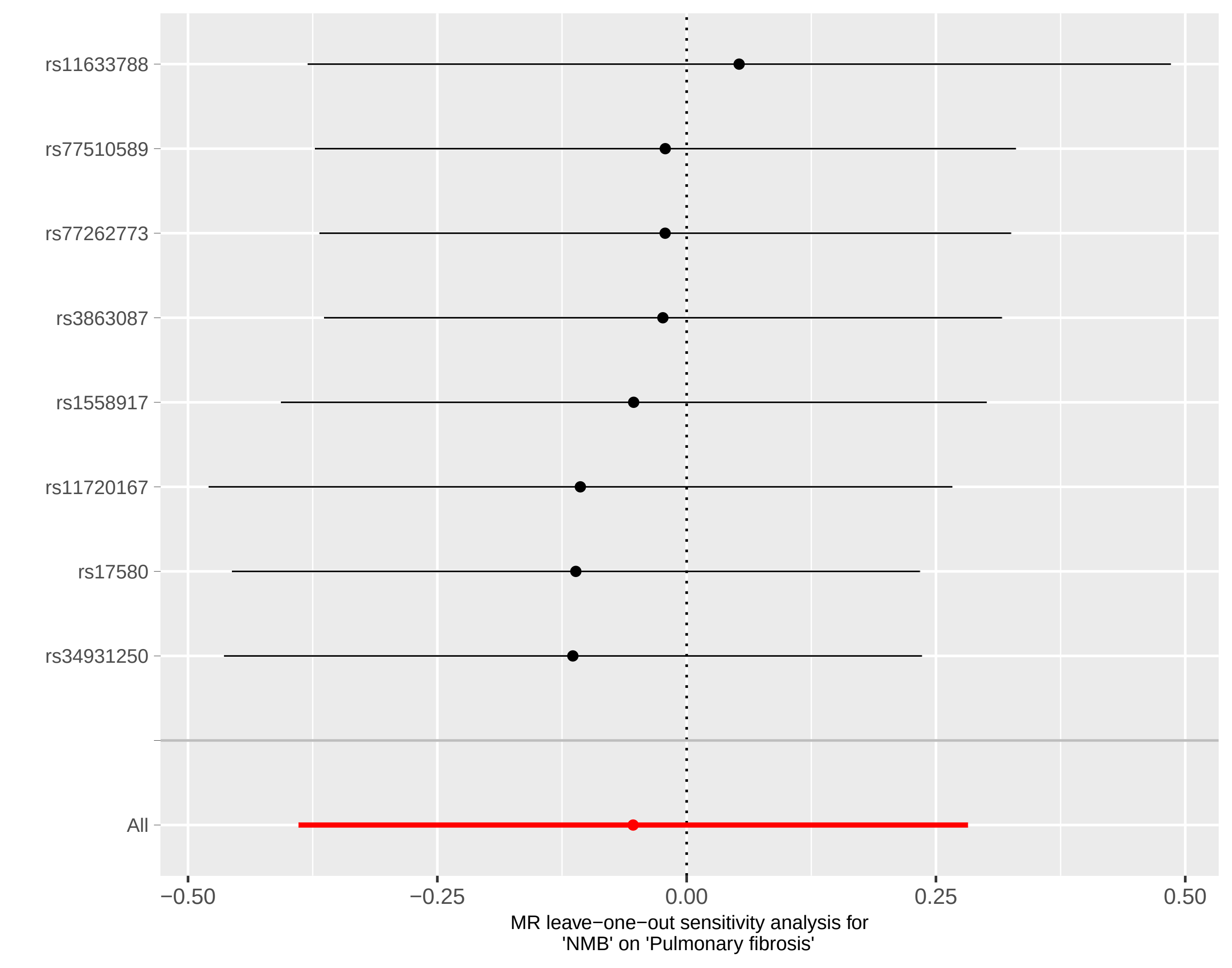
rs11633788
rs77510589
rs77262773
rs3863087
rs1558917
rs11720167
rs17580
rs34931250
All
−0.50
−0.25
0.00
0.25
0.50
MR leave−one−out sensitivity analysis for
'NMB' on 'Pulmonary fibrosis'

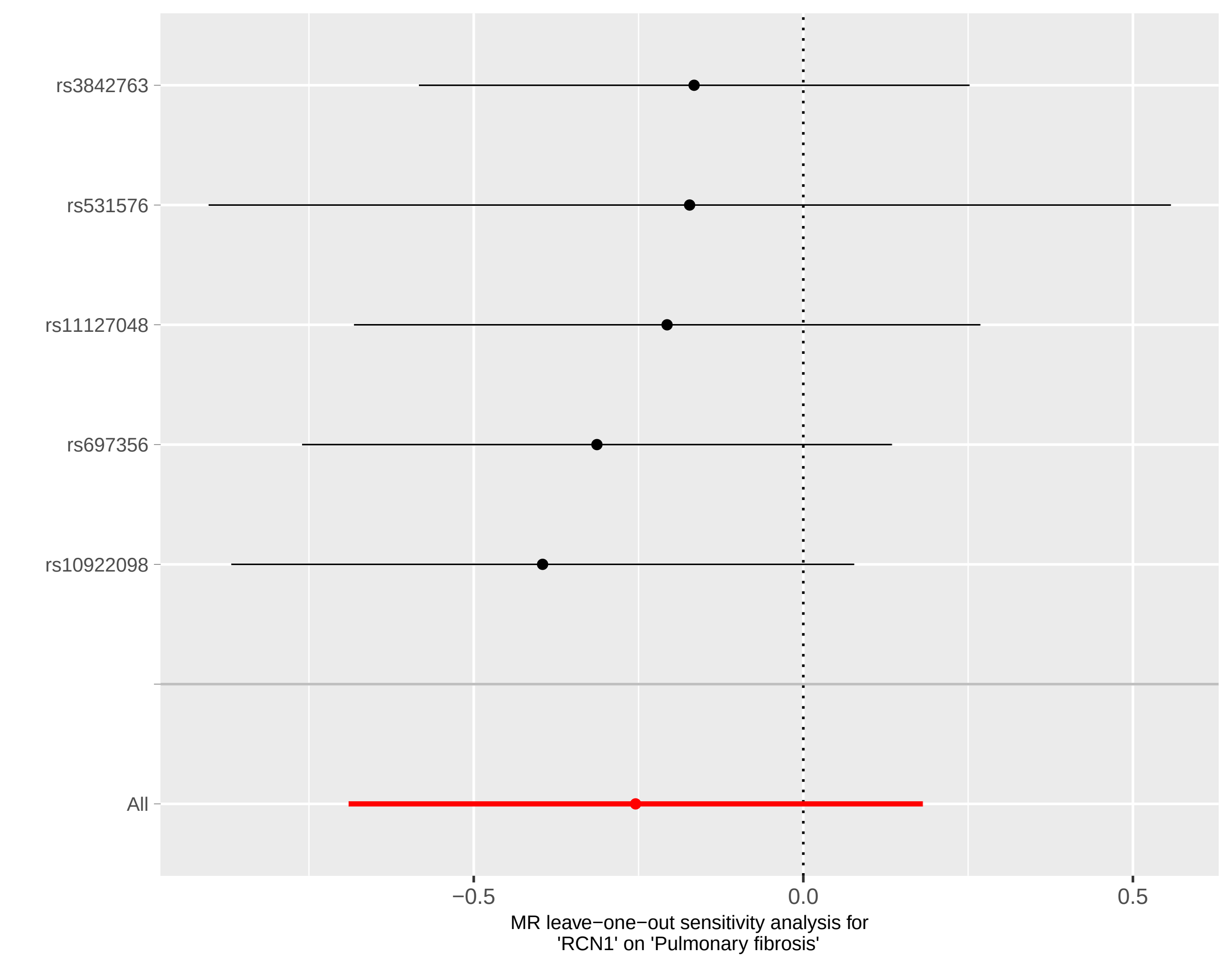
rs3842763
rs531576
rs11127048
rs697356
rs10922098
All
−0.5
0.0
0.5
MR leave−one−out sensitivity analysis for
'RCN1' on 'Pulmonary fibrosis'

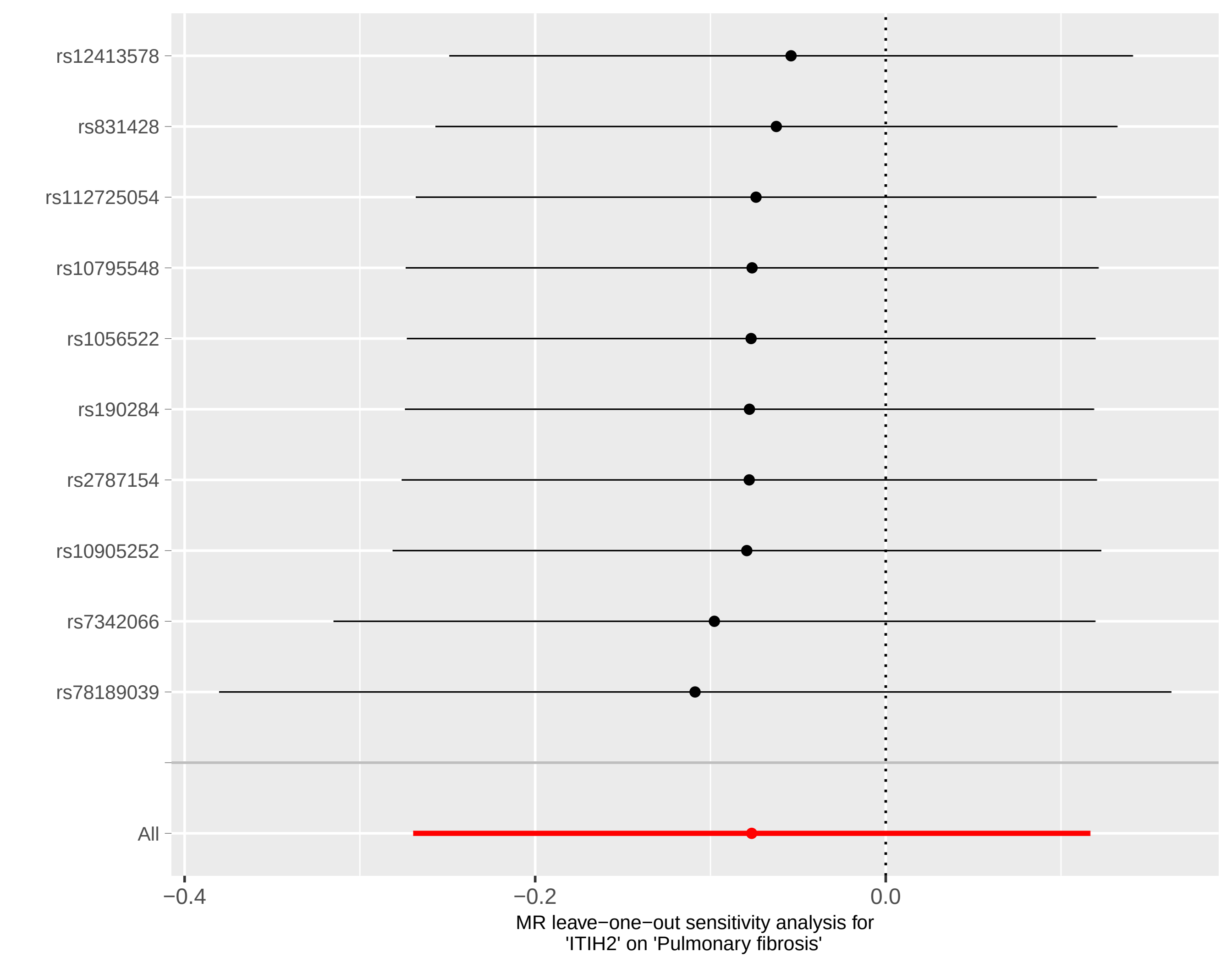
rs12413578
rs831428
rs112725054
rs10795548
rs1056522
rs190284
rs2787154
rs10905252
rs7342066
rs78189039
All
−0.4
−0.2
0.0
MR leave−one−out sensitivity analysis for
'ITIH2' on 'Pulmonary fibrosis'

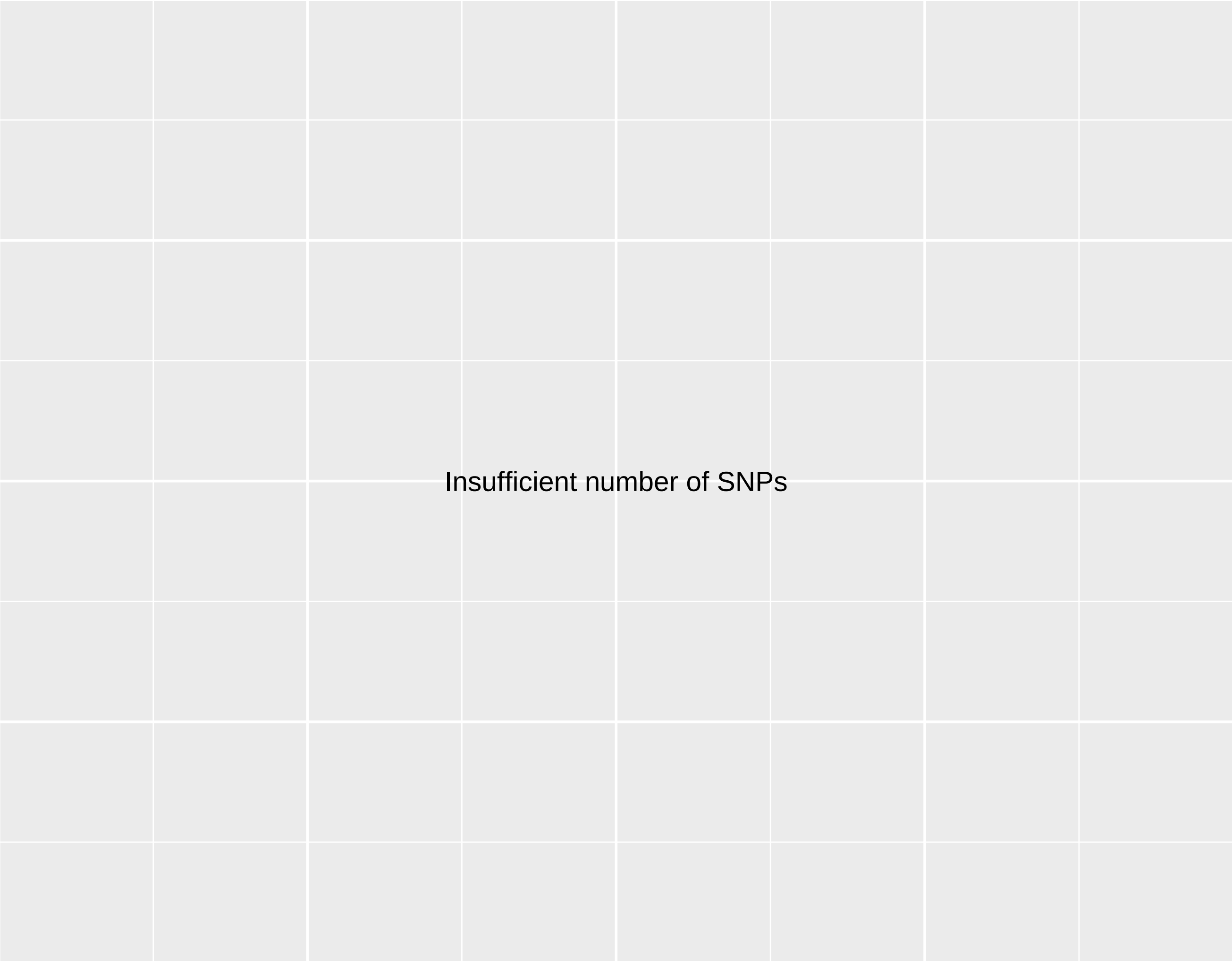
Insufficient number of SNPs

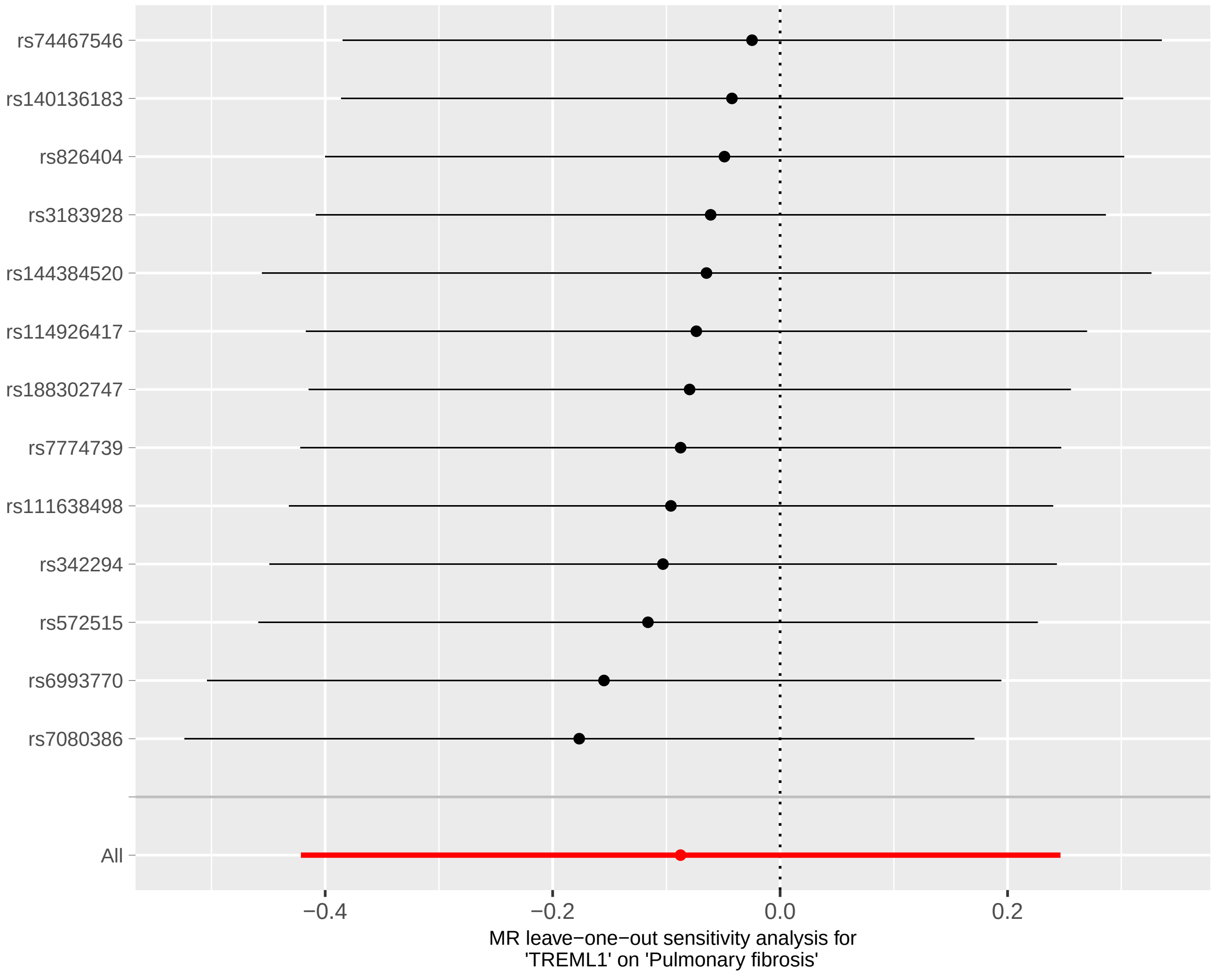

rs74467546
rs140136183
rs826404
rs3183928
rs144384520
rs114926417
rs188302747
rs7774739
rs111638498
rs342294
rs572515
rs6993770
rs7080386
All
−0.4
−0.2
0.0
0.2
MR leave−one−out sensitivity analysis for
'TREML1' on 'Pulmonary fibrosis'

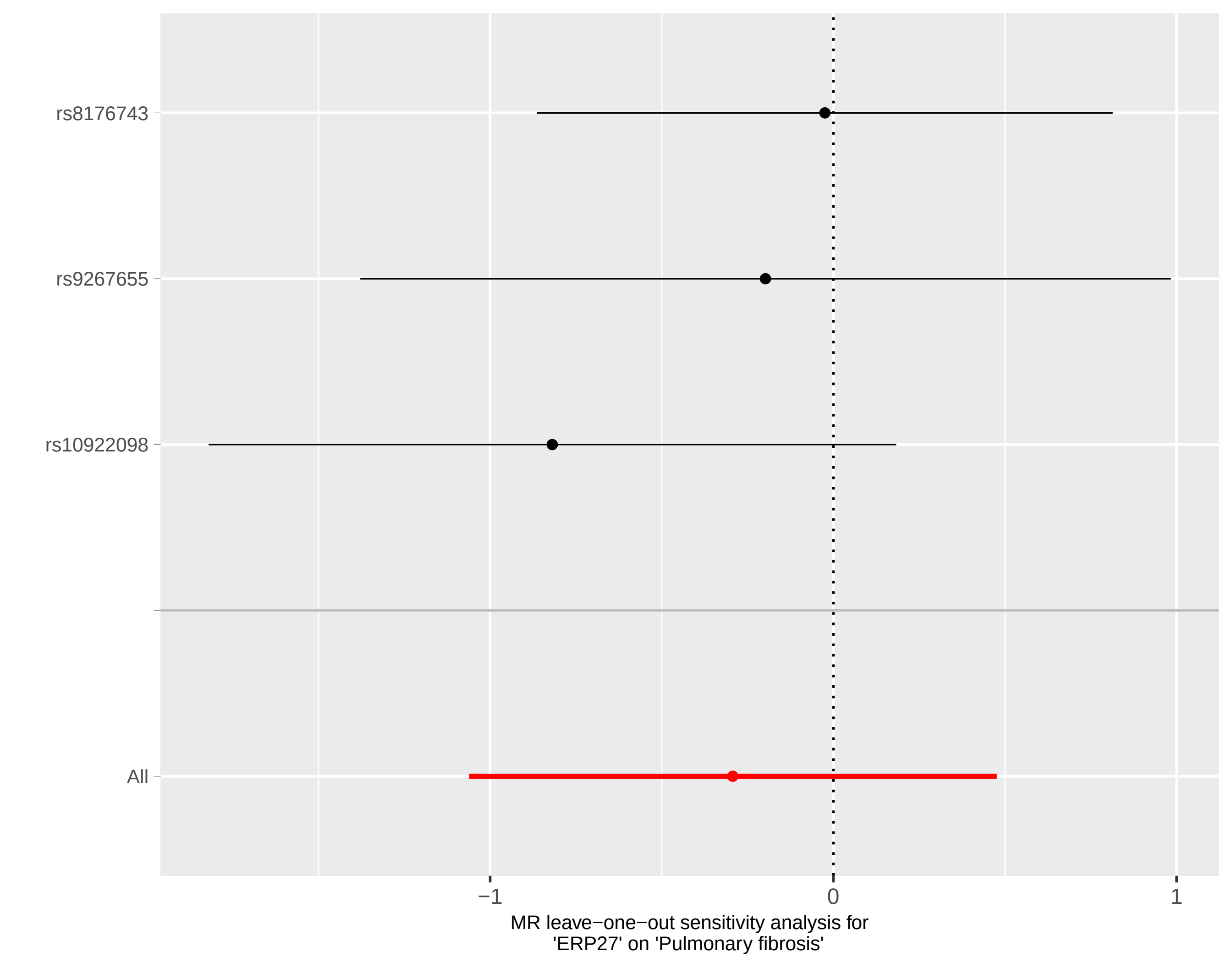
rs8176743
rs9267655
rs10922098
All
−1
0
1
MR leave−one−out sensitivity analysis for
'ERP27' on 'Pulmonary fibrosis'

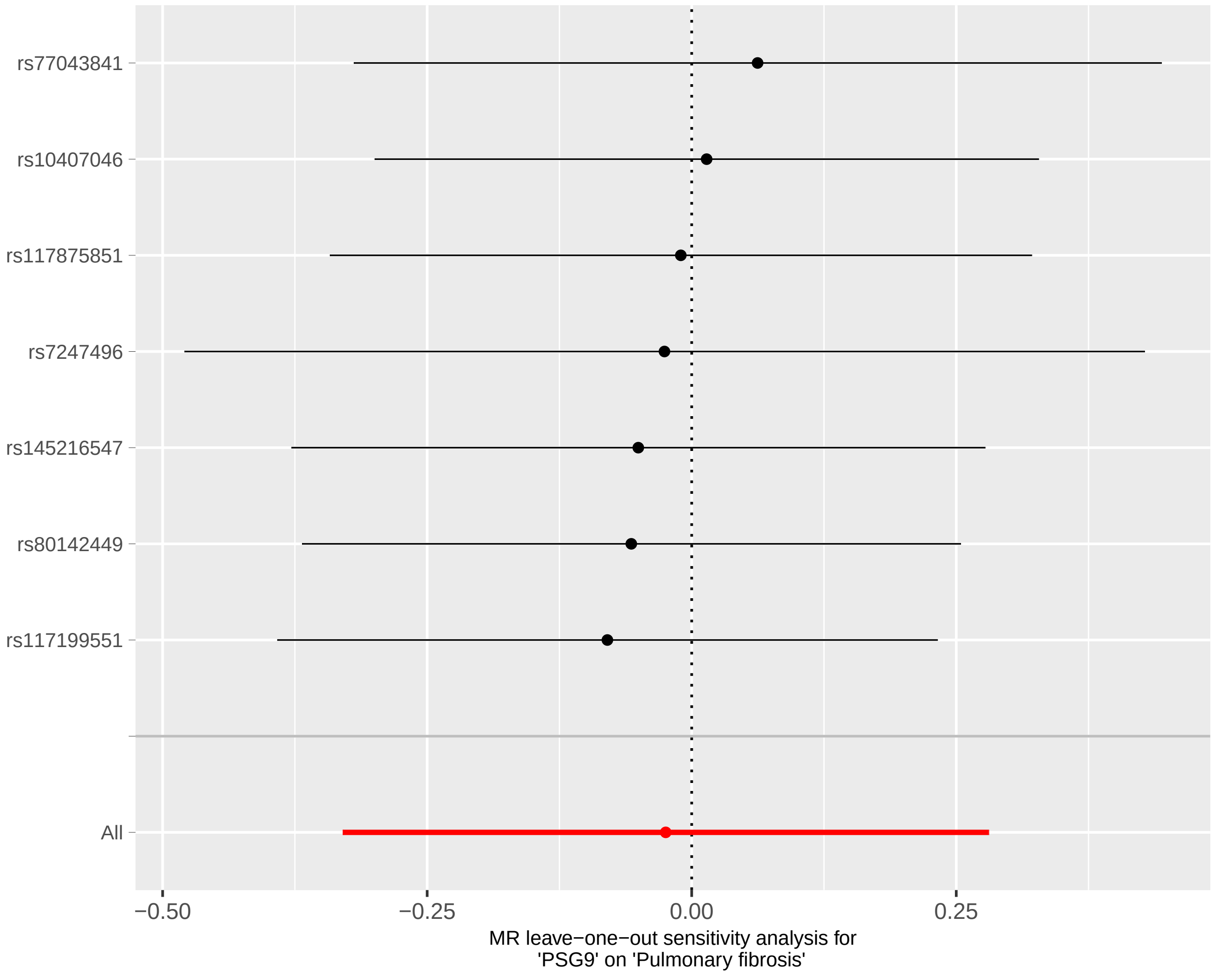

rs77043841
rs10407046
rs117875851
rs7247496
rs145216547
rs80142449
rs117199551
All
−0.50
−0.25
0.00
0.25
MR leave−one−out sensitivity analysis for
'PSG9' on 'Pulmonary fibrosis'

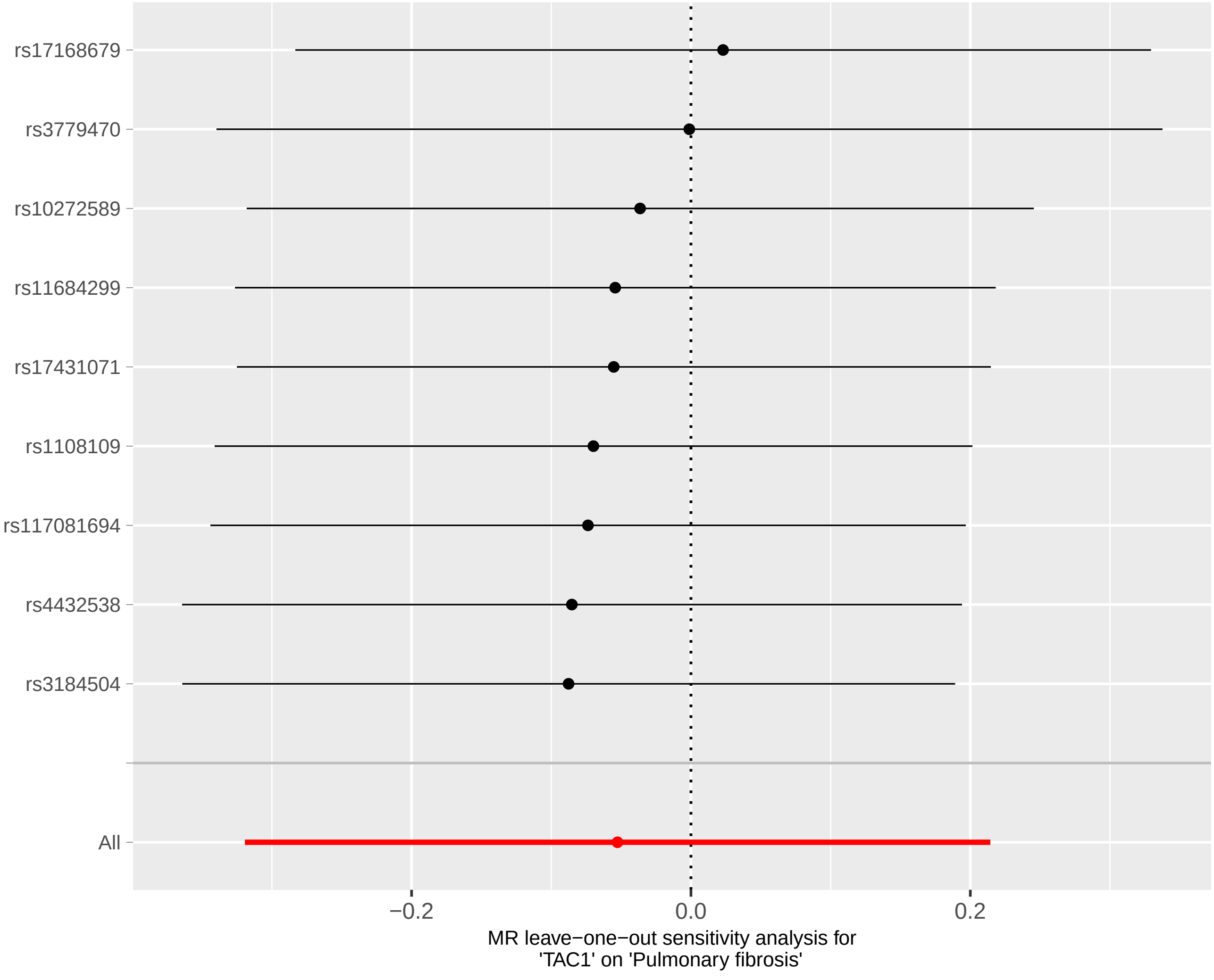

rs17168679
rs3779470
rs10272589
rs11684299
rs17431071
rs1108109
rs117081694
rs4432538
rs3184504
All
−0.2
0.0
0.2
MR leave−one−out sensitivity analysis for
'TAC1' on 'Pulmonary fibrosis'

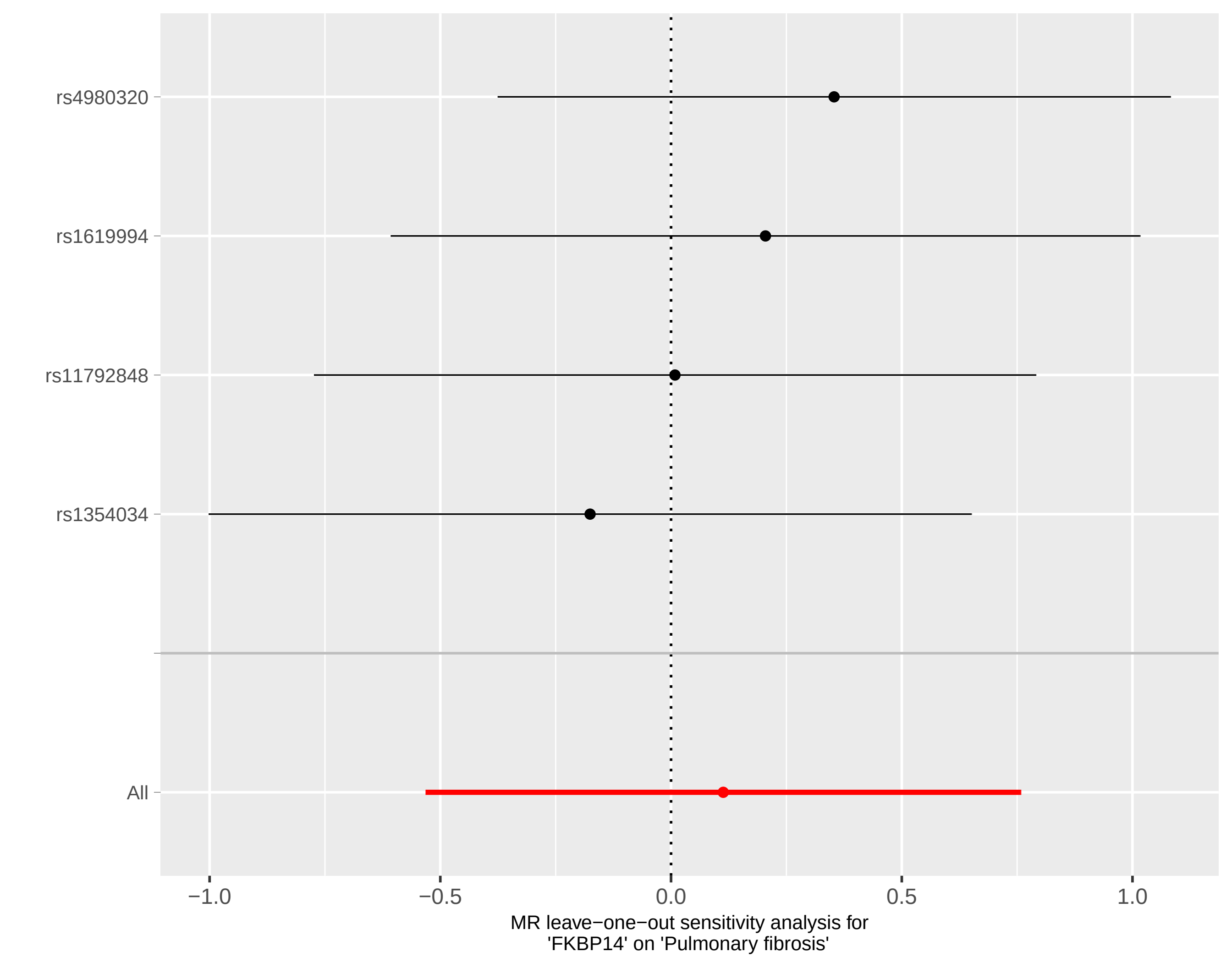
rs4980320
rs1619994
rs11792848
rs1354034
All
−1.0
−0.5
0.0
0.5
1.0
MR leave−one−out sensitivity analysis for
'FKBP14' on 'Pulmonary fibrosis'

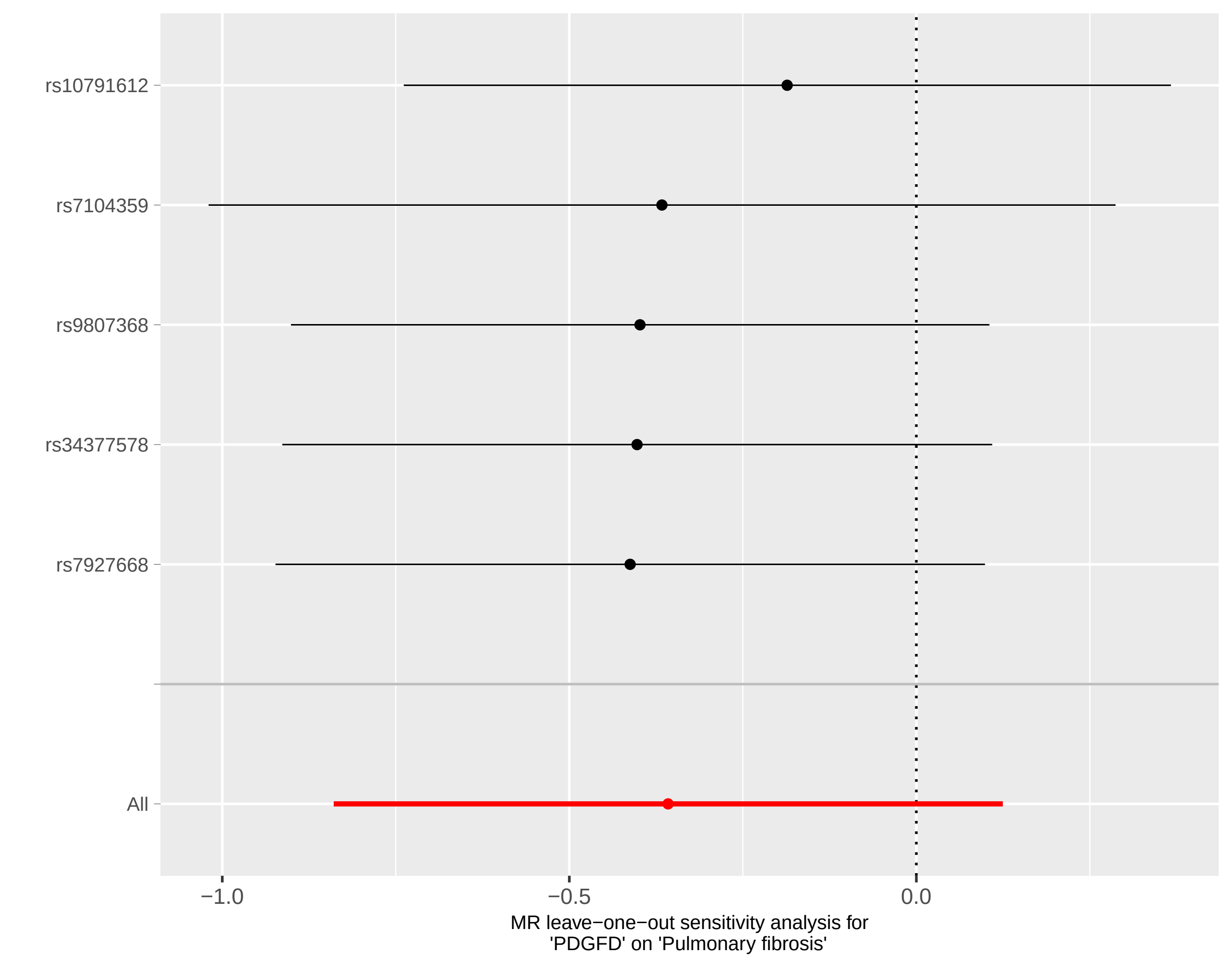
rs10791612
rs7104359
rs9807368
rs34377578
rs7927668
All
−1.0
−0.5
0.0
MR leave−one−out sensitivity analysis for
'PDGFD' on 'Pulmonary fibrosis'

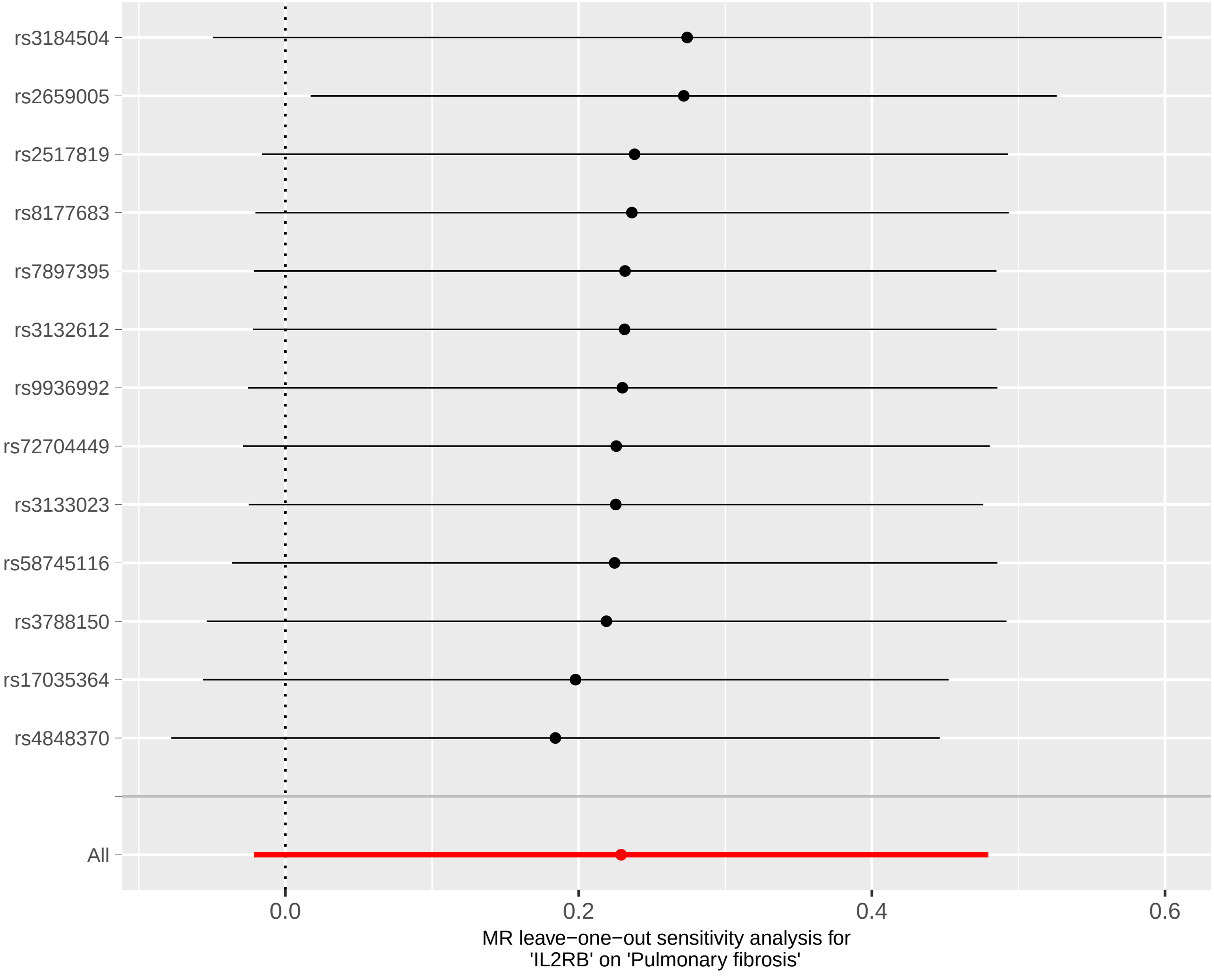
rs3184504
rs2659005
rs2517819
rs8177683
rs7897395
rs3132612
rs9936992
rs72704449
rs3133023
rs58745116
rs3788150
rs17035364
rs4848370
All
0.0
0.2
0.4
0.6
MR leave−one−out sensitivity analysis for
'IL2RB' on 'Pulmonary fibrosis'

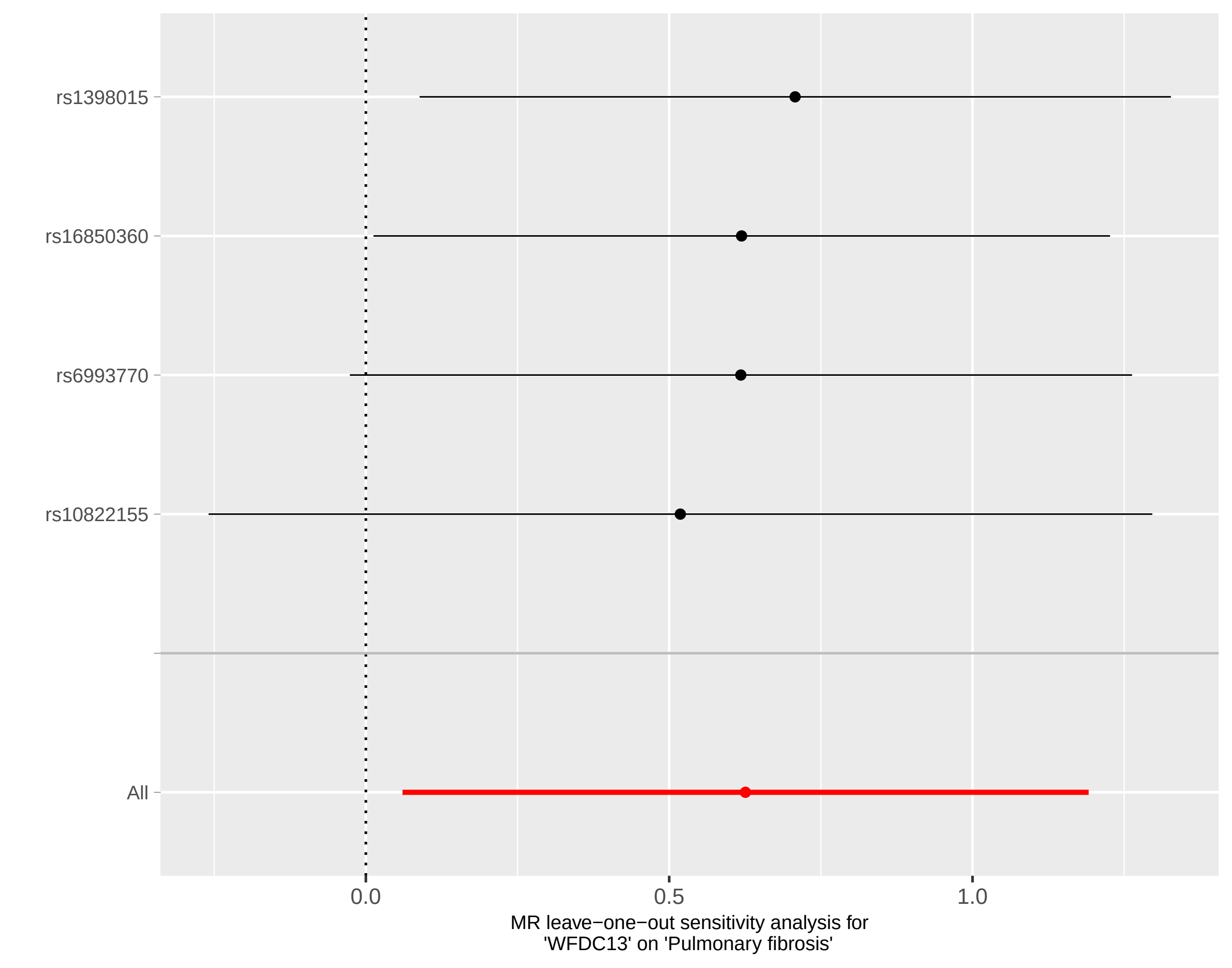

rs1398015
rs16850360
rs6993770
rs10822155
All
0.0
0.5
1.0
MR leave−one−out sensitivity analysis for
'WFDC13' on 'Pulmonary fibrosis'

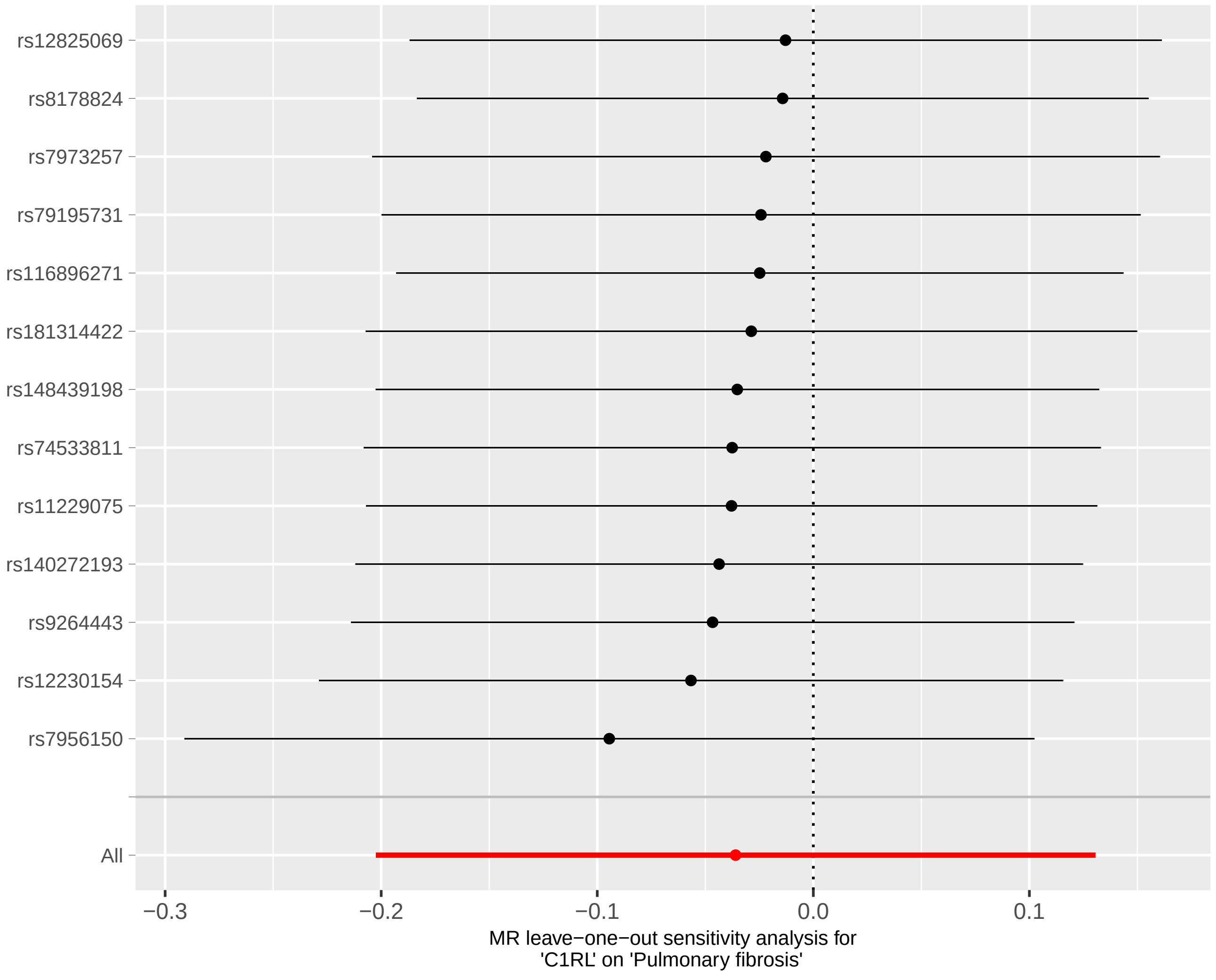
rs12825069
rs8178824
rs7973257
rs79195731
rs116896271
rs181314422
rs148439198
rs74533811
rs11229075
rs140272193
rs9264443
rs12230154
rs7956150
All
−0.3
−0.2
−0.1
0.0
0.1
MR leave−one−out sensitivity analysis for
'C1RL' on 'Pulmonary fibrosis'

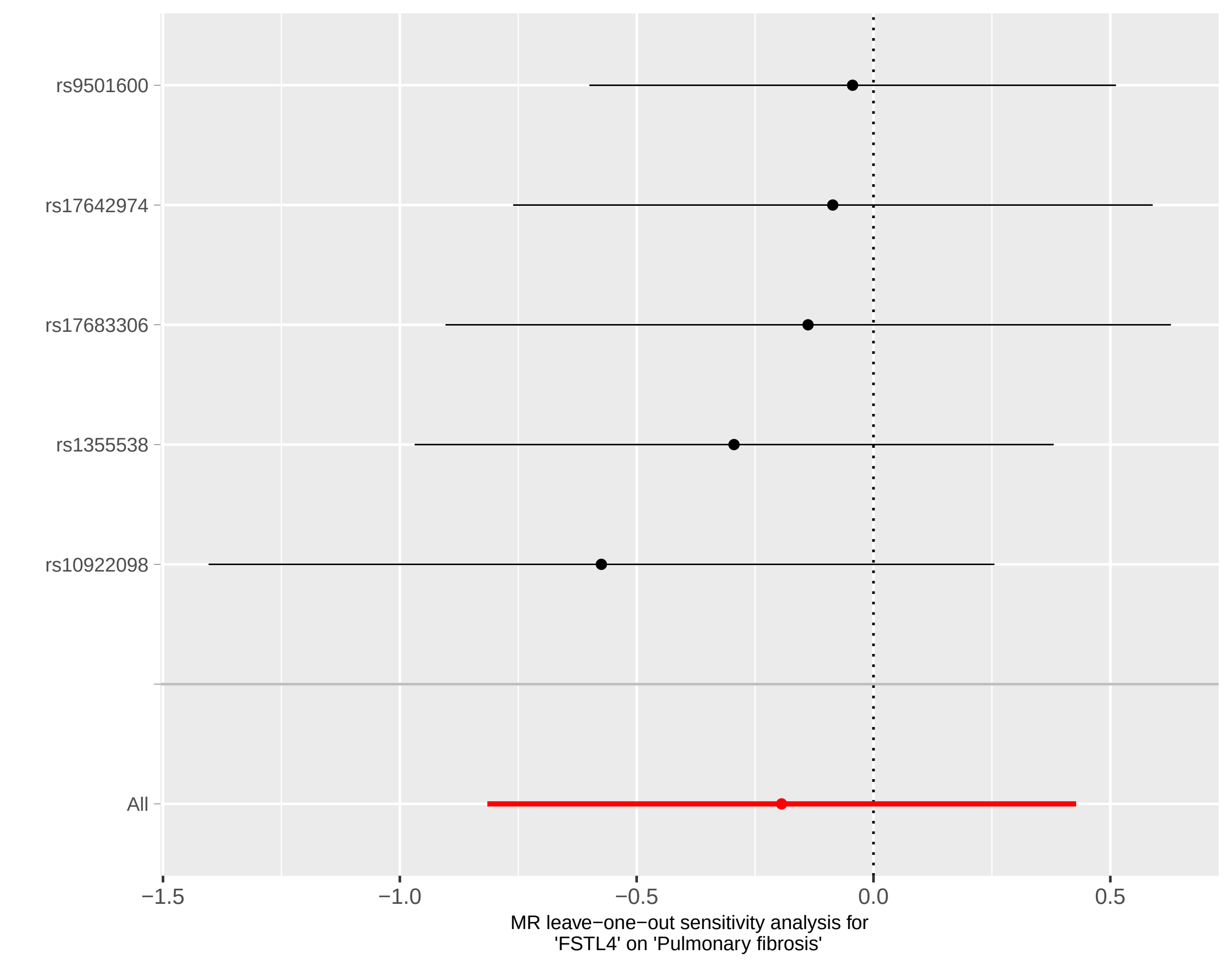

rs9501600
rs17642974
rs17683306
rs1355538
rs10922098
All
−1.5
−1.0
−0.5
0.0
0.5
MR leave−one−out sensitivity analysis for
'FSTL4' on 'Pulmonary fibrosis'

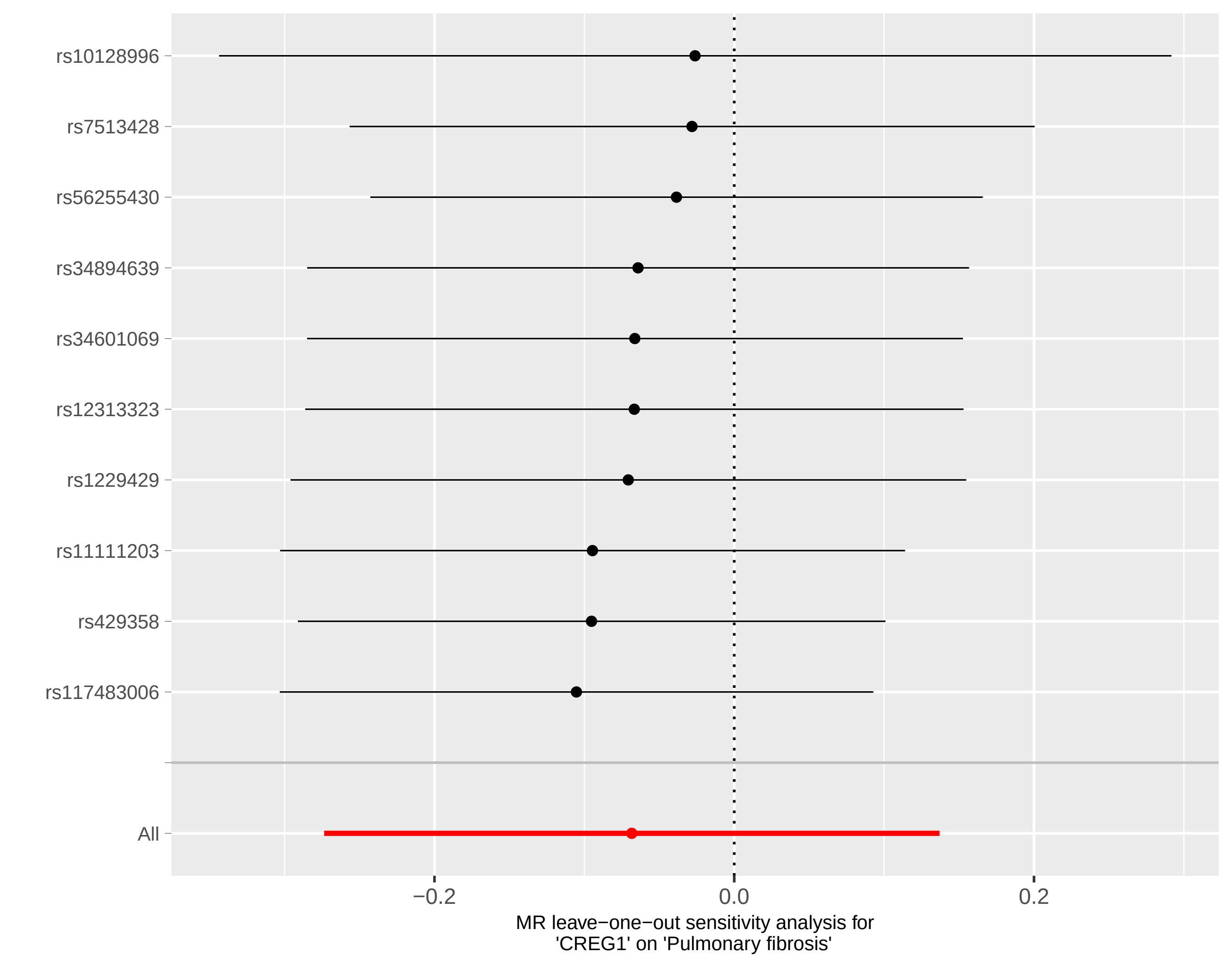

rs10128996
rs7513428
rs56255430
rs34894639
rs34601069
rs12313323
rs1229429
rs11111203
rs429358
rs117483006
All
−0.2
0.0
0.2
MR leave−one−out sensitivity analysis for
'CREG1' on 'Pulmonary fibrosis'

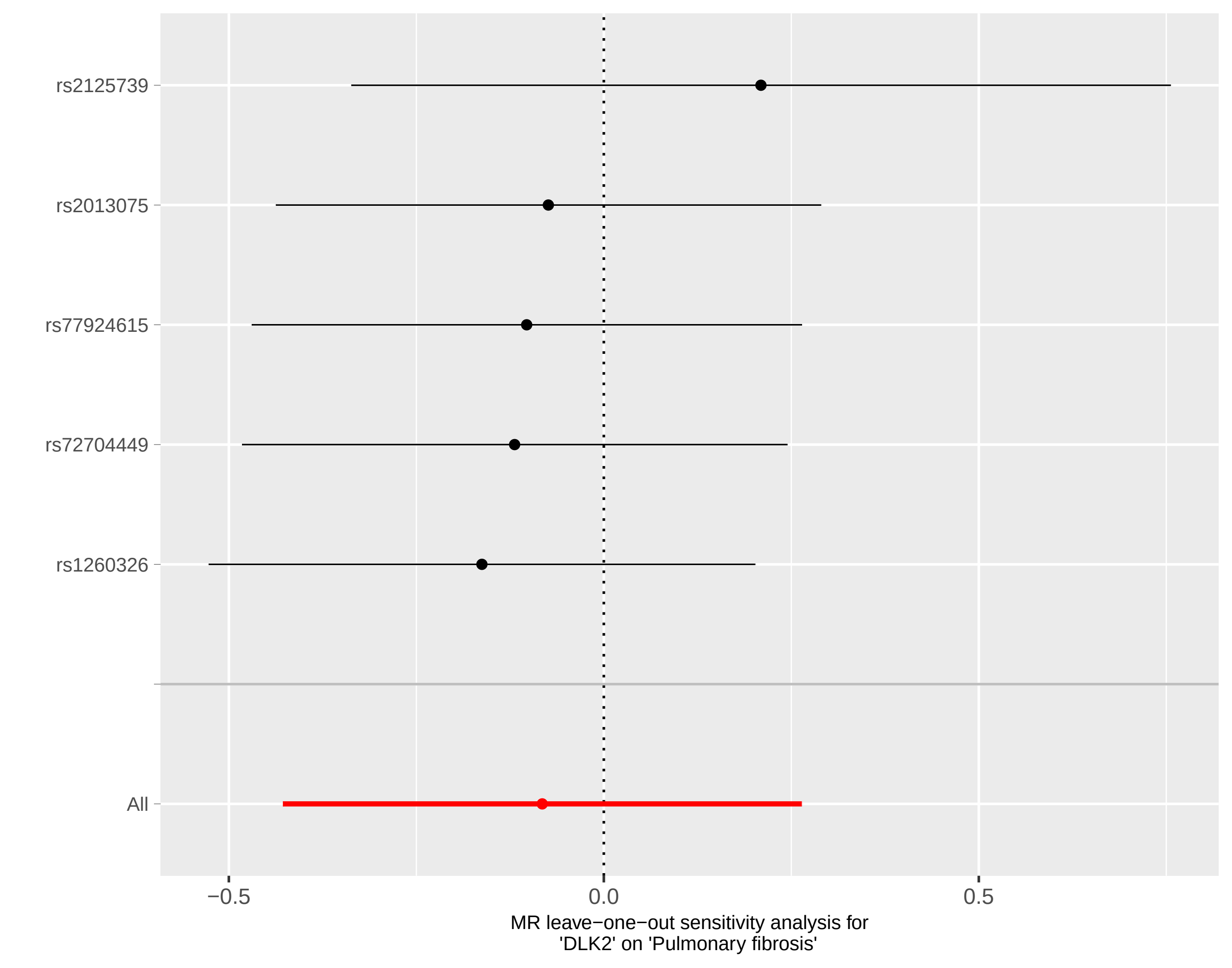
rs2125739
rs2013075
rs77924615
rs72704449
rs1260326
All
−0.5
0.0
0.5
MR leave−one−out sensitivity analysis for
'DLK2' on 'Pulmonary fibrosis'

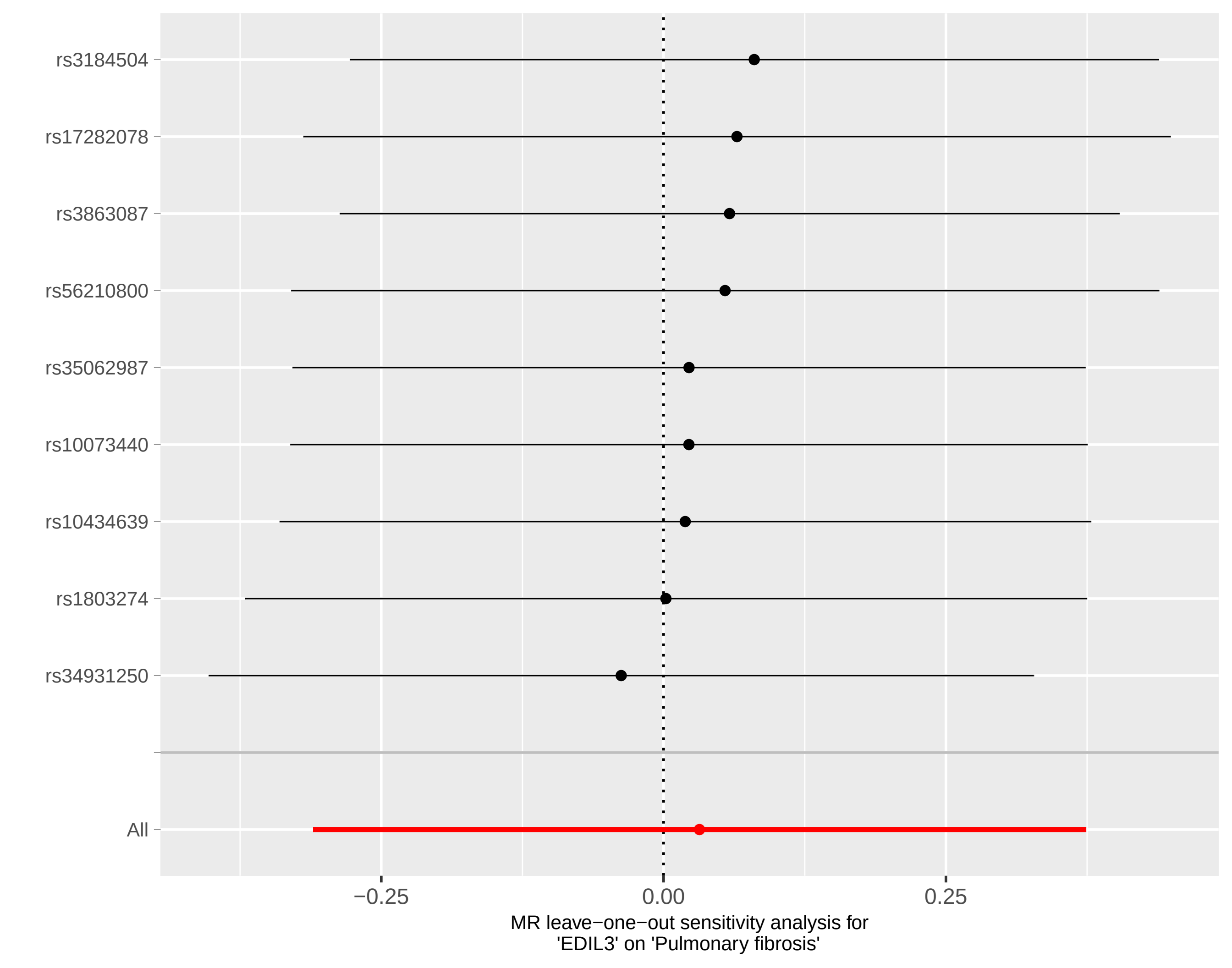
rs3184504
rs17282078
rs3863087
rs56210800
rs35062987
rs10073440
rs10434639
rs1803274
rs34931250
All
−0.25
0.00
0.25
MR leave−one−out sensitivity analysis for
'EDIL3' on 'Pulmonary fibrosis'

Insufficient number of SNPs

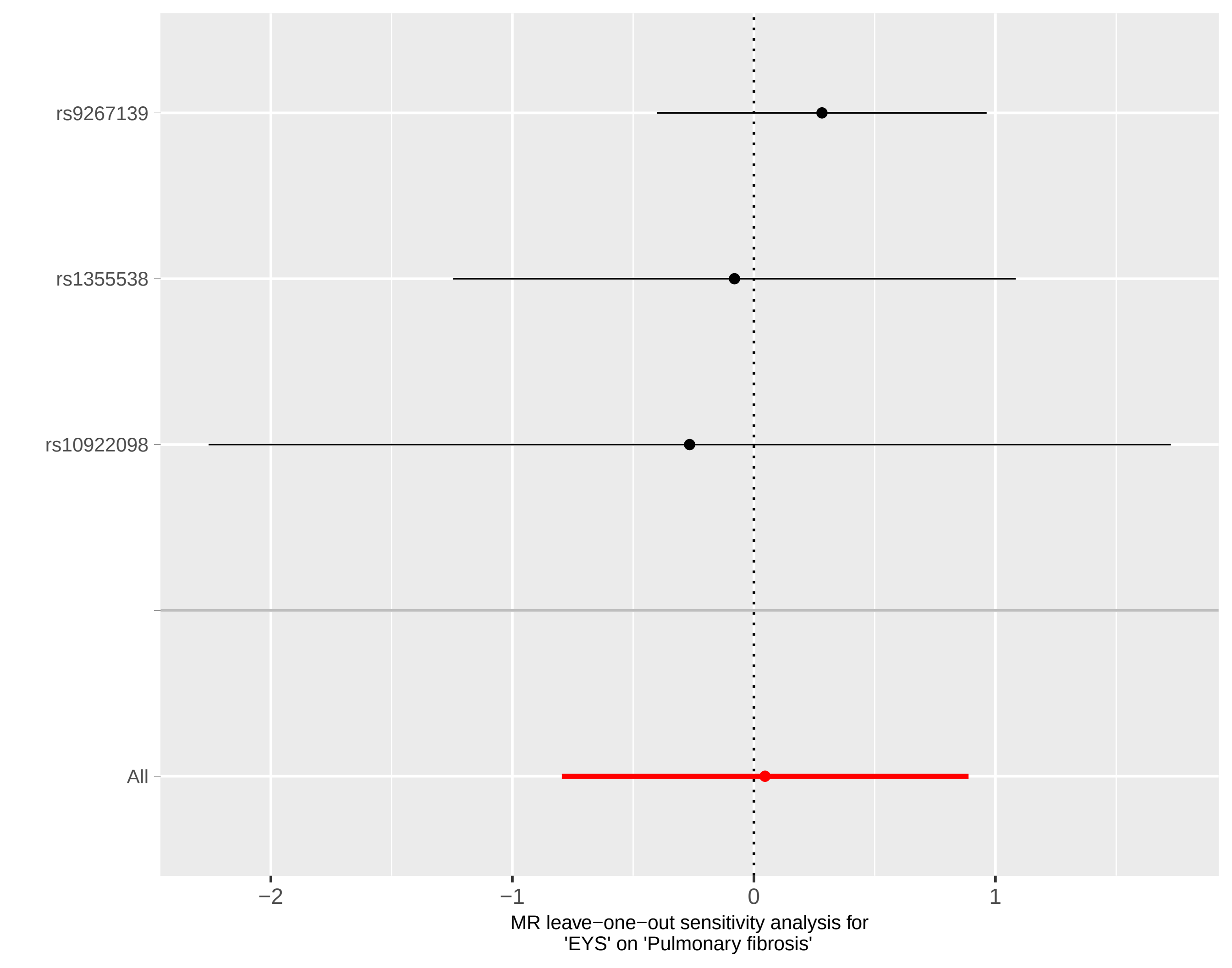
rs9267139
rs1355538
rs10922098
All
−2
−1
0
1
MR leave−one−out sensitivity analysis for
'EYS' on 'Pulmonary fibrosis'

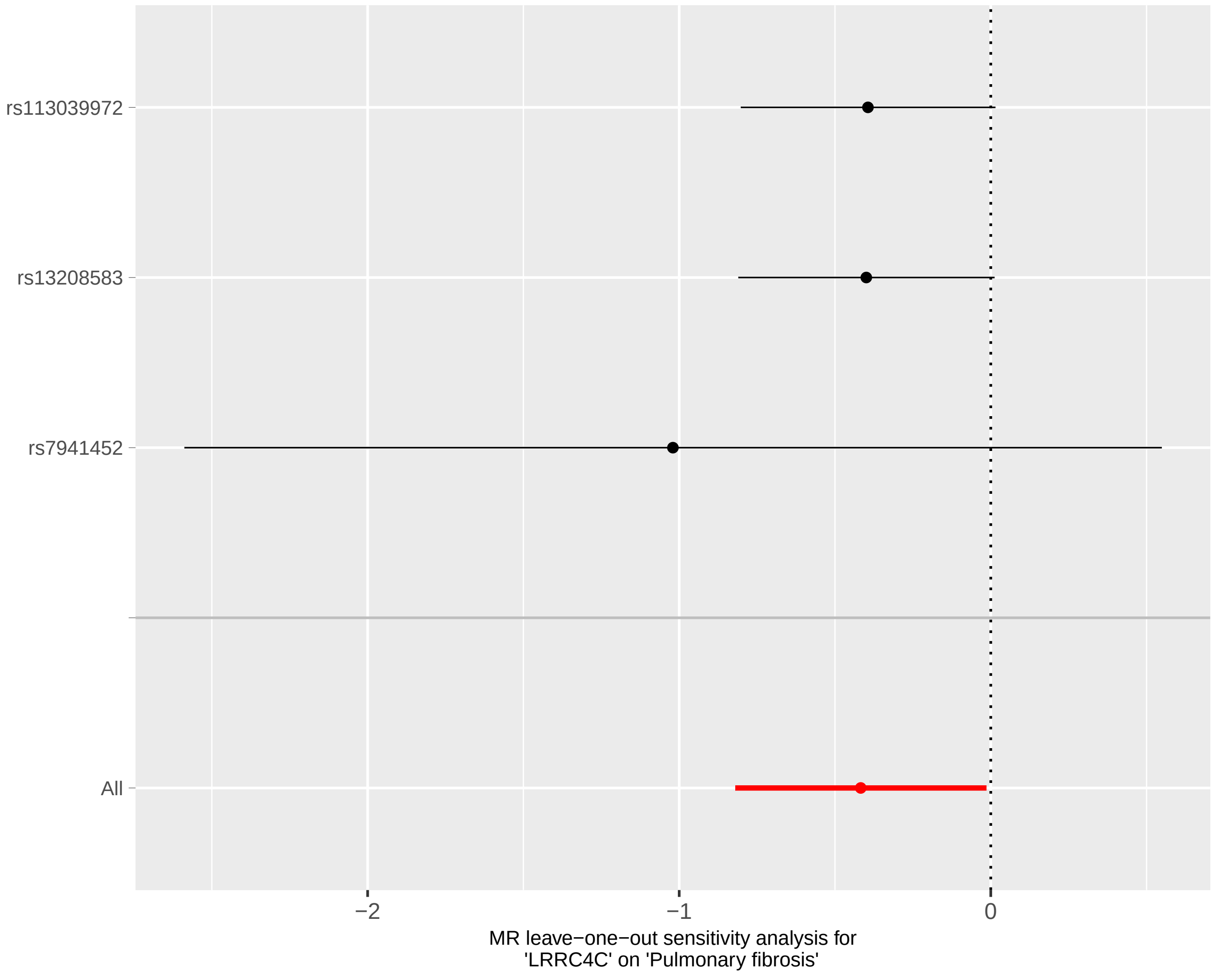
rs113039972
rs13208583
rs7941452
All
−2
−1
0
MR leave−one−out sensitivity analysis for
'LRRC4C' on 'Pulmonary fibrosis'

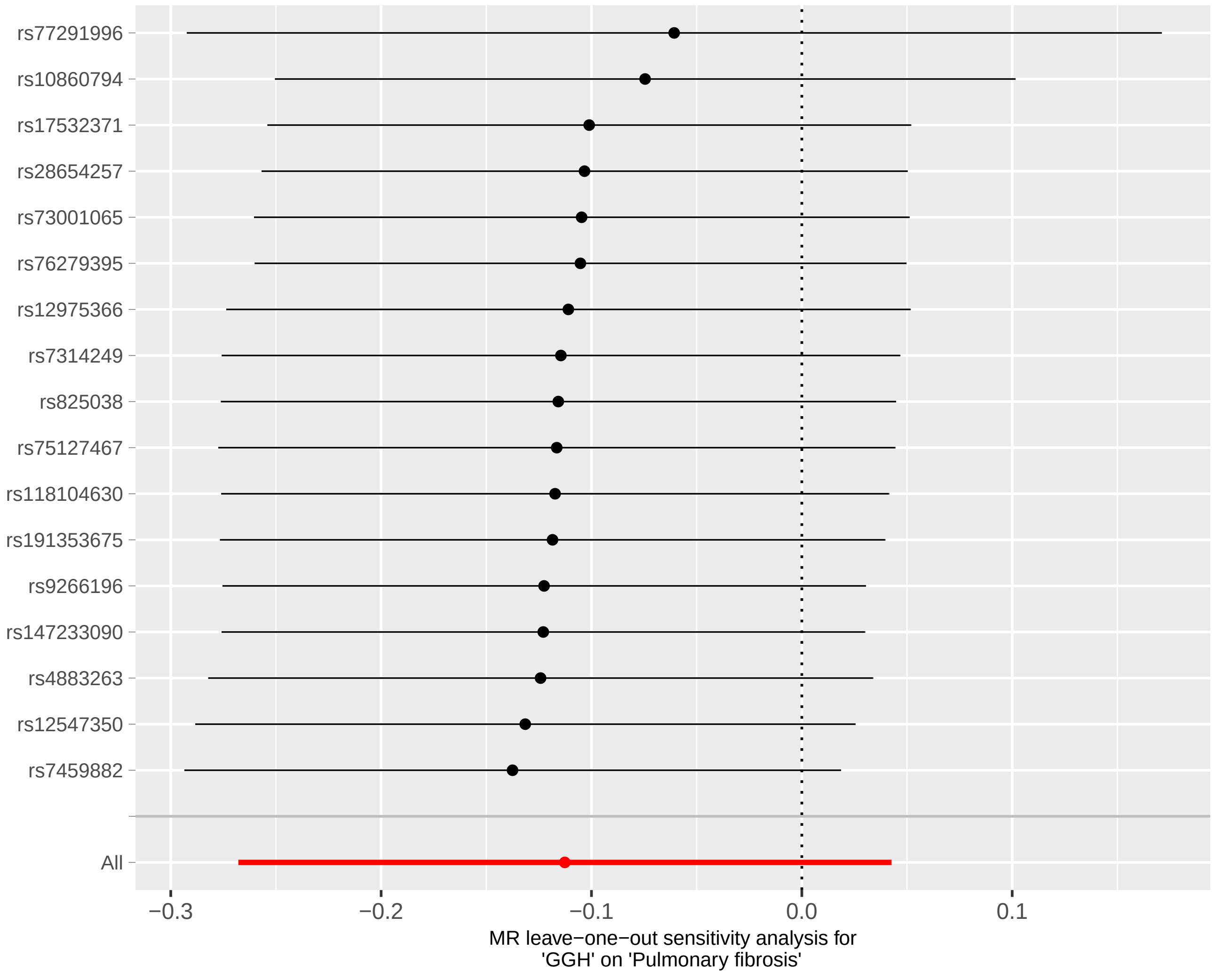

rs77291996
rs10860794
rs17532371
rs28654257
rs73001065
rs76279395
rs12975366
rs7314249
rs825038
rs75127467
rs118104630
rs191353675
rs9266196
rs147233090
rs4883263
rs12547350
rs7459882
All
−0.3
−0.2
−0.1
0.0
0.1
MR leave−one−out sensitivity analysis for
'GGH' on 'Pulmonary fibrosis'

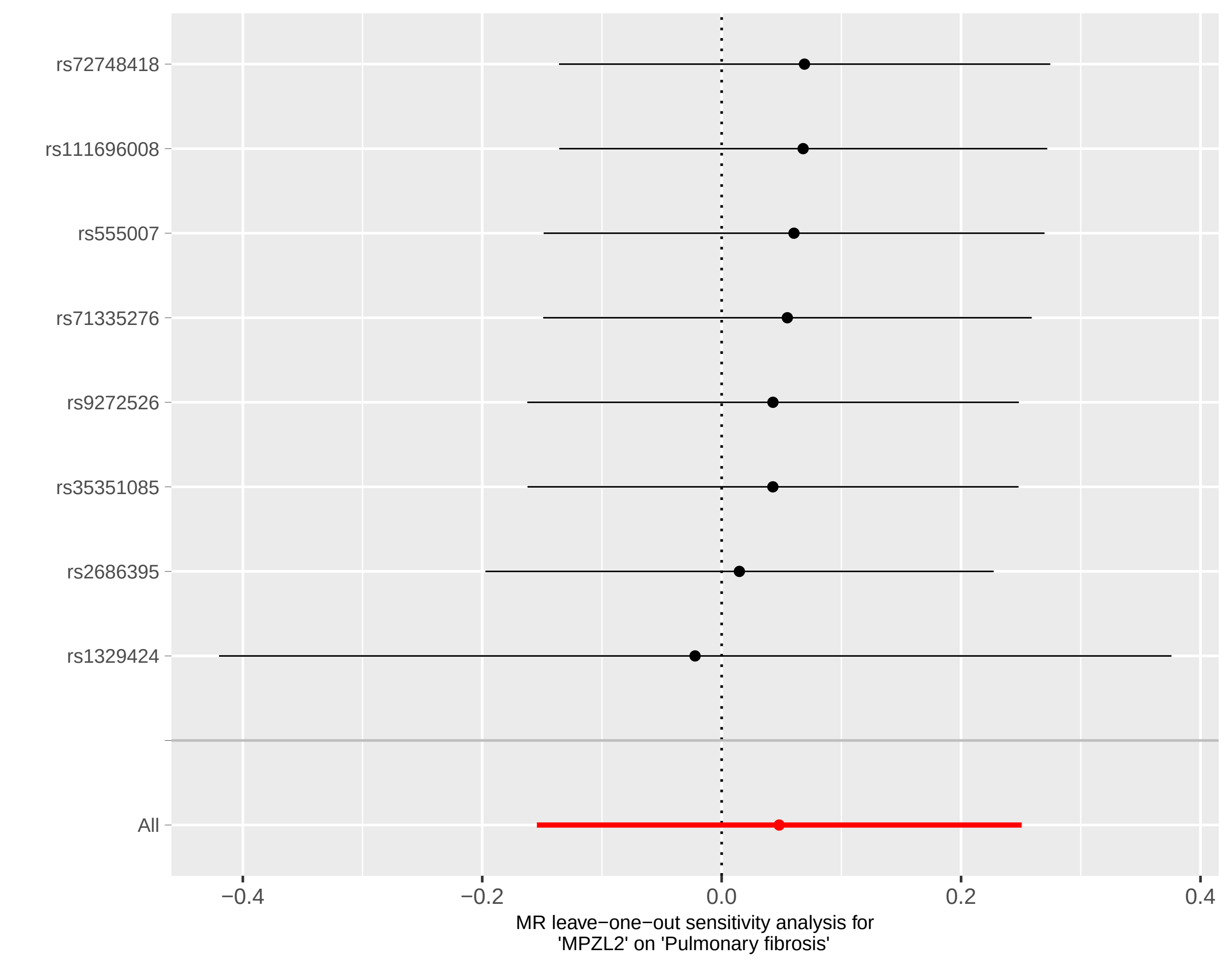
rs72748418
rs111696008
rs555007
rs71335276
rs9272526
rs35351085
rs2686395
rs1329424
All
−0.4
−0.2
0.0
0.2
0.4
MR leave−one−out sensitivity analysis for
'MPZL2' on 'Pulmonary fibrosis'

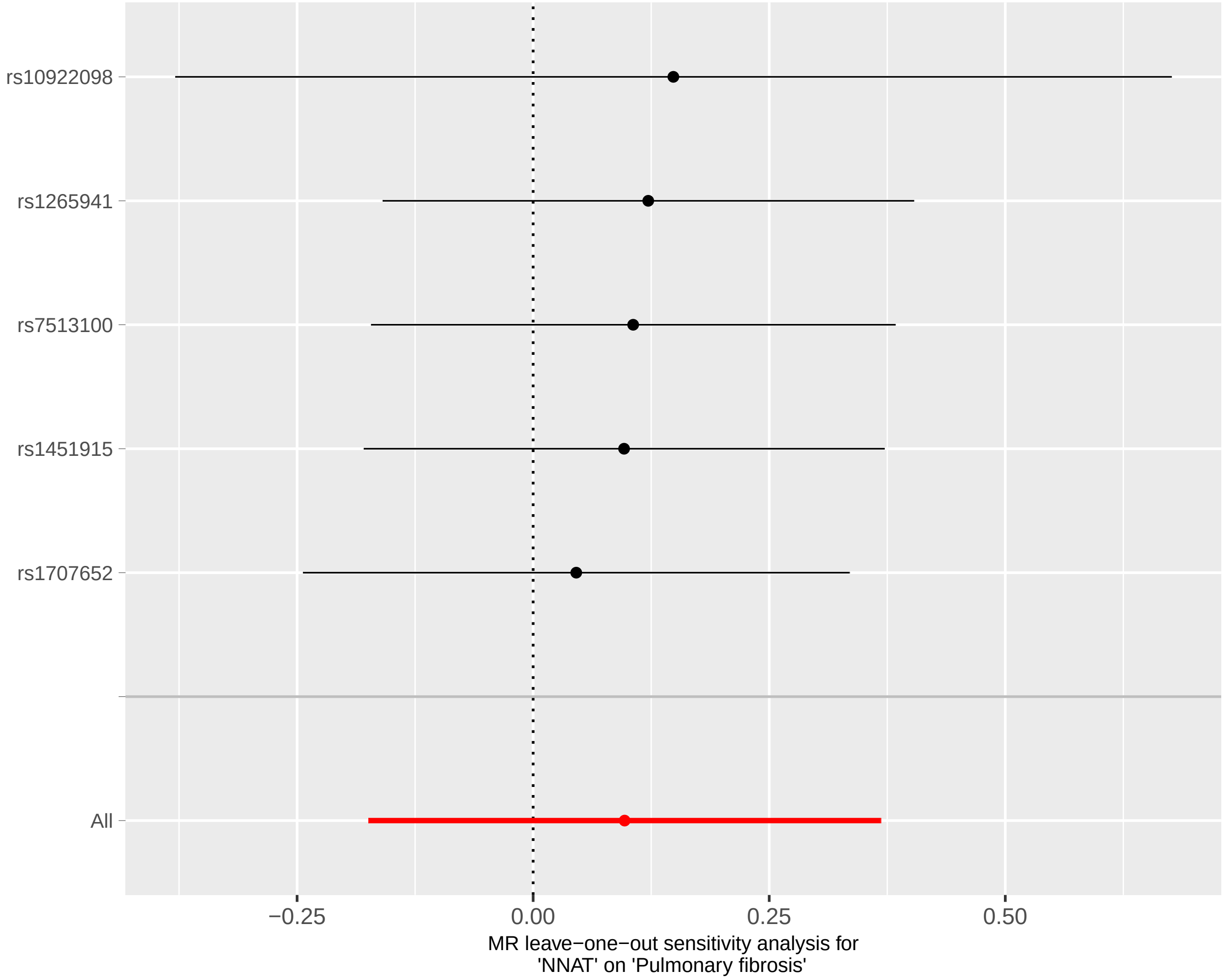
rs10922098
rs1265941
rs7513100
rs1451915
rs1707652
All
−0.25
0.00
0.25
0.50
MR leave−one−out sensitivity analysis for
'NNAT' on 'Pulmonary fibrosis'

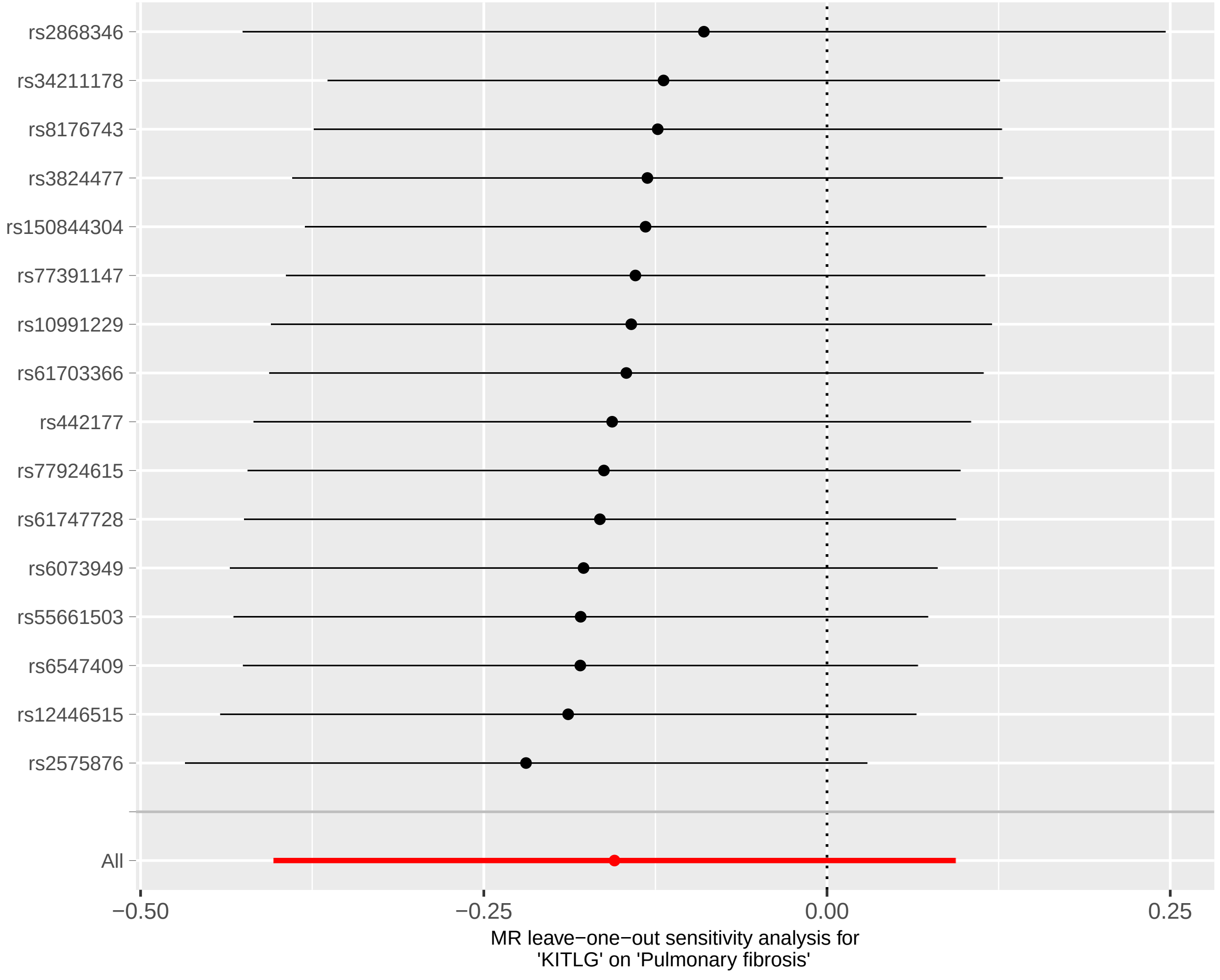

rs2868346
rs34211178
rs8176743
rs3824477
rs150844304
rs77391147
rs10991229
rs61703366
rs442177
rs77924615
rs61747728
rs6073949
rs55661503
rs6547409
rs12446515
rs2575876
All
−0.50
−0.25
0.00
0.25
MR leave−one−out sensitivity analysis for
'KITLG' on 'Pulmonary fibrosis'

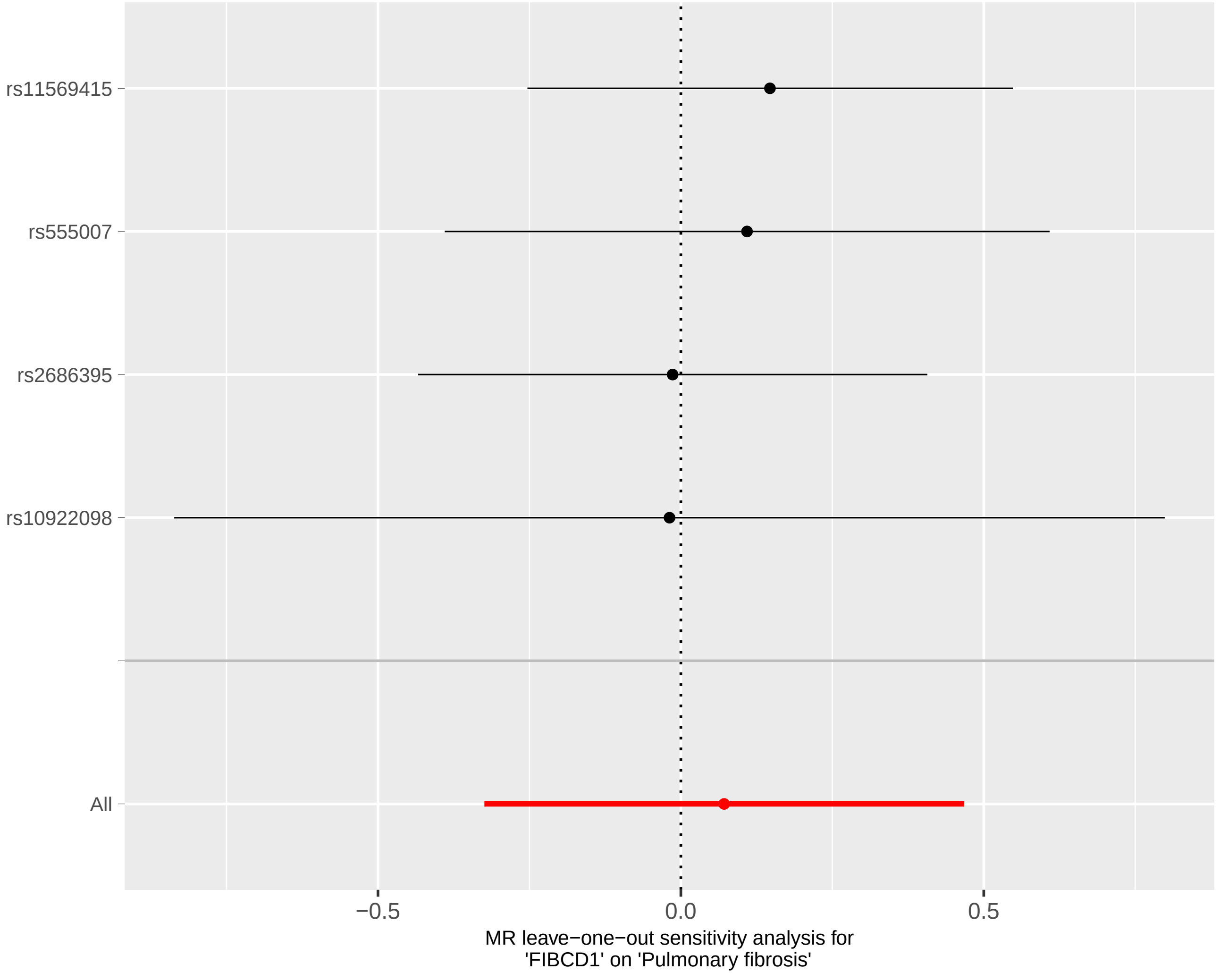
rs11569415
rs555007
rs2686395
rs10922098
All
−0.5
0.0
0.5
MR leave−one−out sensitivity analysis for
'FIBCD1' on 'Pulmonary fibrosis'

Insufficient number of SNPs

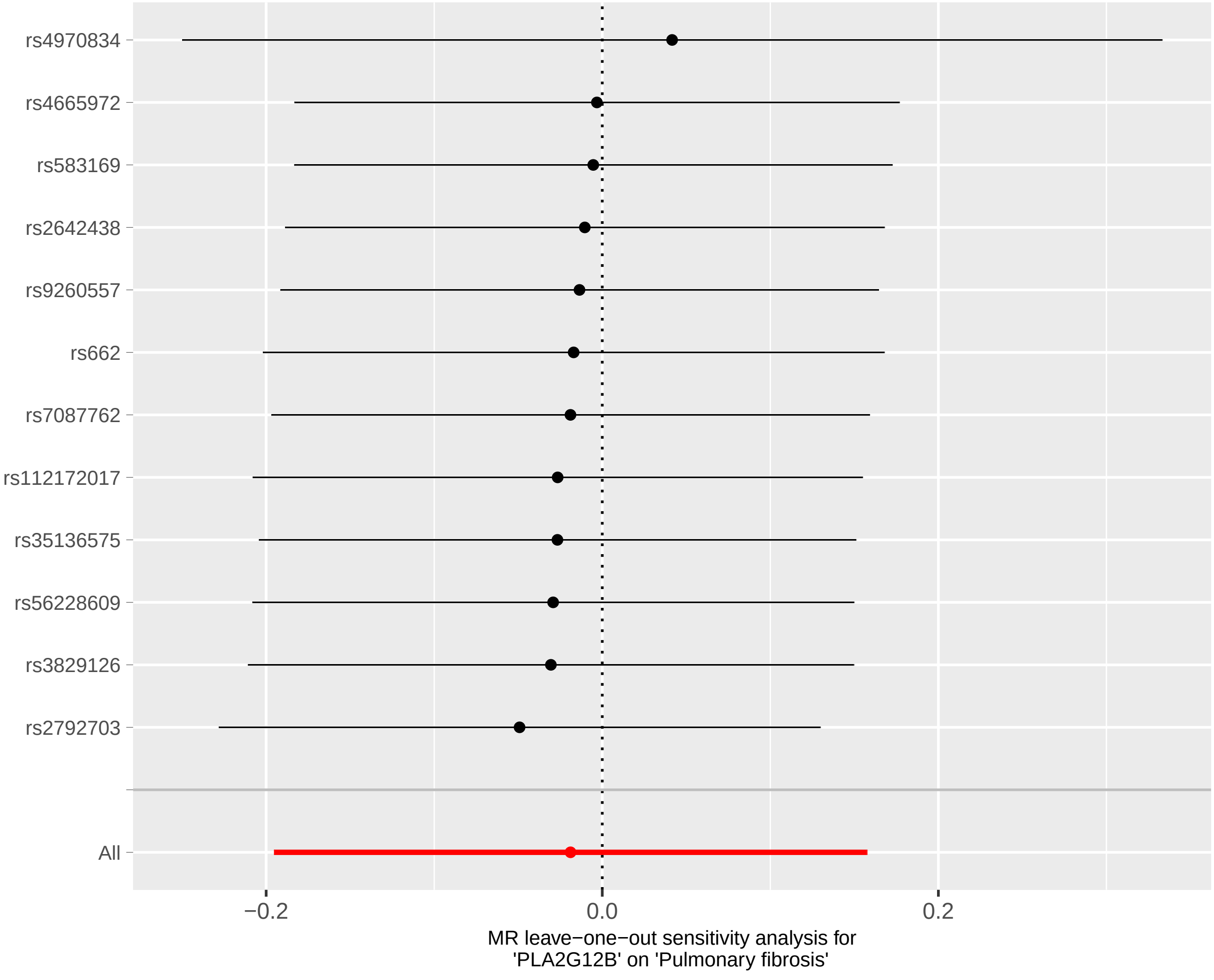
rs4970834
rs4665972
rs583169
rs2642438
rs9260557
rs662
rs7087762
rs112172017
rs35136575
rs56228609
rs3829126
rs2792703
All
−0.2
0.0
0.2
MR leave−one−out sensitivity analysis for
'PLA2G12B' on 'Pulmonary fibrosis'

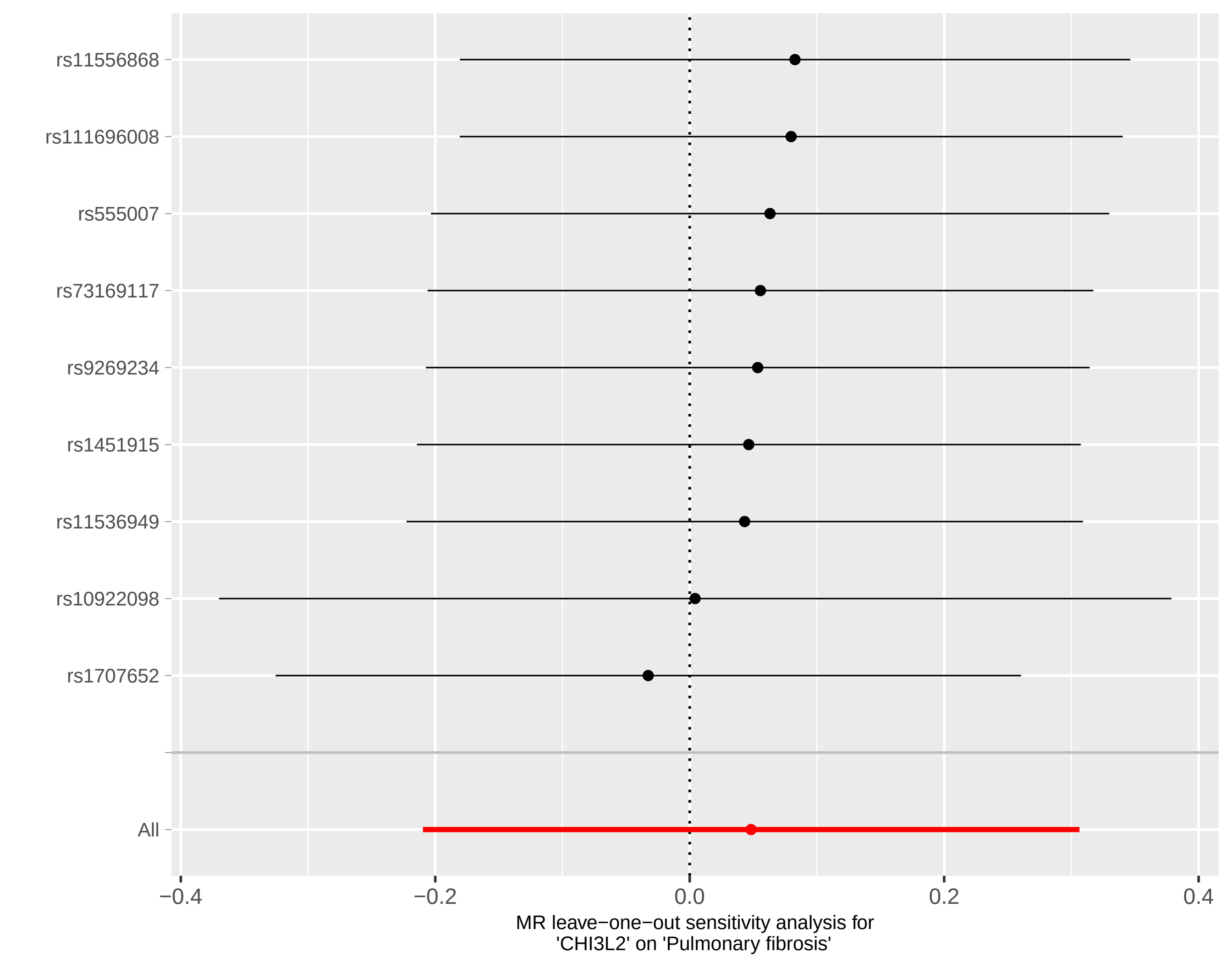
rs11556868
rs111696008
rs555007
rs73169117
rs9269234
rs1451915
rs11536949
rs10922098
rs1707652
All
−0.4
−0.2
0.0
0.2
0.4
MR leave−one−out sensitivity analysis for
'CHI3L2' on 'Pulmonary fibrosis'

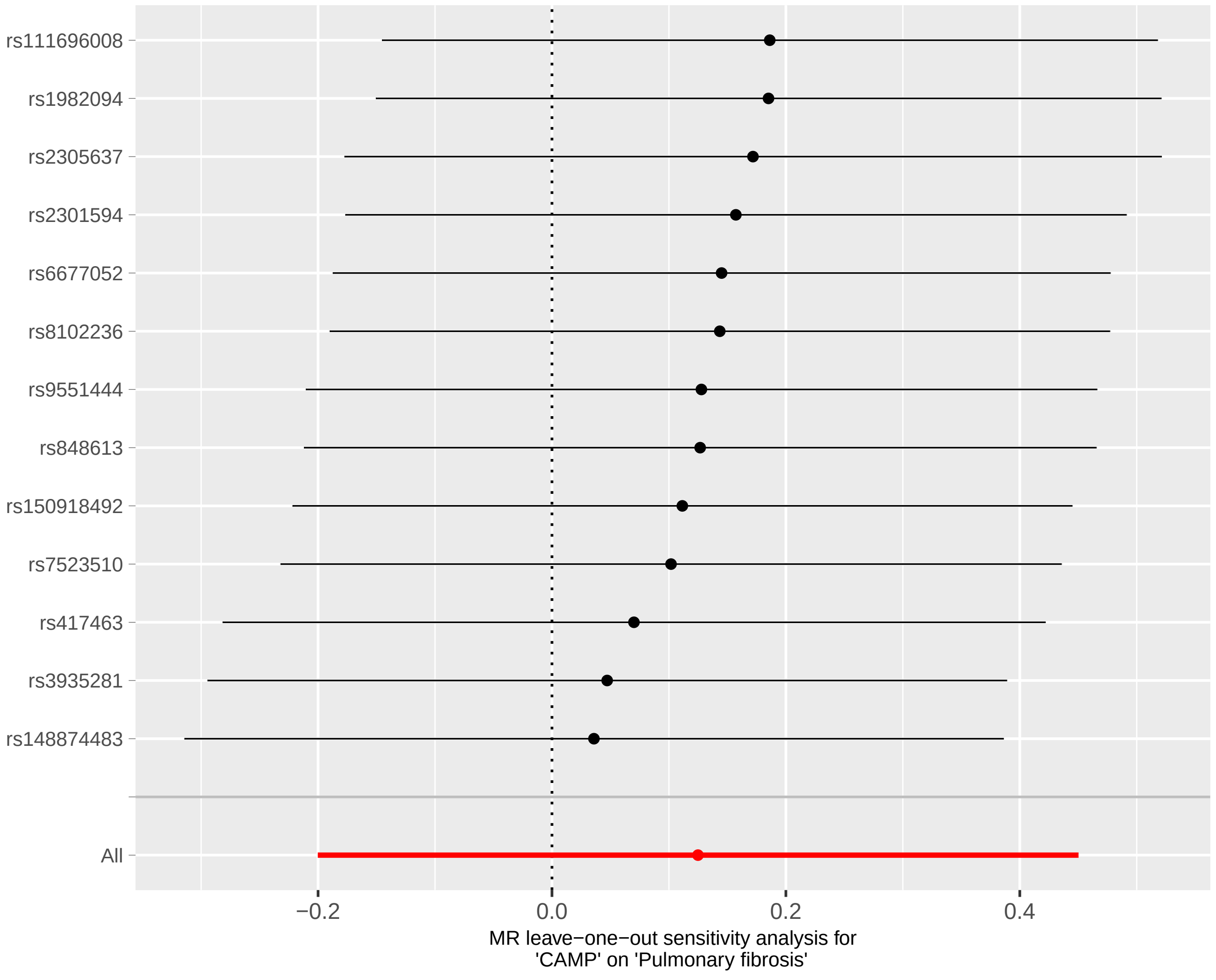
rs111696008
rs1982094
rs2305637
rs2301594
rs6677052
rs8102236
rs9551444
rs848613
rs150918492
rs7523510
rs417463
rs3935281
rs148874483
All
−0.2
0.0
0.2
0.4
MR leave−one−out sensitivity analysis for
'CAMP' on 'Pulmonary fibrosis'

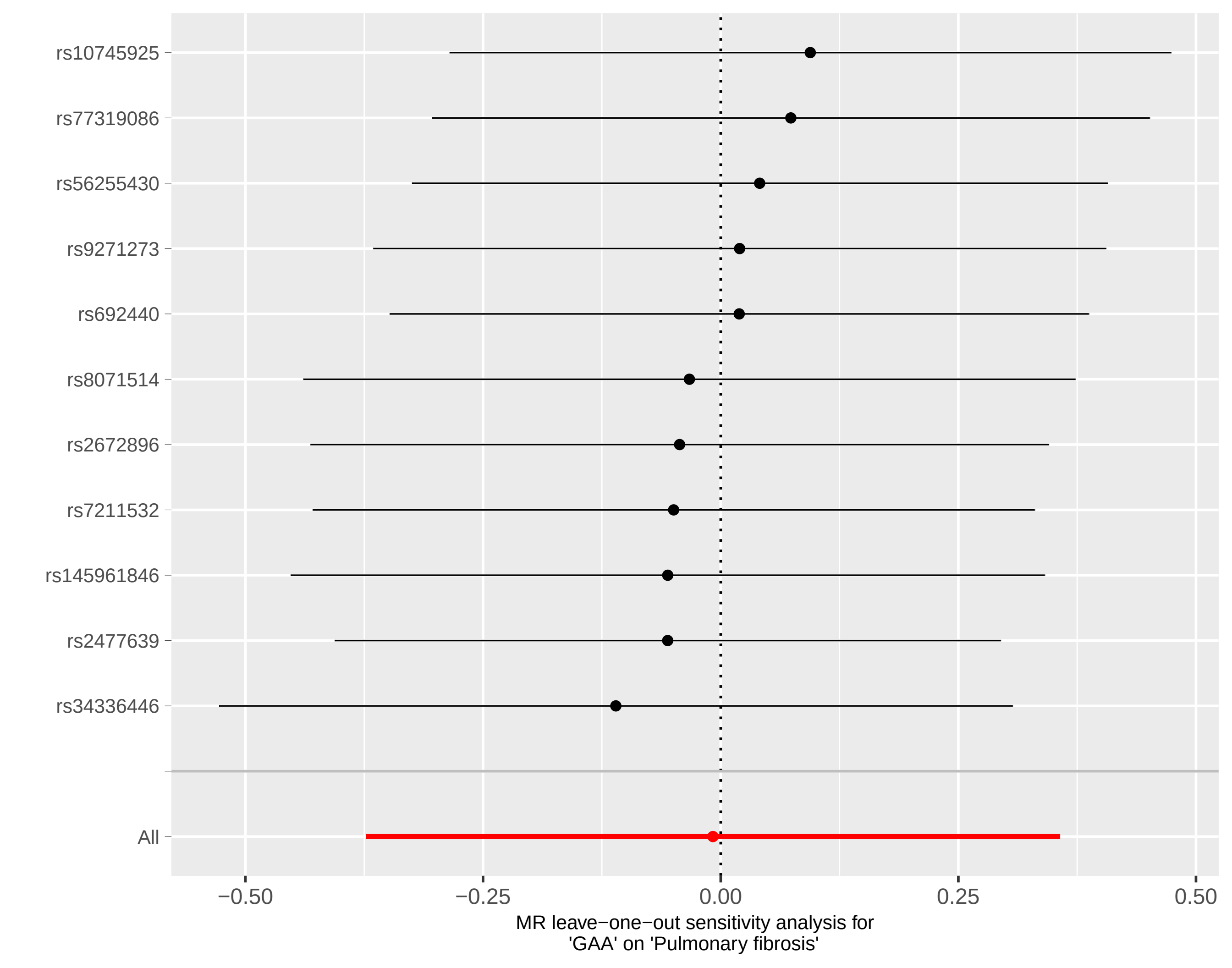
rs10745925
rs77319086
rs56255430
rs9271273
rs692440
rs8071514
rs2672896
rs7211532
rs145961846
rs2477639
rs34336446
All
−0.50
−0.25
0.00
0.25
0.50
MR leave−one−out sensitivity analysis for
'GAA' on 'Pulmonary fibrosis'

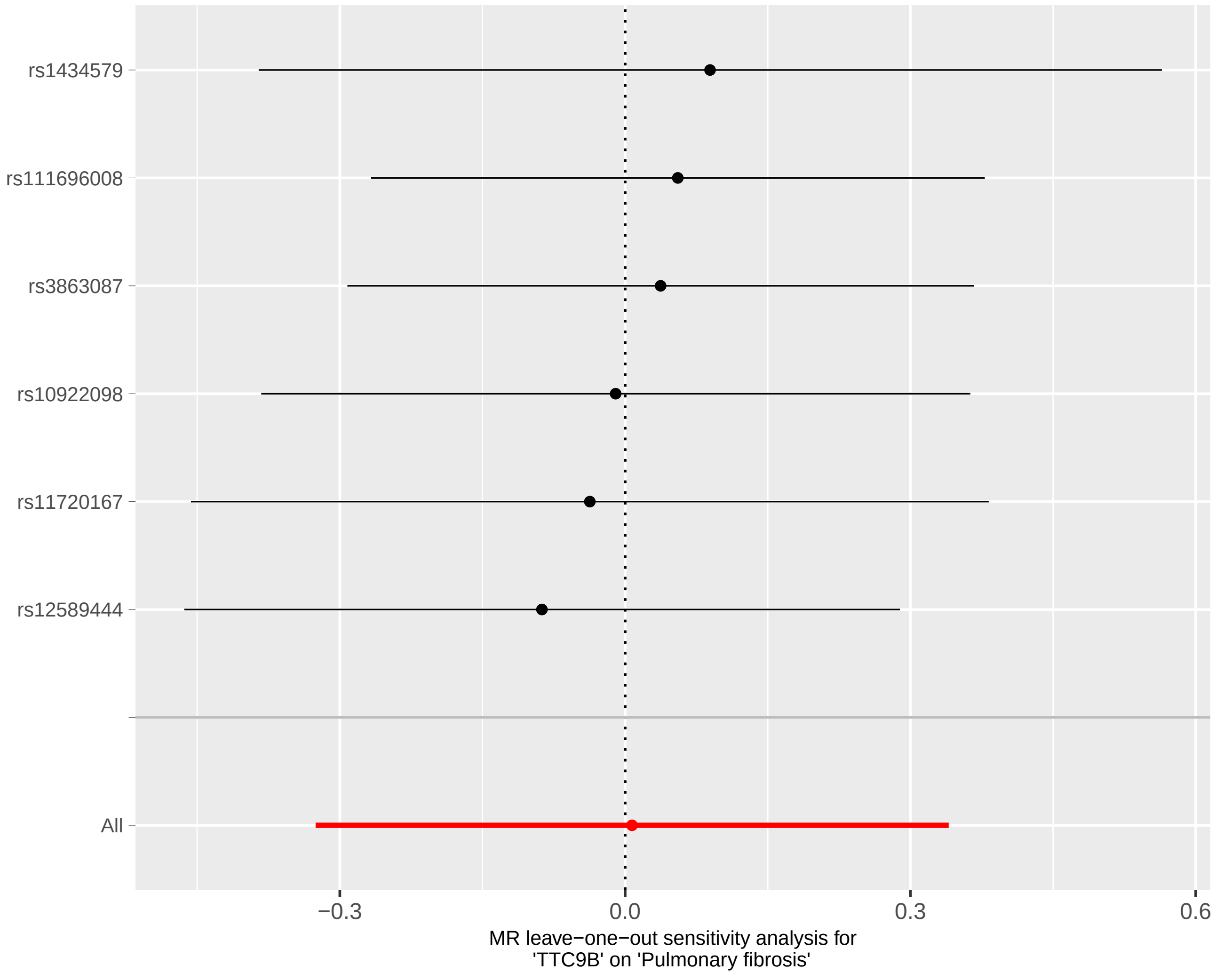
rs1434579
rs111696008
rs3863087
rs10922098
rs11720167
rs12589444
All
−0.3
0.0
0.3
0.6
MR leave−one−out sensitivity analysis for
'TTC9B' on 'Pulmonary fibrosis'

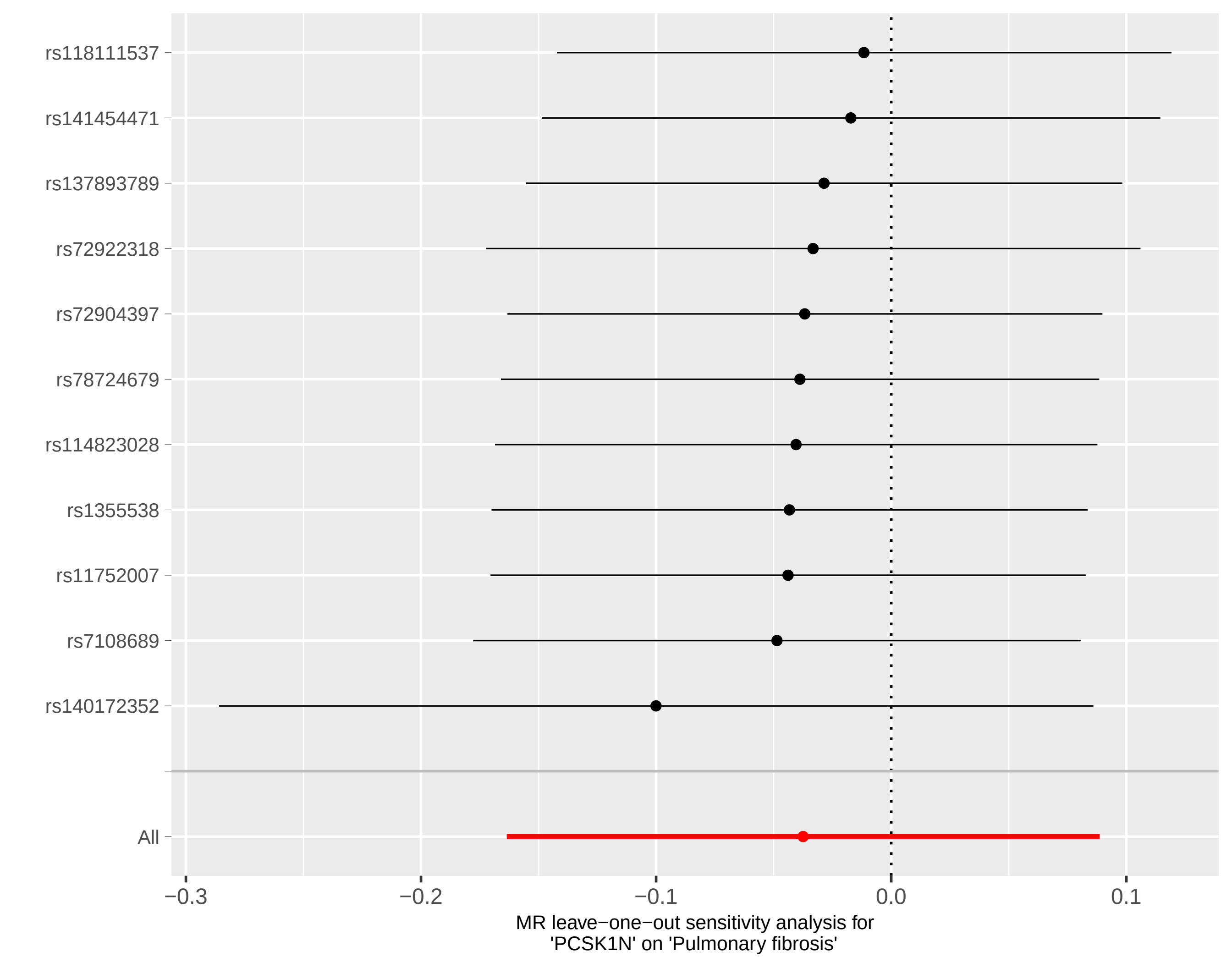
rs118111537
rs141454471
rs137893789
rs72922318
rs72904397
rs78724679
rs114823028
rs1355538
rs11752007
rs7108689
rs140172352
All
−0.3
−0.2
−0.1
0.0
0.1
MR leave−one−out sensitivity analysis for
'PCSK1N' on 'Pulmonary fibrosis'

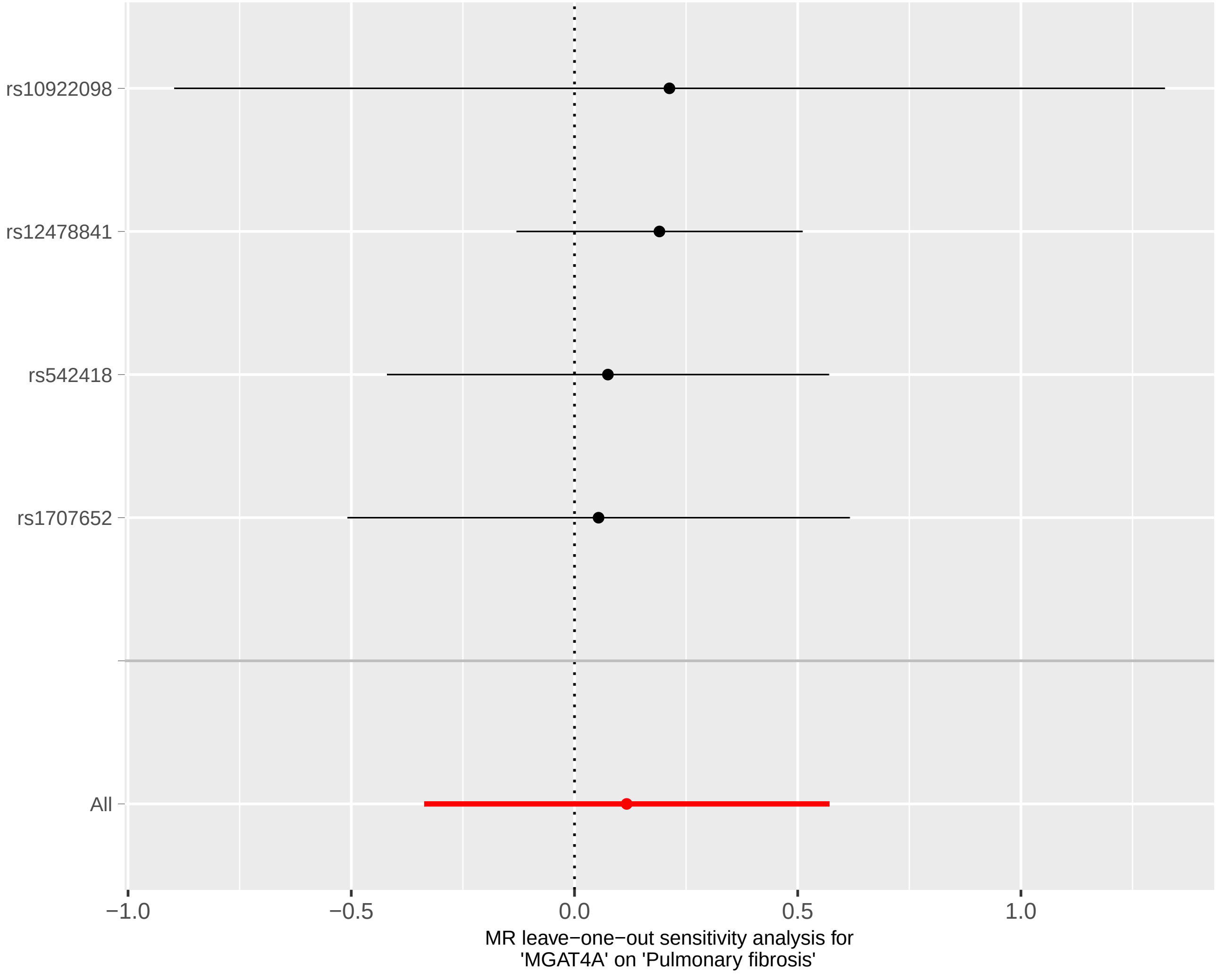
rs10922098
rs12478841
rs542418
rs1707652
All
−1.0
−0.5
0.0
0.5
1.0
MR leave−one−out sensitivity analysis for
'MGAT4A' on 'Pulmonary fibrosis'

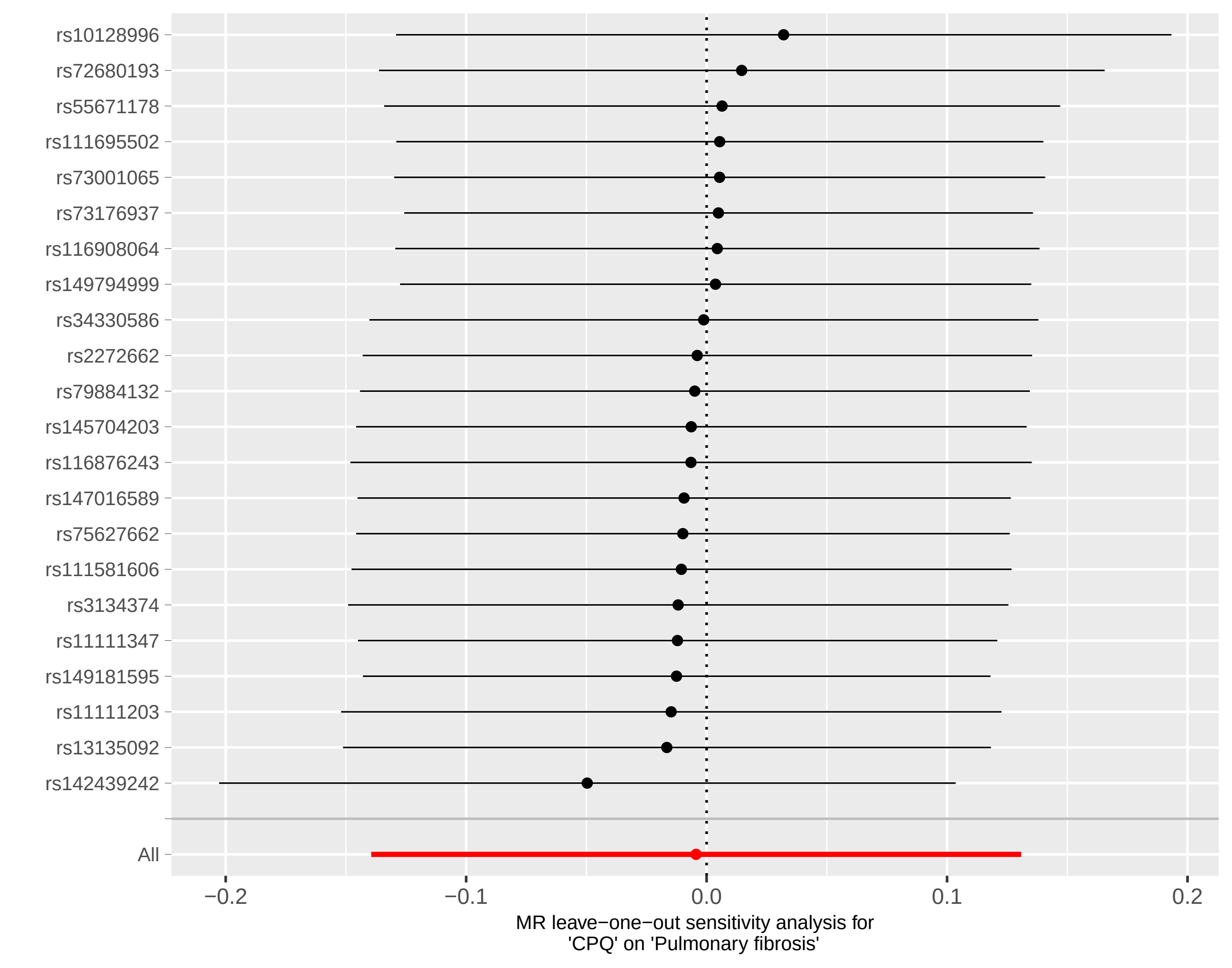

rs10128996
rs72680193
rs55671178
rs111695502
rs73001065
rs73176937
rs116908064
rs149794999
rs34330586
rs2272662
rs79884132
rs145704203
rs116876243
rs147016589
rs75627662
rs111581606
rs3134374
rs11111347
rs149181595
rs11111203
rs13135092
rs142439242
All
−0.2
−0.1
0.0
0.1
0.2
MR leave−one−out sensitivity analysis for
'CPQ' on 'Pulmonary fibrosis'

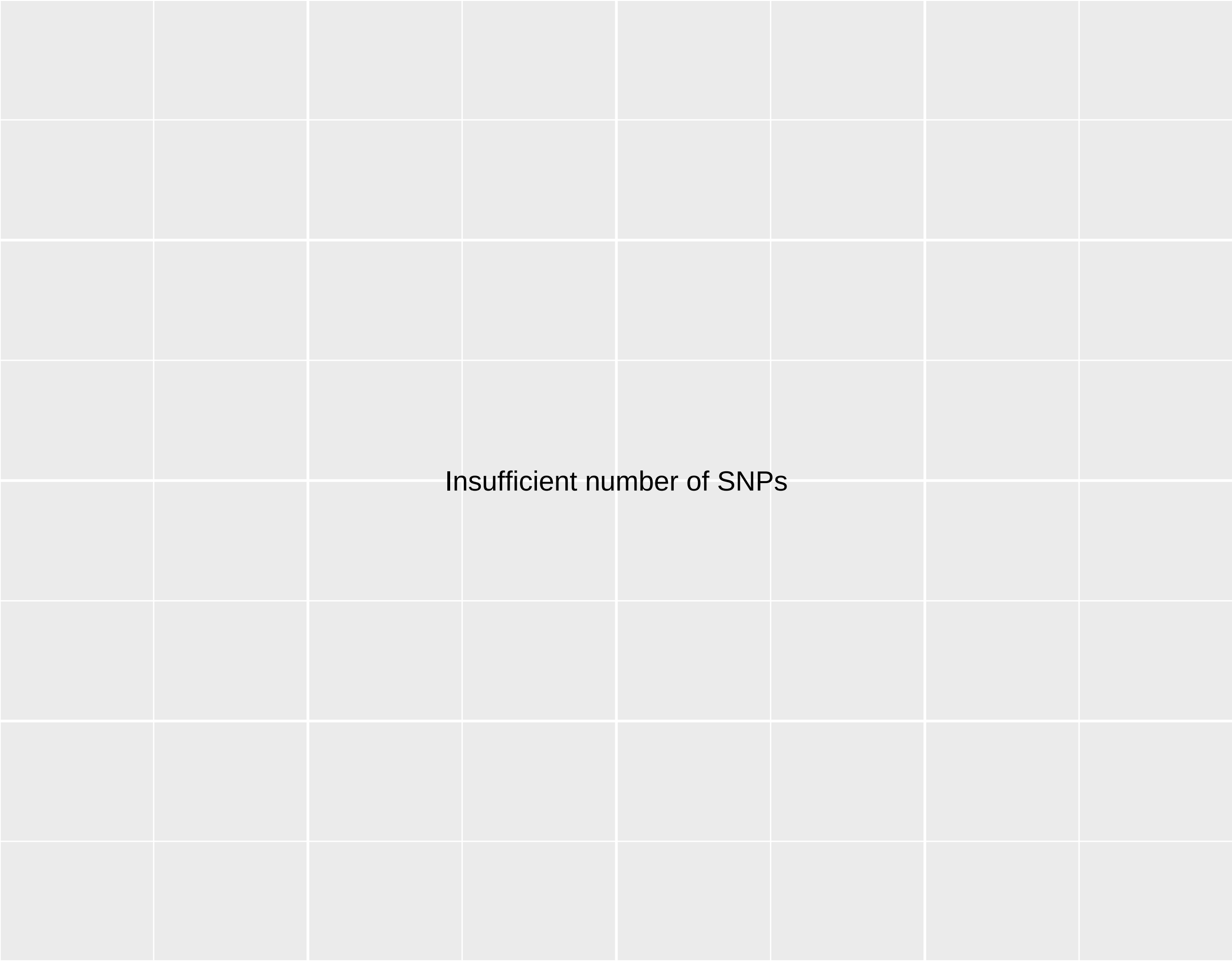
Insufficient number of SNPs

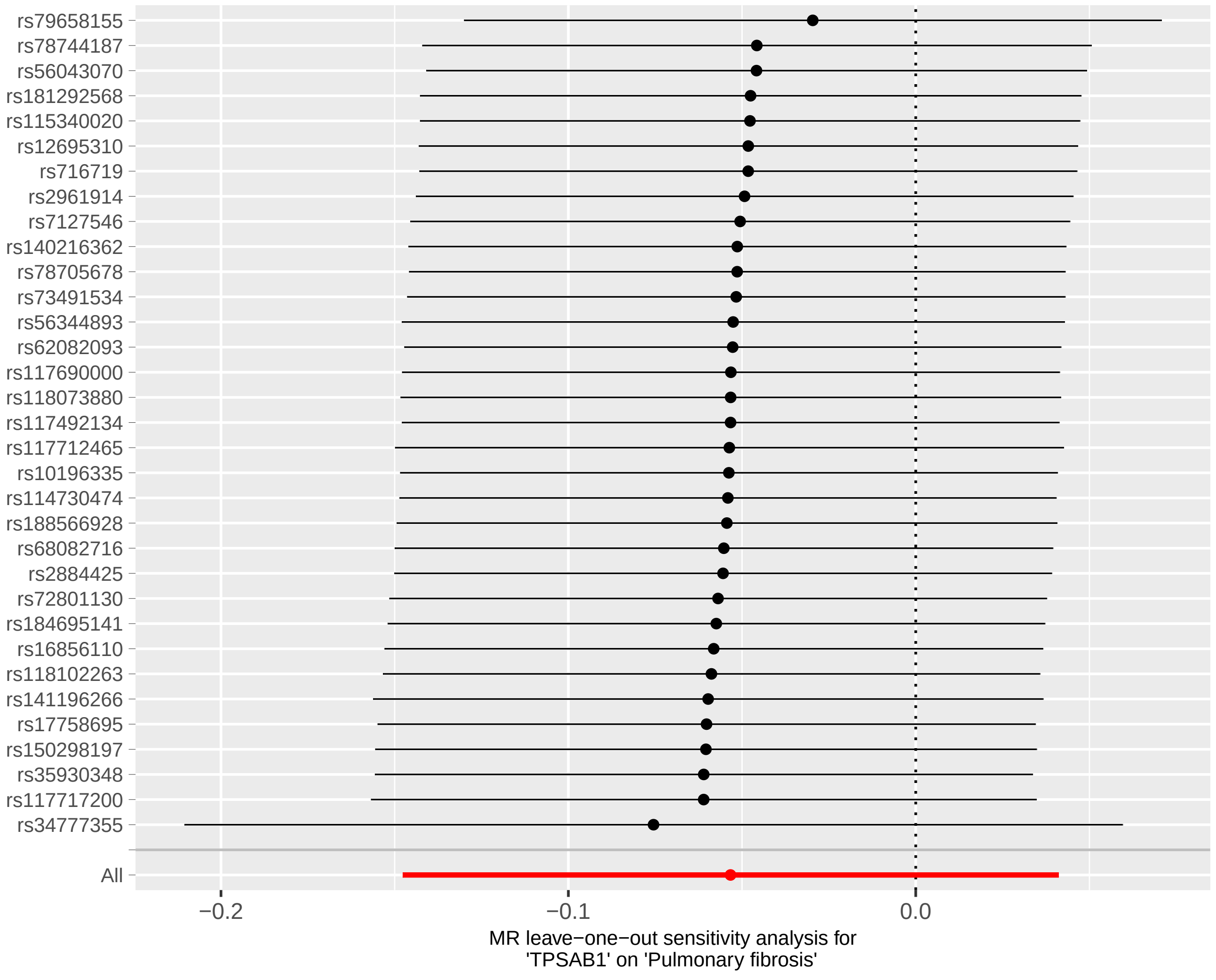

rs79658155
rs78744187
rs56043070
rs181292568
rs115340020
rs12695310
rs716719
rs2961914
rs7127546
rs140216362
rs78705678
rs73491534
rs56344893
rs62082093
rs117690000
rs118073880
rs117492134
rs117712465
rs10196335
rs114730474
rs188566928
rs68082716
rs2884425
rs72801130
rs184695141
rs16856110
rs118102263
rs141196266
rs17758695
rs150298197
rs35930348
rs117717200
rs34777355
All
−0.2
−0.1
0.0
MR leave−one−out sensitivity analysis for
'TPSAB1' on 'Pulmonary fibrosis'

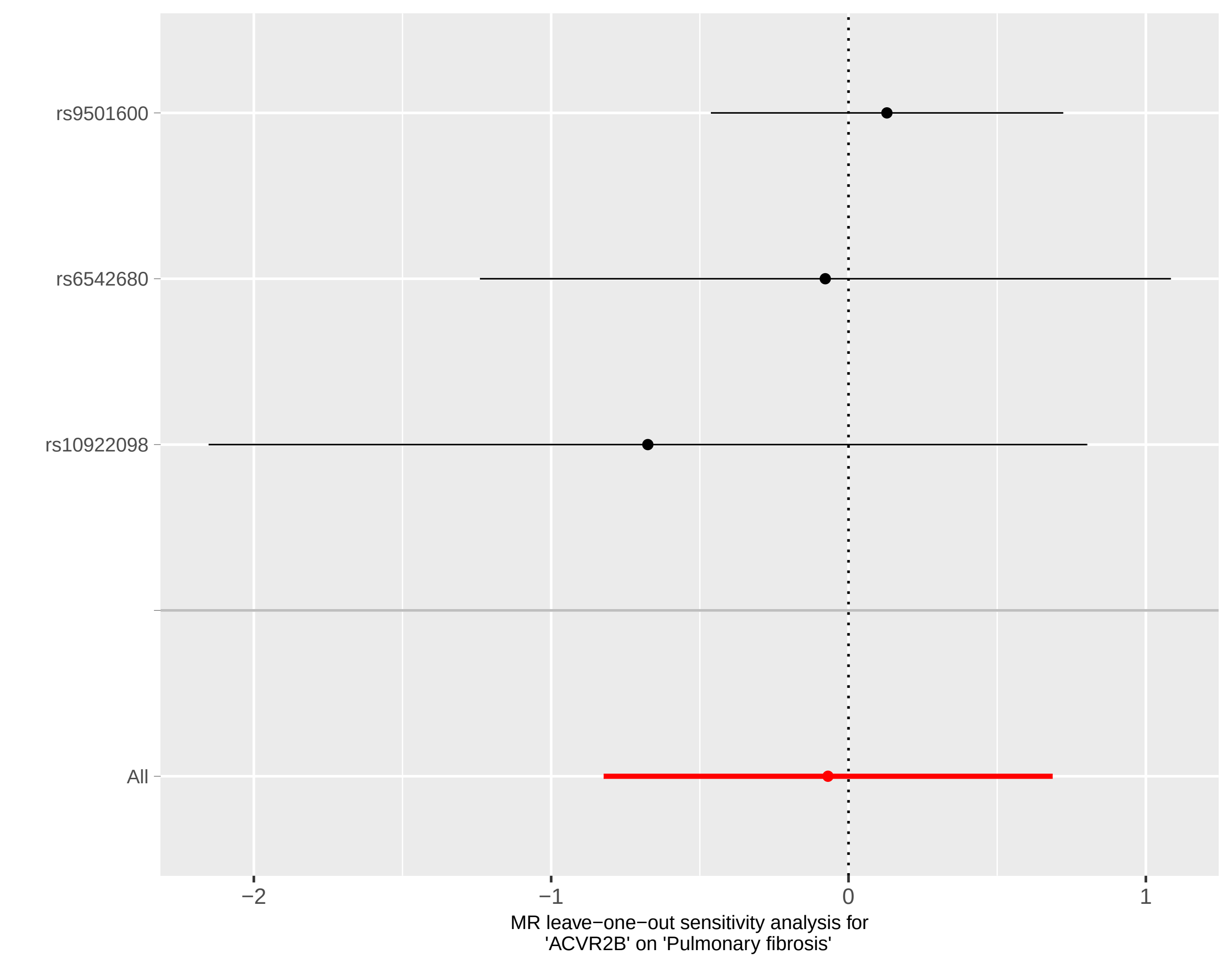
rs9501600
rs6542680
rs10922098
All
−2
−1
0
1
MR leave−one−out sensitivity analysis for
'ACVR2B' on 'Pulmonary fibrosis'

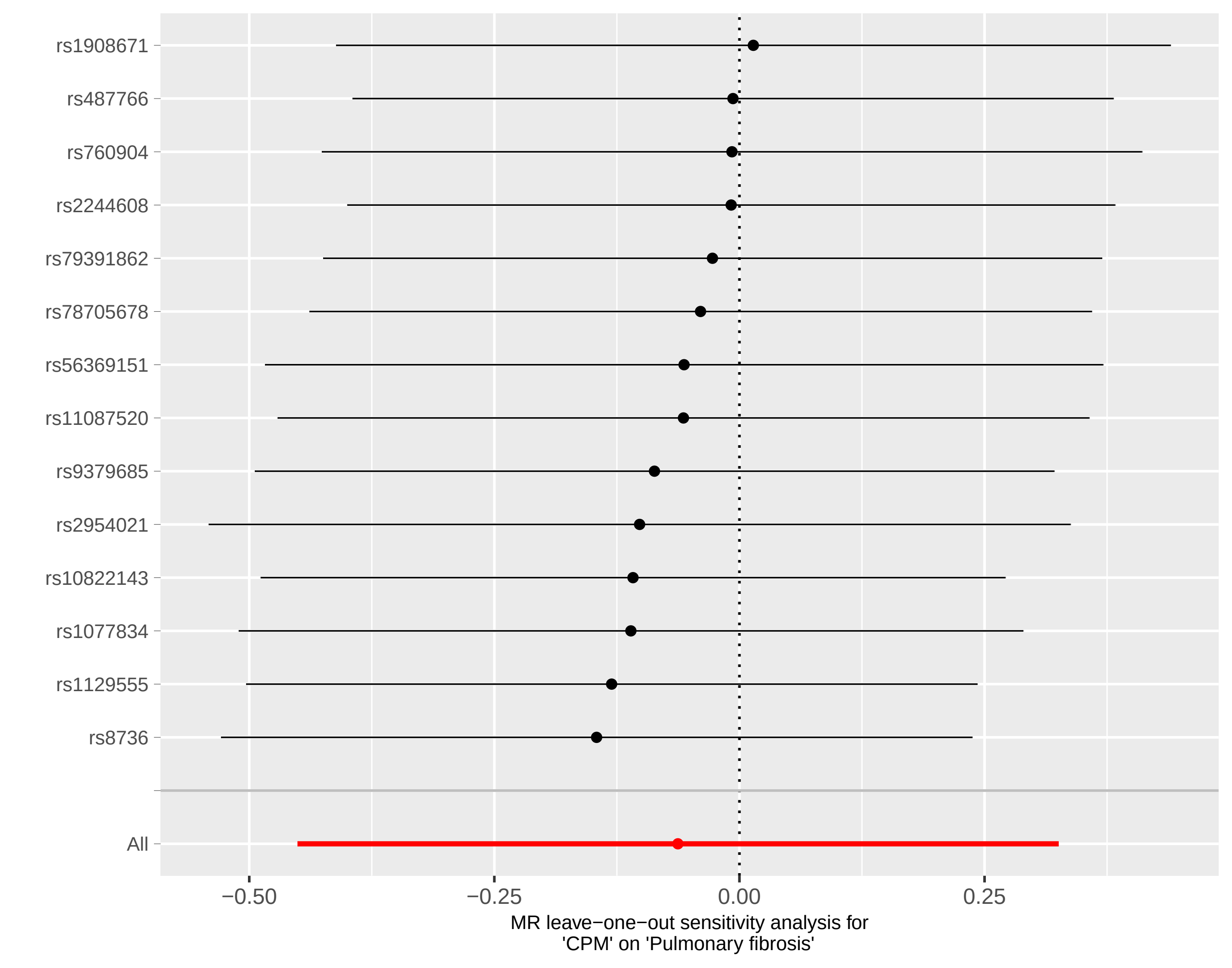
rs1908671
rs487766
rs760904
rs2244608
rs79391862
rs78705678
rs56369151
rs11087520
rs9379685
rs2954021
rs10822143
rs1077834
rs1129555
rs8736
All
−0.50
−0.25
0.00
0.25
MR leave−one−out sensitivity analysis for
'CPM' on 'Pulmonary fibrosis'

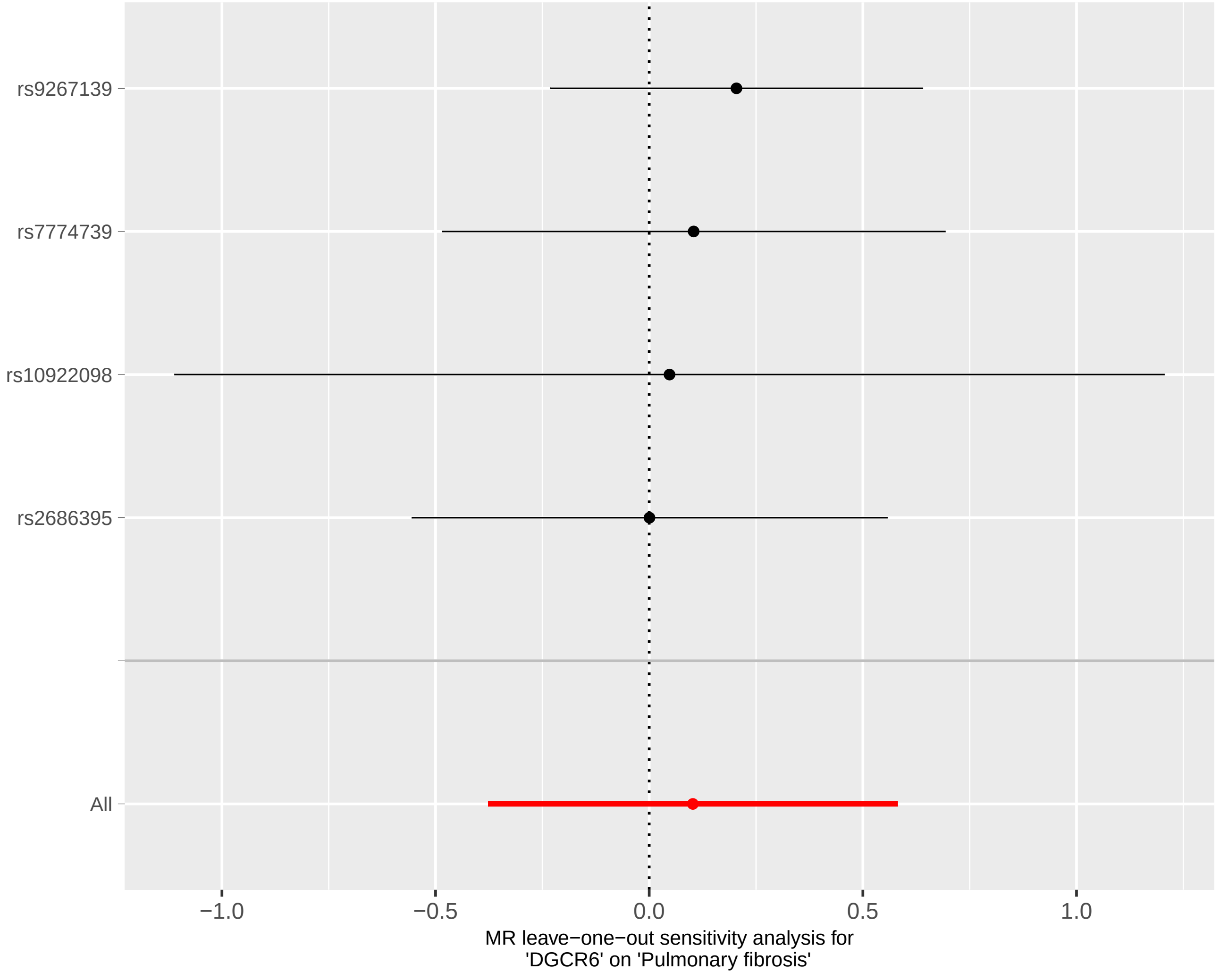

rs9267139
rs7774739
rs10922098
rs2686395
All
−1.0
−0.5
0.0
0.5
1.0
MR leave−one−out sensitivity analysis for
'DGCR6' on 'Pulmonary fibrosis'

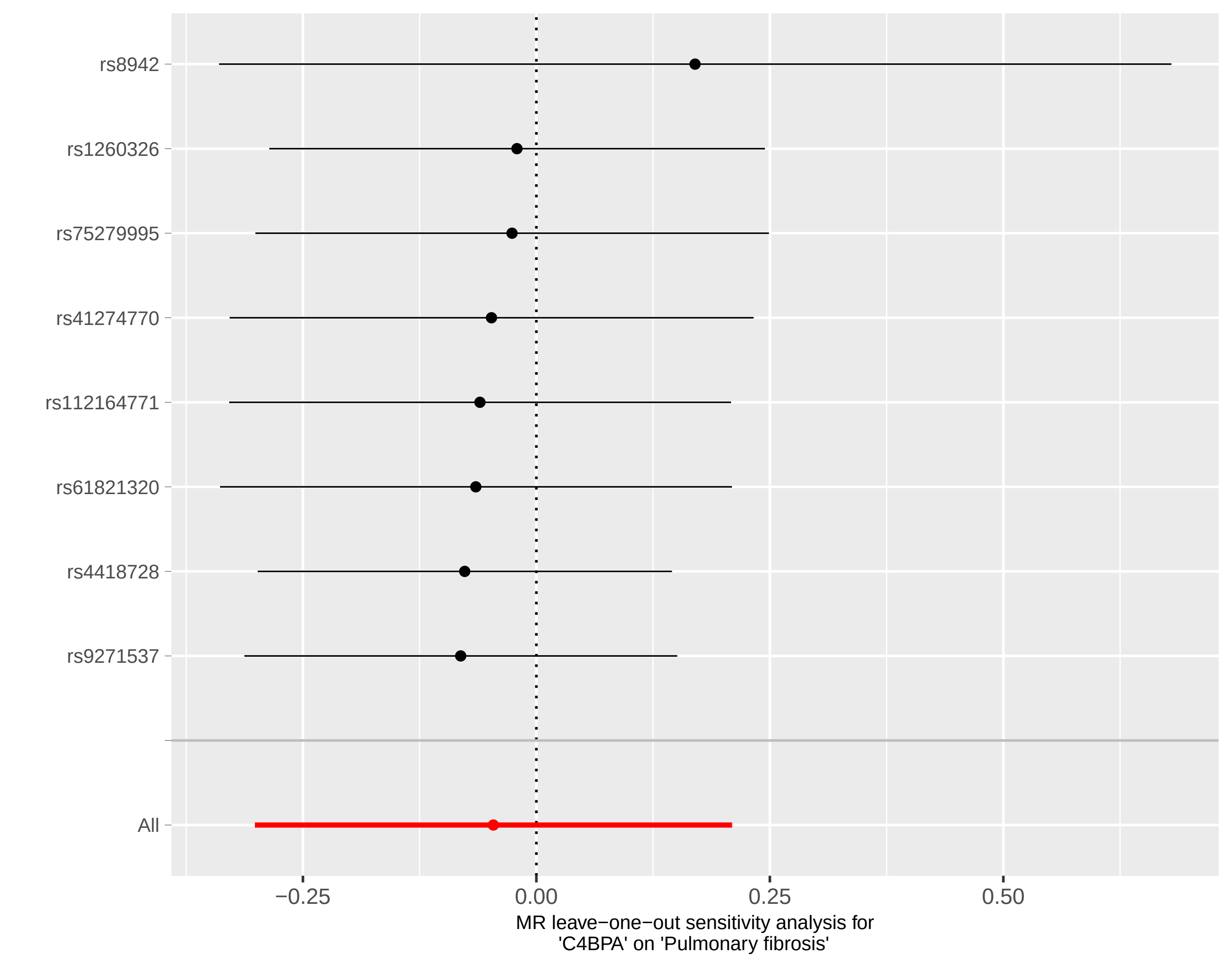
rs8942
rs1260326
rs75279995
rs41274770
rs112164771
rs61821320
rs4418728
rs9271537
All
−0.25
0.00
0.25
0.50
MR leave−one−out sensitivity analysis for
'C4BPA' on 'Pulmonary fibrosis'

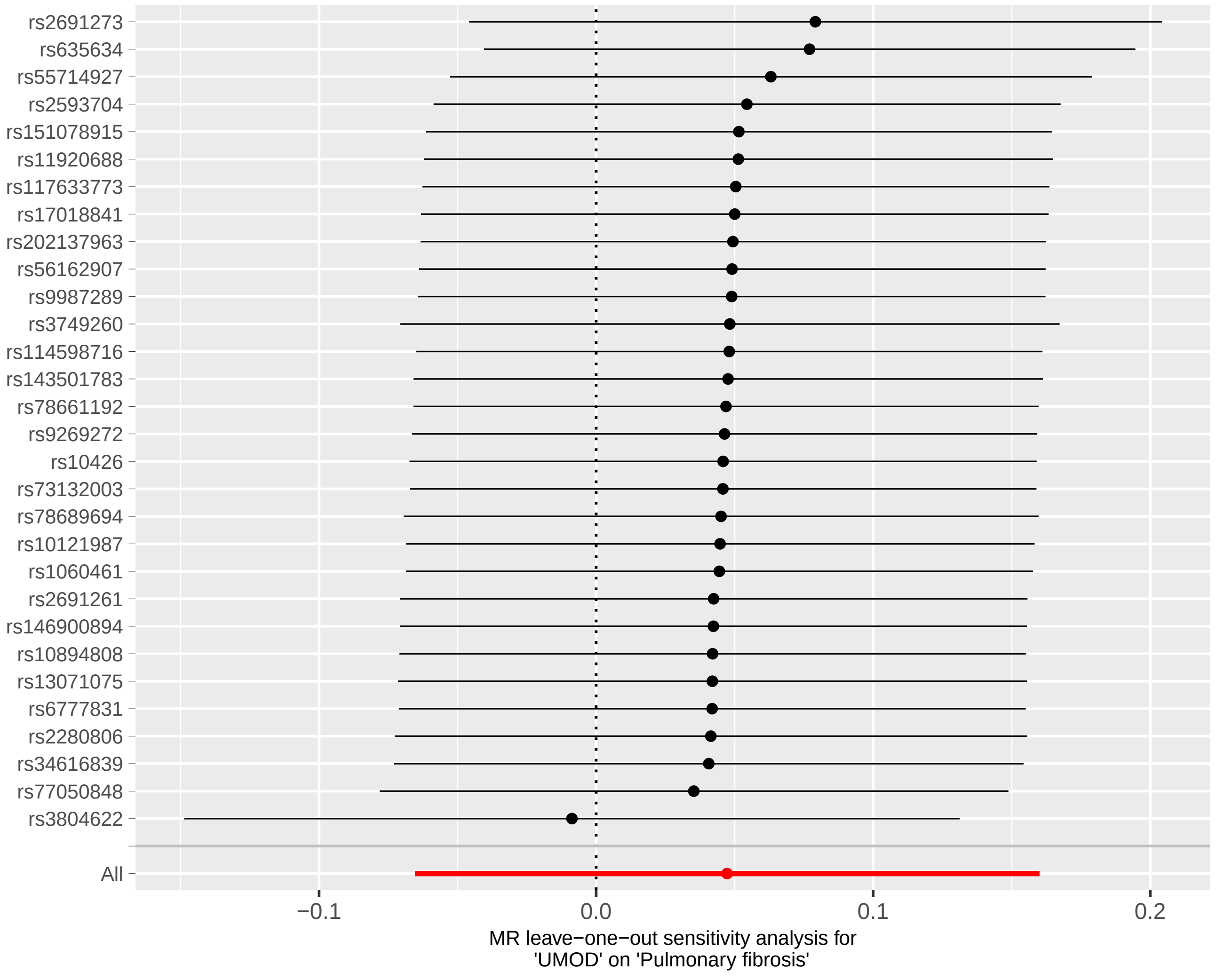
rs2691273
rs635634
rs55714927
rs2593704
rs151078915
rs11920688
rs117633773
rs17018841
rs202137963
rs56162907
rs9987289
rs3749260
rs114598716
rs143501783
rs78661192
rs9269272
rs10426
rs73132003
rs78689694
rs10121987
rs1060461
rs2691261
rs146900894
rs10894808
rs13071075
rs6777831
rs2280806
rs34616839
rs77050848
rs3804622
All
−0.1
0.0
0.1
0.2
MR leave−one−out sensitivity analysis for
'UMOD' on 'Pulmonary fibrosis'

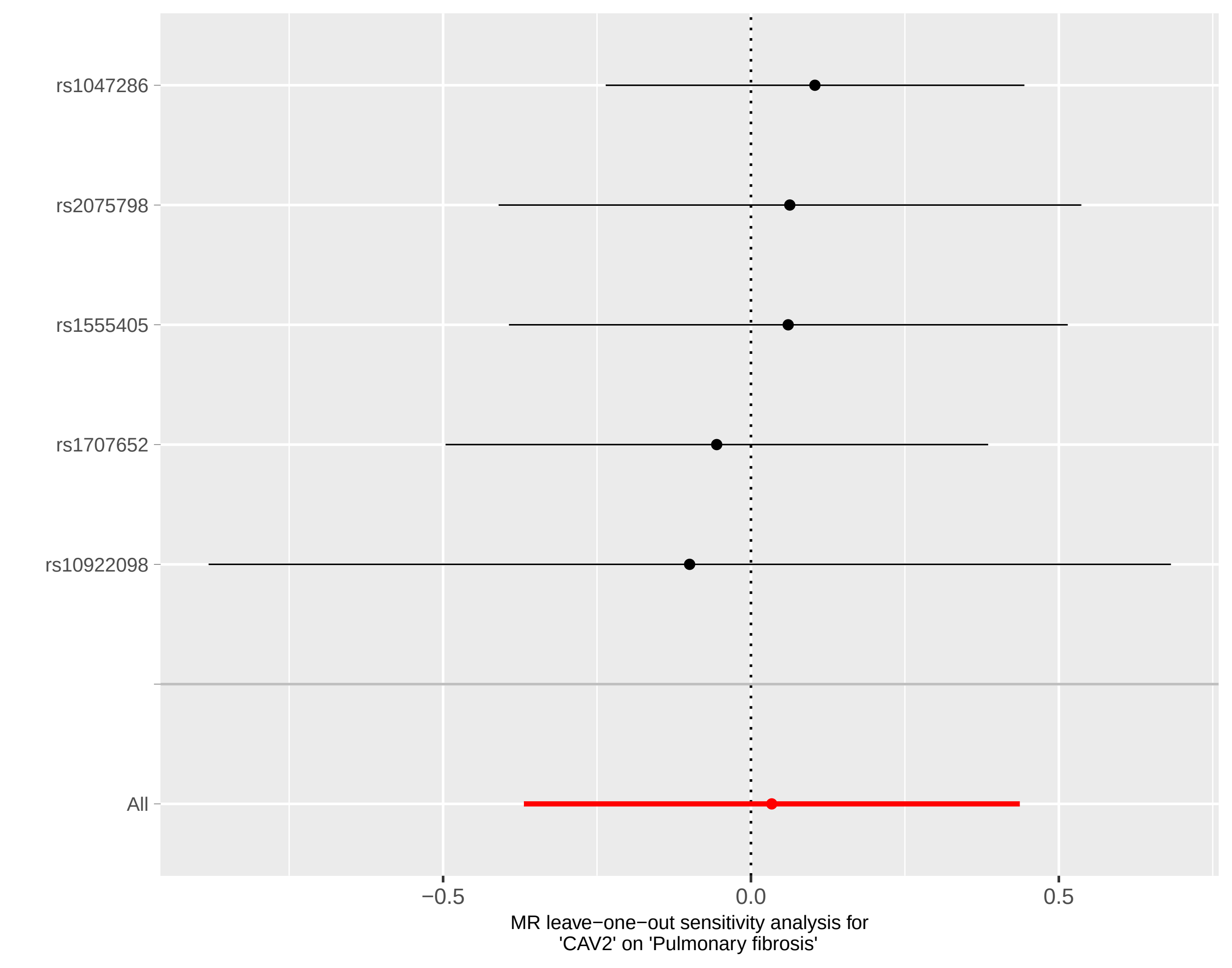
rs1047286
rs2075798
rs1555405
rs1707652
rs10922098
All
−0.5
0.0
0.5
MR leave−one−out sensitivity analysis for
'CAV2' on 'Pulmonary fibrosis'

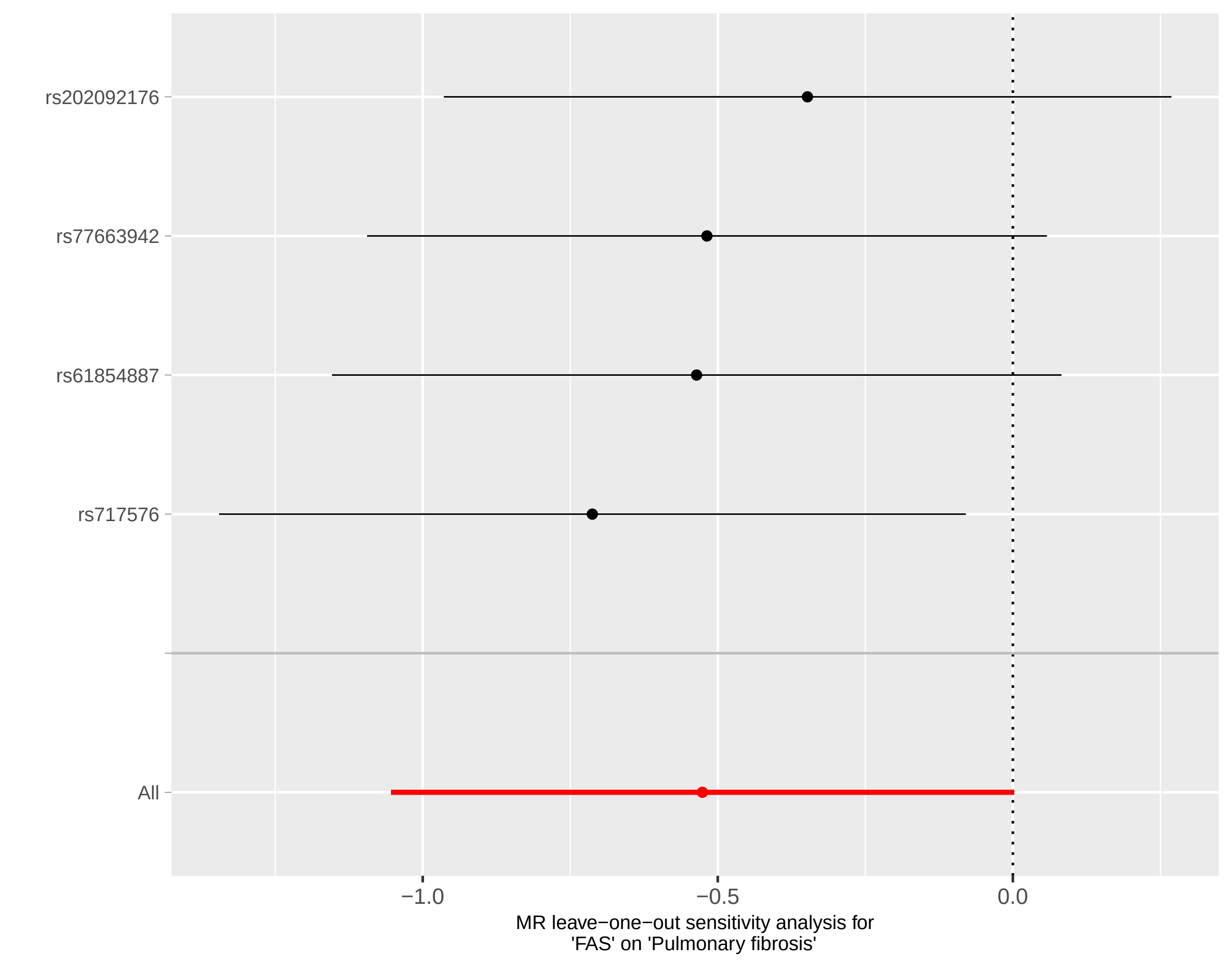
rs202092176
rs77663942
rs61854887
rs717576
All
−1.0
−0.5
0.0
MR leave−one−out sensitivity analysis for
'FAS' on 'Pulmonary fibrosis'

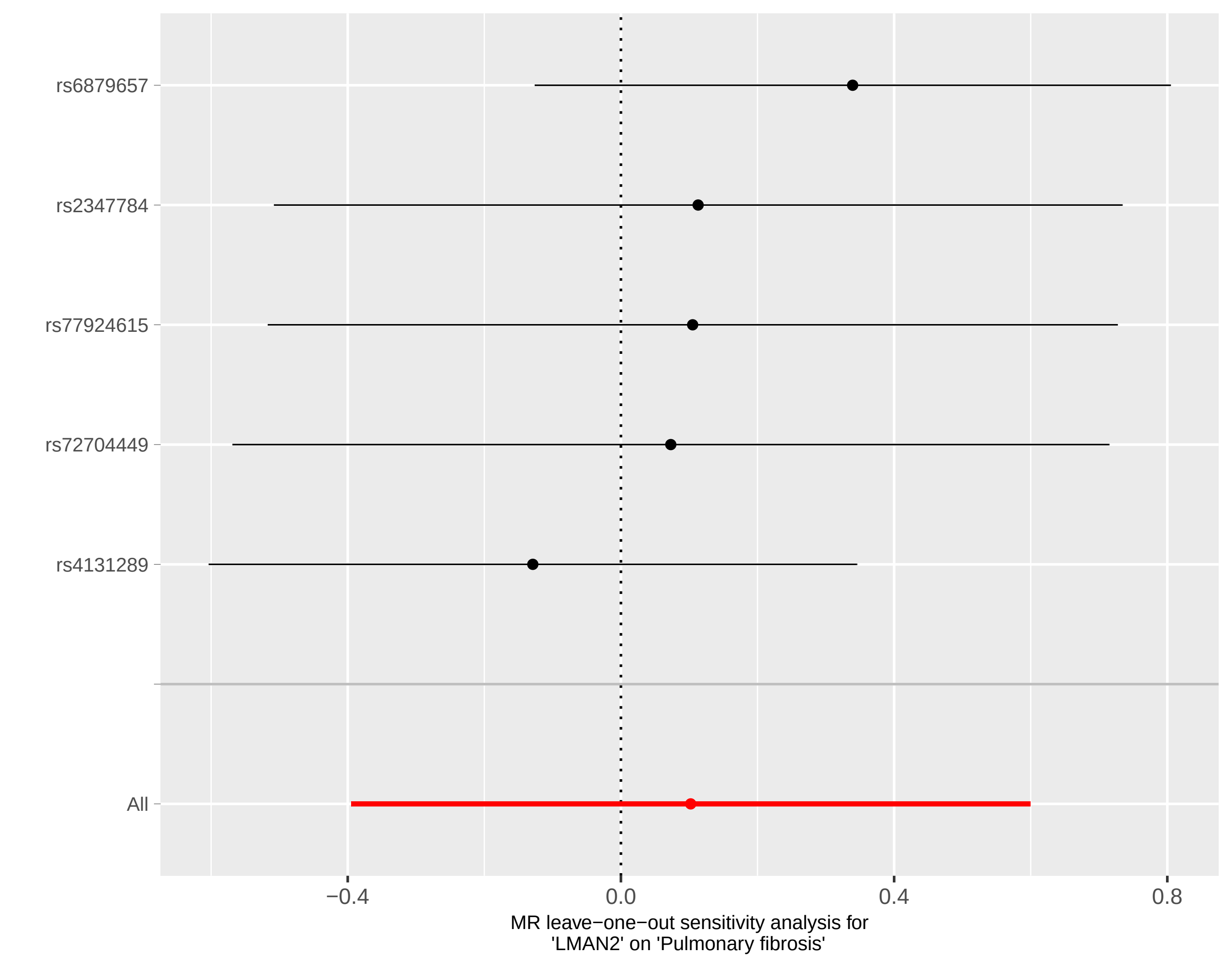

rs6879657
rs2347784
rs77924615
rs72704449
rs4131289
All
−0.4
0.0
0.4
0.8
MR leave−one−out sensitivity analysis for
'LMAN2' on 'Pulmonary fibrosis'

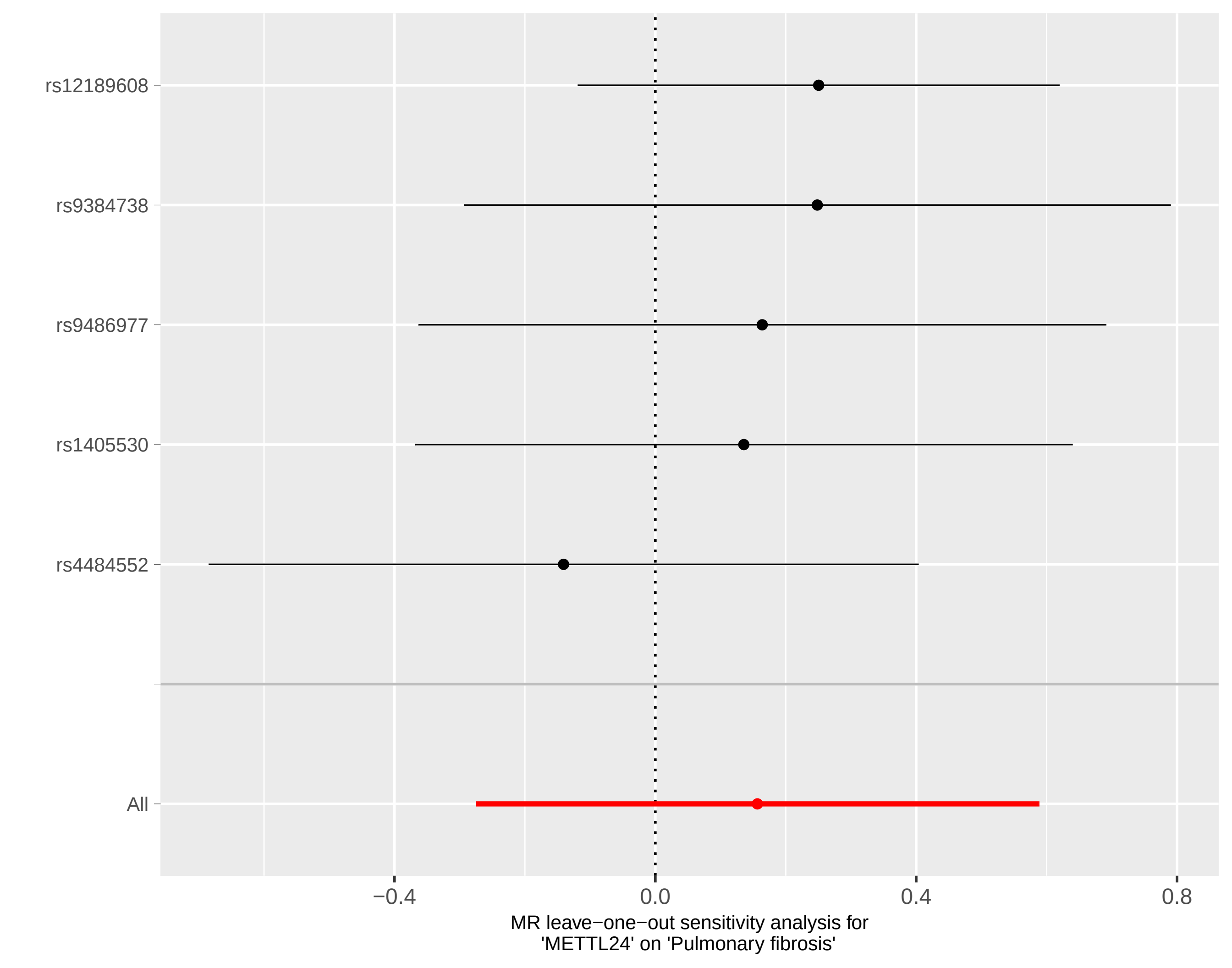
rs12189608
rs9384738
rs9486977
rs1405530
rs4484552
All
−0.4
0.0
0.4
0.8
MR leave−one−out sensitivity analysis for
'METTL24' on 'Pulmonary fibrosis'

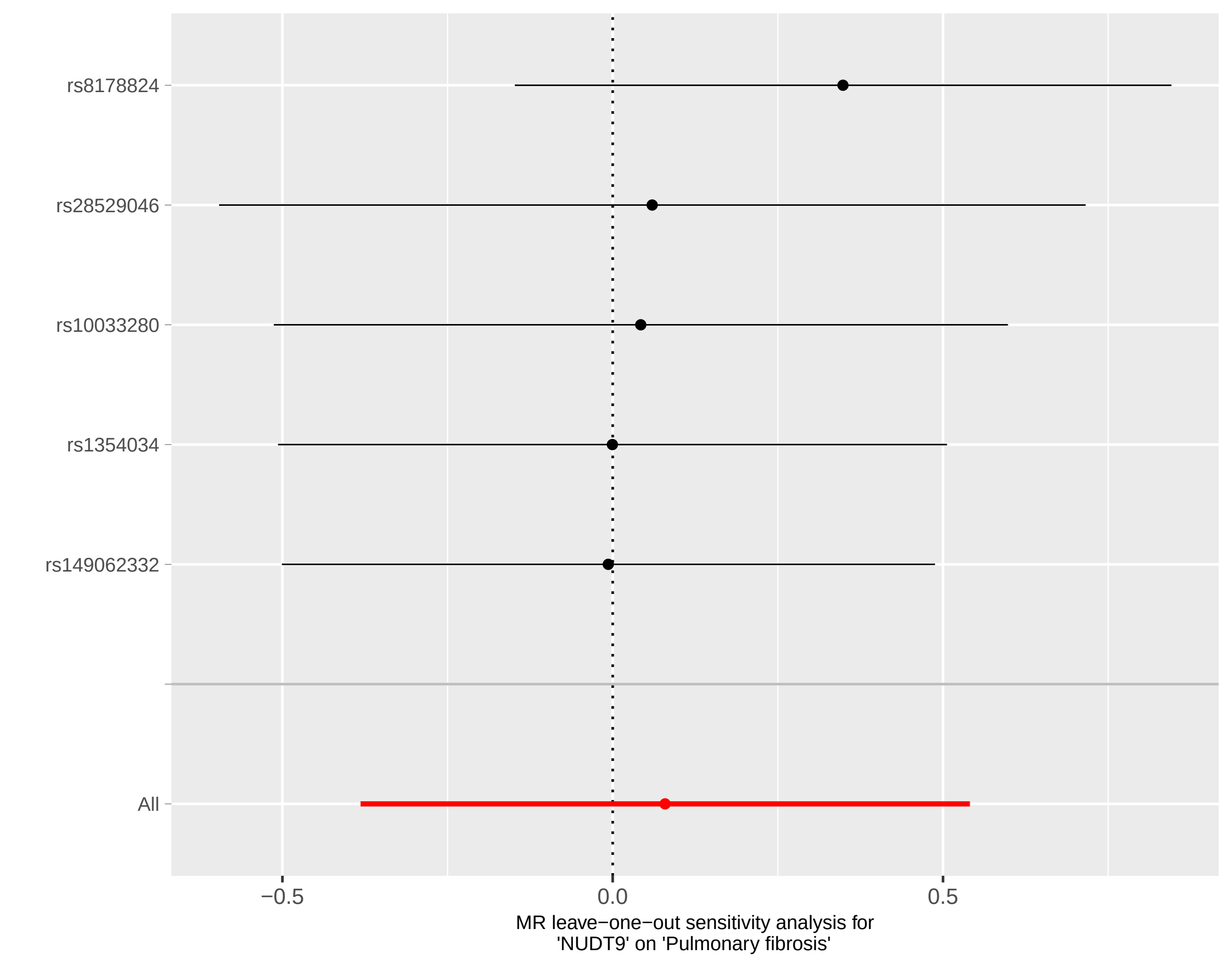
rs8178824
rs28529046
rs10033280
rs1354034
rs149062332
All
−0.5
0.0
0.5
MR leave−one−out sensitivity analysis for
'NUDT9' on 'Pulmonary fibrosis'

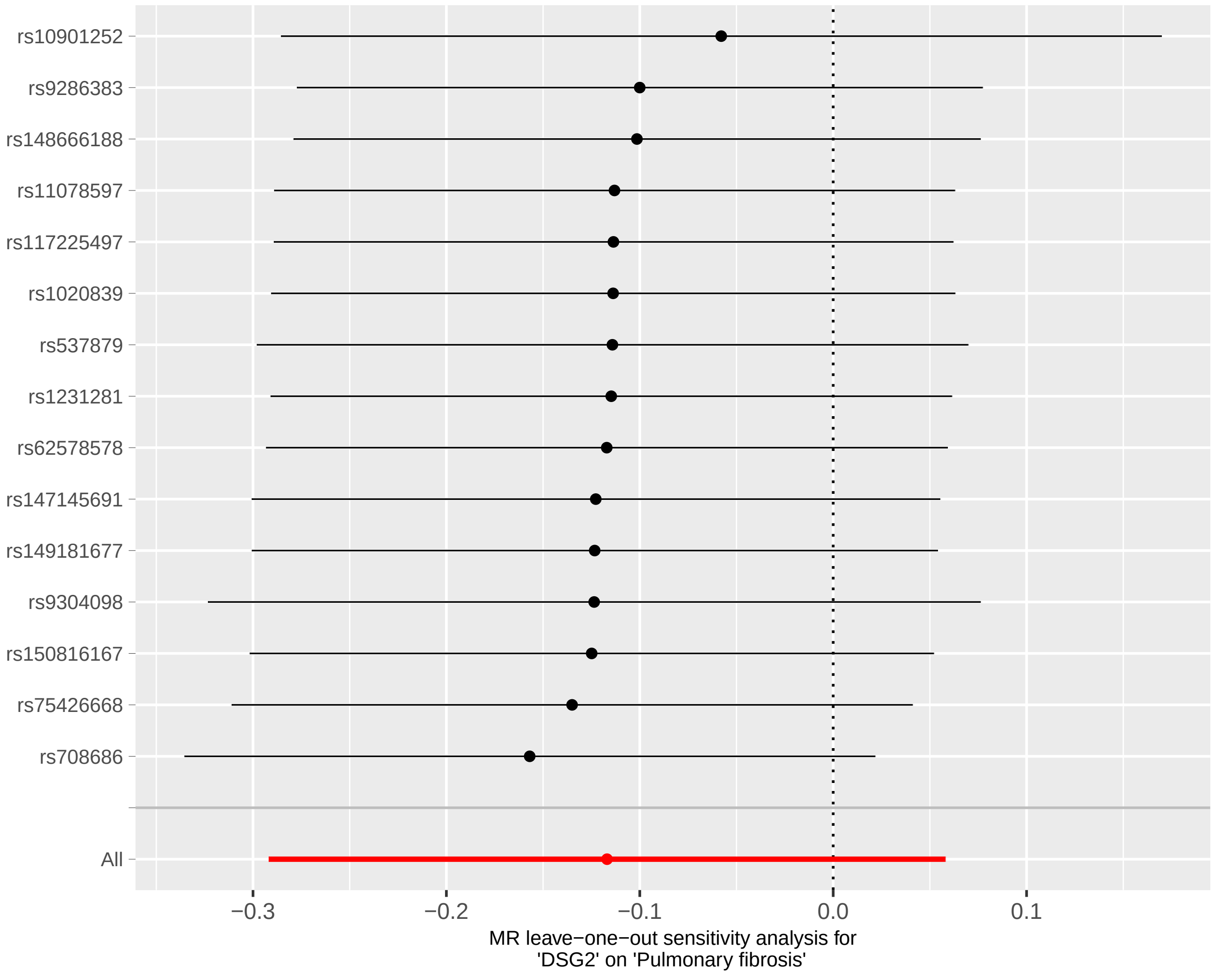

rs10901252
rs9286383
rs148666188
rs11078597
rs117225497
rs1020839
rs537879
rs1231281
rs62578578
rs147145691
rs149181677
rs9304098
rs150816167
rs75426668
rs708686
All
−0.3
−0.2
−0.1
0.0
0.1
MR leave−one−out sensitivity analysis for
'DSG2' on 'Pulmonary fibrosis'

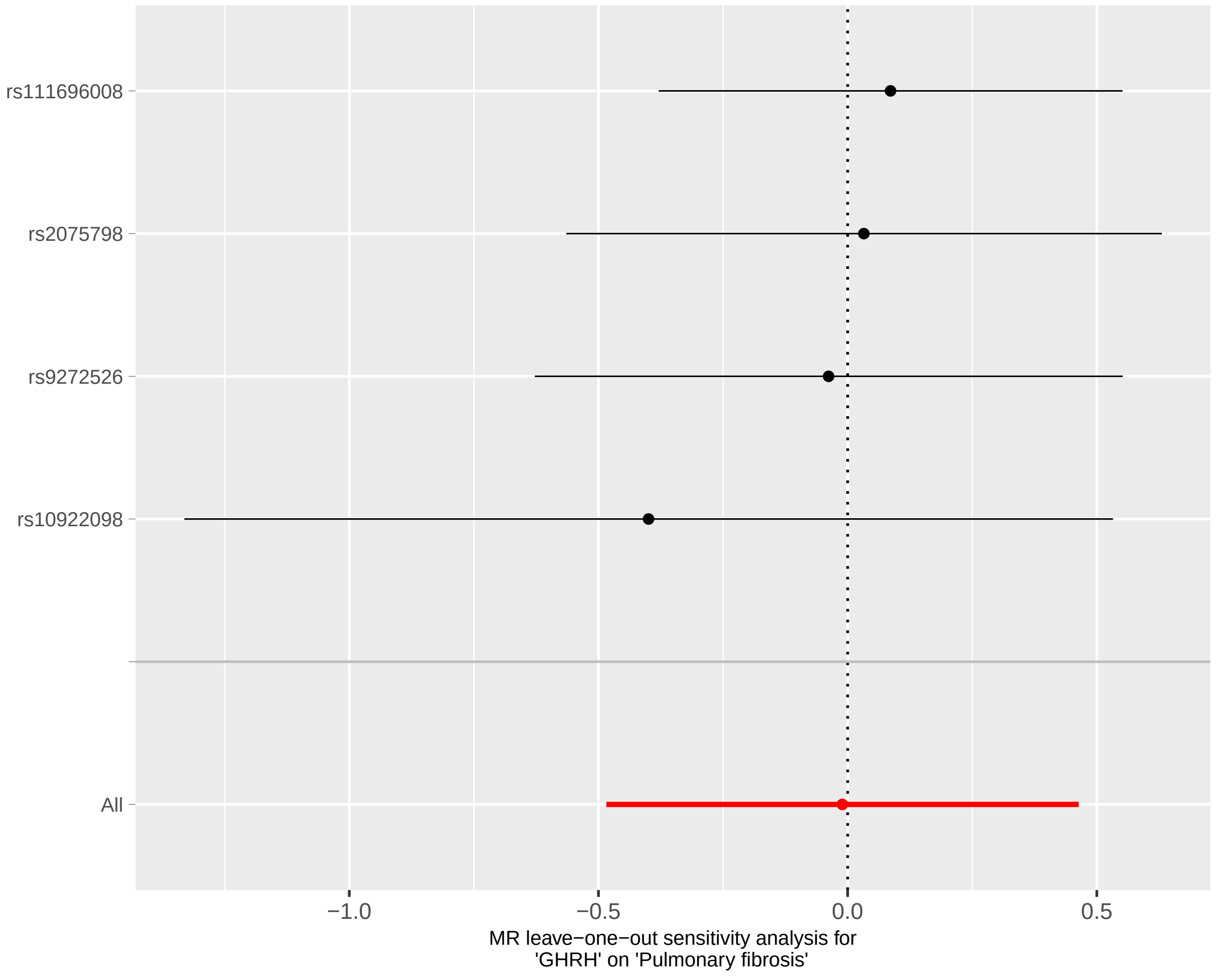
rs111696008
rs2075798
rs9272526
rs10922098
All
−1.0
−0.5
0.0
0.5
MR leave−one−out sensitivity analysis for
'GHRH' on 'Pulmonary fibrosis'

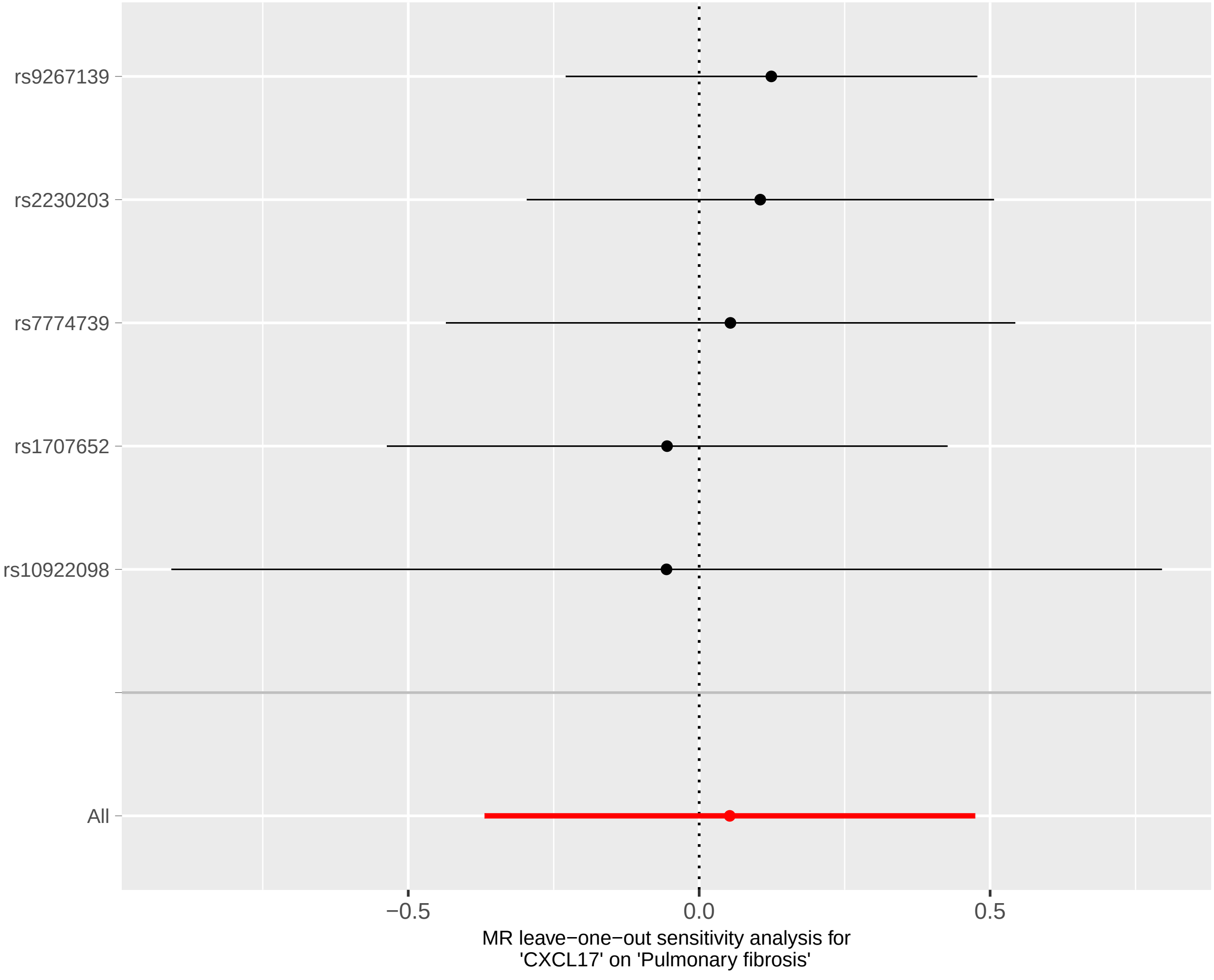

rs9267139
rs2230203
rs7774739
rs1707652
rs10922098
All
−0.5
0.0
0.5
MR leave−one−out sensitivity analysis for
'CXCL17' on 'Pulmonary fibrosis'

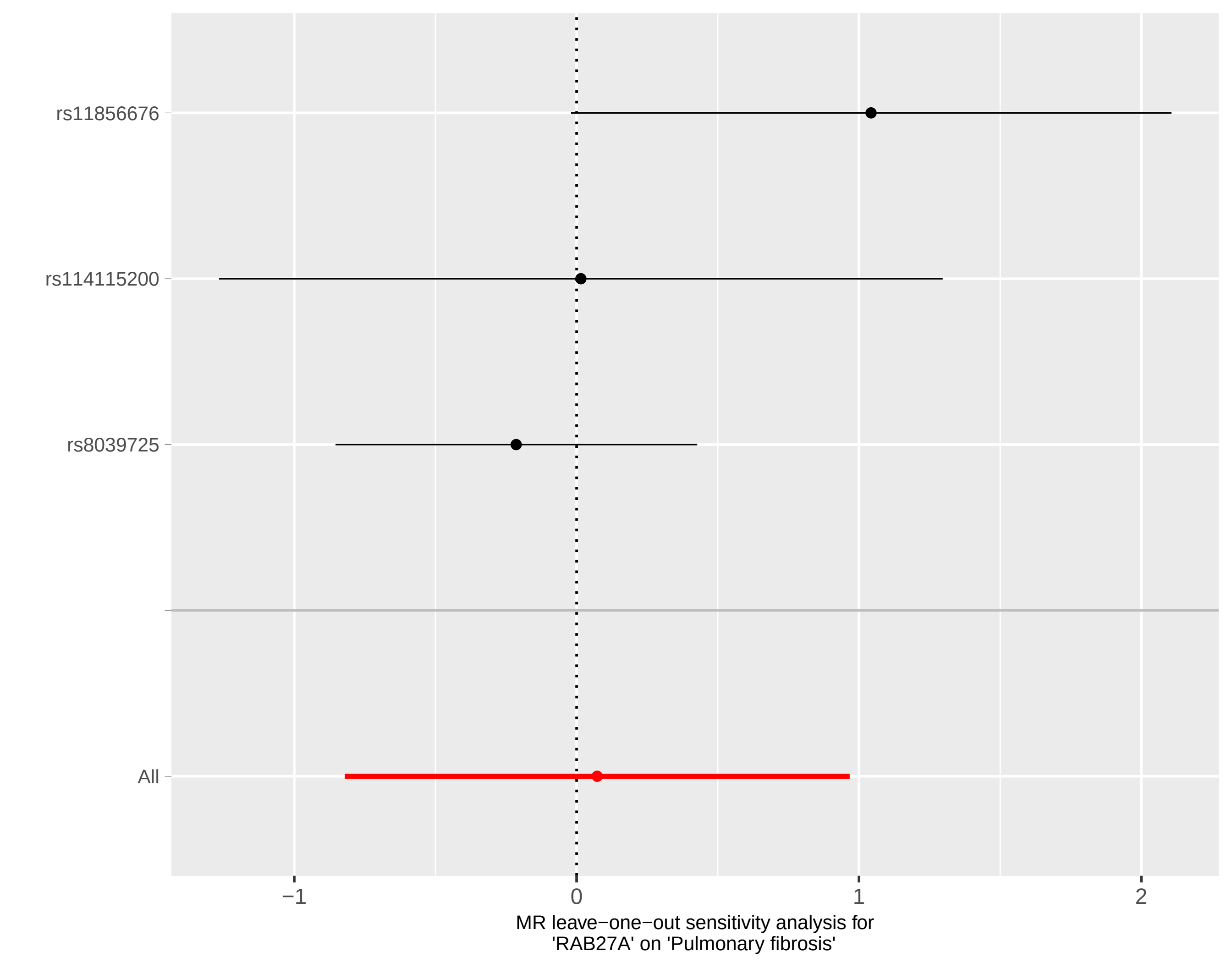
rs11856676
rs114115200
rs8039725
All
−1
0
1
2
MR leave−one−out sensitivity analysis for
'RAB27A' on 'Pulmonary fibrosis'

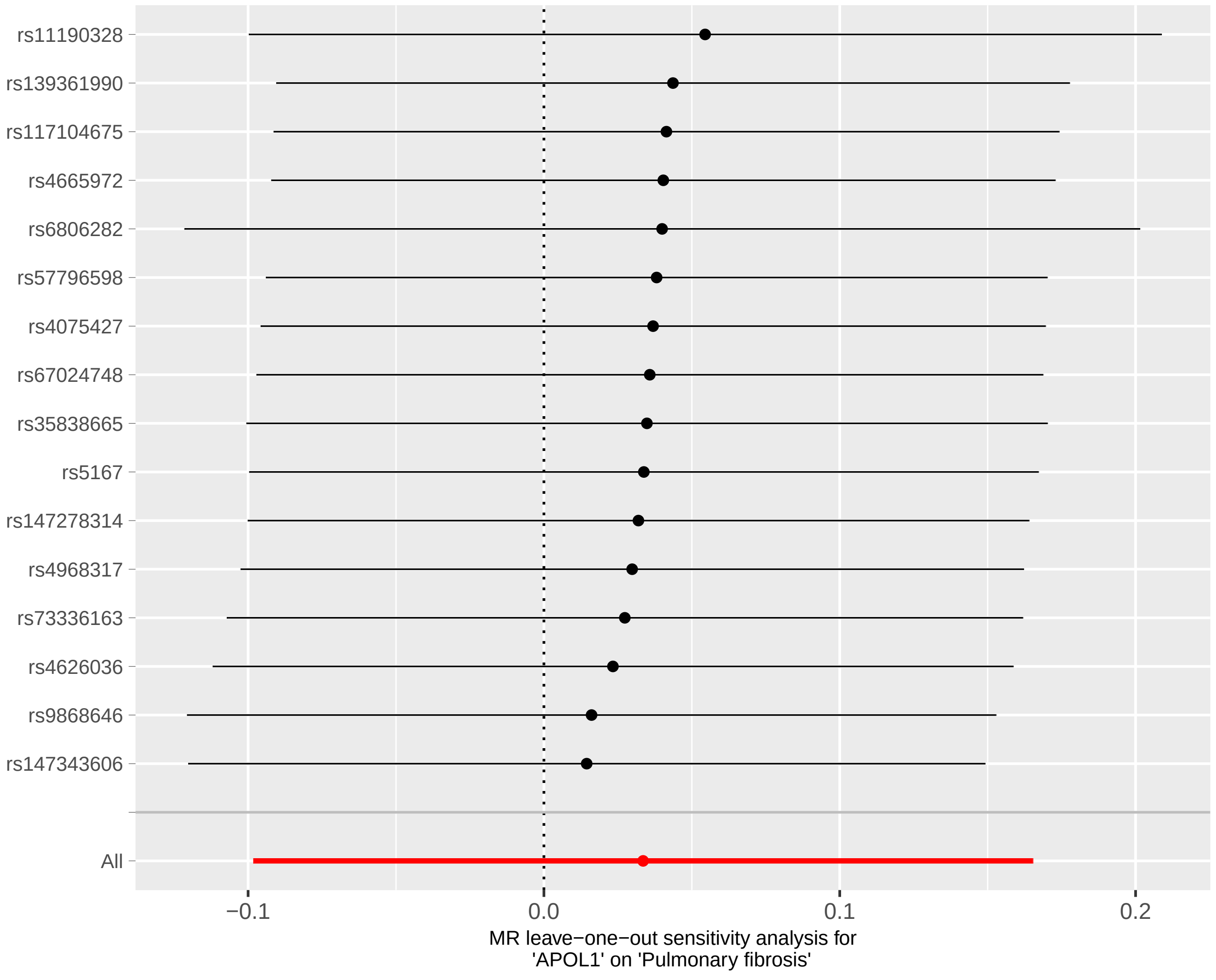

rs11190328
rs139361990
rs117104675
rs4665972
rs6806282
rs57796598
rs4075427
rs67024748
rs35838665
rs5167
rs147278314
rs4968317
rs73336163
rs4626036
rs9868646
rs147343606
All
−0.1
0.0
0.1
0.2
MR leave−one−out sensitivity analysis for
'APOL1' on 'Pulmonary fibrosis'

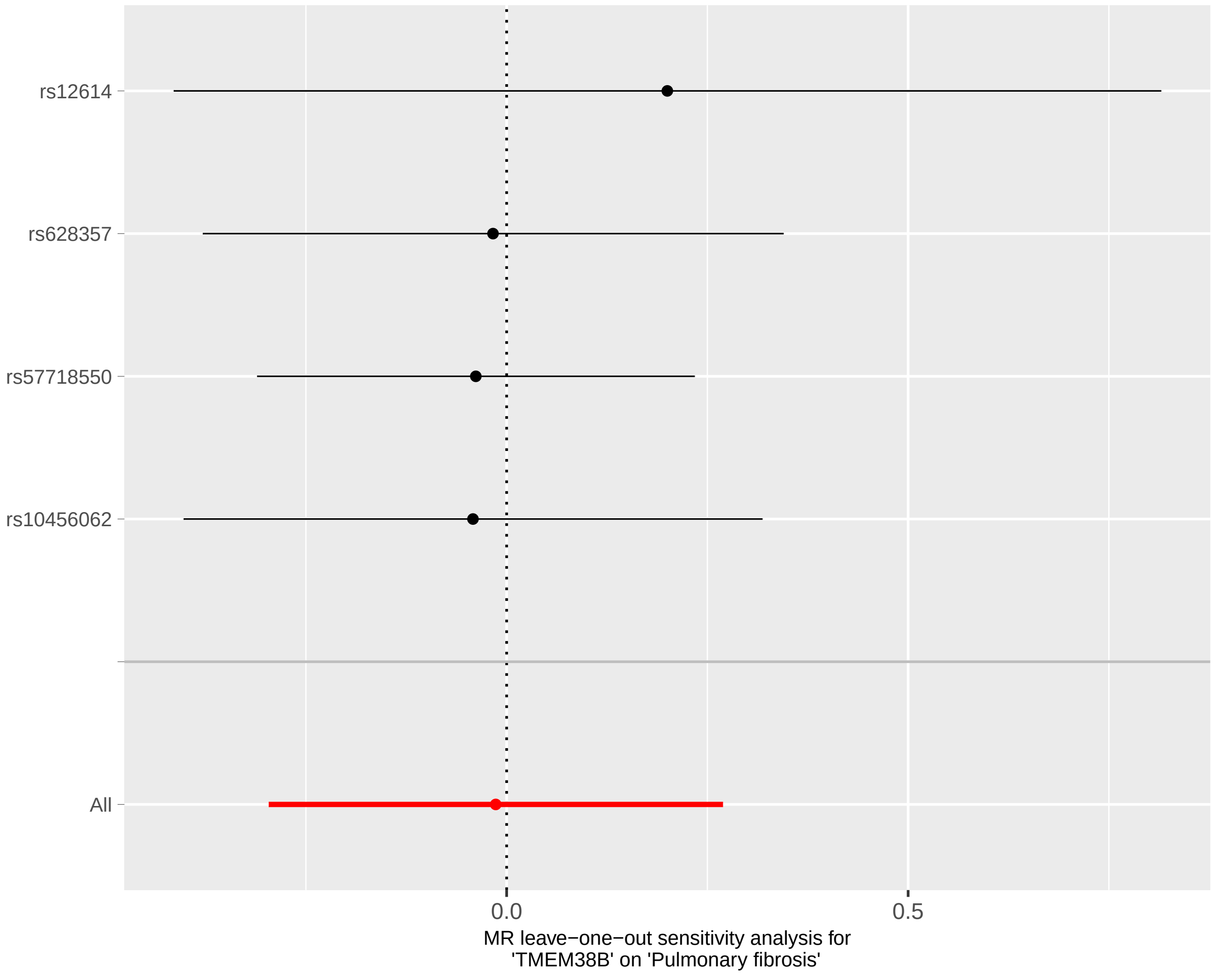
rs12614
rs628357
rs57718550
rs10456062
All
0.0
0.5
MR leave−one−out sensitivity analysis for
'TMEM38B' on 'Pulmonary fibrosis'

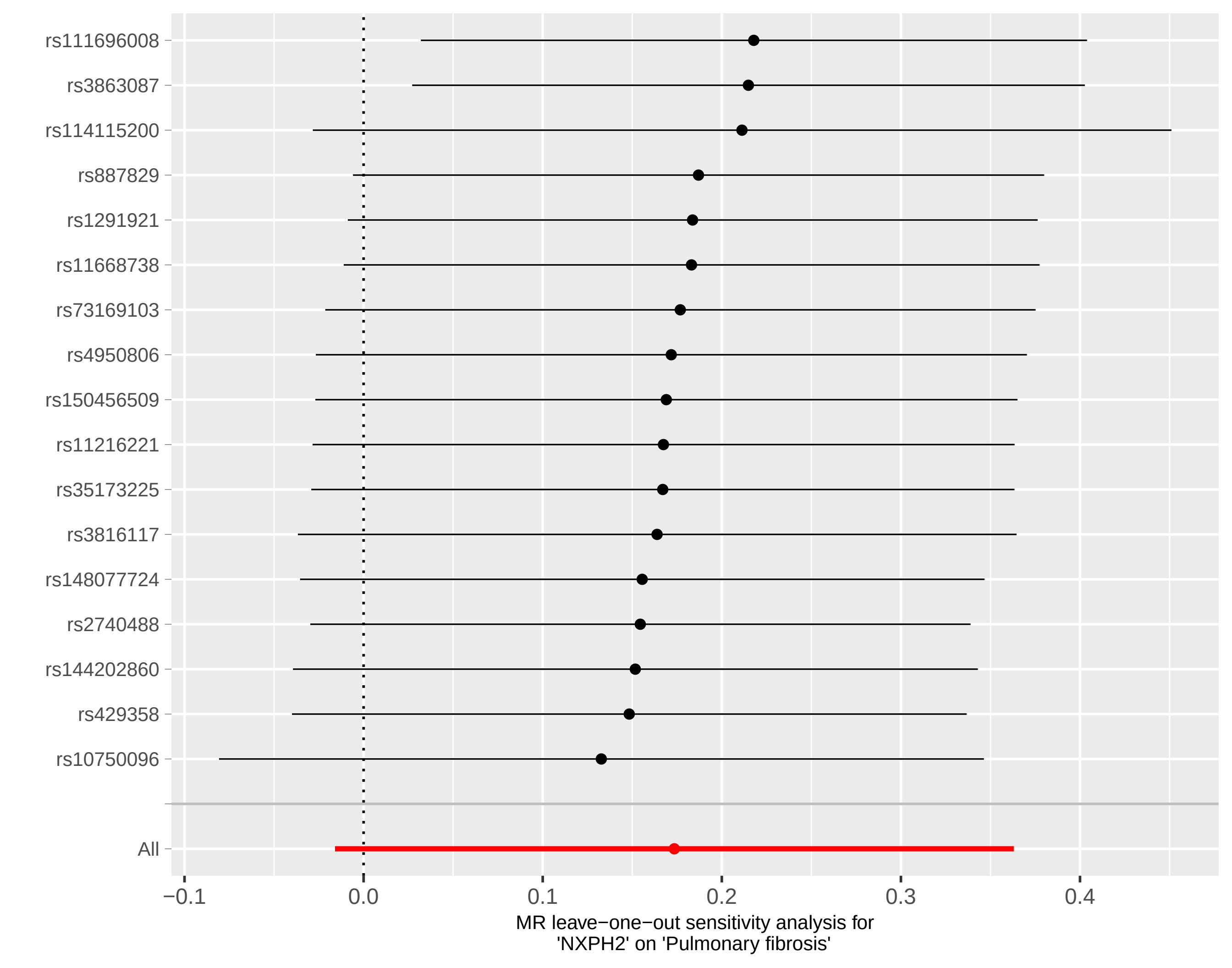
rs111696008
rs3863087
rs114115200
rs887829
rs1291921
rs11668738
rs73169103
rs4950806
rs150456509
rs11216221
rs35173225
rs3816117
rs148077724
rs2740488
rs144202860
rs429358
rs10750096
All
−0.1
0.0
0.1
0.2
0.3
0.4
MR leave−one−out sensitivity analysis for
'NXPH2' on 'Pulmonary fibrosis'

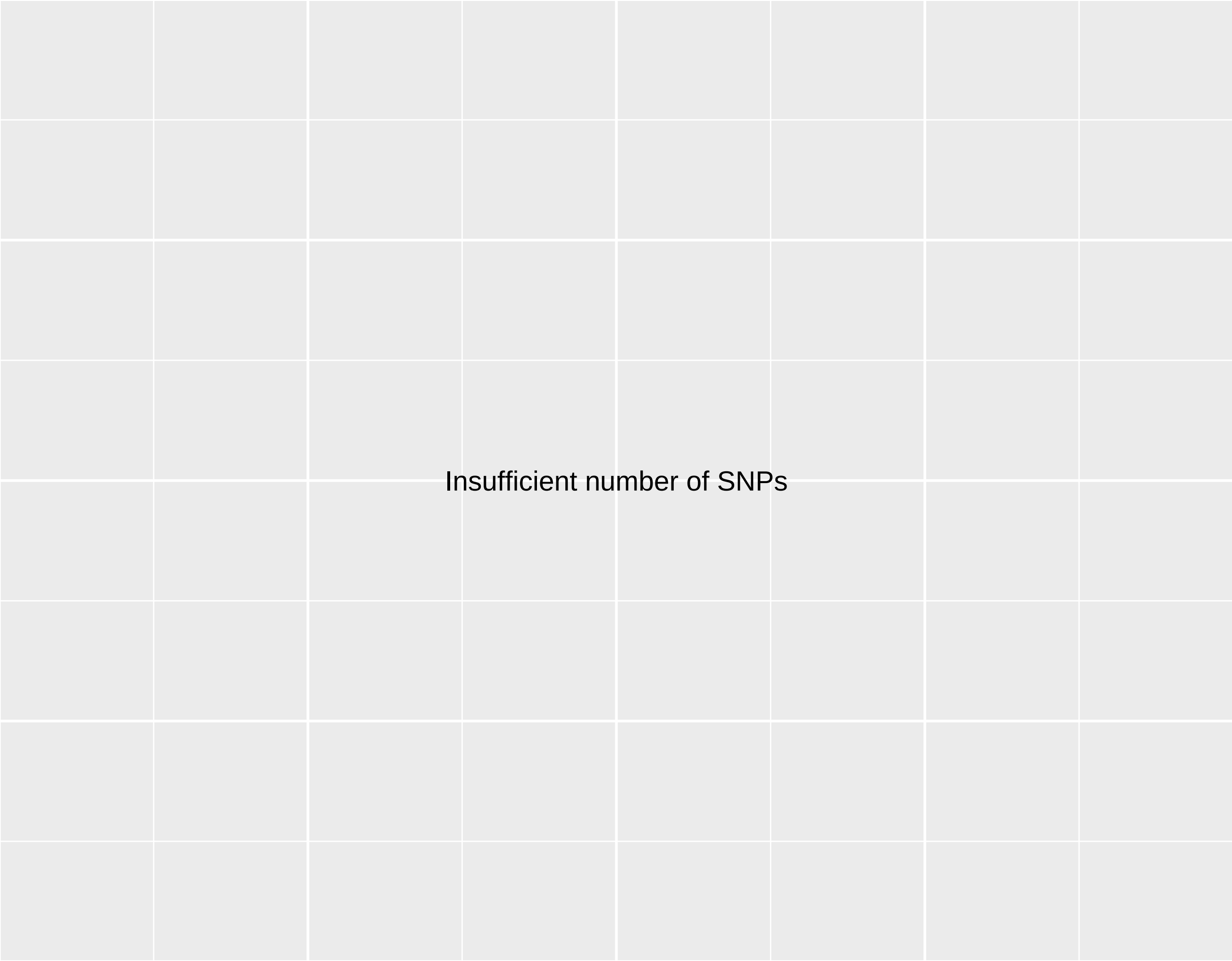
Insufficient number of SNPs

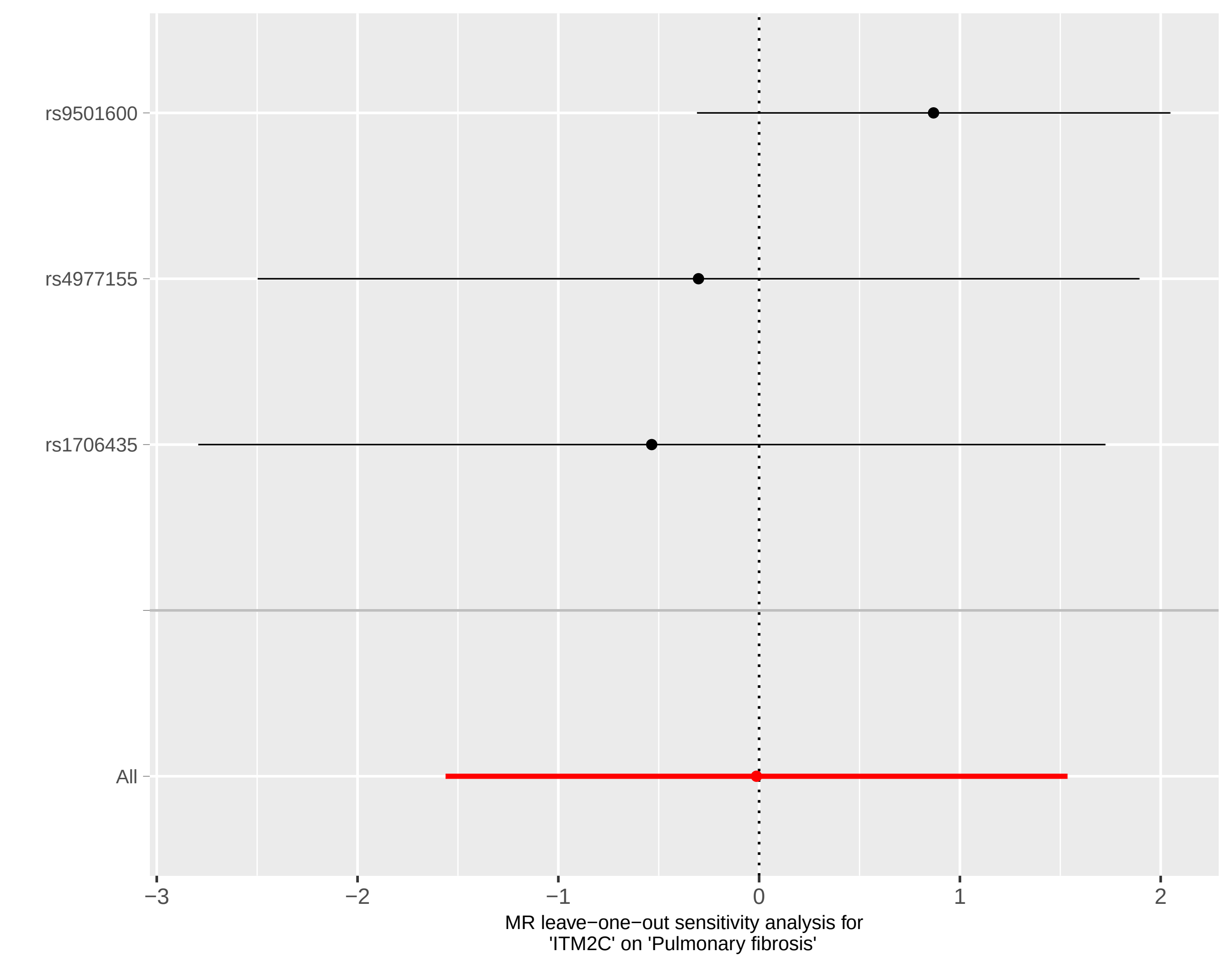
rs9501600
rs4977155
rs1706435
All
−3
−2
−1
0
1
2
MR leave−one−out sensitivity analysis for
'ITM2C' on 'Pulmonary fibrosis'

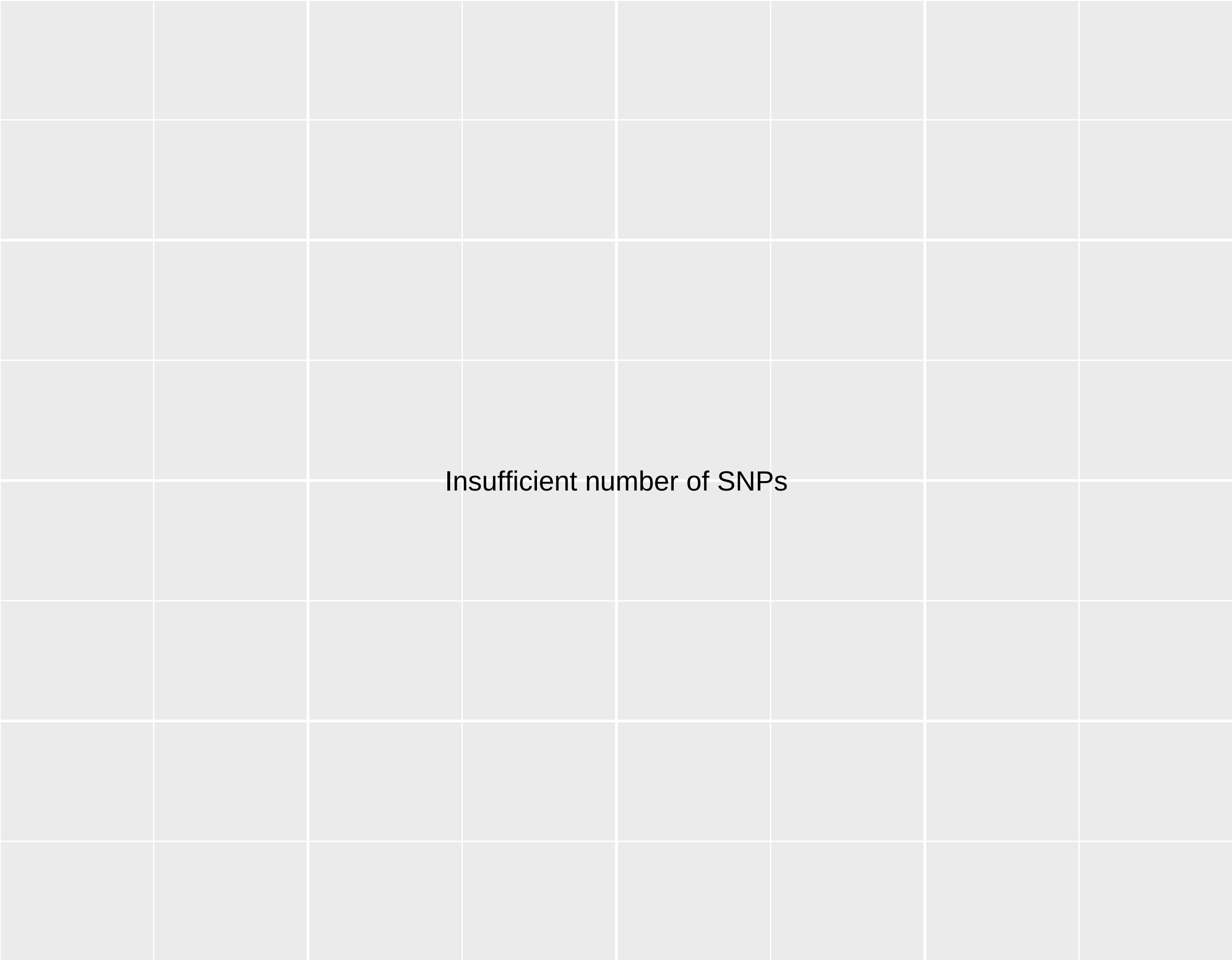
Insufficient number of SNPs

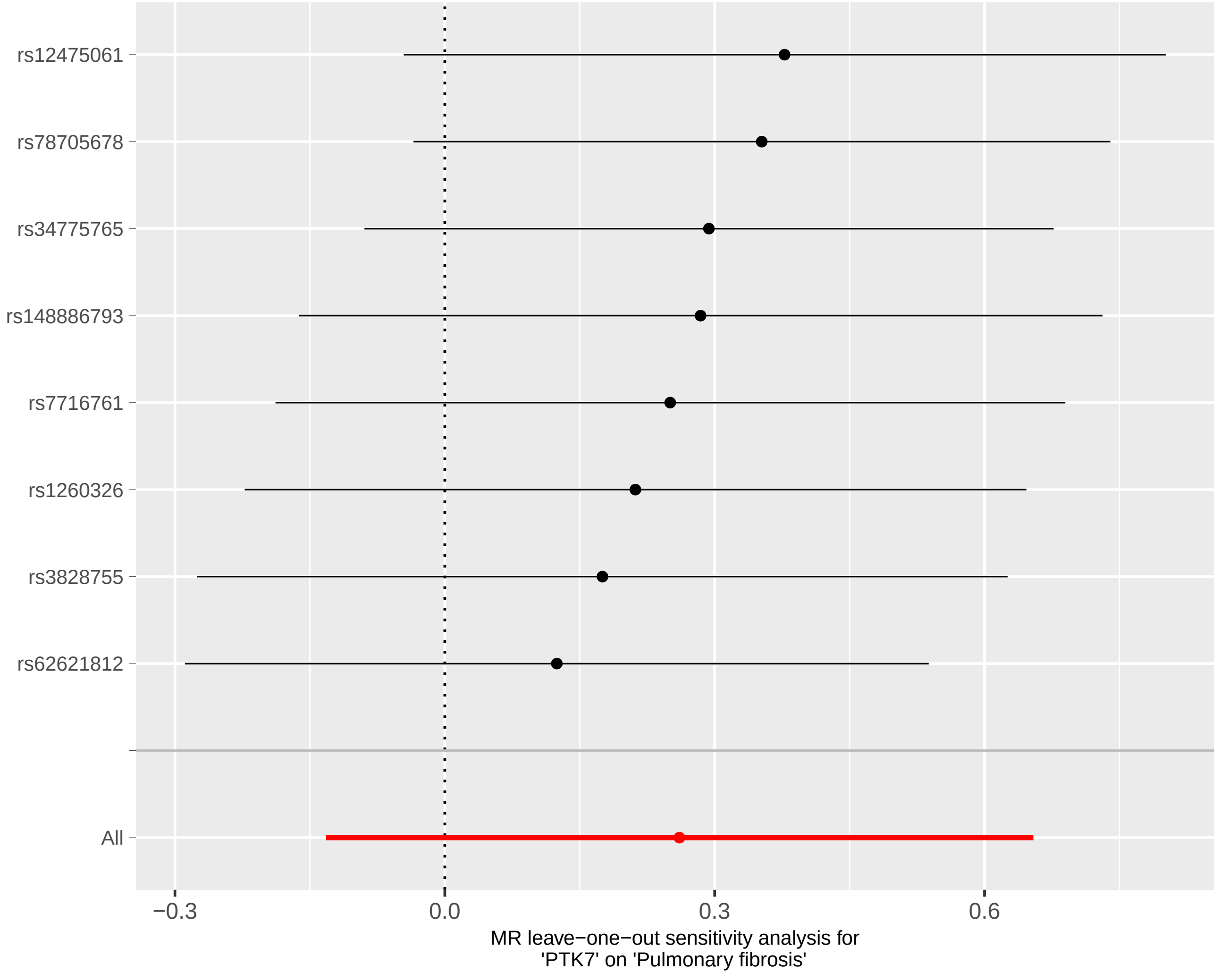
rs12475061
rs78705678
rs34775765
rs148886793
rs7716761
rs1260326
rs3828755
rs62621812
All
−0.3
0.0
0.3
0.6
MR leave−one−out sensitivity analysis for
'PTK7' on 'Pulmonary fibrosis'

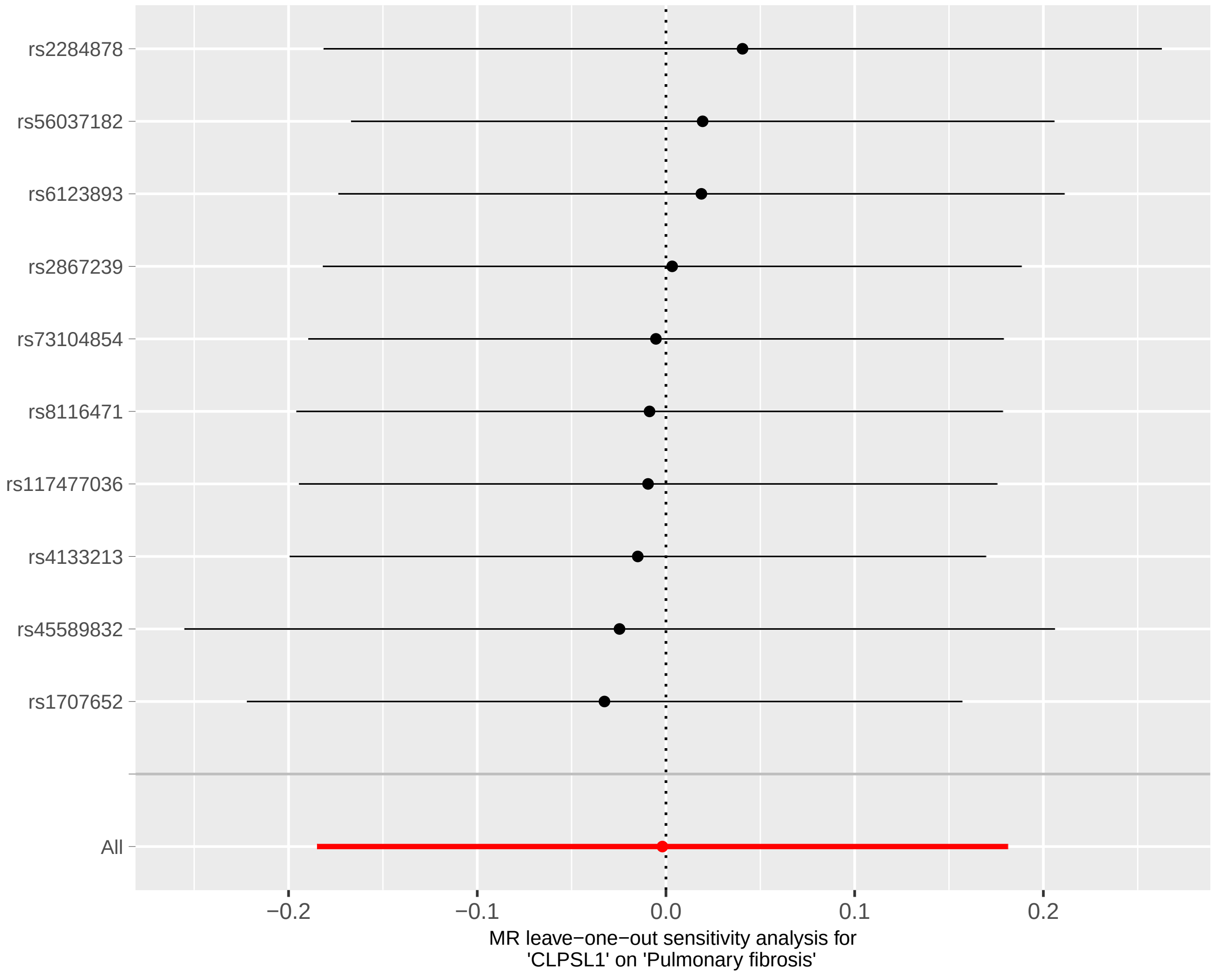
rs2284878
rs56037182
rs6123893
rs2867239
rs73104854
rs8116471
rs117477036
rs4133213
rs45589832
rs1707652
All
−0.2
−0.1
0.0
0.1
0.2
MR leave−one−out sensitivity analysis for
'CLPSL1' on 'Pulmonary fibrosis'

Insufficient number of SNPs

Insufficient number of SNPs

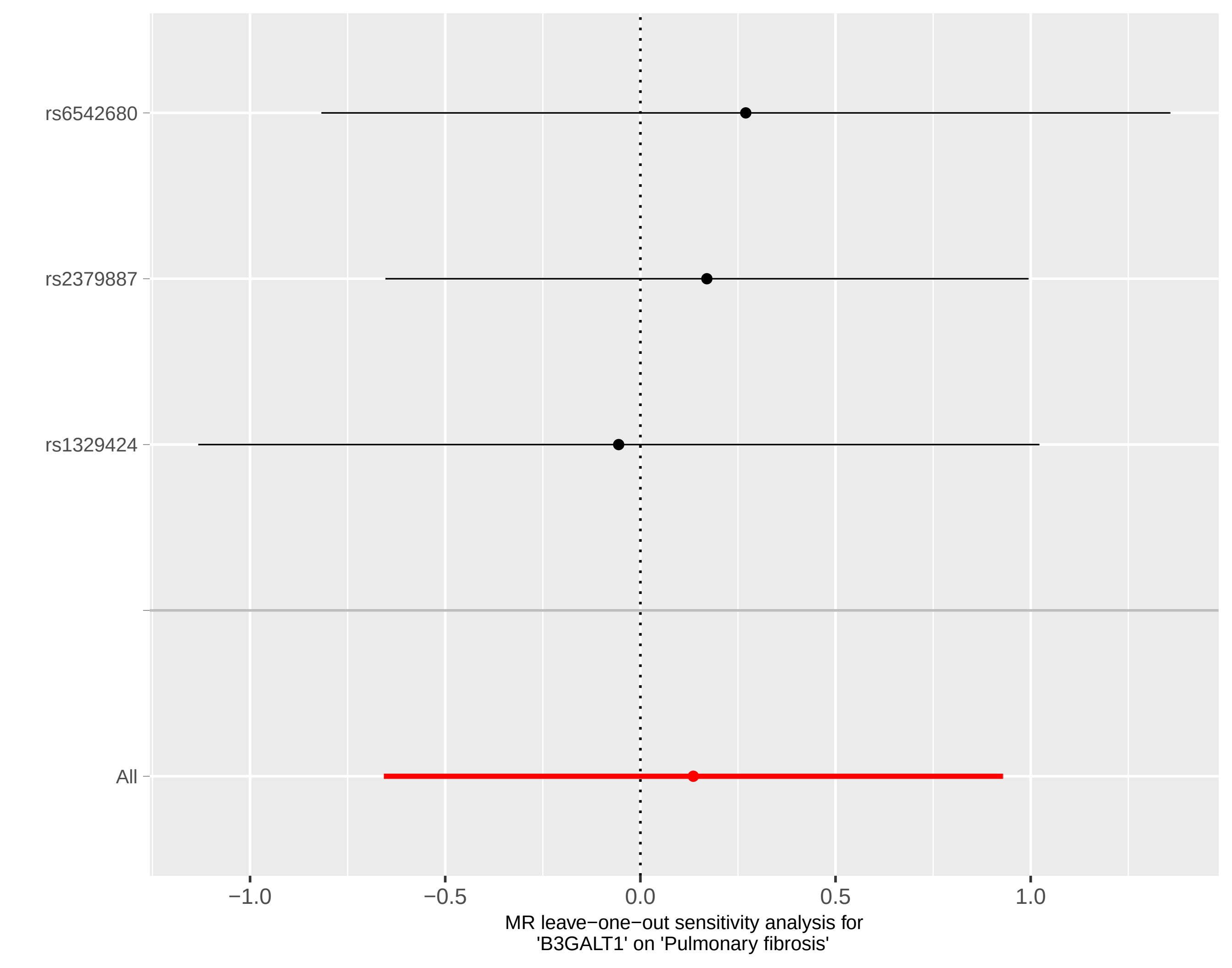
rs6542680
rs2379887
rs1329424
All
−1.0
−0.5
0.0
0.5
1.0
MR leave−one−out sensitivity analysis for
'B3GALT1' on 'Pulmonary fibrosis'

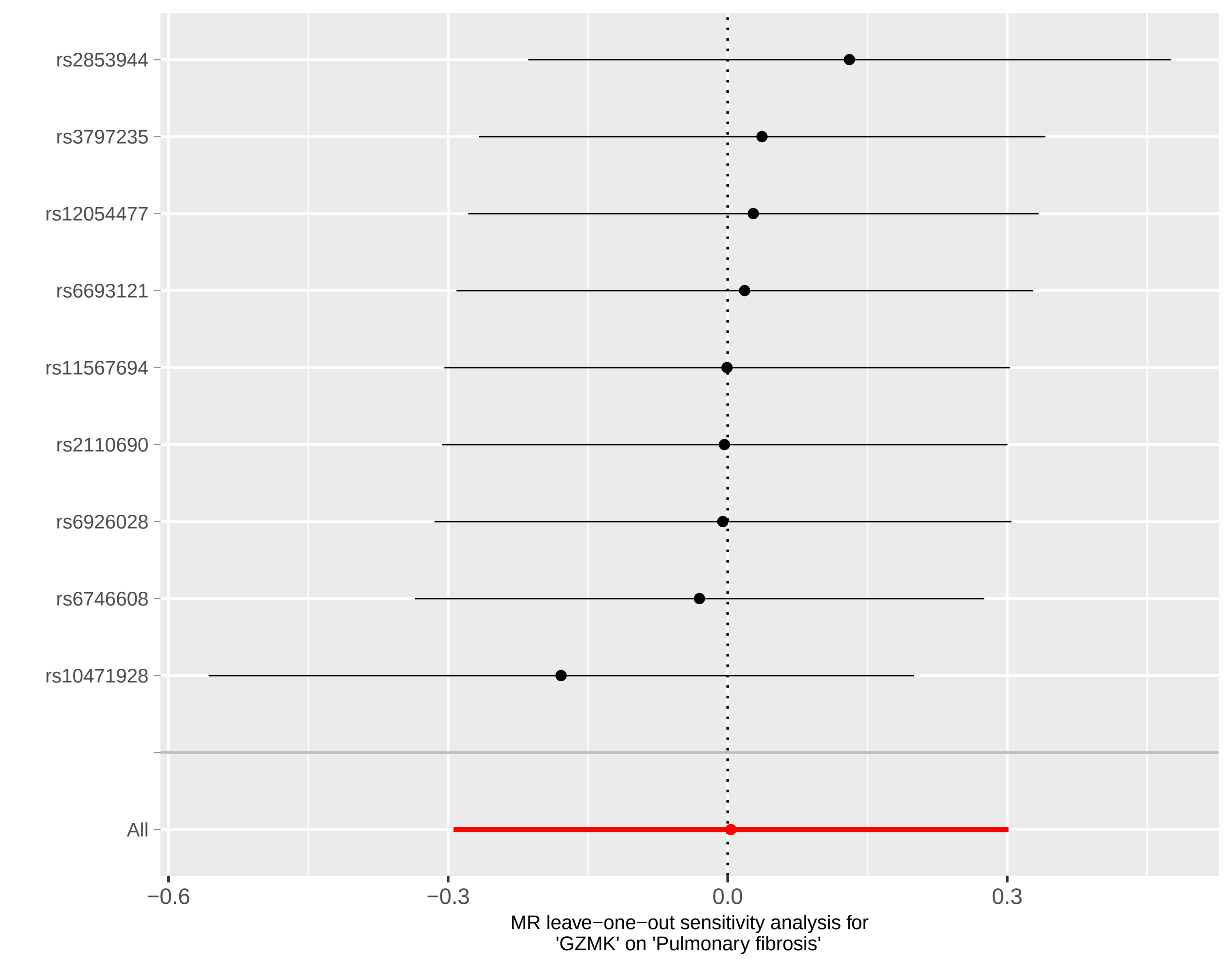
rs2853944
rs3797235
rs12054477
rs6693121
rs11567694
rs2110690
rs6926028
rs6746608
rs10471928
All
−0.6
−0.3
0.0
0.3
MR leave−one−out sensitivity analysis for
'GZMK' on 'Pulmonary fibrosis'

Insufficient number of SNPs

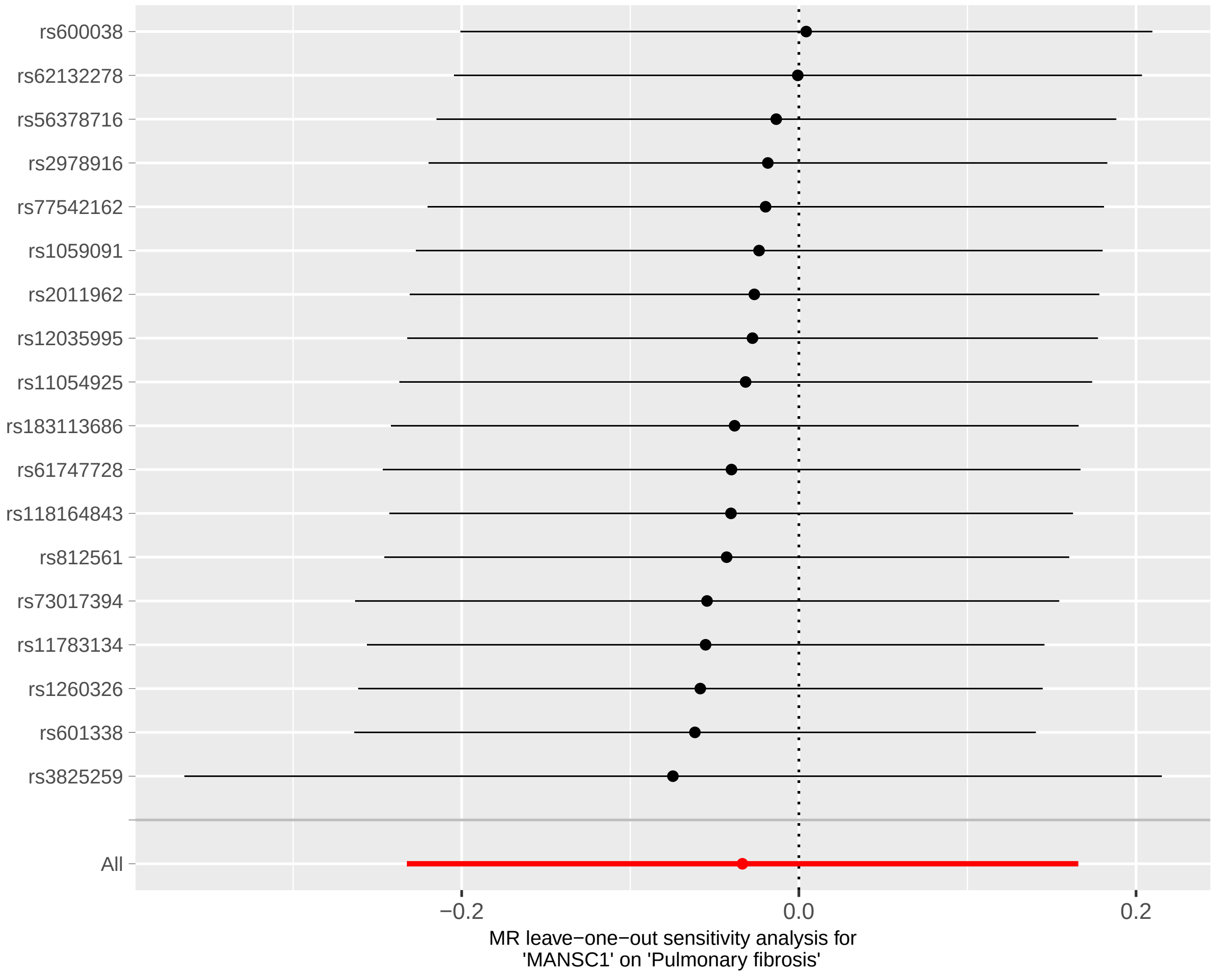
rs600038
rs62132278
rs56378716
rs2978916
rs77542162
rs1059091
rs2011962
rs12035995
rs11054925
rs183113686
rs61747728
rs118164843
rs812561
rs73017394
rs11783134
rs1260326
rs601338
rs3825259
All
−0.2
0.0
0.2
MR leave−one−out sensitivity analysis for
'MANSC1' on 'Pulmonary fibrosis'

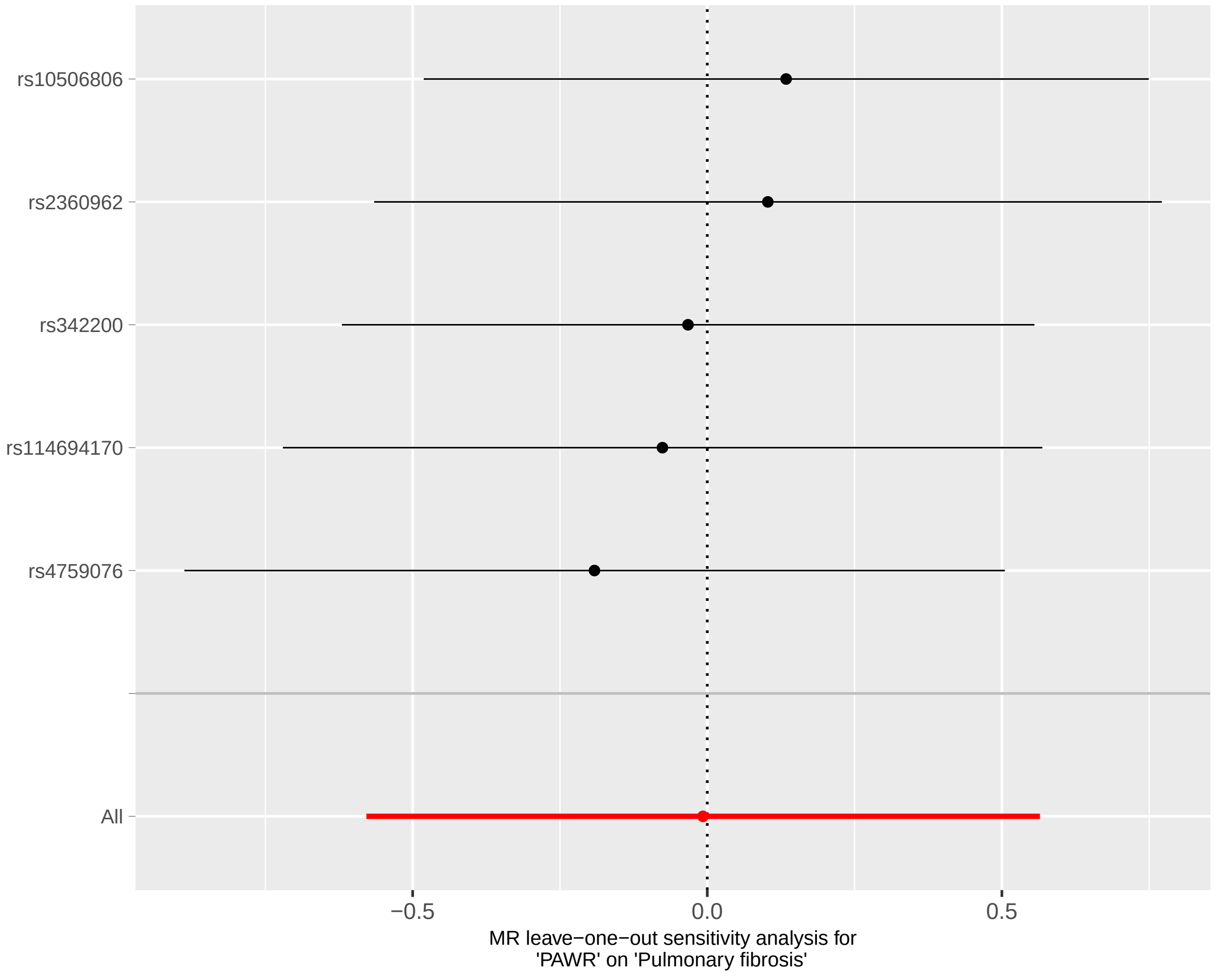
rs10506806
rs2360962
rs342200
rs114694170
rs4759076
All
−0.5
0.0
0.5
MR leave−one−out sensitivity analysis for
'PAWR' on 'Pulmonary fibrosis'

Insufficient number of SNPs

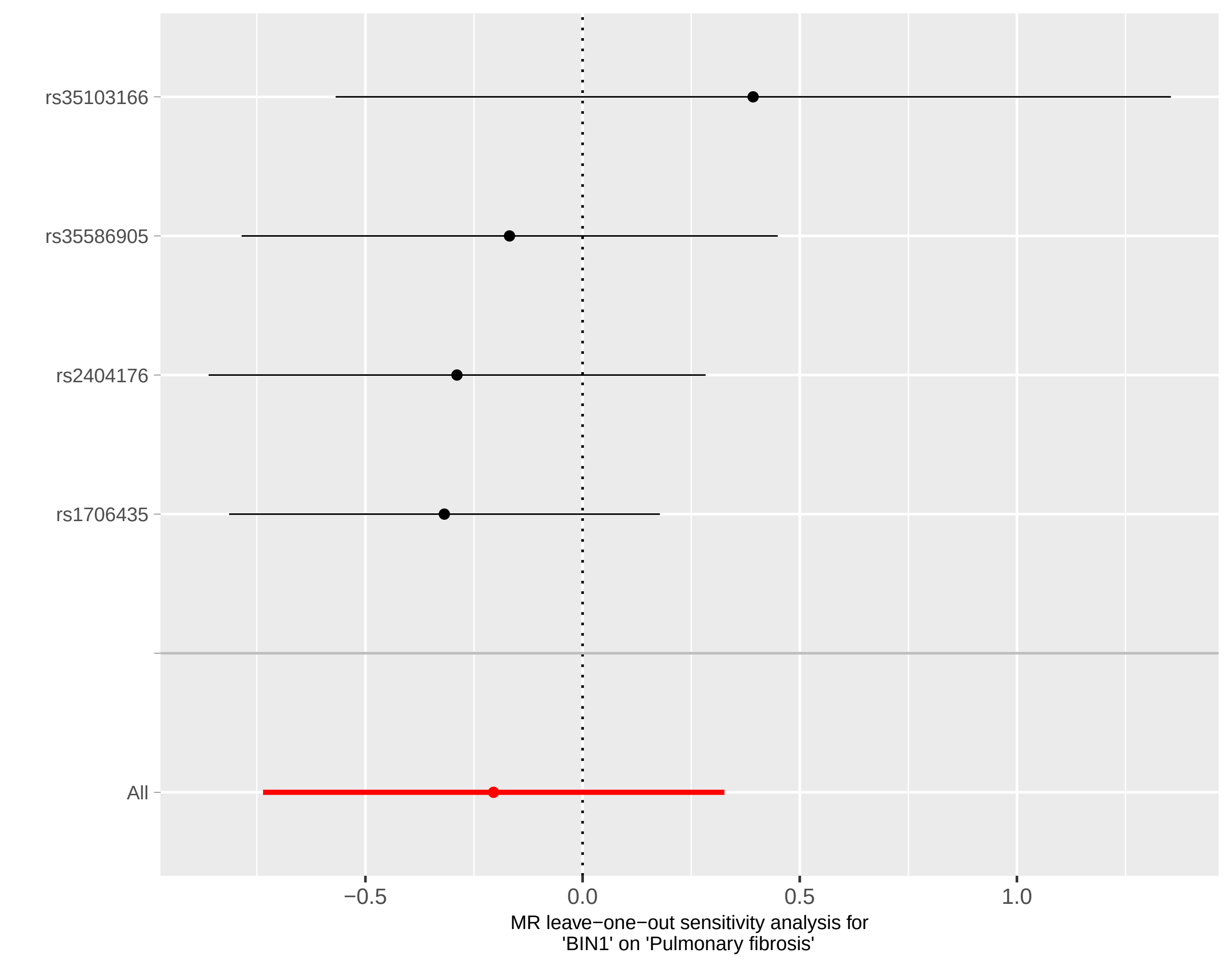
rs35103166
rs35586905
rs2404176
rs1706435
All
−0.5
0.0
0.5
1.0
MR leave−one−out sensitivity analysis for
'BIN1' on 'Pulmonary fibrosis'

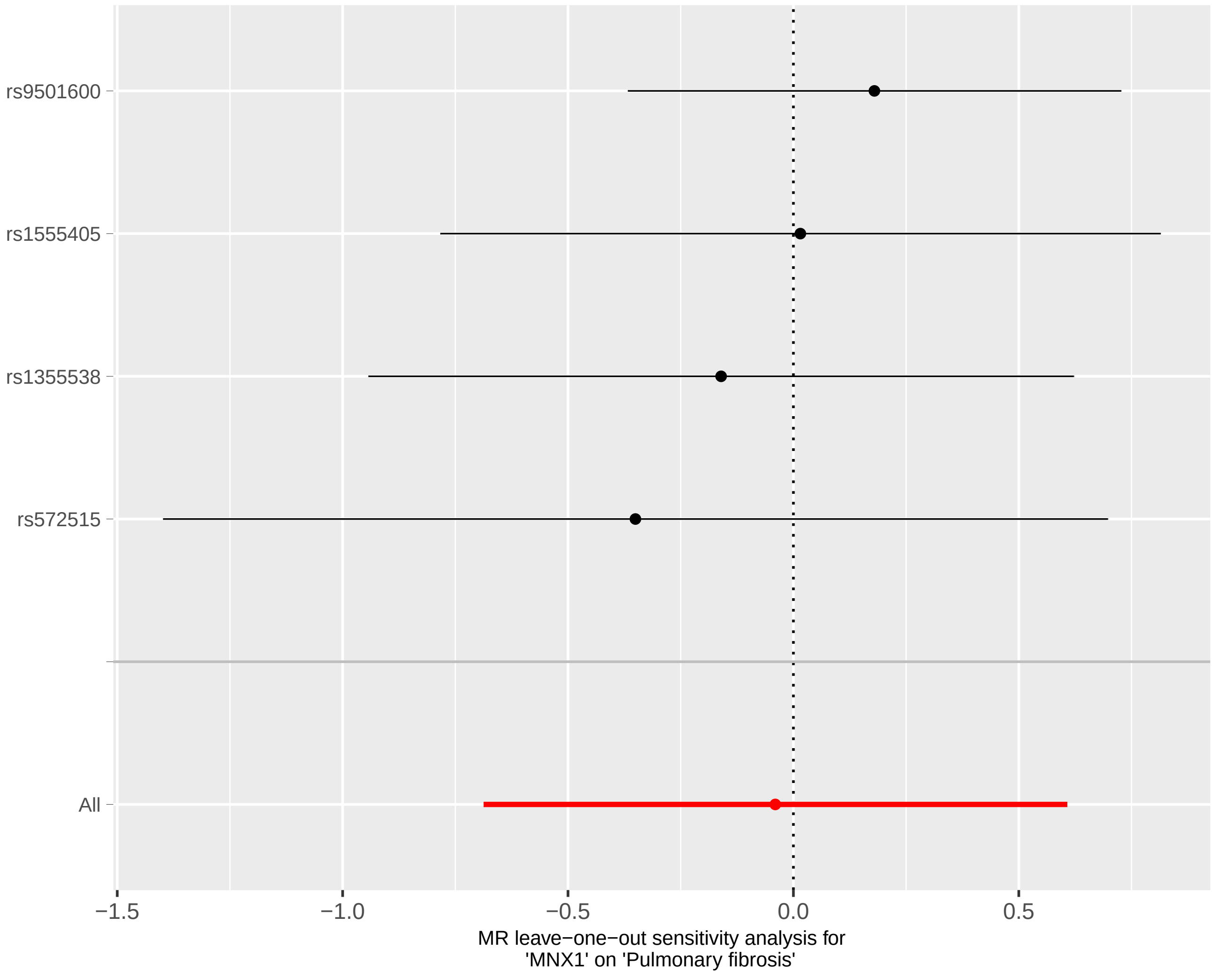
rs9501600
rs1555405
rs1355538
rs572515
All
−1.5
−1.0
−0.5
0.0
0.5
MR leave−one−out sensitivity analysis for
'MNX1' on 'Pulmonary fibrosis'

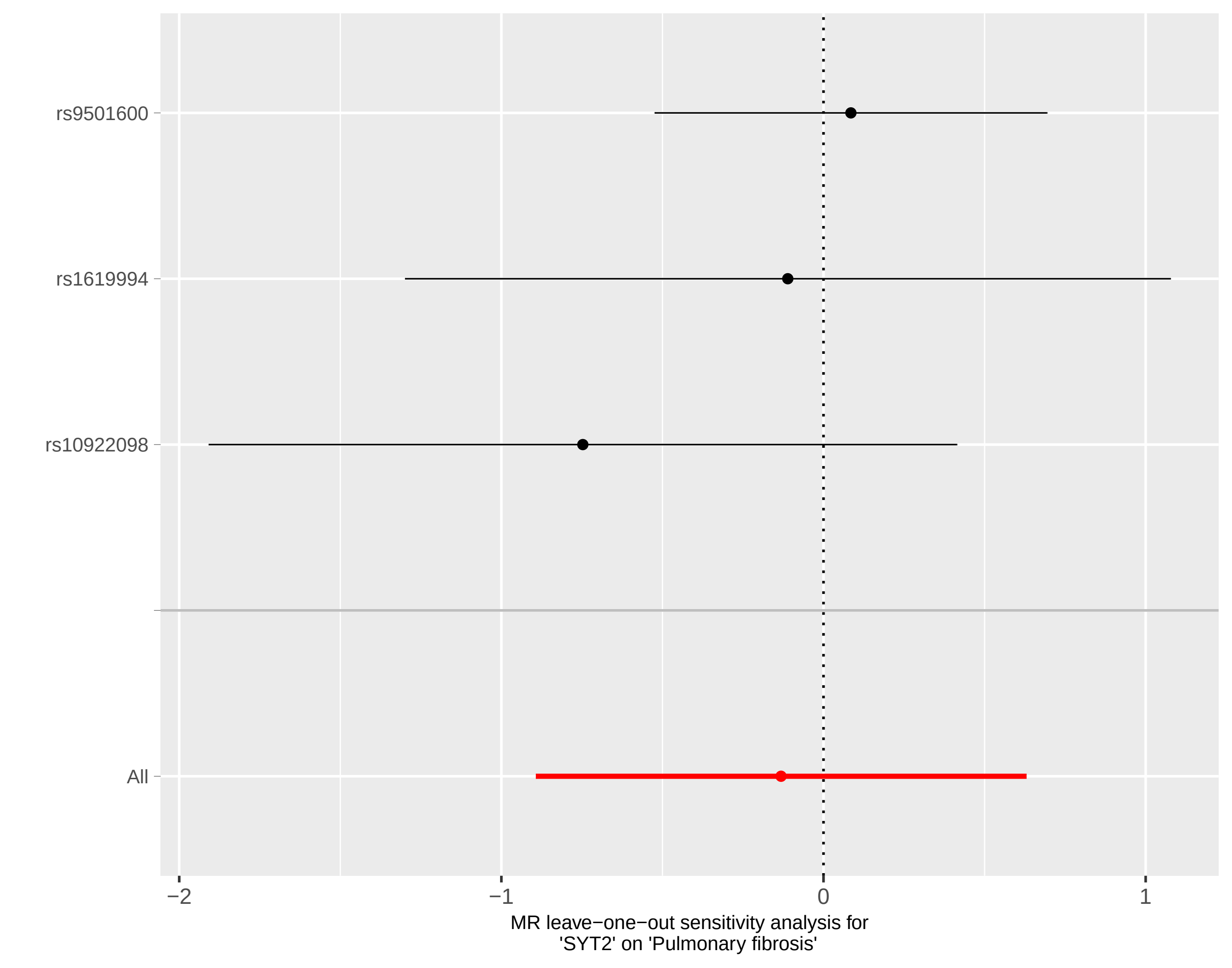
rs9501600
rs1619994
rs10922098
All
−2
−1
0
1
MR leave−one−out sensitivity analysis for
'SYT2' on 'Pulmonary fibrosis'

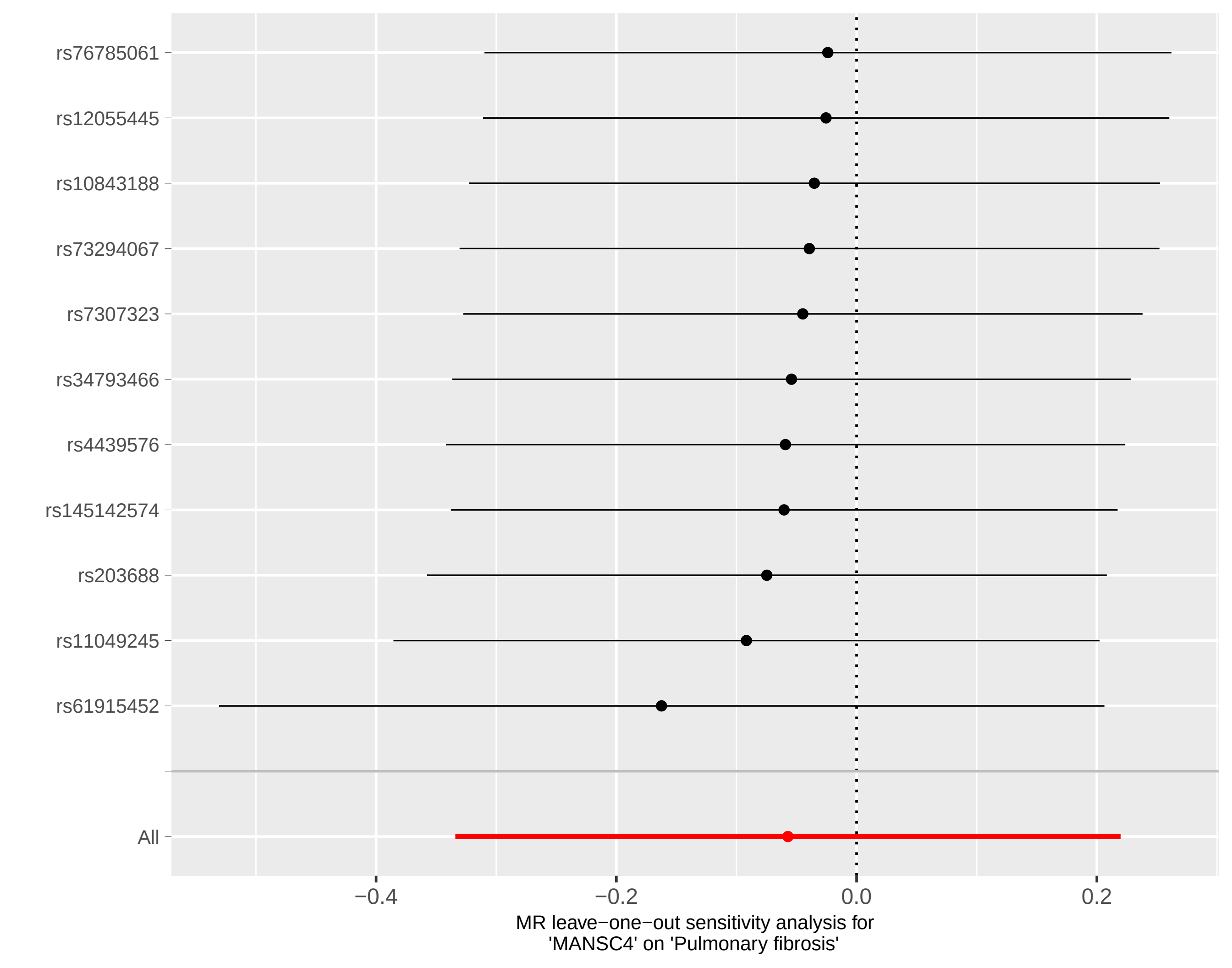
rs76785061
rs12055445
rs10843188
rs73294067
rs7307323
rs34793466
rs4439576
rs145142574
rs203688
rs11049245
rs61915452
All
−0.4
−0.2
0.0
0.2
MR leave−one−out sensitivity analysis for
'MANSC4' on 'Pulmonary fibrosis'

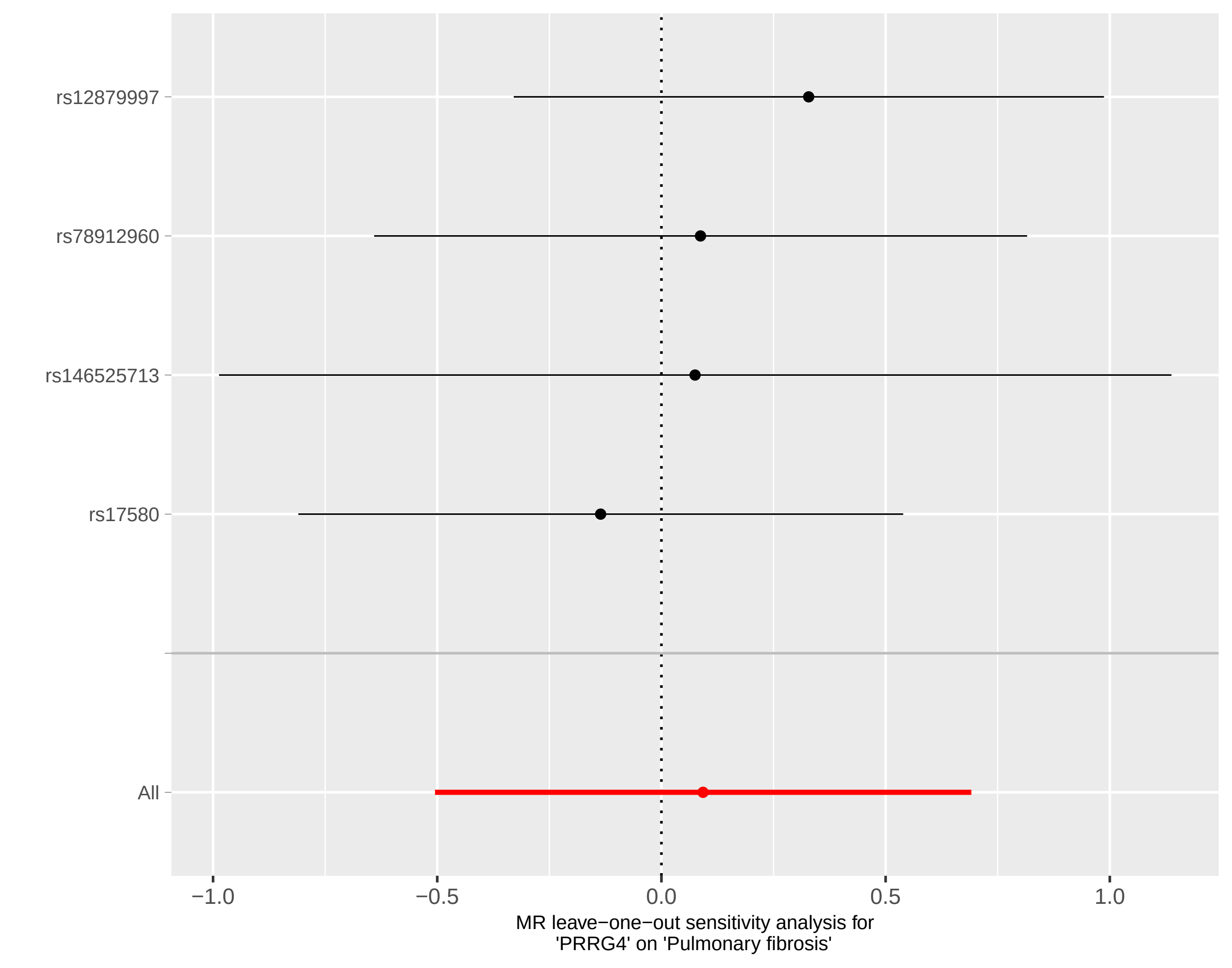

rs12879997
rs78912960
rs146525713
rs17580
All
−1.0
−0.5
0.0
0.5
1.0
MR leave−one−out sensitivity analysis for
'PRRG4' on 'Pulmonary fibrosis'

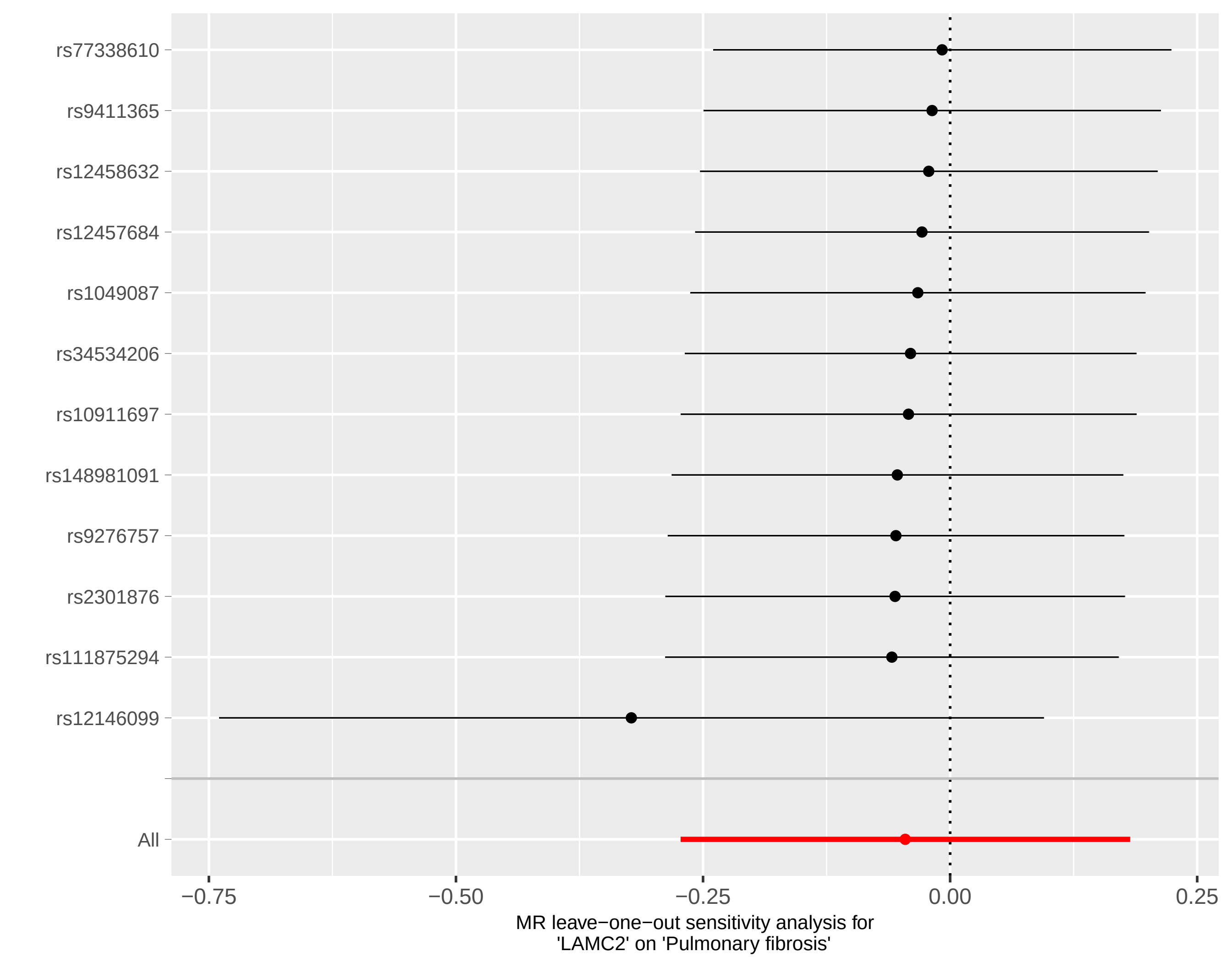
rs77338610
rs9411365
rs12458632
rs12457684
rs1049087
rs34534206
rs10911697
rs148981091
rs9276757
rs2301876
rs111875294
rs12146099
All
−0.75
−0.50
−0.25
0.00
0.25
MR leave−one−out sensitivity analysis for
'LAMC2' on 'Pulmonary fibrosis'

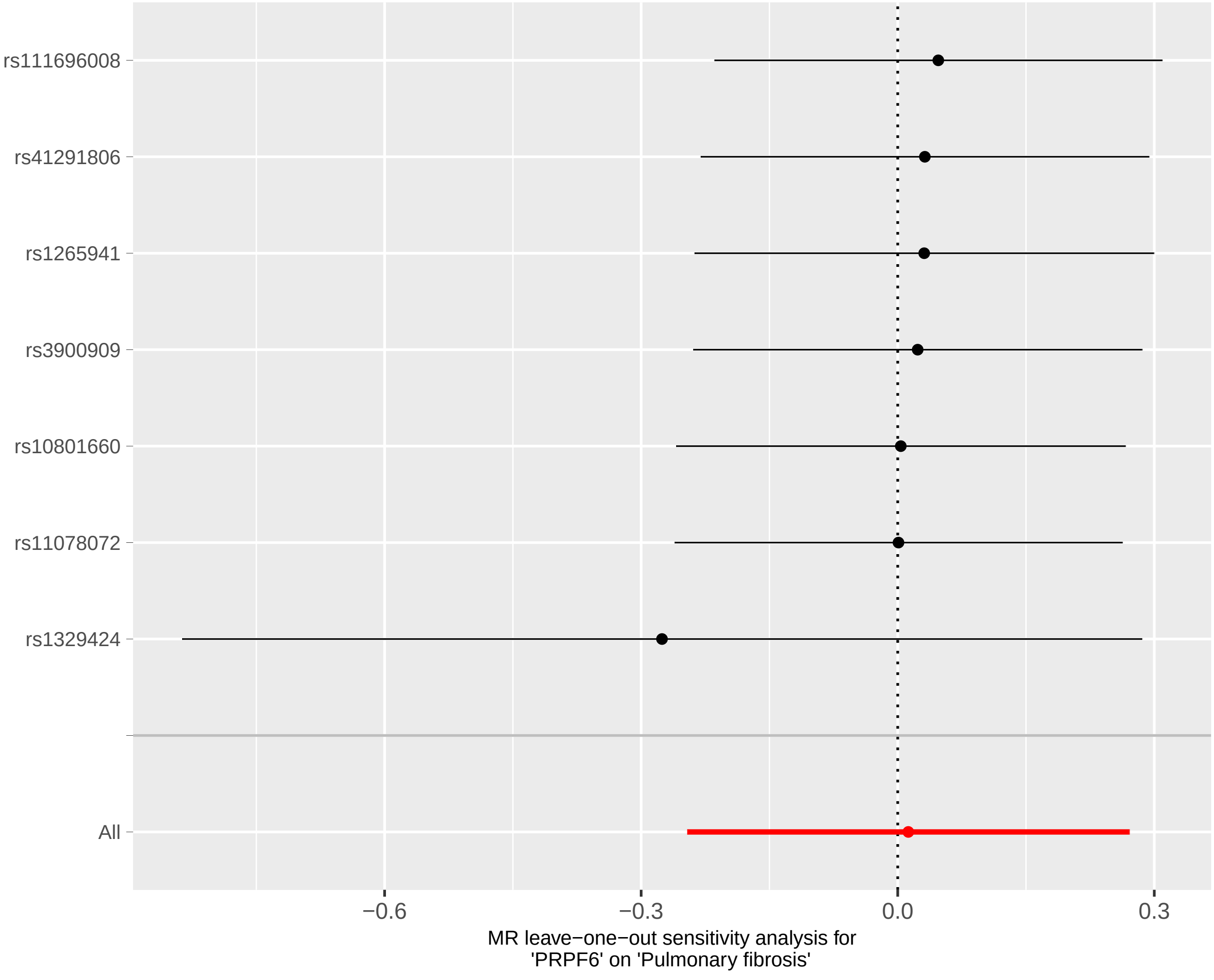

rs111696008
rs41291806
rs1265941
rs3900909
rs10801660
rs11078072
rs1329424
All
−0.6
−0.3
0.0
0.3
MR leave−one−out sensitivity analysis for
'PRPF6' on 'Pulmonary fibrosis'

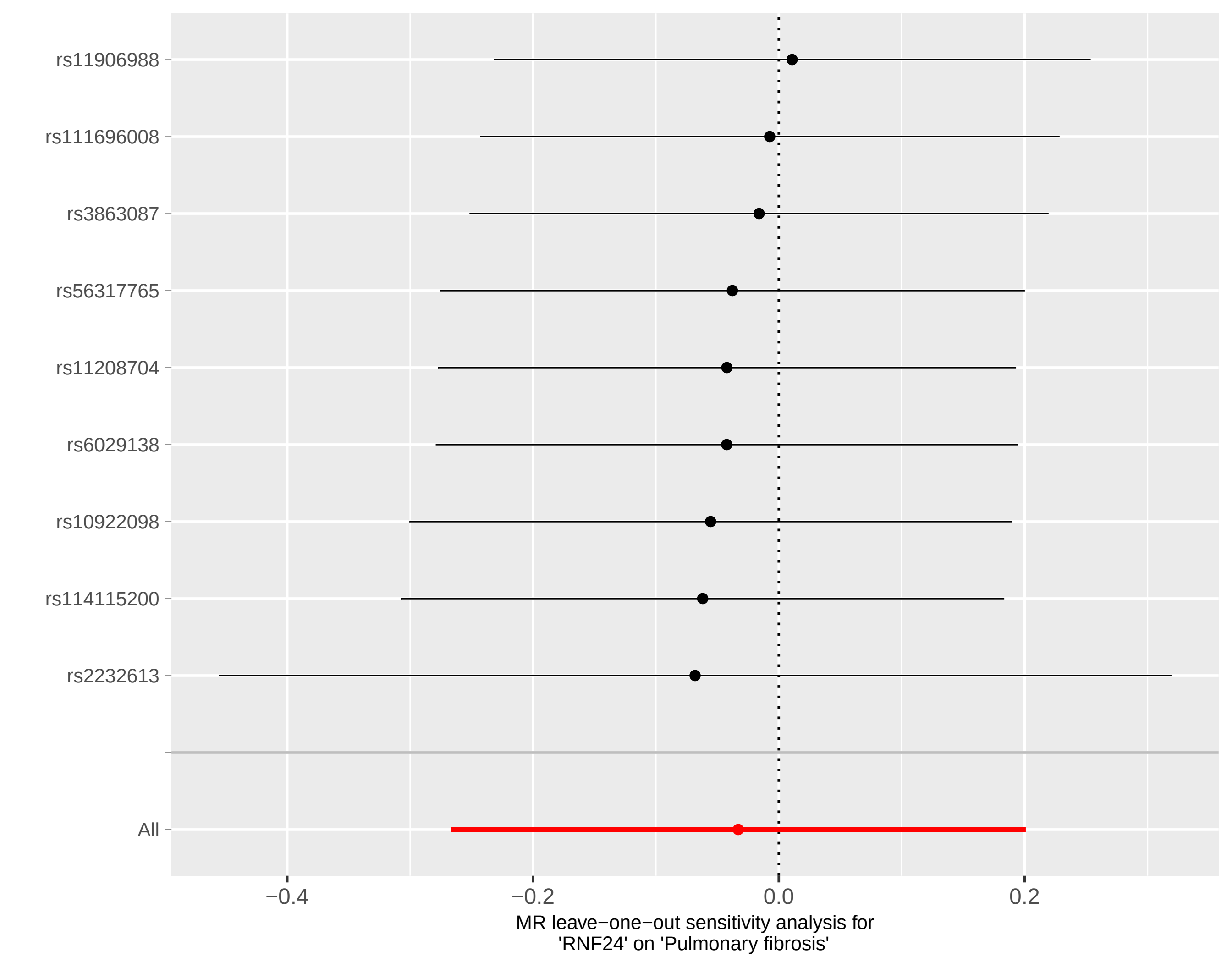
rs11906988
rs111696008
rs3863087
rs56317765
rs11208704
rs6029138
rs10922098
rs114115200
rs2232613
All
−0.4
−0.2
0.0
0.2
MR leave−one−out sensitivity analysis for
'RNF24' on 'Pulmonary fibrosis'

Insufficient number of SNPs

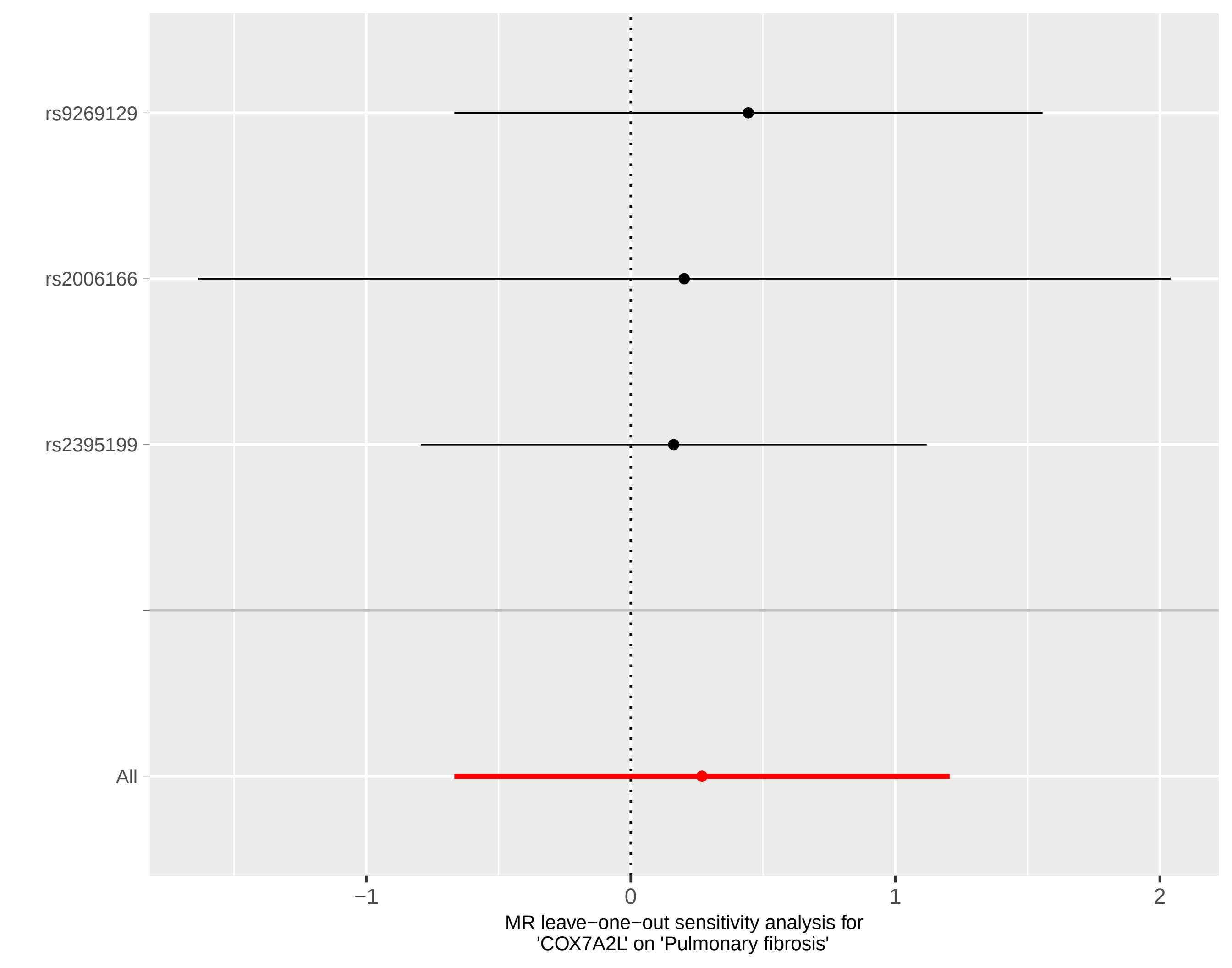
rs9269129
rs2006166
rs2395199
All
−1
0
1
2
MR leave−one−out sensitivity analysis for
'COX7A2L' on 'Pulmonary fibrosis'

Insufficient number of SNPs

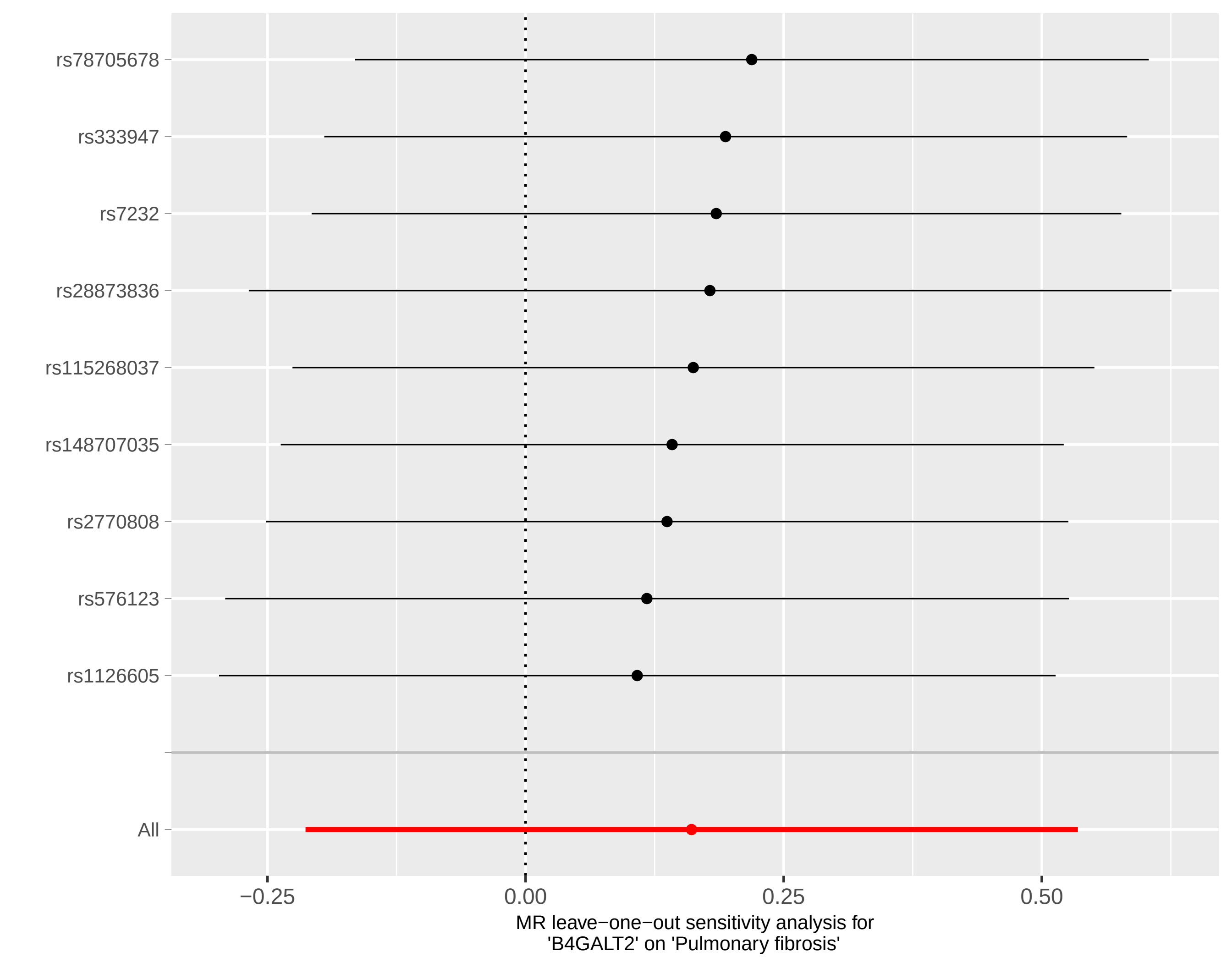

rs78705678
rs333947
rs7232
rs28873836
rs115268037
rs148707035
rs2770808
rs576123
rs1126605
All
−0.25
0.00
0.25
0.50
MR leave−one−out sensitivity analysis for
'B4GALT2' on 'Pulmonary fibrosis'

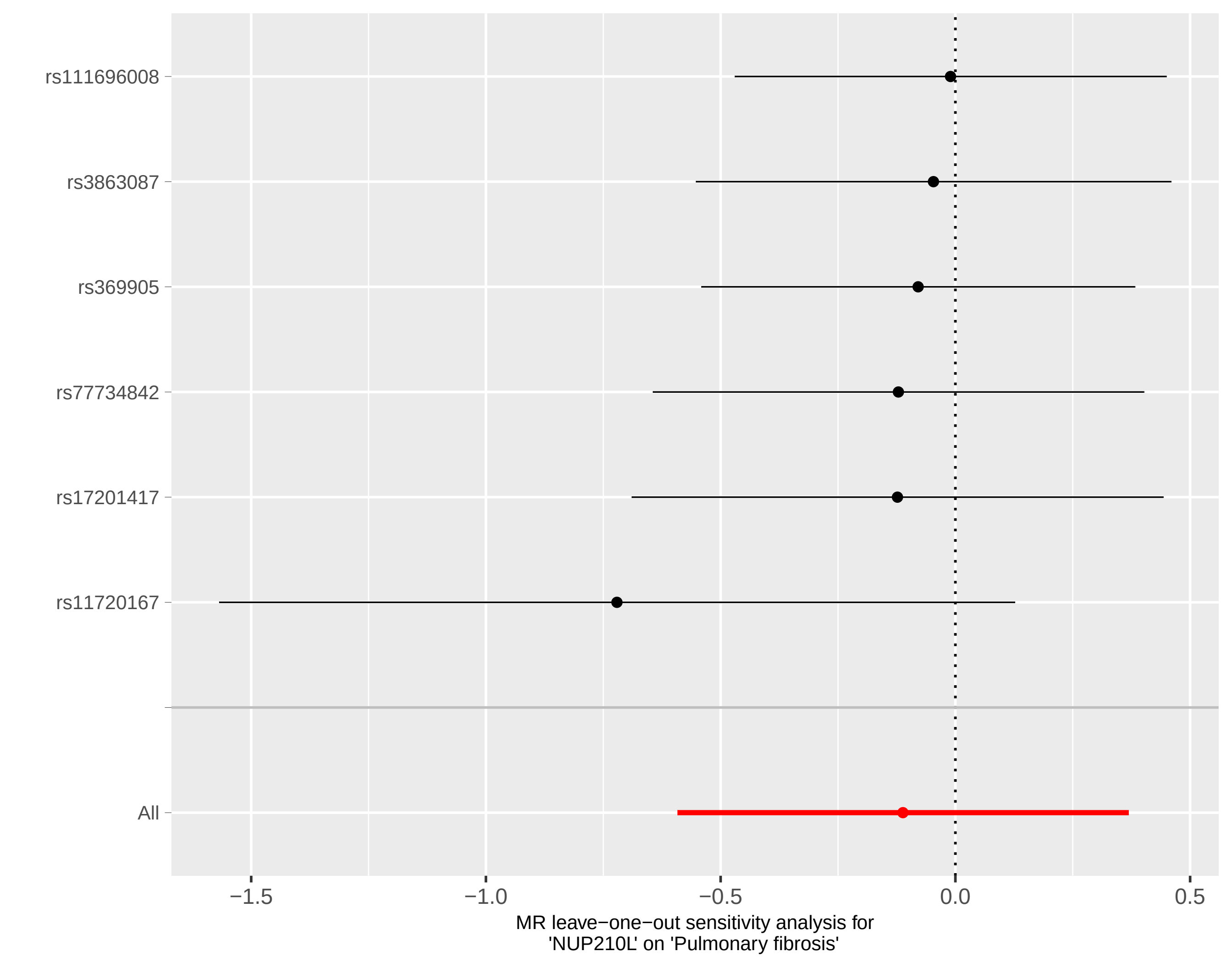

rs111696008
rs3863087
rs369905
rs77734842
rs17201417
rs11720167
All
−1.5
−1.0
−0.5
0.0
0.5
MR leave−one−out sensitivity analysis for
'NUP210L' on 'Pulmonary fibrosis'

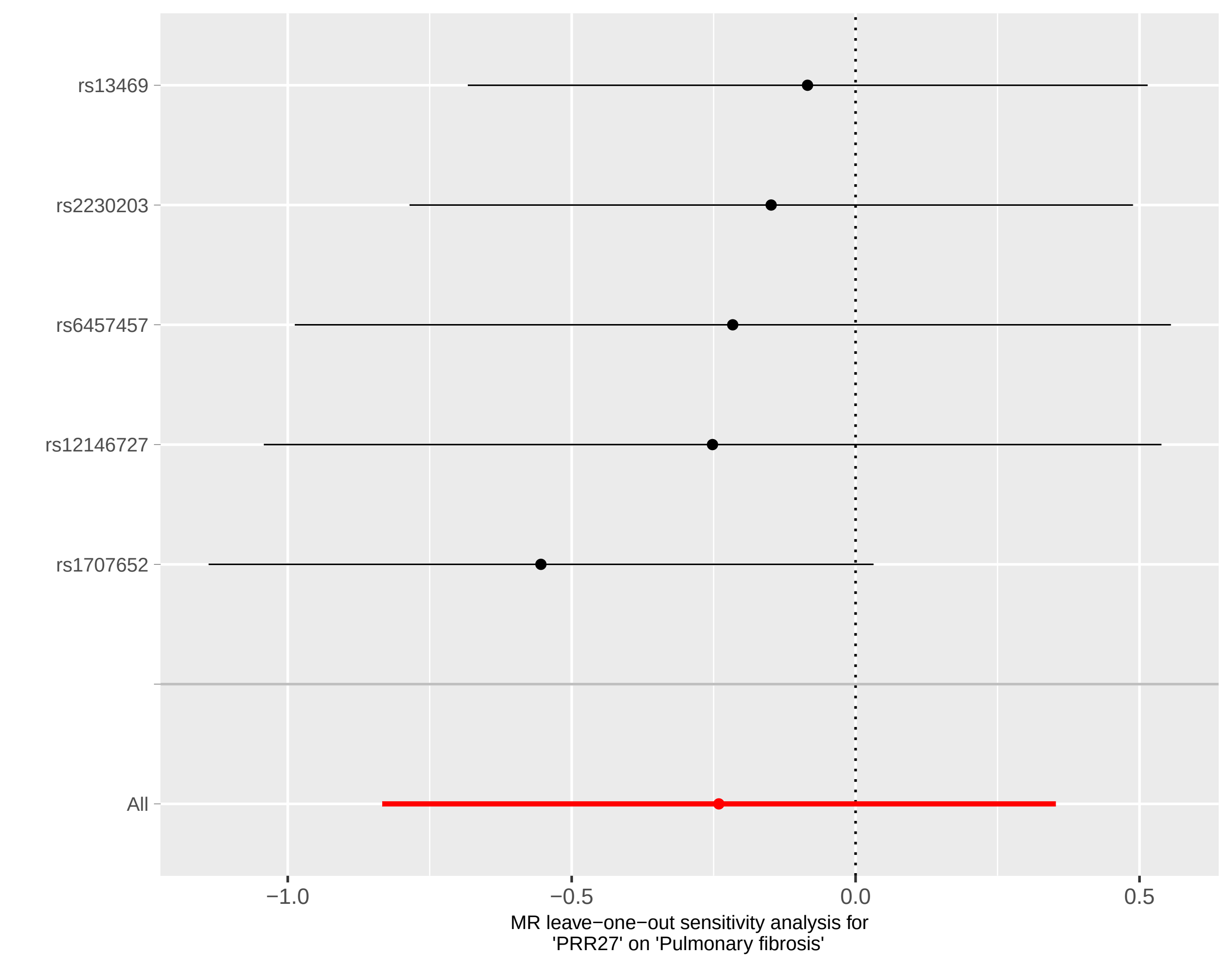

rs13469
rs2230203
rs6457457
rs12146727
rs1707652
All
−1.0
−0.5
0.0
0.5
MR leave−one−out sensitivity analysis for
'PRR27' on 'Pulmonary fibrosis'

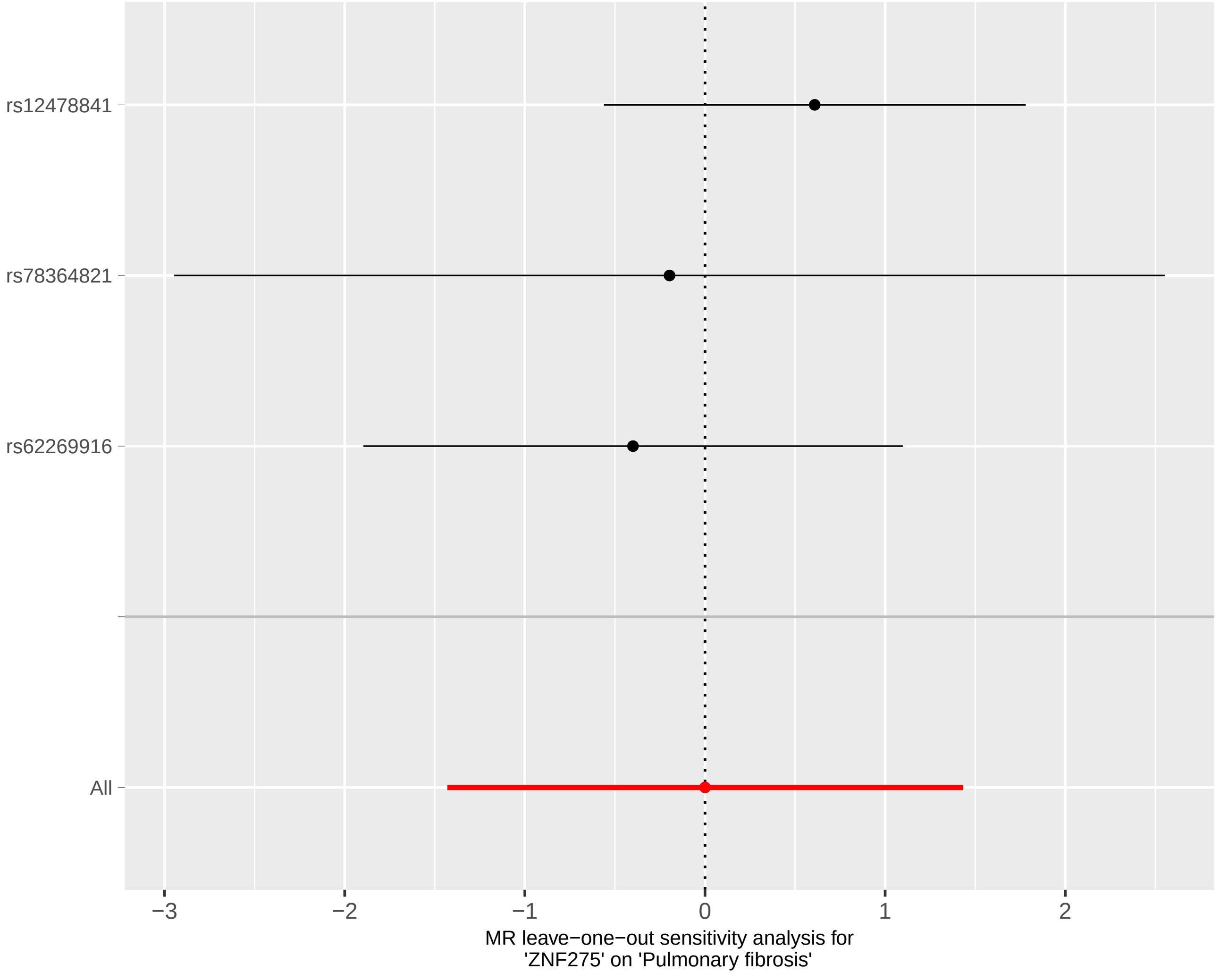

rs12478841
rs78364821
rs62269916
All
−3
−2
−1
0
1
2
MR leave−one−out sensitivity analysis for
'ZNF275' on 'Pulmonary fibrosis'

Insufficient number of SNPs

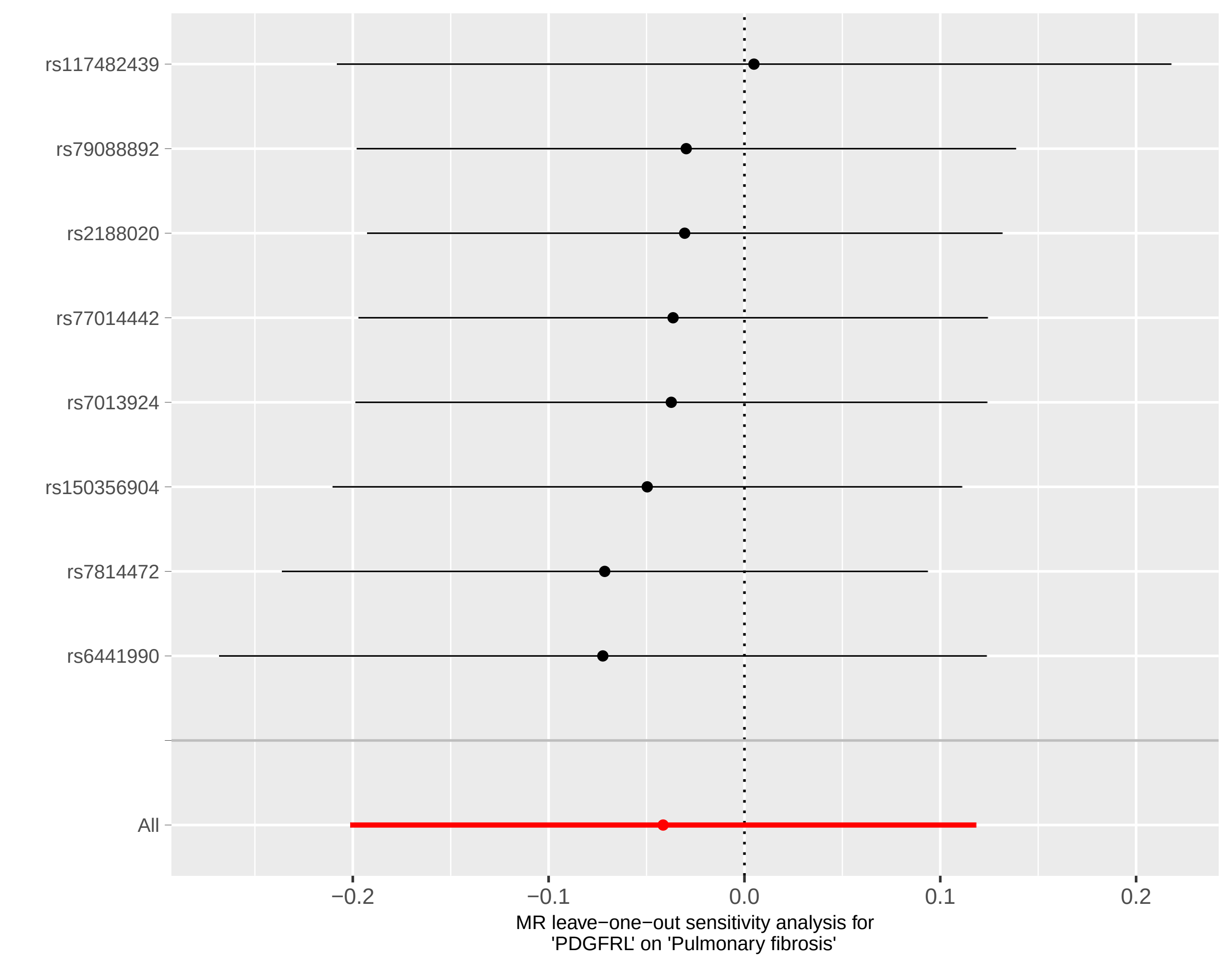
rs117482439
rs79088892
rs2188020
rs77014442
rs7013924
rs150356904
rs7814472
rs6441990
All
−0.2
−0.1
0.0
0.1
0.2
MR leave−one−out sensitivity analysis for
'PDGFRL' on 'Pulmonary fibrosis'

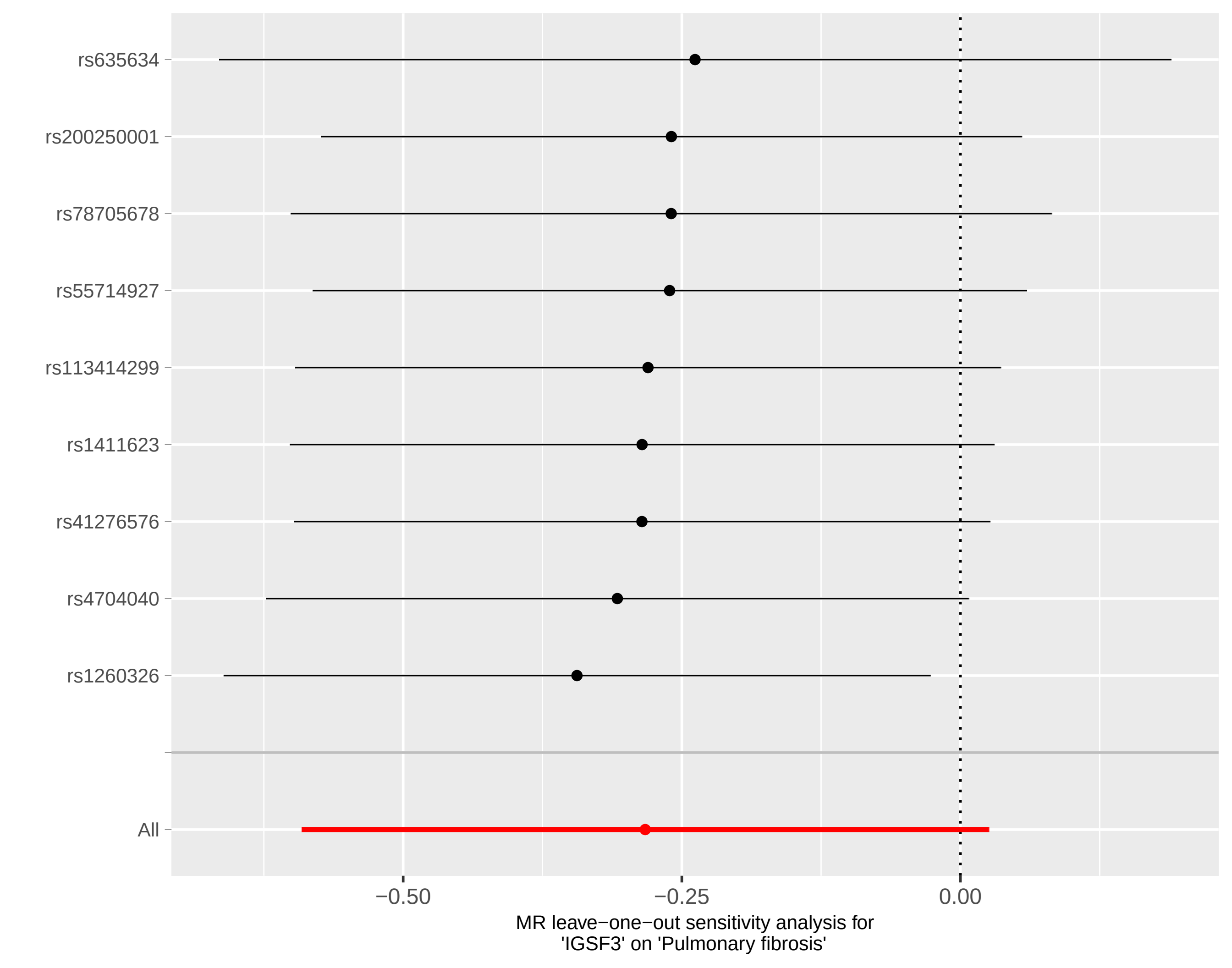
rs635634
rs200250001
rs78705678
rs55714927
rs113414299
rs1411623
rs41276576
rs4704040
rs1260326
All
−0.50
−0.25
0.00
MR leave−one−out sensitivity analysis for
'IGSF3' on 'Pulmonary fibrosis'

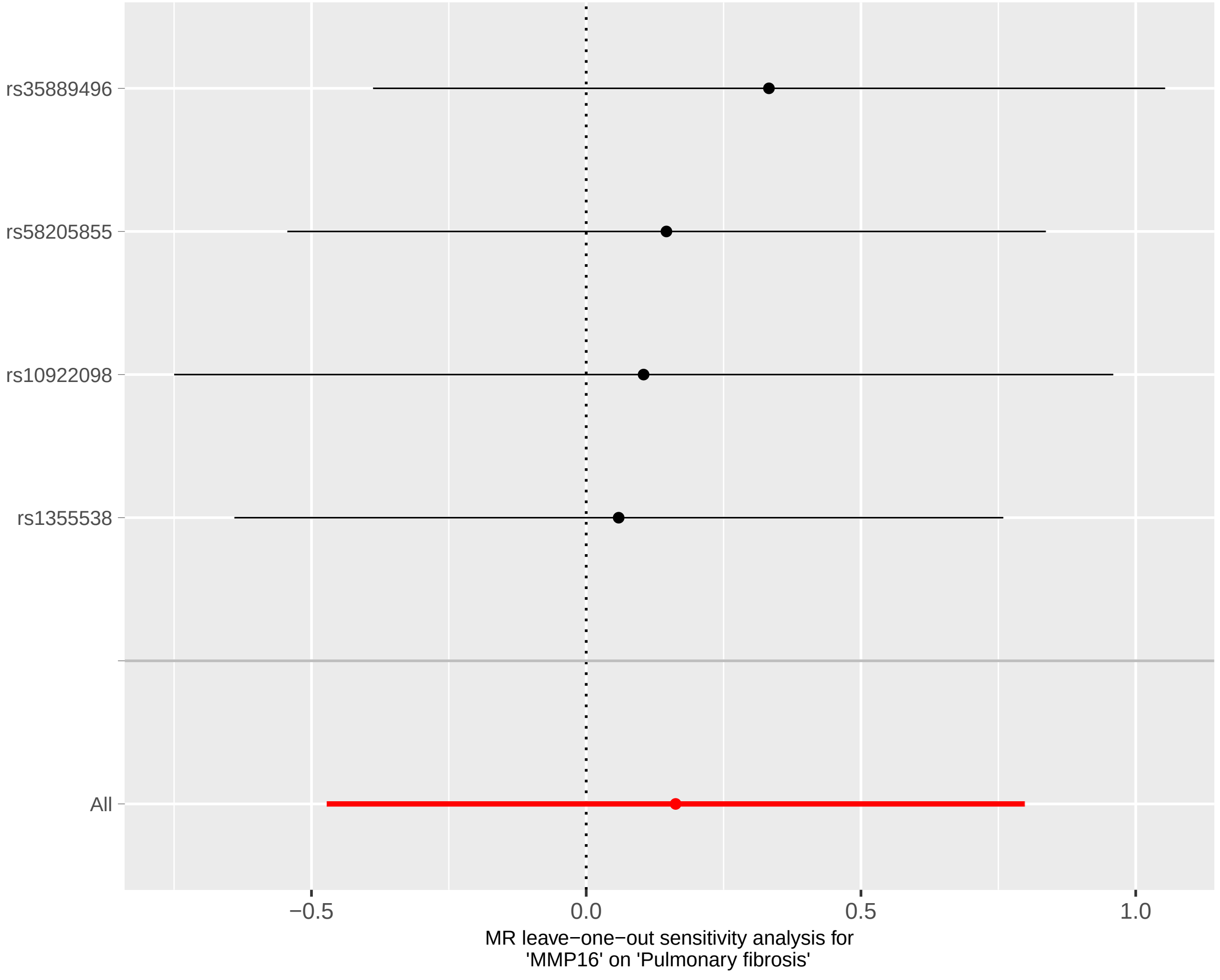
rs35889496
rs58205855
rs10922098
rs1355538
All
−0.5
0.0
0.5
1.0
MR leave−one−out sensitivity analysis for
'MMP16' on 'Pulmonary fibrosis'

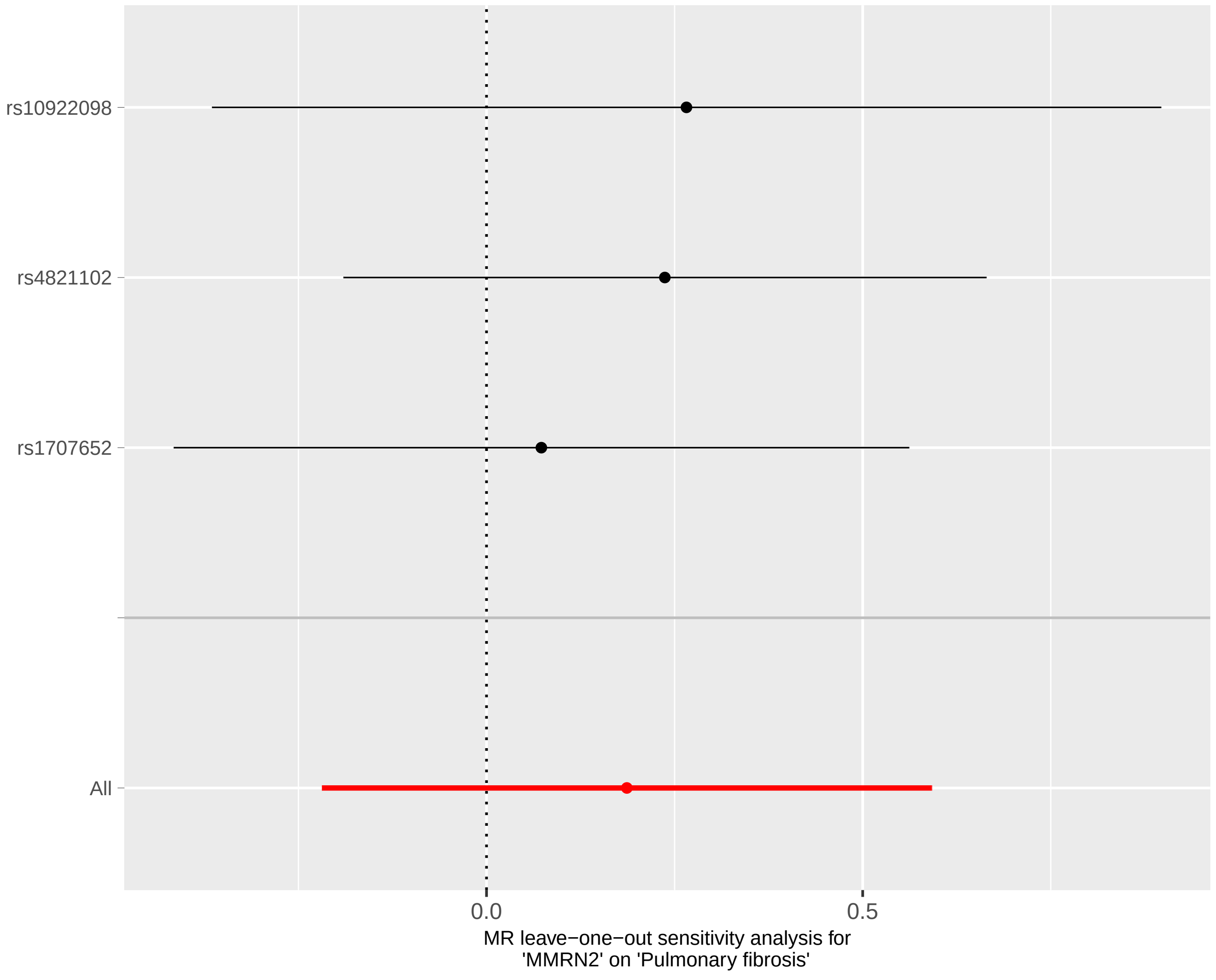

rs10922098
rs4821102
rs1707652
All
0.0
0.5
MR leave−one−out sensitivity analysis for
'MMRN2' on 'Pulmonary fibrosis'

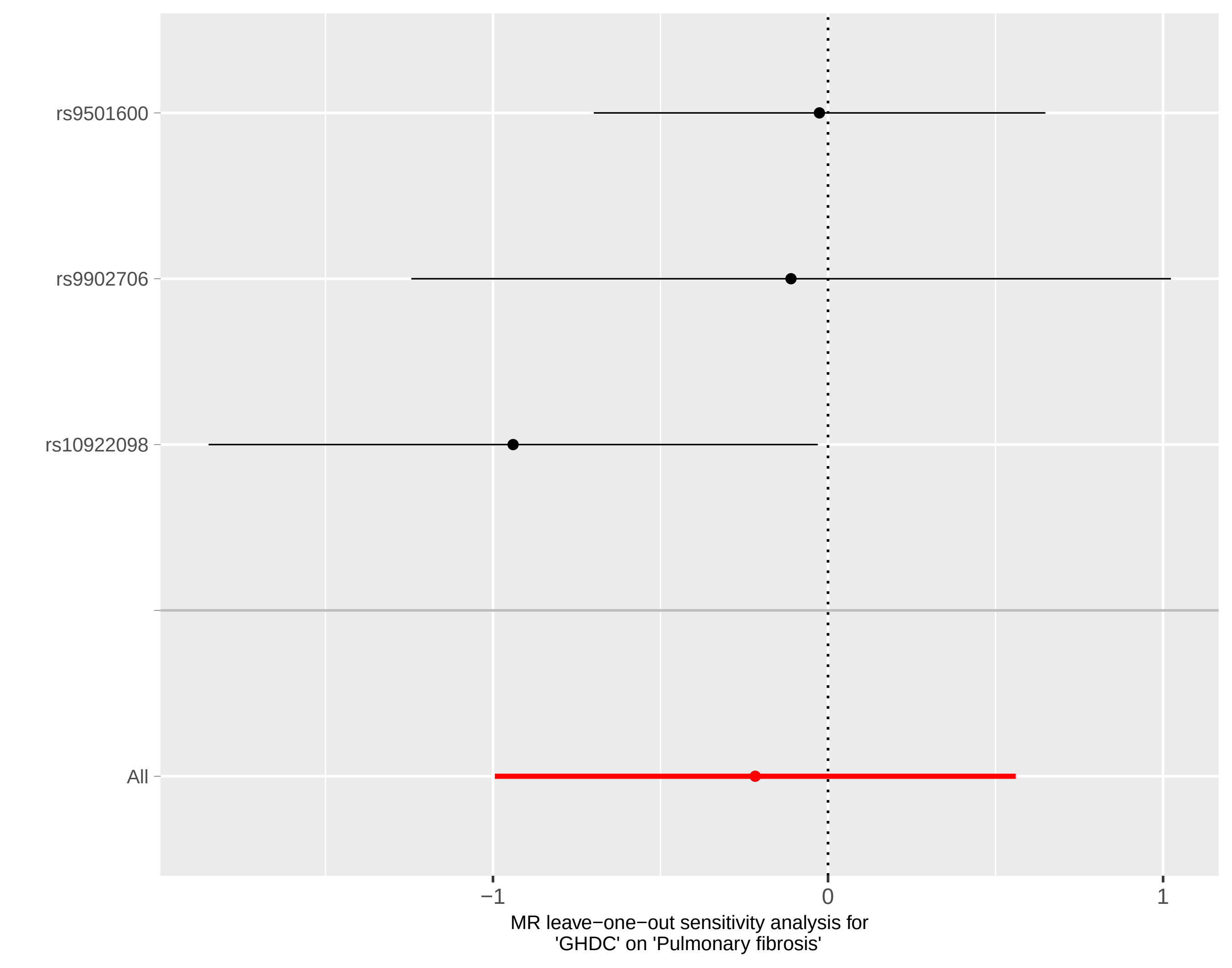
rs9501600
rs9902706
rs10922098
All
−1
0
1
MR leave−one−out sensitivity analysis for
'GHDC' on 'Pulmonary fibrosis'

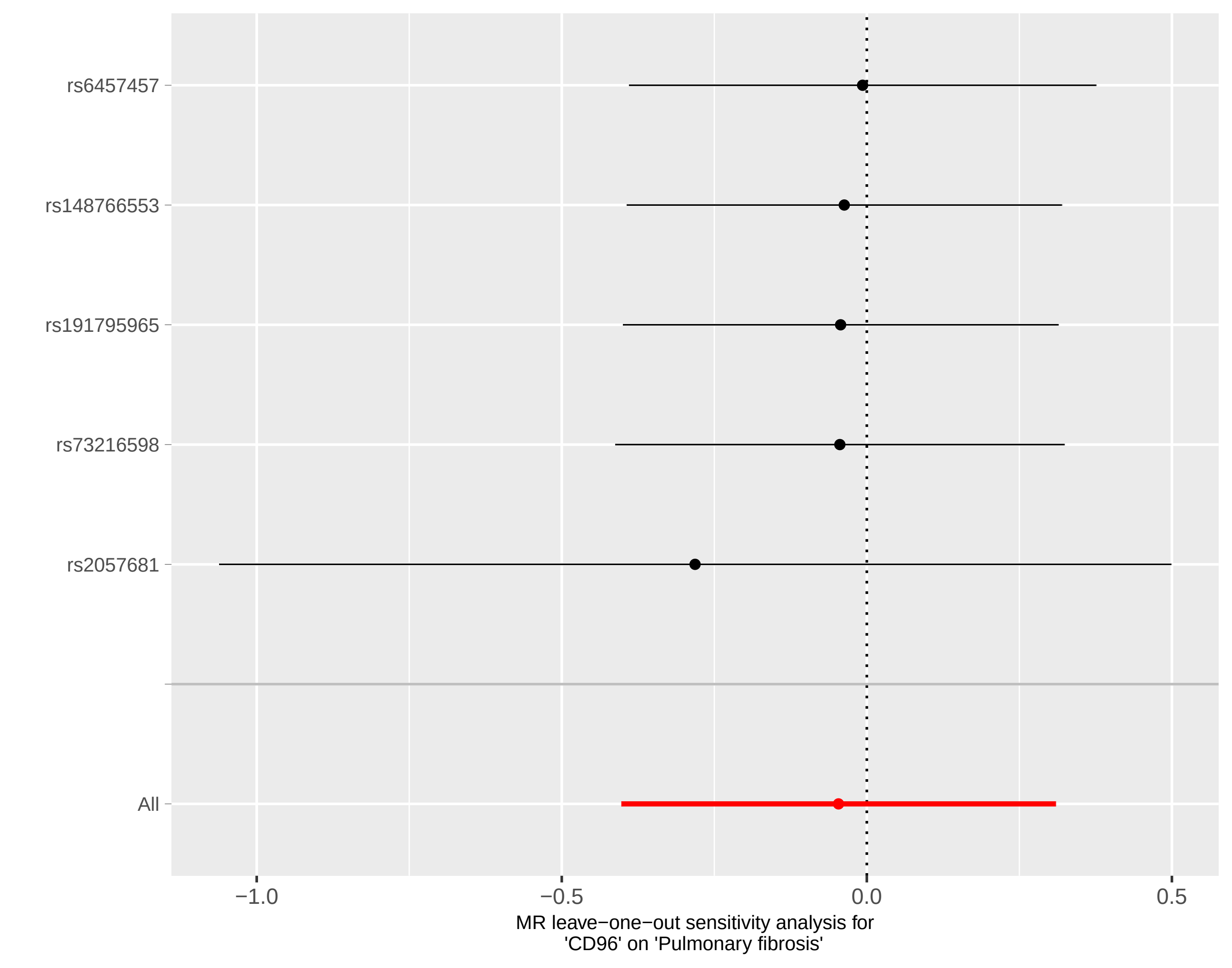
rs6457457
rs148766553
rs191795965
rs73216598
rs2057681
All
−1.0
−0.5
0.0
0.5
MR leave−one−out sensitivity analysis for
'CD96' on 'Pulmonary fibrosis'

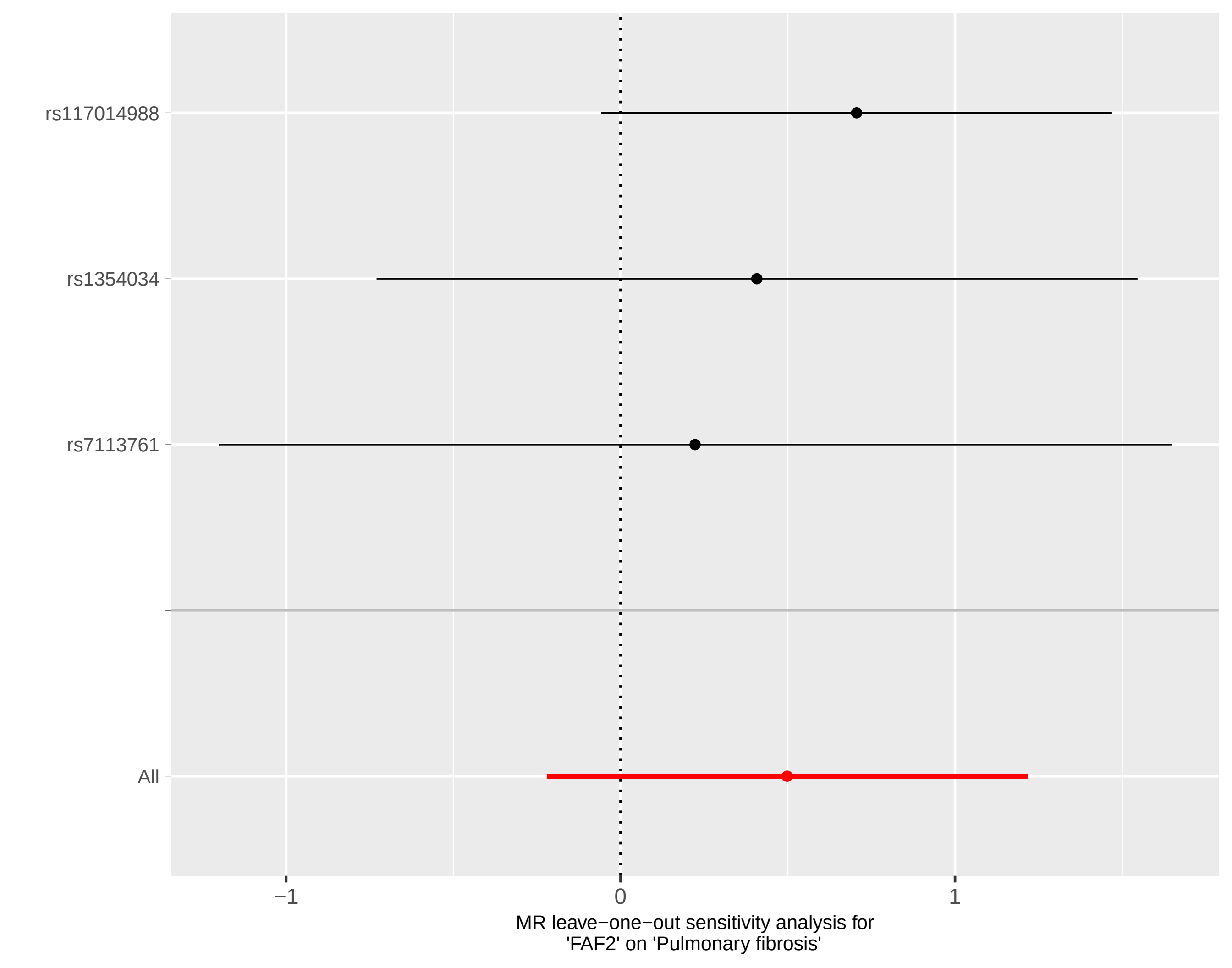
rs117014988
rs1354034
rs7113761
All
−1
0
1
MR leave−one−out sensitivity analysis for
'FAF2' on 'Pulmonary fibrosis'

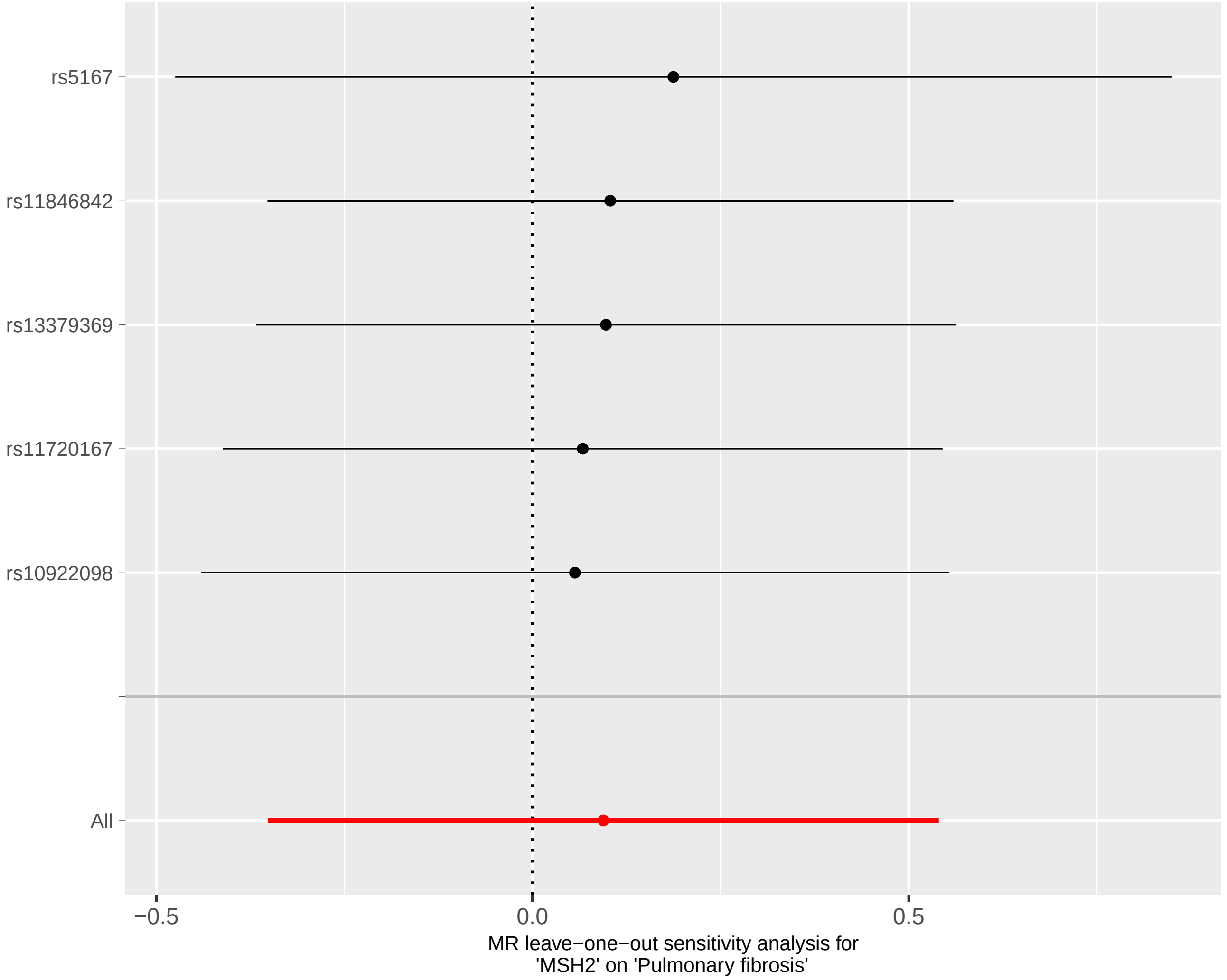

rs5167
rs11846842
rs13379369
rs11720167
rs10922098
All
−0.5
0.0
0.5
MR leave−one−out sensitivity analysis for
'MSH2' on 'Pulmonary fibrosis'

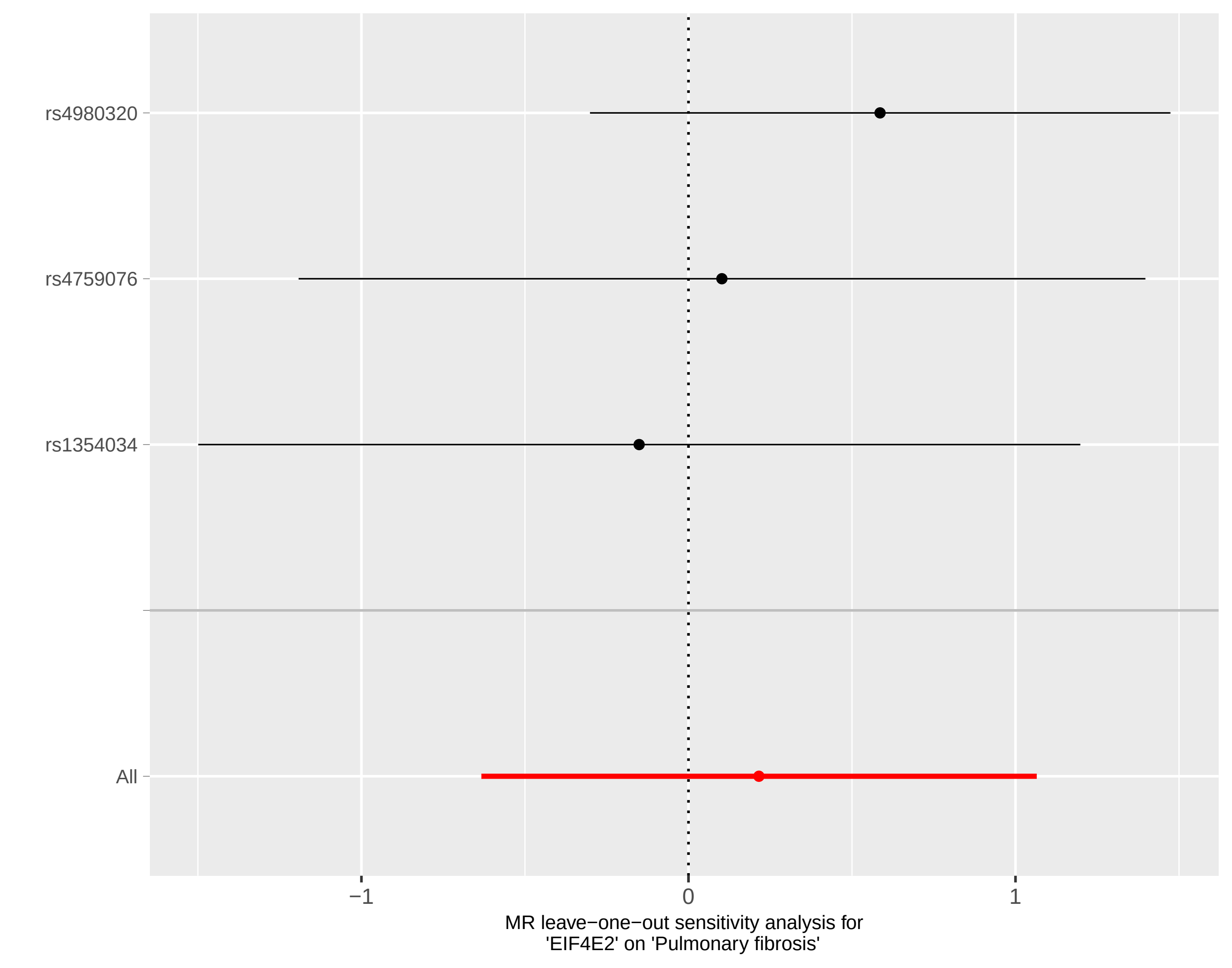
rs4980320
rs4759076
rs1354034
All
−1
0
1
MR leave−one−out sensitivity analysis for
'EIF4E2' on 'Pulmonary fibrosis'

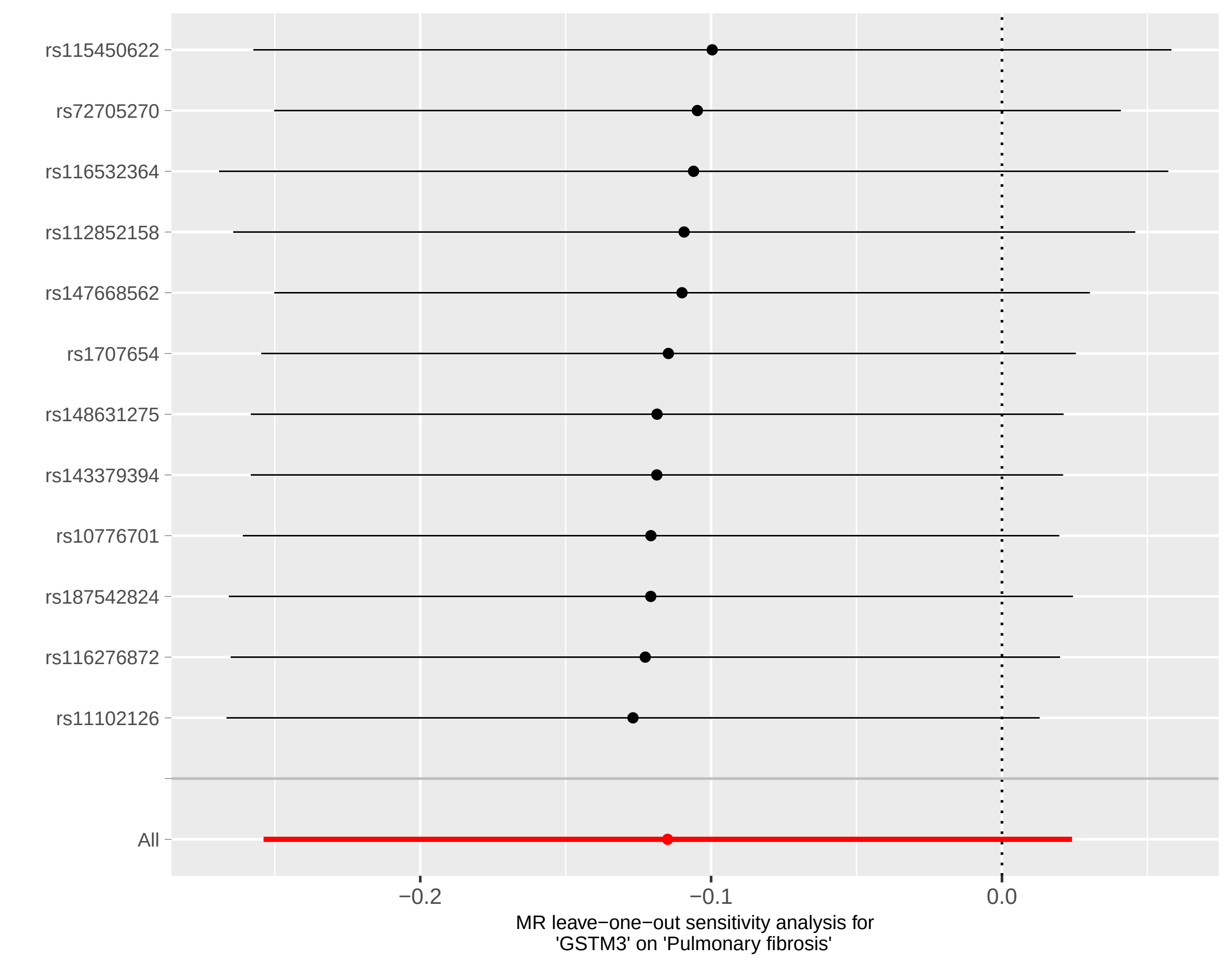

rs115450622
rs72705270
rs116532364
rs112852158
rs147668562
rs1707654
rs148631275
rs143379394
rs10776701
rs187542824
rs116276872
rs11102126
All
−0.2
−0.1
0.0
MR leave−one−out sensitivity analysis for
'GSTM3' on 'Pulmonary fibrosis'

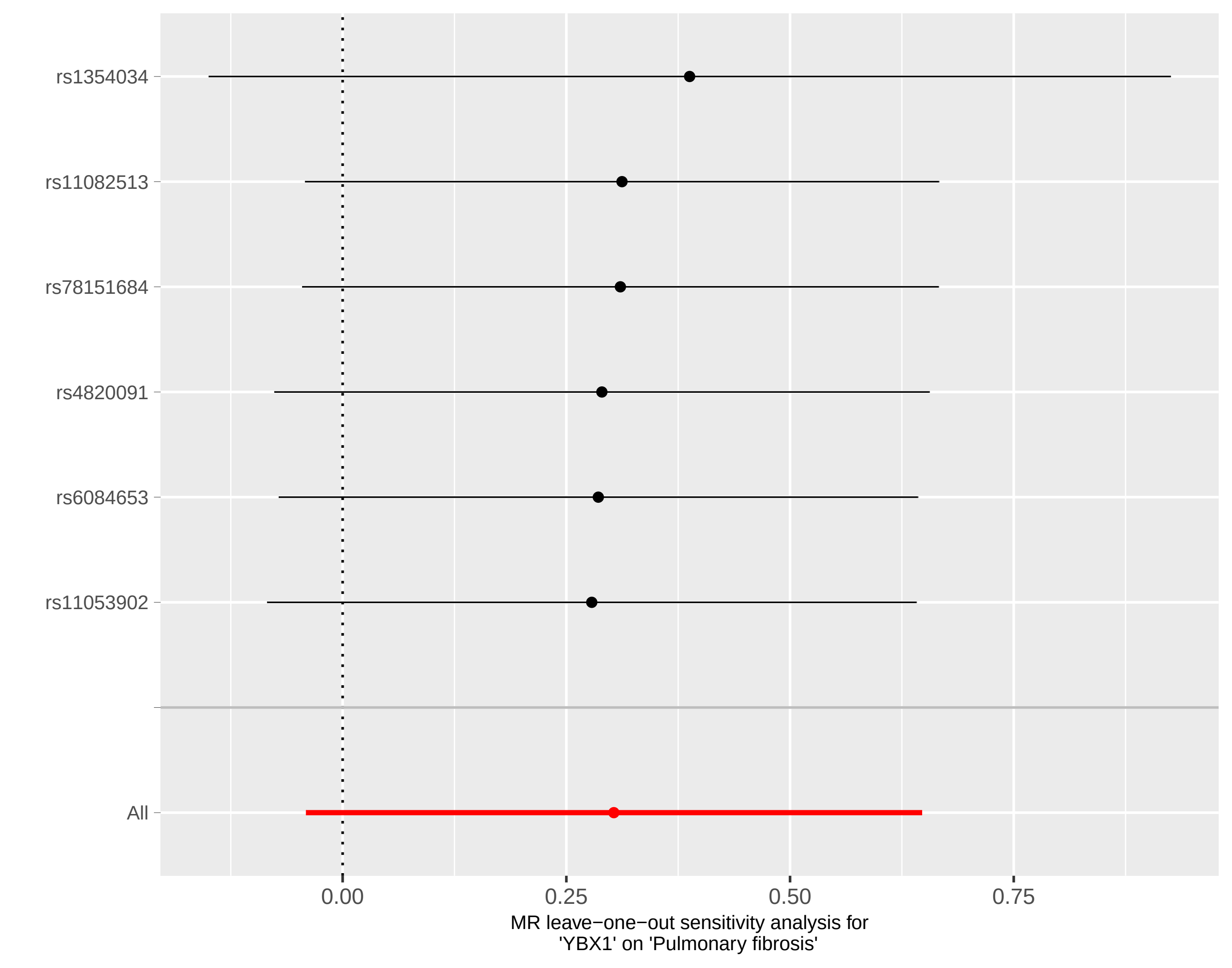
rs1354034
rs11082513
rs78151684
rs4820091
rs6084653
rs11053902
All
0.00
0.25
0.50
0.75
MR leave−one−out sensitivity analysis for
'YBX1' on 'Pulmonary fibrosis'

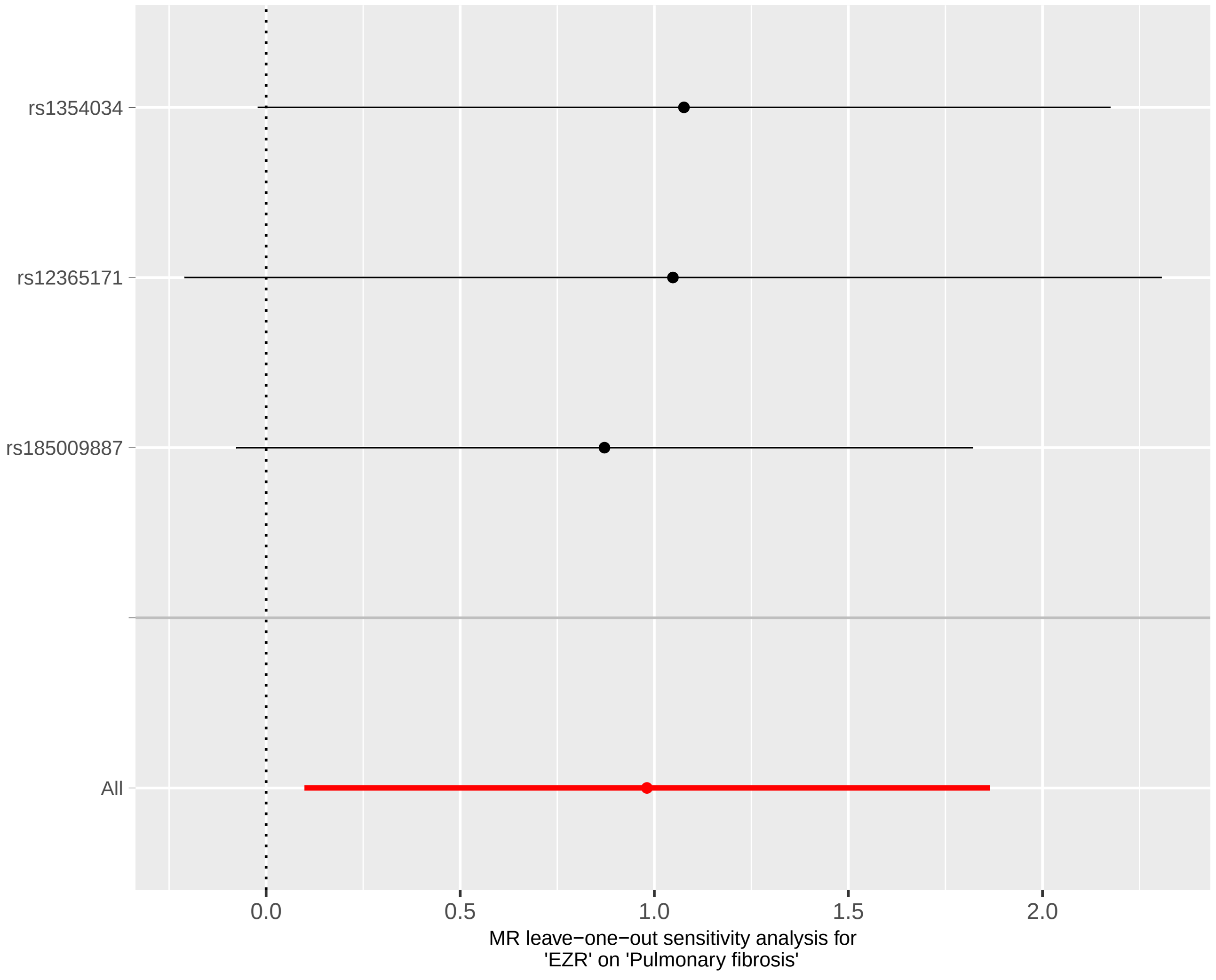

rs1354034
rs12365171
rs185009887
All
0.0
0.5
1.0
1.5
2.0
MR leave−one−out sensitivity analysis for
'EZR' on 'Pulmonary fibrosis'

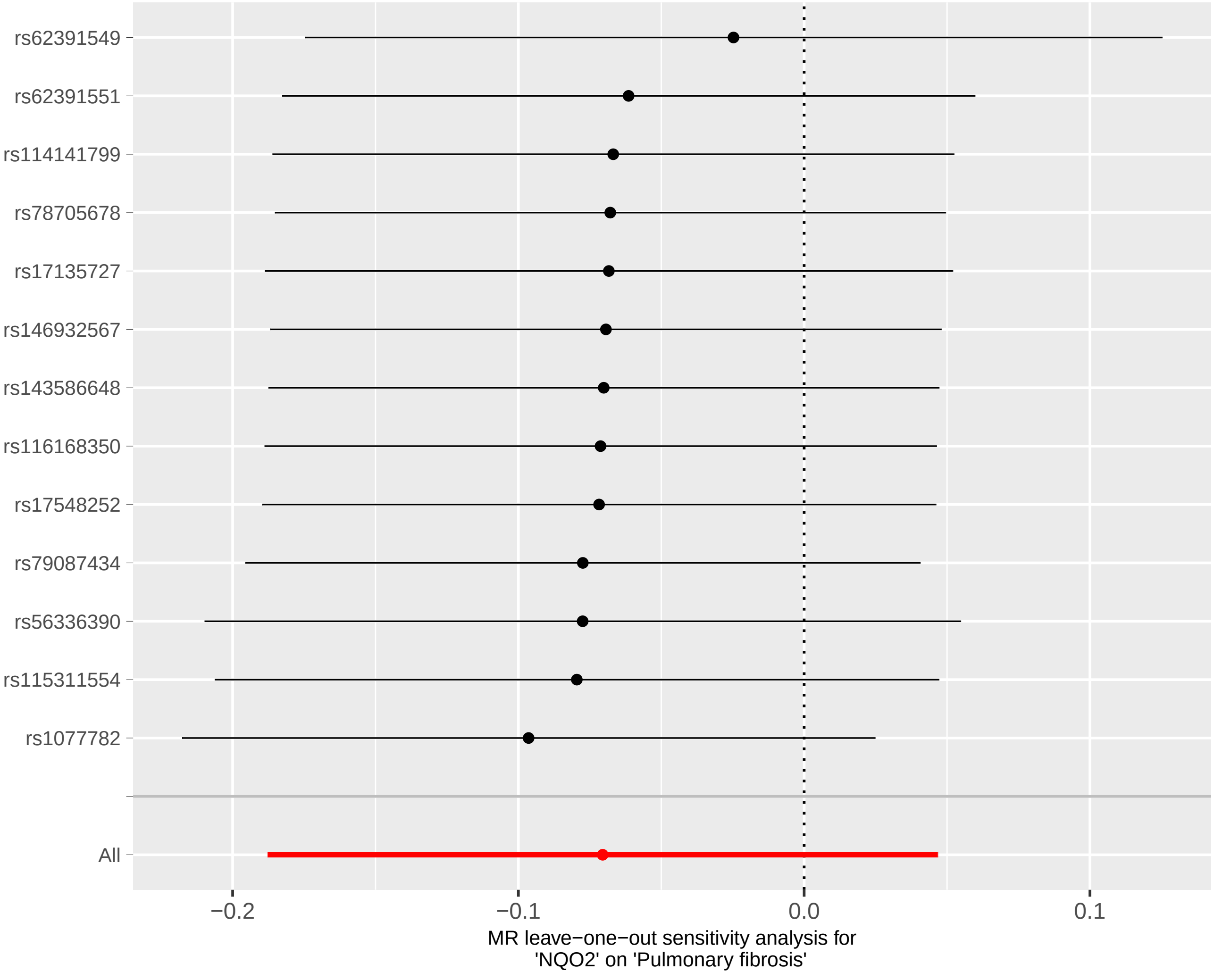

rs62391549
rs62391551
rs114141799
rs78705678
rs17135727
rs146932567
rs143586648
rs116168350
rs17548252
rs79087434
rs56336390
rs115311554
rs1077782
All
−0.2
−0.1
0.0
0.1
MR leave−one−out sensitivity analysis for
'NQO2' on 'Pulmonary fibrosis'

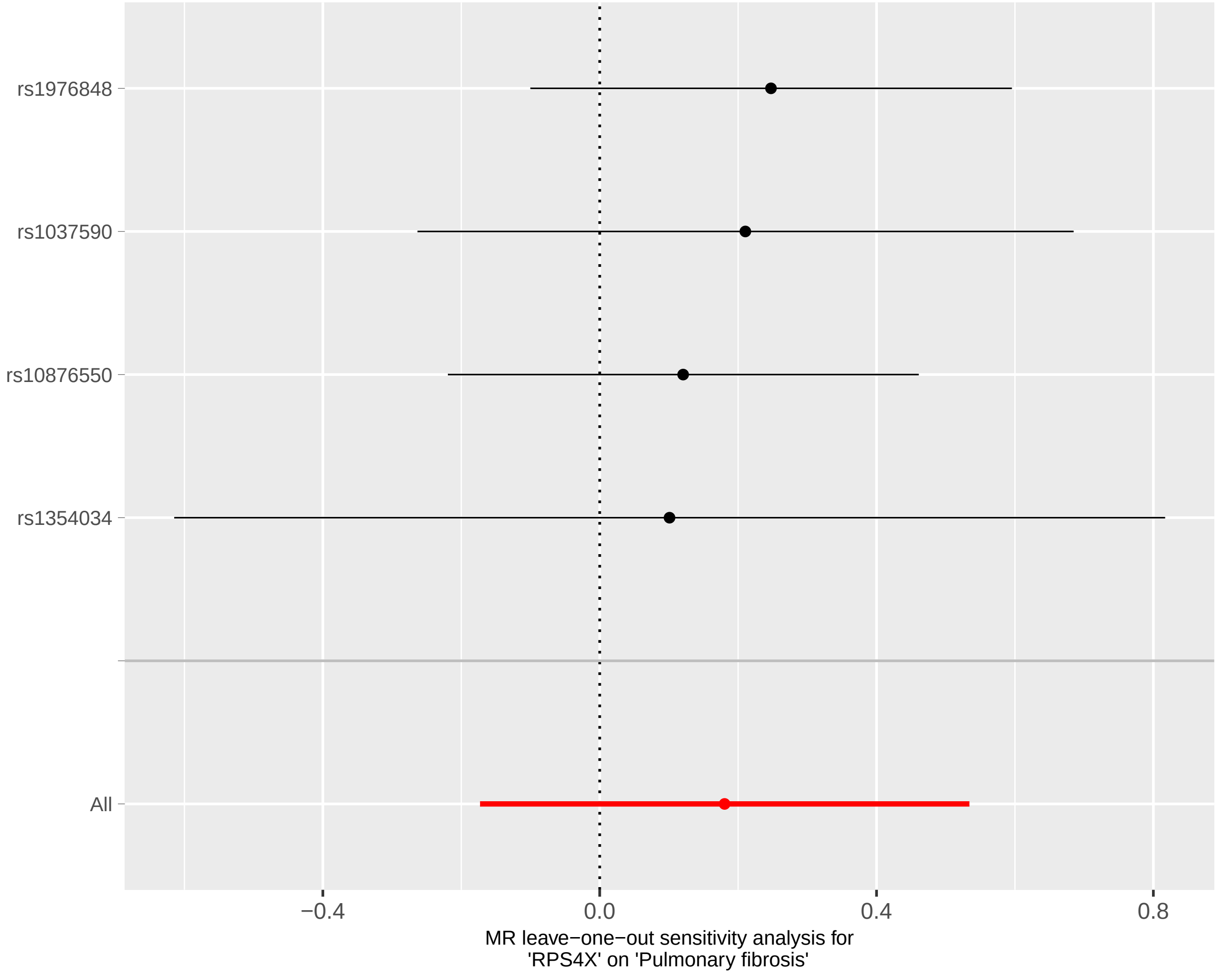
rs1976848
rs1037590
rs10876550
rs1354034
All
−0.4
0.0
0.4
0.8
MR leave−one−out sensitivity analysis for
'RPS4X' on 'Pulmonary fibrosis'

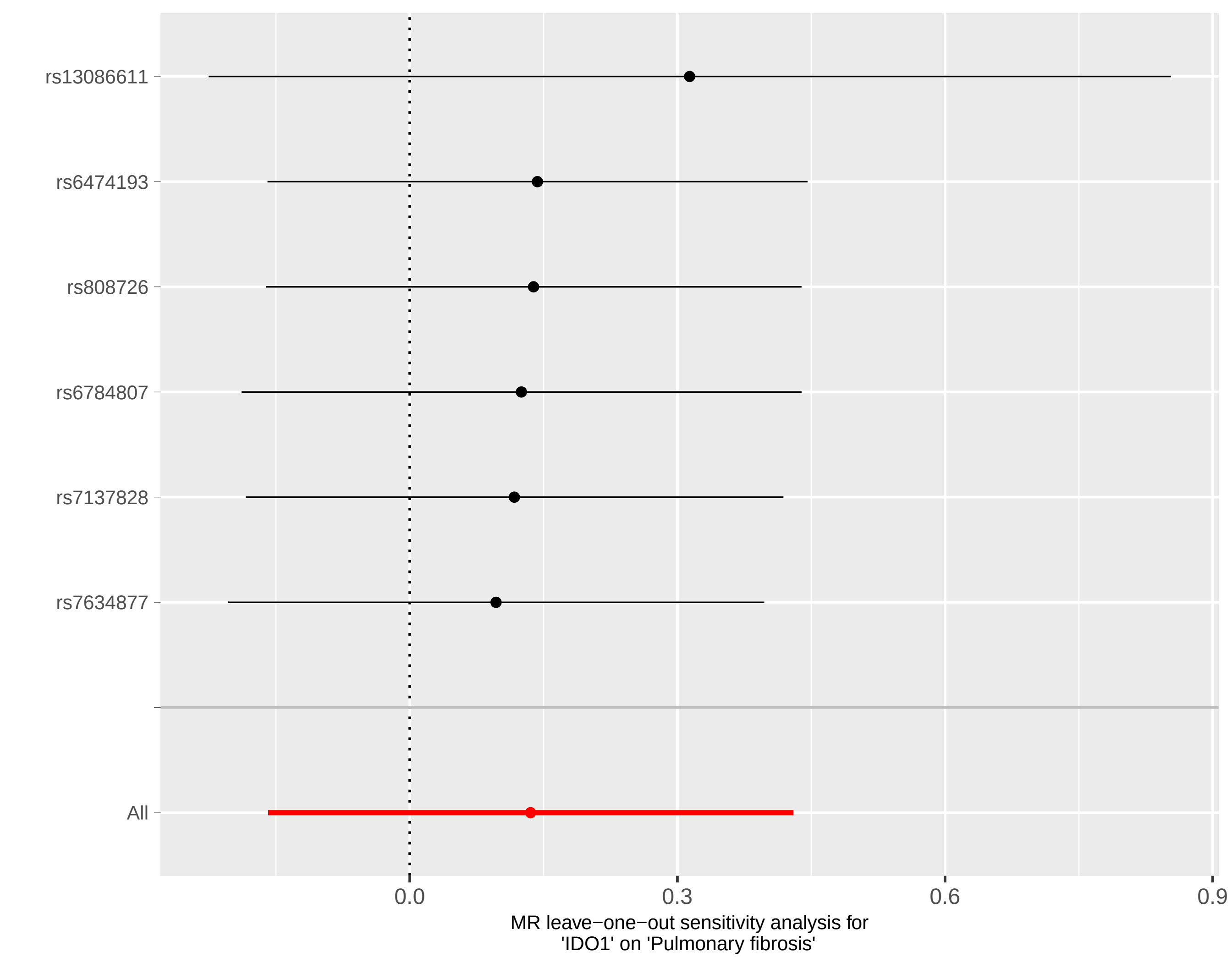
rs13086611
rs6474193
rs808726
rs6784807
rs7137828
rs7634877
All
0.0
0.3
0.6
0.9
MR leave−one−out sensitivity analysis for
'IDO1' on 'Pulmonary fibrosis'

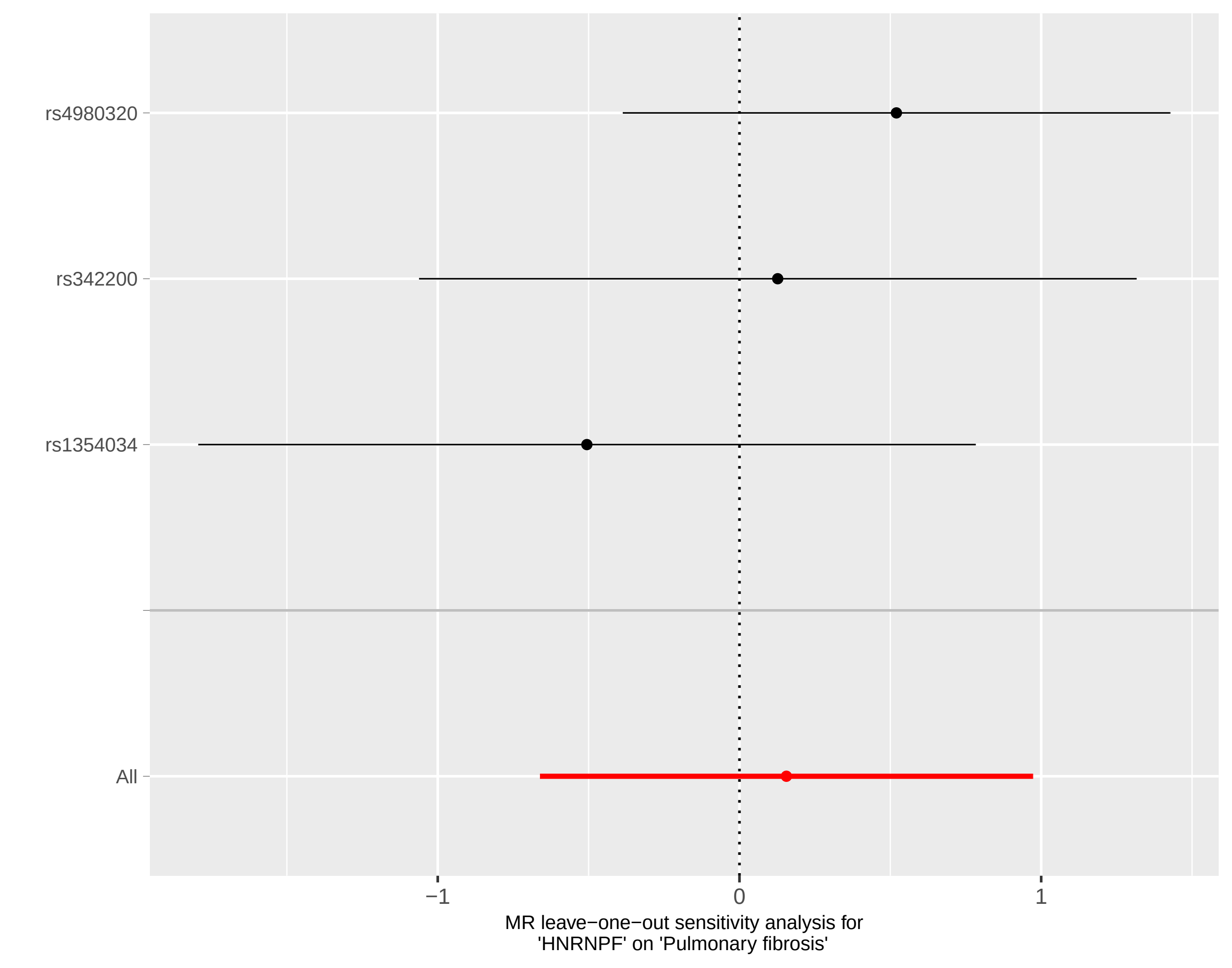
rs4980320
rs342200
rs1354034
All
−1
0
1
MR leave−one−out sensitivity analysis for
'HNRNPF' on 'Pulmonary fibrosis'

Insufficient number of SNPs

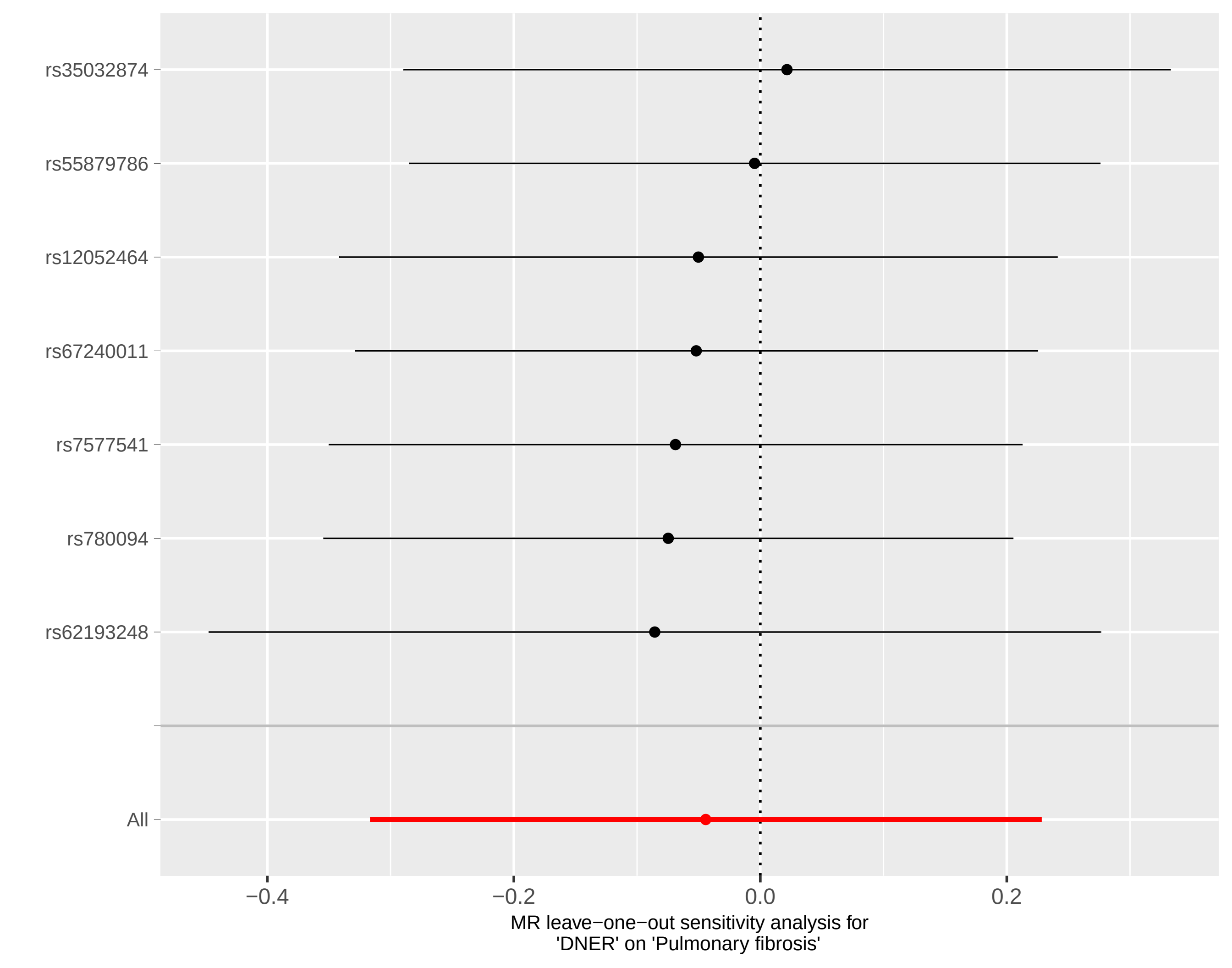

rs35032874
rs55879786
rs12052464
rs67240011
rs7577541
rs780094
rs62193248
All
−0.4
−0.2
0.0
0.2
MR leave−one−out sensitivity analysis for
'DNER' on 'Pulmonary fibrosis'

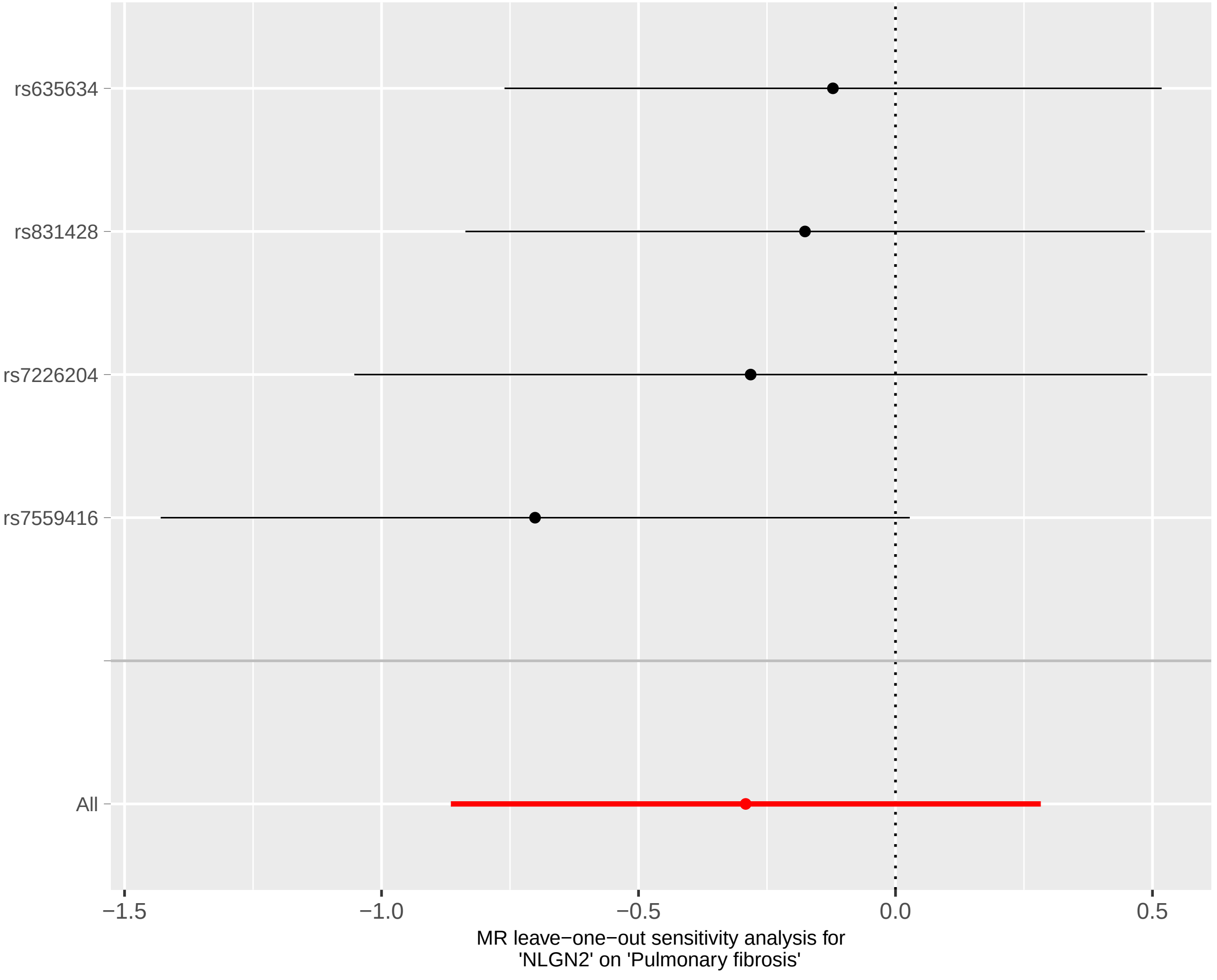
rs635634
rs831428
rs7226204
rs7559416
All
−1.5
−1.0
−0.5
0.0
0.5
MR leave−one−out sensitivity analysis for
'NLGN2' on 'Pulmonary fibrosis'

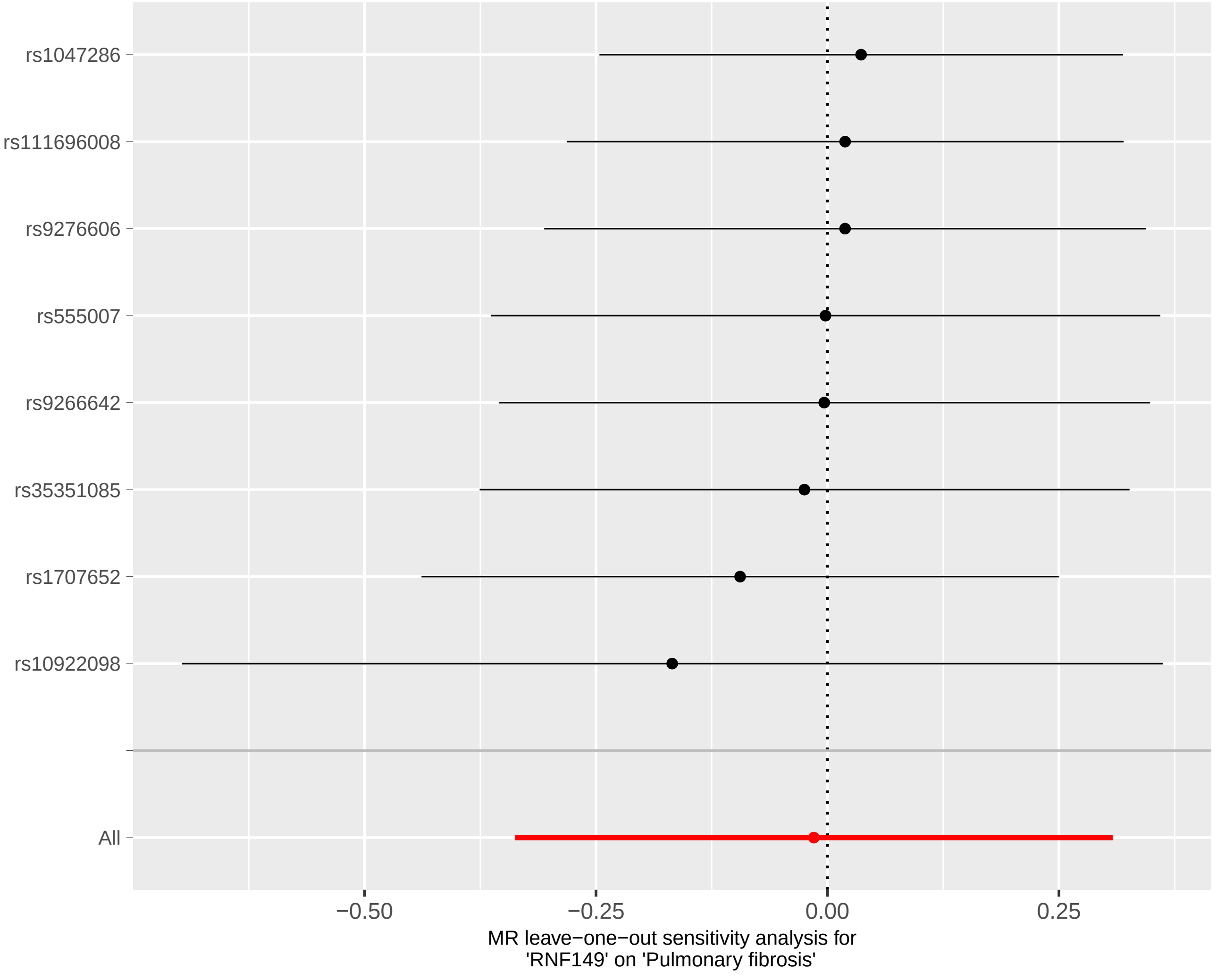
rs1047286
rs111696008
rs9276606
rs555007
rs9266642
rs35351085
rs1707652
rs10922098
All
−0.50
−0.25
0.00
0.25
MR leave−one−out sensitivity analysis for
'RNF149' on 'Pulmonary fibrosis'

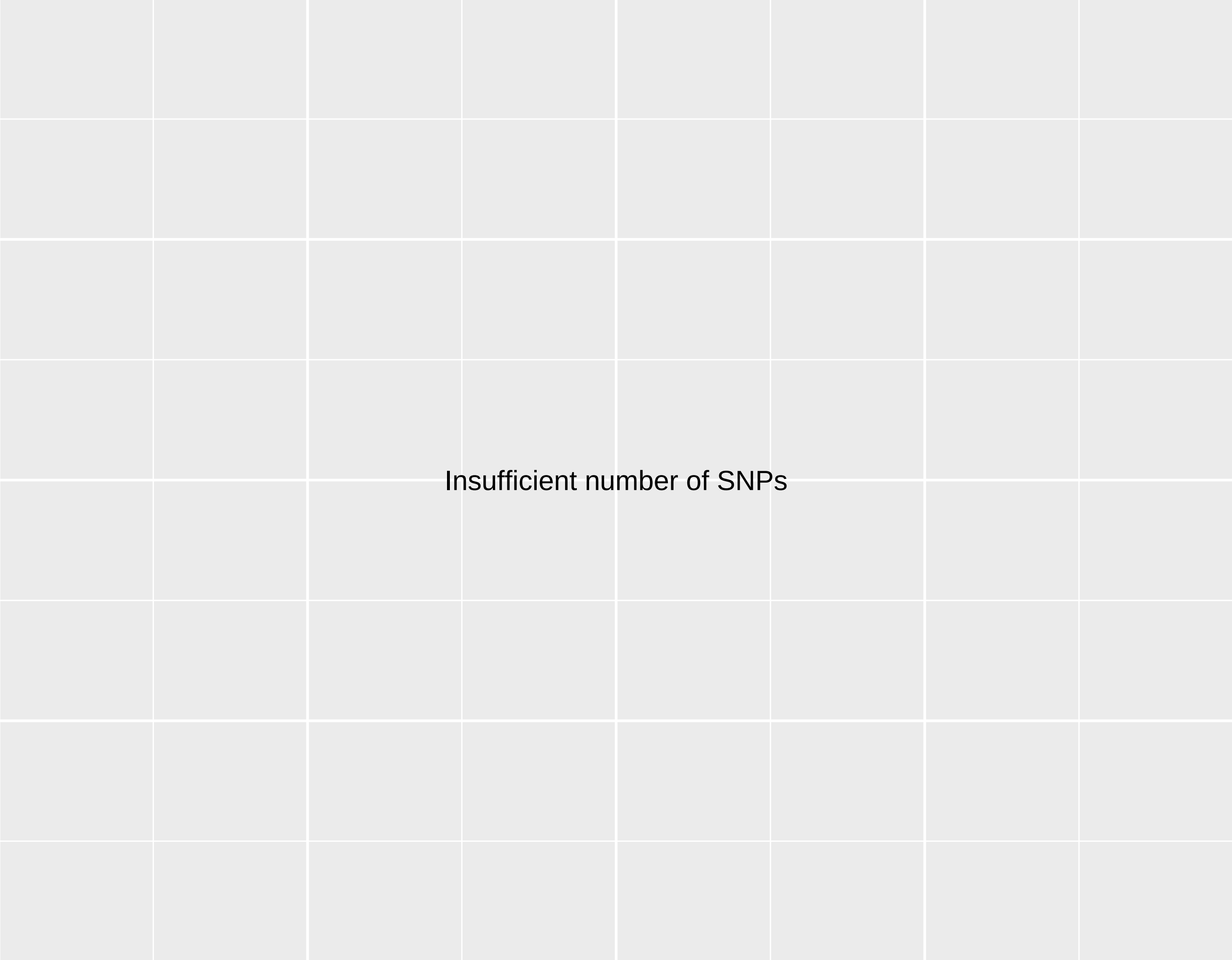
Insufficient number of SNPs

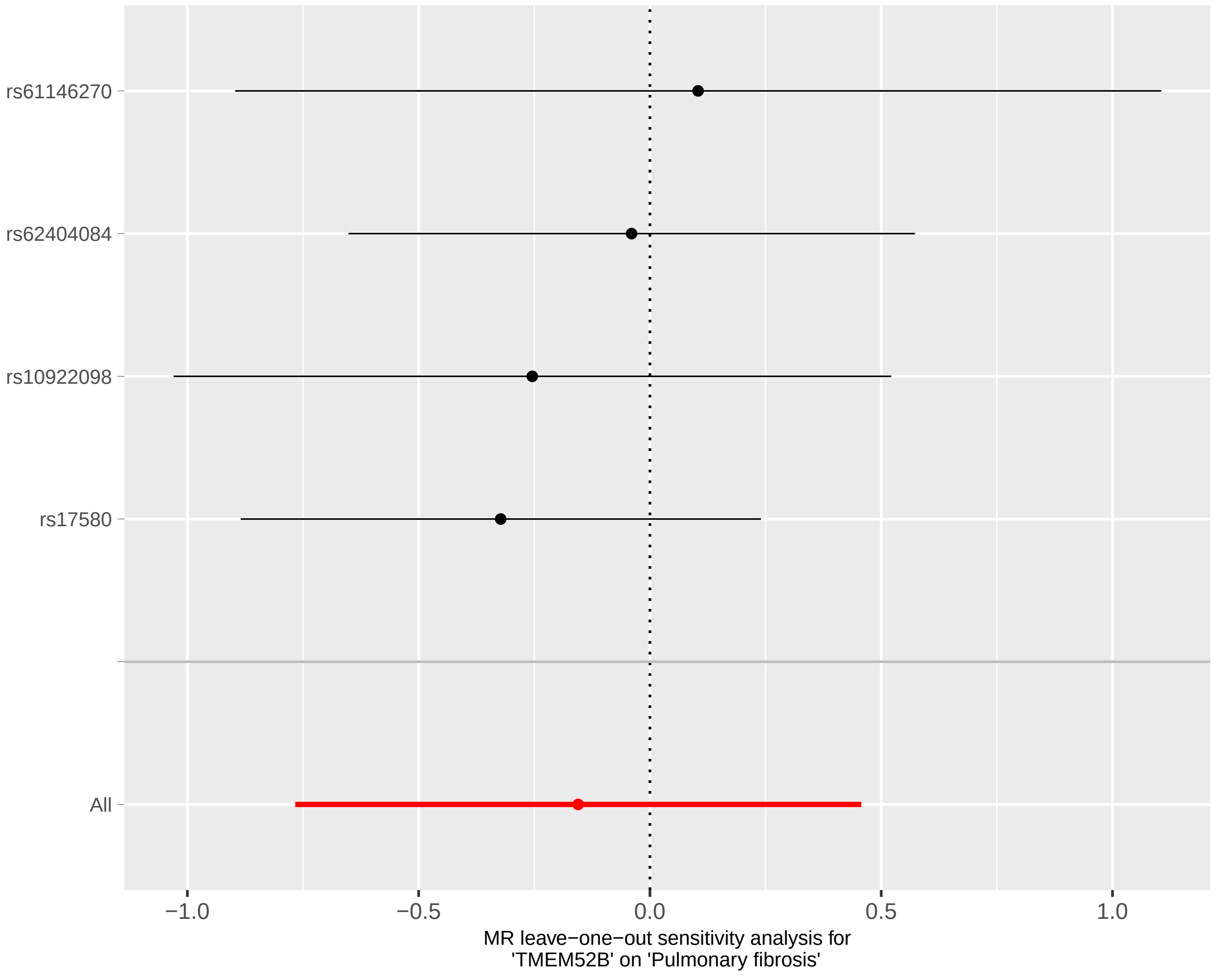

rs61146270
rs62404084
rs10922098
rs17580
All
−1.0
−0.5
0.0
0.5
1.0
MR leave−one−out sensitivity analysis for
'TMEM52B' on 'Pulmonary fibrosis'

Insufficient number of SNPs

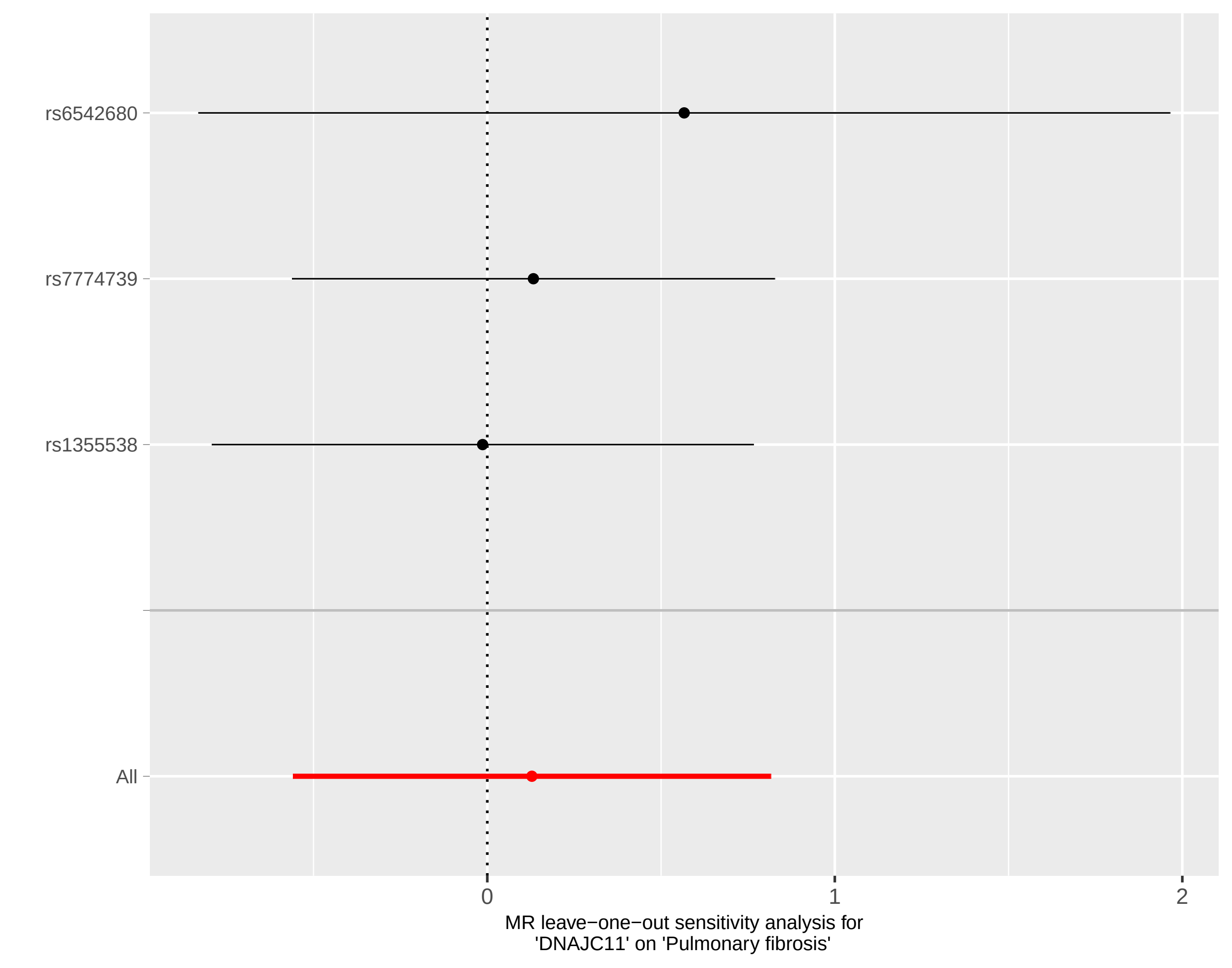
rs6542680
rs7774739
rs1355538
All
0
1
2
MR leave−one−out sensitivity analysis for
'DNAJC11' on 'Pulmonary fibrosis'

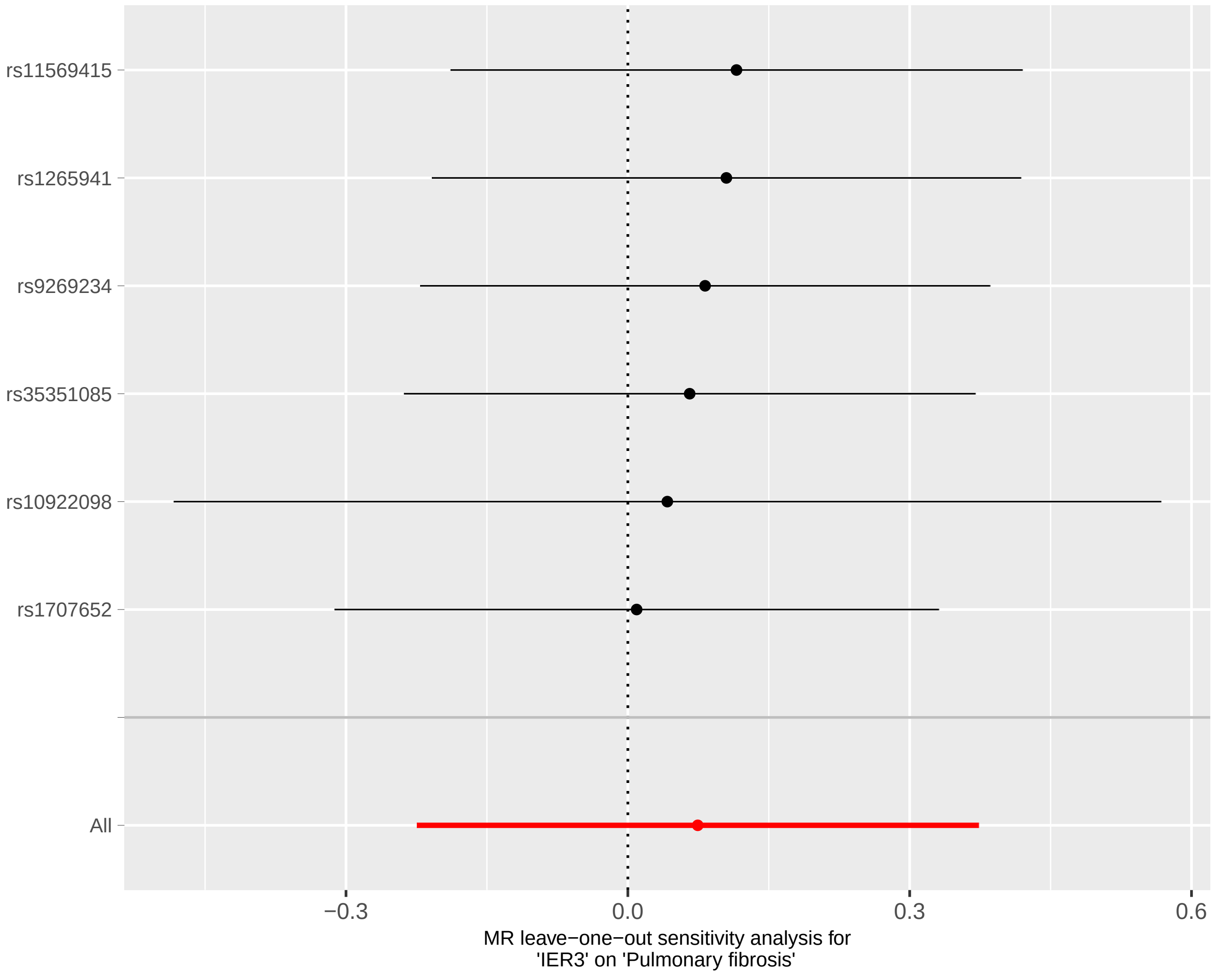
rs11569415
rs1265941
rs9269234
rs35351085
rs10922098
rs1707652
All
−0.3
0.0
0.3
0.6
MR leave−one−out sensitivity analysis for
'IER3' on 'Pulmonary fibrosis'

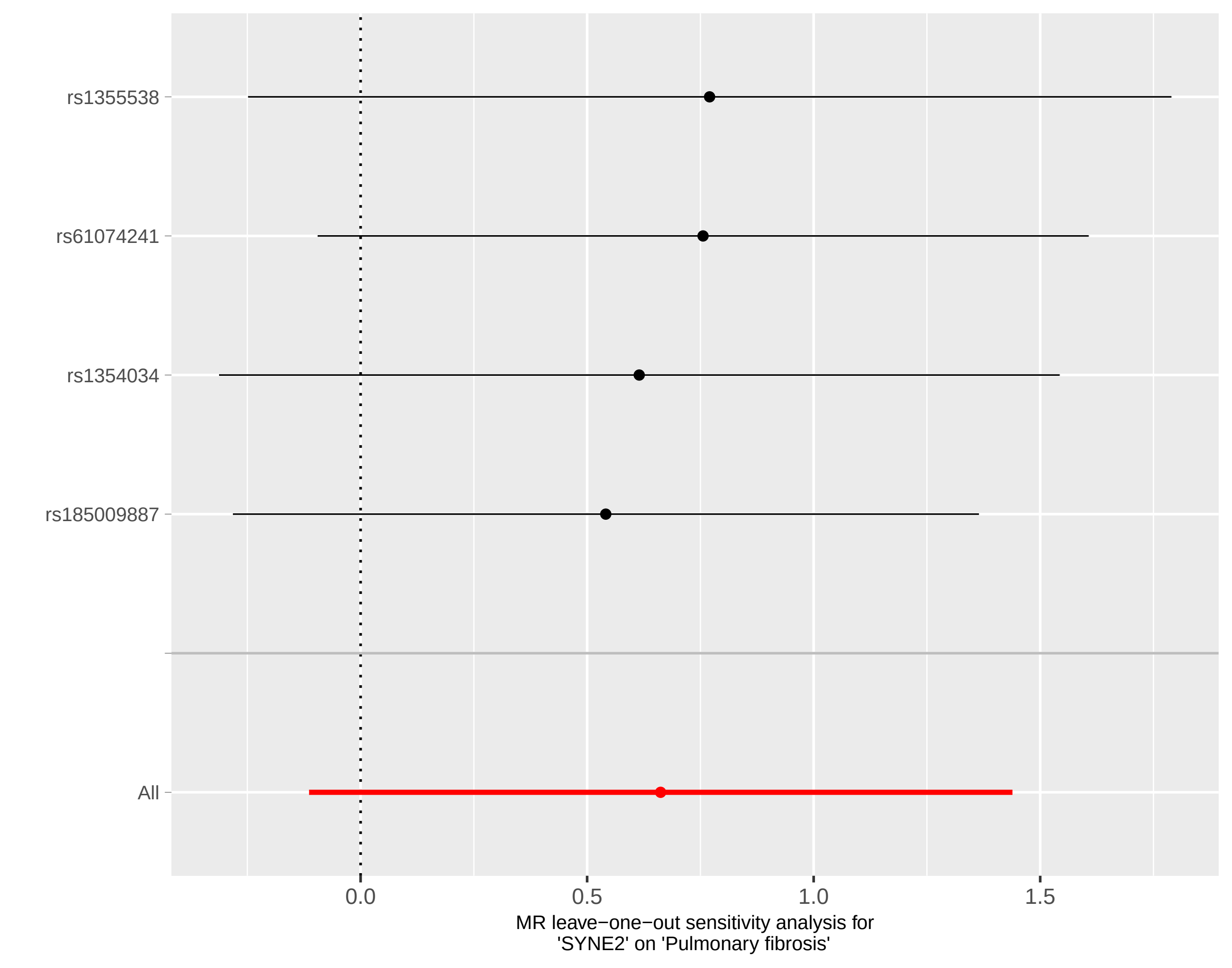
rs1355538
rs61074241
rs1354034
rs185009887
All
0.0
0.5
1.0
1.5
MR leave−one−out sensitivity analysis for
'SYNE2' on 'Pulmonary fibrosis'

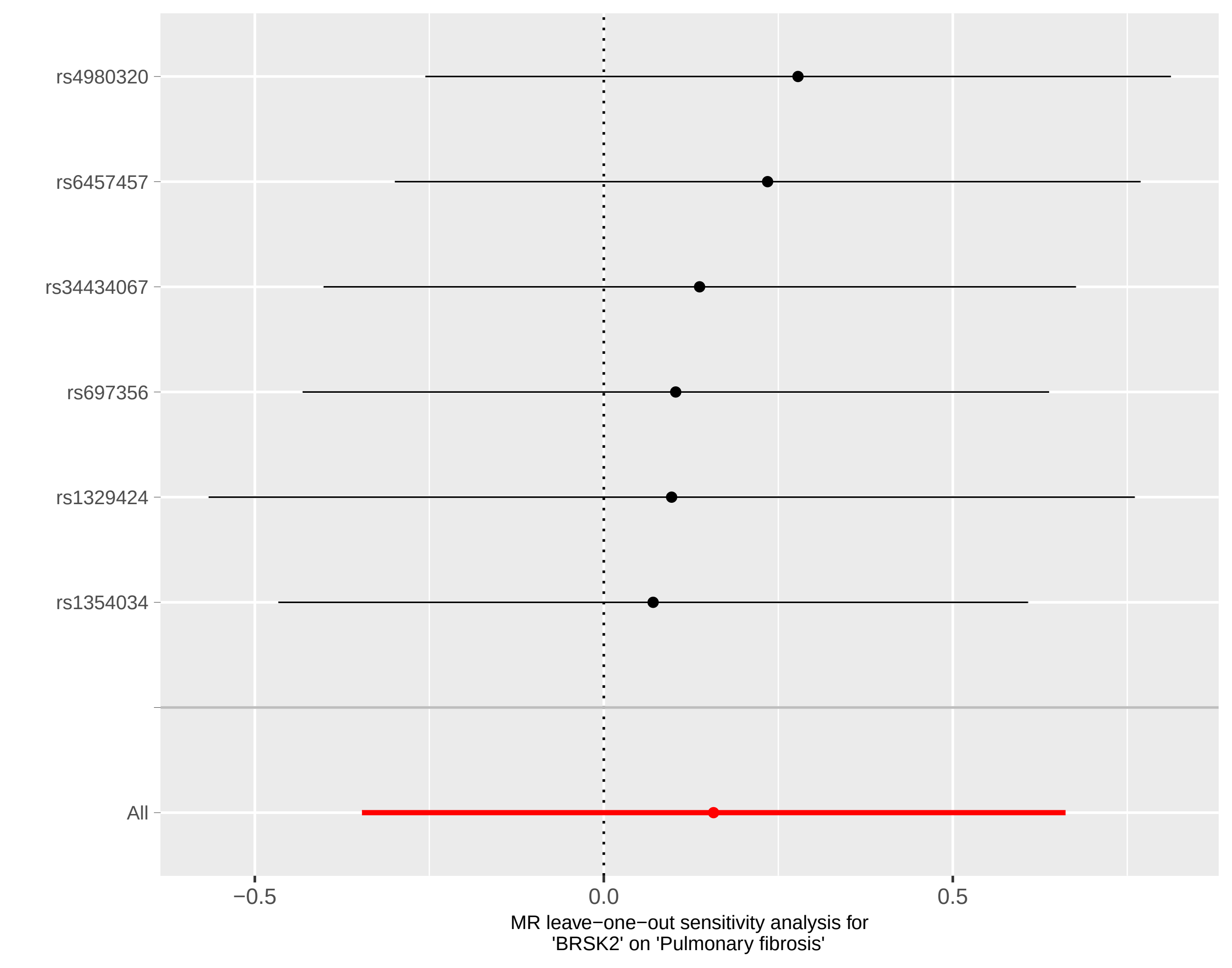
rs4980320
rs6457457
rs34434067
rs697356
rs1329424
rs1354034
All
−0.5
0.0
0.5
MR leave−one−out sensitivity analysis for
'BRSK2' on 'Pulmonary fibrosis'

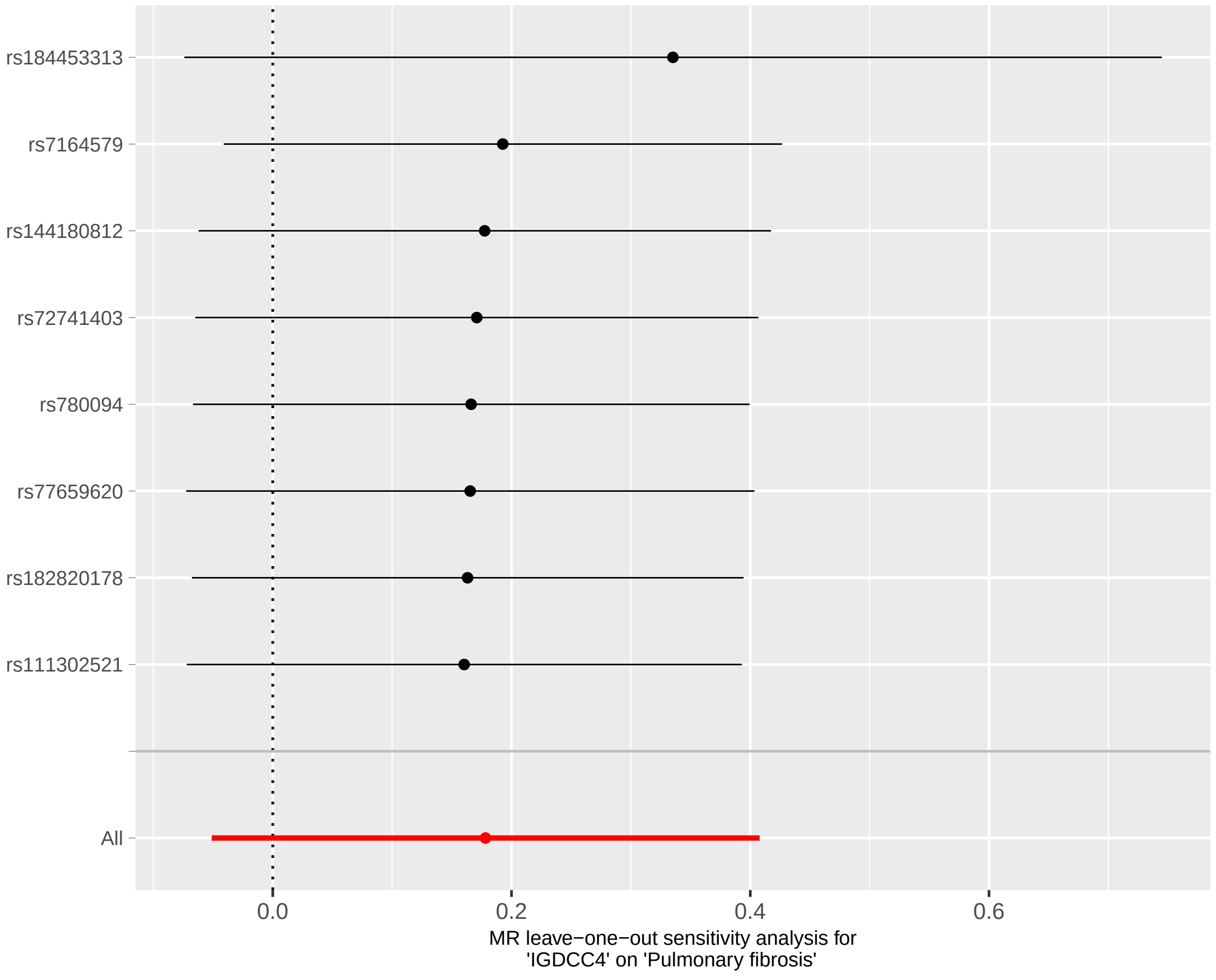

rs184453313
rs7164579
rs144180812
rs72741403
rs780094
rs77659620
rs182820178
rs111302521
All
0.0
0.2
0.4
0.6
MR leave−one−out sensitivity analysis for
'IGDCC4' on 'Pulmonary fibrosis'

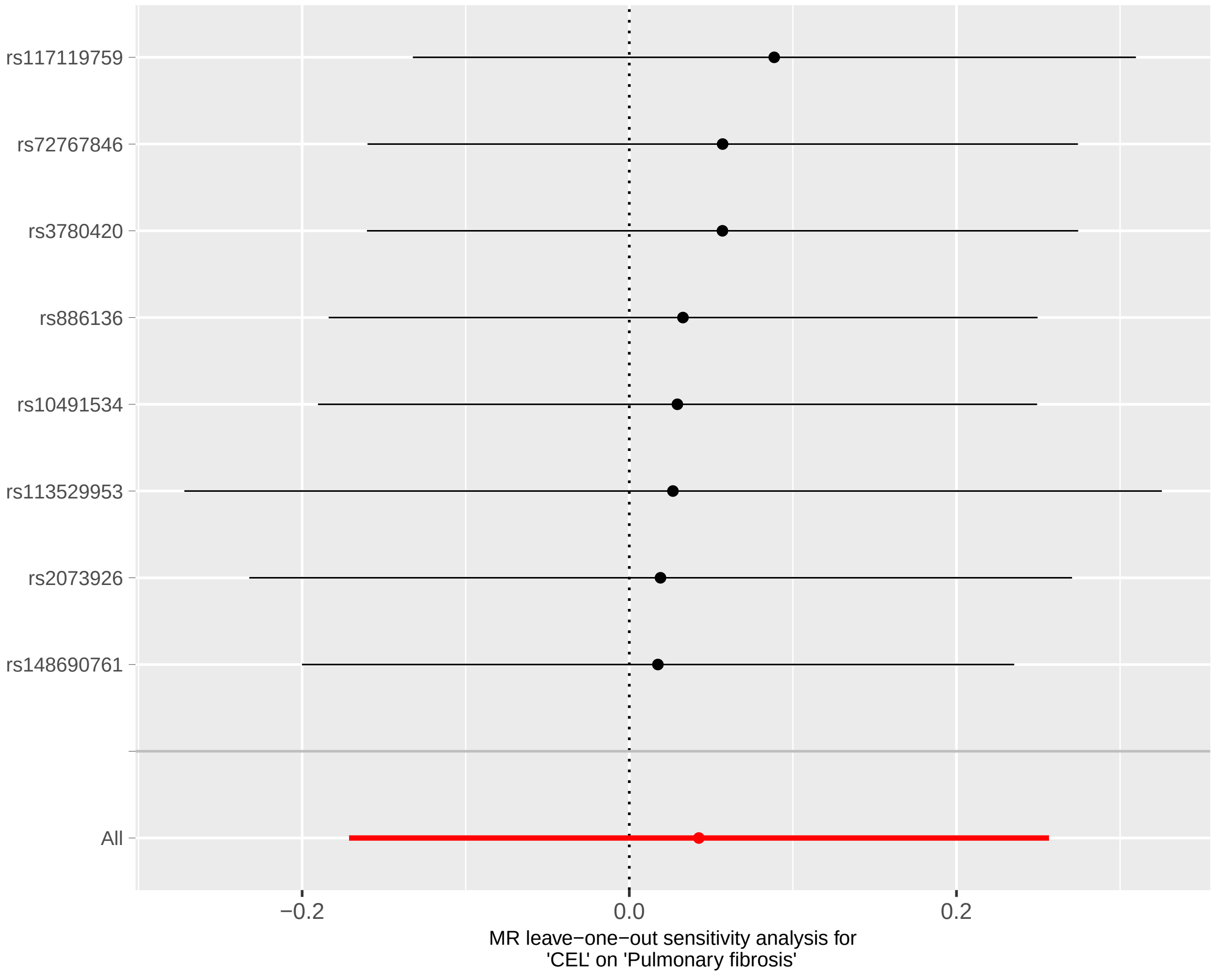
rs117119759
rs72767846
rs3780420
rs886136
rs10491534
rs113529953
rs2073926
rs148690761
All
−0.2
0.0
0.2
MR leave−one−out sensitivity analysis for
'CEL' on 'Pulmonary fibrosis'

Insufficient number of SNPs

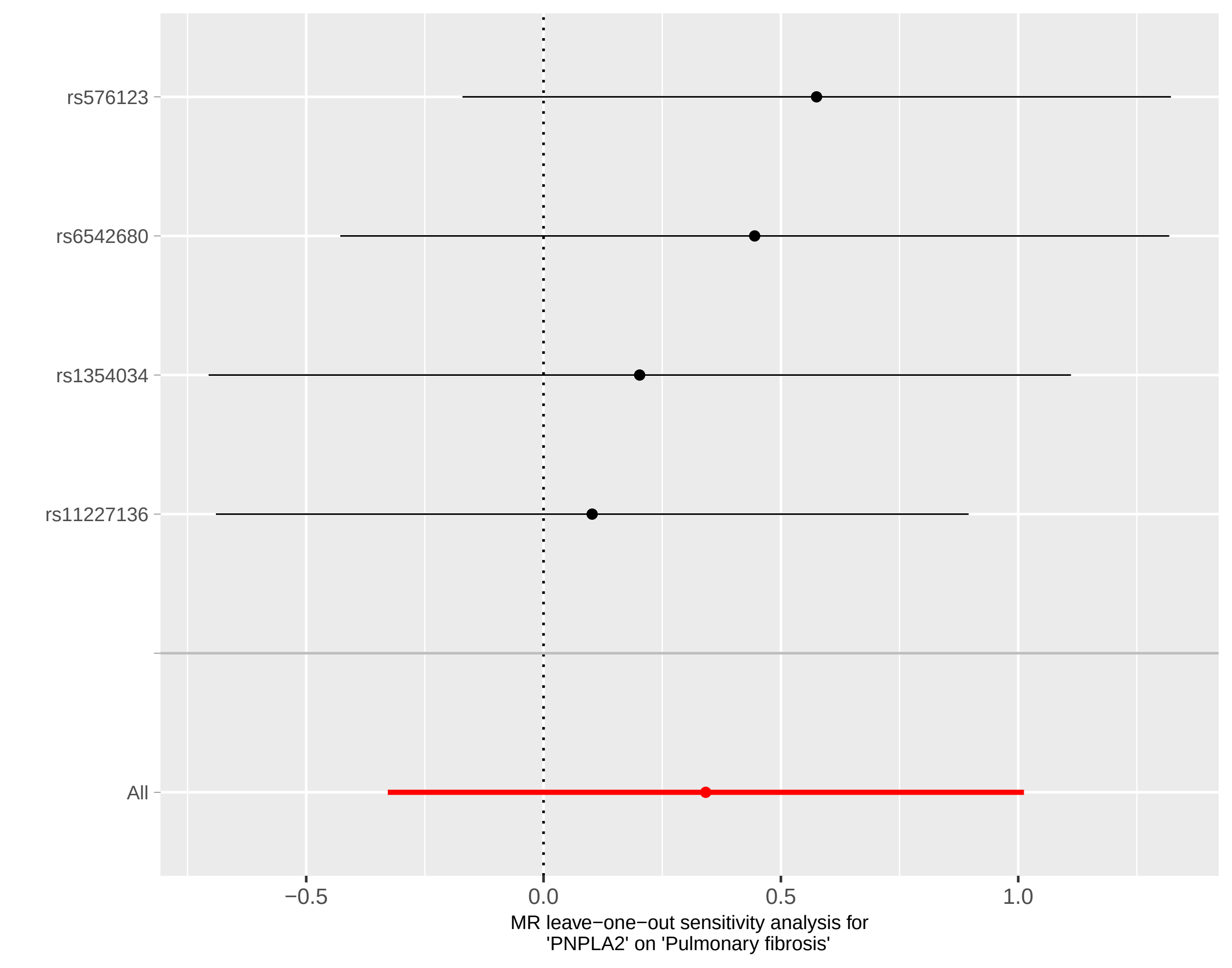
rs576123
rs6542680
rs1354034
rs11227136
All
−0.5
0.0
0.5
1.0
MR leave−one−out sensitivity analysis for
'PNPLA2' on 'Pulmonary fibrosis'

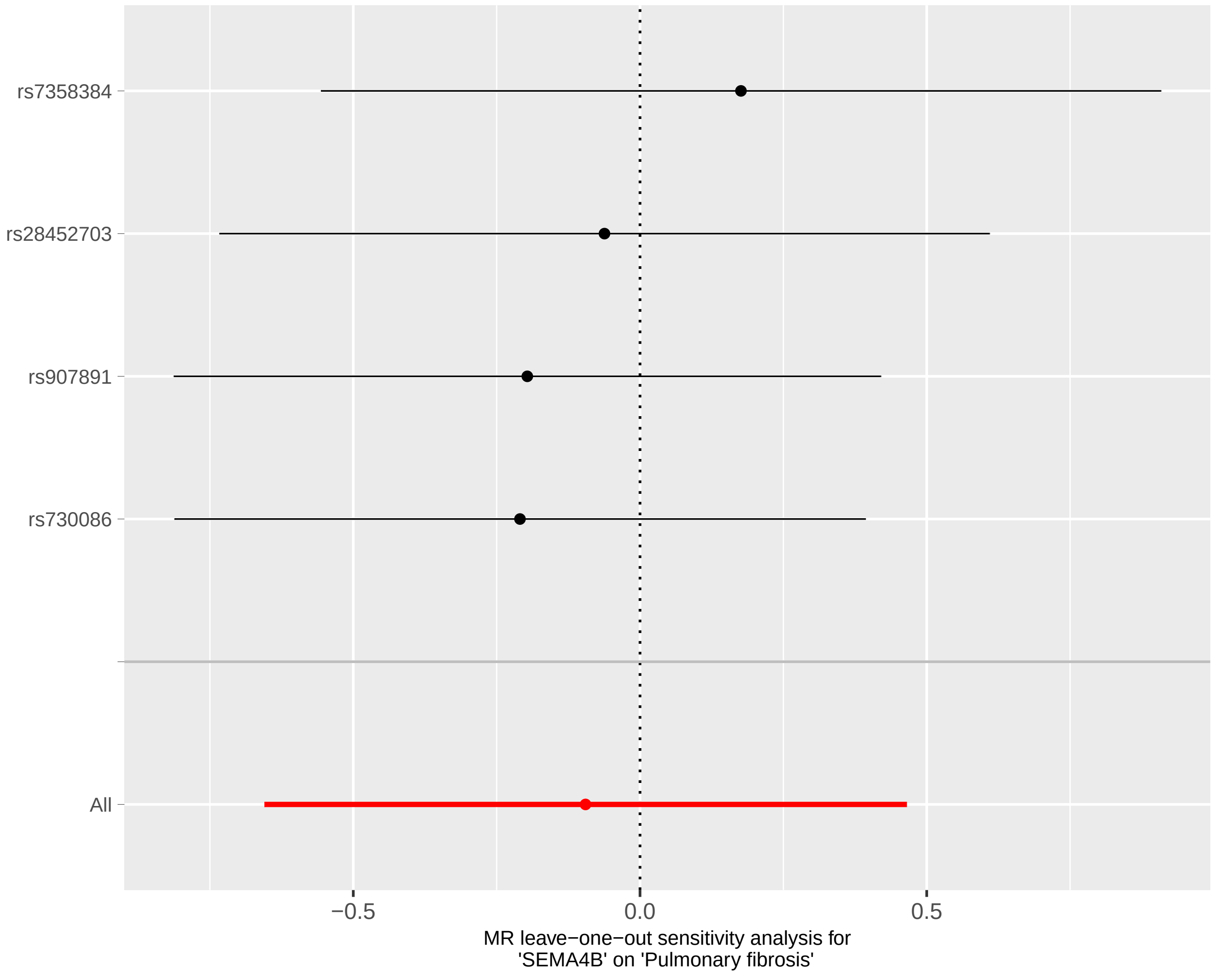
rs7358384
rs28452703
rs907891
rs730086
All
−0.5
0.0
0.5
MR leave−one−out sensitivity analysis for
'SEMA4B' on 'Pulmonary fibrosis'

Insufficient number of SNPs

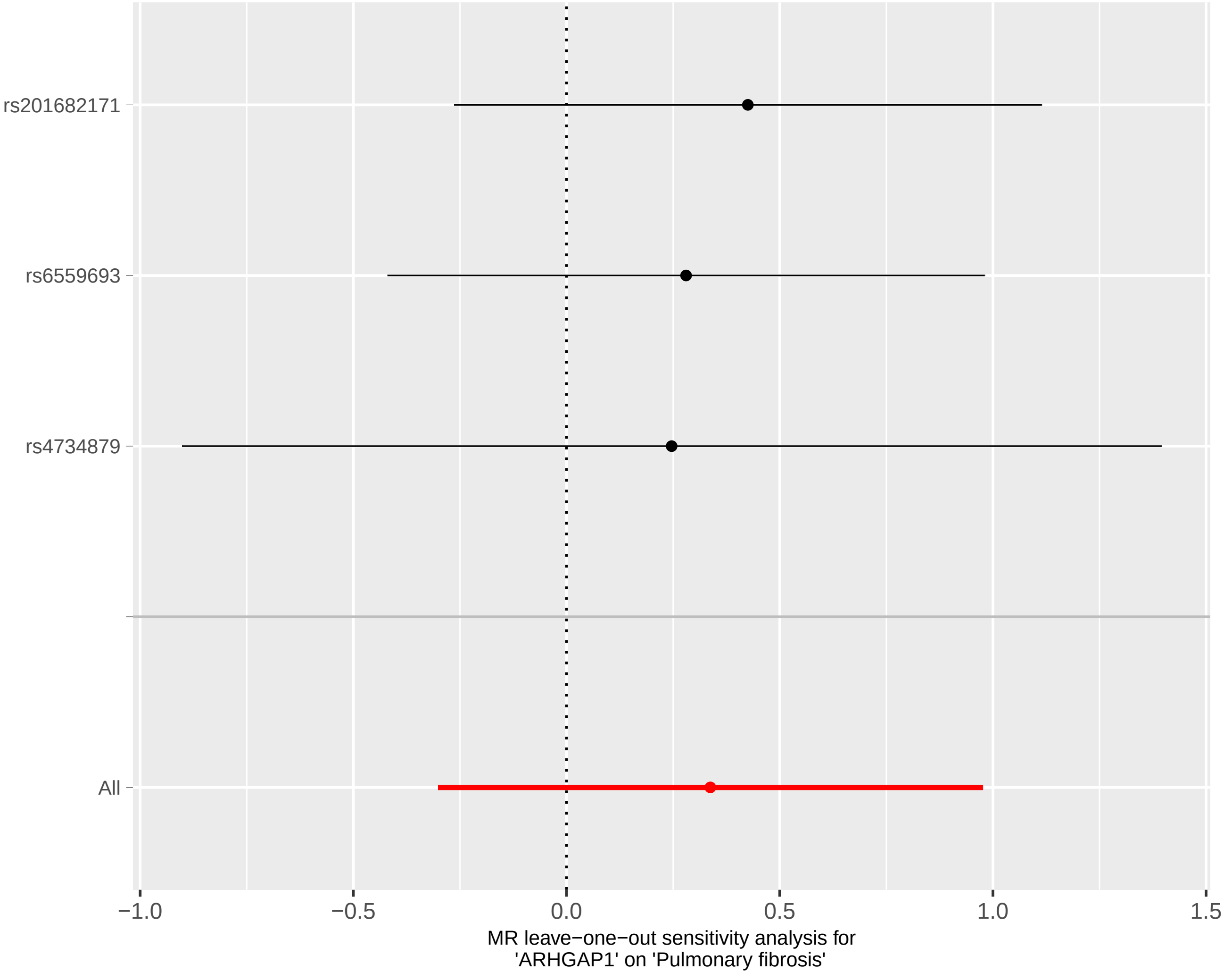
rs201682171
rs6559693
rs4734879
All
−1.0
−0.5
0.0
0.5
1.0
1.5
MR leave−one−out sensitivity analysis for
'ARHGAP1' on 'Pulmonary fibrosis'

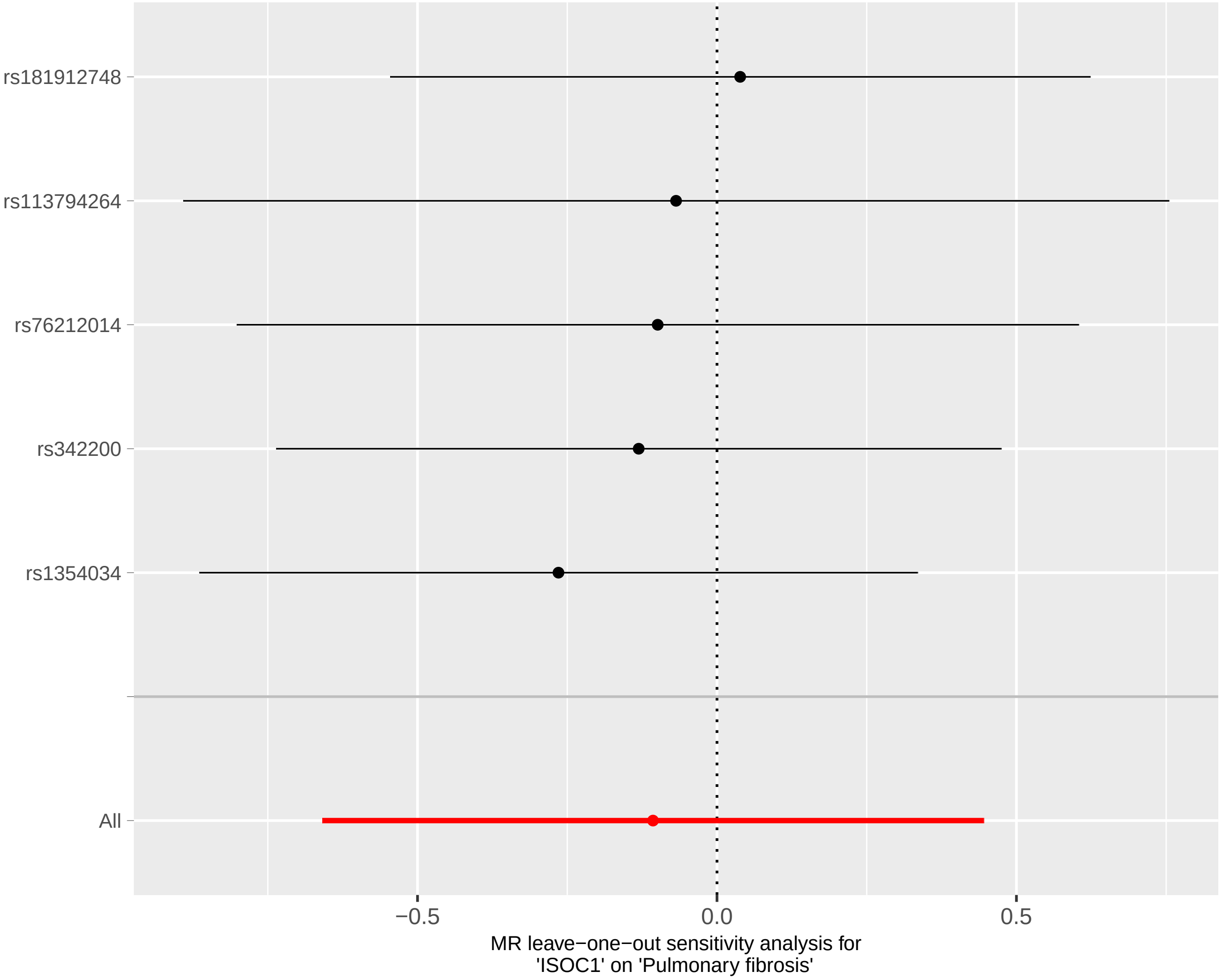
rs181912748
rs113794264
rs76212014
rs342200
rs1354034
All
−0.5
0.0
0.5
MR leave−one−out sensitivity analysis for
'ISOC1' on 'Pulmonary fibrosis'

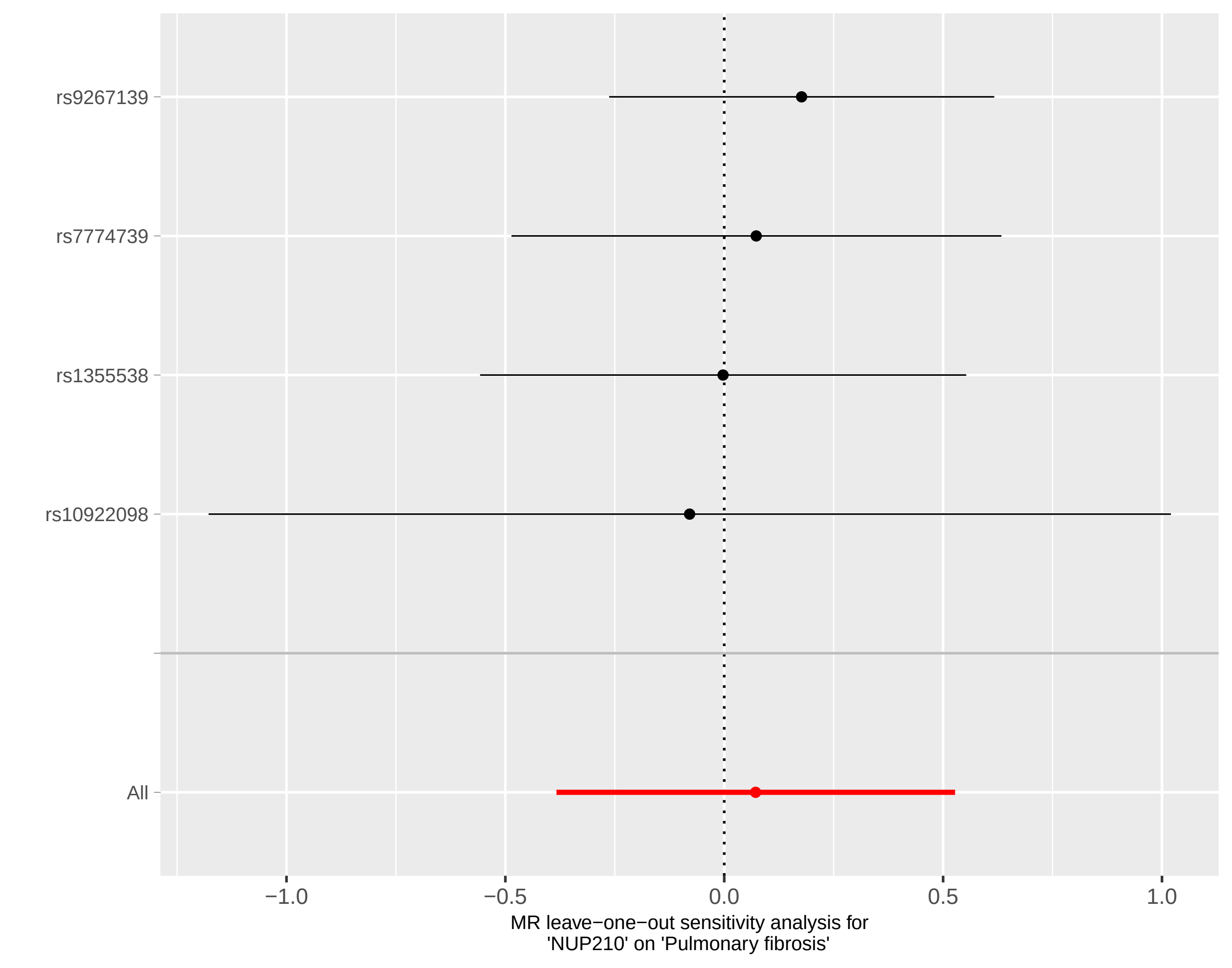
rs9267139
rs7774739
rs1355538
rs10922098
All
−1.0
−0.5
0.0
0.5
1.0
MR leave−one−out sensitivity analysis for
'NUP210' on 'Pulmonary fibrosis'

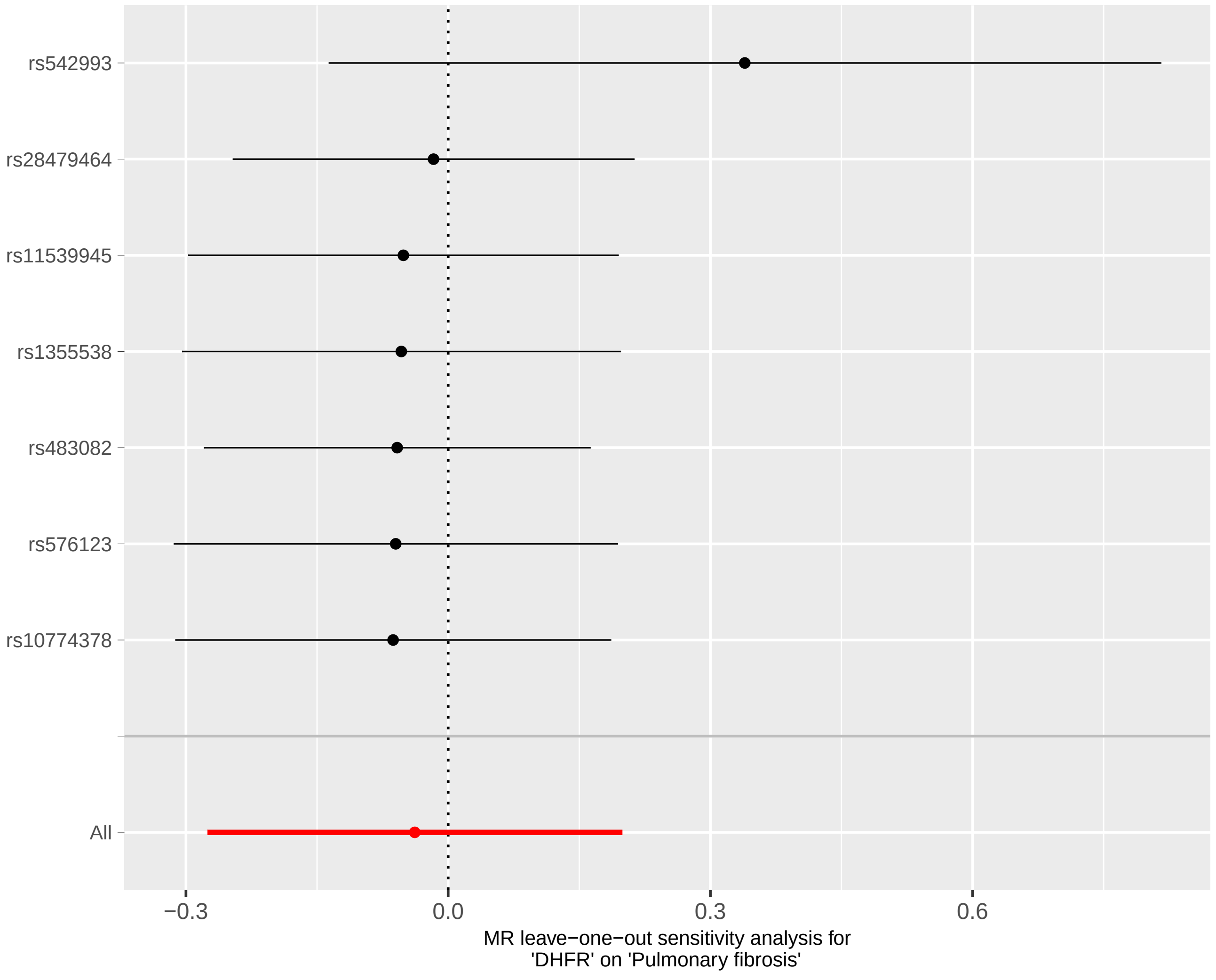
rs542993
rs28479464
rs11539945
rs1355538
rs483082
rs576123
rs10774378
All
−0.3
0.0
0.3
0.6
MR leave−one−out sensitivity analysis for
'DHFR' on 'Pulmonary fibrosis'

Insufficient number of SNPs

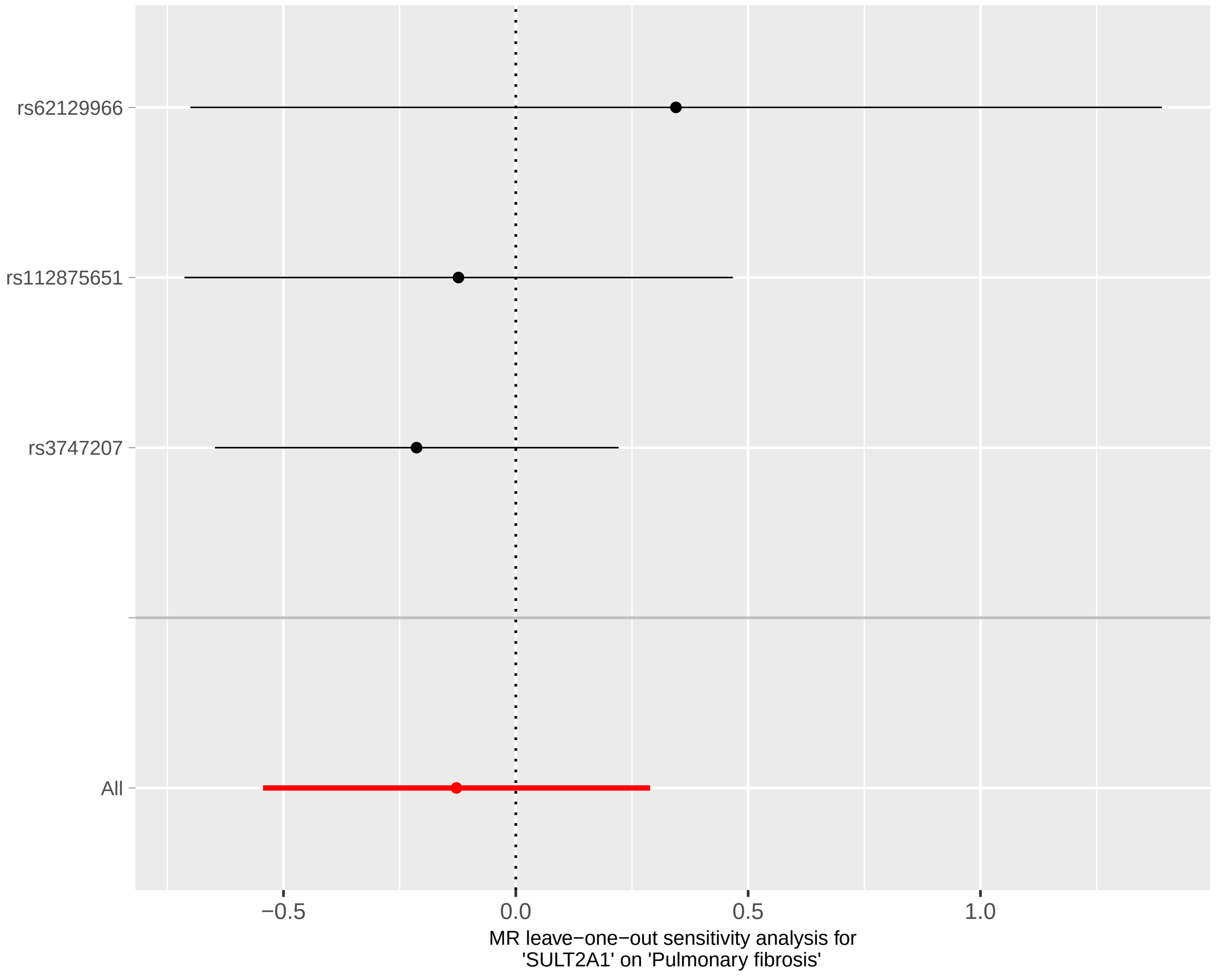
rs62129966
rs112875651
rs3747207
All
−0.5
0.0
0.5
1.0
MR leave−one−out sensitivity analysis for
'SULT2A1' on 'Pulmonary fibrosis'

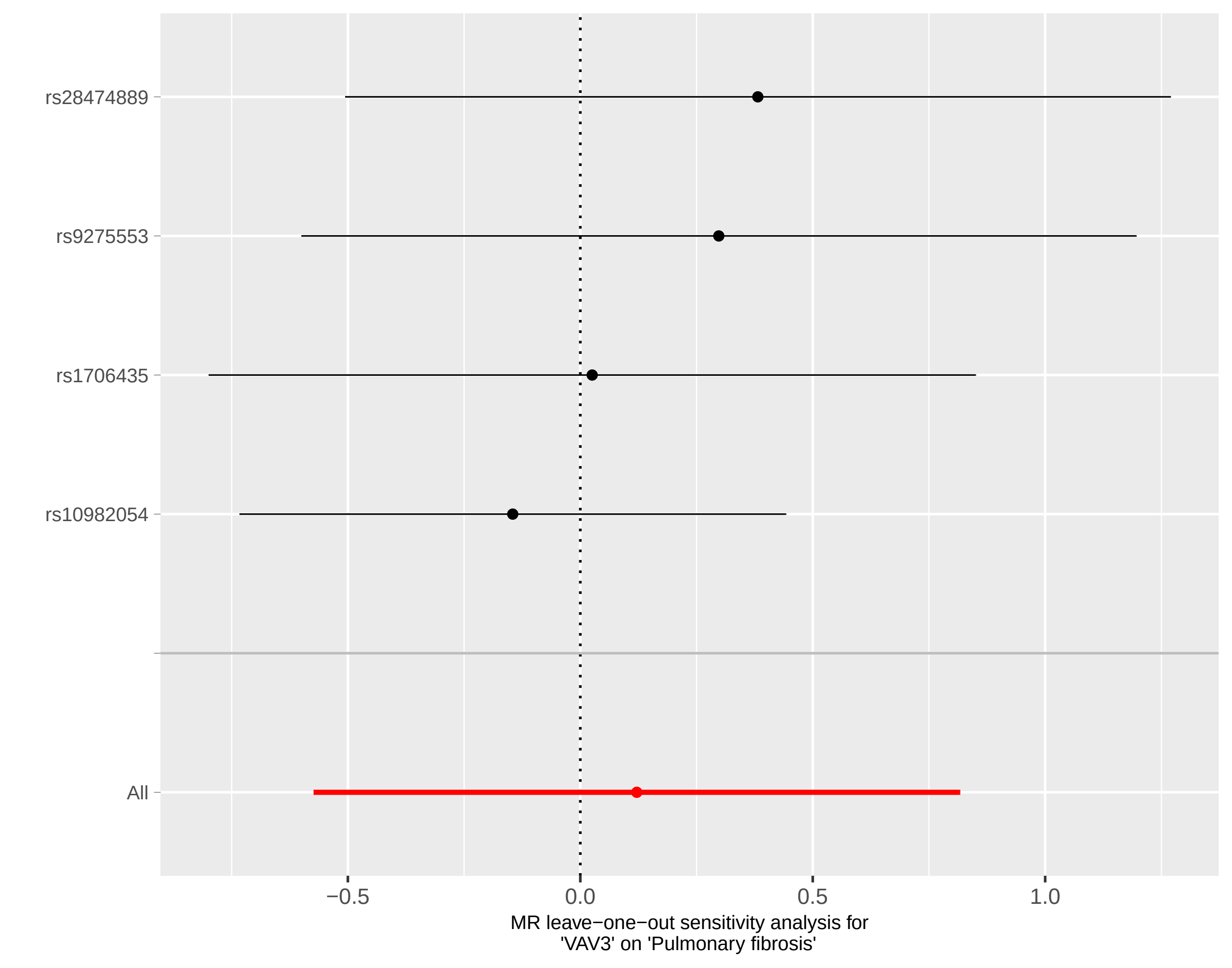
rs28474889
rs9275553
rs1706435
rs10982054
All
−0.5
0.0
0.5
1.0
MR leave−one−out sensitivity analysis for
'VAV3' on 'Pulmonary fibrosis'

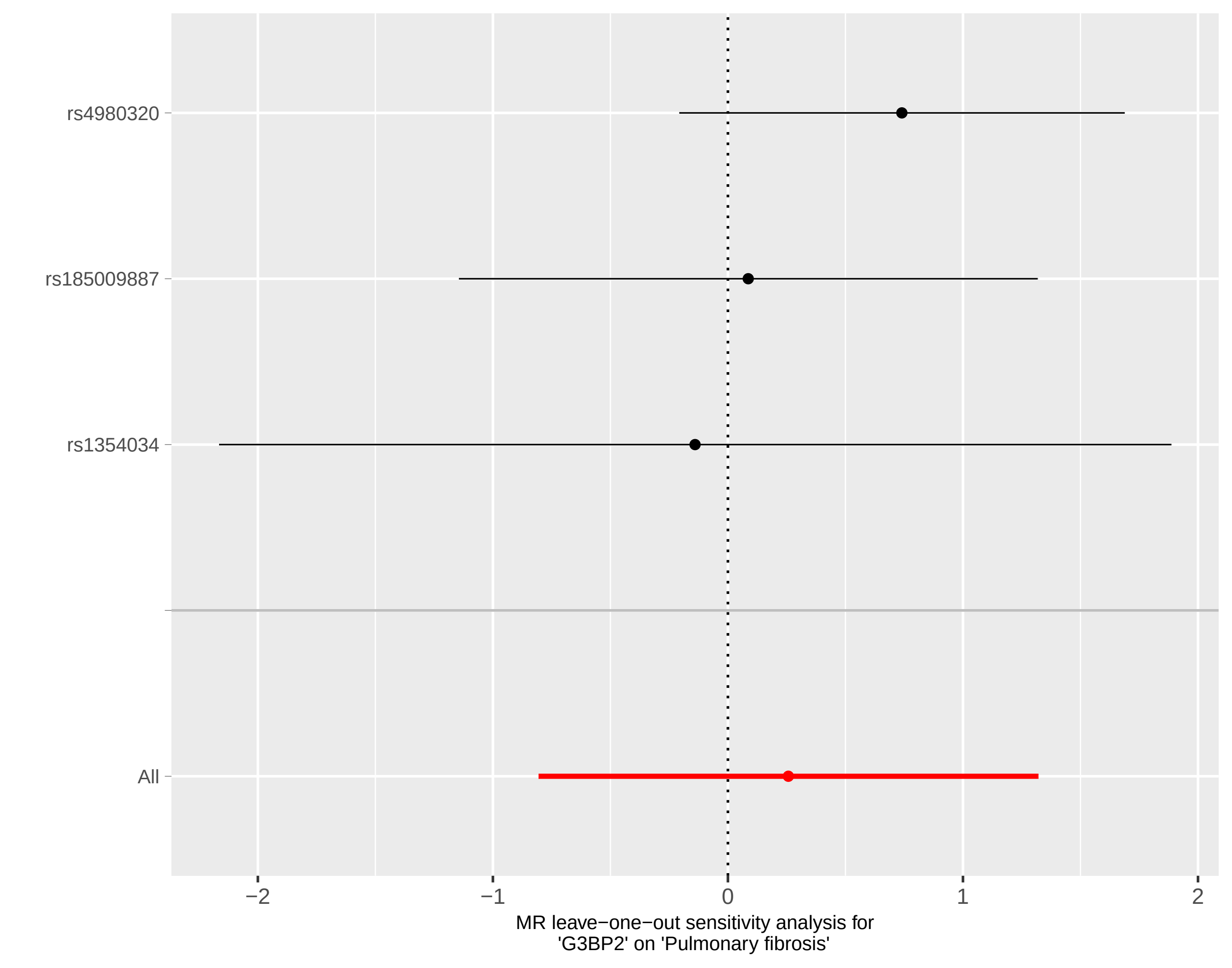
rs4980320
rs185009887
rs1354034
All
−2
−1
0
1
2
MR leave−one−out sensitivity analysis for
'G3BP2' on 'Pulmonary fibrosis'

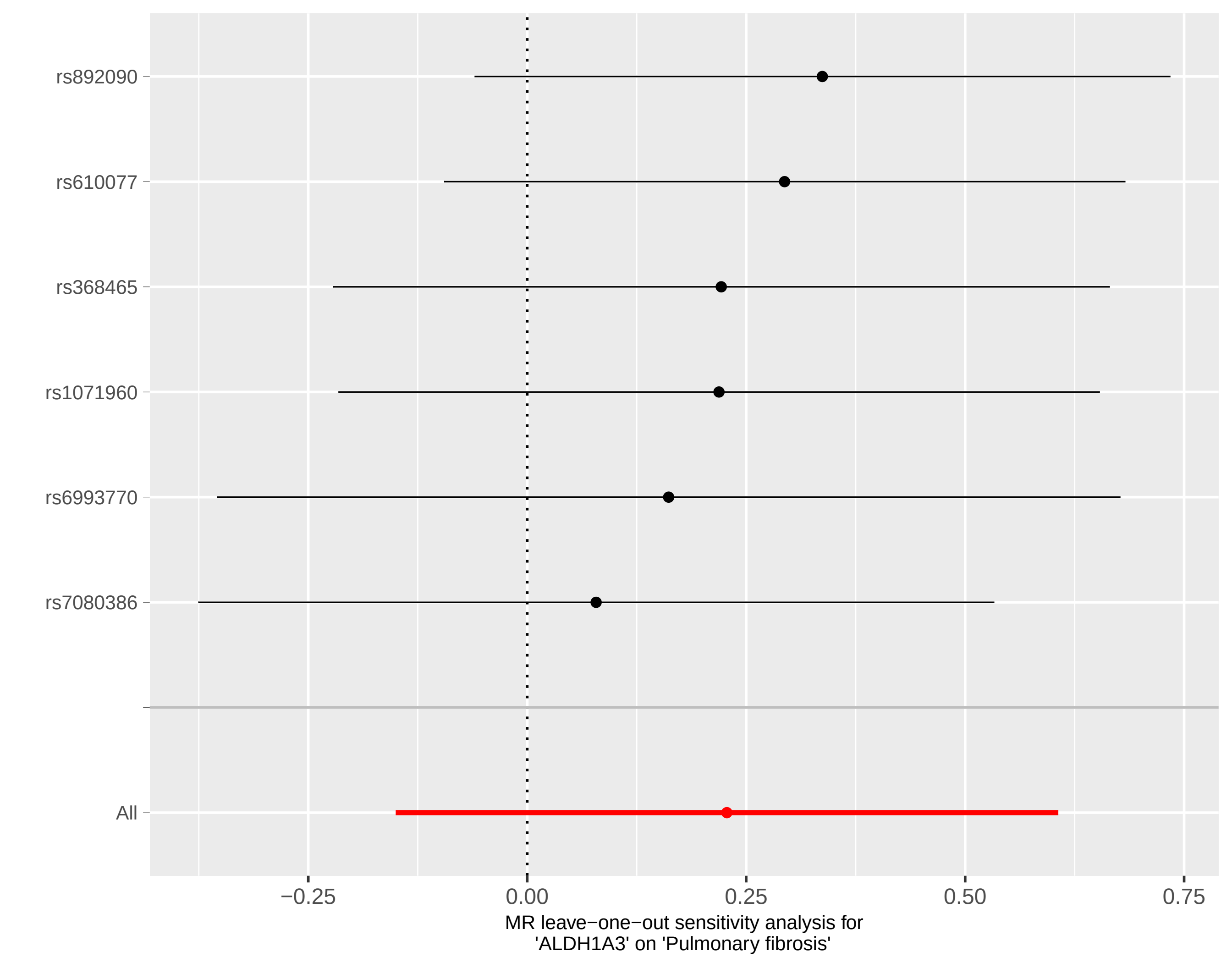

rs892090
rs610077
rs368465
rs1071960
rs6993770
rs7080386
All
−0.25
0.00
0.25
0.50
0.75
MR leave−one−out sensitivity analysis for
'ALDH1A3' on 'Pulmonary fibrosis'

Insufficient number of SNPs

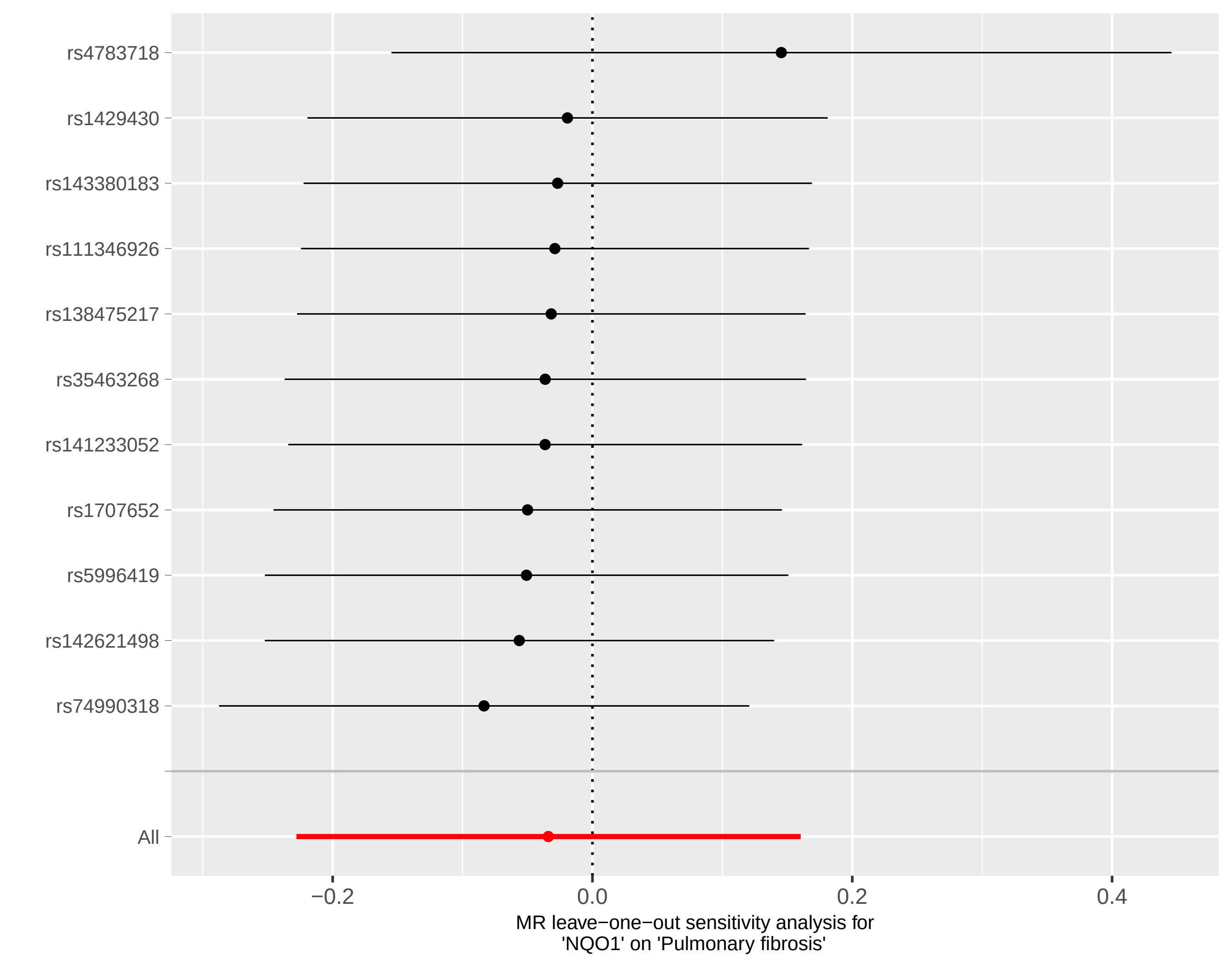

rs4783718
rs1429430
rs143380183
rs111346926
rs138475217
rs35463268
rs141233052
rs1707652
rs5996419
rs142621498
rs74990318
All
−0.2
0.0
0.2
0.4
MR leave−one−out sensitivity analysis for
'NQO1' on 'Pulmonary fibrosis'

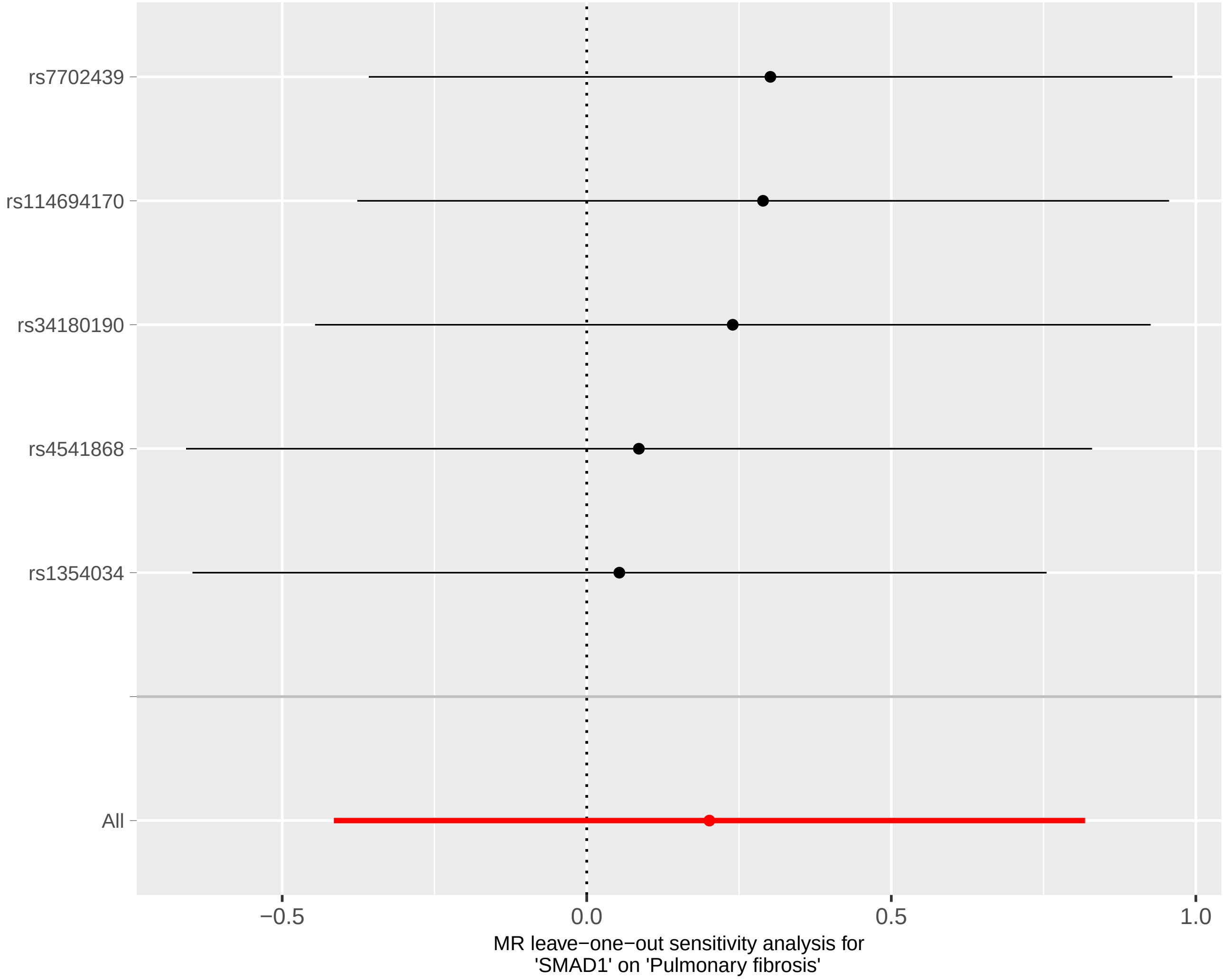
rs7702439
rs114694170
rs34180190
rs4541868
rs1354034
All
−0.5
0.0
0.5
1.0
MR leave−one−out sensitivity analysis for
'SMAD1' on 'Pulmonary fibrosis'

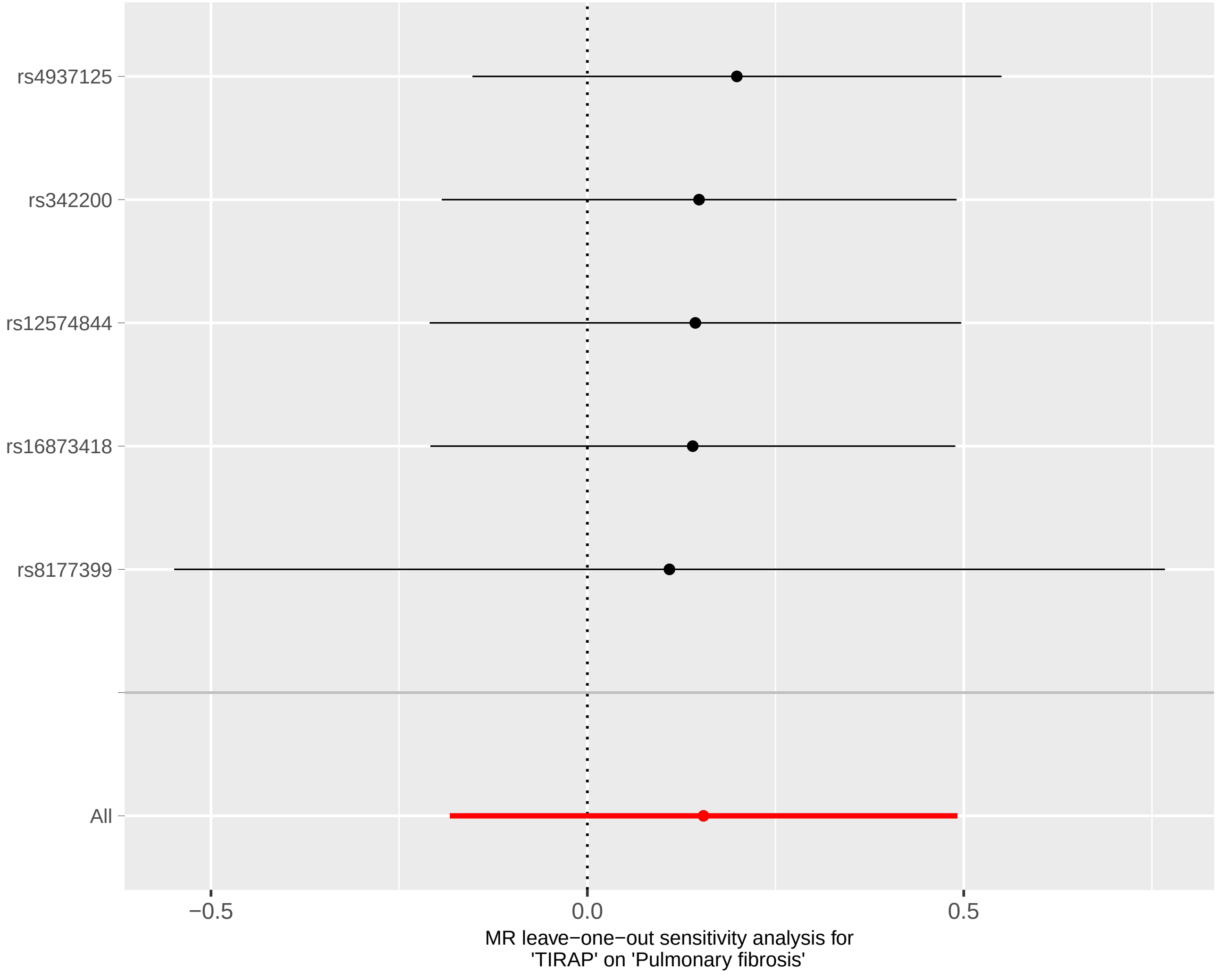

rs4937125
rs342200
rs12574844
rs16873418
rs8177399
All
−0.5
0.0
0.5
MR leave−one−out sensitivity analysis for
'TIRAP' on 'Pulmonary fibrosis'

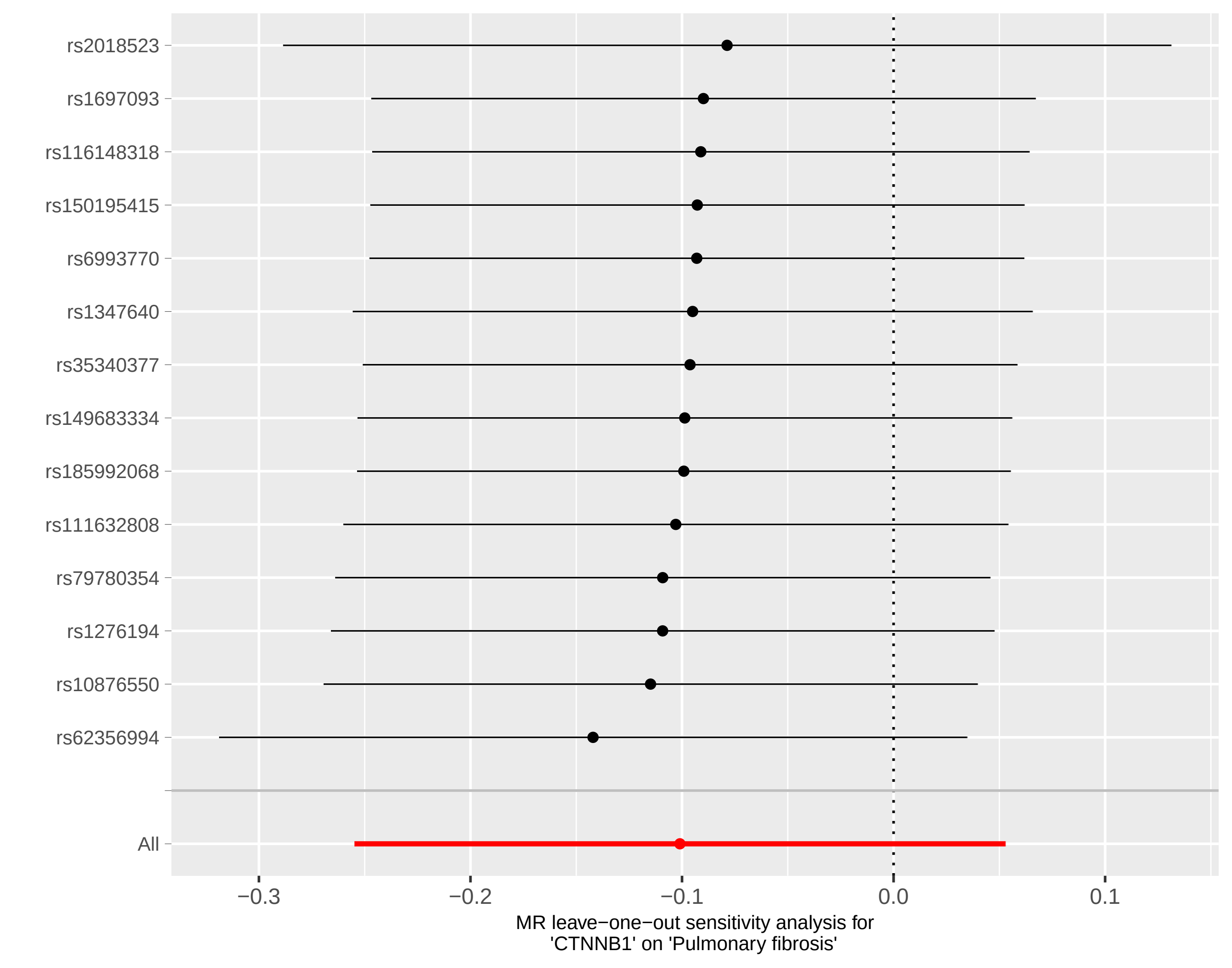
rs2018523
rs1697093
rs116148318
rs150195415
rs6993770
rs1347640
rs35340377
rs149683334
rs185992068
rs111632808
rs79780354
rs1276194
rs10876550
rs62356994
All
−0.3
−0.2
−0.1
0.0
0.1
MR leave−one−out sensitivity analysis for
'CTNNB1' on 'Pulmonary fibrosis'

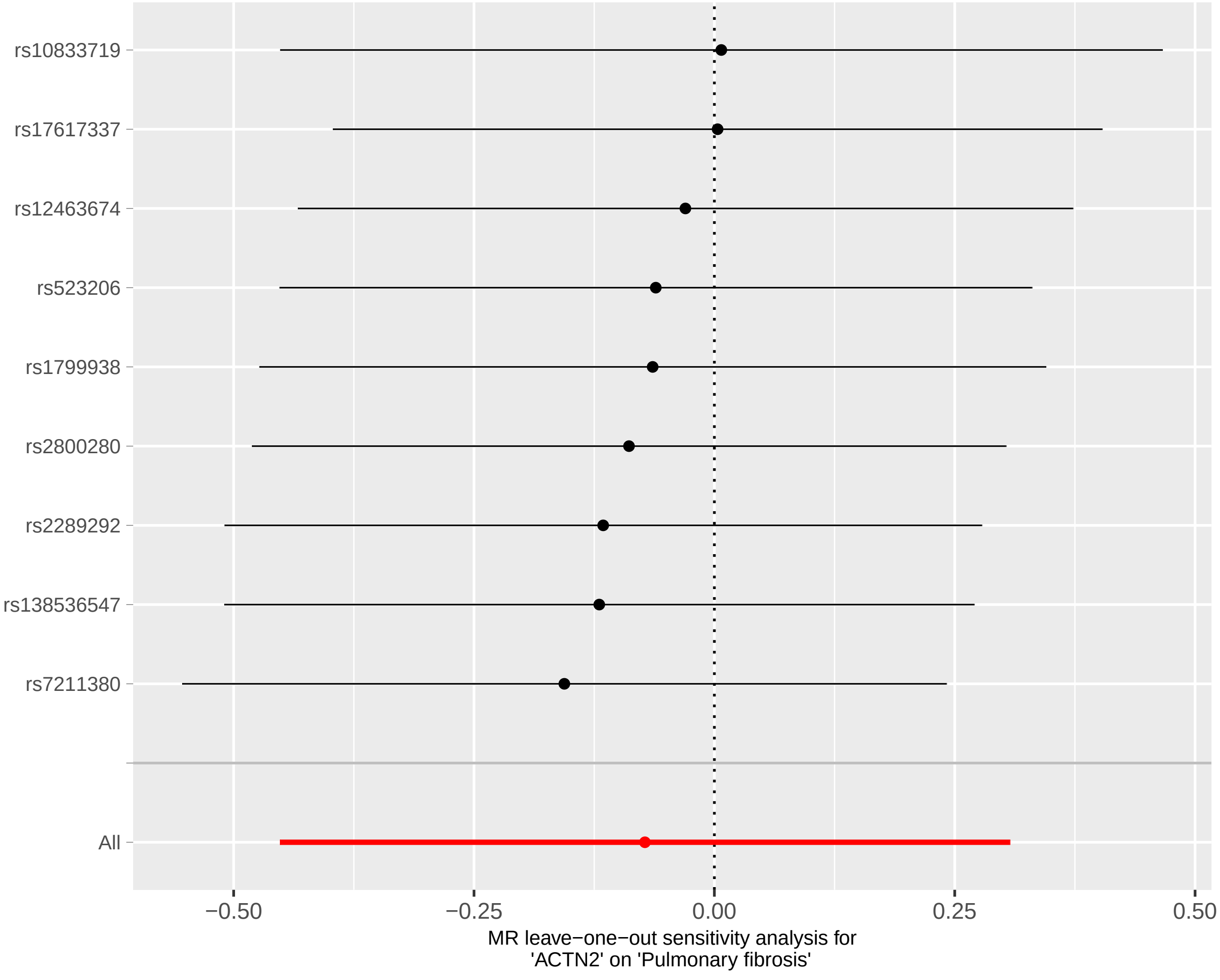
rs10833719
rs17617337
rs12463674
rs523206
rs1799938
rs2800280
rs2289292
rs138536547
rs7211380
All
−0.50
−0.25
0.00
0.25
0.50
MR leave−one−out sensitivity analysis for
'ACTN2' on 'Pulmonary fibrosis'

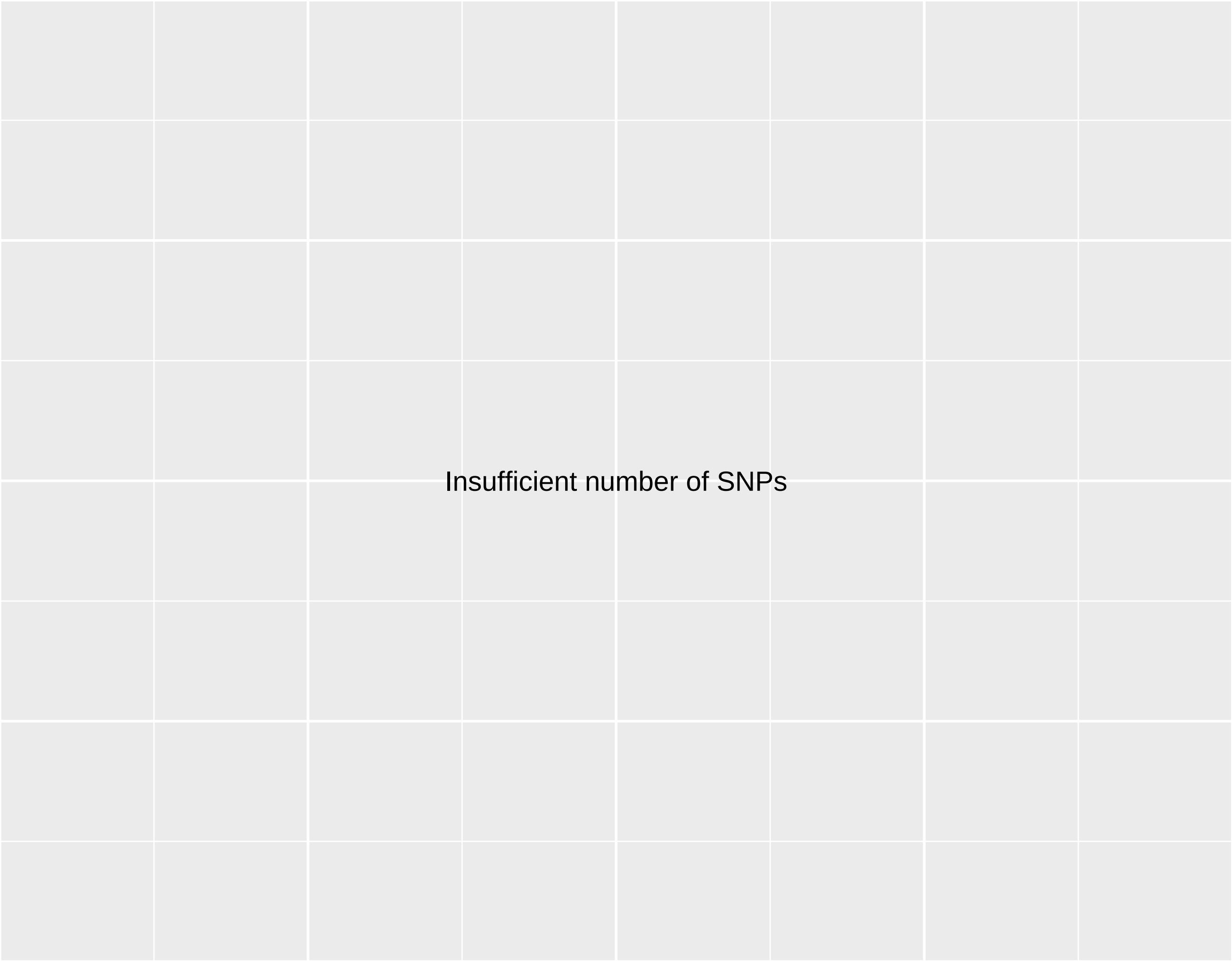
Insufficient number of SNPs

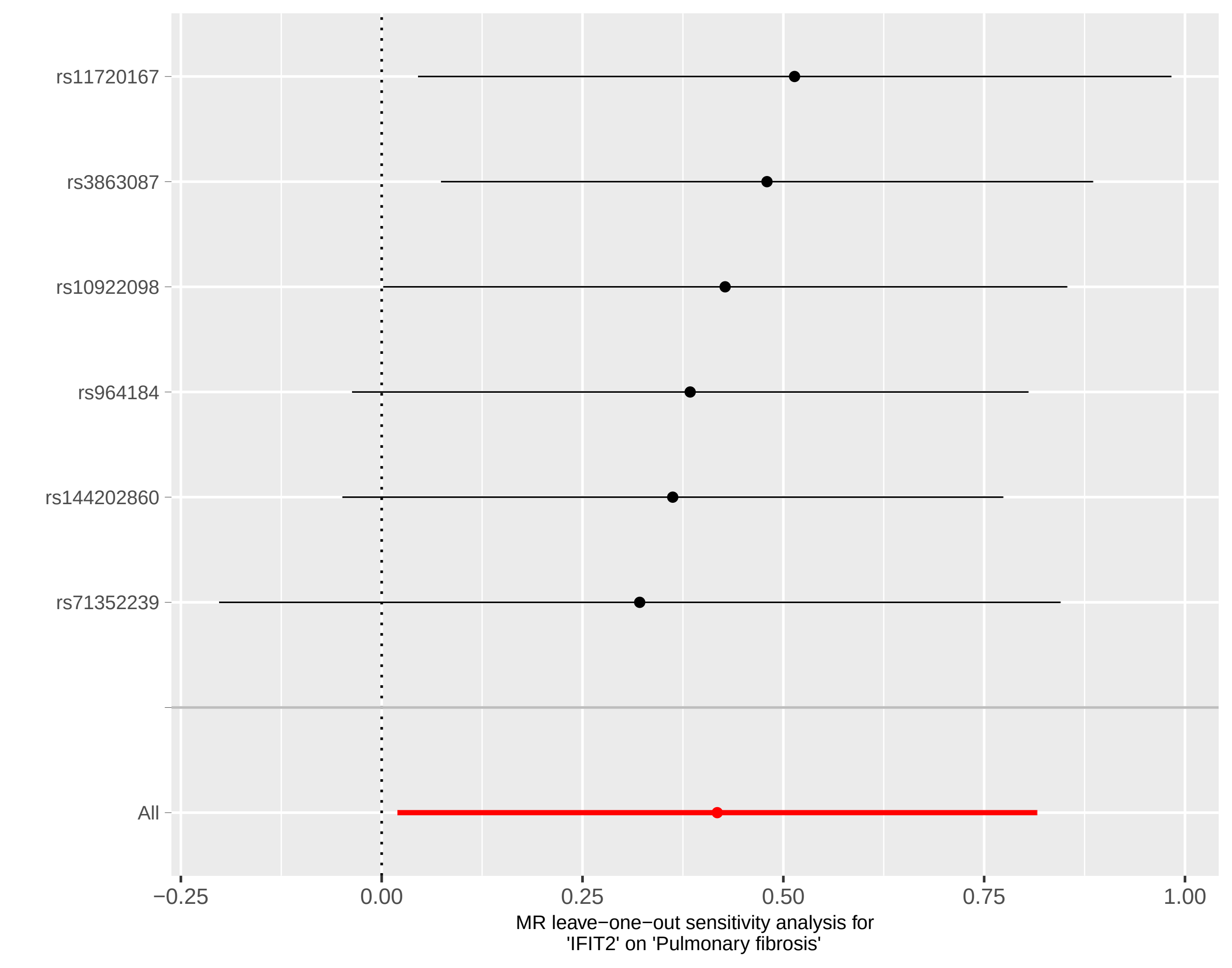

rs11720167
rs3863087
rs10922098
rs964184
rs144202860
rs71352239
All
−0.25
0.00
0.25
0.50
0.75
1.00
MR leave−one−out sensitivity analysis for
'IFIT2' on 'Pulmonary fibrosis'

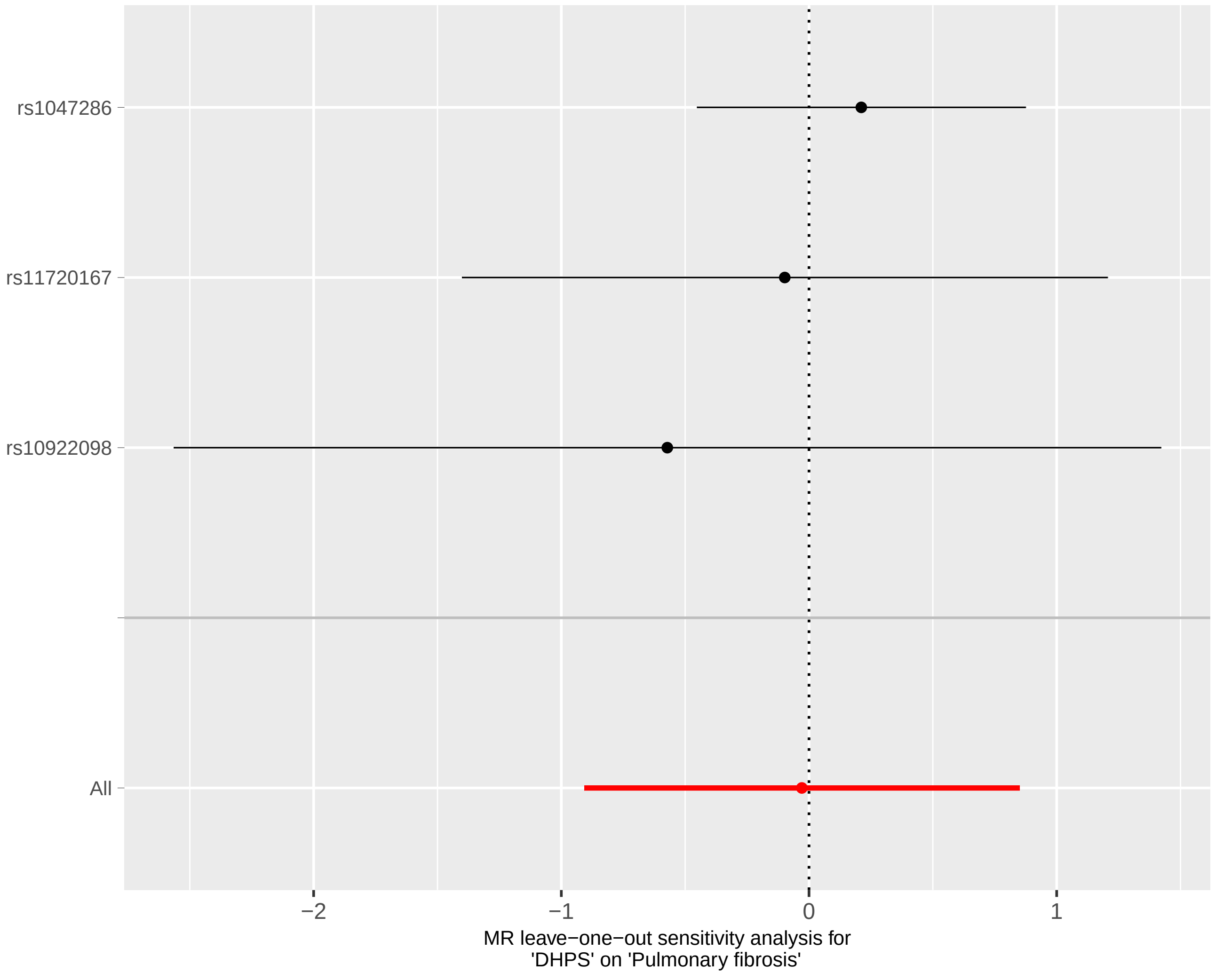
rs1047286
rs11720167
rs10922098
All
−2
−1
0
1
MR leave−one−out sensitivity analysis for
'DHPS' on 'Pulmonary fibrosis'

Insufficient number of SNPs

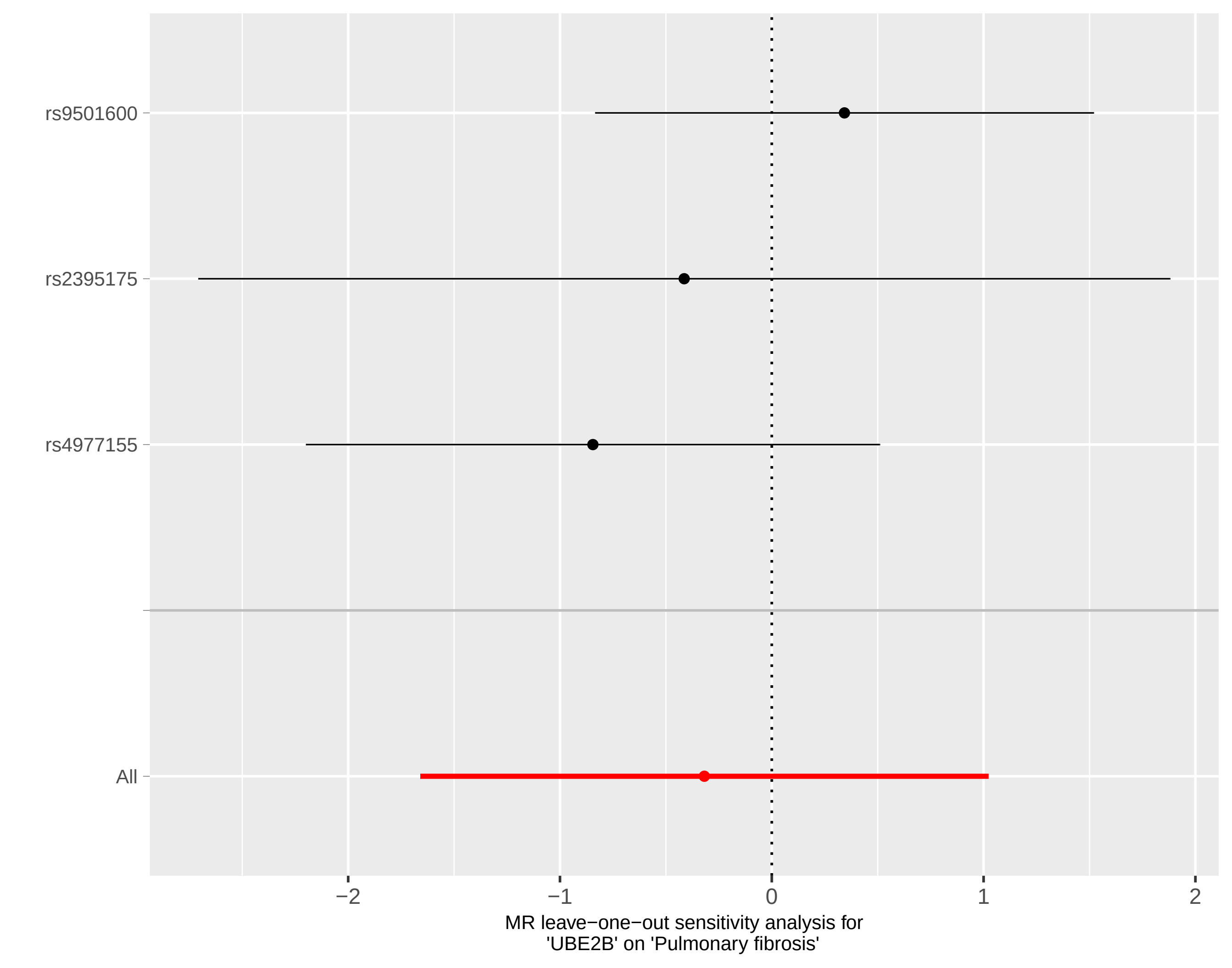
rs9501600
rs2395175
rs4977155
All
−2
−1
0
1
2
MR leave−one−out sensitivity analysis for
'UBE2B' on 'Pulmonary fibrosis'

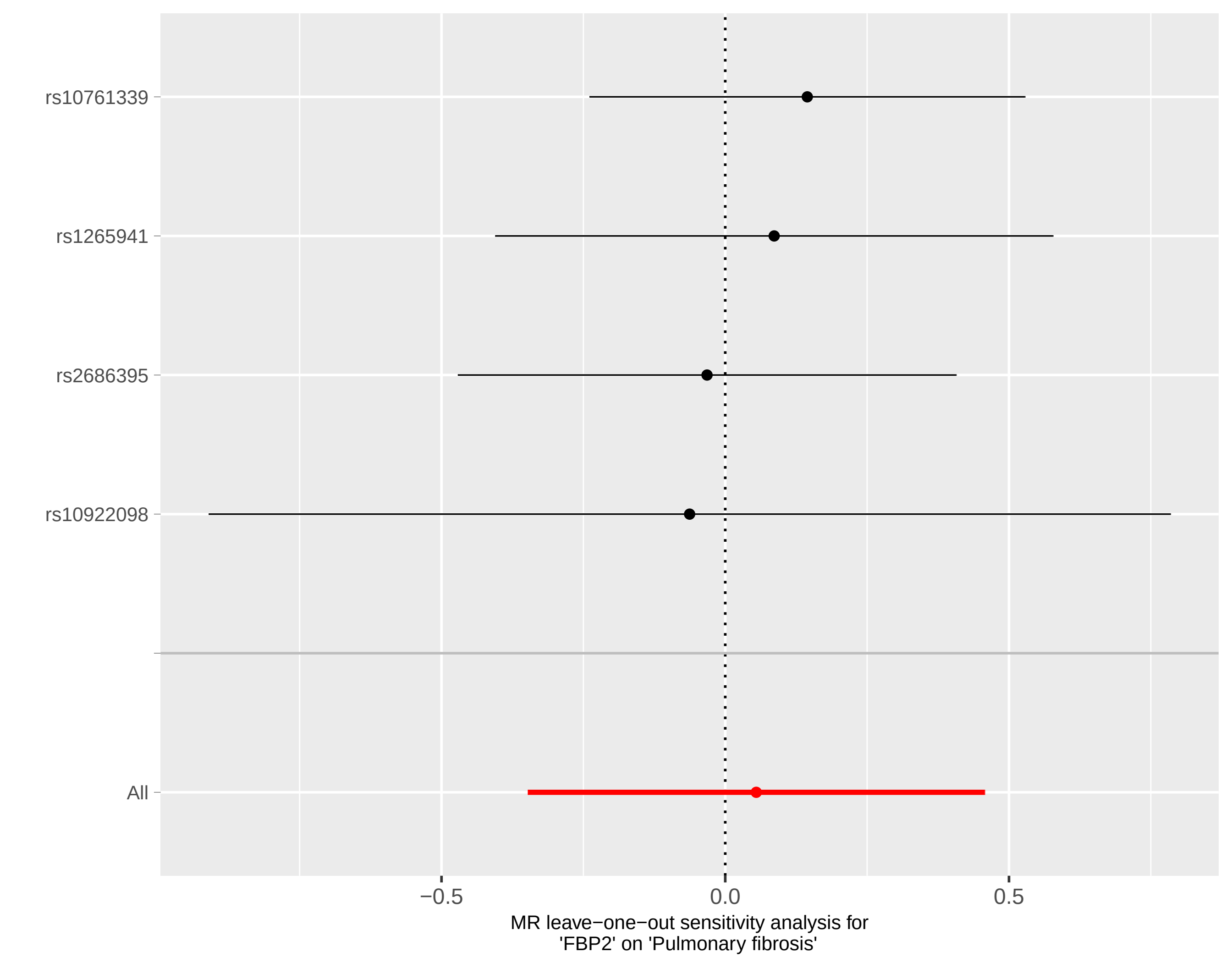
rs10761339
rs1265941
rs2686395
rs10922098
All
−0.5
0.0
0.5
MR leave−one−out sensitivity analysis for
'FBP2' on 'Pulmonary fibrosis'

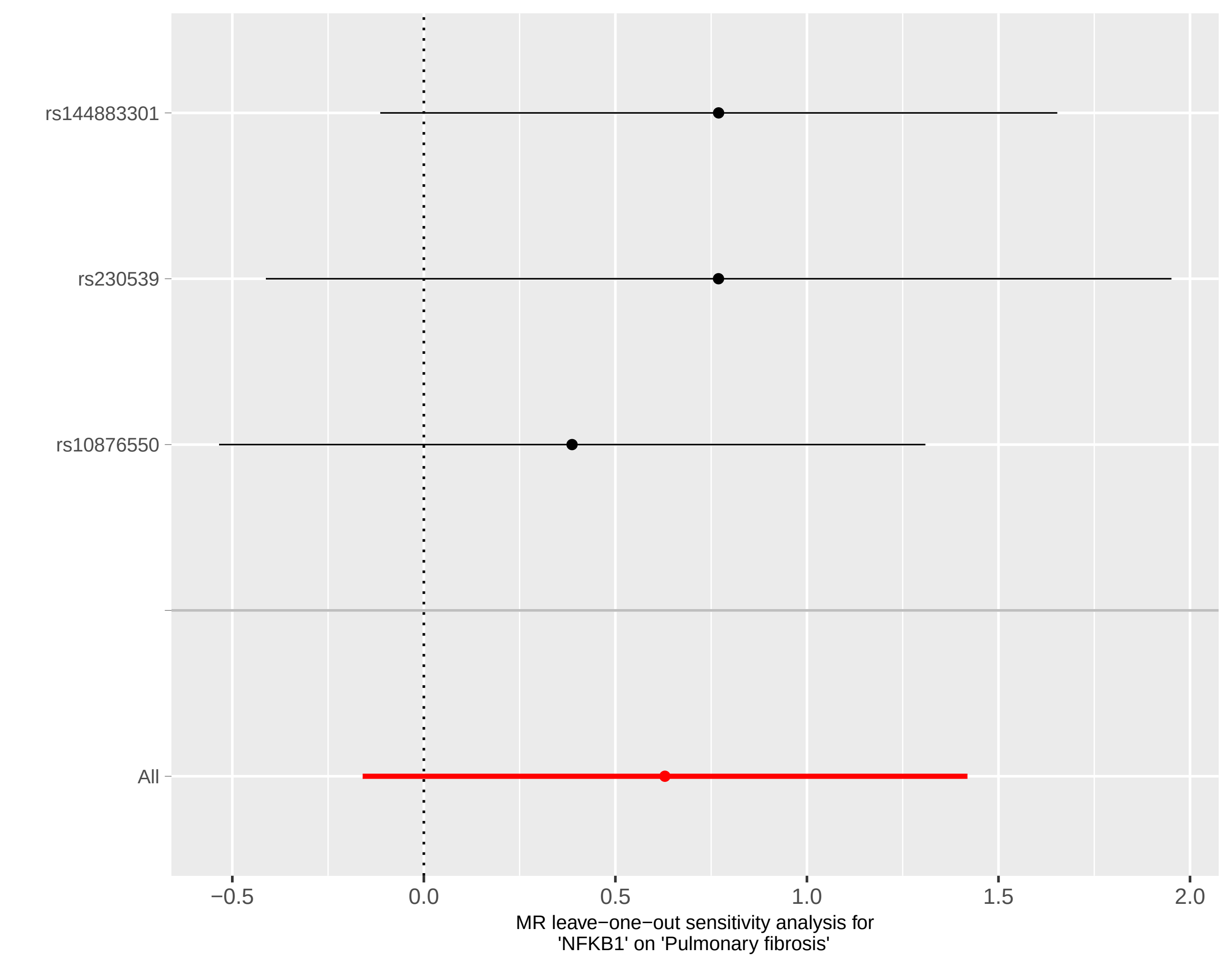
rs144883301
rs230539
rs10876550
All
−0.5
0.0
0.5
1.0
1.5
2.0
MR leave−one−out sensitivity analysis for
'NFKB1' on 'Pulmonary fibrosis'

Insufficient number of SNPs

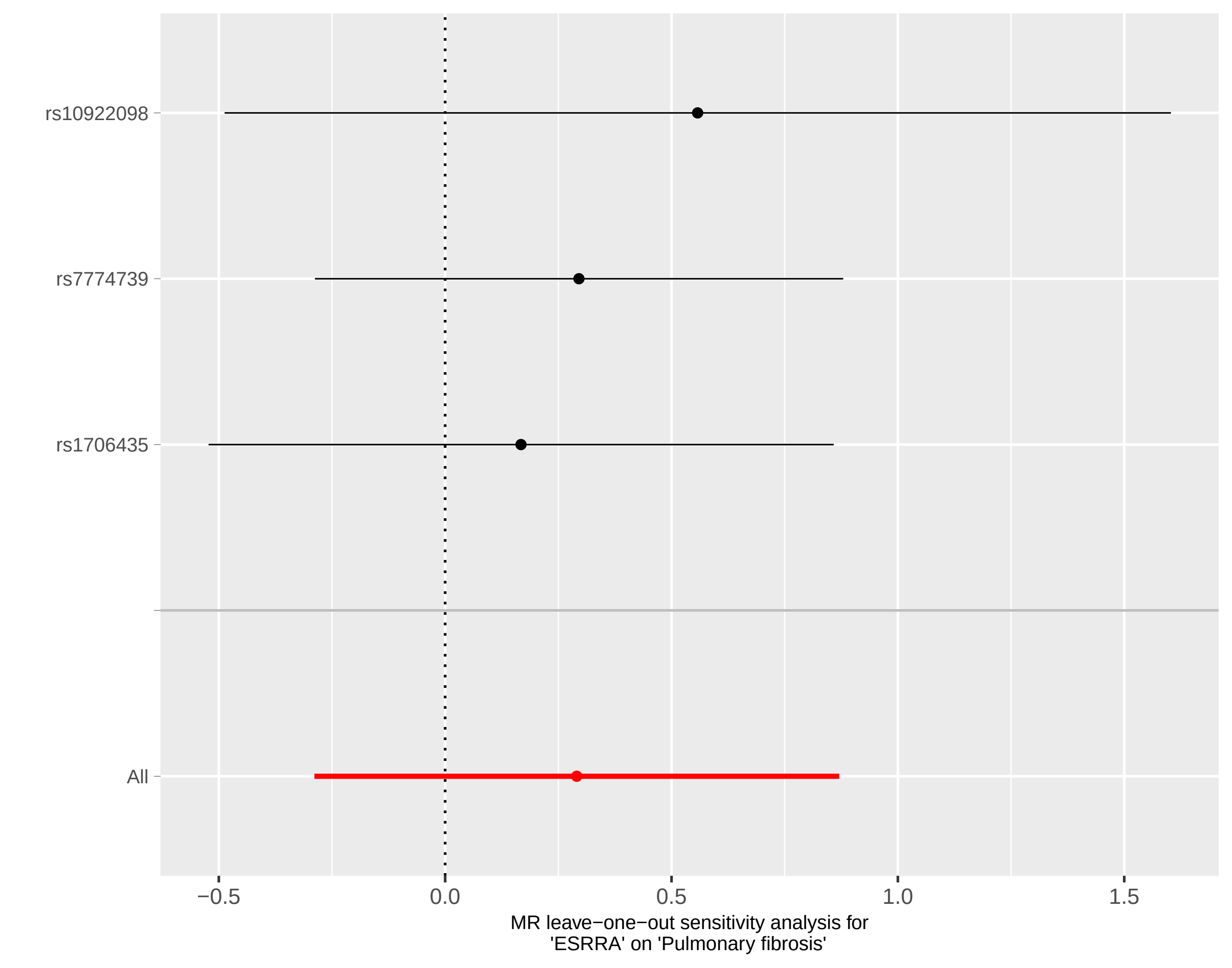
rs10922098
rs7774739
rs1706435
All
−0.5
0.0
0.5
1.0
1.5
MR leave−one−out sensitivity analysis for
'ESRRA' on 'Pulmonary fibrosis'

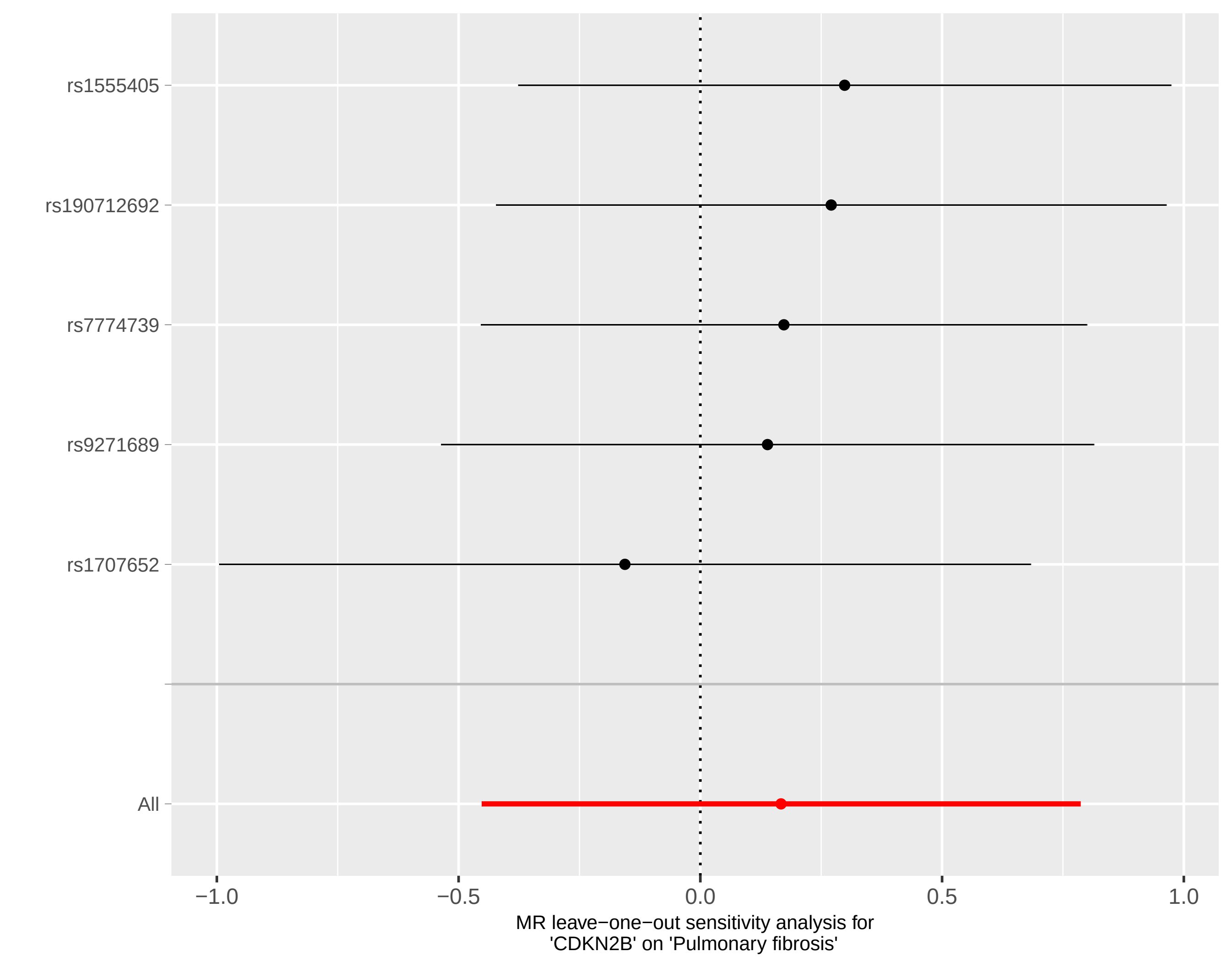

rs1555405
rs190712692
rs7774739
rs9271689
rs1707652
All
−1.0
−0.5
0.0
0.5
1.0
MR leave−one−out sensitivity analysis for
'CDKN2B' on 'Pulmonary fibrosis'

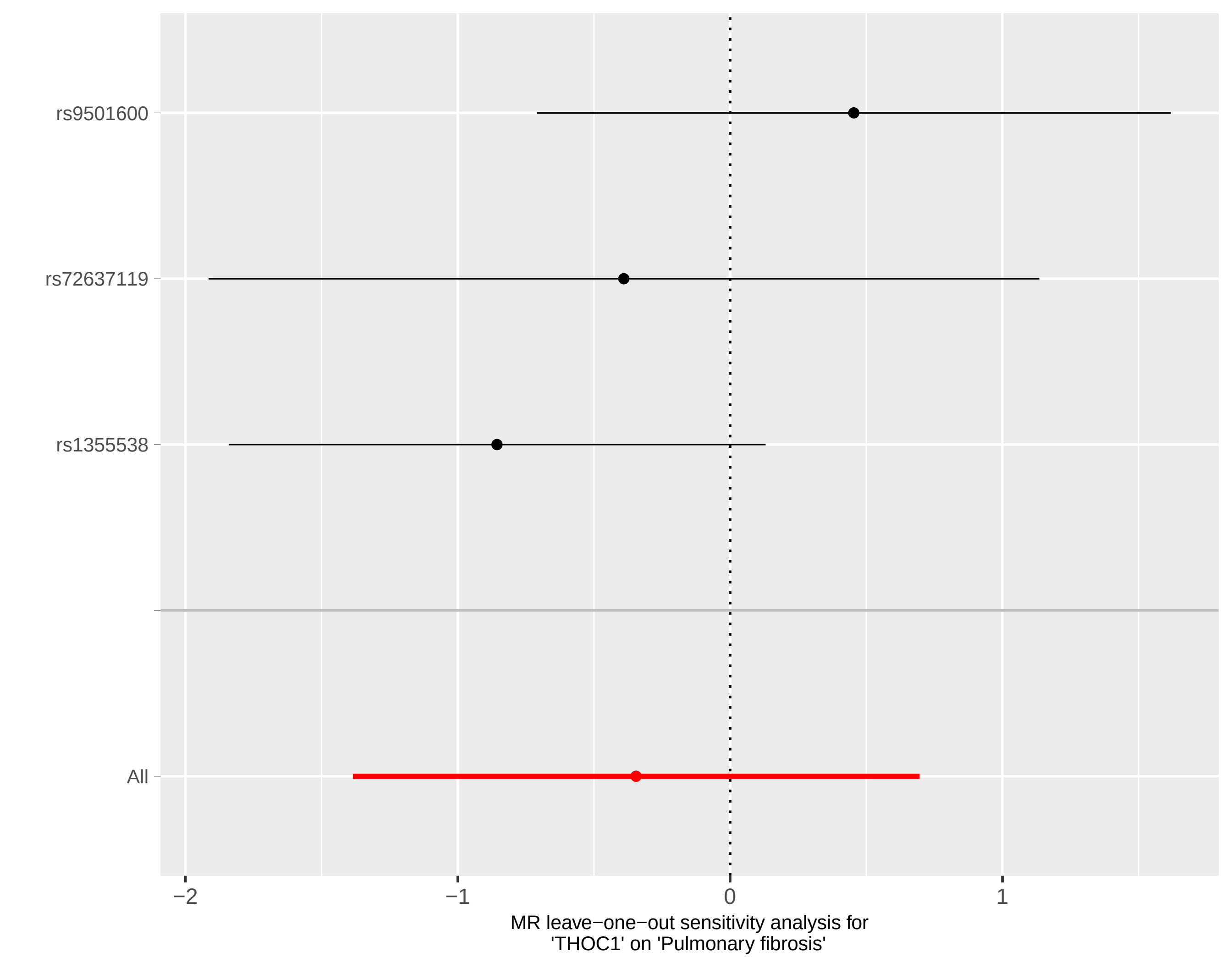
rs9501600
rs72637119
rs1355538
All
−2
−1
0
1
MR leave−one−out sensitivity analysis for
'THOC1' on 'Pulmonary fibrosis'

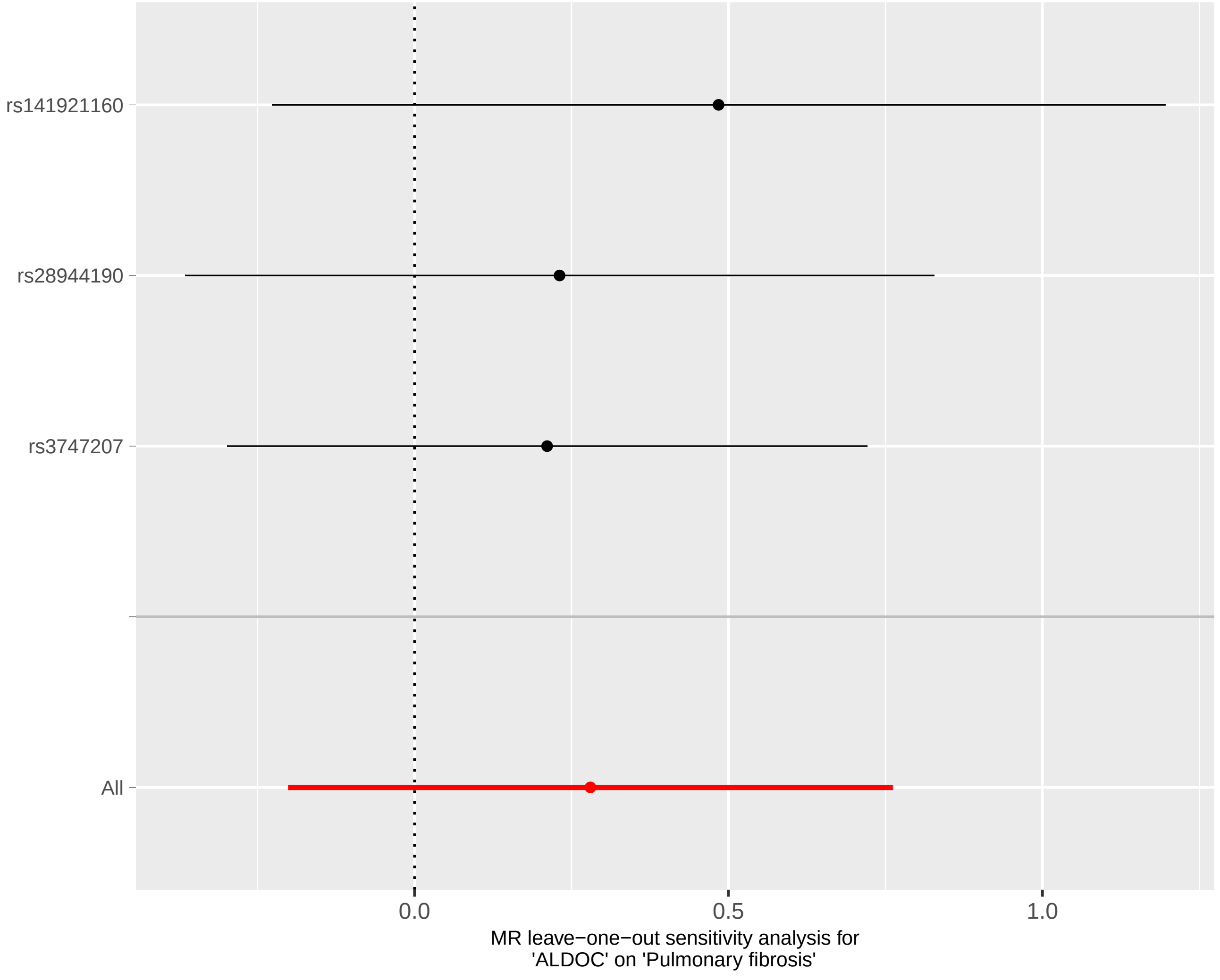
rs141921160
rs28944190
rs3747207
All
0.0
0.5
1.0
MR leave−one−out sensitivity analysis for
'ALDOC' on 'Pulmonary fibrosis'

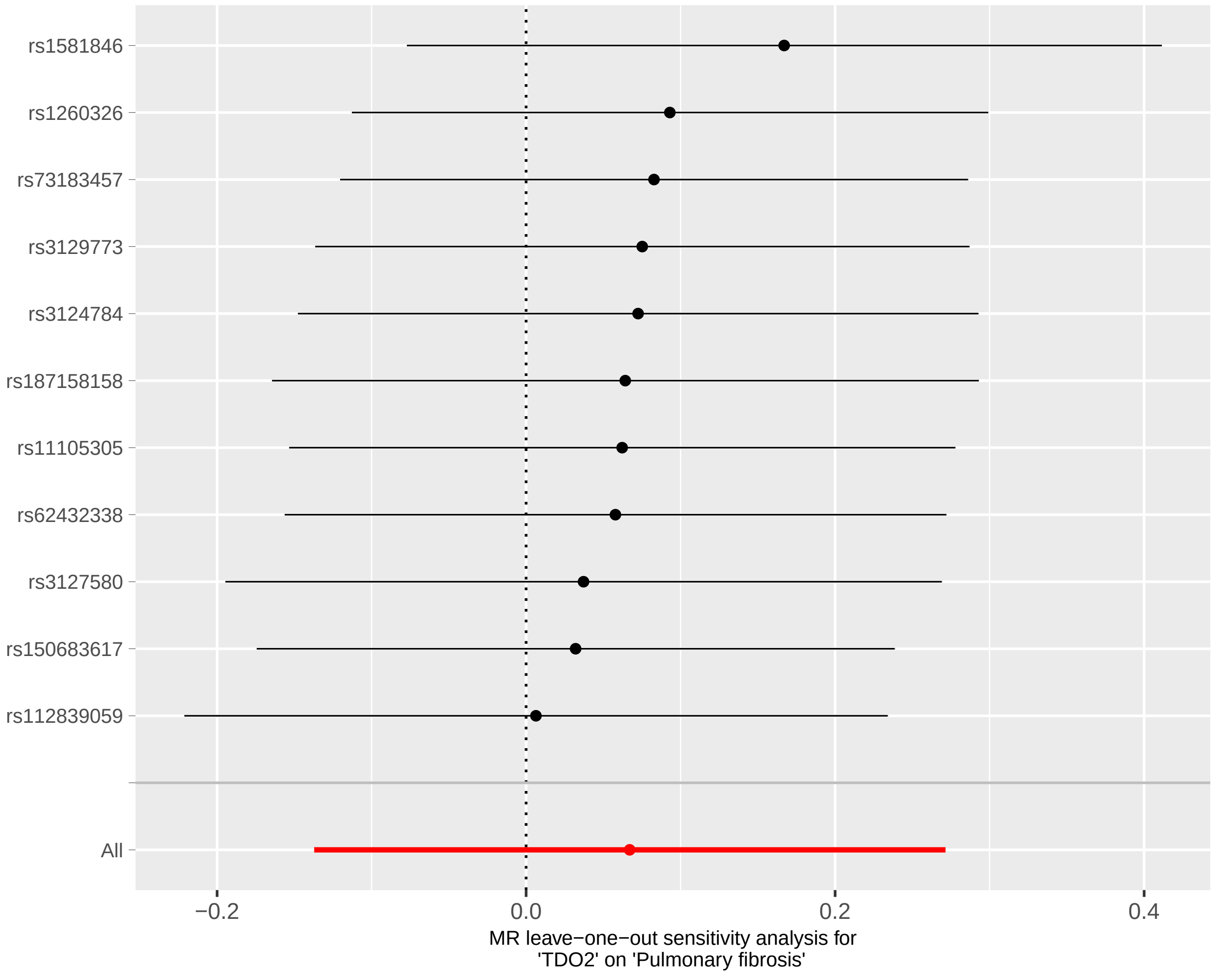
rs1581846
rs1260326
rs73183457
rs3129773
rs3124784
rs187158158
rs11105305
rs62432338
rs3127580
rs150683617
rs112839059
All
−0.2
0.0
0.2
0.4
MR leave−one−out sensitivity analysis for
'TDO2' on 'Pulmonary fibrosis'

Insufficient number of SNPs

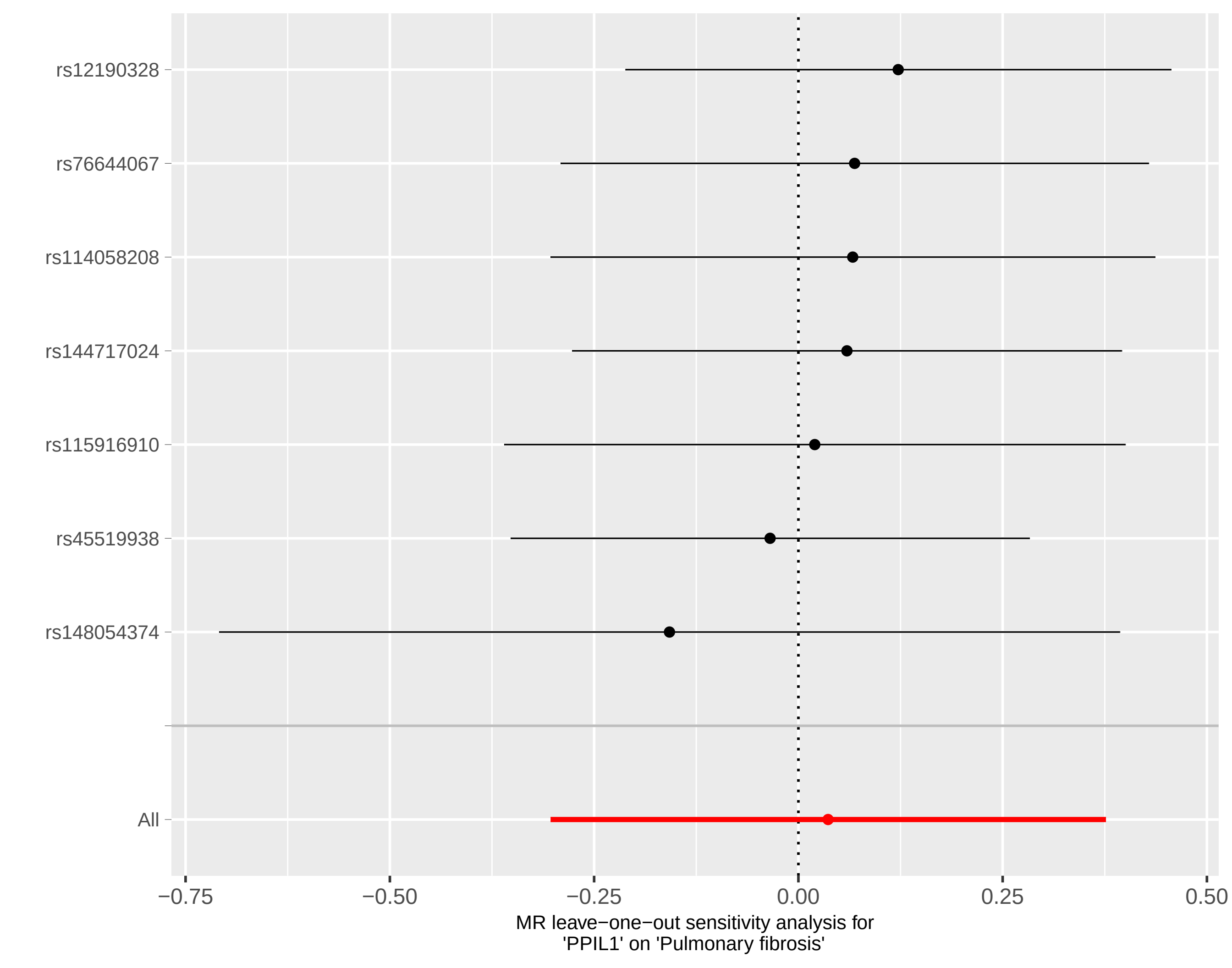
rs12190328
rs76644067
rs114058208
rs144717024
rs115916910
rs45519938
rs148054374
All
−0.75
−0.50
−0.25
0.00
0.25
0.50
MR leave−one−out sensitivity analysis for
'PPIL1' on 'Pulmonary fibrosis'

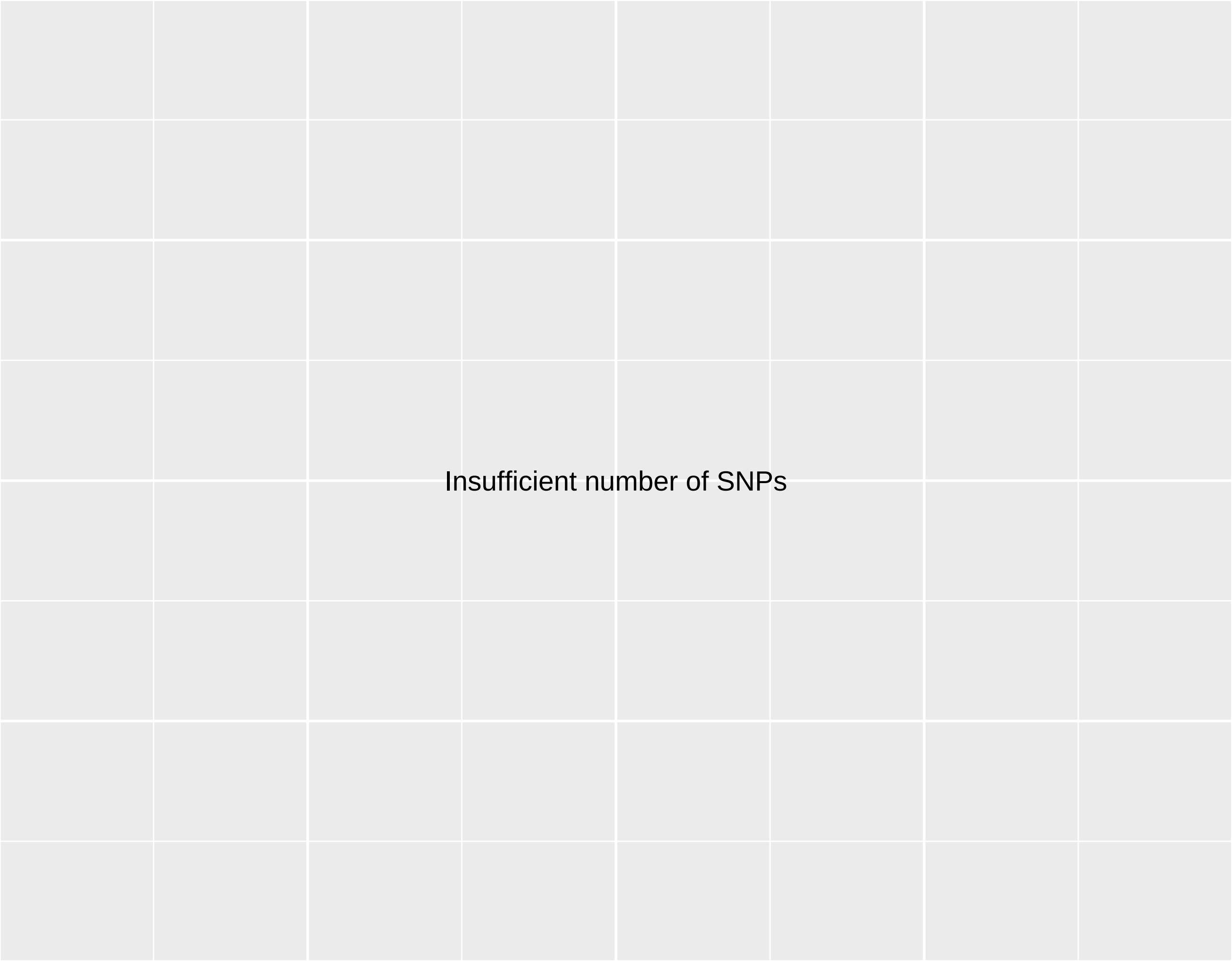
Insufficient number of SNPs

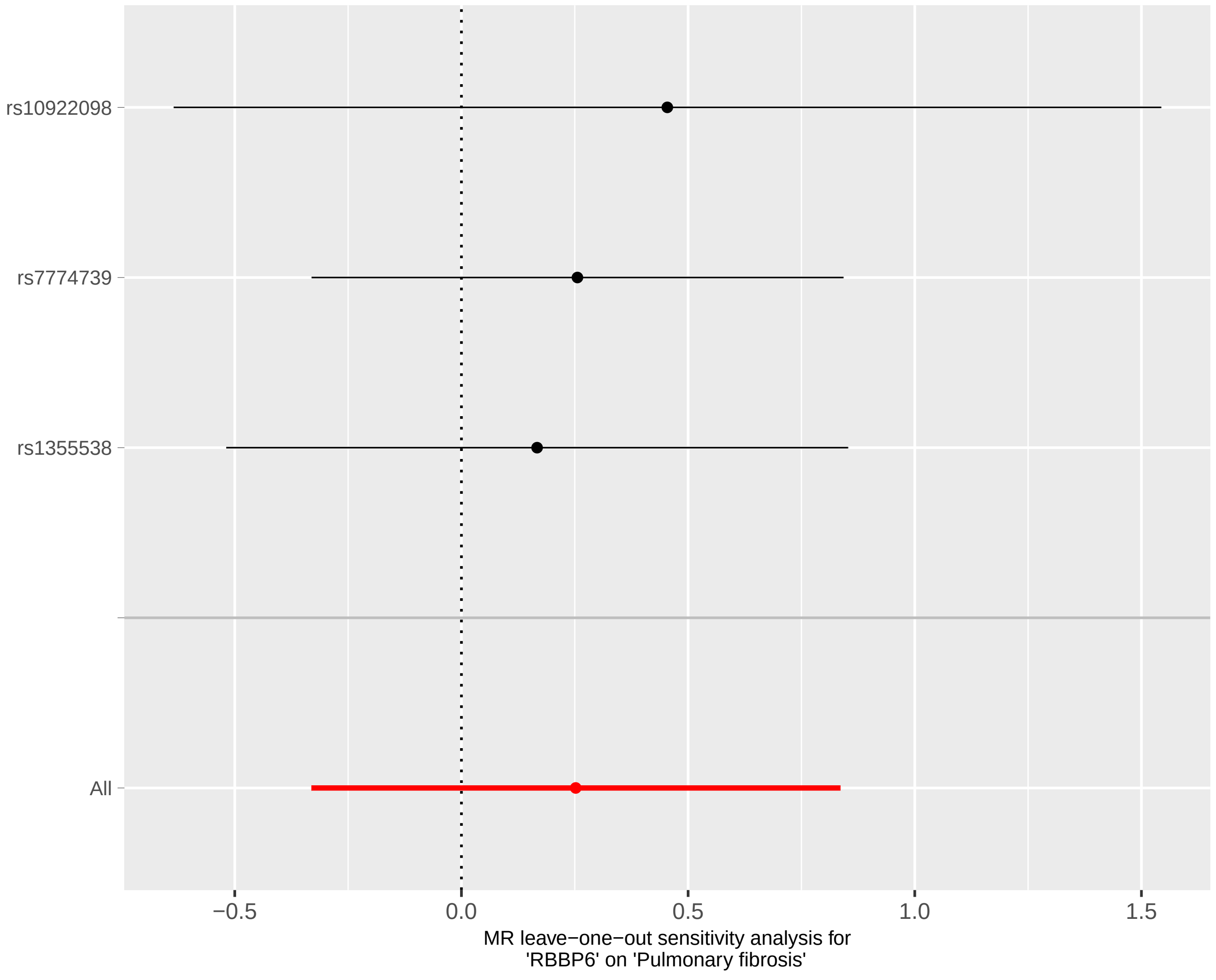
rs10922098
rs7774739
rs1355538
All
−0.5
0.0
0.5
1.0
1.5
MR leave−one−out sensitivity analysis for
'RBBP6' on 'Pulmonary fibrosis'

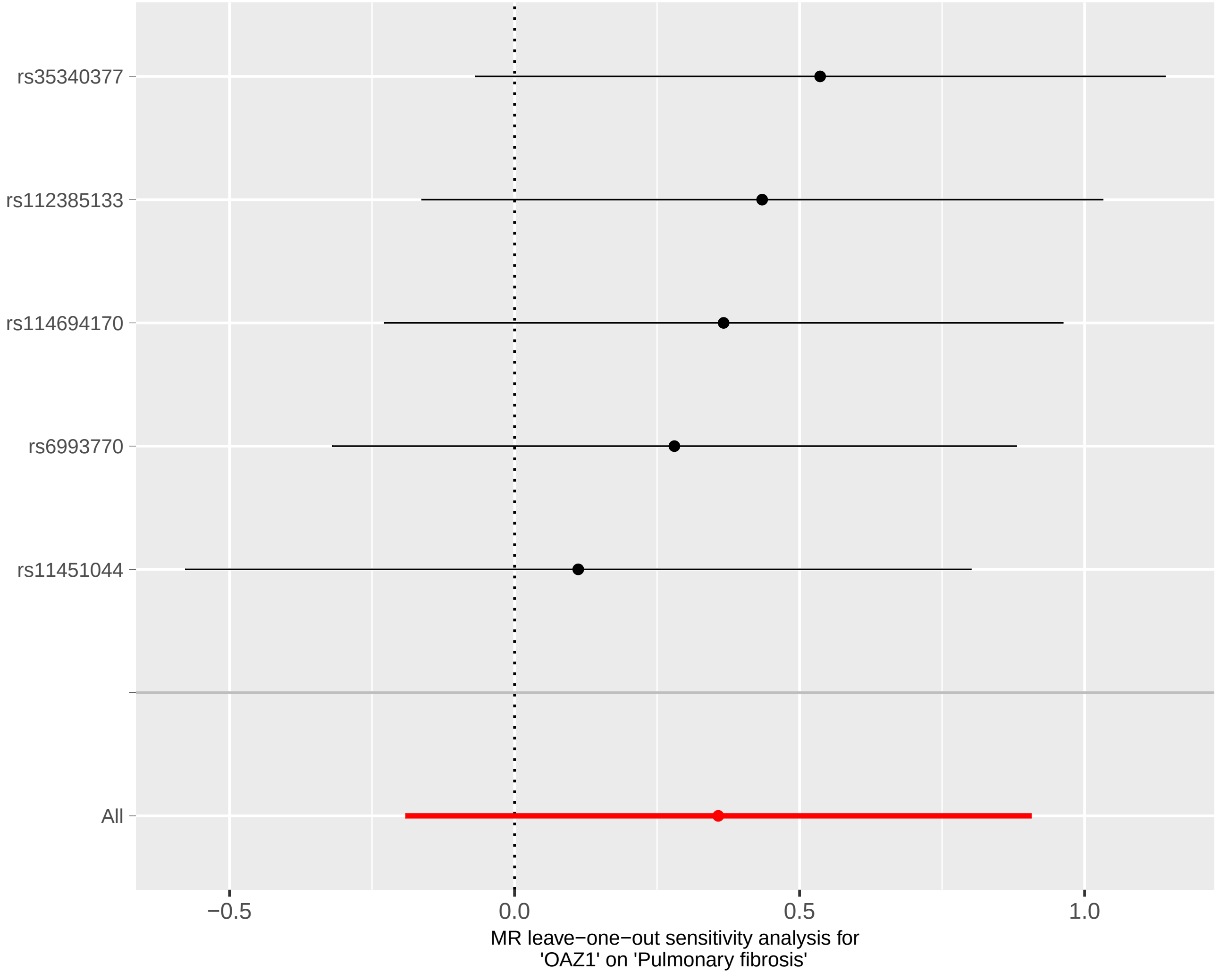

rs35340377
rs112385133
rs114694170
rs6993770
rs11451044
All
−0.5
0.0
0.5
1.0
MR leave−one−out sensitivity analysis for
'OAZ1' on 'Pulmonary fibrosis'

Insufficient number of SNPs

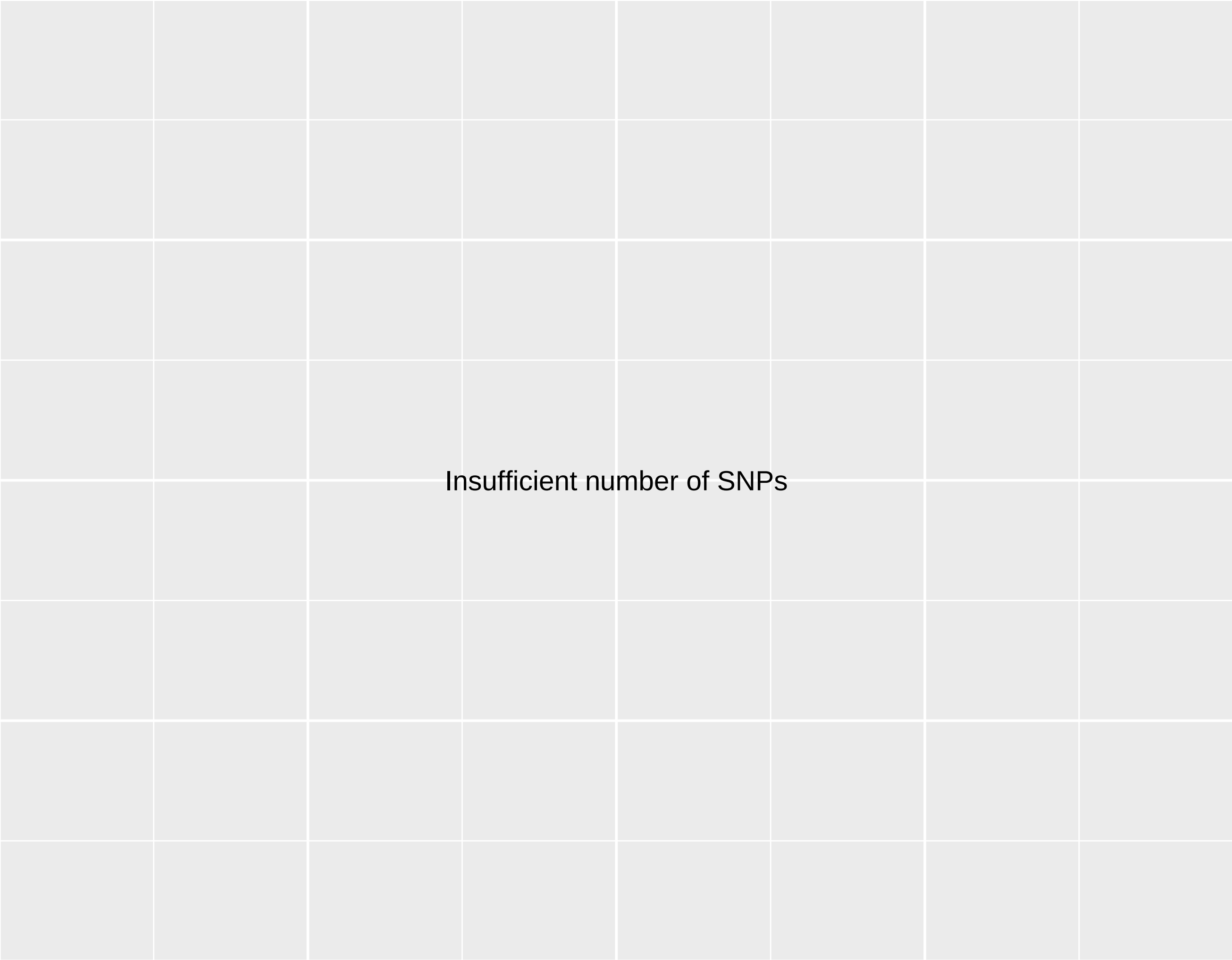
Insufficient number of SNPs

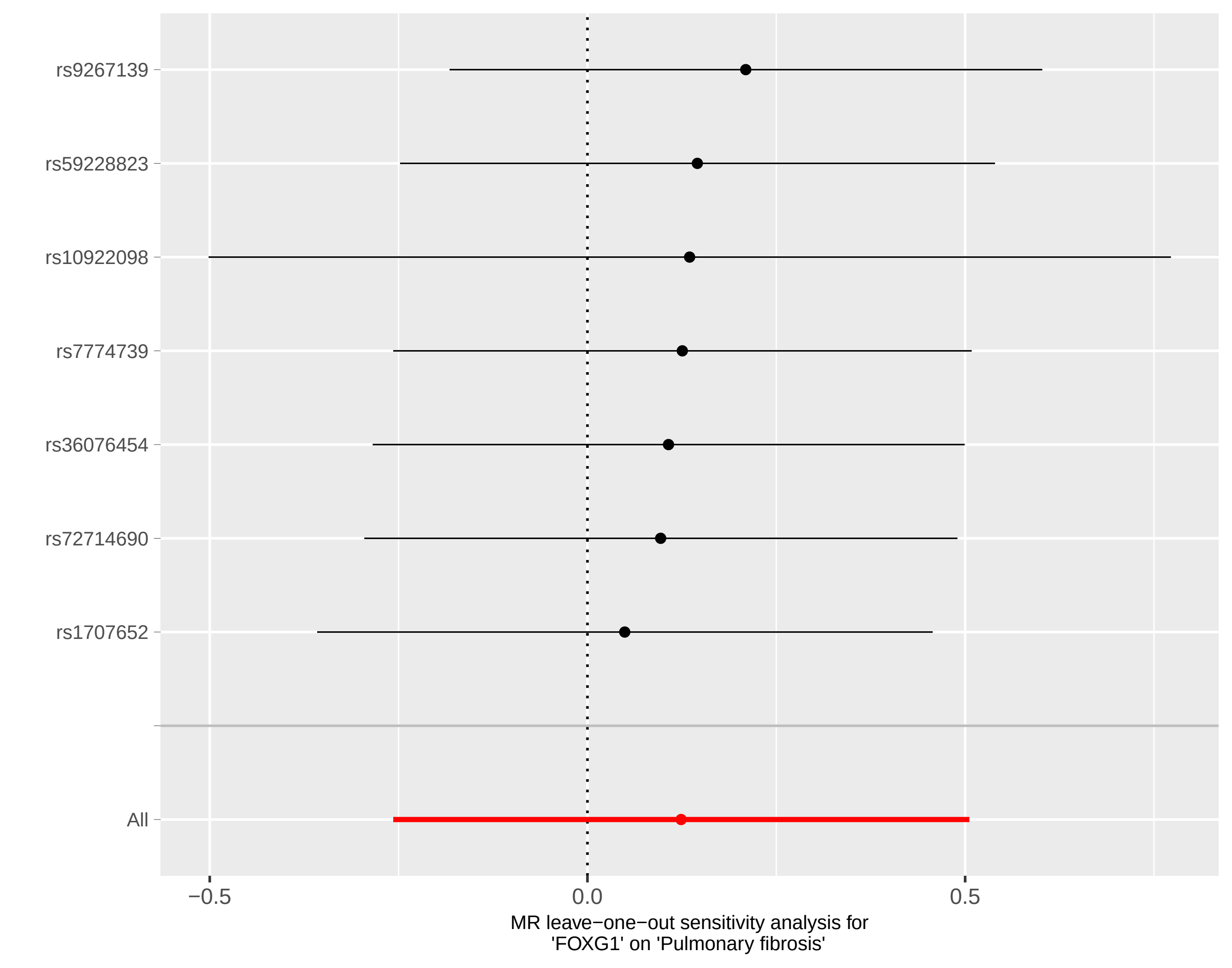
rs9267139
rs59228823
rs10922098
rs7774739
rs36076454
rs72714690
rs1707652
All
−0.5
0.0
0.5
MR leave−one−out sensitivity analysis for
'FOXG1' on 'Pulmonary fibrosis'

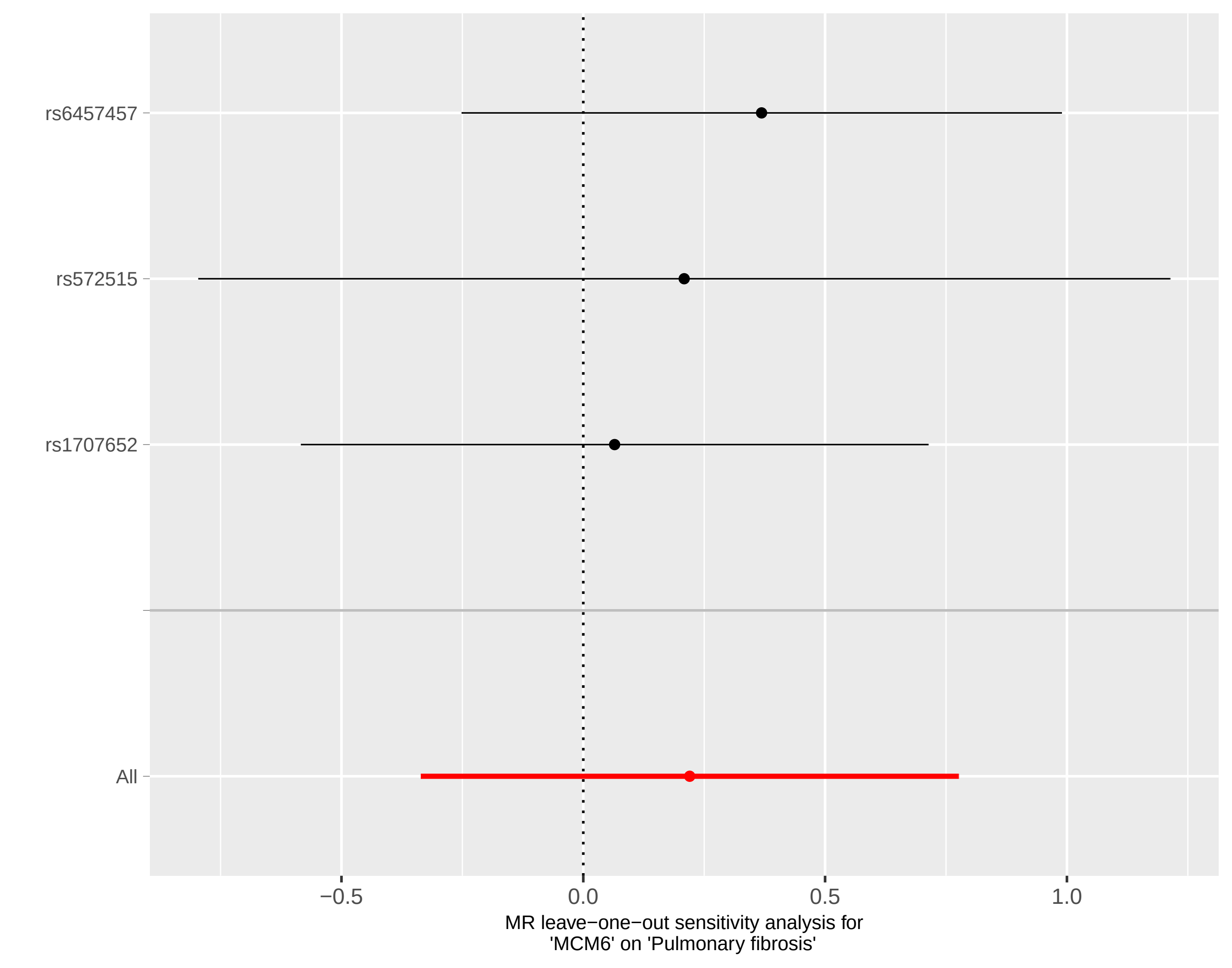
rs6457457
rs572515
rs1707652
All
−0.5
0.0
0.5
1.0
MR leave−one−out sensitivity analysis for
'MCM6' on 'Pulmonary fibrosis'

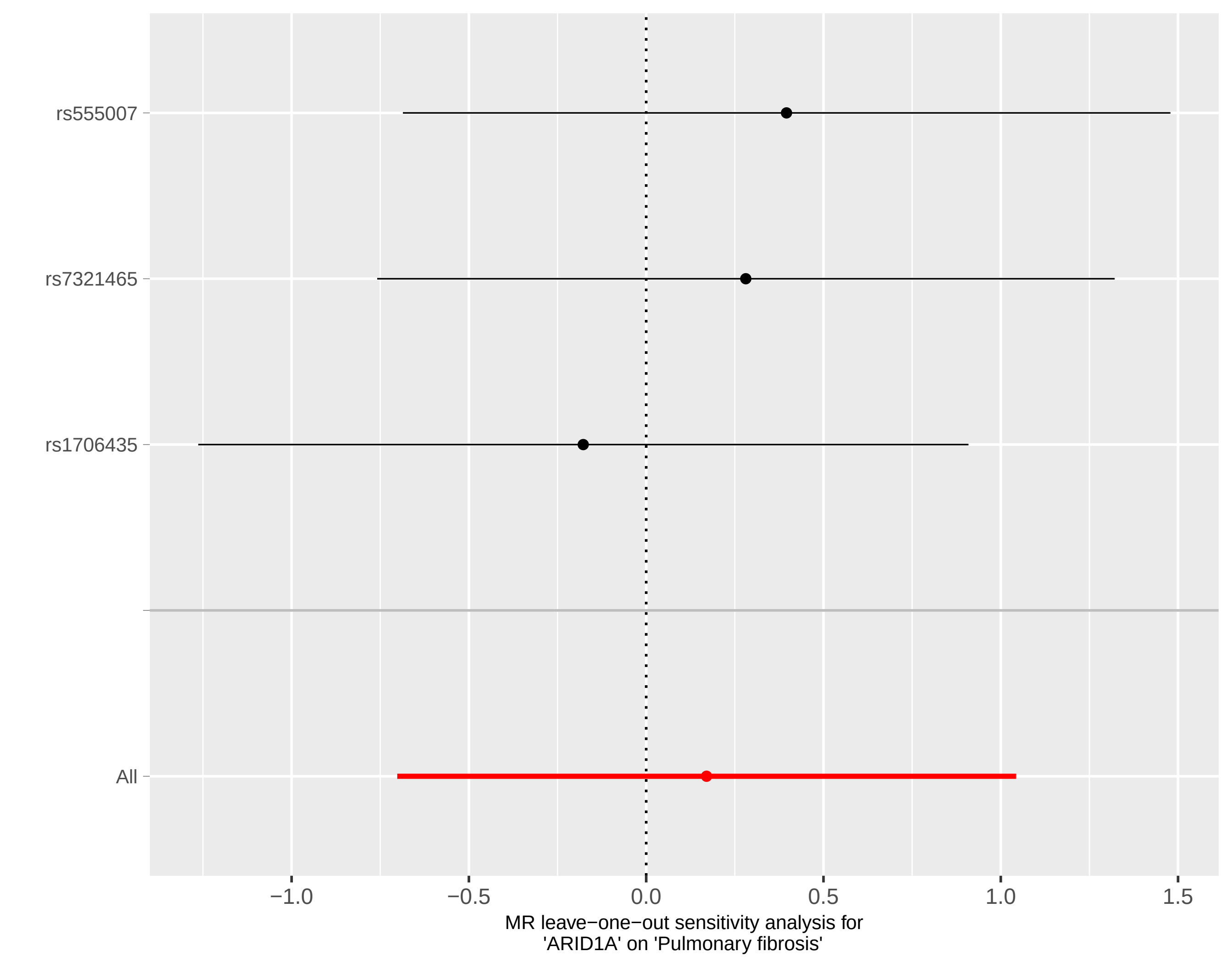
rs555007
rs7321465
rs1706435
All
−1.0
−0.5
0.0
0.5
1.0
1.5
MR leave−one−out sensitivity analysis for
'ARID1A' on 'Pulmonary fibrosis'

Insufficient number of SNPs

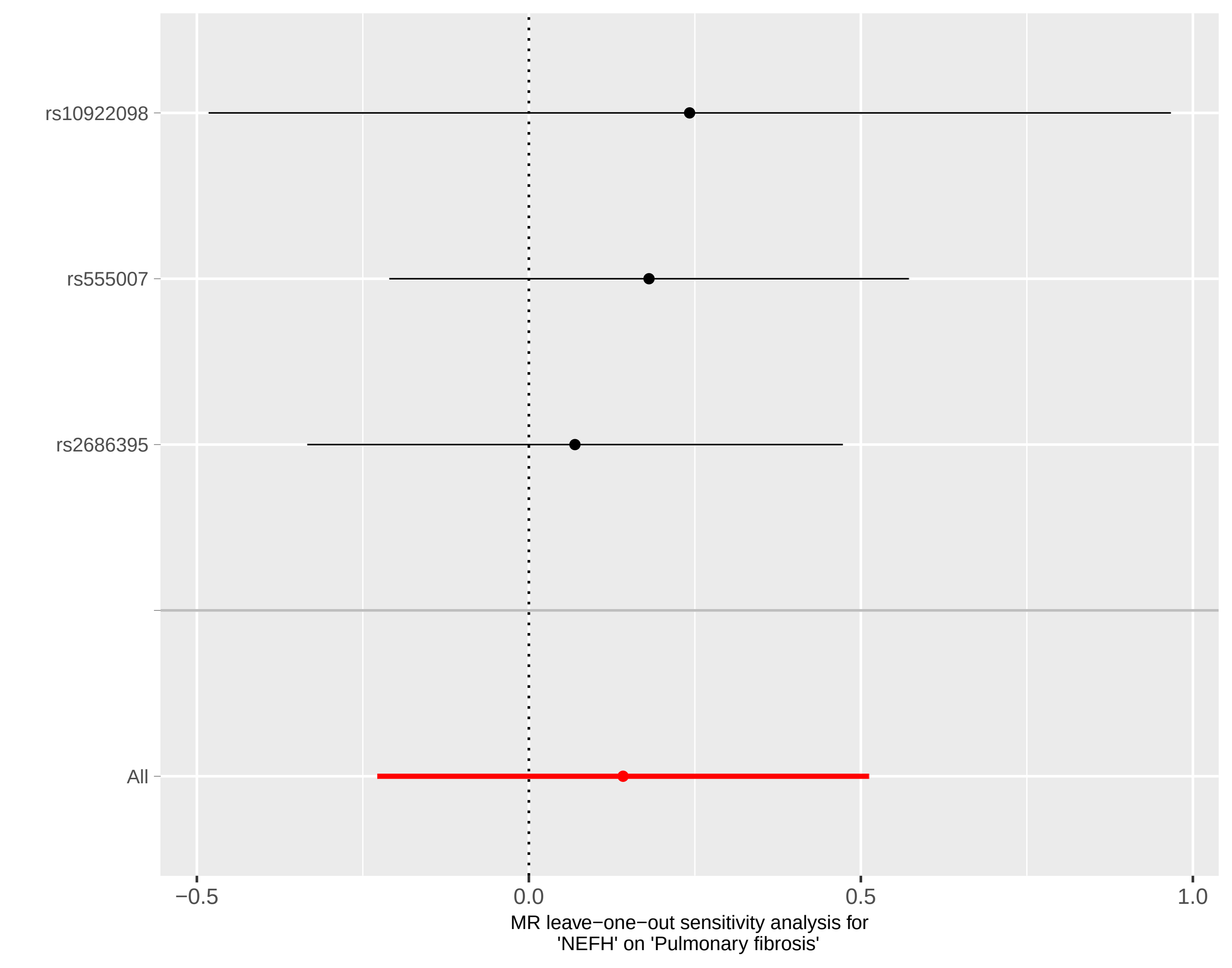
rs10922098
rs555007
rs2686395
All
−0.5
0.0
0.5
1.0
MR leave−one−out sensitivity analysis for
'NEFH' on 'Pulmonary fibrosis'

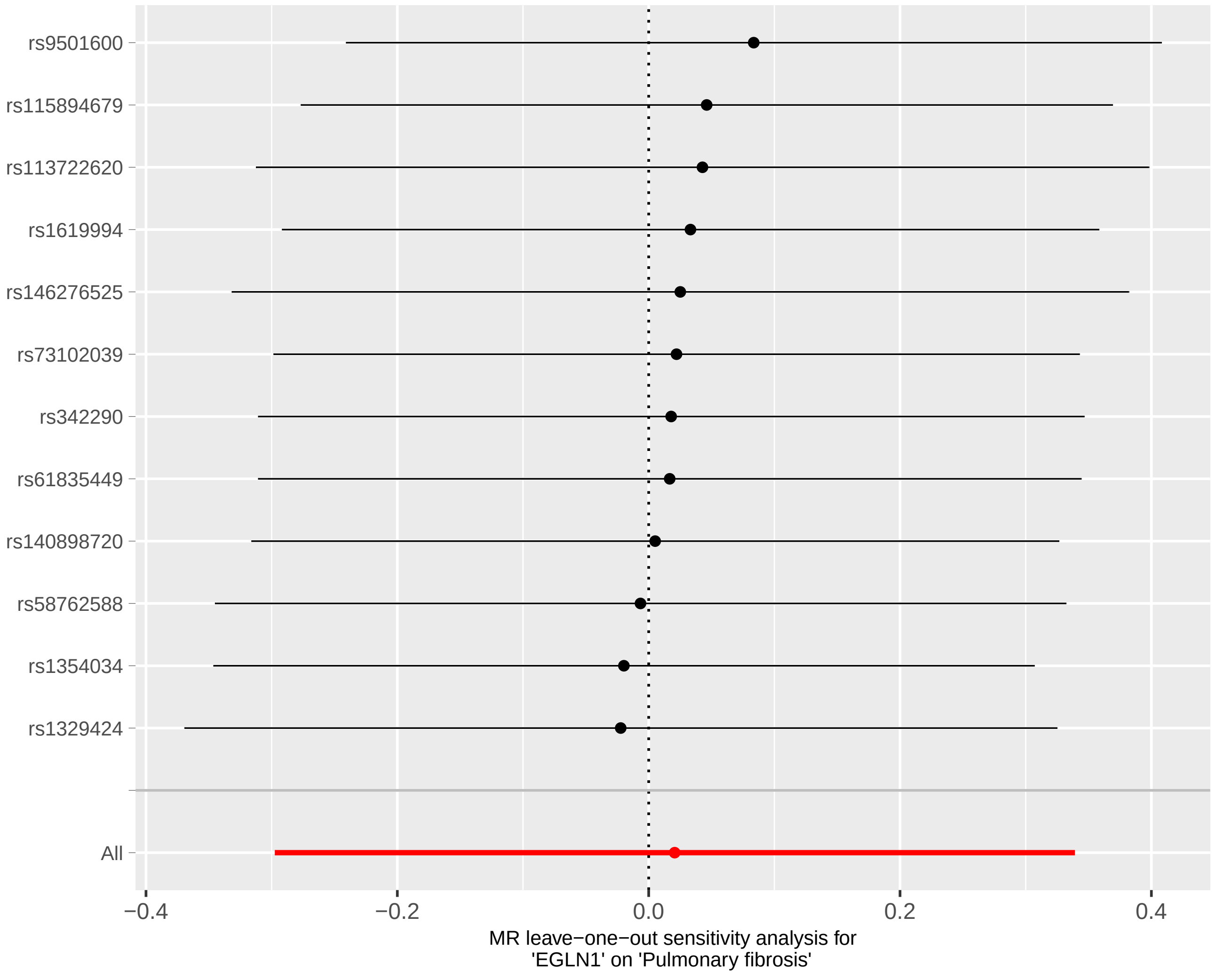
rs9501600
rs115894679
rs113722620
rs1619994
rs146276525
rs73102039
rs342290
rs61835449
rs140898720
rs58762588
rs1354034
rs1329424
All
−0.4
−0.2
0.0
0.2
0.4
MR leave−one−out sensitivity analysis for
'EGLN1' on 'Pulmonary fibrosis'

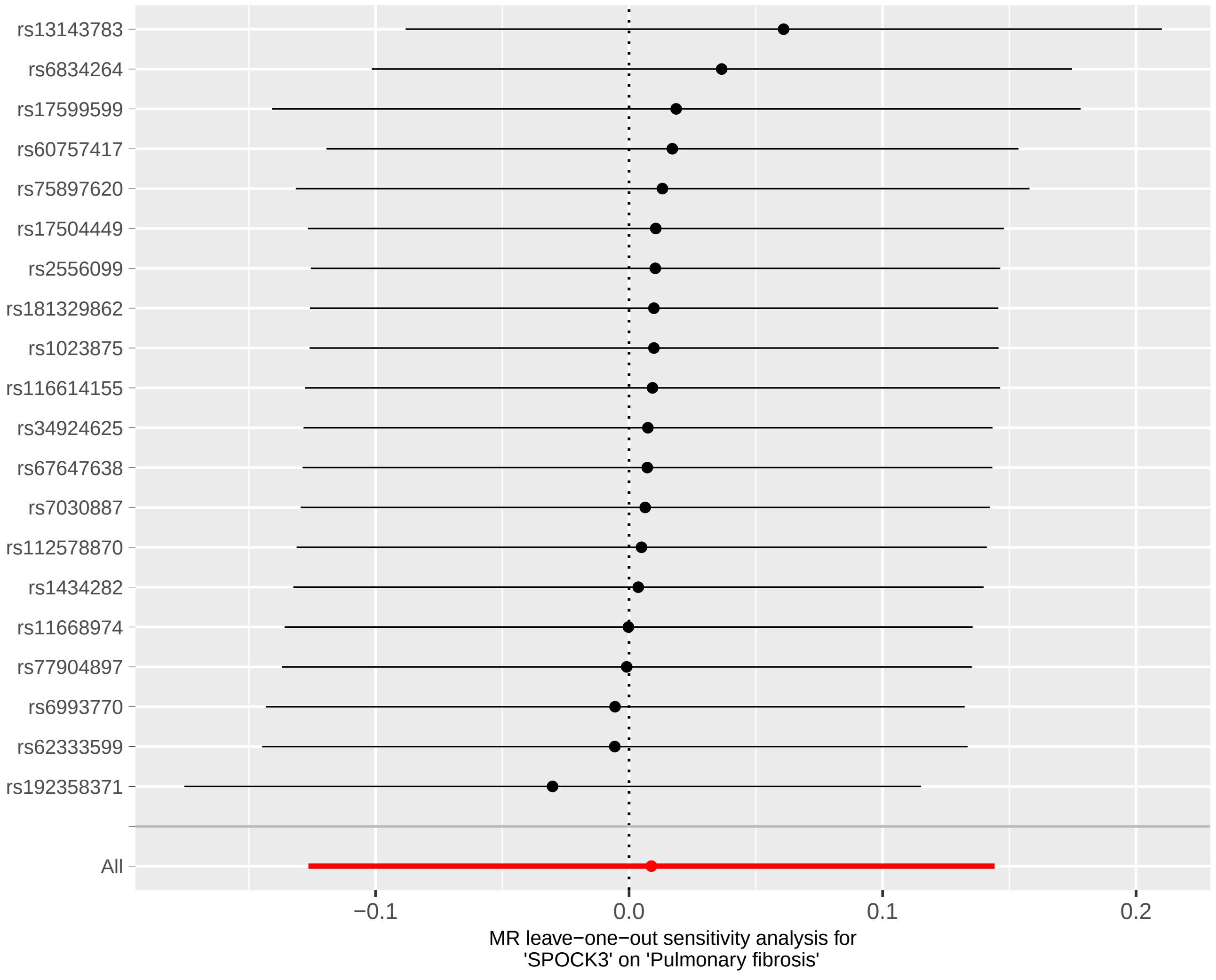
rs13143783
rs6834264
rs17599599
rs60757417
rs75897620
rs17504449
rs2556099
rs181329862
rs1023875
rs116614155
rs34924625
rs67647638
rs7030887
rs112578870
rs1434282
rs11668974
rs77904897
rs6993770
rs62333599
rs192358371
All
−0.1
0.0
0.1
0.2
MR leave−one−out sensitivity analysis for
'SPOCK3' on 'Pulmonary fibrosis'

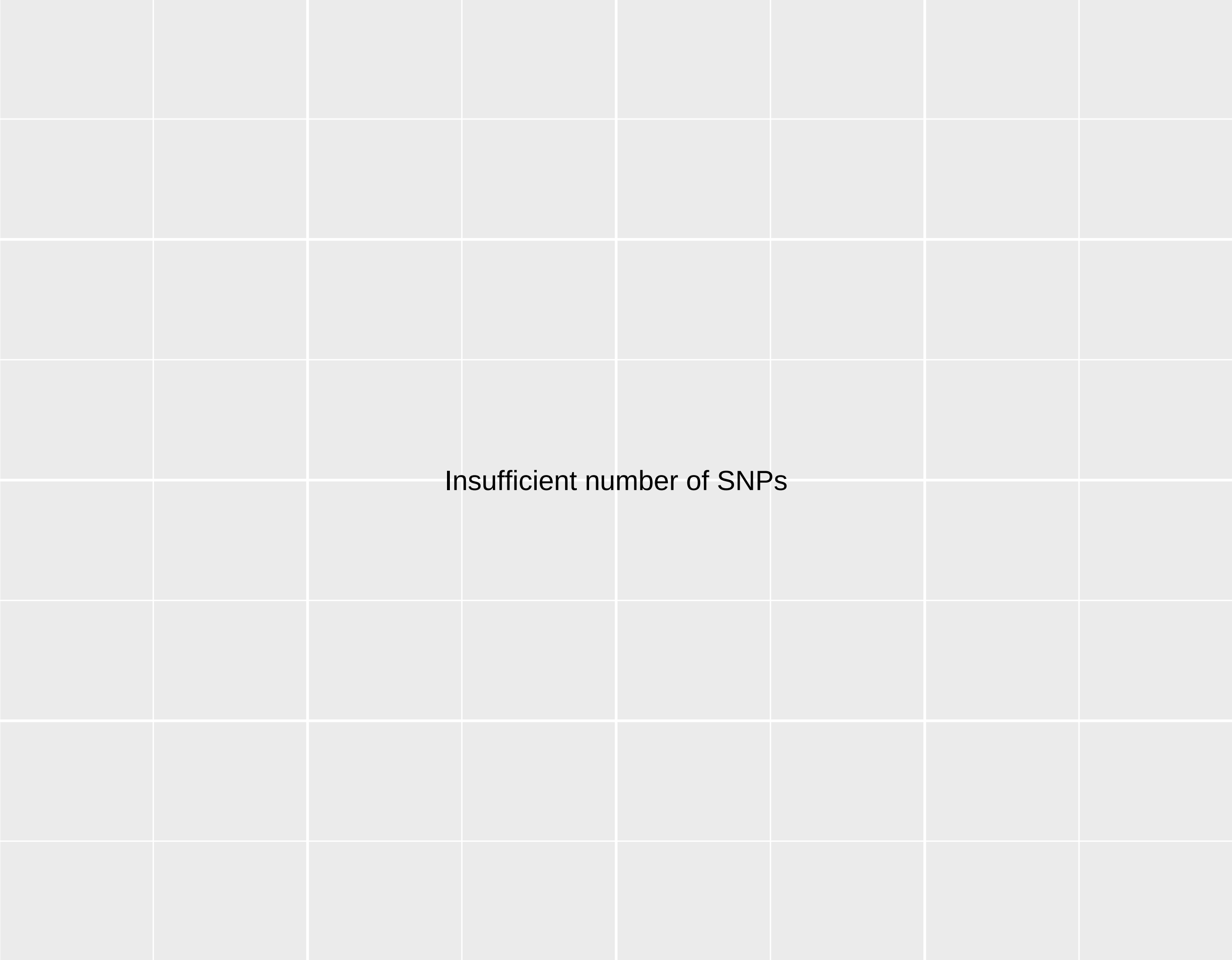
Insufficient number of SNPs

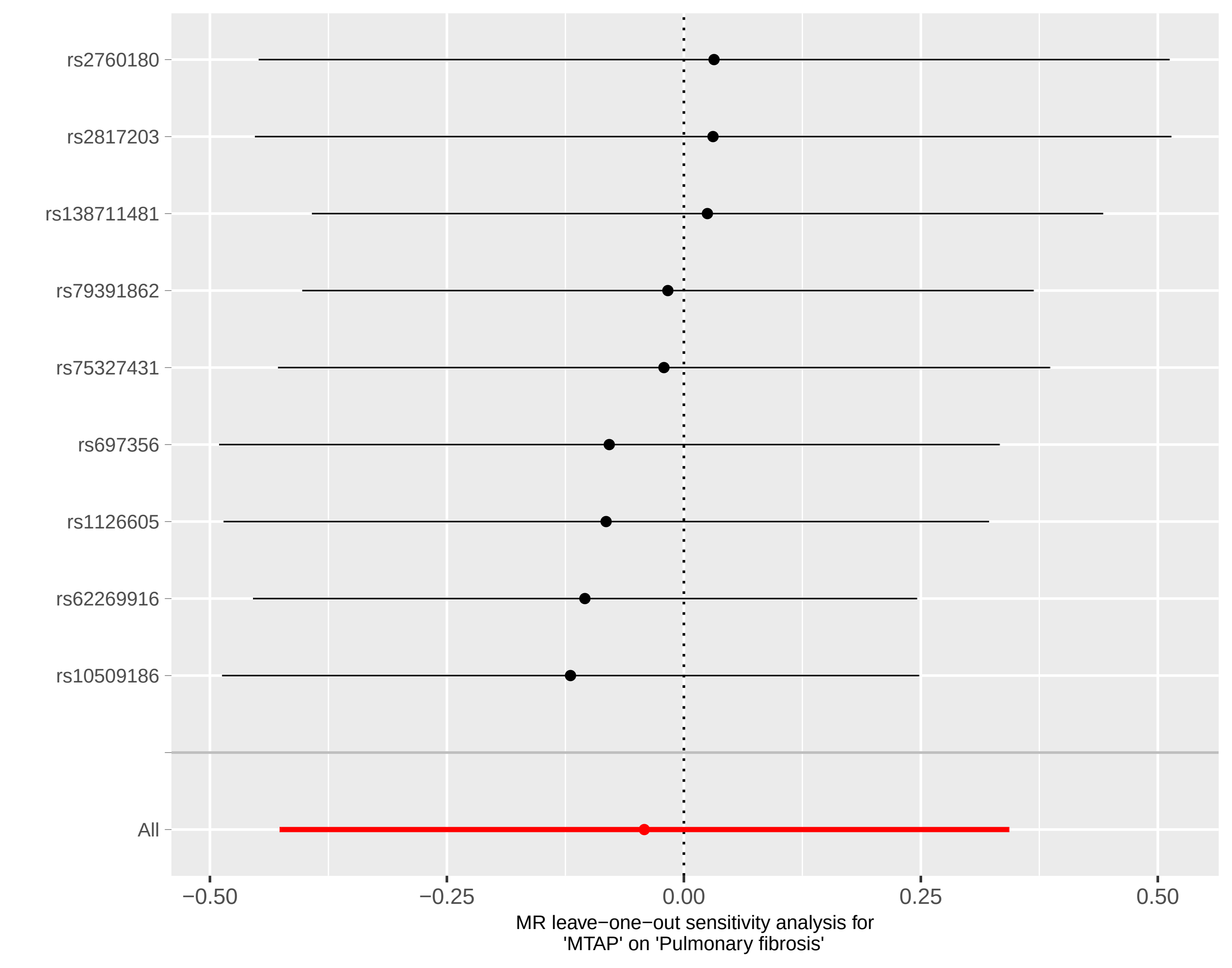
rs2760180
rs2817203
rs138711481
rs79391862
rs75327431
rs697356
rs1126605
rs62269916
rs10509186
All
−0.50
−0.25
0.00
0.25
0.50
MR leave−one−out sensitivity analysis for
'MTAP' on 'Pulmonary fibrosis'

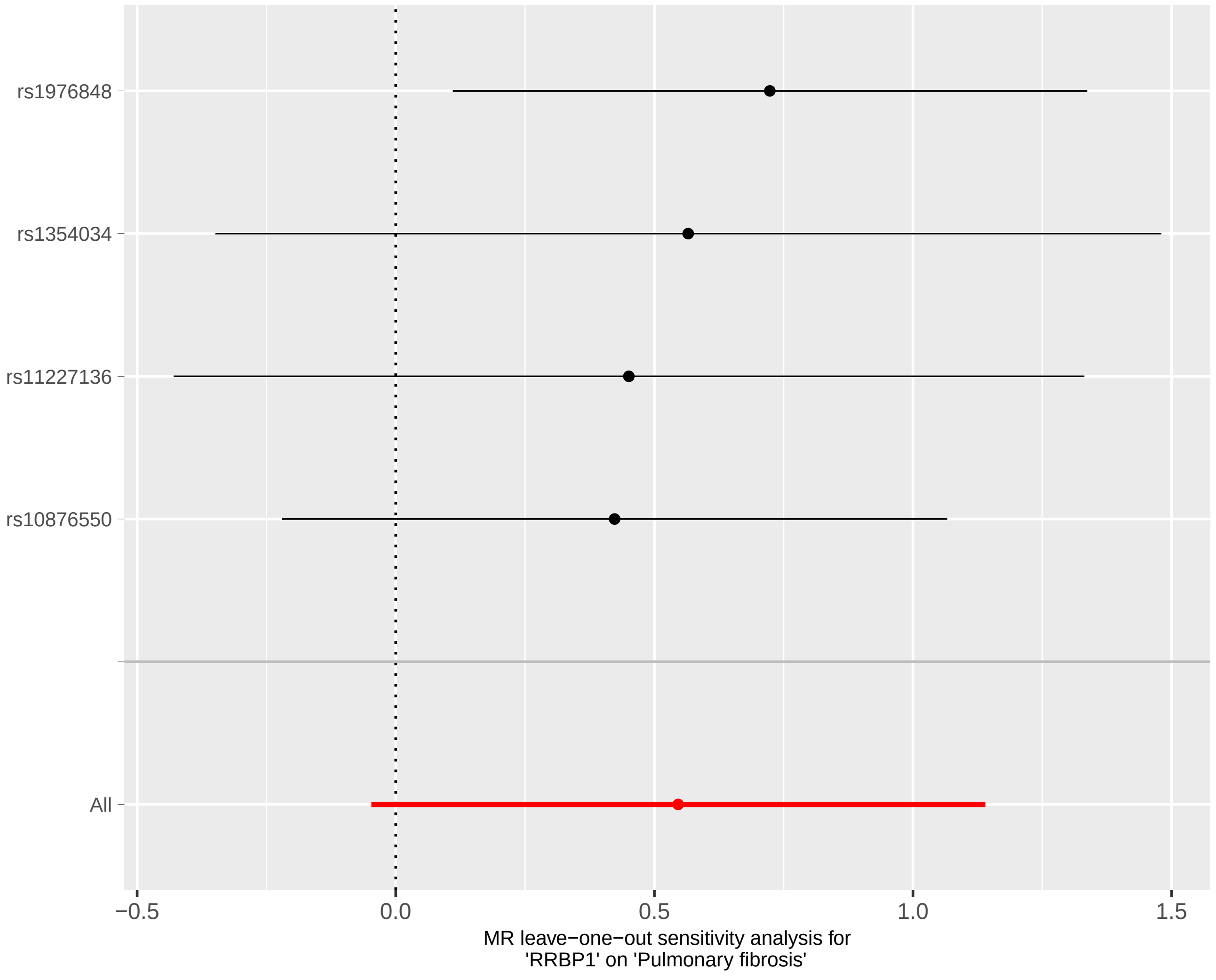
rs1976848
rs1354034
rs11227136
rs10876550
All
−0.5
0.0
0.5
1.0
1.5
MR leave−one−out sensitivity analysis for
'RRBP1' on 'Pulmonary fibrosis'

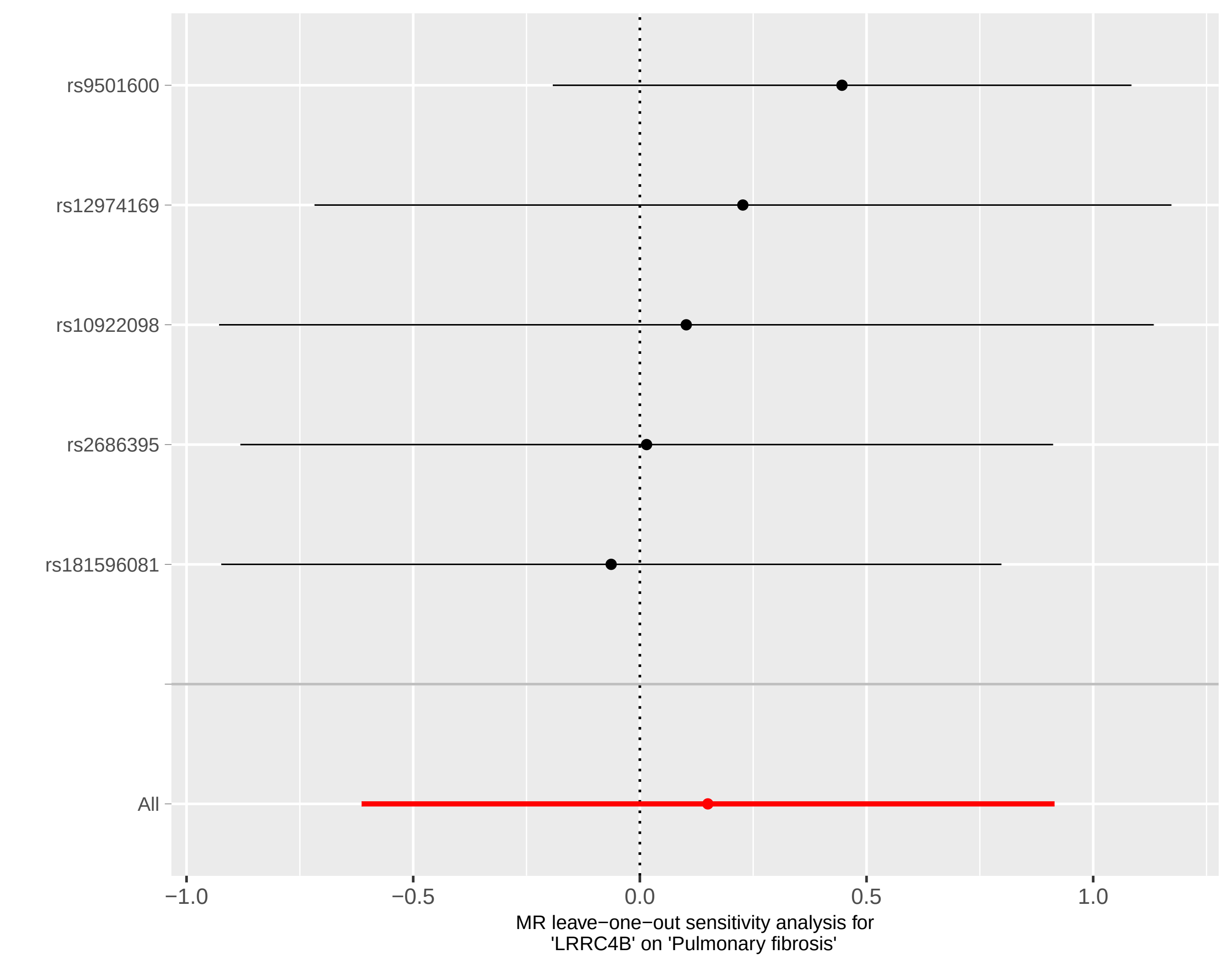
rs9501600
rs12974169
rs10922098
rs2686395
rs181596081
All
−1.0
−0.5
0.0
0.5
1.0
MR leave−one−out sensitivity analysis for
'LRRC4B' on 'Pulmonary fibrosis'

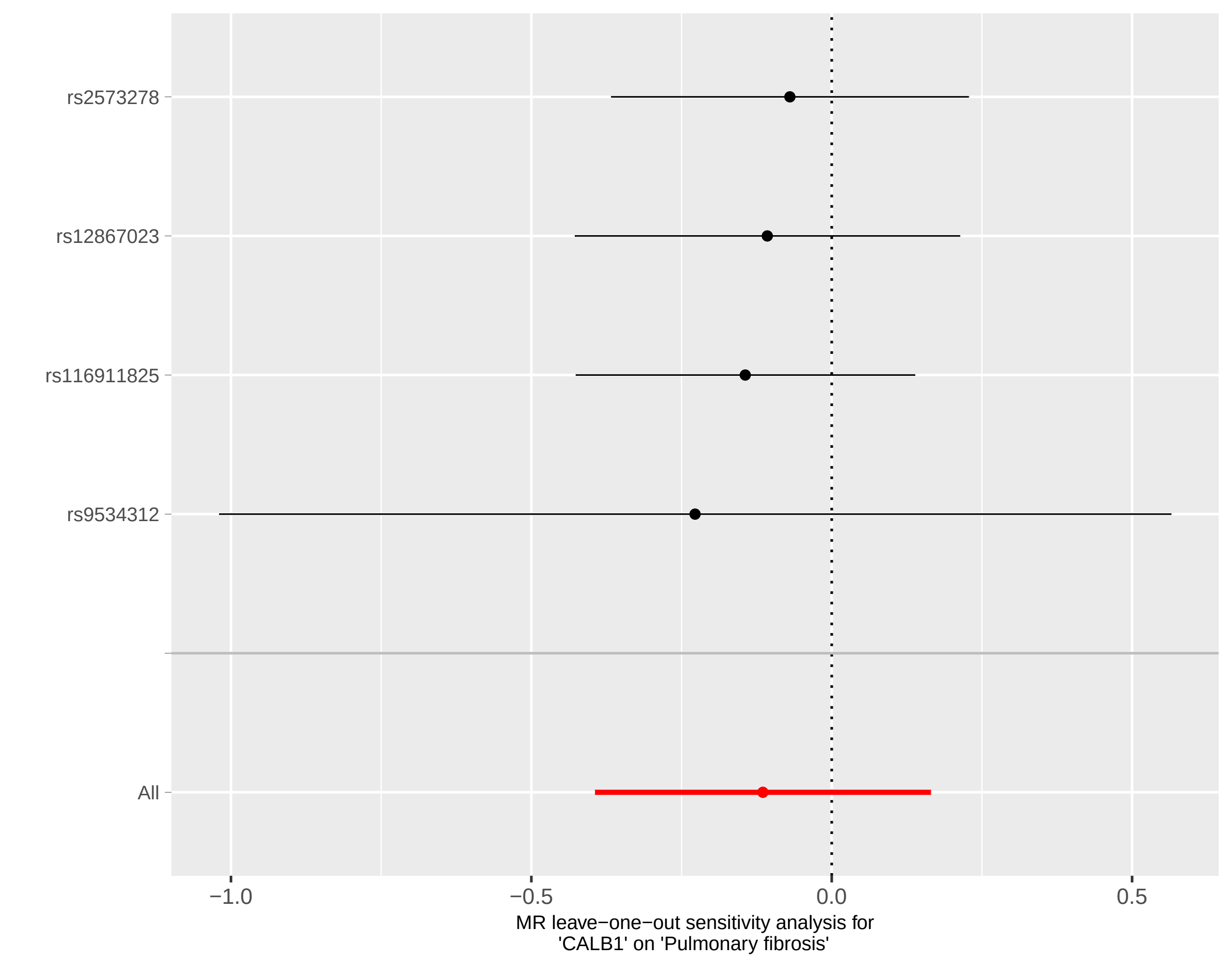
rs2573278
rs12867023
rs116911825
rs9534312
All
−1.0
−0.5
0.0
0.5
MR leave−one−out sensitivity analysis for
'CALB1' on 'Pulmonary fibrosis'

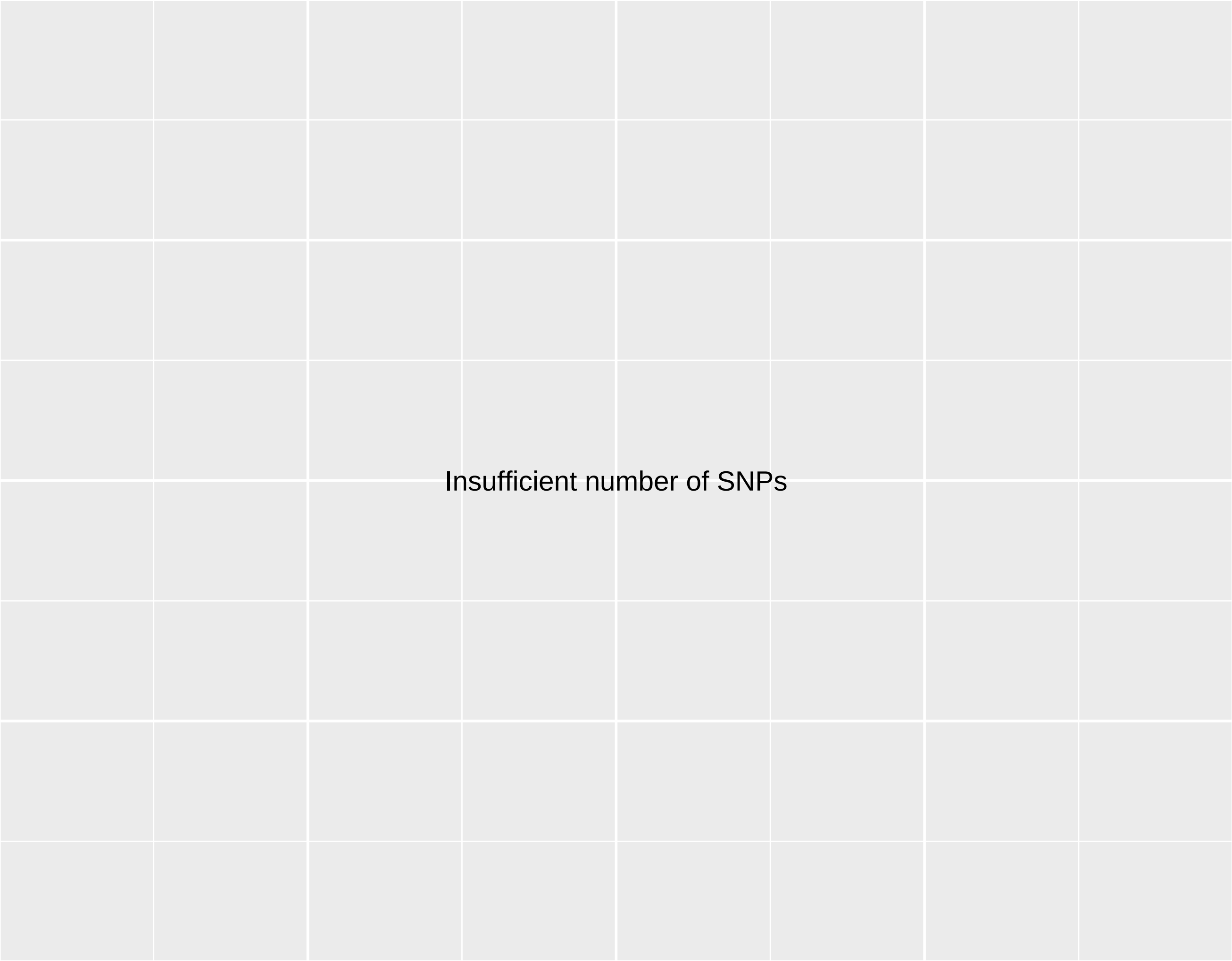
Insufficient number of SNPs

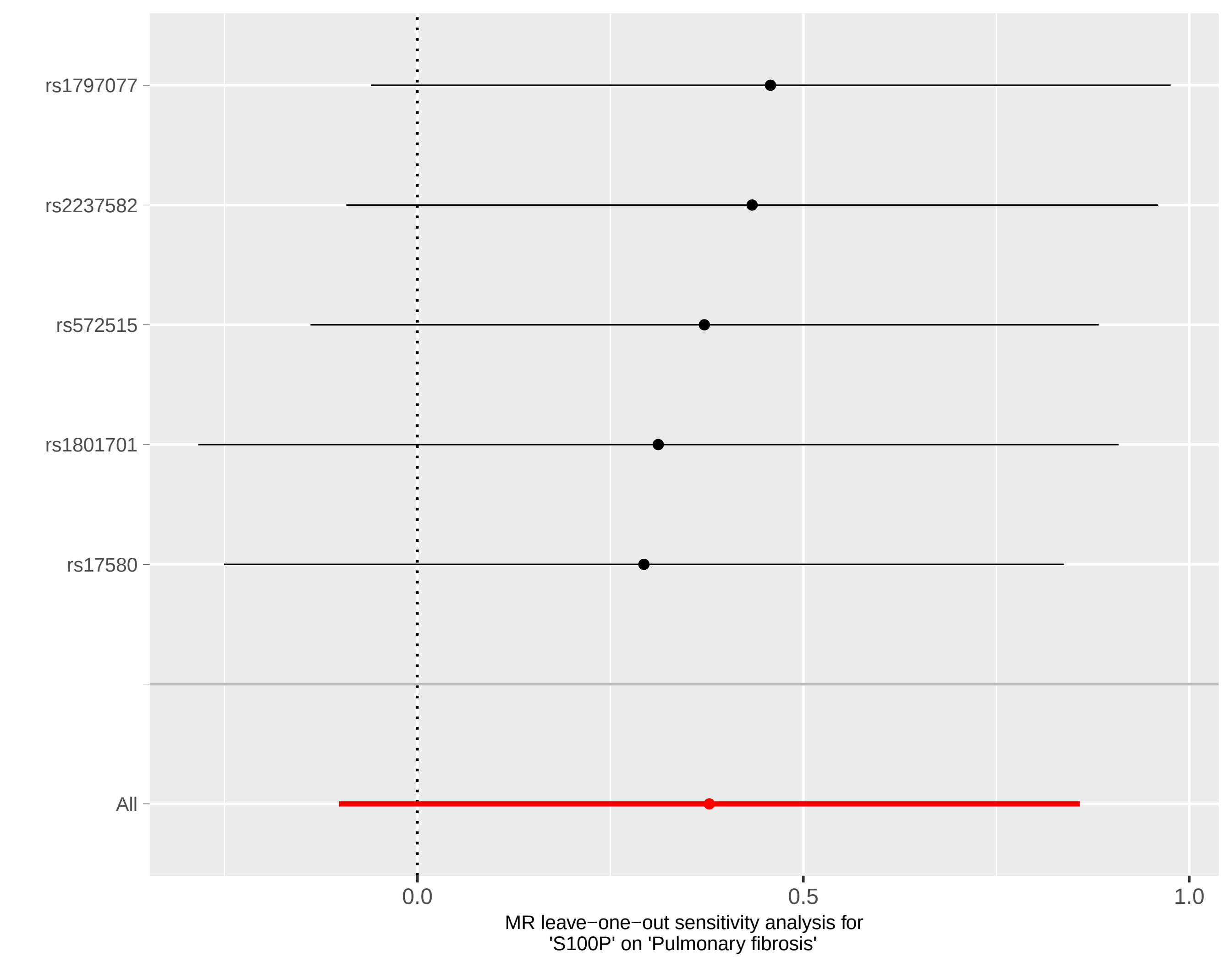
rs1797077
rs2237582
rs572515
rs1801701
rs17580
All
0.0
0.5
1.0
MR leave−one−out sensitivity analysis for
'S100P' on 'Pulmonary fibrosis'

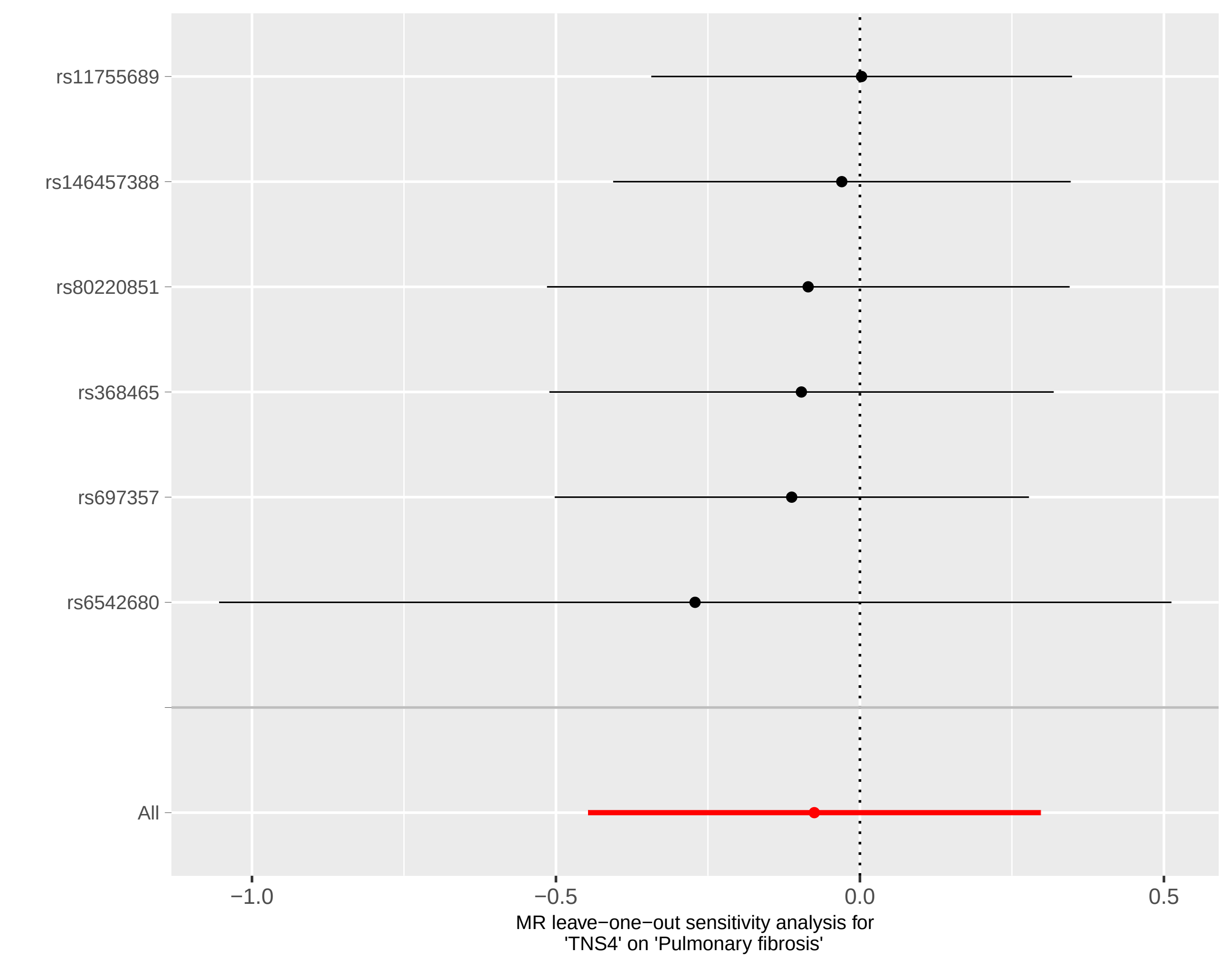
rs11755689
rs146457388
rs80220851
rs368465
rs697357
rs6542680
All
−1.0
−0.5
0.0
0.5
MR leave−one−out sensitivity analysis for
'TNS4' on 'Pulmonary fibrosis'

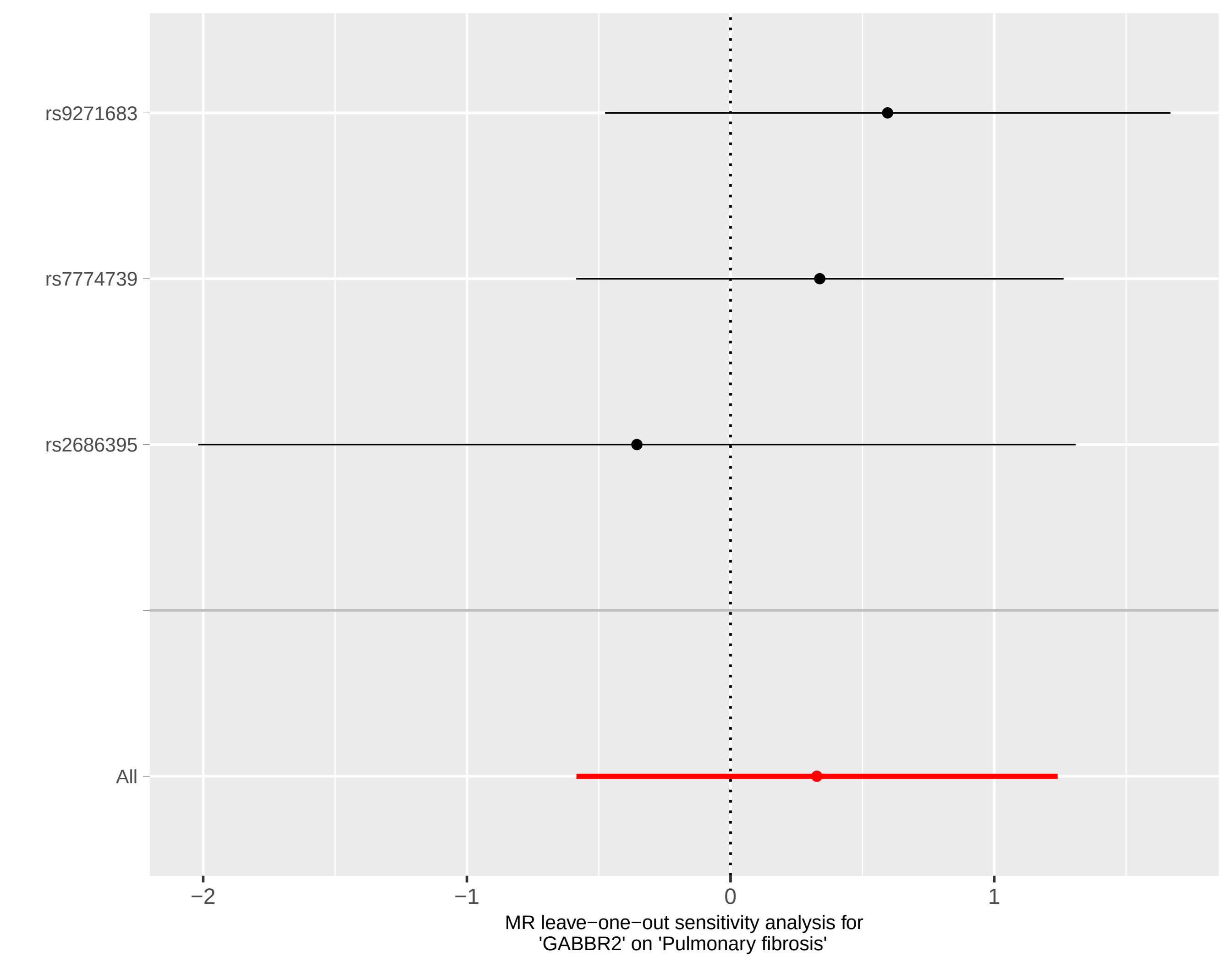
rs9271683
rs7774739
rs2686395
All
−2
−1
0
1
MR leave−one−out sensitivity analysis for
'GABBR2' on 'Pulmonary fibrosis'

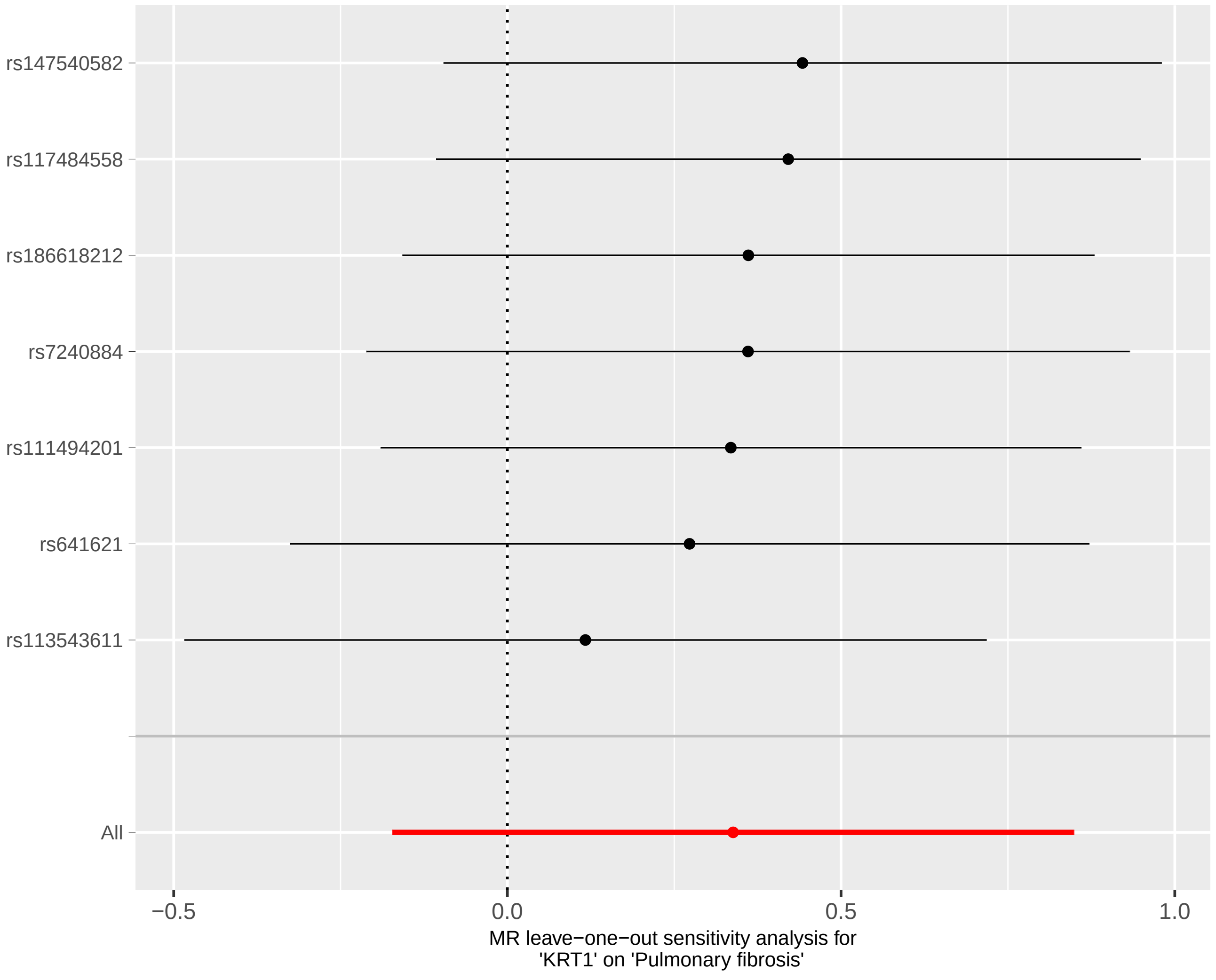
rs147540582
rs117484558
rs186618212
rs7240884
rs111494201
rs641621
rs113543611
All
−0.5
0.0
0.5
1.0
MR leave−one−out sensitivity analysis for
'KRT1' on 'Pulmonary fibrosis'

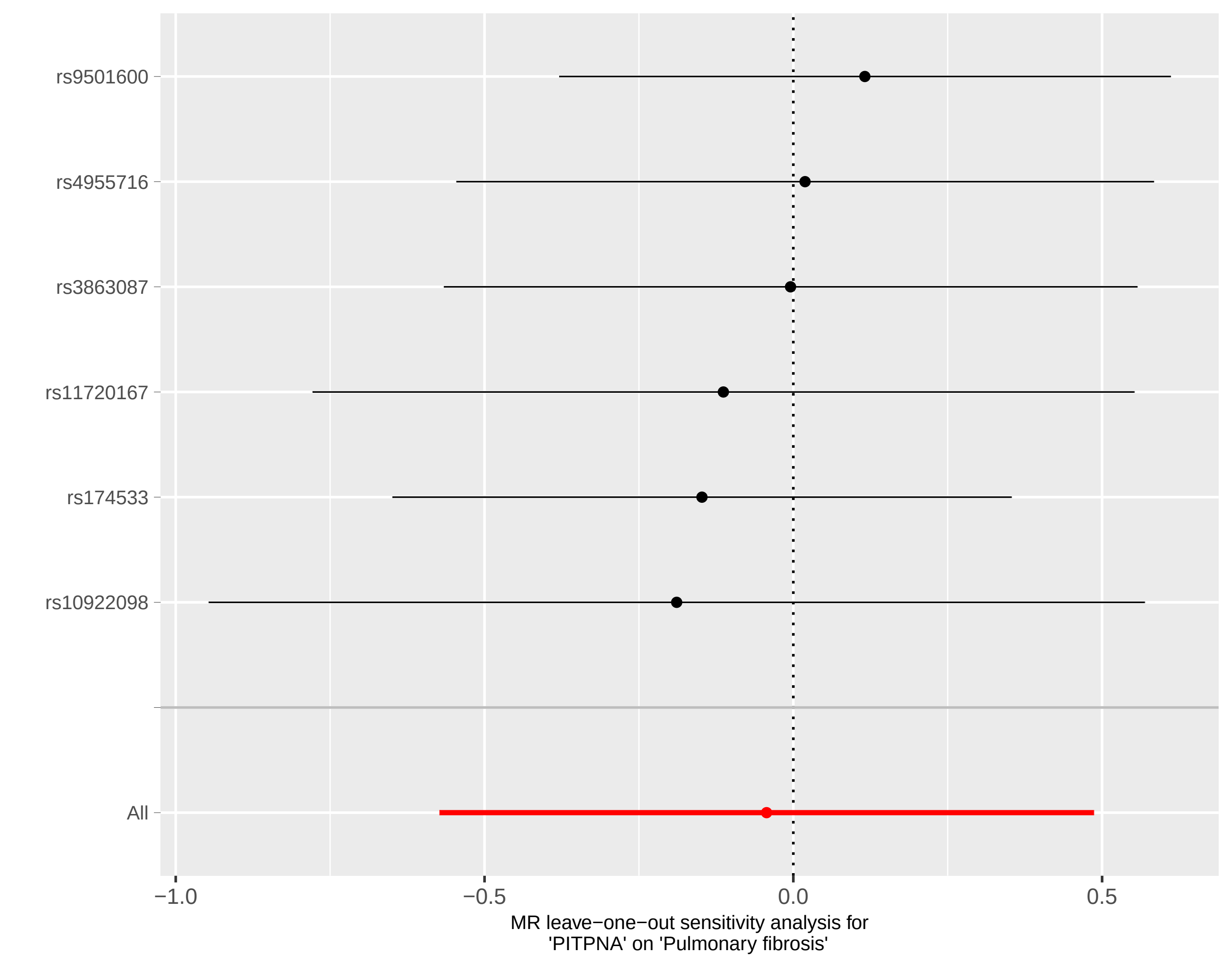

rs9501600
rs4955716
rs3863087
rs11720167
rs174533
rs10922098
All
−1.0
−0.5
0.0
0.5
MR leave−one−out sensitivity analysis for
'PITPNA' on 'Pulmonary fibrosis'

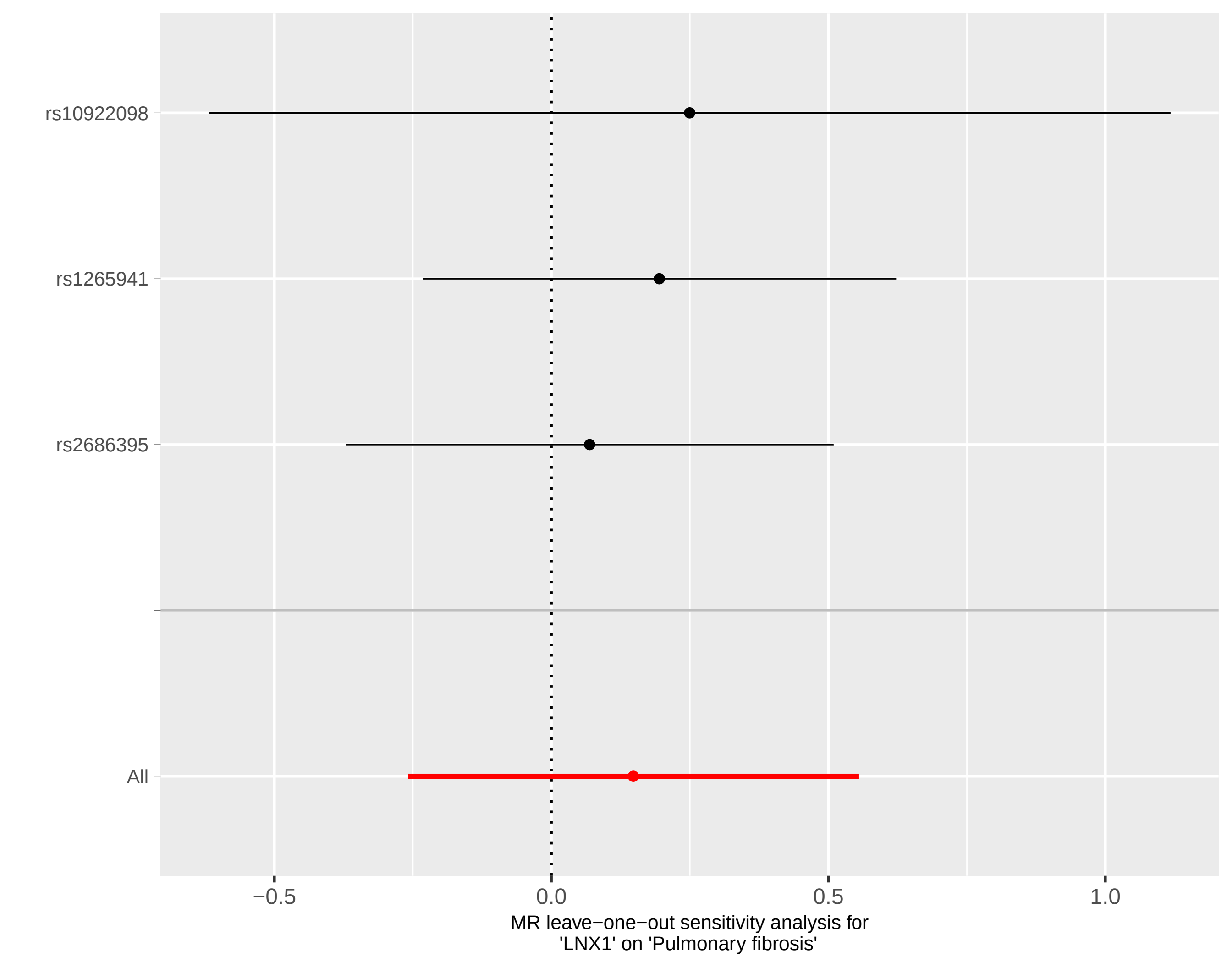
rs10922098
rs1265941
rs2686395
All
−0.5
0.0
0.5
1.0
MR leave−one−out sensitivity analysis for
'LNX1' on 'Pulmonary fibrosis'

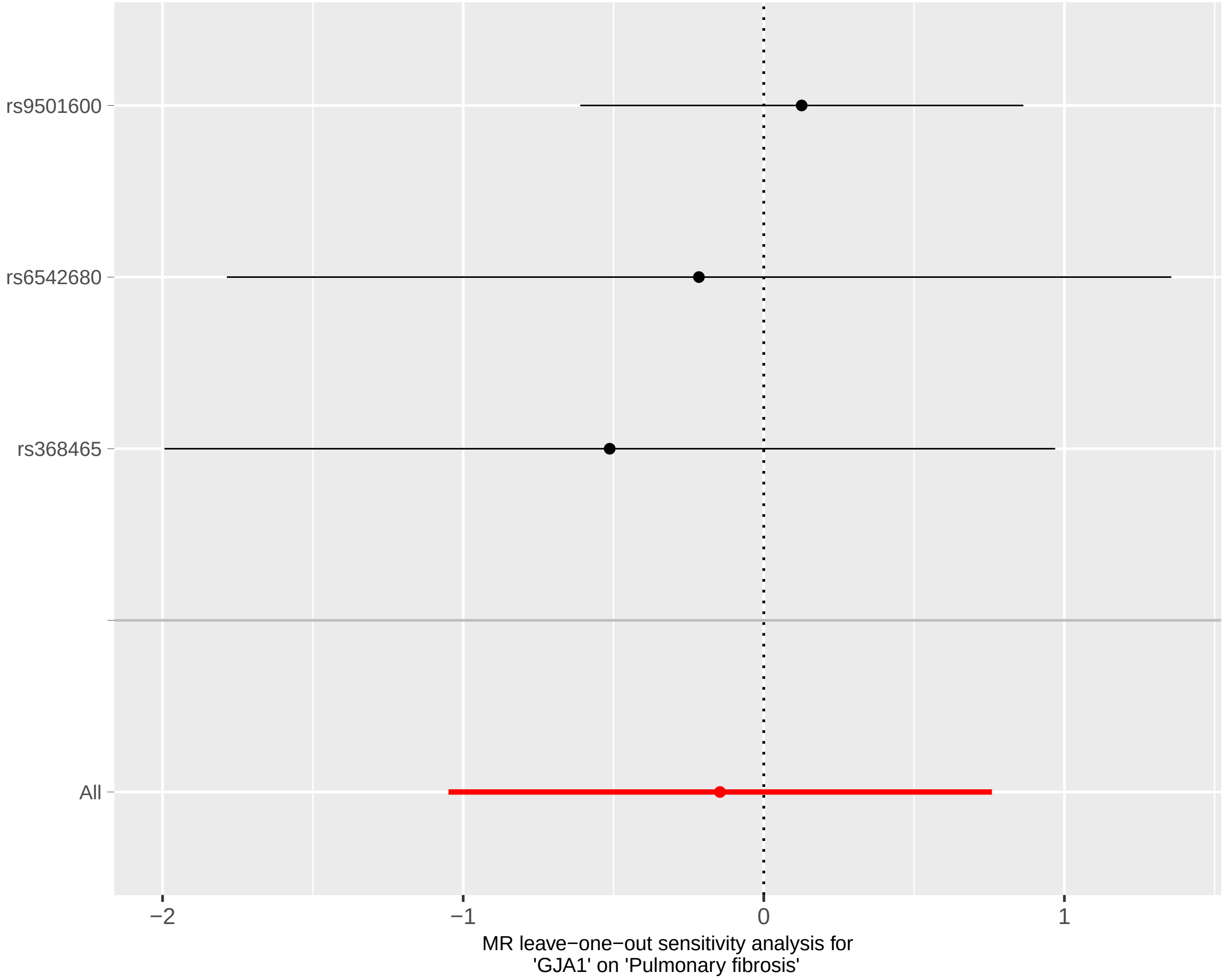
rs9501600
rs6542680
rs368465
All
−2
−1
0
1
MR leave−one−out sensitivity analysis for
'GJA1' on 'Pulmonary fibrosis'

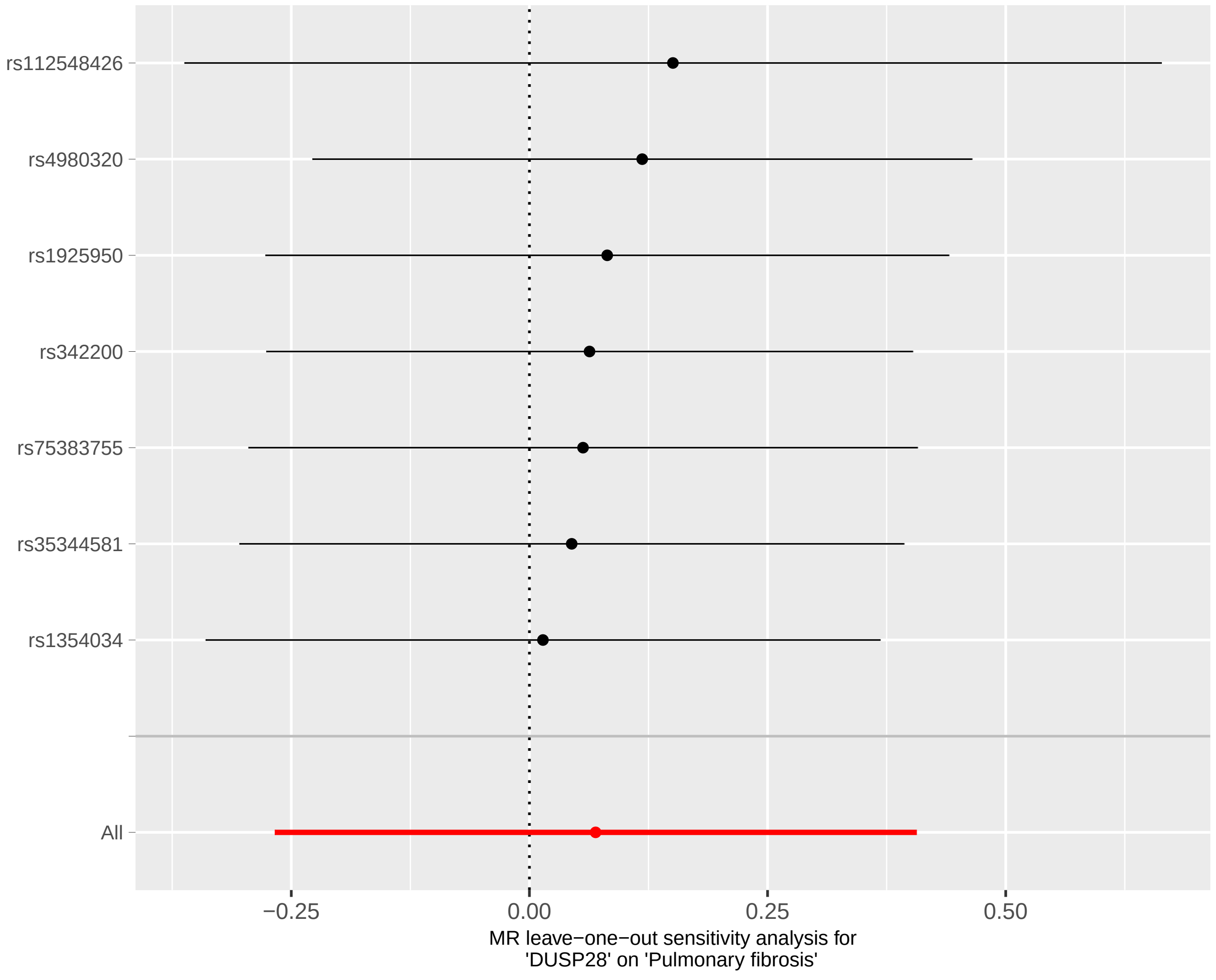
rs112548426
rs4980320
rs1925950
rs342200
rs75383755
rs35344581
rs1354034
All
−0.25
0.00
0.25
0.50
MR leave−one−out sensitivity analysis for
'DUSP28' on 'Pulmonary fibrosis'

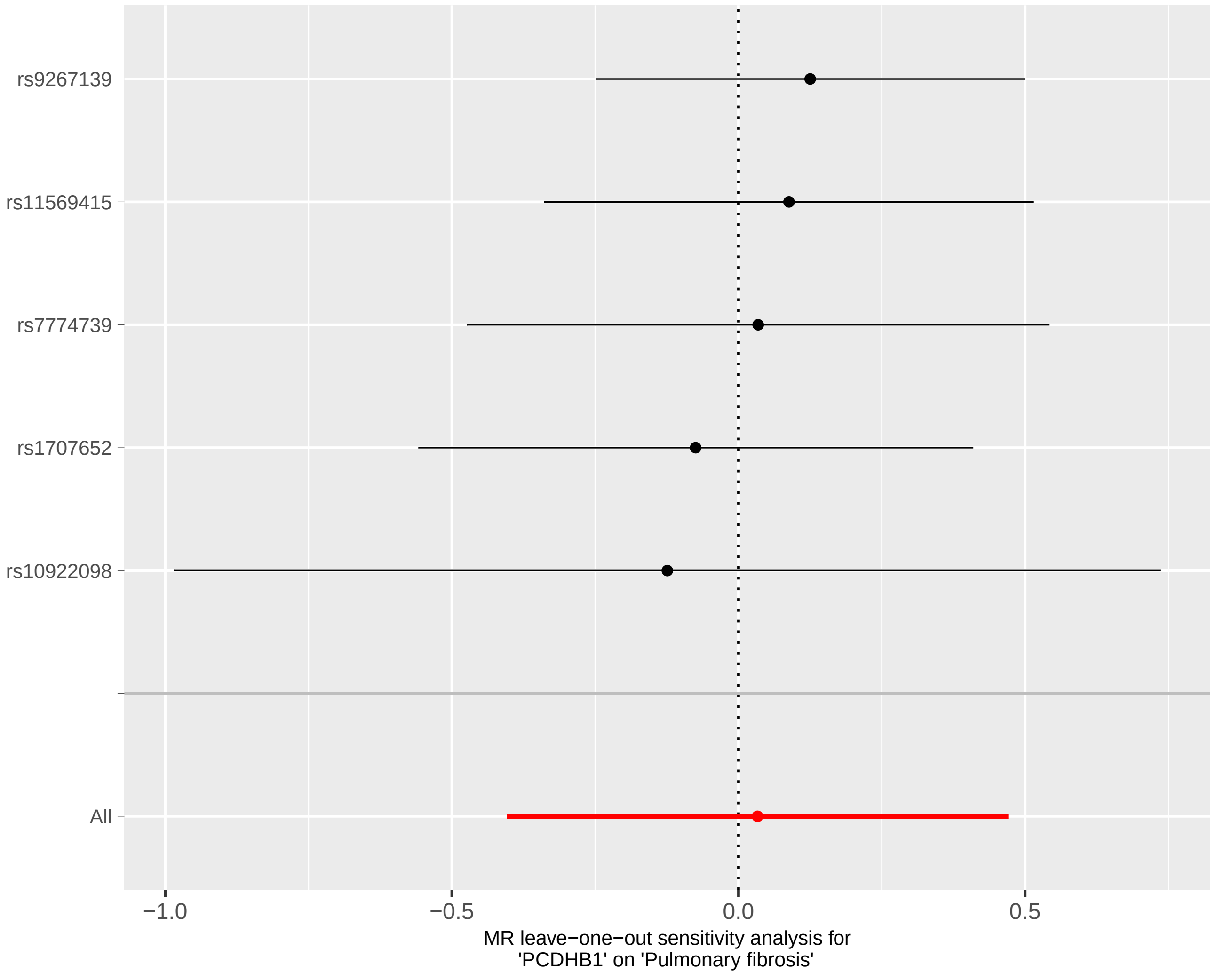
rs9267139
rs11569415
rs7774739
rs1707652
rs10922098
All
−1.0
−0.5
0.0
0.5
MR leave−one−out sensitivity analysis for
'PCDHB1' on 'Pulmonary fibrosis'

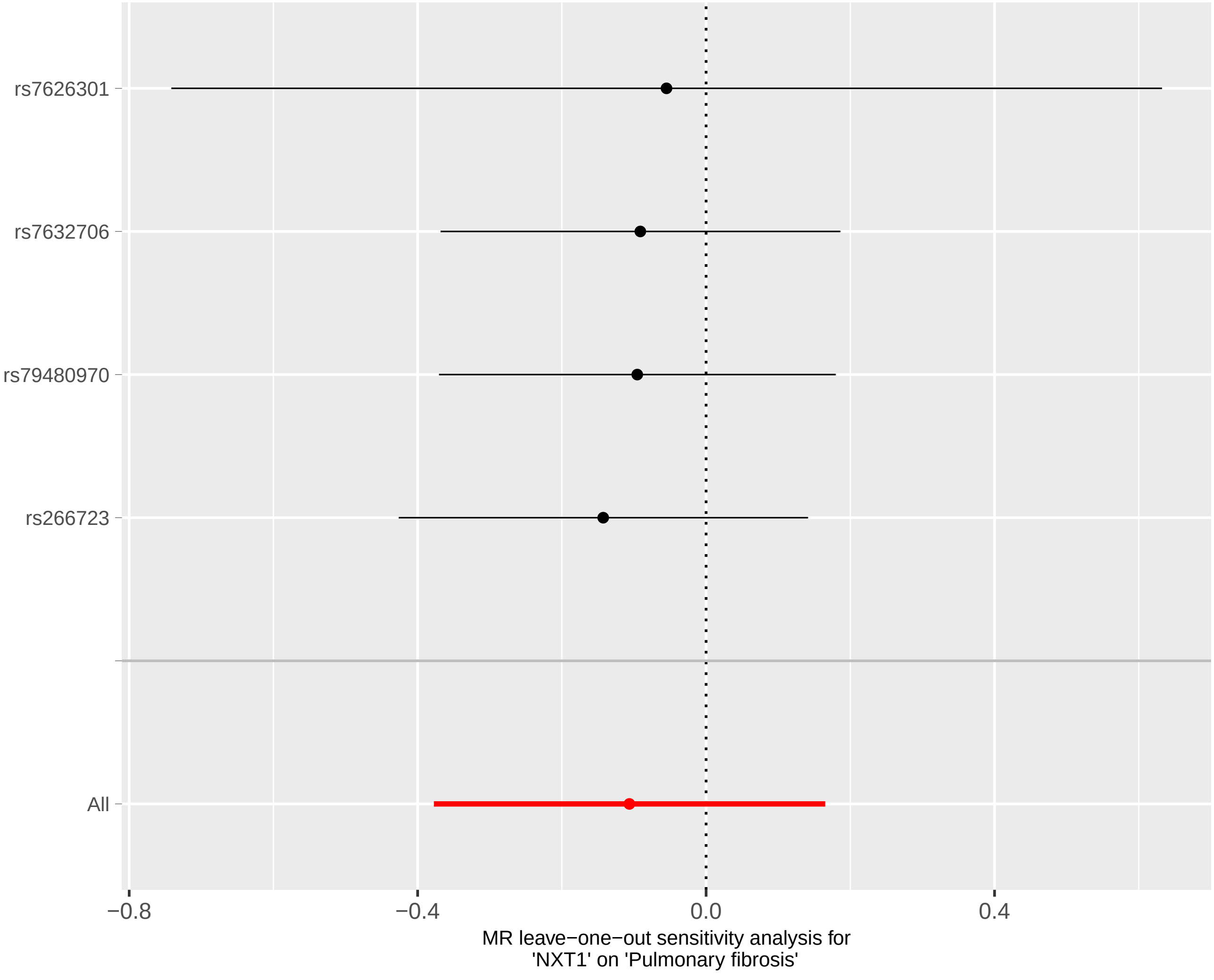
rs7626301
rs7632706
rs79480970
rs266723
All
−0.8
−0.4
0.0
0.4
MR leave−one−out sensitivity analysis for
'NXT1' on 'Pulmonary fibrosis'

Insufficient number of SNPs

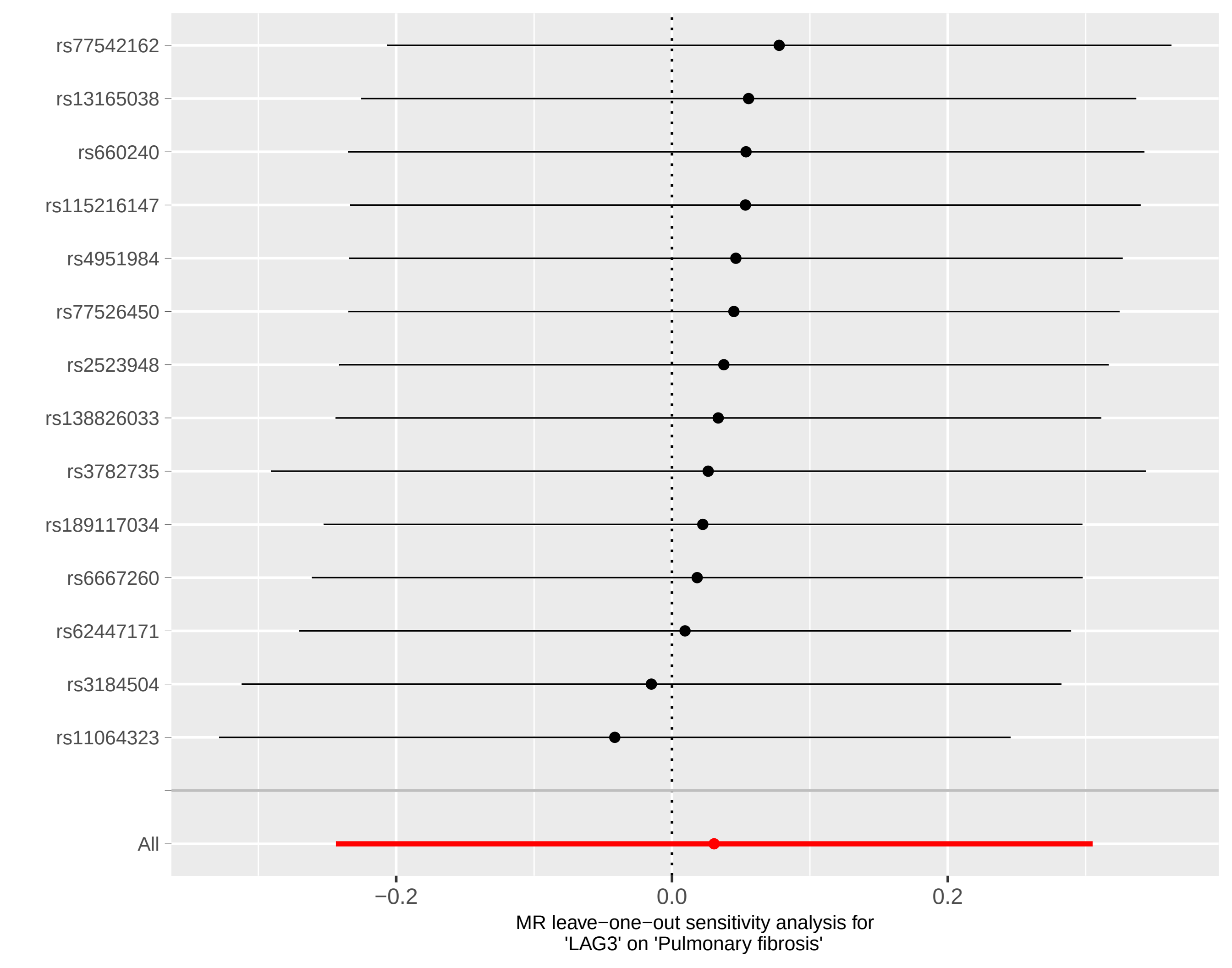
rs77542162
rs13165038
rs660240
rs115216147
rs4951984
rs77526450
rs2523948
rs138826033
rs3782735
rs189117034
rs6667260
rs62447171
rs3184504
rs11064323
All
−0.2
0.0
0.2
MR leave−one−out sensitivity analysis for
'LAG3' on 'Pulmonary fibrosis'

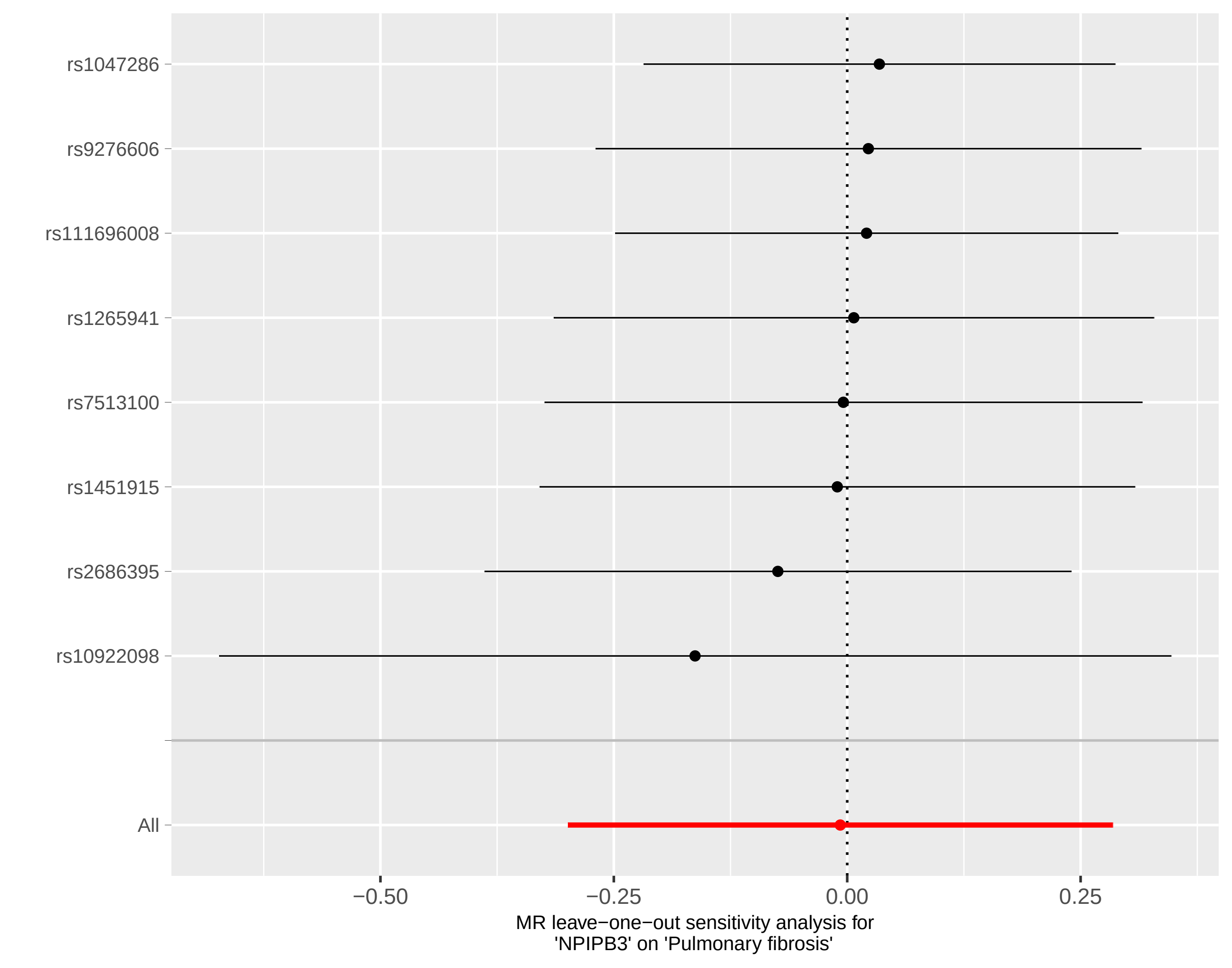
rs1047286
rs9276606
rs111696008
rs1265941
rs7513100
rs1451915
rs2686395
rs10922098
All
−0.50
−0.25
0.00
0.25
MR leave−one−out sensitivity analysis for
'NPIPB3' on 'Pulmonary fibrosis'

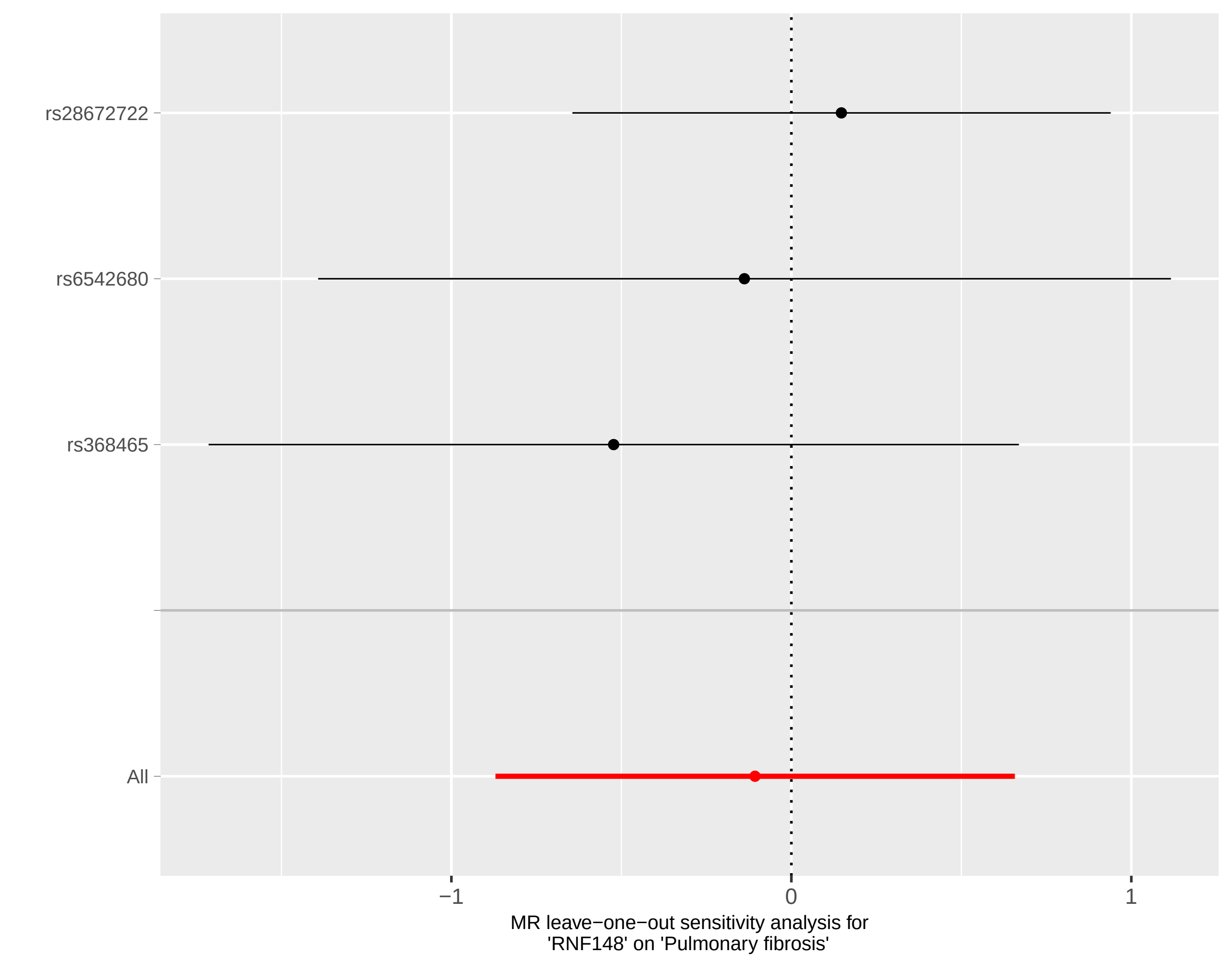

rs28672722
rs6542680
rs368465
All
−1
0
1
MR leave−one−out sensitivity analysis for
'RNF148' on 'Pulmonary fibrosis'

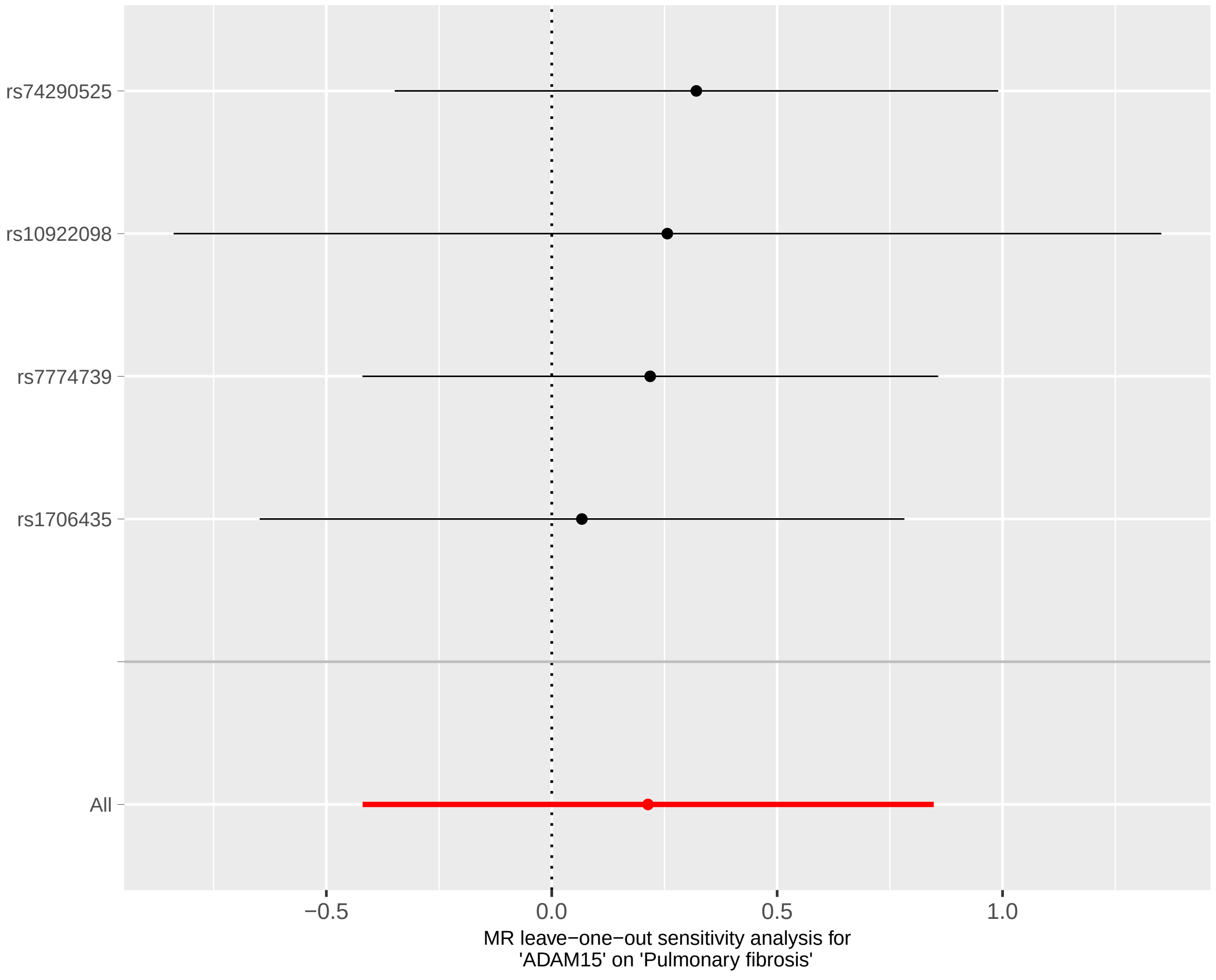
rs74290525
rs10922098
rs7774739
rs1706435
All
−0.5
0.0
0.5
1.0
MR leave−one−out sensitivity analysis for
'ADAM15' on 'Pulmonary fibrosis'

Insufficient number of SNPs

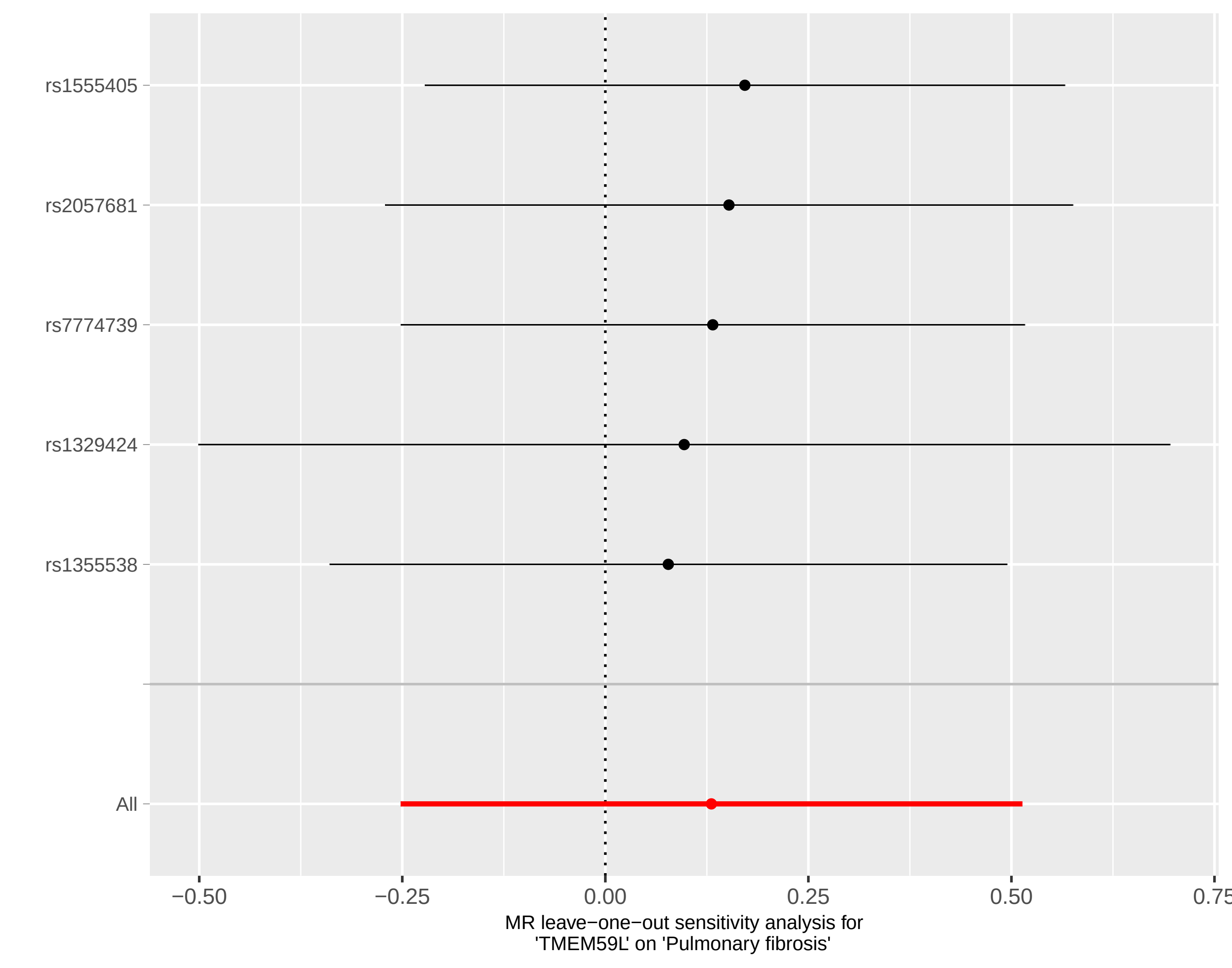
rs1555405
rs2057681
rs7774739
rs1329424
rs1355538
All
−0.50
−0.25
0.00
0.25
0.50
0.75
MR leave−one−out sensitivity analysis for
'TMEM59L' on 'Pulmonary fibrosis'

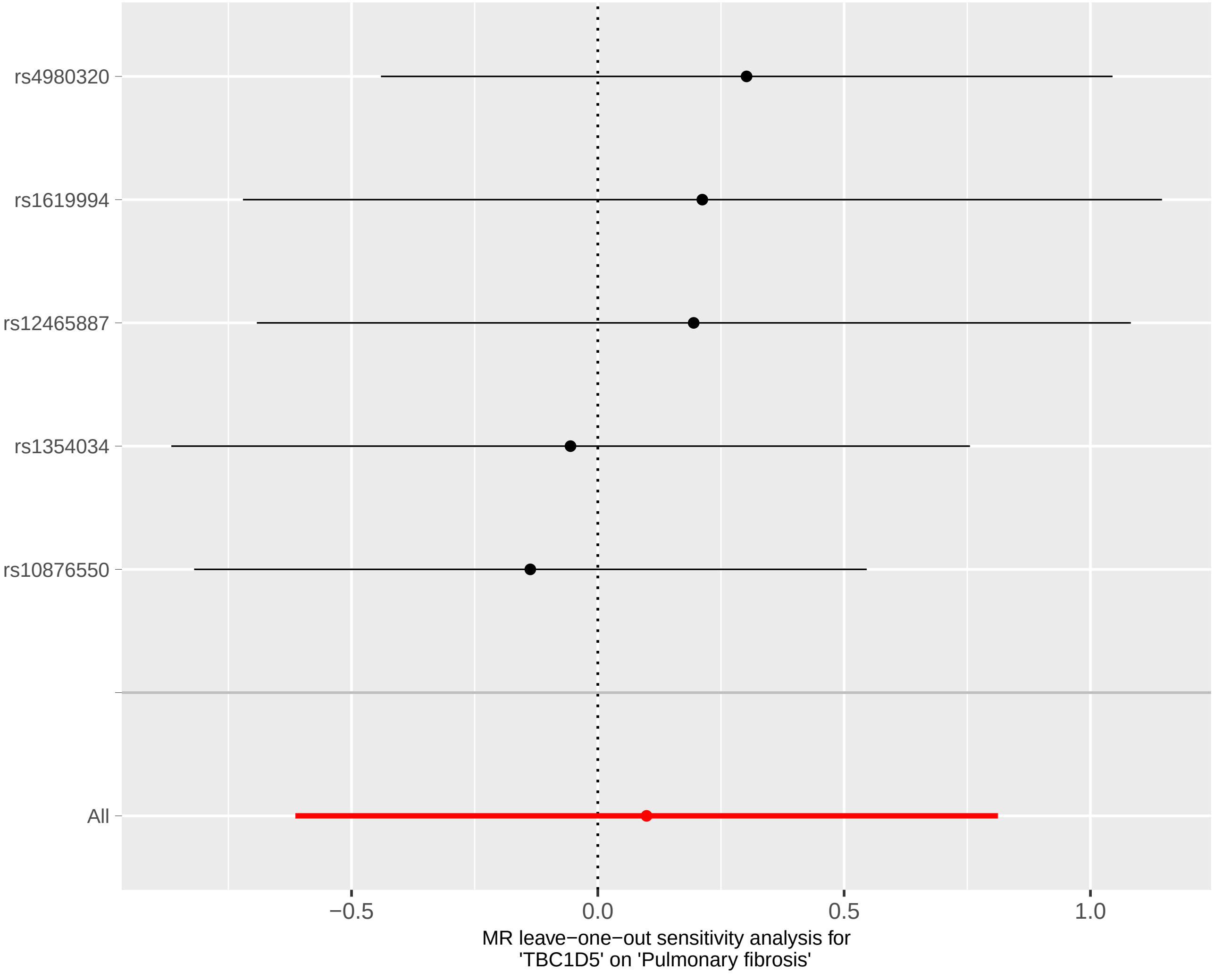

rs4980320
rs1619994
rs12465887
rs1354034
rs10876550
All
−0.5
0.0
0.5
1.0
MR leave−one−out sensitivity analysis for
'TBC1D5' on 'Pulmonary fibrosis'

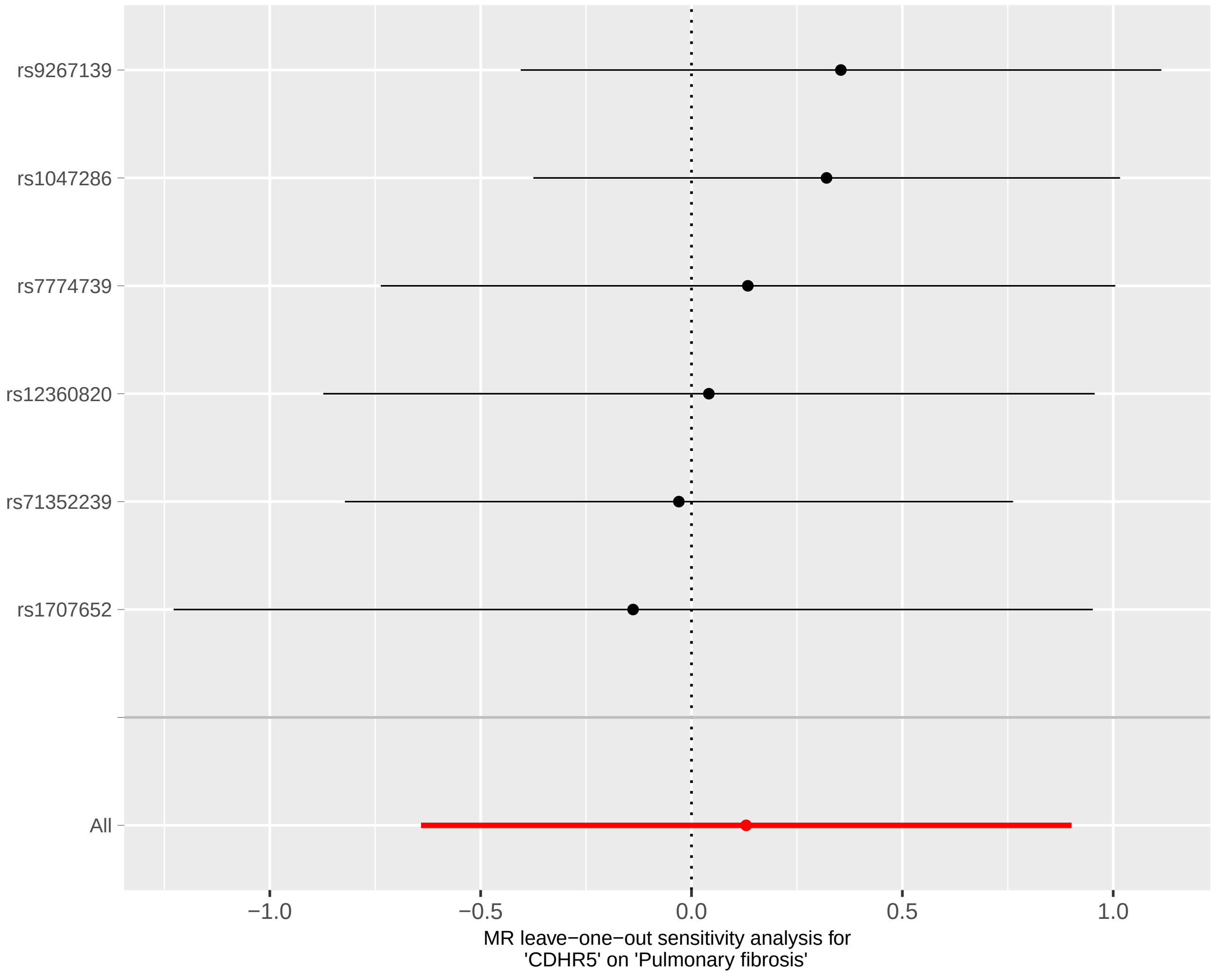
rs9267139
rs1047286
rs7774739
rs12360820
rs71352239
rs1707652
All
−1.0
−0.5
0.0
0.5
1.0
MR leave−one−out sensitivity analysis for
'CDHR5' on 'Pulmonary fibrosis'

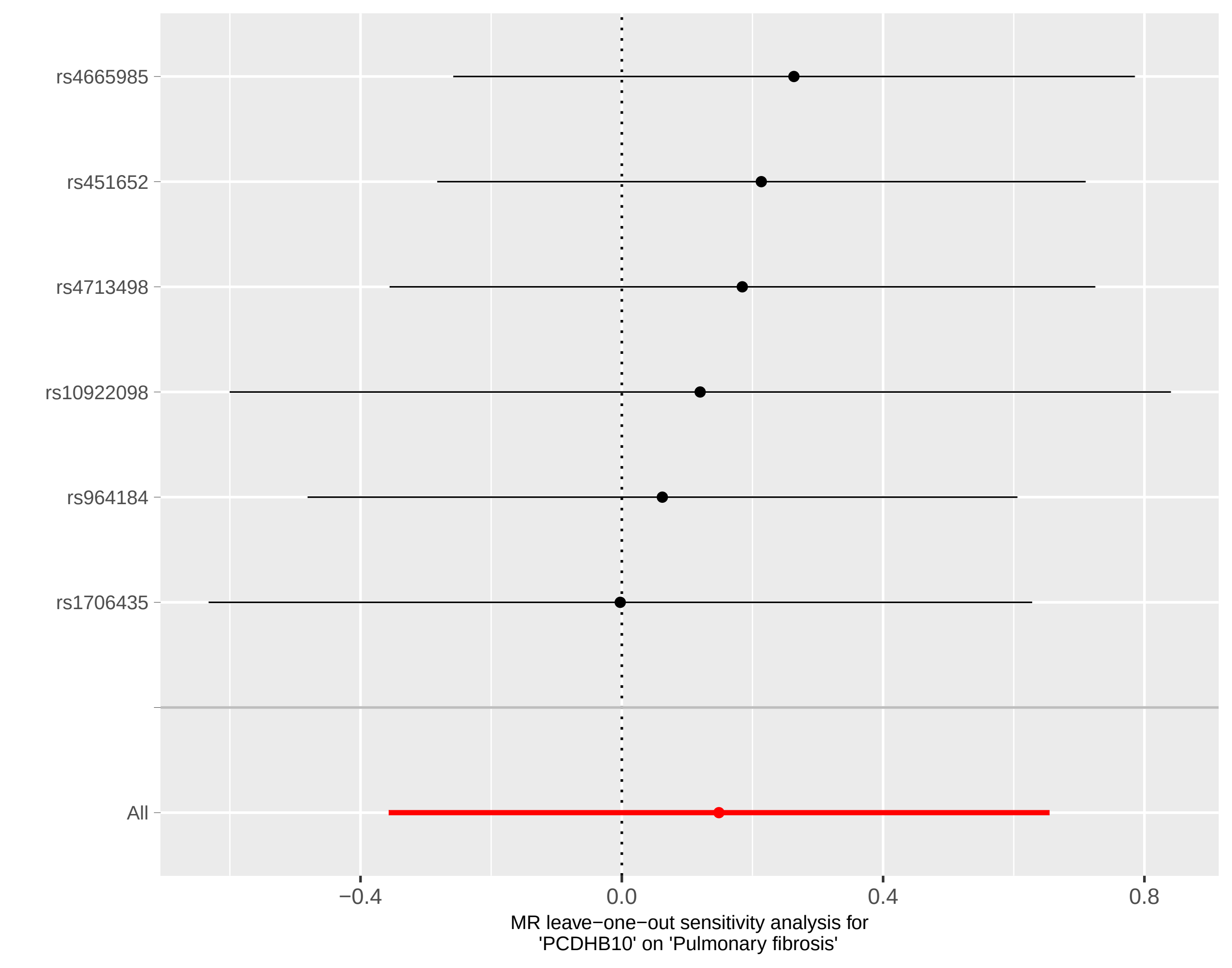

rs4665985
rs451652
rs4713498
rs10922098
rs964184
rs1706435
All
−0.4
0.0
0.4
0.8
MR leave−one−out sensitivity analysis for
'PCDHB10' on 'Pulmonary fibrosis'

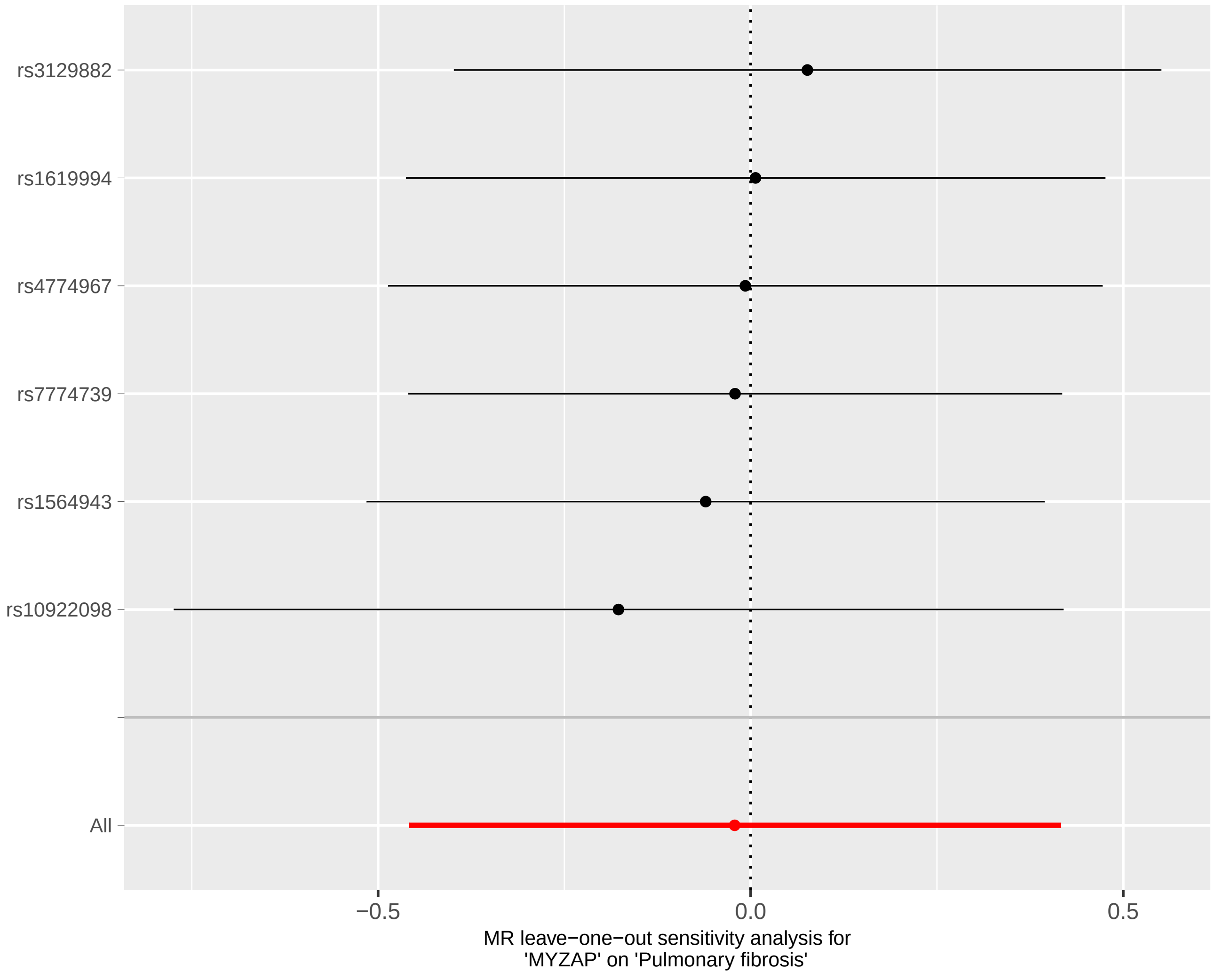

rs3129882
rs1619994
rs4774967
rs7774739
rs1564943
rs10922098
All
−0.5
0.0
0.5
MR leave−one−out sensitivity analysis for
'MYZAP' on 'Pulmonary fibrosis'

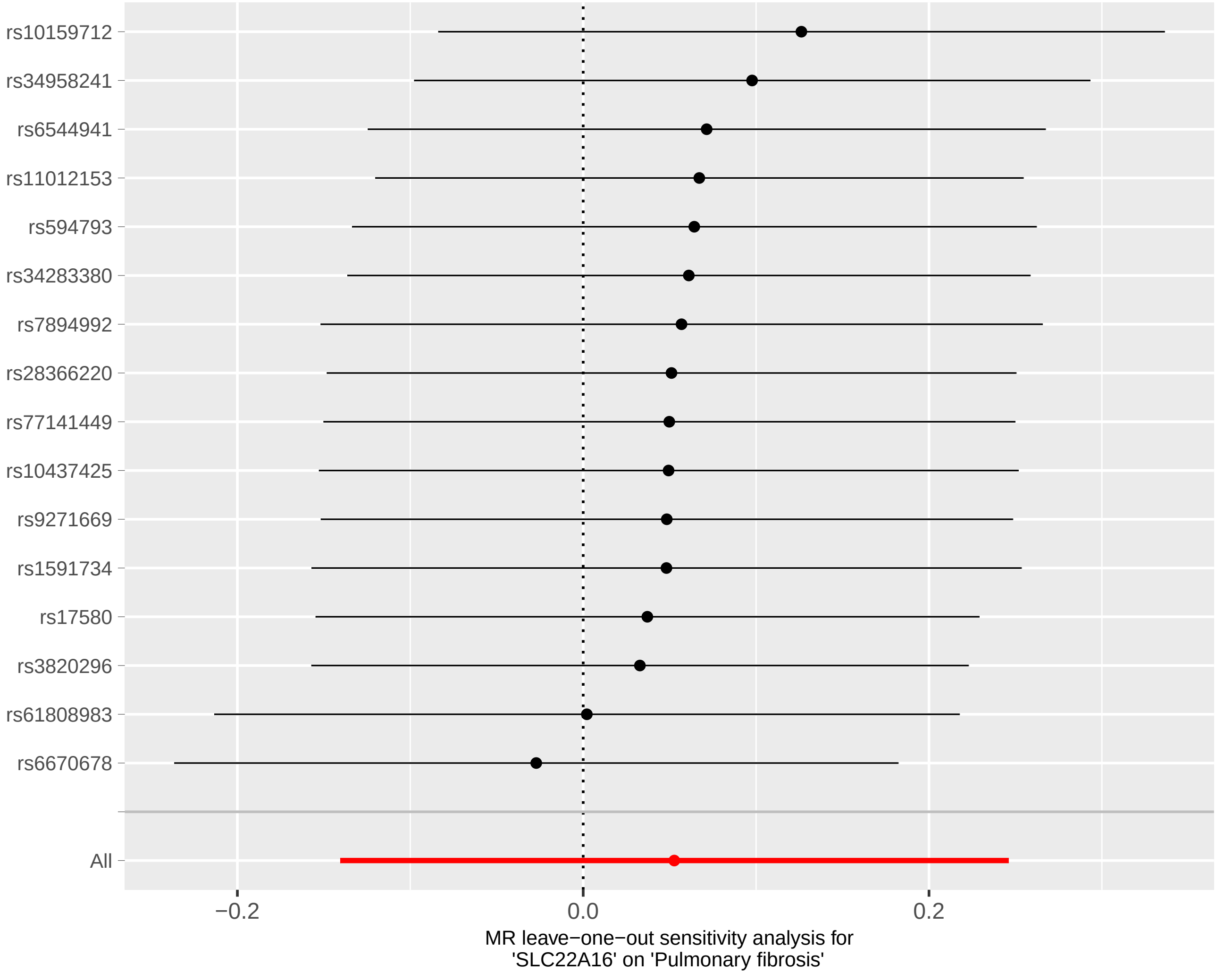
rs10159712
rs34958241
rs6544941
rs11012153
rs594793
rs34283380
rs7894992
rs28366220
rs77141449
rs10437425
rs9271669
rs1591734
rs17580
rs3820296
rs61808983
rs6670678
All
−0.2
0.0
0.2
MR leave−one−out sensitivity analysis for
'SLC22A16' on 'Pulmonary fibrosis'

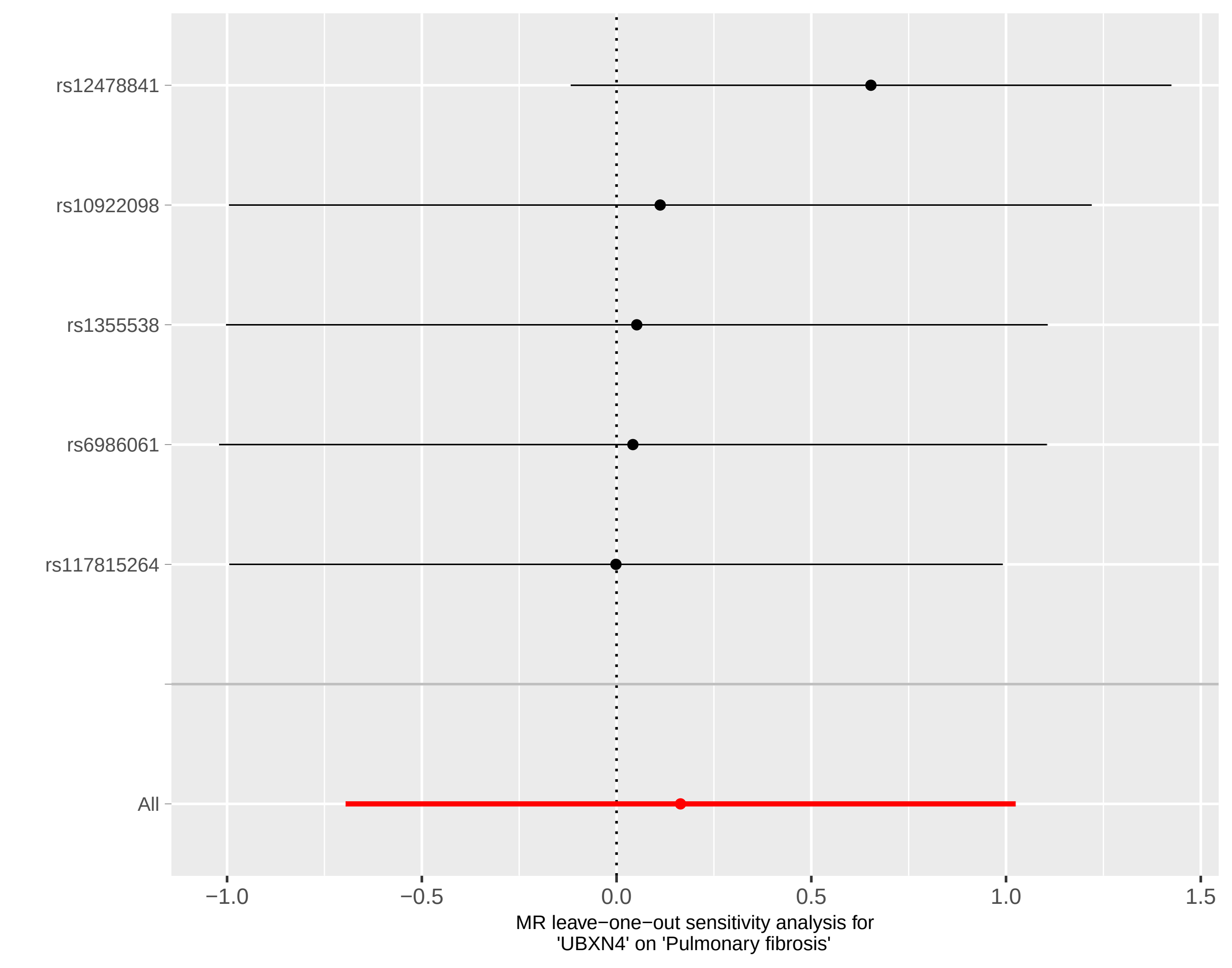
rs12478841
rs10922098
rs1355538
rs6986061
rs117815264
All
−1.0
−0.5
0.0
0.5
1.0
1.5
MR leave−one−out sensitivity analysis for
'UBXN4' on 'Pulmonary fibrosis'

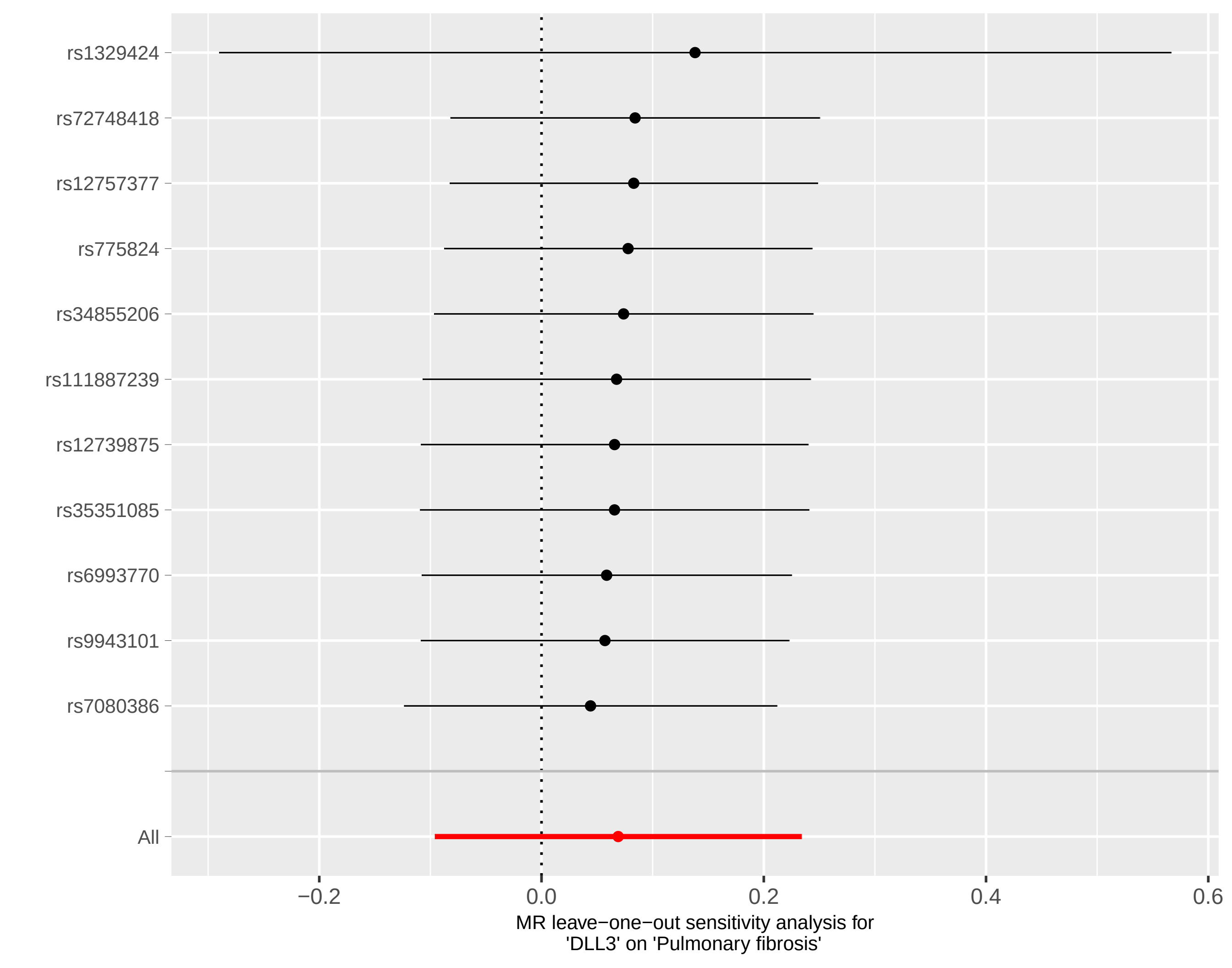
rs1329424
rs72748418
rs12757377
rs775824
rs34855206
rs111887239
rs12739875
rs35351085
rs6993770
rs9943101
rs7080386
All
−0.2
0.0
0.2
0.4
0.6
MR leave−one−out sensitivity analysis for
'DLL3' on 'Pulmonary fibrosis'

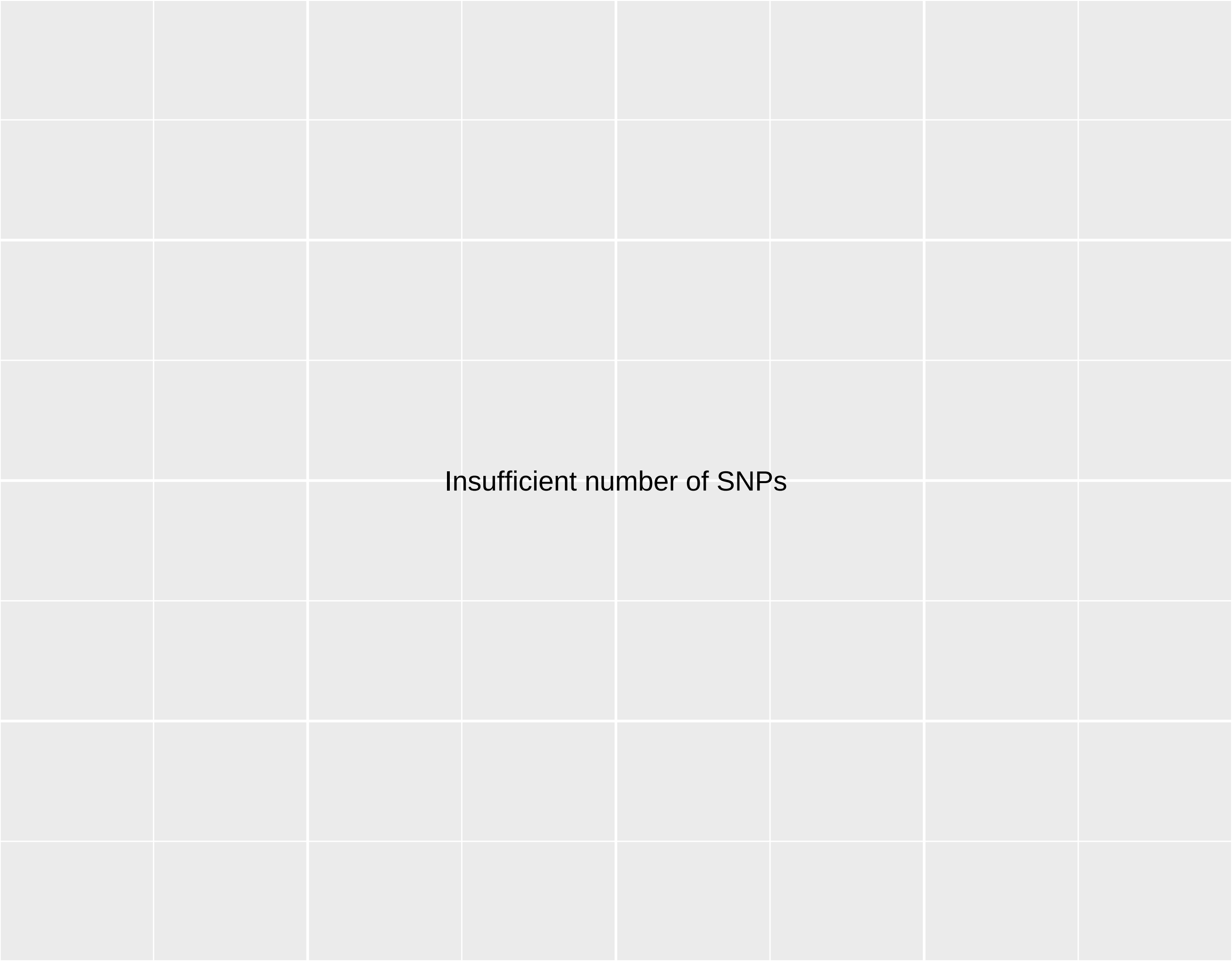
Insufficient number of SNPs

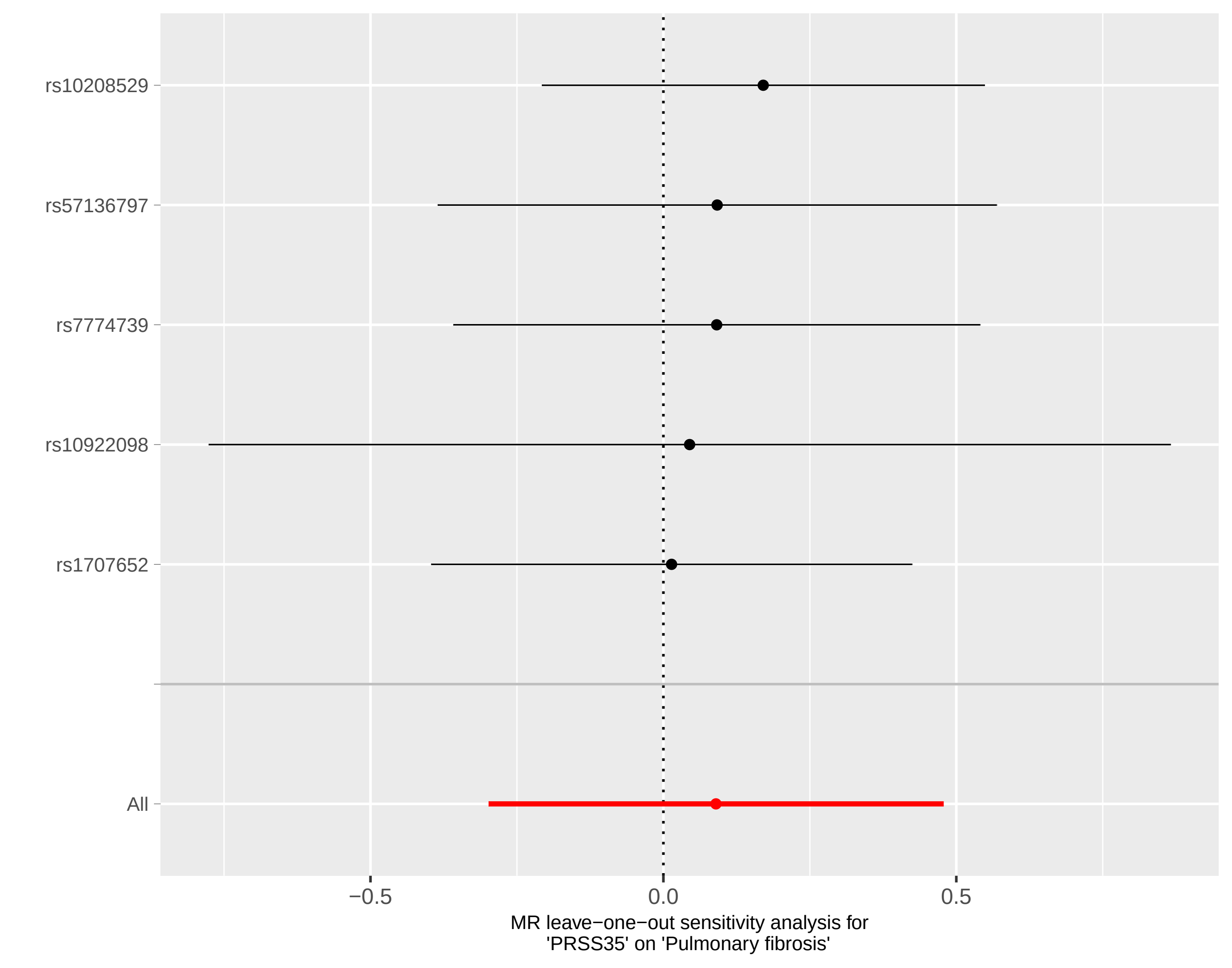
rs10208529
rs57136797
rs7774739
rs10922098
rs1707652
All
−0.5
0.0
0.5
MR leave−one−out sensitivity analysis for
'PRSS35' on 'Pulmonary fibrosis'

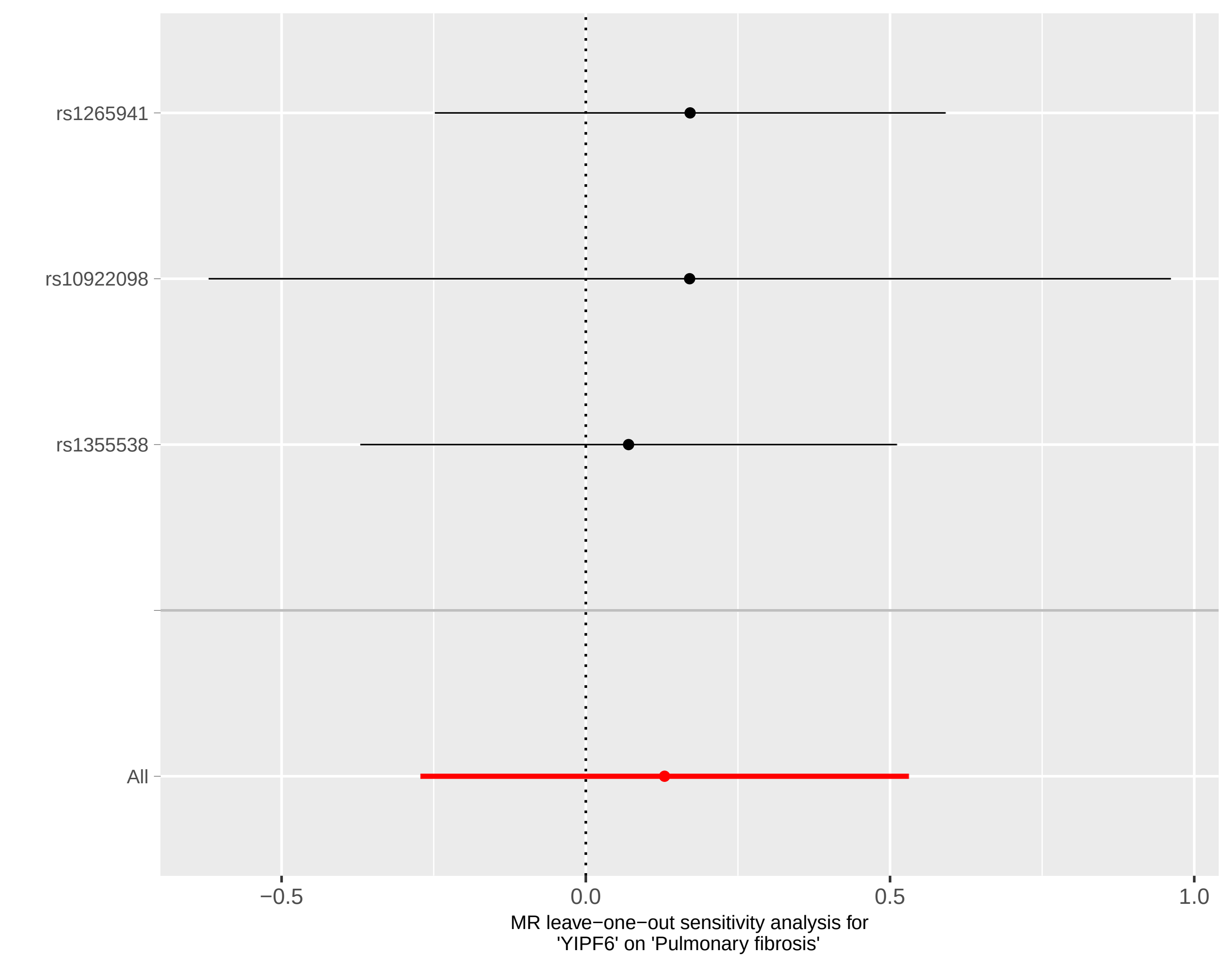
rs1265941
rs10922098
rs1355538
All
−0.5
0.0
0.5
1.0
MR leave−one−out sensitivity analysis for
'YIPF6' on 'Pulmonary fibrosis'

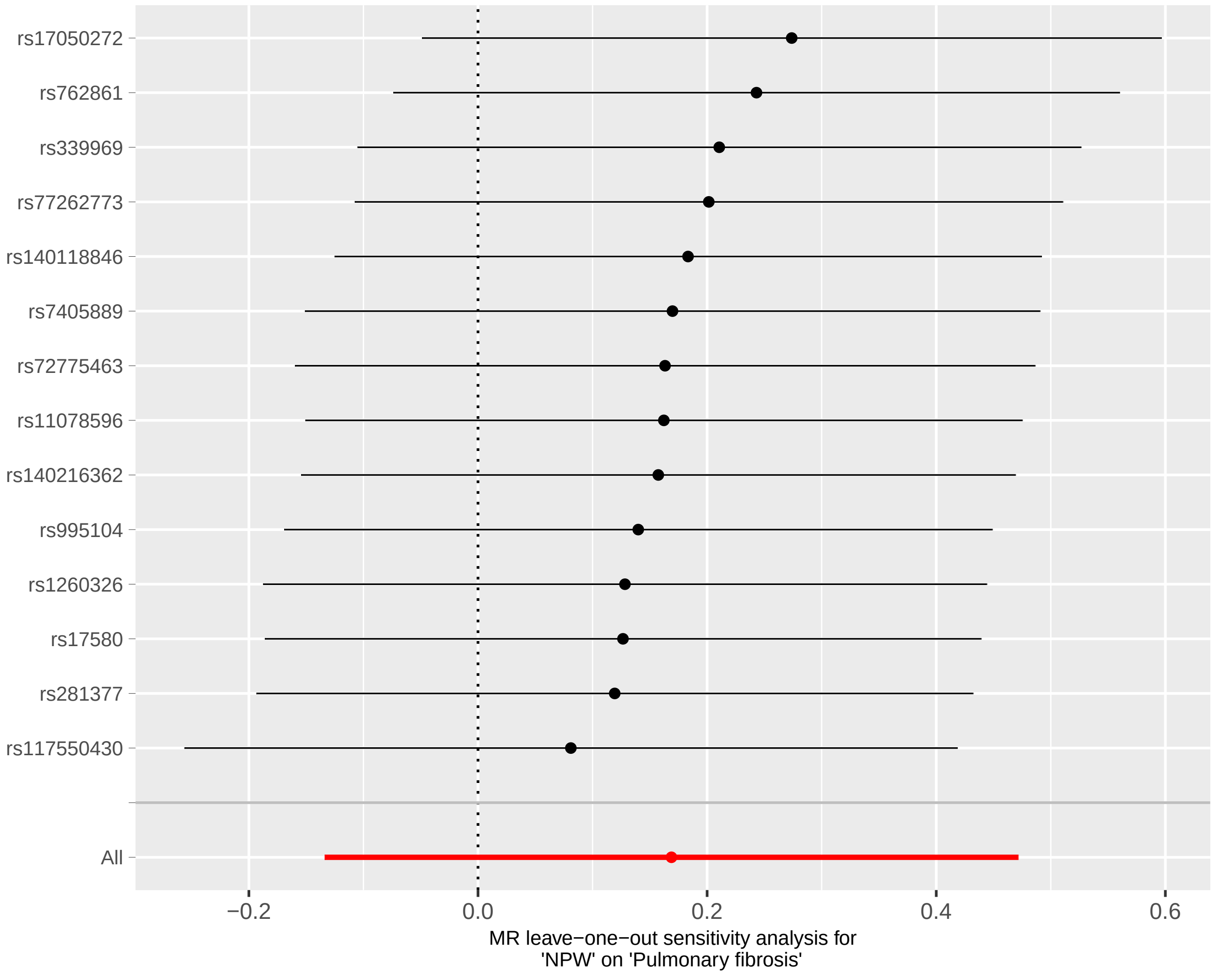
rs17050272
rs762861
rs339969
rs77262773
rs140118846
rs7405889
rs72775463
rs11078596
rs140216362
rs995104
rs1260326
rs17580
rs281377
rs117550430
All
−0.2
0.0
0.2
0.4
0.6
MR leave−one−out sensitivity analysis for
'NPW' on 'Pulmonary fibrosis'

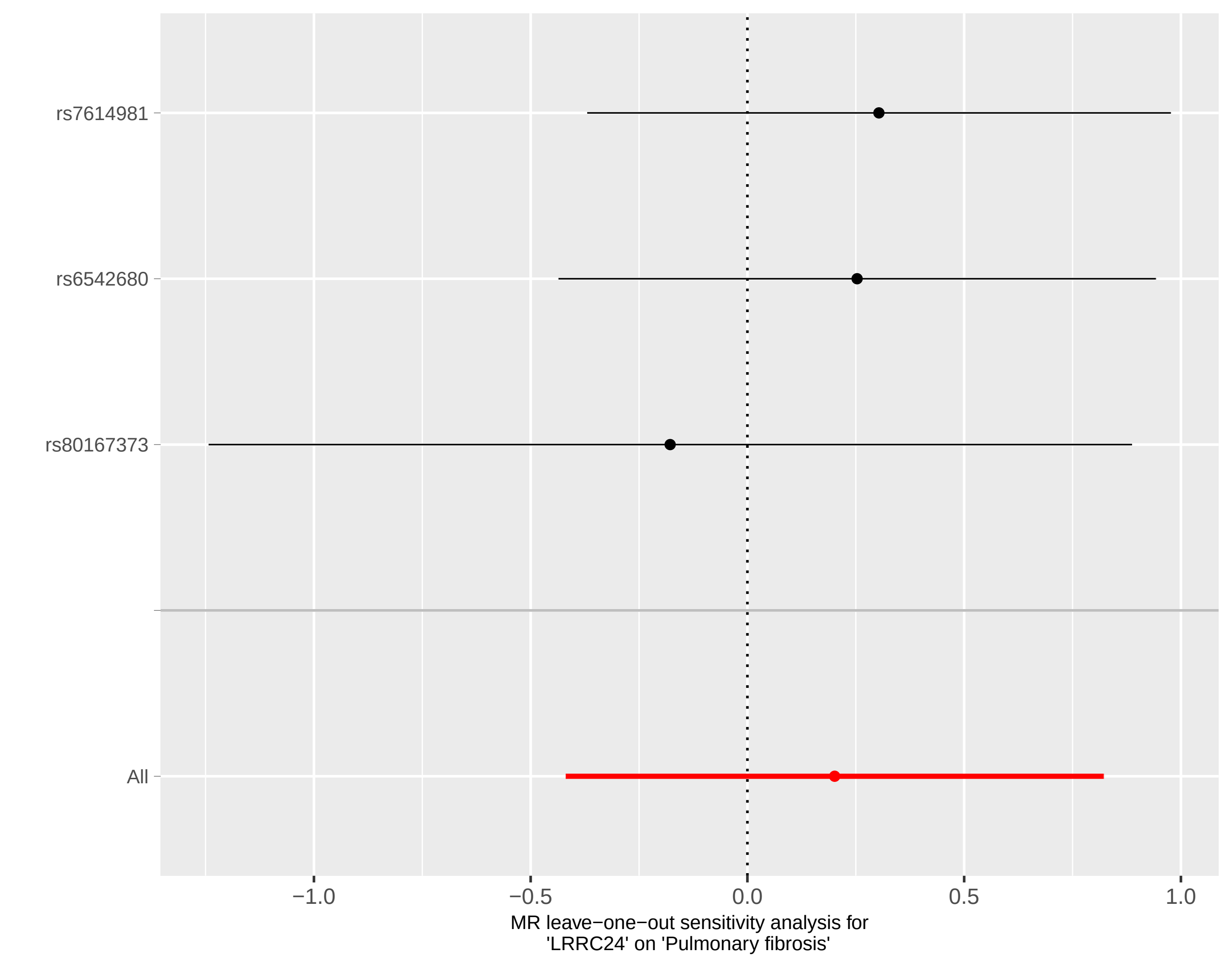
rs7614981
rs6542680
rs80167373
All
−1.0
−0.5
0.0
0.5
1.0
MR leave−one−out sensitivity analysis for
'LRRC24' on 'Pulmonary fibrosis'

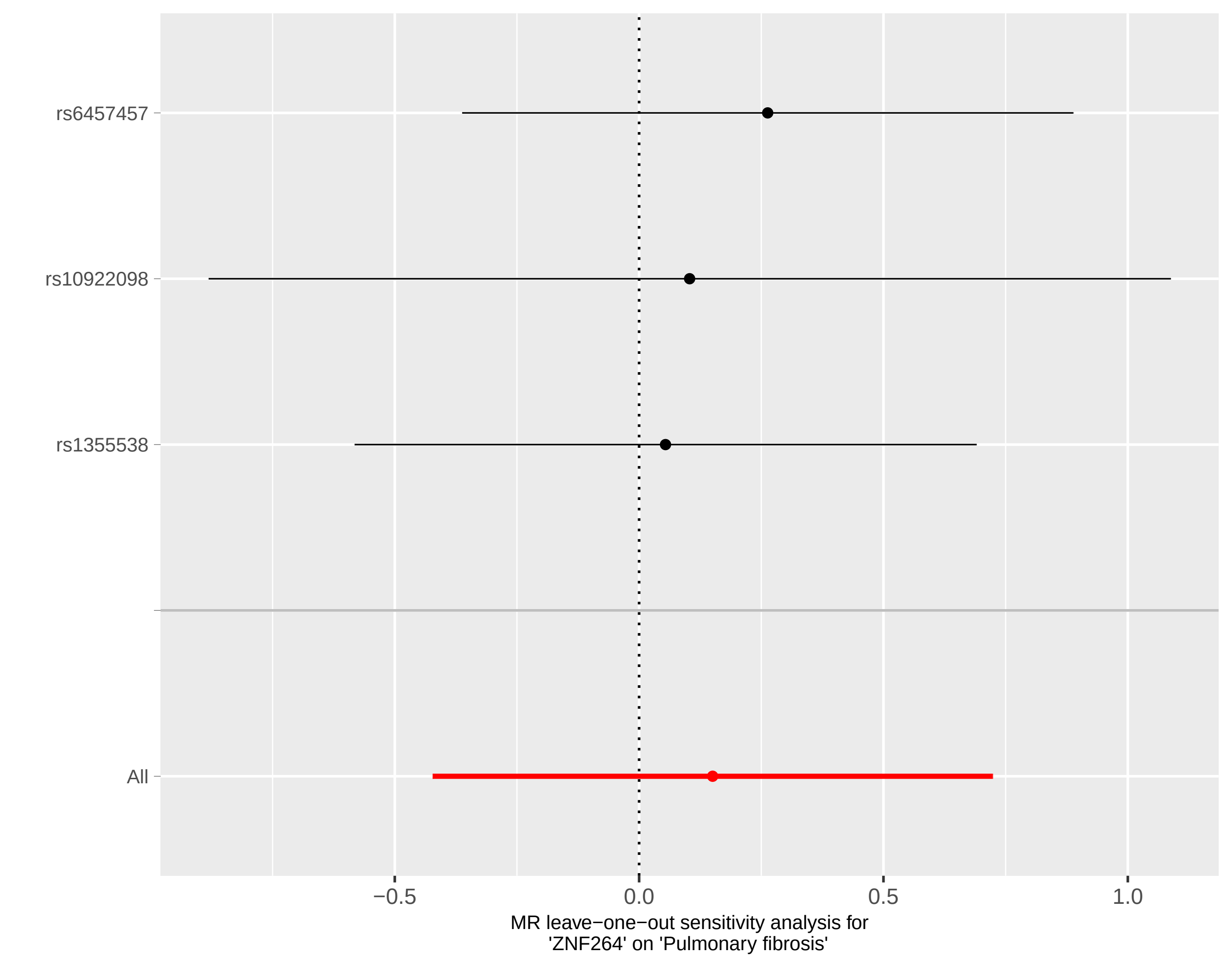
rs6457457
rs10922098
rs1355538
All
−0.5
0.0
0.5
1.0
MR leave−one−out sensitivity analysis for
'ZNF264' on 'Pulmonary fibrosis'

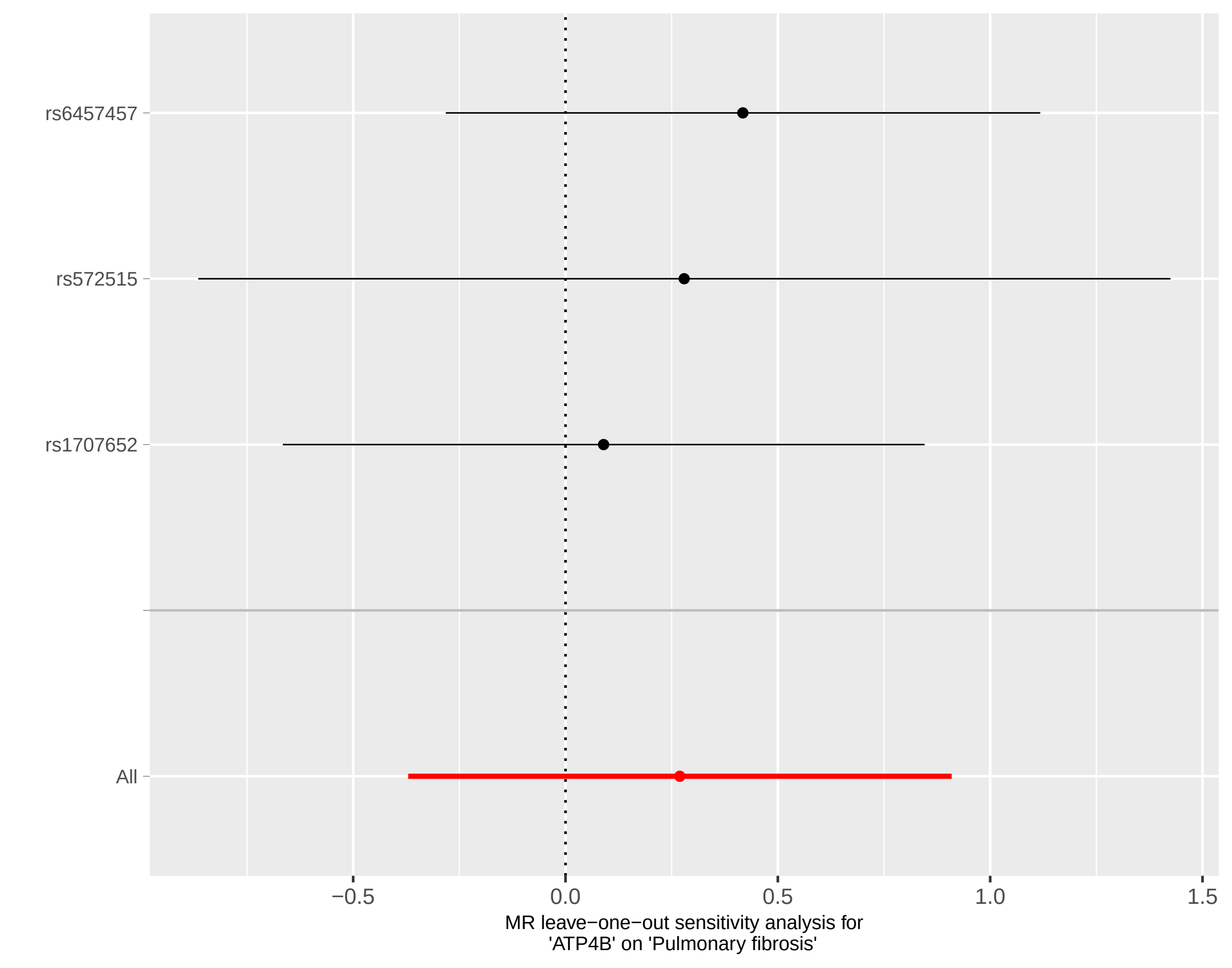
rs6457457
rs572515
rs1707652
All
−0.5
0.0
0.5
1.0
1.5
MR leave−one−out sensitivity analysis for
'ATP4B' on 'Pulmonary fibrosis'

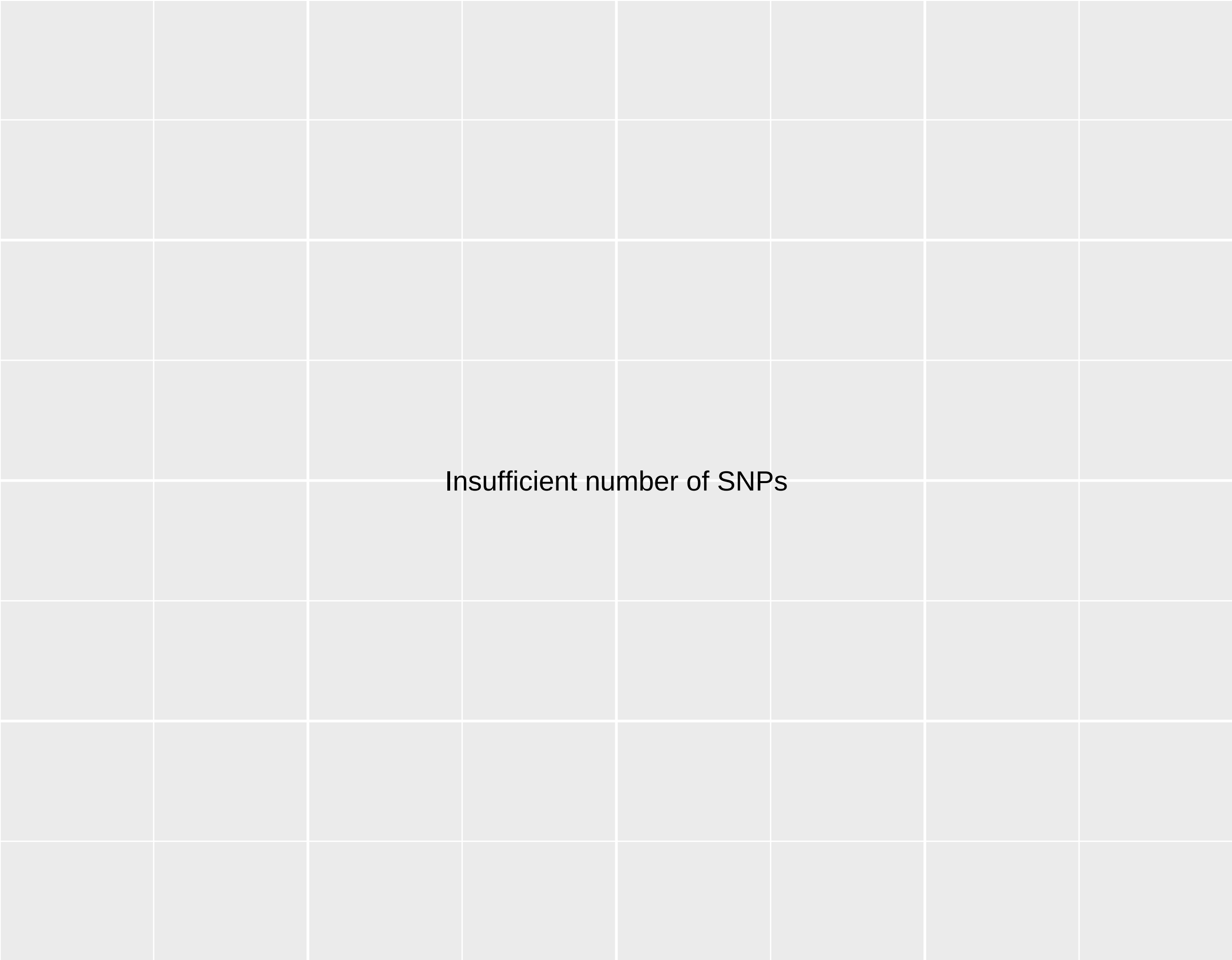
Insufficient number of SNPs
