# Supplementary material for: Assessing the Causal Relationship Between Plasma Proteins and Pulmonary Fibrosis: A Systematic Analysis Based on Mendelian Randomization
Source: Biology (Basel). 2025 Feb 14;14(2):200. doi: 10.3390/biology14020200 (PMC11852313; doi:10.3390/biology14020200)
Supplement: Supplementary file 1 [file Figure S4.pdf › Supplementary Material S1/Figure S4.pdf]

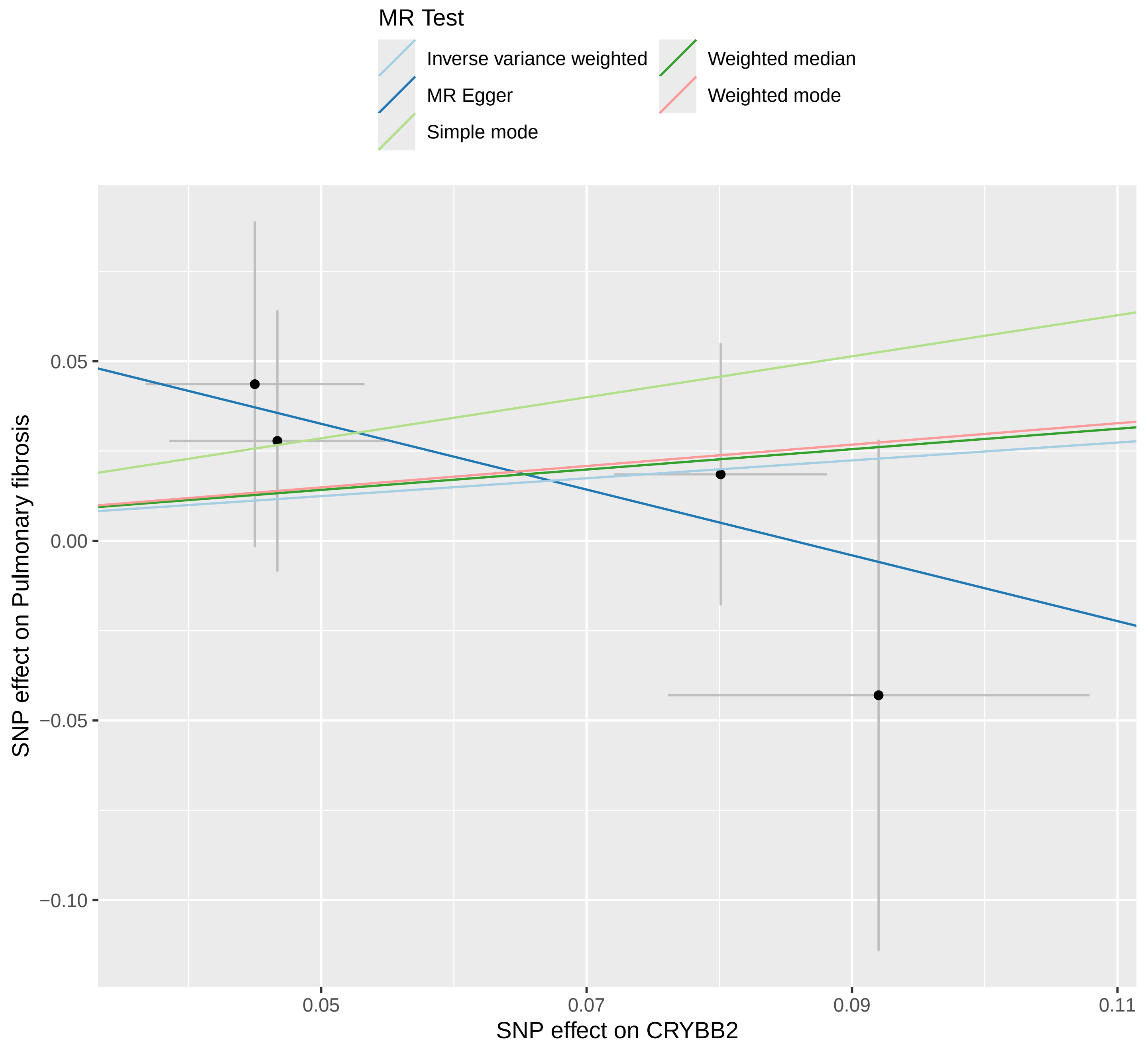
MR Test
Inverse variance weighted
MR Egger
Simple mode
Weighted median
Weighted mode
0.05
0.00
−0.05
−0.10
0.05
0.07
0.09
0.11
SNP effect on Pulmonary fibrosis
SNP effect on CRYBB2

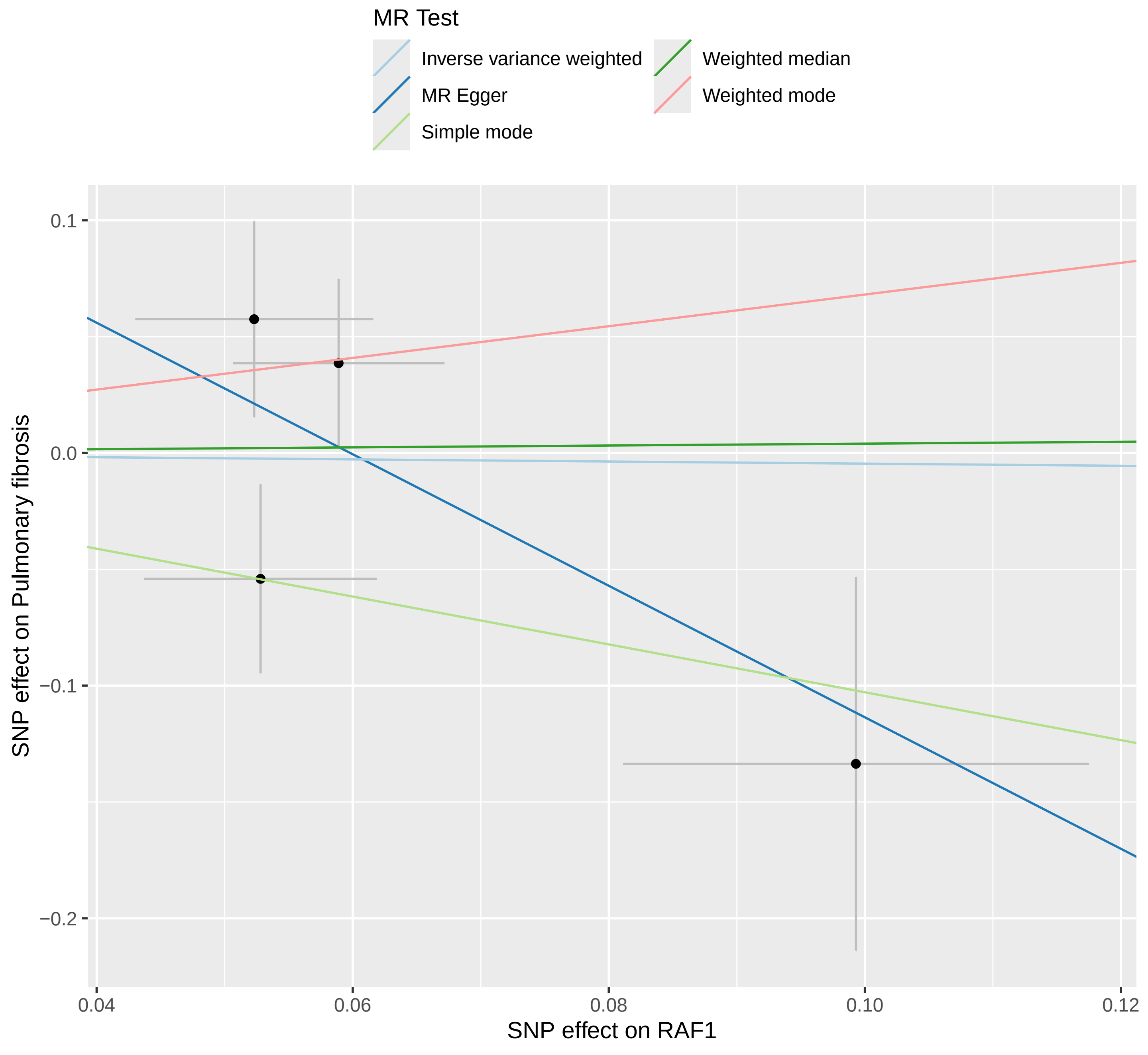

MR Test
Inverse variance weighted
MR Egger
Simple mode
Weighted median
Weighted mode
0.1
0.0
−0.1
−0.2
SNP effect on Pulmonary fibrosis
0.04
0.06
0.08
0.10
0.12
SNP effect on RAF1

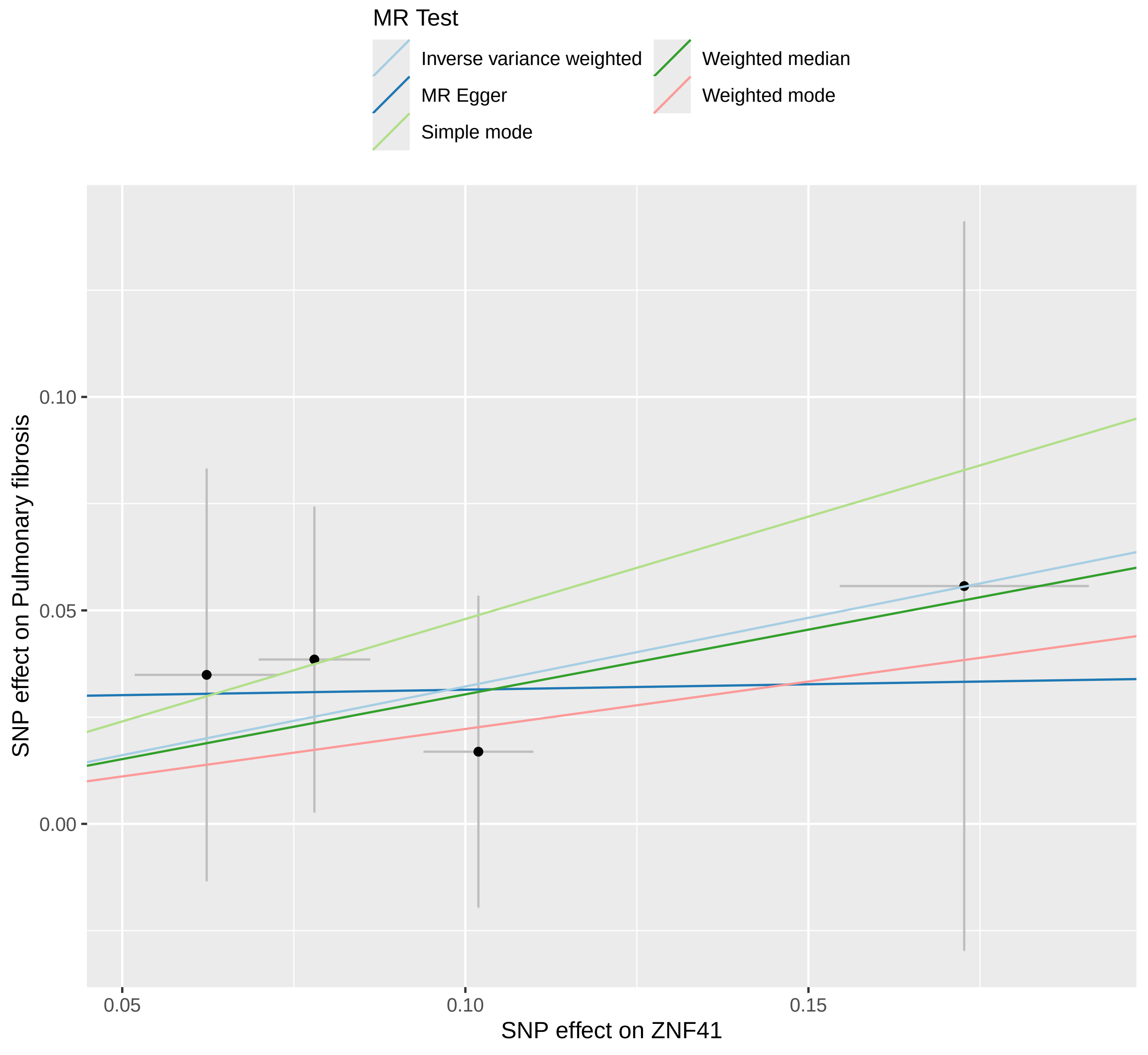
MR Test
Inverse variance weighted
MR Egger
Simple mode
Weighted median
Weighted mode
SNP effect on Pulmonary fibrosis
0.10
0.05
0.00
0.05
0.10
0.15
SNP effect on ZNF41

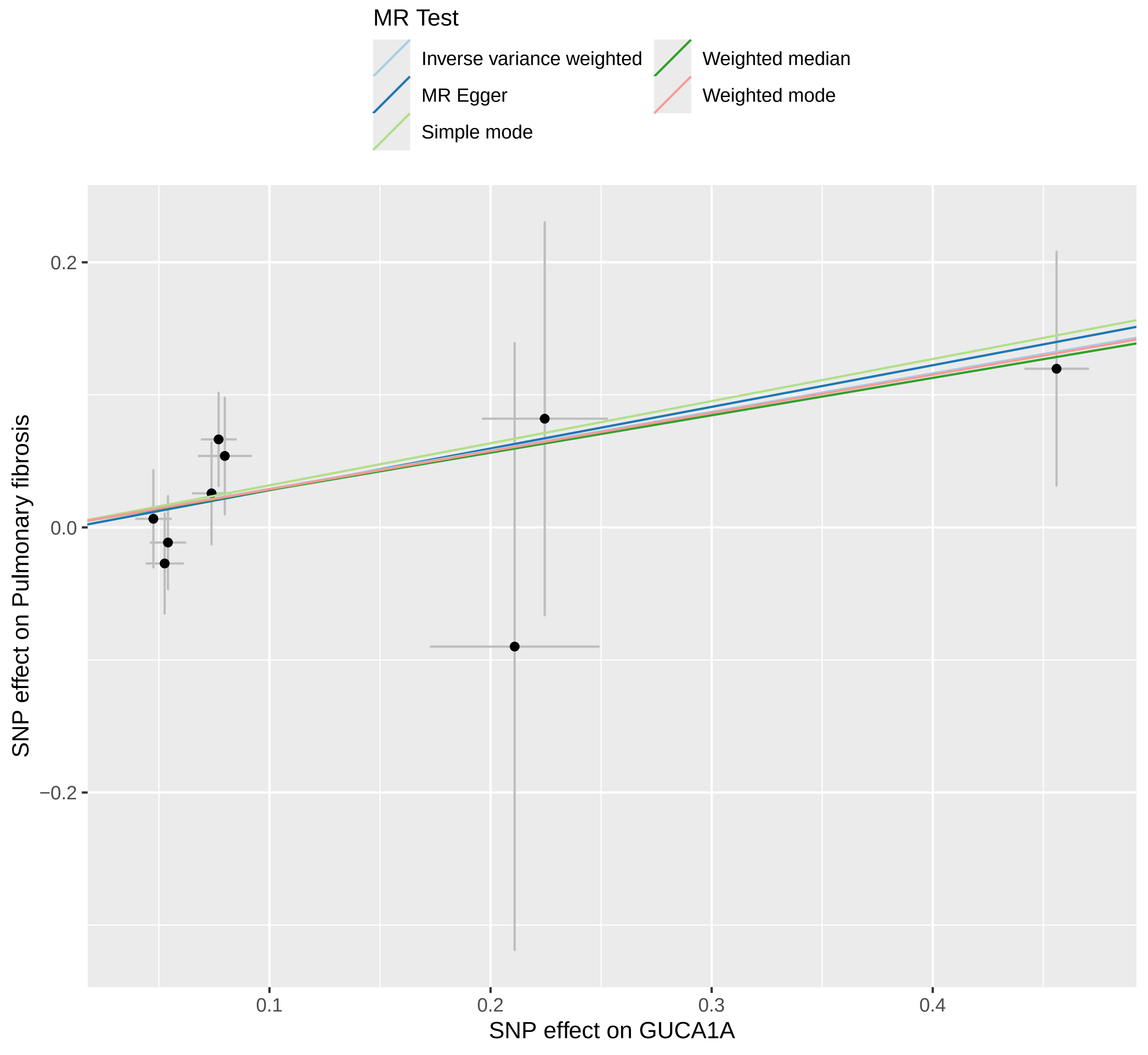

MR Test
Inverse variance weighted
MR Egger
Simple mode
Weighted median
Weighted mode
SNP effect on Pulmonary fibrosis
0.2
0.0
−0.2
0.1
0.2
0.3
0.4
SNP effect on GUCA1A

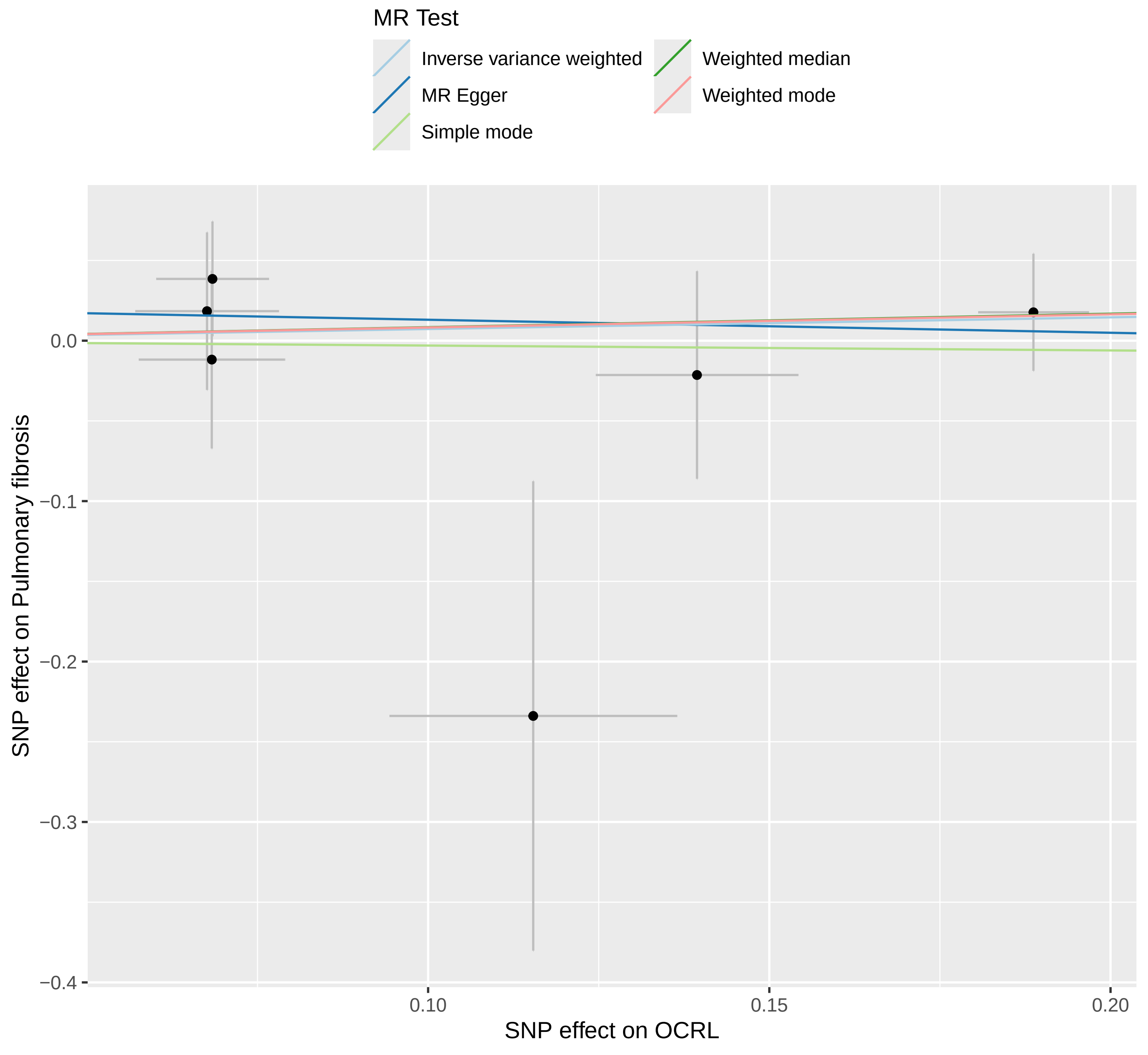
MR Test
Inverse variance weighted
MR Egger
Simple mode
Weighted median
Weighted mode
SNP effect on Pulmonary fibrosis
0.0
−0.1
−0.2
−0.3
−0.4
0.10
0.15
0.20
SNP effect on OCRL

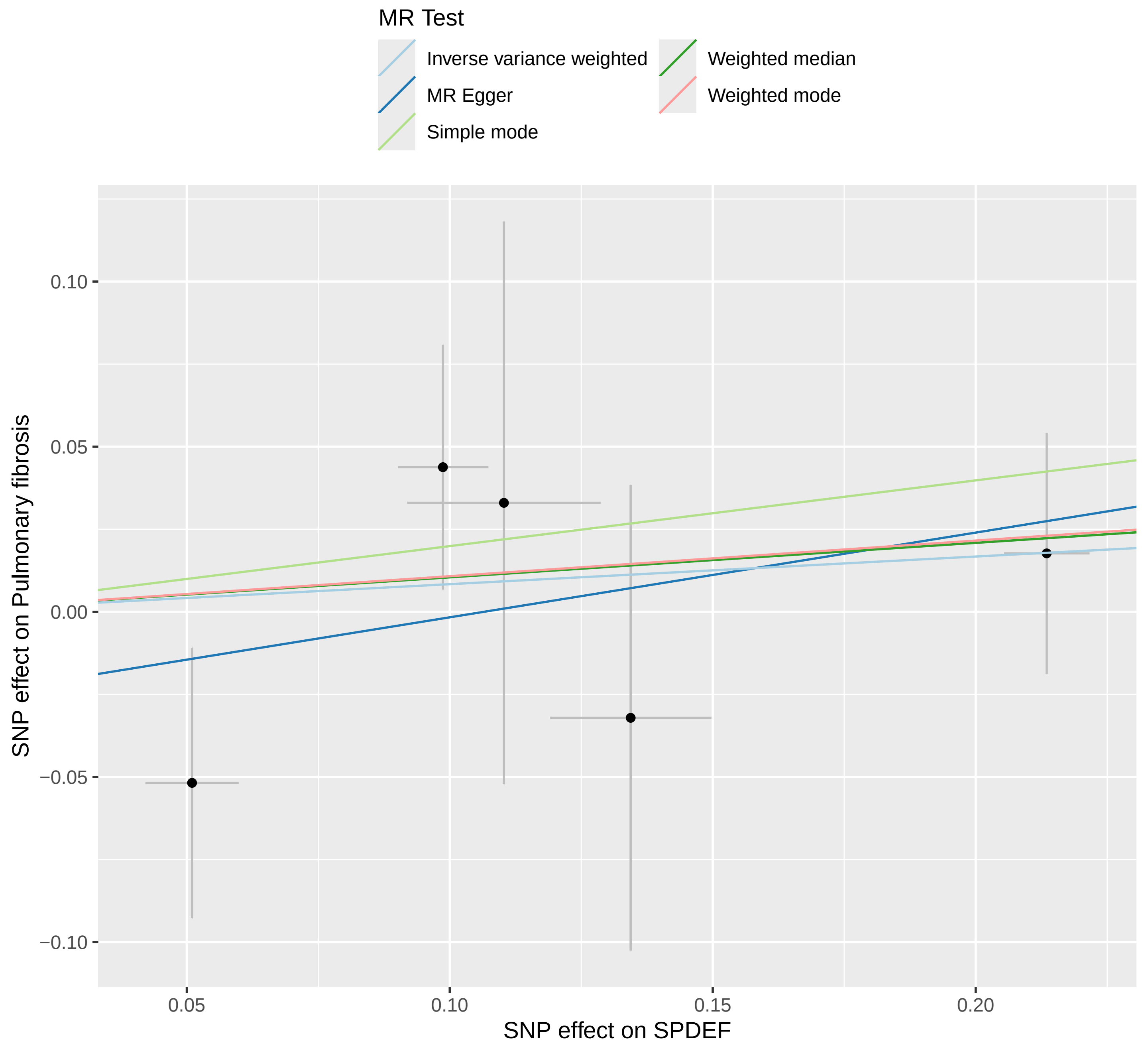
MR Test
Inverse variance weighted
MR Egger
Simple mode
Weighted median
Weighted mode
0.10
0.05
0.00
−0.05
−0.10
SNP effect on Pulmonary fibrosis
0.05
0.10
0.15
0.20
SNP effect on SPDEF

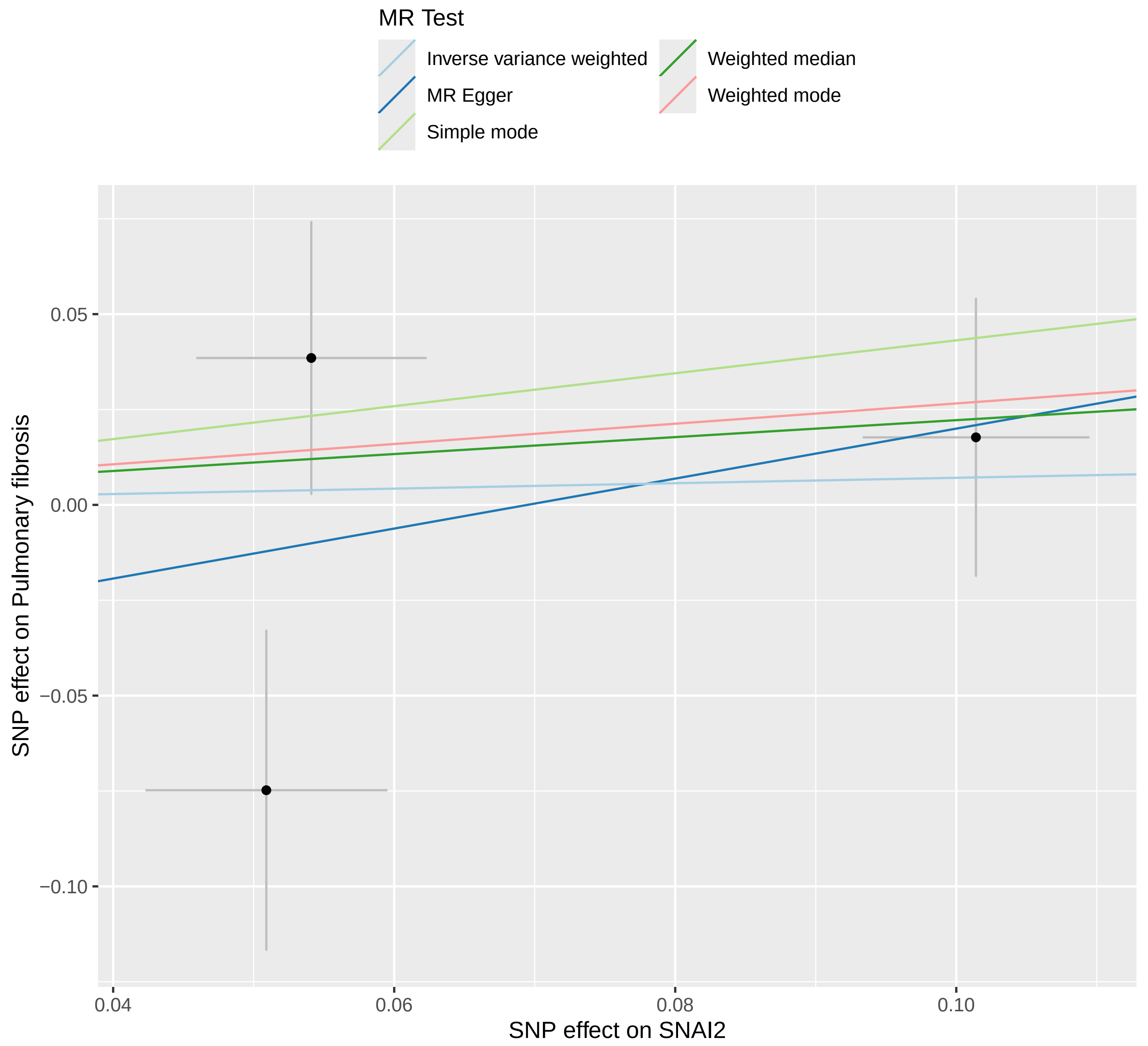
MR Test
Inverse variance weighted
MR Egger
Simple mode
Weighted median
Weighted mode
0.05
0.00
−0.05
−0.10
SNP effect on Pulmonary fibrosis
0.04
0.06
0.08
0.10
SNP effect on SNAI2

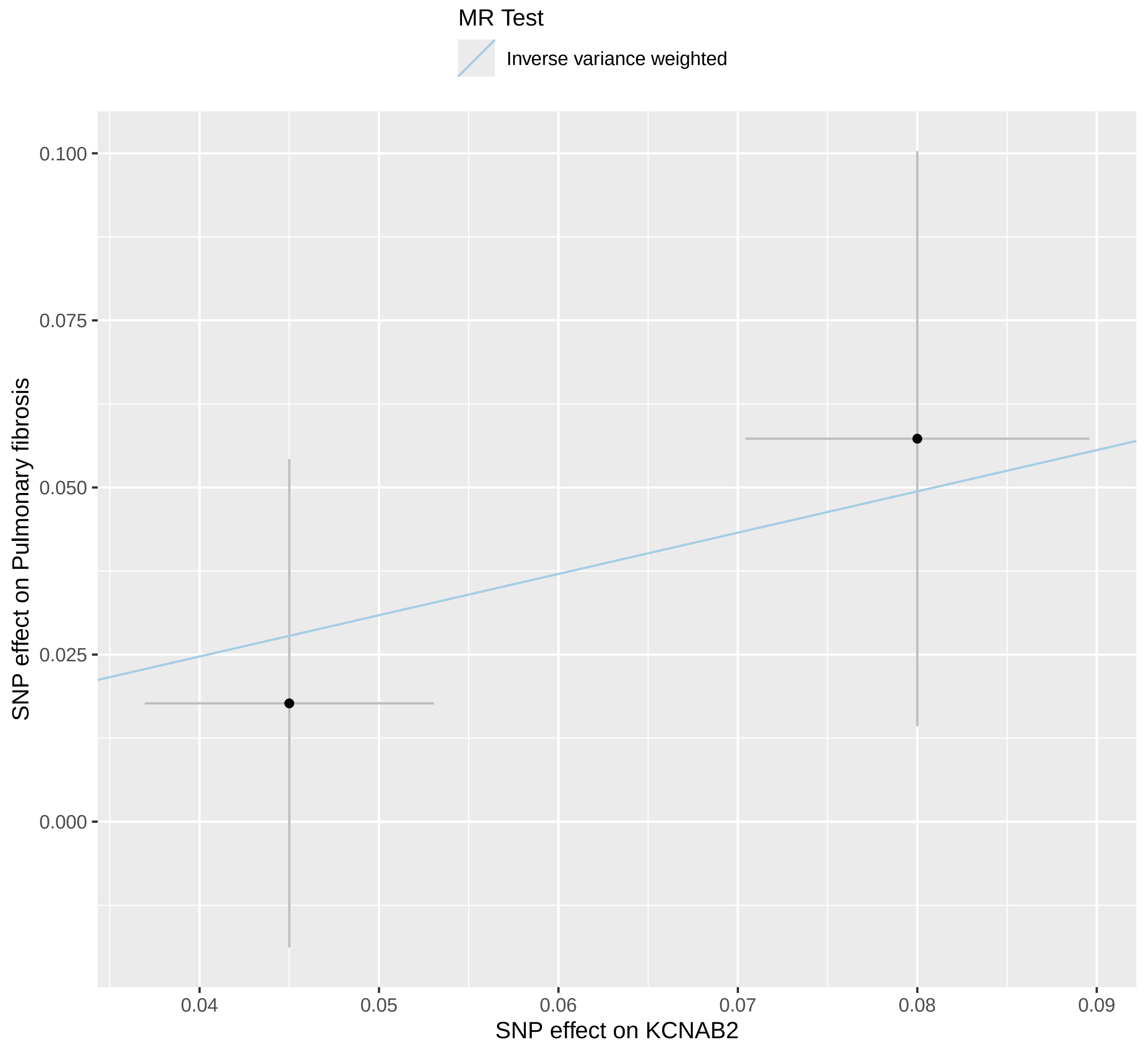
MR Test
Inverse variance weighted
0.100
0.075
0.050
0.025
0.000
SNP effect on Pulmonary fibrosis
0.04
0.05
0.06
0.07
0.08
0.09
SNP effect on KCNAB2

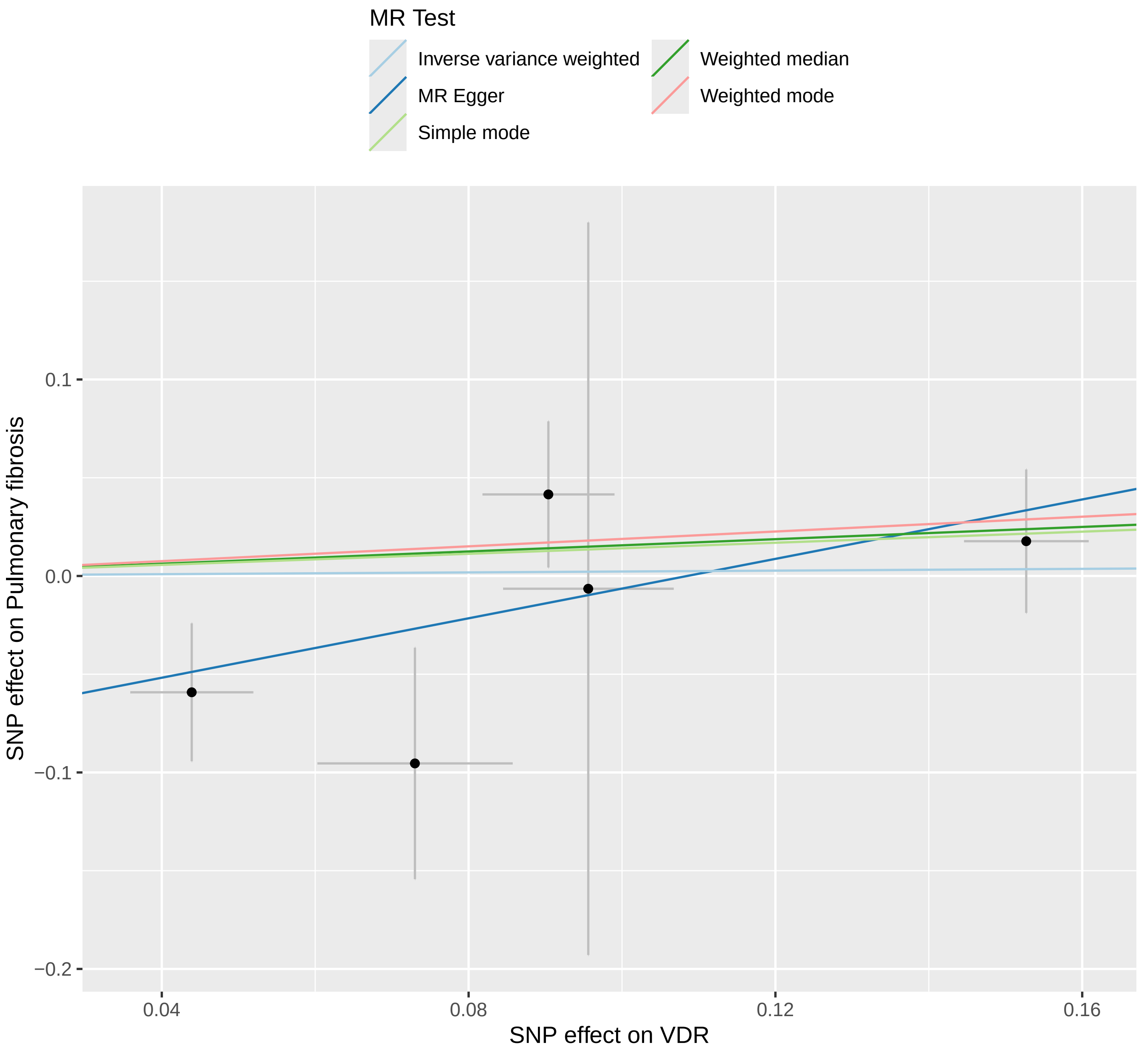
MR Test
Inverse variance weighted
MR Egger
Simple mode
Weighted median
Weighted mode
SNP effect on Pulmonary fibrosis
0.1
0.0
−0.1
−0.2
0.04
0.08
0.12
0.16
SNP effect on VDR

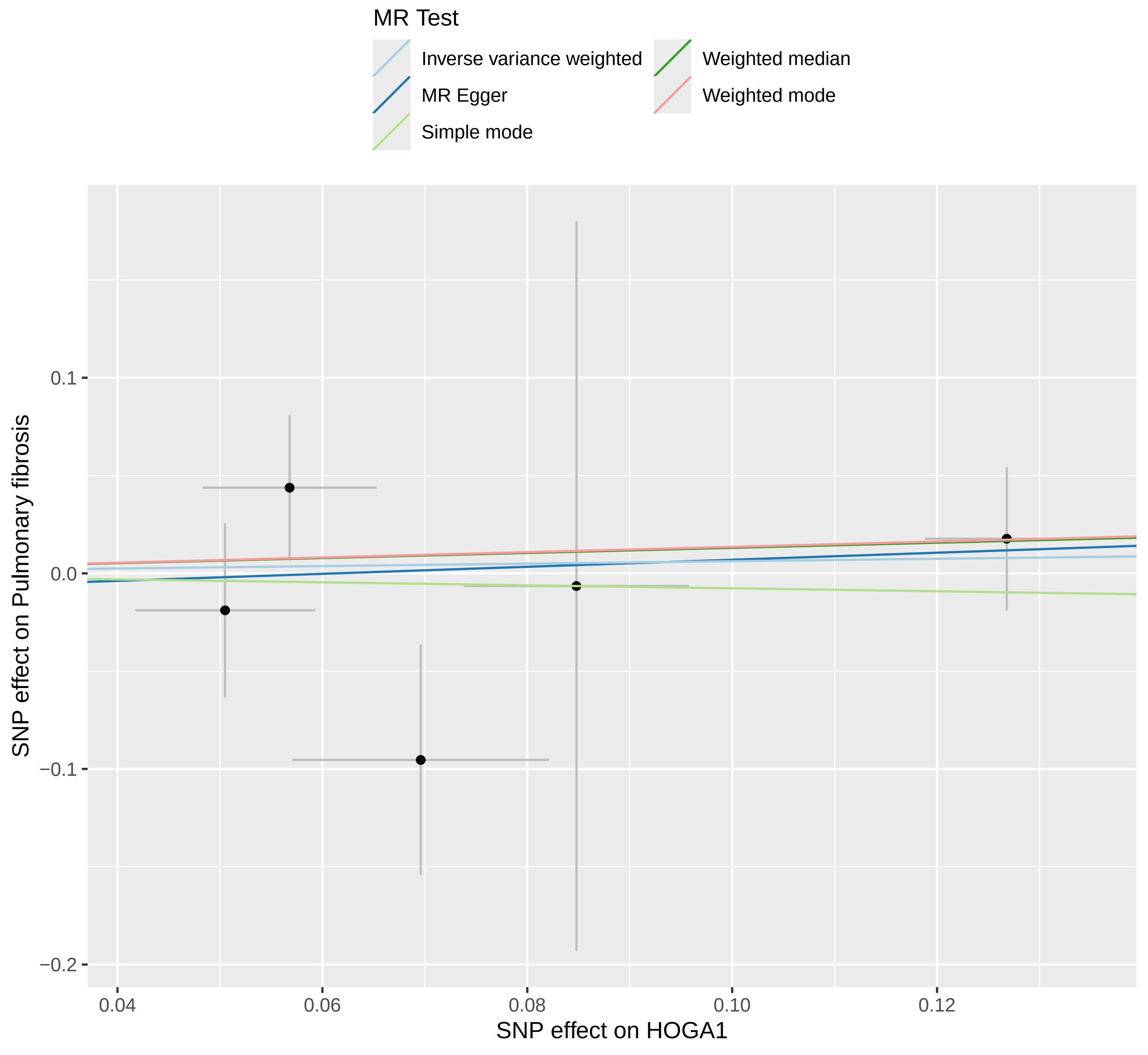

MR Test
Inverse variance weighted
MR Egger
Simple mode
Weighted median
Weighted mode
0.1
0.0
−0.1
−0.2
SNP effect on Pulmonary fibrosis
0.04
0.06
0.08
0.10
0.12
SNP effect on HOGA1

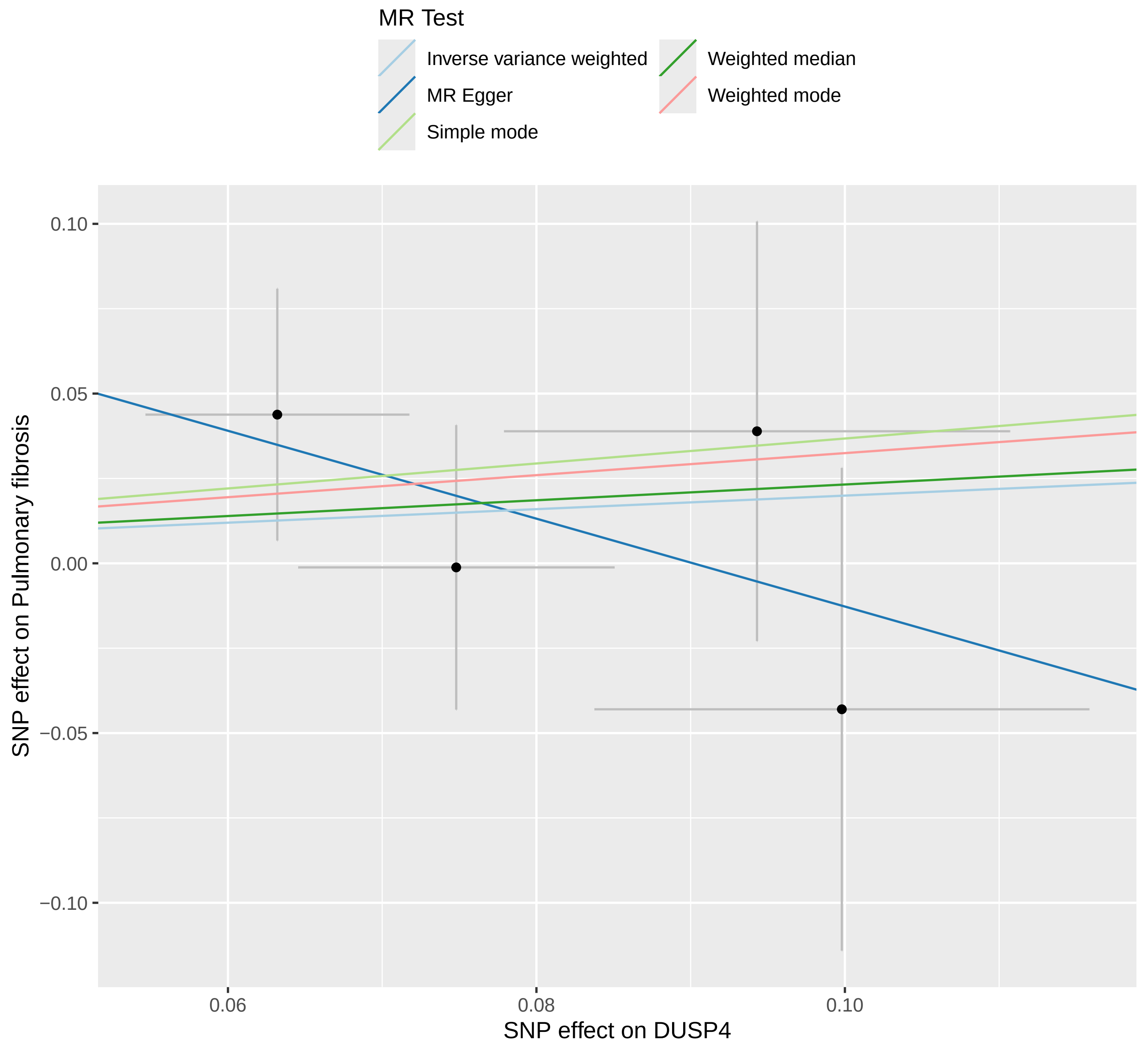
MR Test
Inverse variance weighted
MR Egger
Simple mode
Weighted median
Weighted mode
0.10
0.05
0.00
−0.05
−0.10
SNP effect on Pulmonary fibrosis
0.06
0.08
0.10
SNP effect on DUSP4

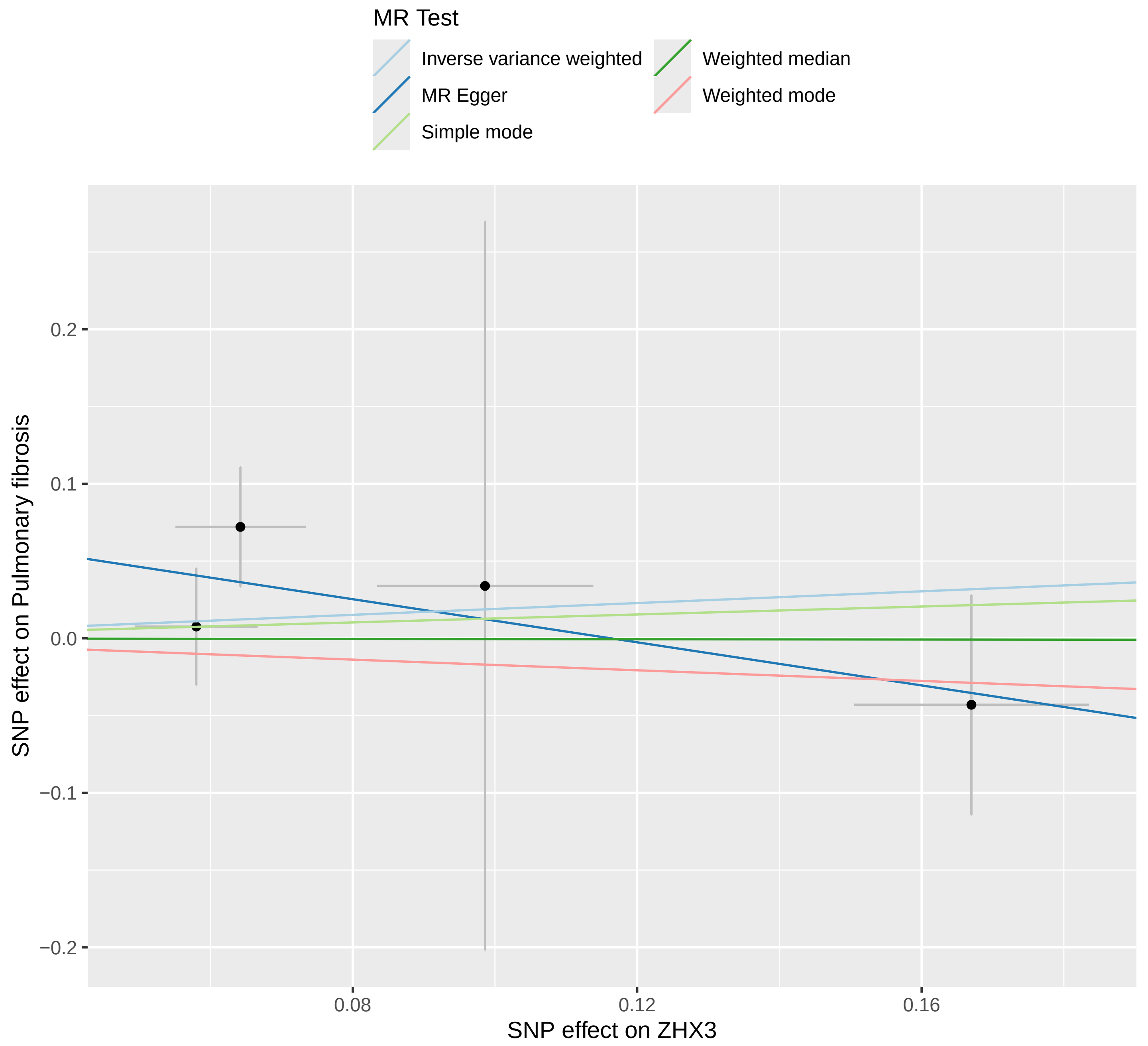
MR Test
Inverse variance weighted
MR Egger
Simple mode
Weighted median
Weighted mode
SNP effect on Pulmonary fibrosis
0.2
0.1
0.0
−0.1
−0.2
0.08
0.12
0.16
SNP effect on ZHX3

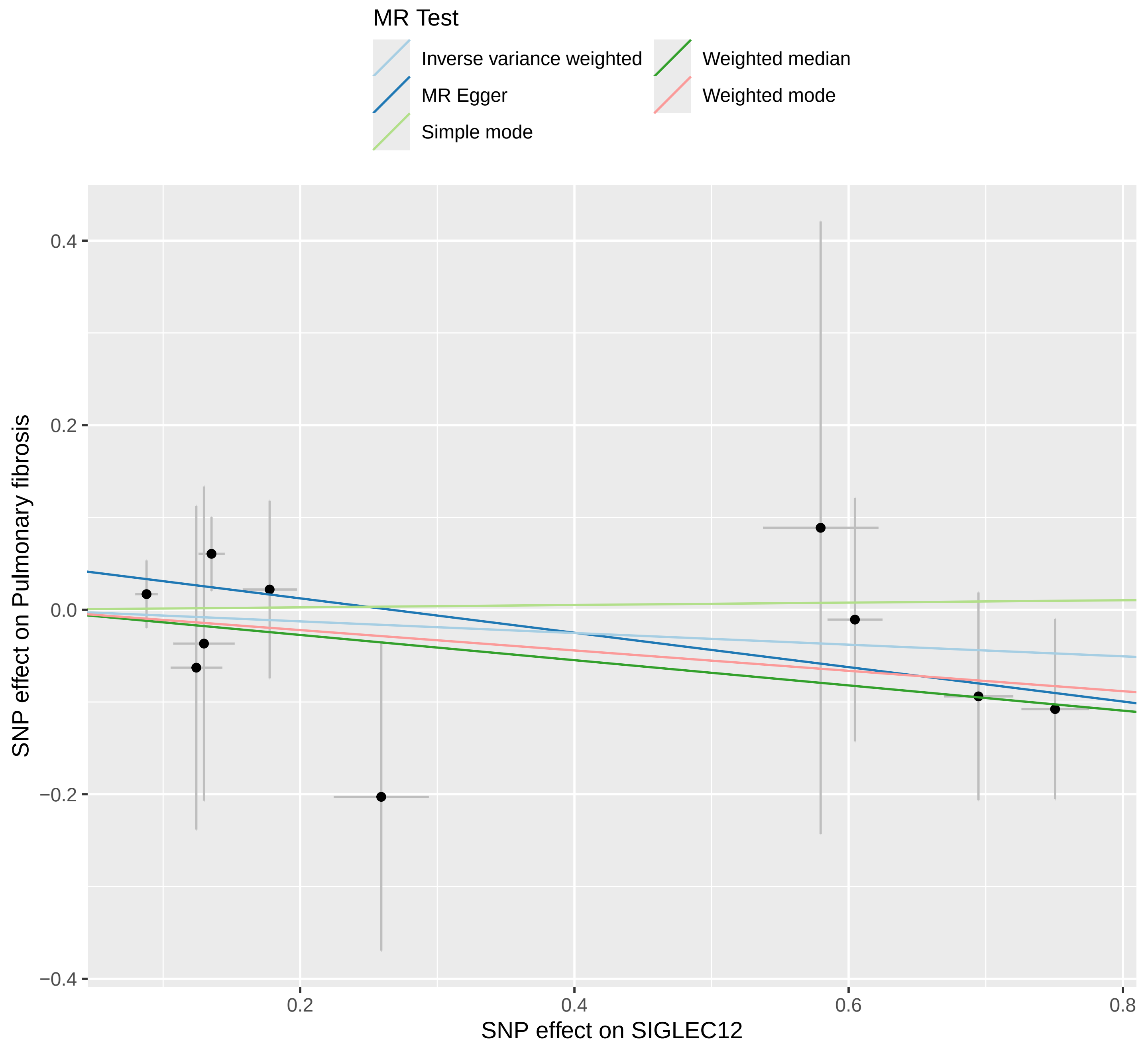
MR Test
Inverse variance weighted
MR Egger
Simple mode
Weighted median
Weighted mode
0.4
0.2
0.0
−0.2
−0.4
0.2
0.4
0.6
0.8
SNP effect on Pulmonary fibrosis
SNP effect on SIGLEC12

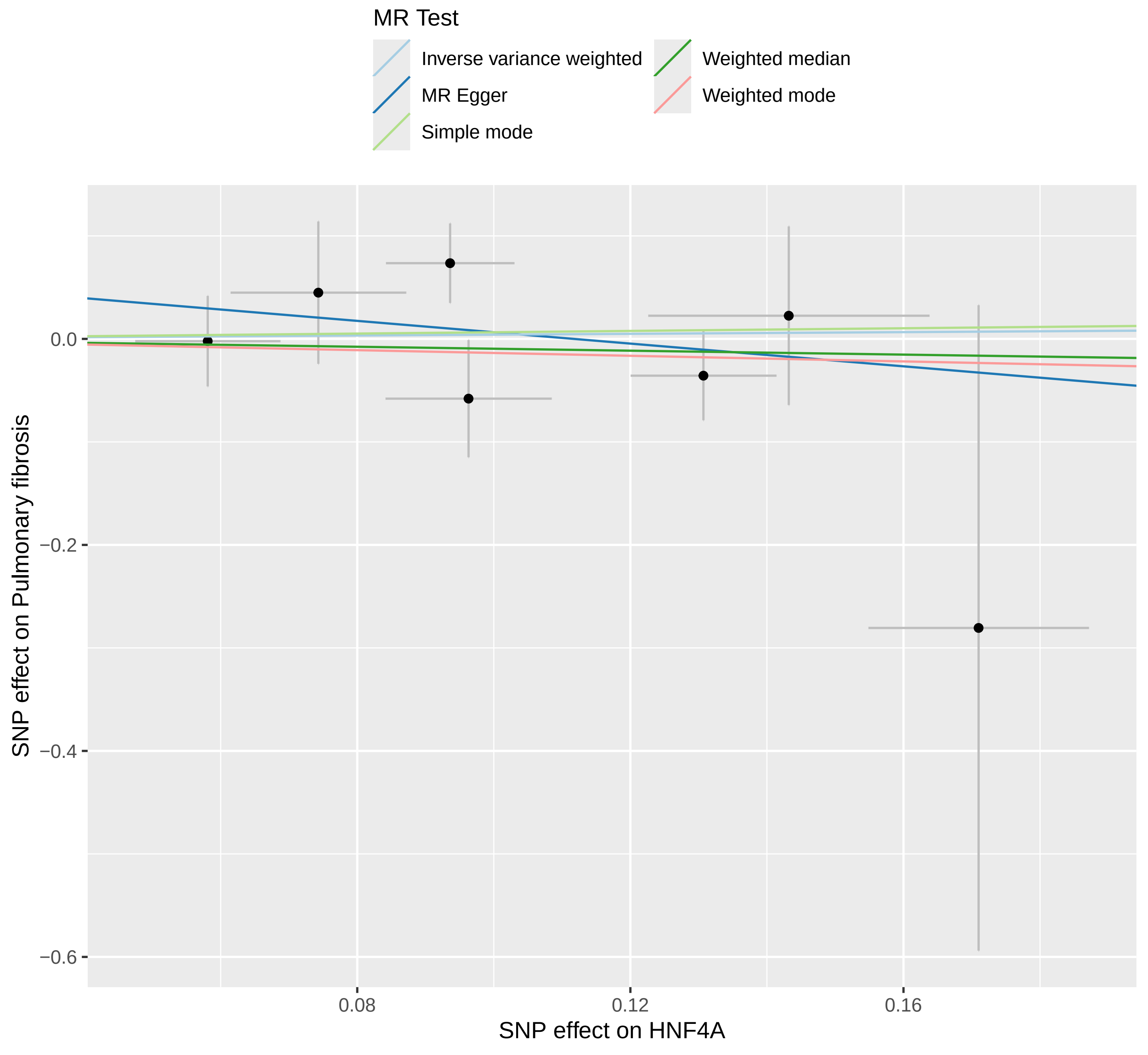

MR Test
Inverse variance weighted
MR Egger
Simple mode
Weighted median
Weighted mode
0.0
−0.2
−0.4
−0.6
0.08
0.12
0.16
SNP effect on HNF4A
SNP effect on Pulmonary fibrosis

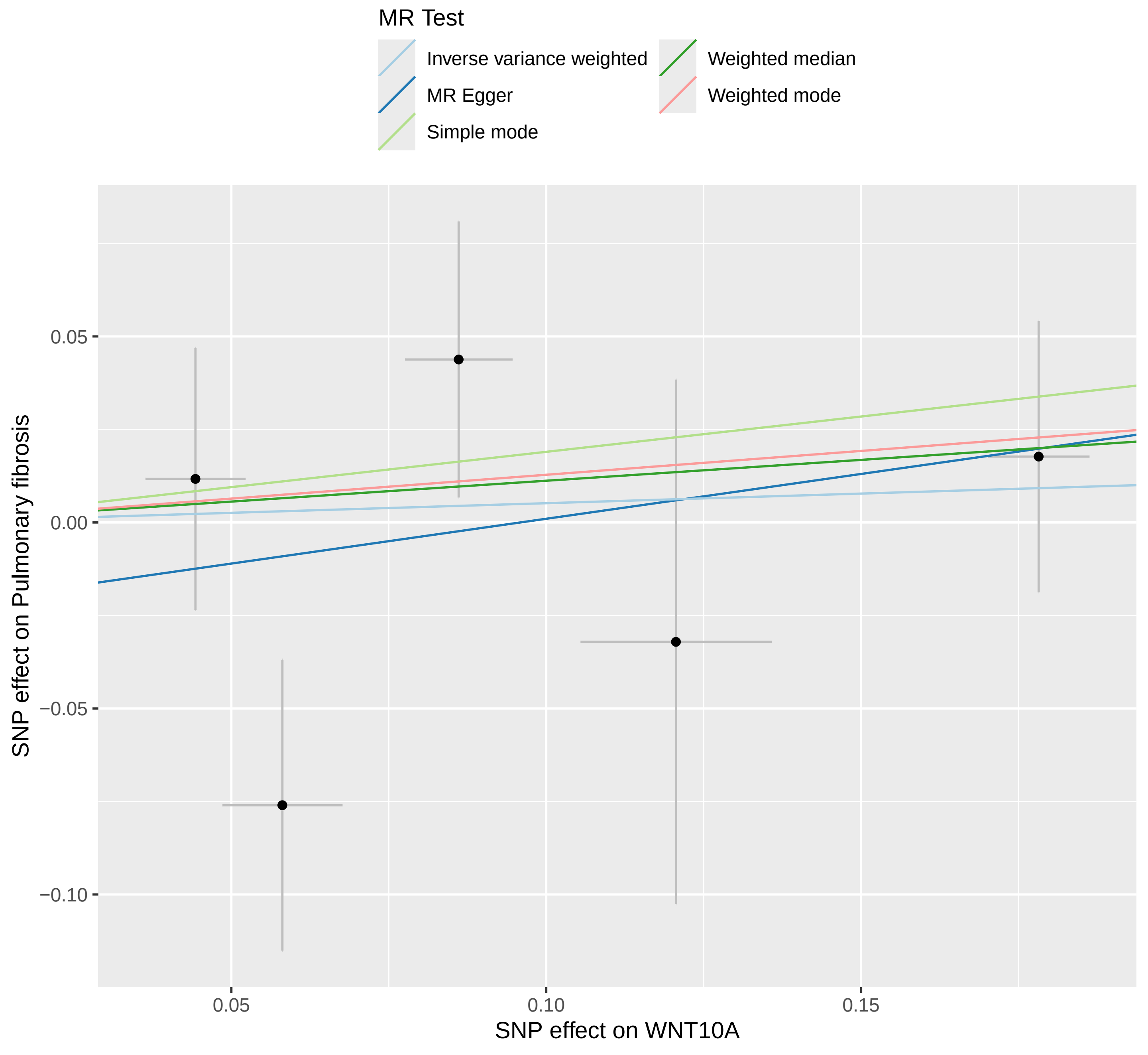
MR Test
Inverse variance weighted
MR Egger
Simple mode
Weighted median
Weighted mode
0.05
0.00
−0.05
−0.10
0.05
0.10
0.15
SNP effect on Pulmonary fibrosis
SNP effect on WNT10A

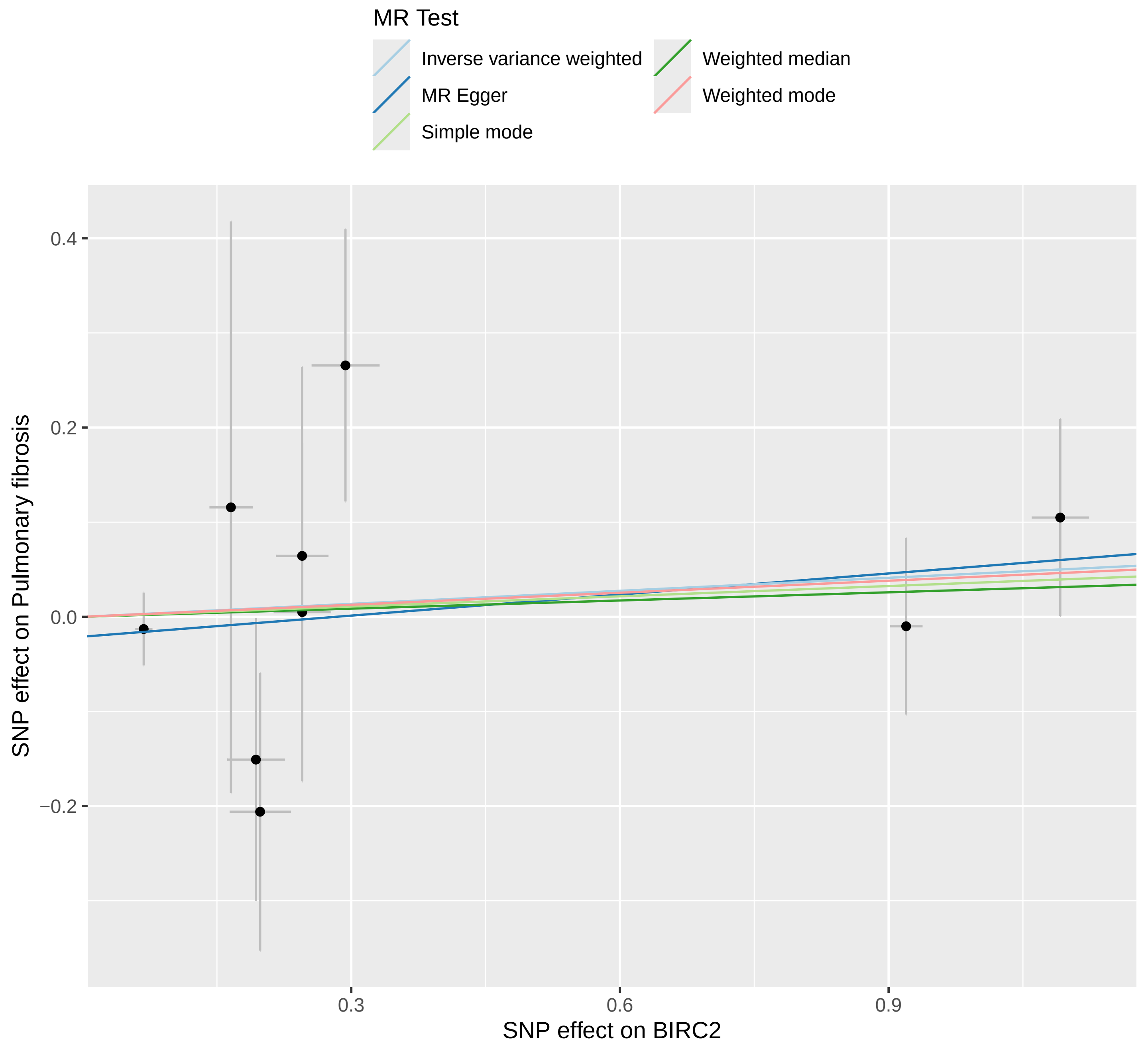
MR Test
Inverse variance weighted
MR Egger
Simple mode
Weighted median
Weighted mode
0.4
0.2
0.0
−0.2
SNP effect on Pulmonary fibrosis
0.3
0.6
0.9
SNP effect on BIRC2

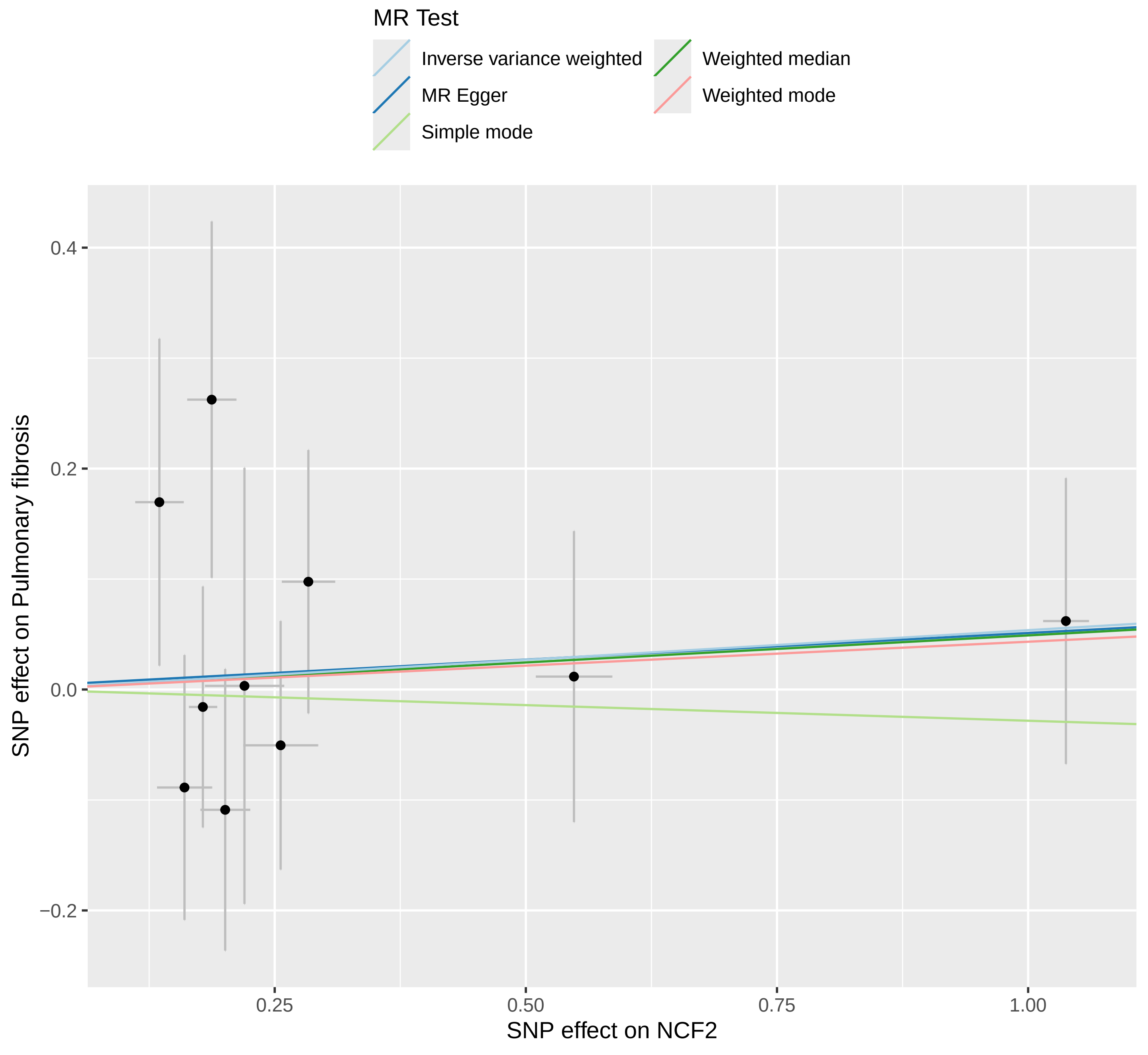
MR Test
Inverse variance weighted
MR Egger
Simple mode
Weighted median
Weighted mode
0.4
0.2
0.0
−0.2
SNP effect on Pulmonary fibrosis
0.25
0.50
0.75
1.00
SNP effect on NCF2

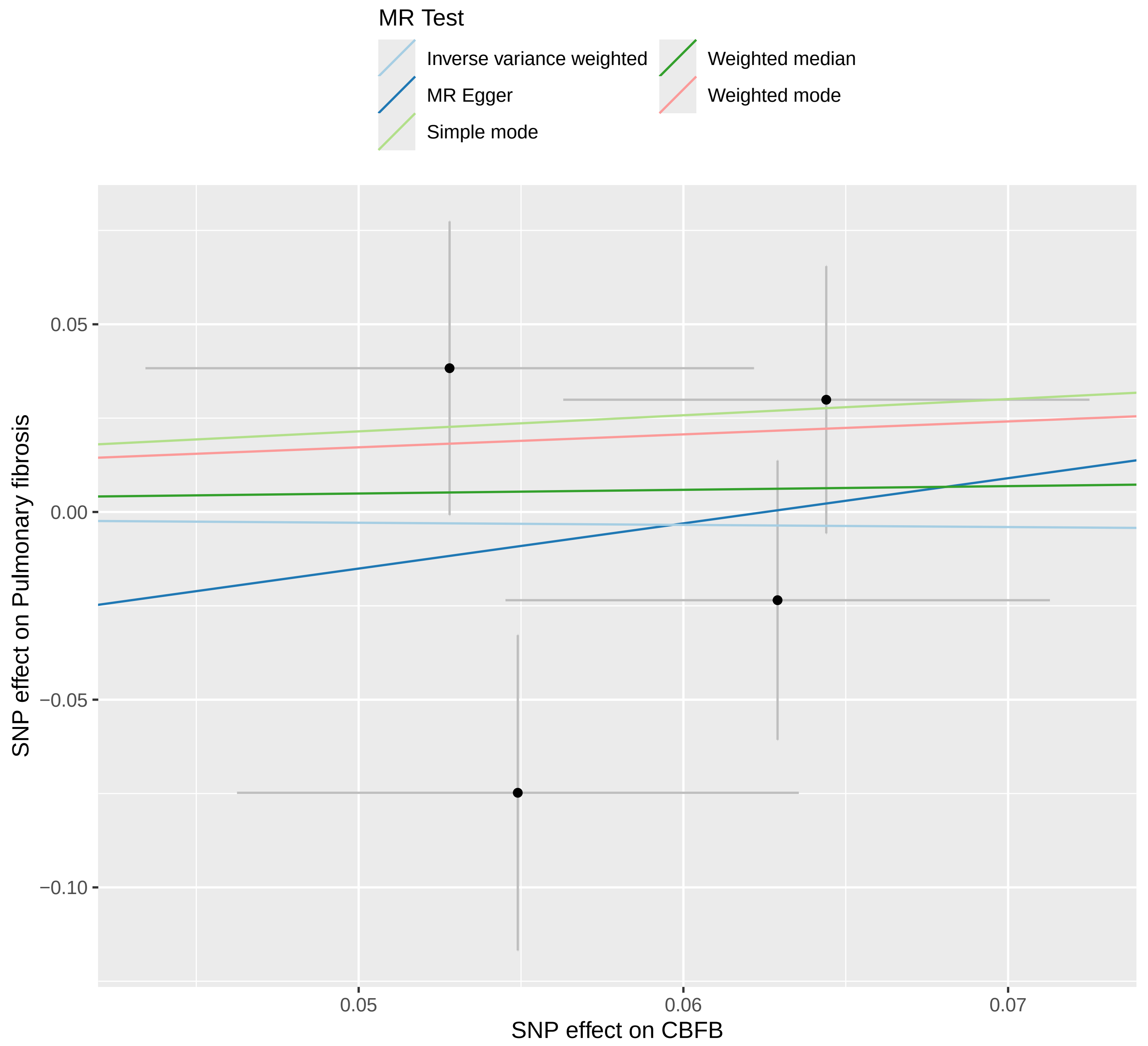
MR Test
Inverse variance weighted
MR Egger
Simple mode
Weighted median
Weighted mode
0.05
0.00
−0.05
−0.10
SNP effect on Pulmonary fibrosis
0.05
0.06
0.07
SNP effect on CBFB

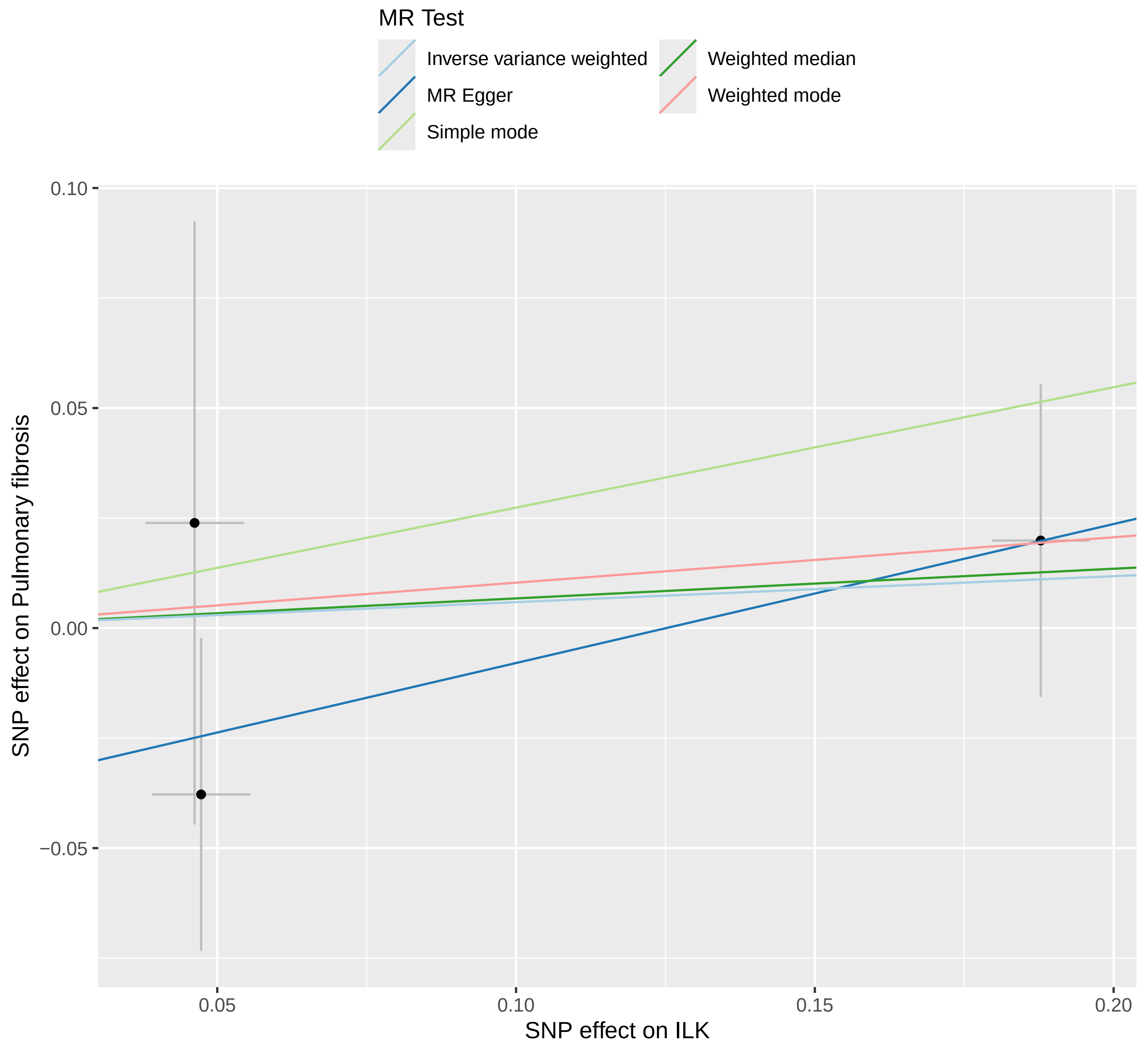

MR Test
Inverse variance weighted
MR Egger
Simple mode
Weighted median
Weighted mode
0.10
0.05
0.00
−0.05
0.05
0.10
0.15
0.20
SNP effect on Pulmonary fibrosis
SNP effect on ILK

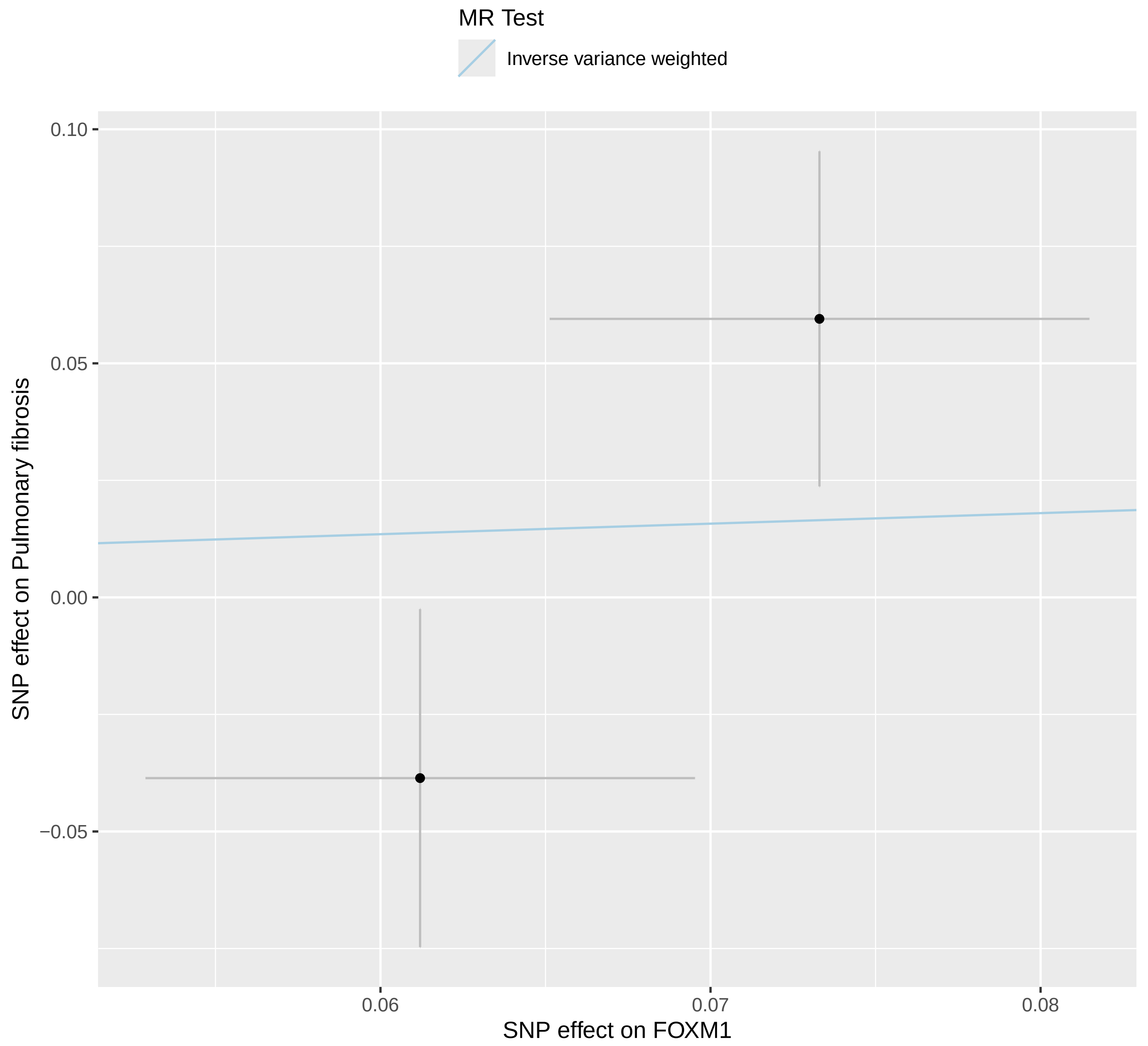
MR Test
Inverse variance weighted
0.10
0.05
0.00
−0.05
0.06
0.07
0.08
SNP effect on Pulmonary fibrosis
SNP effect on FOXM1

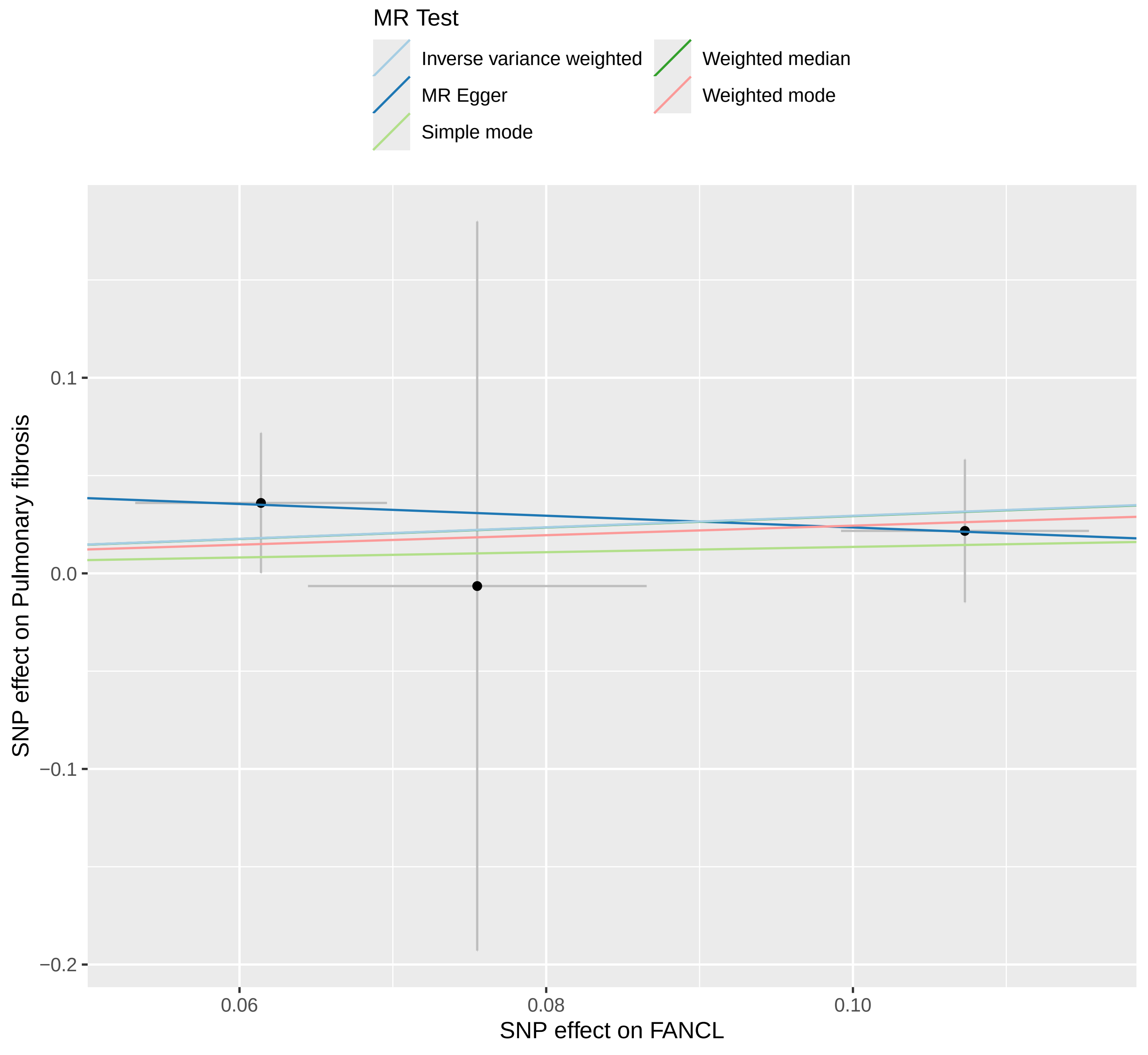

MR Test
Inverse variance weighted
MR Egger
Simple mode
Weighted median
Weighted mode
0.1
0.0
−0.1
−0.2
SNP effect on Pulmonary fibrosis
0.06
0.08
0.10
SNP effect on FANCL

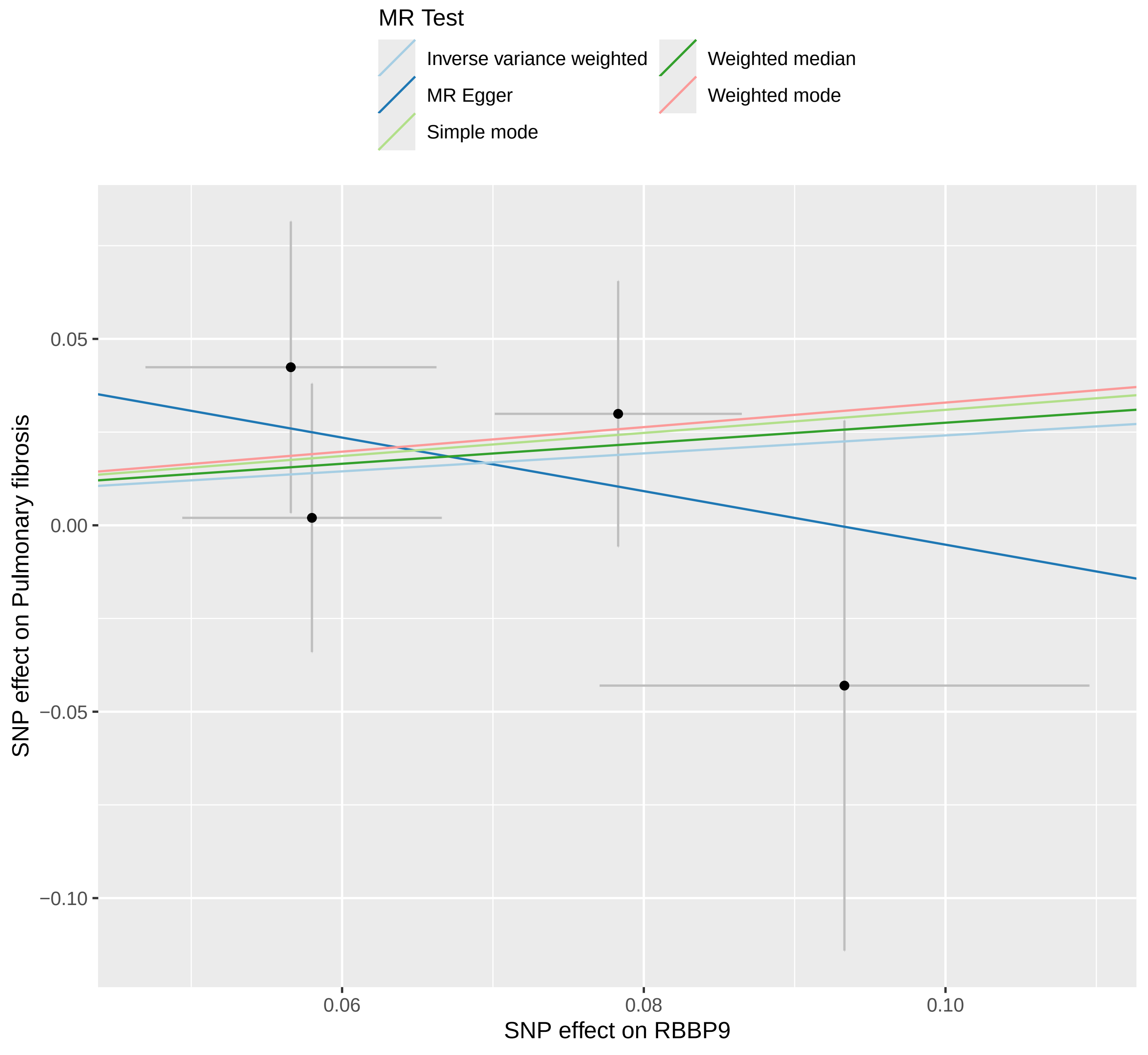
MR Test
Inverse variance weighted
MR Egger
Simple mode
Weighted median
Weighted mode
0.05
0.00
−0.05
−0.10
SNP effect on Pulmonary fibrosis
0.06
0.08
0.10
SNP effect on RBBP9

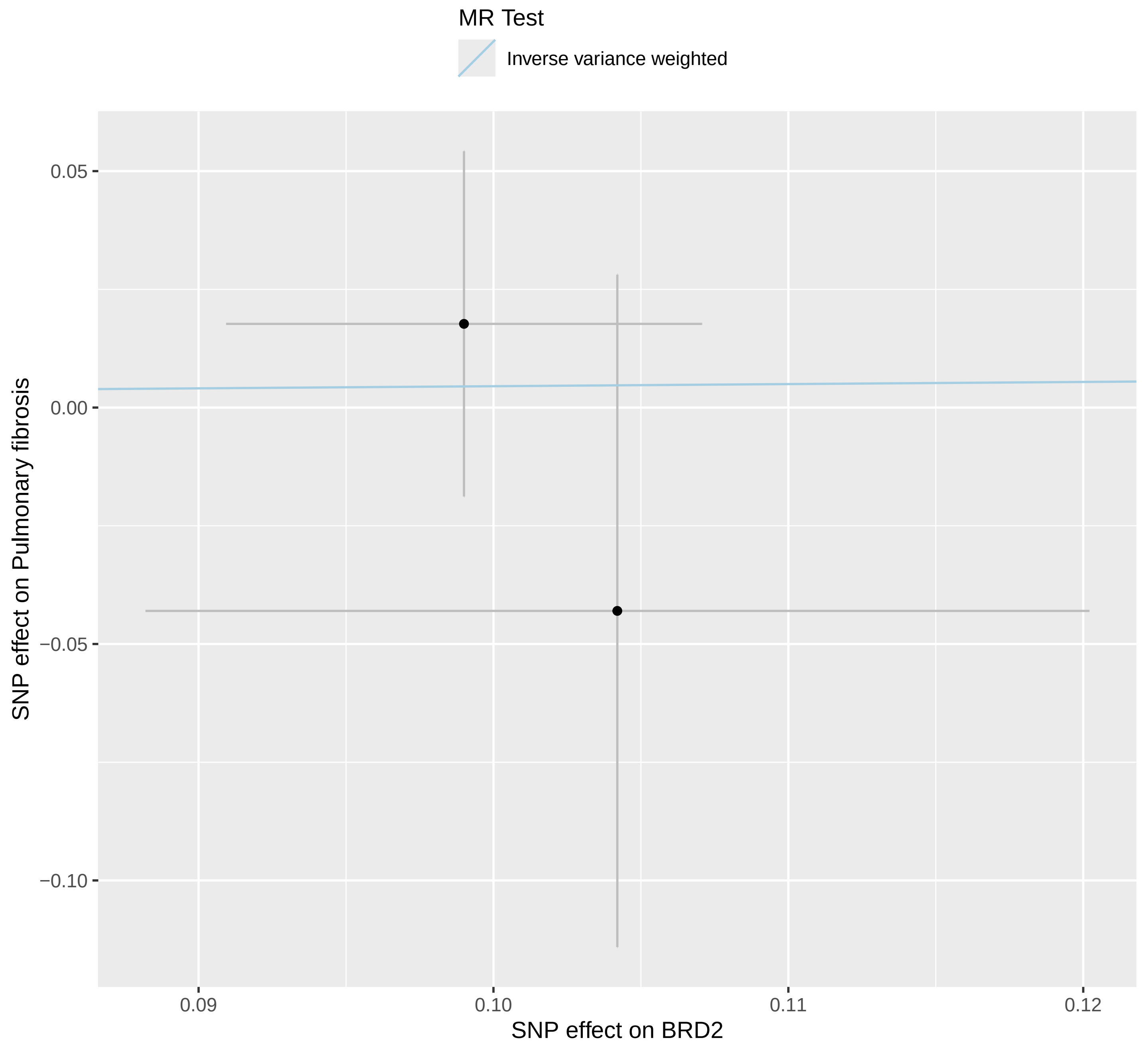
MR Test
Inverse variance weighted
0.05
0.00
−0.05
−0.10
SNP effect on Pulmonary fibrosis
0.09
0.10
0.11
0.12
SNP effect on BRD2

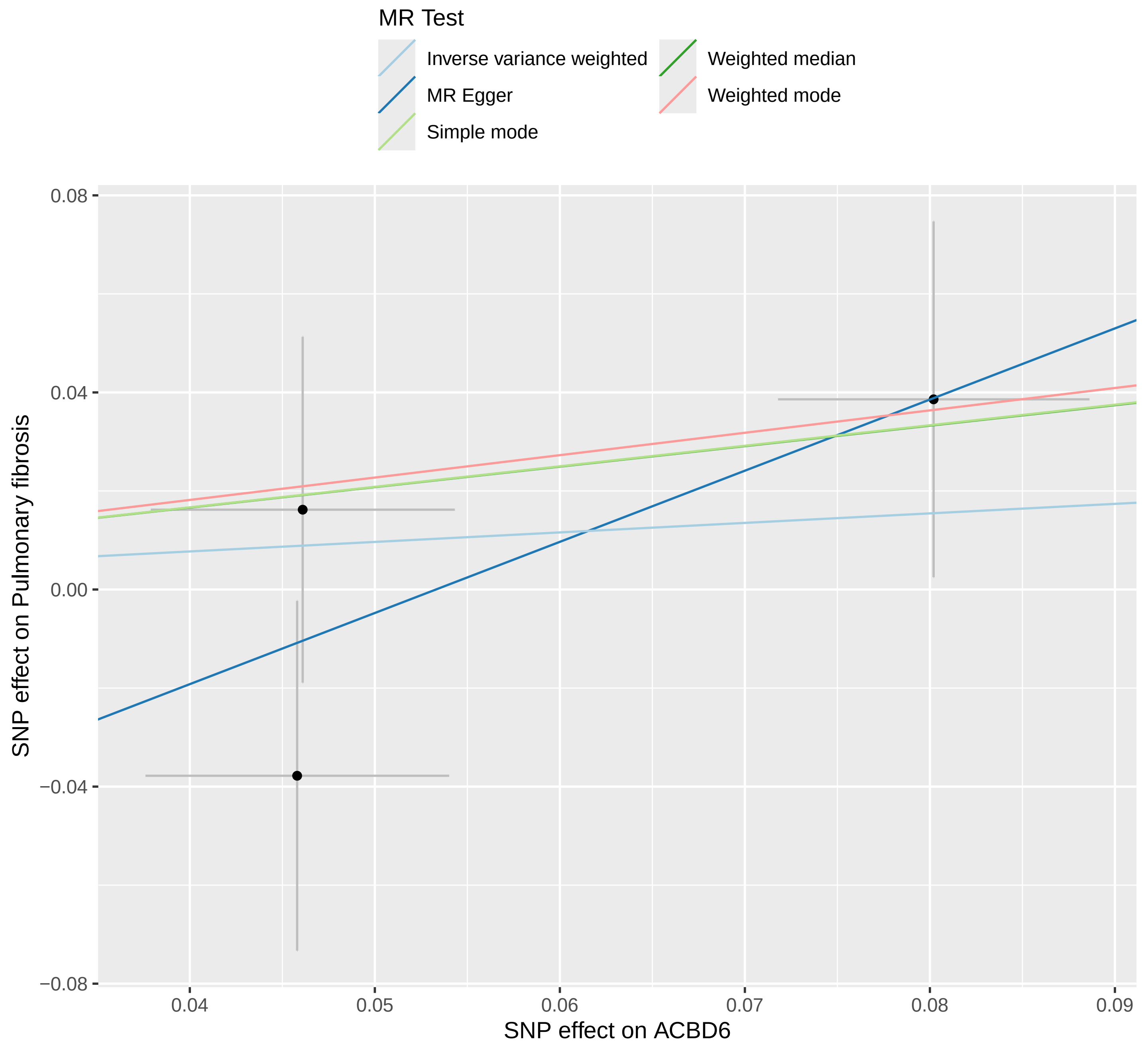
MR Test
Inverse variance weighted
MR Egger
Simple mode
Weighted median
Weighted mode
0.08
0.04
0.00
−0.04
−0.08
0.04
0.05
0.06
0.07
0.08
0.09
SNP effect on Pulmonary fibrosis
SNP effect on ACBD6

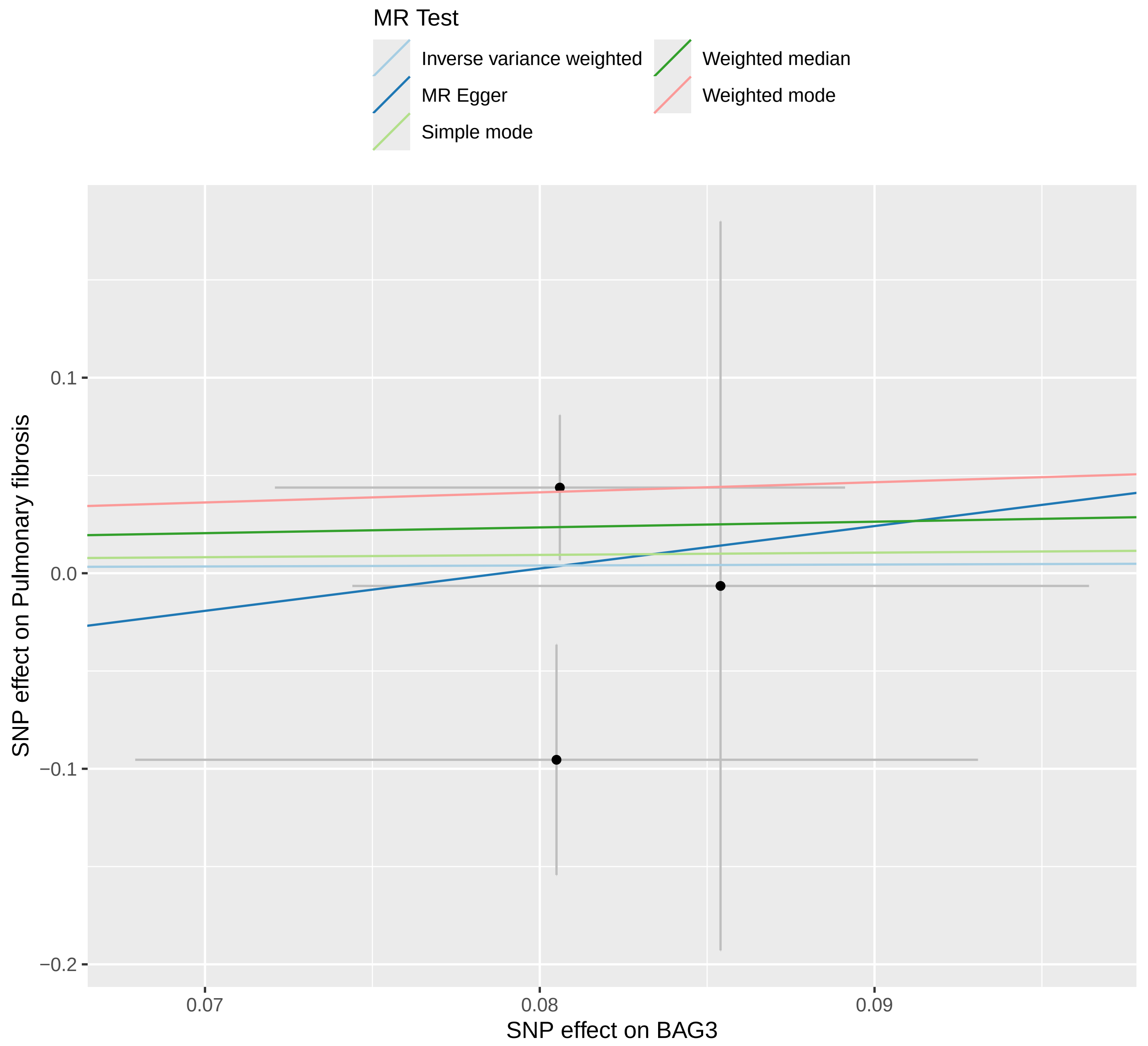
MR Test
Inverse variance weighted
MR Egger
Simple mode
Weighted median
Weighted mode
0.1
0.0
−0.1
−0.2
0.07
0.08
0.09
SNP effect on Pulmonary fibrosis
SNP effect on BAG3

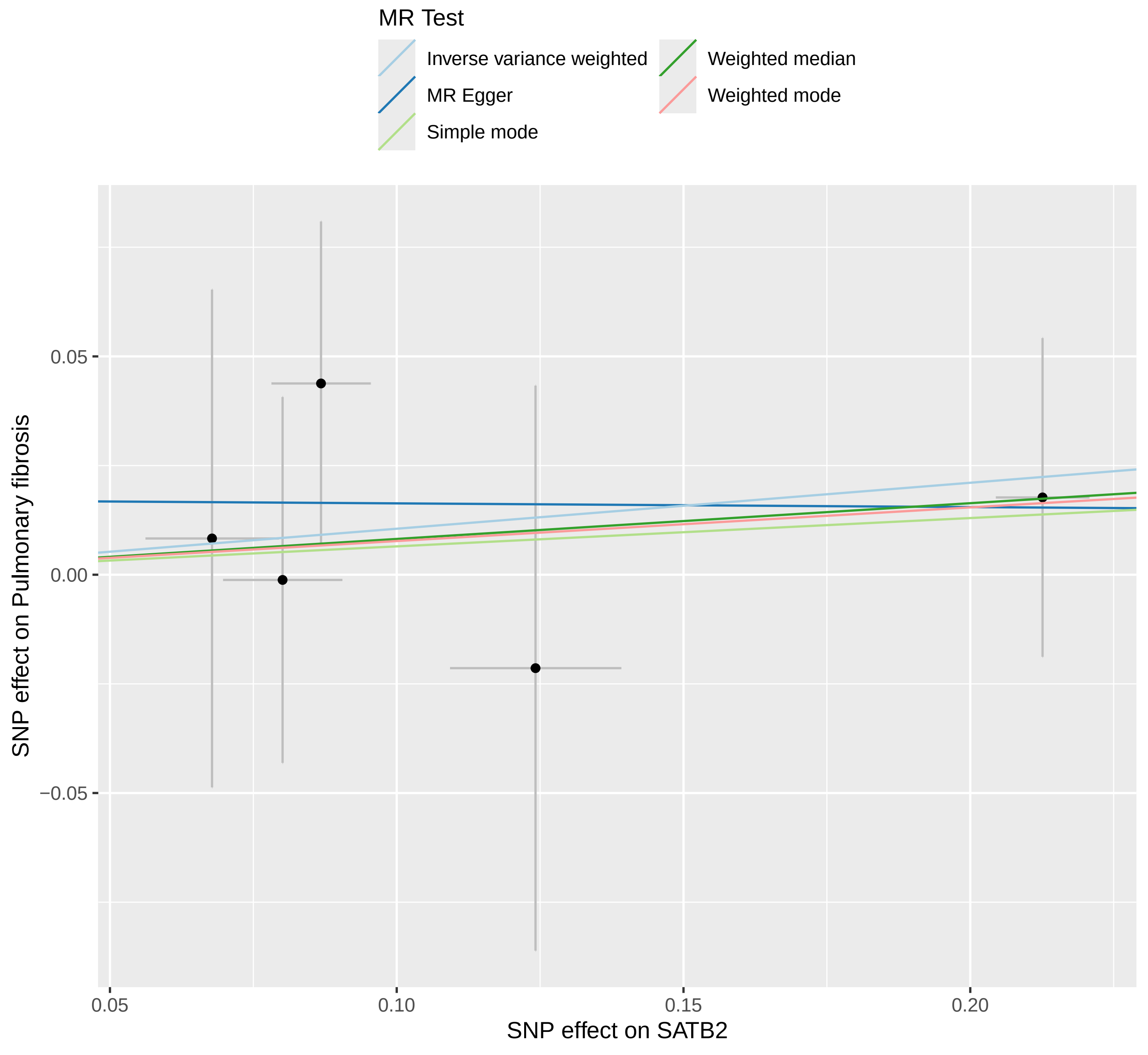
MR Test
Inverse variance weighted
MR Egger
Simple mode
Weighted median
Weighted mode
0.05
0.00
−0.05
SNP effect on Pulmonary fibrosis
0.05
0.10
0.15
0.20
SNP effect on SATB2

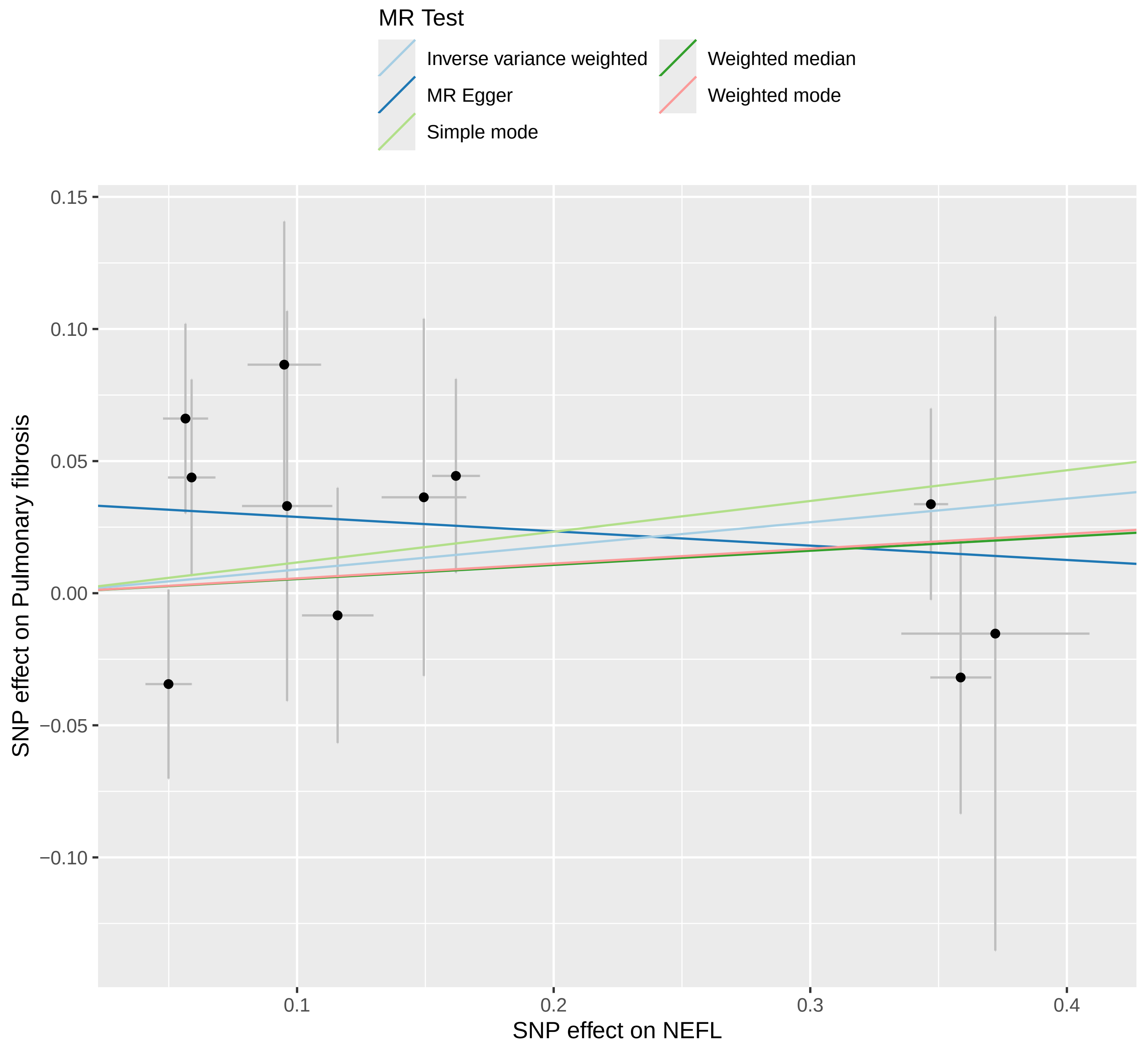
MR Test
Inverse variance weighted
MR Egger
Simple mode
Weighted median
Weighted mode
0.15
0.10
0.05
0.00
−0.05
−0.10
0.1
0.2
0.3
0.4
SNP effect on NEFL
SNP effect on Pulmonary fibrosis

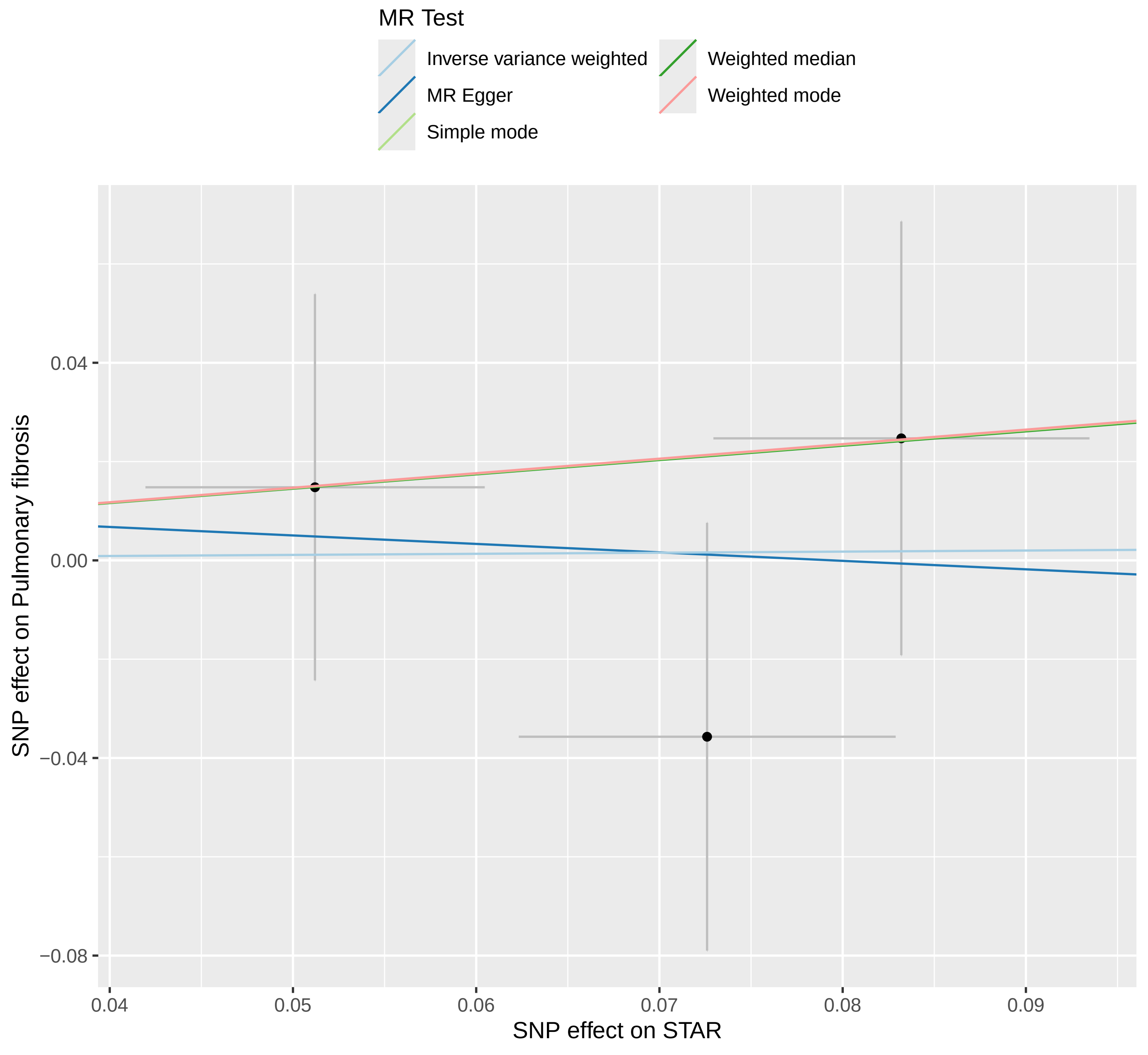
MR Test
Inverse variance weighted
MR Egger
Simple mode
Weighted median
Weighted mode
SNP effect on Pulmonary fibrosis
0.04
0.00
−0.04
−0.08
0.04
0.05
0.06
0.07
0.08
0.09
SNP effect on STAR

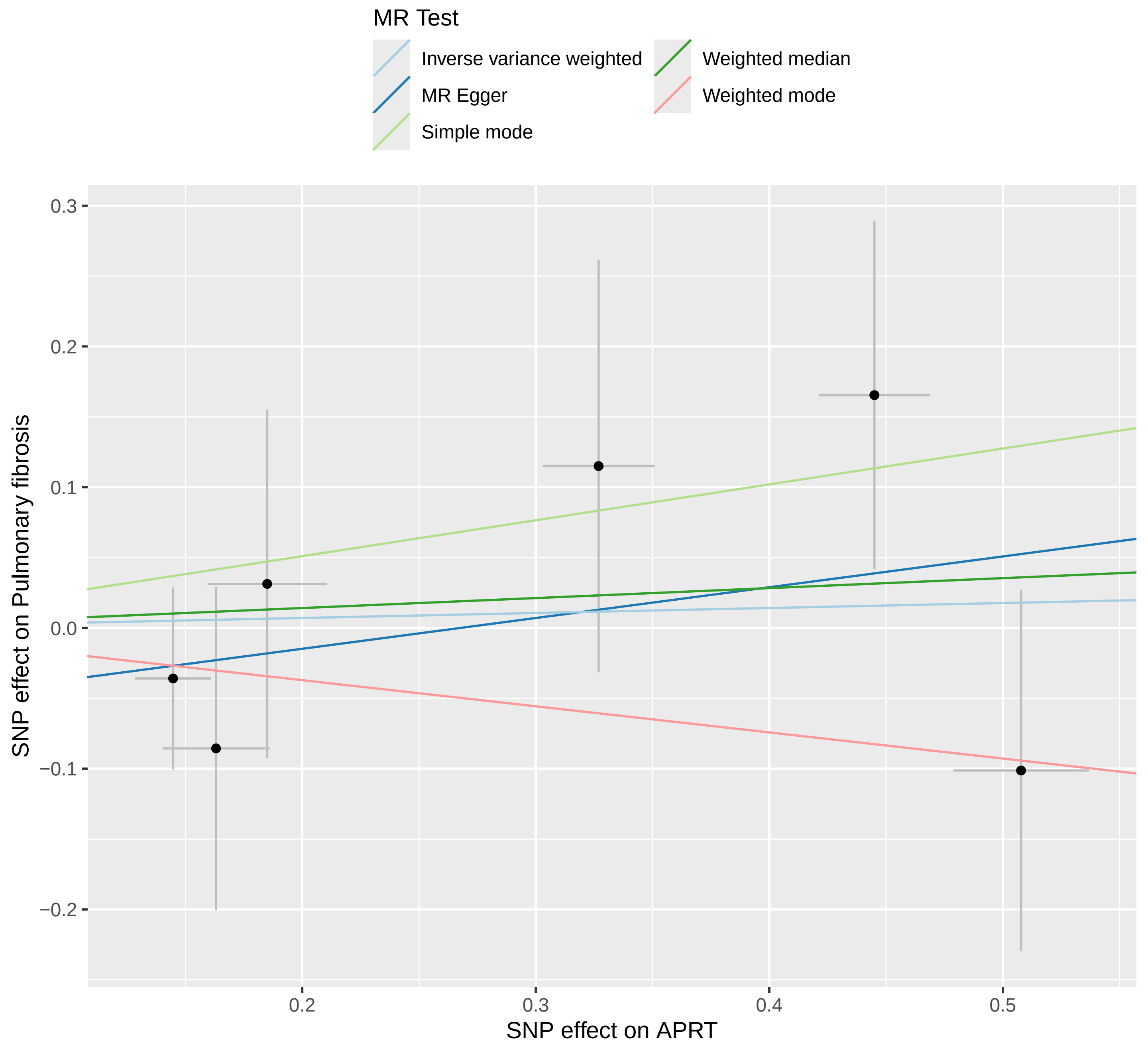
MR Test
Inverse variance weighted
MR Egger
Simple mode
Weighted median
Weighted mode
SNP effect on Pulmonary fibrosis
0.3
0.2
0.1
0.0
−0.1
−0.2
0.2
0.3
0.4
0.5
SNP effect on APRT

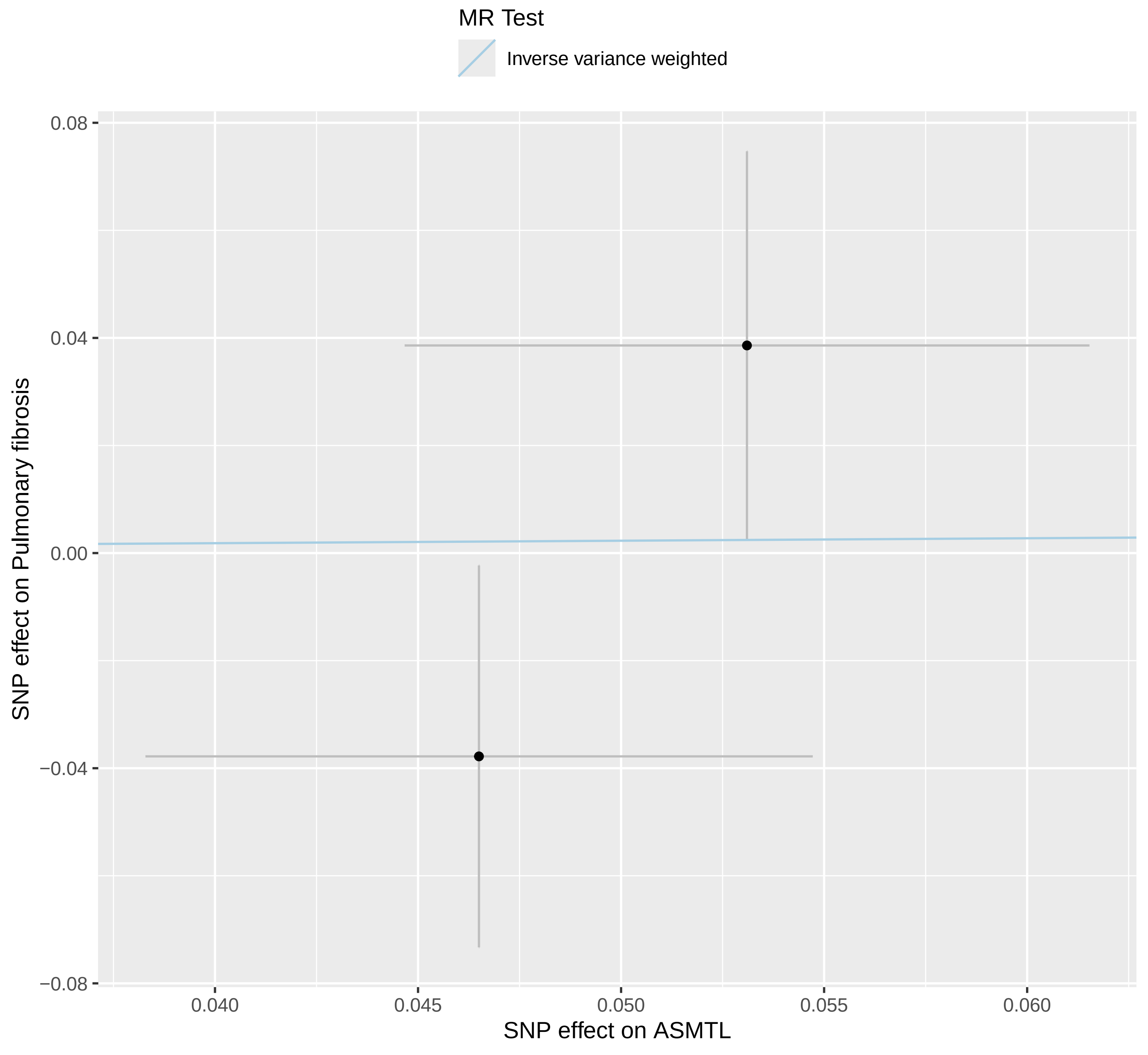
MR Test
Inverse variance weighted
0.08
0.04
0.00
−0.04
−0.08
0.040
0.045
0.050
0.055
0.060
SNP effect on Pulmonary fibrosis
SNP effect on ASMTL

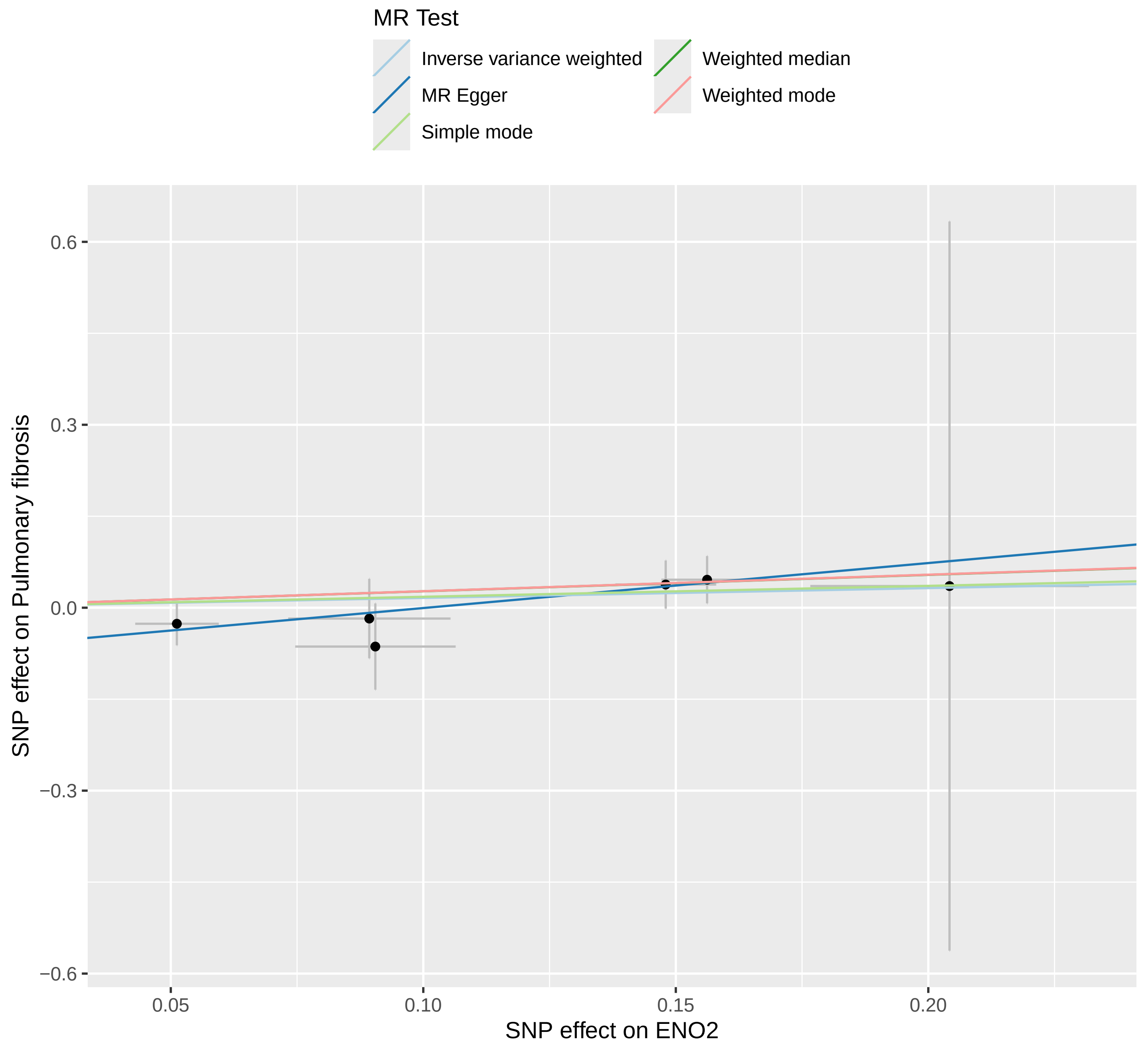
MR Test
Inverse variance weighted
MR Egger
Simple mode
Weighted median
Weighted mode
0.6
0.3
0.0
−0.3
−0.6
SNP effect on Pulmonary fibrosis
0.05
0.10
0.15
0.20
SNP effect on ENO2

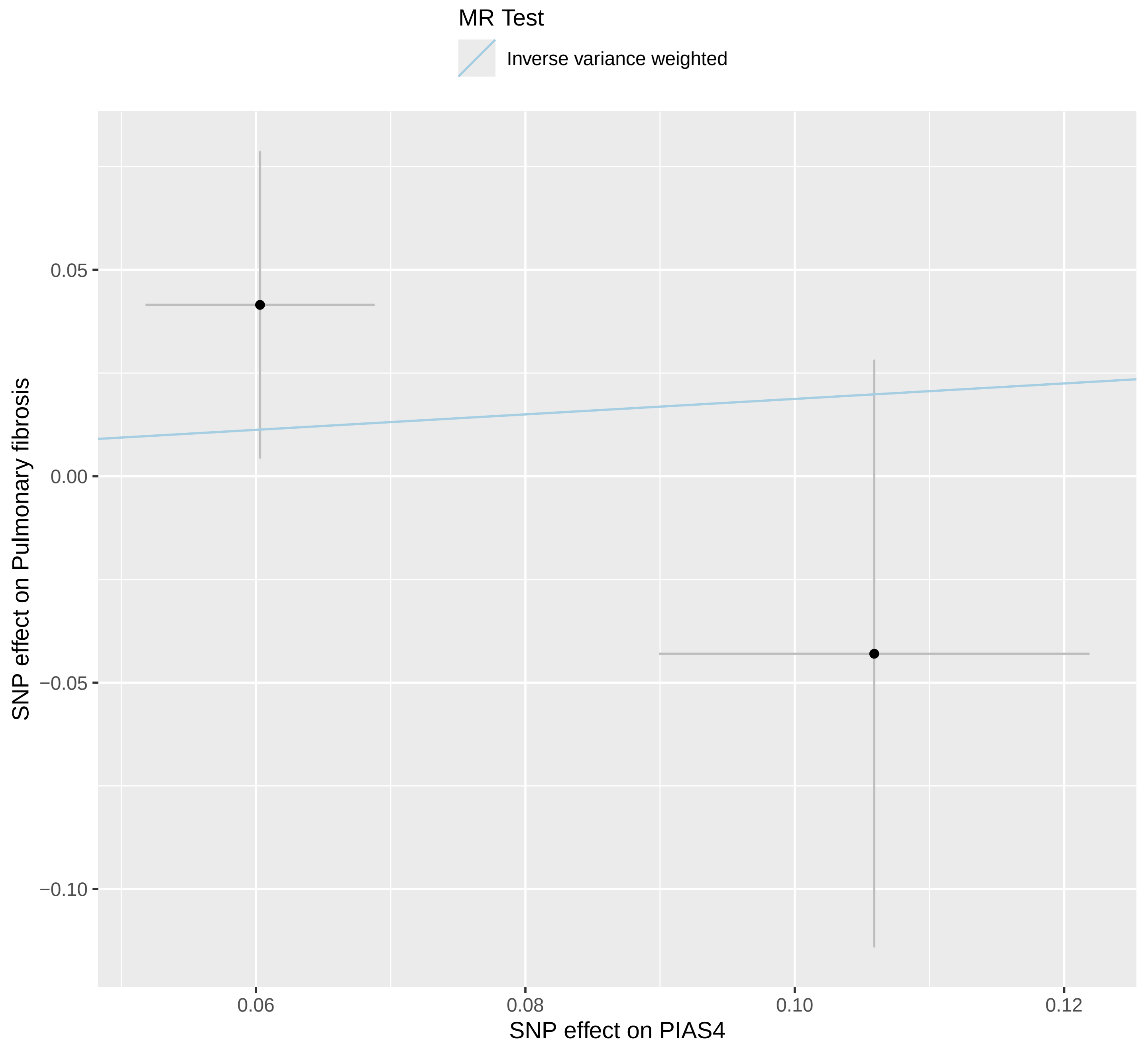
MR Test
Inverse variance weighted
0.05
0.00
−0.05
−0.10
0.06
0.08
0.10
0.12
SNP effect on Pulmonary fibrosis
SNP effect on PIAS4

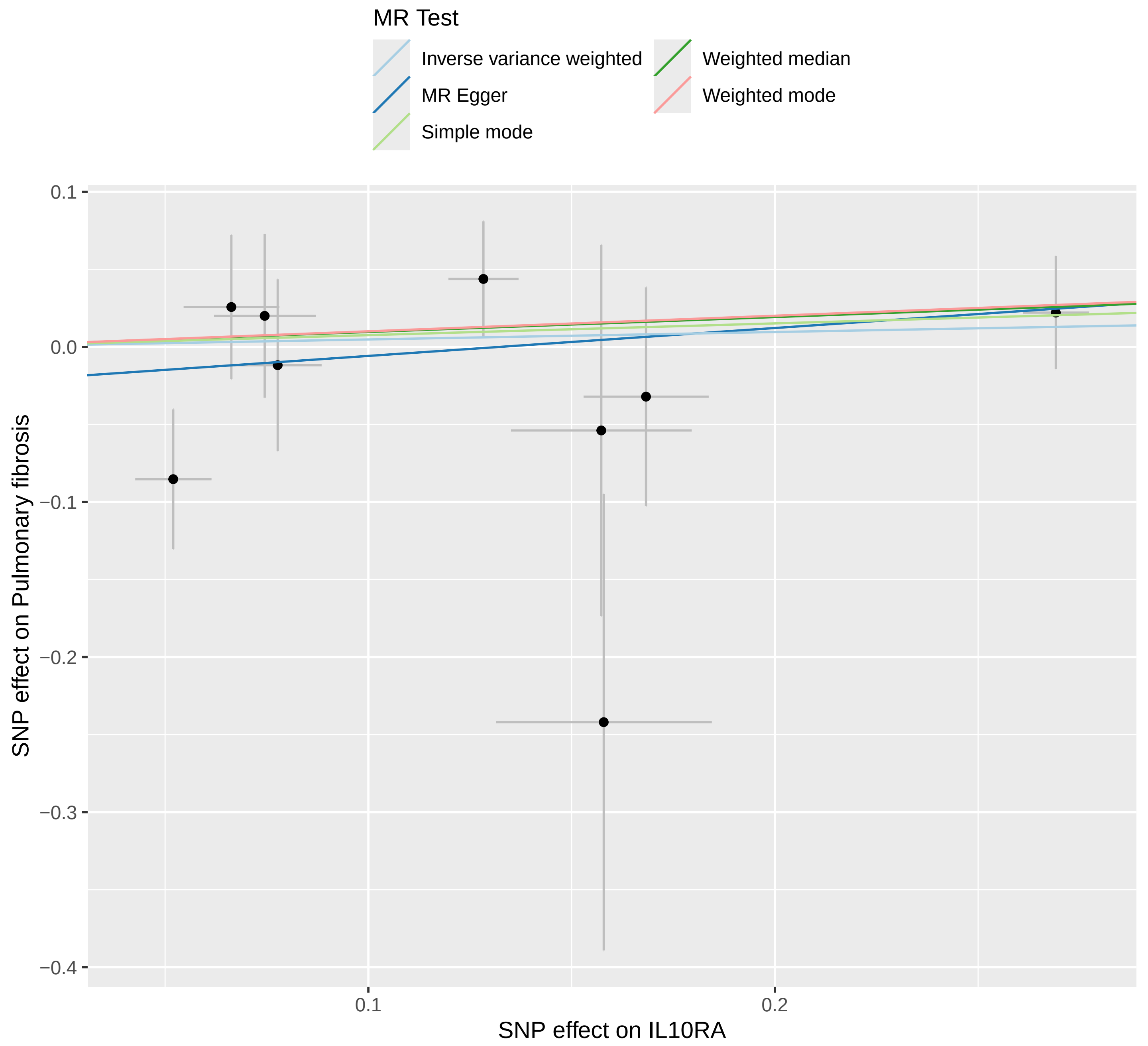
MR Test
Inverse variance weighted
MR Egger
Simple mode
Weighted median
Weighted mode
0.1
0.0
−0.1
−0.2
−0.3
−0.4
SNP effect on Pulmonary fibrosis
0.1
0.2
SNP effect on IL10RA

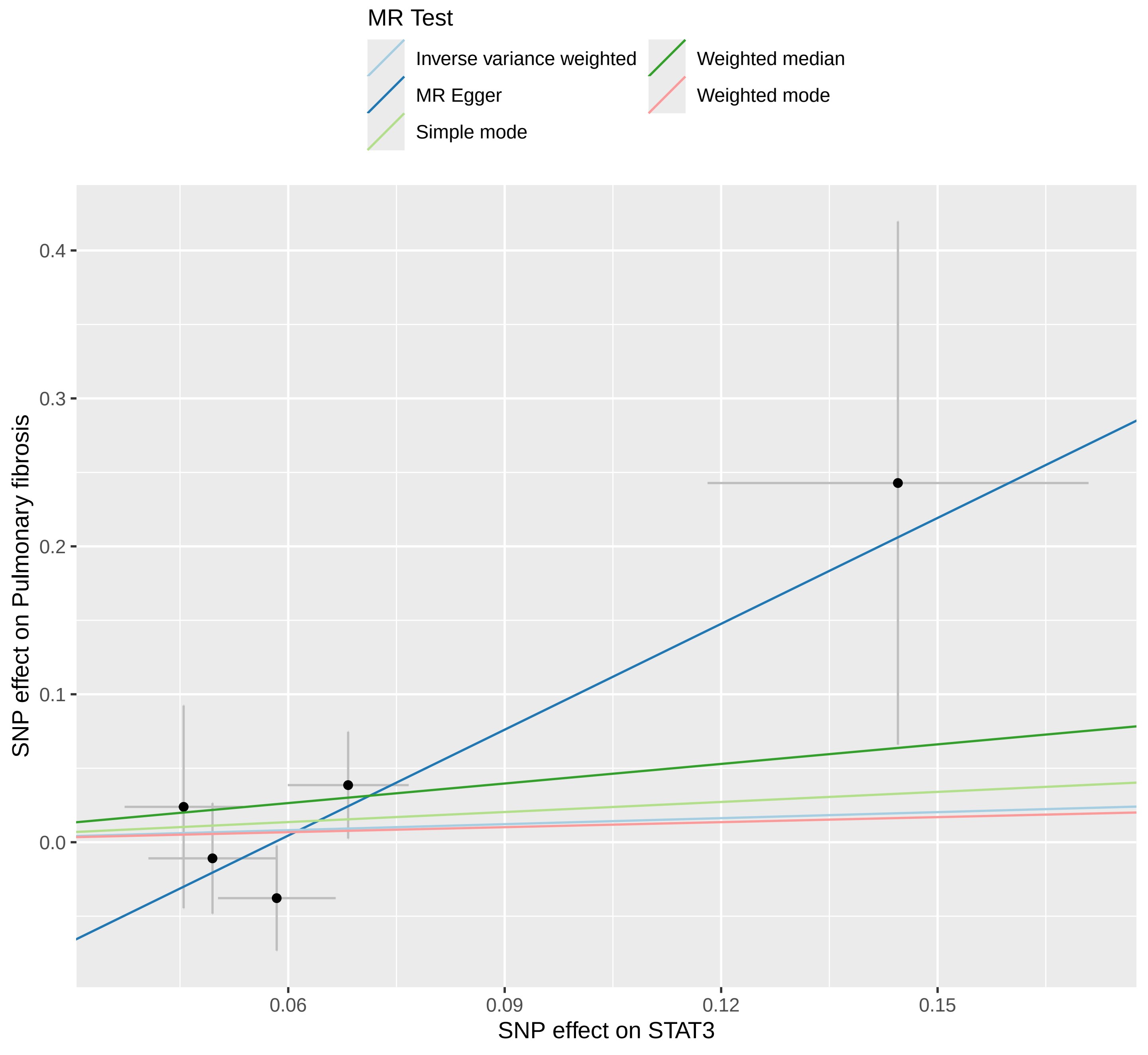
MR Test
Inverse variance weighted
MR Egger
Simple mode
Weighted median
Weighted mode
0.4
0.3
0.2
0.1
0.0
SNP effect on Pulmonary fibrosis
0.06
0.09
0.12
0.15
SNP effect on STAT3

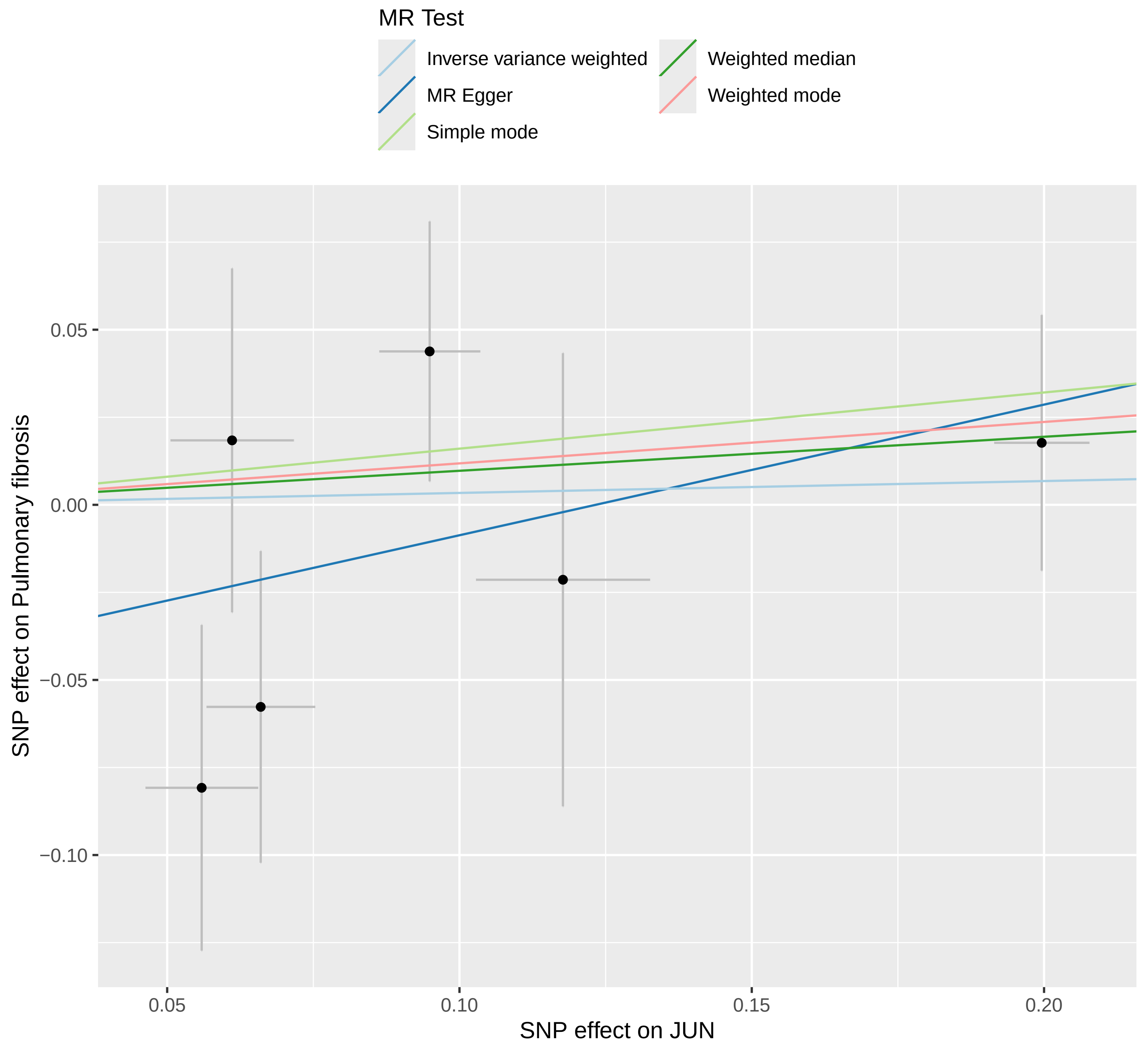
MR Test
Inverse variance weighted
MR Egger
Simple mode
Weighted median
Weighted mode
0.05
0.00
−0.05
−0.10
0.05
0.10
0.15
0.20
SNP effect on JUN
SNP effect on Pulmonary fibrosis

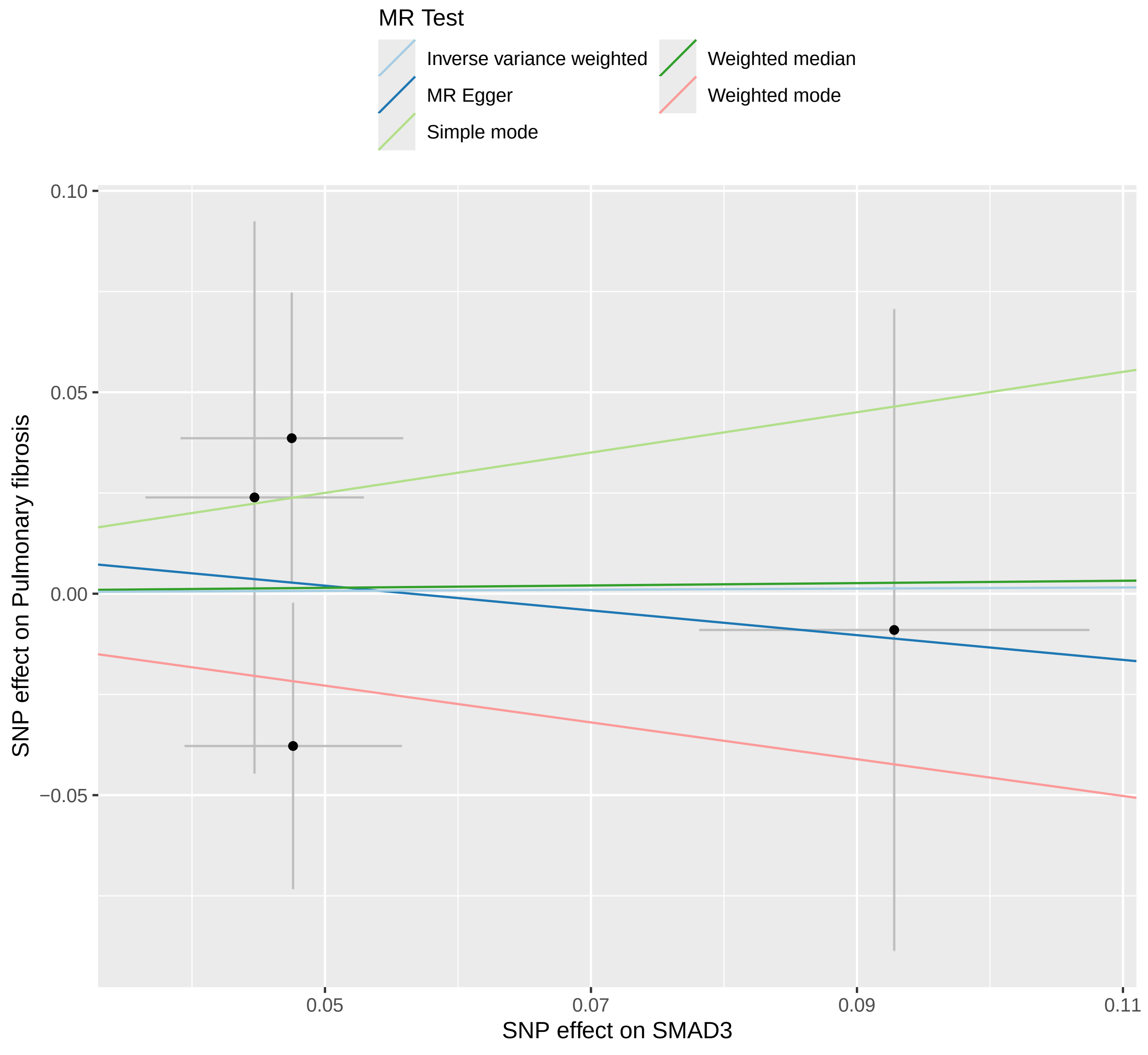
MR Test
Inverse variance weighted
MR Egger
Simple mode
Weighted median
Weighted mode
0.10
0.05
0.00
−0.05
SNP effect on Pulmonary fibrosis
0.05
0.07
0.09
0.11
SNP effect on SMAD3

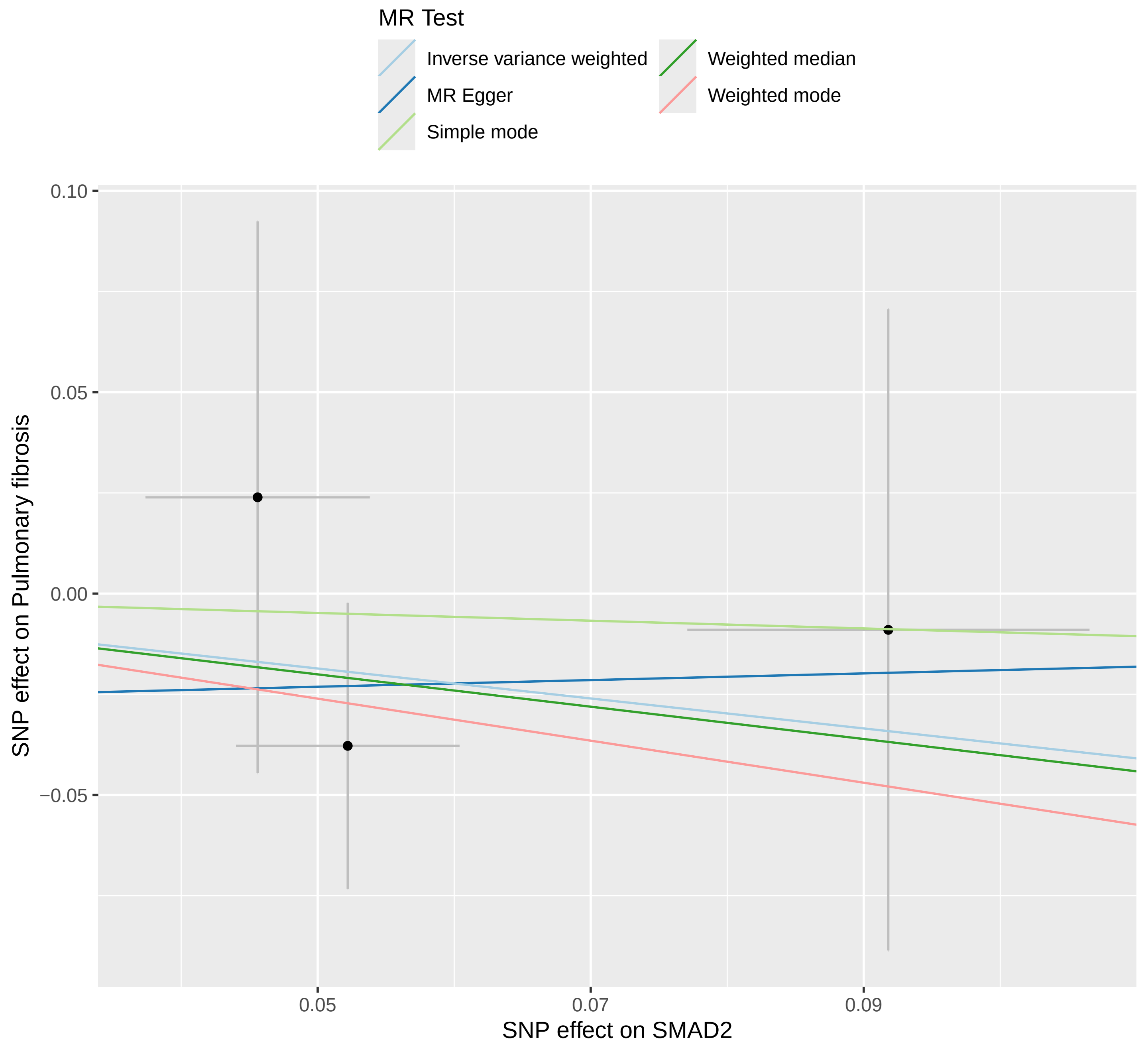
MR Test
Inverse variance weighted
MR Egger
Simple mode
Weighted median
Weighted mode
0.10
0.05
0.00
−0.05
SNP effect on Pulmonary fibrosis
0.05
0.07
0.09
SNP effect on SMAD2

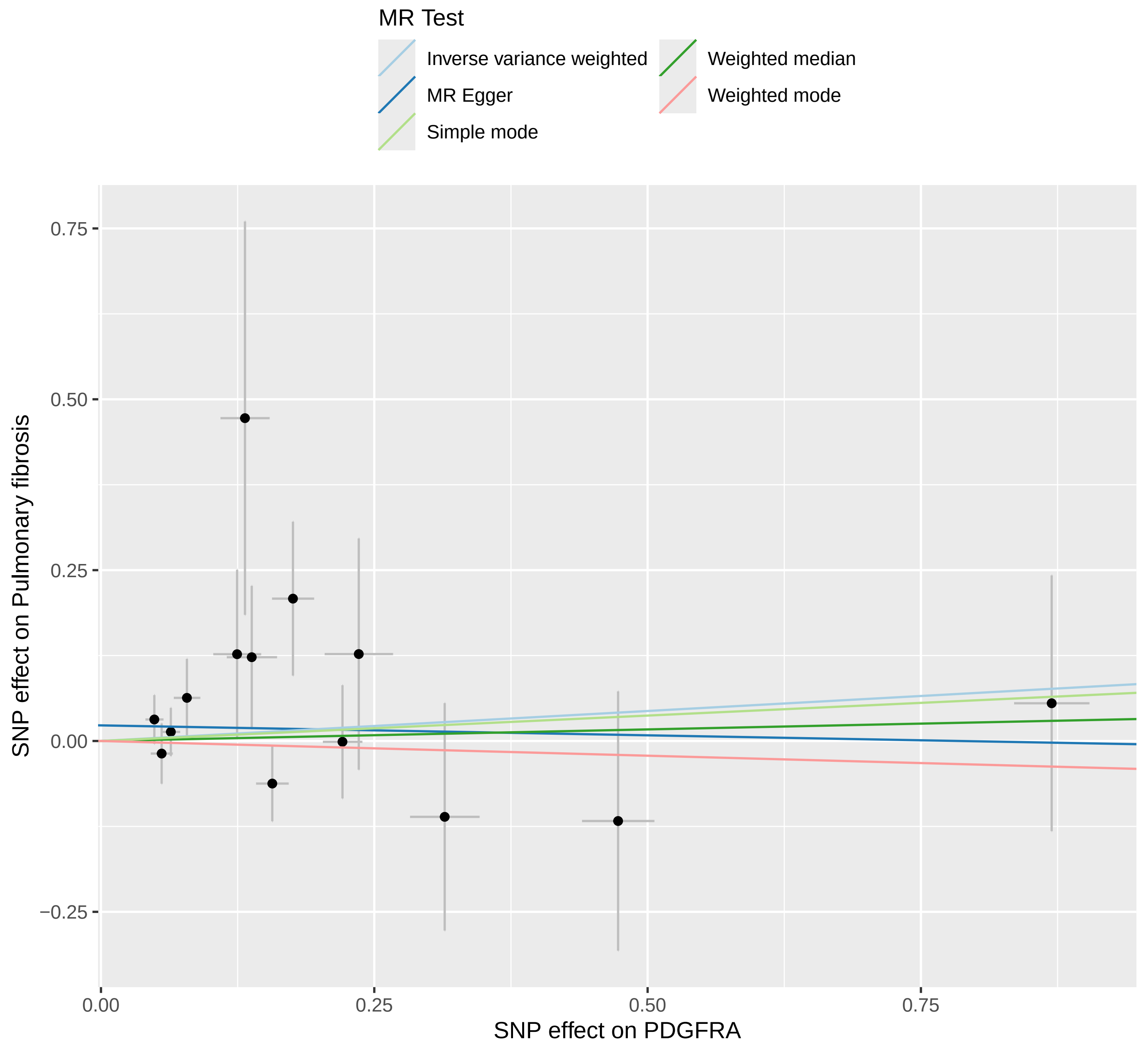

MR Test
Inverse variance weighted
MR Egger
Simple mode
Weighted median
Weighted mode
0.75
0.50
0.25
0.00
−0.25
SNP effect on Pulmonary fibrosis
0.00
0.25
0.50
0.75
SNP effect on PDGFRA

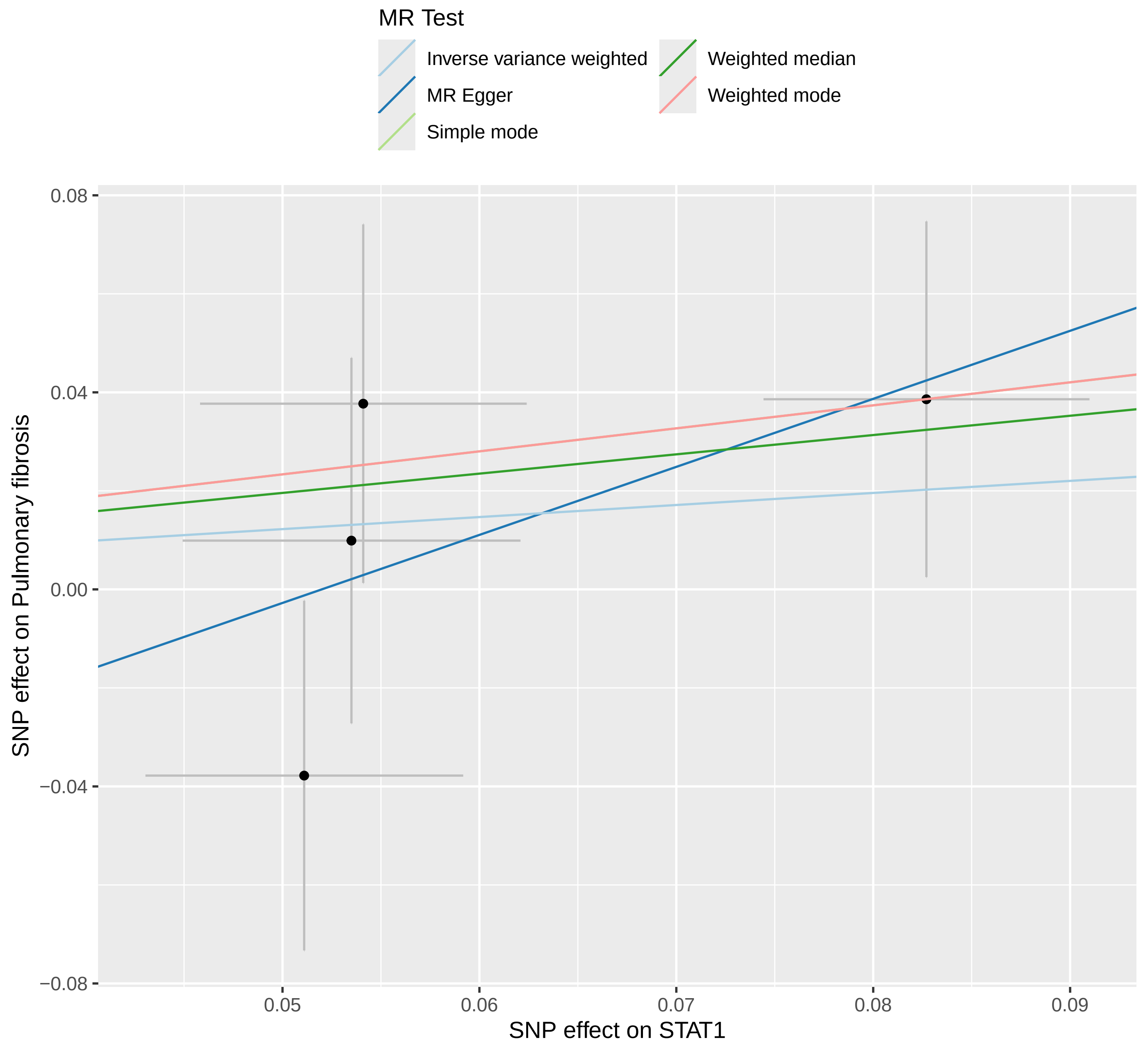

MR Test
Inverse variance weighted
MR Egger
Simple mode
Weighted median
Weighted mode
0.08
0.04
0.00
−0.04
−0.08
SNP effect on Pulmonary fibrosis
0.05
0.06
0.07
0.08
0.09
SNP effect on STAT1

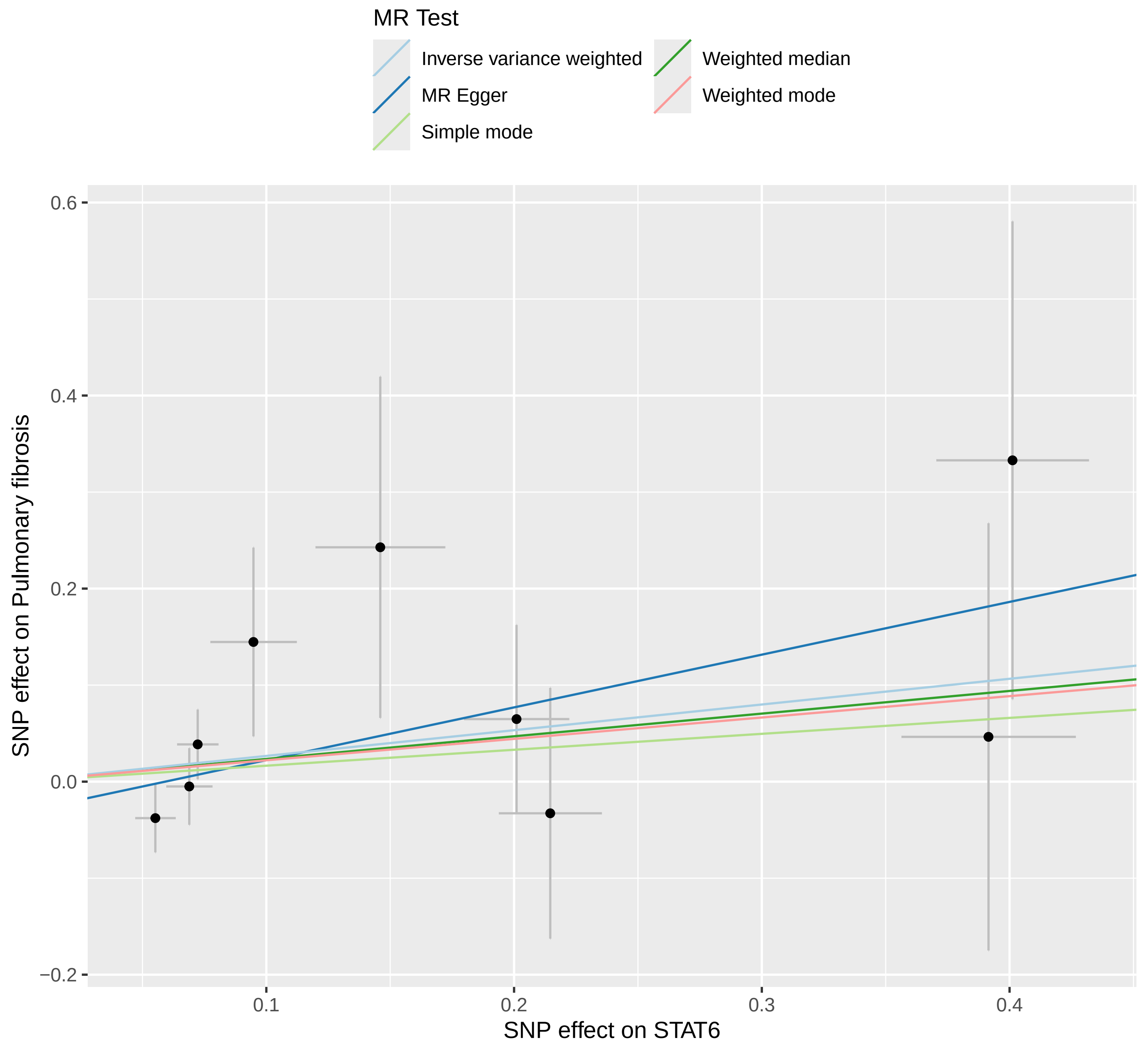
MR Test
Inverse variance weighted
MR Egger
Simple mode
Weighted median
Weighted mode
SNP effect on Pulmonary fibrosis
0.6
0.4
0.2
0.0
−0.2
0.1
0.2
0.3
0.4
SNP effect on STAT6

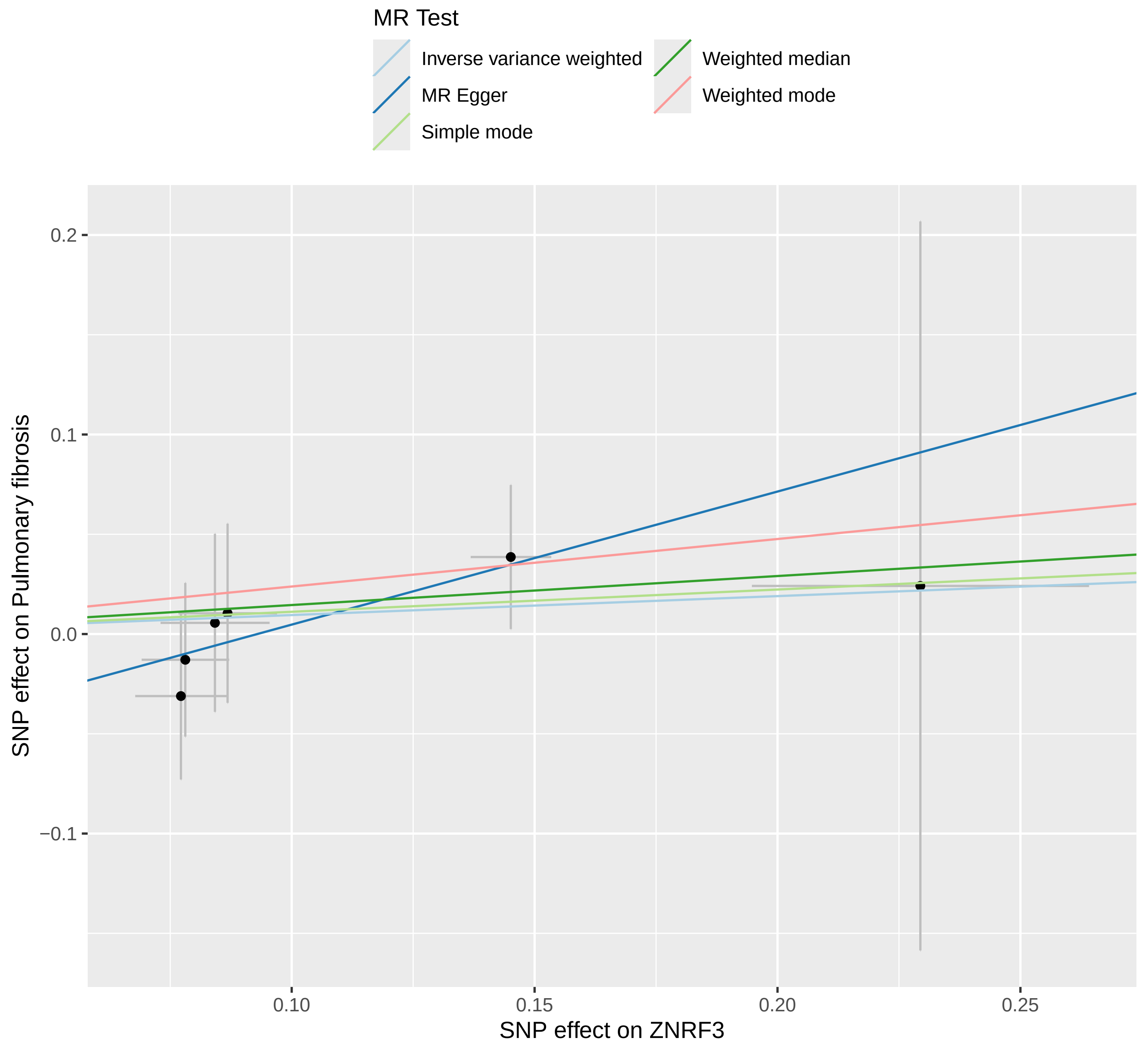
MR Test
Inverse variance weighted
MR Egger
Simple mode
Weighted median
Weighted mode
0.2
0.1
0.0
−0.1
0.10
0.15
0.20
0.25
SNP effect on Pulmonary fibrosis
SNP effect on ZNRF3

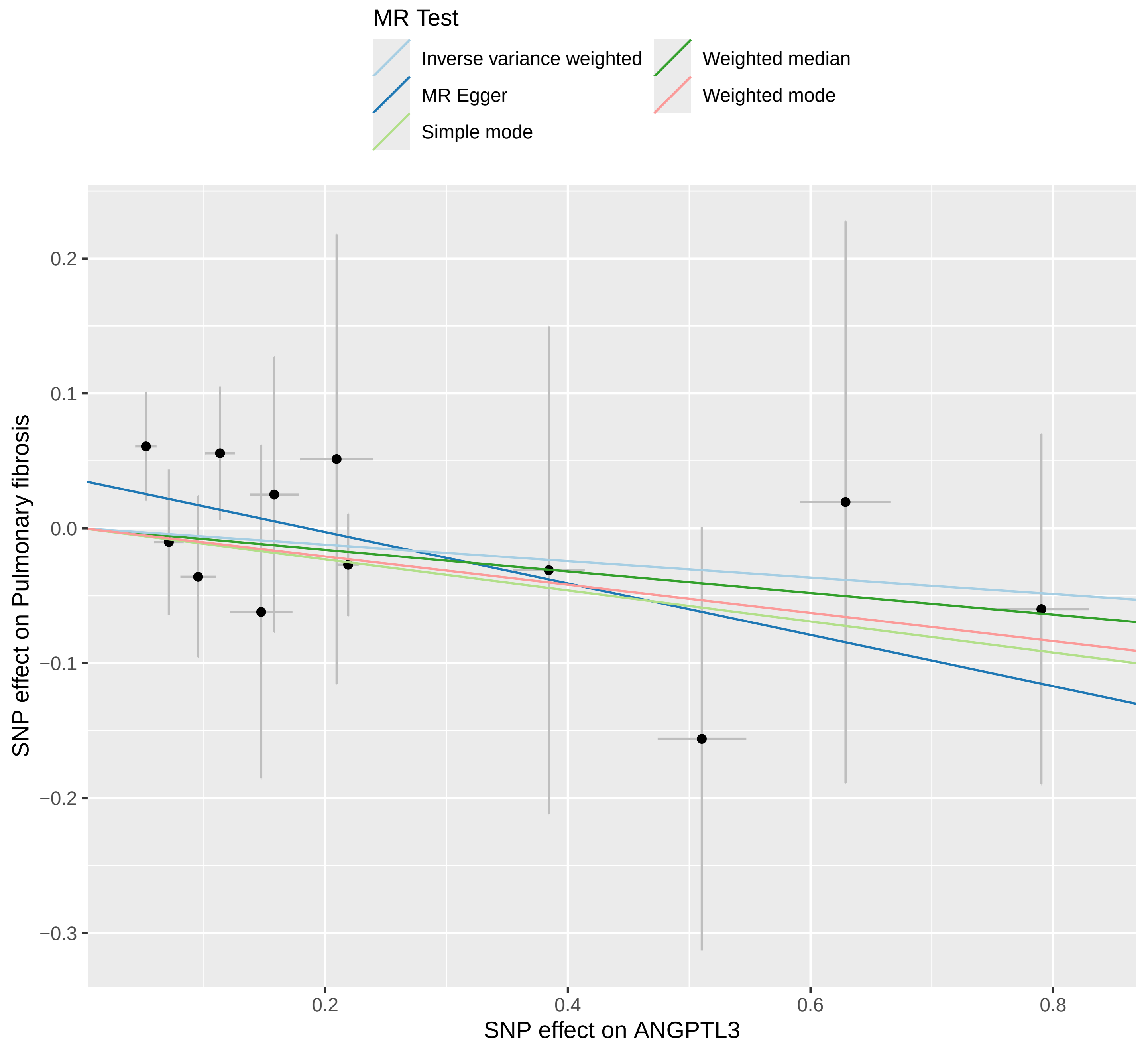
MR Test
Inverse variance weighted
MR Egger
Simple mode
Weighted median
Weighted mode
SNP effect on Pulmonary fibrosis
0.2
0.1
0.0
−0.1
−0.2
−0.3
0.2
0.4
0.6
0.8
SNP effect on ANGPTL3

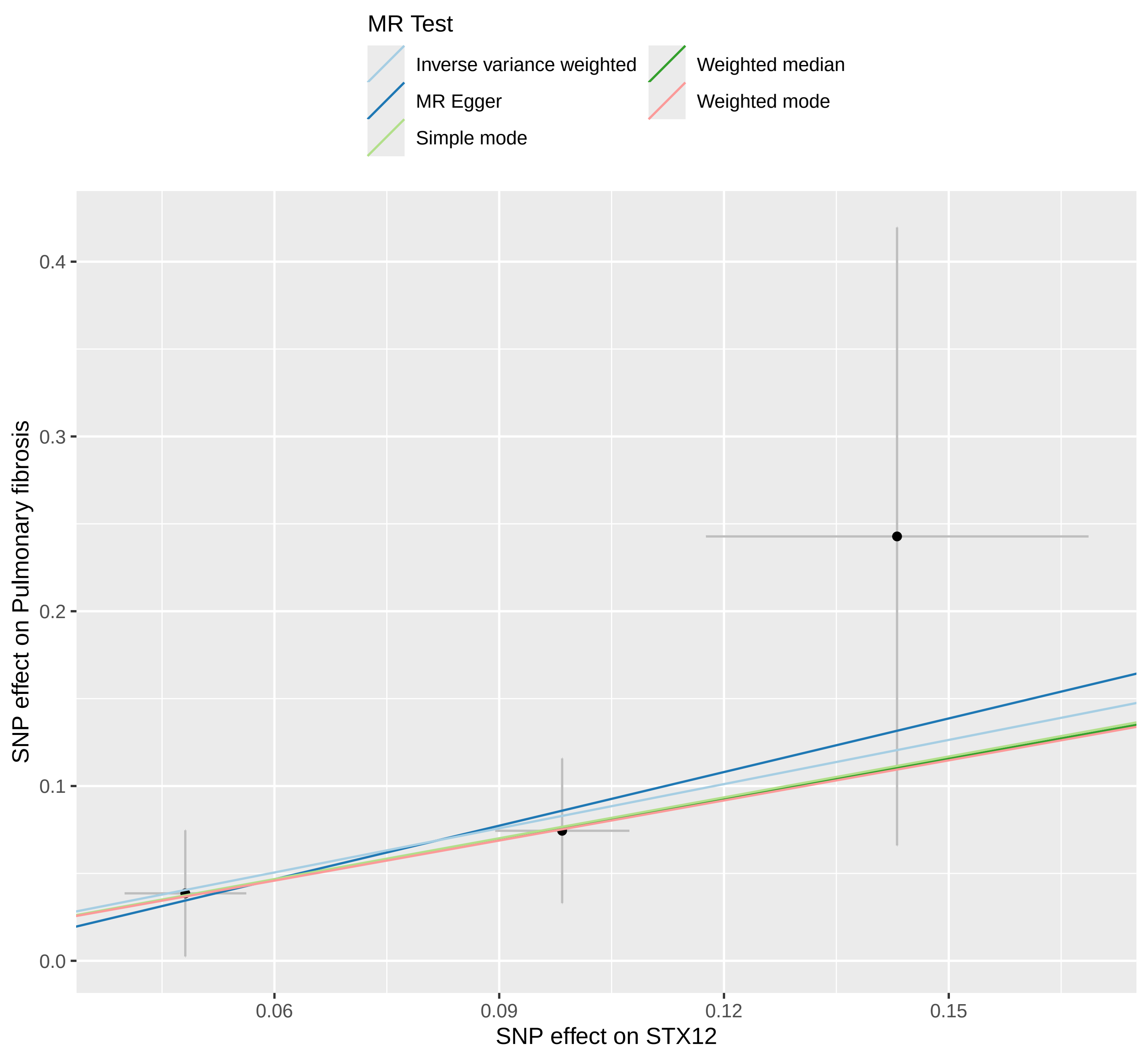
MR Test
Inverse variance weighted
MR Egger
Simple mode
Weighted median
Weighted mode
0.0
0.1
0.2
0.3
0.4
SNP effect on Pulmonary fibrosis
0.06
0.09
0.12
0.15
SNP effect on STX12

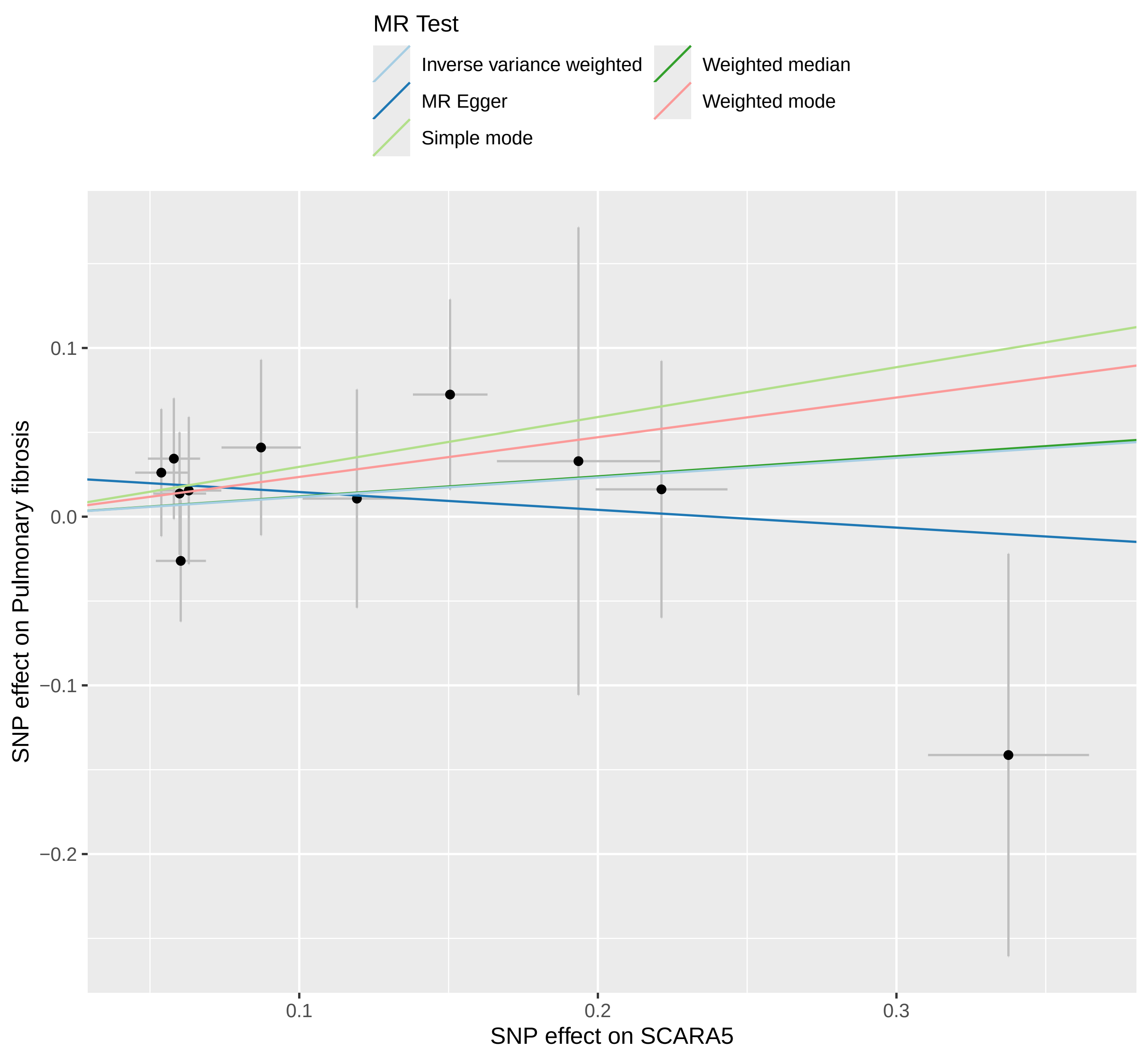
MR Test
Inverse variance weighted
MR Egger
Simple mode
Weighted median
Weighted mode
0.1
0.0
−0.1
−0.2
SNP effect on Pulmonary fibrosis
0.1
0.2
0.3
SNP effect on SCARA5

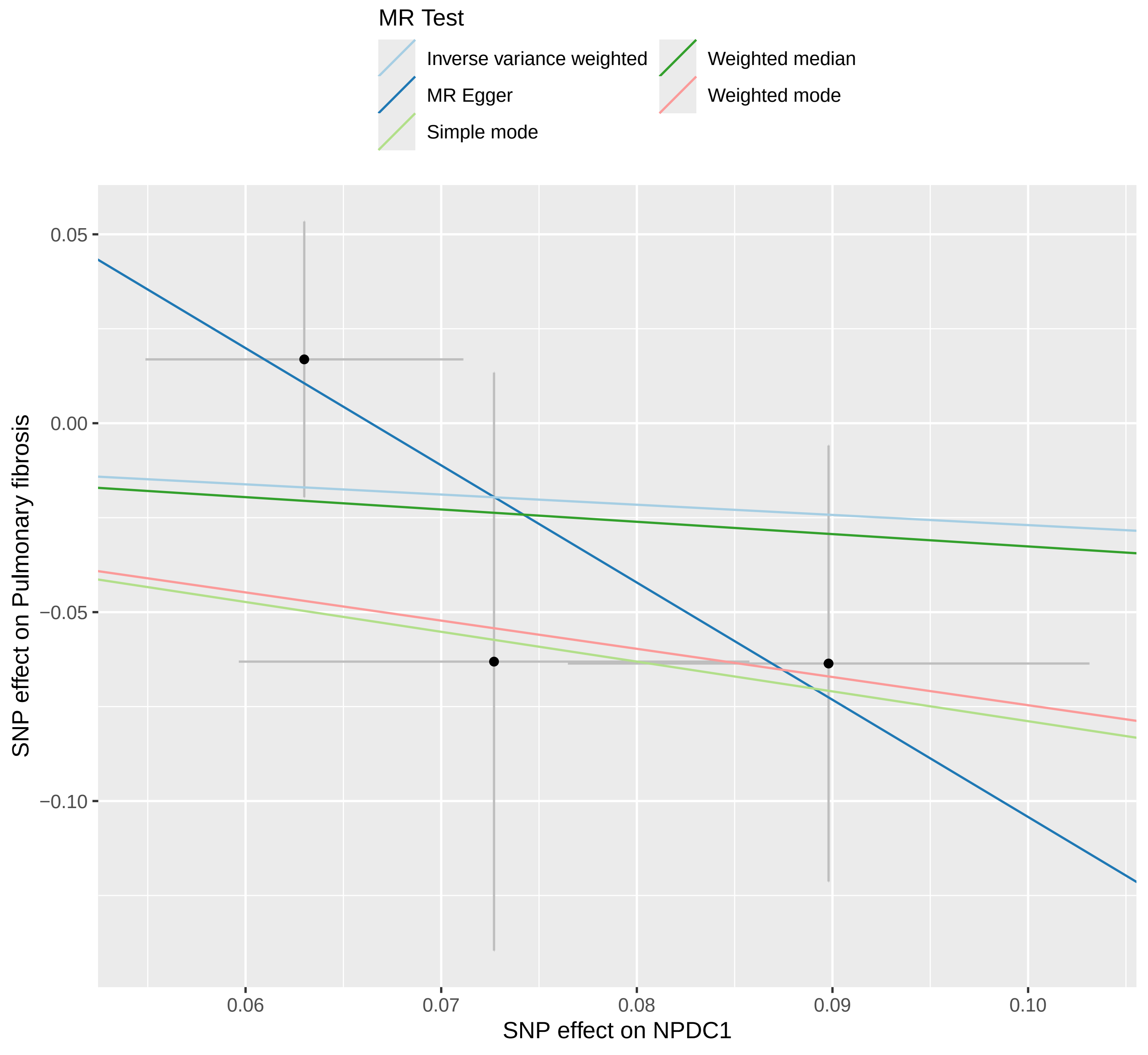
MR Test
Inverse variance weighted
MR Egger
Simple mode
Weighted median
Weighted mode
0.05
0.00
−0.05
−0.10
SNP effect on Pulmonary fibrosis
0.06
0.07
0.08
0.09
0.10
SNP effect on NPDC1

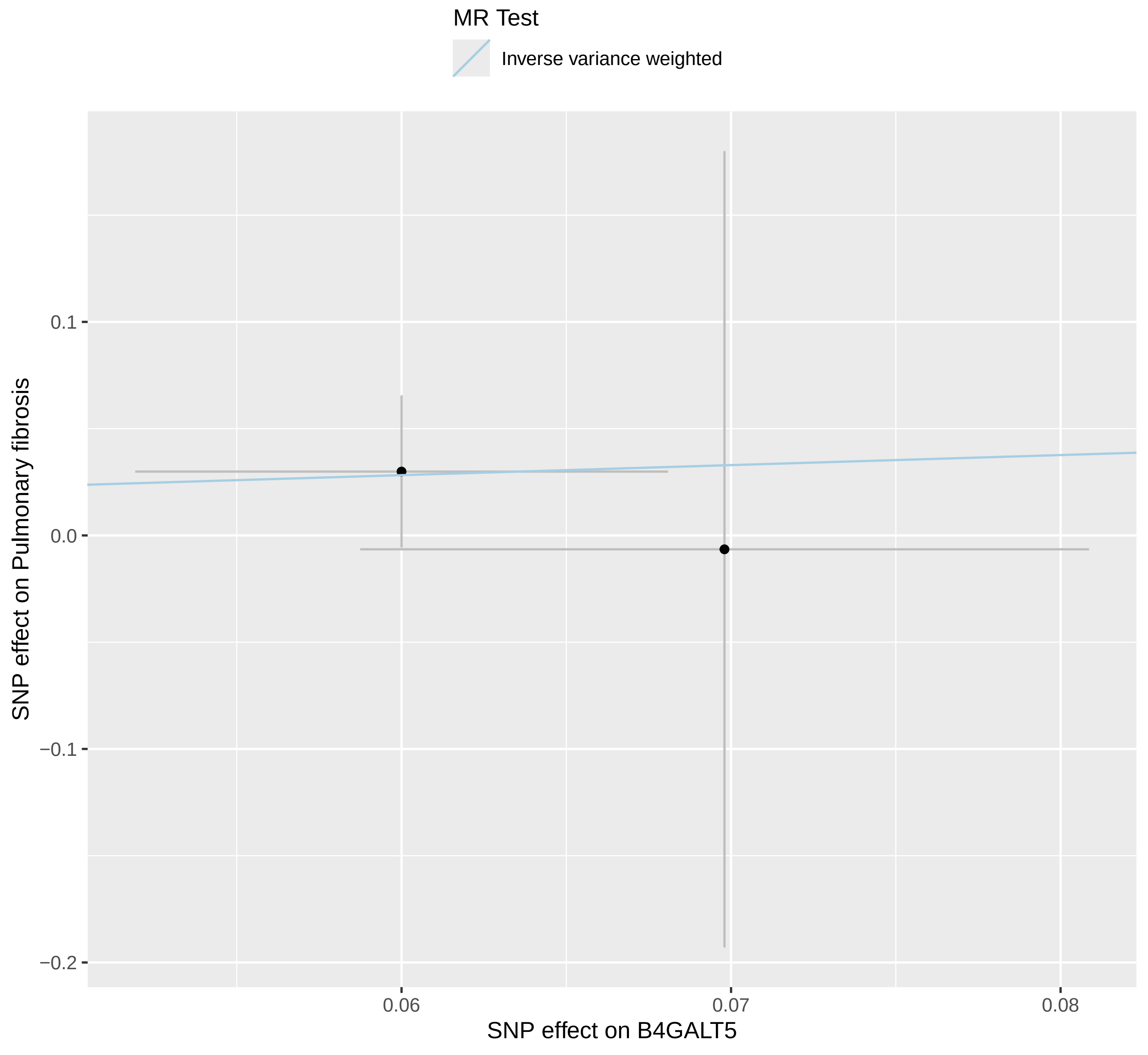
MR Test
Inverse variance weighted
0.1
0.0
−0.1
−0.2
SNP effect on Pulmonary fibrosis
0.06
0.07
0.08
SNP effect on B4GALT5

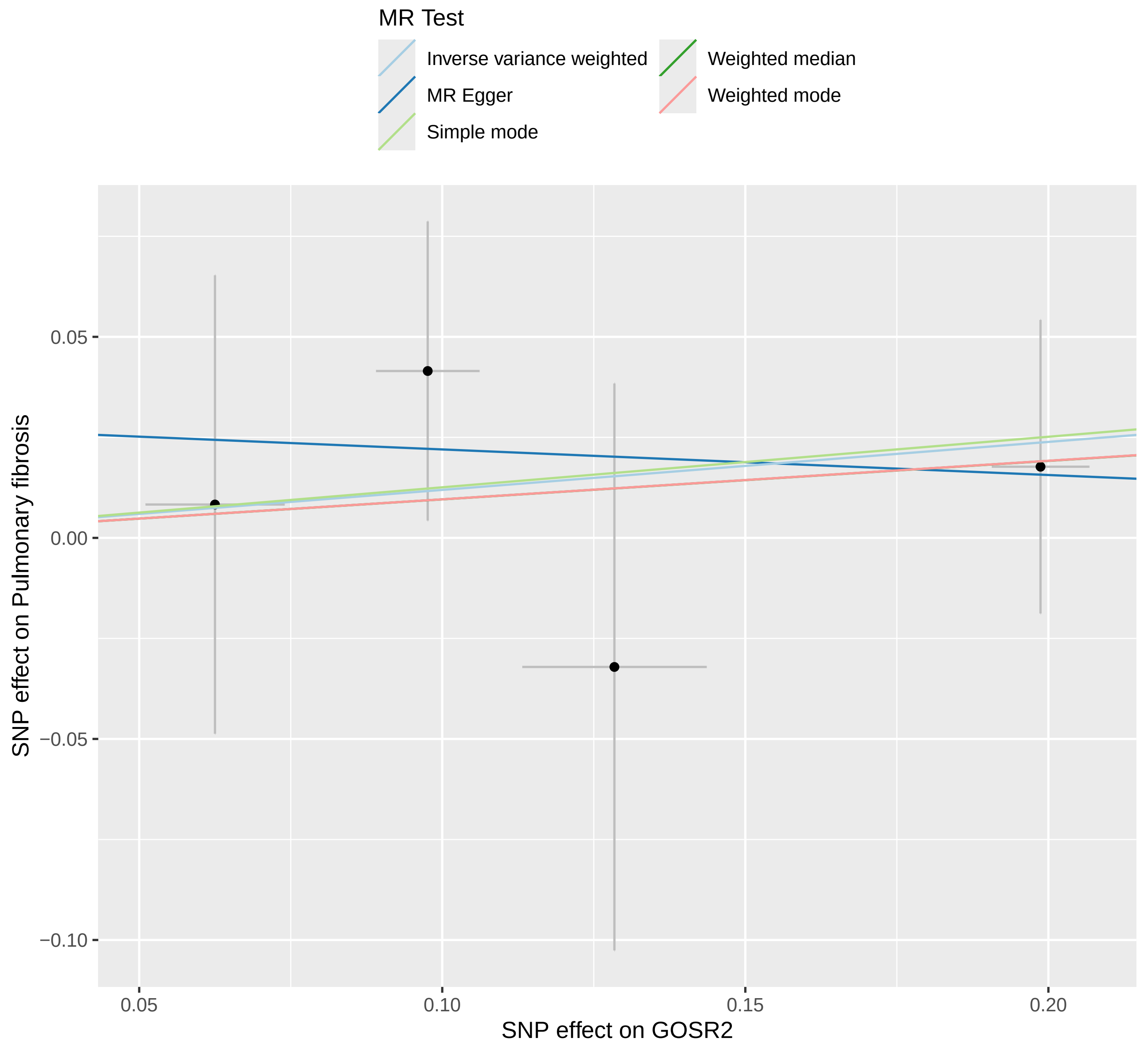
MR Test
Inverse variance weighted
MR Egger
Simple mode
Weighted median
Weighted mode
0.05
0.00
−0.05
−0.10
0.05
0.10
0.15
0.20
SNP effect on Pulmonary fibrosis
SNP effect on GOSR2

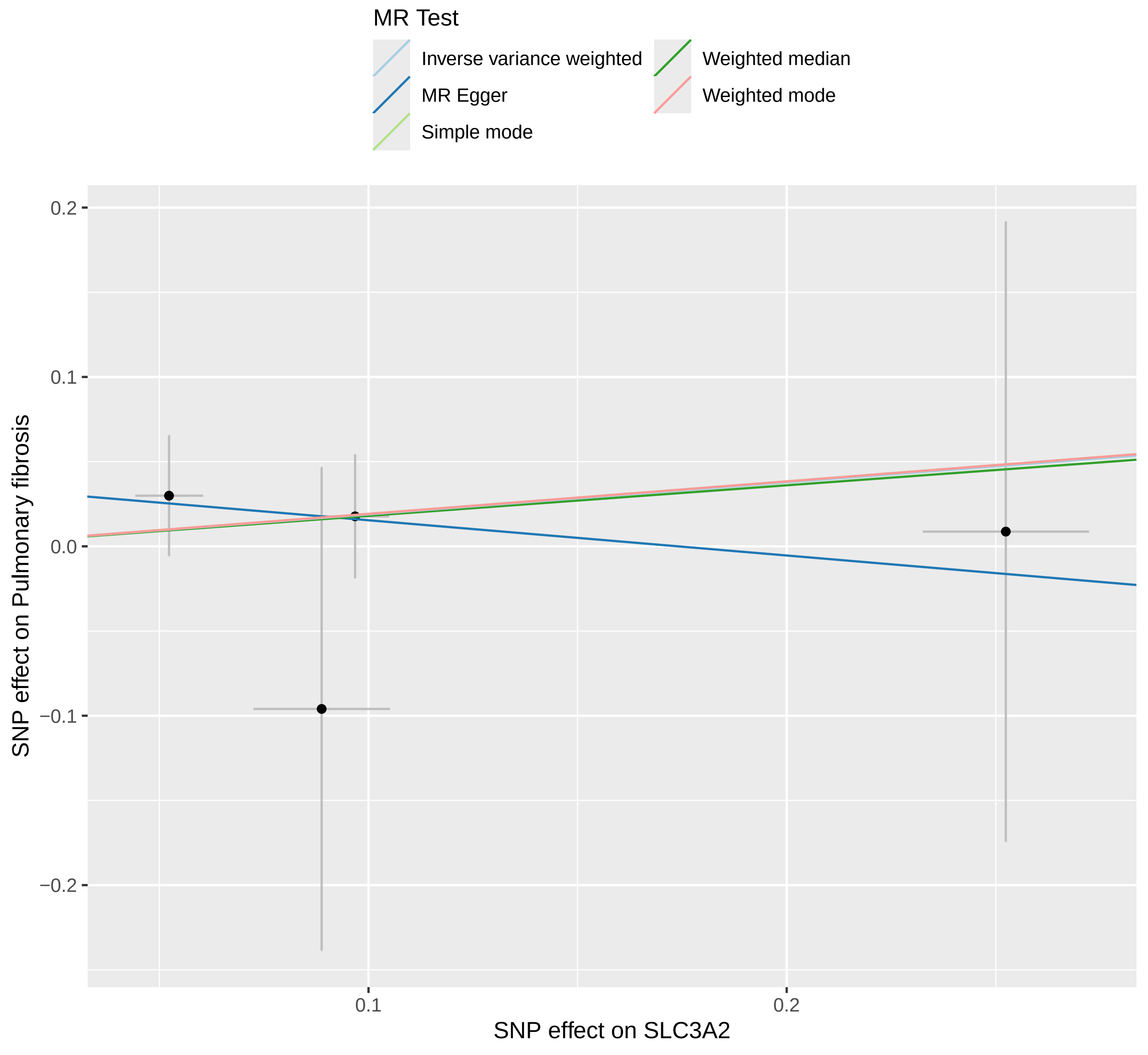
MR Test
Inverse variance weighted
MR Egger
Simple mode
Weighted median
Weighted mode
0.2
0.1
0.0
−0.1
−0.2
0.1
0.2
SNP effect on Pulmonary fibrosis
SNP effect on SLC3A2

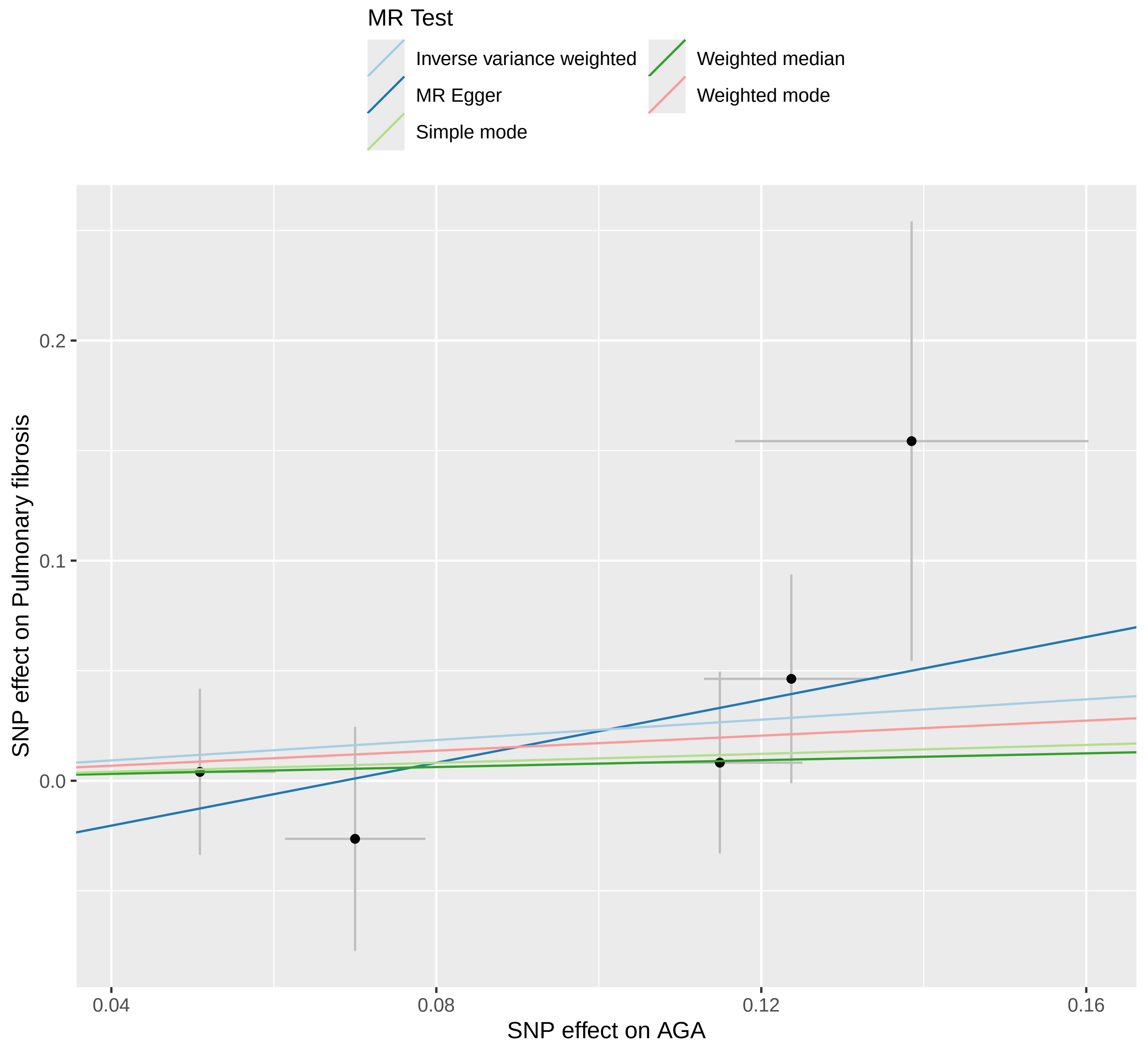
MR Test
Inverse variance weighted
MR Egger
Simple mode
Weighted median
Weighted mode
0.2
0.1
0.0
0.04
0.08
0.12
0.16
SNP effect on Pulmonary fibrosis
SNP effect on AGA

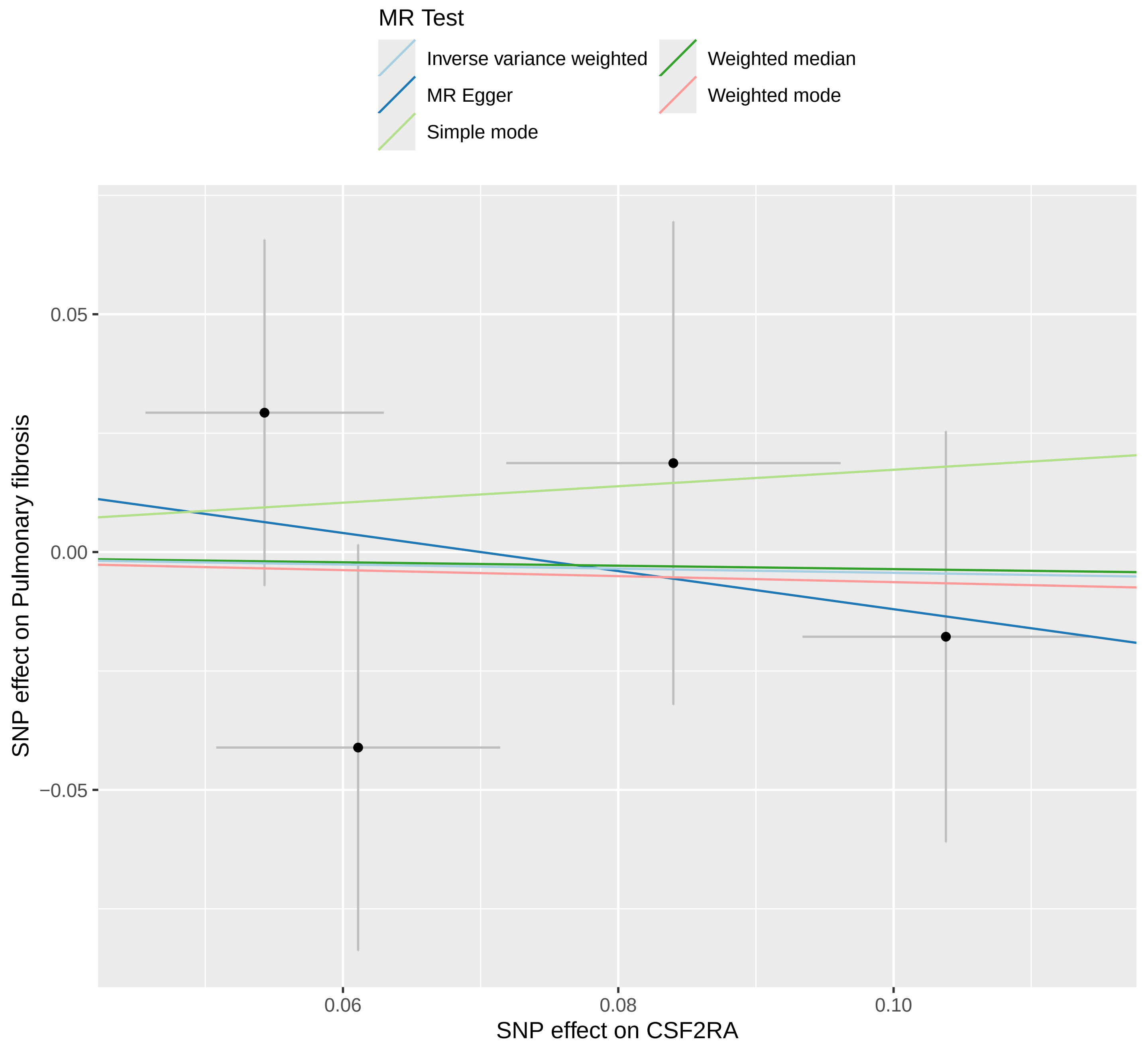

MR Test
Inverse variance weighted
MR Egger
Simple mode
Weighted median
Weighted mode
0.05
0.00
−0.05
0.06
0.08
0.10
SNP effect on Pulmonary fibrosis
SNP effect on CSF2RA

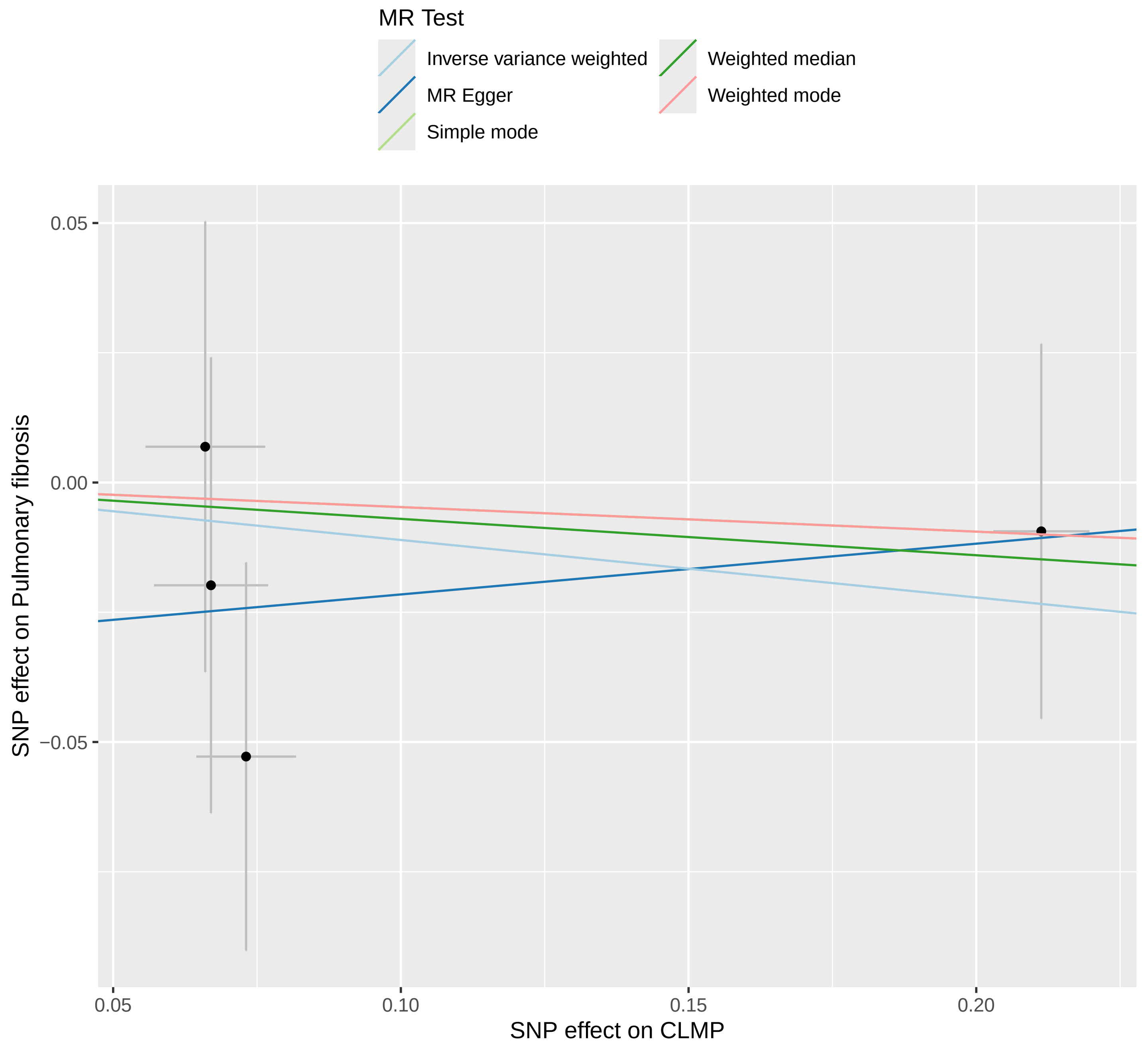
MR Test
Inverse variance weighted
MR Egger
Simple mode
Weighted median
Weighted mode
0.05
0.00
−0.05
SNP effect on Pulmonary fibrosis
0.05
0.10
0.15
0.20
SNP effect on CLMP

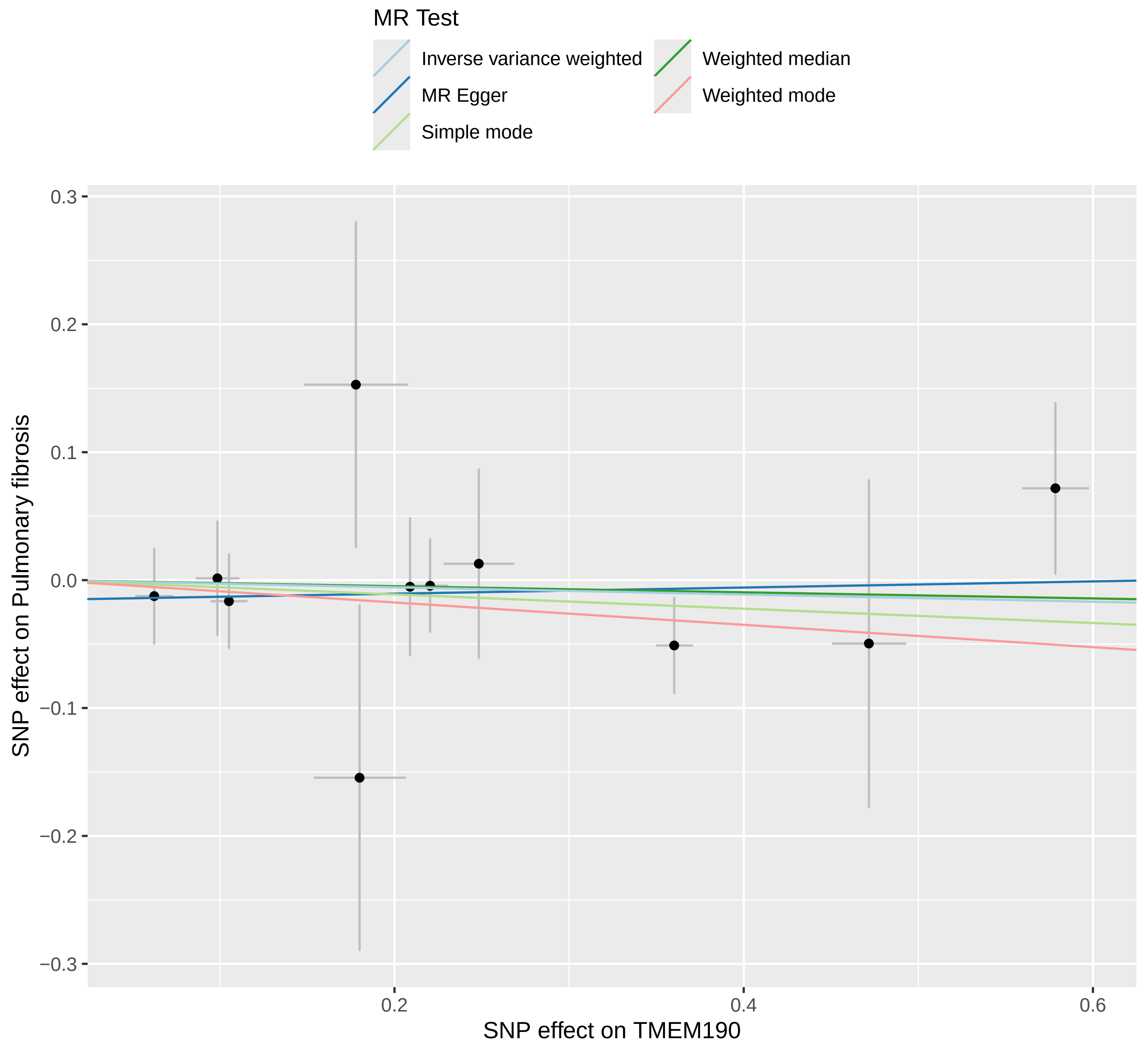
MR Test
Inverse variance weighted
MR Egger
Simple mode
Weighted median
Weighted mode
0.3
0.2
0.1
0.0
−0.1
−0.2
−0.3
SNP effect on Pulmonary fibrosis
0.2
0.4
0.6
SNP effect on TMEM190

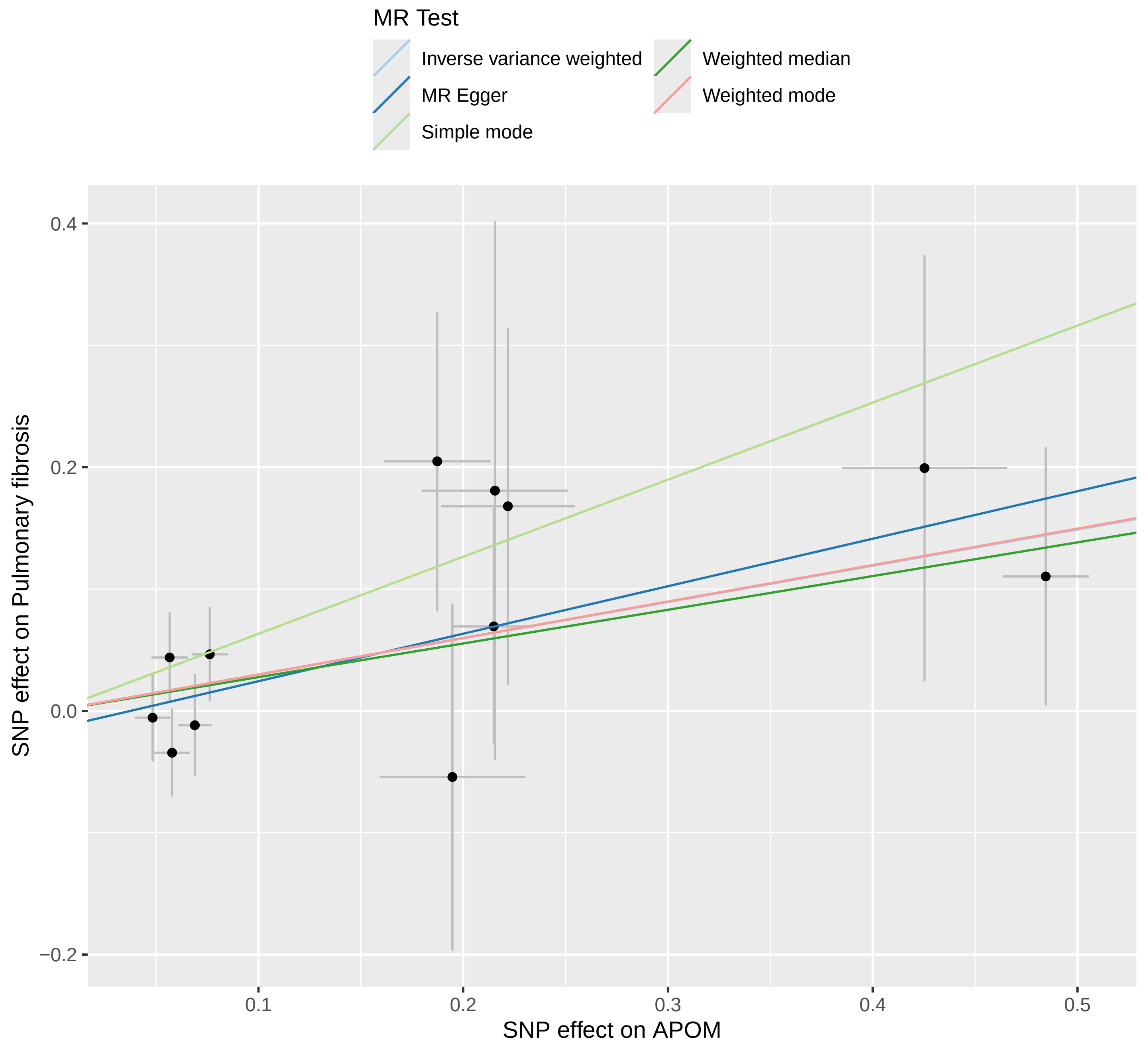
MR Test
Inverse variance weighted
MR Egger
Simple mode
Weighted median
Weighted mode
SNP effect on Pulmonary fibrosis
0.4
0.2
0.0
−0.2
0.1
0.2
0.3
0.4
0.5
SNP effect on APOM

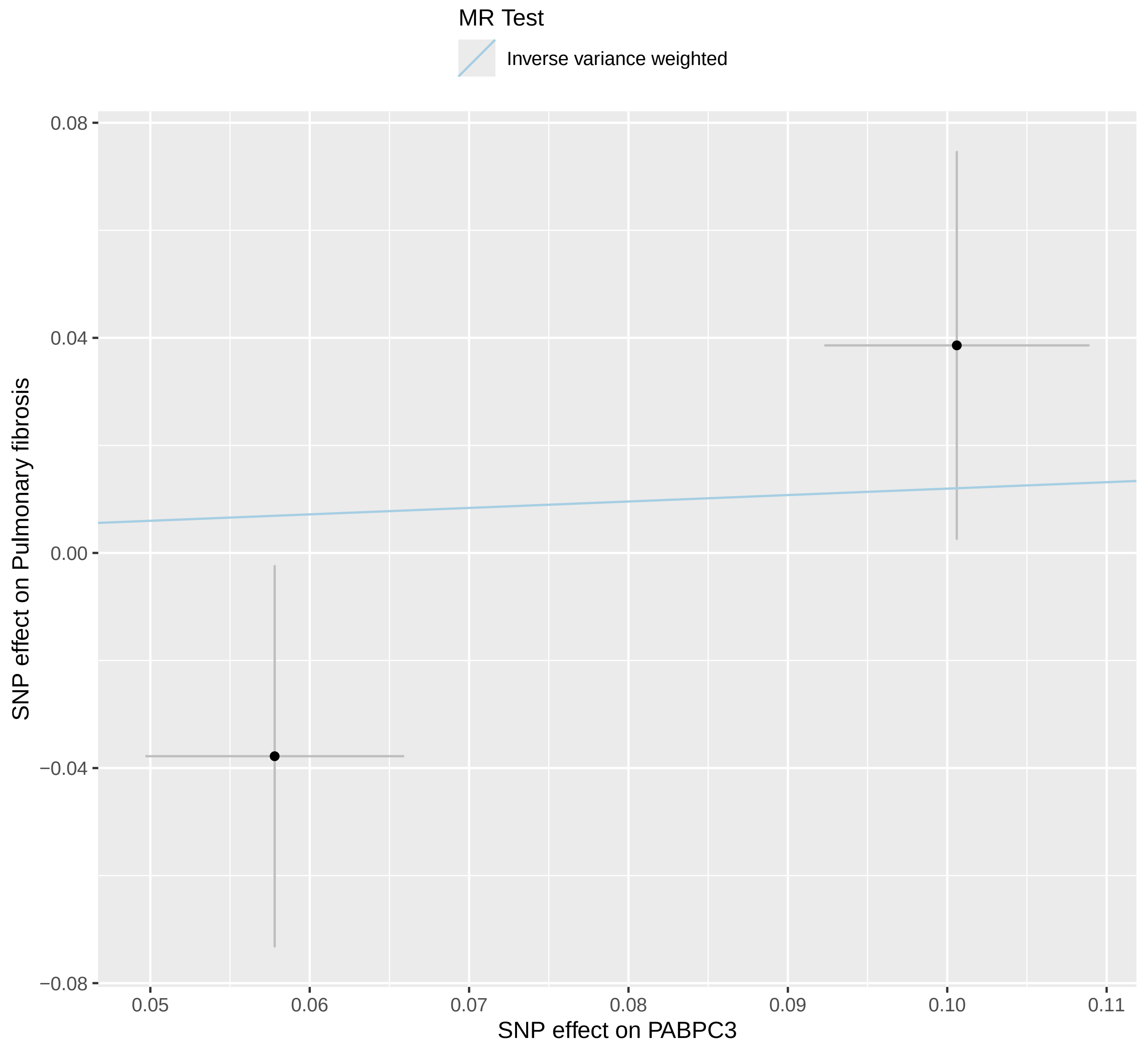

MR Test
Inverse variance weighted
0.08
0.04
0.00
−0.04
−0.08
SNP effect on Pulmonary fibrosis
0.05
0.06
0.07
0.08
0.09
0.10
0.11
SNP effect on PABPC3

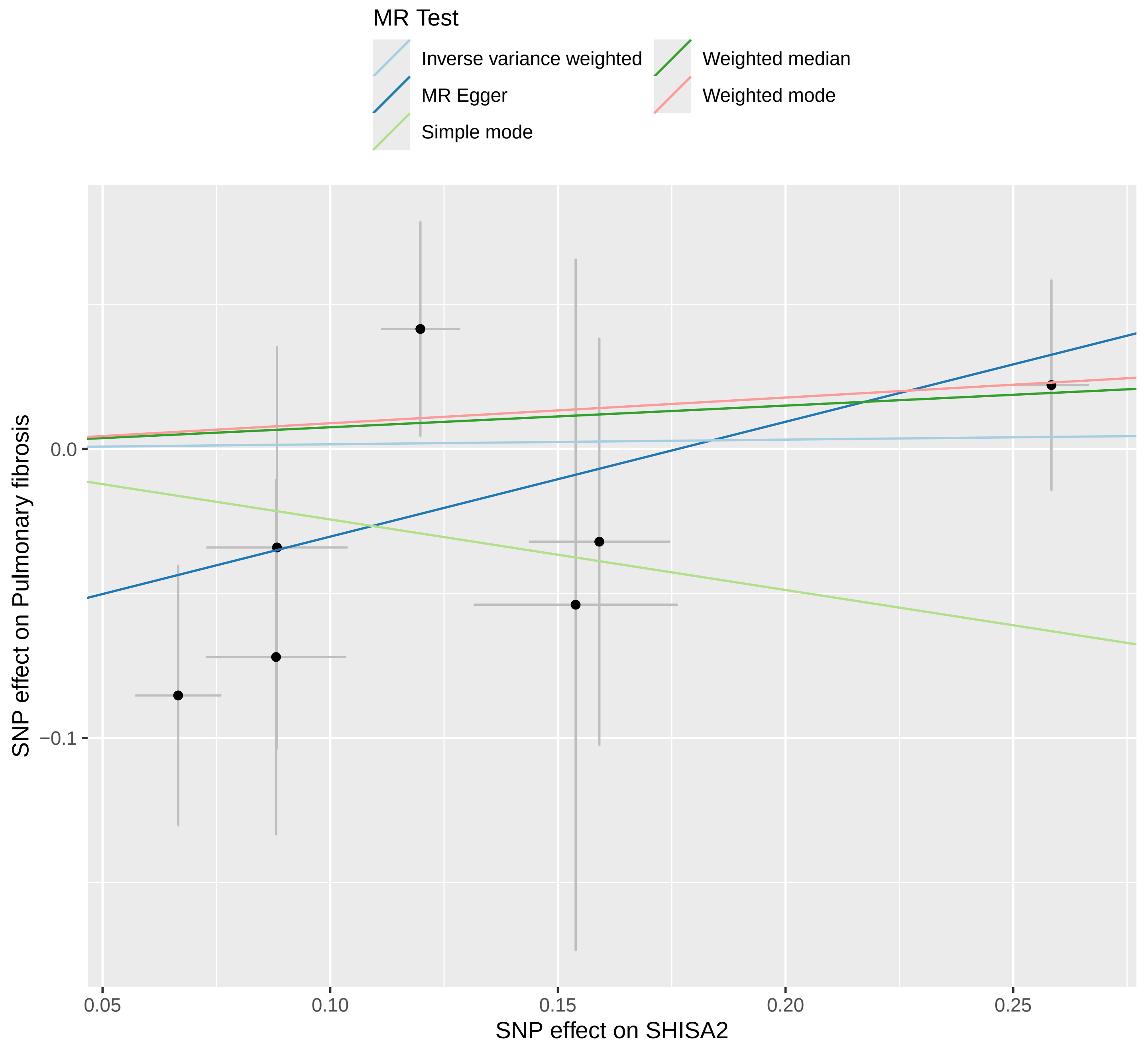
MR Test
Inverse variance weighted
MR Egger
Simple mode
Weighted median
Weighted mode
0.0
−0.1
0.05
0.10
0.15
0.20
0.25
SNP effect on SHISA2
SNP effect on Pulmonary fibrosis

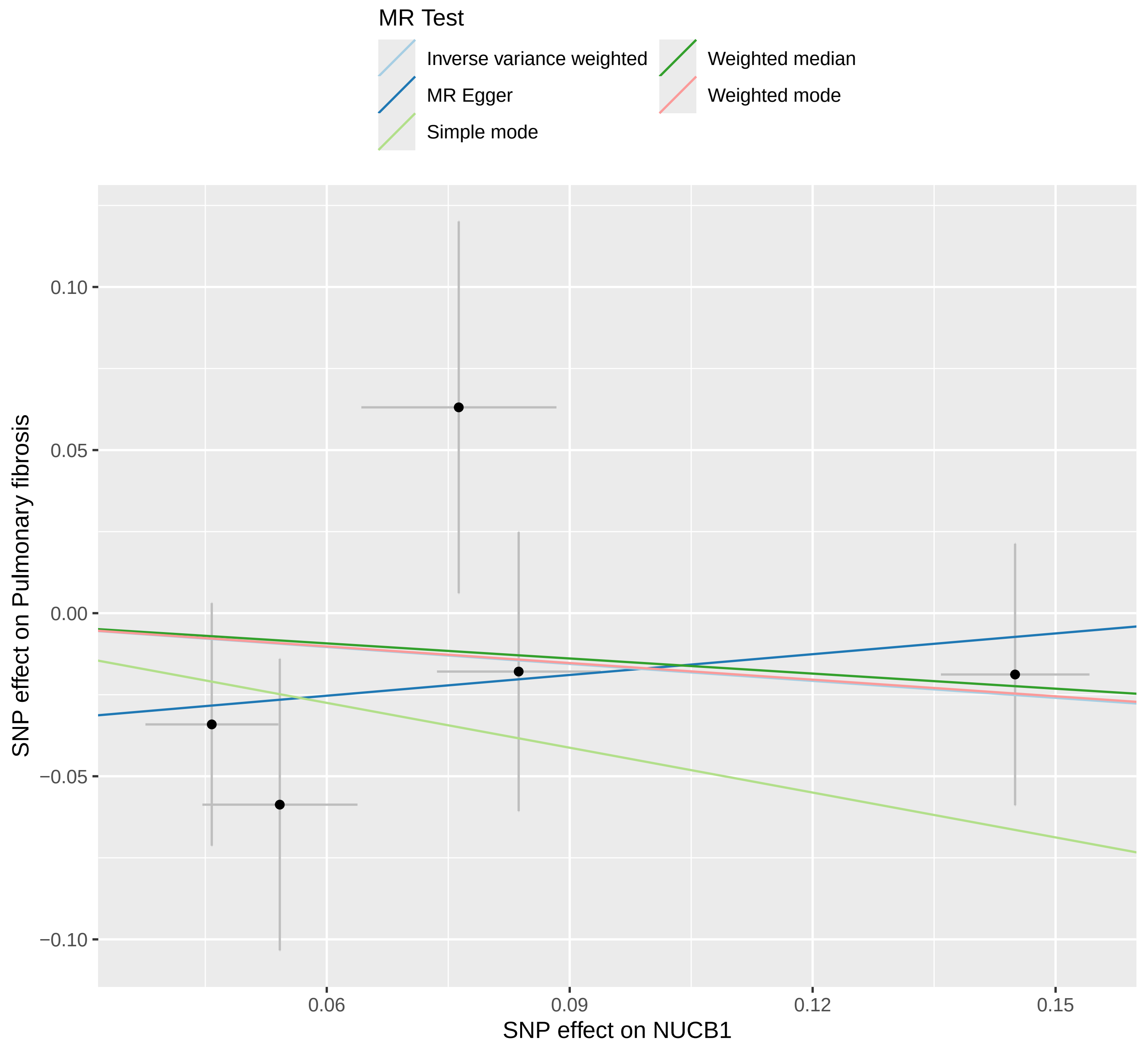
MR Test
Inverse variance weighted
MR Egger
Simple mode
Weighted median
Weighted mode
0.10
0.05
0.00
−0.05
−0.10
SNP effect on Pulmonary fibrosis
0.06
0.09
0.12
0.15
SNP effect on NUCB1

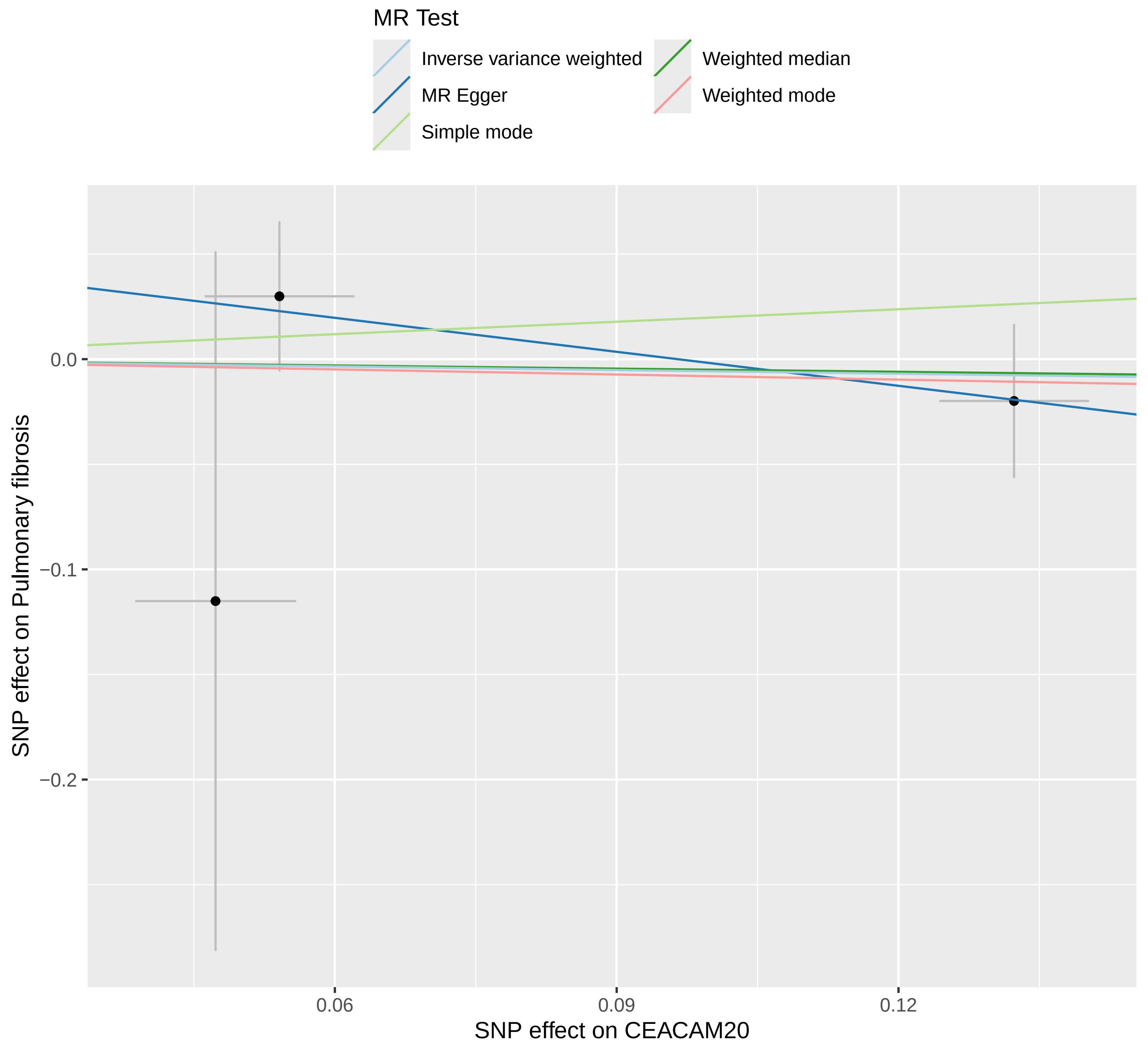
MR Test
Inverse variance weighted
MR Egger
Simple mode
Weighted median
Weighted mode
0.0
−0.1
−0.2
SNP effect on Pulmonary fibrosis
0.06
0.09
0.12
SNP effect on CEACAM20

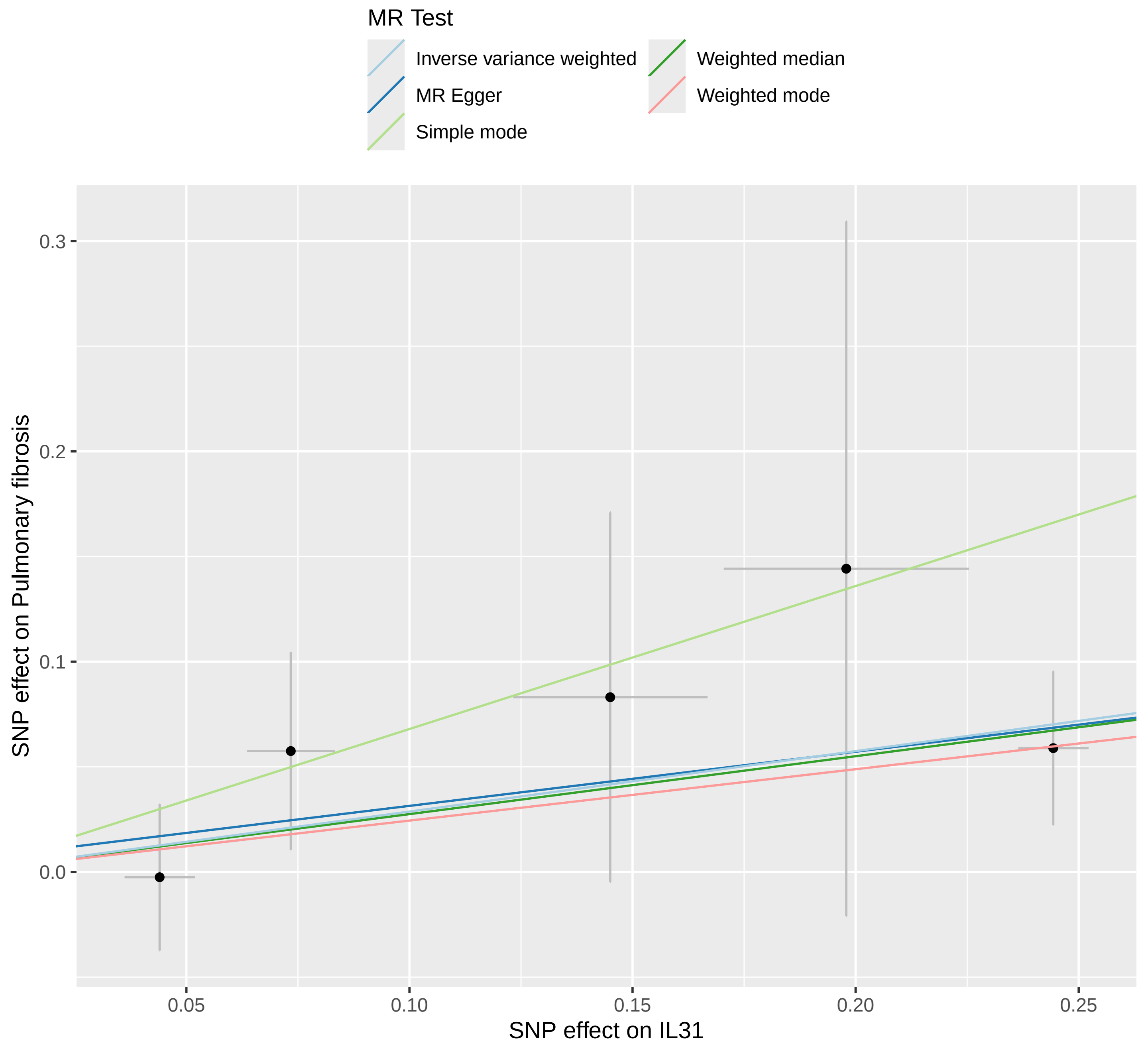
MR Test
Inverse variance weighted
MR Egger
Simple mode
Weighted median
Weighted mode
SNP effect on Pulmonary fibrosis
0.0
0.1
0.2
0.3
0.05
0.10
0.15
0.20
0.25
SNP effect on IL31

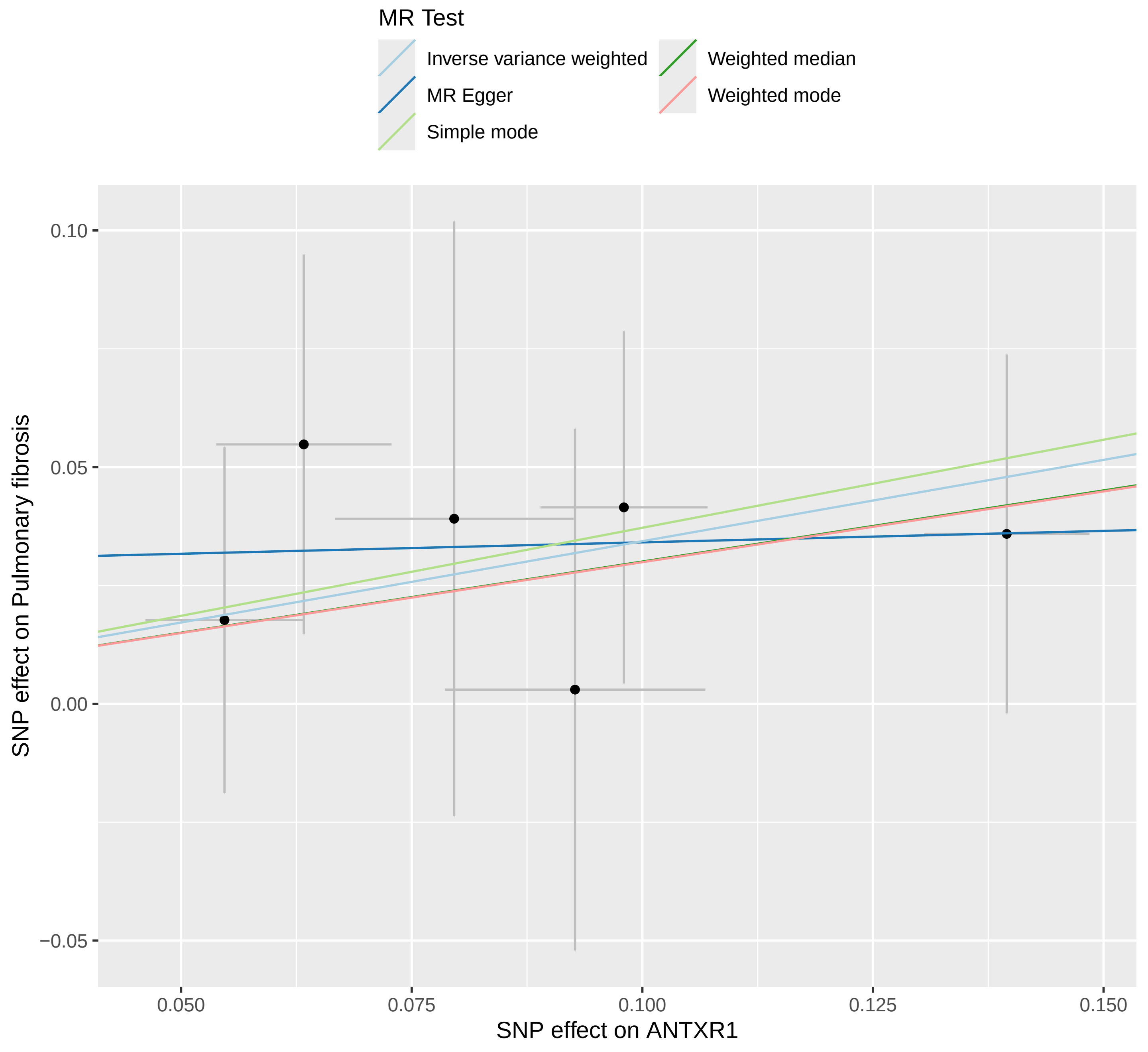
MR Test
Inverse variance weighted
MR Egger
Simple mode
Weighted median
Weighted mode
SNP effect on Pulmonary fibrosis
0.10
0.05
0.00
−0.05
0.050
0.075
0.100
0.125
0.150
SNP effect on ANTXR1

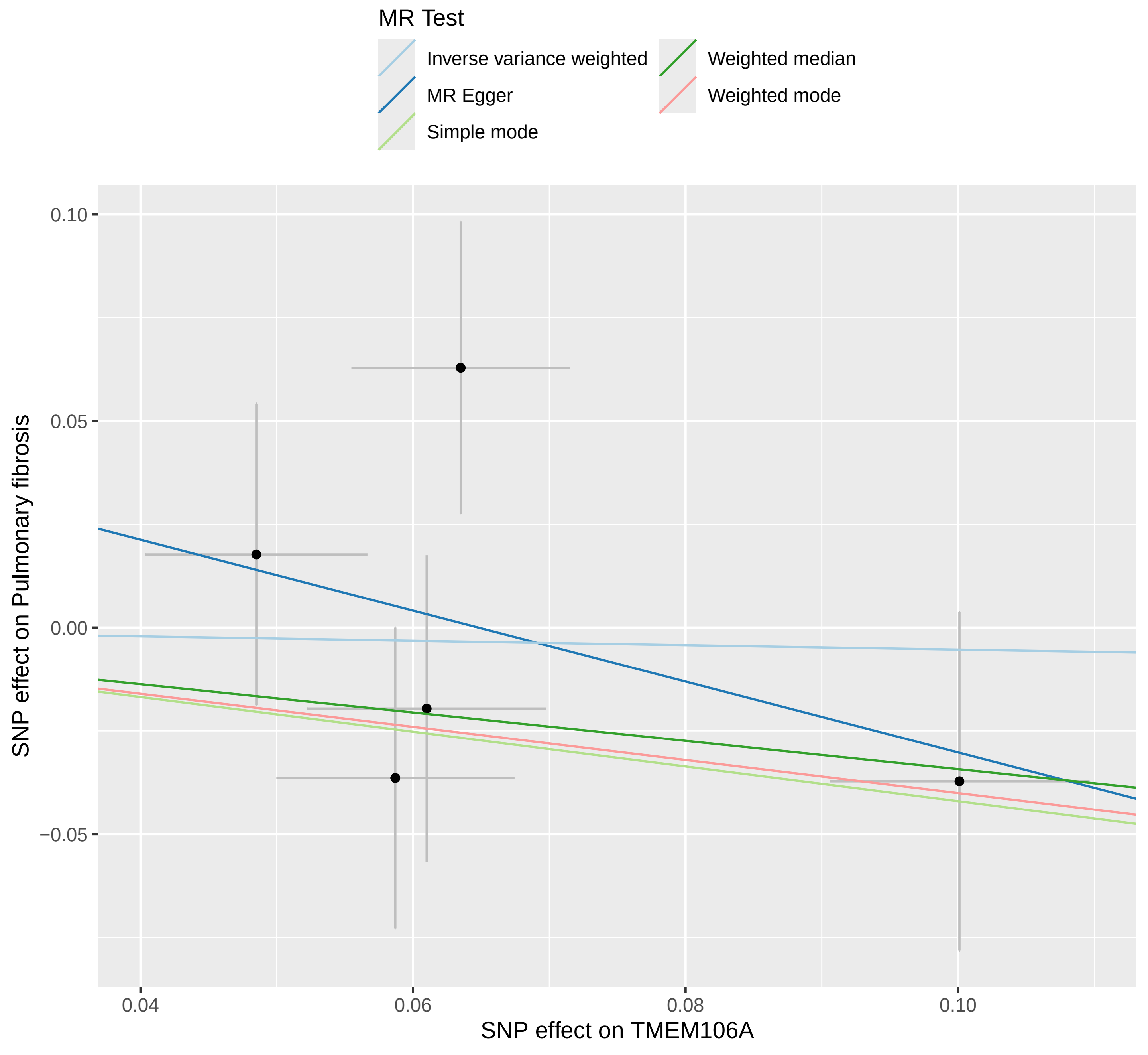
MR Test
Inverse variance weighted
MR Egger
Simple mode
Weighted median
Weighted mode
0.10
0.05
0.00
−0.05
SNP effect on Pulmonary fibrosis
0.04
0.06
0.08
0.10
SNP effect on TMEM106A

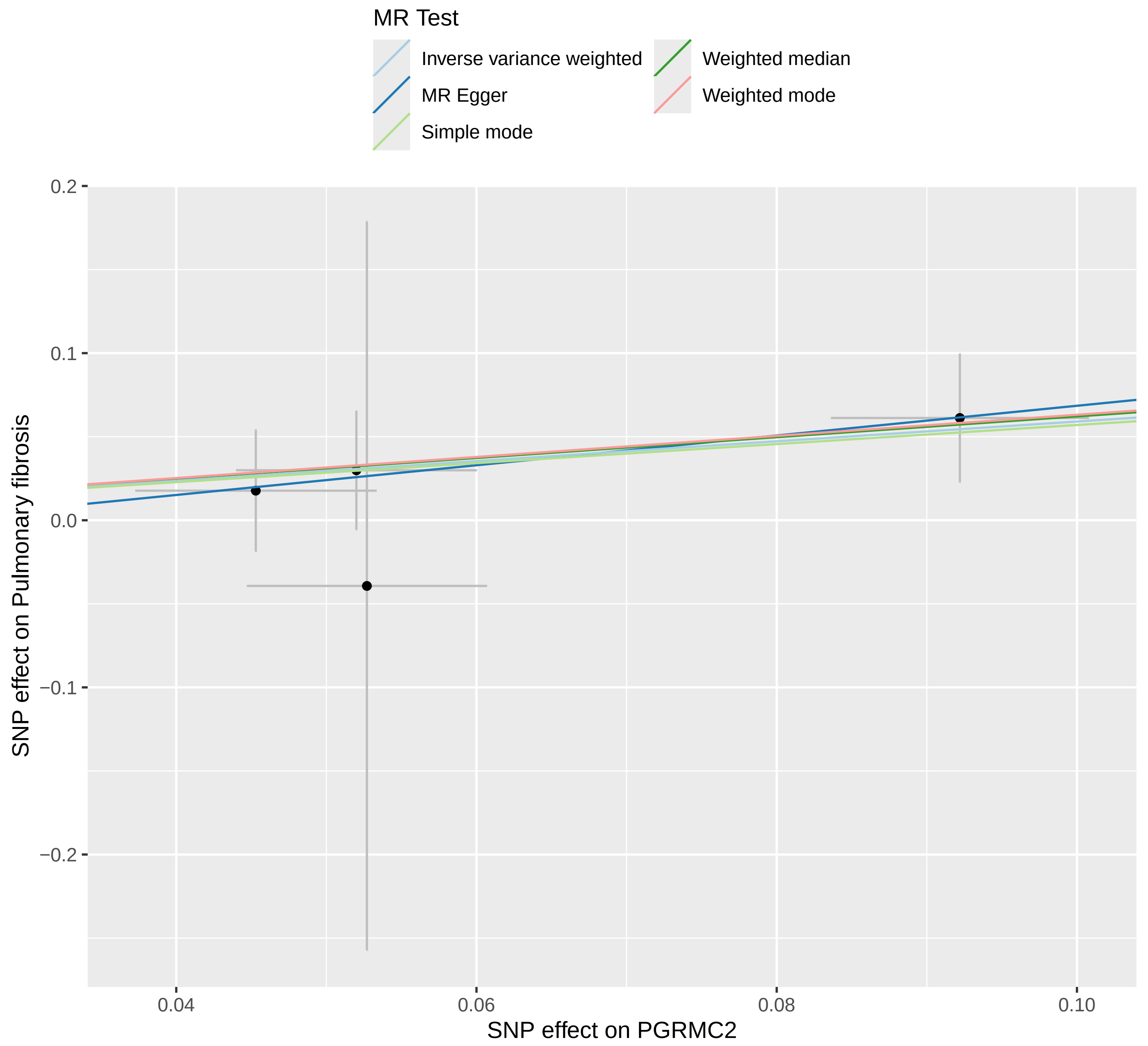
MR Test
Inverse variance weighted
MR Egger
Simple mode
Weighted median
Weighted mode
0.2
0.1
0.0
−0.1
−0.2
SNP effect on Pulmonary fibrosis
0.04
0.06
0.08
0.10
SNP effect on PGRMC2

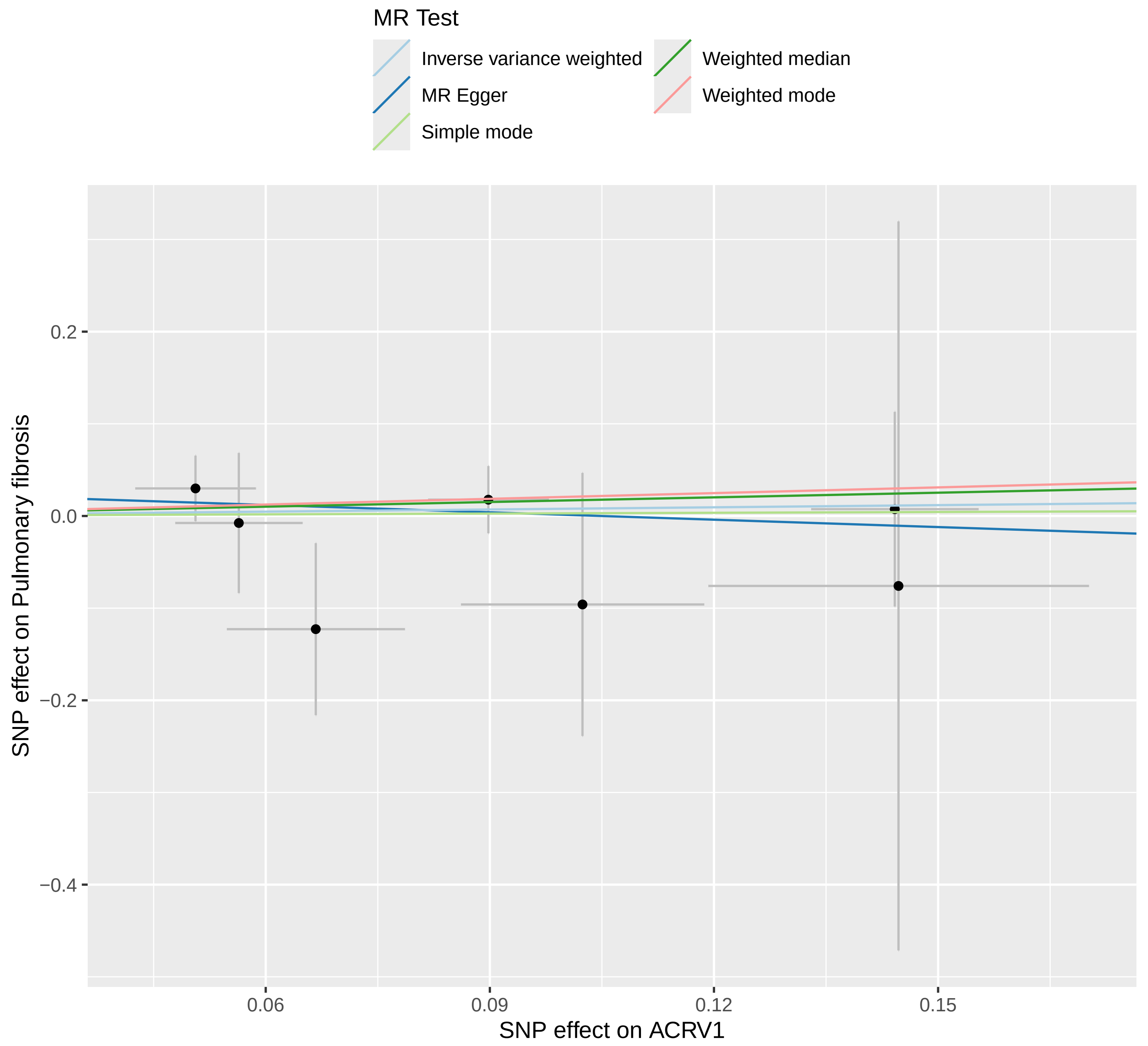
MR Test
Inverse variance weighted
MR Egger
Simple mode
Weighted median
Weighted mode
0.2
0.0
−0.2
−0.4
SNP effect on Pulmonary fibrosis
0.06
0.09
0.12
0.15
SNP effect on ACRV1

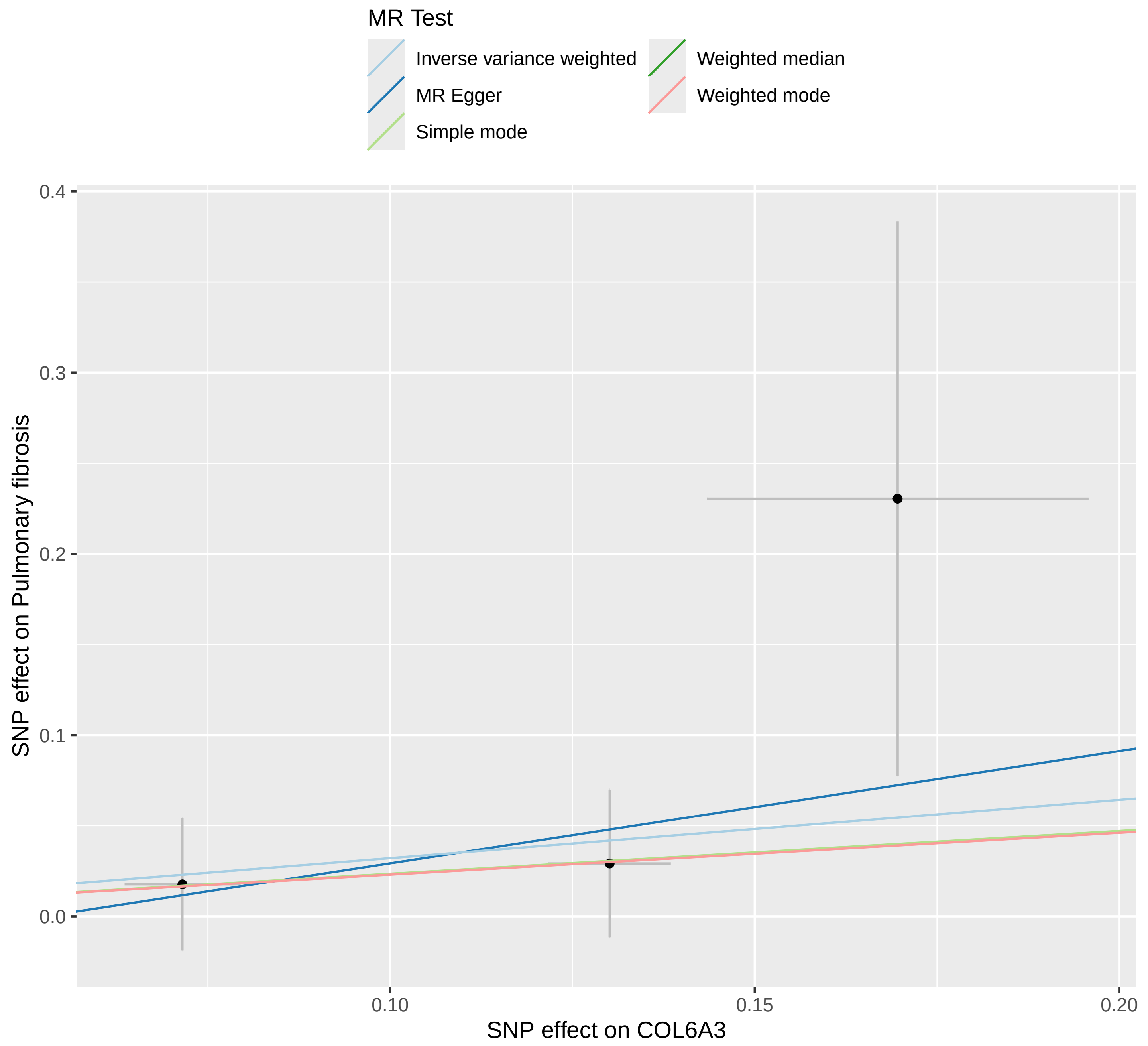
MR Test
Inverse variance weighted
MR Egger
Simple mode
Weighted median
Weighted mode
0.4
0.3
0.2
0.1
0.0
0.10
0.15
0.20
SNP effect on COL6A3
SNP effect on Pulmonary fibrosis

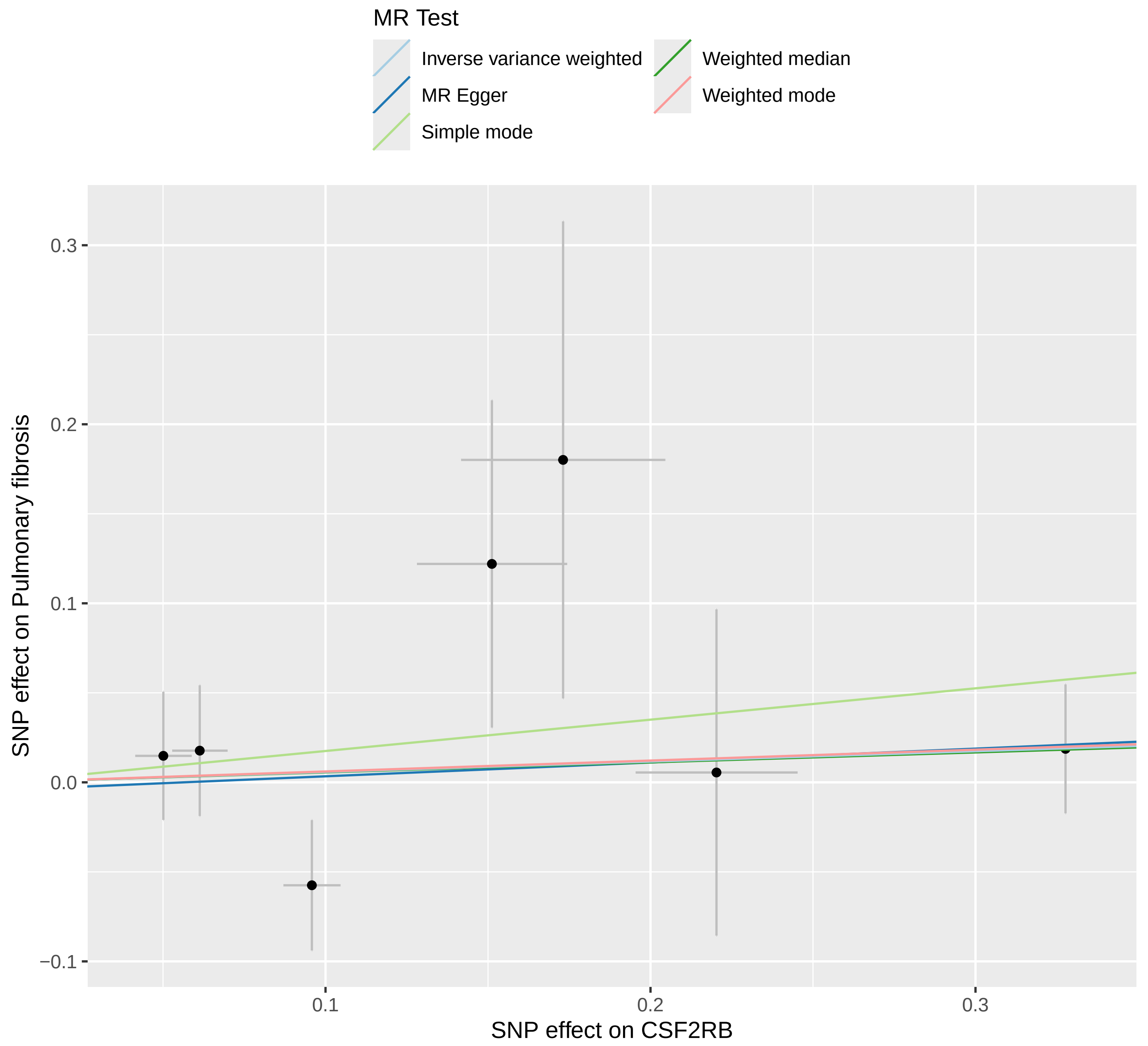

MR Test
Inverse variance weighted
MR Egger
Simple mode
Weighted median
Weighted mode
SNP effect on Pulmonary fibrosis
SNP effect on CSF2RB
0.3
0.2
0.1
0.0
−0.1
0.1
0.2
0.3

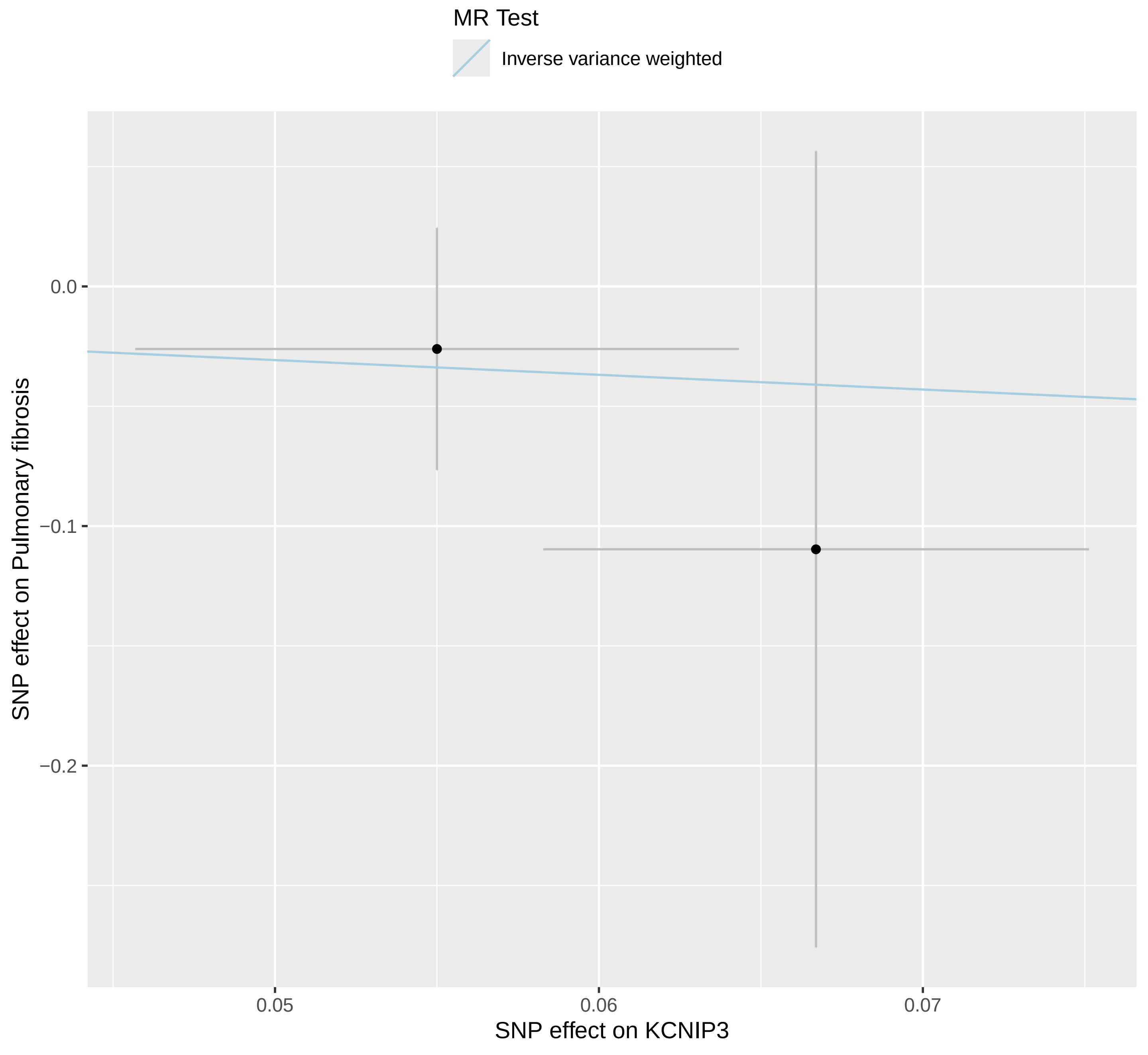
MR Test
Inverse variance weighted
0.0
−0.1
−0.2
SNP effect on Pulmonary fibrosis
0.05
0.06
0.07
SNP effect on KCNIP3

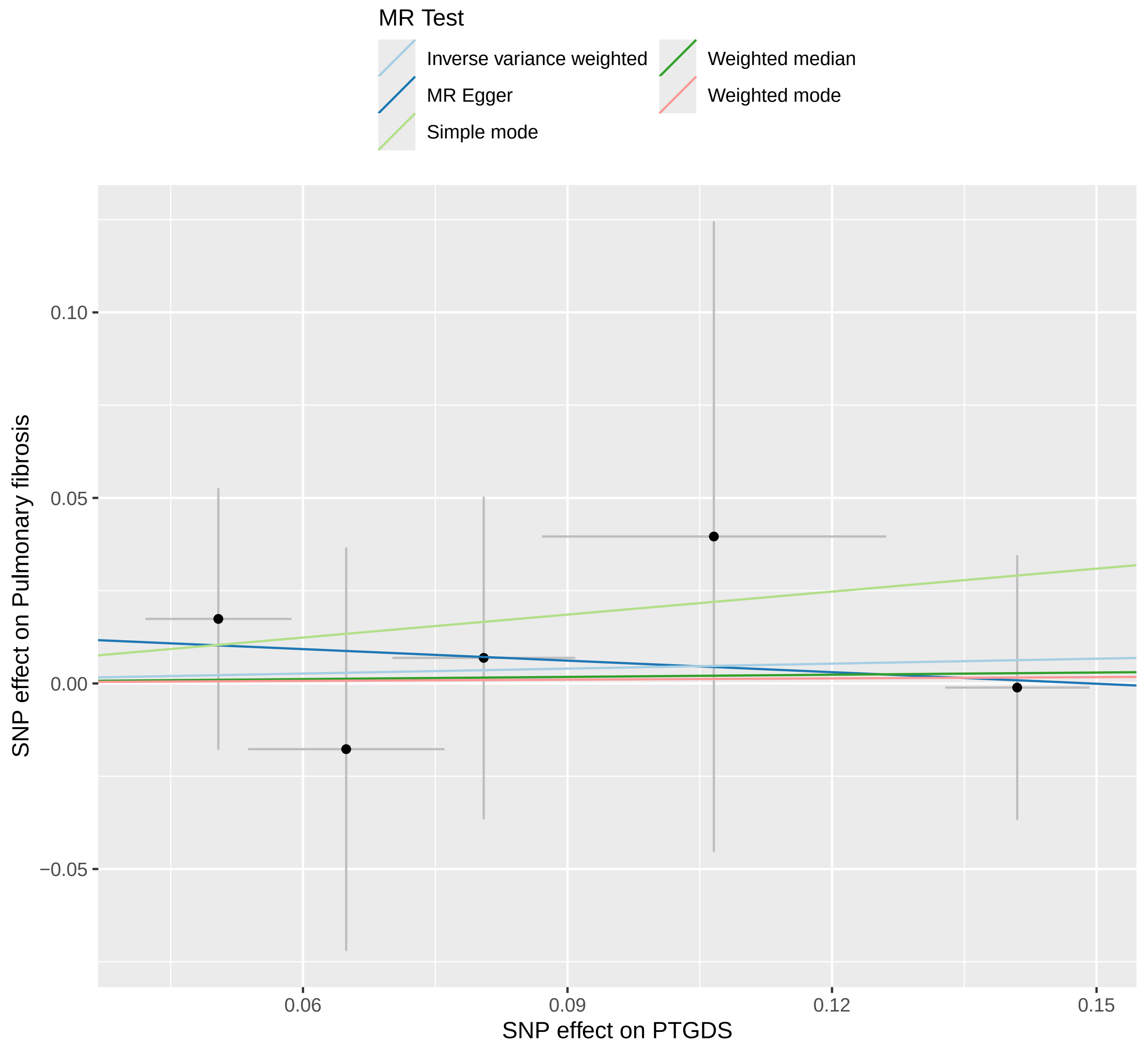
MR Test
Inverse variance weighted
MR Egger
Simple mode
Weighted median
Weighted mode
0.10
0.05
0.00
−0.05
0.06
0.09
0.12
0.15
SNP effect on Pulmonary fibrosis
SNP effect on PTGDS

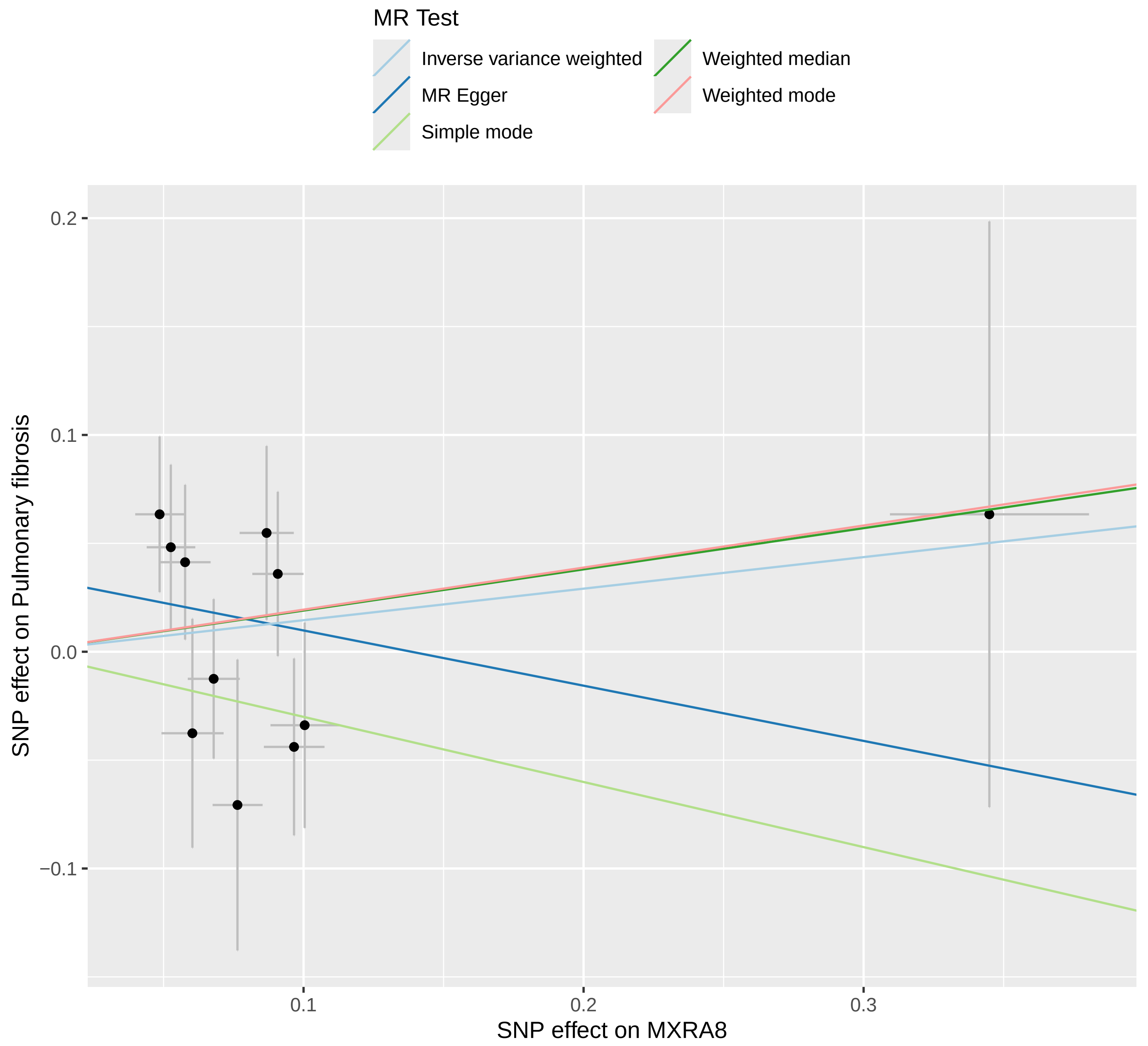

MR Test
Inverse variance weighted
MR Egger
Simple mode
Weighted median
Weighted mode
0.2
0.1
0.0
−0.1
SNP effect on Pulmonary fibrosis
0.1
0.2
0.3
SNP effect on MXRA8

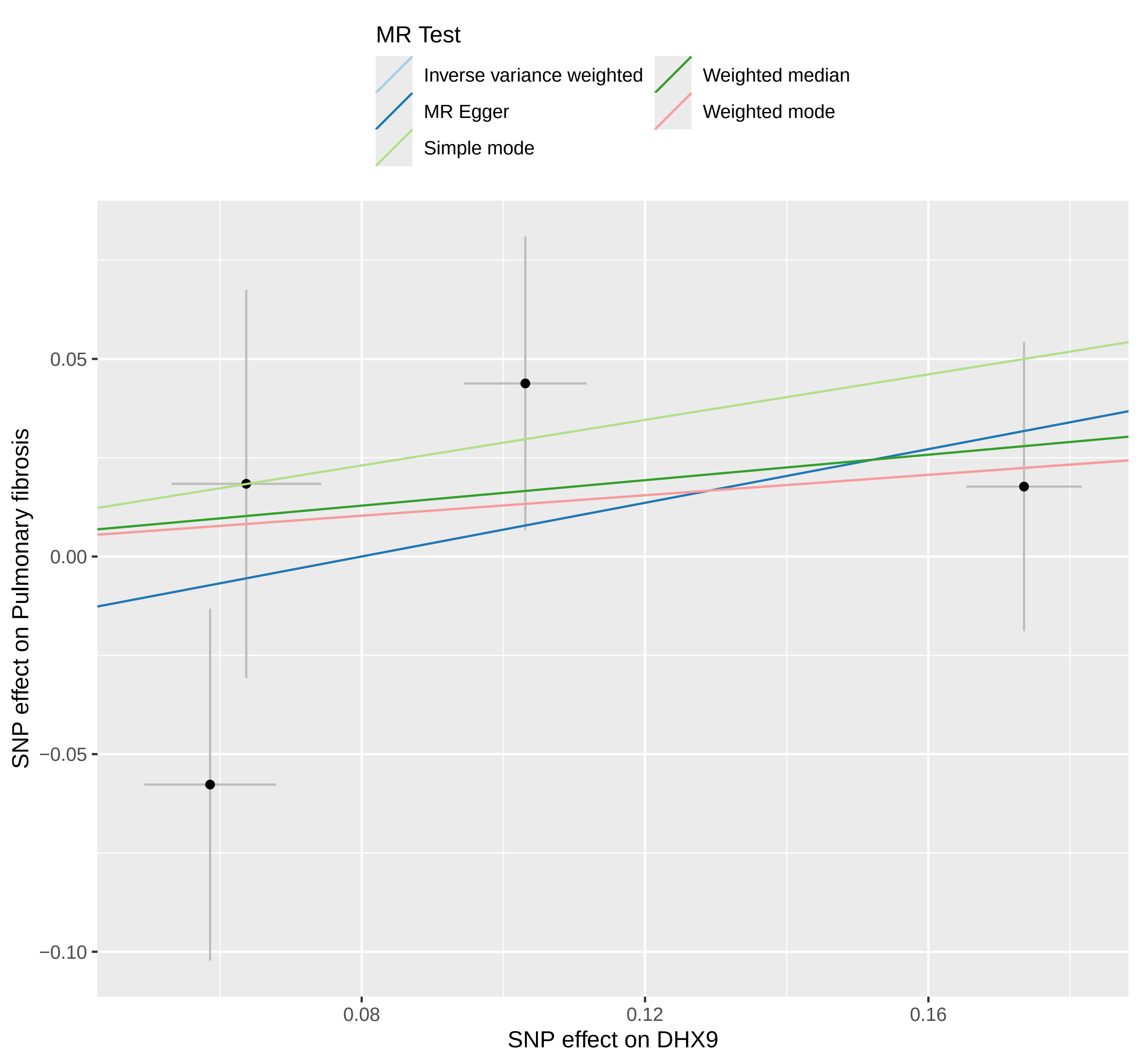
MR Test
Inverse variance weighted
MR Egger
Simple mode
Weighted median
Weighted mode
0.05
0.00
−0.05
−0.10
0.08
0.12
0.16
SNP effect on Pulmonary fibrosis
SNP effect on DHX9

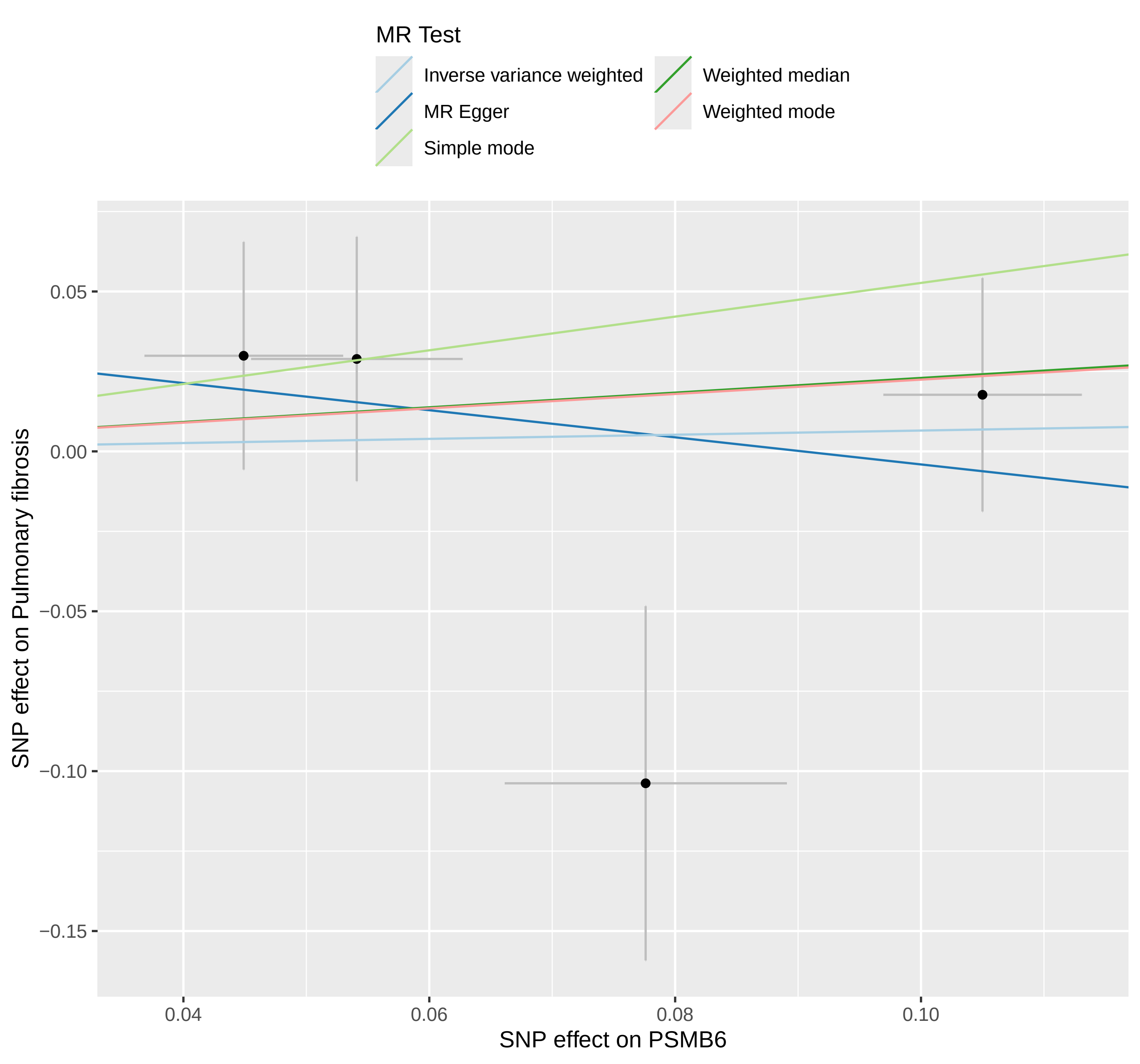
MR Test
Inverse variance weighted
MR Egger
Simple mode
Weighted median
Weighted mode
0.05
0.00
−0.05
−0.10
−0.15
SNP effect on Pulmonary fibrosis
0.04
0.06
0.08
0.10
SNP effect on PSMB6

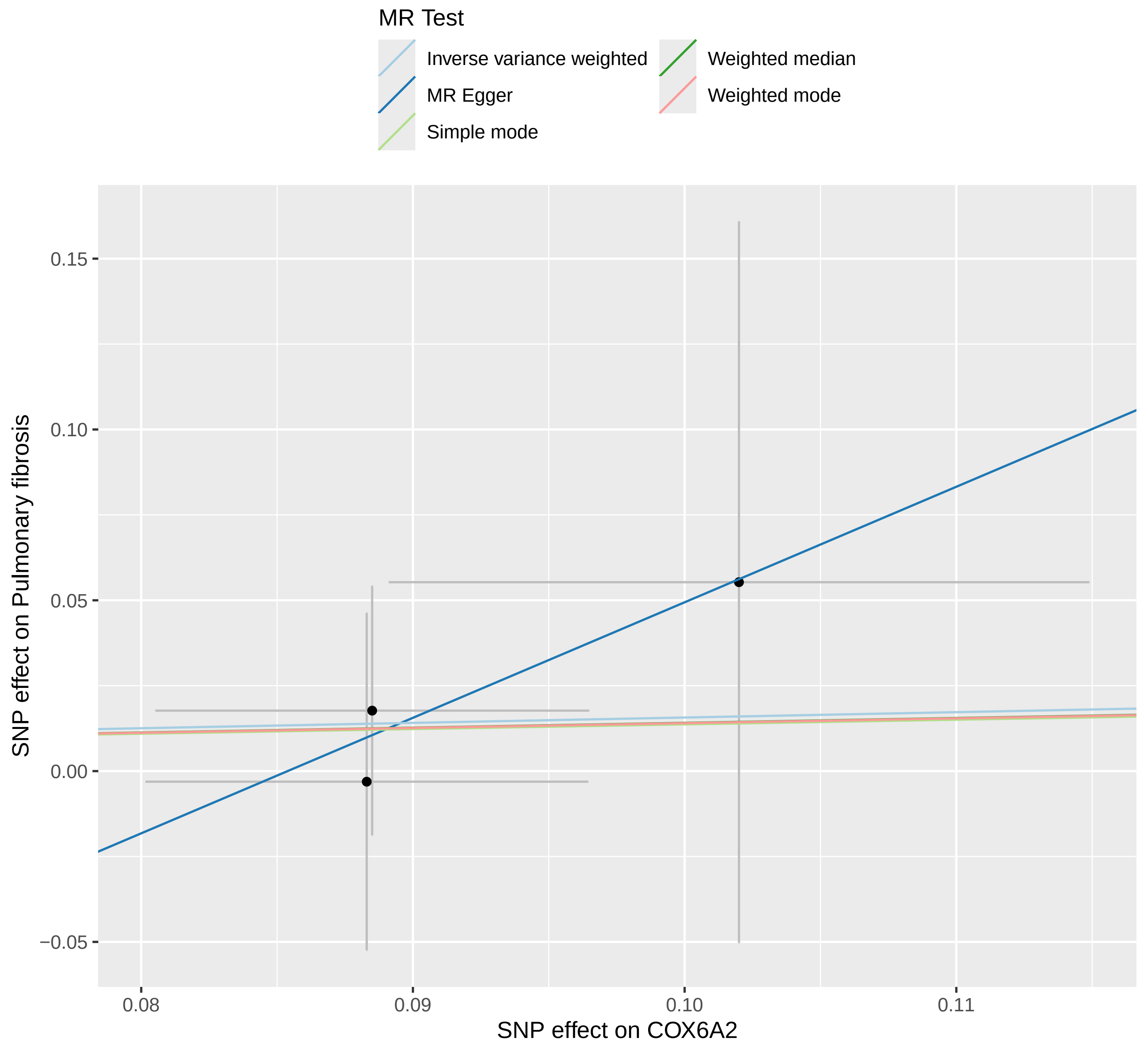
MR Test
Inverse variance weighted
MR Egger
Simple mode
Weighted median
Weighted mode
0.15
0.10
0.05
0.00
−0.05
0.08
0.09
0.10
0.11
SNP effect on Pulmonary fibrosis
SNP effect on COX6A2

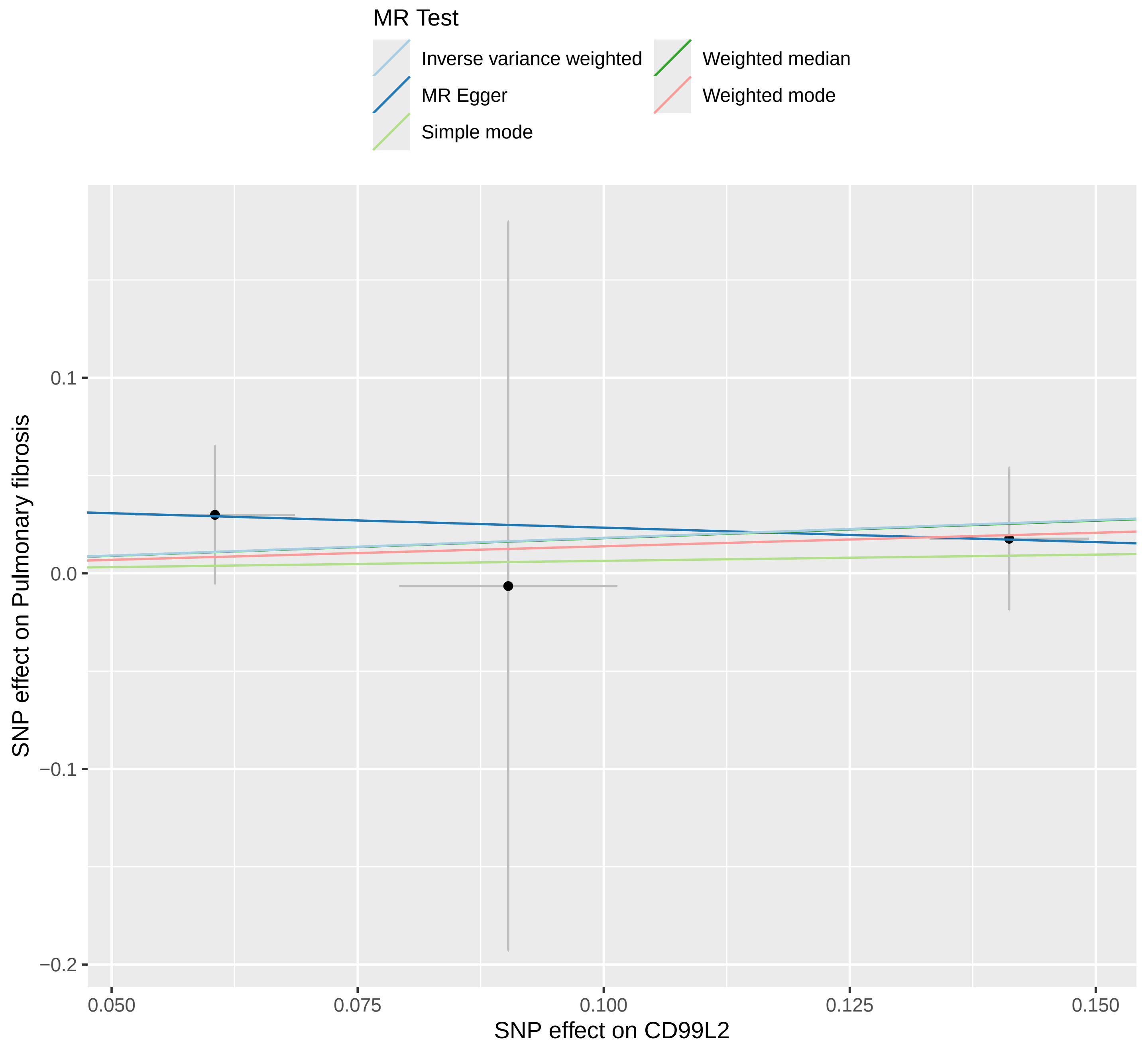
MR Test
Inverse variance weighted
MR Egger
Simple mode
Weighted median
Weighted mode
0.1
0.0
−0.1
−0.2
0.050
0.075
0.100
0.125
0.150
SNP effect on Pulmonary fibrosis
SNP effect on CD99L2

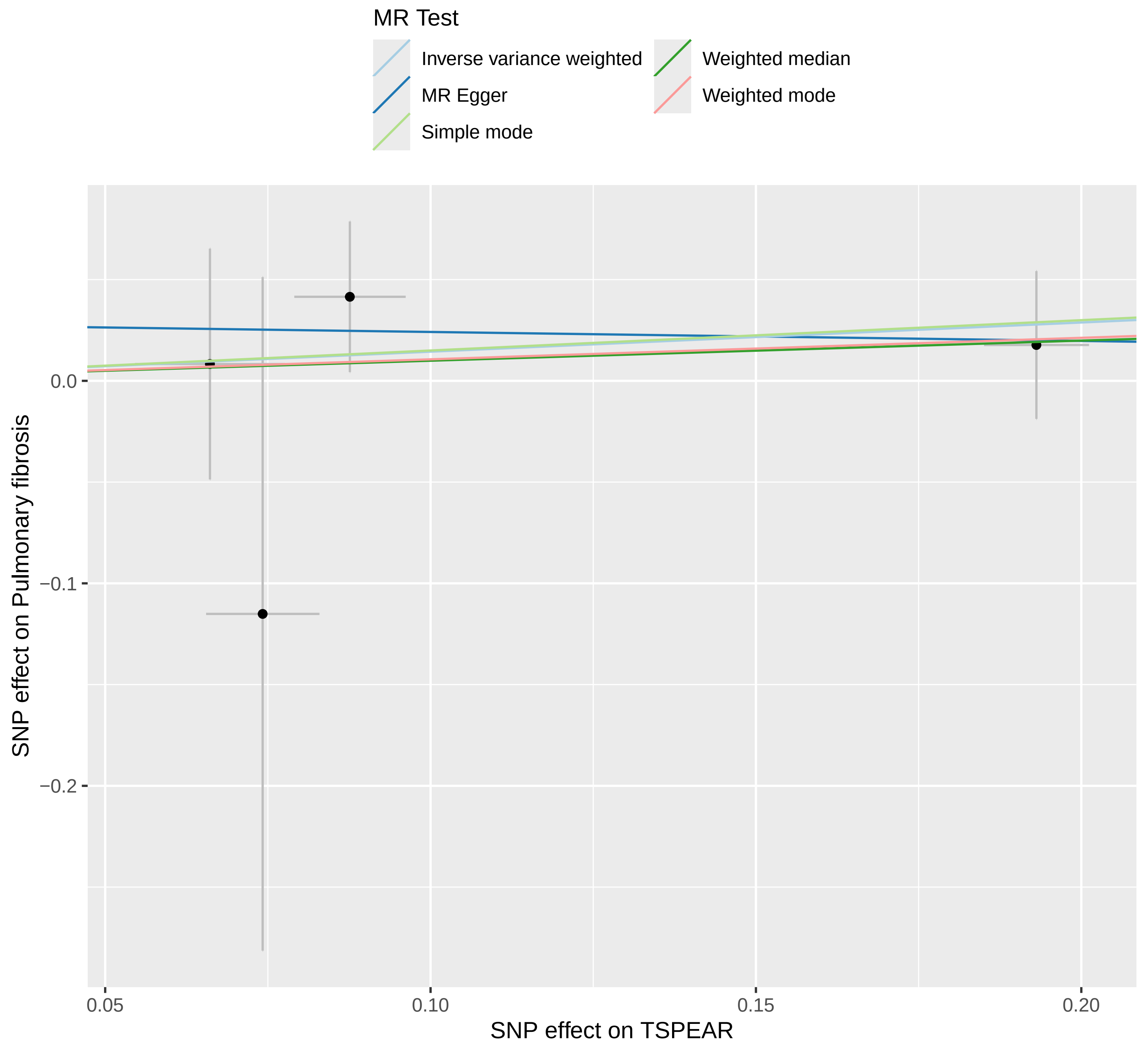

MR Test
Inverse variance weighted
MR Egger
Simple mode
Weighted median
Weighted mode
0.0
−0.1
−0.2
0.05
0.10
0.15
0.20
SNP effect on TSPEAR
SNP effect on Pulmonary fibrosis

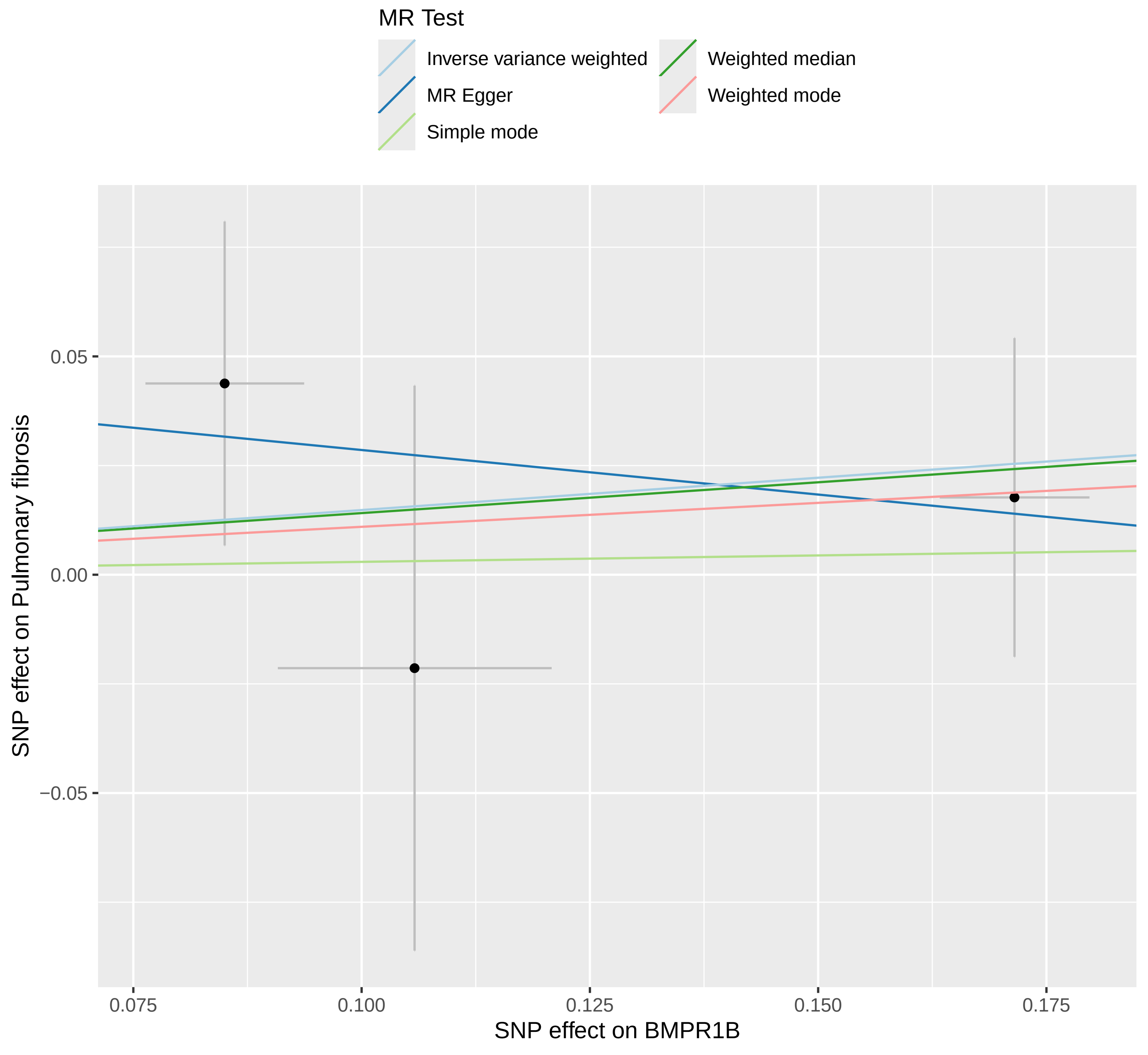
MR Test
Inverse variance weighted
MR Egger
Simple mode
Weighted median
Weighted mode
0.05
0.00
−0.05
SNP effect on Pulmonary fibrosis
0.075
0.100
0.125
0.150
0.175
SNP effect on BMPR1B

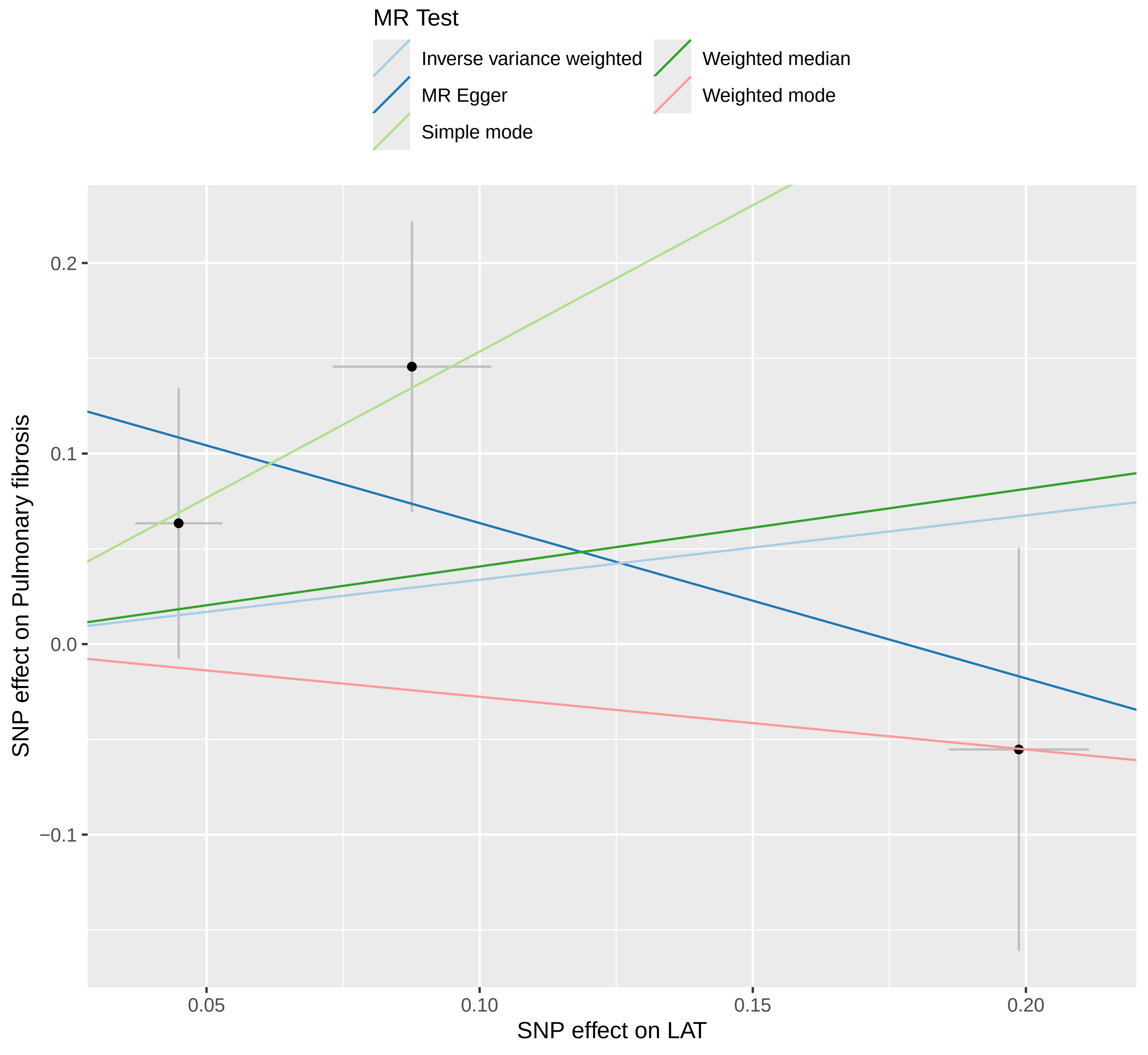

MR Test
Inverse variance weighted
MR Egger
Simple mode
Weighted median
Weighted mode
0.2
0.1
0.0
−0.1
0.05
0.10
0.15
0.20
SNP effect on LAT
SNP effect on Pulmonary fibrosis

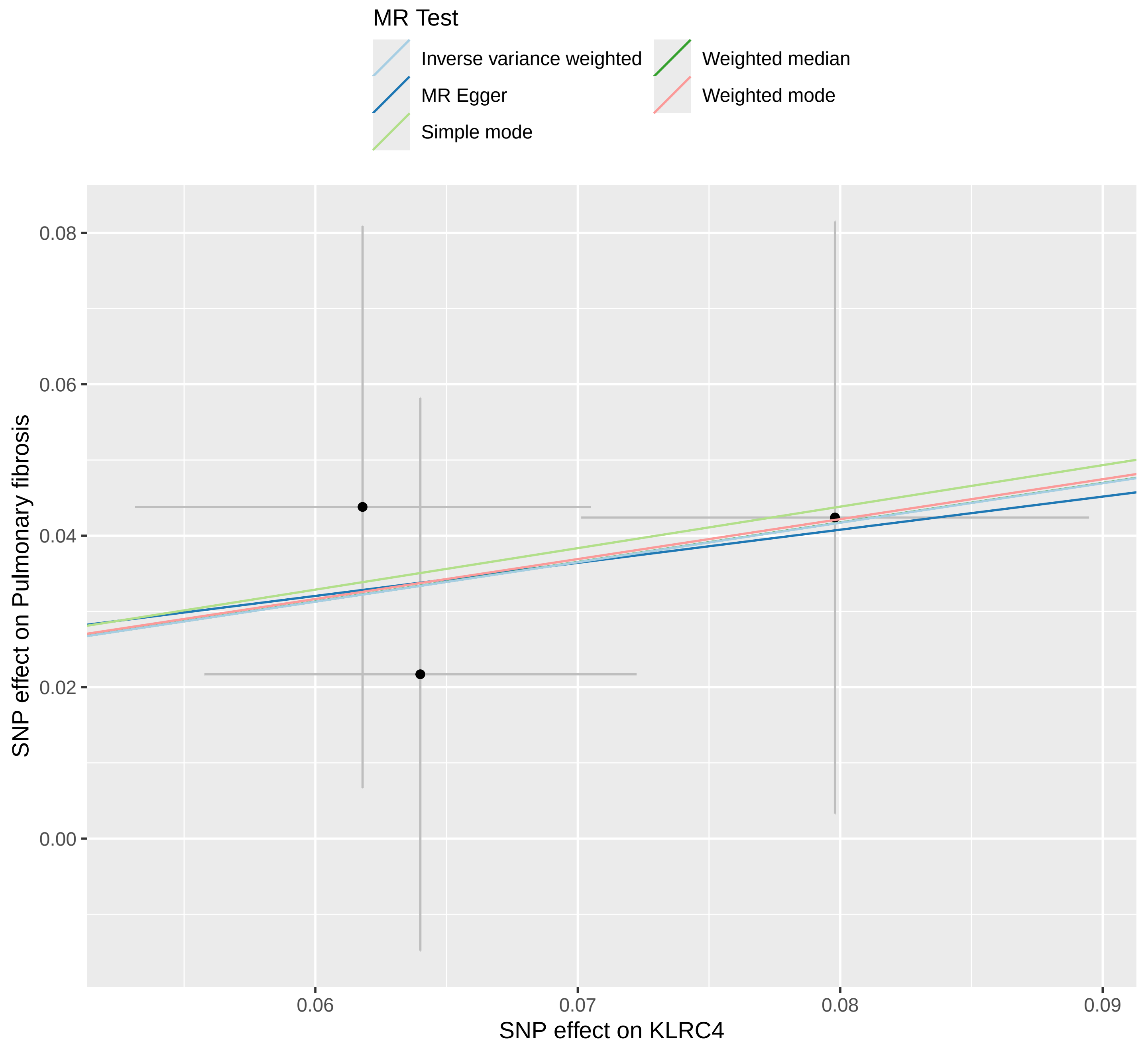
MR Test
Inverse variance weighted
MR Egger
Simple mode
Weighted median
Weighted mode
SNP effect on Pulmonary fibrosis
0.08
0.06
0.04
0.02
0.00
0.06
0.07
0.08
0.09
SNP effect on KLRC4

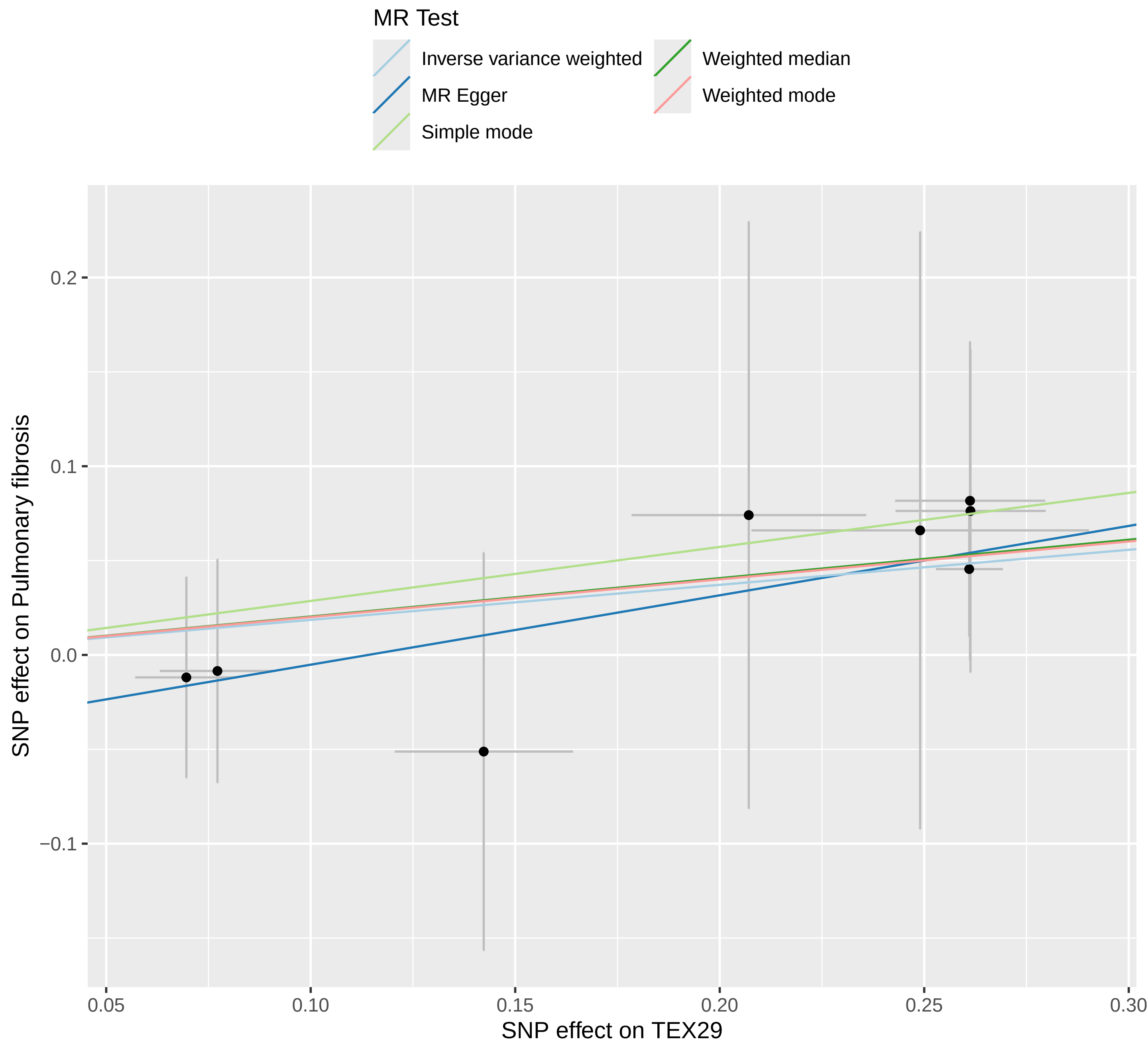
MR Test
Inverse variance weighted
MR Egger
Simple mode
Weighted median
Weighted mode
SNP effect on Pulmonary fibrosis
0.2
0.1
0.0
−0.1
0.05
0.10
0.15
0.20
0.25
0.30
SNP effect on TEX29

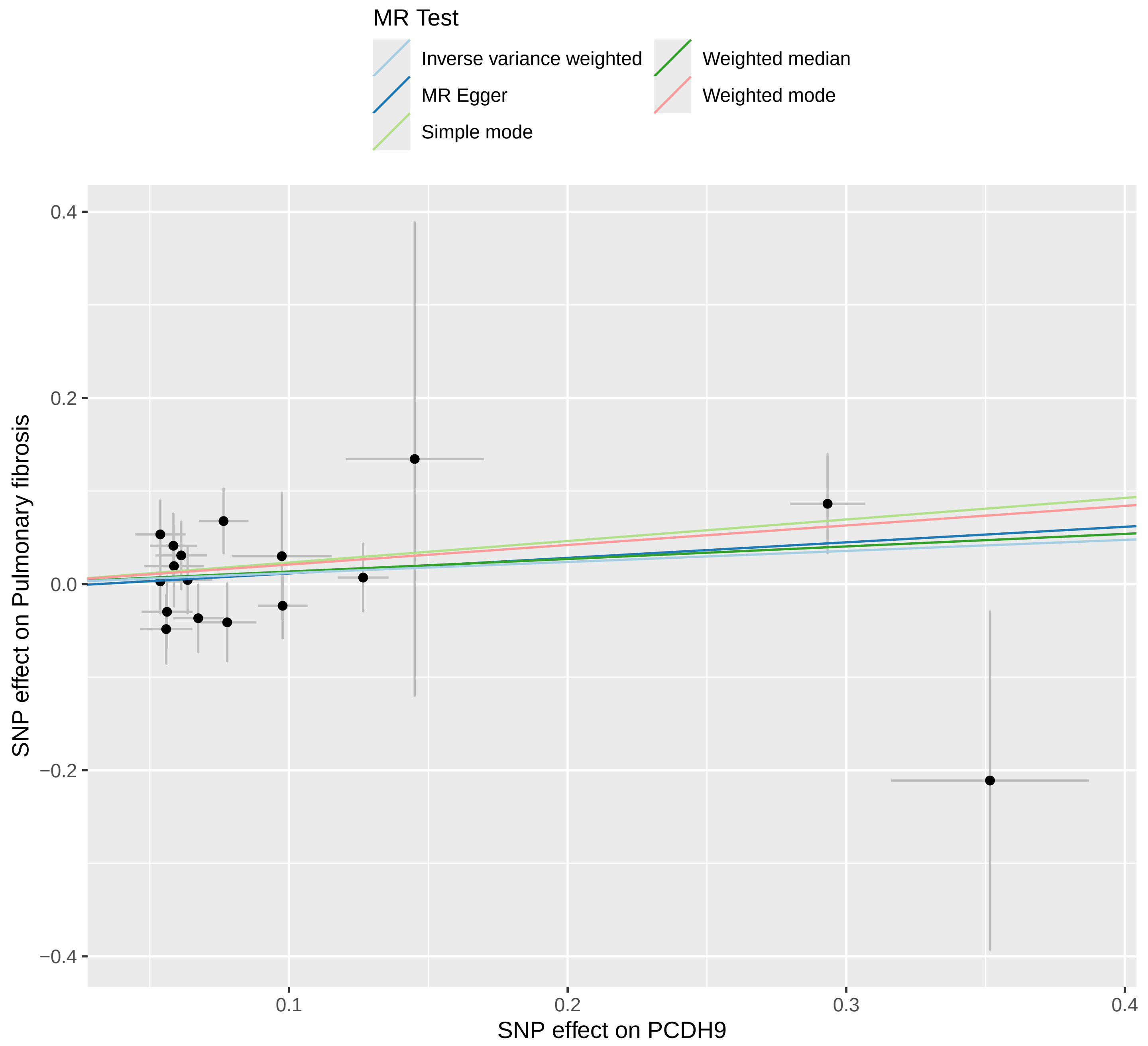
MR Test
Inverse variance weighted
MR Egger
Simple mode
Weighted median
Weighted mode
0.4
0.2
0.0
−0.2
−0.4
SNP effect on Pulmonary fibrosis
0.1
0.2
0.3
0.4
SNP effect on PCDH9

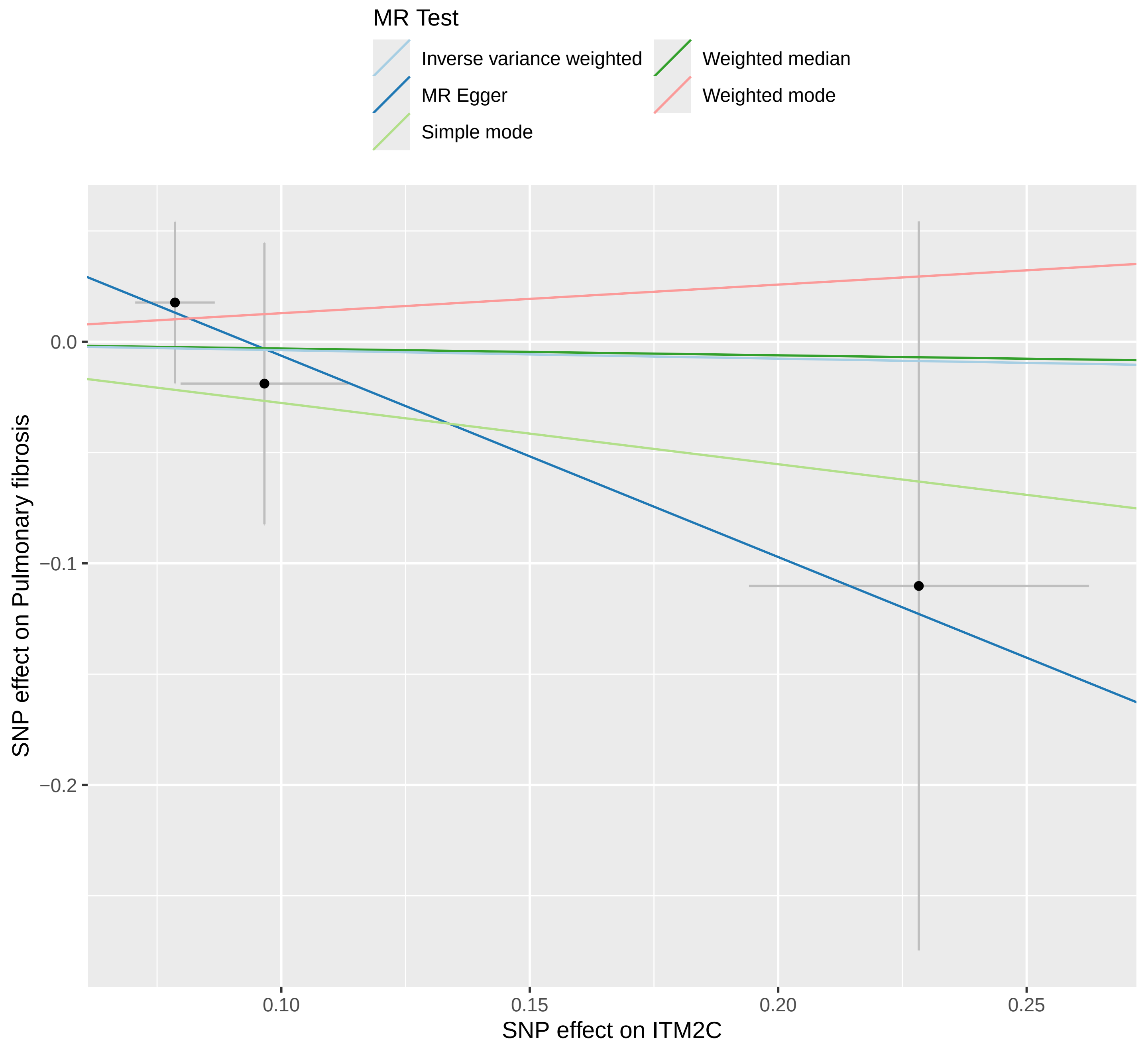
MR Test
Inverse variance weighted
MR Egger
Simple mode
Weighted median
Weighted mode
0.0
−0.1
−0.2
SNP effect on Pulmonary fibrosis
0.10
0.15
0.20
0.25
SNP effect on ITM2C

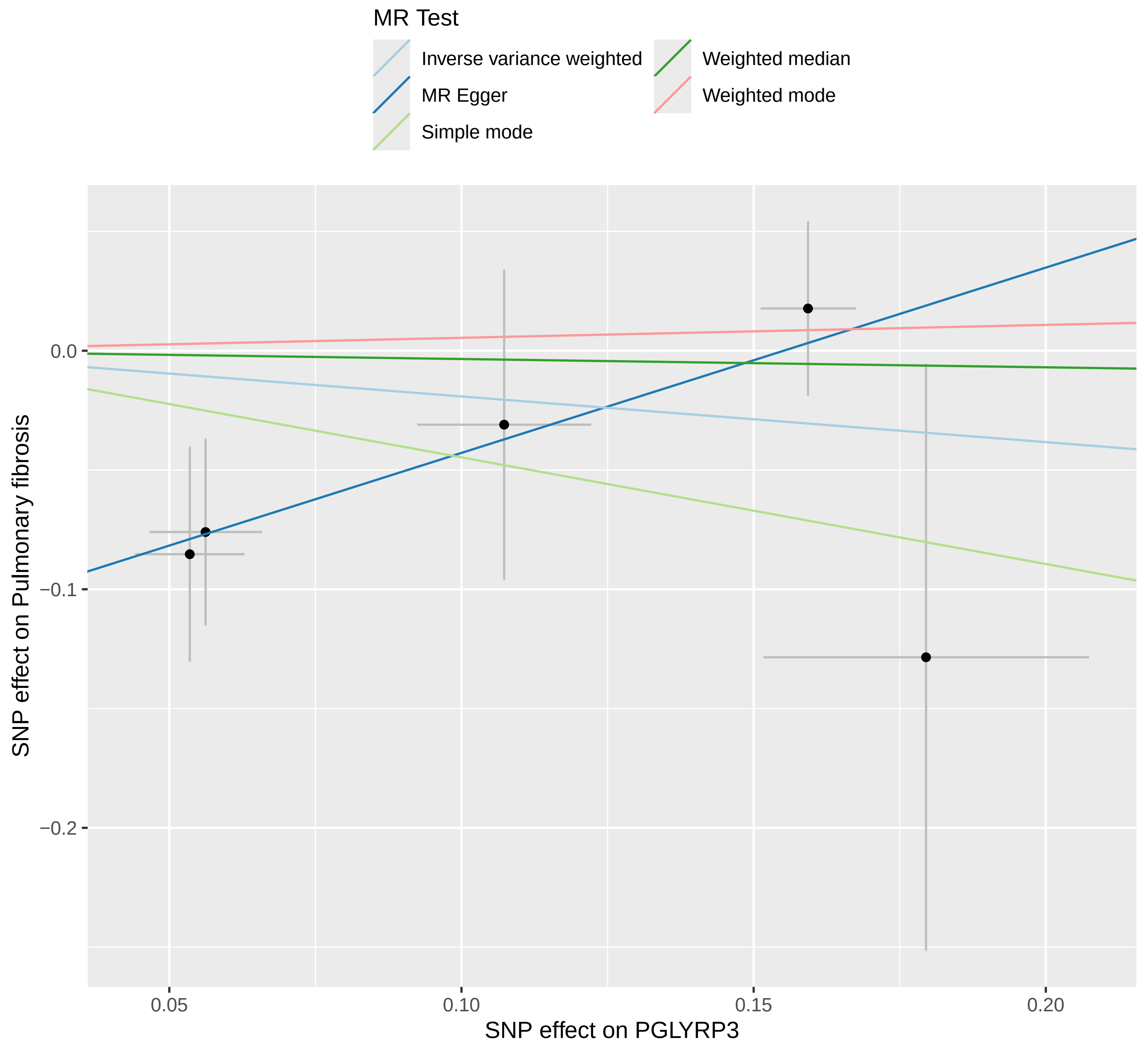

MR Test
Inverse variance weighted
MR Egger
Simple mode
Weighted median
Weighted mode
0.0
−0.1
−0.2
0.05
0.10
0.15
0.20
SNP effect on PGLYRP3
SNP effect on Pulmonary fibrosis

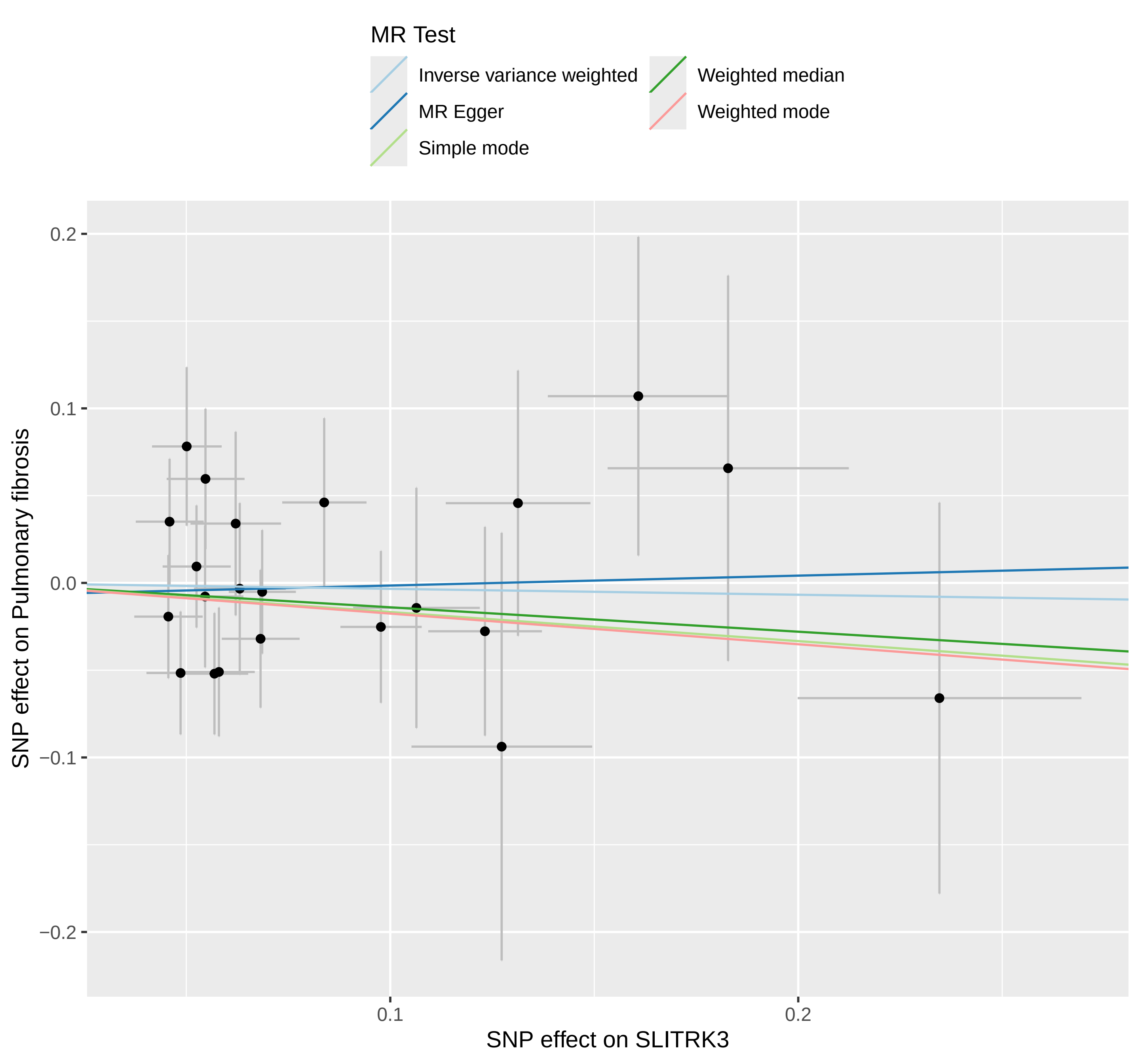
MR Test
Inverse variance weighted
Weighted median
MR Egger
Weighted mode
Simple mode
0.2
0.1
0.0
−0.1
−0.2
SNP effect on Pulmonary fibrosis
0.1
0.2
SNP effect on SLITRK3

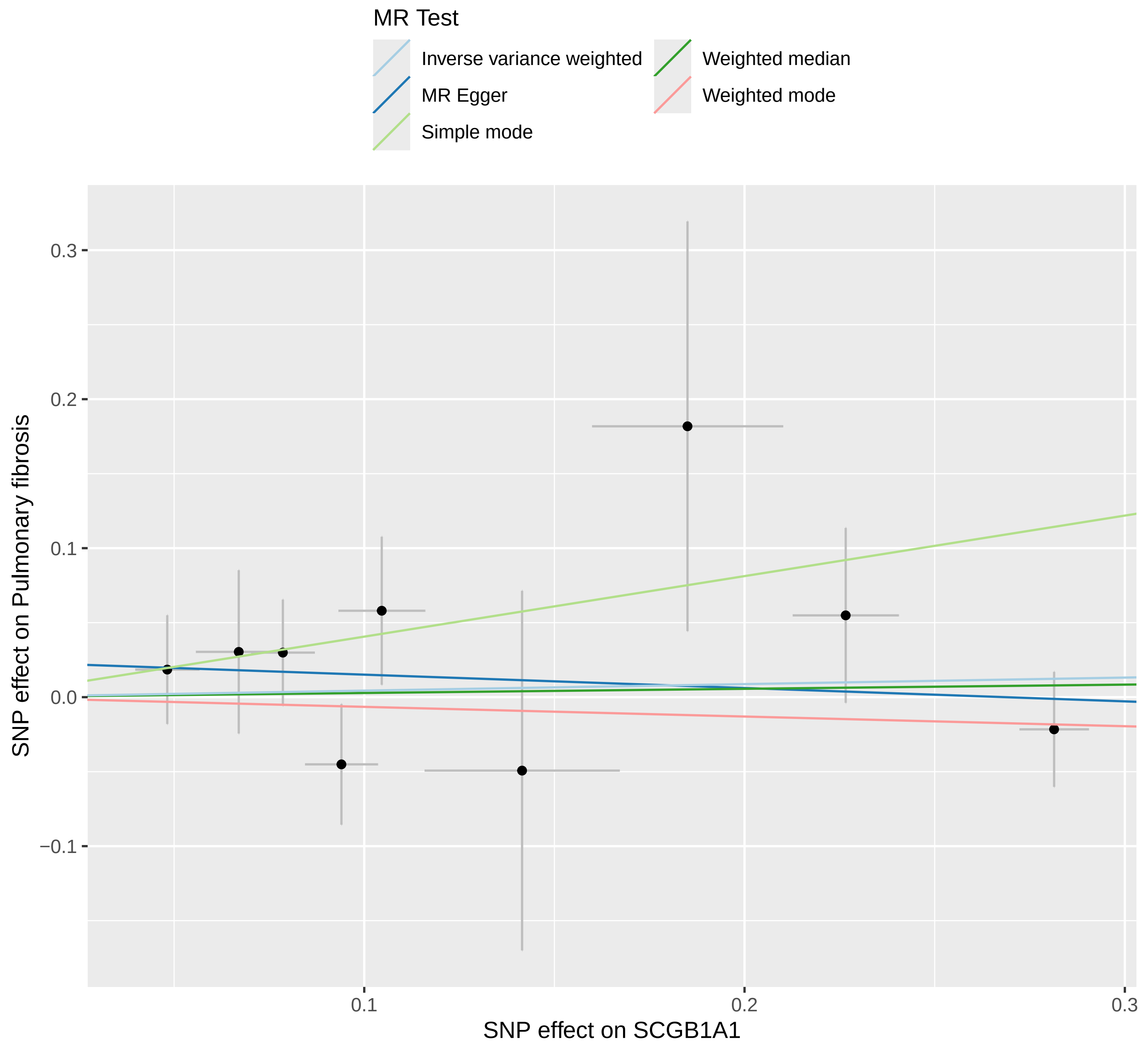
MR Test
Inverse variance weighted
MR Egger
Simple mode
Weighted median
Weighted mode
0.3
0.2
0.1
0.0
−0.1
0.1
0.2
0.3
SNP effect on Pulmonary fibrosis
SNP effect on SCGB1A1

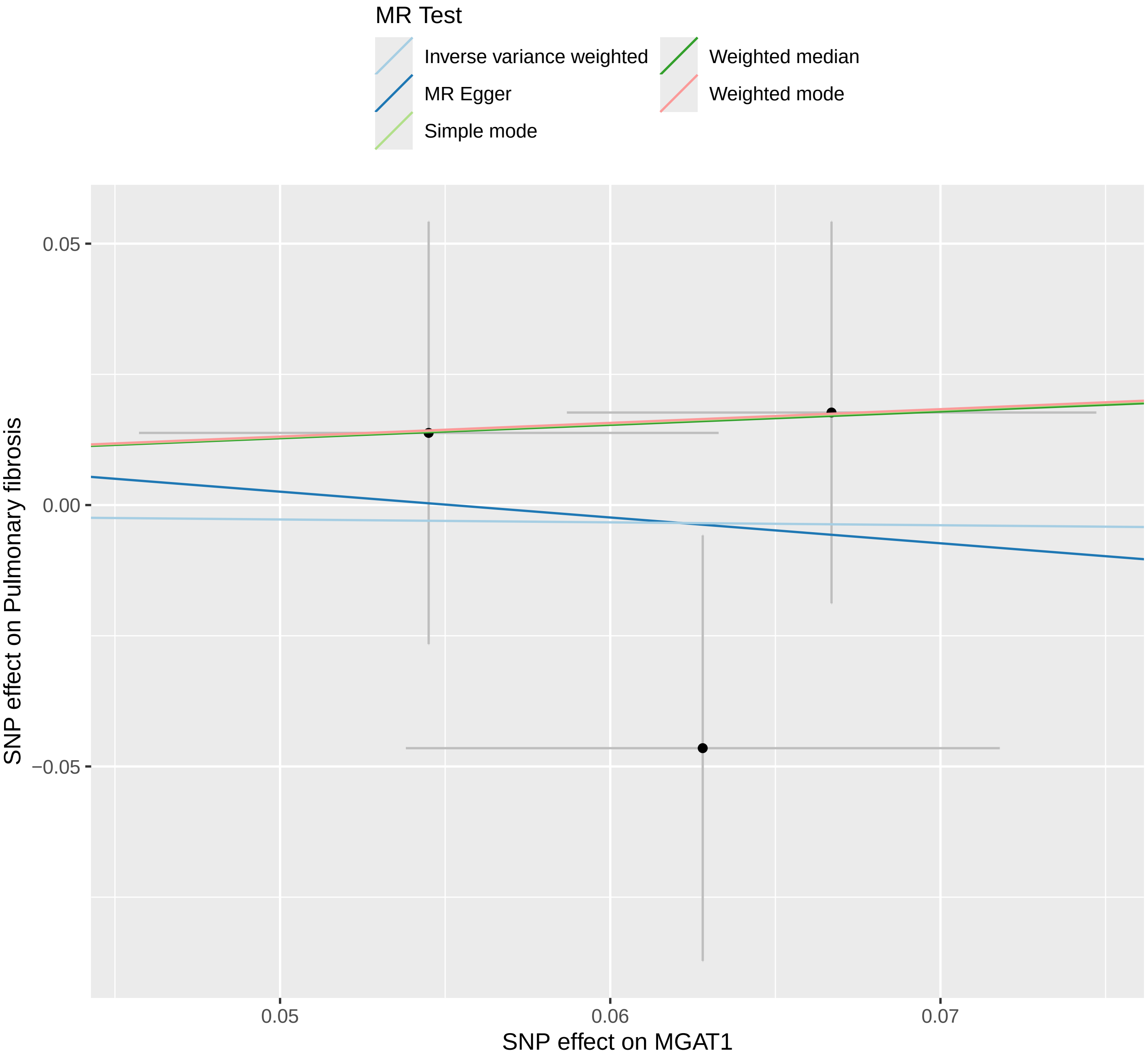
MR Test
Inverse variance weighted
MR Egger
Simple mode
Weighted median
Weighted mode
0.05
0.00
−0.05
SNP effect on Pulmonary fibrosis
0.05
0.06
0.07
SNP effect on MGAT1

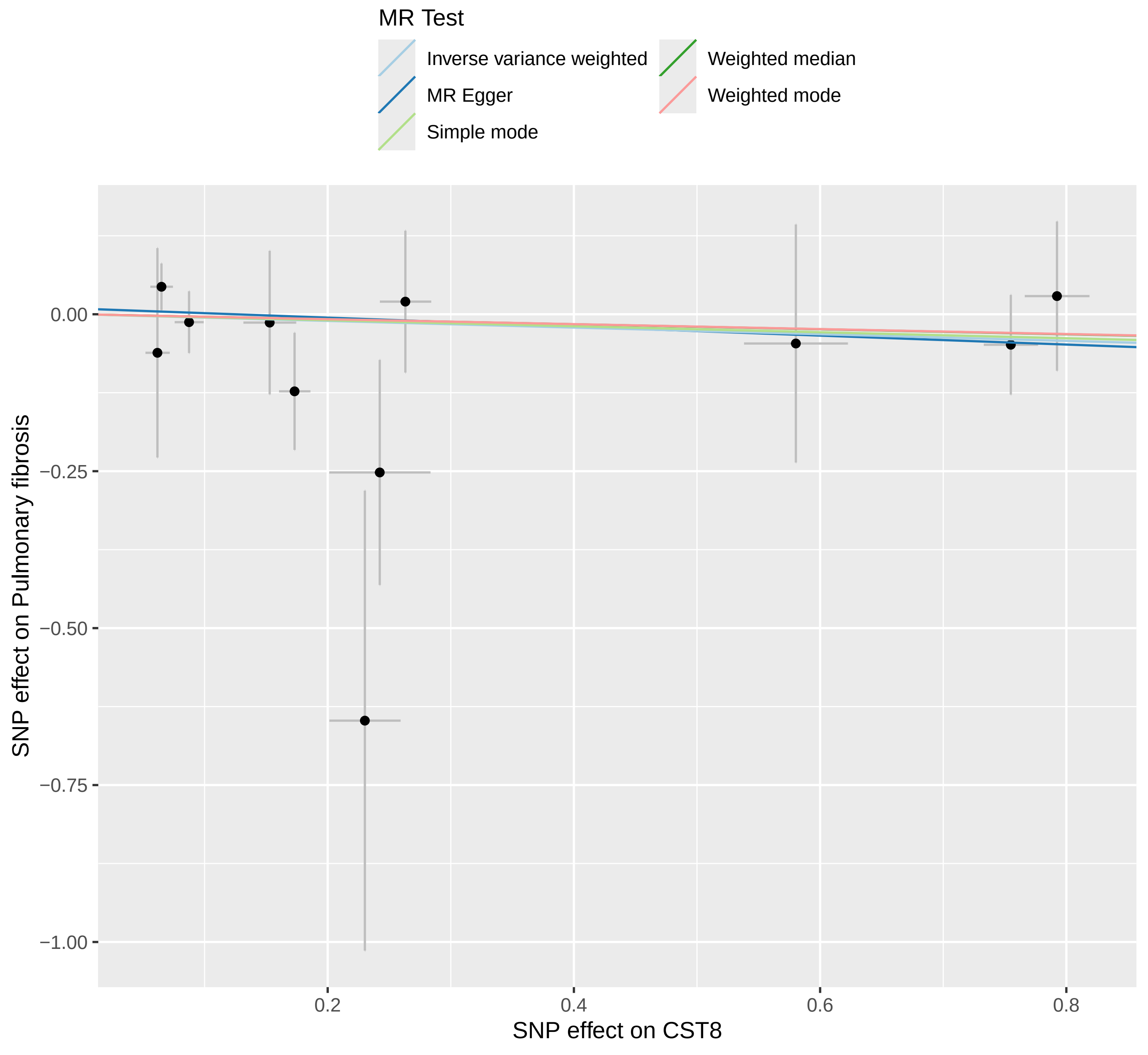

MR Test
Inverse variance weighted
MR Egger
Simple mode
Weighted median
Weighted mode
SNP effect on Pulmonary fibrosis
0.00
−0.25
−0.50
−0.75
−1.00
0.2
0.4
0.6
0.8
SNP effect on CST8

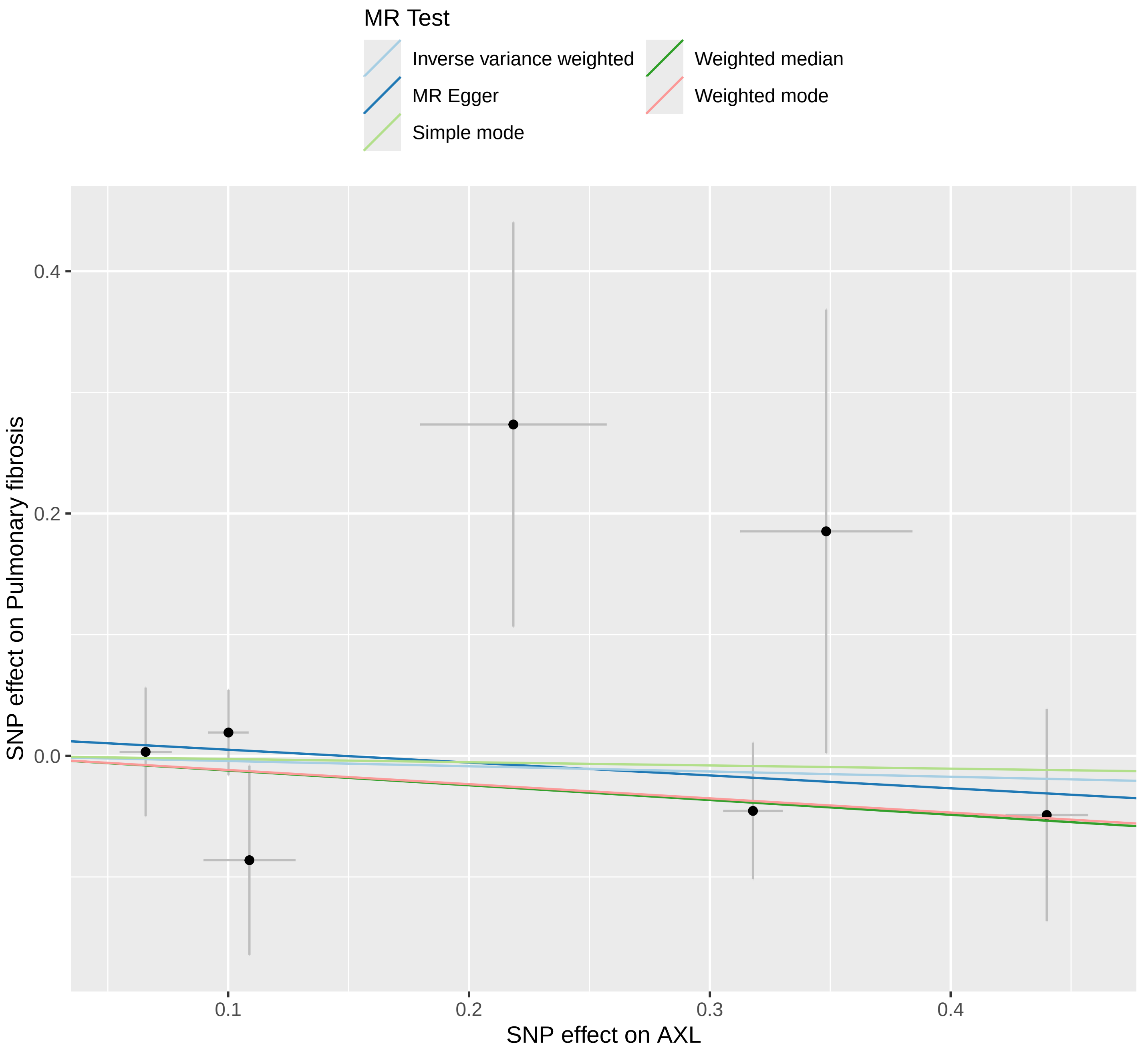
MR Test
Inverse variance weighted
MR Egger
Simple mode
Weighted median
Weighted mode
0.4
0.2
0.0
0.1
0.2
0.3
0.4
SNP effect on AXL
SNP effect on Pulmonary fibrosis

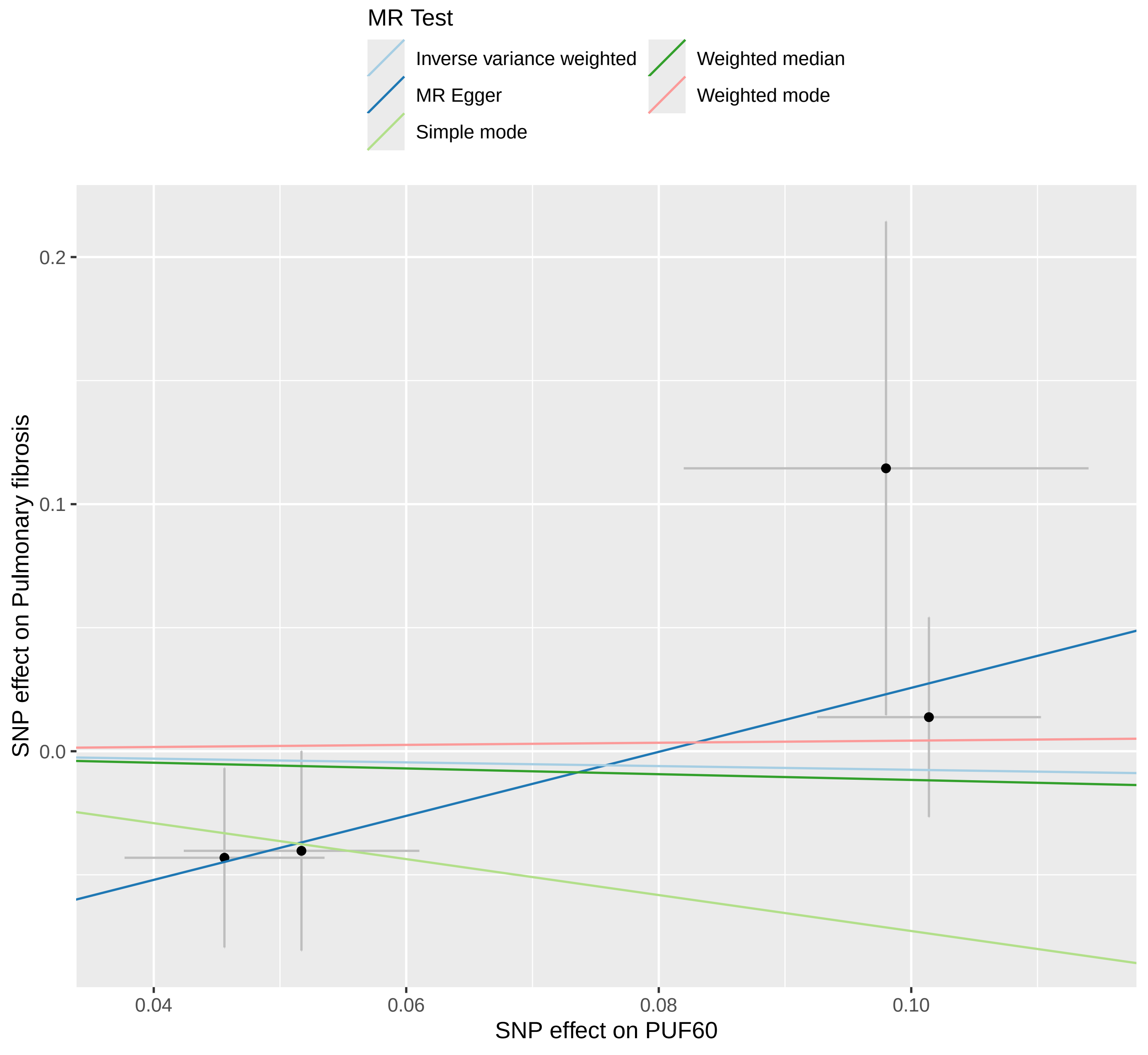
MR Test
Inverse variance weighted
MR Egger
Simple mode
Weighted median
Weighted mode
0.2
0.1
0.0
0.04
0.06
0.08
0.10
SNP effect on Pulmonary fibrosis
SNP effect on PUF60

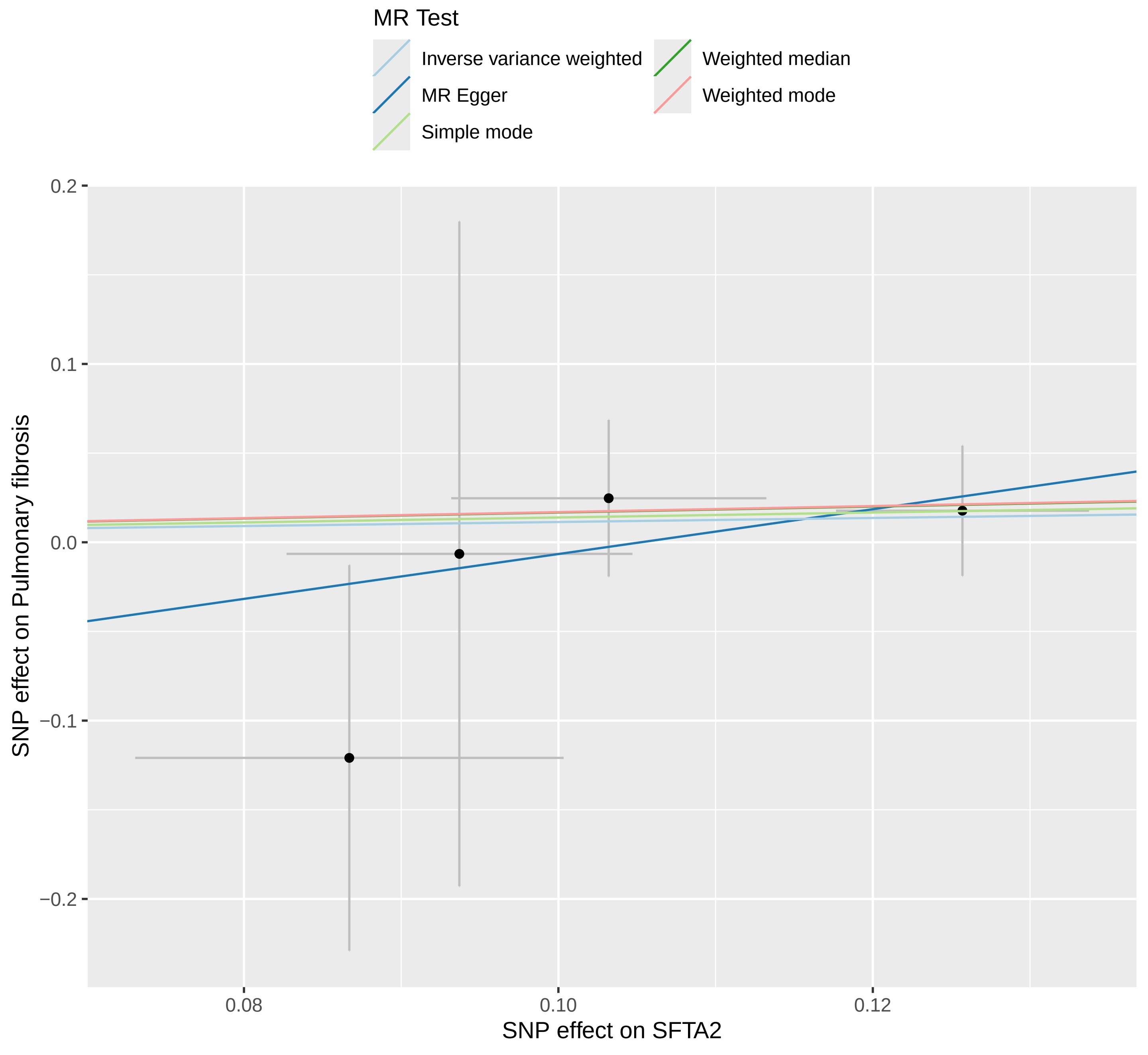
MR Test
Inverse variance weighted
MR Egger
Simple mode
Weighted median
Weighted mode
0.2
0.1
0.0
−0.1
−0.2
SNP effect on Pulmonary fibrosis
0.08
0.10
0.12
SNP effect on SFTA2

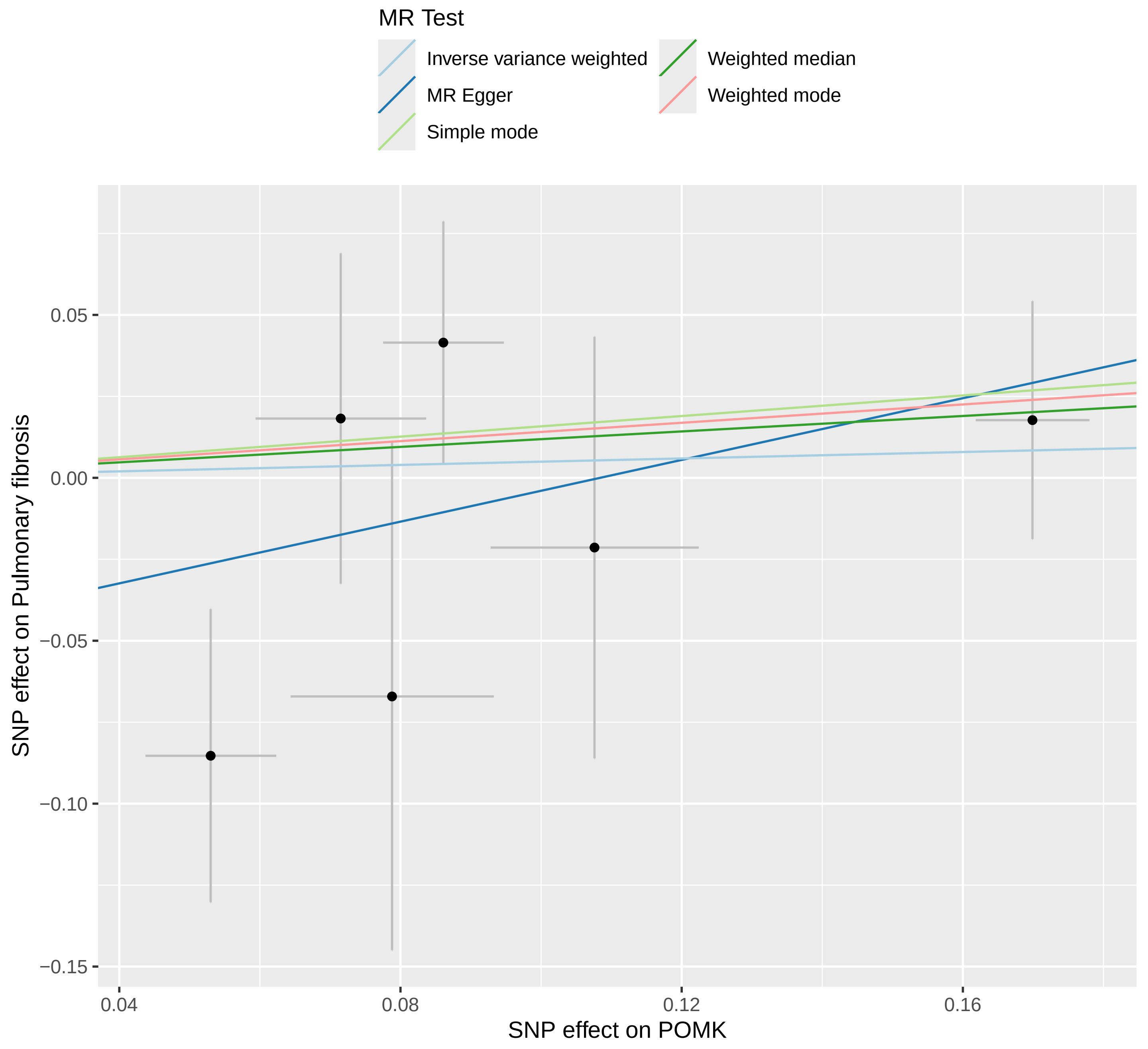
MR Test
Inverse variance weighted
MR Egger
Simple mode
Weighted median
Weighted mode
0.05
0.00
−0.05
−0.10
−0.15
0.04
0.08
0.12
0.16
SNP effect on Pulmonary fibrosis
SNP effect on POMK

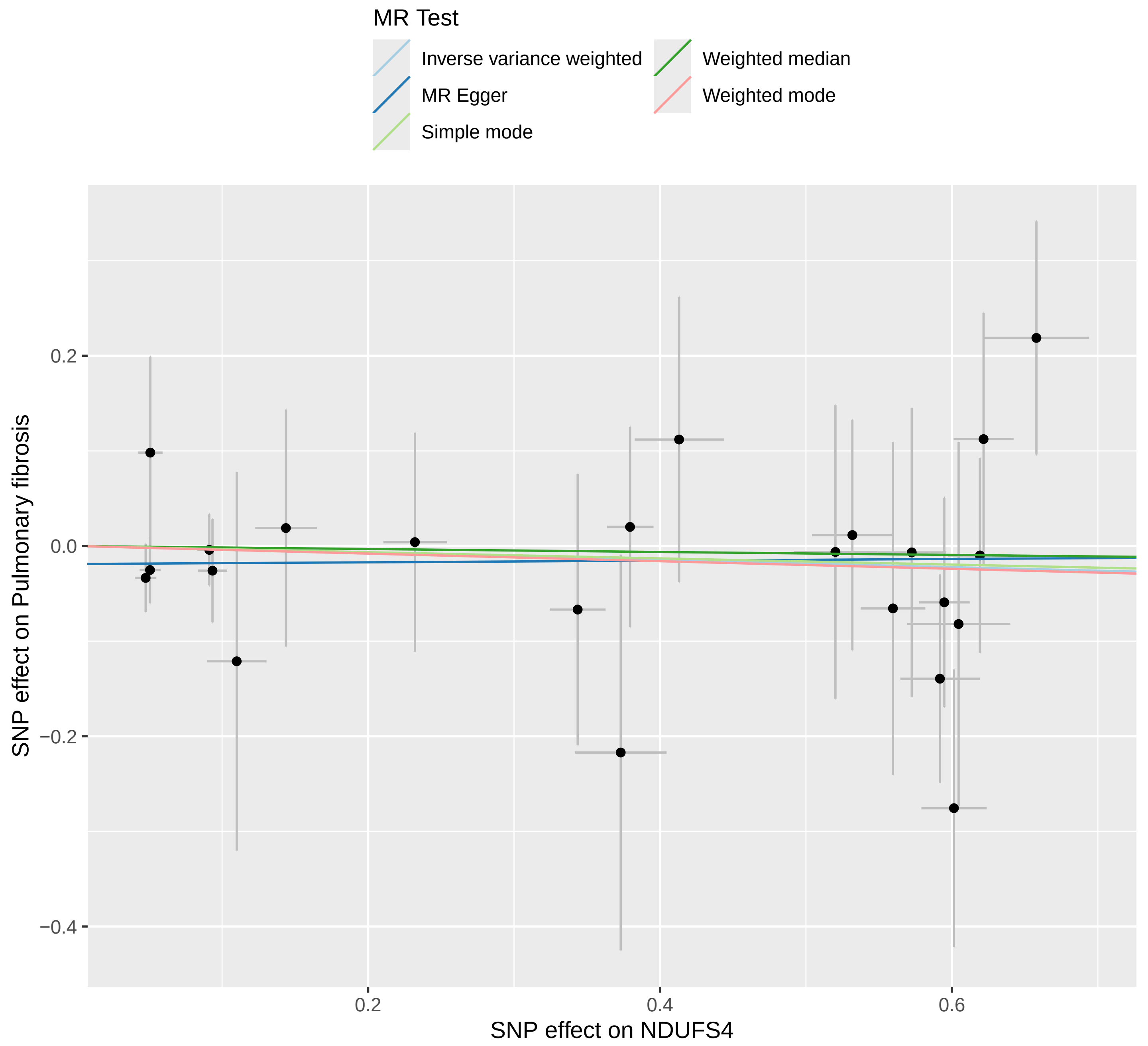
MR Test
Inverse variance weighted
MR Egger
Simple mode
Weighted median
Weighted mode
SNP effect on Pulmonary fibrosis
0.2
0.0
−0.2
−0.4
0.2
0.4
0.6
SNP effect on NDUFS4

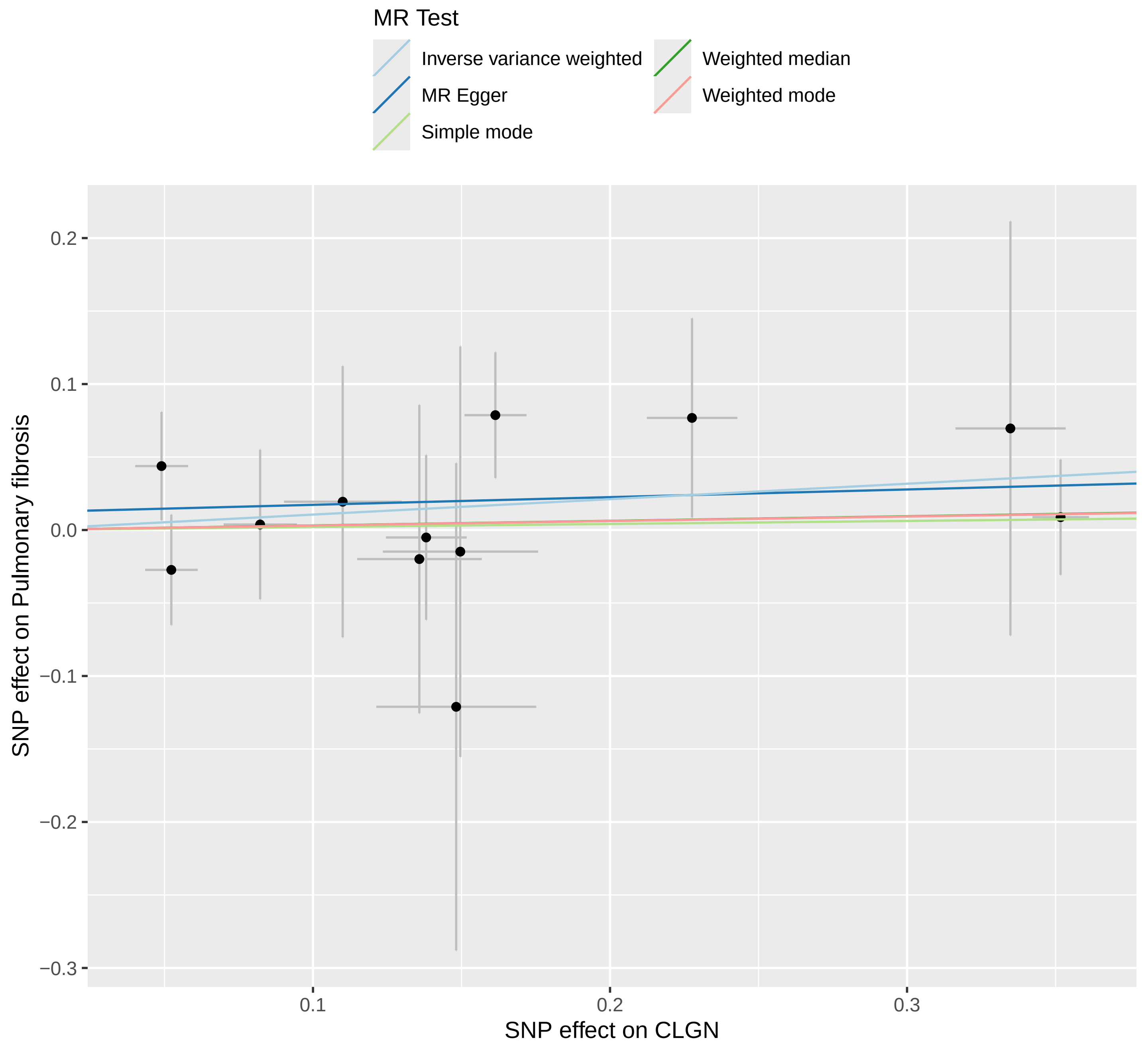
MR Test
Inverse variance weighted
MR Egger
Simple mode
Weighted median
Weighted mode
SNP effect on Pulmonary fibrosis
0.2
0.1
0.0
−0.1
−0.2
−0.3
0.1
0.2
0.3
SNP effect on CLGN

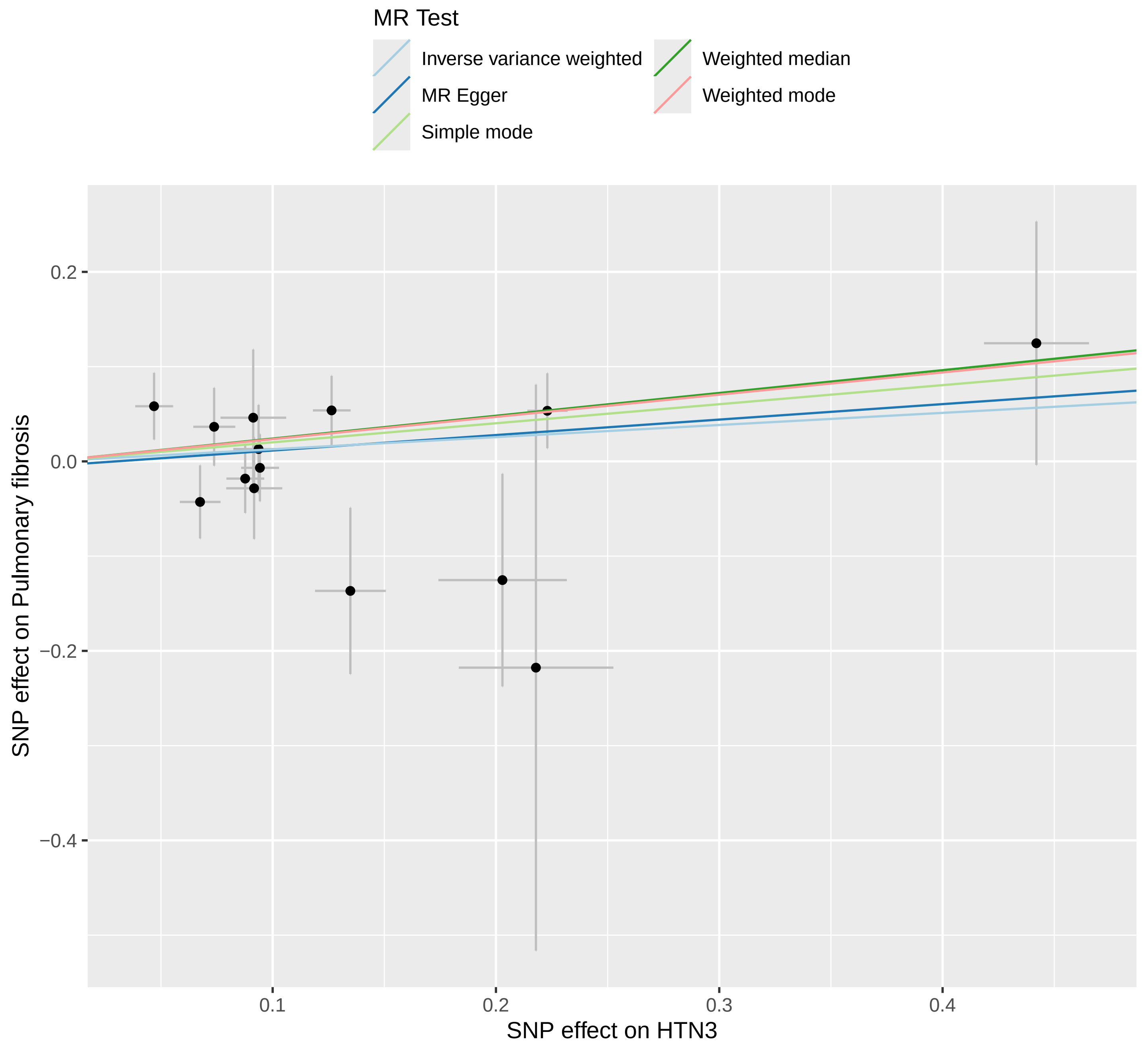
MR Test
Inverse variance weighted
MR Egger
Simple mode
Weighted median
Weighted mode
0.2
0.0
−0.2
−0.4
0.1
0.2
0.3
0.4
SNP effect on Pulmonary fibrosis
SNP effect on HTN3

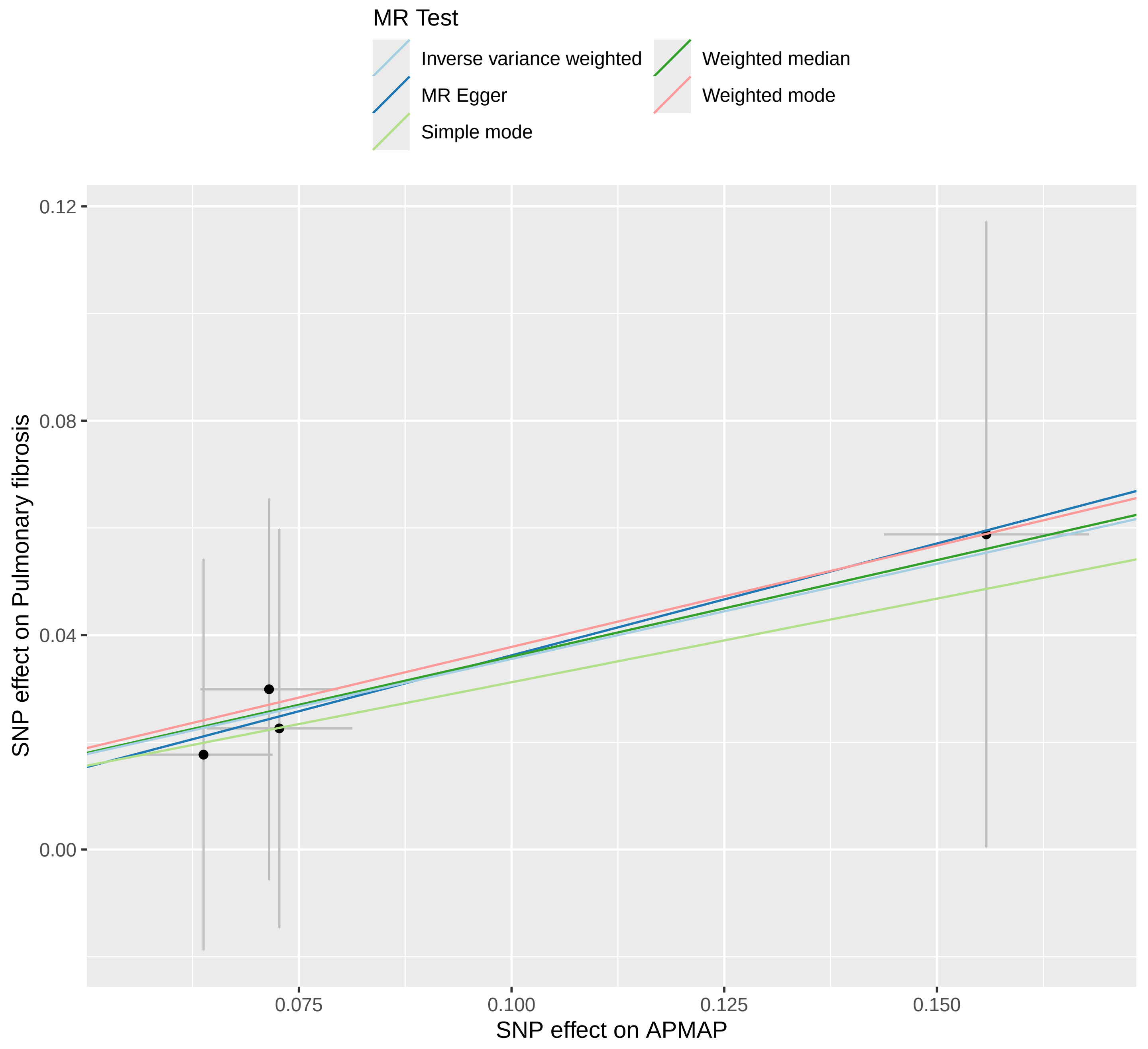
MR Test
Inverse variance weighted
MR Egger
Simple mode
Weighted median
Weighted mode
0.12
0.08
0.04
0.00
0.075
0.100
0.125
0.150
SNP effect on Pulmonary fibrosis
SNP effect on APMAP

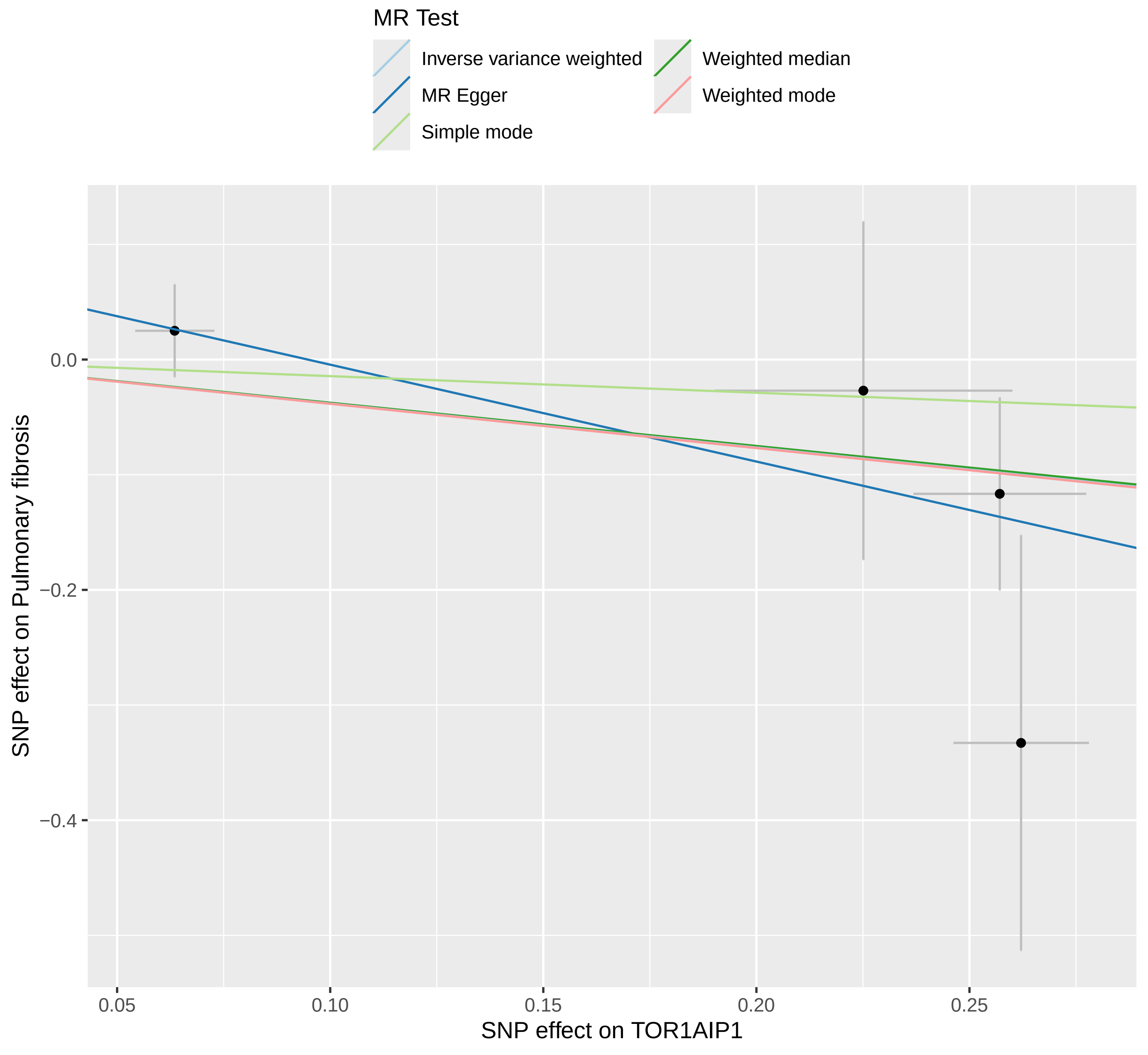
MR Test
Inverse variance weighted
MR Egger
Simple mode
Weighted median
Weighted mode
0.0
−0.2
−0.4
0.05
0.10
0.15
0.20
0.25
SNP effect on Pulmonary fibrosis
SNP effect on TOR1AIP1

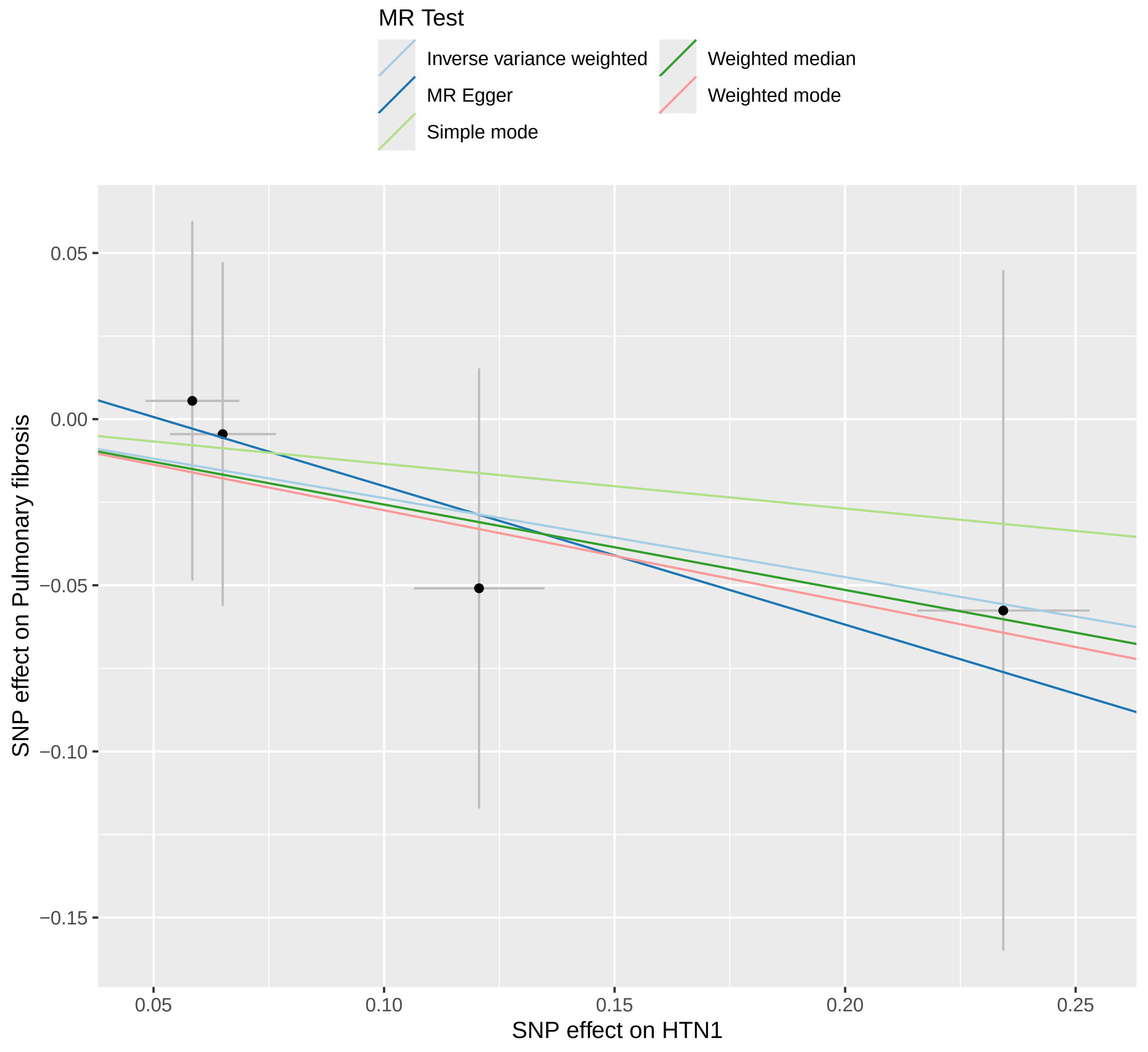
MR Test
Inverse variance weighted
MR Egger
Simple mode
Weighted median
Weighted mode
0.05
0.00
−0.05
−0.10
−0.15
SNP effect on Pulmonary fibrosis
0.05
0.10
0.15
0.20
0.25
SNP effect on HTN1

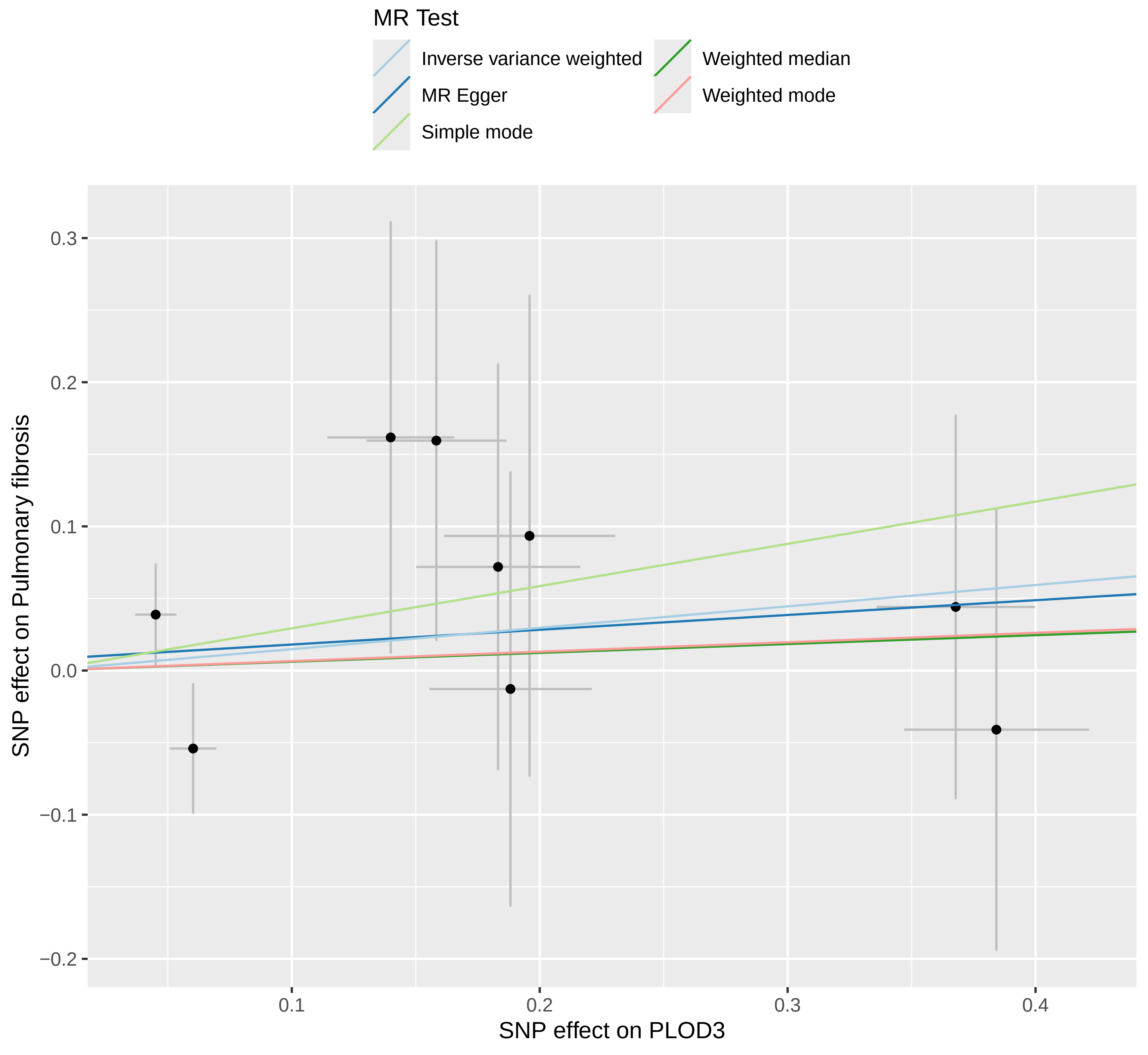
MR Test
Inverse variance weighted
MR Egger
Simple mode
Weighted median
Weighted mode
0.3
0.2
0.1
0.0
−0.1
−0.2
SNP effect on Pulmonary fibrosis
0.1
0.2
0.3
0.4
SNP effect on PLOD3

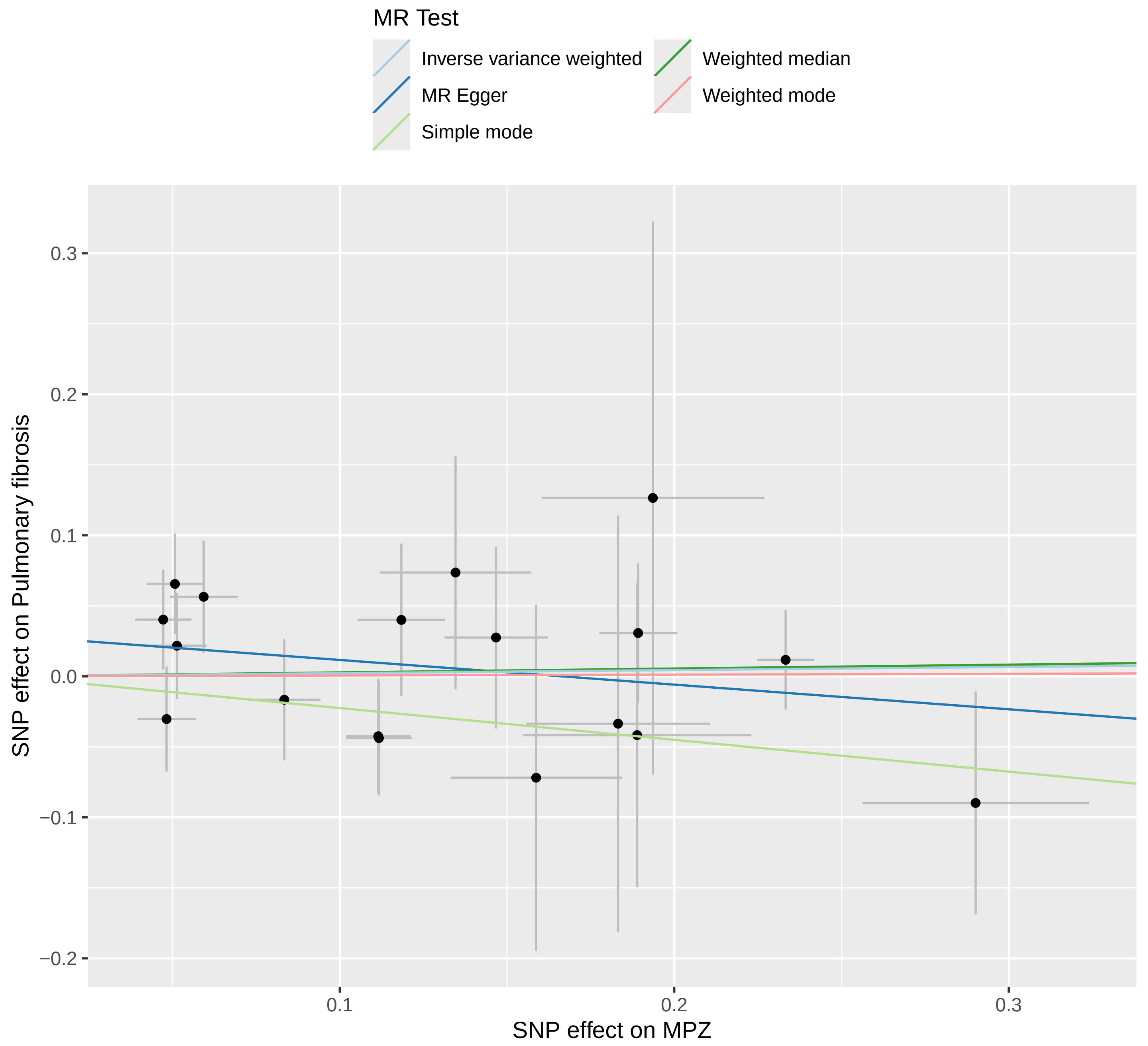
MR Test
Inverse variance weighted
MR Egger
Simple mode
Weighted median
Weighted mode
SNP effect on Pulmonary fibrosis
0.3
0.2
0.1
0.0
−0.1
−0.2
0.1
0.2
0.3
SNP effect on MPZ

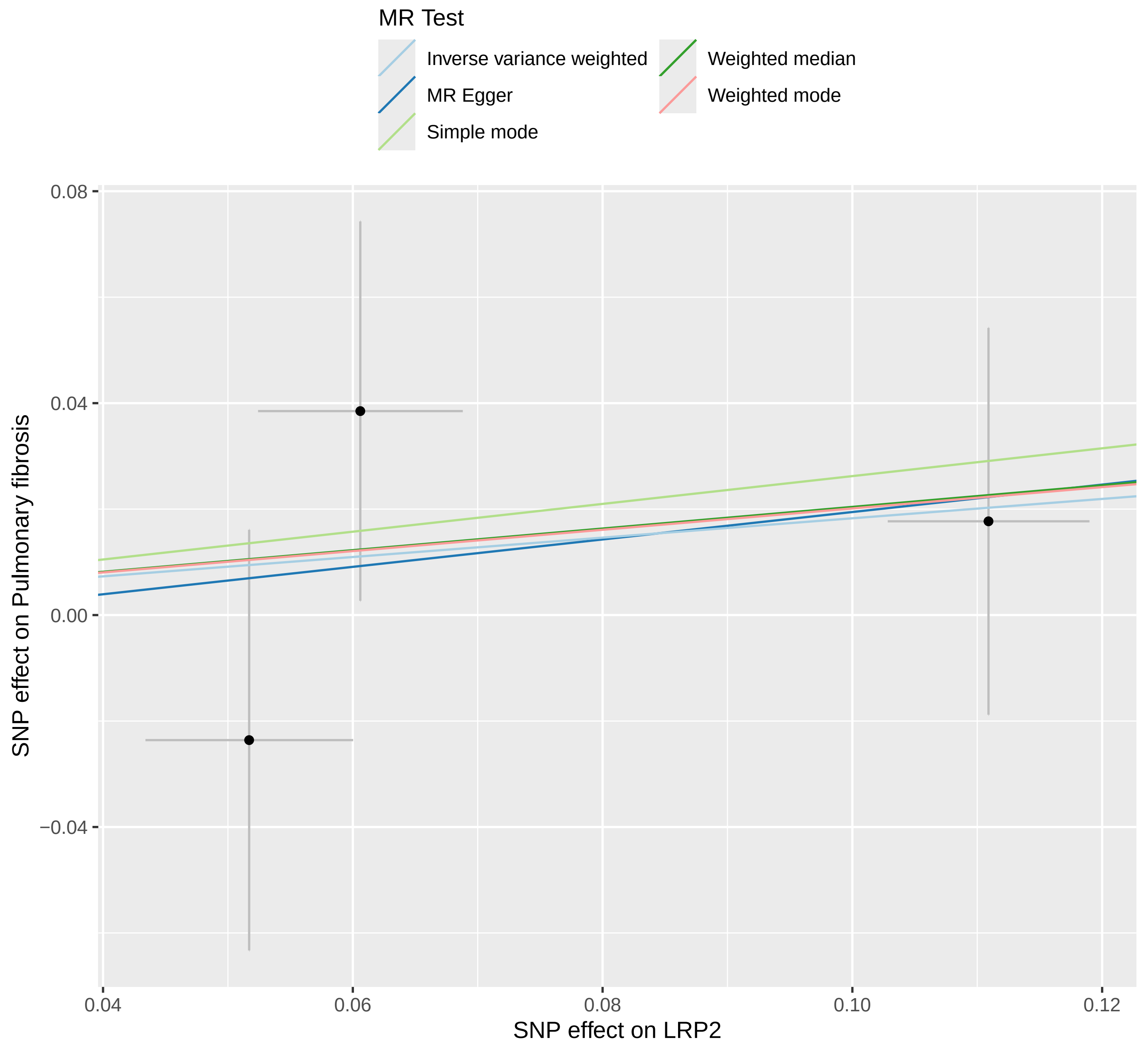
MR Test
Inverse variance weighted
MR Egger
Simple mode
Weighted median
Weighted mode
0.08
0.04
0.00
−0.04
0.04
0.06
0.08
0.10
0.12
SNP effect on Pulmonary fibrosis
SNP effect on LRP2

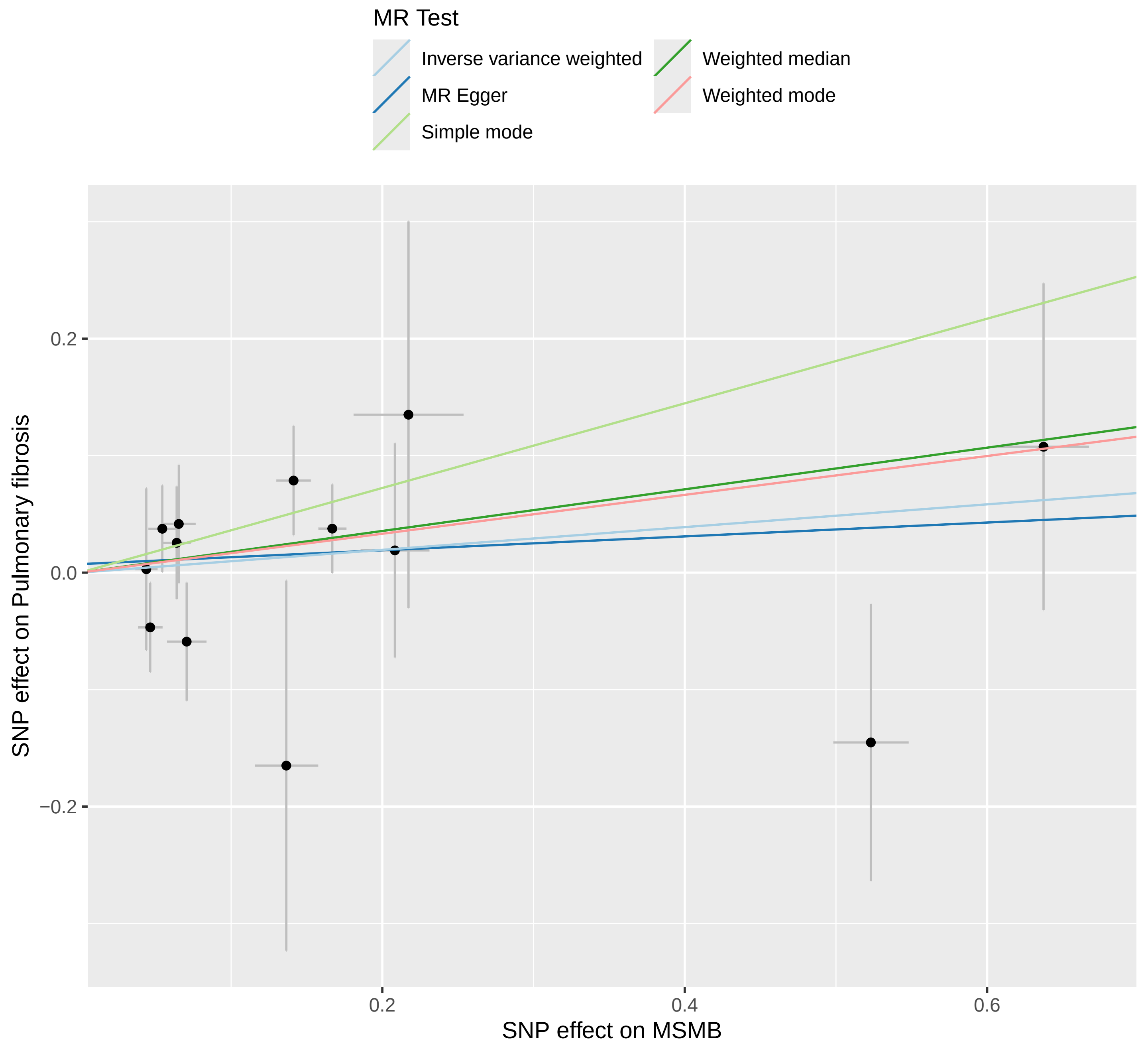
MR Test
Inverse variance weighted
MR Egger
Simple mode
Weighted median
Weighted mode
0.2
0.0
−0.2
SNP effect on Pulmonary fibrosis
0.2
0.4
0.6
SNP effect on MSMB

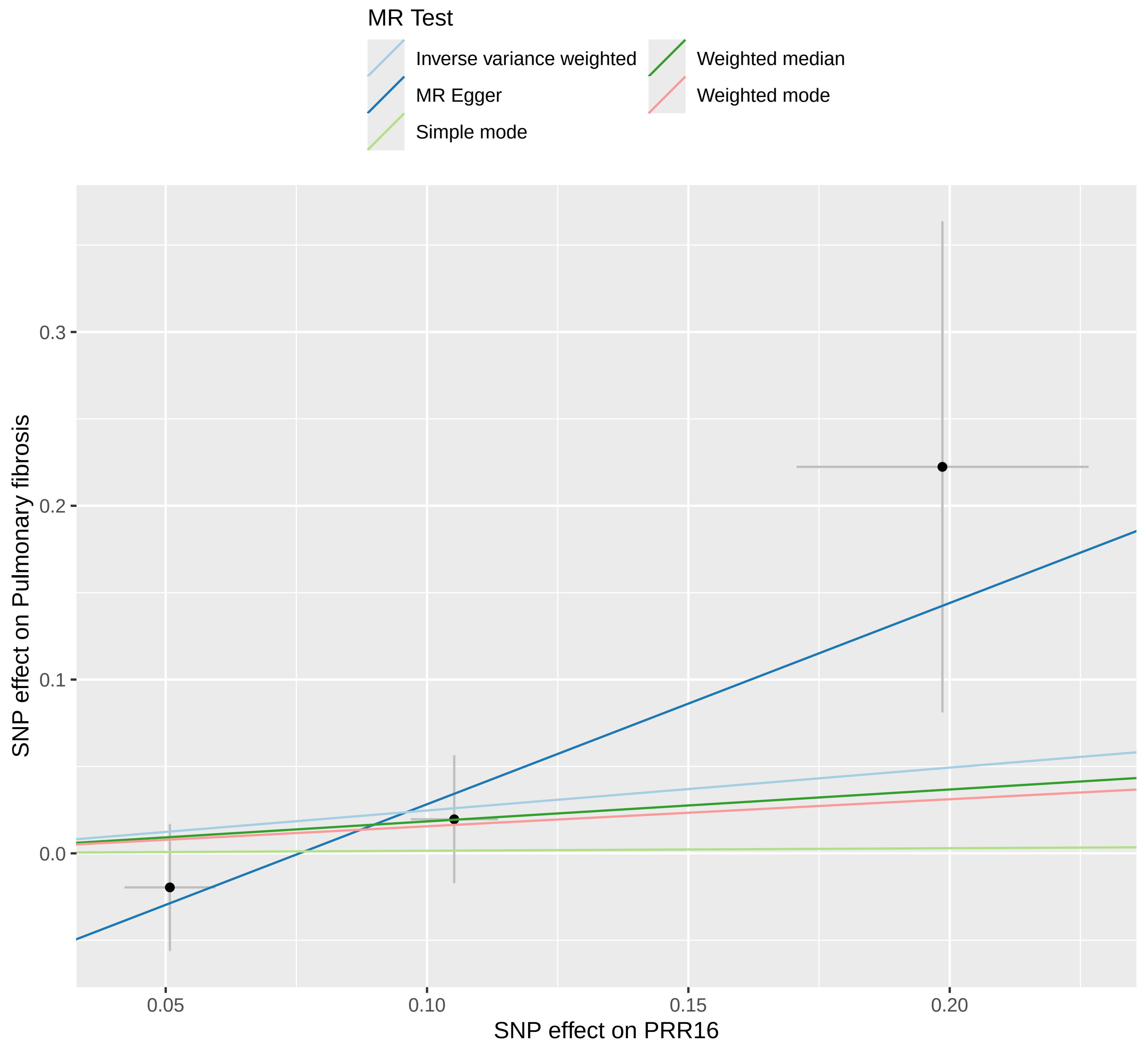
MR Test
Inverse variance weighted
MR Egger
Simple mode
Weighted median
Weighted mode
0.3
0.2
0.1
0.0
0.05
0.10
0.15
0.20
SNP effect on Pulmonary fibrosis
SNP effect on PRR16

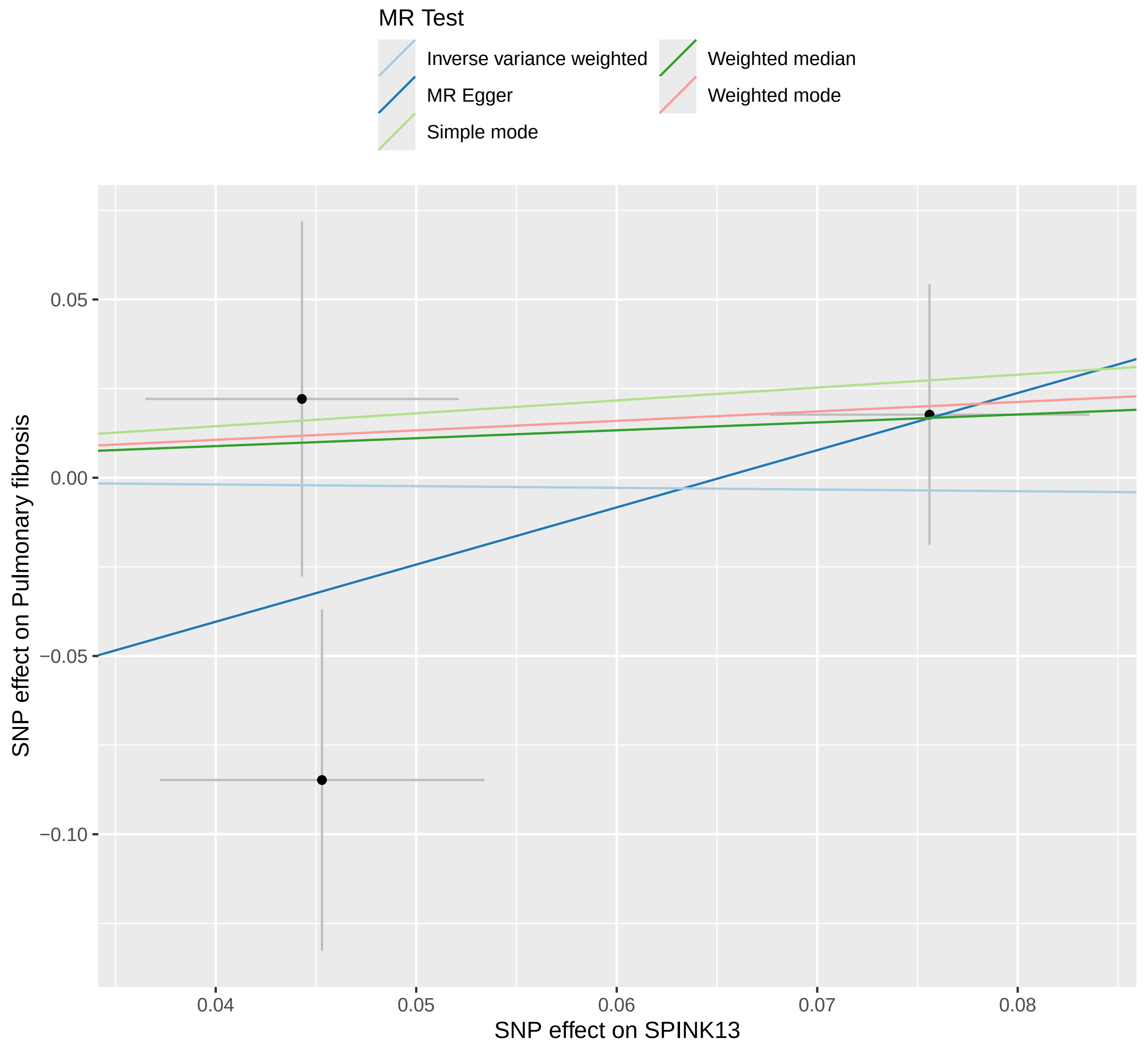

MR Test
Inverse variance weighted
MR Egger
Simple mode
Weighted median
Weighted mode
0.05
0.00
−0.05
−0.10
SNP effect on Pulmonary fibrosis
0.04
0.05
0.06
0.07
0.08
SNP effect on SPINK13

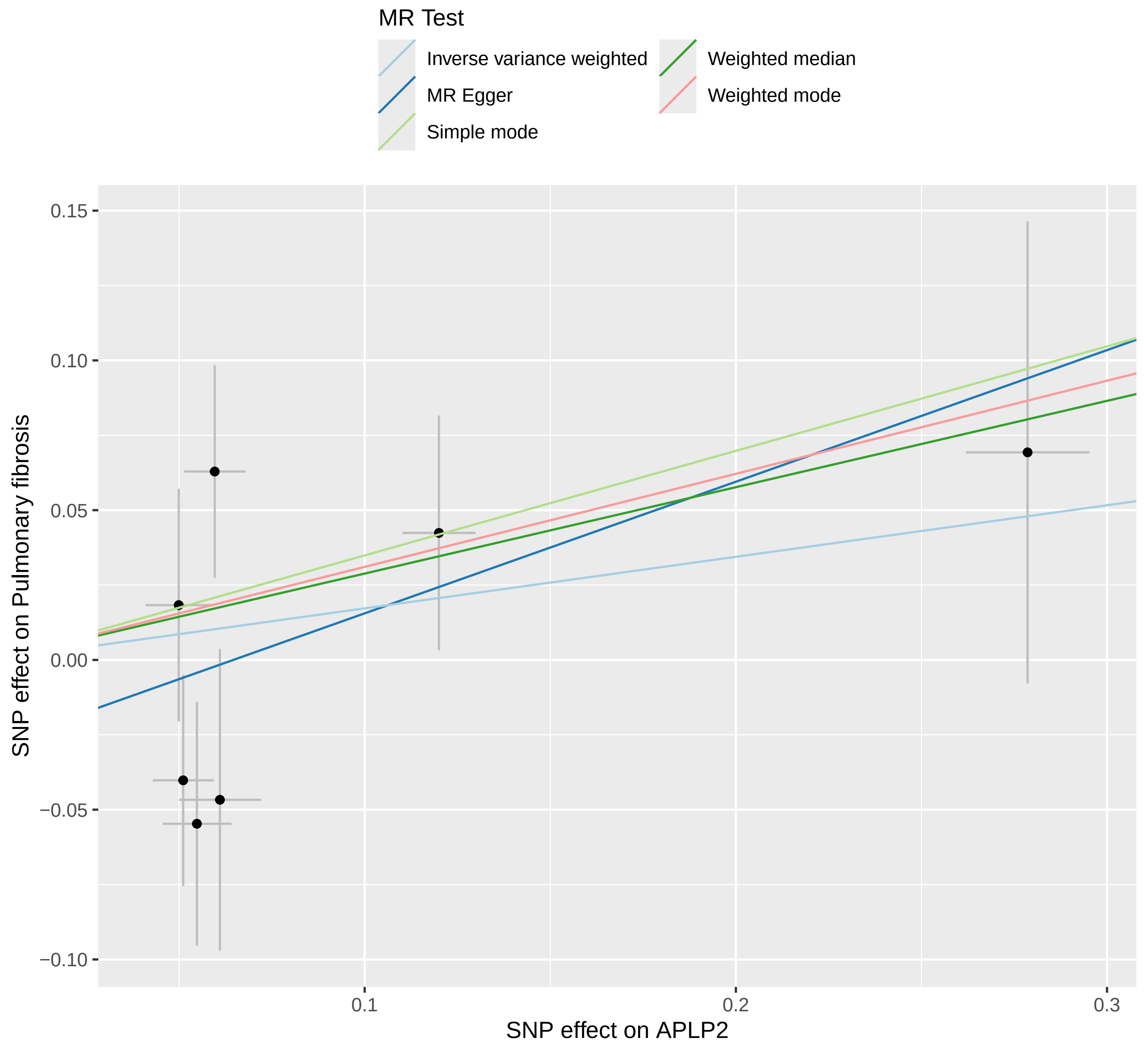
MR Test
Inverse variance weighted
MR Egger
Simple mode
Weighted median
Weighted mode
0.15
0.10
0.05
0.00
−0.05
−0.10
SNP effect on Pulmonary fibrosis
0.1
0.2
0.3
SNP effect on APLP2

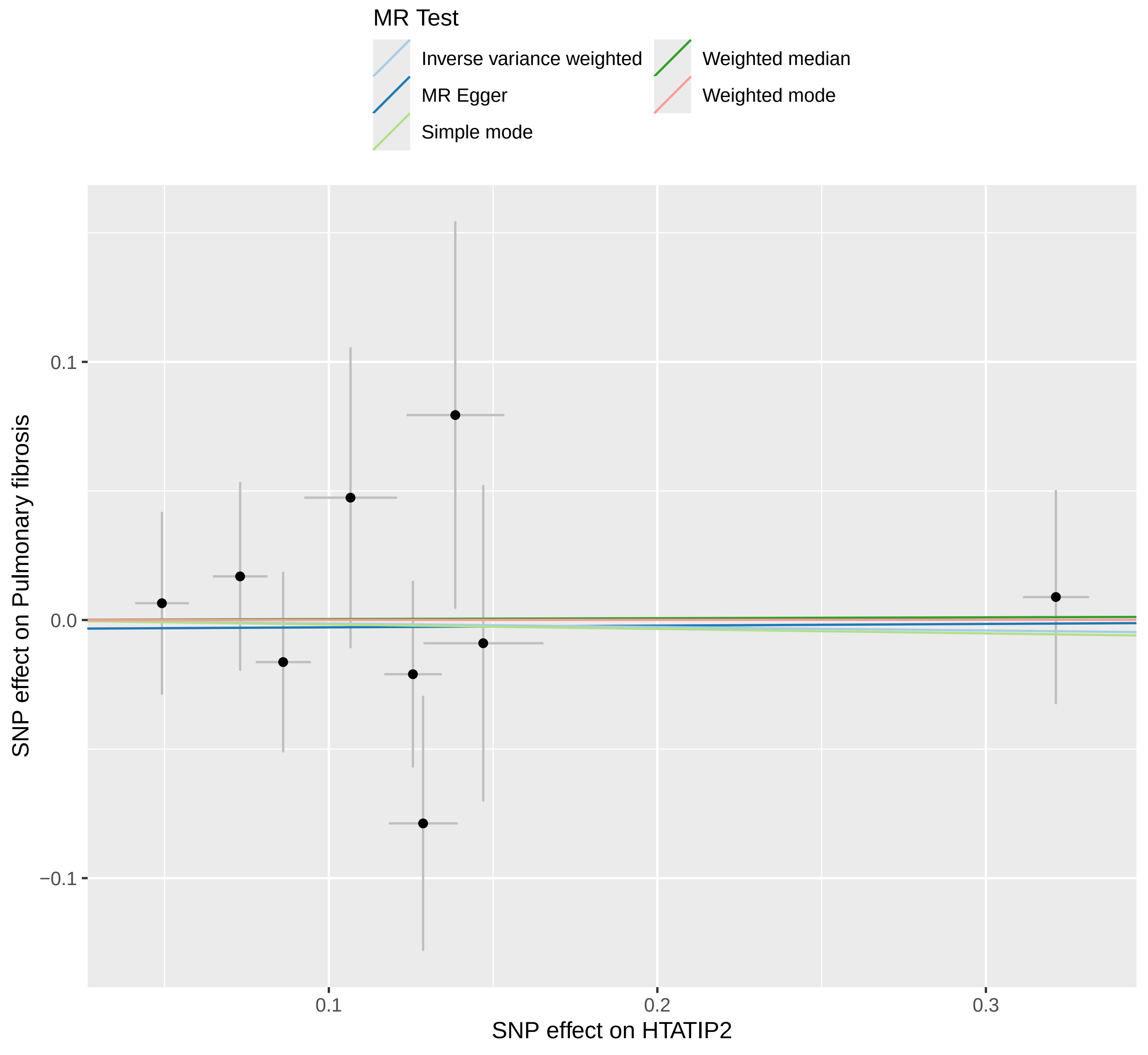

MR Test
Inverse variance weighted
MR Egger
Simple mode
Weighted median
Weighted mode
0.1
0.0
−0.1
SNP effect on Pulmonary fibrosis
0.1
0.2
0.3
SNP effect on HTATIP2

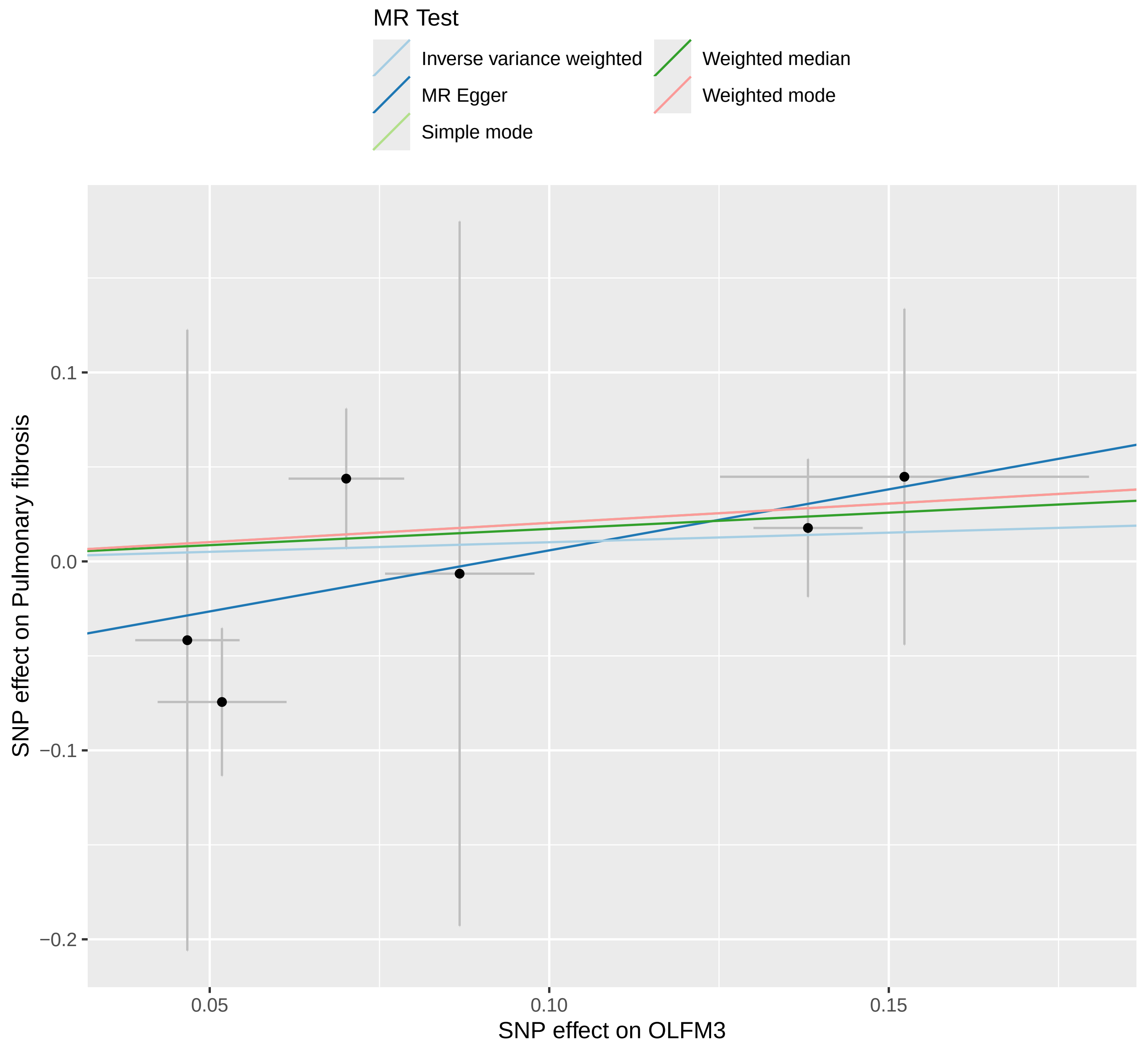
MR Test
Inverse variance weighted
MR Egger
Simple mode
Weighted median
Weighted mode
0.1
0.0
−0.1
−0.2
SNP effect on Pulmonary fibrosis
0.05
0.10
0.15
SNP effect on OLFM3

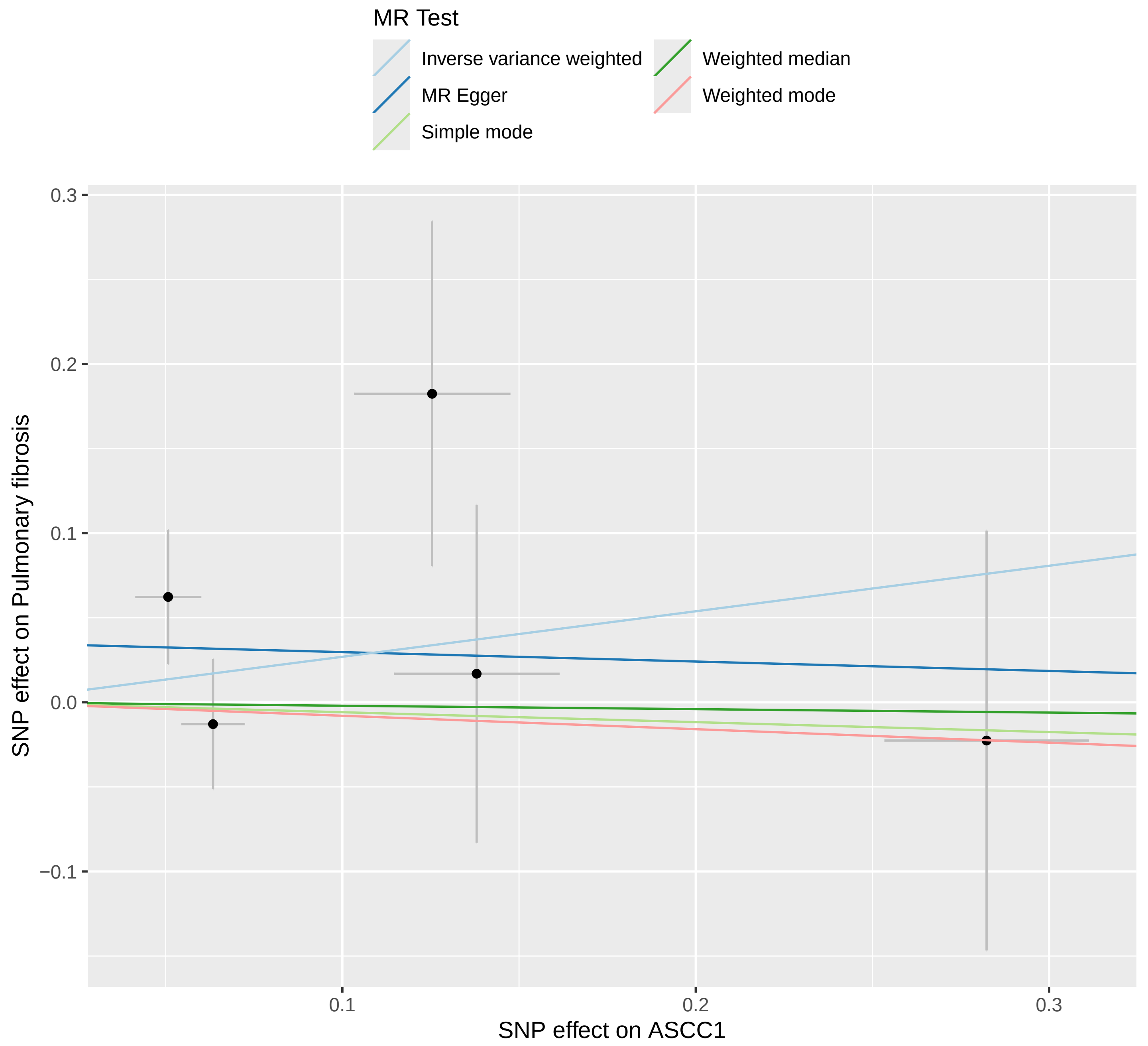

MR Test
Inverse variance weighted
MR Egger
Simple mode
Weighted median
Weighted mode
0.3
0.2
0.1
0.0
−0.1
SNP effect on Pulmonary fibrosis
0.1
0.2
0.3
SNP effect on ASCC1

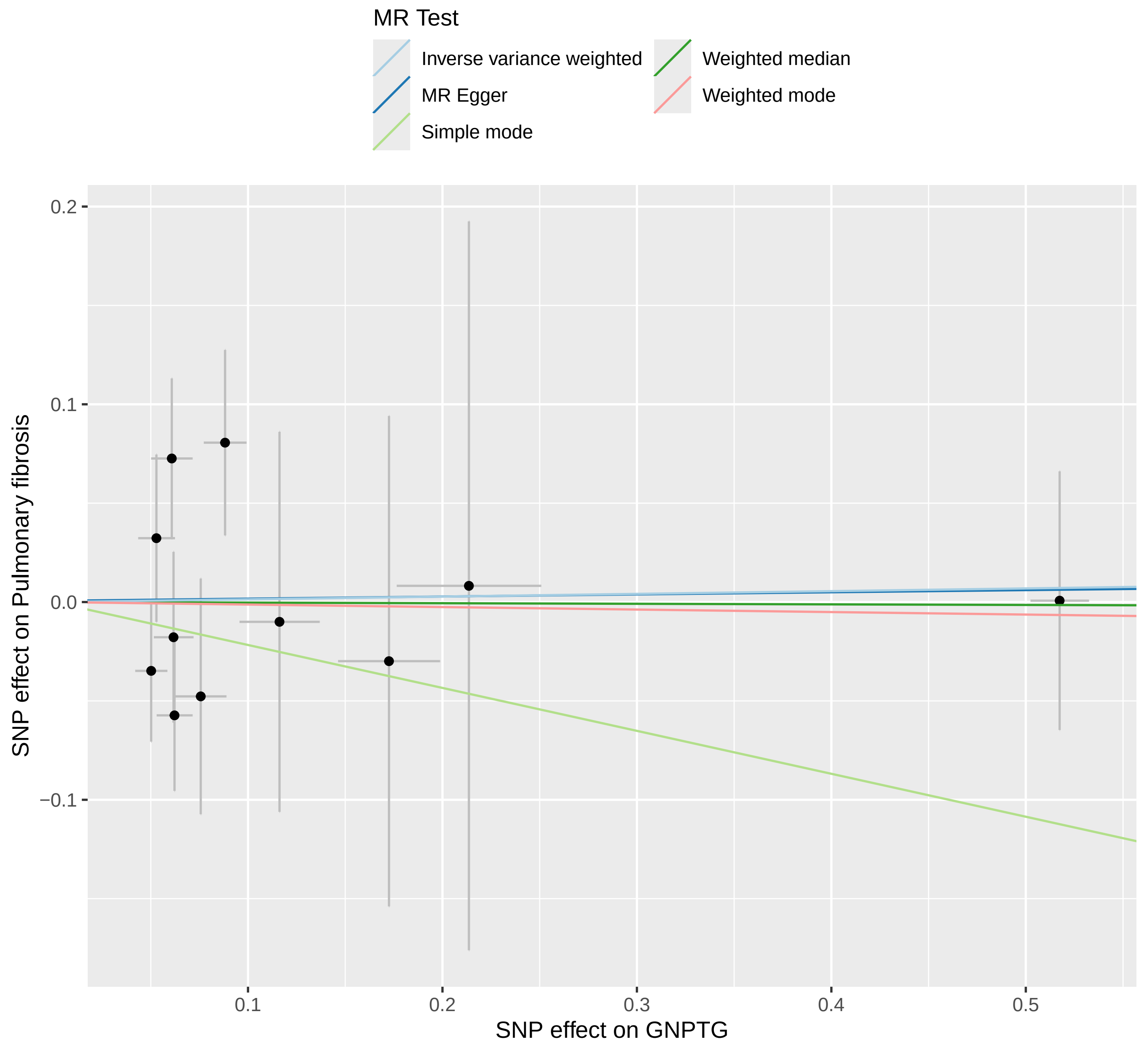

MR Test
Inverse variance weighted
MR Egger
Simple mode
Weighted median
Weighted mode
0.2
0.1
0.0
−0.1
0.1
0.2
0.3
0.4
0.5
SNP effect on Pulmonary fibrosis
SNP effect on GNPTG

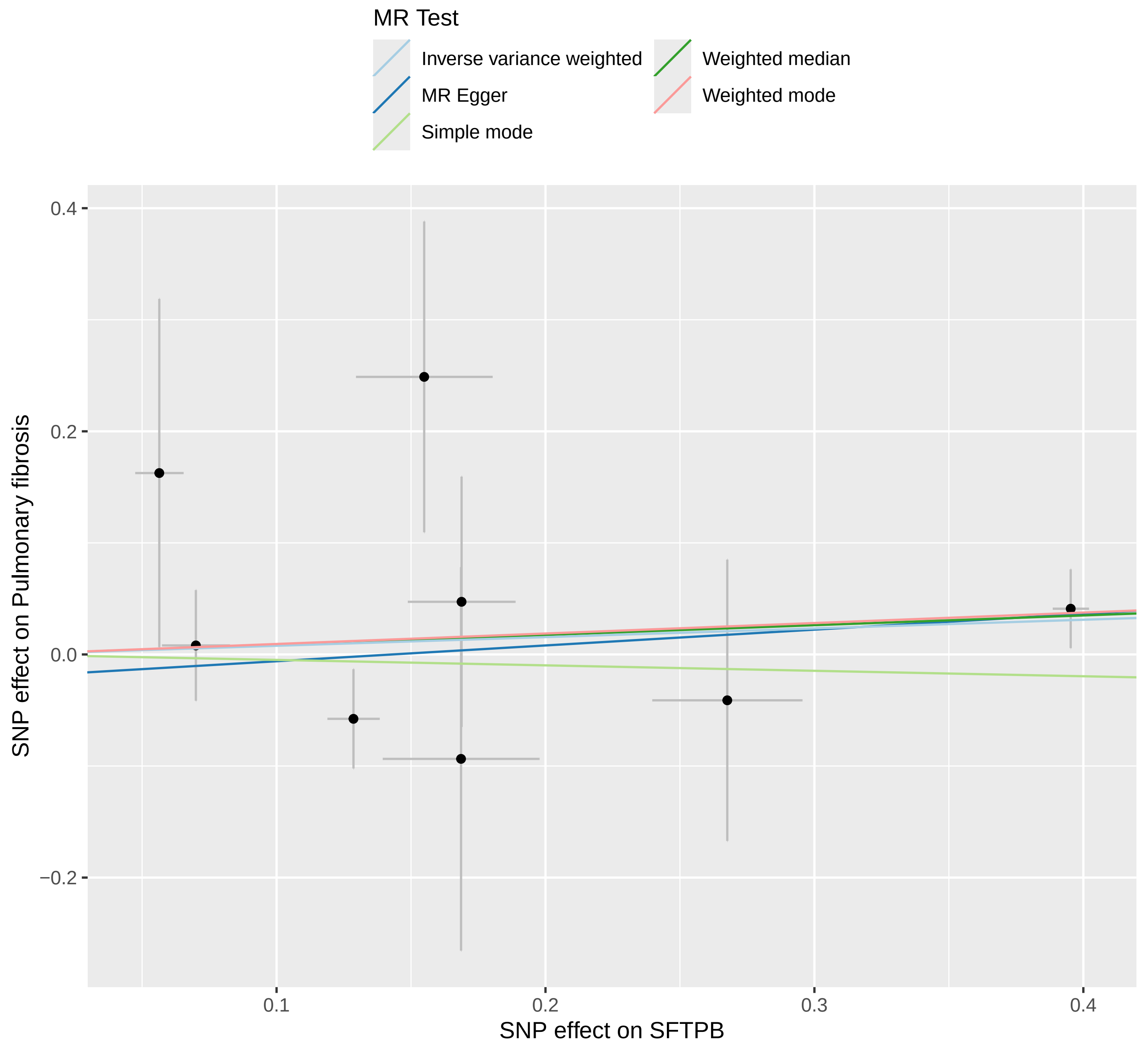

MR Test
Inverse variance weighted
MR Egger
Simple mode
Weighted median
Weighted mode
0.4
0.2
0.0
−0.2
0.1
0.2
0.3
0.4
SNP effect on Pulmonary fibrosis
SNP effect on SFTPB

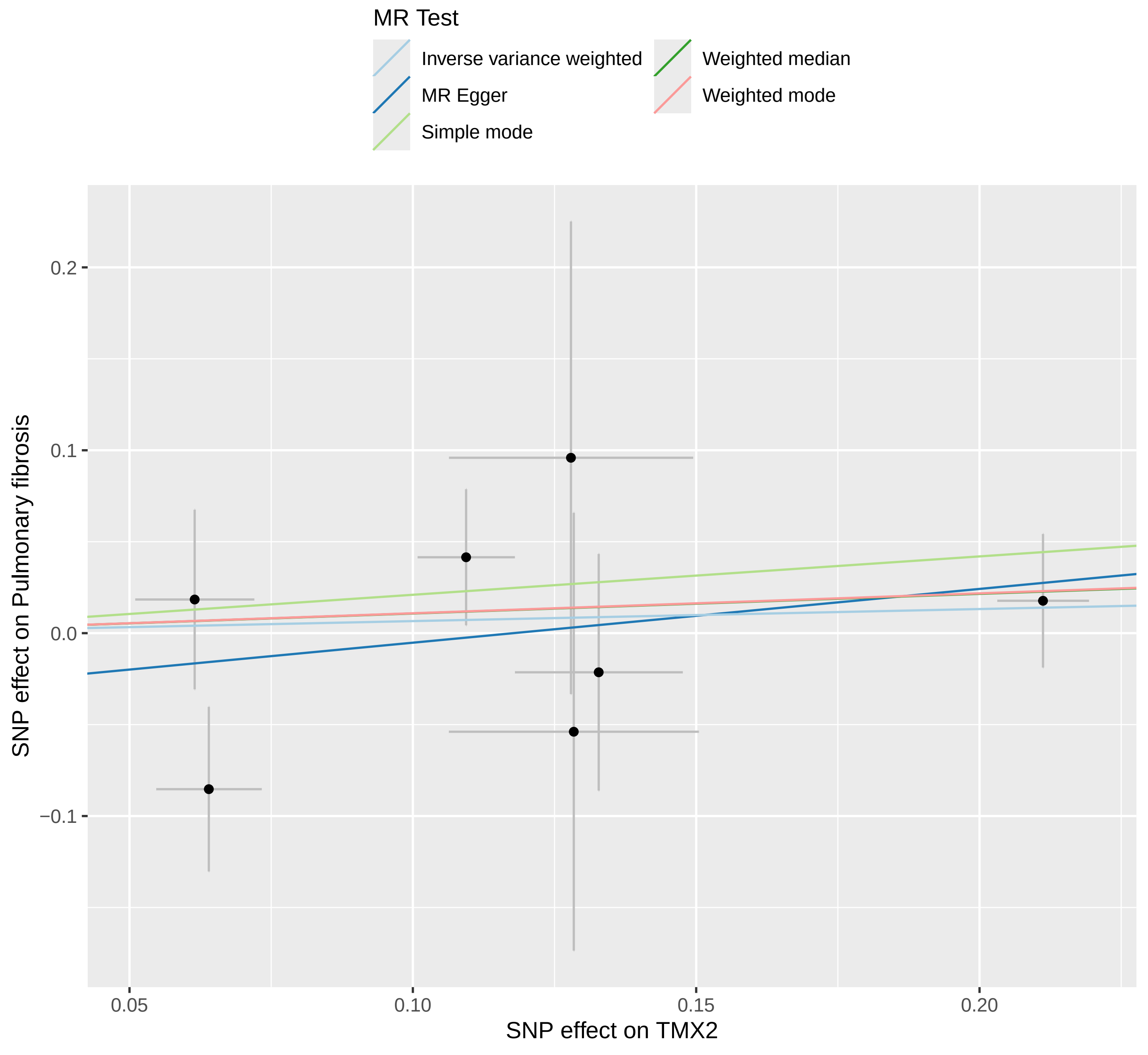
MR Test
Inverse variance weighted
MR Egger
Simple mode
Weighted median
Weighted mode
0.2
0.1
0.0
−0.1
SNP effect on Pulmonary fibrosis
0.05
0.10
0.15
0.20
SNP effect on TMX2

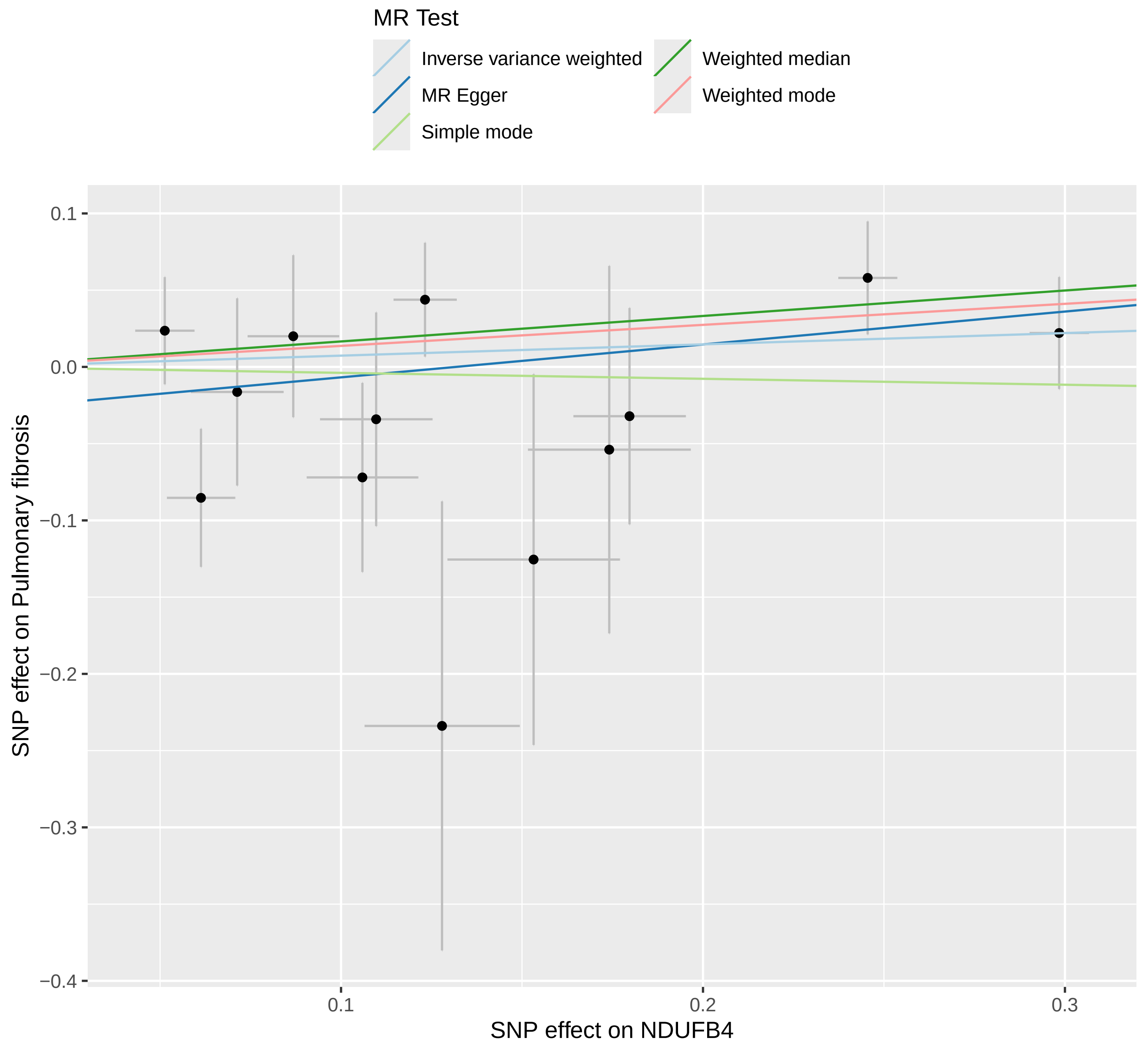

MR Test
Inverse variance weighted
MR Egger
Simple mode
Weighted median
Weighted mode
SNP effect on Pulmonary fibrosis
0.1
0.0
−0.1
−0.2
−0.3
−0.4
0.1
0.2
0.3
SNP effect on NDUFB4

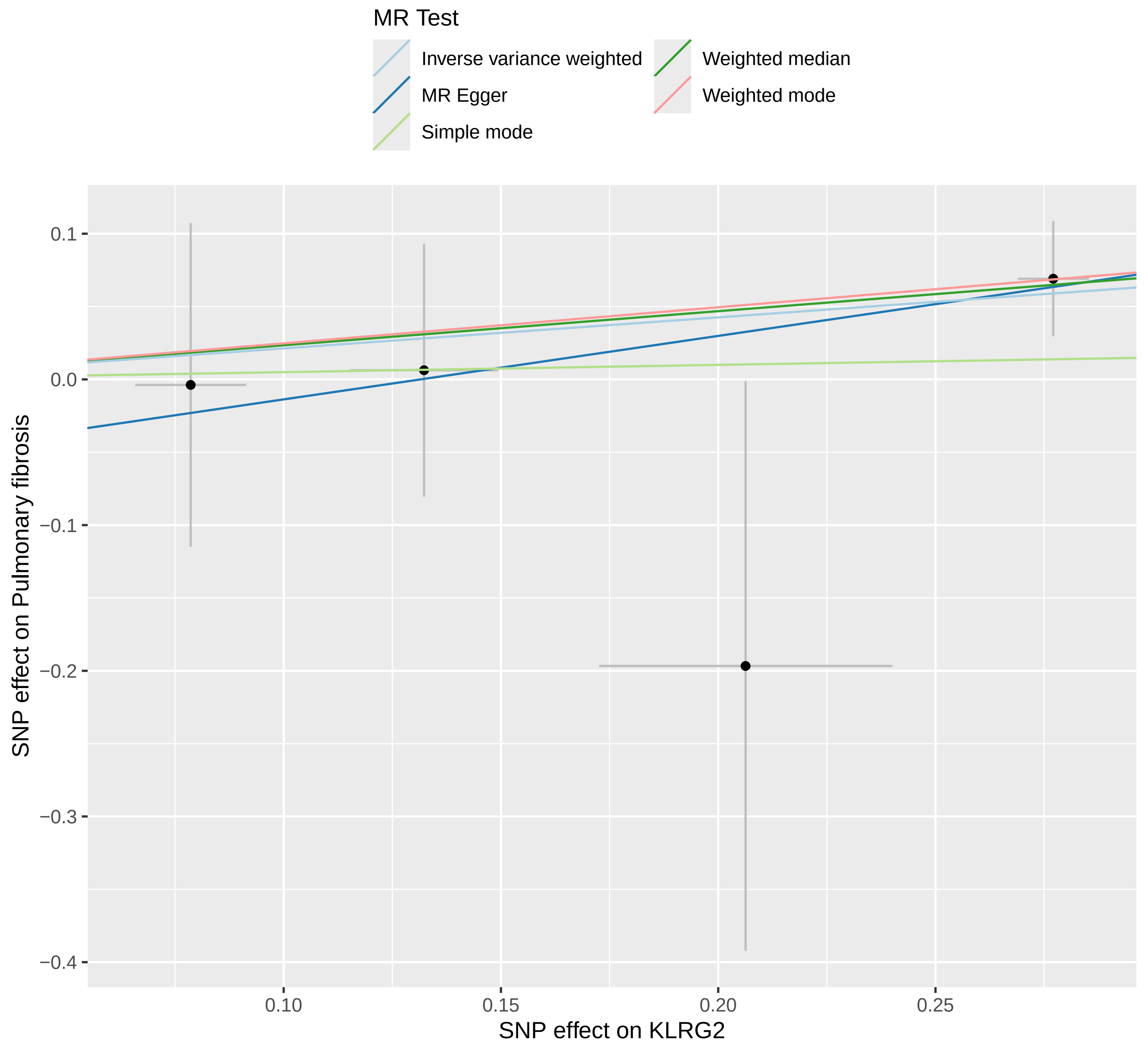
MR Test
Inverse variance weighted
MR Egger
Simple mode
Weighted median
Weighted mode
0.1
0.0
−0.1
−0.2
−0.3
−0.4
0.10
0.15
0.20
0.25
SNP effect on KLRG2
SNP effect on Pulmonary fibrosis

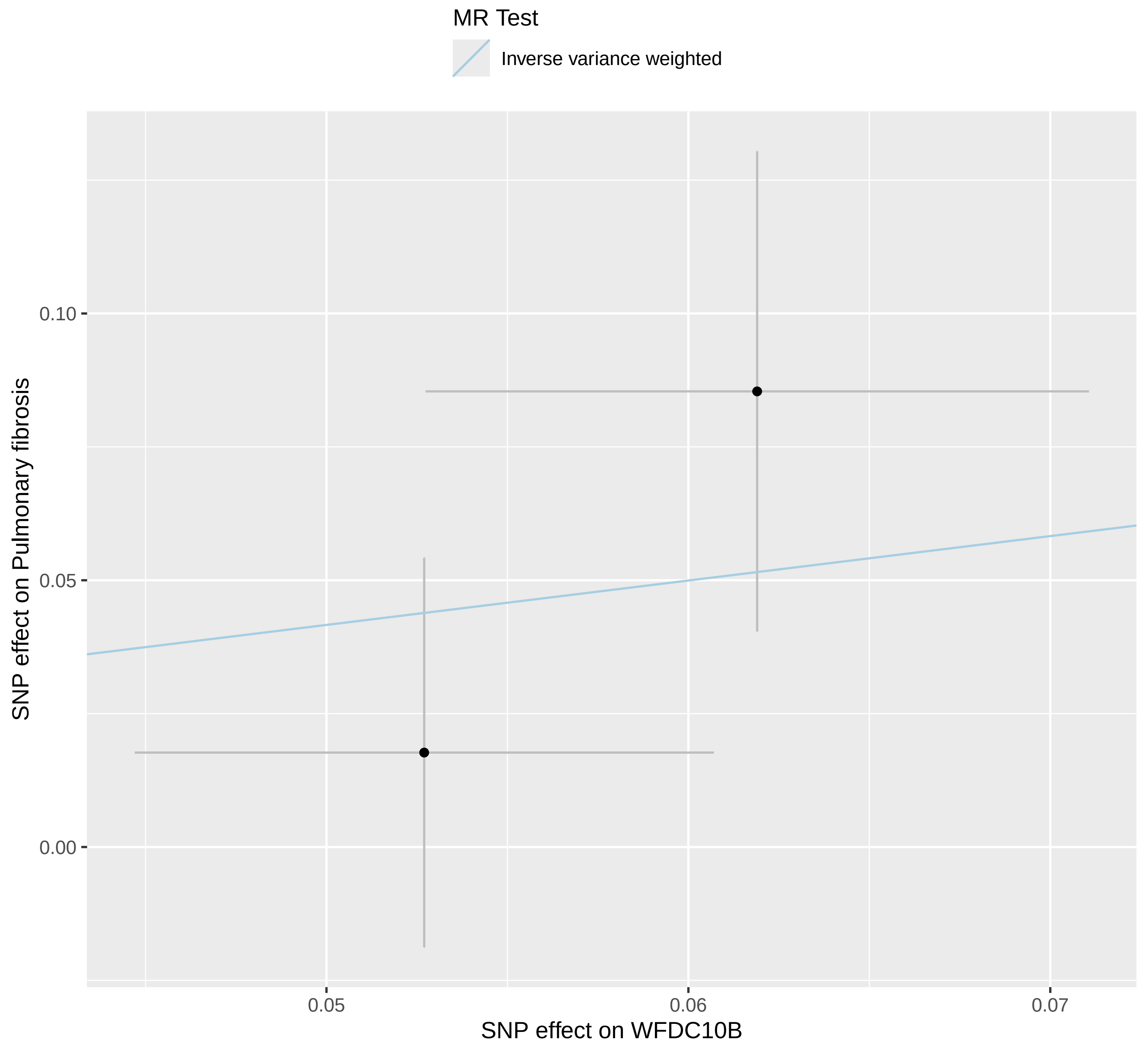

MR Test
Inverse variance weighted
0.10
0.05
0.00
0.05
0.06
0.07
SNP effect on Pulmonary fibrosis
SNP effect on WFDC10B

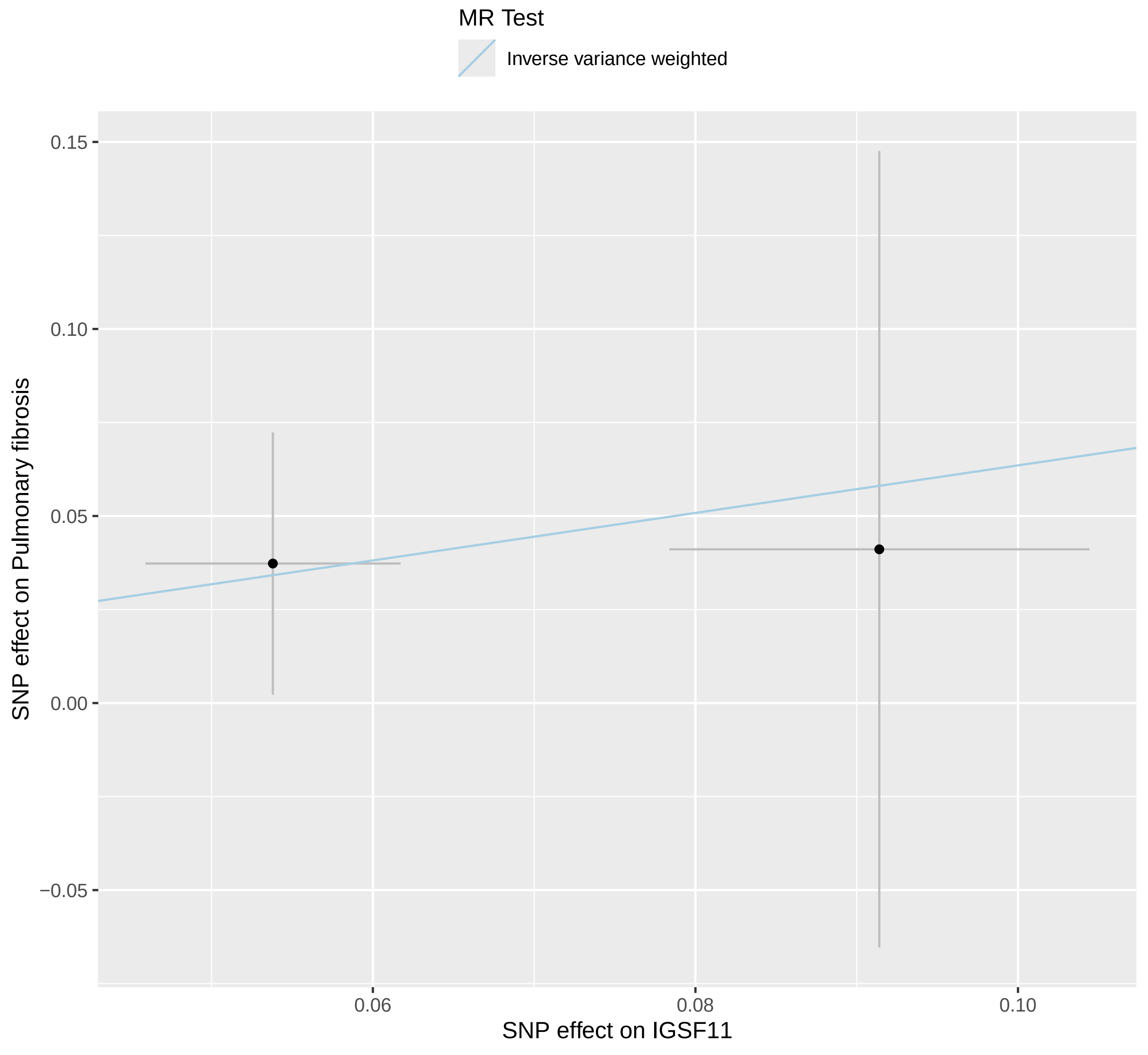
MR Test
Inverse variance weighted
0.15
0.10
0.05
0.00
−0.05
SNP effect on Pulmonary fibrosis
0.06
0.08
0.10
SNP effect on IGSF11

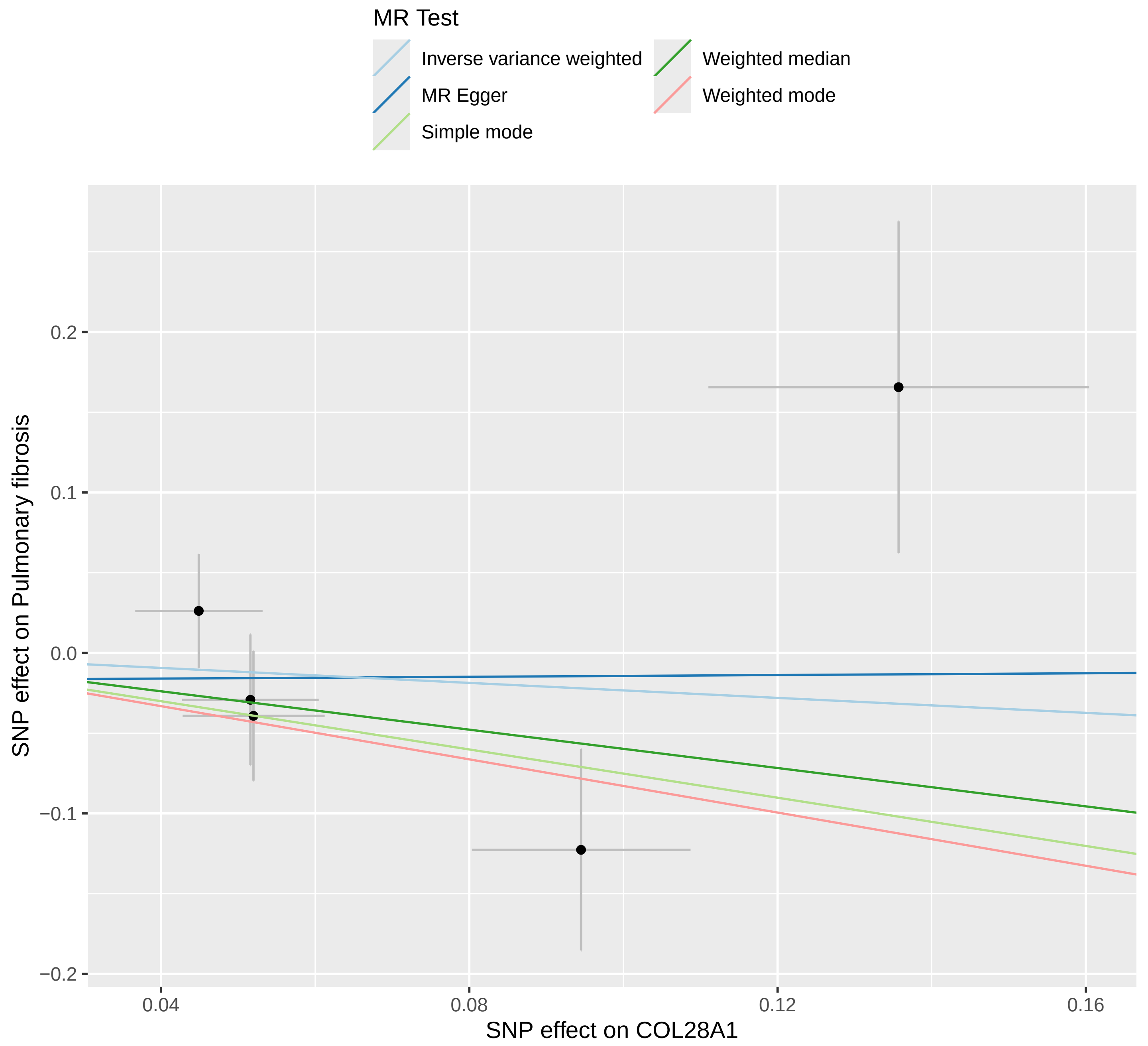

MR Test
Inverse variance weighted
MR Egger
Simple mode
Weighted median
Weighted mode
0.2
0.1
0.0
−0.1
−0.2
0.04
0.08
0.12
0.16
SNP effect on Pulmonary fibrosis
SNP effect on COL28A1

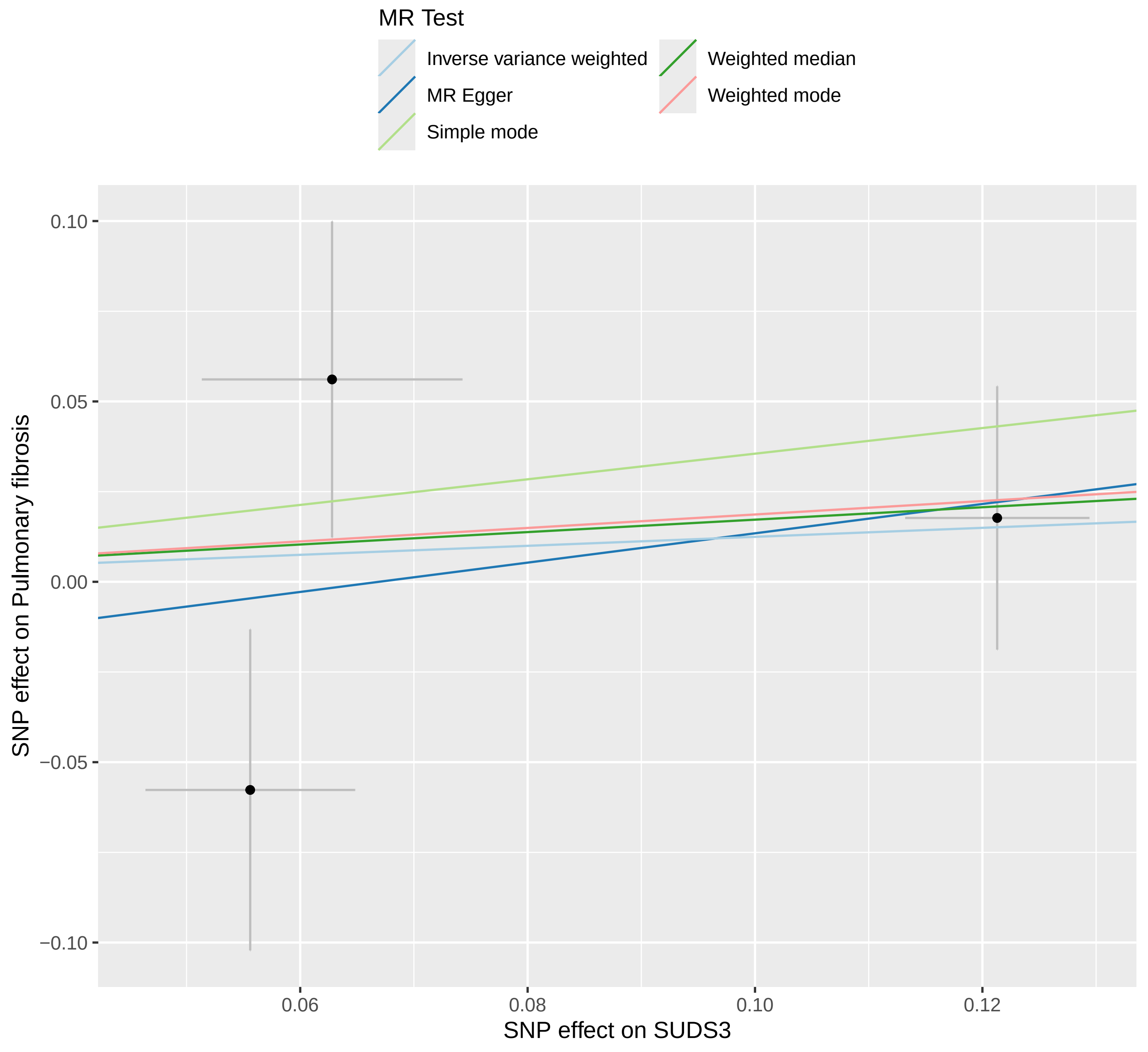

MR Test
Inverse variance weighted
MR Egger
Simple mode
Weighted median
Weighted mode
0.10
0.05
0.00
−0.05
−0.10
SNP effect on Pulmonary fibrosis
0.06
0.08
0.10
0.12
SNP effect on SUDS3

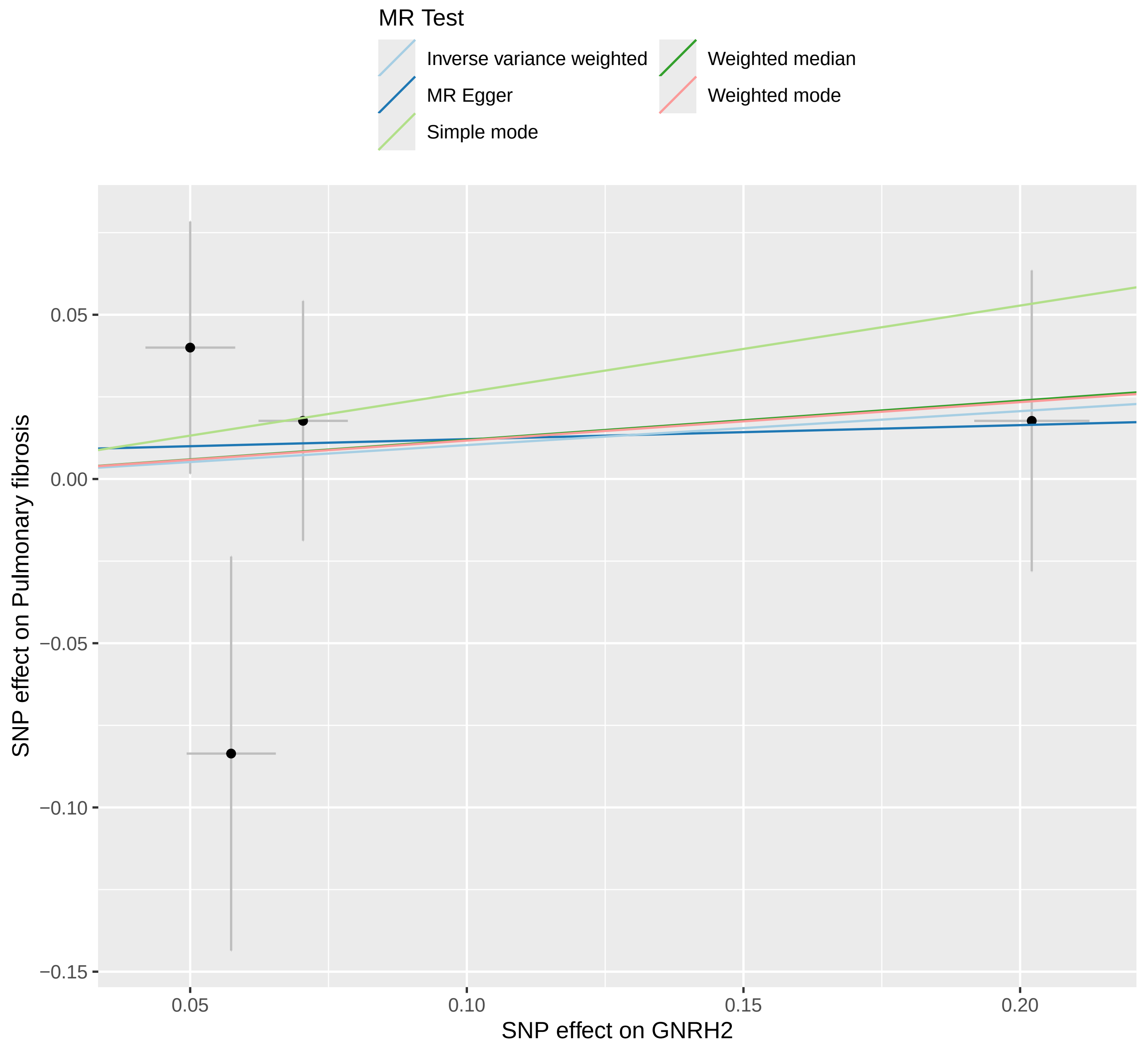
MR Test
Inverse variance weighted
MR Egger
Simple mode
Weighted median
Weighted mode
SNP effect on Pulmonary fibrosis
0.05
0.00
−0.05
−0.10
−0.15
0.05
0.10
0.15
0.20
SNP effect on GNRH2

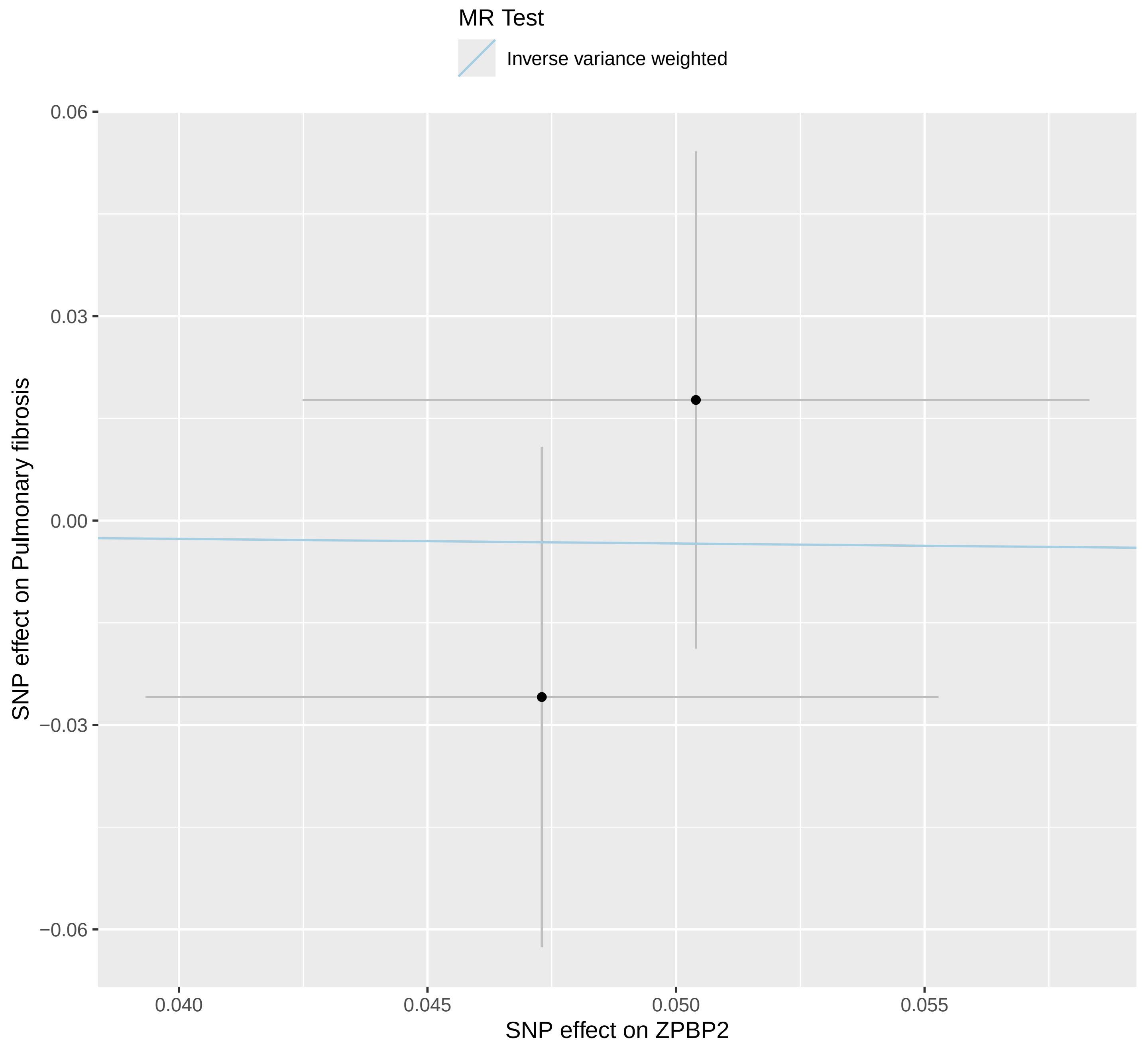

MR Test
Inverse variance weighted
0.06
0.03
0.00
−0.03
−0.06
0.040
0.045
0.050
0.055
SNP effect on Pulmonary fibrosis
SNP effect on ZPBP2

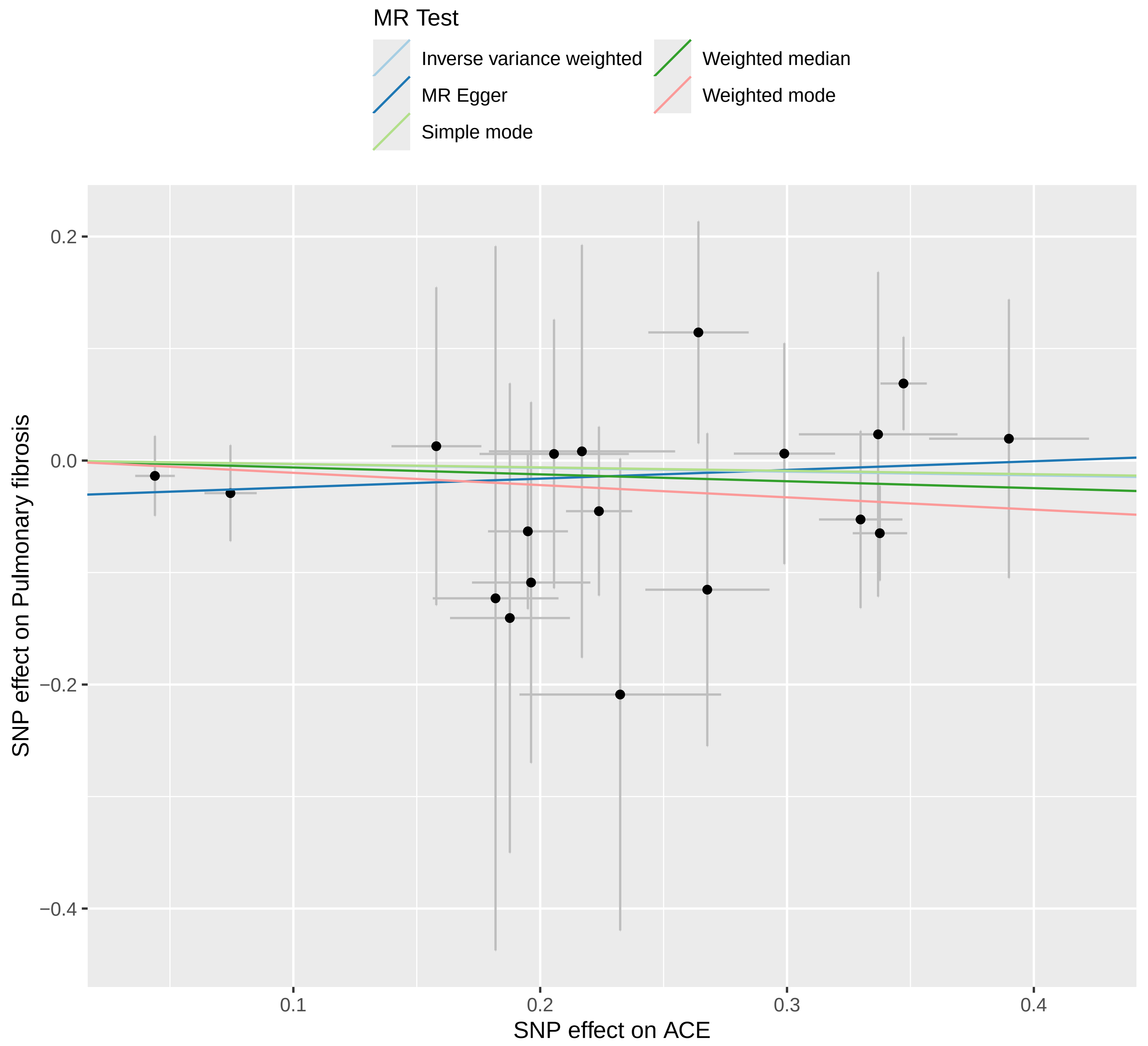

MR Test
Inverse variance weighted
MR Egger
Simple mode
Weighted median
Weighted mode
0.2
0.0
−0.2
−0.4
SNP effect on Pulmonary fibrosis
0.1
0.2
0.3
0.4
SNP effect on ACE

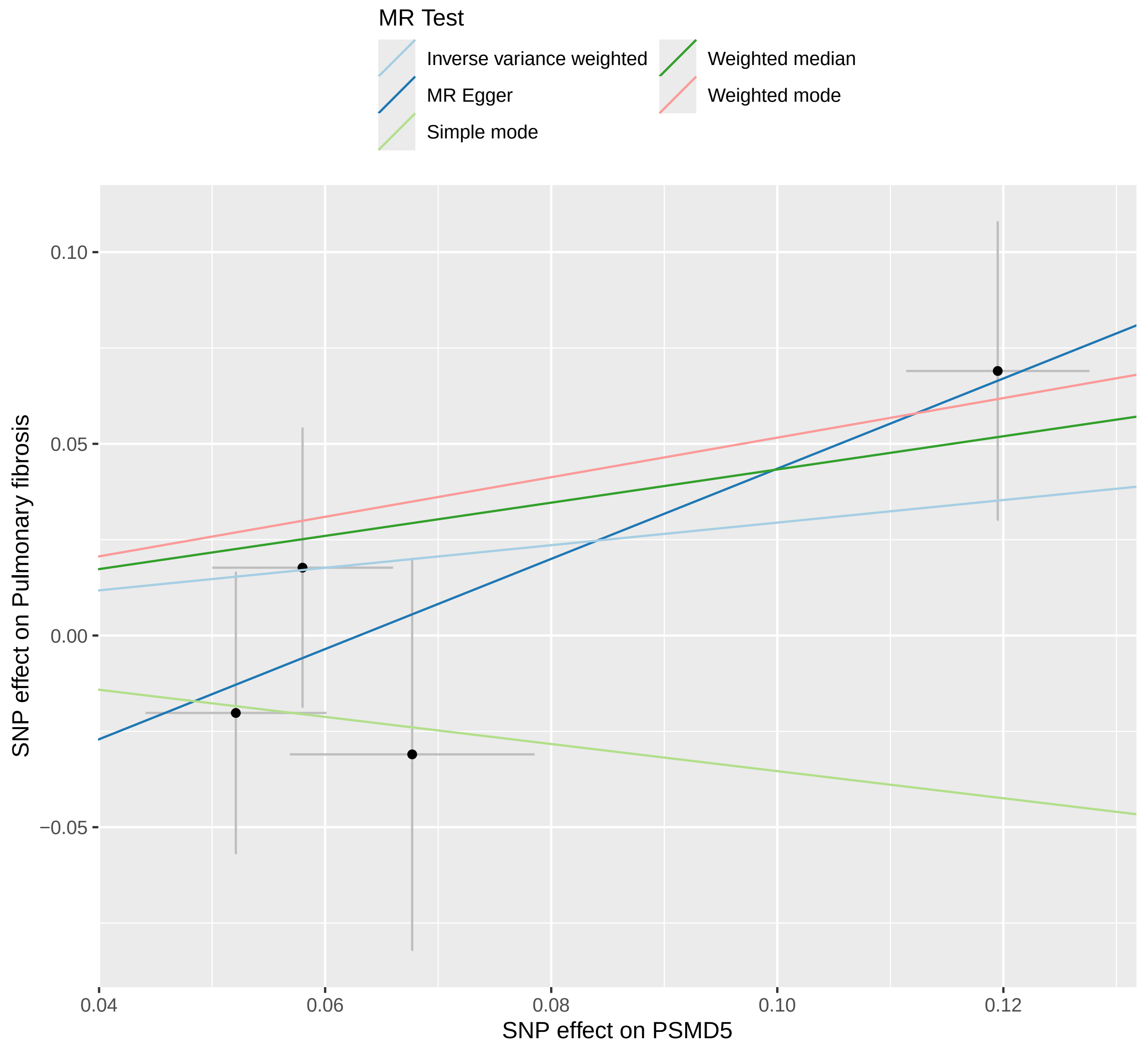

MR Test
Inverse variance weighted
MR Egger
Simple mode
Weighted median
Weighted mode
0.10
0.05
0.00
−0.05
SNP effect on Pulmonary fibrosis
0.04
0.06
0.08
0.10
0.12
SNP effect on PSMD5

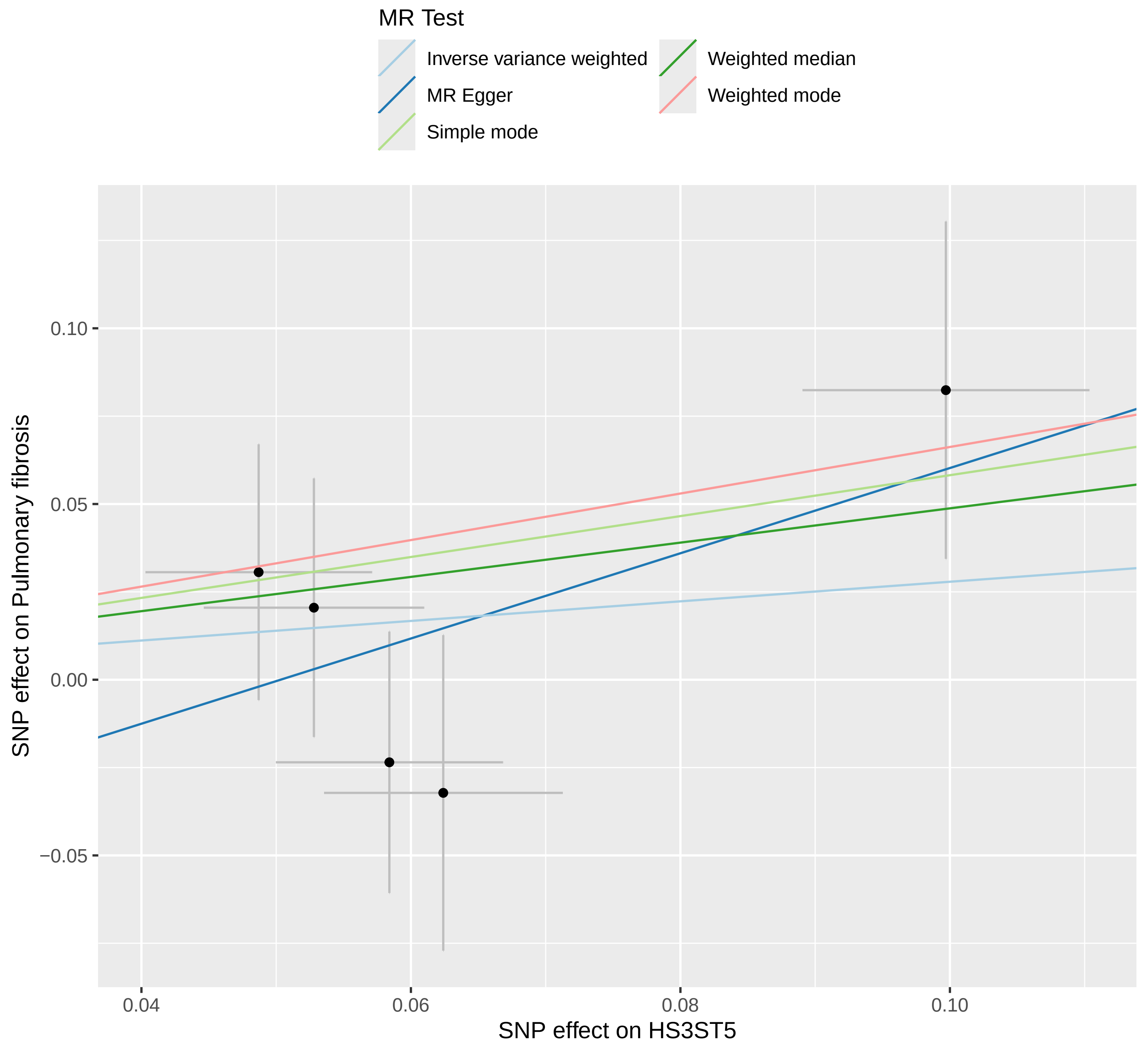
MR Test
Inverse variance weighted
MR Egger
Simple mode
Weighted median
Weighted mode
SNP effect on Pulmonary fibrosis
0.10
0.05
0.00
−0.05
0.04
0.06
0.08
0.10
SNP effect on HS3ST5

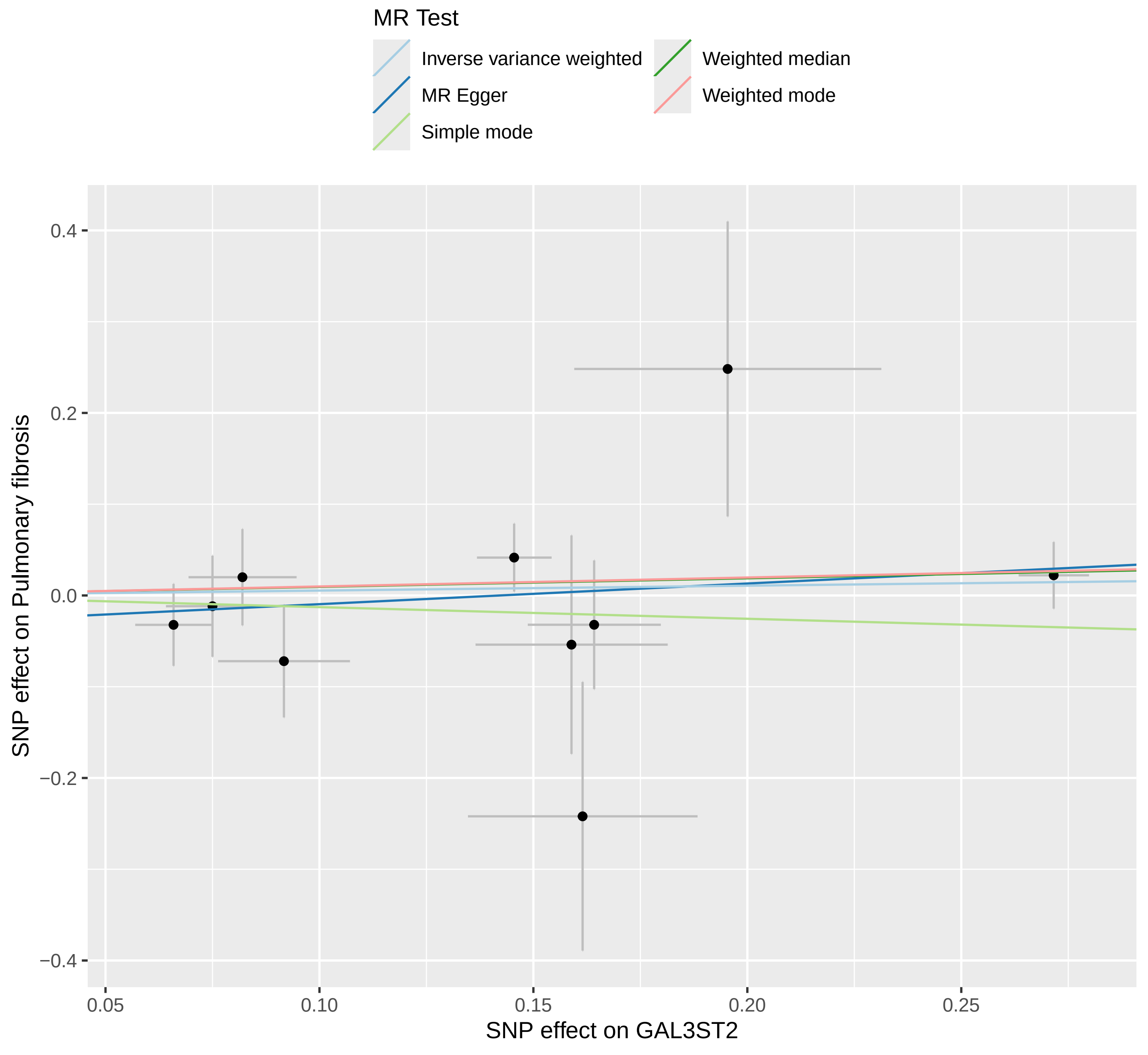
MR Test
Inverse variance weighted
MR Egger
Simple mode
Weighted median
Weighted mode
0.4
0.2
0.0
−0.2
−0.4
SNP effect on Pulmonary fibrosis
0.05
0.10
0.15
0.20
0.25
SNP effect on GAL3ST2

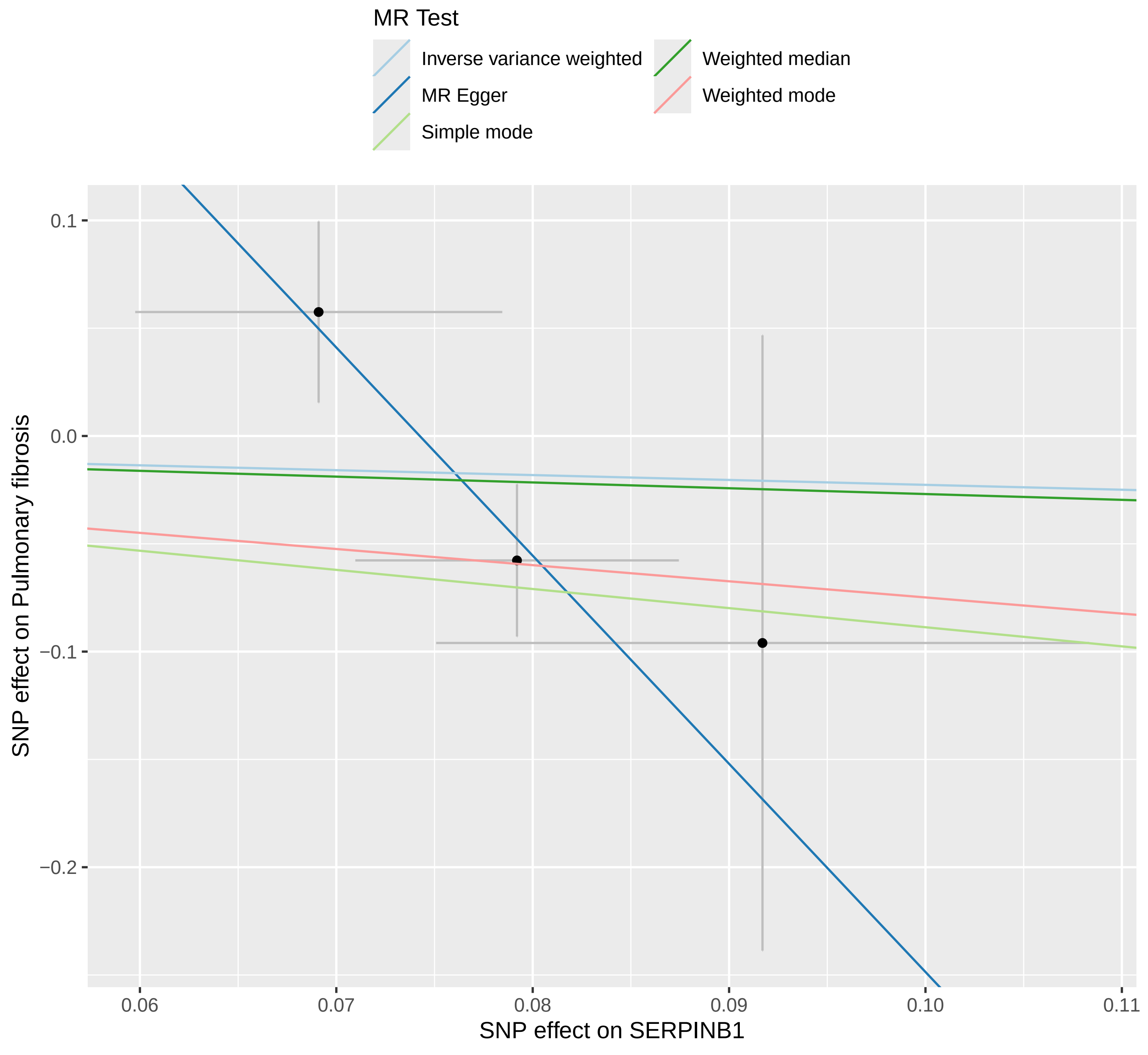
MR Test
Inverse variance weighted
MR Egger
Simple mode
Weighted median
Weighted mode
0.1
0.0
−0.1
−0.2
SNP effect on Pulmonary fibrosis
0.06
0.07
0.08
0.09
0.10
0.11
SNP effect on SERPINB1

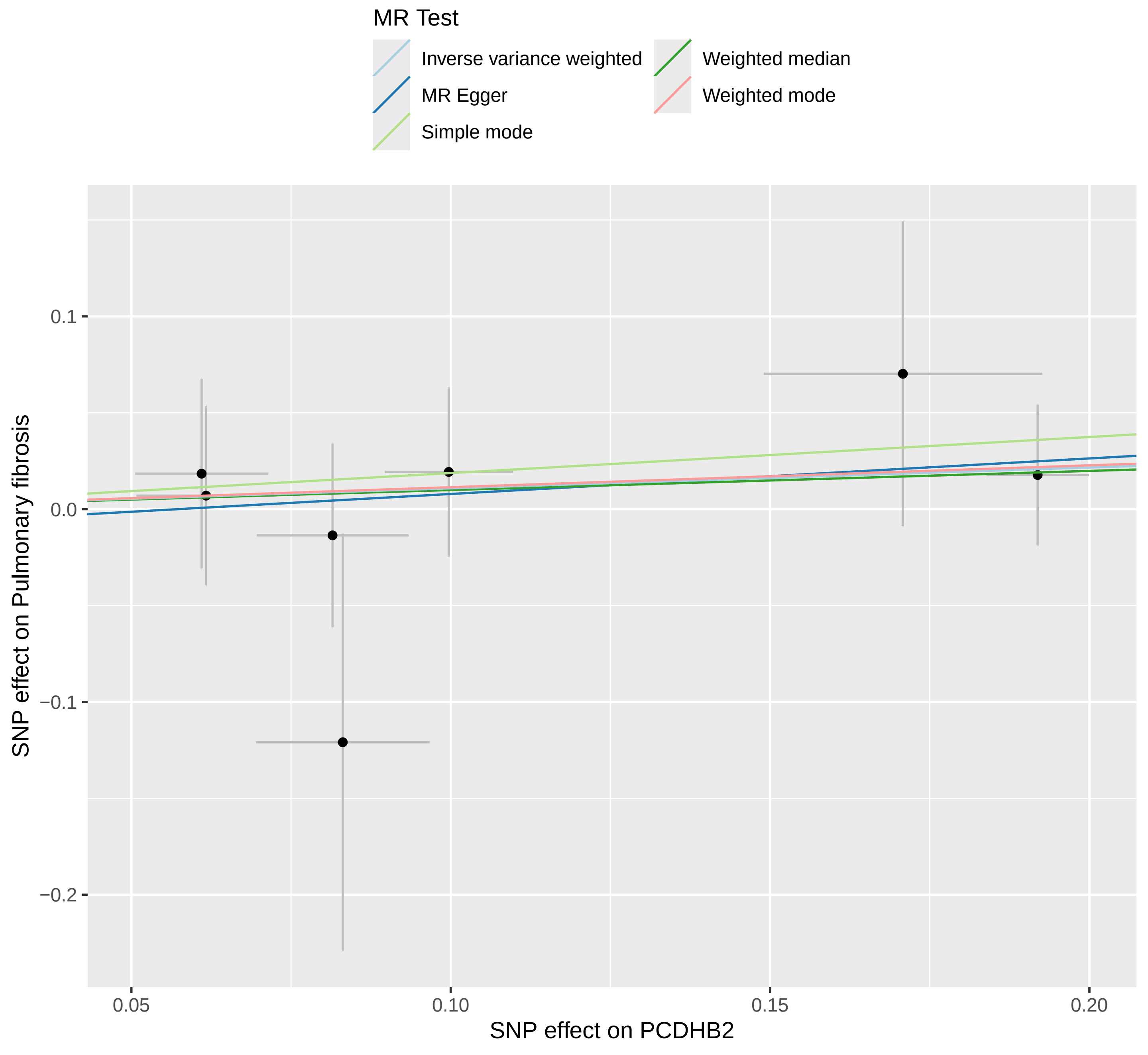
MR Test
Inverse variance weighted
MR Egger
Simple mode
Weighted median
Weighted mode
0.1
0.0
−0.1
−0.2
0.05
0.10
0.15
0.20
SNP effect on Pulmonary fibrosis
SNP effect on PCDHB2

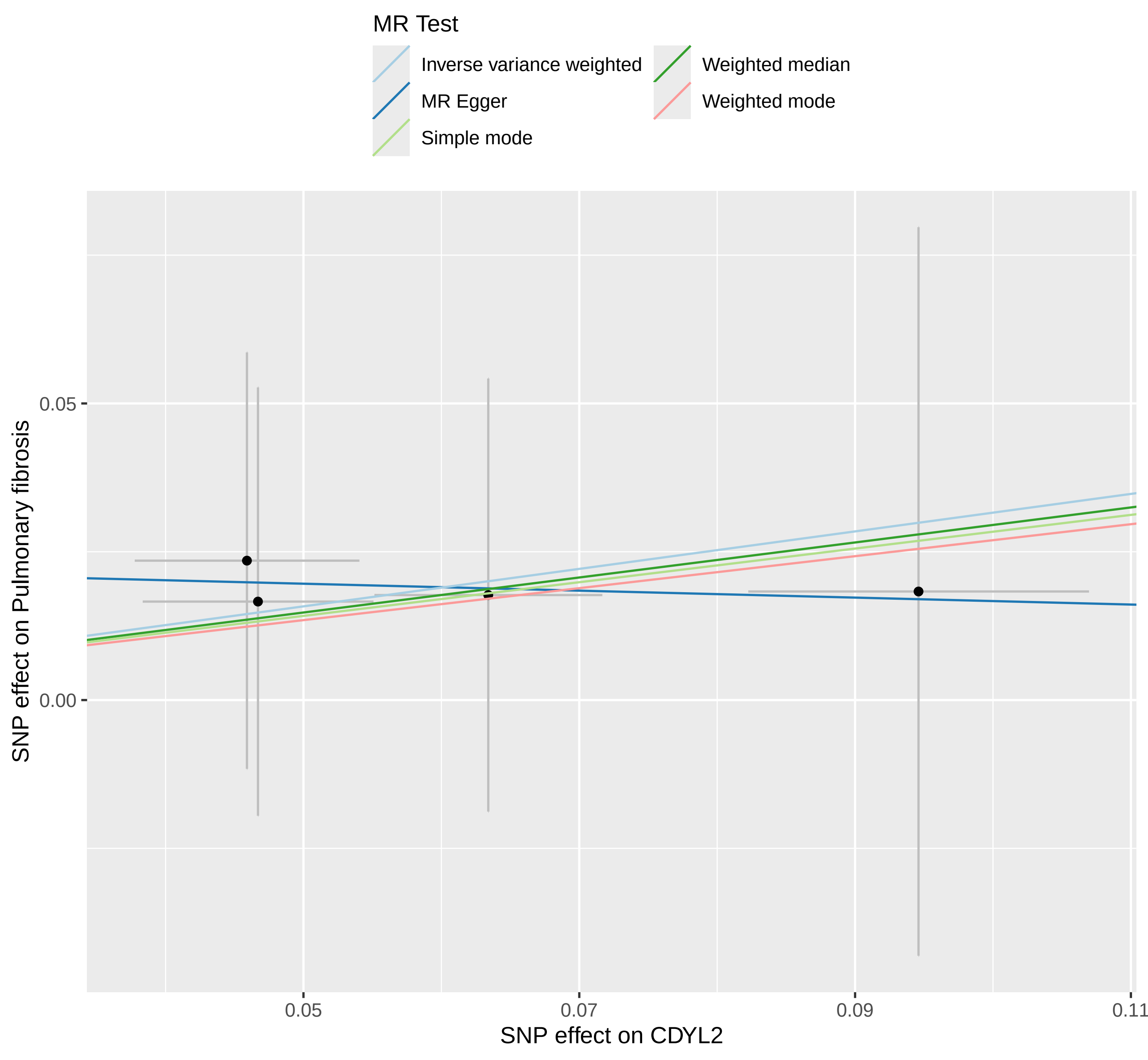

MR Test
Inverse variance weighted
MR Egger
Simple mode
Weighted median
Weighted mode
0.05
0.00
SNP effect on Pulmonary fibrosis
0.05
0.07
0.09
0.11
SNP effect on CDYL2

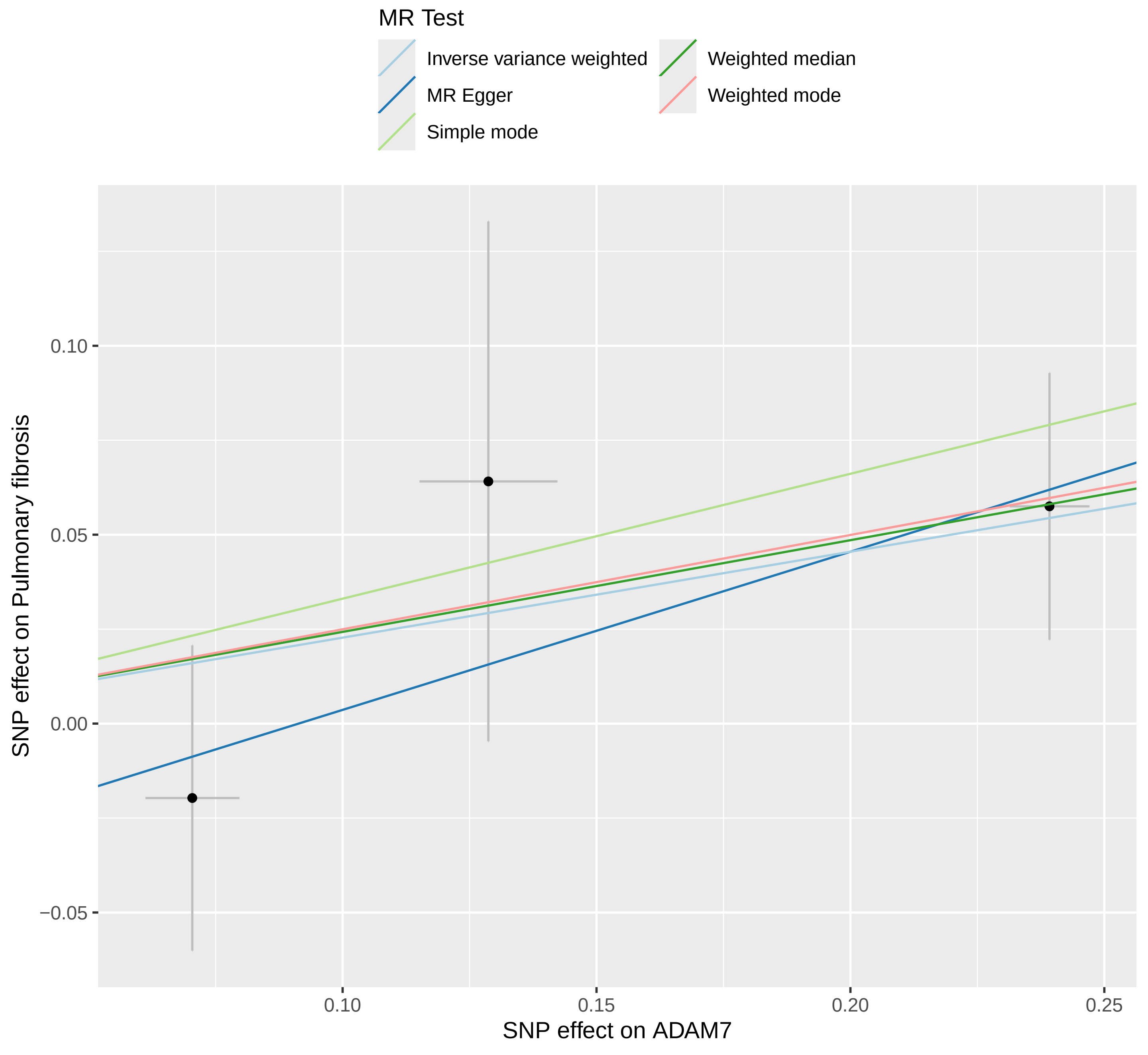
MR Test
Inverse variance weighted
MR Egger
Simple mode
Weighted median
Weighted mode
0.10
0.05
0.00
−0.05
SNP effect on Pulmonary fibrosis
0.10
0.15
0.20
0.25
SNP effect on ADAM7

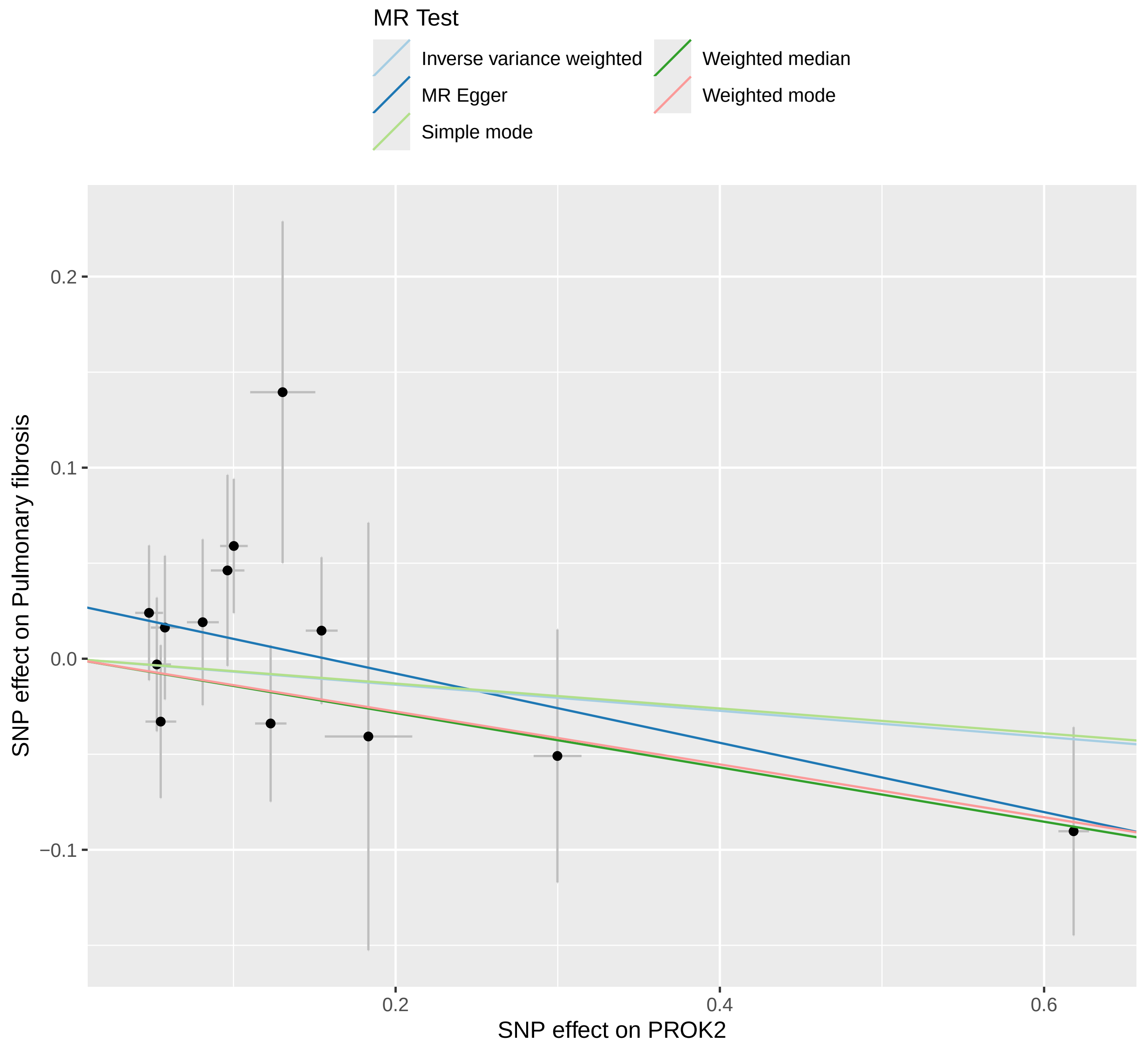
MR Test
Inverse variance weighted
MR Egger
Simple mode
Weighted median
Weighted mode
0.2
0.1
0.0
−0.1
0.2
0.4
0.6
SNP effect on Pulmonary fibrosis
SNP effect on PROK2

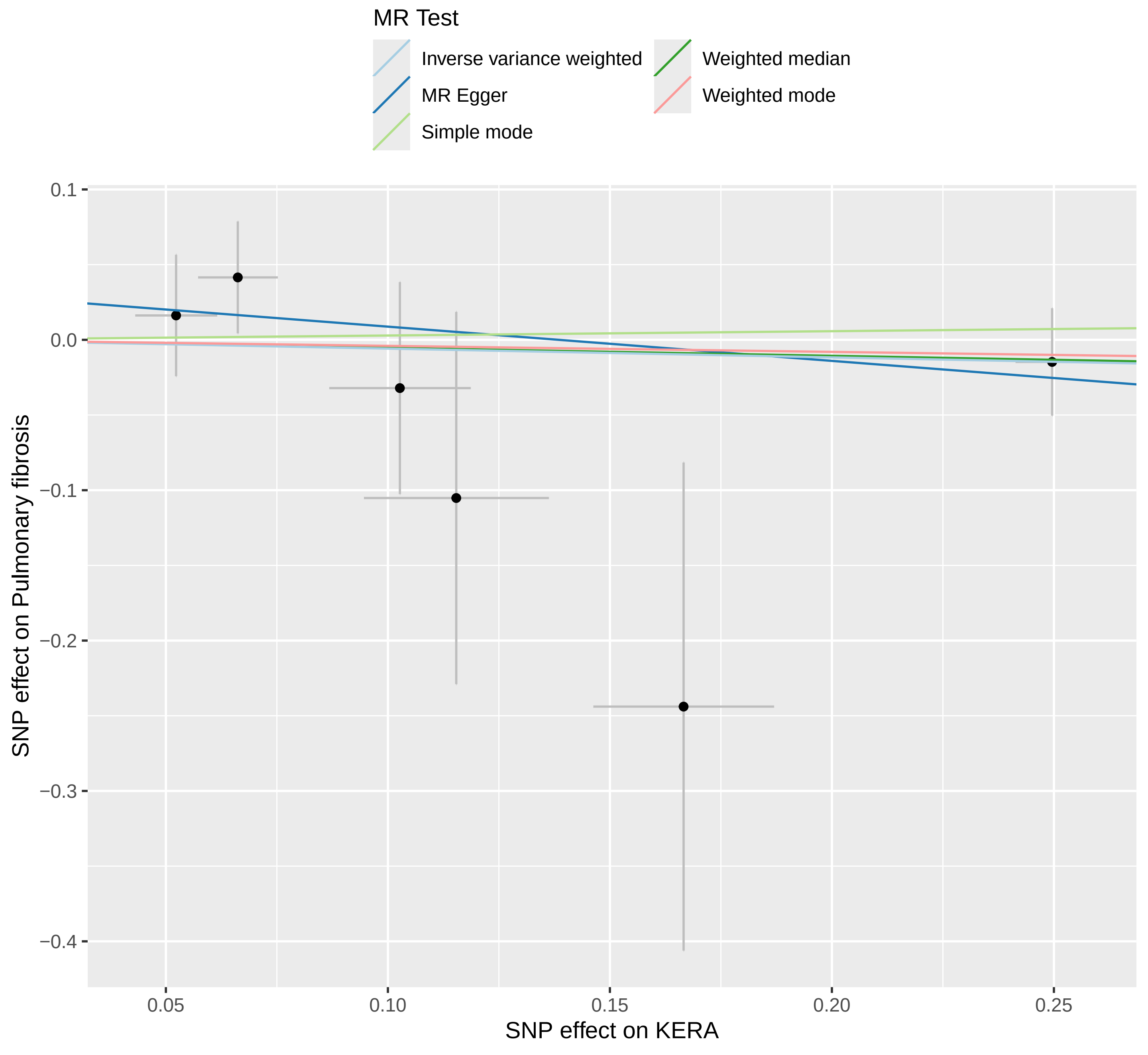

MR Test
Inverse variance weighted
MR Egger
Simple mode
Weighted median
Weighted mode
0.1
0.0
−0.1
−0.2
−0.3
−0.4
0.05
0.10
0.15
0.20
0.25
SNP effect on KERA
SNP effect on Pulmonary fibrosis

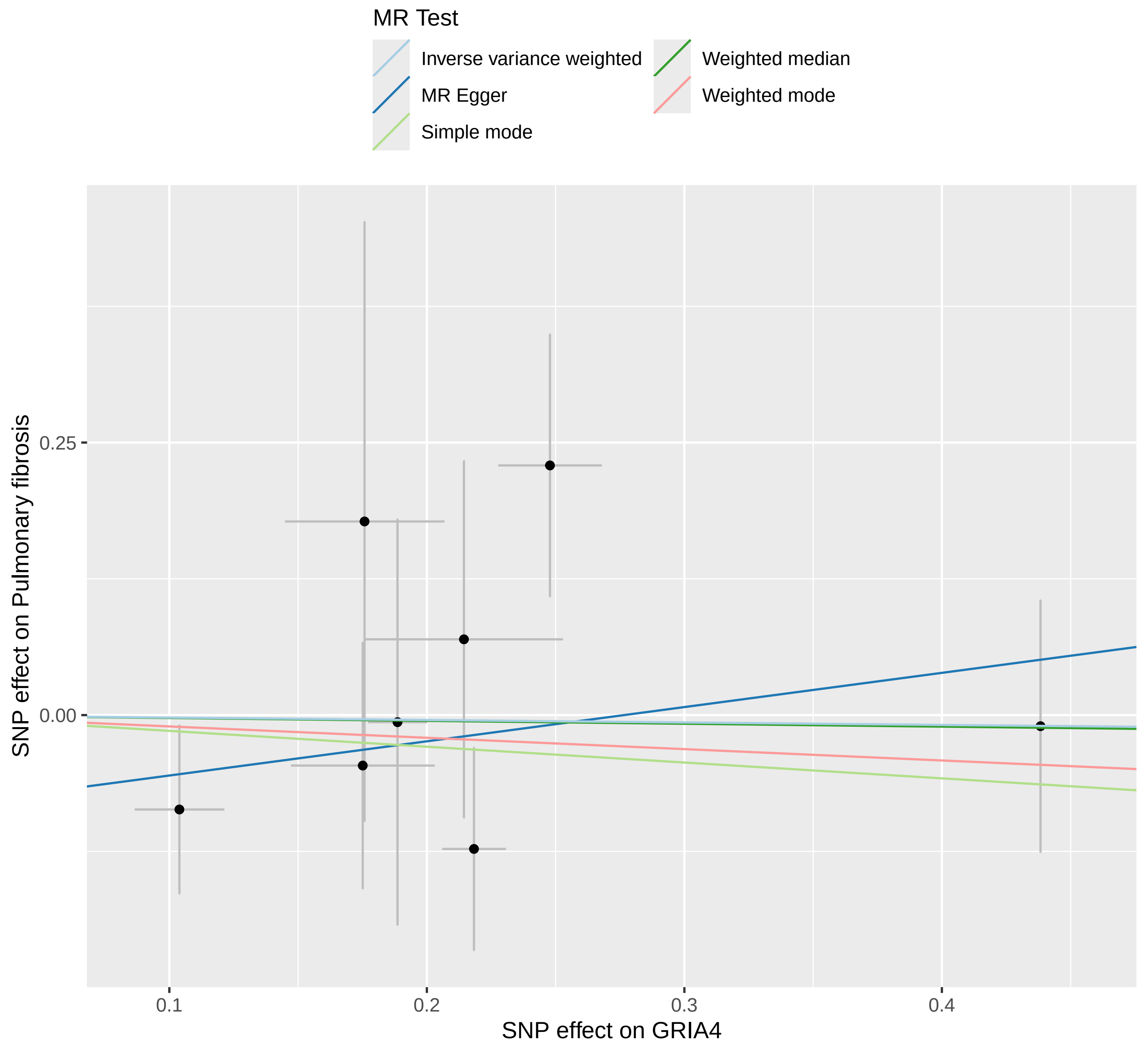
MR Test
Inverse variance weighted
MR Egger
Simple mode
Weighted median
Weighted mode
0.25
0.00
0.1
0.2
0.3
0.4
SNP effect on Pulmonary fibrosis
SNP effect on GRIA4

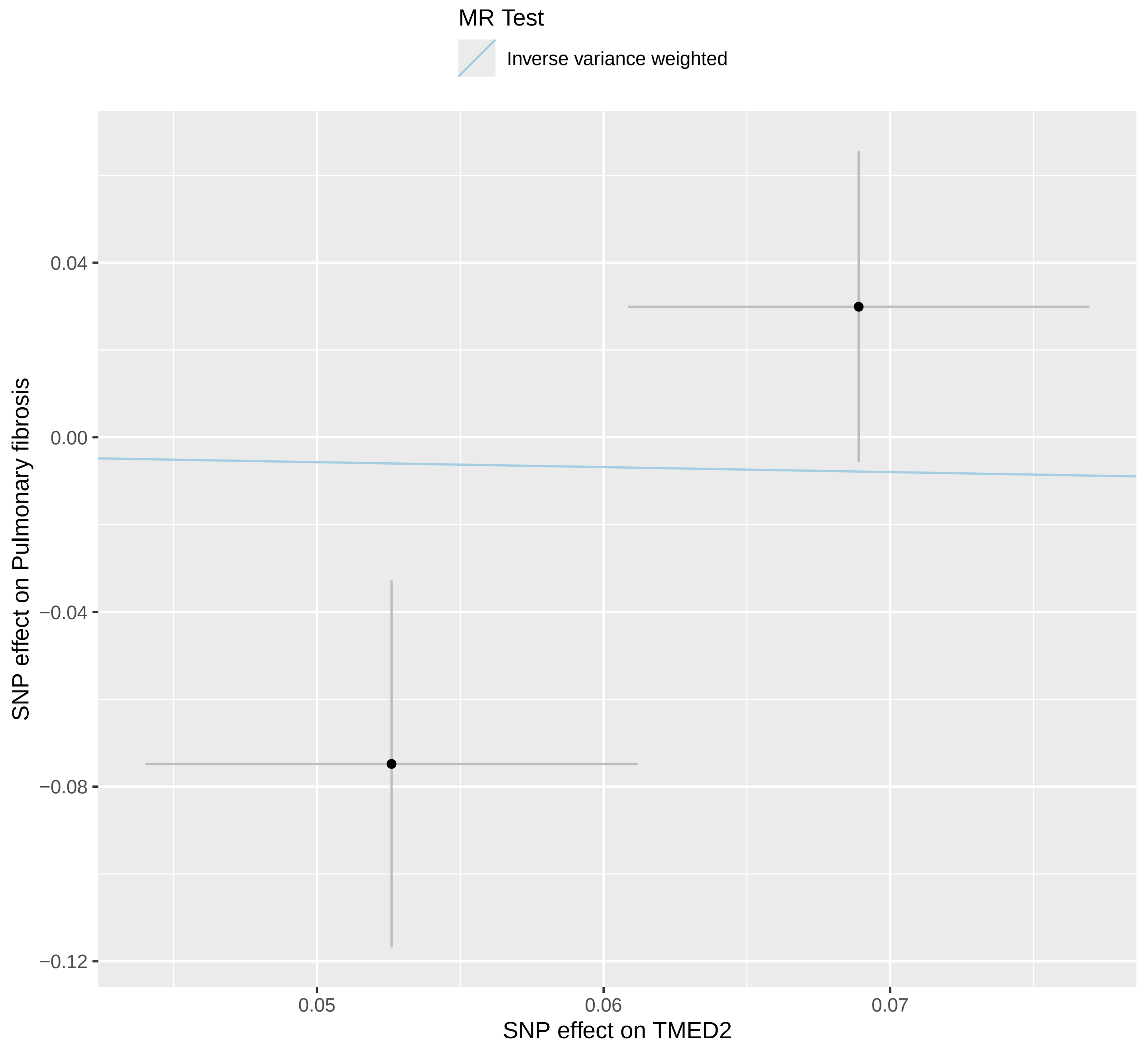

MR Test
Inverse variance weighted
0.04
0.00
−0.04
−0.08
−0.12
SNP effect on Pulmonary fibrosis
0.05
0.06
0.07
SNP effect on TMED2

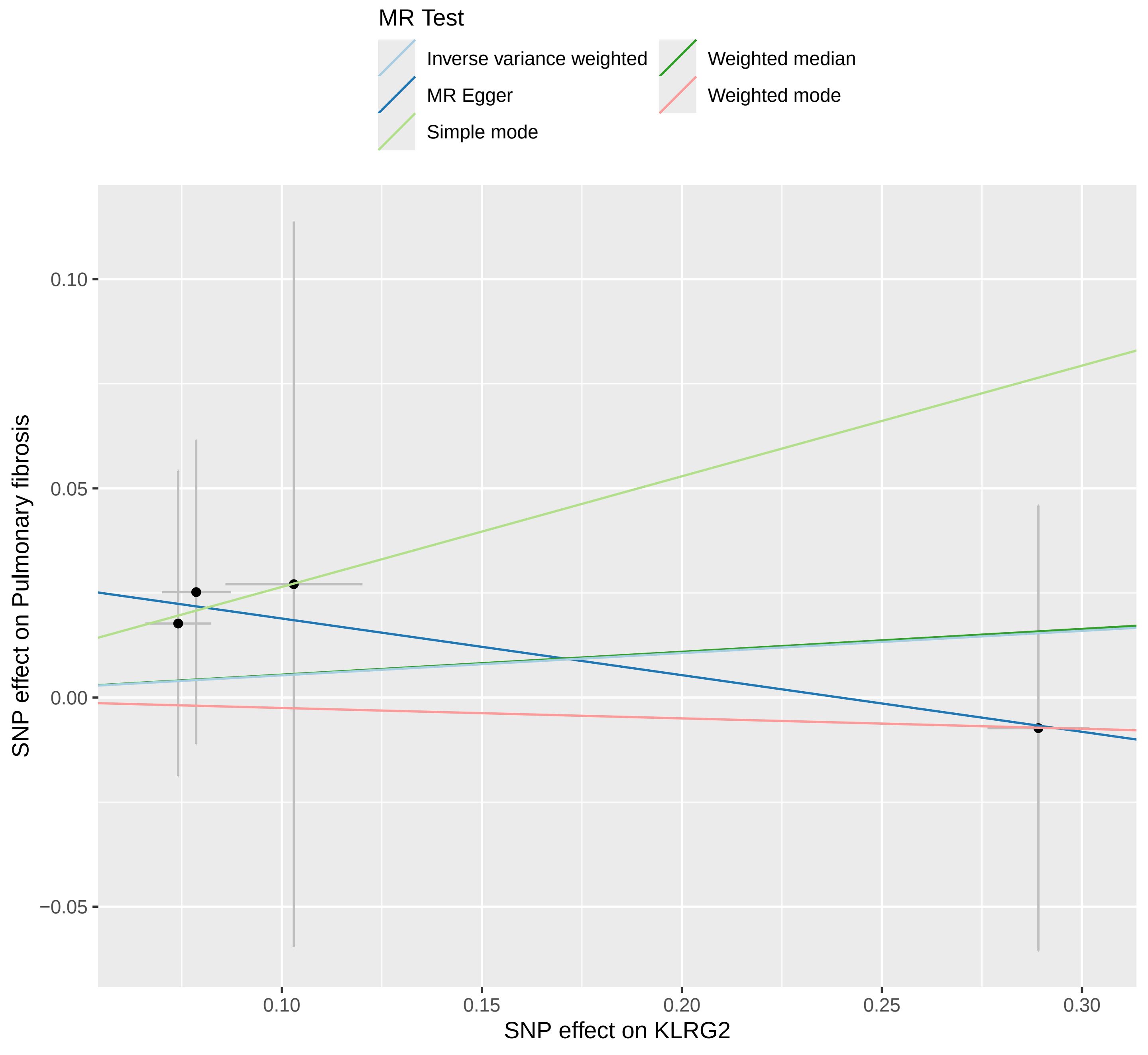

MR Test
Inverse variance weighted
MR Egger
Simple mode
Weighted median
Weighted mode
0.10
0.05
0.00
−0.05
SNP effect on Pulmonary fibrosis
0.10
0.15
0.20
0.25
0.30
SNP effect on KLRG2

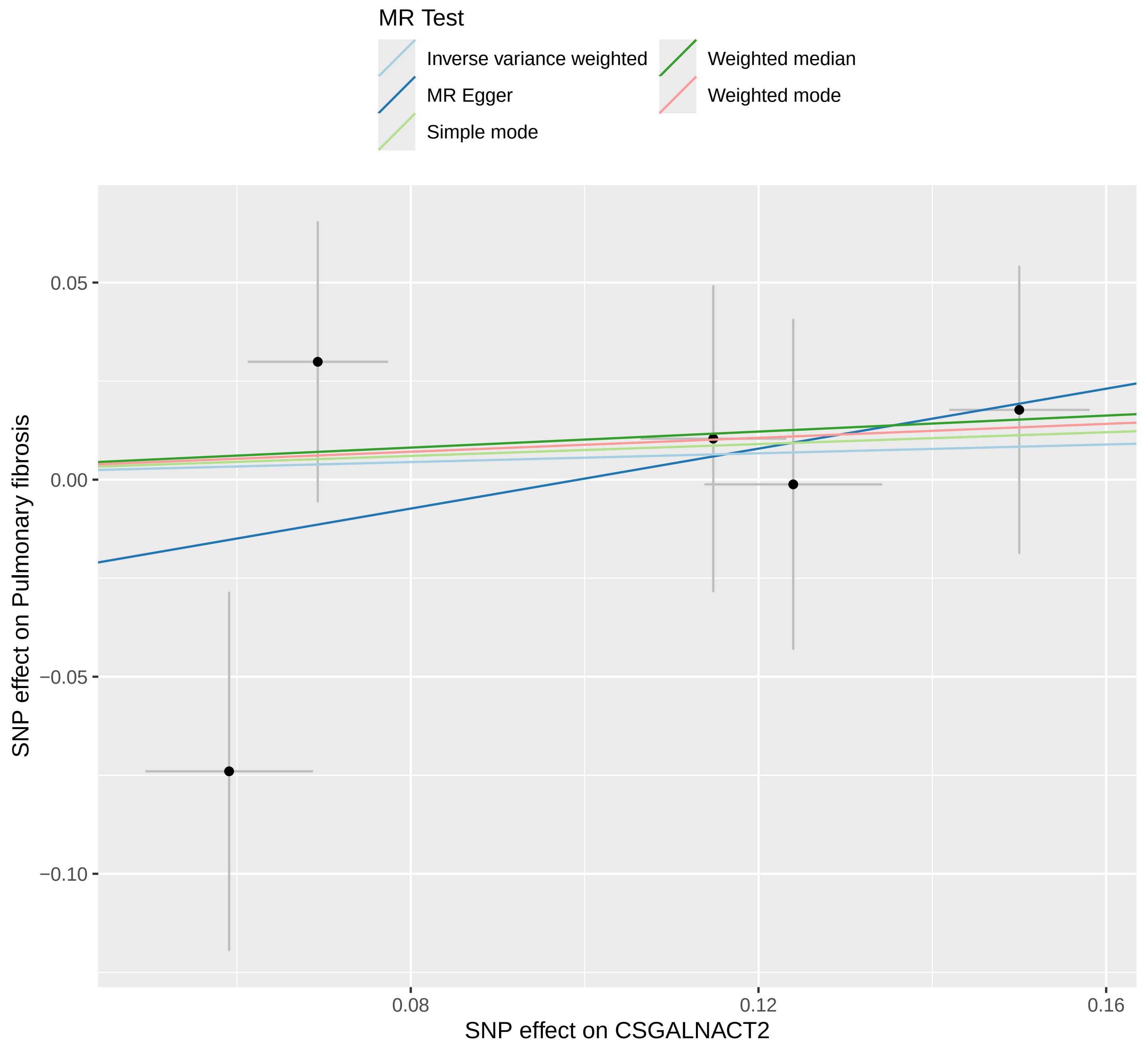
MR Test
Inverse variance weighted
MR Egger
Simple mode
Weighted median
Weighted mode
0.05
0.00
−0.05
−0.10
SNP effect on Pulmonary fibrosis
0.08
0.12
0.16
SNP effect on CSGALNACT2

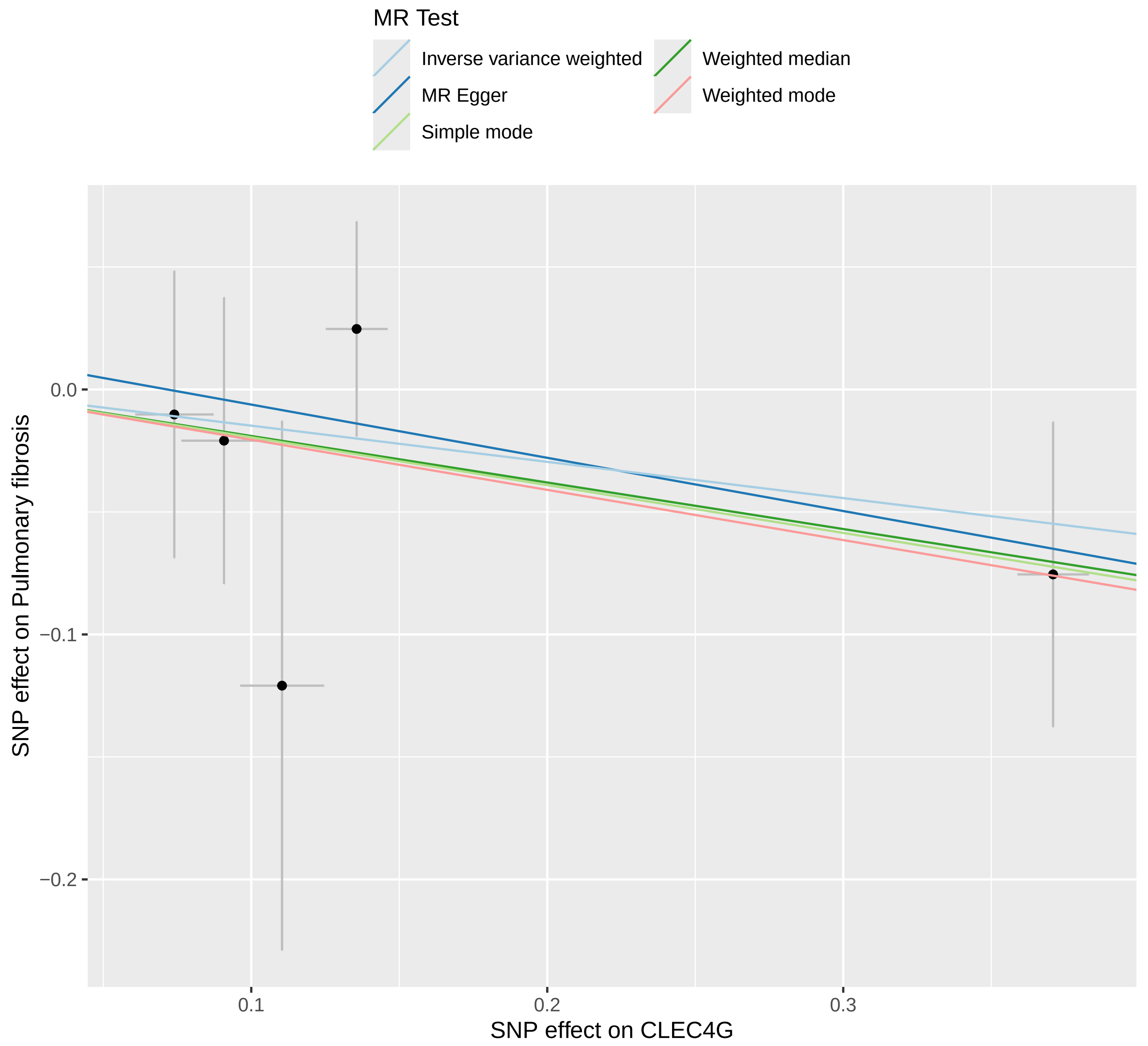
MR Test
Inverse variance weighted
MR Egger
Simple mode
Weighted median
Weighted mode
SNP effect on Pulmonary fibrosis
0.0
−0.1
−0.2
0.1
0.2
0.3
SNP effect on CLEC4G

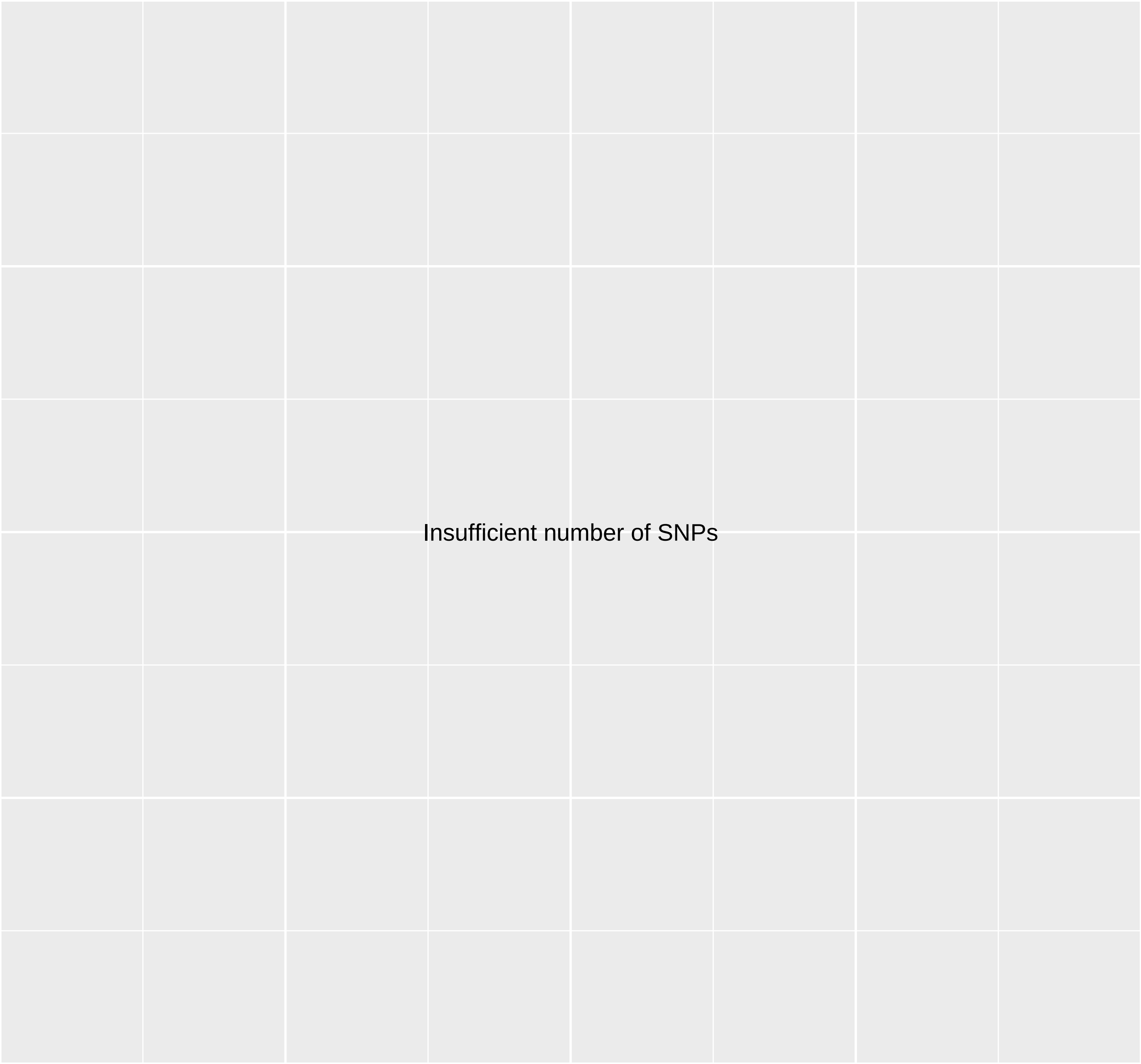
Insufficient number of SNPs

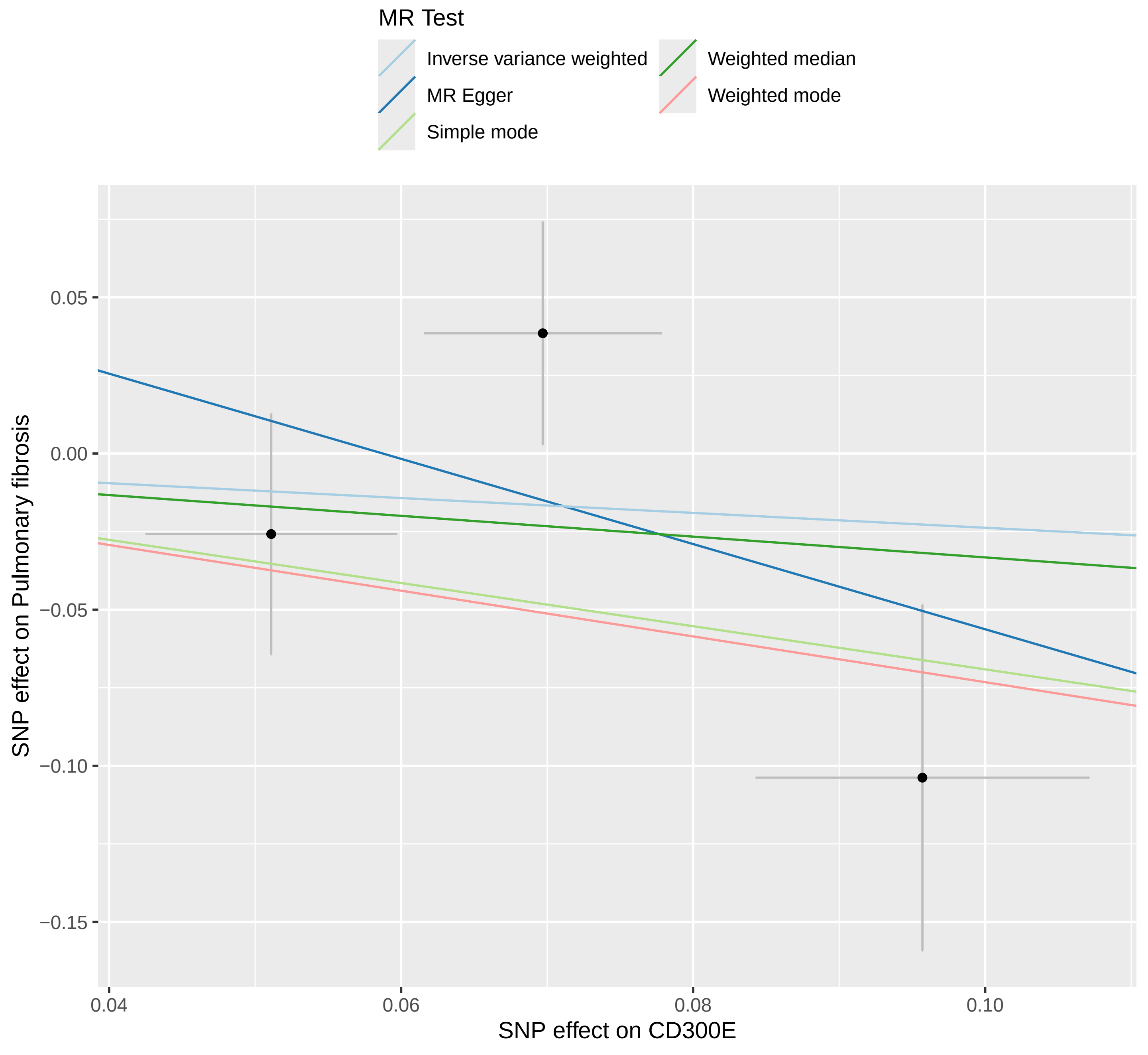
MR Test
Inverse variance weighted
MR Egger
Simple mode
Weighted median
Weighted mode
SNP effect on Pulmonary fibrosis
0.05
0.00
−0.05
−0.10
−0.15
0.04
0.06
0.08
0.10
SNP effect on CD300E

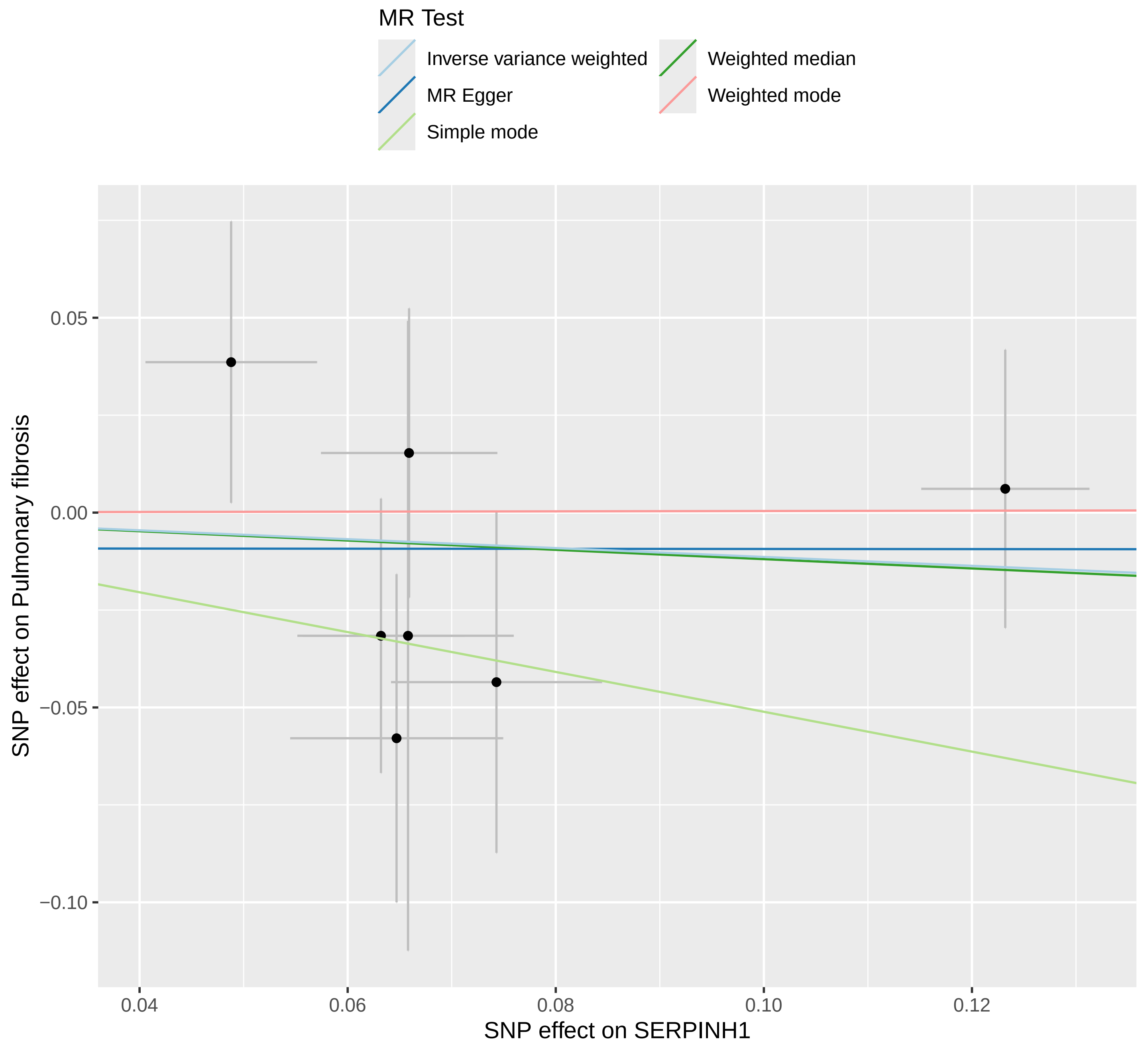
MR Test
Inverse variance weighted
MR Egger
Simple mode
Weighted median
Weighted mode
0.05
0.00
−0.05
−0.10
0.04
0.06
0.08
0.10
0.12
SNP effect on Pulmonary fibrosis
SNP effect on SERPINH1

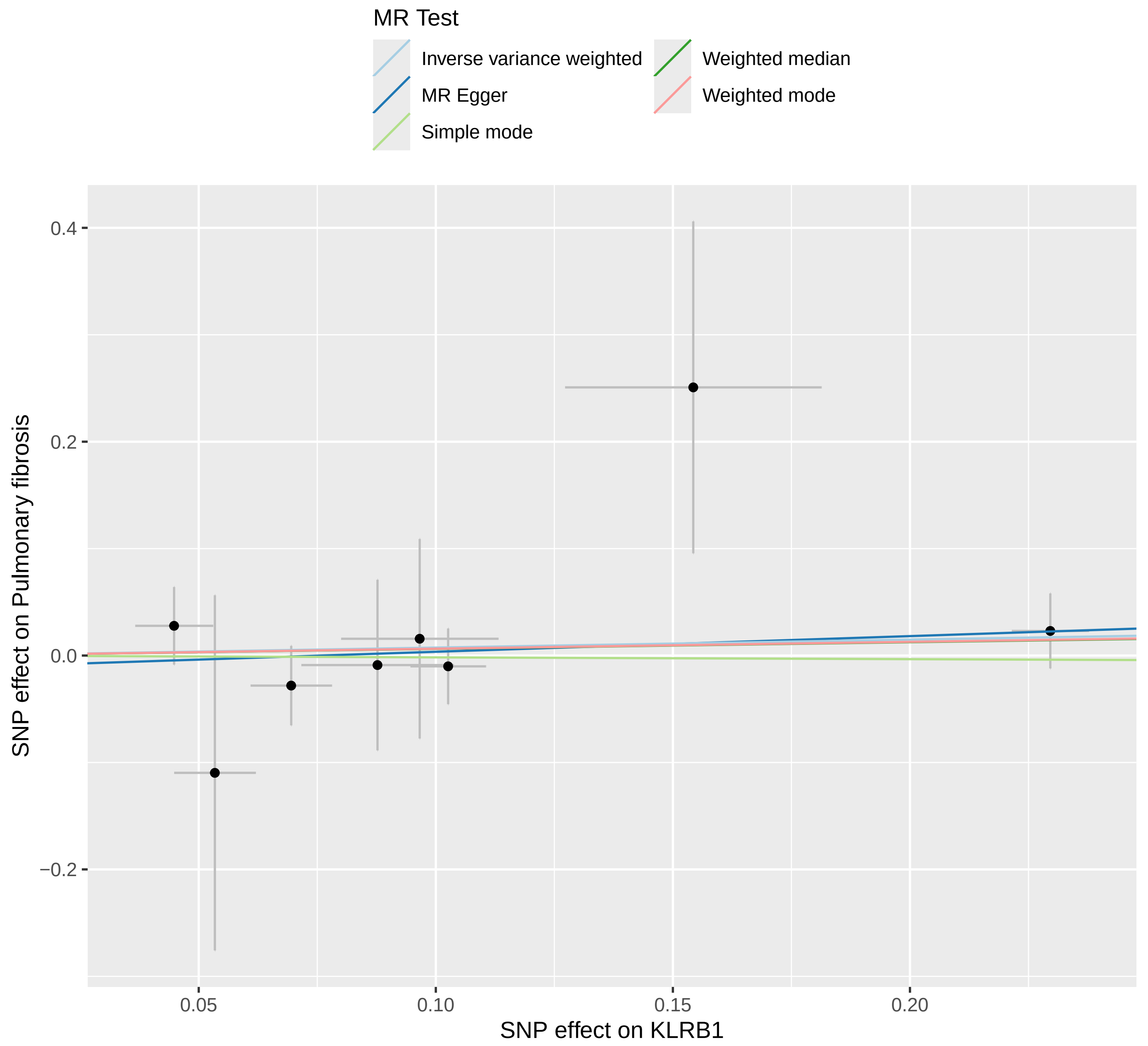
MR Test
Inverse variance weighted
MR Egger
Simple mode
Weighted median
Weighted mode
0.4
0.2
0.0
−0.2
0.05
0.10
0.15
0.20
SNP effect on KLRB1
SNP effect on Pulmonary fibrosis

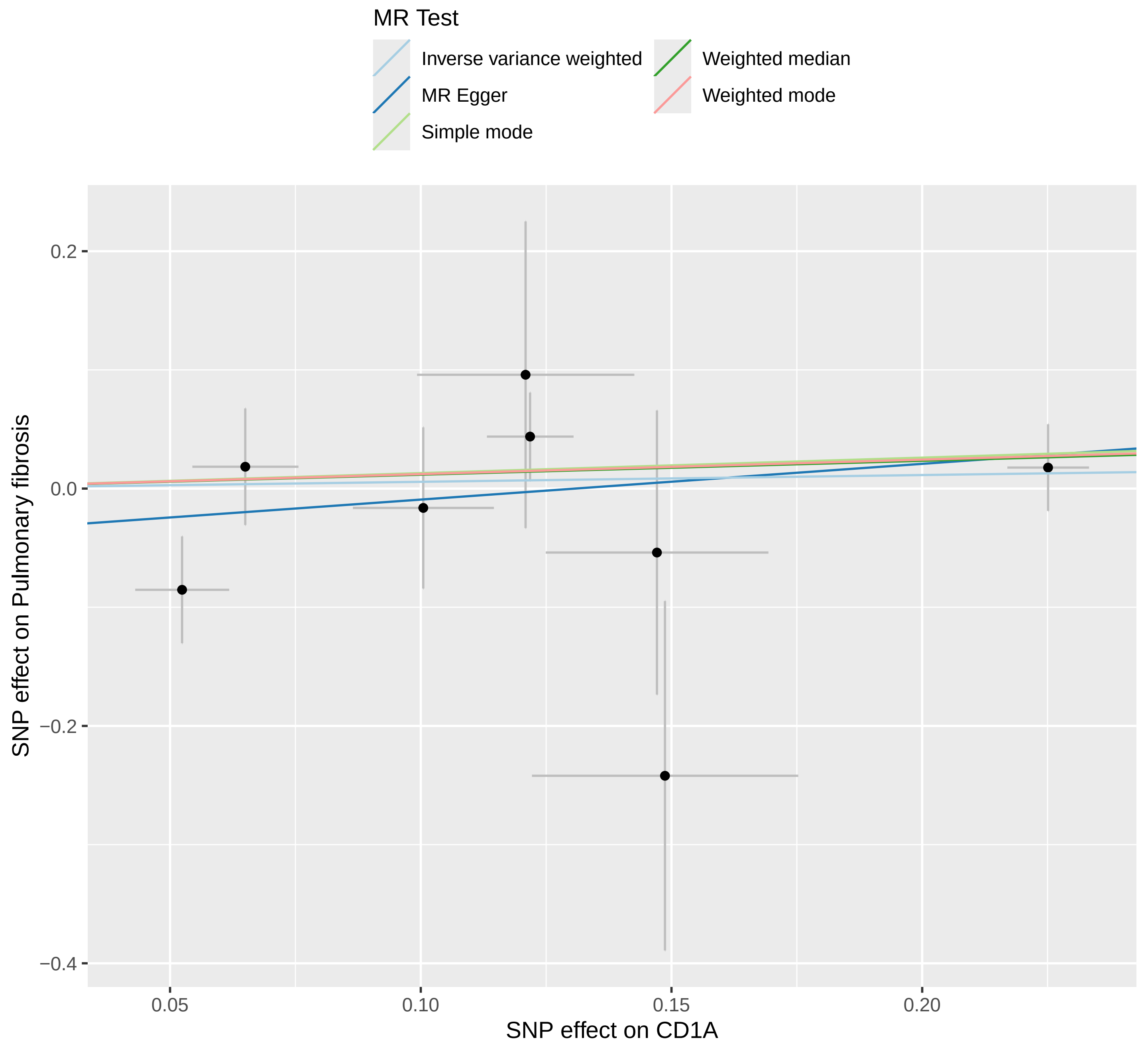
MR Test
Inverse variance weighted
MR Egger
Simple mode
Weighted median
Weighted mode
SNP effect on Pulmonary fibrosis
0.2
0.0
−0.2
−0.4
0.05
0.10
0.15
0.20
SNP effect on CD1A

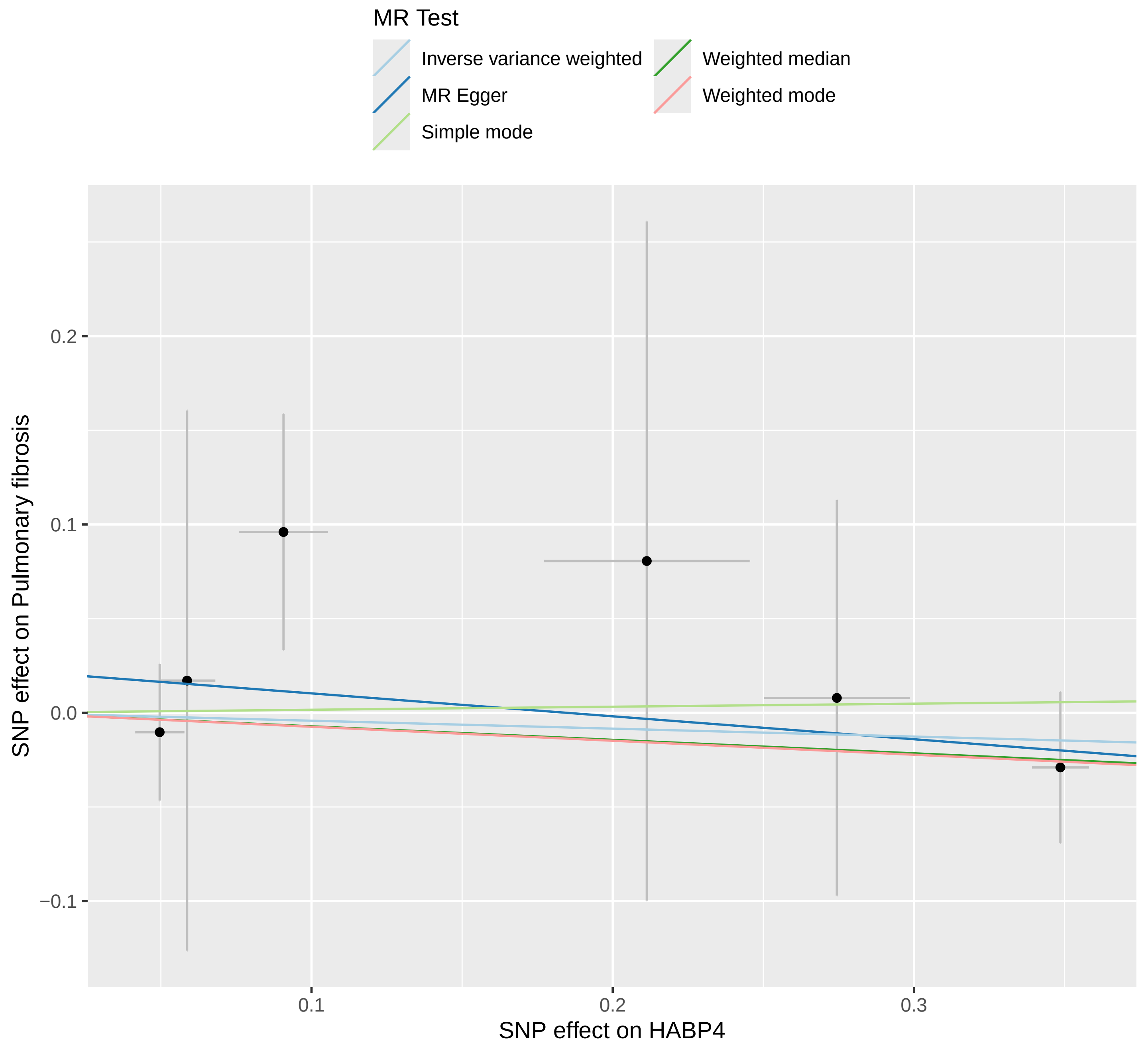
MR Test
Inverse variance weighted
MR Egger
Simple mode
Weighted median
Weighted mode
0.2
0.1
0.0
−0.1
SNP effect on Pulmonary fibrosis
0.1
0.2
0.3
SNP effect on HABP4

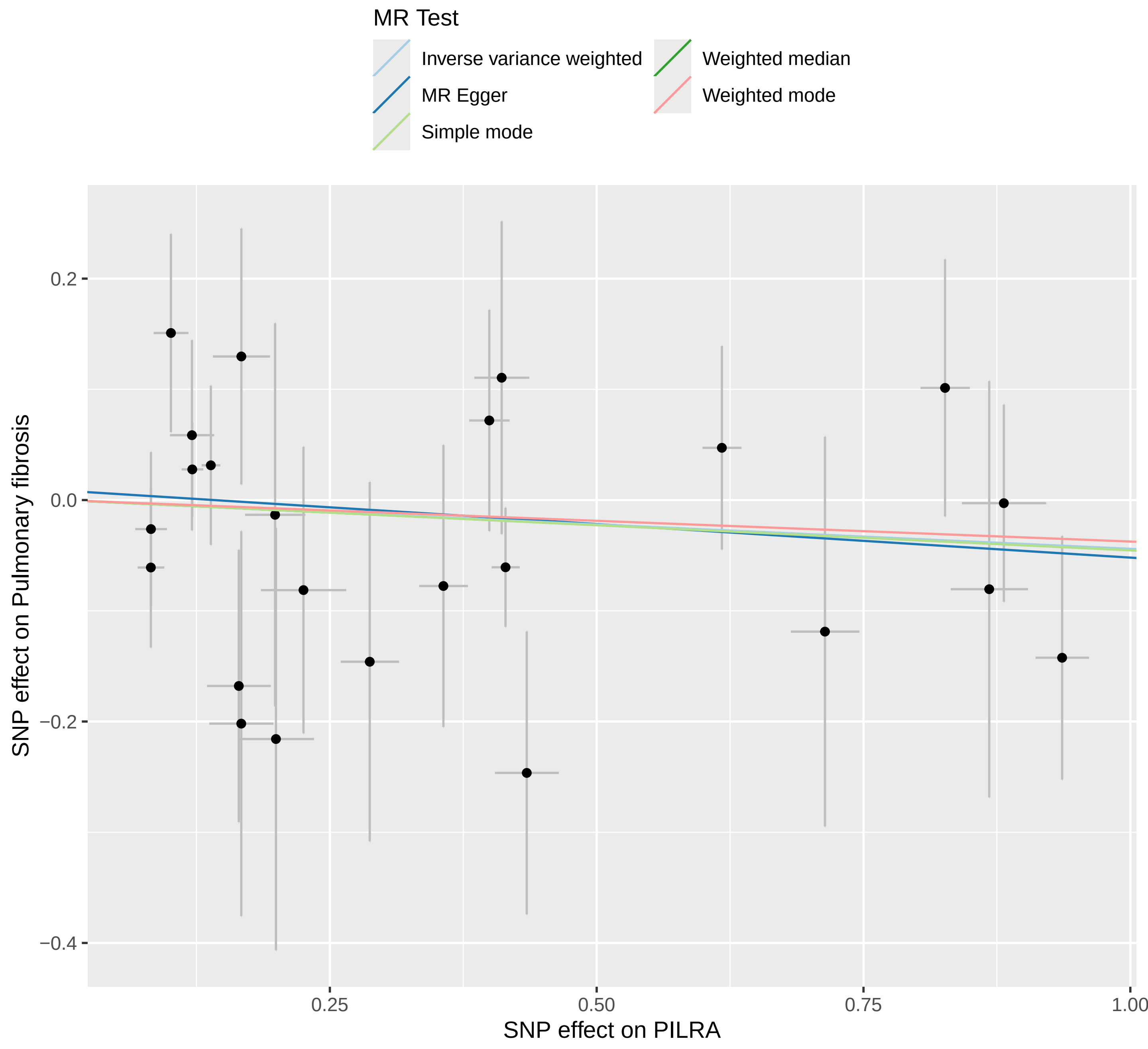

MR Test
Inverse variance weighted
MR Egger
Simple mode
Weighted median
Weighted mode
0.2
0.0
−0.2
−0.4
0.25
0.50
0.75
1.00
SNP effect on Pulmonary fibrosis
SNP effect on PILRA

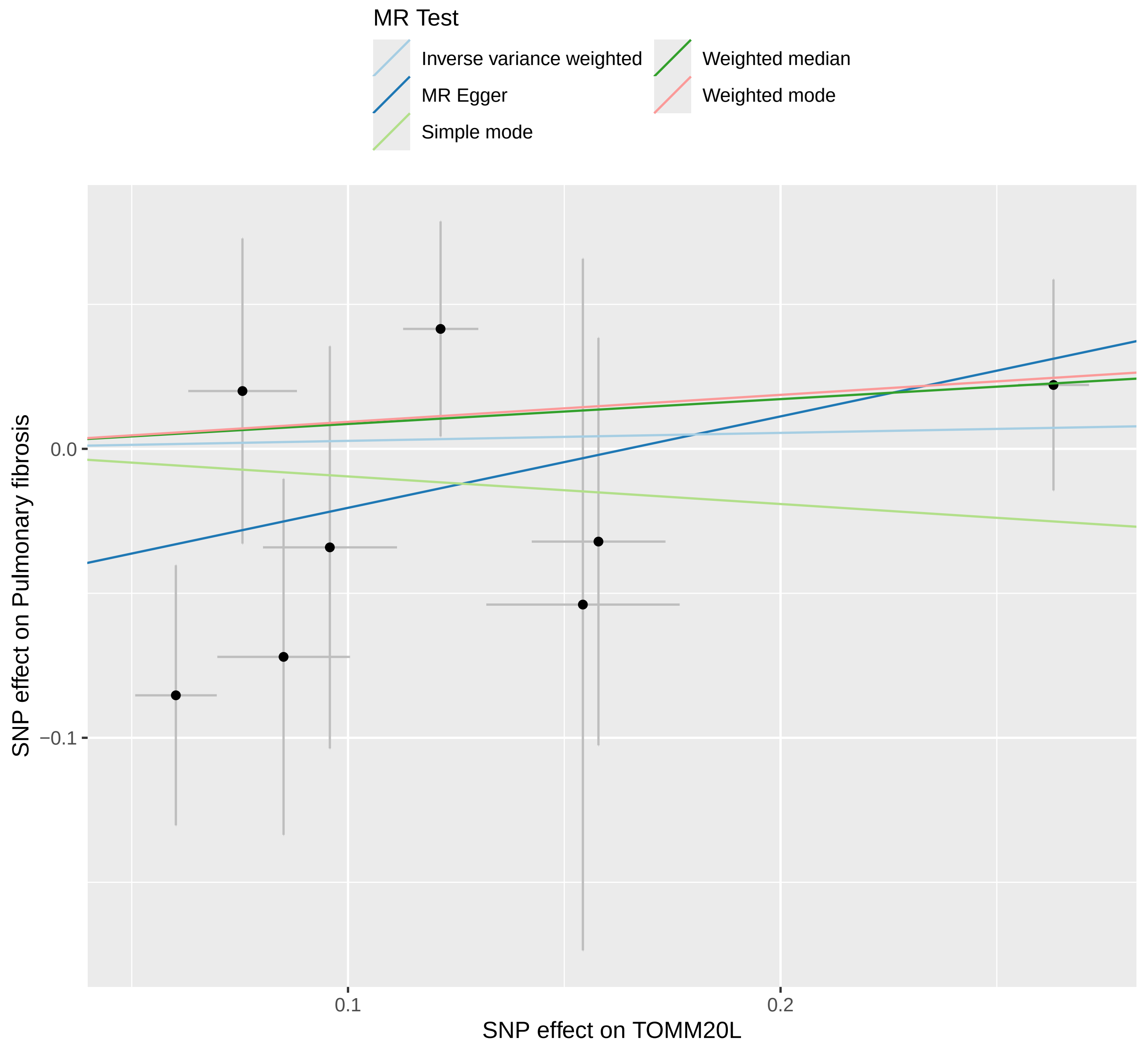
MR Test
Inverse variance weighted
MR Egger
Simple mode
Weighted median
Weighted mode
0.0
−0.1
0.1
0.2
SNP effect on Pulmonary fibrosis
SNP effect on TOMM20L

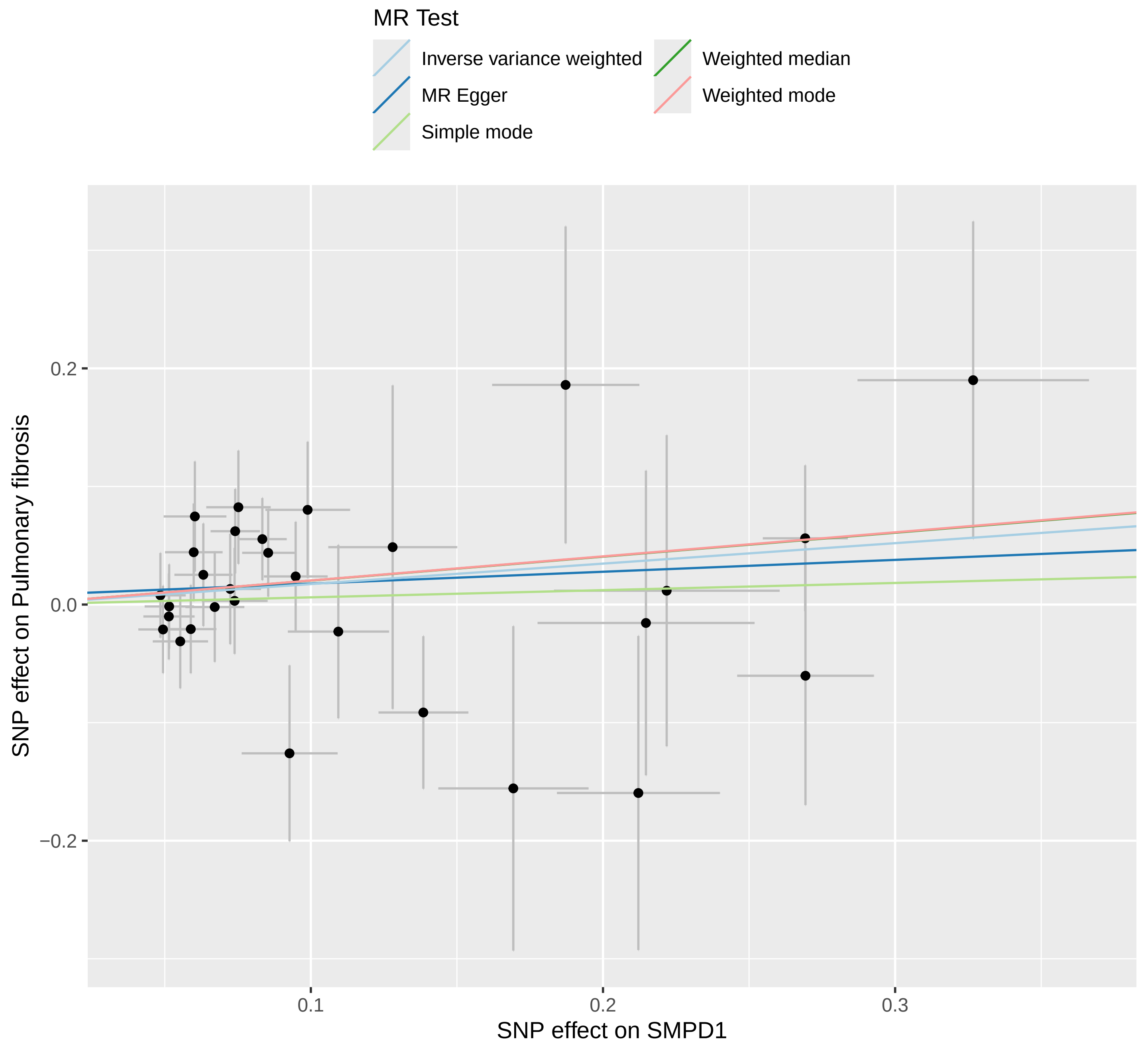
MR Test
Inverse variance weighted
MR Egger
Simple mode
Weighted median
Weighted mode
0.2
0.0
−0.2
0.1
0.2
0.3
SNP effect on Pulmonary fibrosis
SNP effect on SMPD1

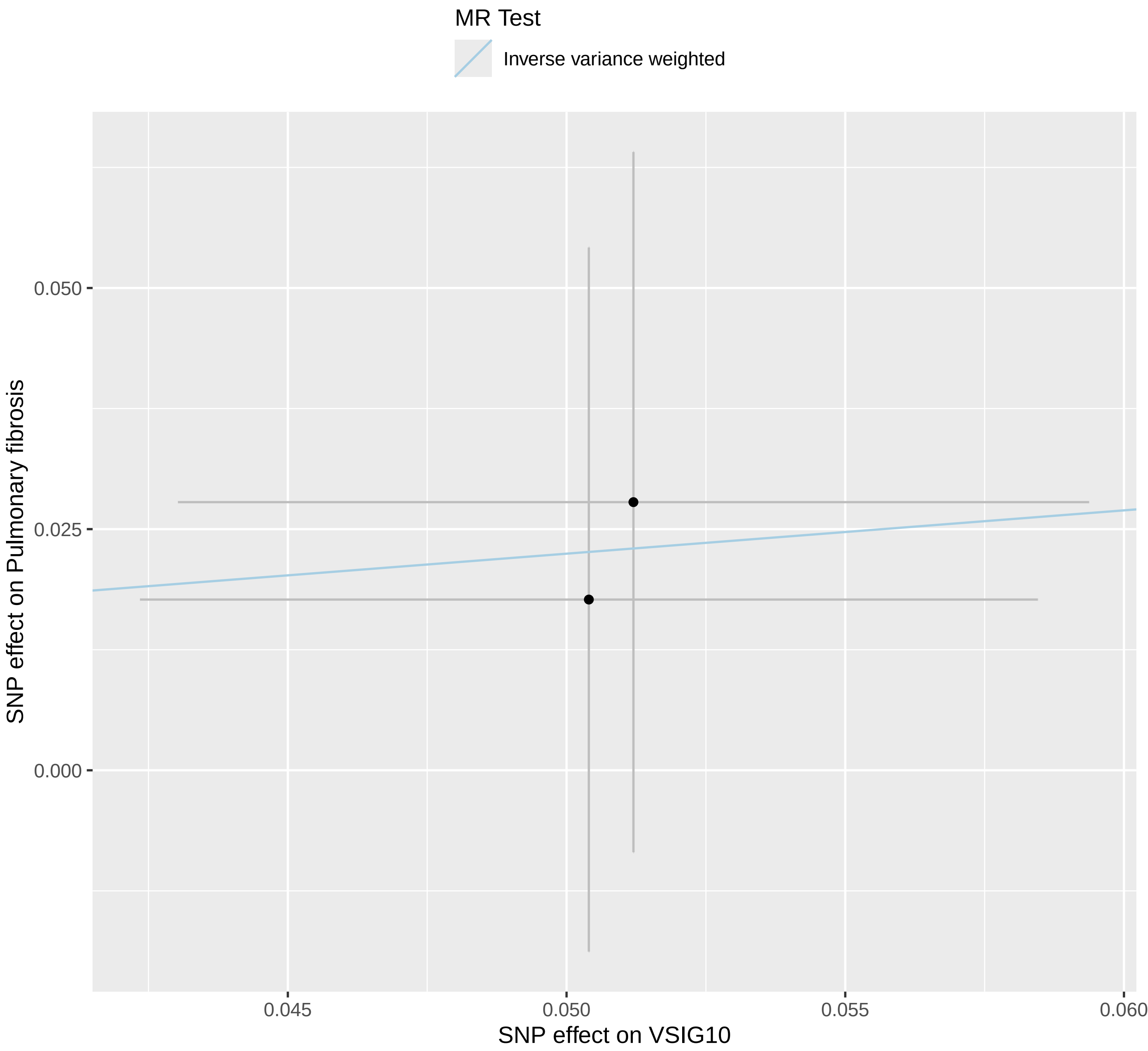

MR Test
Inverse variance weighted
0.050
0.025
0.000
0.045
0.050
0.055
0.060
SNP effect on Pulmonary fibrosis
SNP effect on VSIG10

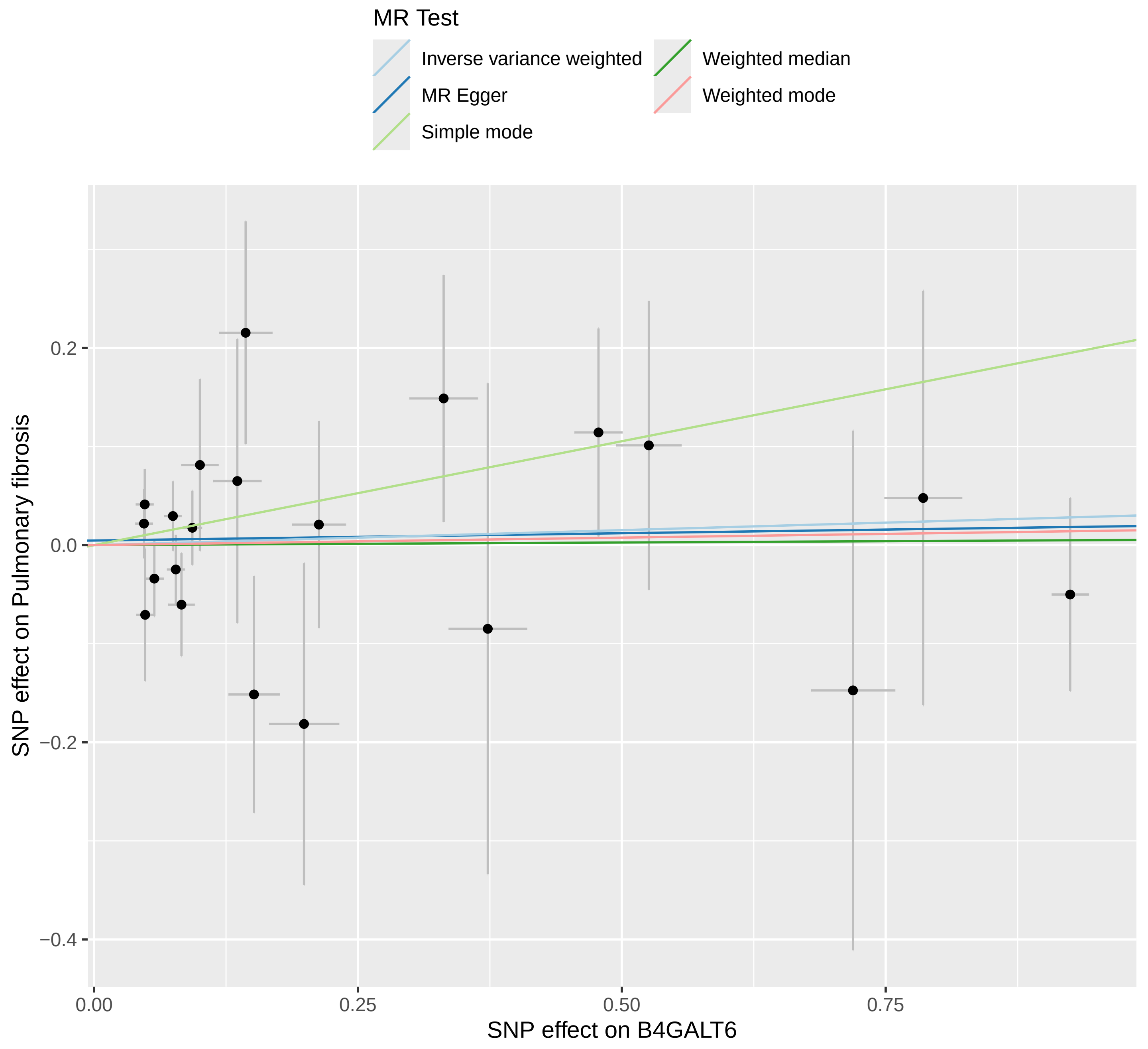
MR Test
Inverse variance weighted
MR Egger
Simple mode
Weighted median
Weighted mode
SNP effect on Pulmonary fibrosis
0.2
0.0
−0.2
−0.4
0.00
0.25
0.50
0.75
SNP effect on B4GALT6

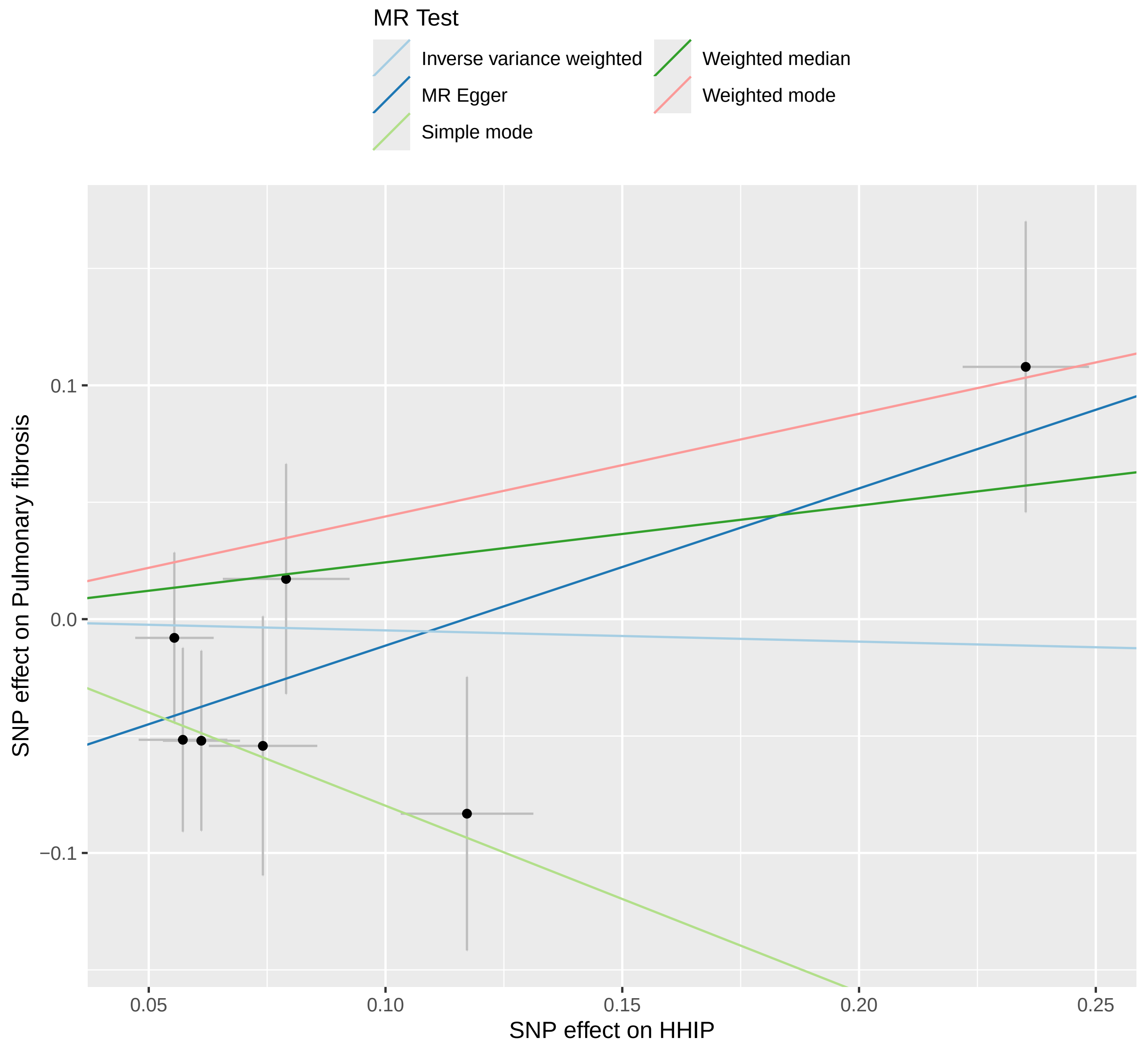

MR Test
Inverse variance weighted
MR Egger
Simple mode
Weighted median
Weighted mode
0.1
0.0
−0.1
0.05
0.10
0.15
0.20
0.25
SNP effect on Pulmonary fibrosis
SNP effect on HHIP

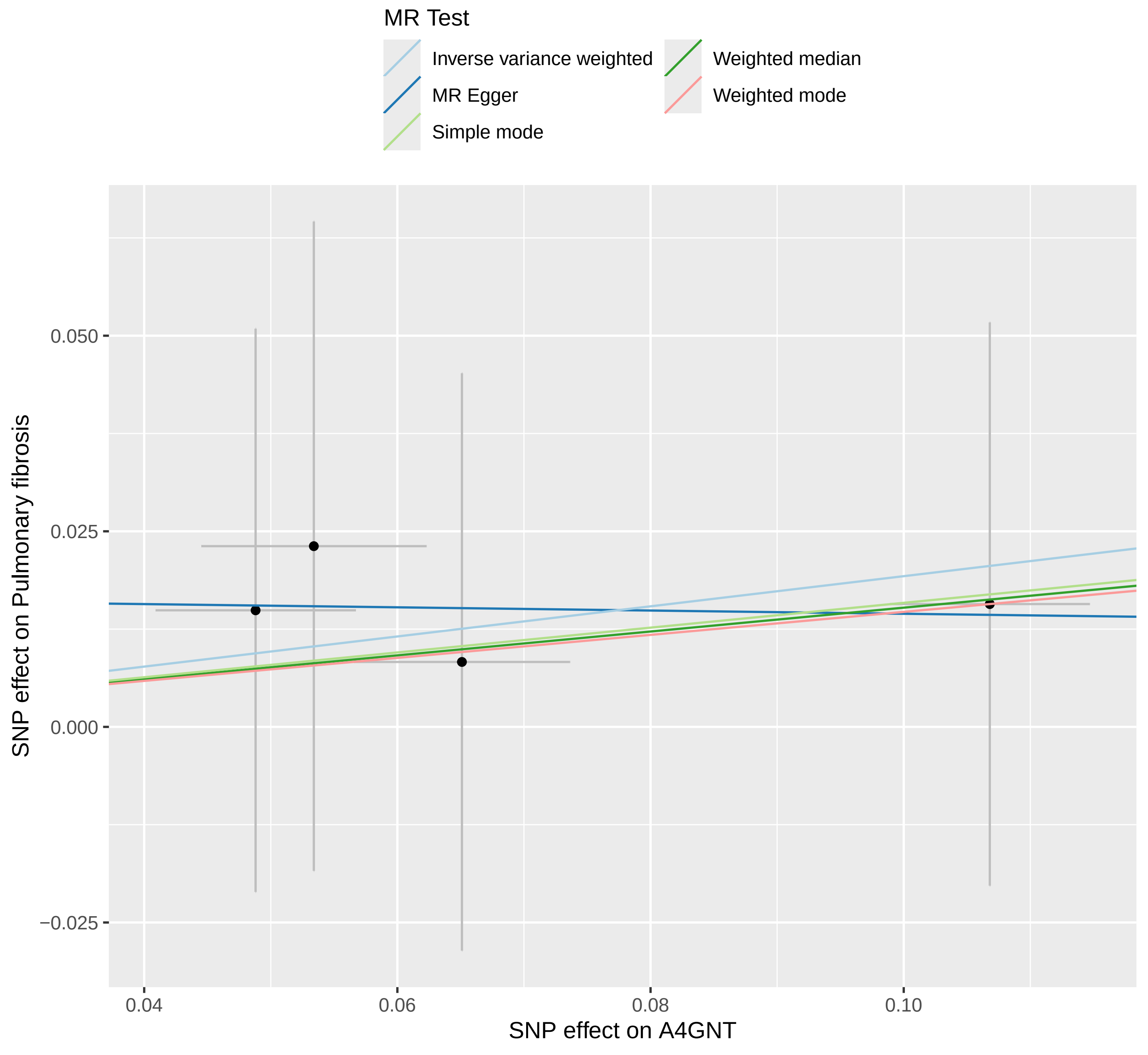
MR Test
Inverse variance weighted
MR Egger
Simple mode
Weighted median
Weighted mode
0.050
0.025
0.000
−0.025
SNP effect on Pulmonary fibrosis
0.04
0.06
0.08
0.10
SNP effect on A4GNT

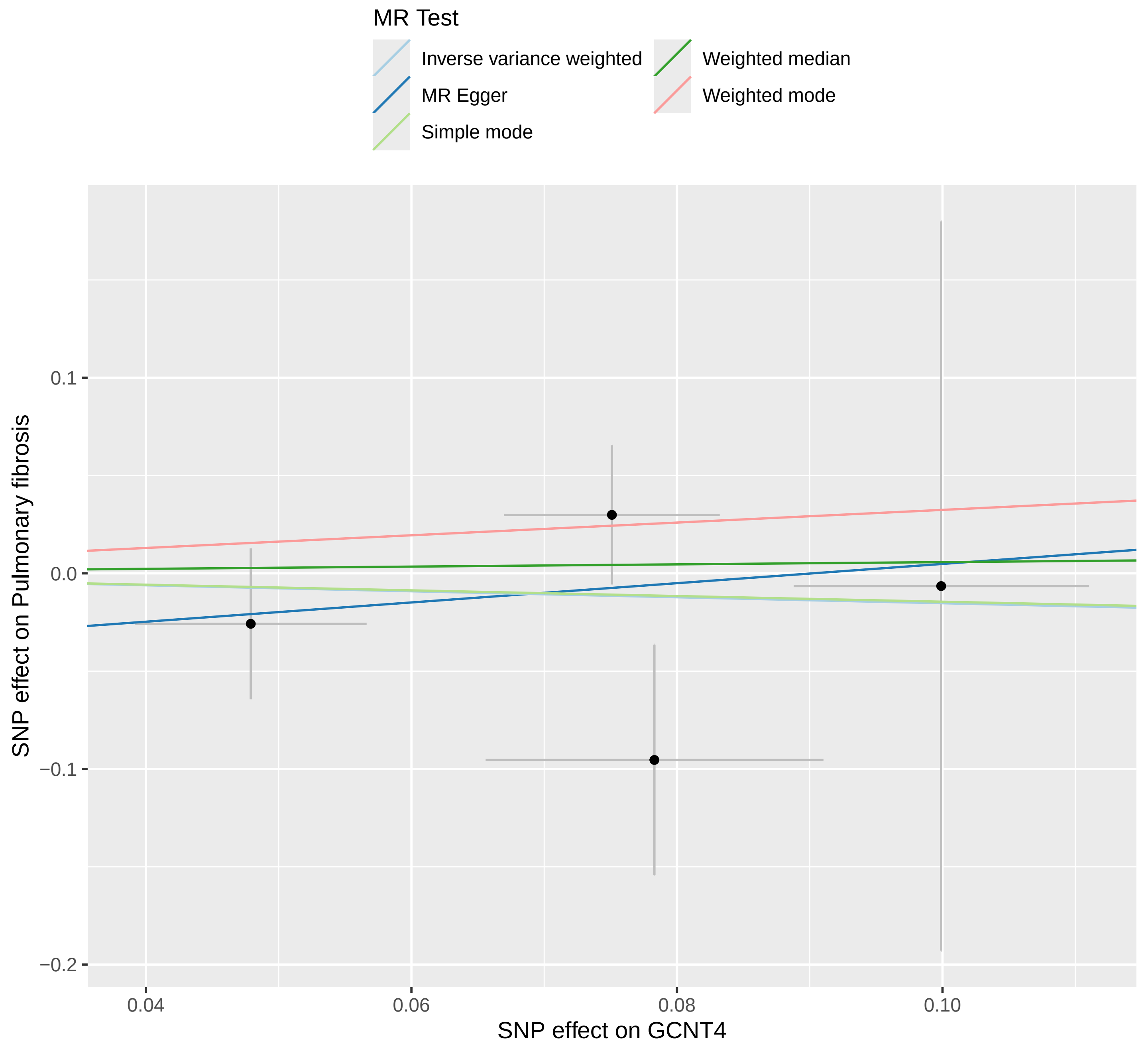
MR Test
Inverse variance weighted
MR Egger
Simple mode
Weighted median
Weighted mode
SNP effect on Pulmonary fibrosis
0.1
0.0
−0.1
−0.2
0.04
0.06
0.08
0.10
SNP effect on GCNT4

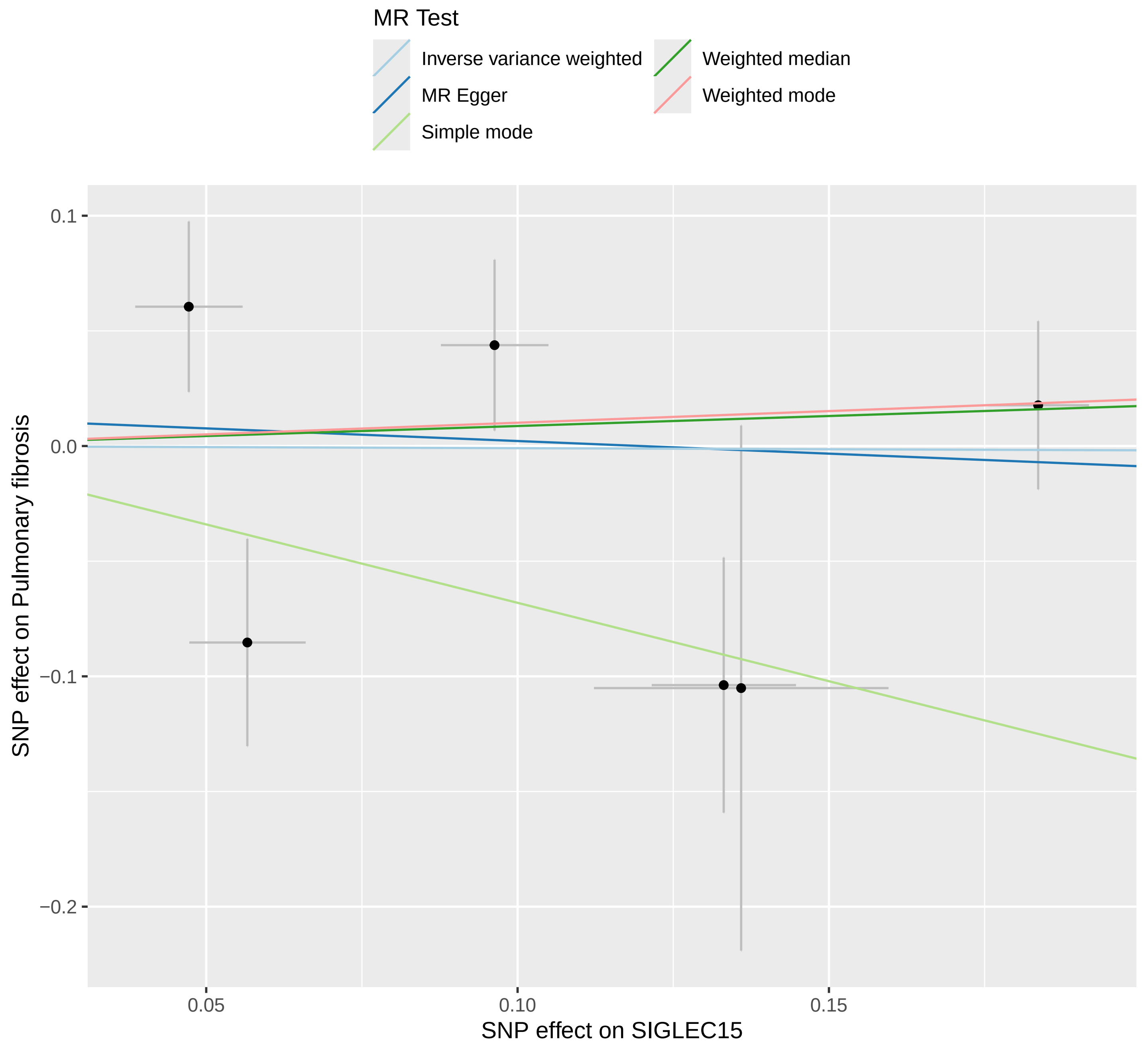
MR Test
Inverse variance weighted
MR Egger
Simple mode
Weighted median
Weighted mode
0.1
0.0
−0.1
−0.2
0.05
0.10
0.15
SNP effect on Pulmonary fibrosis
SNP effect on SIGLEC15

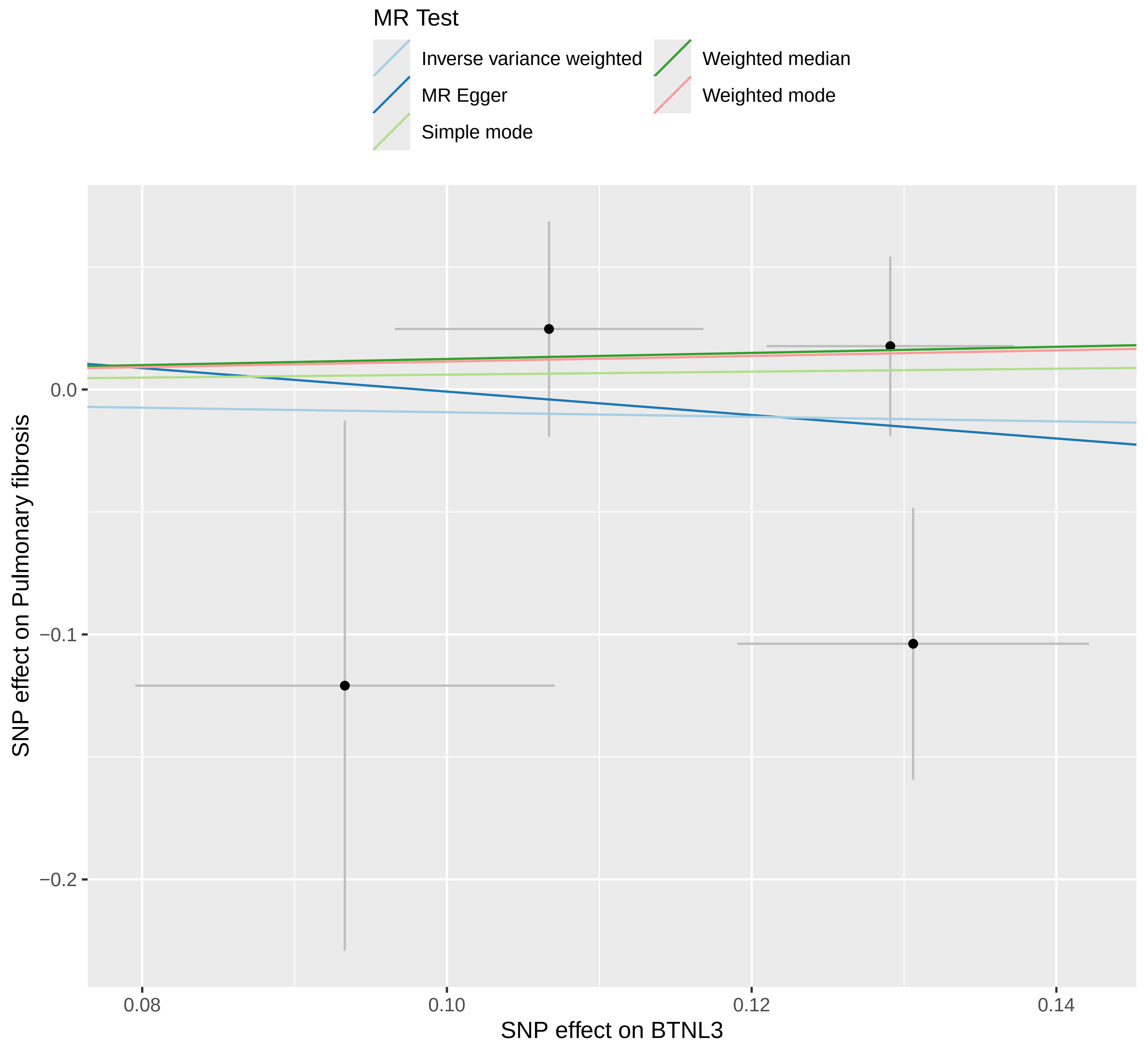
MR Test
Inverse variance weighted
MR Egger
Simple mode
Weighted median
Weighted mode
0.0
−0.1
−0.2
0.08
0.10
0.12
0.14
SNP effect on BTNL3
SNP effect on Pulmonary fibrosis

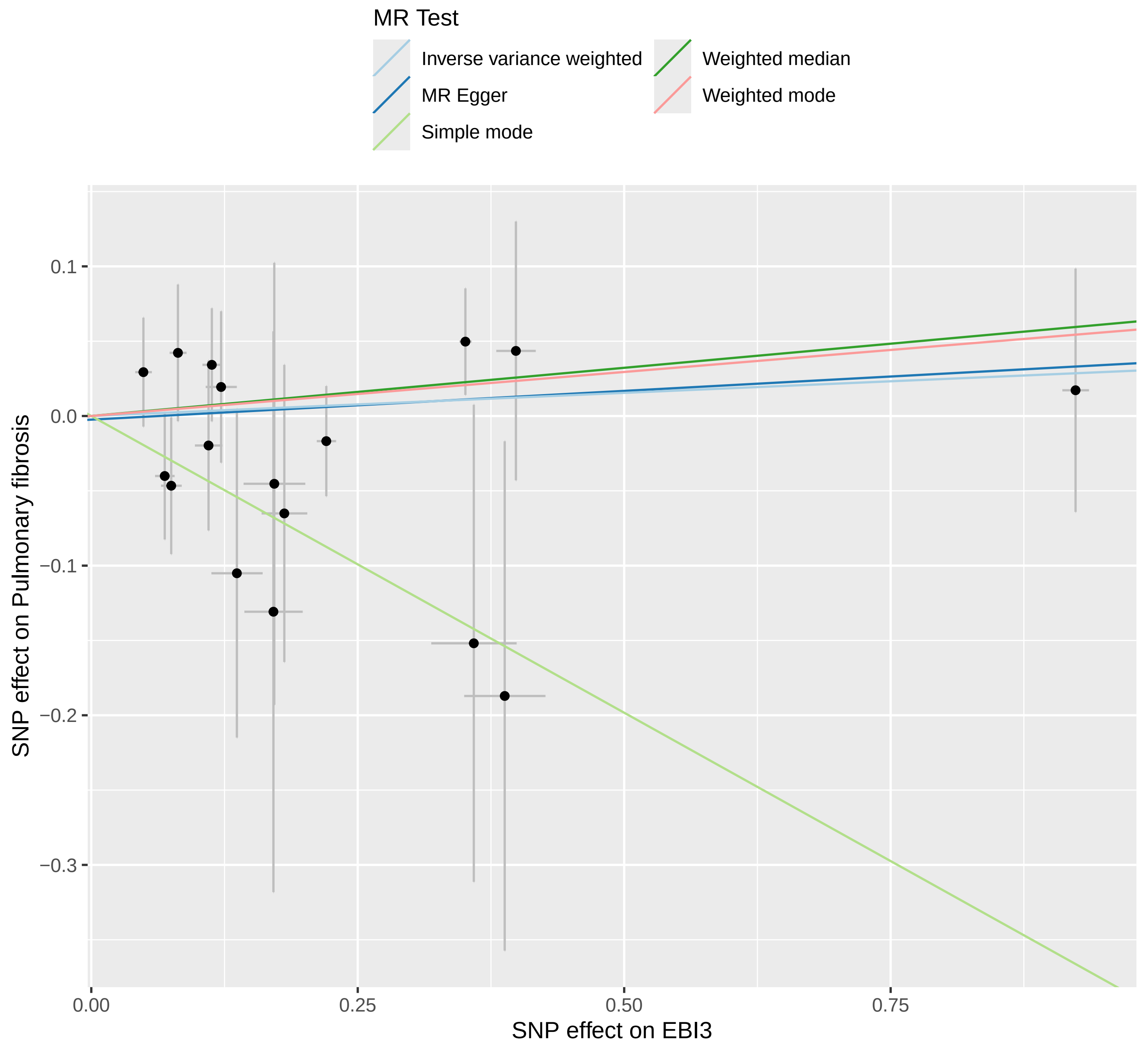
MR Test
Inverse variance weighted
MR Egger
Simple mode
Weighted median
Weighted mode
0.1
0.0
−0.1
−0.2
−0.3
SNP effect on Pulmonary fibrosis
0.00
0.25
0.50
0.75
SNP effect on EBI3

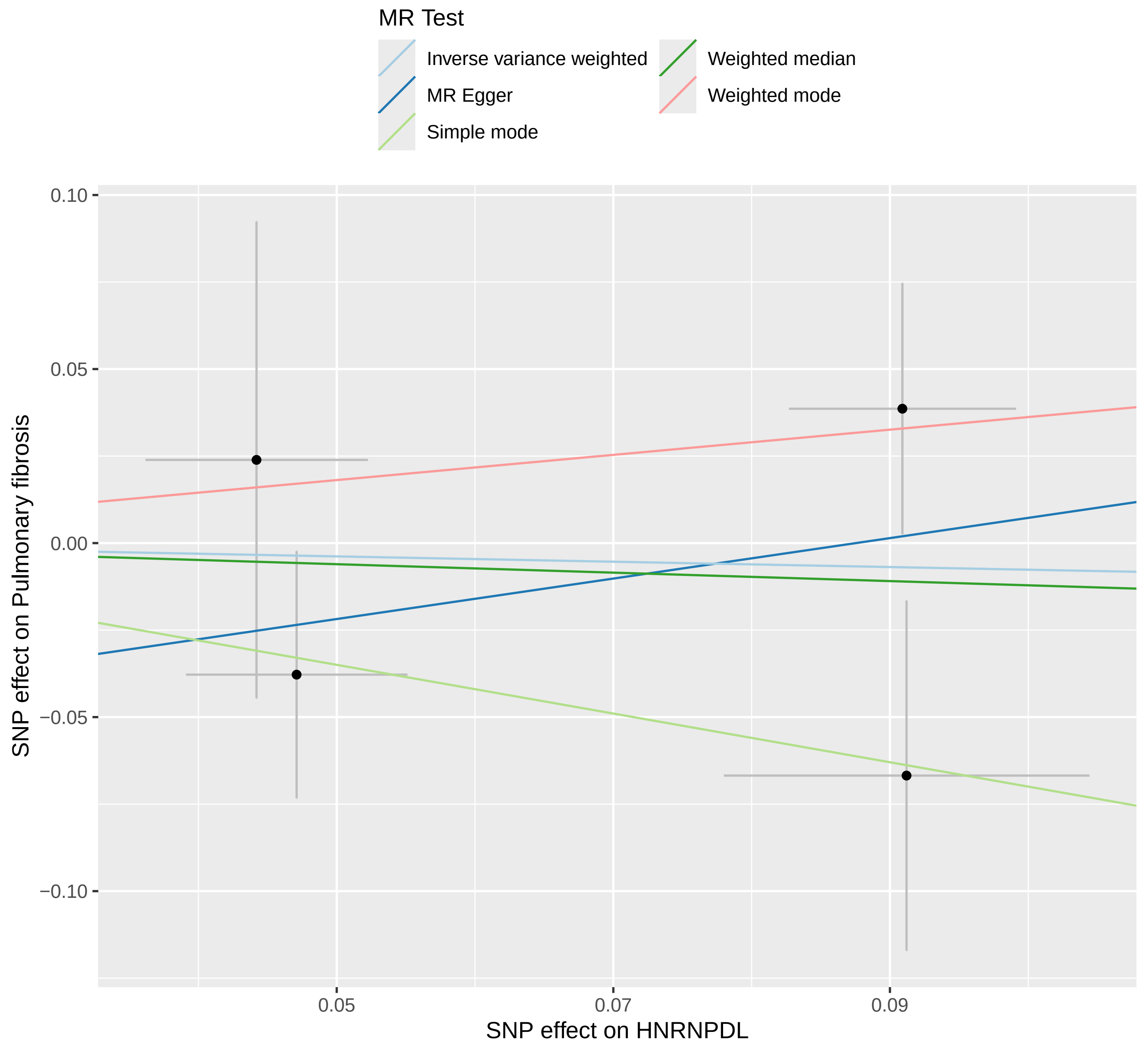
MR Test
Inverse variance weighted
MR Egger
Simple mode
Weighted median
Weighted mode
0.10
0.05
0.00
−0.05
−0.10
0.05
0.07
0.09
SNP effect on Pulmonary fibrosis
SNP effect on HNRNPDL

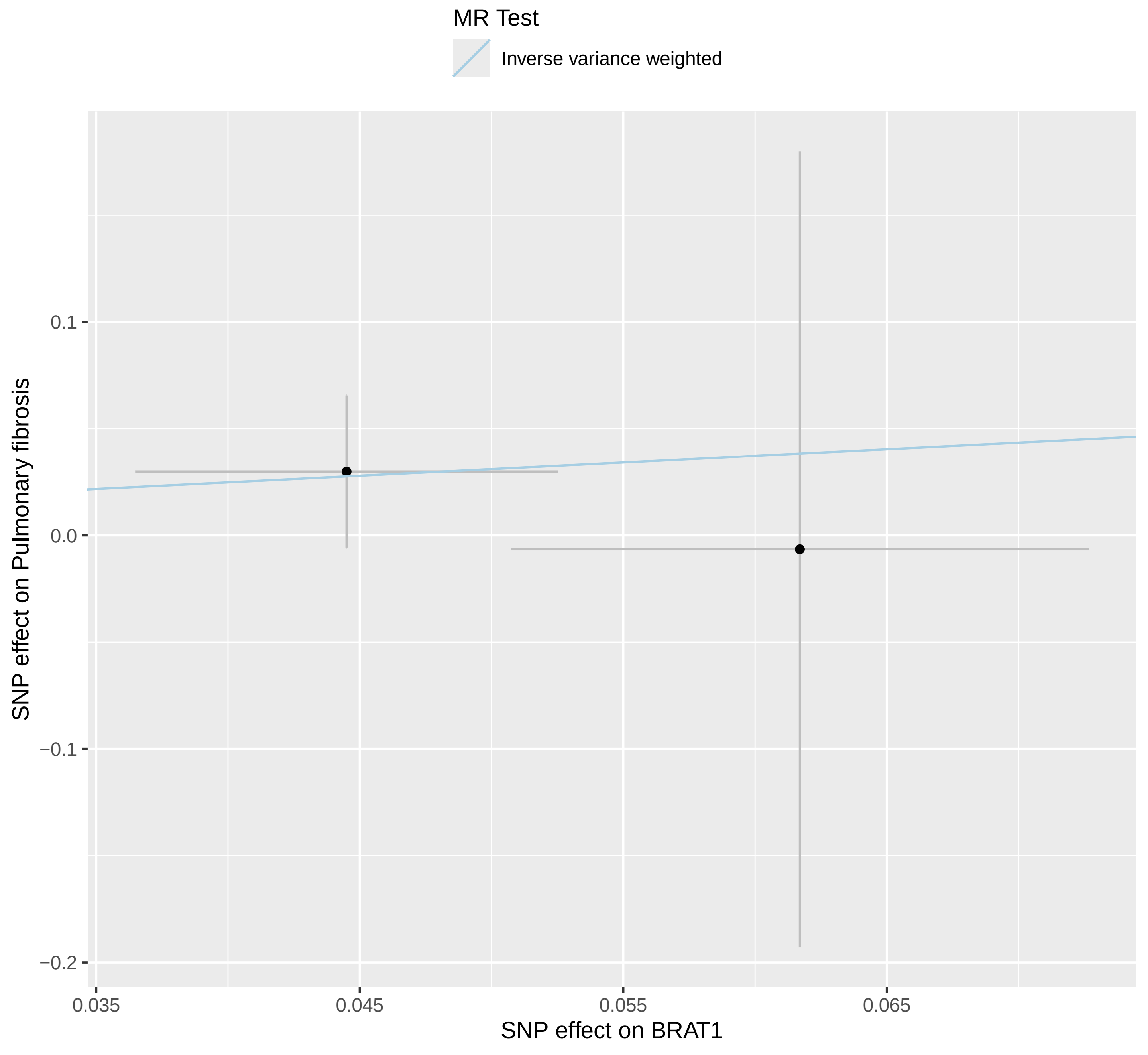
MR Test
Inverse variance weighted
0.1
0.0
−0.1
−0.2
SNP effect on Pulmonary fibrosis
0.035
0.045
0.055
0.065
SNP effect on BRAT1

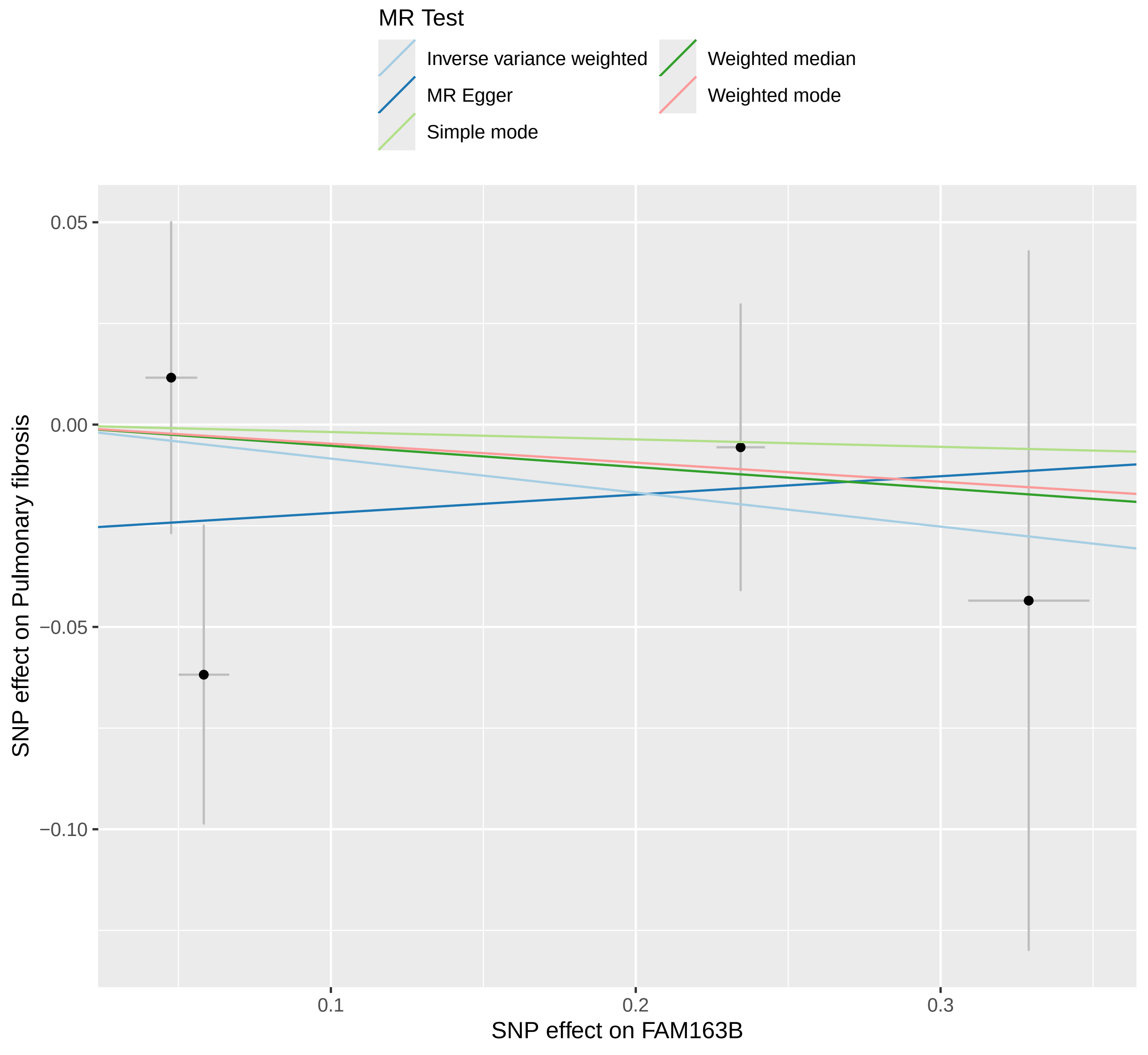
MR Test
Inverse variance weighted
MR Egger
Simple mode
Weighted median
Weighted mode
0.05
0.00
−0.05
−0.10
SNP effect on Pulmonary fibrosis
0.1
0.2
0.3
SNP effect on FAM163B

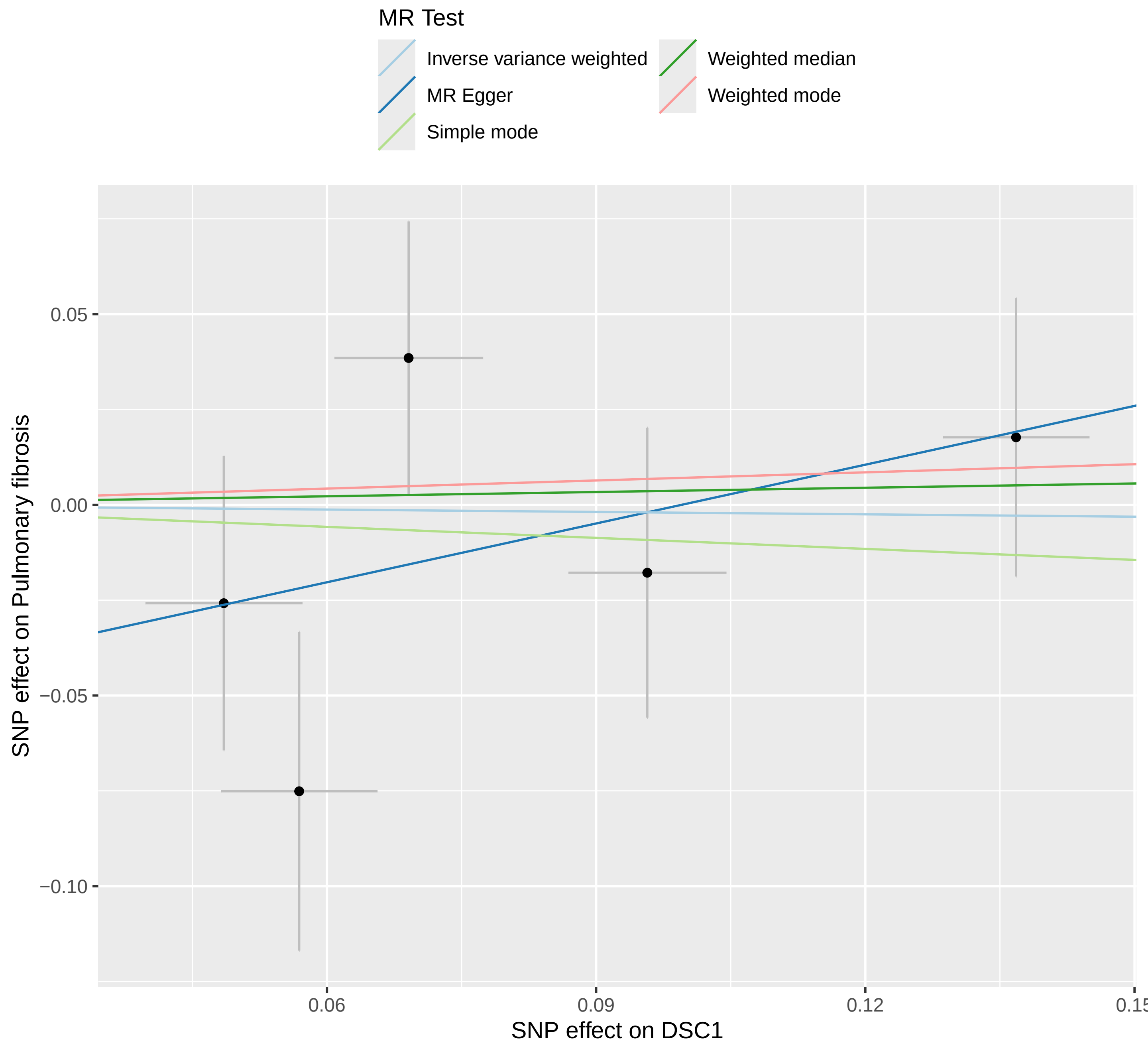
MR Test
Inverse variance weighted
MR Egger
Simple mode
Weighted median
Weighted mode
0.05
0.00
−0.05
−0.10
0.06
0.09
0.12
0.15
SNP effect on Pulmonary fibrosis
SNP effect on DSC1

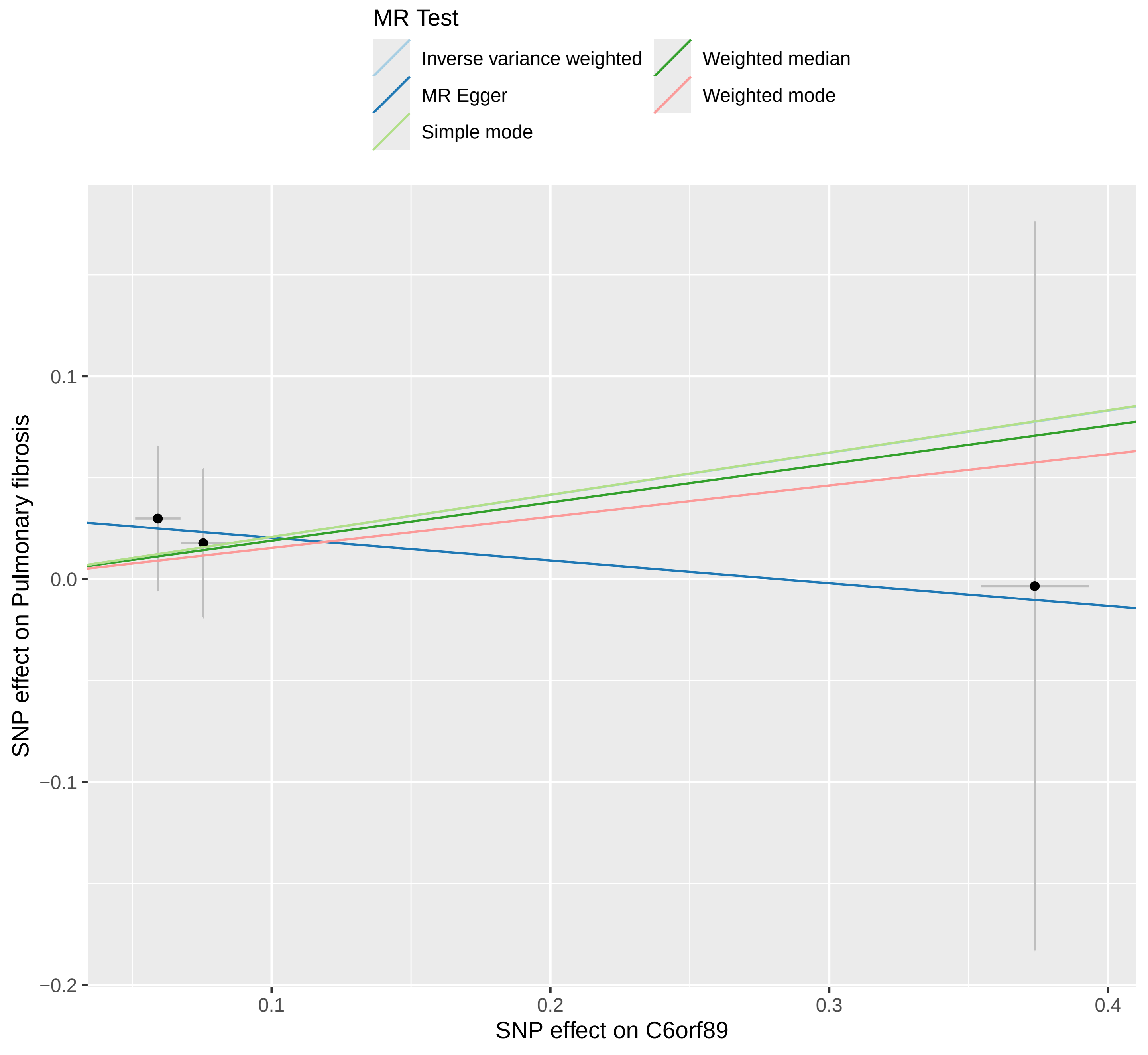
MR Test
Inverse variance weighted
MR Egger
Simple mode
Weighted median
Weighted mode
0.1
0.0
−0.1
−0.2
0.1
0.2
0.3
0.4
SNP effect on Pulmonary fibrosis
SNP effect on C6orf89

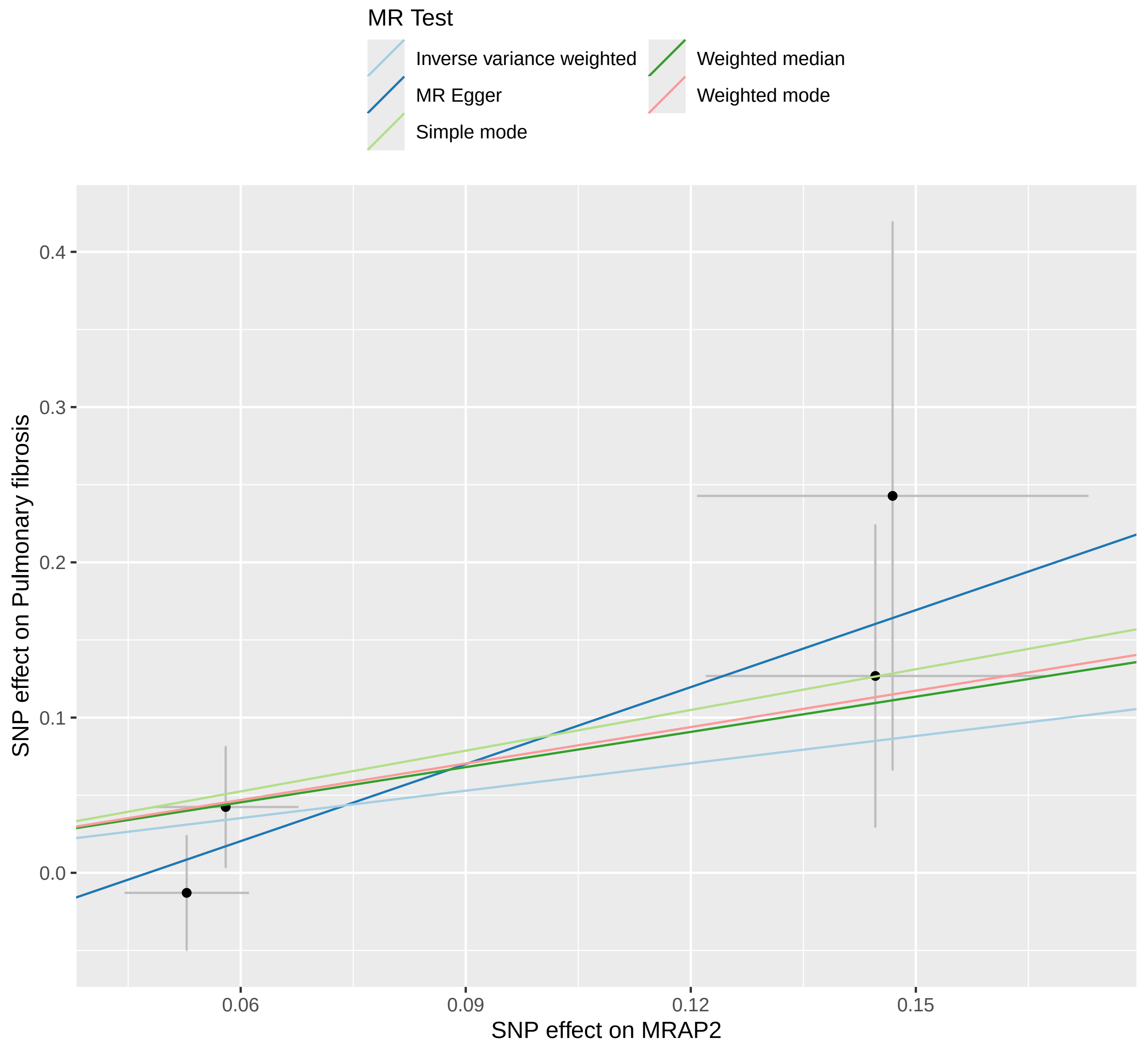

MR Test
Inverse variance weighted
MR Egger
Simple mode
Weighted median
Weighted mode
0.4
0.3
0.2
0.1
0.0
0.06
0.09
0.12
0.15
SNP effect on Pulmonary fibrosis
SNP effect on MRAP2

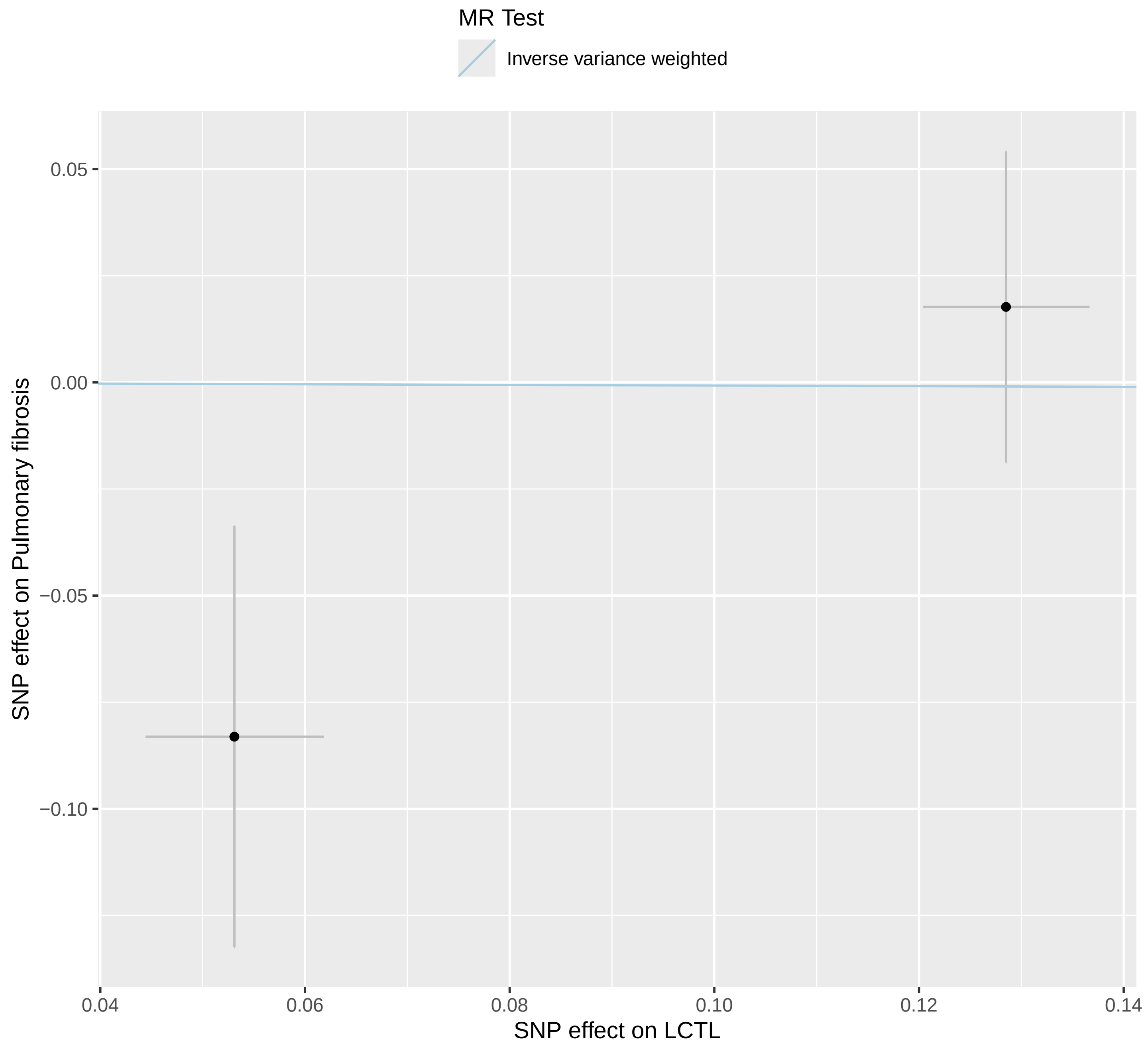

MR Test
Inverse variance weighted
0.05
0.00
−0.05
−0.10
SNP effect on Pulmonary fibrosis
0.04
0.06
0.08
0.10
0.12
0.14
SNP effect on LCTL

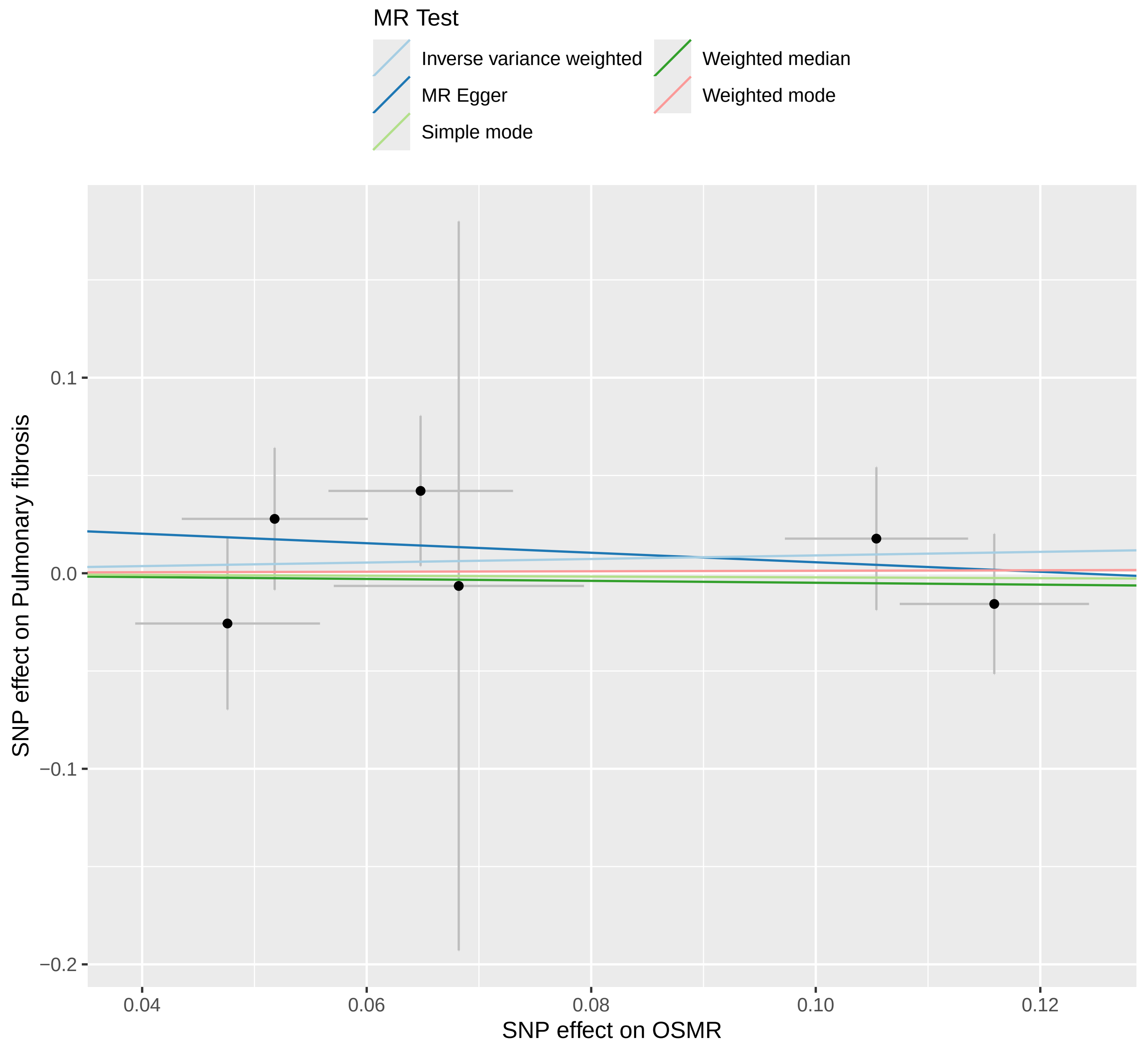

MR Test
Inverse variance weighted
MR Egger
Simple mode
Weighted median
Weighted mode
0.1
0.0
−0.1
−0.2
SNP effect on Pulmonary fibrosis
0.04
0.06
0.08
0.10
0.12
SNP effect on OSMR

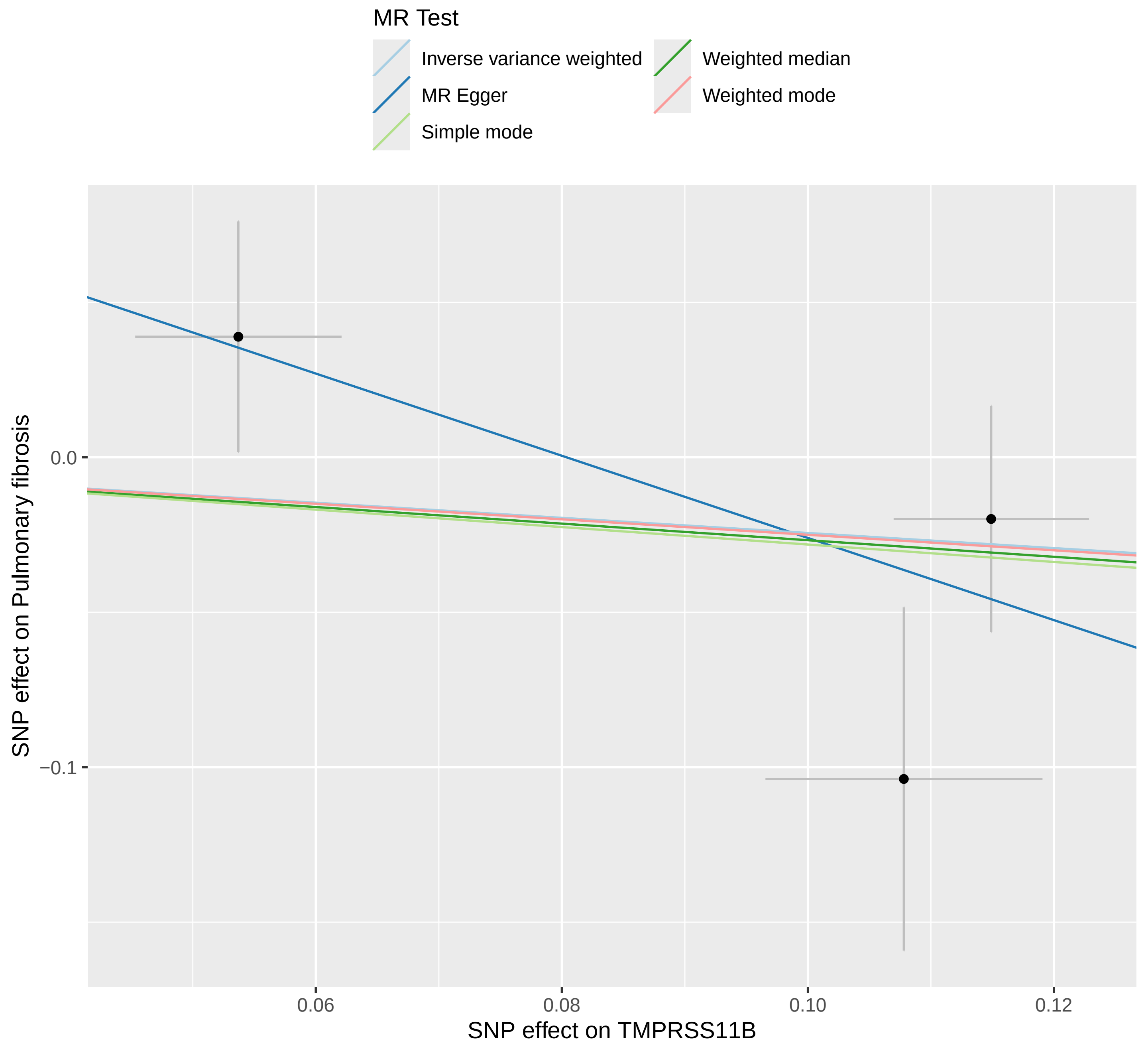
MR Test
Inverse variance weighted
MR Egger
Simple mode
Weighted median
Weighted mode
0.0
−0.1
0.06
0.08
0.10
0.12
SNP effect on TMPRSS11B
SNP effect on Pulmonary fibrosis

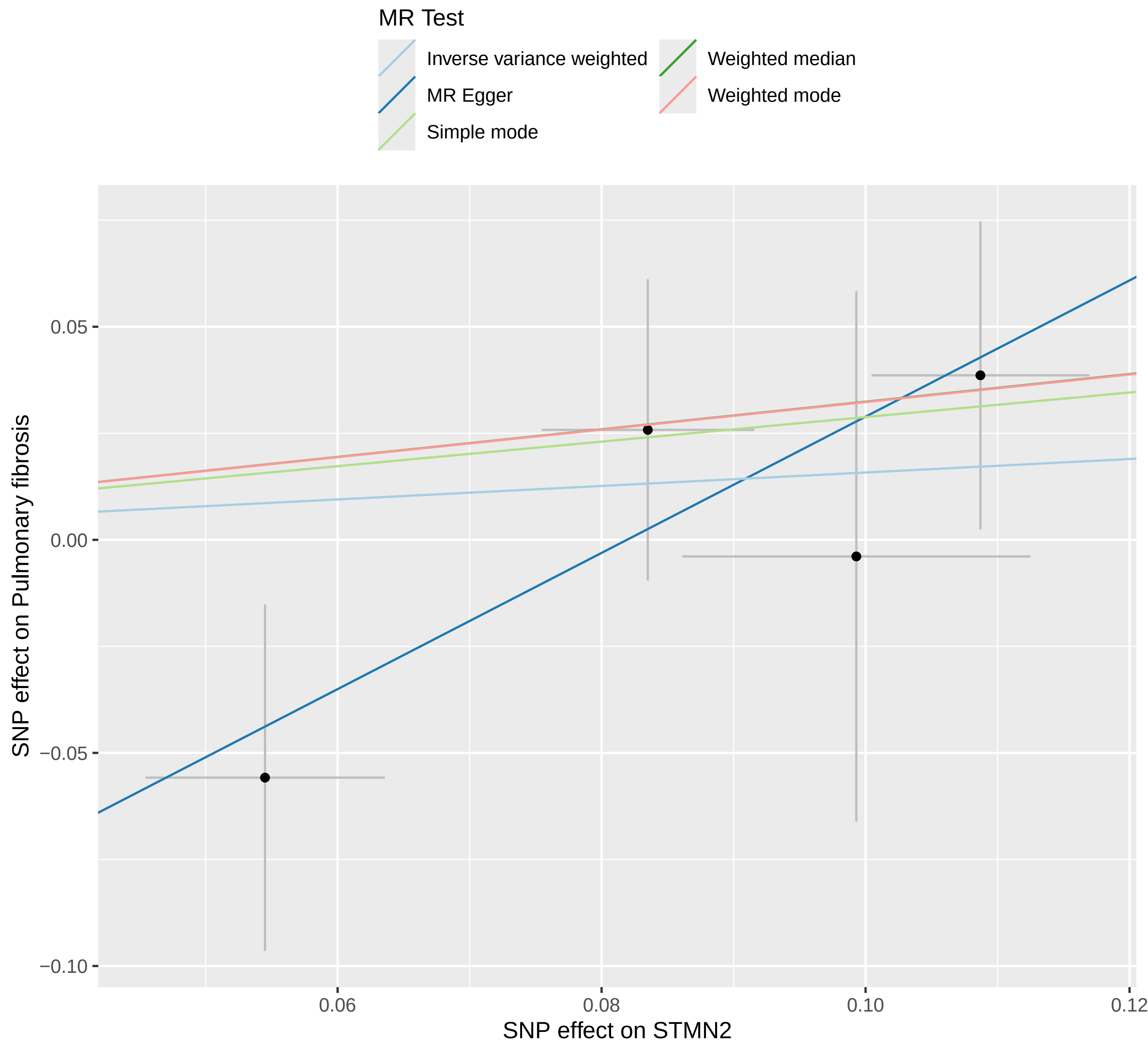

MR Test
Inverse variance weighted
MR Egger
Simple mode
Weighted median
Weighted mode
0.05
0.00
−0.05
−0.10
0.06
0.08
0.10
0.12
SNP effect on STMN2
SNP effect on Pulmonary fibrosis

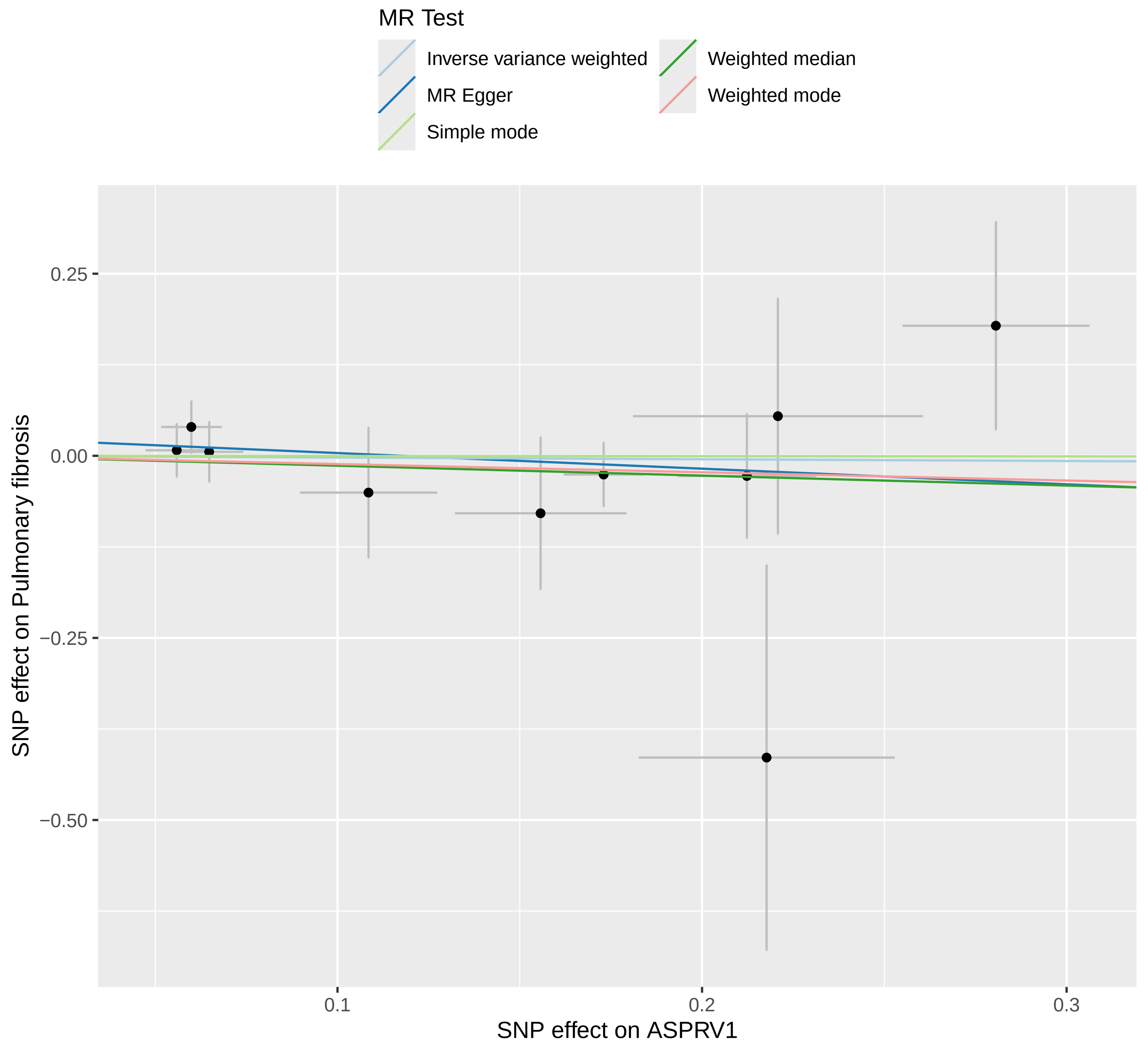

MR Test
Inverse variance weighted
MR Egger
Simple mode
Weighted median
Weighted mode
0.25
0.00
−0.25
−0.50
0.1
0.2
0.3
SNP effect on Pulmonary fibrosis
SNP effect on ASPRV1

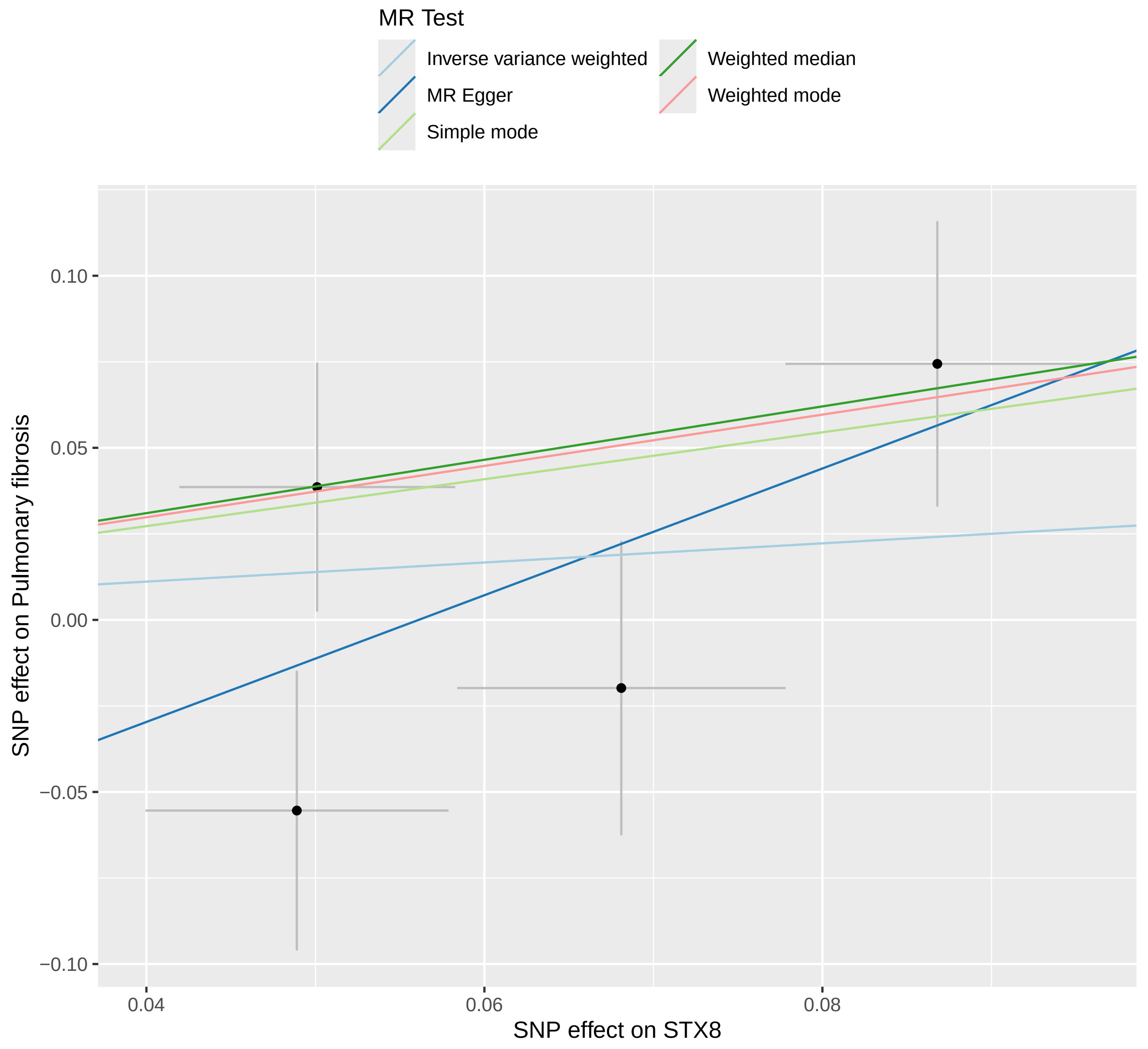
MR Test
Inverse variance weighted
MR Egger
Simple mode
Weighted median
Weighted mode
0.10
0.05
0.00
−0.05
−0.10
SNP effect on Pulmonary fibrosis
0.04
0.06
0.08
SNP effect on STX8

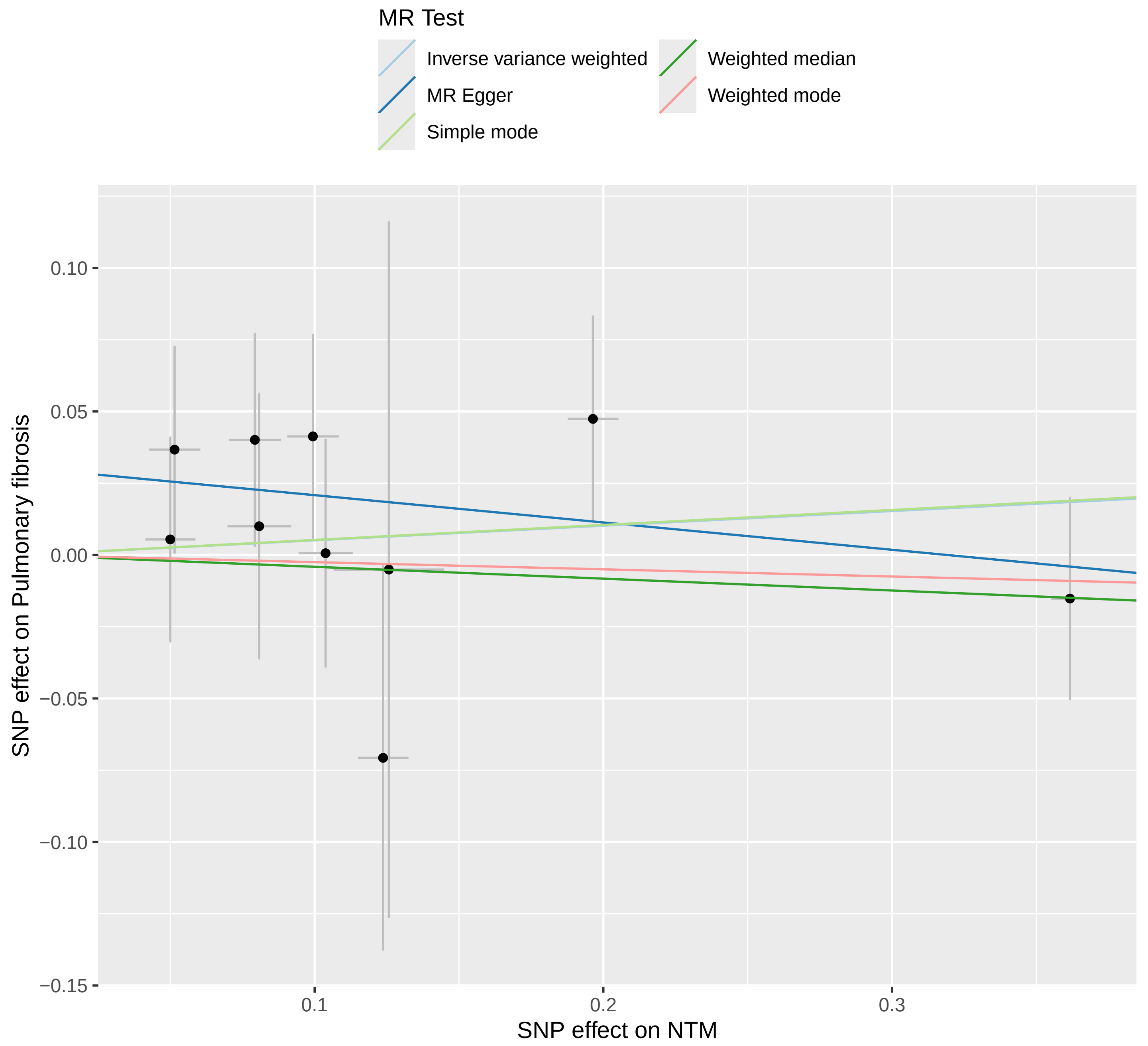

MR Test
Inverse variance weighted
MR Egger
Simple mode
Weighted median
Weighted mode
SNP effect on Pulmonary fibrosis
0.10
0.05
0.00
−0.05
−0.10
−0.15
0.1
0.2
0.3
SNP effect on NTM

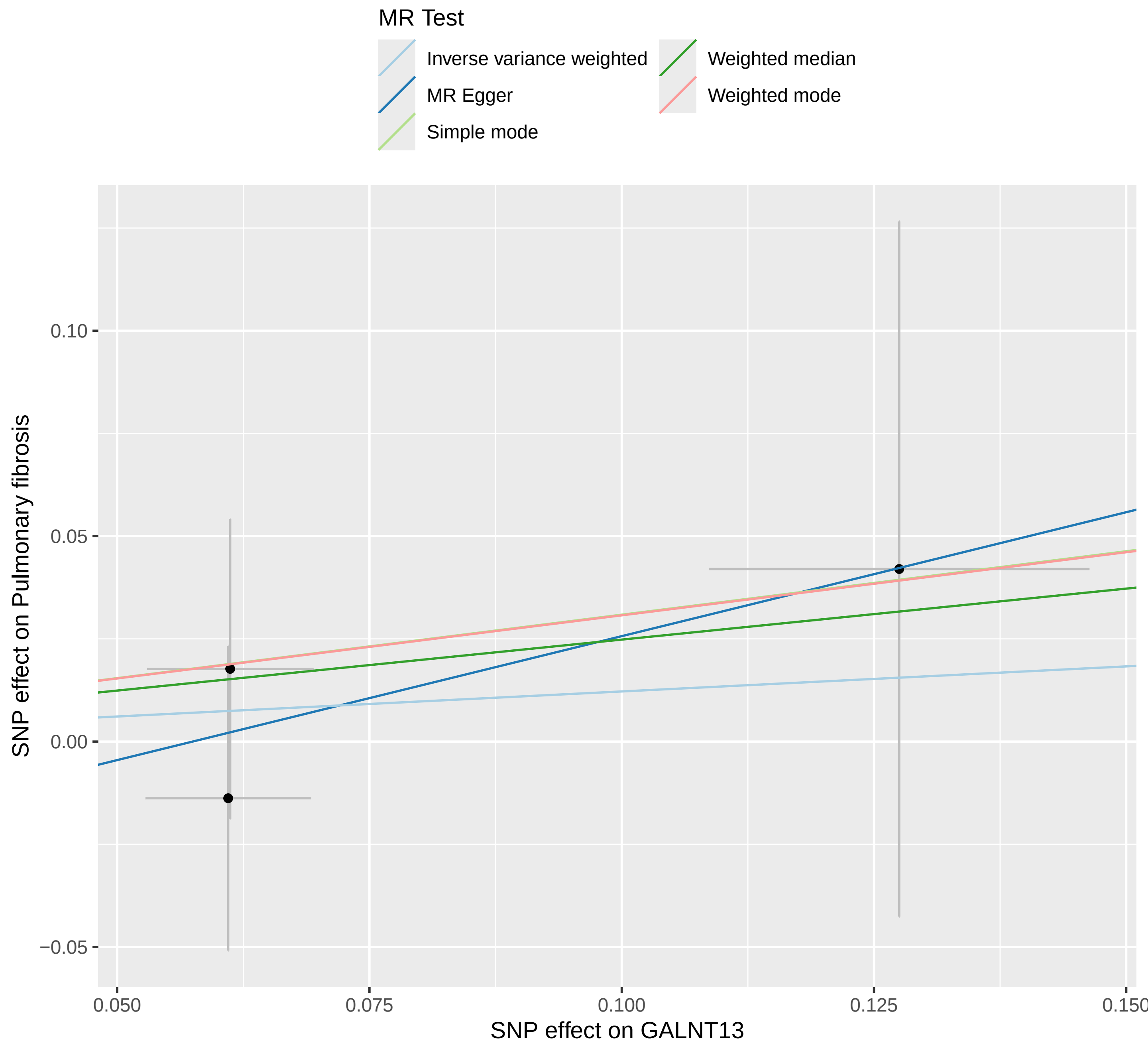
MR Test
Inverse variance weighted
MR Egger
Simple mode
Weighted median
Weighted mode
0.10
0.05
0.00
−0.05
SNP effect on Pulmonary fibrosis
0.050
0.075
0.100
0.125
0.150
SNP effect on GALNT13

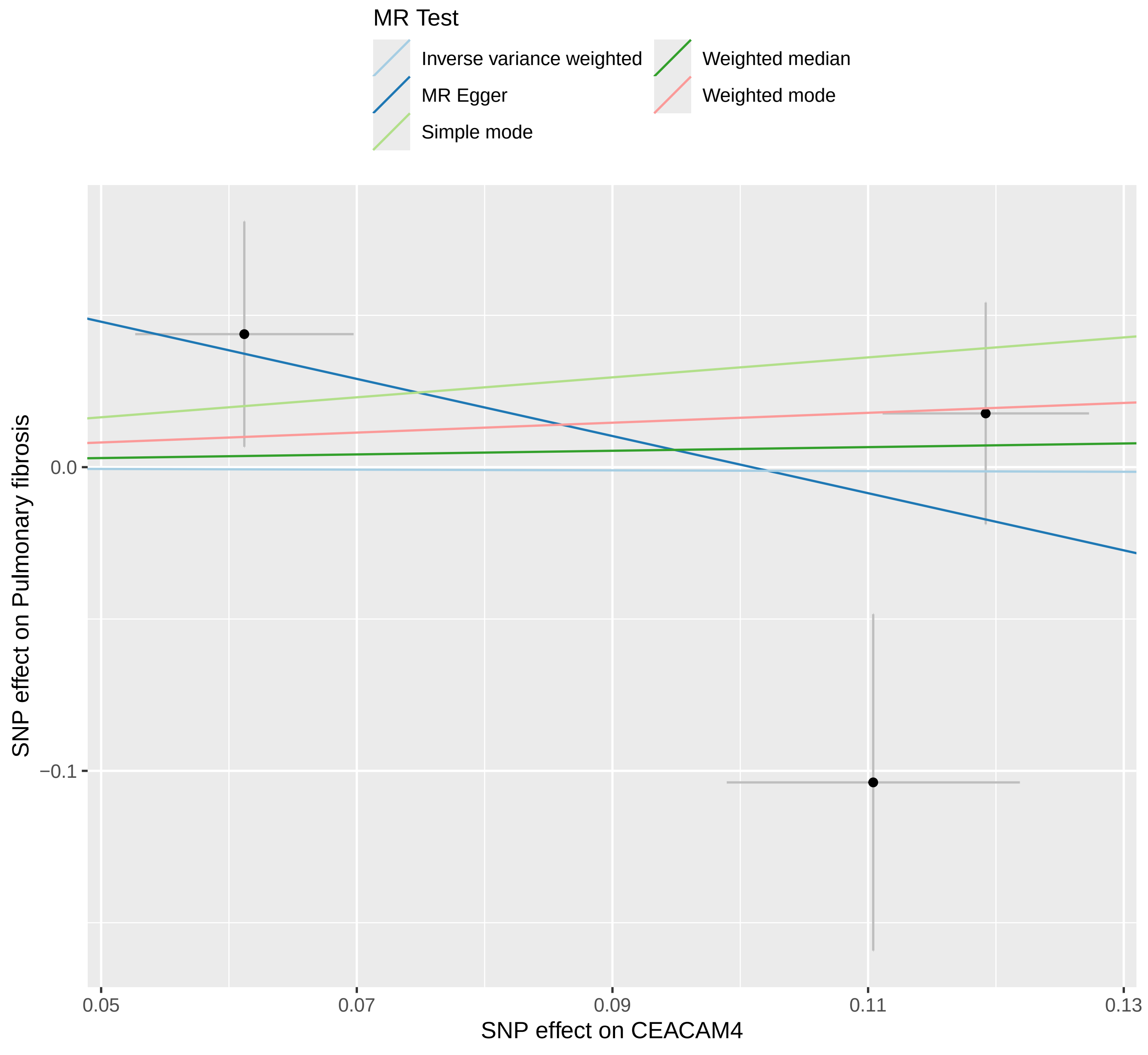
MR Test
Inverse variance weighted
MR Egger
Simple mode
Weighted median
Weighted mode
0.0
−0.1
0.05
0.07
0.09
0.11
0.13
SNP effect on CEACAM4
SNP effect on Pulmonary fibrosis

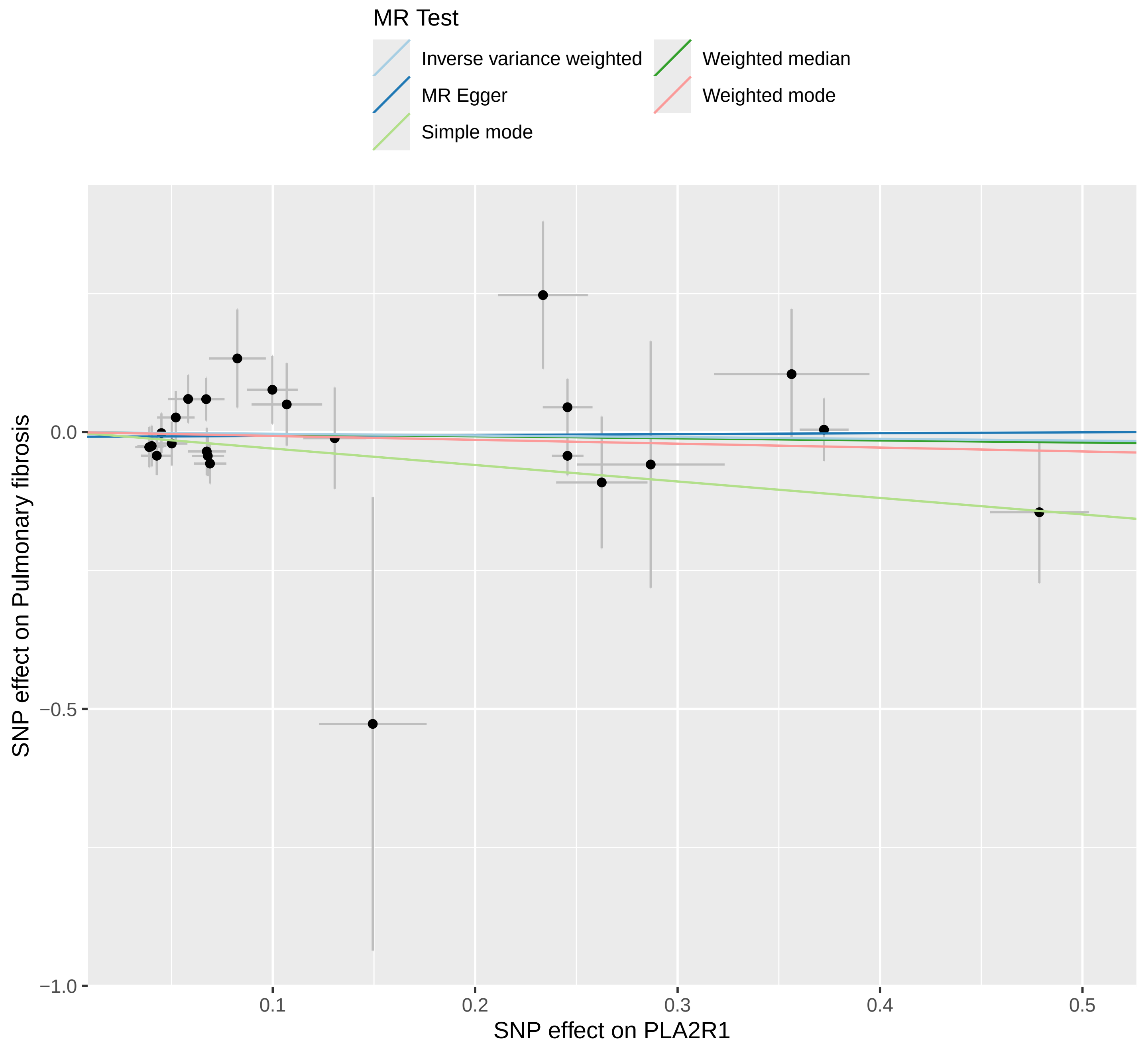

MR Test
Inverse variance weighted
MR Egger
Simple mode
Weighted median
Weighted mode
SNP effect on Pulmonary fibrosis
0.0
−0.5
−1.0
0.1
0.2
0.3
0.4
0.5
SNP effect on PLA2R1

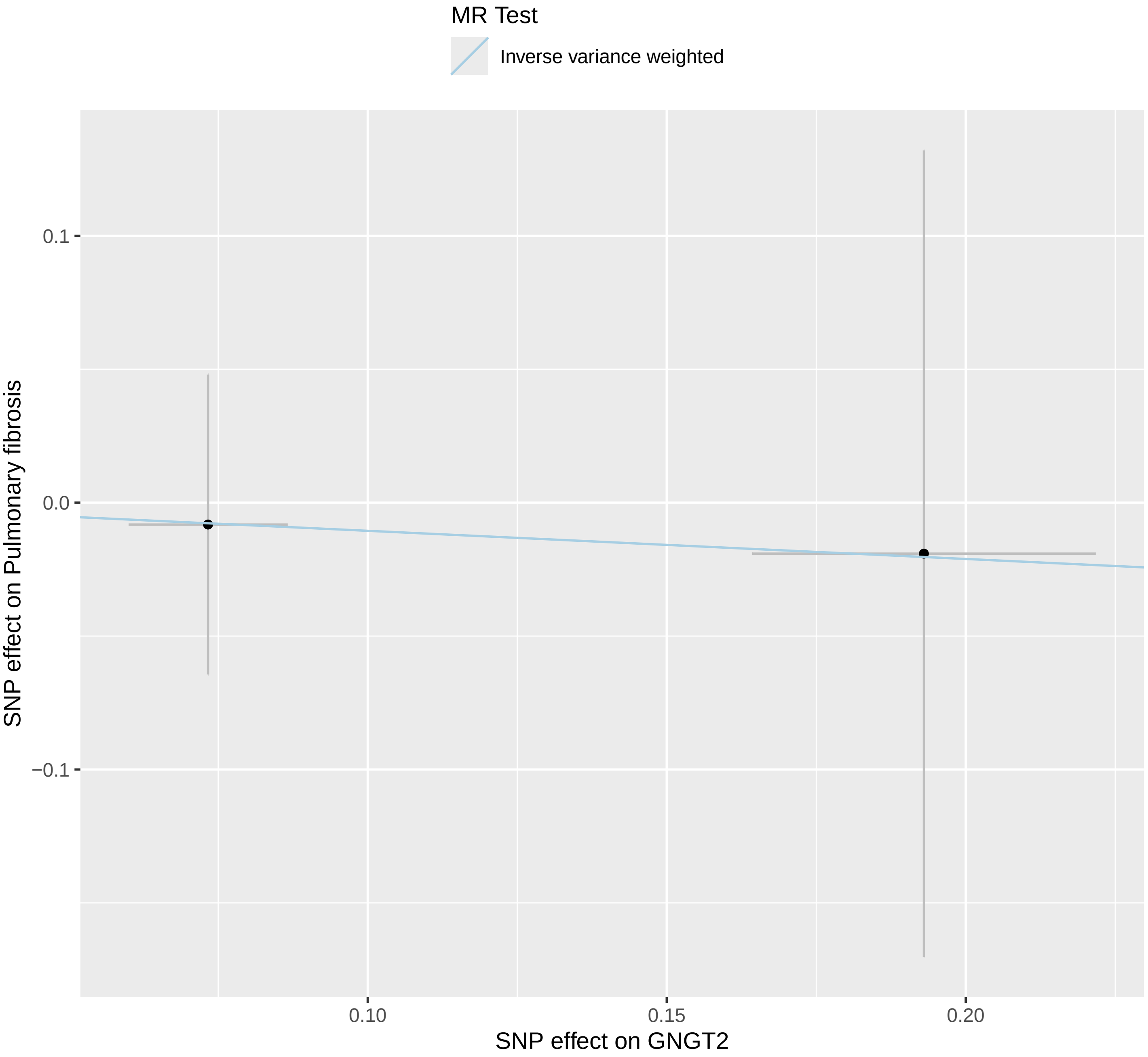

MR Test
Inverse variance weighted
0.1
0.0
−0.1
SNP effect on Pulmonary fibrosis
0.10
0.15
0.20
SNP effect on GNGT2

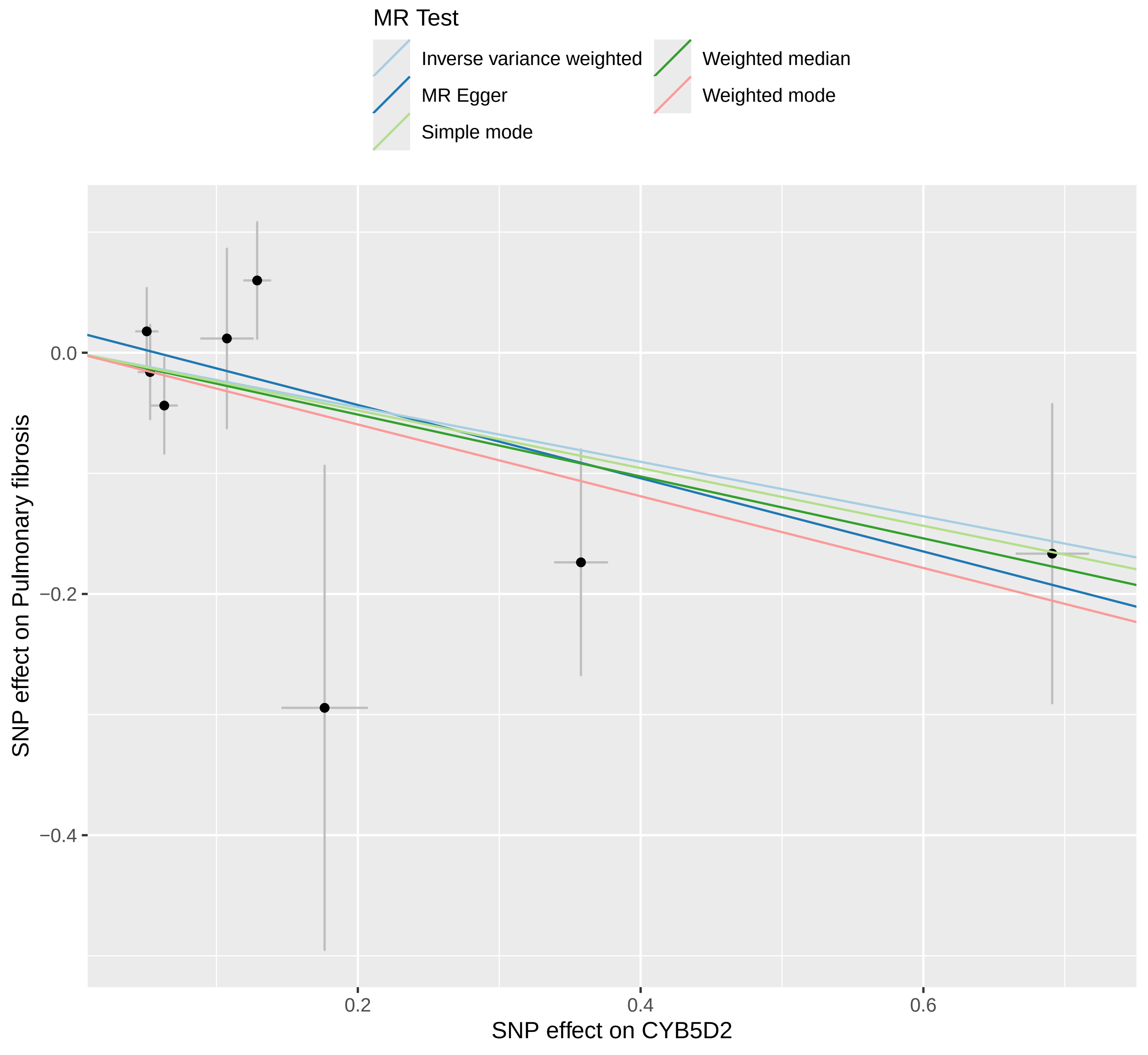
MR Test
Inverse variance weighted
MR Egger
Simple mode
Weighted median
Weighted mode
0.0
−0.2
−0.4
0.2
0.4
0.6
SNP effect on Pulmonary fibrosis
SNP effect on CYB5D2

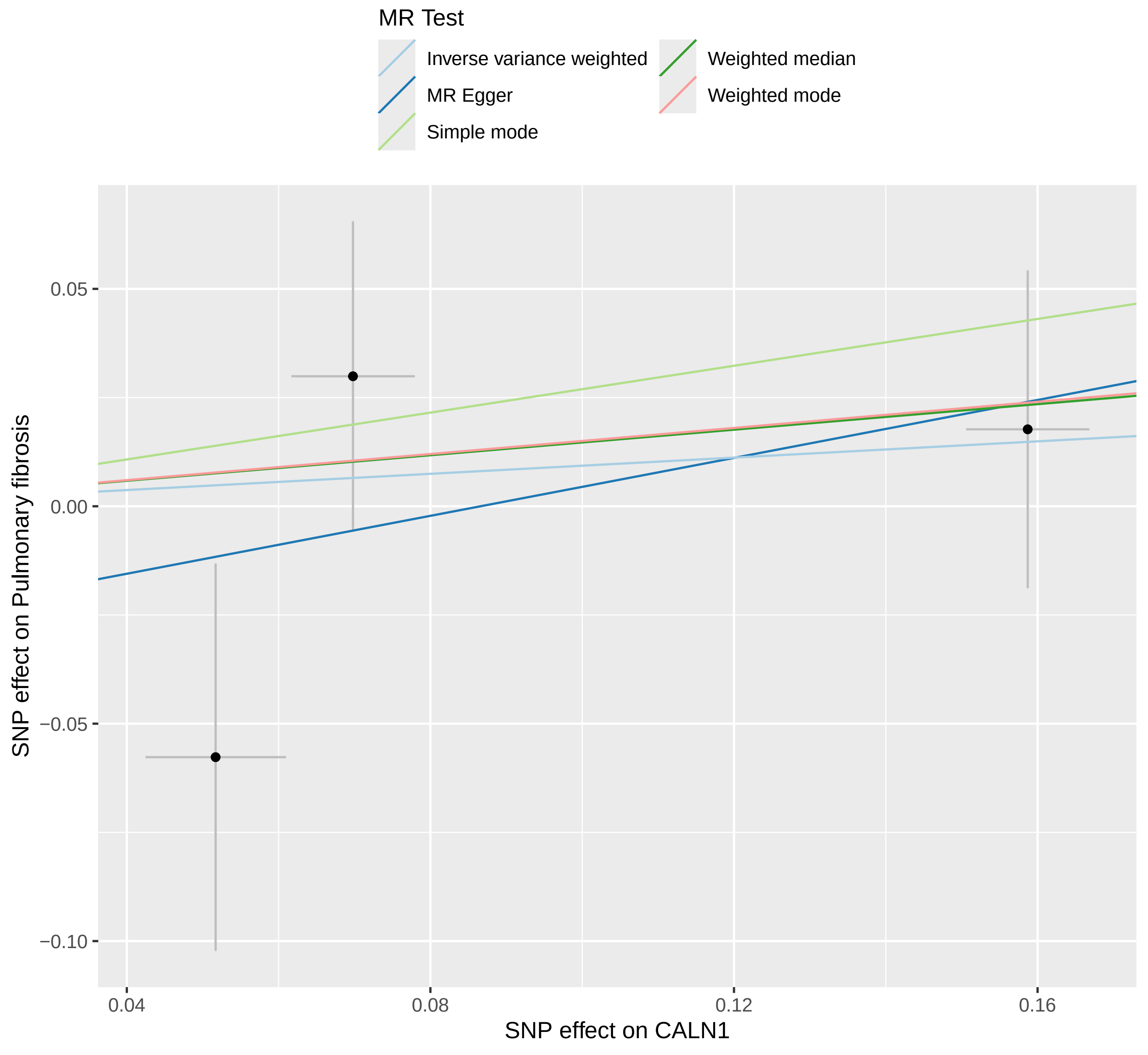
MR Test
Inverse variance weighted
MR Egger
Simple mode
Weighted median
Weighted mode
0.05
0.00
−0.05
−0.10
0.04
0.08
0.12
0.16
SNP effect on Pulmonary fibrosis
SNP effect on CALN1

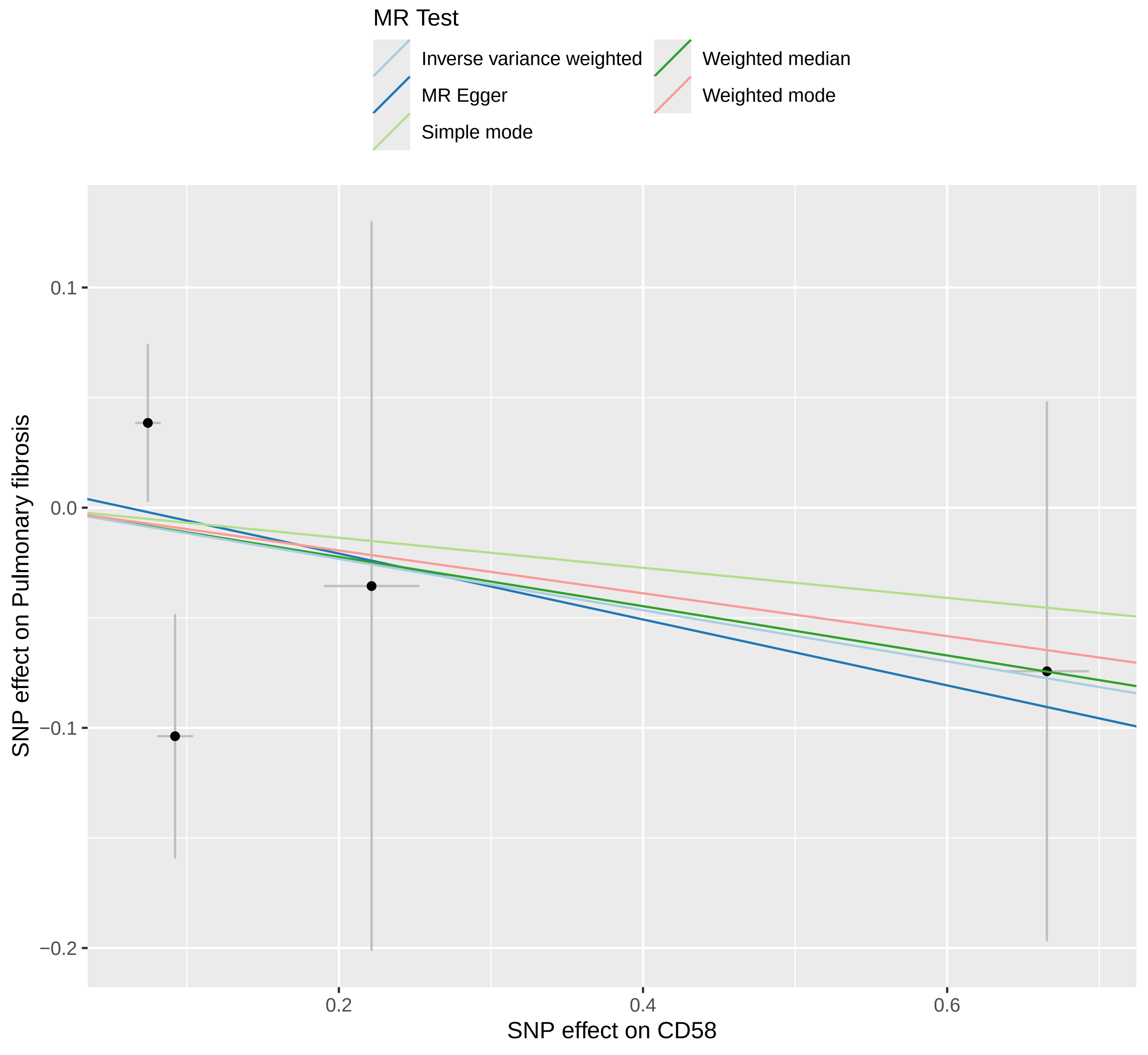
MR Test
Inverse variance weighted
MR Egger
Simple mode
Weighted median
Weighted mode
0.1
0.0
−0.1
−0.2
SNP effect on Pulmonary fibrosis
0.2
0.4
0.6
SNP effect on CD58

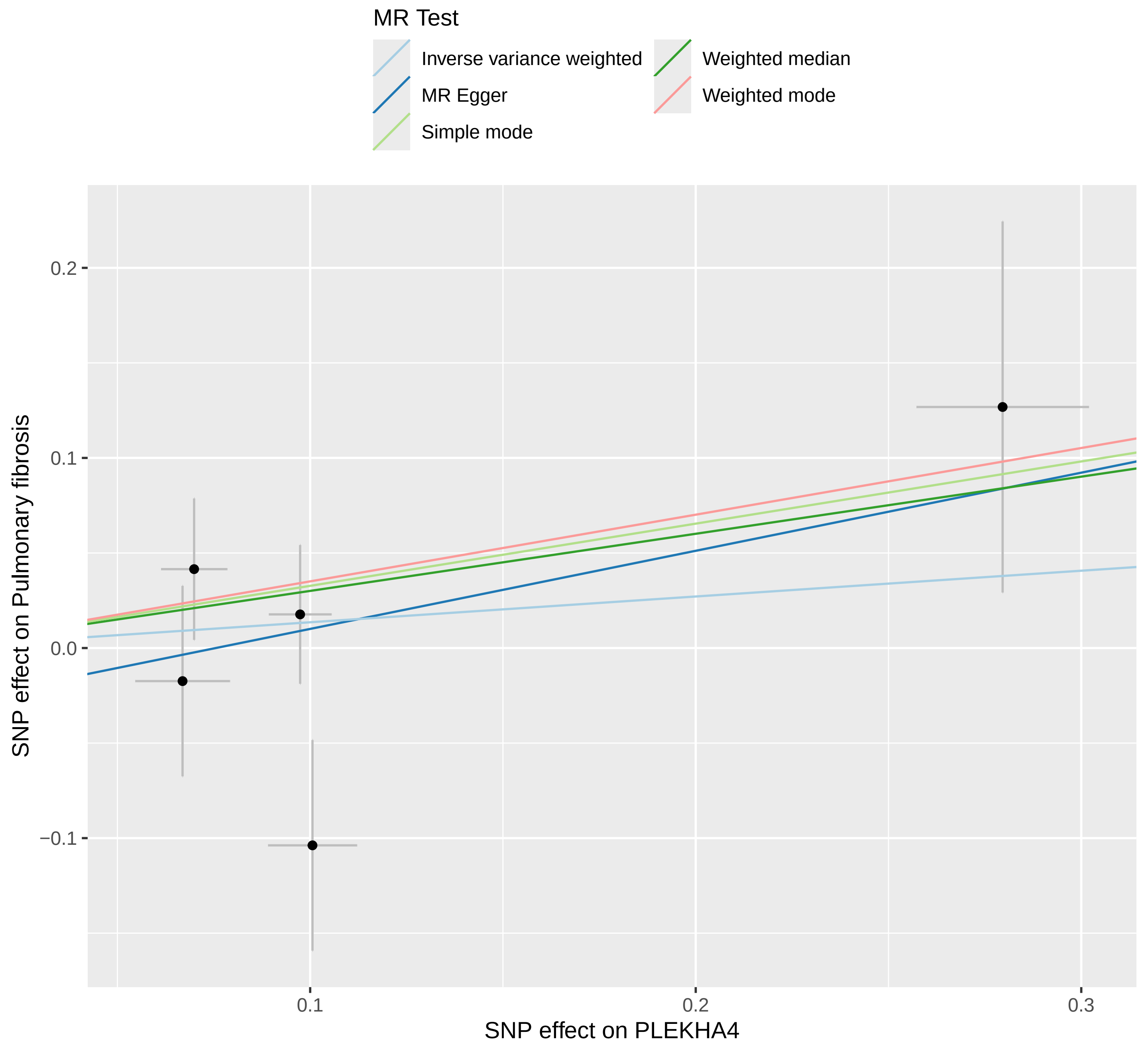
MR Test
Inverse variance weighted
MR Egger
Simple mode
Weighted median
Weighted mode
0.2
0.1
0.0
−0.1
SNP effect on Pulmonary fibrosis
0.1
0.2
0.3
SNP effect on PLEKHA4

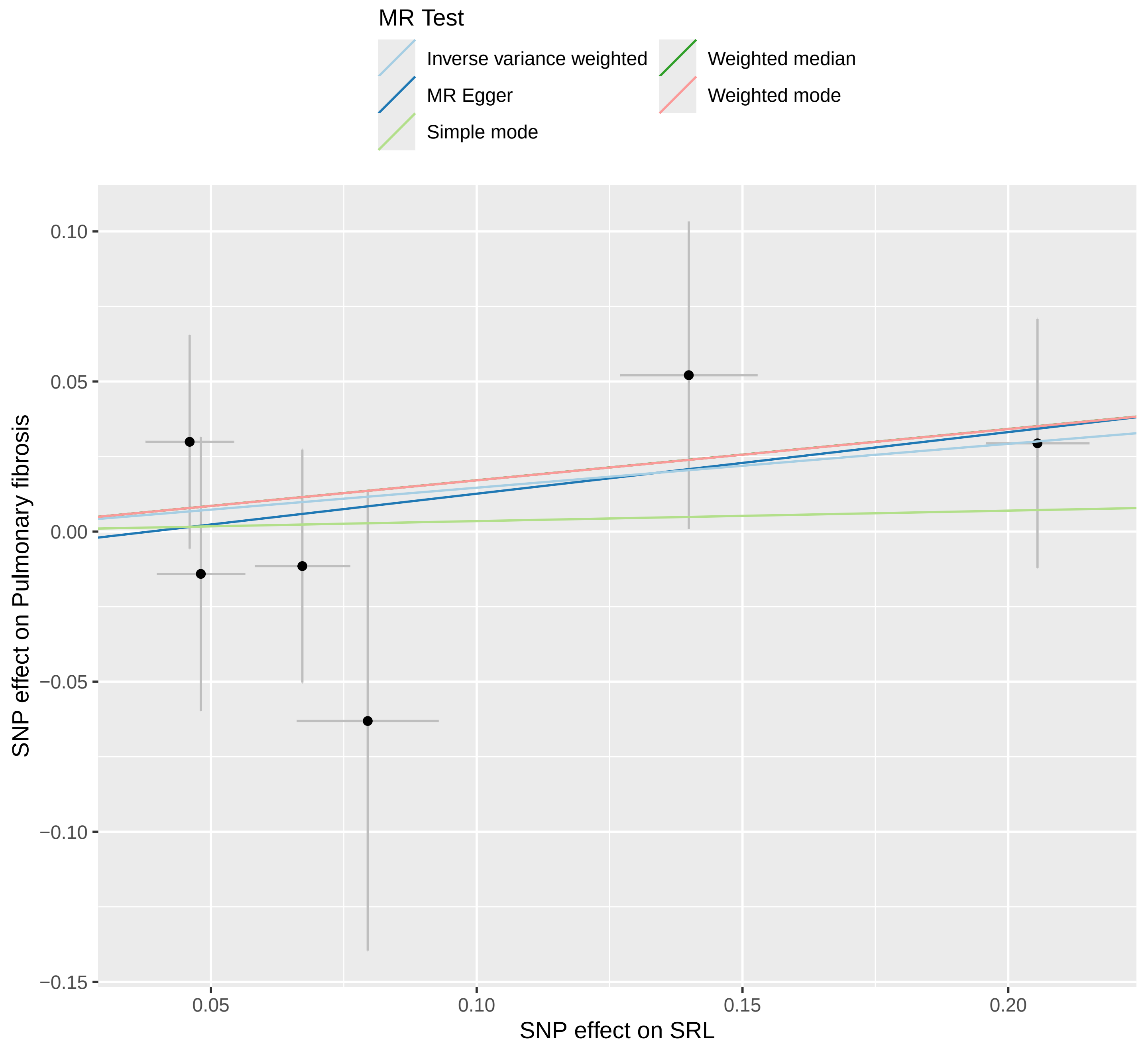

MR Test
Inverse variance weighted
MR Egger
Simple mode
Weighted median
Weighted mode
0.10
0.05
0.00
−0.05
−0.10
−0.15
0.05
0.10
0.15
0.20
SNP effect on SRL
SNP effect on Pulmonary fibrosis

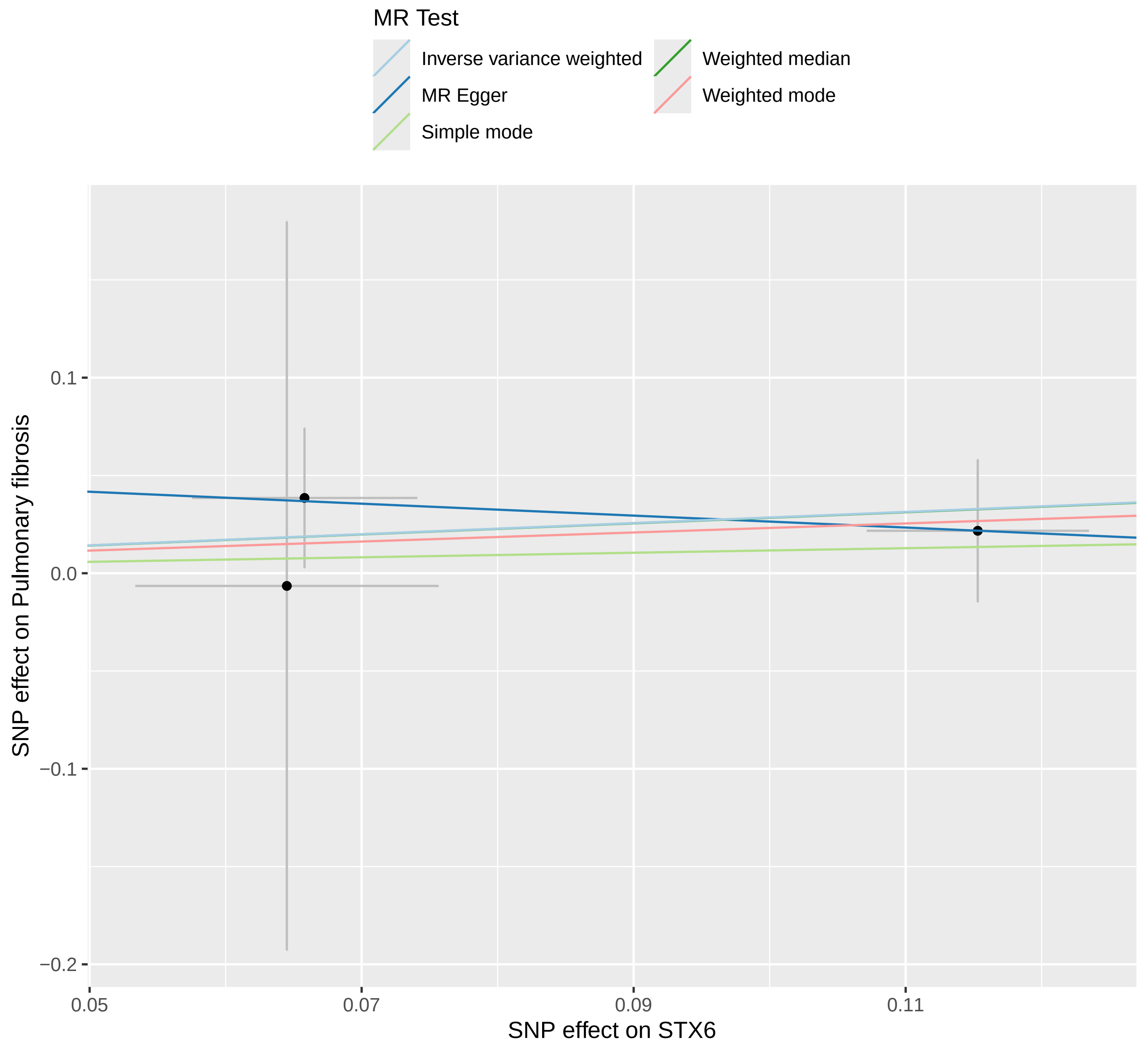
MR Test
Inverse variance weighted
MR Egger
Simple mode
Weighted median
Weighted mode
0.1
0.0
−0.1
−0.2
SNP effect on Pulmonary fibrosis
0.05
0.07
0.09
0.11
SNP effect on STX6

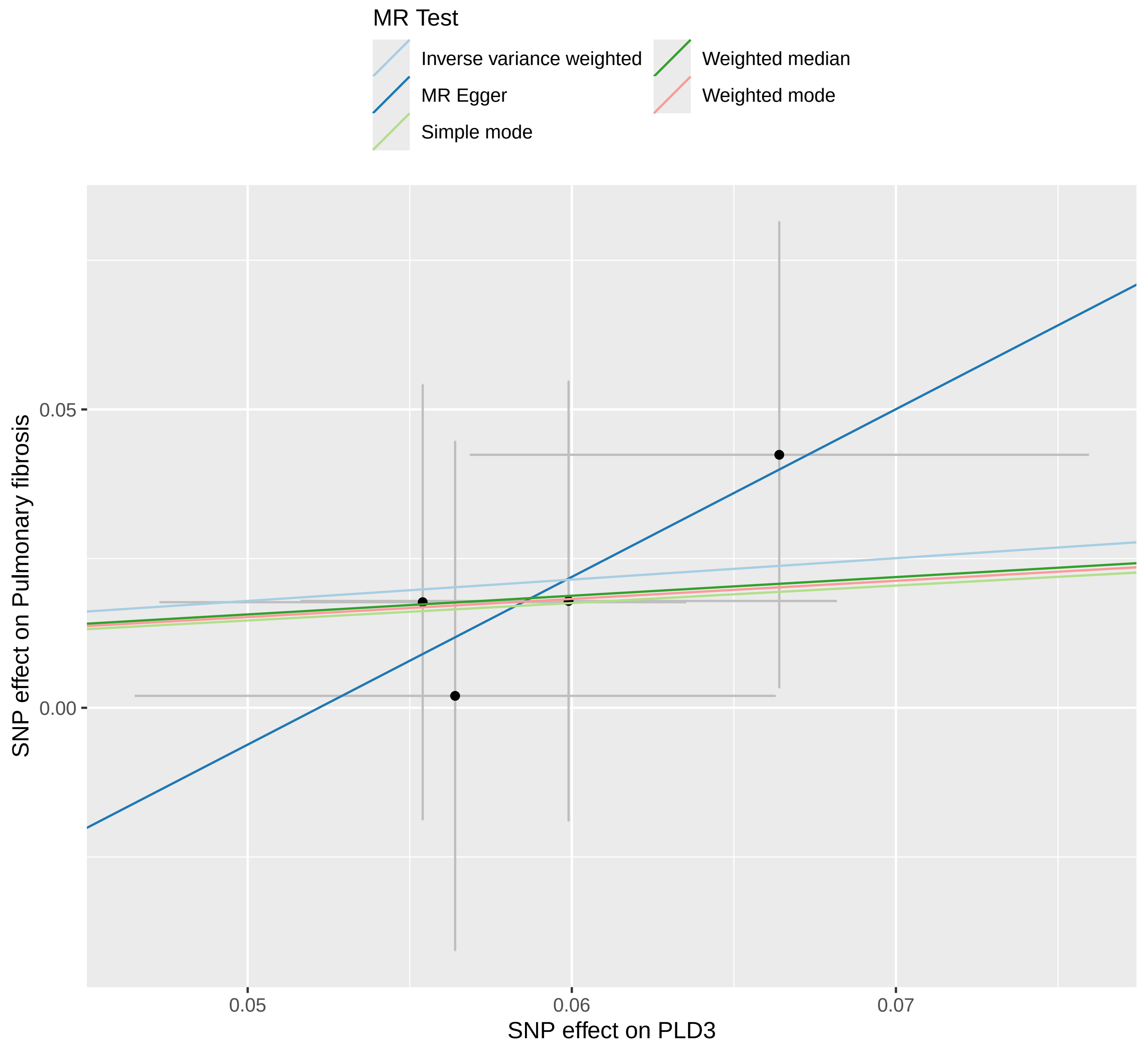
MR Test
Inverse variance weighted
MR Egger
Simple mode
Weighted median
Weighted mode
0.05
0.00
SNP effect on Pulmonary fibrosis
0.05
0.06
0.07
SNP effect on PLD3

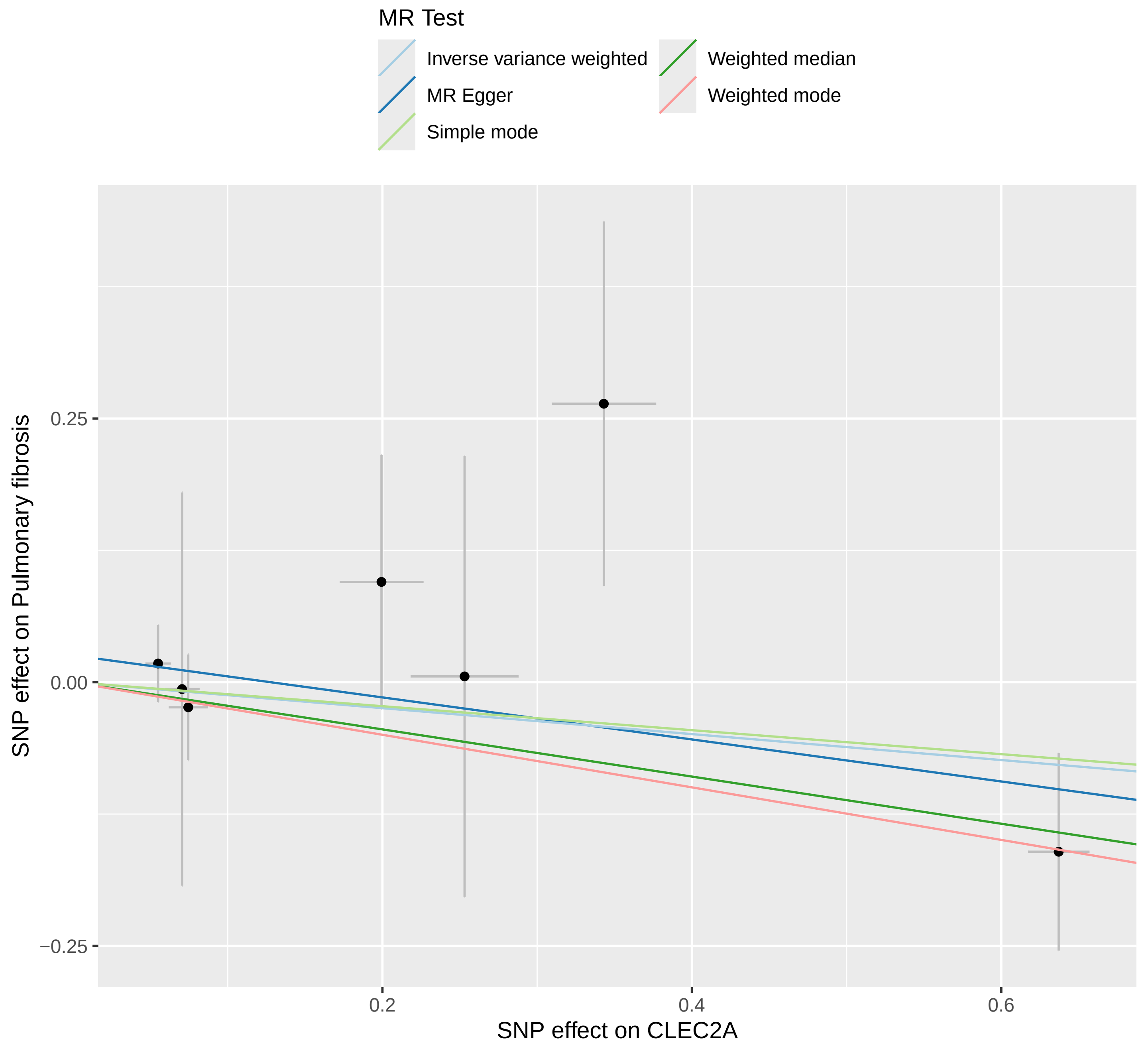
MR Test
Inverse variance weighted
MR Egger
Simple mode
Weighted median
Weighted mode
SNP effect on Pulmonary fibrosis
0.25
0.00
−0.25
0.2
0.4
0.6
SNP effect on CLEC2A

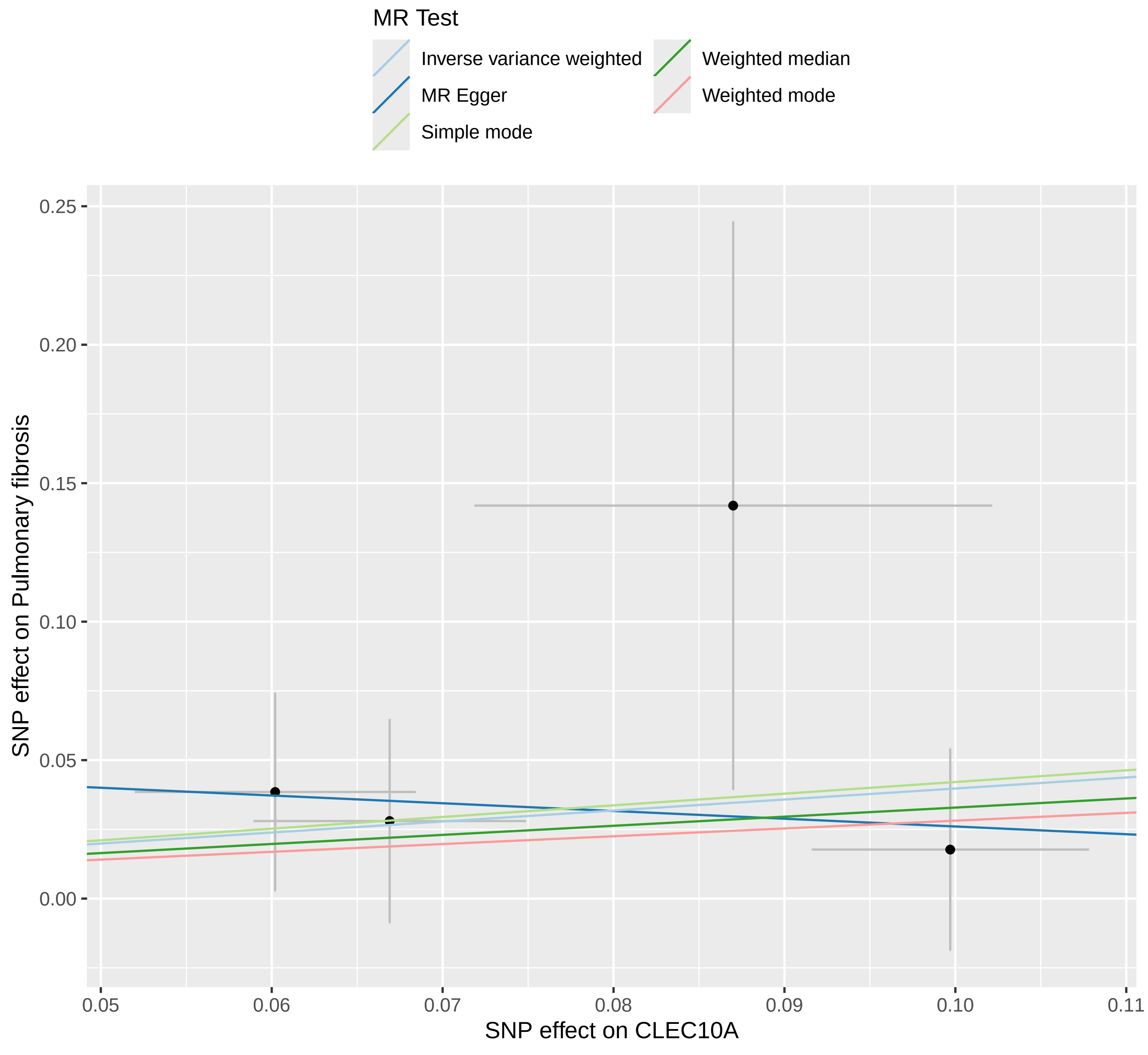
MR Test
Inverse variance weighted
MR Egger
Simple mode
Weighted median
Weighted mode
SNP effect on Pulmonary fibrosis
0.25
0.20
0.15
0.10
0.05
0.00
0.05
0.06
0.07
0.08
0.09
0.10
0.11
SNP effect on CLEC10A

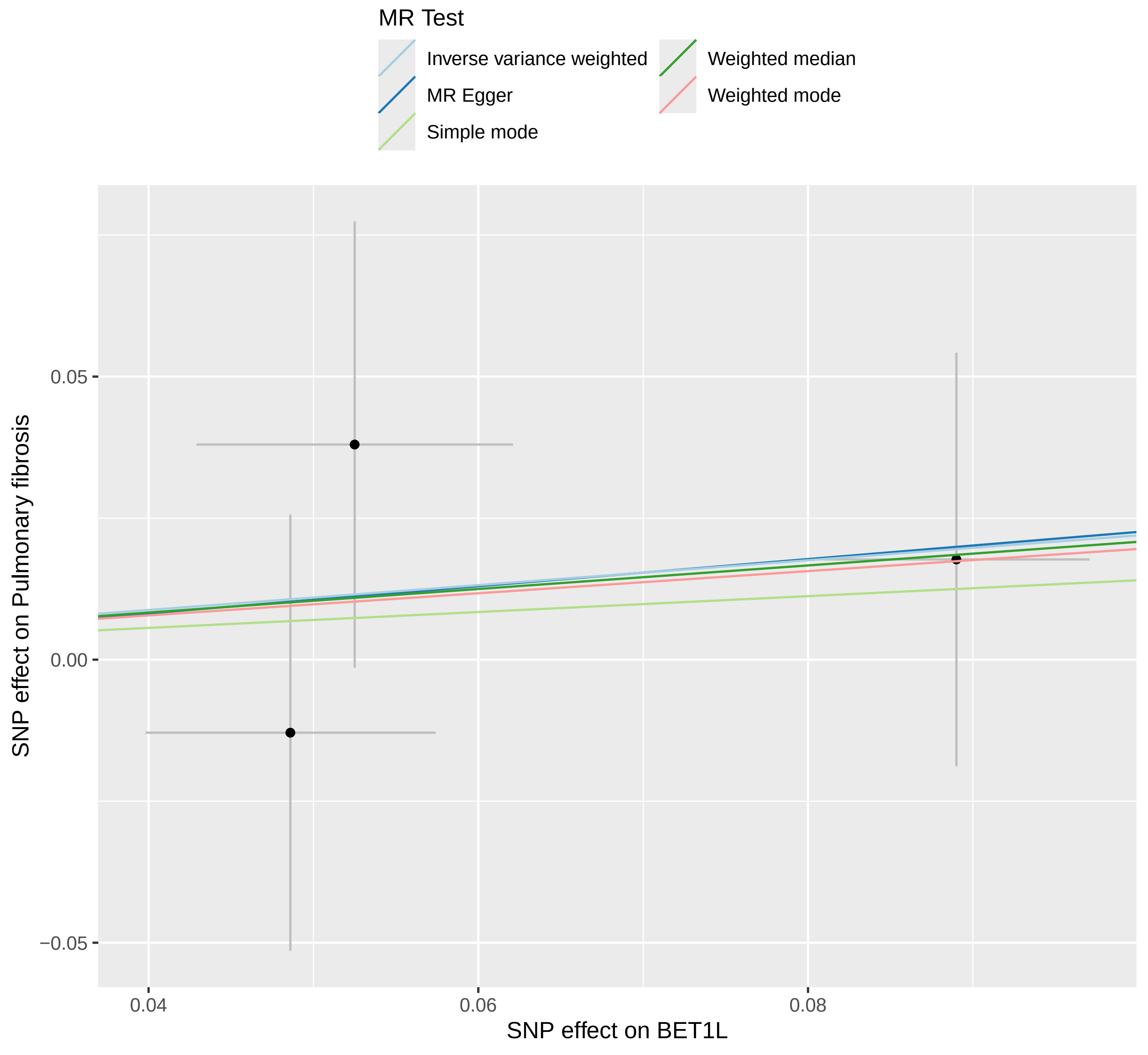

MR Test
Inverse variance weighted
MR Egger
Simple mode
Weighted median
Weighted mode
0.05
0.00
−0.05
0.04
0.06
0.08
SNP effect on Pulmonary fibrosis
SNP effect on BET1L

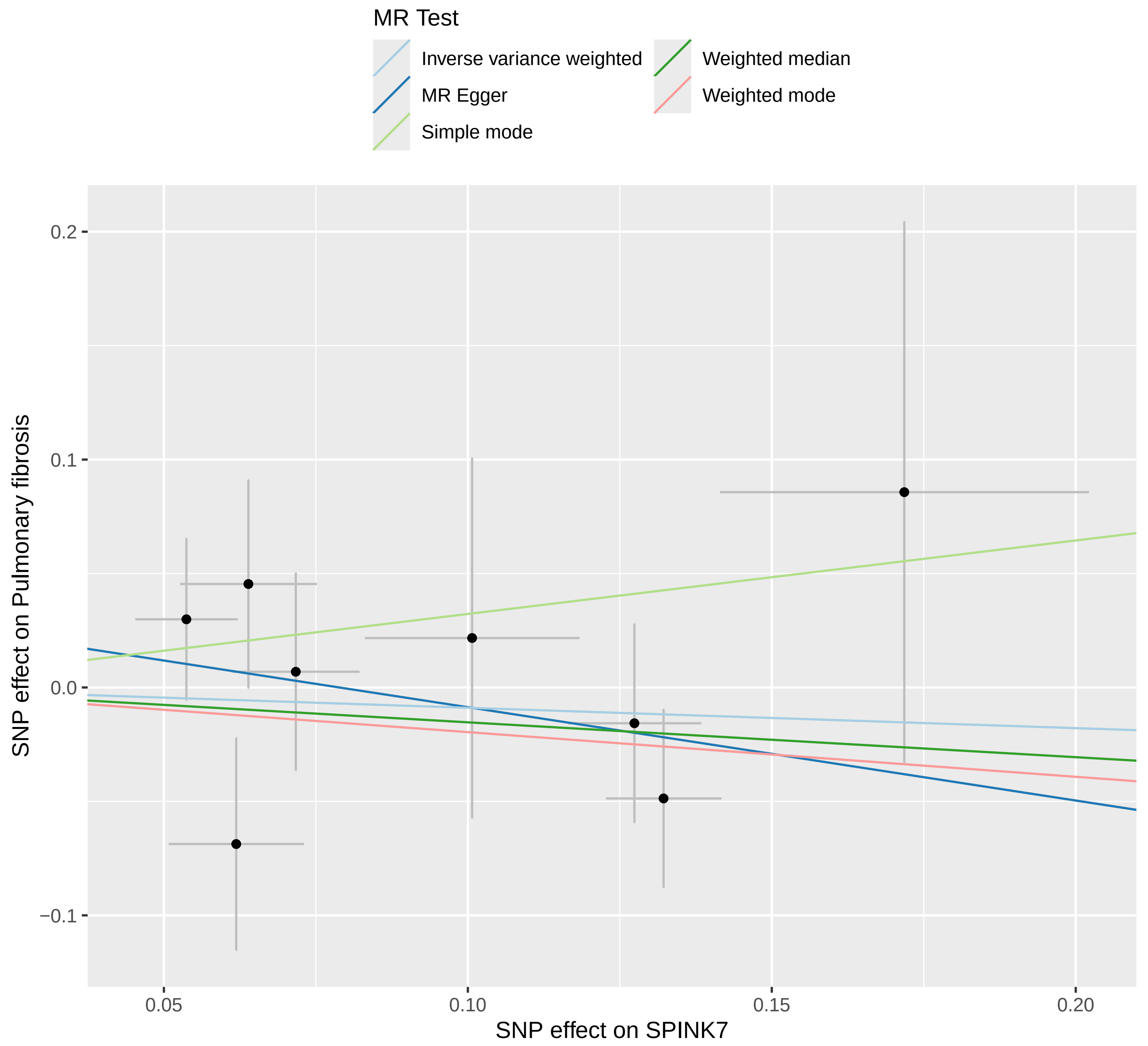

MR Test
Inverse variance weighted
MR Egger
Simple mode
Weighted median
Weighted mode
0.2
0.1
0.0
−0.1
0.05
0.10
0.15
0.20
SNP effect on Pulmonary fibrosis
SNP effect on SPINK7

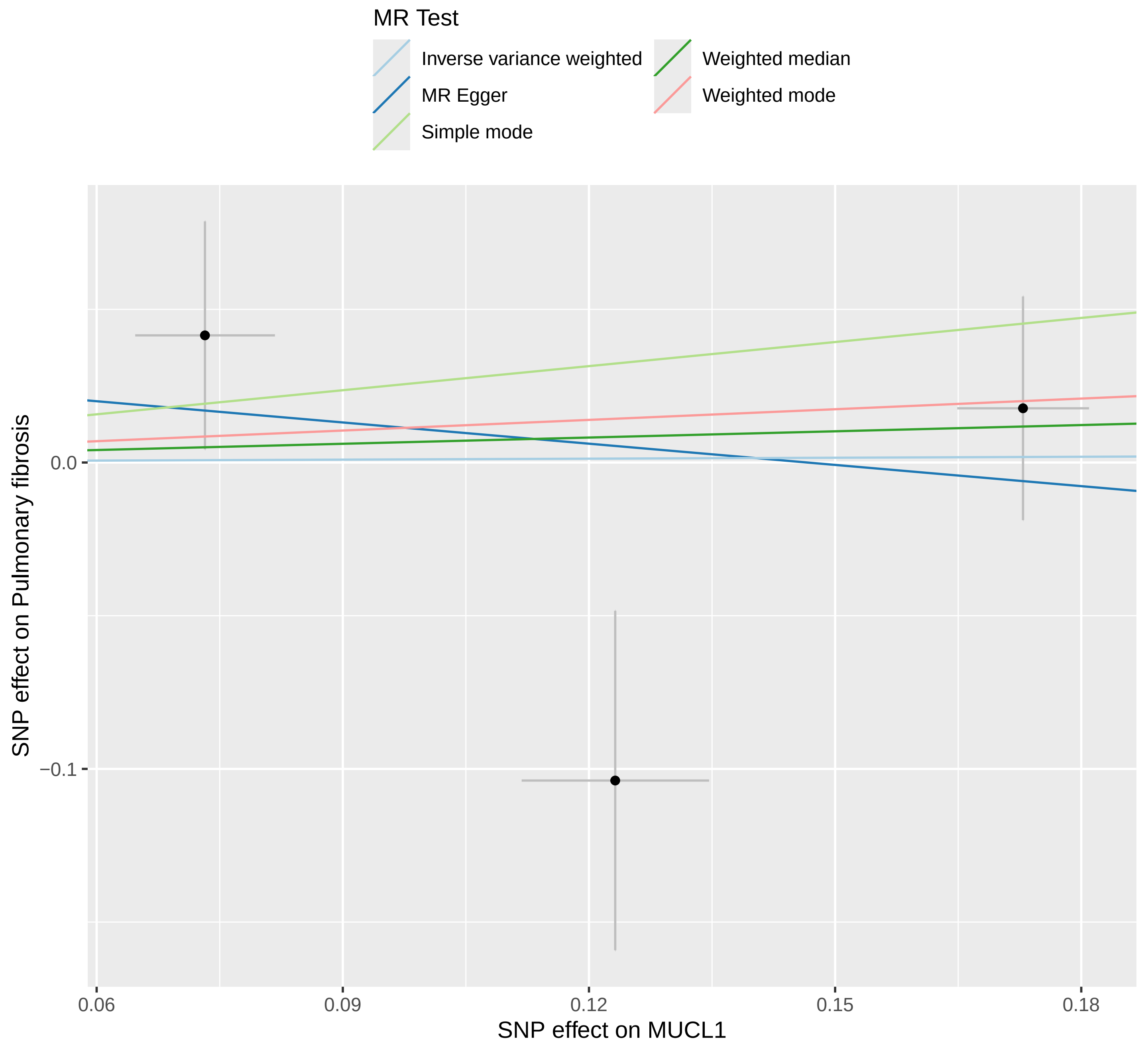

MR Test
Inverse variance weighted
MR Egger
Simple mode
Weighted median
Weighted mode
SNP effect on Pulmonary fibrosis
0.0
−0.1
0.06
0.09
0.12
0.15
0.18
SNP effect on MUCL1

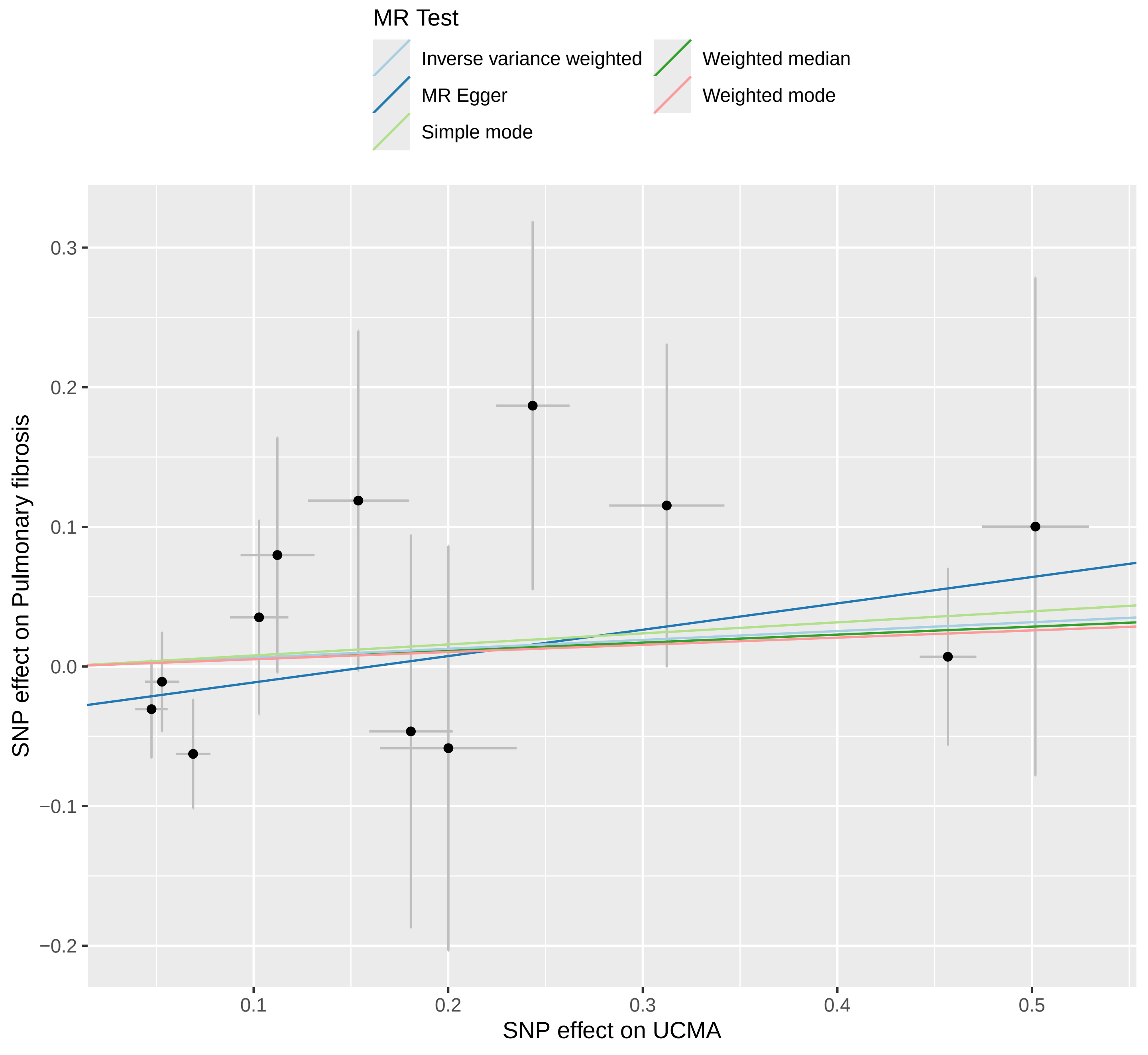
MR Test
Inverse variance weighted
MR Egger
Simple mode
Weighted median
Weighted mode
SNP effect on Pulmonary fibrosis
0.3
0.2
0.1
0.0
−0.1
−0.2
0.1
0.2
0.3
0.4
0.5
SNP effect on UCMA

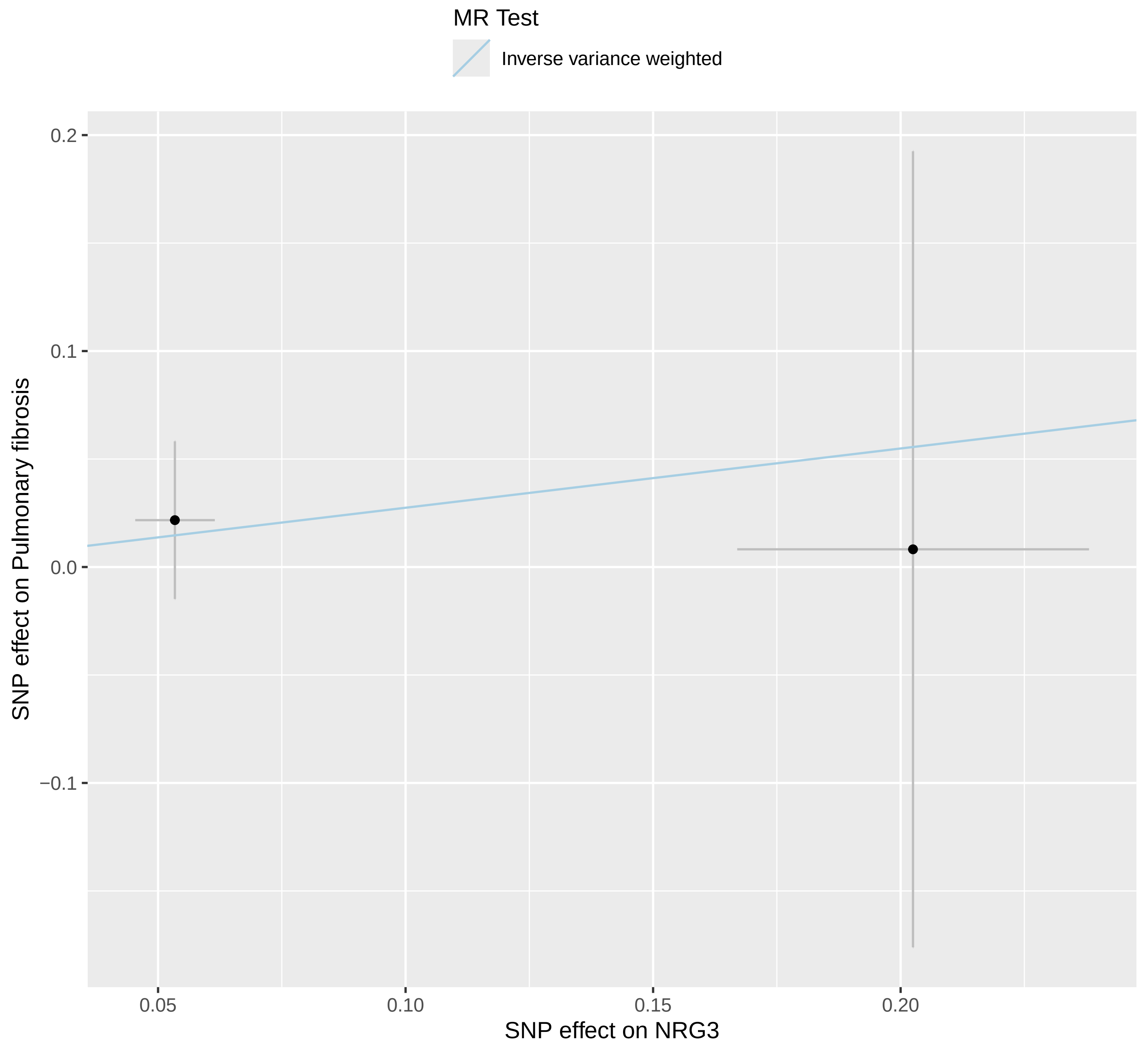
MR Test
Inverse variance weighted
0.2
0.1
0.0
−0.1
0.05
0.10
0.15
0.20
SNP effect on Pulmonary fibrosis
SNP effect on NRG3

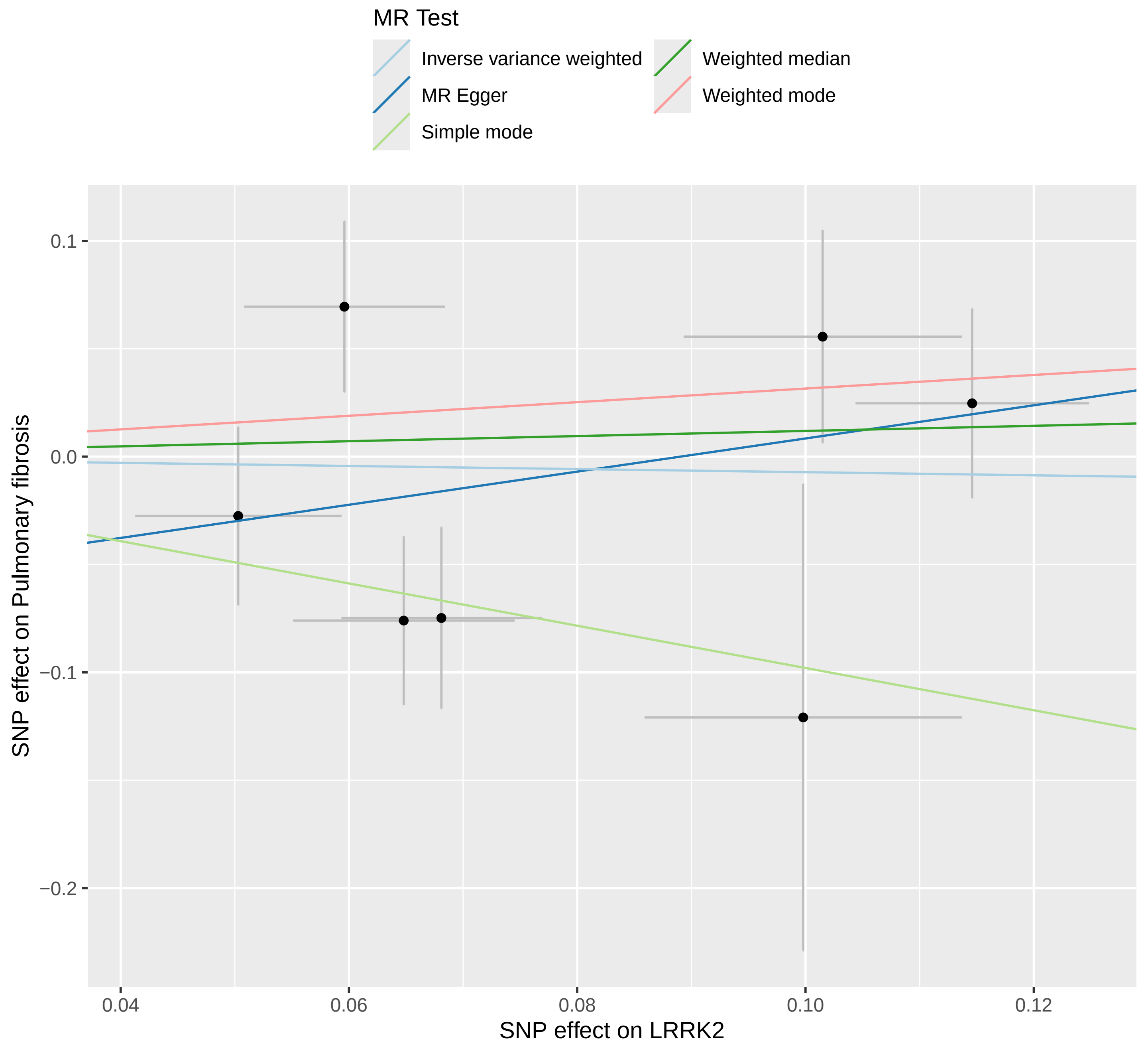
MR Test
Inverse variance weighted
MR Egger
Simple mode
Weighted median
Weighted mode
0.1
0.0
−0.1
−0.2
0.04
0.06
0.08
0.10
0.12
SNP effect on LRRK2
SNP effect on Pulmonary fibrosis

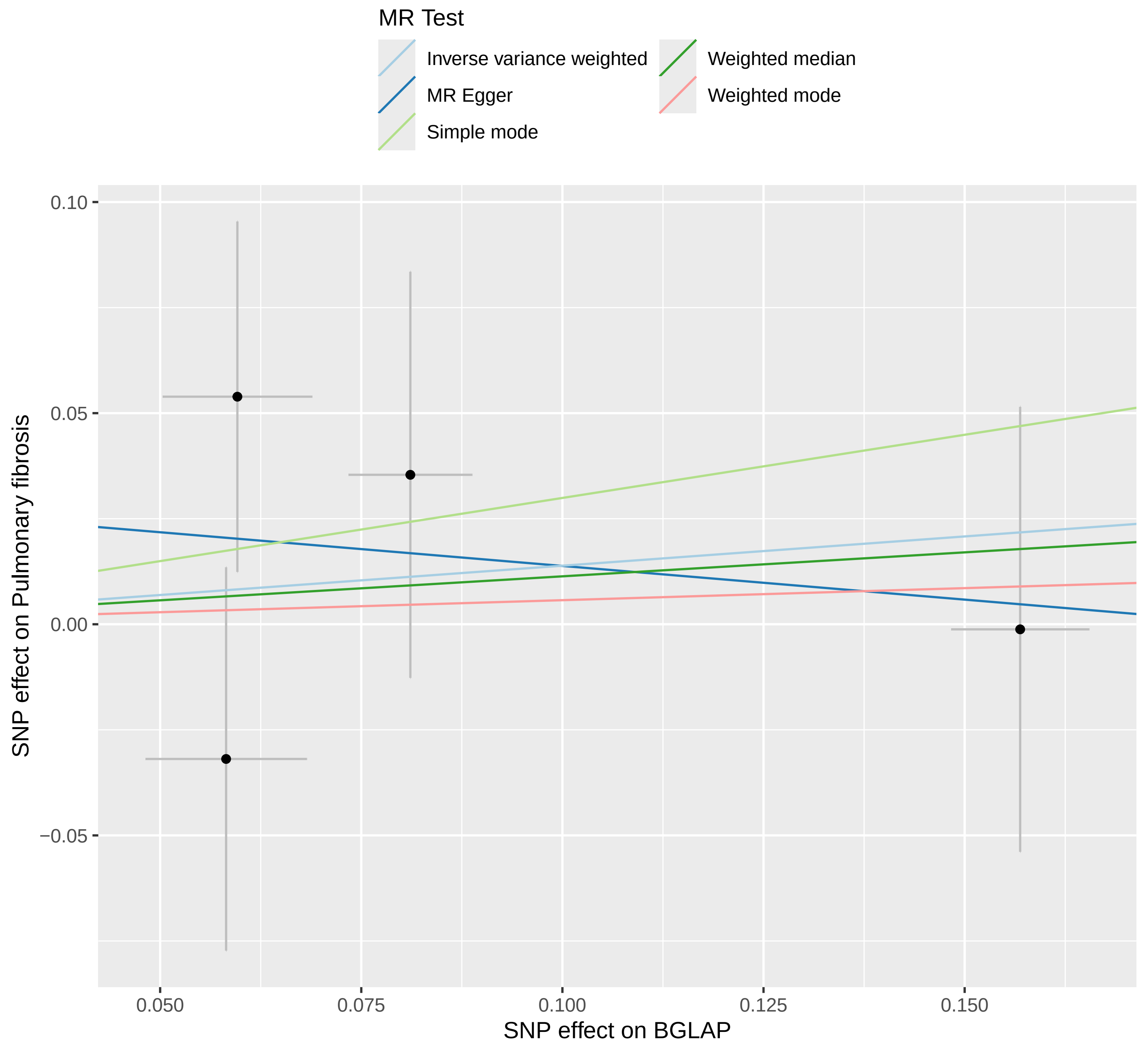
MR Test
Inverse variance weighted
MR Egger
Simple mode
Weighted median
Weighted mode
0.10
0.05
0.00
−0.05
0.050
0.075
0.100
0.125
0.150
SNP effect on BGLAP
SNP effect on Pulmonary fibrosis

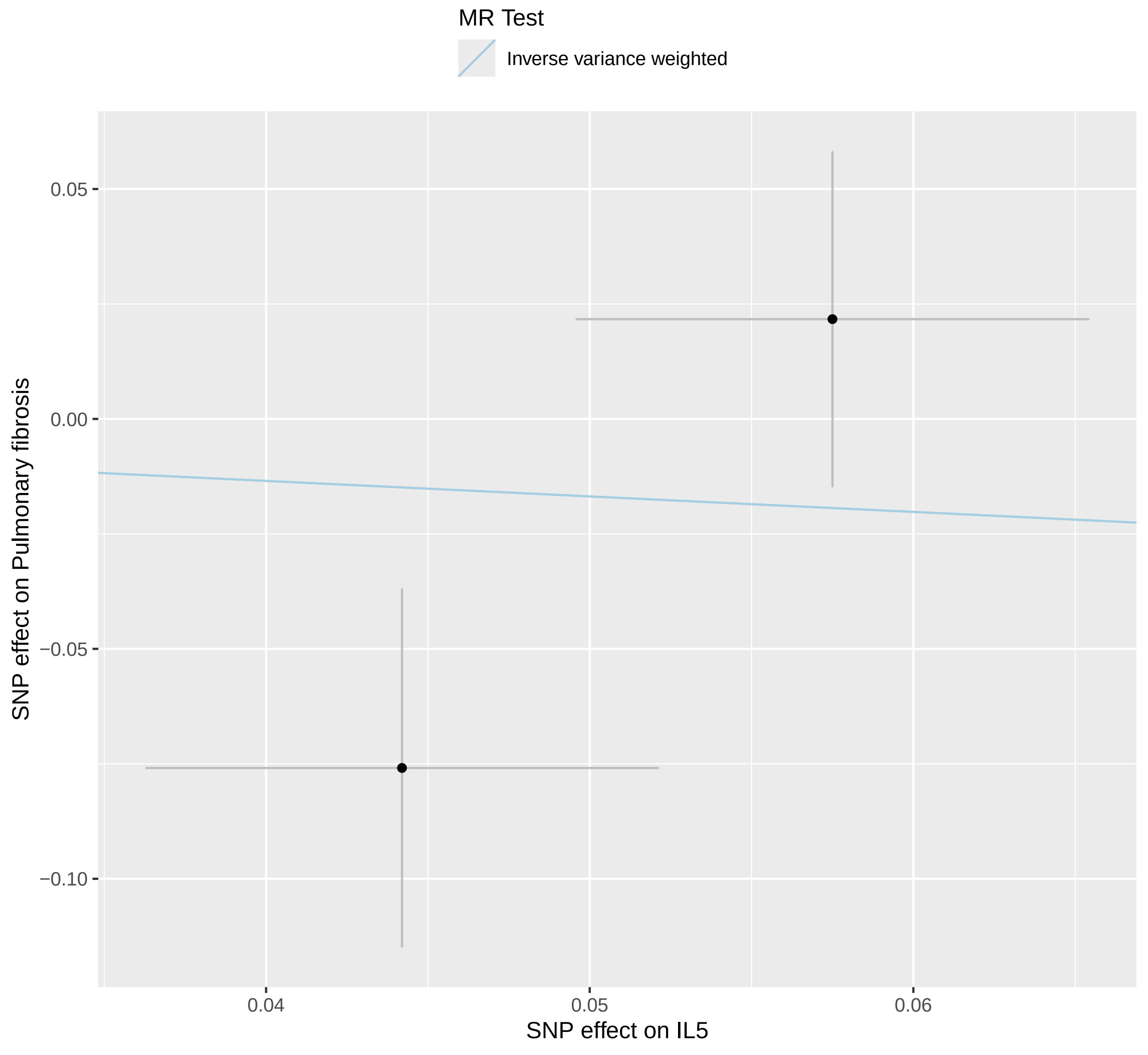
MR Test
Inverse variance weighted
0.05
0.00
−0.05
−0.10
0.04
0.05
0.06
SNP effect on Pulmonary fibrosis
SNP effect on IL5

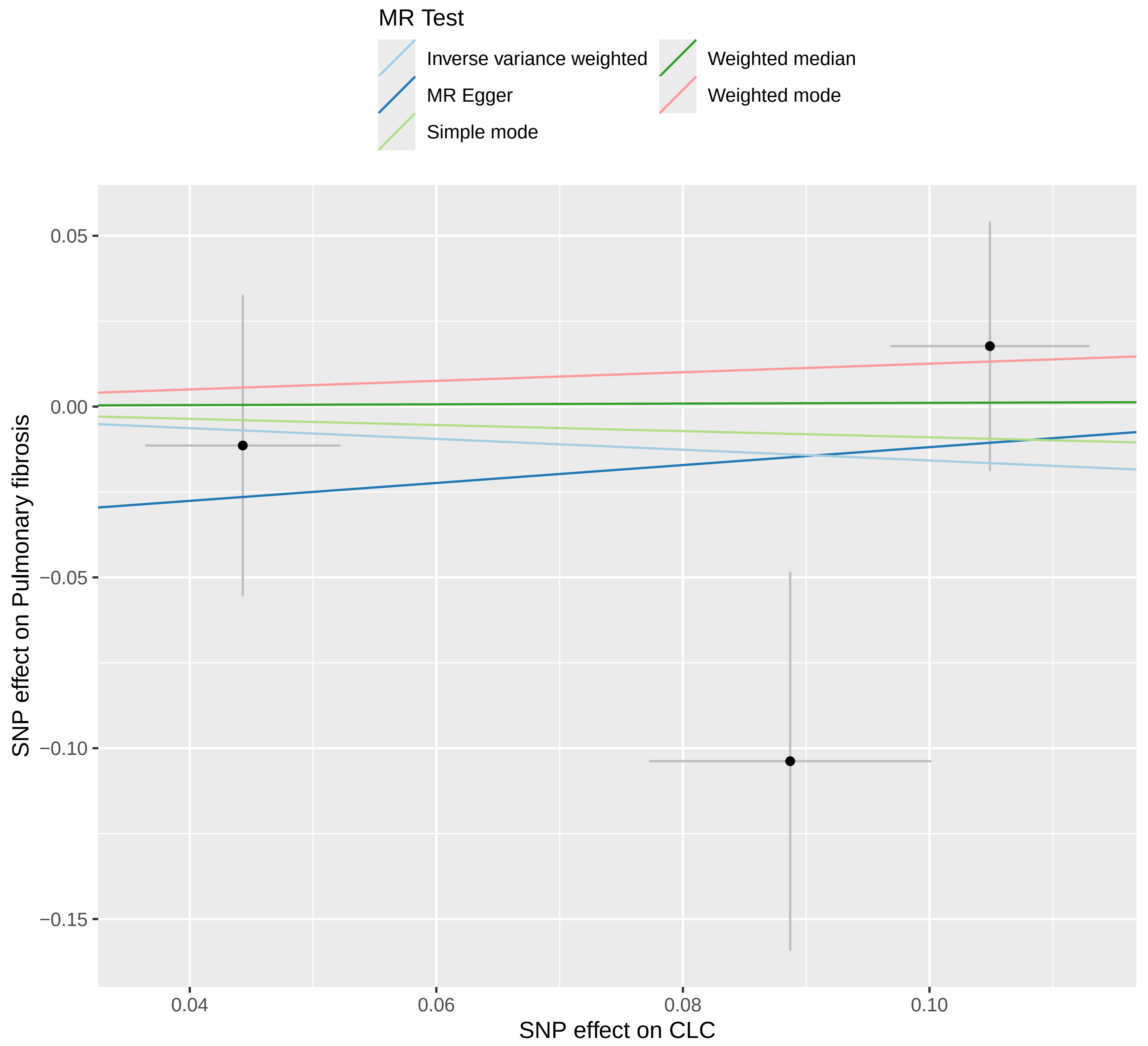
MR Test
Inverse variance weighted
MR Egger
Simple mode
Weighted median
Weighted mode
0.05
0.00
−0.05
−0.10
−0.15
0.04
0.06
0.08
0.10
SNP effect on CLC
SNP effect on Pulmonary fibrosis

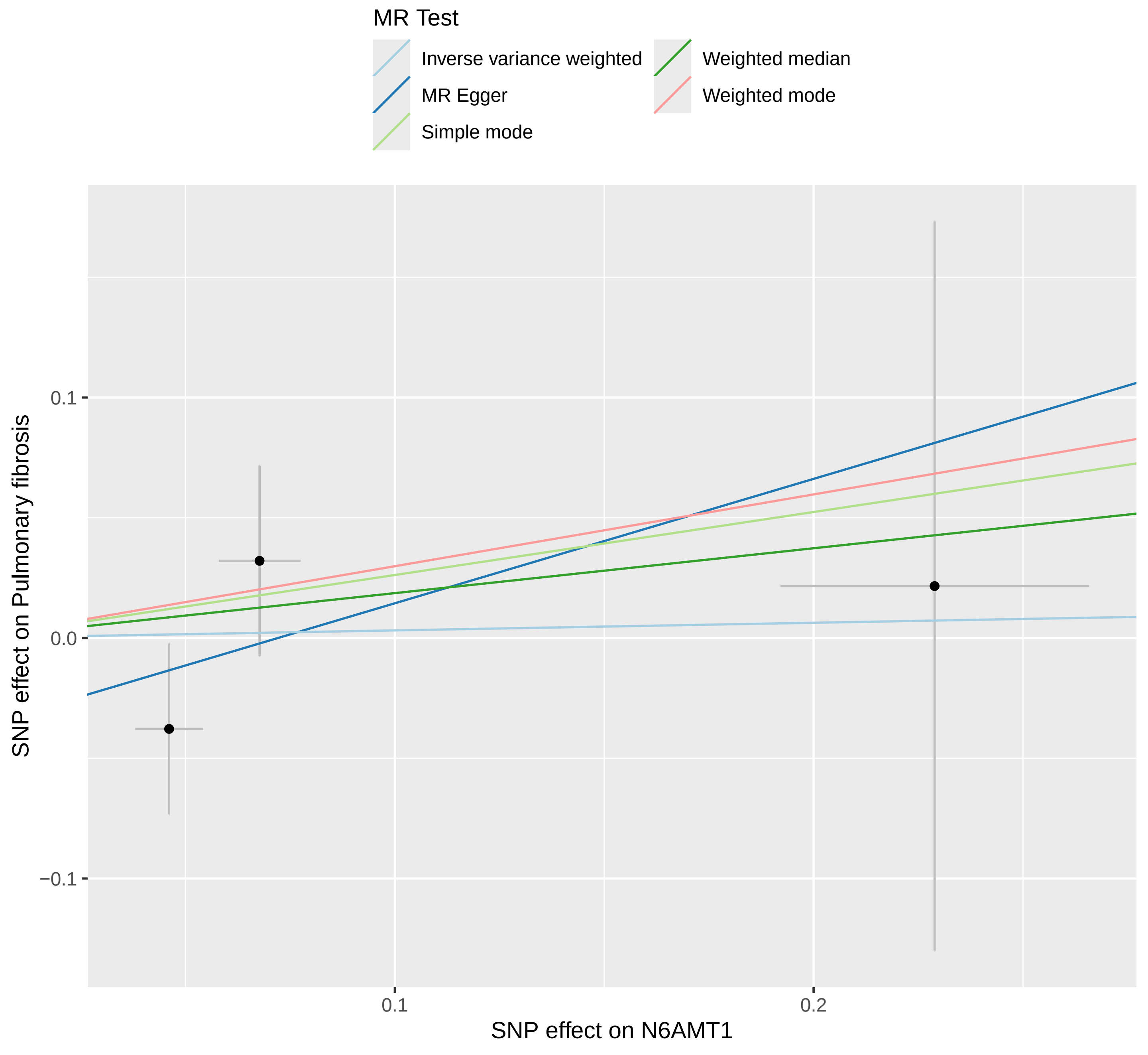

MR Test
Inverse variance weighted
MR Egger
Simple mode
Weighted median
Weighted mode
0.1
0.0
−0.1
SNP effect on Pulmonary fibrosis
0.1
0.2
SNP effect on N6AMT1

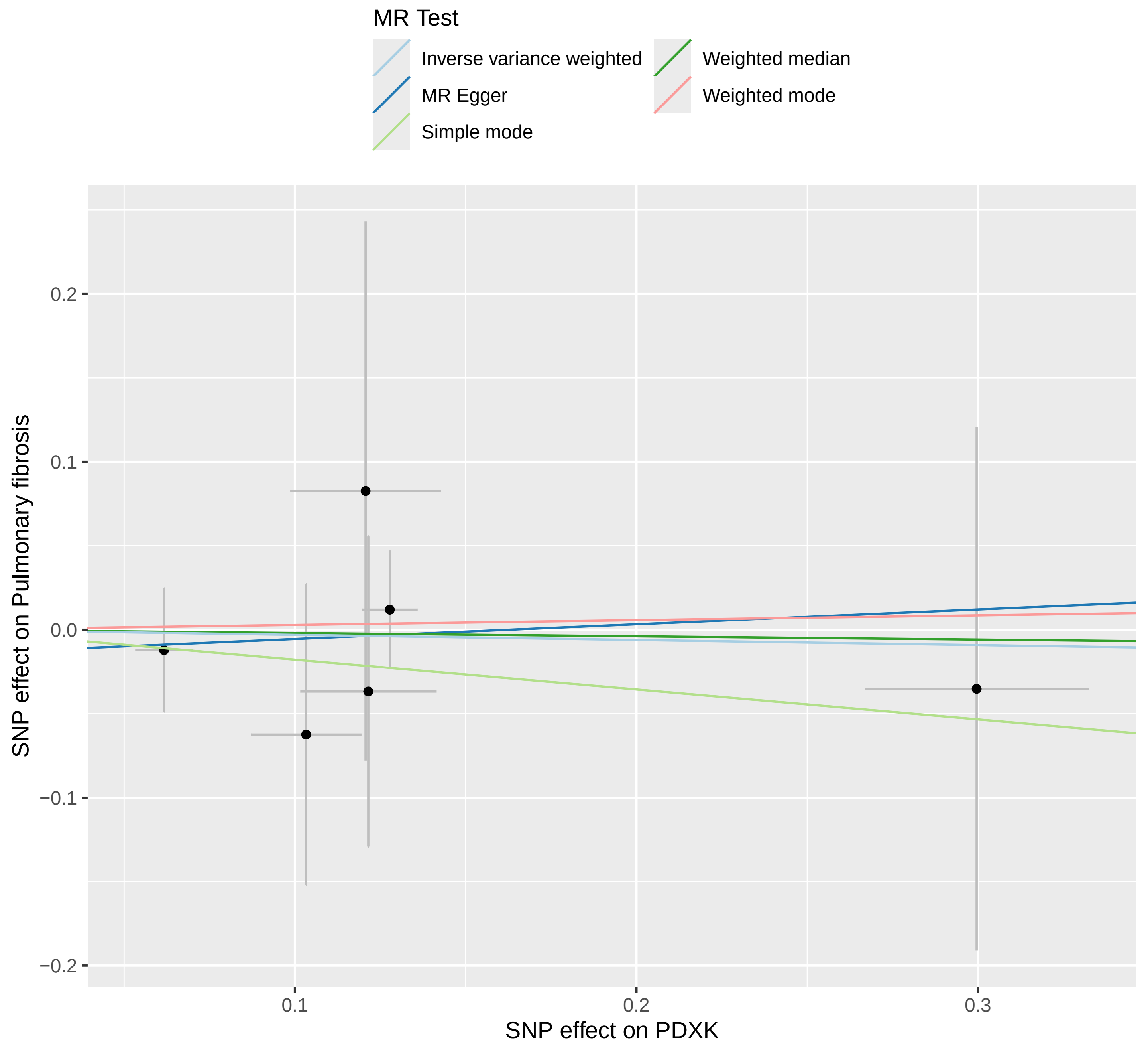
MR Test
Inverse variance weighted
MR Egger
Simple mode
Weighted median
Weighted mode
0.2
0.1
0.0
−0.1
−0.2
SNP effect on Pulmonary fibrosis
0.1
0.2
0.3
SNP effect on PDXK

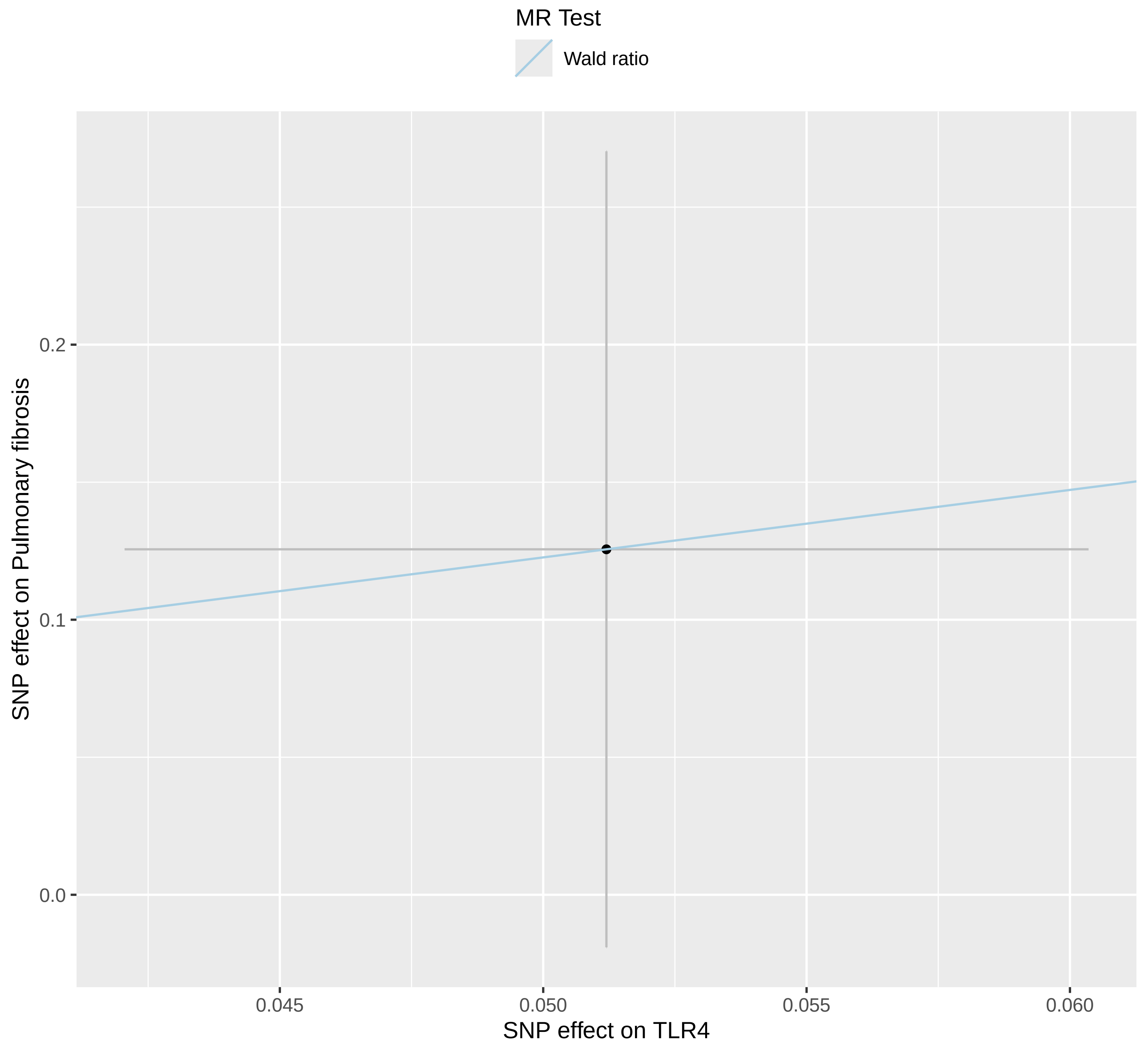
MR Test
Wald ratio
SNP effect on Pulmonary fibrosis
0.2
0.1
0.0
0.045
0.050
0.055
0.060
SNP effect on TLR4

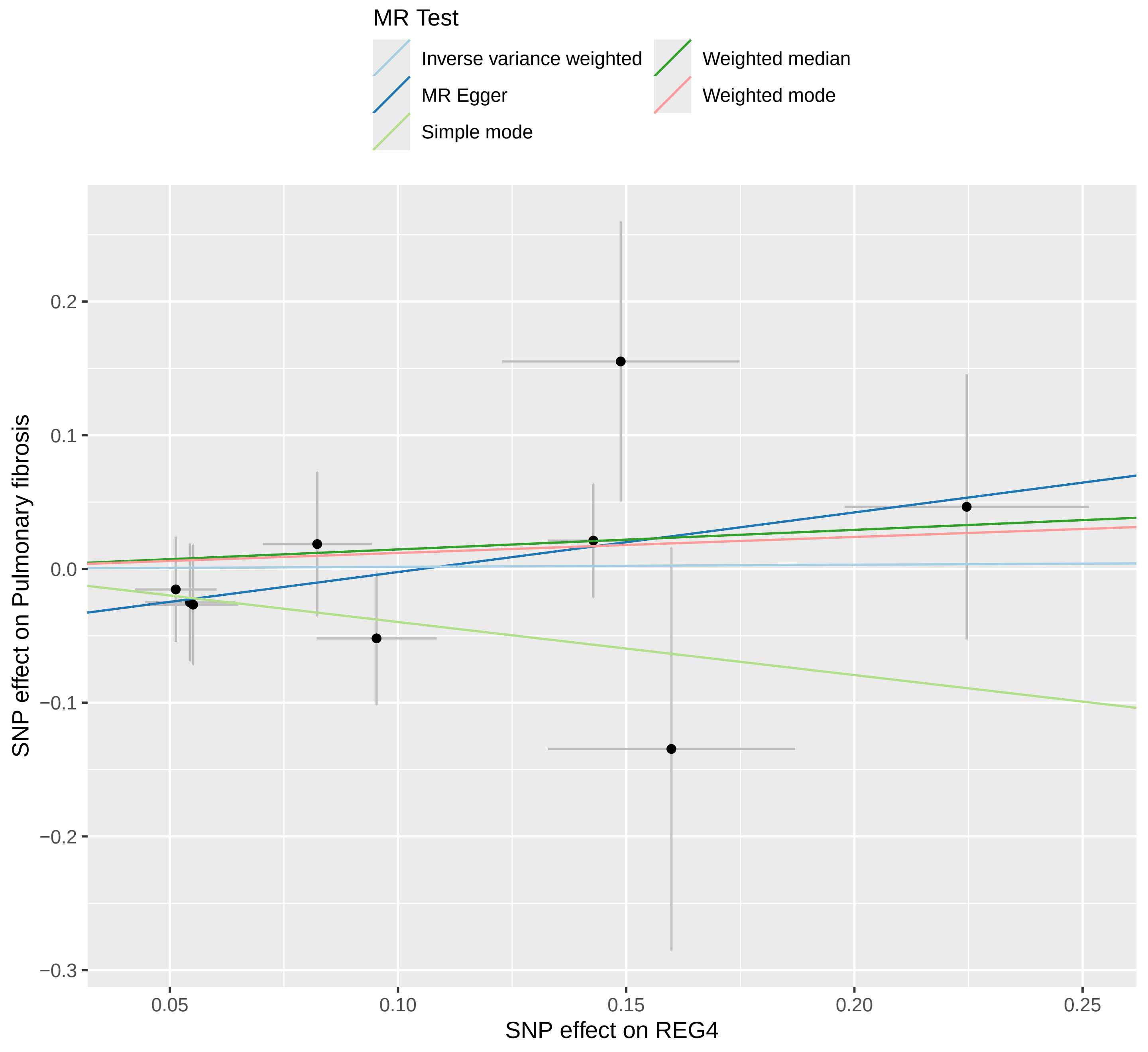
MR Test
Inverse variance weighted
MR Egger
Simple mode
Weighted median
Weighted mode
SNP effect on Pulmonary fibrosis
0.2
0.1
0.0
−0.1
−0.2
−0.3
0.05
0.10
0.15
0.20
0.25
SNP effect on REG4

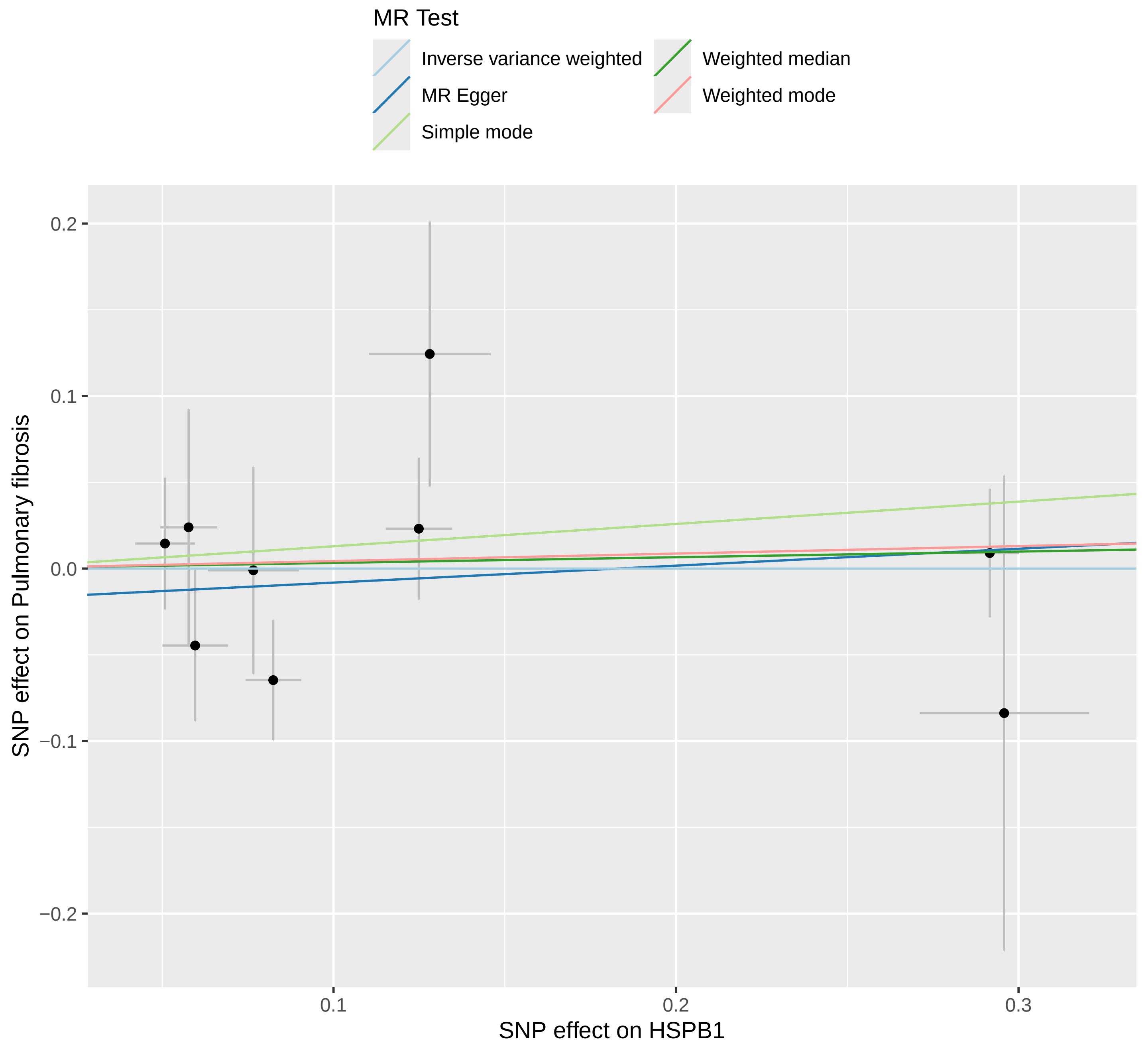

MR Test
Inverse variance weighted
MR Egger
Simple mode
Weighted median
Weighted mode
0.2
0.1
0.0
−0.1
−0.2
0.1
0.2
0.3
SNP effect on Pulmonary fibrosis
SNP effect on HSPB1

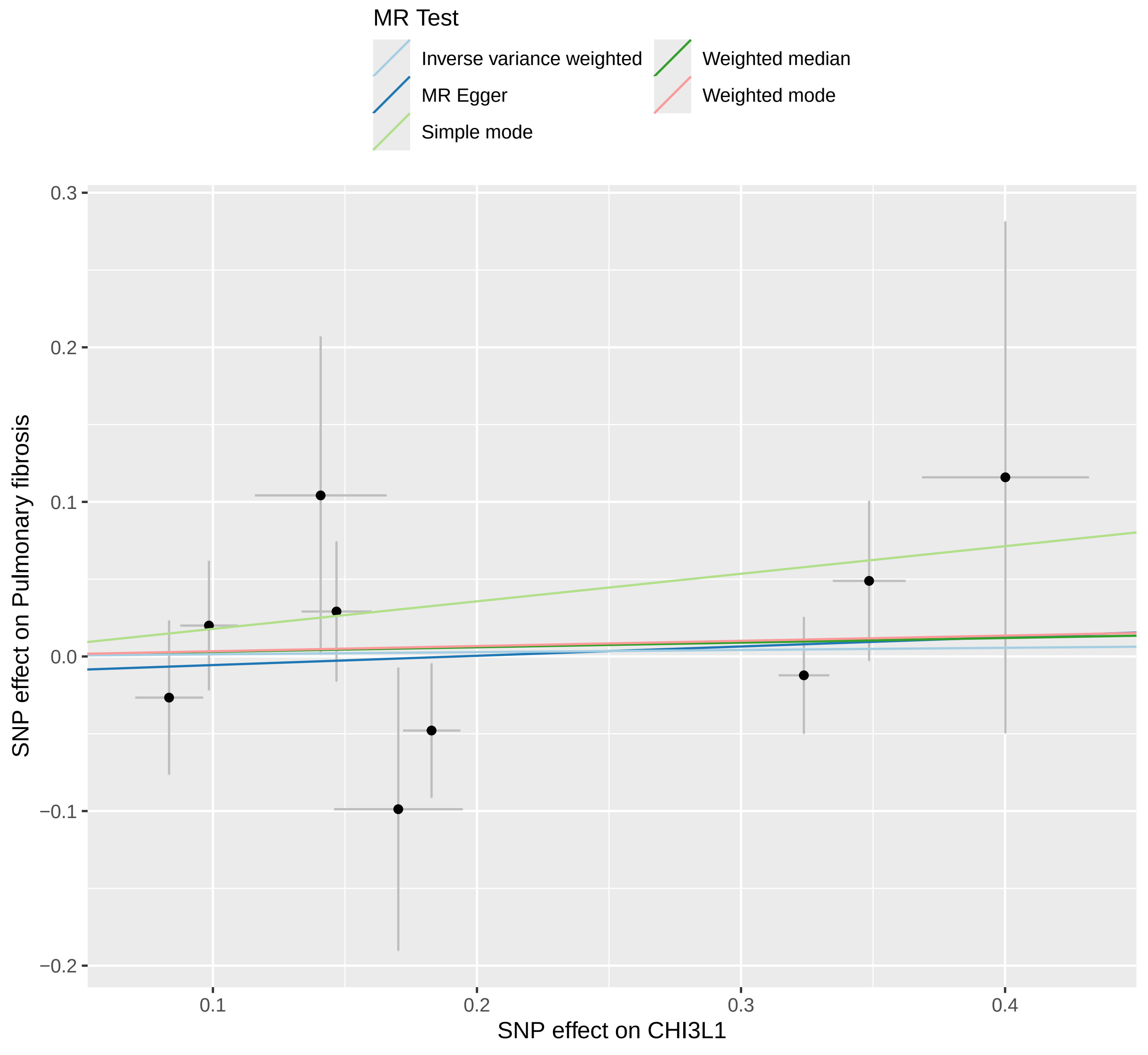

MR Test
Inverse variance weighted
MR Egger
Simple mode
Weighted median
Weighted mode
0.3
0.2
0.1
0.0
−0.1
−0.2
0.1
0.2
0.3
0.4
SNP effect on CHI3L1
SNP effect on Pulmonary fibrosis

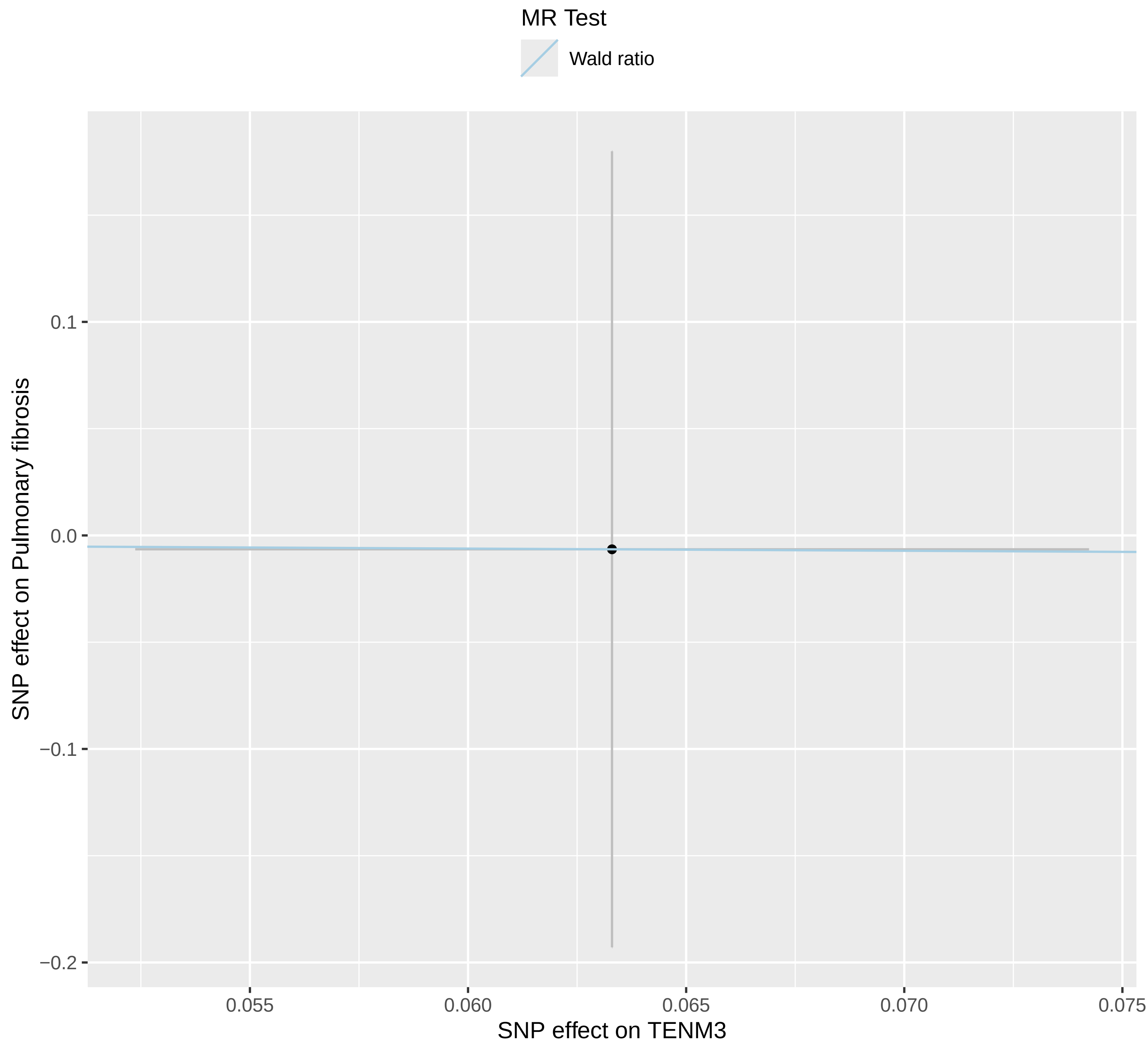
MR Test
Wald ratio
SNP effect on Pulmonary fibrosis
0.1
0.0
−0.1
−0.2
0.055
0.060
0.065
0.070
0.075
SNP effect on TENM3

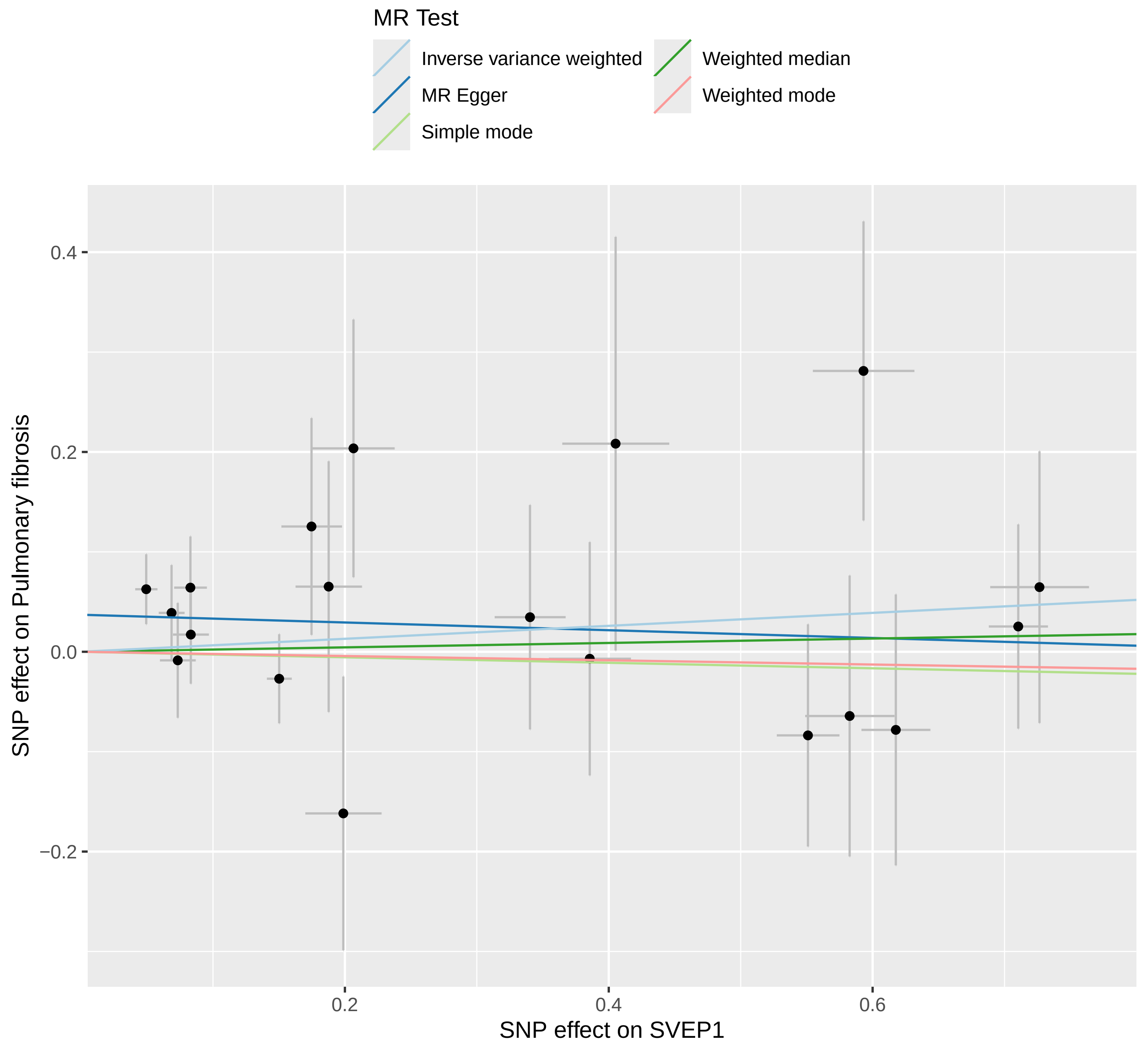

MR Test
Inverse variance weighted
MR Egger
Simple mode
Weighted median
Weighted mode
0.4
0.2
0.0
−0.2
0.2
0.4
0.6
SNP effect on Pulmonary fibrosis
SNP effect on SVEP1

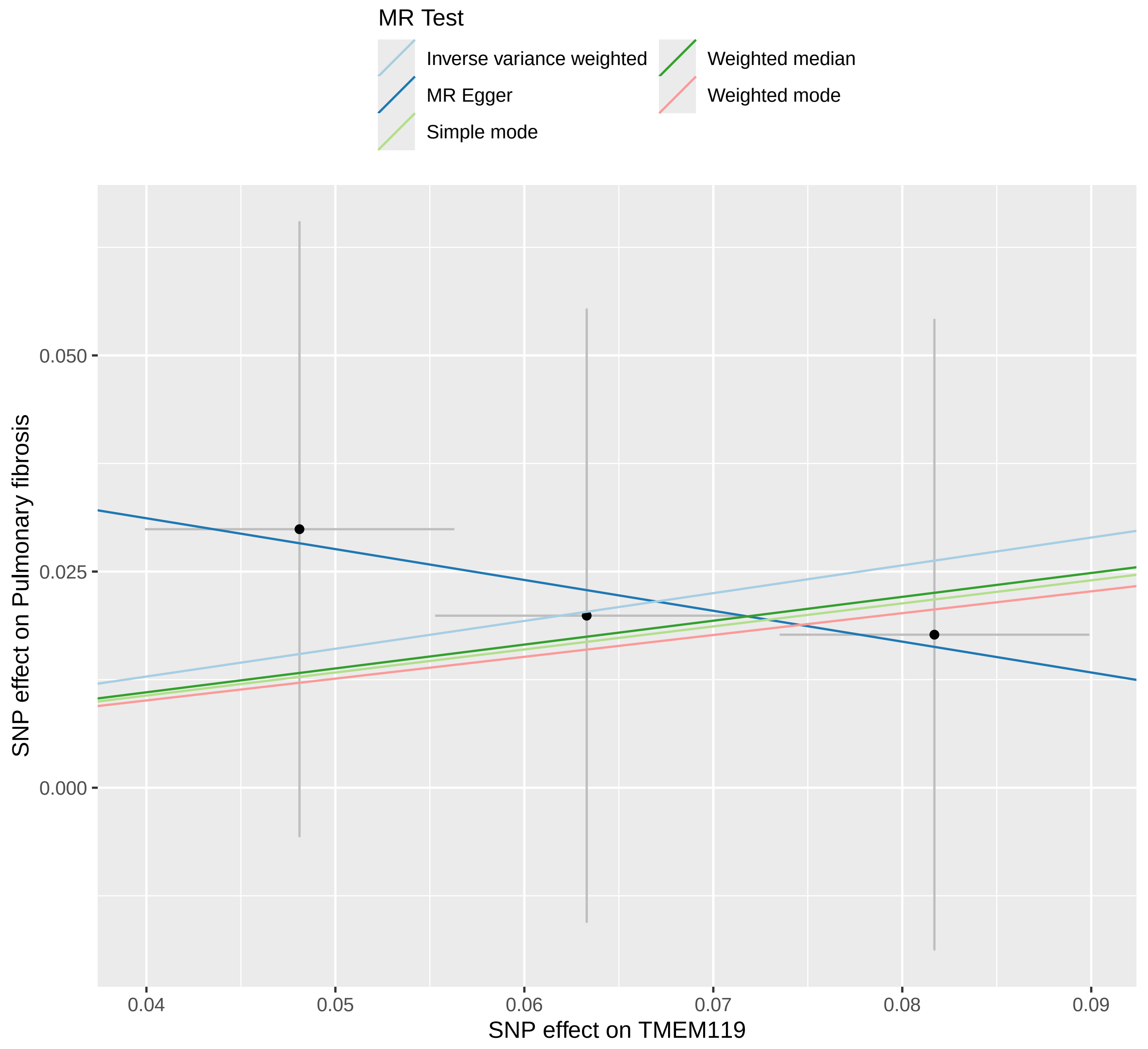

MR Test
Inverse variance weighted
MR Egger
Simple mode
Weighted median
Weighted mode
0.050
0.025
0.000
SNP effect on Pulmonary fibrosis
0.04
0.05
0.06
0.07
0.08
0.09
SNP effect on TMEM119

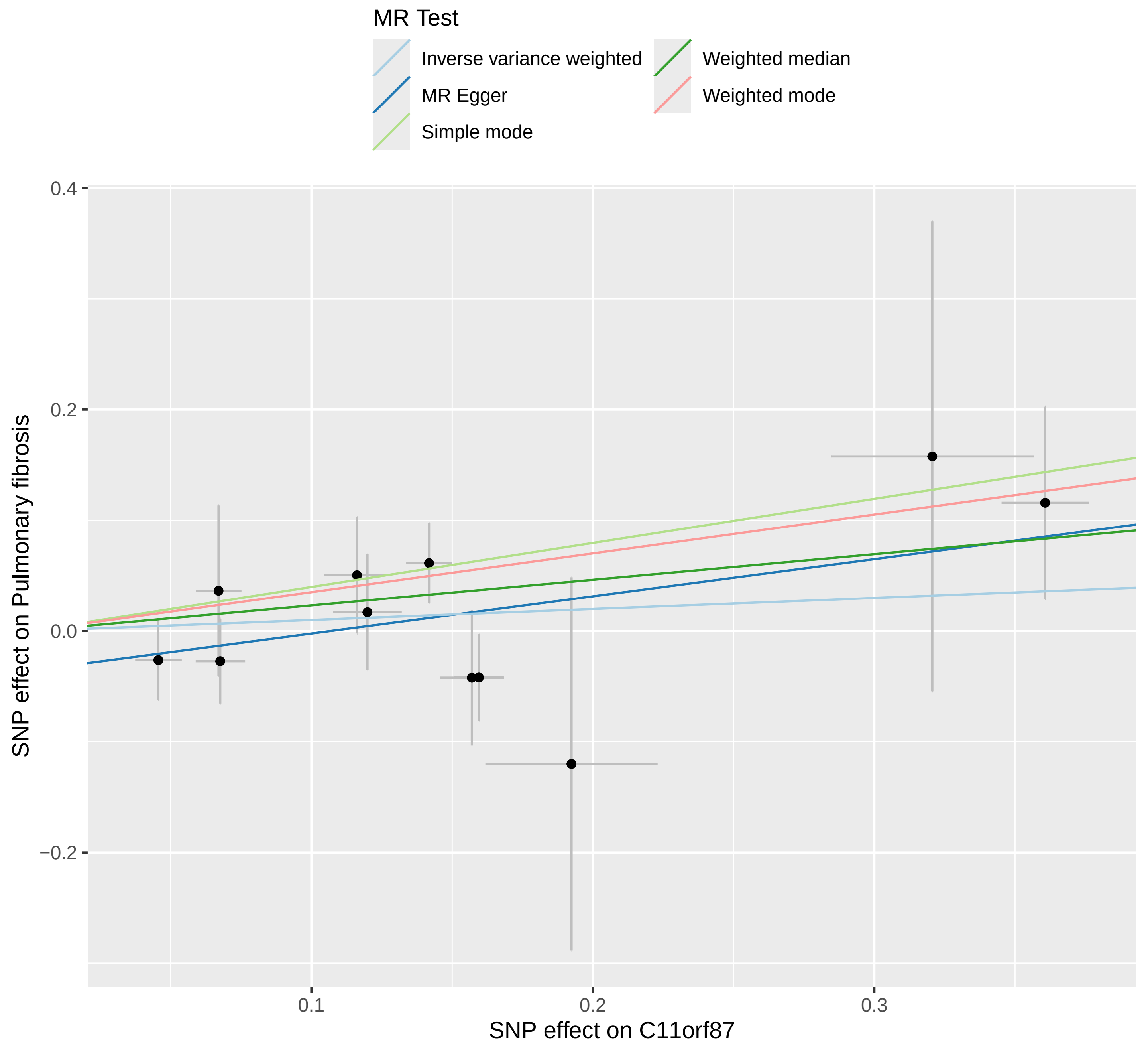
MR Test
Inverse variance weighted
MR Egger
Simple mode
Weighted median
Weighted mode
0.4
0.2
0.0
−0.2
0.1
0.2
0.3
SNP effect on Pulmonary fibrosis
SNP effect on C11orf87

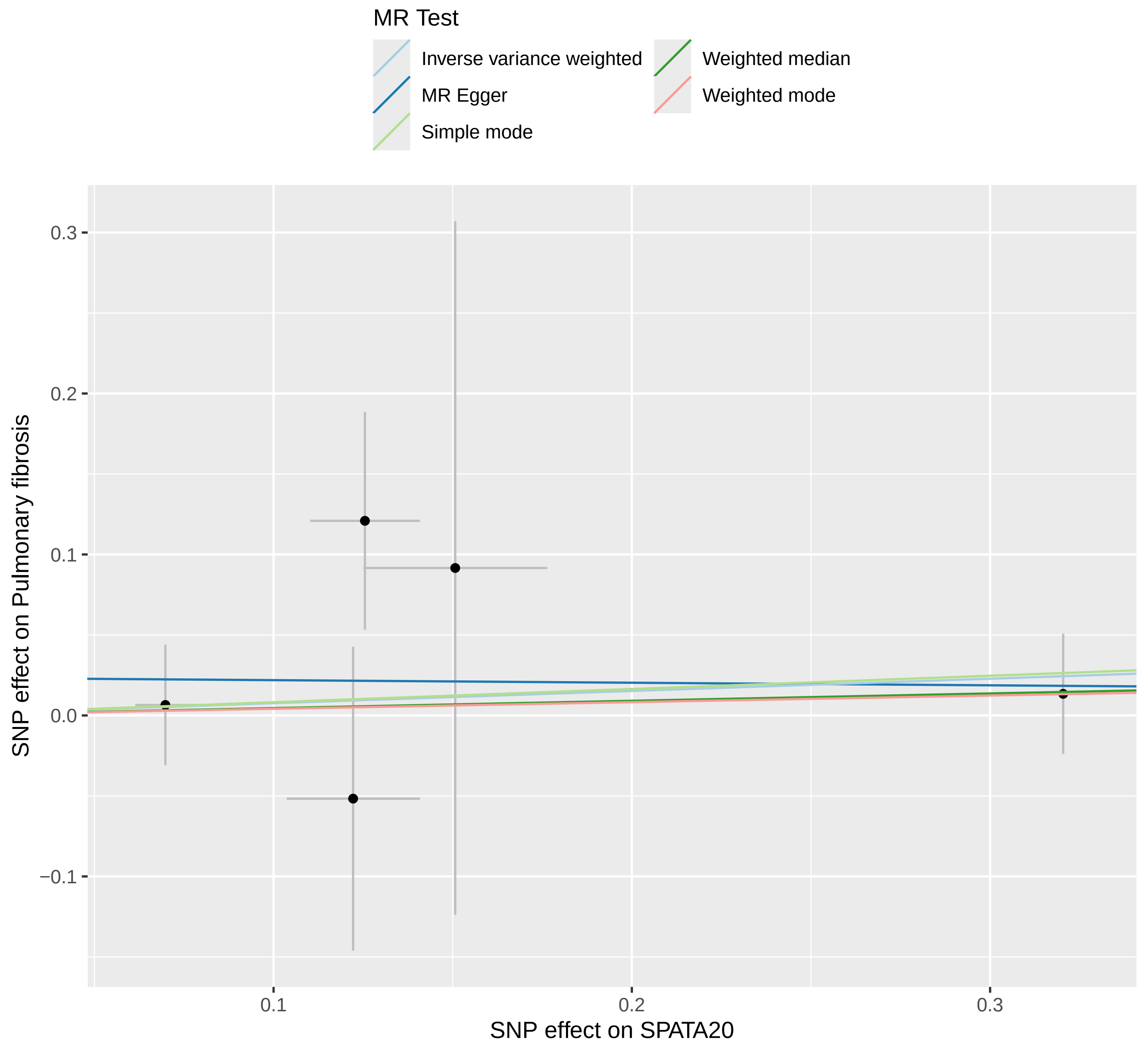
MR Test
Inverse variance weighted
MR Egger
Simple mode
Weighted median
Weighted mode
SNP effect on Pulmonary fibrosis
0.3
0.2
0.1
0.0
−0.1
0.1
0.2
0.3
SNP effect on SPATA20

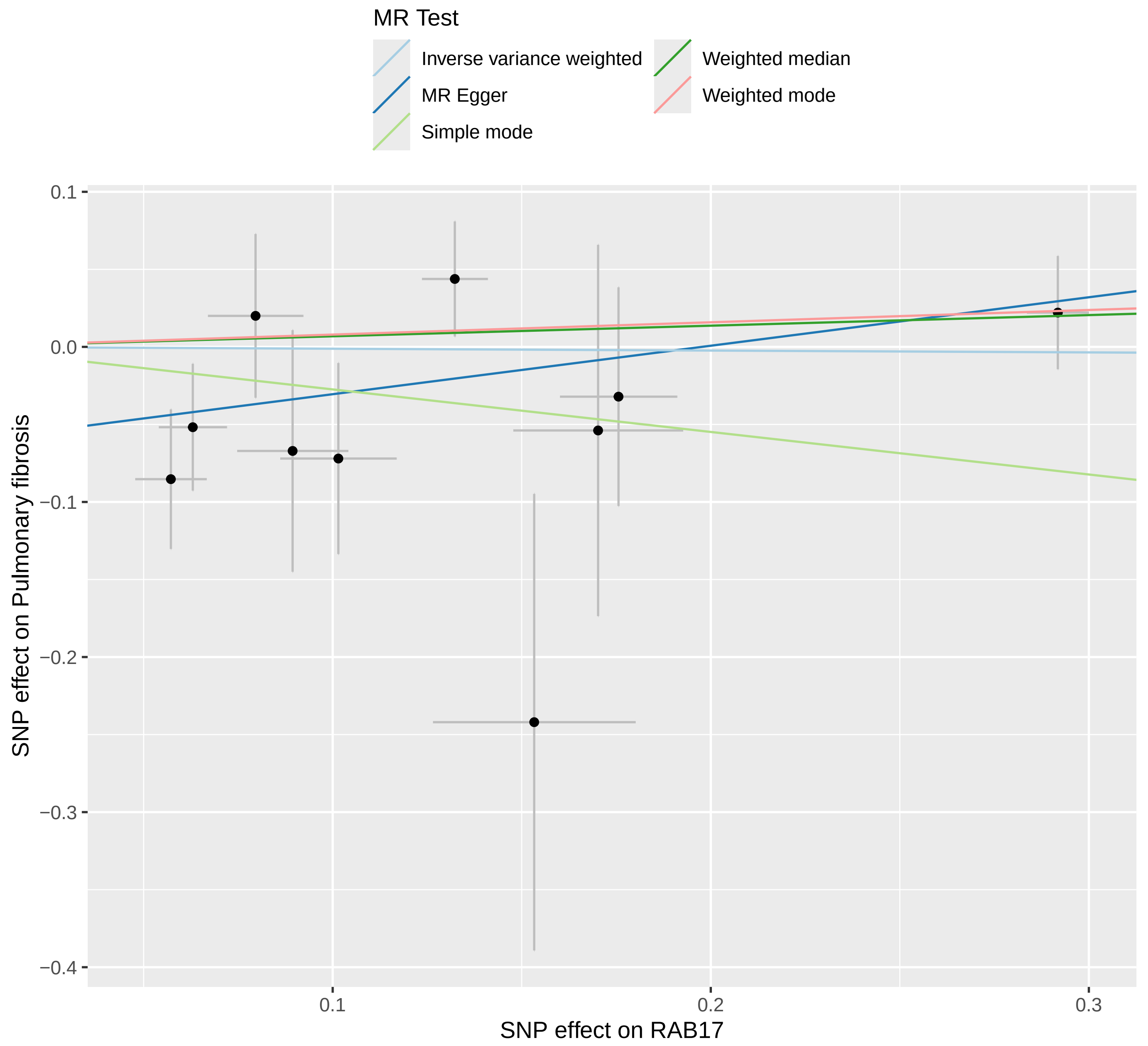
MR Test
Inverse variance weighted
MR Egger
Simple mode
Weighted median
Weighted mode
0.1
0.0
−0.1
−0.2
−0.3
−0.4
SNP effect on Pulmonary fibrosis
0.1
0.2
0.3
SNP effect on RAB17

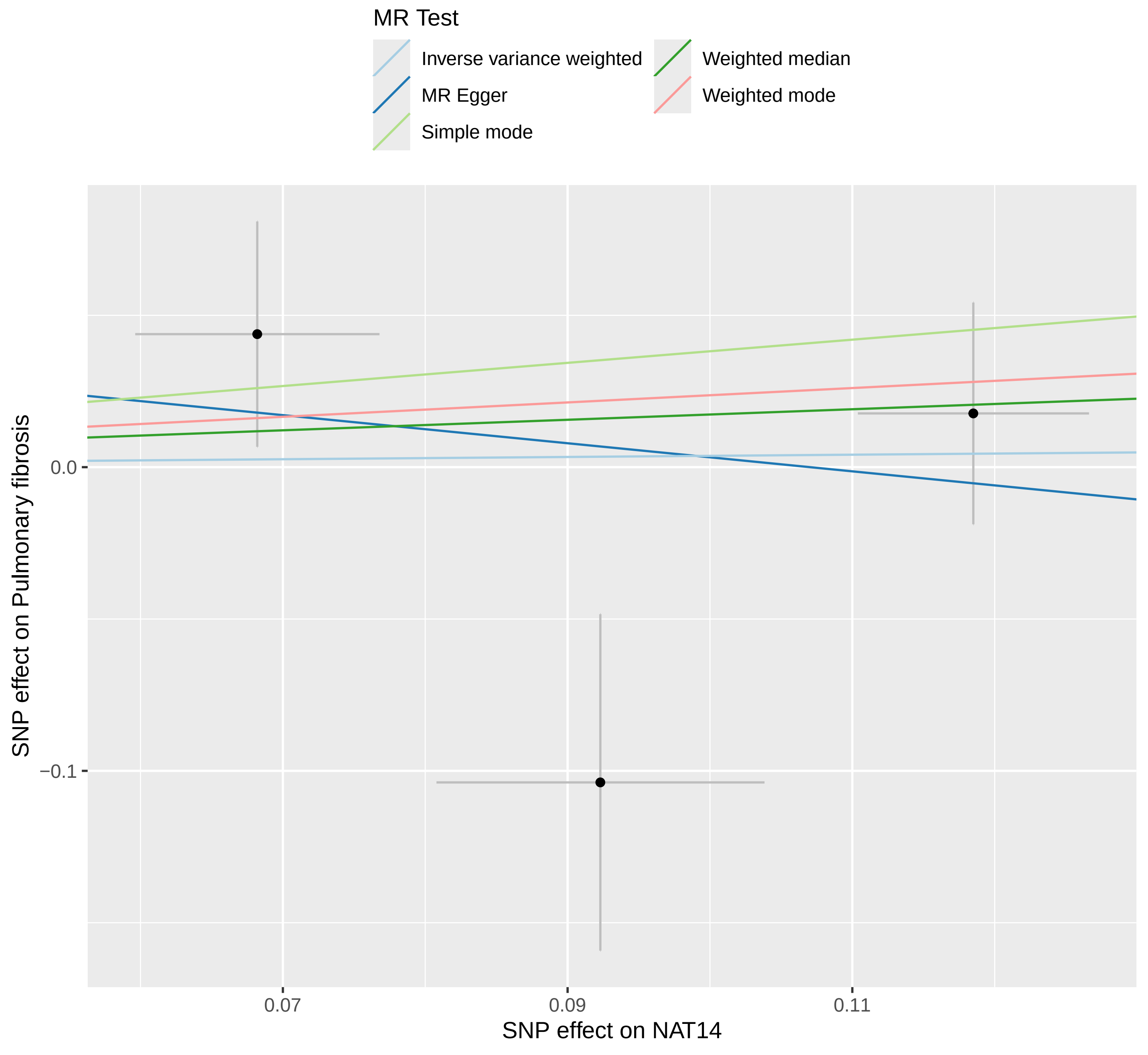

MR Test
Inverse variance weighted
MR Egger
Simple mode
Weighted median
Weighted mode
0.0
−0.1
0.07
0.09
0.11
SNP effect on NAT14
SNP effect on Pulmonary fibrosis

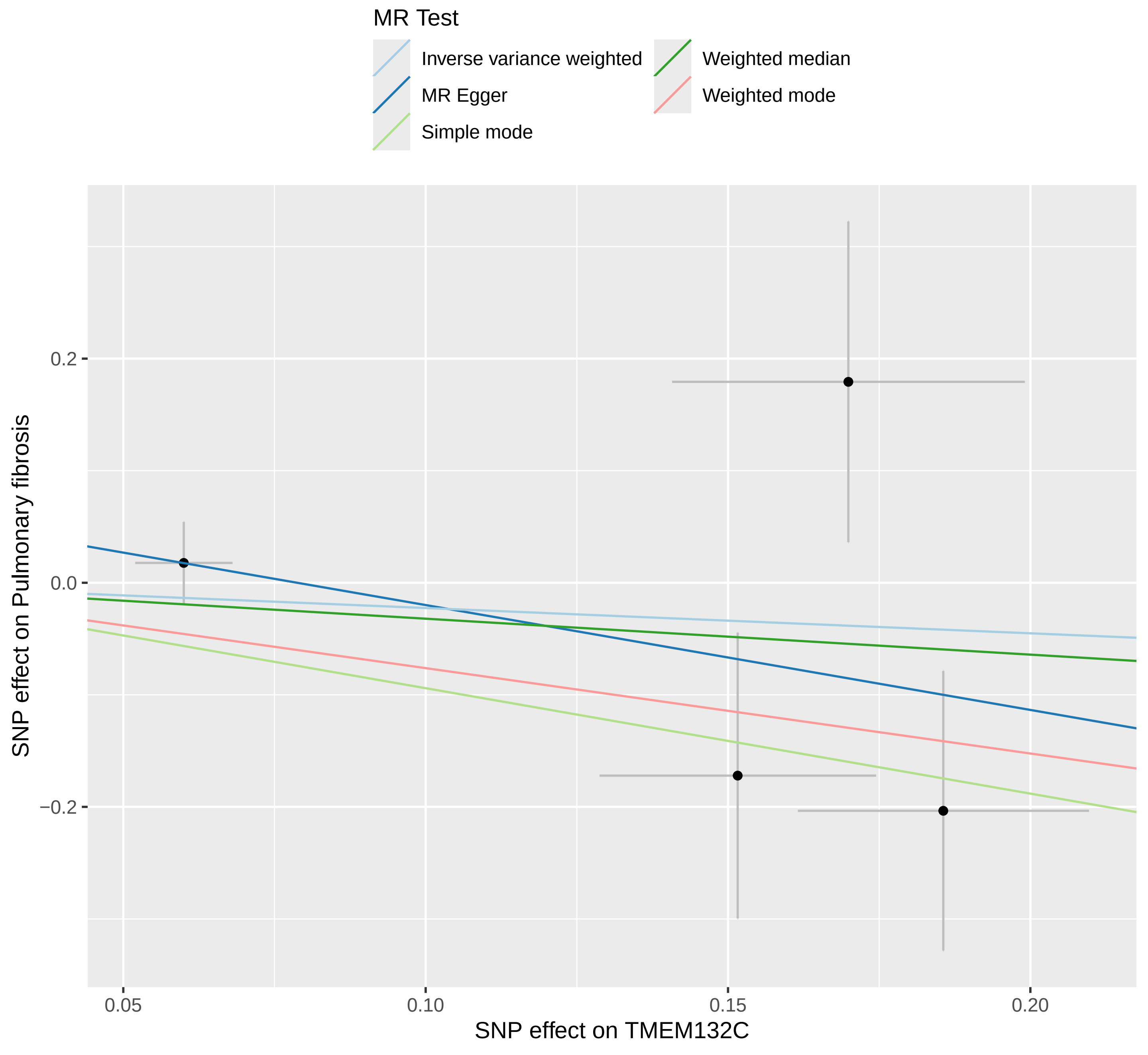
MR Test
Inverse variance weighted
MR Egger
Simple mode
Weighted median
Weighted mode
0.2
0.0
−0.2
SNP effect on Pulmonary fibrosis
0.05
0.10
0.15
0.20
SNP effect on TMEM132C

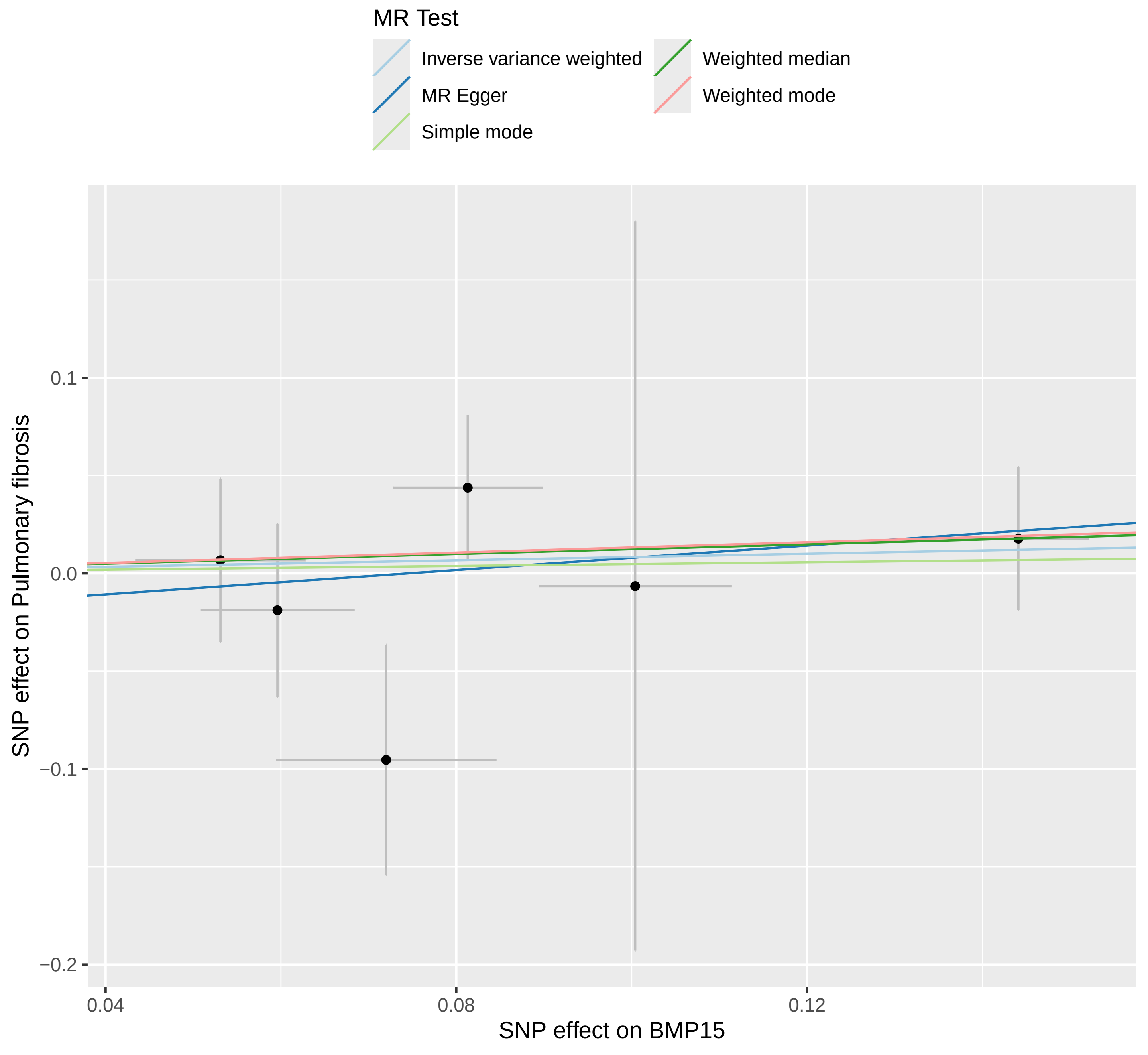

MR Test
Inverse variance weighted
MR Egger
Simple mode
Weighted median
Weighted mode
SNP effect on Pulmonary fibrosis
0.1
0.0
−0.1
−0.2
0.04
0.08
0.12
SNP effect on BMP15

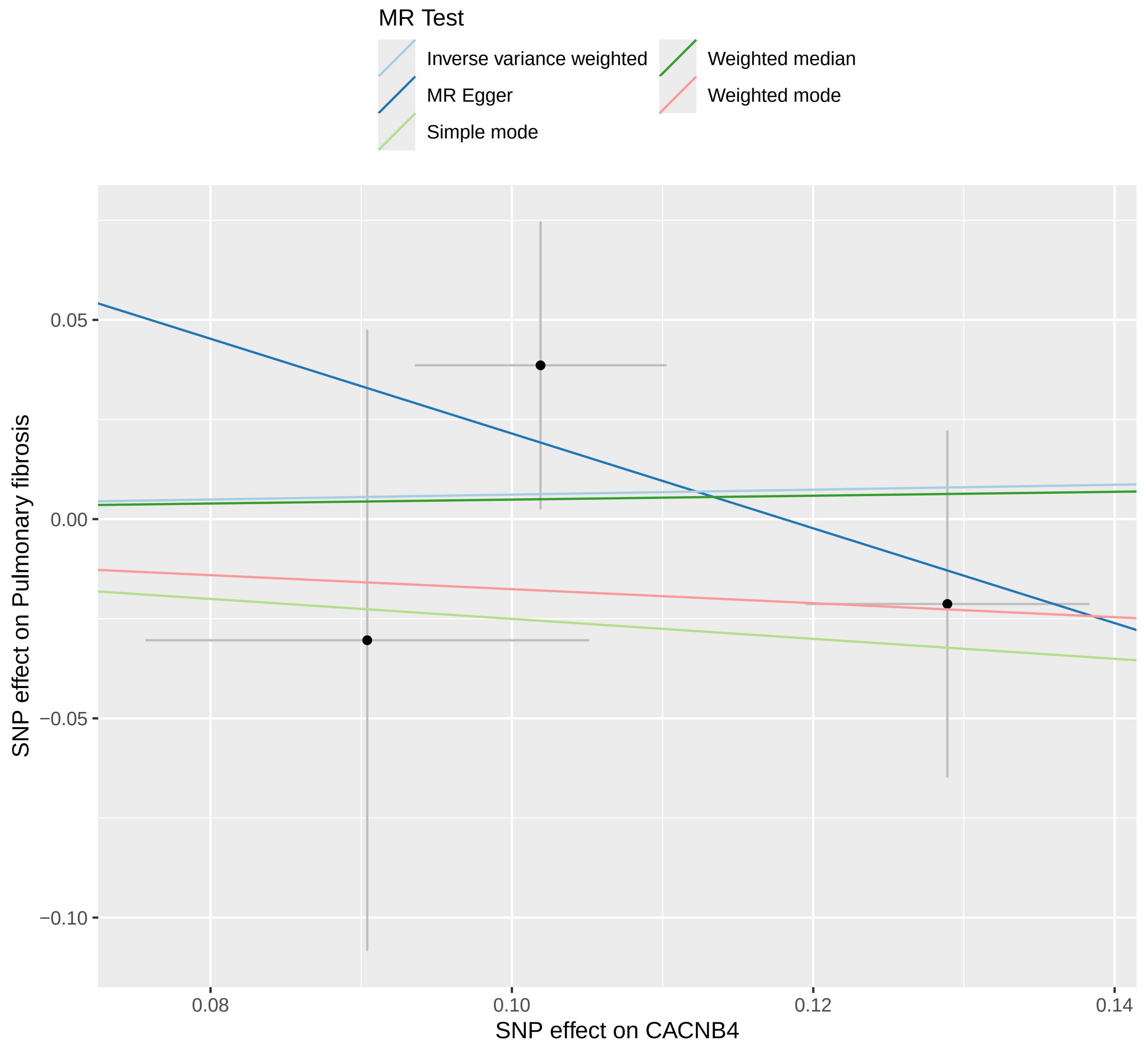
MR Test
Inverse variance weighted
MR Egger
Simple mode
Weighted median
Weighted mode
SNP effect on Pulmonary fibrosis
0.05
0.00
−0.05
−0.10
0.08
0.10
0.12
0.14
SNP effect on CACNB4

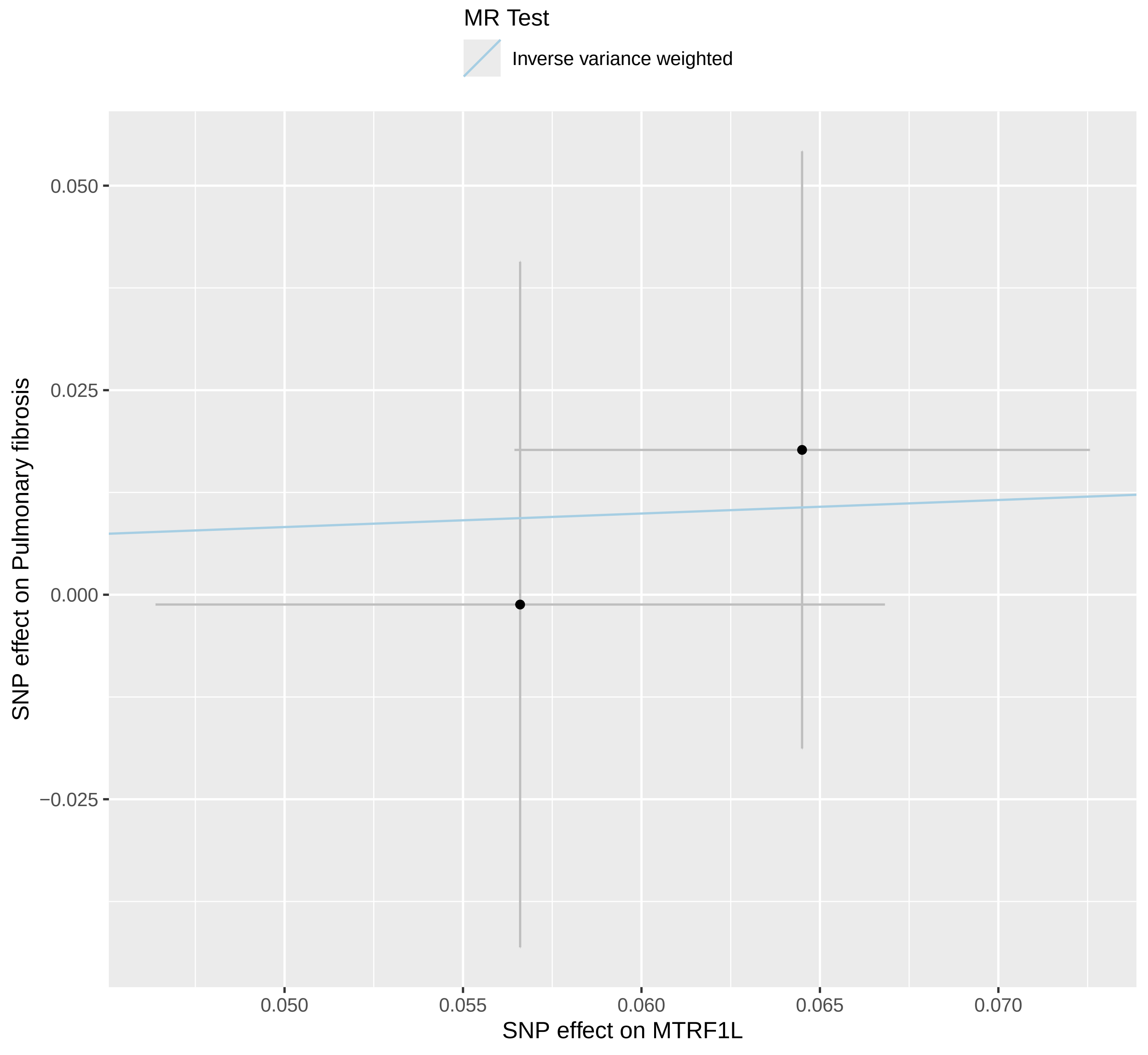

MR Test
Inverse variance weighted
0.050
0.025
0.000
−0.025
SNP effect on Pulmonary fibrosis
0.050
0.055
0.060
0.065
0.070
SNP effect on MTRF1L

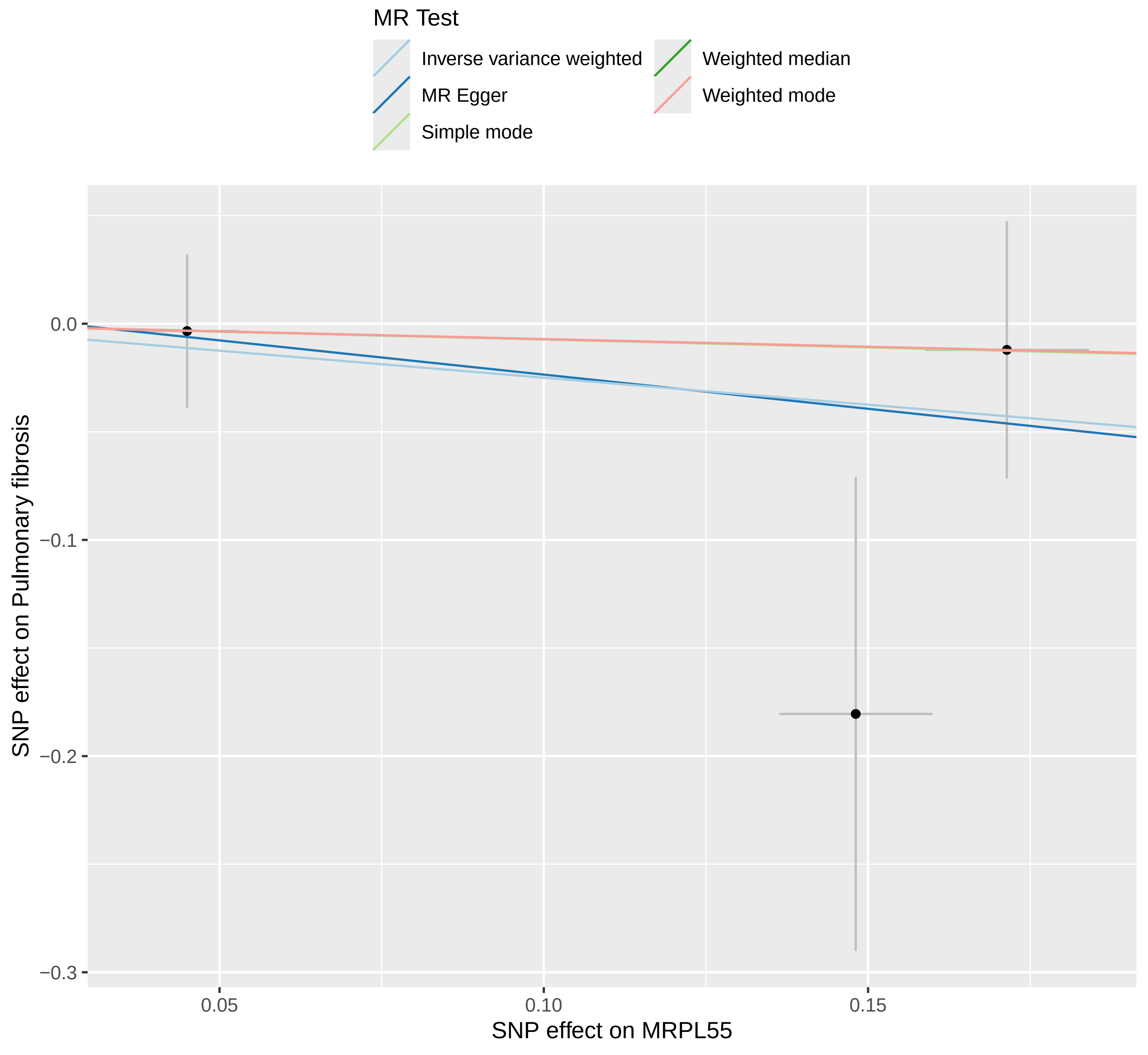

MR Test
Inverse variance weighted
MR Egger
Simple mode
Weighted median
Weighted mode
0.0
−0.1
−0.2
−0.3
0.05
0.10
0.15
SNP effect on MRPL55
SNP effect on Pulmonary fibrosis

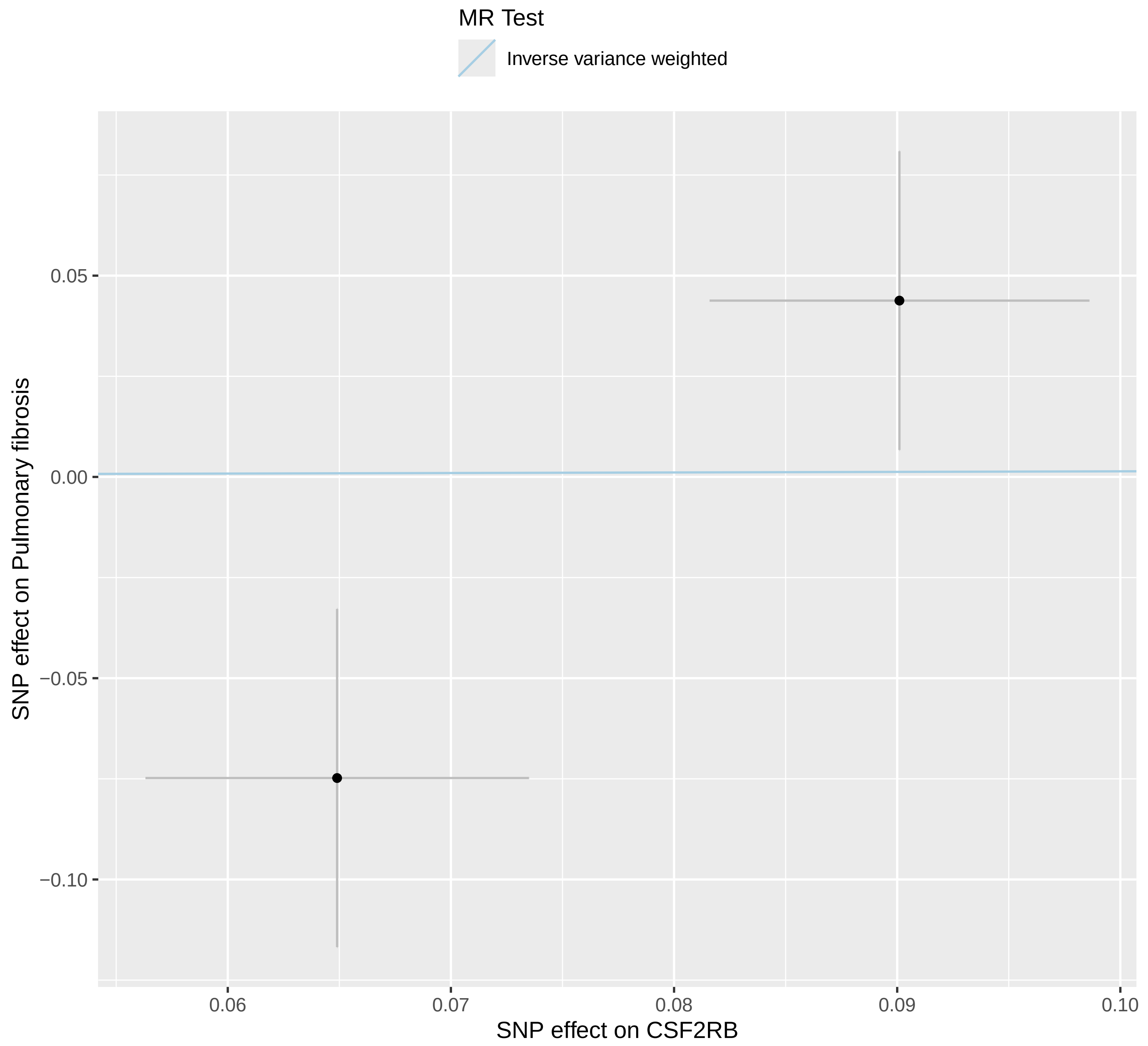

MR Test
Inverse variance weighted
SNP effect on Pulmonary fibrosis
0.05
0.00
−0.05
−0.10
0.06
0.07
0.08
0.09
0.10
SNP effect on CSF2RB

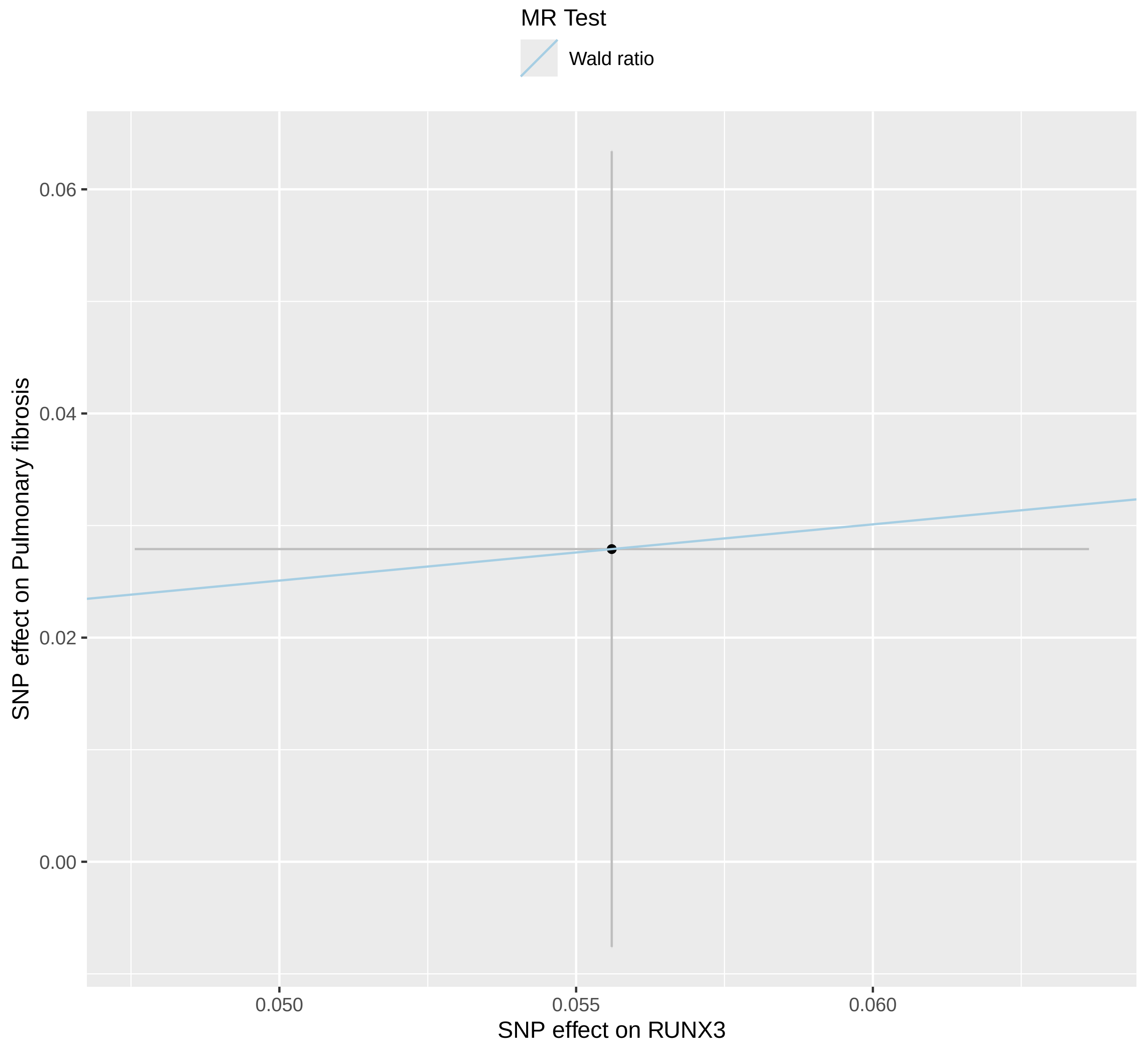
MR Test
Wald ratio
SNP effect on Pulmonary fibrosis
0.06
0.04
0.02
0.00
0.050
0.055
0.060
SNP effect on RUNX3

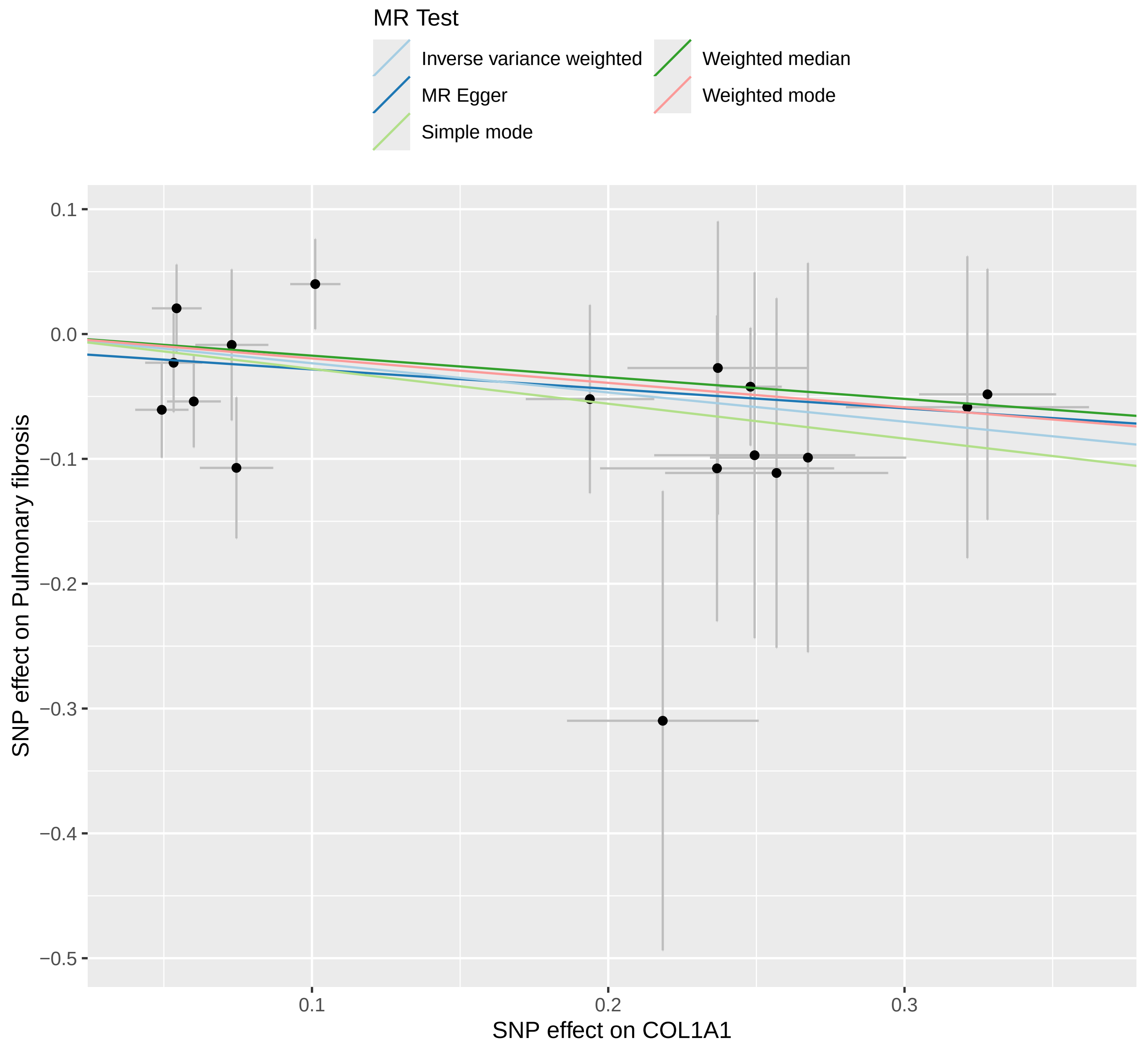
MR Test
Inverse variance weighted
MR Egger
Simple mode
Weighted median
Weighted mode
SNP effect on Pulmonary fibrosis
0.1
0.0
−0.1
−0.2
−0.3
−0.4
−0.5
0.1
0.2
0.3
SNP effect on COL1A1

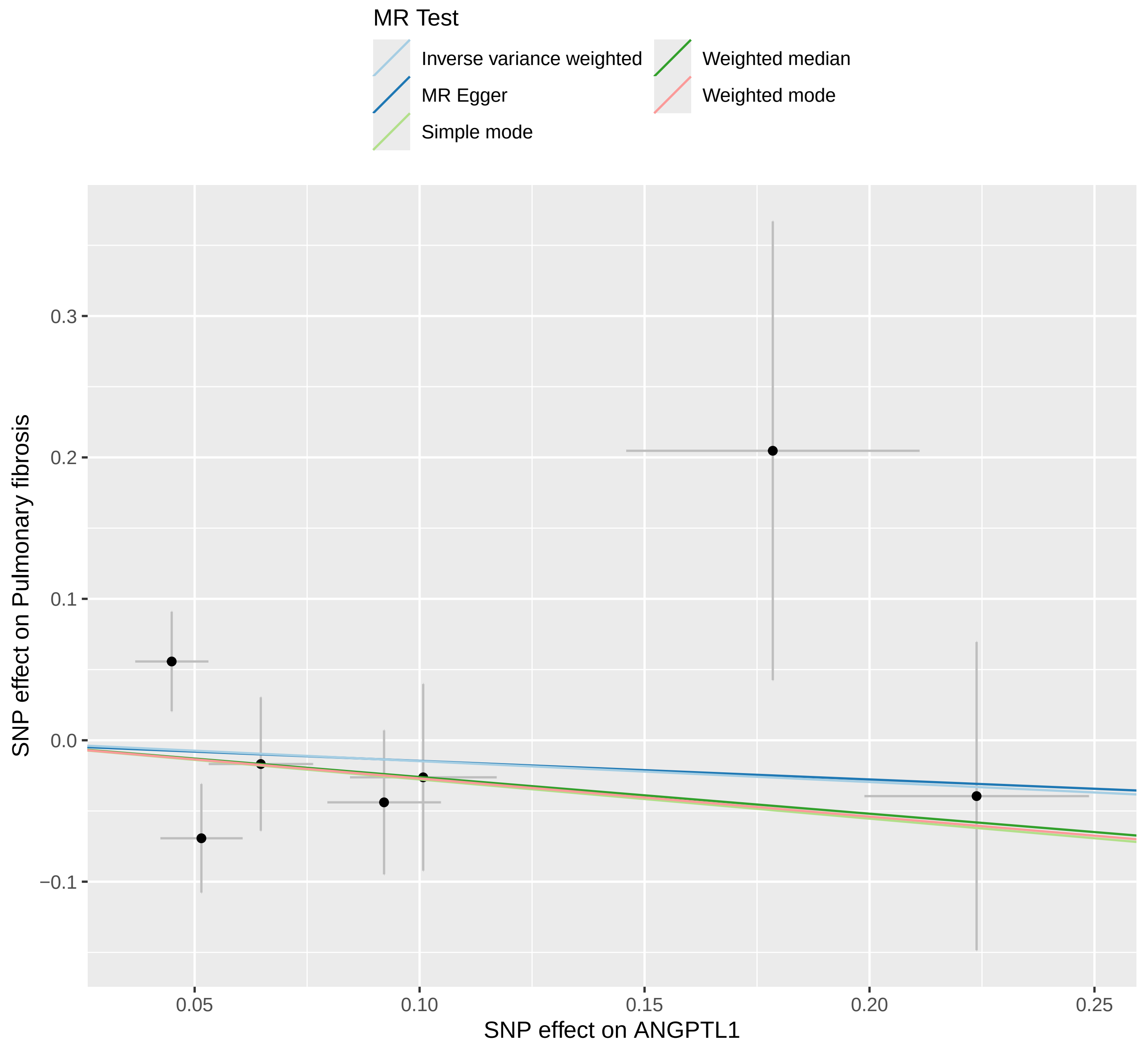
MR Test
Inverse variance weighted
MR Egger
Simple mode
Weighted median
Weighted mode
0.3
0.2
0.1
0.0
−0.1
0.05
0.10
0.15
0.20
0.25
SNP effect on Pulmonary fibrosis
SNP effect on ANGPTL1

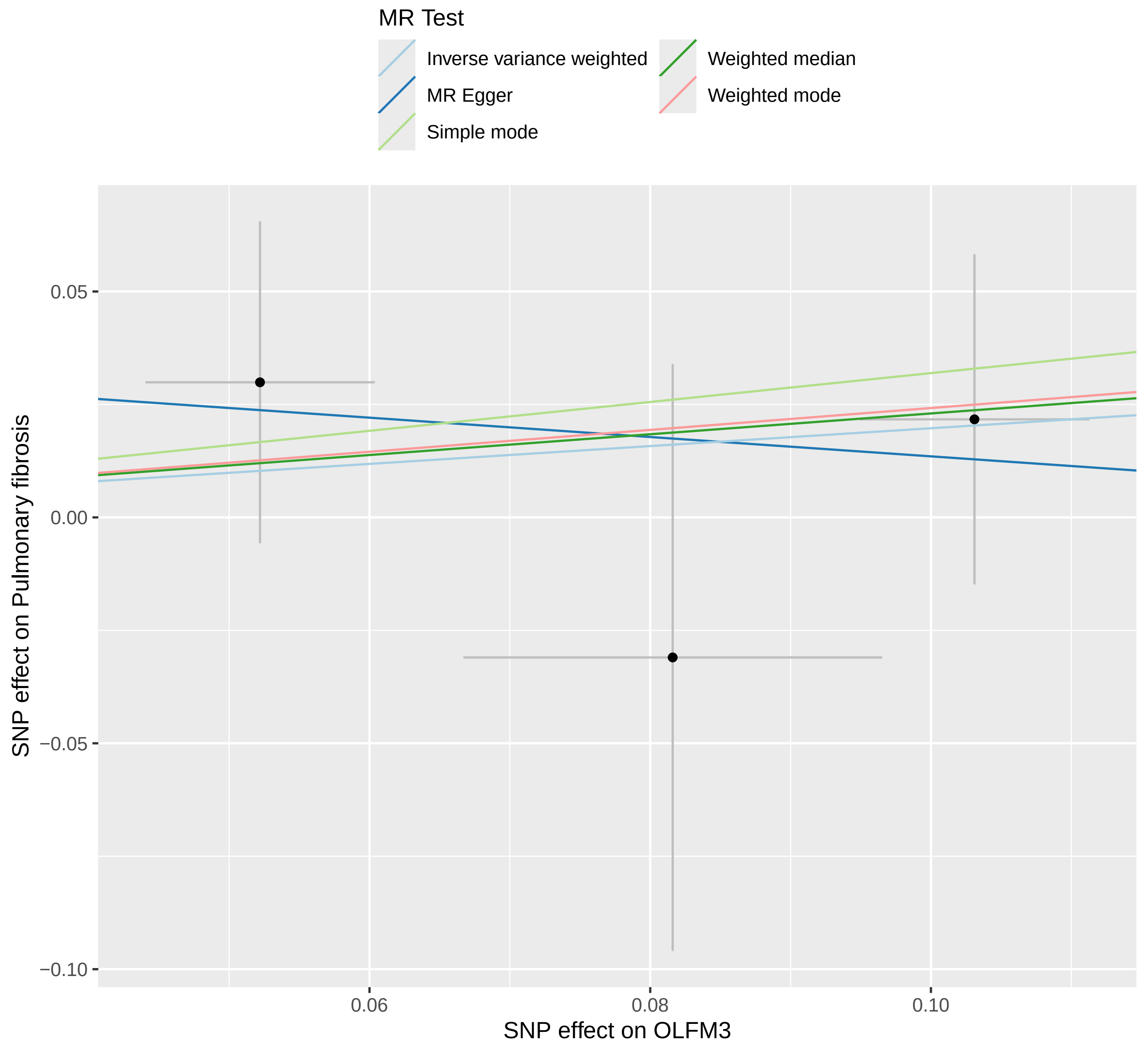

MR Test
Inverse variance weighted
MR Egger
Simple mode
Weighted median
Weighted mode
0.05
0.00
−0.05
−0.10
0.06
0.08
0.10
SNP effect on Pulmonary fibrosis
SNP effect on OLFM3

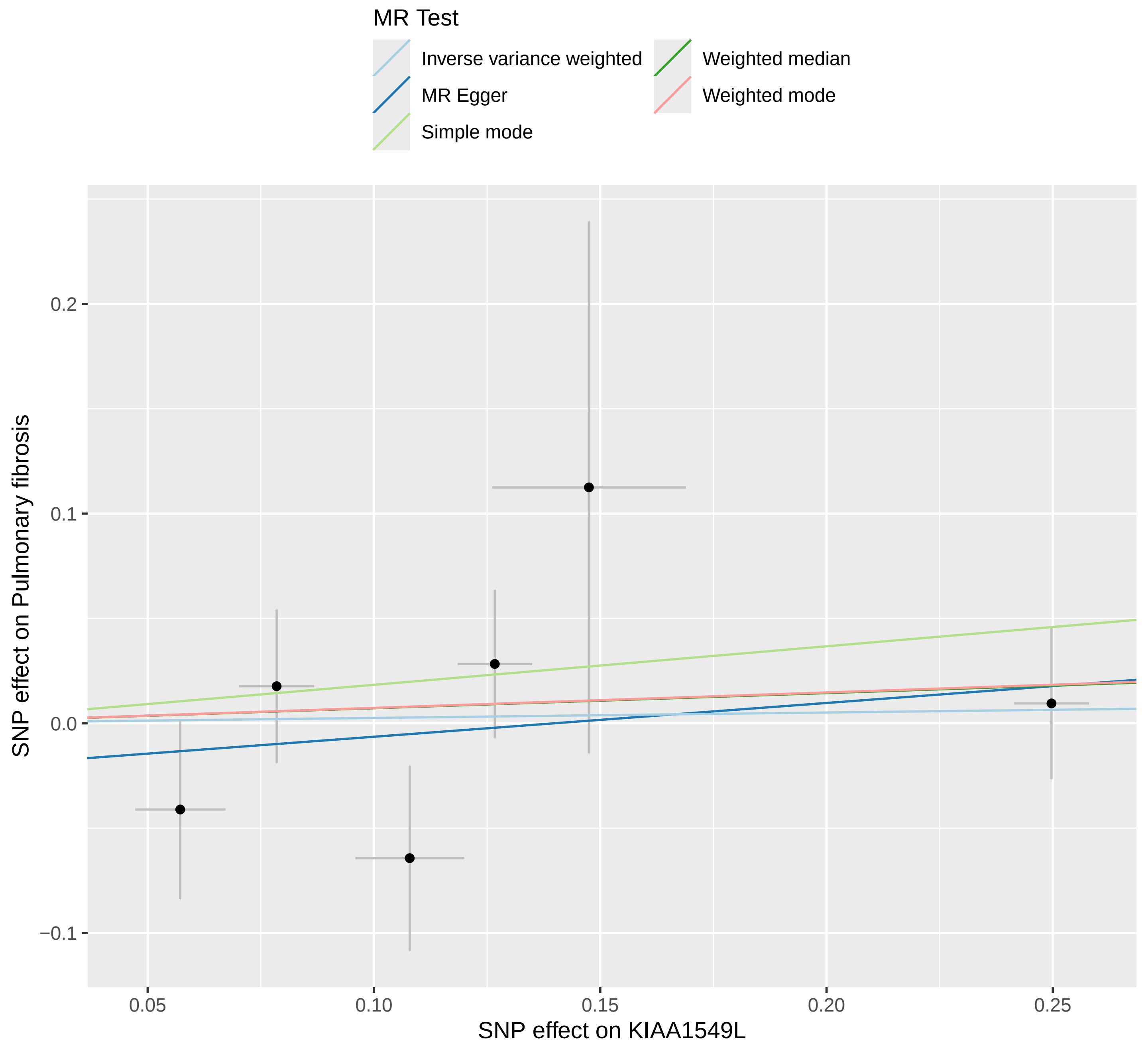
MR Test
Inverse variance weighted
MR Egger
Simple mode
Weighted median
Weighted mode
0.2
0.1
0.0
−0.1
0.05
0.10
0.15
0.20
0.25
SNP effect on KIAA1549L
SNP effect on Pulmonary fibrosis

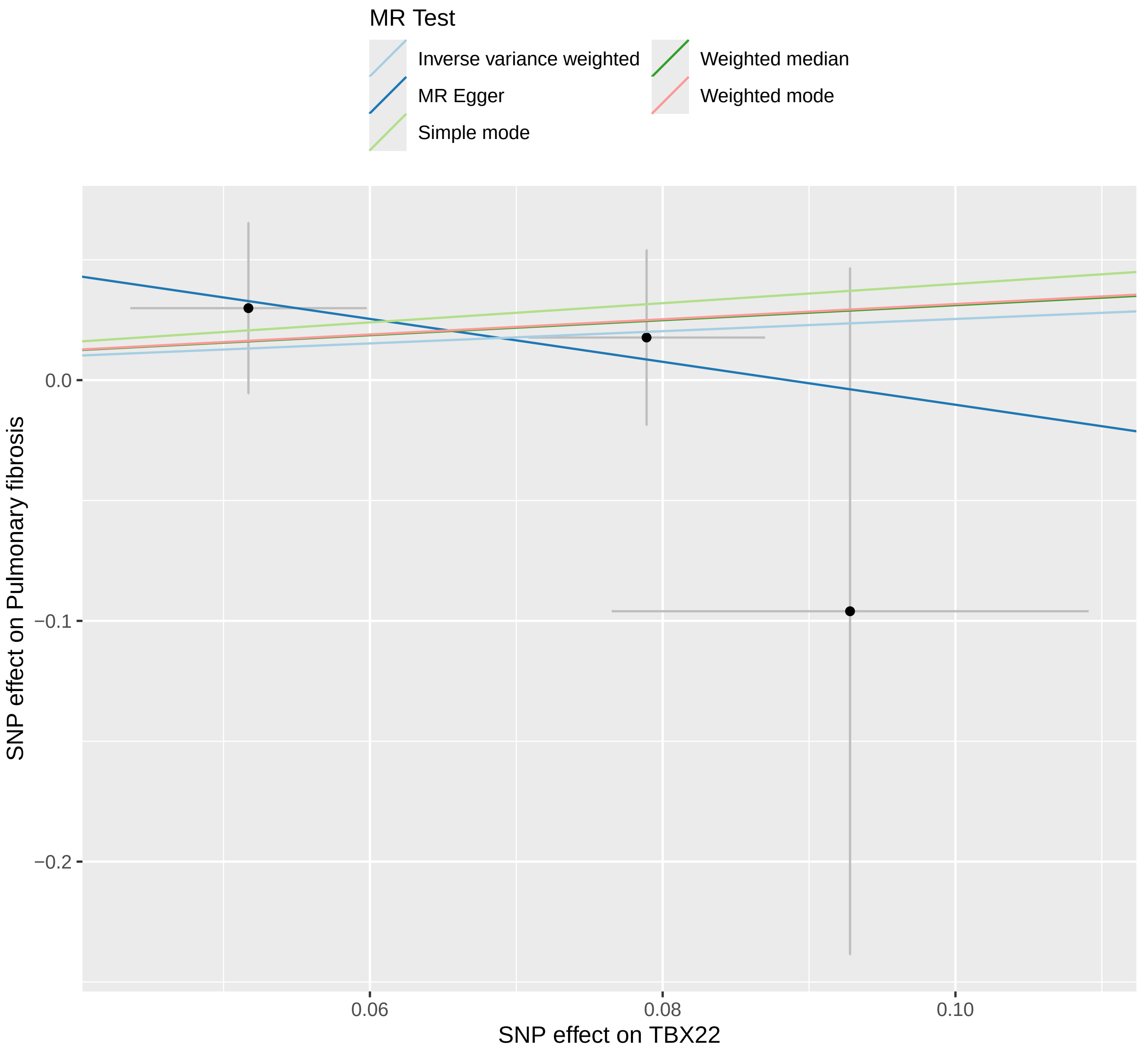

MR Test
Inverse variance weighted
MR Egger
Simple mode
Weighted median
Weighted mode
SNP effect on Pulmonary fibrosis
0.0
−0.1
−0.2
0.06
0.08
0.10
SNP effect on TBX22

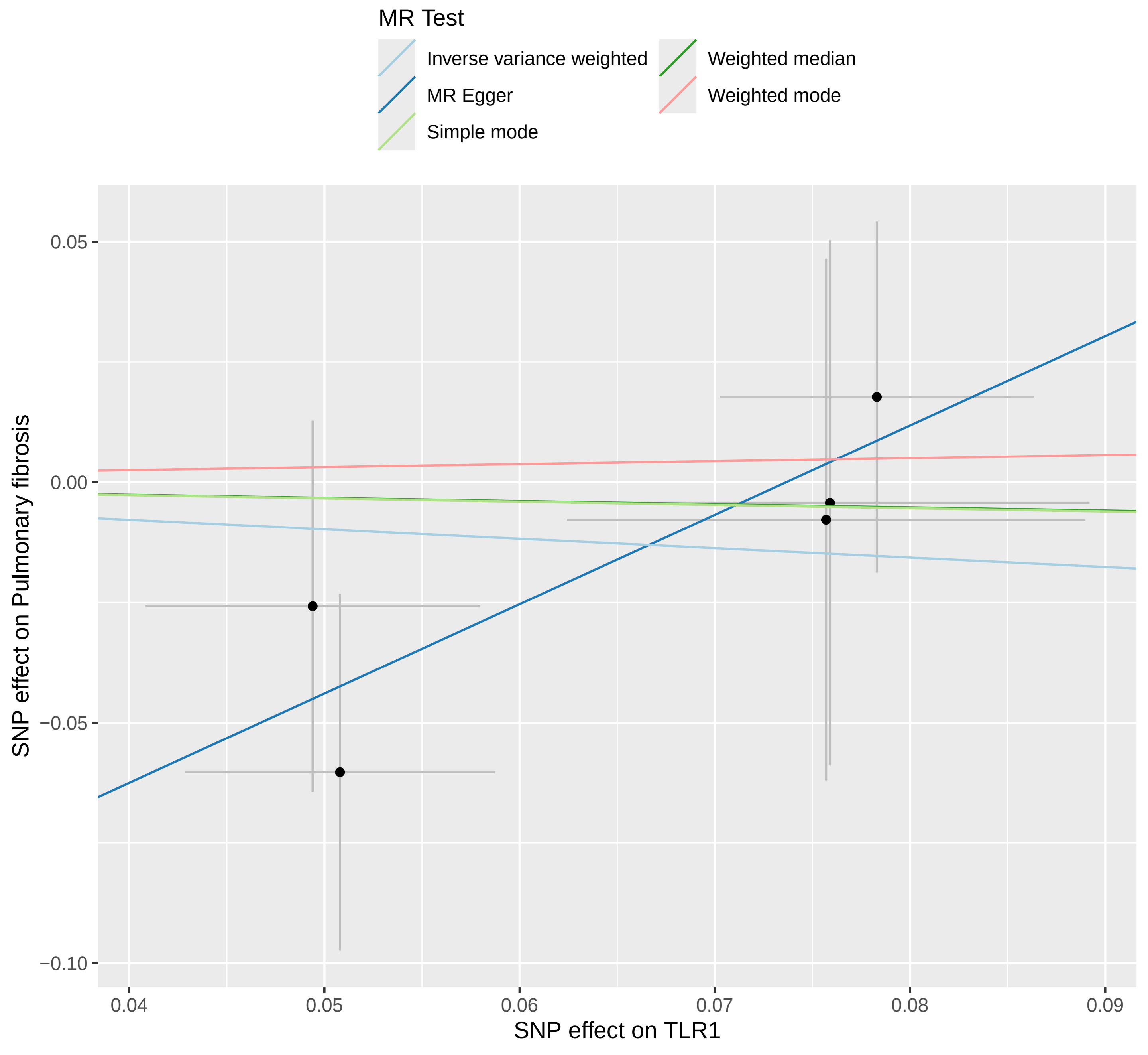
MR Test
Inverse variance weighted
MR Egger
Simple mode
Weighted median
Weighted mode
0.05
0.00
−0.05
−0.10
0.04
0.05
0.06
0.07
0.08
0.09
SNP effect on TLR1
SNP effect on Pulmonary fibrosis

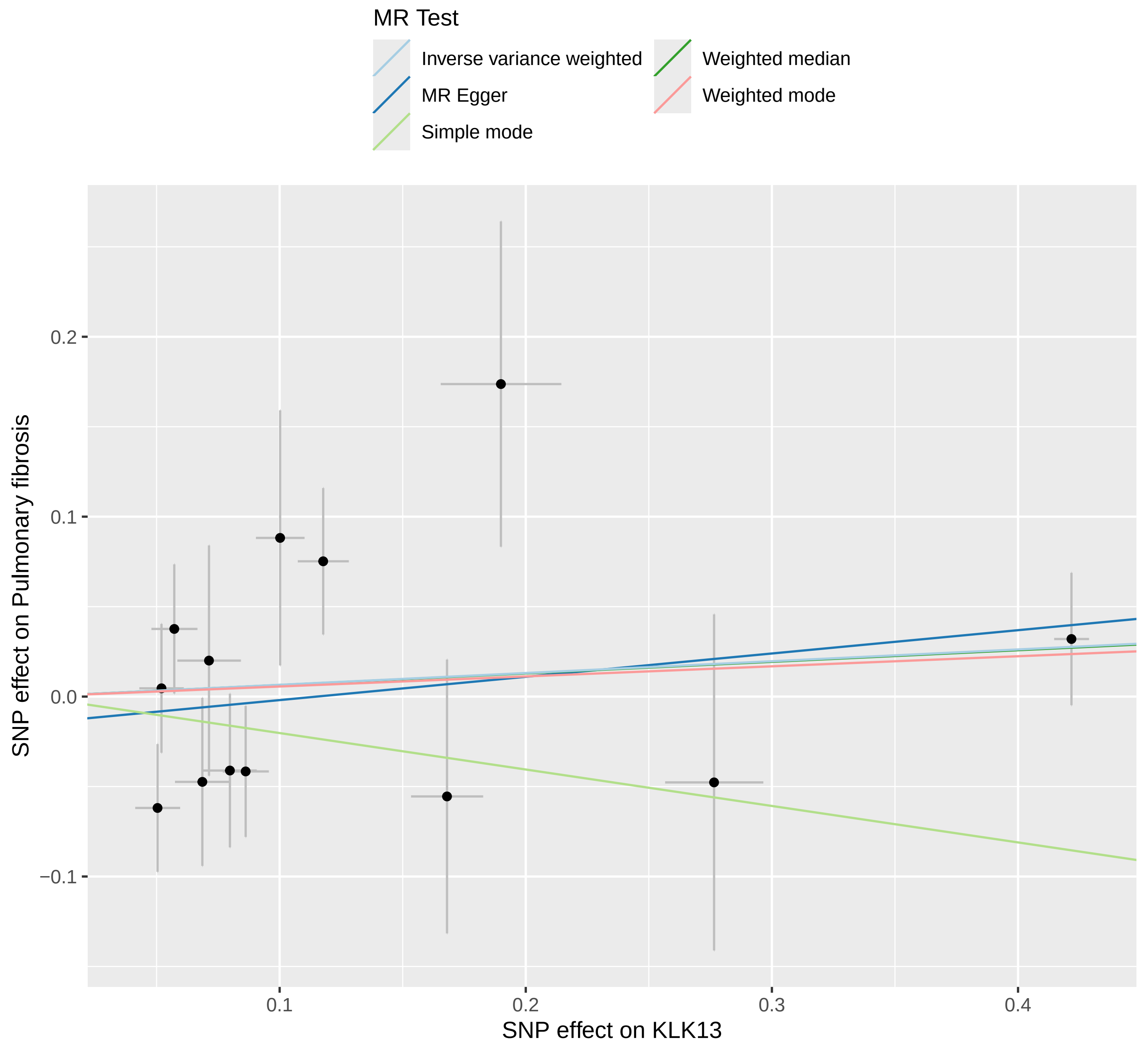

MR Test
Inverse variance weighted
MR Egger
Simple mode
Weighted median
Weighted mode
SNP effect on Pulmonary fibrosis
0.2
0.1
0.0
−0.1
0.1
0.2
0.3
0.4
SNP effect on KLK13

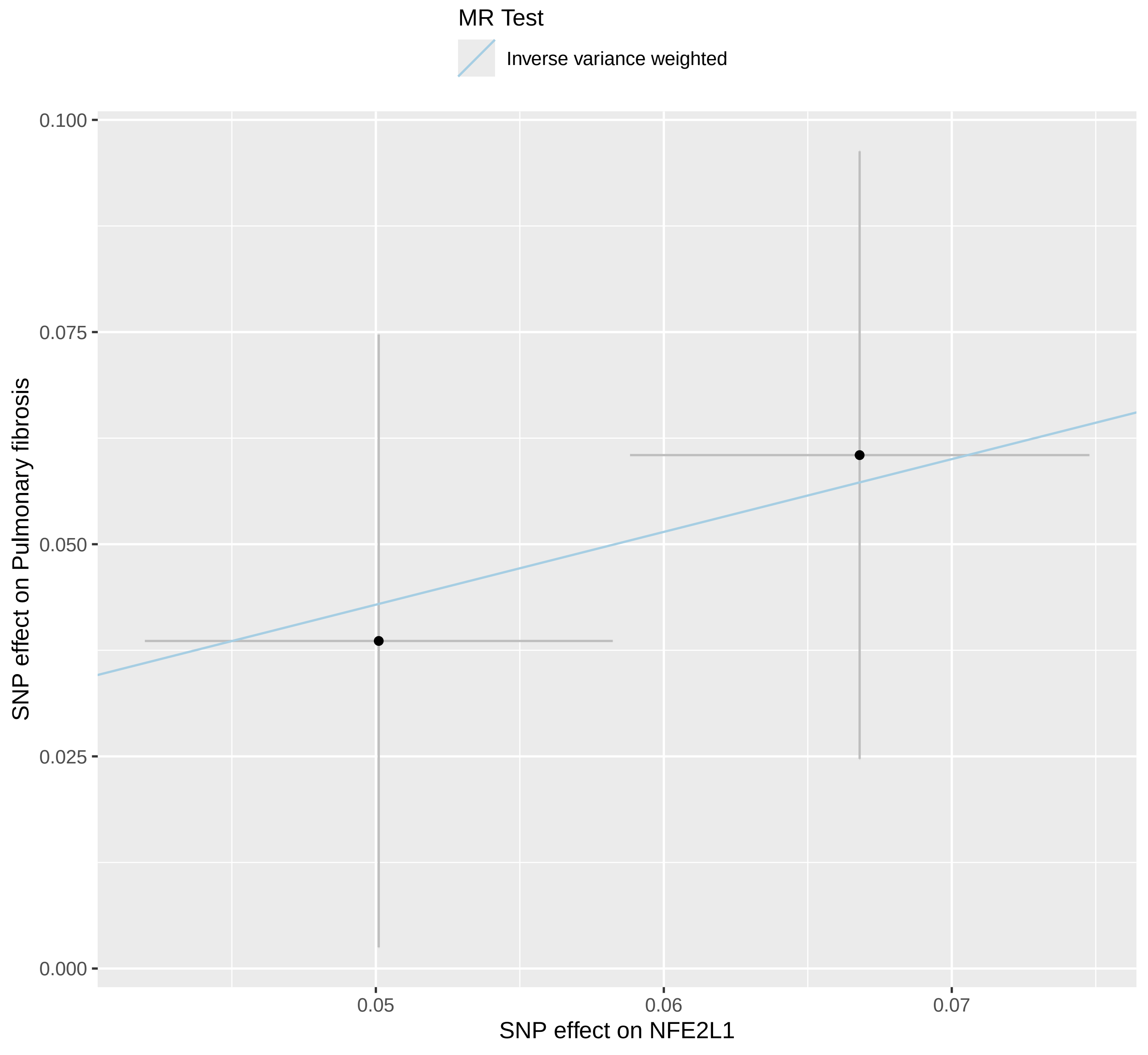
MR Test
Inverse variance weighted
0.100
0.075
0.050
0.025
0.000
SNP effect on Pulmonary fibrosis
0.05
0.06
0.07
SNP effect on NFE2L1

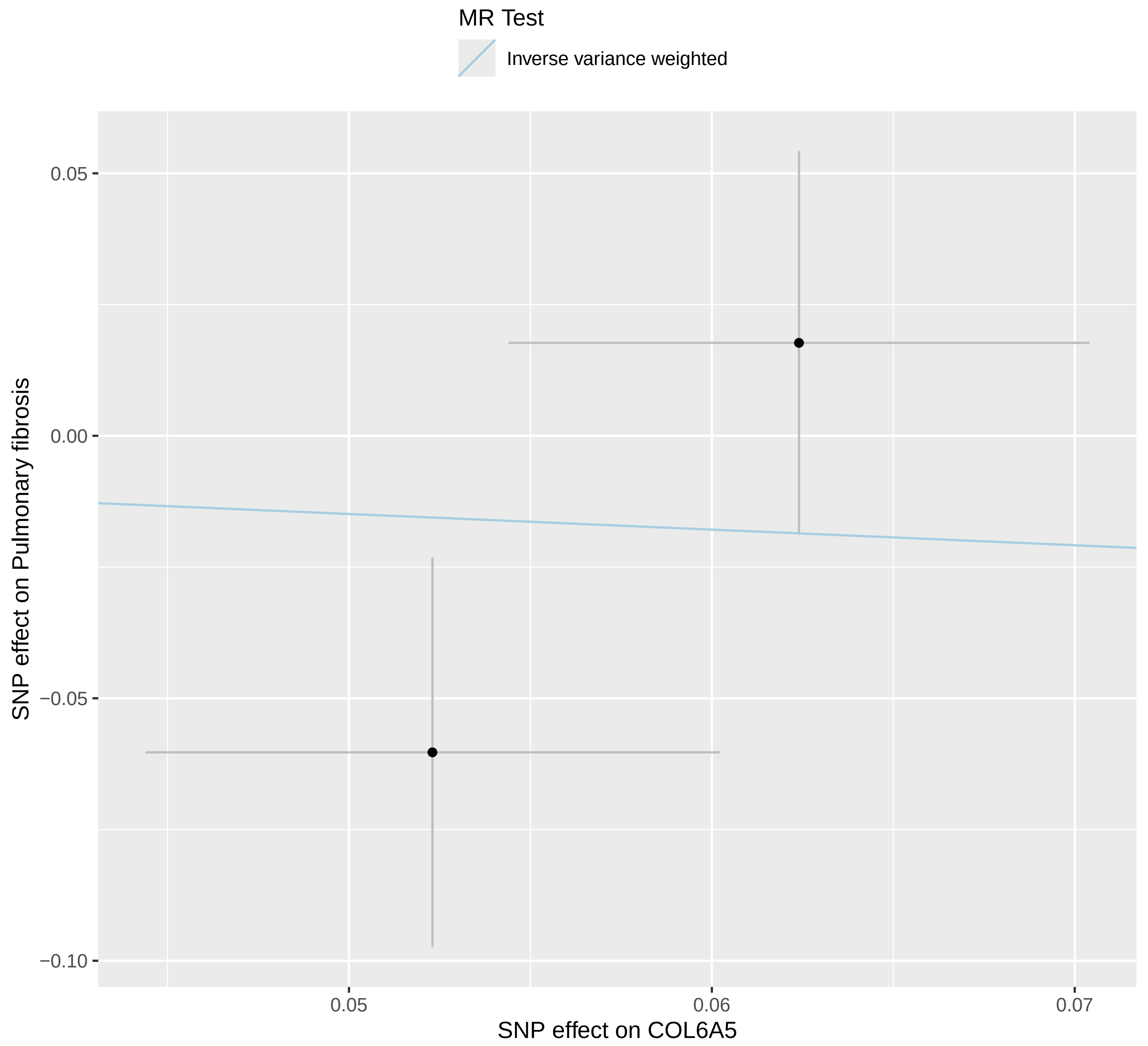
MR Test
Inverse variance weighted
0.05
0.00
−0.05
−0.10
SNP effect on Pulmonary fibrosis
0.05
0.06
0.07
SNP effect on COL6A5

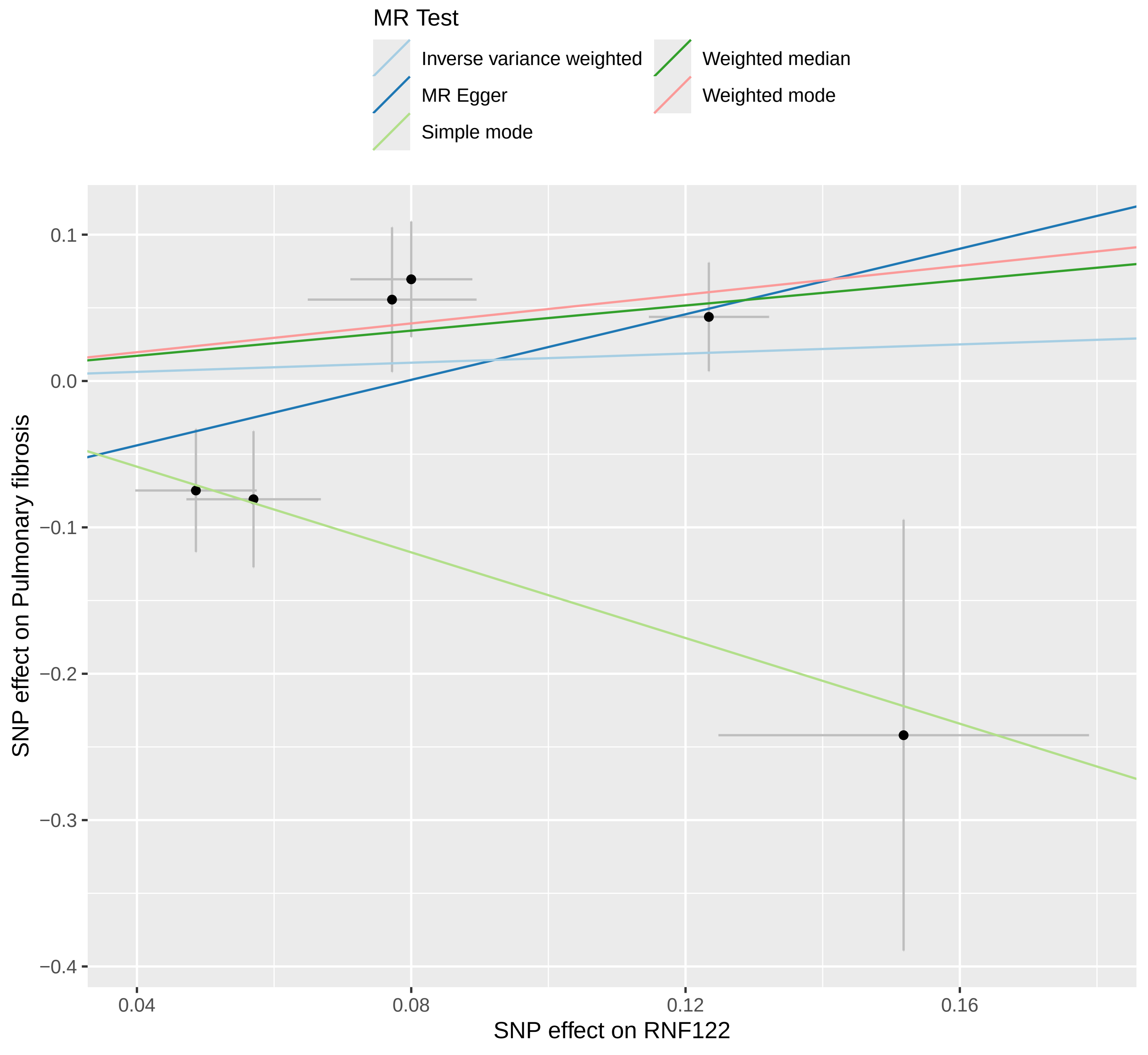

MR Test
Inverse variance weighted
MR Egger
Simple mode
Weighted median
Weighted mode
SNP effect on Pulmonary fibrosis
0.1
0.0
−0.1
−0.2
−0.3
−0.4
0.04
0.08
0.12
0.16
SNP effect on RNF122

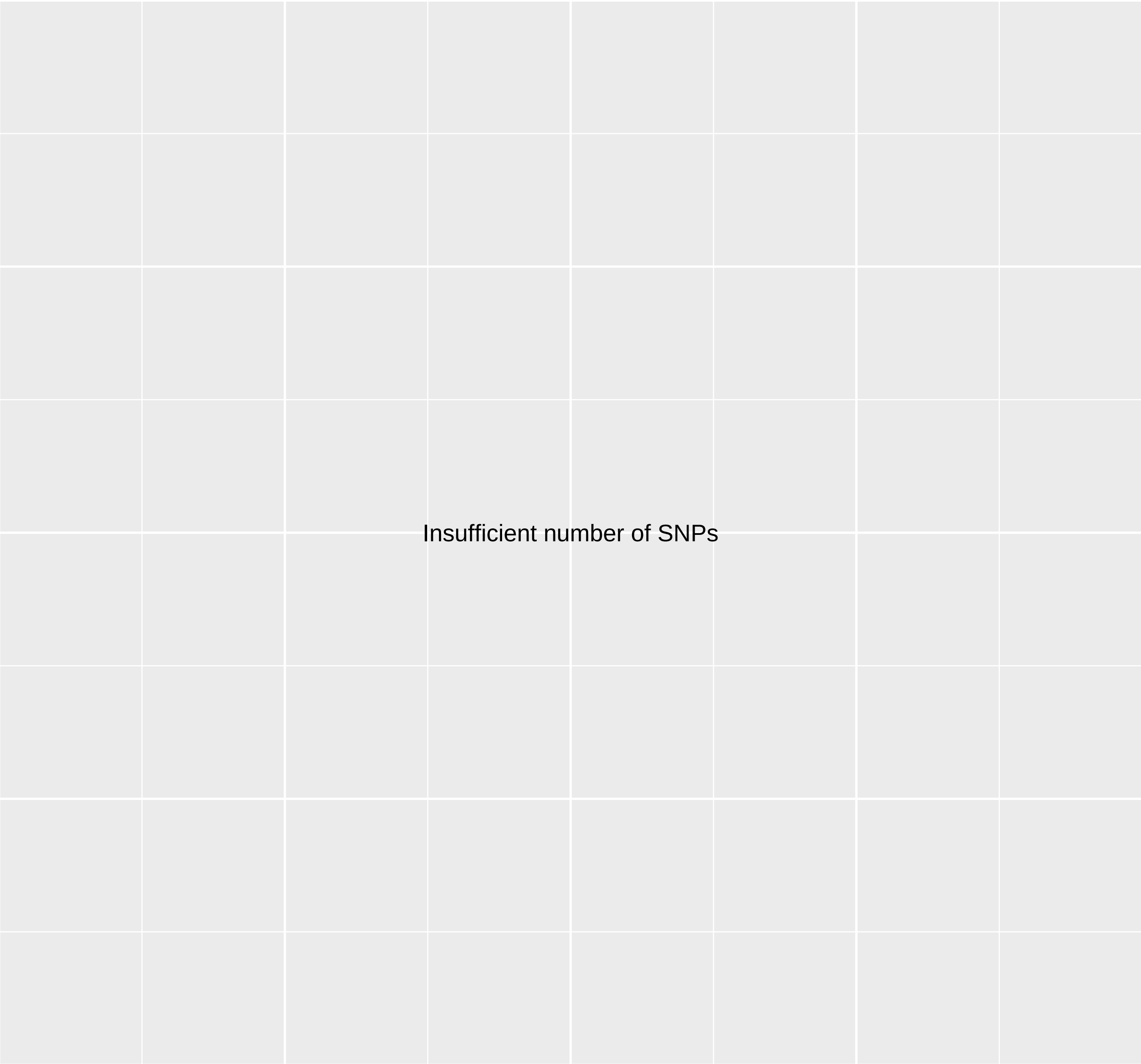
Insufficient number of SNPs

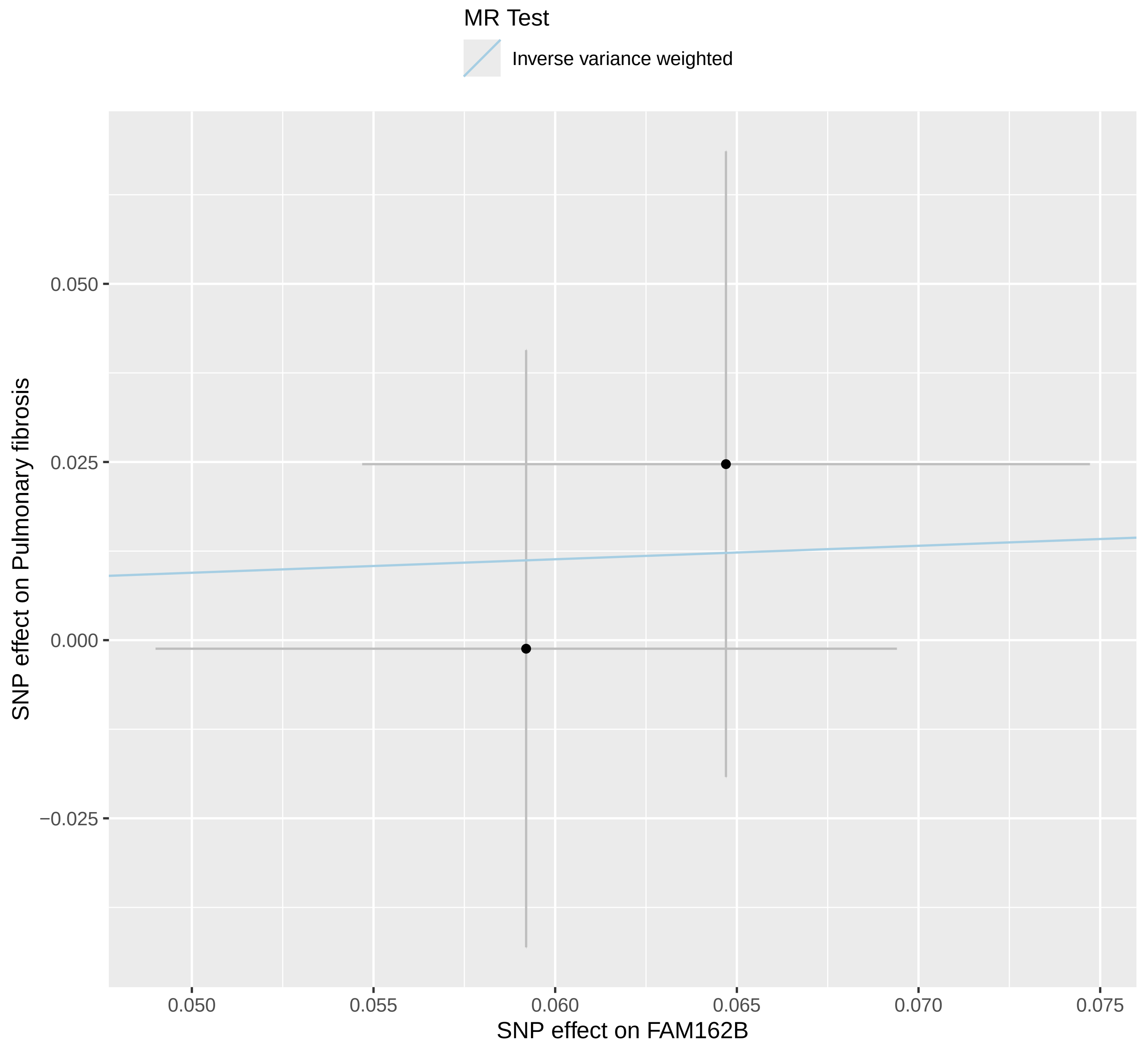
MR Test
Inverse variance weighted
0.050
0.025
0.000
−0.025
SNP effect on Pulmonary fibrosis
0.050
0.055
0.060
0.065
0.070
0.075
SNP effect on FAM162B

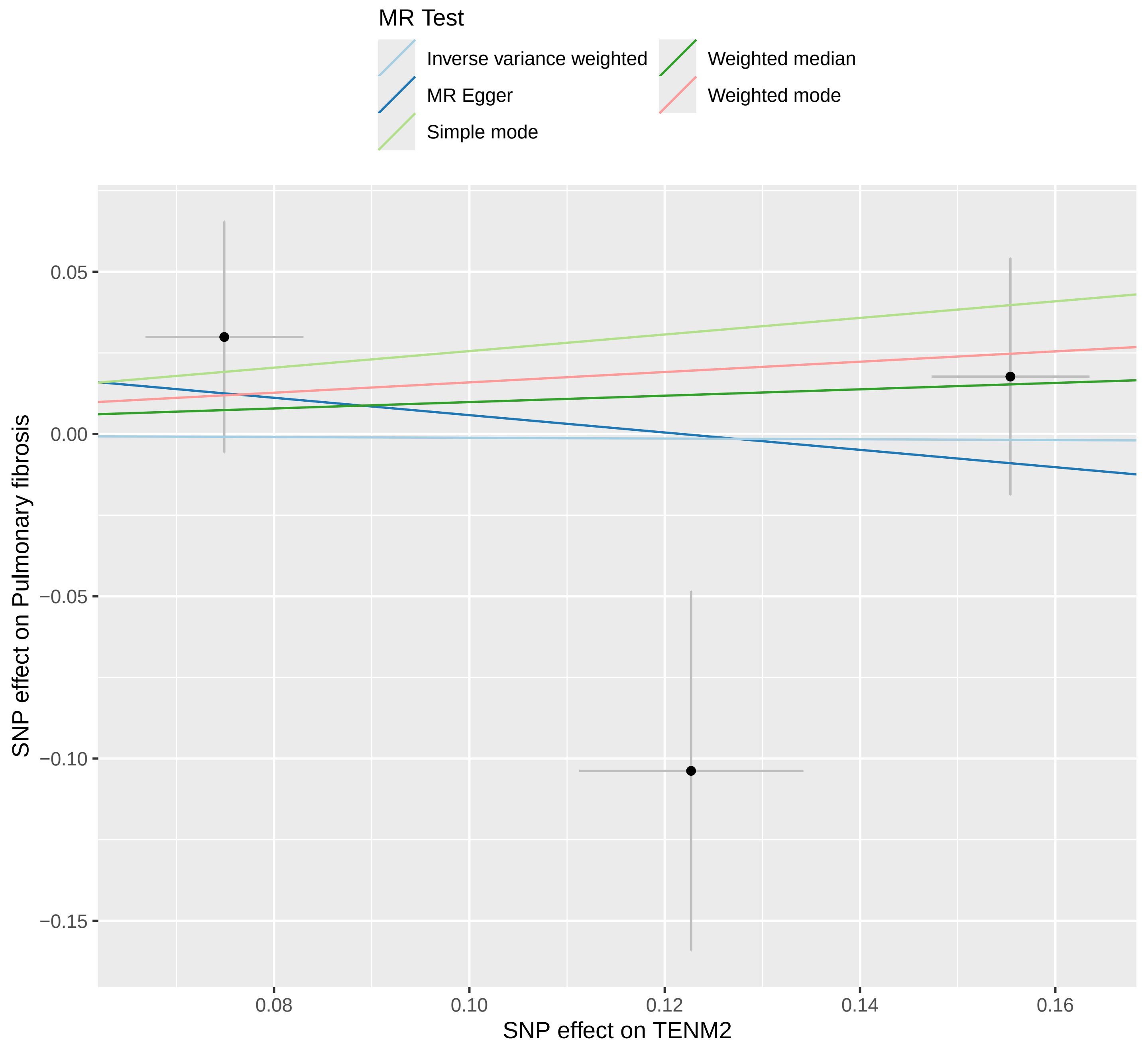

MR Test
Inverse variance weighted
MR Egger
Simple mode
Weighted median
Weighted mode
0.05
0.00
−0.05
−0.10
−0.15
0.08
0.10
0.12
0.14
0.16
SNP effect on Pulmonary fibrosis
SNP effect on TENM2

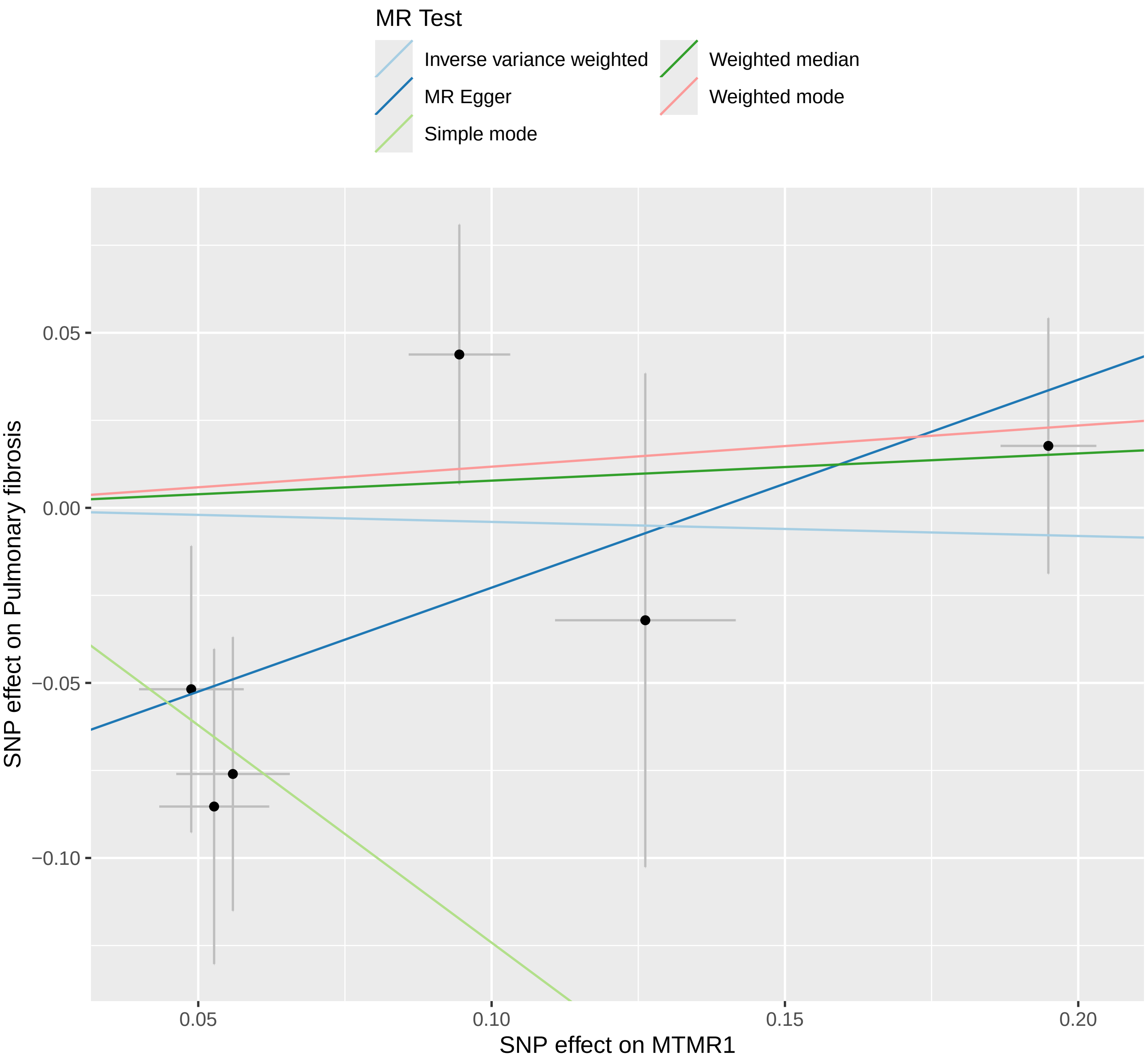

MR Test
Inverse variance weighted
MR Egger
Simple mode
Weighted median
Weighted mode
0.05
0.00
−0.05
−0.10
0.05
0.10
0.15
0.20
SNP effect on Pulmonary fibrosis
SNP effect on MTMR1

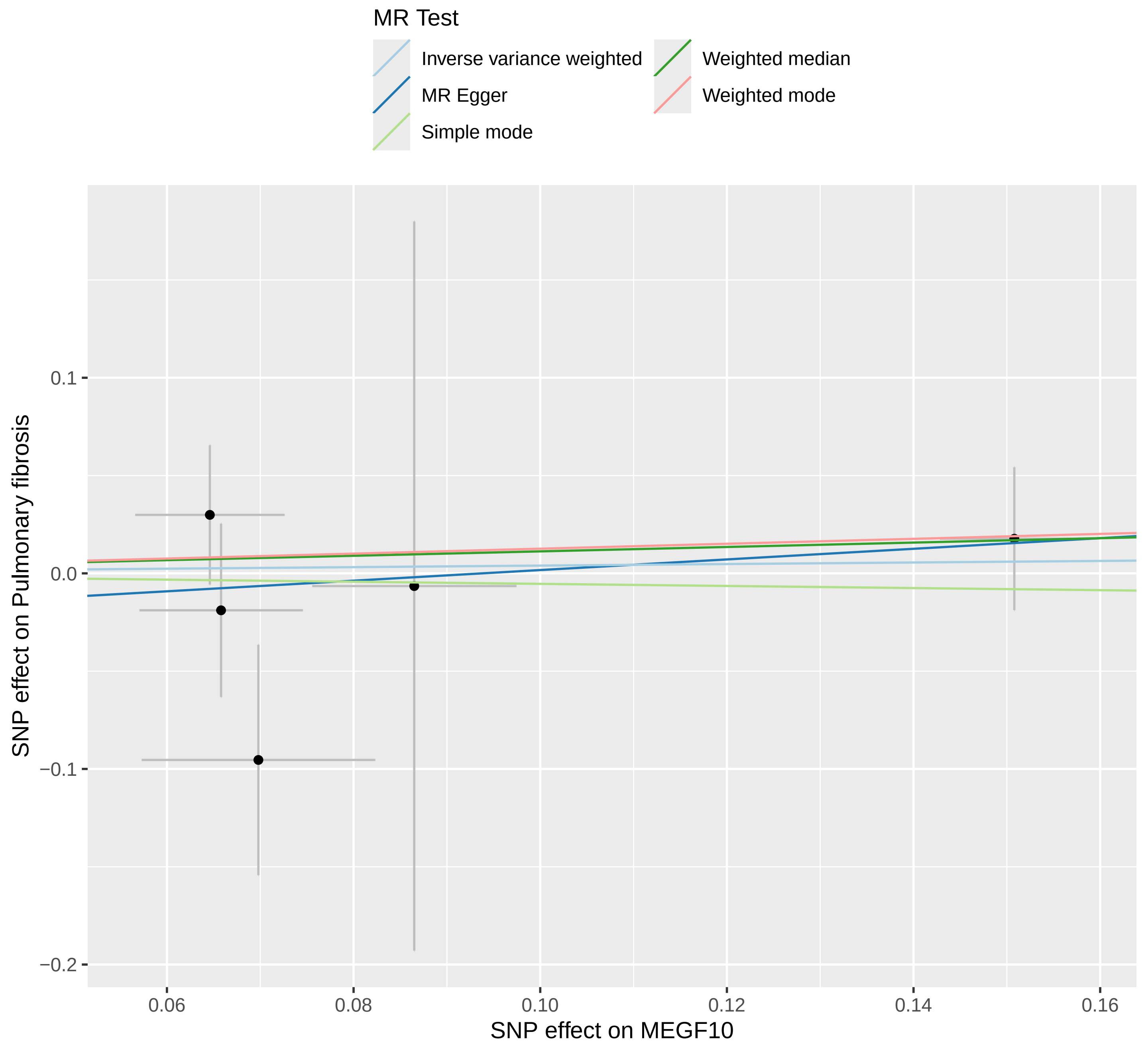
MR Test
Inverse variance weighted
MR Egger
Simple mode
Weighted median
Weighted mode
0.1
0.0
−0.1
−0.2
SNP effect on Pulmonary fibrosis
0.06
0.08
0.10
0.12
0.14
0.16
SNP effect on MEGF10

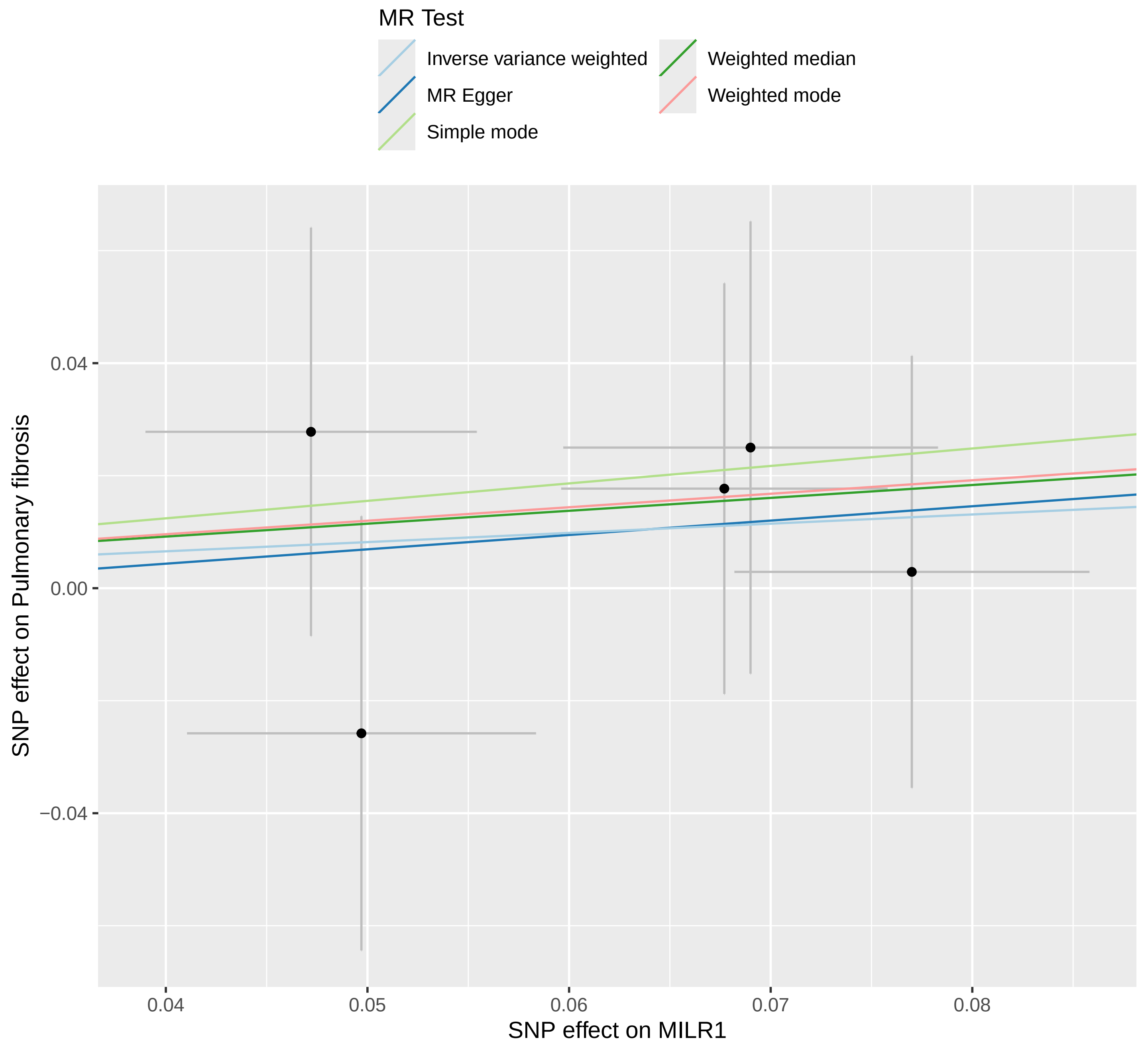
MR Test
Inverse variance weighted
MR Egger
Simple mode
Weighted median
Weighted mode
0.04
0.00
−0.04
SNP effect on Pulmonary fibrosis
0.04
0.05
0.06
0.07
0.08
SNP effect on MILR1

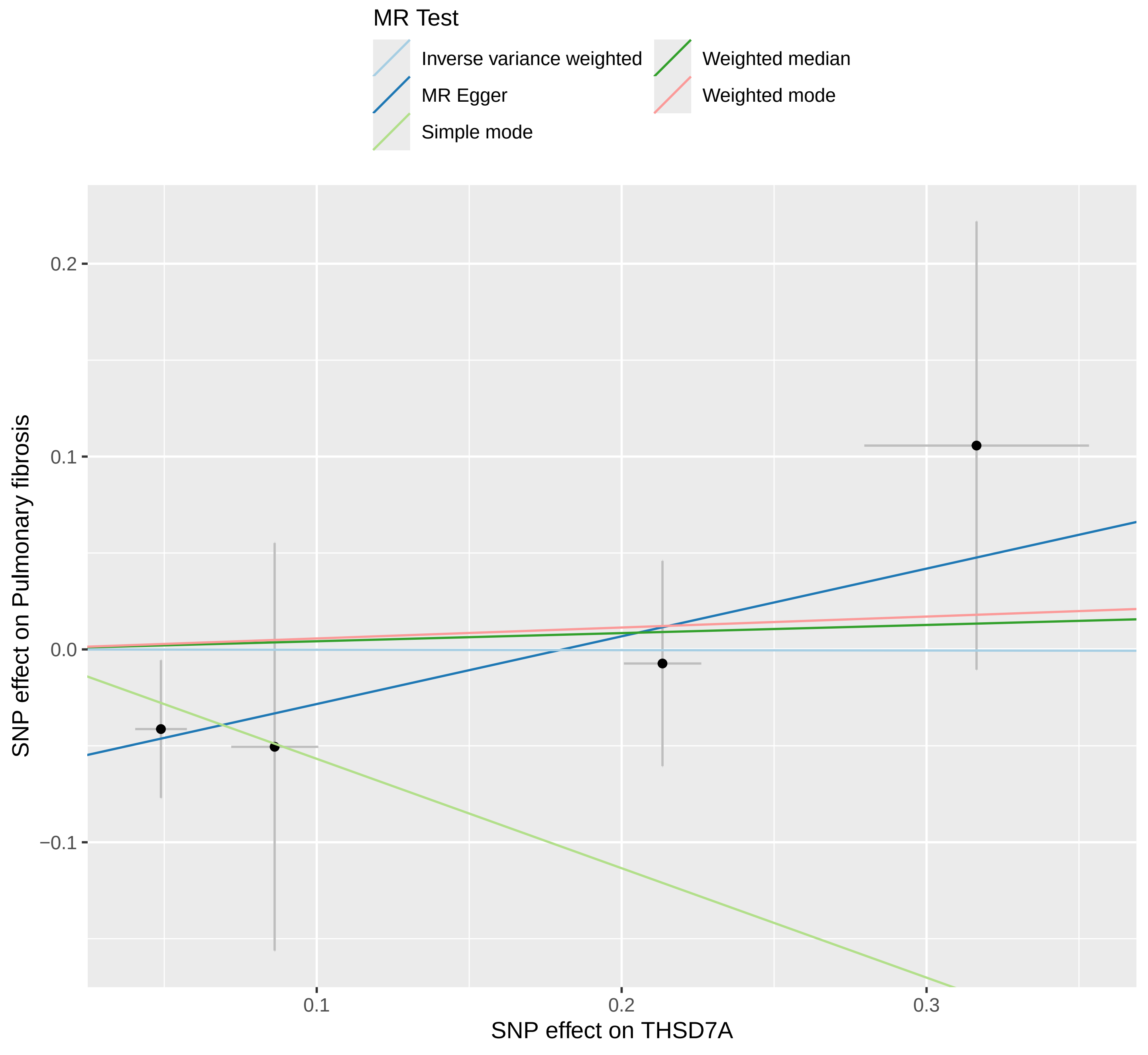

MR Test
Inverse variance weighted
MR Egger
Simple mode
Weighted median
Weighted mode
SNP effect on Pulmonary fibrosis
0.2
0.1
0.0
−0.1
0.1
0.2
0.3
SNP effect on THSD7A

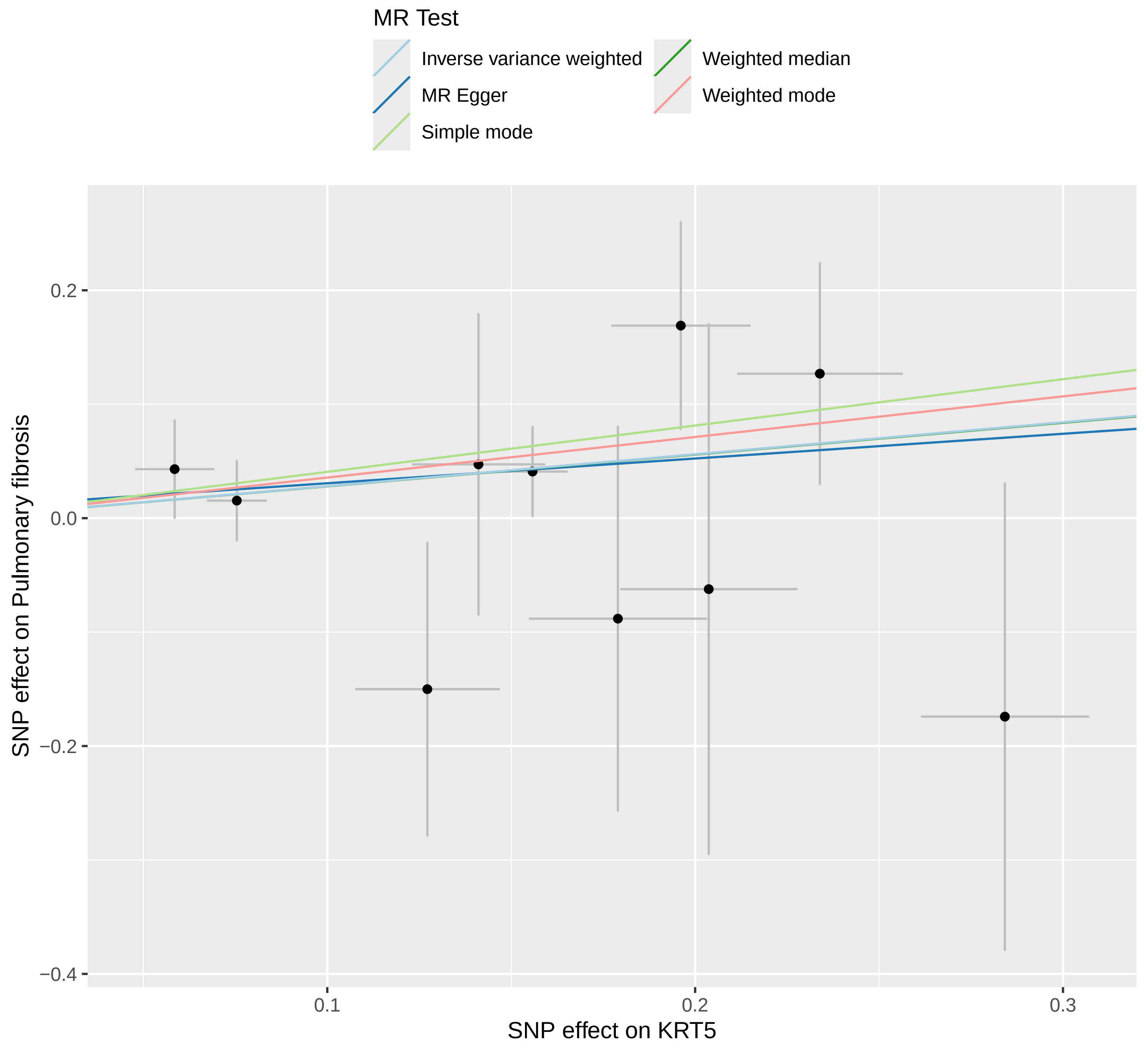
MR Test
Inverse variance weighted
MR Egger
Simple mode
Weighted median
Weighted mode
0.2
0.0
−0.2
−0.4
SNP effect on Pulmonary fibrosis
0.1
0.2
0.3
SNP effect on KRT5

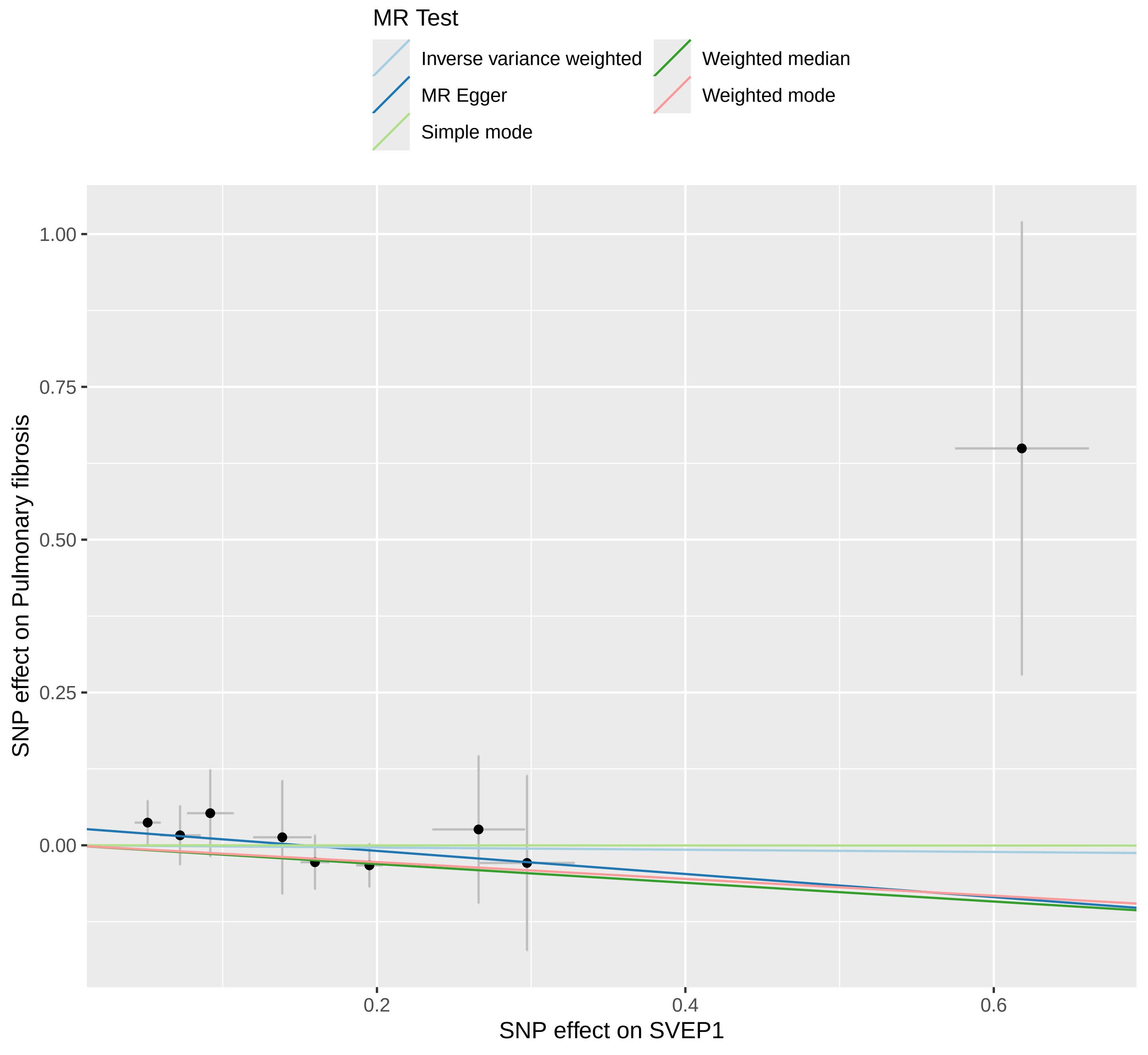
MR Test
Inverse variance weighted
MR Egger
Simple mode
Weighted median
Weighted mode
1.00
0.75
0.50
0.25
0.00
0.2
0.4
0.6
SNP effect on Pulmonary fibrosis
SNP effect on SVEP1

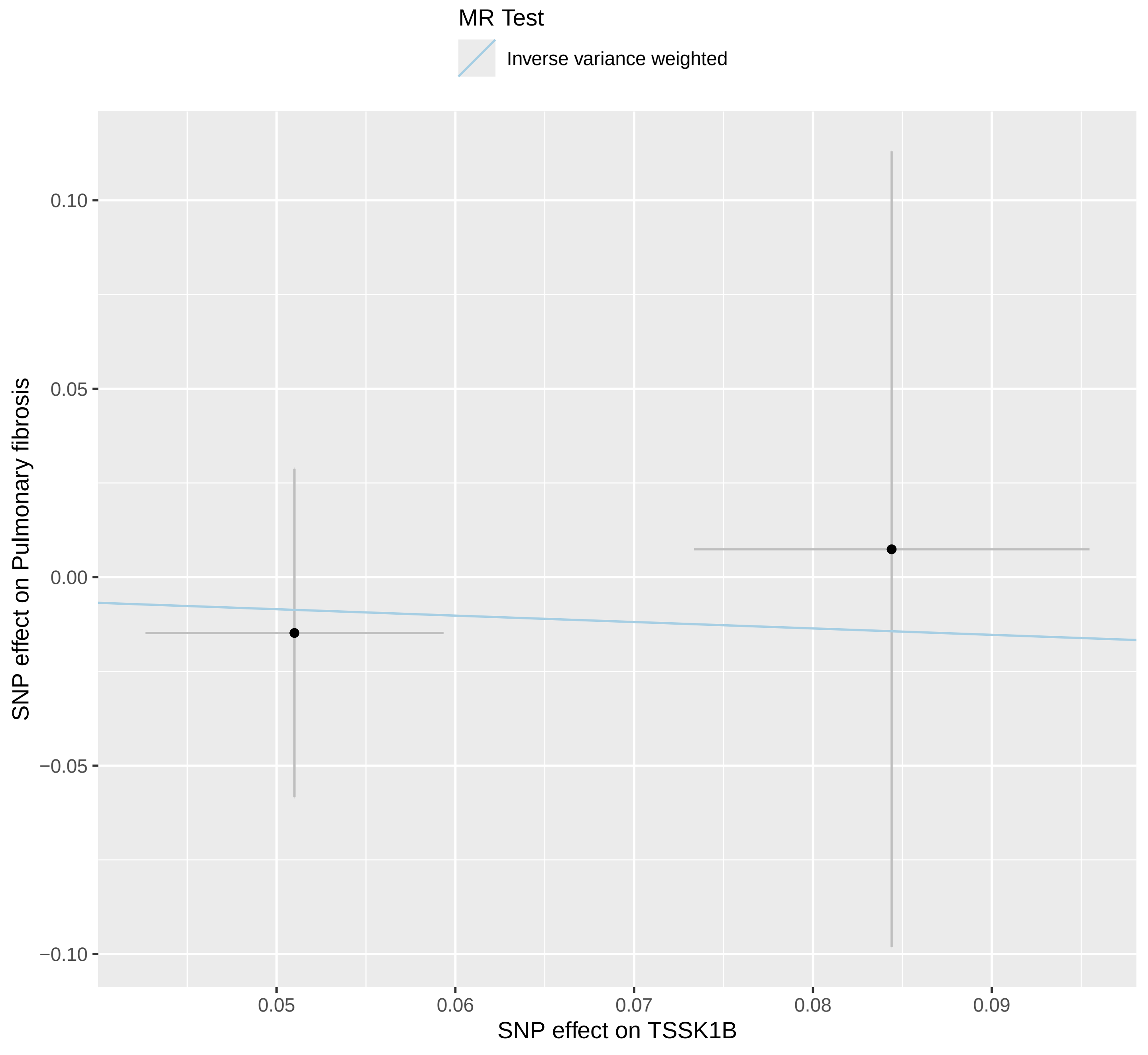

MR Test
Inverse variance weighted
0.10
0.05
0.00
−0.05
−0.10
SNP effect on Pulmonary fibrosis
0.05
0.06
0.07
0.08
0.09
SNP effect on TSSK1B

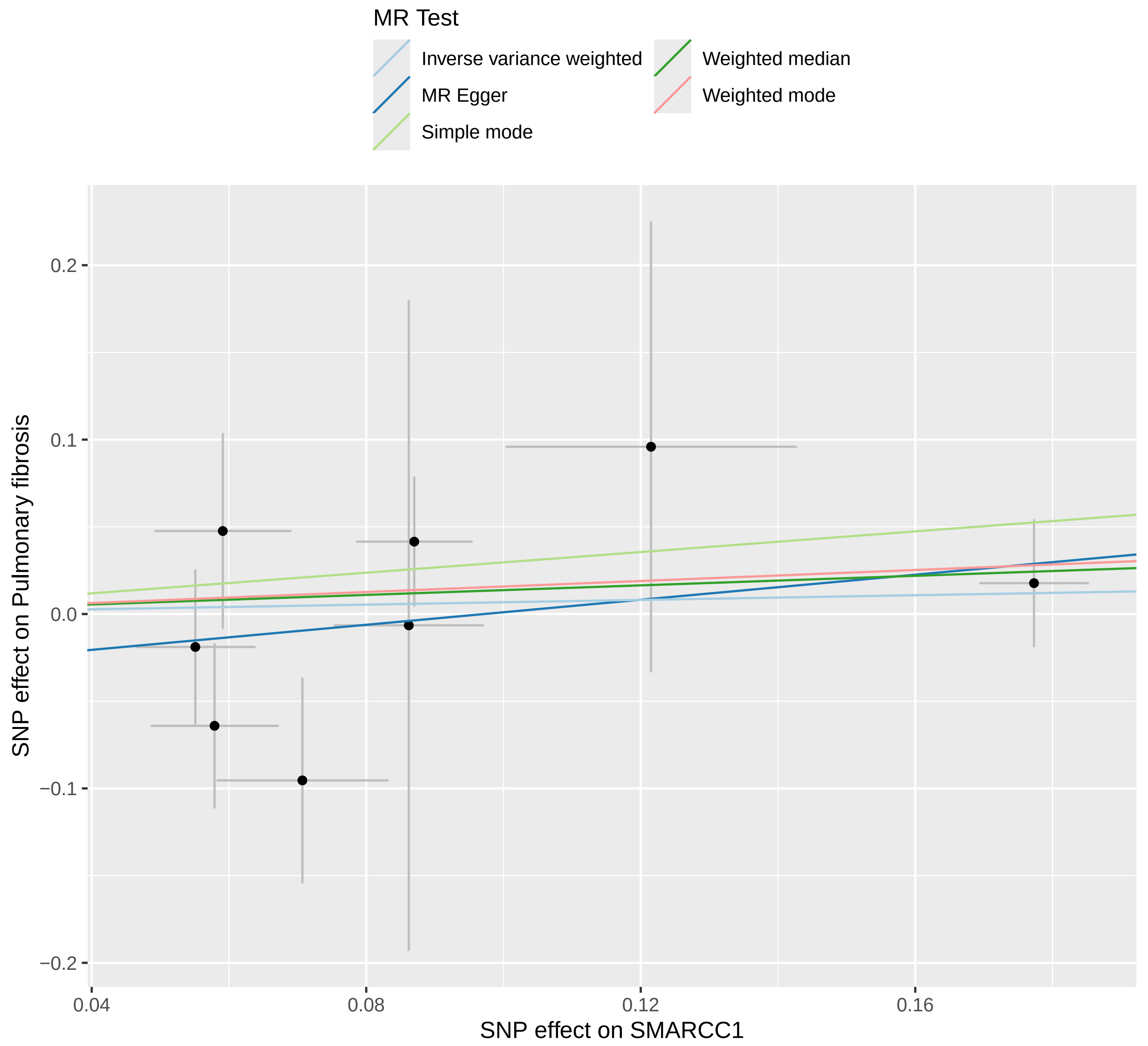
MR Test
Inverse variance weighted
MR Egger
Simple mode
Weighted median
Weighted mode
SNP effect on Pulmonary fibrosis
0.2
0.1
0.0
−0.1
−0.2
0.04
0.08
0.12
0.16
SNP effect on SMARCC1

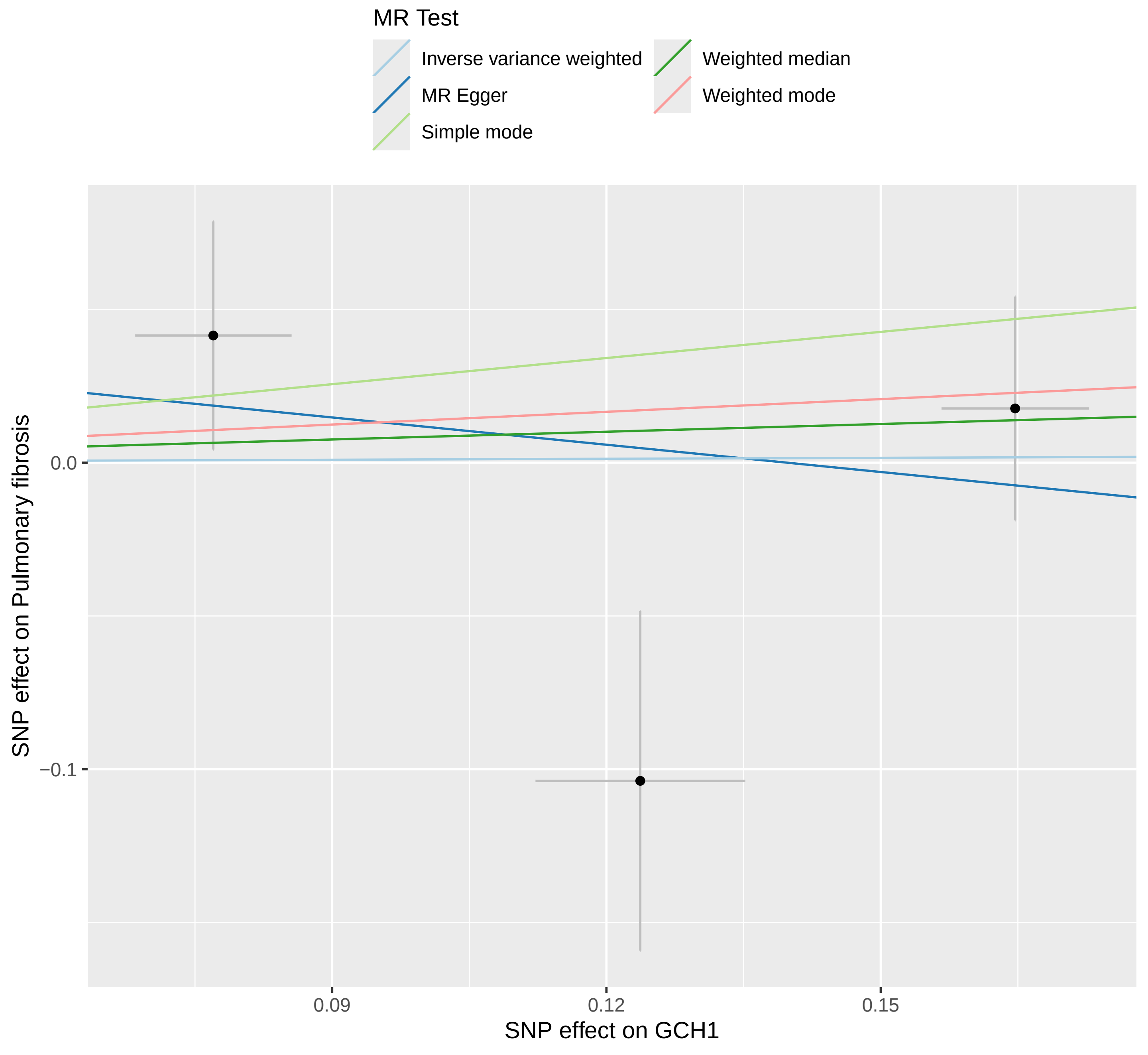

MR Test
Inverse variance weighted
MR Egger
Simple mode
Weighted median
Weighted mode
0.0
−0.1
0.09
0.12
0.15
SNP effect on GCH1
SNP effect on Pulmonary fibrosis

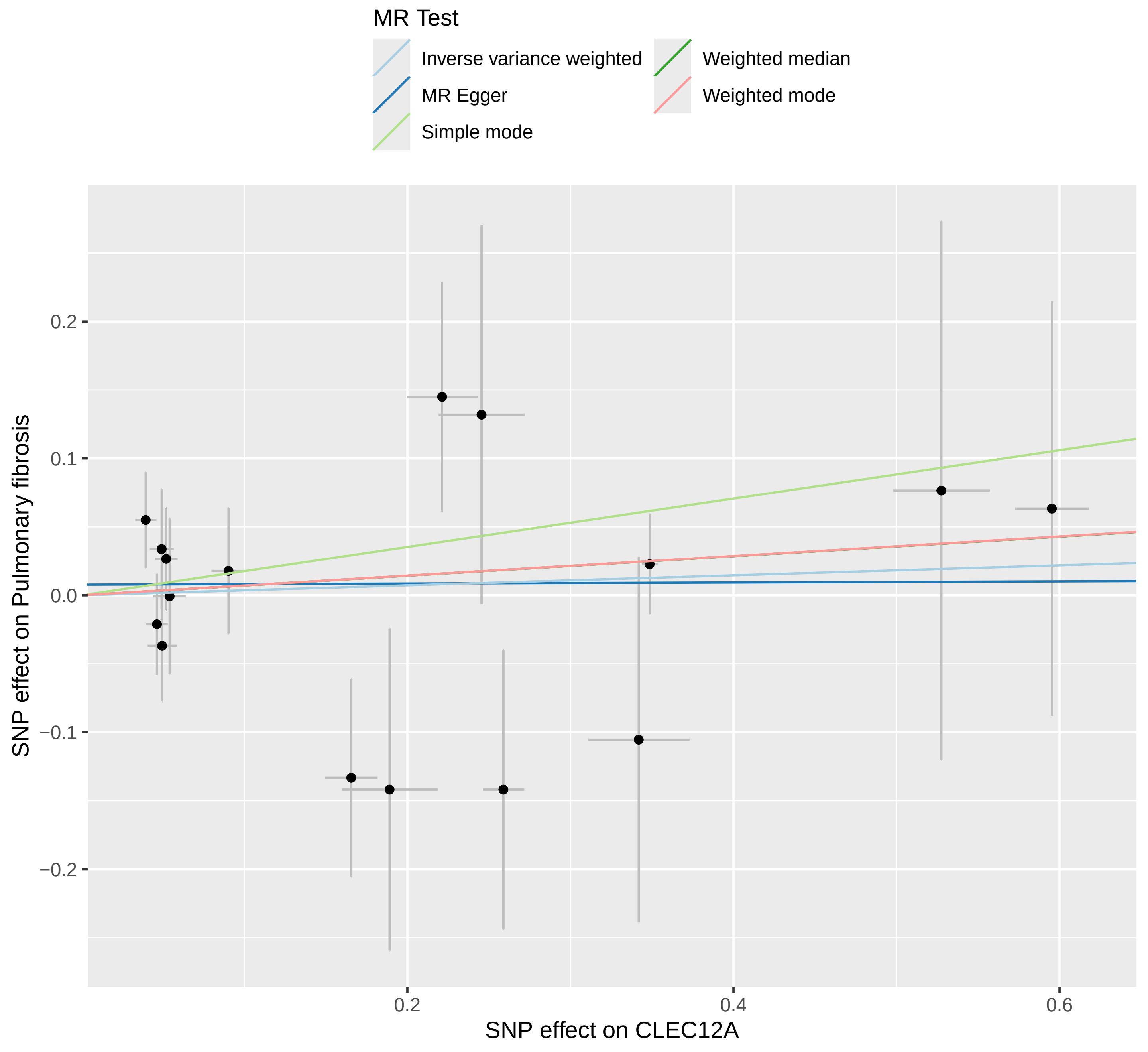
MR Test
Inverse variance weighted
MR Egger
Simple mode
Weighted median
Weighted mode
SNP effect on Pulmonary fibrosis
0.2
0.1
0.0
−0.1
−0.2
0.2
0.4
0.6
SNP effect on CLEC12A

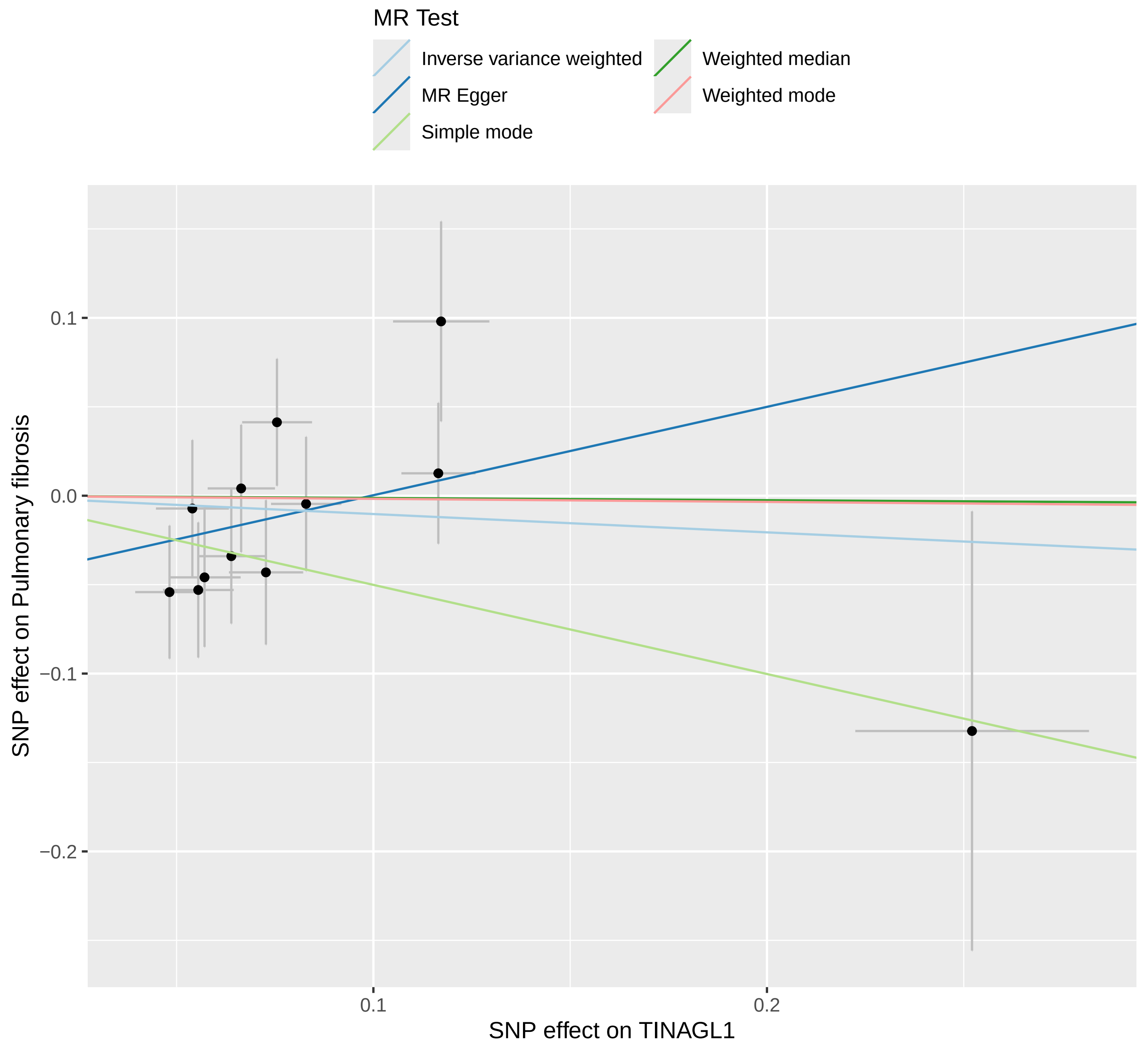
MR Test
Inverse variance weighted
MR Egger
Simple mode
Weighted median
Weighted mode
0.1
0.0
−0.1
−0.2
0.1
0.2
SNP effect on Pulmonary fibrosis
SNP effect on TINAGL1

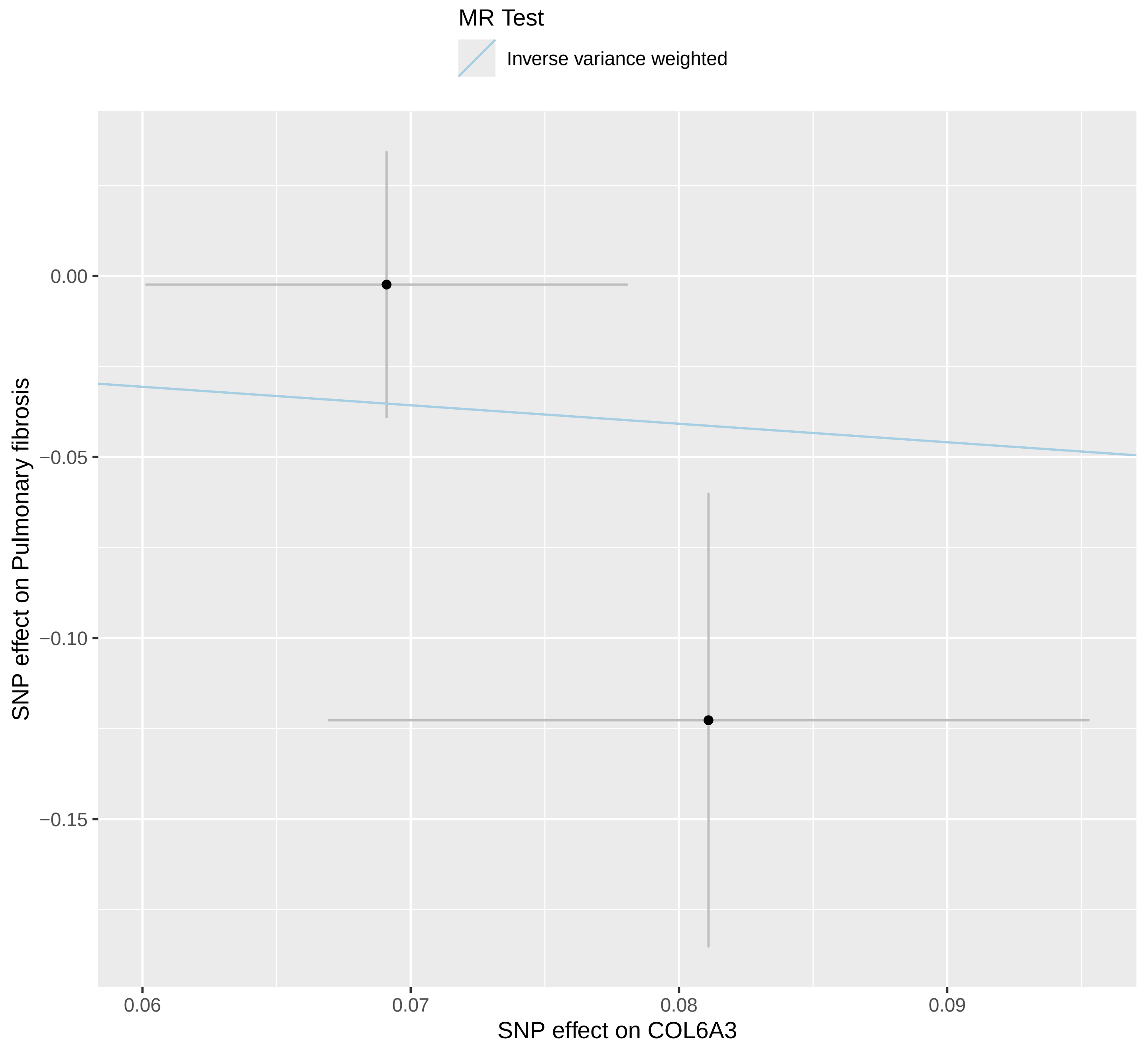
MR Test
Inverse variance weighted
0.00
−0.05
−0.10
−0.15
0.06
0.07
0.08
0.09
SNP effect on COL6A3
SNP effect on Pulmonary fibrosis

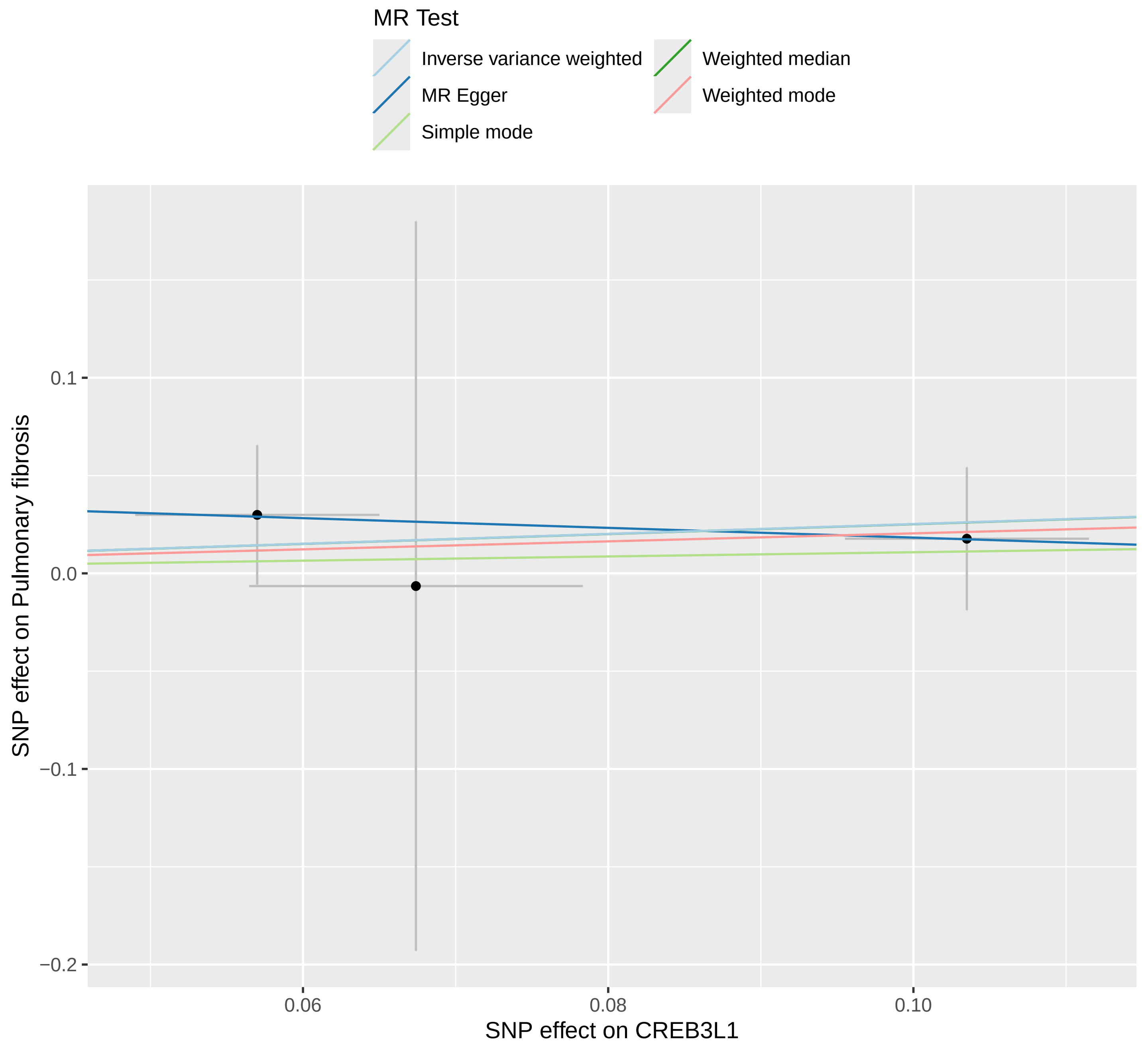
MR Test
Inverse variance weighted
MR Egger
Simple mode
Weighted median
Weighted mode
SNP effect on Pulmonary fibrosis
0.1
0.0
−0.1
−0.2
0.06
0.08
0.10
SNP effect on CREB3L1

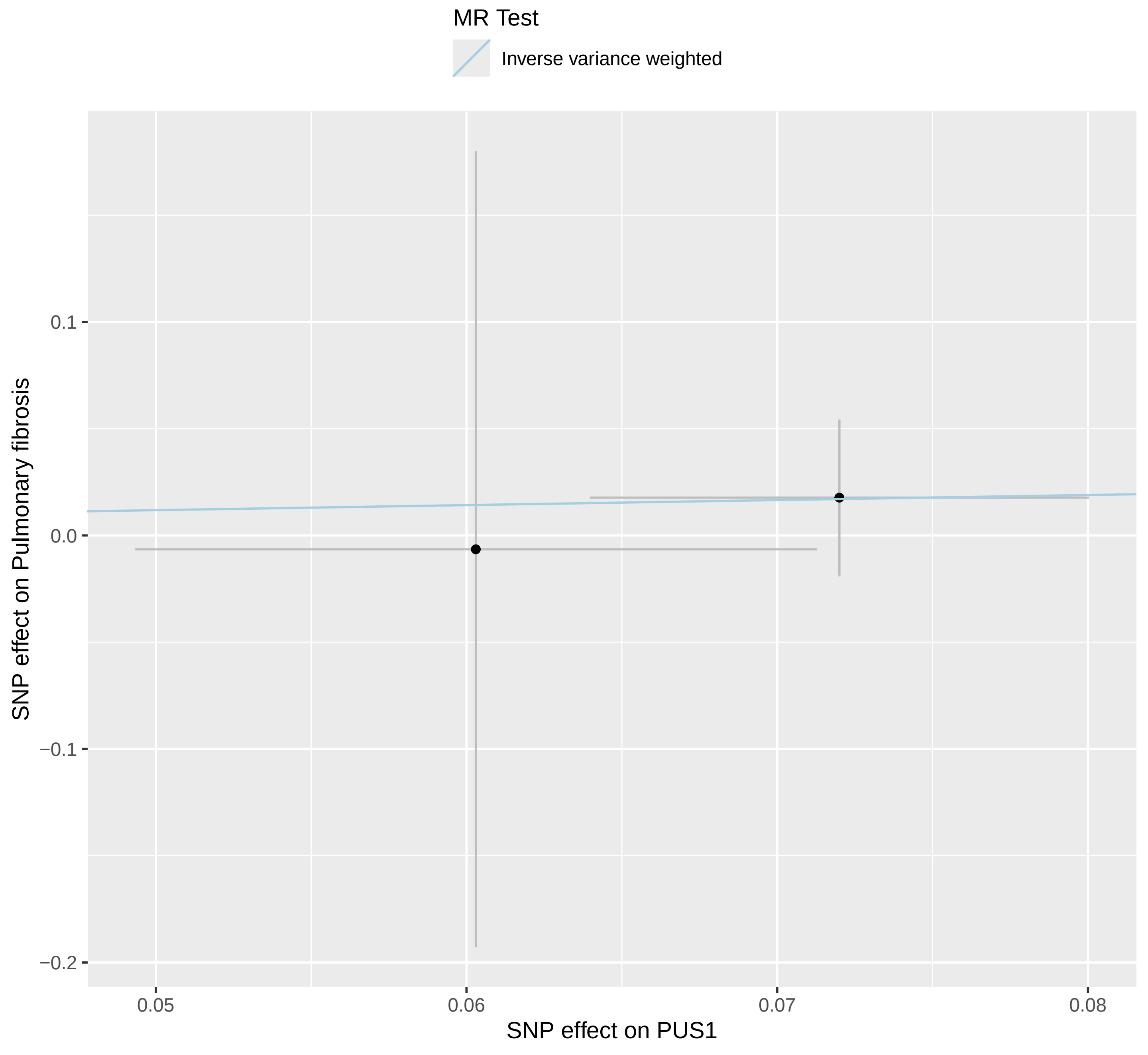

MR Test
Inverse variance weighted
SNP effect on Pulmonary fibrosis
0.1
0.0
−0.1
−0.2
0.05
0.06
0.07
0.08
SNP effect on PUS1

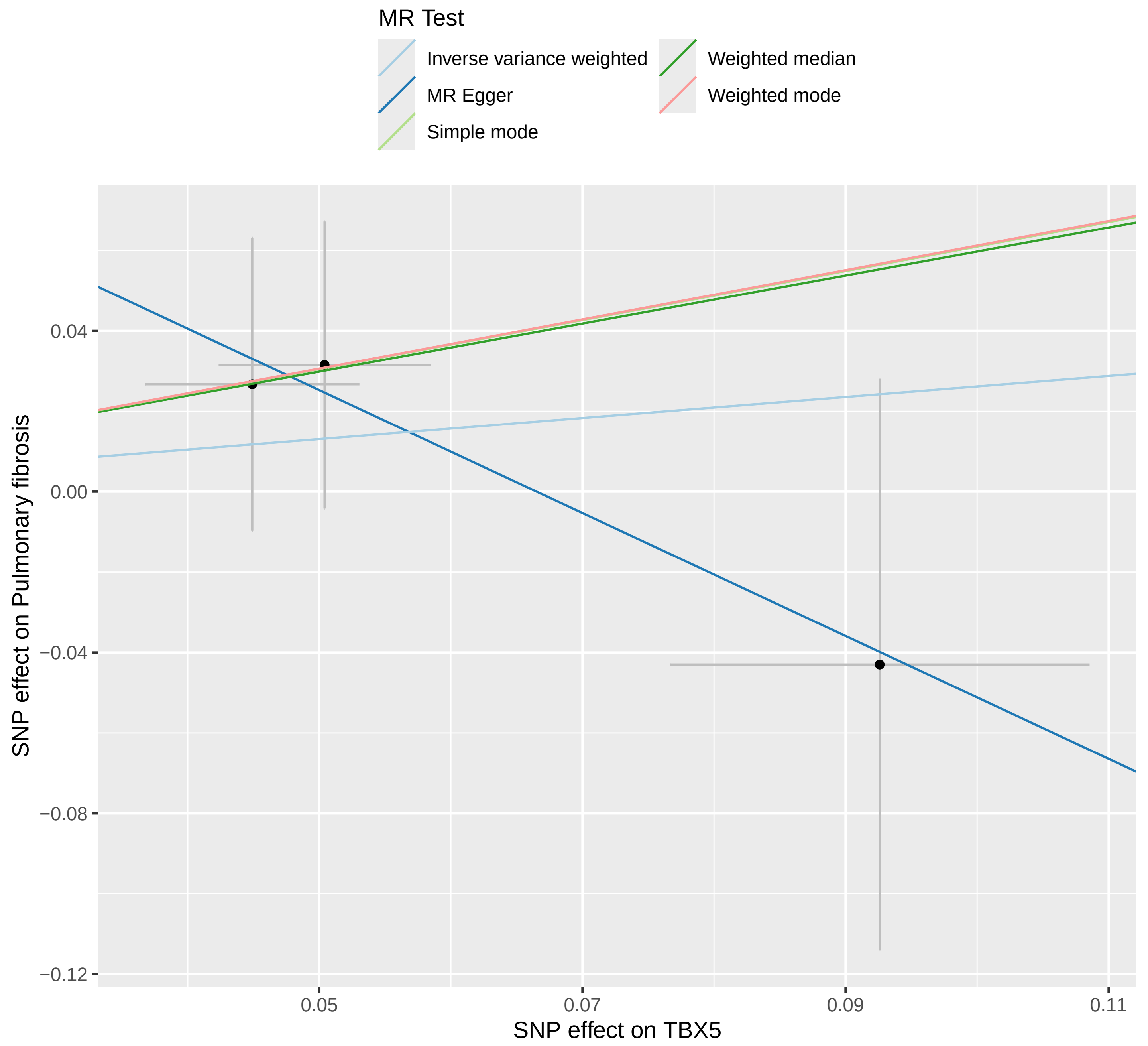
MR Test
Inverse variance weighted
MR Egger
Simple mode
Weighted median
Weighted mode
SNP effect on Pulmonary fibrosis
0.04
0.00
−0.04
−0.08
−0.12
0.05
0.07
0.09
0.11
SNP effect on TBX5

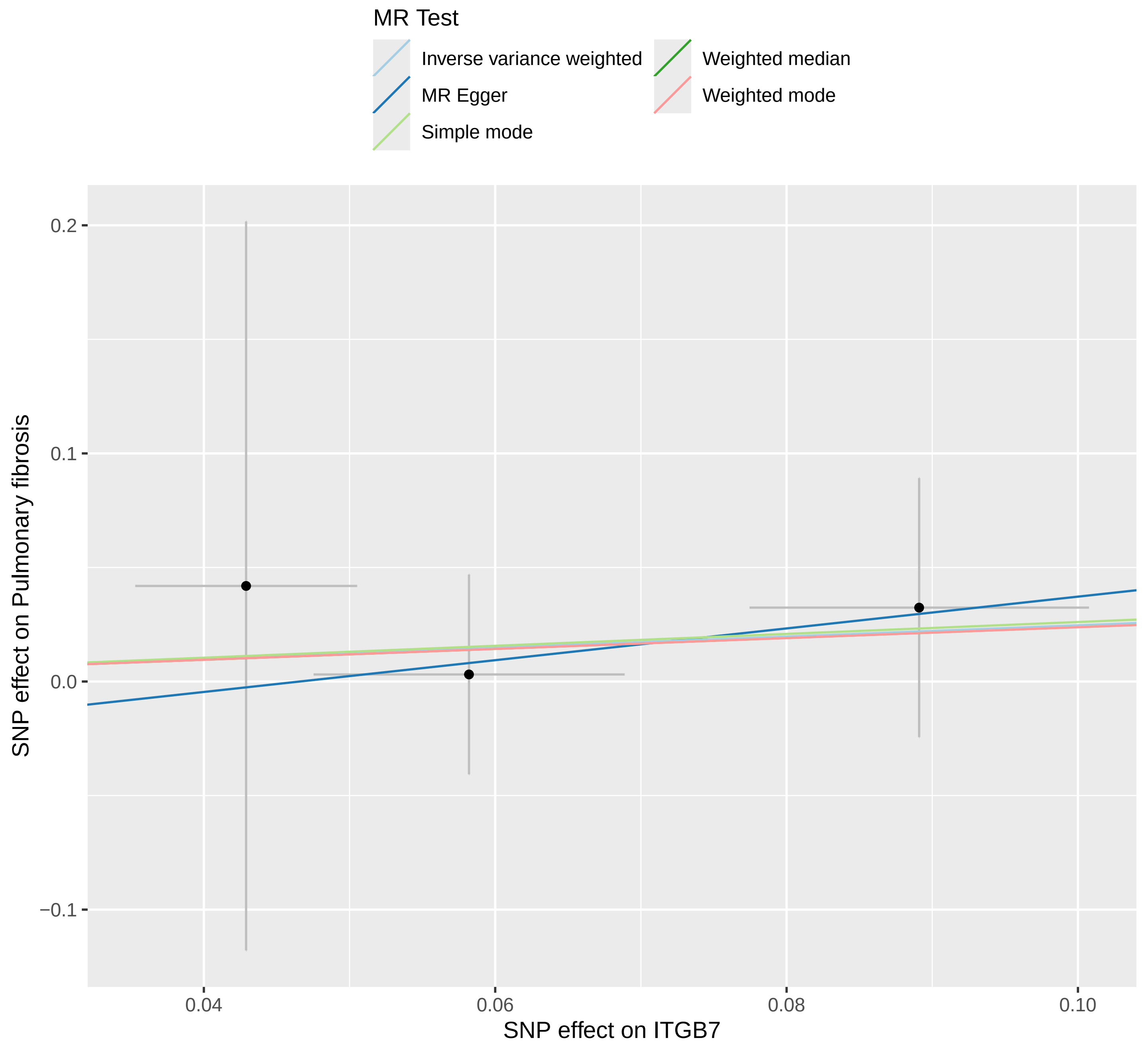
MR Test
Inverse variance weighted
MR Egger
Simple mode
Weighted median
Weighted mode
0.2
0.1
0.0
−0.1
0.04
0.06
0.08
0.10
SNP effect on Pulmonary fibrosis
SNP effect on ITGB7

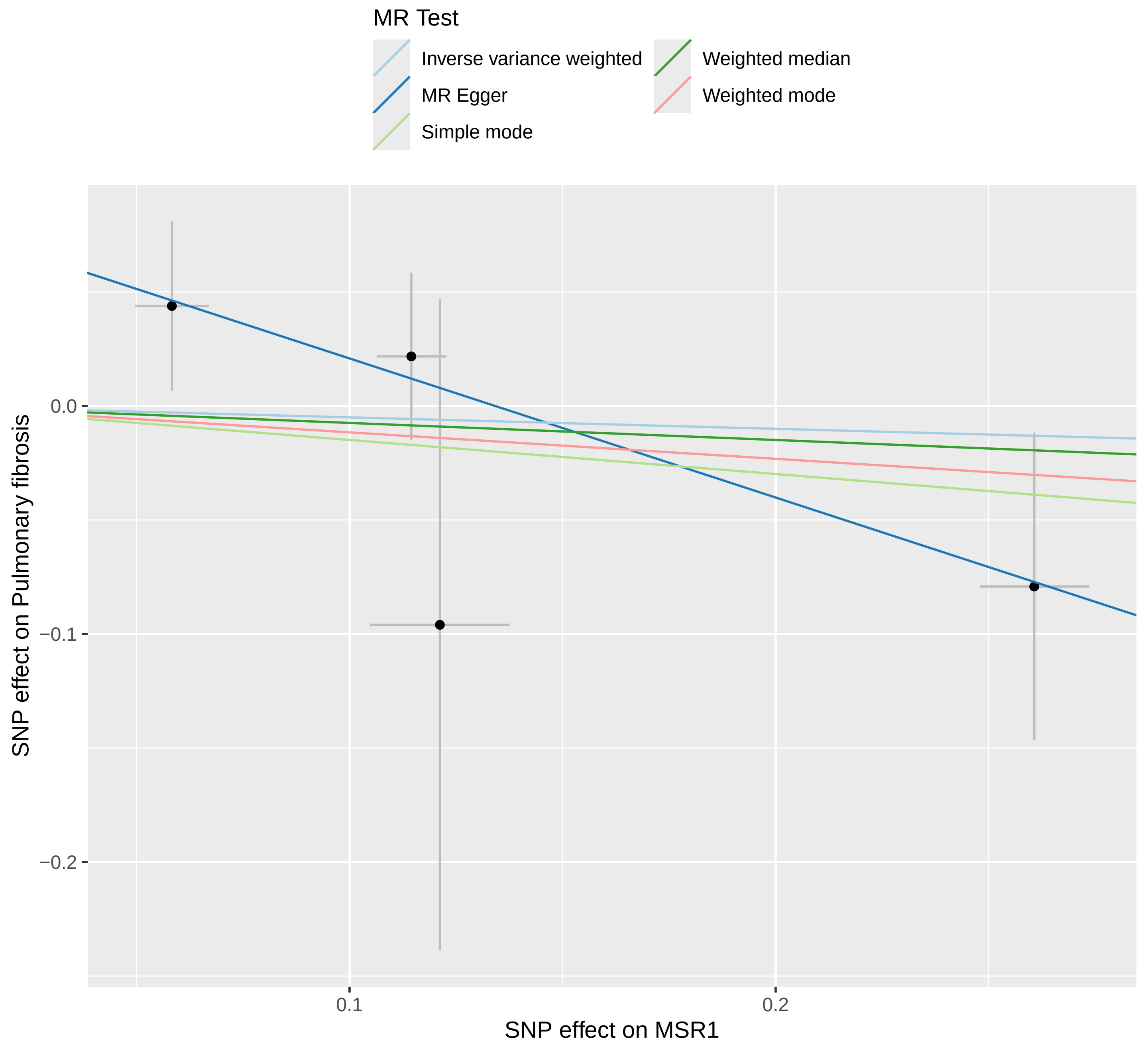
MR Test
Inverse variance weighted
MR Egger
Simple mode
Weighted median
Weighted mode
SNP effect on Pulmonary fibrosis
0.0
−0.1
−0.2
0.1
0.2
SNP effect on MSR1

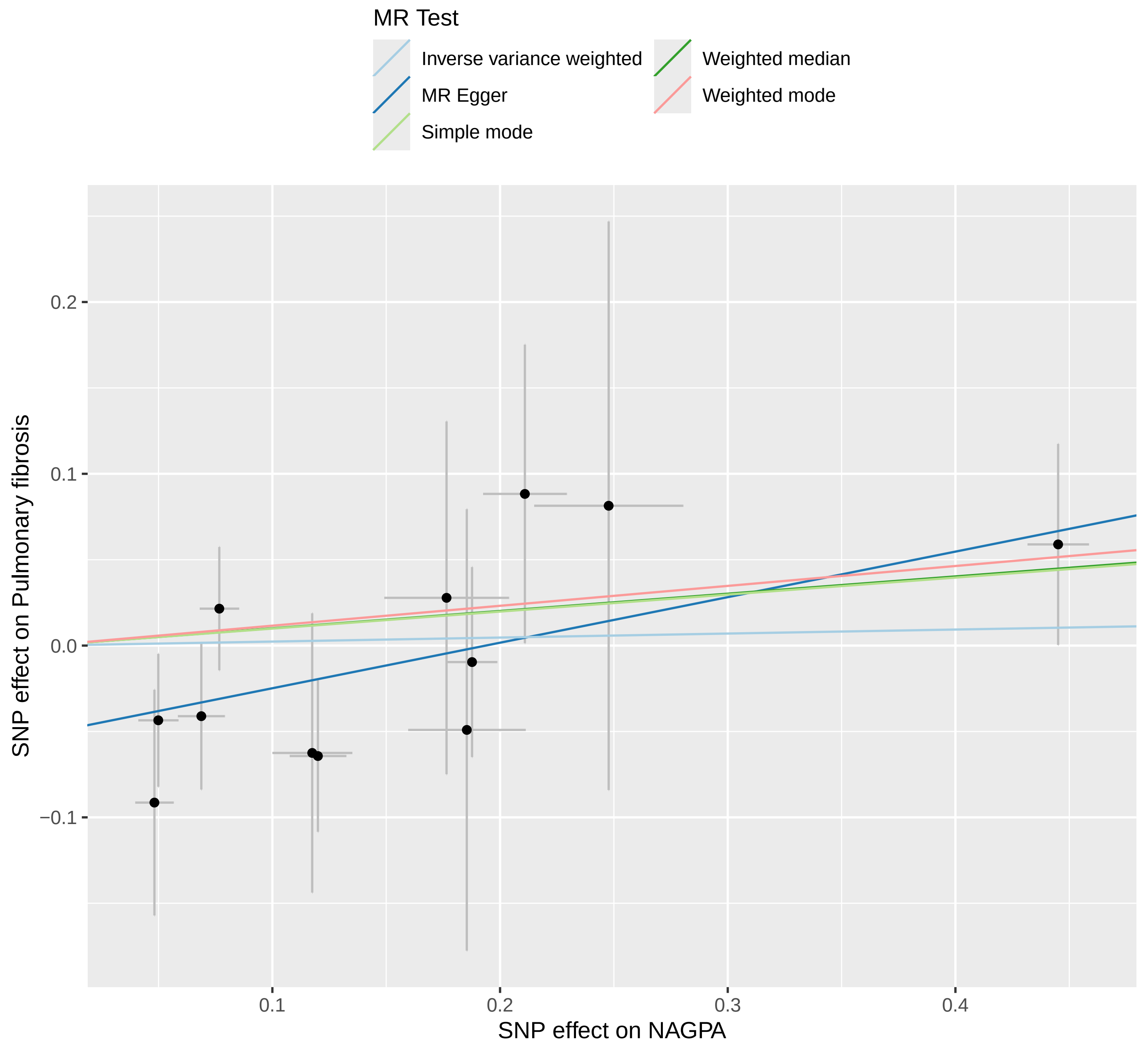
MR Test
Inverse variance weighted
MR Egger
Simple mode
Weighted median
Weighted mode
SNP effect on Pulmonary fibrosis
0.2
0.1
0.0
−0.1
0.1
0.2
0.3
0.4
SNP effect on NAGPA

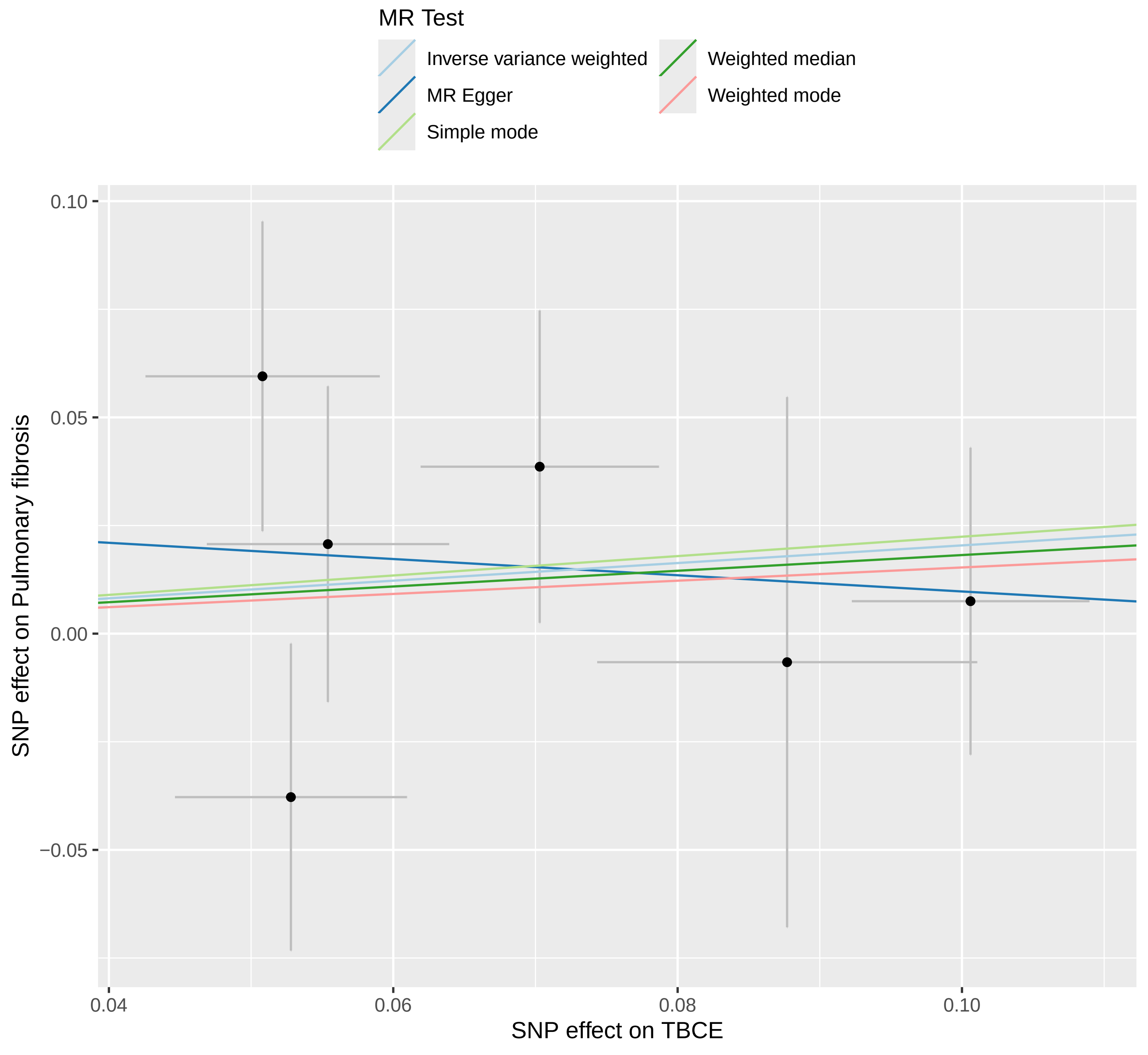
MR Test
Inverse variance weighted
MR Egger
Simple mode
Weighted median
Weighted mode
0.10
0.05
0.00
−0.05
0.04
0.06
0.08
0.10
SNP effect on Pulmonary fibrosis
SNP effect on TBCE

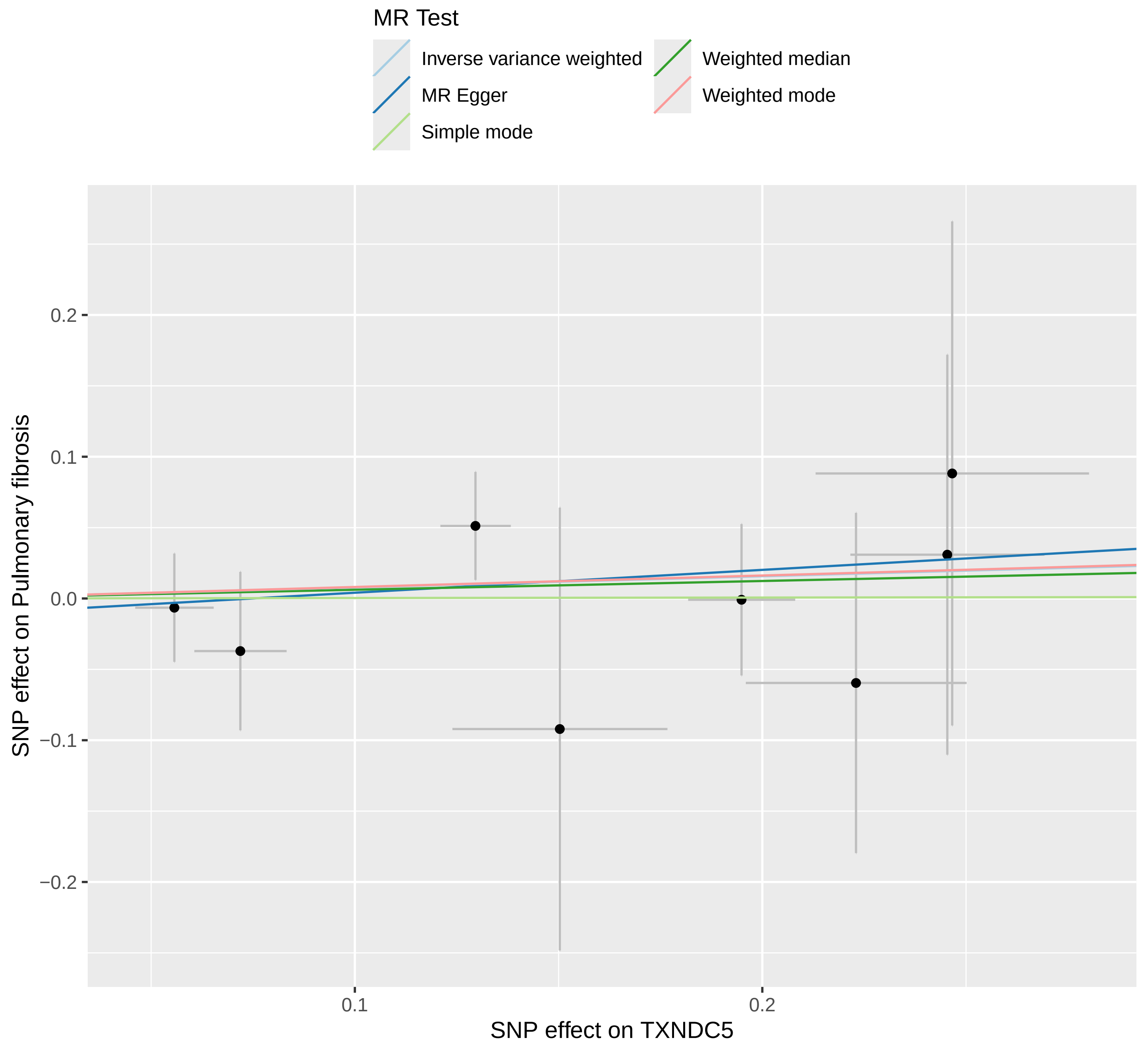
MR Test
Inverse variance weighted
MR Egger
Simple mode
Weighted median
Weighted mode
SNP effect on Pulmonary fibrosis
0.2
0.1
0.0
−0.1
−0.2
0.1
0.2
SNP effect on TXNDC5

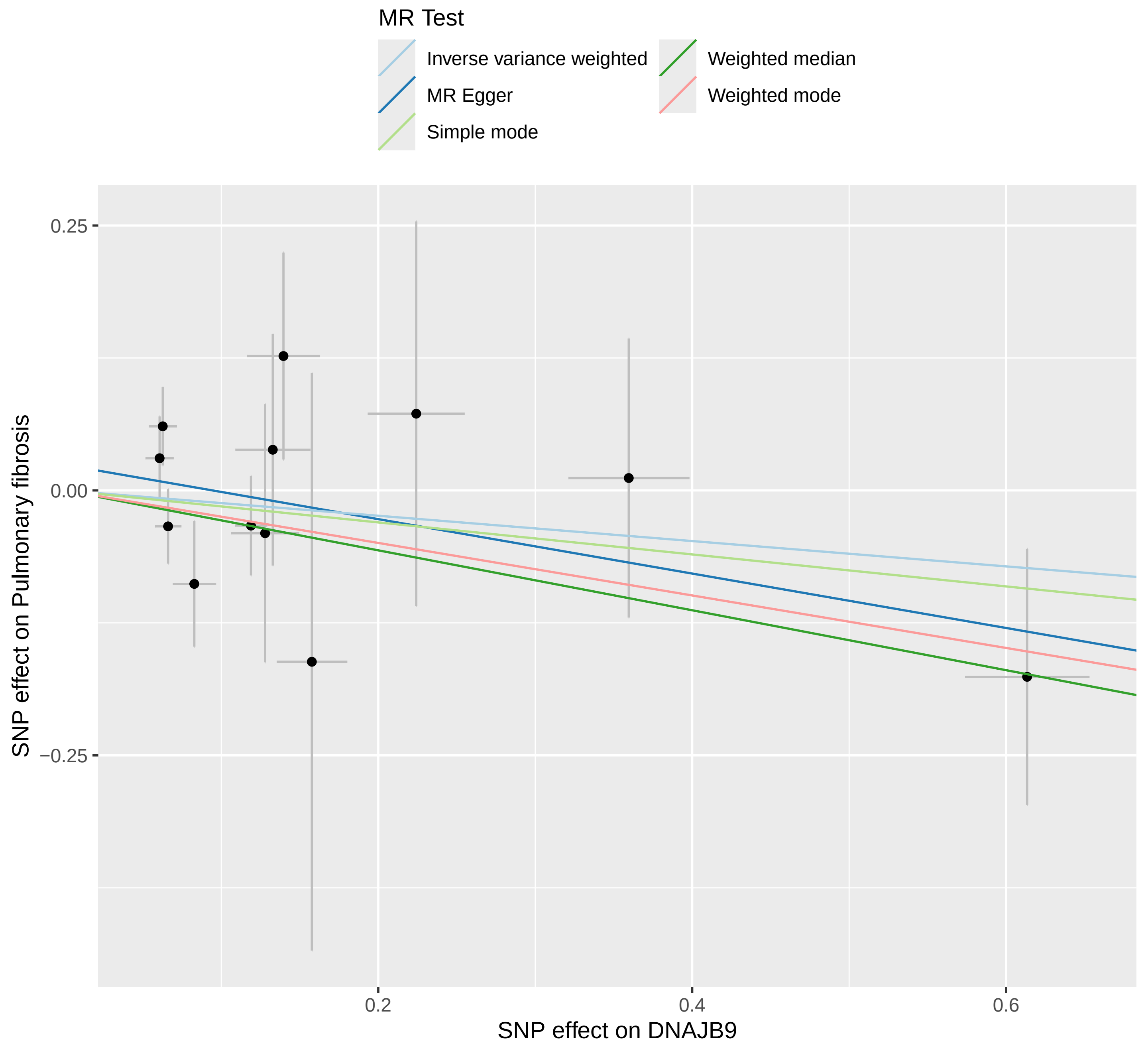
MR Test
Inverse variance weighted
MR Egger
Simple mode
Weighted median
Weighted mode
0.25
0.00
−0.25
0.2
0.4
0.6
SNP effect on Pulmonary fibrosis
SNP effect on DNAJB9

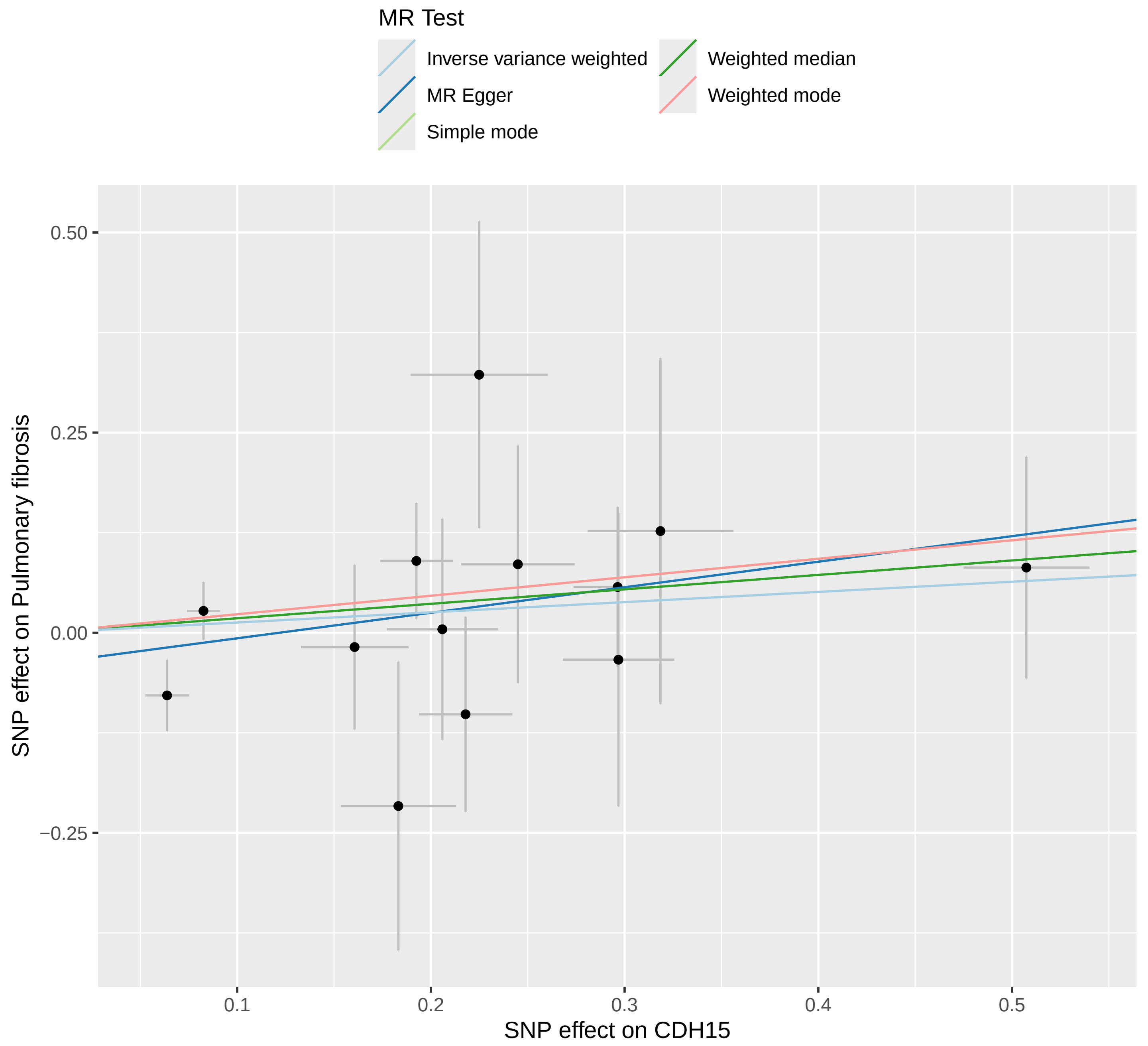

MR Test
Inverse variance weighted
MR Egger
Simple mode
Weighted median
Weighted mode
0.50
0.25
0.00
−0.25
SNP effect on Pulmonary fibrosis
0.1
0.2
0.3
0.4
0.5
SNP effect on CDH15

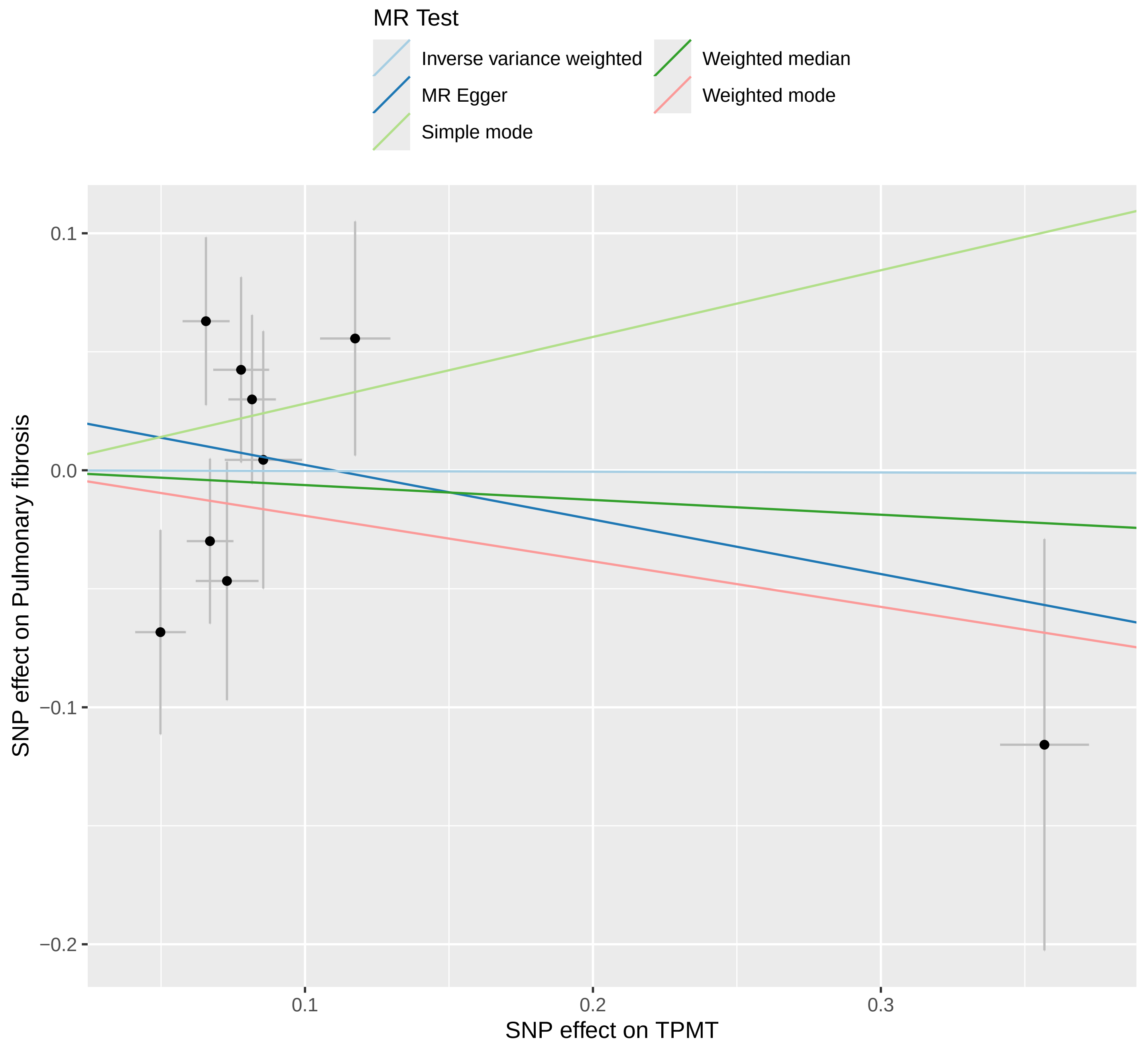
MR Test
Inverse variance weighted
MR Egger
Simple mode
Weighted median
Weighted mode
0.1
0.0
−0.1
−0.2
SNP effect on Pulmonary fibrosis
0.1
0.2
0.3
SNP effect on TPMT

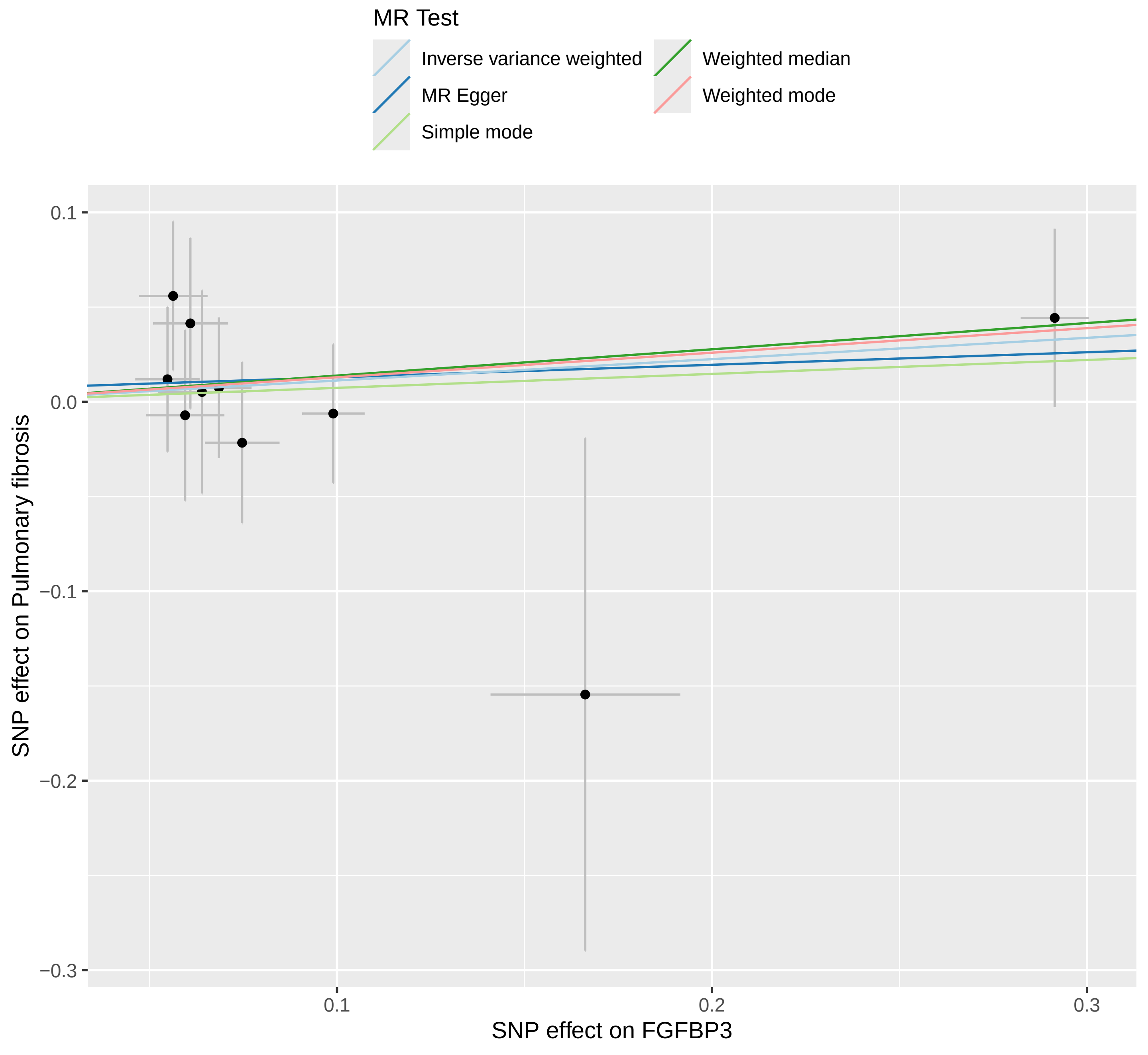

MR Test
Inverse variance weighted
MR Egger
Simple mode
Weighted median
Weighted mode
0.1
0.0
−0.1
−0.2
−0.3
SNP effect on Pulmonary fibrosis
0.1
0.2
0.3
SNP effect on FGFBP3

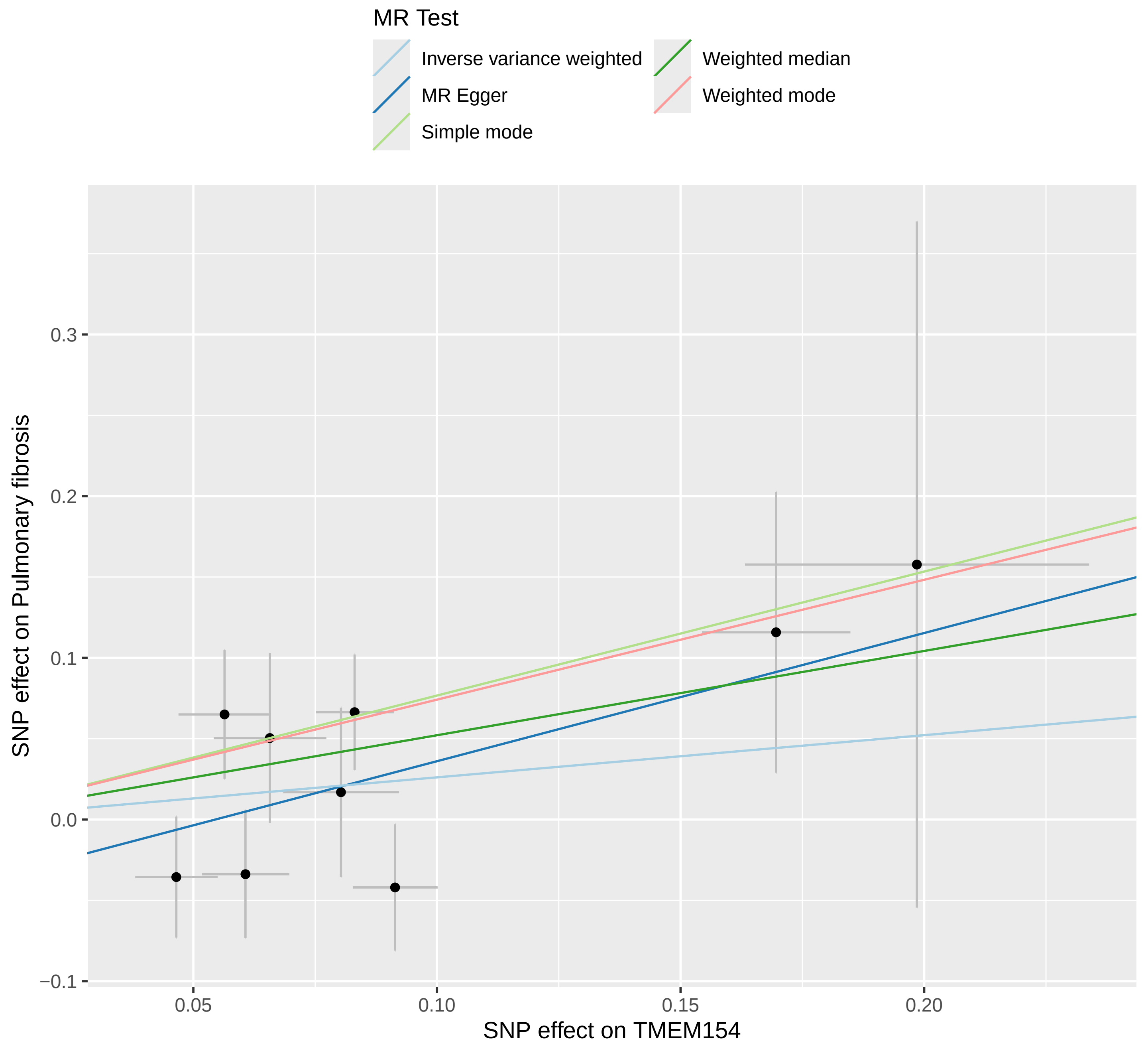
MR Test
Inverse variance weighted
MR Egger
Simple mode
Weighted median
Weighted mode
0.3
0.2
0.1
0.0
−0.1
0.05
0.10
0.15
0.20
SNP effect on Pulmonary fibrosis
SNP effect on TMEM154

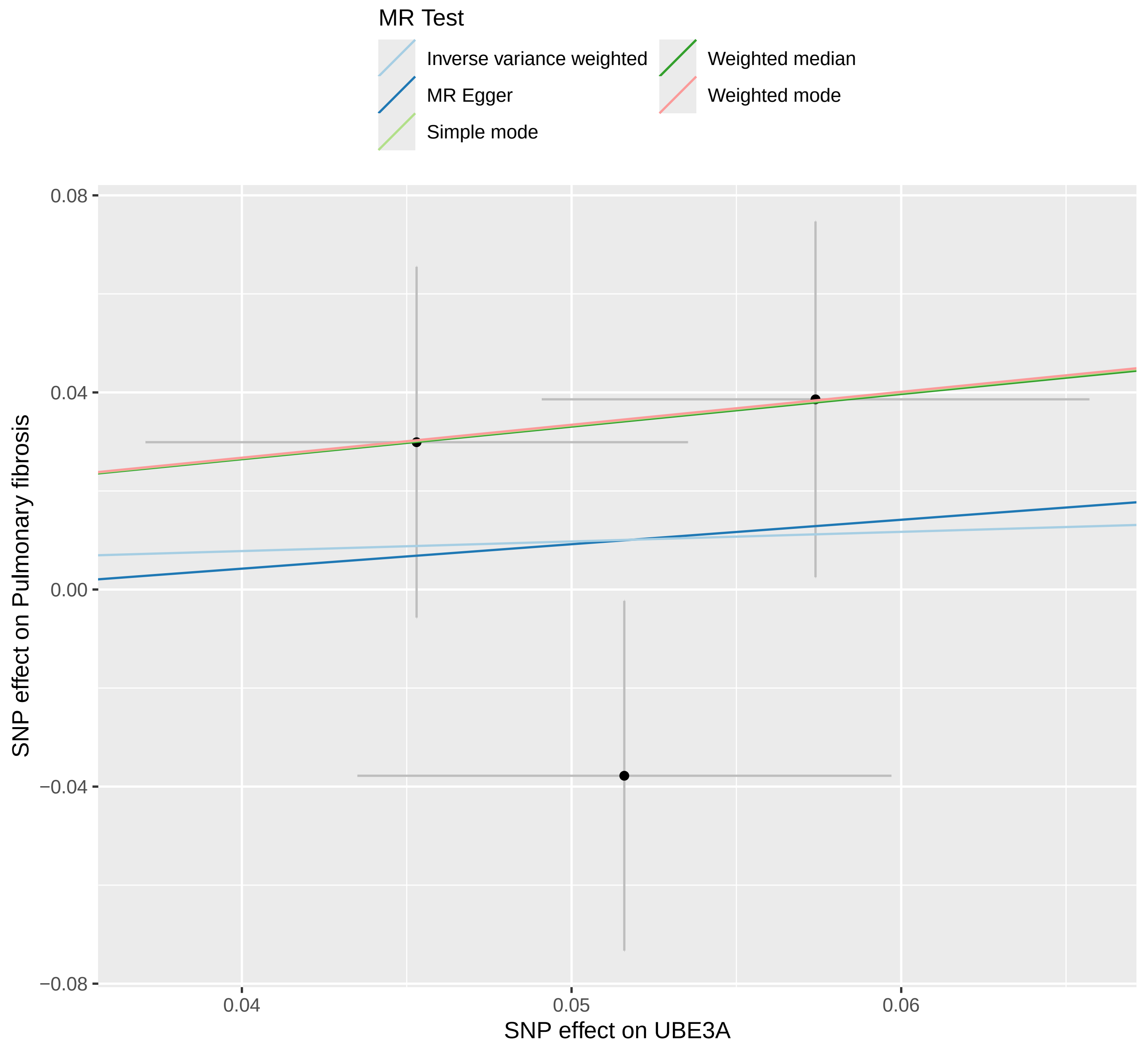
MR Test
Inverse variance weighted
MR Egger
Simple mode
Weighted median
Weighted mode
0.08
0.04
0.00
−0.04
−0.08
SNP effect on Pulmonary fibrosis
0.04
0.05
0.06
SNP effect on UBE3A

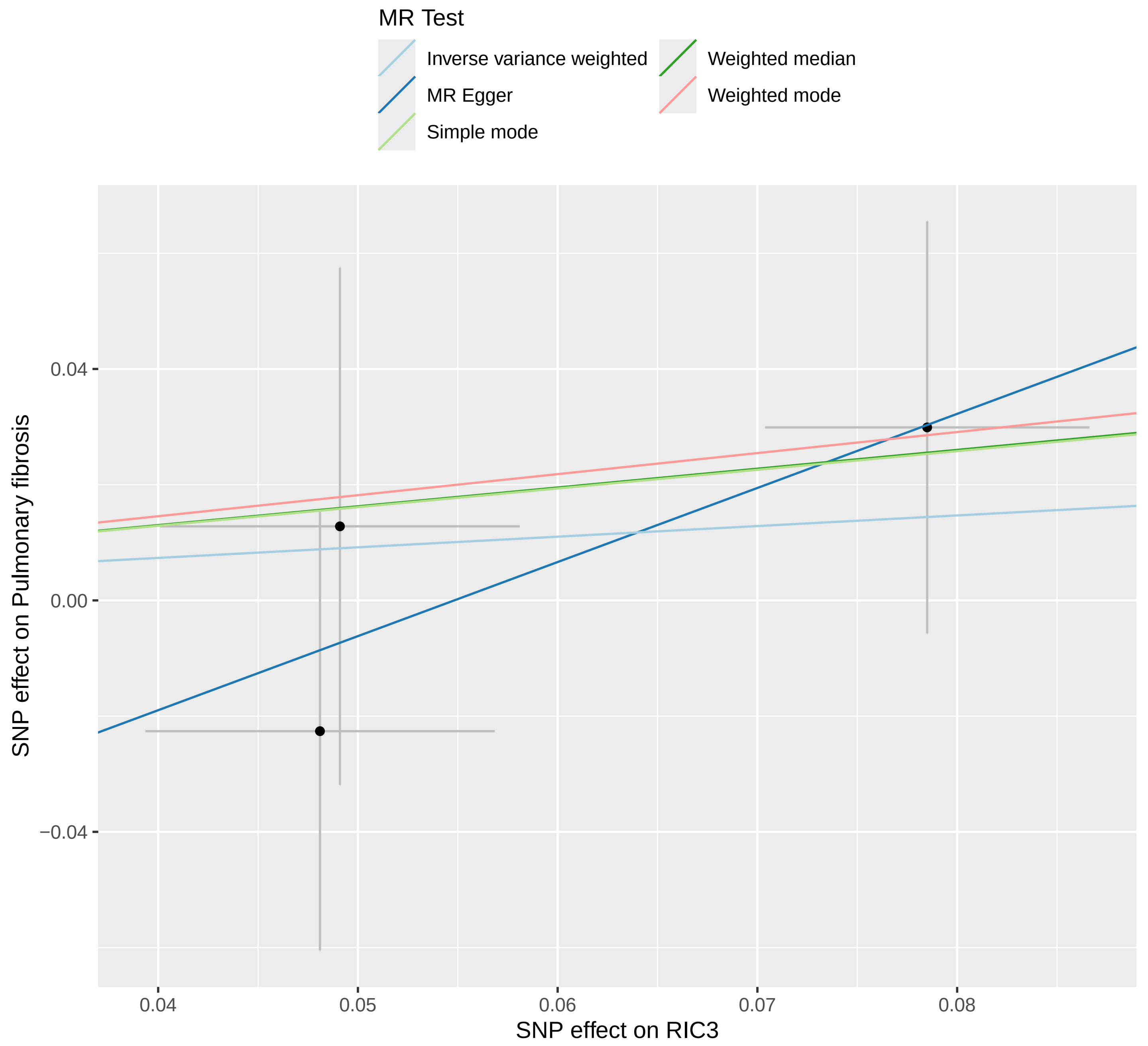

MR Test
Inverse variance weighted
MR Egger
Simple mode
Weighted median
Weighted mode
0.04
0.00
−0.04
SNP effect on Pulmonary fibrosis
0.04
0.05
0.06
0.07
0.08
SNP effect on RIC3

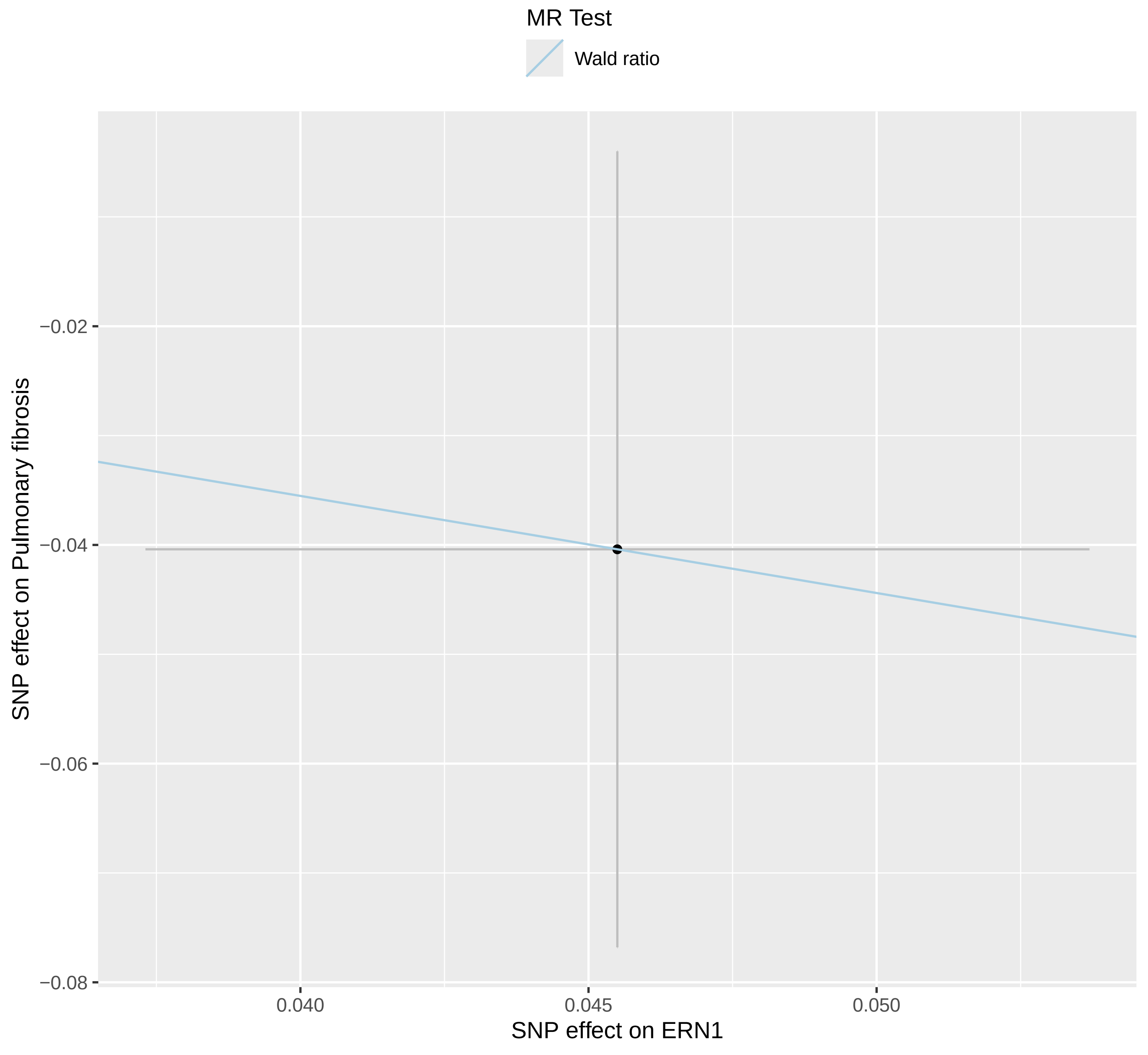
MR Test
Wald ratio
−0.02
−0.04
−0.06
−0.08
0.040
0.045
0.050
SNP effect on ERN1
SNP effect on Pulmonary fibrosis

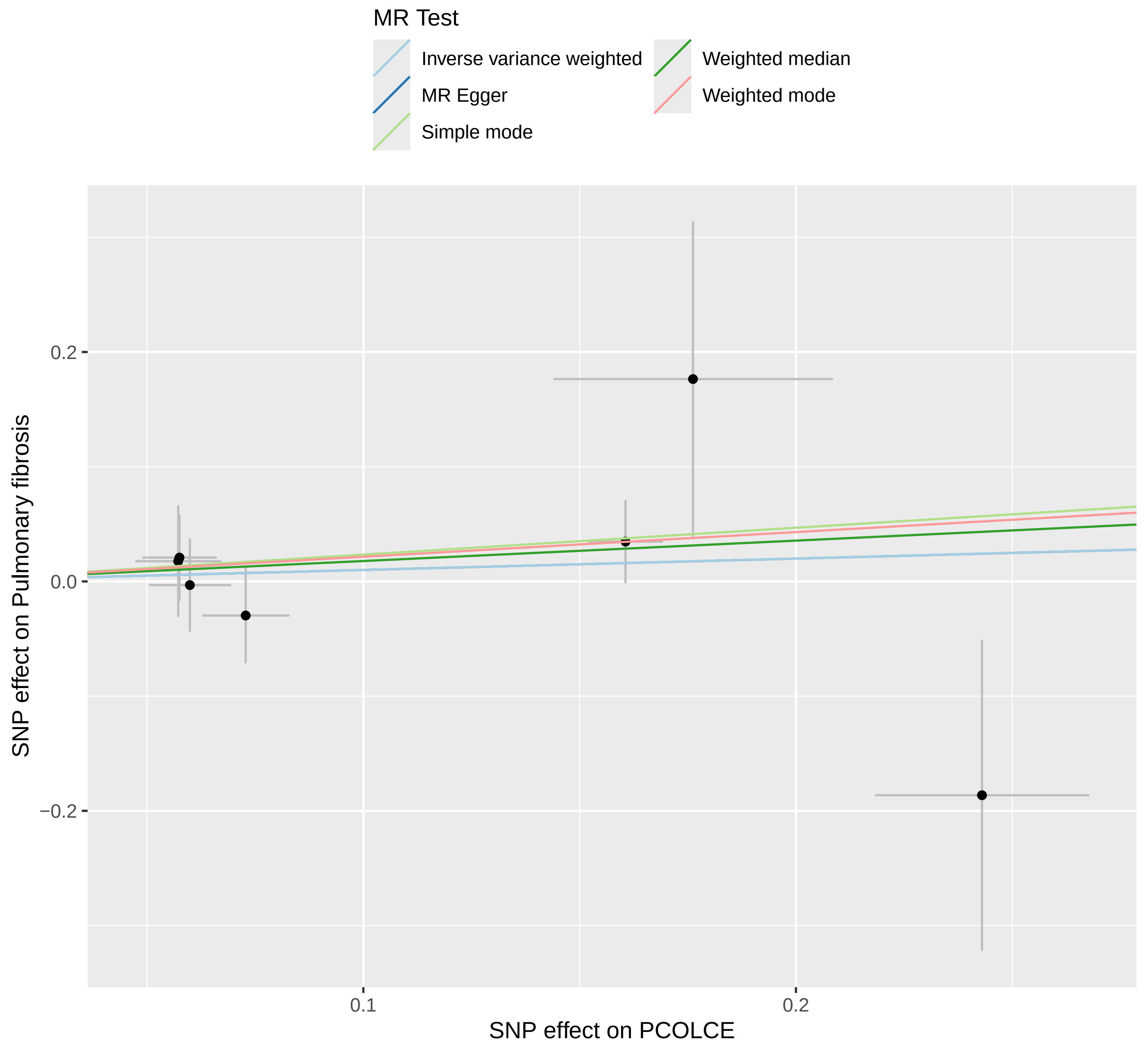
MR Test
Inverse variance weighted
MR Egger
Simple mode
Weighted median
Weighted mode
0.2
0.0
−0.2
0.1
0.2
SNP effect on Pulmonary fibrosis
SNP effect on PCOLCE

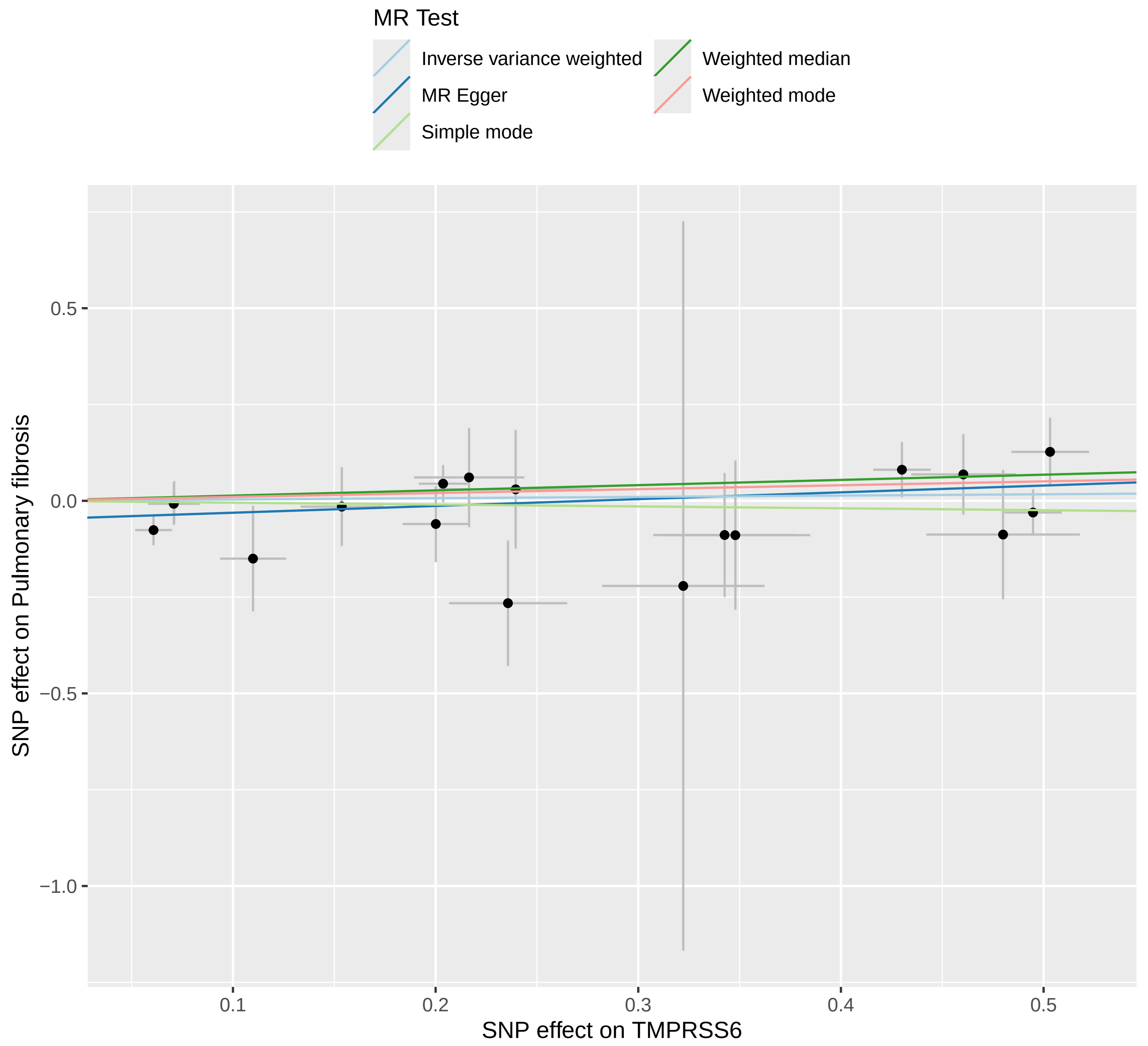
MR Test
Inverse variance weighted
MR Egger
Simple mode
Weighted median
Weighted mode
SNP effect on Pulmonary fibrosis
0.5
0.0
−0.5
−1.0
0.1
0.2
0.3
0.4
0.5
SNP effect on TMPRSS6

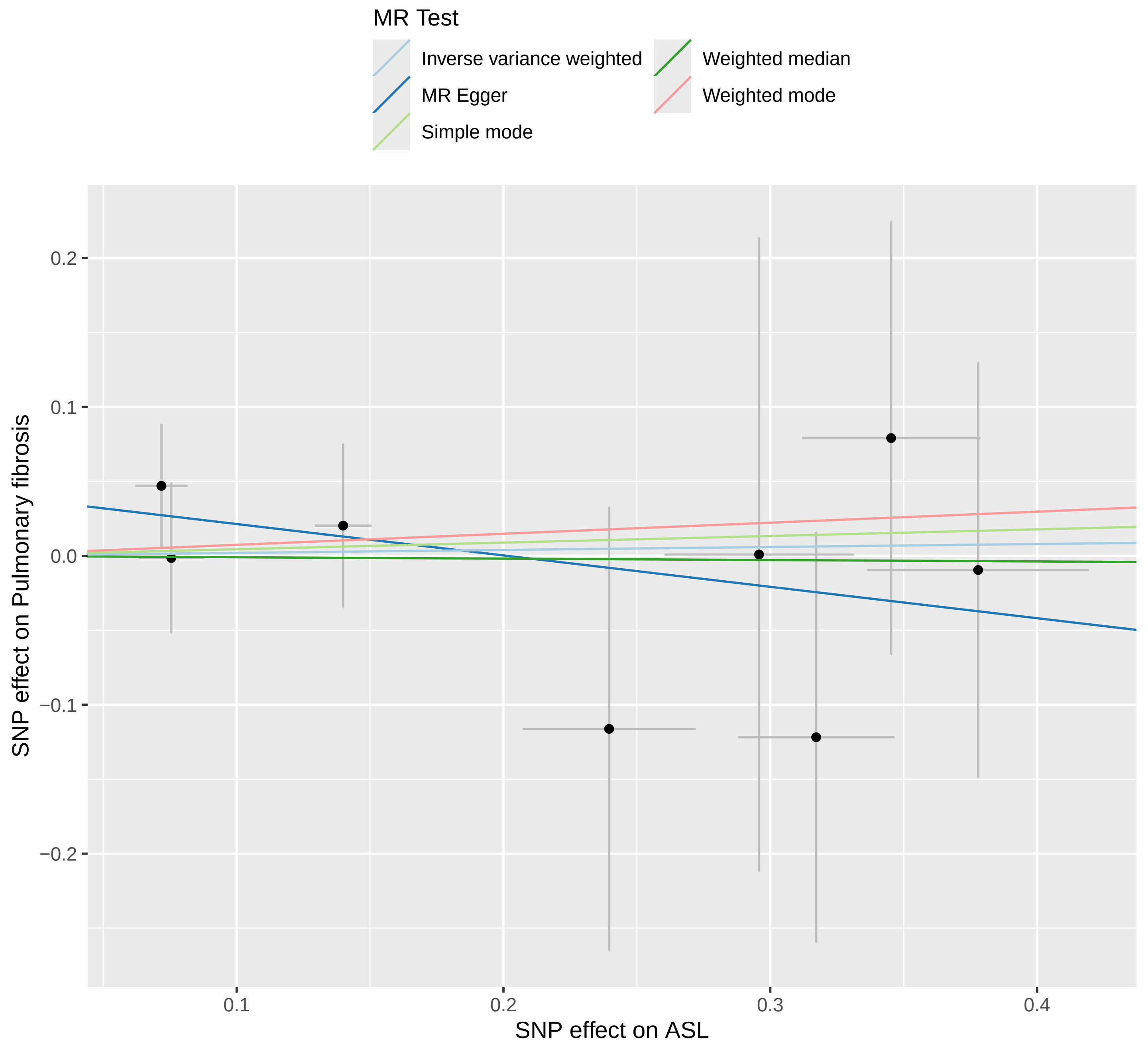

MR Test
Inverse variance weighted
MR Egger
Simple mode
Weighted median
Weighted mode
SNP effect on Pulmonary fibrosis
0.2
0.1
0.0
−0.1
−0.2
0.1
0.2
0.3
0.4
SNP effect on ASL

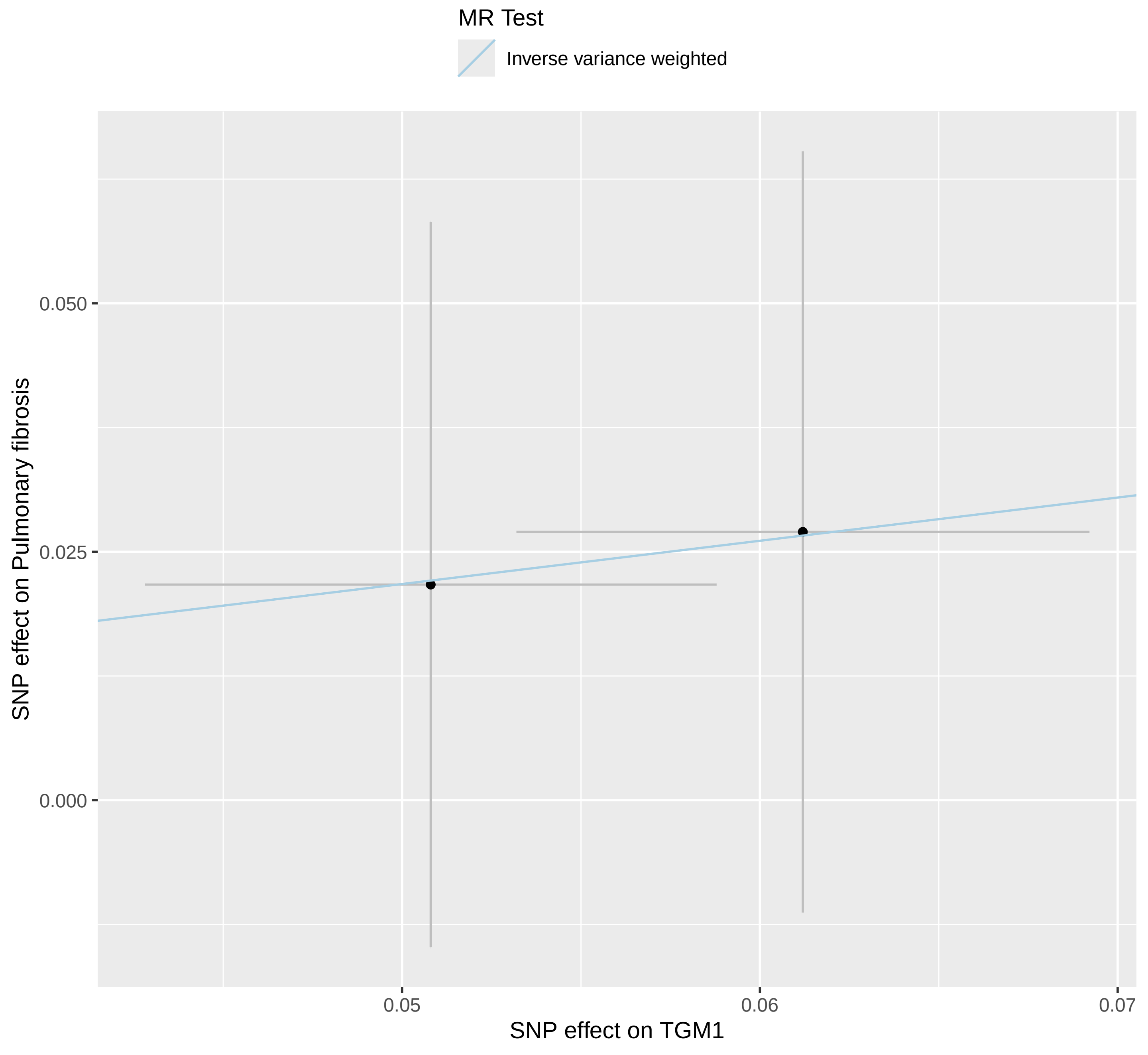
MR Test
Inverse variance weighted
0.050
0.025
0.000
SNP effect on Pulmonary fibrosis
0.05
0.06
0.07
SNP effect on TGM1

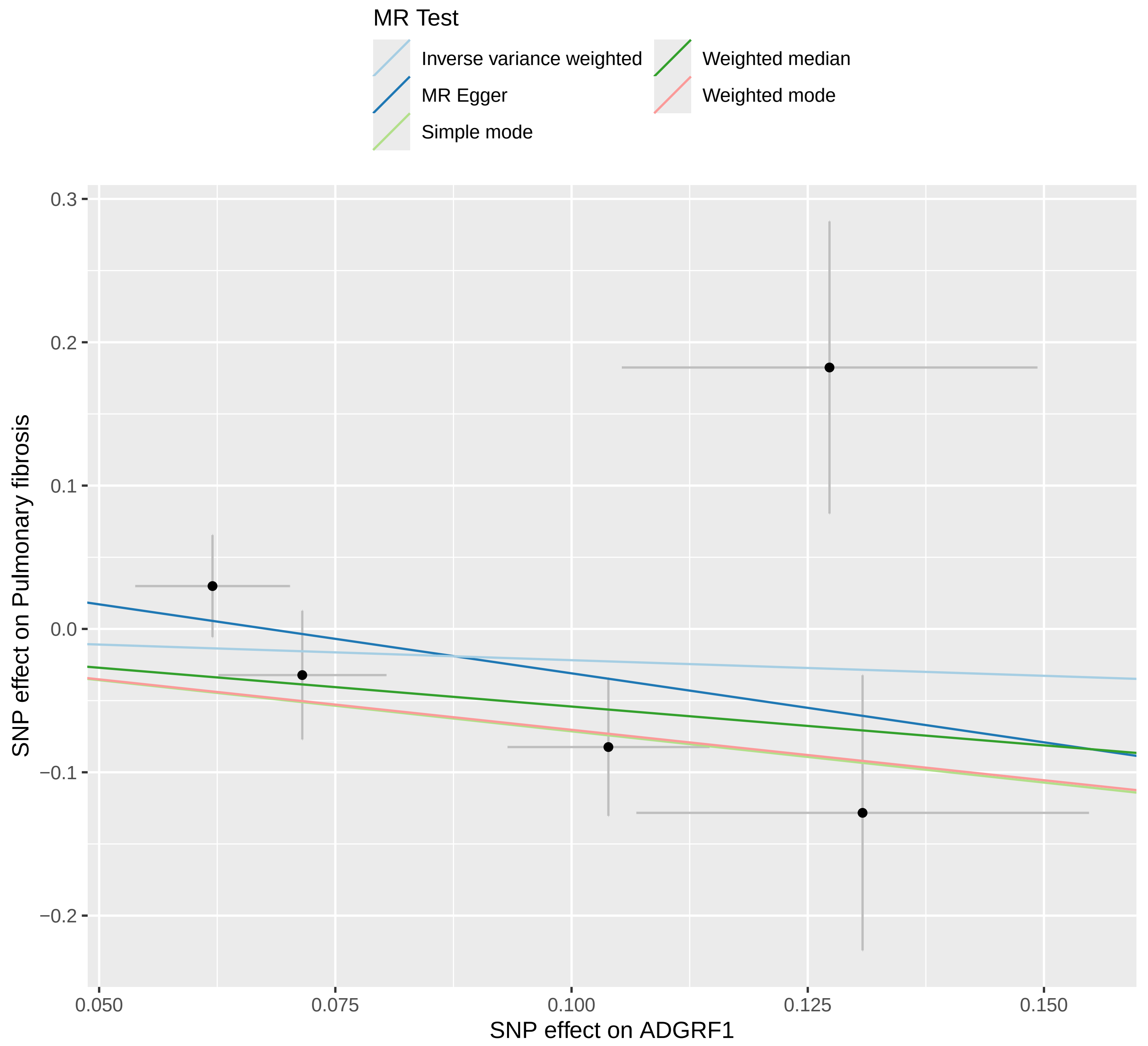
MR Test
Inverse variance weighted
MR Egger
Simple mode
Weighted median
Weighted mode
SNP effect on Pulmonary fibrosis
0.3
0.2
0.1
0.0
−0.1
−0.2
0.050
0.075
0.100
0.125
0.150
SNP effect on ADGRF1

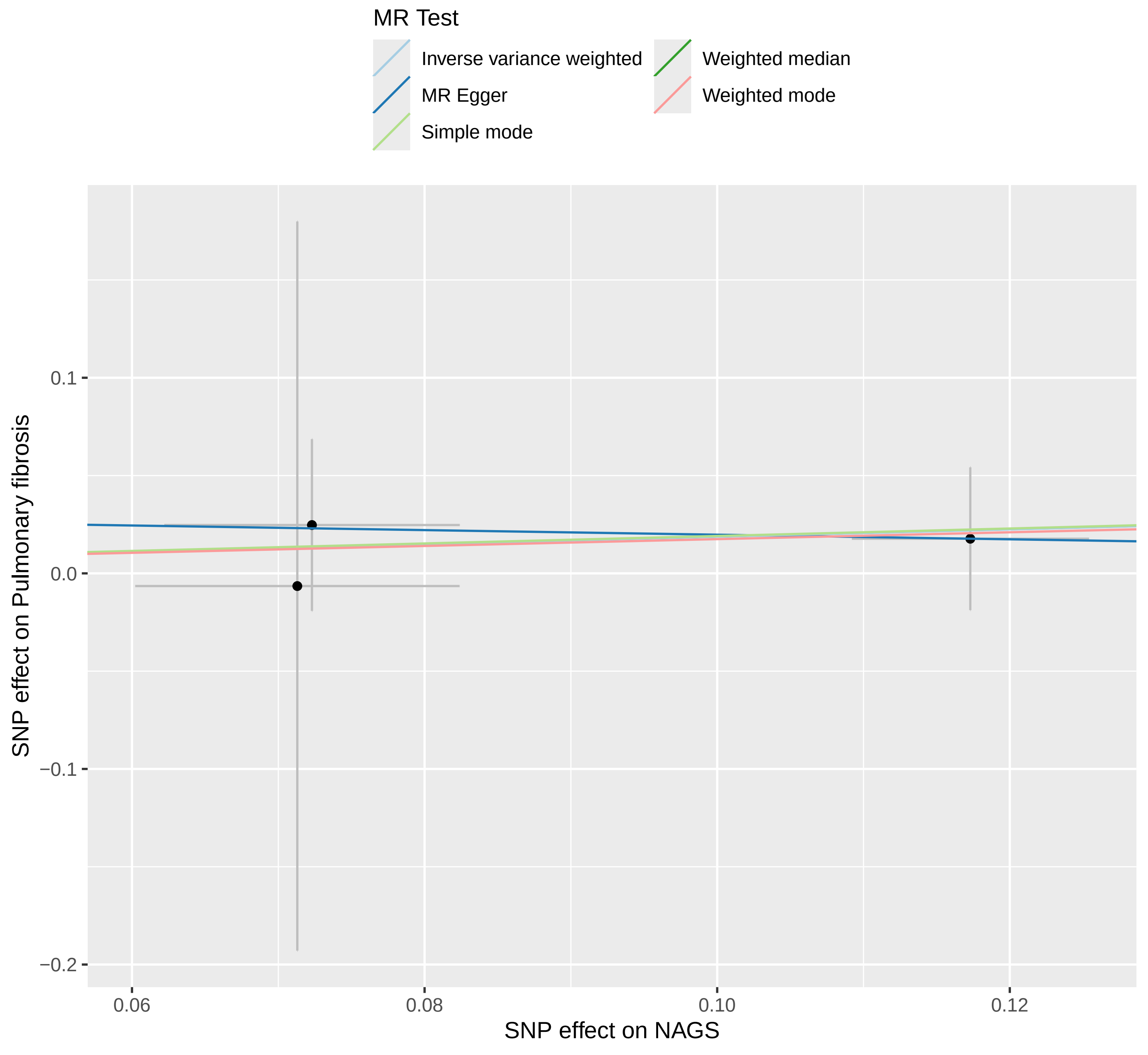

MR Test
Inverse variance weighted
MR Egger
Simple mode
Weighted median
Weighted mode
SNP effect on Pulmonary fibrosis
0.1
0.0
−0.1
−0.2
0.06
0.08
0.10
0.12
SNP effect on NAGS

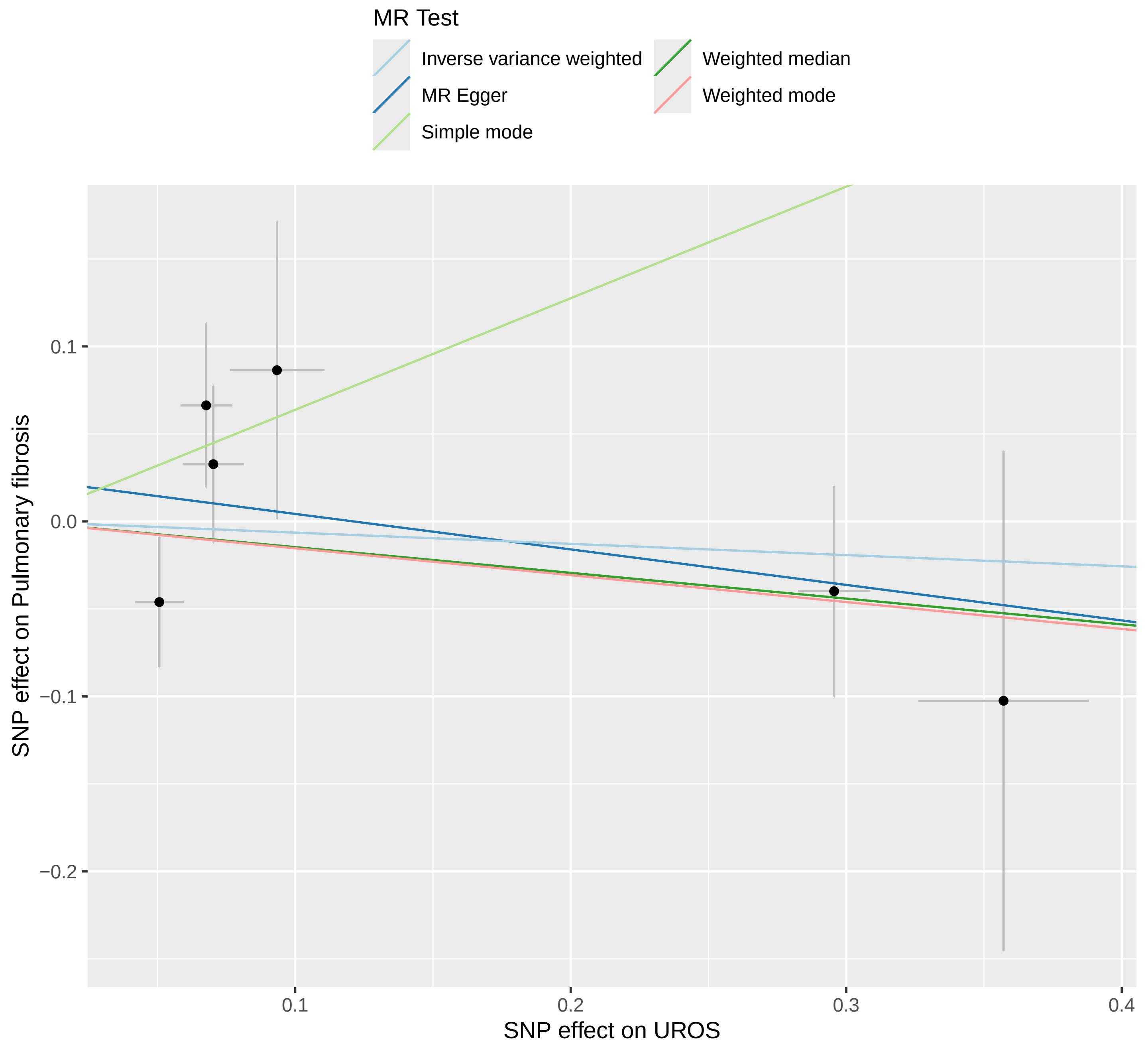

MR Test
Inverse variance weighted
MR Egger
Simple mode
Weighted median
Weighted mode
0.1
0.0
−0.1
−0.2
SNP effect on Pulmonary fibrosis
0.1
0.2
0.3
0.4
SNP effect on UROS

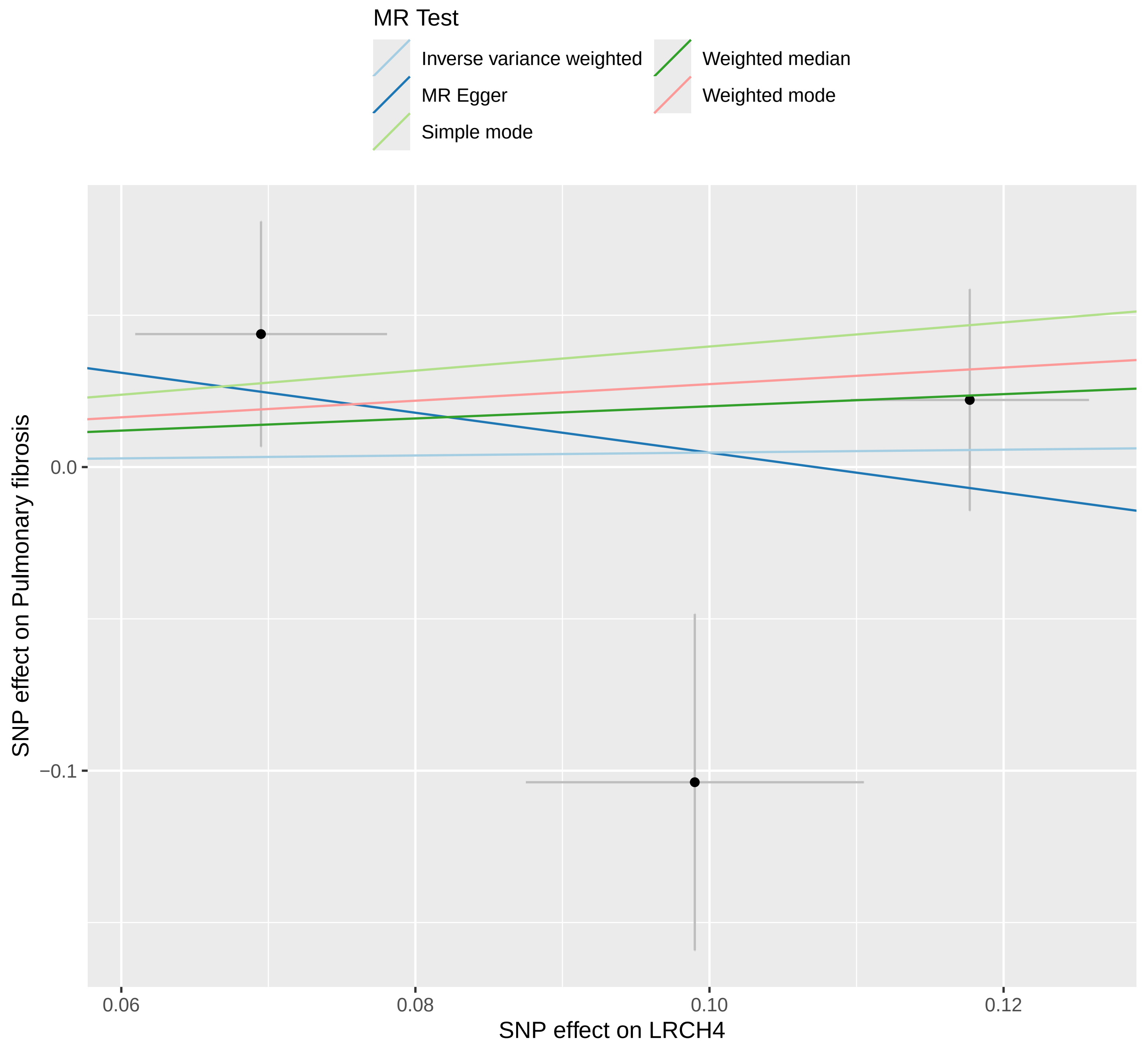

MR Test
Inverse variance weighted
MR Egger
Simple mode
Weighted median
Weighted mode
SNP effect on Pulmonary fibrosis
0.0
−0.1
0.06
0.08
0.10
0.12
SNP effect on LRCH4

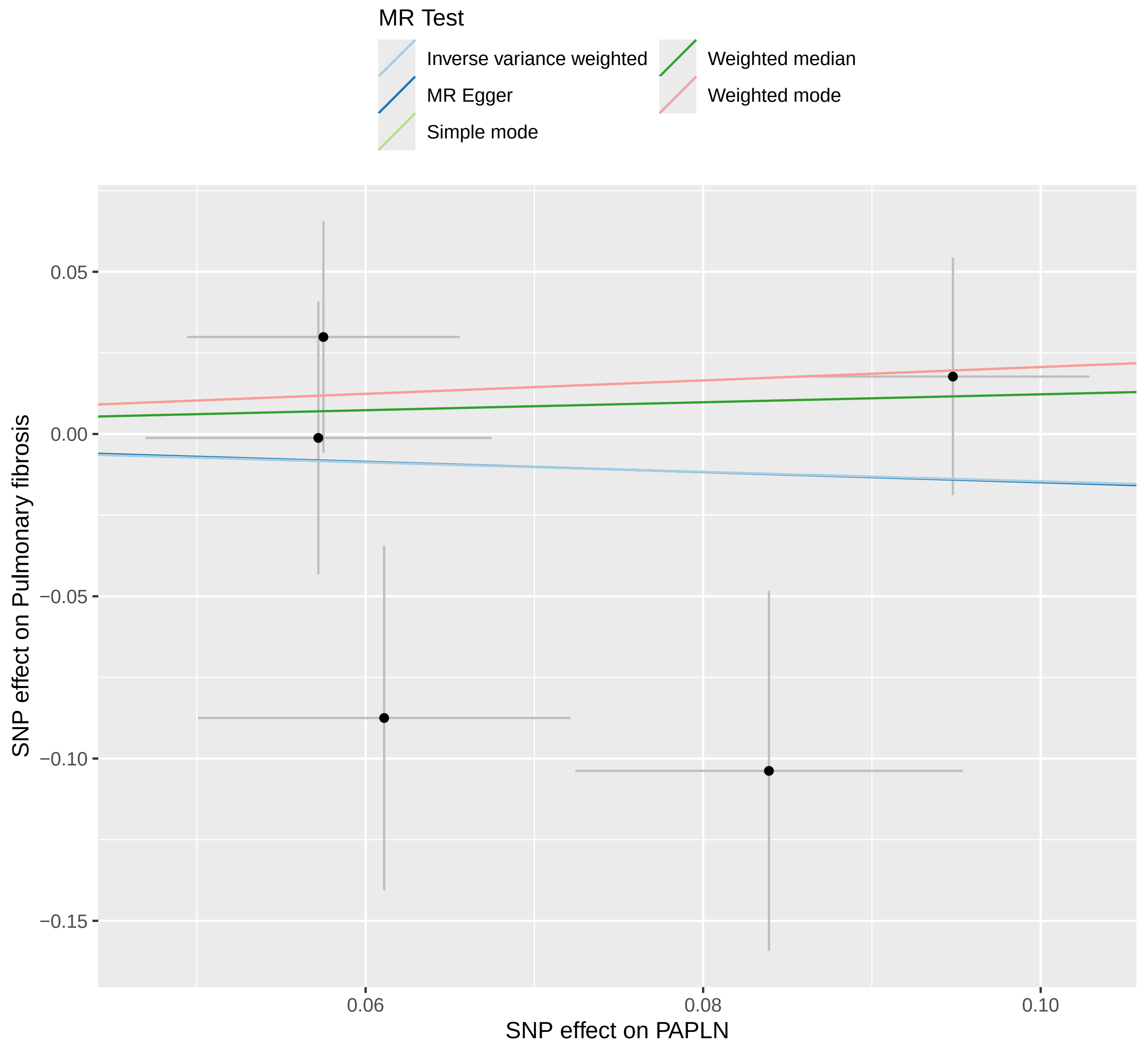
MR Test
Inverse variance weighted
MR Egger
Simple mode
Weighted median
Weighted mode
SNP effect on Pulmonary fibrosis
0.05
0.00
−0.05
−0.10
−0.15
0.06
0.08
0.10
SNP effect on PAPLN

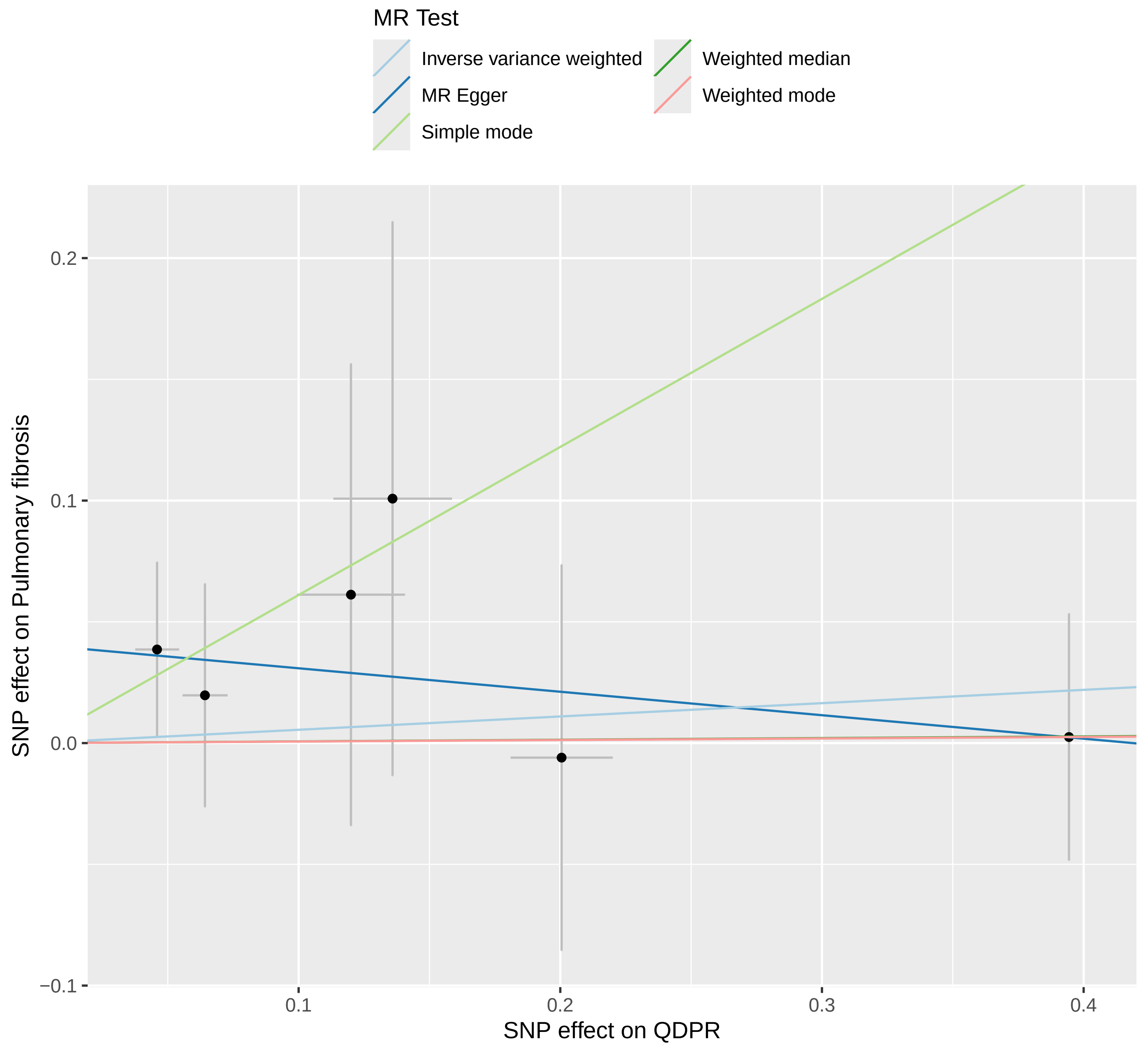
MR Test
Inverse variance weighted
MR Egger
Simple mode
Weighted median
Weighted mode
SNP effect on Pulmonary fibrosis
SNP effect on QDPR
0.2
0.1
0.0
−0.1
0.1
0.2
0.3
0.4

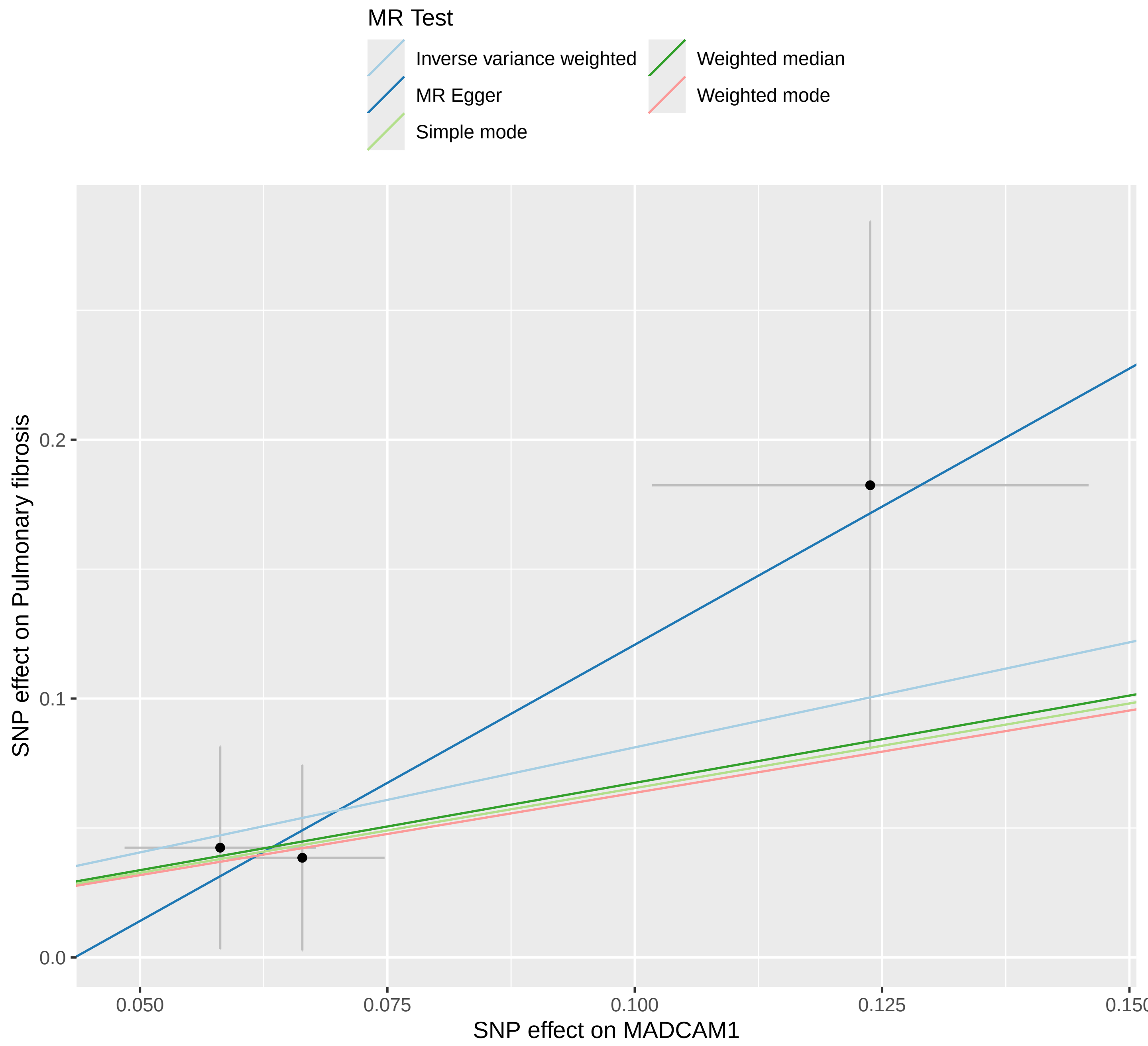
MR Test
Inverse variance weighted
MR Egger
Simple mode
Weighted median
Weighted mode
SNP effect on Pulmonary fibrosis
0.0
0.1
0.2
0.050
0.075
0.100
0.125
0.150
SNP effect on MADCAM1

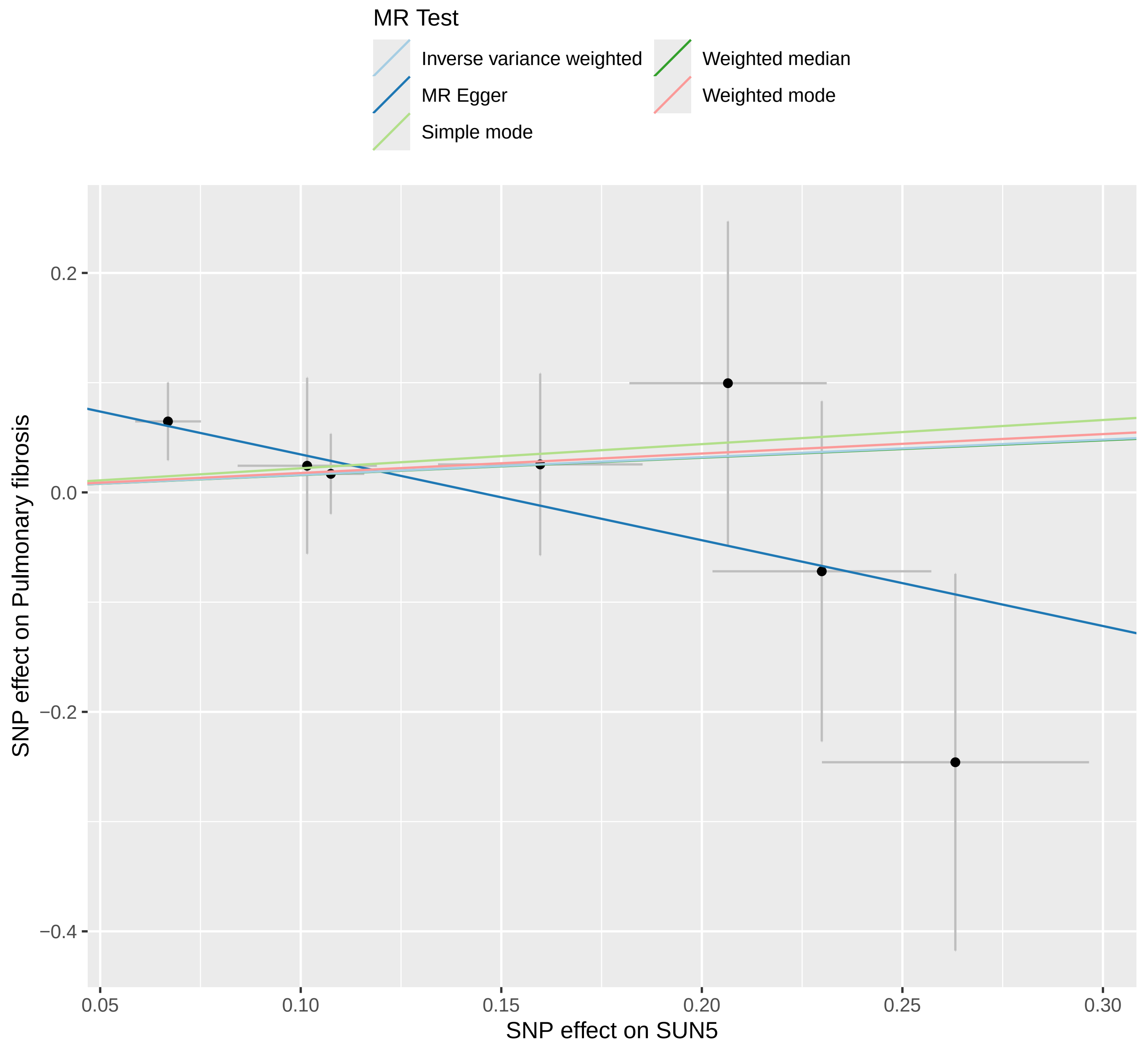
MR Test
Inverse variance weighted
MR Egger
Simple mode
Weighted median
Weighted mode
SNP effect on Pulmonary fibrosis
0.2
0.0
−0.2
−0.4
0.05
0.10
0.15
0.20
0.25
0.30
SNP effect on SUN5

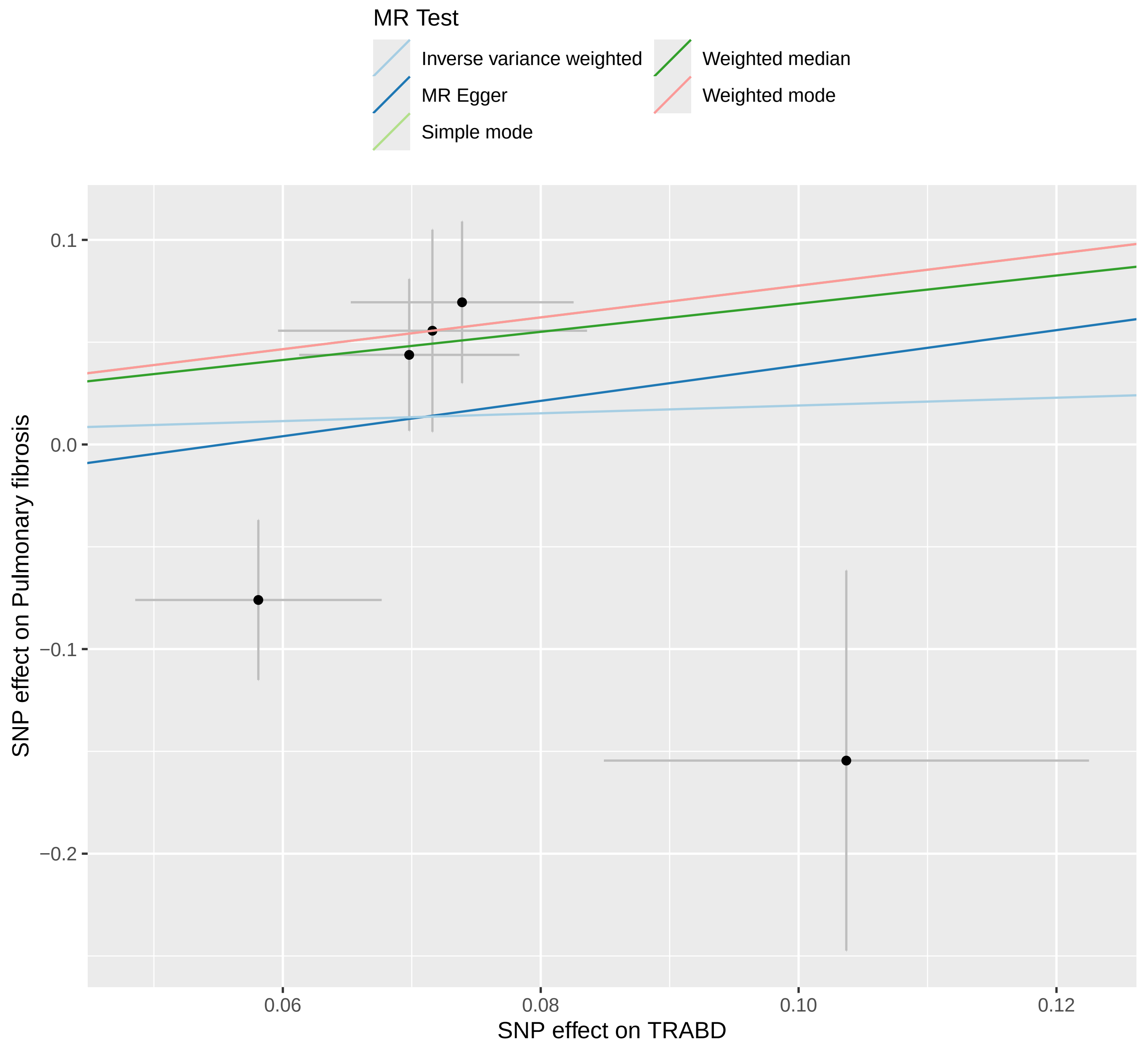
MR Test
Inverse variance weighted
MR Egger
Simple mode
Weighted median
Weighted mode
0.1
0.0
−0.1
−0.2
0.06
0.08
0.10
0.12
SNP effect on TRABD
SNP effect on Pulmonary fibrosis

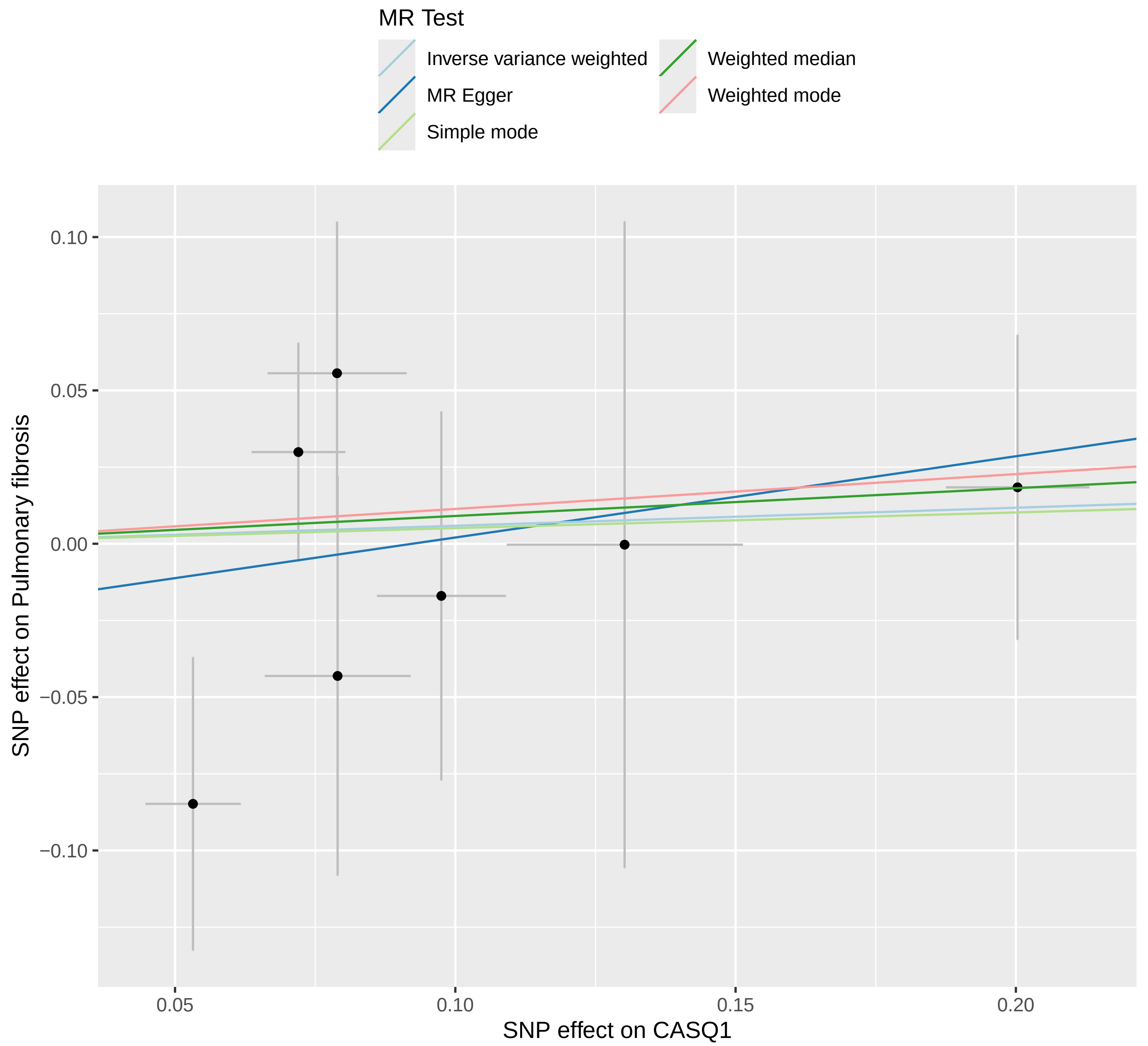

MR Test
Inverse variance weighted
MR Egger
Simple mode
Weighted median
Weighted mode
0.10
0.05
0.00
−0.05
−0.10
SNP effect on Pulmonary fibrosis
0.05
0.10
0.15
0.20
SNP effect on CASQ1

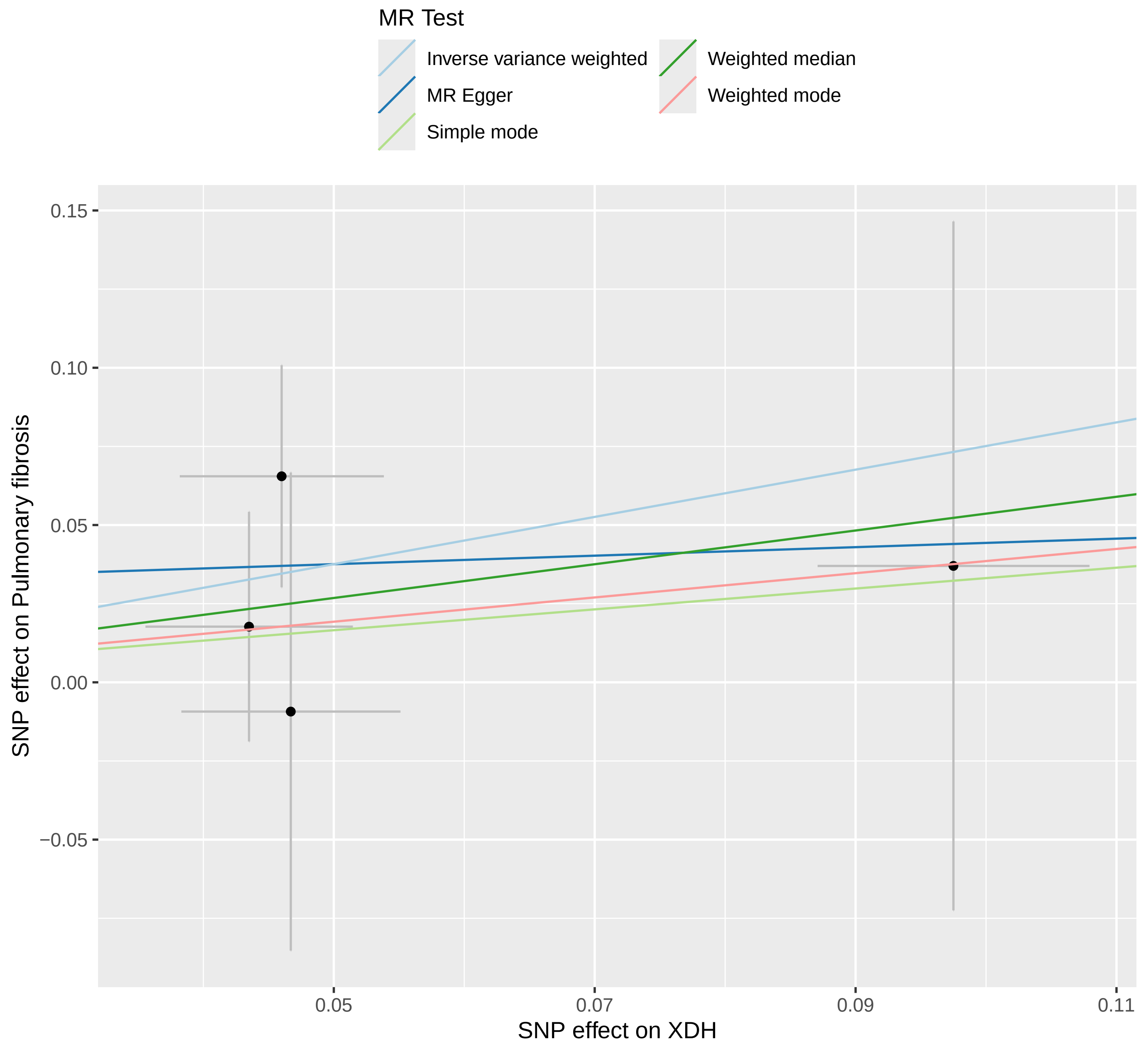
MR Test
Inverse variance weighted
MR Egger
Simple mode
Weighted median
Weighted mode
0.15
0.10
0.05
0.00
−0.05
SNP effect on Pulmonary fibrosis
0.05
0.07
0.09
0.11
SNP effect on XDH

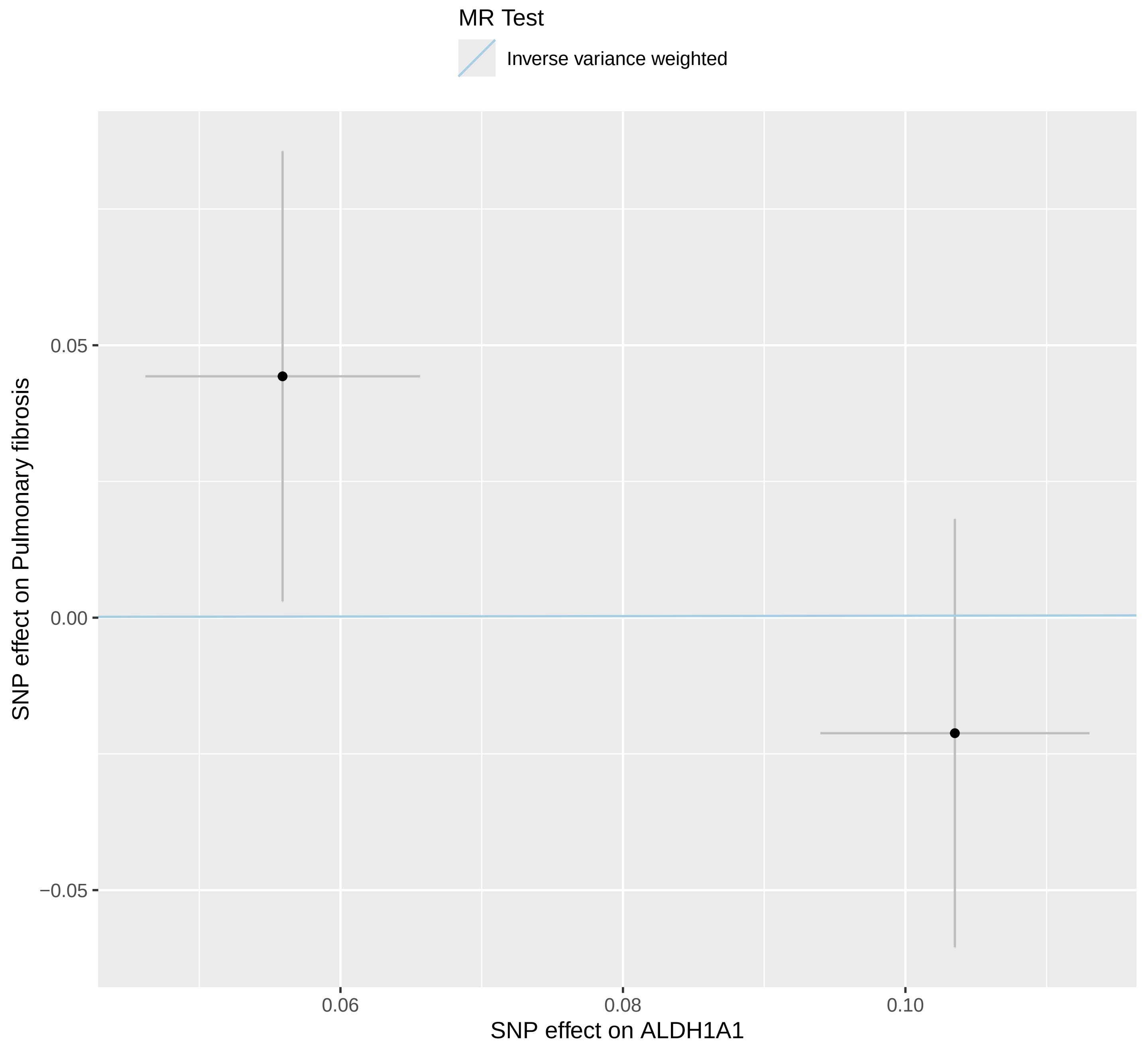

MR Test
Inverse variance weighted
SNP effect on Pulmonary fibrosis
0.05
0.00
−0.05
0.06
0.08
0.10
SNP effect on ALDH1A1

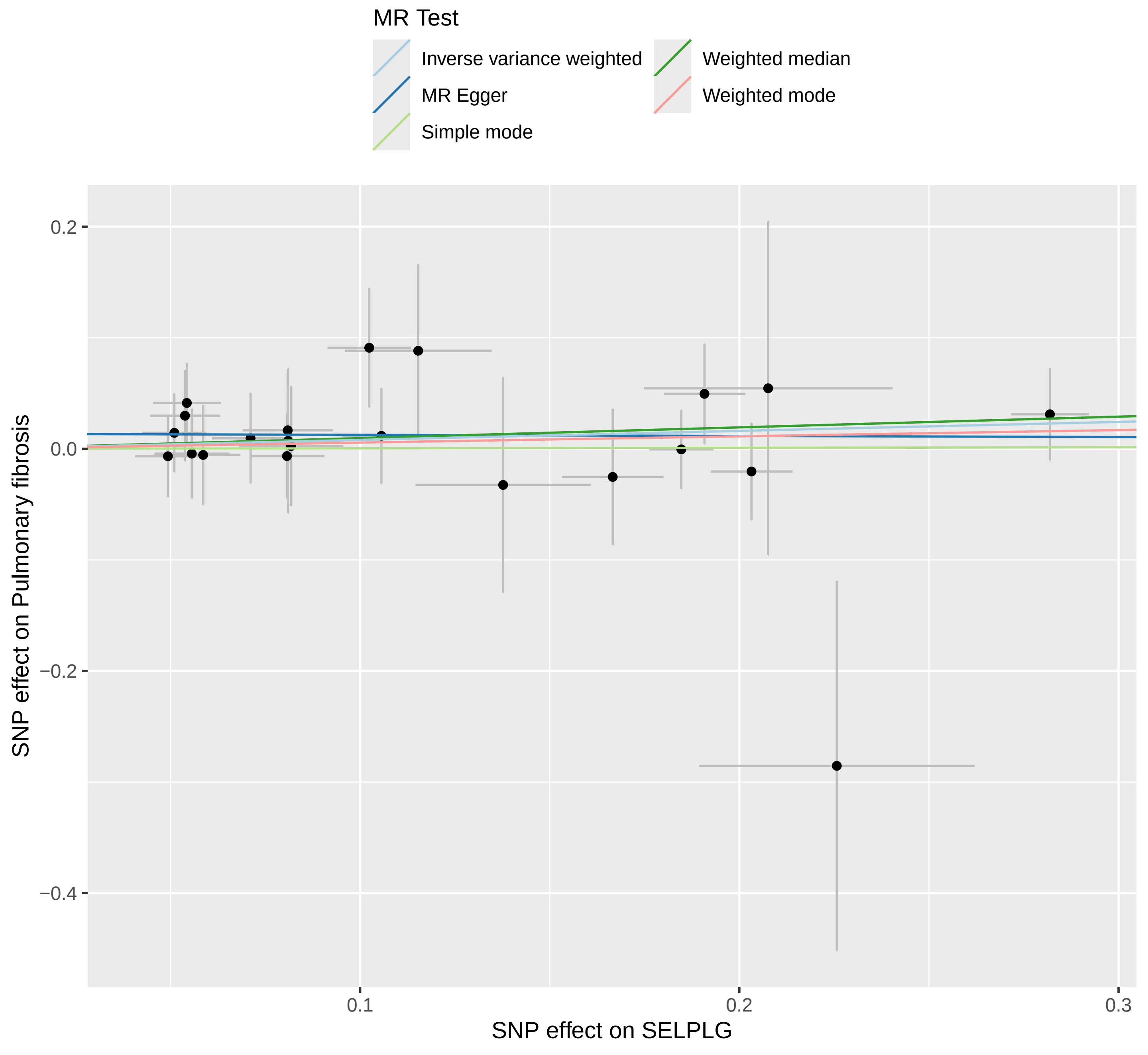

MR Test
Inverse variance weighted
MR Egger
Simple mode
Weighted median
Weighted mode
0.2
0.0
−0.2
−0.4
0.1
0.2
0.3
SNP effect on Pulmonary fibrosis
SNP effect on SELPLG

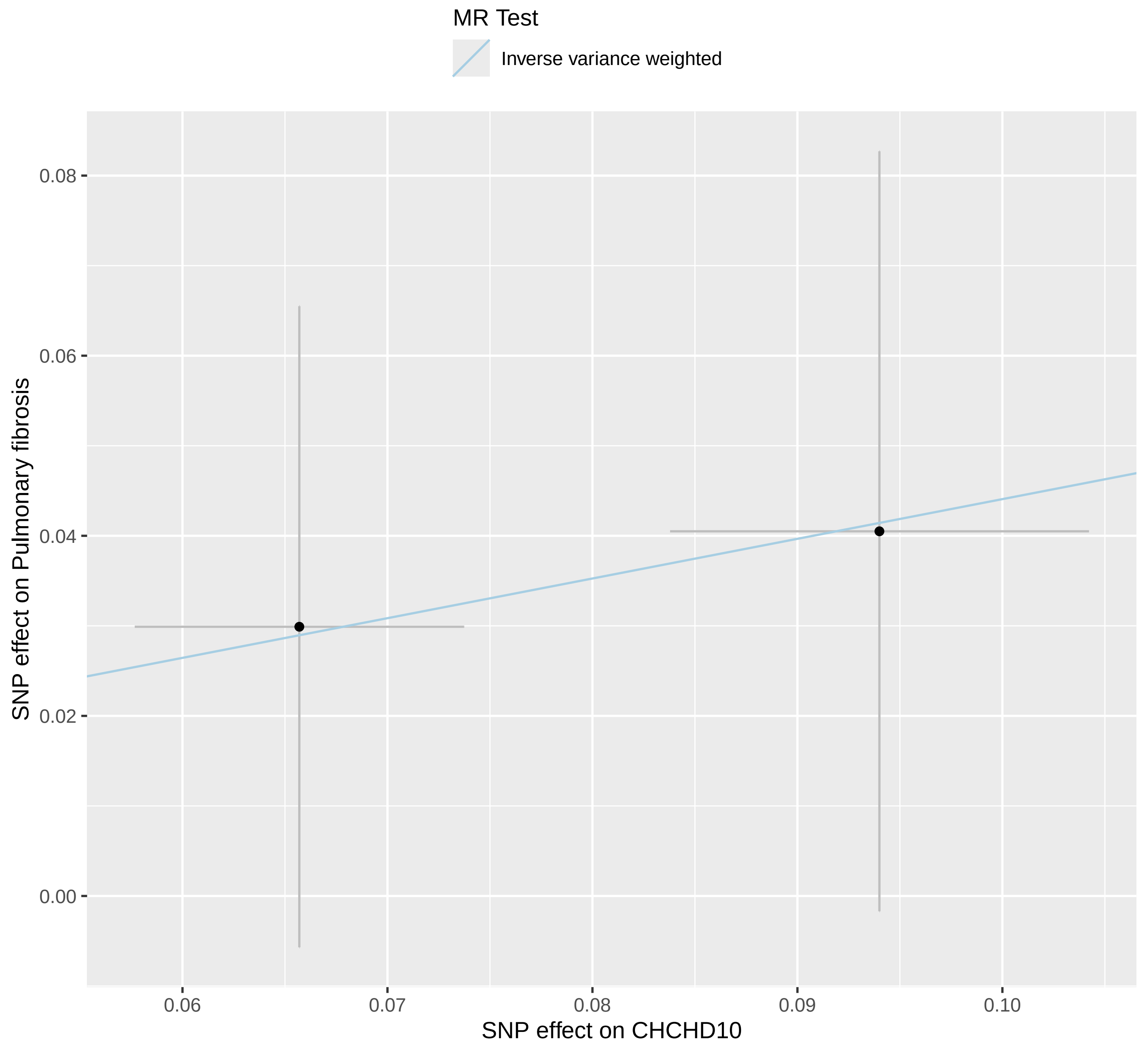
MR Test
Inverse variance weighted
0.08
0.06
0.04
0.02
0.00
SNP effect on Pulmonary fibrosis
0.06
0.07
0.08
0.09
0.10
SNP effect on CHCHD10

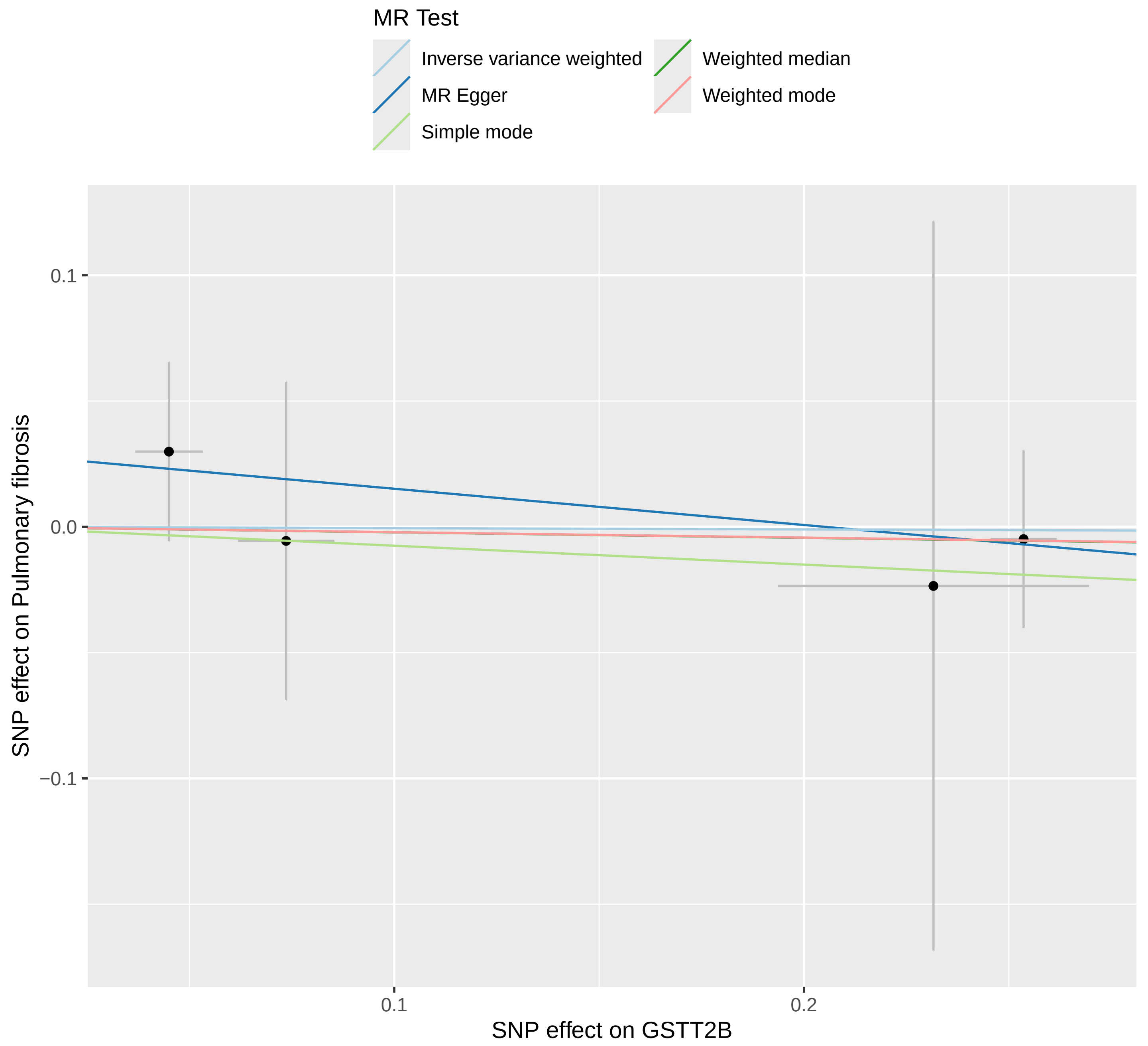
MR Test
Inverse variance weighted
MR Egger
Simple mode
Weighted median
Weighted mode
0.1
0.0
−0.1
SNP effect on Pulmonary fibrosis
0.1
0.2
SNP effect on GSTT2B

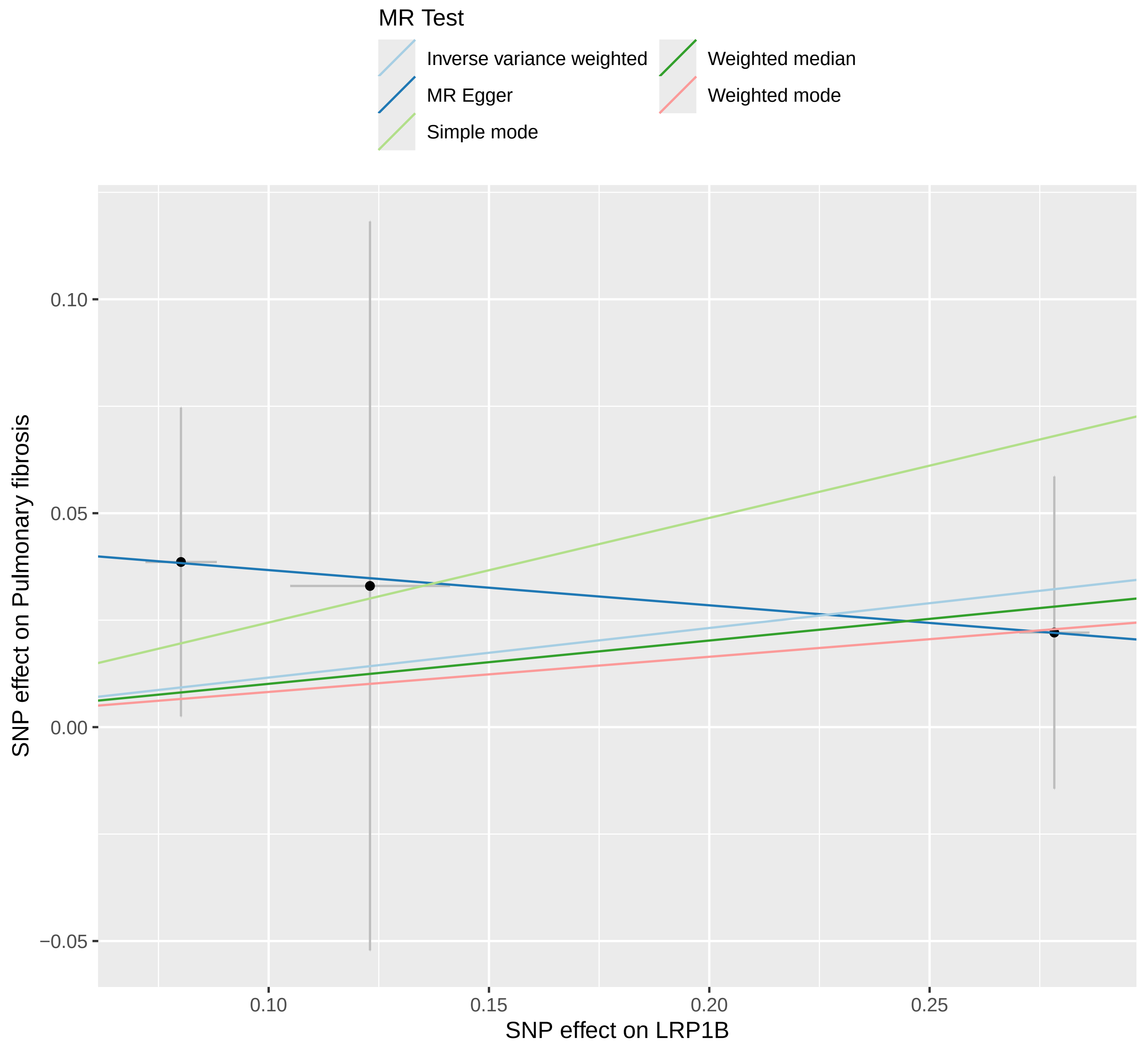
MR Test
Inverse variance weighted
MR Egger
Simple mode
Weighted median
Weighted mode
0.10
0.05
0.00
−0.05
SNP effect on Pulmonary fibrosis
0.10
0.15
0.20
0.25
SNP effect on LRP1B

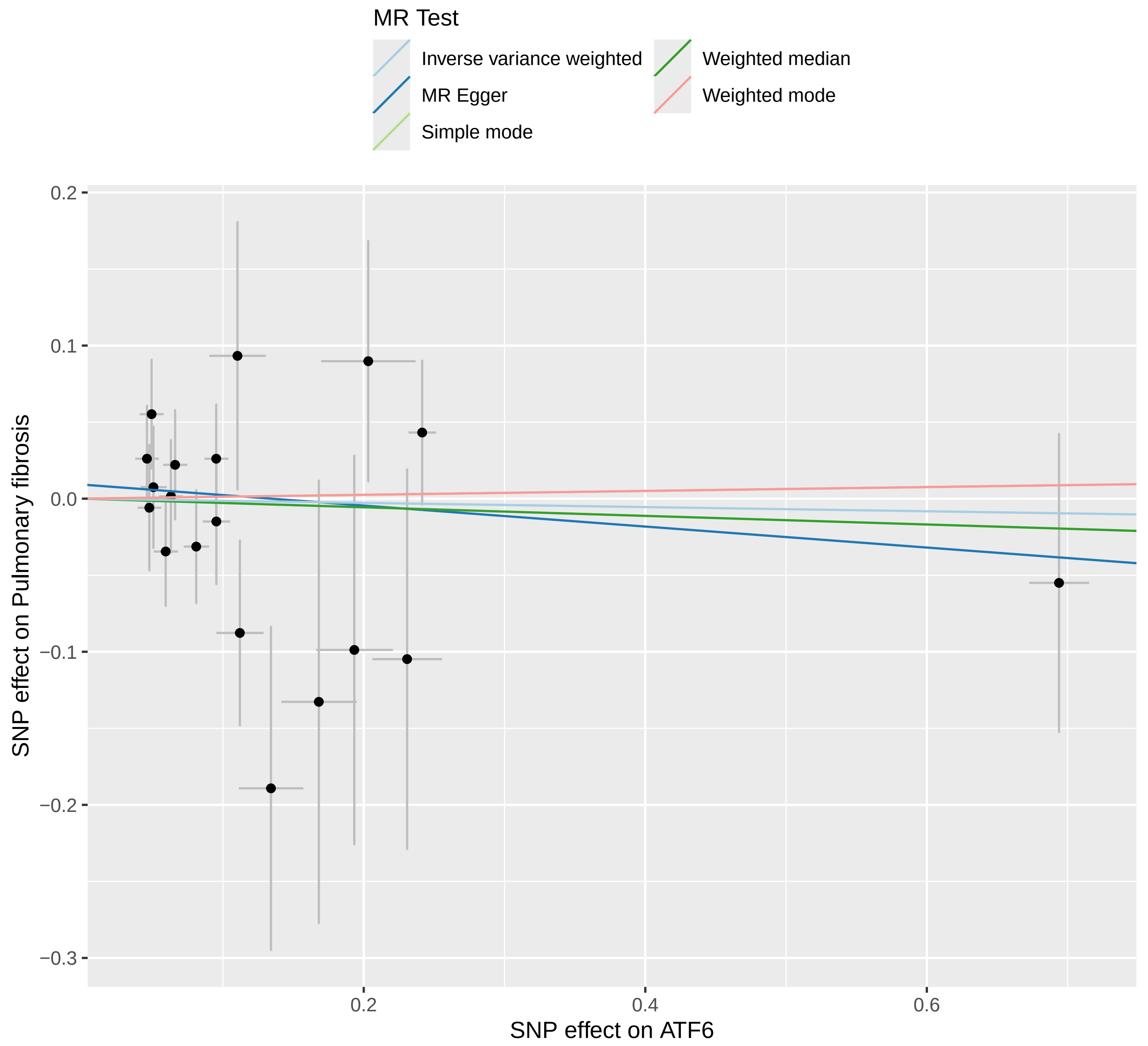

MR Test
Inverse variance weighted
MR Egger
Simple mode
Weighted median
Weighted mode
0.2
0.1
0.0
−0.1
−0.2
−0.3
SNP effect on Pulmonary fibrosis
0.2
0.4
0.6
SNP effect on ATF6

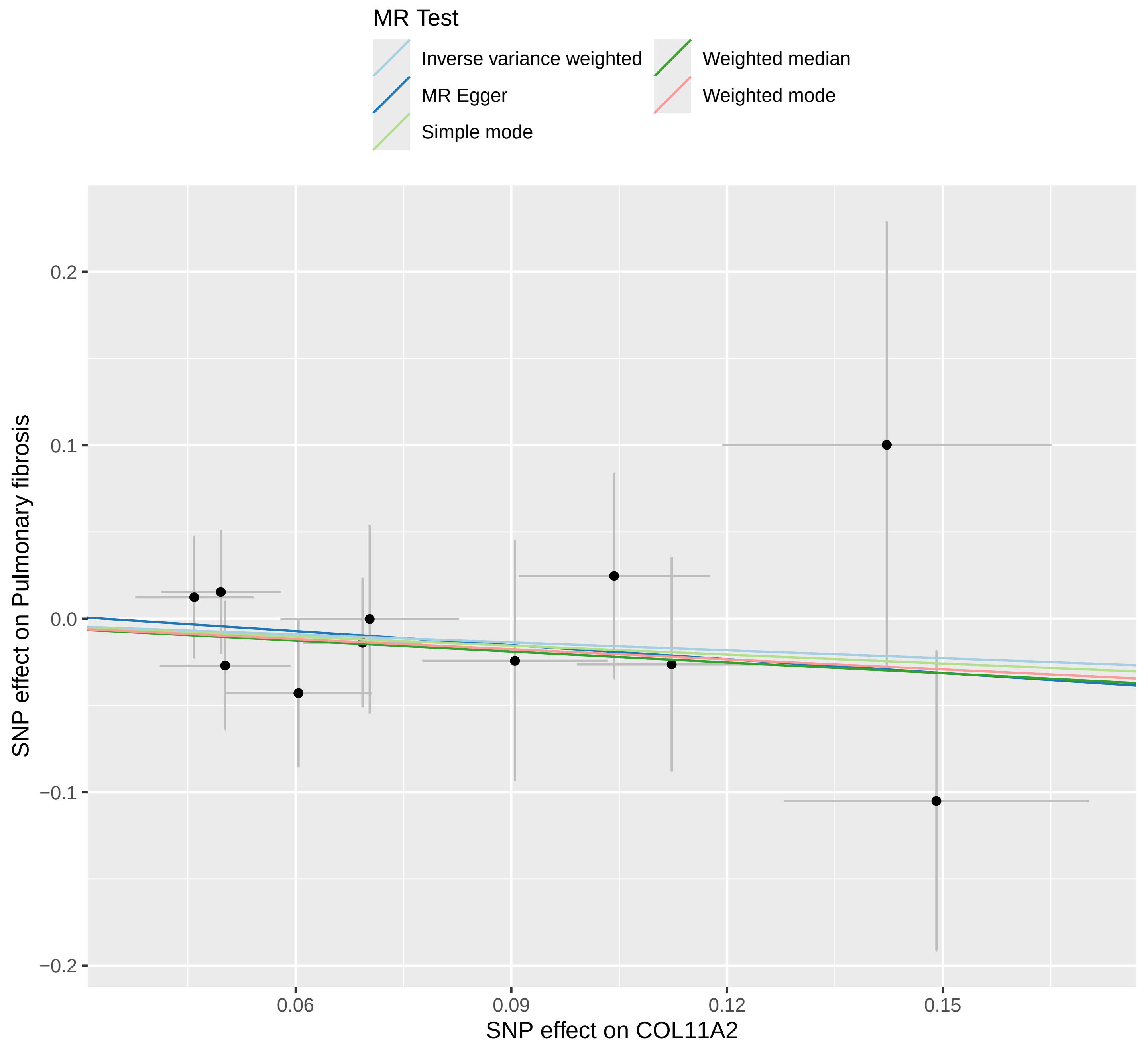
MR Test
Inverse variance weighted
MR Egger
Simple mode
Weighted median
Weighted mode
SNP effect on Pulmonary fibrosis
0.2
0.1
0.0
−0.1
−0.2
0.06
0.09
0.12
0.15
SNP effect on COL11A2

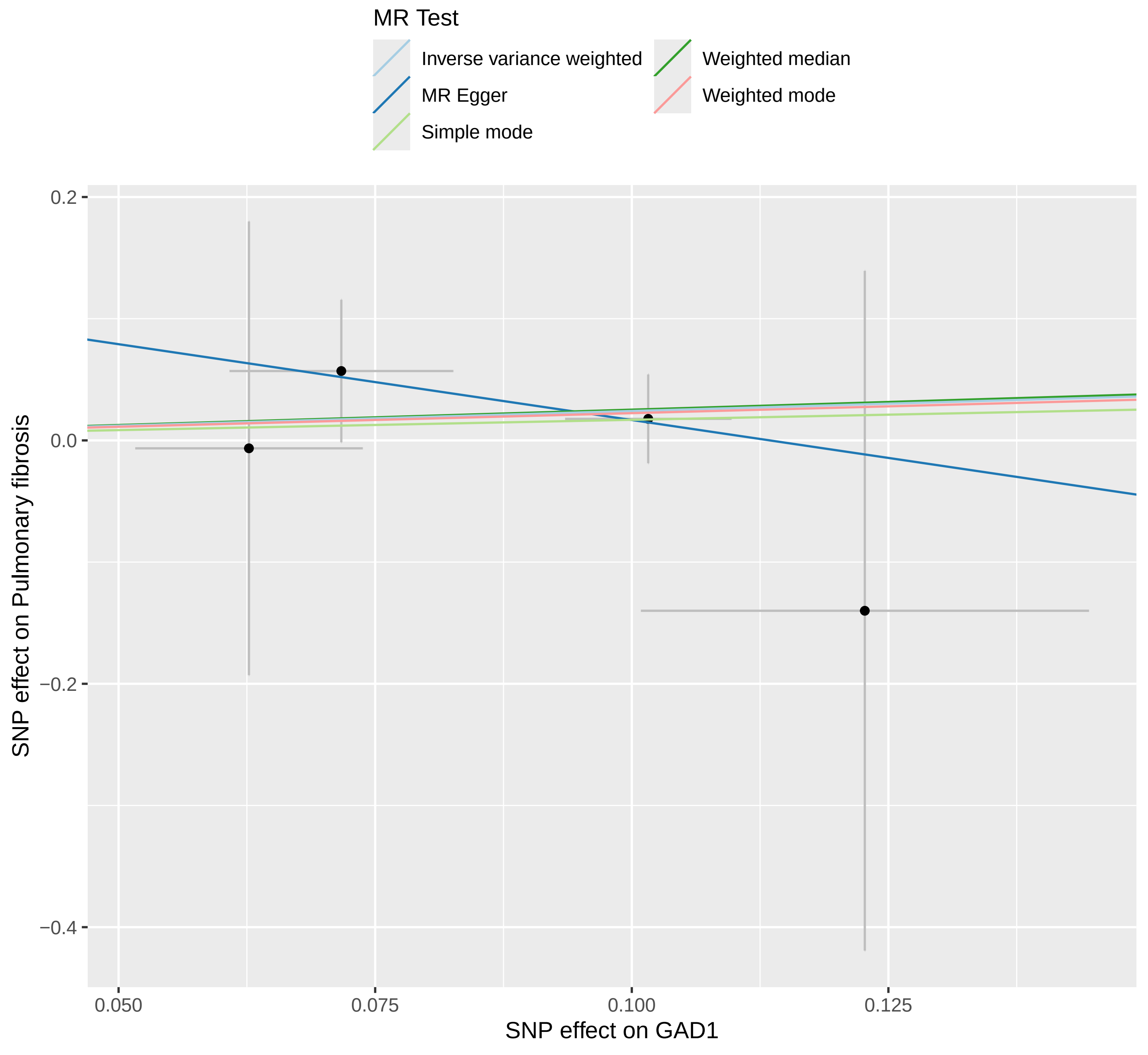
MR Test
Inverse variance weighted
MR Egger
Simple mode
Weighted median
Weighted mode
0.2
0.0
−0.2
−0.4
0.050
0.075
0.100
0.125
SNP effect on GAD1
SNP effect on Pulmonary fibrosis

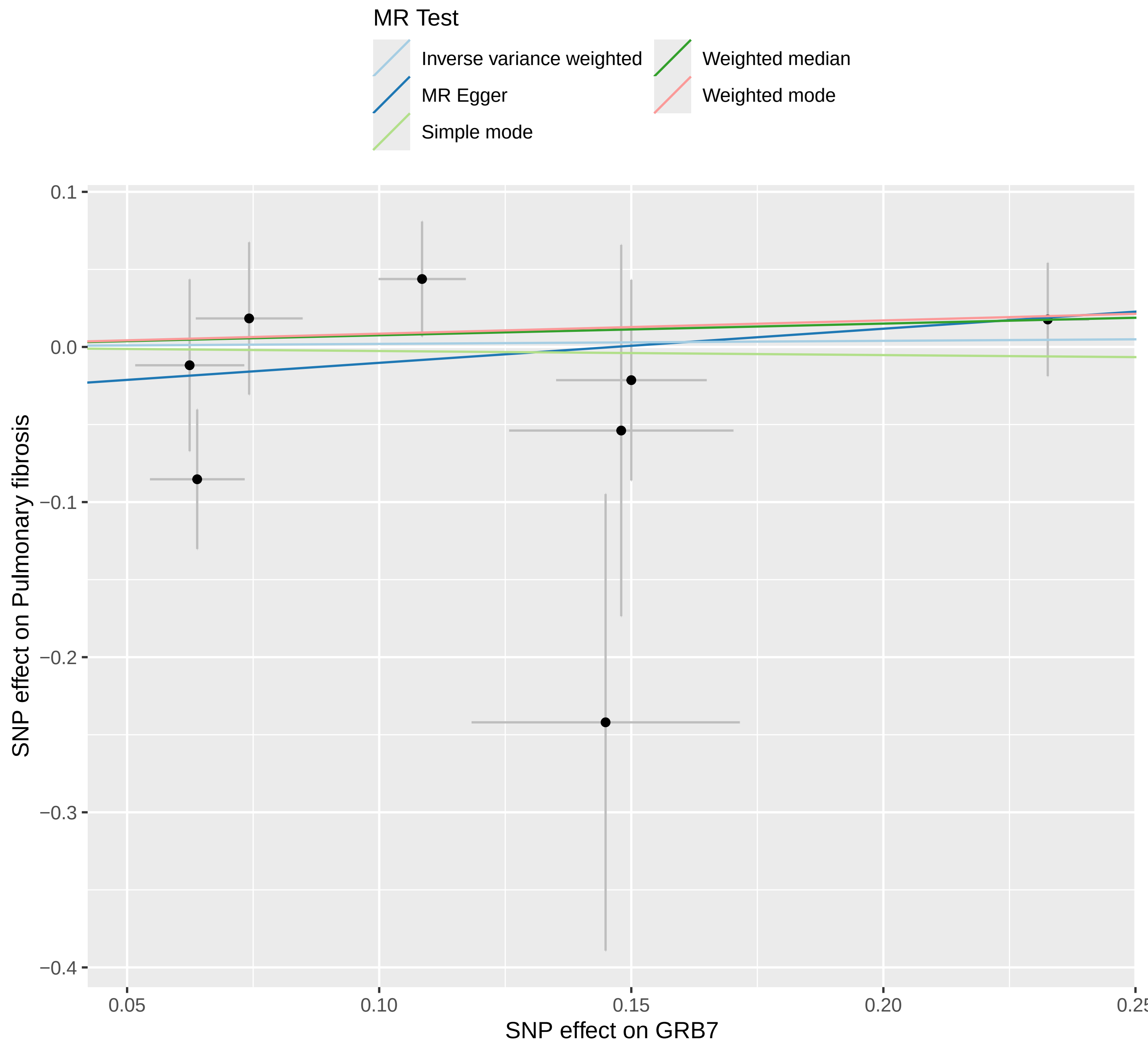
MR Test
Inverse variance weighted
MR Egger
Simple mode
Weighted median
Weighted mode
0.1
0.0
−0.1
−0.2
−0.3
−0.4
SNP effect on Pulmonary fibrosis
0.05
0.10
0.15
0.20
0.25
SNP effect on GRB7

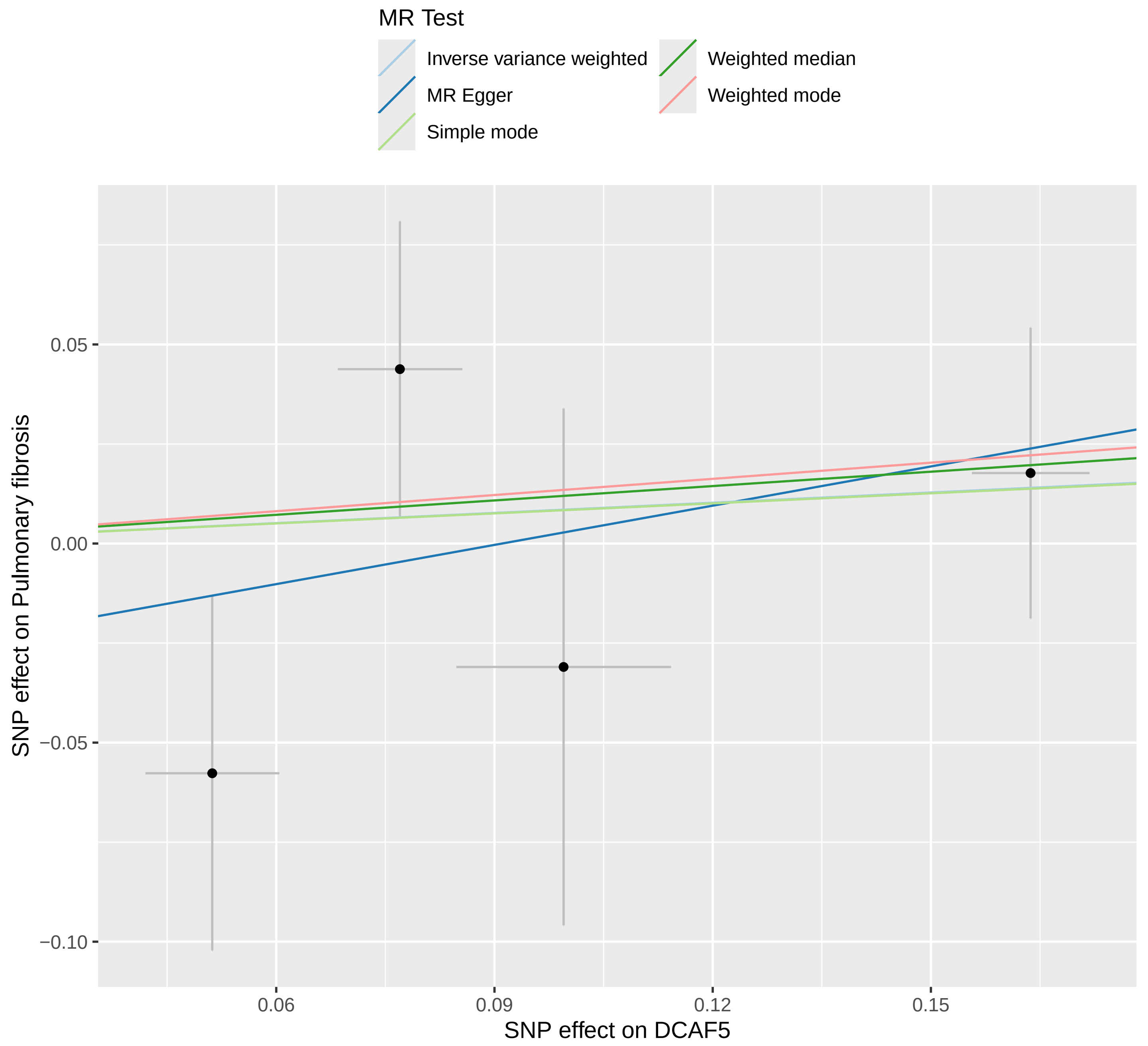

MR Test
Inverse variance weighted
MR Egger
Simple mode
Weighted median
Weighted mode
0.05
0.00
−0.05
−0.10
SNP effect on Pulmonary fibrosis
0.06
0.09
0.12
0.15
SNP effect on DCAF5

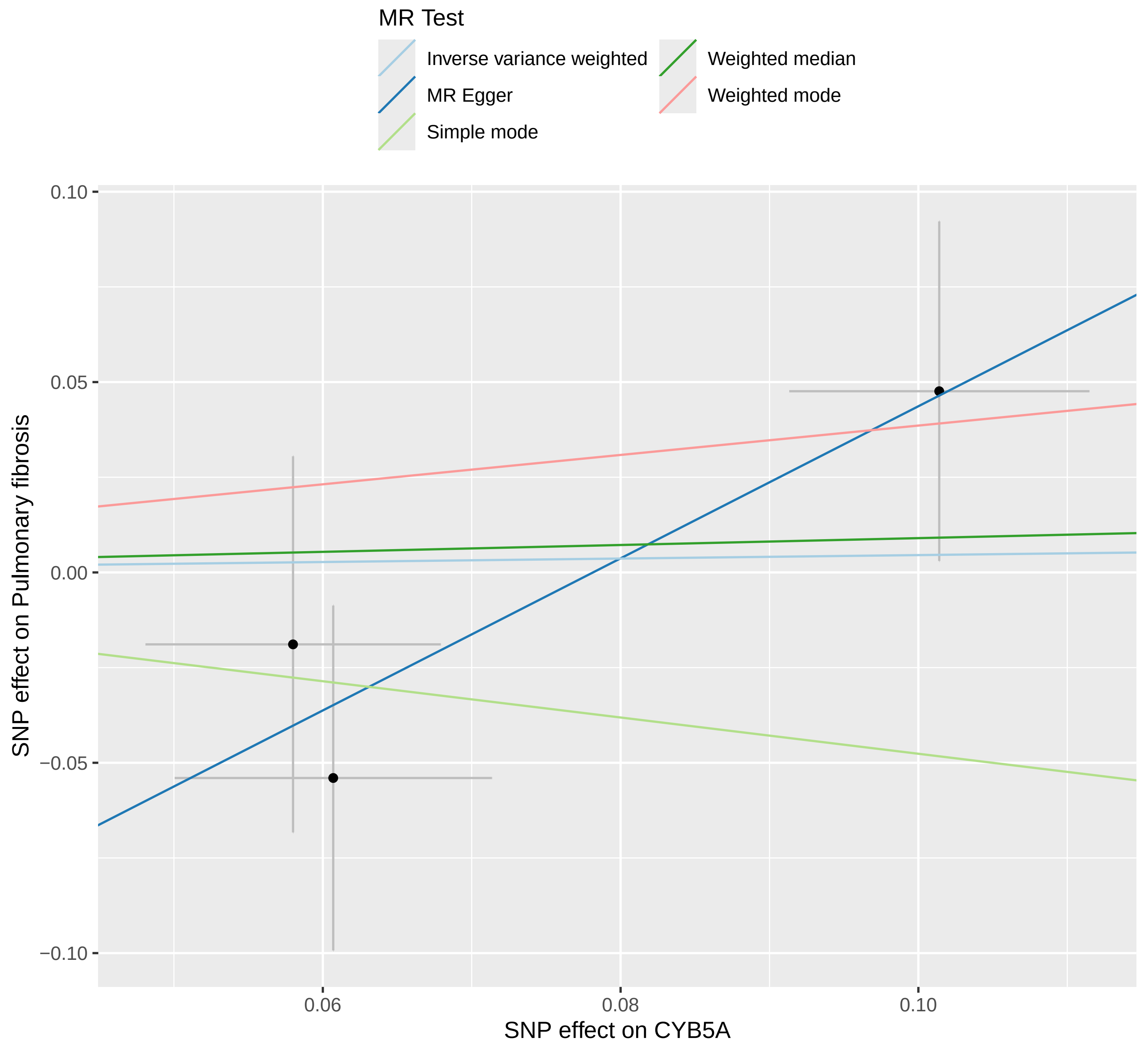
MR Test
Inverse variance weighted
MR Egger
Simple mode
Weighted median
Weighted mode
0.10
0.05
0.00
−0.05
−0.10
0.06
0.08
0.10
SNP effect on Pulmonary fibrosis
SNP effect on CYB5A

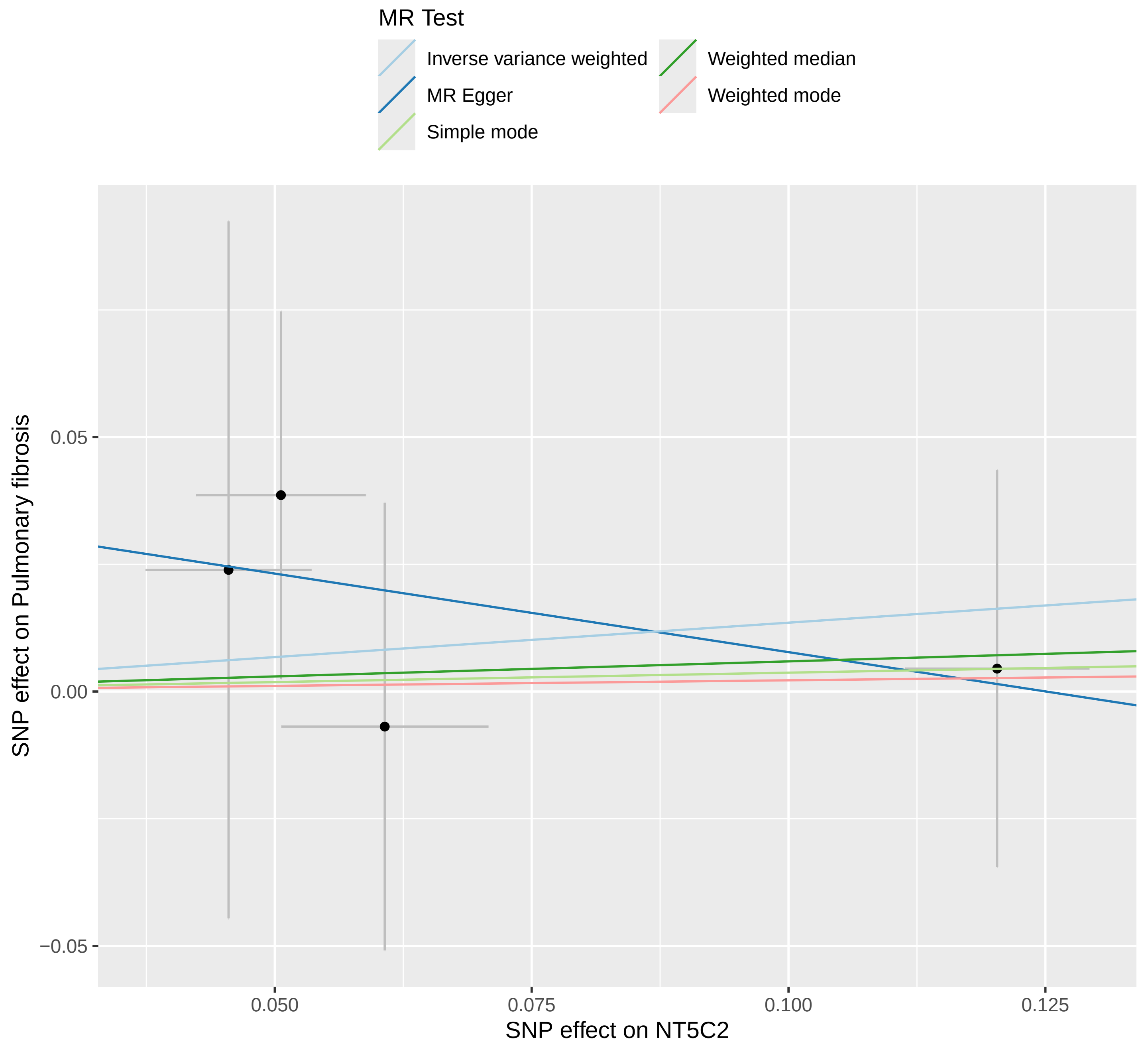
MR Test
Inverse variance weighted
MR Egger
Simple mode
Weighted median
Weighted mode
0.05
0.00
−0.05
0.050
0.075
0.100
0.125
SNP effect on Pulmonary fibrosis
SNP effect on NT5C2

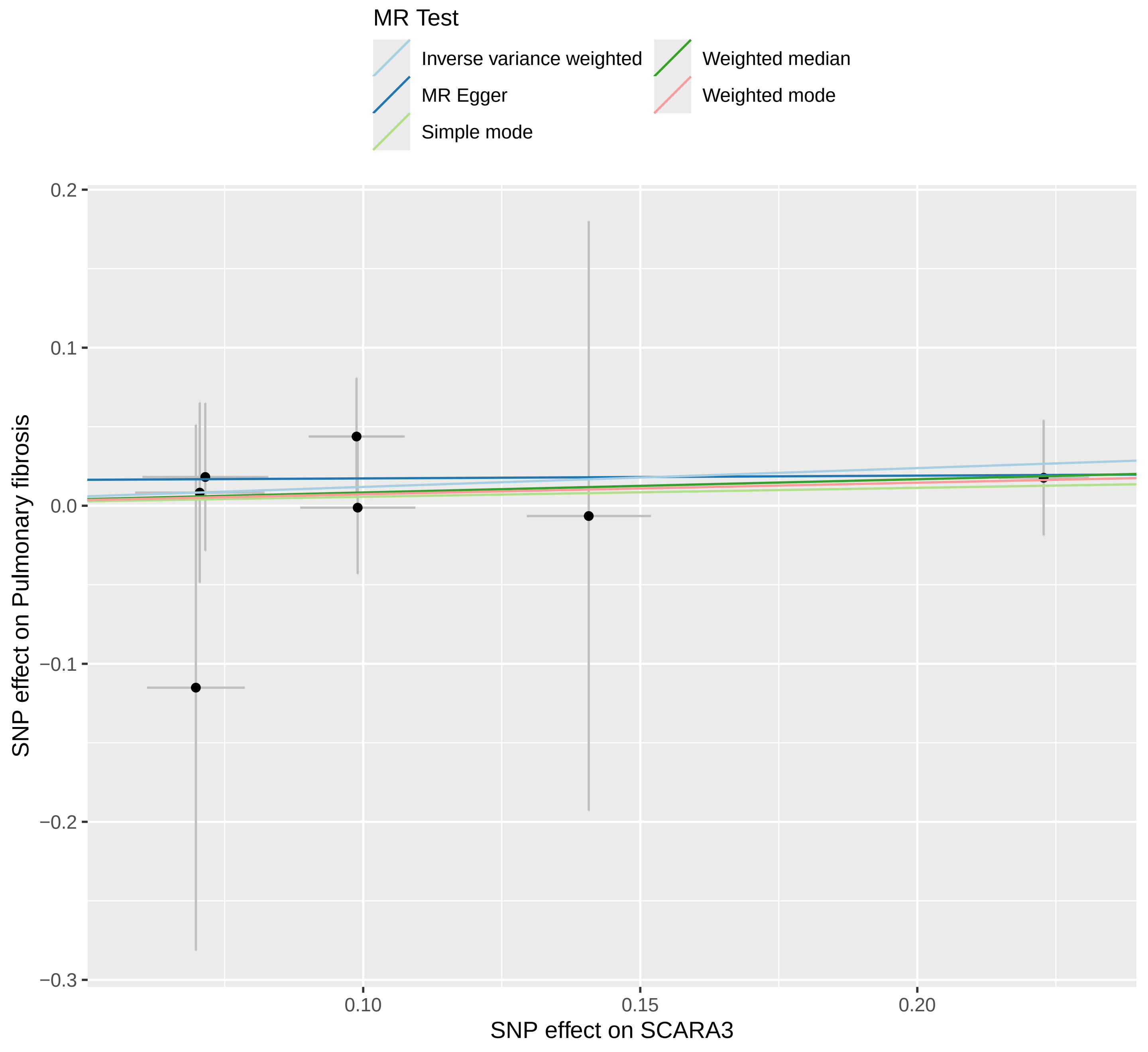

MR Test
Inverse variance weighted
MR Egger
Simple mode
Weighted median
Weighted mode
0.2
0.1
0.0
−0.1
−0.2
−0.3
SNP effect on Pulmonary fibrosis
0.10
0.15
0.20
SNP effect on SCARA3

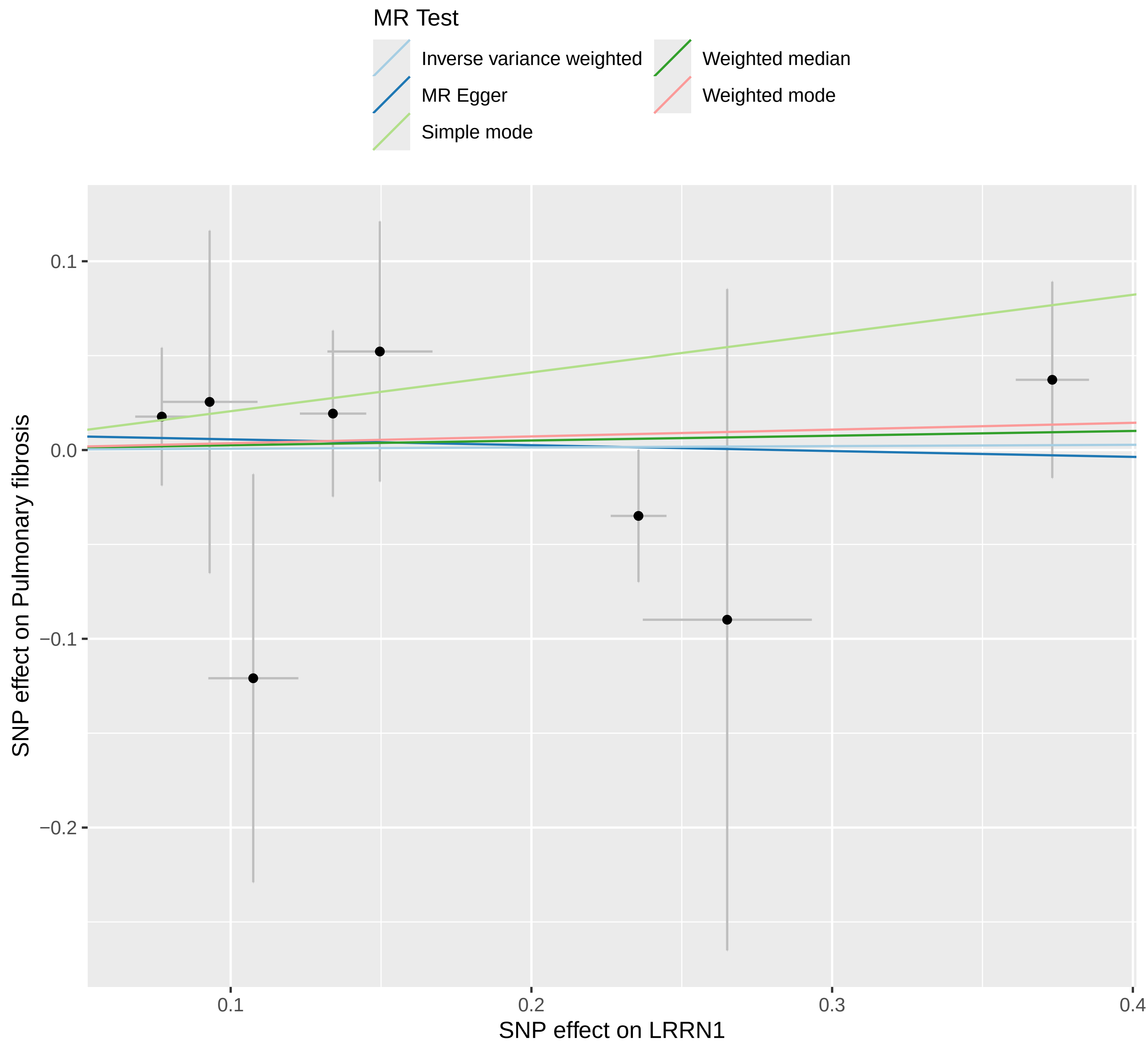
MR Test
Inverse variance weighted
MR Egger
Simple mode
Weighted median
Weighted mode
SNP effect on Pulmonary fibrosis
0.1
0.0
−0.1
−0.2
0.1
0.2
0.3
0.4
SNP effect on LRRN1

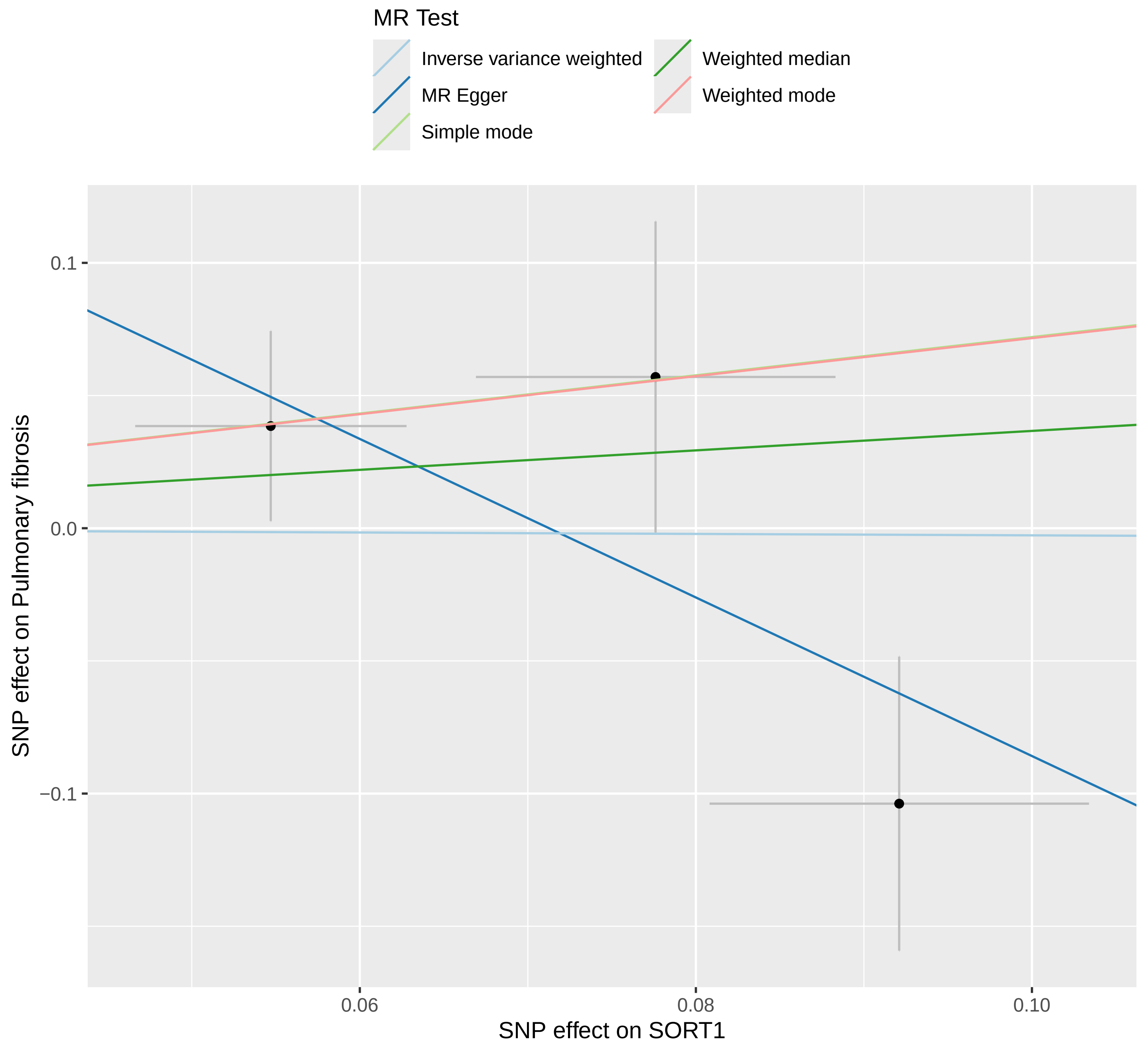
MR Test
Inverse variance weighted
MR Egger
Simple mode
Weighted median
Weighted mode
0.1
0.0
−0.1
SNP effect on Pulmonary fibrosis
0.06
0.08
0.10
SNP effect on SORT1

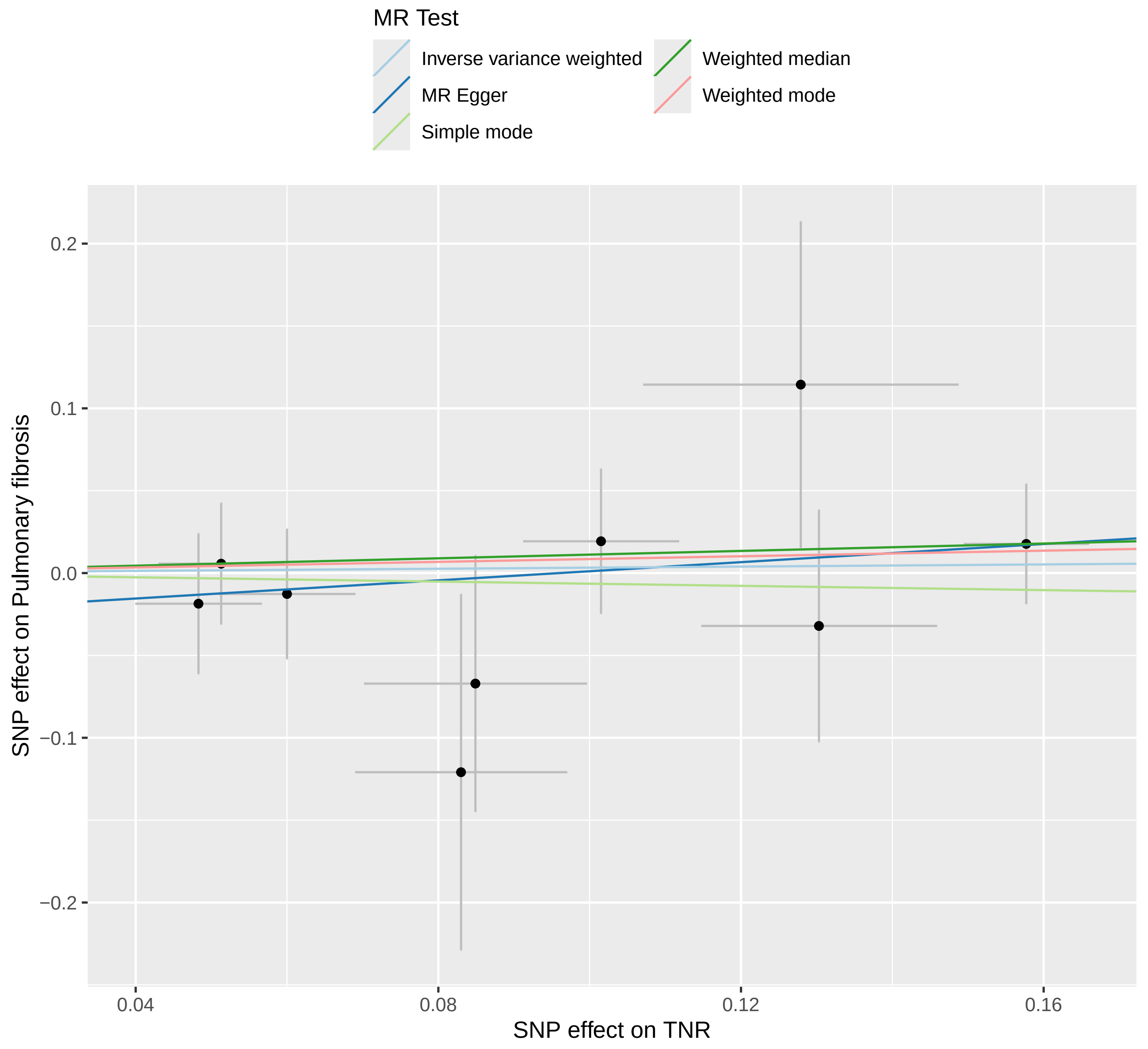
MR Test
Inverse variance weighted
MR Egger
Simple mode
Weighted median
Weighted mode
0.2
0.1
0.0
−0.1
−0.2
SNP effect on Pulmonary fibrosis
0.04
0.08
0.12
0.16
SNP effect on TNR

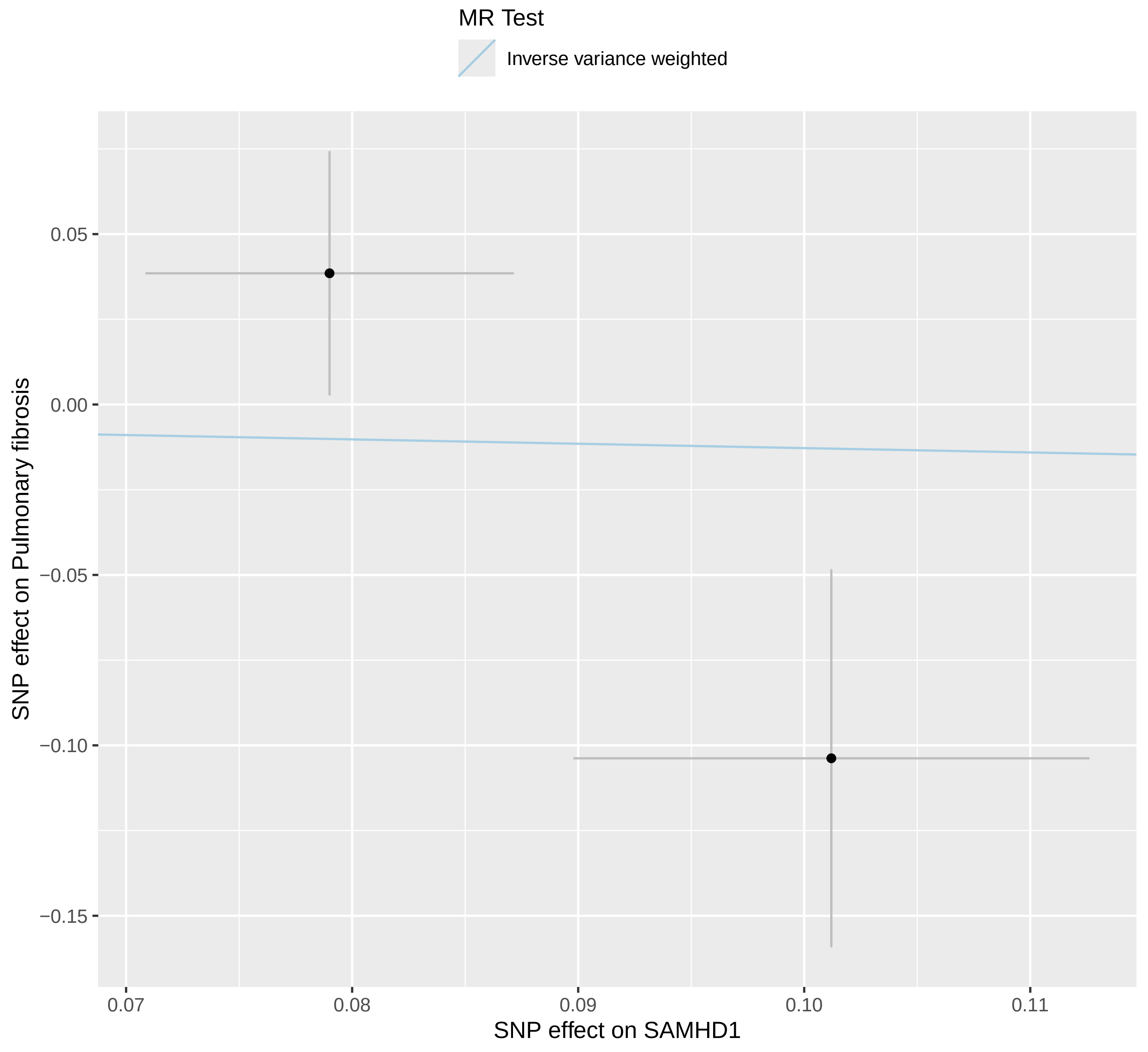

MR Test
Inverse variance weighted
0.05
0.00
−0.05
−0.10
−0.15
SNP effect on Pulmonary fibrosis
0.07
0.08
0.09
0.10
0.11
SNP effect on SAMHD1

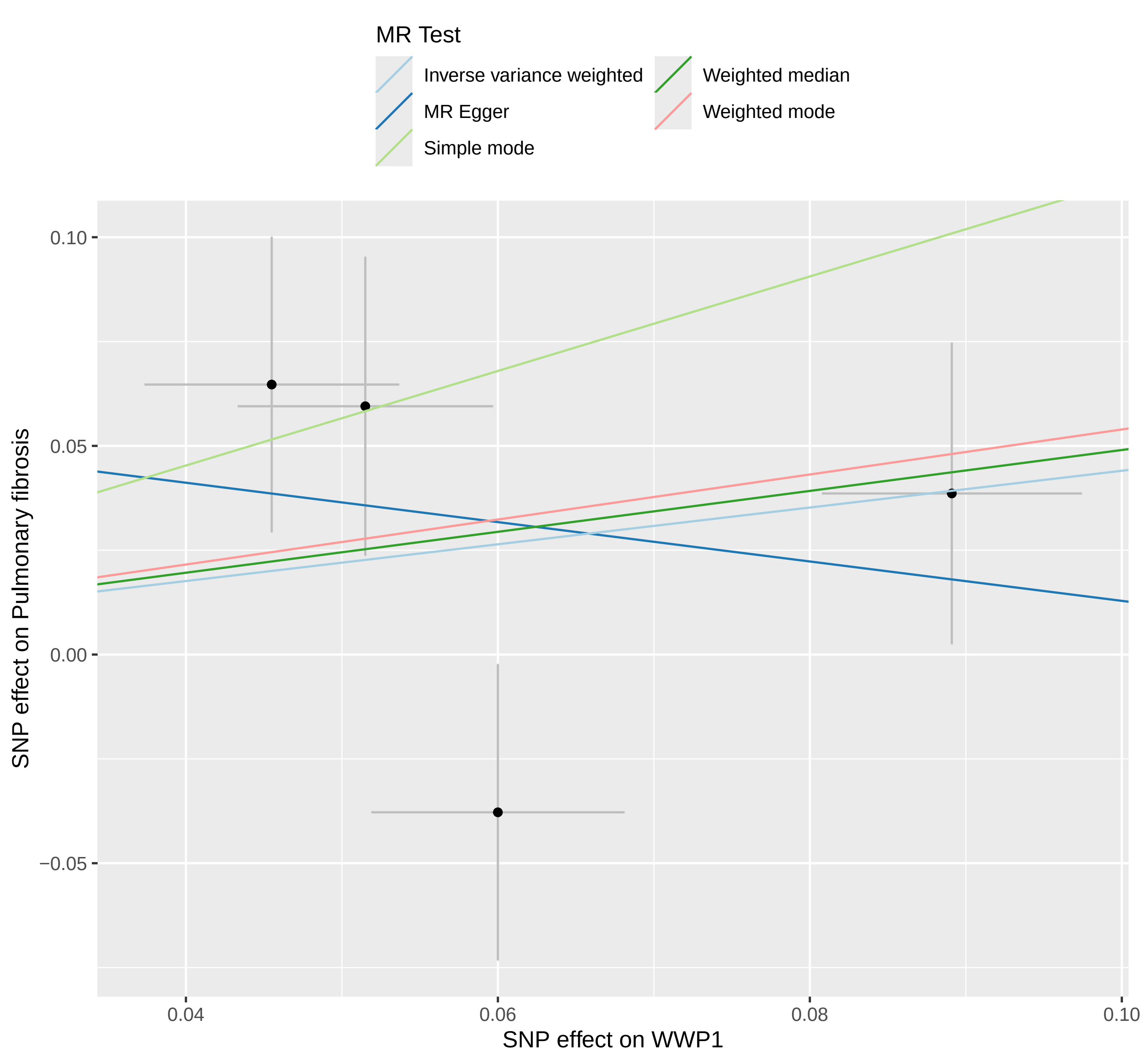
MR Test
Inverse variance weighted
MR Egger
Simple mode
Weighted median
Weighted mode
0.10
0.05
0.00
−0.05
SNP effect on Pulmonary fibrosis
0.04
0.06
0.08
0.10
SNP effect on WWP1

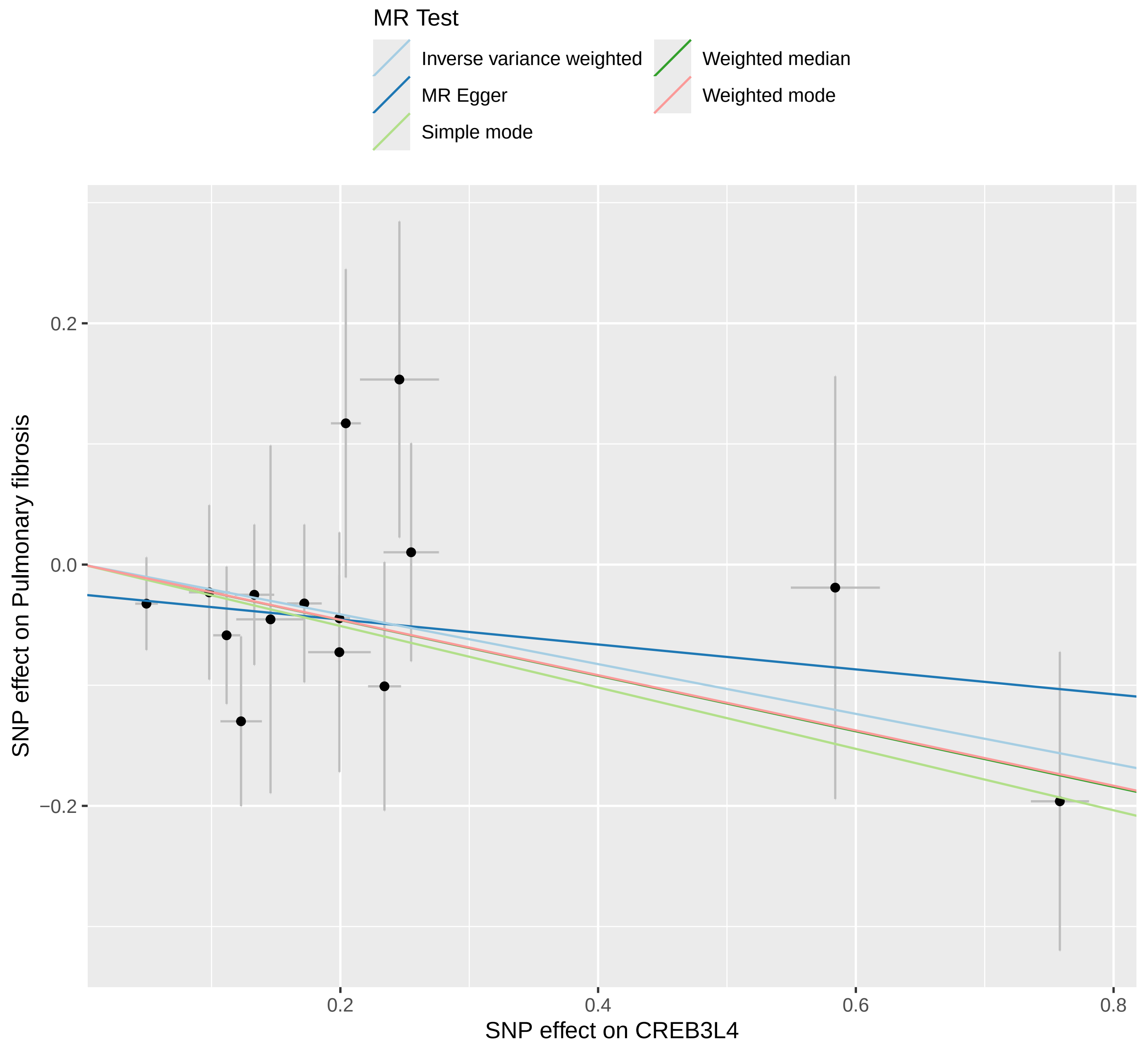
MR Test
Inverse variance weighted
MR Egger
Simple mode
Weighted median
Weighted mode
0.2
0.0
−0.2
0.2
0.4
0.6
0.8
SNP effect on Pulmonary fibrosis
SNP effect on CREB3L4

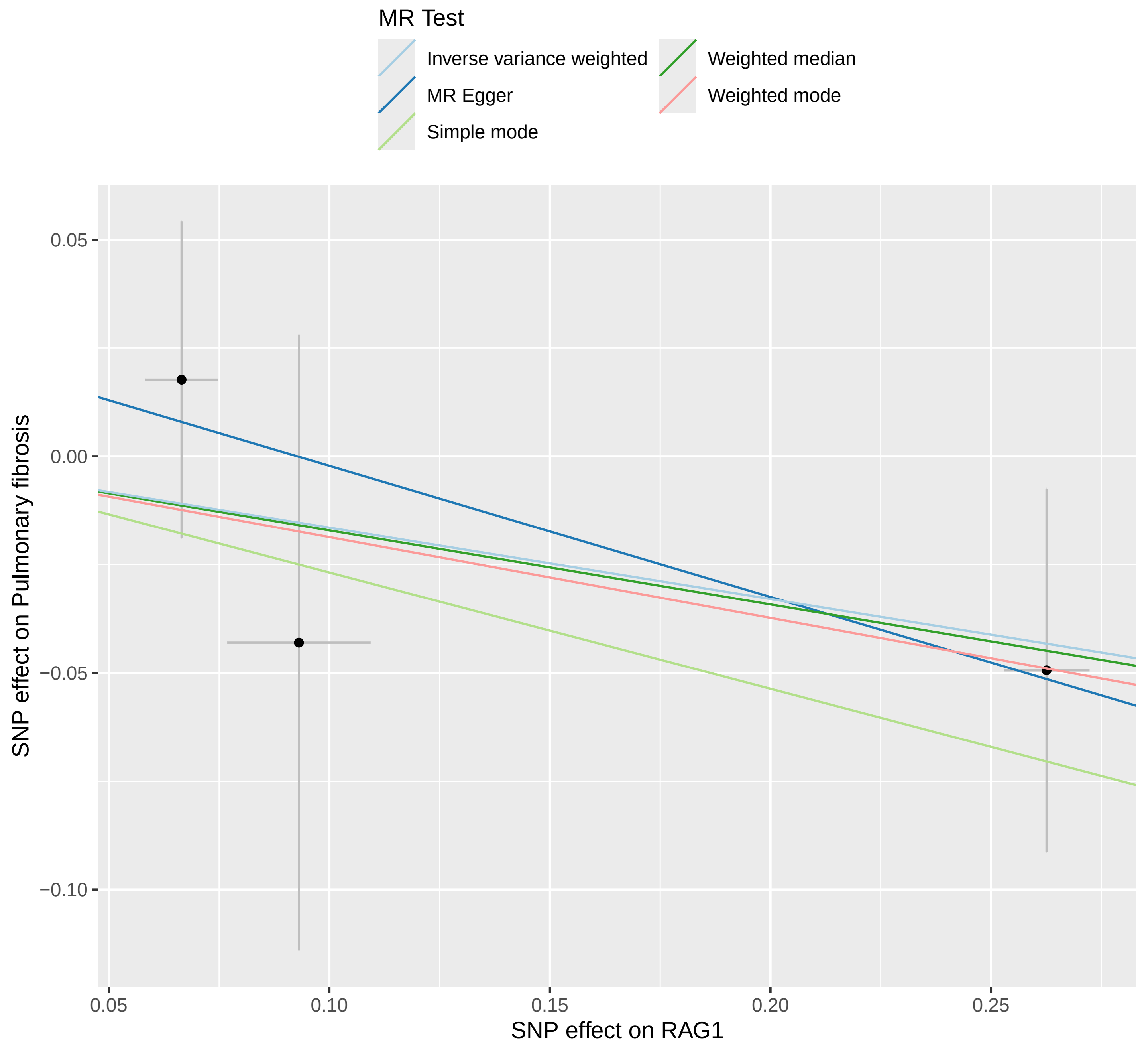
MR Test
Inverse variance weighted
MR Egger
Simple mode
Weighted median
Weighted mode
SNP effect on Pulmonary fibrosis
0.05
0.00
−0.05
−0.10
0.05
0.10
0.15
0.20
0.25
SNP effect on RAG1

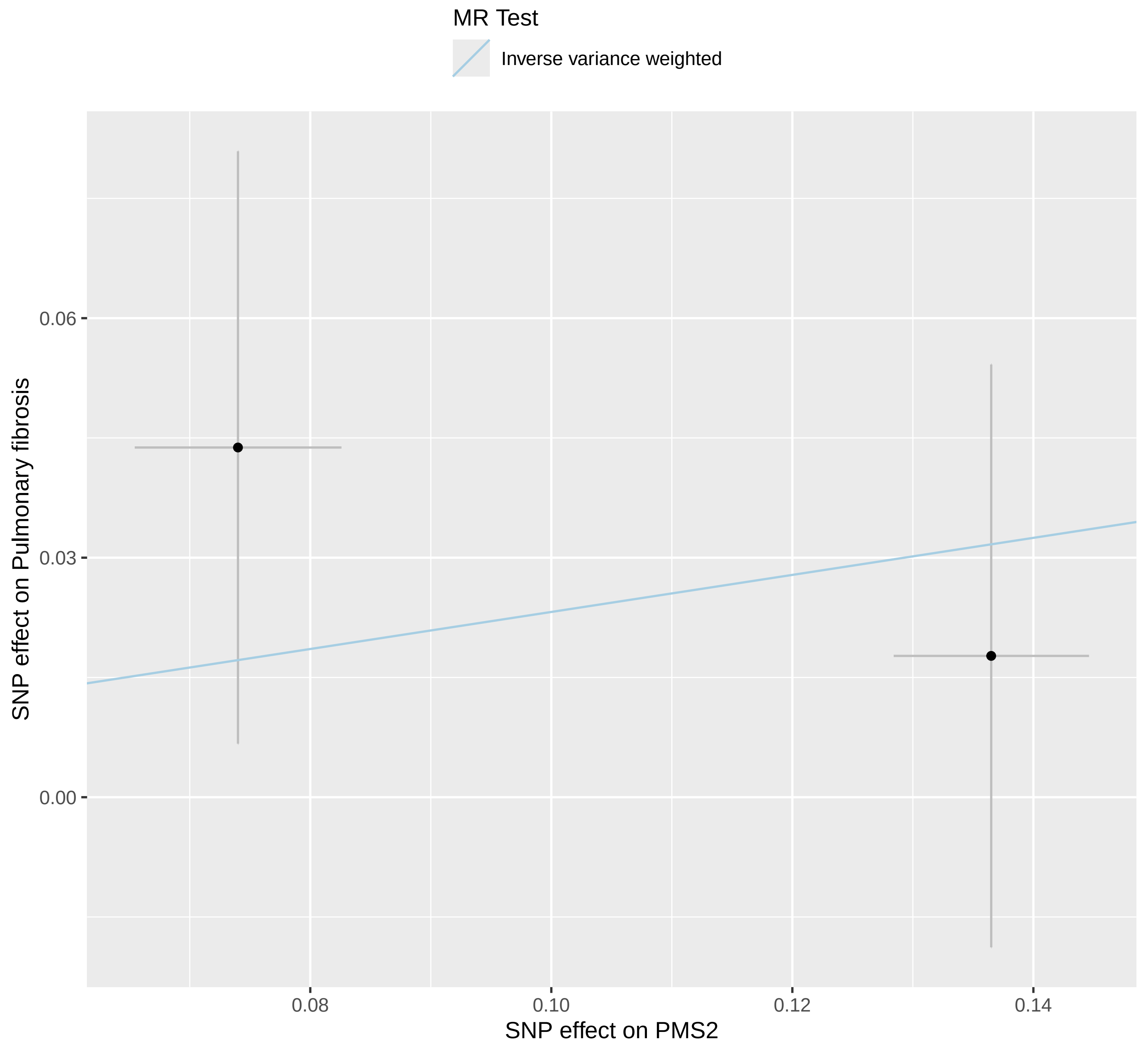
MR Test
Inverse variance weighted
0.06
0.03
0.00
0.08
0.10
0.12
0.14
SNP effect on Pulmonary fibrosis
SNP effect on PMS2

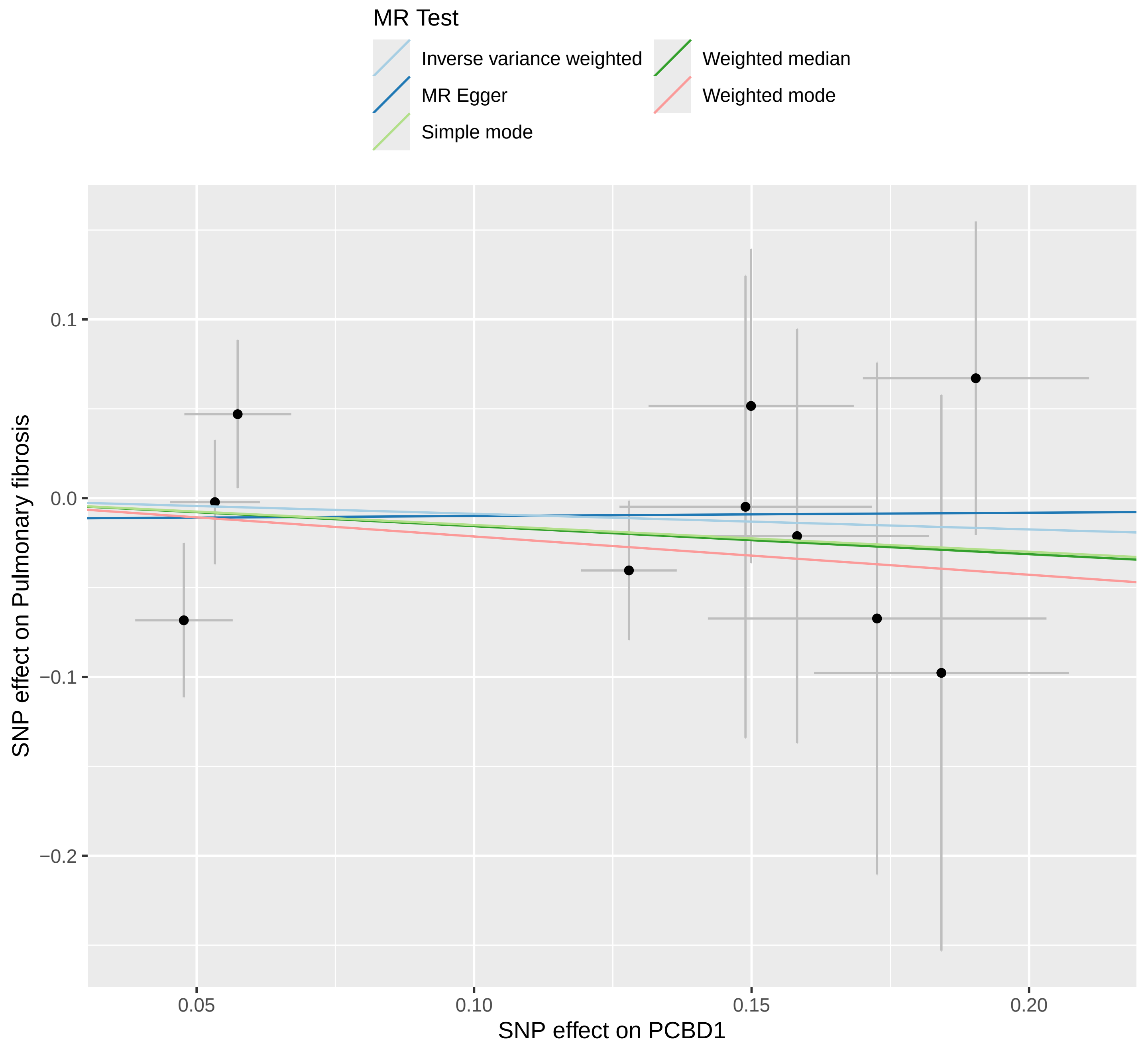
MR Test
Inverse variance weighted
MR Egger
Simple mode
Weighted median
Weighted mode
0.1
0.0
−0.1
−0.2
0.05
0.10
0.15
0.20
SNP effect on Pulmonary fibrosis
SNP effect on PCBD1

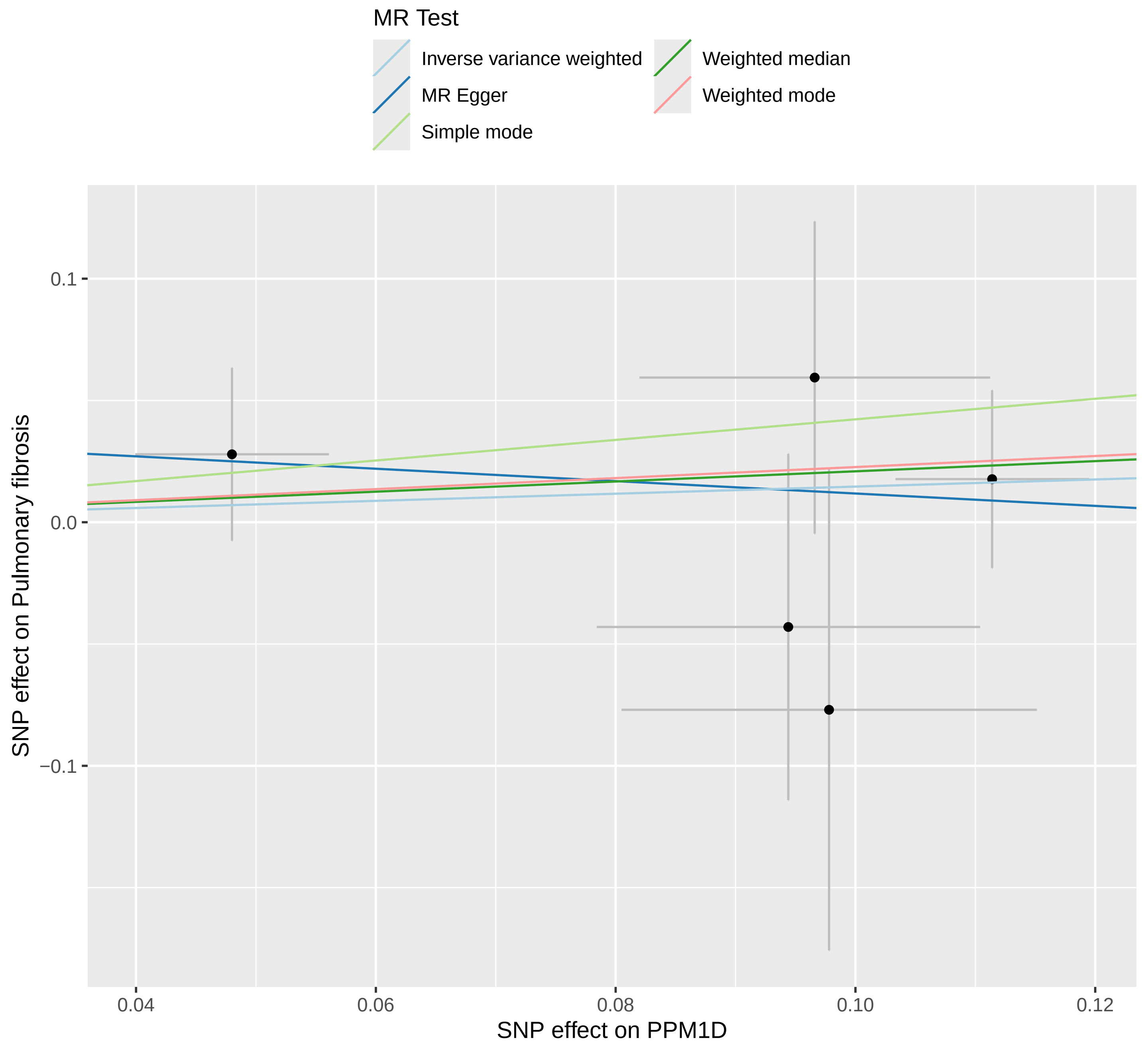
MR Test
Inverse variance weighted
MR Egger
Simple mode
Weighted median
Weighted mode
0.1
0.0
−0.1
0.04
0.06
0.08
0.10
0.12
SNP effect on PPM1D
SNP effect on Pulmonary fibrosis

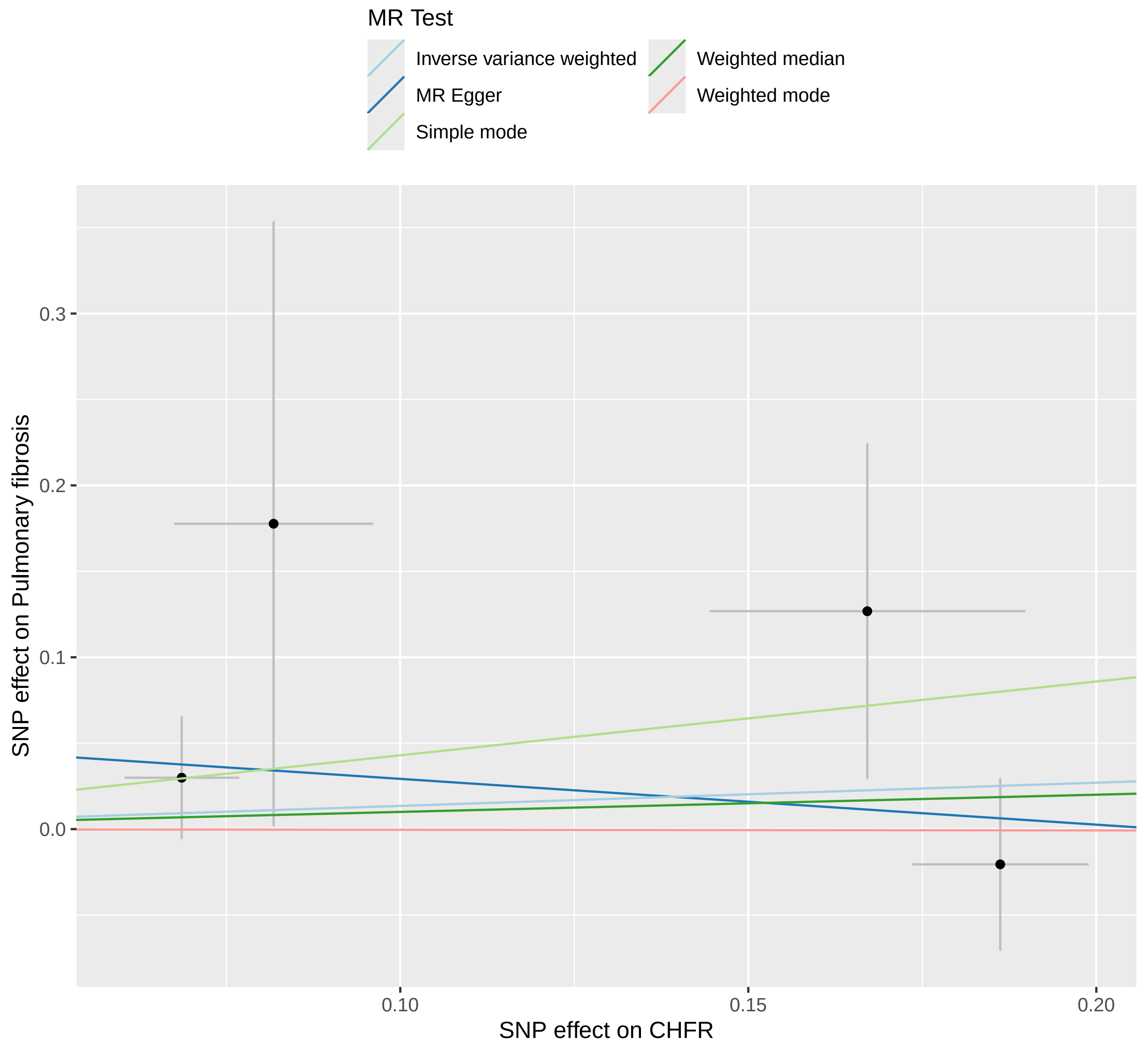
MR Test
Inverse variance weighted
MR Egger
Simple mode
Weighted median
Weighted mode
0.3
0.2
0.1
0.0
0.10
0.15
0.20
SNP effect on Pulmonary fibrosis
SNP effect on CHFR

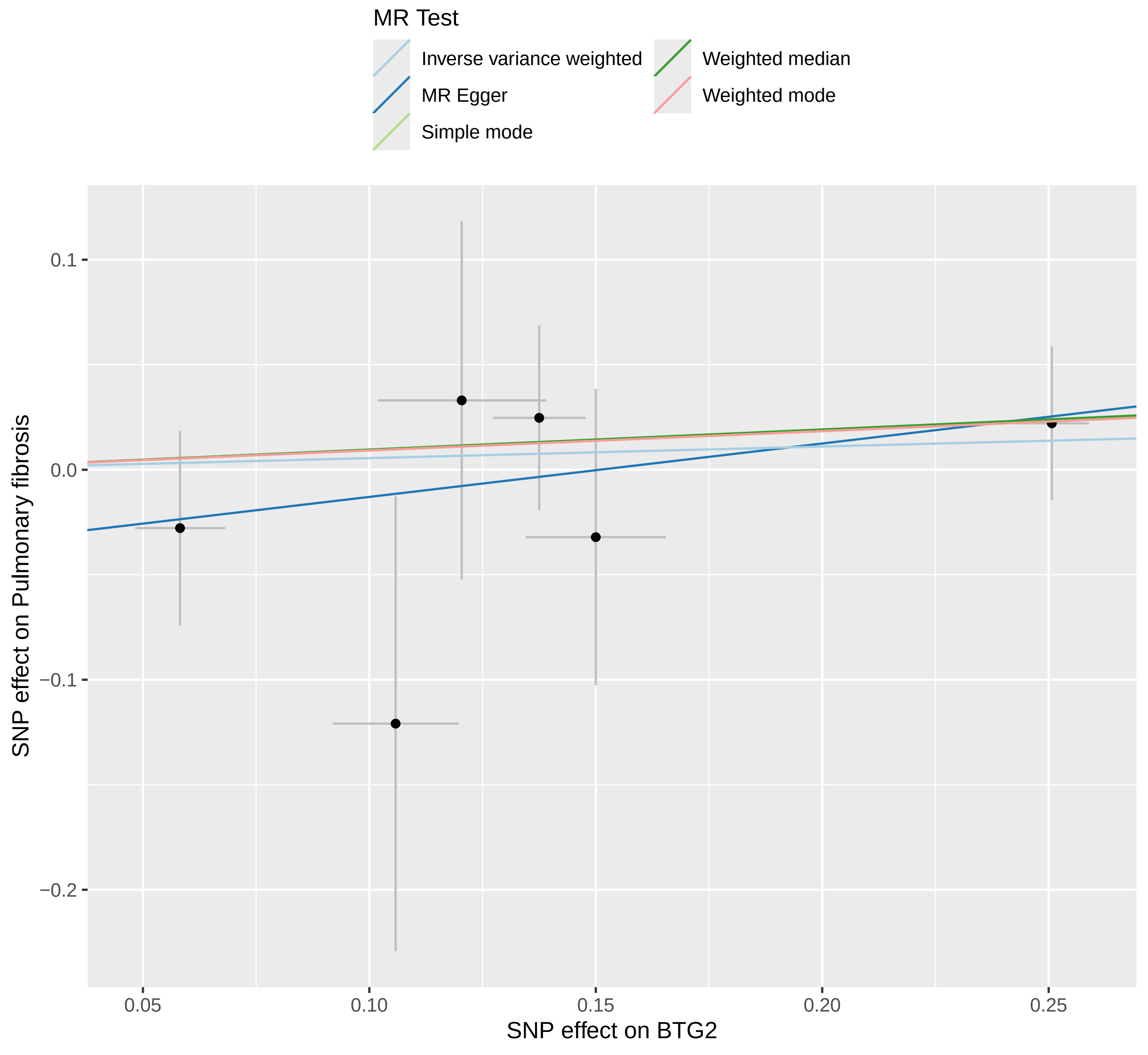
MR Test
Inverse variance weighted
MR Egger
Simple mode
Weighted median
Weighted mode
0.1
0.0
−0.1
−0.2
SNP effect on Pulmonary fibrosis
0.05
0.10
0.15
0.20
0.25
SNP effect on BTG2

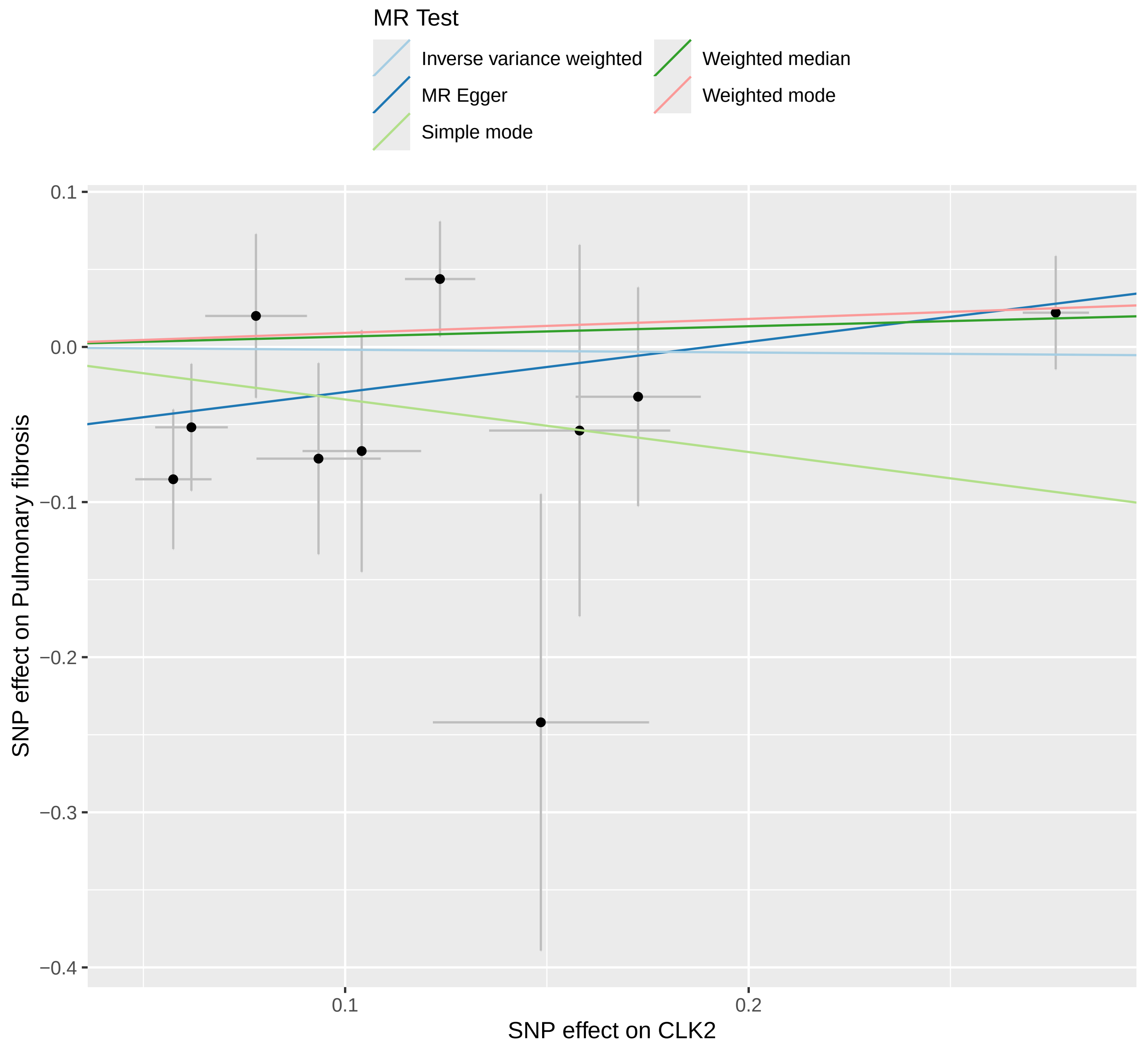
MR Test
Inverse variance weighted
MR Egger
Simple mode
Weighted median
Weighted mode
0.1
0.0
−0.1
−0.2
−0.3
−0.4
SNP effect on Pulmonary fibrosis
0.1
0.2
SNP effect on CLK2

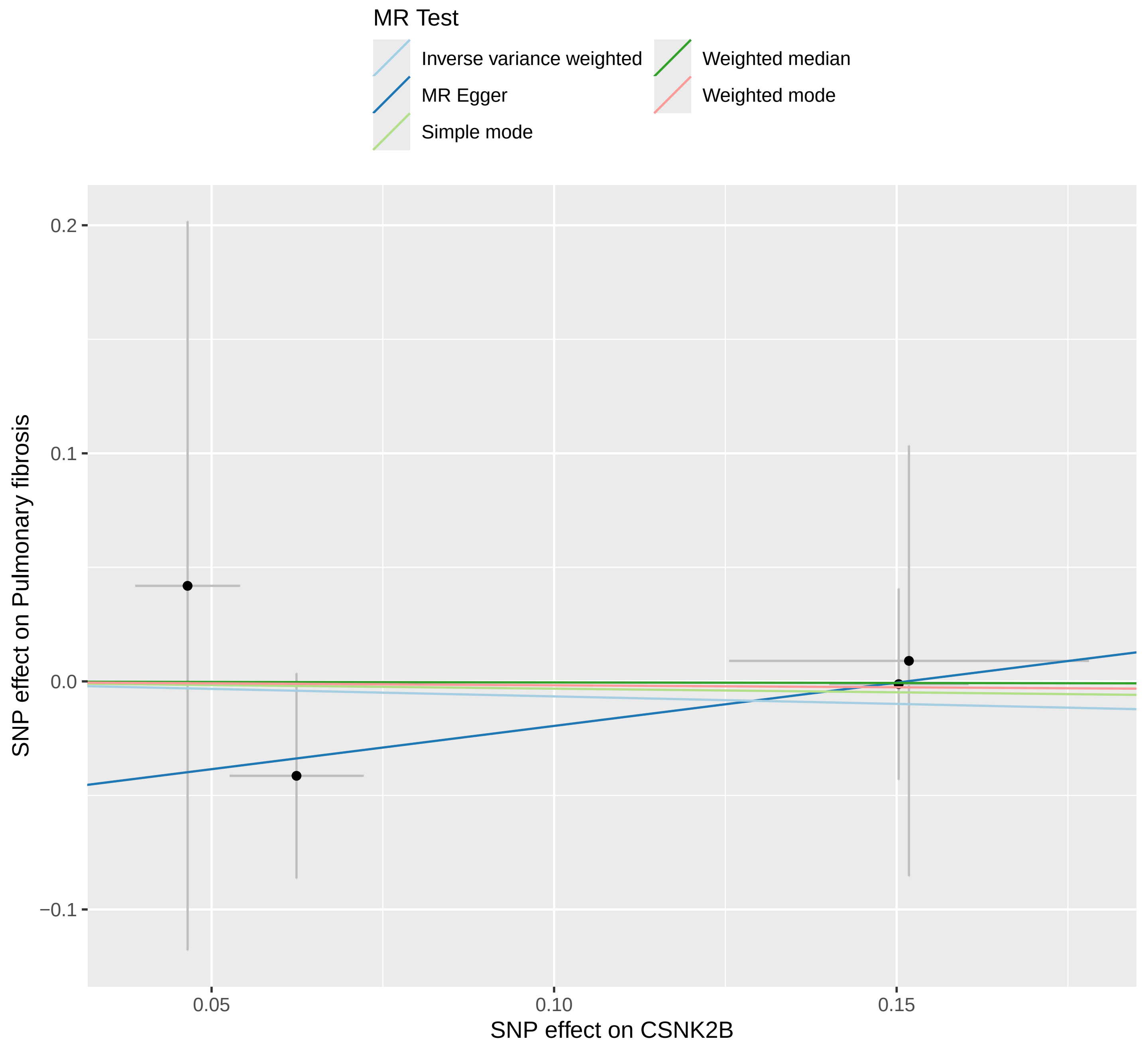
MR Test
Inverse variance weighted
MR Egger
Simple mode
Weighted median
Weighted mode
0.2
0.1
0.0
−0.1
SNP effect on Pulmonary fibrosis
0.05
0.10
0.15
SNP effect on CSNK2B

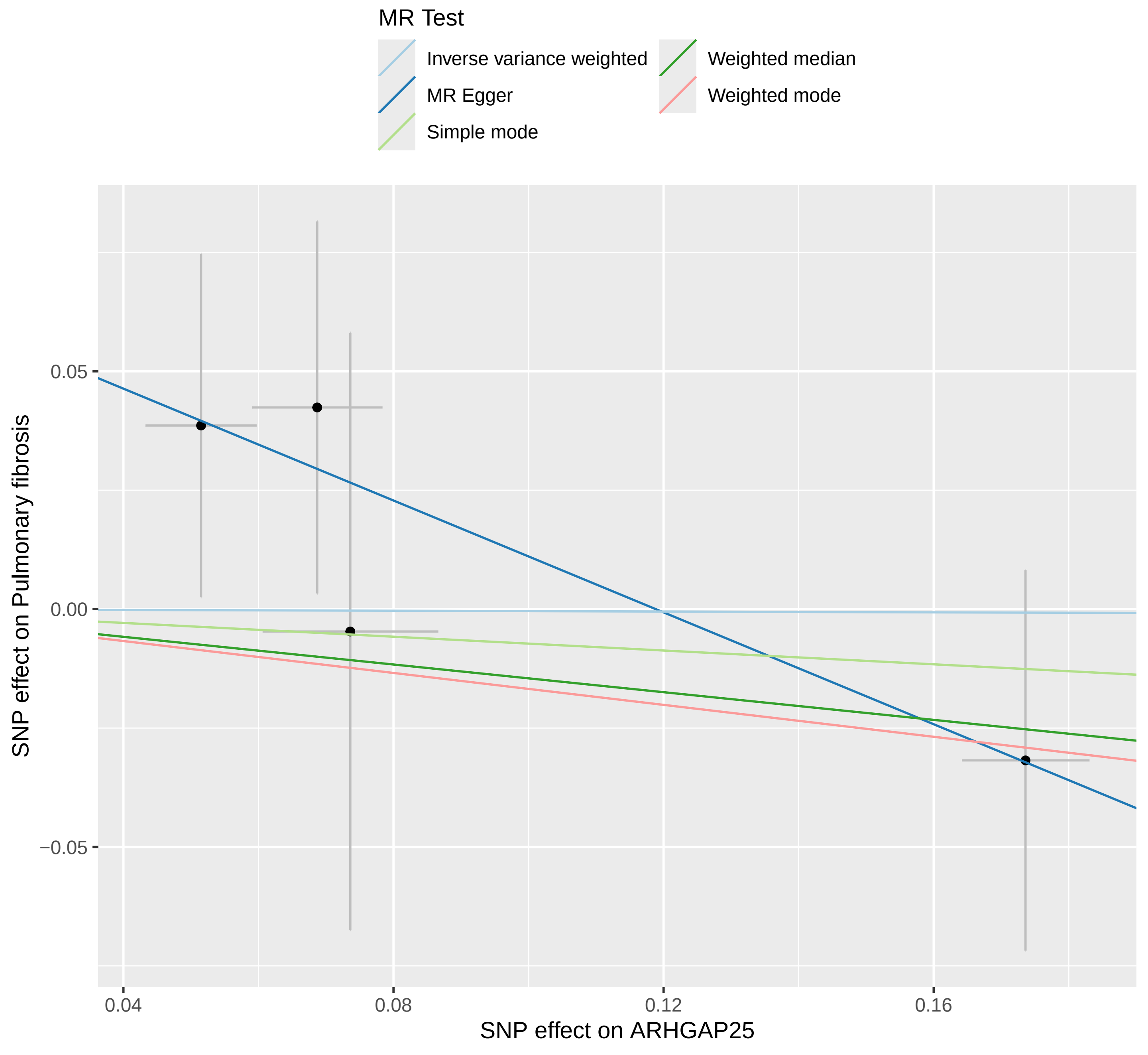
MR Test
Inverse variance weighted
MR Egger
Simple mode
Weighted median
Weighted mode
SNP effect on Pulmonary fibrosis
0.05
0.00
−0.05
0.04
0.08
0.12
0.16
SNP effect on ARHGAP25

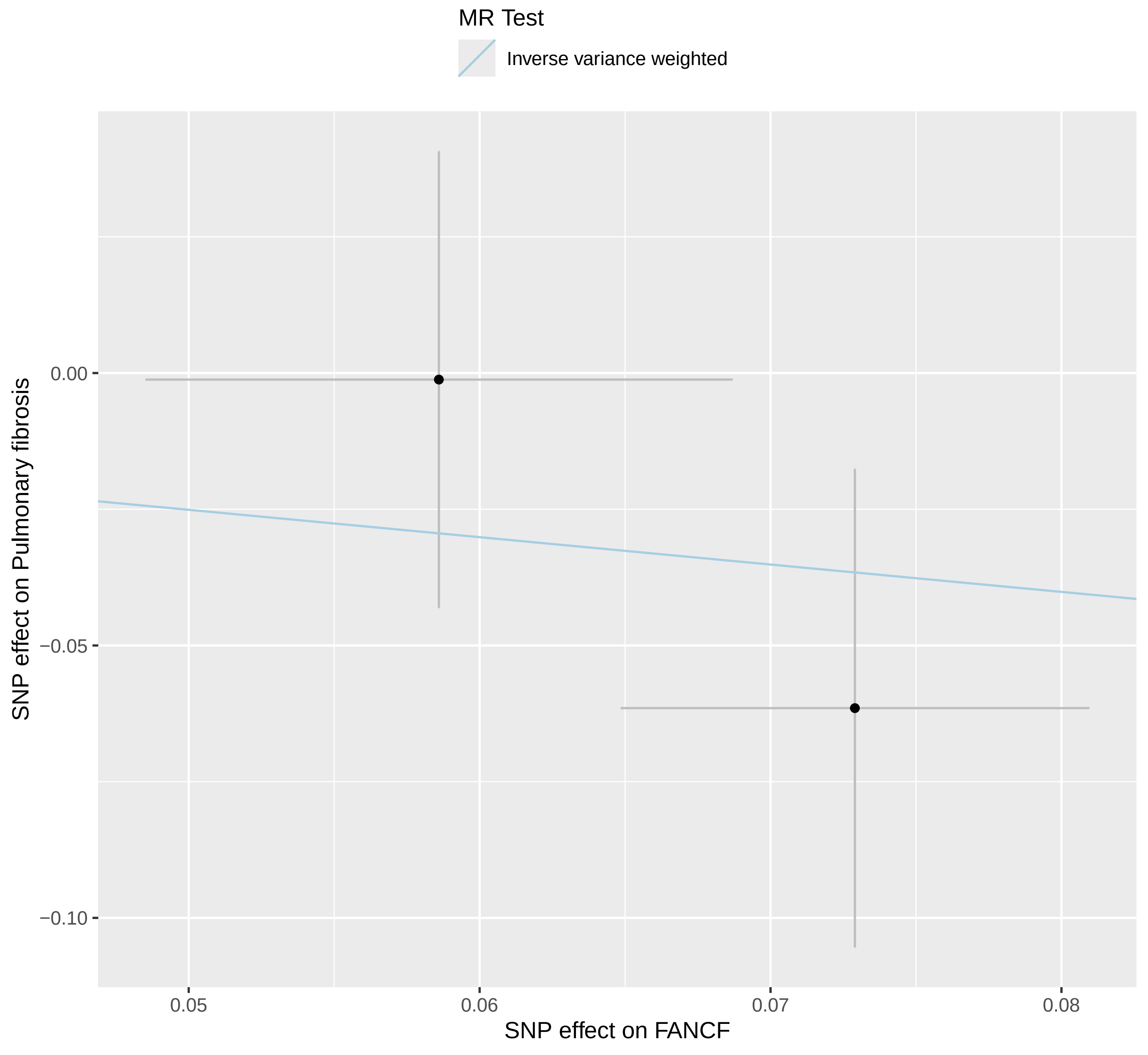

MR Test
Inverse variance weighted
0.00
−0.05
−0.10
0.05
0.06
0.07
0.08
SNP effect on FANCF
SNP effect on Pulmonary fibrosis

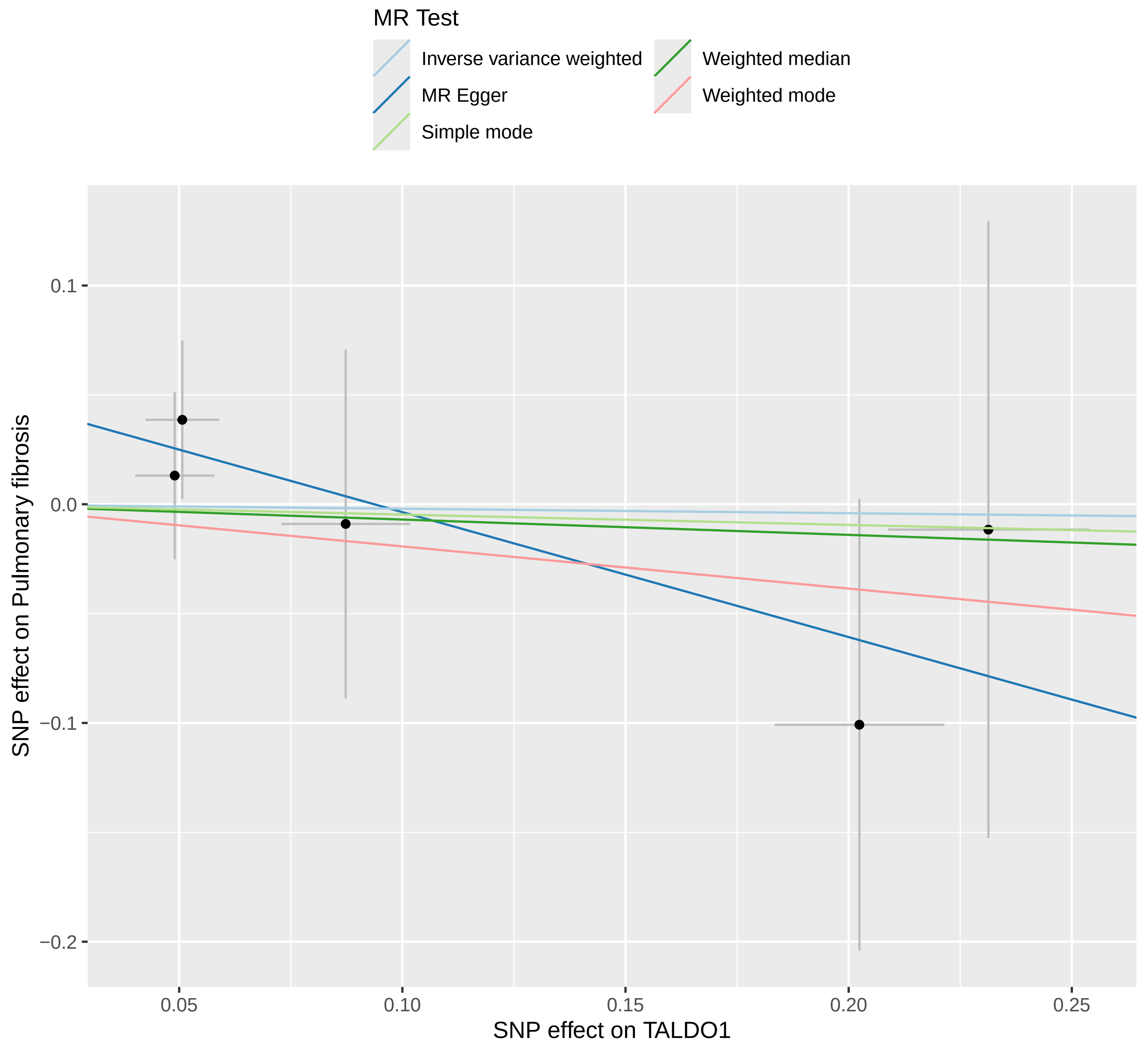
MR Test
Inverse variance weighted
MR Egger
Simple mode
Weighted median
Weighted mode
0.1
0.0
−0.1
−0.2
SNP effect on Pulmonary fibrosis
0.05
0.10
0.15
0.20
0.25
SNP effect on TALDO1

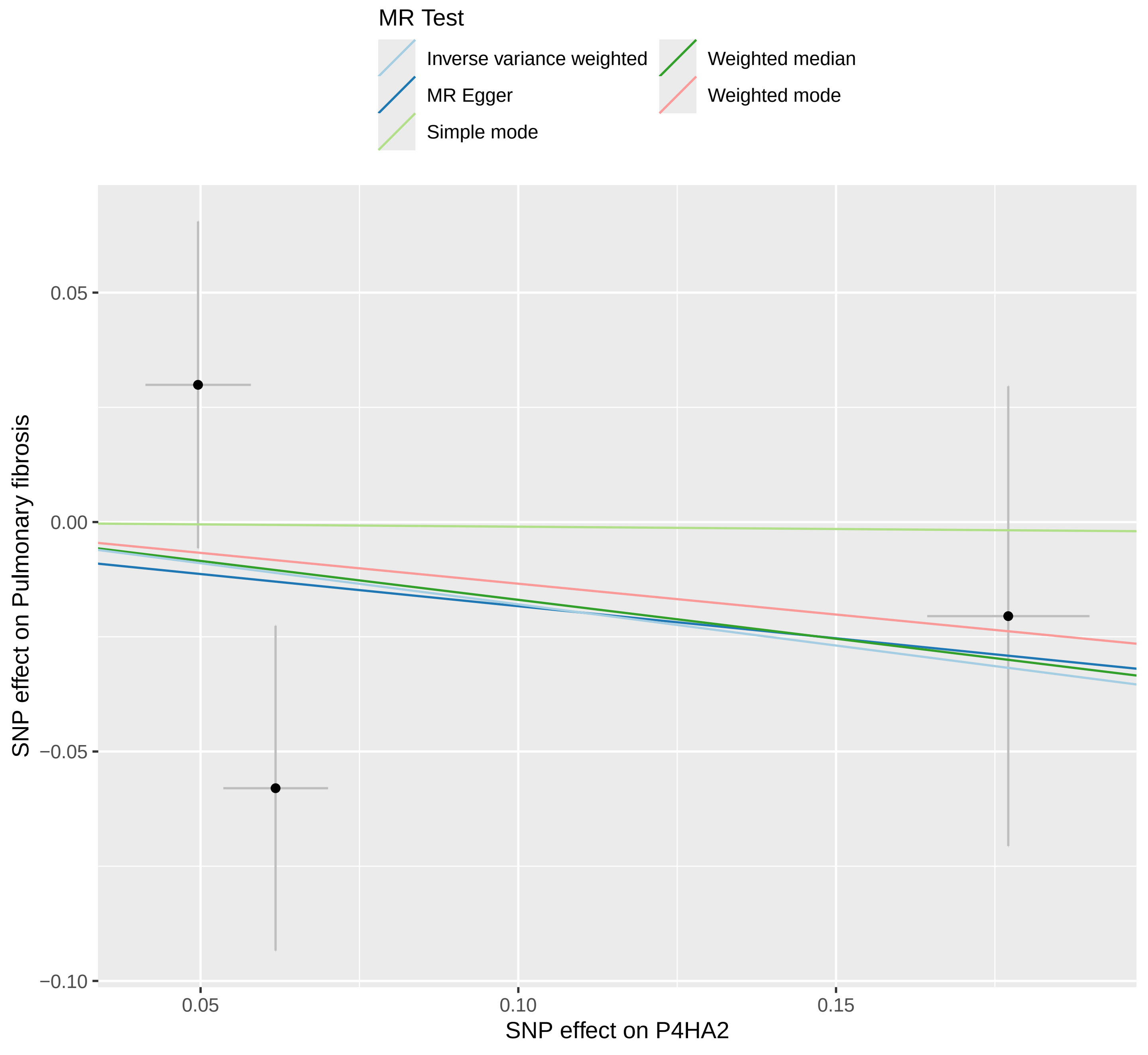
MR Test
Inverse variance weighted
MR Egger
Simple mode
Weighted median
Weighted mode
0.05
0.00
−0.05
−0.10
0.05
0.10
0.15
SNP effect on Pulmonary fibrosis
SNP effect on P4HA2

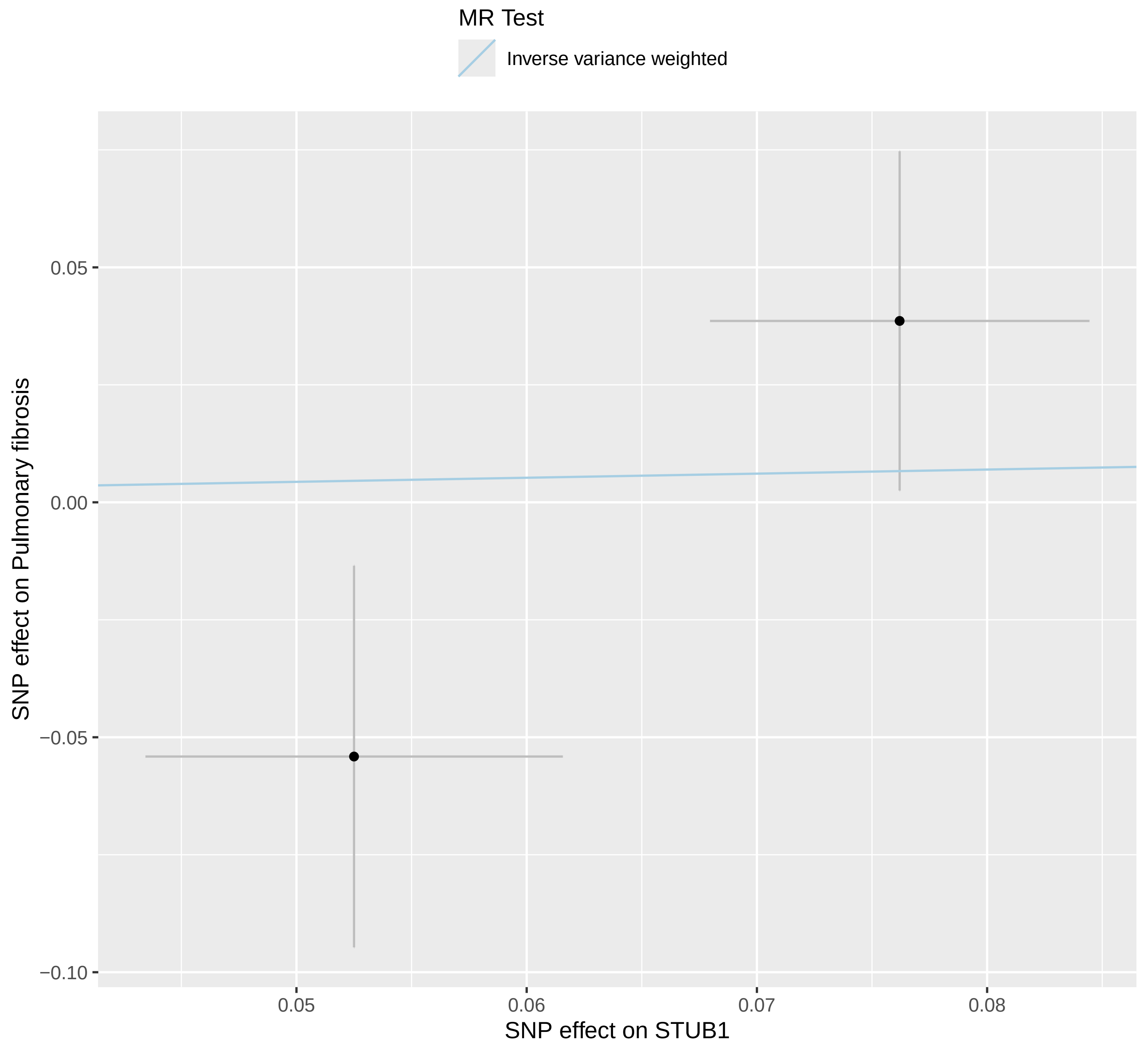

MR Test
Inverse variance weighted
0.05
0.00
−0.05
−0.10
0.05
0.06
0.07
0.08
SNP effect on STUB1
SNP effect on Pulmonary fibrosis

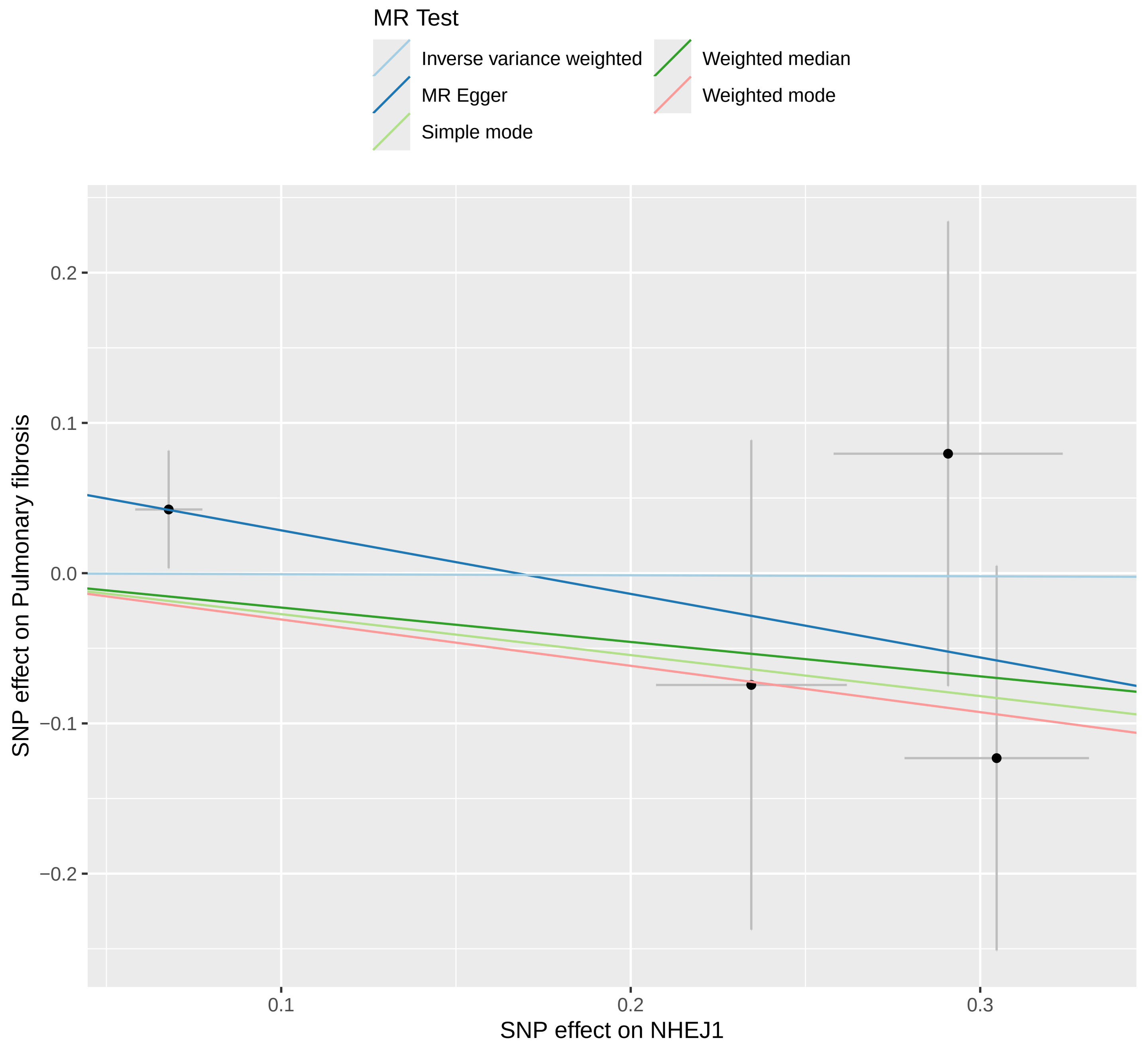
MR Test
Inverse variance weighted
MR Egger
Simple mode
Weighted median
Weighted mode
SNP effect on Pulmonary fibrosis
0.2
0.1
0.0
−0.1
−0.2
0.1
0.2
0.3
SNP effect on NHEJ1

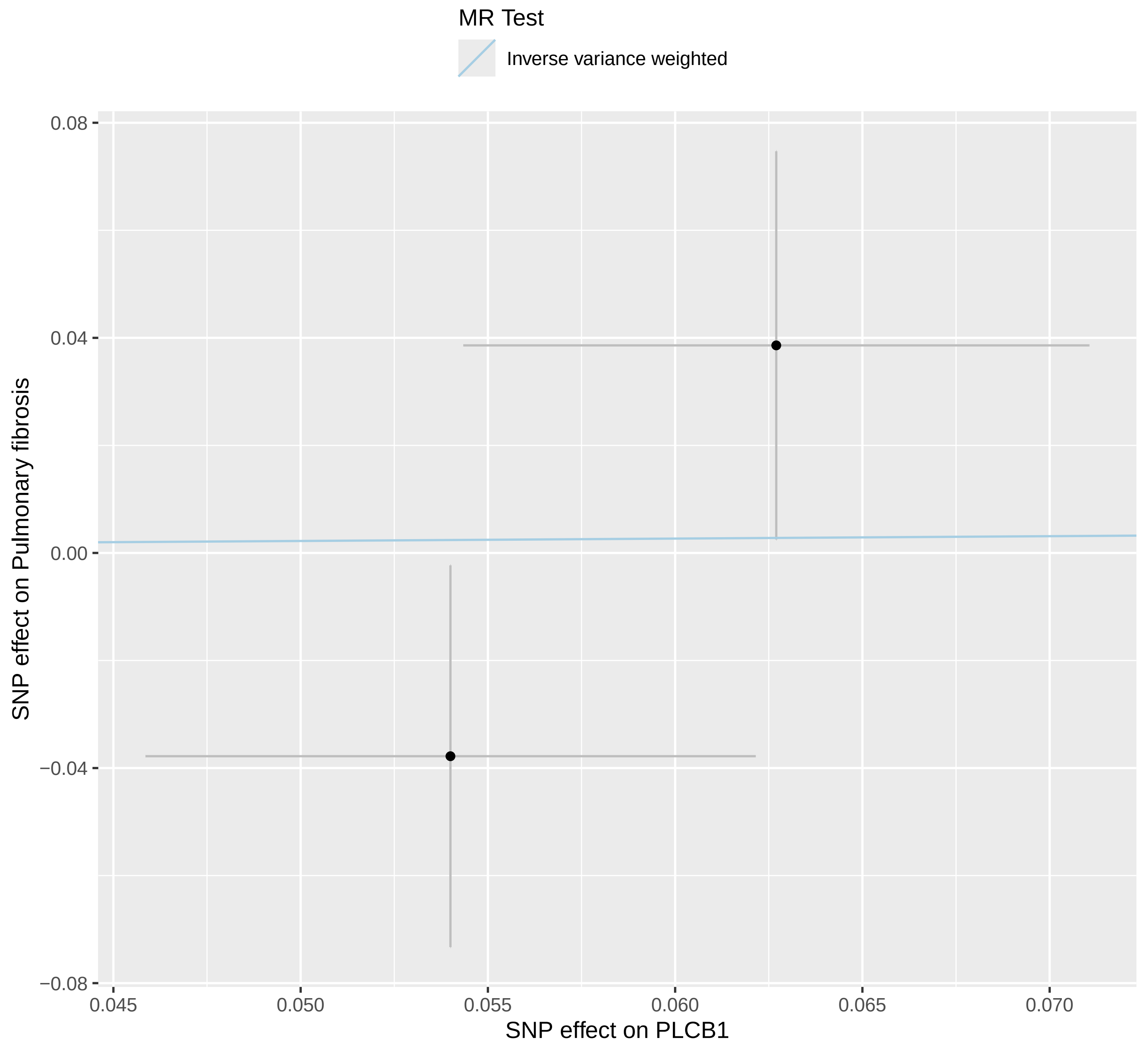
MR Test
Inverse variance weighted
0.08
0.04
0.00
−0.04
−0.08
0.045
0.050
0.055
0.060
0.065
0.070
SNP effect on PLCB1
SNP effect on Pulmonary fibrosis

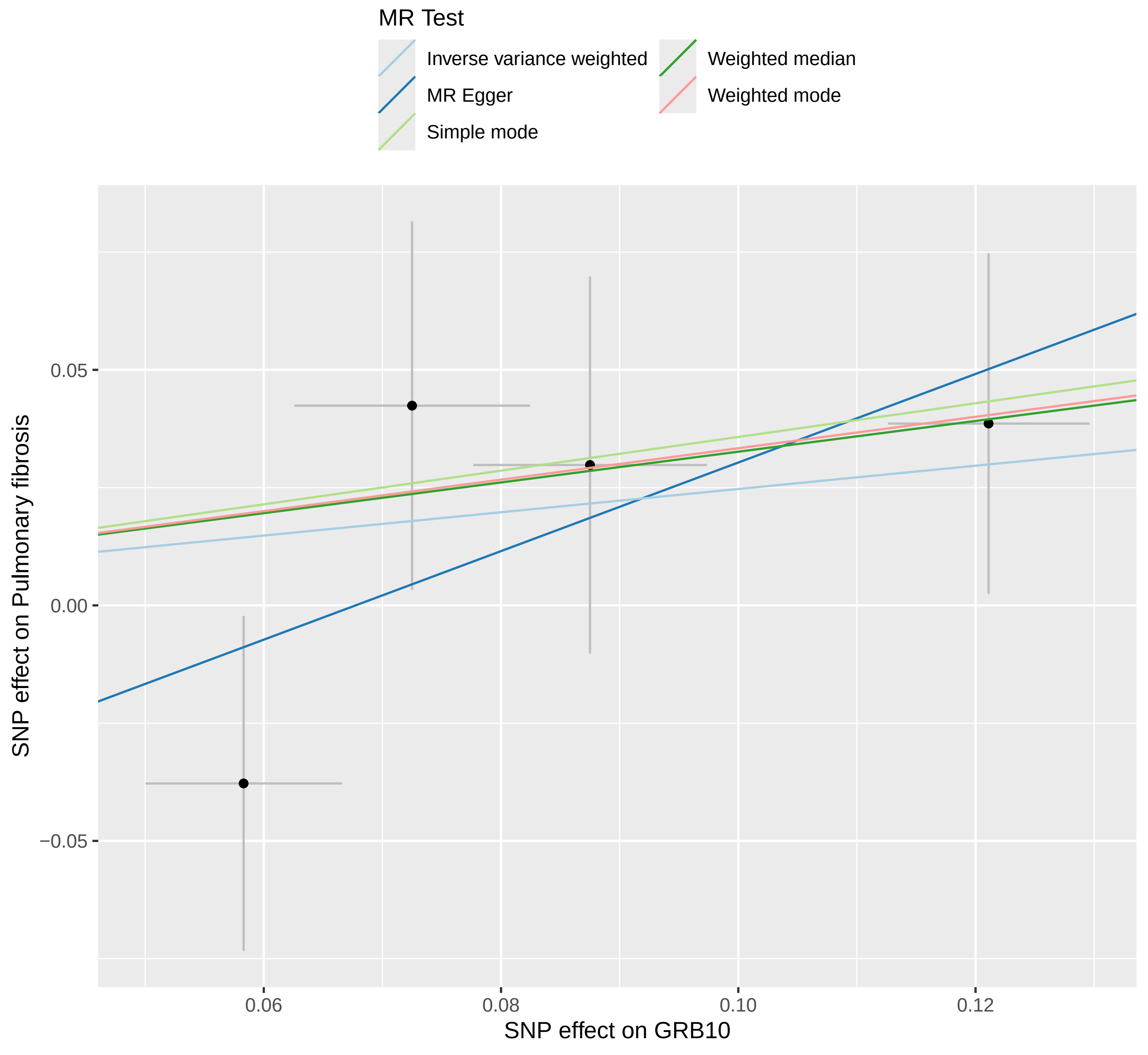

MR Test
Inverse variance weighted
MR Egger
Simple mode
Weighted median
Weighted mode
SNP effect on Pulmonary fibrosis
0.05
0.00
−0.05
0.06
0.08
0.10
0.12
SNP effect on GRB10

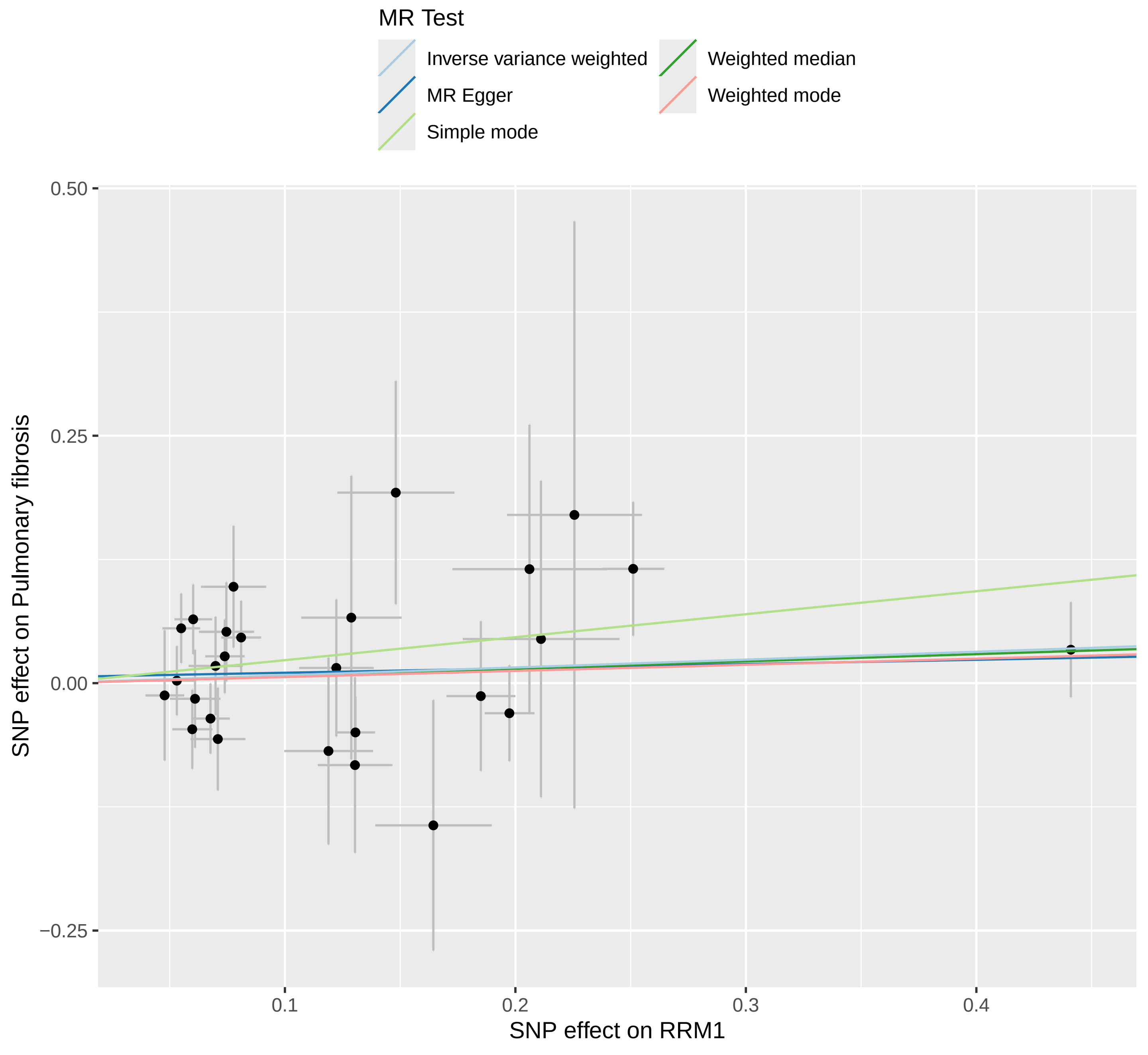
MR Test
Inverse variance weighted
MR Egger
Simple mode
Weighted median
Weighted mode
0.50
0.25
0.00
−0.25
0.1
0.2
0.3
0.4
SNP effect on RRM1
SNP effect on Pulmonary fibrosis

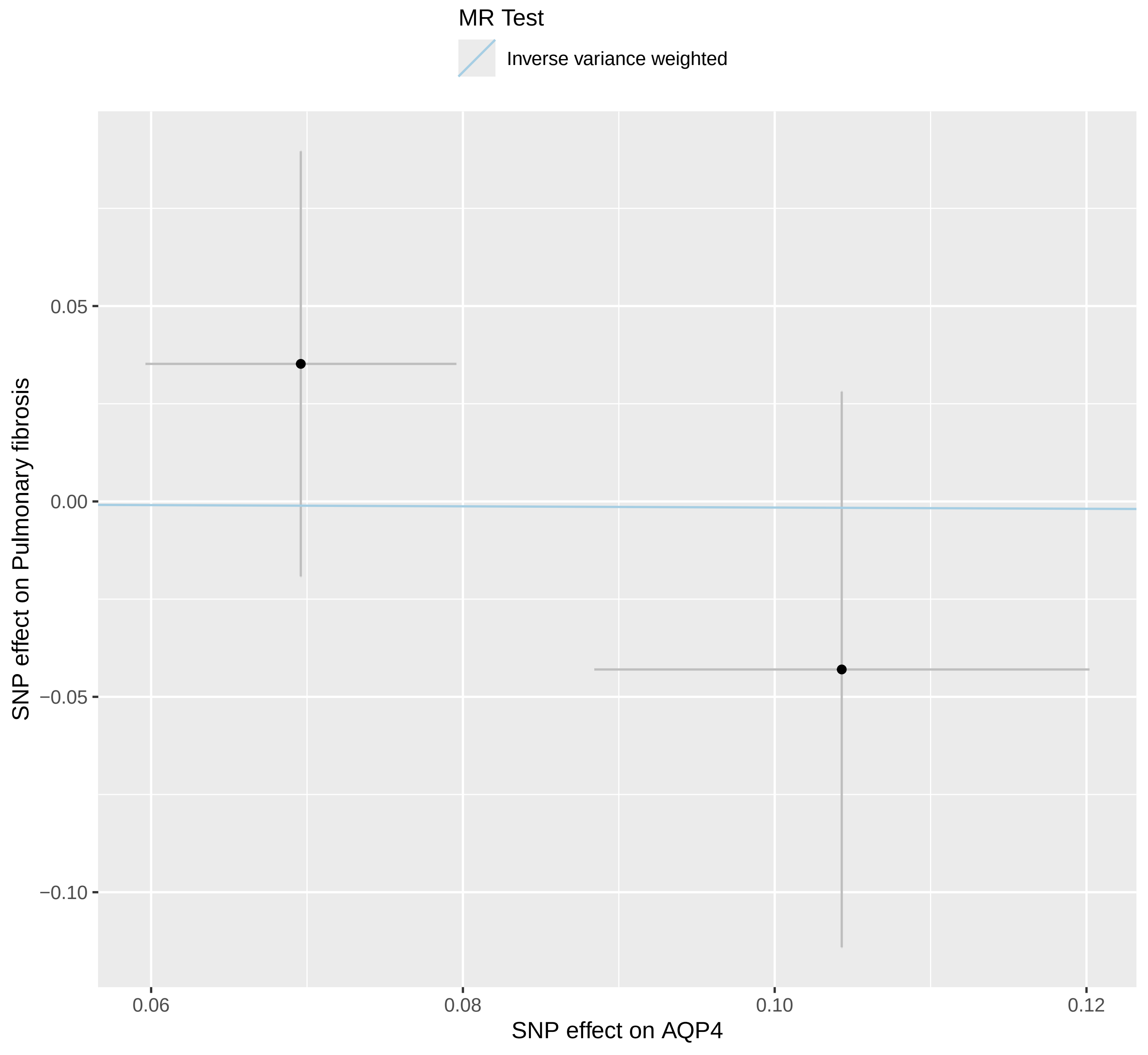

MR Test
Inverse variance weighted
0.05
0.00
−0.05
−0.10
0.06
0.08
0.10
0.12
SNP effect on Pulmonary fibrosis
SNP effect on AQP4

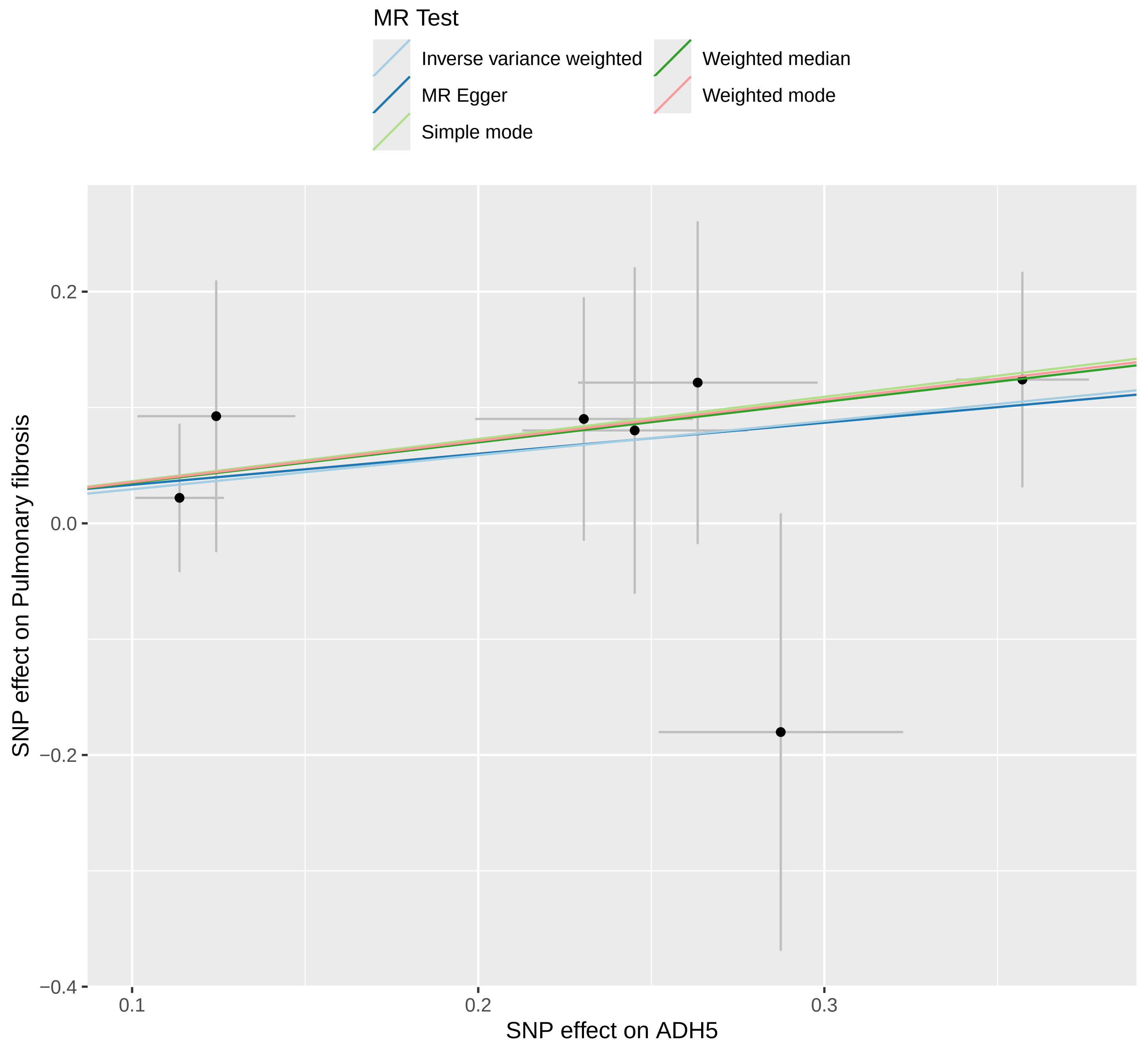
MR Test
Inverse variance weighted
MR Egger
Simple mode
Weighted median
Weighted mode
0.2
0.0
−0.2
−0.4
0.1
0.2
0.3
SNP effect on Pulmonary fibrosis
SNP effect on ADH5

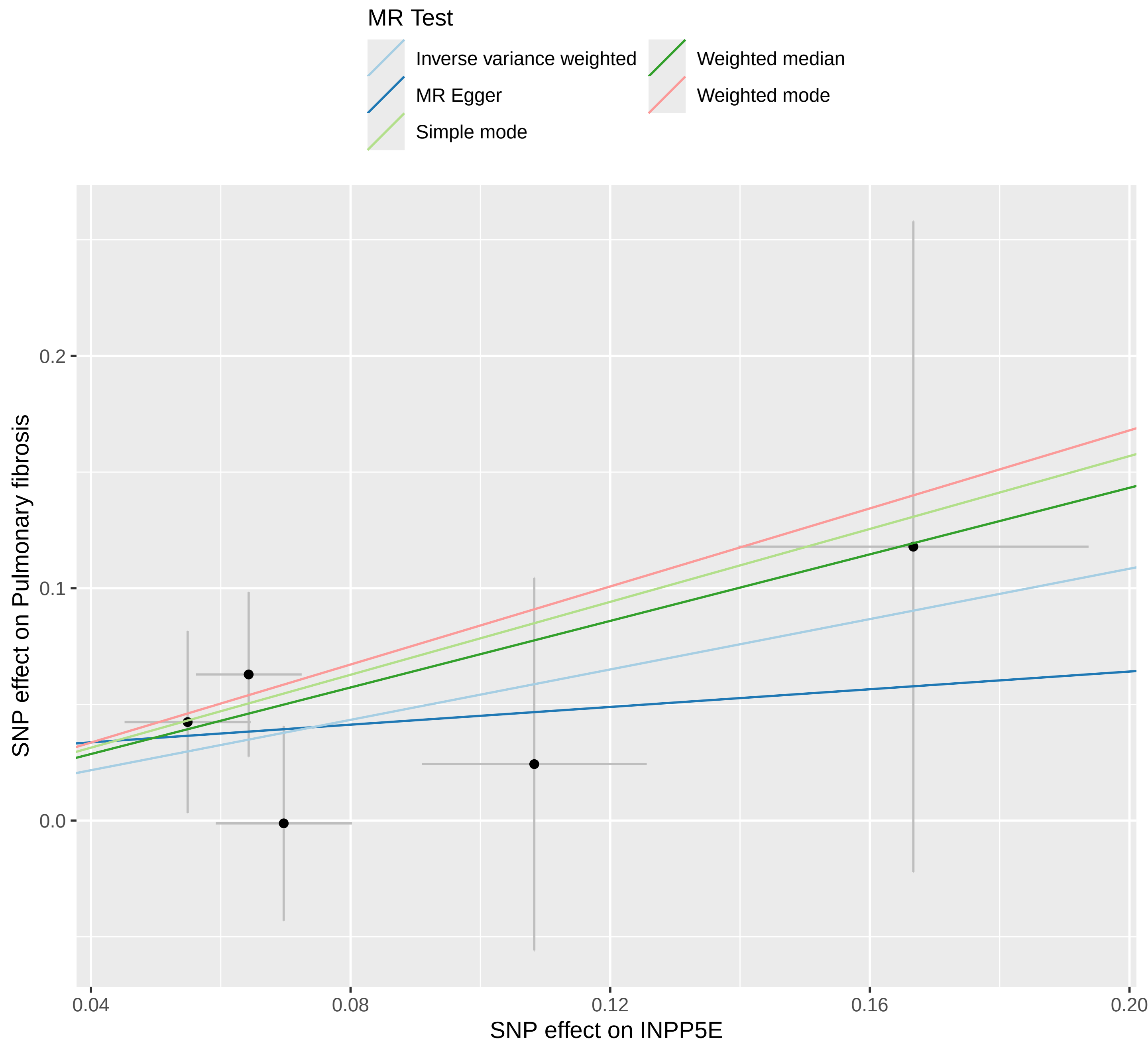

MR Test
Inverse variance weighted
MR Egger
Simple mode
Weighted median
Weighted mode
SNP effect on Pulmonary fibrosis
0.2
0.1
0.0
0.04
0.08
0.12
0.16
0.20
SNP effect on INPP5E

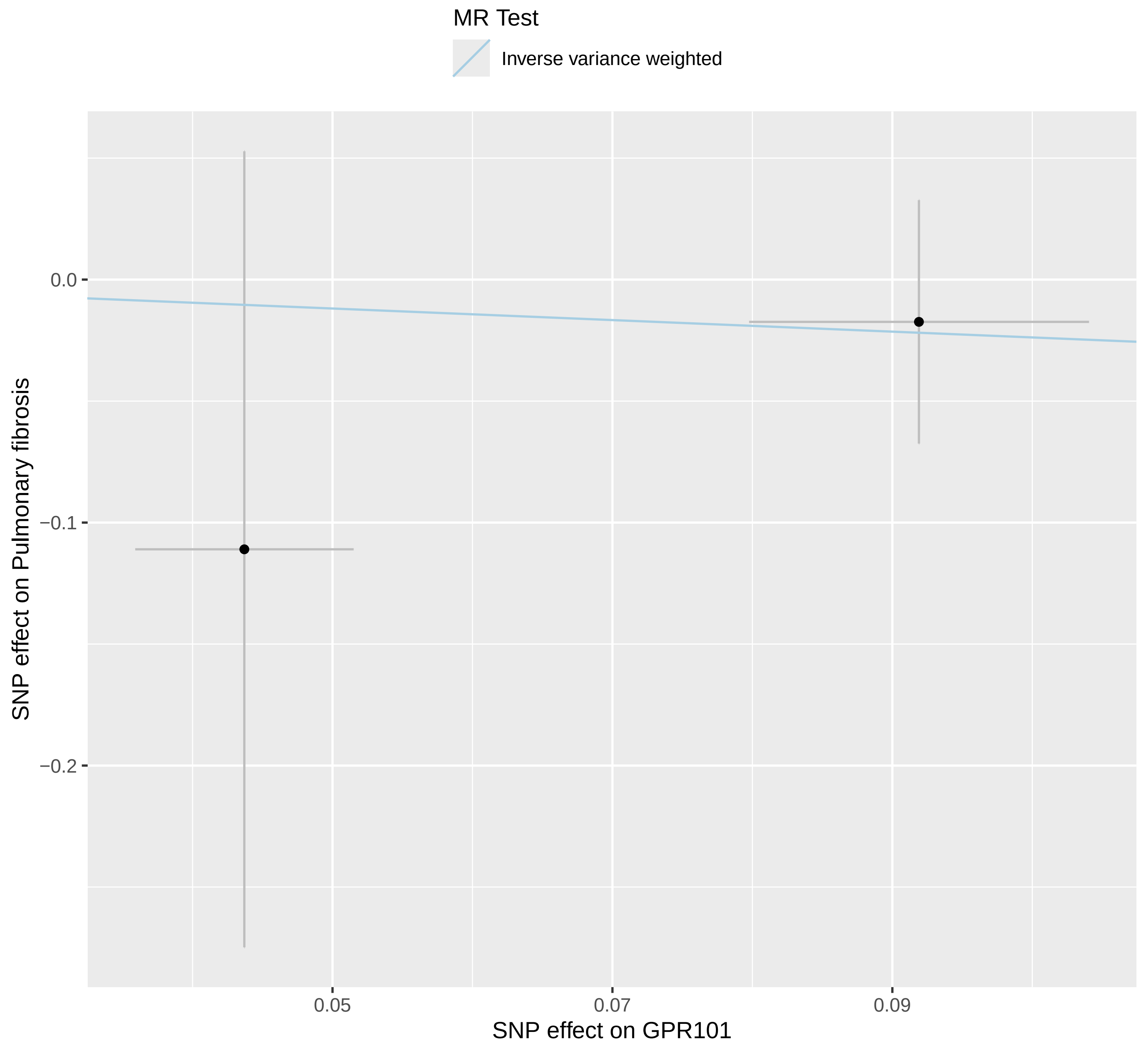

MR Test
Inverse variance weighted
0.0
−0.1
−0.2
0.05
0.07
0.09
SNP effect on GPR101
SNP effect on Pulmonary fibrosis

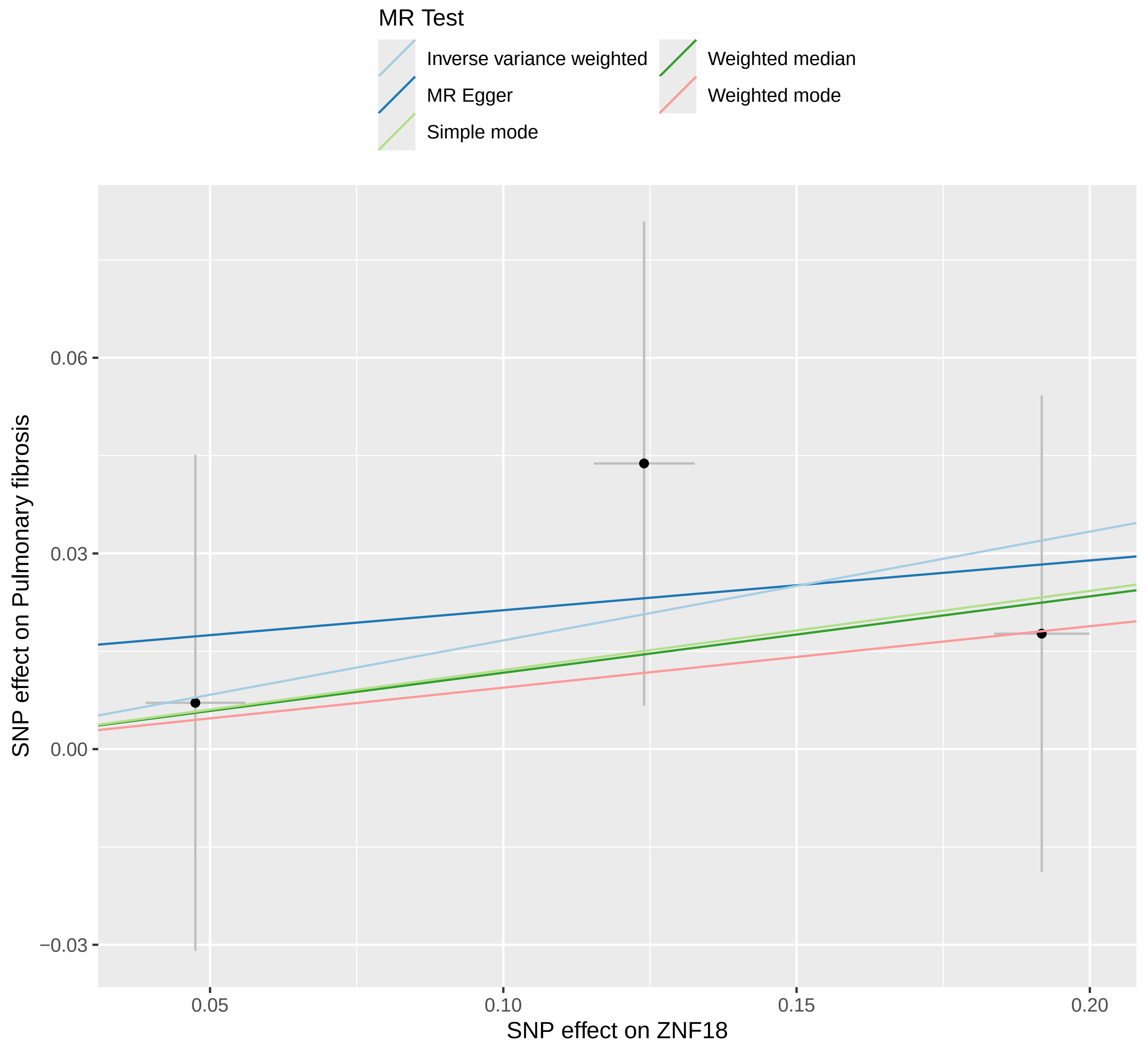

MR Test
Inverse variance weighted
MR Egger
Simple mode
Weighted median
Weighted mode
0.06
0.03
0.00
−0.03
0.05
0.10
0.15
0.20
SNP effect on Pulmonary fibrosis
SNP effect on ZNF18

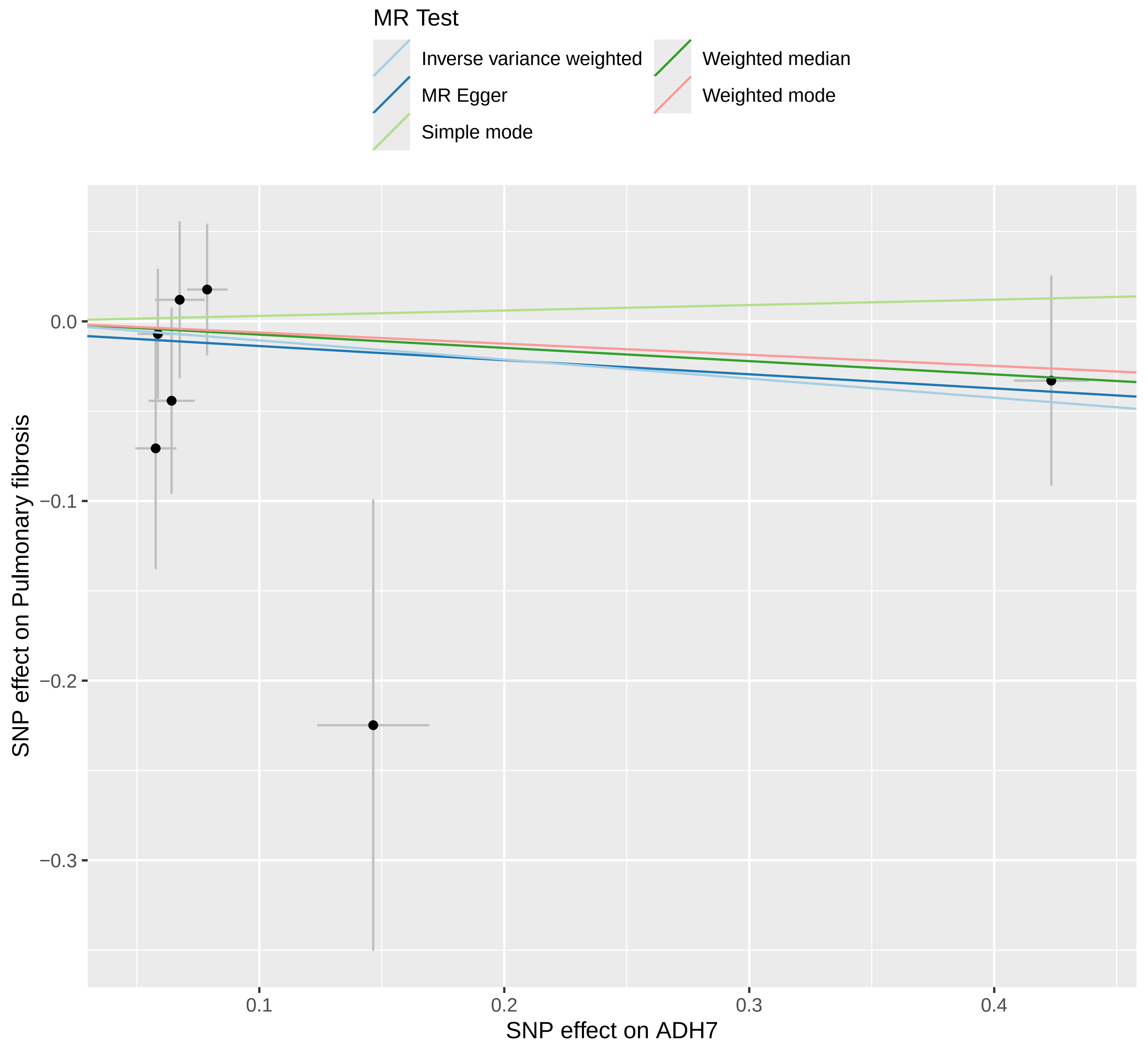

MR Test
Inverse variance weighted
MR Egger
Simple mode
Weighted median
Weighted mode
0.0
−0.1
−0.2
−0.3
0.1
0.2
0.3
0.4
SNP effect on Pulmonary fibrosis
SNP effect on ADH7

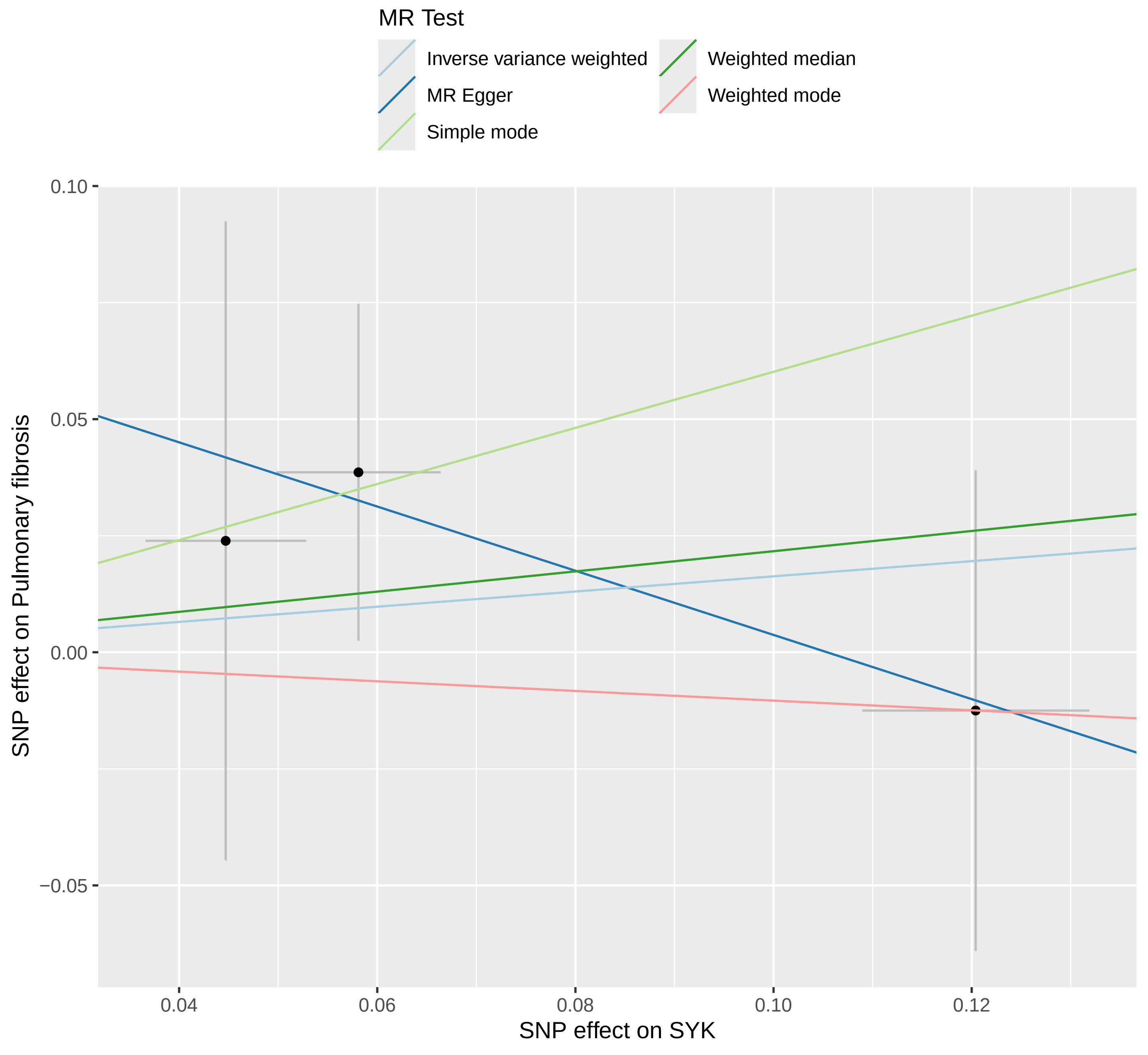

MR Test
Inverse variance weighted
MR Egger
Simple mode
Weighted median
Weighted mode
0.10
0.05
0.00
−0.05
SNP effect on Pulmonary fibrosis
0.04
0.06
0.08
0.10
0.12
SNP effect on SYK

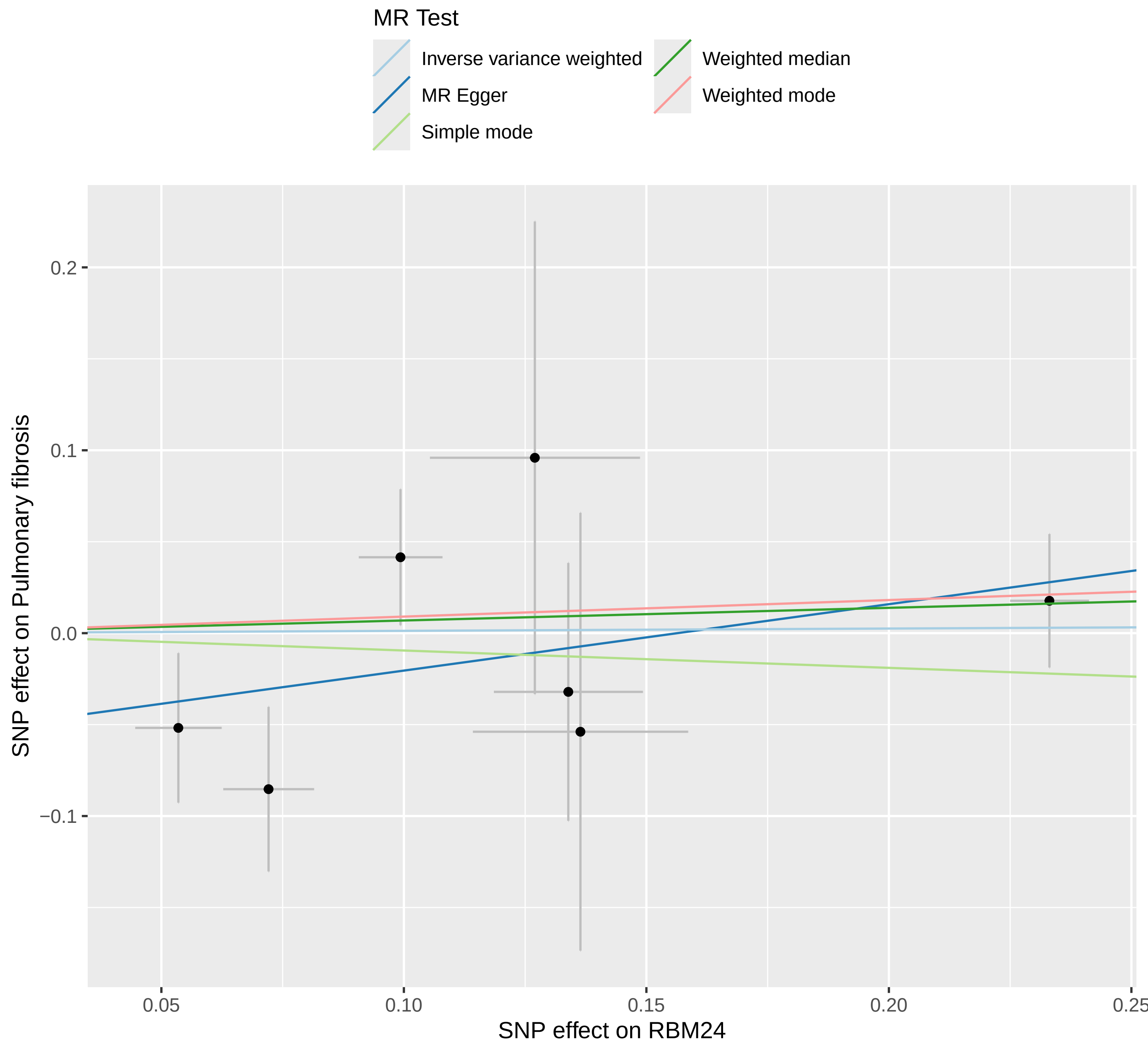

MR Test
Inverse variance weighted
MR Egger
Simple mode
Weighted median
Weighted mode
0.2
0.1
0.0
−0.1
0.05
0.10
0.15
0.20
0.25
SNP effect on RBM24
SNP effect on Pulmonary fibrosis

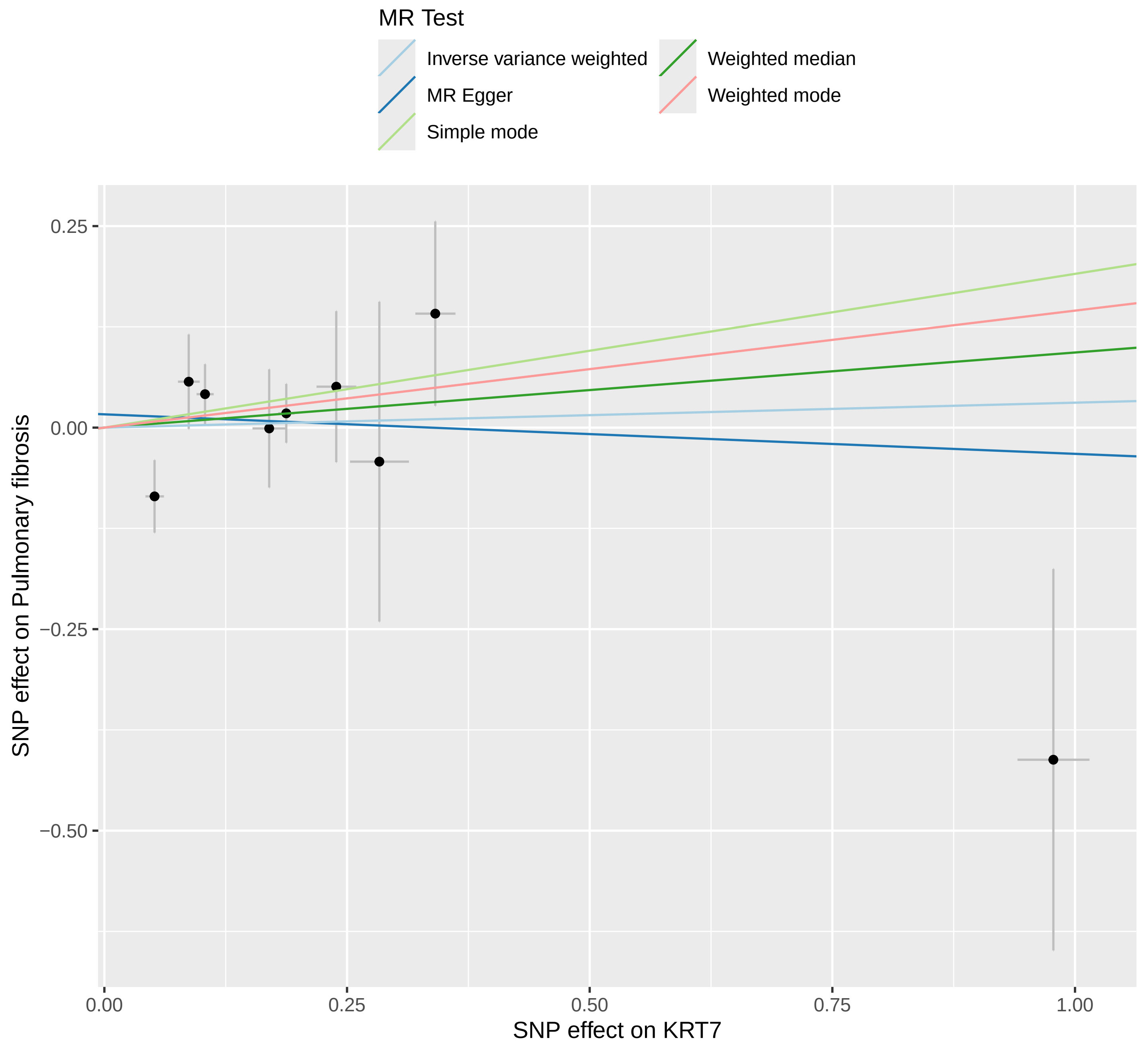
MR Test
Inverse variance weighted
MR Egger
Simple mode
Weighted median
Weighted mode
0.25
0.00
−0.25
−0.50
SNP effect on Pulmonary fibrosis
0.00
0.25
0.50
0.75
1.00
SNP effect on KRT7

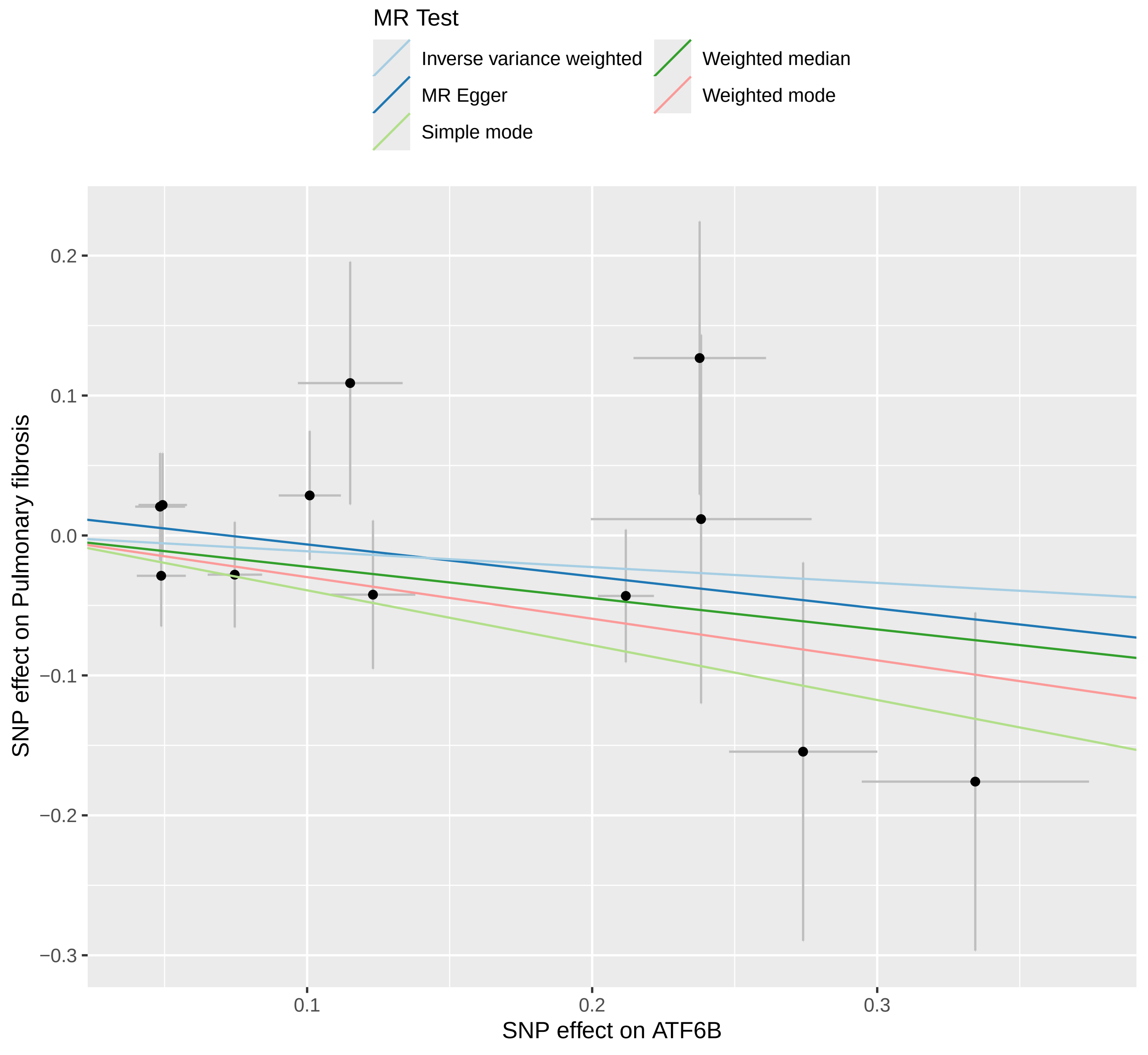
MR Test
Inverse variance weighted
MR Egger
Simple mode
Weighted median
Weighted mode
0.2
0.1
0.0
−0.1
−0.2
−0.3
0.1
0.2
0.3
SNP effect on Pulmonary fibrosis
SNP effect on ATF6B

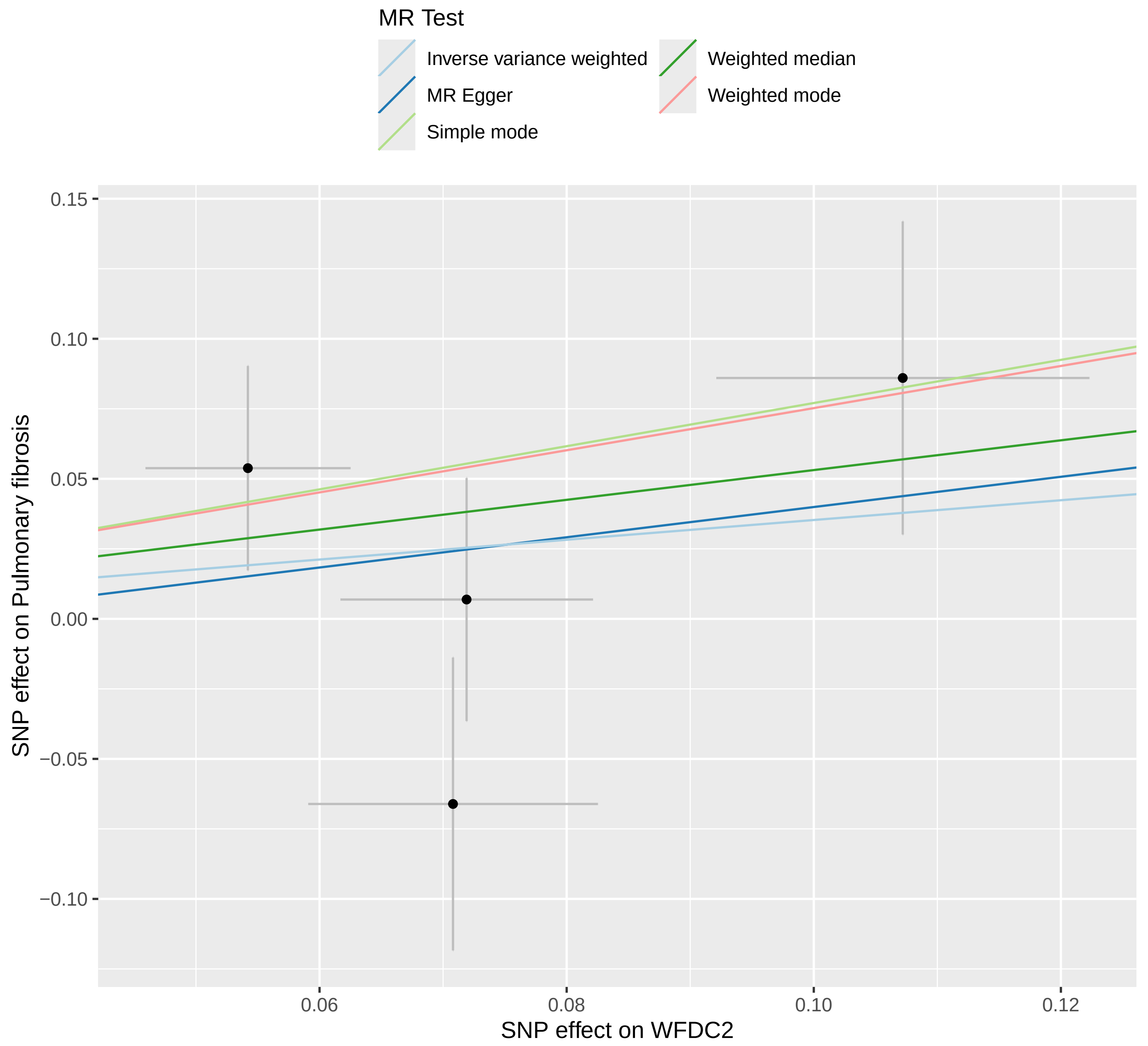

MR Test
Inverse variance weighted
MR Egger
Simple mode
Weighted median
Weighted mode
0.15
0.10
0.05
0.00
−0.05
−0.10
0.06
0.08
0.10
0.12
SNP effect on Pulmonary fibrosis
SNP effect on WFDC2

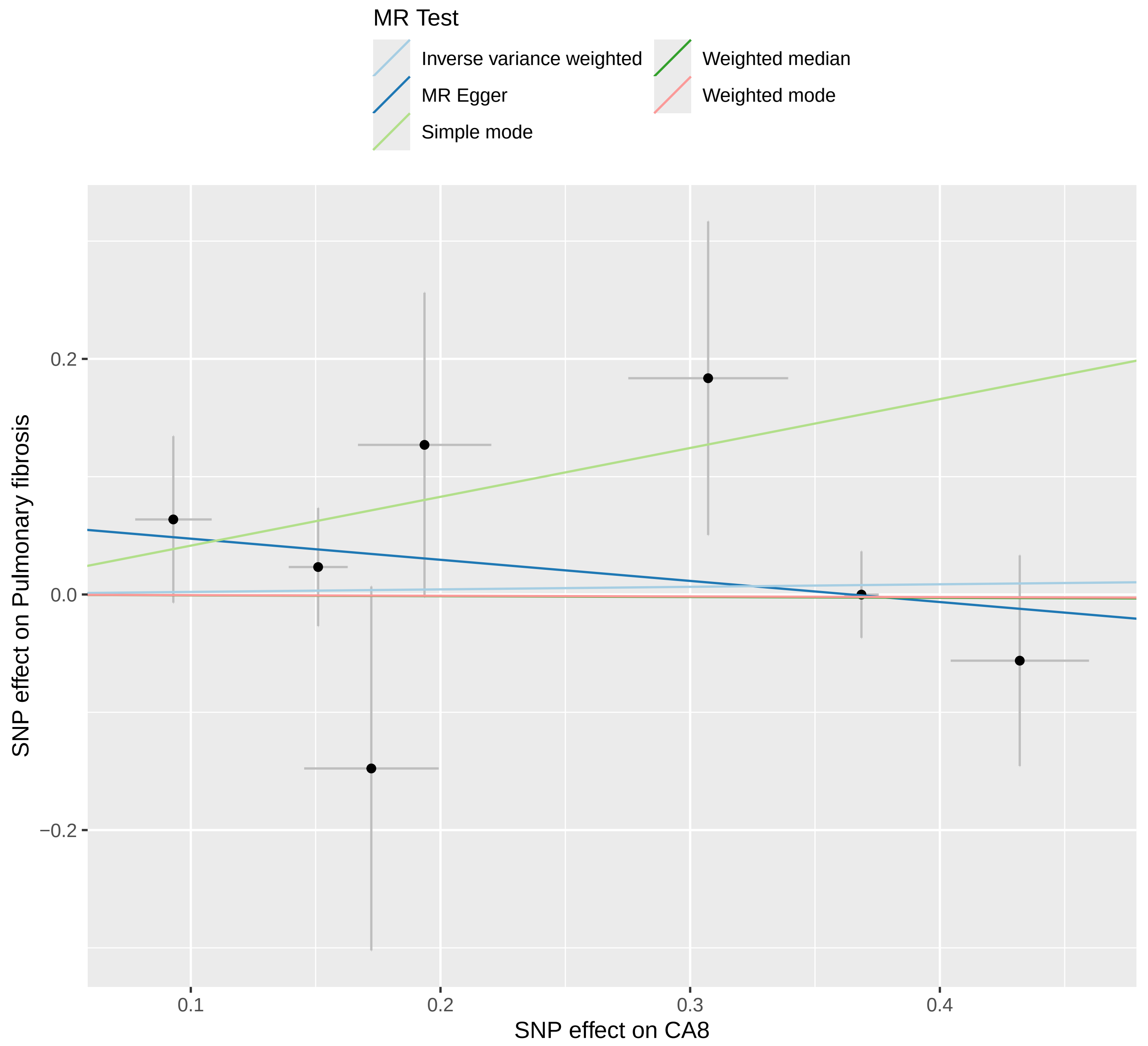
MR Test
Inverse variance weighted
MR Egger
Simple mode
Weighted median
Weighted mode
0.2
0.0
−0.2
0.1
0.2
0.3
0.4
SNP effect on Pulmonary fibrosis
SNP effect on CA8

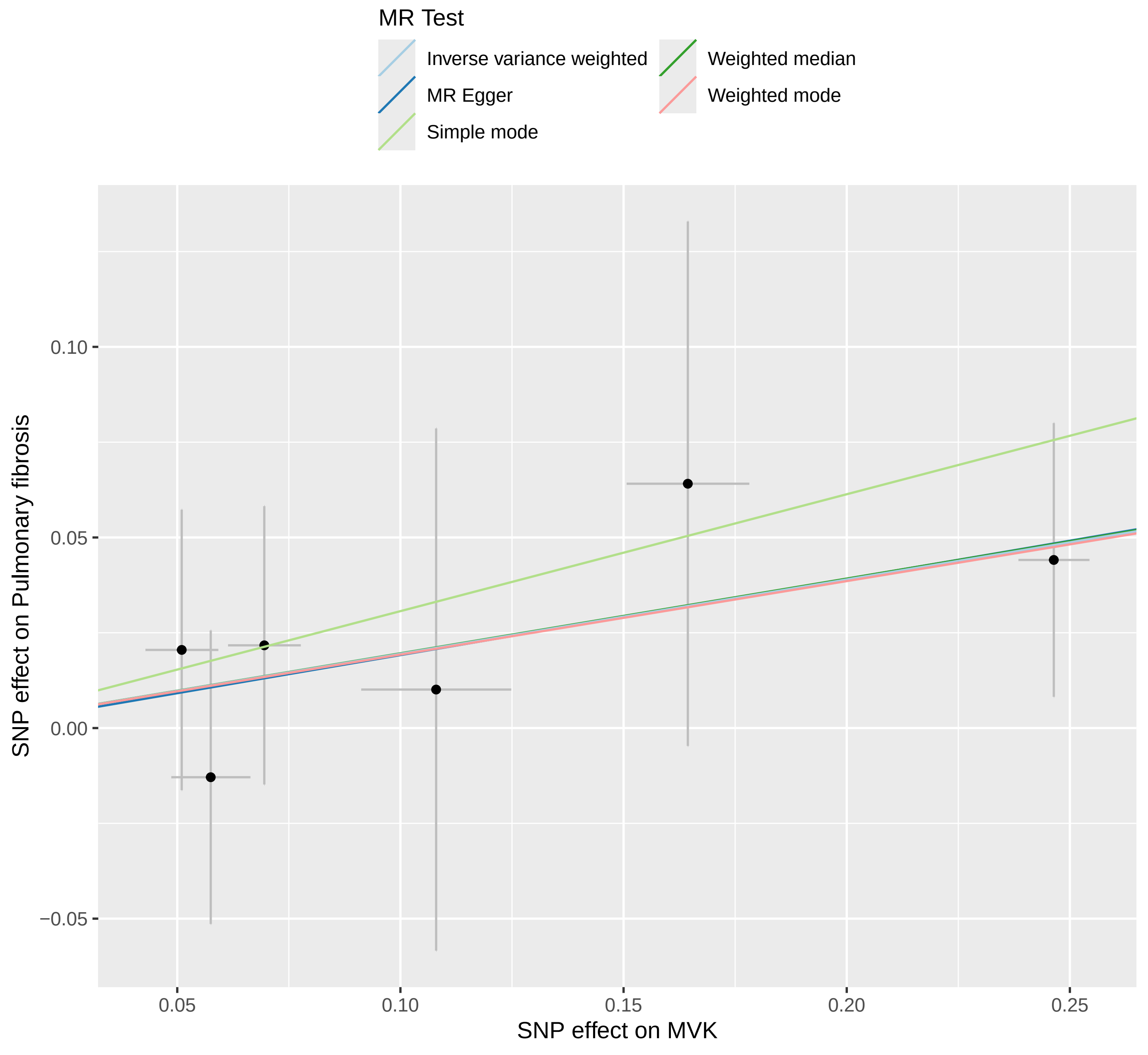
MR Test
Inverse variance weighted
MR Egger
Simple mode
Weighted median
Weighted mode
0.10
0.05
0.00
−0.05
0.05
0.10
0.15
0.20
0.25
SNP effect on Pulmonary fibrosis
SNP effect on MVK

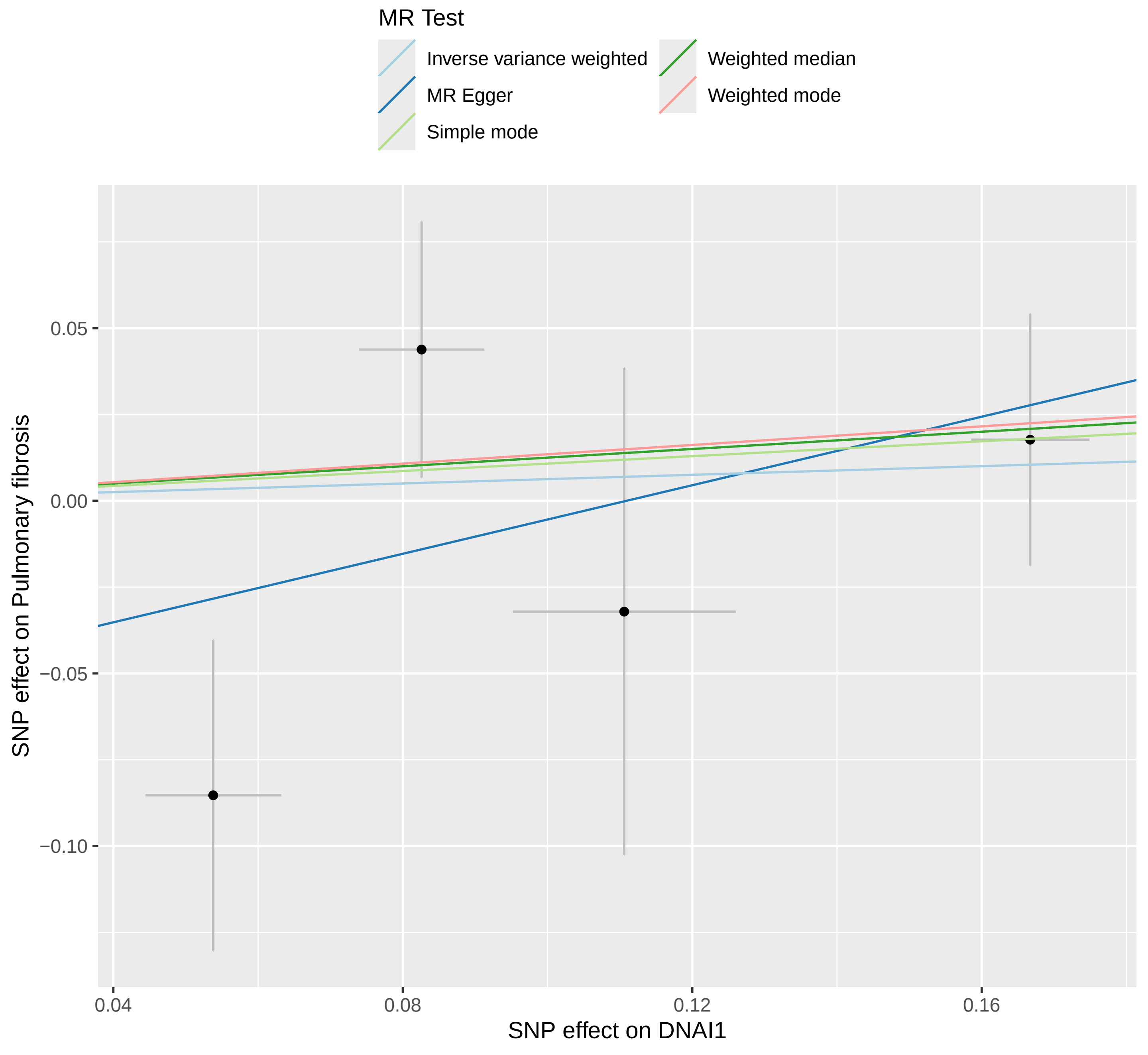

MR Test
Inverse variance weighted
MR Egger
Simple mode
Weighted median
Weighted mode
0.05
0.00
−0.05
−0.10
SNP effect on Pulmonary fibrosis
0.04
0.08
0.12
0.16
SNP effect on DNAI1

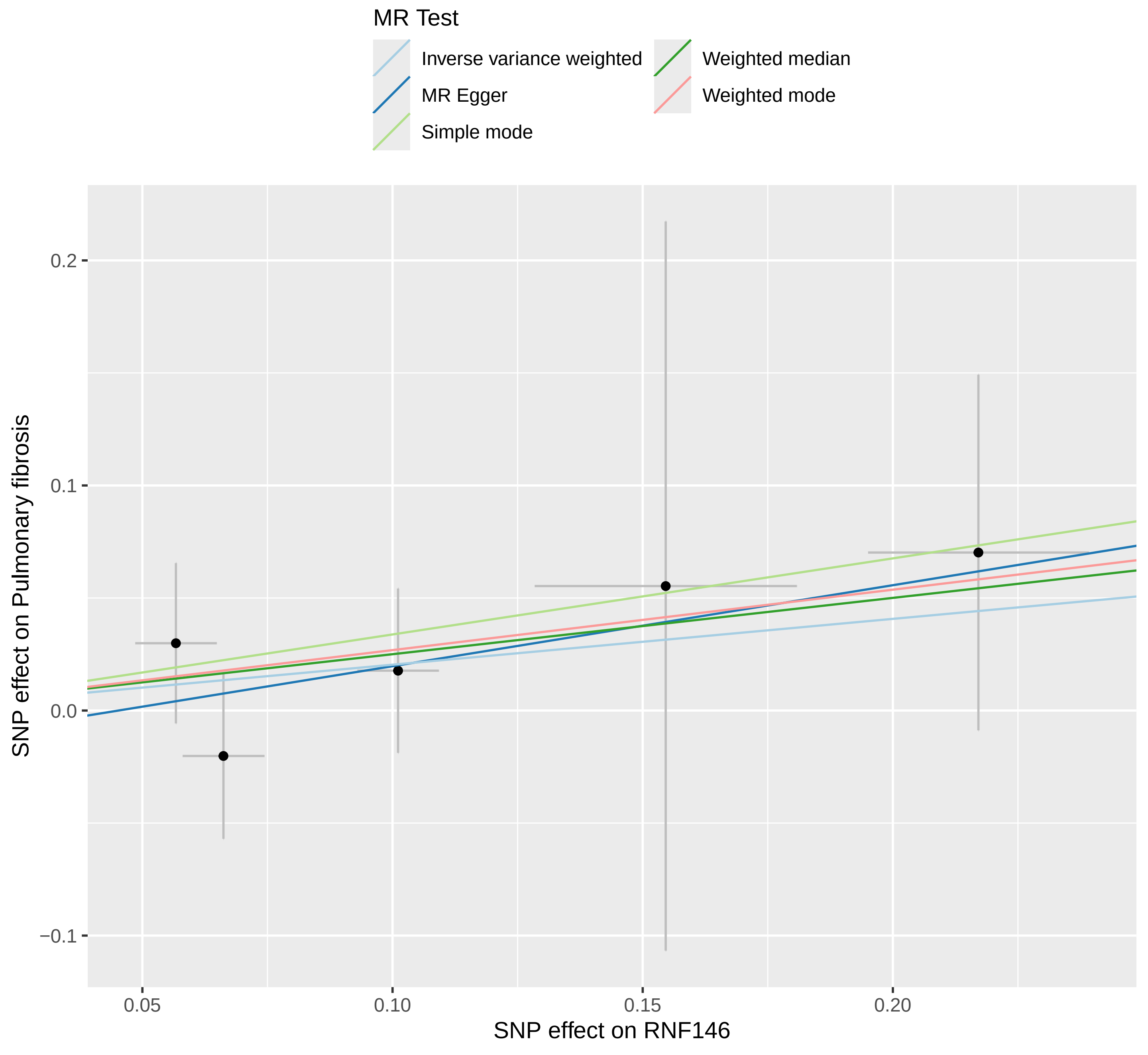
MR Test
Inverse variance weighted
MR Egger
Simple mode
Weighted median
Weighted mode
SNP effect on Pulmonary fibrosis
0.2
0.1
0.0
−0.1
0.05
0.10
0.15
0.20
SNP effect on RNF146

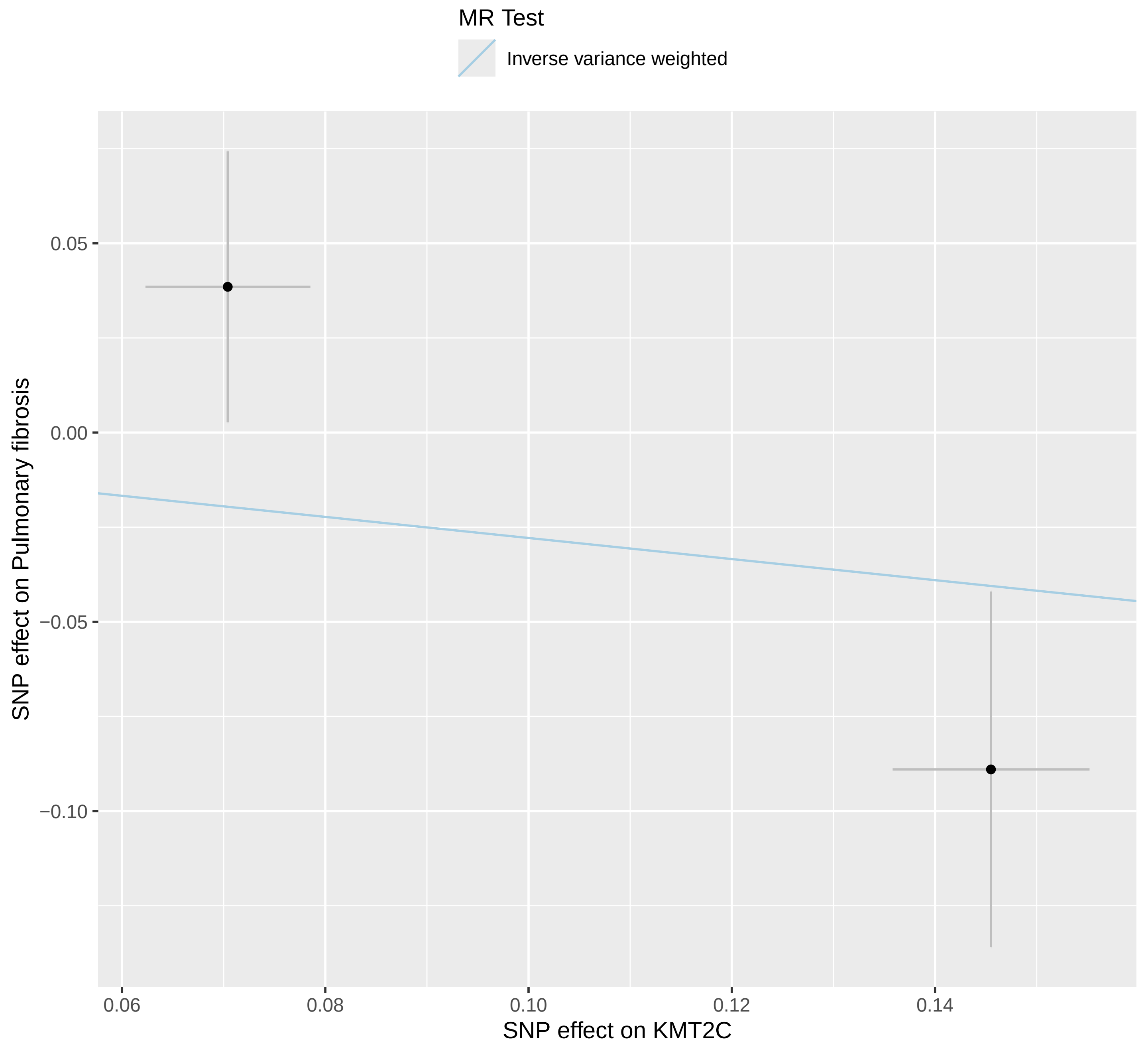
MR Test
Inverse variance weighted
SNP effect on Pulmonary fibrosis
0.05
0.00
−0.05
−0.10
0.06
0.08
0.10
0.12
0.14
SNP effect on KMT2C

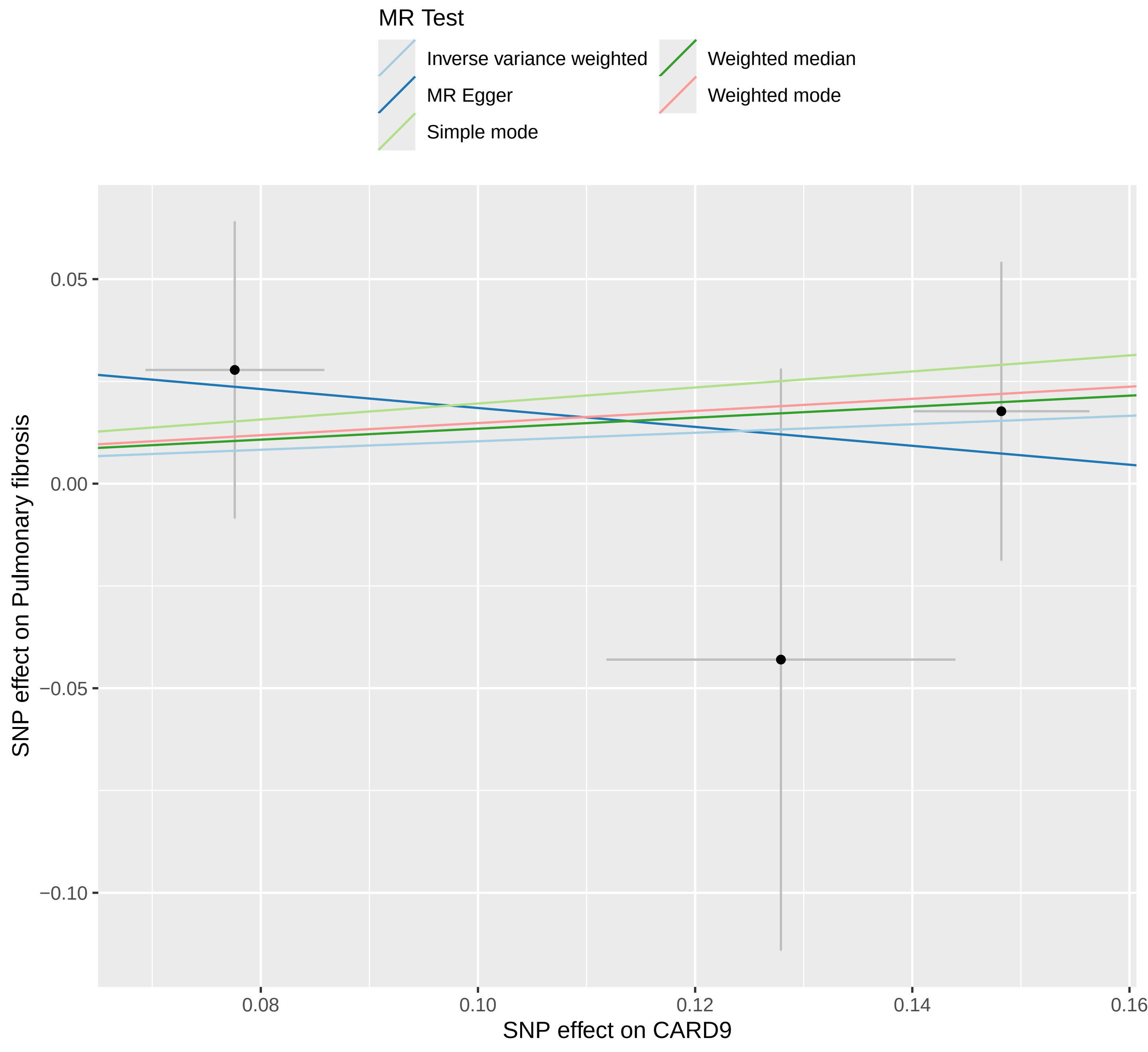
MR Test
Inverse variance weighted
MR Egger
Simple mode
Weighted median
Weighted mode
0.05
0.00
−0.05
−0.10
0.08
0.10
0.12
0.14
0.16
SNP effect on CARD9
SNP effect on Pulmonary fibrosis

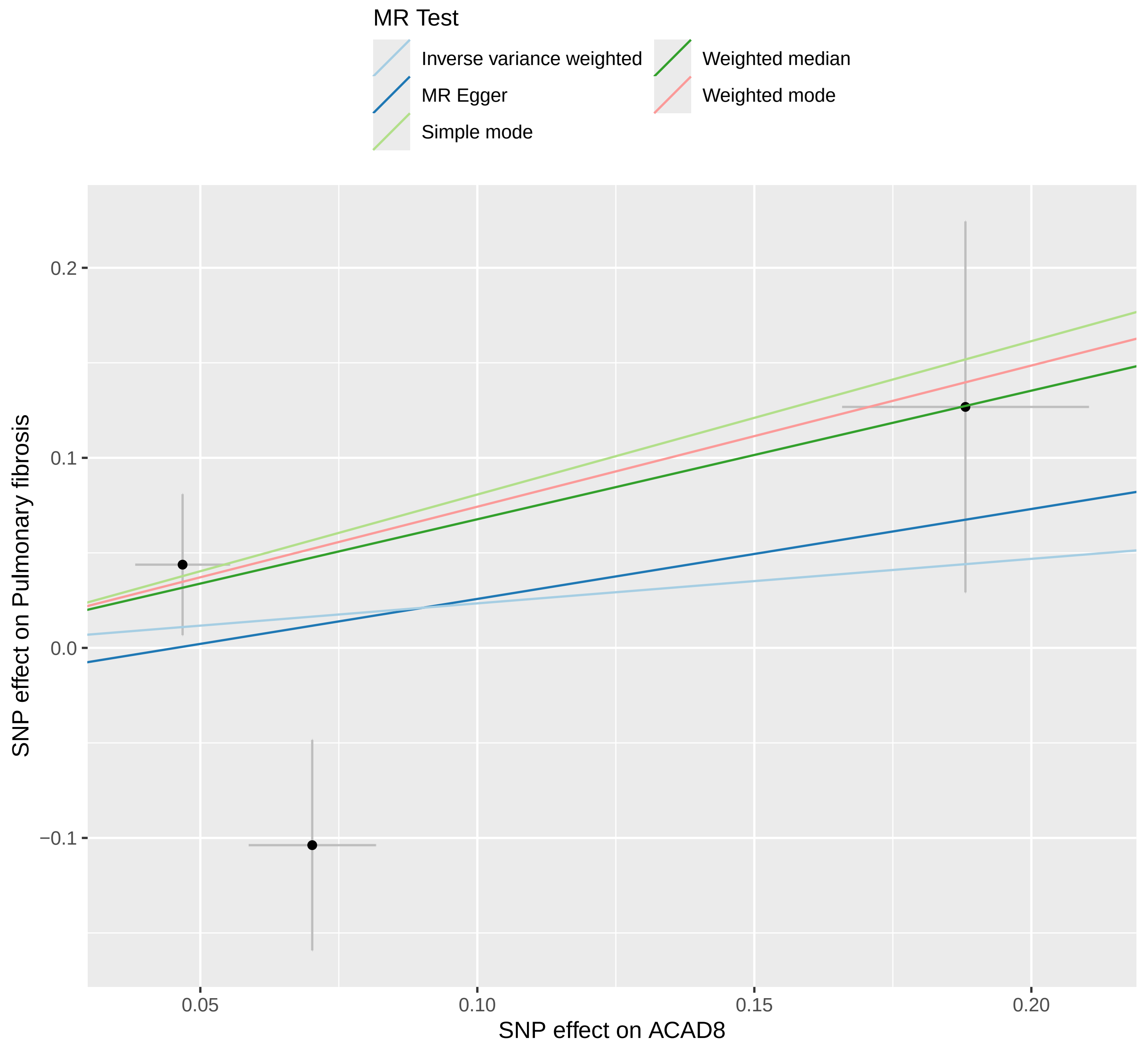
MR Test
Inverse variance weighted
MR Egger
Simple mode
Weighted median
Weighted mode
SNP effect on Pulmonary fibrosis
0.2
0.1
0.0
−0.1
0.05
0.10
0.15
0.20
SNP effect on ACAD8

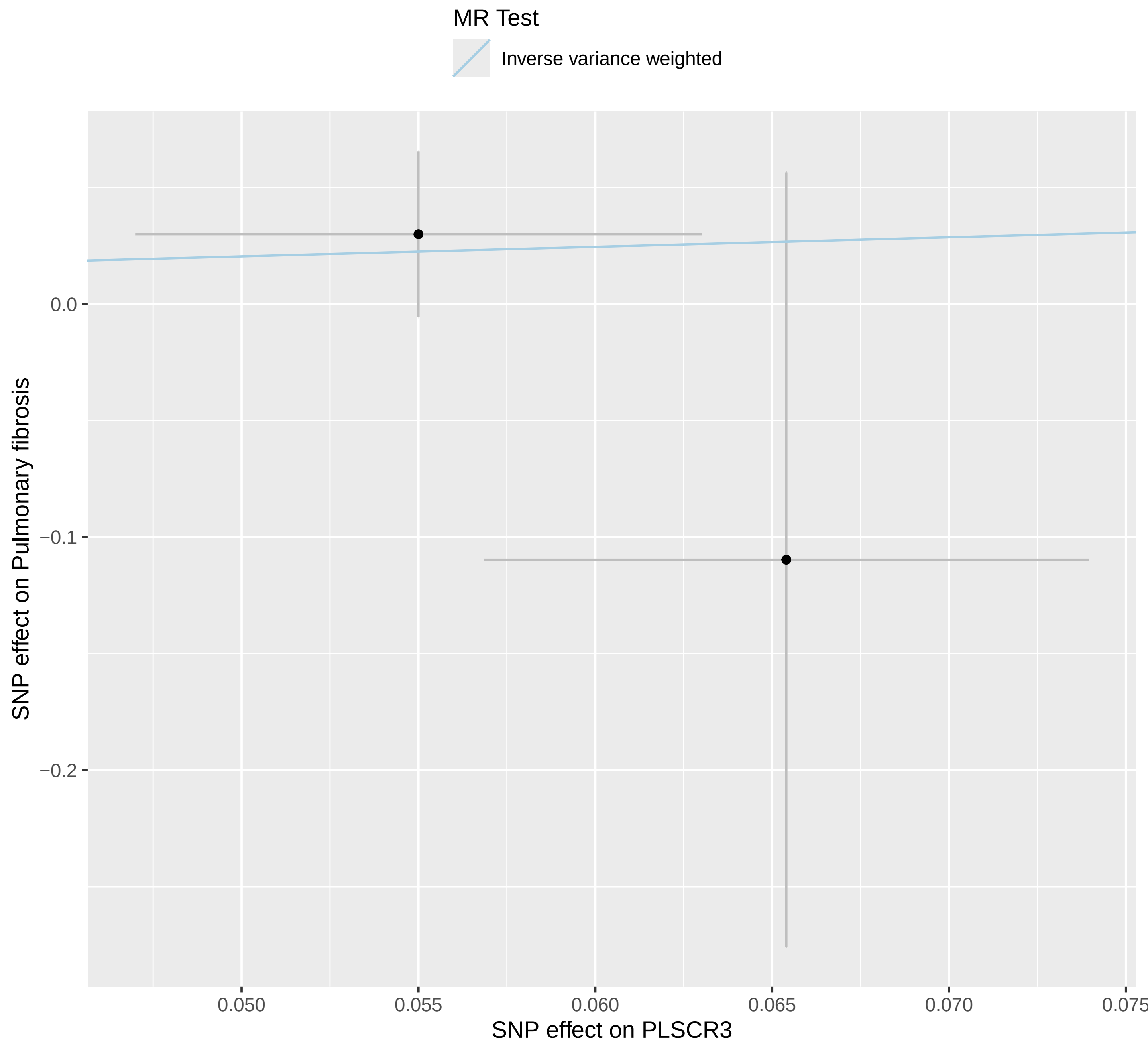
MR Test
Inverse variance weighted
0.0
−0.1
−0.2
SNP effect on Pulmonary fibrosis
0.050
0.055
0.060
0.065
0.070
0.075
SNP effect on PLSCR3

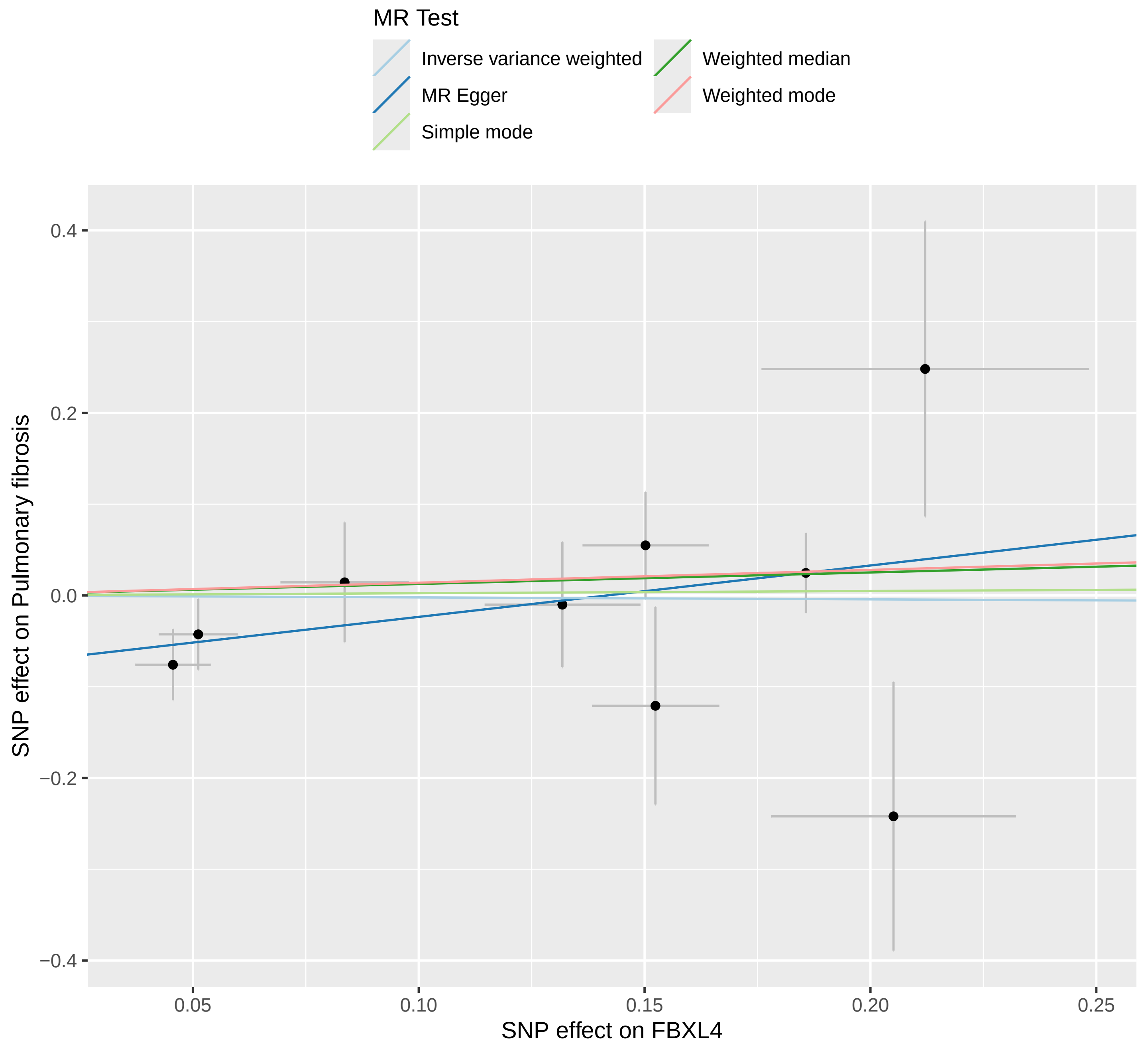
MR Test
Inverse variance weighted
MR Egger
Simple mode
Weighted median
Weighted mode
SNP effect on Pulmonary fibrosis
0.4
0.2
0.0
−0.2
−0.4
0.05
0.10
0.15
0.20
0.25
SNP effect on FBXL4

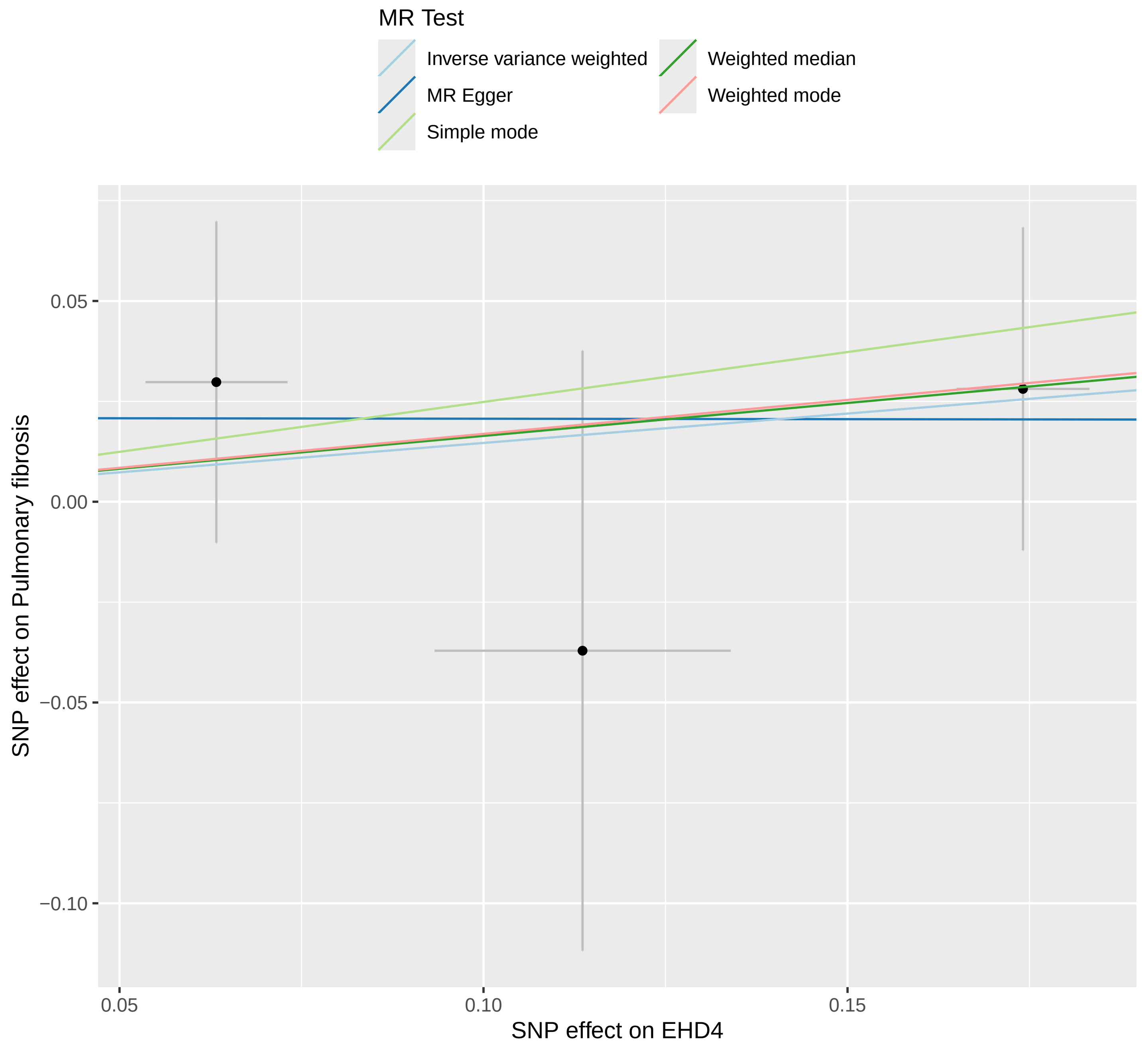
MR Test
Inverse variance weighted
MR Egger
Simple mode
Weighted median
Weighted mode
SNP effect on Pulmonary fibrosis
0.05
0.00
−0.05
−0.10
0.05
0.10
0.15
SNP effect on EHD4

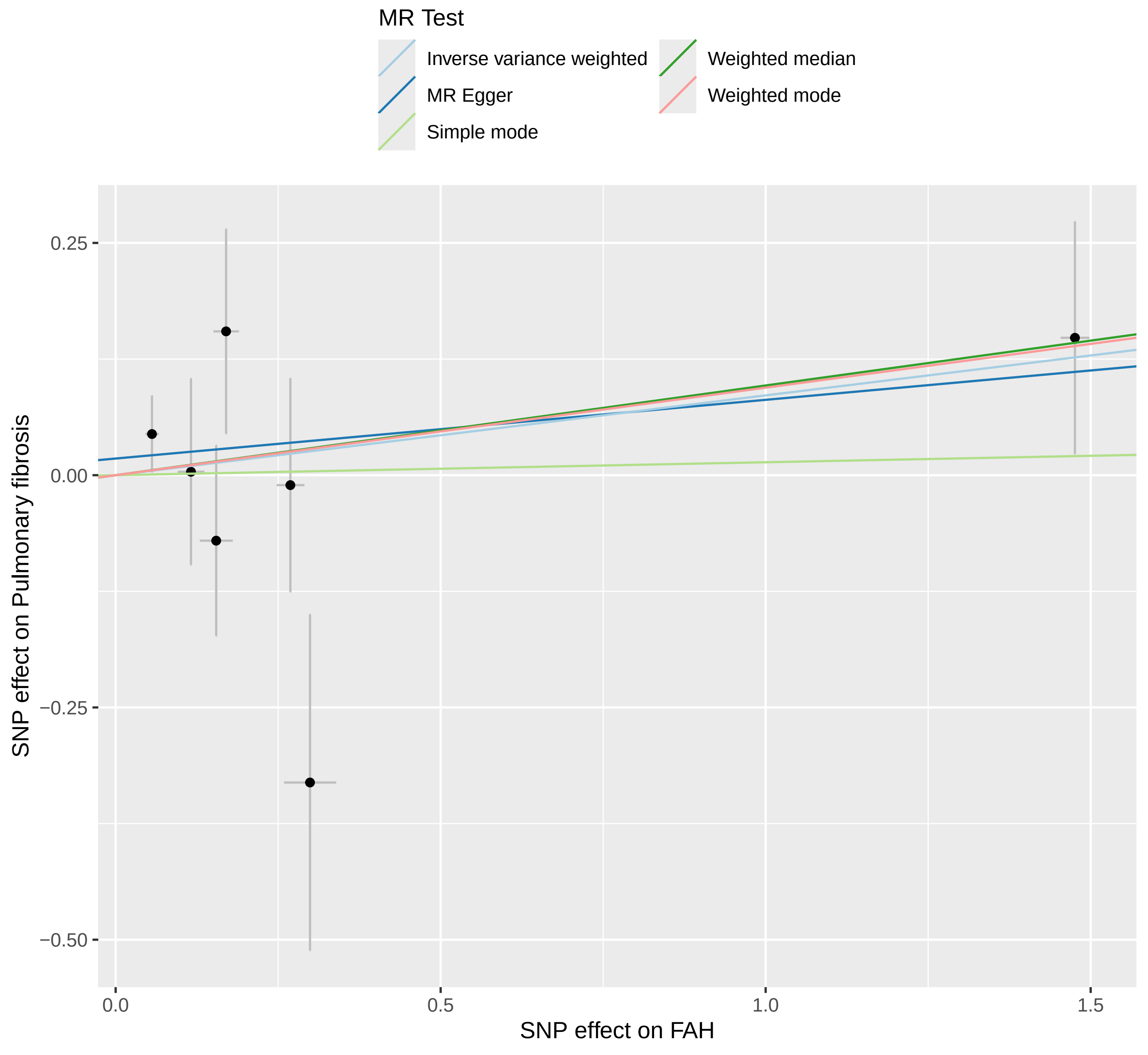
MR Test
Inverse variance weighted
MR Egger
Simple mode
Weighted median
Weighted mode
0.25
0.00
−0.25
−0.50
0.0
0.5
1.0
1.5
SNP effect on Pulmonary fibrosis
SNP effect on FAH

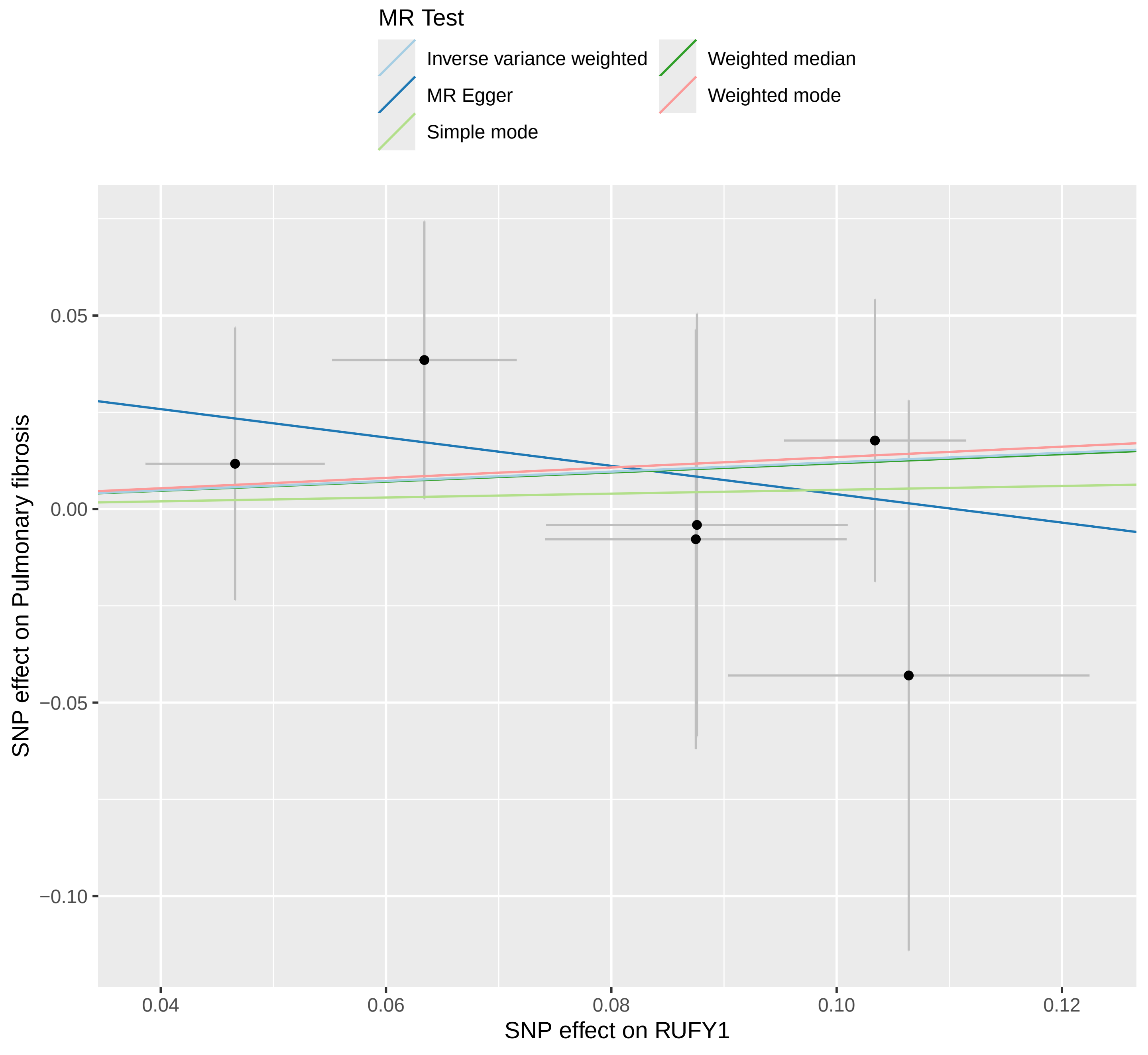
MR Test
Inverse variance weighted
MR Egger
Simple mode
Weighted median
Weighted mode
0.05
0.00
−0.05
−0.10
0.04
0.06
0.08
0.10
0.12
SNP effect on Pulmonary fibrosis
SNP effect on RUFY1

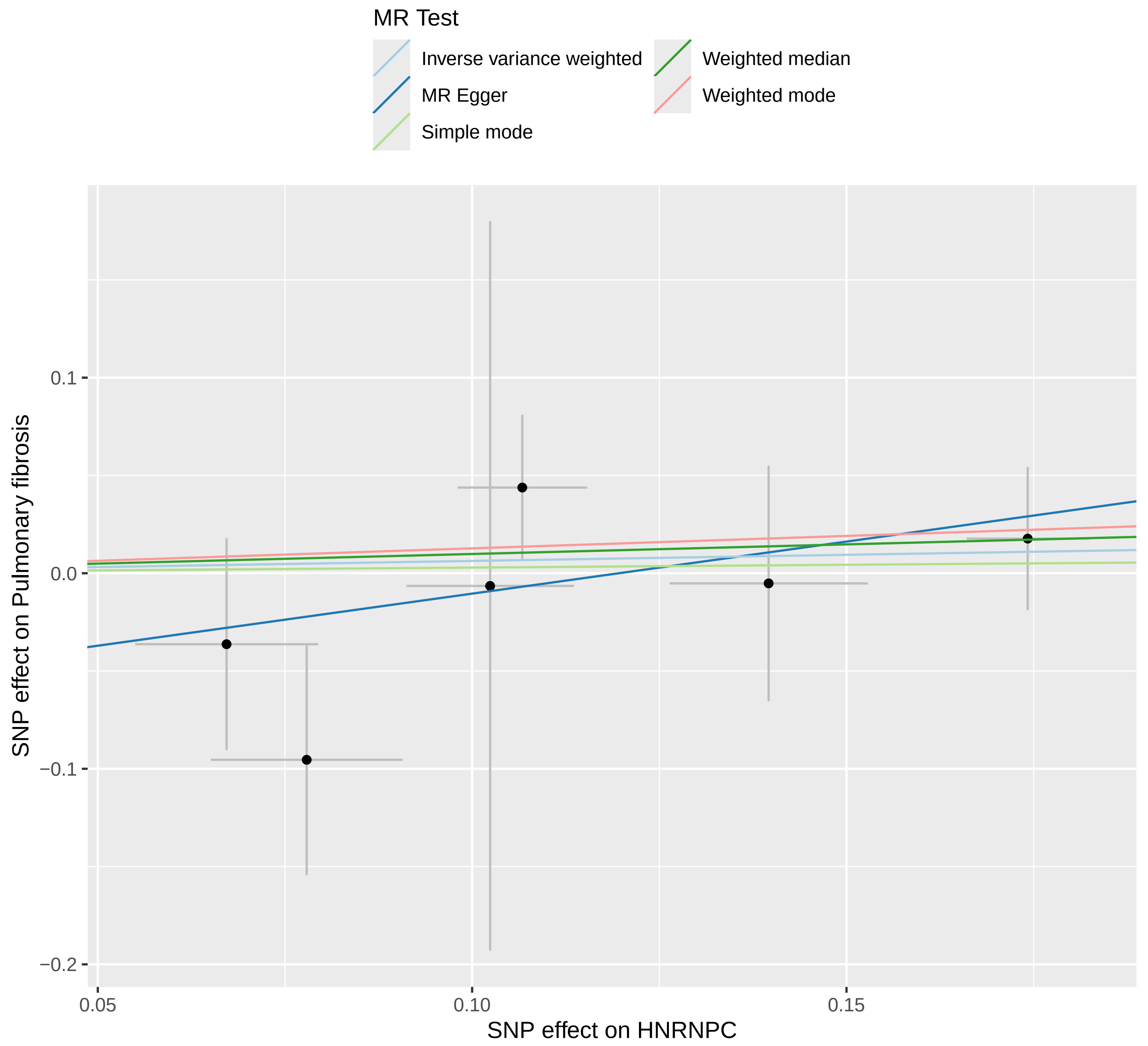
MR Test
Inverse variance weighted
MR Egger
Simple mode
Weighted median
Weighted mode
0.1
0.0
−0.1
−0.2
SNP effect on Pulmonary fibrosis
0.05
0.10
0.15
SNP effect on HNRNPC

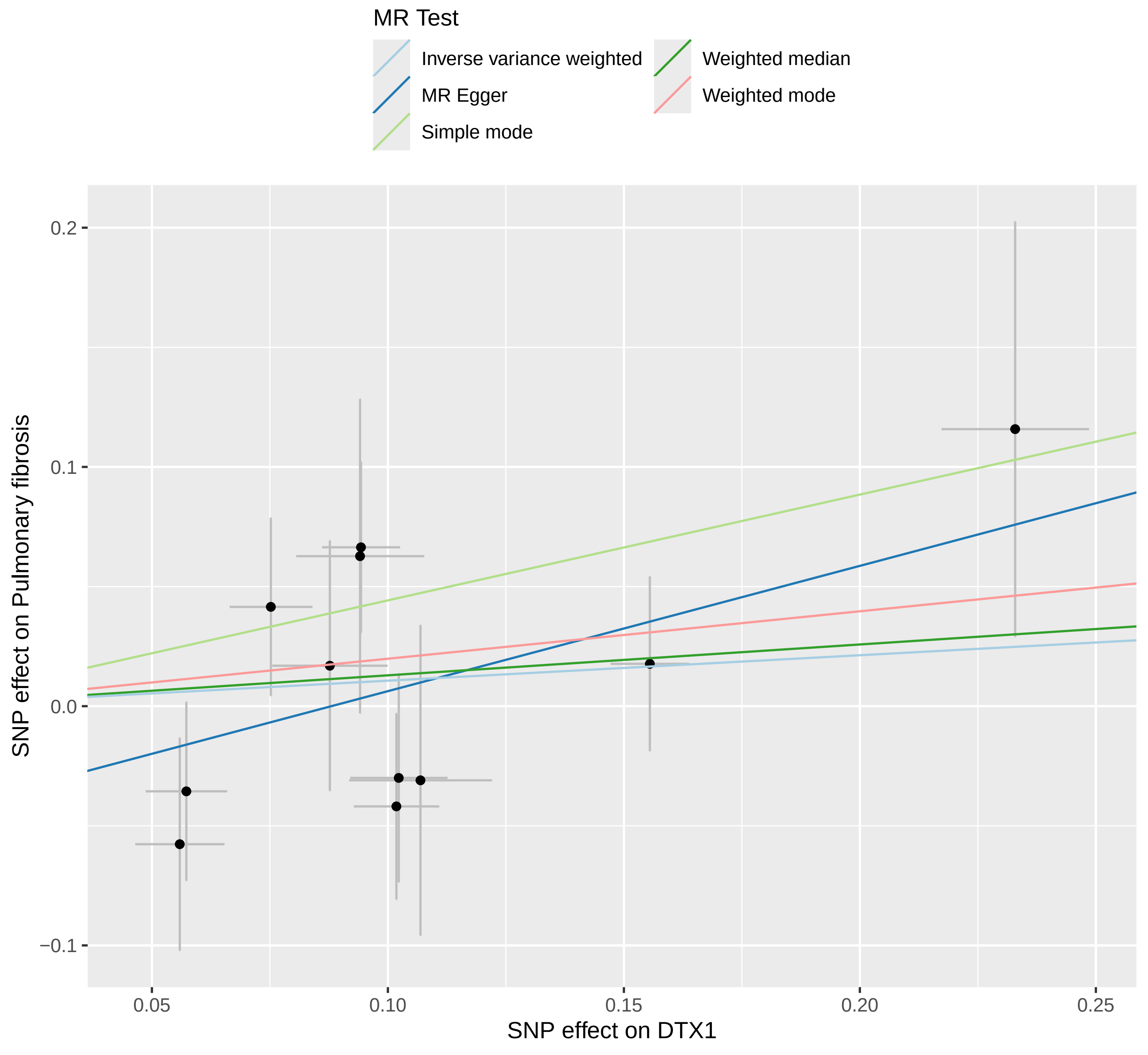
MR Test
Inverse variance weighted
MR Egger
Simple mode
Weighted median
Weighted mode
SNP effect on Pulmonary fibrosis
0.2
0.1
0.0
−0.1
0.05
0.10
0.15
0.20
0.25
SNP effect on DTX1

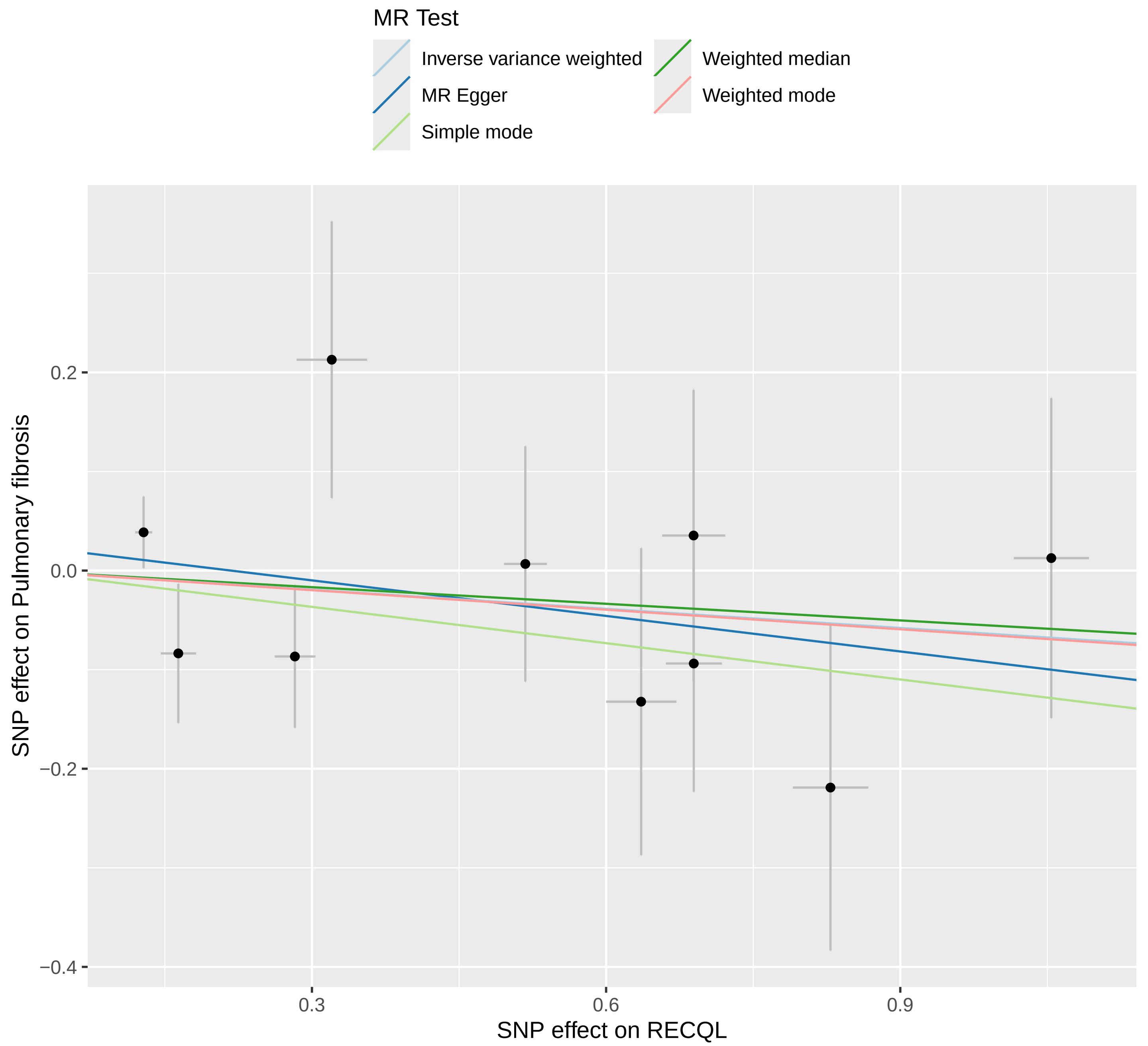
MR Test
Inverse variance weighted
MR Egger
Simple mode
Weighted median
Weighted mode
SNP effect on Pulmonary fibrosis
0.2
0.0
−0.2
−0.4
0.3
0.6
0.9
SNP effect on RECQL

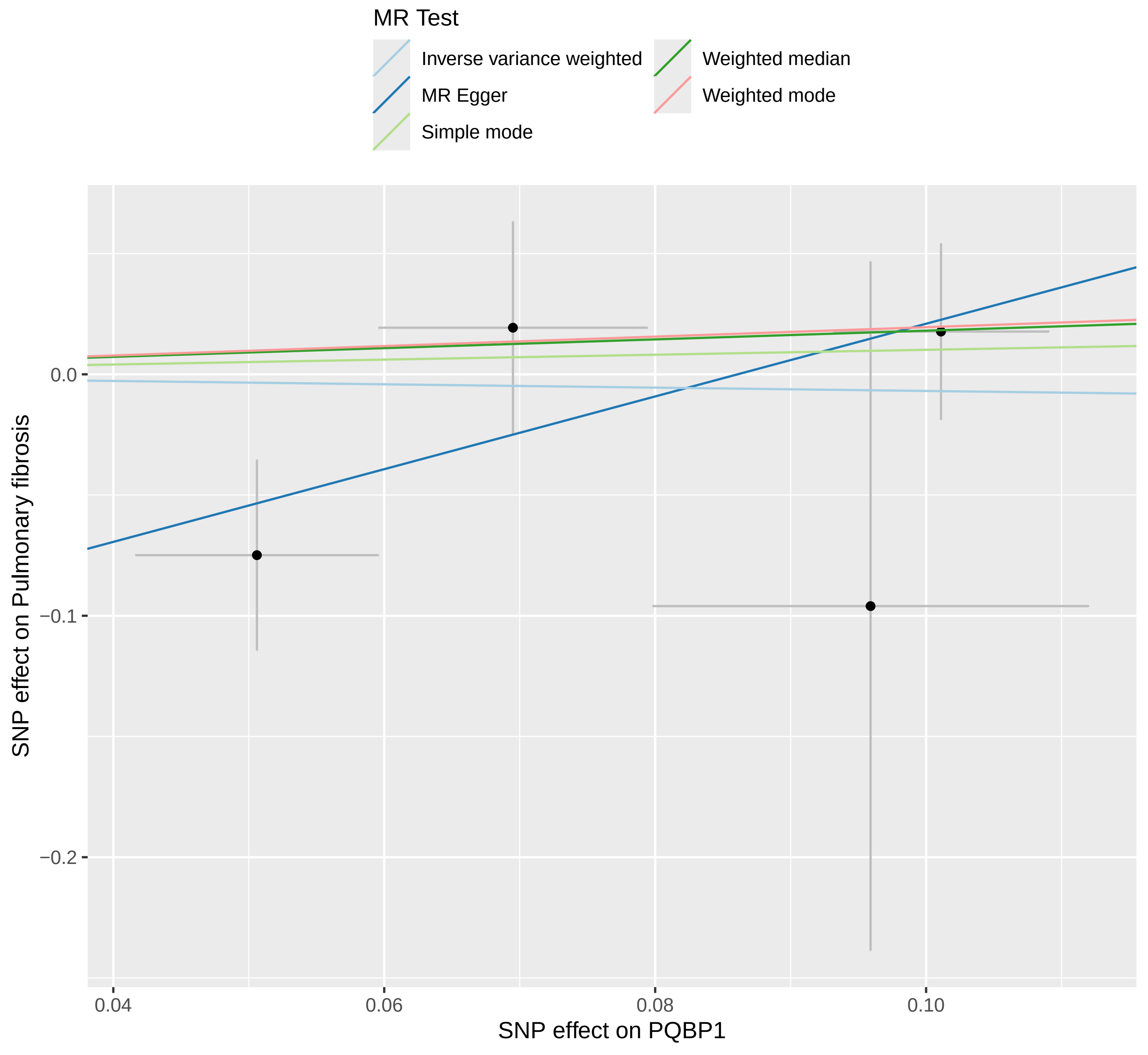

MR Test
Inverse variance weighted
MR Egger
Simple mode
Weighted median
Weighted mode
SNP effect on Pulmonary fibrosis
0.0
−0.1
−0.2
0.04
0.06
0.08
0.10
SNP effect on PQBP1

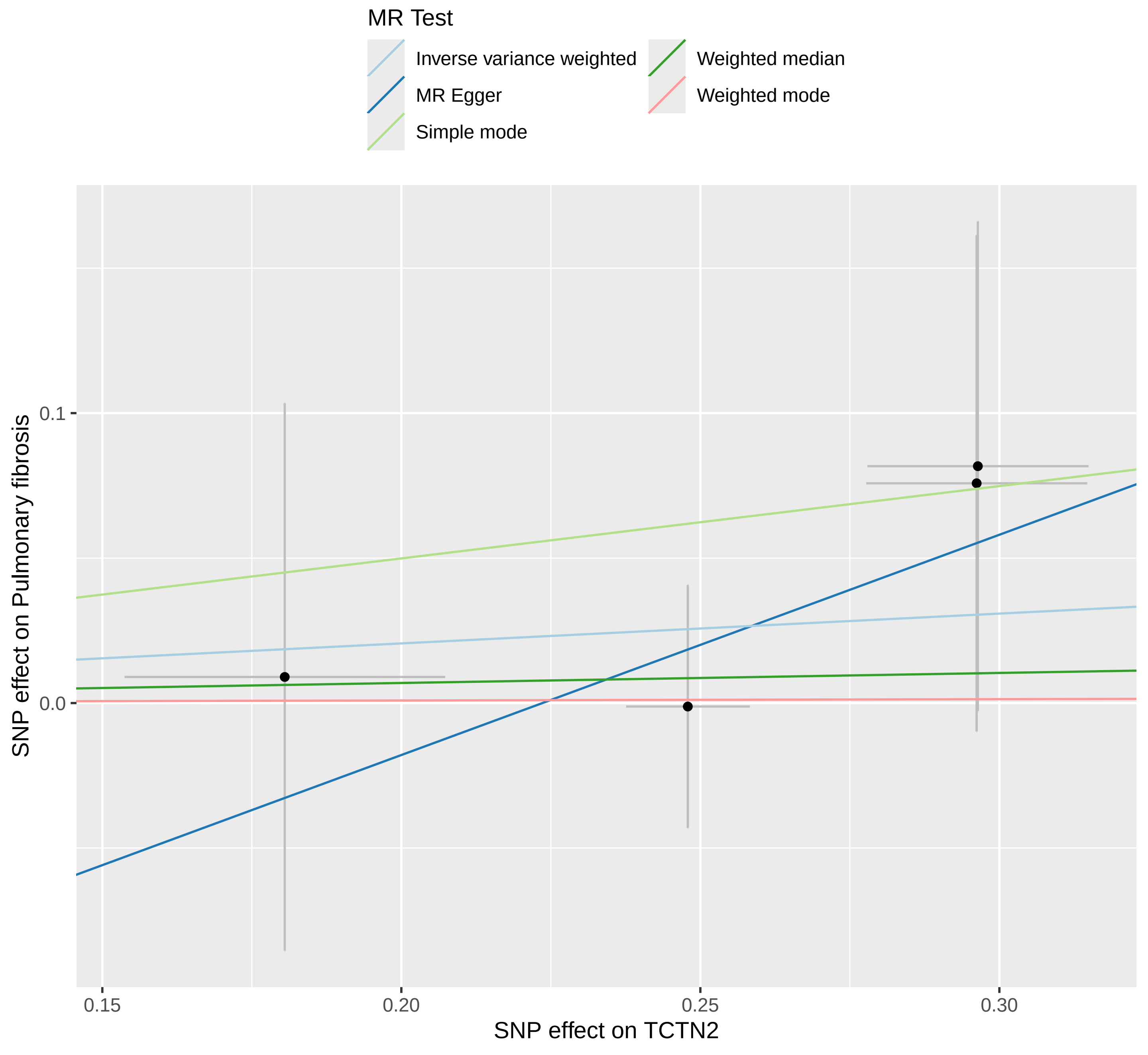

MR Test
Inverse variance weighted
MR Egger
Simple mode
Weighted median
Weighted mode
SNP effect on Pulmonary fibrosis
0.1
0.0
0.15
0.20
0.25
0.30
SNP effect on TCTN2

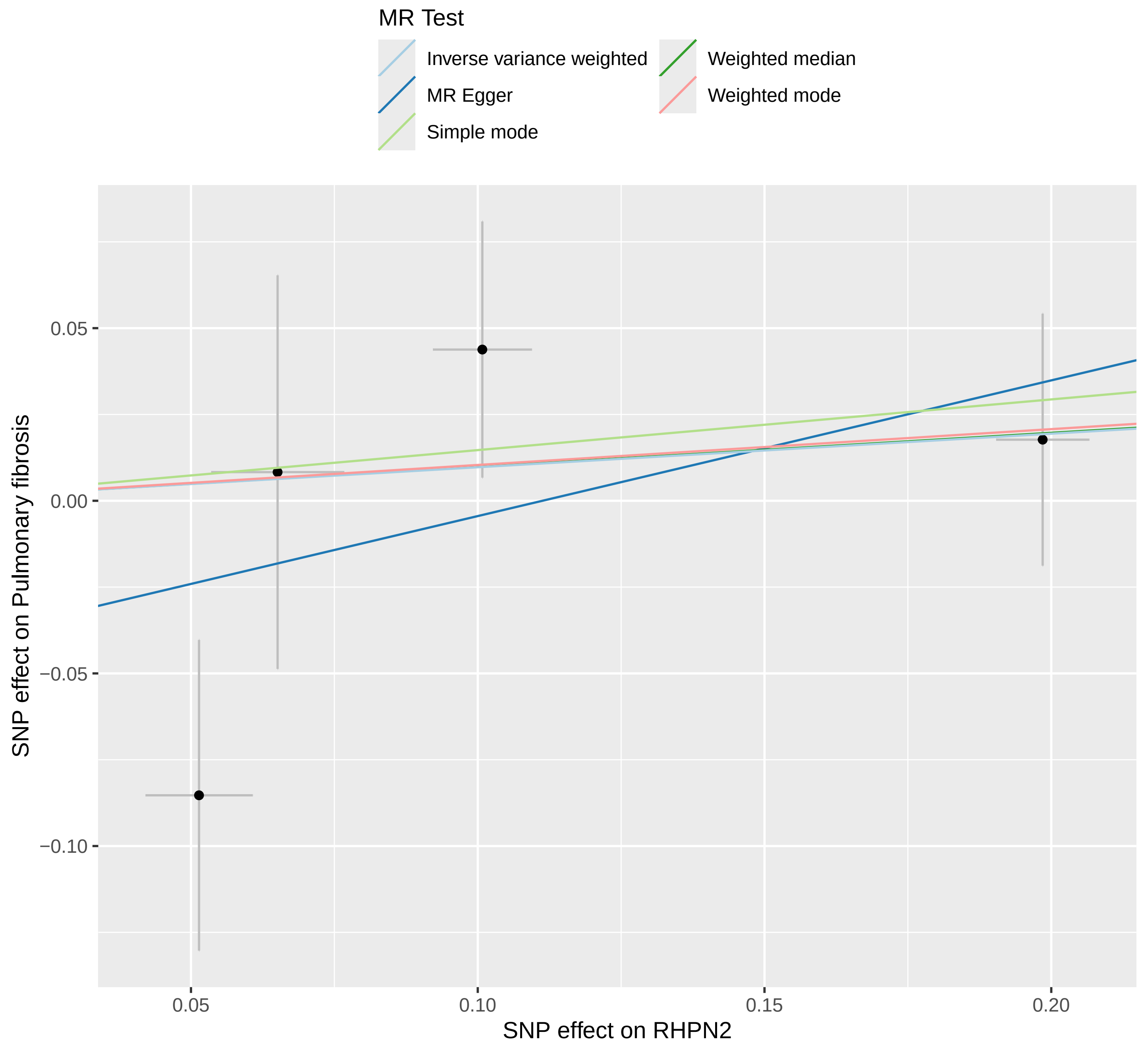
MR Test
Inverse variance weighted
MR Egger
Simple mode
Weighted median
Weighted mode
0.05
0.00
−0.05
−0.10
SNP effect on Pulmonary fibrosis
0.05
0.10
0.15
0.20
SNP effect on RHPN2

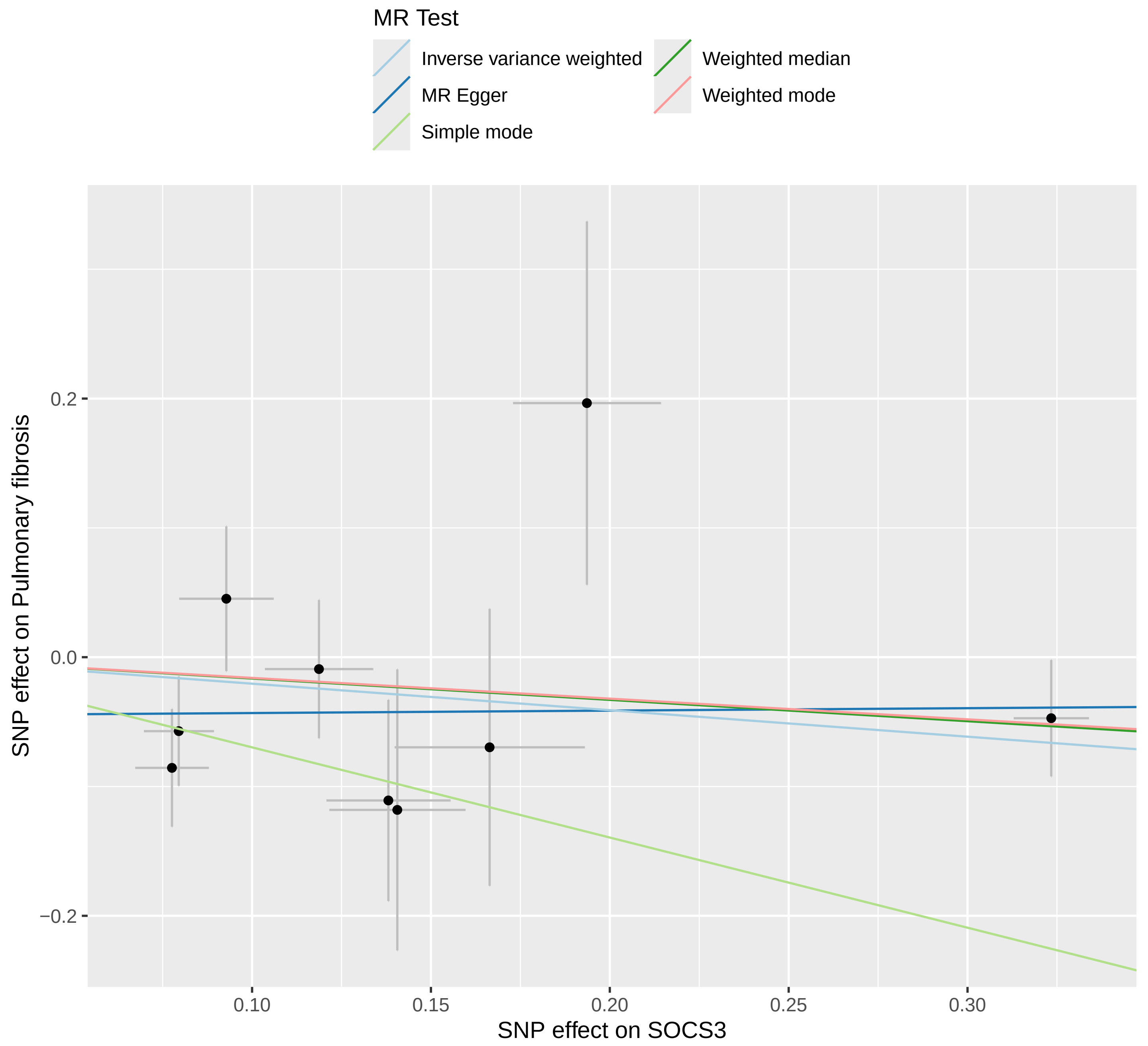

MR Test
Inverse variance weighted
MR Egger
Simple mode
Weighted median
Weighted mode
0.2
0.0
−0.2
0.10
0.15
0.20
0.25
0.30
SNP effect on Pulmonary fibrosis
SNP effect on SOCS3

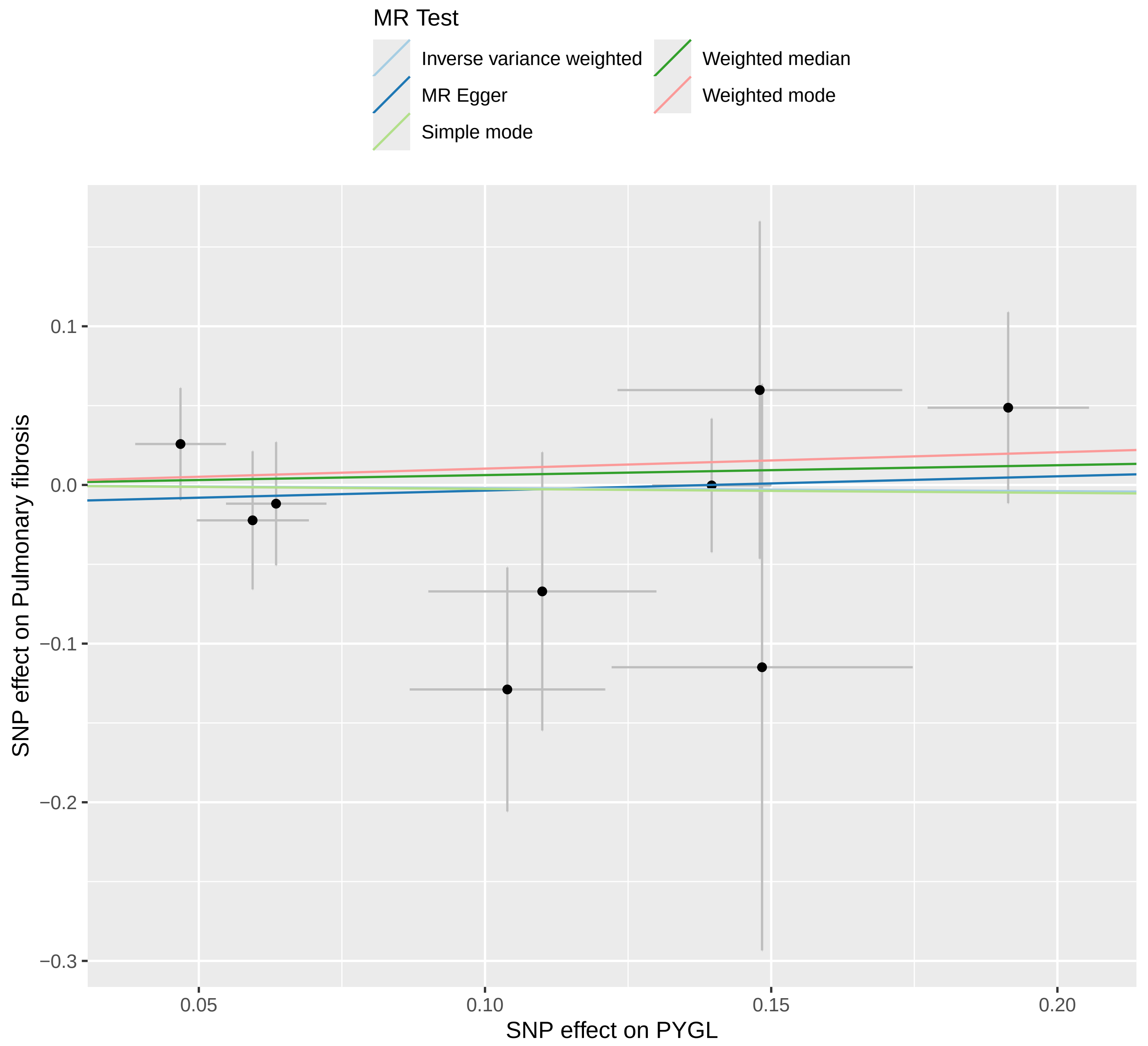
MR Test
Inverse variance weighted
MR Egger
Simple mode
Weighted median
Weighted mode
0.1
0.0
−0.1
−0.2
−0.3
0.05
0.10
0.15
0.20
SNP effect on Pulmonary fibrosis
SNP effect on PYGL

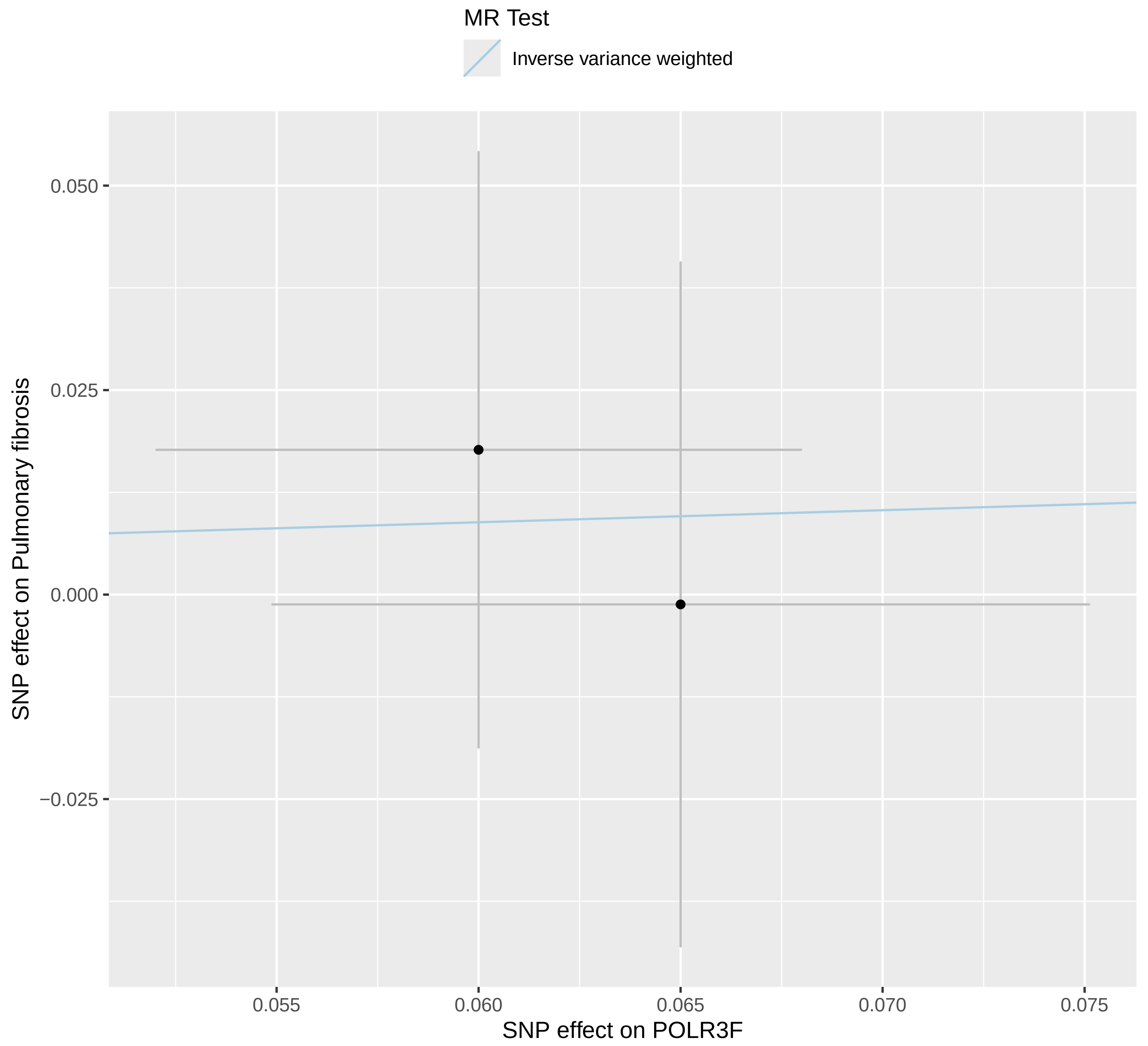
MR Test
Inverse variance weighted
0.050
0.025
0.000
−0.025
SNP effect on Pulmonary fibrosis
0.055
0.060
0.065
0.070
0.075
SNP effect on POLR3F

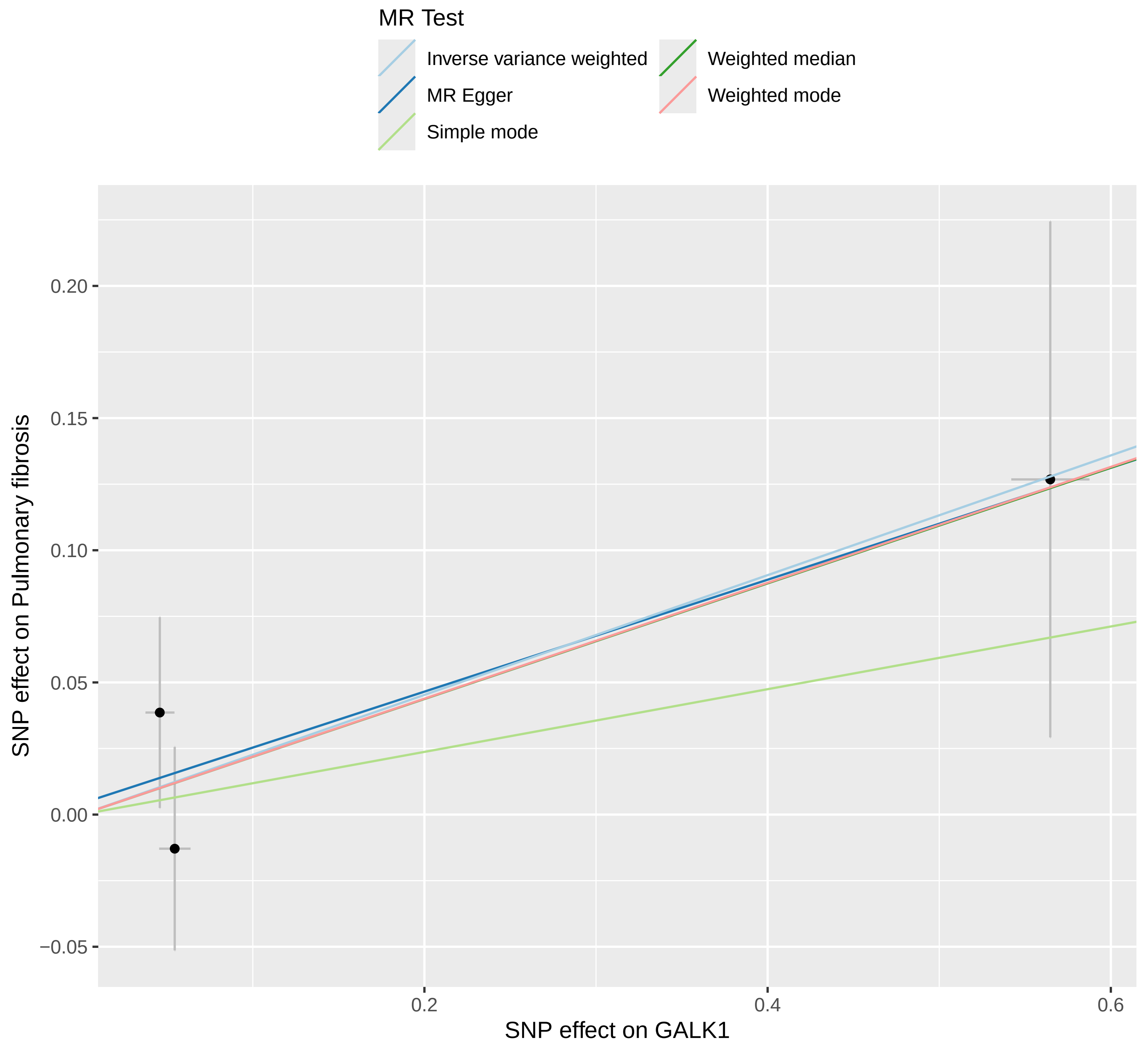
MR Test
Inverse variance weighted
MR Egger
Simple mode
Weighted median
Weighted mode
0.20
0.15
0.10
0.05
0.00
−0.05
0.2
0.4
0.6
SNP effect on Pulmonary fibrosis
SNP effect on GALK1

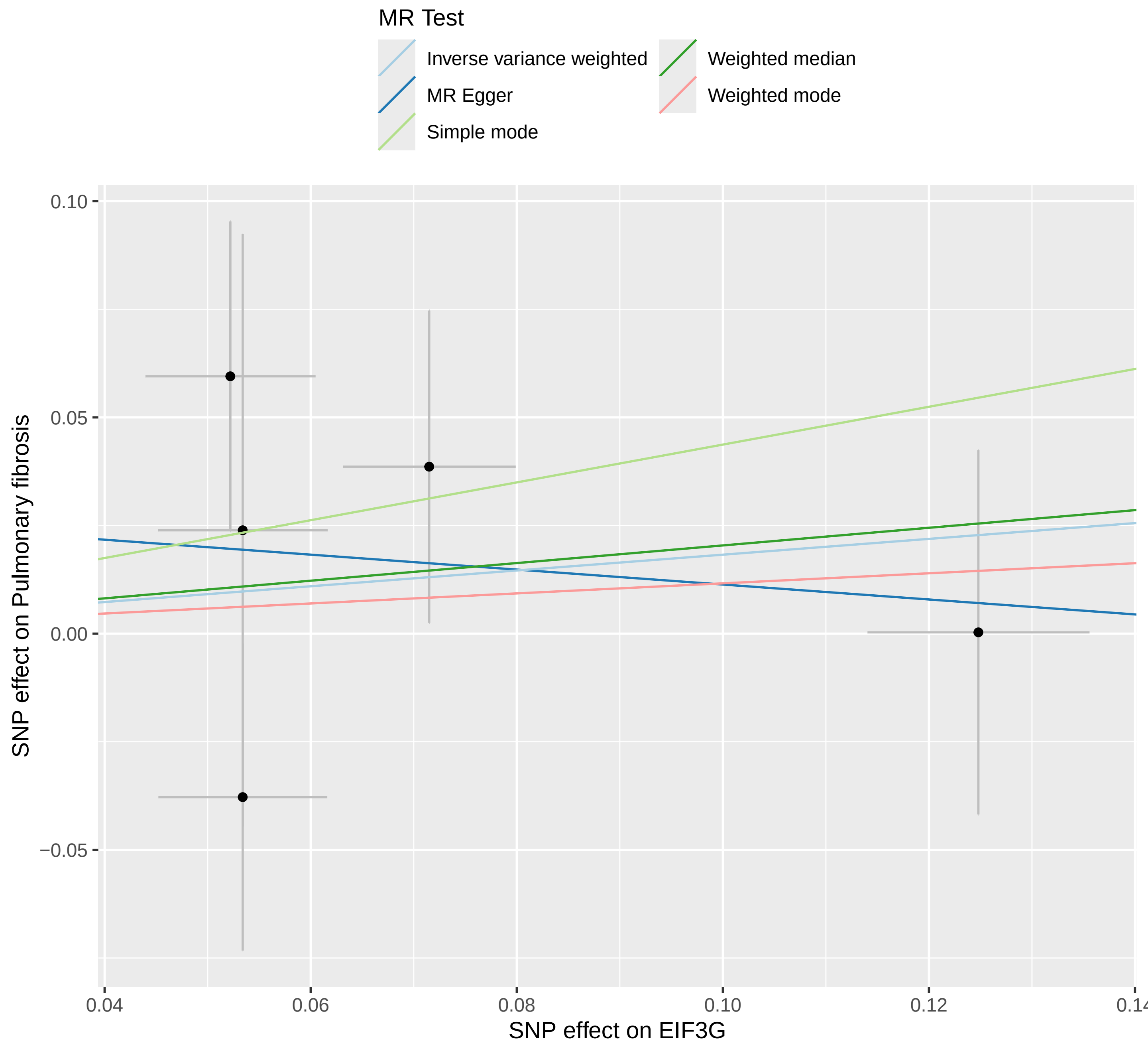
MR Test
Inverse variance weighted
MR Egger
Simple mode
Weighted median
Weighted mode
0.10
0.05
0.00
−0.05
SNP effect on Pulmonary fibrosis
0.04
0.06
0.08
0.10
0.12
0.14
SNP effect on EIF3G

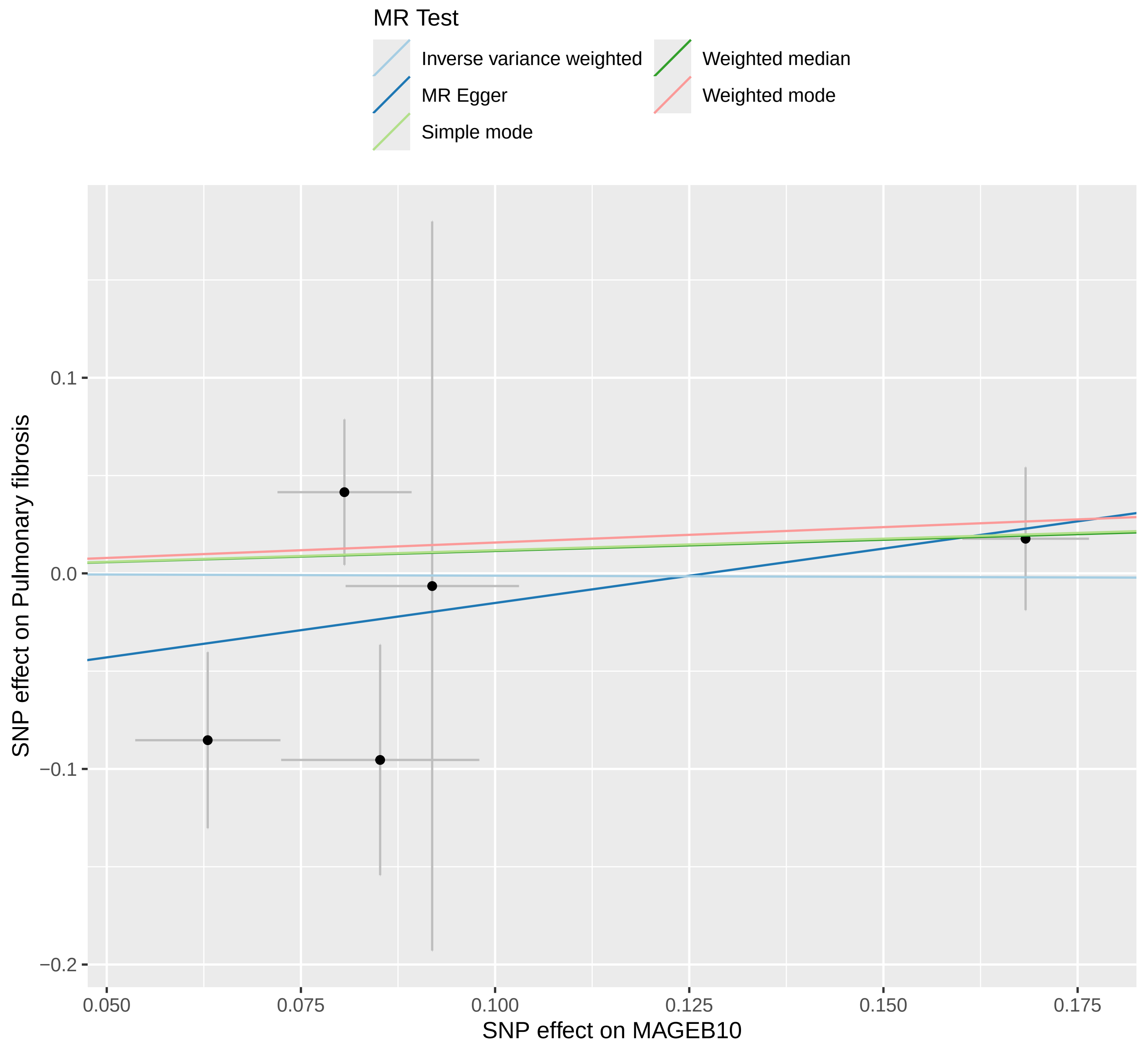
MR Test
Inverse variance weighted
MR Egger
Simple mode
Weighted median
Weighted mode
0.1
0.0
−0.1
−0.2
SNP effect on Pulmonary fibrosis
0.050
0.075
0.100
0.125
0.150
0.175
SNP effect on MAGEB10

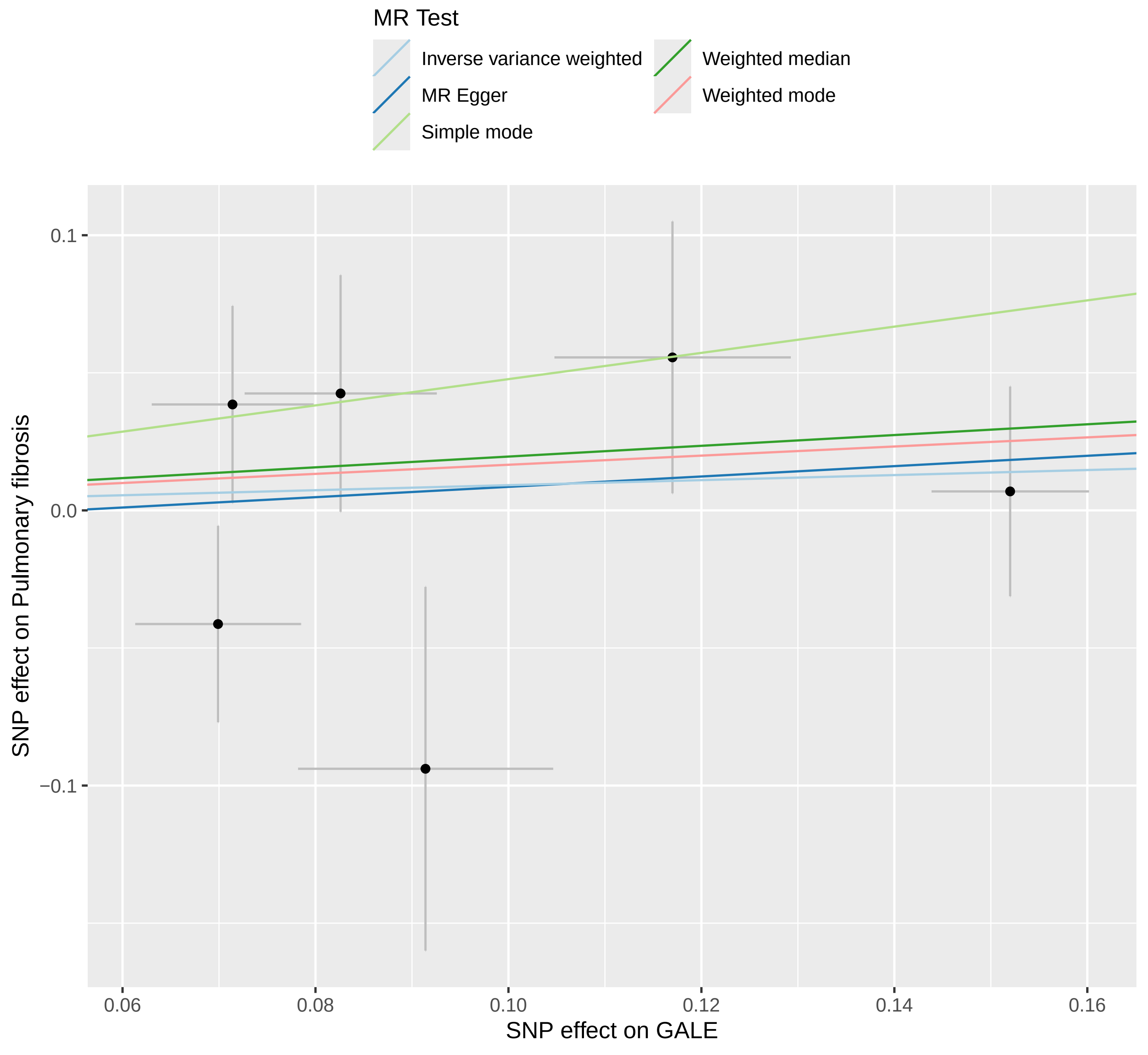
MR Test
Inverse variance weighted
MR Egger
Simple mode
Weighted median
Weighted mode
0.1
0.0
−0.1
0.06
0.08
0.10
0.12
0.14
0.16
SNP effect on GALE
SNP effect on Pulmonary fibrosis

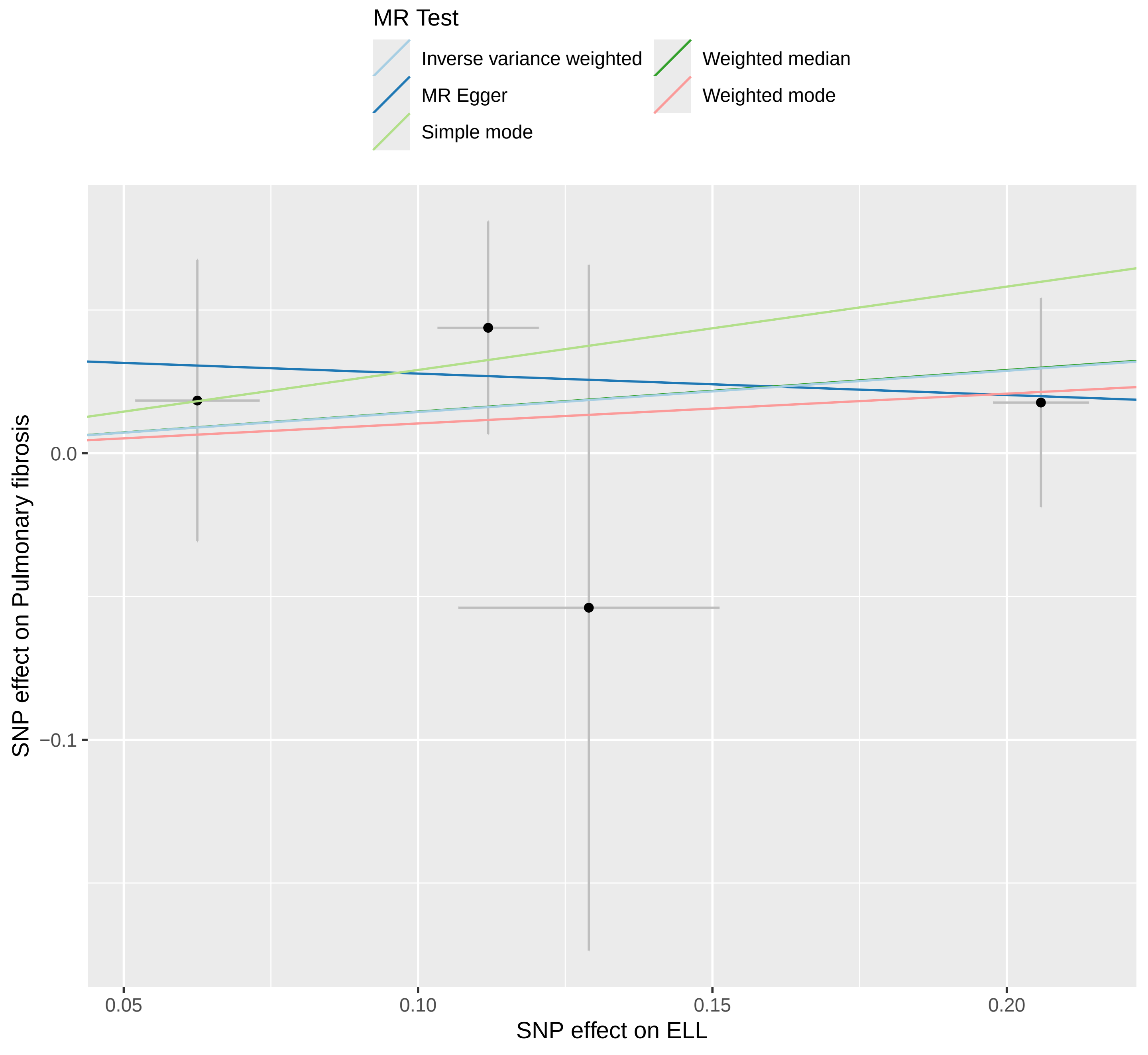

MR Test
Inverse variance weighted
MR Egger
Simple mode
Weighted median
Weighted mode
SNP effect on Pulmonary fibrosis
0.0
−0.1
0.05
0.10
0.15
0.20
SNP effect on ELL

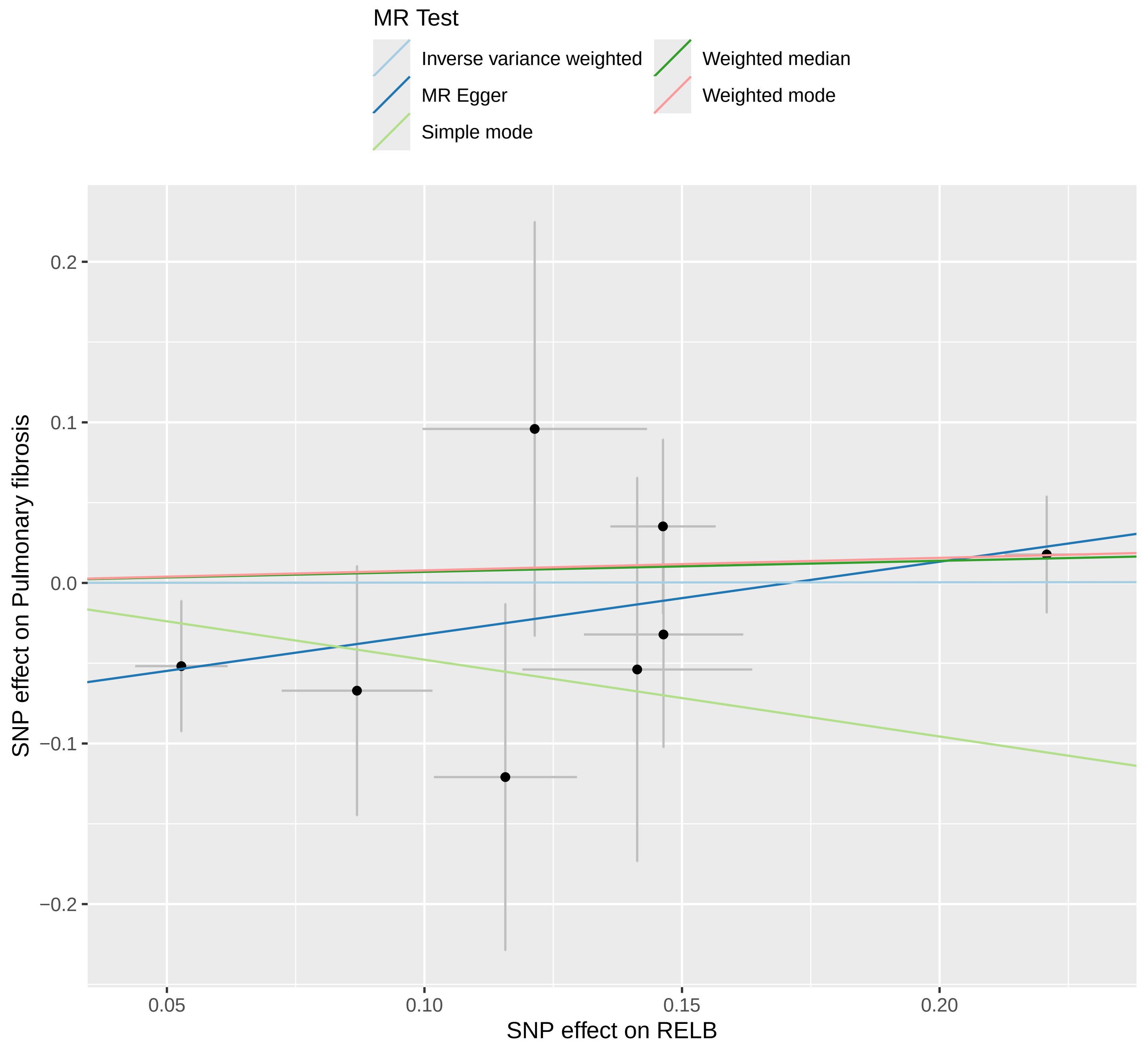

MR Test
Inverse variance weighted
MR Egger
Simple mode
Weighted median
Weighted mode
SNP effect on Pulmonary fibrosis
0.2
0.1
0.0
−0.1
−0.2
0.05
0.10
0.15
0.20
SNP effect on RELB

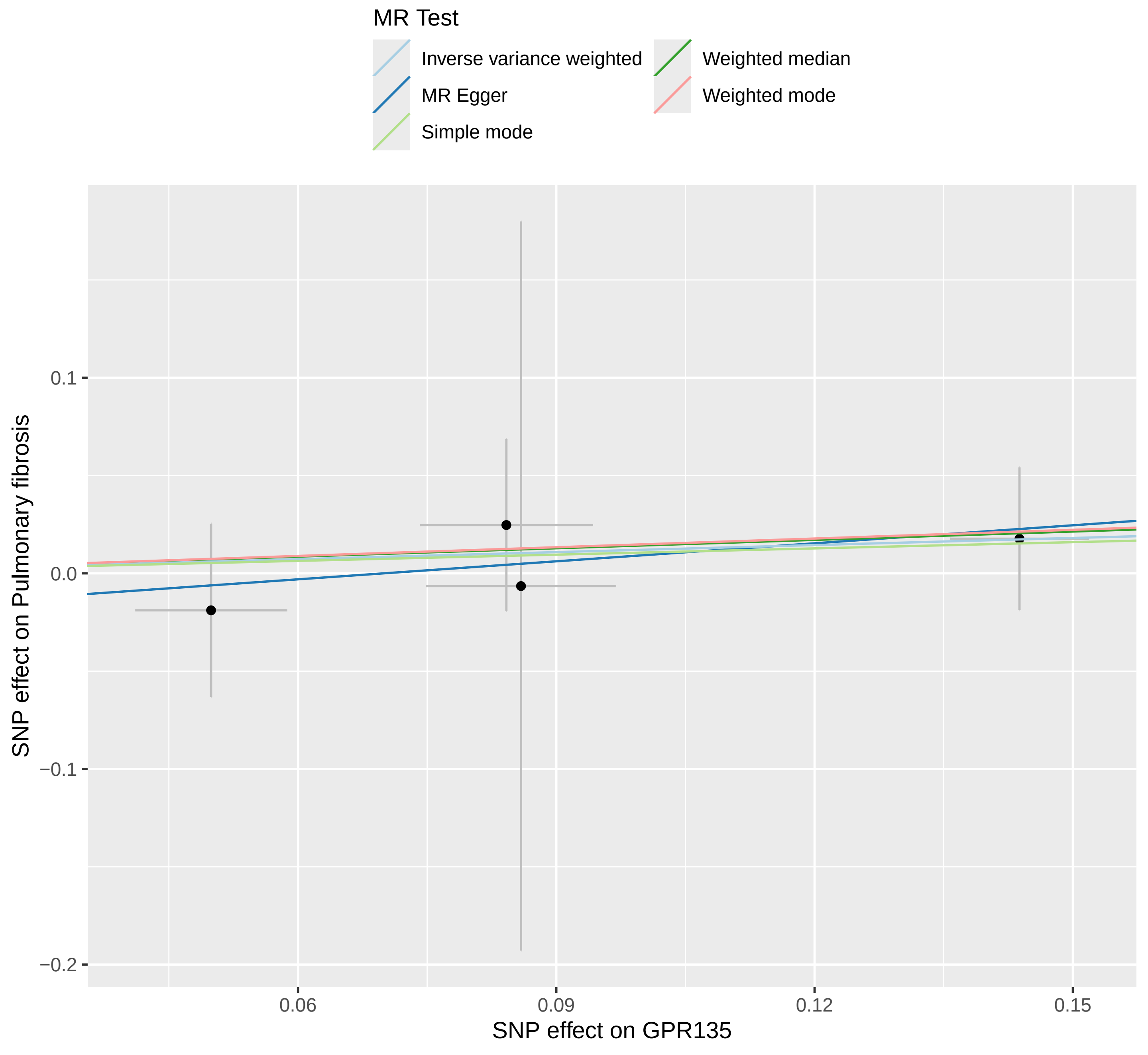
MR Test
Inverse variance weighted
MR Egger
Simple mode
Weighted median
Weighted mode
0.1
0.0
−0.1
−0.2
SNP effect on Pulmonary fibrosis
0.06
0.09
0.12
0.15
SNP effect on GPR135

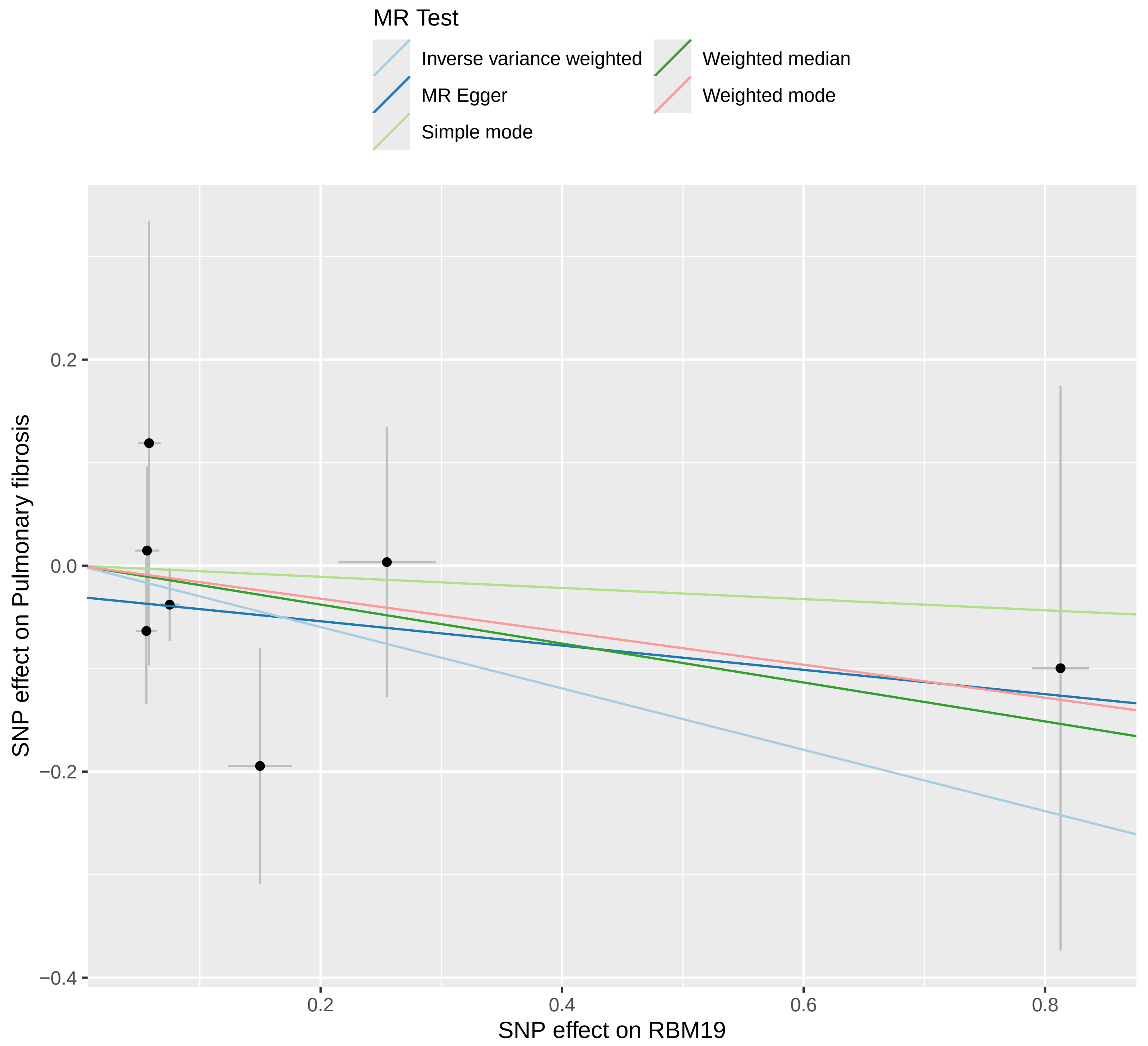
MR Test
Inverse variance weighted
MR Egger
Simple mode
Weighted median
Weighted mode
SNP effect on Pulmonary fibrosis
0.2
0.0
−0.2
−0.4
0.2
0.4
0.6
0.8
SNP effect on RBM19

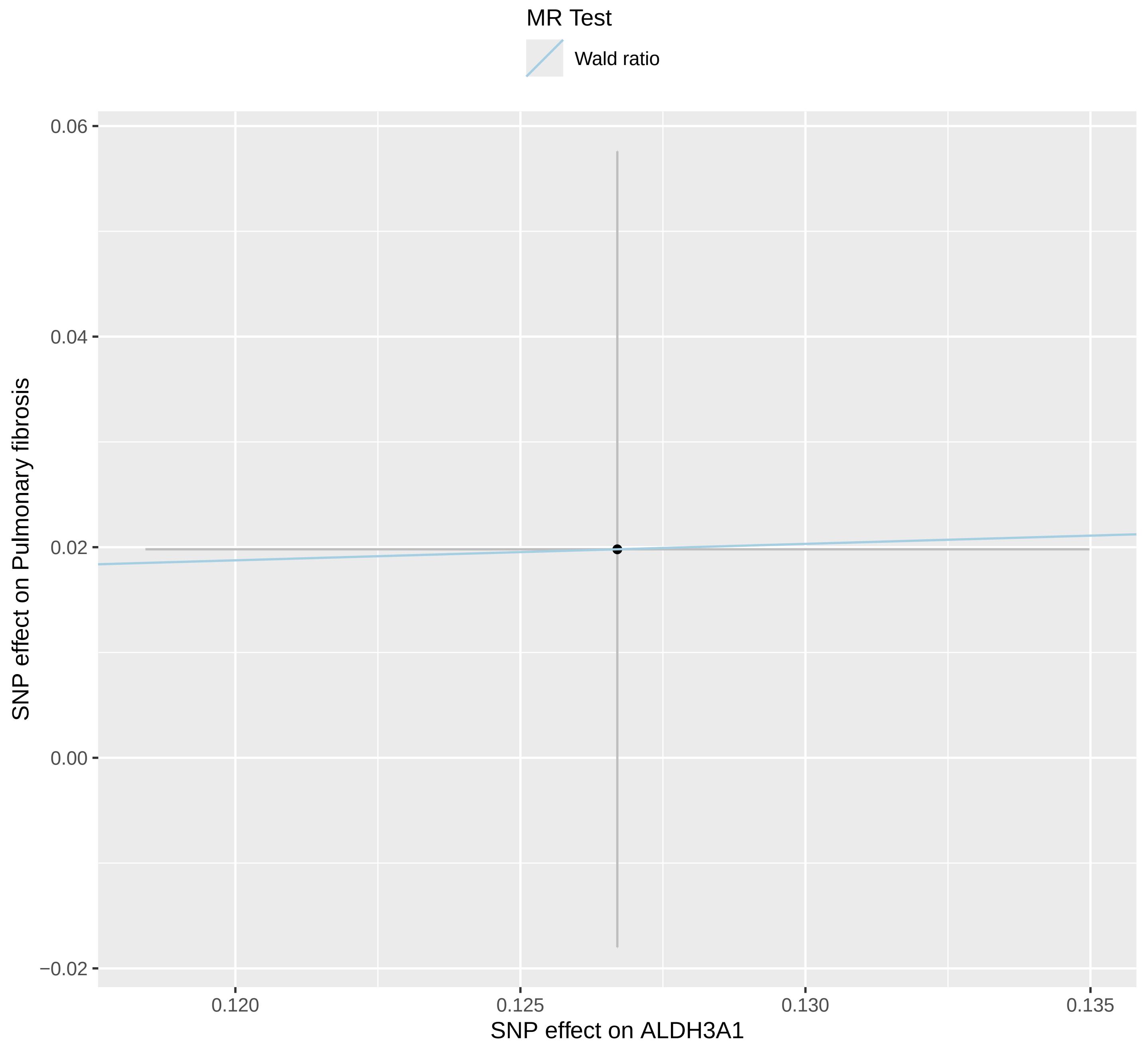
MR Test
Wald ratio
0.06
0.04
0.02
0.00
−0.02
0.120
0.125
0.130
0.135
SNP effect on Pulmonary fibrosis
SNP effect on ALDH3A1

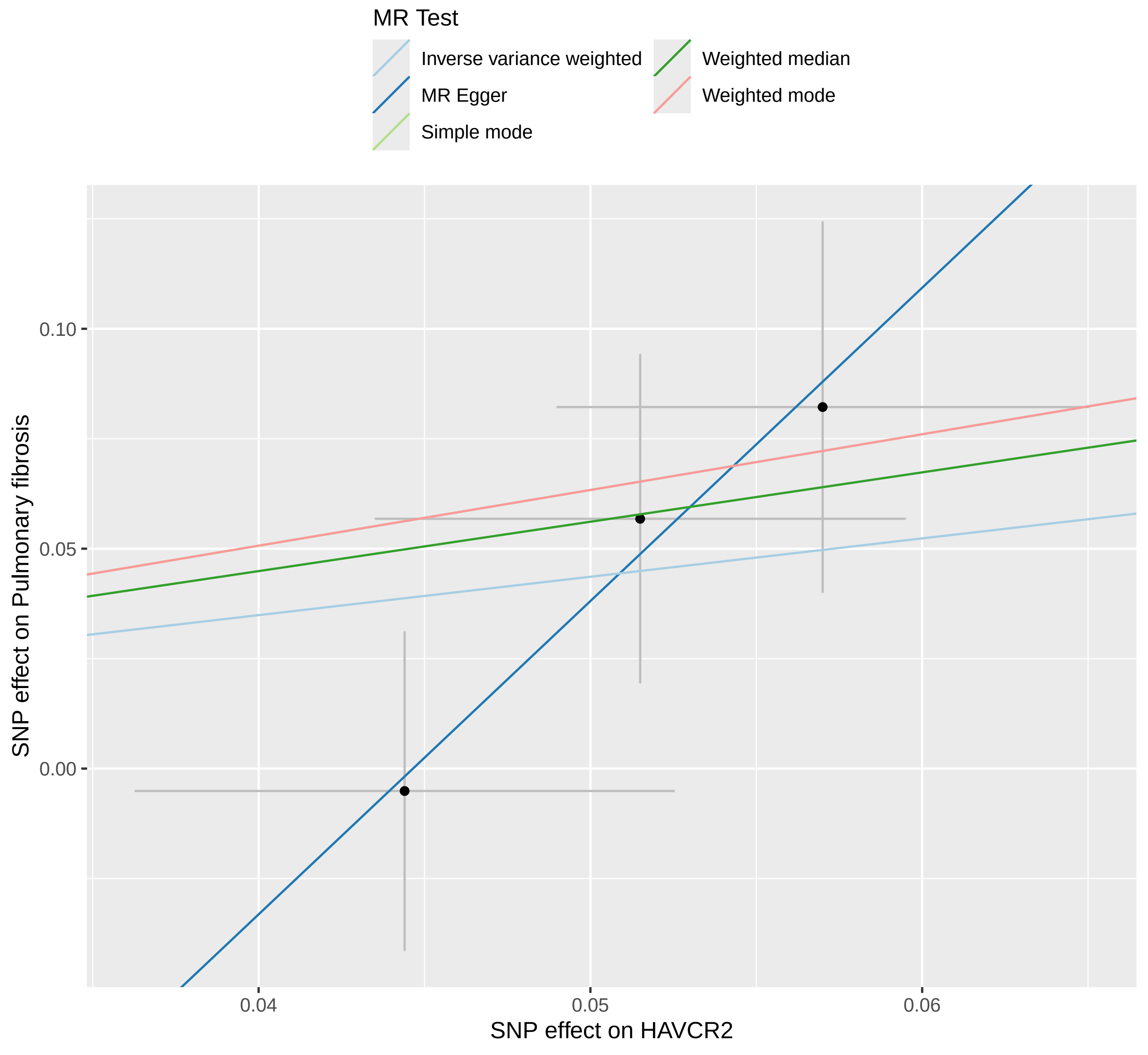

MR Test
Inverse variance weighted
MR Egger
Simple mode
Weighted median
Weighted mode
0.10
0.05
0.00
0.04
0.05
0.06
SNP effect on Pulmonary fibrosis
SNP effect on HAVCR2

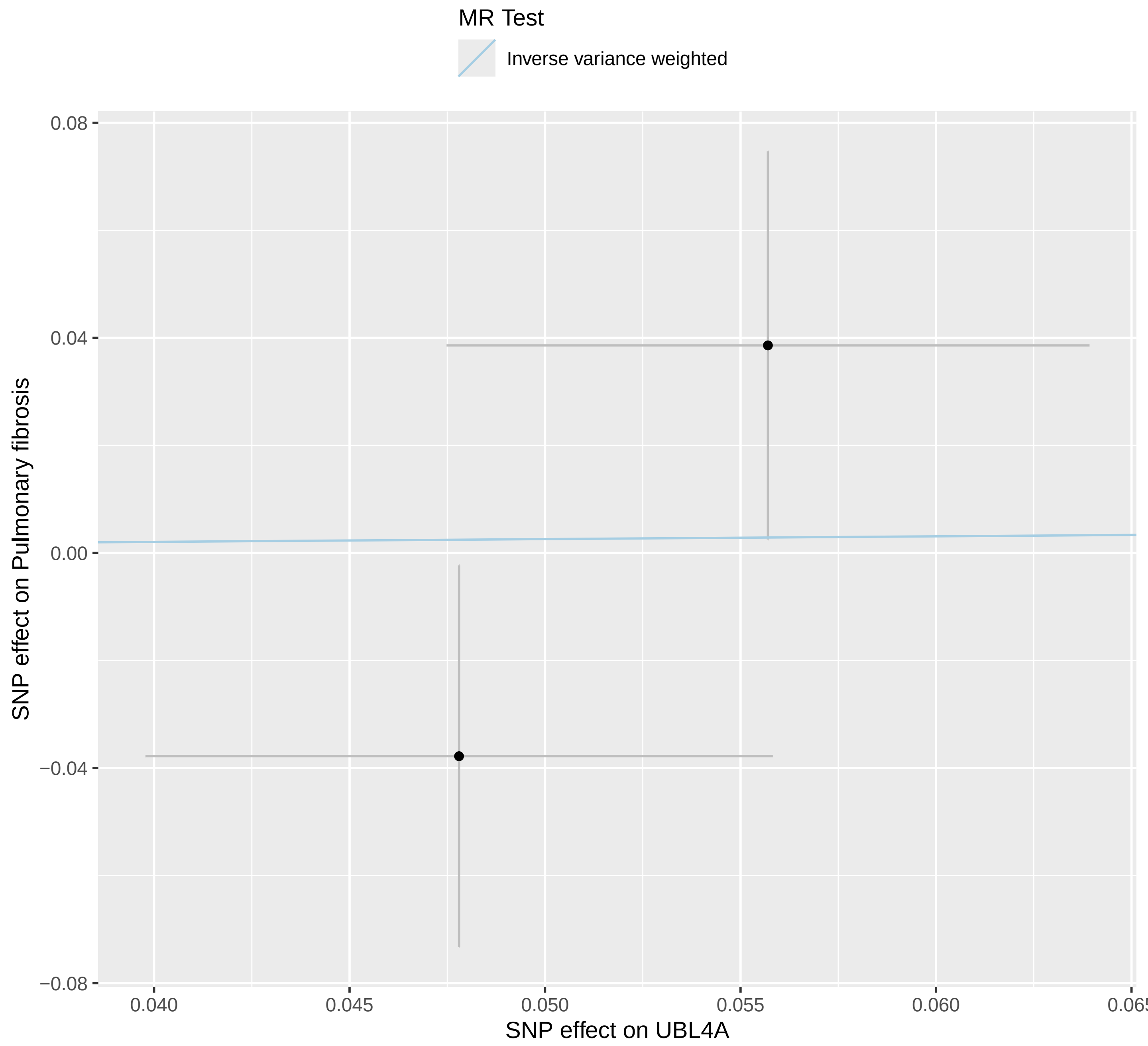

MR Test
Inverse variance weighted
0.08
0.04
0.00
−0.04
−0.08
0.040
0.045
0.050
0.055
0.060
0.065
SNP effect on UBL4A
SNP effect on Pulmonary fibrosis

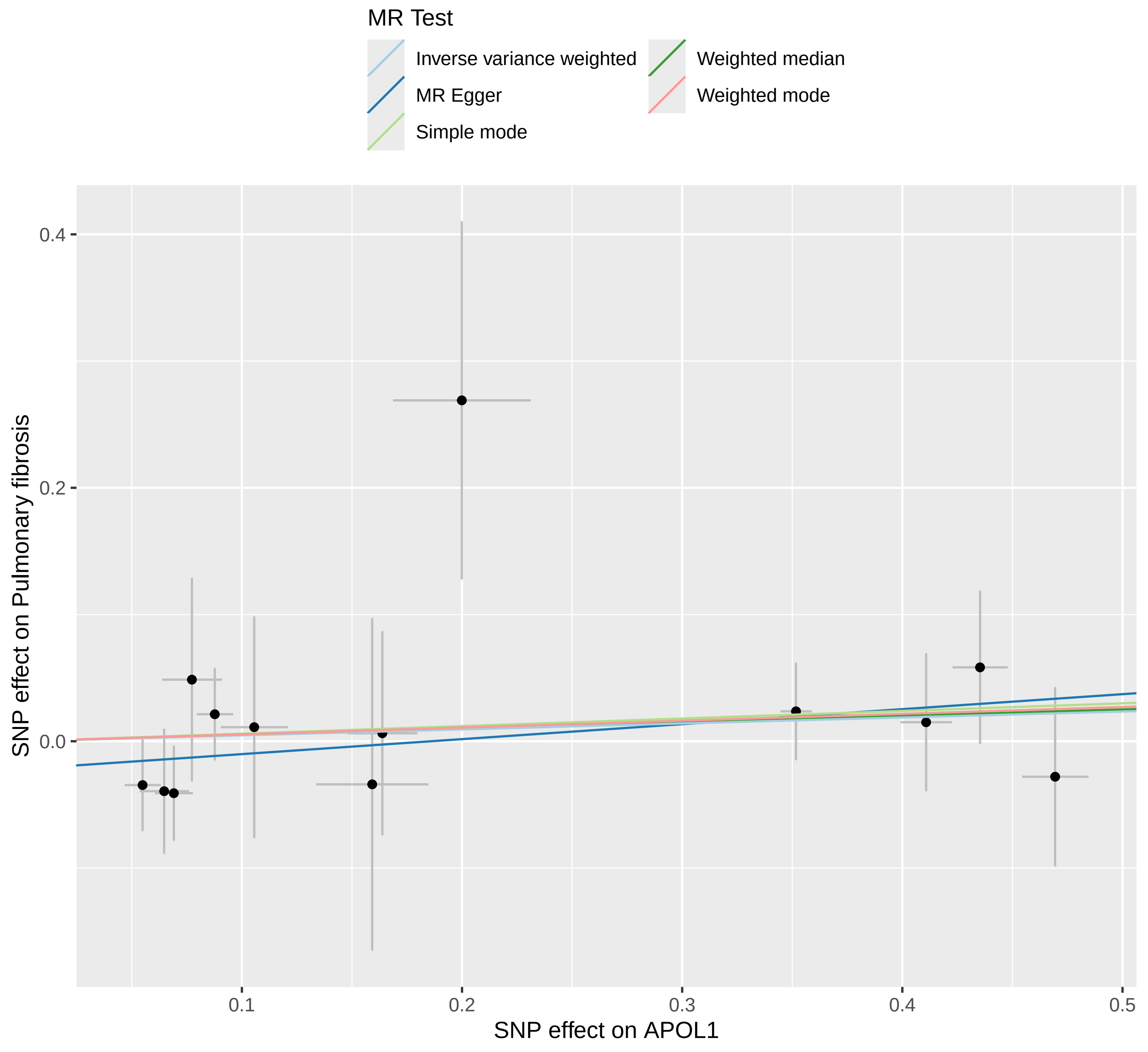
MR Test
Inverse variance weighted
MR Egger
Simple mode
Weighted median
Weighted mode
SNP effect on Pulmonary fibrosis
0.4
0.2
0.0
0.1
0.2
0.3
0.4
0.5
SNP effect on APOL1

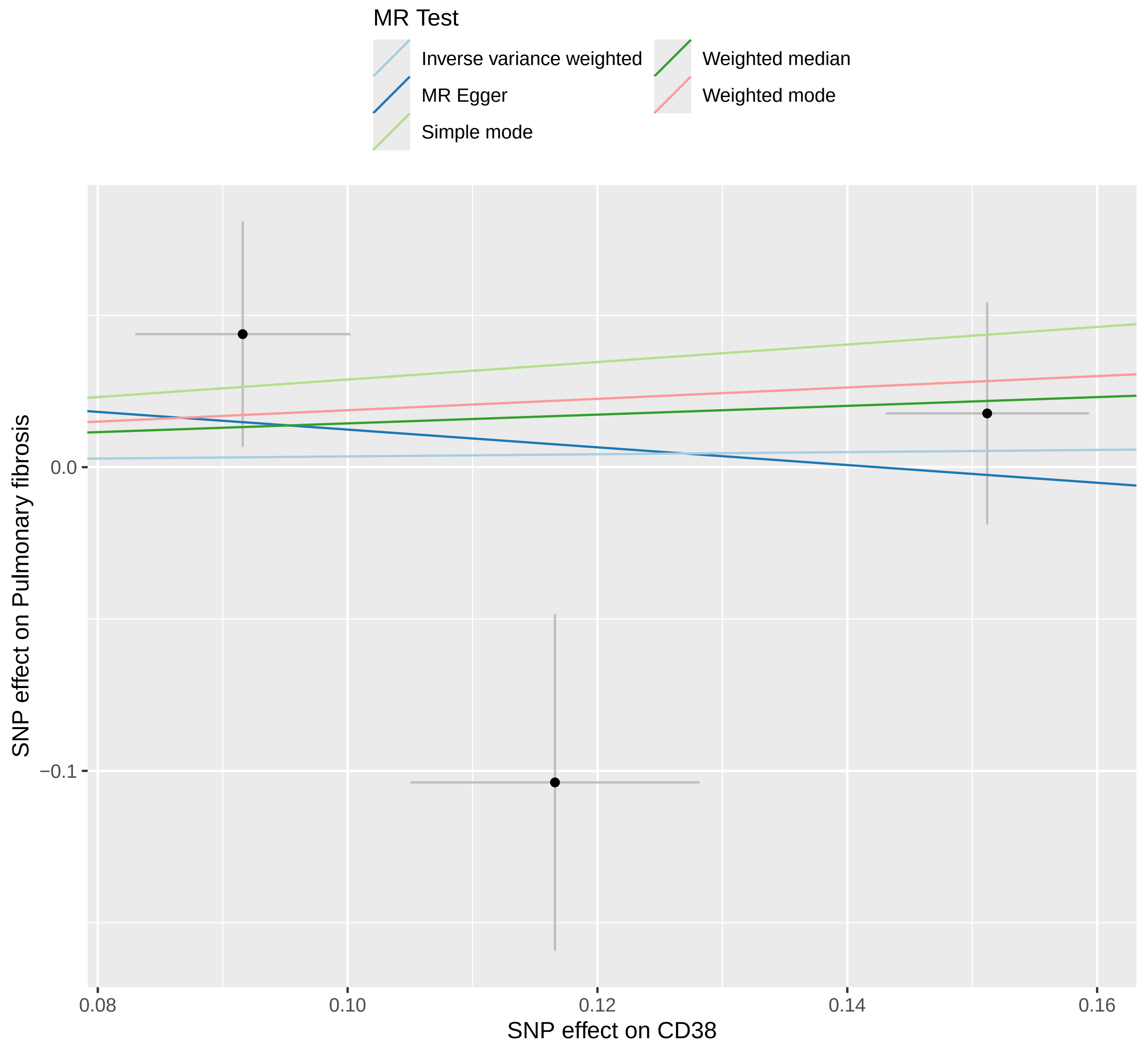
MR Test
Inverse variance weighted
MR Egger
Simple mode
Weighted median
Weighted mode
SNP effect on Pulmonary fibrosis
0.0
−0.1
0.08
0.10
0.12
0.14
0.16
SNP effect on CD38

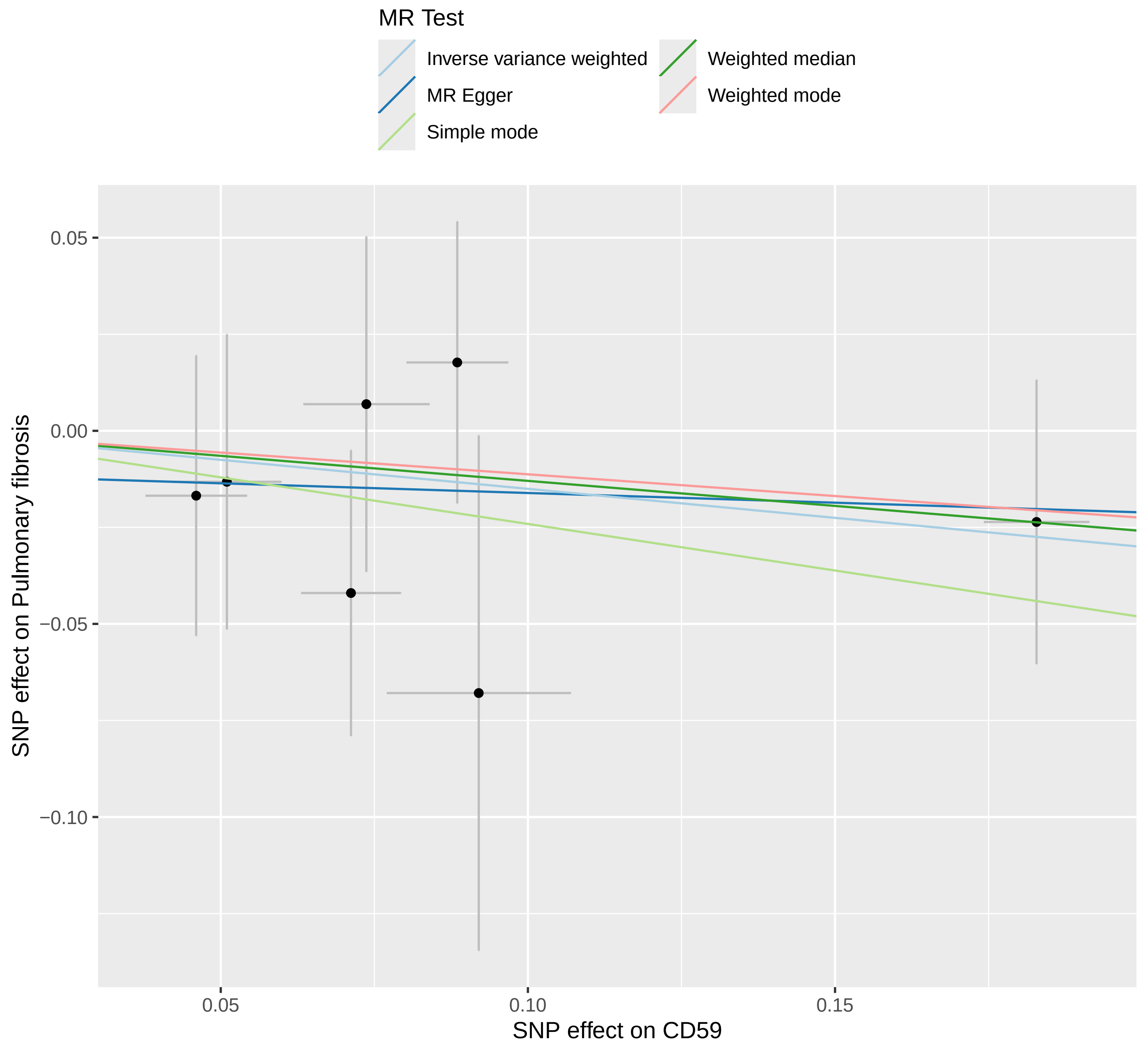
MR Test
Inverse variance weighted
MR Egger
Simple mode
Weighted median
Weighted mode
0.05
0.00
−0.05
−0.10
SNP effect on Pulmonary fibrosis
0.05
0.10
0.15
SNP effect on CD59

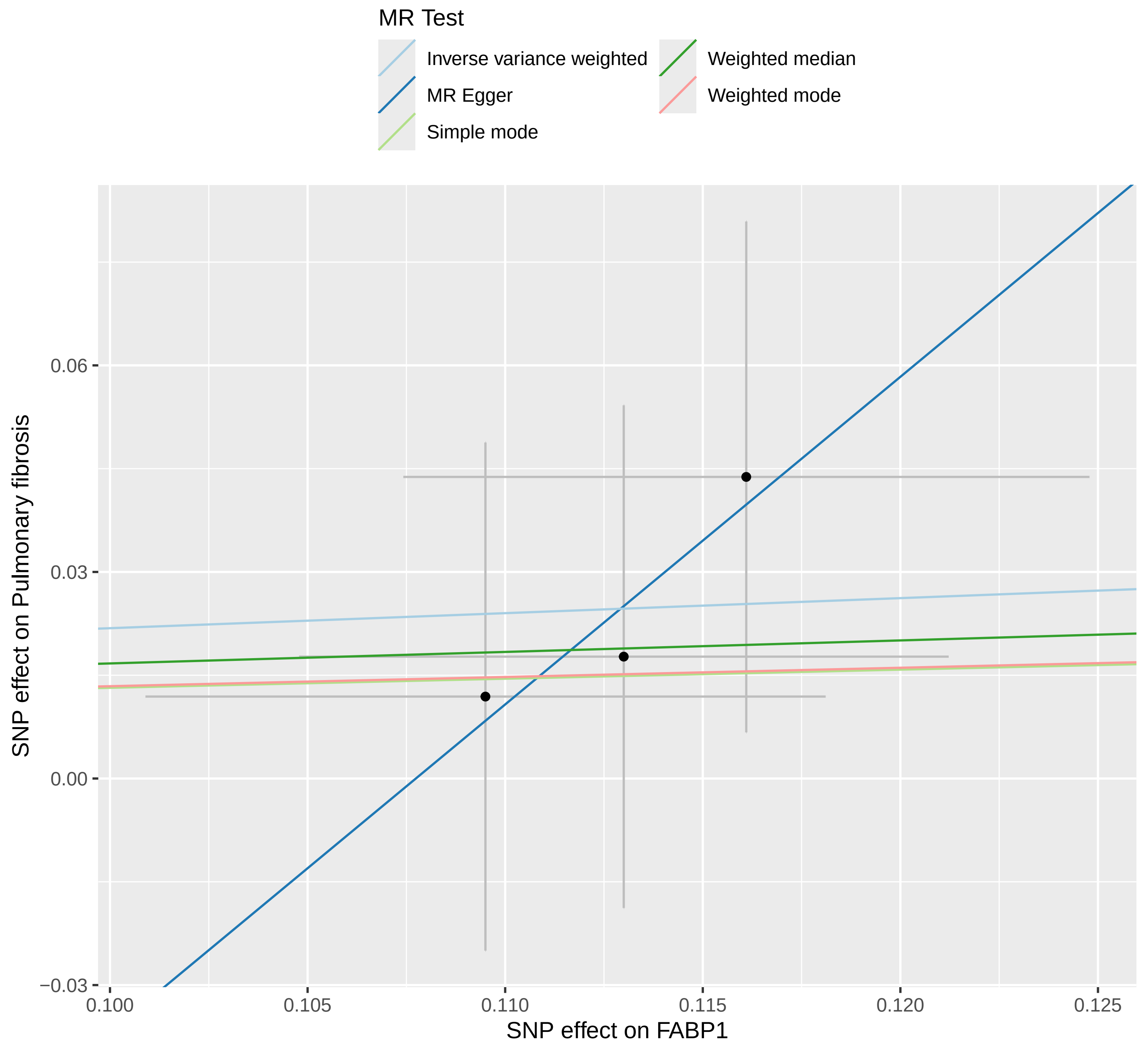
MR Test
Inverse variance weighted
MR Egger
Simple mode
Weighted median
Weighted mode
SNP effect on Pulmonary fibrosis
0.06
0.03
0.00
−0.03
0.100
0.105
0.110
0.115
0.120
0.125
SNP effect on FABP1

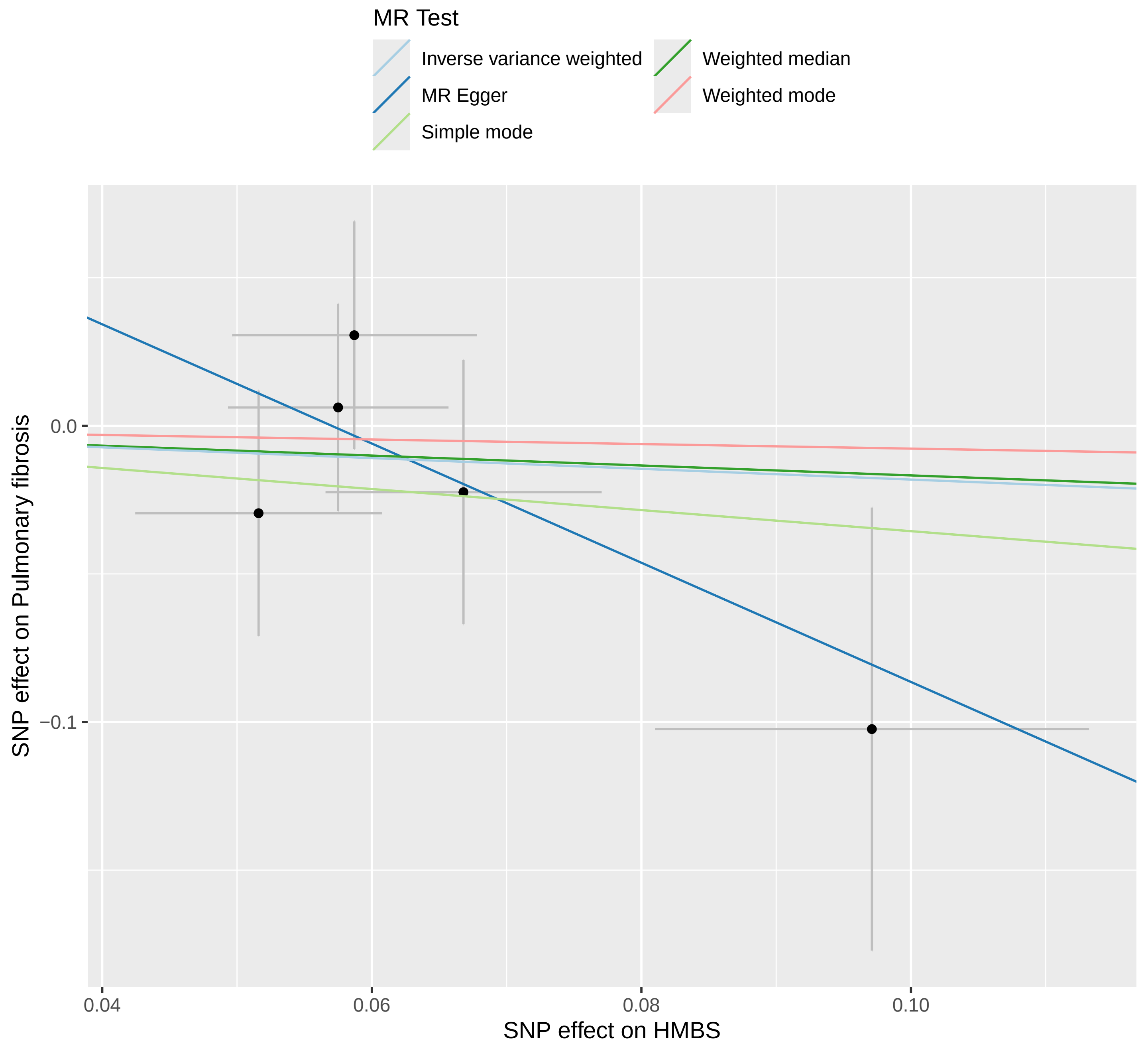
MR Test
Inverse variance weighted
MR Egger
Simple mode
Weighted median
Weighted mode
0.0
−0.1
SNP effect on Pulmonary fibrosis
0.04
0.06
0.08
0.10
SNP effect on HMBS

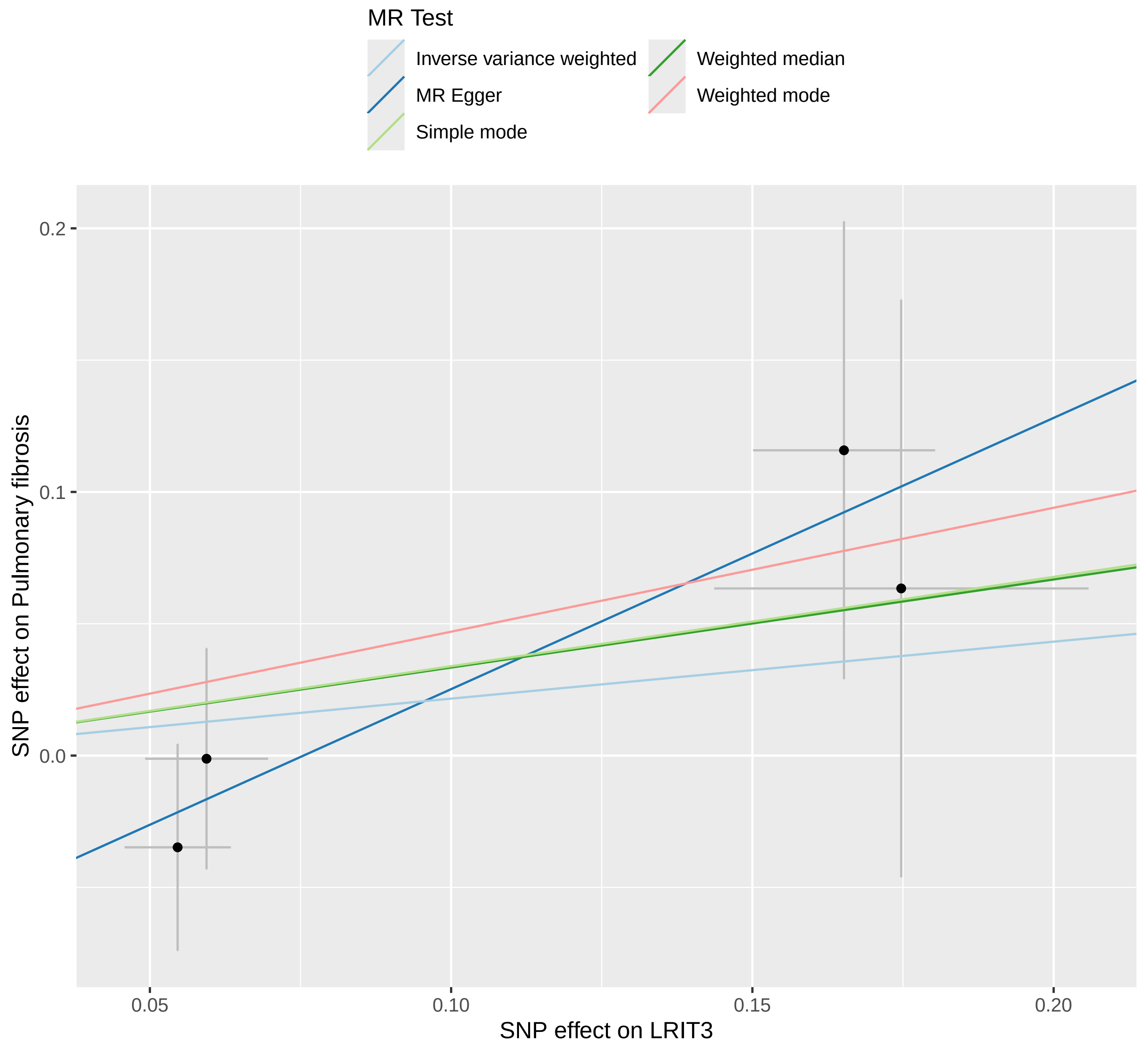
MR Test
Inverse variance weighted
MR Egger
Simple mode
Weighted median
Weighted mode
SNP effect on Pulmonary fibrosis
0.2
0.1
0.0
0.05
0.10
0.15
0.20
SNP effect on LRIT3

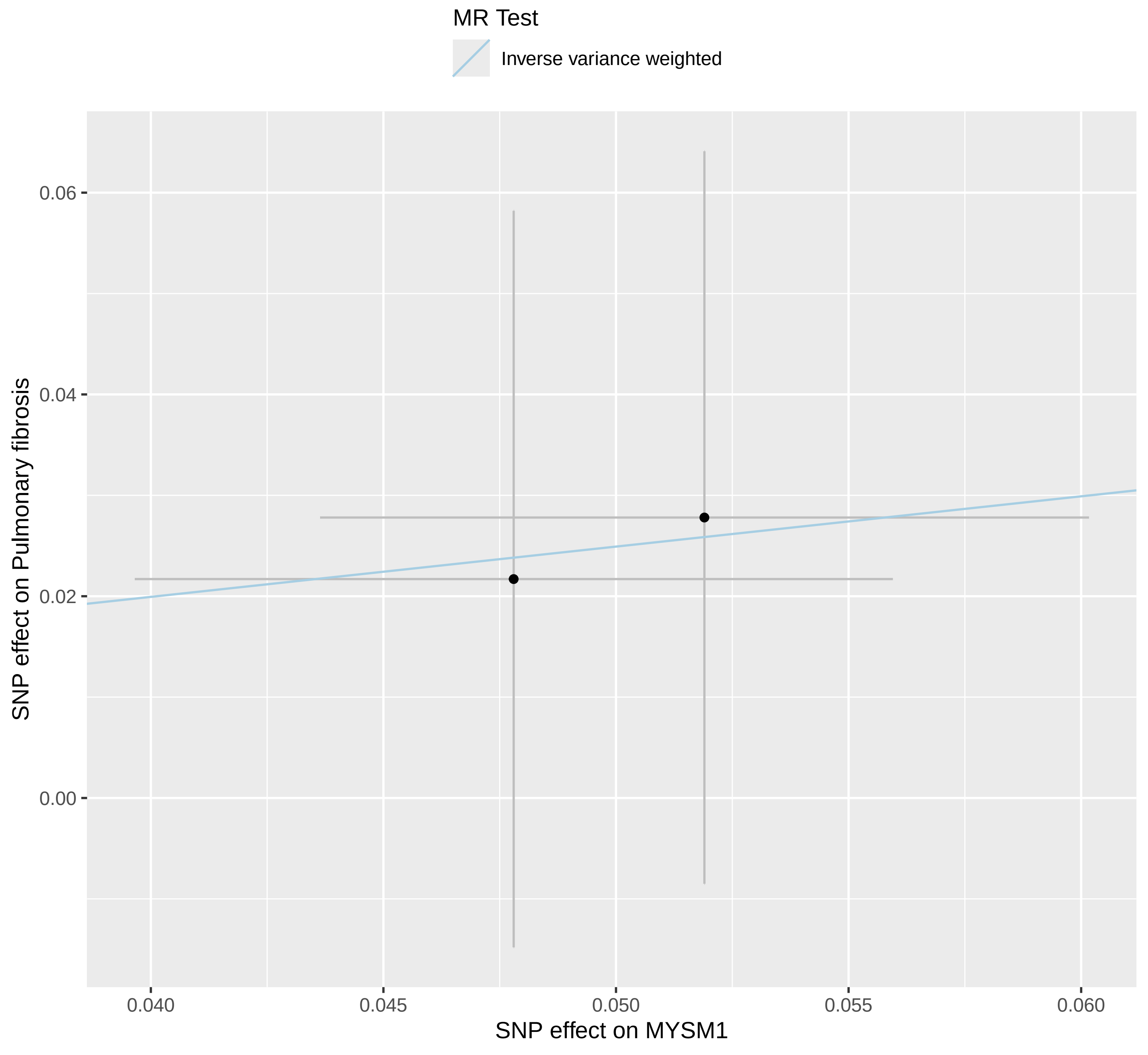
MR Test
Inverse variance weighted
SNP effect on Pulmonary fibrosis
0.06
0.04
0.02
0.00
0.040
0.045
0.050
0.055
0.060
SNP effect on MYSM1

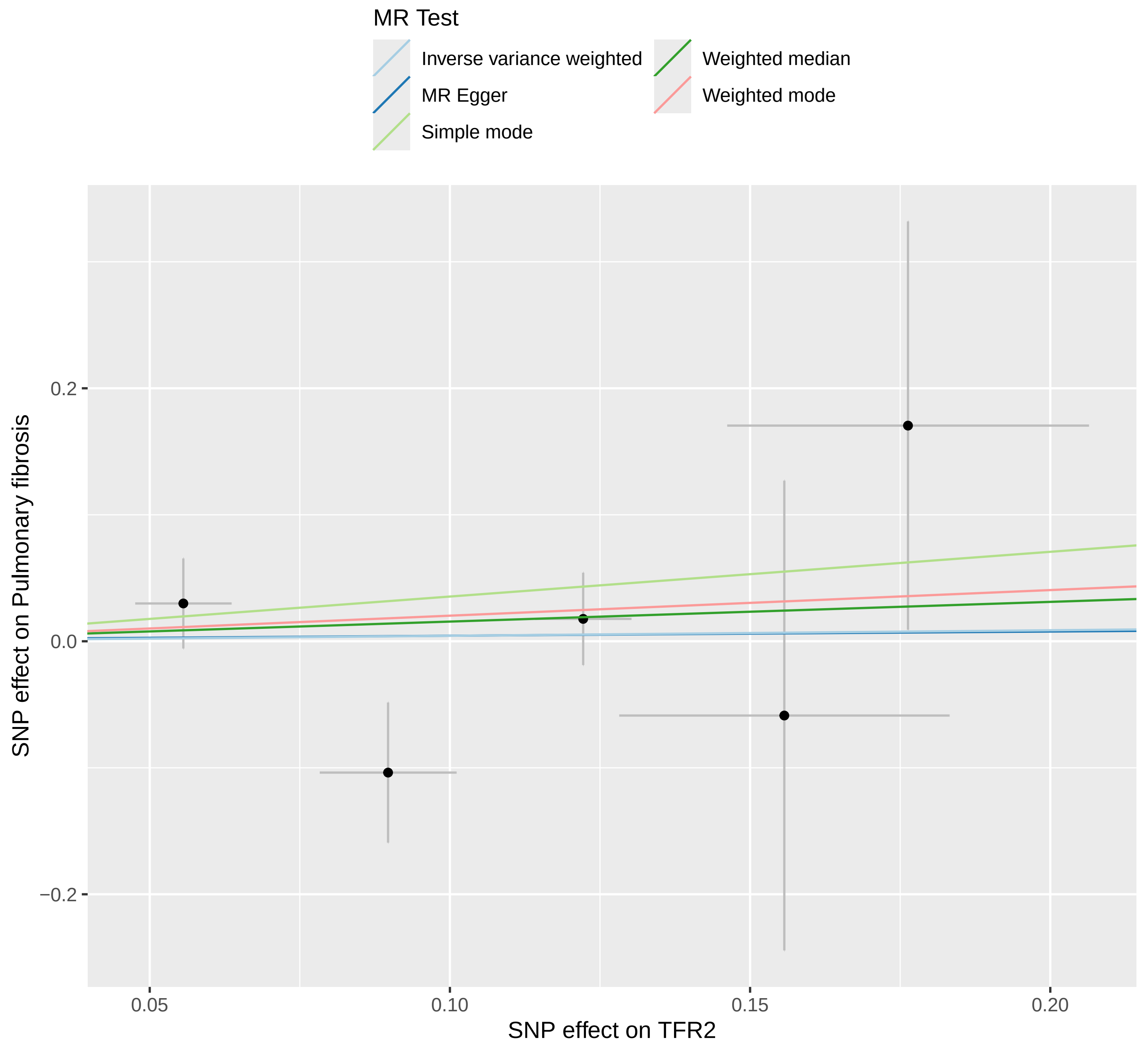
MR Test
Inverse variance weighted
MR Egger
Simple mode
Weighted median
Weighted mode
0.2
0.0
−0.2
0.05
0.10
0.15
0.20
SNP effect on Pulmonary fibrosis
SNP effect on TFR2

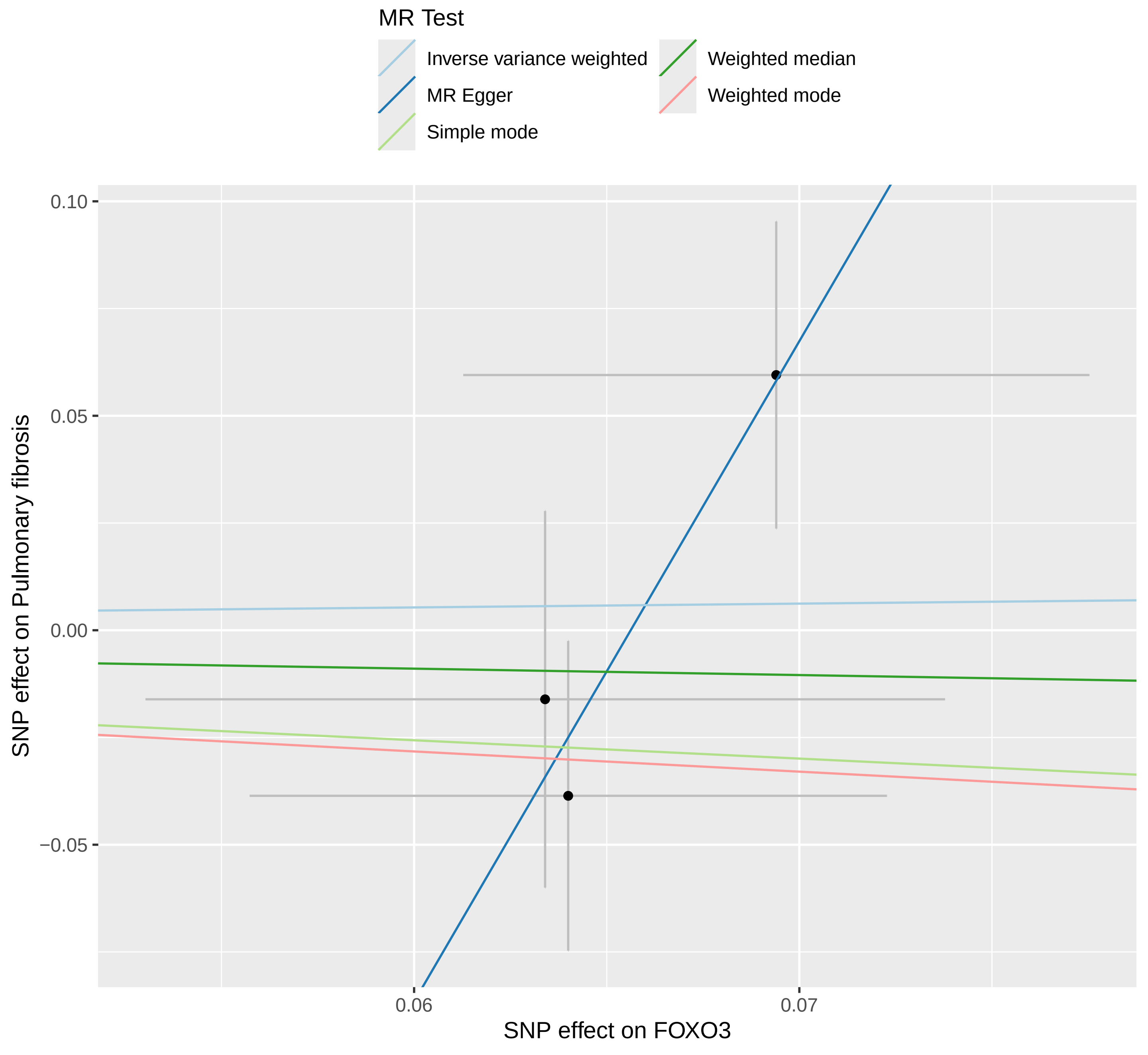
MR Test
Inverse variance weighted
MR Egger
Simple mode
Weighted median
Weighted mode
0.10
0.05
0.00
−0.05
0.06
0.07
SNP effect on FOXO3
SNP effect on Pulmonary fibrosis

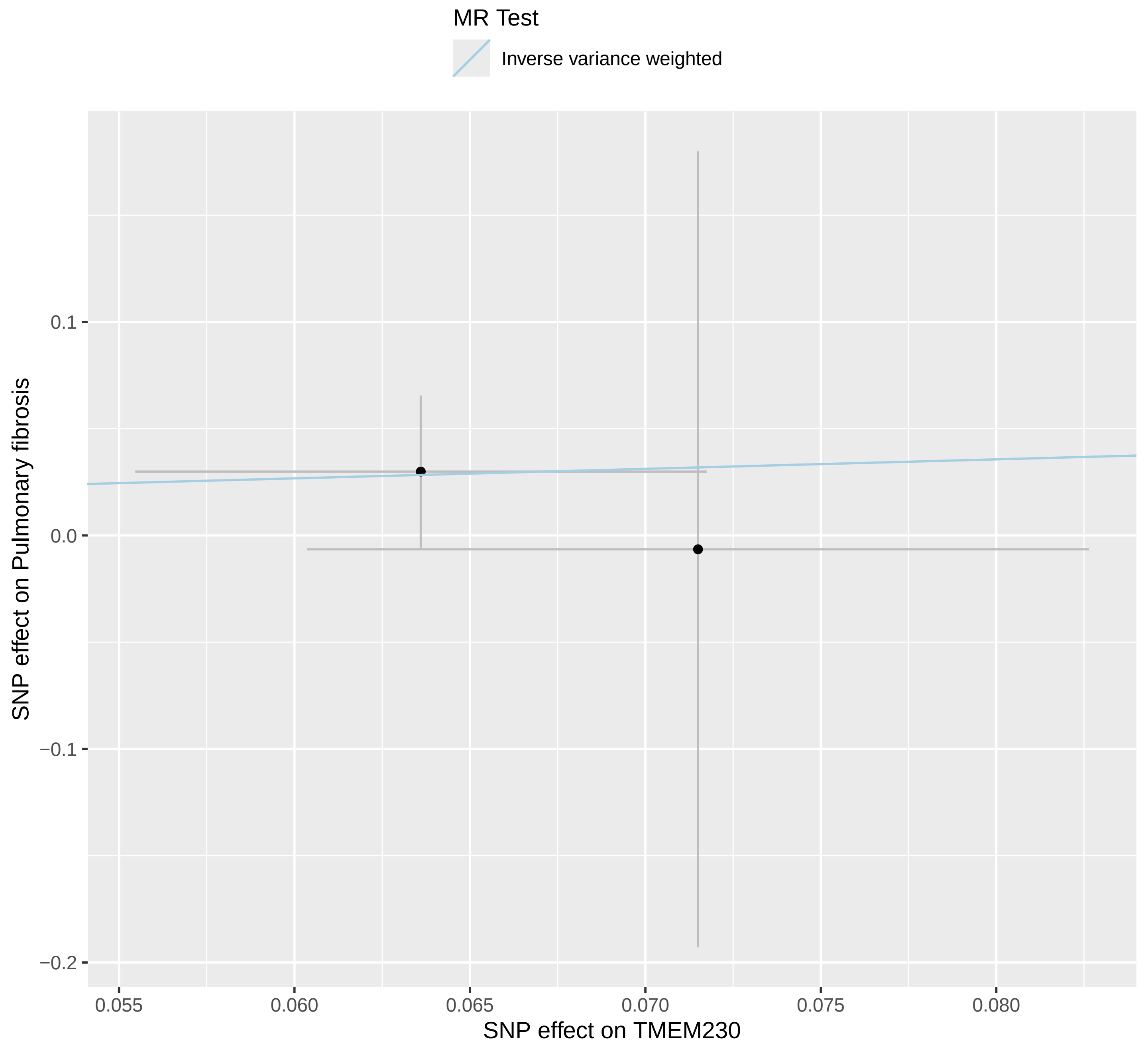

MR Test
Inverse variance weighted
0.1
0.0
−0.1
−0.2
SNP effect on Pulmonary fibrosis
0.055
0.060
0.065
0.070
0.075
0.080
SNP effect on TMEM230

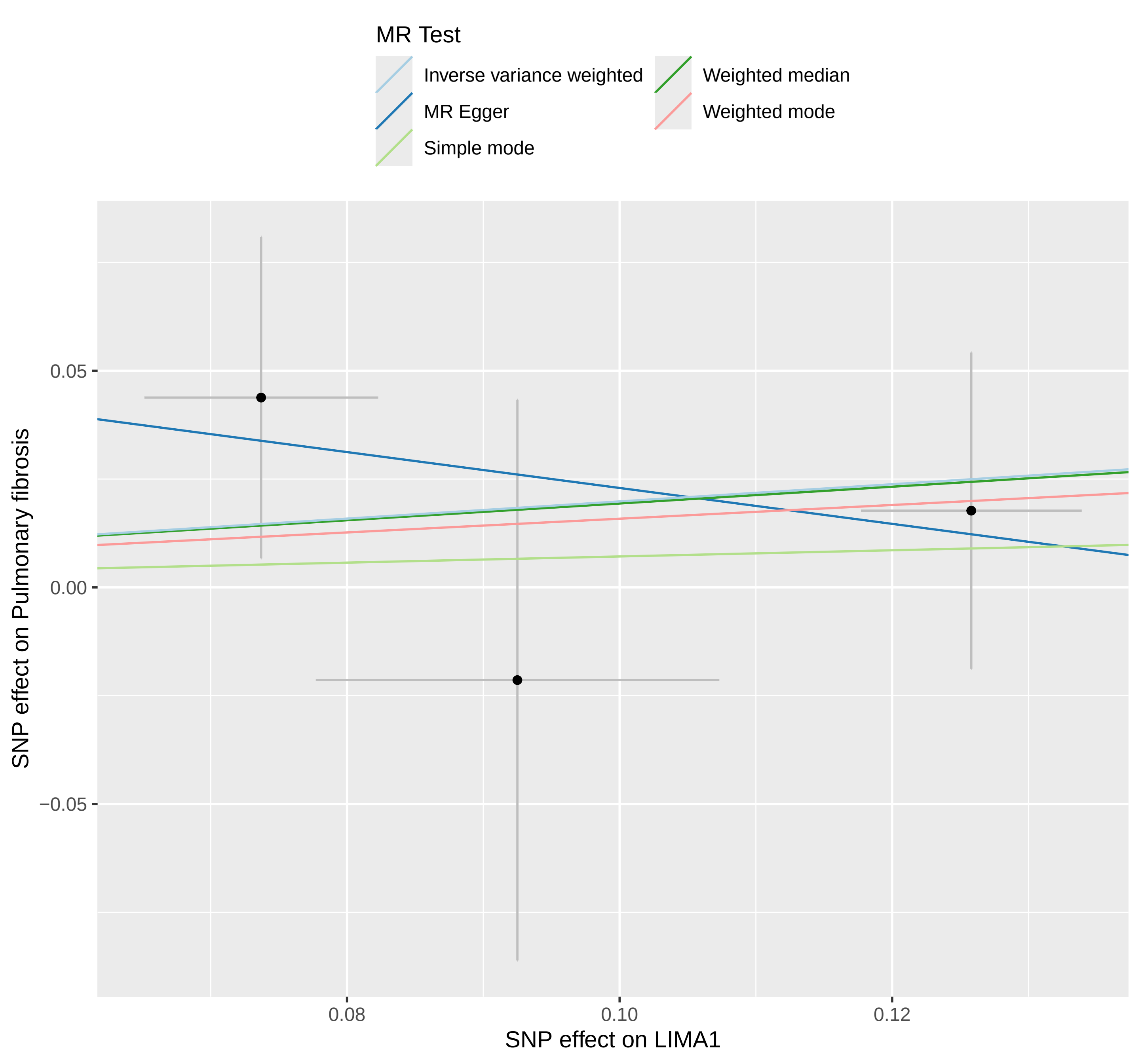
MR Test
Inverse variance weighted
MR Egger
Simple mode
Weighted median
Weighted mode
0.05
0.00
−0.05
SNP effect on Pulmonary fibrosis
0.08
0.10
0.12
SNP effect on LIMA1

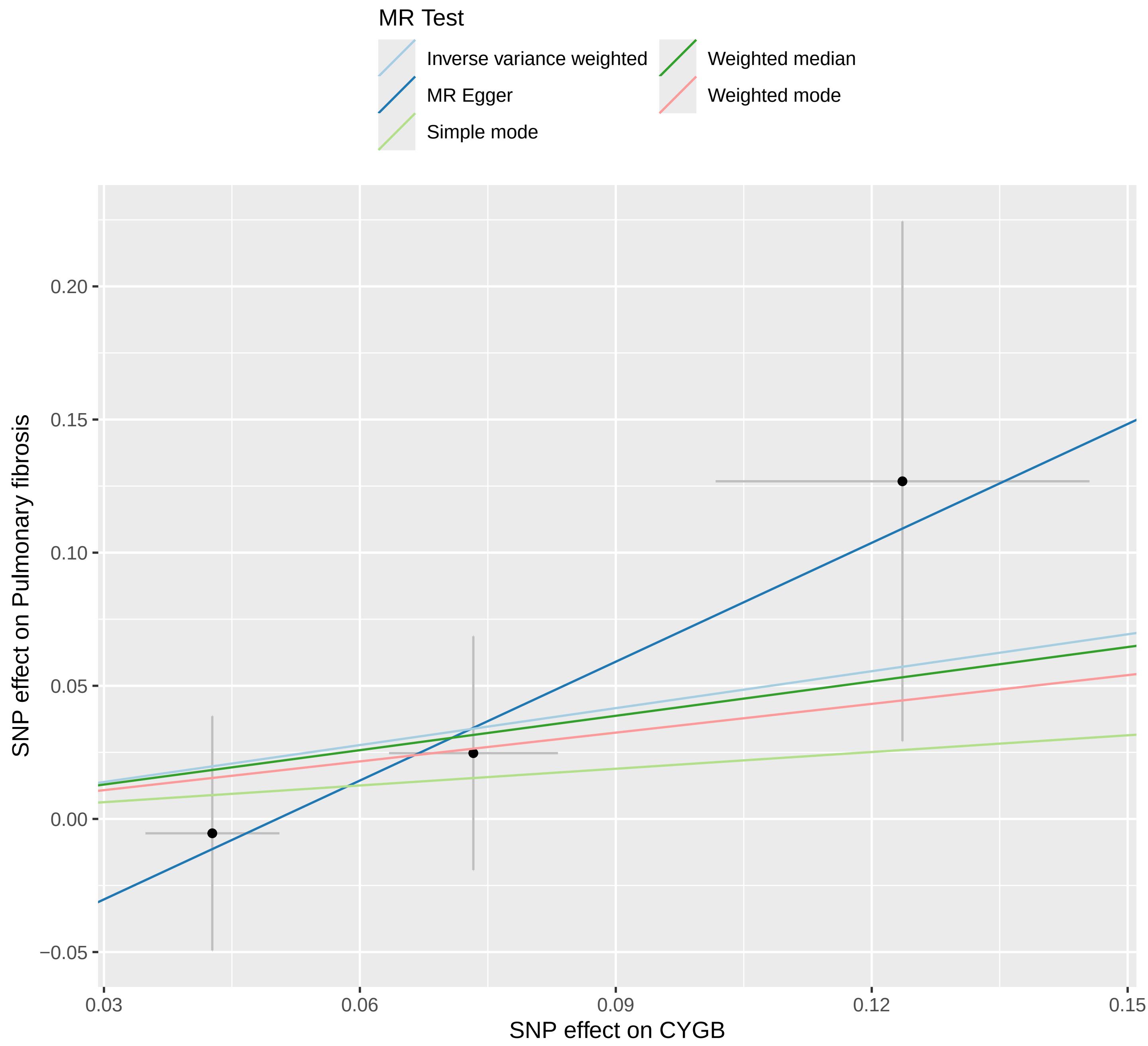
MR Test
Inverse variance weighted
MR Egger
Simple mode
Weighted median
Weighted mode
0.20
0.15
0.10
0.05
0.00
−0.05
SNP effect on Pulmonary fibrosis
0.03
0.06
0.09
0.12
0.15
SNP effect on CYGB

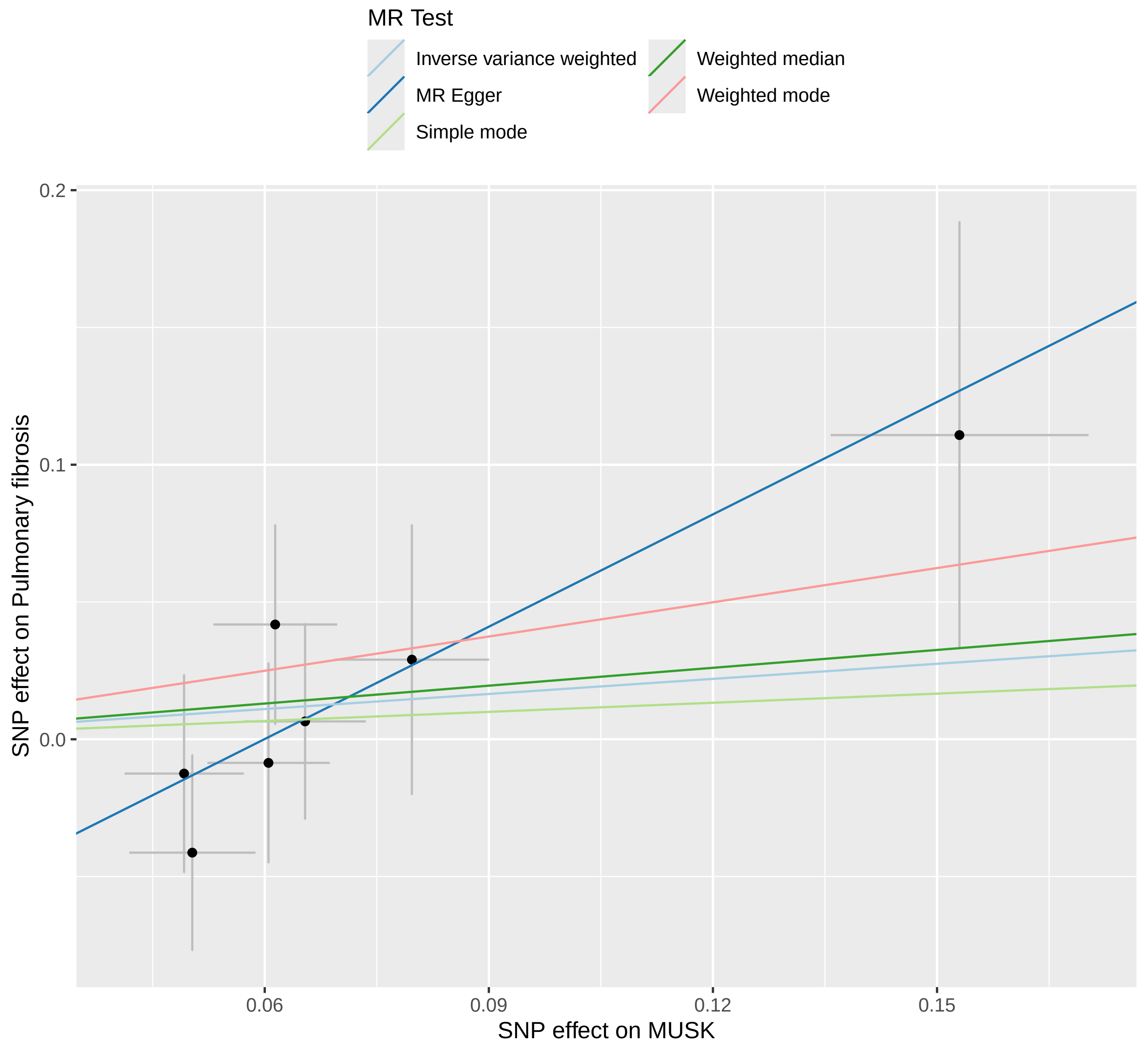

MR Test
Inverse variance weighted
MR Egger
Simple mode
Weighted median
Weighted mode
0.2
0.1
0.0
0.06
0.09
0.12
0.15
SNP effect on Pulmonary fibrosis
SNP effect on MUSK

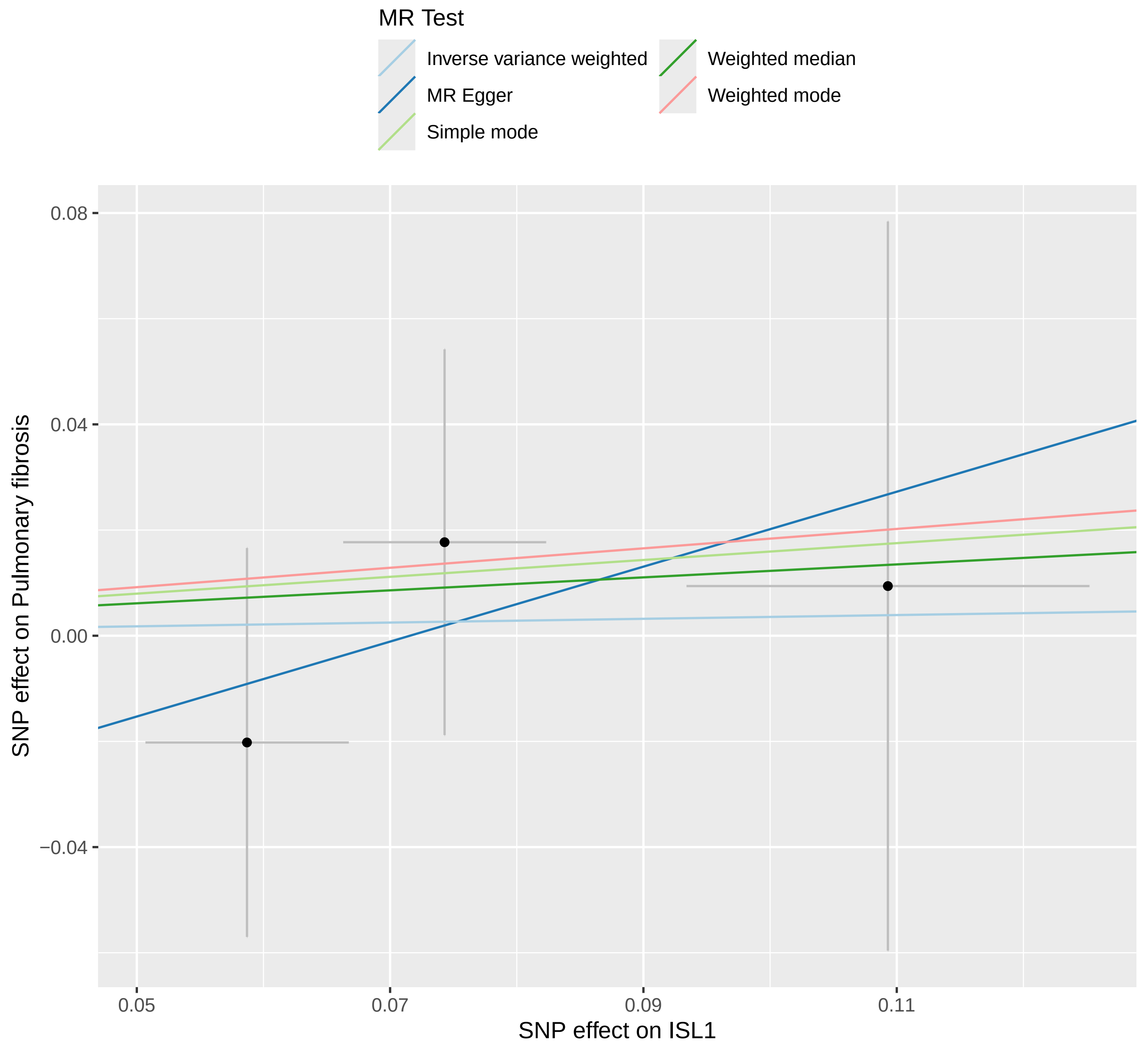
MR Test
Inverse variance weighted
MR Egger
Simple mode
Weighted median
Weighted mode
0.08
0.04
0.00
−0.04
SNP effect on Pulmonary fibrosis
0.05
0.07
0.09
0.11
SNP effect on ISL1

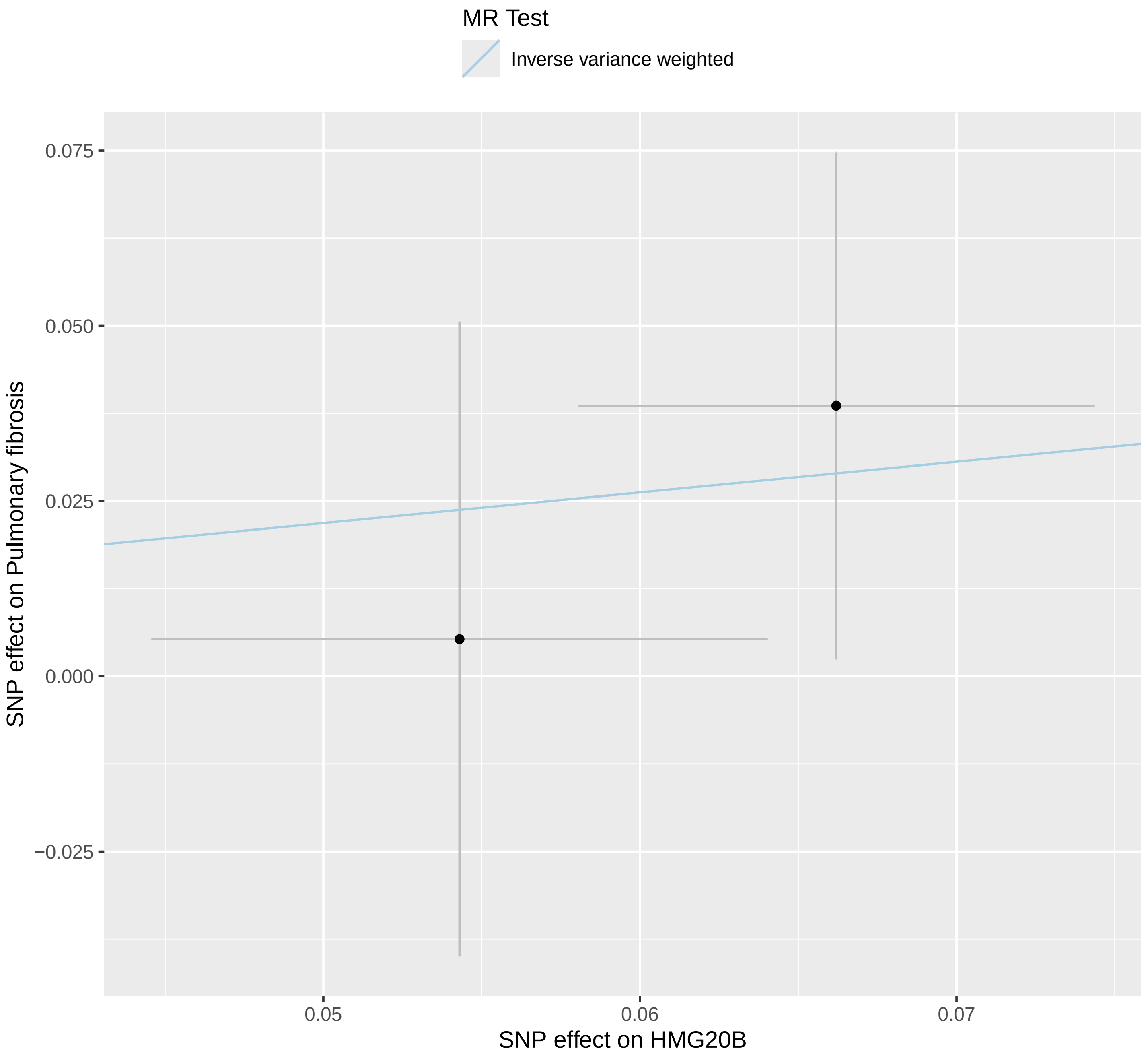
MR Test
Inverse variance weighted
0.075
0.050
0.025
0.000
−0.025
SNP effect on Pulmonary fibrosis
0.05
0.06
0.07
SNP effect on HMG20B

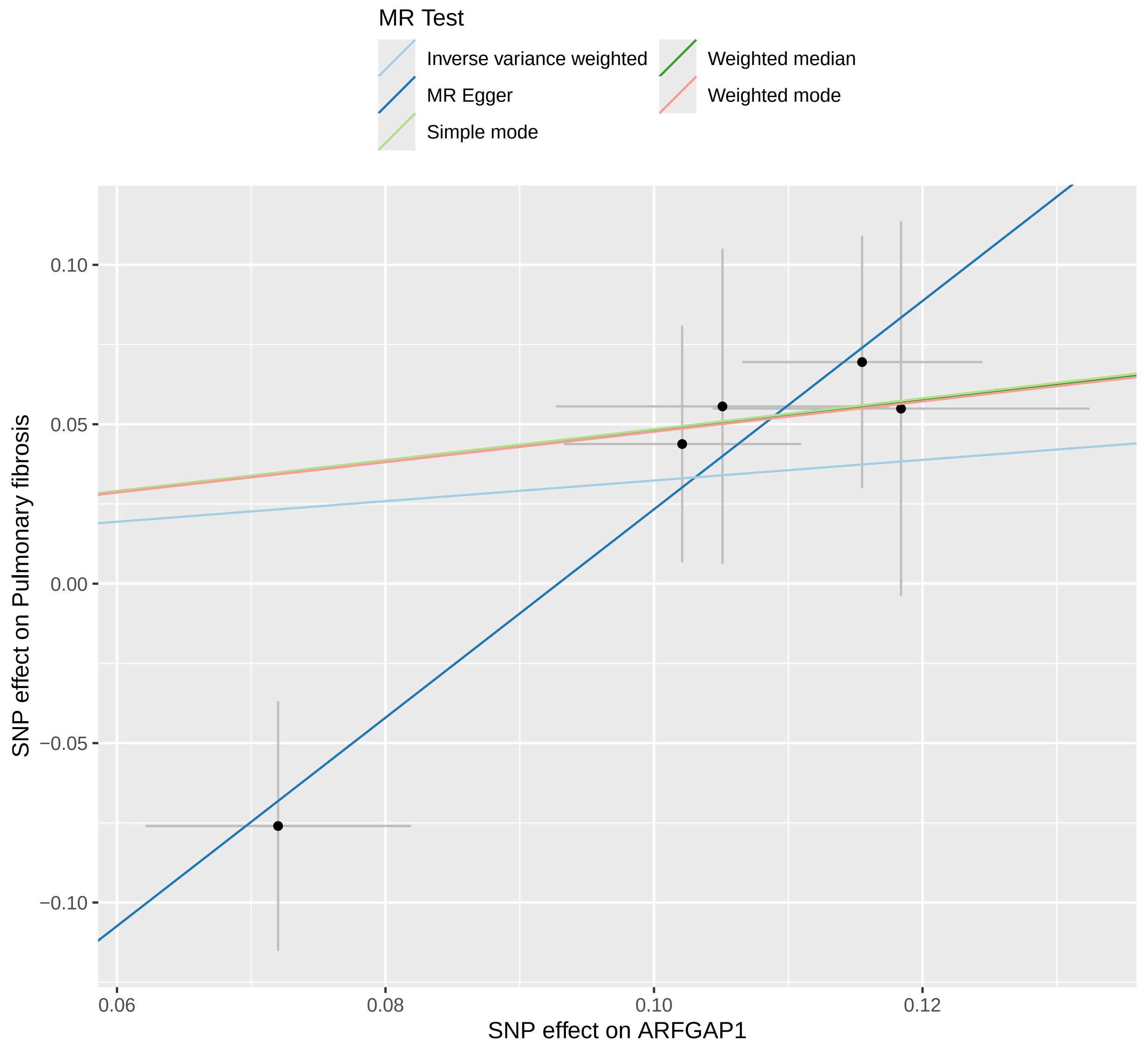
MR Test
Inverse variance weighted
MR Egger
Simple mode
Weighted median
Weighted mode
0.10
0.05
0.00
−0.05
−0.10
SNP effect on Pulmonary fibrosis
0.06
0.08
0.10
0.12
SNP effect on ARFGAP1

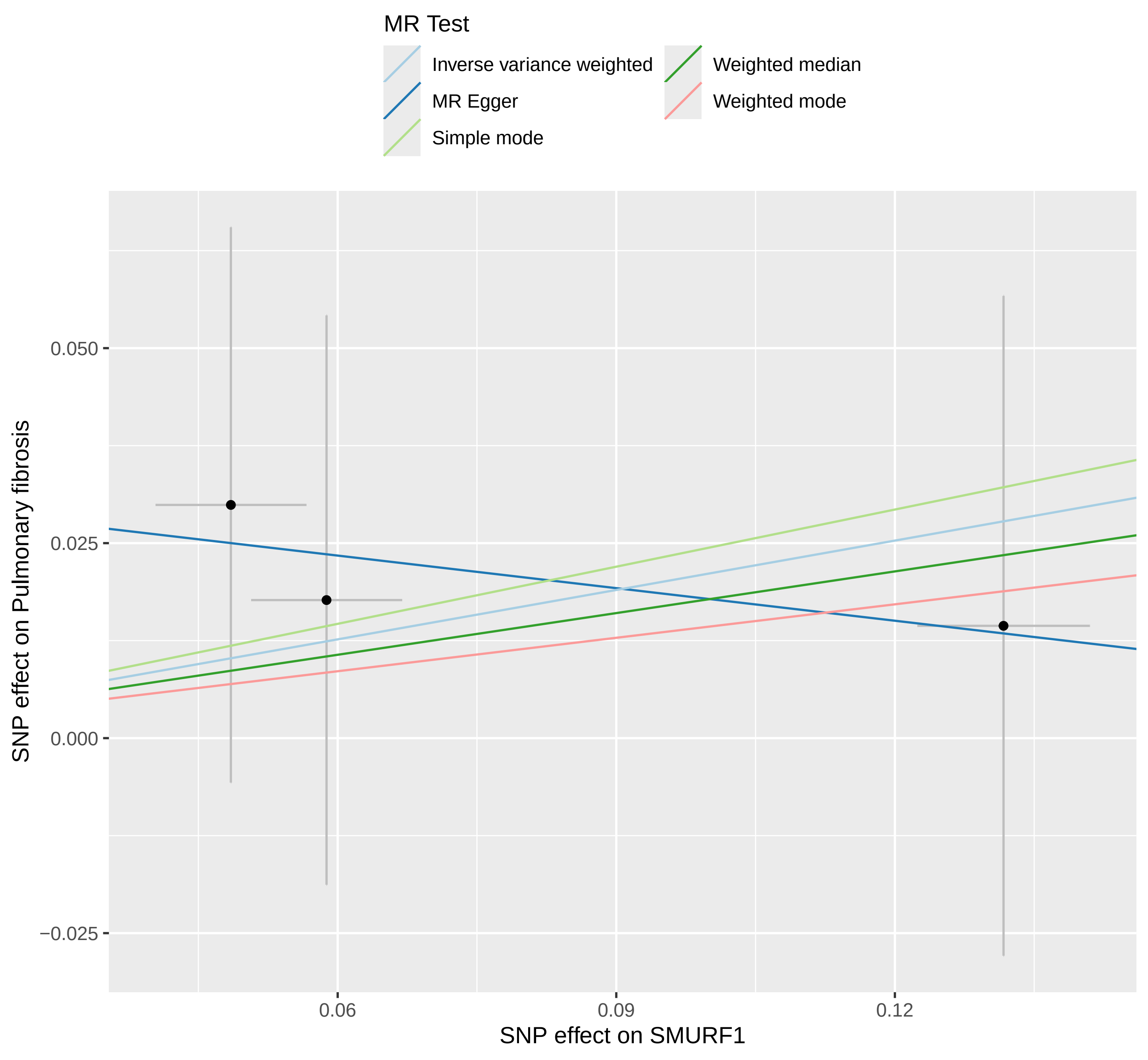
MR Test
Inverse variance weighted
MR Egger
Simple mode
Weighted median
Weighted mode
SNP effect on Pulmonary fibrosis
0.050
0.025
0.000
−0.025
0.06
0.09
0.12
SNP effect on SMURF1

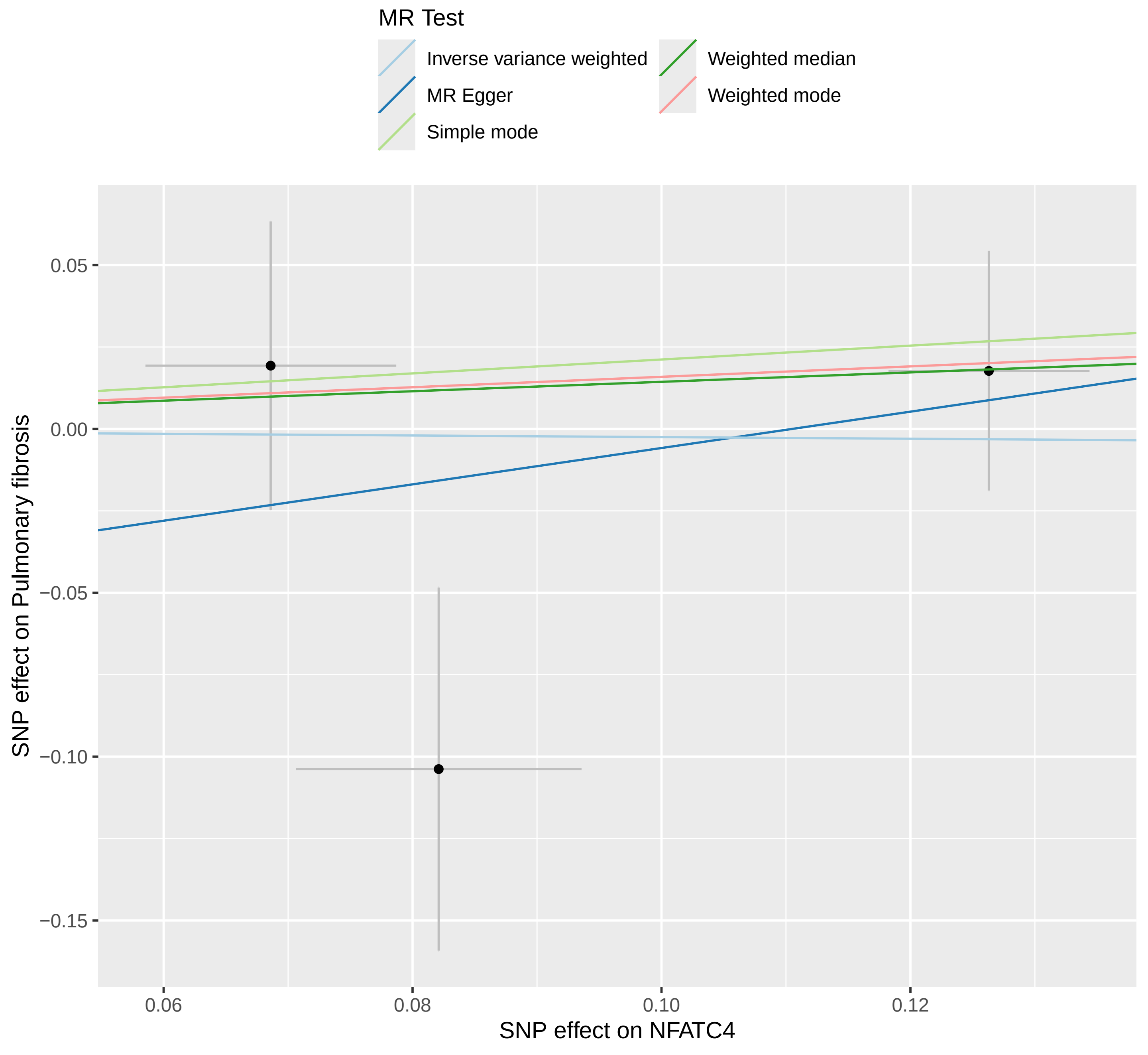
MR Test
Inverse variance weighted
MR Egger
Simple mode
Weighted median
Weighted mode
0.05
0.00
−0.05
−0.10
−0.15
0.06
0.08
0.10
0.12
SNP effect on Pulmonary fibrosis
SNP effect on NFATC4

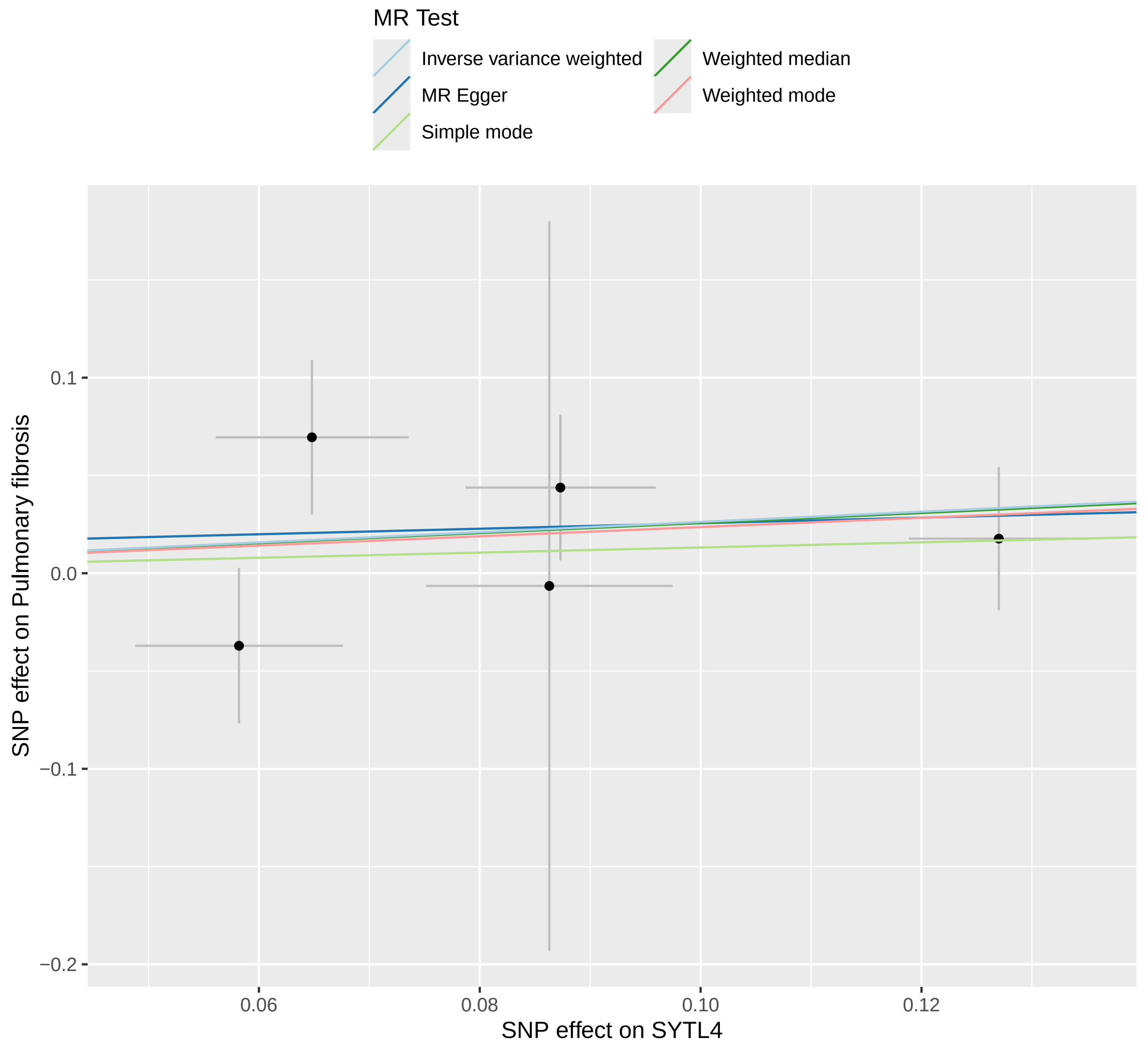
MR Test
Inverse variance weighted
MR Egger
Simple mode
Weighted median
Weighted mode
SNP effect on Pulmonary fibrosis
0.1
0.0
−0.1
−0.2
0.06
0.08
0.10
0.12
SNP effect on SYTL4

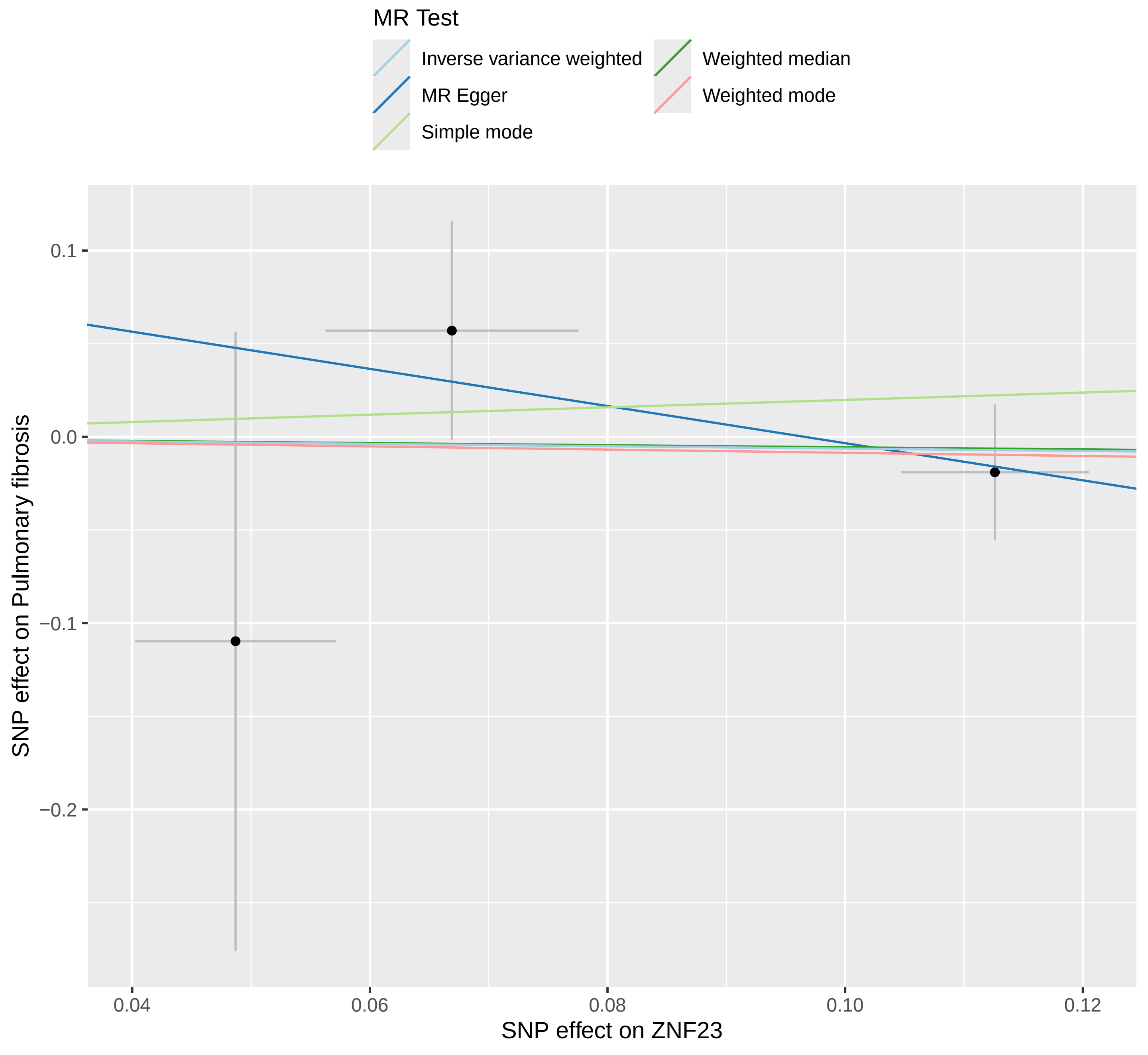
MR Test
Inverse variance weighted
MR Egger
Simple mode
Weighted median
Weighted mode
0.1
0.0
−0.1
−0.2
0.04
0.06
0.08
0.10
0.12
SNP effect on ZNF23
SNP effect on Pulmonary fibrosis

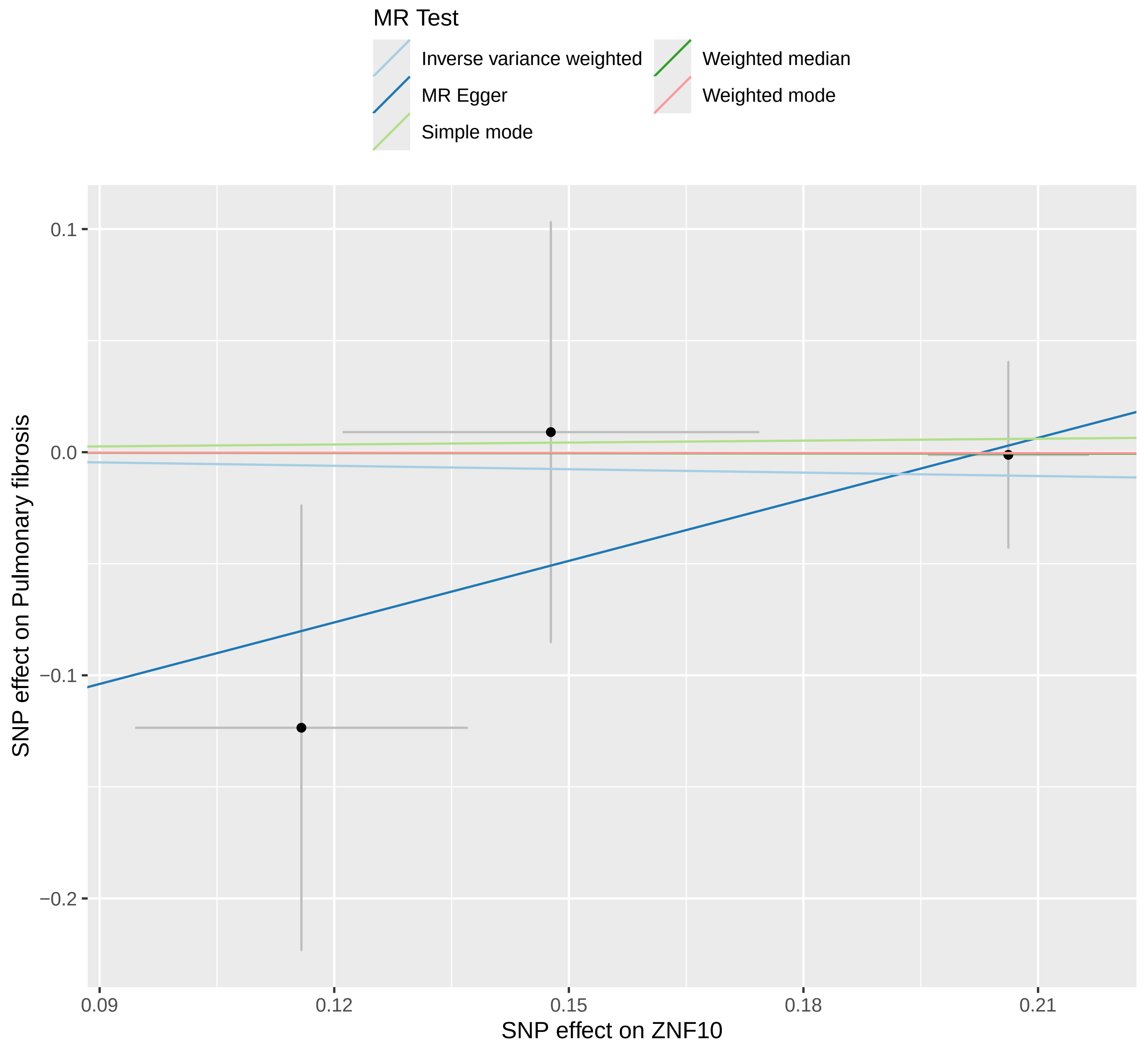
MR Test
Inverse variance weighted
MR Egger
Simple mode
Weighted median
Weighted mode
0.1
0.0
−0.1
−0.2
SNP effect on Pulmonary fibrosis
0.09
0.12
0.15
0.18
0.21
SNP effect on ZNF10

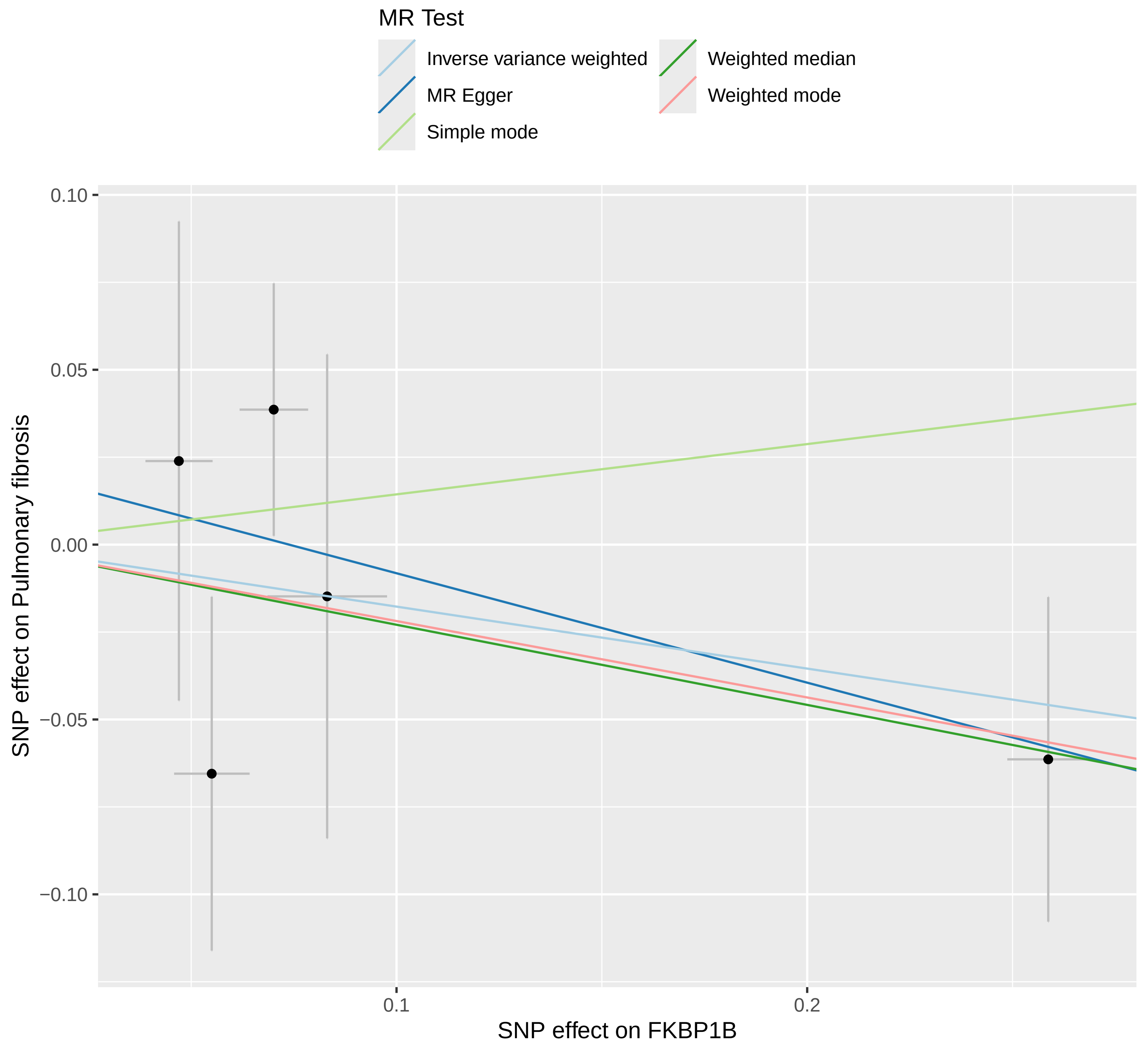
MR Test
Inverse variance weighted
MR Egger
Simple mode
Weighted median
Weighted mode
0.10
0.05
0.00
−0.05
−0.10
SNP effect on Pulmonary fibrosis
0.1
0.2
SNP effect on FKBP1B

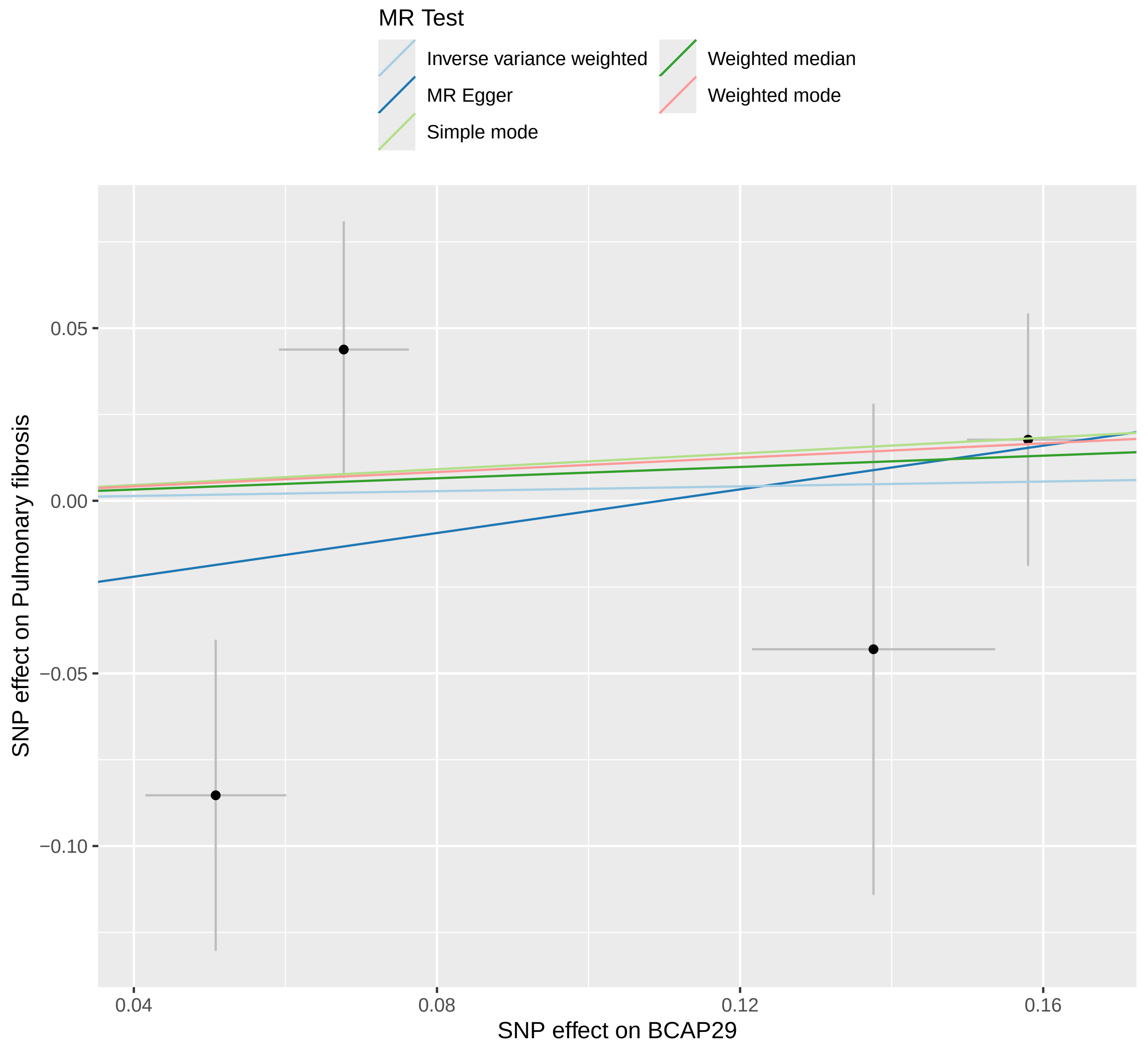
MR Test
Inverse variance weighted
MR Egger
Simple mode
Weighted median
Weighted mode
0.05
0.00
−0.05
−0.10
0.04
0.08
0.12
0.16
SNP effect on Pulmonary fibrosis
SNP effect on BCAP29

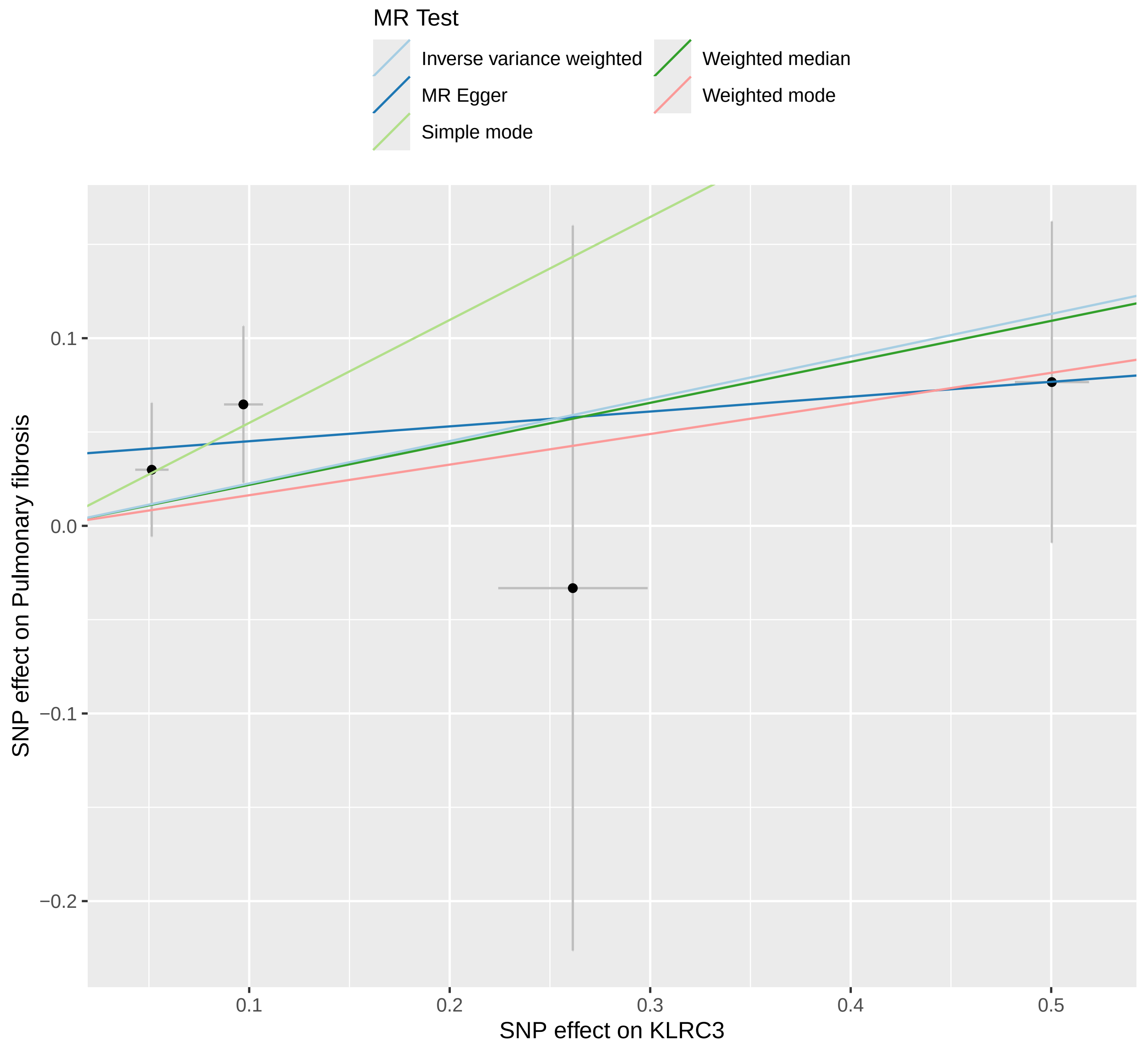
MR Test
Inverse variance weighted
MR Egger
Simple mode
Weighted median
Weighted mode
0.1
0.0
−0.1
−0.2
SNP effect on Pulmonary fibrosis
0.1
0.2
0.3
0.4
0.5
SNP effect on KLRC3

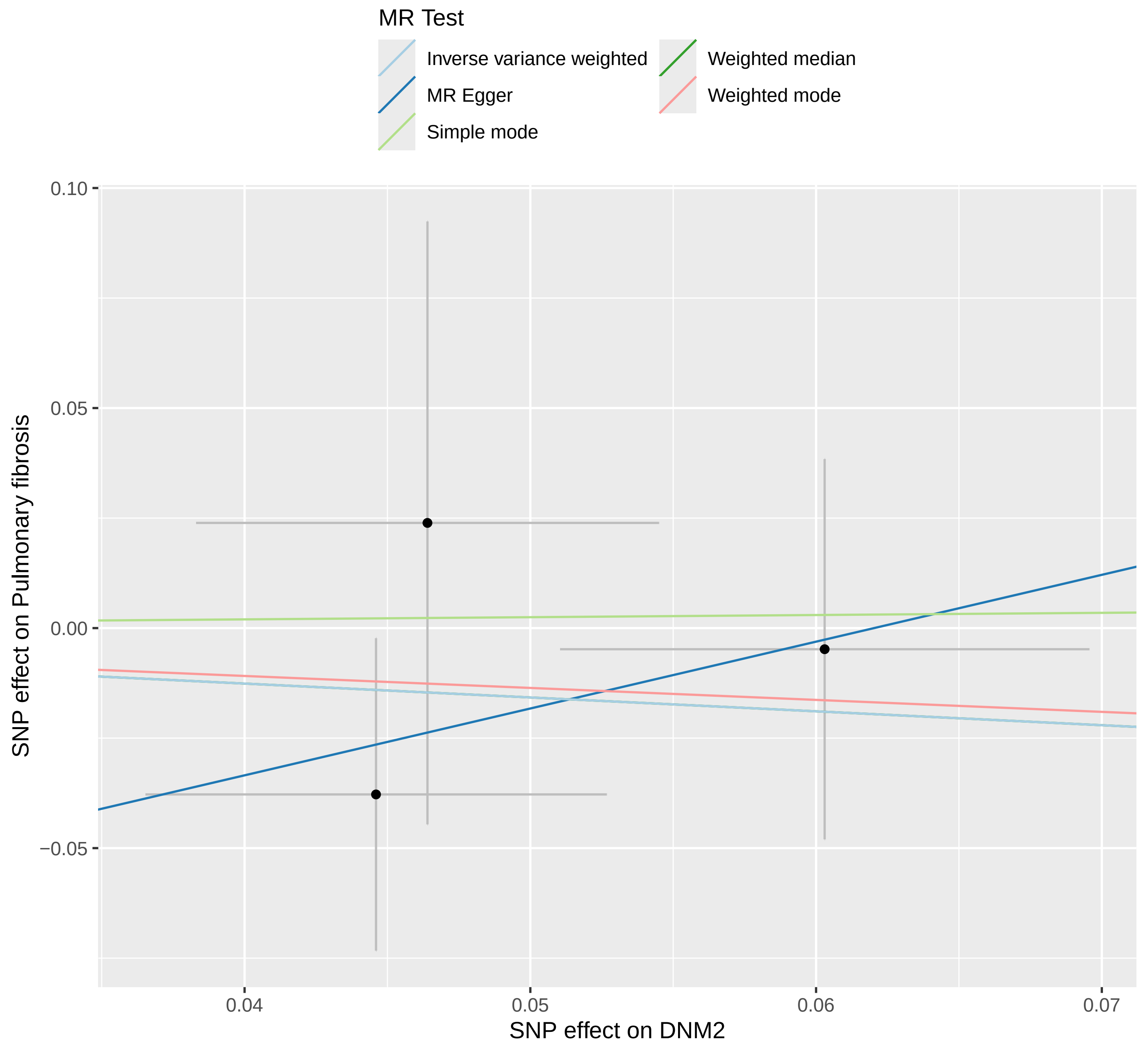
MR Test
Inverse variance weighted
MR Egger
Simple mode
Weighted median
Weighted mode
0.10
0.05
0.00
−0.05
SNP effect on Pulmonary fibrosis
0.04
0.05
0.06
0.07
SNP effect on DNM2

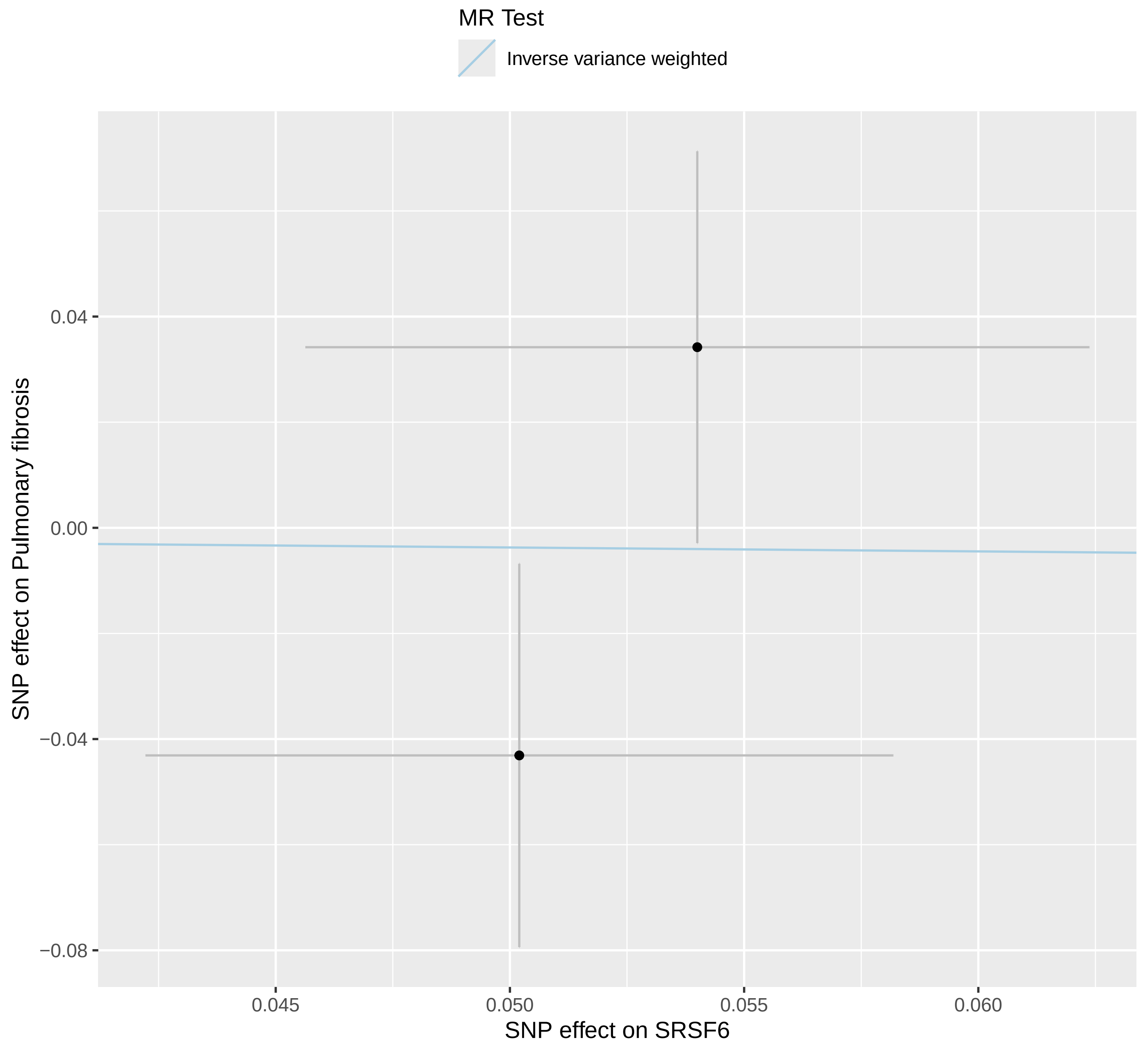

MR Test
Inverse variance weighted
0.04
0.00
−0.04
−0.08
0.045
0.050
0.055
0.060
SNP effect on SRSF6
SNP effect on Pulmonary fibrosis

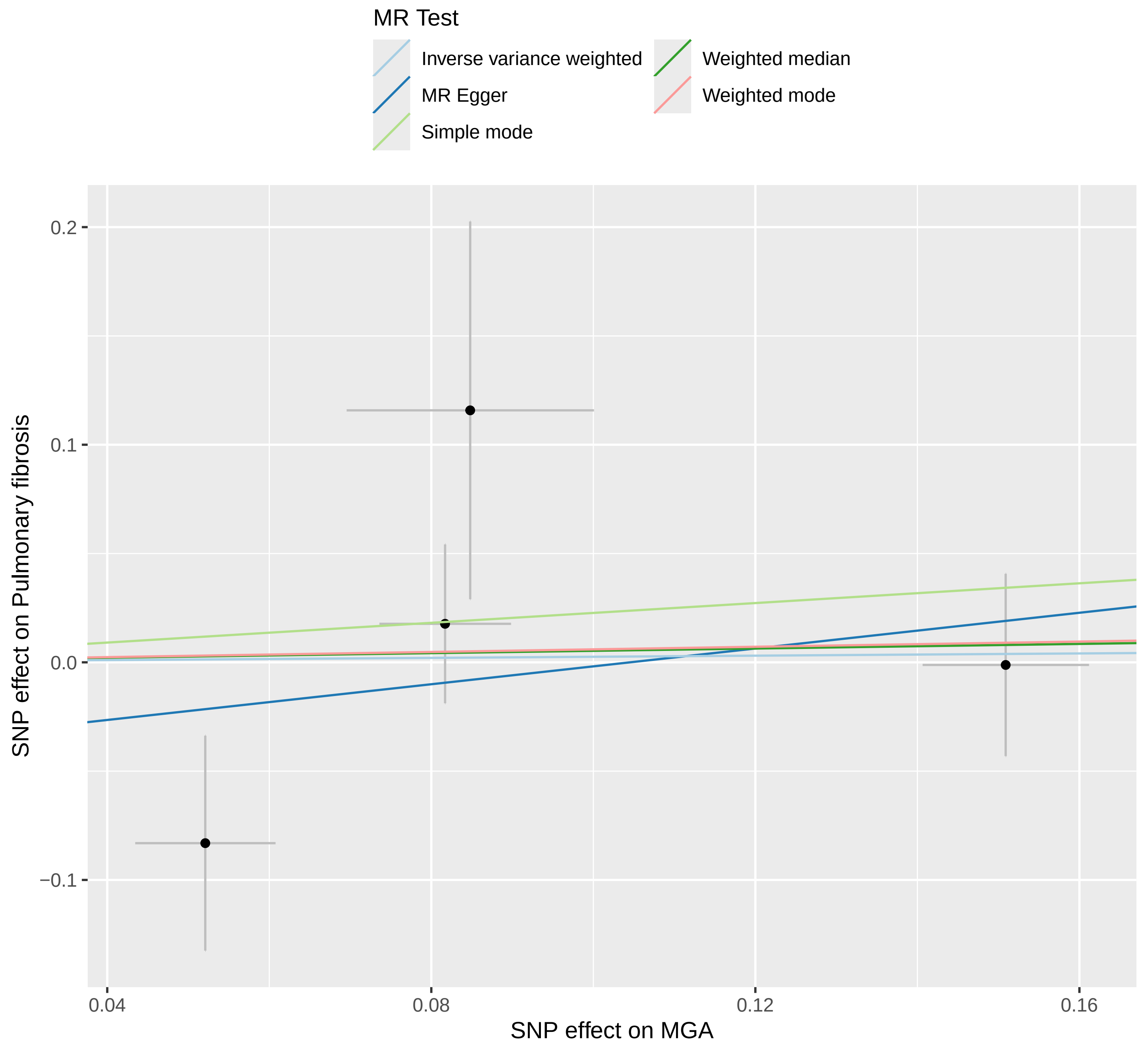
MR Test
Inverse variance weighted
MR Egger
Simple mode
Weighted median
Weighted mode
SNP effect on Pulmonary fibrosis
0.2
0.1
0.0
−0.1
0.04
0.08
0.12
0.16
SNP effect on MGA

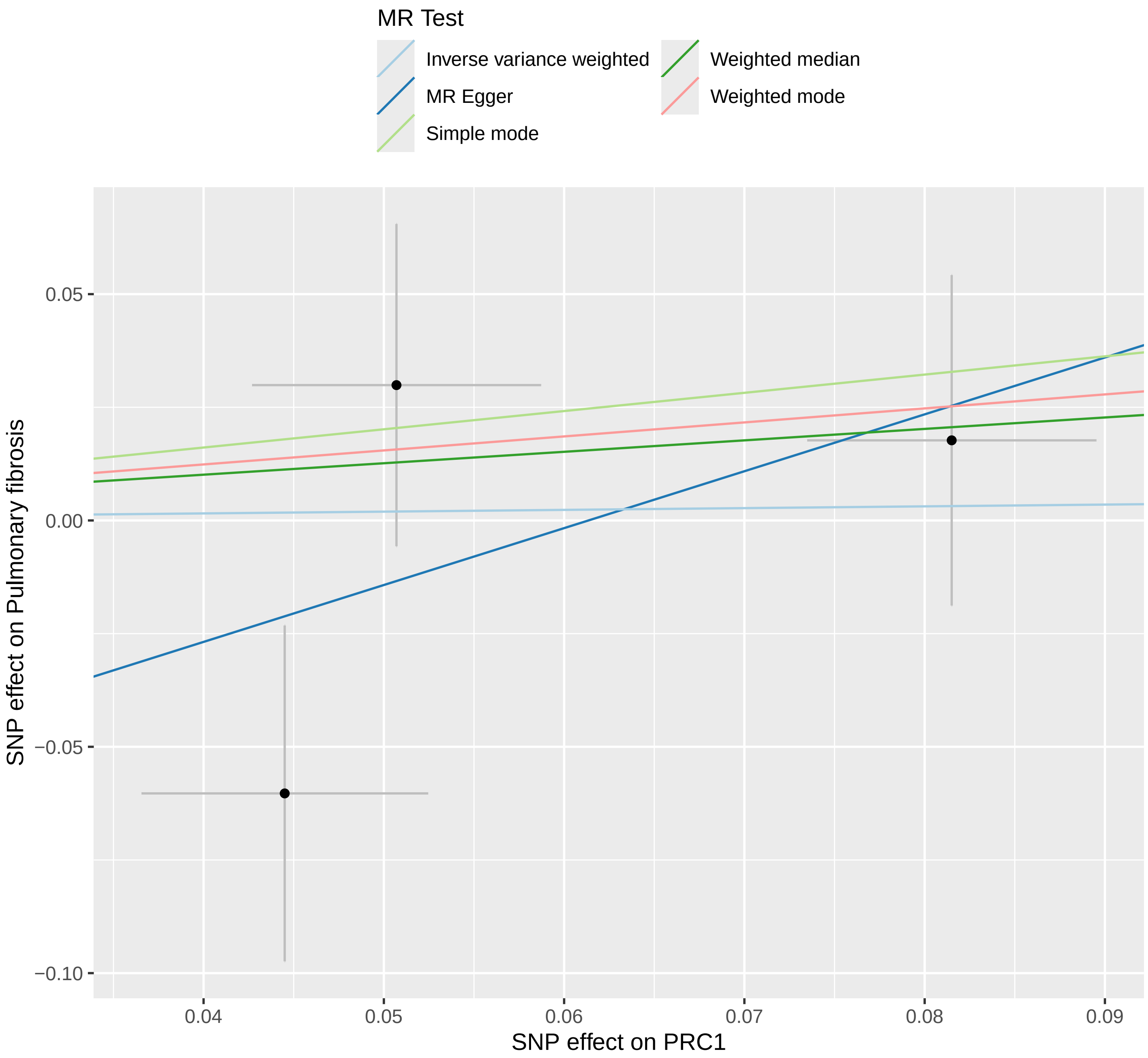

MR Test
Inverse variance weighted
MR Egger
Simple mode
Weighted median
Weighted mode
0.05
0.00
−0.05
−0.10
0.04
0.05
0.06
0.07
0.08
0.09
SNP effect on Pulmonary fibrosis
SNP effect on PRC1

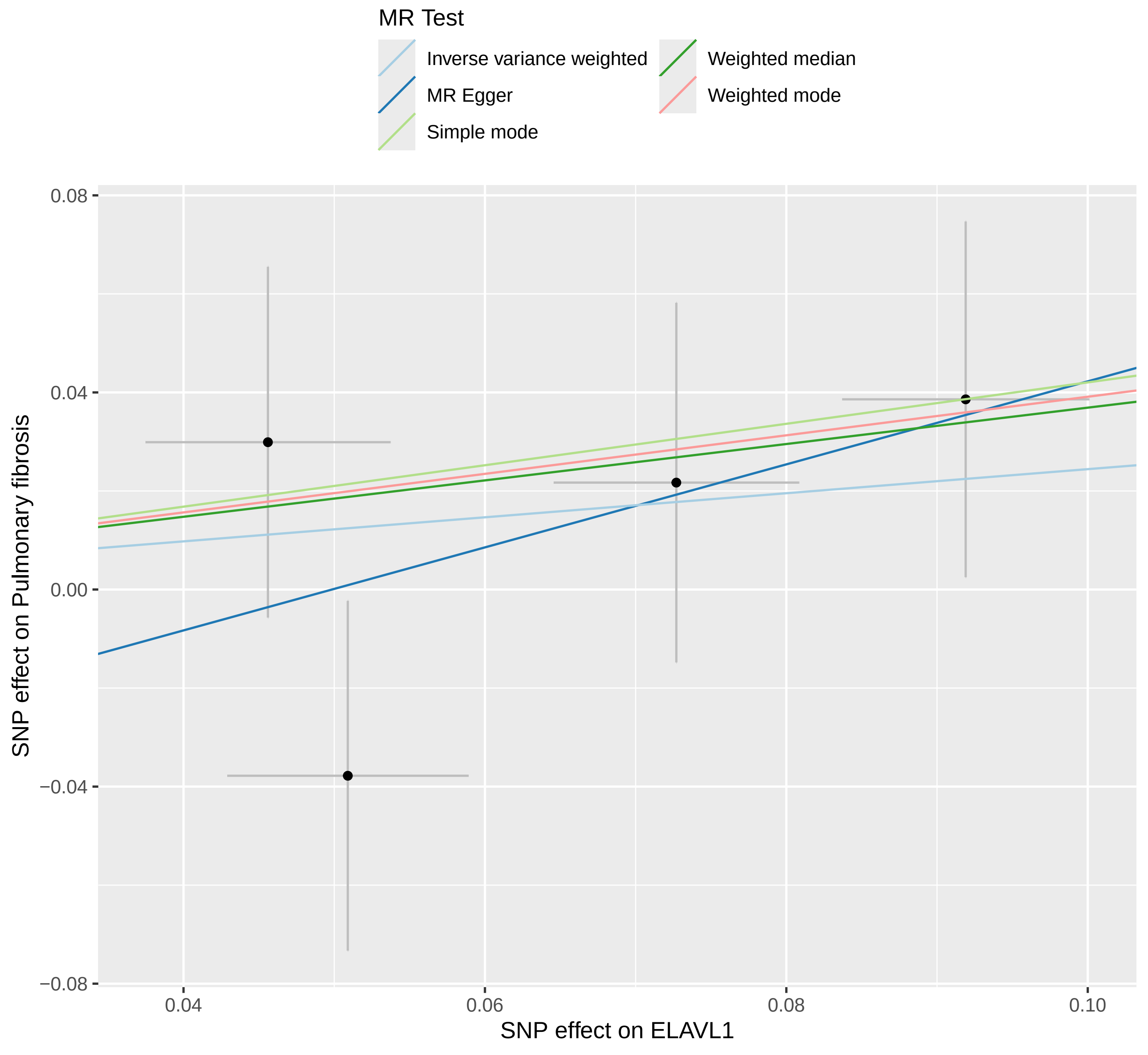
MR Test
Inverse variance weighted
MR Egger
Simple mode
Weighted median
Weighted mode
0.08
0.04
0.00
−0.04
−0.08
SNP effect on Pulmonary fibrosis
0.04
0.06
0.08
0.10
SNP effect on ELAVL1

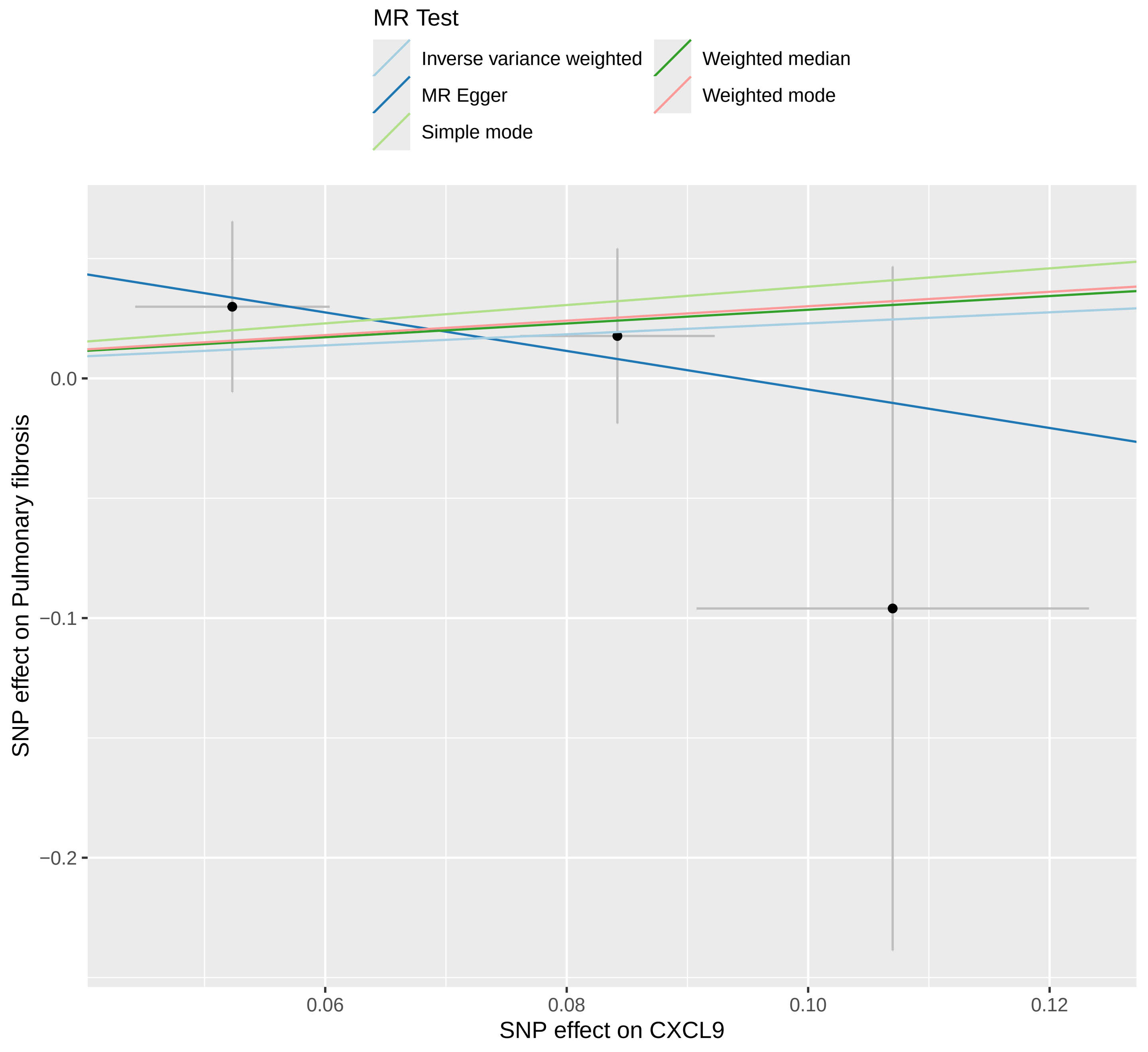
MR Test
Inverse variance weighted
MR Egger
Simple mode
Weighted median
Weighted mode
0.0
−0.1
−0.2
SNP effect on Pulmonary fibrosis
0.06
0.08
0.10
0.12
SNP effect on CXCL9

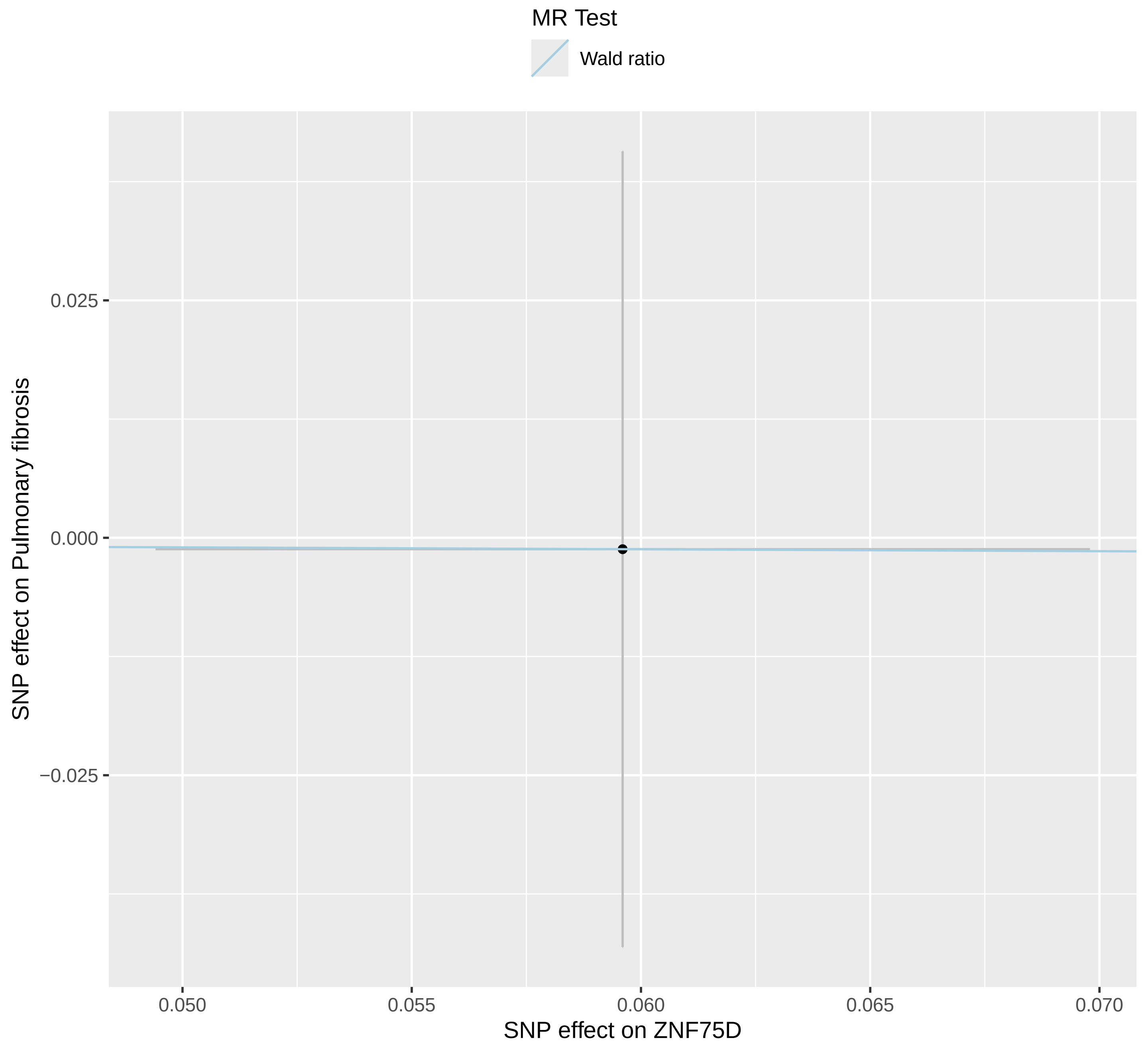

MR Test
Wald ratio
0.025
0.000
−0.025
SNP effect on Pulmonary fibrosis
0.050
0.055
0.060
0.065
0.070
SNP effect on ZNF75D

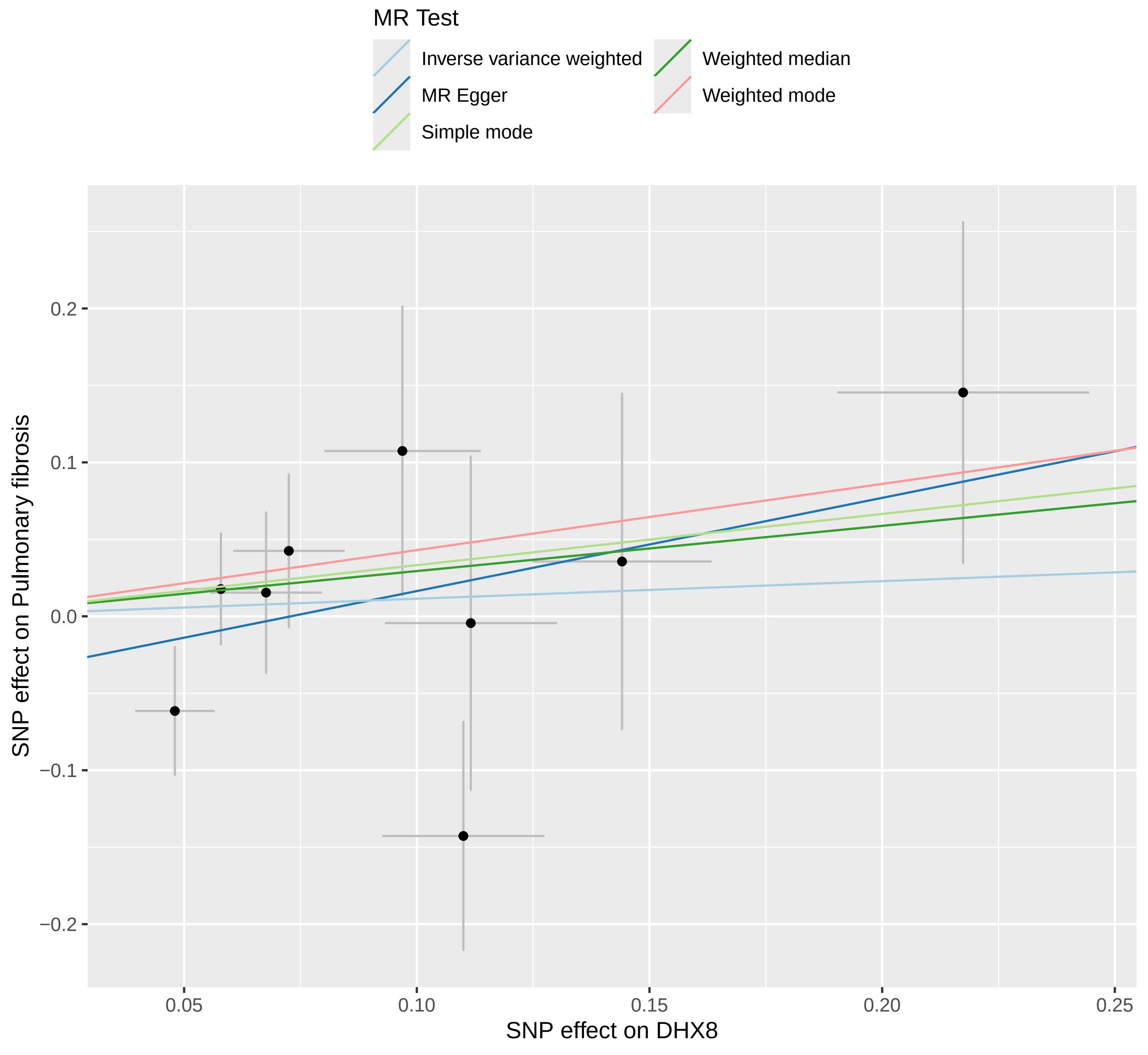
MR Test
Inverse variance weighted
MR Egger
Simple mode
Weighted median
Weighted mode
SNP effect on Pulmonary fibrosis
0.2
0.1
0.0
−0.1
−0.2
0.05
0.10
0.15
0.20
0.25
SNP effect on DHX8

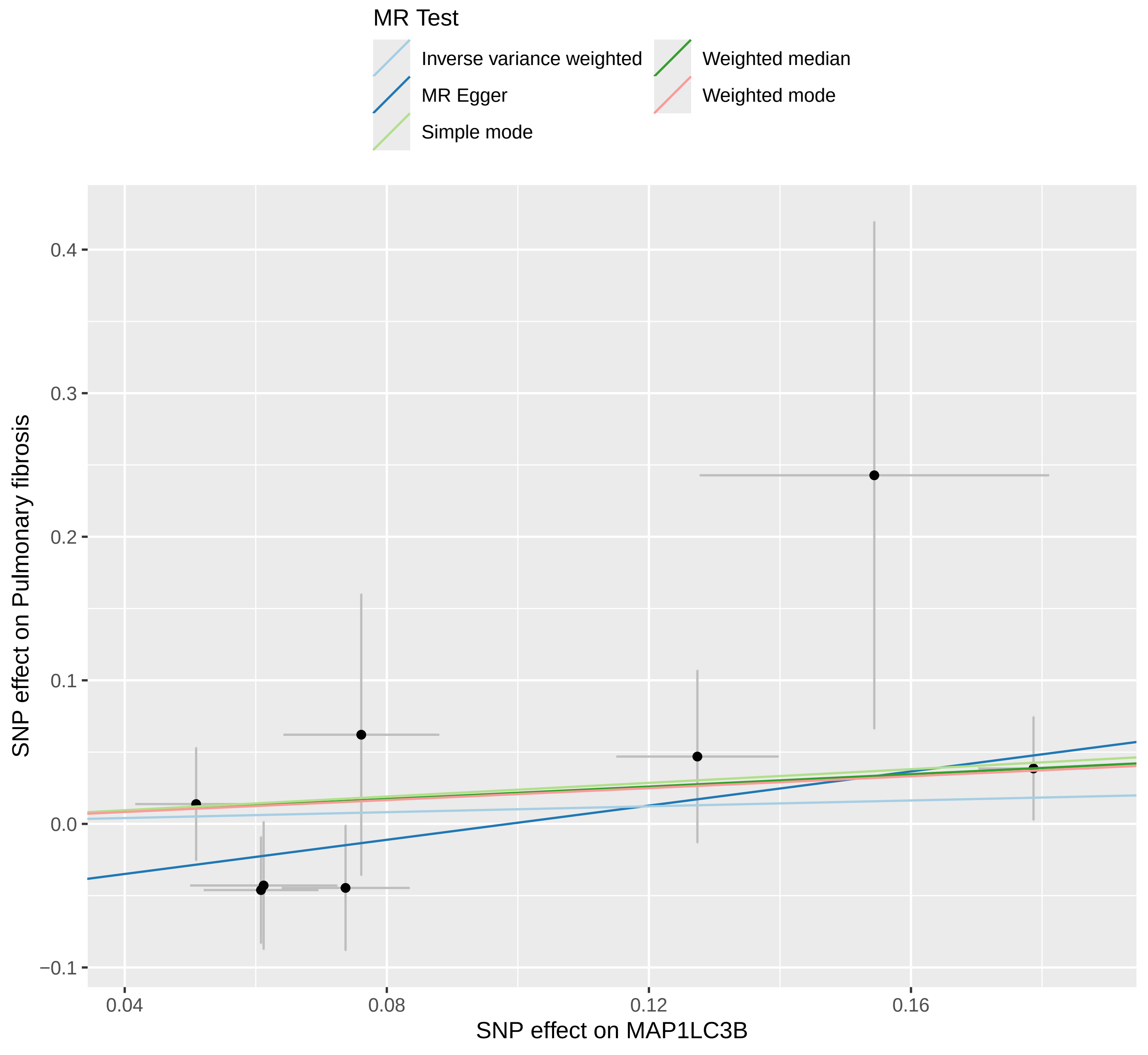

MR Test
Inverse variance weighted
MR Egger
Simple mode
Weighted median
Weighted mode
0.4
0.3
0.2
0.1
0.0
−0.1
SNP effect on Pulmonary fibrosis
0.04
0.08
0.12
0.16
SNP effect on MAP1LC3B

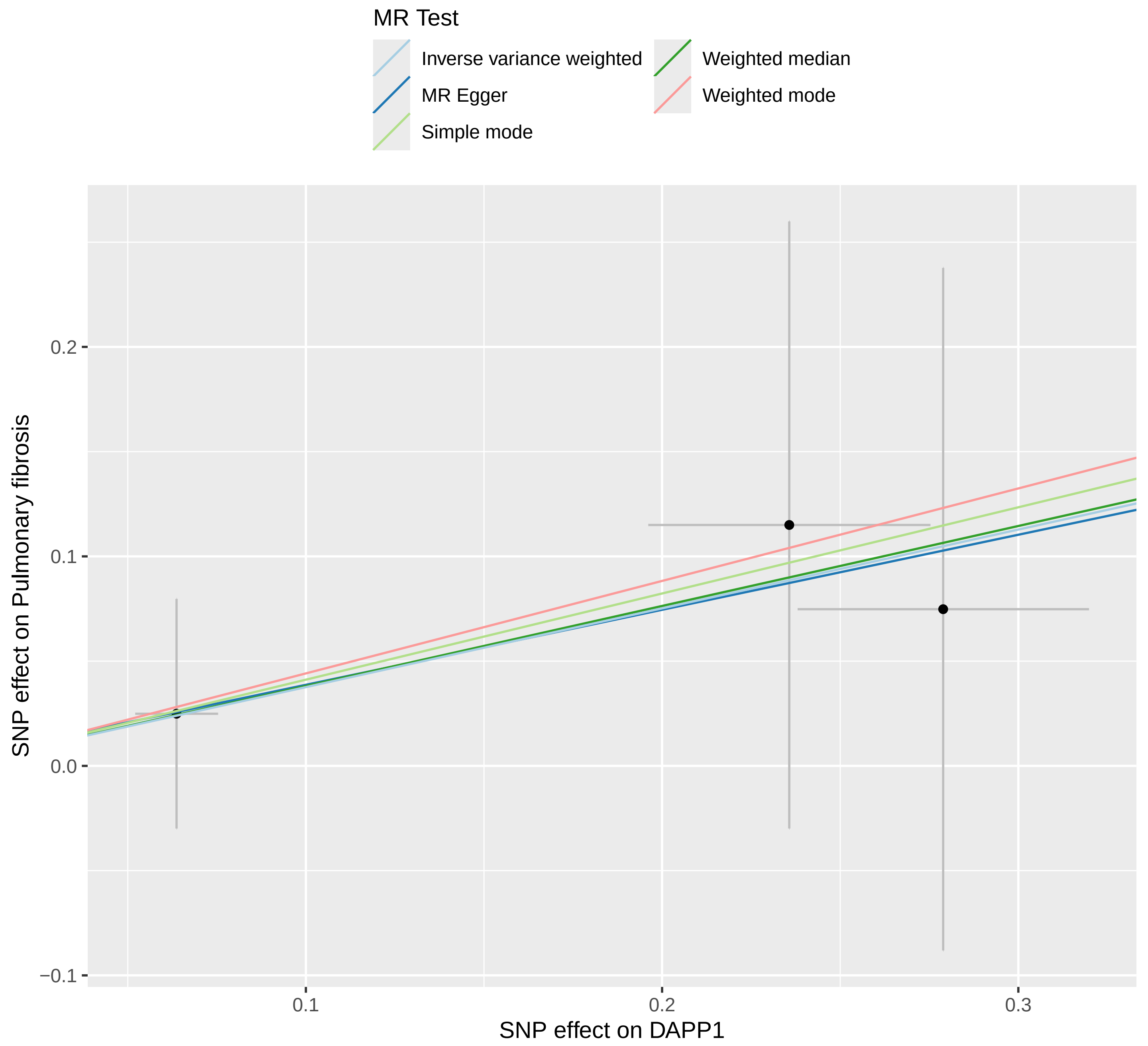
MR Test
Inverse variance weighted
MR Egger
Simple mode
Weighted median
Weighted mode
0.2
0.1
0.0
−0.1
SNP effect on Pulmonary fibrosis
0.1
0.2
0.3
SNP effect on DAPP1

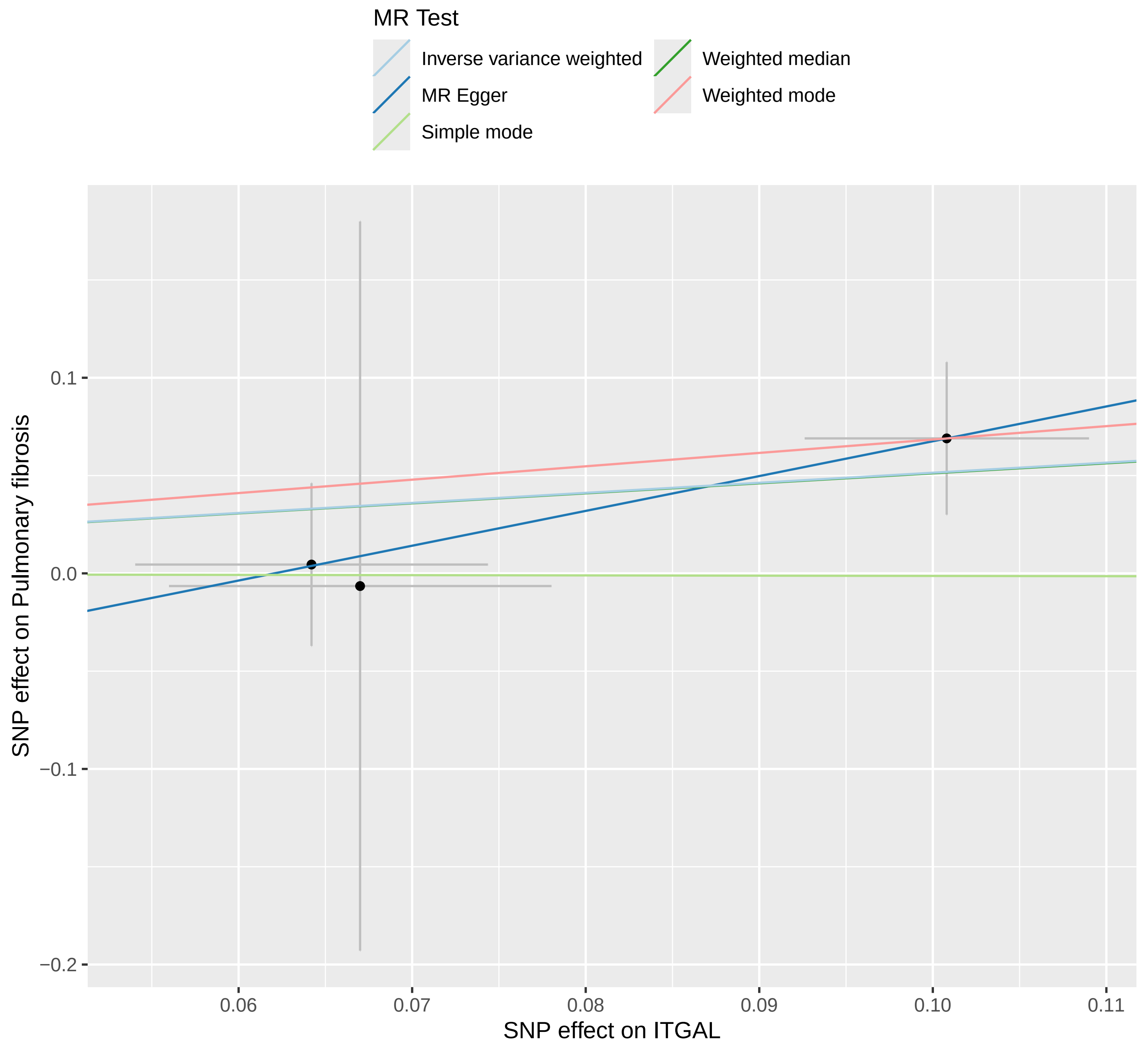

MR Test
Inverse variance weighted
MR Egger
Simple mode
Weighted median
Weighted mode
0.1
0.0
−0.1
−0.2
SNP effect on Pulmonary fibrosis
0.06
0.07
0.08
0.09
0.10
0.11
SNP effect on ITGAL

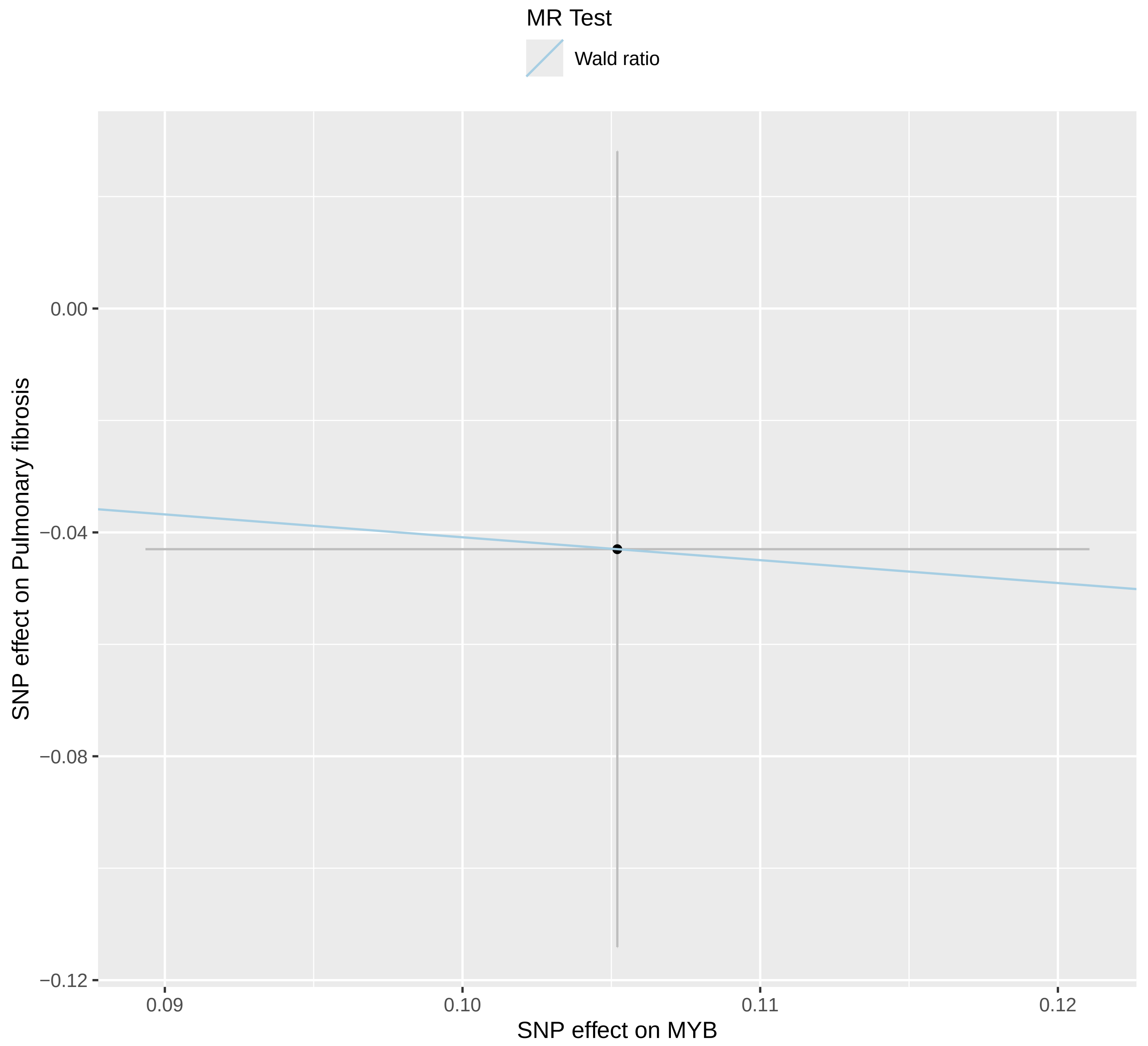
MR Test
Wald ratio
0.00
−0.04
−0.08
−0.12
SNP effect on Pulmonary fibrosis
0.09
0.10
0.11
0.12
SNP effect on MYB

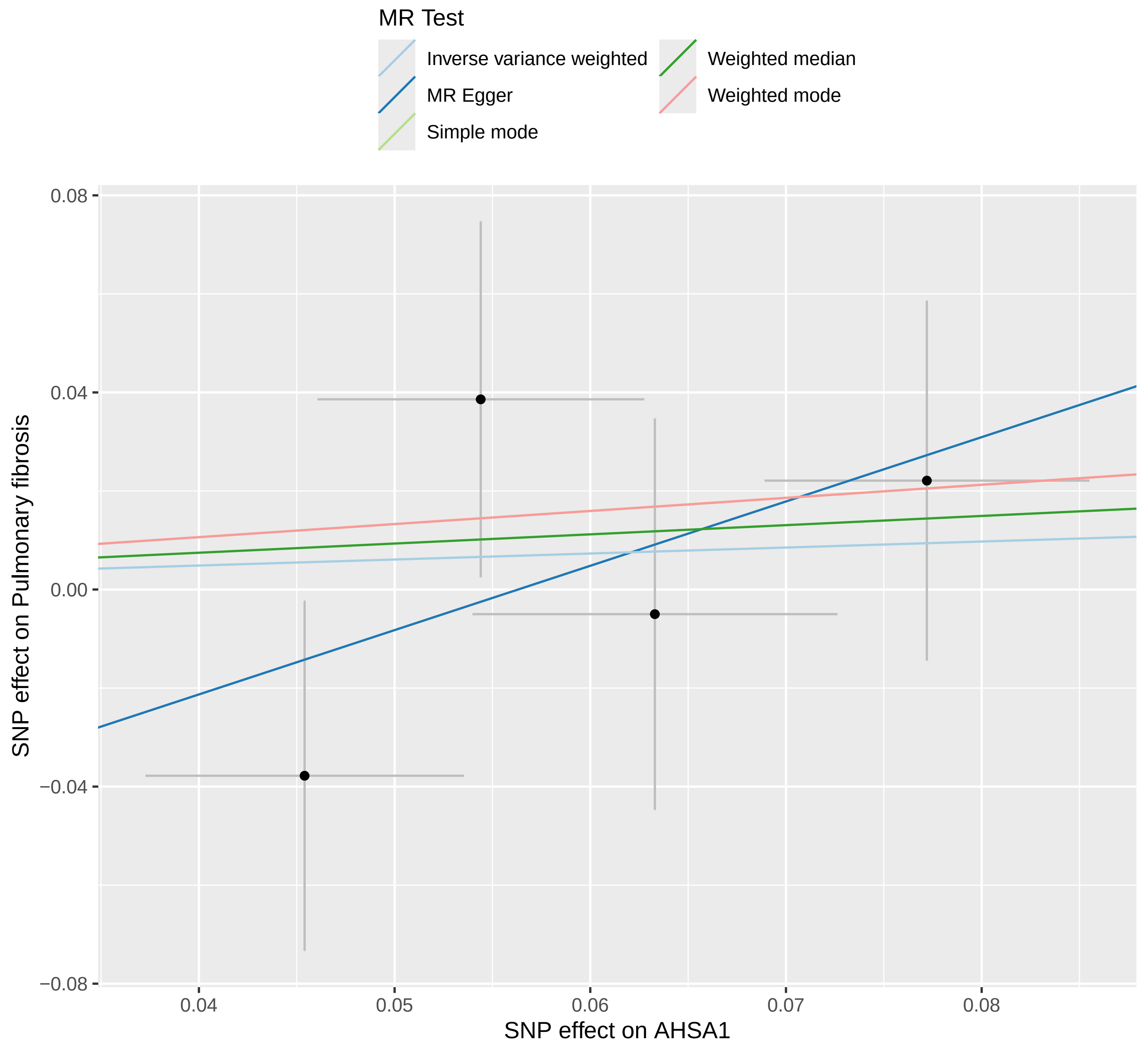

MR Test
Inverse variance weighted
MR Egger
Simple mode
Weighted median
Weighted mode
0.08
0.04
0.00
−0.04
−0.08
SNP effect on Pulmonary fibrosis
0.04
0.05
0.06
0.07
0.08
SNP effect on AHSA1

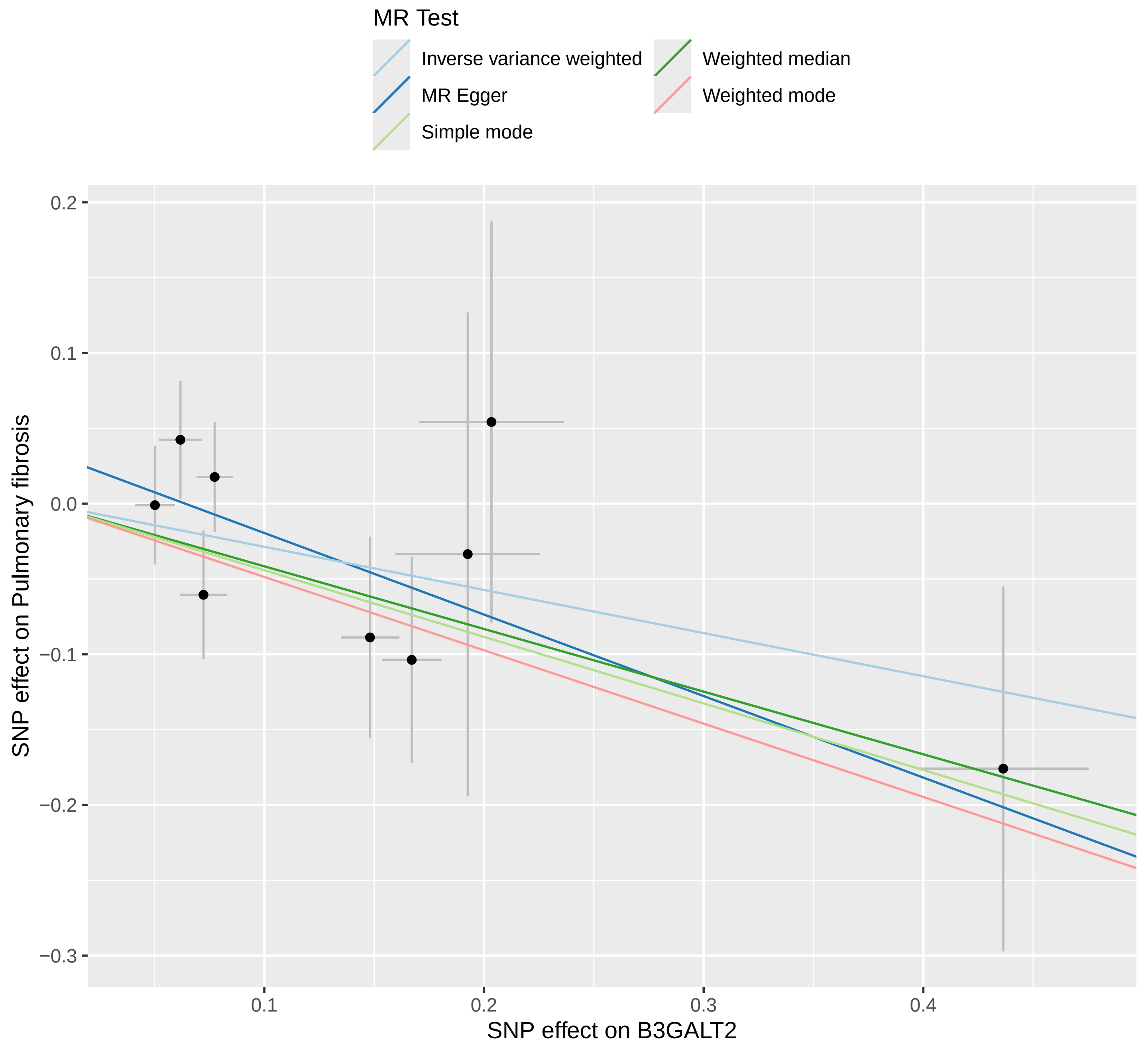

MR Test
Inverse variance weighted
MR Egger
Simple mode
Weighted median
Weighted mode
0.2
0.1
0.0
−0.1
−0.2
−0.3
SNP effect on Pulmonary fibrosis
0.1
0.2
0.3
0.4
SNP effect on B3GALT2

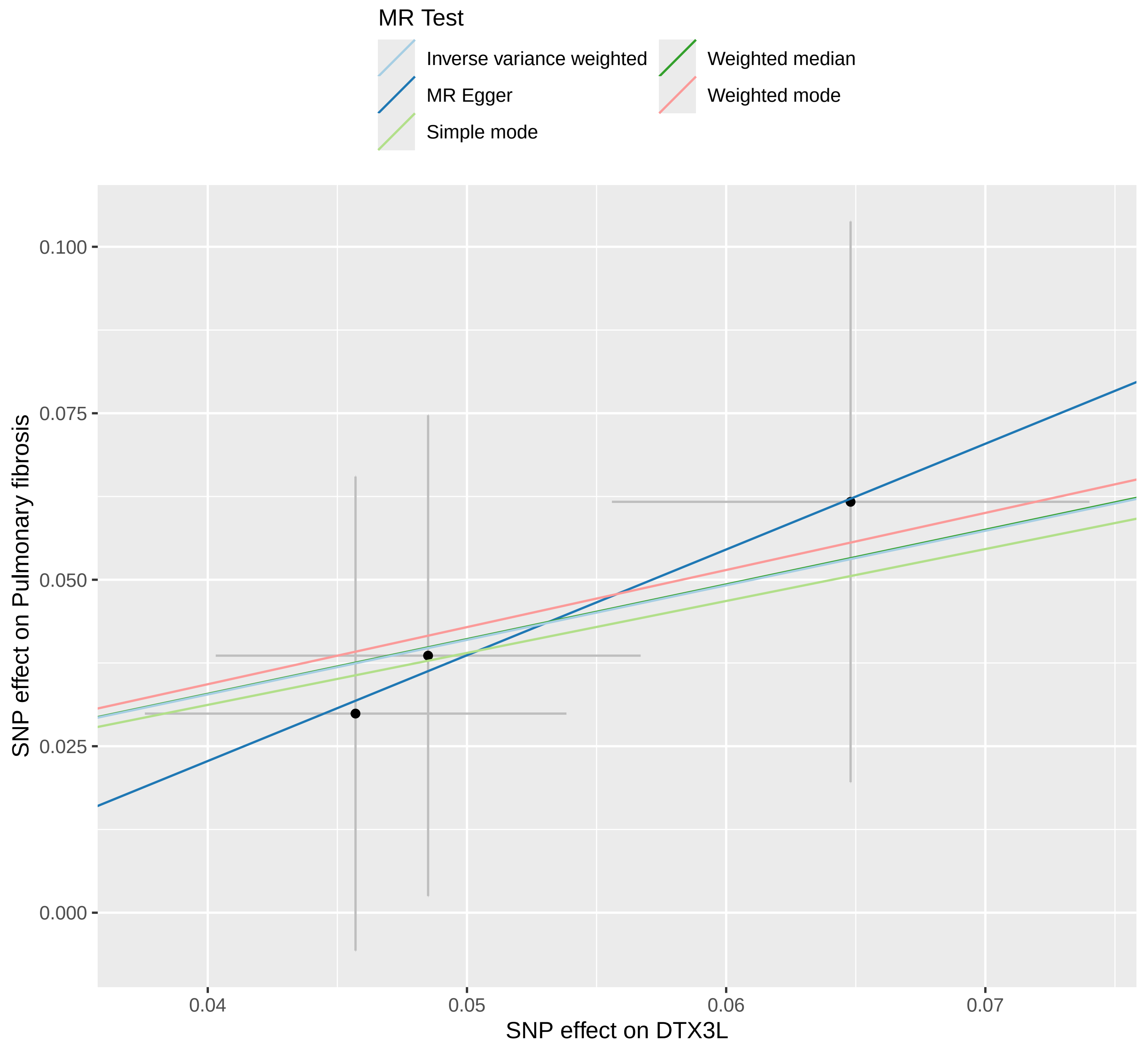

MR Test
Inverse variance weighted
MR Egger
Simple mode
Weighted median
Weighted mode
0.100
0.075
0.050
0.025
0.000
SNP effect on Pulmonary fibrosis
0.04
0.05
0.06
0.07
SNP effect on DTX3L

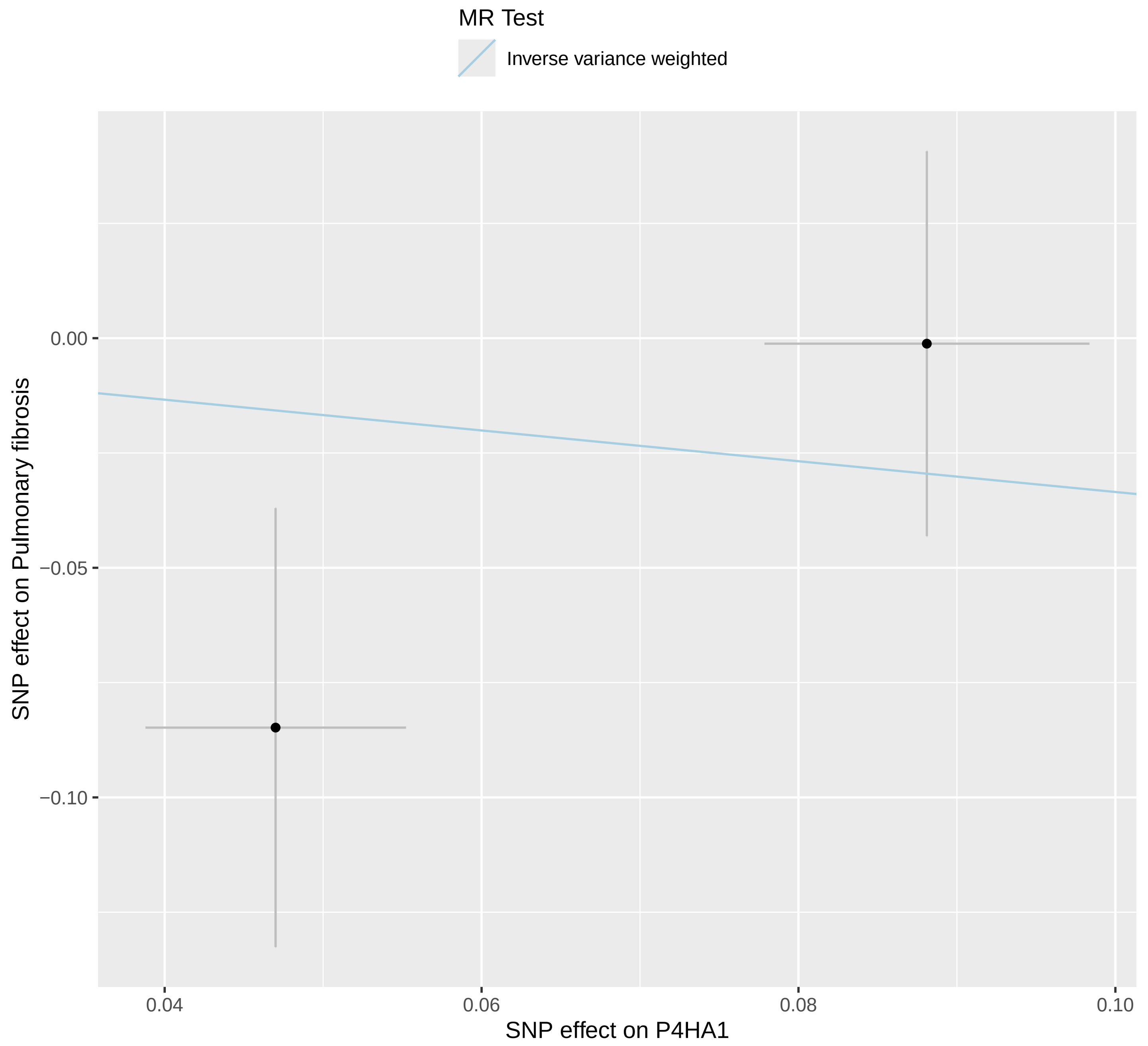

MR Test
Inverse variance weighted
0.00
−0.05
−0.10
0.04
0.06
0.08
0.10
SNP effect on Pulmonary fibrosis
SNP effect on P4HA1

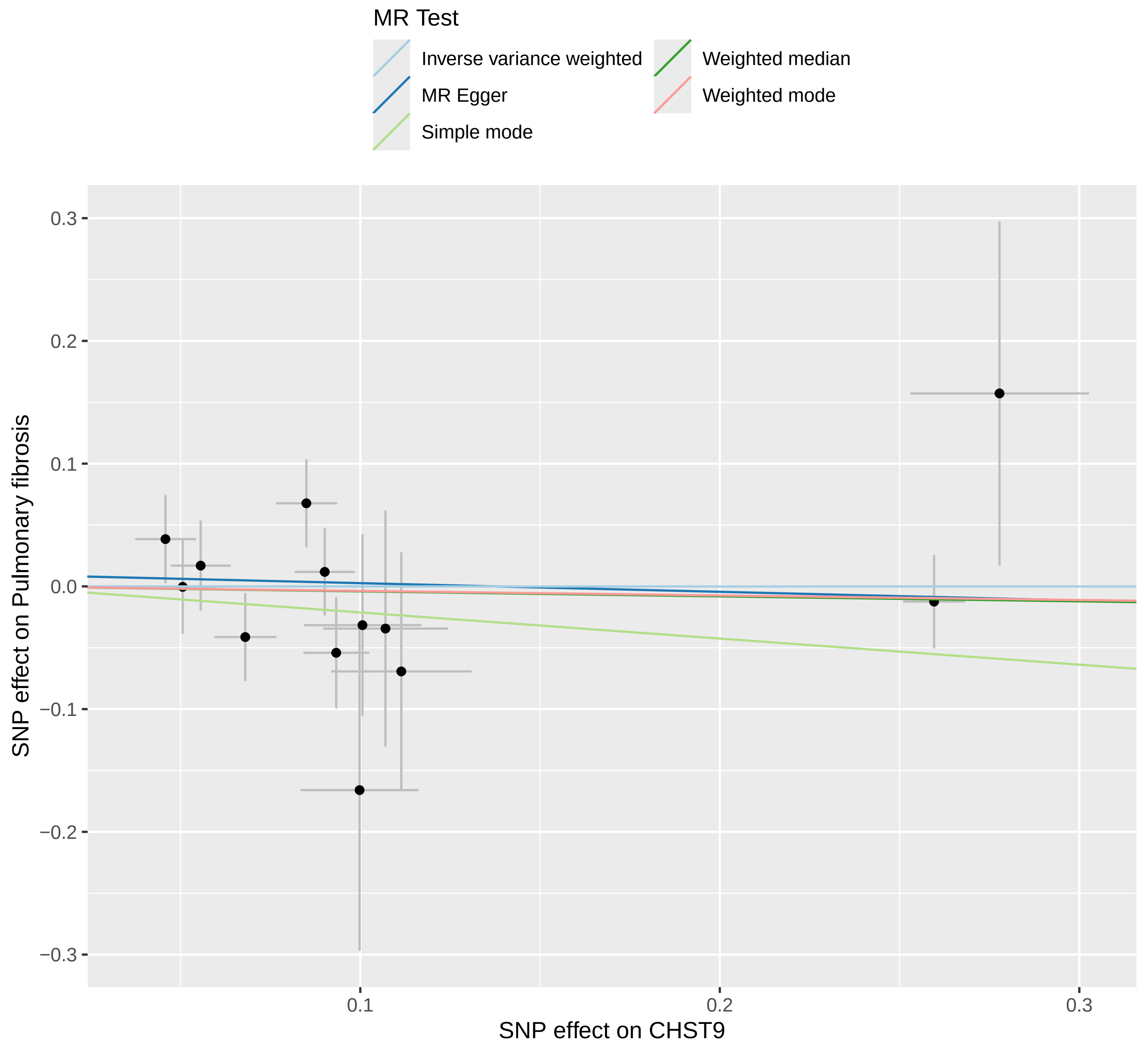

MR Test
Inverse variance weighted
MR Egger
Simple mode
Weighted median
Weighted mode
SNP effect on Pulmonary fibrosis
SNP effect on CHST9
0.3
0.2
0.1
0.0
−0.1
−0.2
−0.3
0.1
0.2
0.3

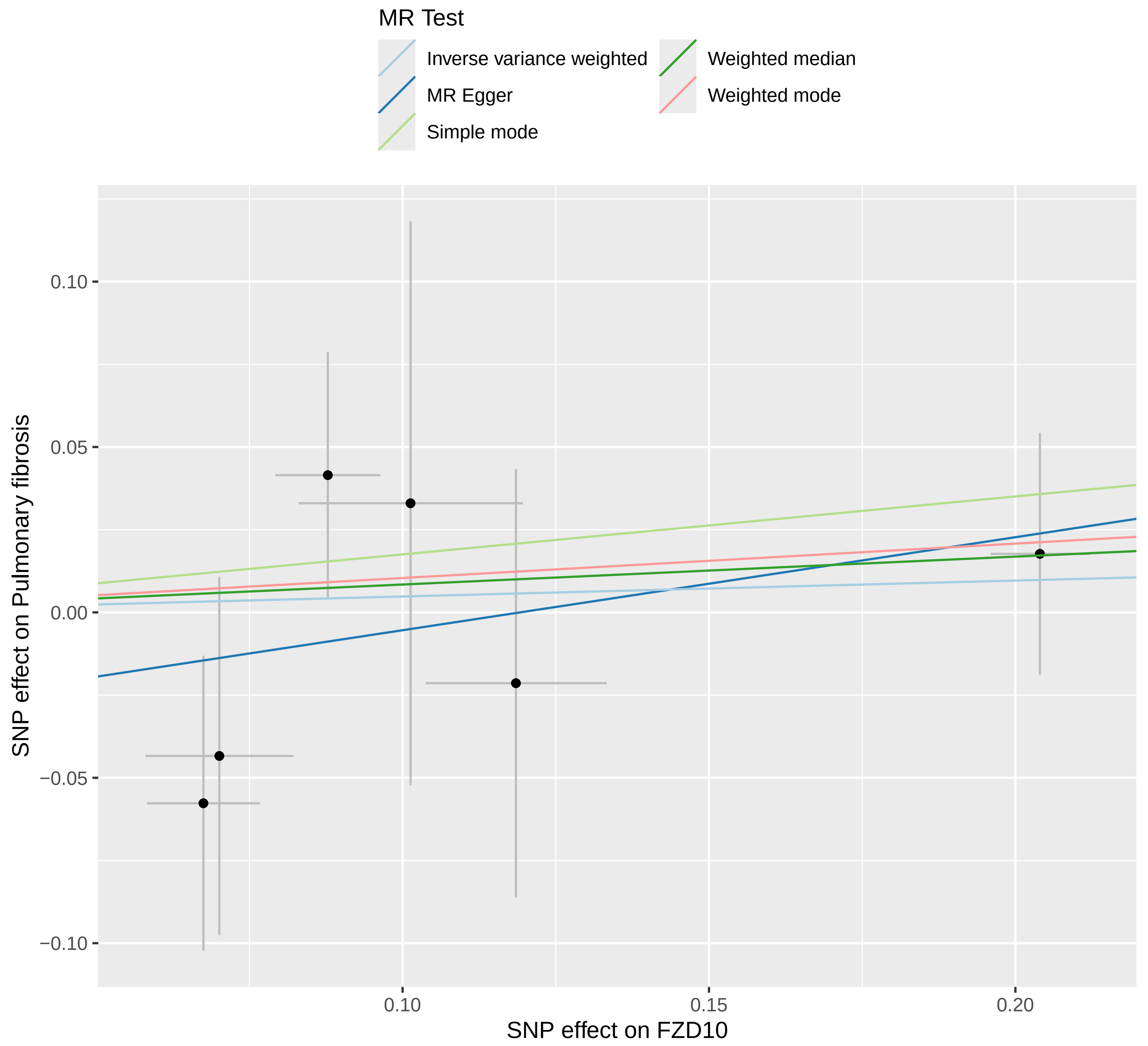

MR Test
Inverse variance weighted
MR Egger
Simple mode
Weighted median
Weighted mode
0.10
0.05
0.00
−0.05
−0.10
SNP effect on Pulmonary fibrosis
0.10
0.15
0.20
SNP effect on FZD10

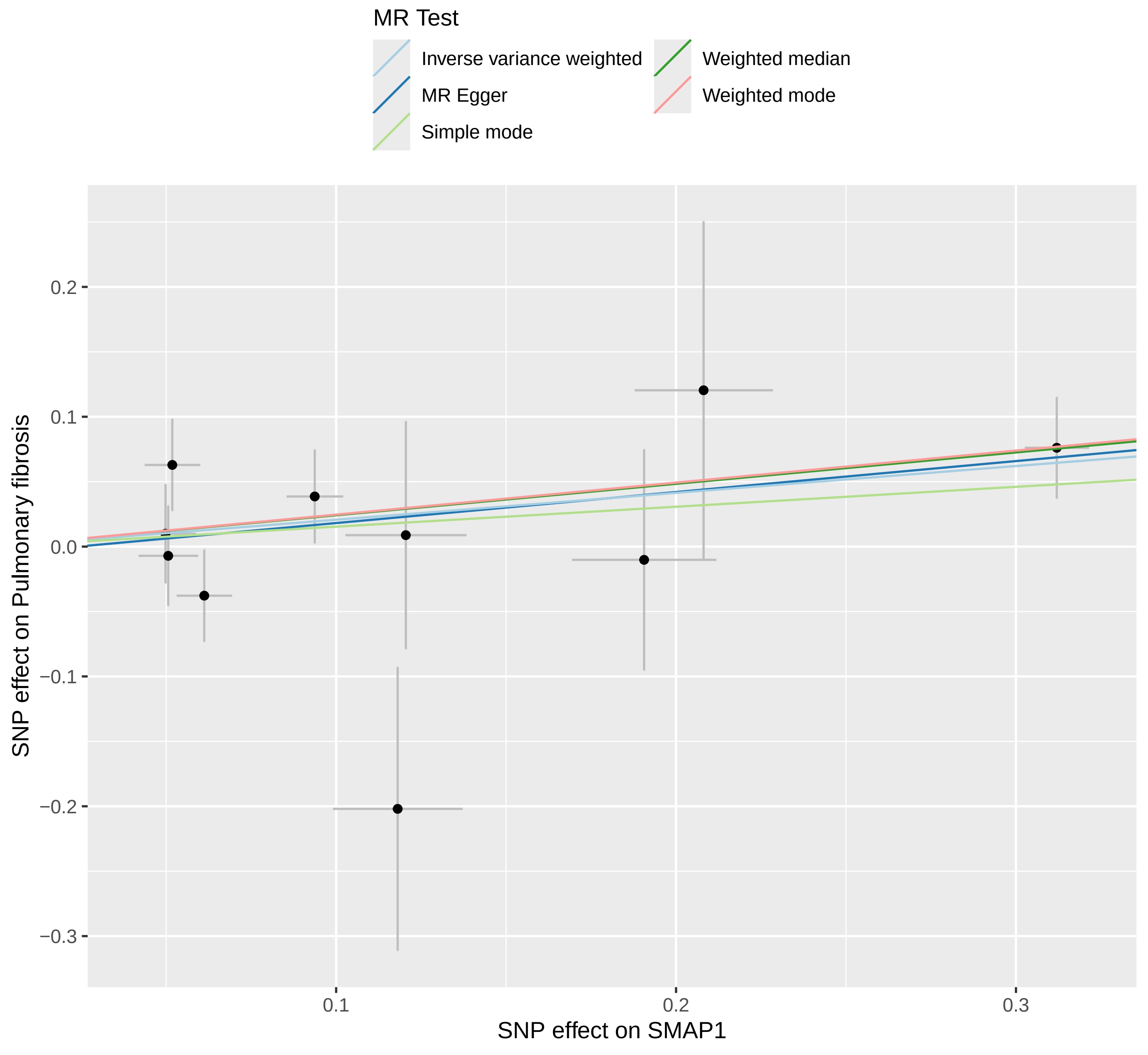

MR Test
Inverse variance weighted
MR Egger
Simple mode
Weighted median
Weighted mode
0.2
0.1
0.0
−0.1
−0.2
−0.3
SNP effect on Pulmonary fibrosis
0.1
0.2
0.3
SNP effect on SMAP1

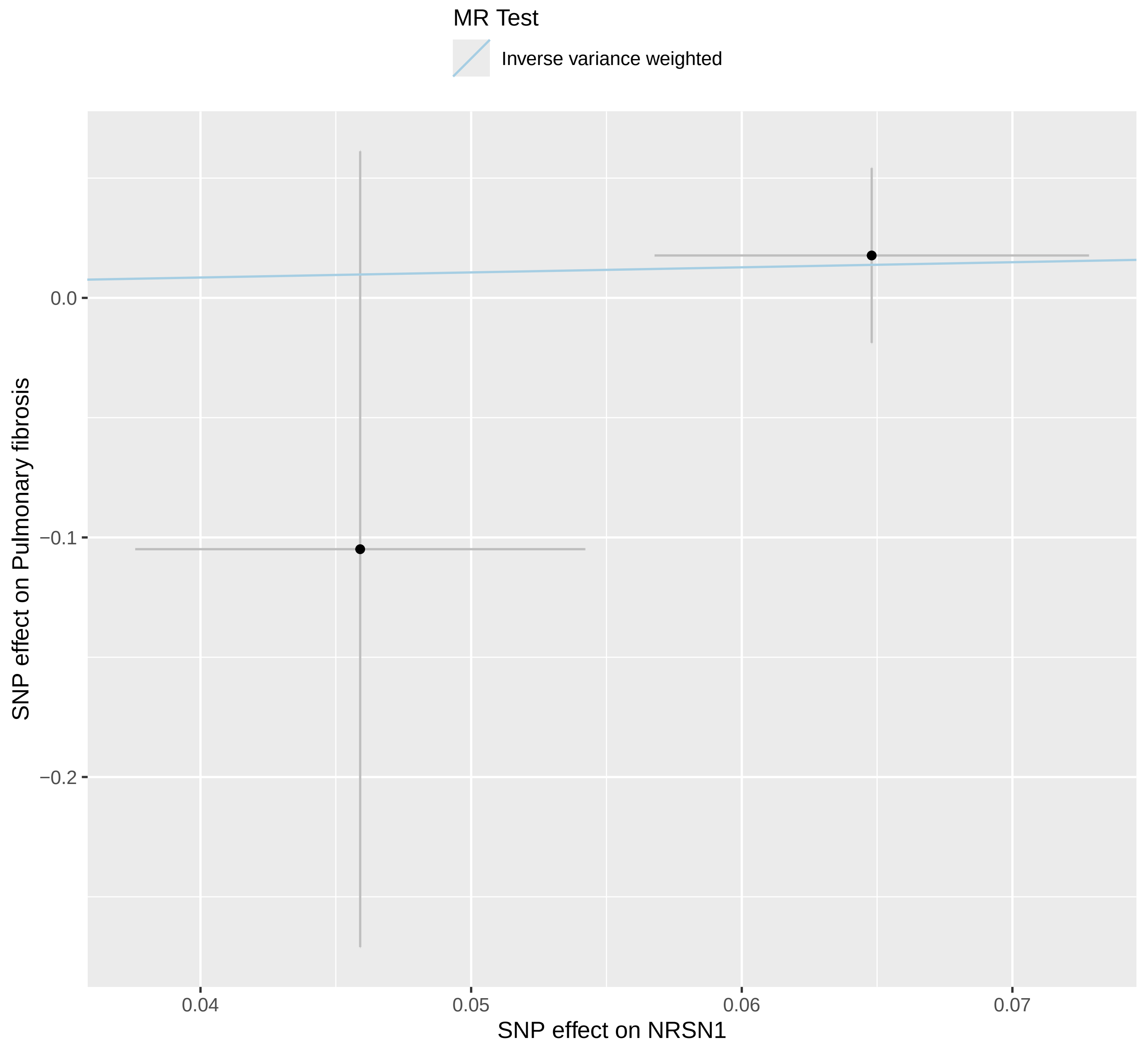

MR Test
Inverse variance weighted
0.0
−0.1
−0.2
SNP effect on Pulmonary fibrosis
0.04
0.05
0.06
0.07
SNP effect on NRSN1

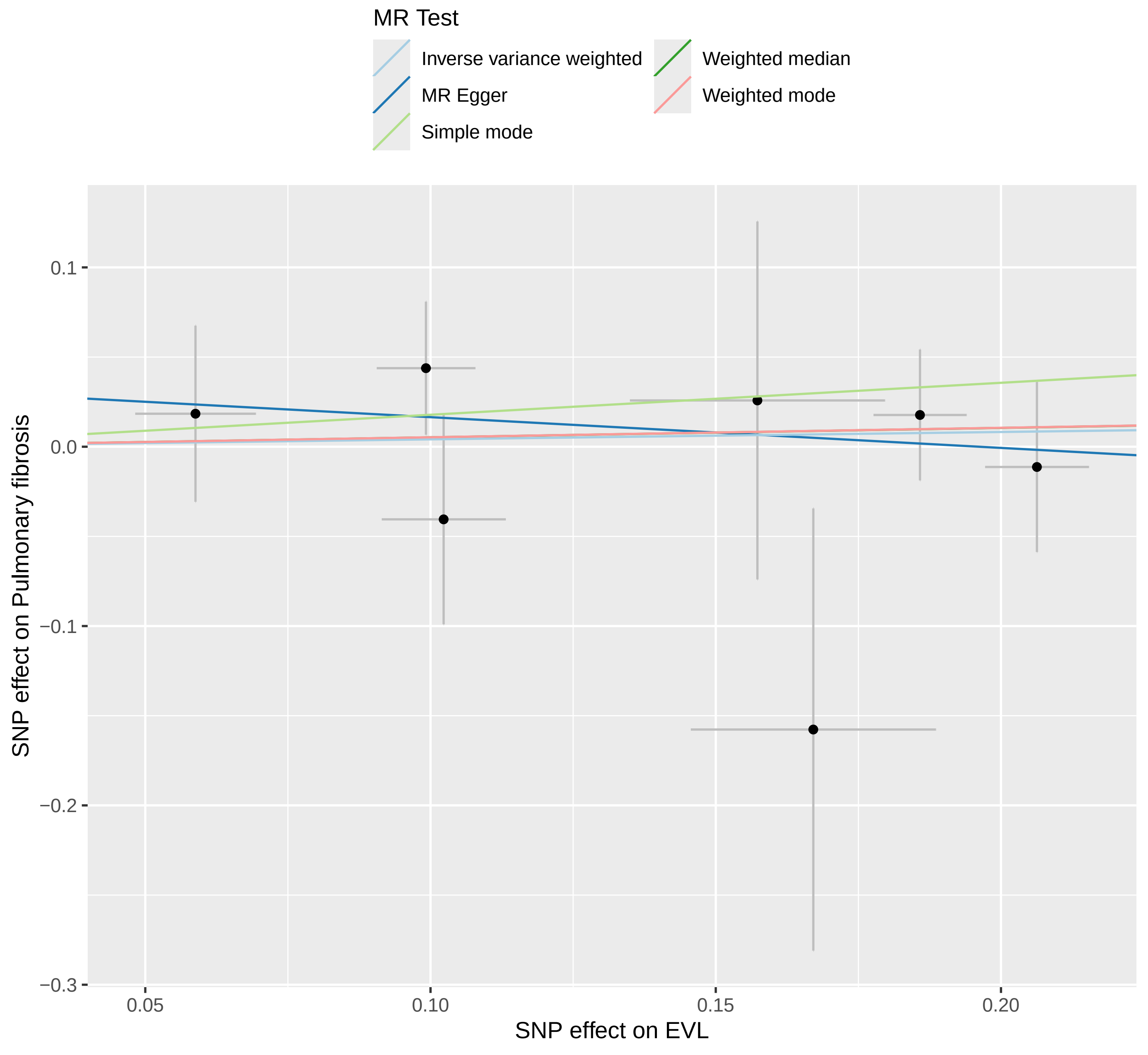

MR Test
Inverse variance weighted
MR Egger
Simple mode
Weighted median
Weighted mode
SNP effect on Pulmonary fibrosis
0.1
0.0
−0.1
−0.2
−0.3
0.05
0.10
0.15
0.20
SNP effect on EVL

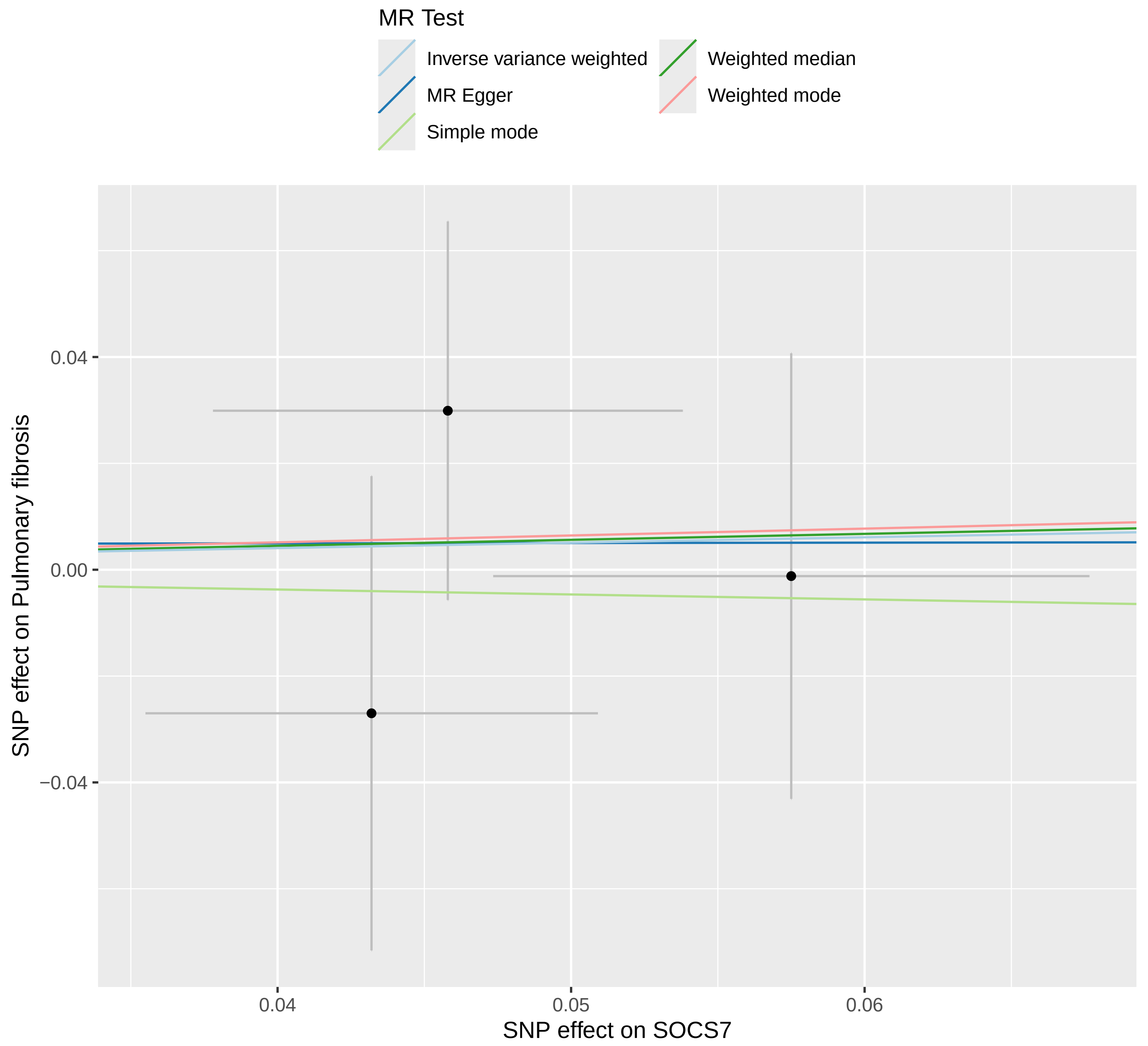

MR Test
Inverse variance weighted
MR Egger
Simple mode
Weighted median
Weighted mode
SNP effect on Pulmonary fibrosis
0.04
0.00
−0.04
0.04
0.05
0.06
SNP effect on SOCS7

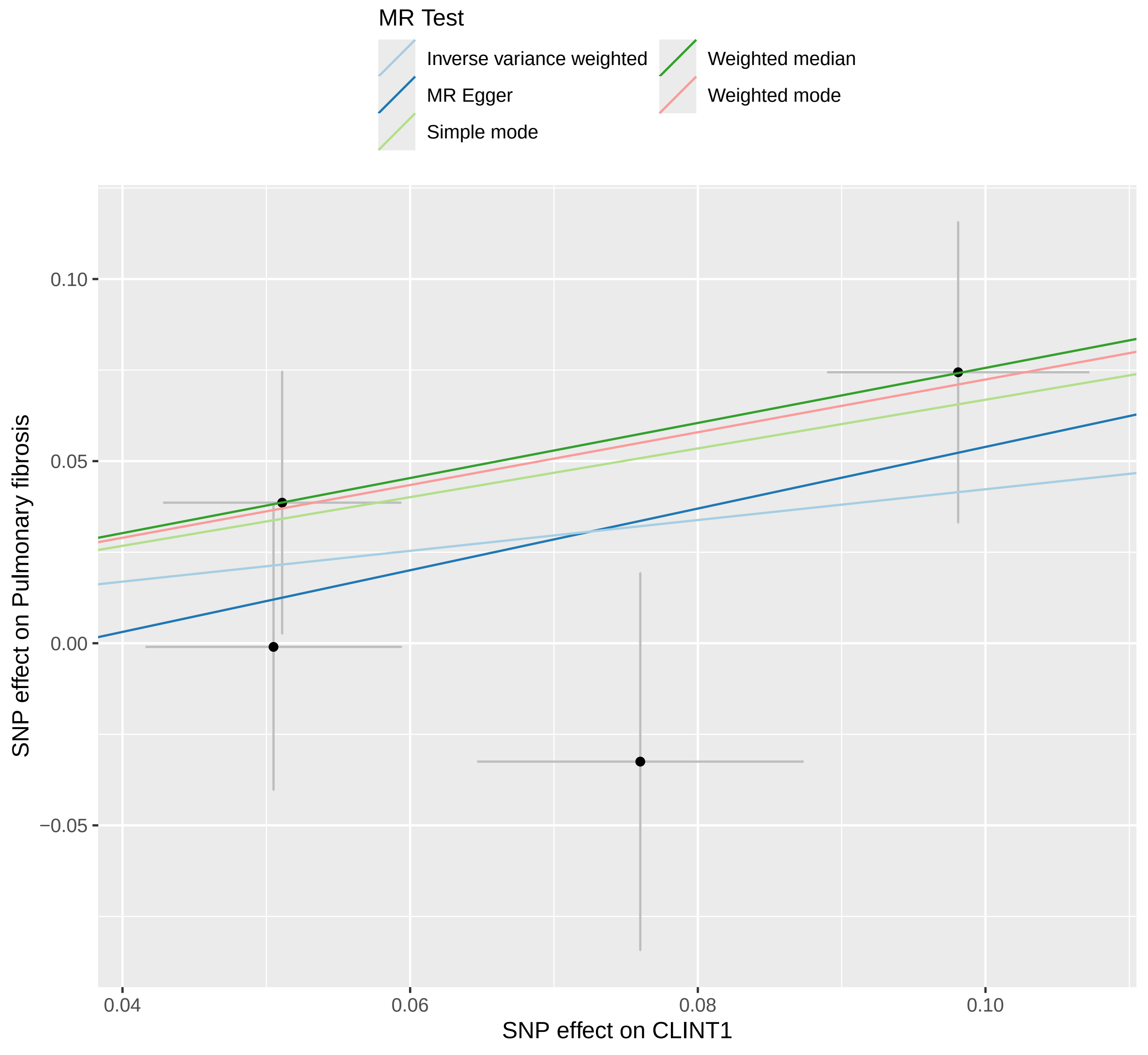

MR Test
Inverse variance weighted
MR Egger
Simple mode
Weighted median
Weighted mode
0.10
0.05
0.00
−0.05
0.04
0.06
0.08
0.10
SNP effect on Pulmonary fibrosis
SNP effect on CLINT1

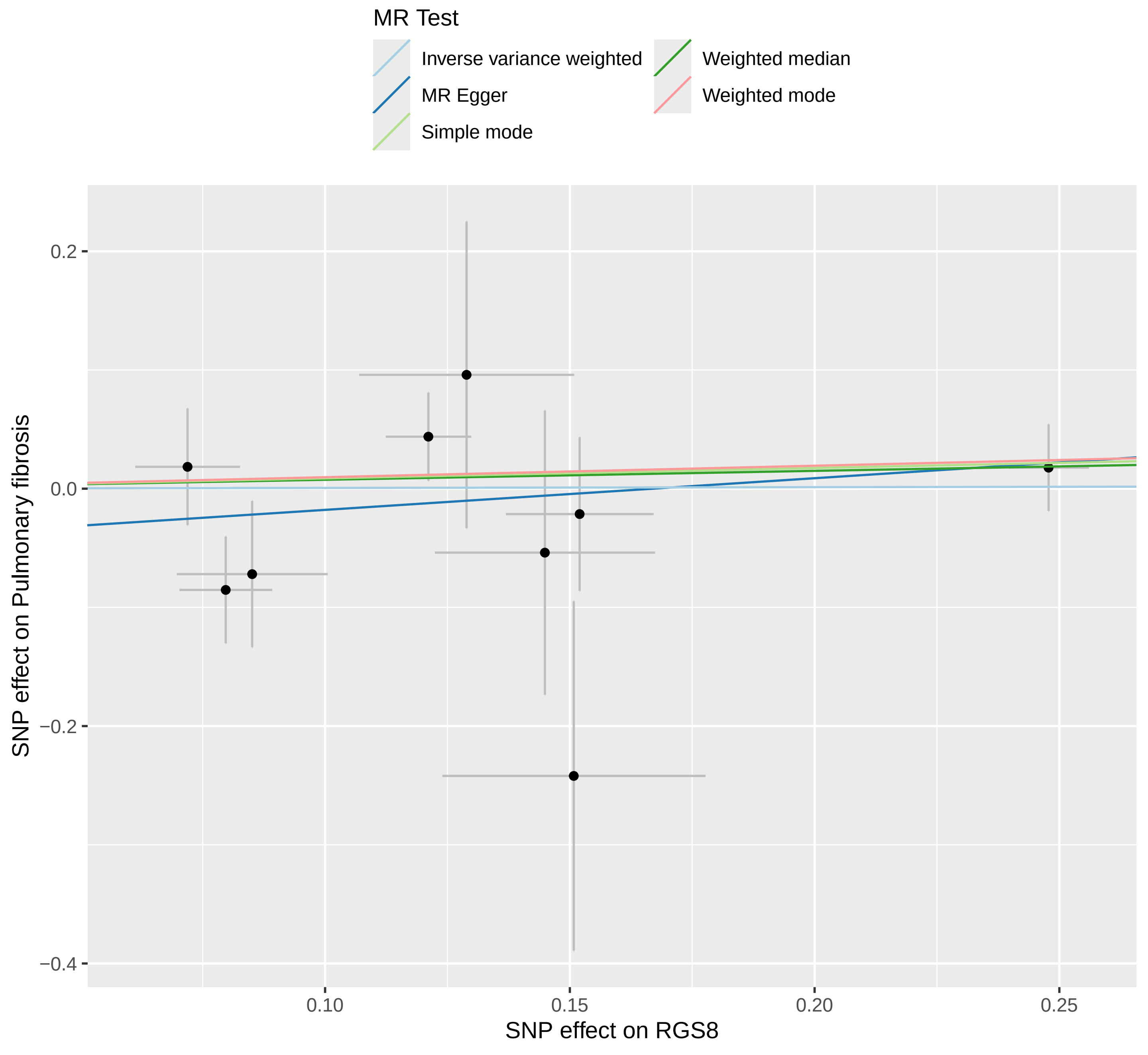
MR Test
Inverse variance weighted
MR Egger
Simple mode
Weighted median
Weighted mode
0.2
0.0
−0.2
−0.4
0.10
0.15
0.20
0.25
SNP effect on RGS8
SNP effect on Pulmonary fibrosis

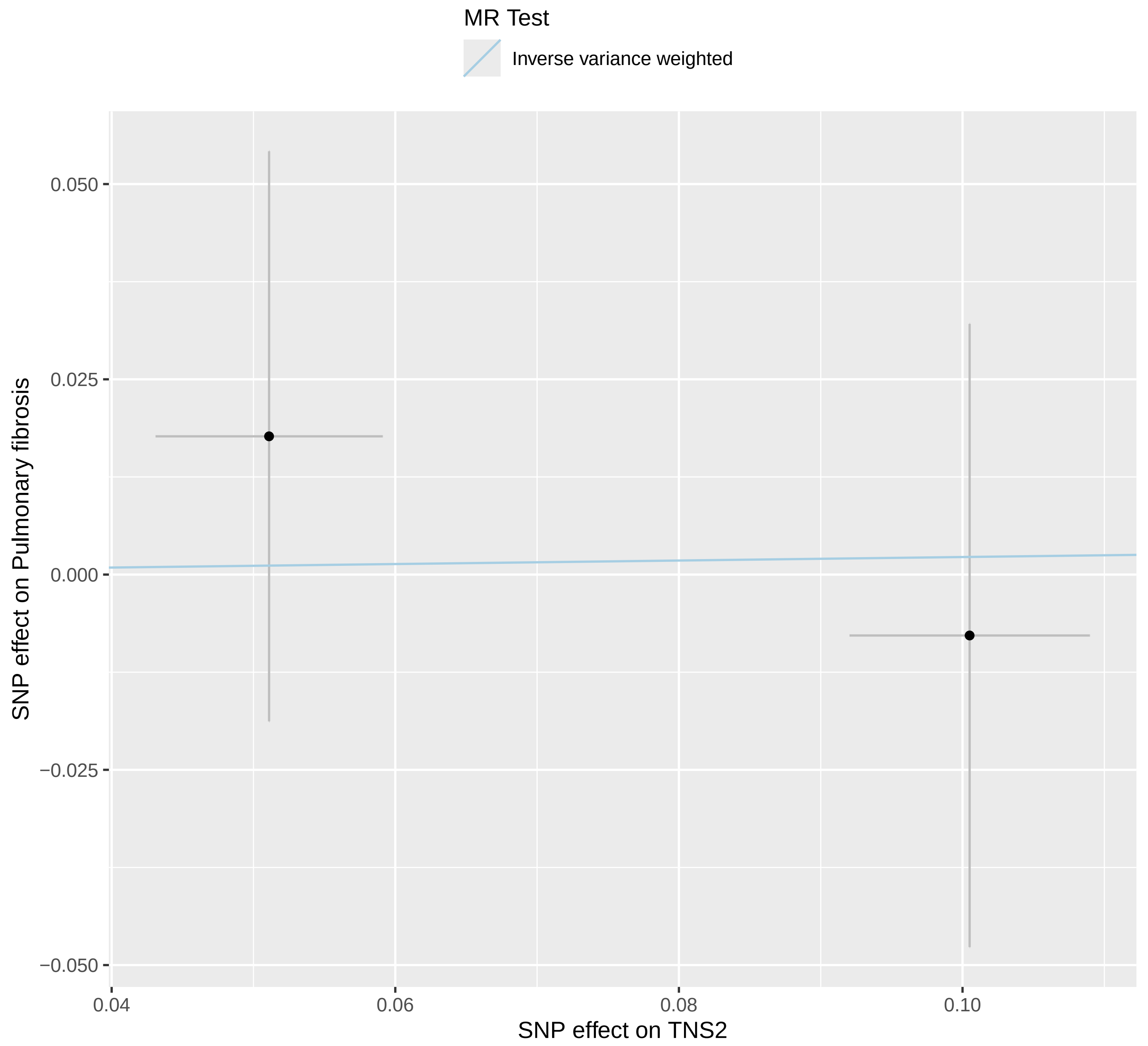

MR Test
Inverse variance weighted
0.050
0.025
0.000
−0.025
−0.050
SNP effect on Pulmonary fibrosis
0.04
0.06
0.08
0.10
SNP effect on TNS2

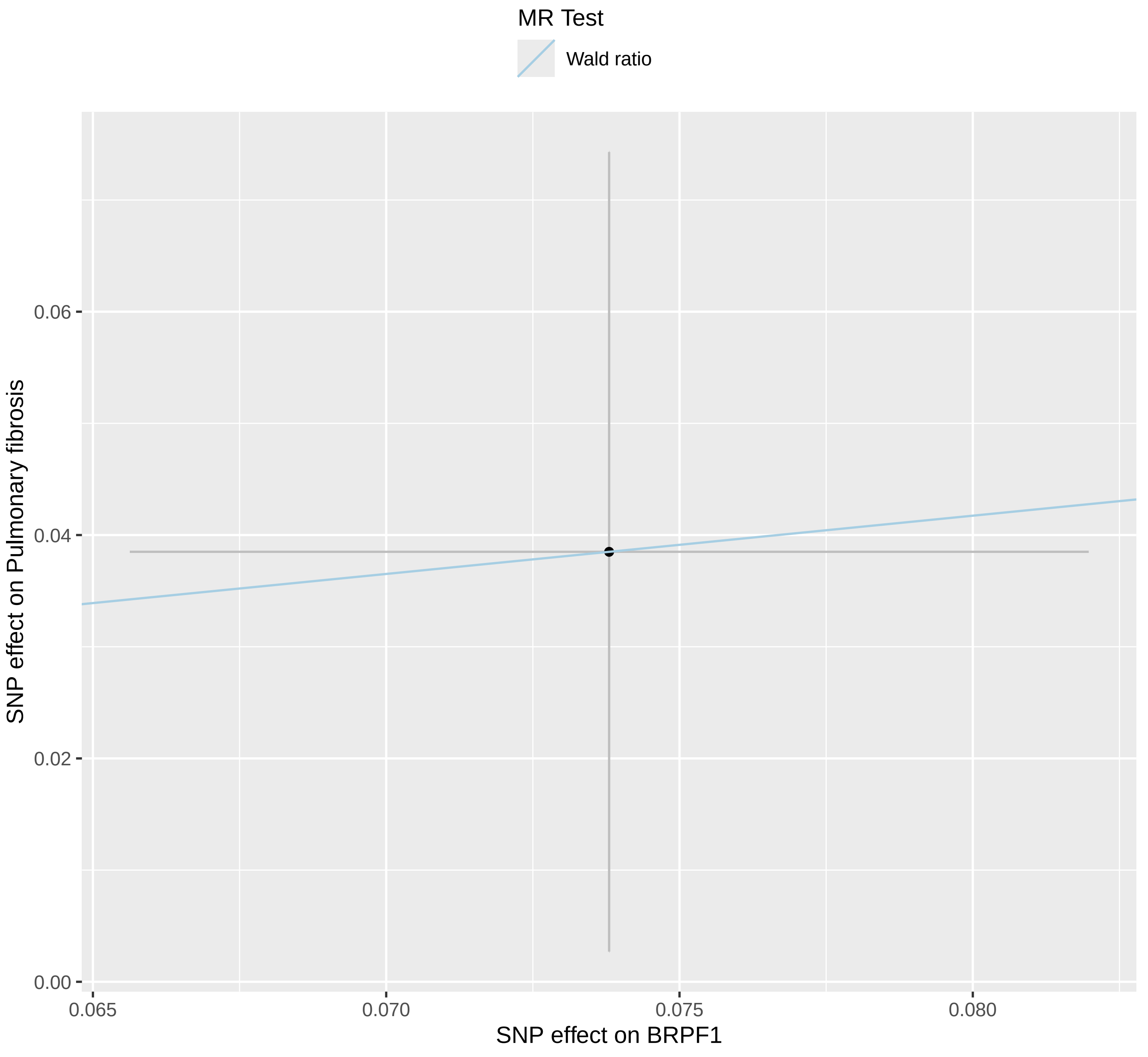
MR Test
Wald ratio
0.06
0.04
0.02
0.00
0.065
0.070
0.075
0.080
SNP effect on Pulmonary fibrosis
SNP effect on BRPF1

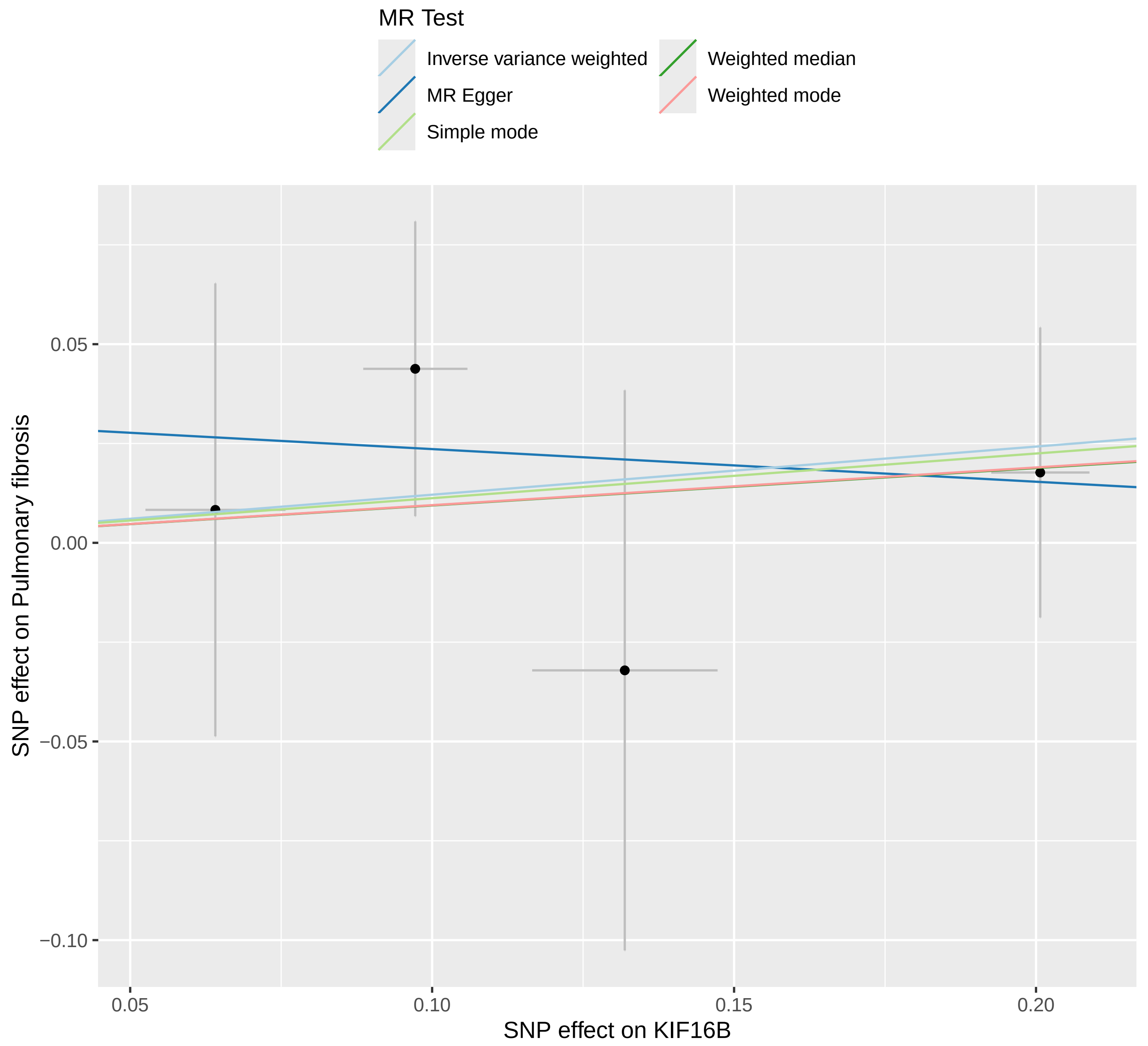

MR Test
Inverse variance weighted
MR Egger
Simple mode
Weighted median
Weighted mode
0.05
0.00
−0.05
−0.10
SNP effect on Pulmonary fibrosis
0.05
0.10
0.15
0.20
SNP effect on KIF16B

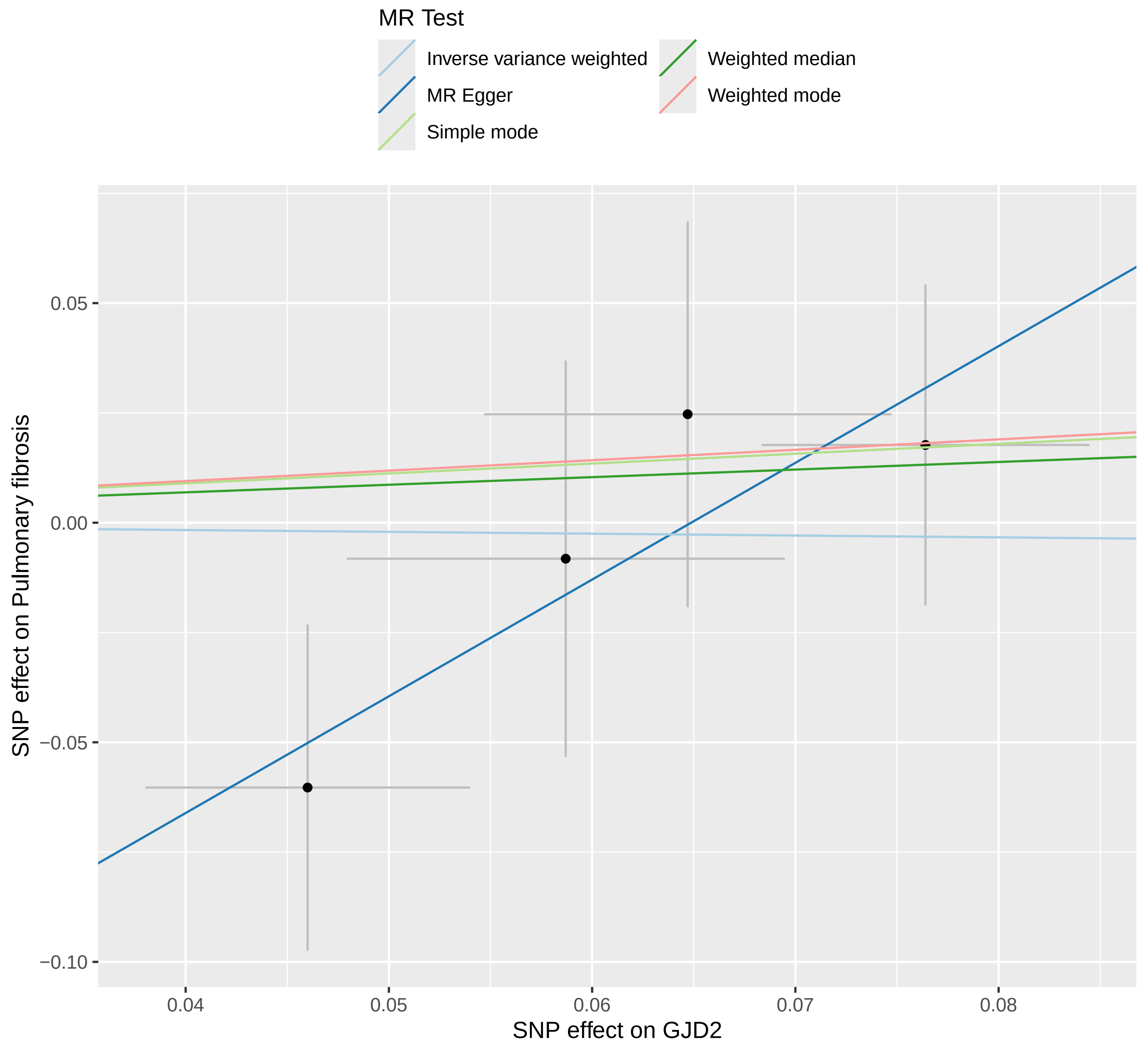
MR Test
Inverse variance weighted
MR Egger
Simple mode
Weighted median
Weighted mode
0.05
0.00
−0.05
−0.10
SNP effect on Pulmonary fibrosis
0.04
0.05
0.06
0.07
0.08
SNP effect on GJD2

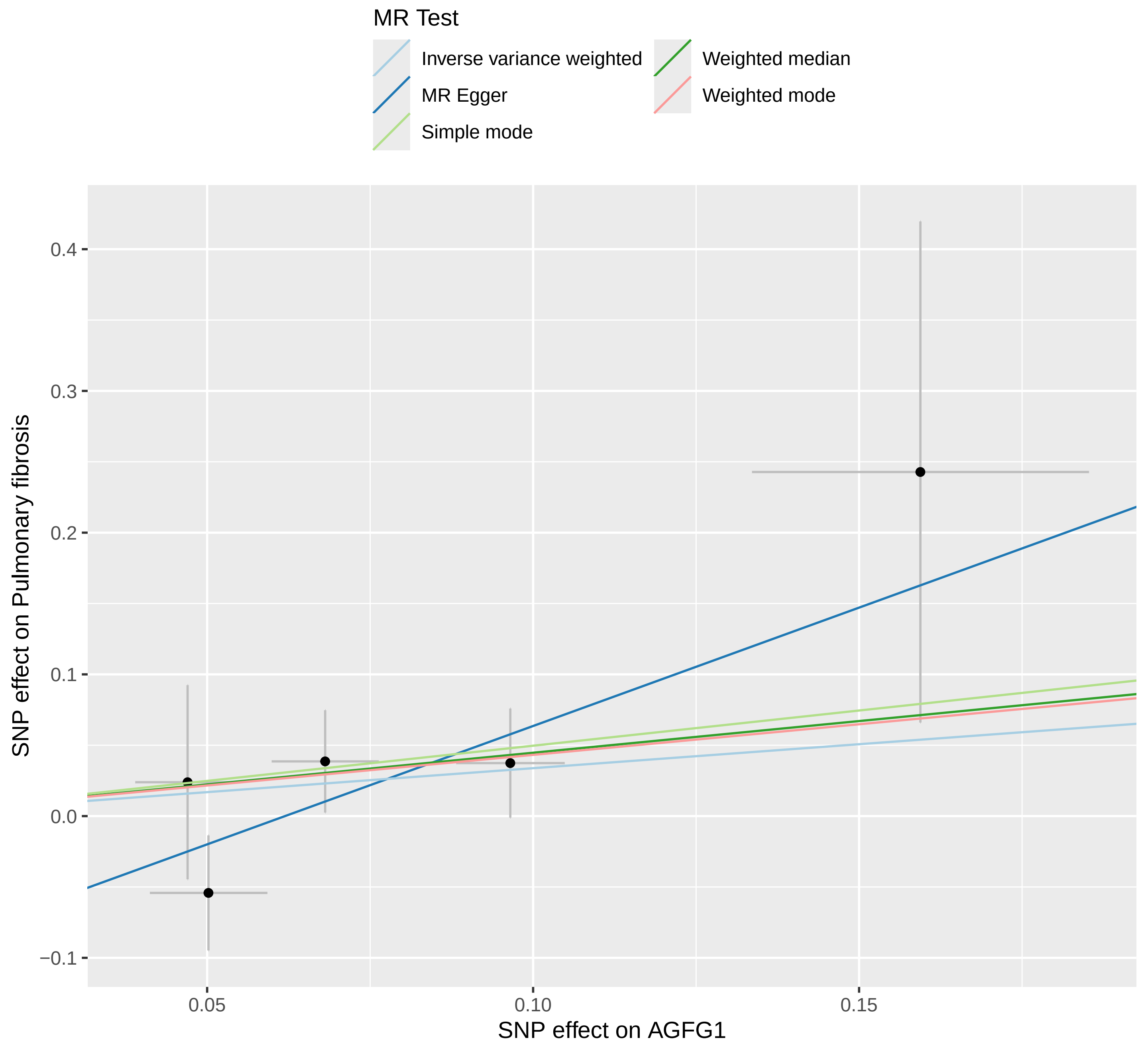

MR Test
Inverse variance weighted
MR Egger
Simple mode
Weighted median
Weighted mode
0.4
0.3
0.2
0.1
0.0
−0.1
0.05
0.10
0.15
SNP effect on Pulmonary fibrosis
SNP effect on AGFG1

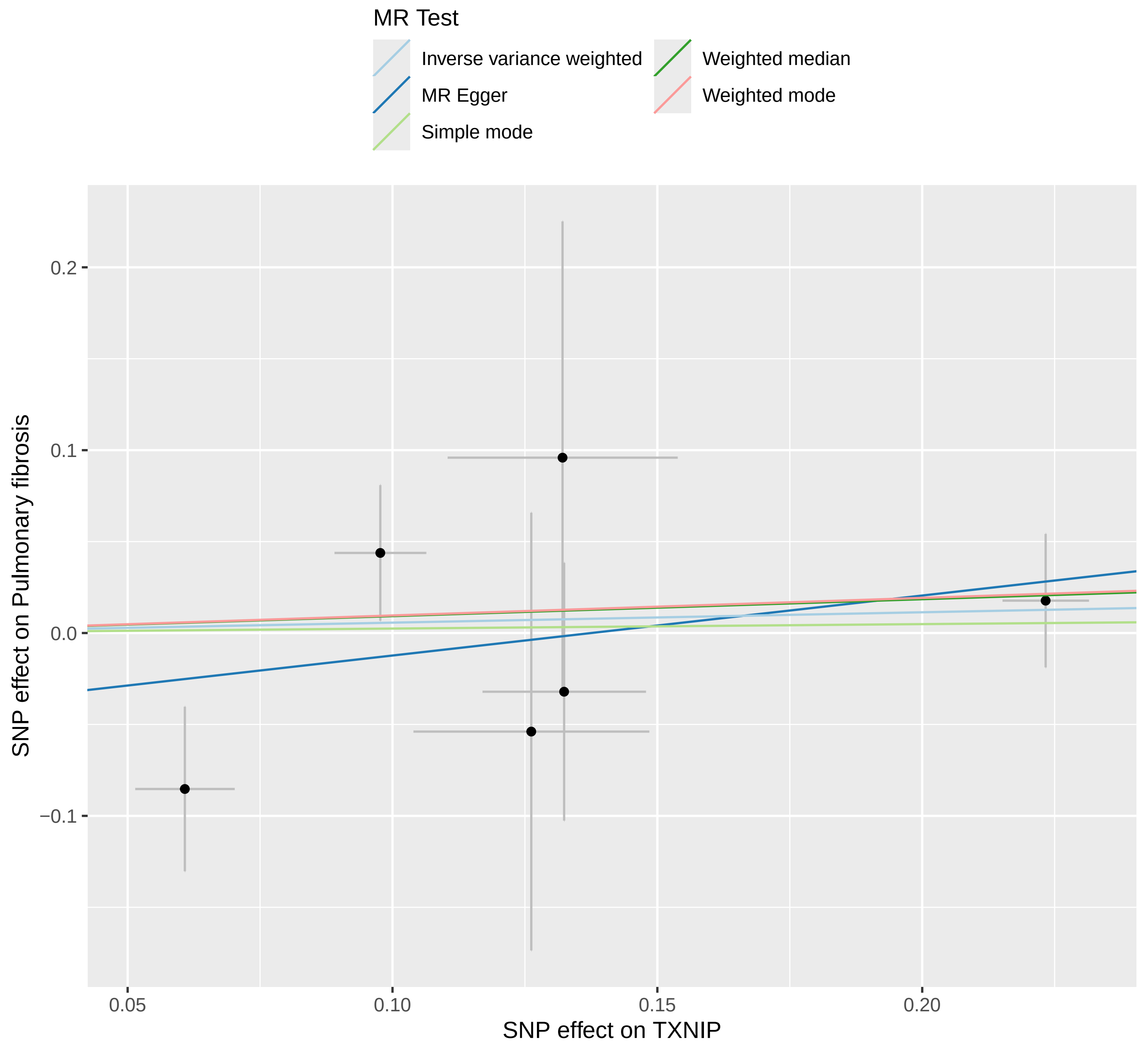
MR Test
Inverse variance weighted
MR Egger
Simple mode
Weighted median
Weighted mode
0.2
0.1
0.0
−0.1
0.05
0.10
0.15
0.20
SNP effect on TXNIP
SNP effect on Pulmonary fibrosis

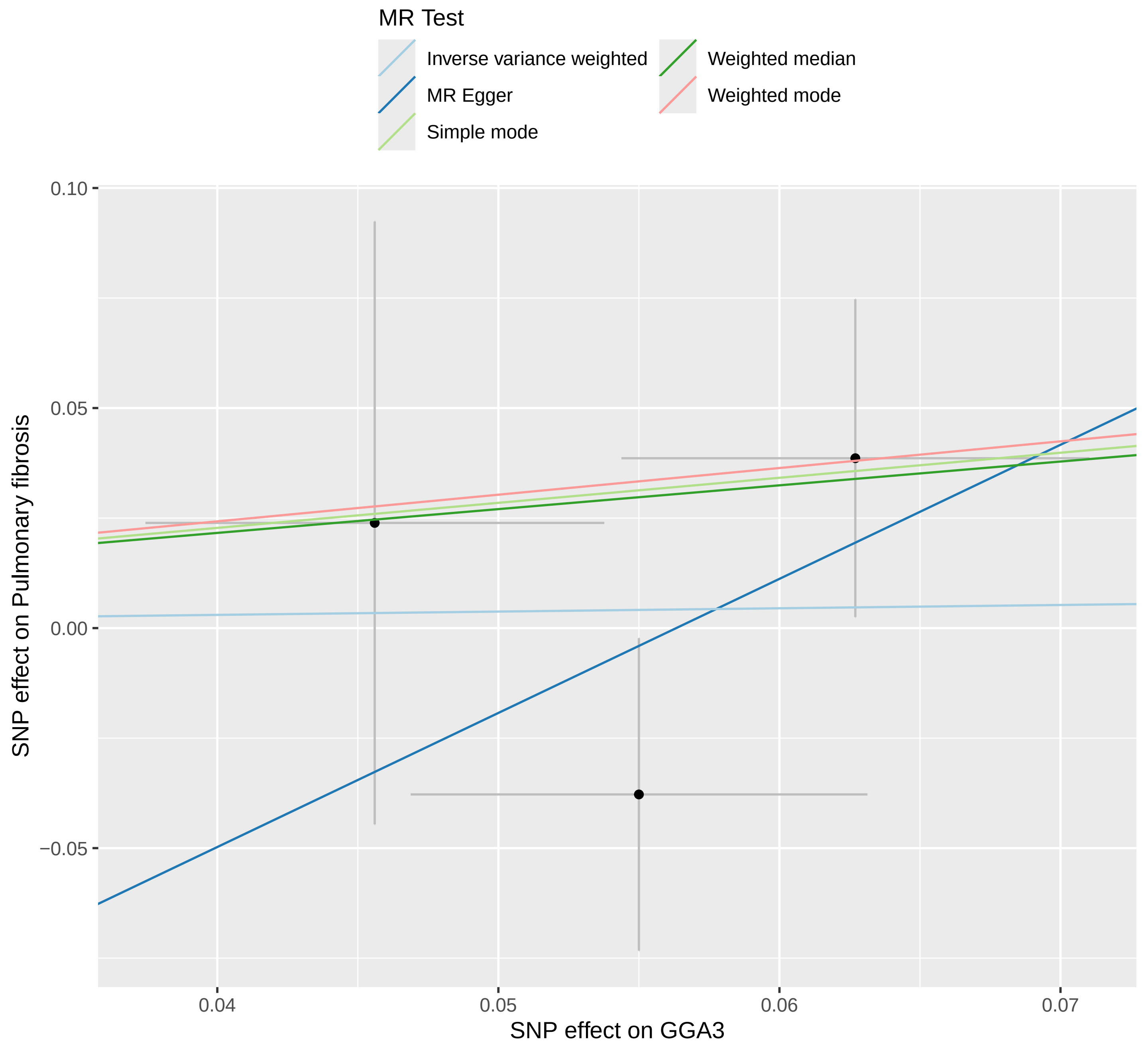
MR Test
Inverse variance weighted
MR Egger
Simple mode
Weighted median
Weighted mode
0.10
0.05
0.00
−0.05
0.04
0.05
0.06
0.07
SNP effect on Pulmonary fibrosis
SNP effect on GGA3

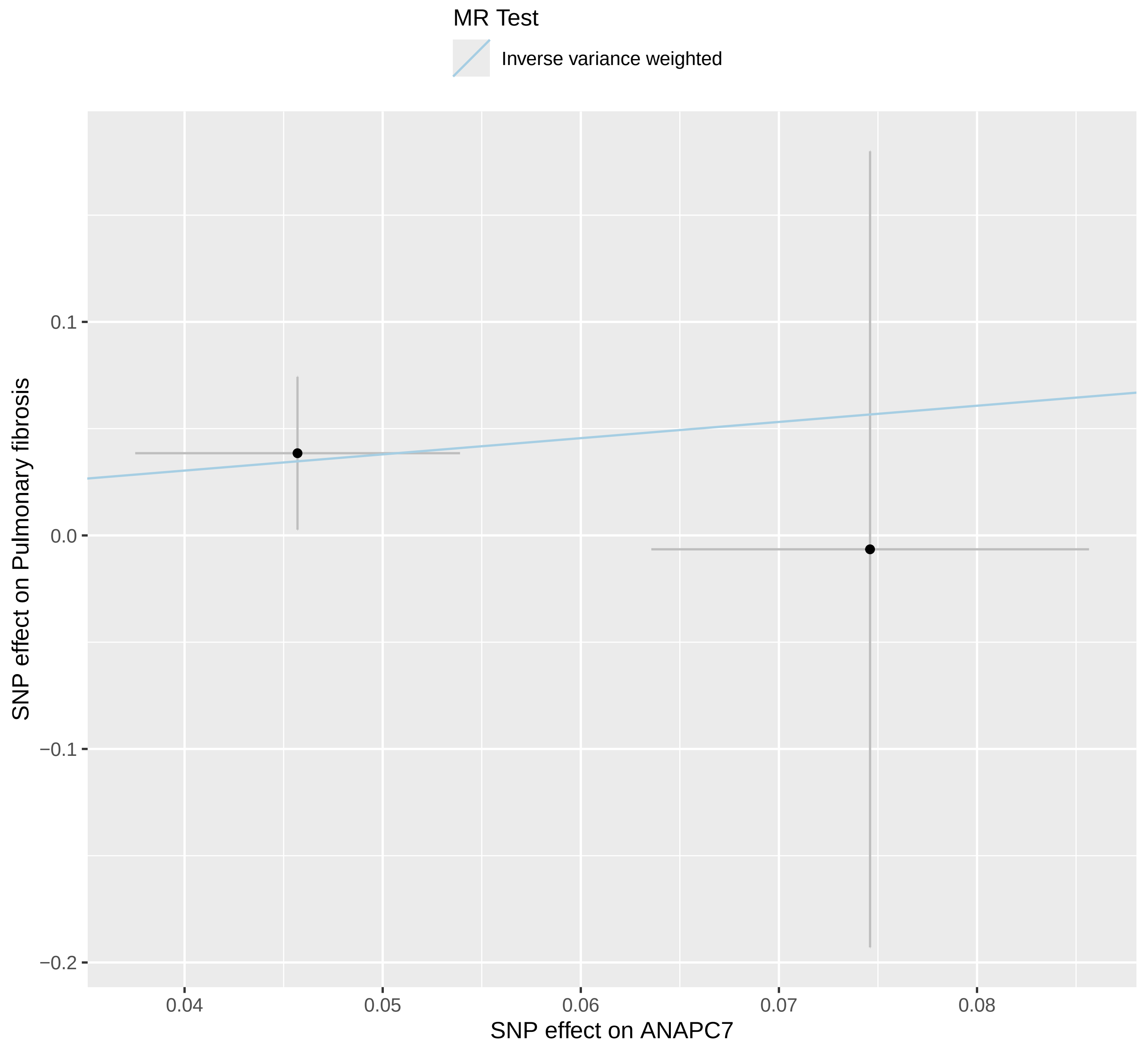
MR Test
Inverse variance weighted
SNP effect on Pulmonary fibrosis
0.1
0.0
−0.1
−0.2
0.04
0.05
0.06
0.07
0.08
SNP effect on ANAPC7

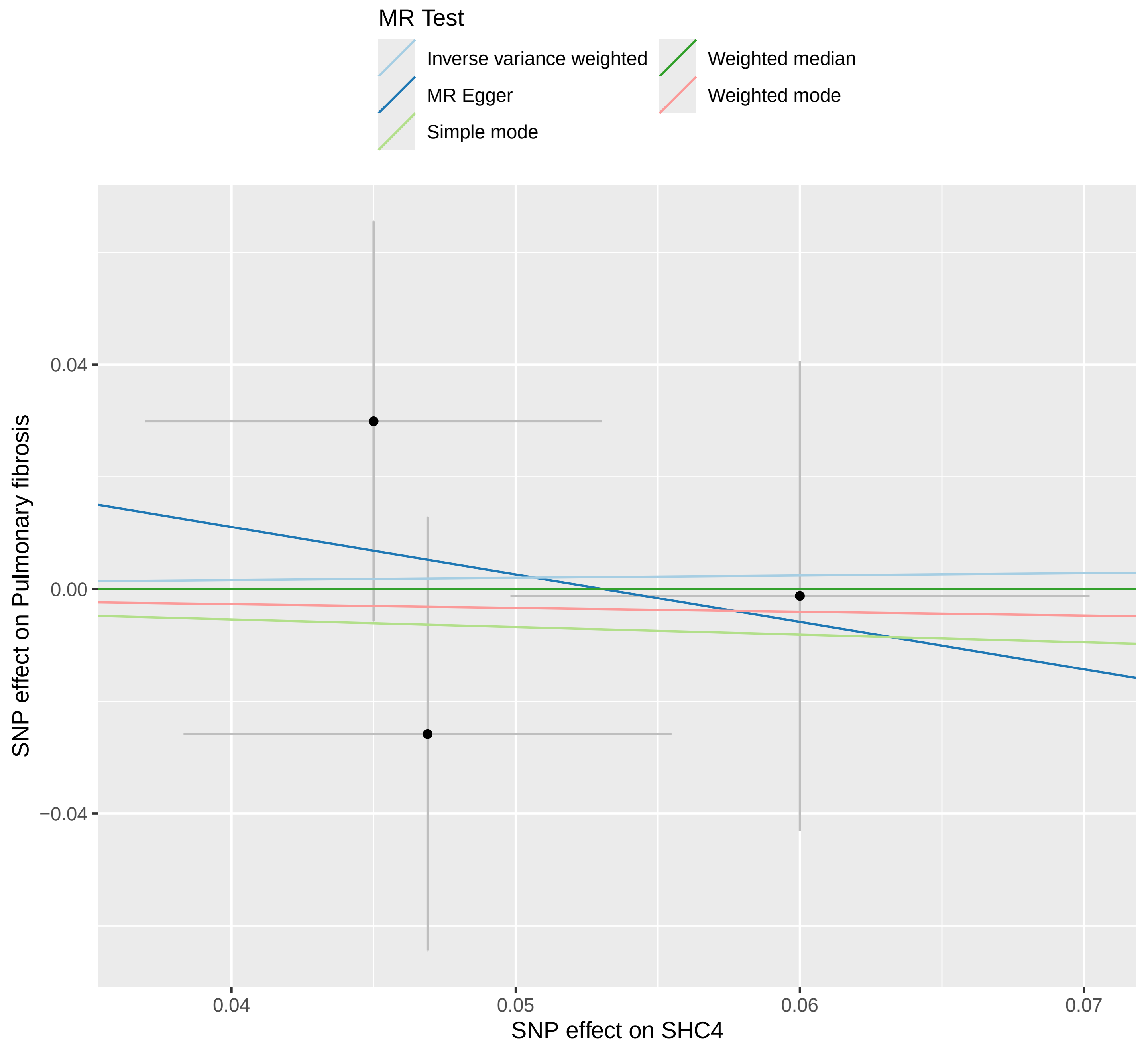

MR Test
Inverse variance weighted
MR Egger
Simple mode
Weighted median
Weighted mode
0.04
0.00
−0.04
0.04
0.05
0.06
0.07
SNP effect on SHC4
SNP effect on Pulmonary fibrosis

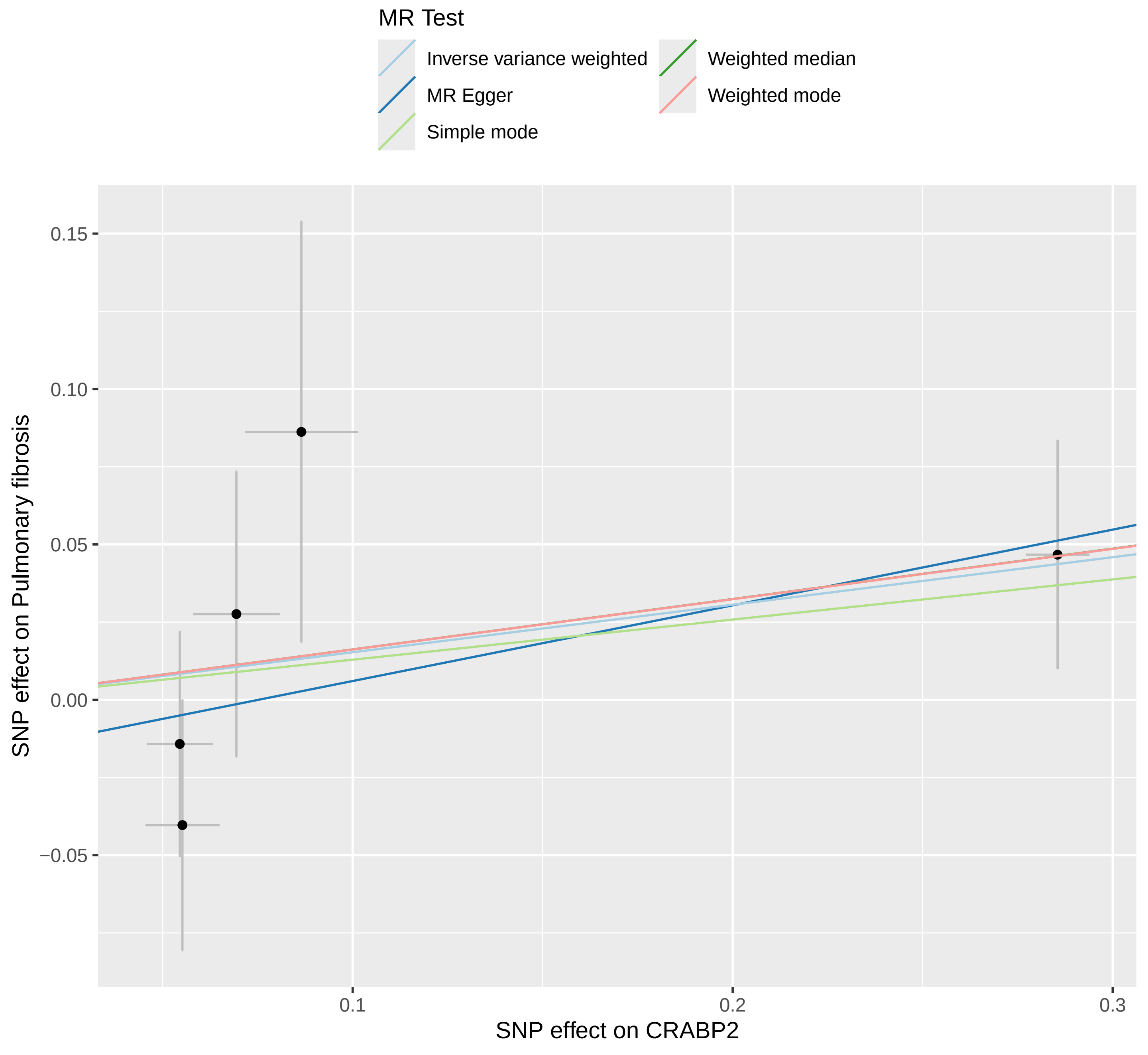

MR Test
Inverse variance weighted
MR Egger
Simple mode
Weighted median
Weighted mode
0.15
0.10
0.05
0.00
−0.05
SNP effect on Pulmonary fibrosis
0.1
0.2
0.3
SNP effect on CRABP2

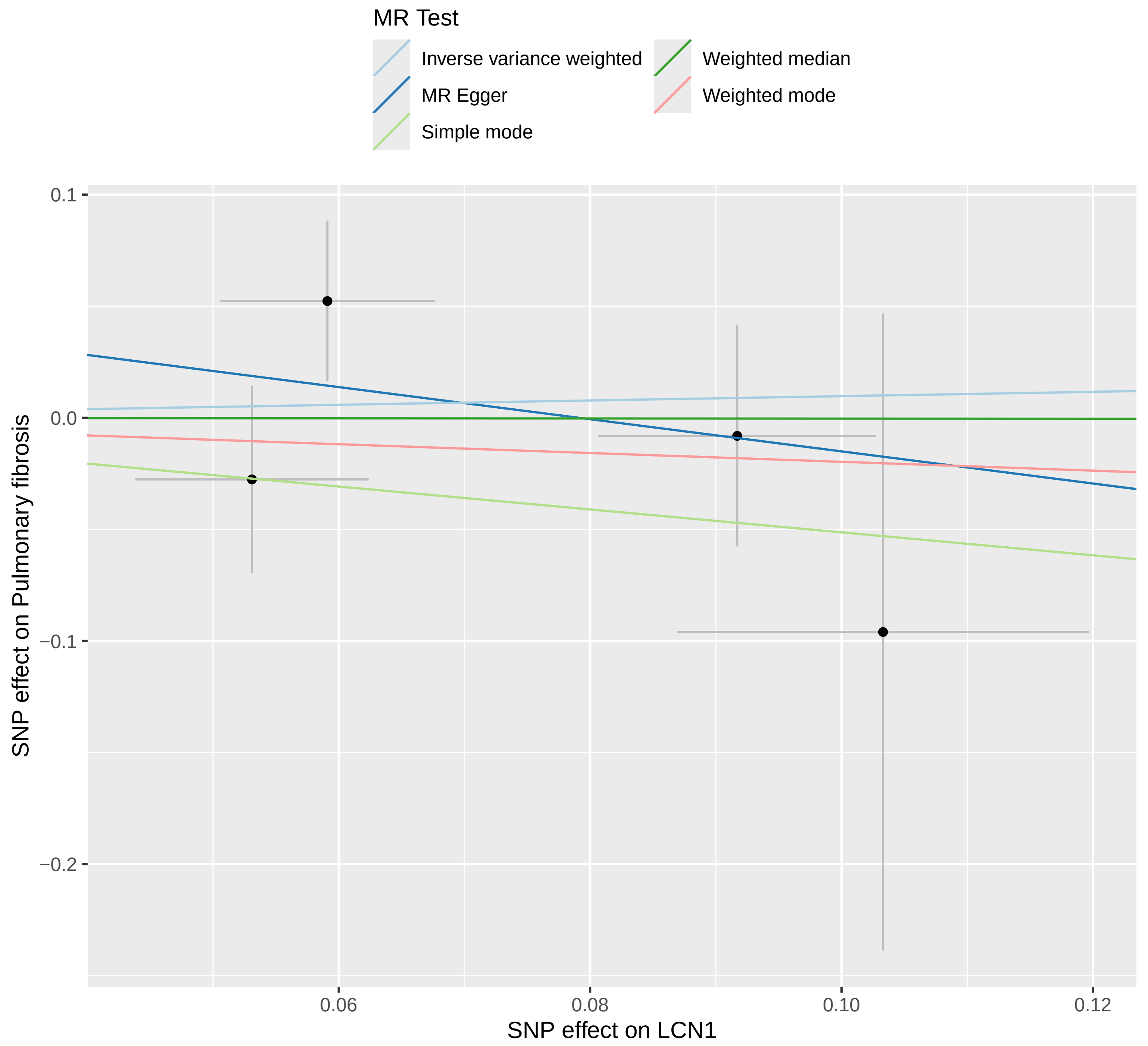
MR Test
Inverse variance weighted
MR Egger
Simple mode
Weighted median
Weighted mode
0.1
0.0
−0.1
−0.2
SNP effect on Pulmonary fibrosis
0.06
0.08
0.10
0.12
SNP effect on LCN1

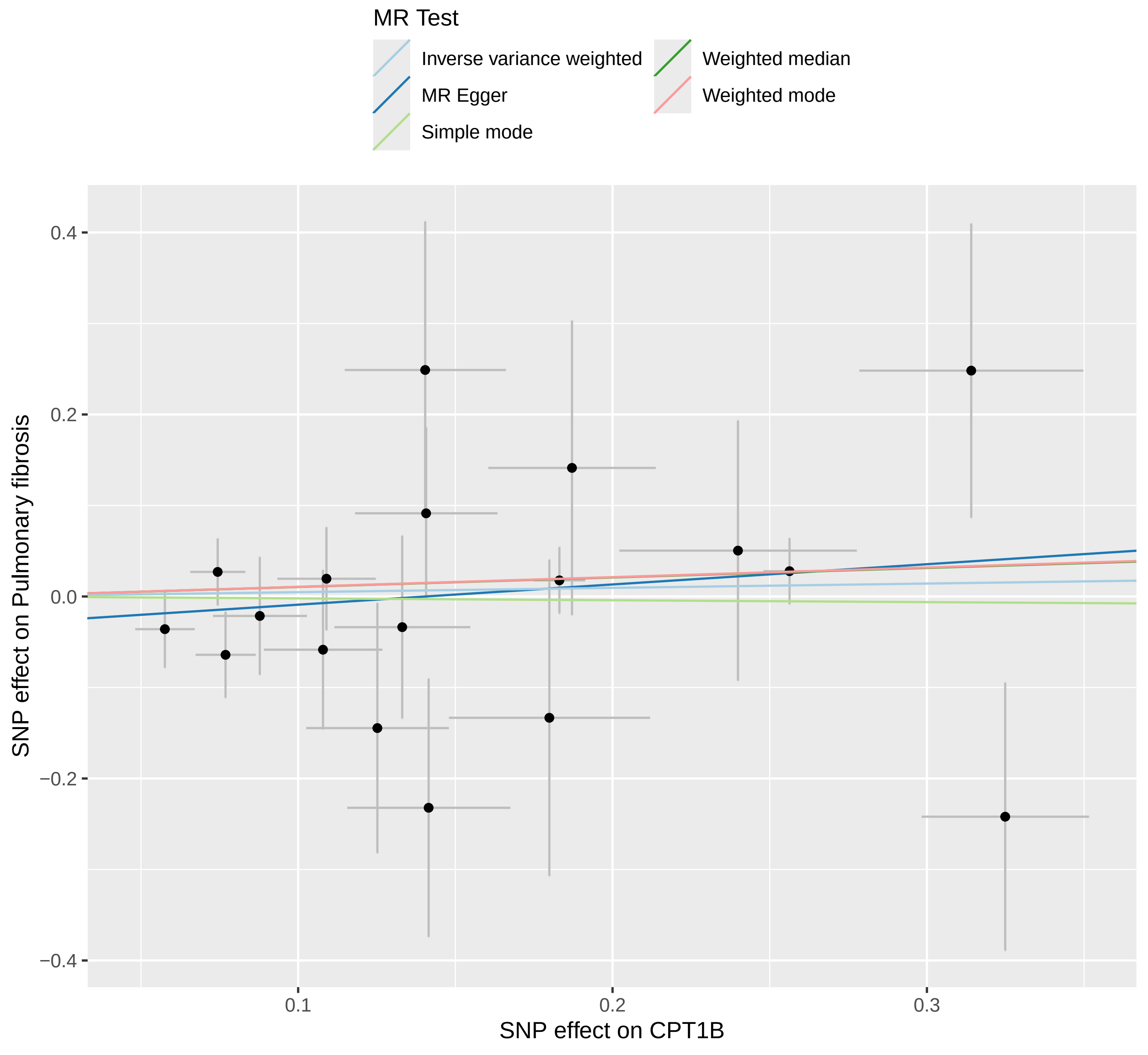
MR Test
Inverse variance weighted
MR Egger
Simple mode
Weighted median
Weighted mode
SNP effect on Pulmonary fibrosis
0.4
0.2
0.0
−0.2
−0.4
0.1
0.2
0.3
SNP effect on CPT1B

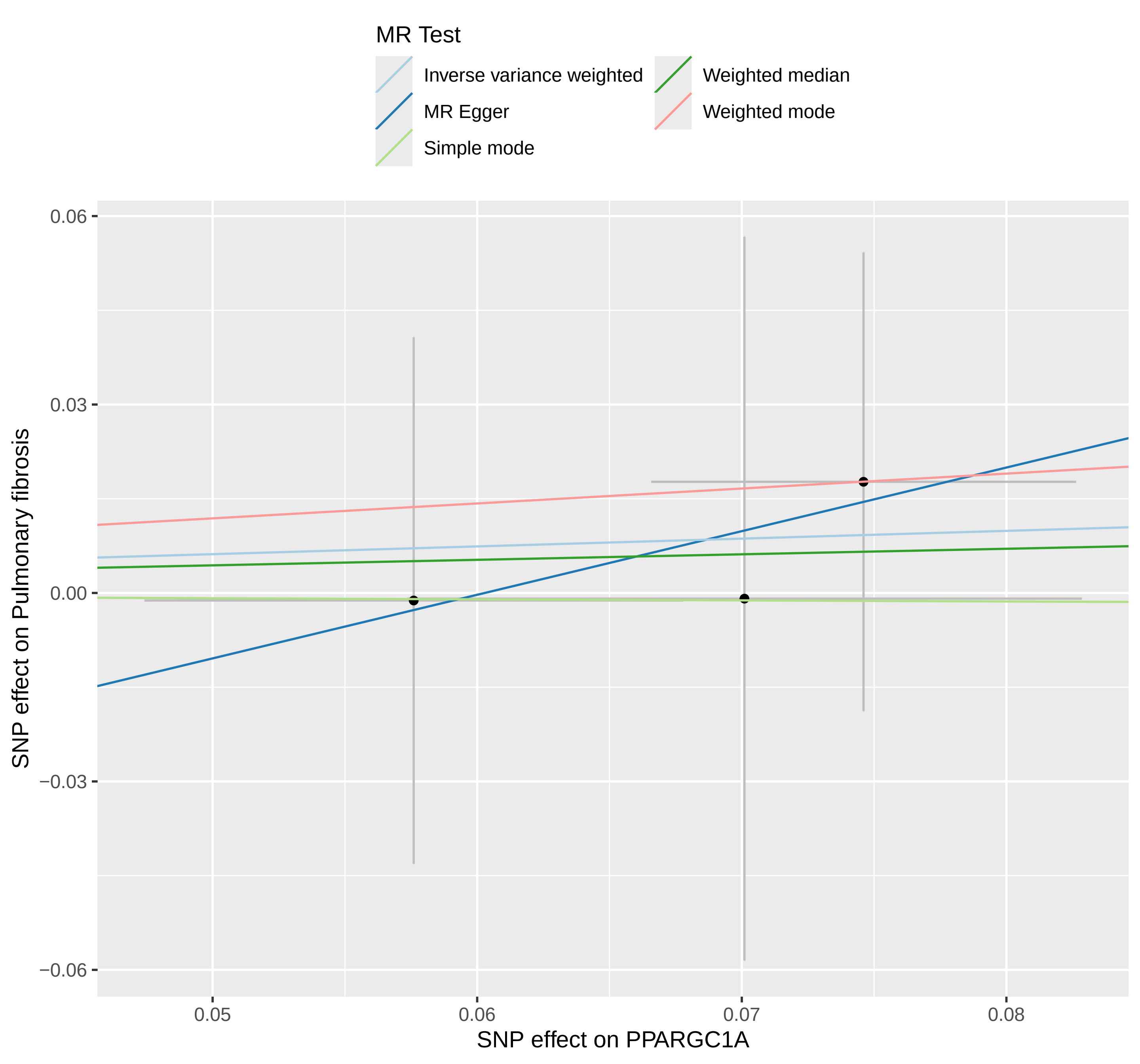

MR Test
Inverse variance weighted
MR Egger
Simple mode
Weighted median
Weighted mode
0.06
0.03
0.00
−0.03
−0.06
SNP effect on Pulmonary fibrosis
0.05
0.06
0.07
0.08
SNP effect on PPARGC1A

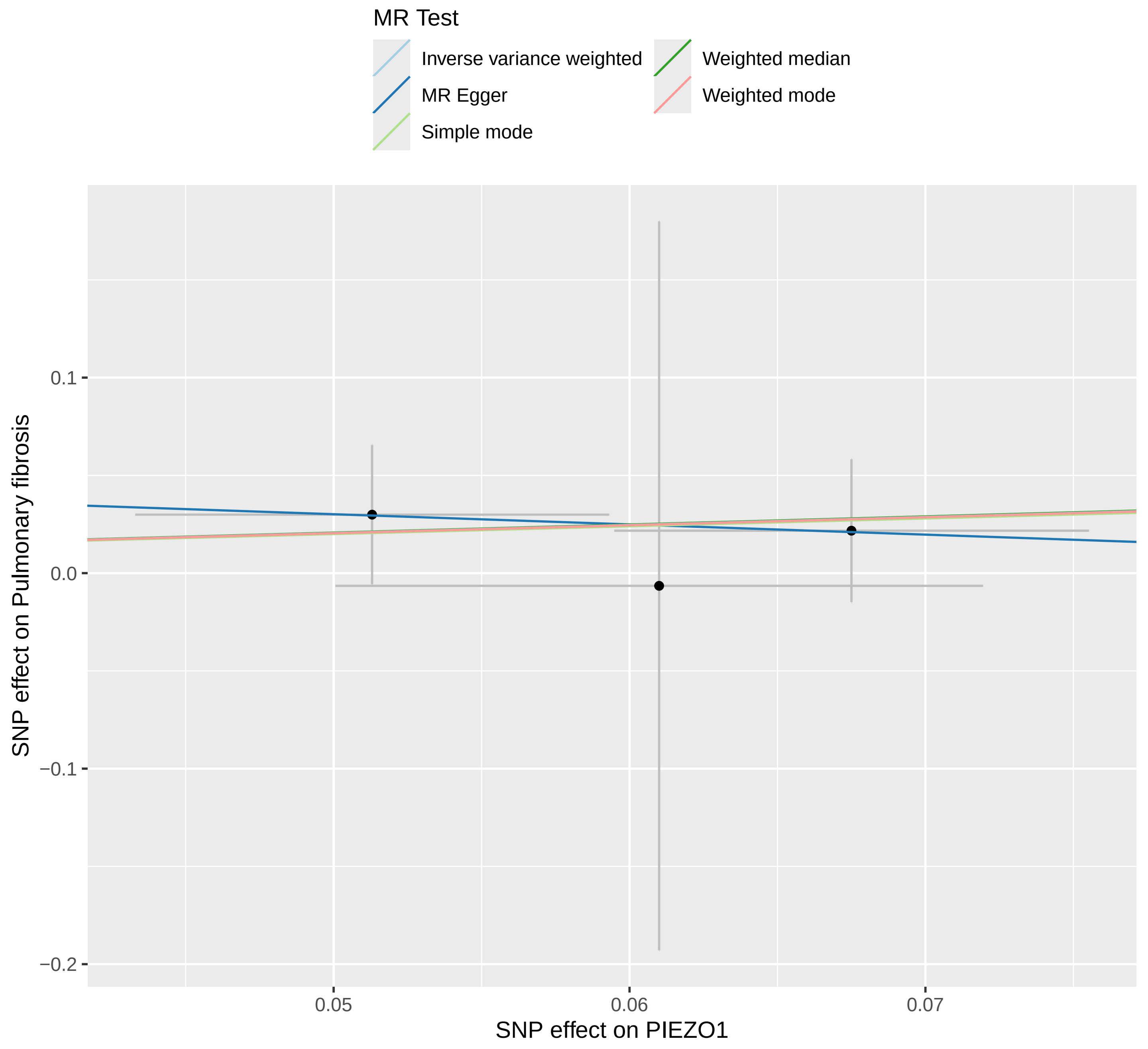

MR Test
Inverse variance weighted
MR Egger
Simple mode
Weighted median
Weighted mode
0.1
0.0
−0.1
−0.2
SNP effect on Pulmonary fibrosis
0.05
0.06
0.07
SNP effect on PIEZO1

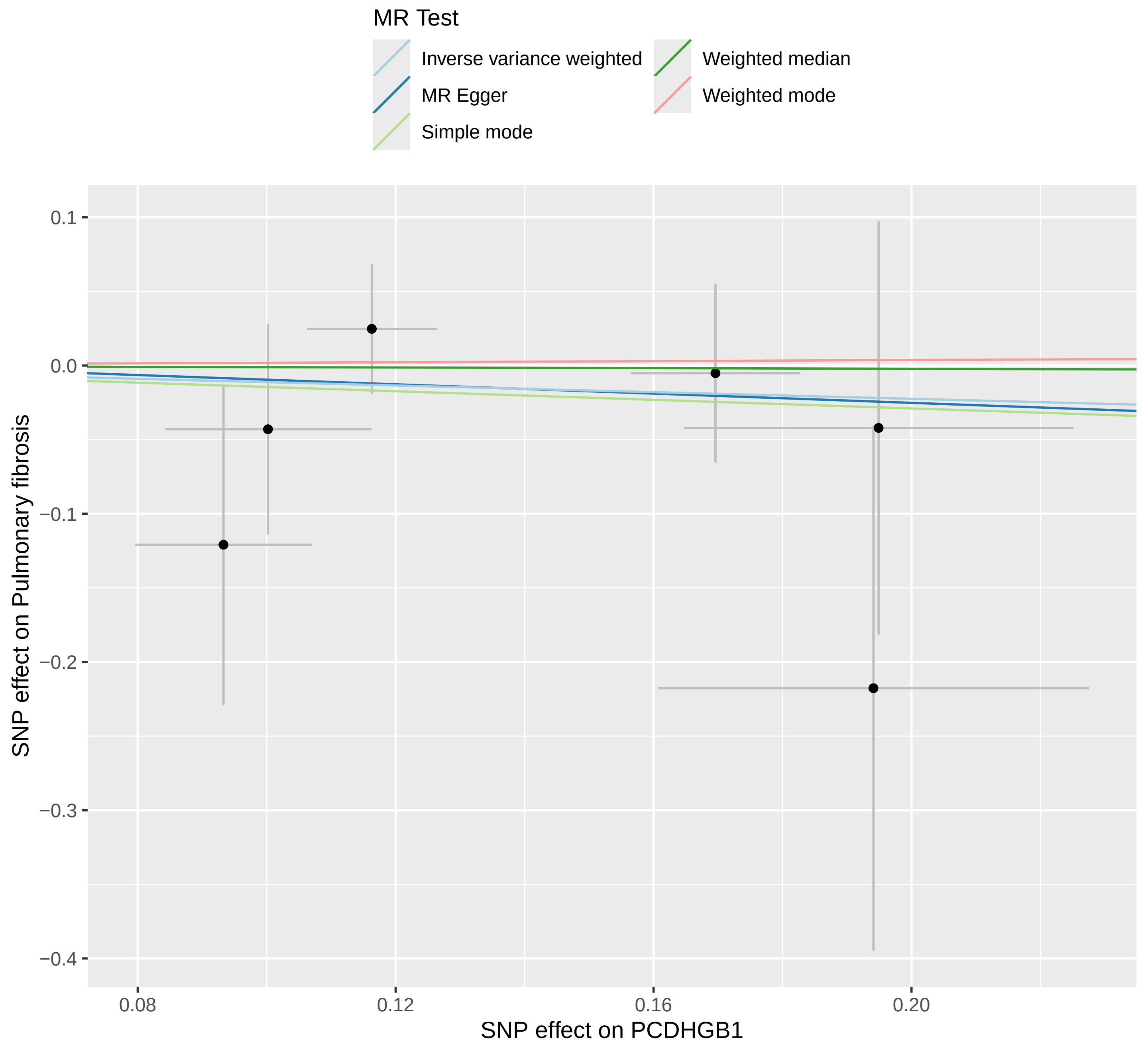
MR Test
Inverse variance weighted
MR Egger
Simple mode
Weighted median
Weighted mode
0.1
0.0
−0.1
−0.2
−0.3
−0.4
SNP effect on Pulmonary fibrosis
0.08
0.12
0.16
0.20
SNP effect on PCDHGB1

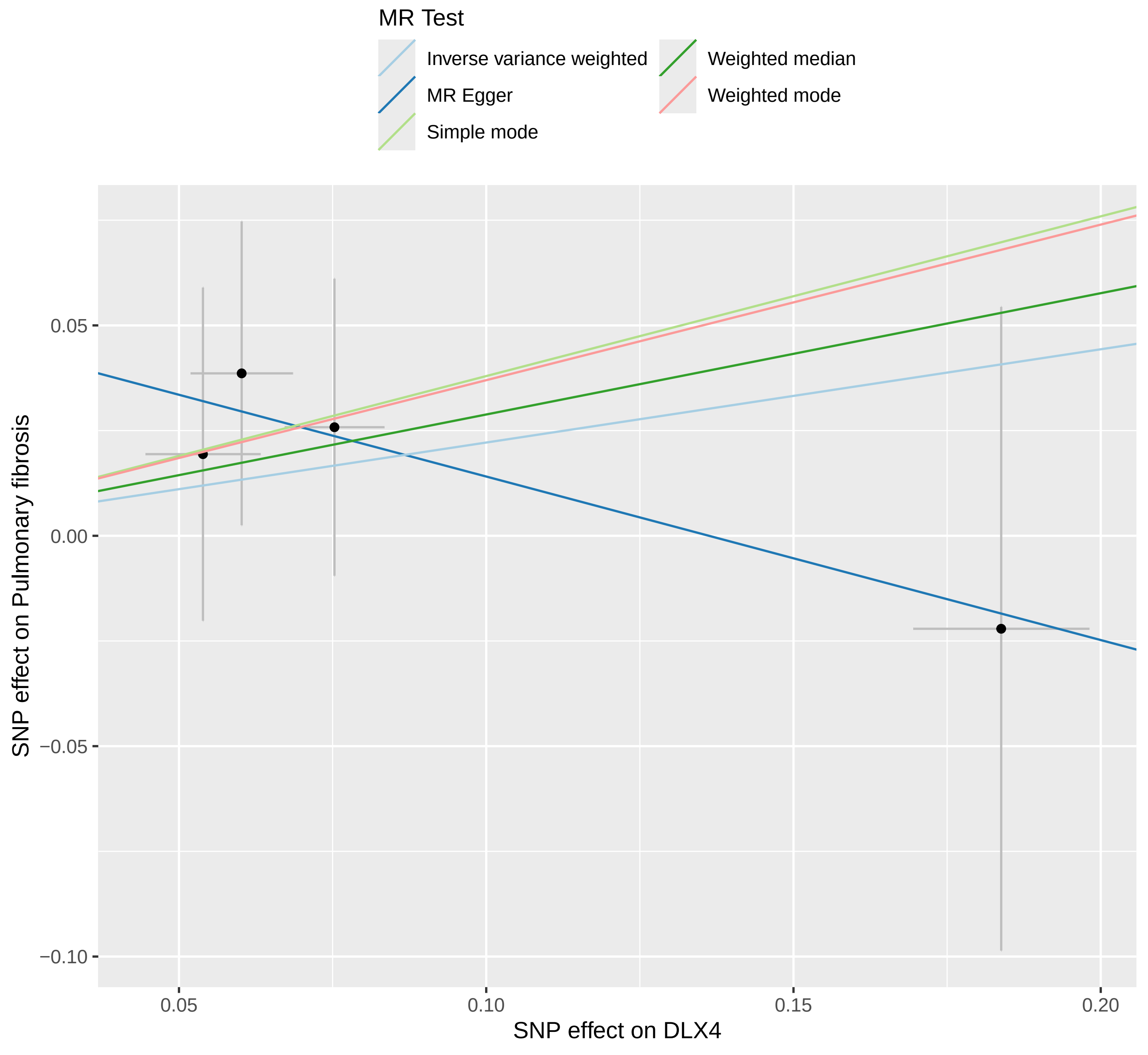
MR Test
Inverse variance weighted
MR Egger
Simple mode
Weighted median
Weighted mode
SNP effect on Pulmonary fibrosis
0.05
0.00
−0.05
−0.10
0.05
0.10
0.15
0.20
SNP effect on DLX4

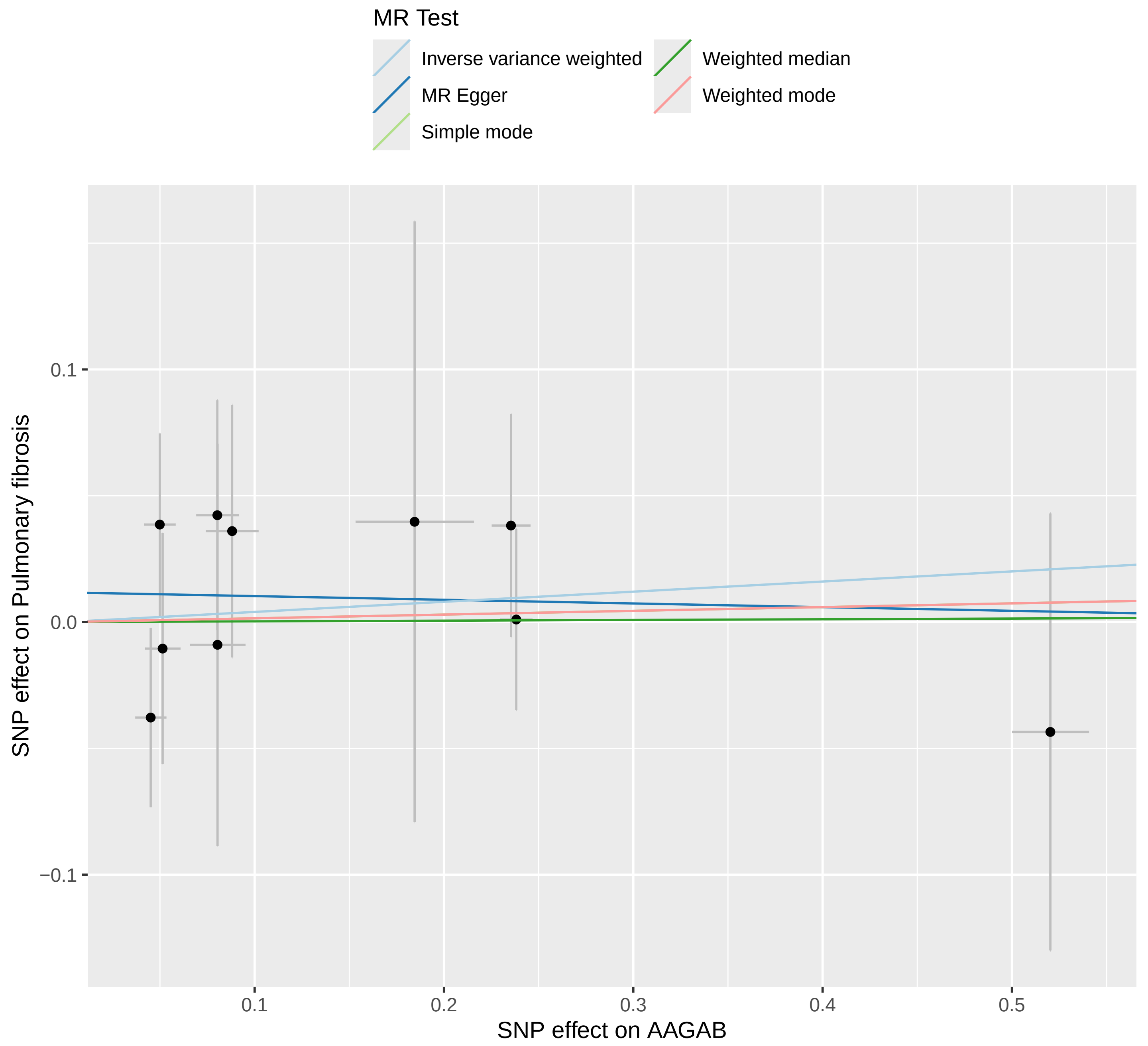
MR Test
Inverse variance weighted
MR Egger
Simple mode
Weighted median
Weighted mode
0.1
0.0
−0.1
SNP effect on Pulmonary fibrosis
0.1
0.2
0.3
0.4
0.5
SNP effect on AAGAB

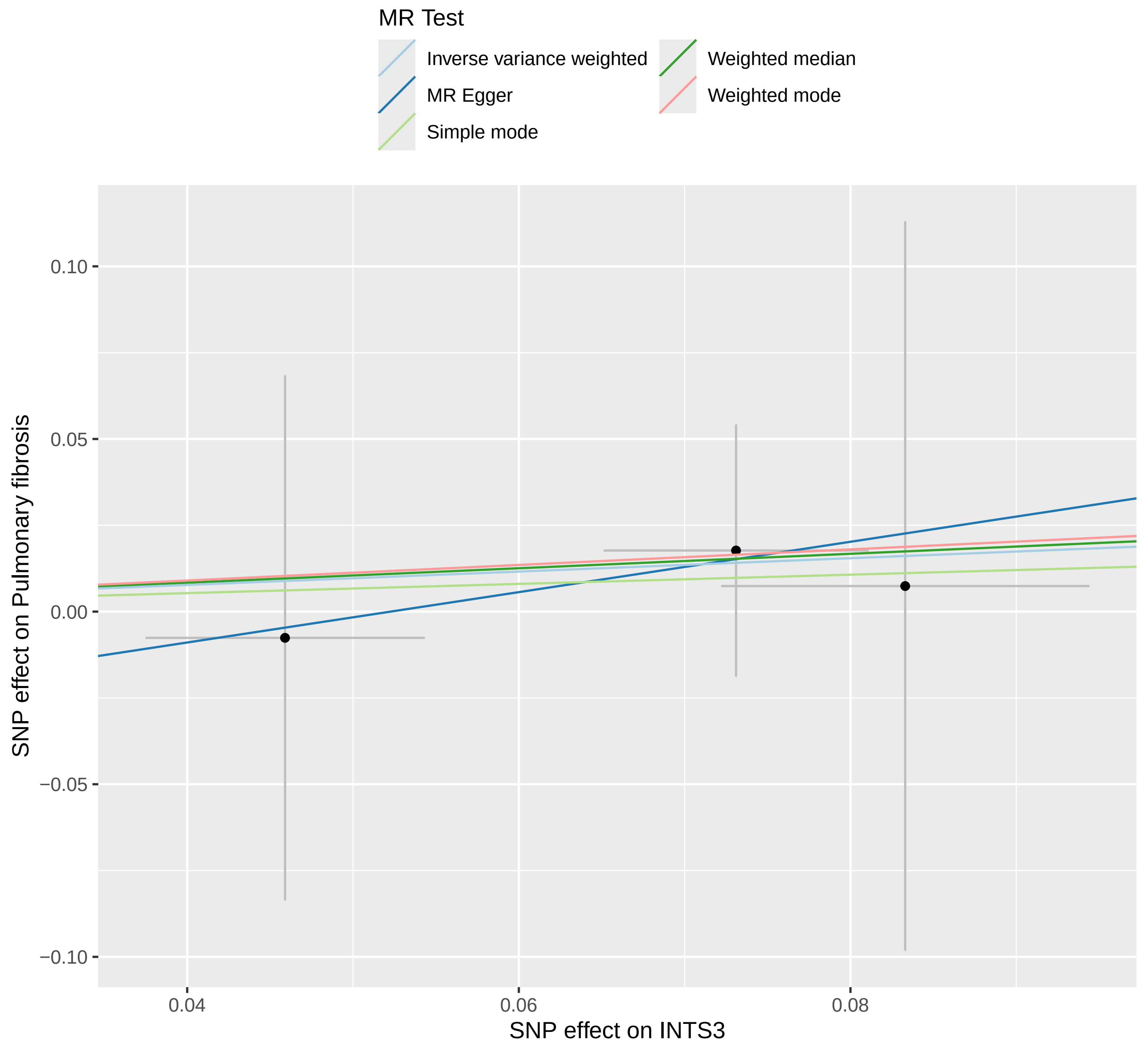
MR Test
Inverse variance weighted
MR Egger
Simple mode
Weighted median
Weighted mode
0.10
0.05
0.00
−0.05
−0.10
SNP effect on Pulmonary fibrosis
0.04
0.06
0.08
SNP effect on INTS3

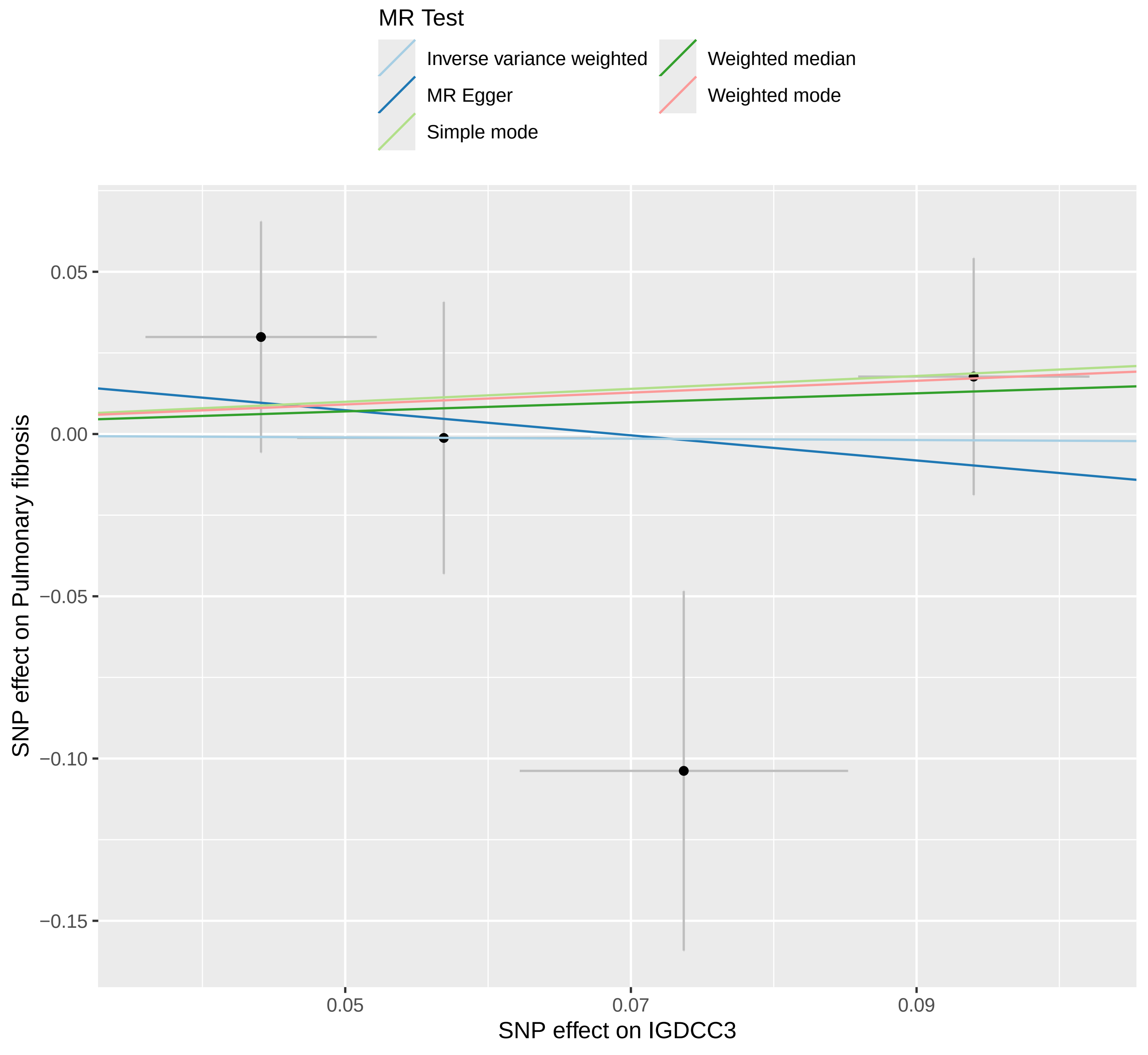

MR Test
Inverse variance weighted
MR Egger
Simple mode
Weighted median
Weighted mode
0.05
0.00
−0.05
−0.10
−0.15
SNP effect on Pulmonary fibrosis
0.05
0.07
0.09
SNP effect on IGDCC3

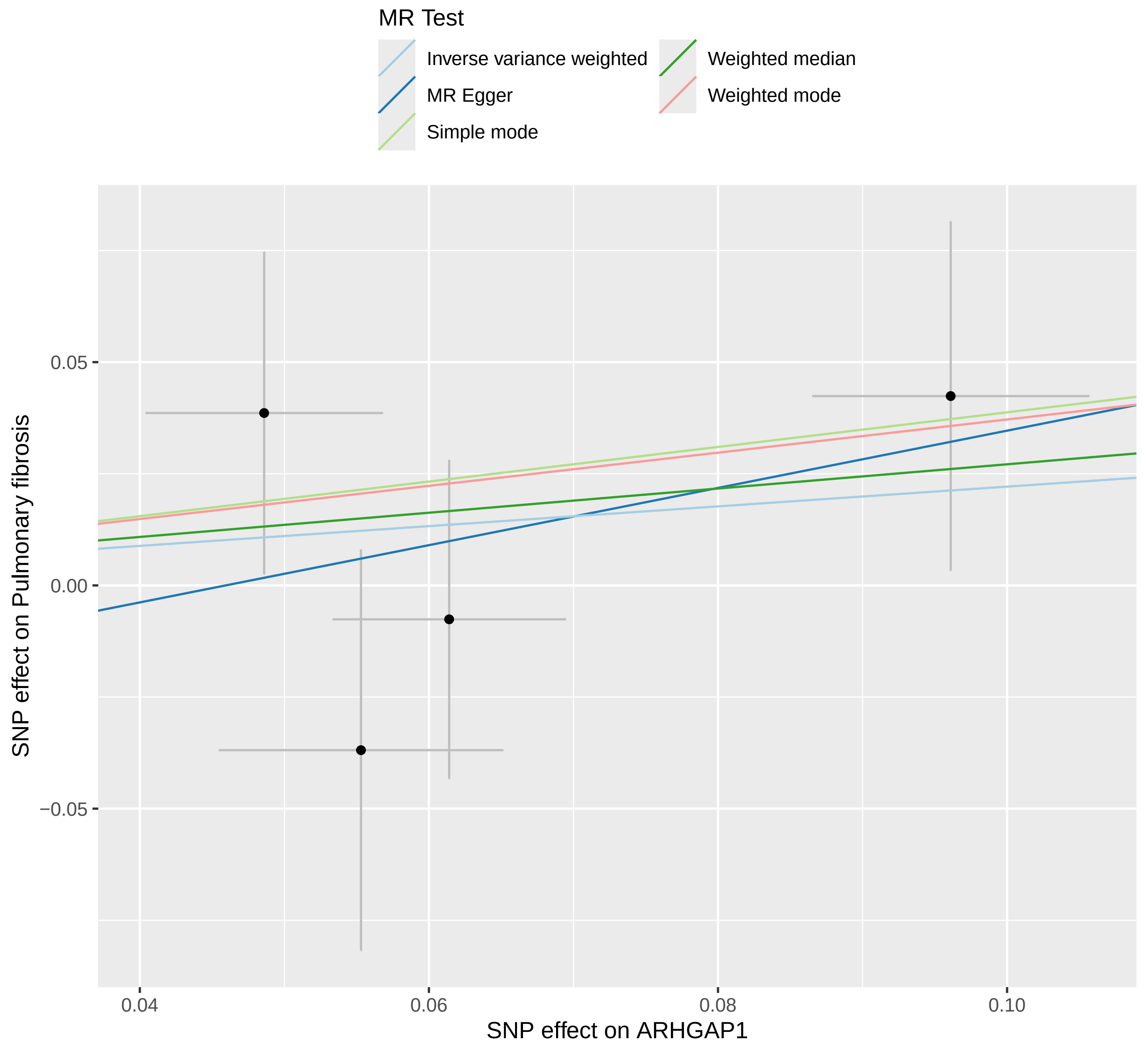
MR Test
Inverse variance weighted
MR Egger
Simple mode
Weighted median
Weighted mode
SNP effect on Pulmonary fibrosis
0.05
0.00
−0.05
0.04
0.06
0.08
0.10
SNP effect on ARHGAP1

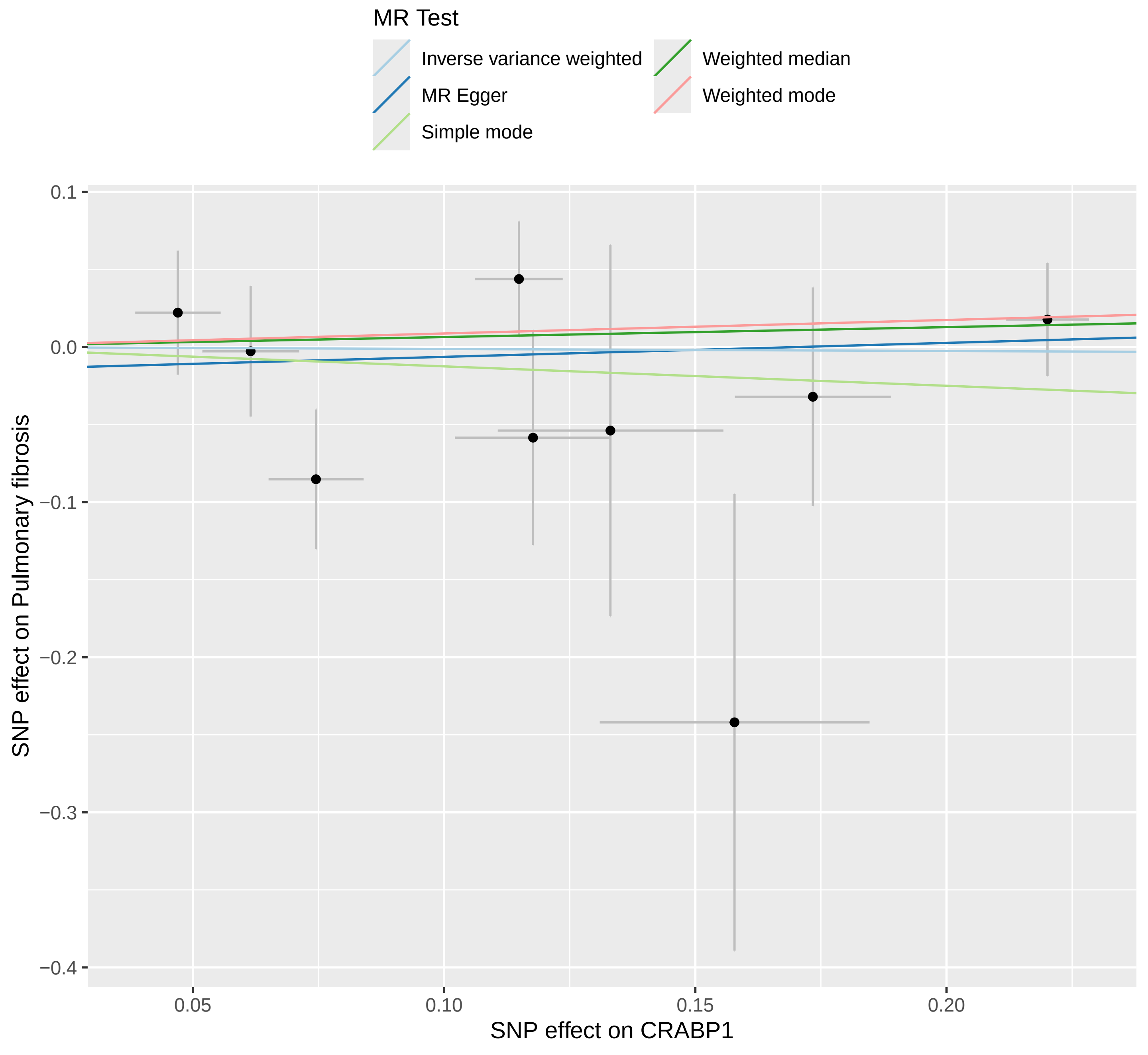

MR Test
Inverse variance weighted
MR Egger
Simple mode
Weighted median
Weighted mode
SNP effect on Pulmonary fibrosis
0.1
0.0
−0.1
−0.2
−0.3
−0.4
0.05
0.10
0.15
0.20
SNP effect on CRABP1

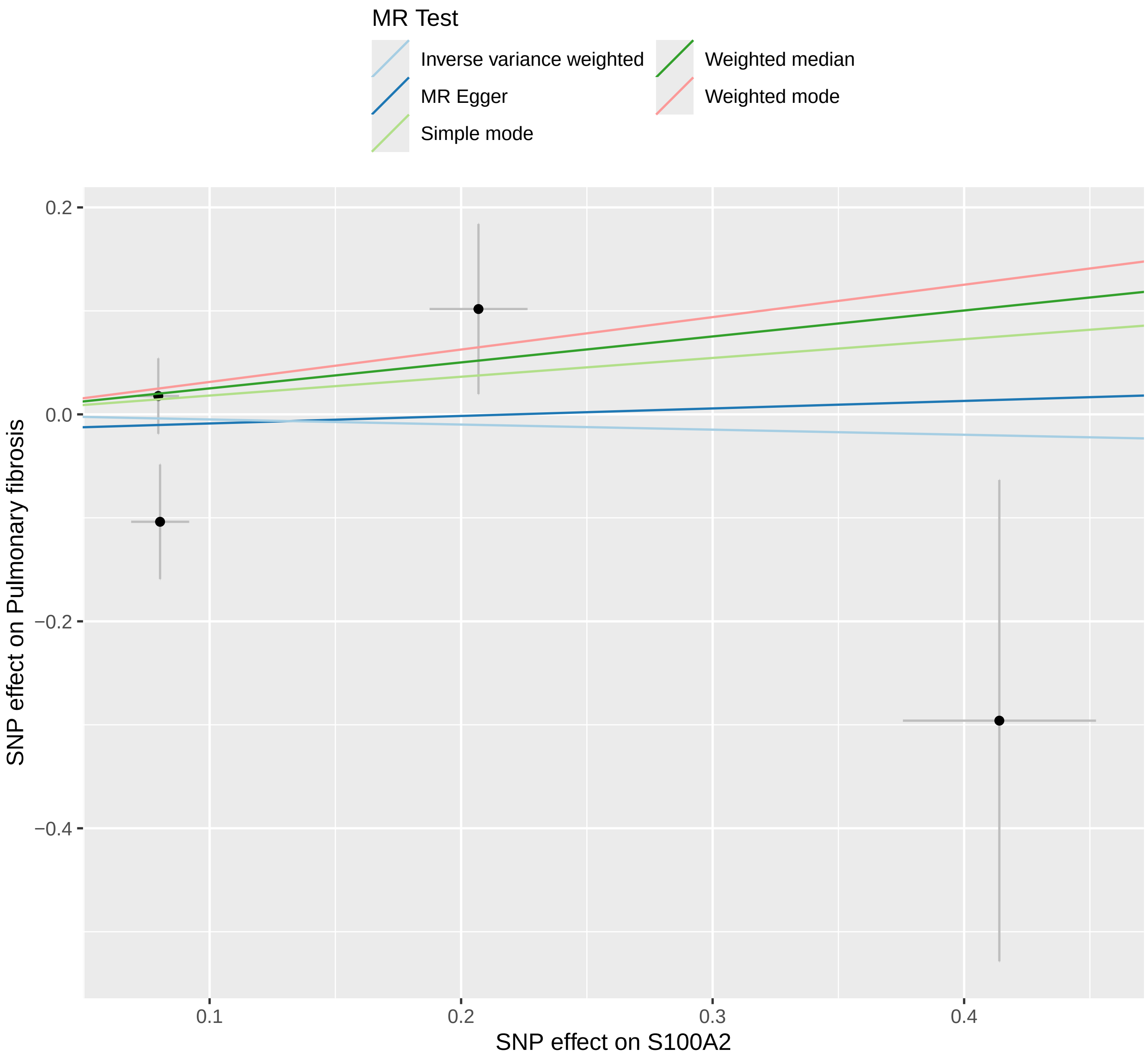
MR Test
Inverse variance weighted
MR Egger
Simple mode
Weighted median
Weighted mode
0.2
0.0
−0.2
−0.4
0.1
0.2
0.3
0.4
SNP effect on Pulmonary fibrosis
SNP effect on S100A2

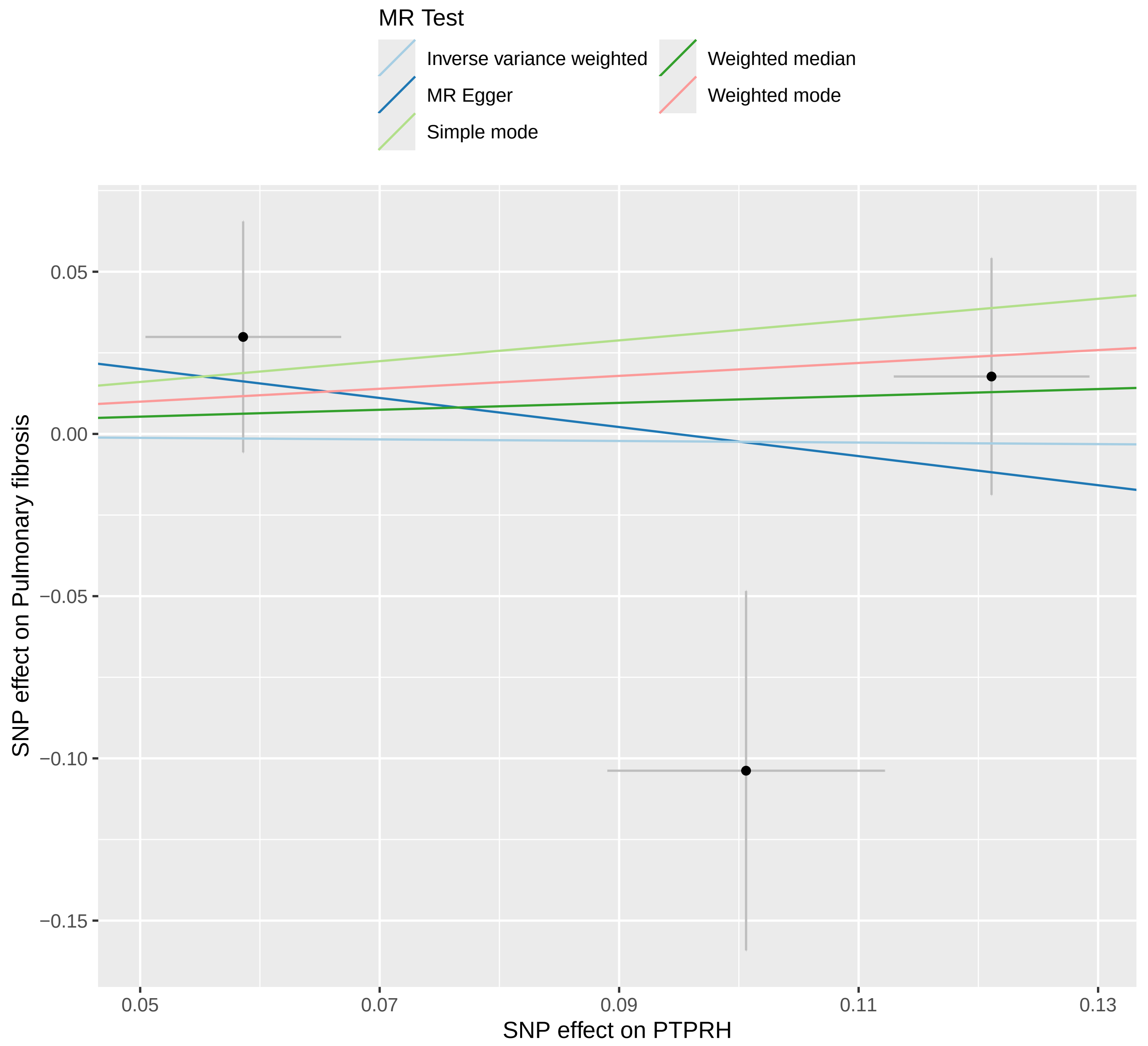
MR Test
Inverse variance weighted
MR Egger
Simple mode
Weighted median
Weighted mode
0.05
0.00
−0.05
−0.10
−0.15
SNP effect on Pulmonary fibrosis
0.05
0.07
0.09
0.11
0.13
SNP effect on PTPRH

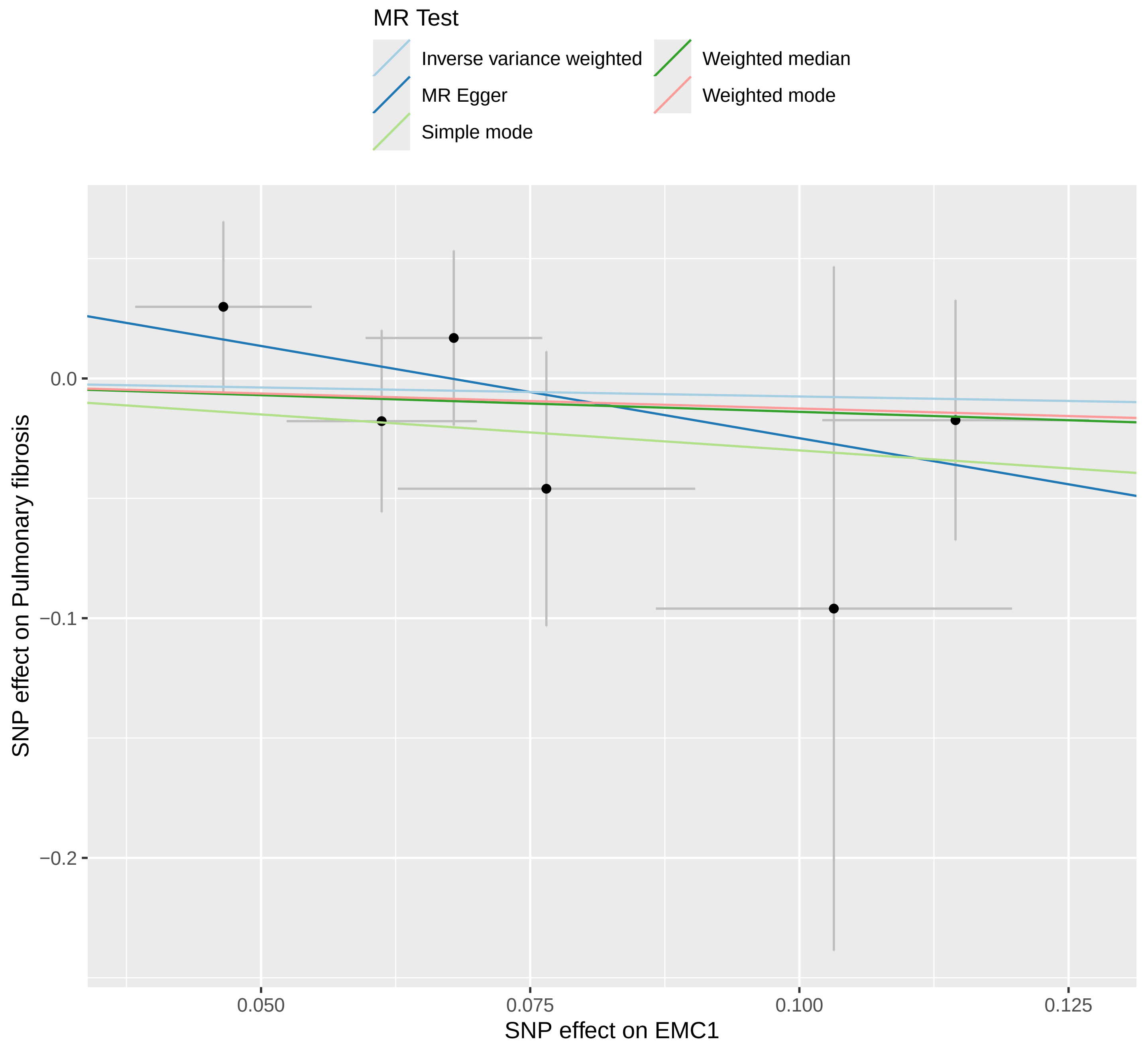

MR Test
Inverse variance weighted
MR Egger
Simple mode
Weighted median
Weighted mode
0.0
−0.1
−0.2
SNP effect on Pulmonary fibrosis
0.050
0.075
0.100
0.125
SNP effect on EMC1

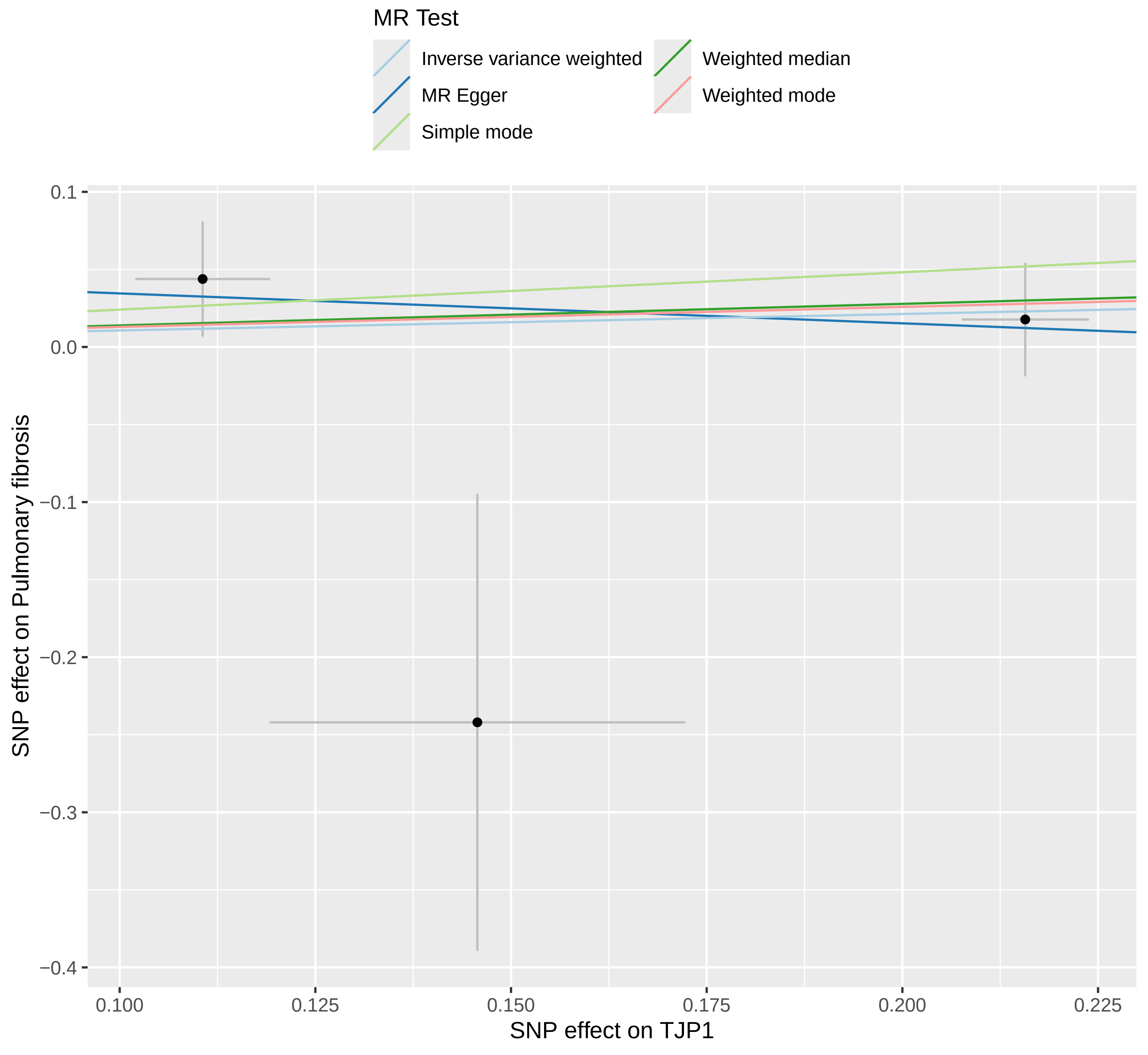
MR Test
Inverse variance weighted
MR Egger
Simple mode
Weighted median
Weighted mode
0.1
0.0
−0.1
−0.2
−0.3
−0.4
SNP effect on Pulmonary fibrosis
0.100
0.125
0.150
0.175
0.200
0.225
SNP effect on TJP1

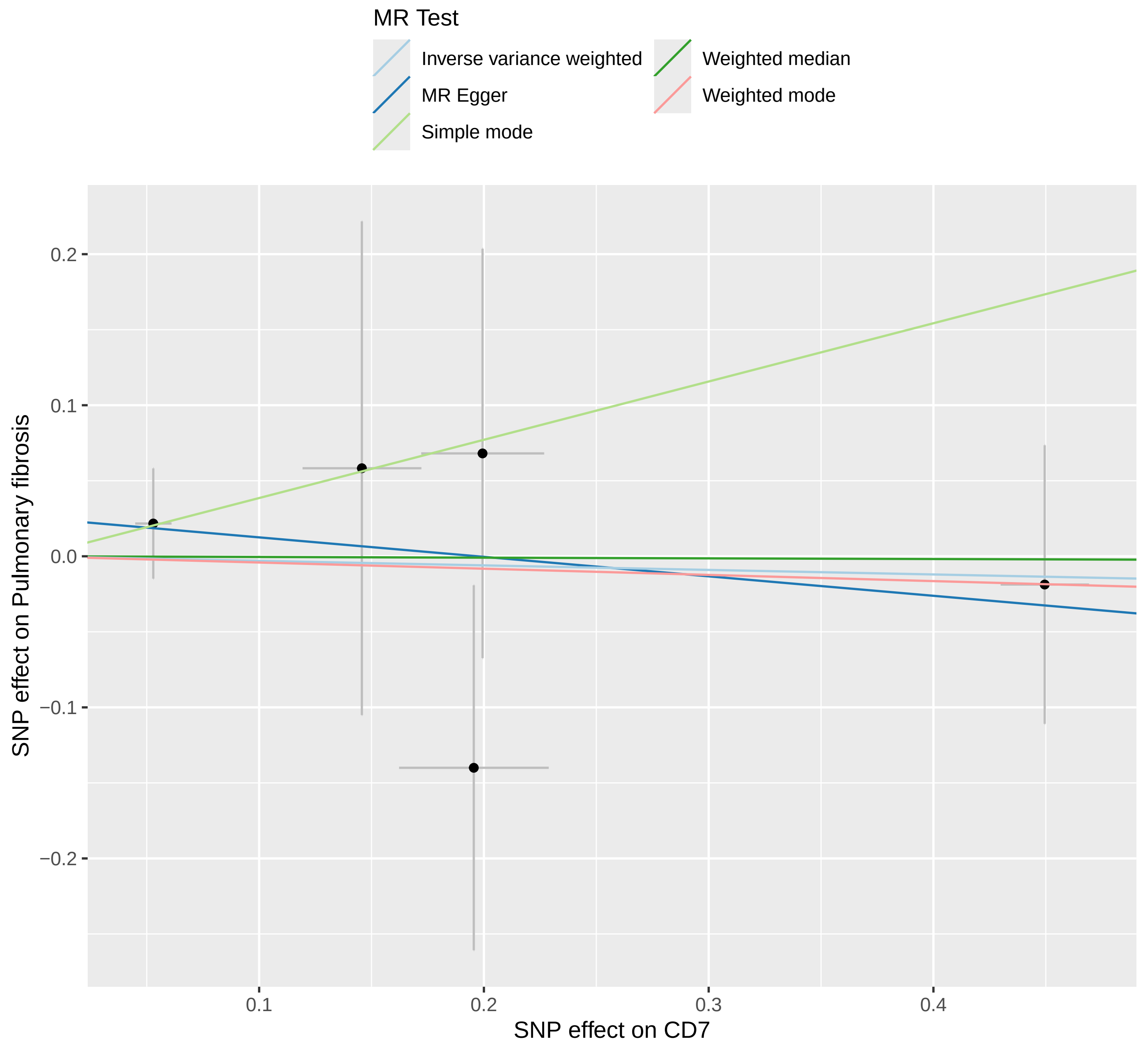

MR Test
Inverse variance weighted
MR Egger
Simple mode
Weighted median
Weighted mode
0.2
0.1
0.0
−0.1
−0.2
SNP effect on Pulmonary fibrosis
0.1
0.2
0.3
0.4
SNP effect on CD7

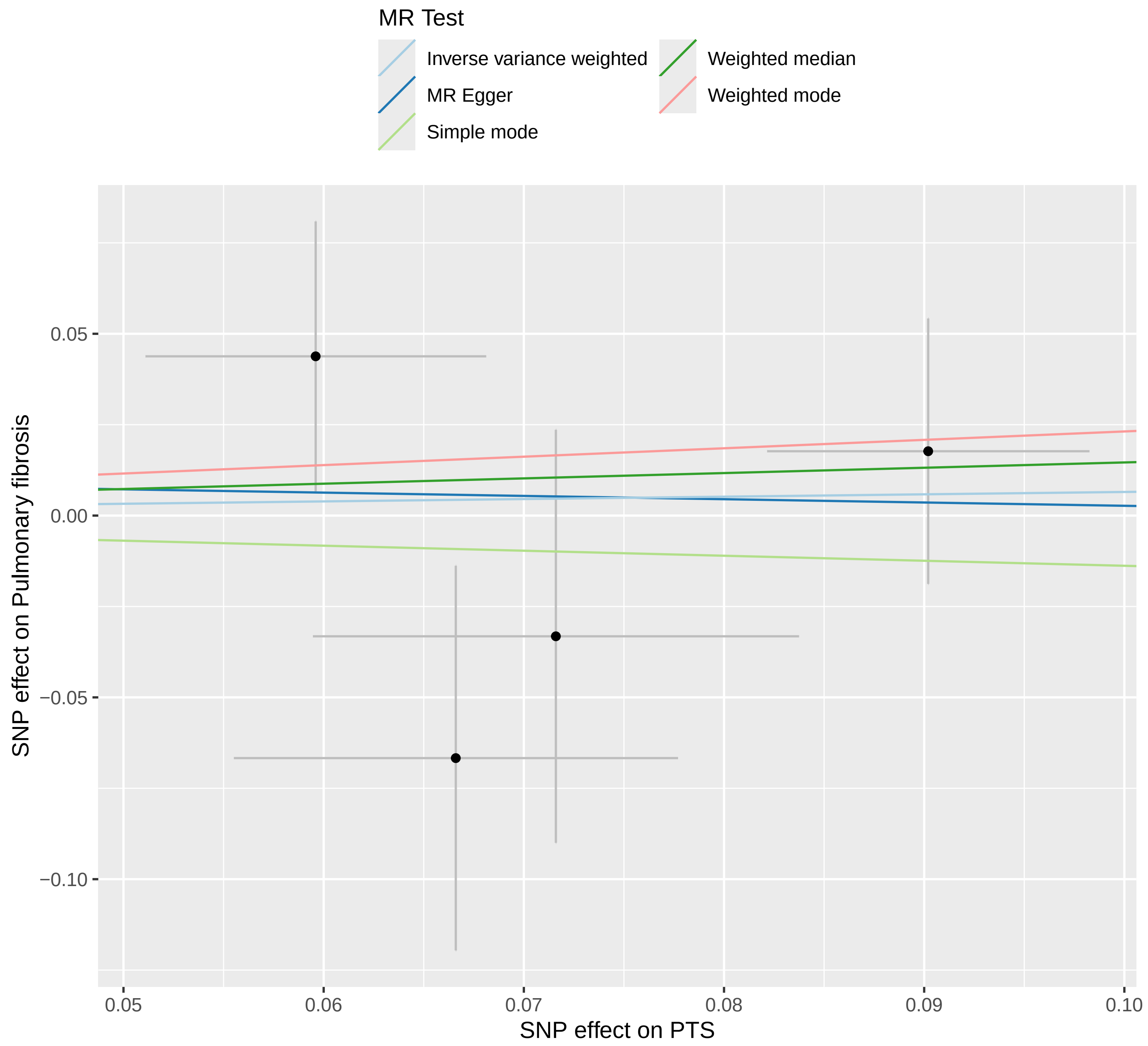

MR Test
Inverse variance weighted
MR Egger
Simple mode
Weighted median
Weighted mode
0.05
0.00
−0.05
−0.10
SNP effect on Pulmonary fibrosis
0.05
0.06
0.07
0.08
0.09
0.10
SNP effect on PTS

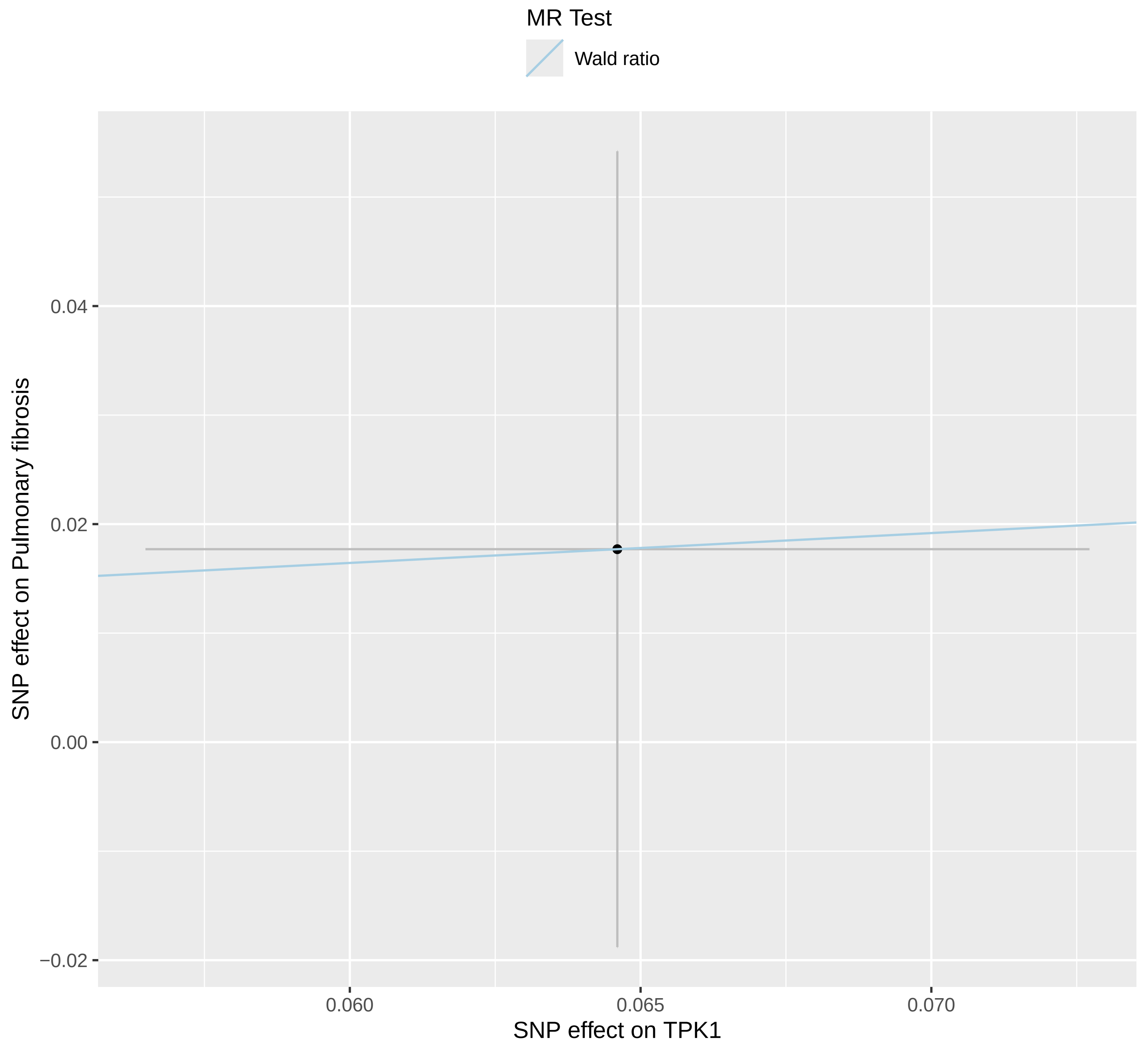
MR Test
Wald ratio
0.04
0.02
0.00
−0.02
SNP effect on Pulmonary fibrosis
0.060
0.065
0.070
SNP effect on TPK1

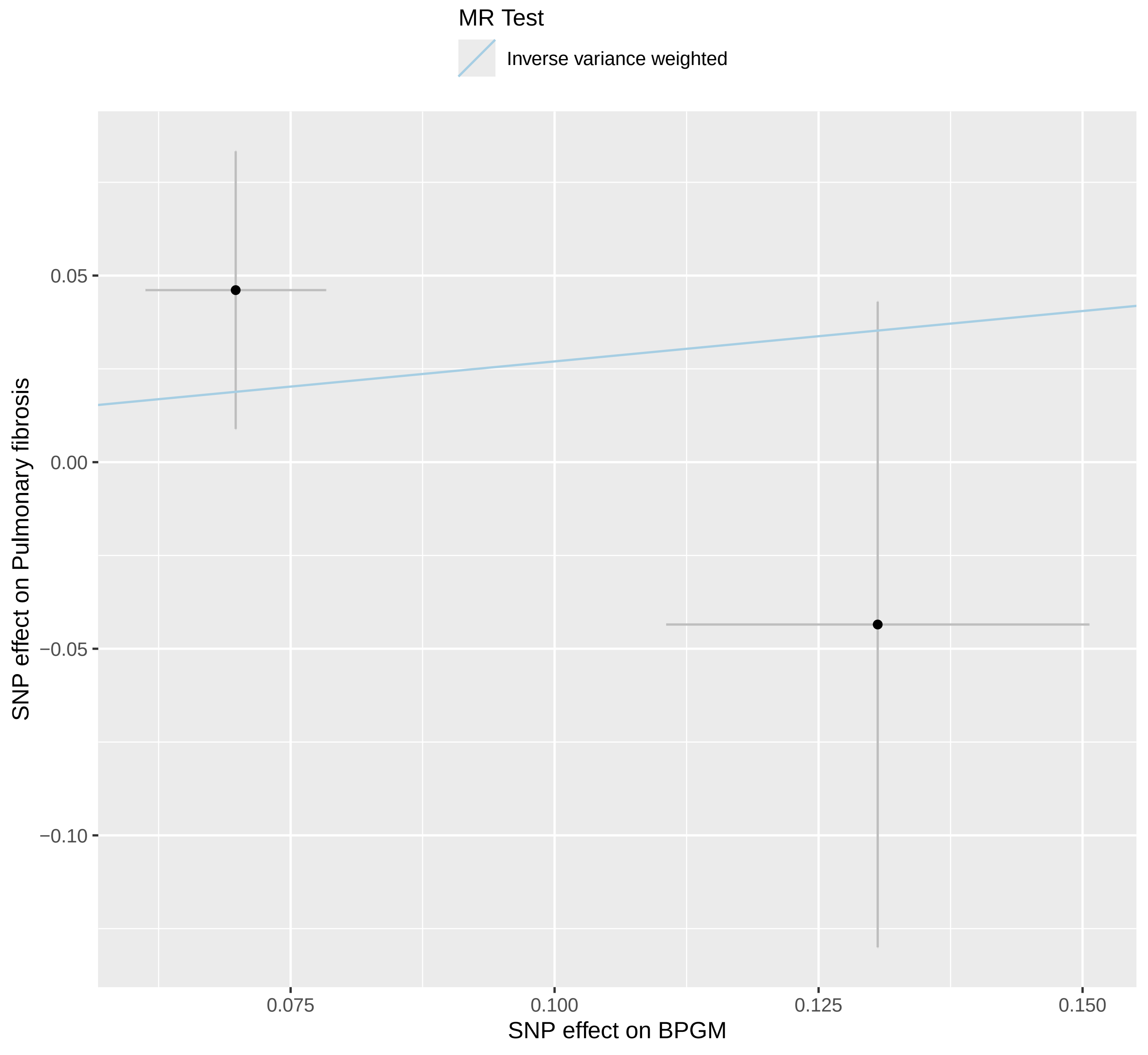

MR Test
Inverse variance weighted
0.05
0.00
−0.05
−0.10
0.075
0.100
0.125
0.150
SNP effect on BPGM
SNP effect on Pulmonary fibrosis

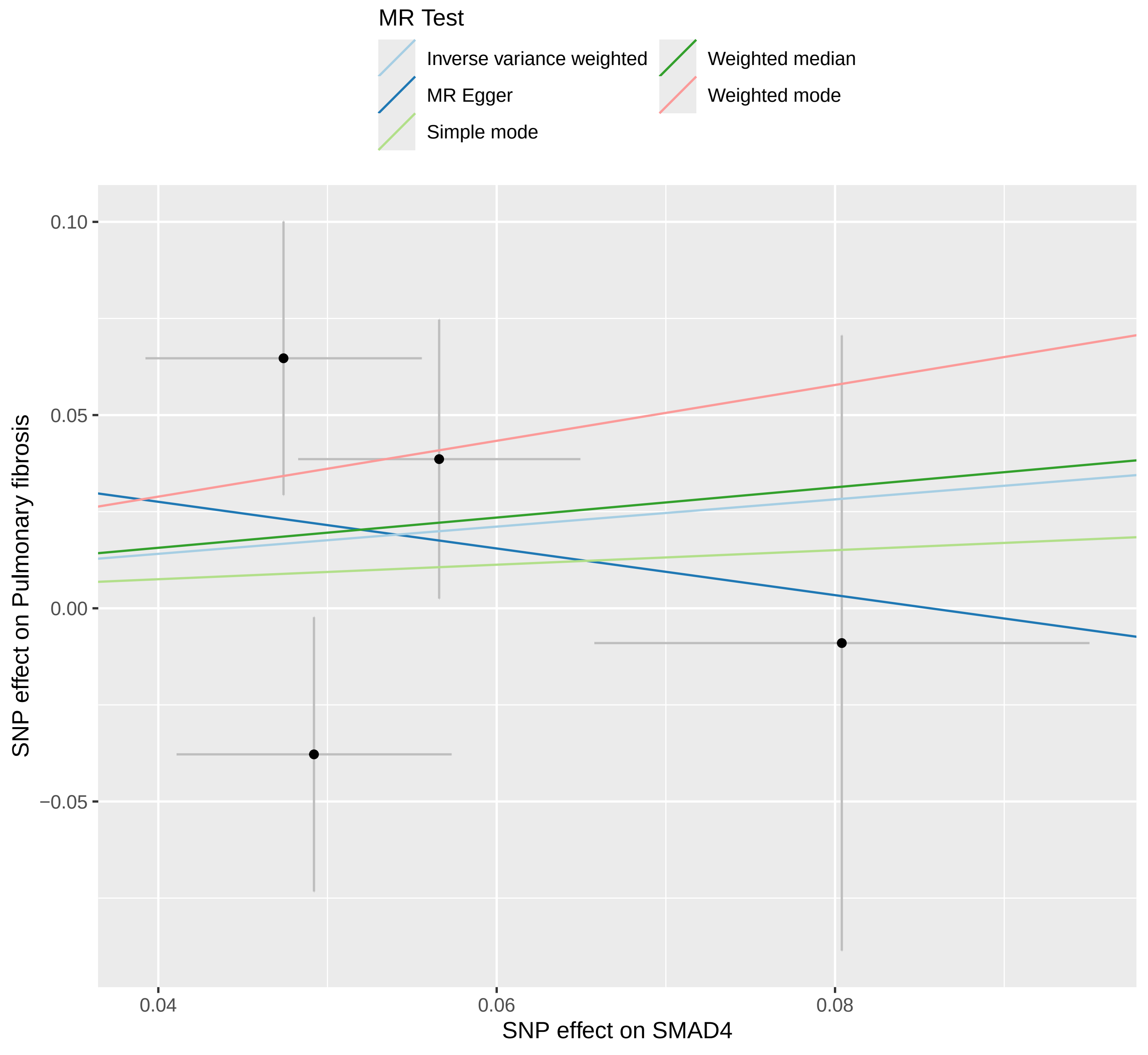
MR Test
Inverse variance weighted
MR Egger
Simple mode
Weighted median
Weighted mode
SNP effect on Pulmonary fibrosis
0.10
0.05
0.00
−0.05
0.04
0.06
0.08
SNP effect on SMAD4

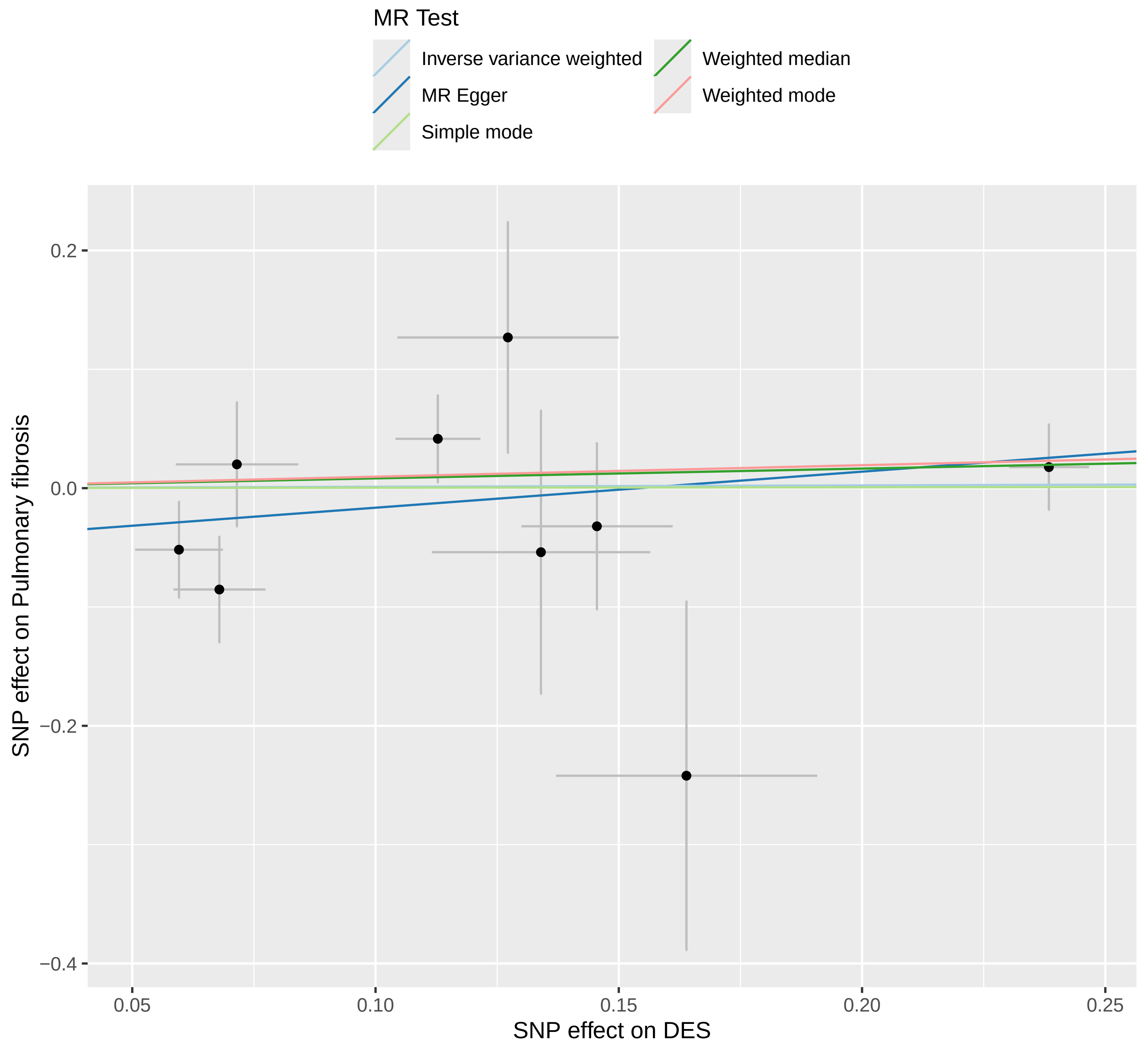
MR Test
Inverse variance weighted
MR Egger
Simple mode
Weighted median
Weighted mode
0.2
0.0
−0.2
−0.4
0.05
0.10
0.15
0.20
0.25
SNP effect on Pulmonary fibrosis
SNP effect on DES

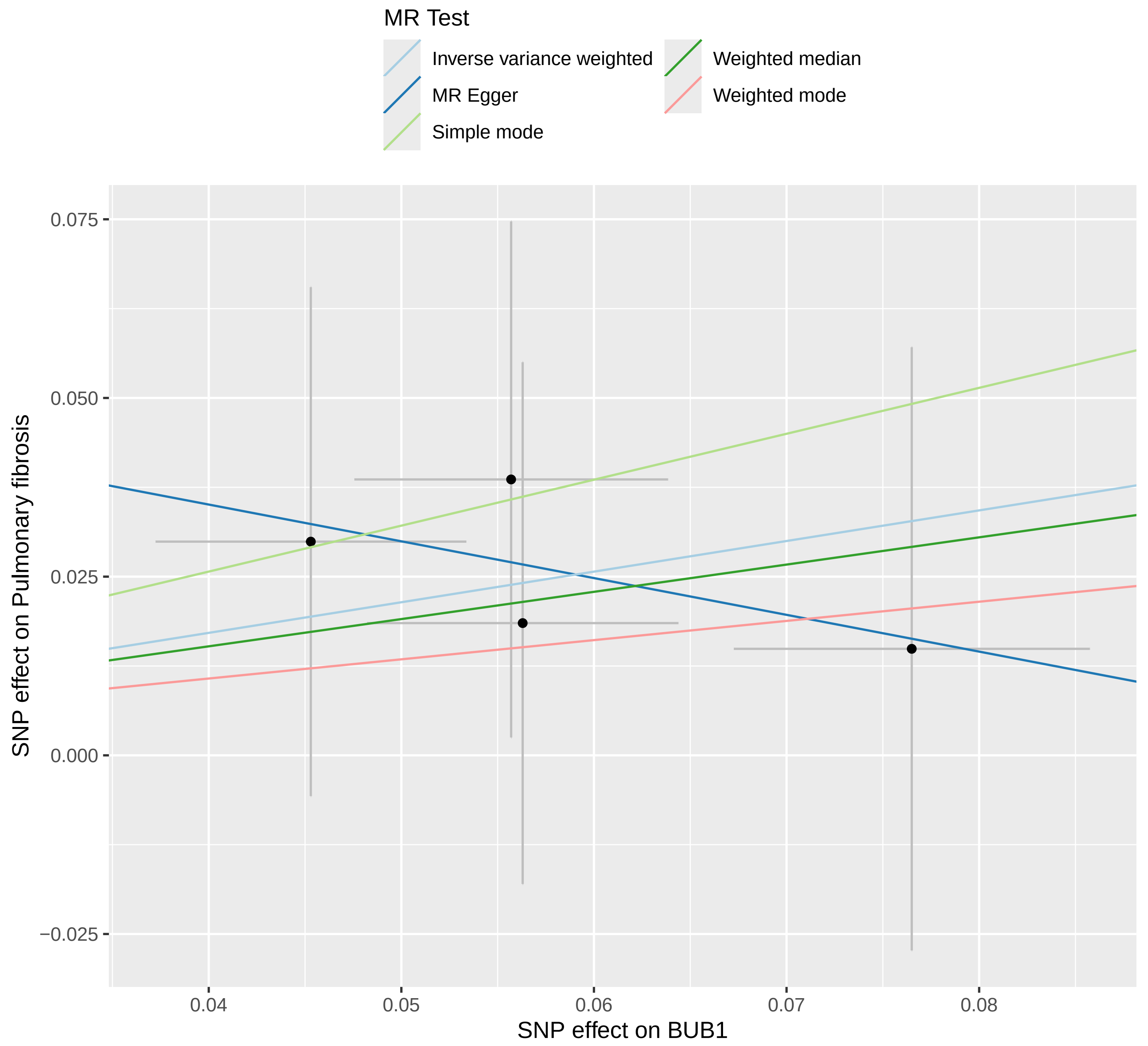
MR Test
Inverse variance weighted
MR Egger
Simple mode
Weighted median
Weighted mode
0.075
0.050
0.025
0.000
−0.025
SNP effect on Pulmonary fibrosis
0.04
0.05
0.06
0.07
0.08
SNP effect on BUB1

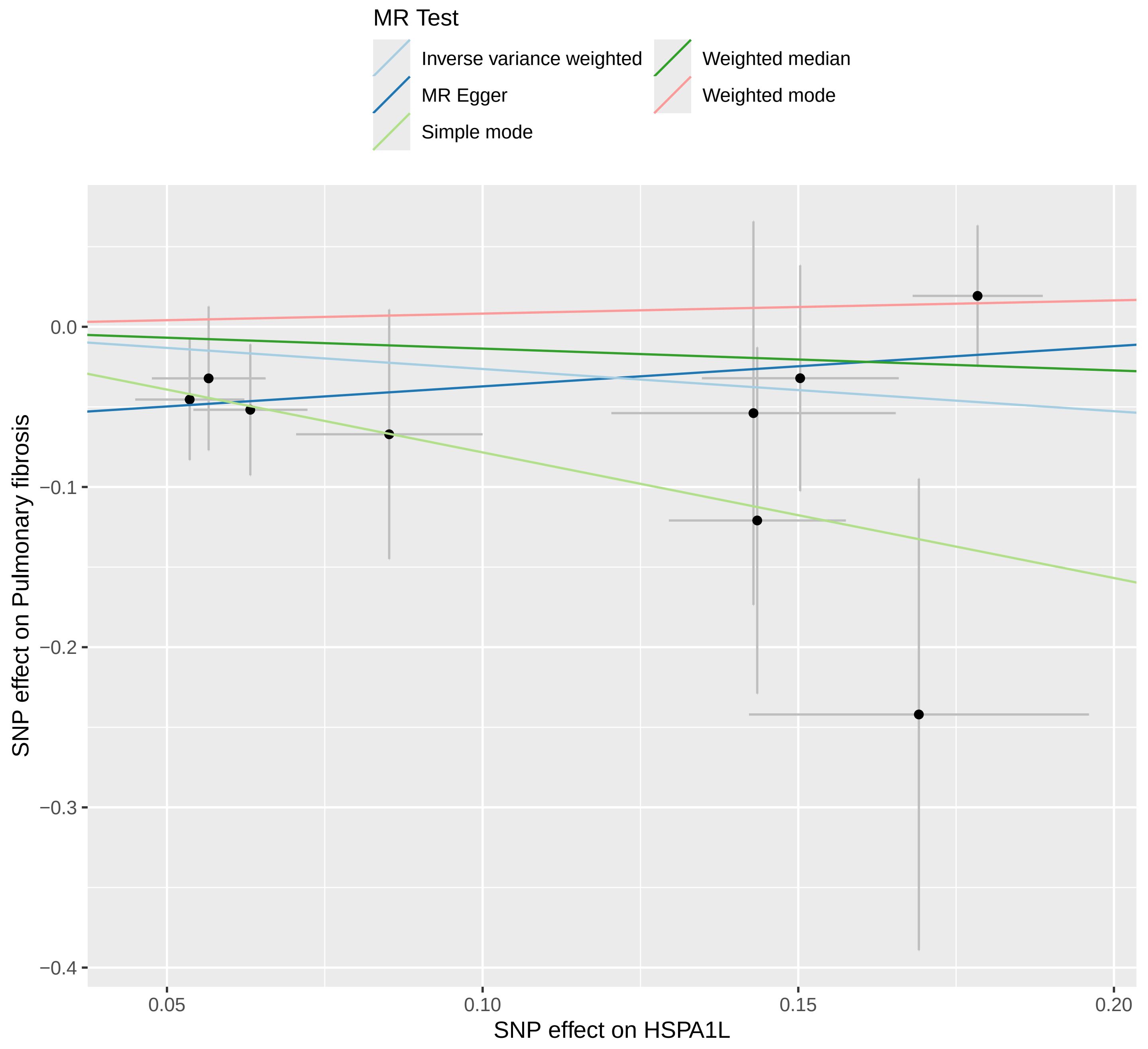

MR Test
Inverse variance weighted
MR Egger
Simple mode
Weighted median
Weighted mode
0.0
−0.1
−0.2
−0.3
−0.4
SNP effect on Pulmonary fibrosis
0.05
0.10
0.15
0.20
SNP effect on HSPA1L

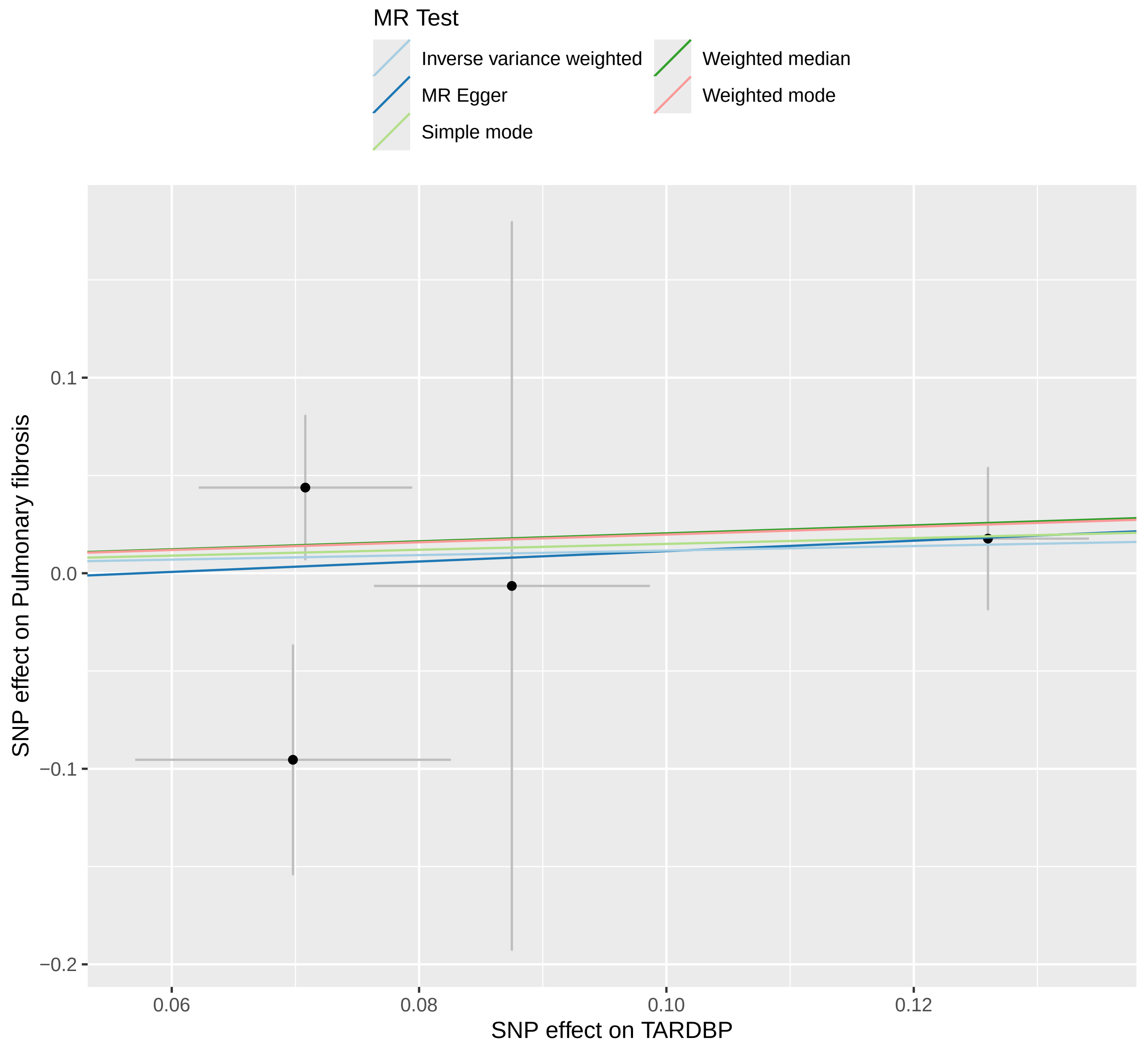
MR Test
Inverse variance weighted
MR Egger
Simple mode
Weighted median
Weighted mode
0.1
0.0
−0.1
−0.2
SNP effect on Pulmonary fibrosis
0.06
0.08
0.10
0.12
SNP effect on TARDBP

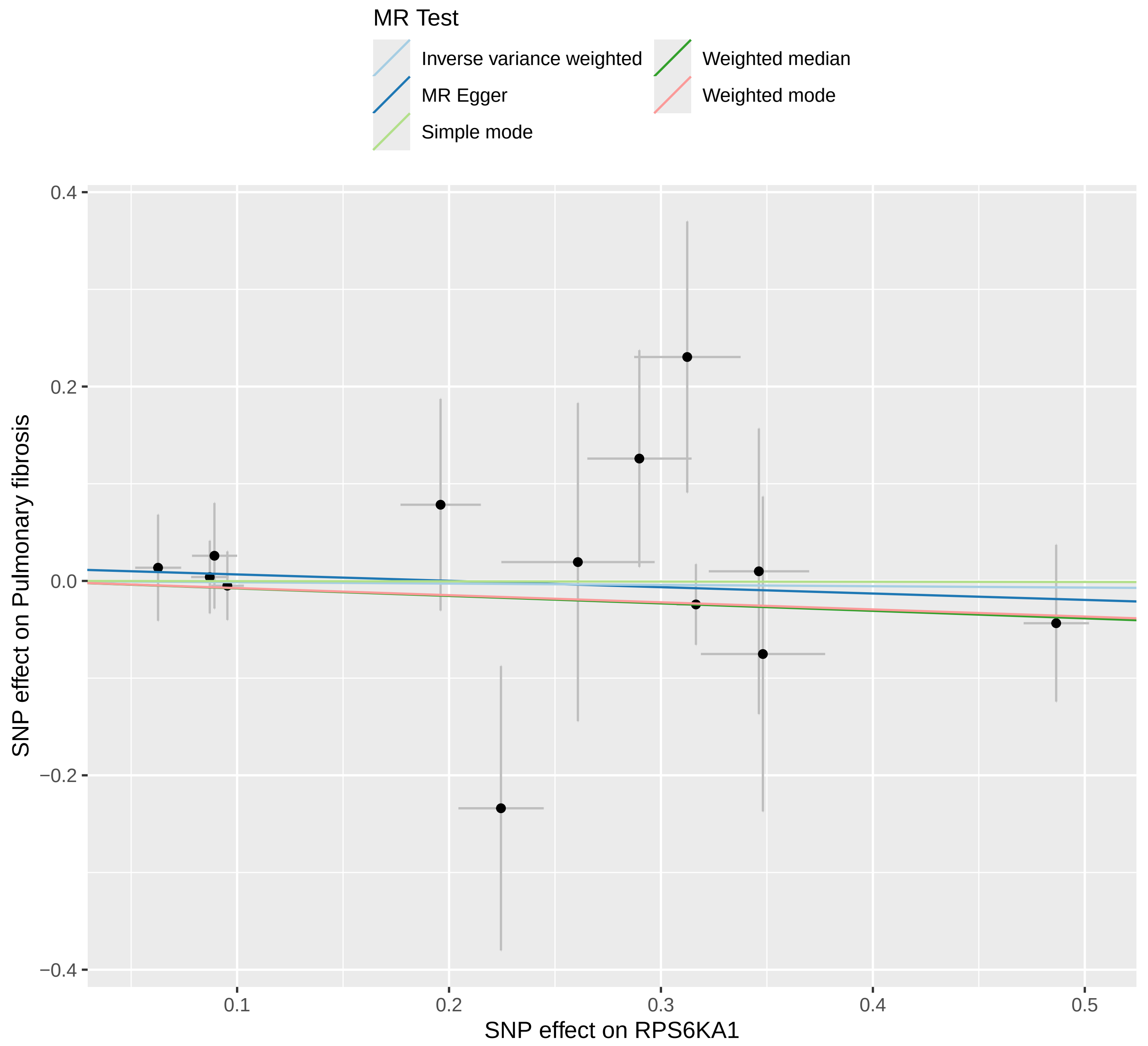
MR Test
Inverse variance weighted
MR Egger
Simple mode
Weighted median
Weighted mode
0.4
0.2
0.0
−0.2
−0.4
SNP effect on Pulmonary fibrosis
0.1
0.2
0.3
0.4
0.5
SNP effect on RPS6KA1

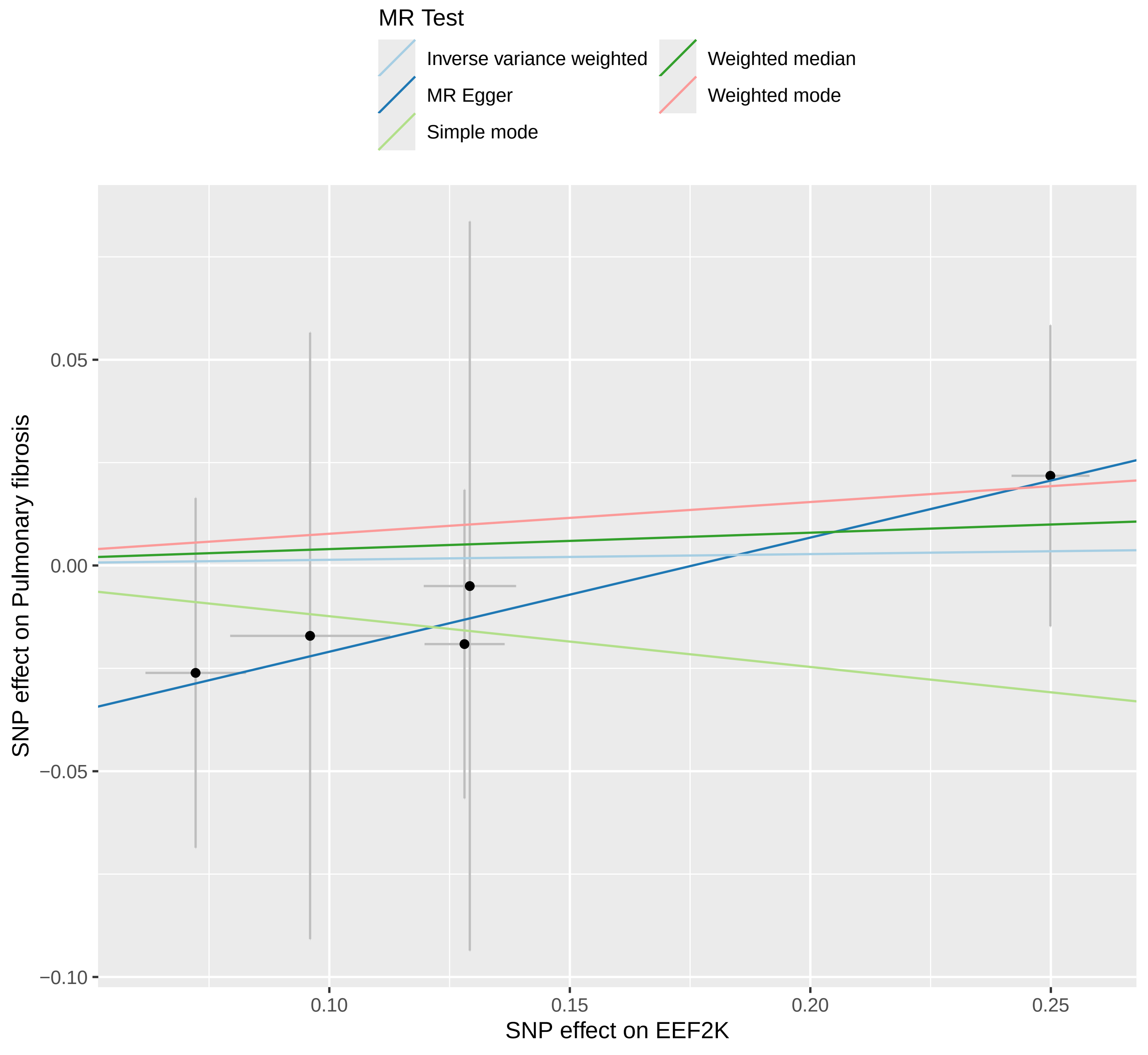
MR Test
Inverse variance weighted
MR Egger
Simple mode
Weighted median
Weighted mode
0.05
0.00
−0.05
−0.10
0.10
0.15
0.20
0.25
SNP effect on EEF2K
SNP effect on Pulmonary fibrosis

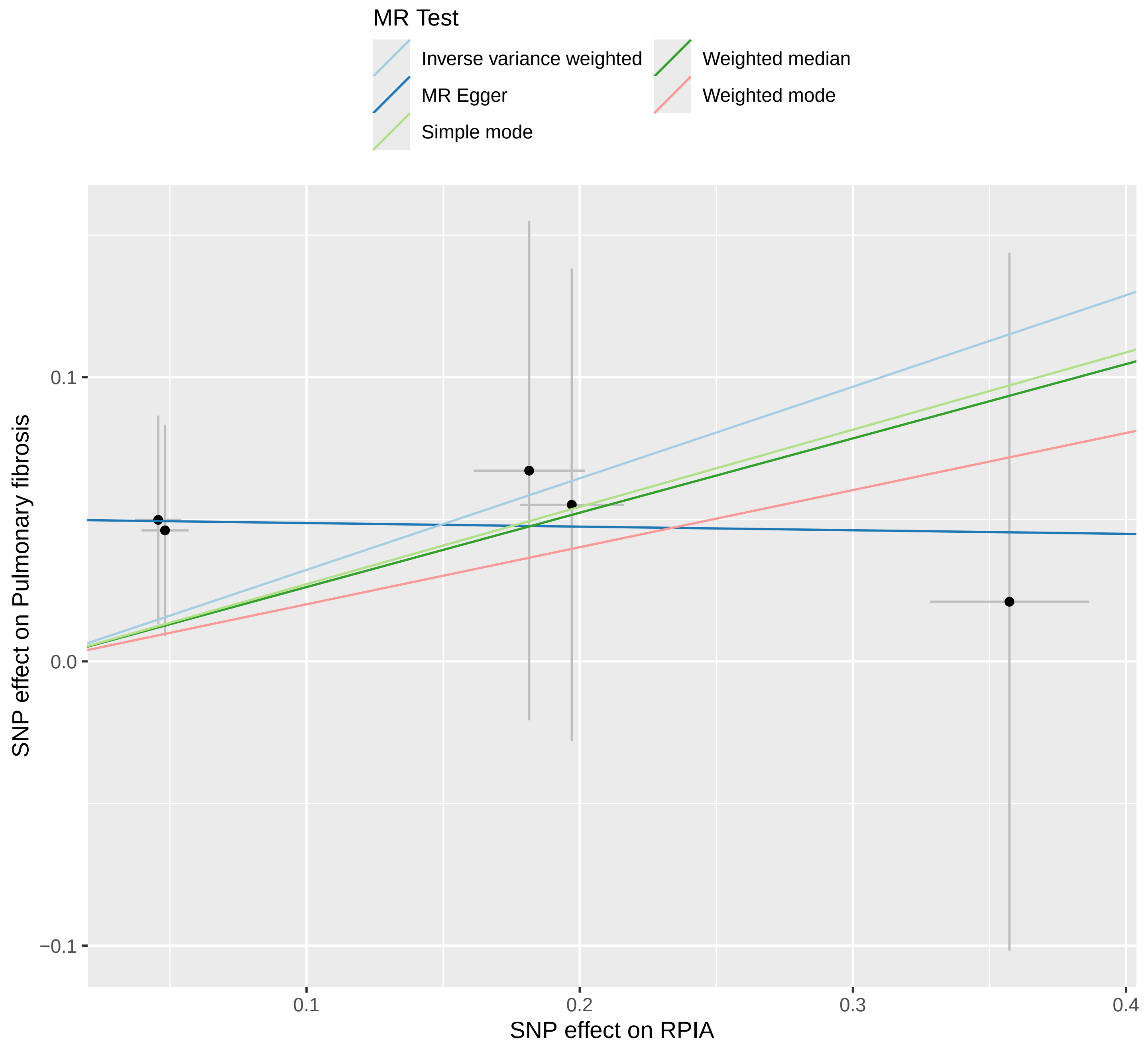
MR Test
Inverse variance weighted
MR Egger
Simple mode
Weighted median
Weighted mode
0.1
0.0
−0.1
SNP effect on Pulmonary fibrosis
0.1
0.2
0.3
0.4
SNP effect on RPIA

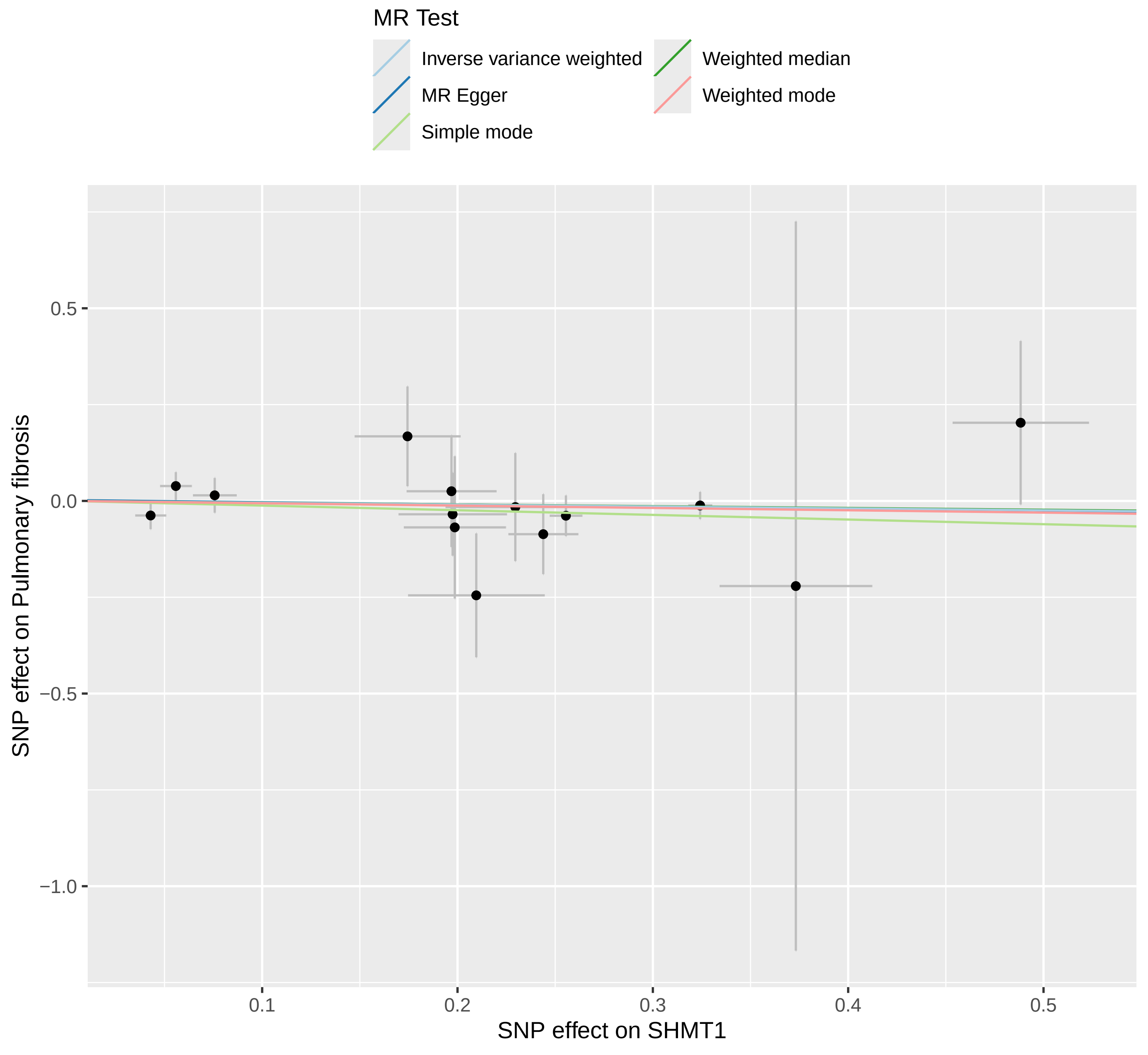

MR Test
Inverse variance weighted
MR Egger
Simple mode
Weighted median
Weighted mode
0.5
0.0
−0.5
−1.0
0.1
0.2
0.3
0.4
0.5
SNP effect on Pulmonary fibrosis
SNP effect on SHMT1

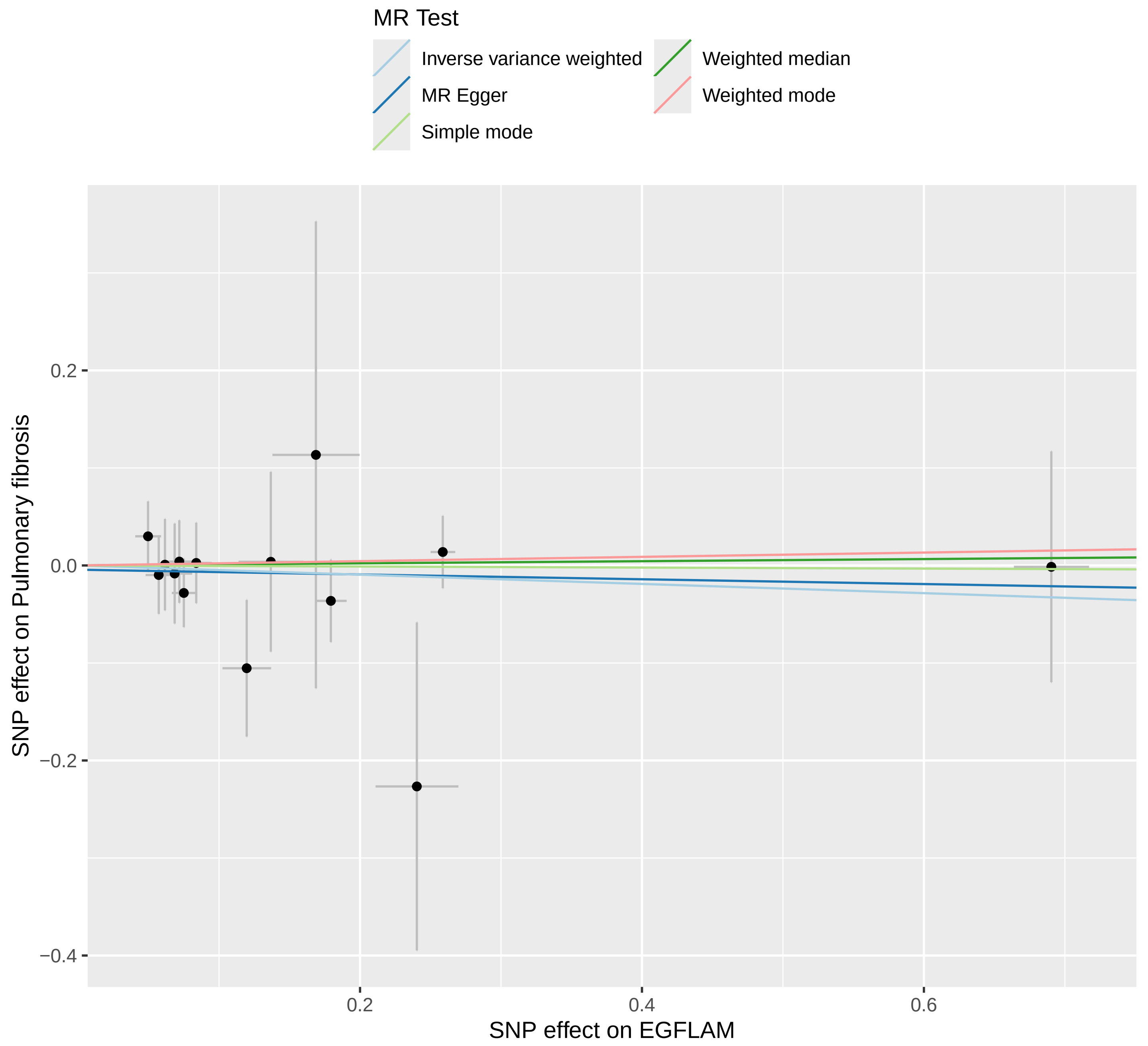
MR Test
Inverse variance weighted
MR Egger
Simple mode
Weighted median
Weighted mode
0.2
0.0
−0.2
−0.4
SNP effect on Pulmonary fibrosis
0.2
0.4
0.6
SNP effect on EGFLAM

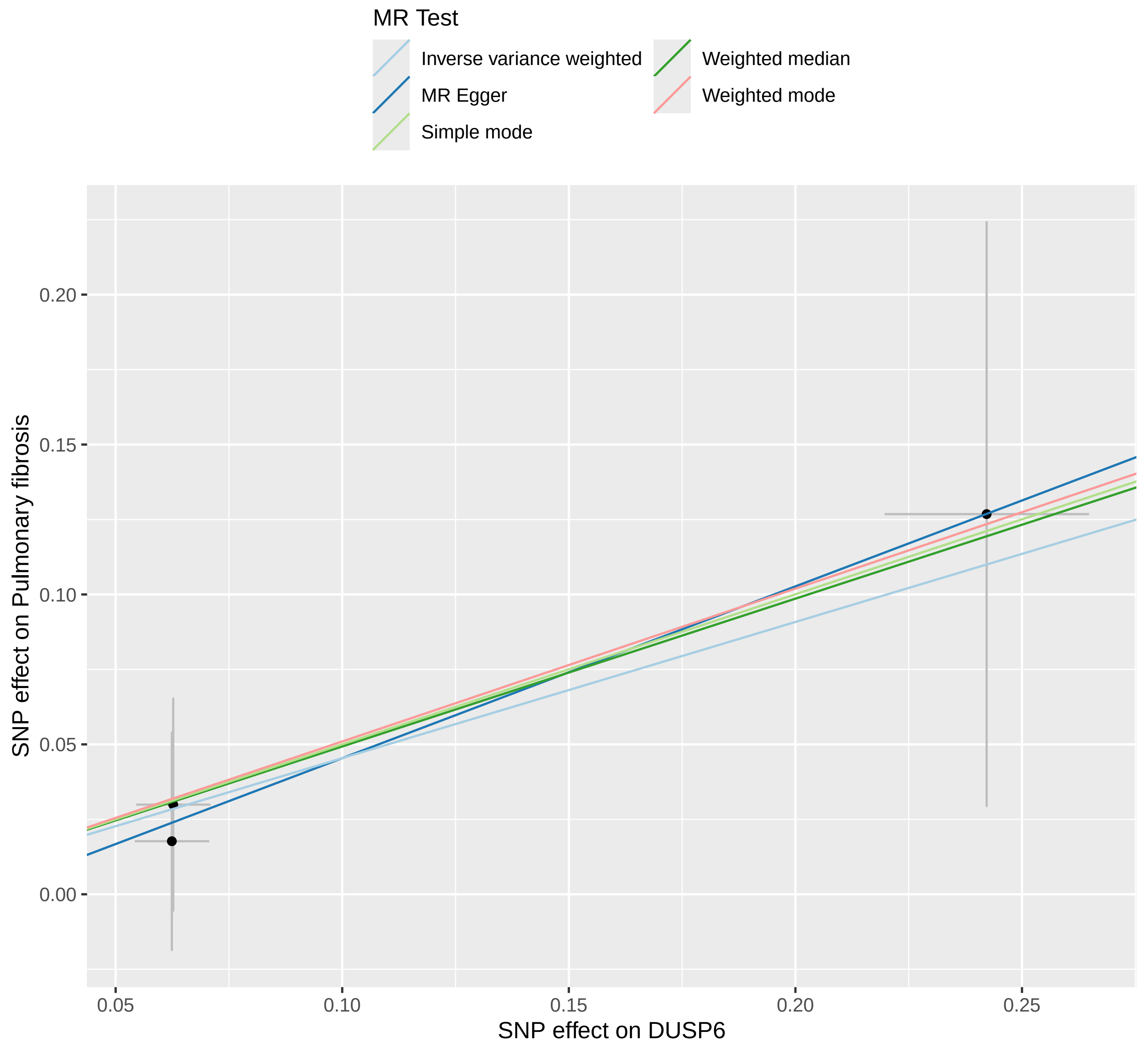
MR Test
Inverse variance weighted
MR Egger
Simple mode
Weighted median
Weighted mode
0.20
0.15
0.10
0.05
0.00
SNP effect on Pulmonary fibrosis
0.05
0.10
0.15
0.20
0.25
SNP effect on DUSP6

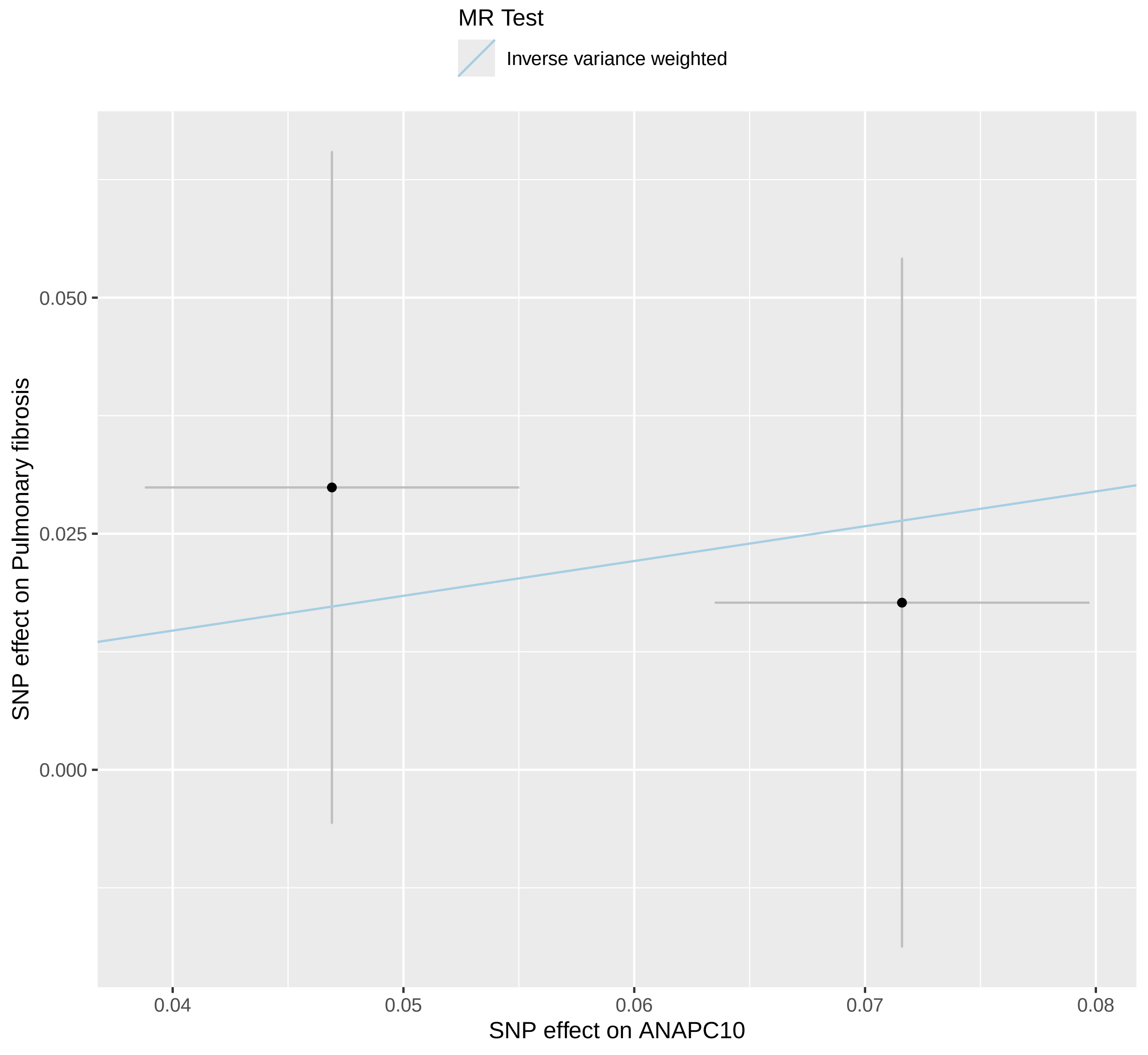
MR Test
Inverse variance weighted
0.050
0.025
0.000
SNP effect on Pulmonary fibrosis
0.04
0.05
0.06
0.07
0.08
SNP effect on ANAPC10

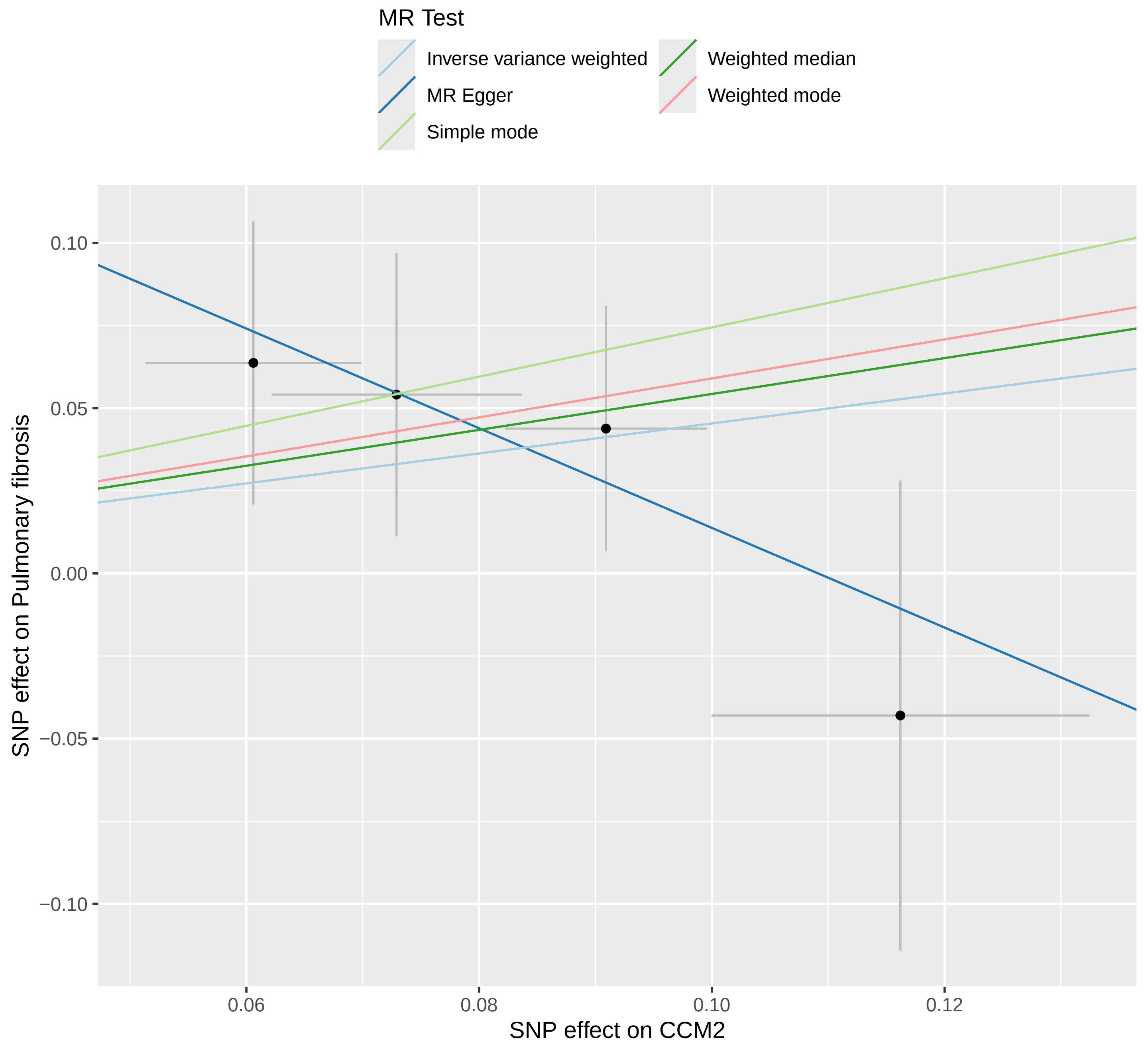
MR Test
Inverse variance weighted
MR Egger
Simple mode
Weighted median
Weighted mode
0.10
0.05
0.00
−0.05
−0.10
SNP effect on Pulmonary fibrosis
0.06
0.08
0.10
0.12
SNP effect on CCM2

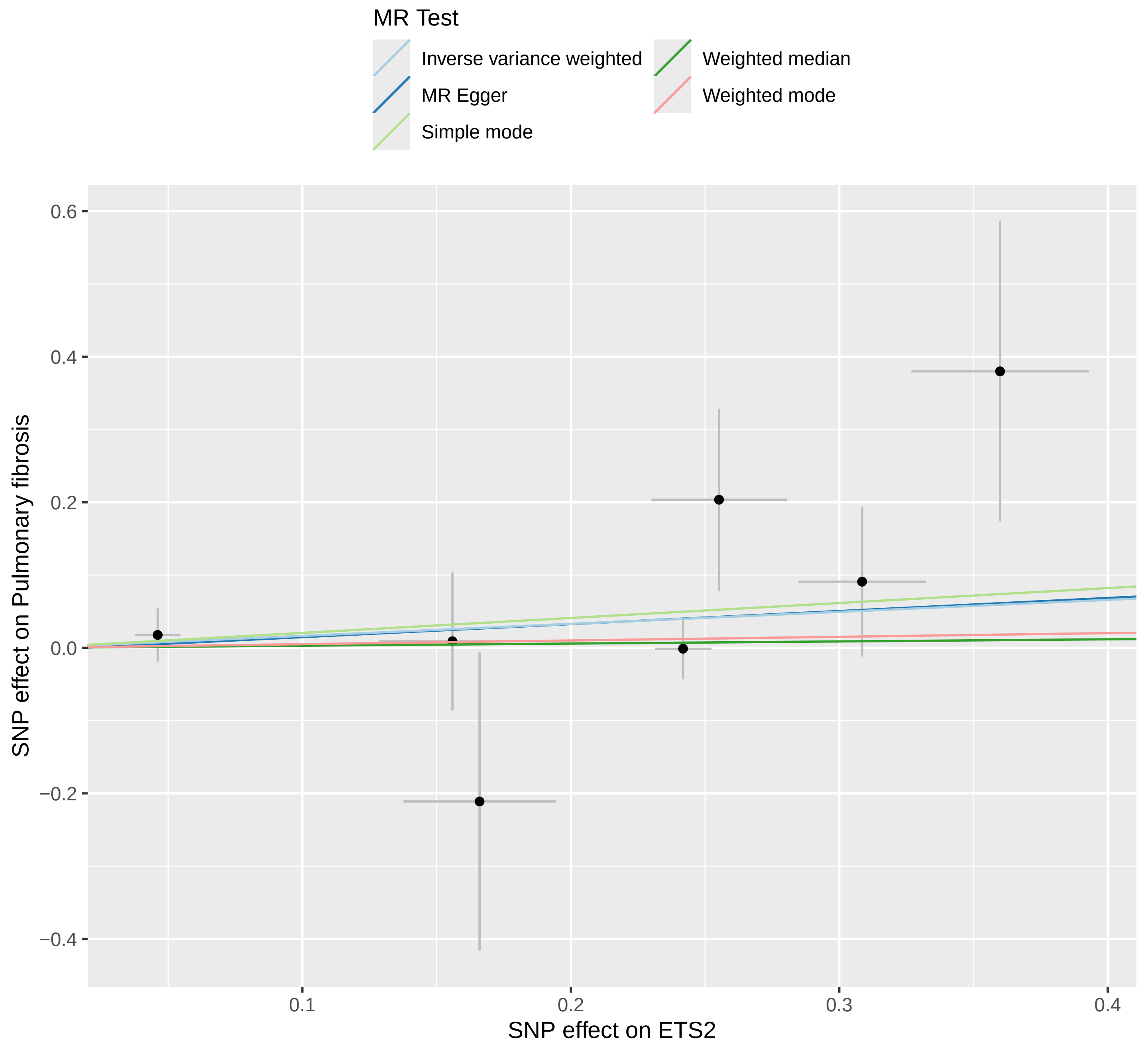
MR Test
Inverse variance weighted
MR Egger
Simple mode
Weighted median
Weighted mode
SNP effect on Pulmonary fibrosis
0.6
0.4
0.2
0.0
−0.2
−0.4
0.1
0.2
0.3
0.4
SNP effect on ETS2

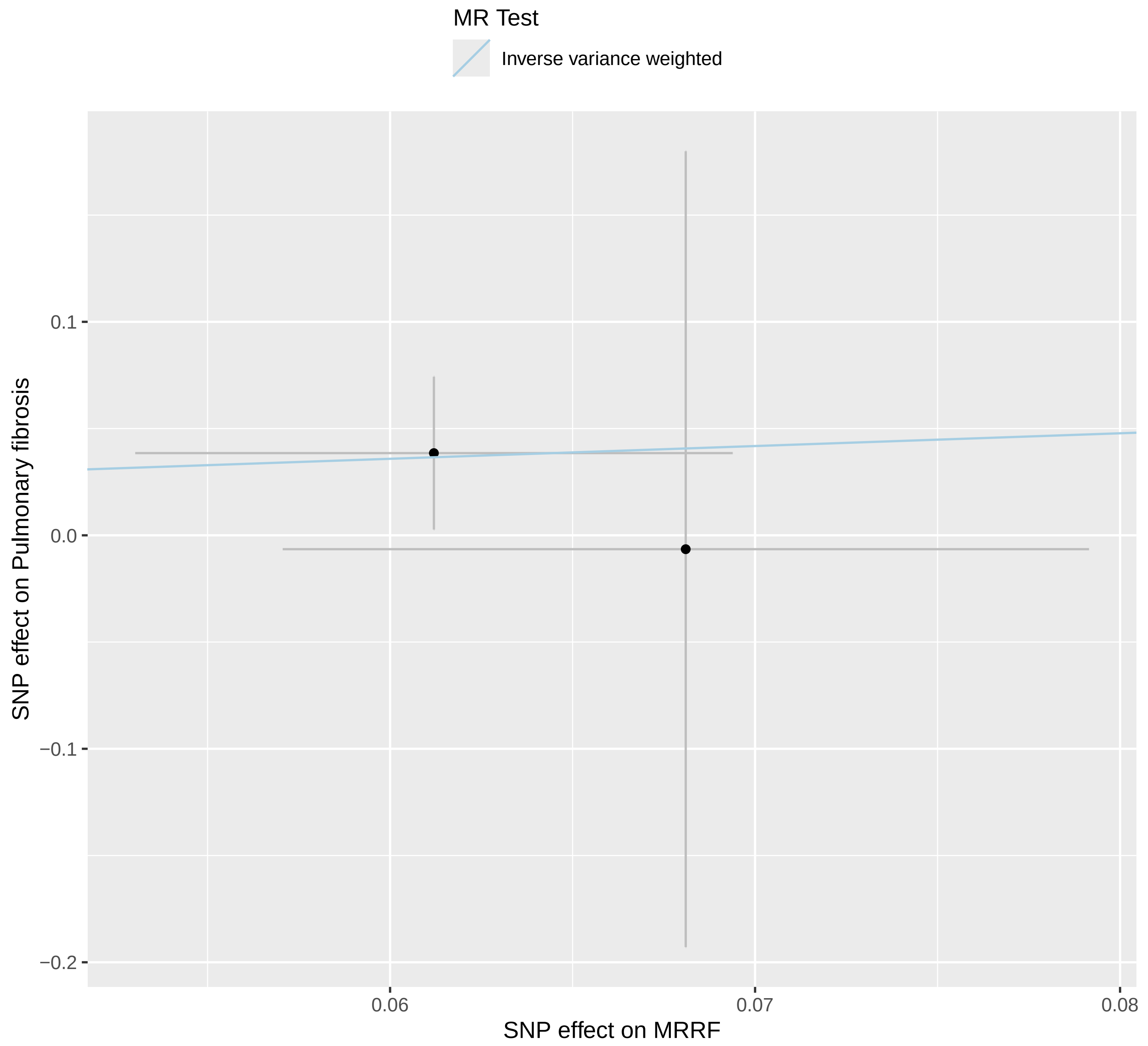
MR Test
Inverse variance weighted
0.1
0.0
−0.1
−0.2
SNP effect on Pulmonary fibrosis
0.06
0.07
0.08
SNP effect on MRRF

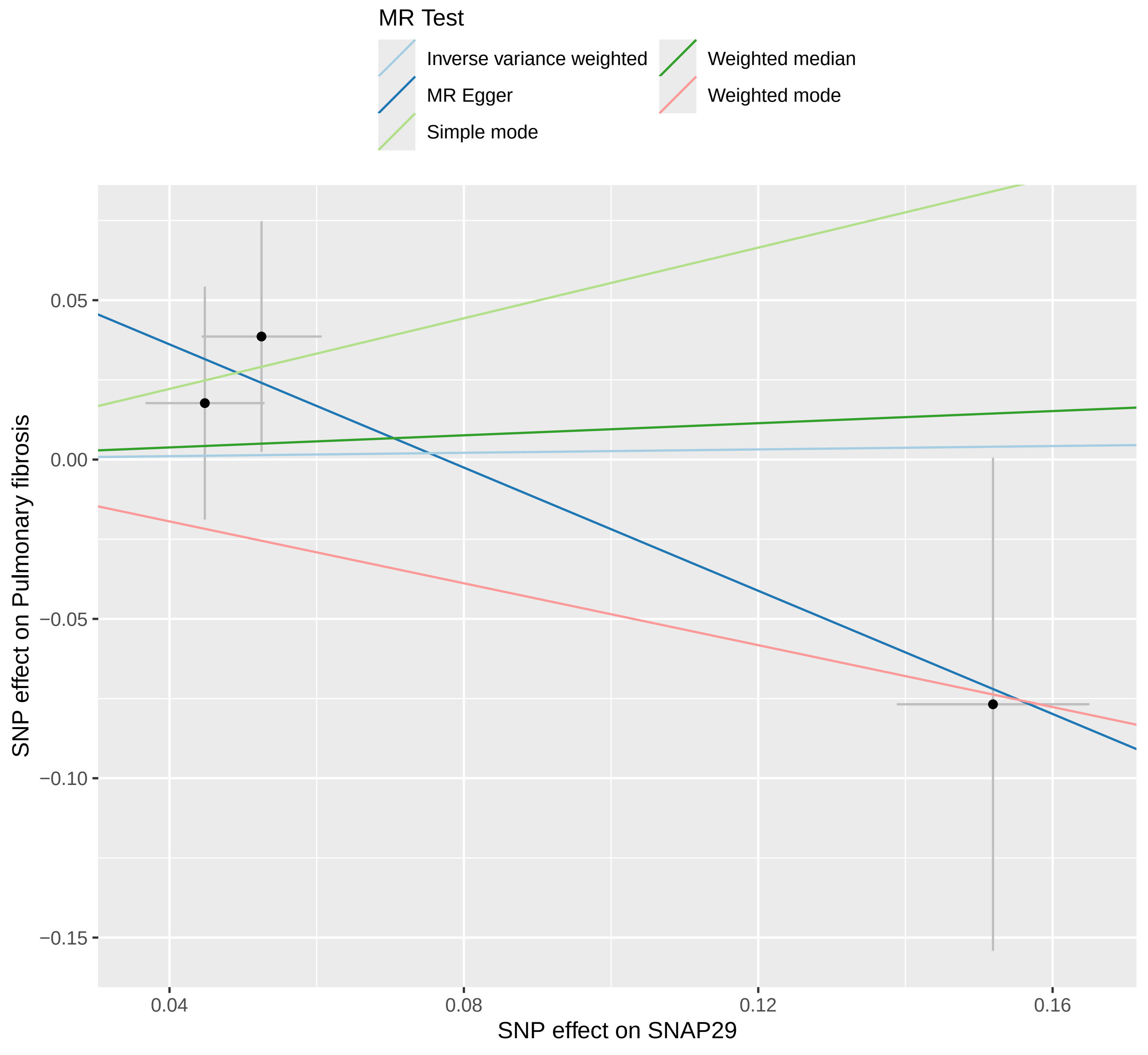
MR Test
Inverse variance weighted
MR Egger
Simple mode
Weighted median
Weighted mode
SNP effect on Pulmonary fibrosis
0.05
0.00
−0.05
−0.10
−0.15
0.04
0.08
0.12
0.16
SNP effect on SNAP29

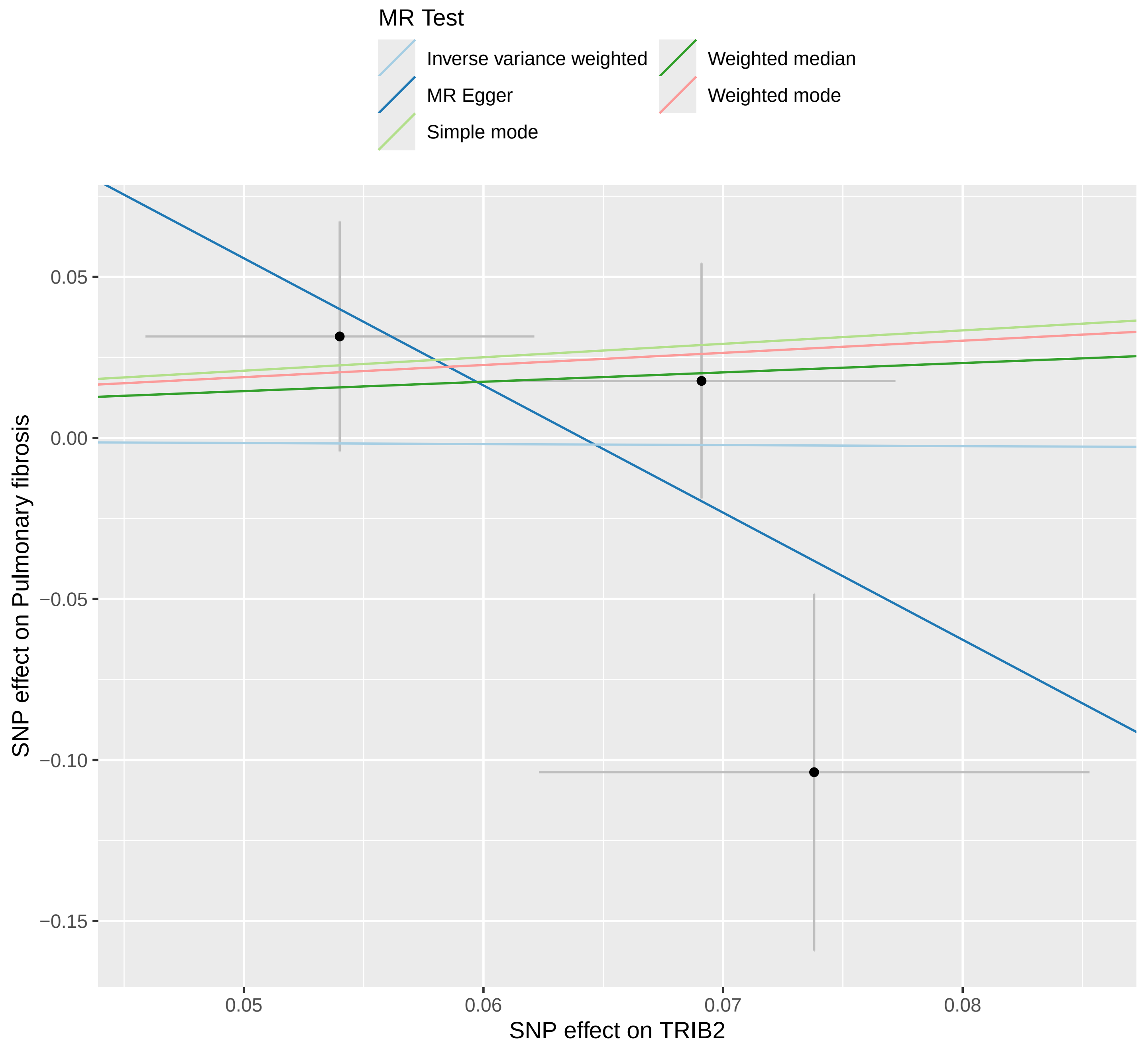
MR Test
Inverse variance weighted
MR Egger
Simple mode
Weighted median
Weighted mode
0.05
0.00
−0.05
−0.10
−0.15
SNP effect on Pulmonary fibrosis
0.05
0.06
0.07
0.08
SNP effect on TRIB2

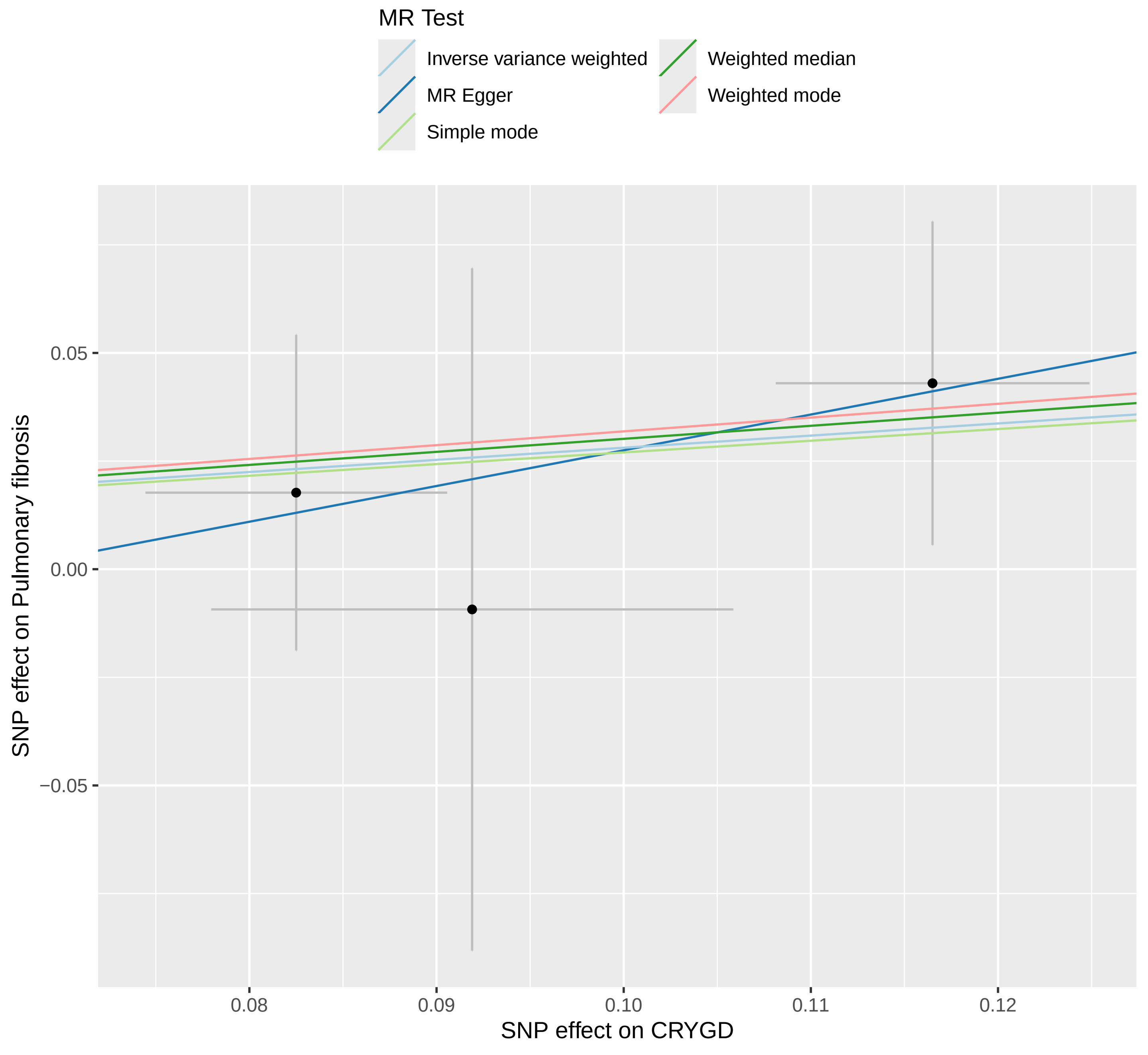
MR Test
Inverse variance weighted
MR Egger
Simple mode
Weighted median
Weighted mode
0.05
0.00
−0.05
0.08
0.09
0.10
0.11
0.12
SNP effect on CRYGD
SNP effect on Pulmonary fibrosis

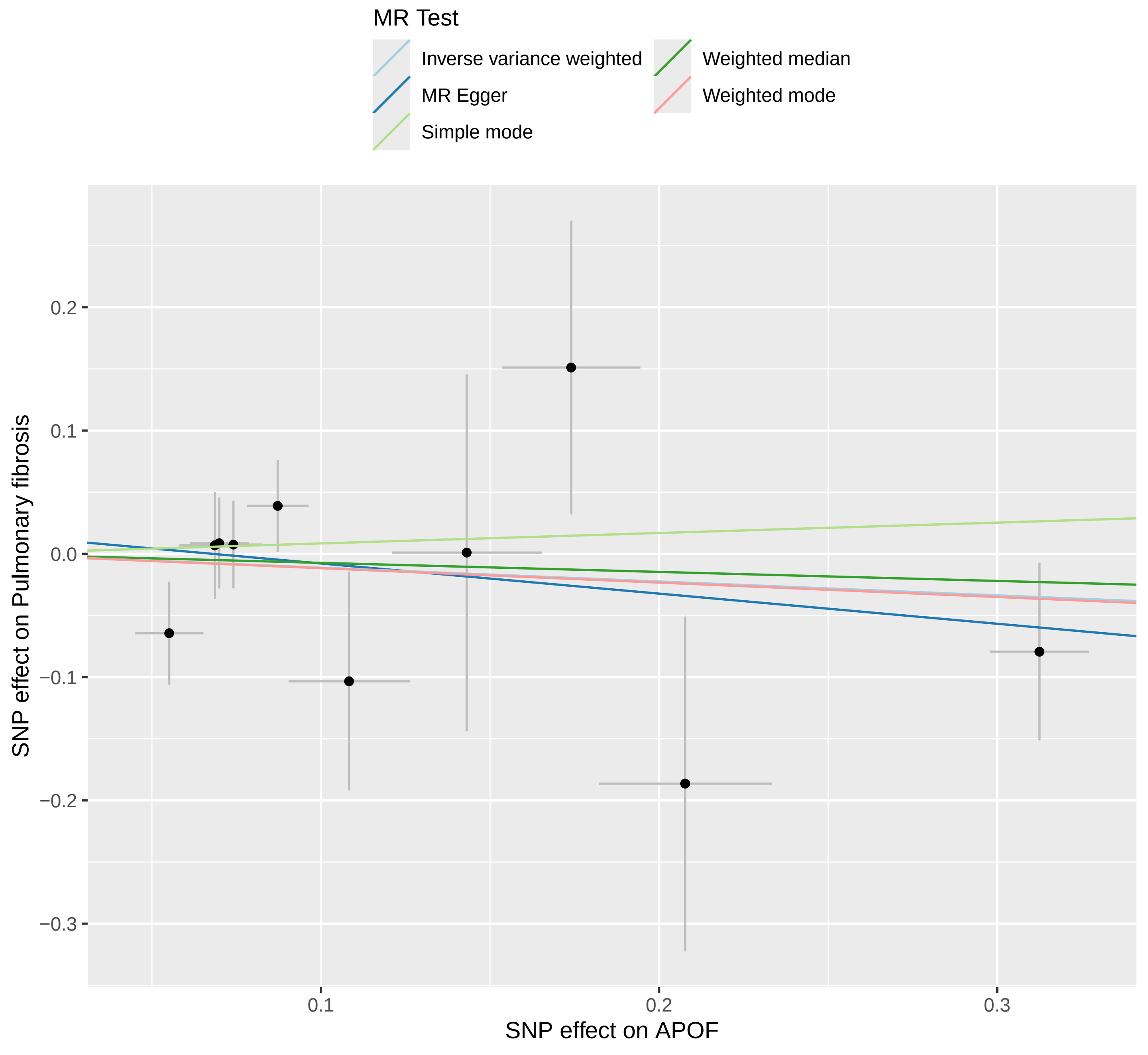
MR Test
Inverse variance weighted
MR Egger
Simple mode
Weighted median
Weighted mode
0.2
0.1
0.0
−0.1
−0.2
−0.3
SNP effect on Pulmonary fibrosis
0.1
0.2
0.3
SNP effect on APOF

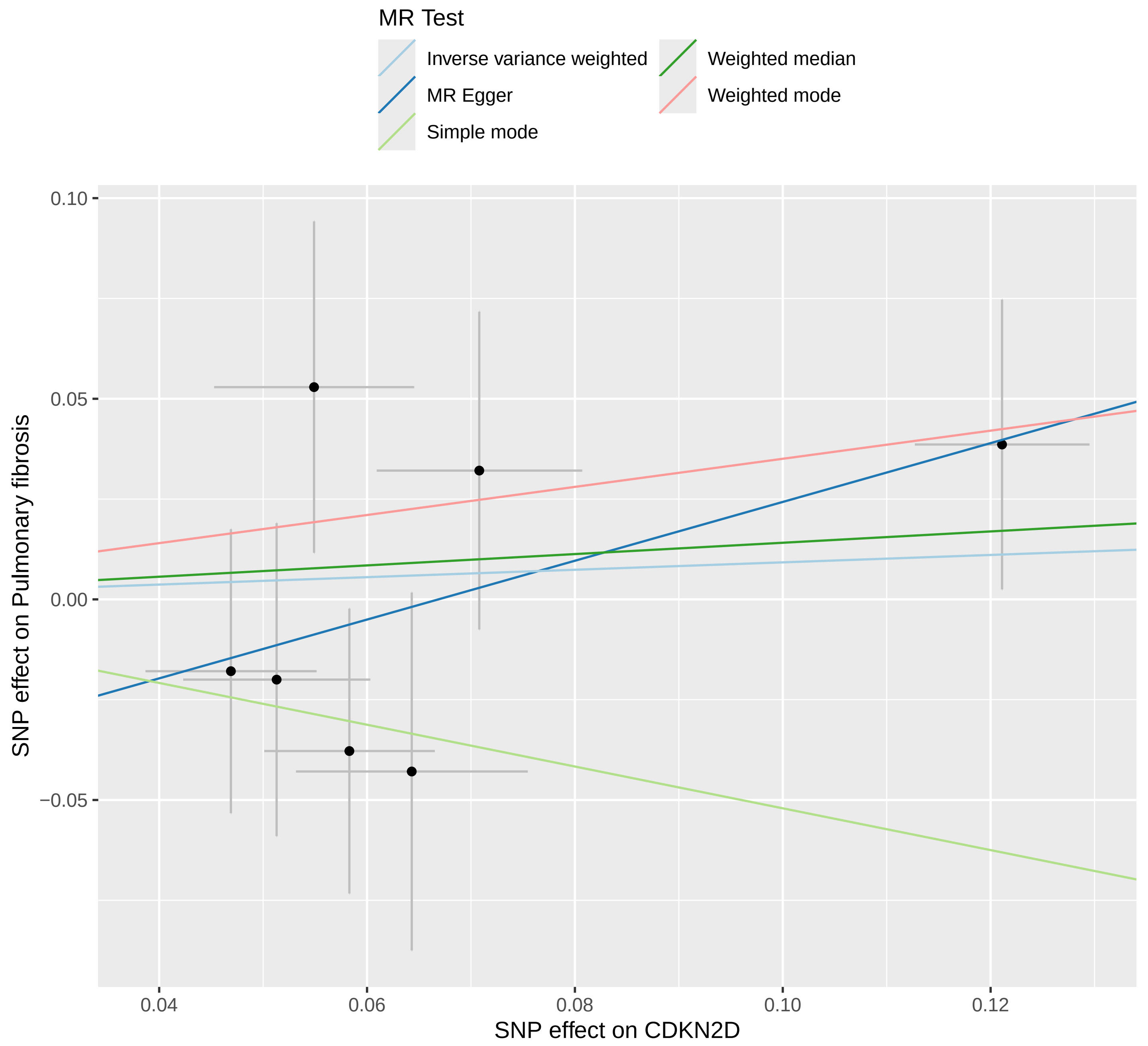
MR Test
Inverse variance weighted
MR Egger
Simple mode
Weighted median
Weighted mode
0.10
0.05
0.00
−0.05
0.04
0.06
0.08
0.10
0.12
SNP effect on CDKN2D
SNP effect on Pulmonary fibrosis

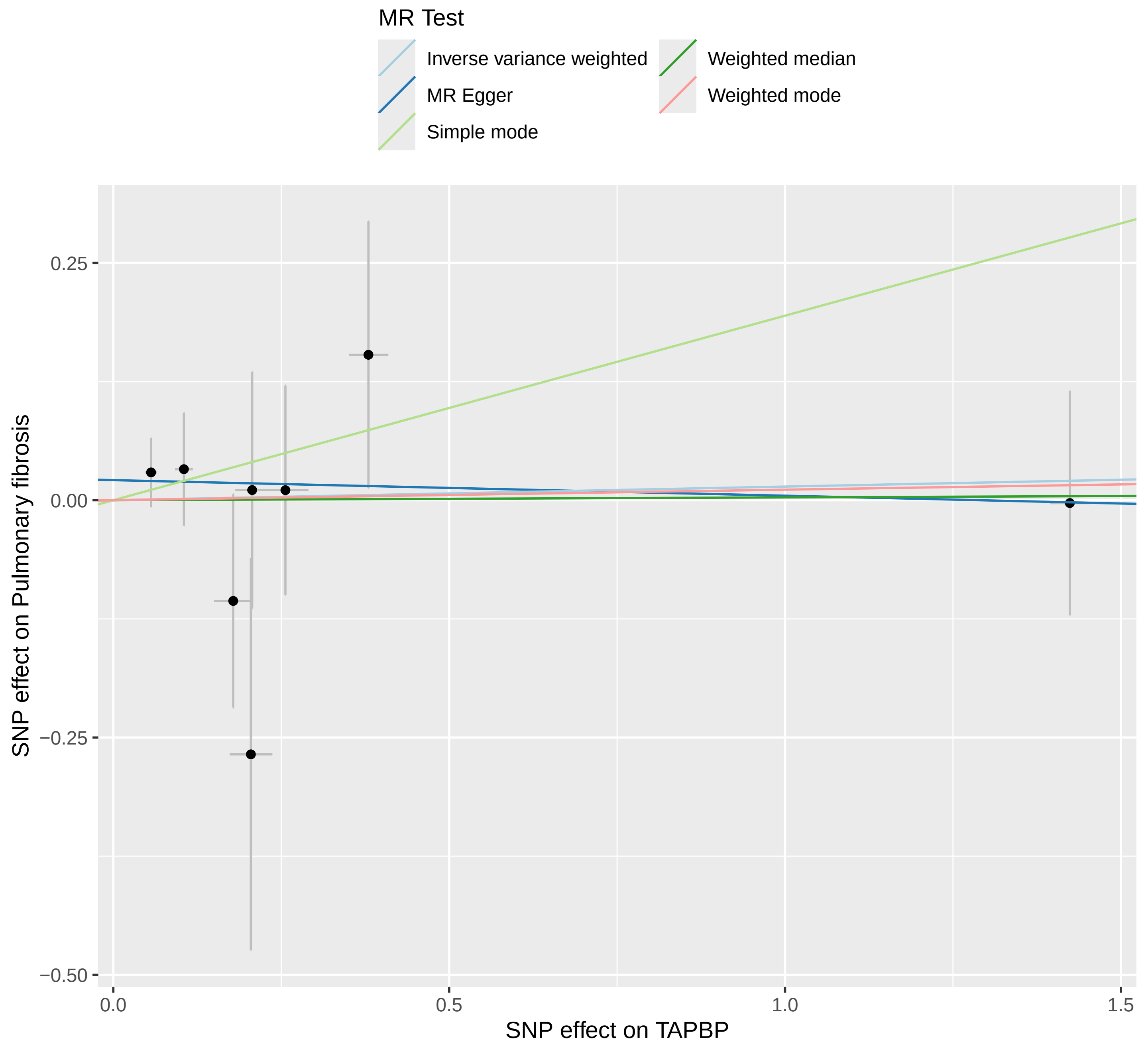
MR Test
Inverse variance weighted
MR Egger
Simple mode
Weighted median
Weighted mode
0.25
0.00
−0.25
−0.50
0.0
0.5
1.0
1.5
SNP effect on TAPBP
SNP effect on Pulmonary fibrosis

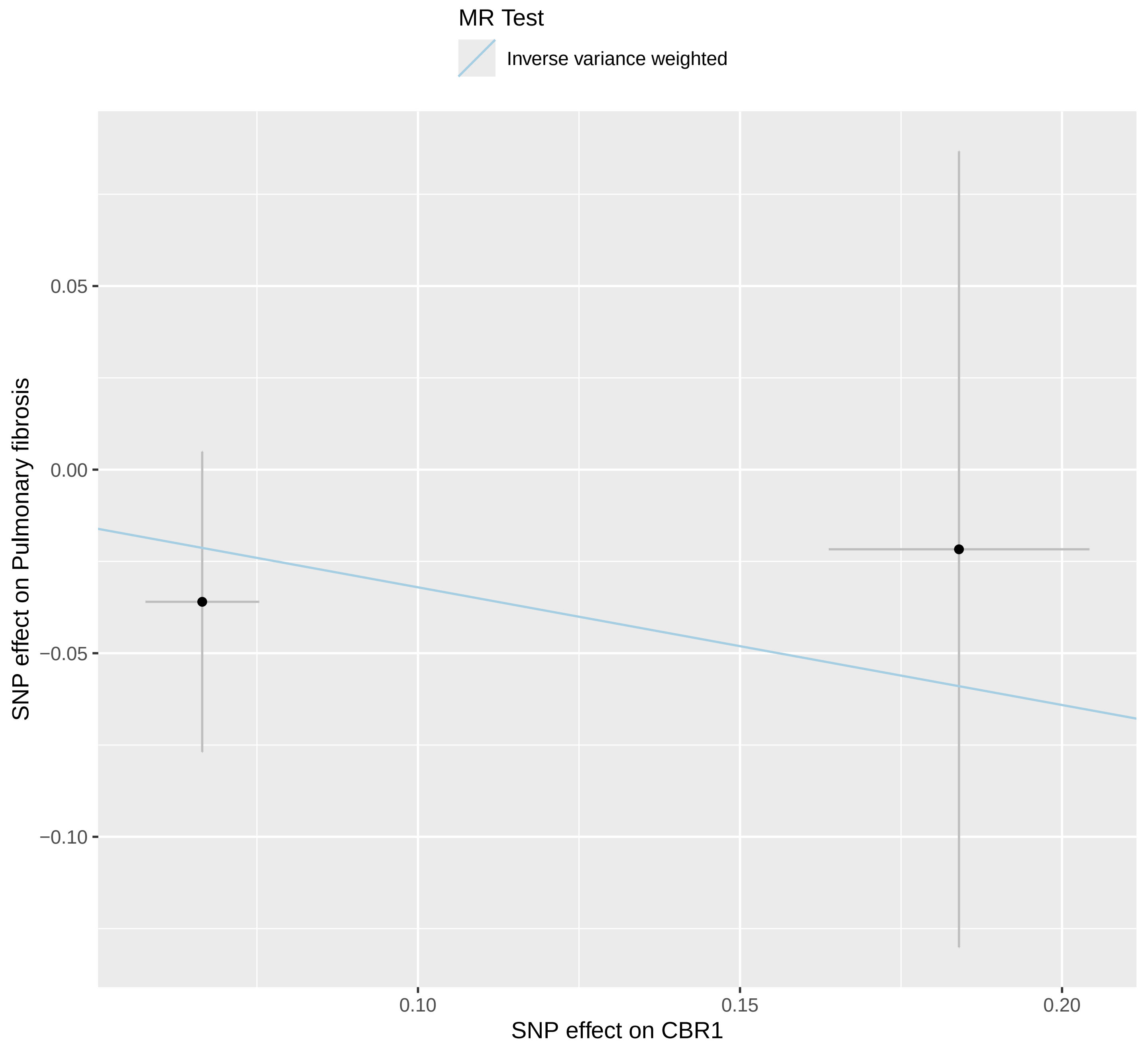
MR Test
Inverse variance weighted
0.05
0.00
−0.05
−0.10
SNP effect on Pulmonary fibrosis
0.10
0.15
0.20
SNP effect on CBR1

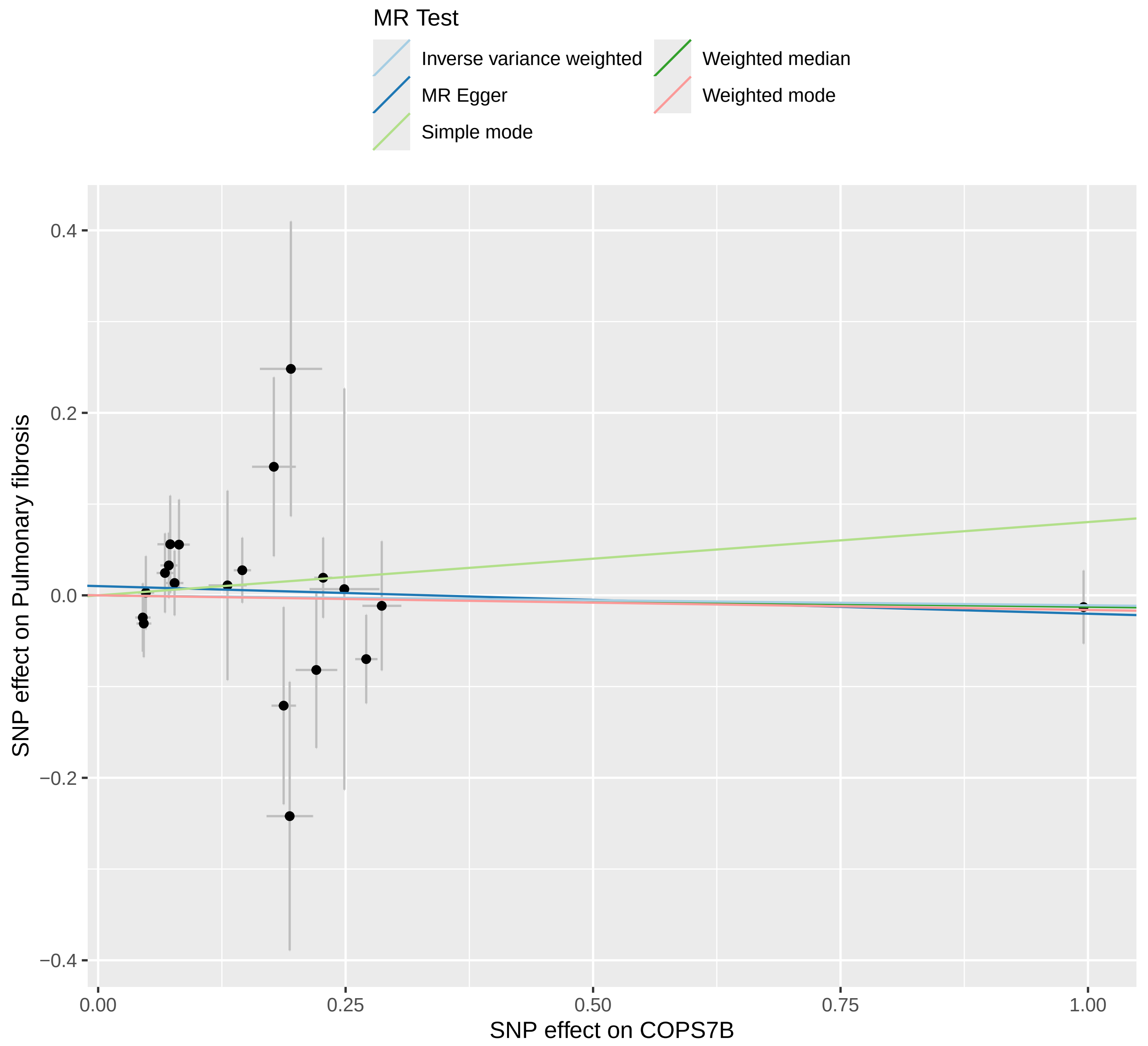

MR Test
Inverse variance weighted
MR Egger
Simple mode
Weighted median
Weighted mode
0.4
0.2
0.0
−0.2
−0.4
SNP effect on Pulmonary fibrosis
0.00
0.25
0.50
0.75
1.00
SNP effect on COPS7B

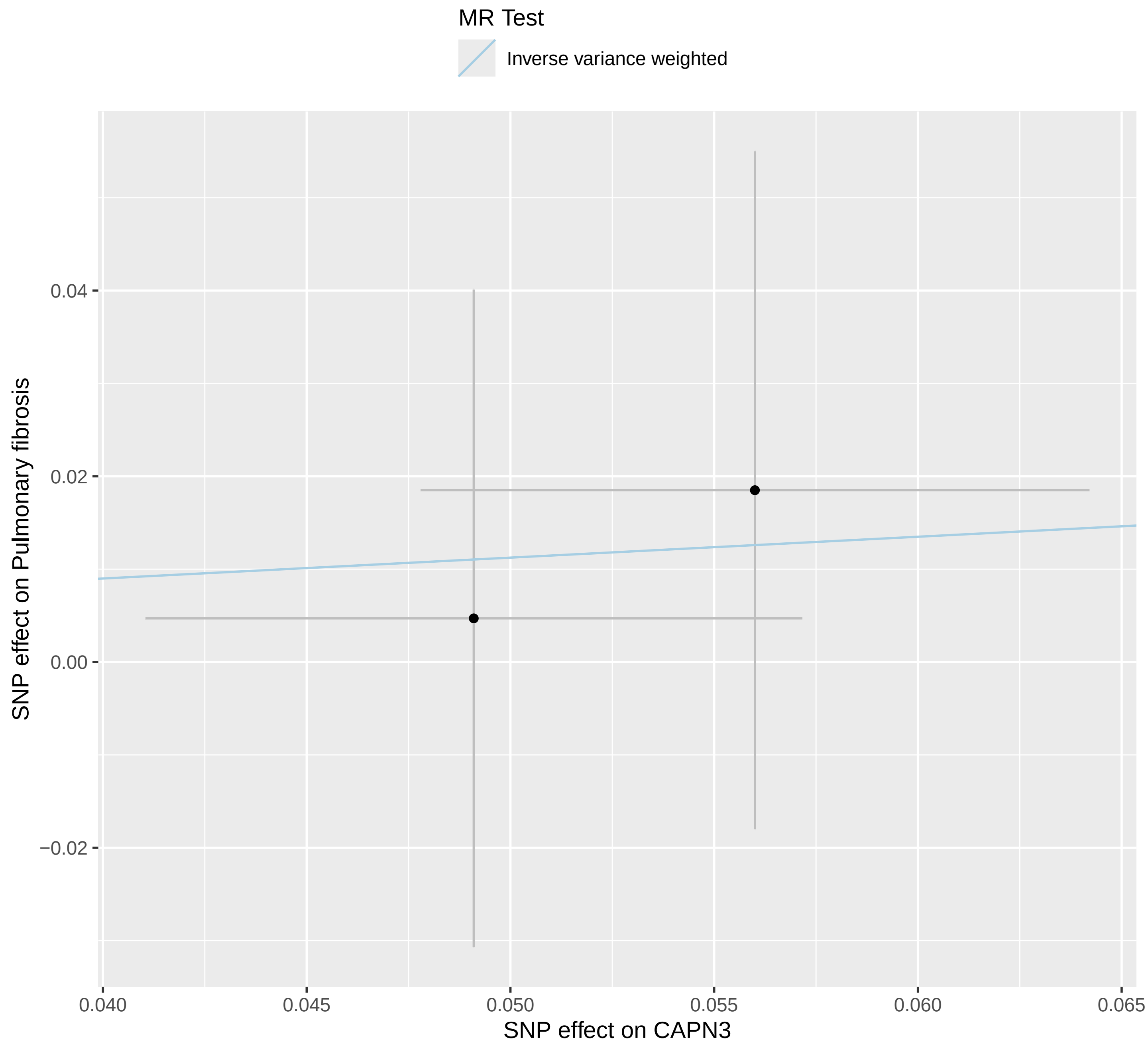
MR Test
Inverse variance weighted
0.04
0.02
0.00
−0.02
SNP effect on Pulmonary fibrosis
0.040
0.045
0.050
0.055
0.060
0.065
SNP effect on CAPN3

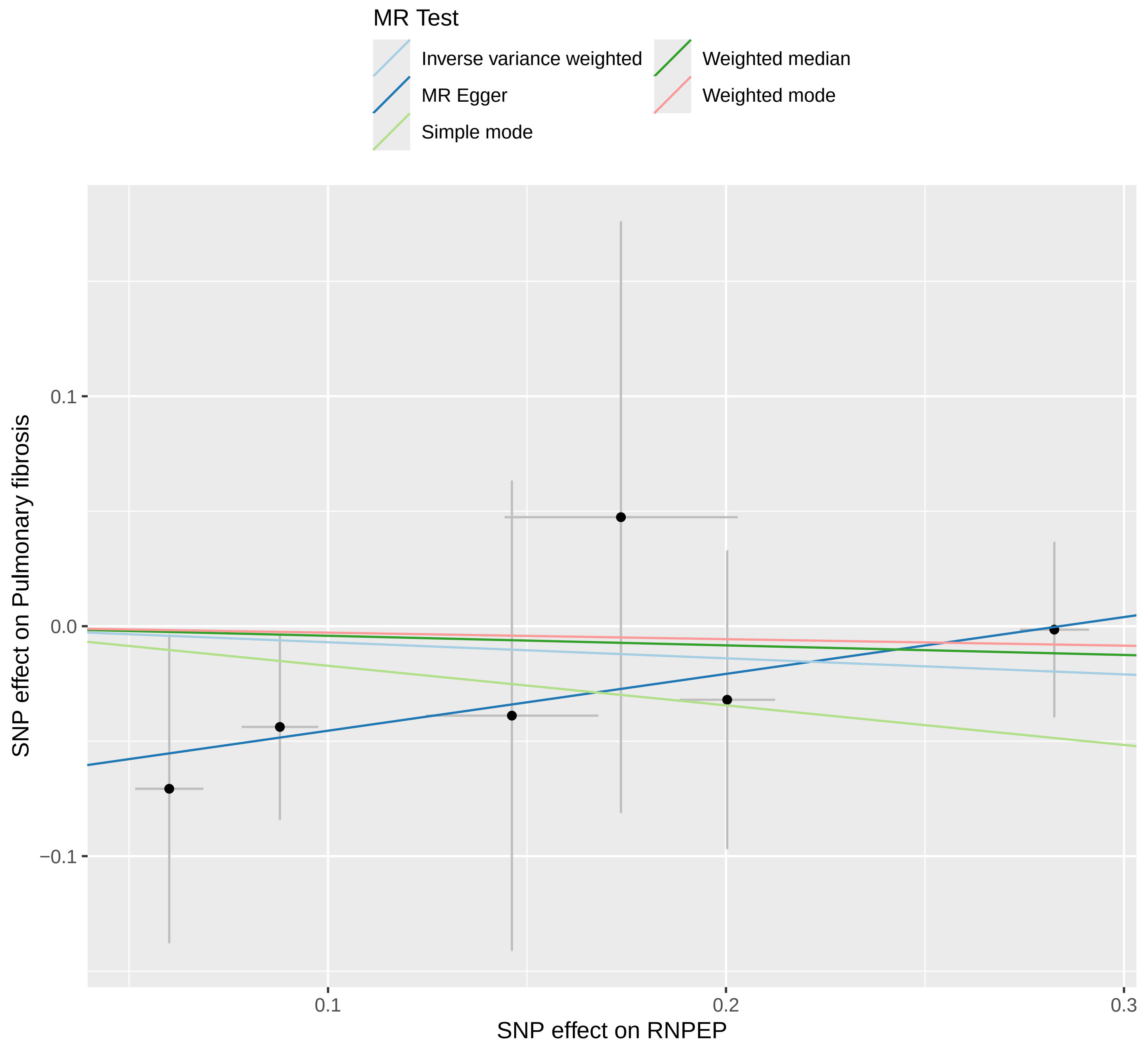
MR Test
Inverse variance weighted
MR Egger
Simple mode
Weighted median
Weighted mode
0.1
0.0
−0.1
SNP effect on Pulmonary fibrosis
0.1
0.2
0.3
SNP effect on RNPEP

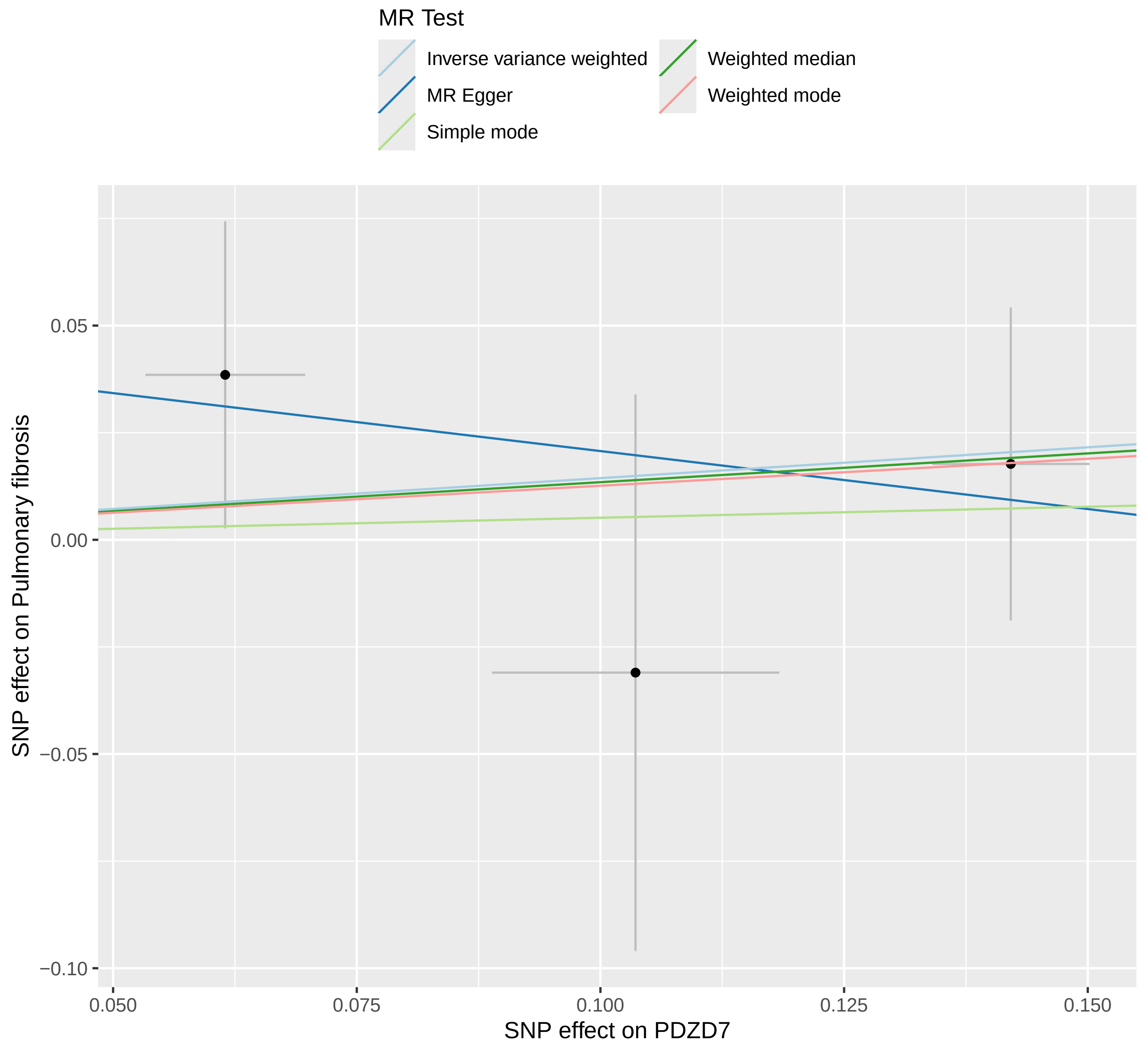
MR Test
Inverse variance weighted
MR Egger
Simple mode
Weighted median
Weighted mode
0.05
0.00
−0.05
−0.10
0.050
0.075
0.100
0.125
0.150
SNP effect on Pulmonary fibrosis
SNP effect on PDZD7

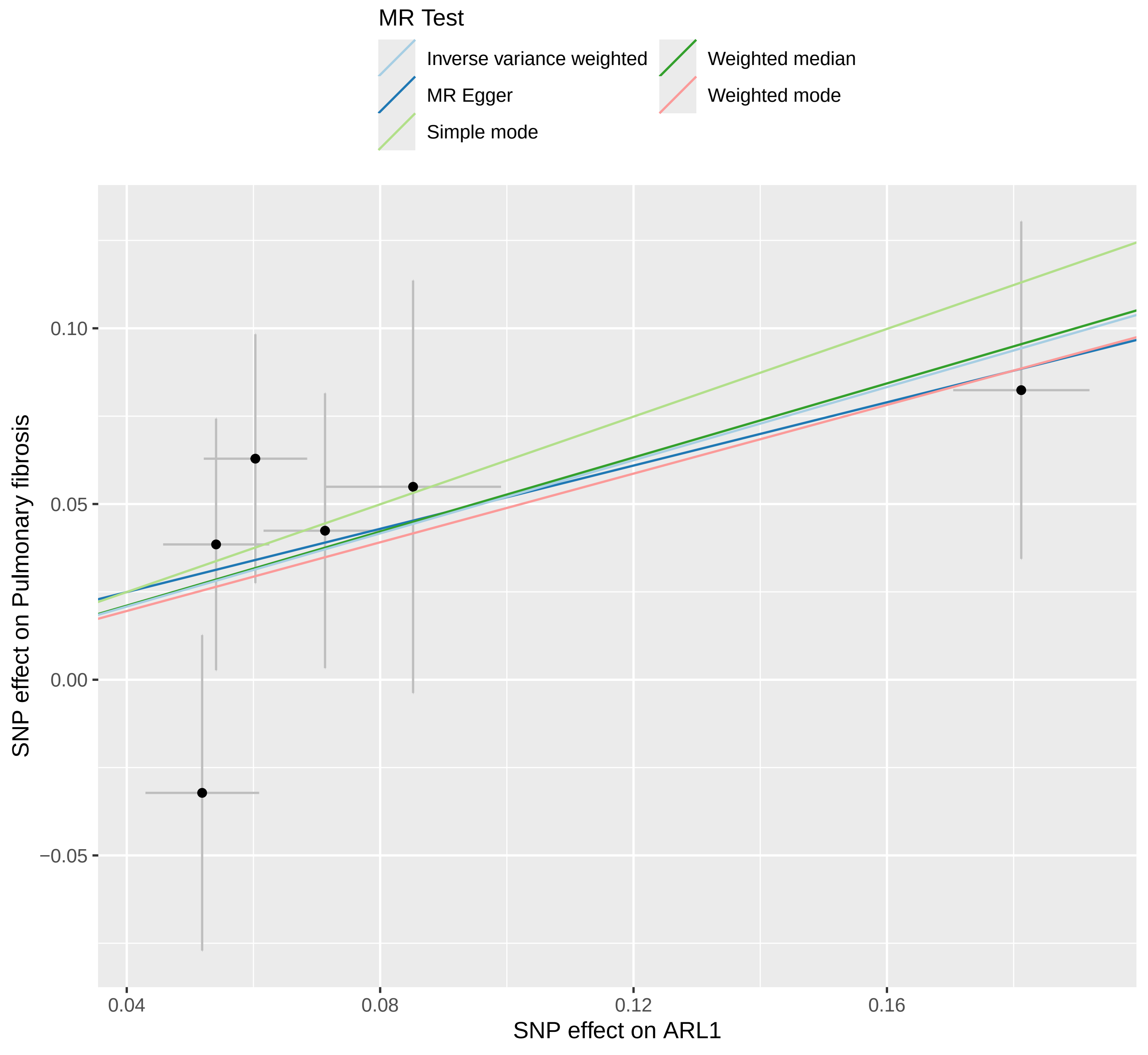
MR Test
Inverse variance weighted
MR Egger
Simple mode
Weighted median
Weighted mode
0.10
0.05
0.00
−0.05
0.04
0.08
0.12
0.16
SNP effect on ARL1
SNP effect on Pulmonary fibrosis

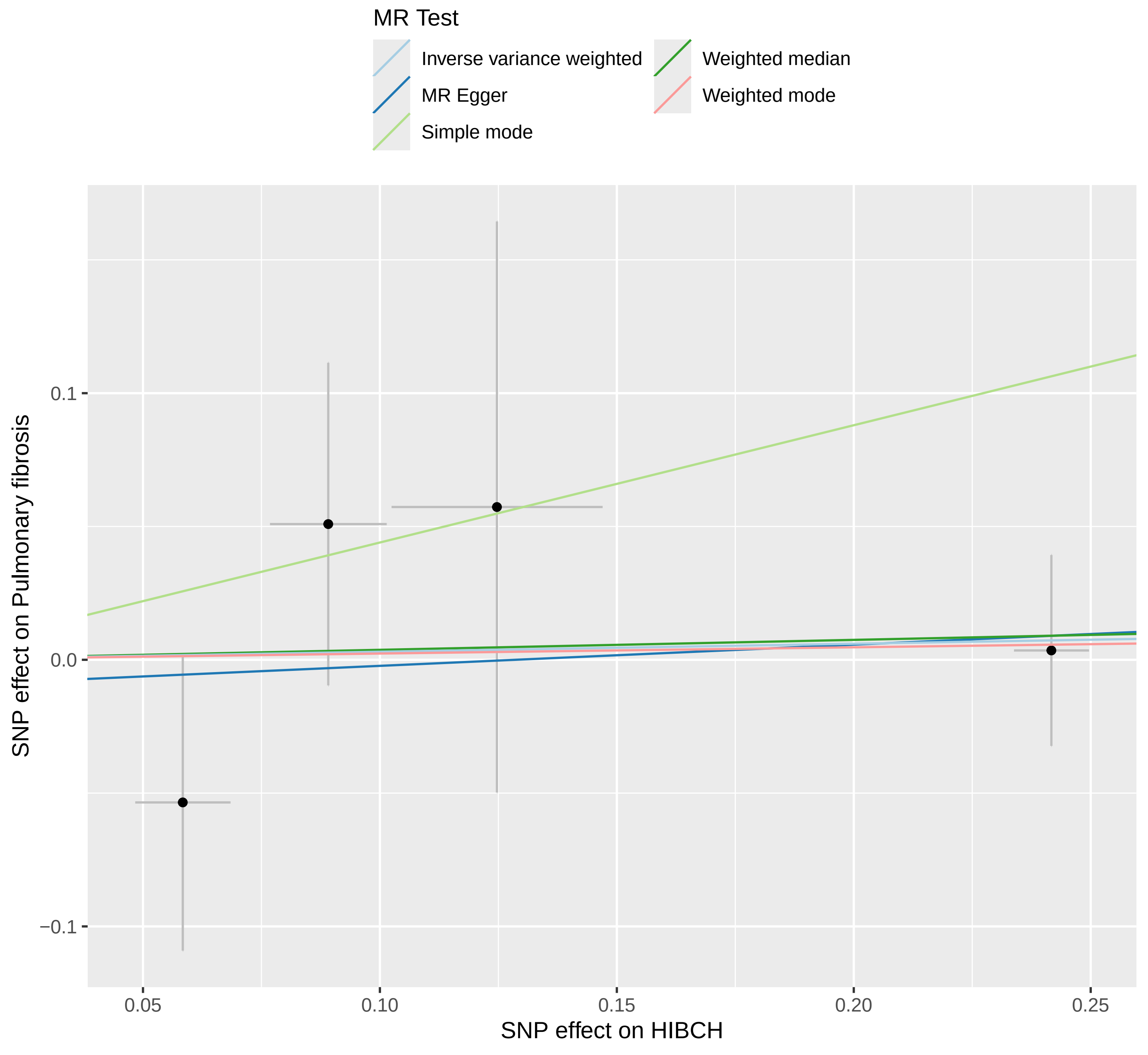
MR Test
Inverse variance weighted
MR Egger
Simple mode
Weighted median
Weighted mode
0.1
0.0
−0.1
SNP effect on Pulmonary fibrosis
0.05
0.10
0.15
0.20
0.25
SNP effect on HIBCH

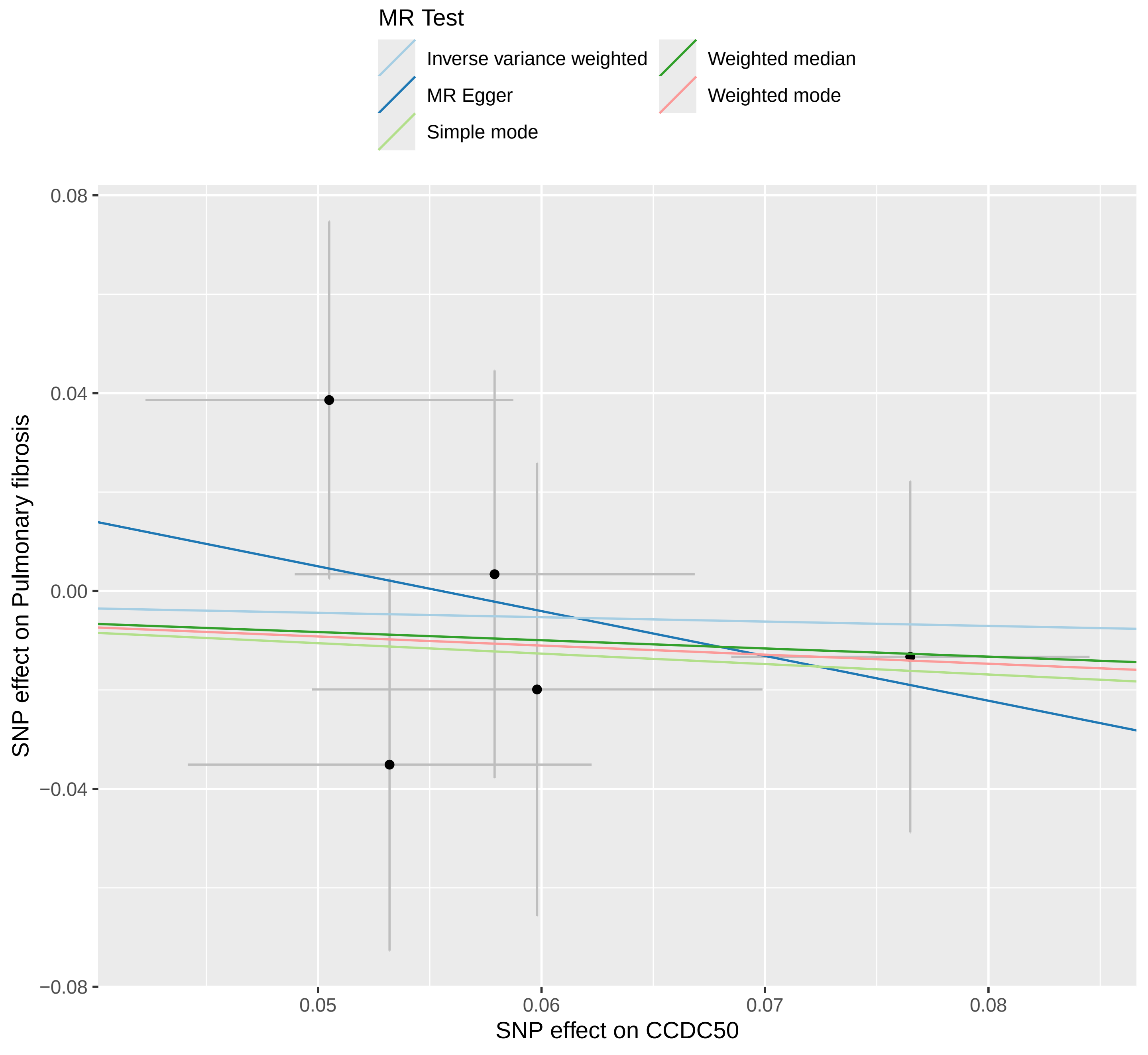
MR Test
Inverse variance weighted
MR Egger
Simple mode
Weighted median
Weighted mode
SNP effect on Pulmonary fibrosis
0.08
0.04
0.00
−0.04
−0.08
0.05
0.06
0.07
0.08
SNP effect on CCDC50

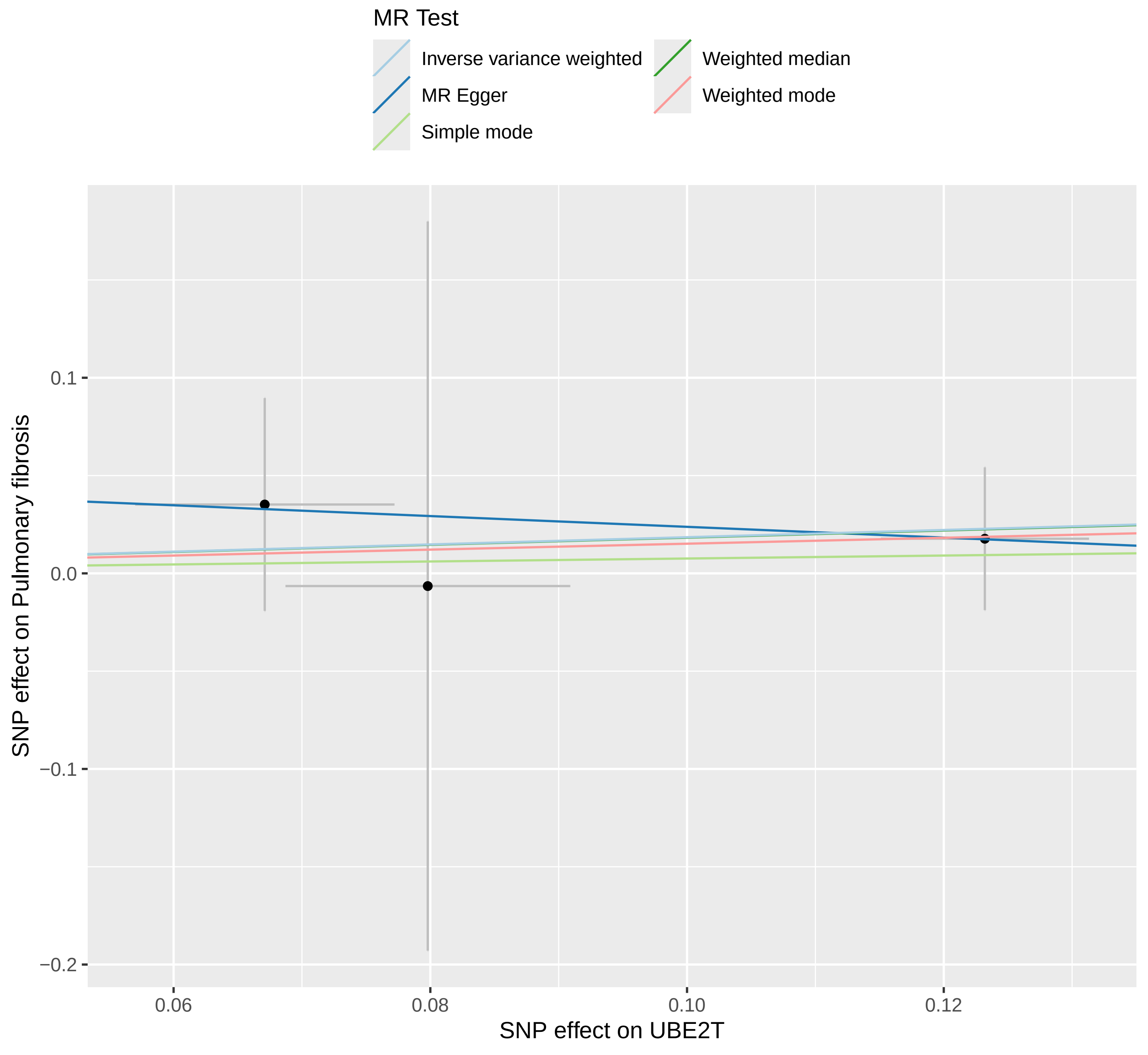
MR Test
Inverse variance weighted
MR Egger
Simple mode
Weighted median
Weighted mode
0.1
0.0
−0.1
−0.2
SNP effect on Pulmonary fibrosis
0.06
0.08
0.10
0.12
SNP effect on UBE2T

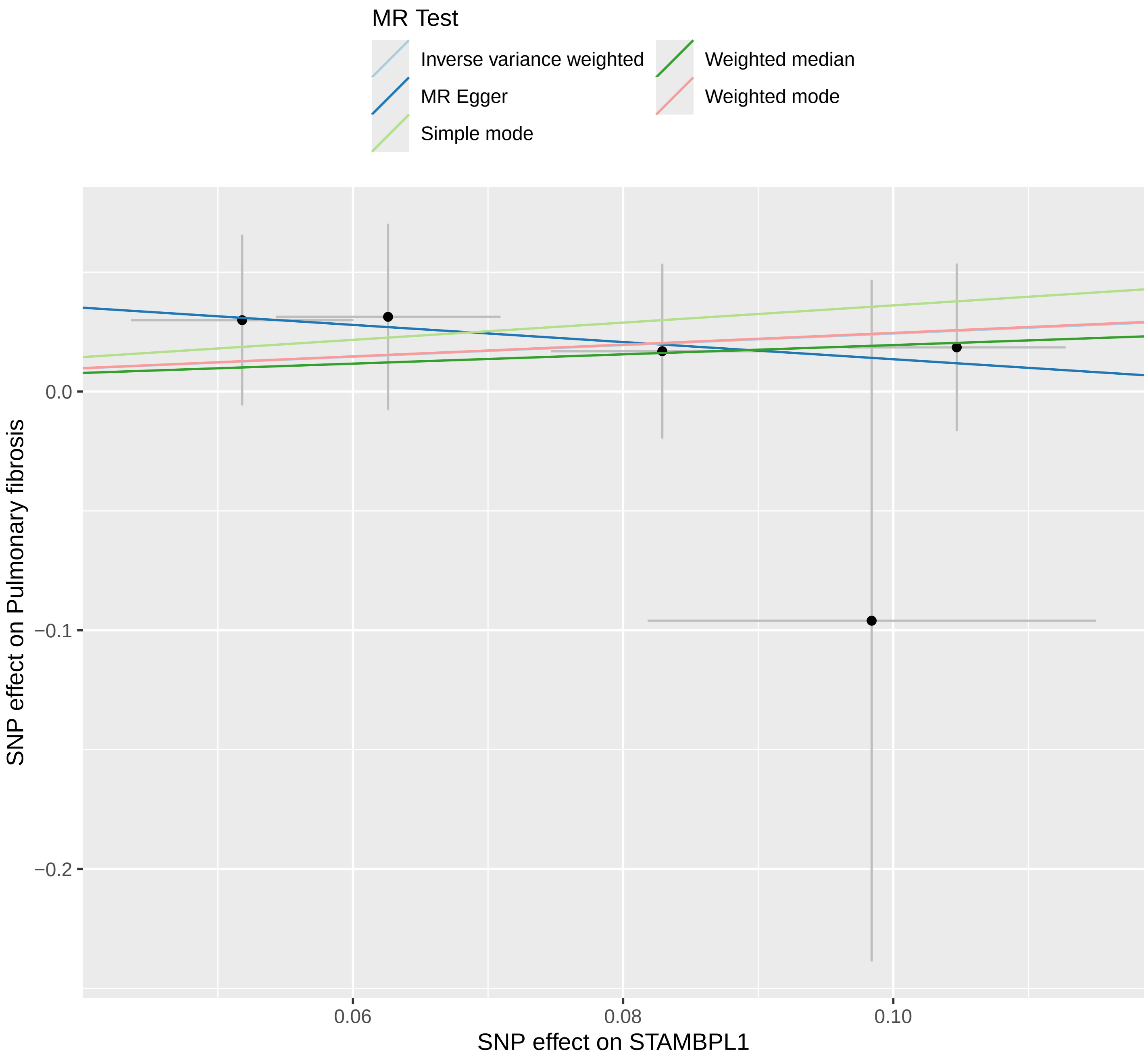

MR Test
Inverse variance weighted
MR Egger
Simple mode
Weighted median
Weighted mode
0.0
−0.1
−0.2
SNP effect on Pulmonary fibrosis
0.06
0.08
0.10
SNP effect on STAMBPL1

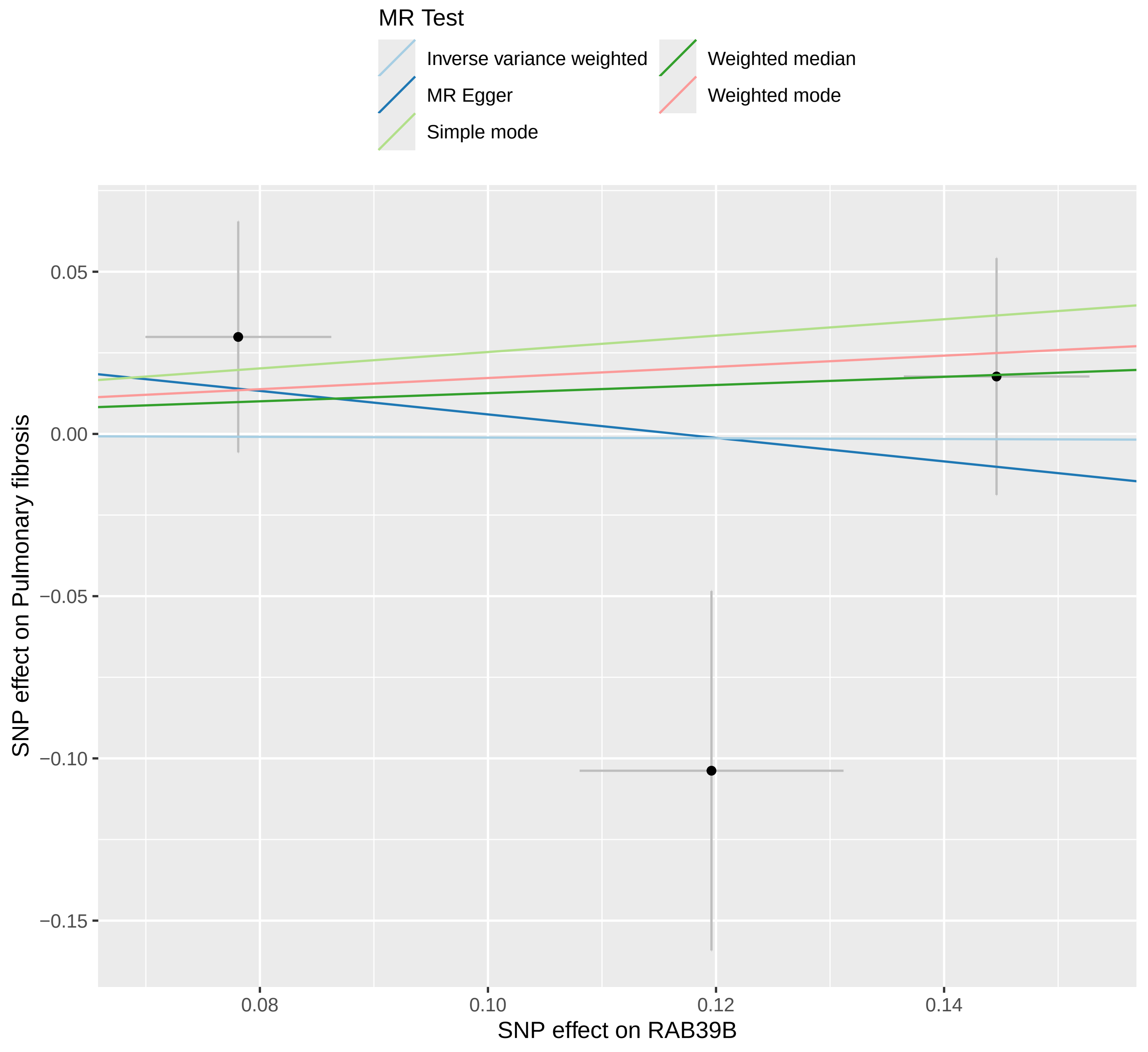
MR Test
Inverse variance weighted
MR Egger
Simple mode
Weighted median
Weighted mode
0.05
0.00
−0.05
−0.10
−0.15
SNP effect on Pulmonary fibrosis
0.08
0.10
0.12
0.14
SNP effect on RAB39B

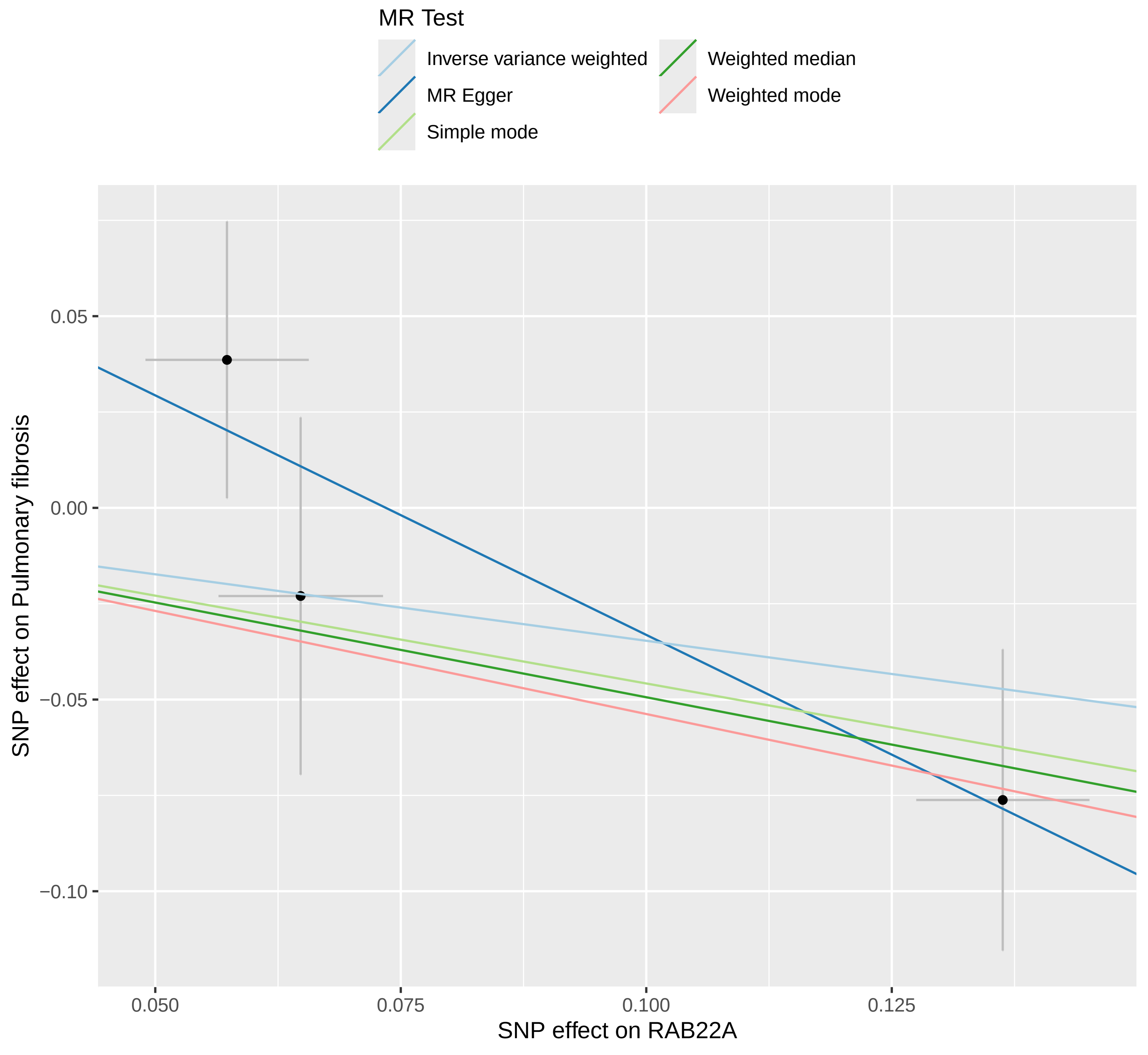
MR Test
Inverse variance weighted
MR Egger
Simple mode
Weighted median
Weighted mode
SNP effect on Pulmonary fibrosis
0.05
0.00
−0.05
−0.10
0.050
0.075
0.100
0.125
SNP effect on RAB22A

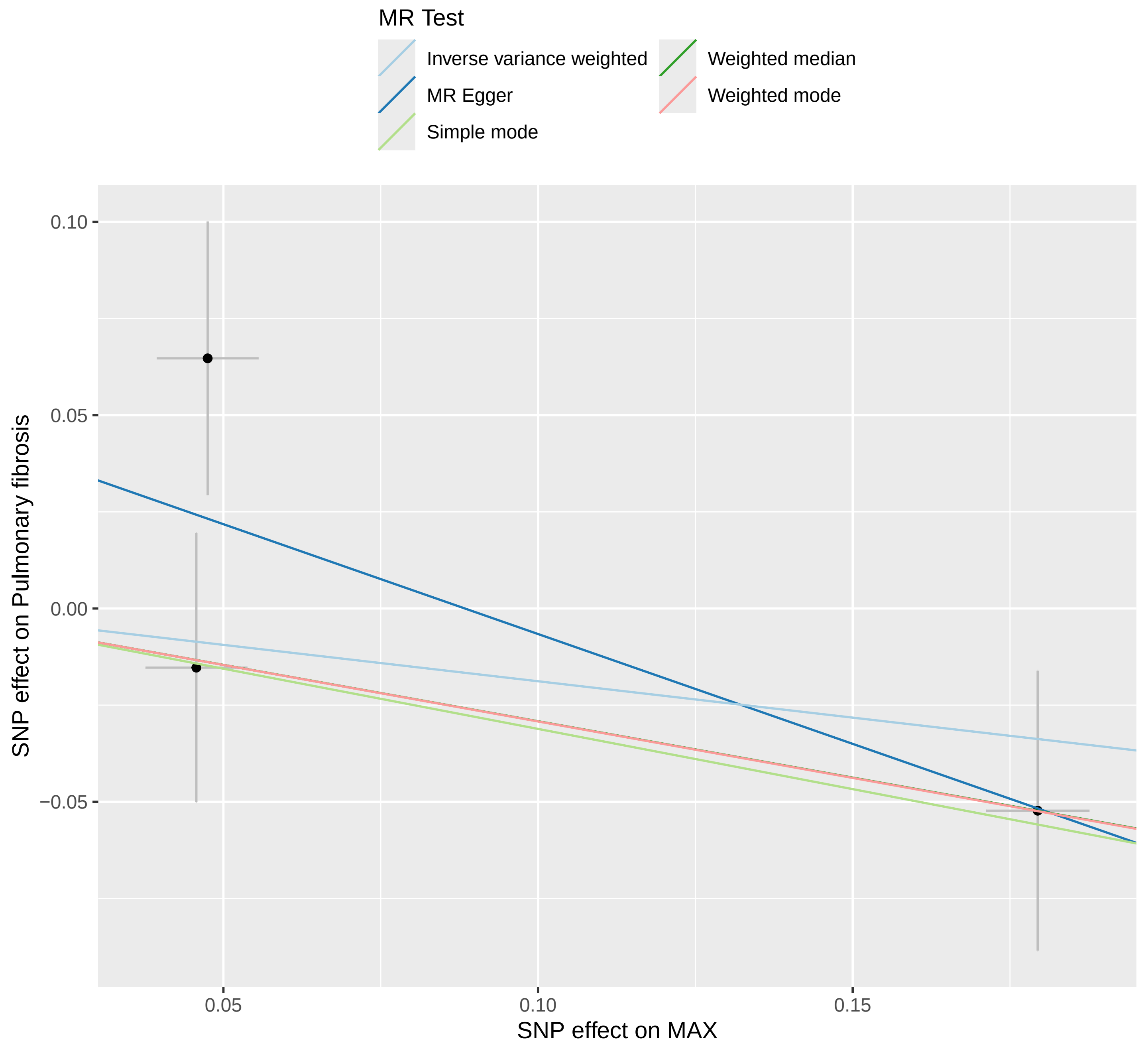
MR Test
Inverse variance weighted
MR Egger
Simple mode
Weighted median
Weighted mode
0.10
0.05
0.00
−0.05
SNP effect on Pulmonary fibrosis
0.05
0.10
0.15
SNP effect on MAX

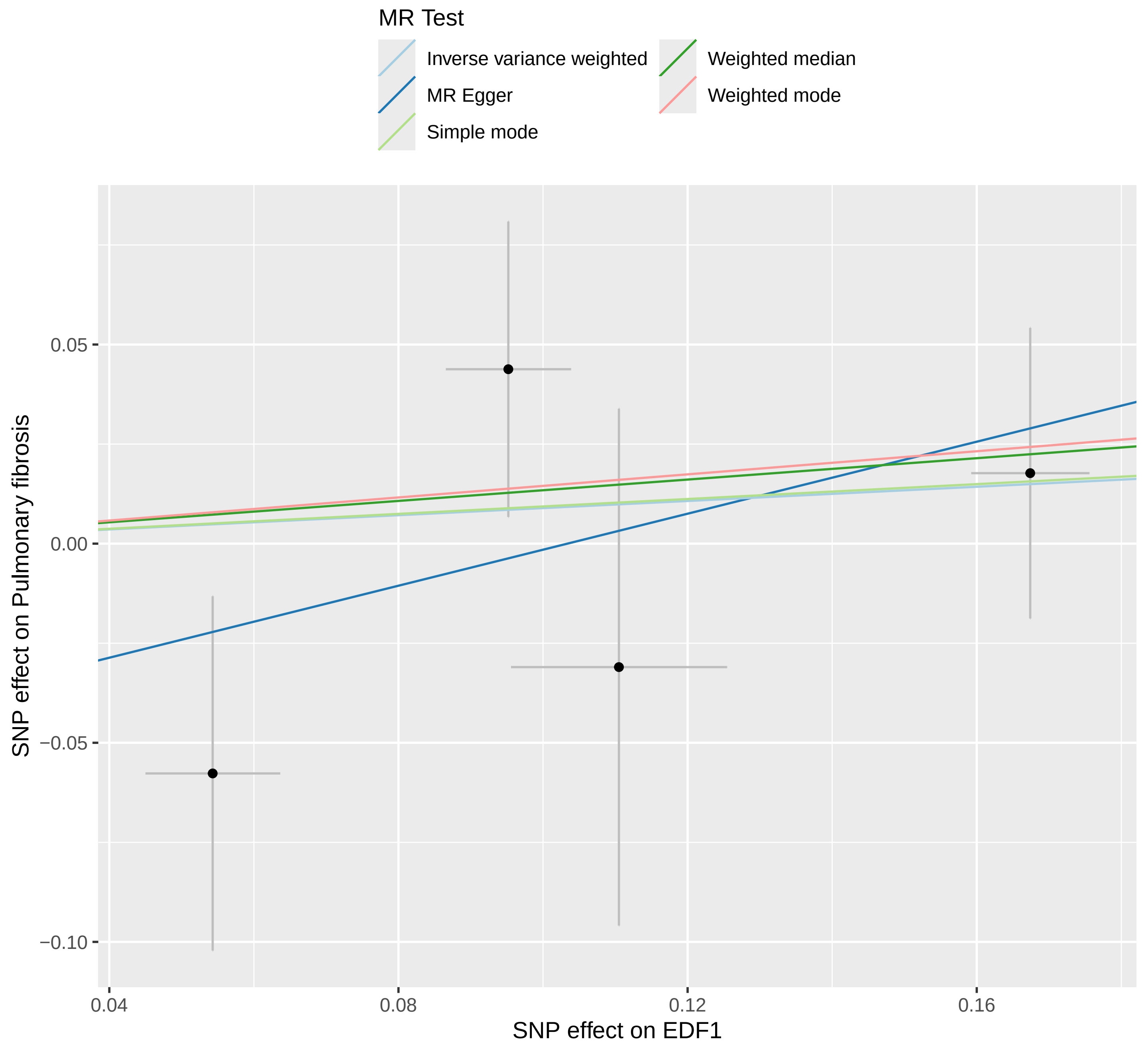
MR Test
Inverse variance weighted
MR Egger
Simple mode
Weighted median
Weighted mode
0.05
0.00
−0.05
−0.10
0.04
0.08
0.12
0.16
SNP effect on Pulmonary fibrosis
SNP effect on EDF1

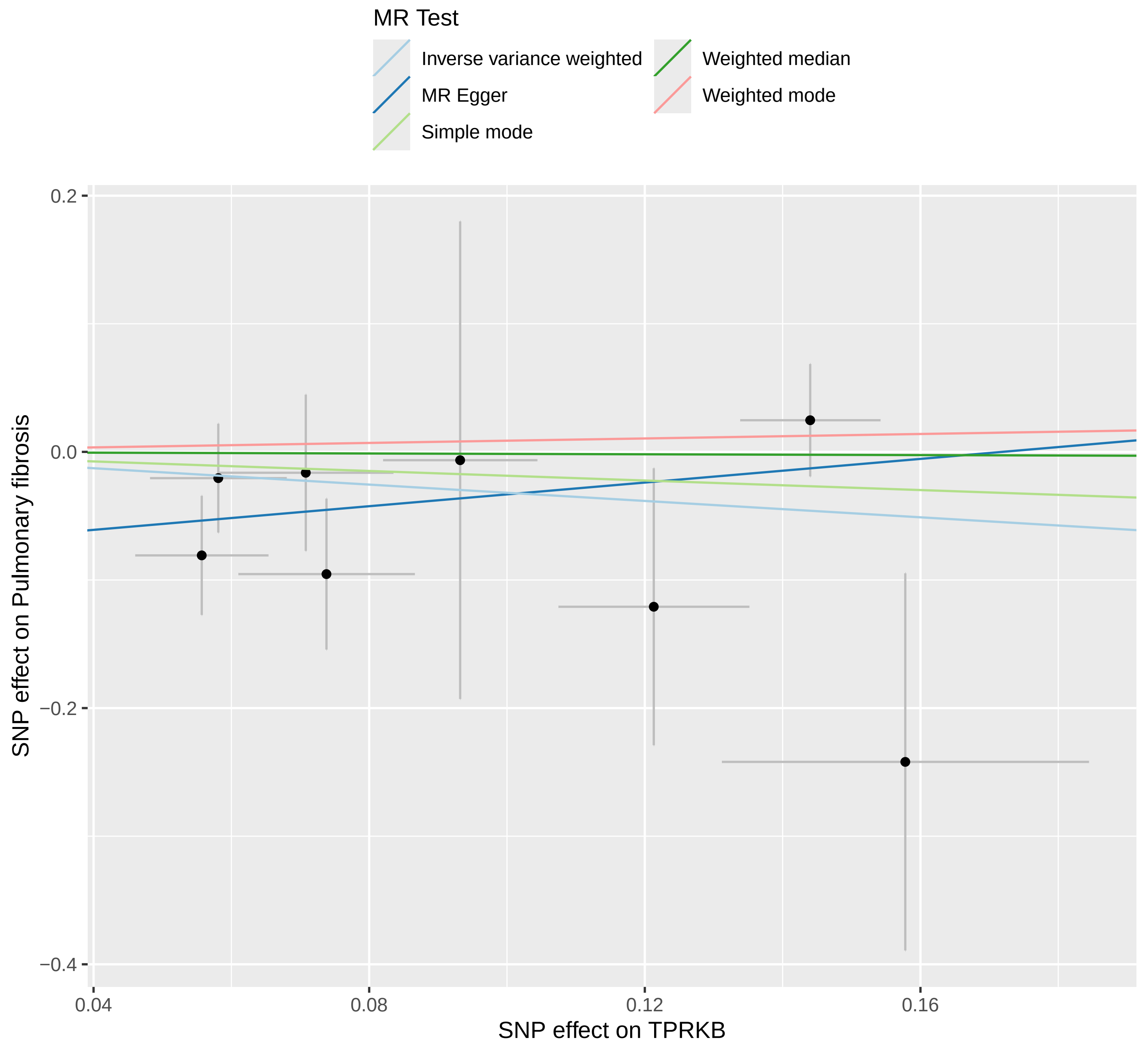
MR Test
Inverse variance weighted
MR Egger
Simple mode
Weighted median
Weighted mode
0.2
0.0
−0.2
−0.4
0.04
0.08
0.12
0.16
SNP effect on Pulmonary fibrosis
SNP effect on TPRKB

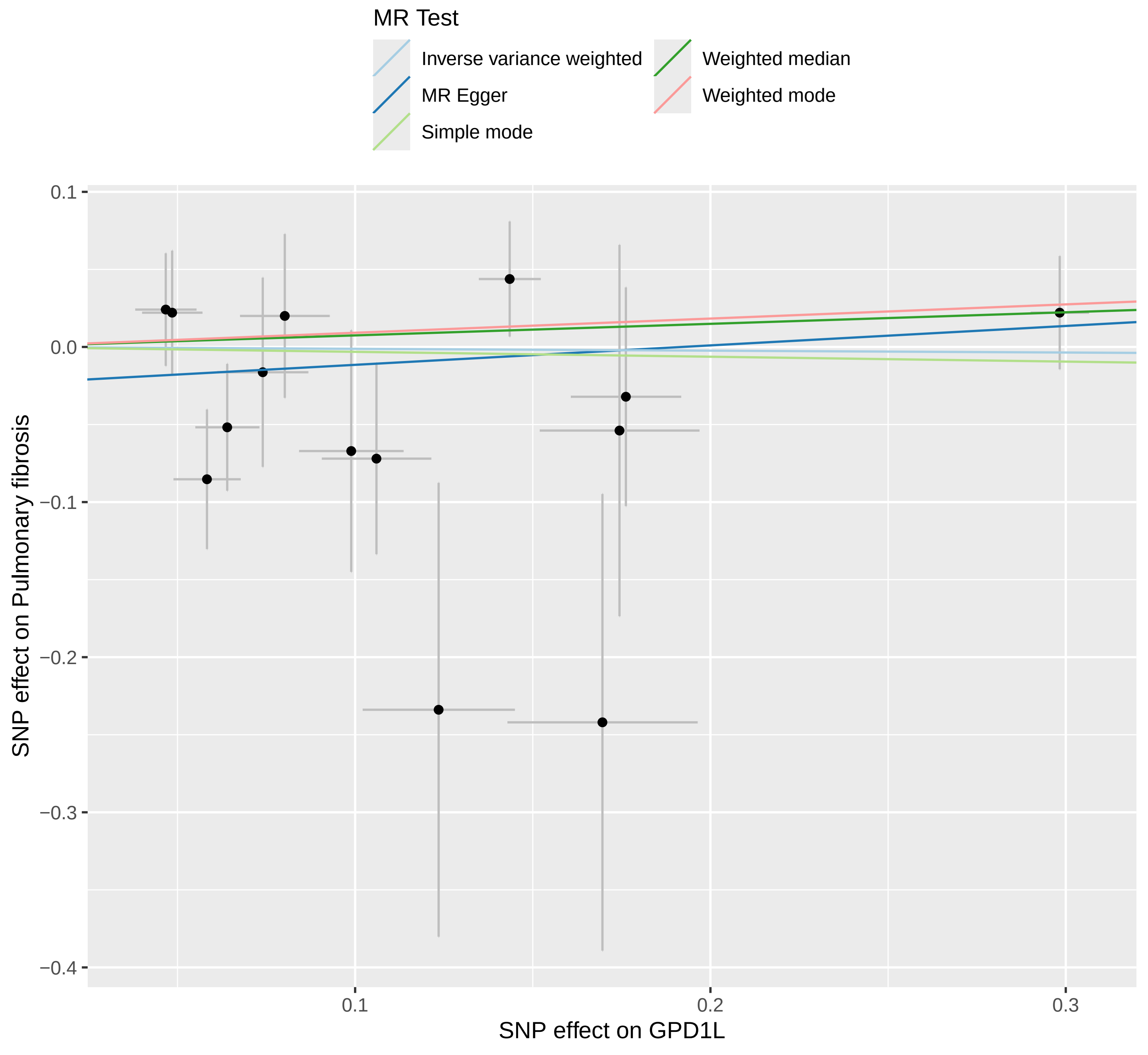
MR Test
Inverse variance weighted
MR Egger
Simple mode
Weighted median
Weighted mode
0.1
0.0
−0.1
−0.2
−0.3
−0.4
SNP effect on Pulmonary fibrosis
0.1
0.2
0.3
SNP effect on GPD1L

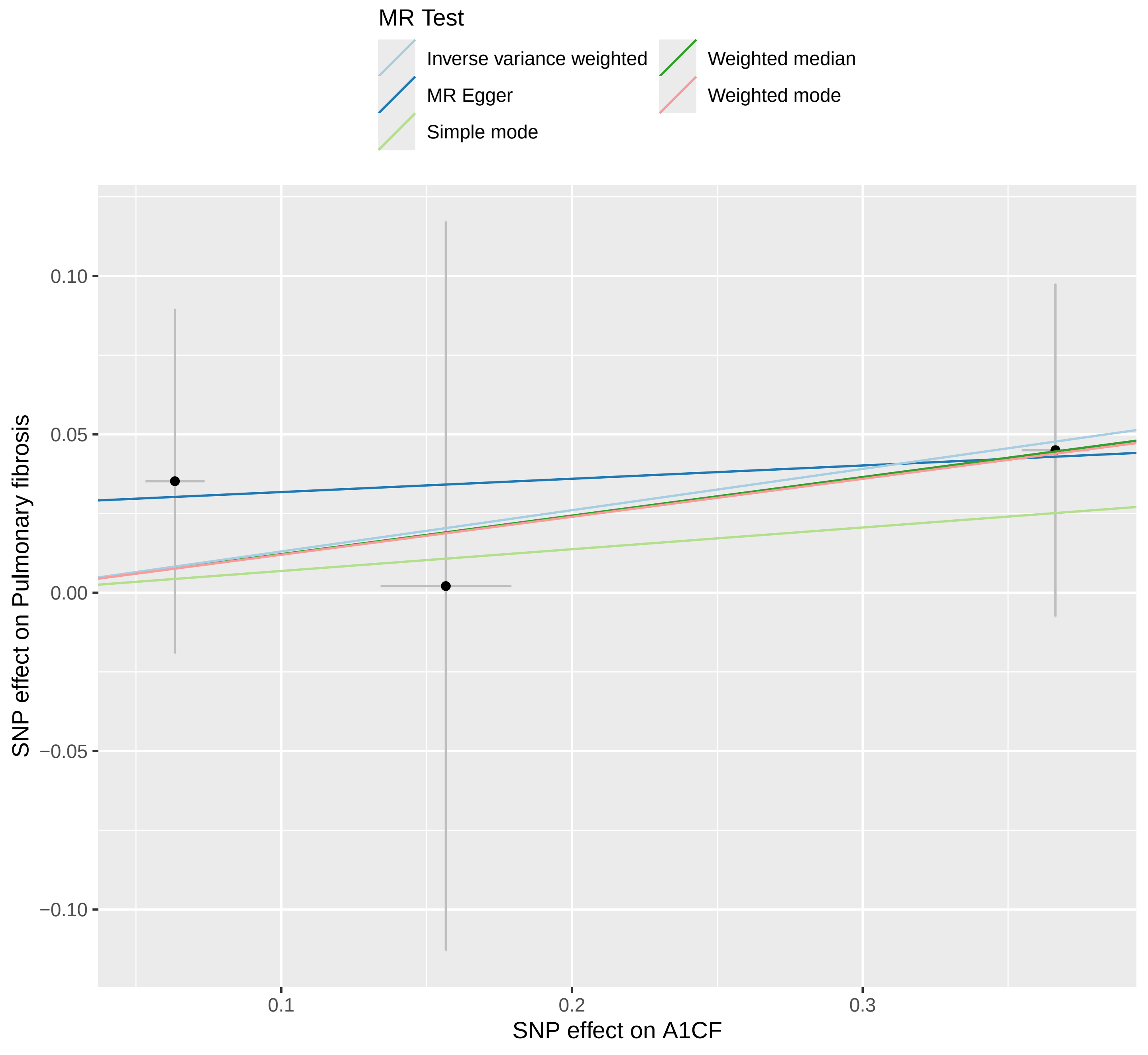
MR Test
Inverse variance weighted
MR Egger
Simple mode
Weighted median
Weighted mode
0.10
0.05
0.00
−0.05
−0.10
SNP effect on Pulmonary fibrosis
0.1
0.2
0.3
SNP effect on A1CF

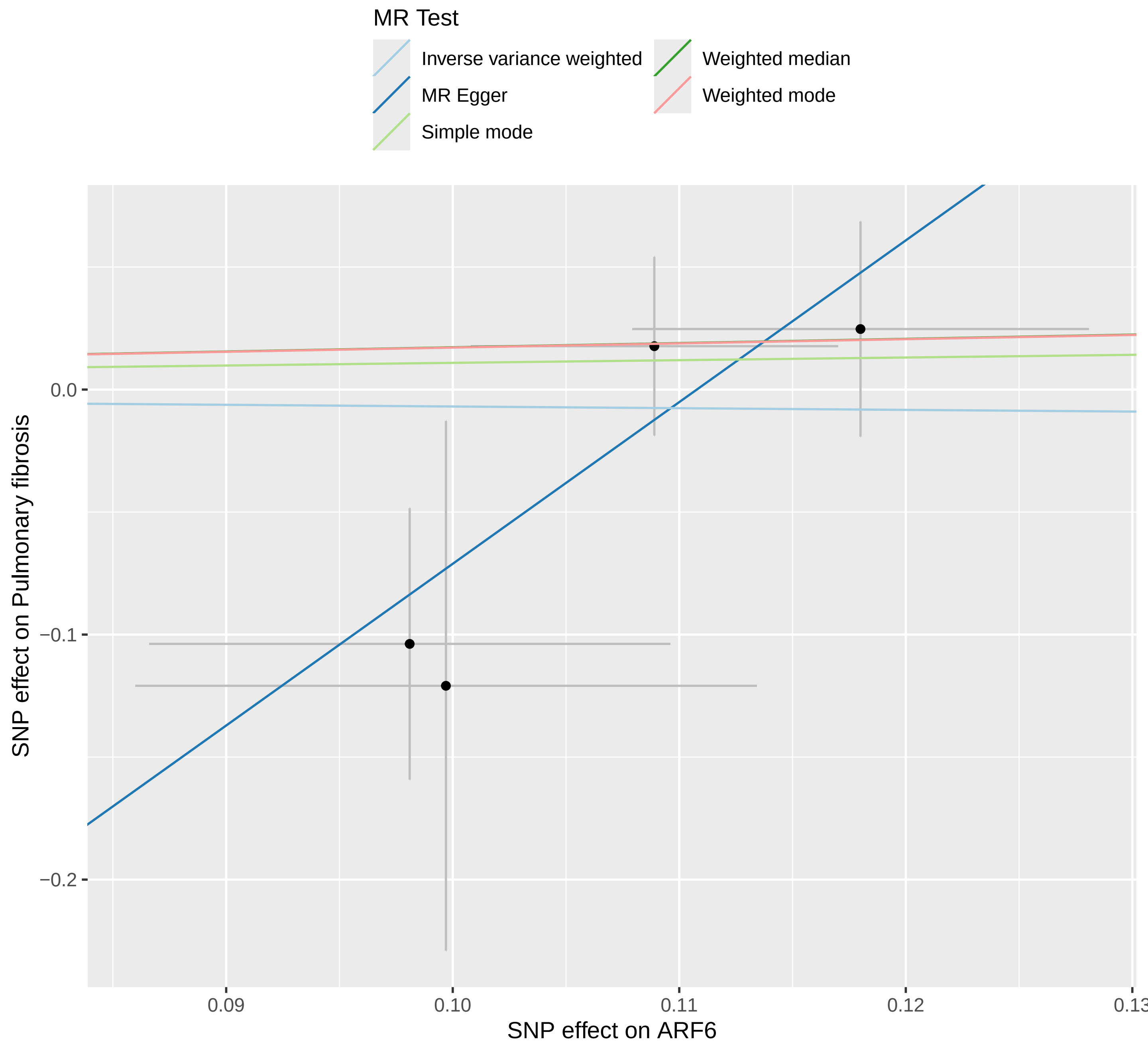
MR Test
Inverse variance weighted
MR Egger
Simple mode
Weighted median
Weighted mode
0.0
−0.1
−0.2
0.09
0.10
0.11
0.12
0.13
SNP effect on ARF6
SNP effect on Pulmonary fibrosis

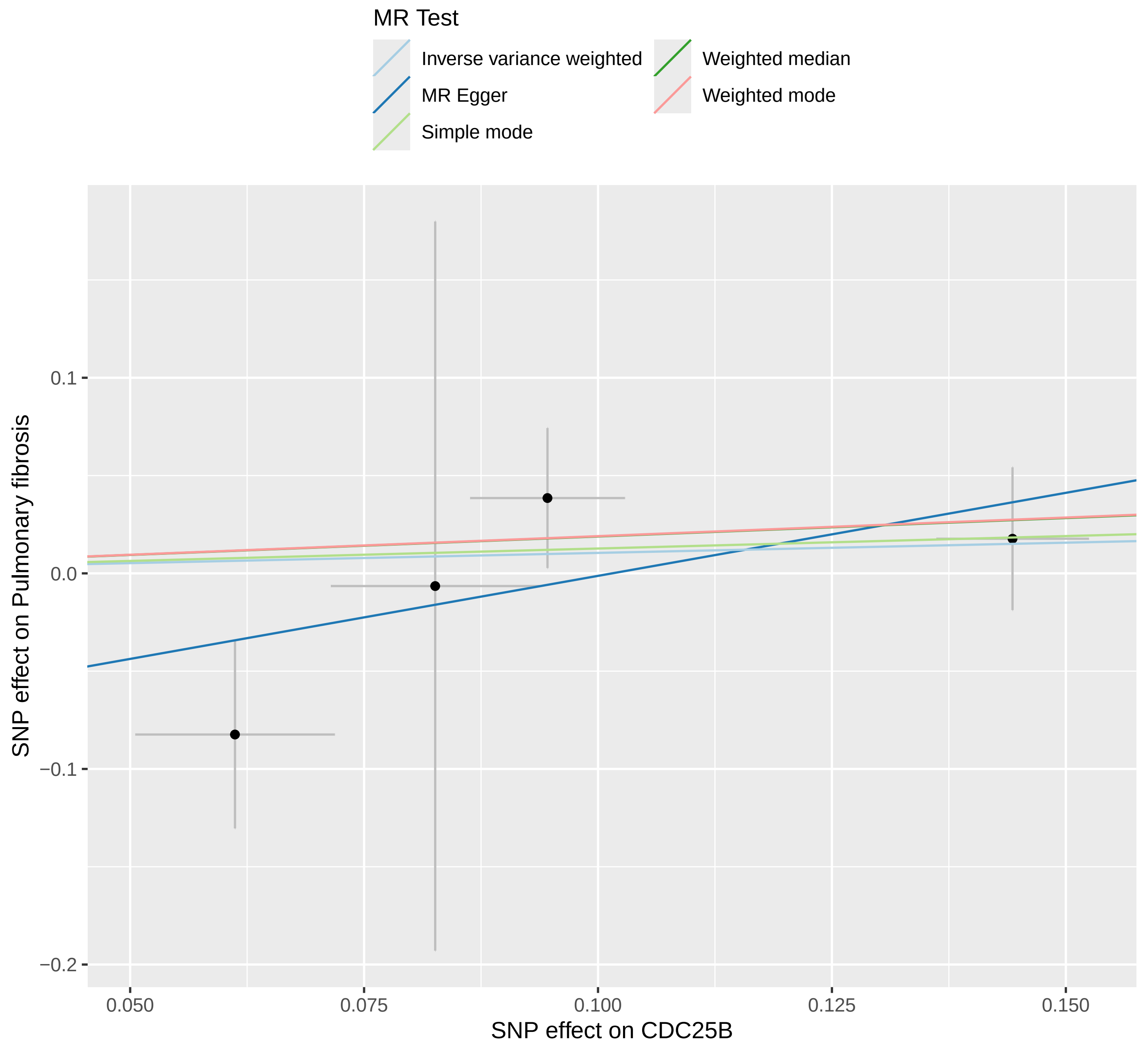
MR Test
Inverse variance weighted
MR Egger
Simple mode
Weighted median
Weighted mode
SNP effect on Pulmonary fibrosis
0.1
0.0
−0.1
−0.2
0.050
0.075
0.100
0.125
0.150
SNP effect on CDC25B

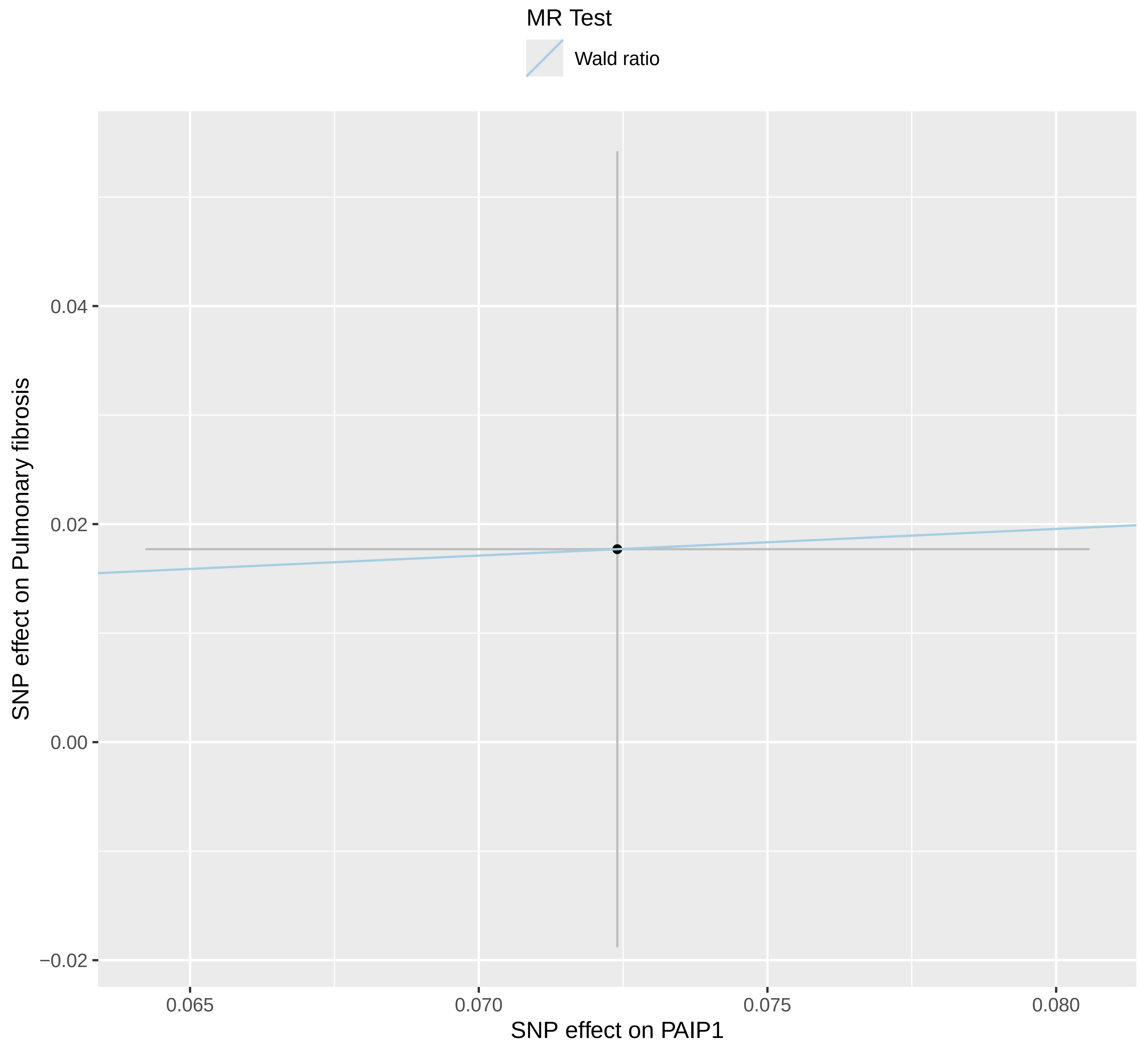
MR Test
Wald ratio
0.04
0.02
0.00
−0.02
SNP effect on Pulmonary fibrosis
0.065
0.070
0.075
0.080
SNP effect on PAIP1

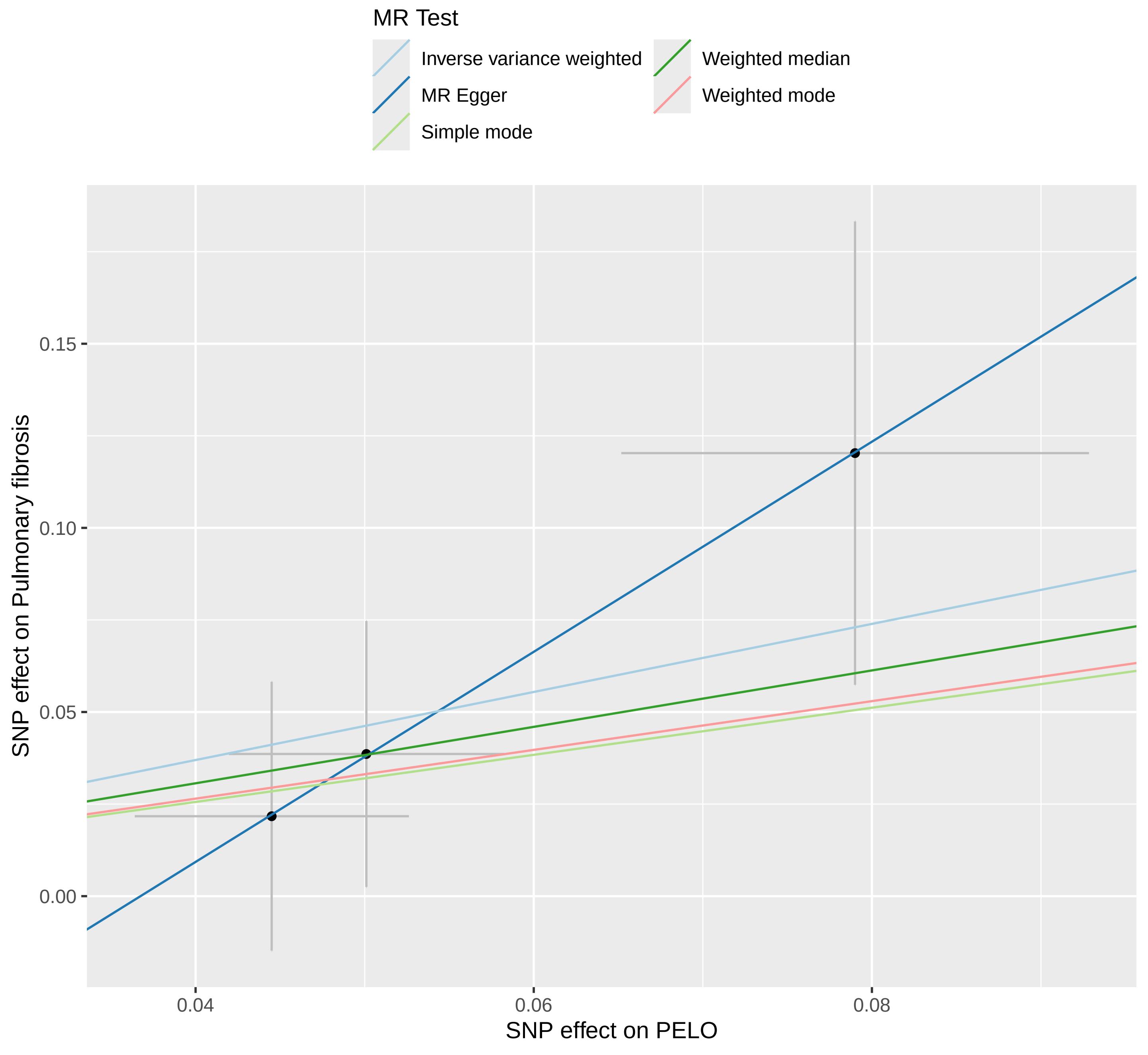
MR Test
Inverse variance weighted
MR Egger
Simple mode
Weighted median
Weighted mode
0.15
0.10
0.05
0.00
SNP effect on Pulmonary fibrosis
0.04
0.06
0.08
SNP effect on PELO

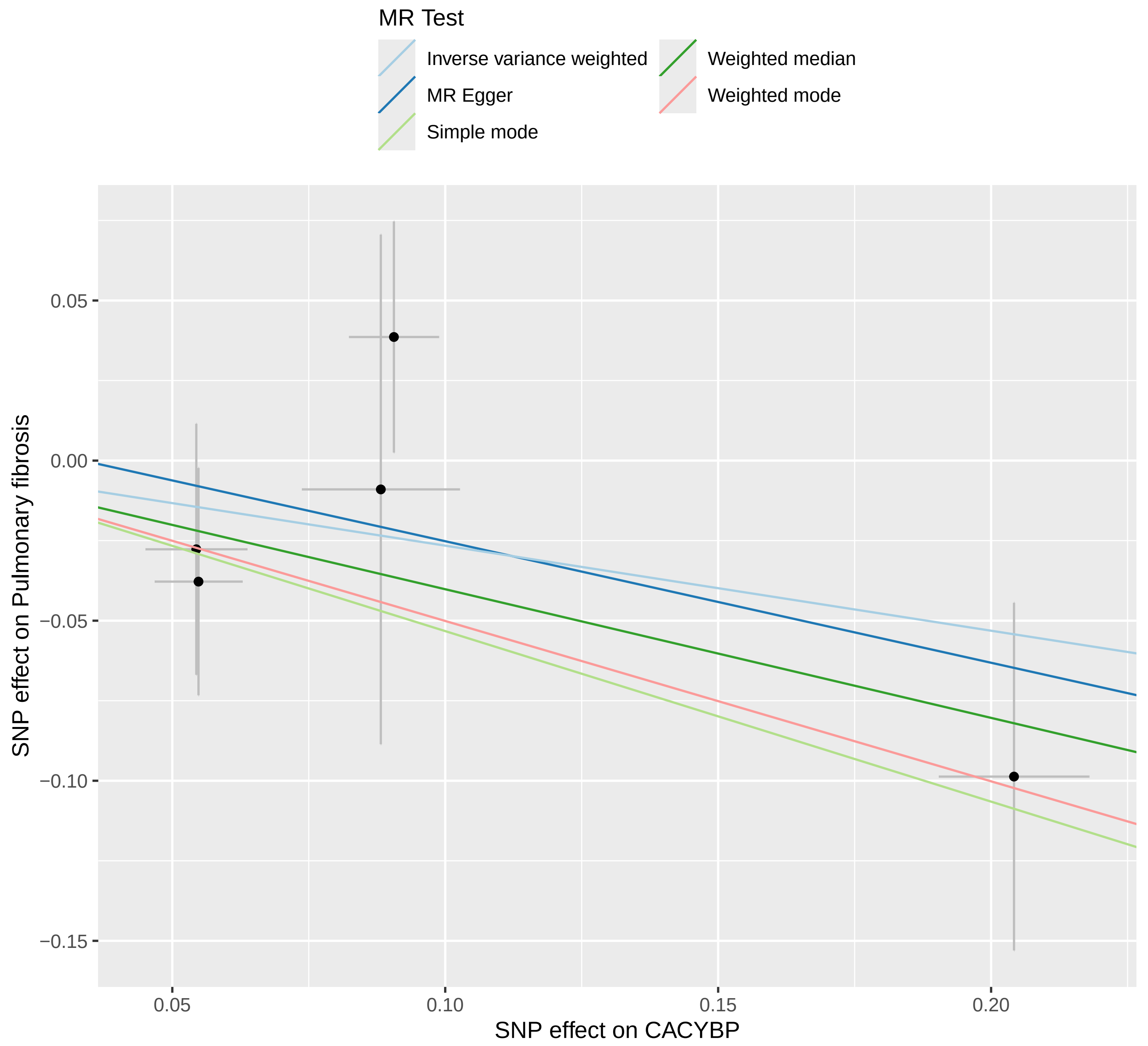

MR Test
Inverse variance weighted
MR Egger
Simple mode
Weighted median
Weighted mode
0.05
0.00
−0.05
−0.10
−0.15
0.05
0.10
0.15
0.20
SNP effect on Pulmonary fibrosis
SNP effect on CACYBP

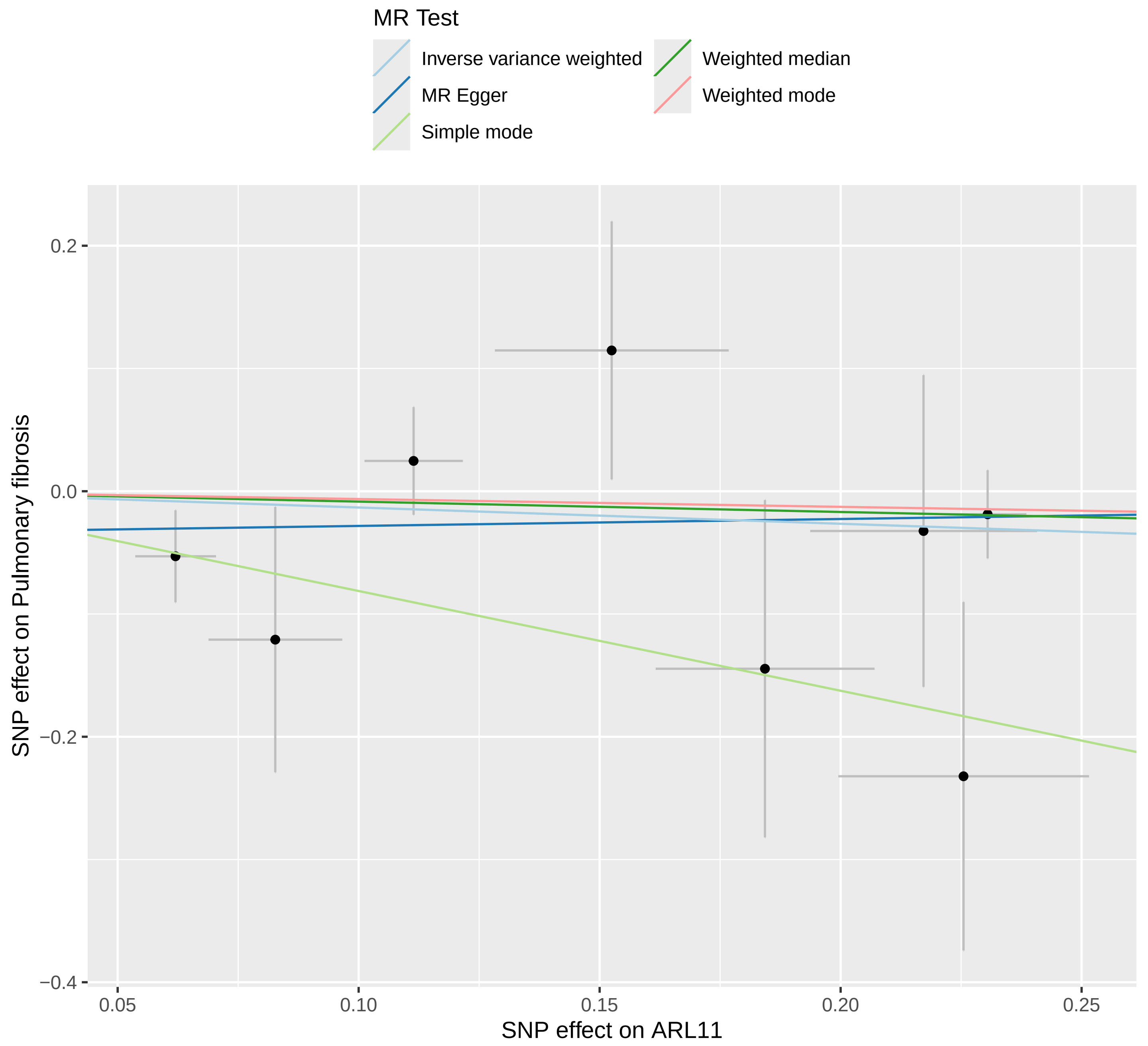
MR Test
Inverse variance weighted
MR Egger
Simple mode
Weighted median
Weighted mode
0.2
0.0
−0.2
−0.4
0.05
0.10
0.15
0.20
0.25
SNP effect on ARL11
SNP effect on Pulmonary fibrosis

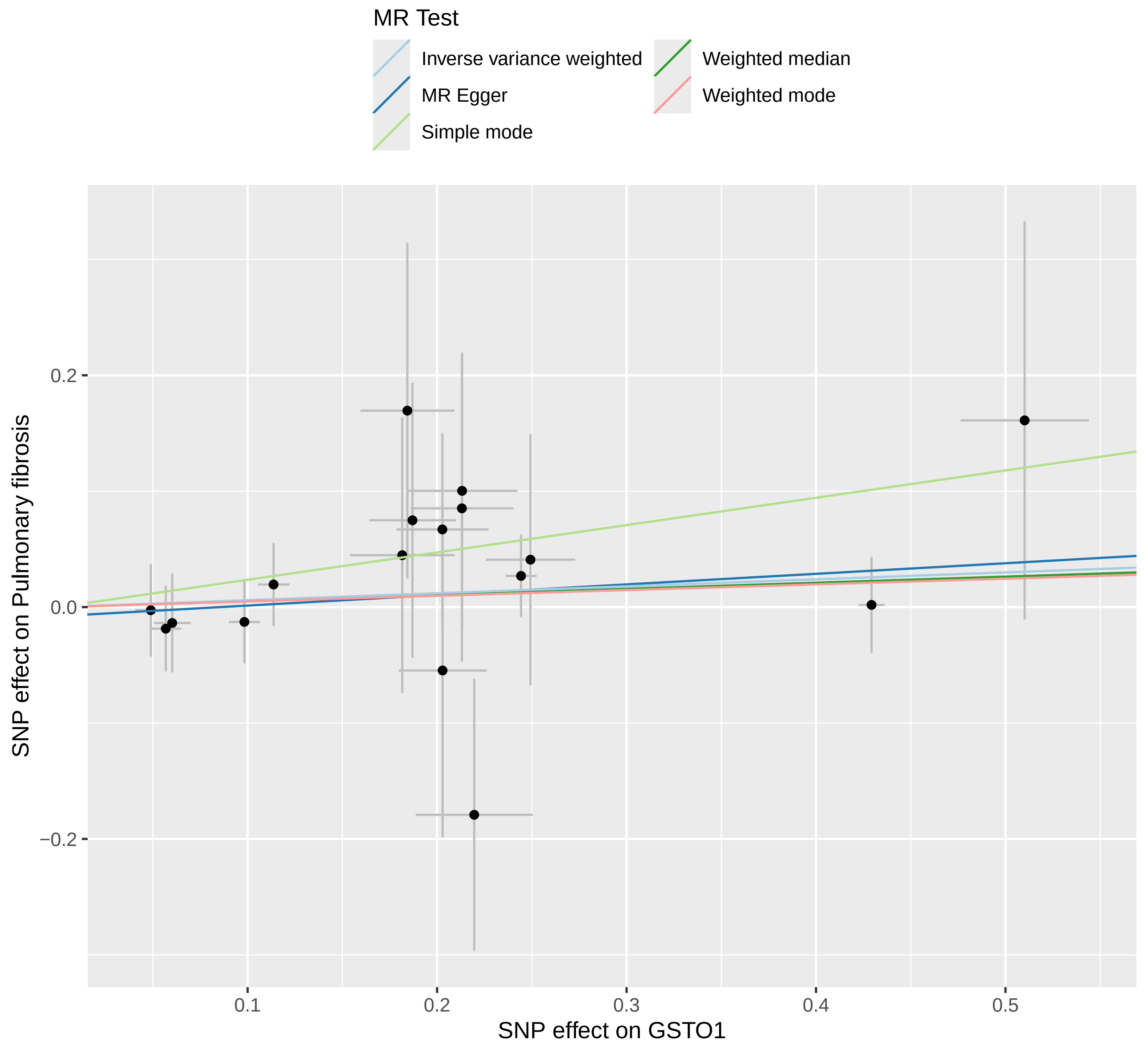
MR Test
Inverse variance weighted
MR Egger
Simple mode
Weighted median
Weighted mode
0.2
0.0
−0.2
SNP effect on Pulmonary fibrosis
0.1
0.2
0.3
0.4
0.5
SNP effect on GSTO1

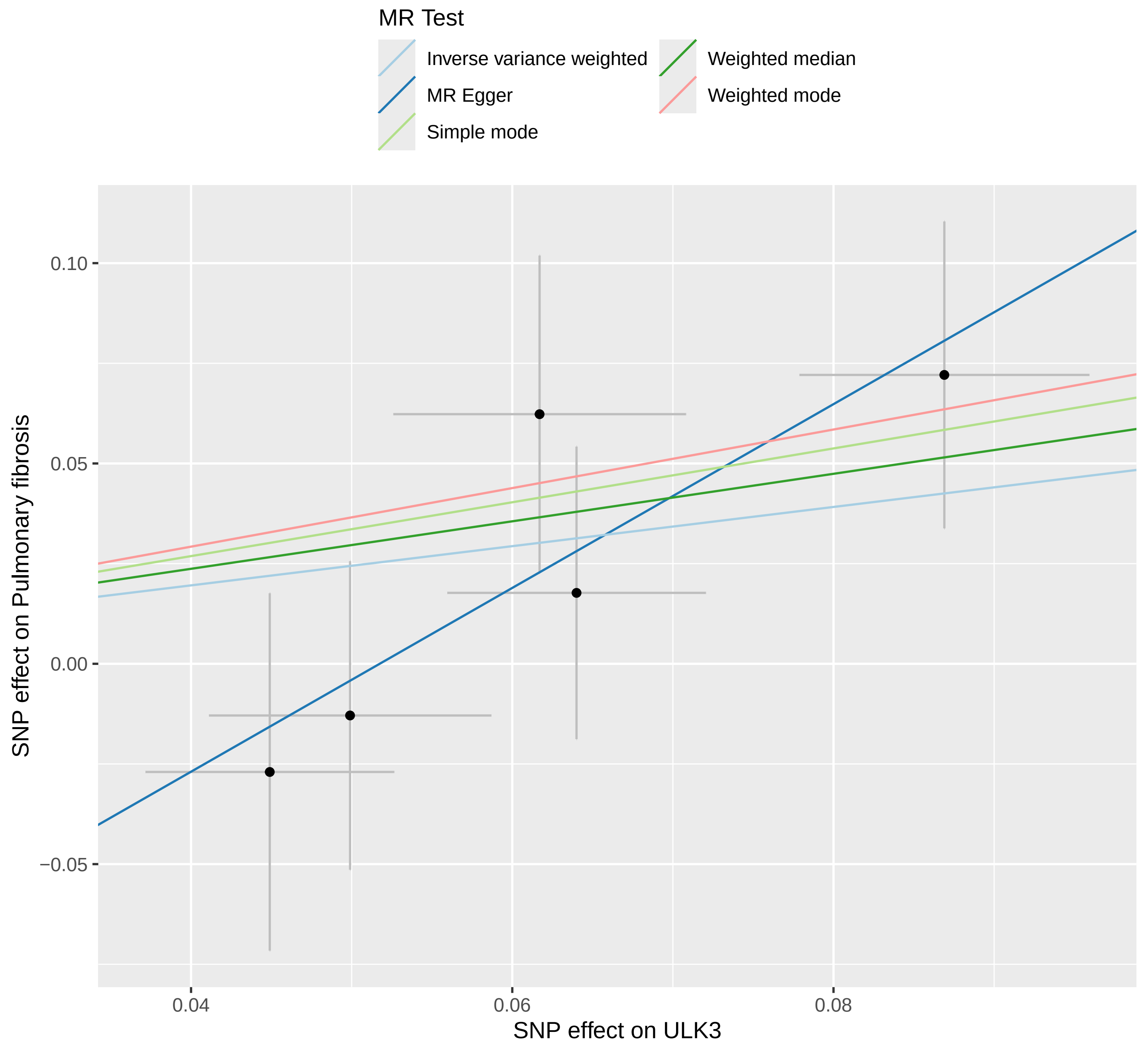

MR Test
Inverse variance weighted
MR Egger
Simple mode
Weighted median
Weighted mode
0.10
0.05
0.00
−0.05
0.04
0.06
0.08
SNP effect on Pulmonary fibrosis
SNP effect on ULK3

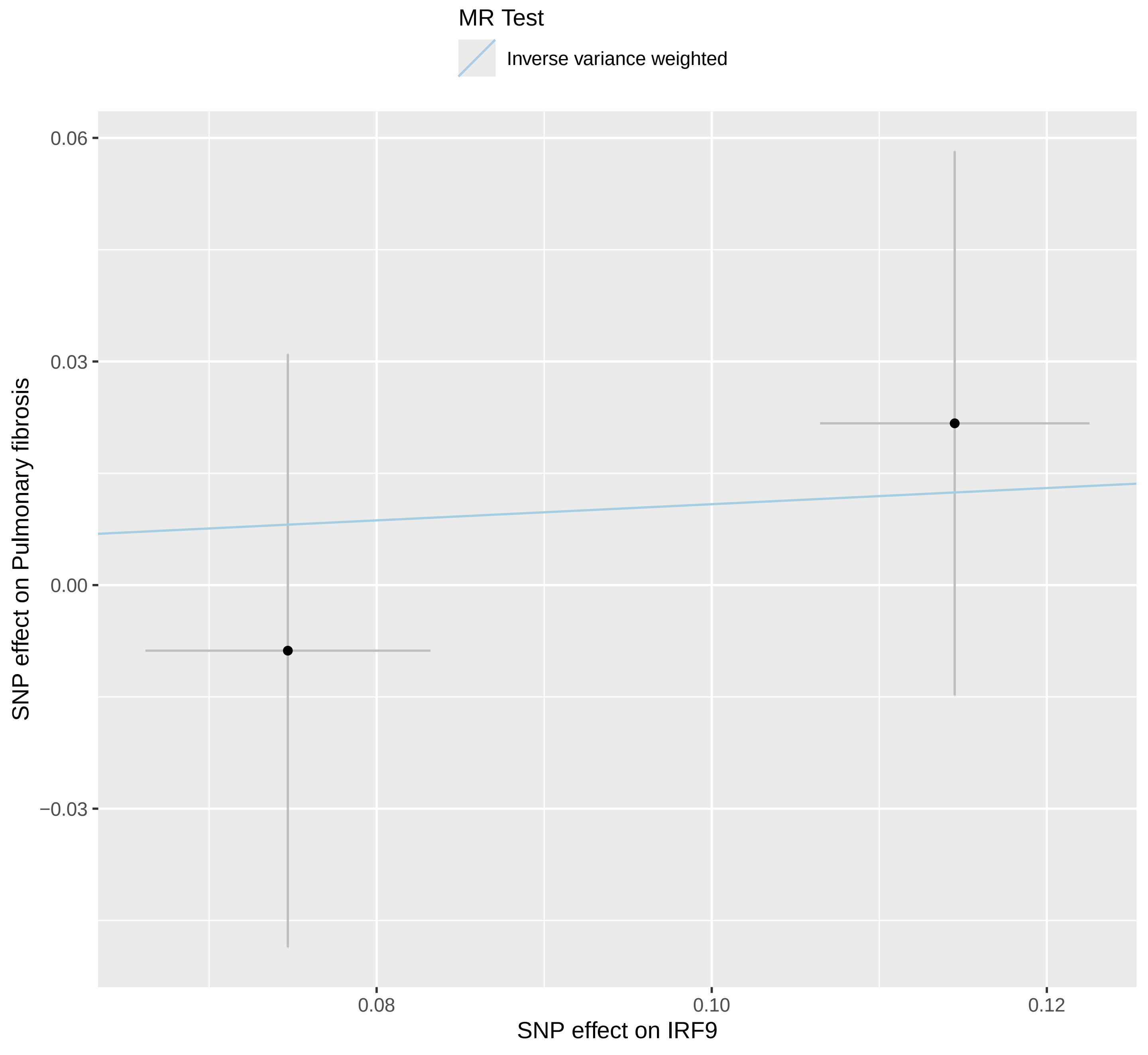
MR Test
Inverse variance weighted
0.06
0.03
0.00
−0.03
SNP effect on Pulmonary fibrosis
0.08
0.10
0.12
SNP effect on IRF9

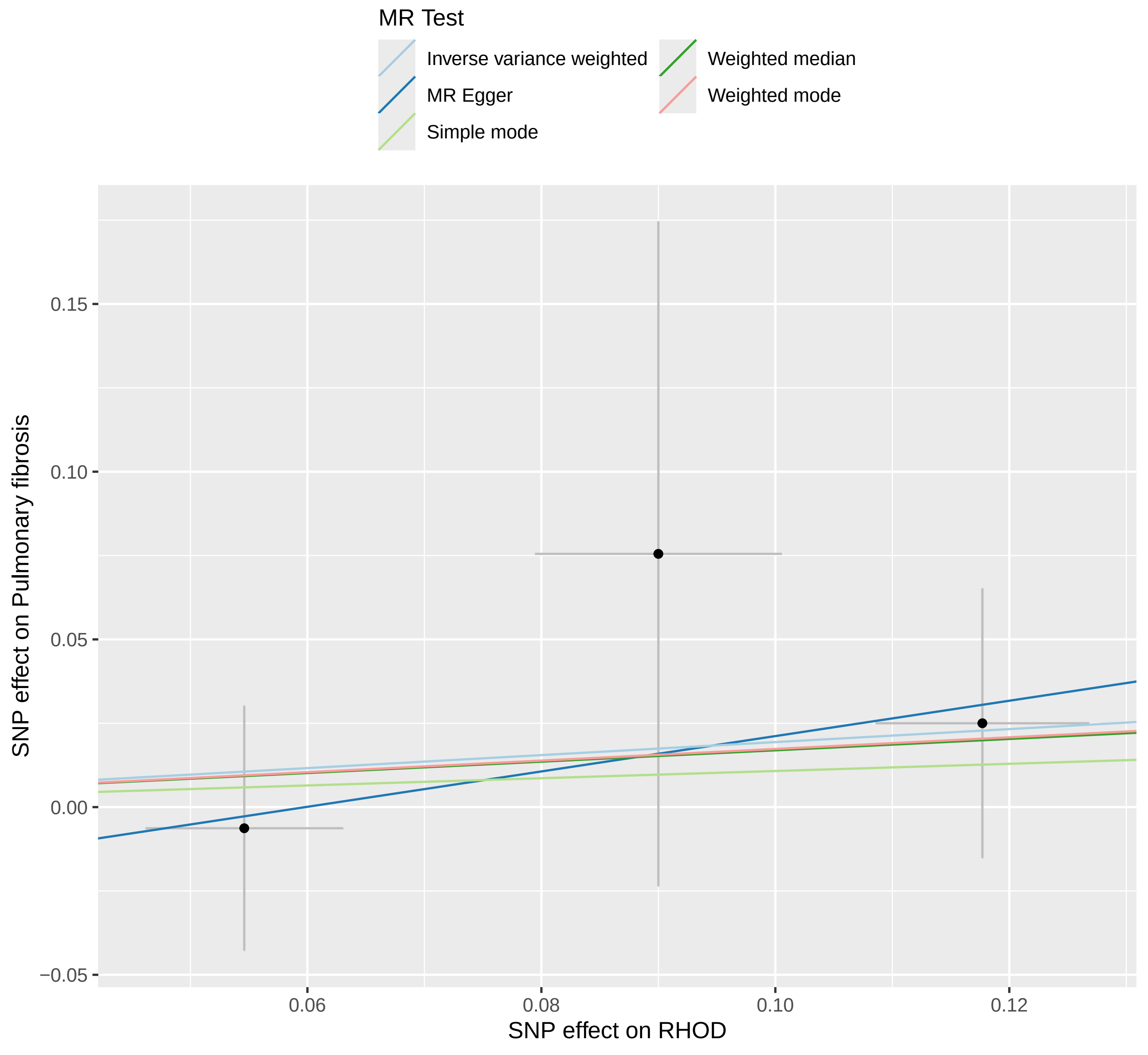

MR Test
Inverse variance weighted
MR Egger
Simple mode
Weighted median
Weighted mode
0.15
0.10
0.05
0.00
−0.05
0.06
0.08
0.10
0.12
SNP effect on Pulmonary fibrosis
SNP effect on RHOD

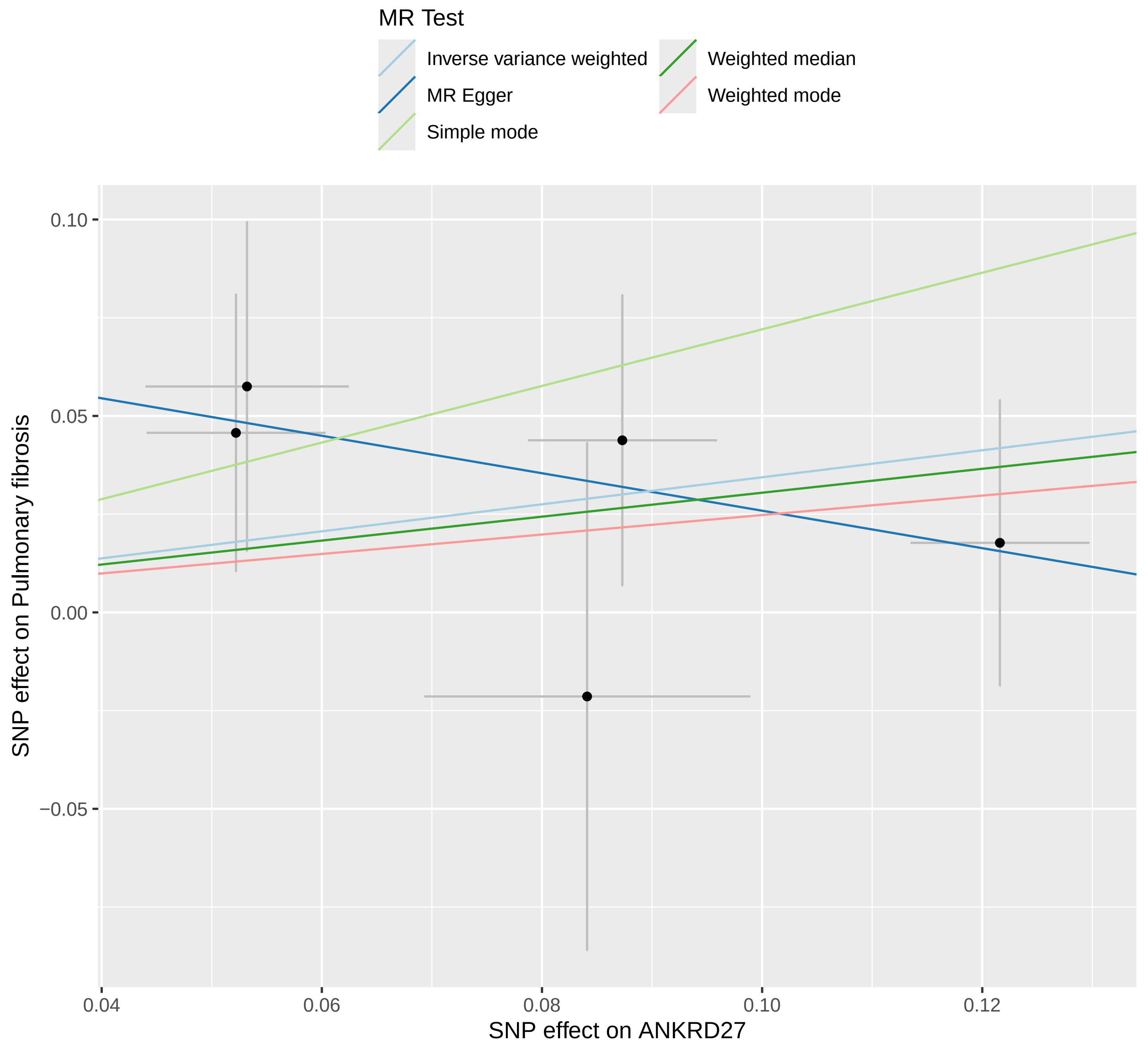
MR Test
Inverse variance weighted
MR Egger
Simple mode
Weighted median
Weighted mode
0.10
0.05
0.00
−0.05
SNP effect on Pulmonary fibrosis
0.04
0.06
0.08
0.10
0.12
SNP effect on ANKRD27

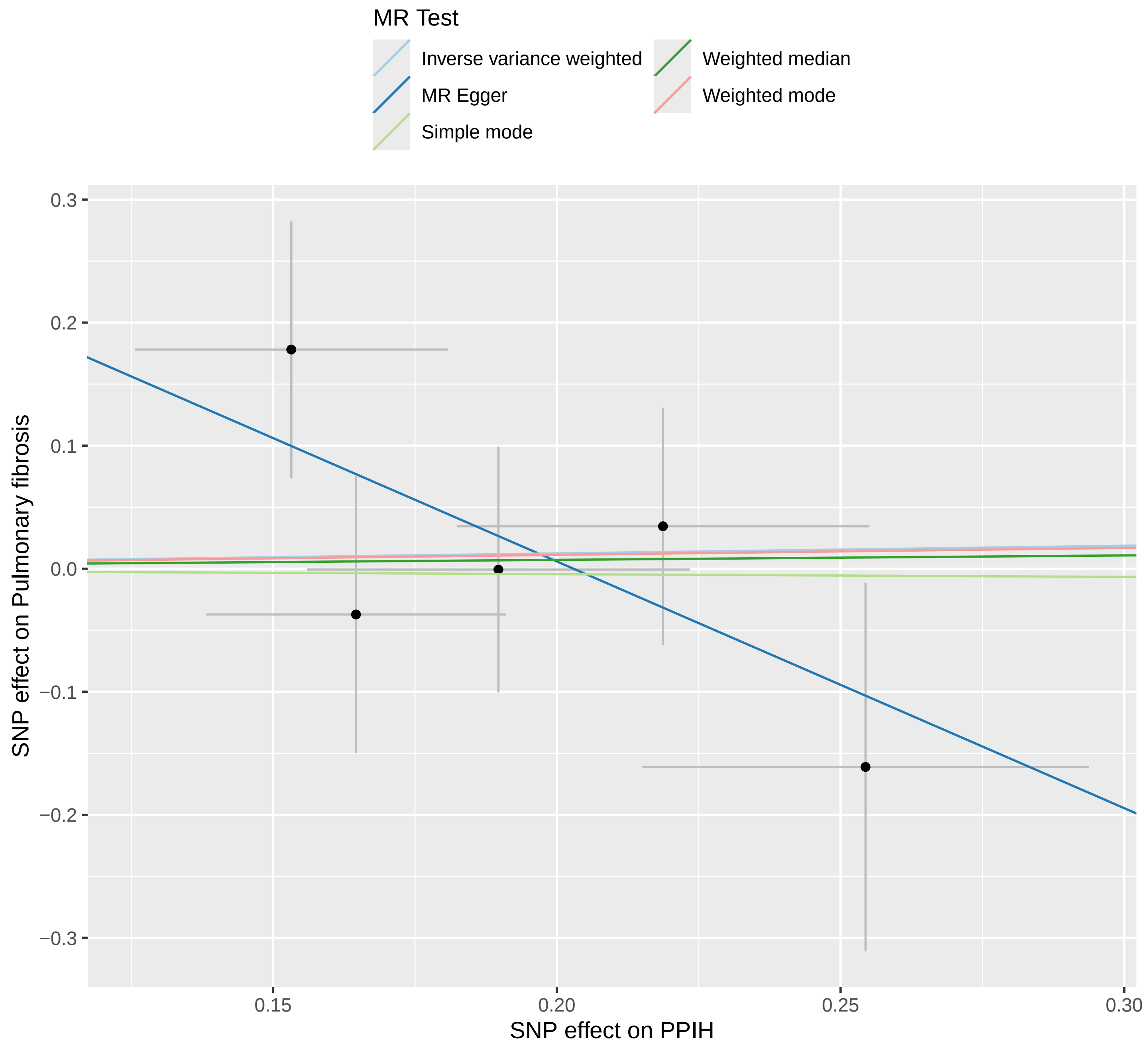
MR Test
Inverse variance weighted
MR Egger
Simple mode
Weighted median
Weighted mode
0.3
0.2
0.1
0.0
−0.1
−0.2
−0.3
SNP effect on Pulmonary fibrosis
0.15
0.20
0.25
0.30
SNP effect on PPIH

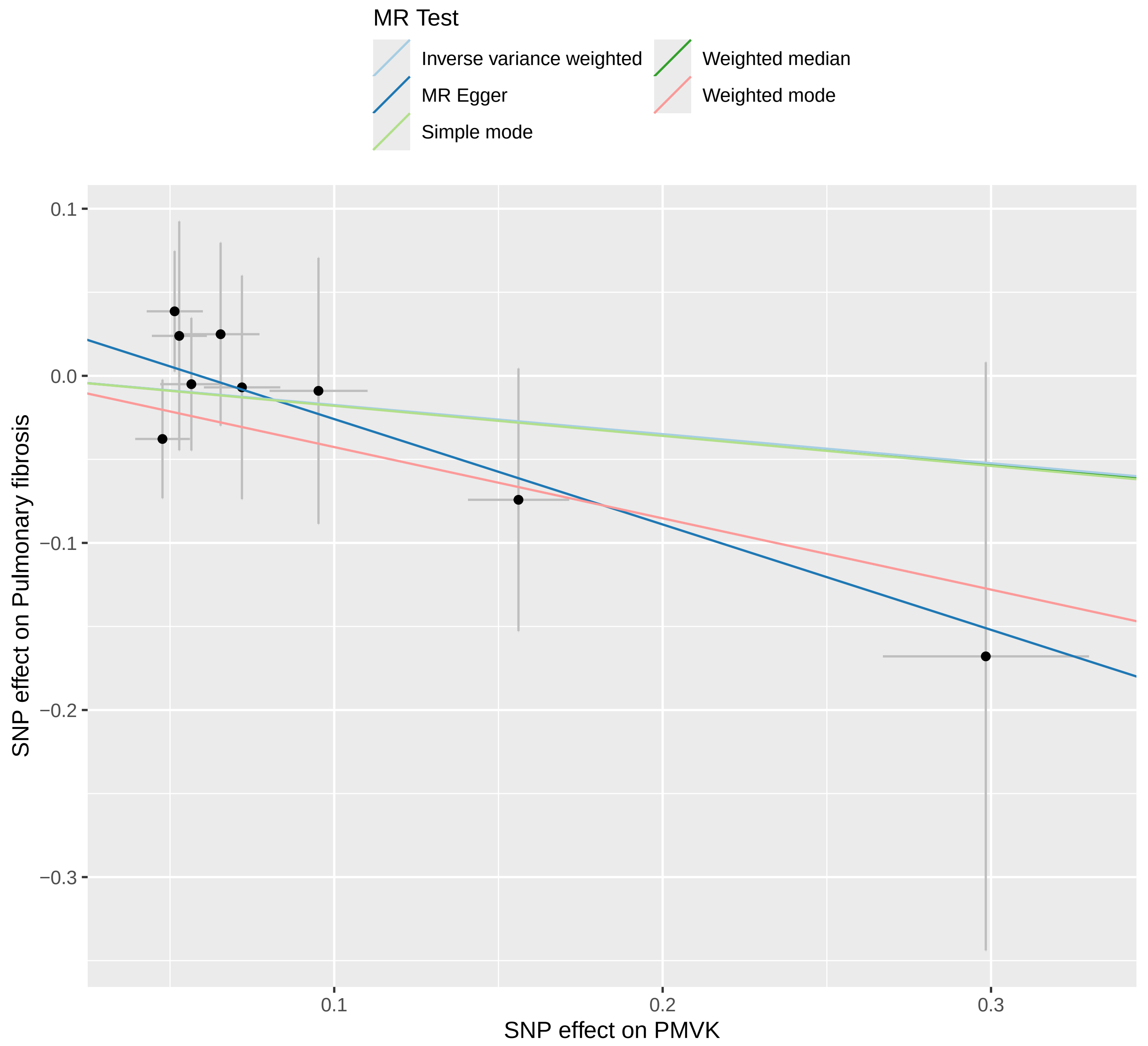

MR Test
Inverse variance weighted
MR Egger
Simple mode
Weighted median
Weighted mode
0.1
0.0
−0.1
−0.2
−0.3
0.1
0.2
0.3
SNP effect on Pulmonary fibrosis
SNP effect on PMVK

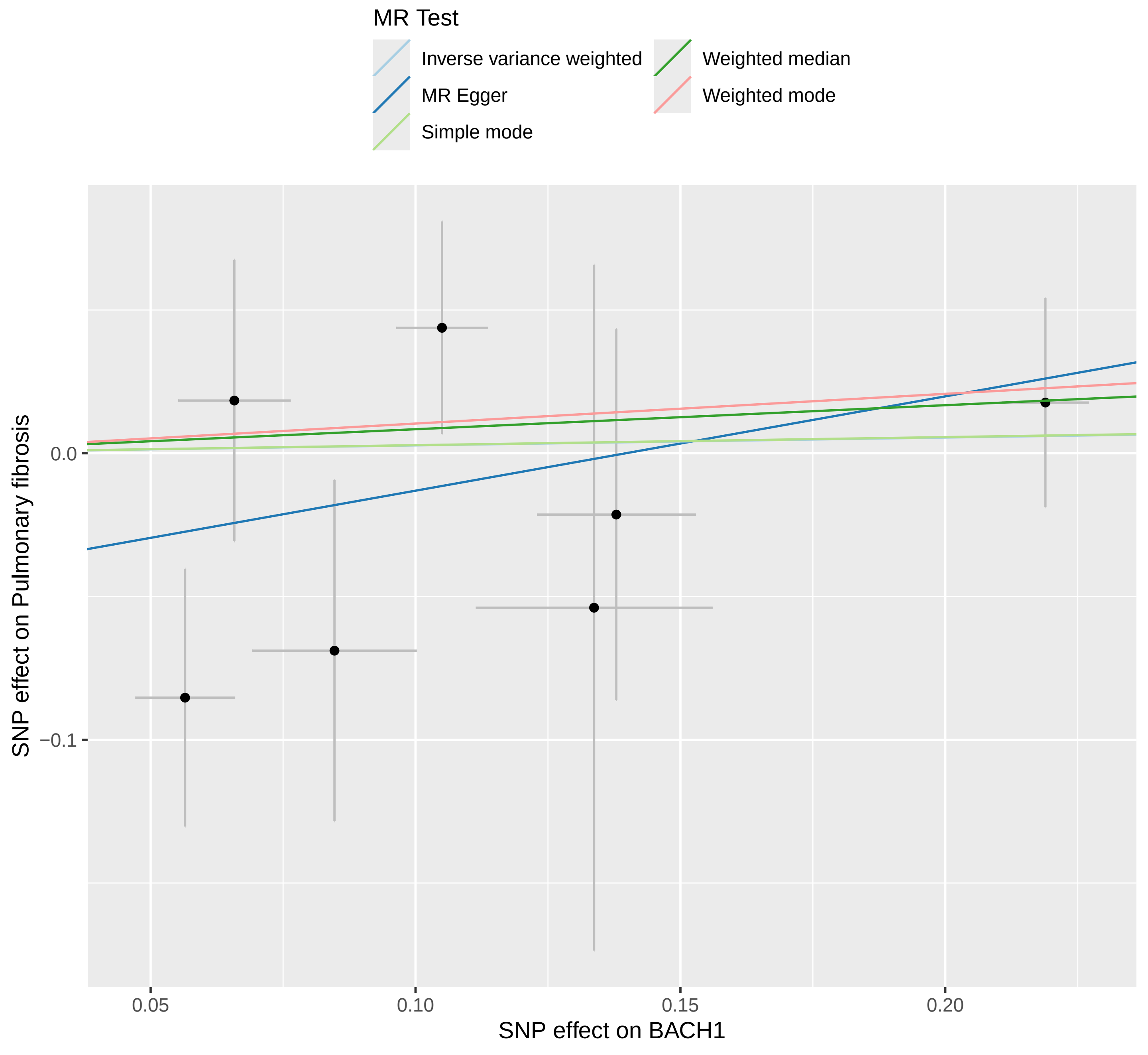

MR Test
Inverse variance weighted
MR Egger
Simple mode
Weighted median
Weighted mode
0.0
−0.1
SNP effect on Pulmonary fibrosis
0.05
0.10
0.15
0.20
SNP effect on BACH1

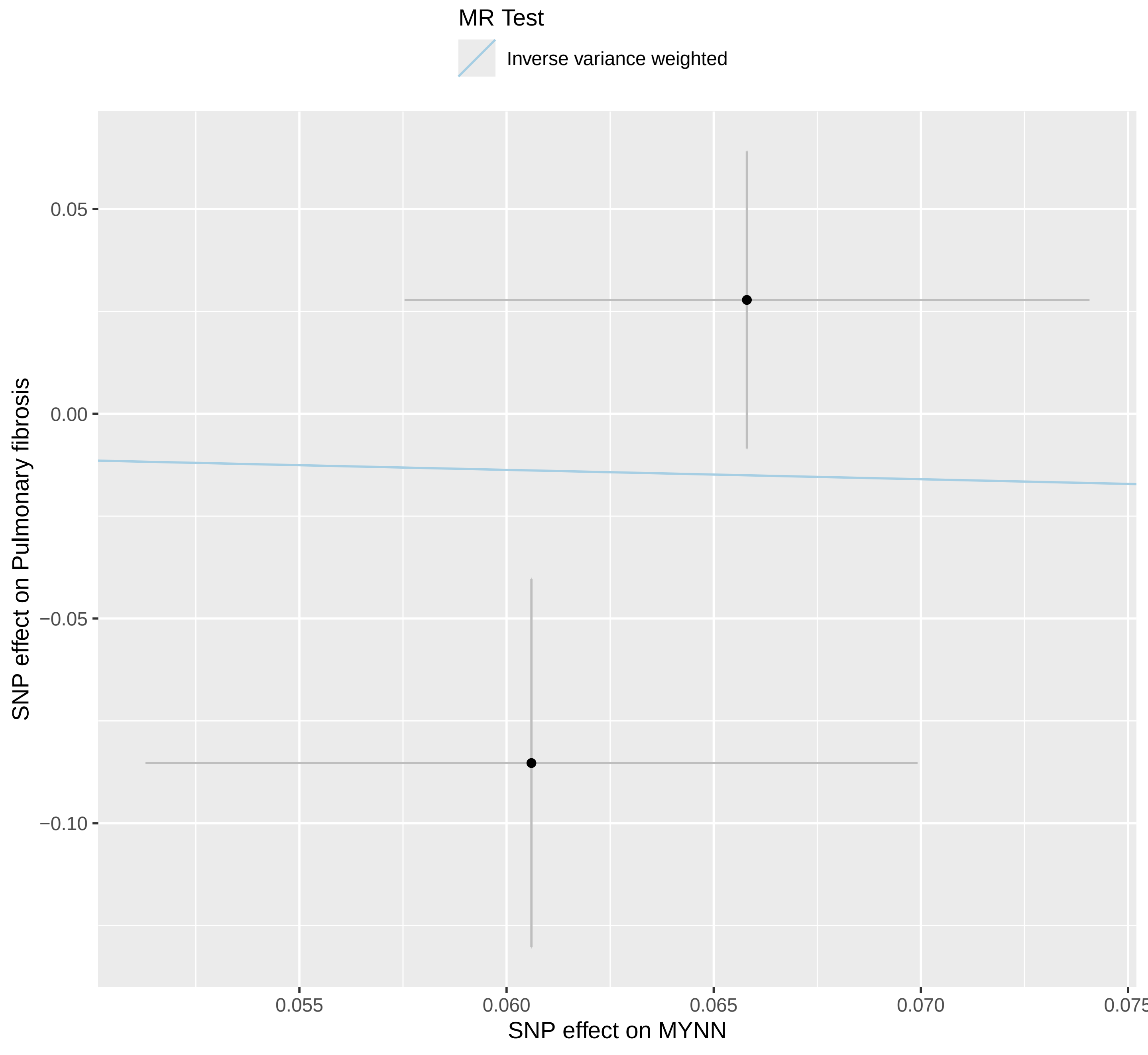
MR Test
Inverse variance weighted
SNP effect on Pulmonary fibrosis
0.05
0.00
−0.05
−0.10
0.055
0.060
0.065
0.070
0.075
SNP effect on MYNN

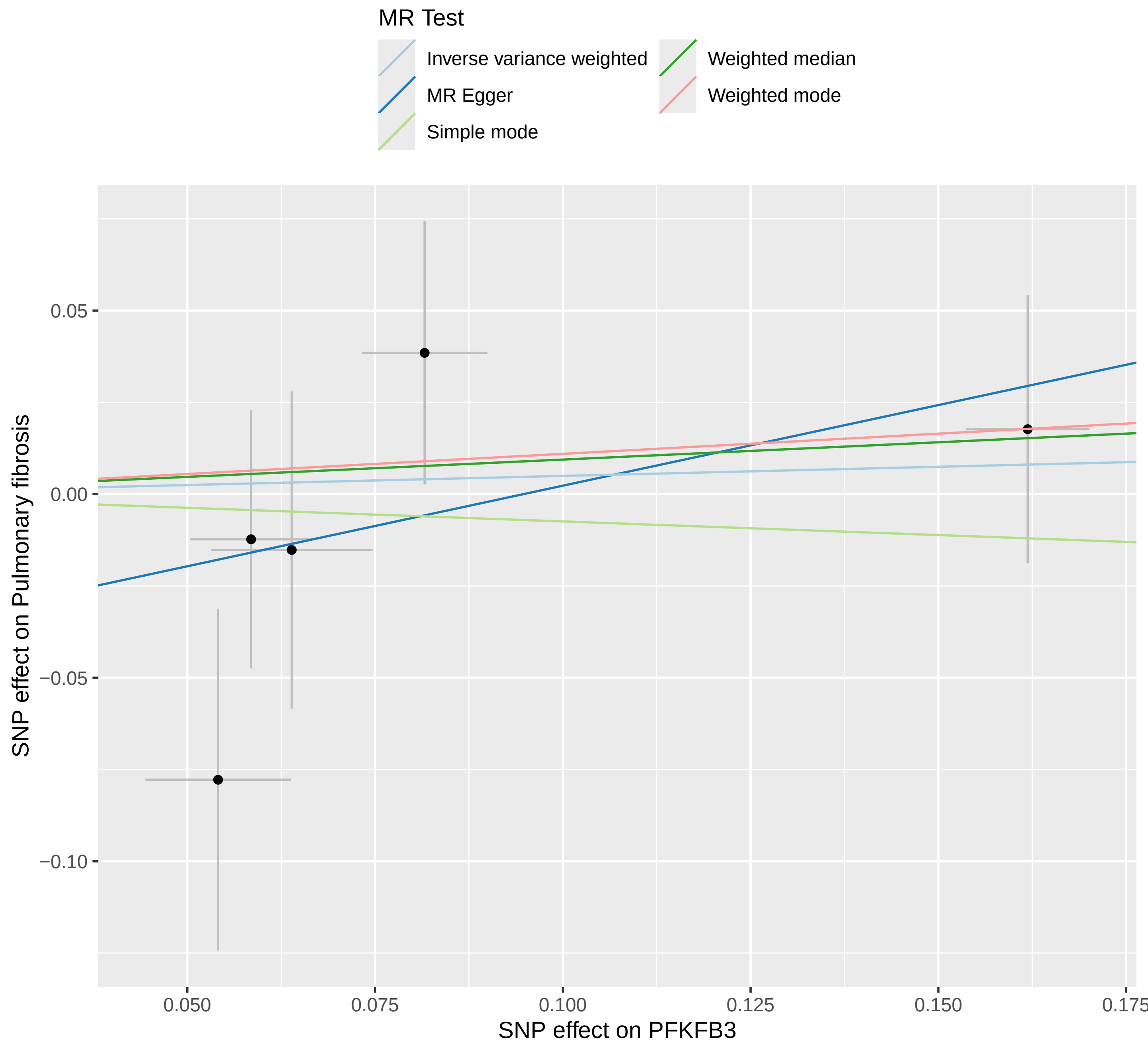
MR Test
Inverse variance weighted
MR Egger
Simple mode
Weighted median
Weighted mode
0.05
0.00
−0.05
−0.10
0.050
0.075
0.100
0.125
0.150
0.175
SNP effect on Pulmonary fibrosis
SNP effect on PFKFB3

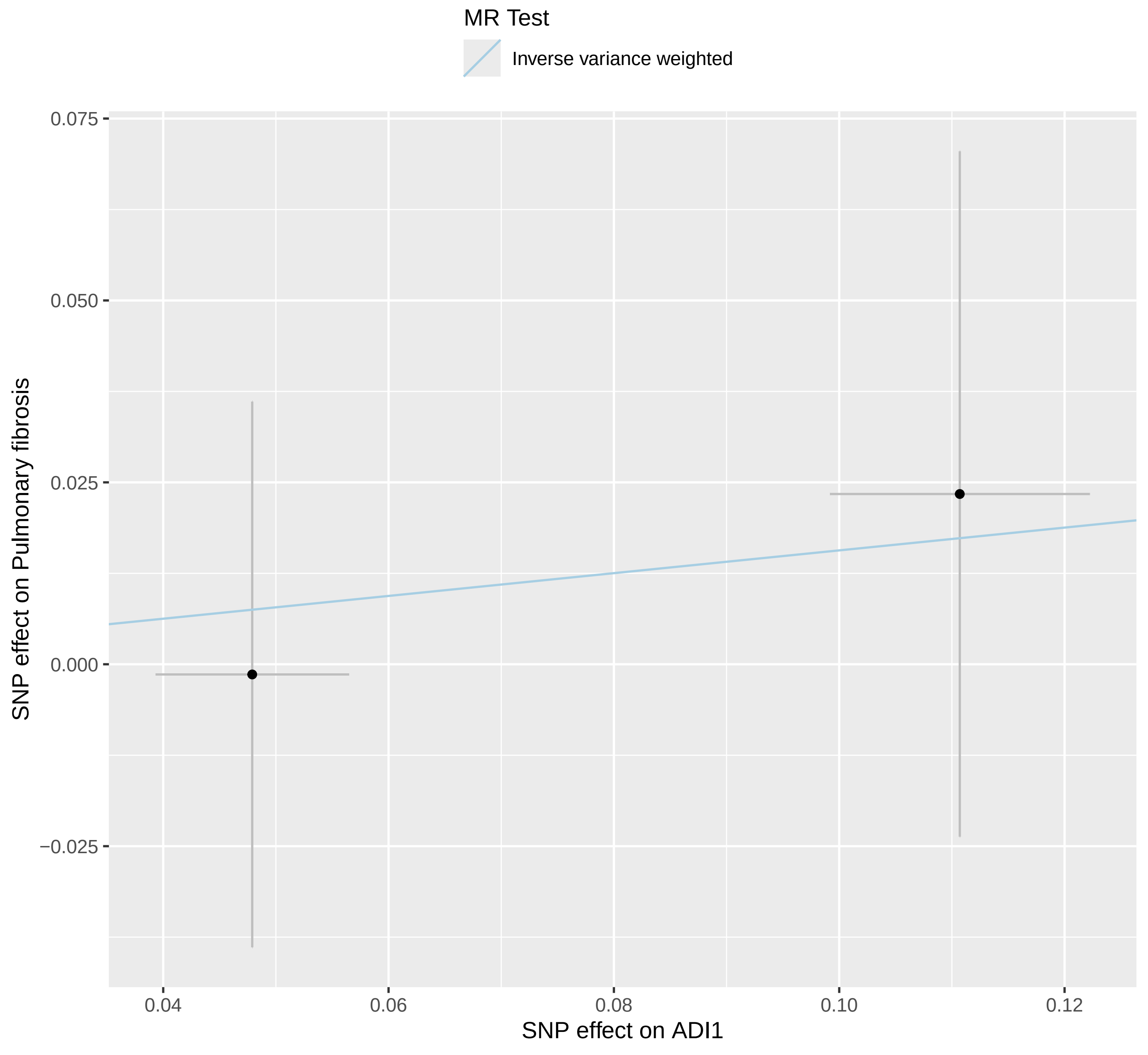
MR Test
Inverse variance weighted
SNP effect on Pulmonary fibrosis
0.075
0.050
0.025
0.000
−0.025
0.04
0.06
0.08
0.10
0.12
SNP effect on ADI1

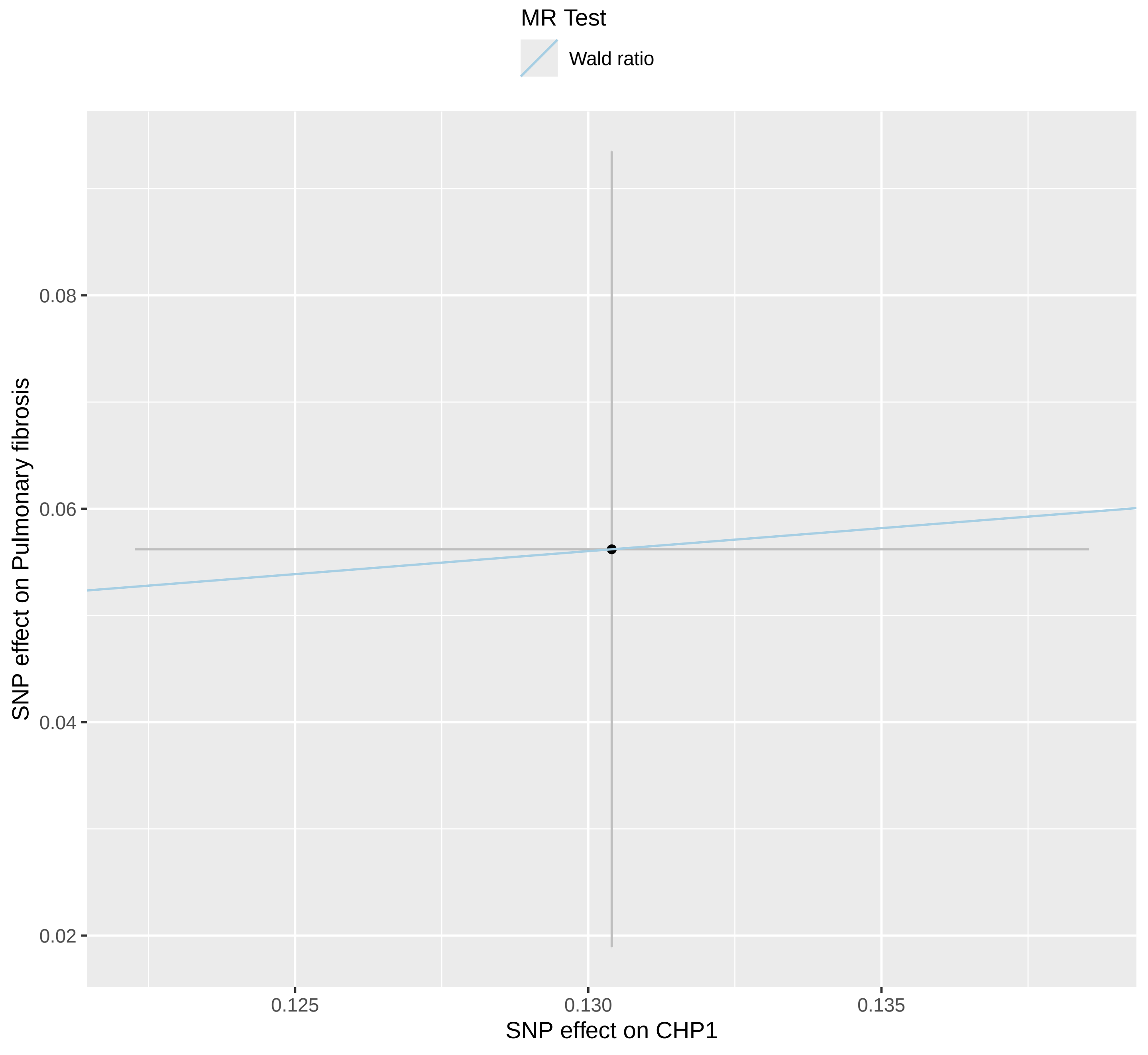
MR Test
Wald ratio
0.08
0.06
0.04
0.02
SNP effect on Pulmonary fibrosis
0.125
0.130
0.135
SNP effect on CHP1

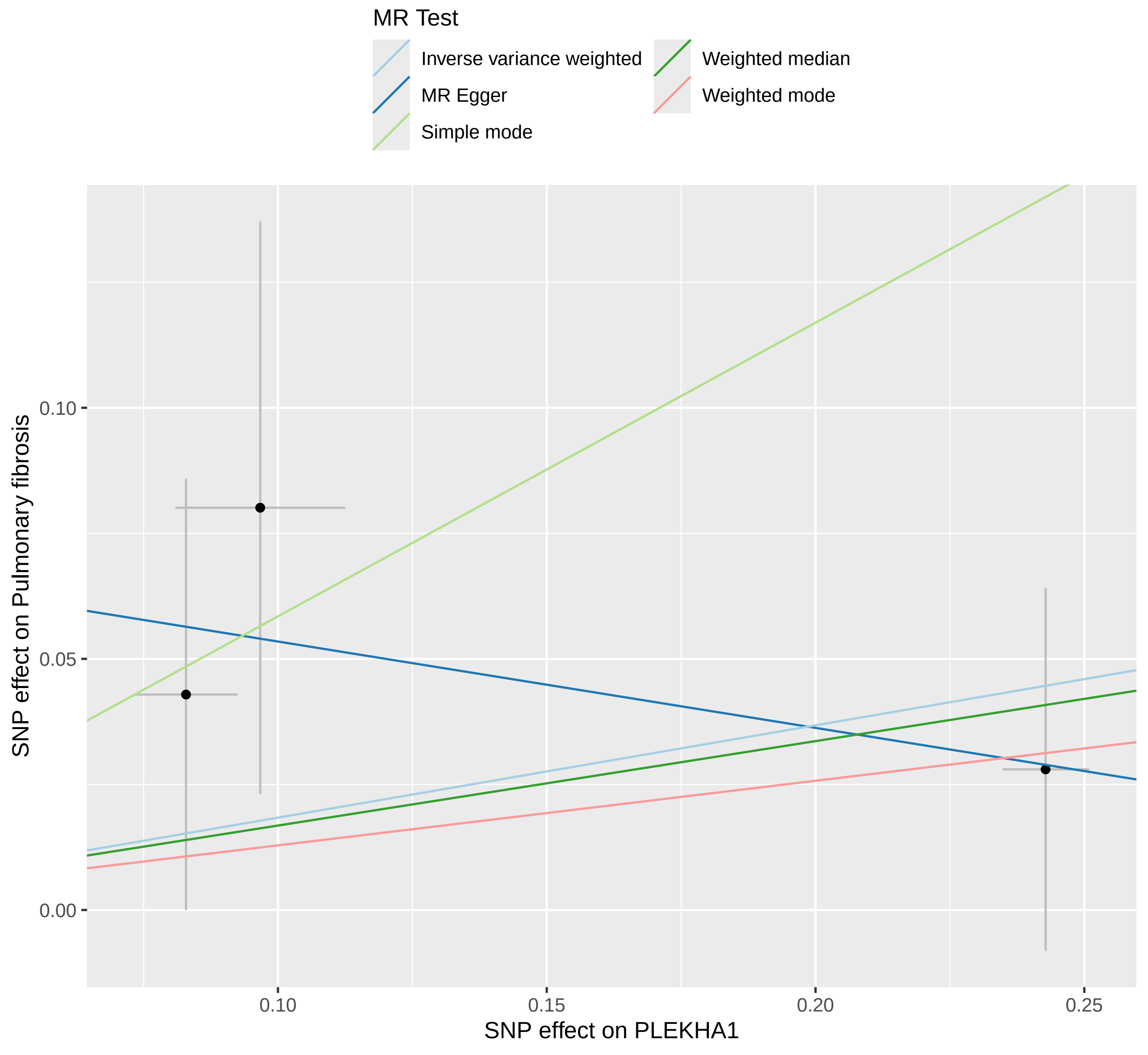

MR Test
Inverse variance weighted
MR Egger
Simple mode
Weighted median
Weighted mode
0.10
0.05
0.00
SNP effect on Pulmonary fibrosis
0.10
0.15
0.20
0.25
SNP effect on PLEKHA1

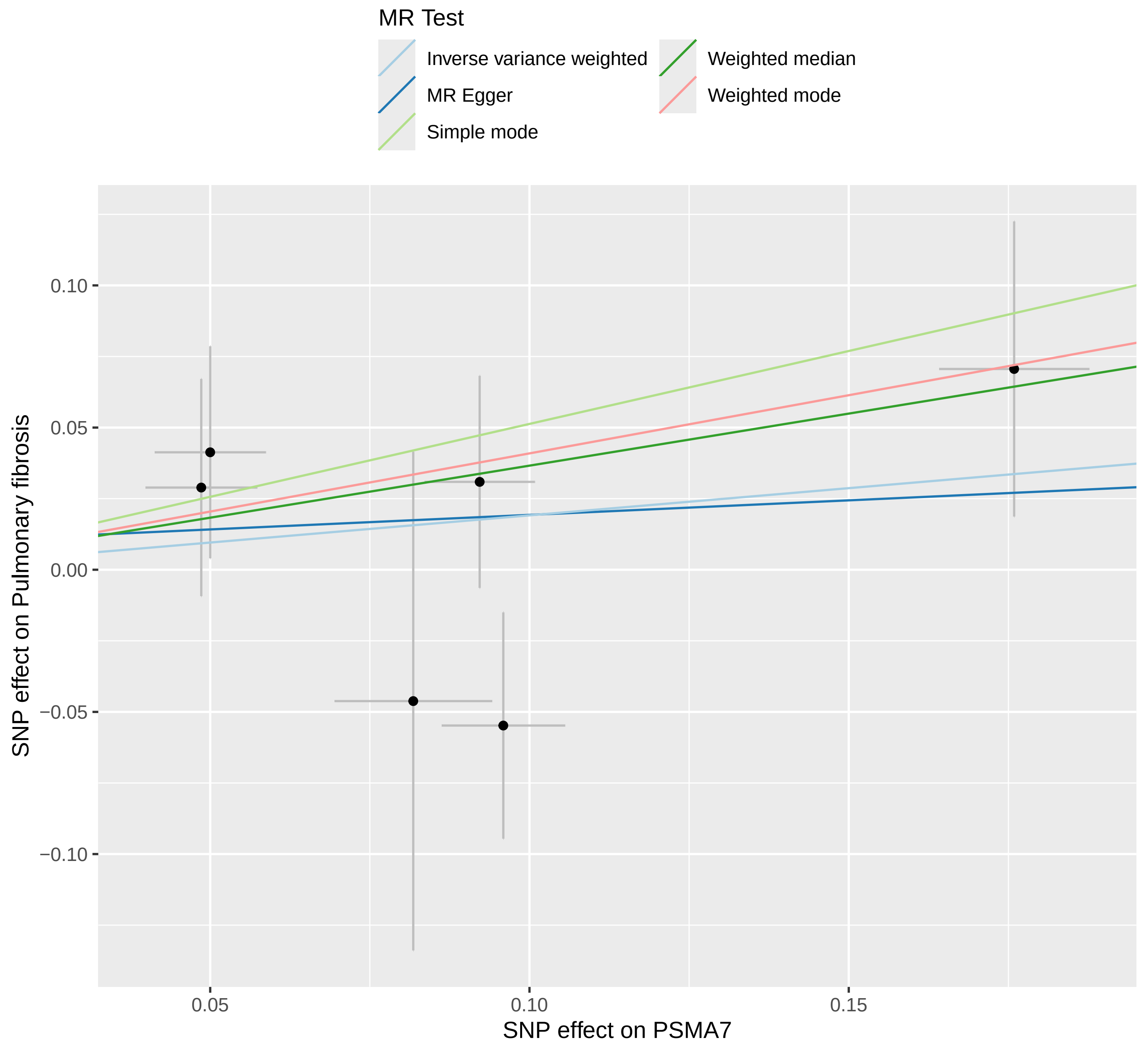
MR Test
Inverse variance weighted
MR Egger
Simple mode
Weighted median
Weighted mode
SNP effect on Pulmonary fibrosis
0.10
0.05
0.00
−0.05
−0.10
0.05
0.10
0.15
SNP effect on PSMA7

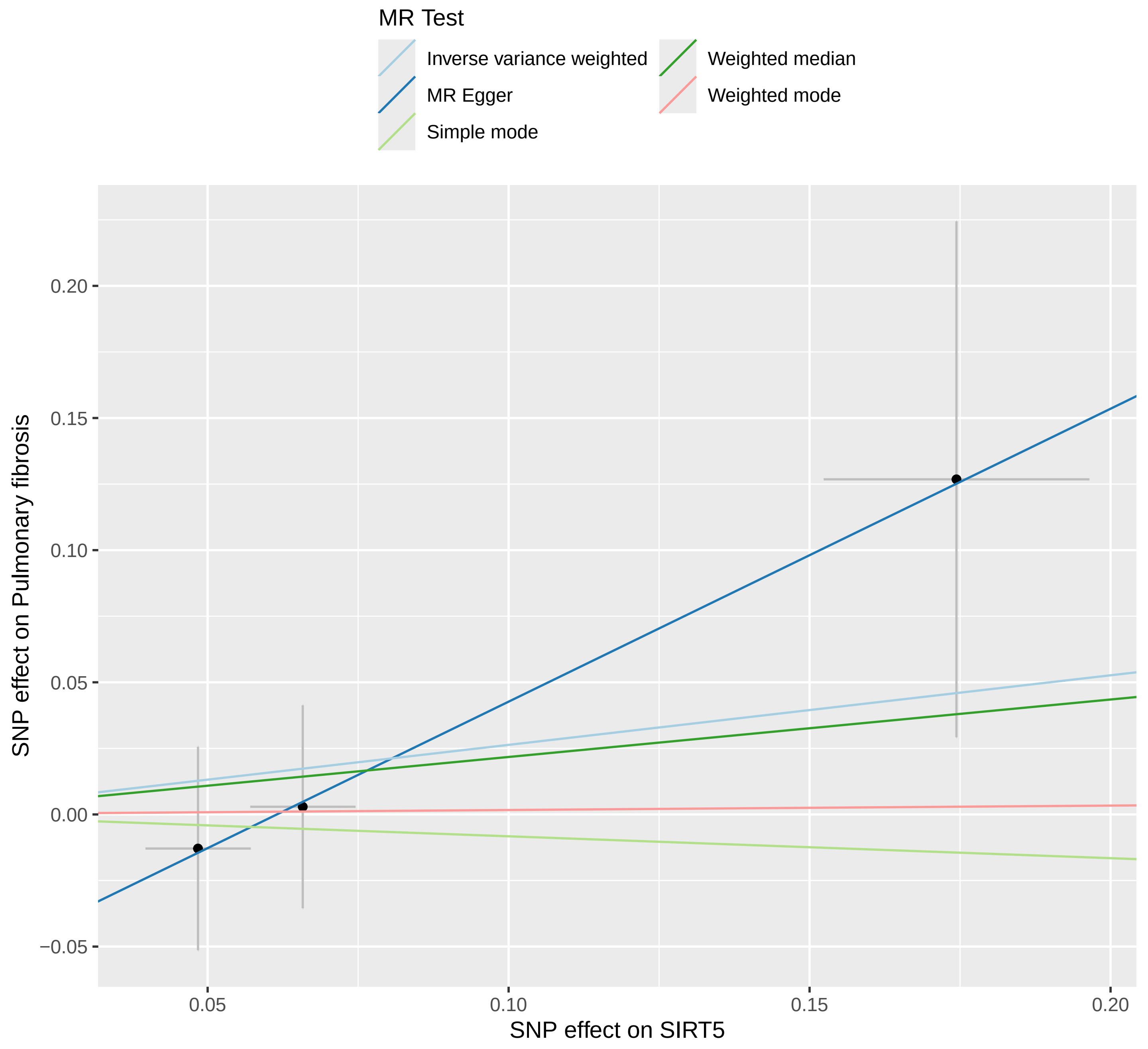
MR Test
Inverse variance weighted
MR Egger
Simple mode
Weighted median
Weighted mode
0.20
0.15
0.10
0.05
0.00
−0.05
SNP effect on Pulmonary fibrosis
0.05
0.10
0.15
0.20
SNP effect on SIRT5

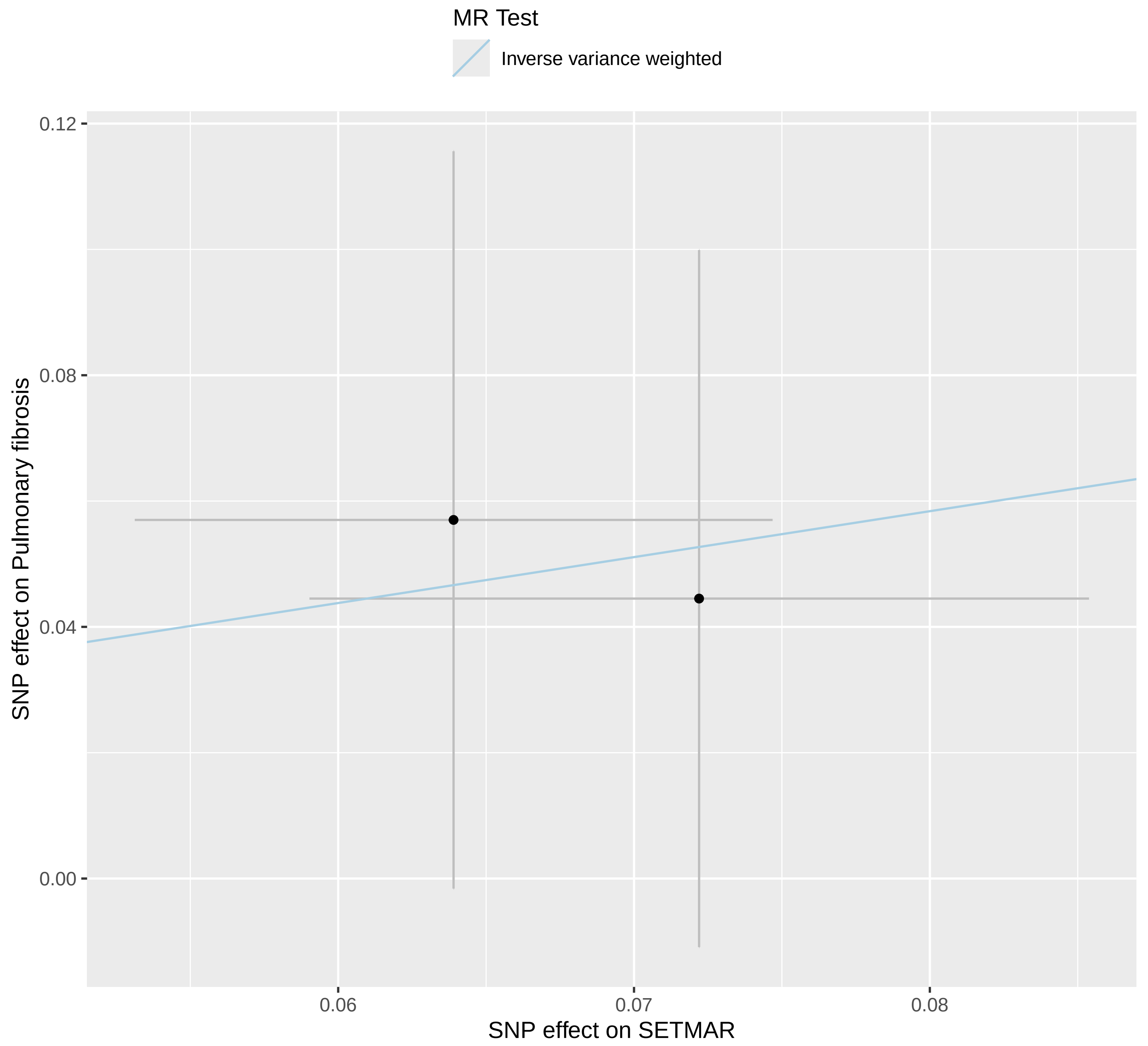
MR Test
Inverse variance weighted
0.12
0.08
0.04
0.00
0.06
0.07
0.08
SNP effect on SETMAR
SNP effect on Pulmonary fibrosis

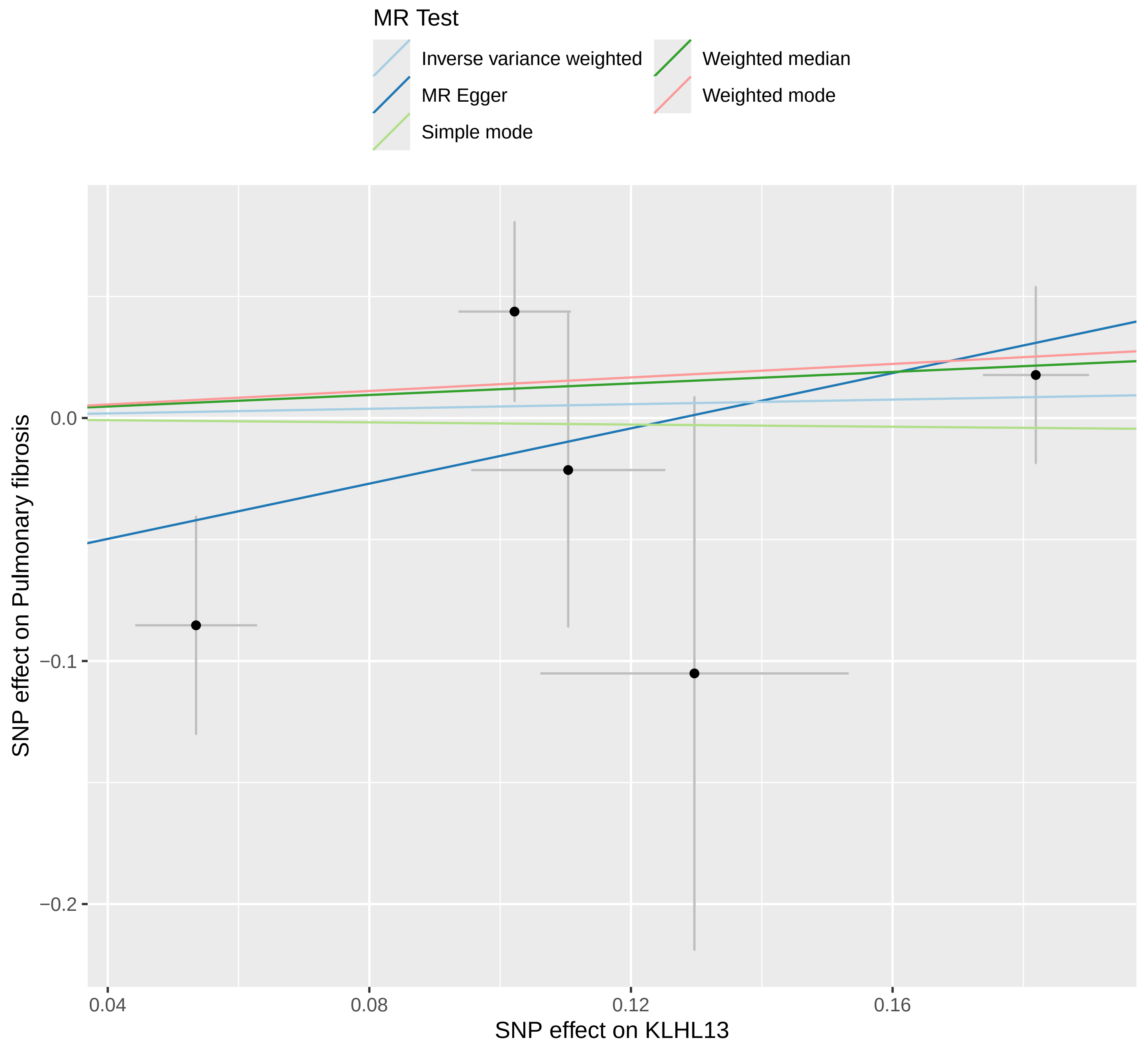
MR Test
Inverse variance weighted
MR Egger
Simple mode
Weighted median
Weighted mode
SNP effect on Pulmonary fibrosis
0.0
−0.1
−0.2
0.04
0.08
0.12
0.16
SNP effect on KLHL13

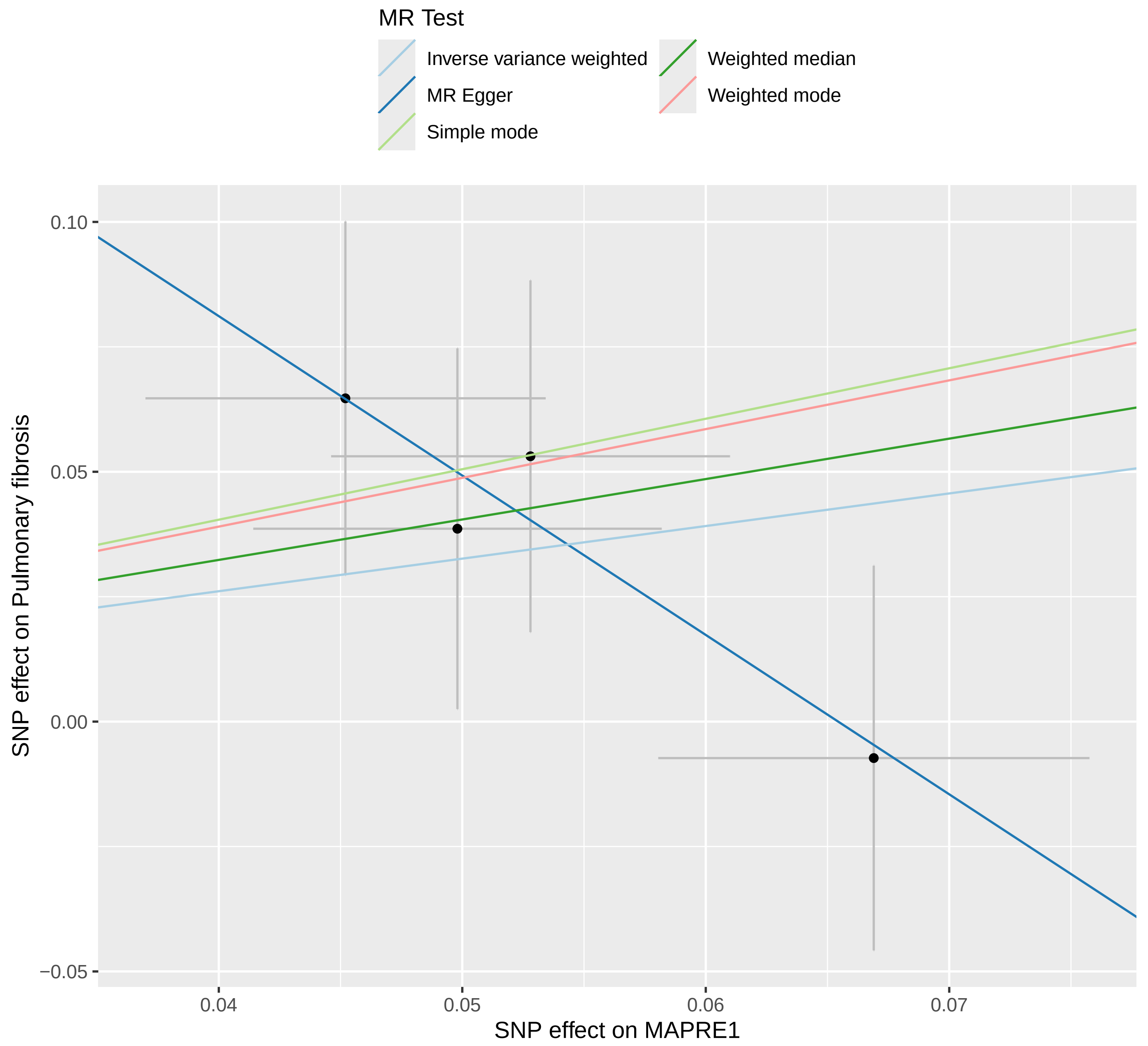
MR Test
Inverse variance weighted
MR Egger
Simple mode
Weighted median
Weighted mode
0.10
0.05
0.00
−0.05
0.04
0.05
0.06
0.07
SNP effect on Pulmonary fibrosis
SNP effect on MAPRE1

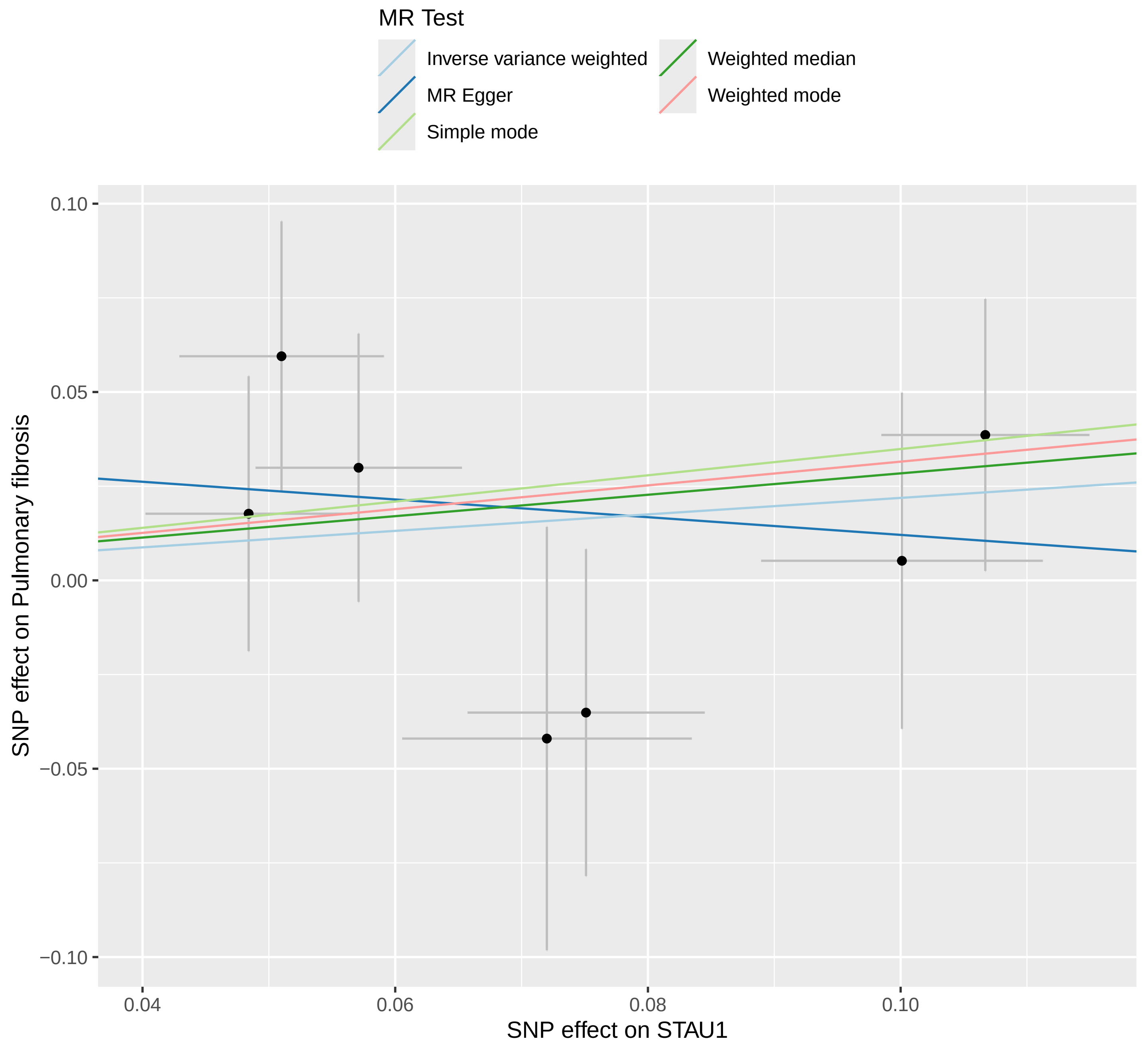
MR Test
Inverse variance weighted
MR Egger
Simple mode
Weighted median
Weighted mode
0.10
0.05
0.00
−0.05
−0.10
SNP effect on Pulmonary fibrosis
0.04
0.06
0.08
0.10
SNP effect on STAU1

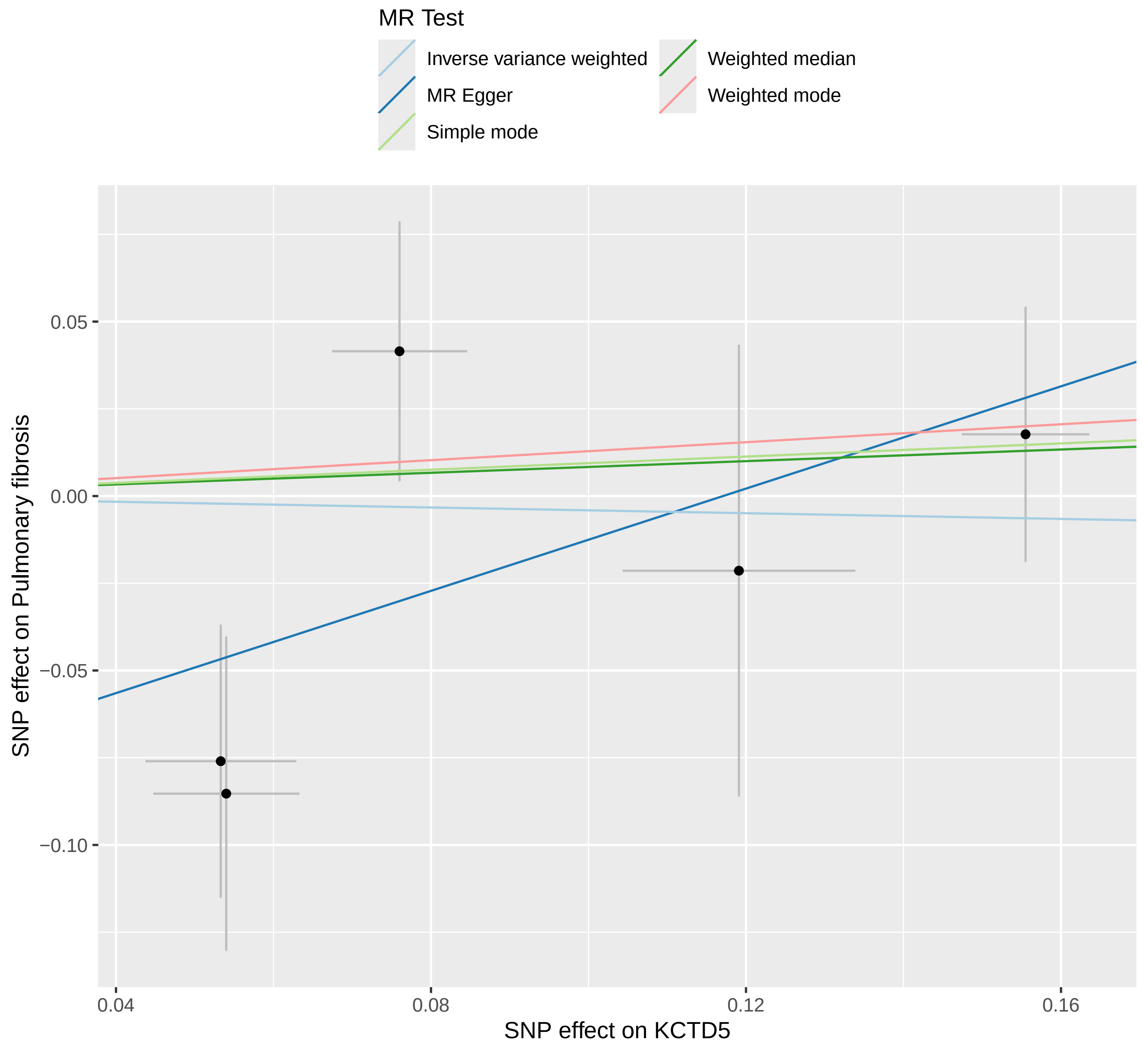
MR Test
Inverse variance weighted
MR Egger
Simple mode
Weighted median
Weighted mode
0.05
0.00
−0.05
−0.10
SNP effect on Pulmonary fibrosis
0.04
0.08
0.12
0.16
SNP effect on KCTD5

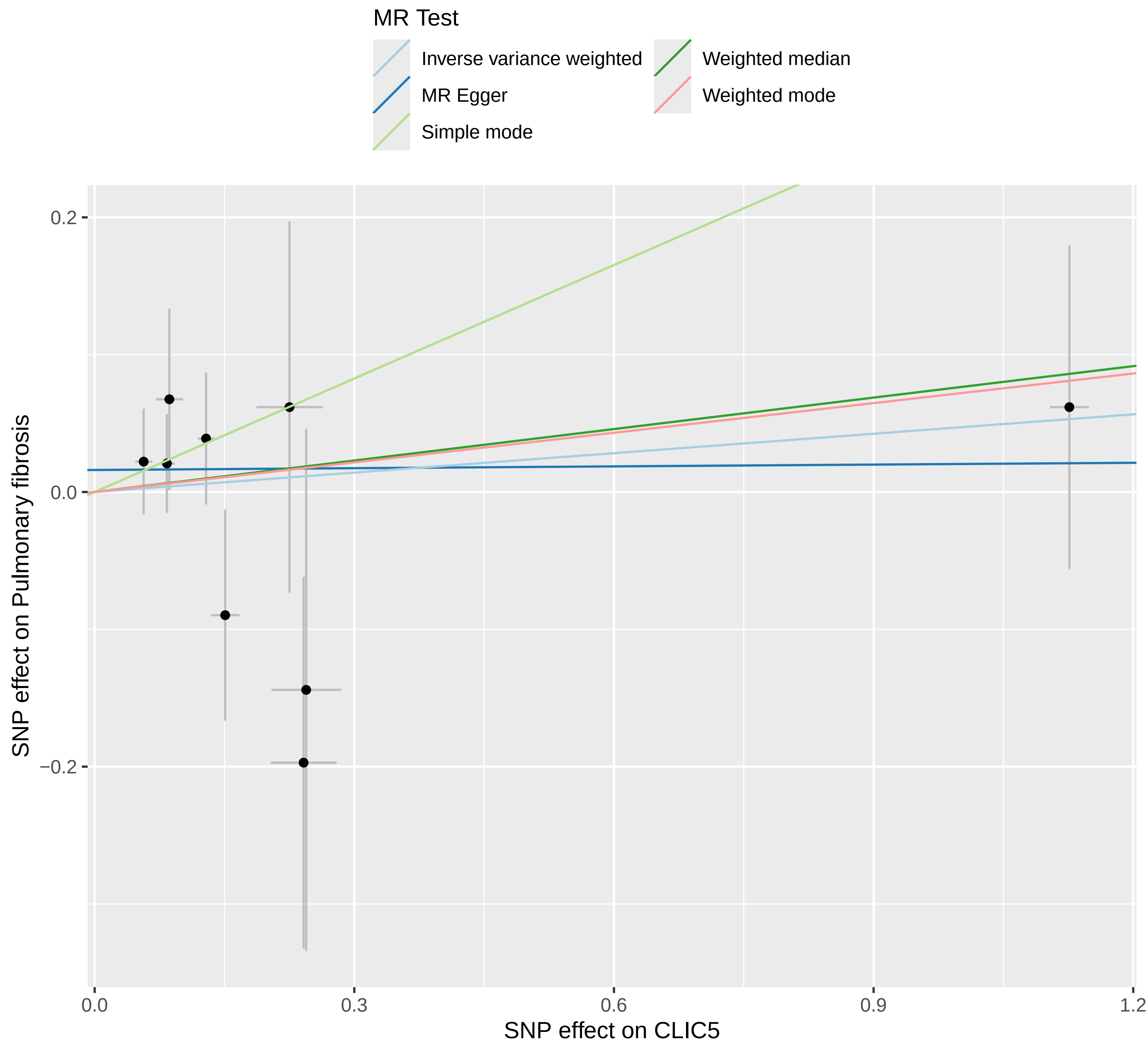
MR Test
Inverse variance weighted
MR Egger
Simple mode
Weighted median
Weighted mode
SNP effect on Pulmonary fibrosis
0.2
0.0
−0.2
0.0
0.3
0.6
0.9
1.2
SNP effect on CLIC5

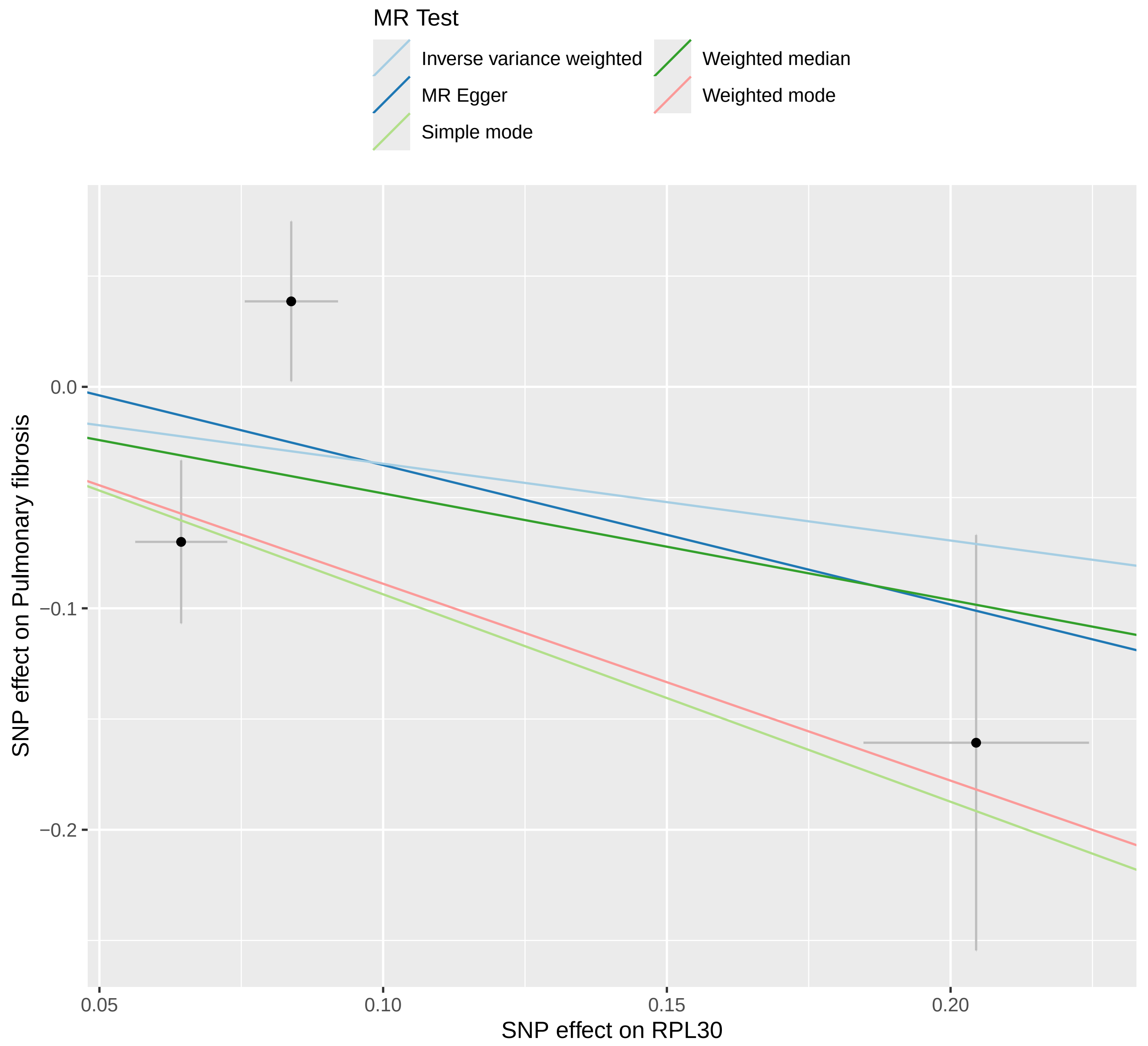

MR Test
Inverse variance weighted
MR Egger
Simple mode
Weighted median
Weighted mode
0.0
−0.1
−0.2
0.05
0.10
0.15
0.20
SNP effect on Pulmonary fibrosis
SNP effect on RPL30

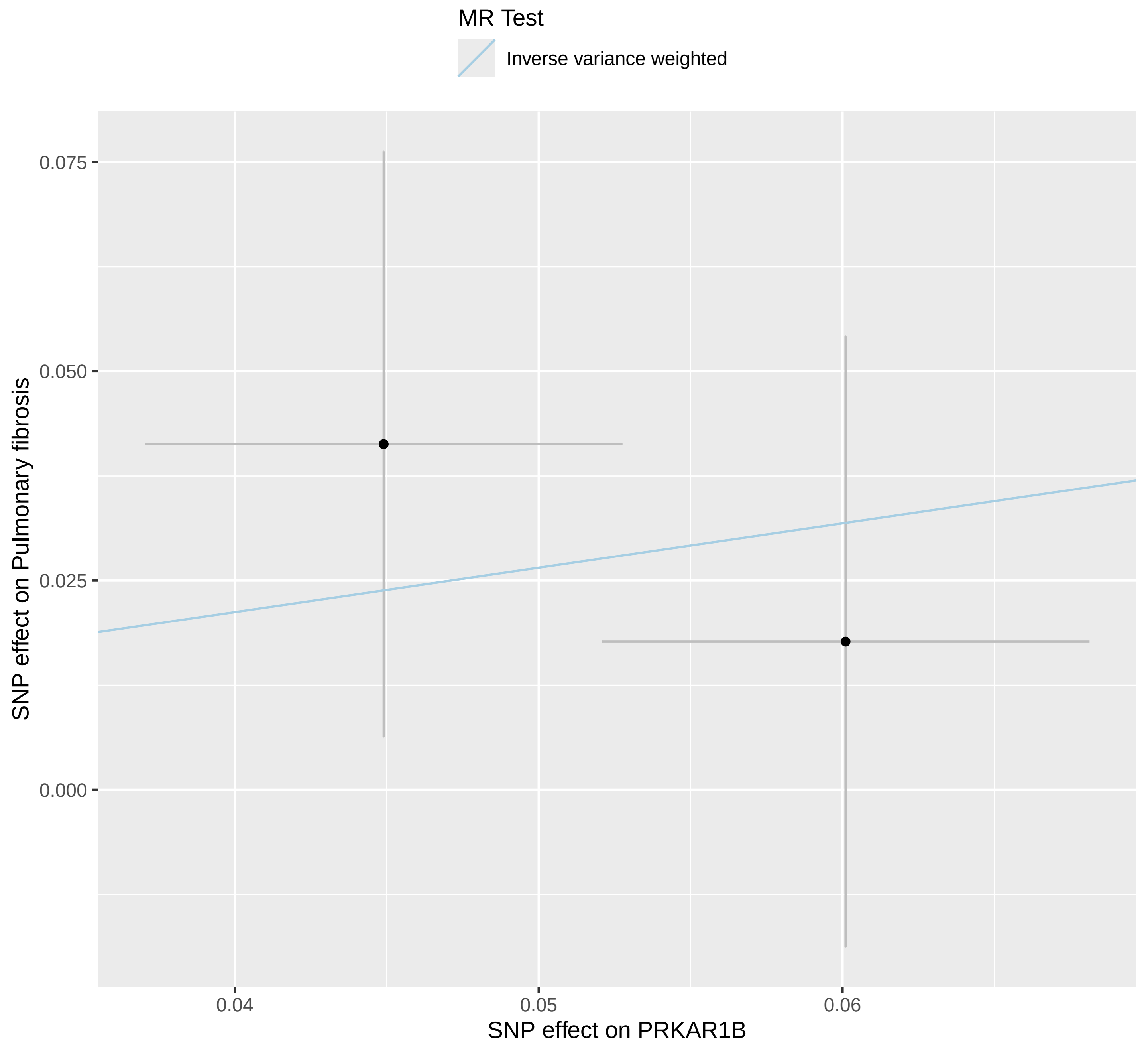
MR Test
Inverse variance weighted
0.075
0.050
0.025
0.000
SNP effect on Pulmonary fibrosis
0.04
0.05
0.06
SNP effect on PRKAR1B

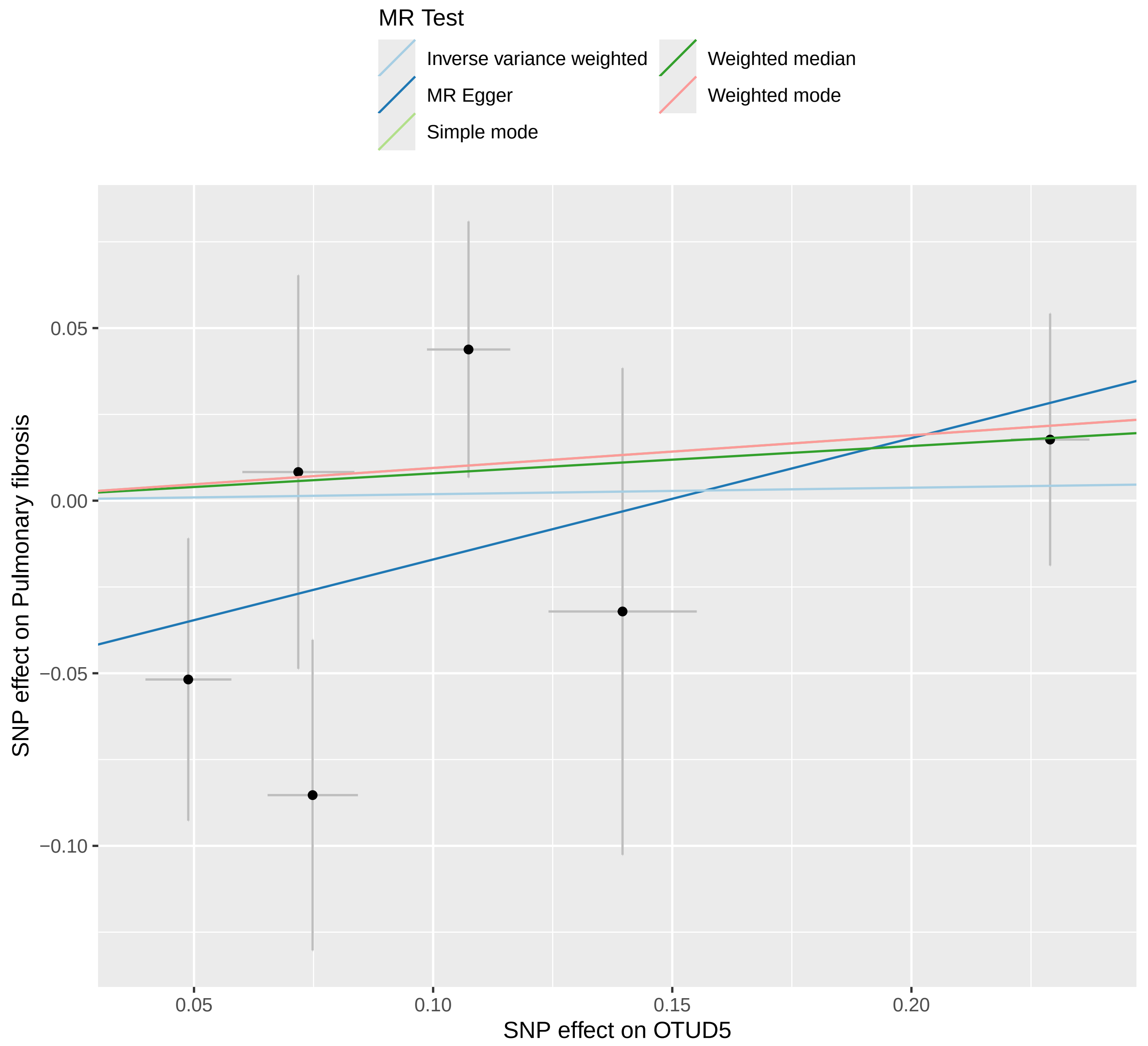

MR Test
Inverse variance weighted
MR Egger
Simple mode
Weighted median
Weighted mode
0.05
0.00
−0.05
−0.10
SNP effect on Pulmonary fibrosis
0.05
0.10
0.15
0.20
SNP effect on OTUD5

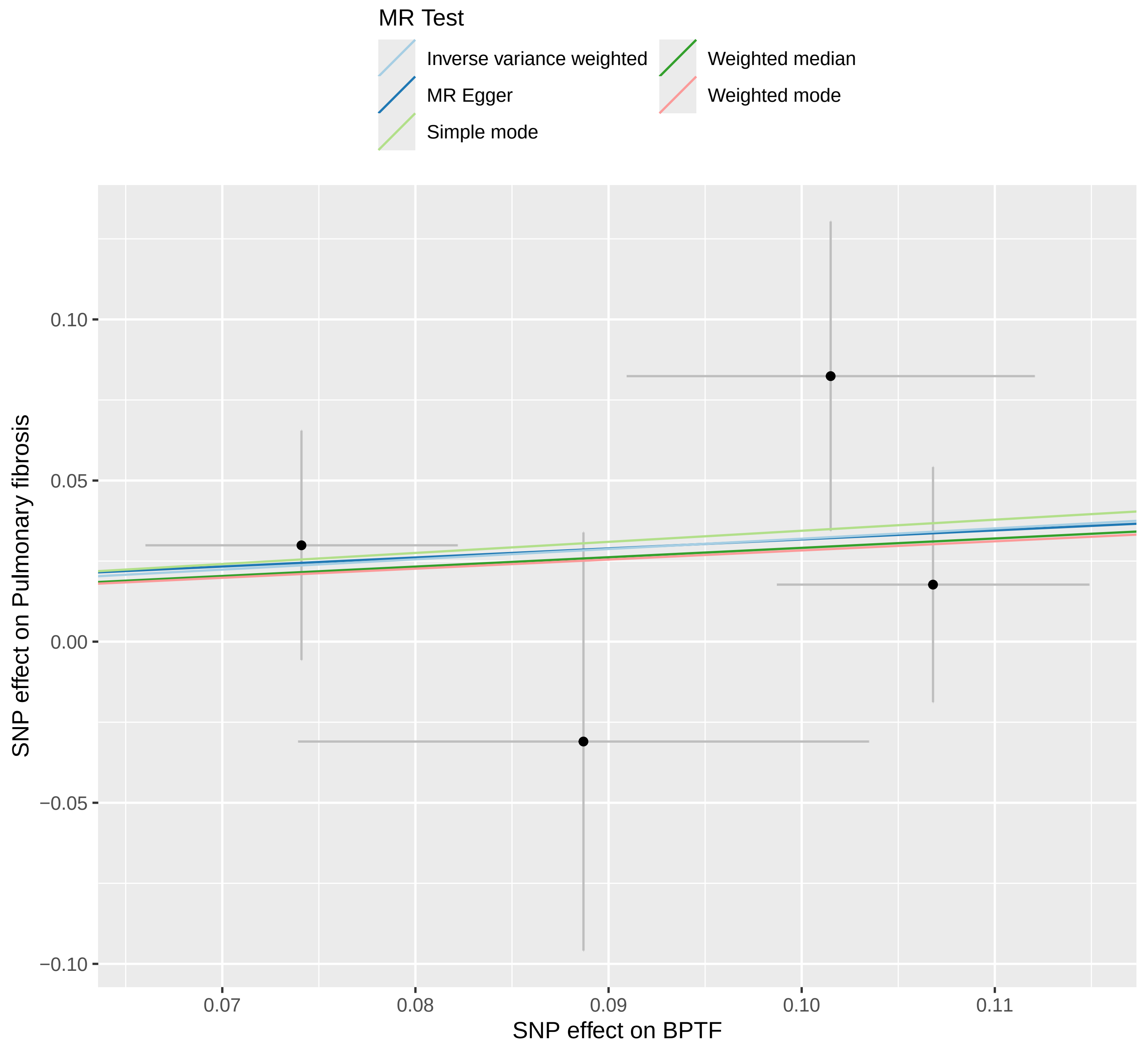

MR Test
Inverse variance weighted
MR Egger
Simple mode
Weighted median
Weighted mode
0.10
0.05
0.00
−0.05
−0.10
SNP effect on Pulmonary fibrosis
0.07
0.08
0.09
0.10
0.11
SNP effect on BPTF

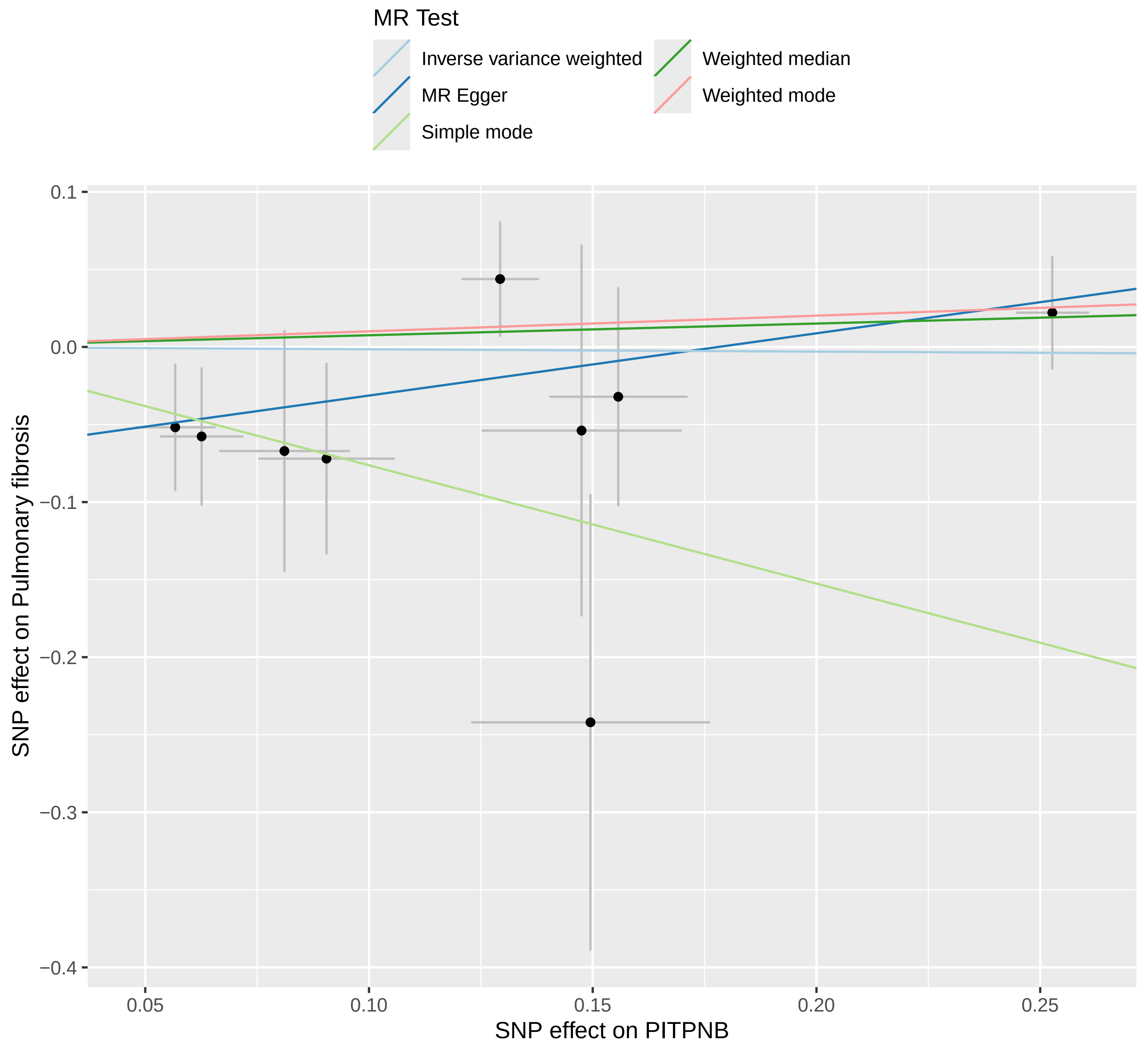
MR Test
Inverse variance weighted
MR Egger
Simple mode
Weighted median
Weighted mode
0.1
0.0
−0.1
−0.2
−0.3
−0.4
SNP effect on Pulmonary fibrosis
0.05
0.10
0.15
0.20
0.25
SNP effect on PITPNB

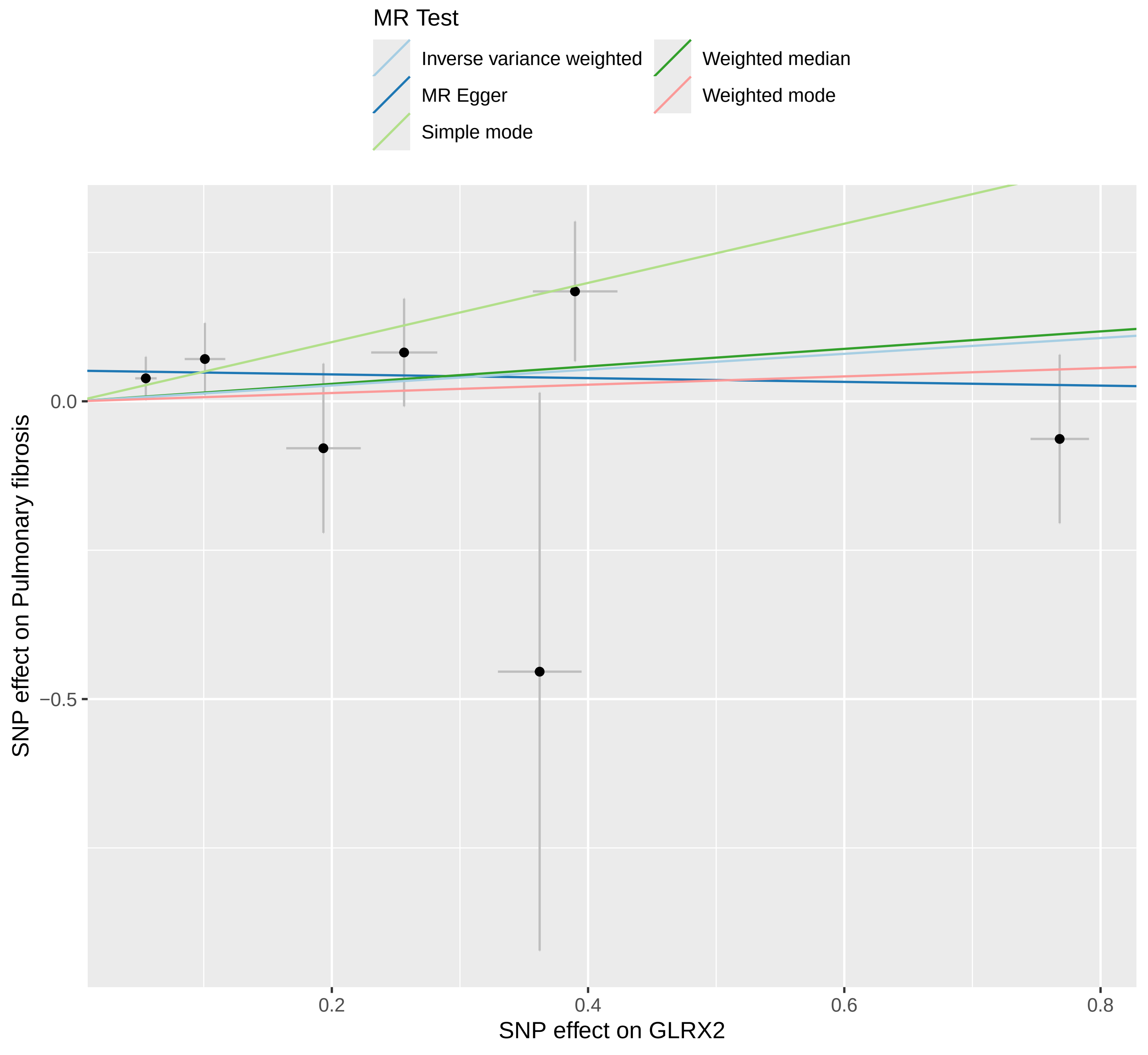
MR Test
Inverse variance weighted
MR Egger
Simple mode
Weighted median
Weighted mode
0.0
−0.5
0.2
0.4
0.6
0.8
SNP effect on Pulmonary fibrosis
SNP effect on GLRX2

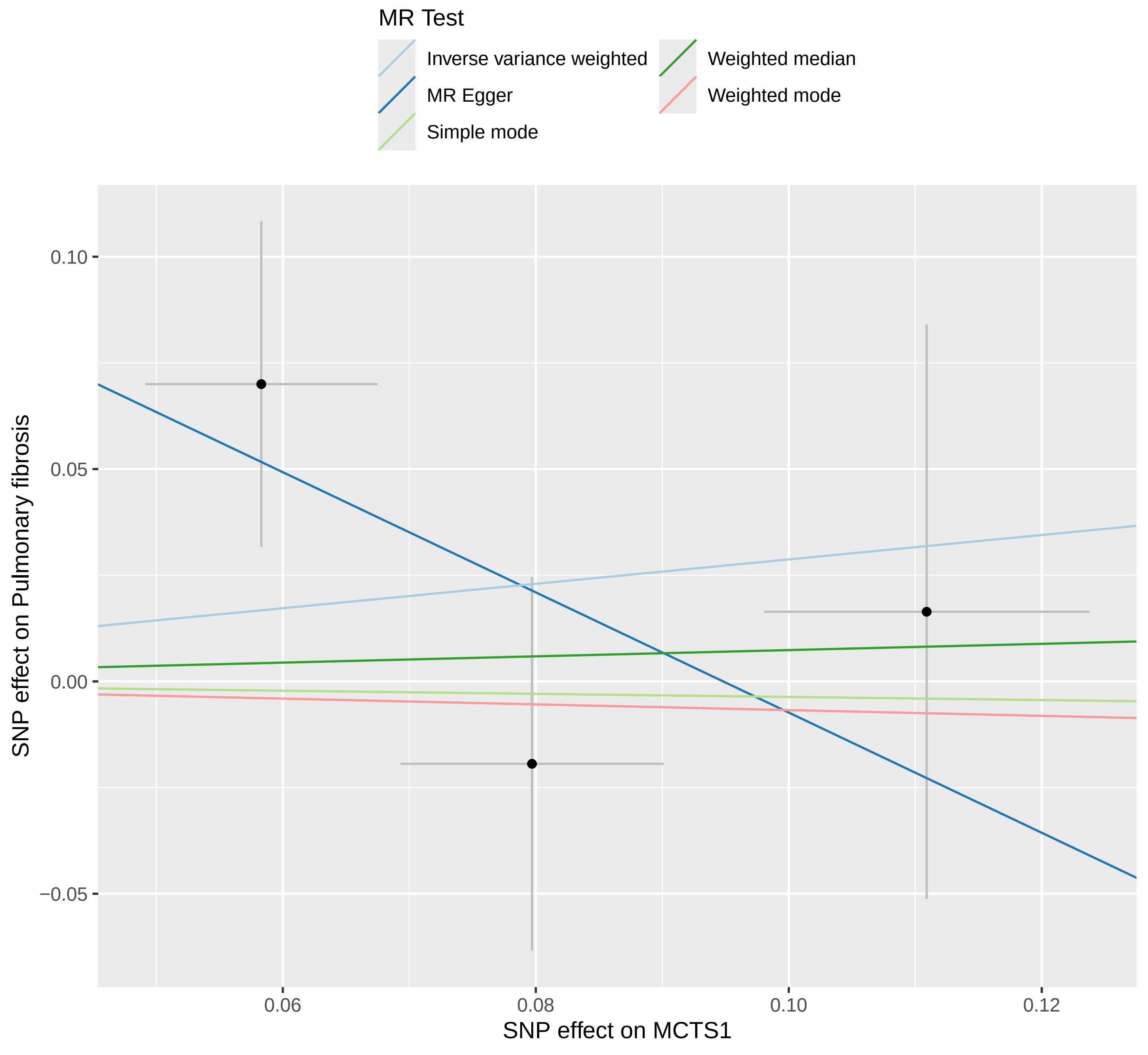
MR Test
Inverse variance weighted
MR Egger
Simple mode
Weighted median
Weighted mode
SNP effect on Pulmonary fibrosis
0.10
0.05
0.00
−0.05
0.06
0.08
0.10
0.12
SNP effect on MCTS1

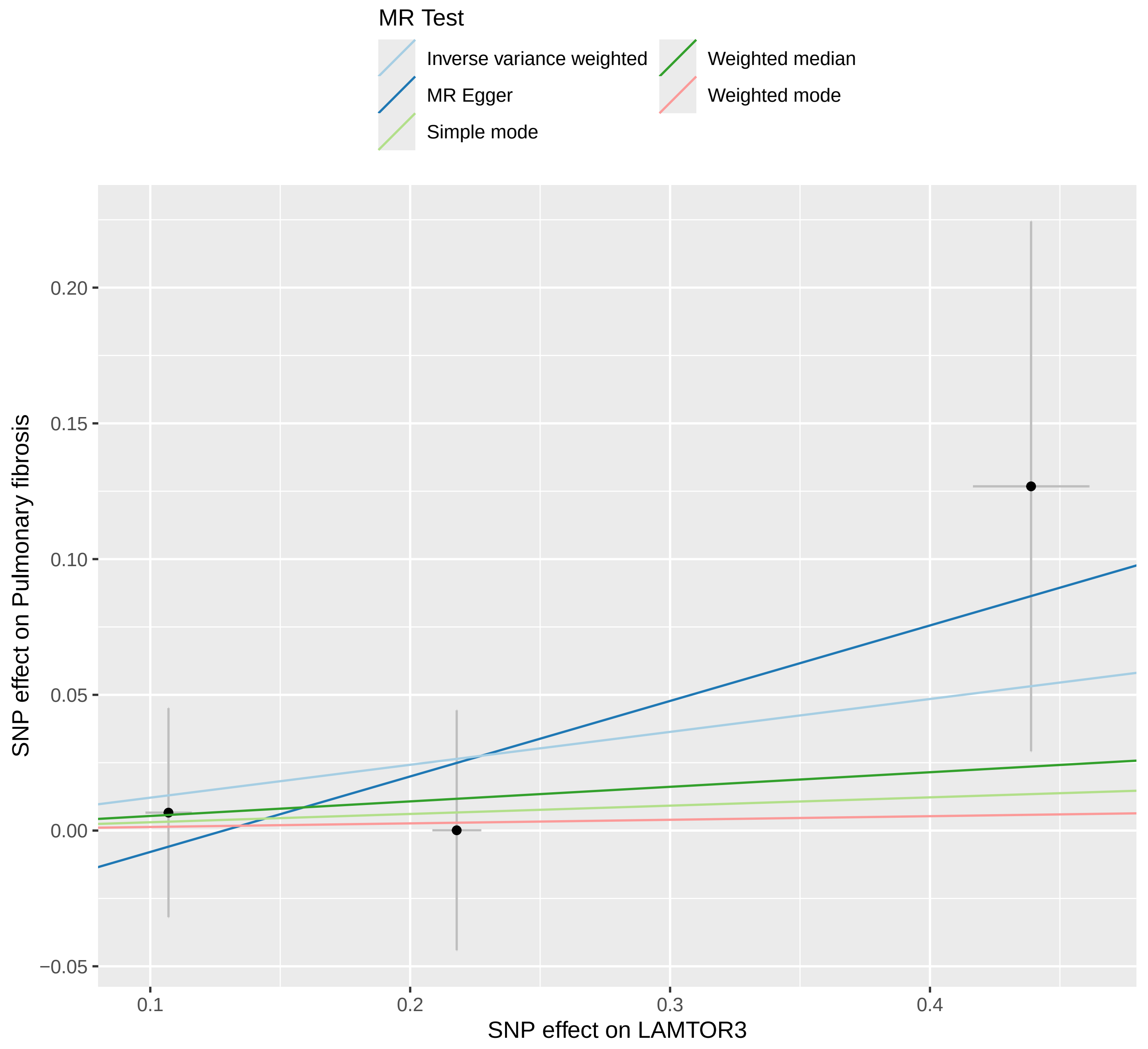
MR Test
Inverse variance weighted
MR Egger
Simple mode
Weighted median
Weighted mode
SNP effect on Pulmonary fibrosis
0.20
0.15
0.10
0.05
0.00
−0.05
0.1
0.2
0.3
0.4
SNP effect on LAMTOR3

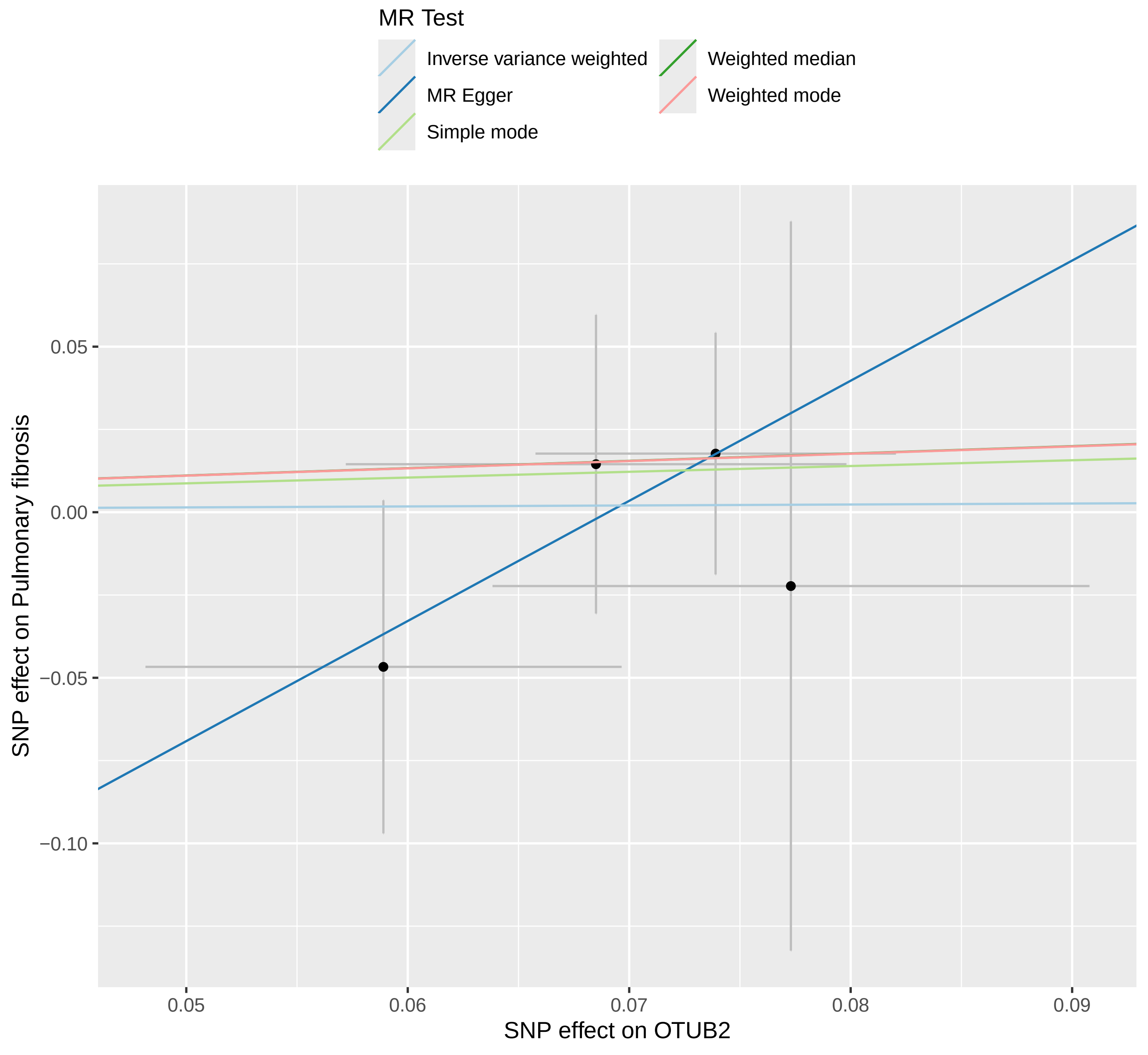
MR Test
Inverse variance weighted
MR Egger
Simple mode
Weighted median
Weighted mode
SNP effect on Pulmonary fibrosis
0.05
0.00
−0.05
−0.10
0.05
0.06
0.07
0.08
0.09
SNP effect on OTUB2

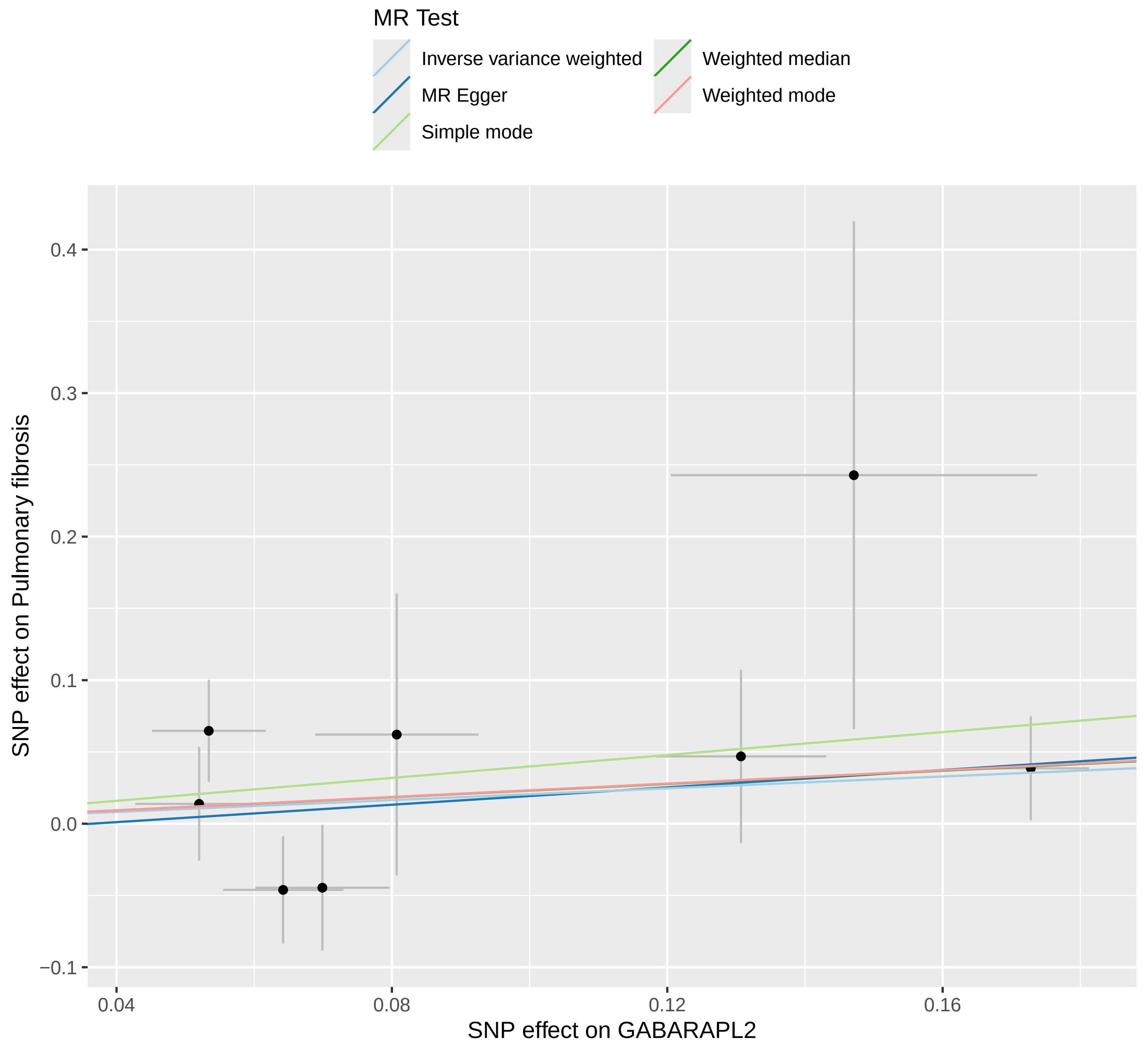
MR Test
Inverse variance weighted
MR Egger
Simple mode
Weighted median
Weighted mode
SNP effect on Pulmonary fibrosis
0.4
0.3
0.2
0.1
0.0
−0.1
0.04
0.08
0.12
0.16
SNP effect on GABARAPL2

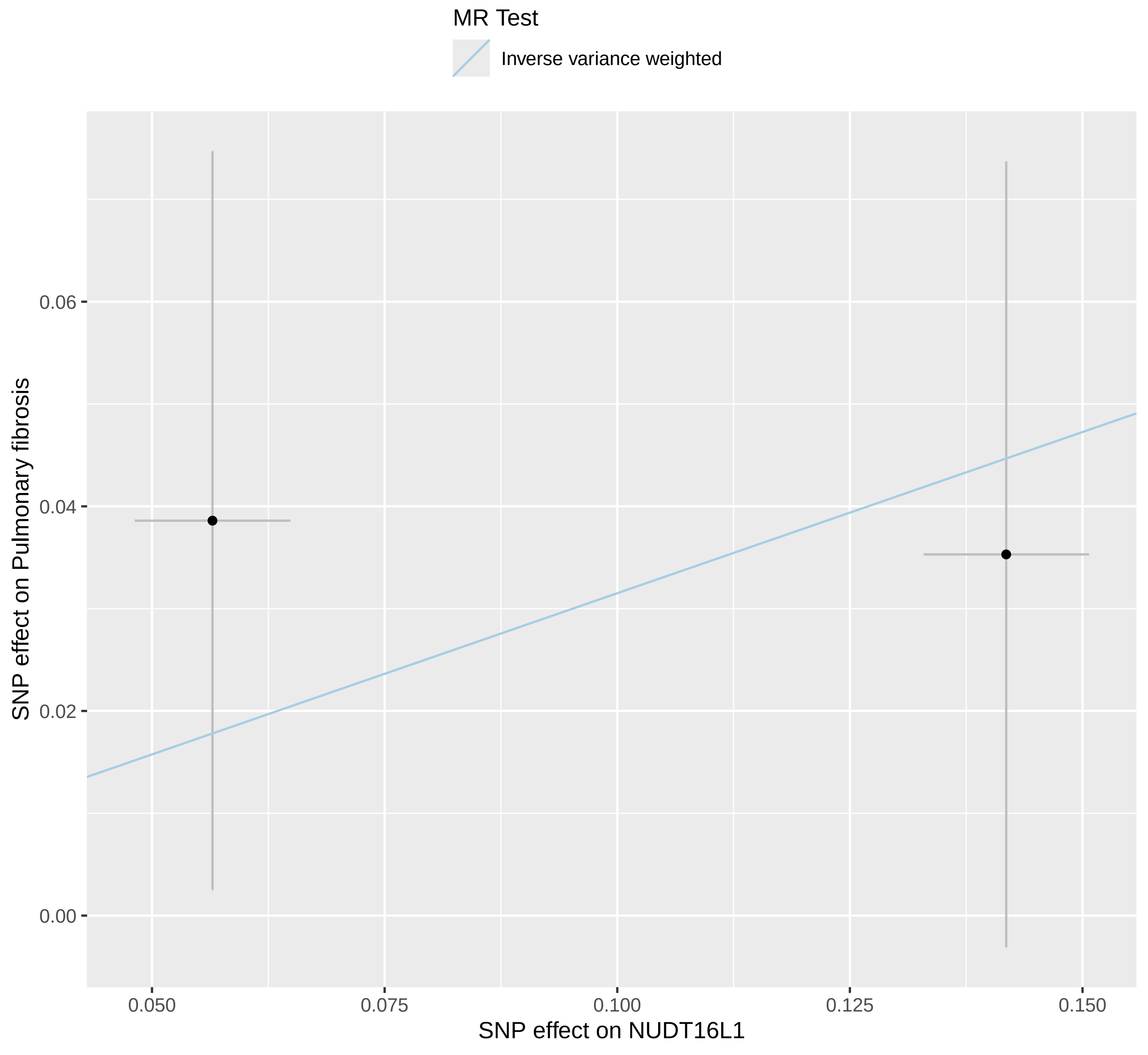

MR Test
Inverse variance weighted
SNP effect on Pulmonary fibrosis
0.06
0.04
0.02
0.00
0.050
0.075
0.100
0.125
0.150
SNP effect on NUDT16L1

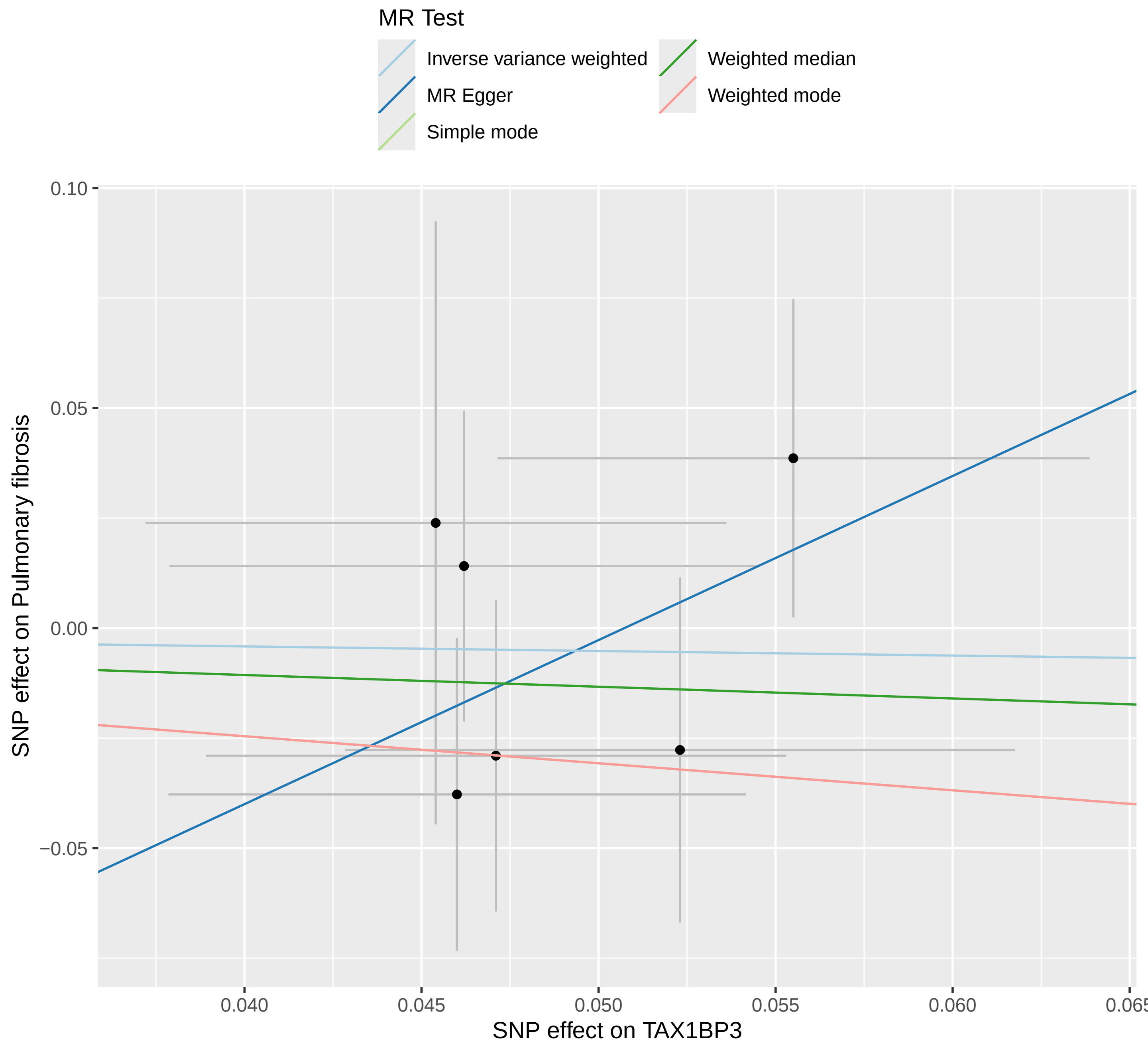
MR Test
Inverse variance weighted
MR Egger
Simple mode
Weighted median
Weighted mode
0.10
0.05
0.00
−0.05
SNP effect on Pulmonary fibrosis
0.040
0.045
0.050
0.055
0.060
0.065
SNP effect on TAX1BP3

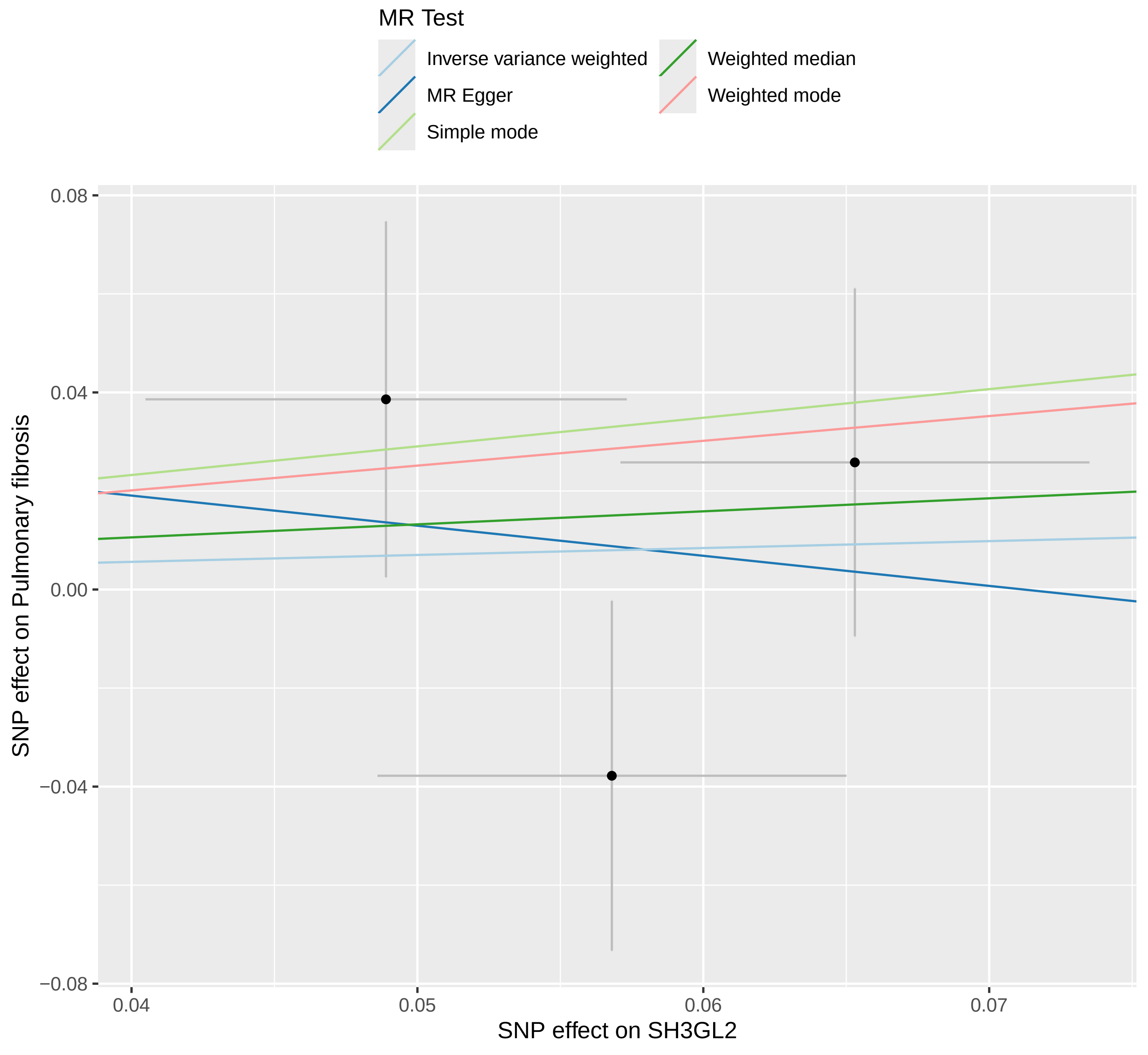
MR Test
Inverse variance weighted
MR Egger
Simple mode
Weighted median
Weighted mode
0.08
0.04
0.00
−0.04
−0.08
0.04
0.05
0.06
0.07
SNP effect on Pulmonary fibrosis
SNP effect on SH3GL2

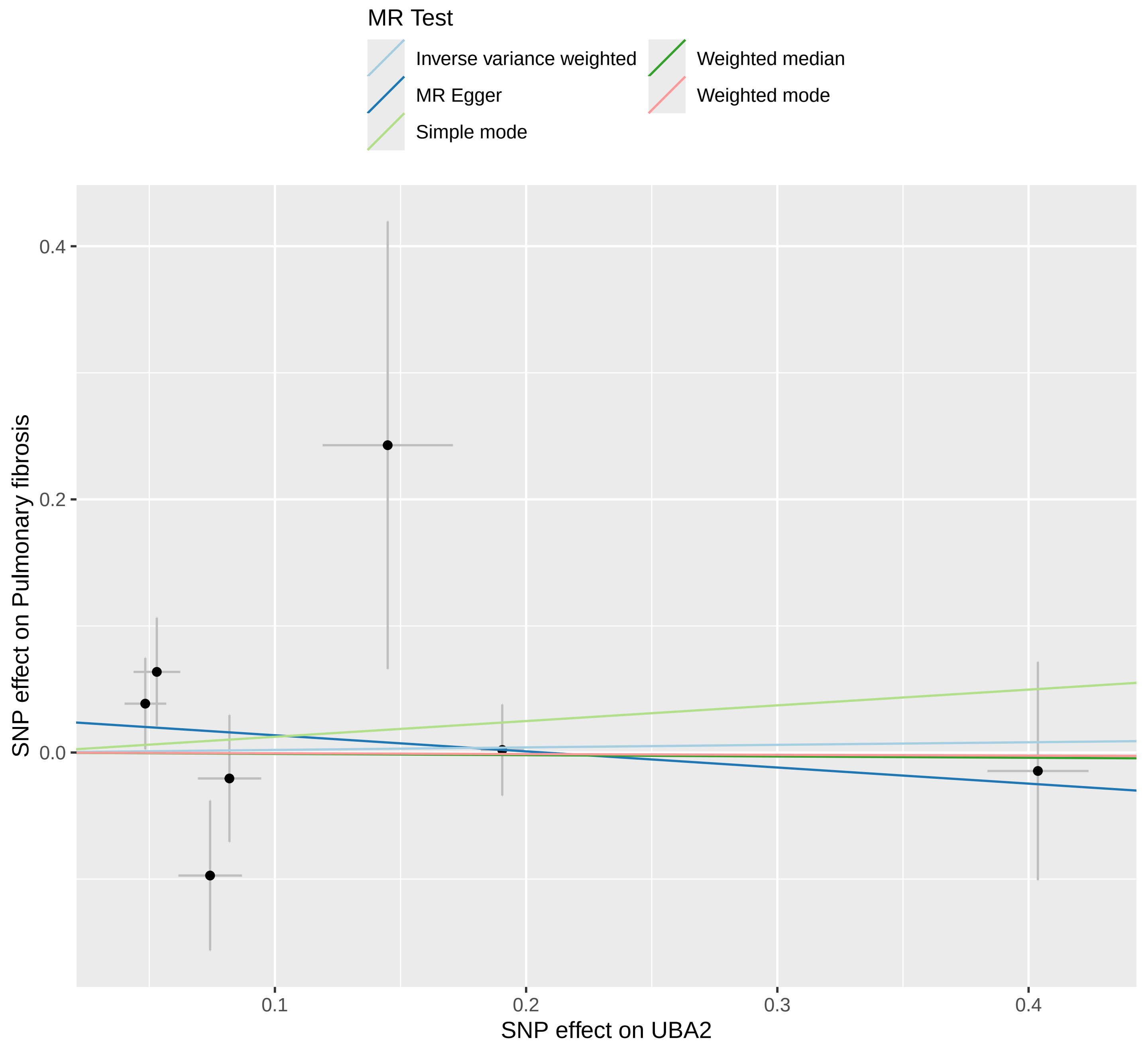
MR Test
Inverse variance weighted
MR Egger
Simple mode
Weighted median
Weighted mode
SNP effect on Pulmonary fibrosis
0.4
0.2
0.0
0.1
0.2
0.3
0.4
SNP effect on UBA2

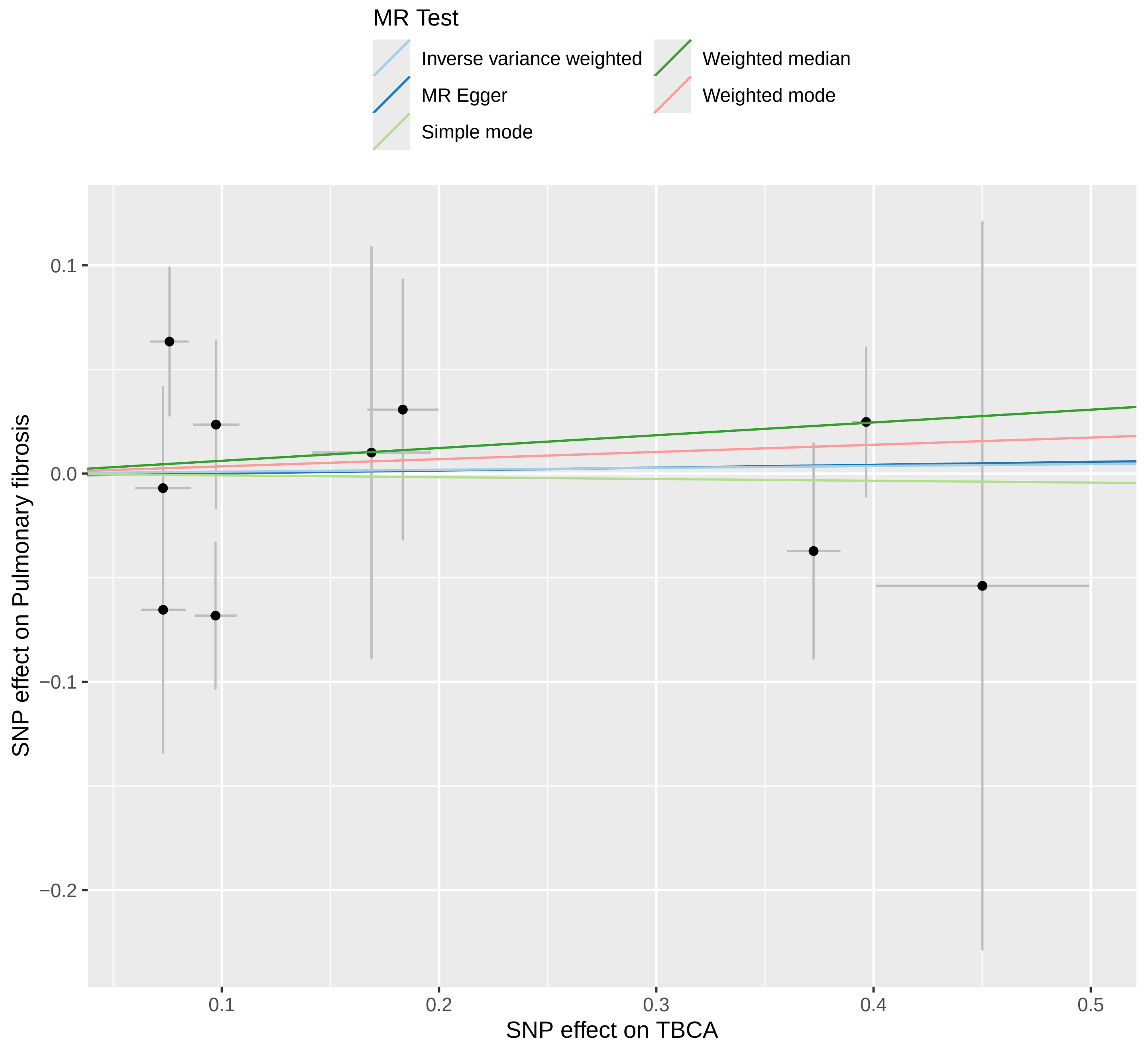
MR Test
Inverse variance weighted
MR Egger
Simple mode
Weighted median
Weighted mode
0.1
0.0
−0.1
−0.2
SNP effect on Pulmonary fibrosis
0.1
0.2
0.3
0.4
0.5
SNP effect on TBCA

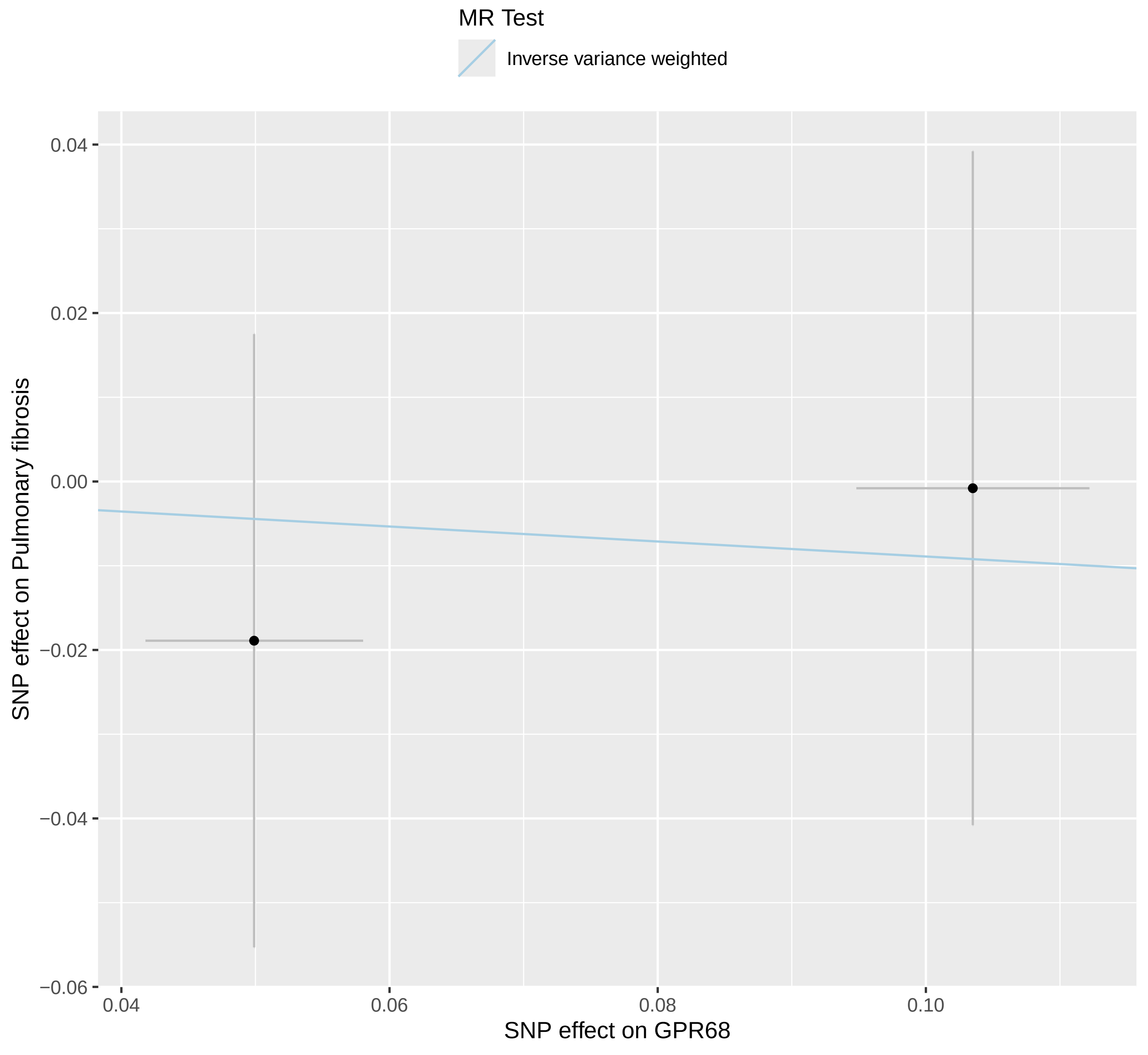

MR Test
Inverse variance weighted
SNP effect on Pulmonary fibrosis
0.04
0.02
0.00
−0.02
−0.04
−0.06
0.04
0.06
0.08
0.10
SNP effect on GPR68

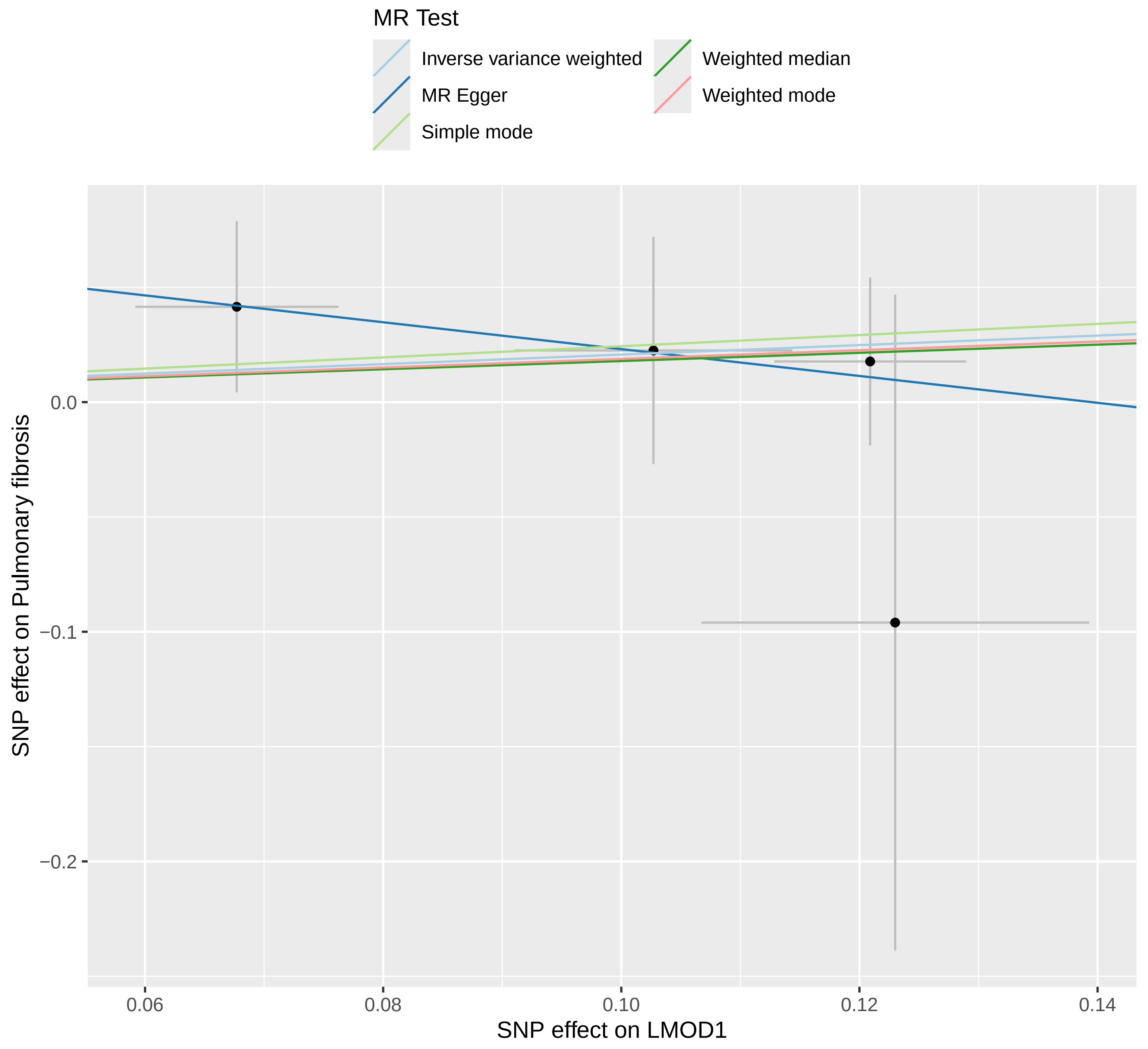
MR Test
Inverse variance weighted
MR Egger
Simple mode
Weighted median
Weighted mode
SNP effect on Pulmonary fibrosis
0.0
−0.1
−0.2
0.06
0.08
0.10
0.12
0.14
SNP effect on LMOD1

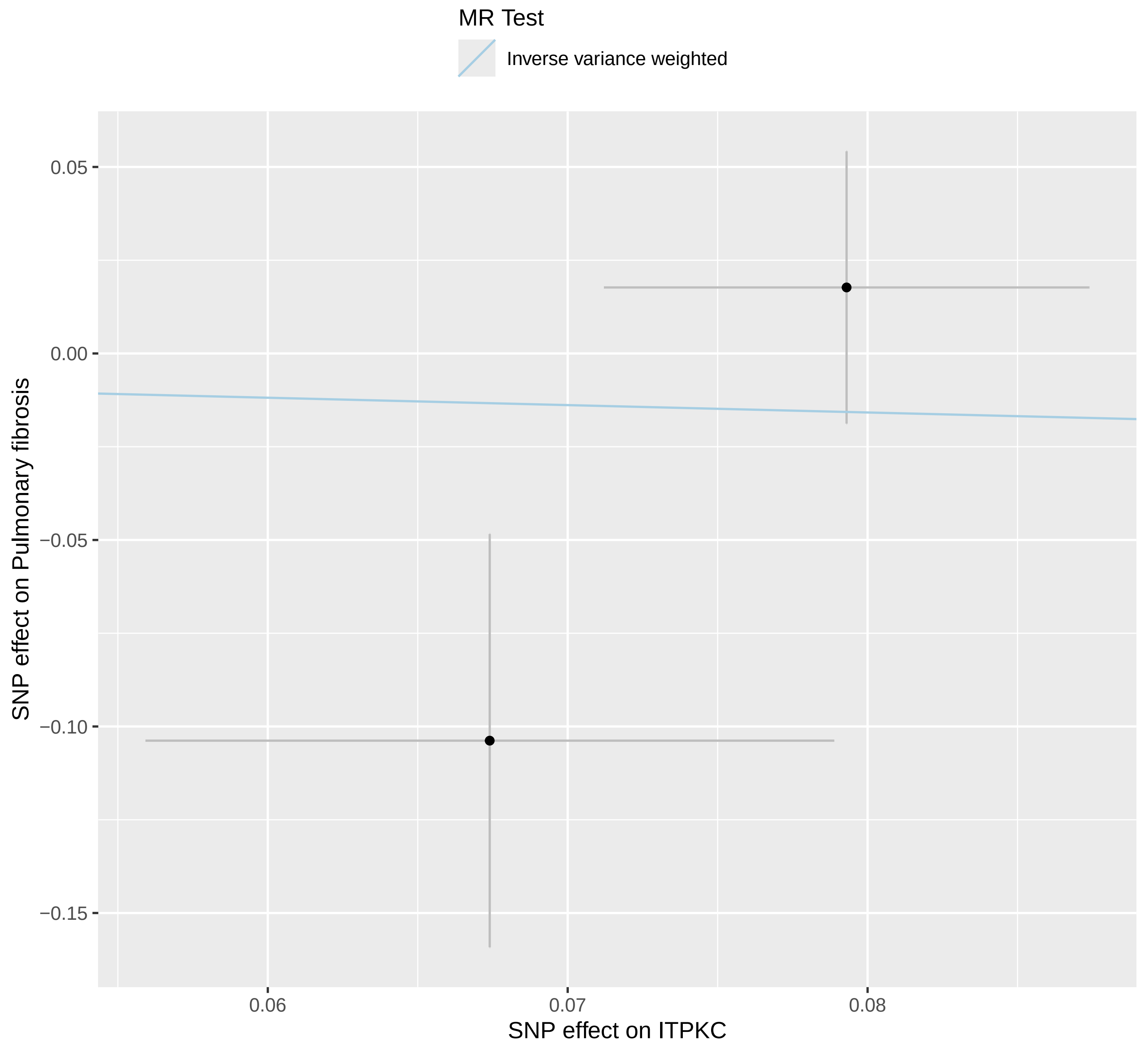
MR Test
Inverse variance weighted
0.05
0.00
−0.05
−0.10
−0.15
0.06
0.07
0.08
SNP effect on Pulmonary fibrosis
SNP effect on ITPKC

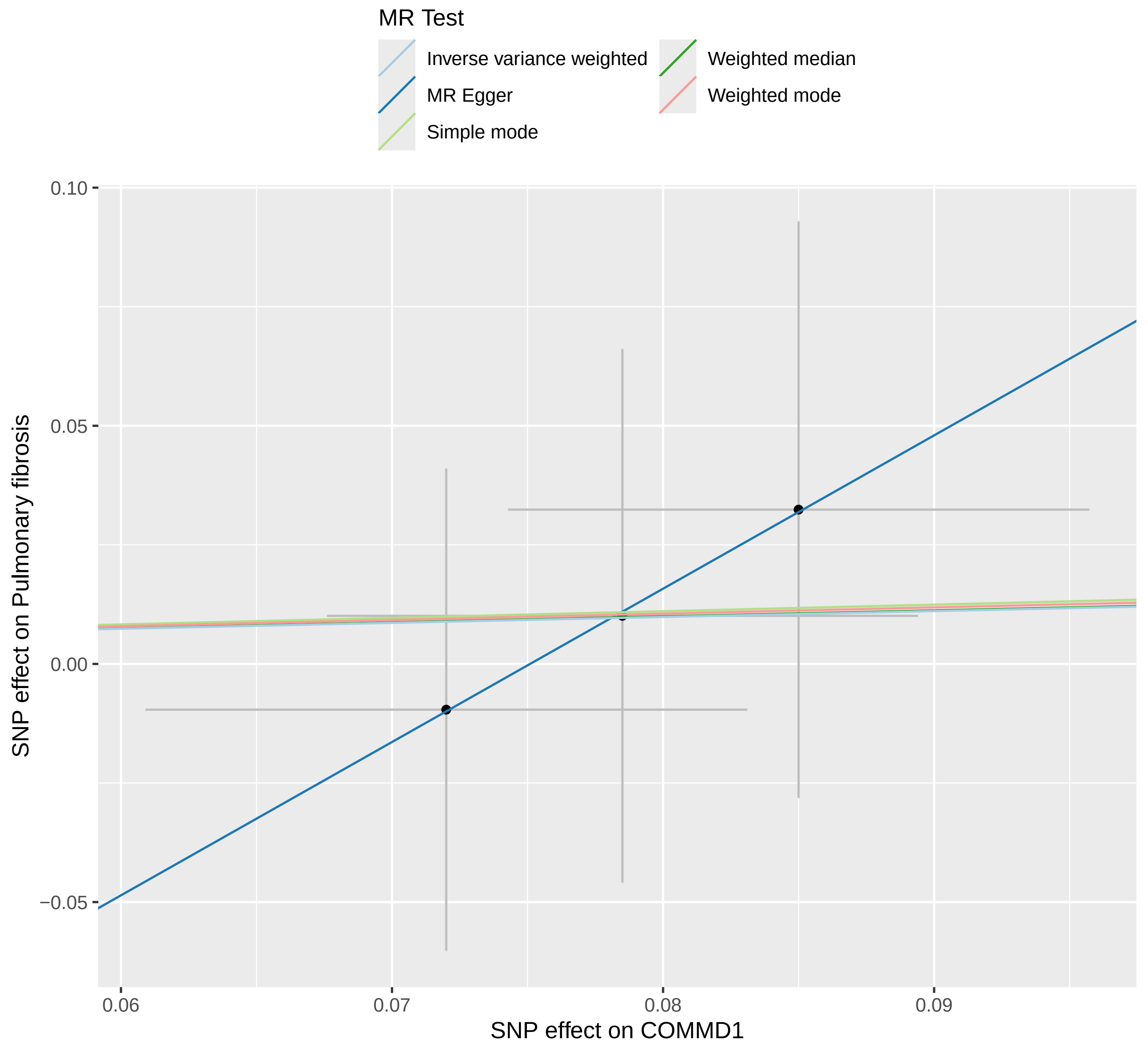
MR Test
Inverse variance weighted
MR Egger
Simple mode
Weighted median
Weighted mode
0.10
0.05
0.00
−0.05
0.06
0.07
0.08
0.09
SNP effect on Pulmonary fibrosis
SNP effect on COMMD1

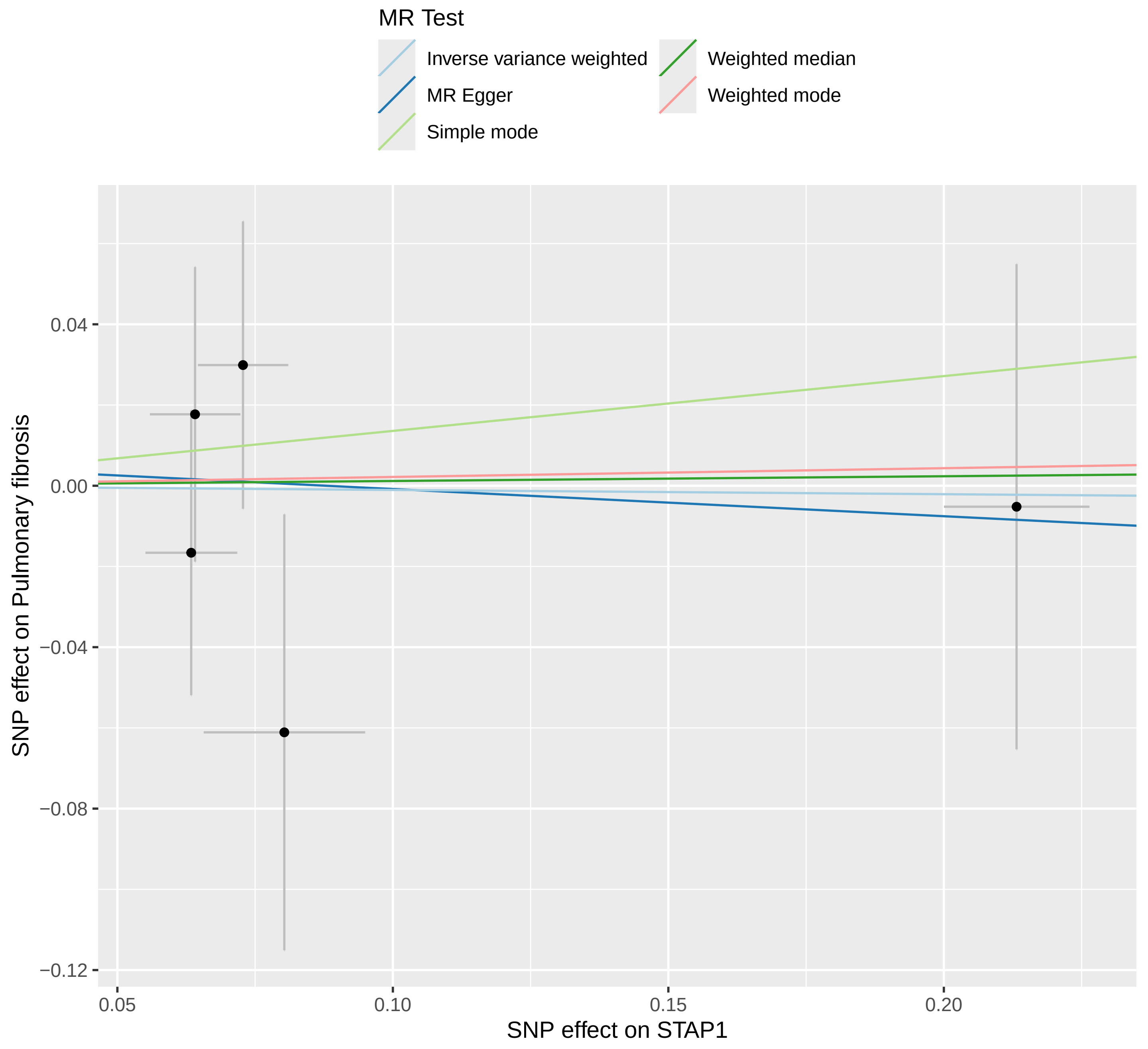
MR Test
Inverse variance weighted
MR Egger
Simple mode
Weighted median
Weighted mode
0.04
0.00
−0.04
−0.08
−0.12
SNP effect on Pulmonary fibrosis
0.05
0.10
0.15
0.20
SNP effect on STAP1

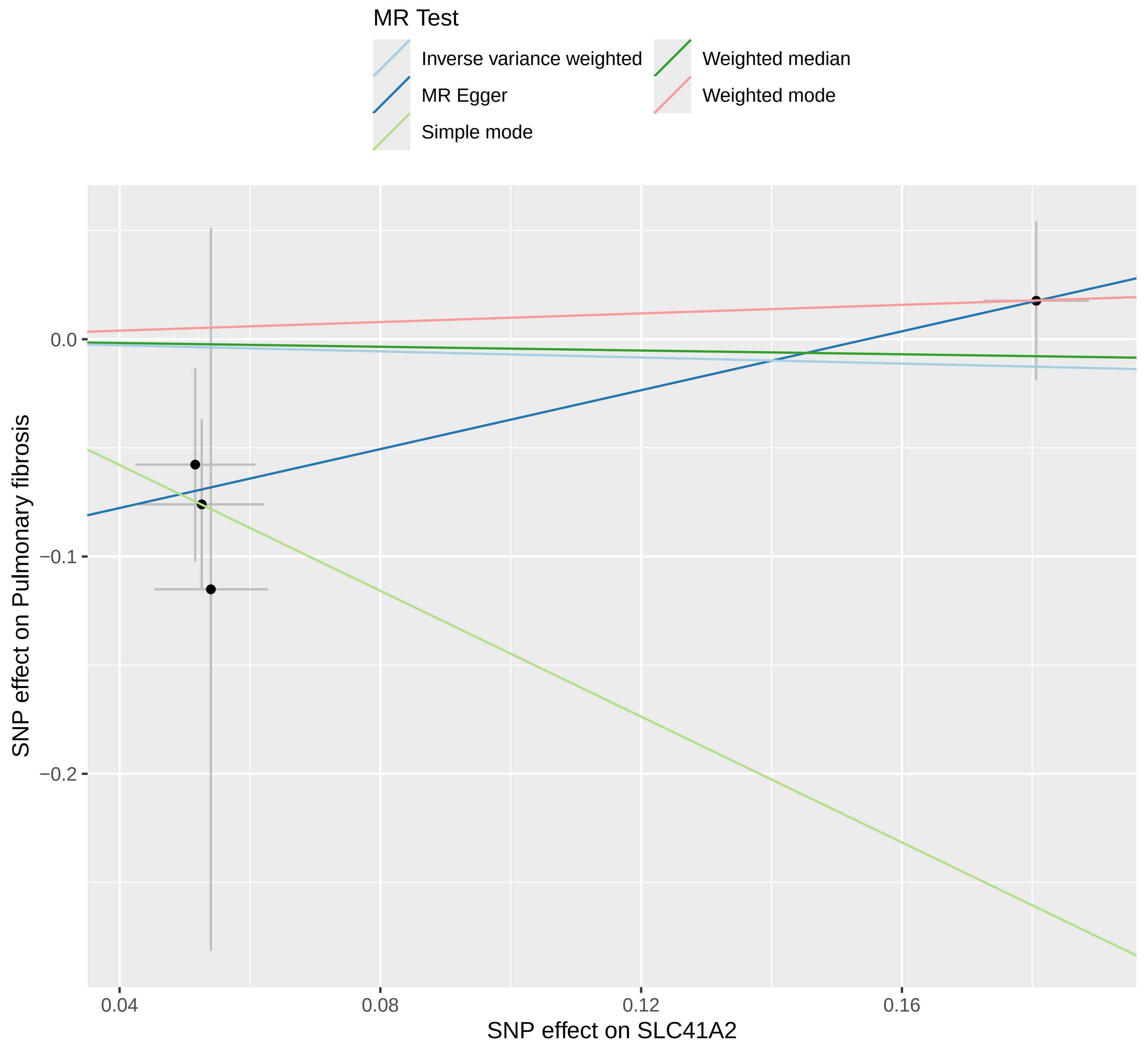
MR Test
Inverse variance weighted
MR Egger
Simple mode
Weighted median
Weighted mode
0.0
−0.1
−0.2
SNP effect on Pulmonary fibrosis
0.04
0.08
0.12
0.16
SNP effect on SLC41A2

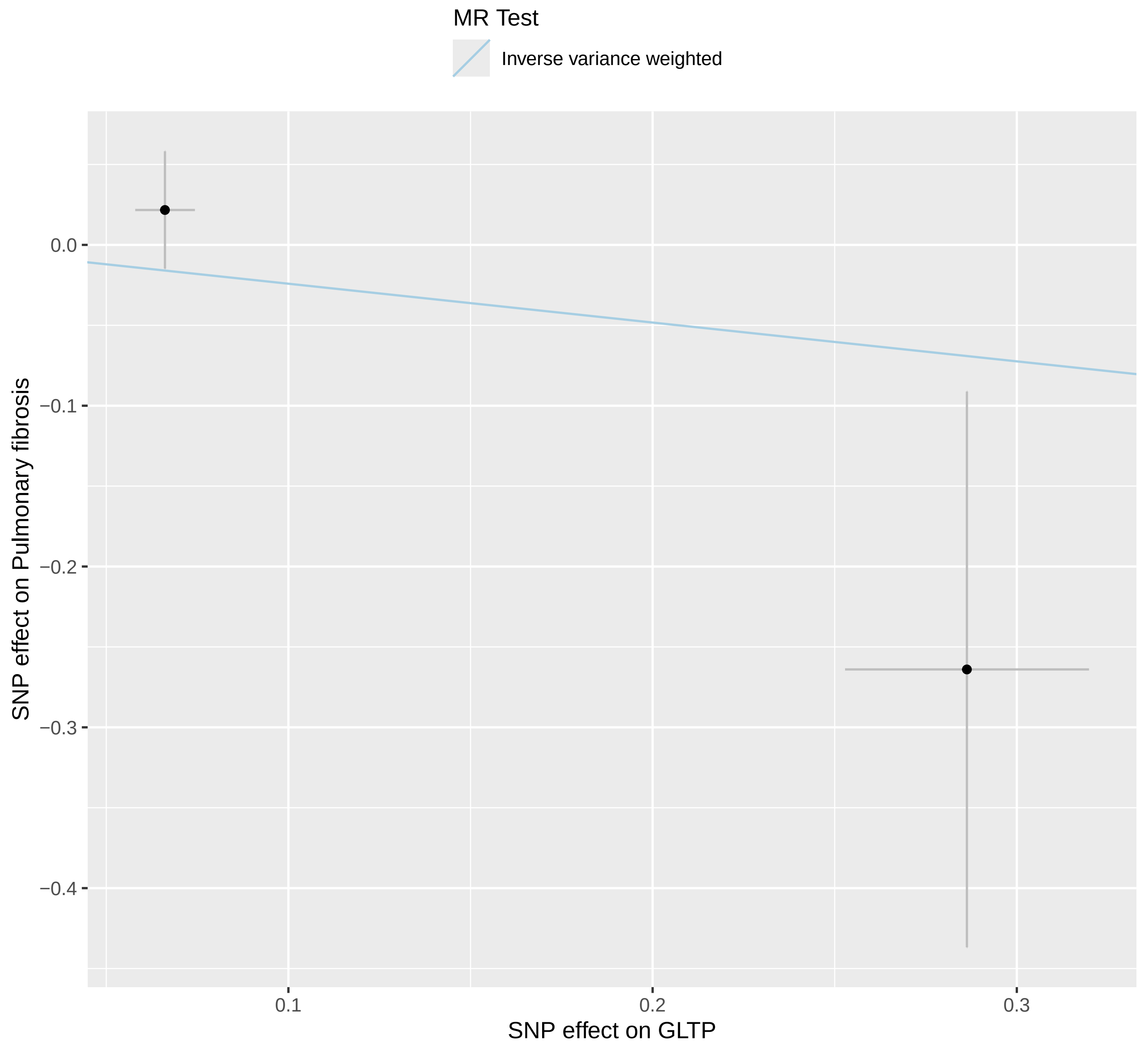
MR Test
Inverse variance weighted
0.0
−0.1
−0.2
−0.3
−0.4
SNP effect on Pulmonary fibrosis
0.1
0.2
0.3
SNP effect on GLTP

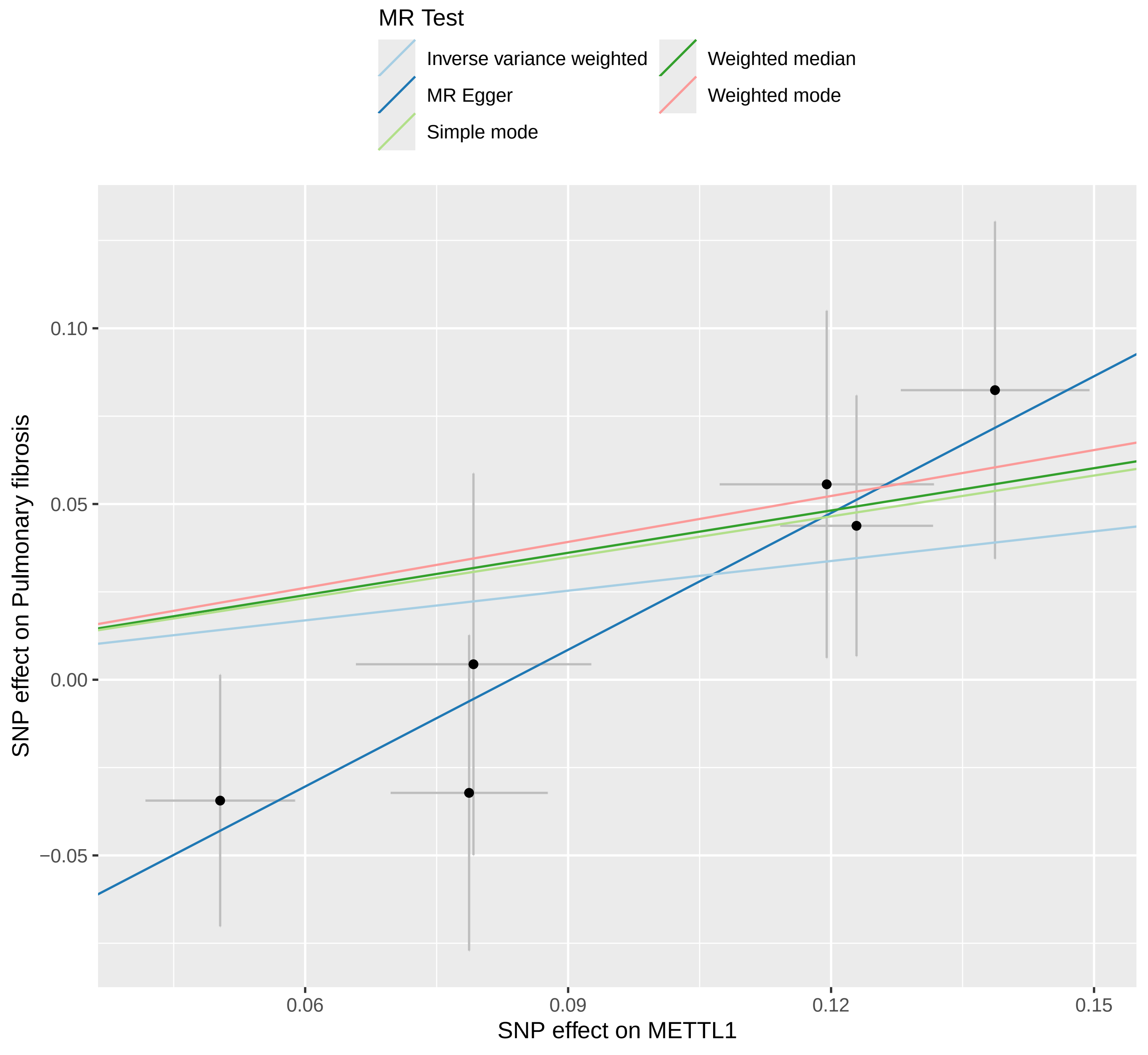
MR Test
Inverse variance weighted
MR Egger
Simple mode
Weighted median
Weighted mode
SNP effect on Pulmonary fibrosis
0.10
0.05
0.00
−0.05
0.06
0.09
0.12
0.15
SNP effect on METTL1

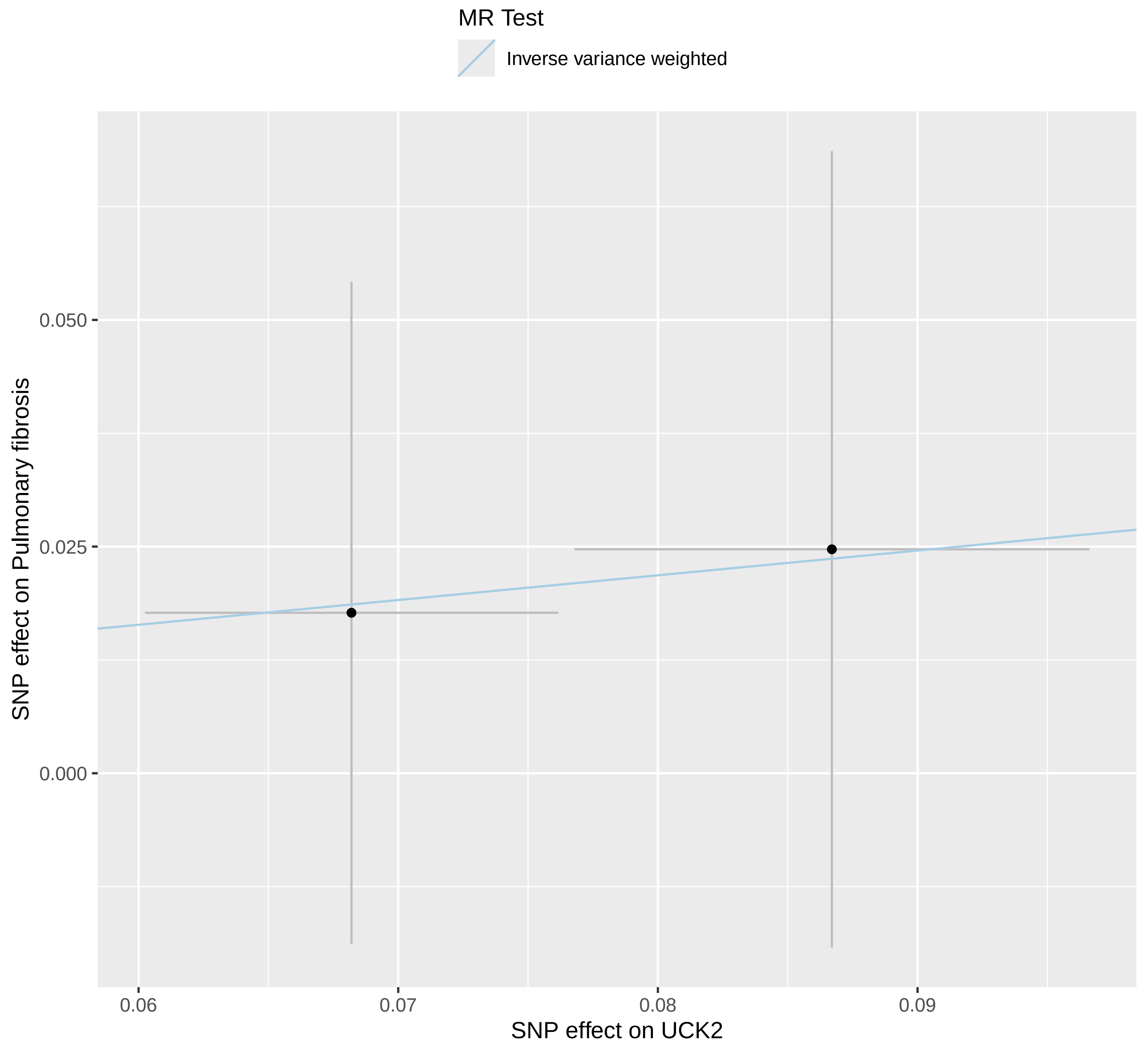

MR Test
Inverse variance weighted
SNP effect on Pulmonary fibrosis
0.050
0.025
0.000
0.06
0.07
0.08
0.09
SNP effect on UCK2

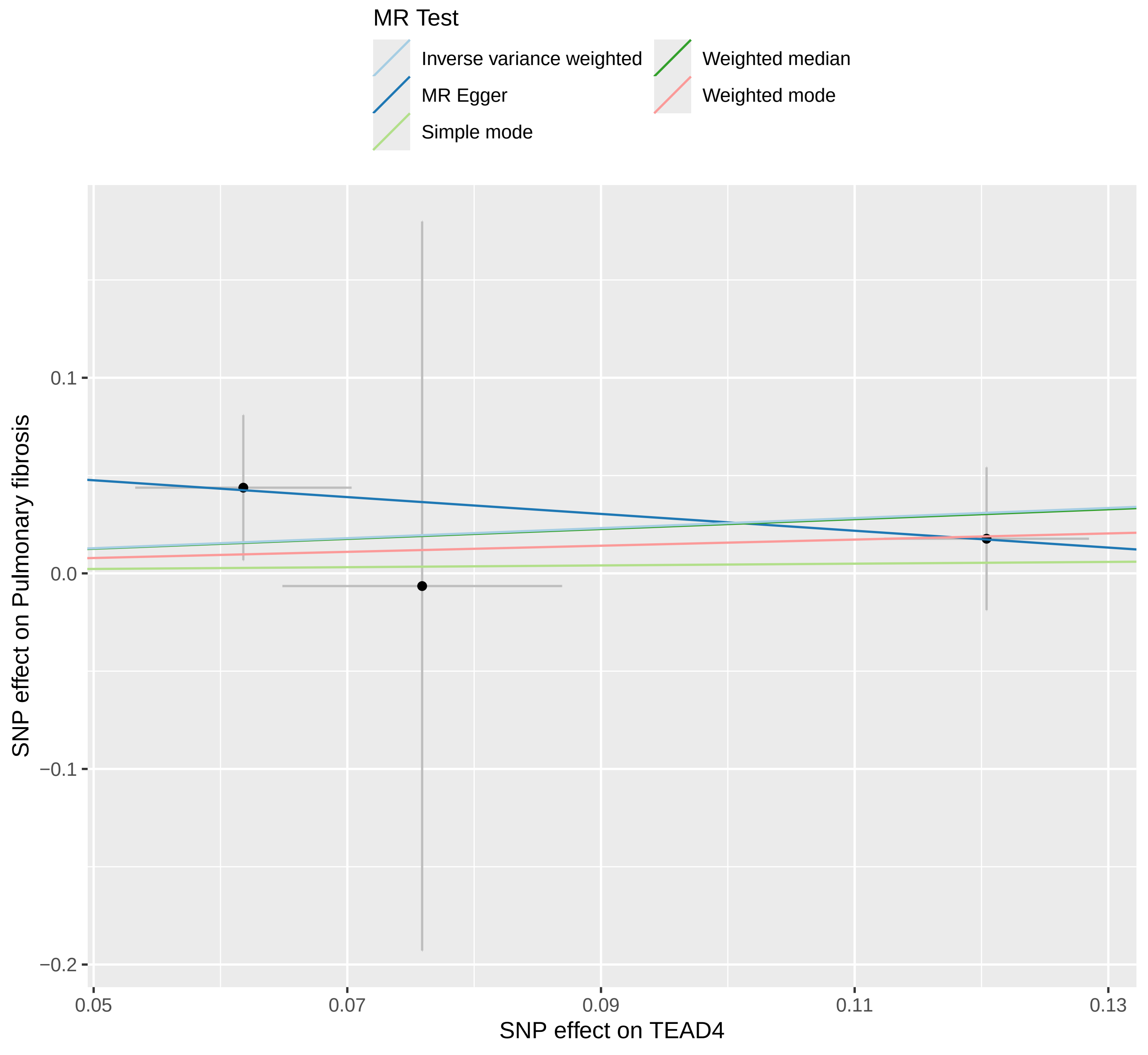

MR Test
Inverse variance weighted
MR Egger
Simple mode
Weighted median
Weighted mode
0.1
0.0
−0.1
−0.2
SNP effect on Pulmonary fibrosis
0.05
0.07
0.09
0.11
0.13
SNP effect on TEAD4

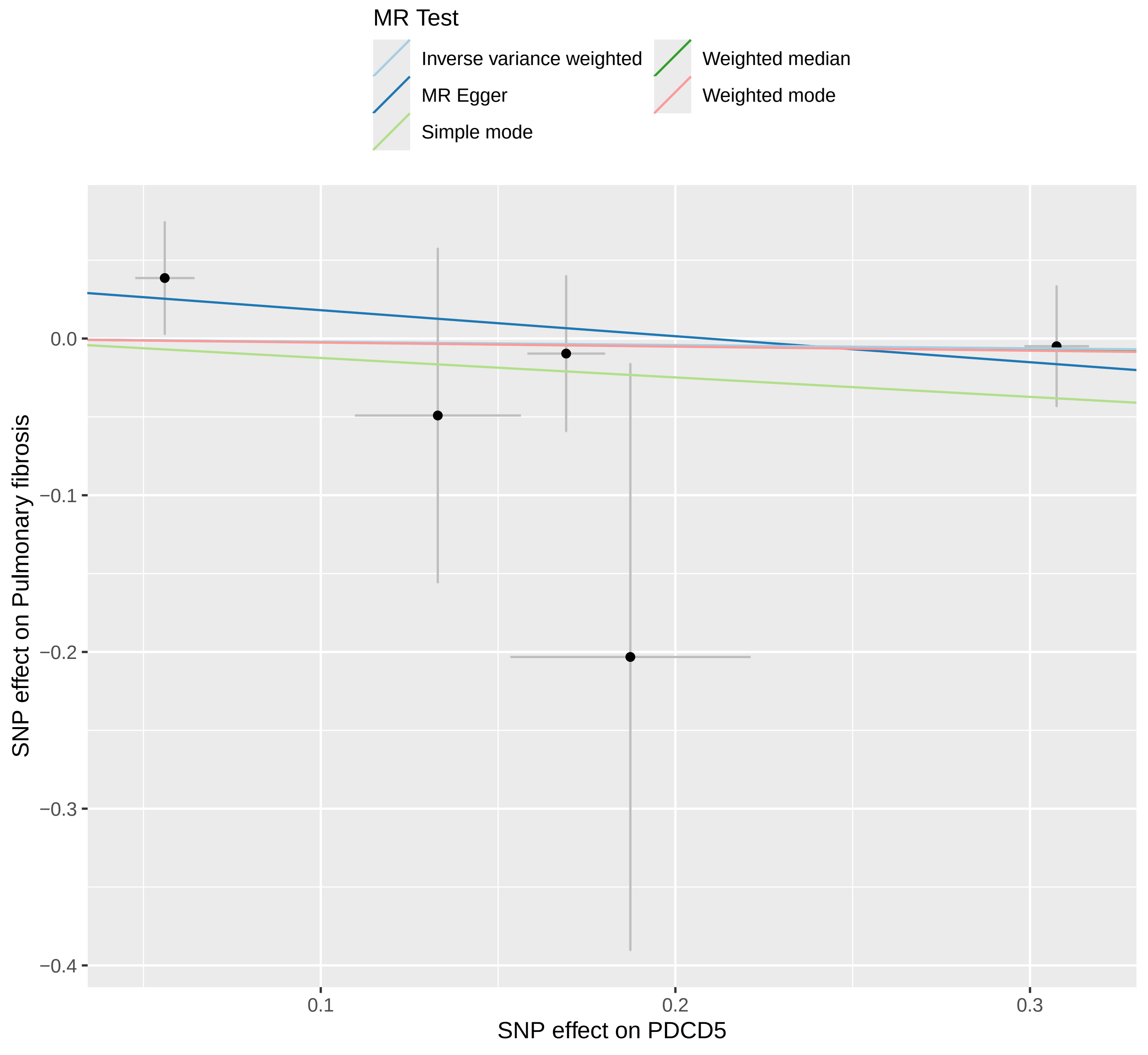
MR Test
Inverse variance weighted
MR Egger
Simple mode
Weighted median
Weighted mode
0.0
−0.1
−0.2
−0.3
−0.4
0.1
0.2
0.3
SNP effect on Pulmonary fibrosis
SNP effect on PDCD5

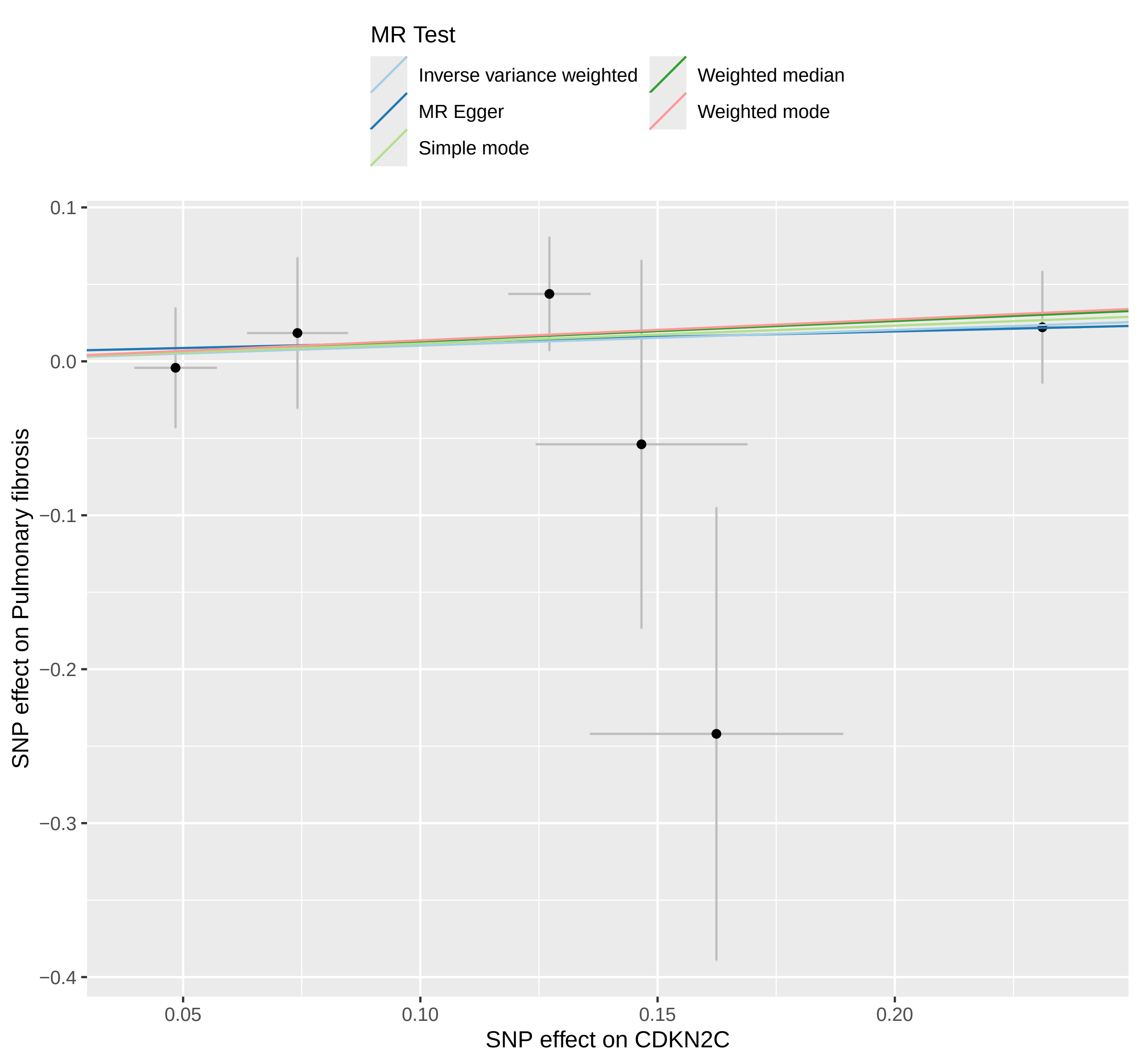
MR Test
Inverse variance weighted
MR Egger
Simple mode
Weighted median
Weighted mode
0.1
0.0
−0.1
−0.2
−0.3
−0.4
SNP effect on Pulmonary fibrosis
0.05
0.10
0.15
0.20
SNP effect on CDKN2C

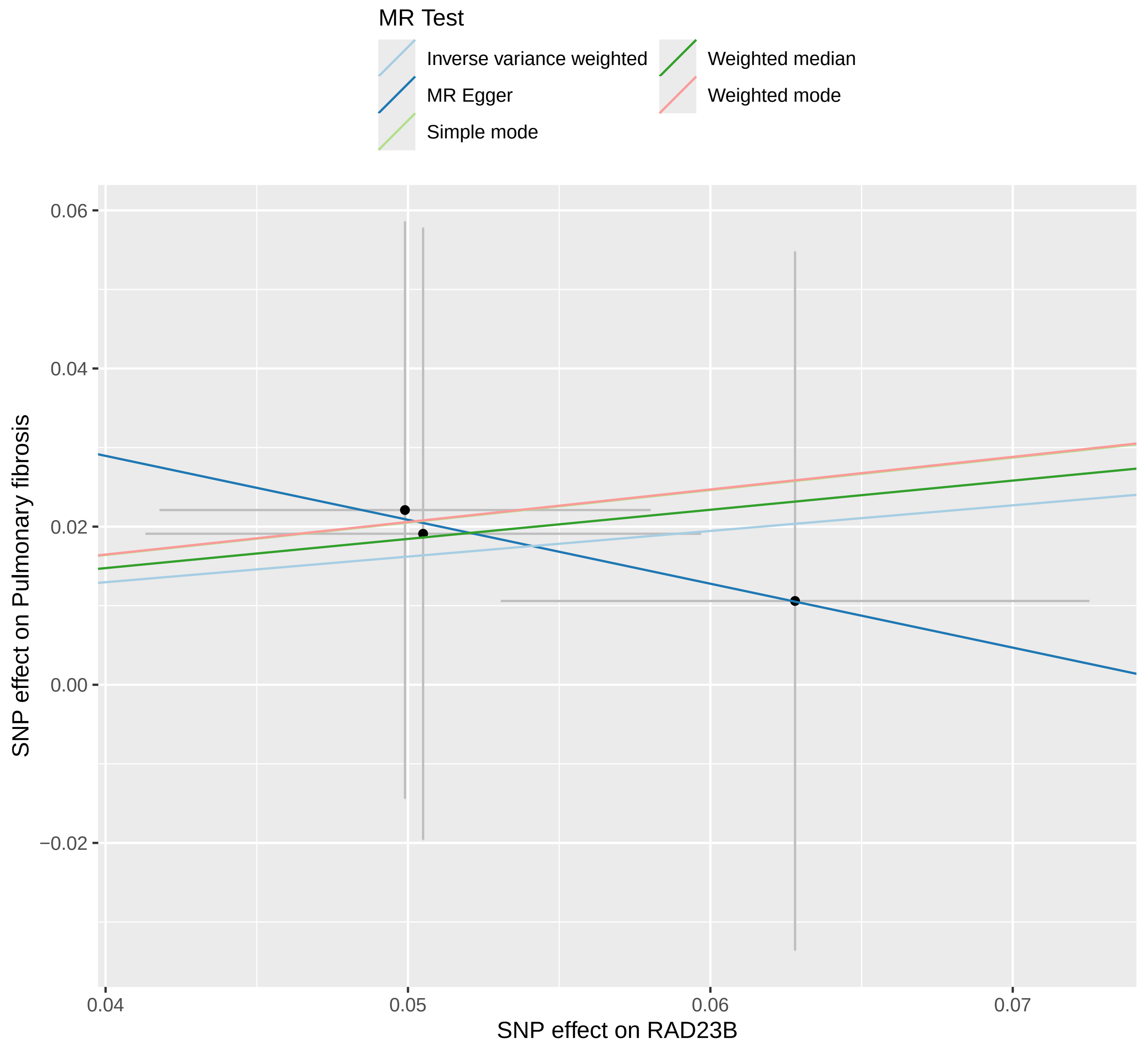
MR Test
Inverse variance weighted
MR Egger
Simple mode
Weighted median
Weighted mode
0.06
0.04
0.02
0.00
−0.02
SNP effect on Pulmonary fibrosis
0.04
0.05
0.06
0.07
SNP effect on RAD23B

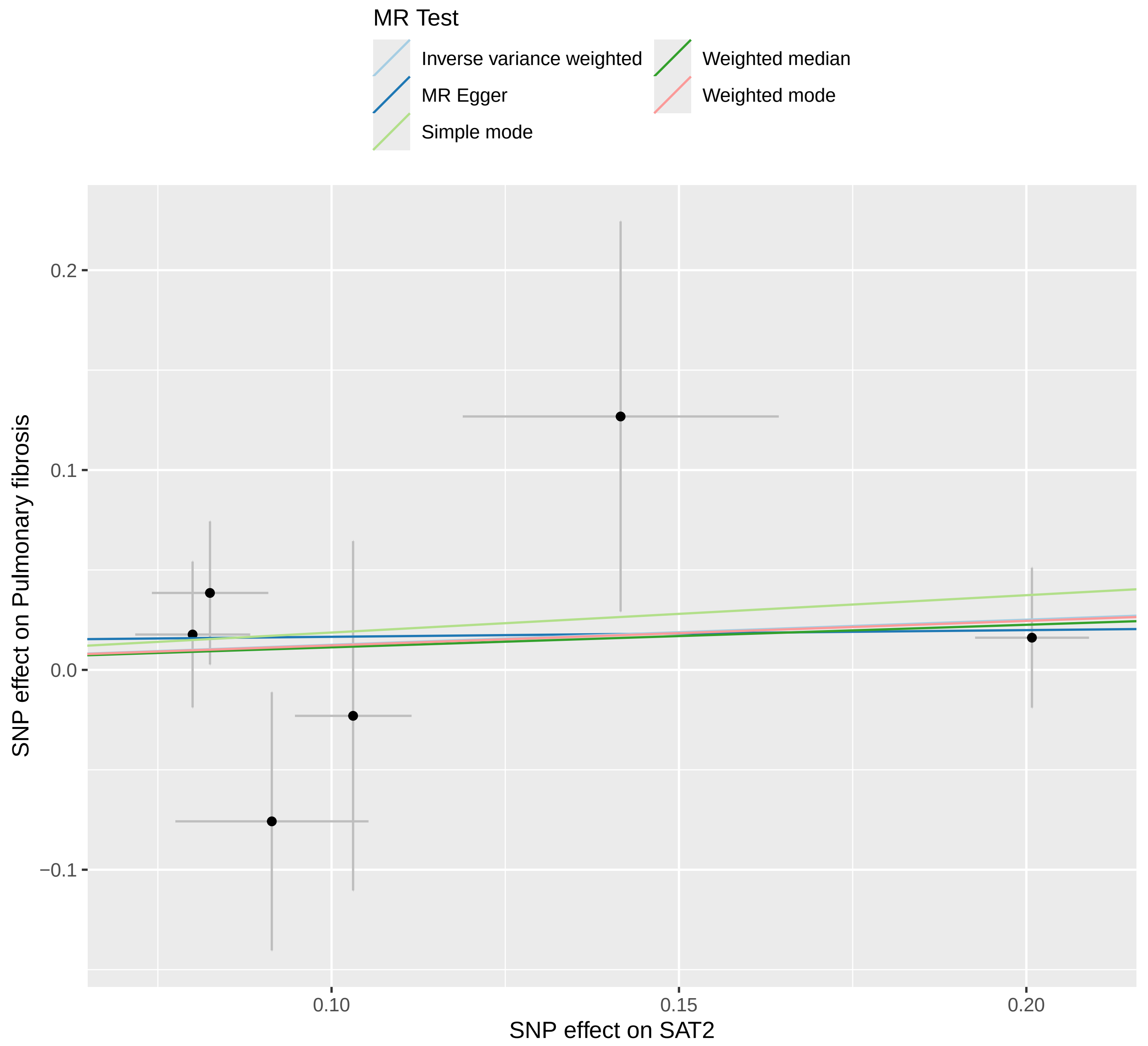
MR Test
Inverse variance weighted
MR Egger
Simple mode
Weighted median
Weighted mode
0.2
0.1
0.0
−0.1
0.10
0.15
0.20
SNP effect on Pulmonary fibrosis
SNP effect on SAT2

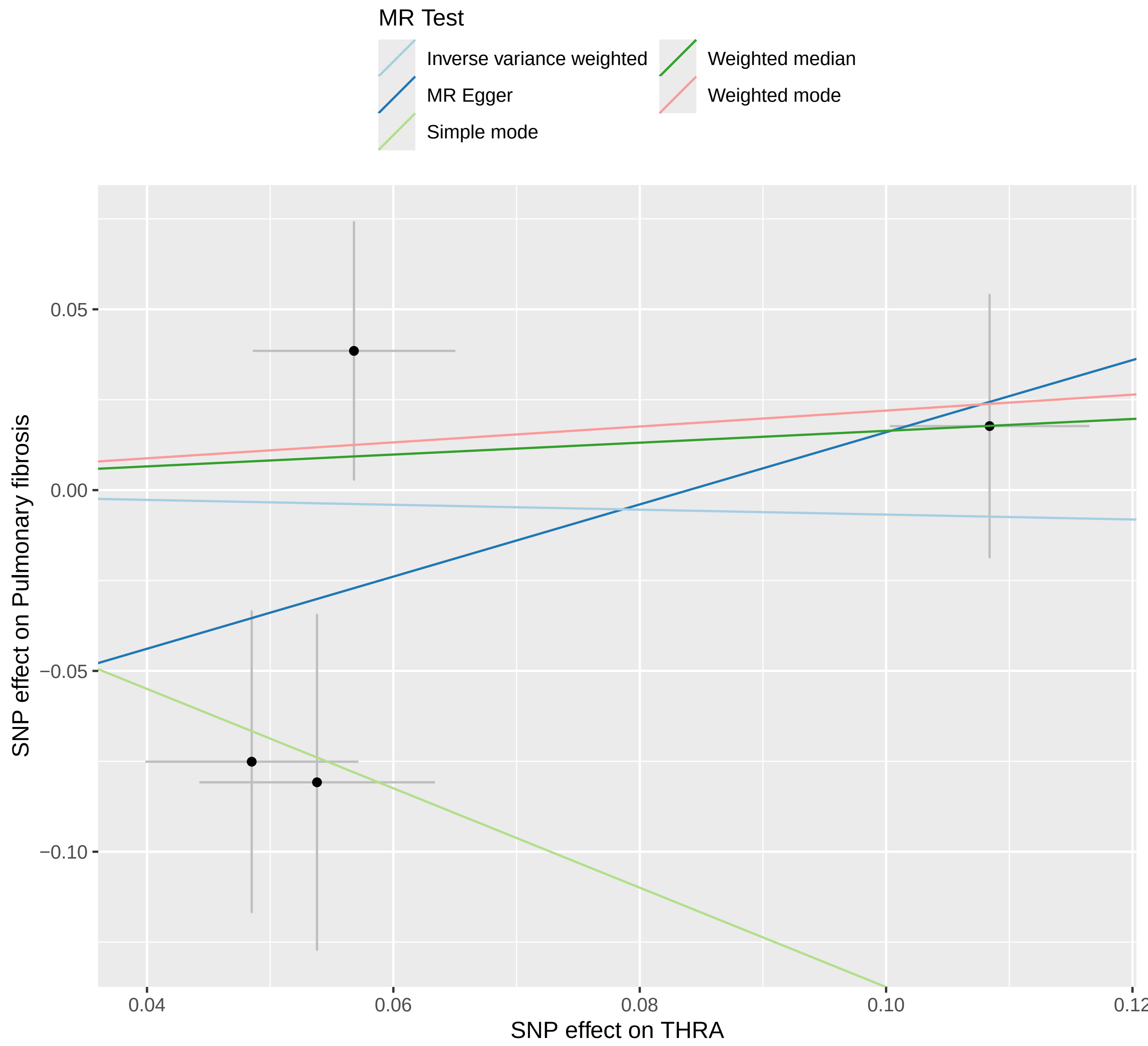
MR Test
Inverse variance weighted
MR Egger
Simple mode
Weighted median
Weighted mode
SNP effect on Pulmonary fibrosis
0.05
0.00
−0.05
−0.10
0.04
0.06
0.08
0.10
0.12
SNP effect on THRA

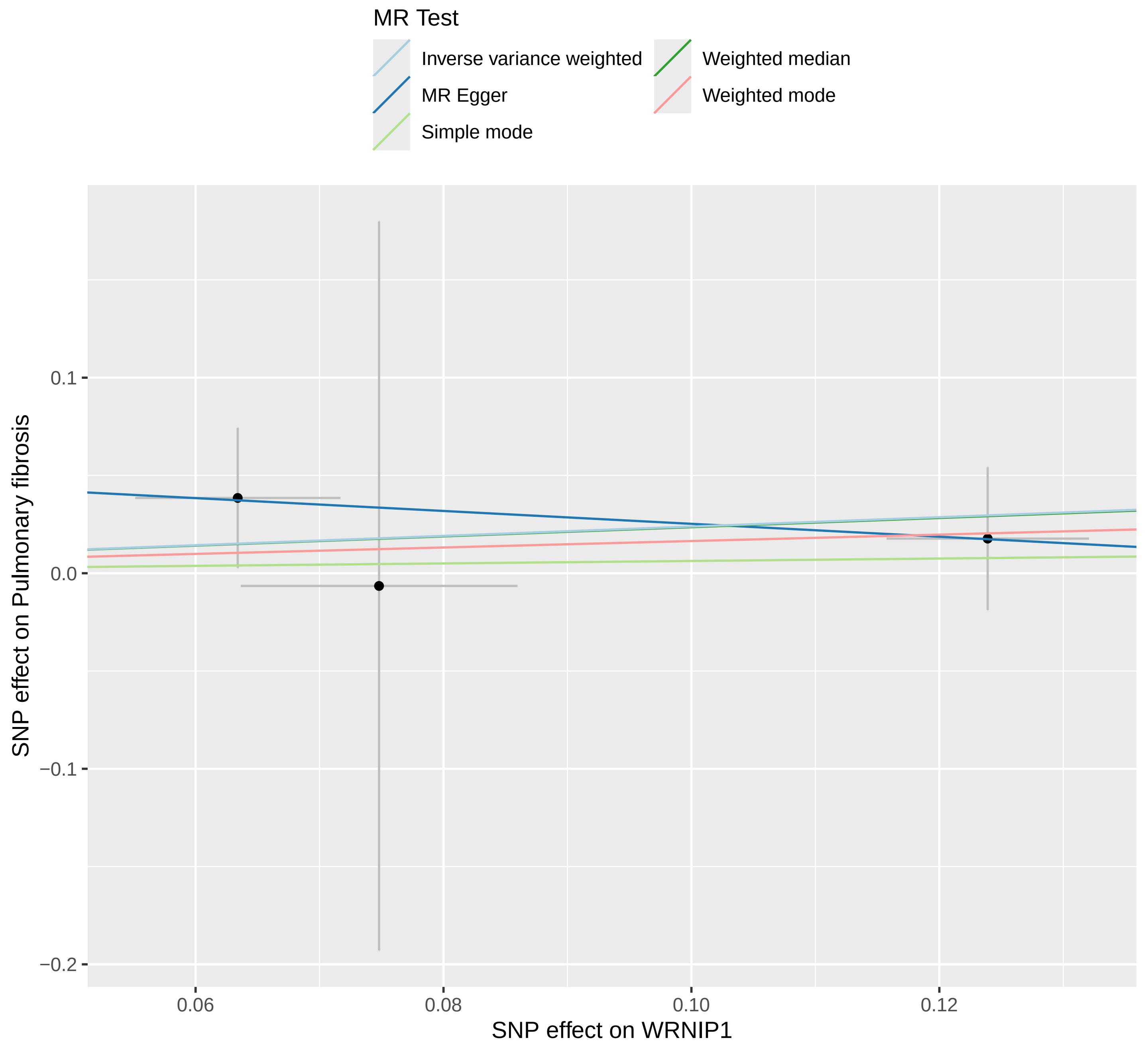

MR Test
Inverse variance weighted
MR Egger
Simple mode
Weighted median
Weighted mode
0.1
0.0
−0.1
−0.2
SNP effect on Pulmonary fibrosis
0.06
0.08
0.10
0.12
SNP effect on WRNIP1

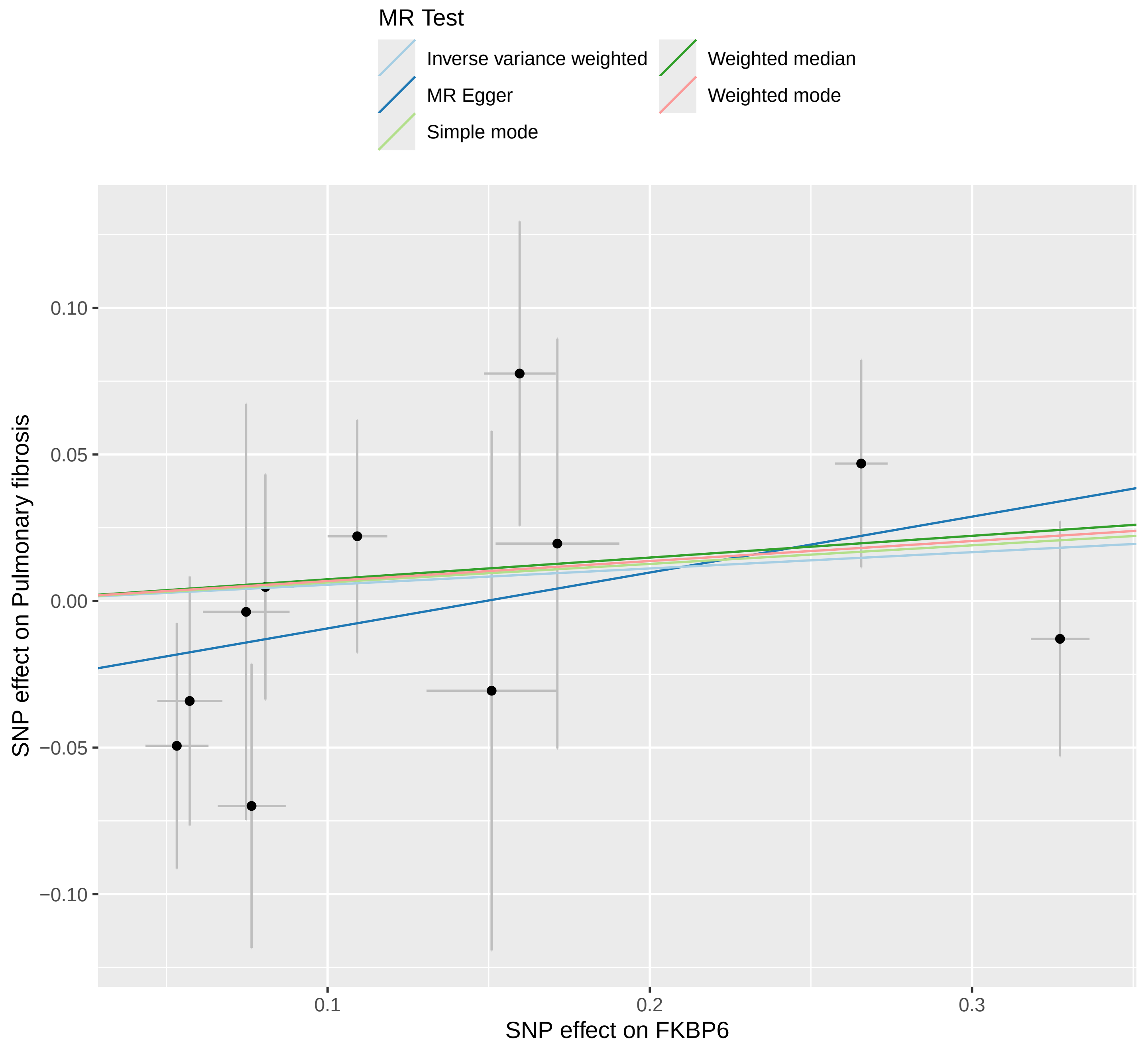
MR Test
Inverse variance weighted
MR Egger
Simple mode
Weighted median
Weighted mode
0.10
0.05
0.00
−0.05
−0.10
SNP effect on Pulmonary fibrosis
0.1
0.2
0.3
SNP effect on FKBP6

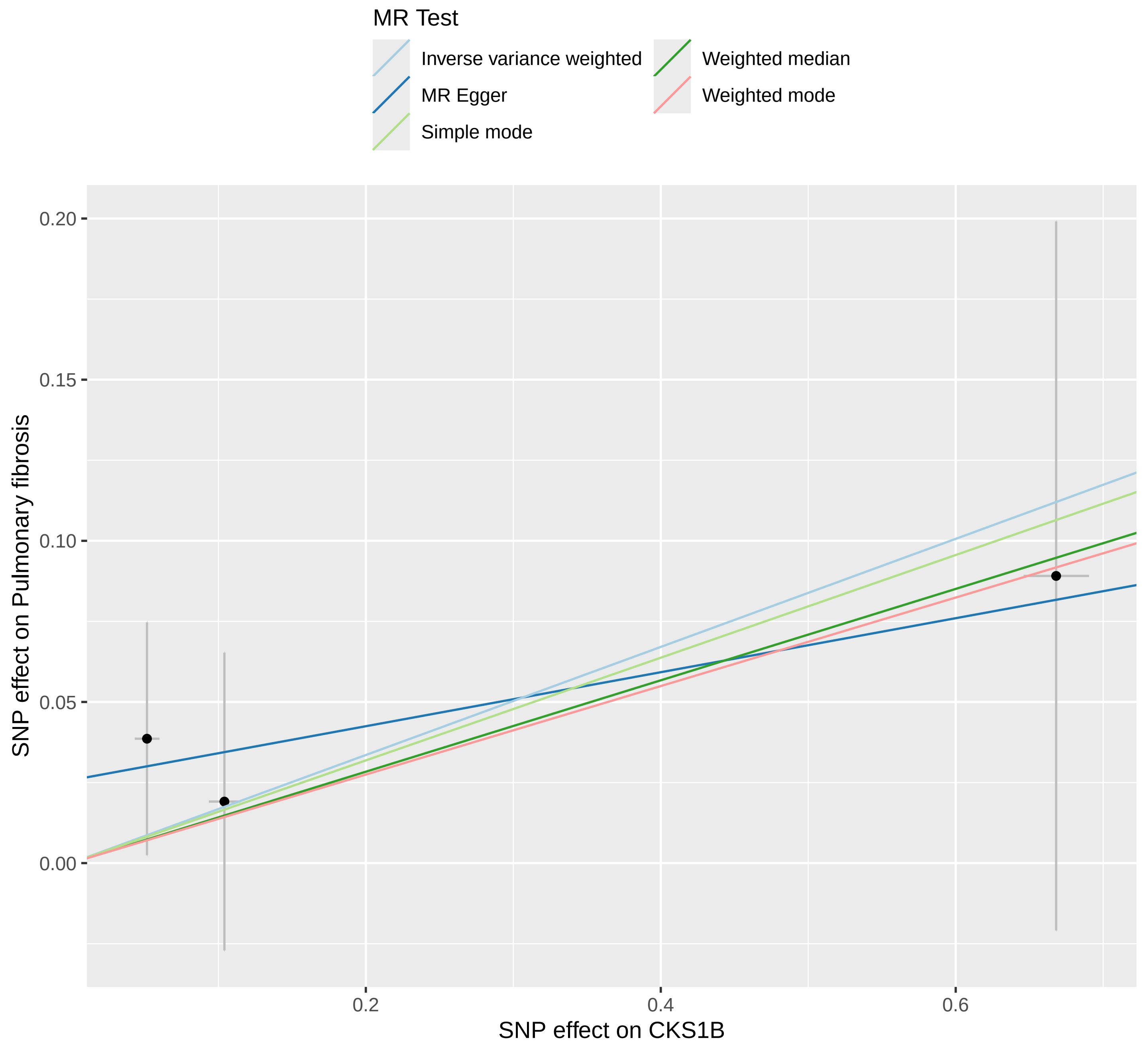

MR Test
Inverse variance weighted
MR Egger
Simple mode
Weighted median
Weighted mode
0.20
0.15
0.10
0.05
0.00
SNP effect on Pulmonary fibrosis
0.2
0.4
0.6
SNP effect on CKS1B

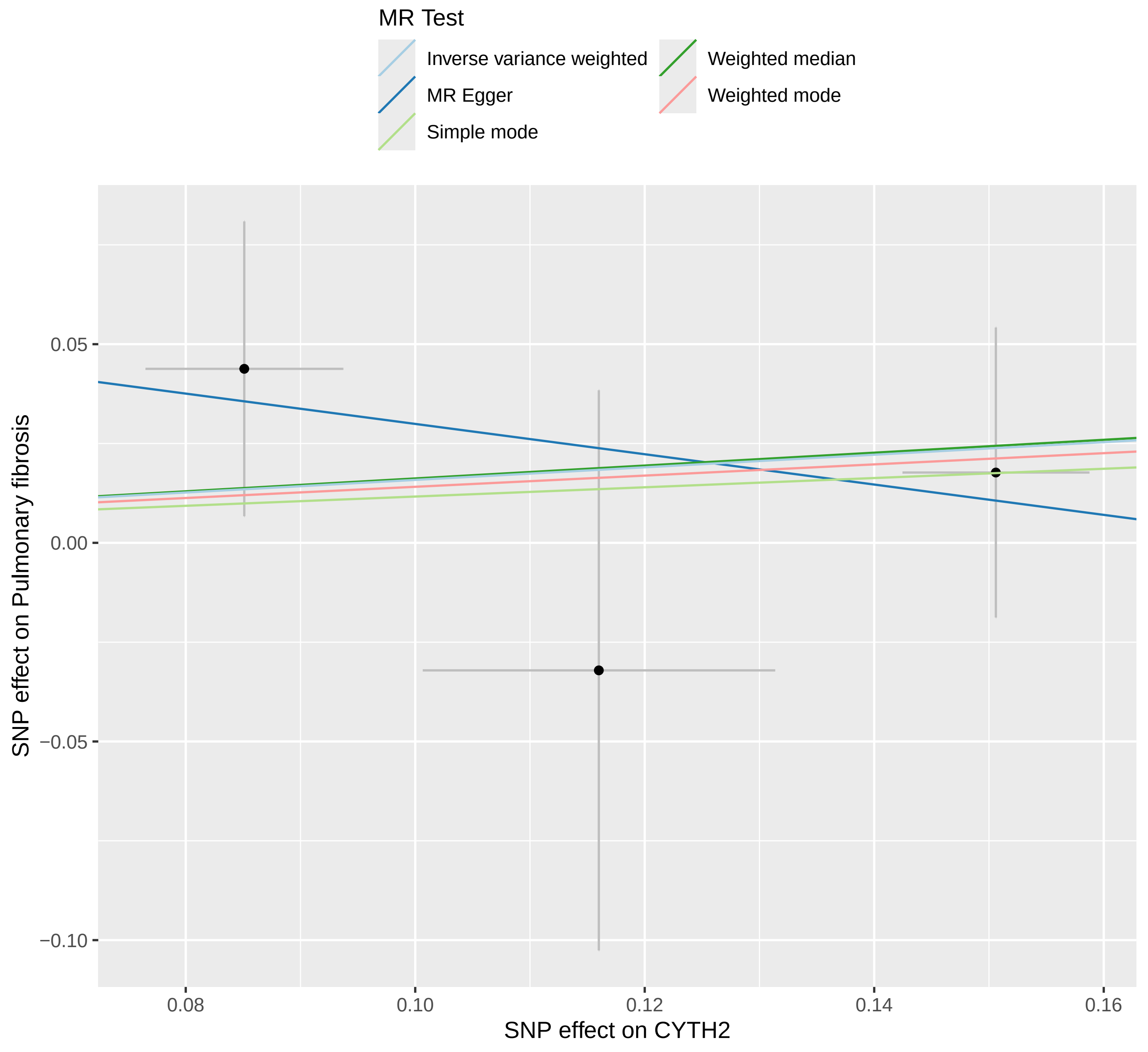
MR Test
Inverse variance weighted
MR Egger
Simple mode
Weighted median
Weighted mode
0.05
0.00
−0.05
−0.10
SNP effect on Pulmonary fibrosis
0.08
0.10
0.12
0.14
0.16
SNP effect on CYTH2

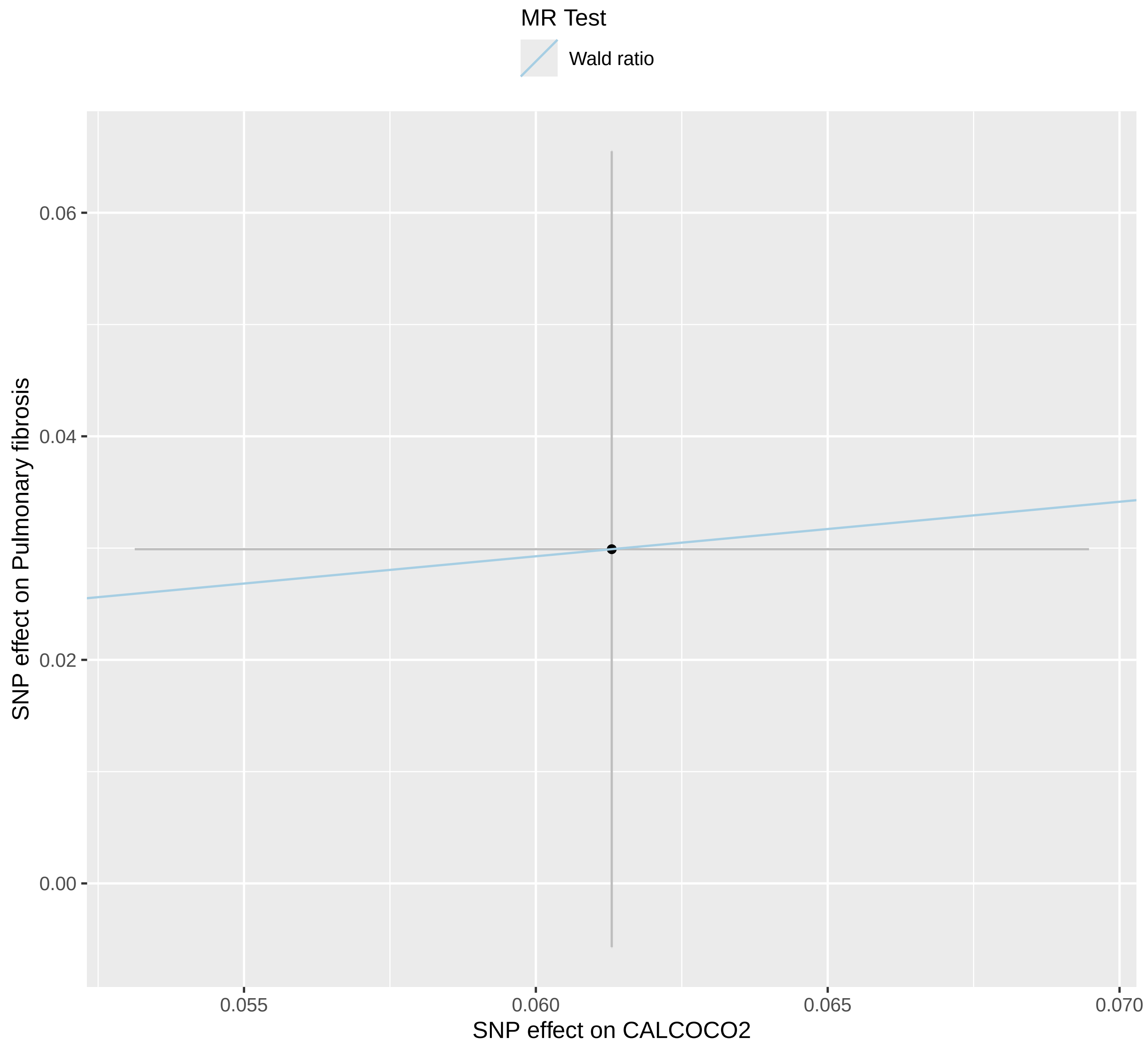
MR Test
Wald ratio
SNP effect on Pulmonary fibrosis
0.06
0.04
0.02
0.00
0.055
0.060
0.065
0.070
SNP effect on CALCOCO2

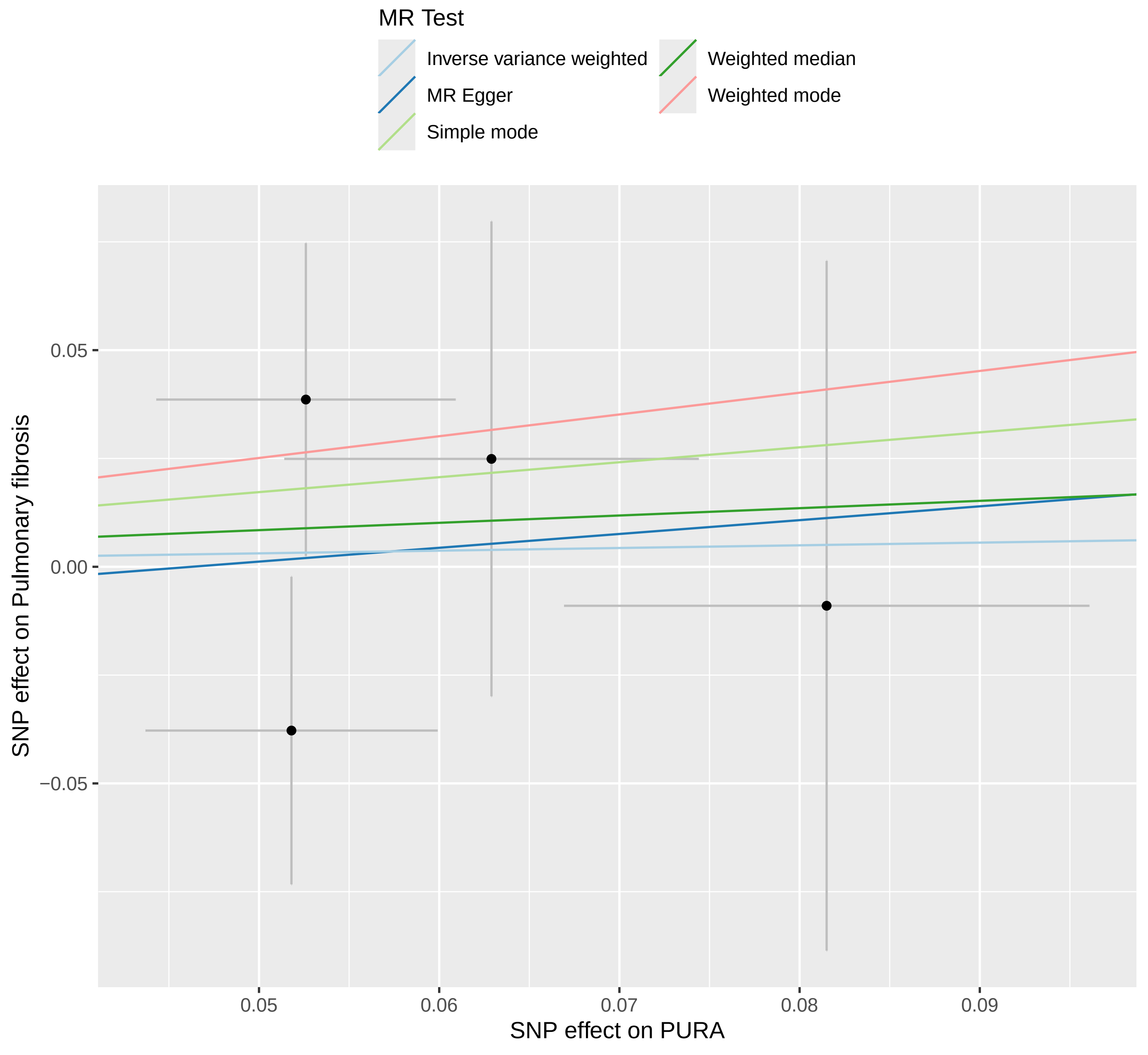
MR Test
Inverse variance weighted
MR Egger
Simple mode
Weighted median
Weighted mode
0.05
0.00
−0.05
0.05
0.06
0.07
0.08
0.09
SNP effect on Pulmonary fibrosis
SNP effect on PURA

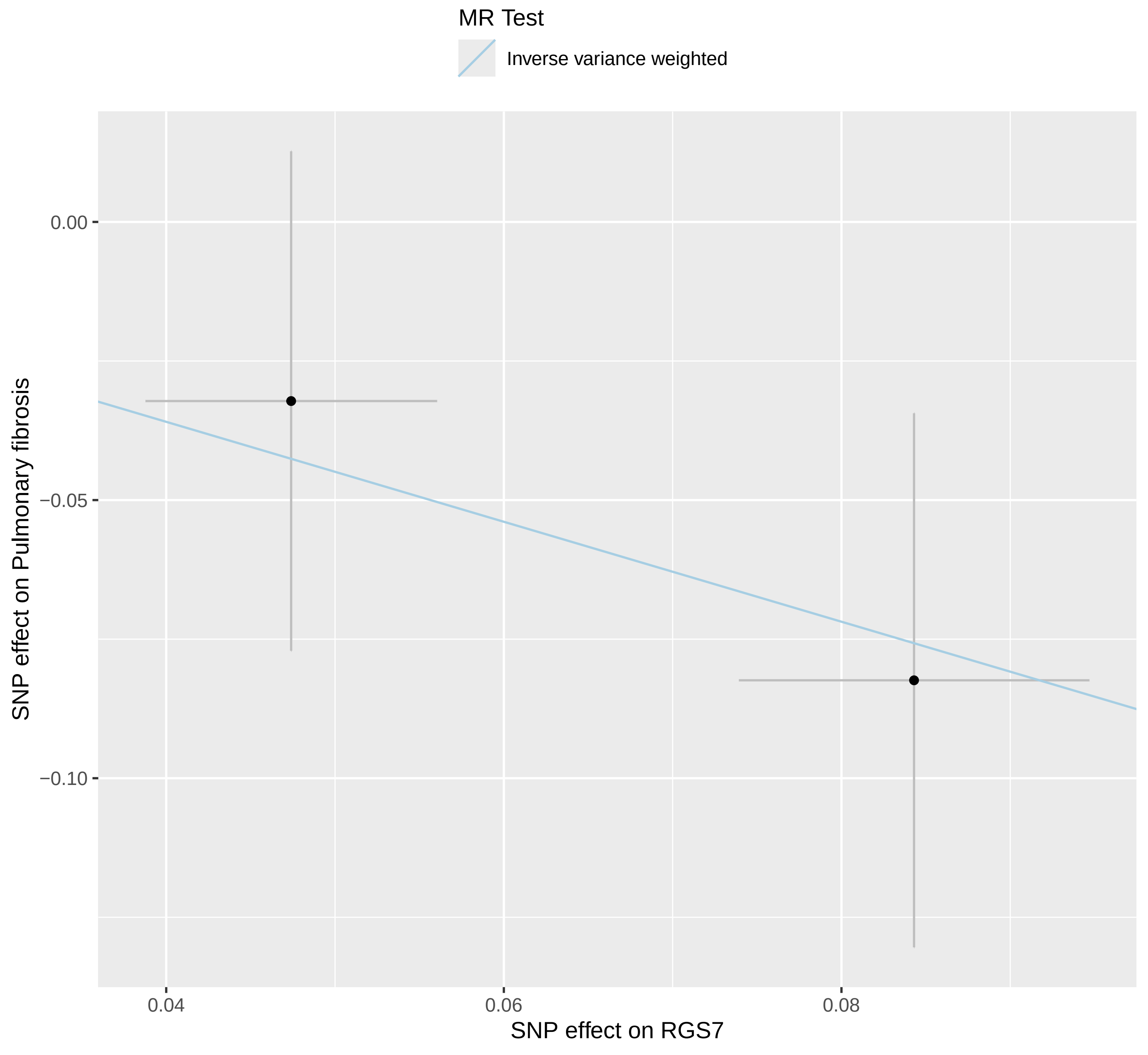
MR Test
Inverse variance weighted
0.00
−0.05
−0.10
SNP effect on Pulmonary fibrosis
0.04
0.06
0.08
SNP effect on RGS7

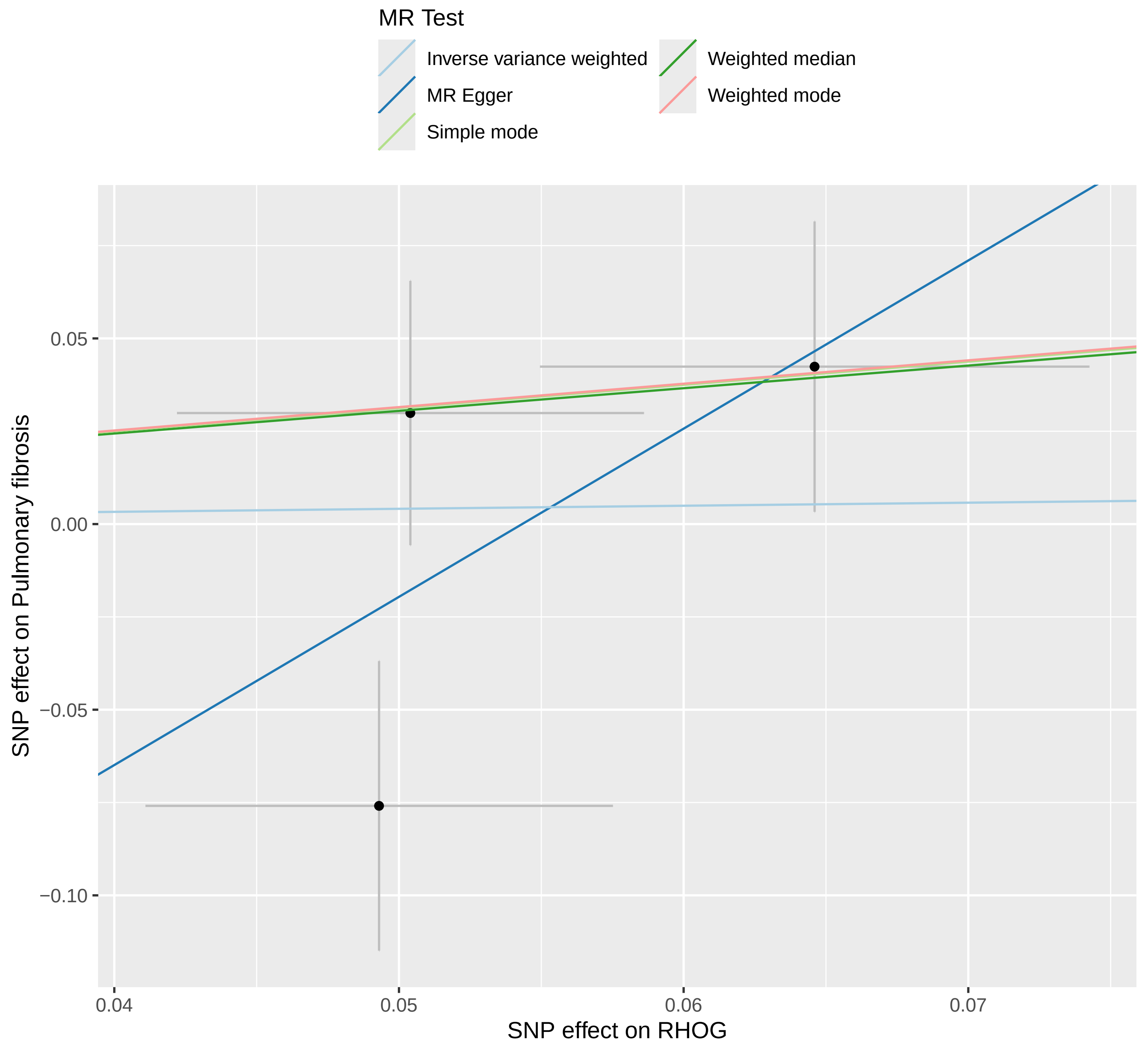

MR Test
Inverse variance weighted
MR Egger
Simple mode
Weighted median
Weighted mode
0.05
0.00
−0.05
−0.10
SNP effect on Pulmonary fibrosis
0.04
0.05
0.06
0.07
SNP effect on RHOG

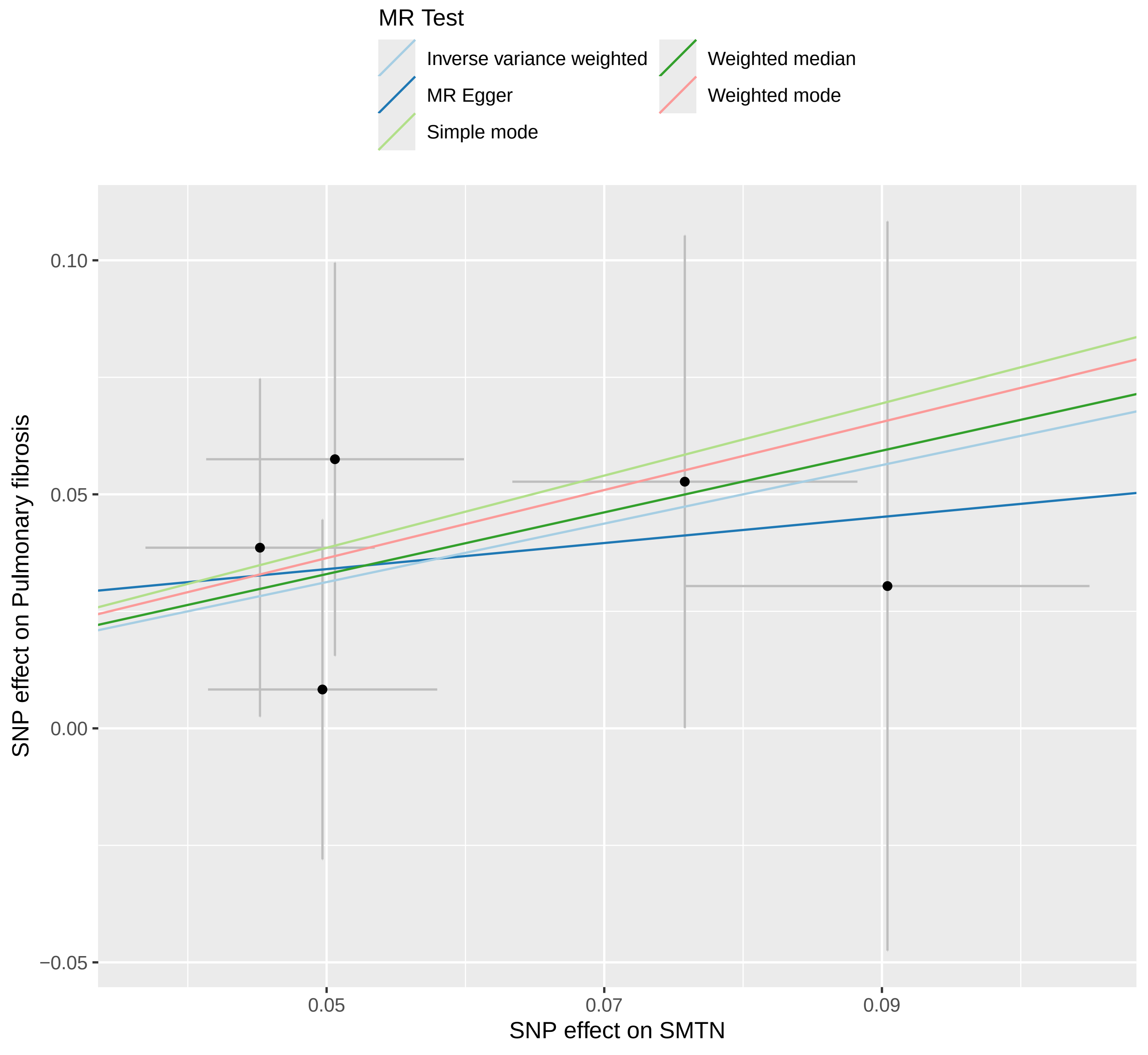
MR Test
Inverse variance weighted
MR Egger
Simple mode
Weighted median
Weighted mode
SNP effect on Pulmonary fibrosis
0.10
0.05
0.00
−0.05
0.05
0.07
0.09
SNP effect on SMTN

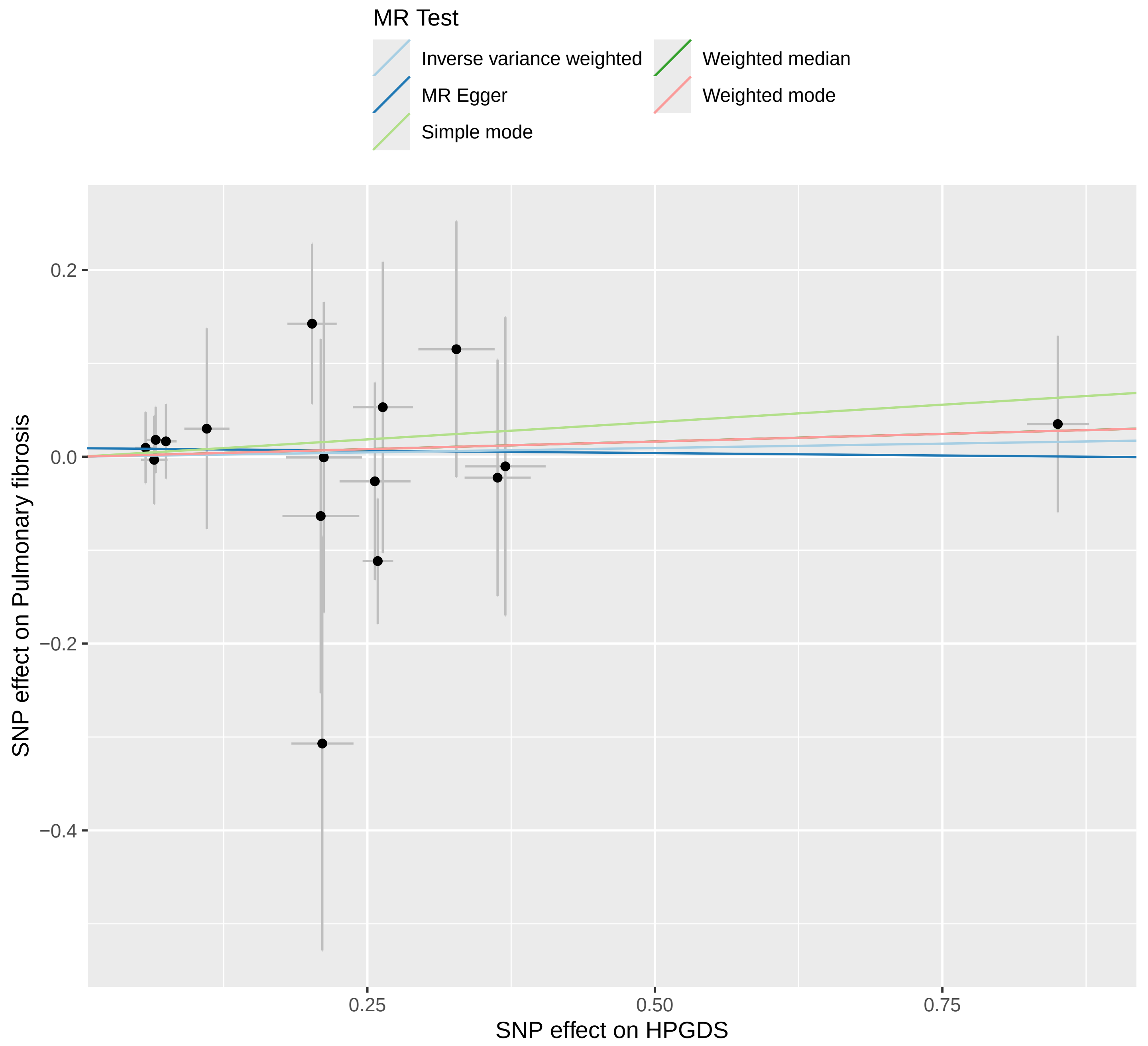
MR Test
Inverse variance weighted
MR Egger
Simple mode
Weighted median
Weighted mode
SNP effect on Pulmonary fibrosis
0.2
0.0
−0.2
−0.4
0.25
0.50
0.75
SNP effect on HPGDS

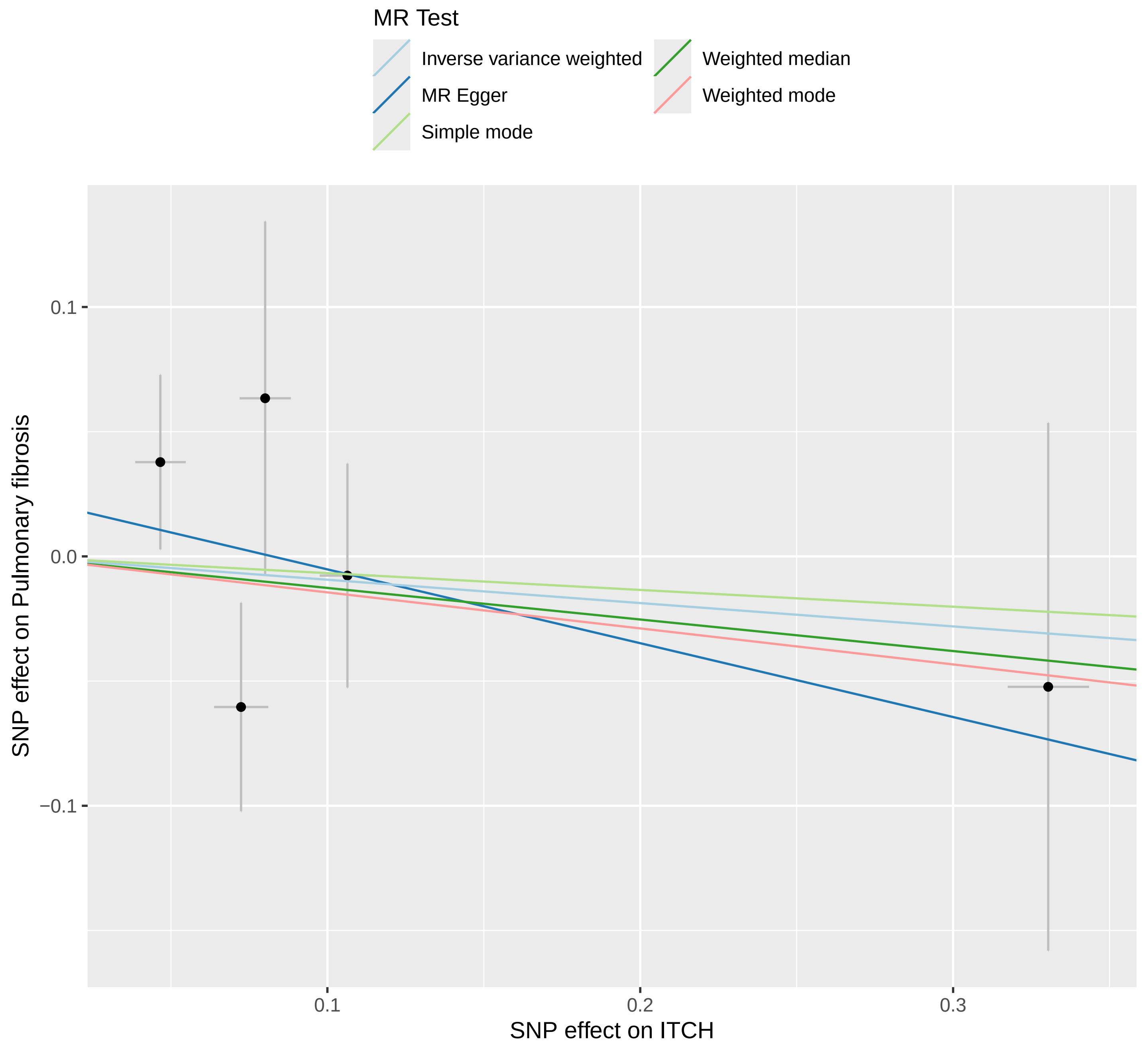
MR Test
Inverse variance weighted
MR Egger
Simple mode
Weighted median
Weighted mode
0.1
0.0
−0.1
0.1
0.2
0.3
SNP effect on Pulmonary fibrosis
SNP effect on ITCH

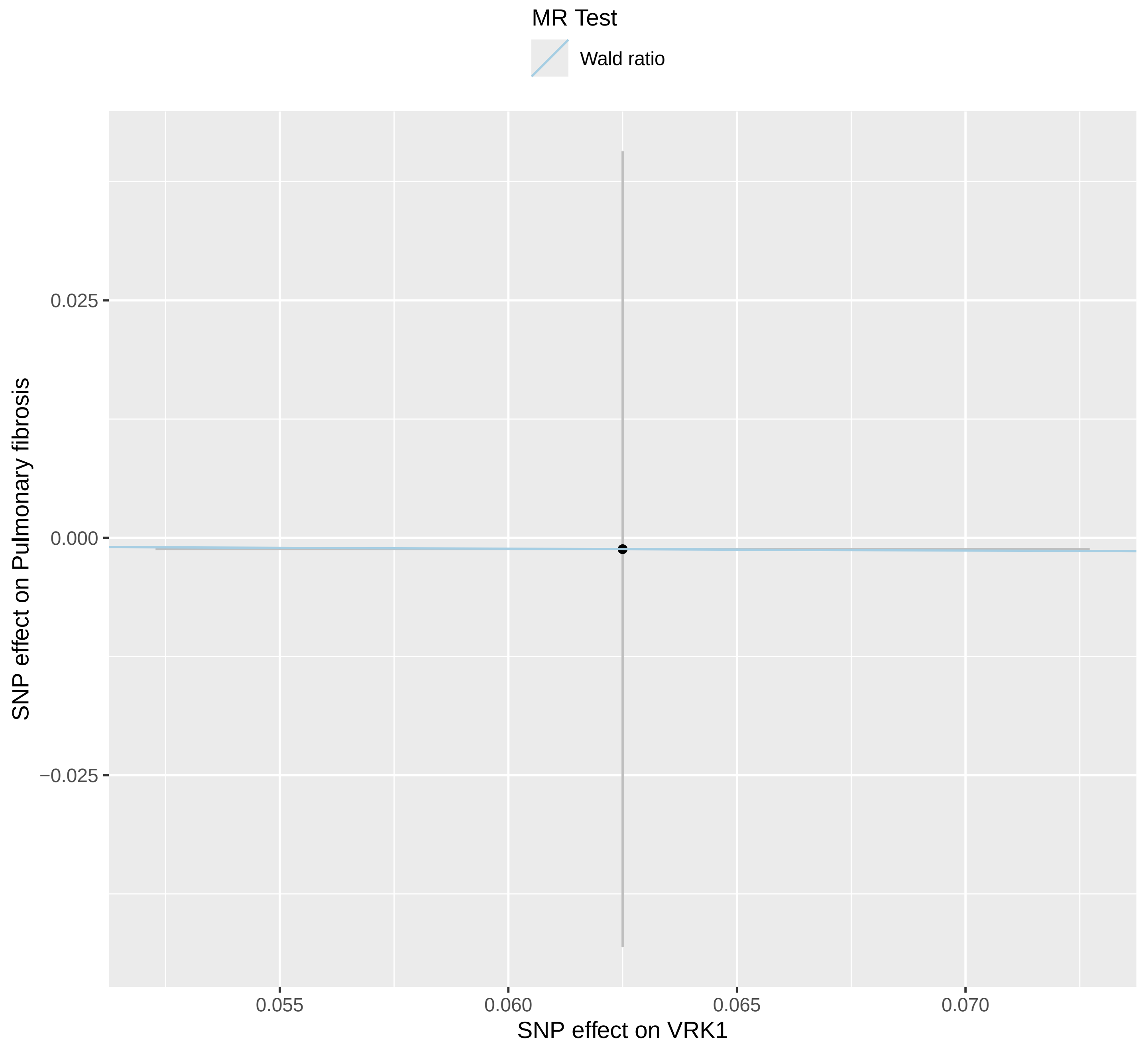
MR Test
Wald ratio
0.025
0.000
−0.025
0.055
0.060
0.065
0.070
SNP effect on Pulmonary fibrosis
SNP effect on VRK1

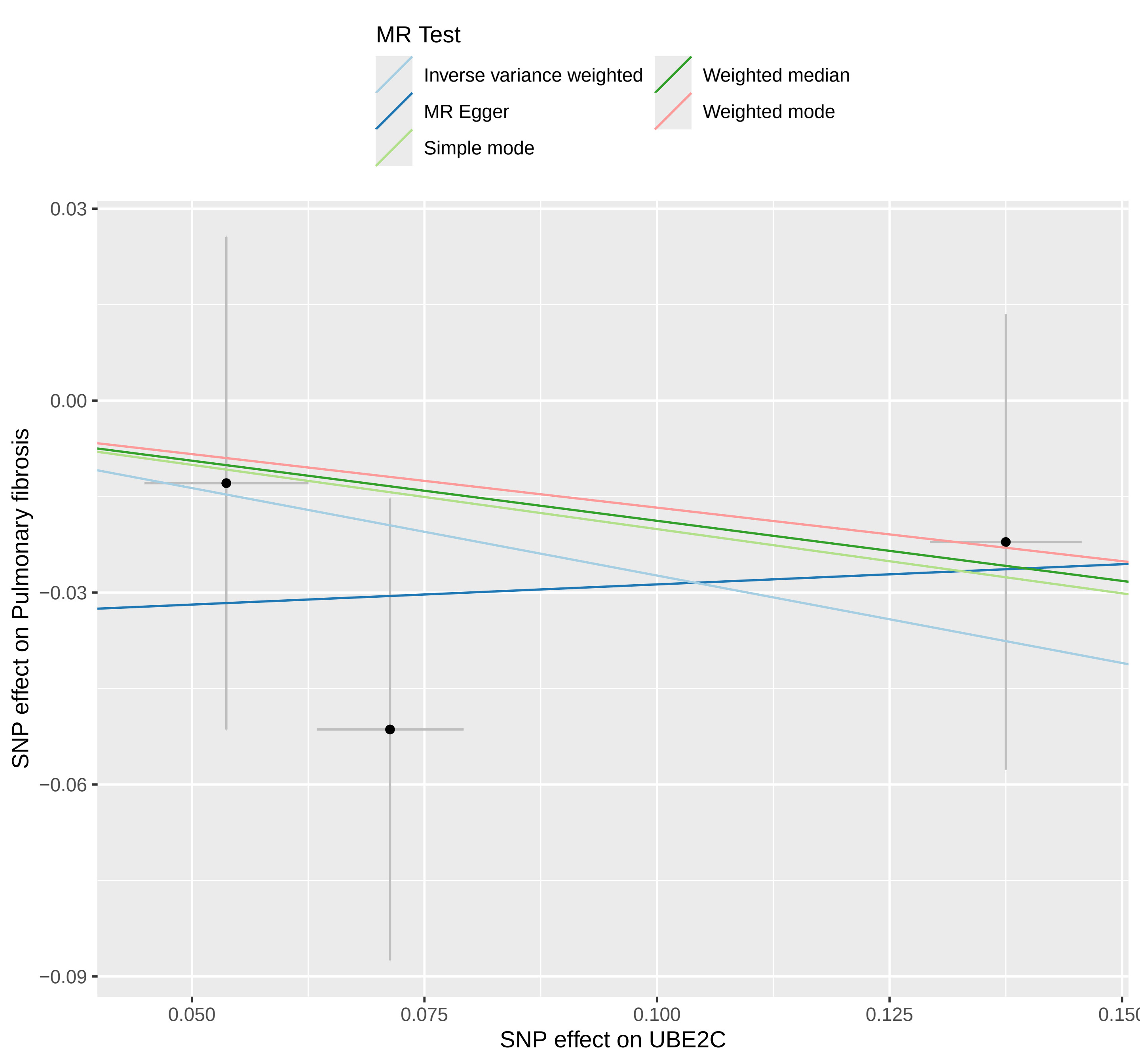

MR Test
Inverse variance weighted
MR Egger
Simple mode
Weighted median
Weighted mode
0.03
0.00
−0.03
−0.06
−0.09
0.050
0.075
0.100
0.125
0.150
SNP effect on Pulmonary fibrosis
SNP effect on UBE2C

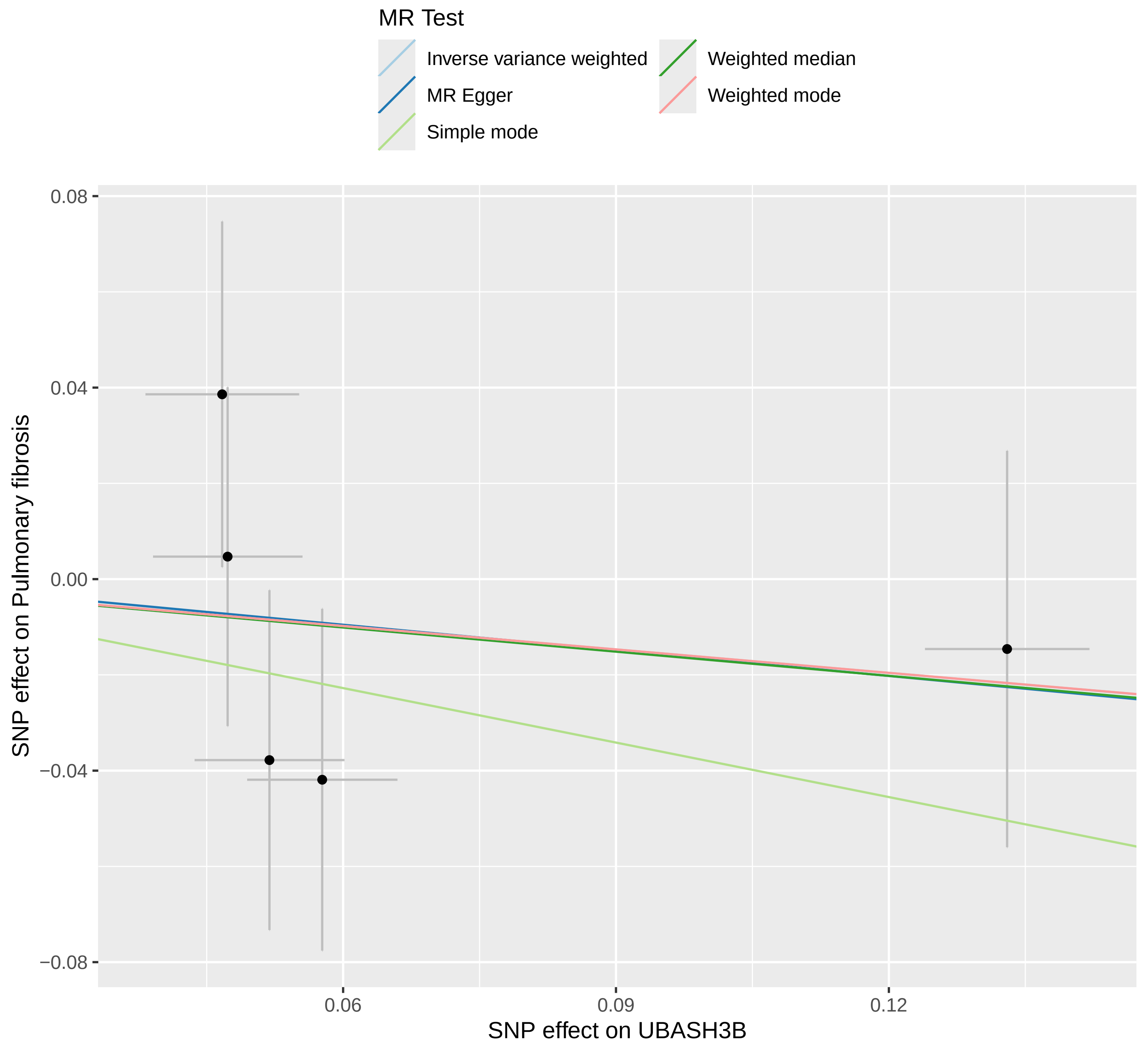
MR Test
Inverse variance weighted
MR Egger
Simple mode
Weighted median
Weighted mode
0.08
0.04
0.00
−0.04
−0.08
SNP effect on Pulmonary fibrosis
0.06
0.09
0.12
SNP effect on UBASH3B

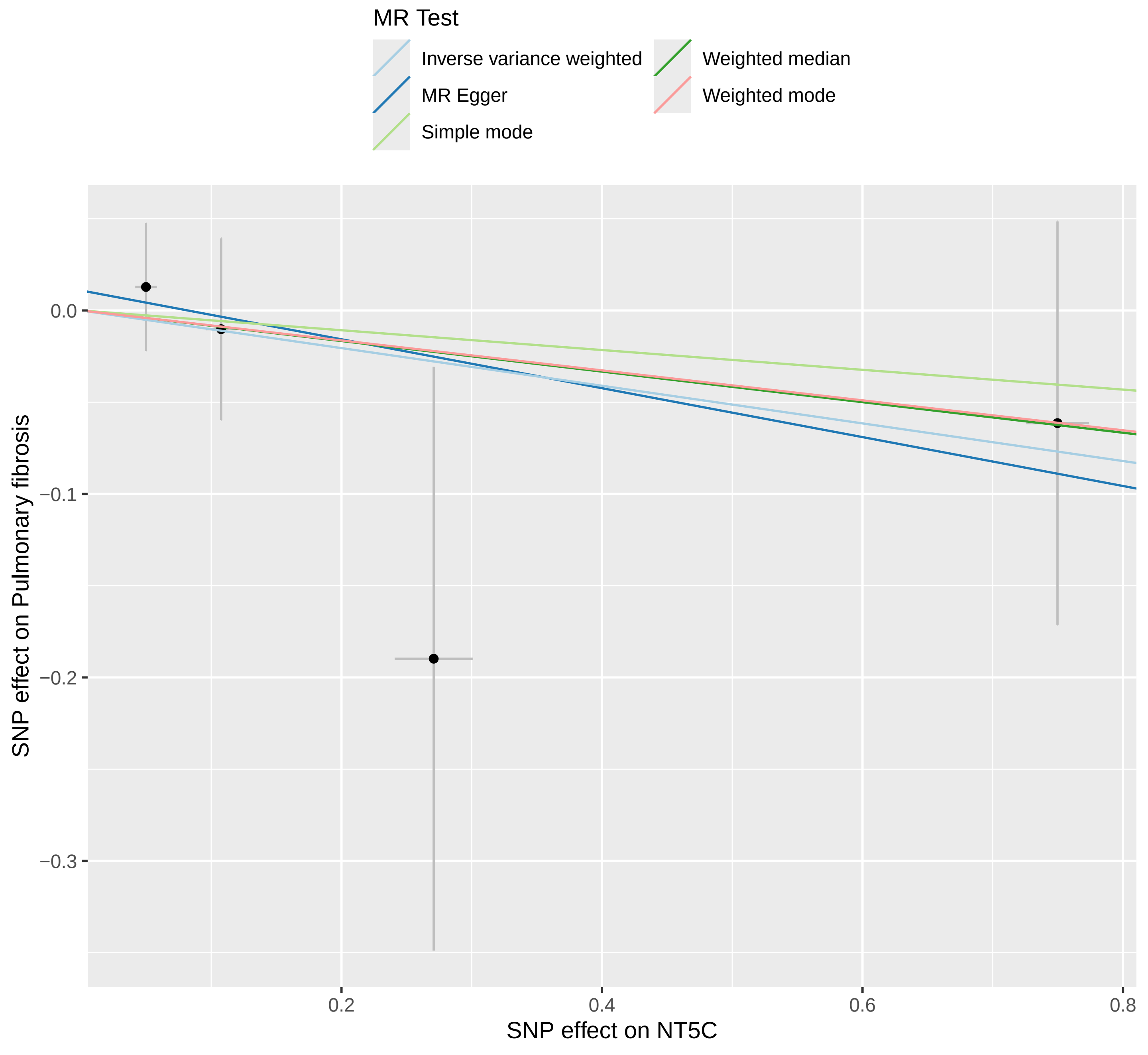

MR Test
Inverse variance weighted
MR Egger
Simple mode
Weighted median
Weighted mode
0.0
−0.1
−0.2
−0.3
0.2
0.4
0.6
0.8
SNP effect on Pulmonary fibrosis
SNP effect on NT5C

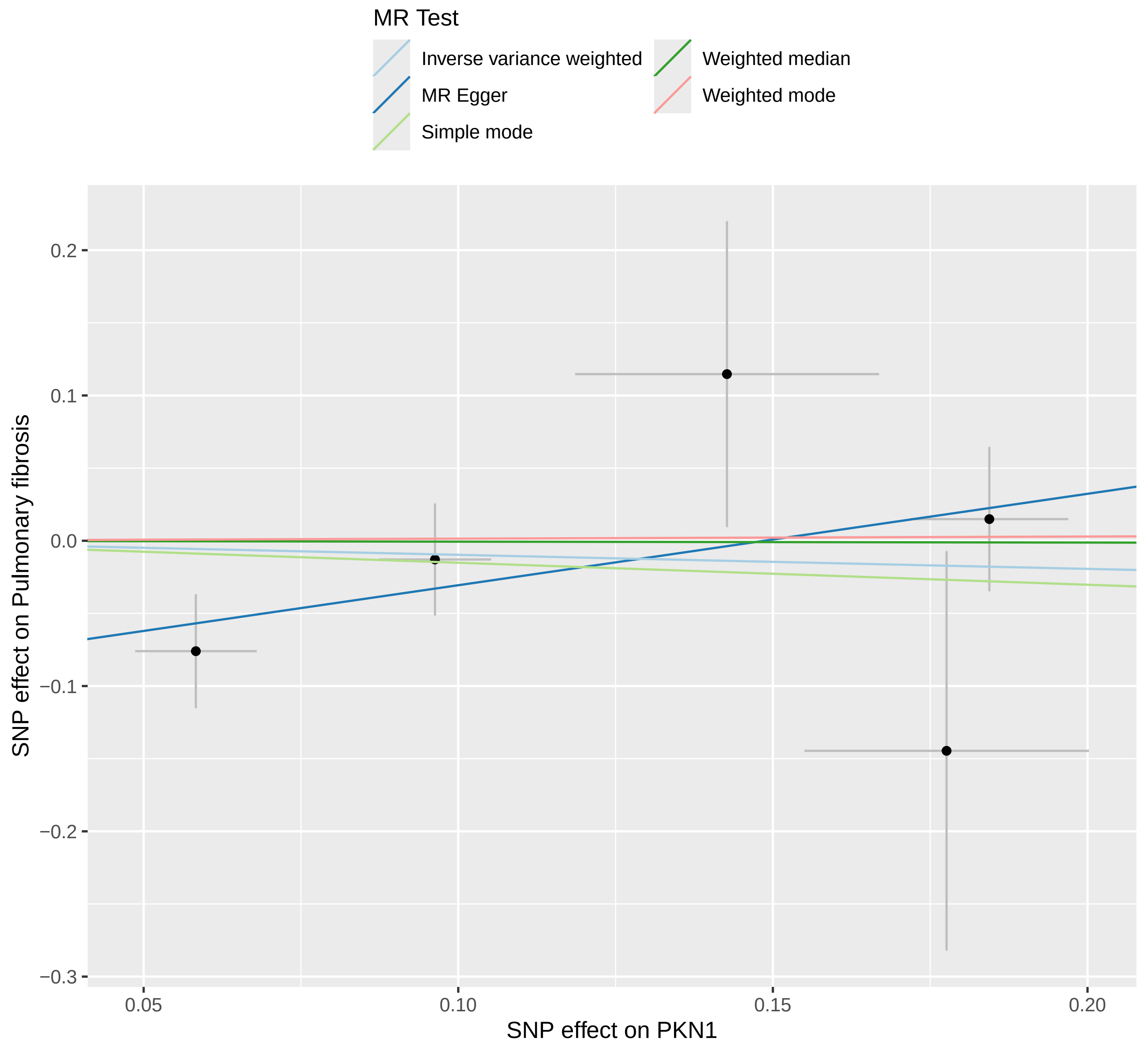
MR Test
Inverse variance weighted
MR Egger
Simple mode
Weighted median
Weighted mode
SNP effect on Pulmonary fibrosis
0.2
0.1
0.0
−0.1
−0.2
−0.3
0.05
0.10
0.15
0.20
SNP effect on PKN1

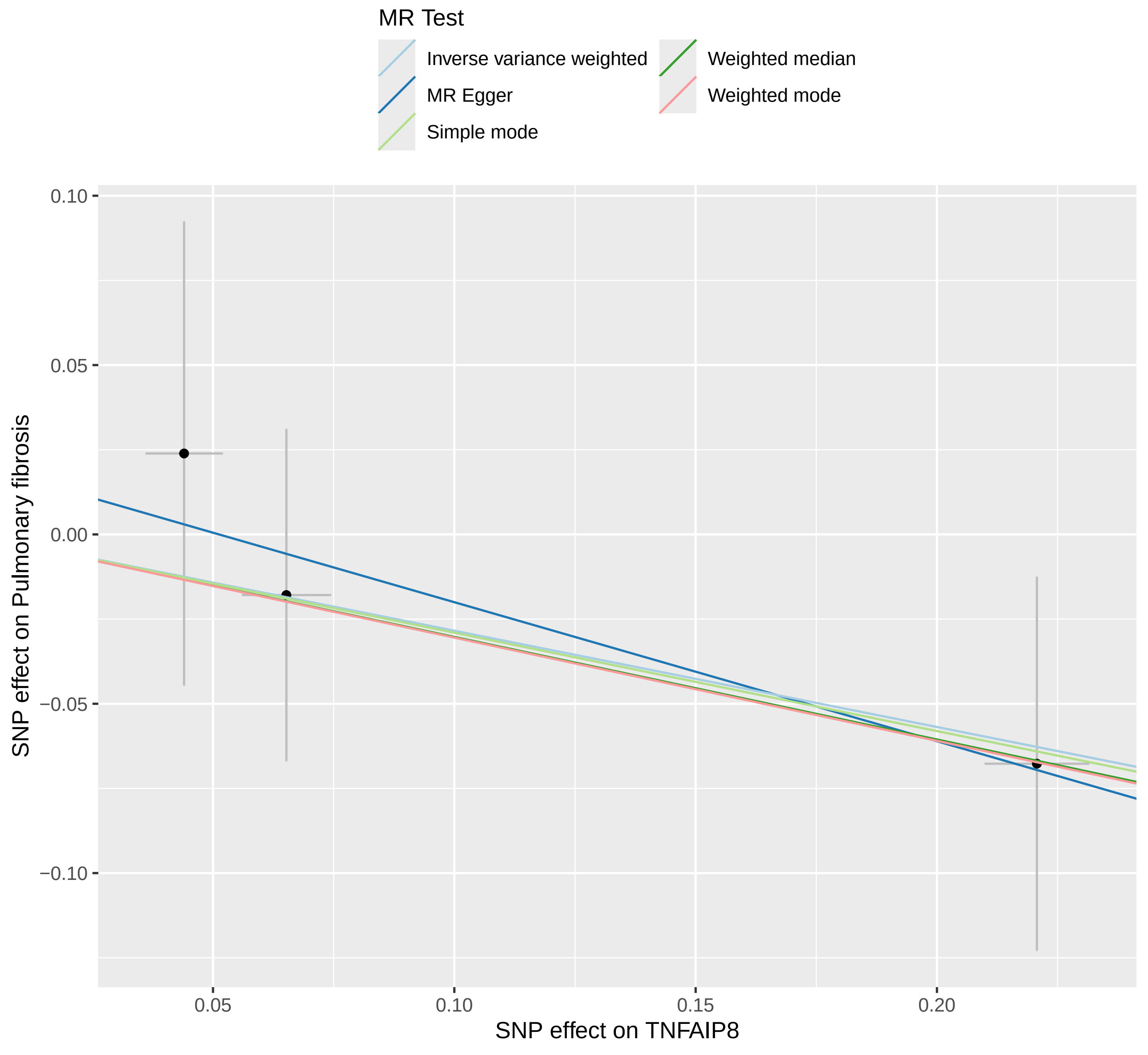
MR Test
Inverse variance weighted
MR Egger
Simple mode
Weighted median
Weighted mode
0.10
0.05
0.00
−0.05
−0.10
SNP effect on Pulmonary fibrosis
0.05
0.10
0.15
0.20
SNP effect on TNFAIP8

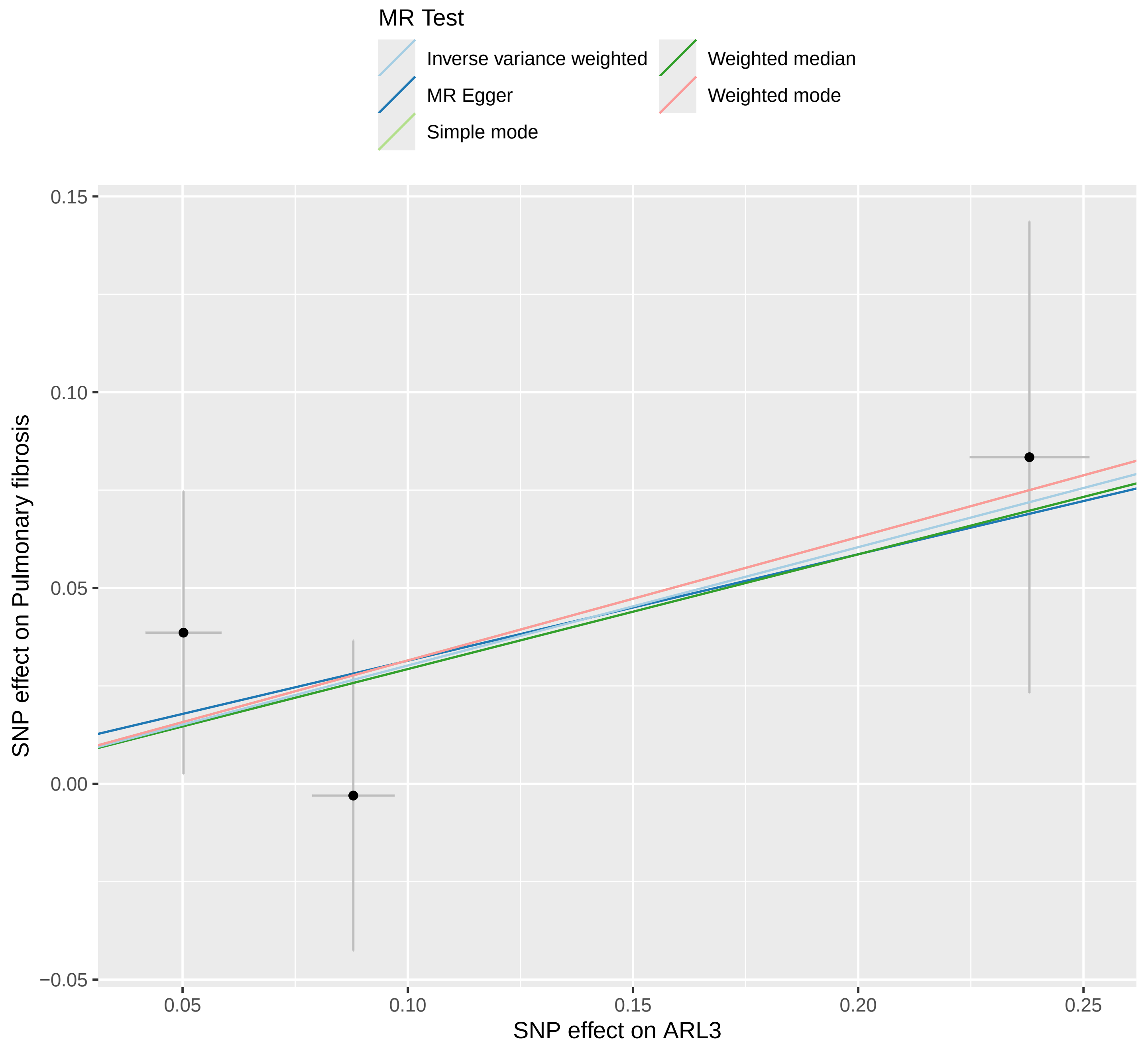
MR Test
Inverse variance weighted
MR Egger
Simple mode
Weighted median
Weighted mode
0.15
0.10
0.05
0.00
−0.05
SNP effect on Pulmonary fibrosis
0.05
0.10
0.15
0.20
0.25
SNP effect on ARL3

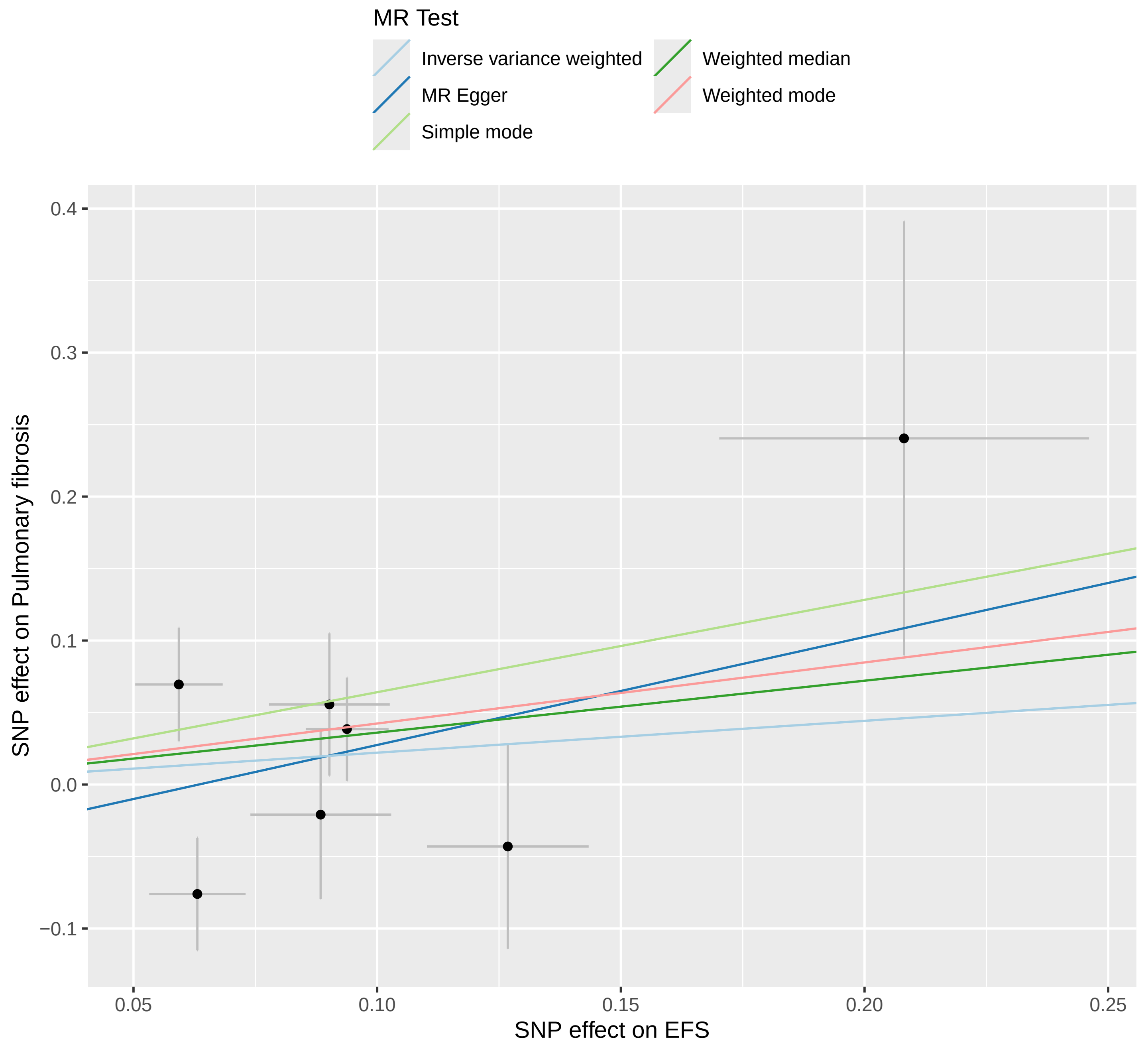

MR Test
Inverse variance weighted
MR Egger
Simple mode
Weighted median
Weighted mode
SNP effect on Pulmonary fibrosis
0.4
0.3
0.2
0.1
0.0
−0.1
0.05
0.10
0.15
0.20
0.25
SNP effect on EFS

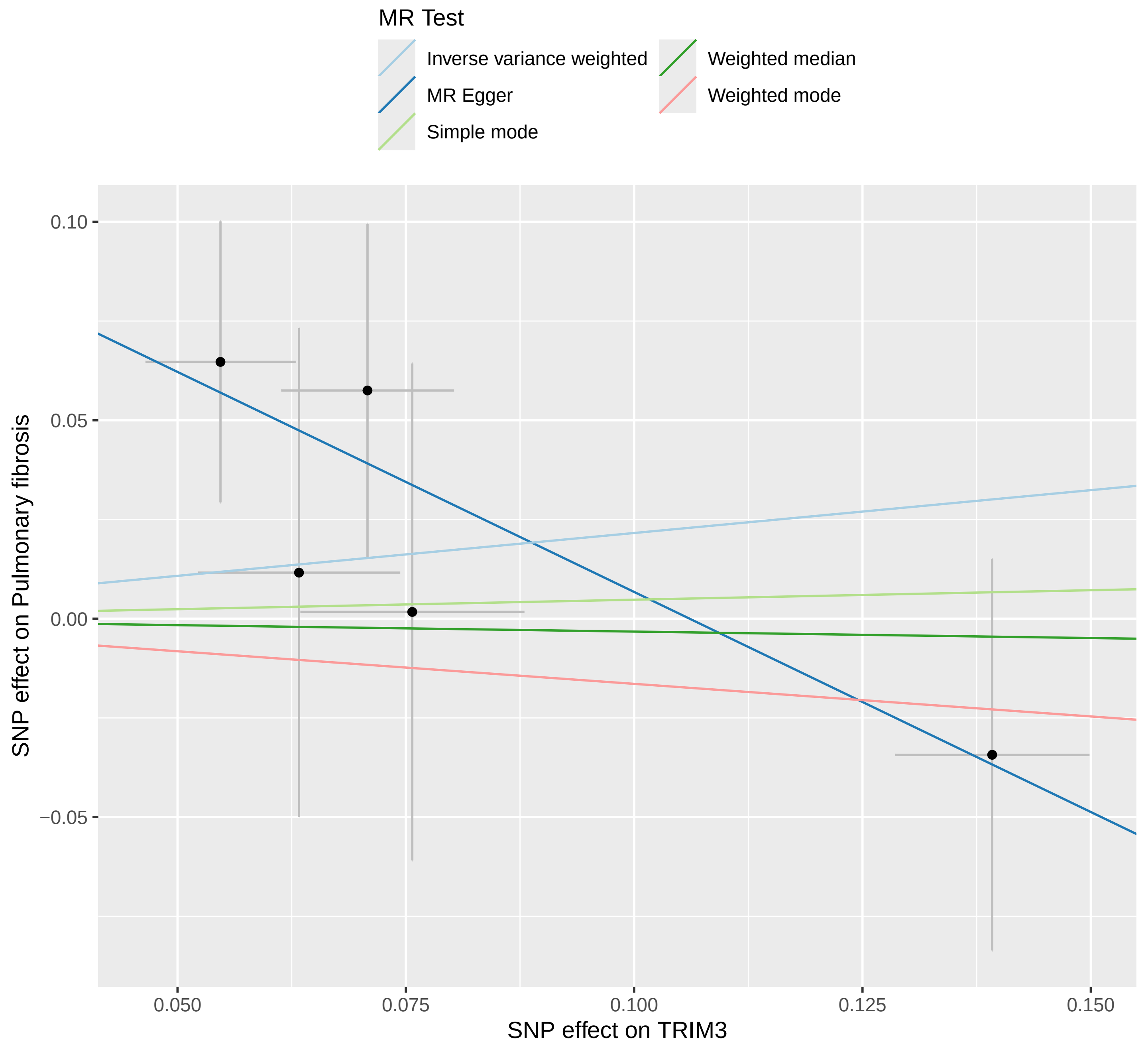
MR Test
Inverse variance weighted
MR Egger
Simple mode
Weighted median
Weighted mode
SNP effect on Pulmonary fibrosis
0.10
0.05
0.00
−0.05
0.050
0.075
0.100
0.125
0.150
SNP effect on TRIM3

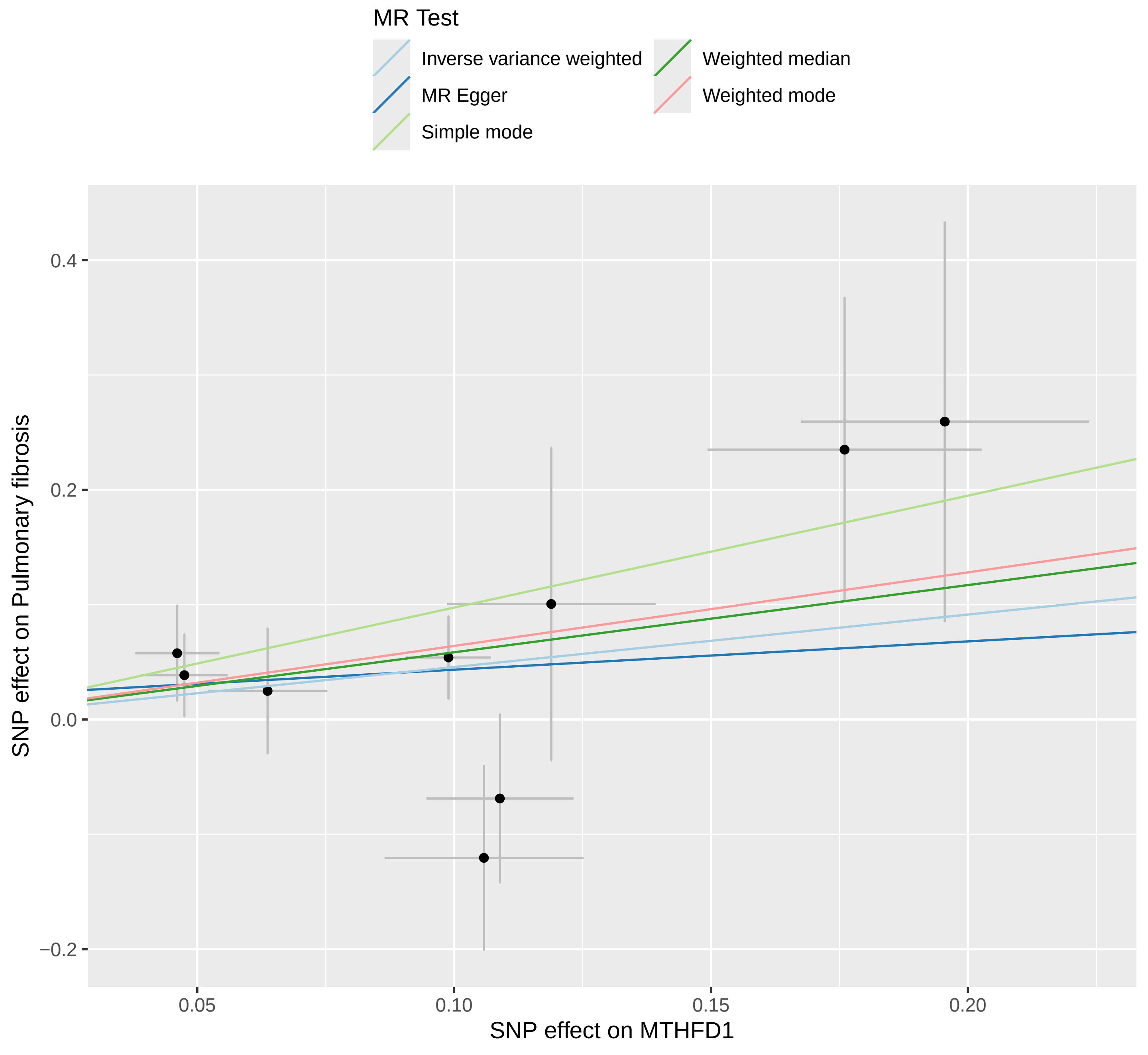
MR Test
Inverse variance weighted
MR Egger
Simple mode
Weighted median
Weighted mode
SNP effect on Pulmonary fibrosis
0.4
0.2
0.0
−0.2
0.05
0.10
0.15
0.20
SNP effect on MTHFD1

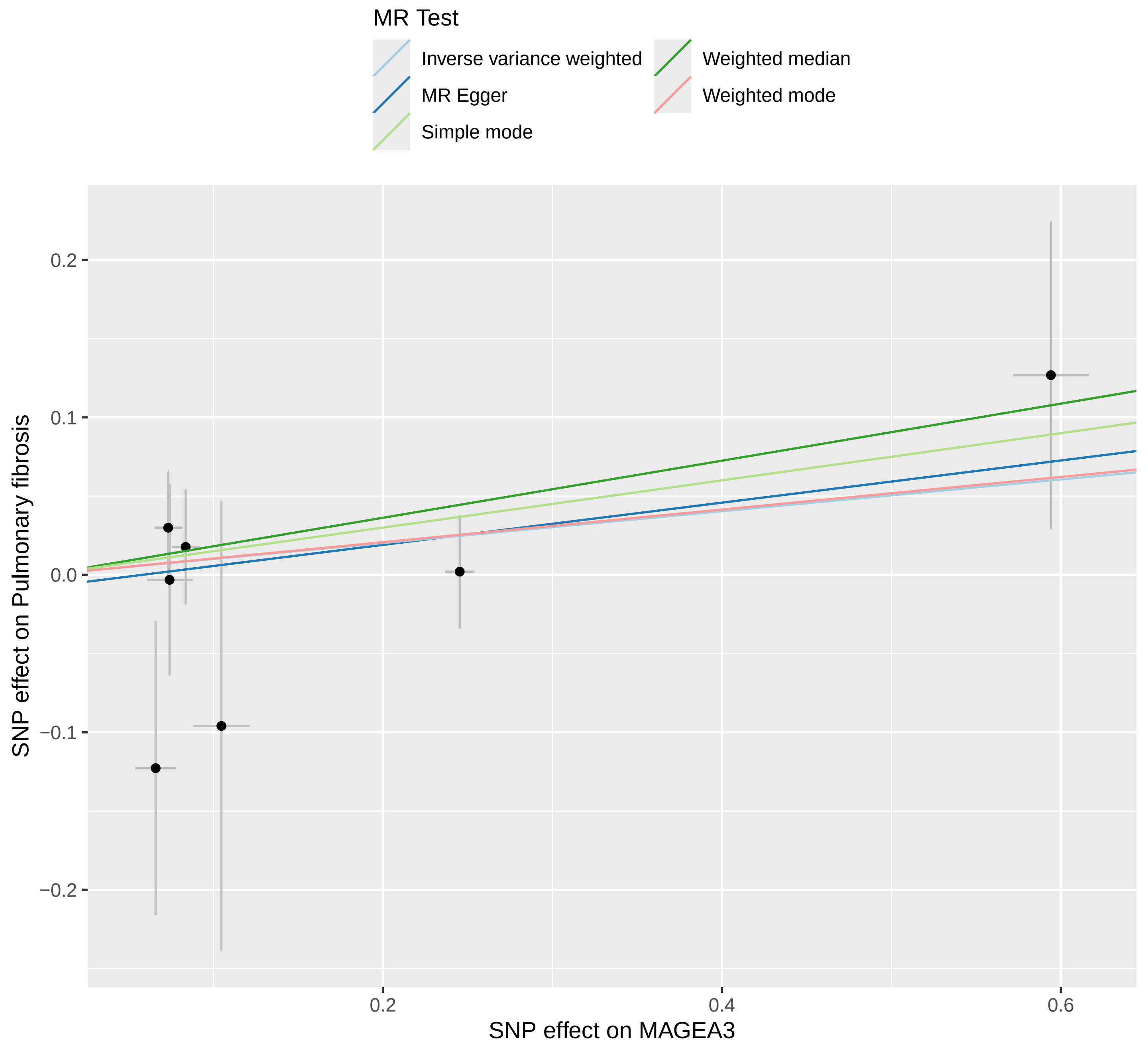

MR Test
Inverse variance weighted
MR Egger
Simple mode
Weighted median
Weighted mode
SNP effect on Pulmonary fibrosis
0.2
0.1
0.0
−0.1
−0.2
0.2
0.4
0.6
SNP effect on MAGEA3

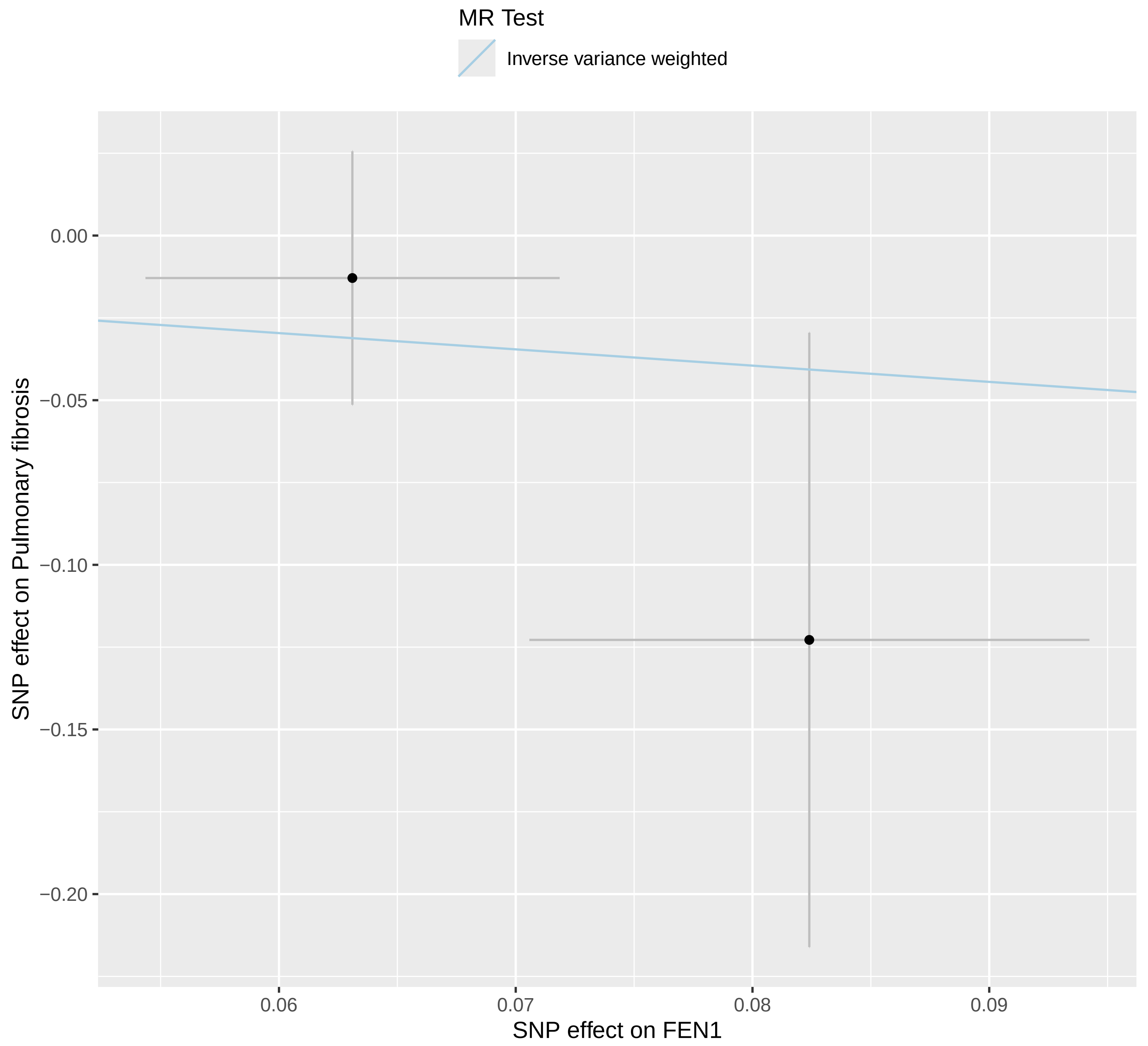
MR Test
Inverse variance weighted
0.00
−0.05
−0.10
−0.15
−0.20
0.06
0.07
0.08
0.09
SNP effect on Pulmonary fibrosis
SNP effect on FEN1

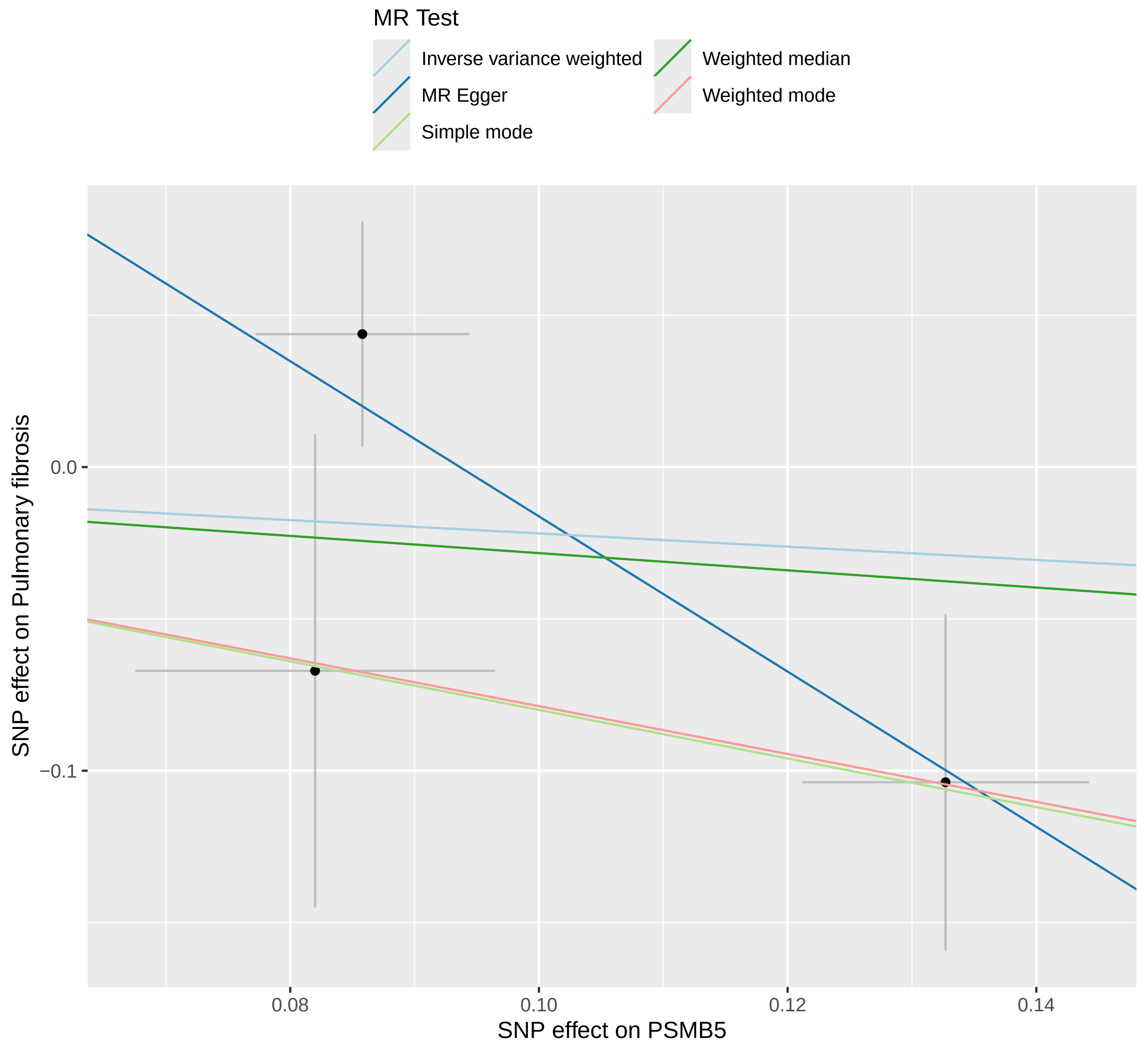
MR Test
Inverse variance weighted
MR Egger
Simple mode
Weighted median
Weighted mode
SNP effect on Pulmonary fibrosis
0.0
−0.1
0.08
0.10
0.12
0.14
SNP effect on PSMB5

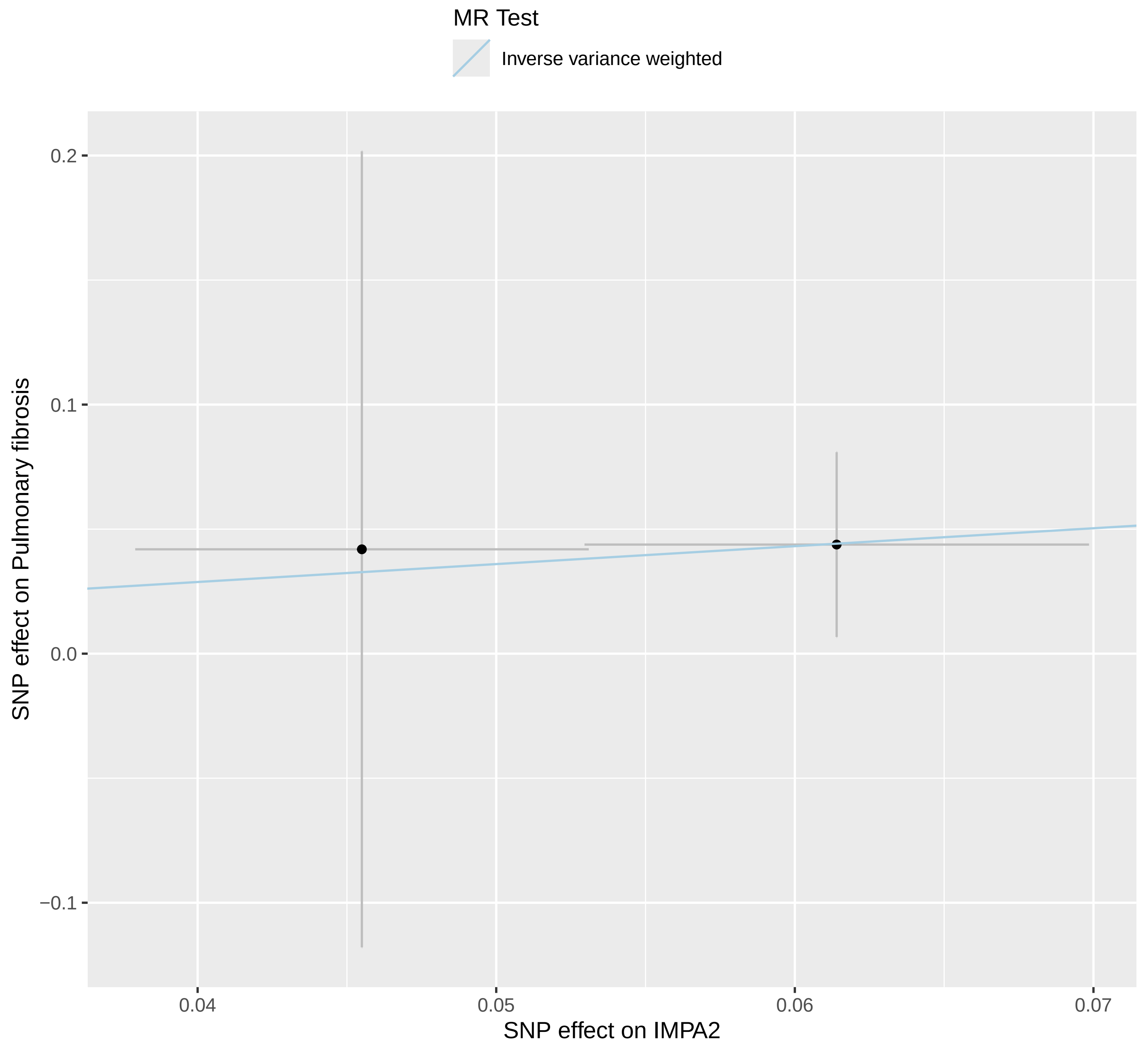
MR Test
Inverse variance weighted
0.2
0.1
0.0
−0.1
SNP effect on Pulmonary fibrosis
0.04
0.05
0.06
0.07
SNP effect on IMPA2

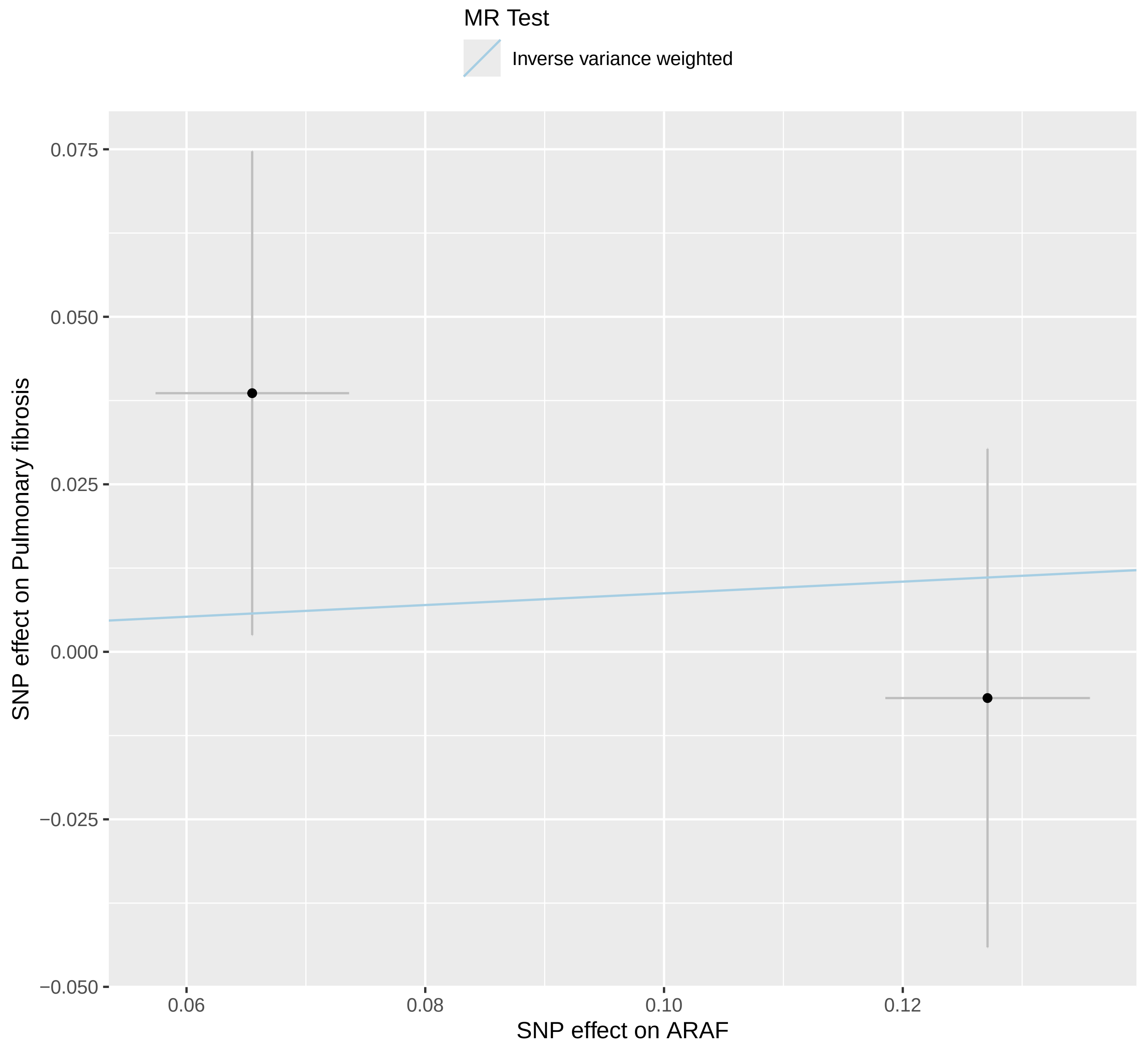
MR Test
Inverse variance weighted
0.075
0.050
0.025
0.000
−0.025
−0.050
SNP effect on Pulmonary fibrosis
0.06
0.08
0.10
0.12
SNP effect on ARAF

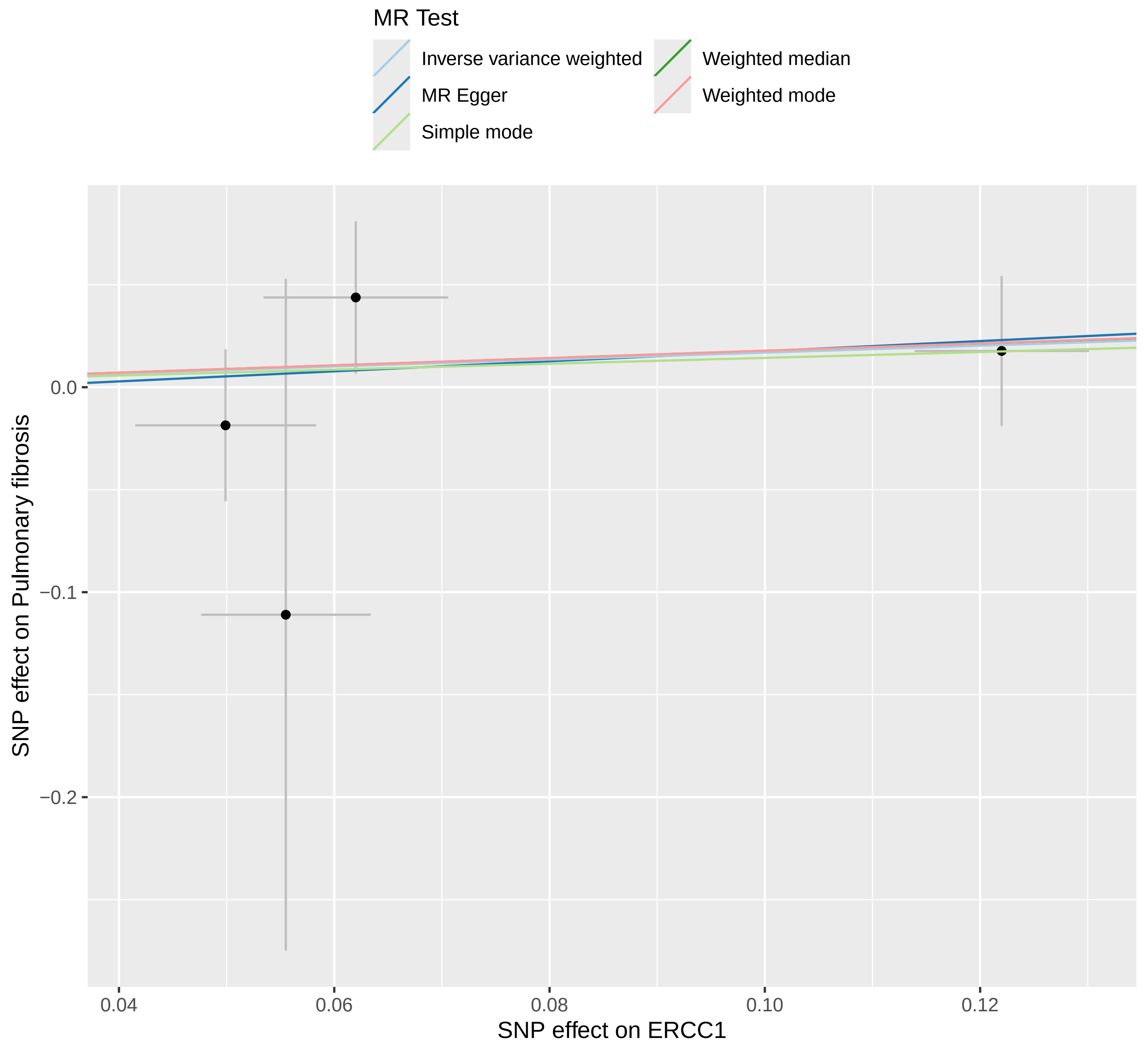
MR Test
Inverse variance weighted
MR Egger
Simple mode
Weighted median
Weighted mode
0.0
−0.1
−0.2
SNP effect on Pulmonary fibrosis
0.04
0.06
0.08
0.10
0.12
SNP effect on ERCC1

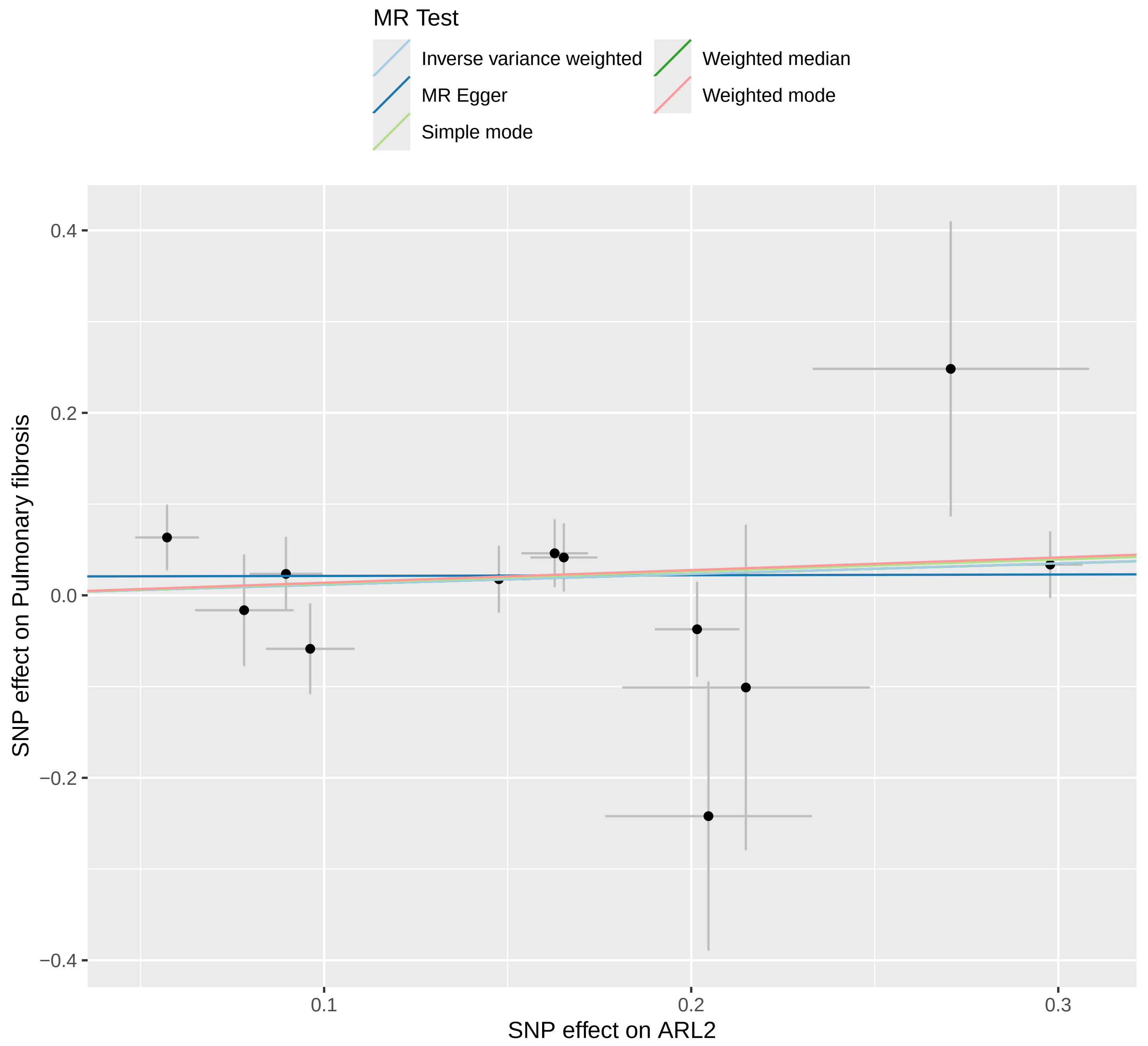

MR Test
Inverse variance weighted
MR Egger
Simple mode
Weighted median
Weighted mode
0.4
0.2
0.0
−0.2
−0.4
0.1
0.2
0.3
SNP effect on Pulmonary fibrosis
SNP effect on ARL2

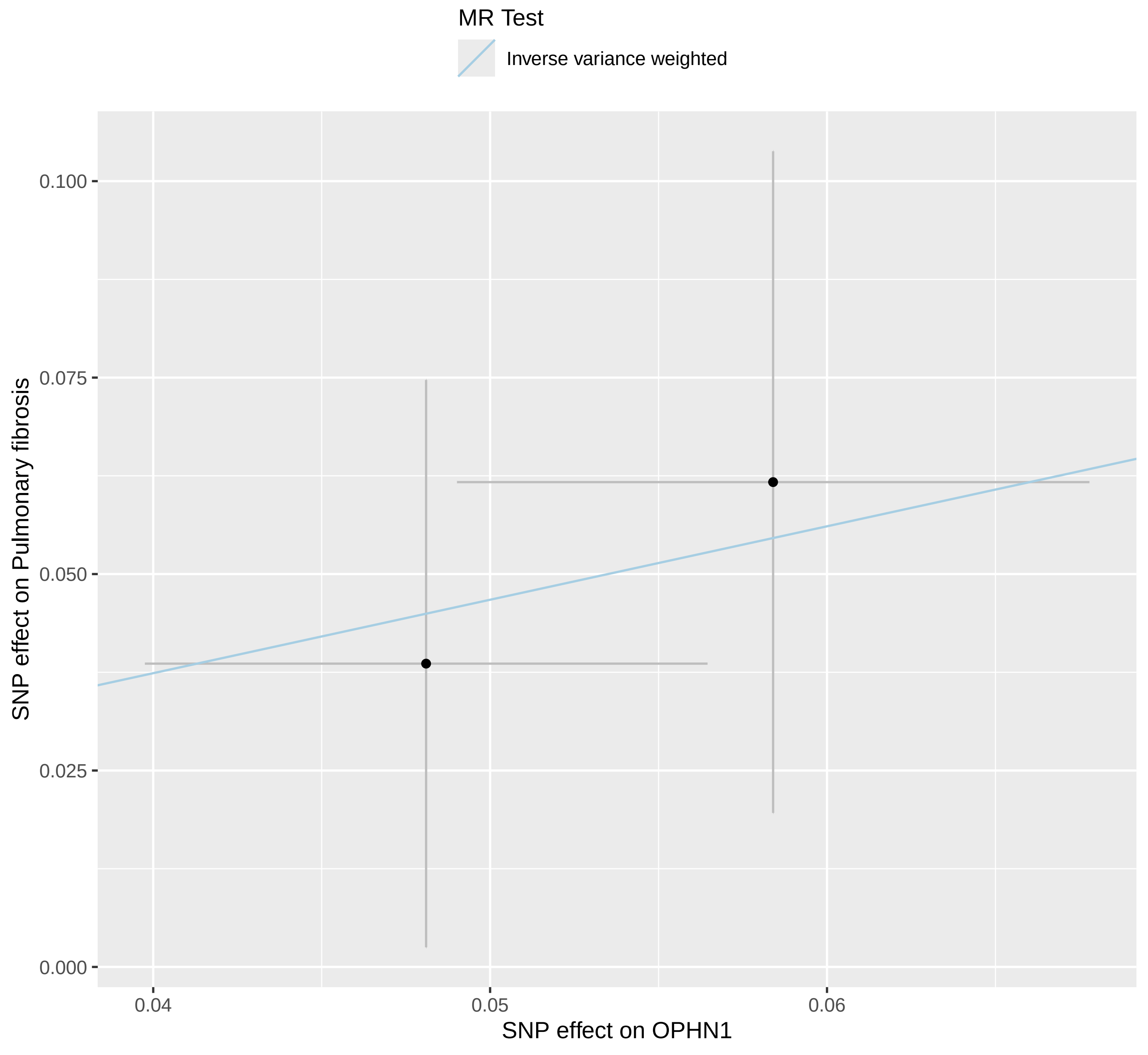

MR Test
Inverse variance weighted
0.100
0.075
0.050
0.025
0.000
SNP effect on Pulmonary fibrosis
0.04
0.05
0.06
SNP effect on OPHN1

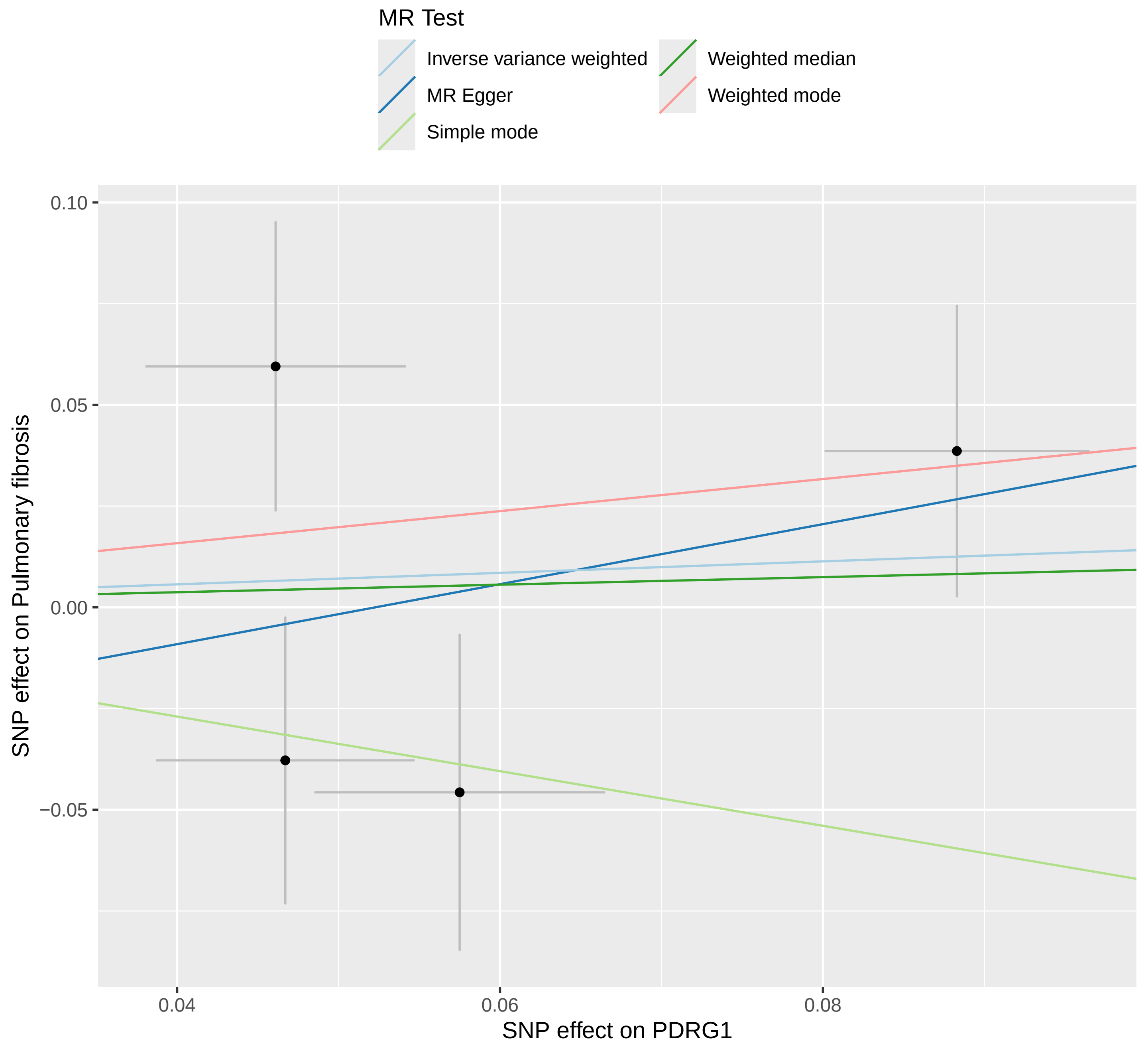
MR Test
Inverse variance weighted
MR Egger
Simple mode
Weighted median
Weighted mode
0.10
0.05
0.00
−0.05
SNP effect on Pulmonary fibrosis
0.04
0.06
0.08
SNP effect on PDRG1

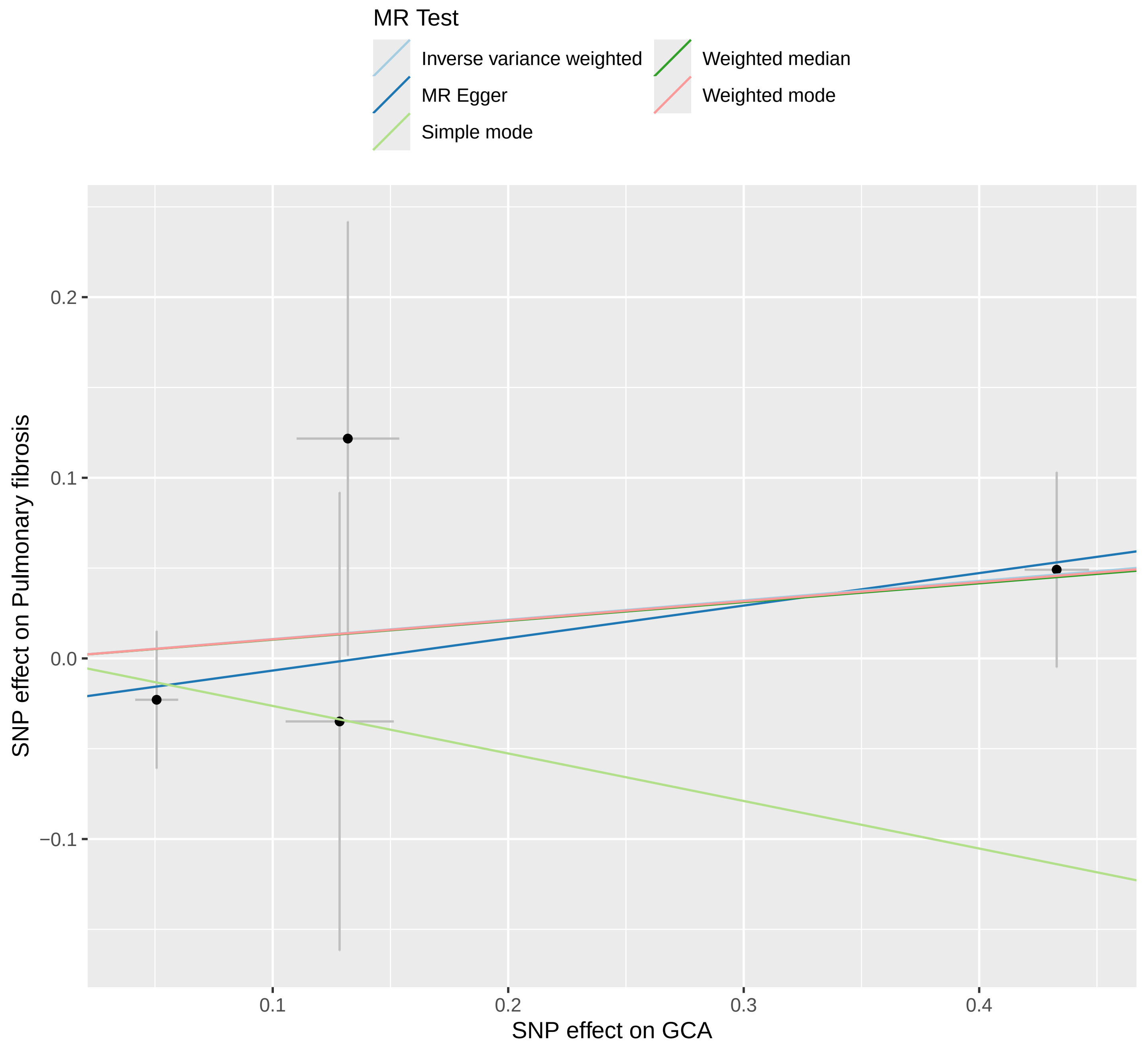
MR Test
Inverse variance weighted
MR Egger
Simple mode
Weighted median
Weighted mode
SNP effect on Pulmonary fibrosis
0.2
0.1
0.0
−0.1
0.1
0.2
0.3
0.4
SNP effect on GCA

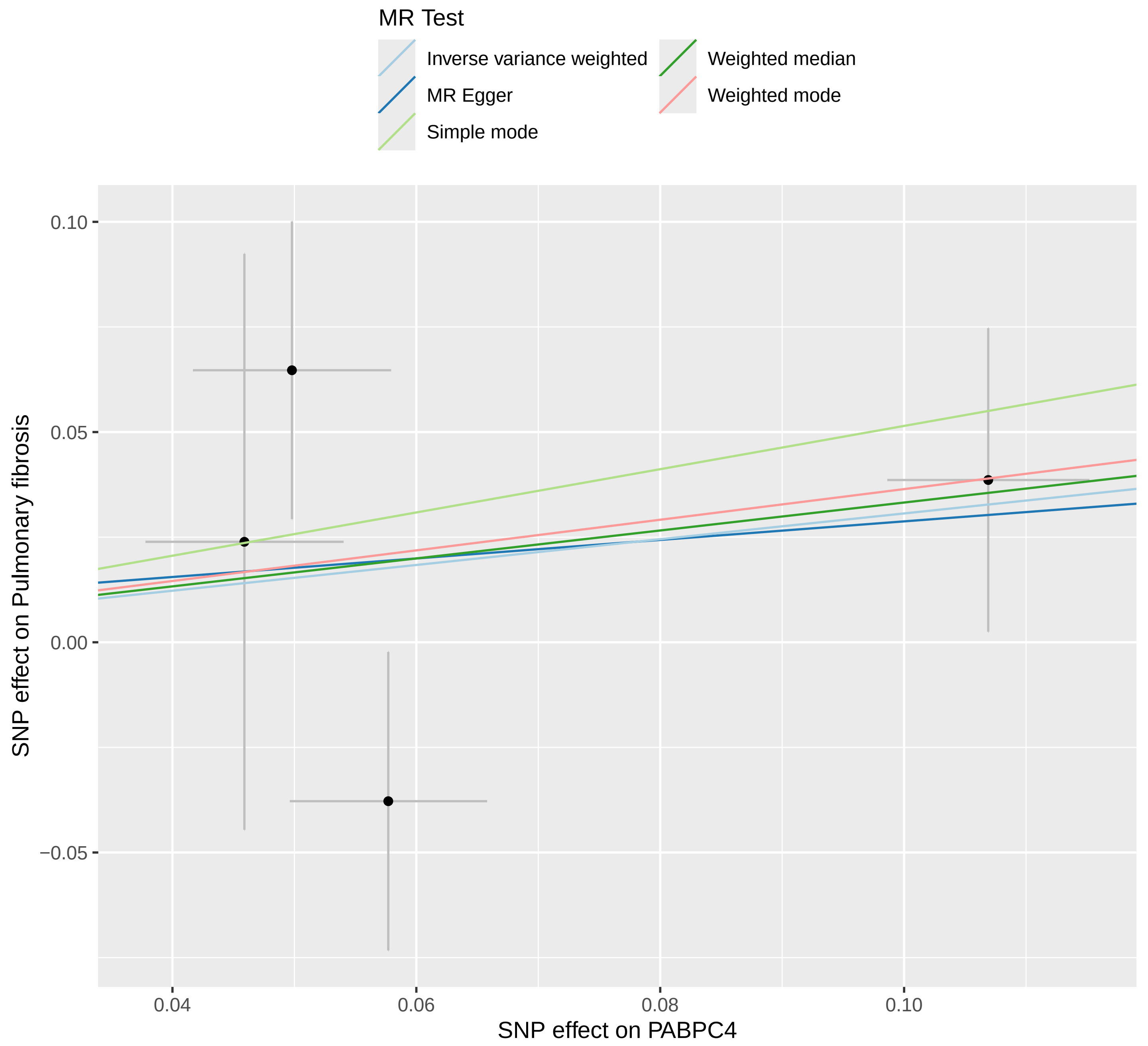
MR Test
Inverse variance weighted
MR Egger
Simple mode
Weighted median
Weighted mode
0.10
0.05
0.00
−0.05
0.04
0.06
0.08
0.10
SNP effect on Pulmonary fibrosis
SNP effect on PABPC4

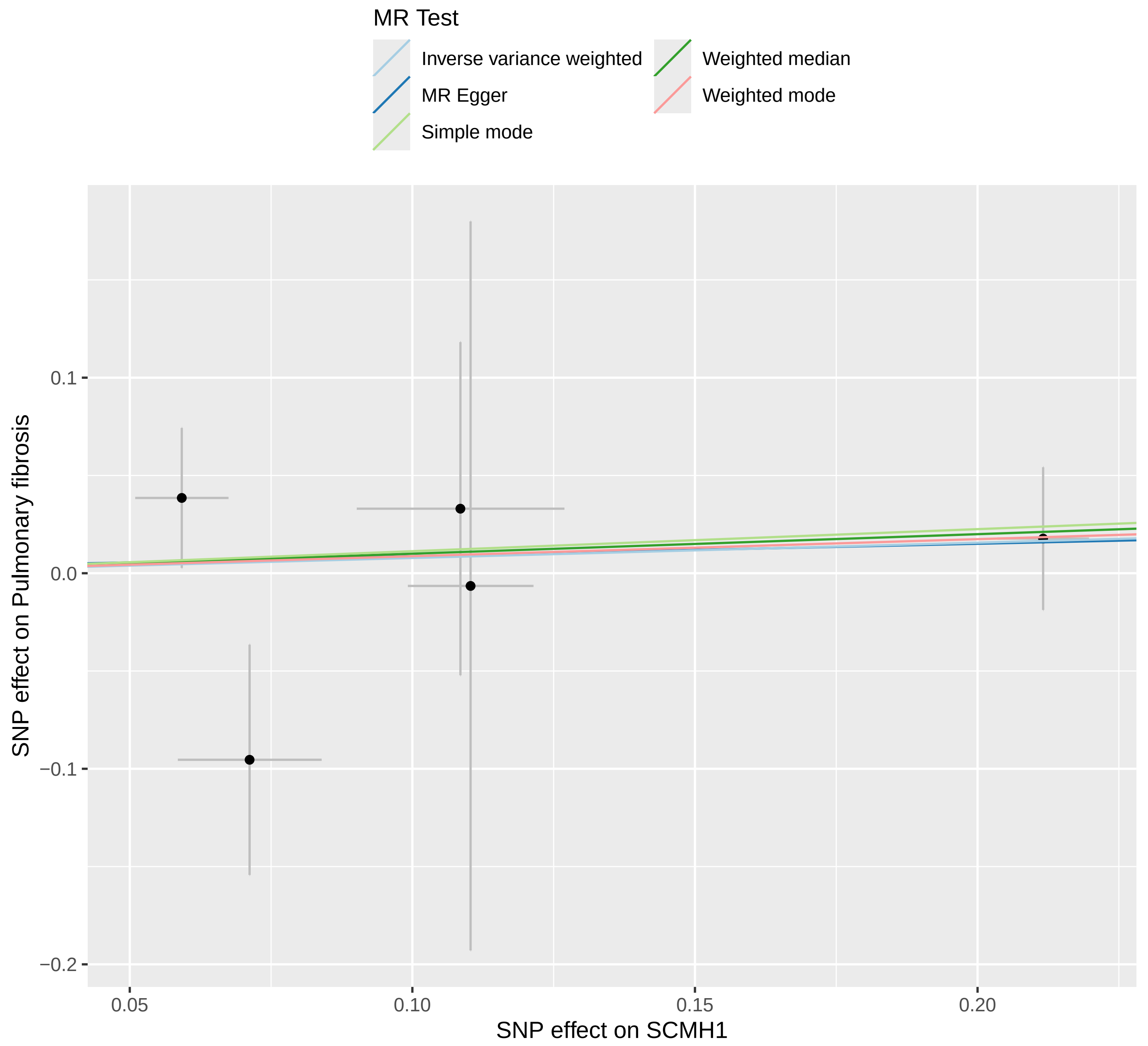

MR Test
Inverse variance weighted
MR Egger
Simple mode
Weighted median
Weighted mode
SNP effect on Pulmonary fibrosis
0.1
0.0
−0.1
−0.2
0.05
0.10
0.15
0.20
SNP effect on SCMH1

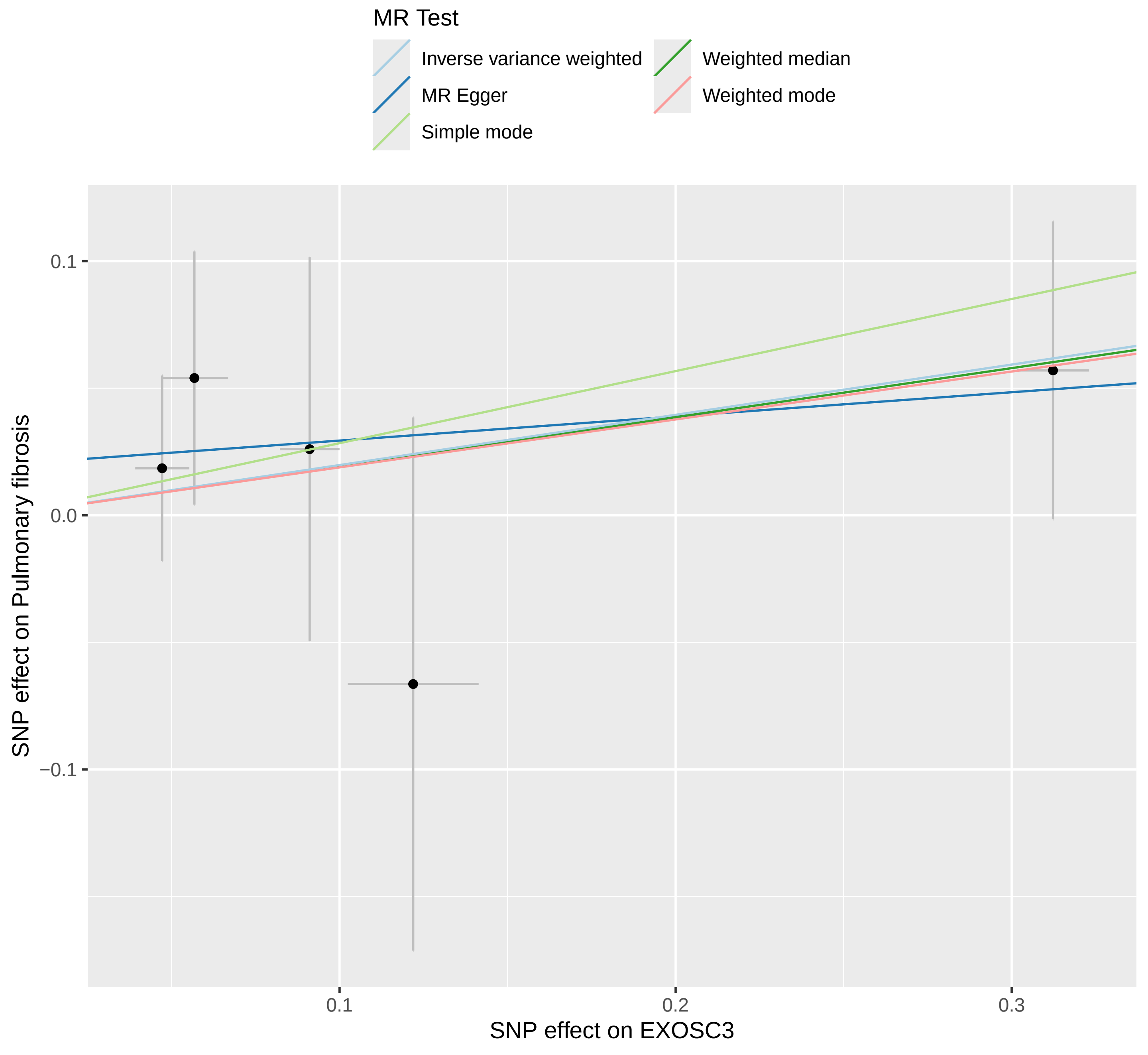

MR Test
Inverse variance weighted
MR Egger
Simple mode
Weighted median
Weighted mode
0.1
0.0
−0.1
0.1
0.2
0.3
SNP effect on Pulmonary fibrosis
SNP effect on EXOSC3

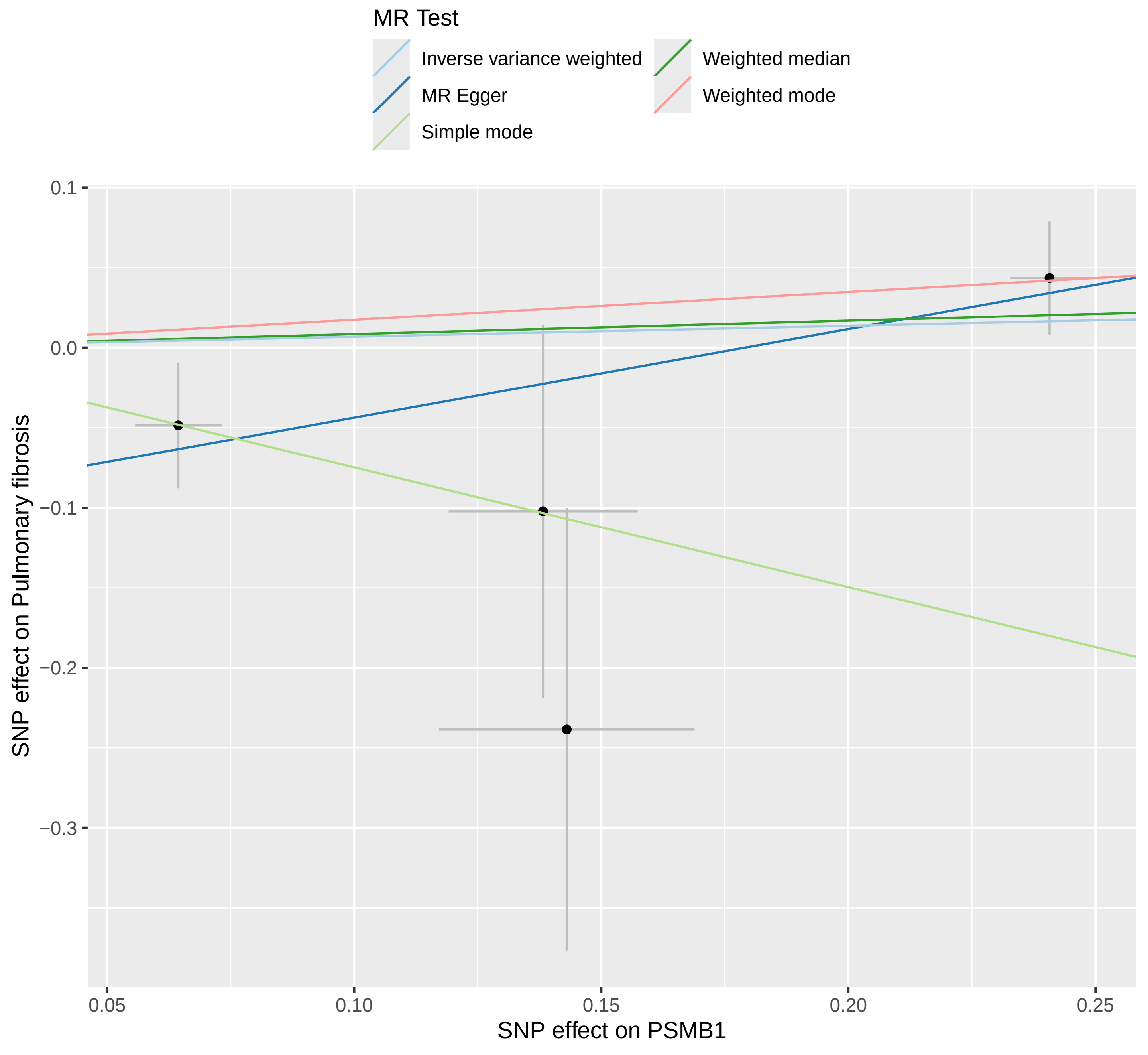

MR Test
Inverse variance weighted
MR Egger
Simple mode
Weighted median
Weighted mode
0.1
0.0
−0.1
−0.2
−0.3
SNP effect on Pulmonary fibrosis
0.05
0.10
0.15
0.20
0.25
SNP effect on PSMB1

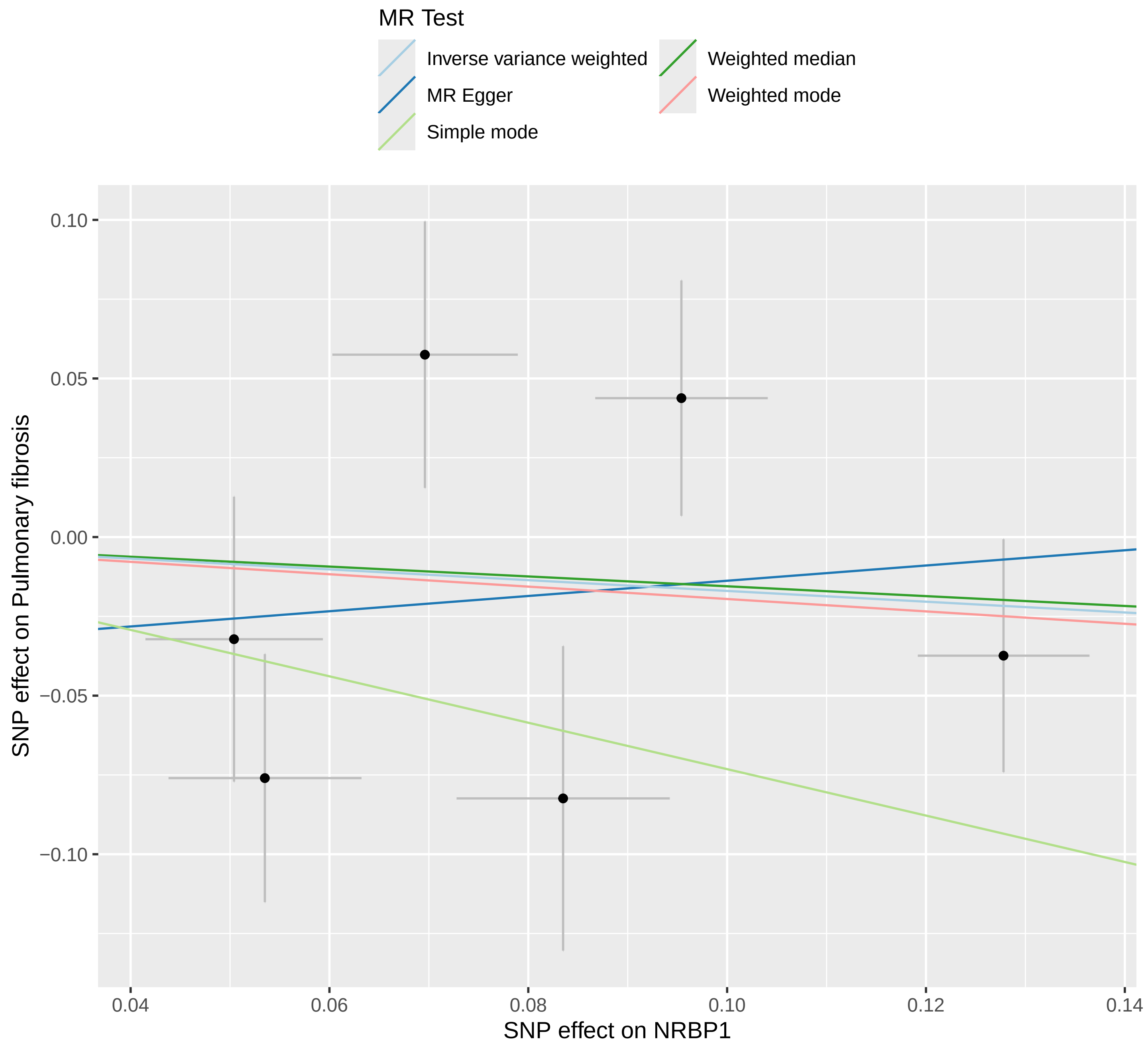

MR Test
Inverse variance weighted
MR Egger
Simple mode
Weighted median
Weighted mode
0.10
0.05
0.00
−0.05
−0.10
SNP effect on Pulmonary fibrosis
0.04
0.06
0.08
0.10
0.12
0.14
SNP effect on NRBP1

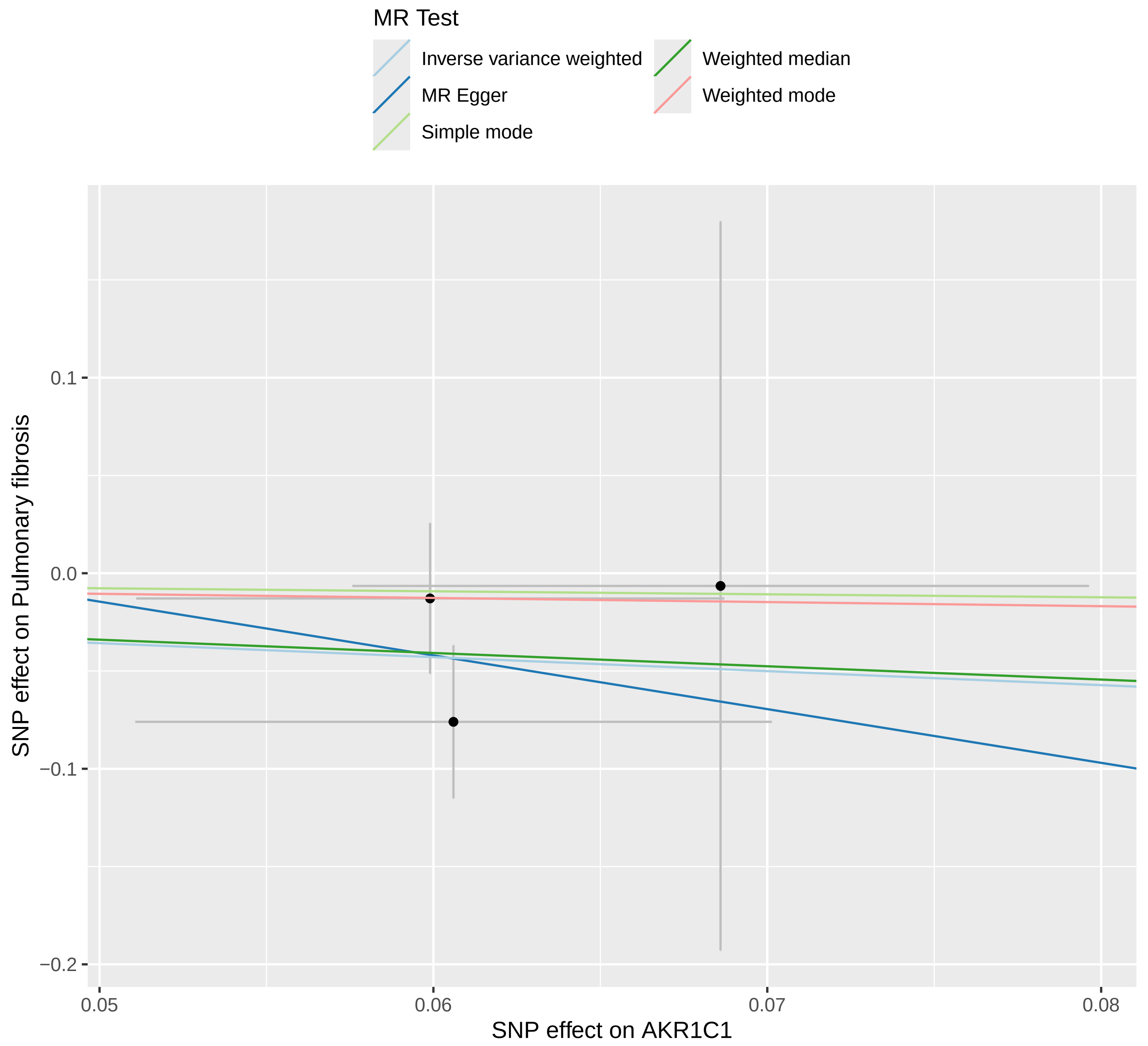
MR Test
Inverse variance weighted
MR Egger
Simple mode
Weighted median
Weighted mode
SNP effect on Pulmonary fibrosis
0.1
0.0
−0.1
−0.2
0.05
0.06
0.07
0.08
SNP effect on AKR1C1

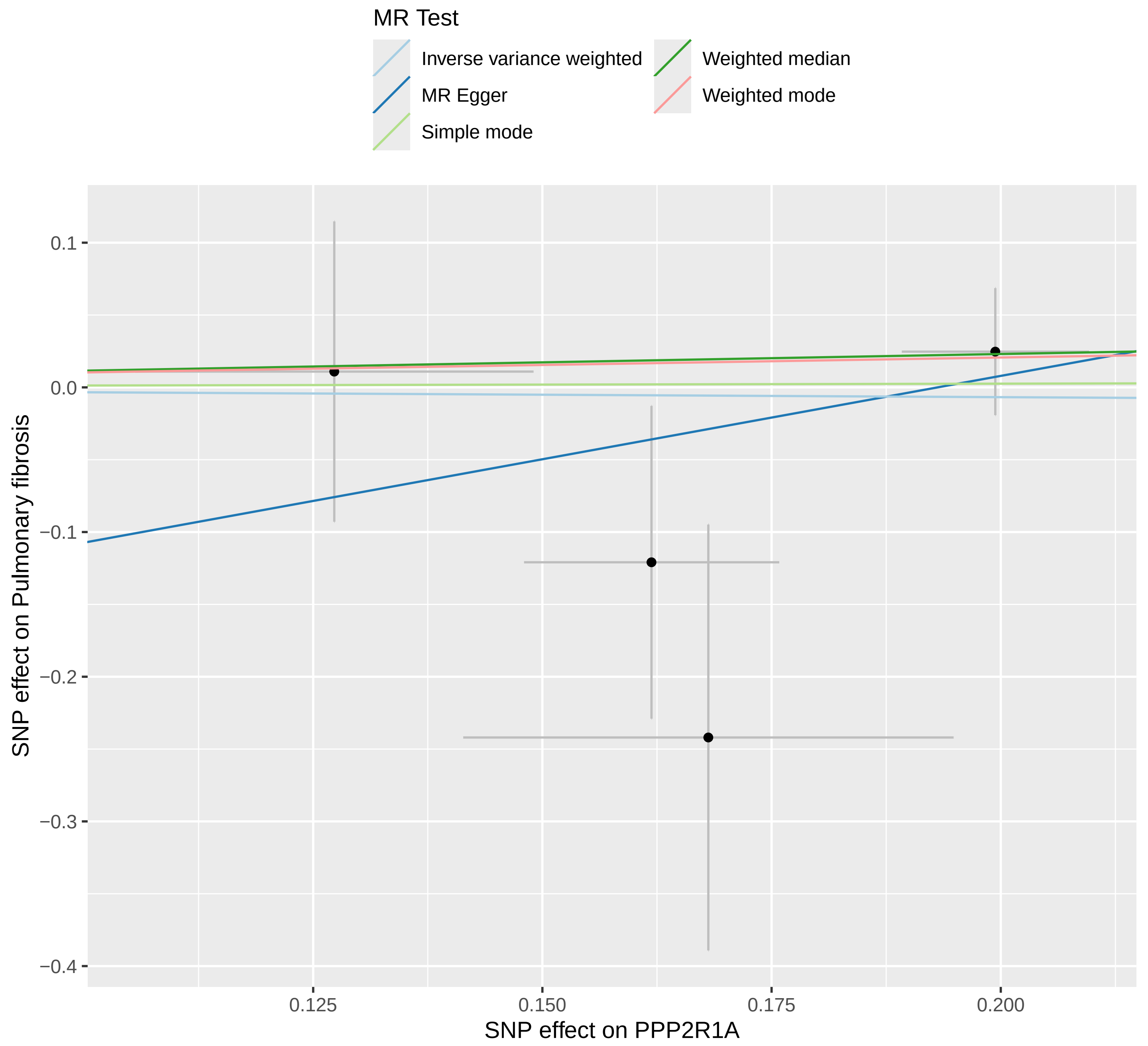
MR Test
Inverse variance weighted
MR Egger
Simple mode
Weighted median
Weighted mode
0.1
0.0
−0.1
−0.2
−0.3
−0.4
SNP effect on Pulmonary fibrosis
0.125
0.150
0.175
0.200
SNP effect on PPP2R1A

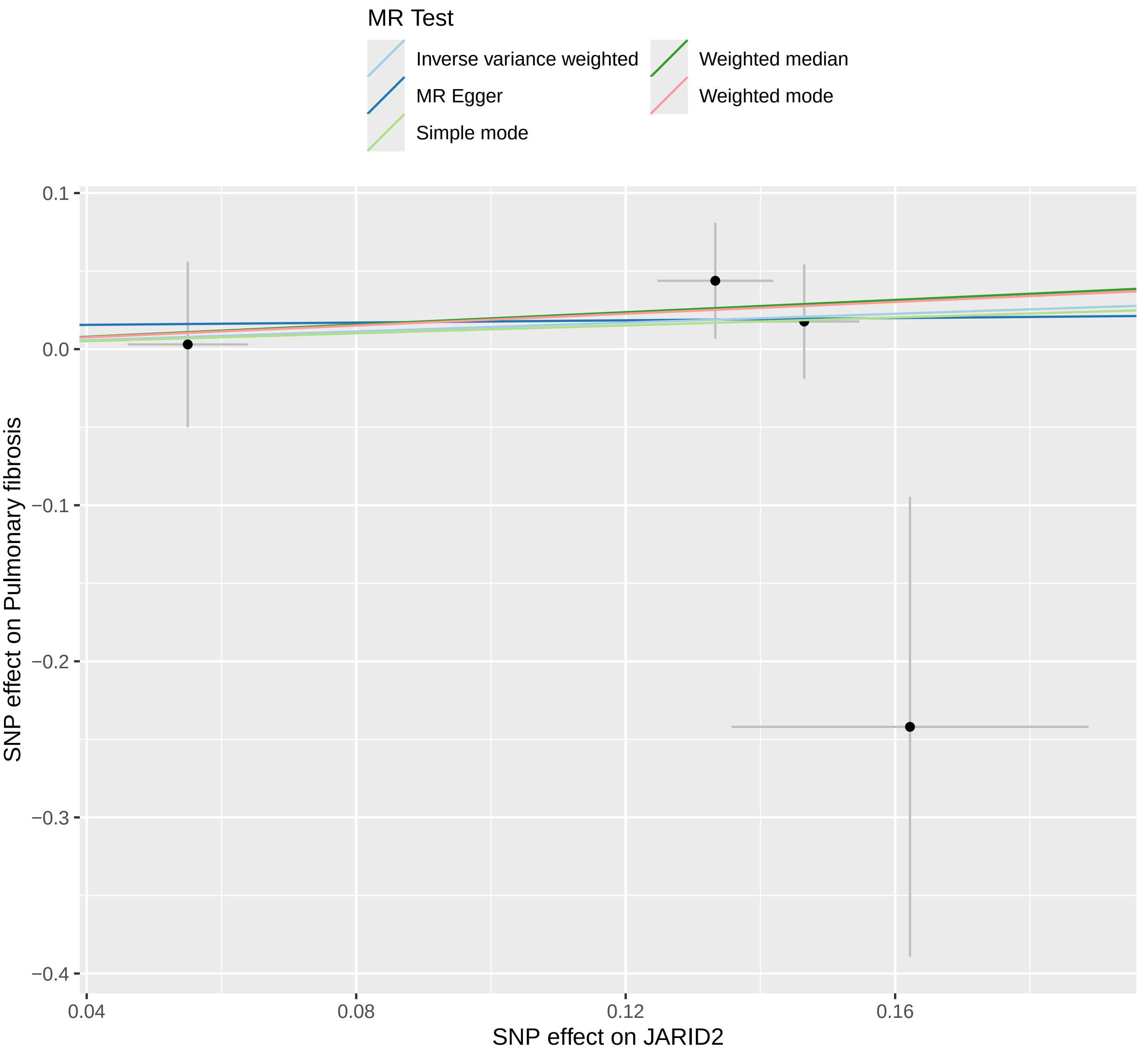
MR Test
Inverse variance weighted
MR Egger
Simple mode
Weighted median
Weighted mode
0.1
0.0
−0.1
−0.2
−0.3
−0.4
SNP effect on Pulmonary fibrosis
0.04
0.08
0.12
0.16
SNP effect on JARID2

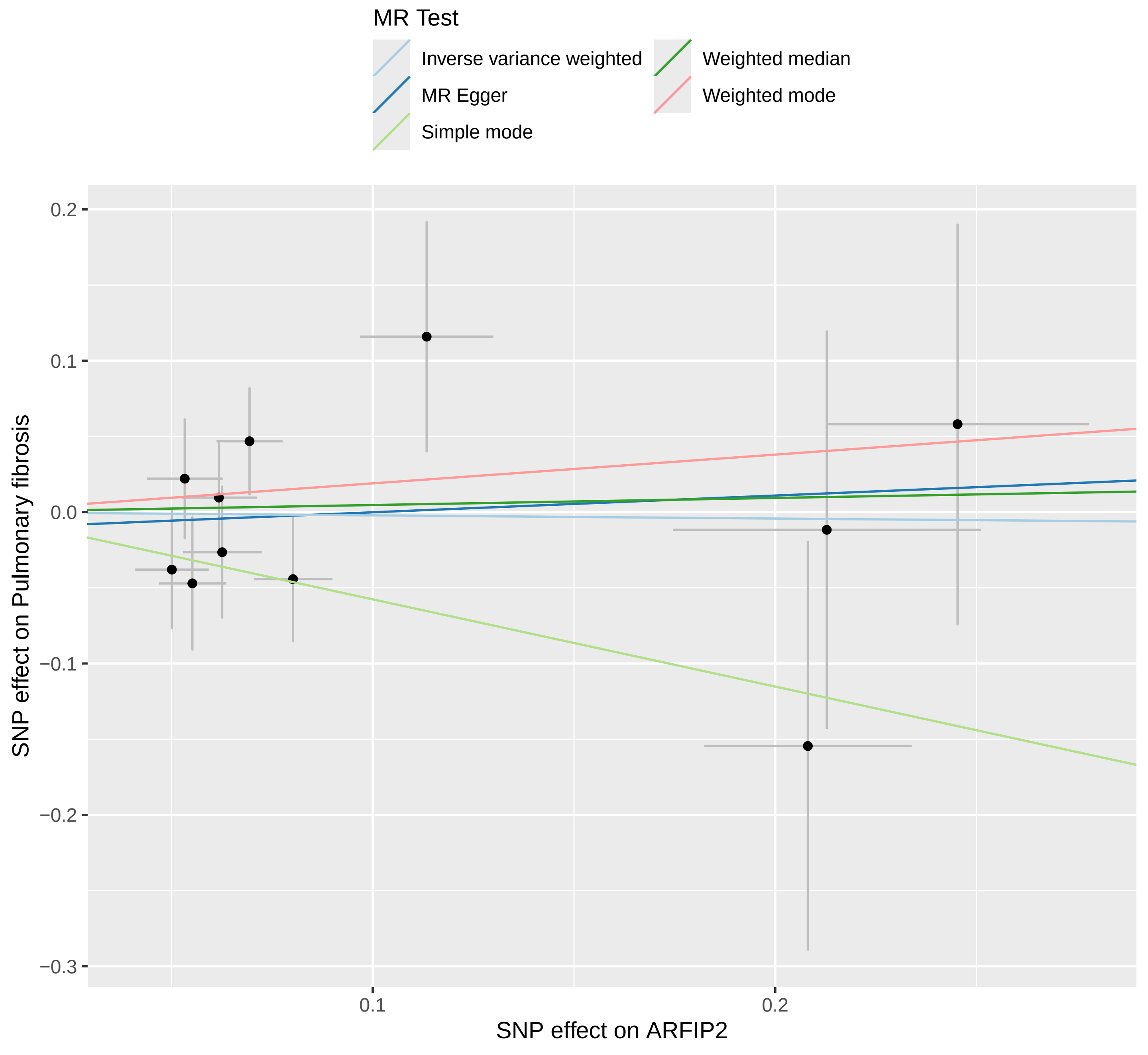
MR Test
Inverse variance weighted
MR Egger
Simple mode
Weighted median
Weighted mode
0.2
0.1
0.0
−0.1
−0.2
−0.3
SNP effect on Pulmonary fibrosis
0.1
0.2
SNP effect on ARFIP2

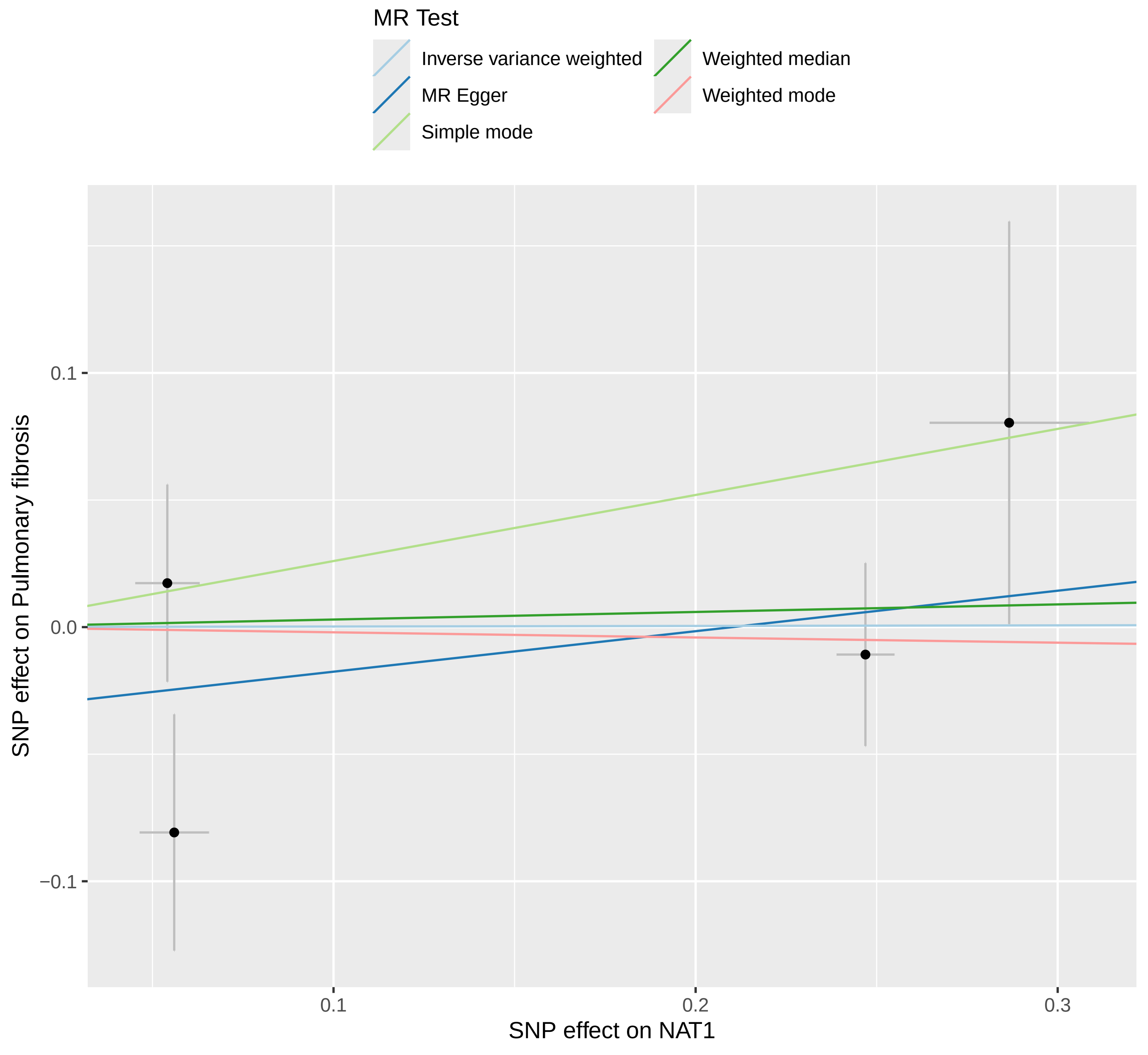

MR Test
Inverse variance weighted
MR Egger
Simple mode
Weighted median
Weighted mode
0.1
0.0
−0.1
SNP effect on Pulmonary fibrosis
0.1
0.2
0.3
SNP effect on NAT1

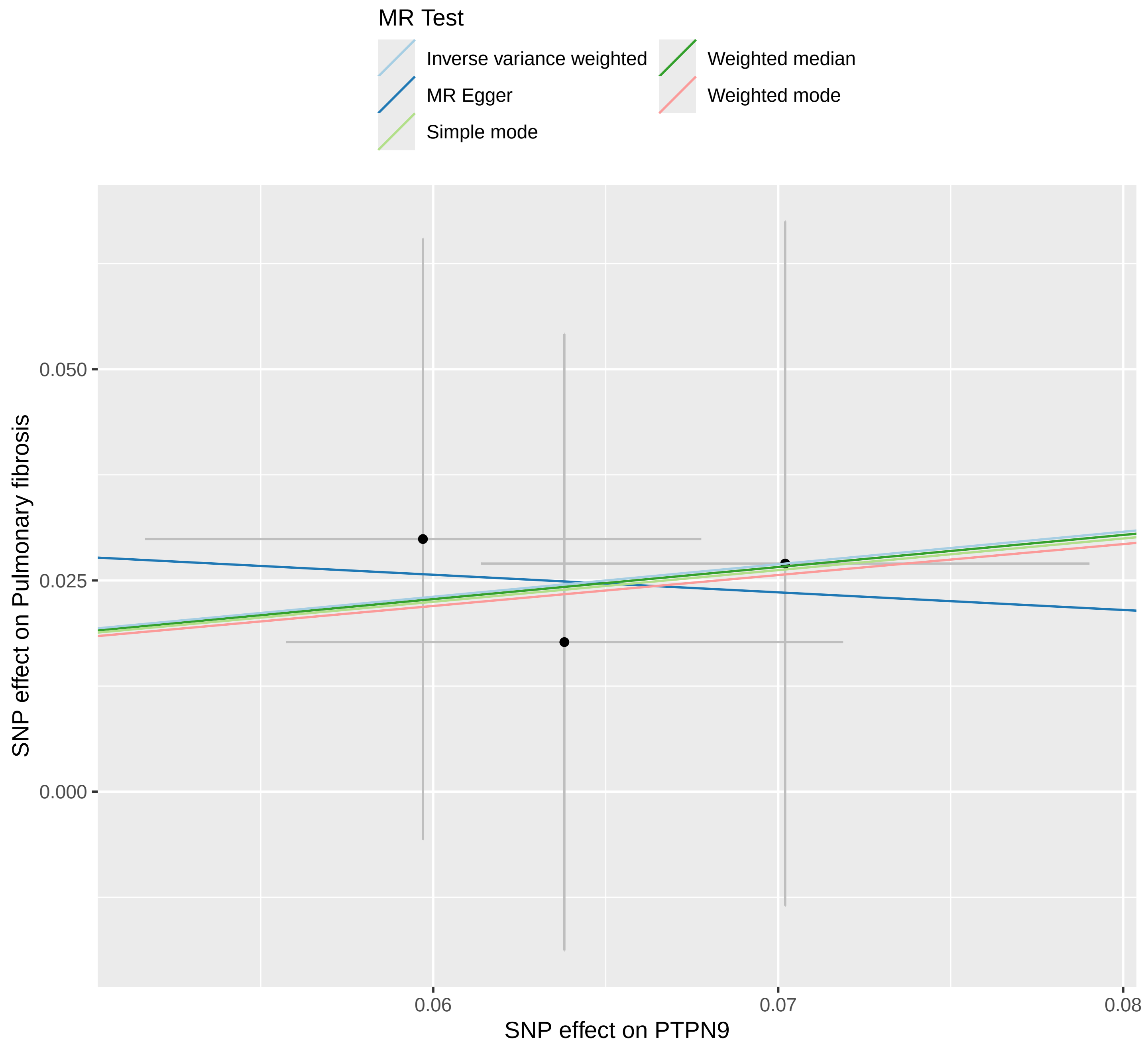

MR Test
Inverse variance weighted
MR Egger
Simple mode
Weighted median
Weighted mode
0.050
0.025
0.000
SNP effect on Pulmonary fibrosis
0.06
0.07
0.08
SNP effect on PTPN9

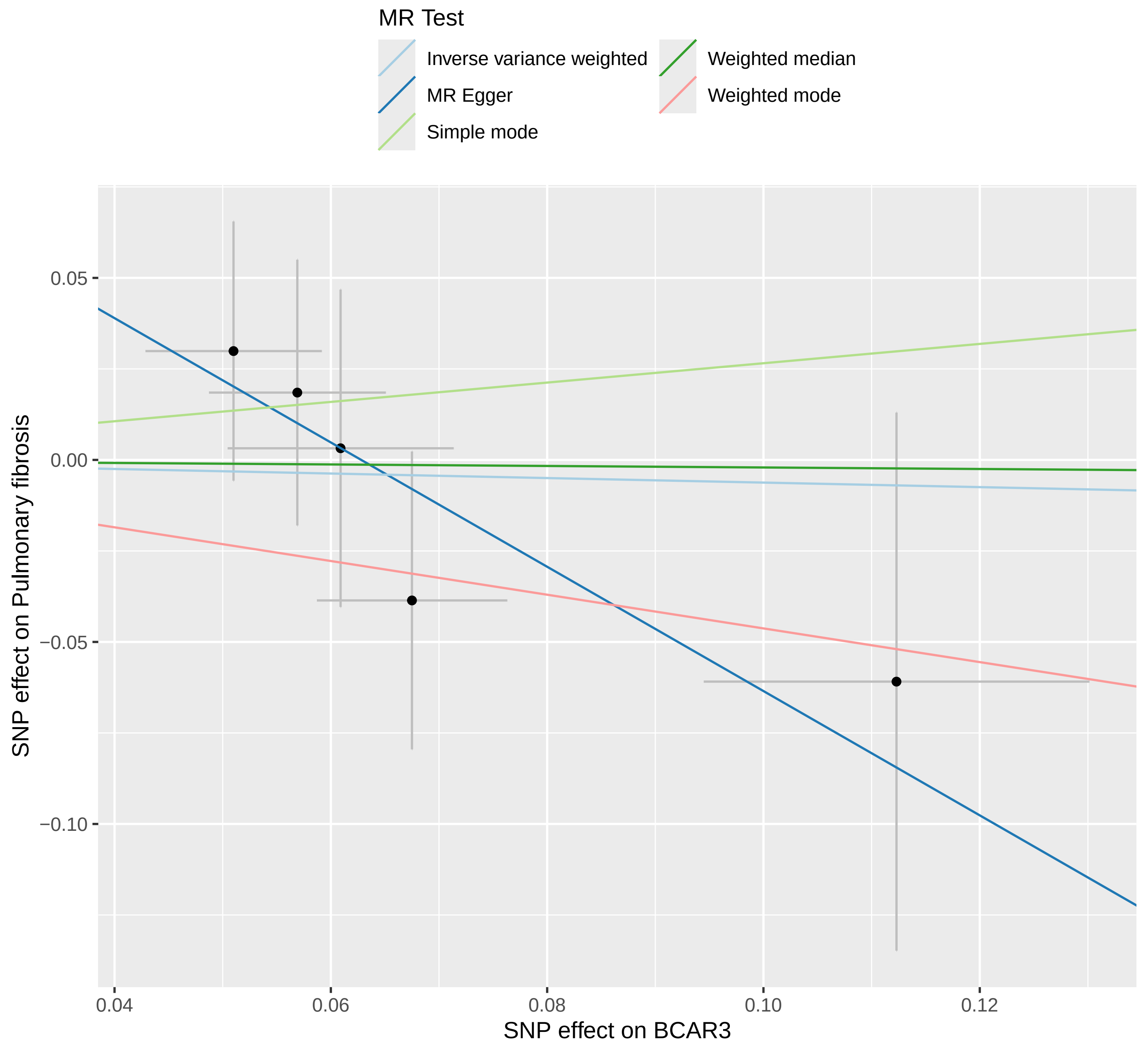
MR Test
Inverse variance weighted
MR Egger
Simple mode
Weighted median
Weighted mode
0.05
0.00
−0.05
−0.10
SNP effect on Pulmonary fibrosis
0.04
0.06
0.08
0.10
0.12
SNP effect on BCAR3

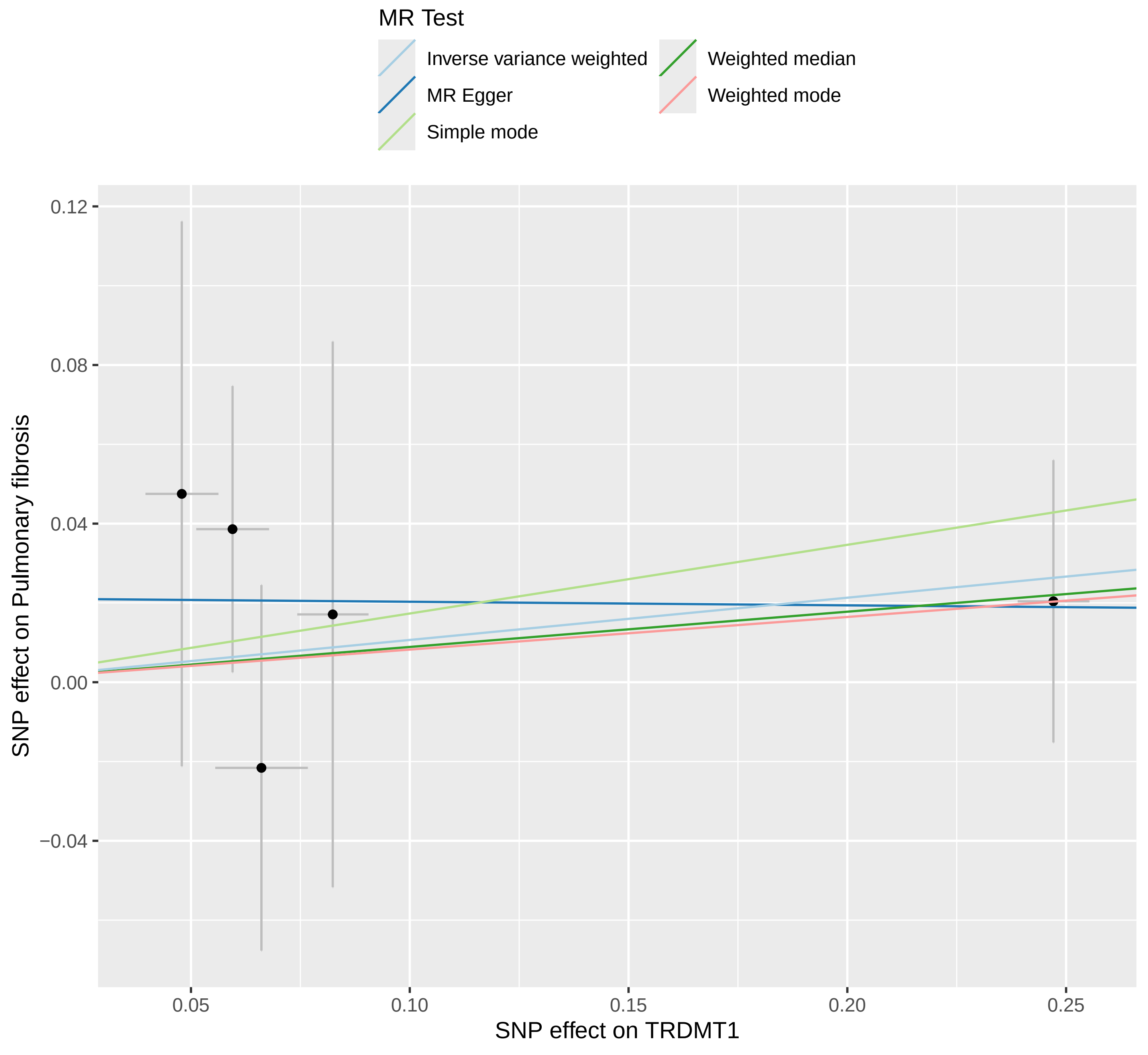
MR Test
Inverse variance weighted
MR Egger
Simple mode
Weighted median
Weighted mode
0.12
0.08
0.04
0.00
−0.04
SNP effect on Pulmonary fibrosis
0.05
0.10
0.15
0.20
0.25
SNP effect on TRDMT1

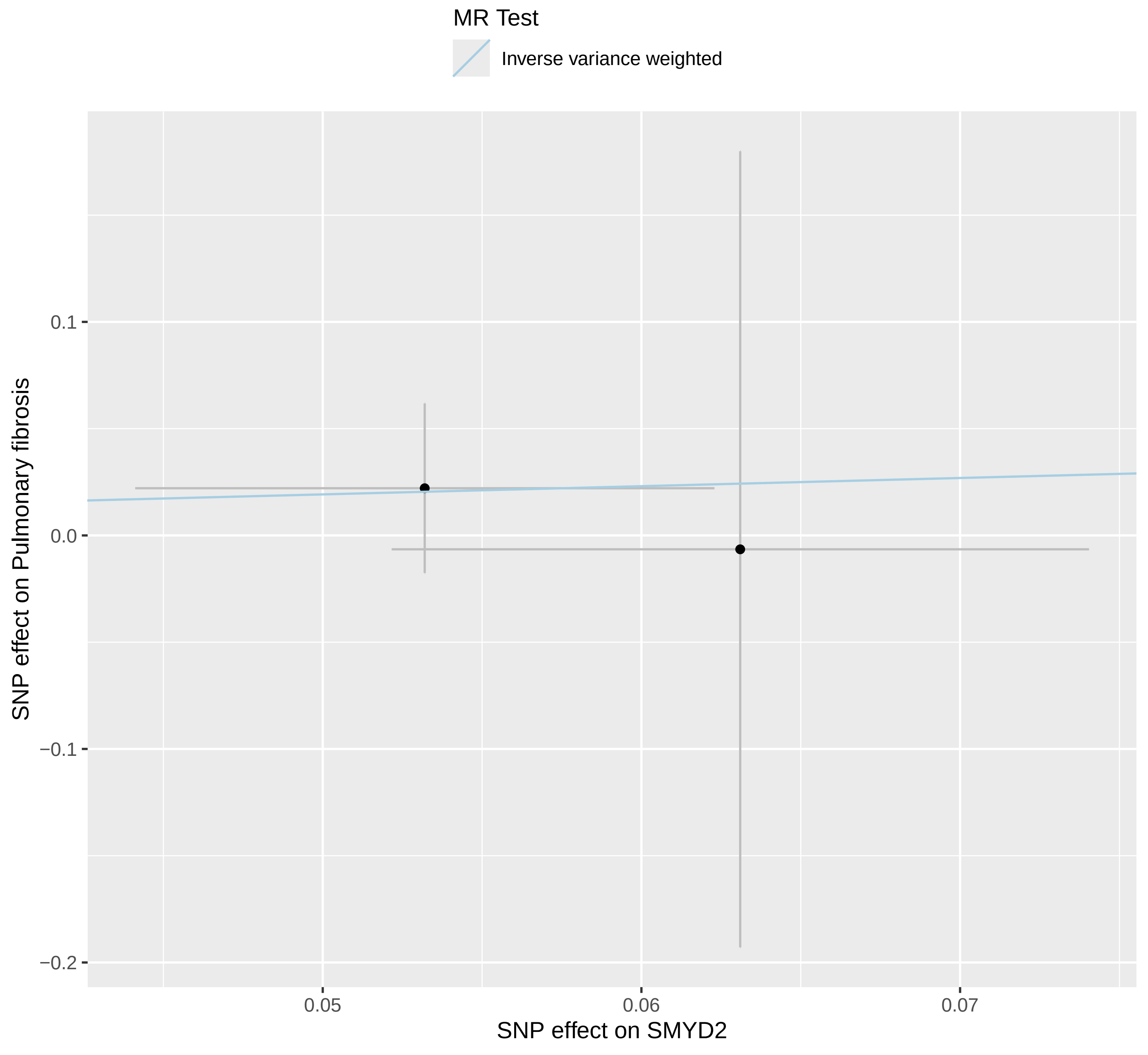
MR Test
Inverse variance weighted
0.1
0.0
−0.1
−0.2
SNP effect on Pulmonary fibrosis
0.05
0.06
0.07
SNP effect on SMYD2

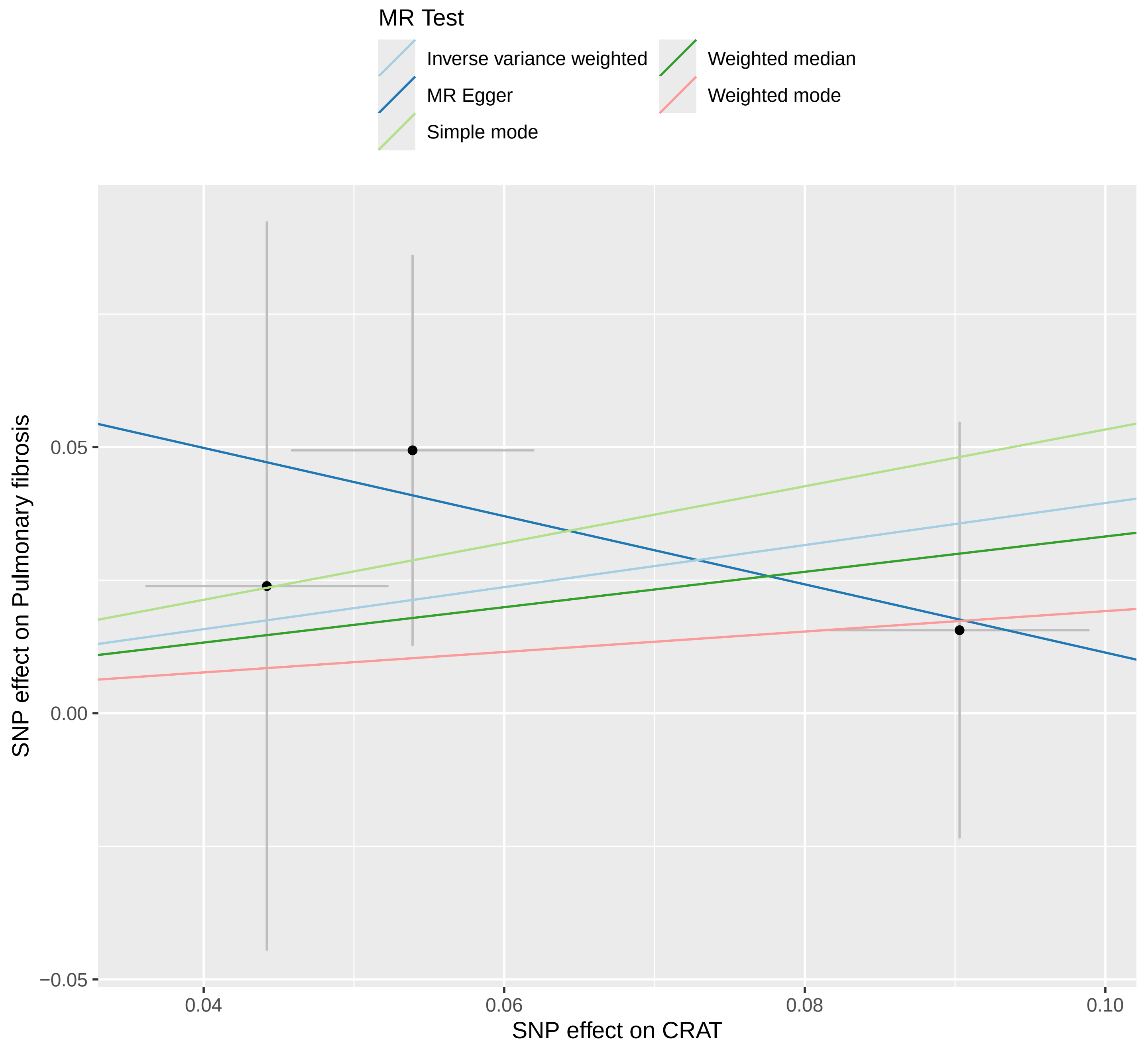

MR Test
Inverse variance weighted
MR Egger
Simple mode
Weighted median
Weighted mode
0.05
0.00
−0.05
SNP effect on Pulmonary fibrosis
0.04
0.06
0.08
0.10
SNP effect on CRAT

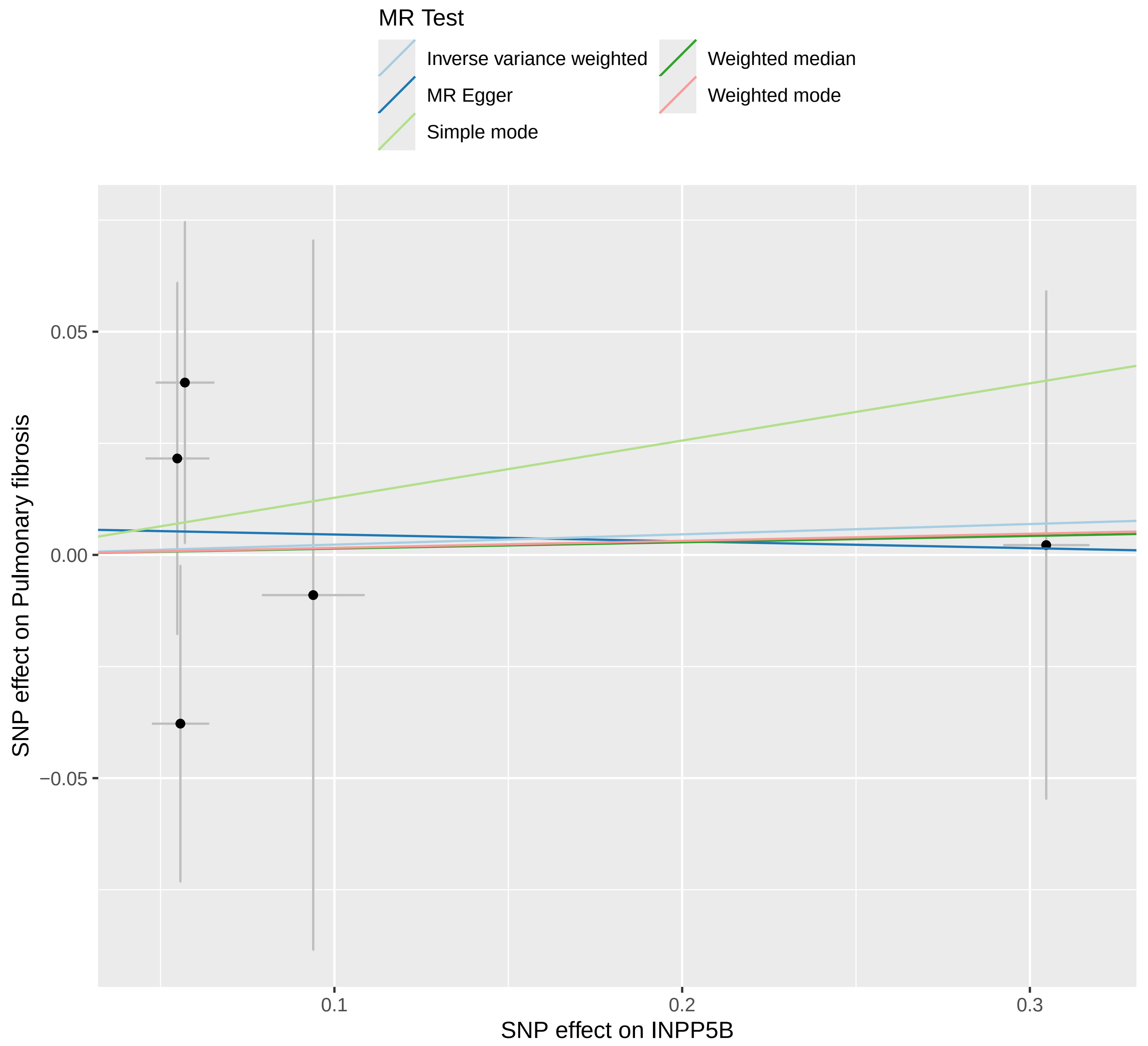
MR Test
Inverse variance weighted
MR Egger
Simple mode
Weighted median
Weighted mode
0.05
0.00
−0.05
0.1
0.2
0.3
SNP effect on Pulmonary fibrosis
SNP effect on INPP5B

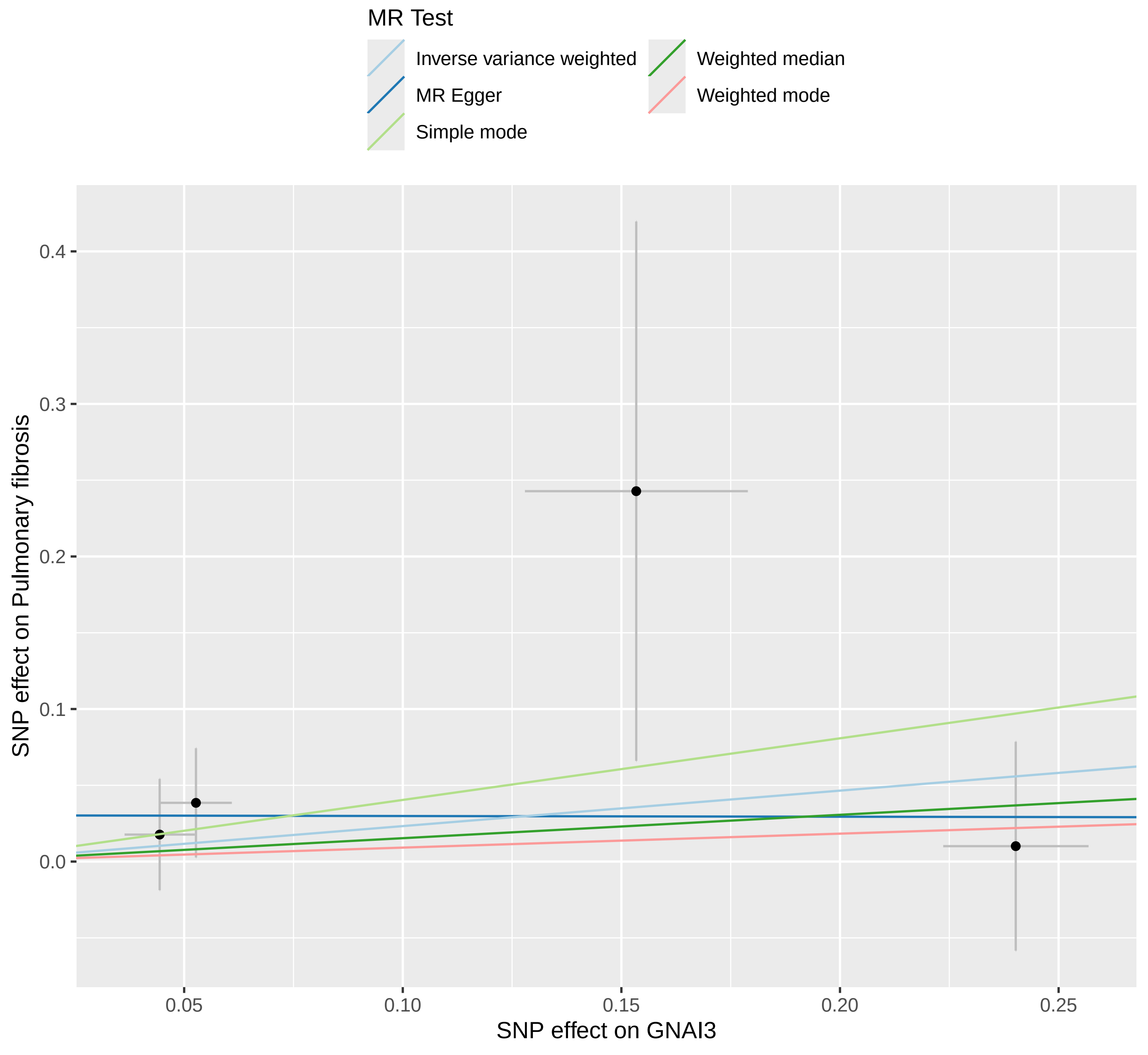
MR Test
Inverse variance weighted
MR Egger
Simple mode
Weighted median
Weighted mode
SNP effect on Pulmonary fibrosis
0.4
0.3
0.2
0.1
0.0
0.05
0.10
0.15
0.20
0.25
SNP effect on GNAI3

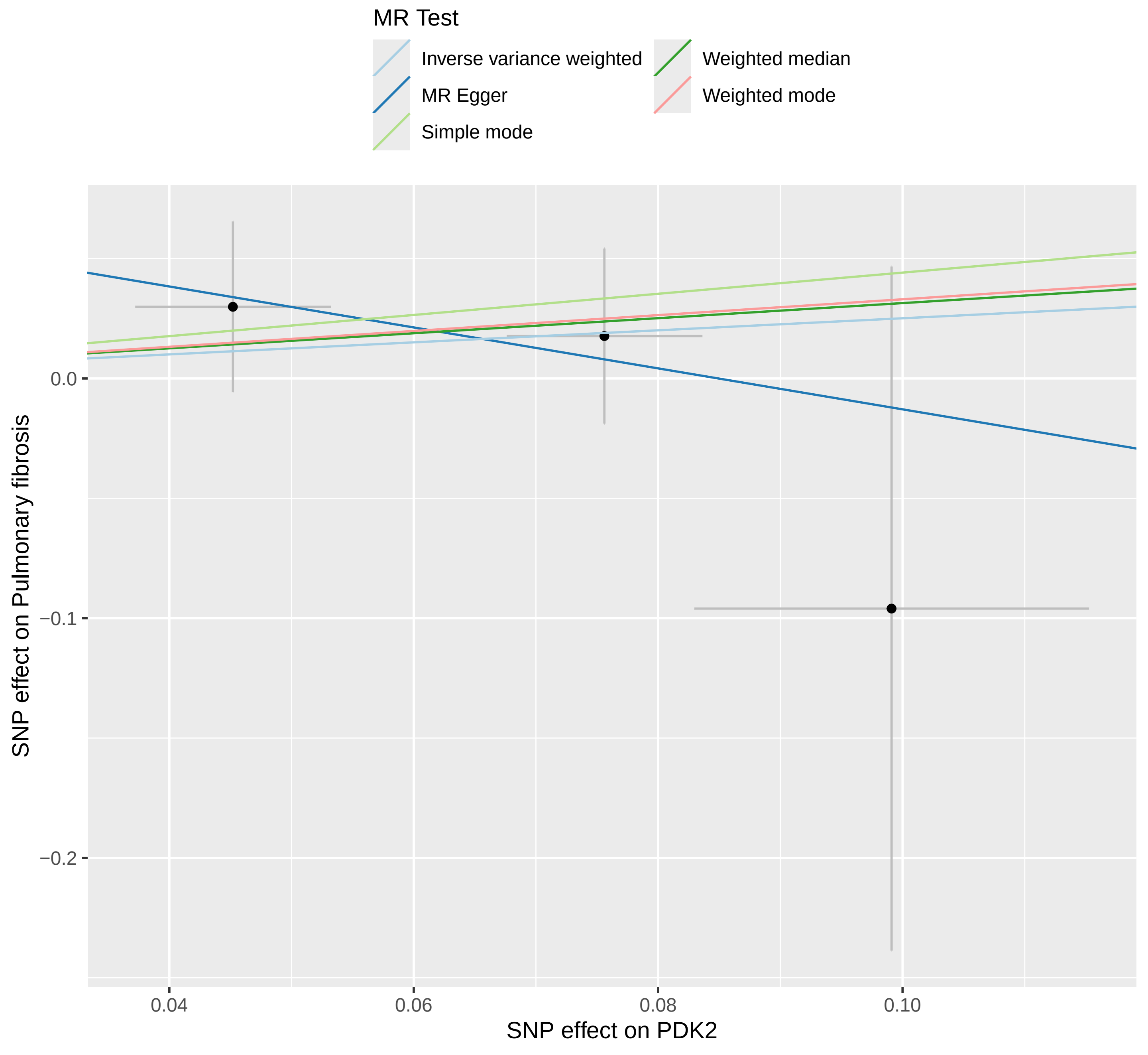
MR Test
Inverse variance weighted
MR Egger
Simple mode
Weighted median
Weighted mode
SNP effect on Pulmonary fibrosis
0.0
−0.1
−0.2
0.04
0.06
0.08
0.10
SNP effect on PDK2

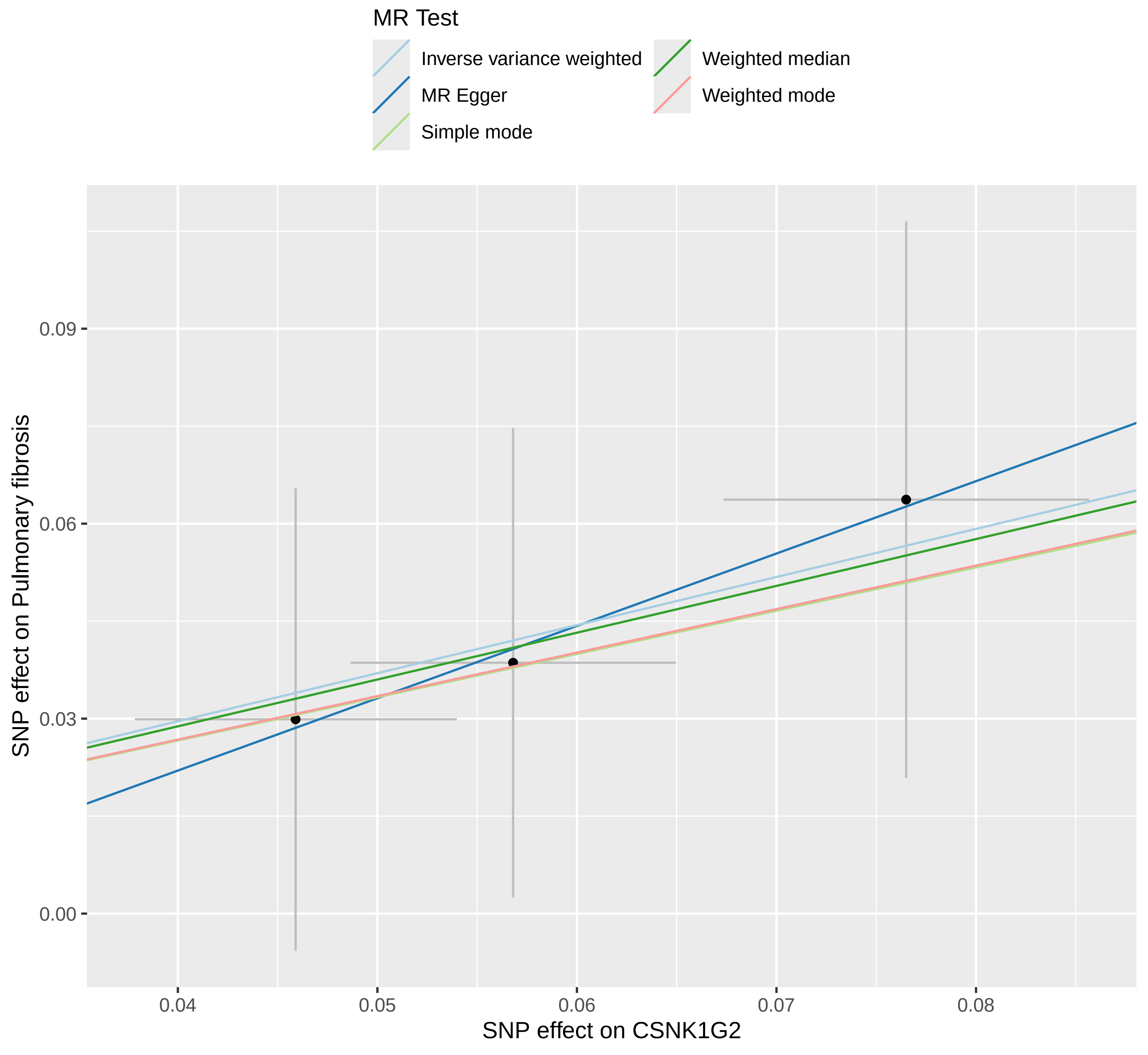
MR Test
Inverse variance weighted
MR Egger
Simple mode
Weighted median
Weighted mode
0.09
0.06
0.03
0.00
0.04
0.05
0.06
0.07
0.08
SNP effect on Pulmonary fibrosis
SNP effect on CSNK1G2

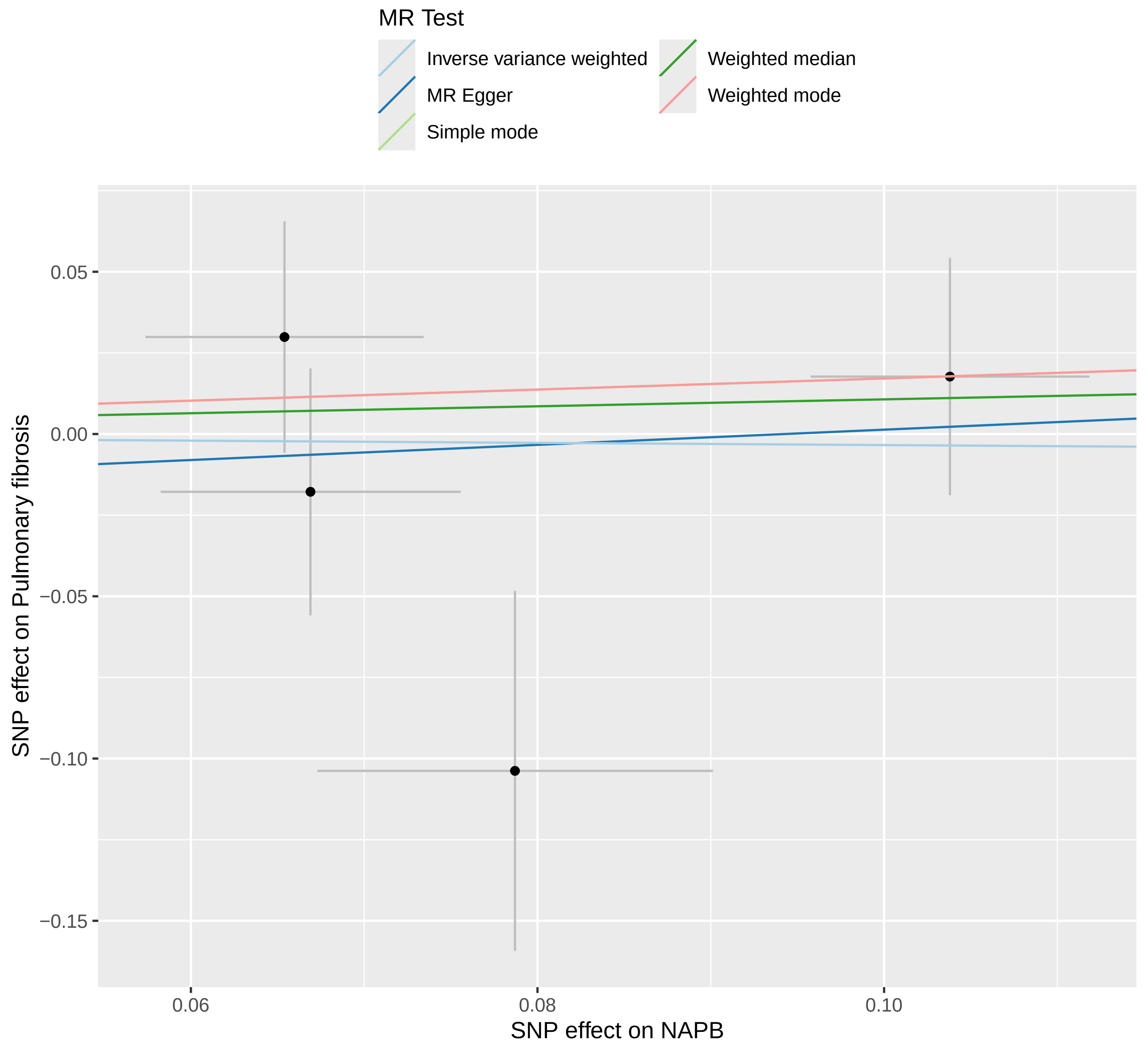

MR Test
Inverse variance weighted
MR Egger
Simple mode
Weighted median
Weighted mode
0.05
0.00
−0.05
−0.10
−0.15
SNP effect on Pulmonary fibrosis
0.06
0.08
0.10
SNP effect on NAPB

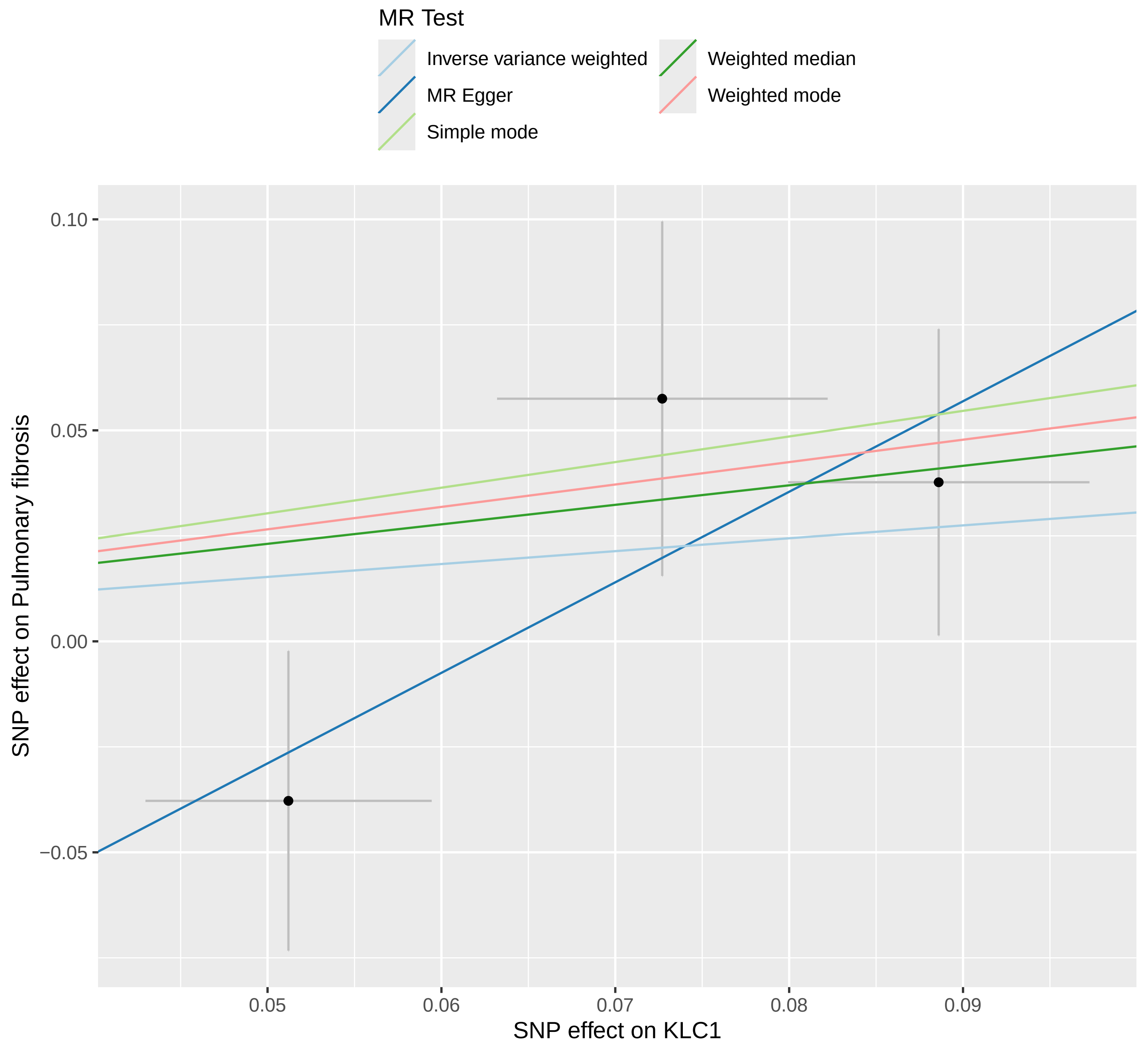

MR Test
Inverse variance weighted
MR Egger
Simple mode
Weighted median
Weighted mode
0.10
0.05
0.00
−0.05
0.05
0.06
0.07
0.08
0.09
SNP effect on Pulmonary fibrosis
SNP effect on KLC1

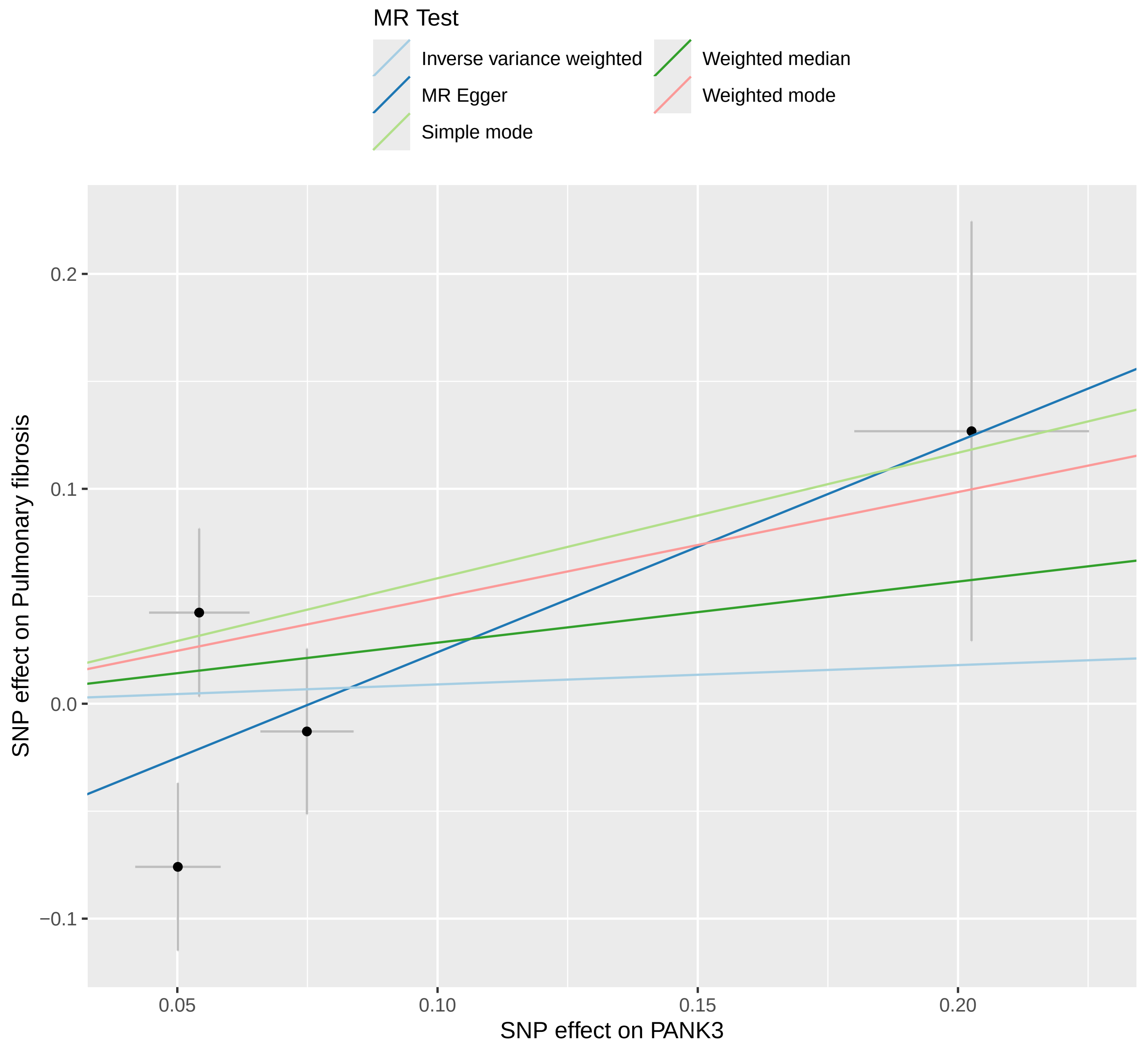
MR Test
Inverse variance weighted
MR Egger
Simple mode
Weighted median
Weighted mode
SNP effect on Pulmonary fibrosis
0.2
0.1
0.0
−0.1
0.05
0.10
0.15
0.20
SNP effect on PANK3

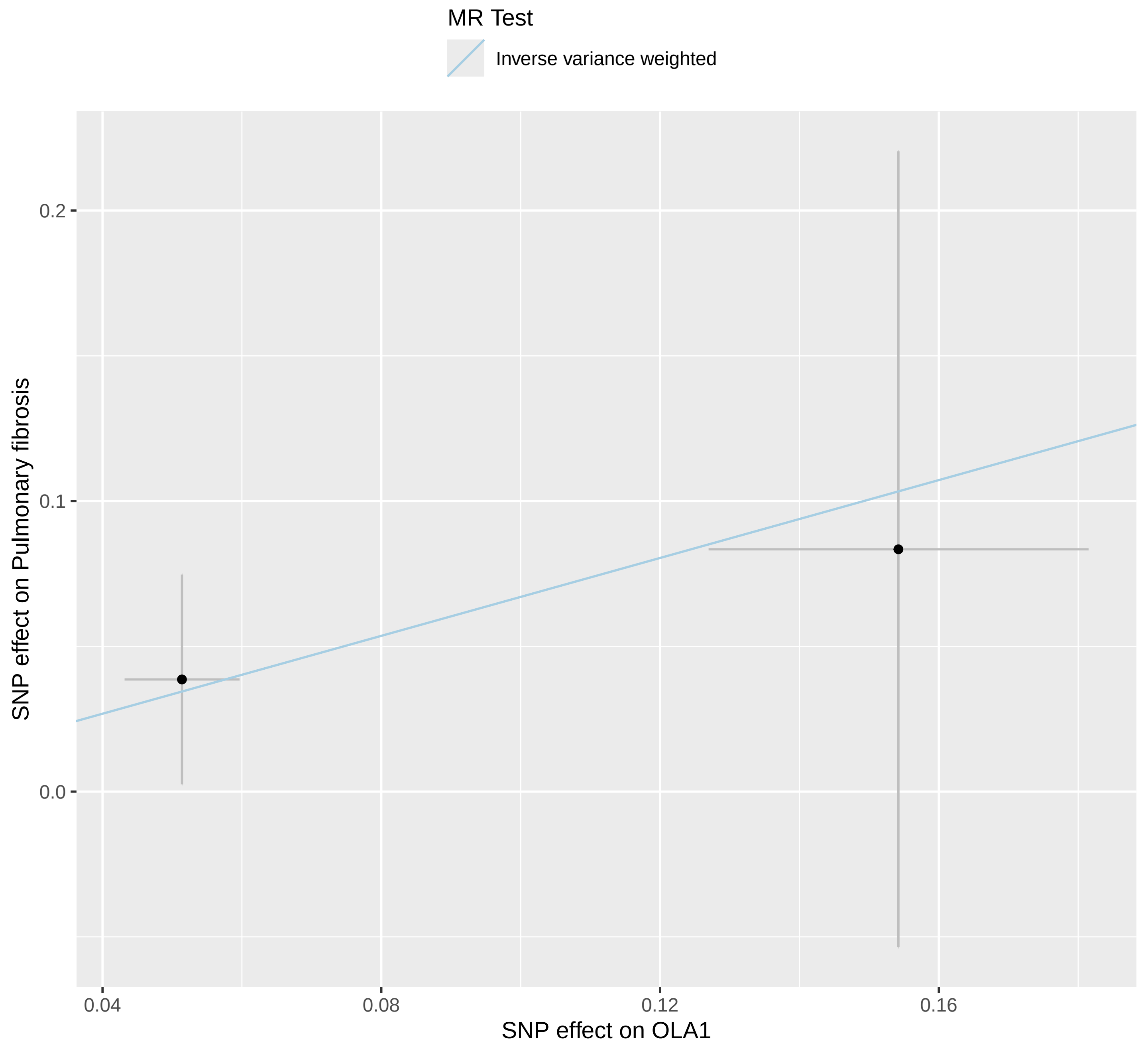
MR Test
Inverse variance weighted
SNP effect on Pulmonary fibrosis
0.2
0.1
0.0
0.04
0.08
0.12
0.16
SNP effect on OLA1

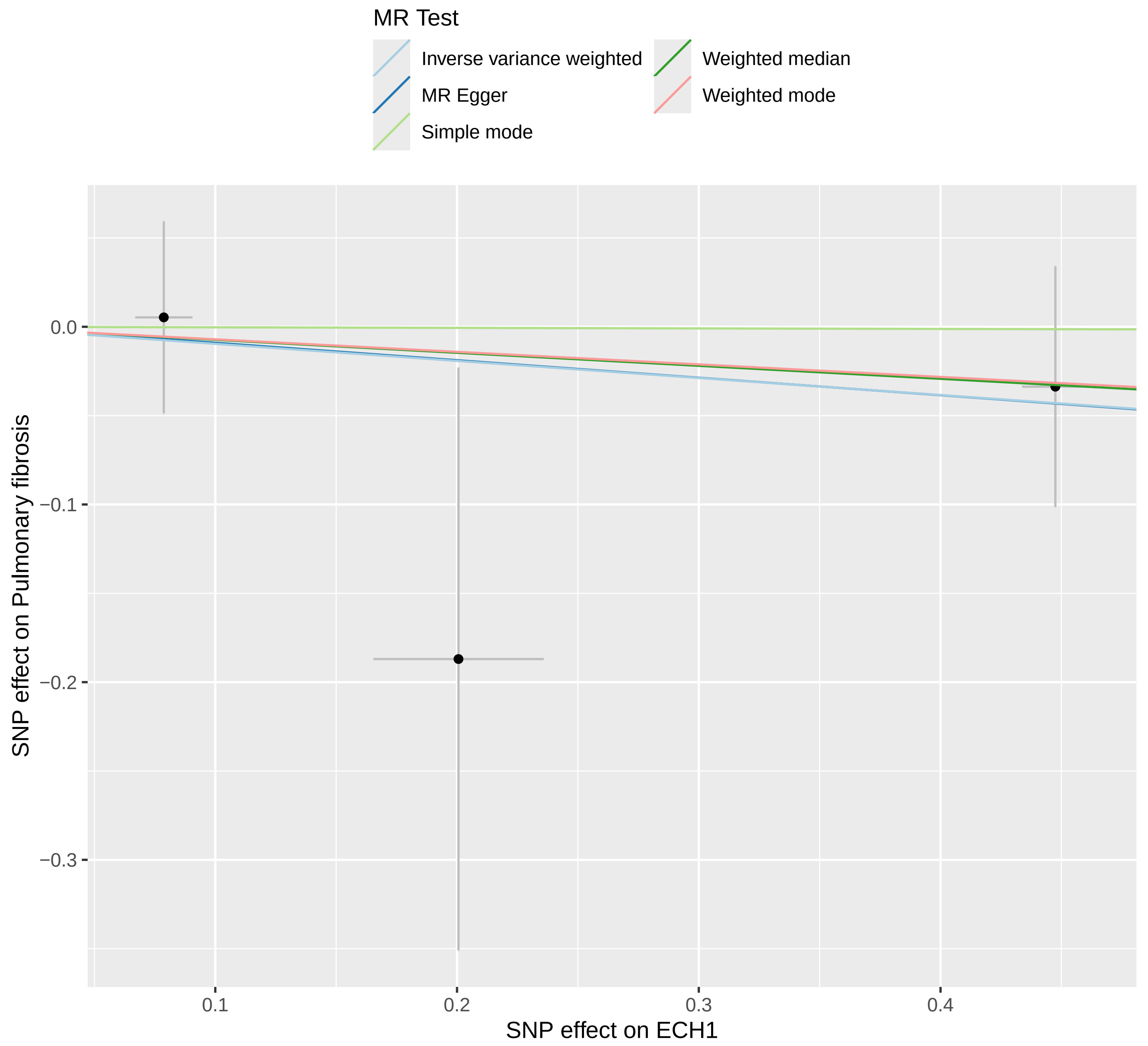
MR Test
Inverse variance weighted
MR Egger
Simple mode
Weighted median
Weighted mode
0.0
−0.1
−0.2
−0.3
0.1
0.2
0.3
0.4
SNP effect on ECH1
SNP effect on Pulmonary fibrosis

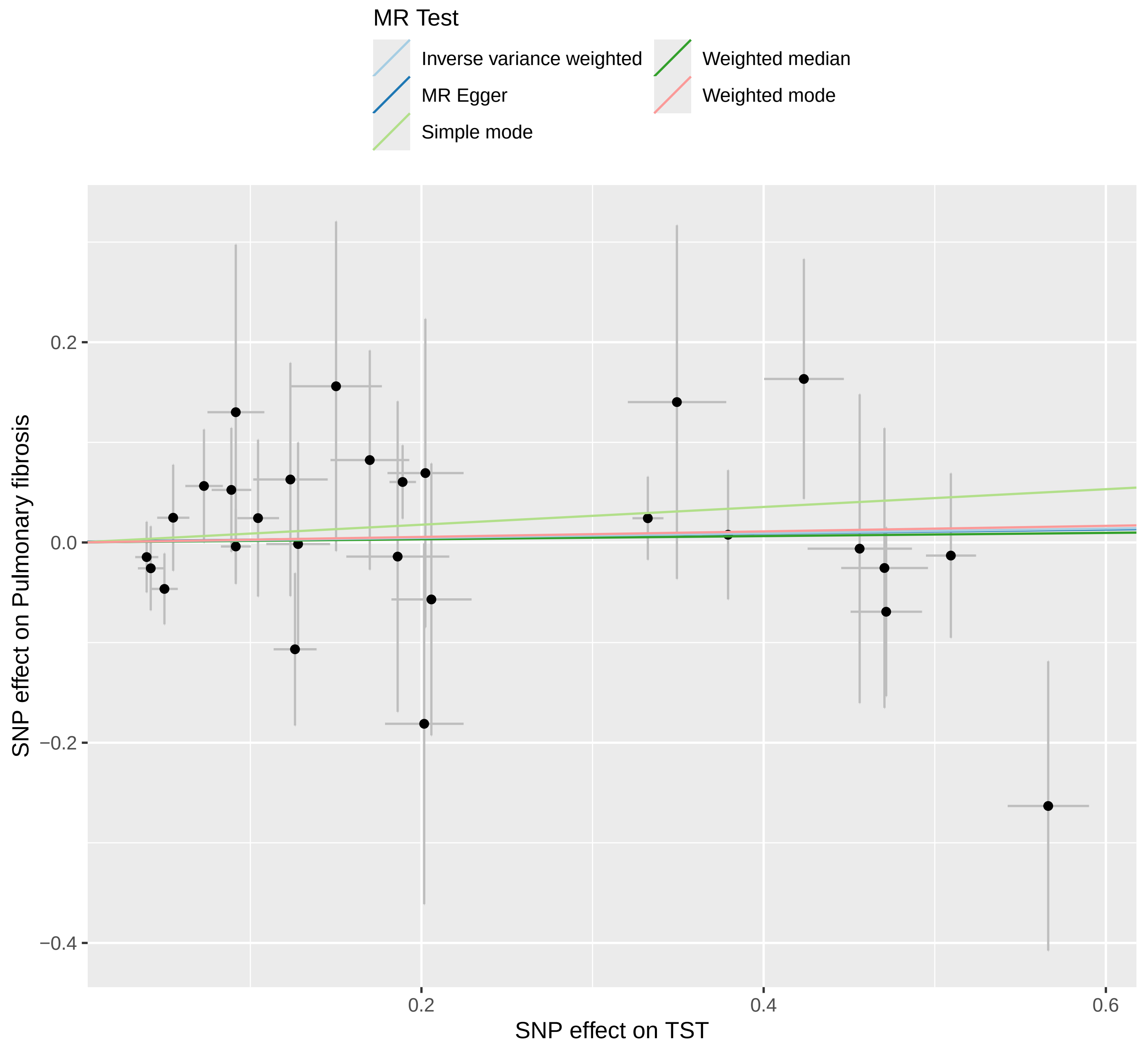

MR Test
Inverse variance weighted
MR Egger
Simple mode
Weighted median
Weighted mode
SNP effect on Pulmonary fibrosis
0.2
0.0
−0.2
−0.4
0.2
0.4
0.6
SNP effect on TST

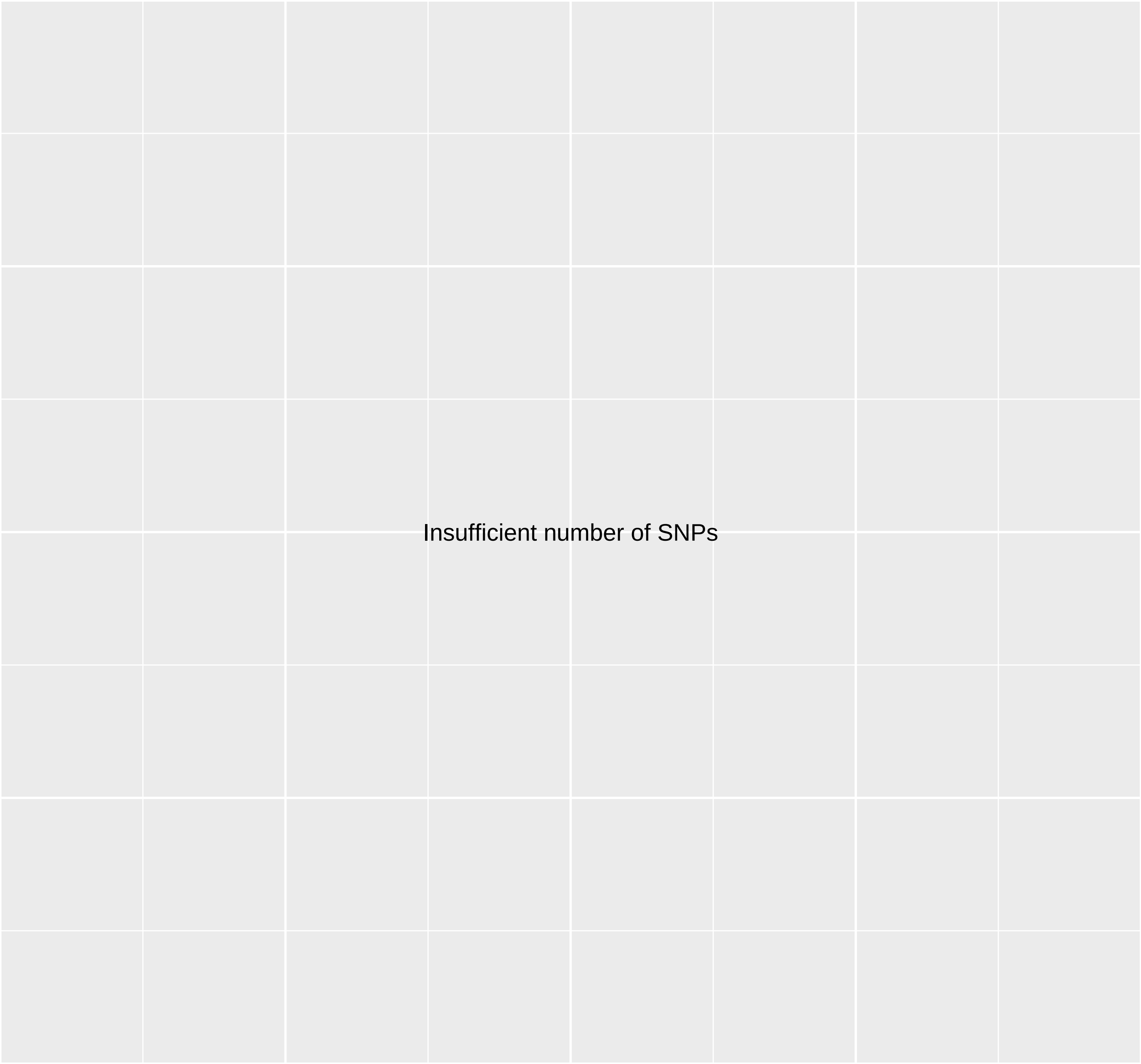
Insufficient number of SNPs

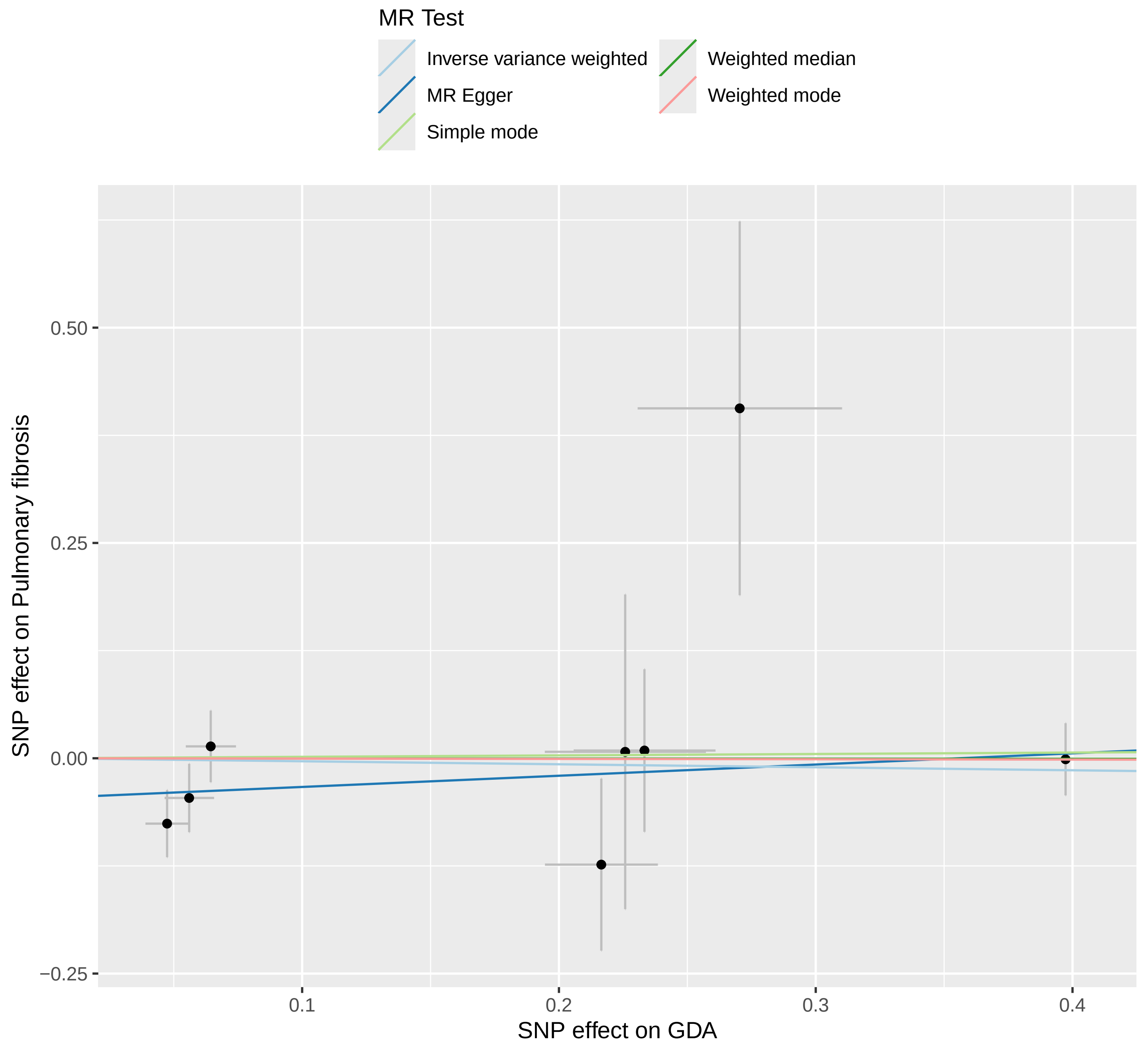
MR Test
Inverse variance weighted
MR Egger
Simple mode
Weighted median
Weighted mode
0.50
0.25
0.00
−0.25
0.1
0.2
0.3
0.4
SNP effect on Pulmonary fibrosis
SNP effect on GDA

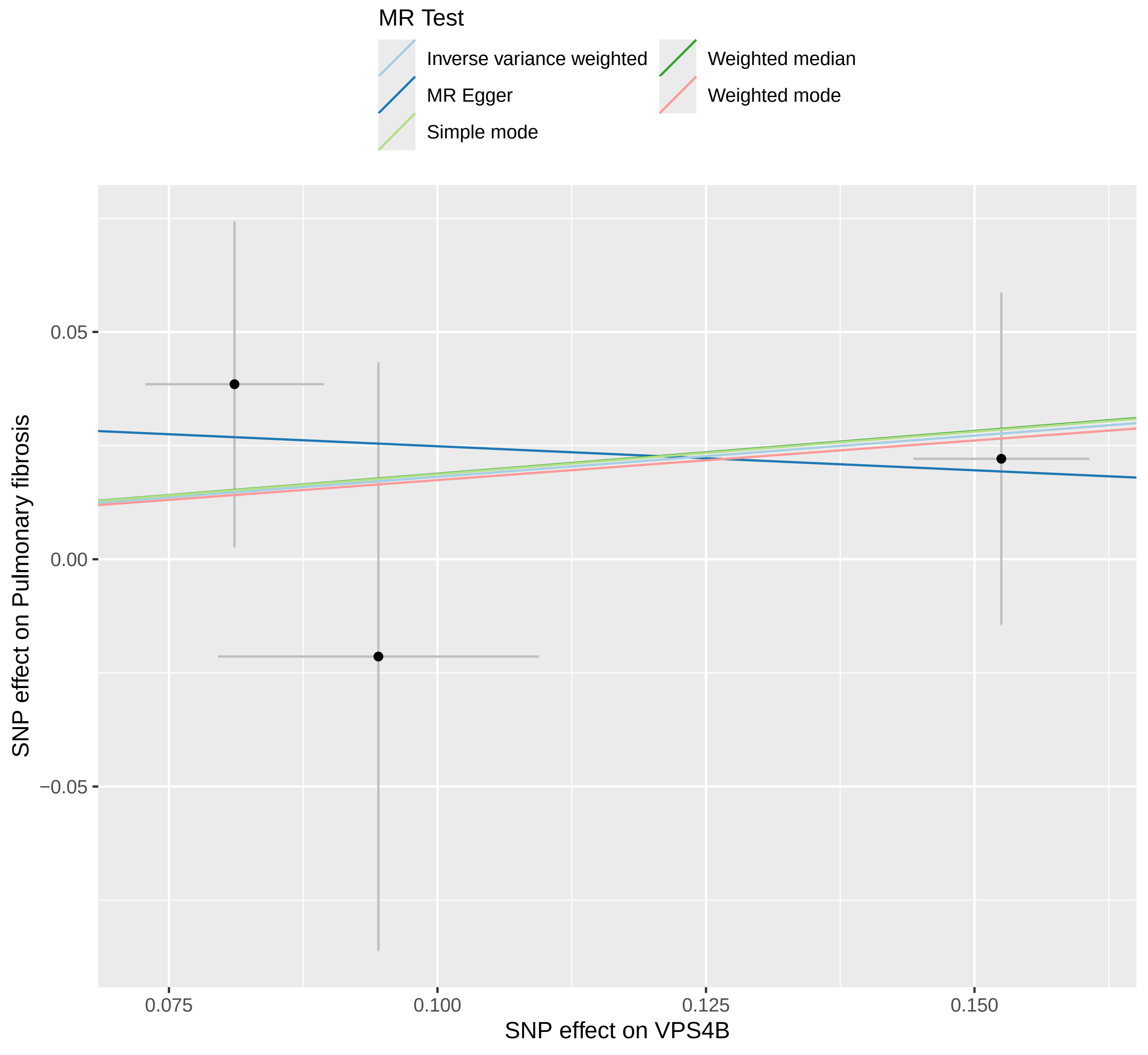
MR Test
Inverse variance weighted
MR Egger
Simple mode
Weighted median
Weighted mode
0.05
0.00
−0.05
0.075
0.100
0.125
0.150
SNP effect on VPS4B
SNP effect on Pulmonary fibrosis

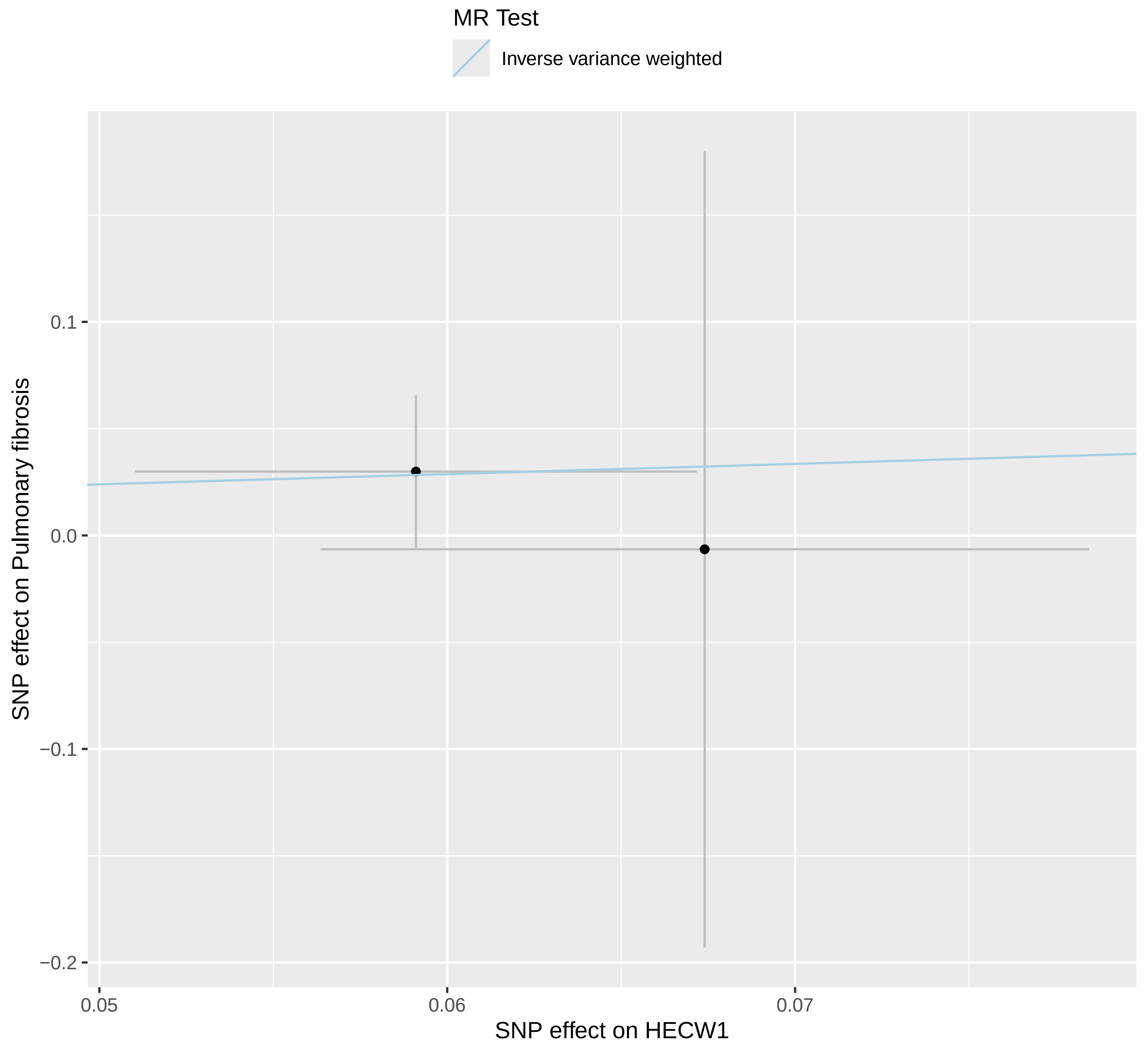

MR Test
Inverse variance weighted
0.1
0.0
−0.1
−0.2
0.05
0.06
0.07
SNP effect on Pulmonary fibrosis
SNP effect on HECW1

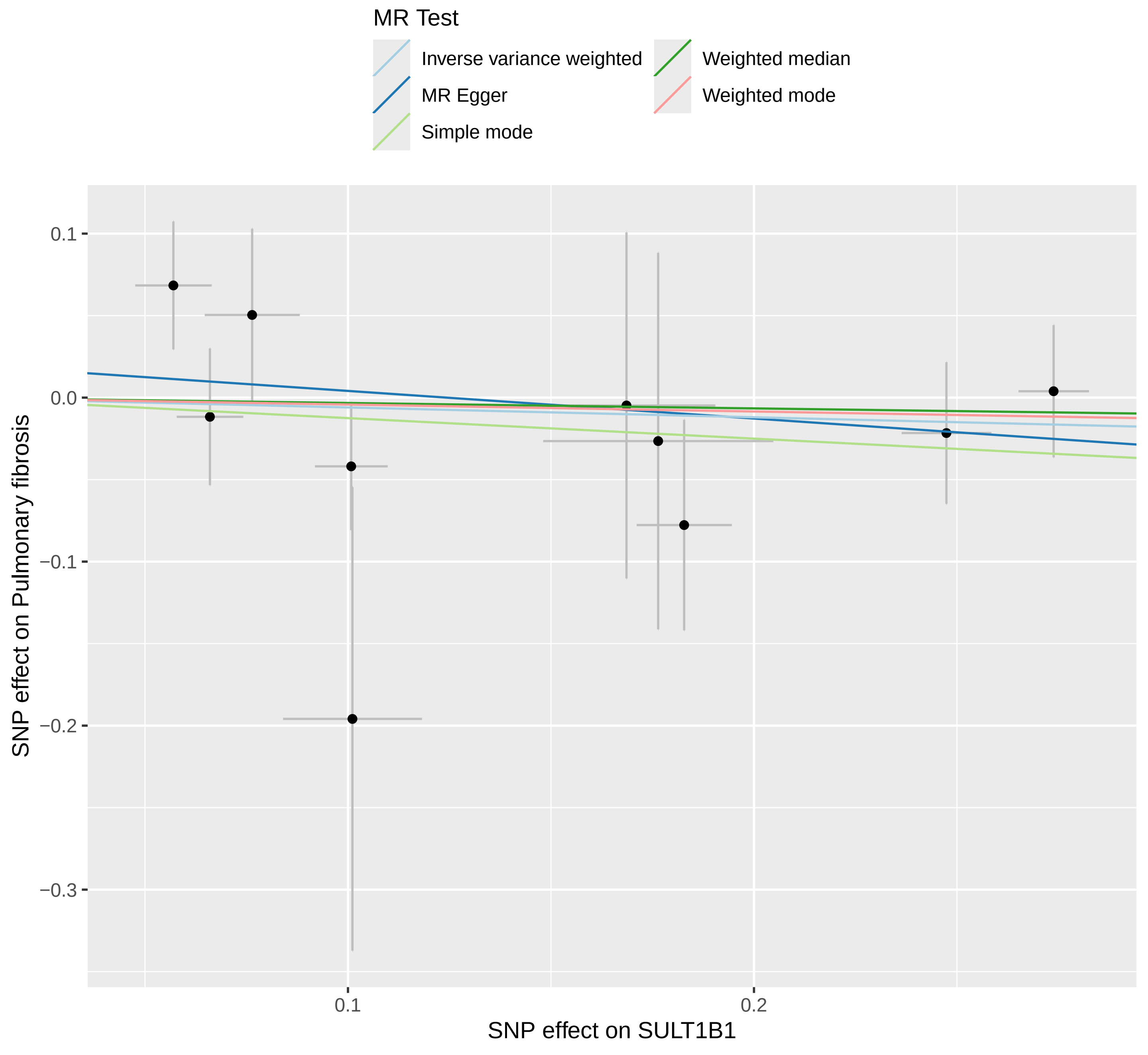
MR Test
Inverse variance weighted
MR Egger
Simple mode
Weighted median
Weighted mode
0.1
0.0
−0.1
−0.2
−0.3
SNP effect on Pulmonary fibrosis
0.1
0.2
SNP effect on SULT1B1

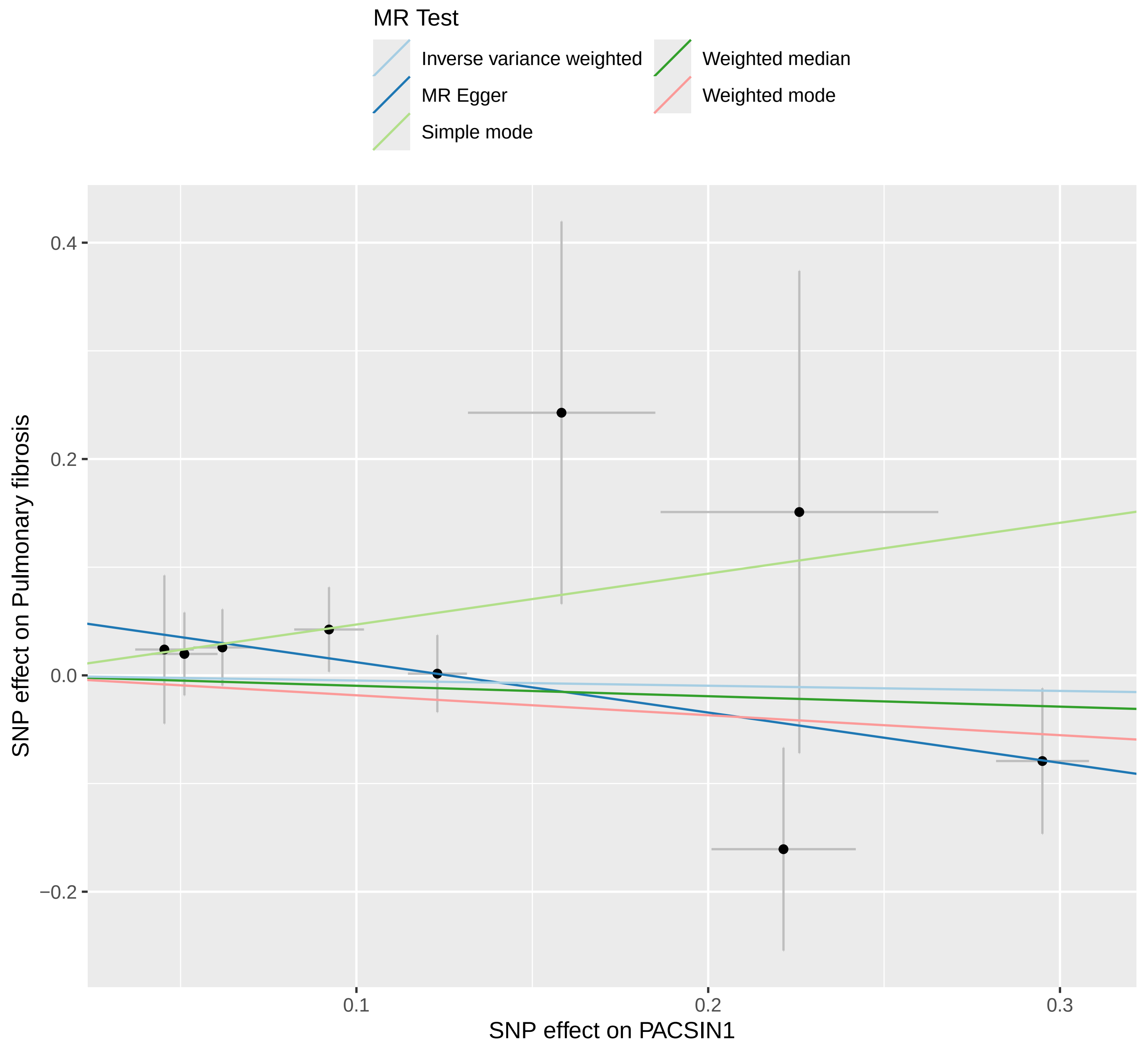

MR Test
Inverse variance weighted
MR Egger
Simple mode
Weighted median
Weighted mode
0.4
0.2
0.0
−0.2
0.1
0.2
0.3
SNP effect on Pulmonary fibrosis
SNP effect on PACSIN1

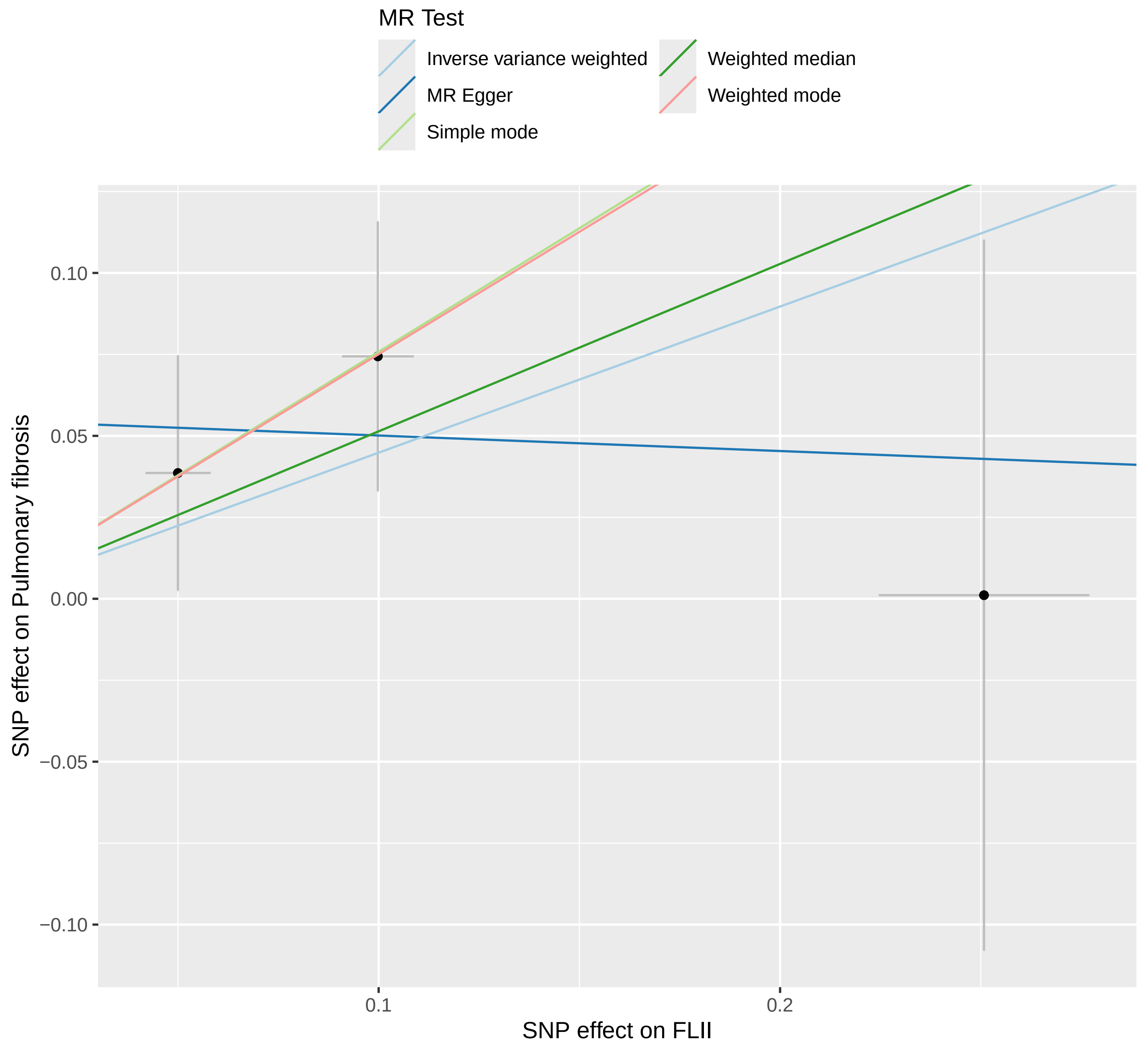

MR Test
Inverse variance weighted
MR Egger
Simple mode
Weighted median
Weighted mode
0.10
0.05
0.00
−0.05
−0.10
SNP effect on Pulmonary fibrosis
0.1
0.2
SNP effect on FLII

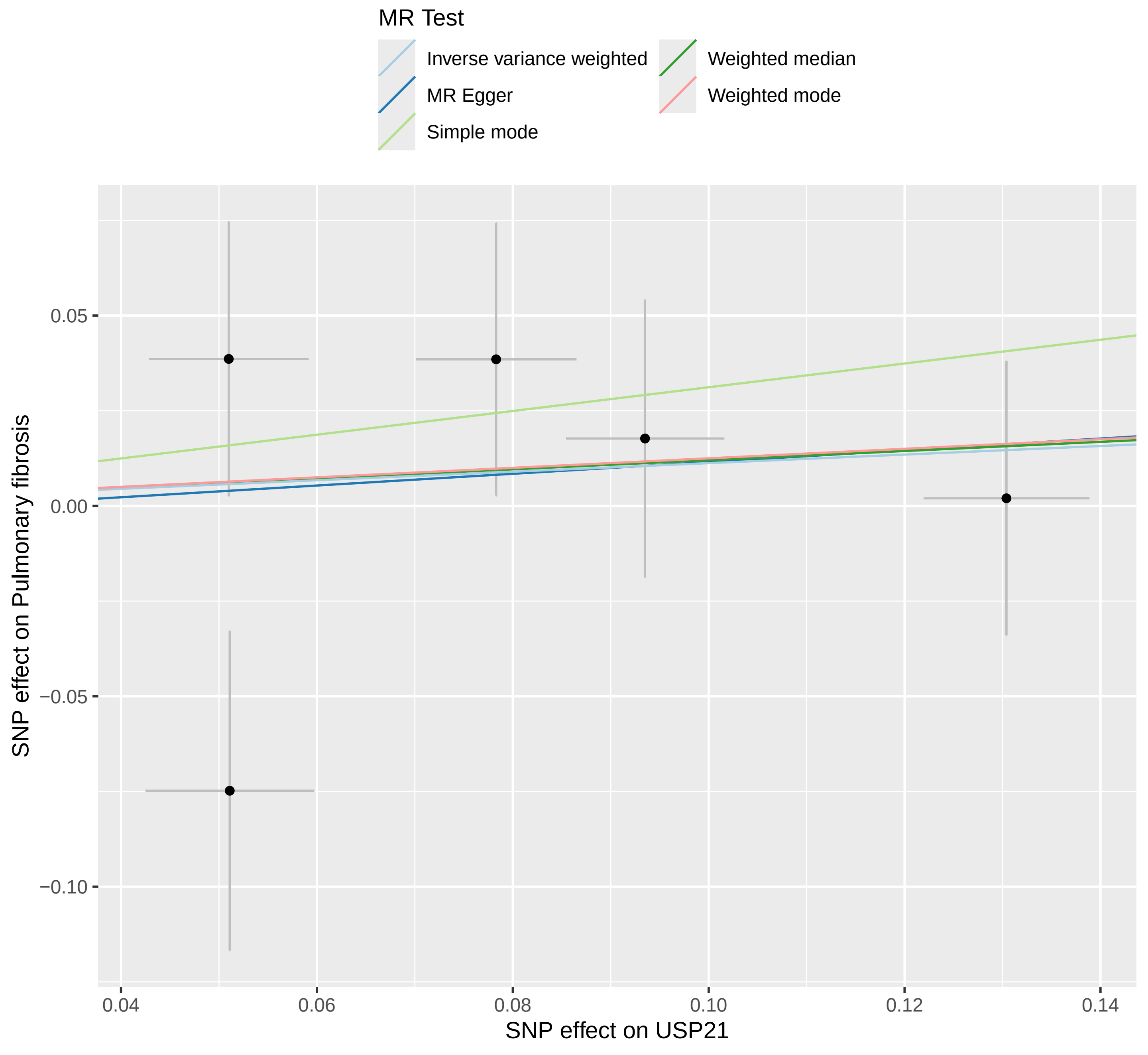
MR Test
Inverse variance weighted
MR Egger
Simple mode
Weighted median
Weighted mode
0.05
0.00
−0.05
−0.10
0.04
0.06
0.08
0.10
0.12
0.14
SNP effect on USP21
SNP effect on Pulmonary fibrosis

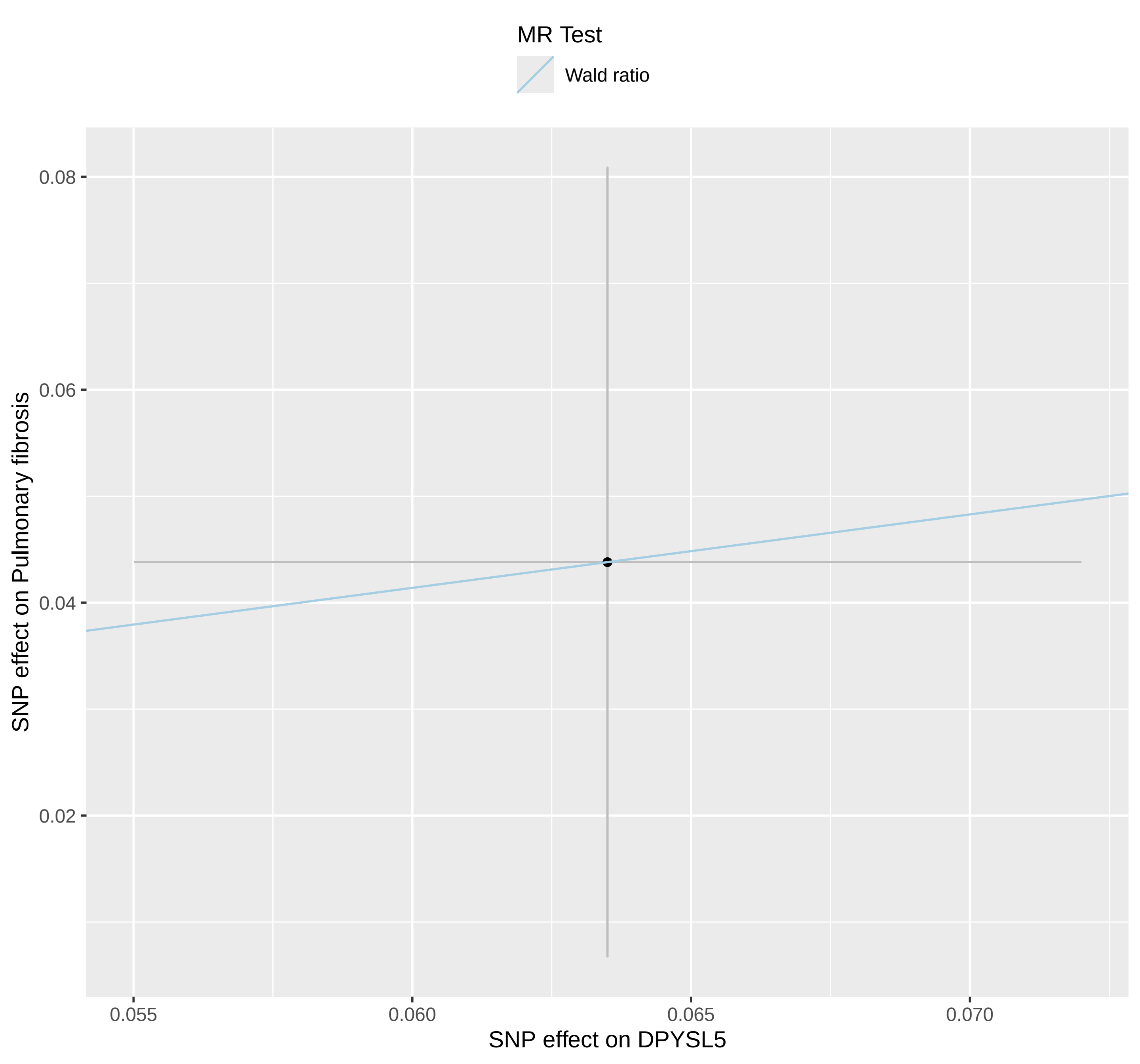
MR Test
Wald ratio
0.08
0.06
0.04
0.02
SNP effect on Pulmonary fibrosis
0.055
0.060
0.065
0.070
SNP effect on DPYSL5

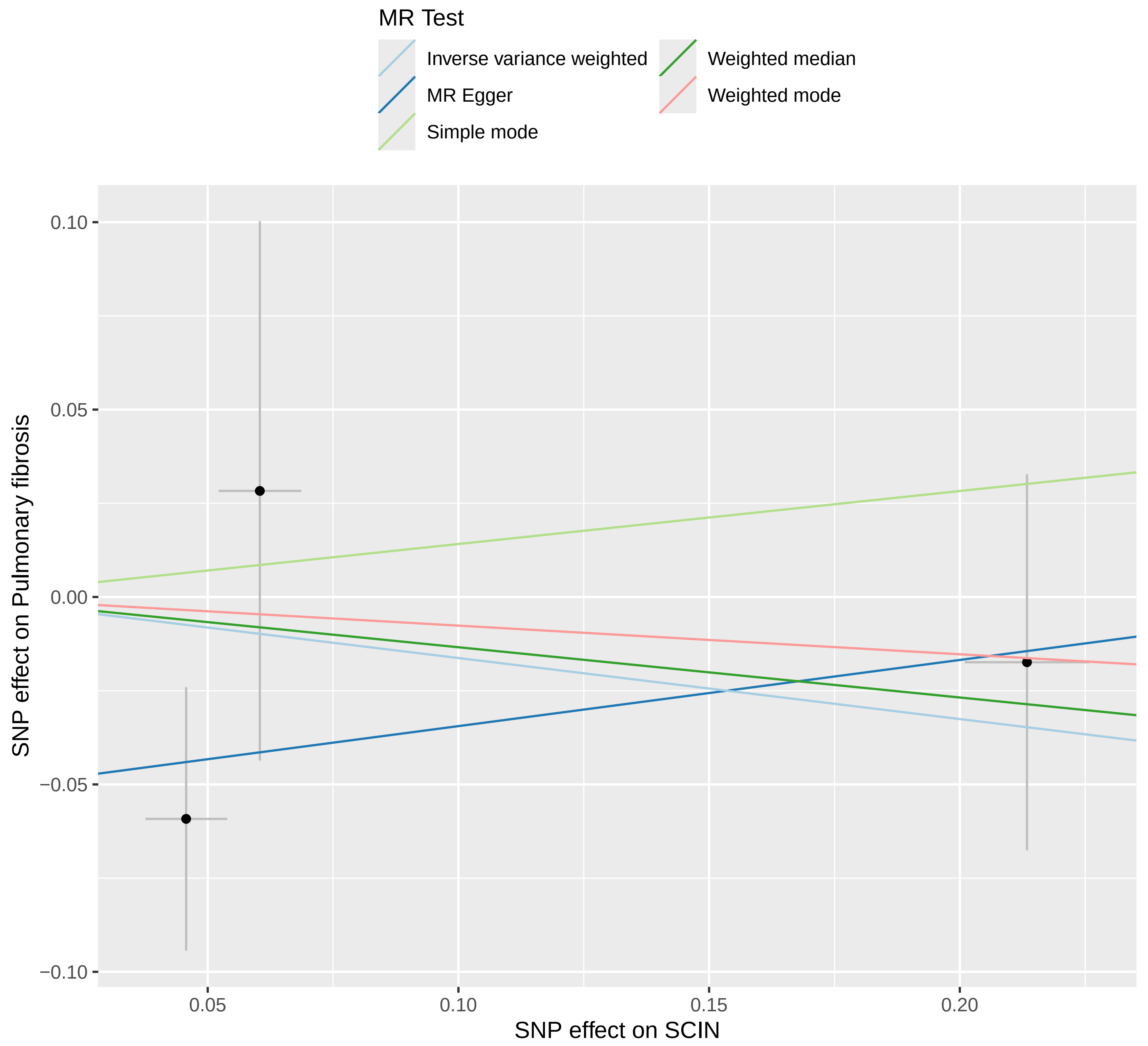
MR Test
Inverse variance weighted
MR Egger
Simple mode
Weighted median
Weighted mode
0.10
0.05
0.00
−0.05
−0.10
SNP effect on Pulmonary fibrosis
0.05
0.10
0.15
0.20
SNP effect on SCIN

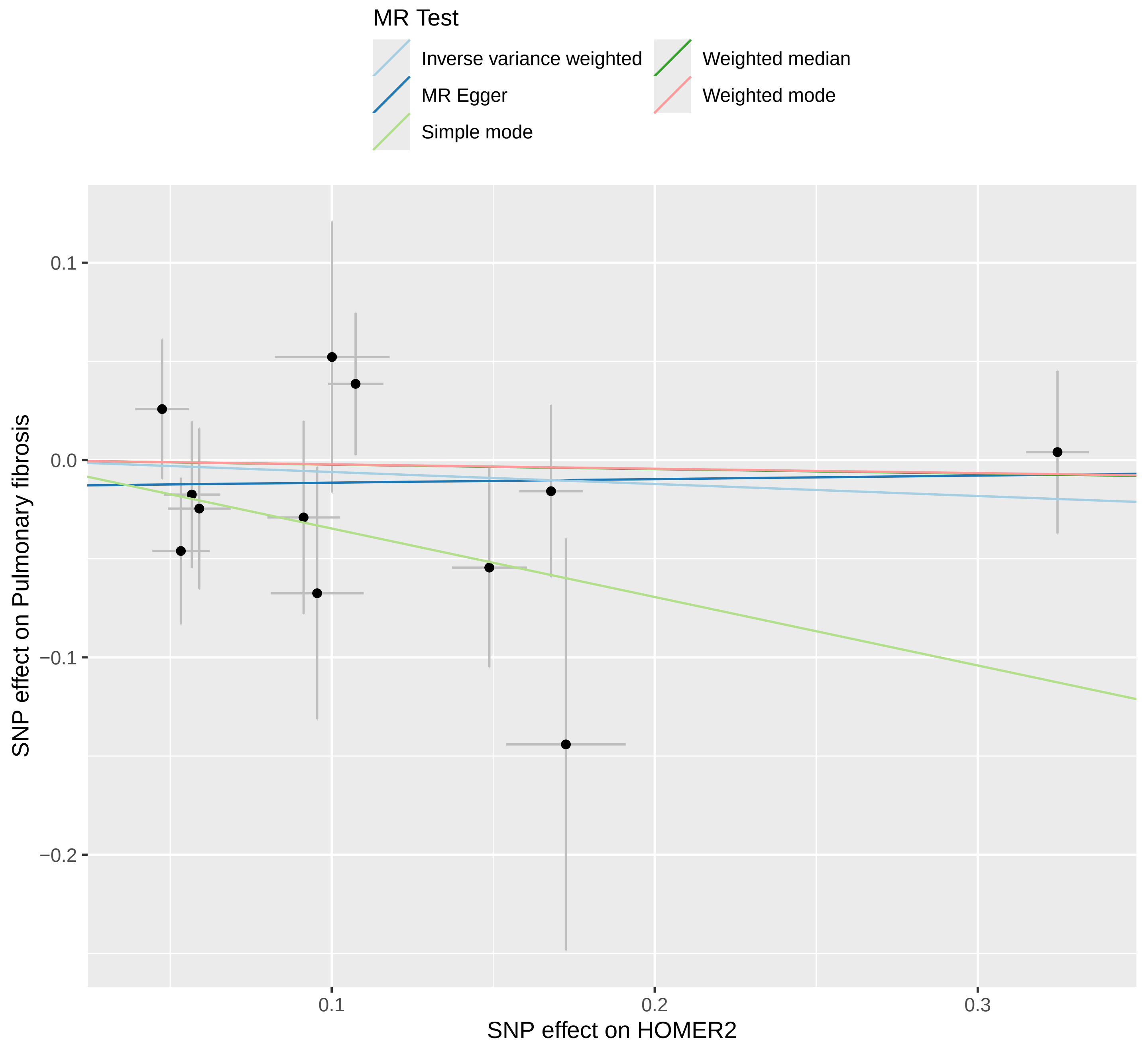

MR Test
Inverse variance weighted
MR Egger
Simple mode
Weighted median
Weighted mode
0.1
0.0
−0.1
−0.2
SNP effect on Pulmonary fibrosis
0.1
0.2
0.3
SNP effect on HOMER2

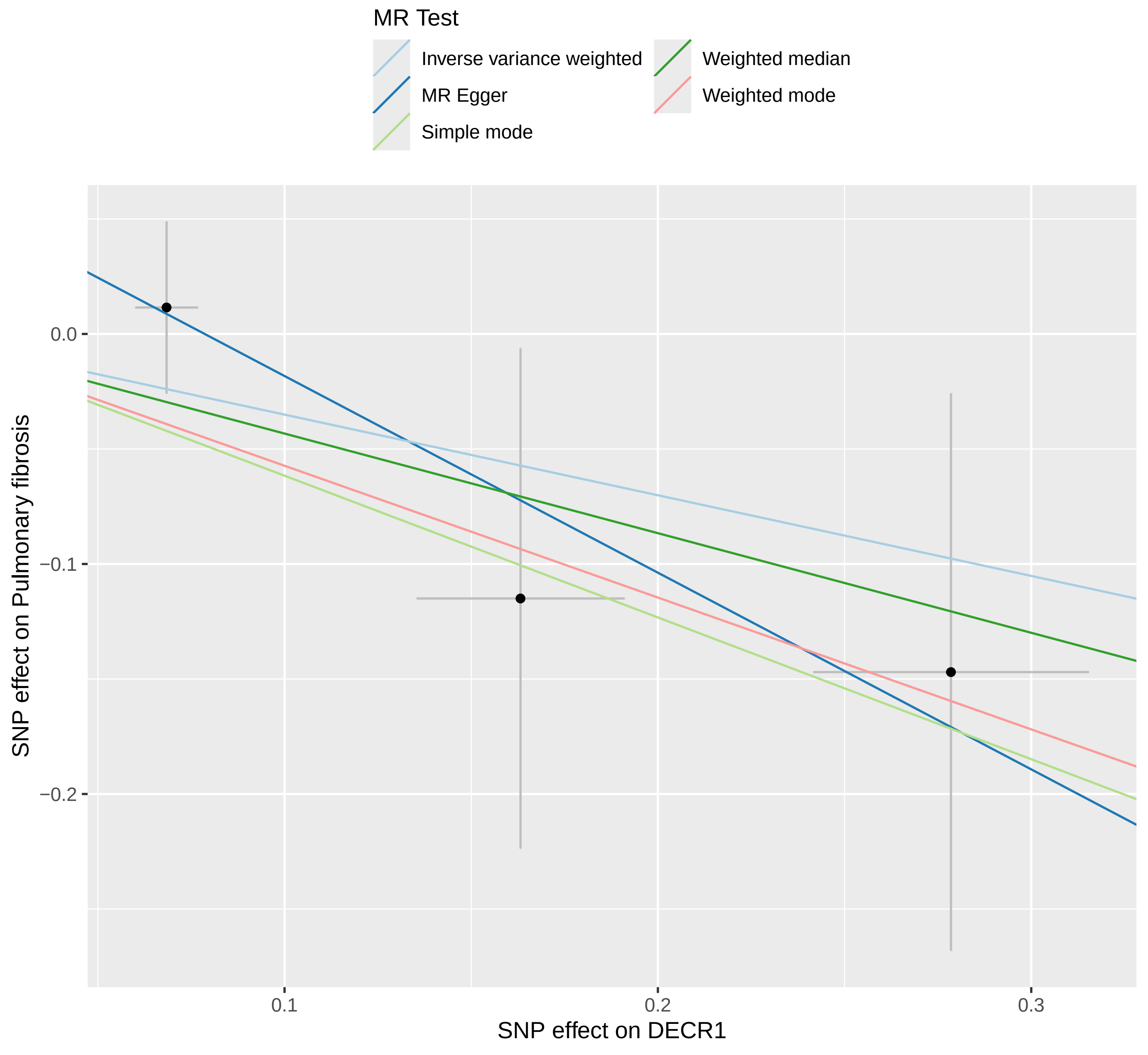

MR Test
Inverse variance weighted
MR Egger
Simple mode
Weighted median
Weighted mode
SNP effect on Pulmonary fibrosis
0.0
−0.1
−0.2
0.1
0.2
0.3
SNP effect on DECR1

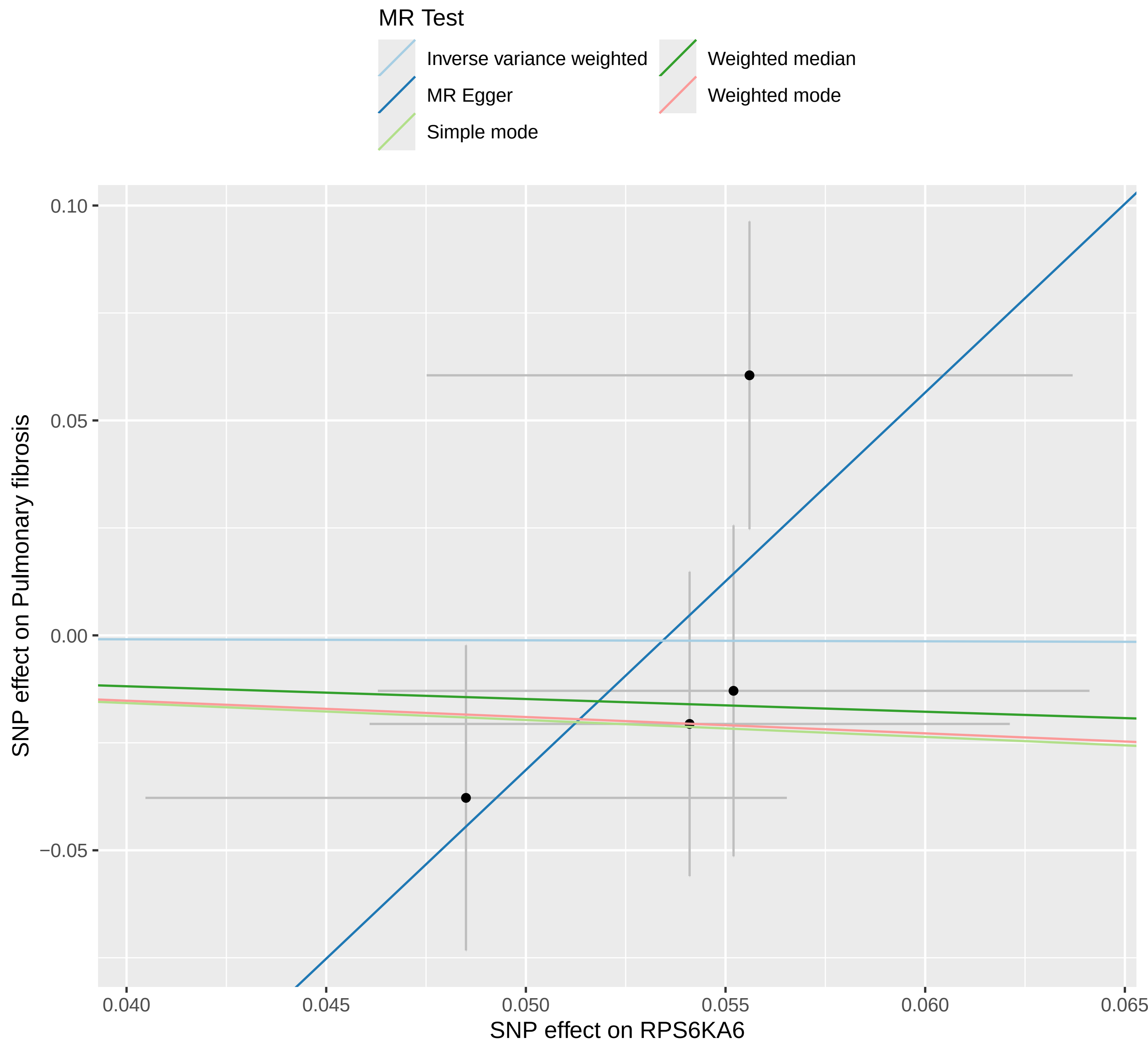
MR Test
Inverse variance weighted
MR Egger
Simple mode
Weighted median
Weighted mode
0.10
0.05
0.00
−0.05
SNP effect on Pulmonary fibrosis
0.040
0.045
0.050
0.055
0.060
0.065
SNP effect on RPS6KA6

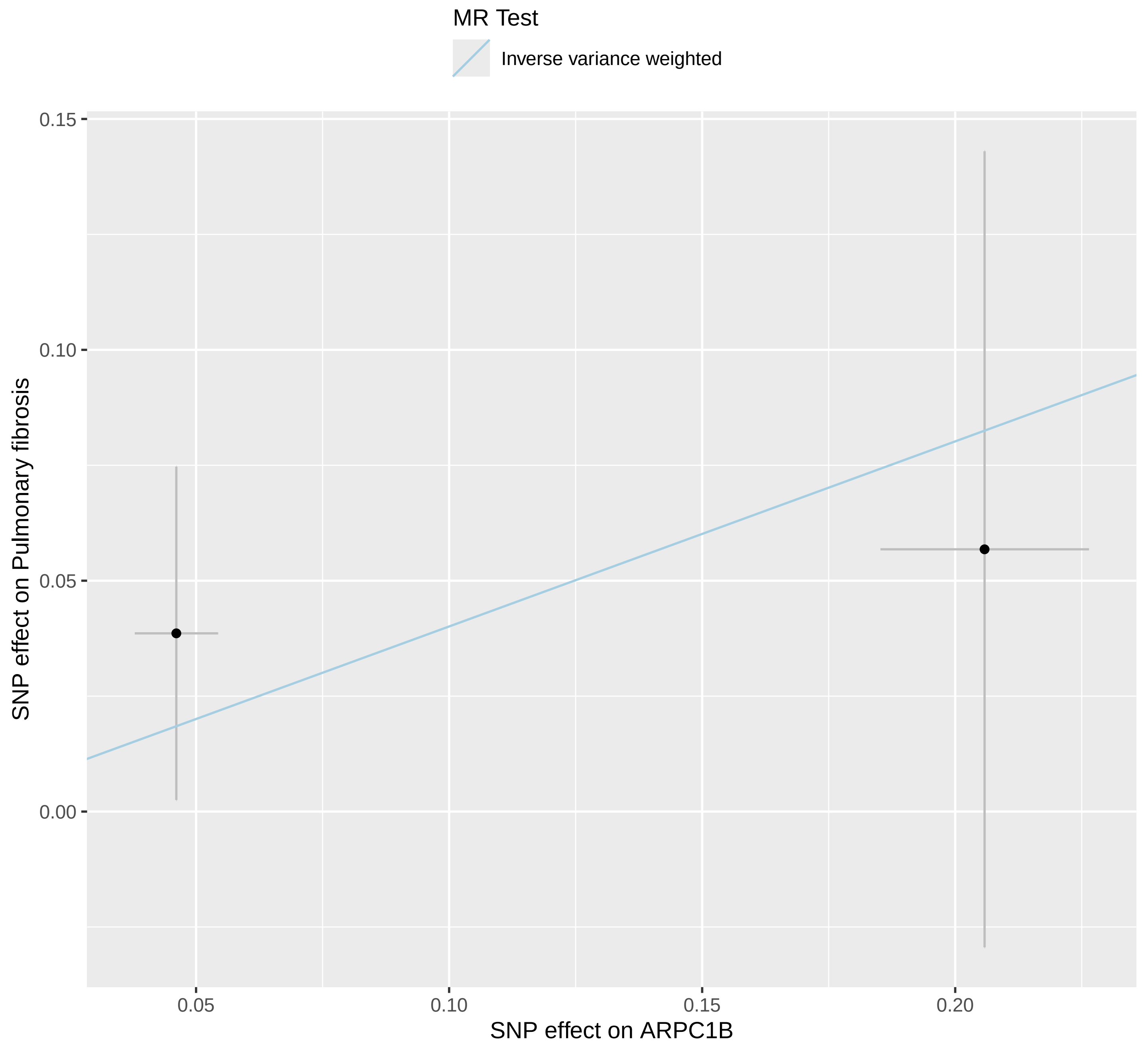
MR Test
Inverse variance weighted
0.15
0.10
0.05
0.00
SNP effect on Pulmonary fibrosis
0.05
0.10
0.15
0.20
SNP effect on ARPC1B

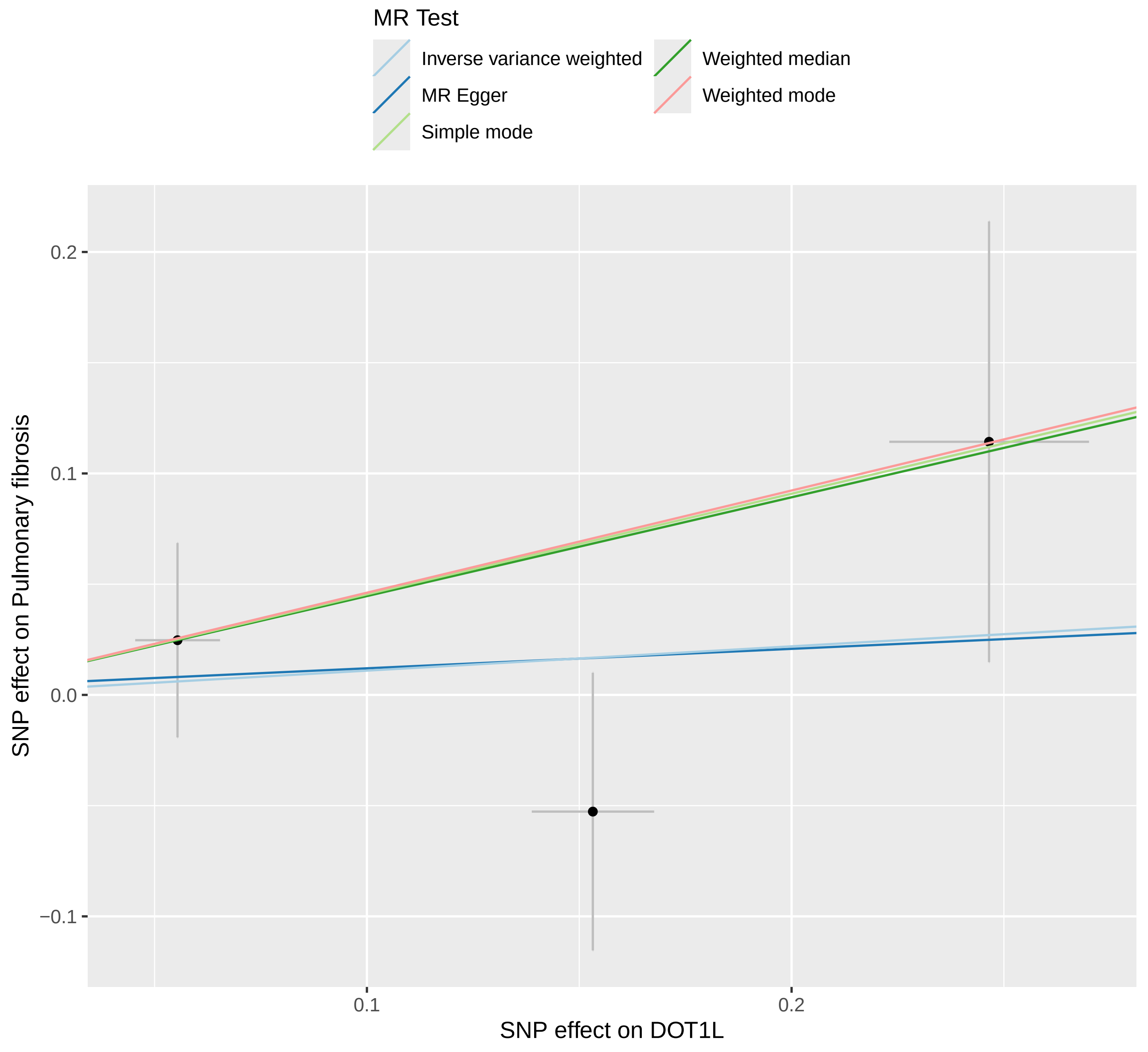
MR Test
Inverse variance weighted
MR Egger
Simple mode
Weighted median
Weighted mode
0.2
0.1
0.0
−0.1
0.1
0.2
SNP effect on Pulmonary fibrosis
SNP effect on DOT1L

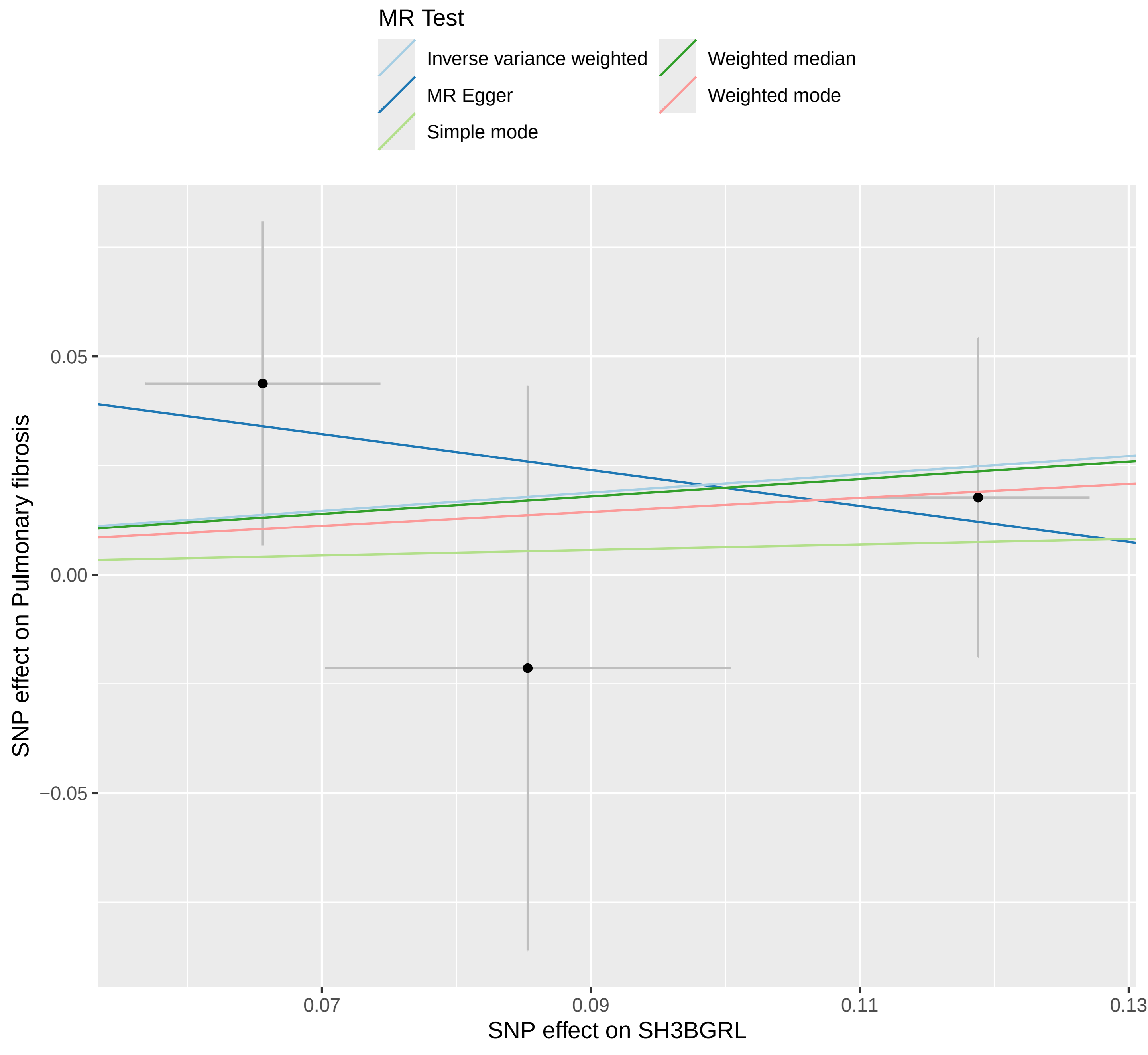

MR Test
Inverse variance weighted
MR Egger
Simple mode
Weighted median
Weighted mode
SNP effect on Pulmonary fibrosis
0.05
0.00
−0.05
0.07
0.09
0.11
0.13
SNP effect on SH3BGRL

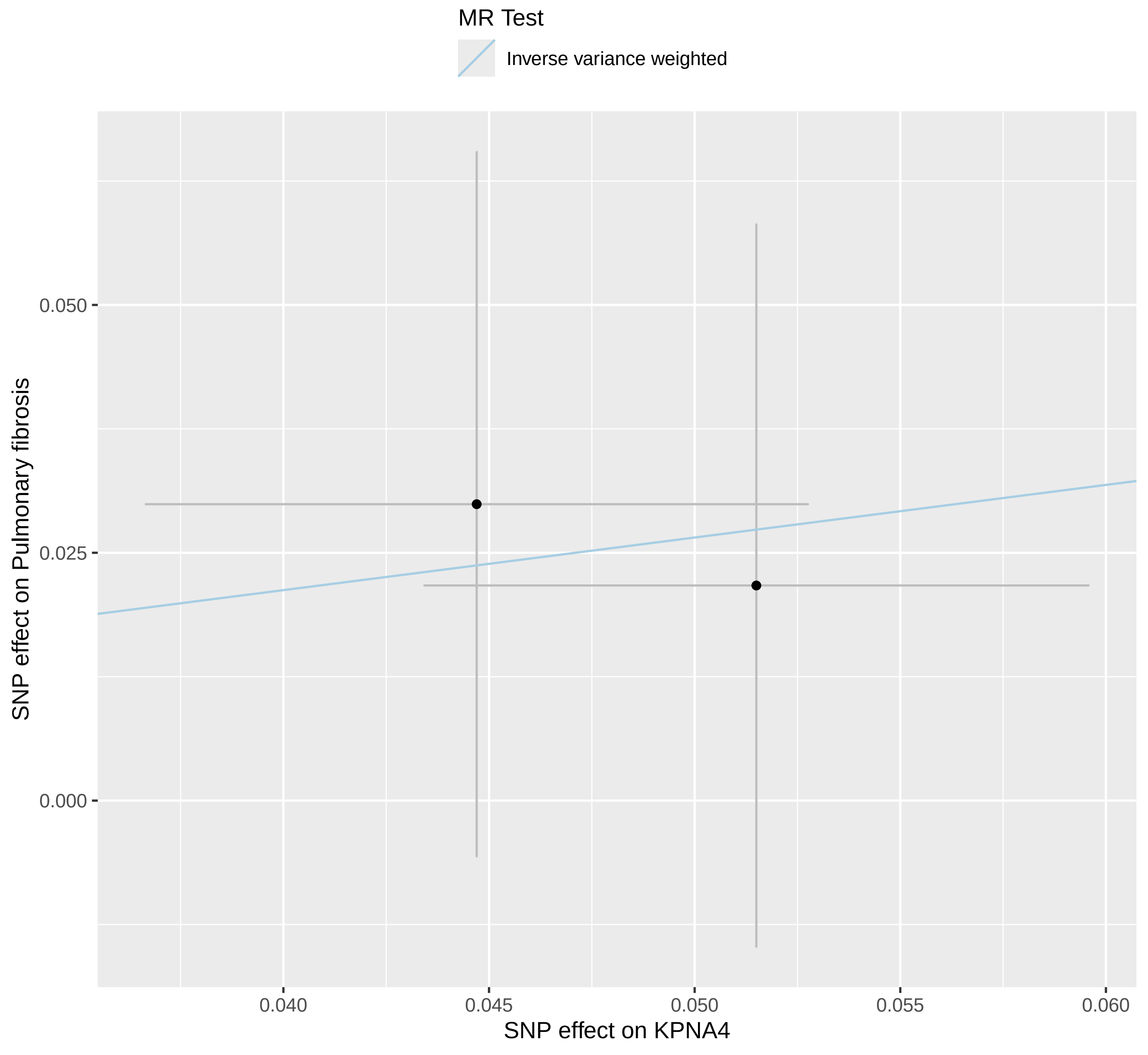

MR Test
Inverse variance weighted
0.050
0.025
0.000
SNP effect on Pulmonary fibrosis
0.040
0.045
0.050
0.055
0.060
SNP effect on KPNA4

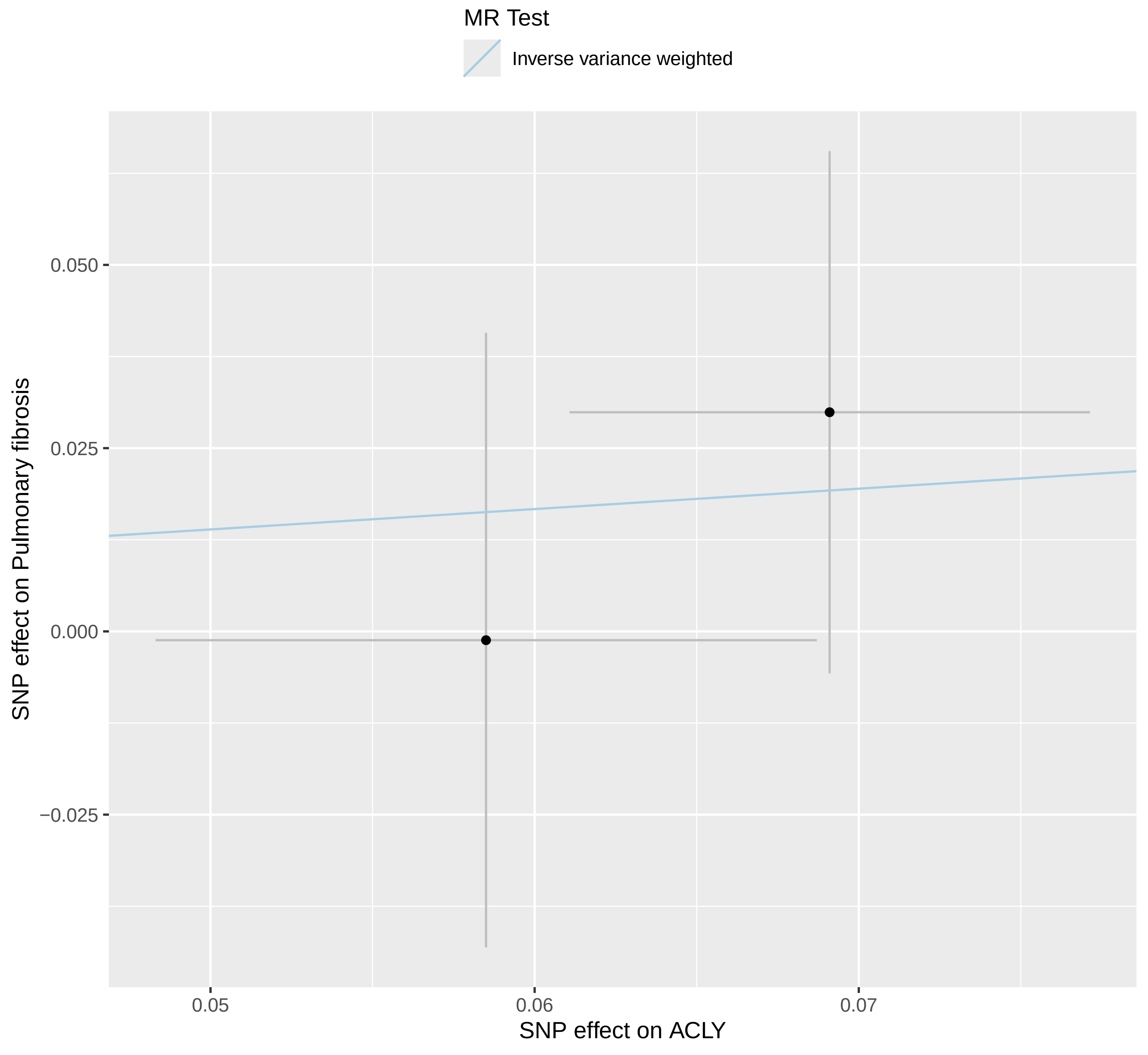
MR Test
Inverse variance weighted
SNP effect on Pulmonary fibrosis
0.050
0.025
0.000
−0.025
0.05
0.06
0.07
SNP effect on ACLY

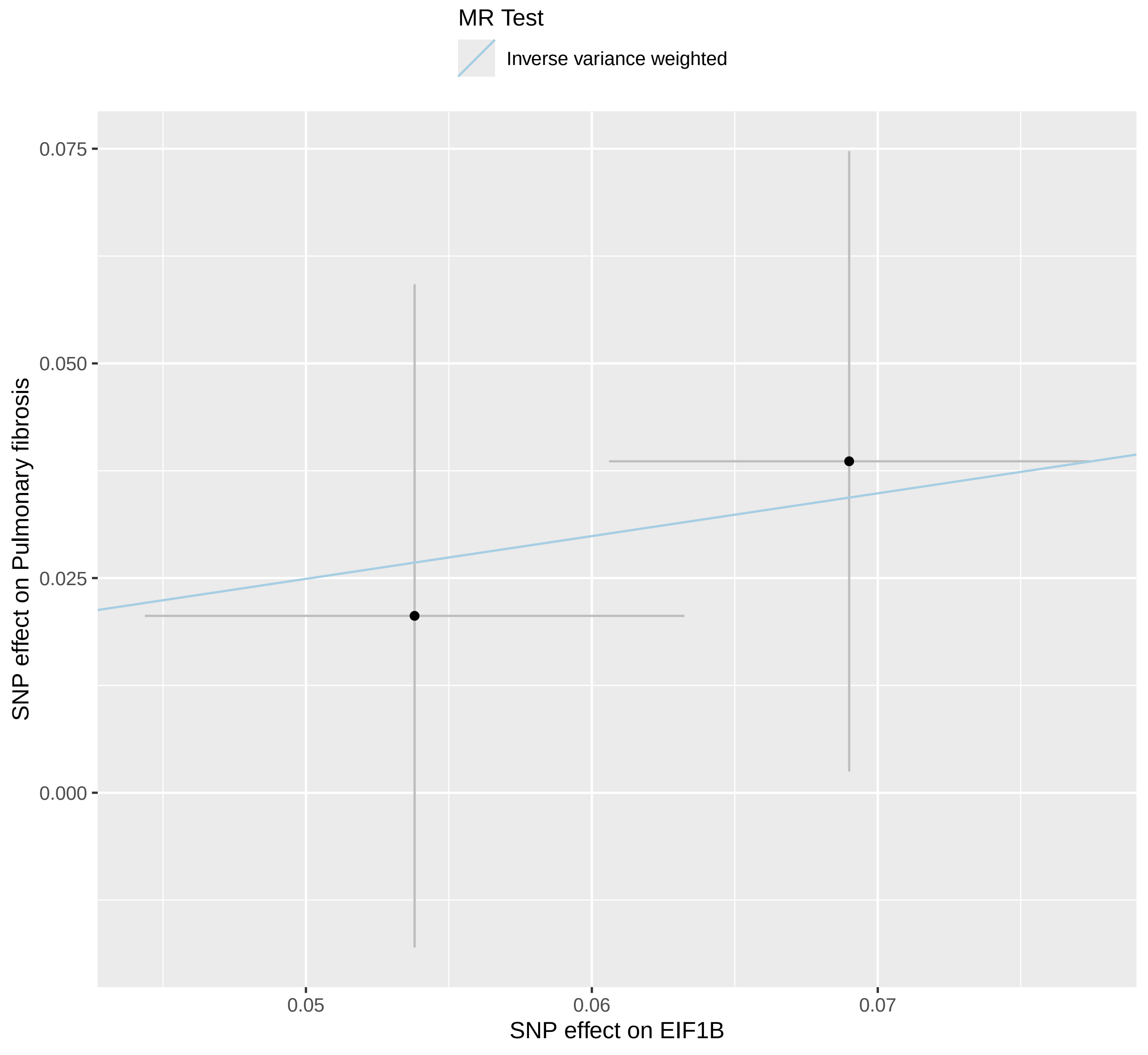
MR Test
Inverse variance weighted
0.075
0.050
0.025
0.000
0.05
0.06
0.07
SNP effect on Pulmonary fibrosis
SNP effect on EIF1B

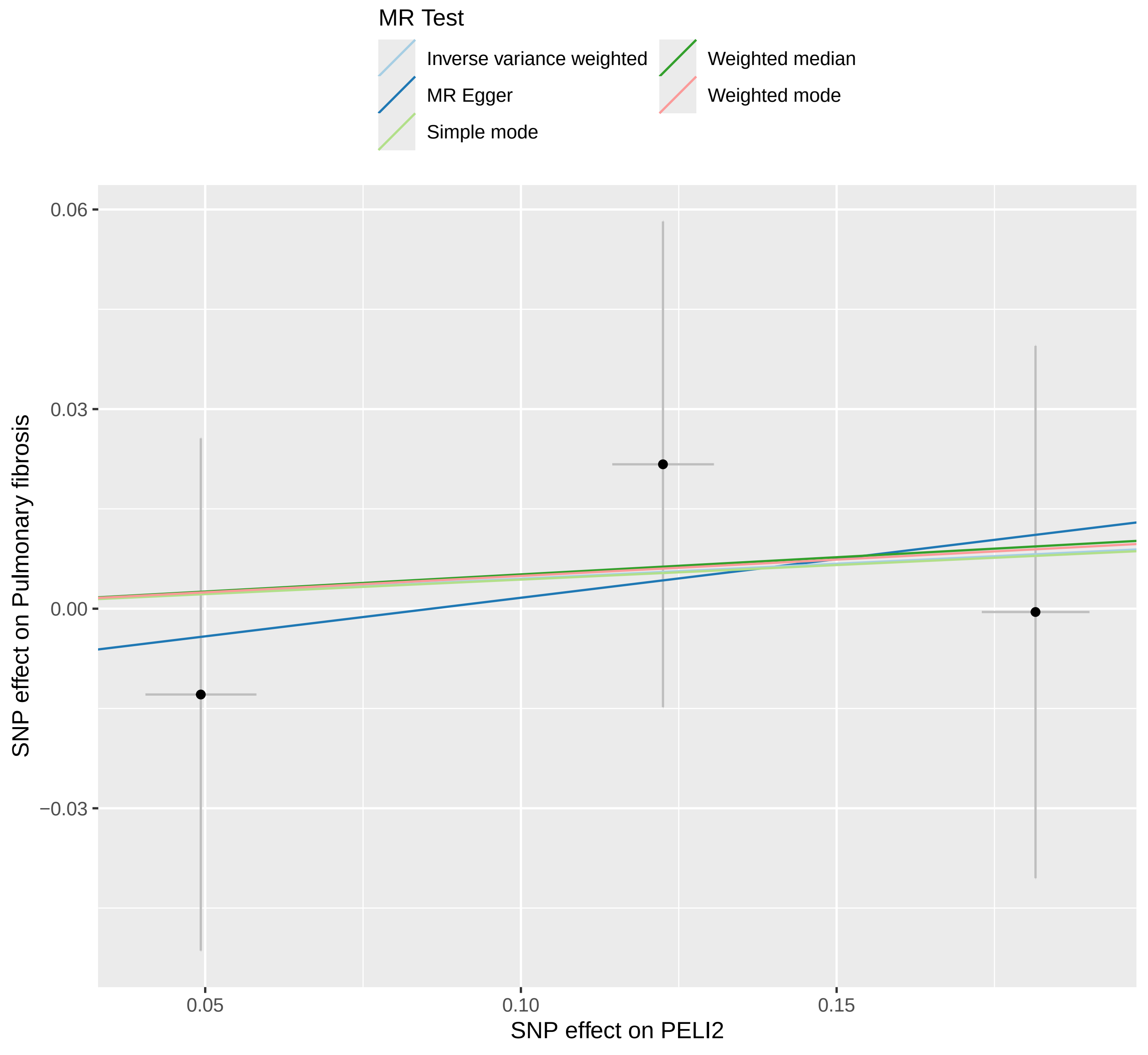
MR Test
Inverse variance weighted
MR Egger
Simple mode
Weighted median
Weighted mode
0.06
0.03
0.00
−0.03
SNP effect on Pulmonary fibrosis
0.05
0.10
0.15
SNP effect on PELI2

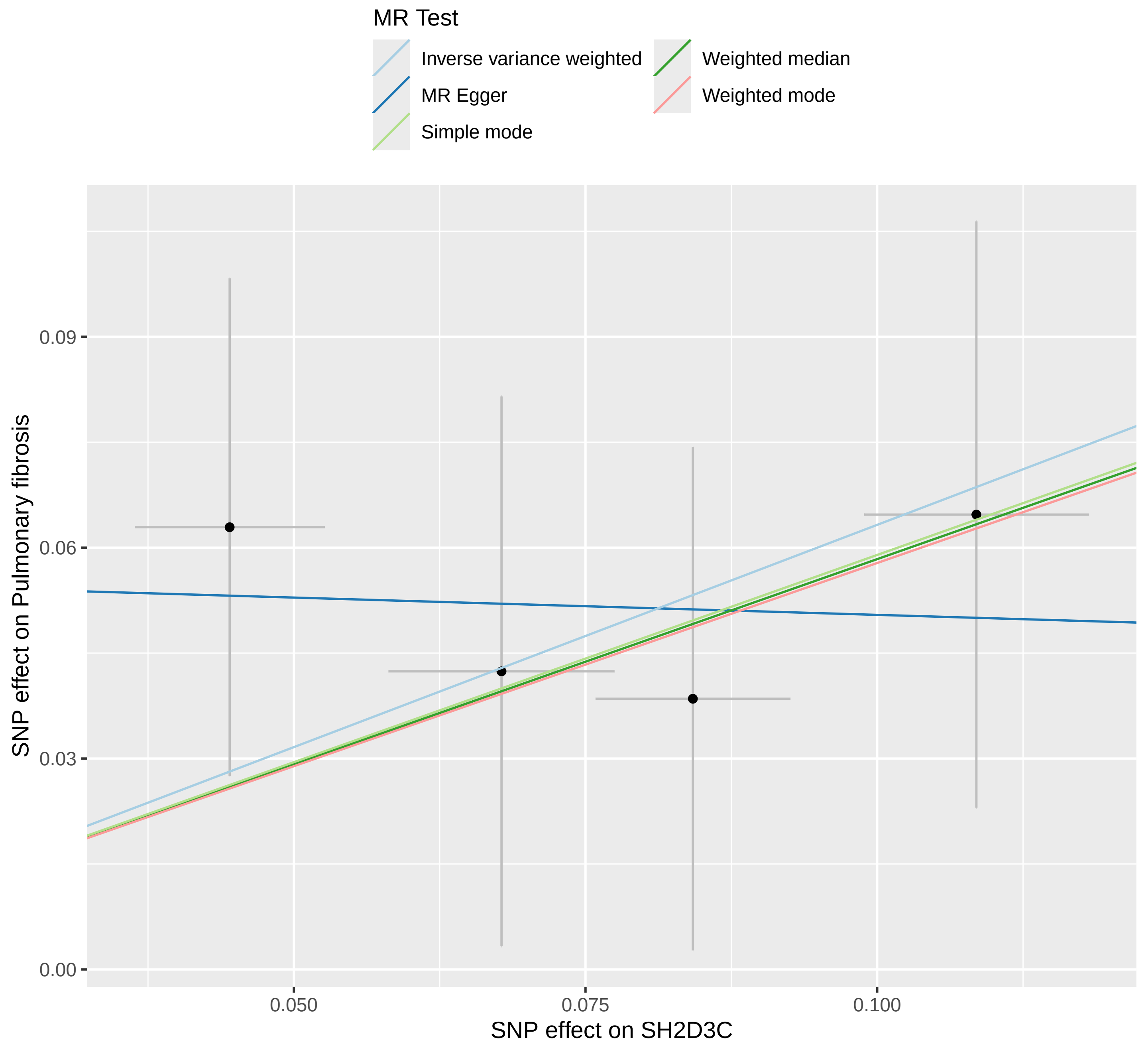

MR Test
Inverse variance weighted
MR Egger
Simple mode
Weighted median
Weighted mode
SNP effect on Pulmonary fibrosis
0.09
0.06
0.03
0.00
0.050
0.075
0.100
SNP effect on SH2D3C

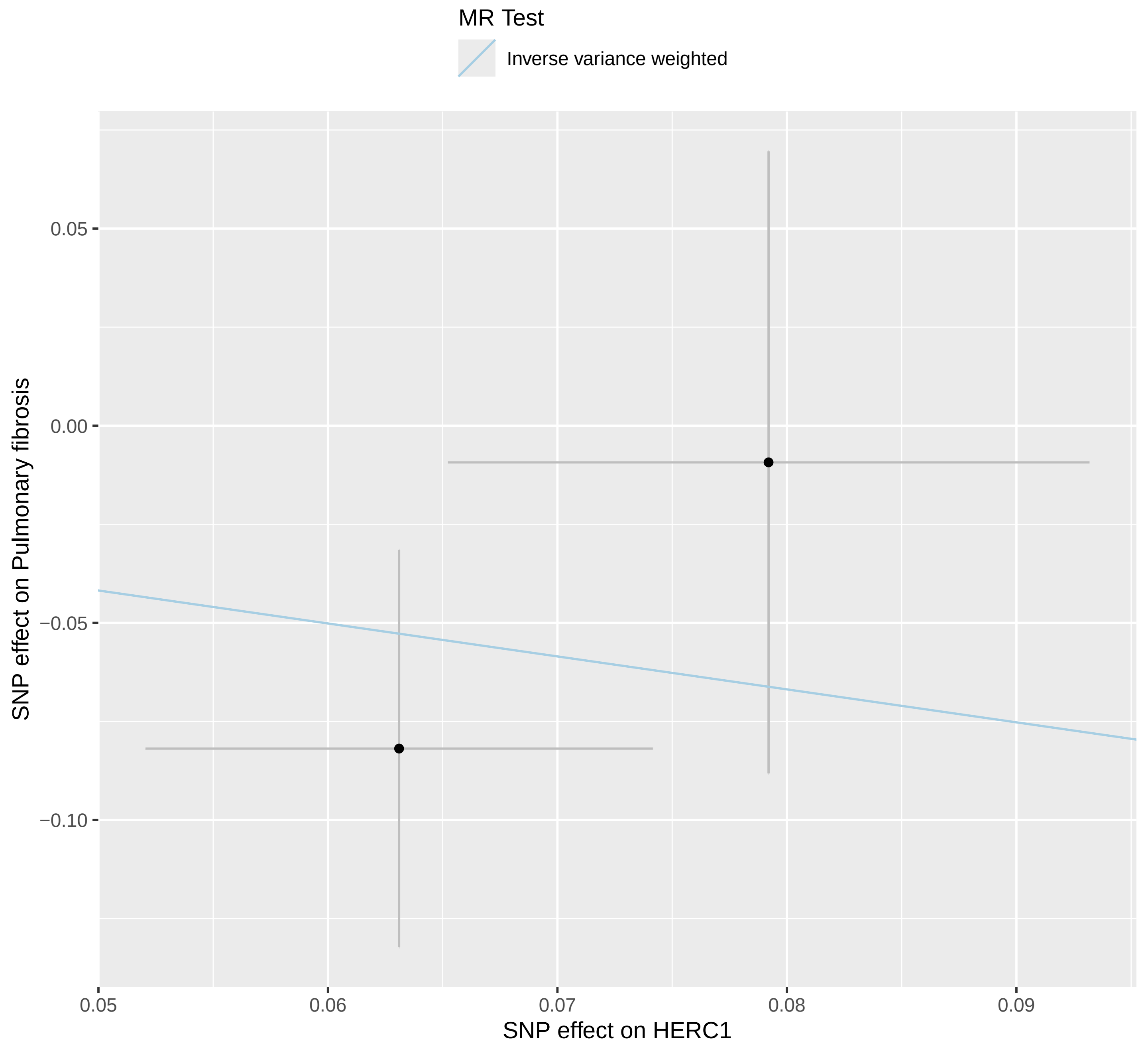

MR Test
Inverse variance weighted
0.05
0.00
−0.05
−0.10
SNP effect on Pulmonary fibrosis
0.05
0.06
0.07
0.08
0.09
SNP effect on HERC1

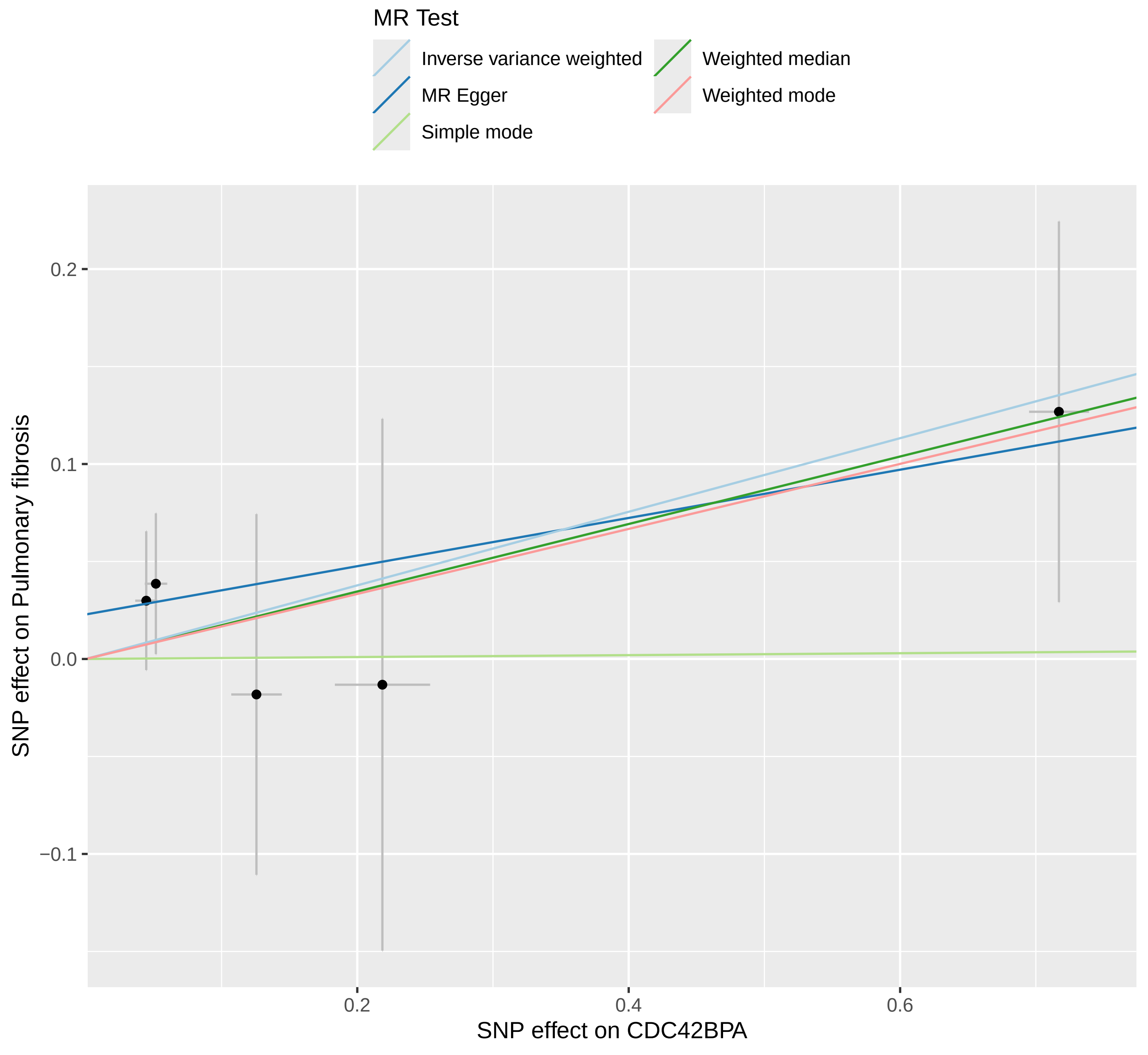
MR Test
Inverse variance weighted
MR Egger
Simple mode
Weighted median
Weighted mode
0.2
0.1
0.0
−0.1
0.2
0.4
0.6
SNP effect on Pulmonary fibrosis
SNP effect on CDC42BPA

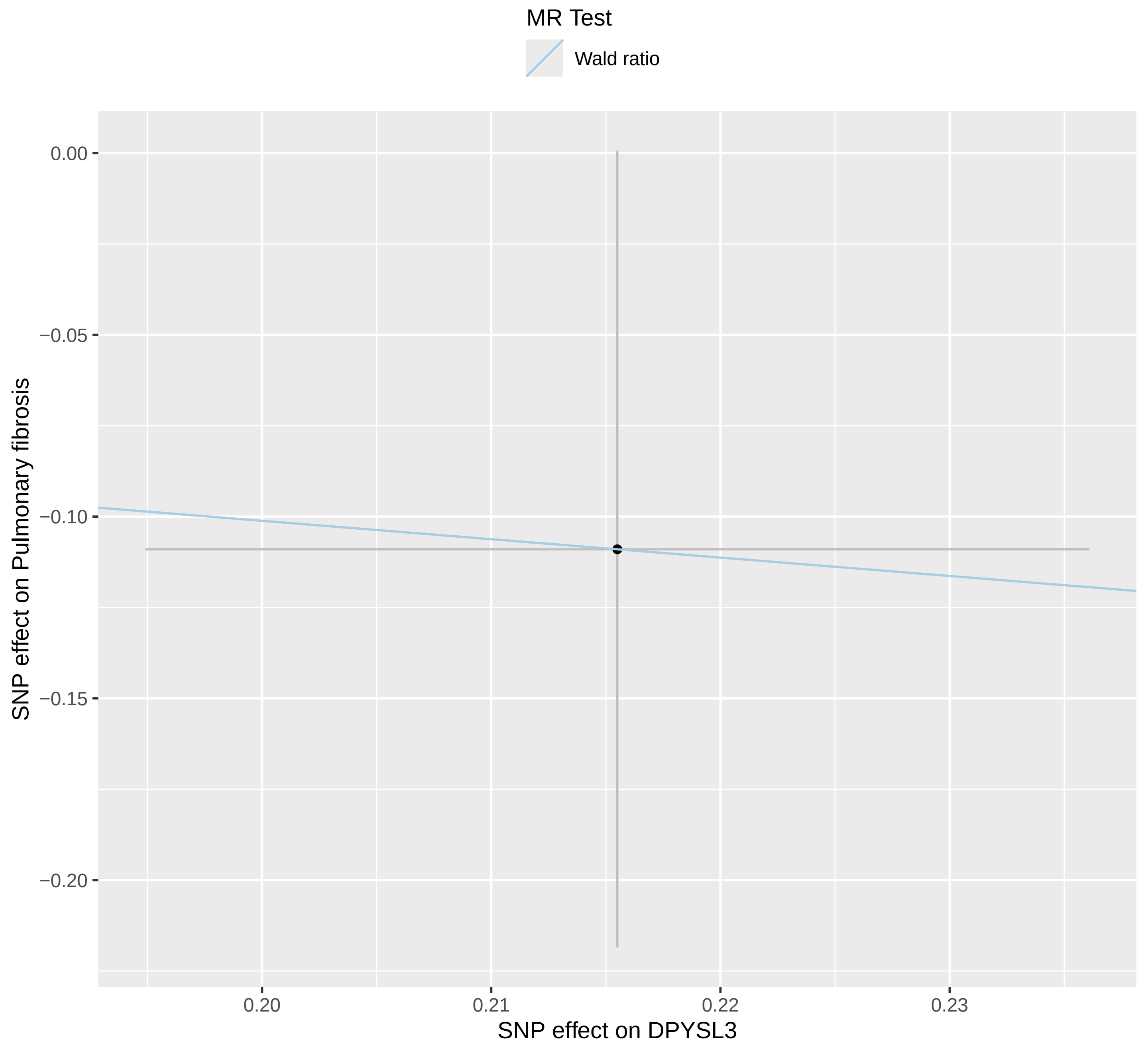
MR Test
Wald ratio
0.00
−0.05
−0.10
−0.15
−0.20
SNP effect on Pulmonary fibrosis
0.20
0.21
0.22
0.23
SNP effect on DPYSL3

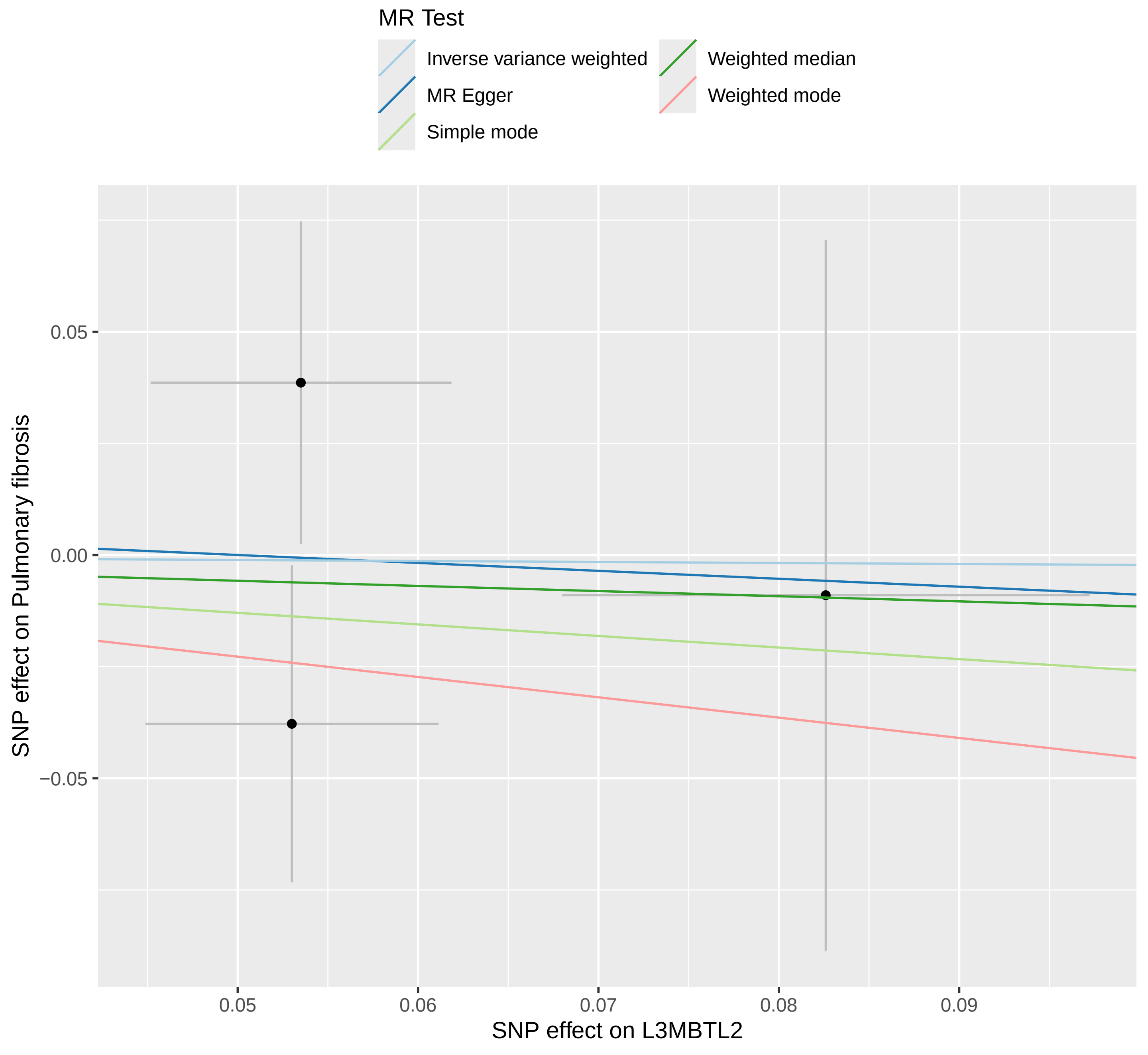
MR Test
Inverse variance weighted
MR Egger
Simple mode
Weighted median
Weighted mode
0.05
0.00
−0.05
0.05
0.06
0.07
0.08
0.09
SNP effect on Pulmonary fibrosis
SNP effect on L3MBTL2

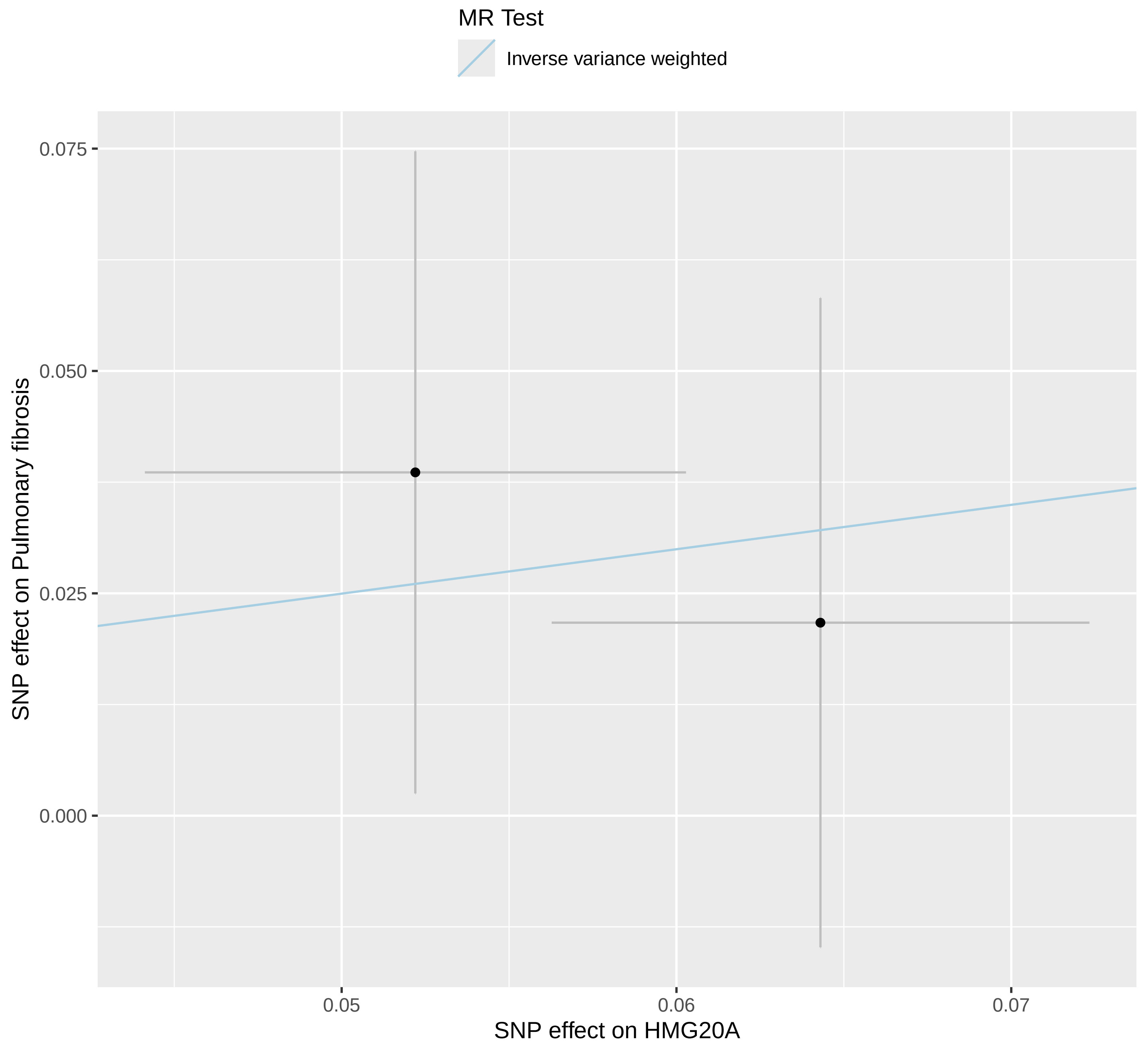
MR Test
Inverse variance weighted
0.075
0.050
0.025
0.000
SNP effect on Pulmonary fibrosis
0.05
0.06
0.07
SNP effect on HMG20A

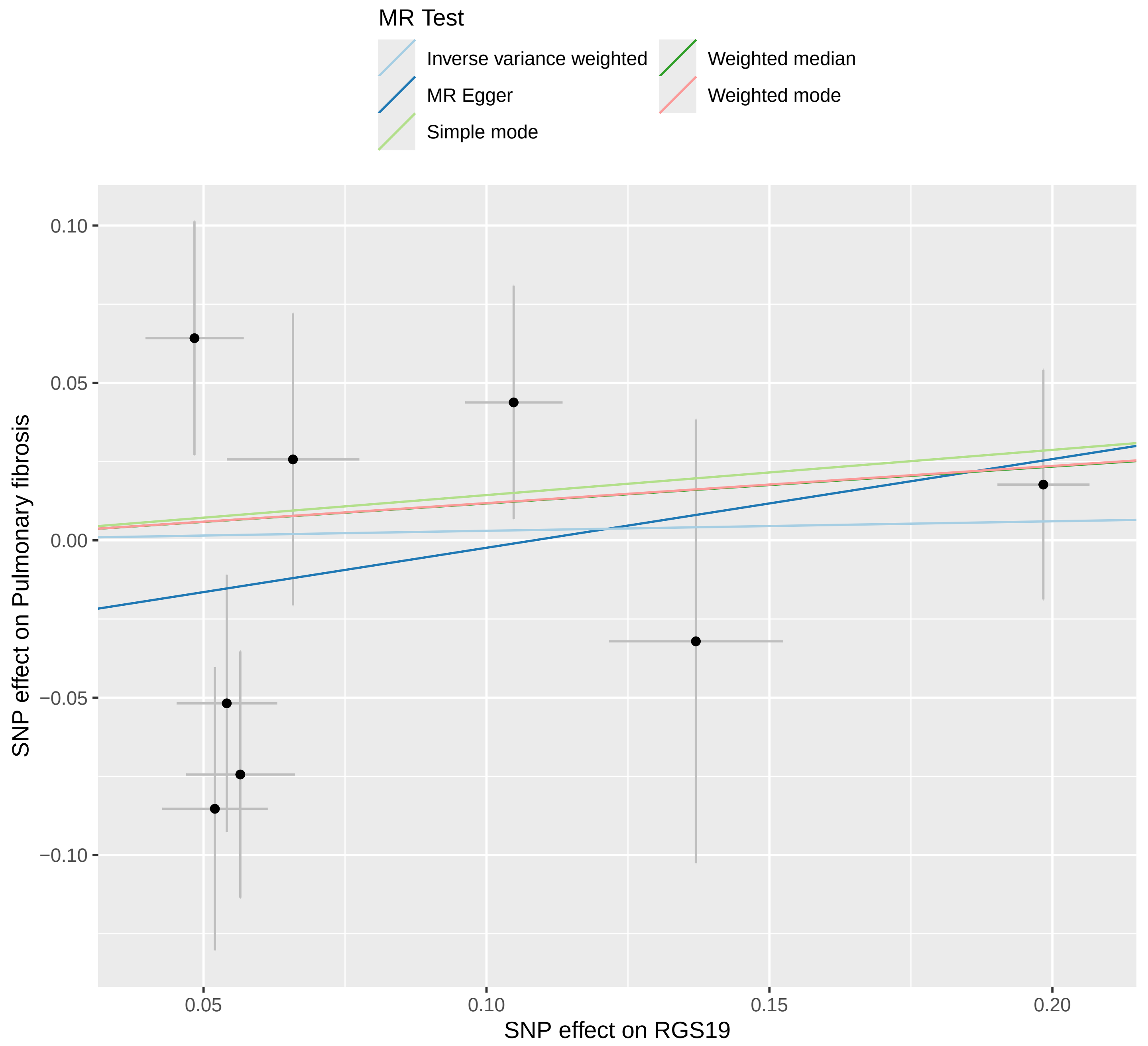
MR Test
Inverse variance weighted
MR Egger
Simple mode
Weighted median
Weighted mode
0.10
0.05
0.00
−0.05
−0.10
SNP effect on Pulmonary fibrosis
0.05
0.10
0.15
0.20
SNP effect on RGS19

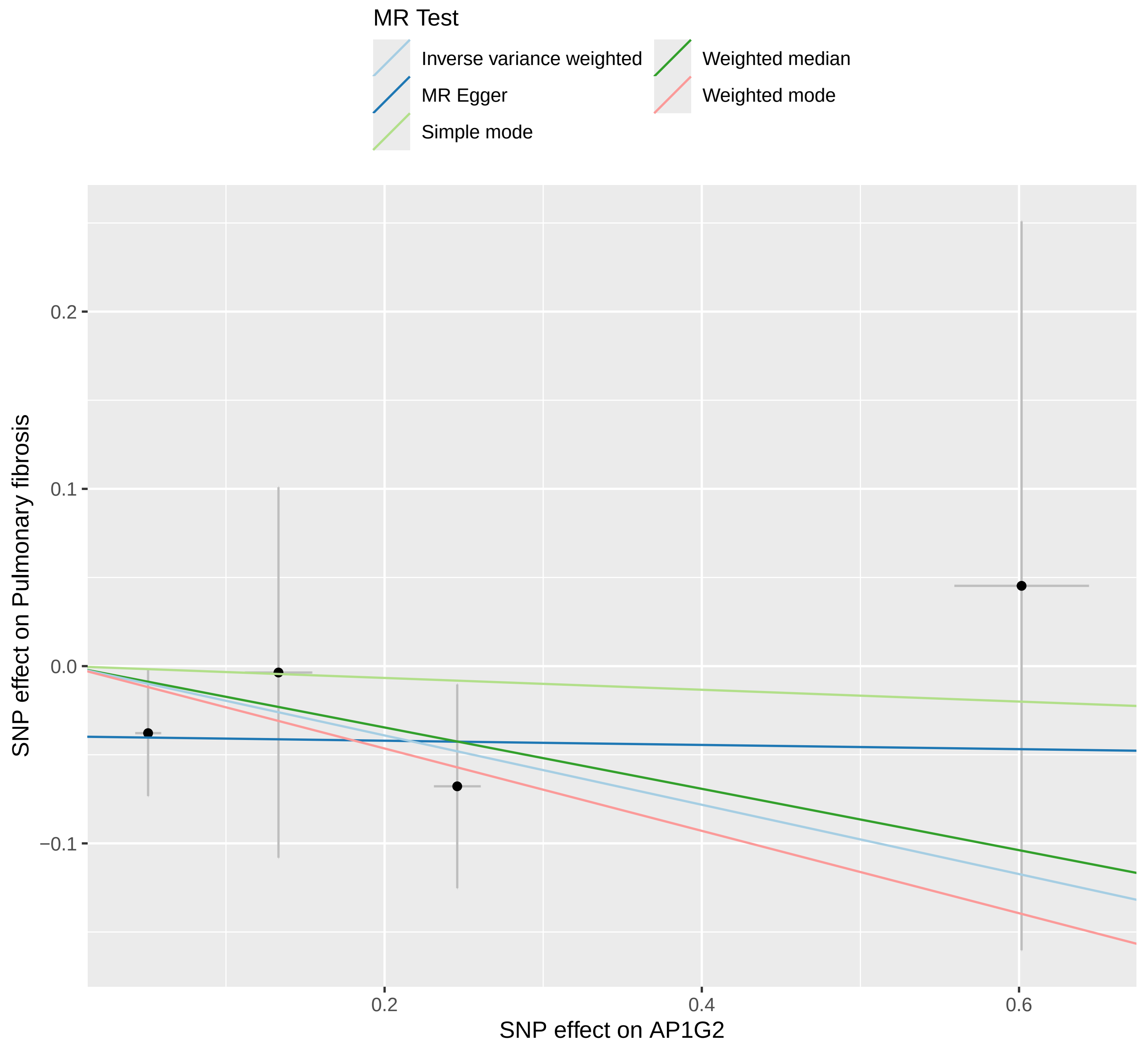
MR Test
Inverse variance weighted
MR Egger
Simple mode
Weighted median
Weighted mode
0.2
0.1
0.0
−0.1
0.2
0.4
0.6
SNP effect on Pulmonary fibrosis
SNP effect on AP1G2

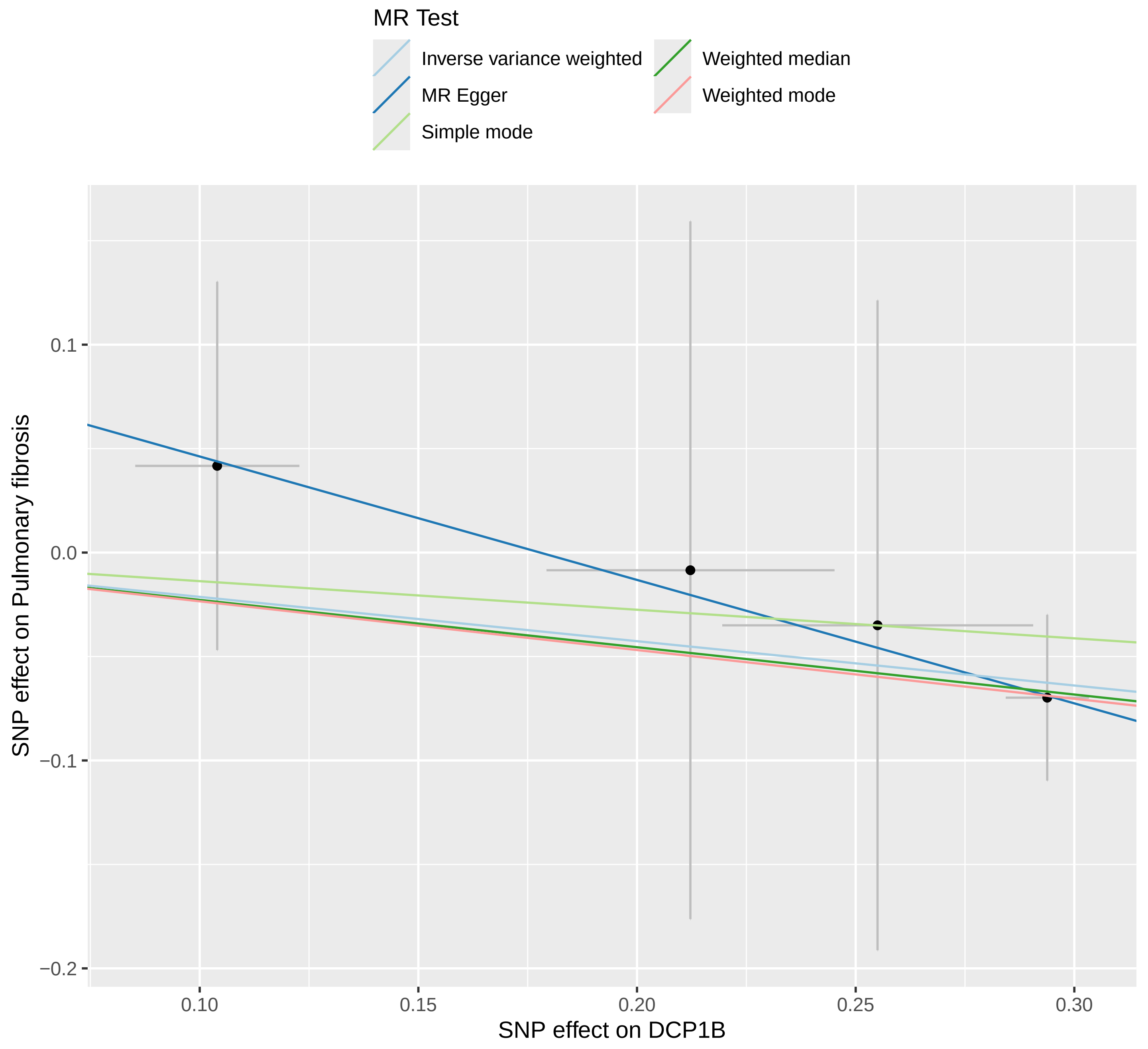
MR Test
Inverse variance weighted
MR Egger
Simple mode
Weighted median
Weighted mode
0.1
0.0
−0.1
−0.2
SNP effect on Pulmonary fibrosis
0.10
0.15
0.20
0.25
0.30
SNP effect on DCP1B

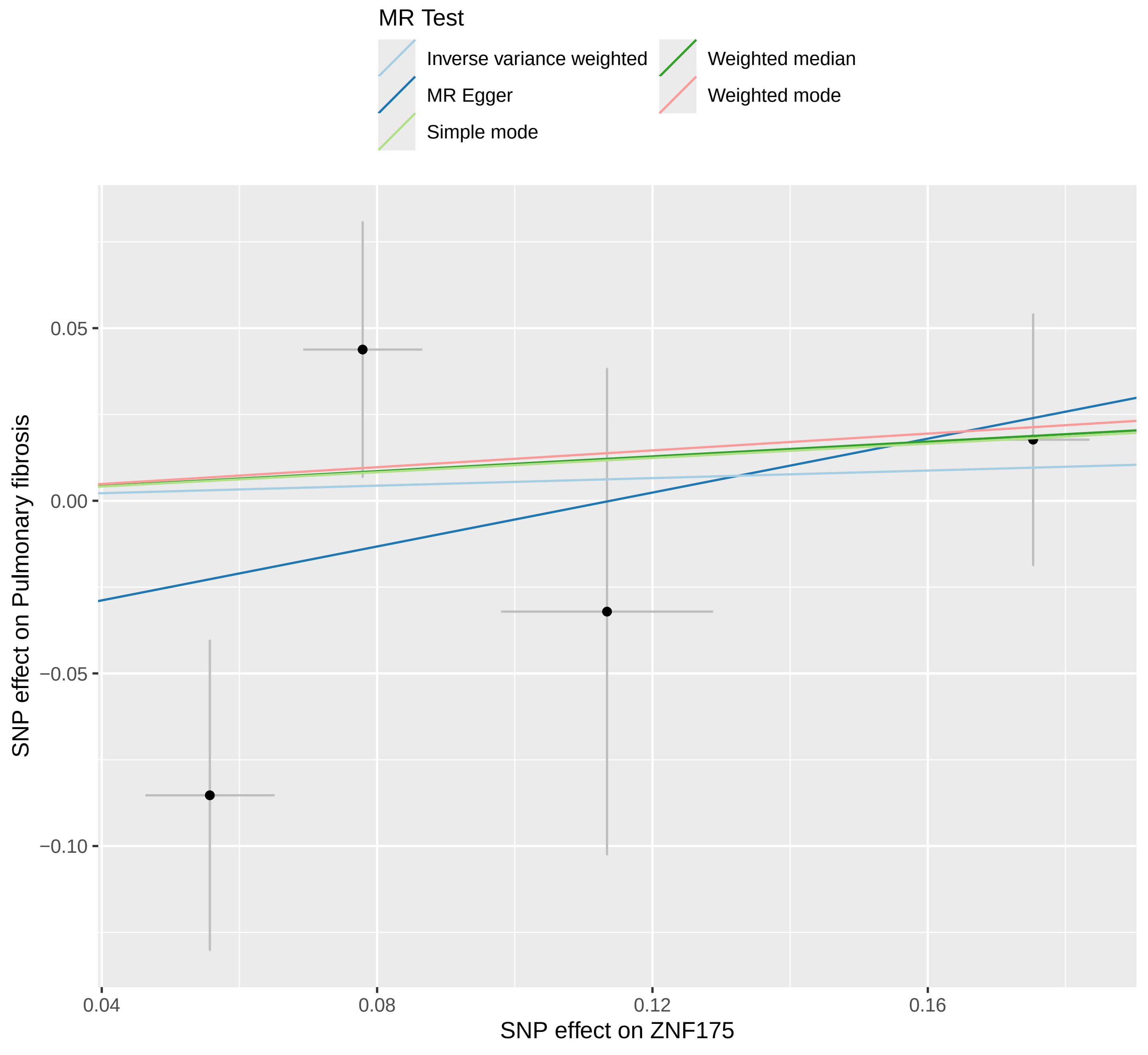
MR Test
Inverse variance weighted
MR Egger
Simple mode
Weighted median
Weighted mode
SNP effect on Pulmonary fibrosis
0.05
0.00
−0.05
−0.10
0.04
0.08
0.12
0.16
SNP effect on ZNF175

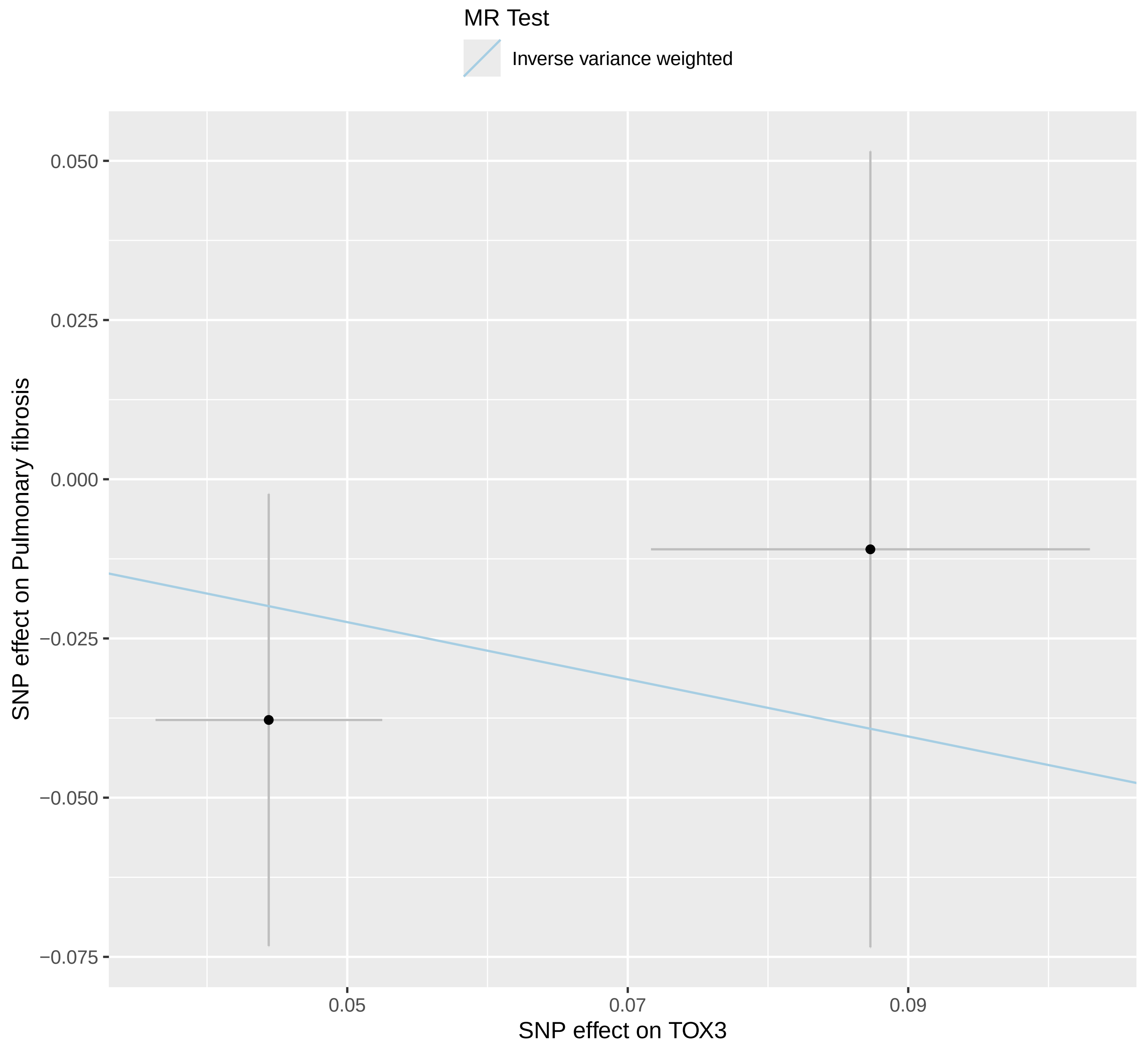

MR Test
Inverse variance weighted
SNP effect on Pulmonary fibrosis
0.050
0.025
0.000
−0.025
−0.050
−0.075
0.05
0.07
0.09
SNP effect on TOX3

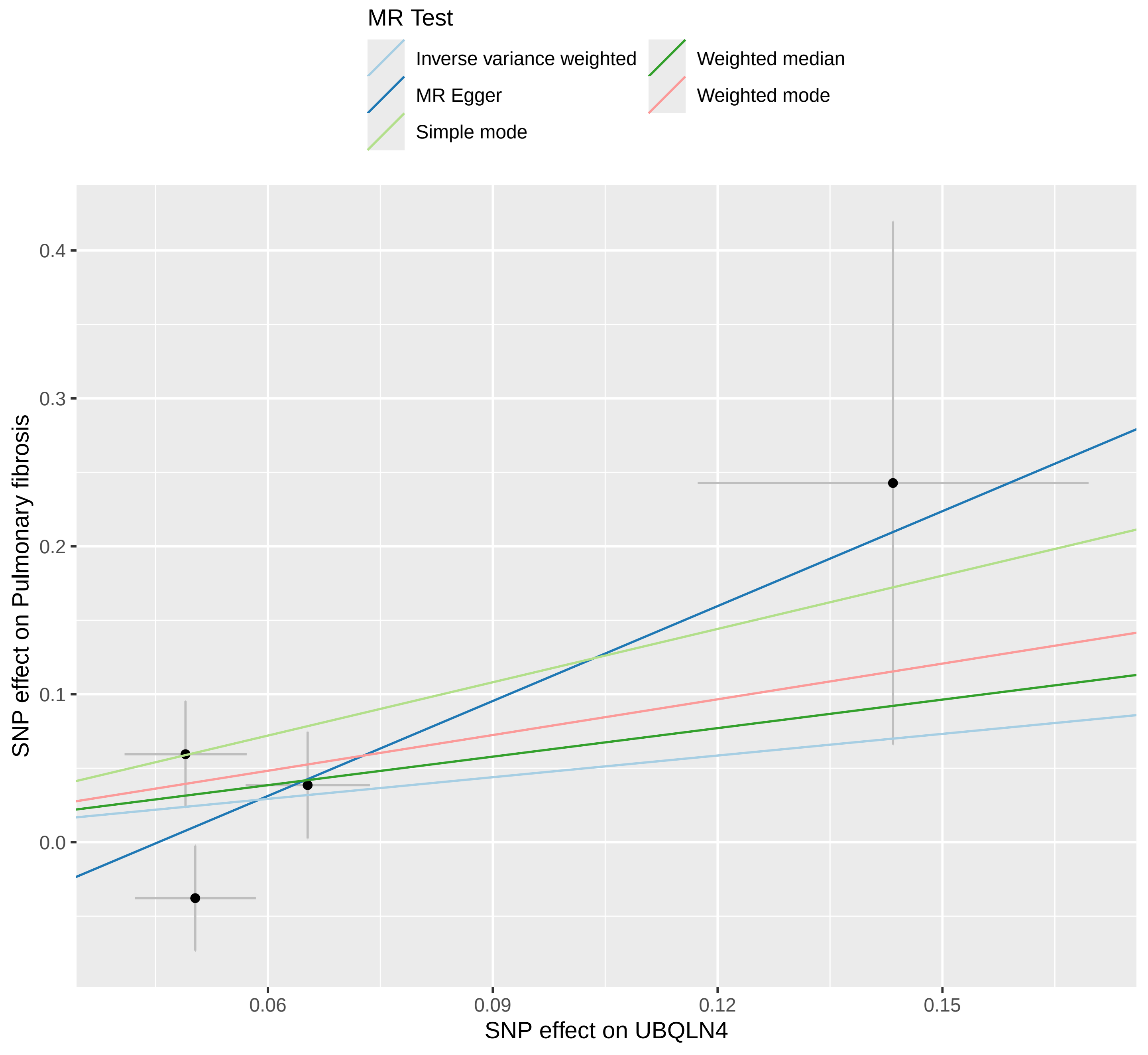

MR Test
Inverse variance weighted
MR Egger
Simple mode
Weighted median
Weighted mode
0.4
0.3
0.2
0.1
0.0
SNP effect on Pulmonary fibrosis
0.06
0.09
0.12
0.15
SNP effect on UBQLN4

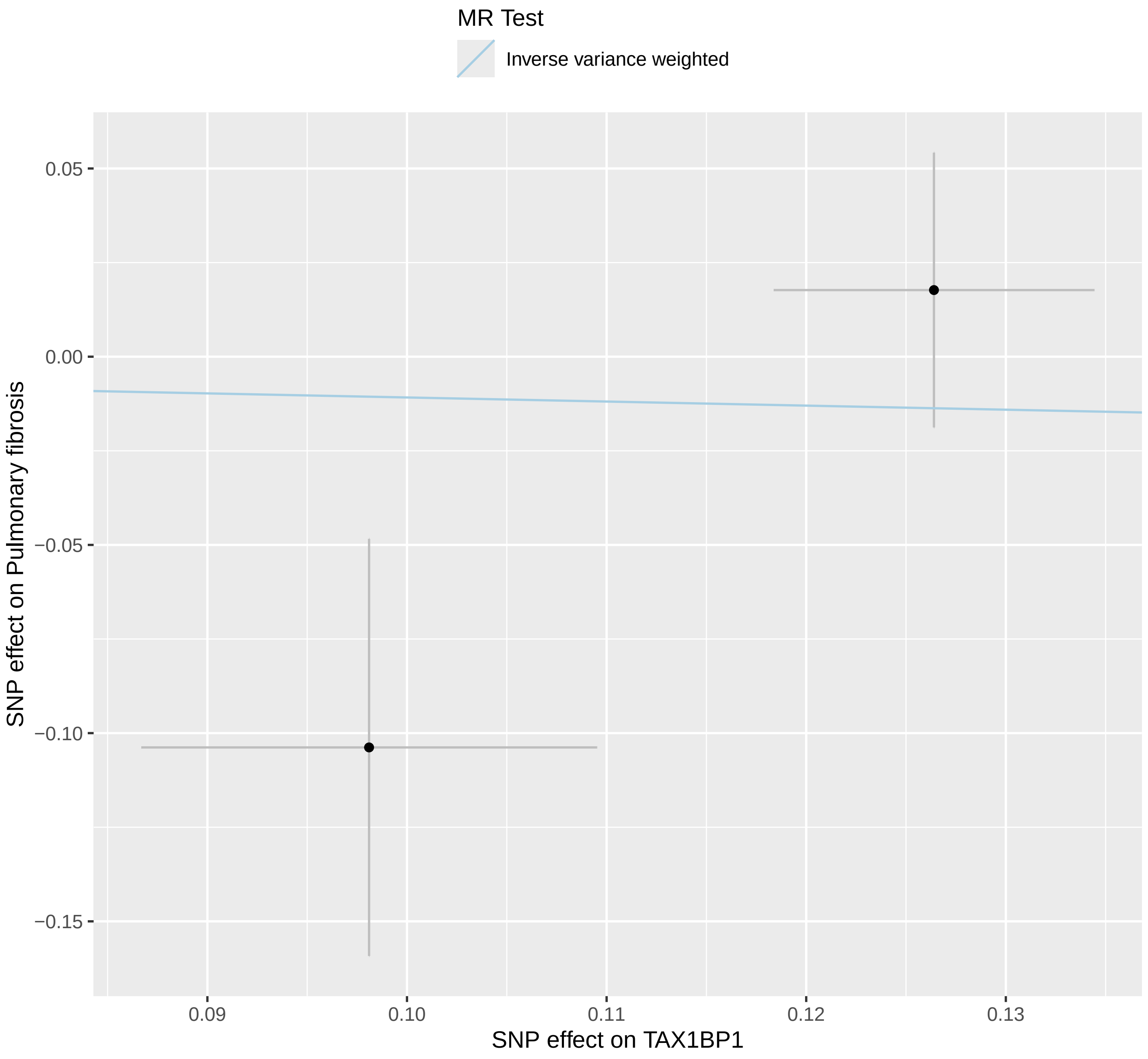
MR Test
Inverse variance weighted
0.05
0.00
−0.05
−0.10
−0.15
SNP effect on Pulmonary fibrosis
0.09
0.10
0.11
0.12
0.13
SNP effect on TAX1BP1

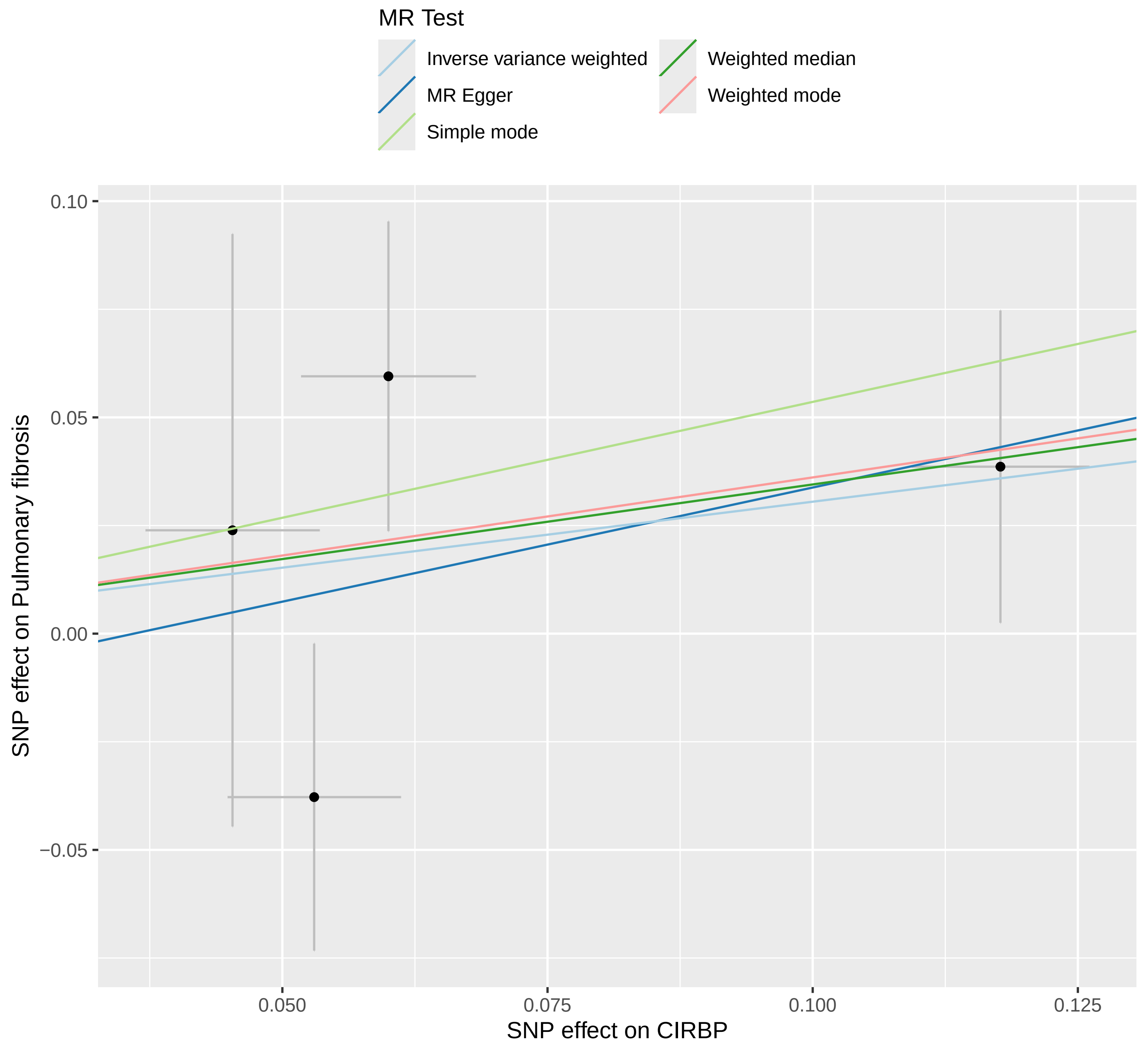
MR Test
Inverse variance weighted
MR Egger
Simple mode
Weighted median
Weighted mode
0.10
0.05
0.00
−0.05
SNP effect on Pulmonary fibrosis
0.050
0.075
0.100
0.125
SNP effect on CIRBP

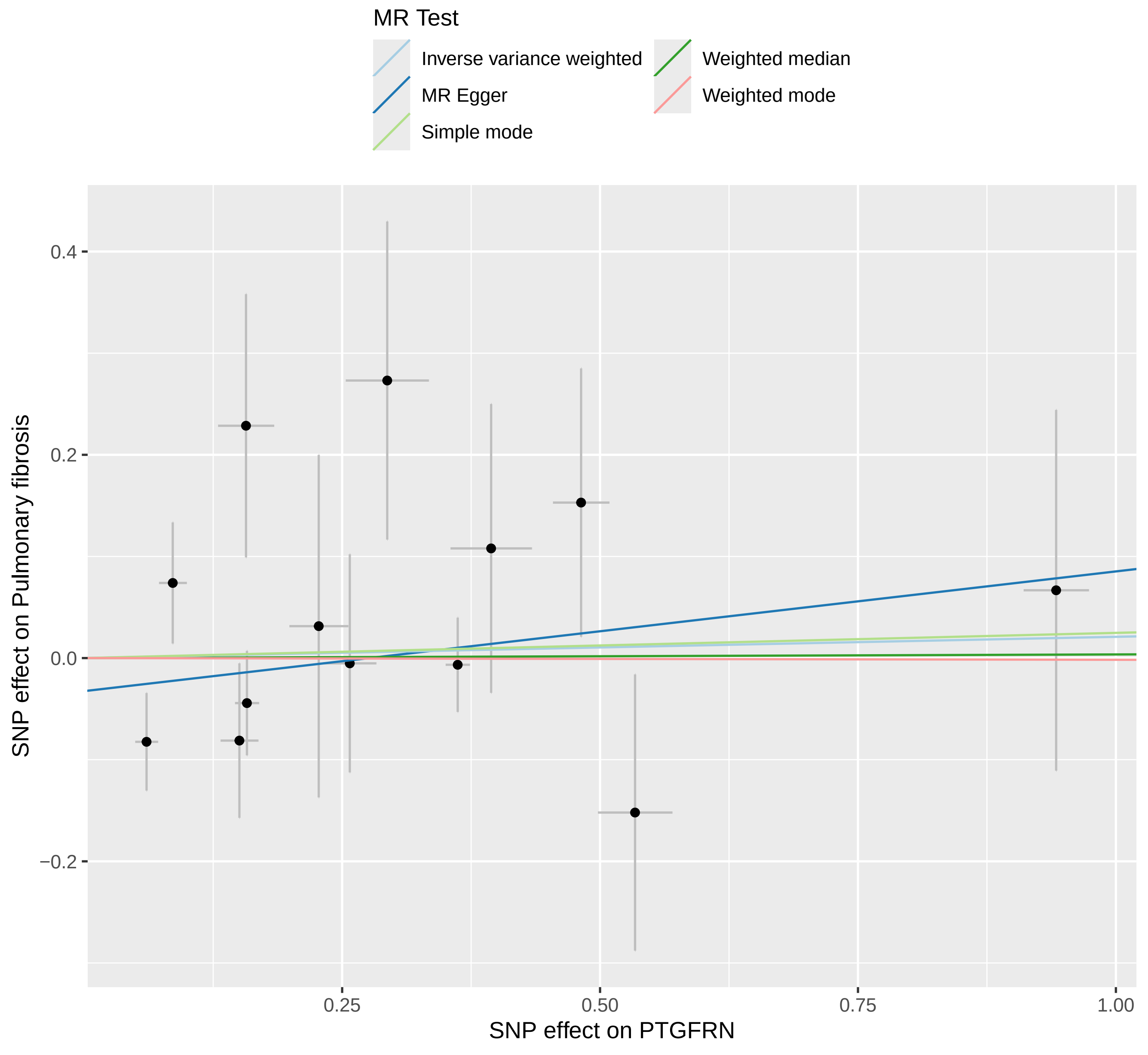
MR Test
Inverse variance weighted
MR Egger
Simple mode
Weighted median
Weighted mode
0.4
0.2
0.0
−0.2
SNP effect on Pulmonary fibrosis
0.25
0.50
0.75
1.00
SNP effect on PTGFRN

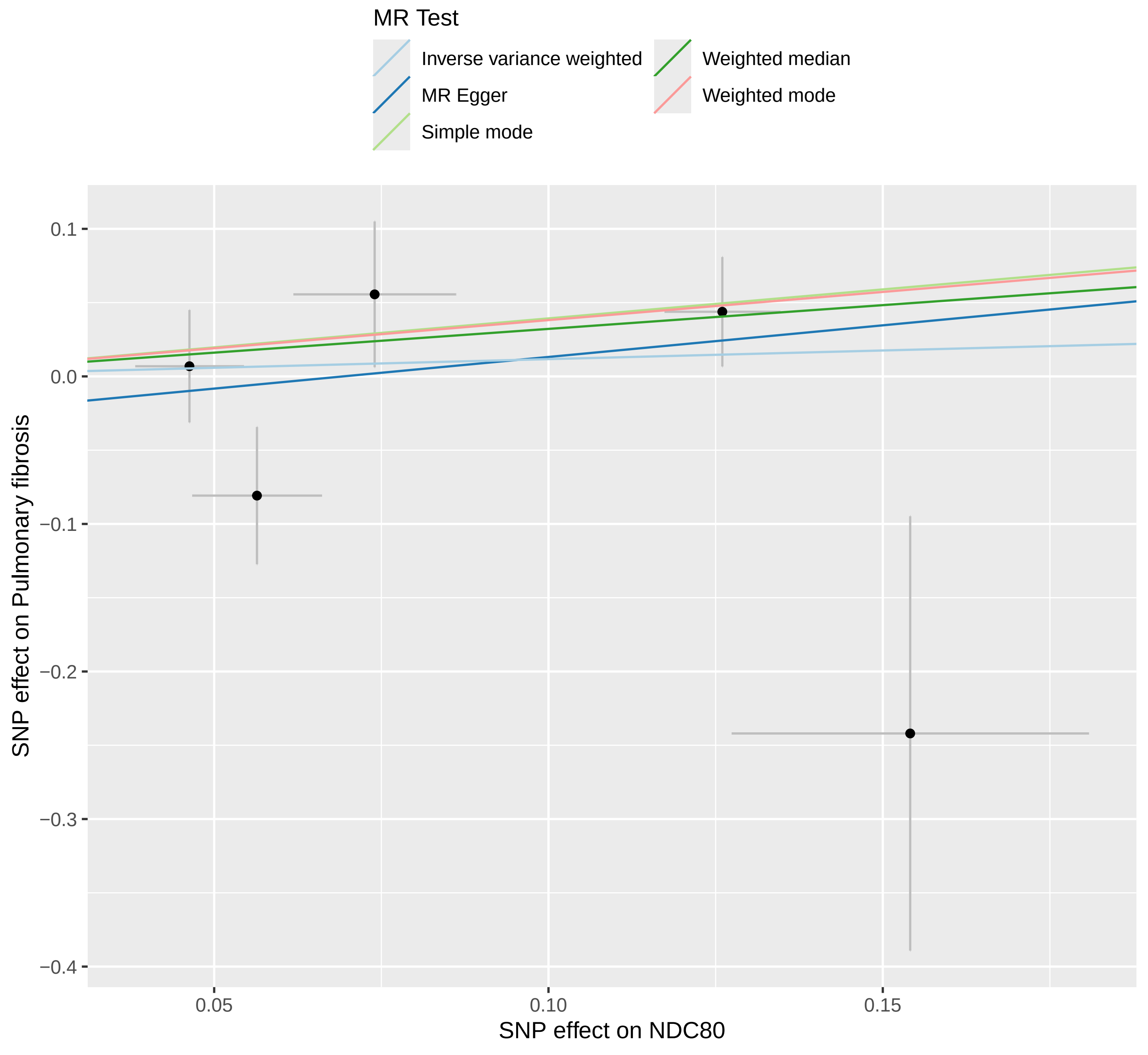
MR Test
Inverse variance weighted
MR Egger
Simple mode
Weighted median
Weighted mode
0.1
0.0
−0.1
−0.2
−0.3
−0.4
0.05
0.10
0.15
SNP effect on Pulmonary fibrosis
SNP effect on NDC80

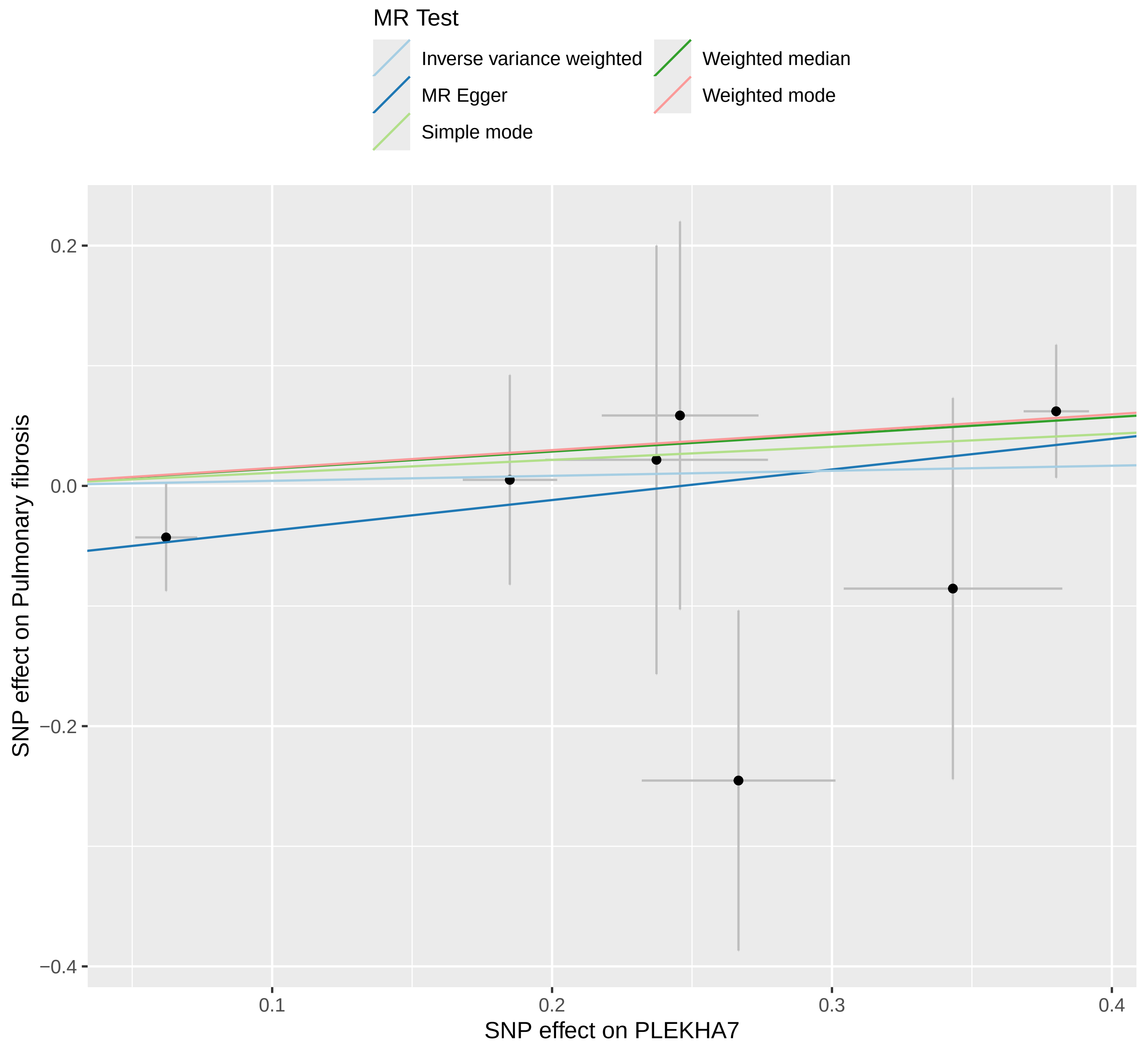
MR Test
Inverse variance weighted
MR Egger
Simple mode
Weighted median
Weighted mode
0.2
0.0
−0.2
−0.4
0.1
0.2
0.3
0.4
SNP effect on Pulmonary fibrosis
SNP effect on PLEKHA7

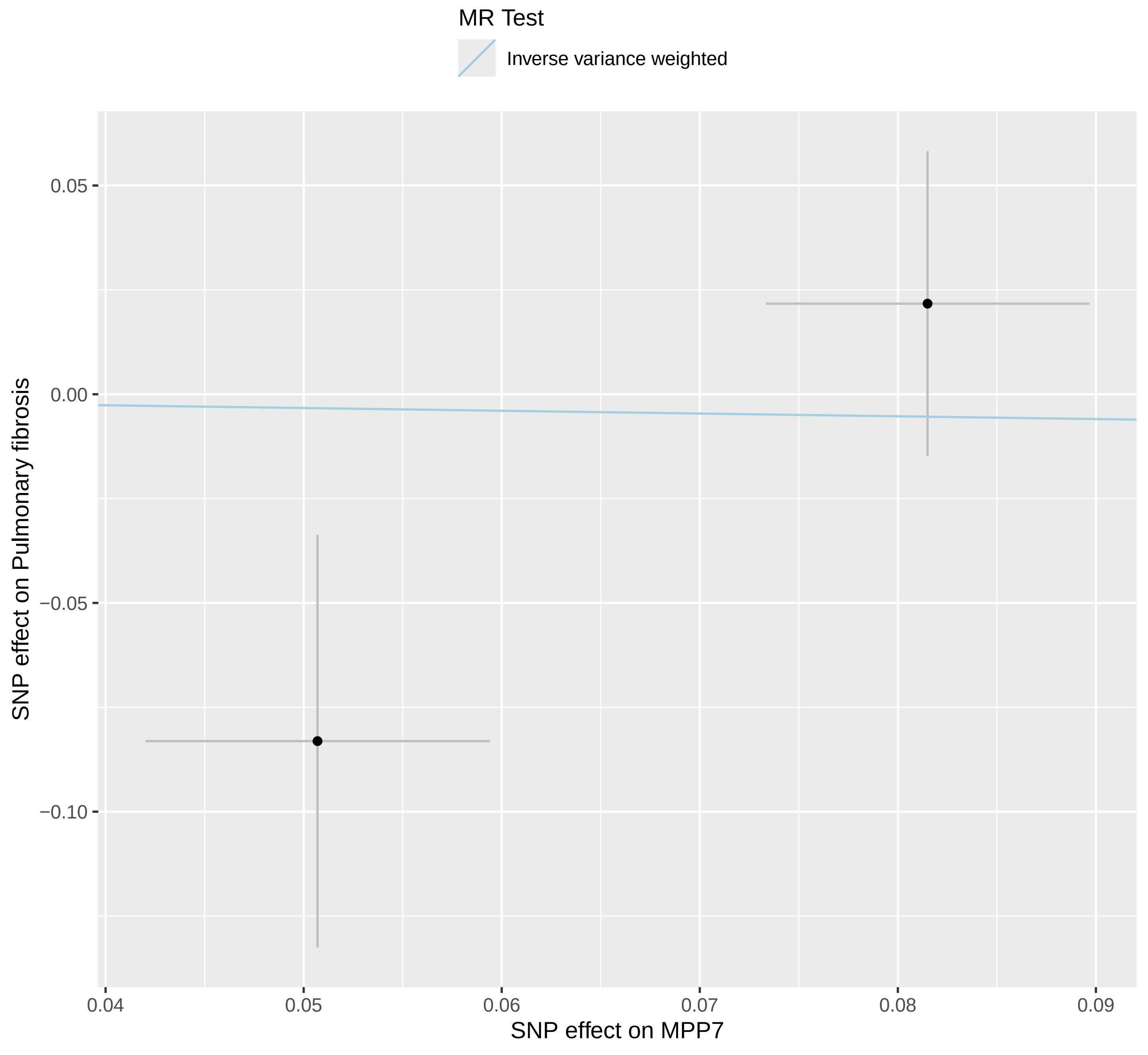
MR Test
Inverse variance weighted
0.05
0.00
−0.05
−0.10
SNP effect on Pulmonary fibrosis
0.04
0.05
0.06
0.07
0.08
0.09
SNP effect on MPP7

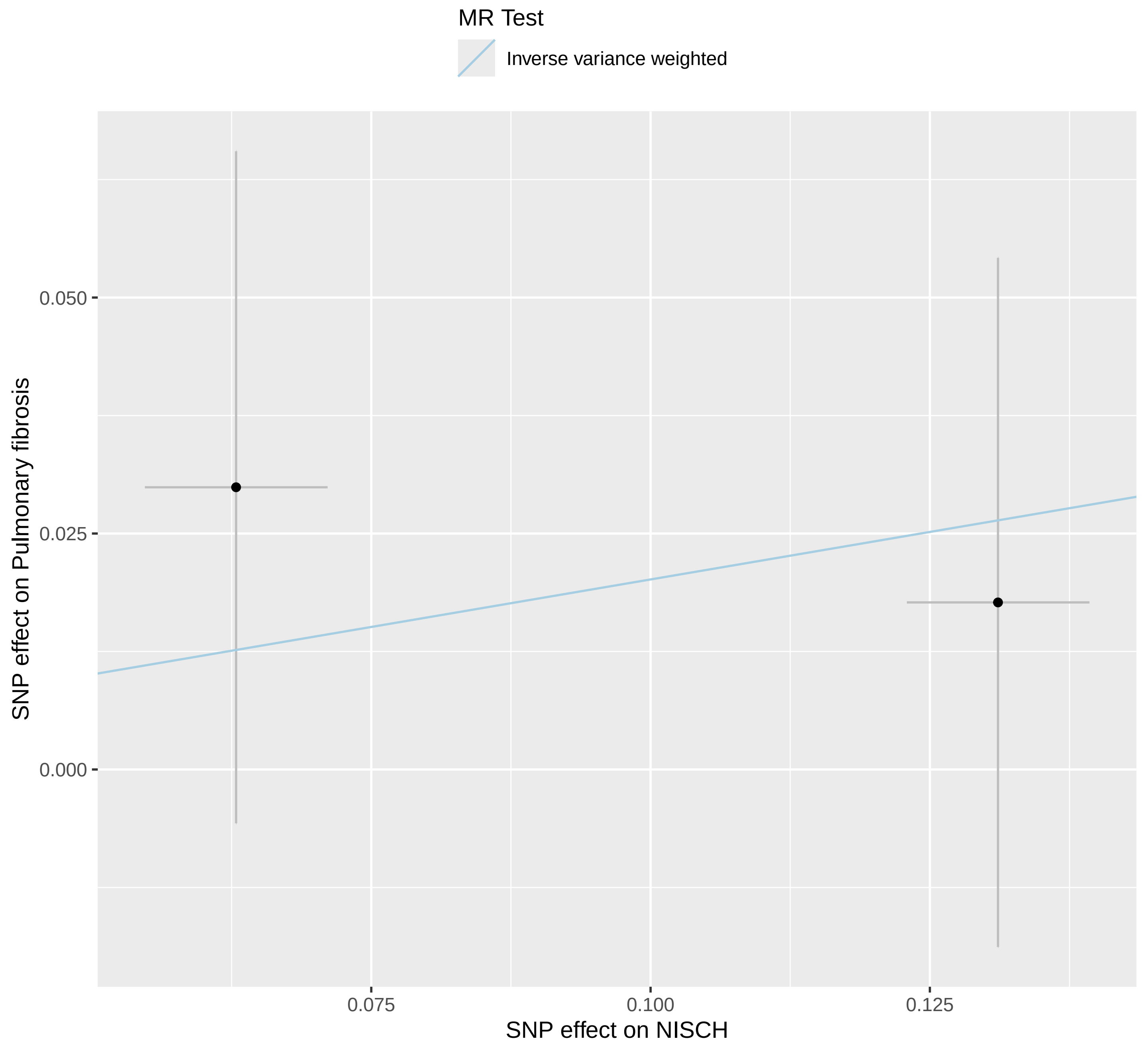
MR Test
Inverse variance weighted
SNP effect on Pulmonary fibrosis
0.050
0.025
0.000
0.075
0.100
0.125
SNP effect on NISCH

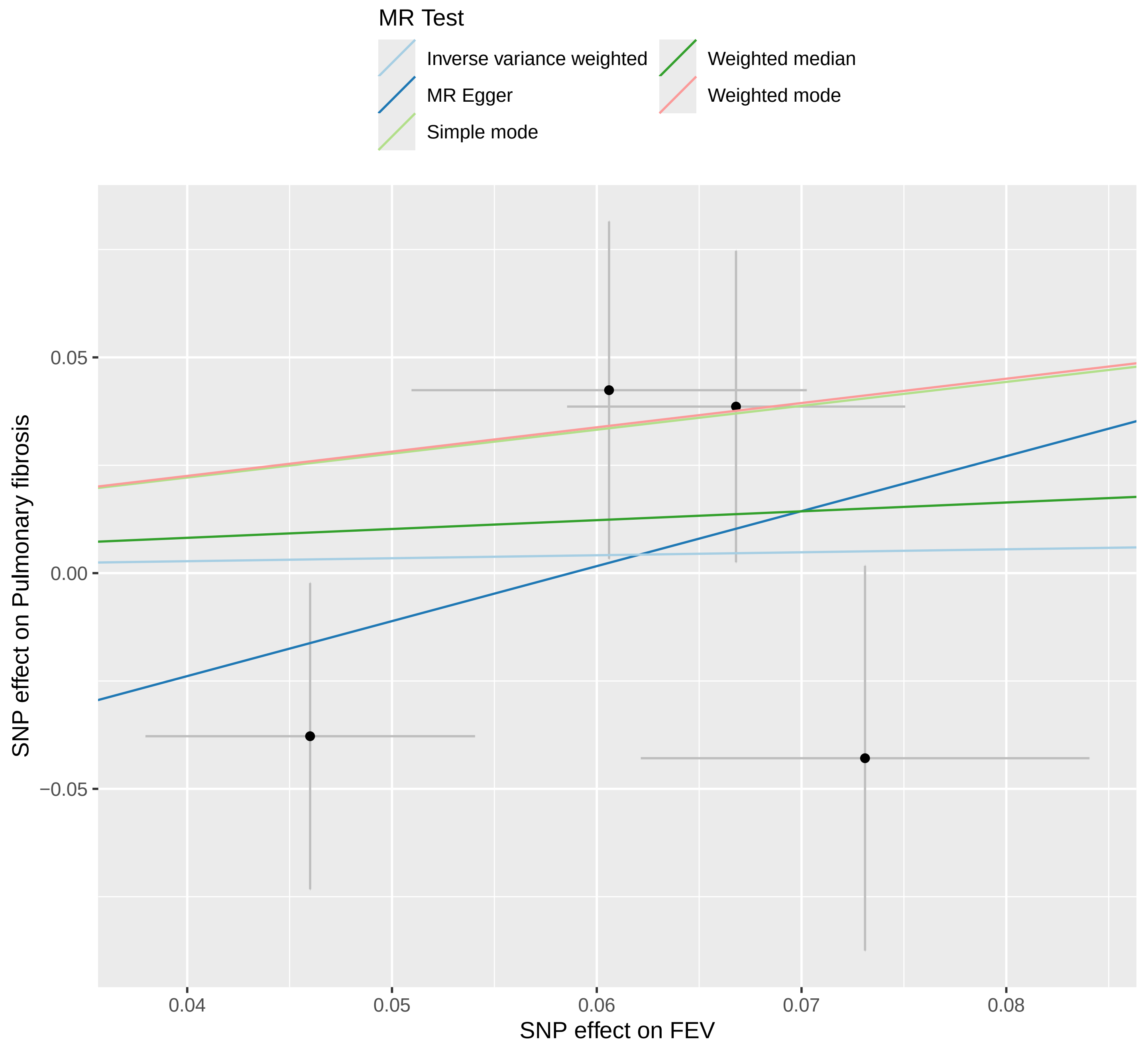
MR Test
Inverse variance weighted
MR Egger
Simple mode
Weighted median
Weighted mode
SNP effect on Pulmonary fibrosis
0.05
0.00
−0.05
0.04
0.05
0.06
0.07
0.08
SNP effect on FEV

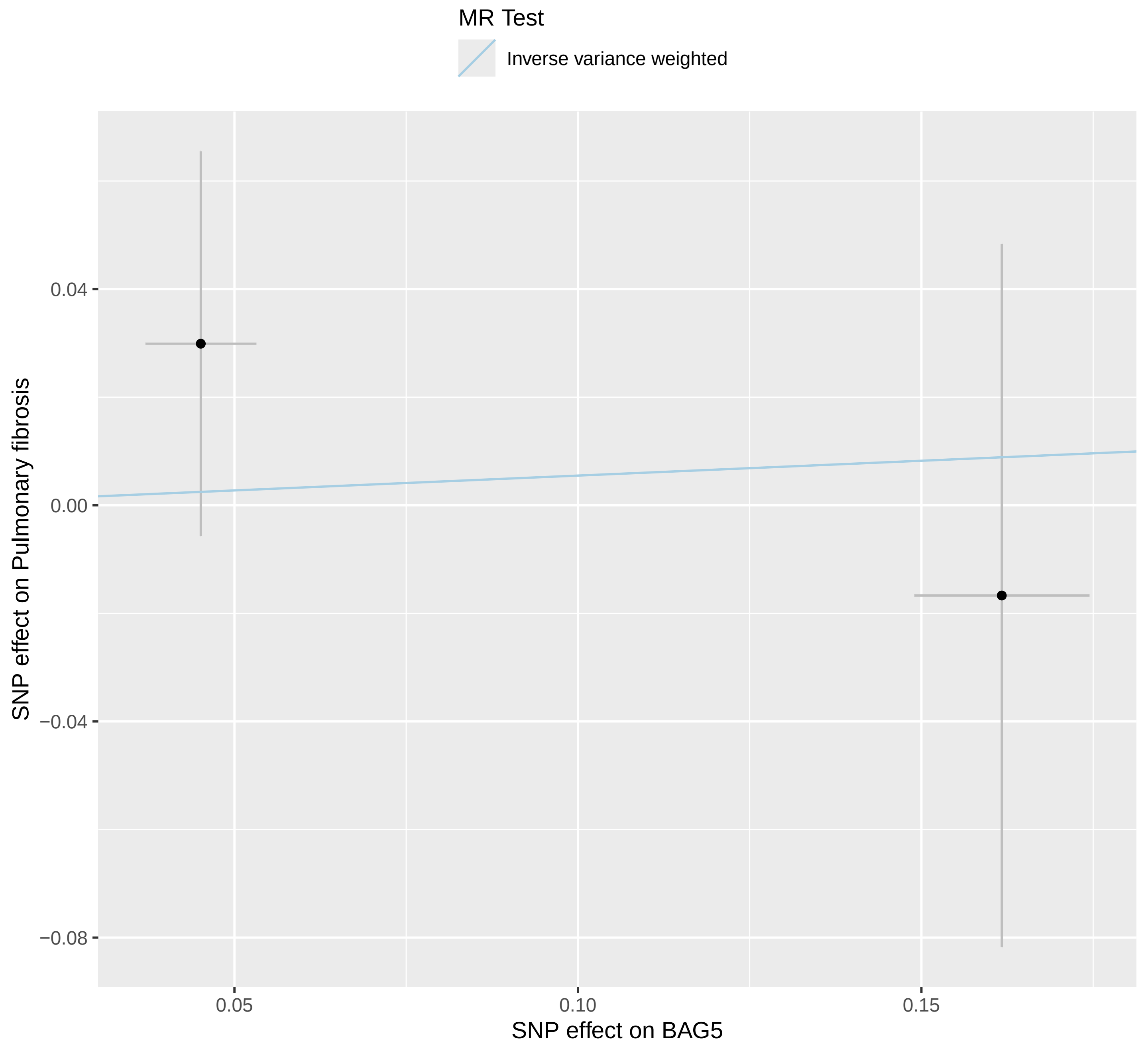
MR Test
Inverse variance weighted
0.04
0.00
−0.04
−0.08
SNP effect on Pulmonary fibrosis
0.05
0.10
0.15
SNP effect on BAG5

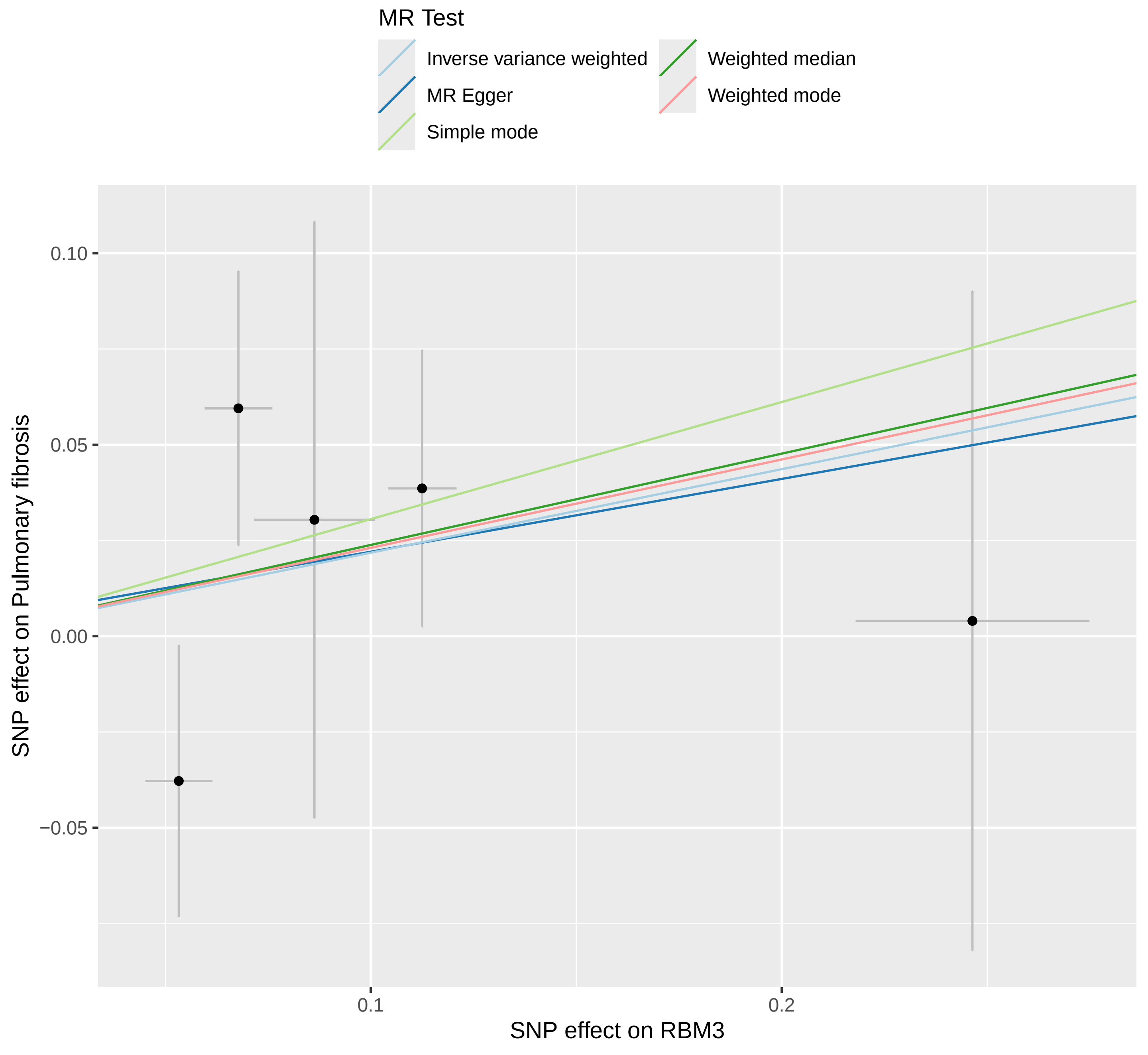
MR Test
Inverse variance weighted
MR Egger
Simple mode
Weighted median
Weighted mode
0.10
0.05
0.00
−0.05
0.1
0.2
SNP effect on Pulmonary fibrosis
SNP effect on RBM3

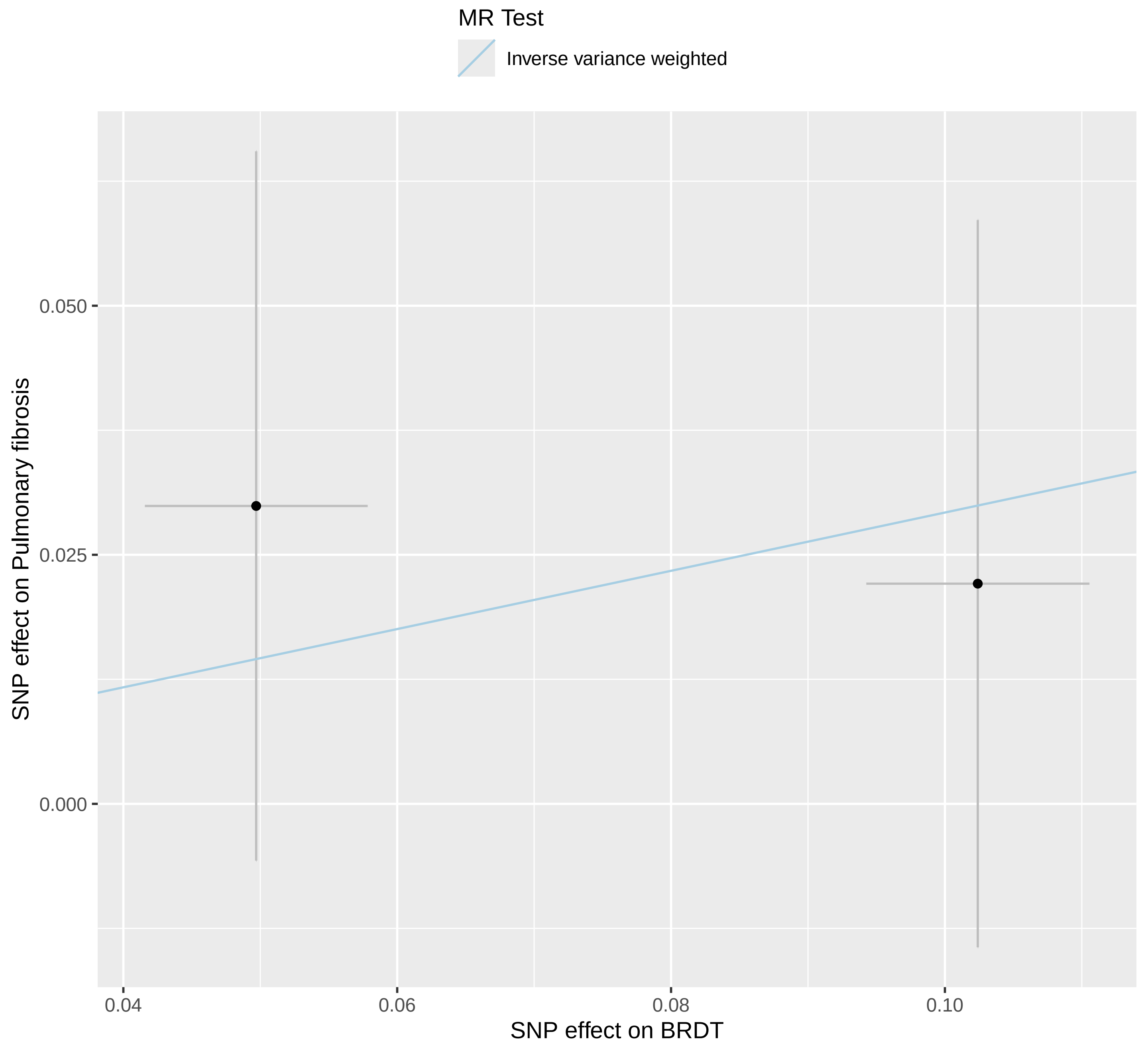

MR Test
Inverse variance weighted
0.050
0.025
0.000
SNP effect on Pulmonary fibrosis
0.04
0.06
0.08
0.10
SNP effect on BRDT

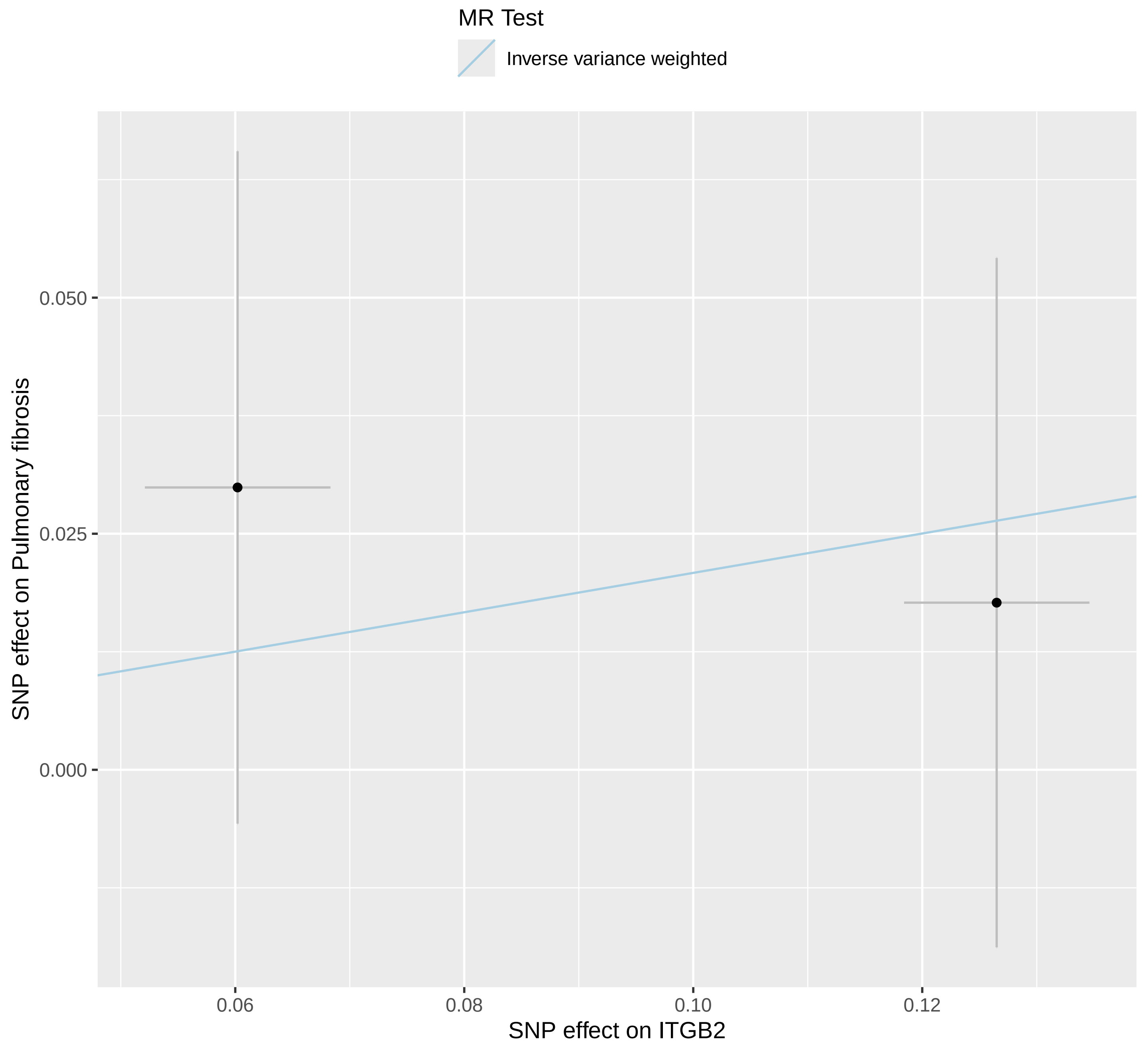
MR Test
Inverse variance weighted
0.050
0.025
0.000
0.06
0.08
0.10
0.12
SNP effect on Pulmonary fibrosis
SNP effect on ITGB2

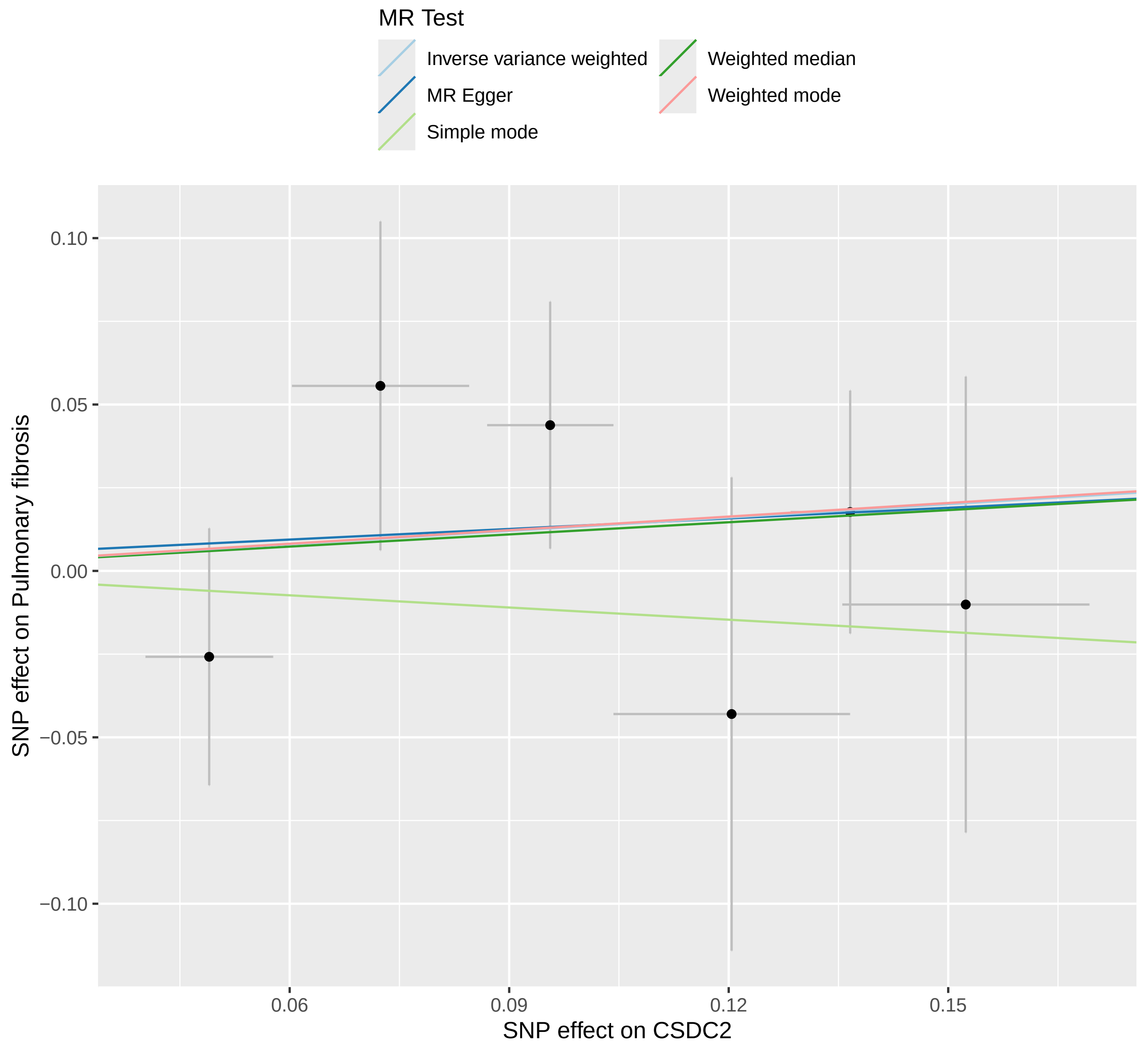

MR Test
Inverse variance weighted
MR Egger
Simple mode
Weighted median
Weighted mode
0.10
0.05
0.00
−0.05
−0.10
SNP effect on Pulmonary fibrosis
0.06
0.09
0.12
0.15
SNP effect on CSDC2

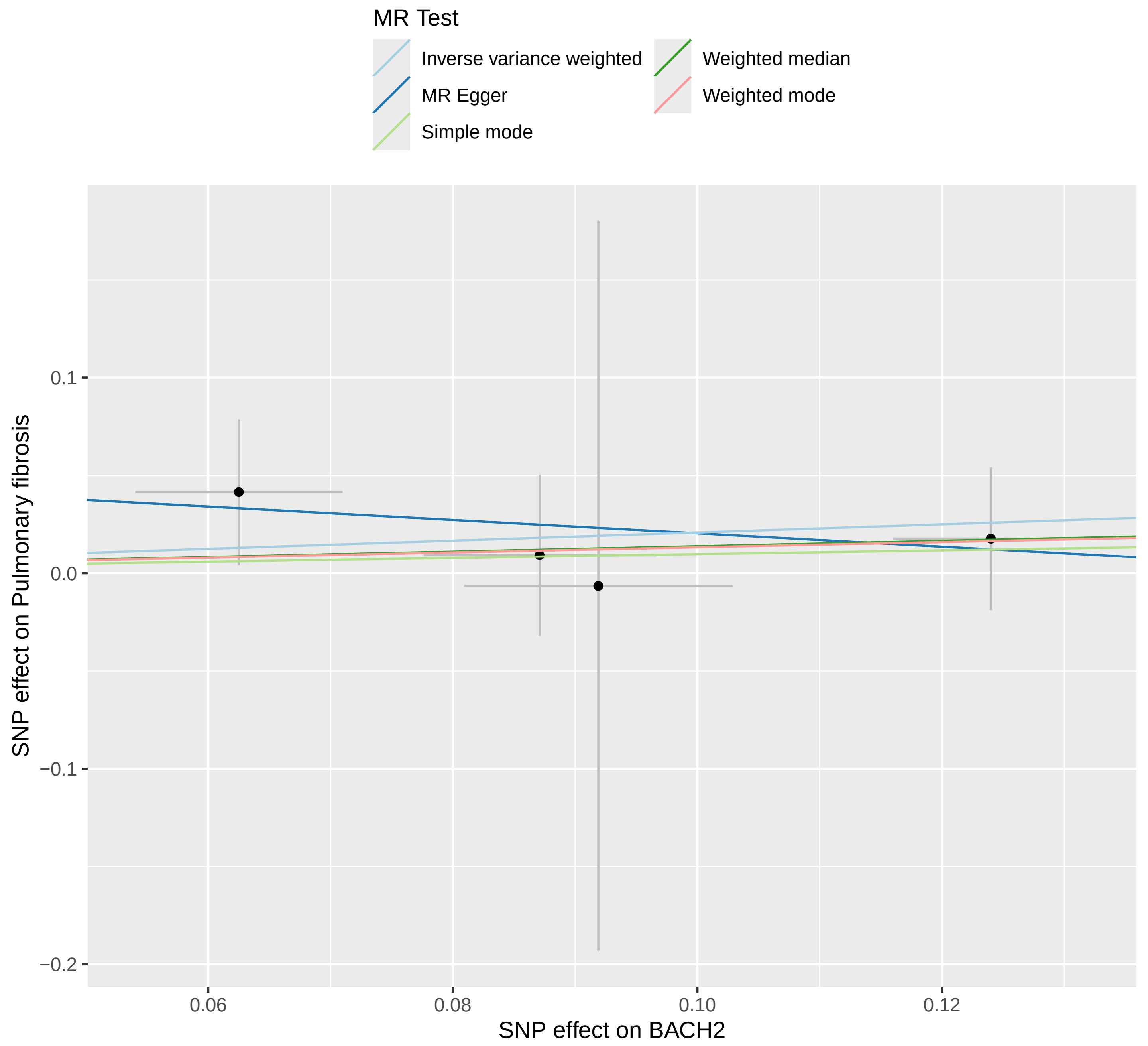

MR Test
Inverse variance weighted
MR Egger
Simple mode
Weighted median
Weighted mode
0.1
0.0
−0.1
−0.2
0.06
0.08
0.10
0.12
SNP effect on BACH2
SNP effect on Pulmonary fibrosis

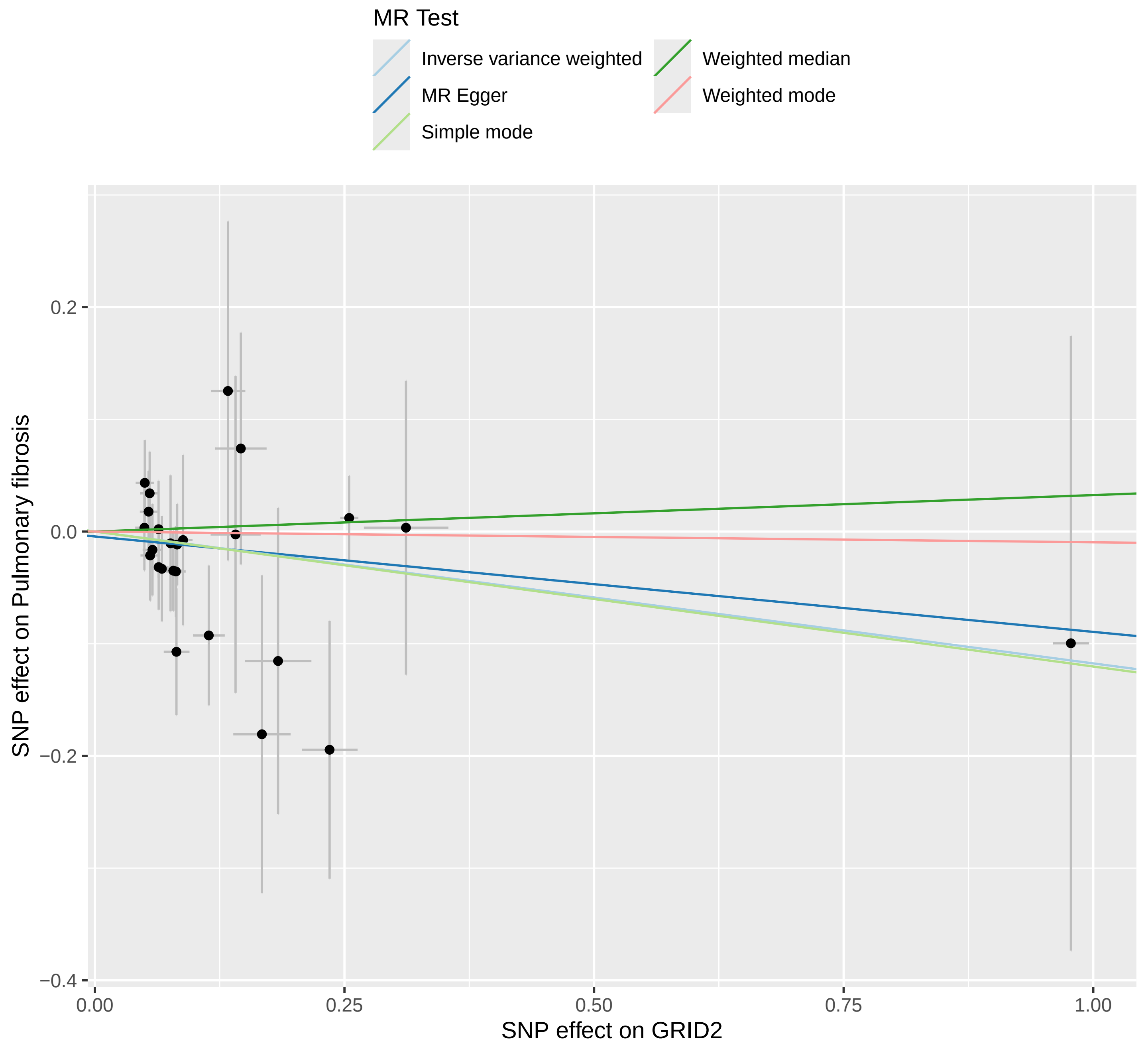
MR Test
Inverse variance weighted
MR Egger
Simple mode
Weighted median
Weighted mode
0.2
0.0
−0.2
−0.4
0.00
0.25
0.50
0.75
1.00
SNP effect on Pulmonary fibrosis
SNP effect on GRID2

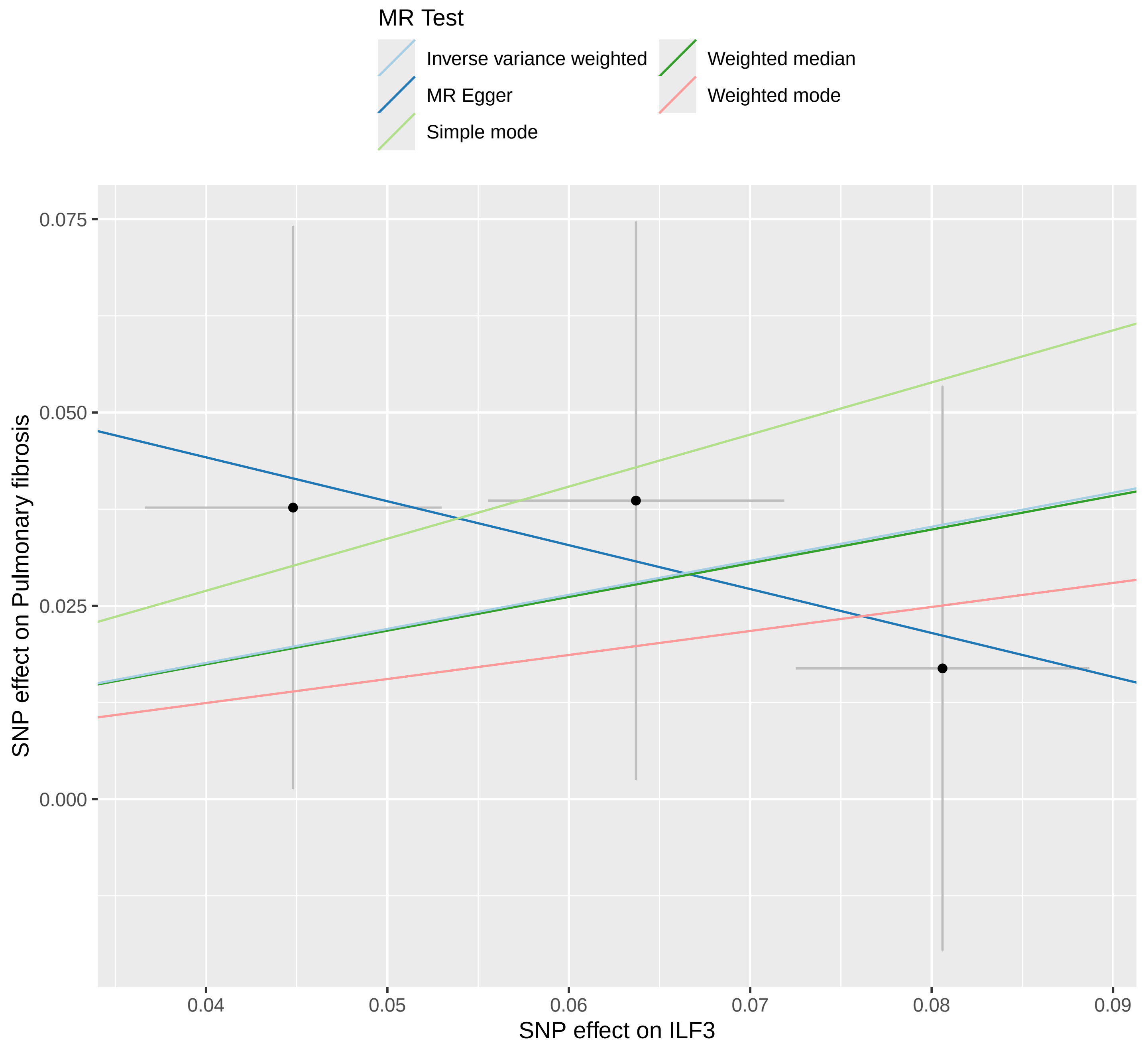
MR Test
Inverse variance weighted
MR Egger
Simple mode
Weighted median
Weighted mode
SNP effect on Pulmonary fibrosis
0.075
0.050
0.025
0.000
0.04
0.05
0.06
0.07
0.08
0.09
SNP effect on ILF3

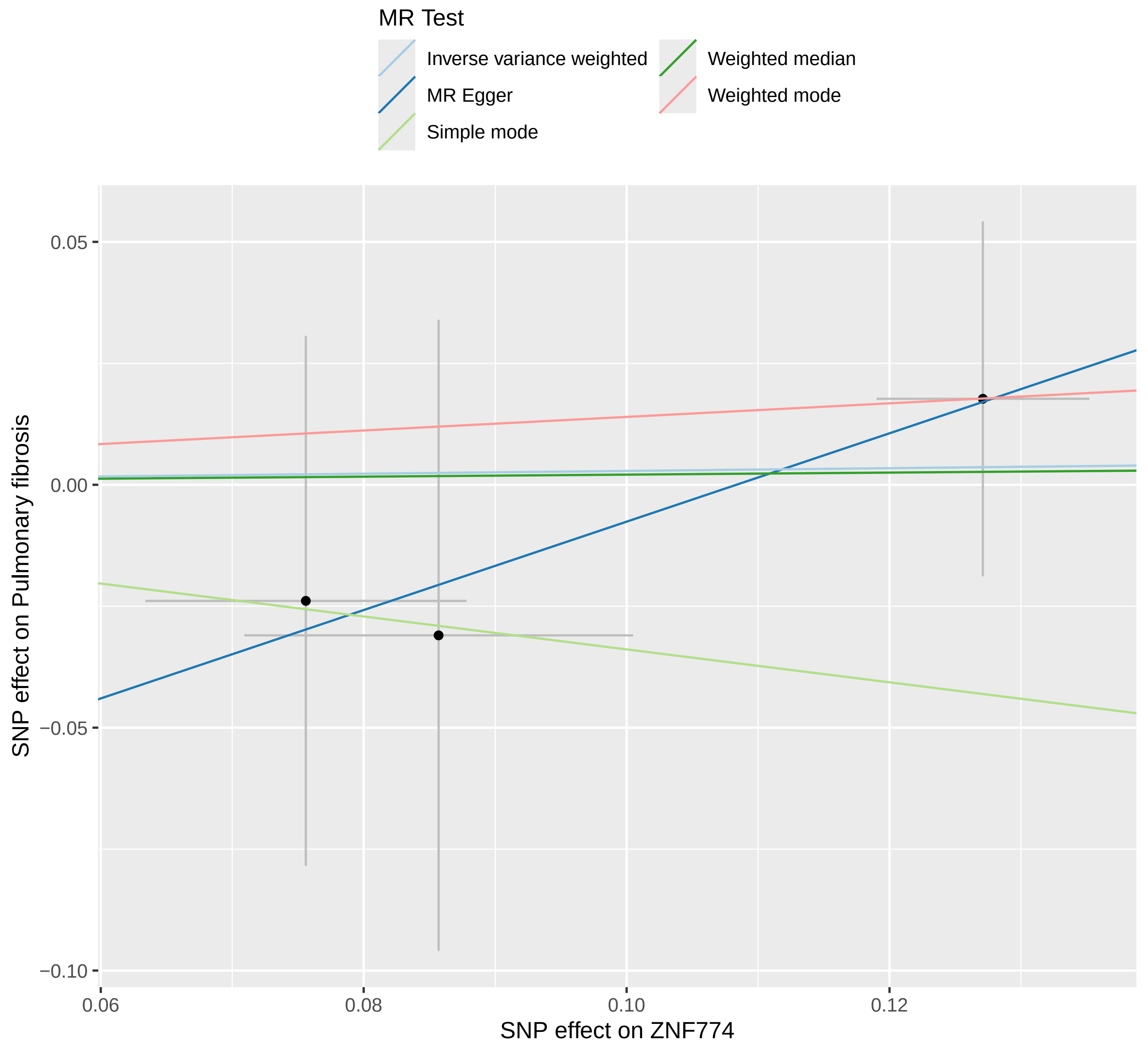
MR Test
Inverse variance weighted
MR Egger
Simple mode
Weighted median
Weighted mode
SNP effect on Pulmonary fibrosis
0.05
0.00
−0.05
−0.10
0.06
0.08
0.10
0.12
SNP effect on ZNF774

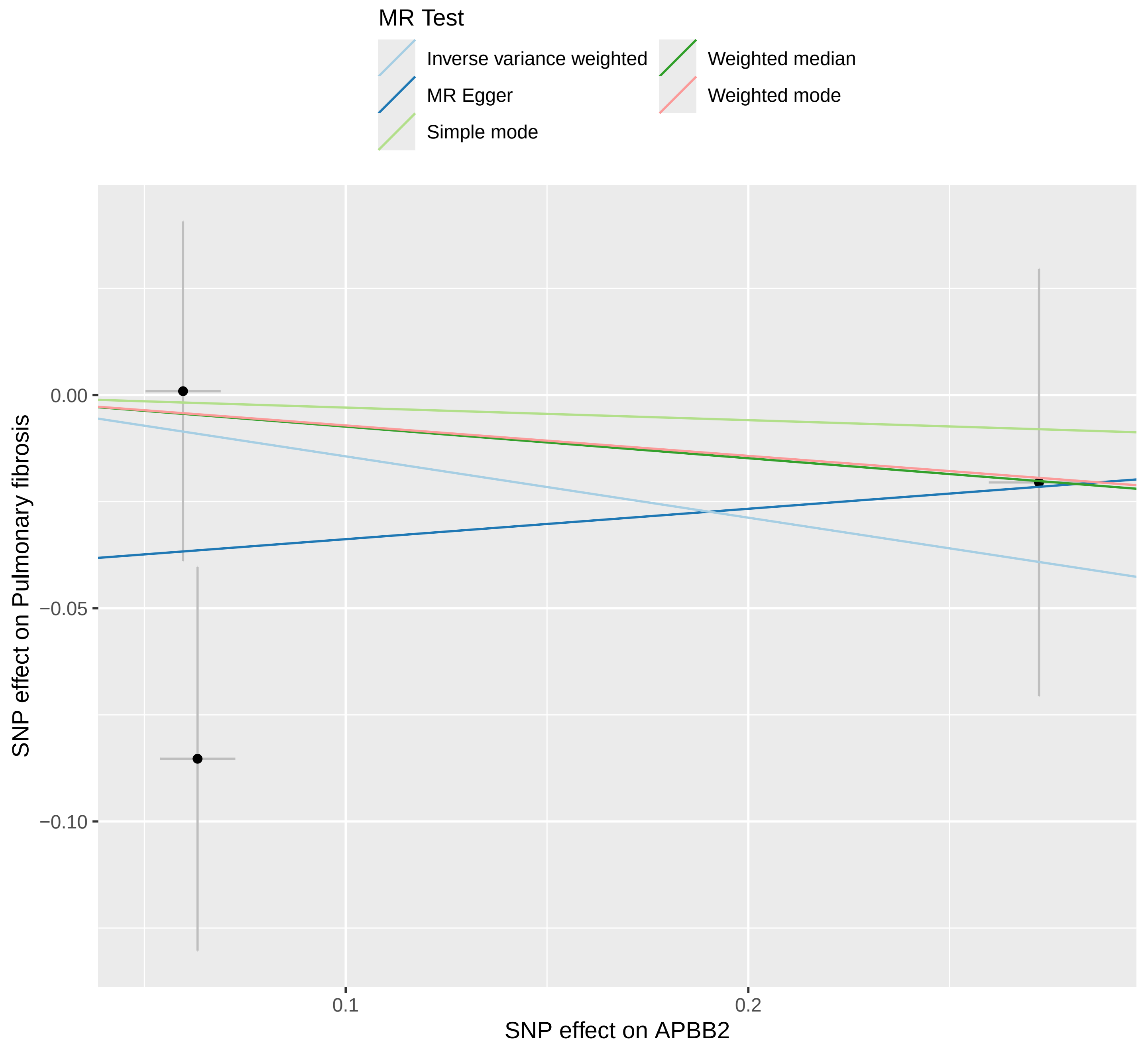

MR Test
Inverse variance weighted
MR Egger
Simple mode
Weighted median
Weighted mode
0.00
−0.05
−0.10
0.1
0.2
SNP effect on Pulmonary fibrosis
SNP effect on APBB2

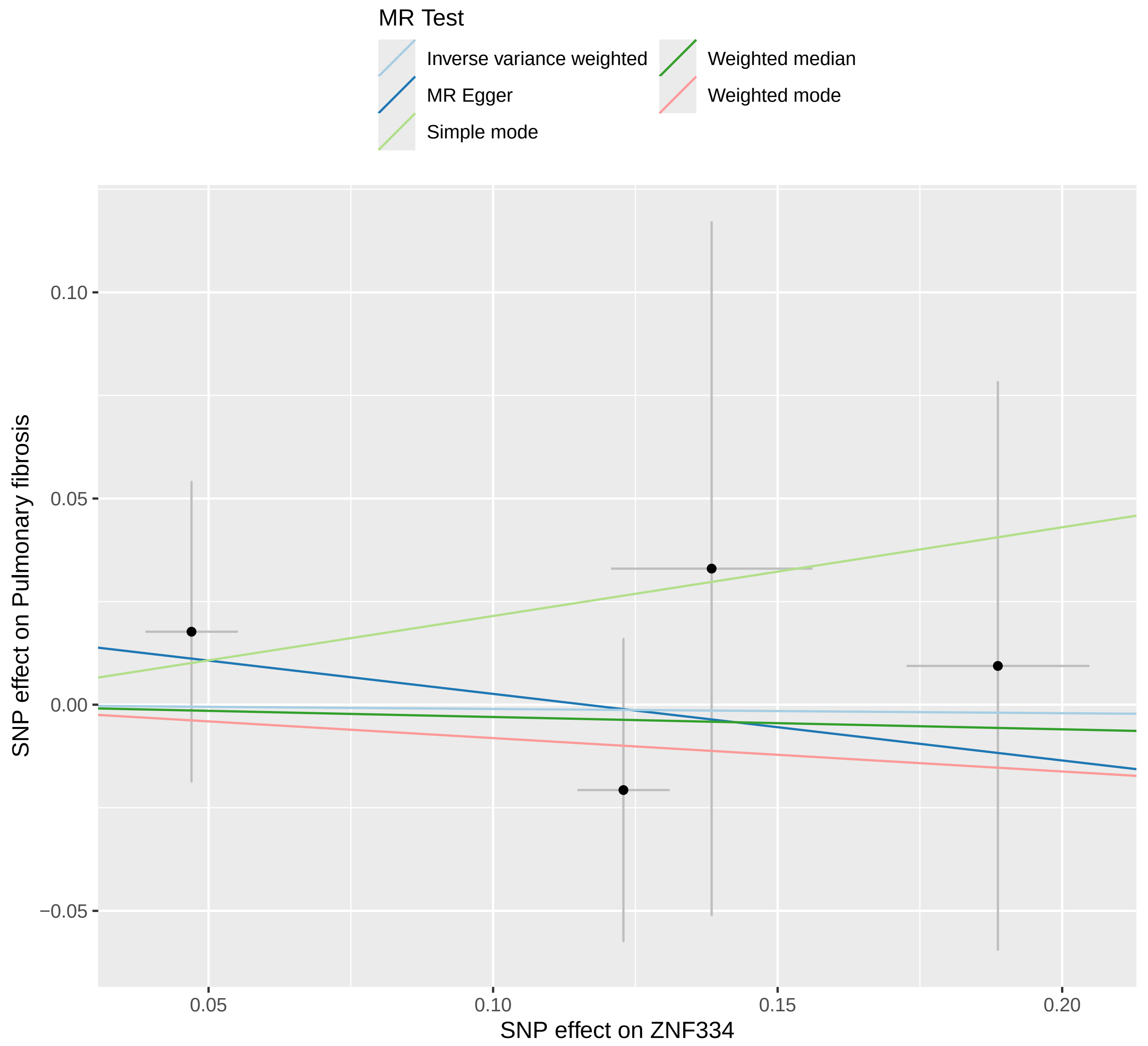
MR Test
Inverse variance weighted
MR Egger
Simple mode
Weighted median
Weighted mode
0.10
0.05
0.00
−0.05
SNP effect on Pulmonary fibrosis
0.05
0.10
0.15
0.20
SNP effect on ZNF334

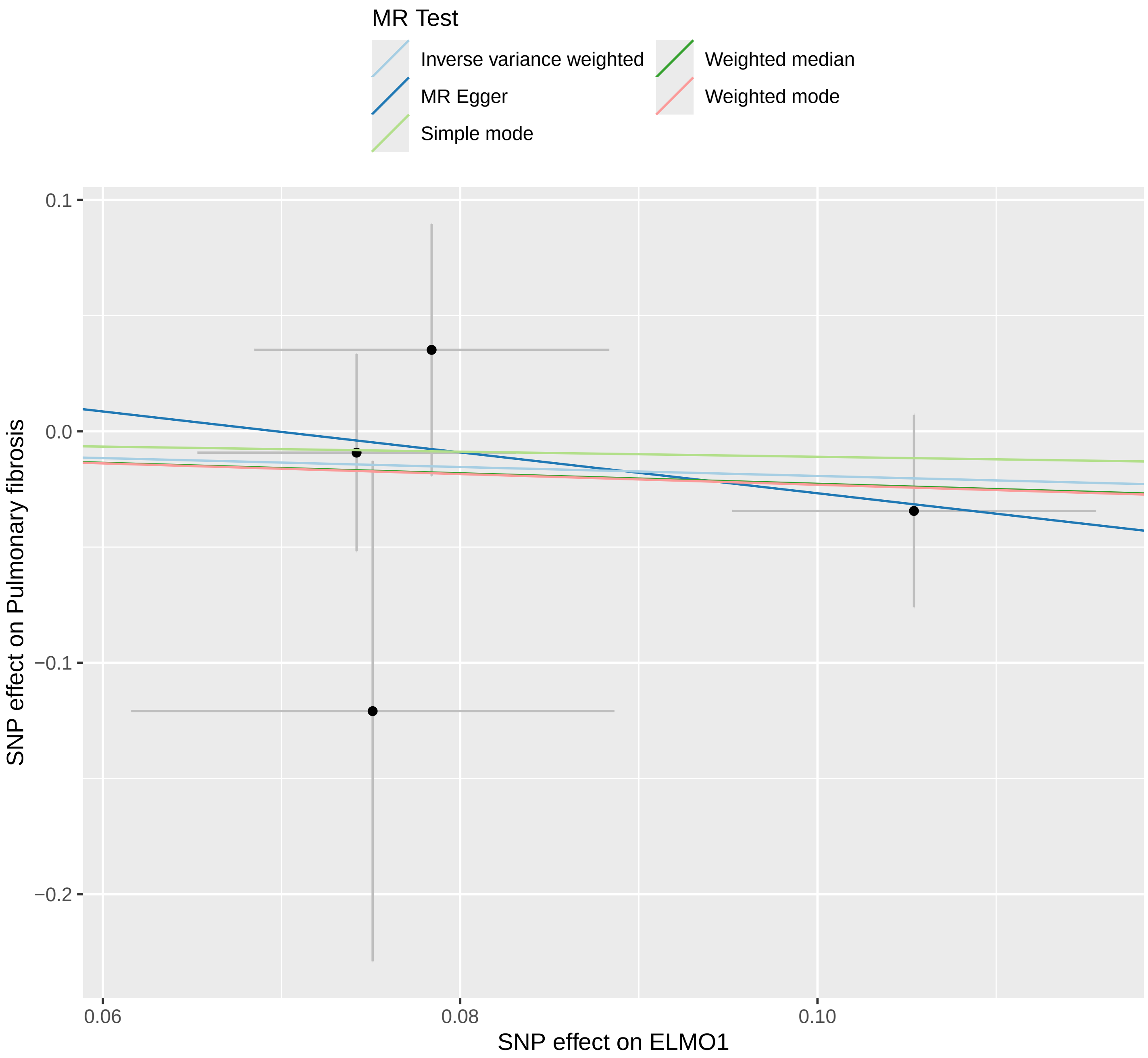
MR Test
Inverse variance weighted
MR Egger
Simple mode
Weighted median
Weighted mode
0.1
0.0
−0.1
−0.2
0.06
0.08
0.10
SNP effect on ELMO1
SNP effect on Pulmonary fibrosis

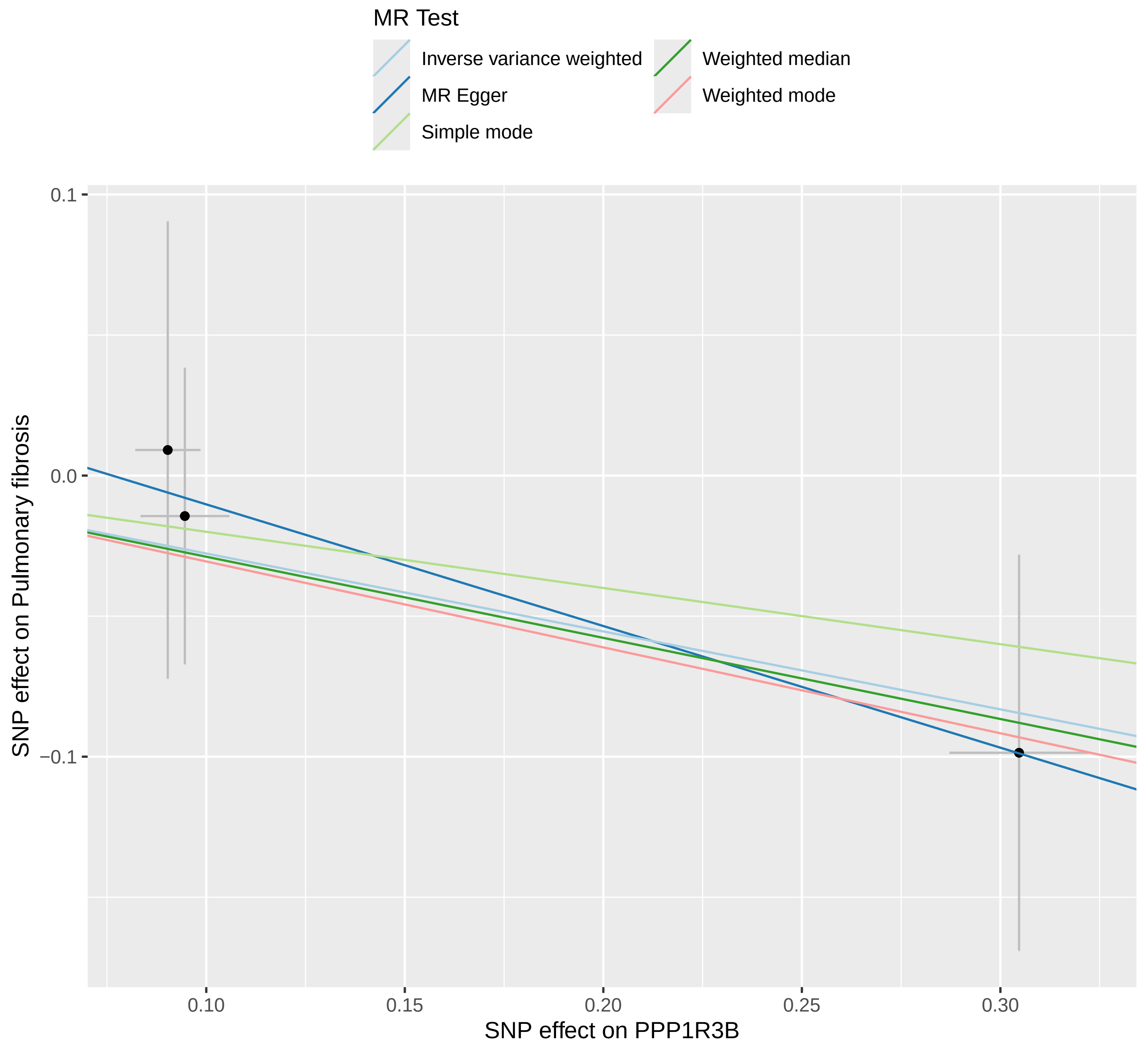
MR Test
Inverse variance weighted
MR Egger
Simple mode
Weighted median
Weighted mode
0.1
0.0
−0.1
SNP effect on Pulmonary fibrosis
0.10
0.15
0.20
0.25
0.30
SNP effect on PPP1R3B

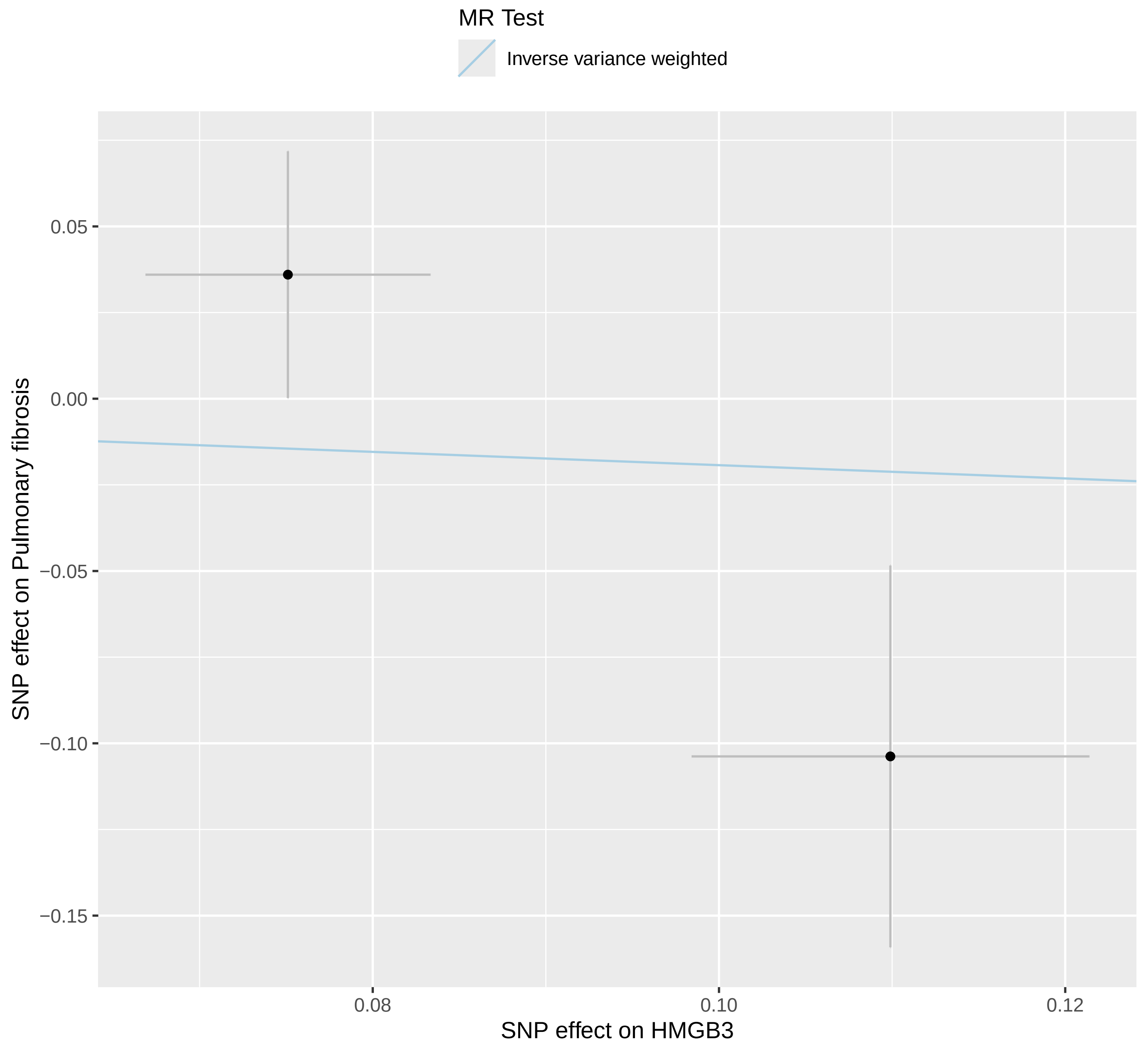
MR Test
Inverse variance weighted
0.05
0.00
−0.05
−0.10
−0.15
SNP effect on Pulmonary fibrosis
0.08
0.10
0.12
SNP effect on HMGB3

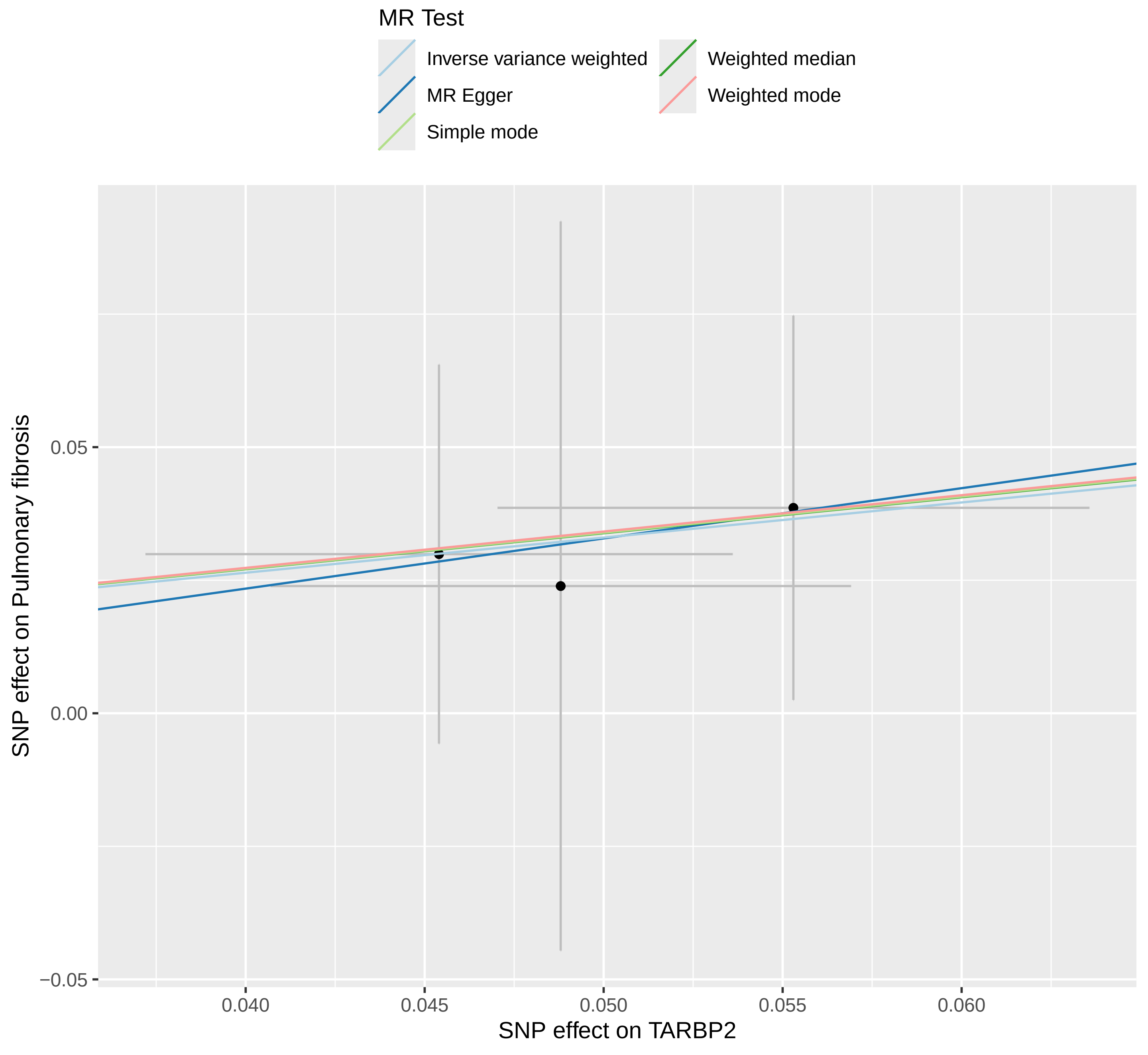

MR Test
Inverse variance weighted
MR Egger
Simple mode
Weighted median
Weighted mode
SNP effect on Pulmonary fibrosis
0.05
0.00
−0.05
0.040
0.045
0.050
0.055
0.060
SNP effect on TARBP2

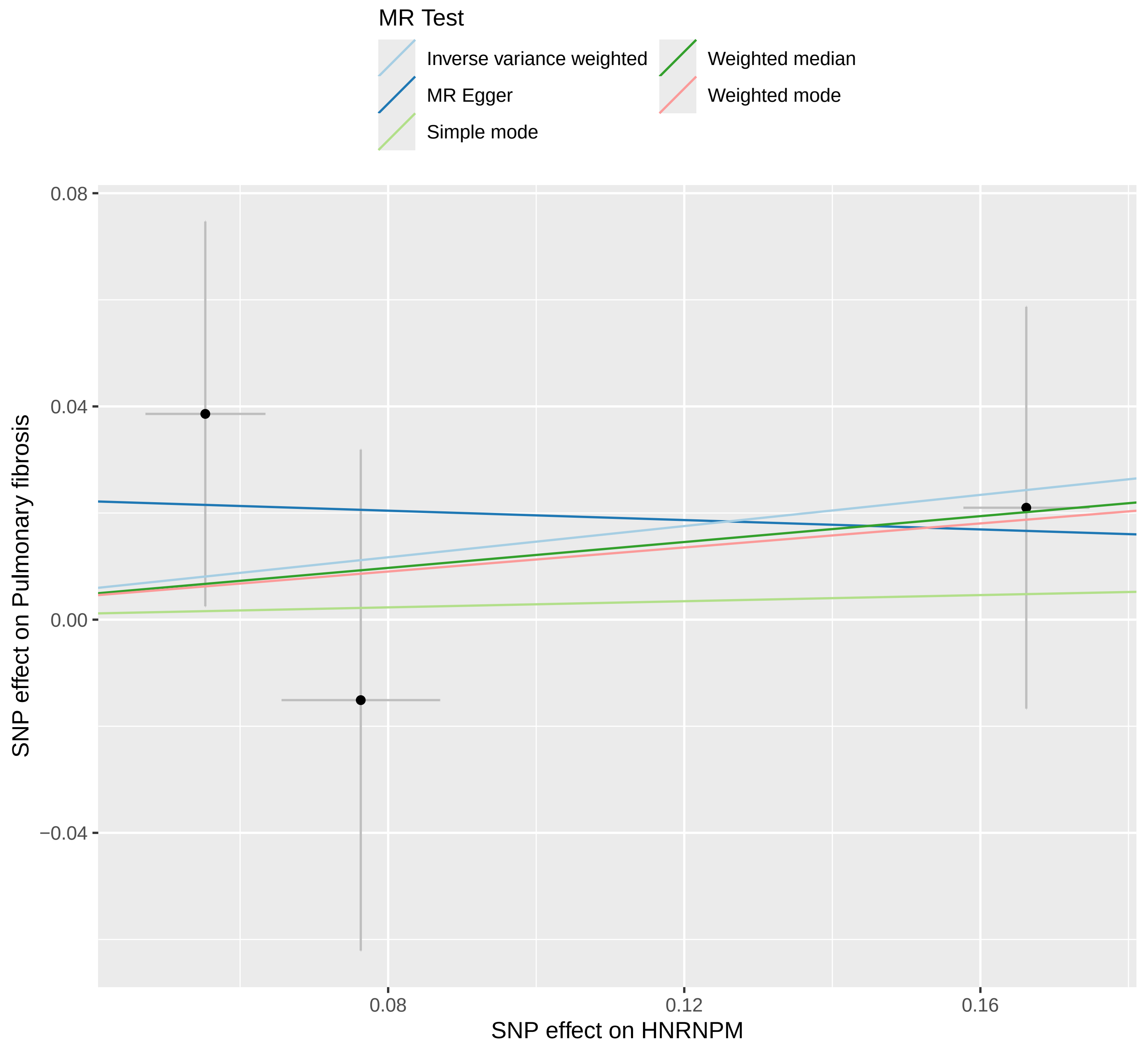

MR Test
Inverse variance weighted
MR Egger
Simple mode
Weighted median
Weighted mode
0.08
0.04
0.00
−0.04
0.08
0.12
0.16
SNP effect on Pulmonary fibrosis
SNP effect on HNRNPM

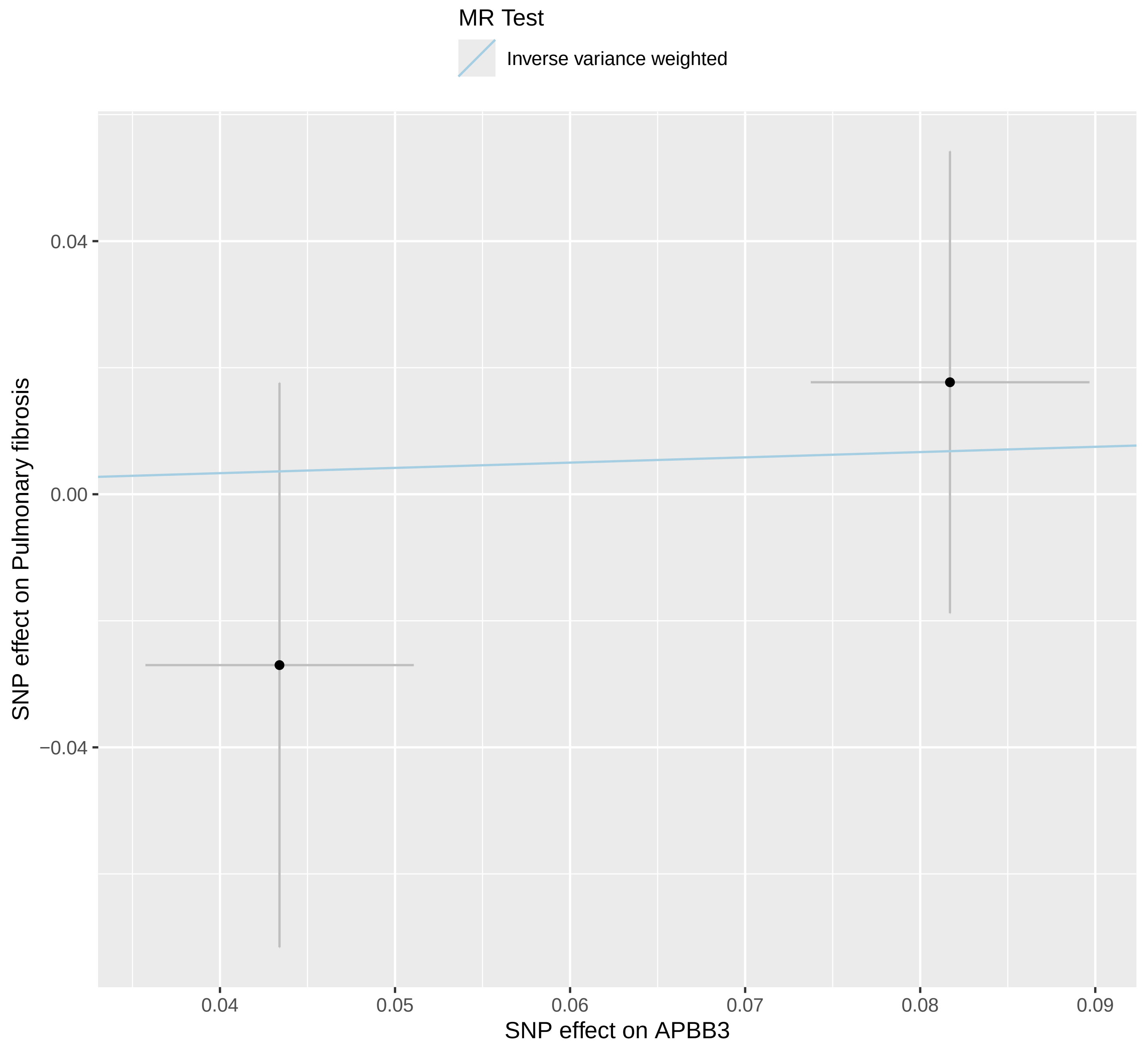

MR Test
Inverse variance weighted
0.04
0.00
−0.04
SNP effect on Pulmonary fibrosis
0.04
0.05
0.06
0.07
0.08
0.09
SNP effect on APBB3

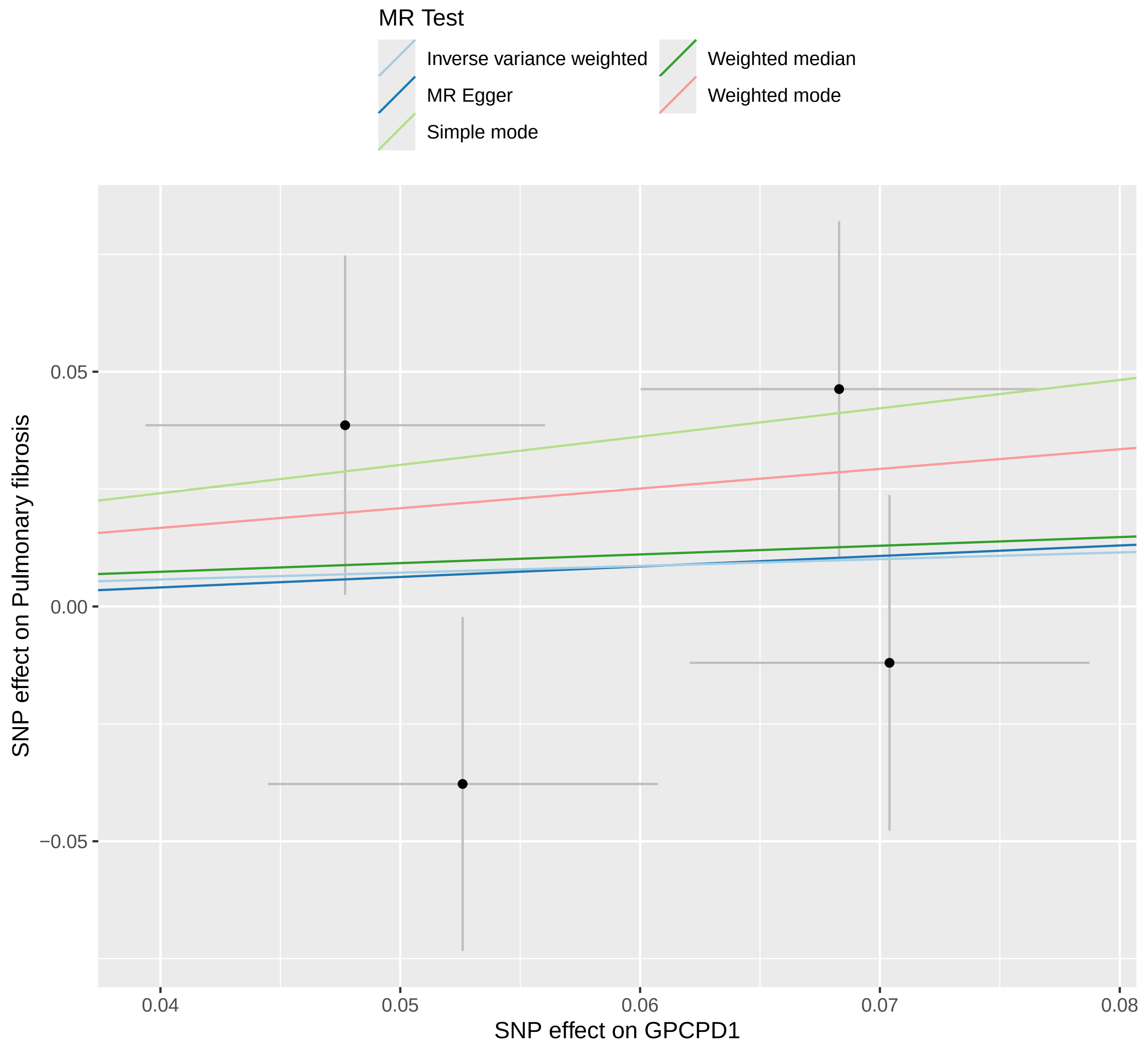
MR Test
Inverse variance weighted
MR Egger
Simple mode
Weighted median
Weighted mode
SNP effect on Pulmonary fibrosis
0.05
0.00
−0.05
0.04
0.05
0.06
0.07
0.08
SNP effect on GPCPD1

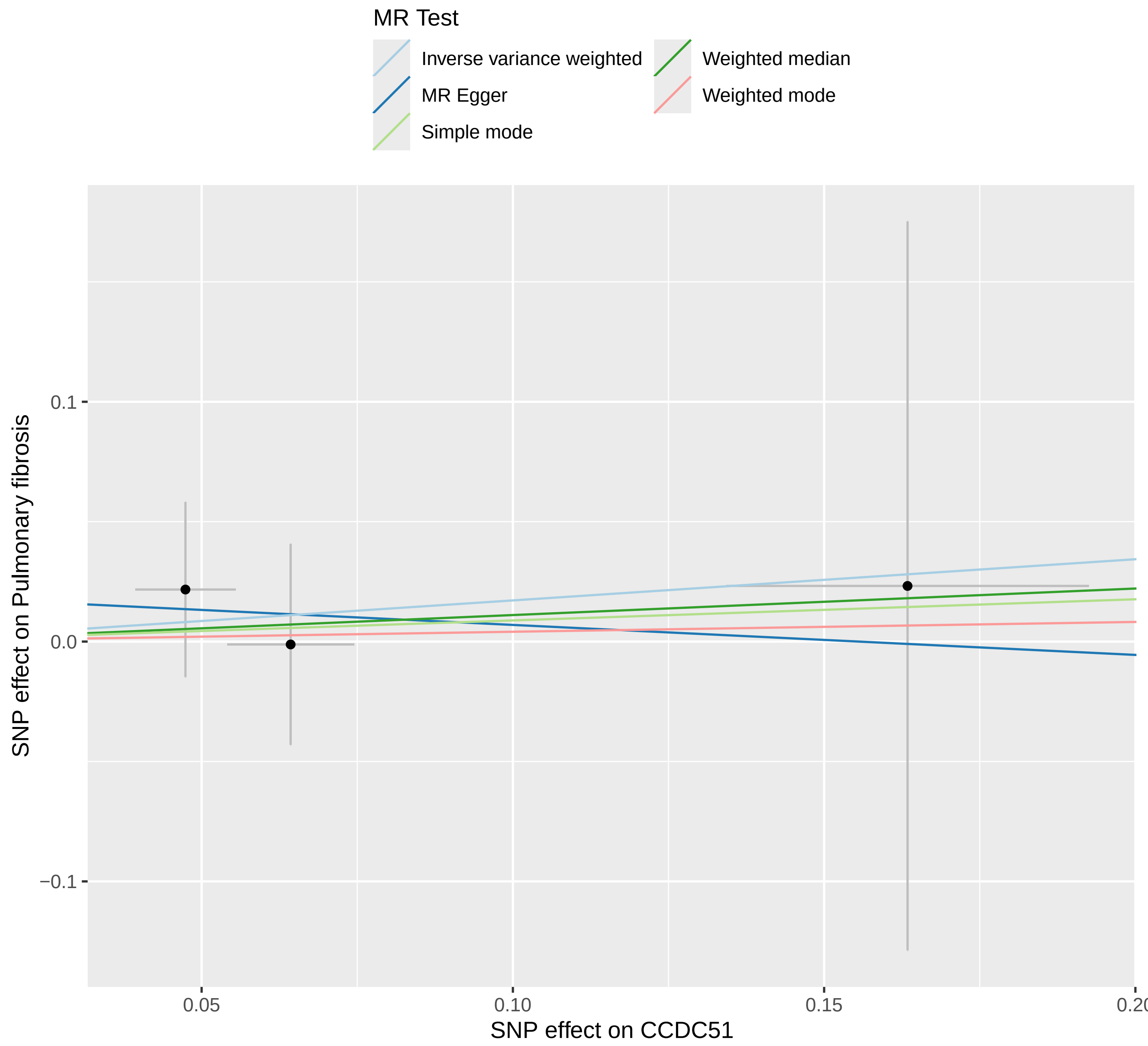
MR Test
Inverse variance weighted
MR Egger
Simple mode
Weighted median
Weighted mode
0.1
0.0
−0.1
0.05
0.10
0.15
0.20
SNP effect on CCDC51
SNP effect on Pulmonary fibrosis

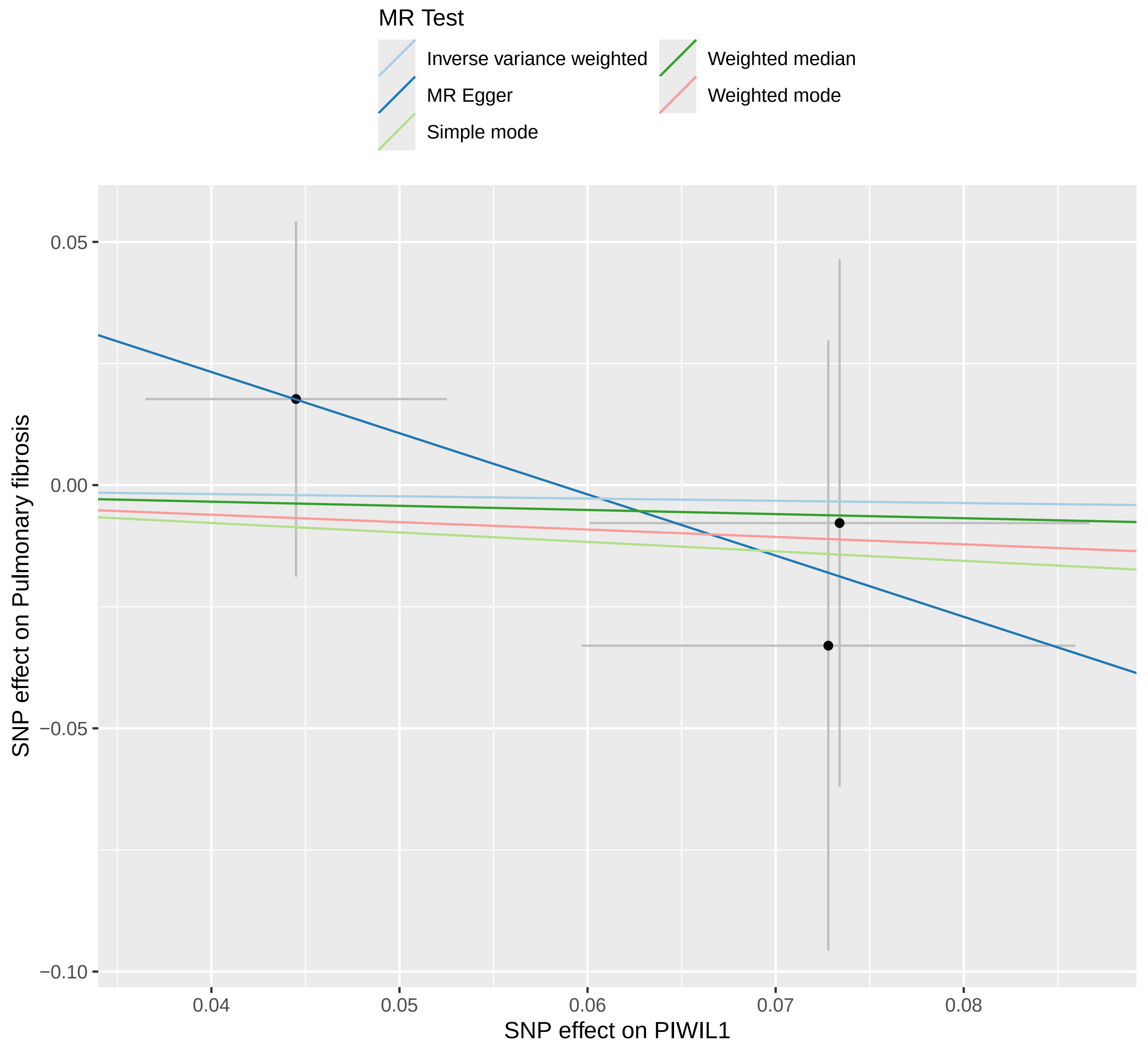
MR Test
Inverse variance weighted
MR Egger
Simple mode
Weighted median
Weighted mode
0.05
0.00
−0.05
−0.10
0.04
0.05
0.06
0.07
0.08
SNP effect on Pulmonary fibrosis
SNP effect on PIWIL1

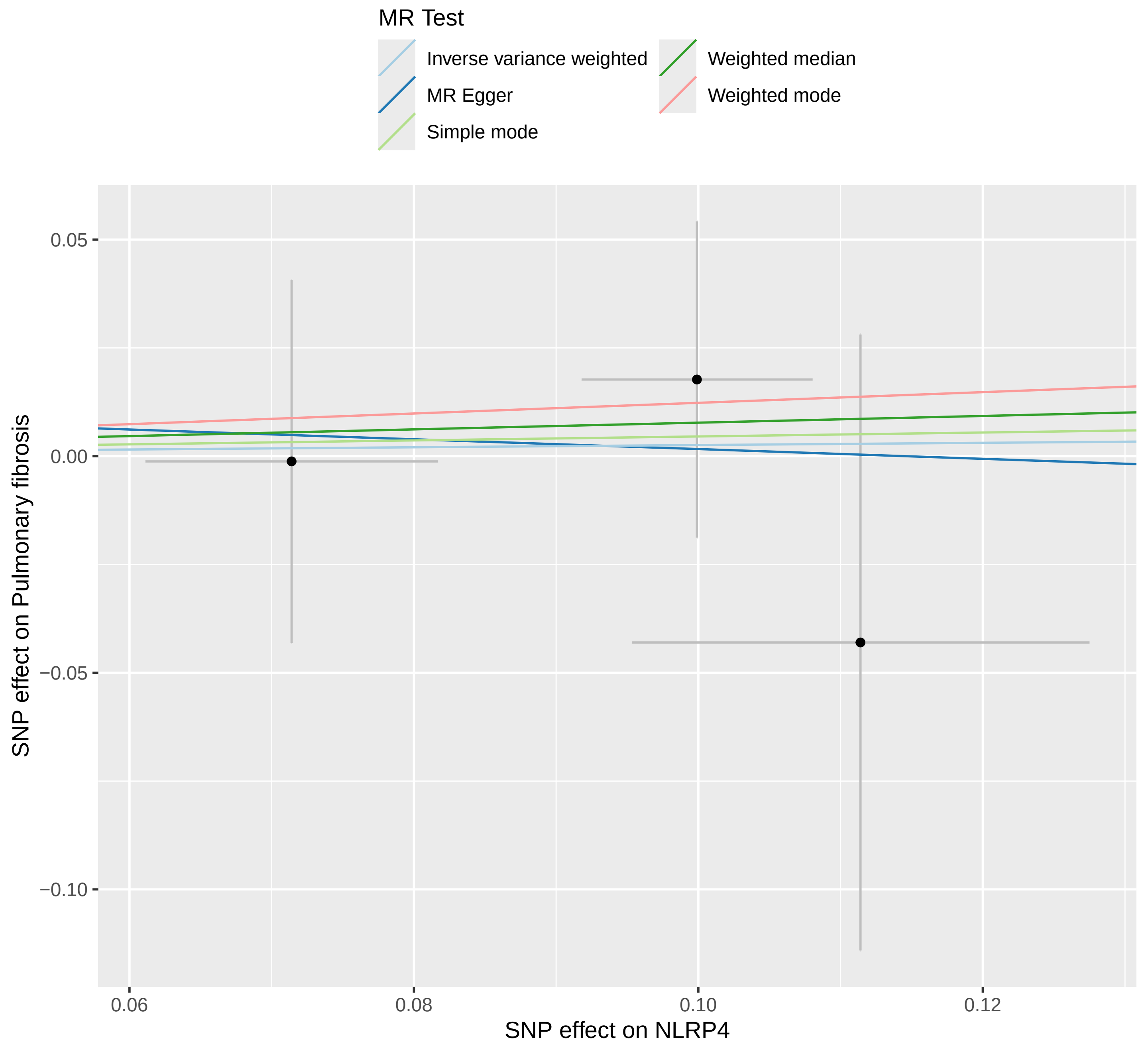
MR Test
Inverse variance weighted
MR Egger
Simple mode
Weighted median
Weighted mode
0.05
0.00
−0.05
−0.10
SNP effect on Pulmonary fibrosis
0.06
0.08
0.10
0.12
SNP effect on NLRP4

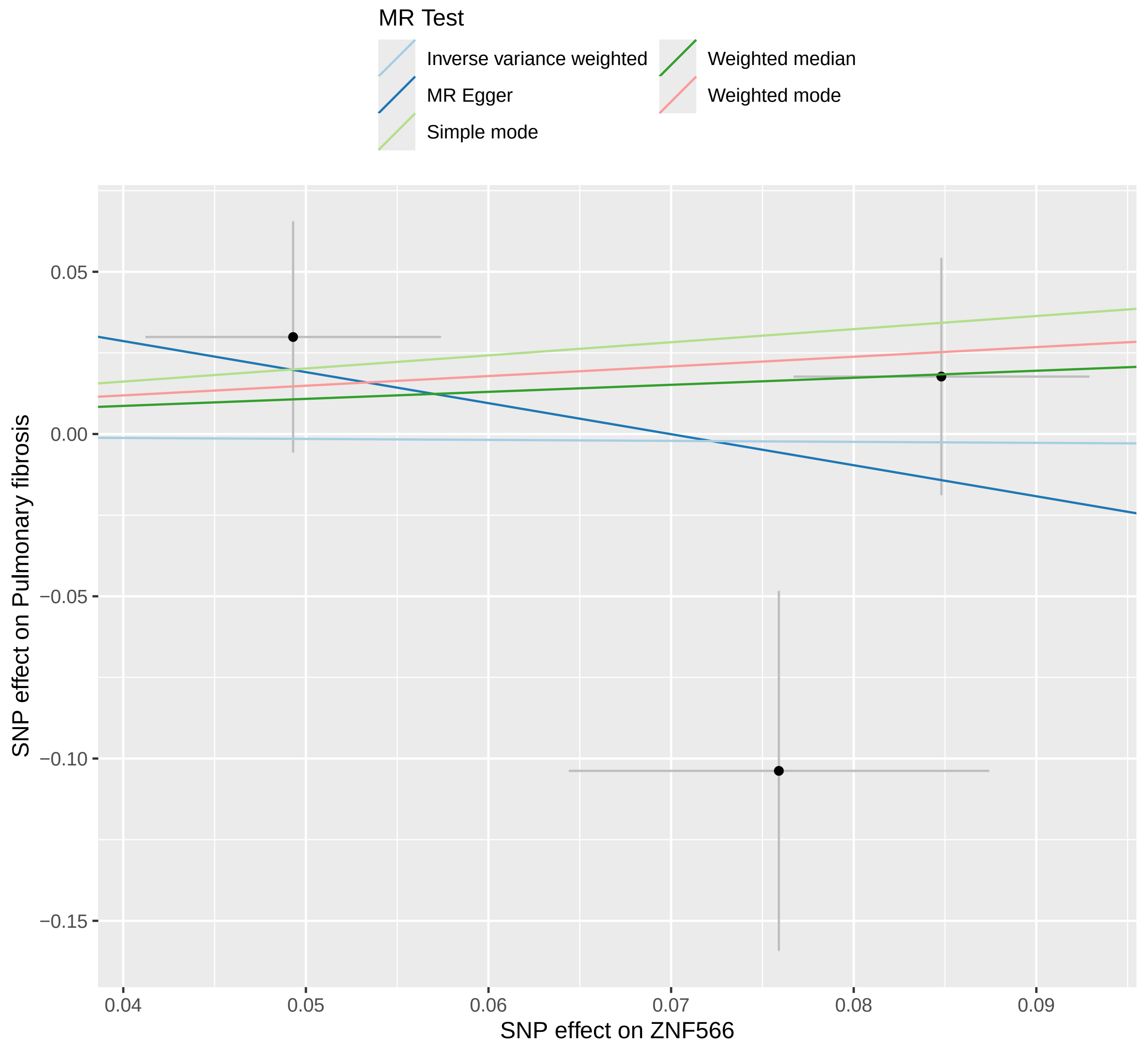
MR Test
Inverse variance weighted
MR Egger
Simple mode
Weighted median
Weighted mode
0.05
0.00
−0.05
−0.10
−0.15
SNP effect on Pulmonary fibrosis
0.04
0.05
0.06
0.07
0.08
0.09
SNP effect on ZNF566

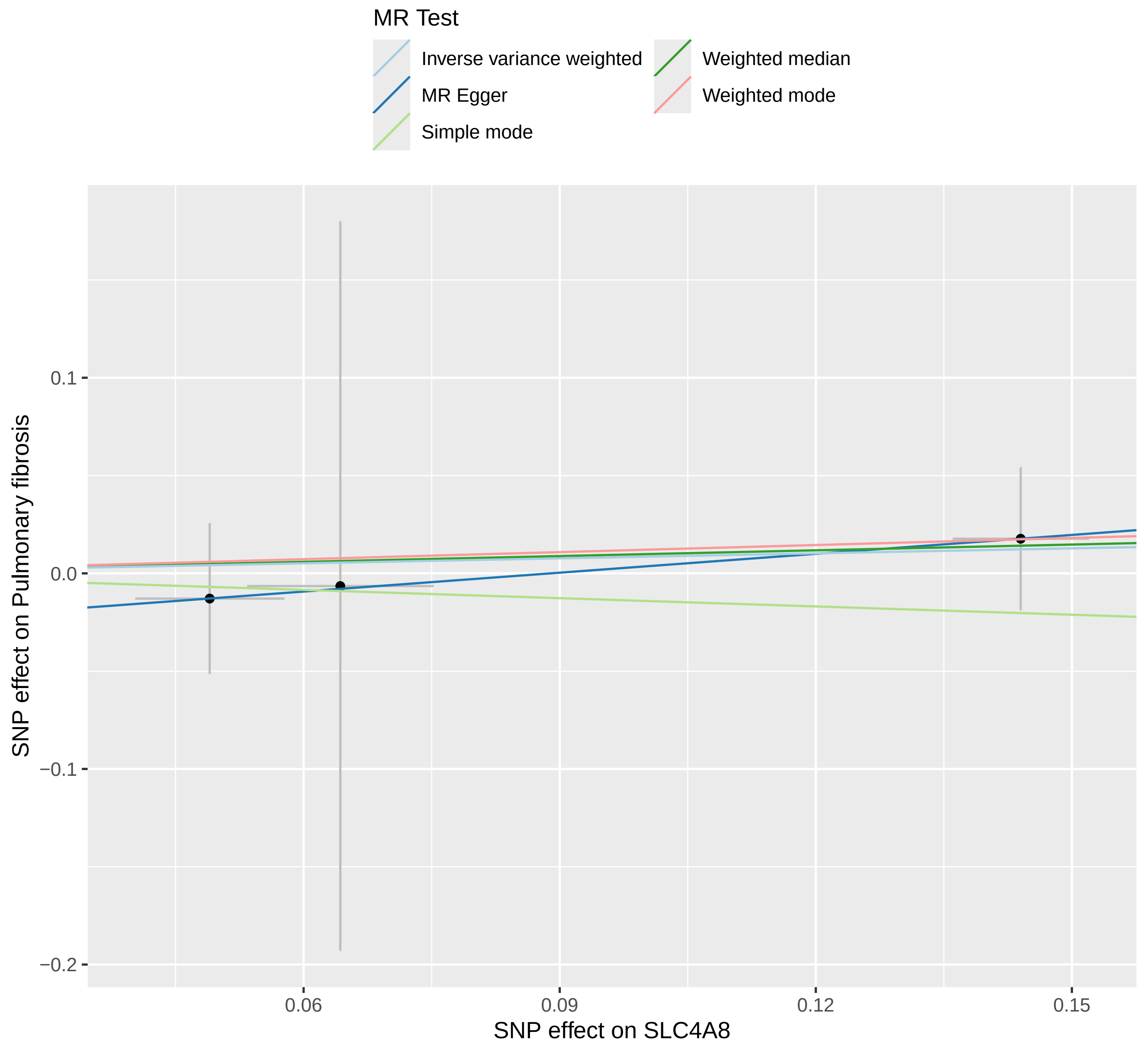

MR Test
Inverse variance weighted
MR Egger
Simple mode
Weighted median
Weighted mode
0.1
0.0
−0.1
−0.2
SNP effect on Pulmonary fibrosis
0.06
0.09
0.12
0.15
SNP effect on SLC4A8

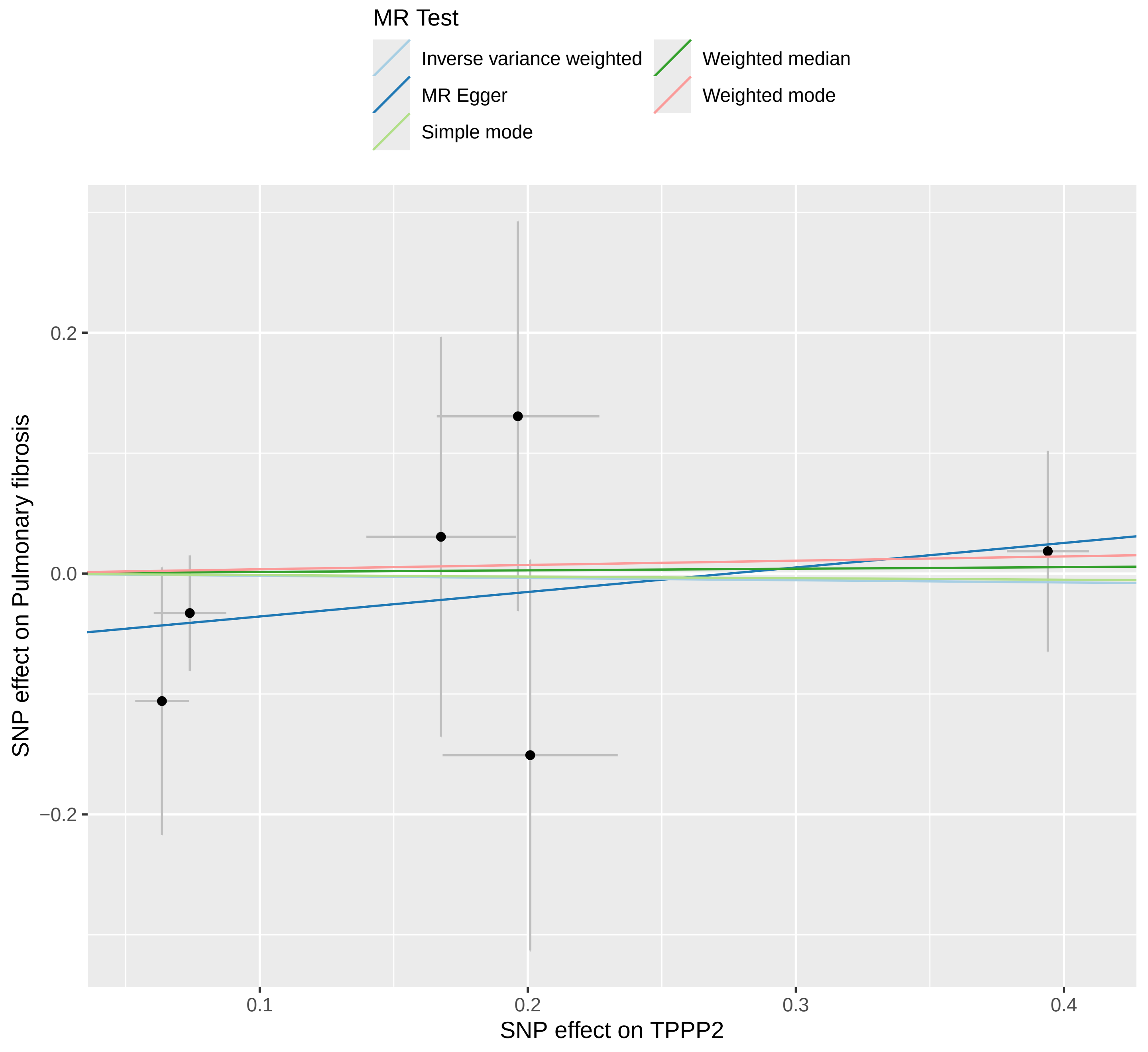

MR Test
Inverse variance weighted
MR Egger
Simple mode
Weighted median
Weighted mode
0.2
0.0
−0.2
0.1
0.2
0.3
0.4
SNP effect on Pulmonary fibrosis
SNP effect on TPPP2

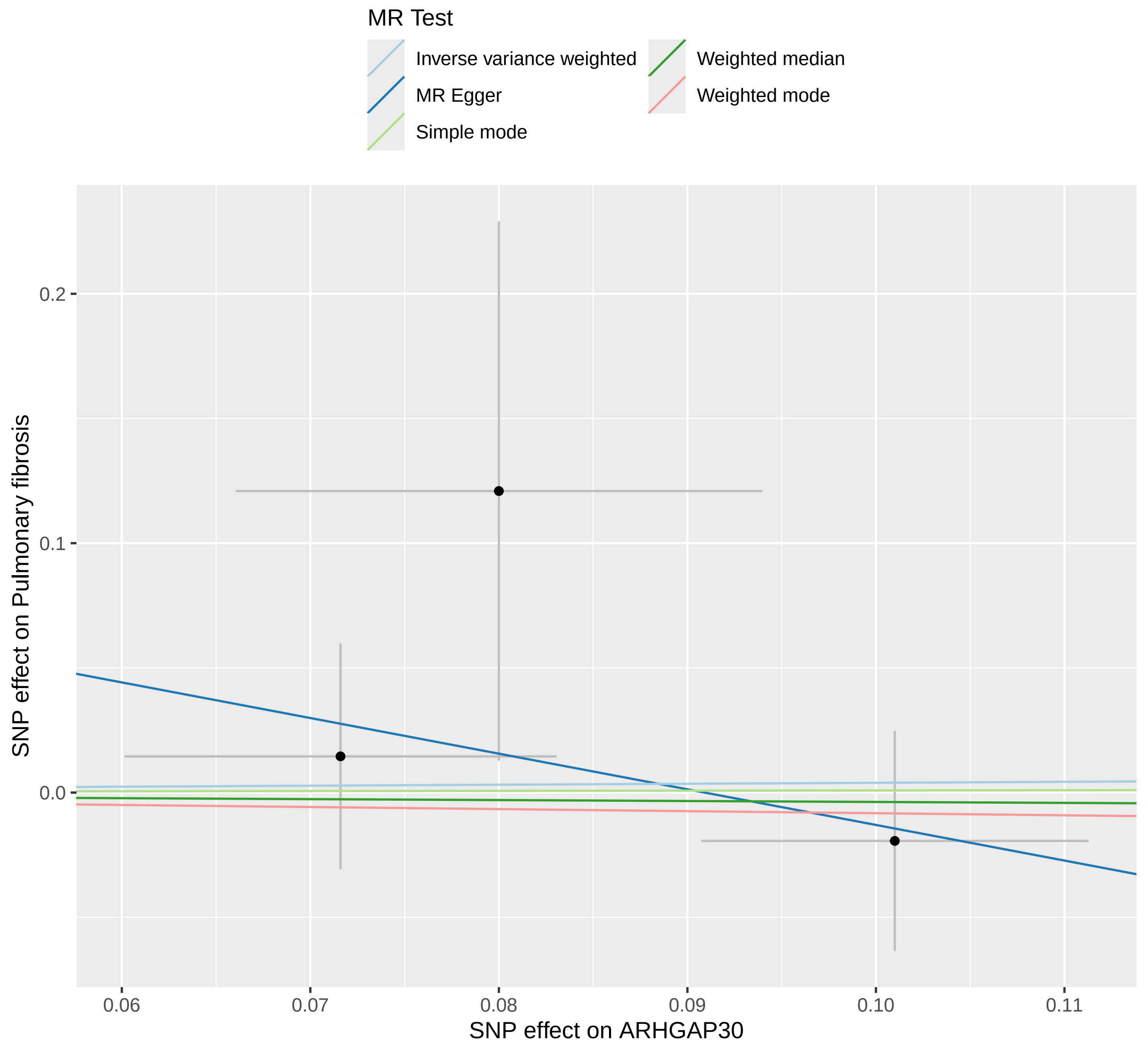
MR Test
Inverse variance weighted
MR Egger
Simple mode
Weighted median
Weighted mode
0.2
0.1
0.0
SNP effect on Pulmonary fibrosis
0.06
0.07
0.08
0.09
0.10
0.11
SNP effect on ARHGAP30

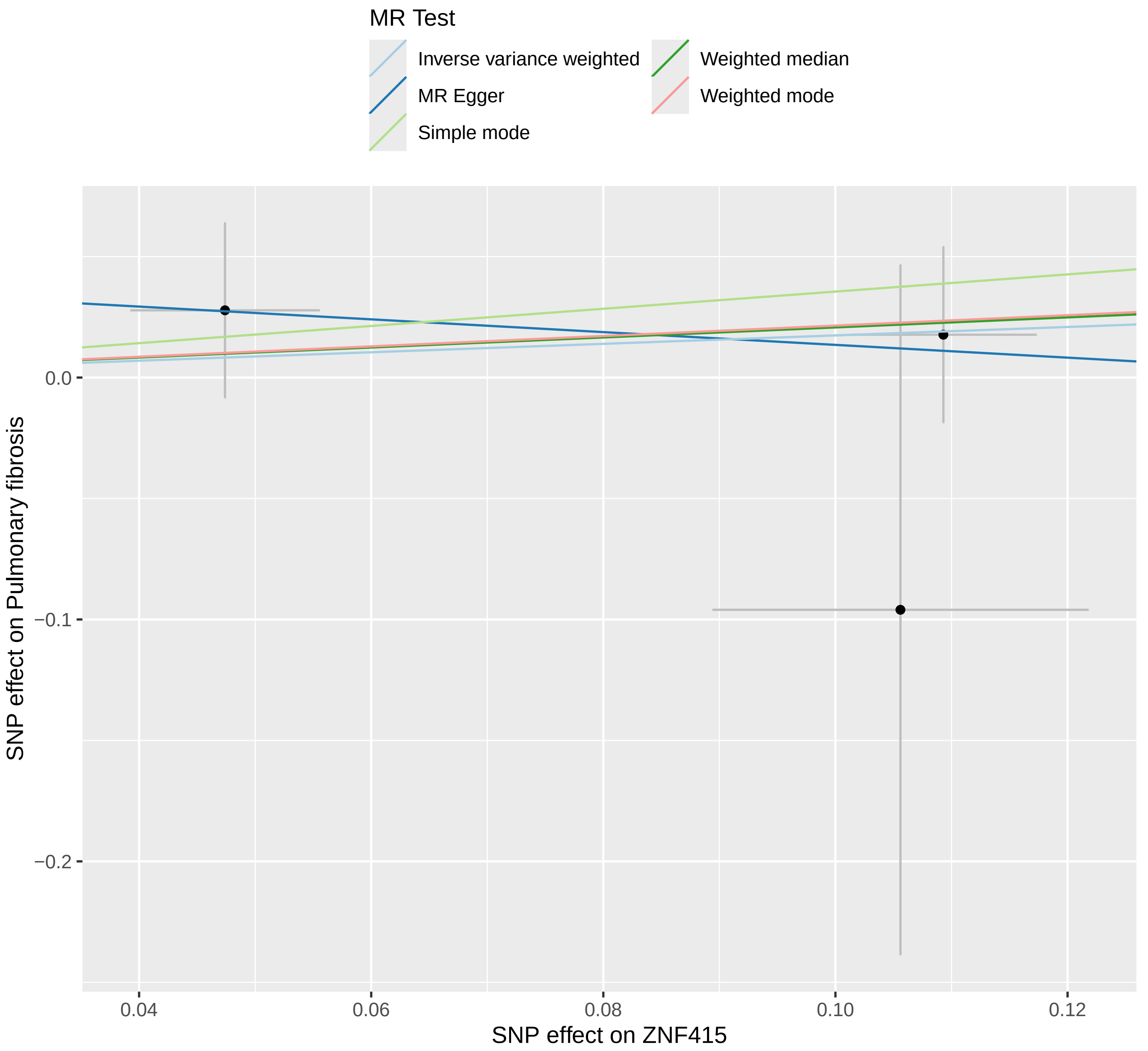
MR Test
Inverse variance weighted
MR Egger
Simple mode
Weighted median
Weighted mode
0.0
−0.1
−0.2
0.04
0.06
0.08
0.10
0.12
SNP effect on ZNF415
SNP effect on Pulmonary fibrosis

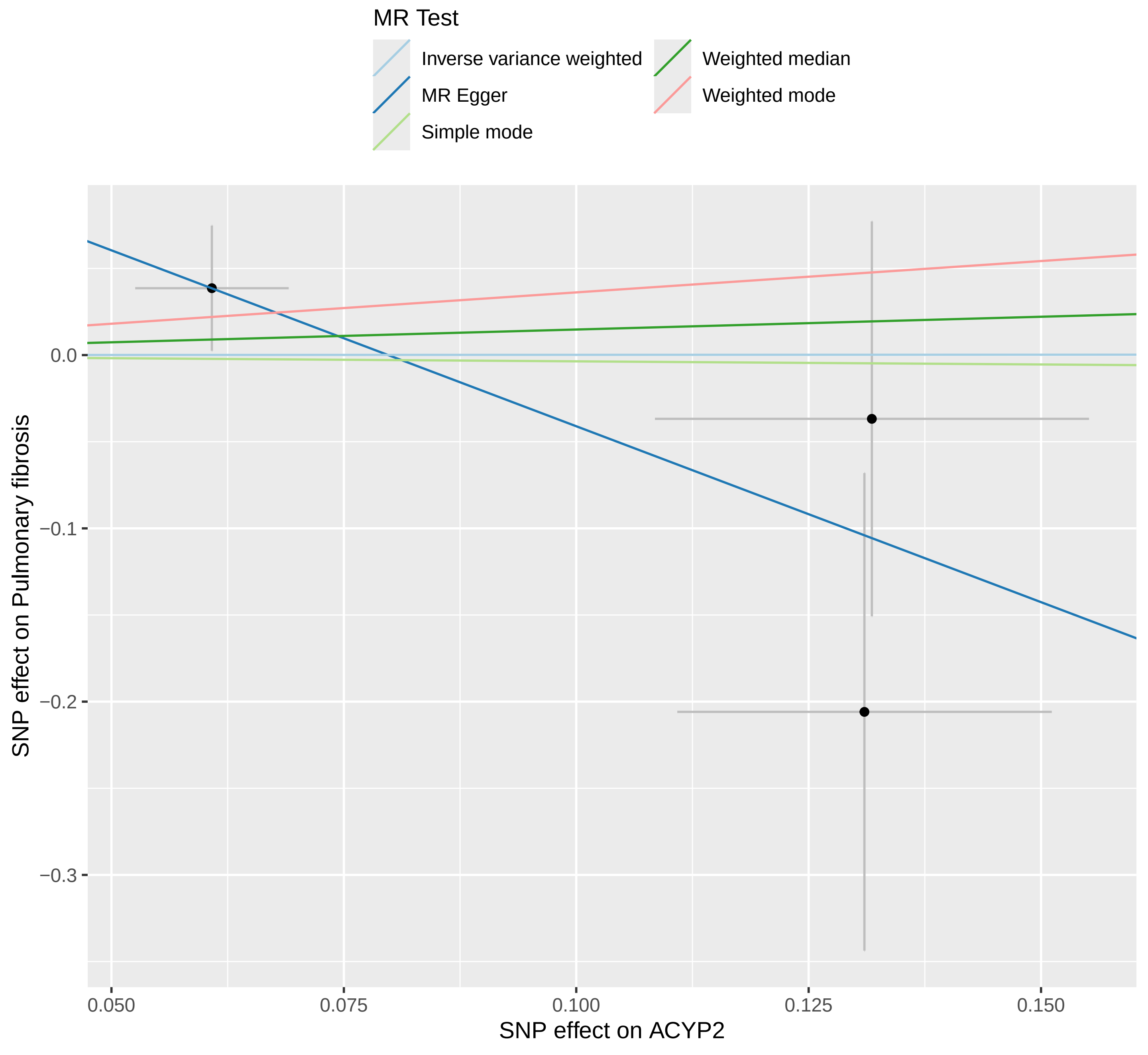
MR Test
Inverse variance weighted
MR Egger
Simple mode
Weighted median
Weighted mode
SNP effect on Pulmonary fibrosis
0.0
−0.1
−0.2
−0.3
0.050
0.075
0.100
0.125
0.150
SNP effect on ACYP2

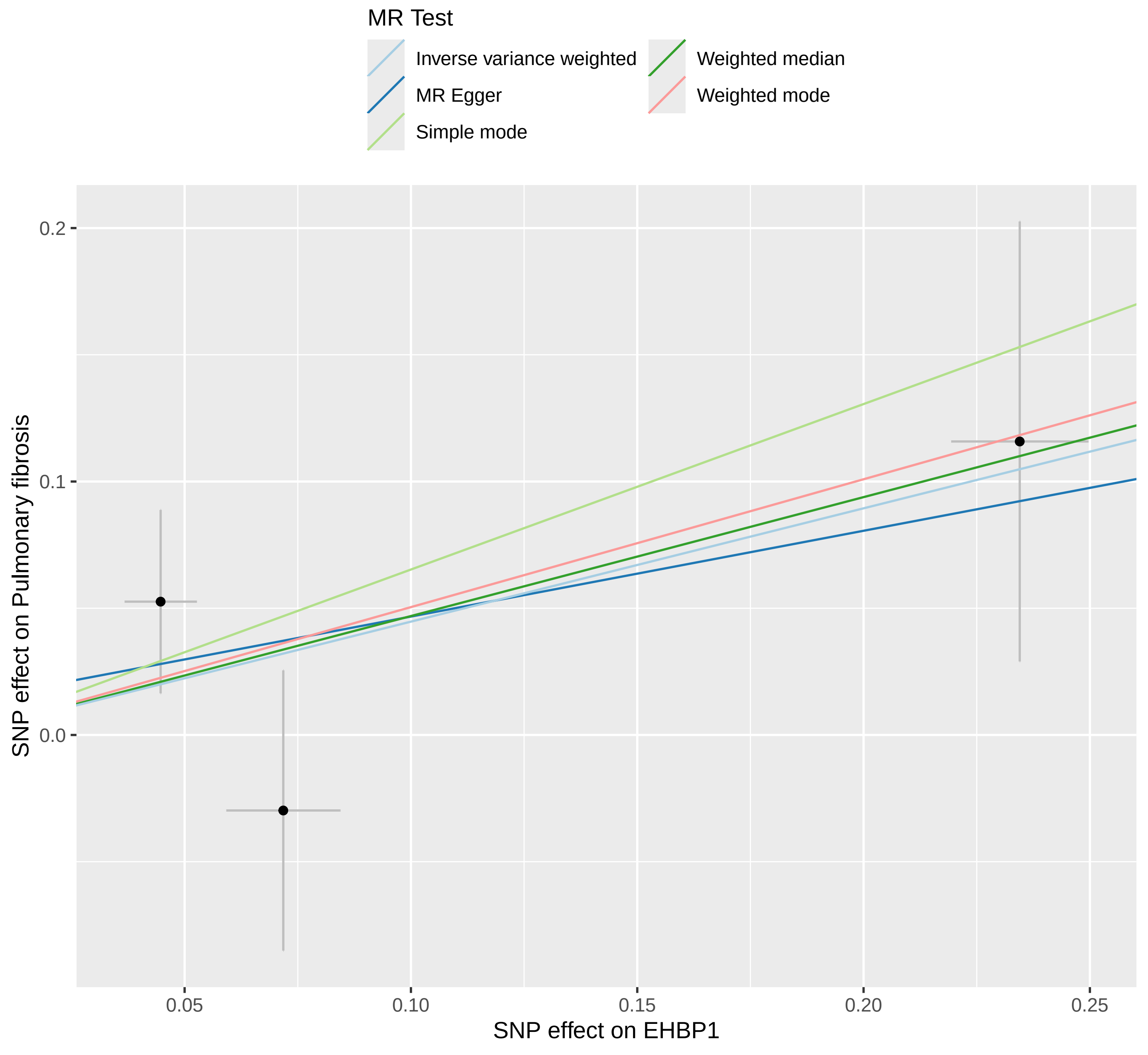
MR Test
Inverse variance weighted
MR Egger
Simple mode
Weighted median
Weighted mode
0.2
0.1
0.0
0.05
0.10
0.15
0.20
0.25
SNP effect on EHBP1
SNP effect on Pulmonary fibrosis

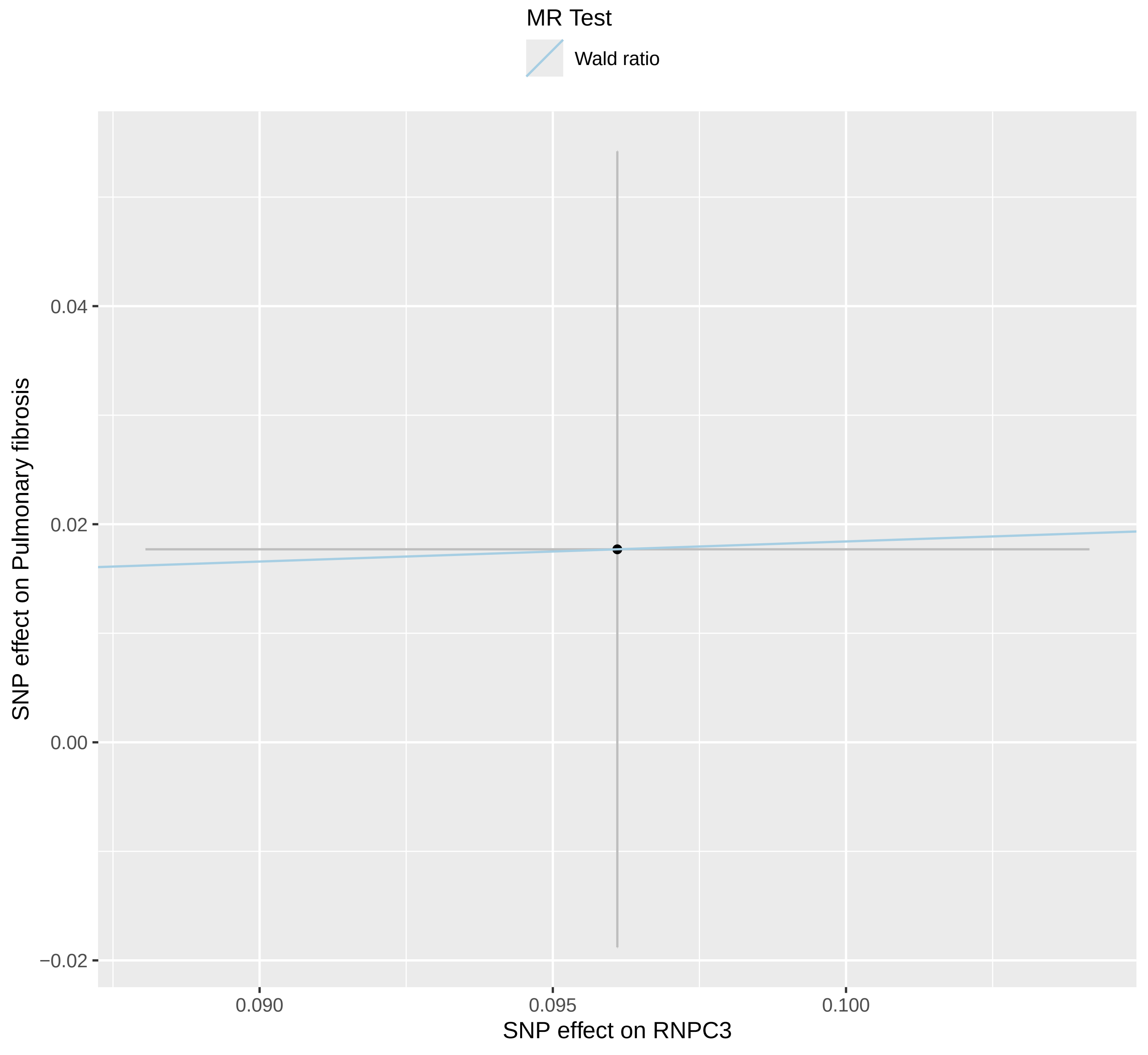

MR Test
Wald ratio
0.04
0.02
0.00
−0.02
SNP effect on Pulmonary fibrosis
0.090
0.095
0.100
SNP effect on RNPC3

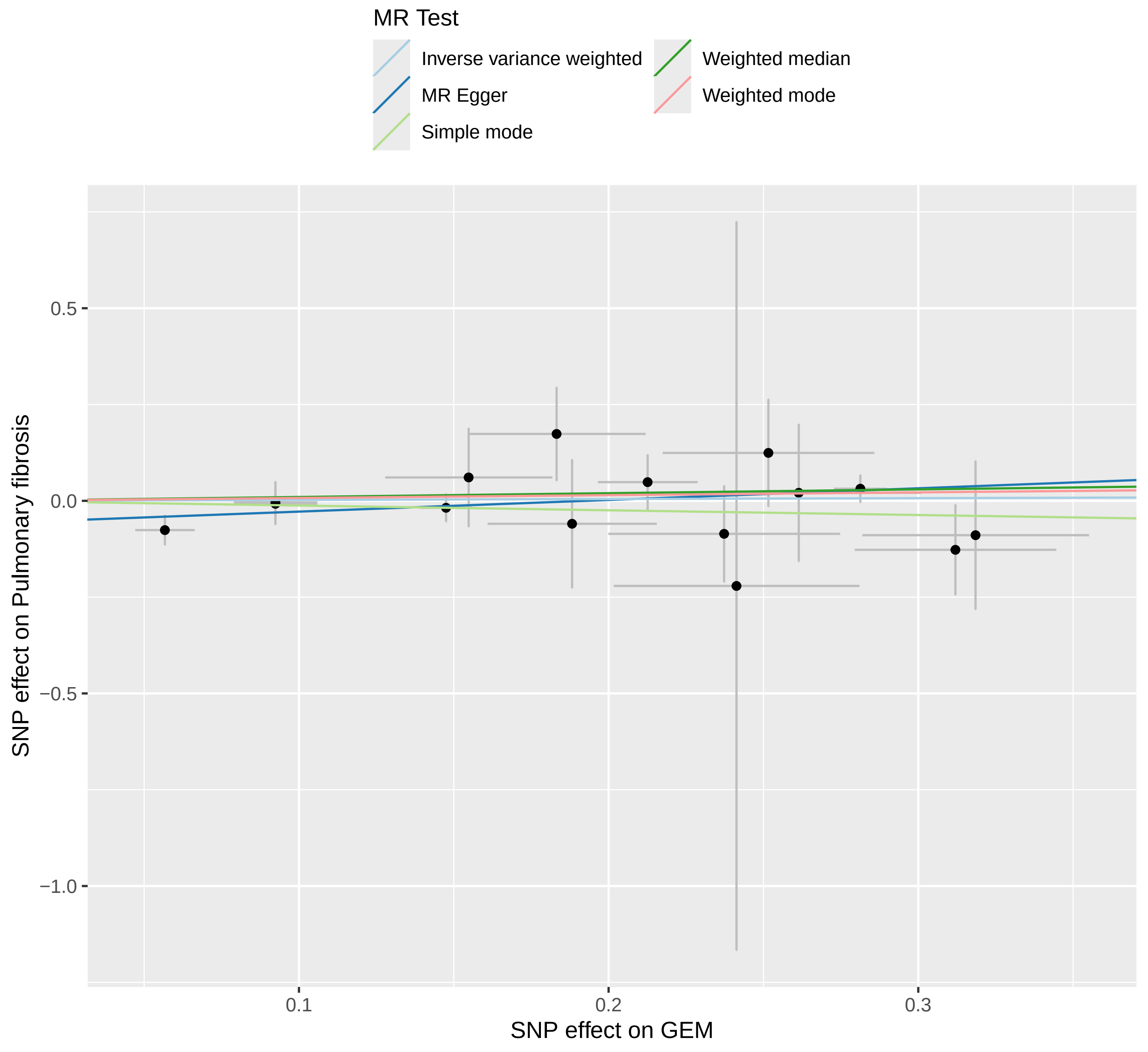
MR Test
Inverse variance weighted
MR Egger
Simple mode
Weighted median
Weighted mode
0.5
0.0
−0.5
−1.0
SNP effect on Pulmonary fibrosis
0.1
0.2
0.3
SNP effect on GEM

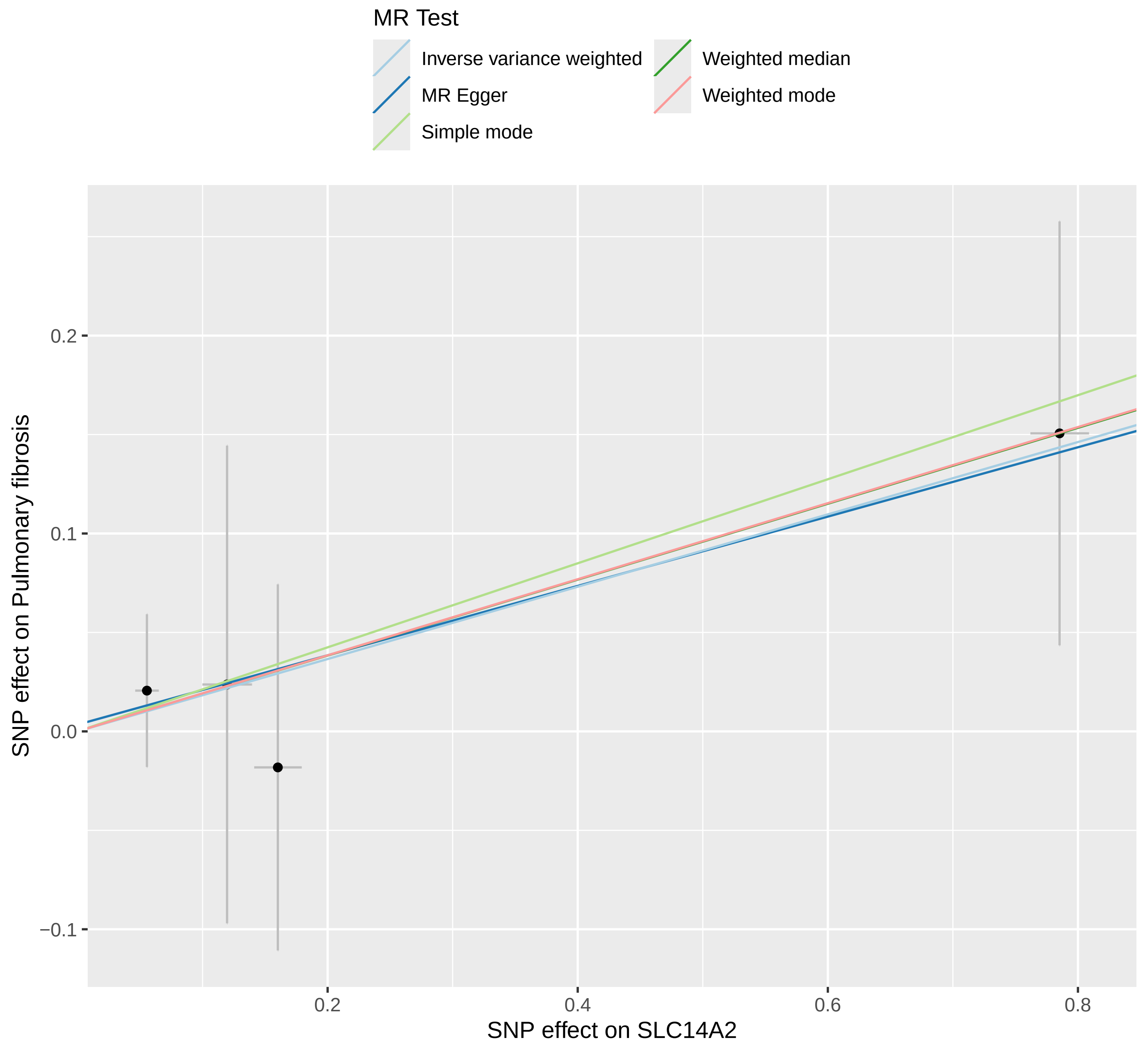
MR Test
Inverse variance weighted
MR Egger
Simple mode
Weighted median
Weighted mode
0.2
0.1
0.0
−0.1
0.2
0.4
0.6
0.8
SNP effect on Pulmonary fibrosis
SNP effect on SLC14A2

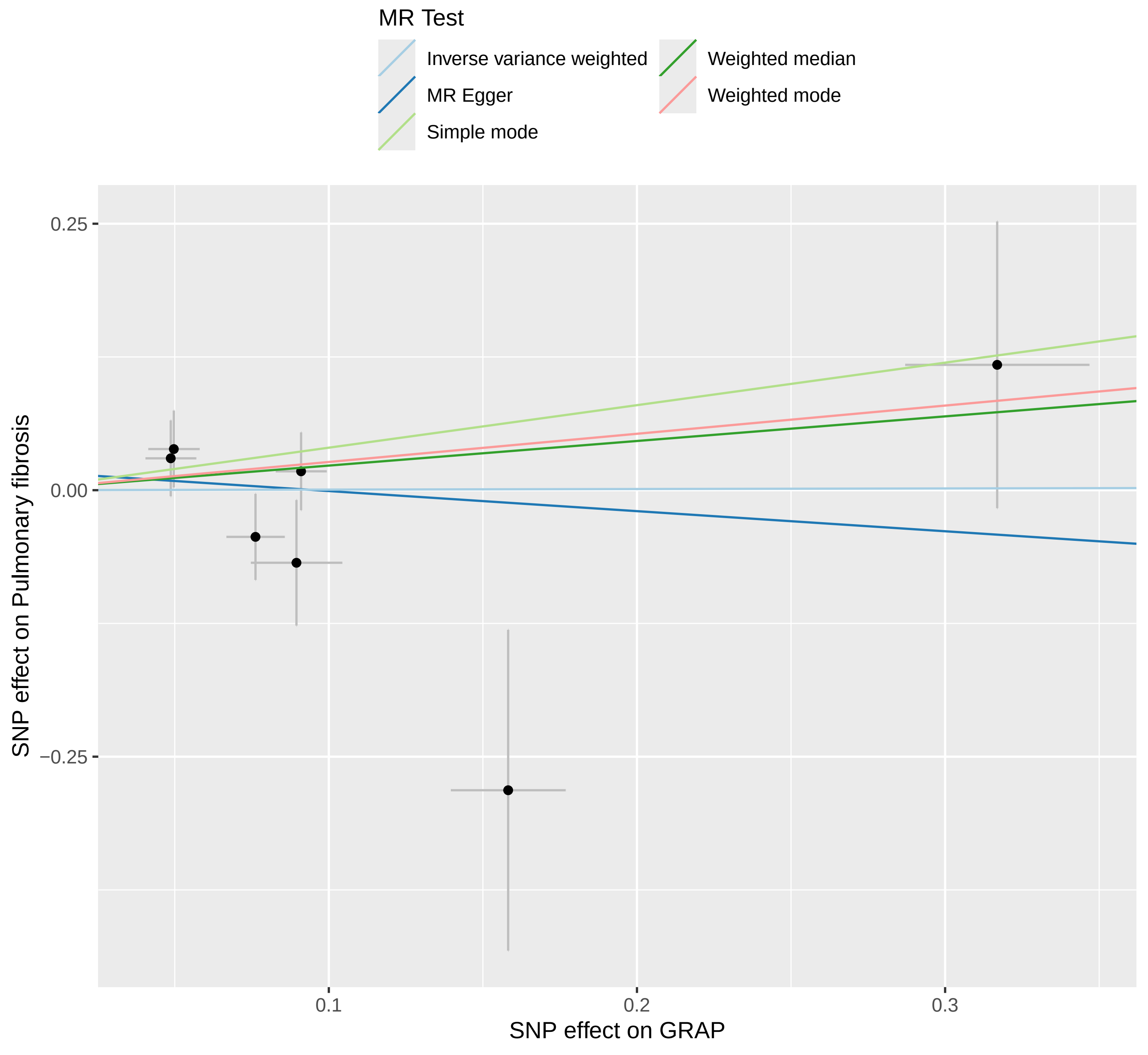

MR Test
Inverse variance weighted
MR Egger
Simple mode
Weighted median
Weighted mode
0.25
0.00
−0.25
0.1
0.2
0.3
SNP effect on Pulmonary fibrosis
SNP effect on GRAP

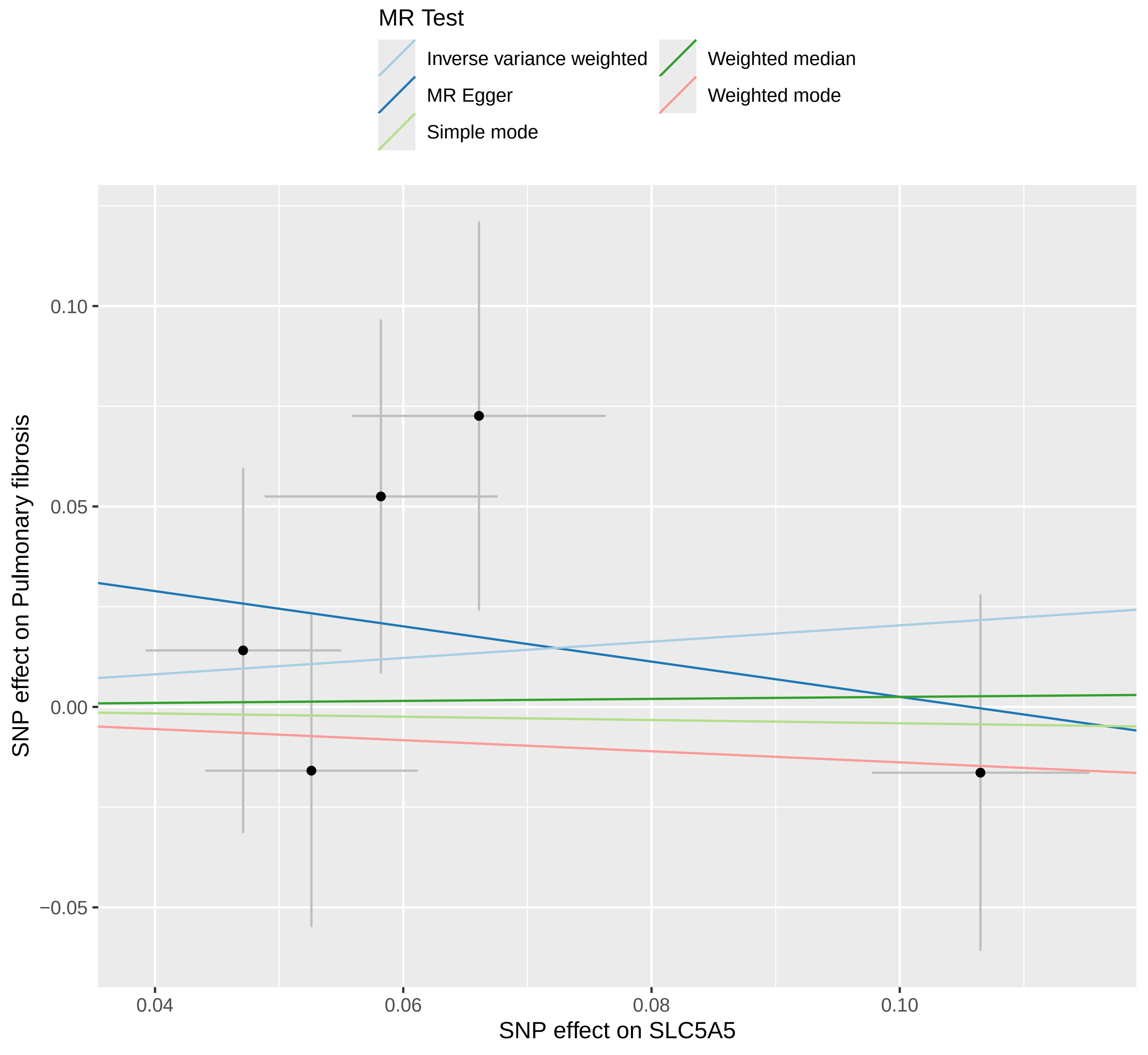
MR Test
Inverse variance weighted
MR Egger
Simple mode
Weighted median
Weighted mode
0.10
0.05
0.00
−0.05
0.04
0.06
0.08
0.10
SNP effect on Pulmonary fibrosis
SNP effect on SLC5A5

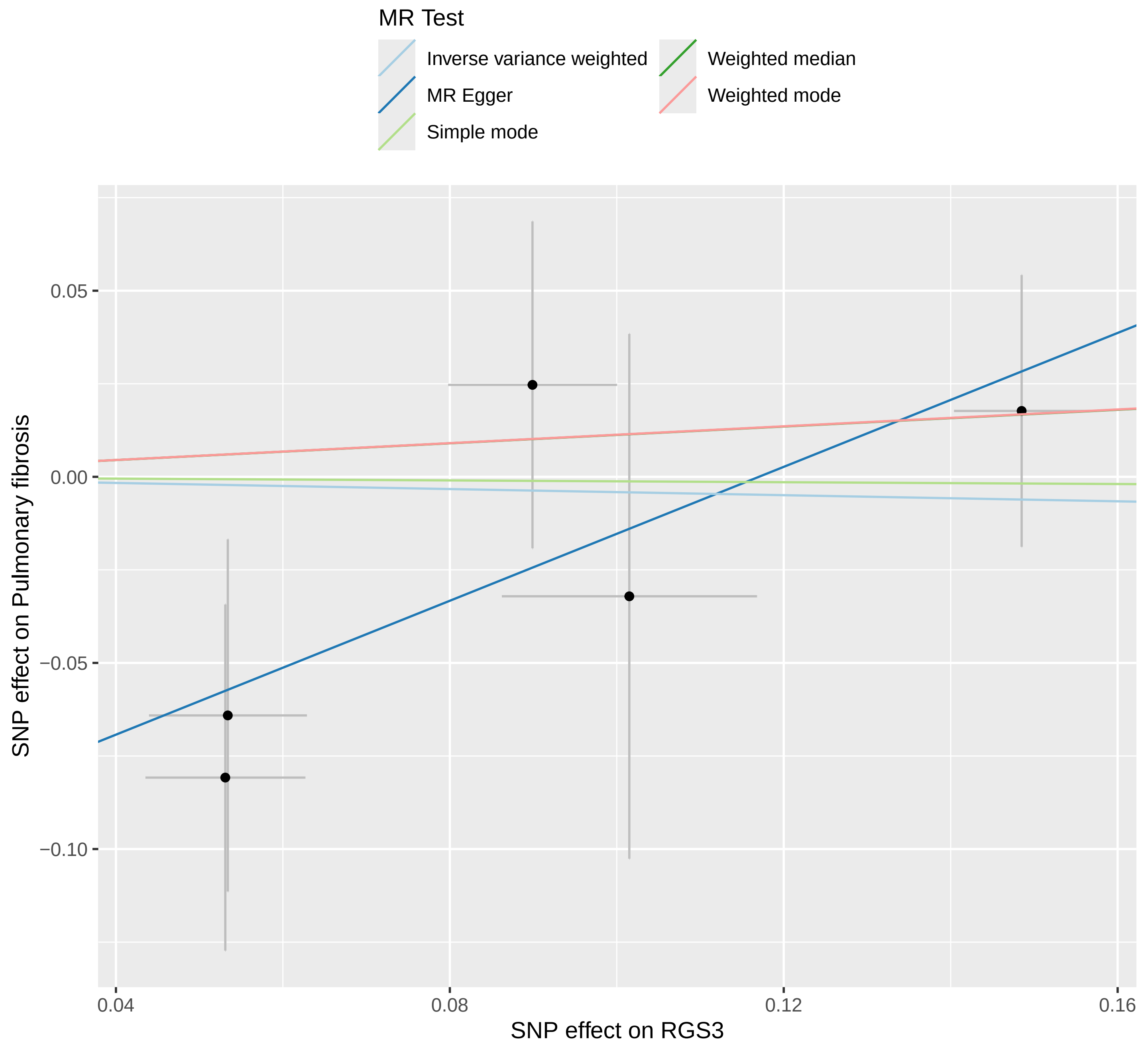
MR Test
Inverse variance weighted
MR Egger
Simple mode
Weighted median
Weighted mode
0.05
0.00
−0.05
−0.10
0.04
0.08
0.12
0.16
SNP effect on Pulmonary fibrosis
SNP effect on RGS3

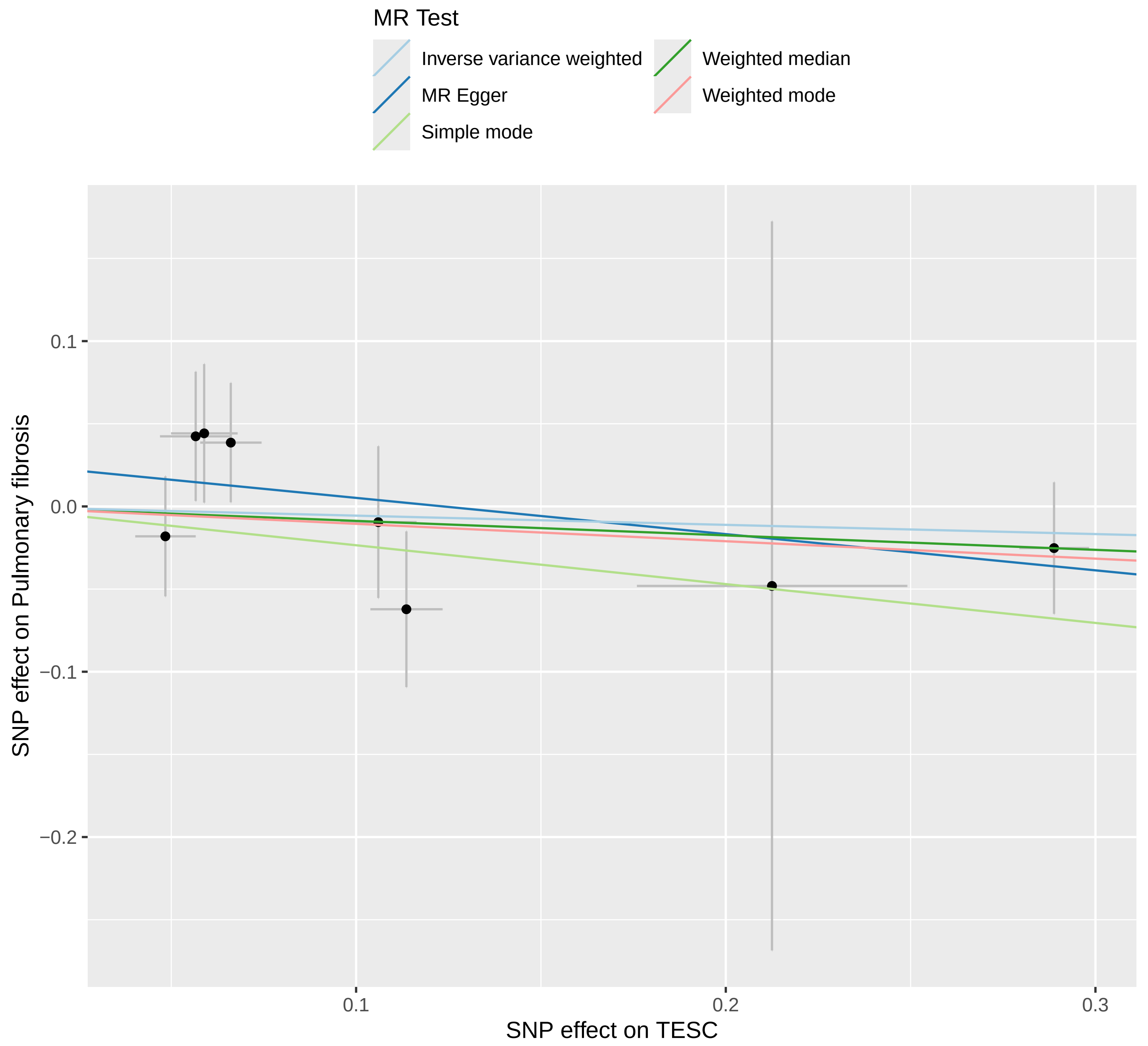
MR Test
Inverse variance weighted
MR Egger
Simple mode
Weighted median
Weighted mode
0.1
0.0
−0.1
−0.2
SNP effect on Pulmonary fibrosis
0.1
0.2
0.3
SNP effect on TESC

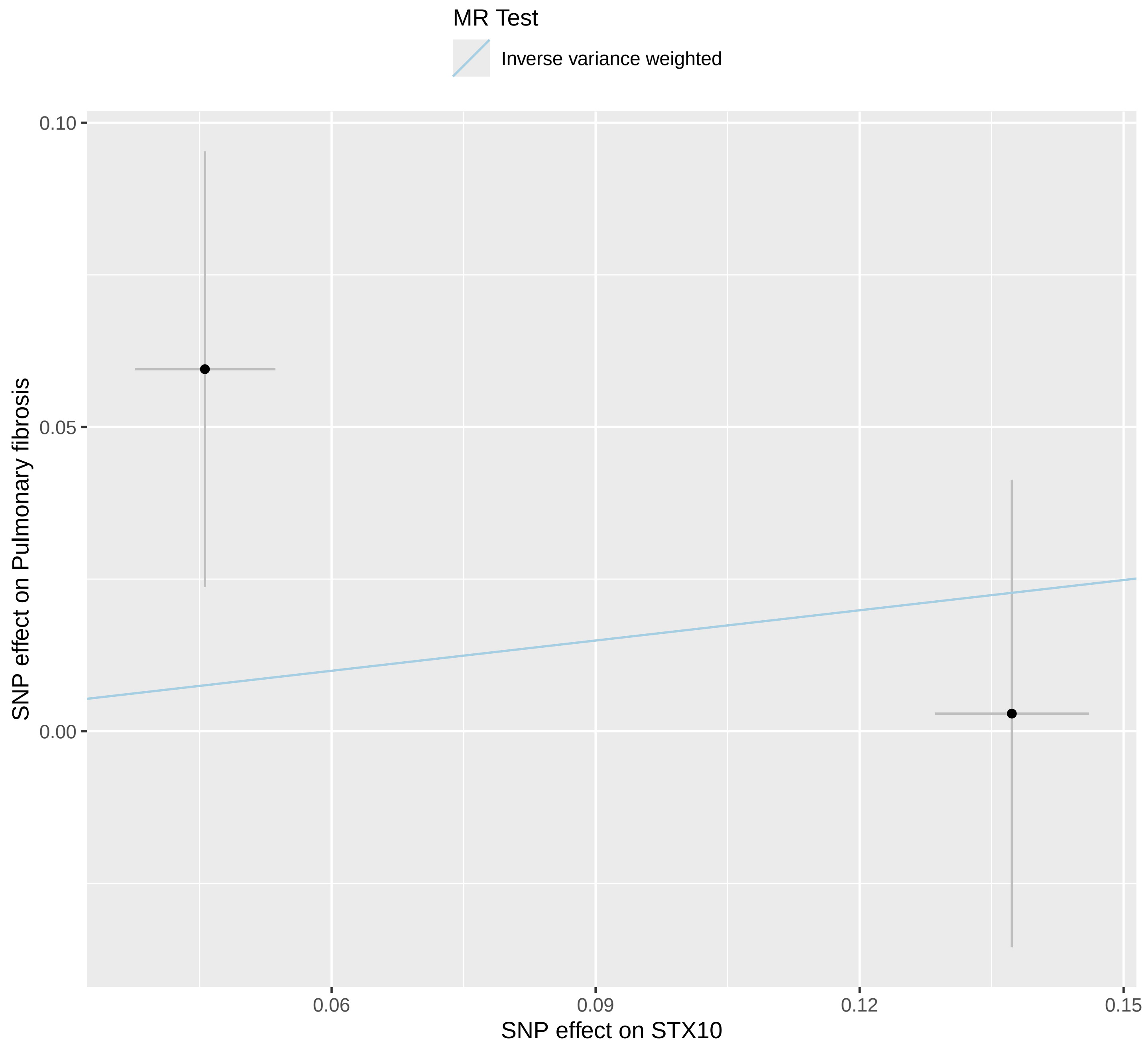
MR Test
Inverse variance weighted
0.10
0.05
0.00
0.06
0.09
0.12
0.15
SNP effect on Pulmonary fibrosis
SNP effect on STX10

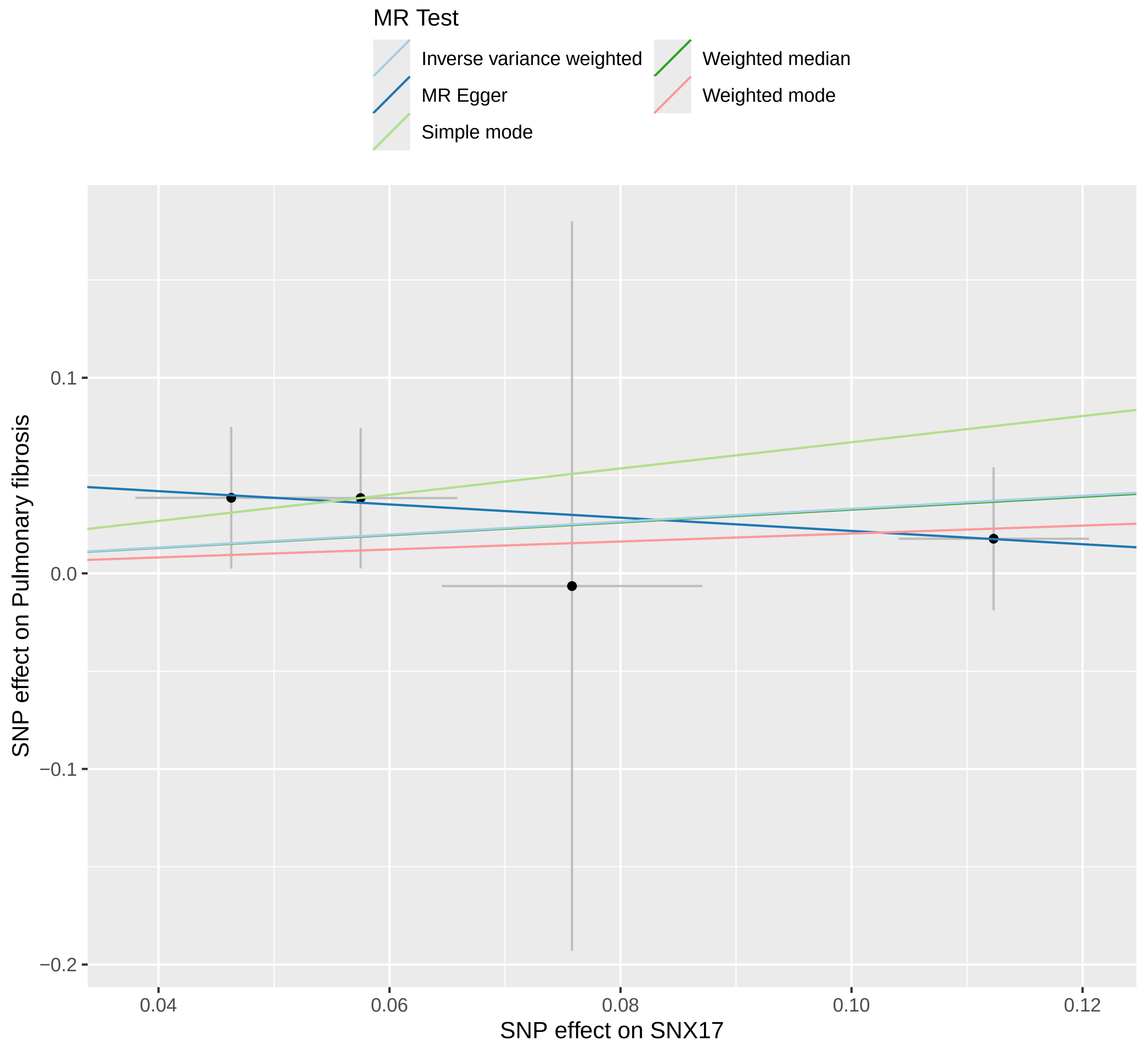
MR Test
Inverse variance weighted
MR Egger
Simple mode
Weighted median
Weighted mode
0.1
0.0
−0.1
−0.2
0.04
0.06
0.08
0.10
0.12
SNP effect on Pulmonary fibrosis
SNP effect on SNX17

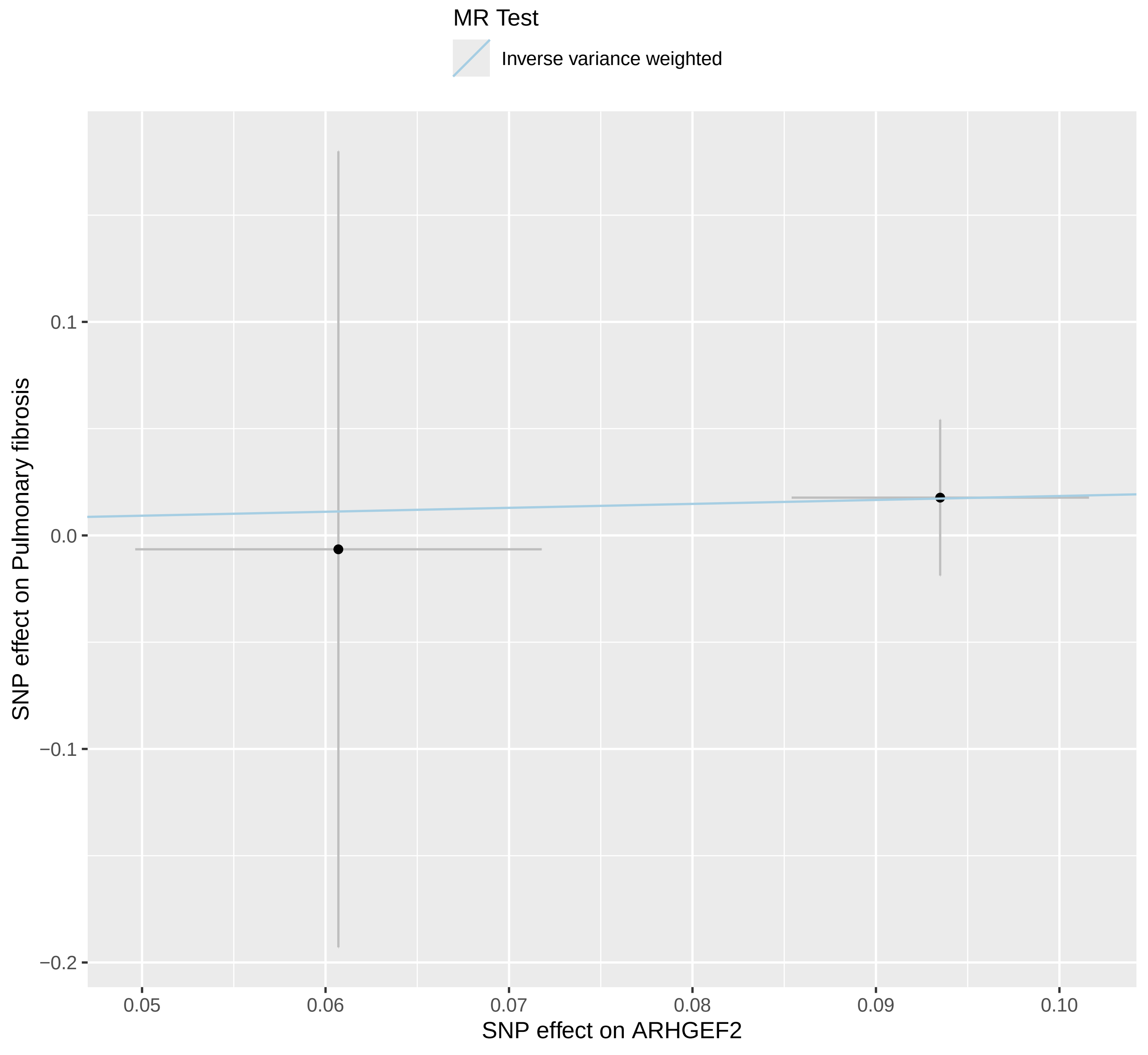

MR Test
Inverse variance weighted
SNP effect on Pulmonary fibrosis
0.1
0.0
−0.1
−0.2
0.05
0.06
0.07
0.08
0.09
0.10
SNP effect on ARHGEF2

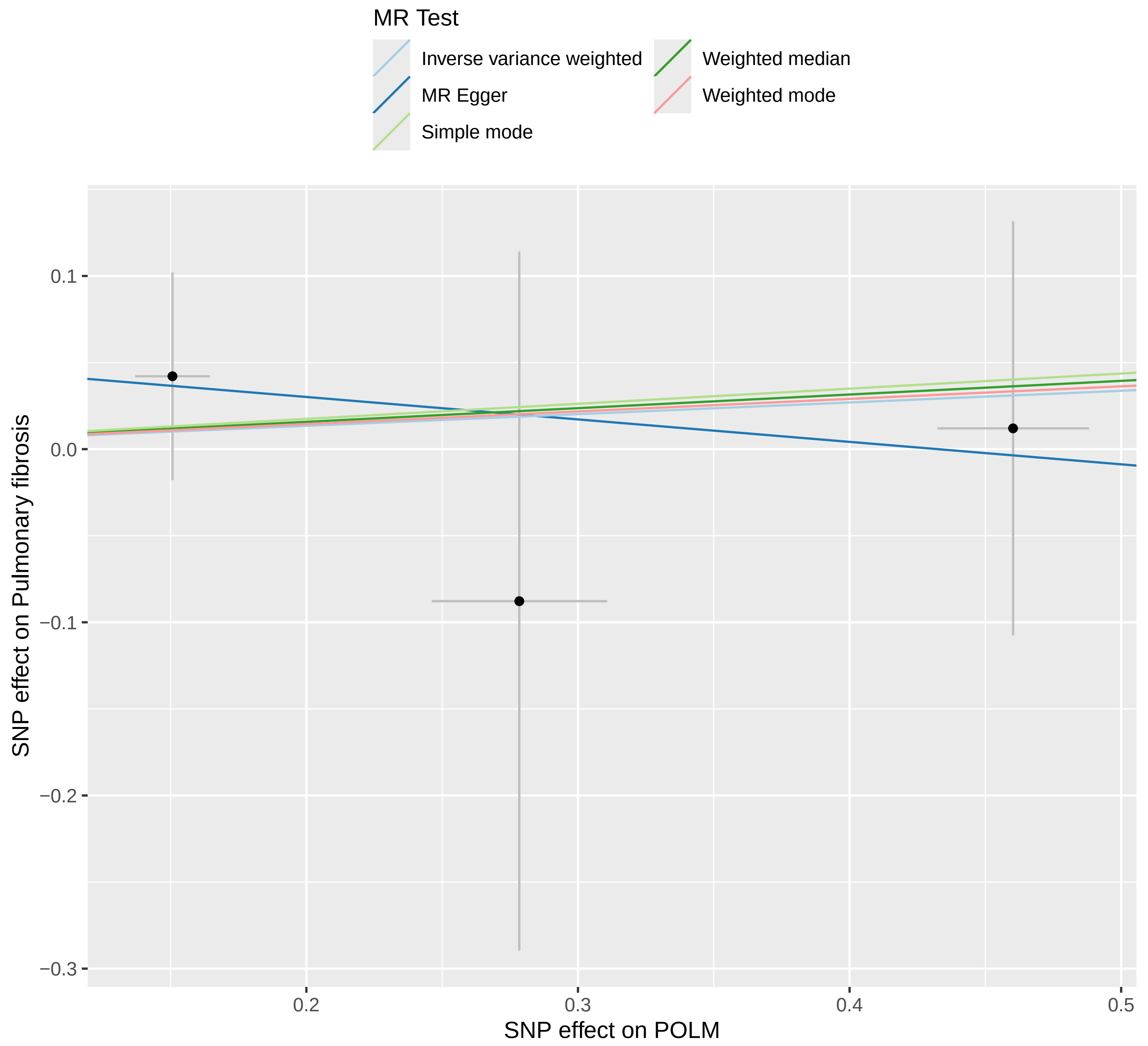

MR Test
Inverse variance weighted
MR Egger
Simple mode
Weighted median
Weighted mode
0.1
0.0
−0.1
−0.2
−0.3
SNP effect on Pulmonary fibrosis
0.2
0.3
0.4
0.5
SNP effect on POLM

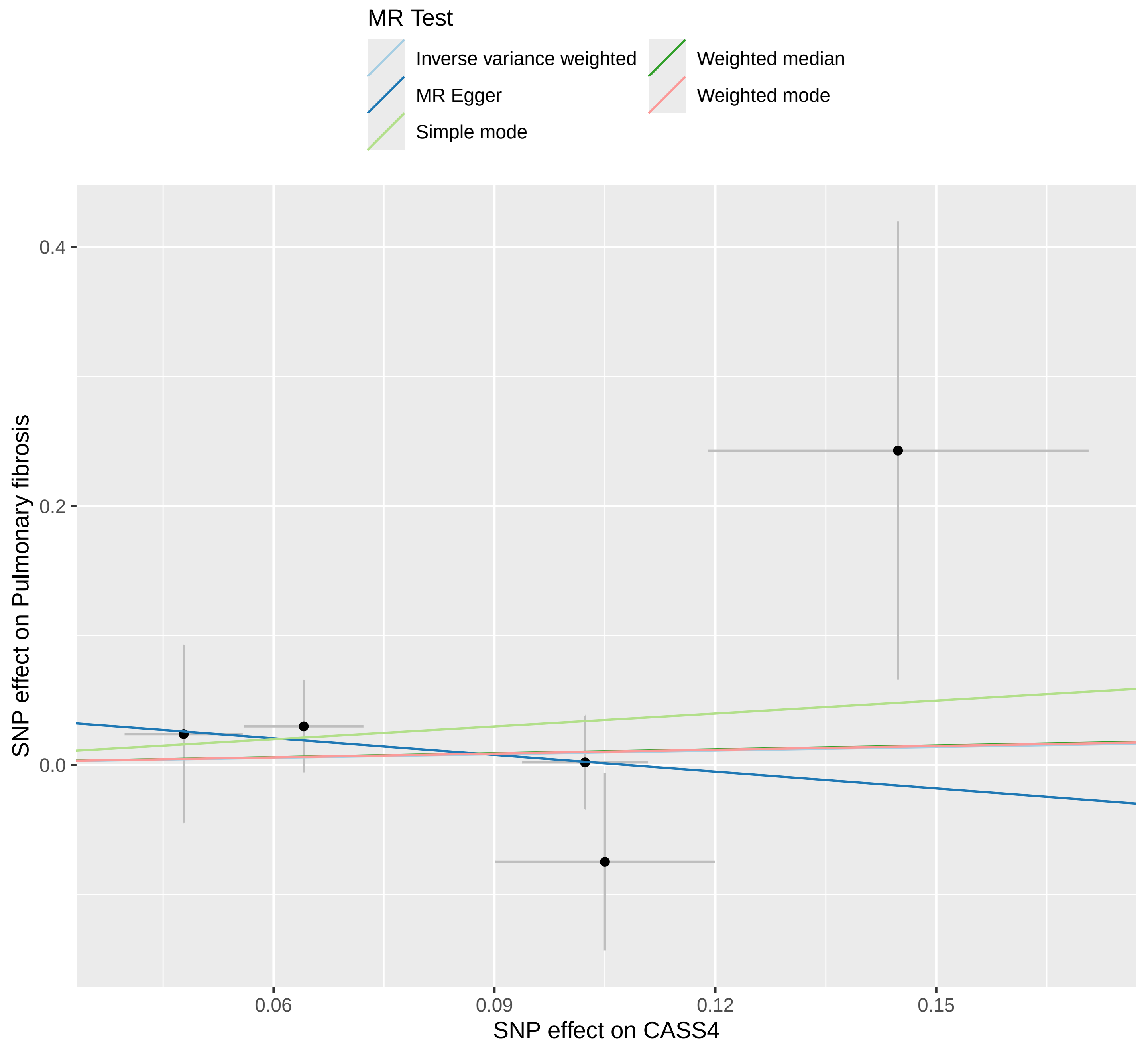

MR Test
Inverse variance weighted
MR Egger
Simple mode
Weighted median
Weighted mode
0.4
0.2
0.0
0.06
0.09
0.12
0.15
SNP effect on CASS4
SNP effect on Pulmonary fibrosis

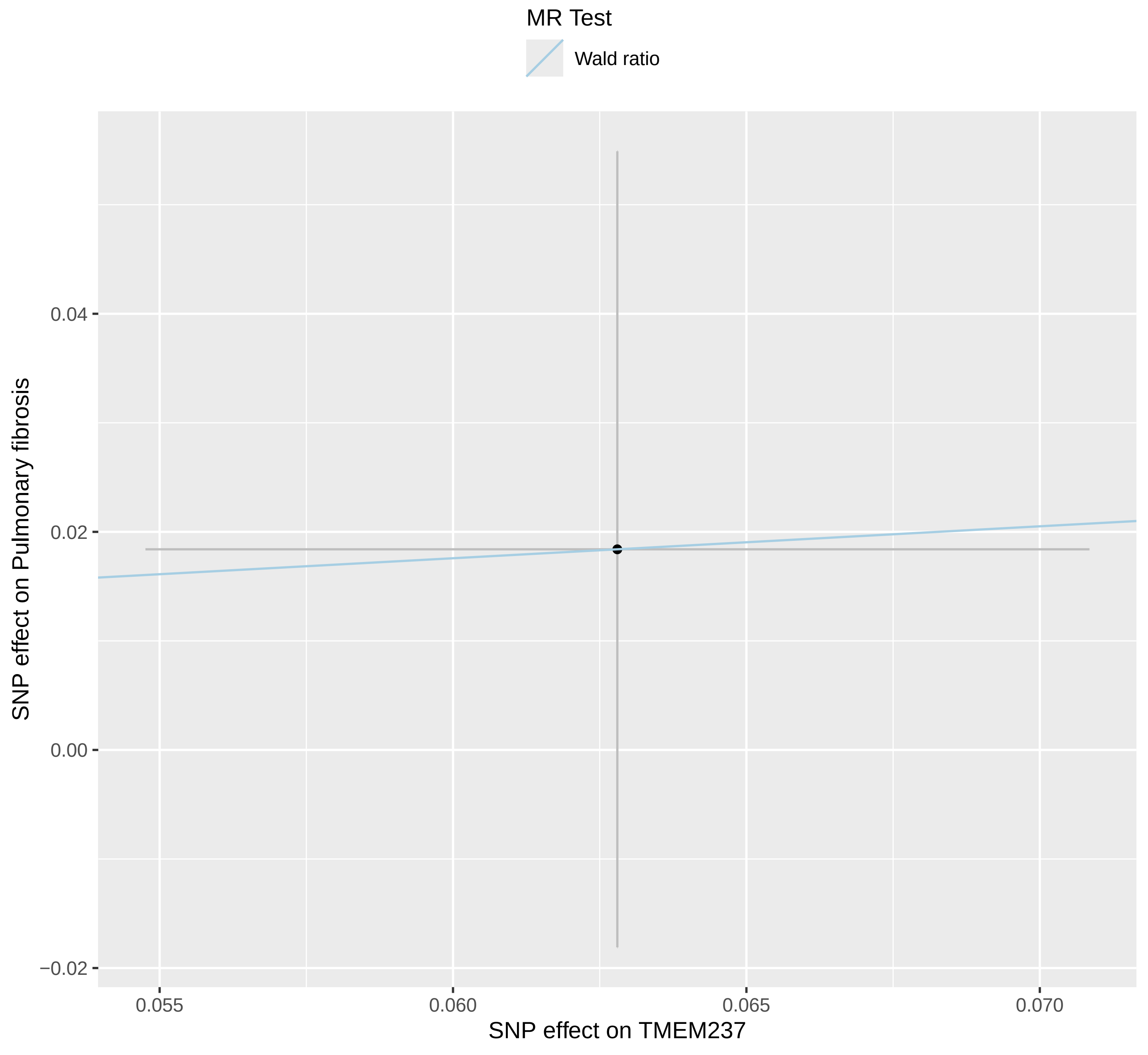
MR Test
Wald ratio
SNP effect on Pulmonary fibrosis
0.04
0.02
0.00
−0.02
0.055
0.060
0.065
0.070
SNP effect on TMEM237

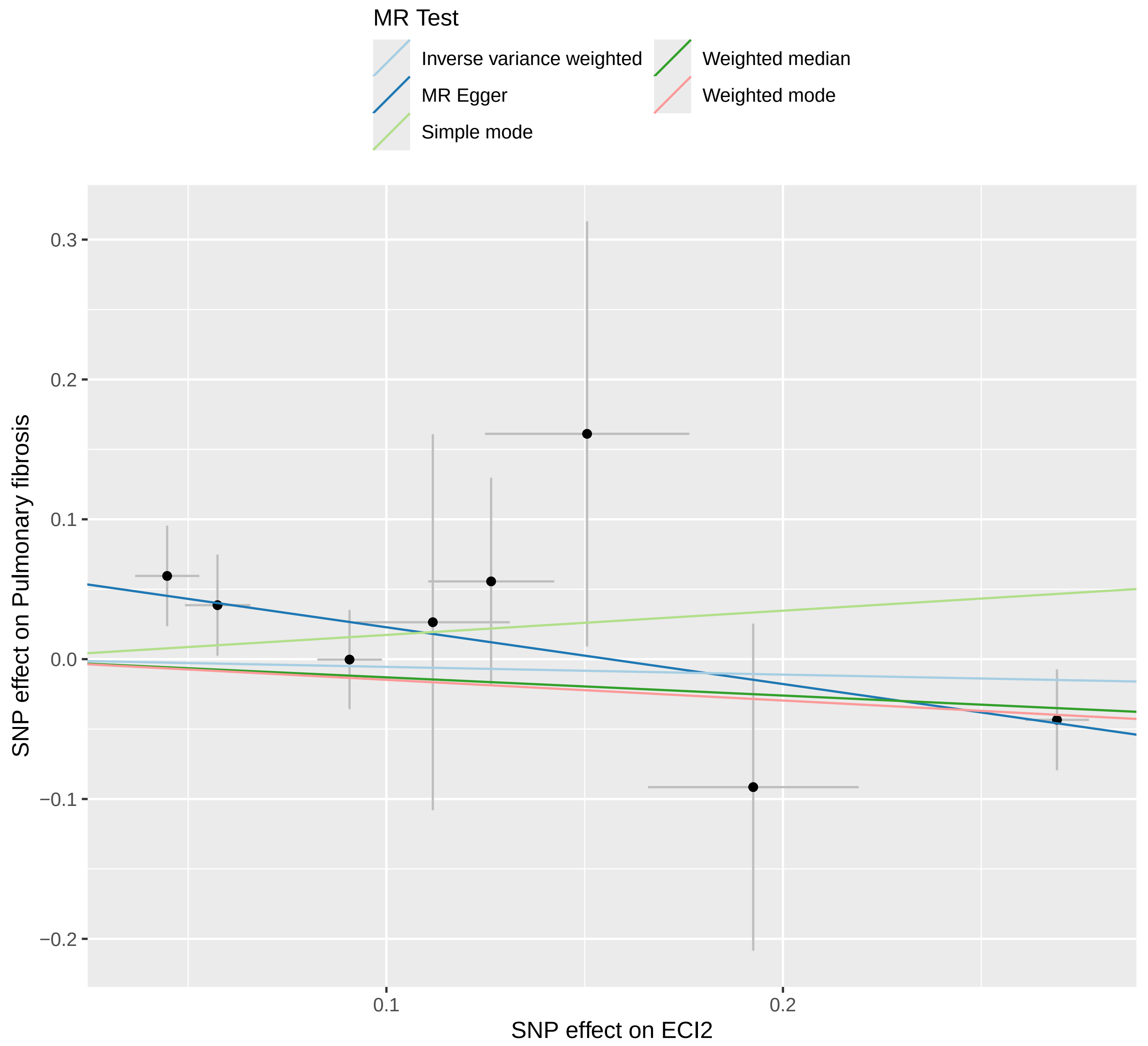

MR Test
Inverse variance weighted
MR Egger
Simple mode
Weighted median
Weighted mode
SNP effect on Pulmonary fibrosis
0.3
0.2
0.1
0.0
−0.1
−0.2
0.1
0.2
SNP effect on ECI2

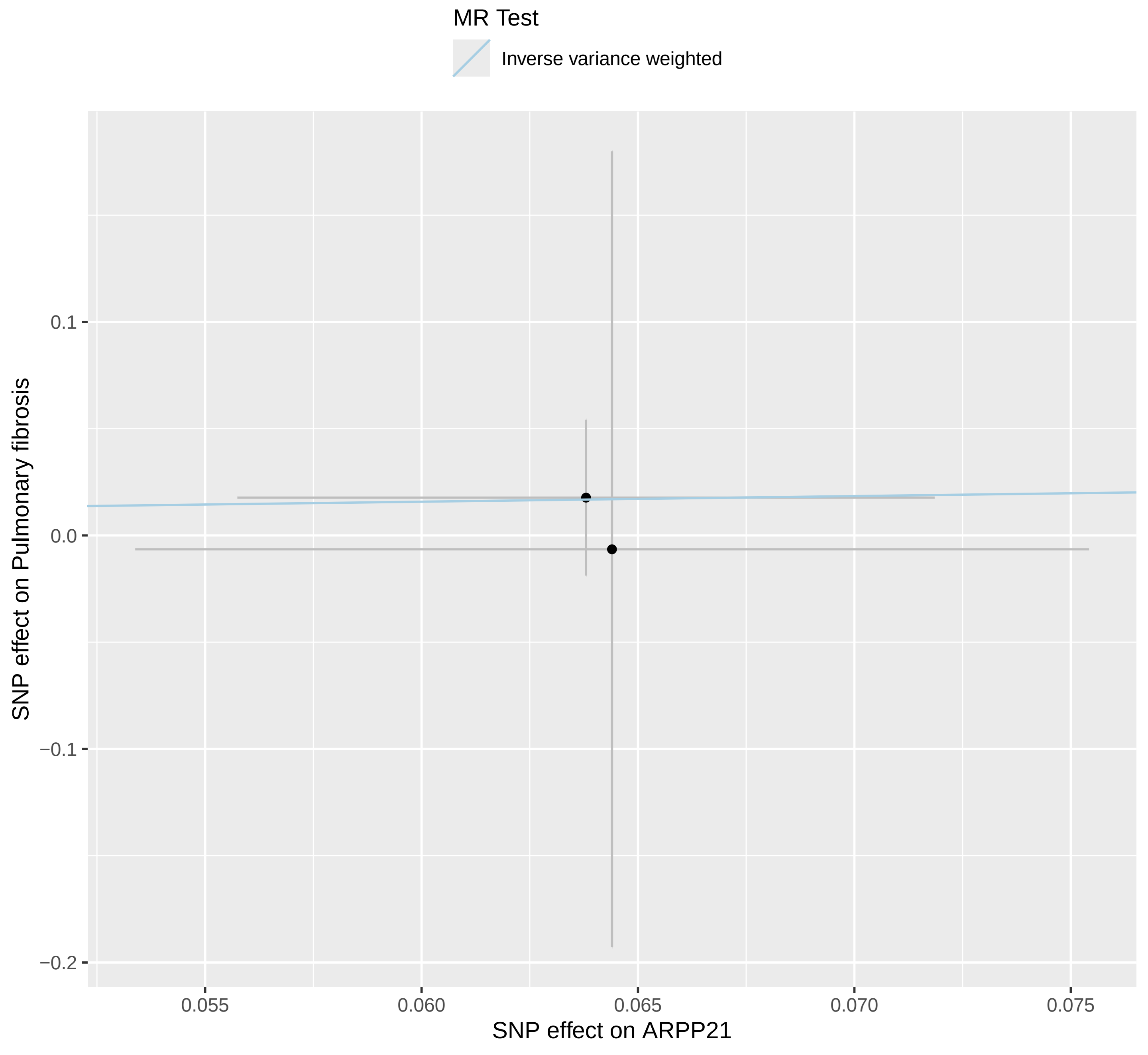

MR Test
Inverse variance weighted
0.1
0.0
−0.1
−0.2
SNP effect on Pulmonary fibrosis
0.055
0.060
0.065
0.070
0.075
SNP effect on ARPP21

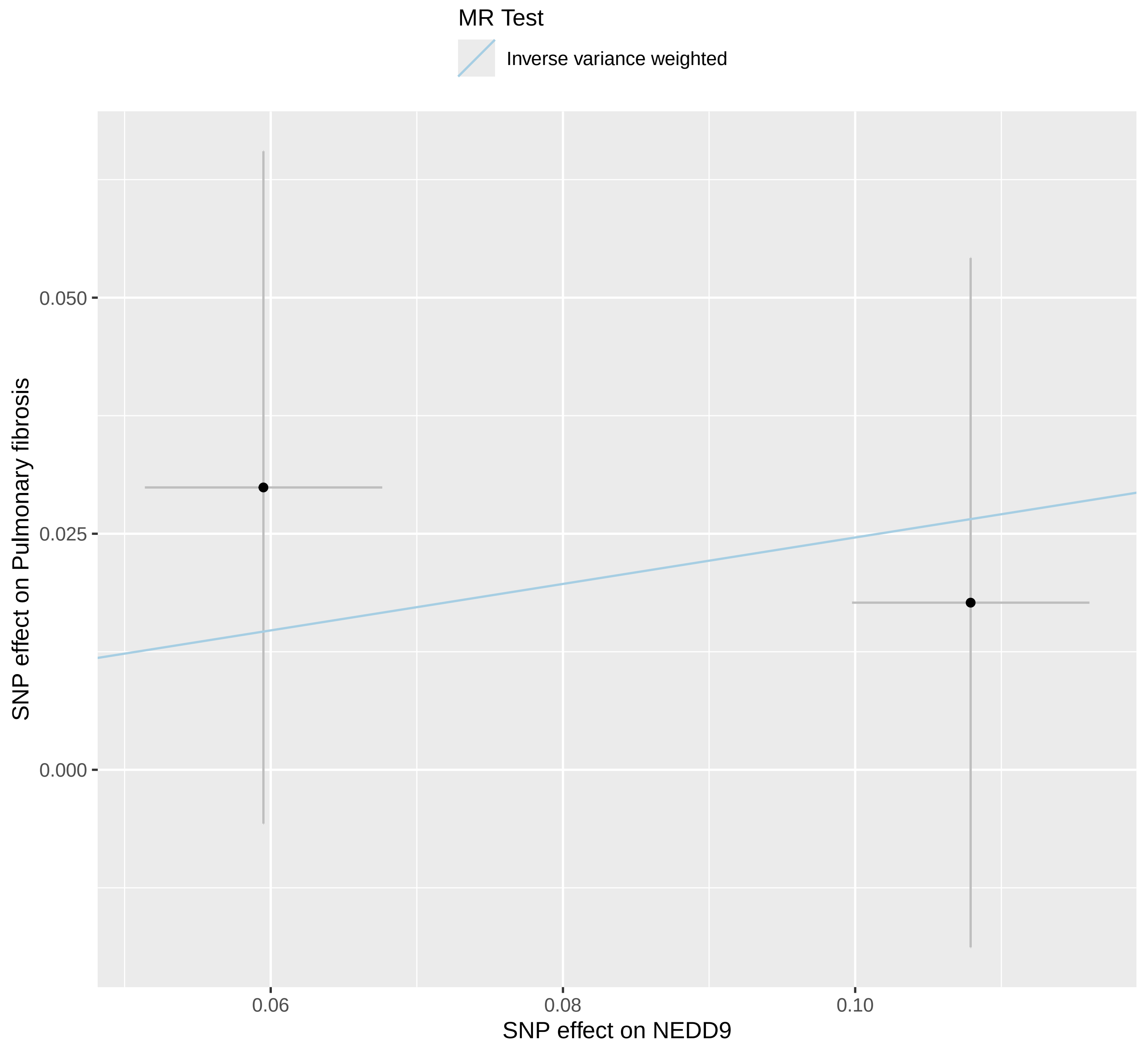

MR Test
Inverse variance weighted
SNP effect on Pulmonary fibrosis
0.050
0.025
0.000
0.06
0.08
0.10
SNP effect on NEDD9

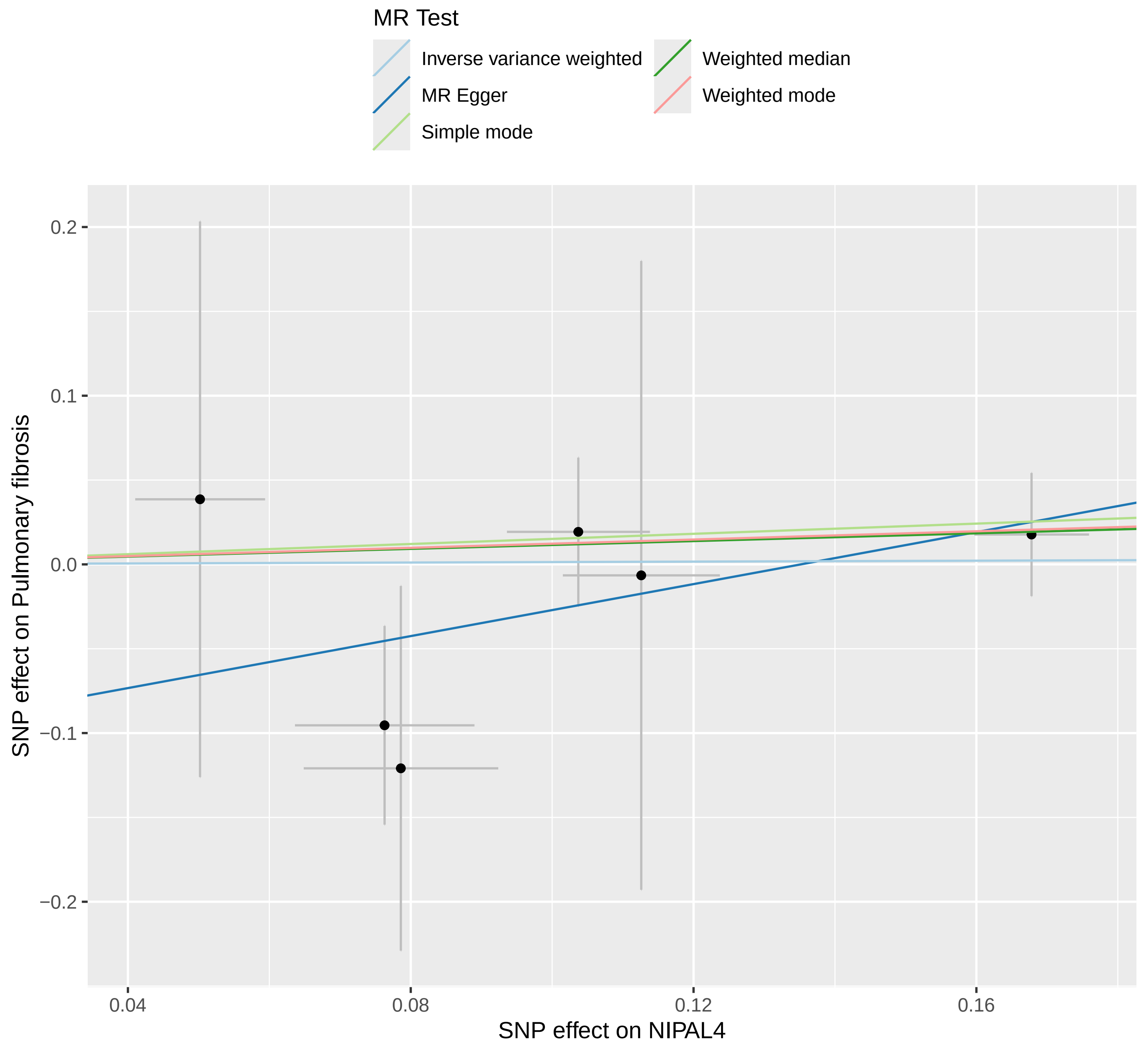

MR Test
Inverse variance weighted
MR Egger
Simple mode
Weighted median
Weighted mode
0.2
0.1
0.0
−0.1
−0.2
0.04
0.08
0.12
0.16
SNP effect on NIPAL4
SNP effect on Pulmonary fibrosis

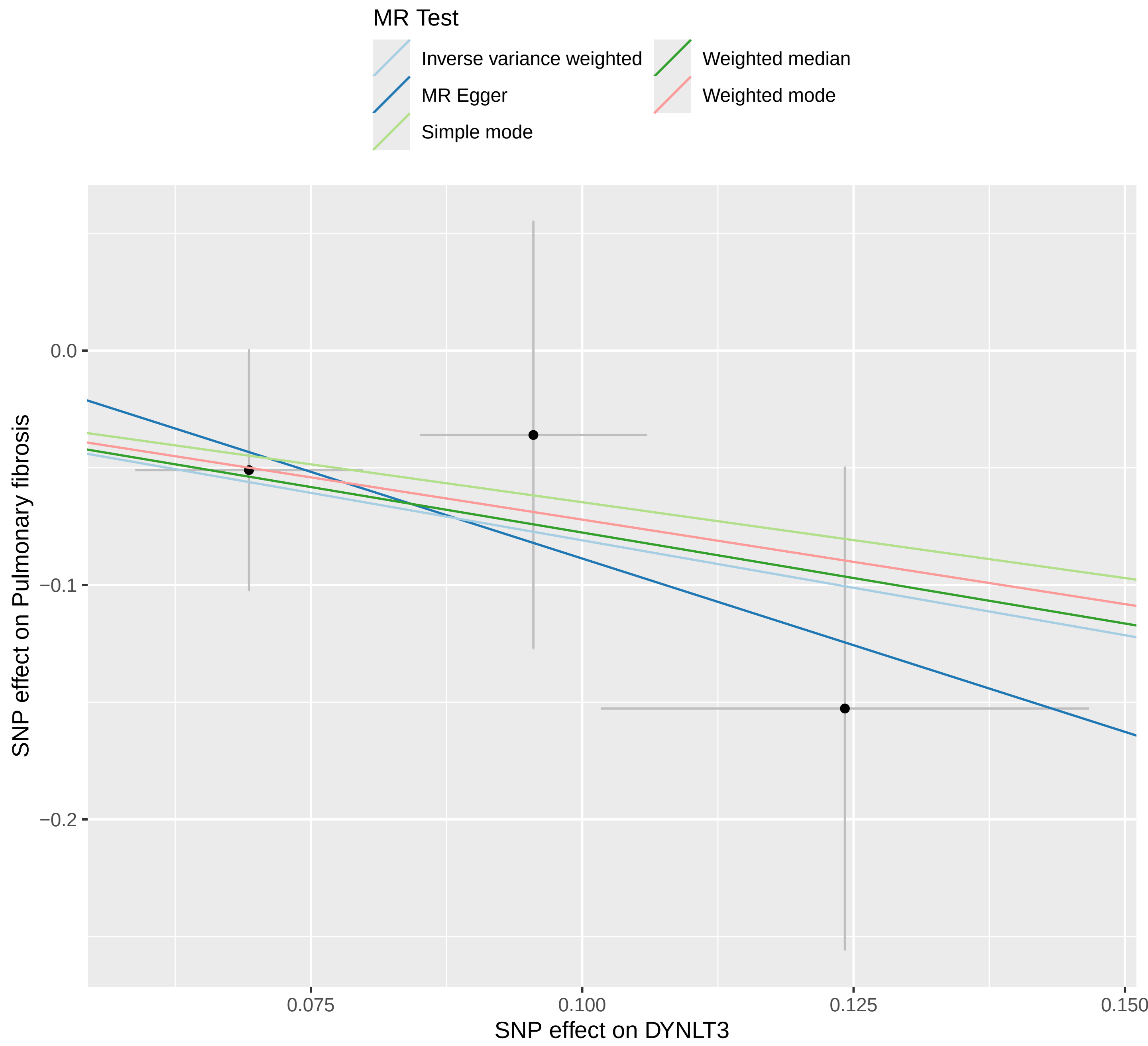
MR Test
Inverse variance weighted
MR Egger
Simple mode
Weighted median
Weighted mode
0.0
−0.1
−0.2
0.075
0.100
0.125
0.150
SNP effect on DYNLT3
SNP effect on Pulmonary fibrosis

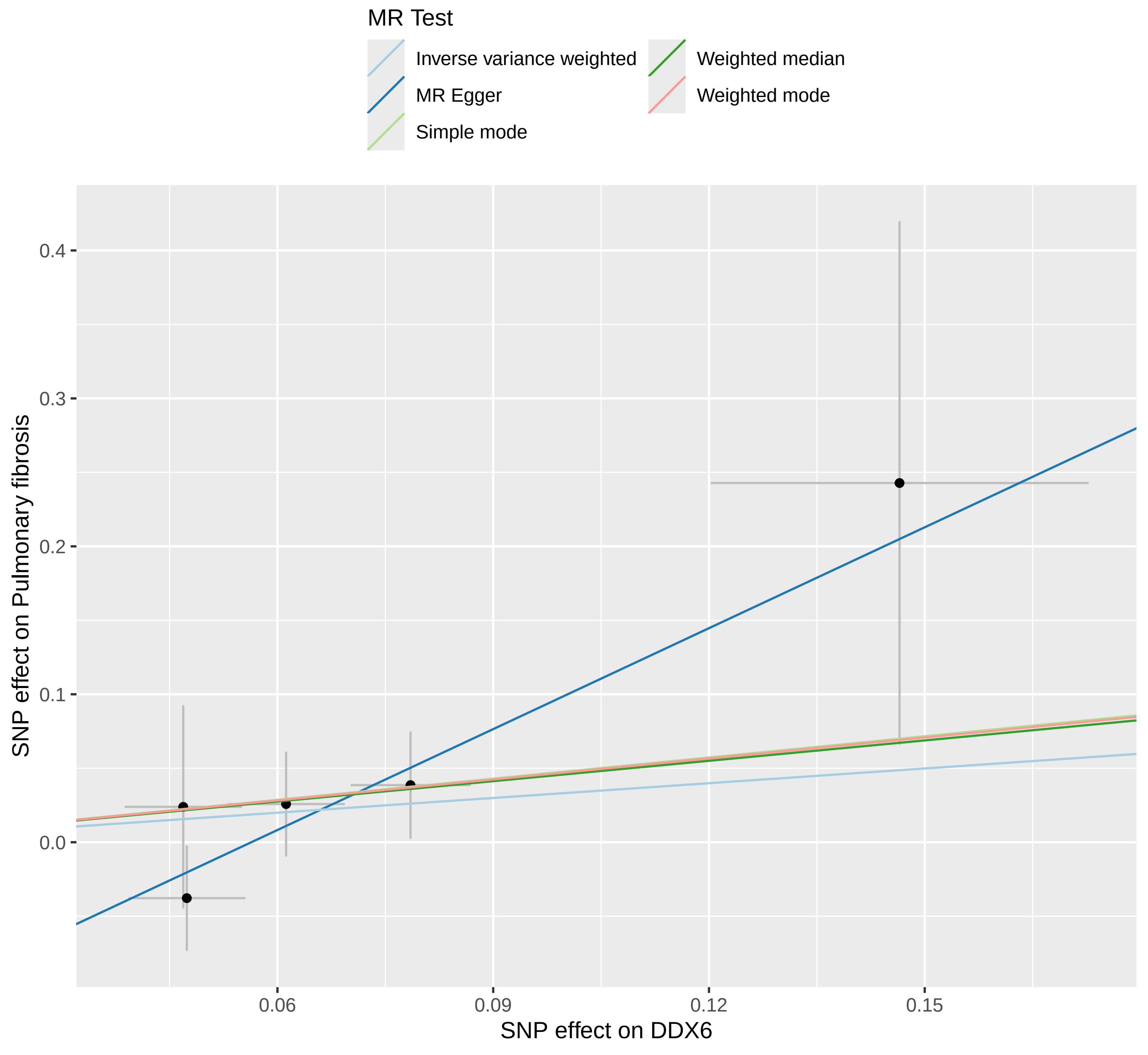
MR Test
Inverse variance weighted
MR Egger
Simple mode
Weighted median
Weighted mode
0.4
0.3
0.2
0.1
0.0
SNP effect on Pulmonary fibrosis
0.06
0.09
0.12
0.15
SNP effect on DDX6

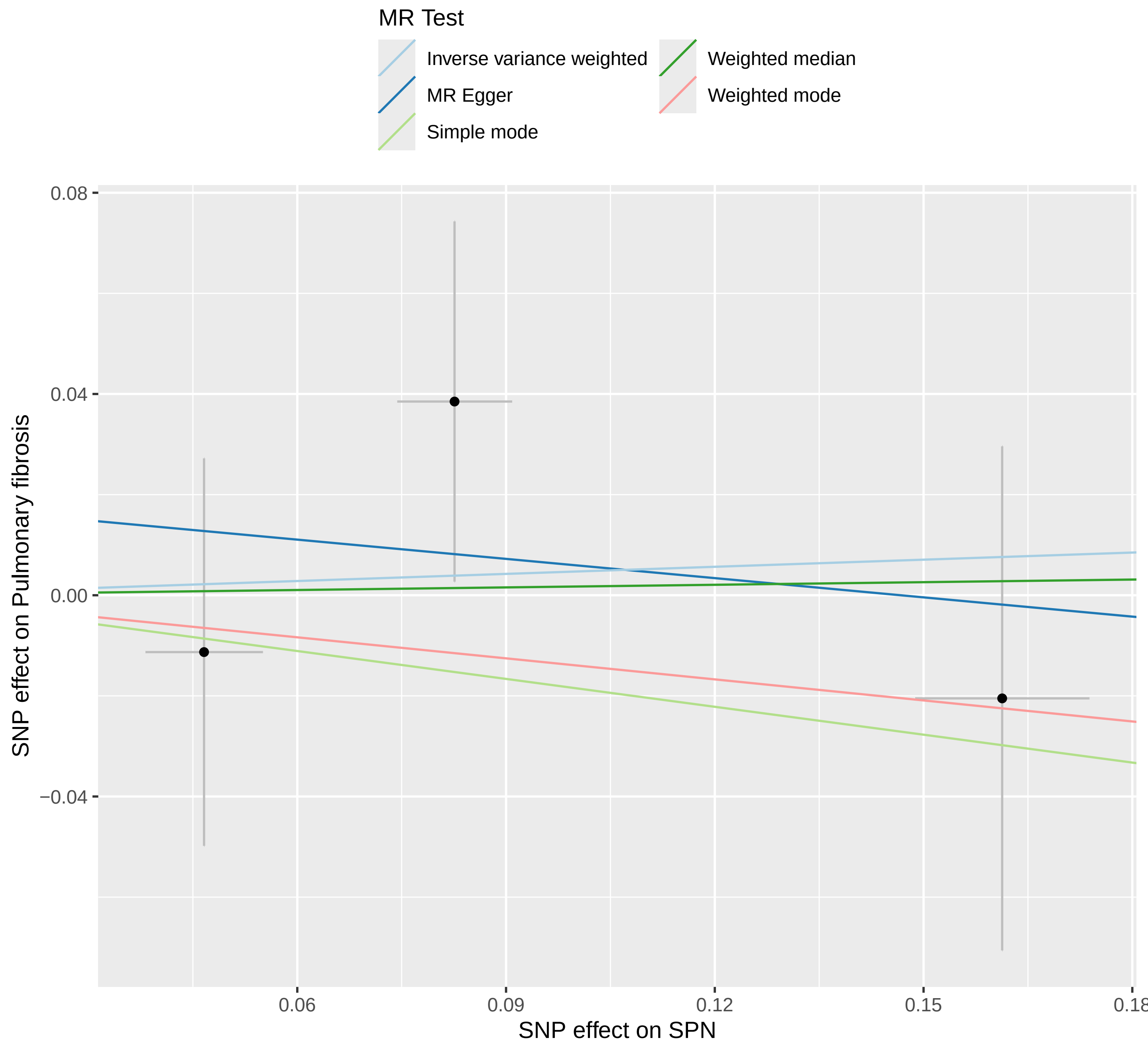
MR Test
Inverse variance weighted
MR Egger
Simple mode
Weighted median
Weighted mode
0.08
0.04
0.00
−0.04
0.06
0.09
0.12
0.15
0.18
SNP effect on Pulmonary fibrosis
SNP effect on SPN

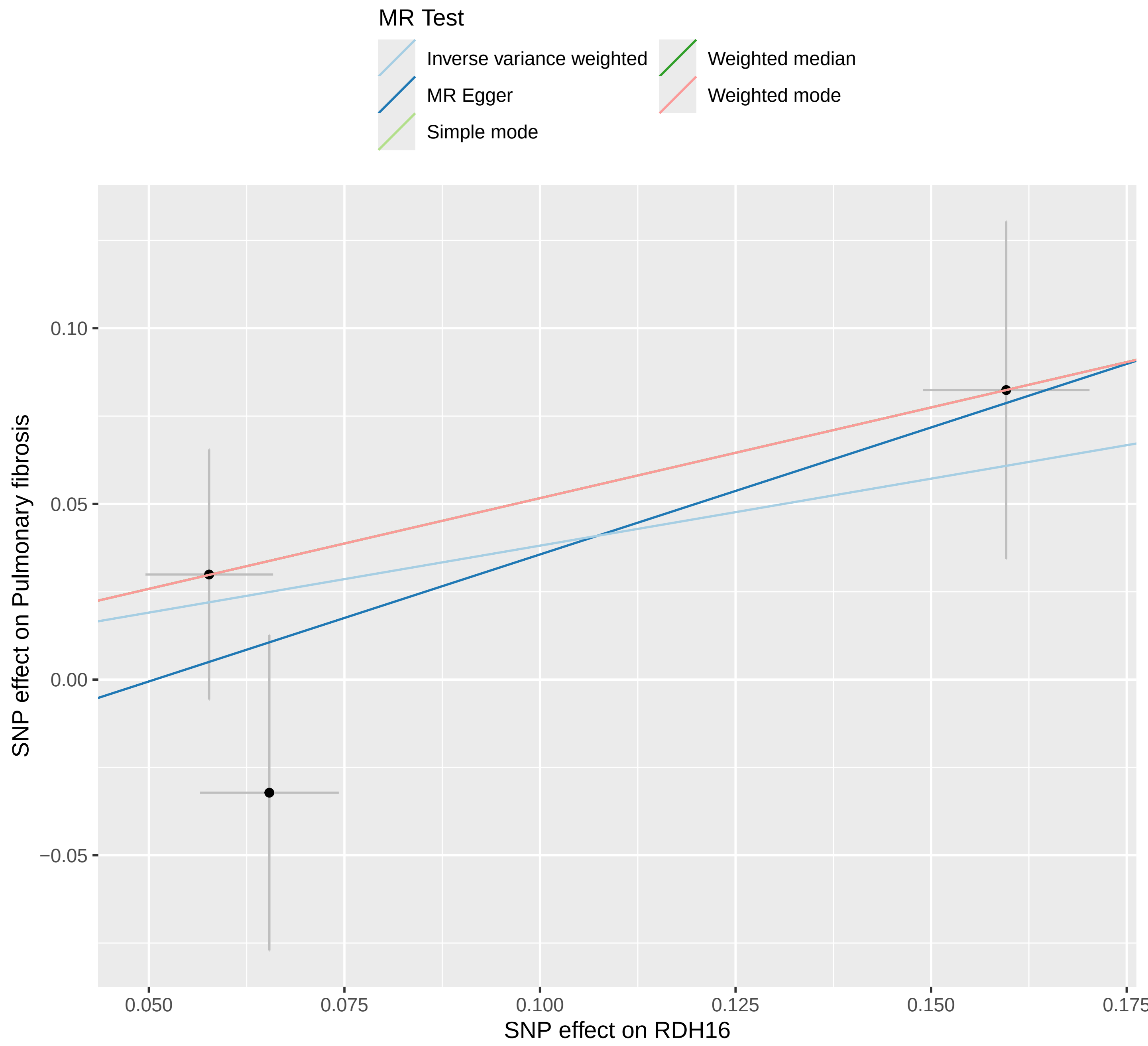
MR Test
Inverse variance weighted
MR Egger
Simple mode
Weighted median
Weighted mode
0.10
0.05
0.00
−0.05
SNP effect on Pulmonary fibrosis
0.050
0.075
0.100
0.125
0.150
0.175
SNP effect on RDH16

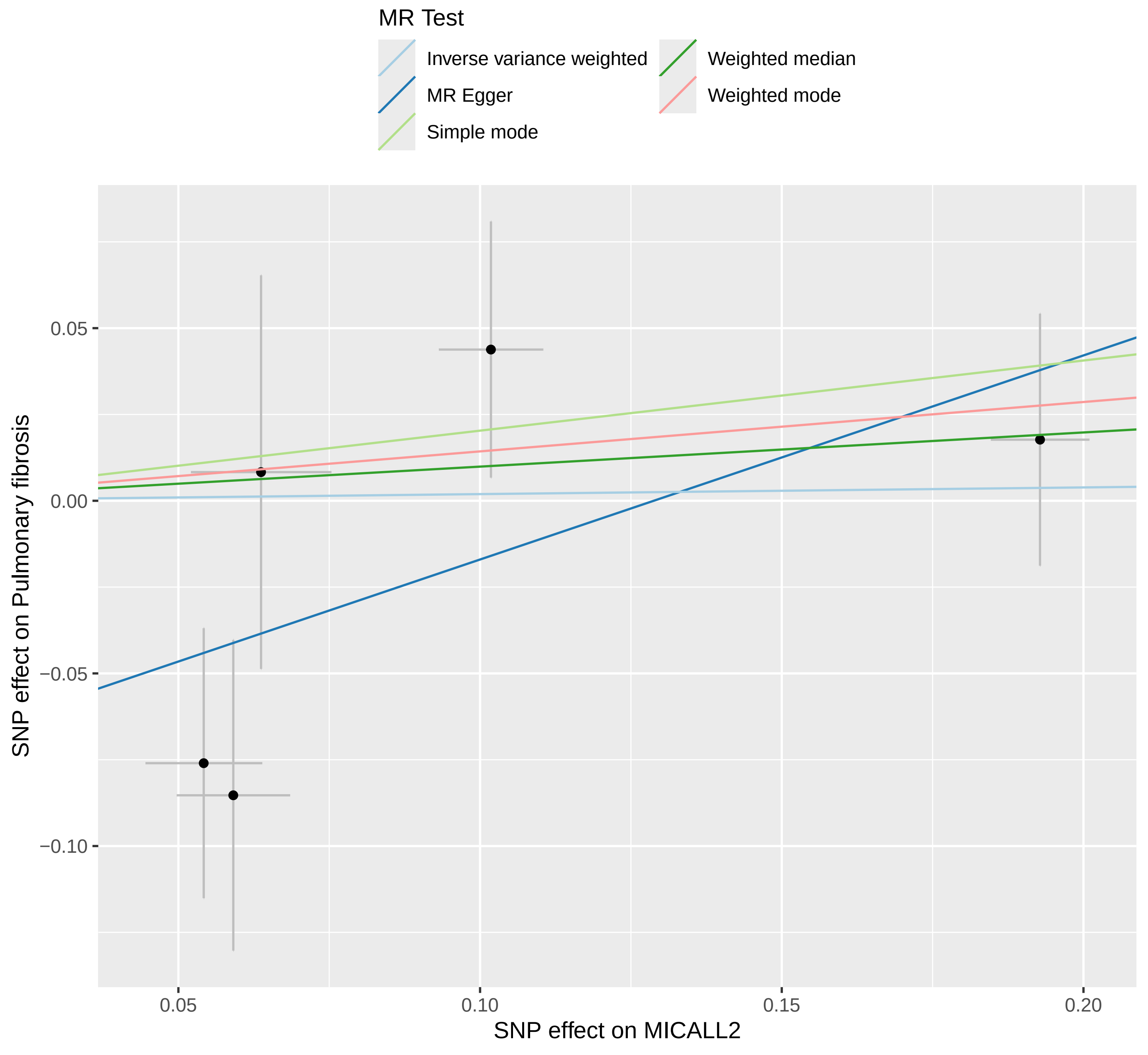
MR Test
Inverse variance weighted
MR Egger
Simple mode
Weighted median
Weighted mode
0.05
0.00
−0.05
−0.10
SNP effect on Pulmonary fibrosis
0.05
0.10
0.15
0.20
SNP effect on MICALL2

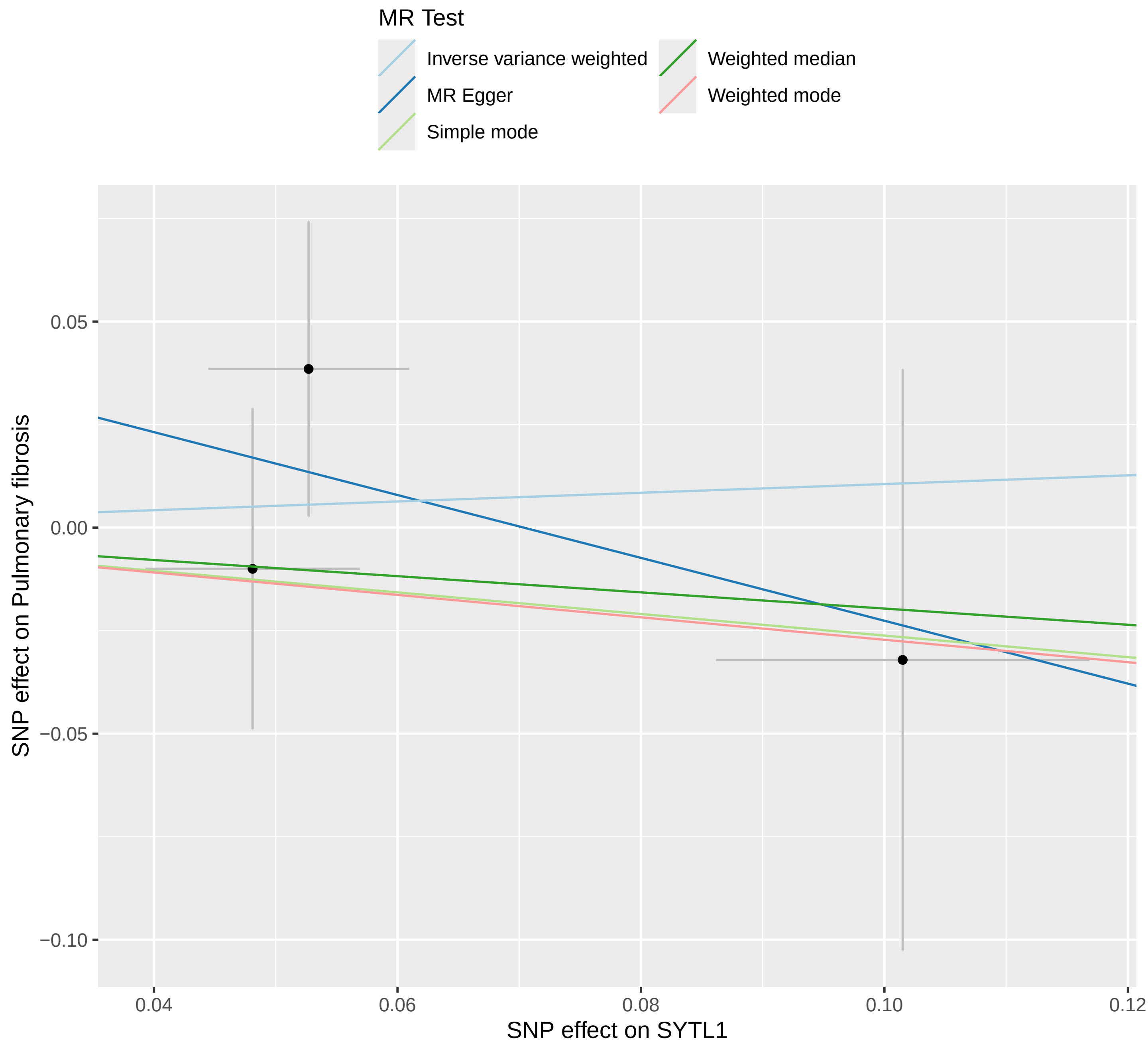
MR Test
Inverse variance weighted
MR Egger
Simple mode
Weighted median
Weighted mode
0.05
0.00
−0.05
−0.10
0.04
0.06
0.08
0.10
0.12
SNP effect on Pulmonary fibrosis
SNP effect on SYTL1

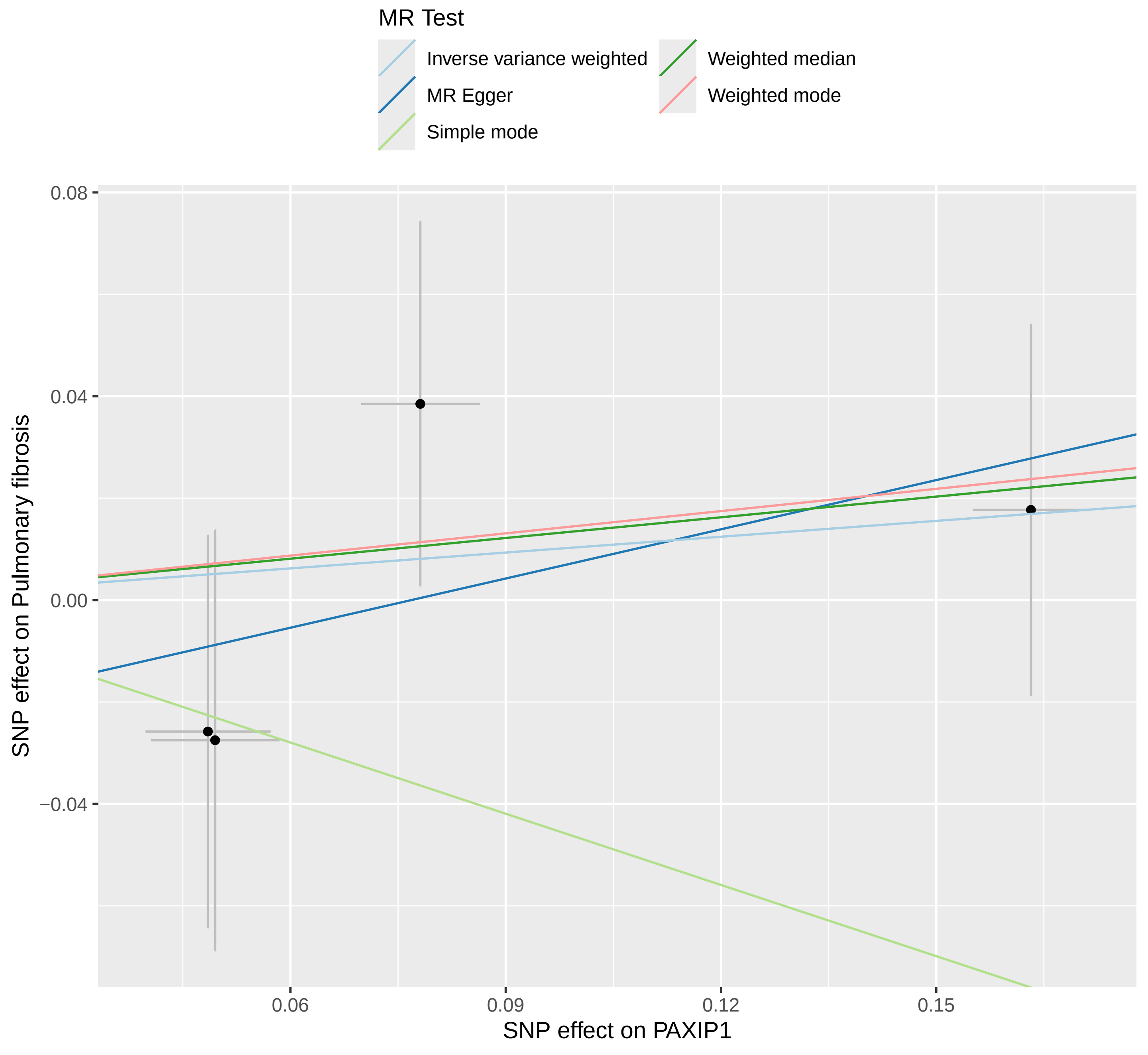

MR Test
Inverse variance weighted
MR Egger
Simple mode
Weighted median
Weighted mode
0.08
0.04
0.00
−0.04
0.06
0.09
0.12
0.15
SNP effect on Pulmonary fibrosis
SNP effect on PAXIP1

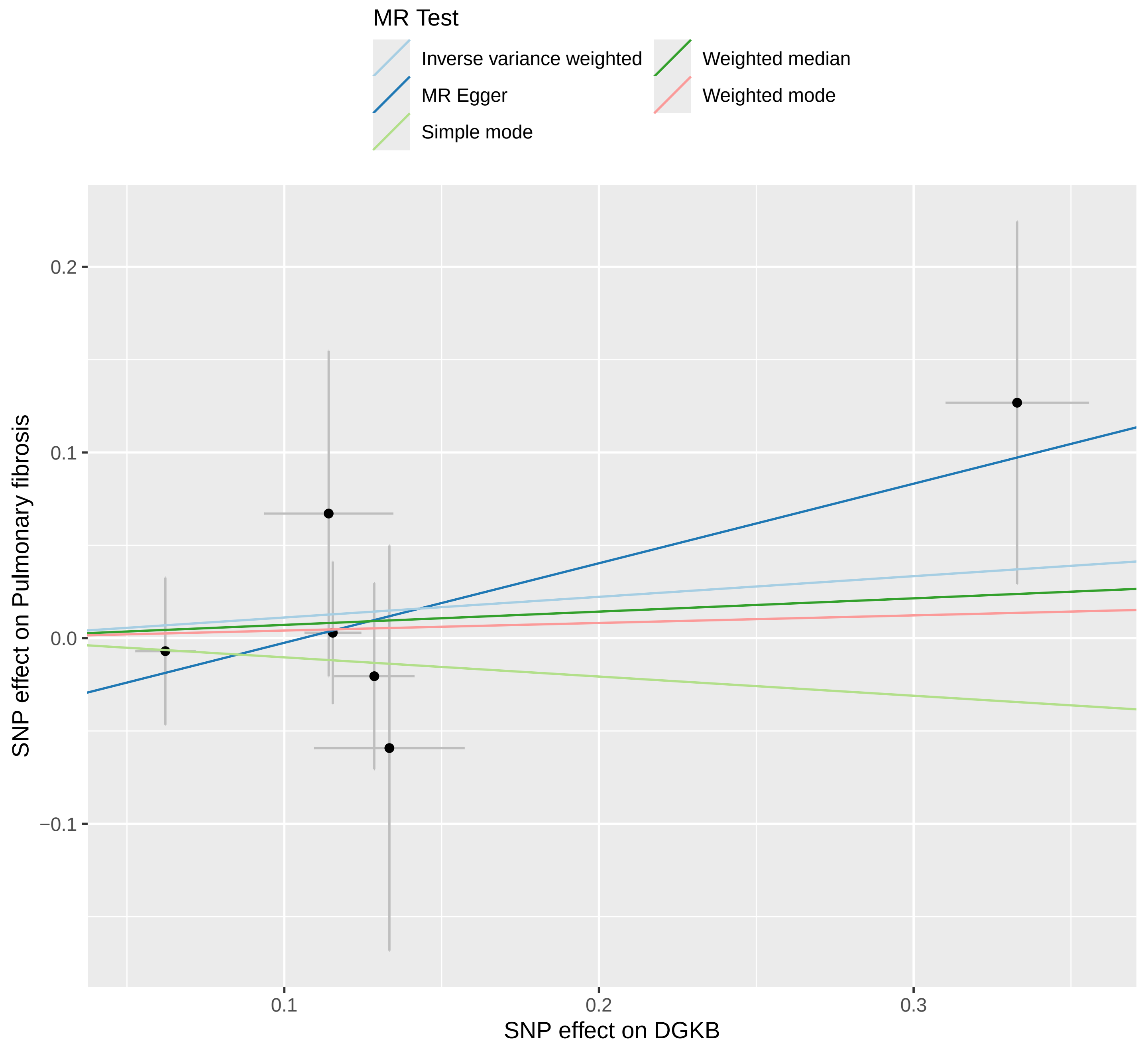

MR Test
Inverse variance weighted
MR Egger
Simple mode
Weighted median
Weighted mode
0.2
0.1
0.0
−0.1
0.1
0.2
0.3
SNP effect on Pulmonary fibrosis
SNP effect on DGKB

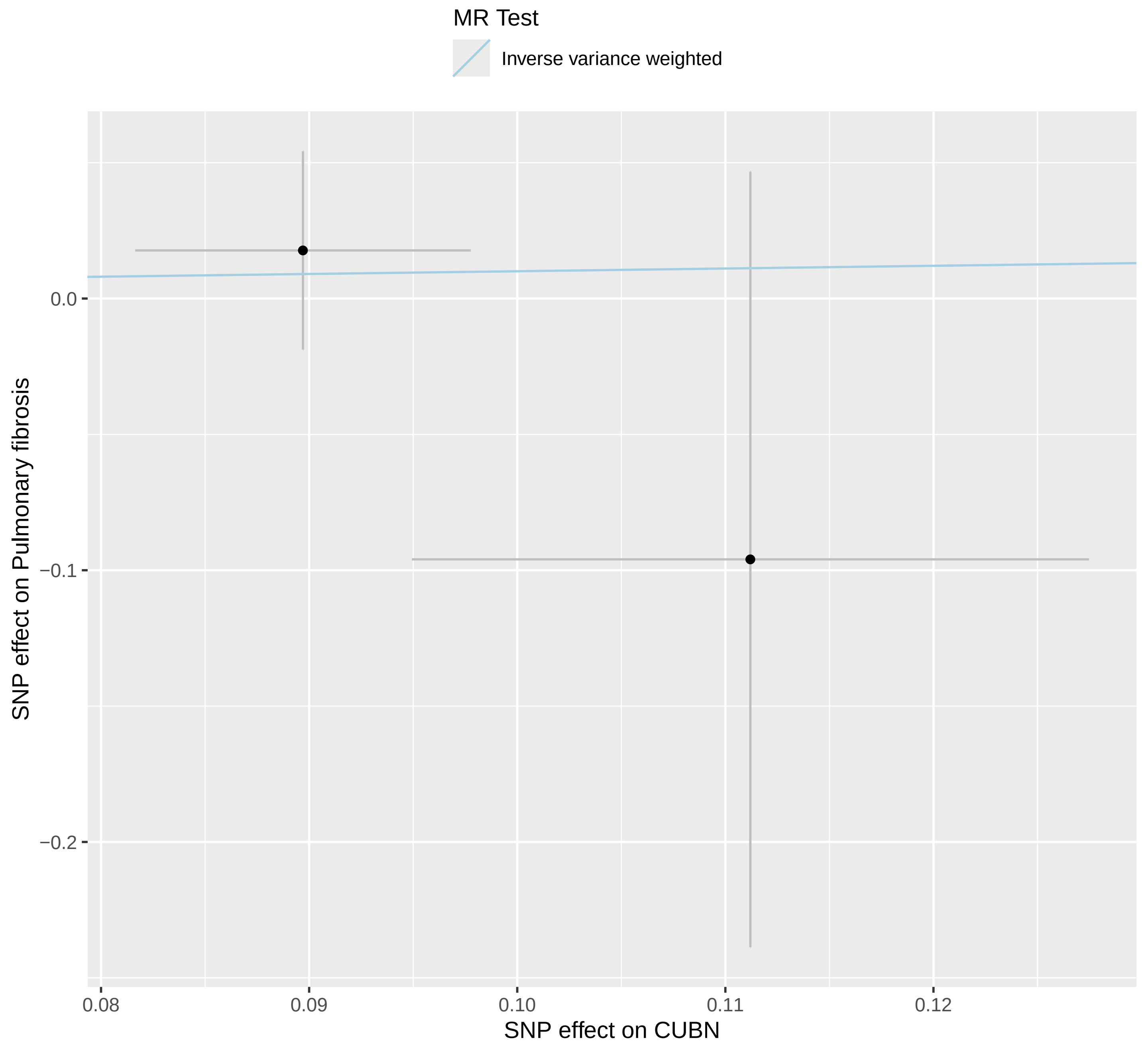

MR Test
Inverse variance weighted
SNP effect on Pulmonary fibrosis
0.0
−0.1
−0.2
0.08
0.09
0.10
0.11
0.12
SNP effect on CUBN

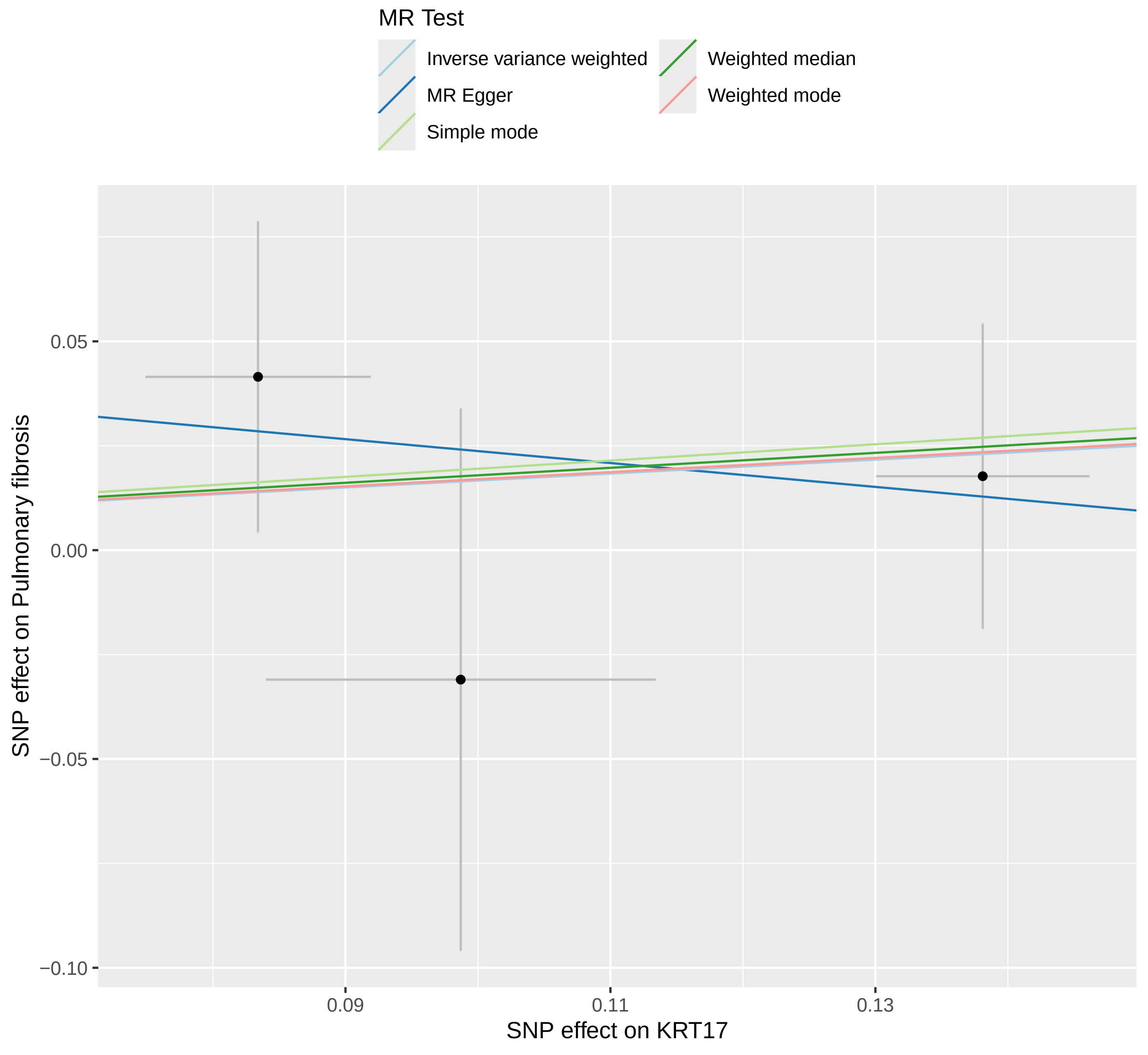
MR Test
Inverse variance weighted
MR Egger
Simple mode
Weighted median
Weighted mode
SNP effect on Pulmonary fibrosis
0.05
0.00
−0.05
−0.10
0.09
0.11
0.13
SNP effect on KRT17

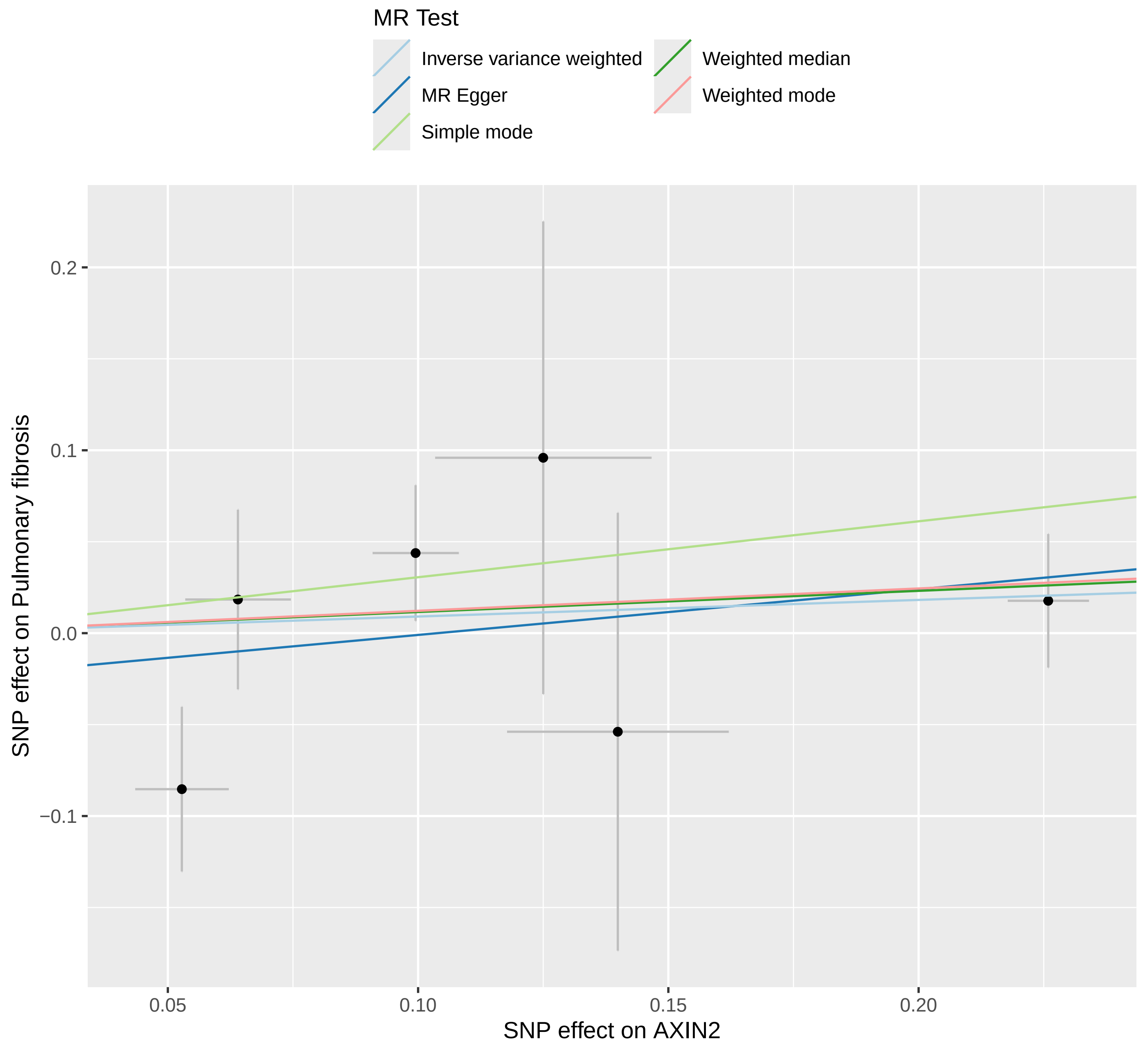

MR Test
Inverse variance weighted
MR Egger
Simple mode
Weighted median
Weighted mode
0.2
0.1
0.0
−0.1
0.05
0.10
0.15
0.20
SNP effect on AXIN2
SNP effect on Pulmonary fibrosis

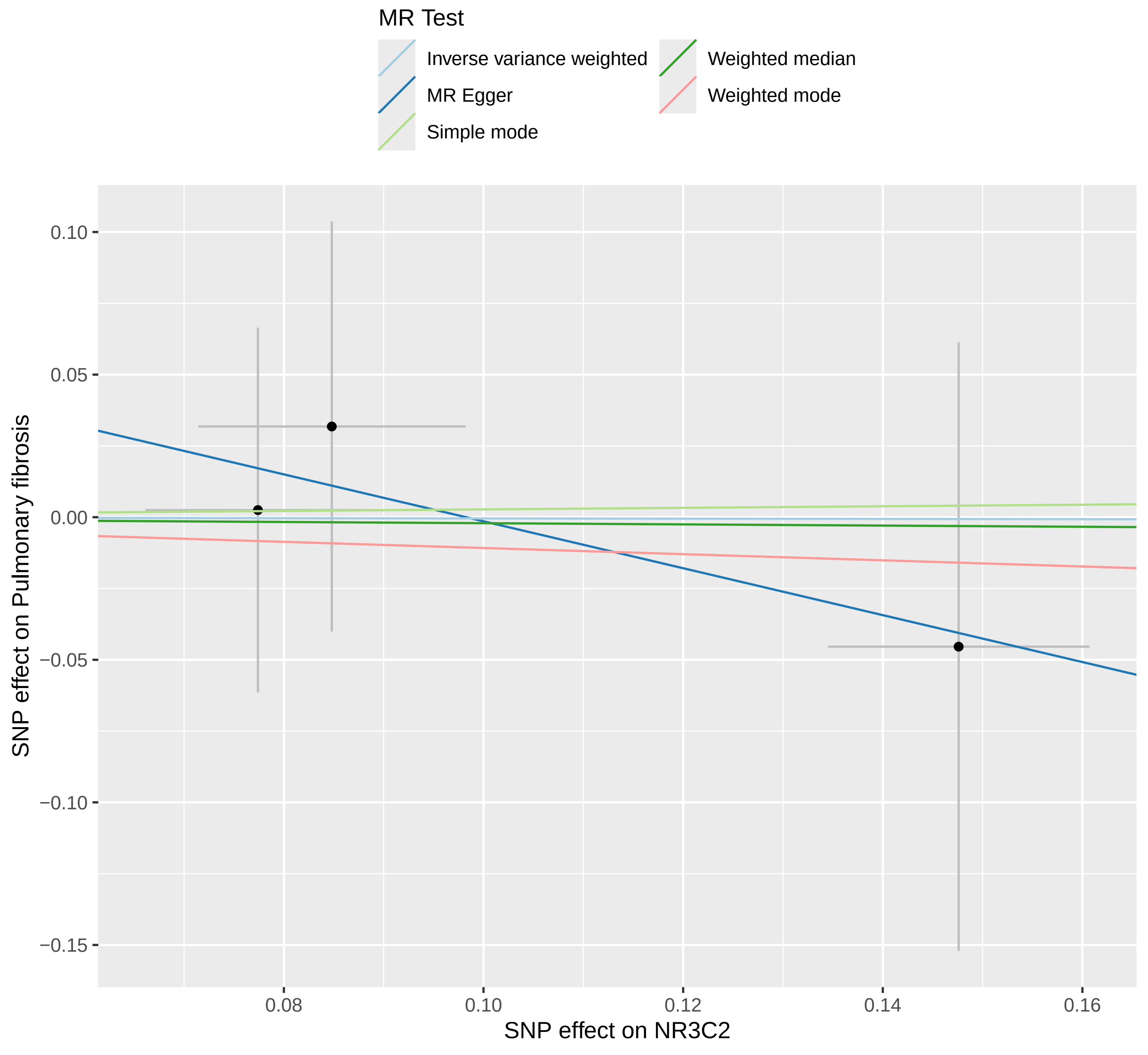

MR Test
Inverse variance weighted
MR Egger
Simple mode
Weighted median
Weighted mode
0.10
0.05
0.00
−0.05
−0.10
−0.15
SNP effect on Pulmonary fibrosis
0.08
0.10
0.12
0.14
0.16
SNP effect on NR3C2

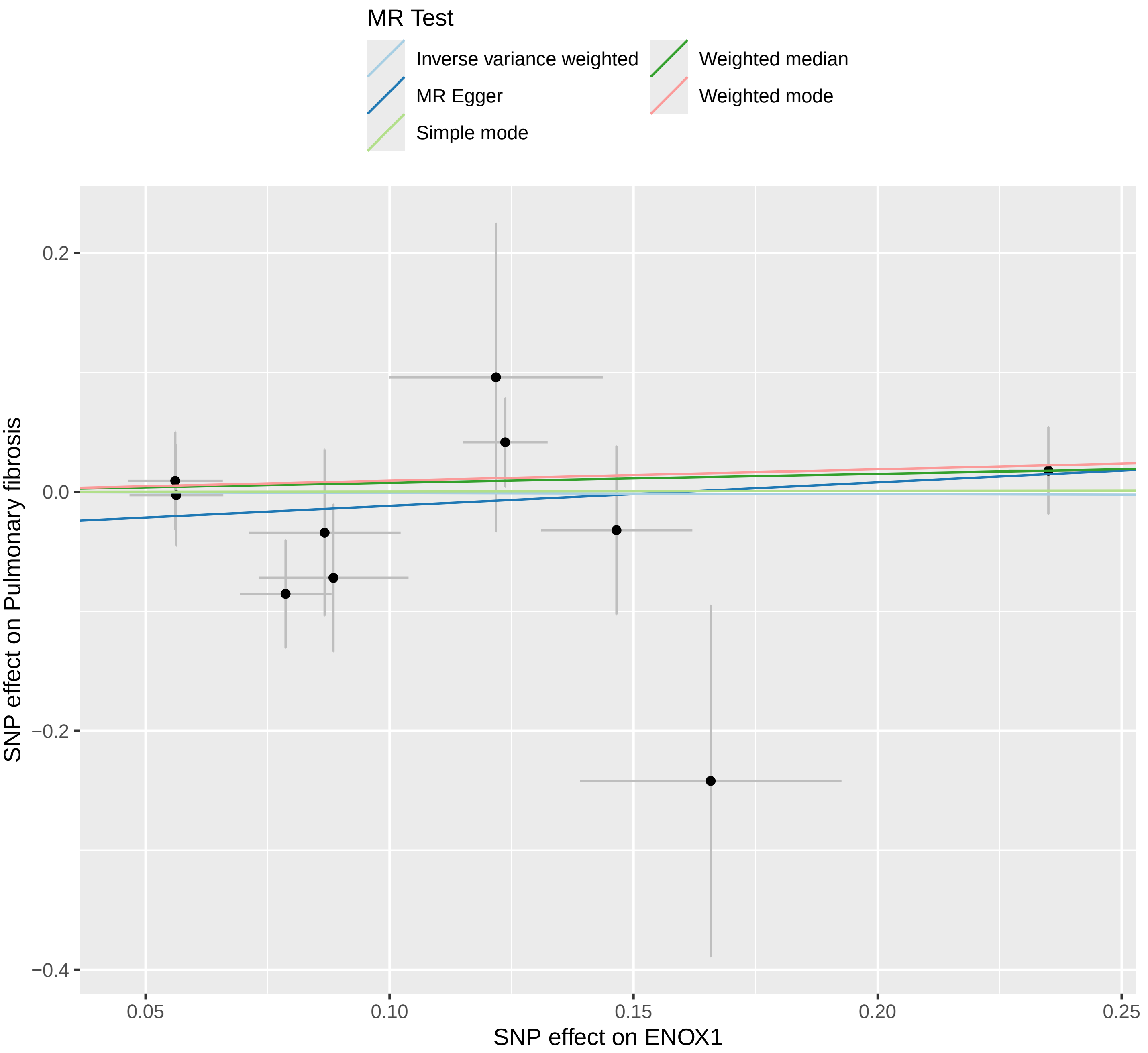
MR Test
Inverse variance weighted
MR Egger
Simple mode
Weighted median
Weighted mode
0.2
0.0
−0.2
−0.4
0.05
0.10
0.15
0.20
0.25
SNP effect on ENOX1
SNP effect on Pulmonary fibrosis

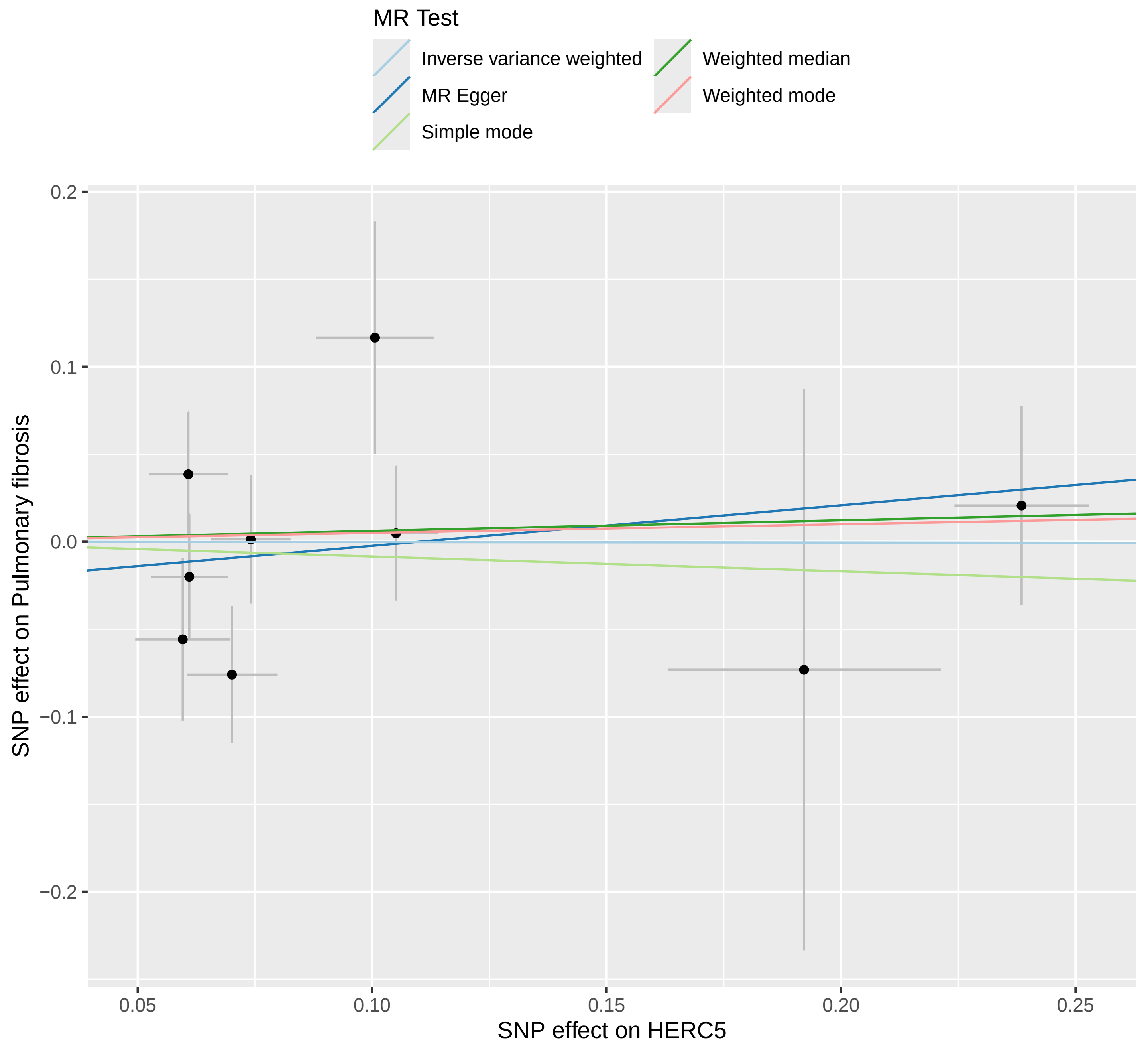
MR Test
Inverse variance weighted
MR Egger
Simple mode
Weighted median
Weighted mode
0.2
0.1
0.0
−0.1
−0.2
SNP effect on Pulmonary fibrosis
0.05
0.10
0.15
0.20
0.25
SNP effect on HERC5

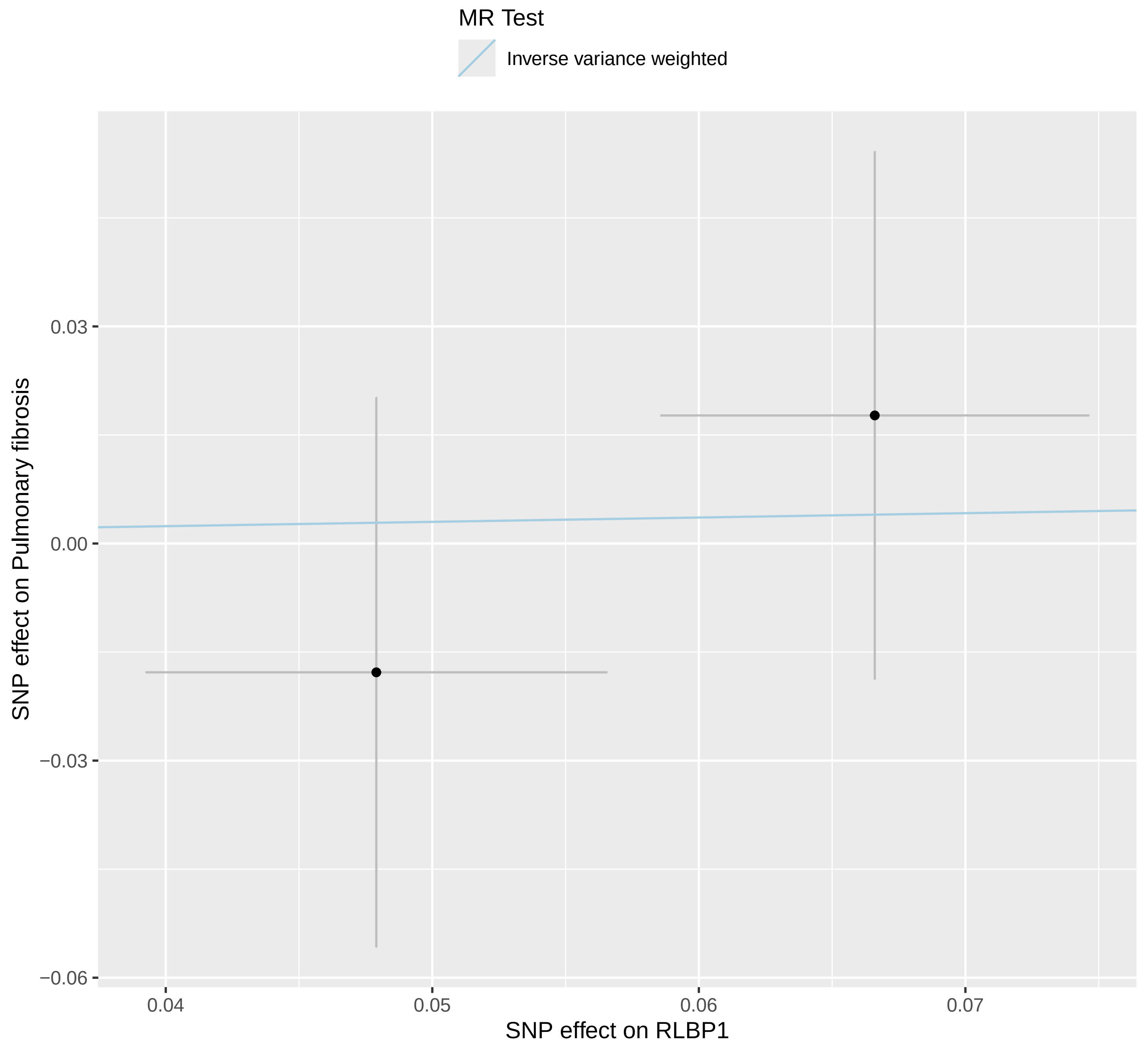

MR Test
Inverse variance weighted
0.03
0.00
−0.03
−0.06
0.04
0.05
0.06
0.07
SNP effect on Pulmonary fibrosis
SNP effect on RLBP1

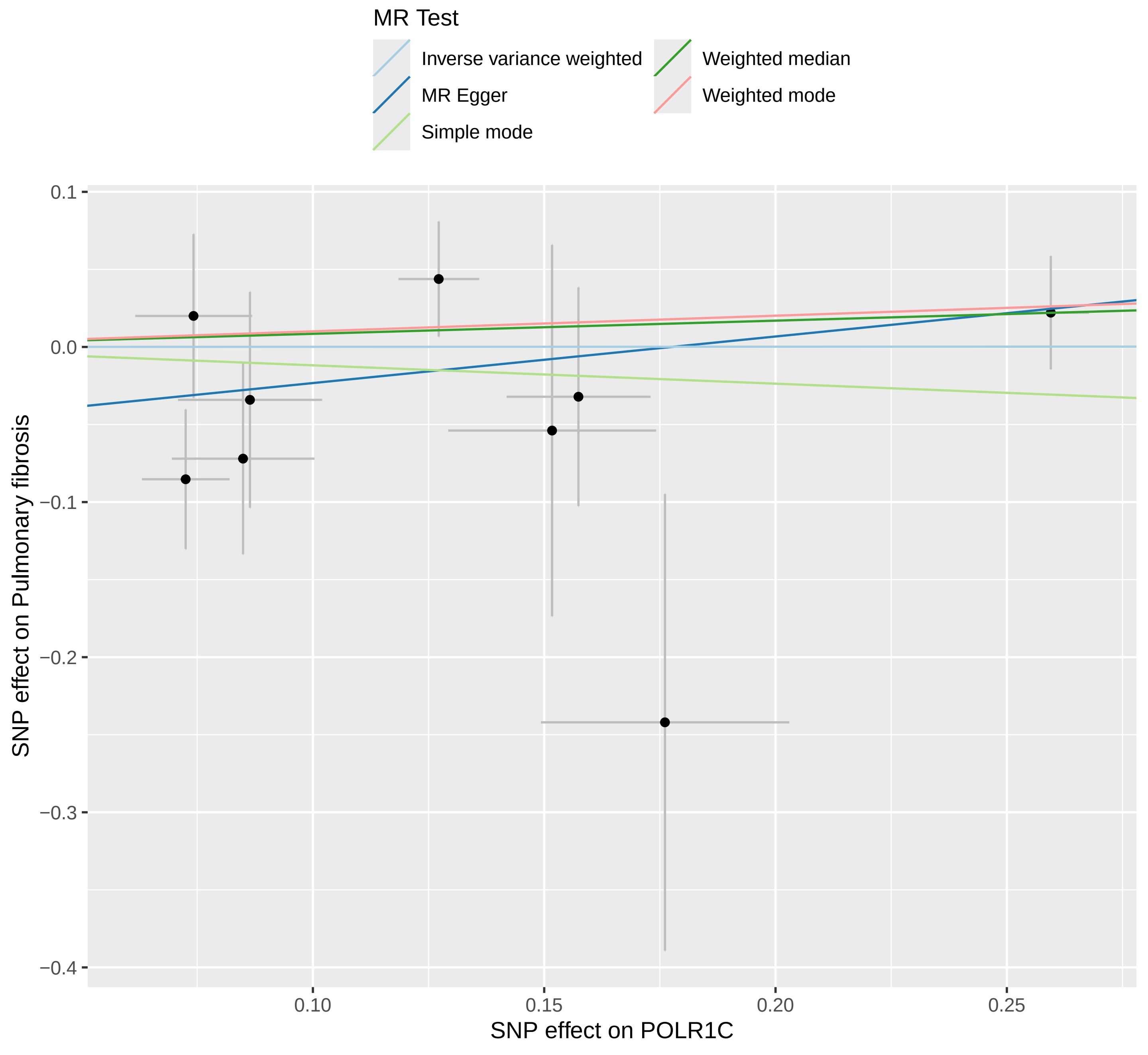

MR Test
Inverse variance weighted
MR Egger
Simple mode
Weighted median
Weighted mode
0.1
0.0
−0.1
−0.2
−0.3
−0.4
0.10
0.15
0.20
0.25
SNP effect on Pulmonary fibrosis
SNP effect on POLR1C

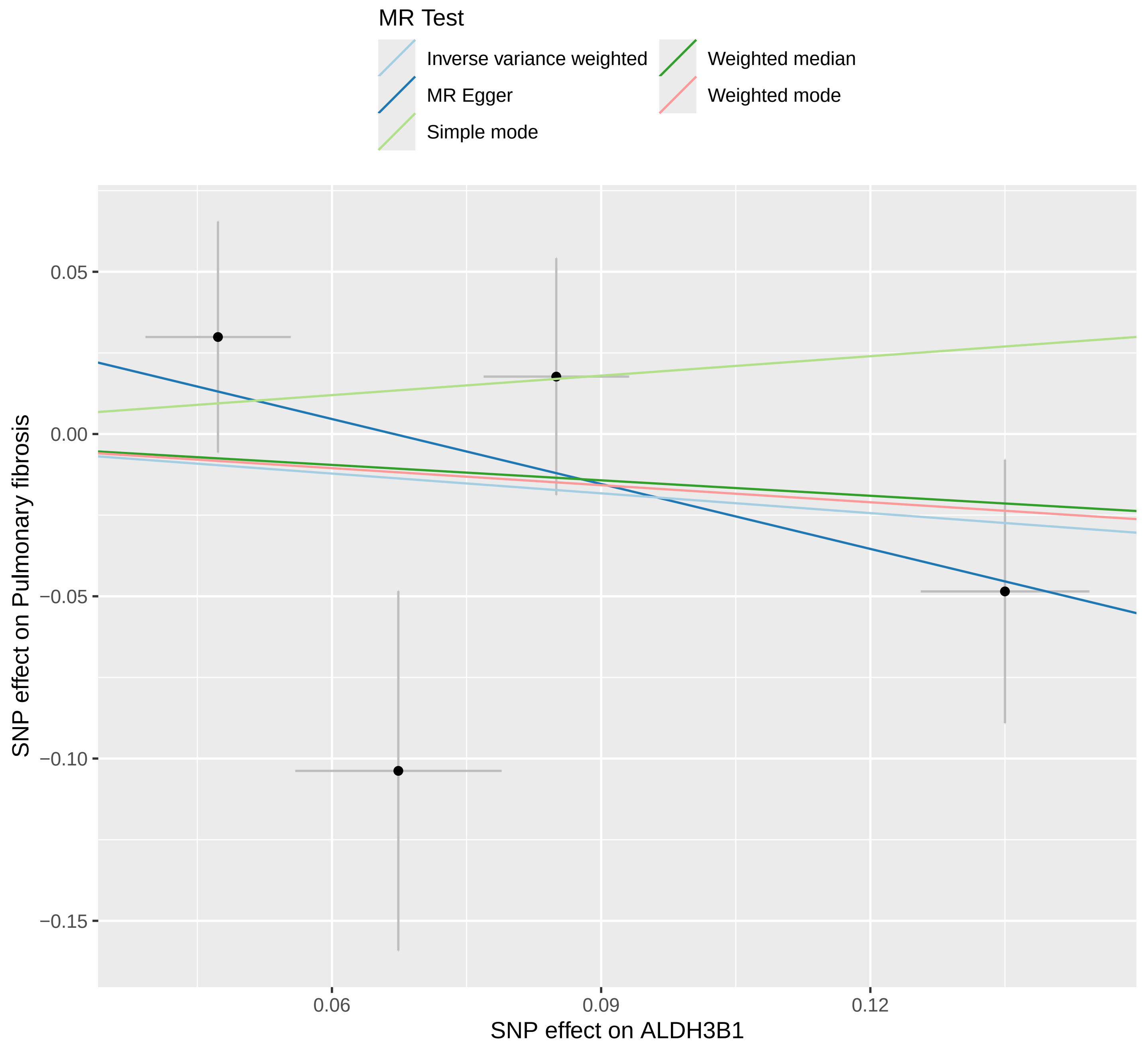
MR Test
Inverse variance weighted
MR Egger
Simple mode
Weighted median
Weighted mode
0.05
0.00
−0.05
−0.10
−0.15
0.06
0.09
0.12
SNP effect on Pulmonary fibrosis
SNP effect on ALDH3B1

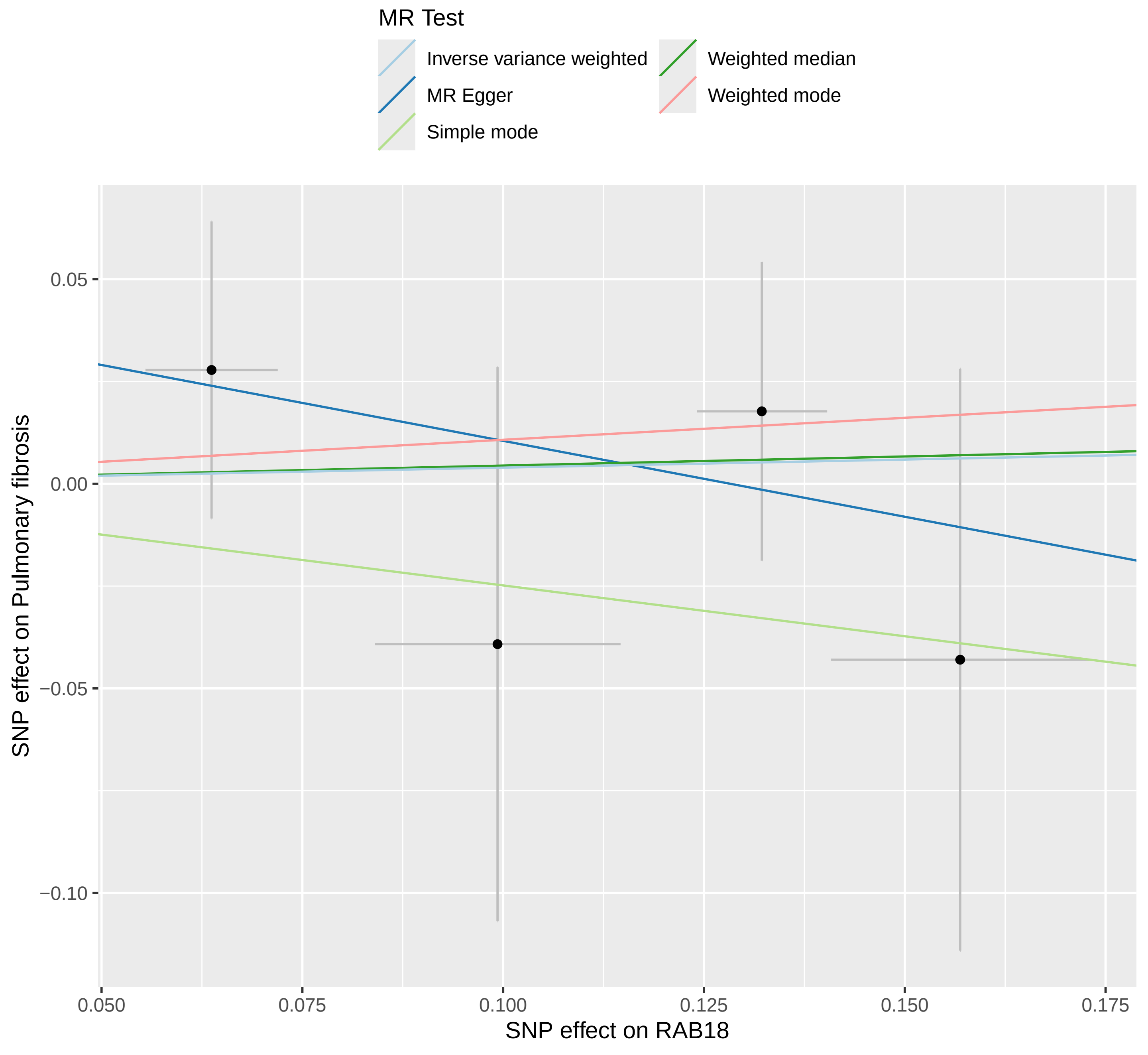
MR Test
Inverse variance weighted
MR Egger
Simple mode
Weighted median
Weighted mode
0.05
0.00
−0.05
−0.10
SNP effect on Pulmonary fibrosis
0.050
0.075
0.100
0.125
0.150
0.175
SNP effect on RAB18

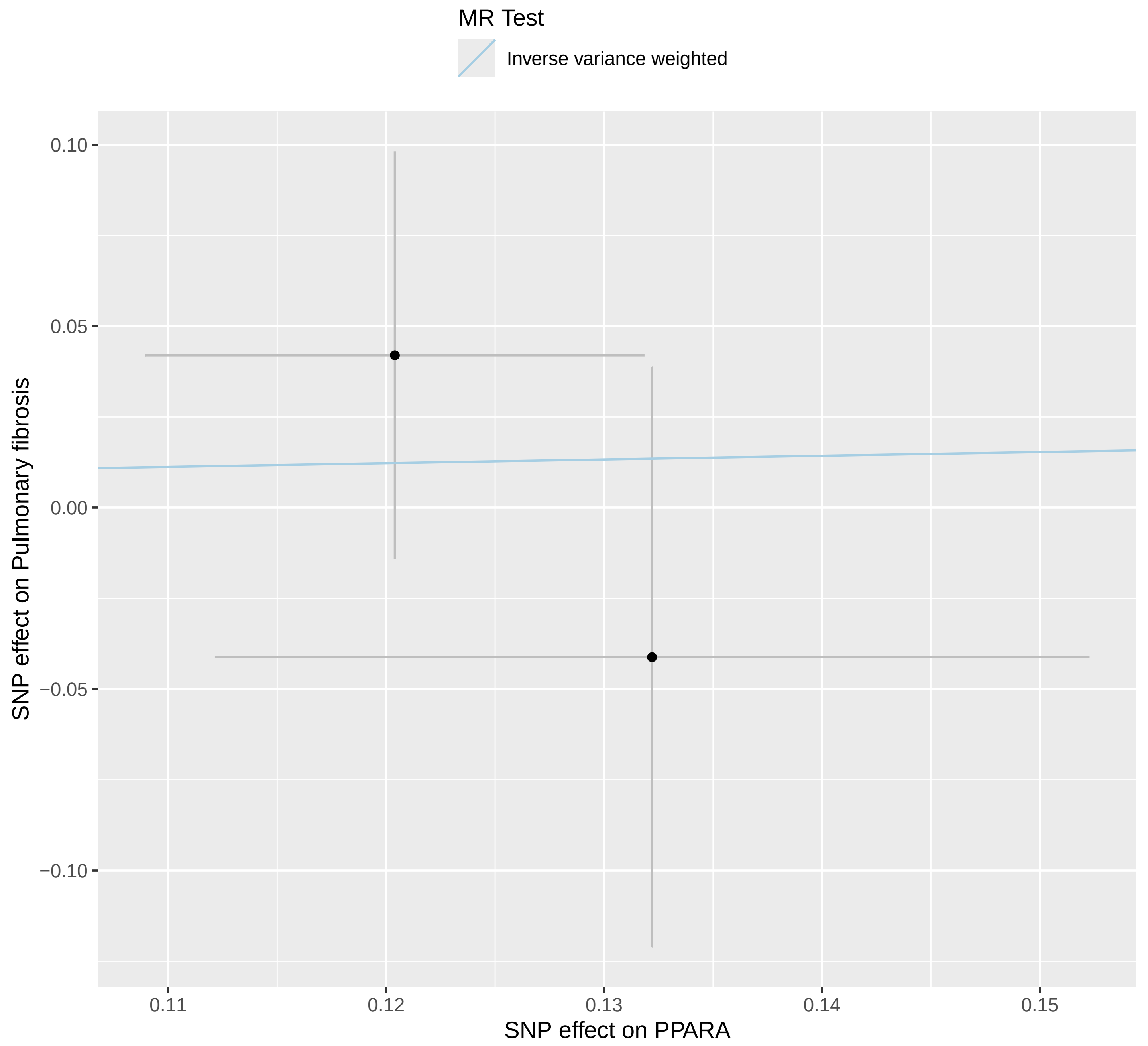

MR Test
Inverse variance weighted
0.10
0.05
0.00
−0.05
−0.10
0.11
0.12
0.13
0.14
0.15
SNP effect on PPARA
SNP effect on Pulmonary fibrosis

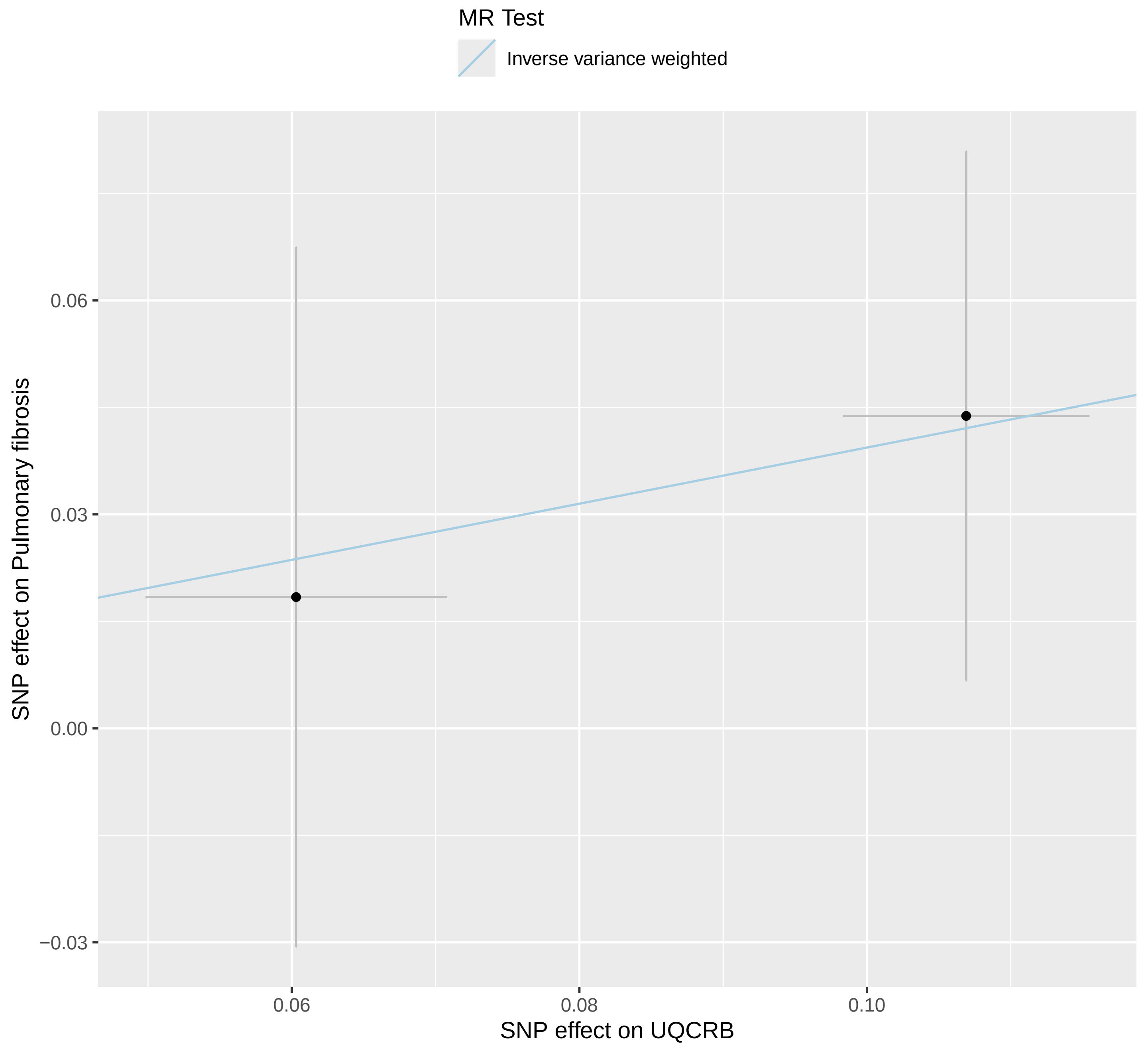
MR Test
Inverse variance weighted
0.06
0.03
0.00
−0.03
SNP effect on Pulmonary fibrosis
0.06
0.08
0.10
SNP effect on UQCRB

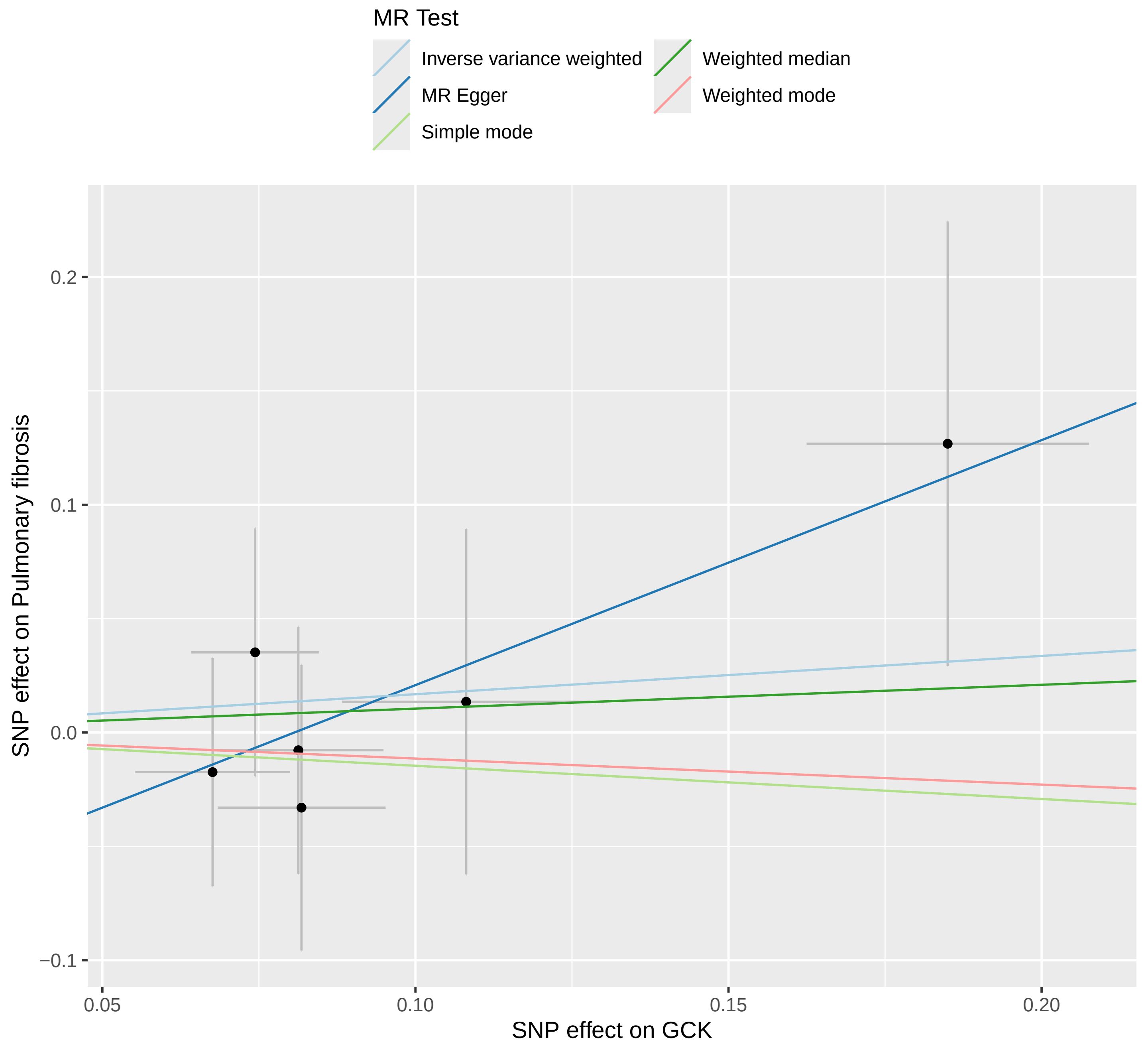
MR Test
Inverse variance weighted
MR Egger
Simple mode
Weighted median
Weighted mode
SNP effect on Pulmonary fibrosis
0.2
0.1
0.0
−0.1
0.05
0.10
0.15
0.20
SNP effect on GCK

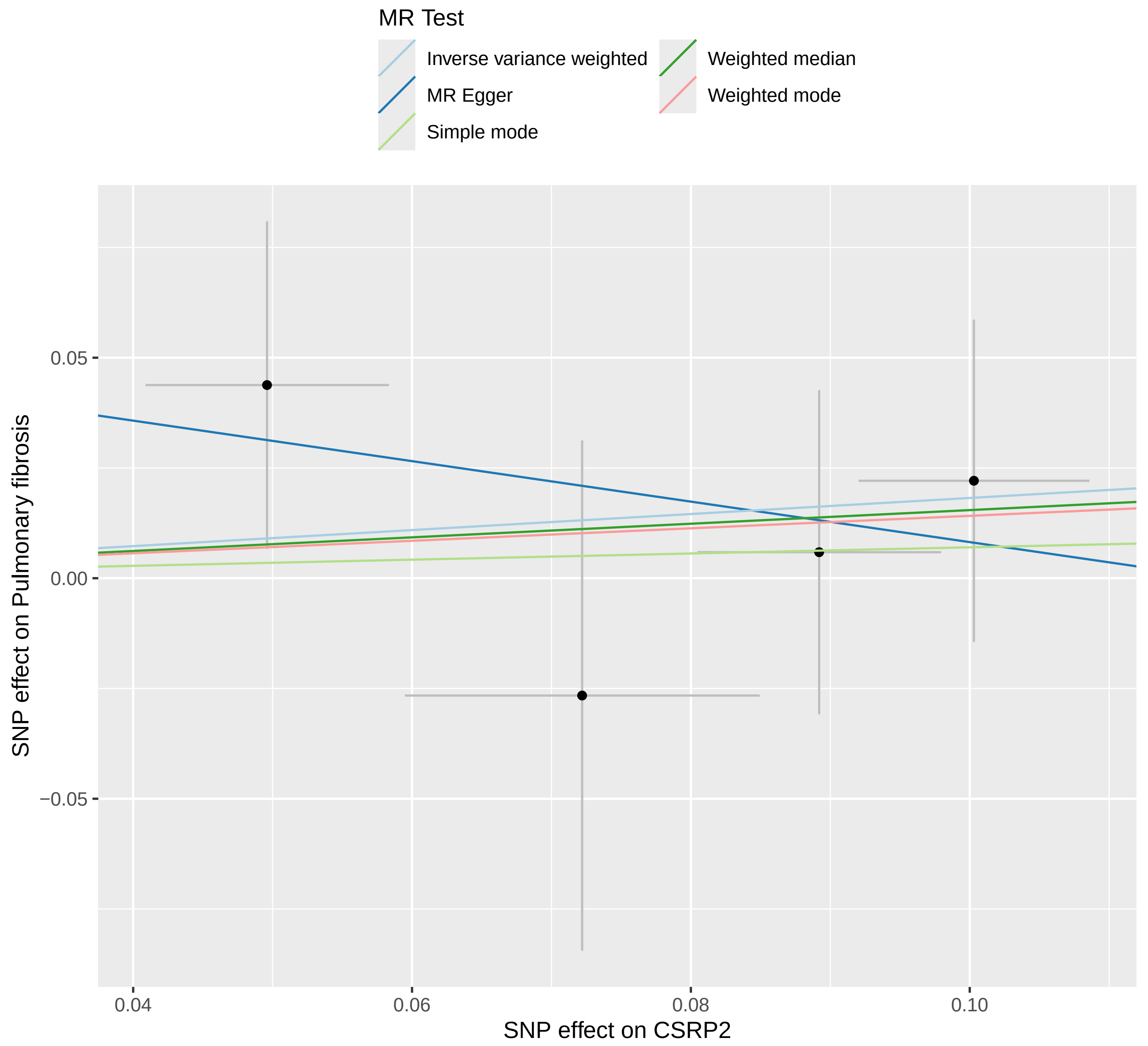
MR Test
Inverse variance weighted
MR Egger
Simple mode
Weighted median
Weighted mode
0.05
0.00
−0.05
SNP effect on Pulmonary fibrosis
0.04
0.06
0.08
0.10
SNP effect on CSRP2

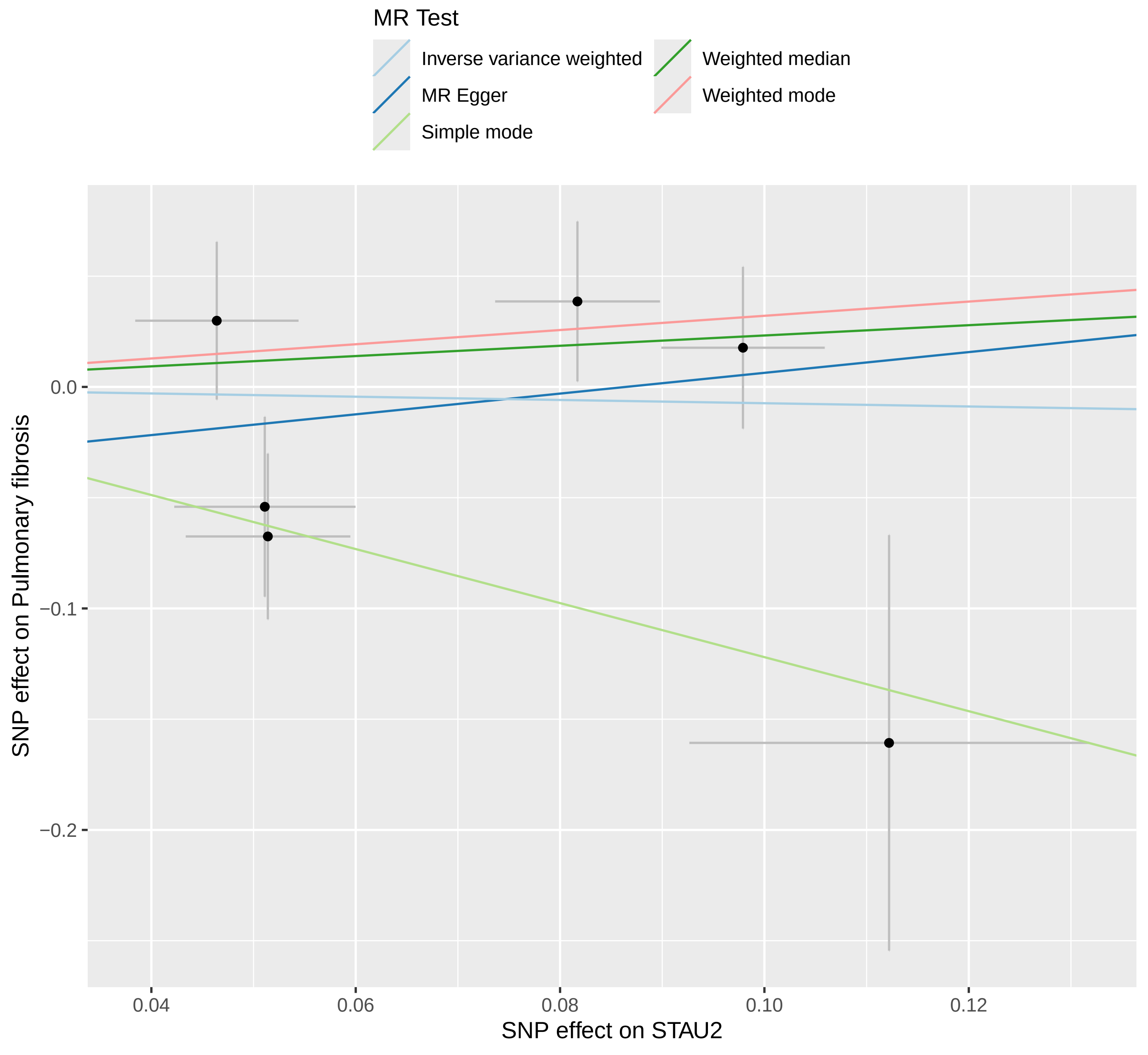

MR Test
Inverse variance weighted
MR Egger
Simple mode
Weighted median
Weighted mode
0.0
−0.1
−0.2
SNP effect on Pulmonary fibrosis
0.04
0.06
0.08
0.10
0.12
SNP effect on STAU2

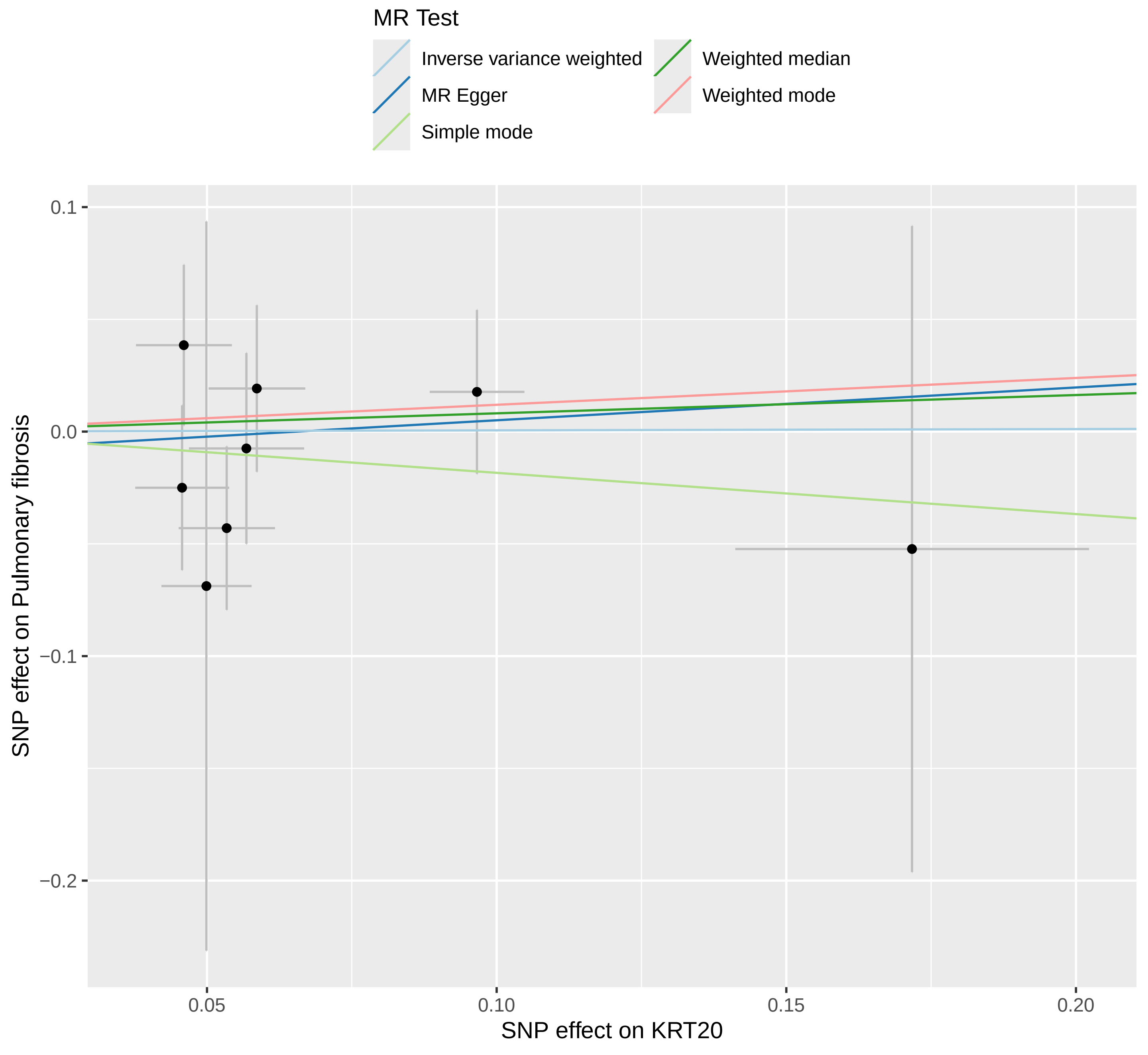
MR Test
Inverse variance weighted
MR Egger
Simple mode
Weighted median
Weighted mode
0.1
0.0
−0.1
−0.2
SNP effect on Pulmonary fibrosis
0.05
0.10
0.15
0.20
SNP effect on KRT20

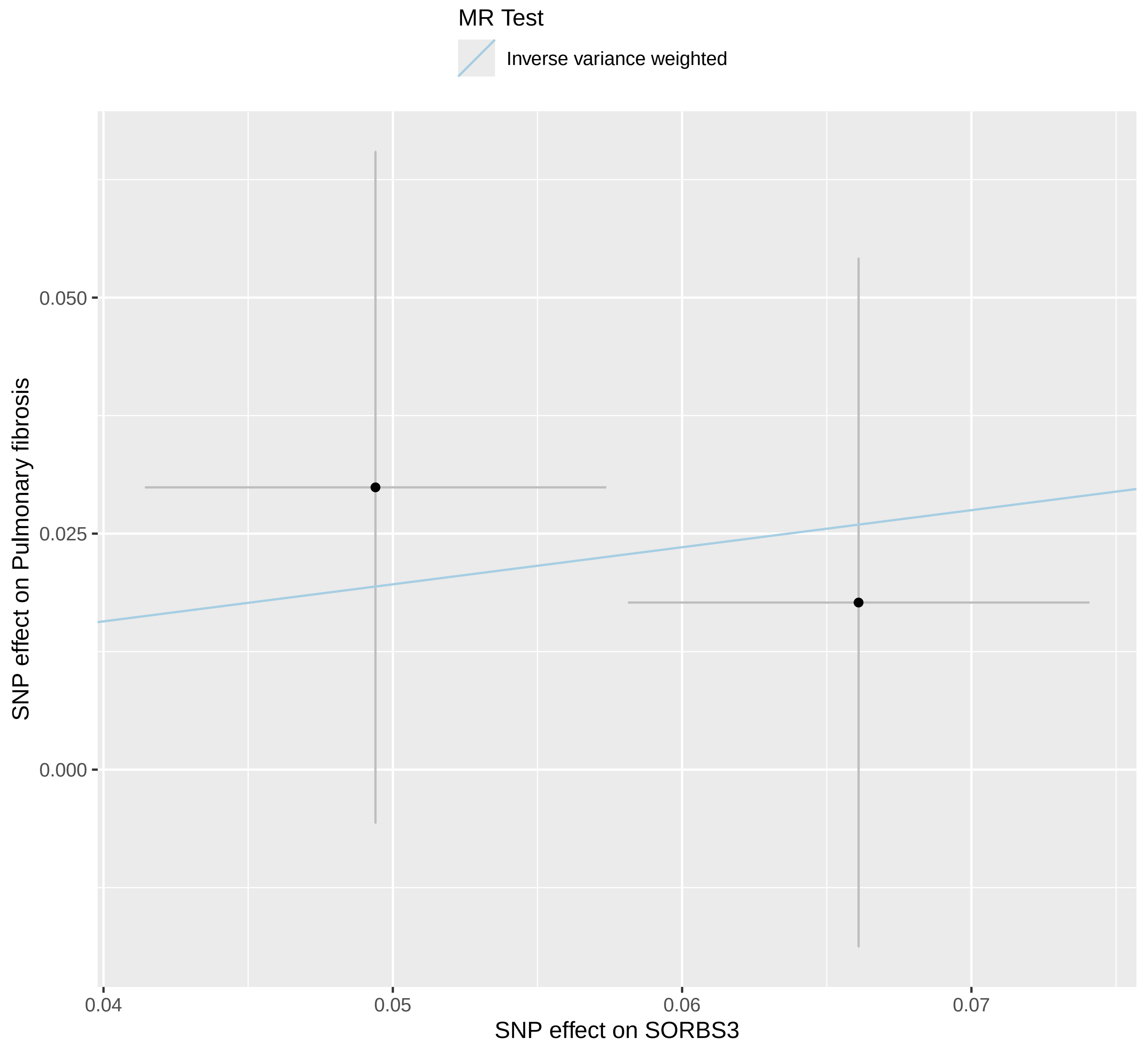

MR Test
Inverse variance weighted
0.050
0.025
0.000
0.04
0.05
0.06
0.07
SNP effect on SORBS3
SNP effect on Pulmonary fibrosis

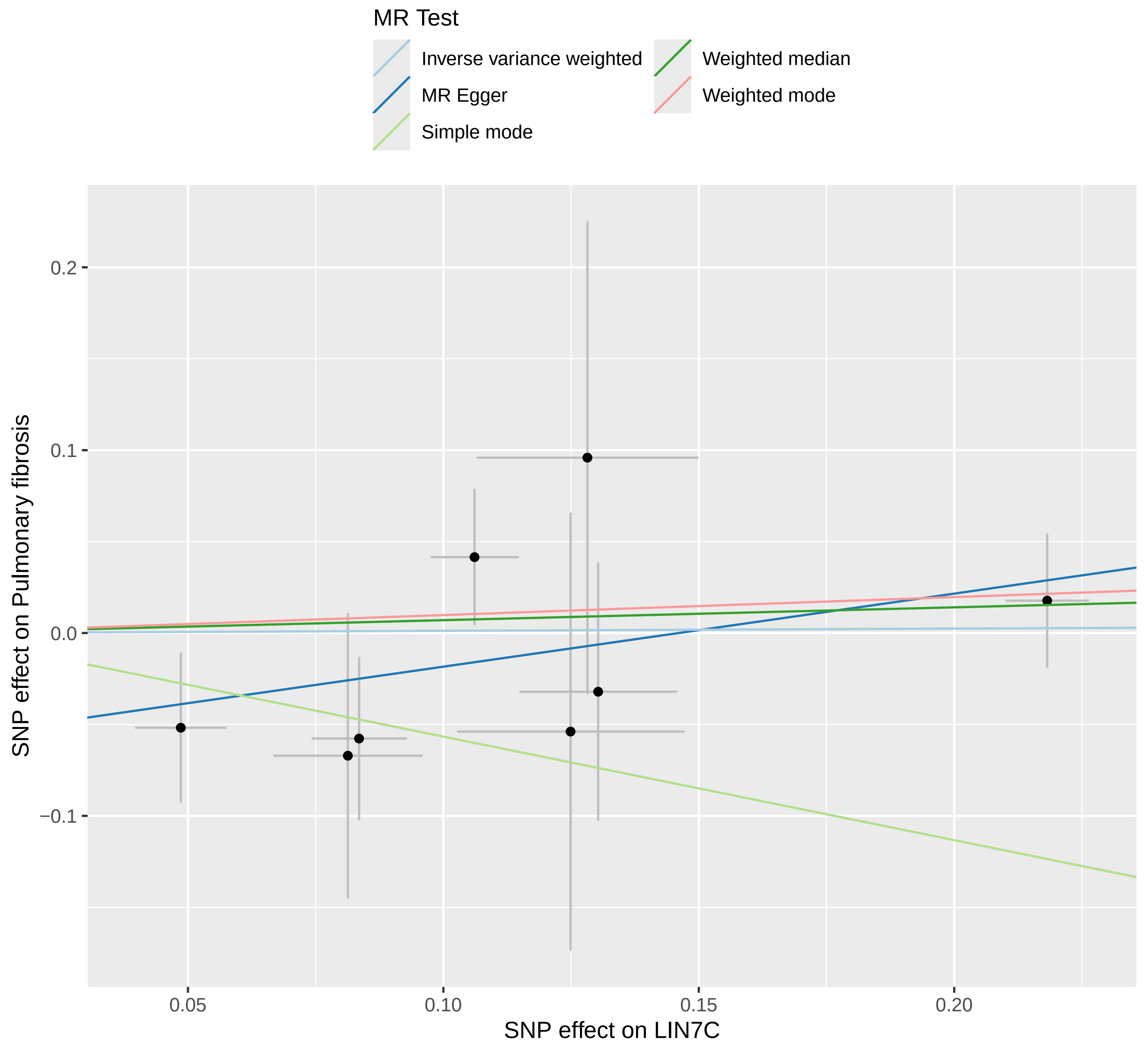
MR Test
Inverse variance weighted
MR Egger
Simple mode
Weighted median
Weighted mode
0.2
0.1
0.0
−0.1
0.05
0.10
0.15
0.20
SNP effect on Pulmonary fibrosis
SNP effect on LIN7C

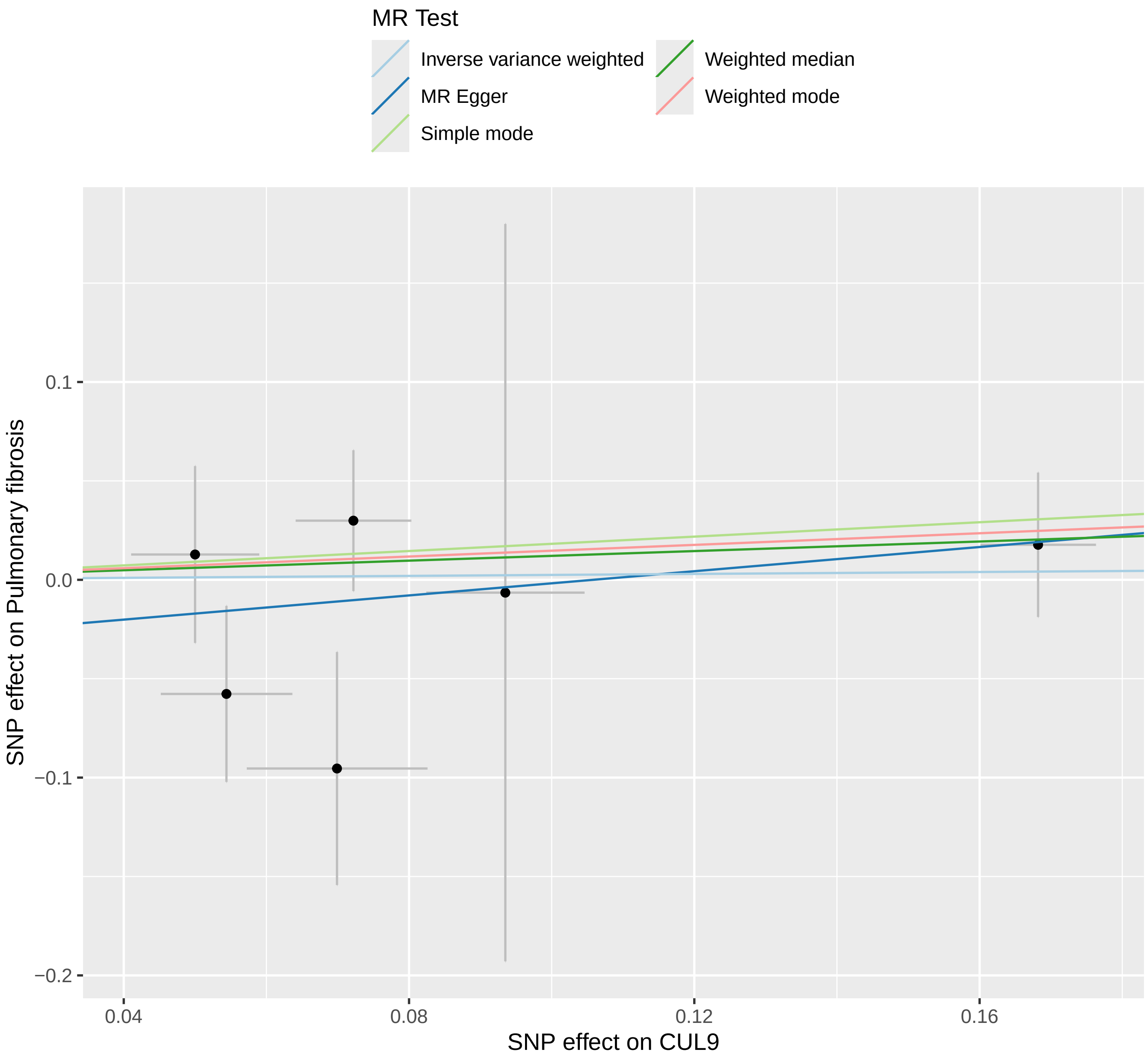

MR Test
Inverse variance weighted
MR Egger
Simple mode
Weighted median
Weighted mode
0.1
0.0
−0.1
−0.2
SNP effect on Pulmonary fibrosis
0.04
0.08
0.12
0.16
SNP effect on CUL9

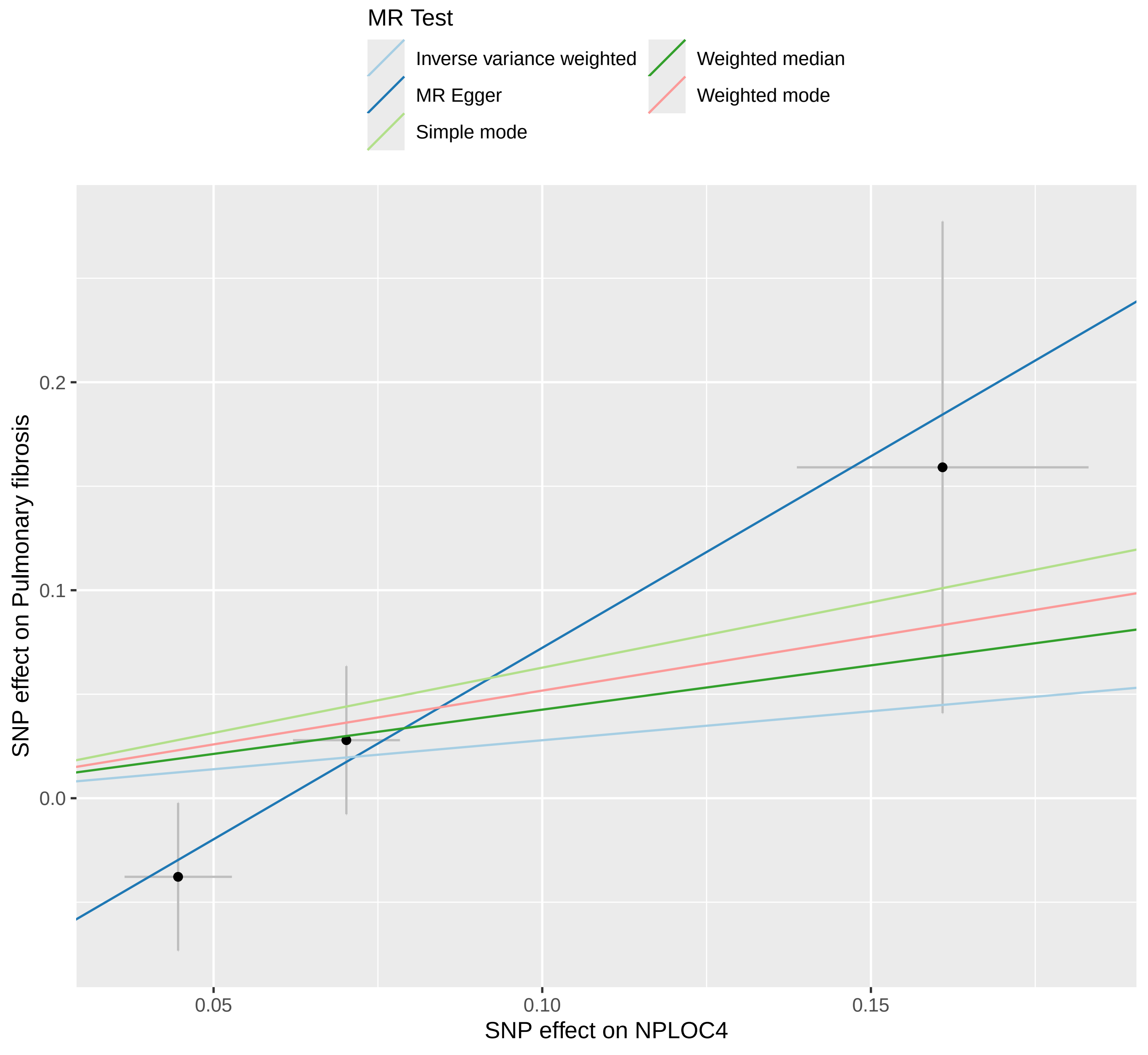
MR Test
Inverse variance weighted
MR Egger
Simple mode
Weighted median
Weighted mode
0.2
0.1
0.0
0.05
0.10
0.15
SNP effect on Pulmonary fibrosis
SNP effect on NPLOC4

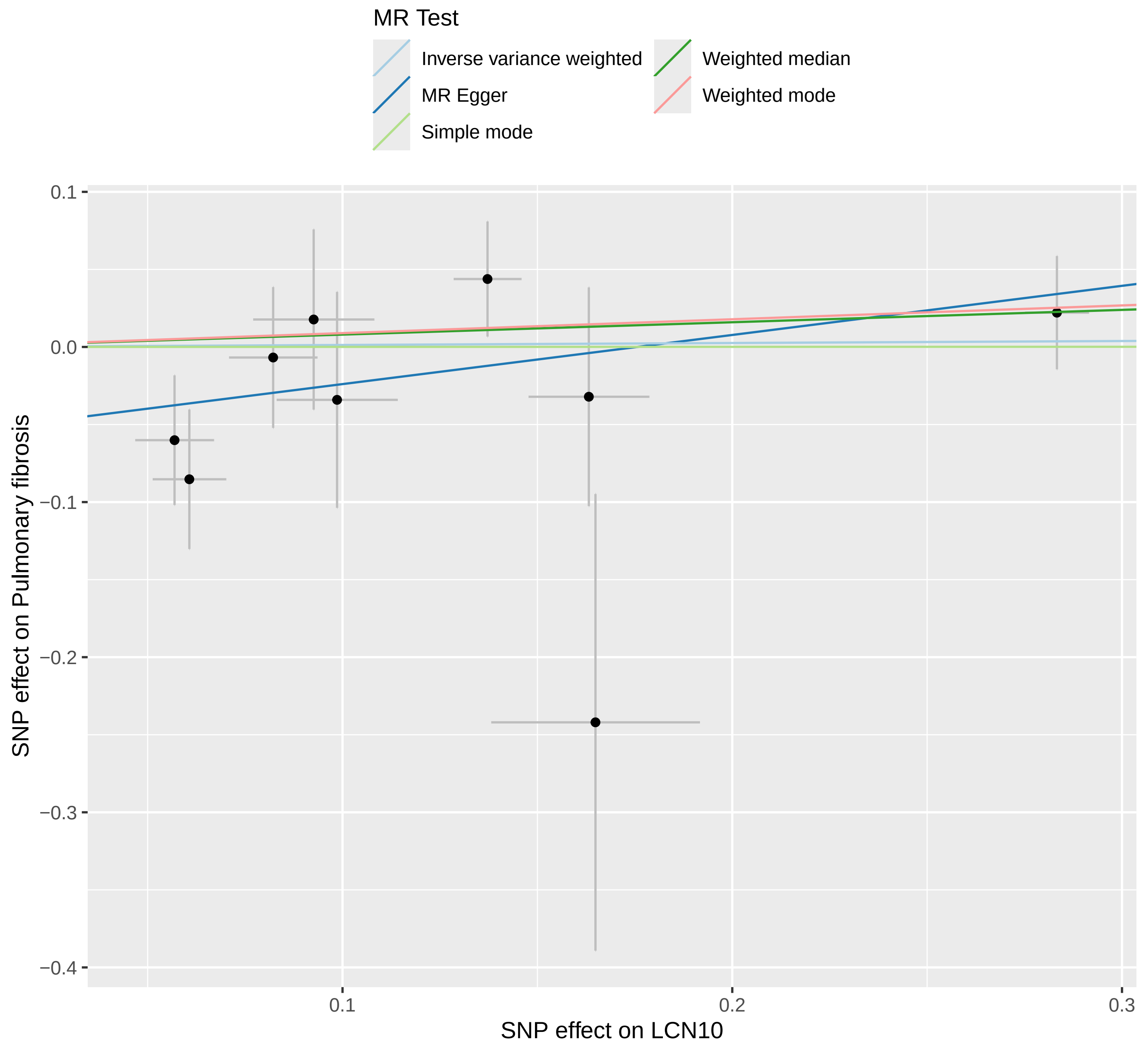

MR Test
Inverse variance weighted
MR Egger
Simple mode
Weighted median
Weighted mode
0.1
0.0
−0.1
−0.2
−0.3
−0.4
0.1
0.2
0.3
SNP effect on Pulmonary fibrosis
SNP effect on LCN10

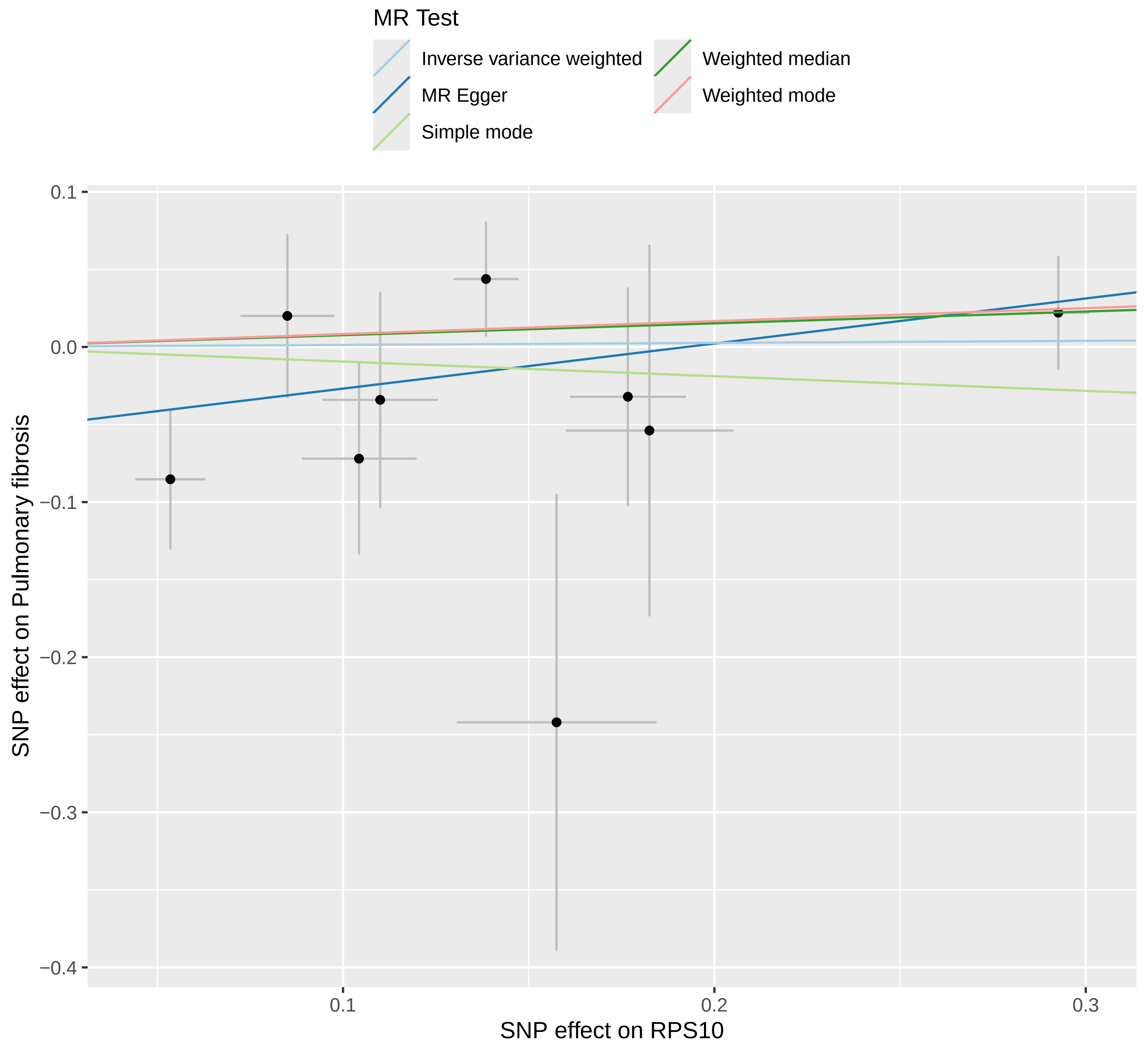
MR Test
Inverse variance weighted
MR Egger
Simple mode
Weighted median
Weighted mode
0.1
0.0
−0.1
−0.2
−0.3
−0.4
SNP effect on Pulmonary fibrosis
0.1
0.2
0.3
SNP effect on RPS10

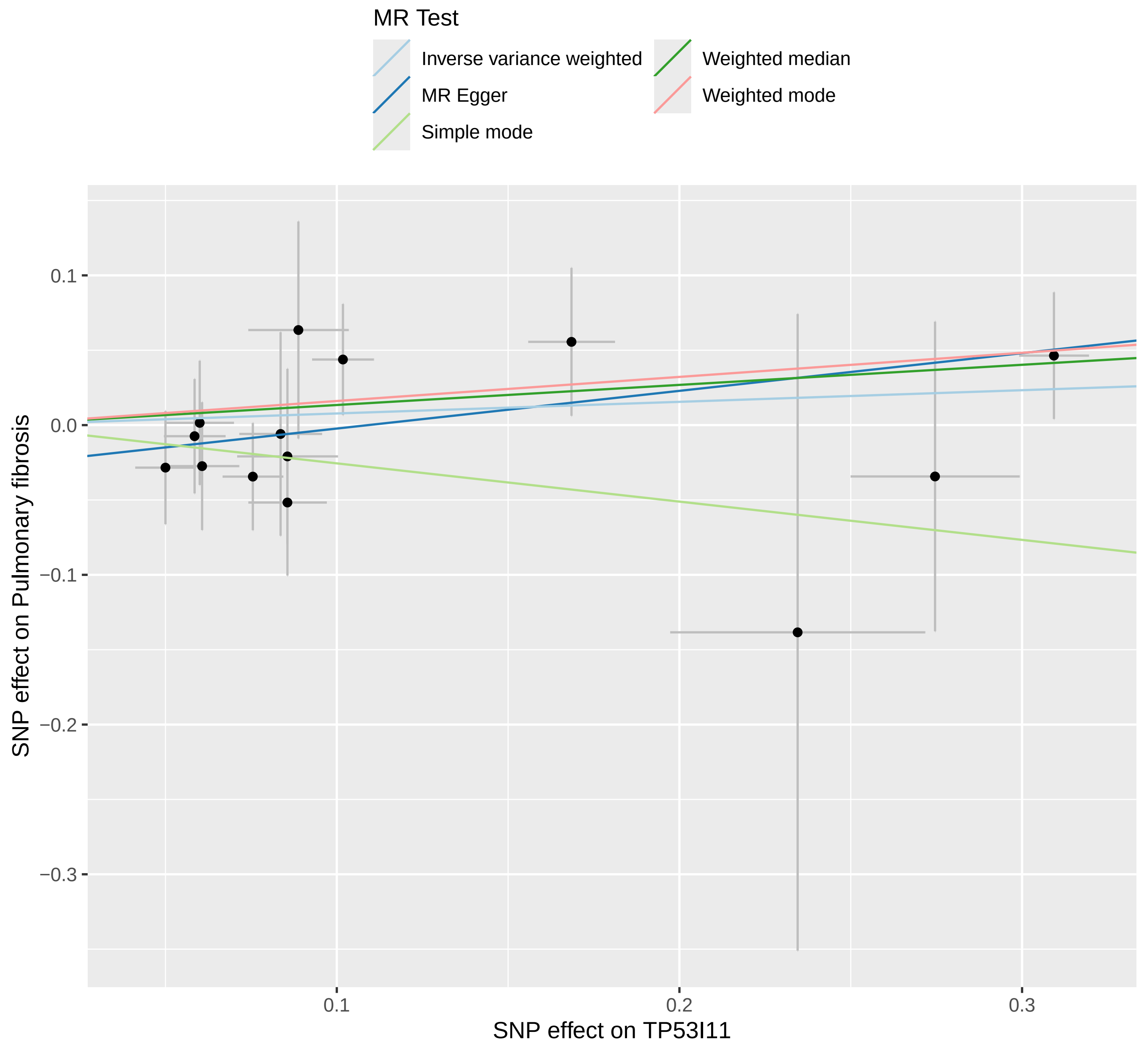
MR Test
Inverse variance weighted
MR Egger
Simple mode
Weighted median
Weighted mode
0.1
0.0
−0.1
−0.2
−0.3
SNP effect on Pulmonary fibrosis
0.1
0.2
0.3
SNP effect on TP53I11

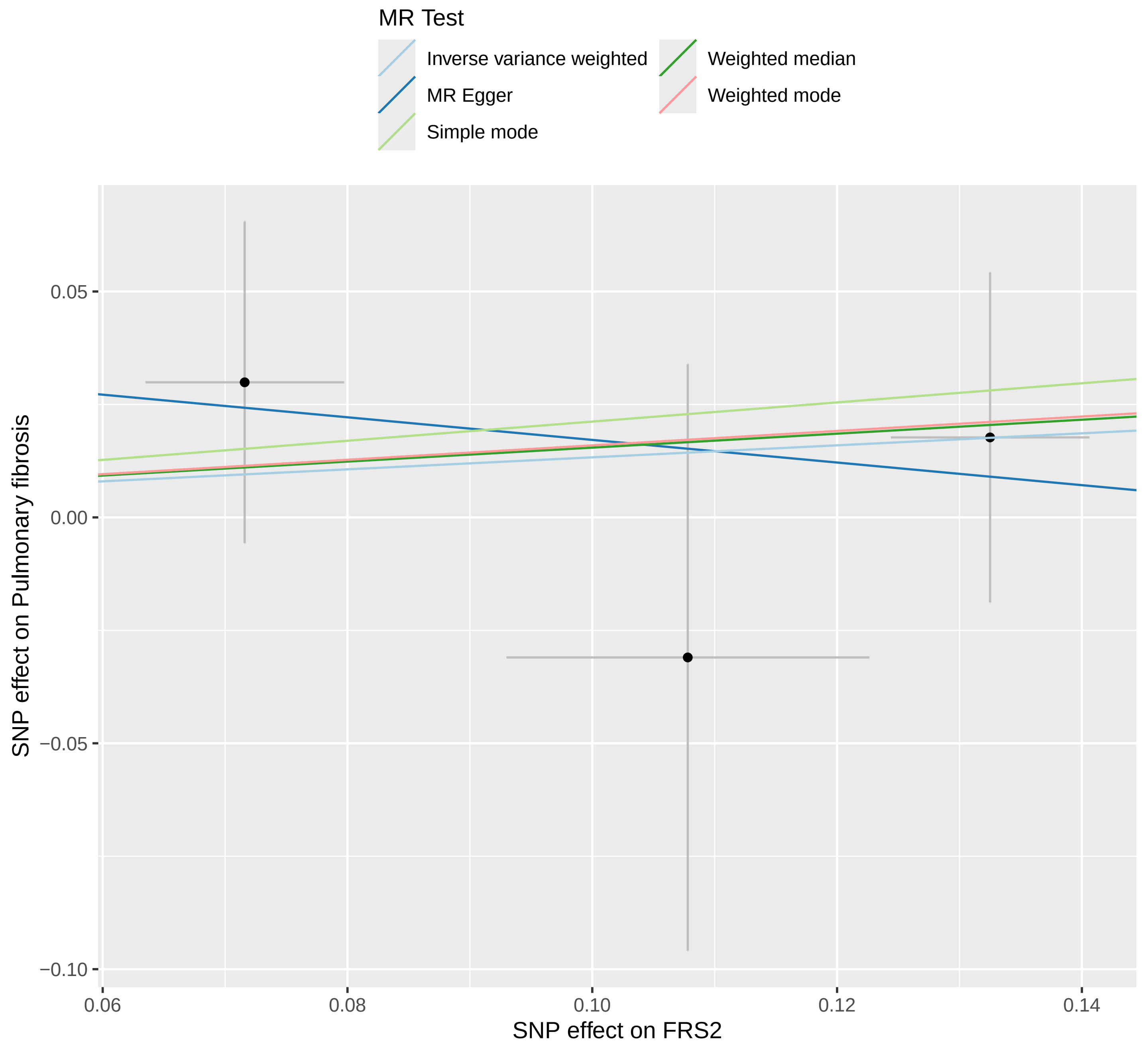
MR Test
Inverse variance weighted
MR Egger
Simple mode
Weighted median
Weighted mode
0.05
0.00
−0.05
−0.10
0.06
0.08
0.10
0.12
0.14
SNP effect on Pulmonary fibrosis
SNP effect on FRS2

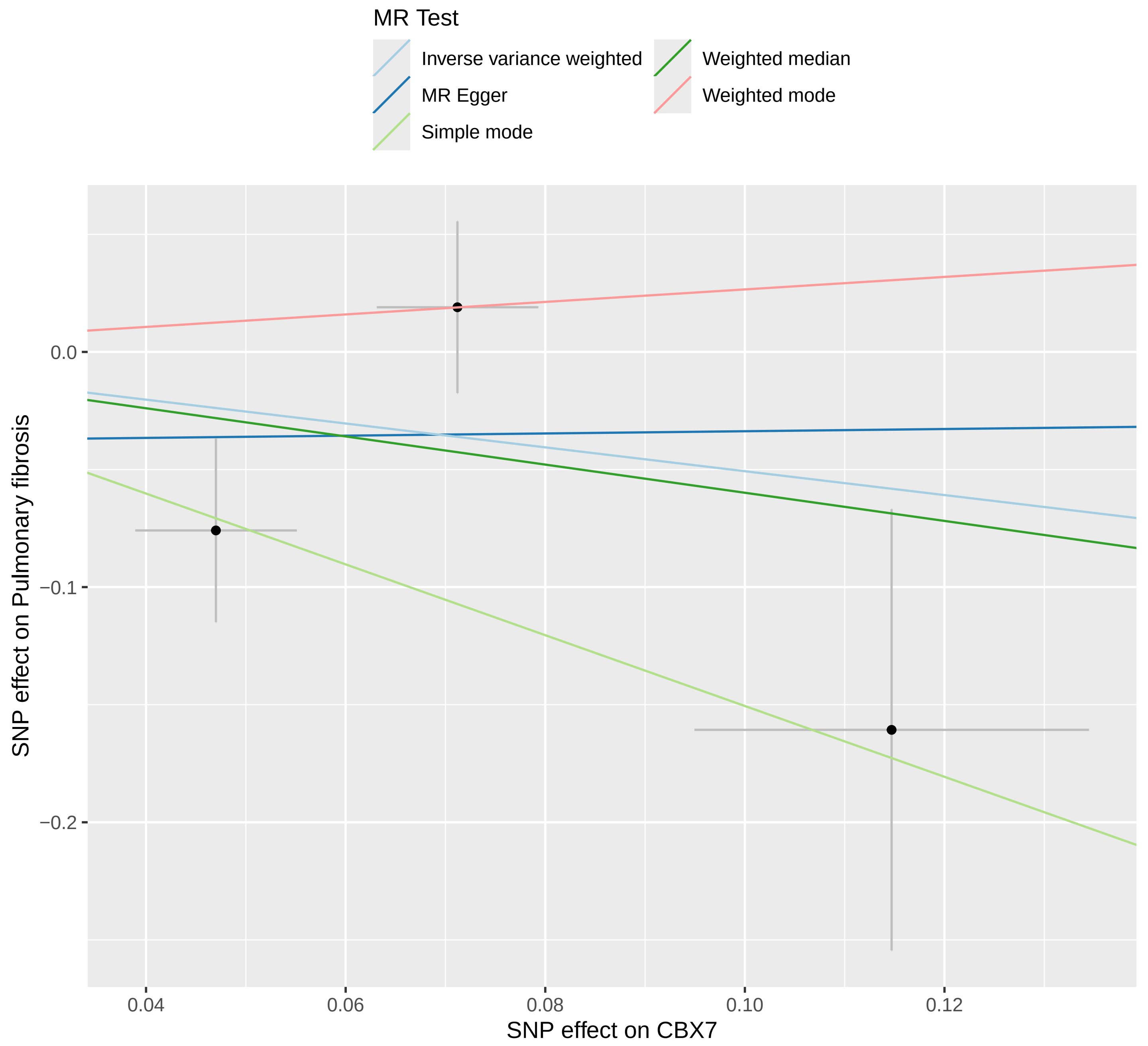
MR Test
Inverse variance weighted
MR Egger
Simple mode
Weighted median
Weighted mode
0.0
−0.1
−0.2
SNP effect on Pulmonary fibrosis
0.04
0.06
0.08
0.10
0.12
SNP effect on CBX7

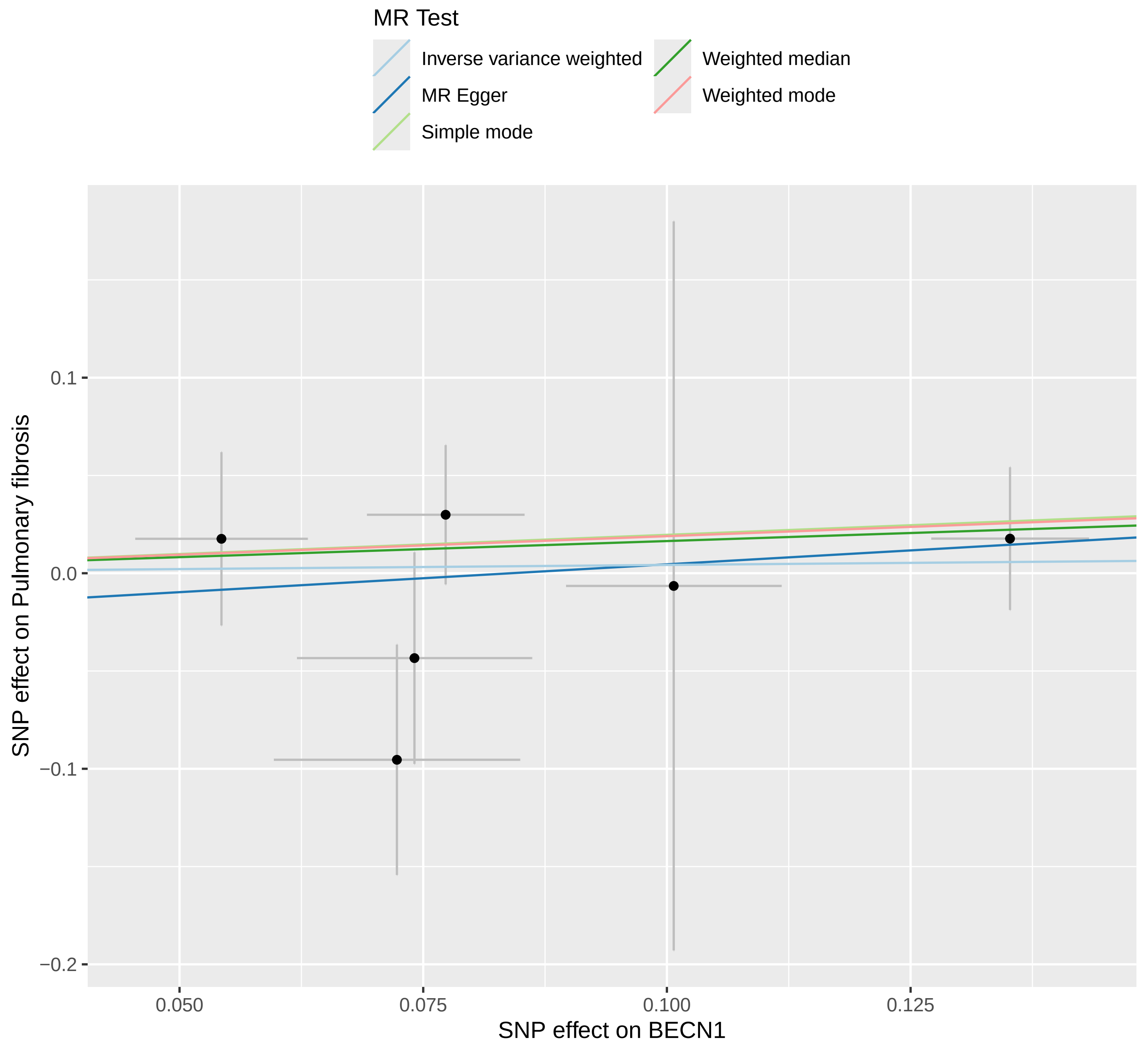
MR Test
Inverse variance weighted
MR Egger
Simple mode
Weighted median
Weighted mode
0.1
0.0
−0.1
−0.2
SNP effect on Pulmonary fibrosis
0.050
0.075
0.100
0.125
SNP effect on BECN1

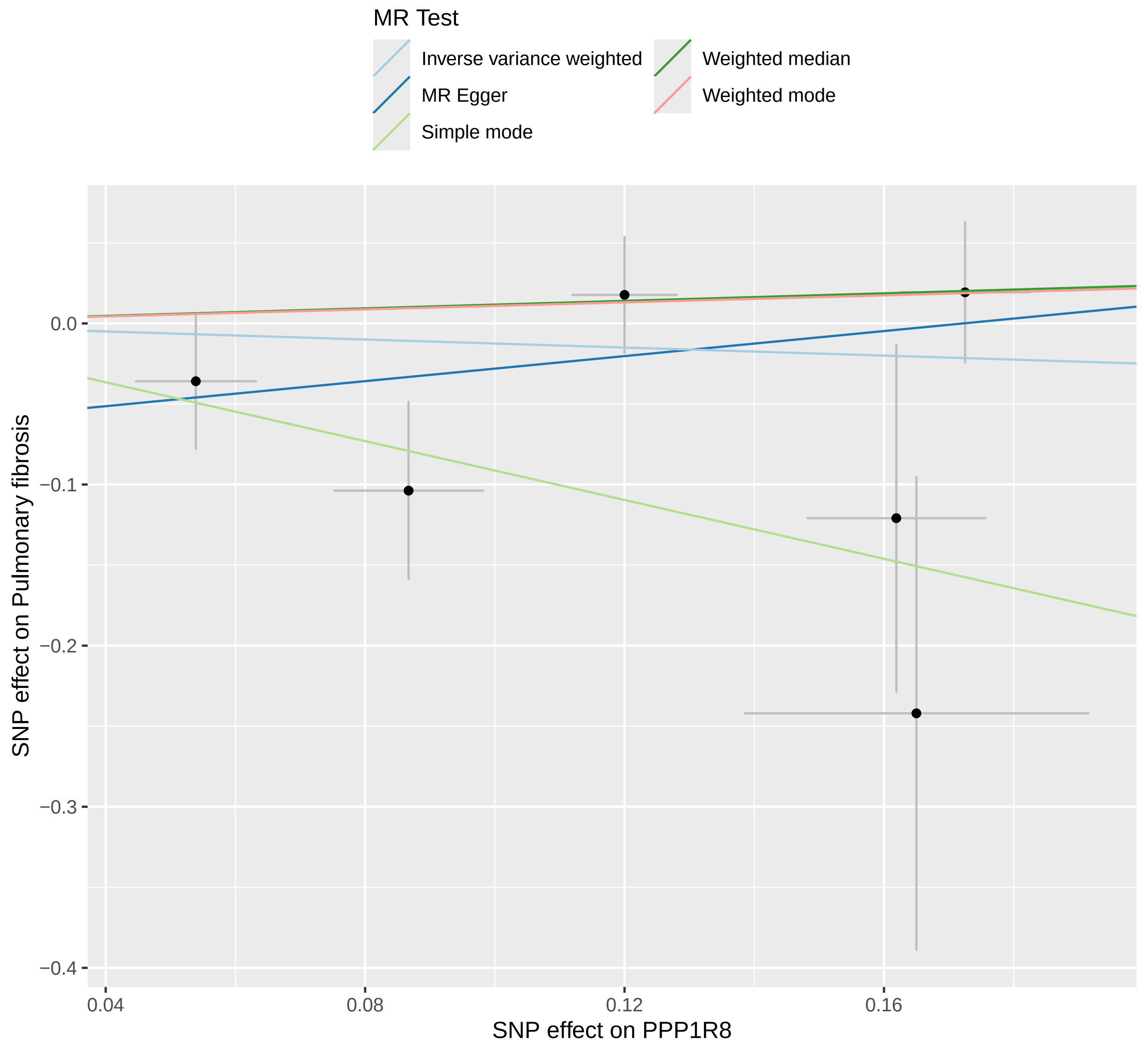
MR Test
Inverse variance weighted
MR Egger
Simple mode
Weighted median
Weighted mode
SNP effect on Pulmonary fibrosis
0.0
−0.1
−0.2
−0.3
−0.4
0.04
0.08
0.12
0.16
SNP effect on PPP1R8

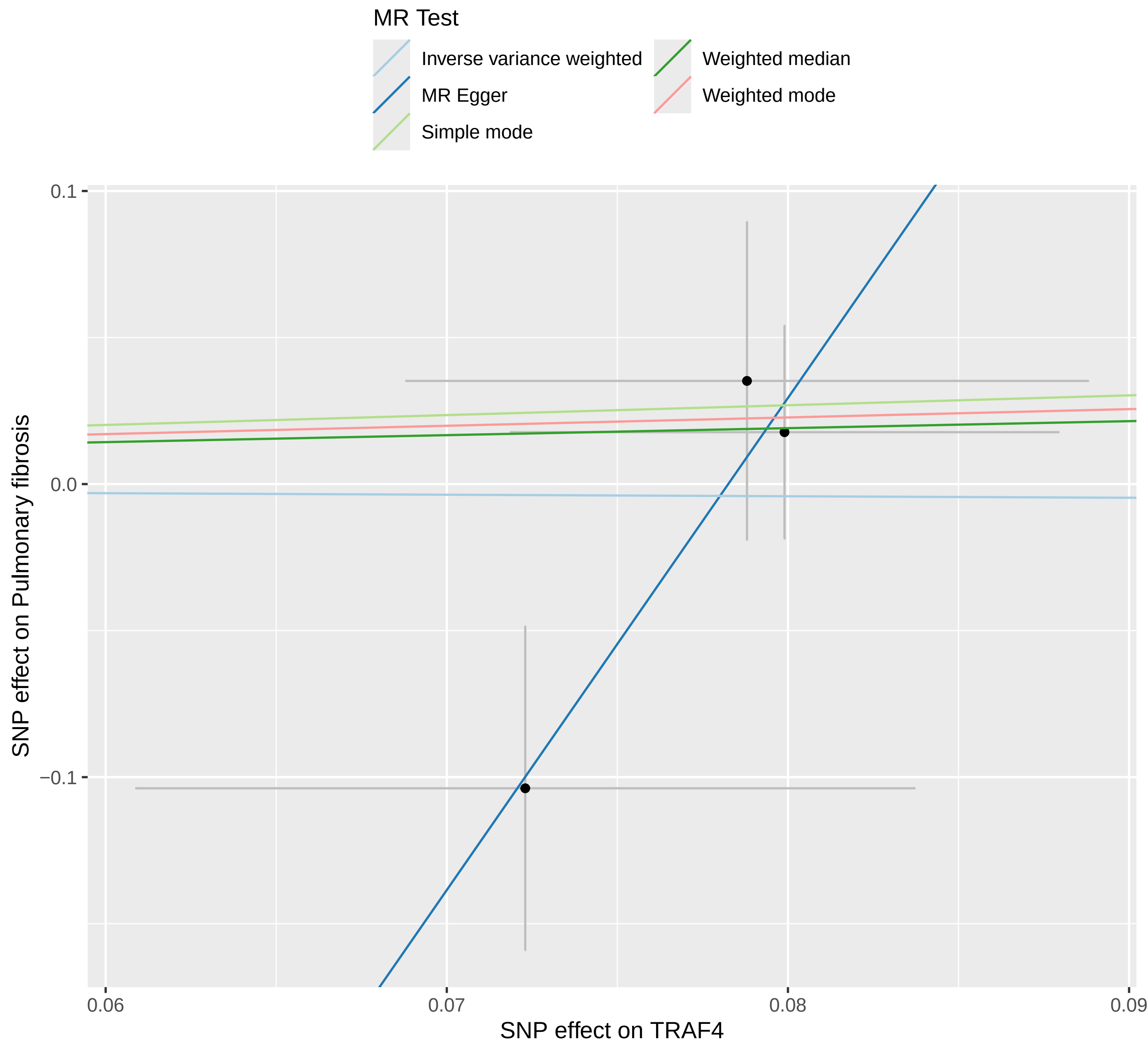

MR Test
Inverse variance weighted
MR Egger
Simple mode
Weighted median
Weighted mode
0.1
0.0
−0.1
0.06
0.07
0.08
0.09
SNP effect on Pulmonary fibrosis
SNP effect on TRAF4

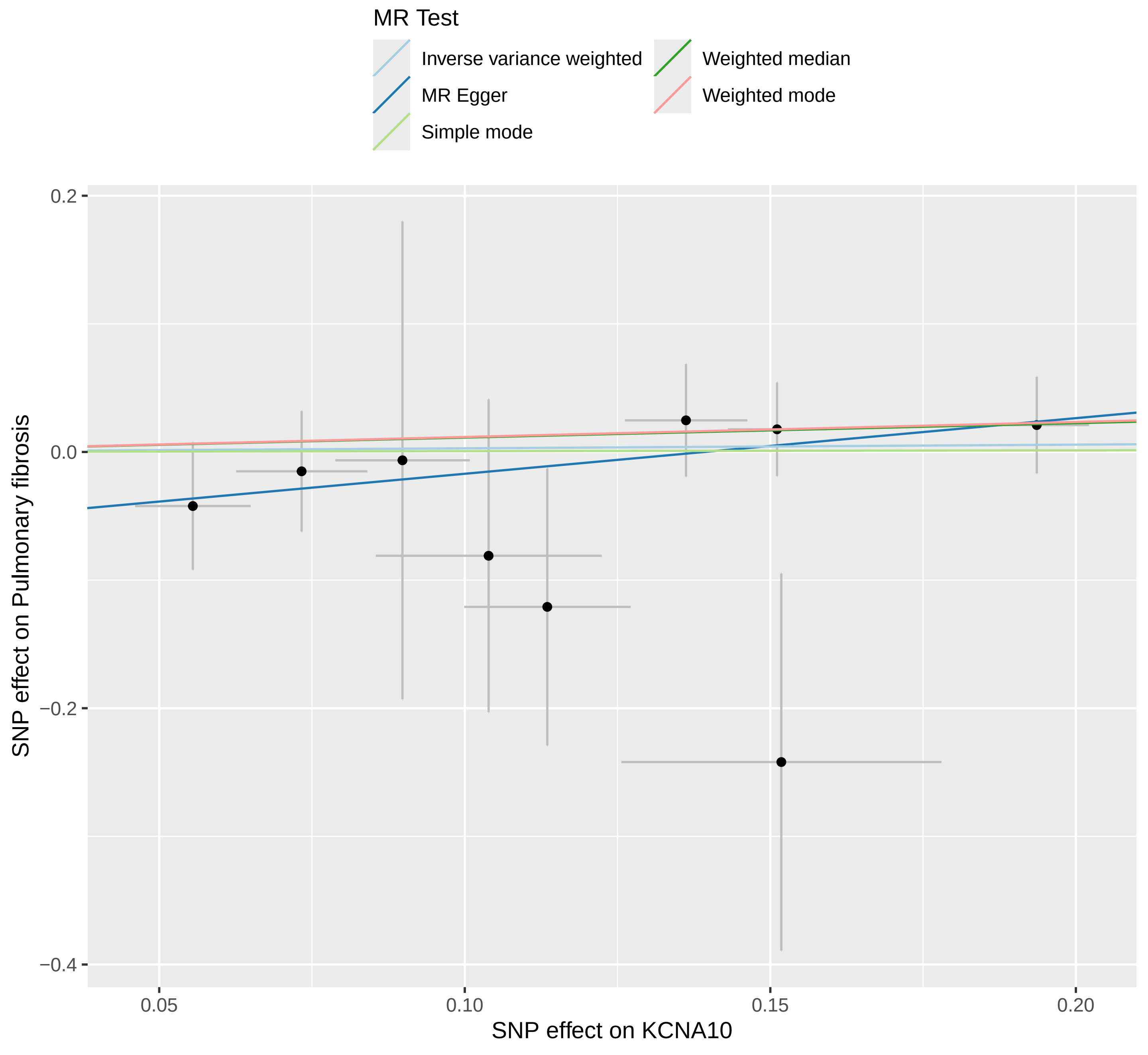
MR Test
Inverse variance weighted
MR Egger
Simple mode
Weighted median
Weighted mode
0.2
0.0
−0.2
−0.4
0.05
0.10
0.15
0.20
SNP effect on Pulmonary fibrosis
SNP effect on KCNA10

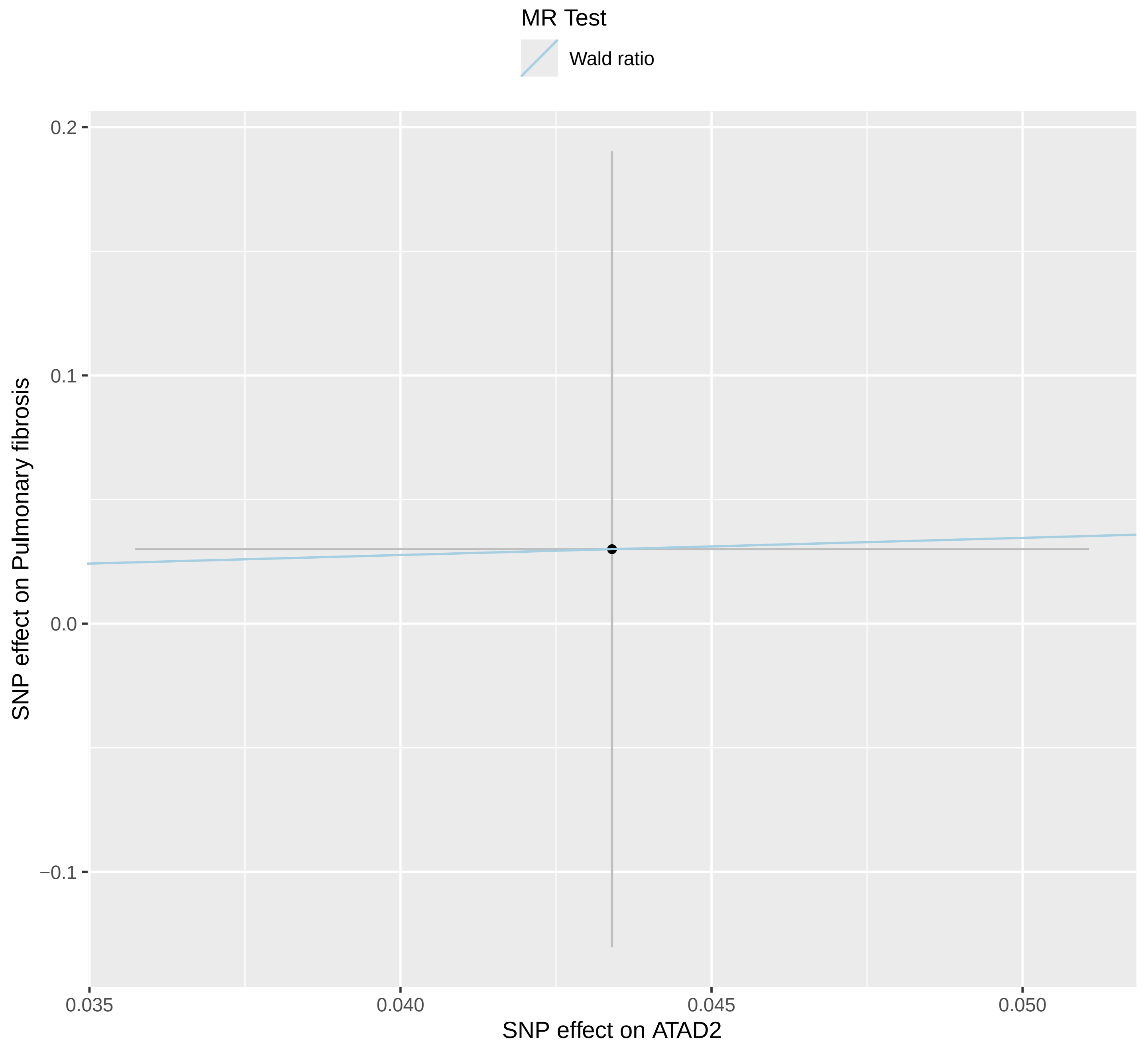
MR Test
Wald ratio
0.2
0.1
0.0
−0.1
SNP effect on Pulmonary fibrosis
0.035
0.040
0.045
0.050
SNP effect on ATAD2

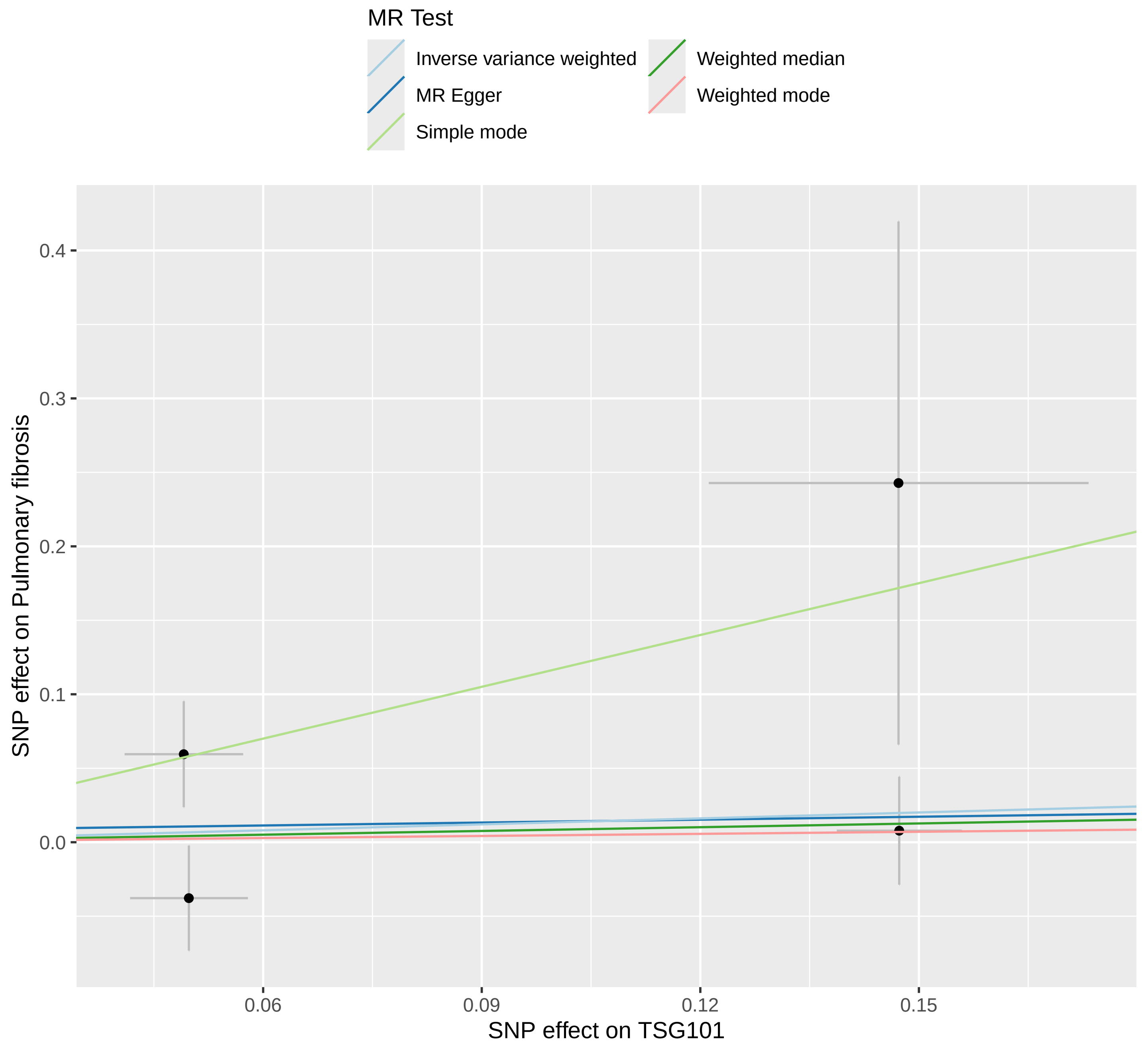
MR Test
Inverse variance weighted
MR Egger
Simple mode
Weighted median
Weighted mode
0.4
0.3
0.2
0.1
0.0
SNP effect on Pulmonary fibrosis
0.06
0.09
0.12
0.15
SNP effect on TSG101

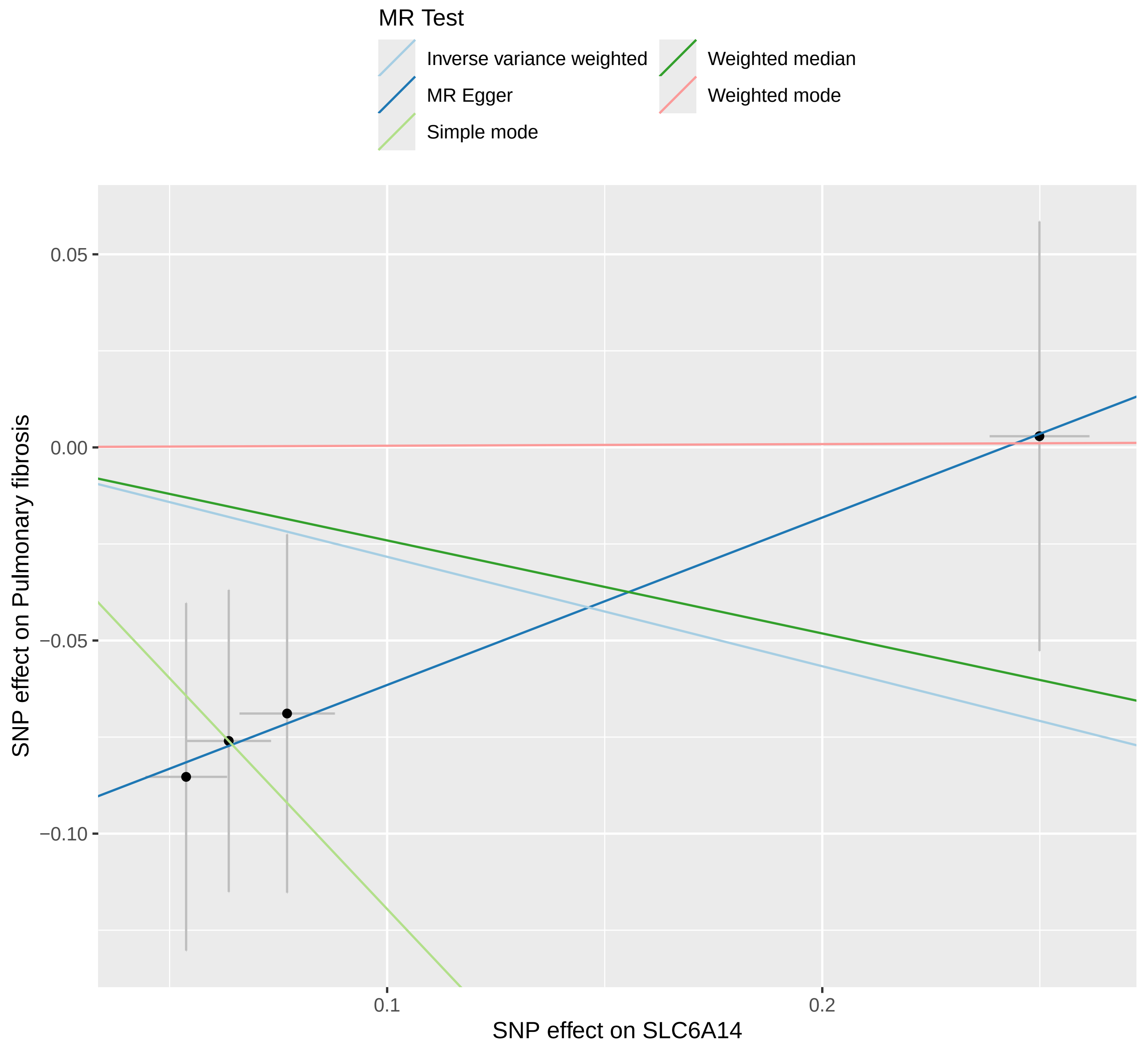
MR Test
Inverse variance weighted
MR Egger
Simple mode
Weighted median
Weighted mode
0.05
0.00
−0.05
−0.10
0.1
0.2
SNP effect on Pulmonary fibrosis
SNP effect on SLC6A14

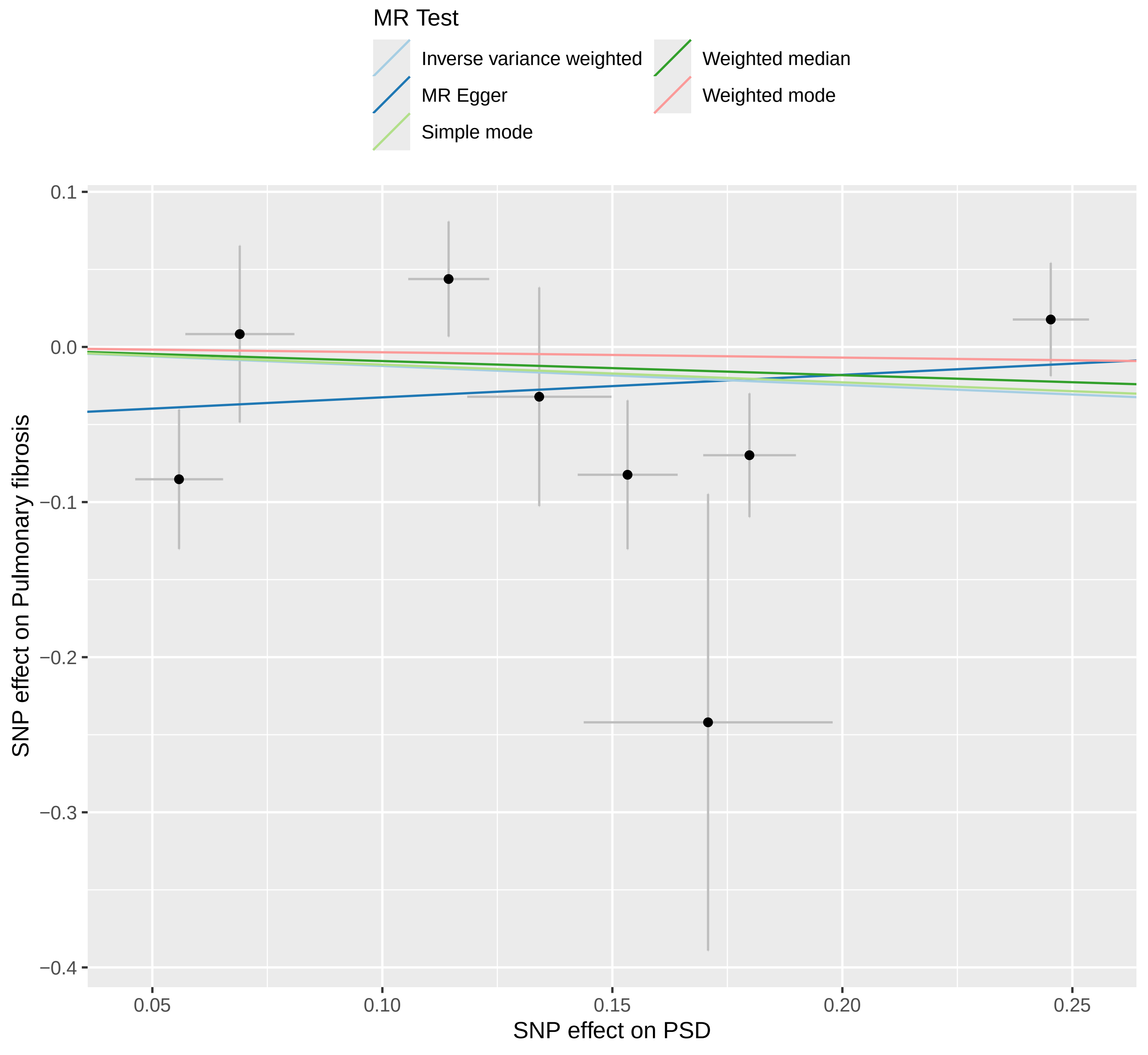
MR Test
Inverse variance weighted
MR Egger
Simple mode
Weighted median
Weighted mode
0.1
0.0
−0.1
−0.2
−0.3
−0.4
0.05
0.10
0.15
0.20
0.25
SNP effect on Pulmonary fibrosis
SNP effect on PSD

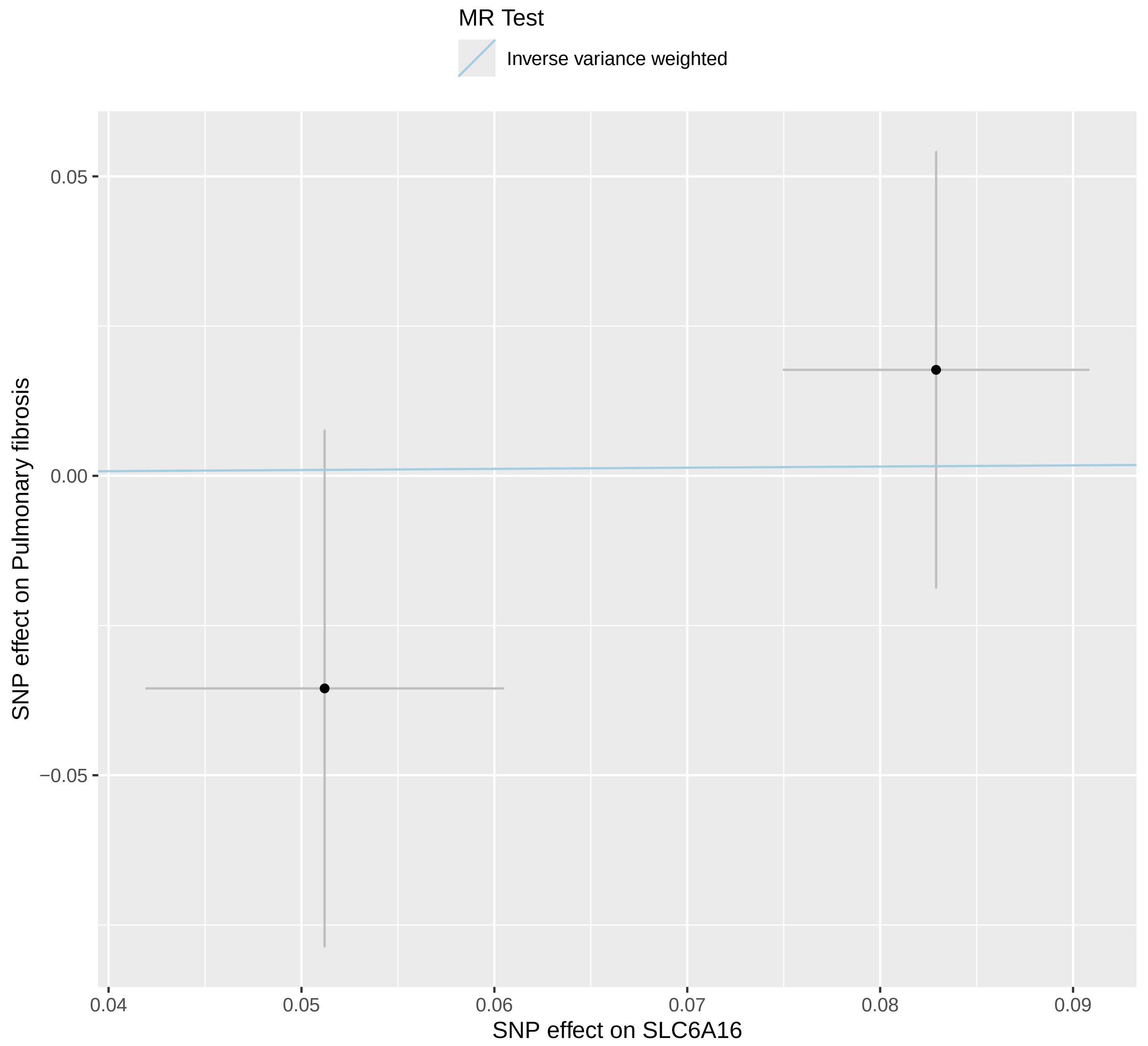
MR Test
Inverse variance weighted
0.05
0.00
−0.05
0.04
0.05
0.06
0.07
0.08
0.09
SNP effect on Pulmonary fibrosis
SNP effect on SLC6A16

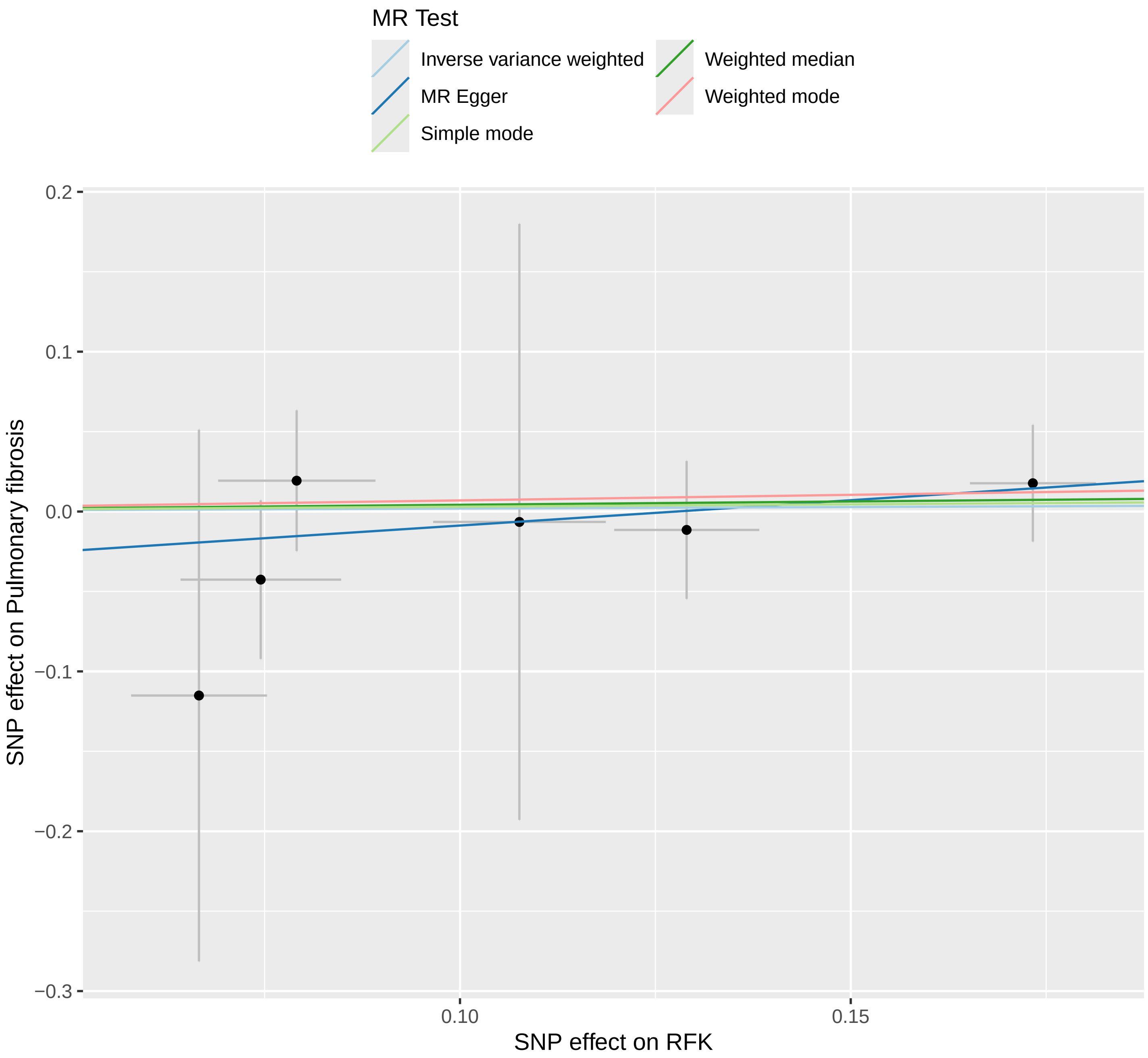

MR Test
Inverse variance weighted
MR Egger
Simple mode
Weighted median
Weighted mode
0.2
0.1
0.0
−0.1
−0.2
−0.3
SNP effect on Pulmonary fibrosis
0.10
0.15
SNP effect on RFK

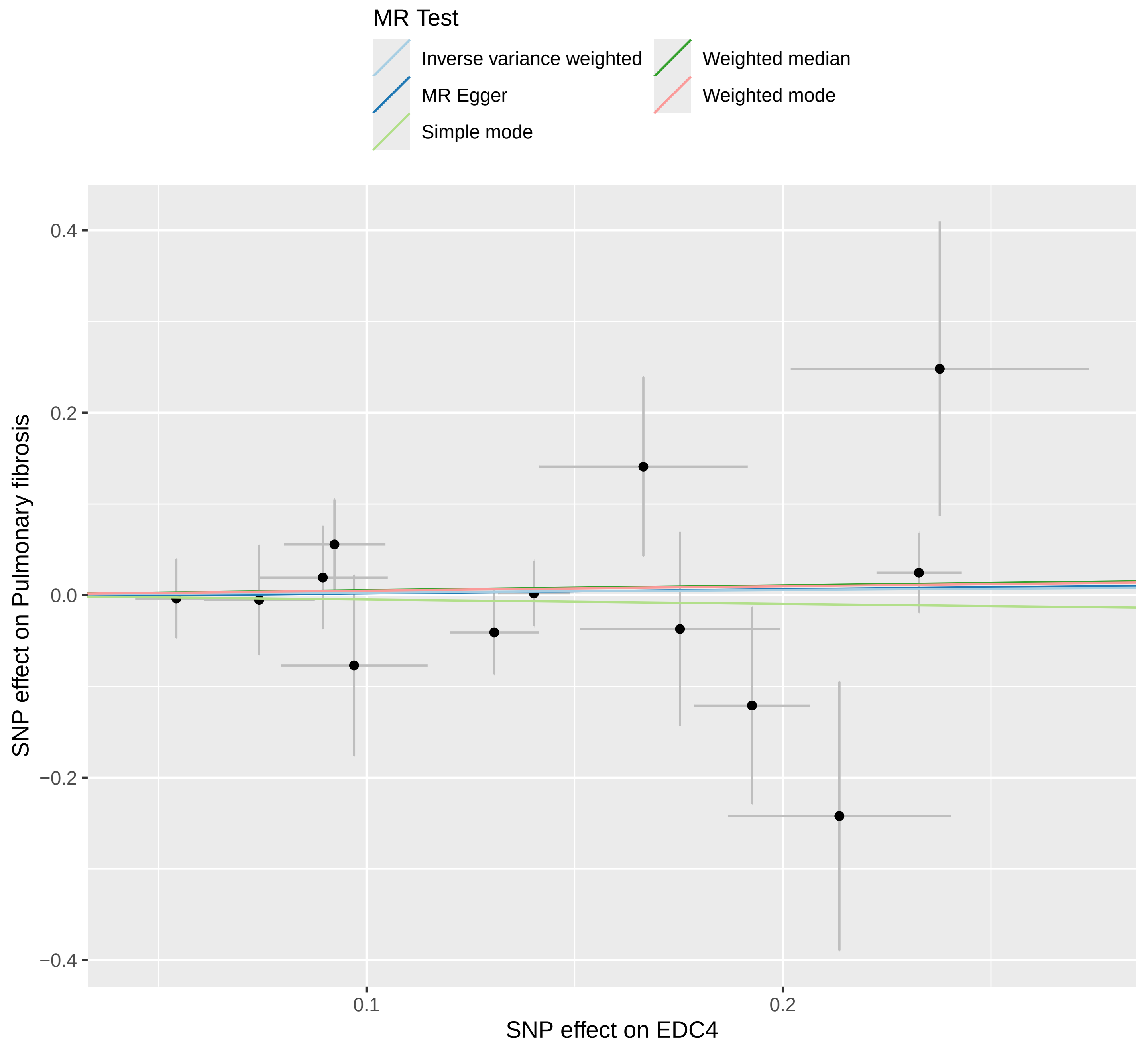
MR Test
Inverse variance weighted
MR Egger
Simple mode
Weighted median
Weighted mode
0.4
0.2
0.0
−0.2
−0.4
0.1
0.2
SNP effect on Pulmonary fibrosis
SNP effect on EDC4

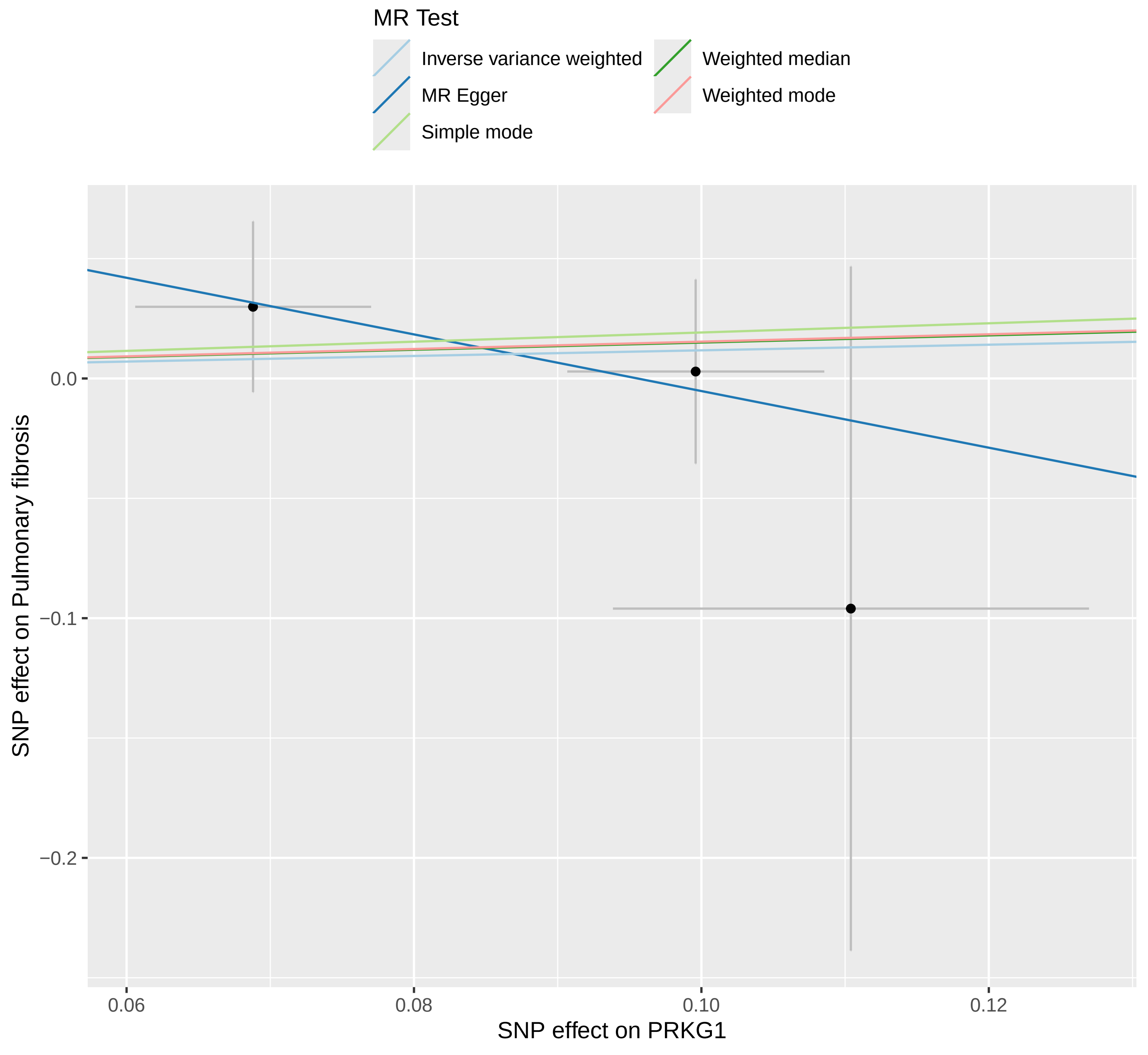
MR Test
Inverse variance weighted
MR Egger
Simple mode
Weighted median
Weighted mode
0.0
−0.1
−0.2
SNP effect on Pulmonary fibrosis
0.06
0.08
0.10
0.12
SNP effect on PRKG1

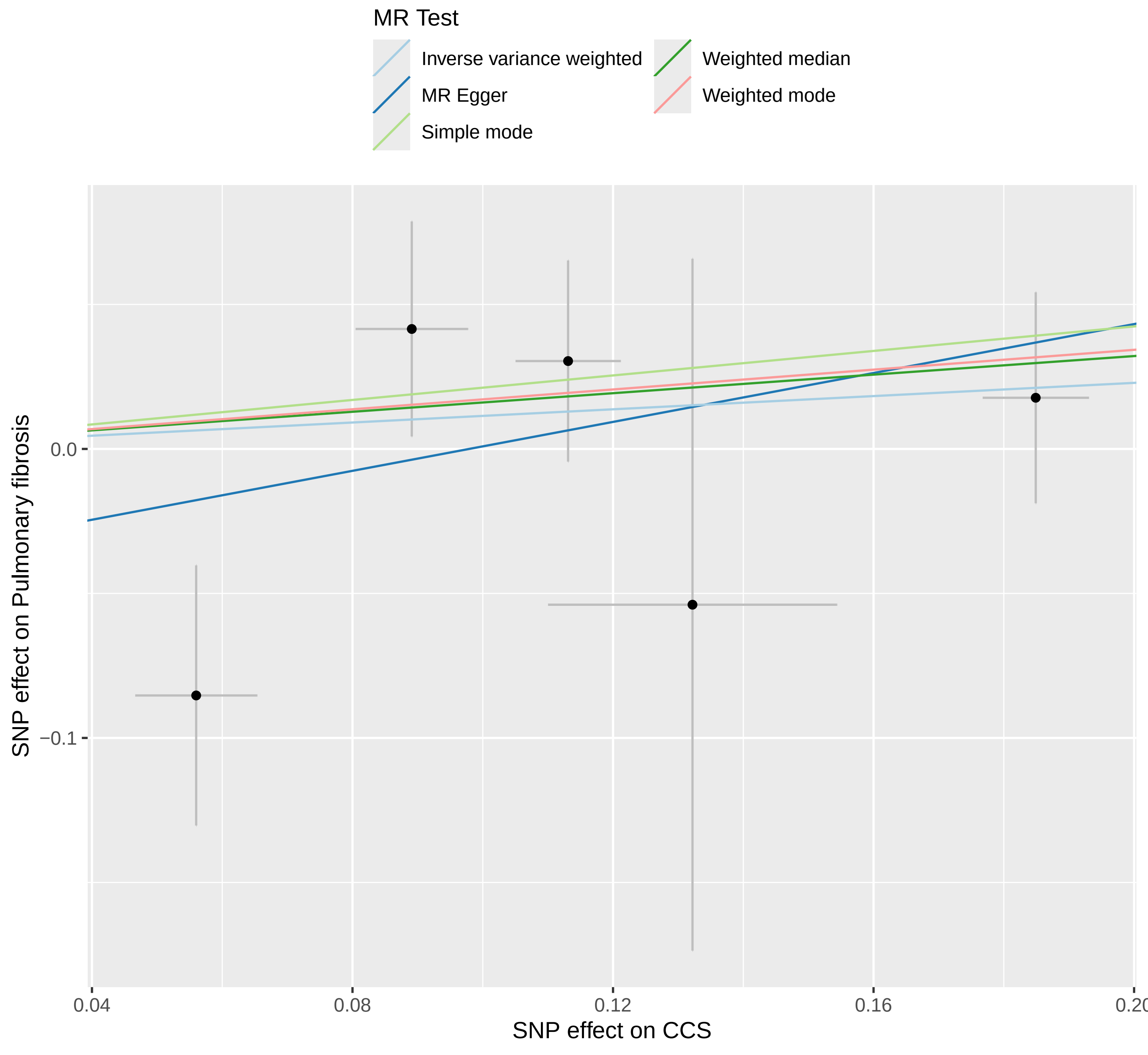

MR Test
Inverse variance weighted
MR Egger
Simple mode
Weighted median
Weighted mode
0.0
−0.1
SNP effect on Pulmonary fibrosis
0.04
0.08
0.12
0.16
0.20
SNP effect on CCS

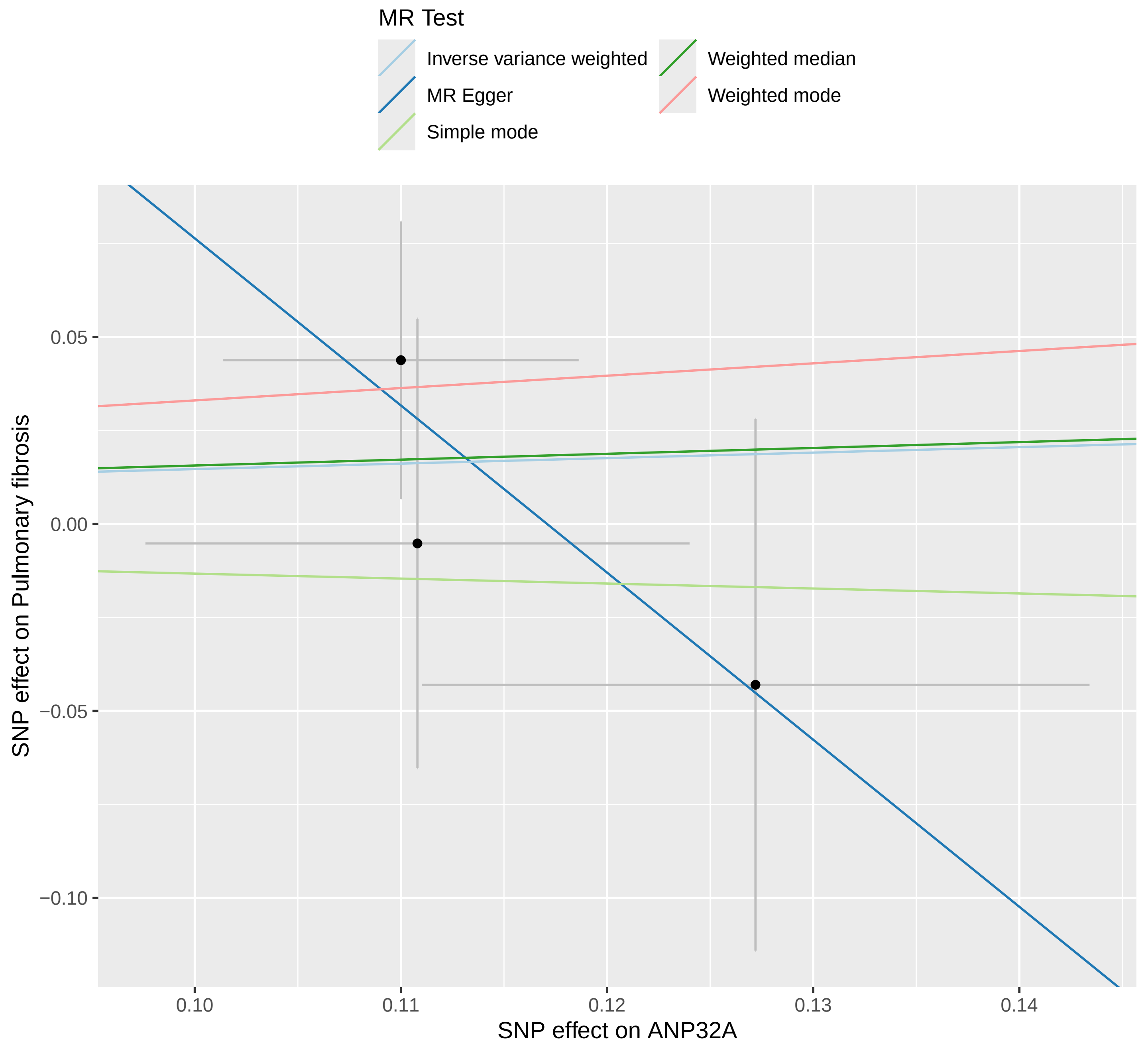

MR Test
Inverse variance weighted
MR Egger
Simple mode
Weighted median
Weighted mode
SNP effect on Pulmonary fibrosis
0.05
0.00
−0.05
−0.10
0.10
0.11
0.12
0.13
0.14
SNP effect on ANP32A

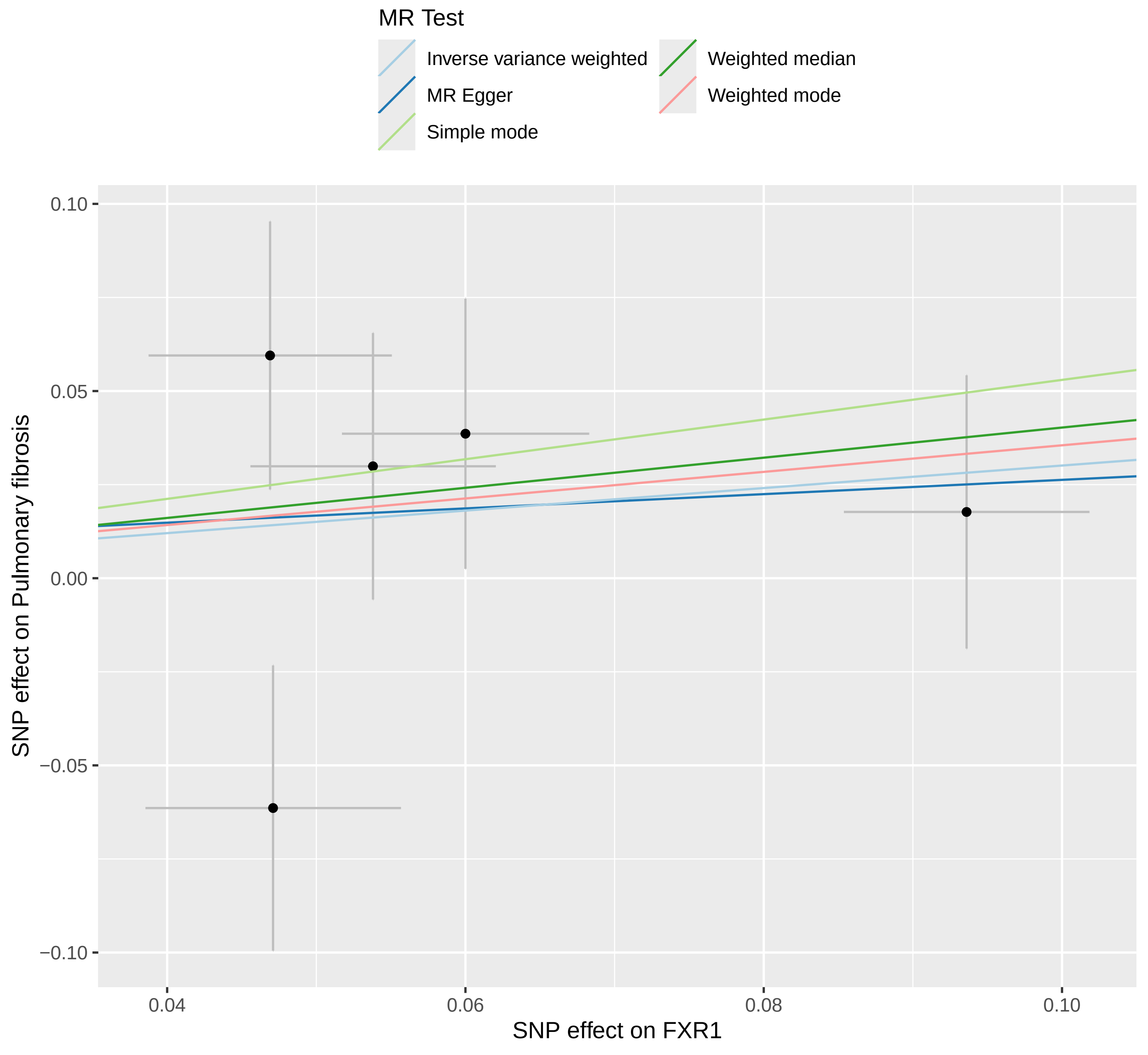
MR Test
Inverse variance weighted
MR Egger
Simple mode
Weighted median
Weighted mode
0.10
0.05
0.00
−0.05
−0.10
SNP effect on Pulmonary fibrosis
0.04
0.06
0.08
0.10
SNP effect on FXR1

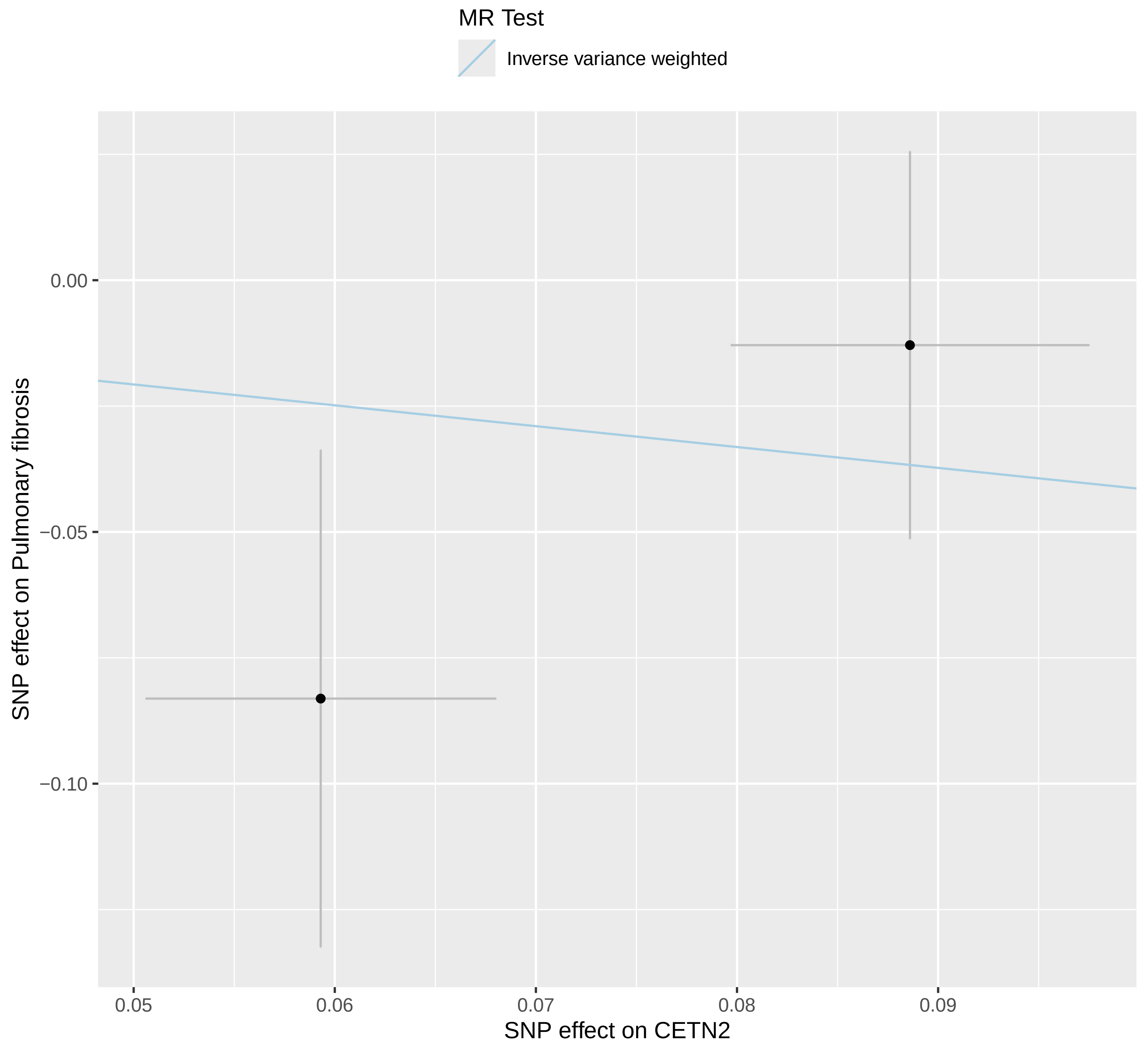
MR Test
Inverse variance weighted
0.00
−0.05
−0.10
0.05
0.06
0.07
0.08
0.09
SNP effect on CETN2
SNP effect on Pulmonary fibrosis

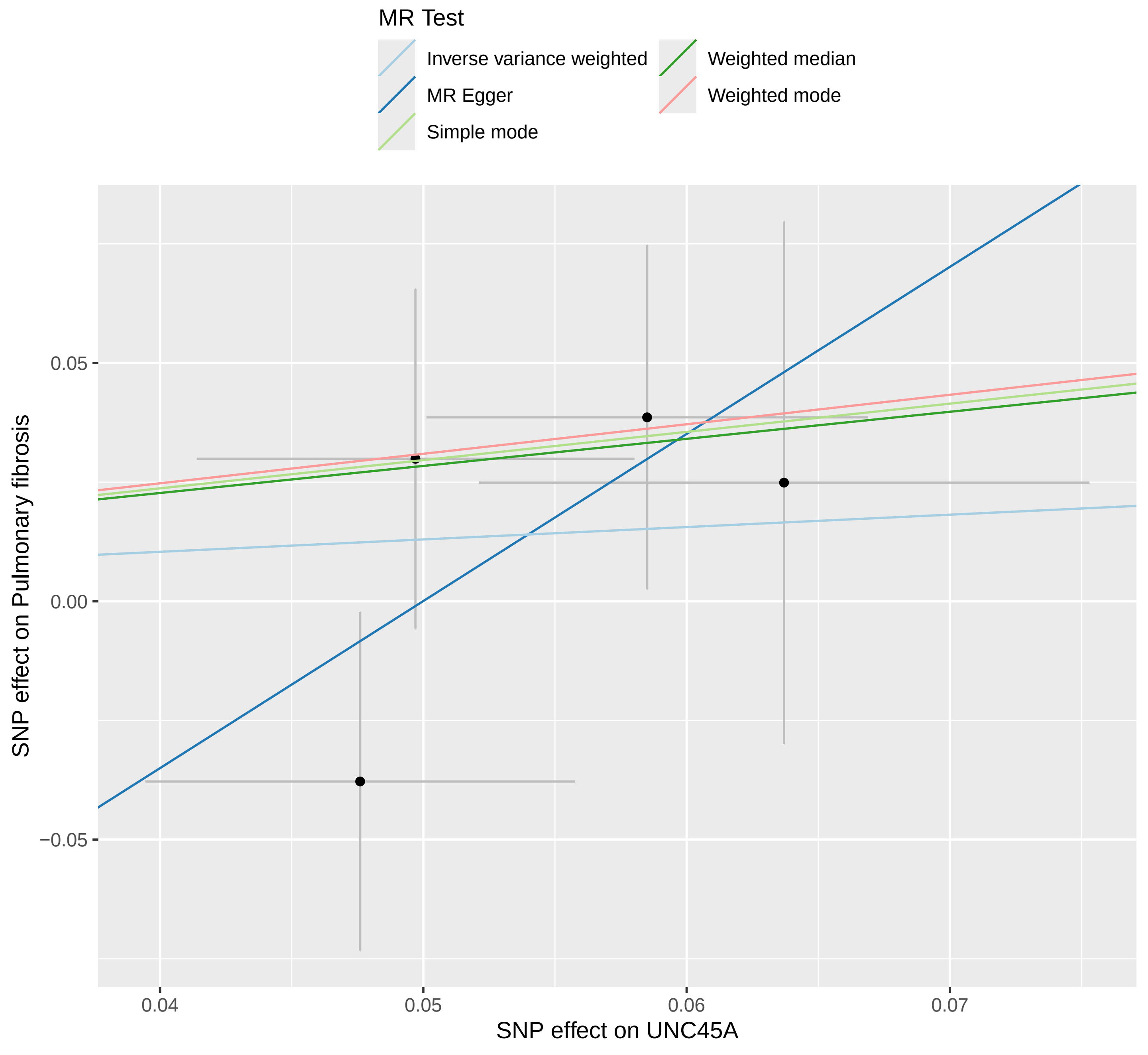

MR Test
Inverse variance weighted
MR Egger
Simple mode
Weighted median
Weighted mode
0.05
0.00
−0.05
0.04
0.05
0.06
0.07
SNP effect on Pulmonary fibrosis
SNP effect on UNC45A

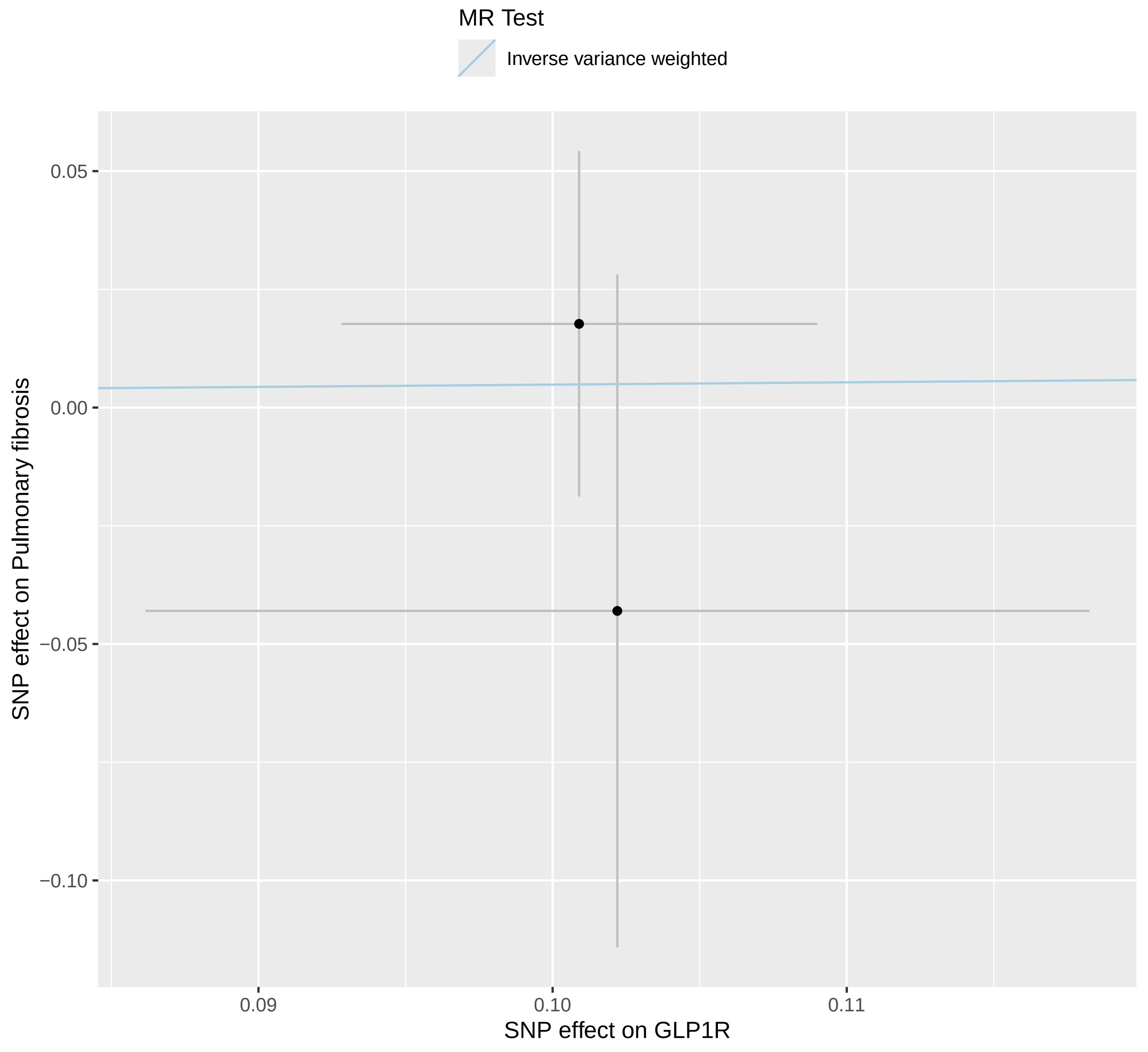
MR Test
Inverse variance weighted
0.05
0.00
−0.05
−0.10
0.09
0.10
0.11
SNP effect on Pulmonary fibrosis
SNP effect on GLP1R

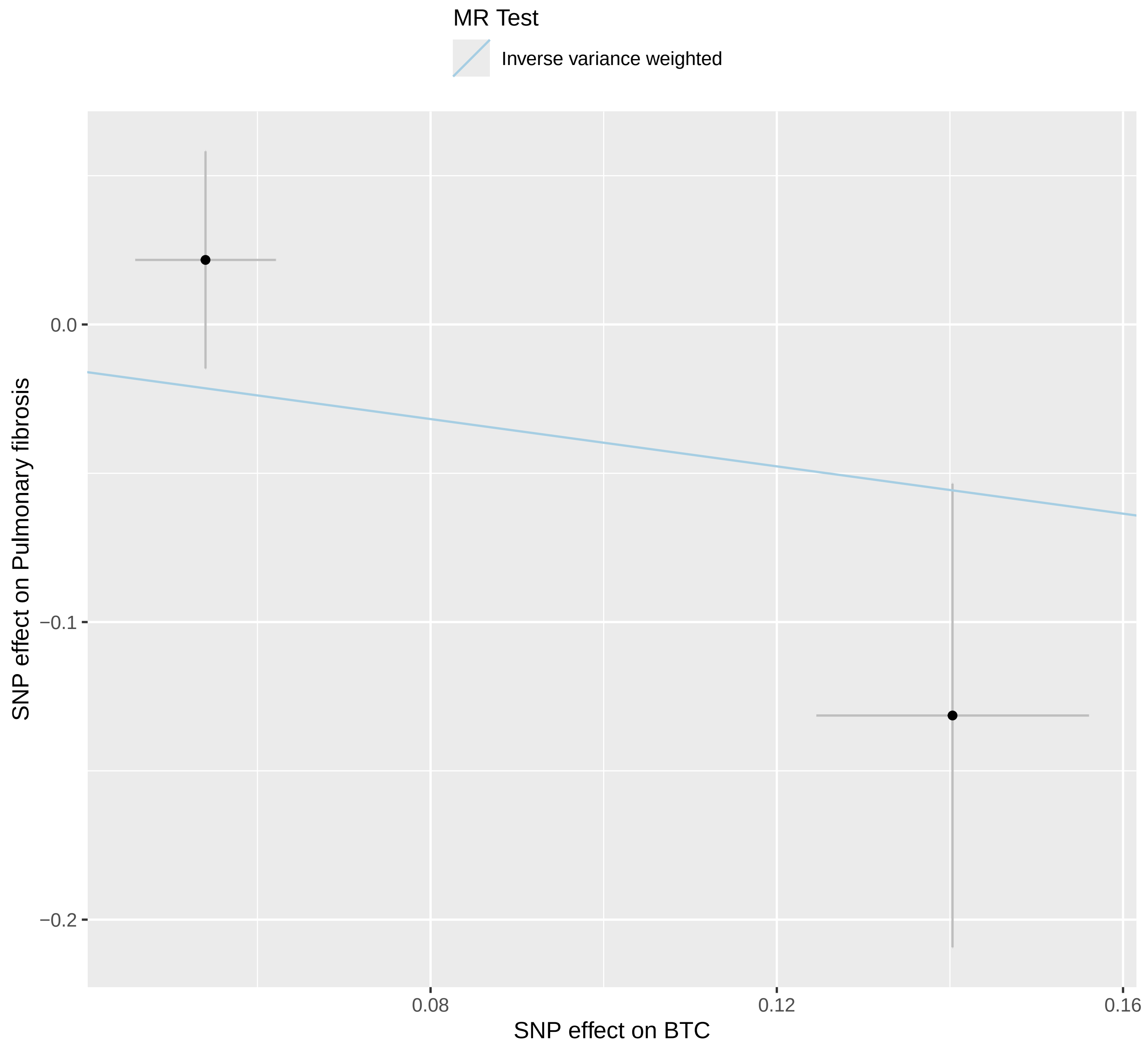
MR Test
Inverse variance weighted
0.0
−0.1
−0.2
SNP effect on Pulmonary fibrosis
0.08
0.12
0.16
SNP effect on BTC

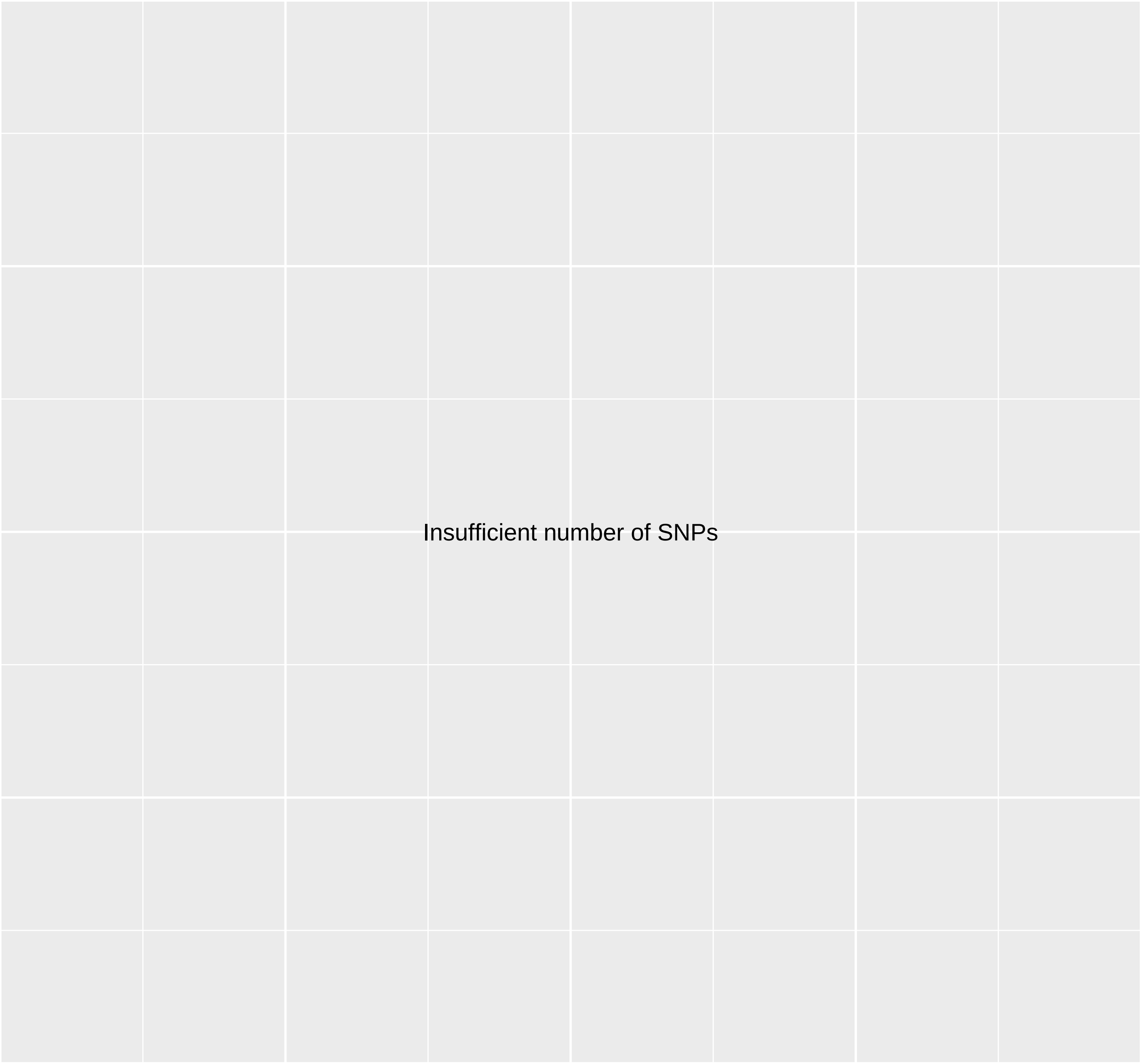
Insufficient number of SNPs

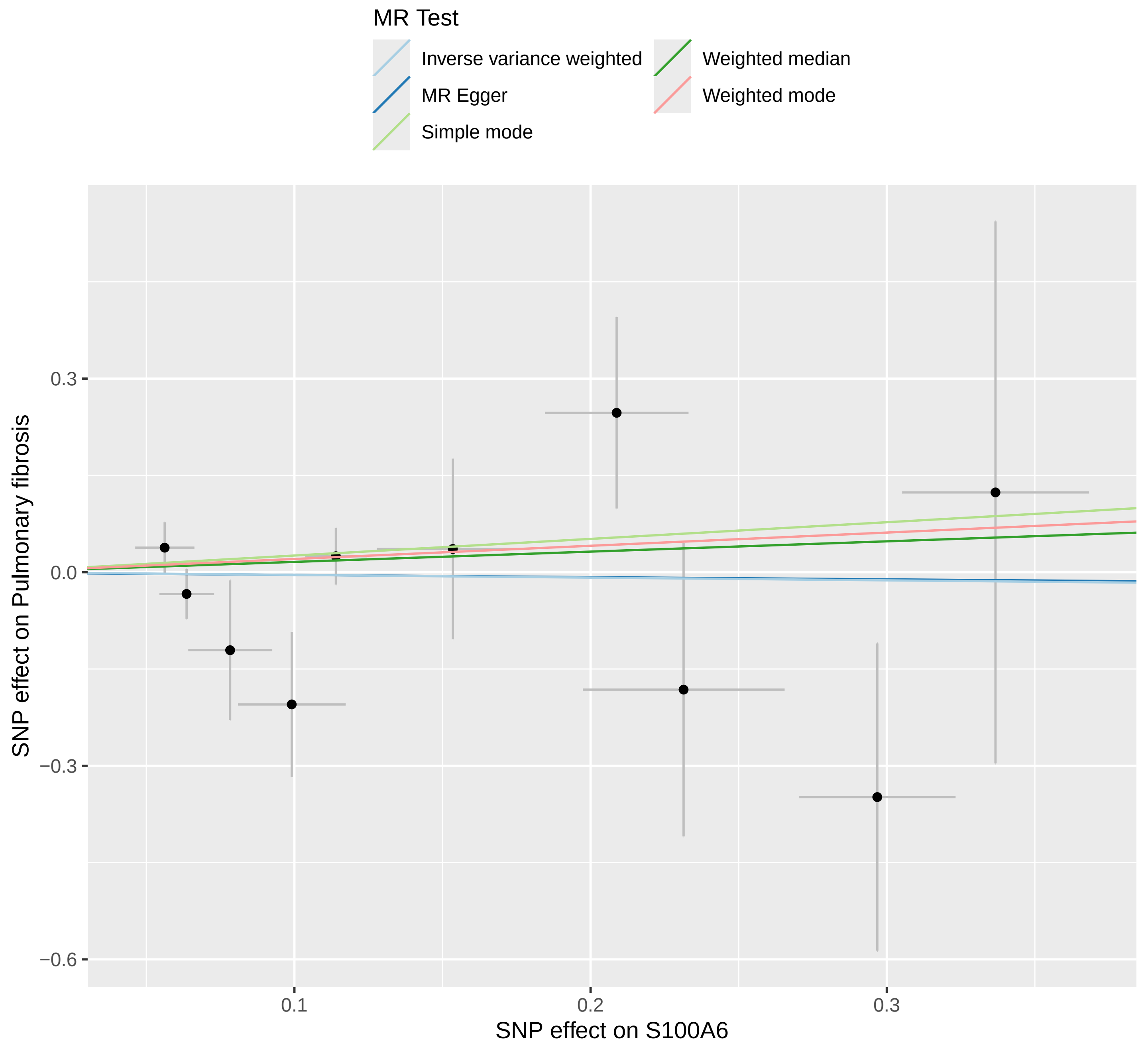
MR Test
Inverse variance weighted
MR Egger
Simple mode
Weighted median
Weighted mode
0.3
0.0
−0.3
−0.6
SNP effect on Pulmonary fibrosis
0.1
0.2
0.3
SNP effect on S100A6

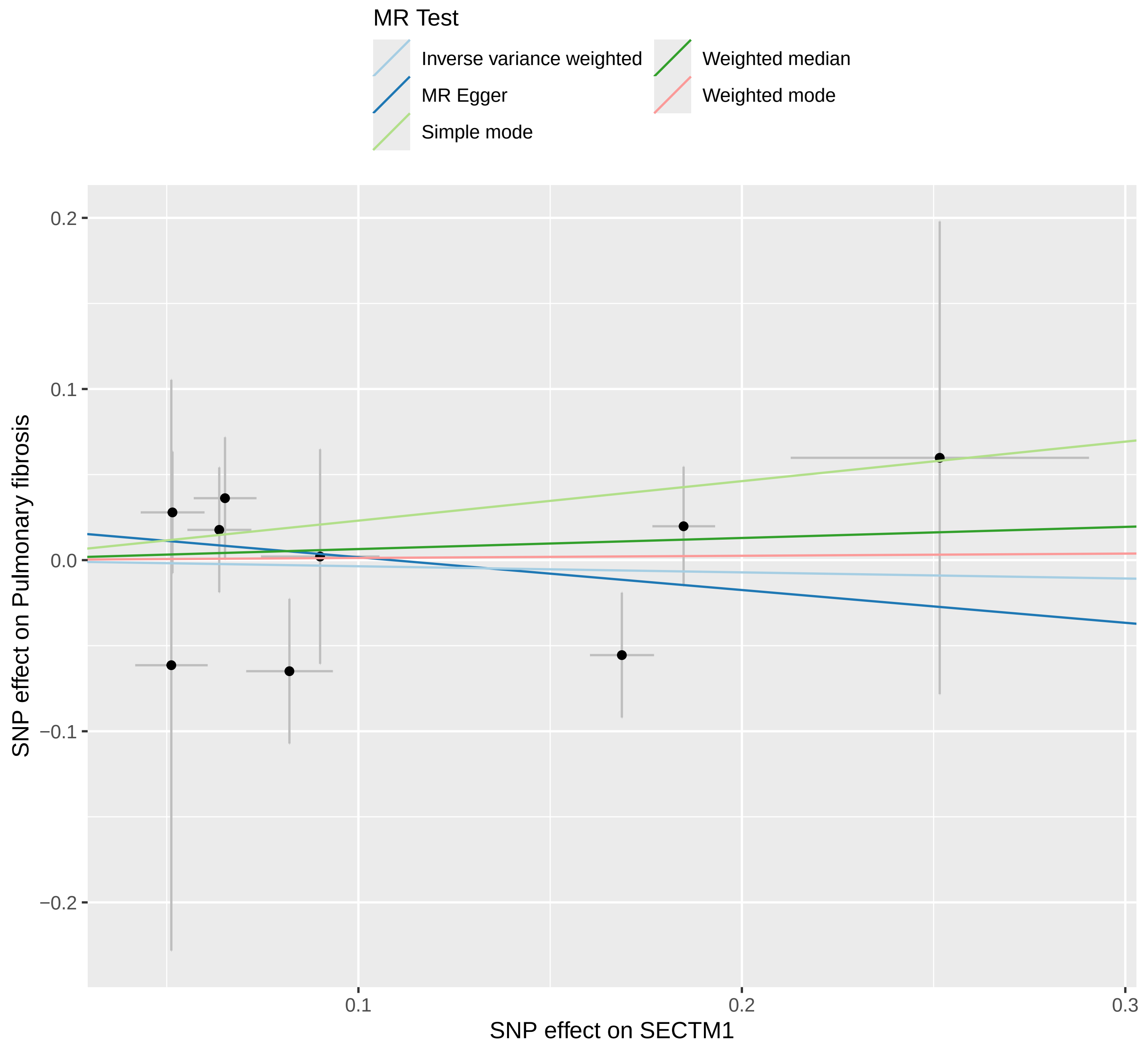
MR Test
Inverse variance weighted
MR Egger
Simple mode
Weighted median
Weighted mode
0.2
0.1
0.0
−0.1
−0.2
SNP effect on Pulmonary fibrosis
0.1
0.2
0.3
SNP effect on SECTM1

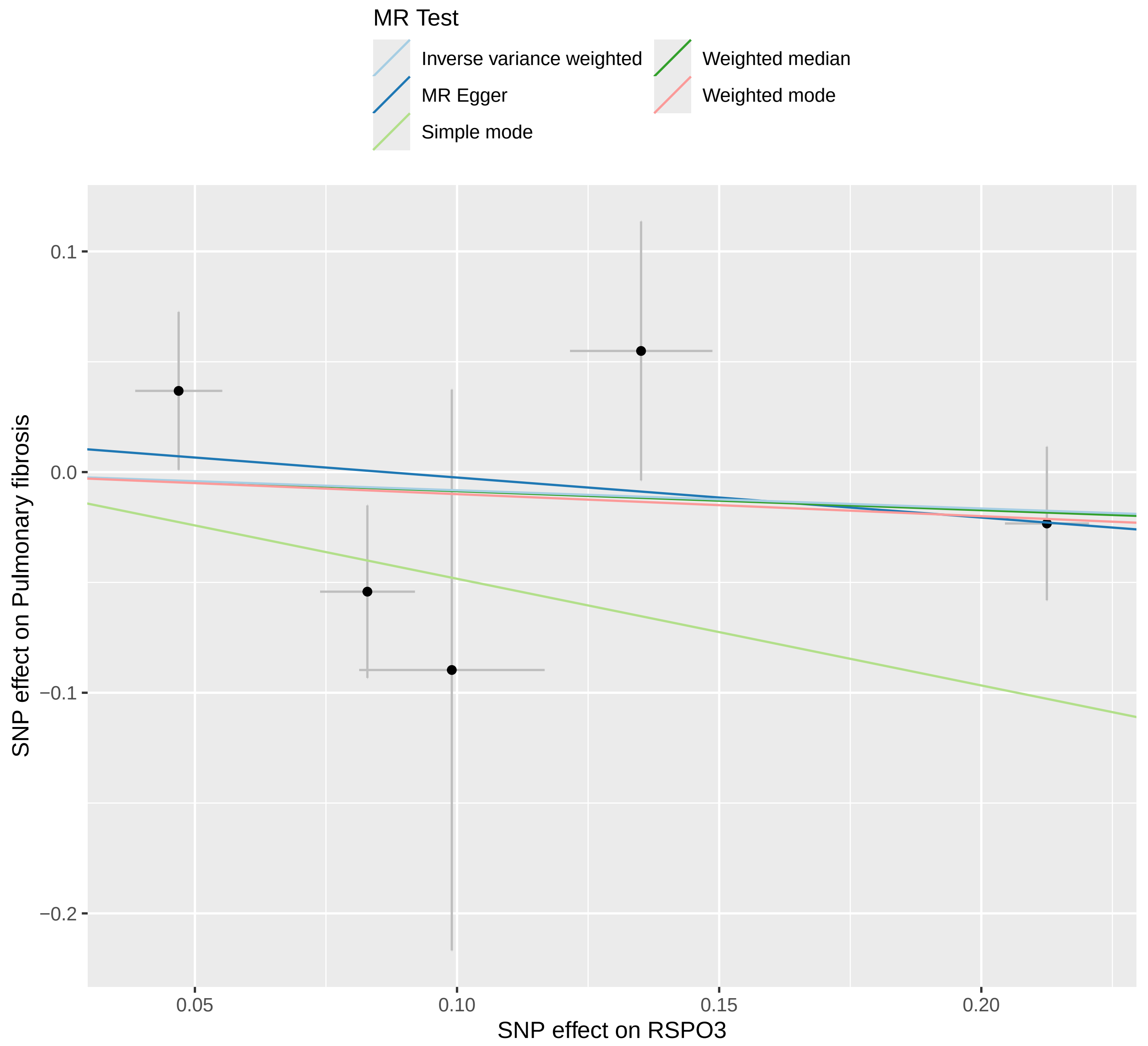

MR Test
Inverse variance weighted
MR Egger
Simple mode
Weighted median
Weighted mode
0.1
0.0
−0.1
−0.2
0.05
0.10
0.15
0.20
SNP effect on Pulmonary fibrosis
SNP effect on RSPO3

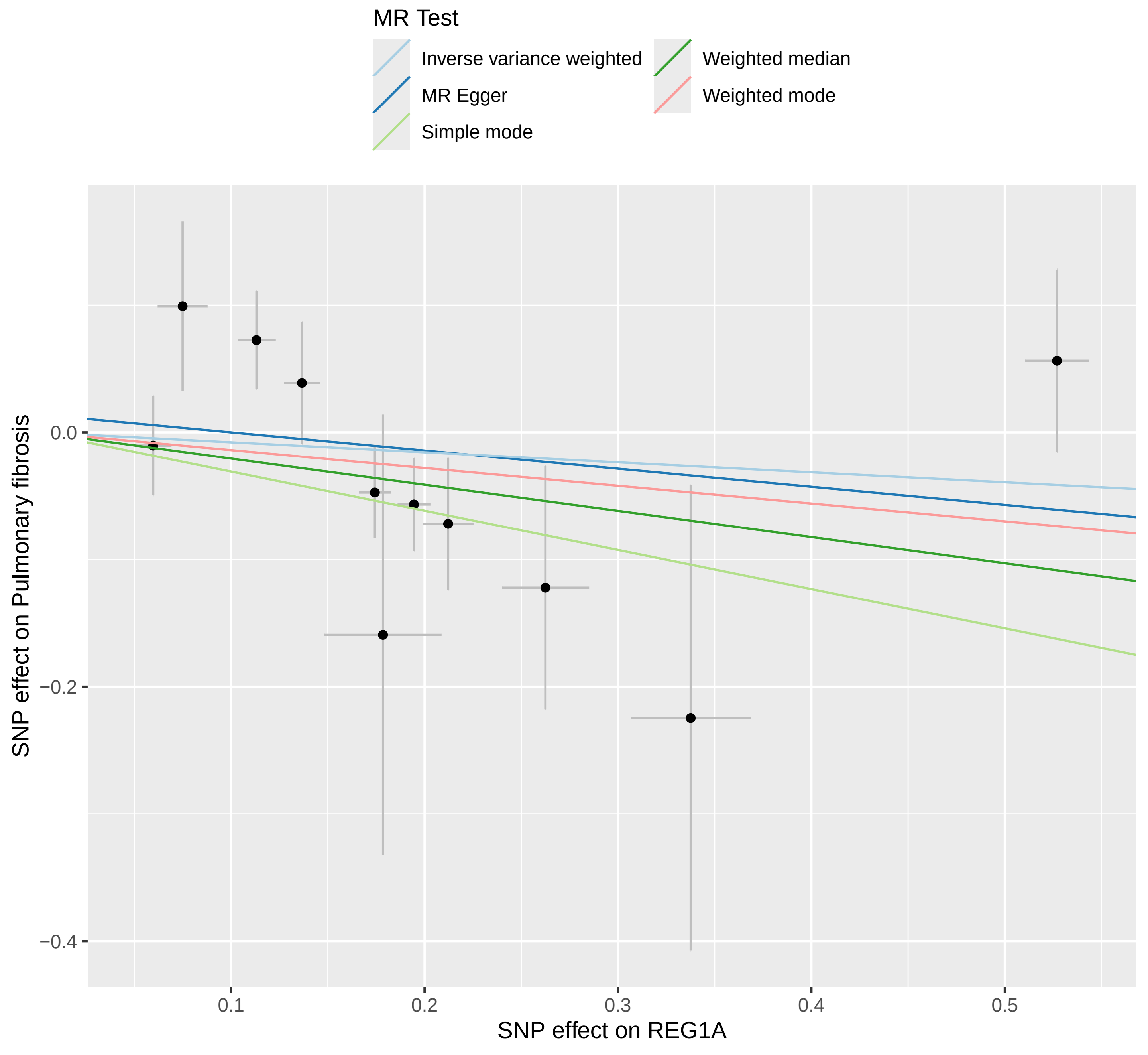
MR Test
Inverse variance weighted
MR Egger
Simple mode
Weighted median
Weighted mode
0.0
−0.2
−0.4
0.1
0.2
0.3
0.4
0.5
SNP effect on REG1A
SNP effect on Pulmonary fibrosis

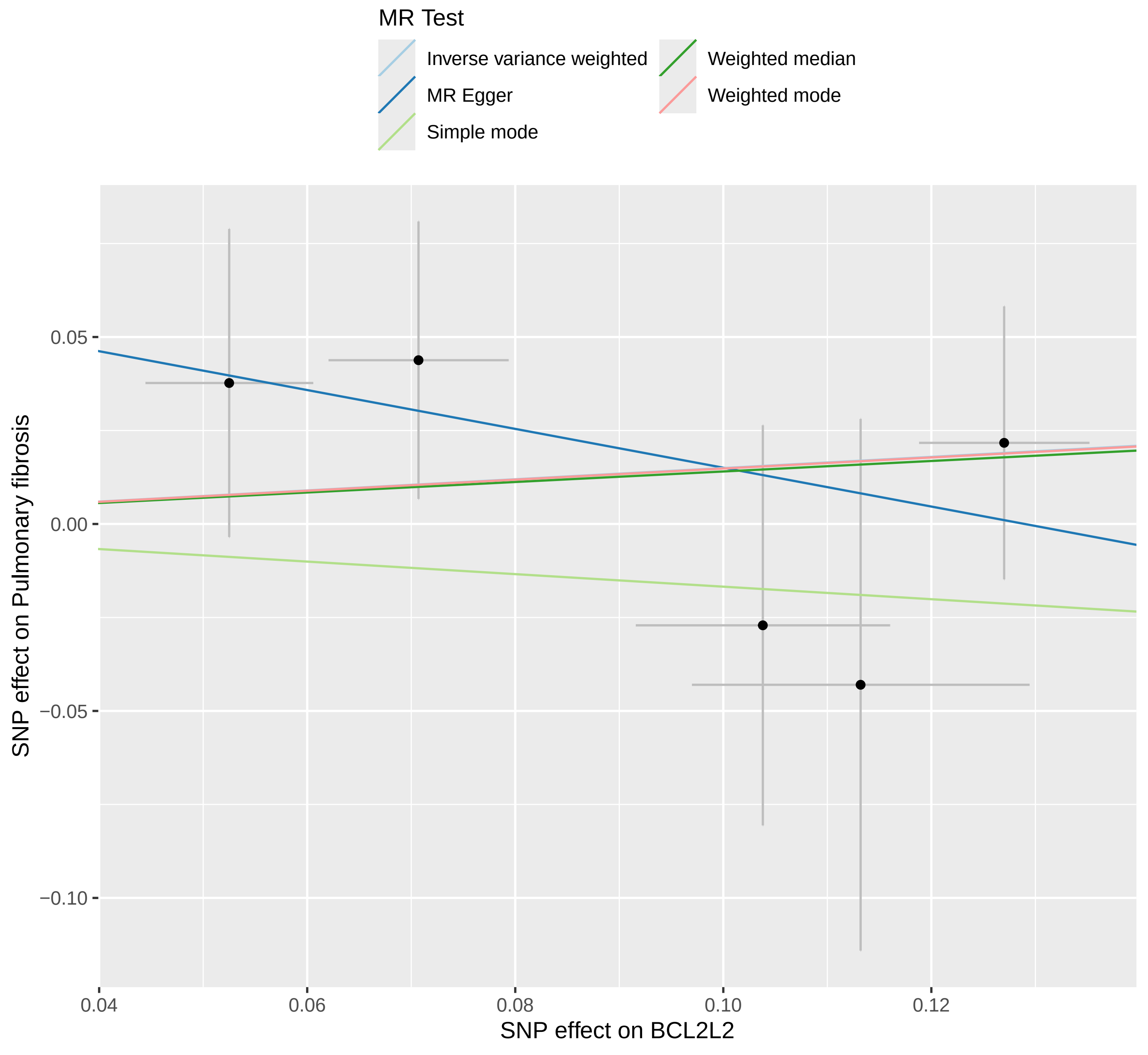
MR Test
Inverse variance weighted
MR Egger
Simple mode
Weighted median
Weighted mode
0.05
0.00
−0.05
−0.10
SNP effect on Pulmonary fibrosis
0.04
0.06
0.08
0.10
0.12
SNP effect on BCL2L2

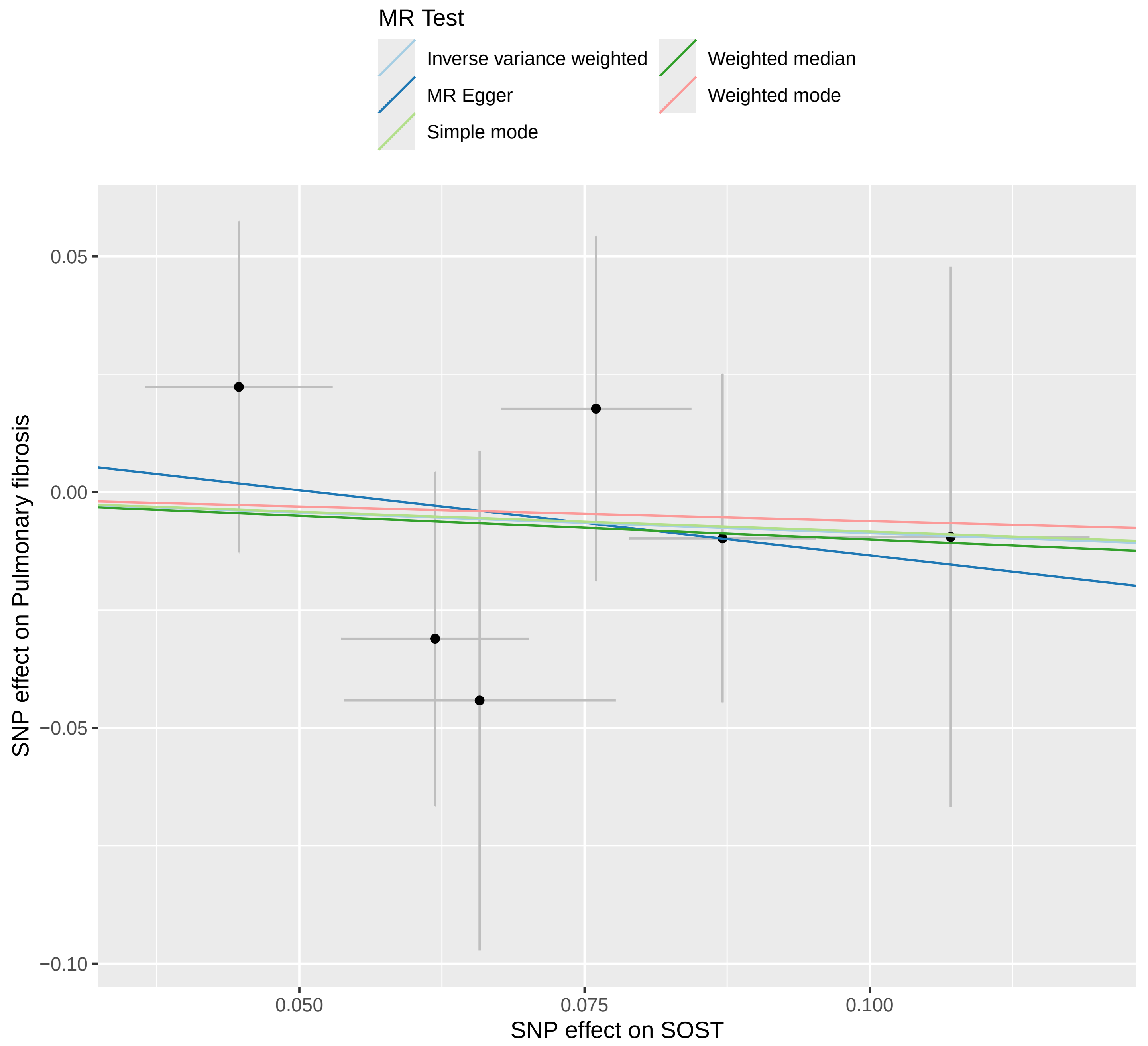
MR Test
Inverse variance weighted
MR Egger
Simple mode
Weighted median
Weighted mode
0.05
0.00
−0.05
−0.10
0.050
0.075
0.100
SNP effect on Pulmonary fibrosis
SNP effect on SOST

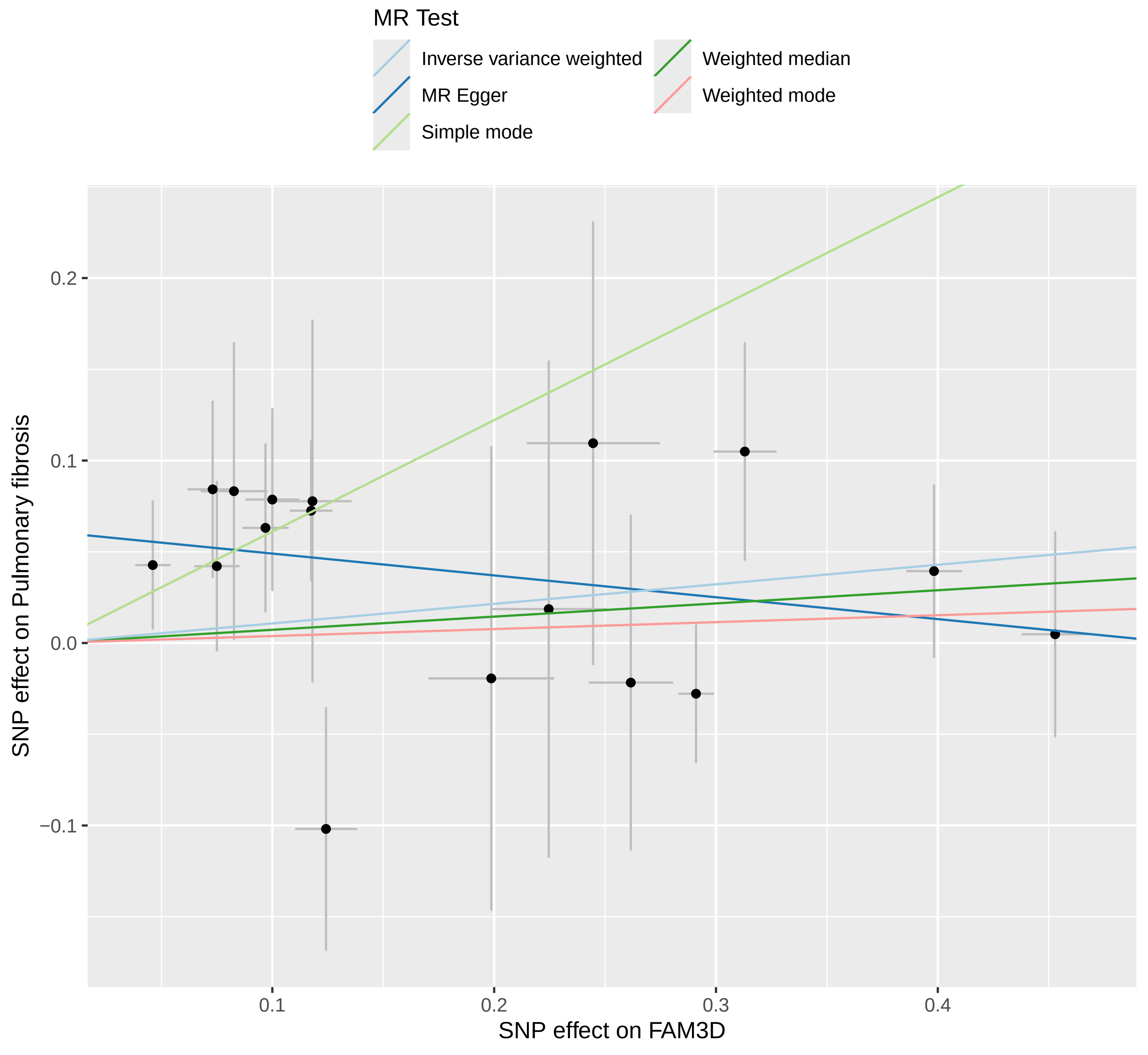

MR Test
Inverse variance weighted
MR Egger
Simple mode
Weighted median
Weighted mode
0.2
0.1
0.0
−0.1
0.1
0.2
0.3
0.4
SNP effect on Pulmonary fibrosis
SNP effect on FAM3D

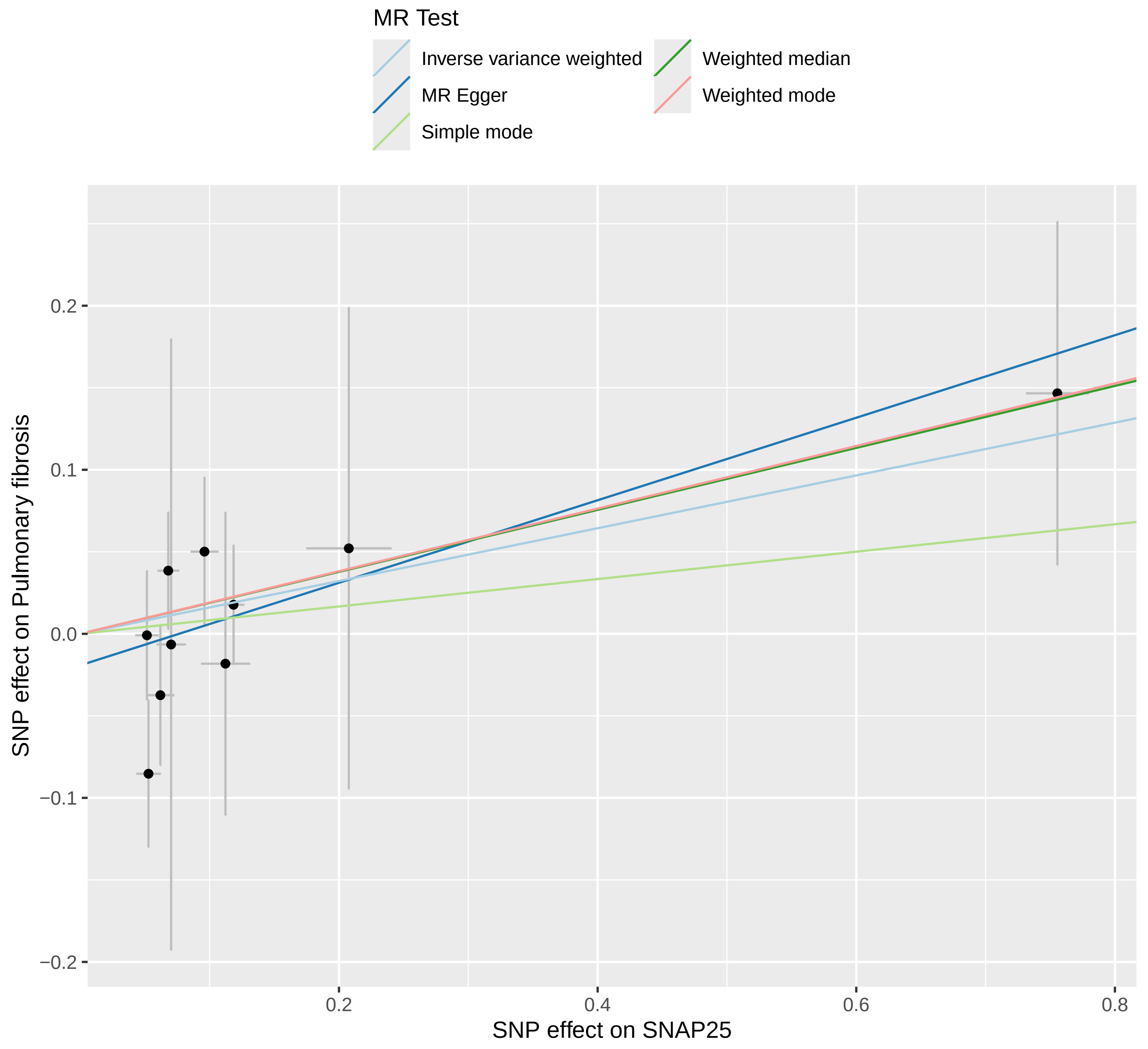
MR Test
Inverse variance weighted
MR Egger
Simple mode
Weighted median
Weighted mode
SNP effect on Pulmonary fibrosis
0.2
0.1
0.0
−0.1
−0.2
0.2
0.4
0.6
0.8
SNP effect on SNAP25

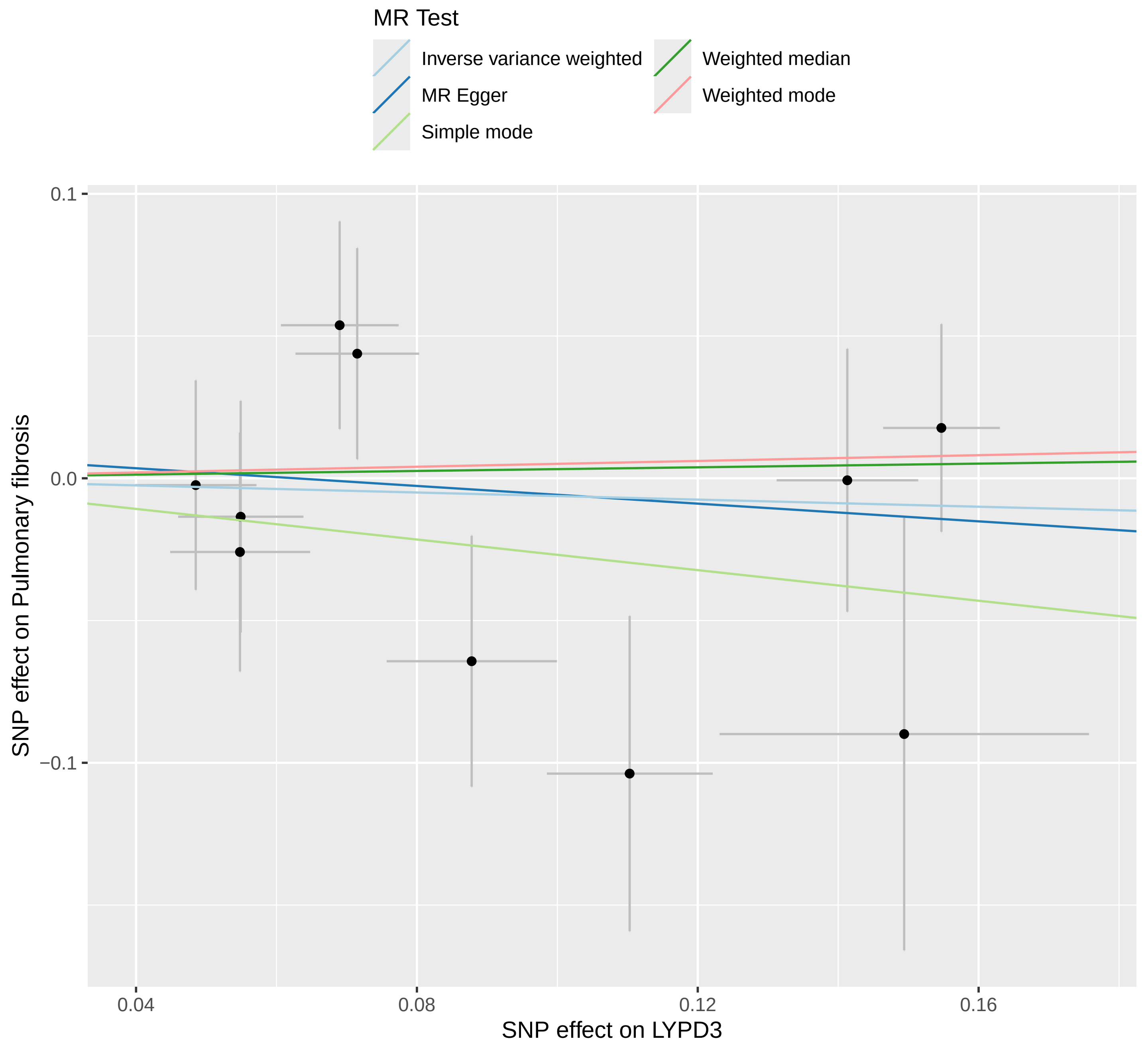

MR Test
Inverse variance weighted
MR Egger
Simple mode
Weighted median
Weighted mode
0.1
0.0
−0.1
0.04
0.08
0.12
0.16
SNP effect on LYPD3
SNP effect on Pulmonary fibrosis

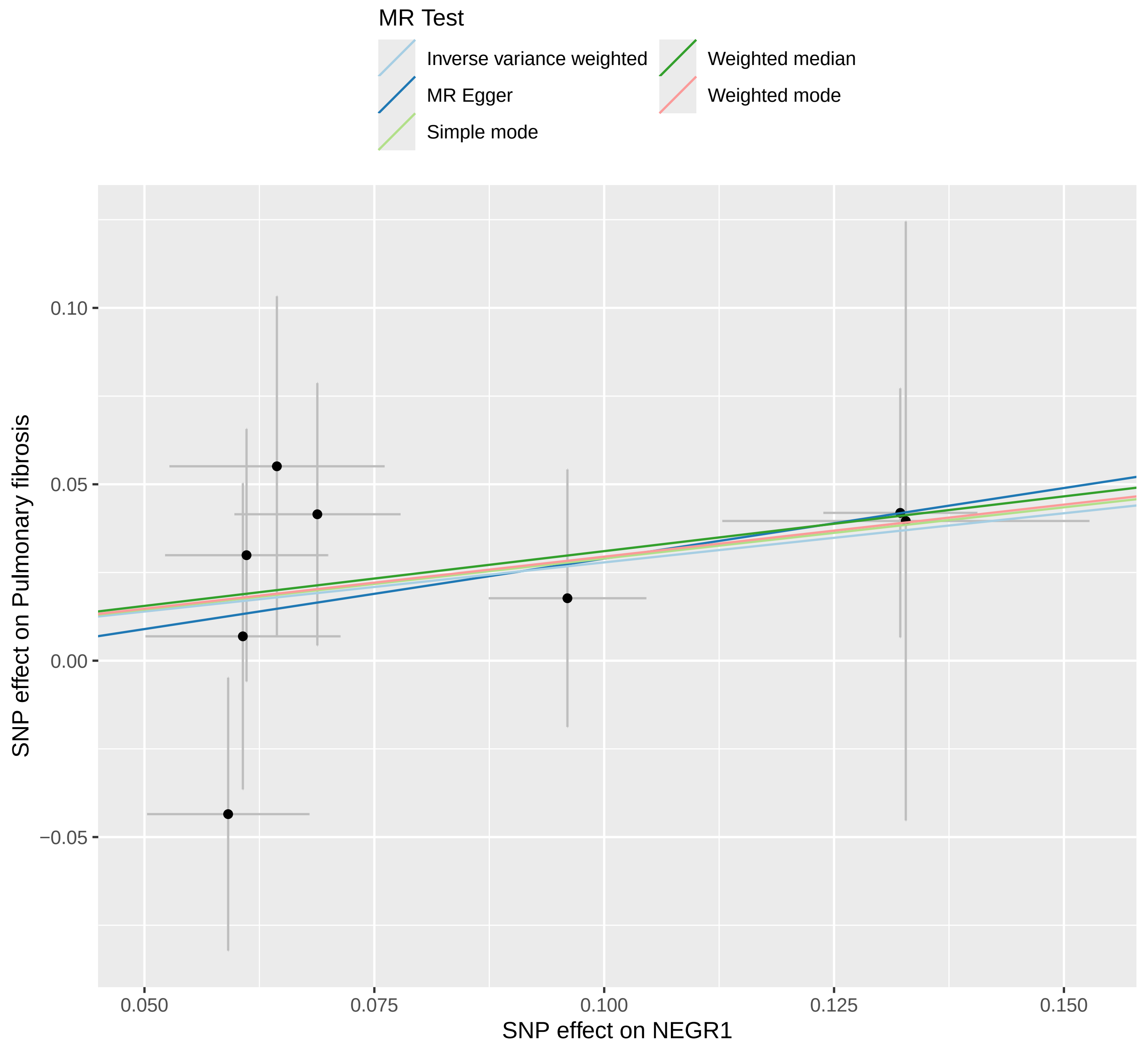

MR Test
Inverse variance weighted
MR Egger
Simple mode
Weighted median
Weighted mode
0.10
0.05
0.00
−0.05
SNP effect on Pulmonary fibrosis
0.050
0.075
0.100
0.125
0.150
SNP effect on NEGR1

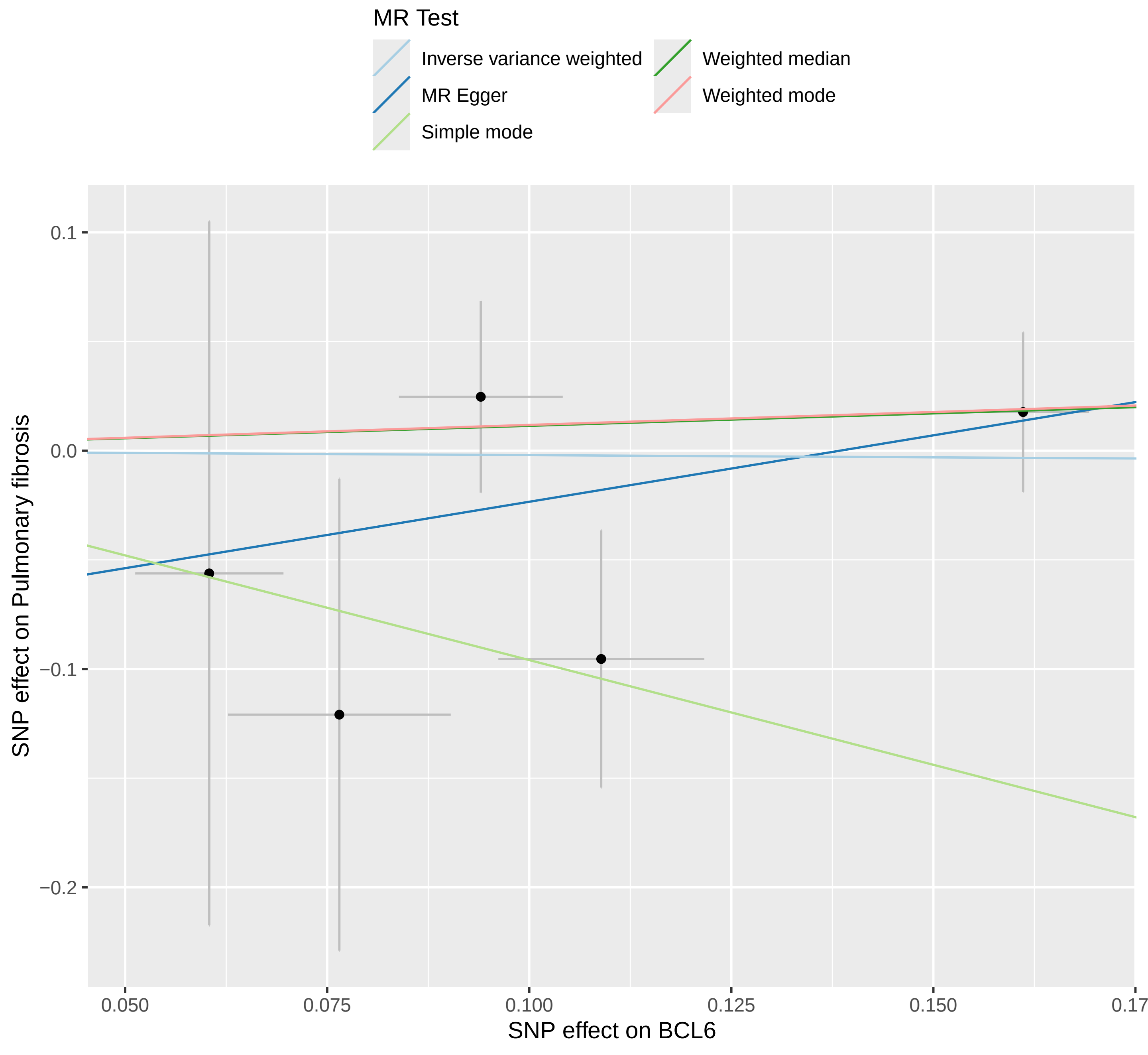
MR Test
Inverse variance weighted
MR Egger
Simple mode
Weighted median
Weighted mode
0.1
0.0
−0.1
−0.2
SNP effect on Pulmonary fibrosis
0.050
0.075
0.100
0.125
0.150
SNP effect on BCL6

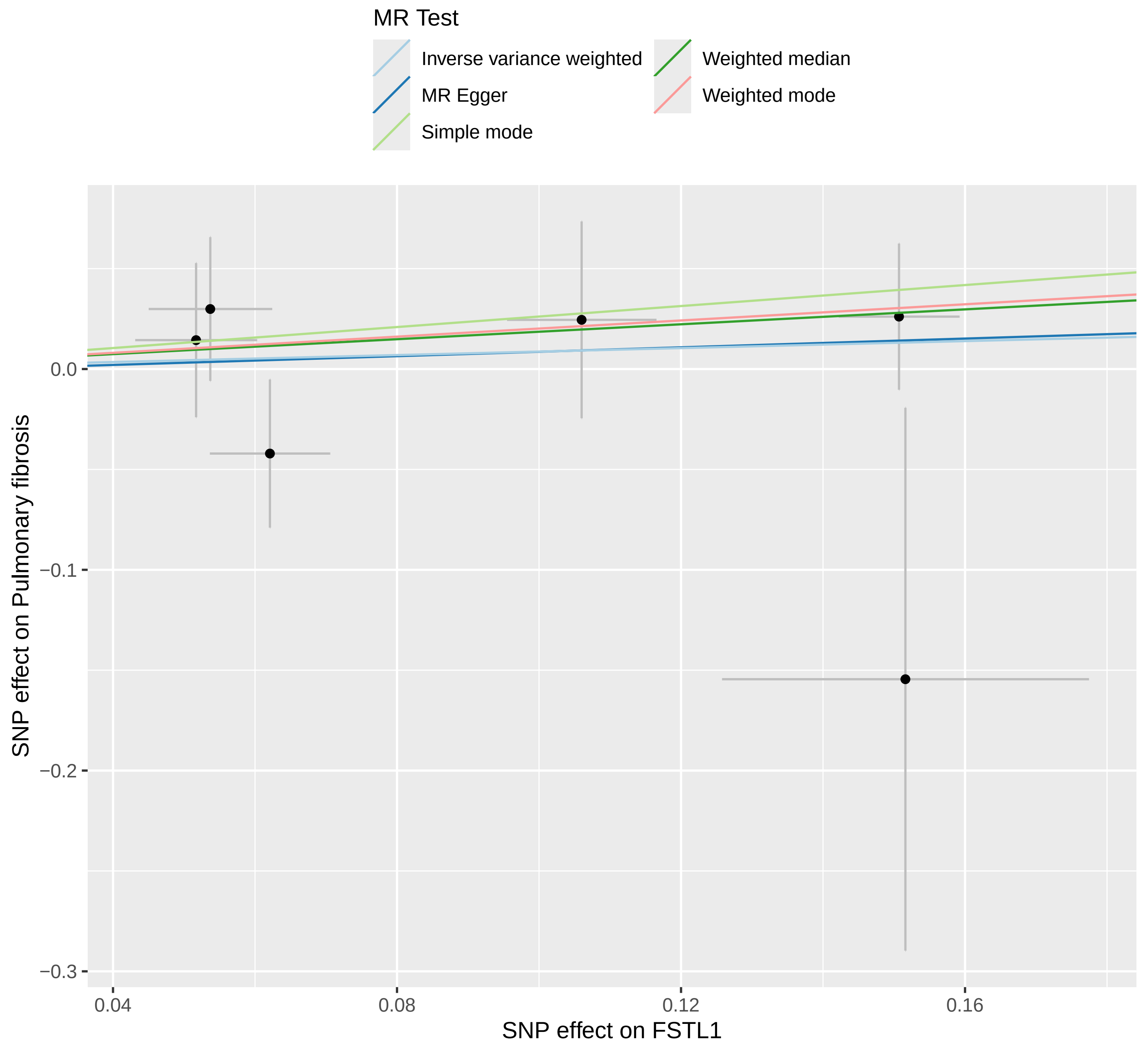
MR Test
Inverse variance weighted
MR Egger
Simple mode
Weighted median
Weighted mode
0.0
−0.1
−0.2
−0.3
0.04
0.08
0.12
0.16
SNP effect on FSTL1
SNP effect on Pulmonary fibrosis

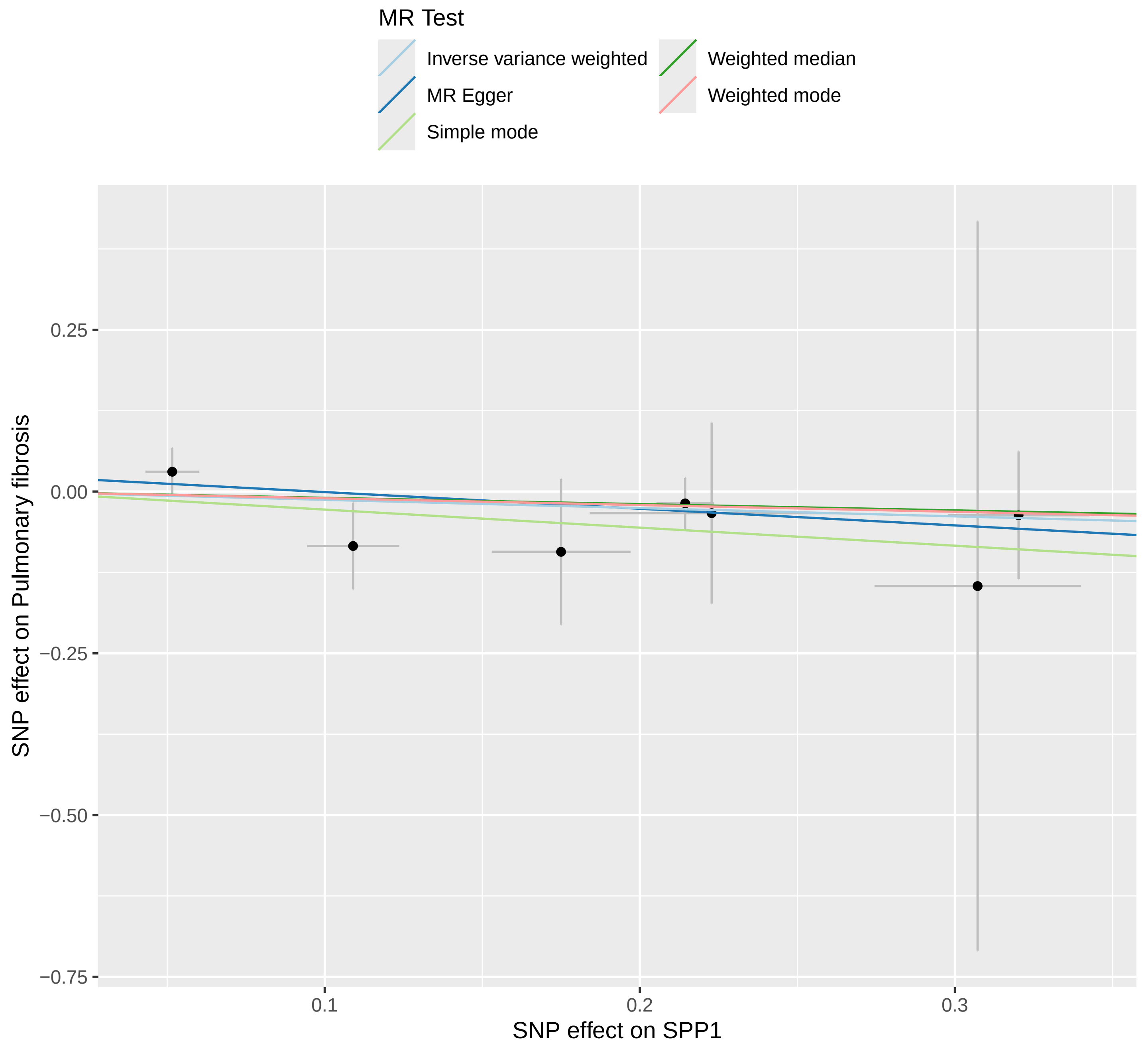

MR Test
Inverse variance weighted
MR Egger
Simple mode
Weighted median
Weighted mode
0.25
0.00
−0.25
−0.50
−0.75
SNP effect on Pulmonary fibrosis
0.1
0.2
0.3
SNP effect on SPP1

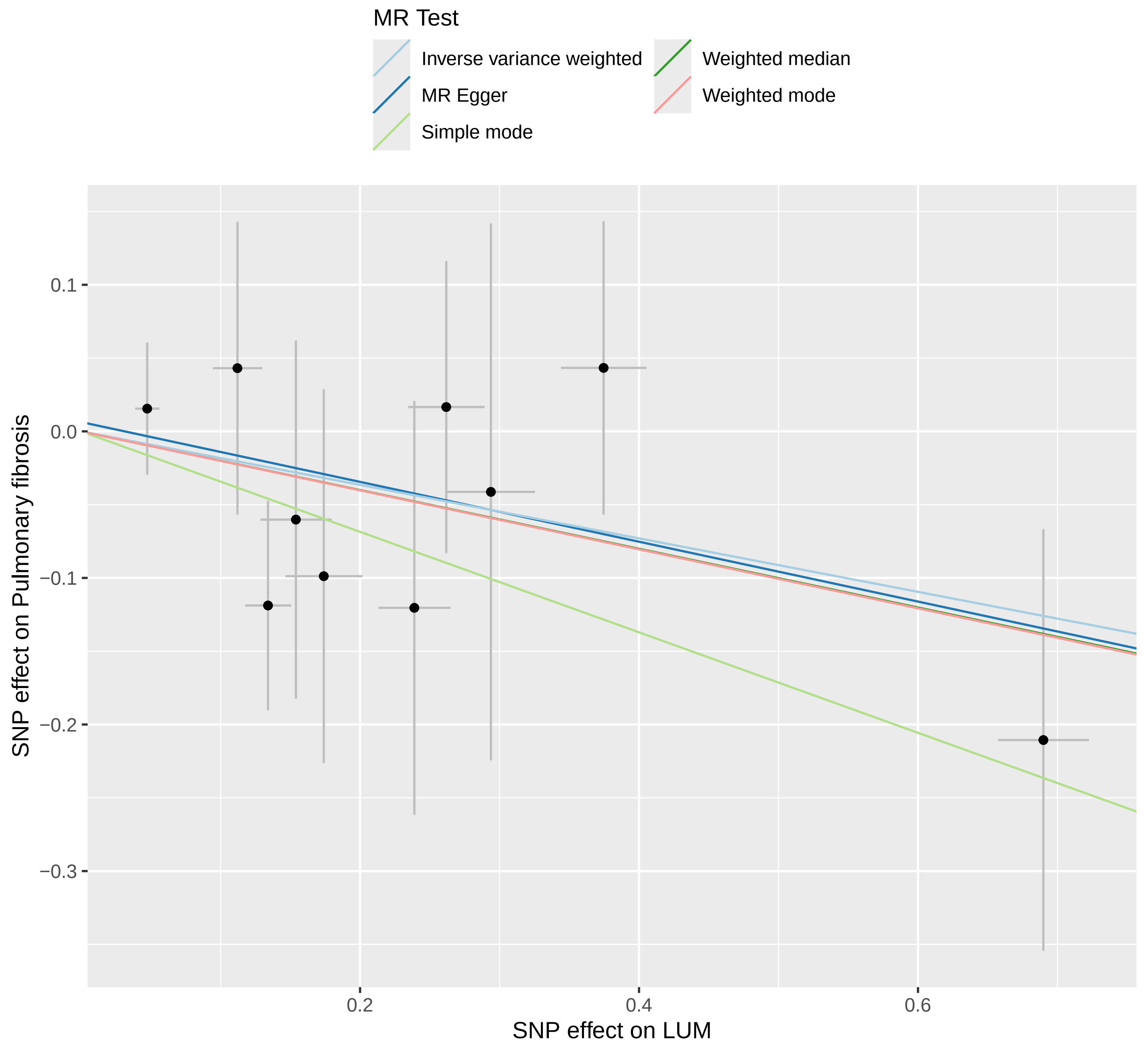
MR Test
Inverse variance weighted
MR Egger
Simple mode
Weighted median
Weighted mode
SNP effect on Pulmonary fibrosis
0.1
0.0
−0.1
−0.2
−0.3
0.2
0.4
0.6
SNP effect on LUM

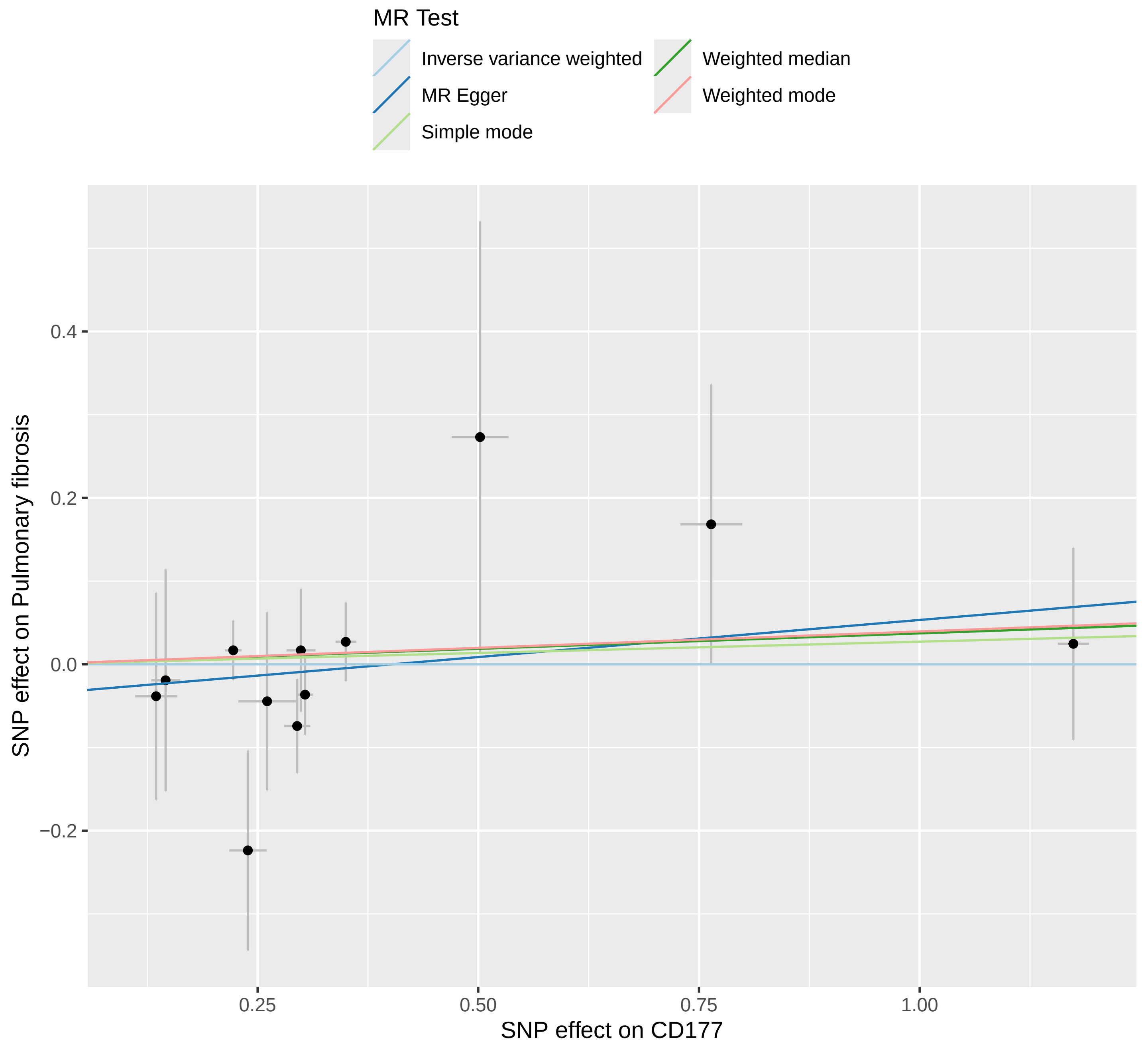
MR Test
Inverse variance weighted
MR Egger
Simple mode
Weighted median
Weighted mode
SNP effect on Pulmonary fibrosis
0.4
0.2
0.0
−0.2
0.25
0.50
0.75
1.00
SNP effect on CD177

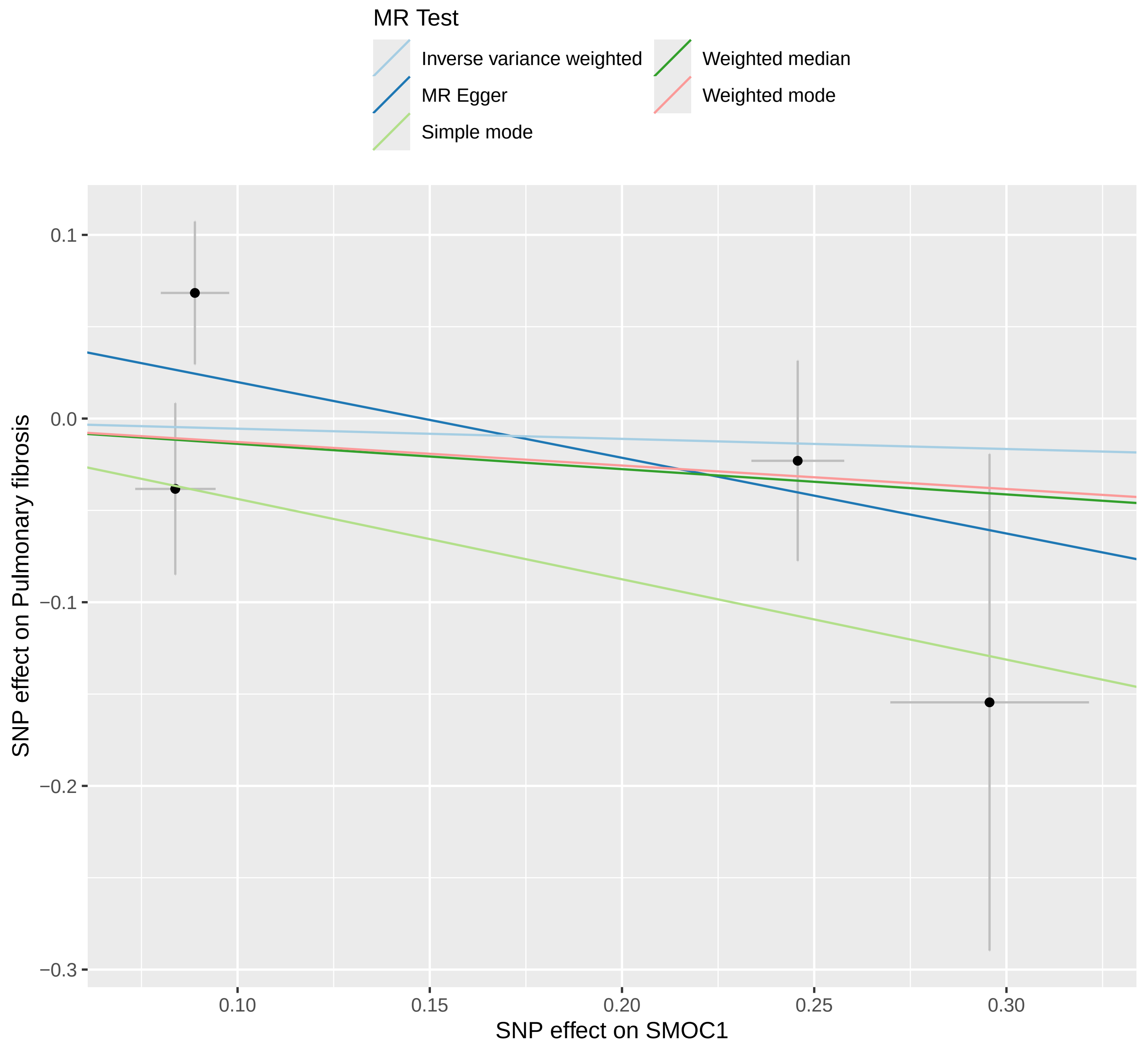
MR Test
Inverse variance weighted
MR Egger
Simple mode
Weighted median
Weighted mode
SNP effect on Pulmonary fibrosis
0.1
0.0
−0.1
−0.2
−0.3
0.10
0.15
0.20
0.25
0.30
SNP effect on SMOC1

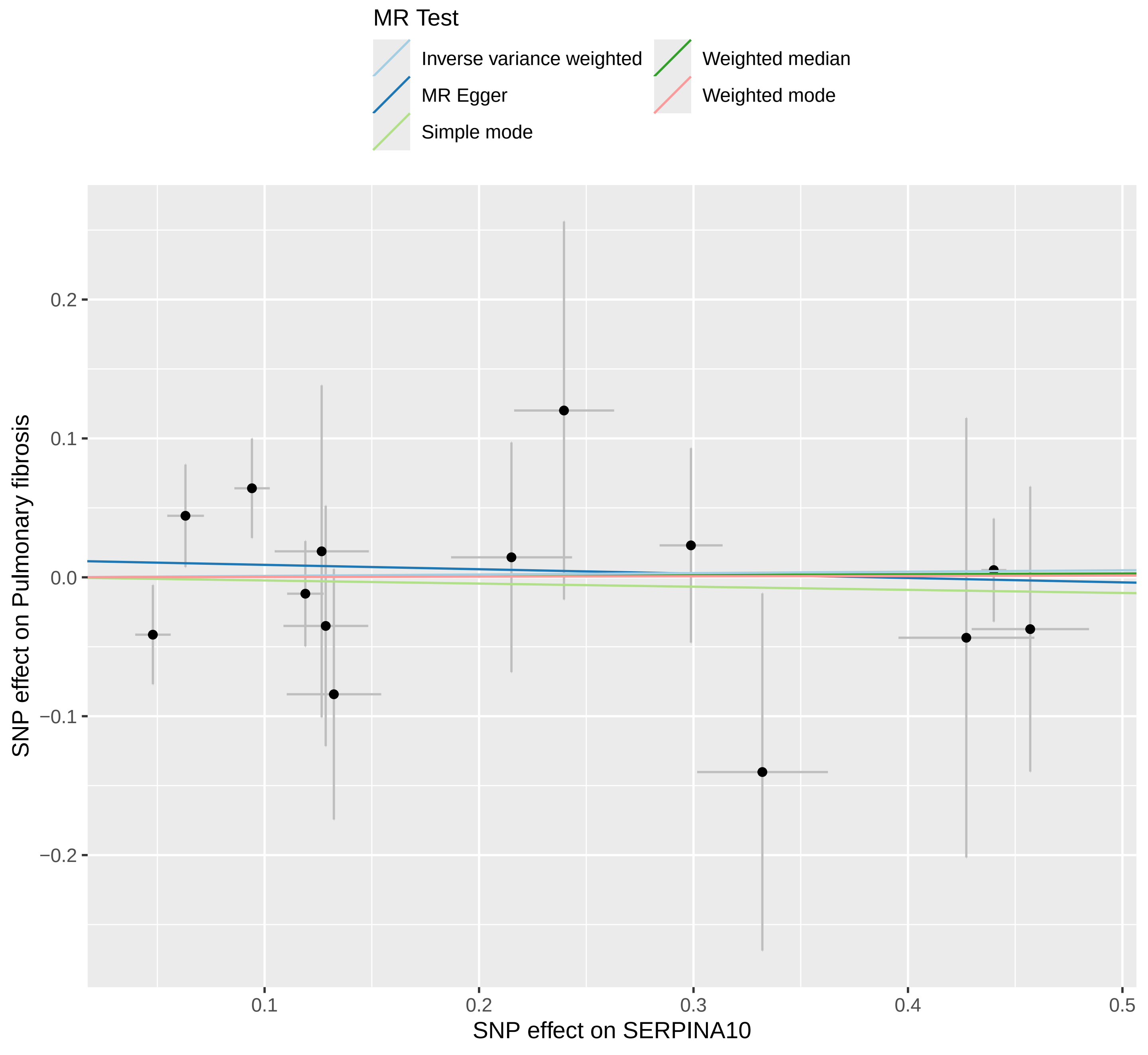

MR Test
Inverse variance weighted
Weighted median
MR Egger
Weighted mode
Simple mode
0.2
0.1
0.0
−0.1
−0.2
SNP effect on Pulmonary fibrosis
0.1
0.2
0.3
0.4
0.5
SNP effect on SERPINA10

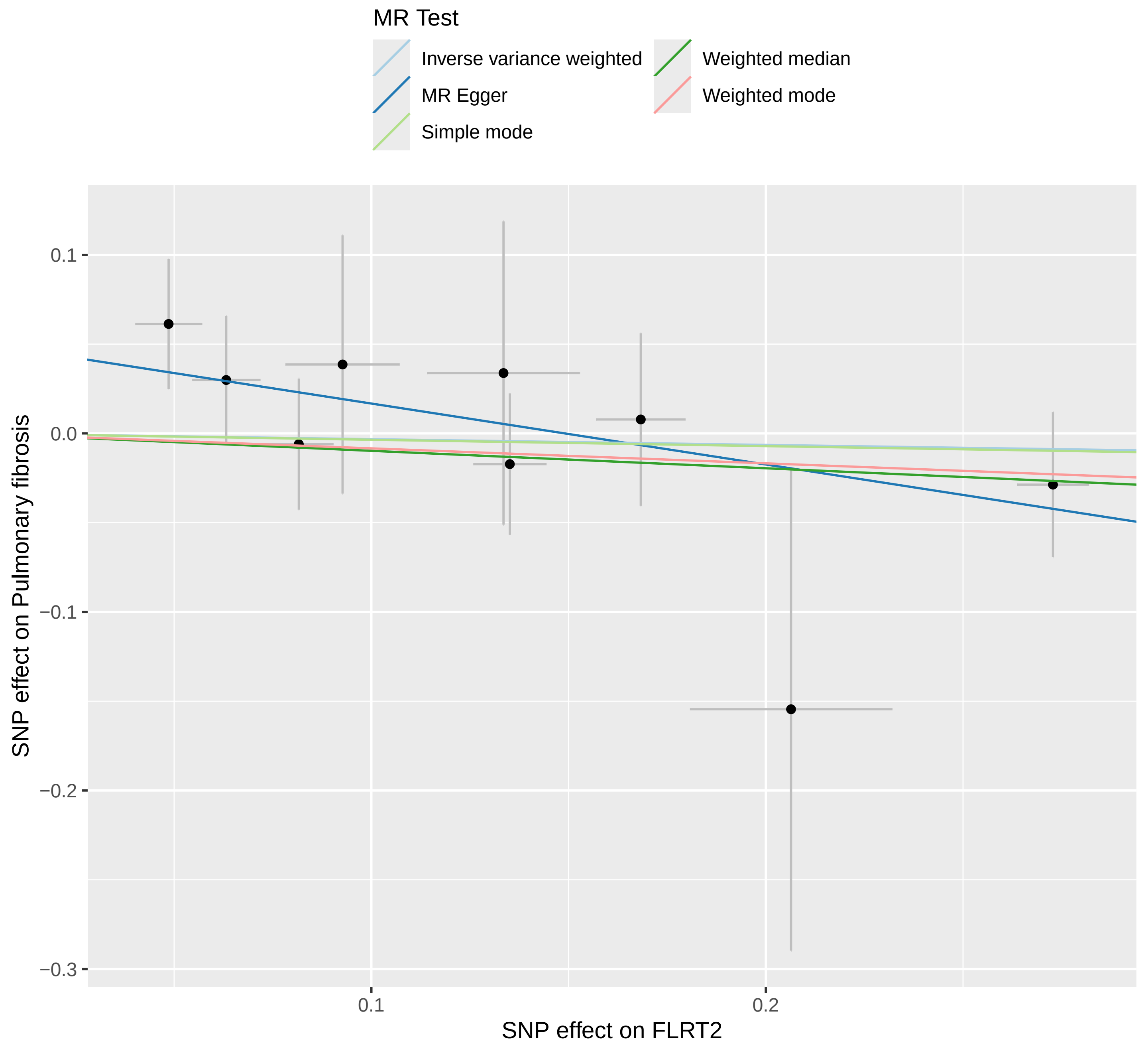
MR Test
Inverse variance weighted
MR Egger
Simple mode
Weighted median
Weighted mode
0.1
0.0
−0.1
−0.2
−0.3
SNP effect on Pulmonary fibrosis
0.1
0.2
SNP effect on FLRT2

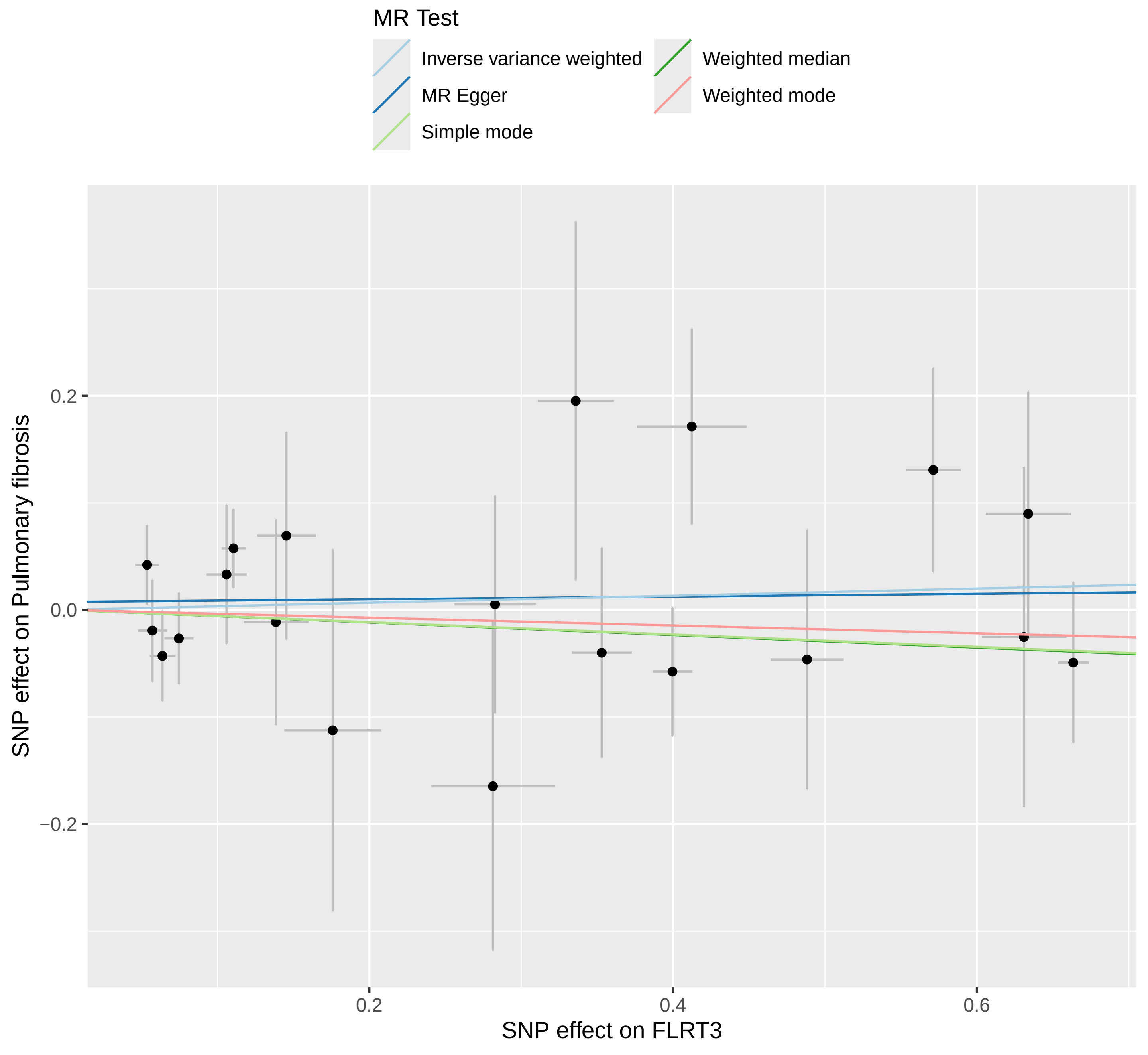
MR Test
Inverse variance weighted
MR Egger
Simple mode
Weighted median
Weighted mode
0.2
0.0
−0.2
SNP effect on Pulmonary fibrosis
0.2
0.4
0.6
SNP effect on FLRT3

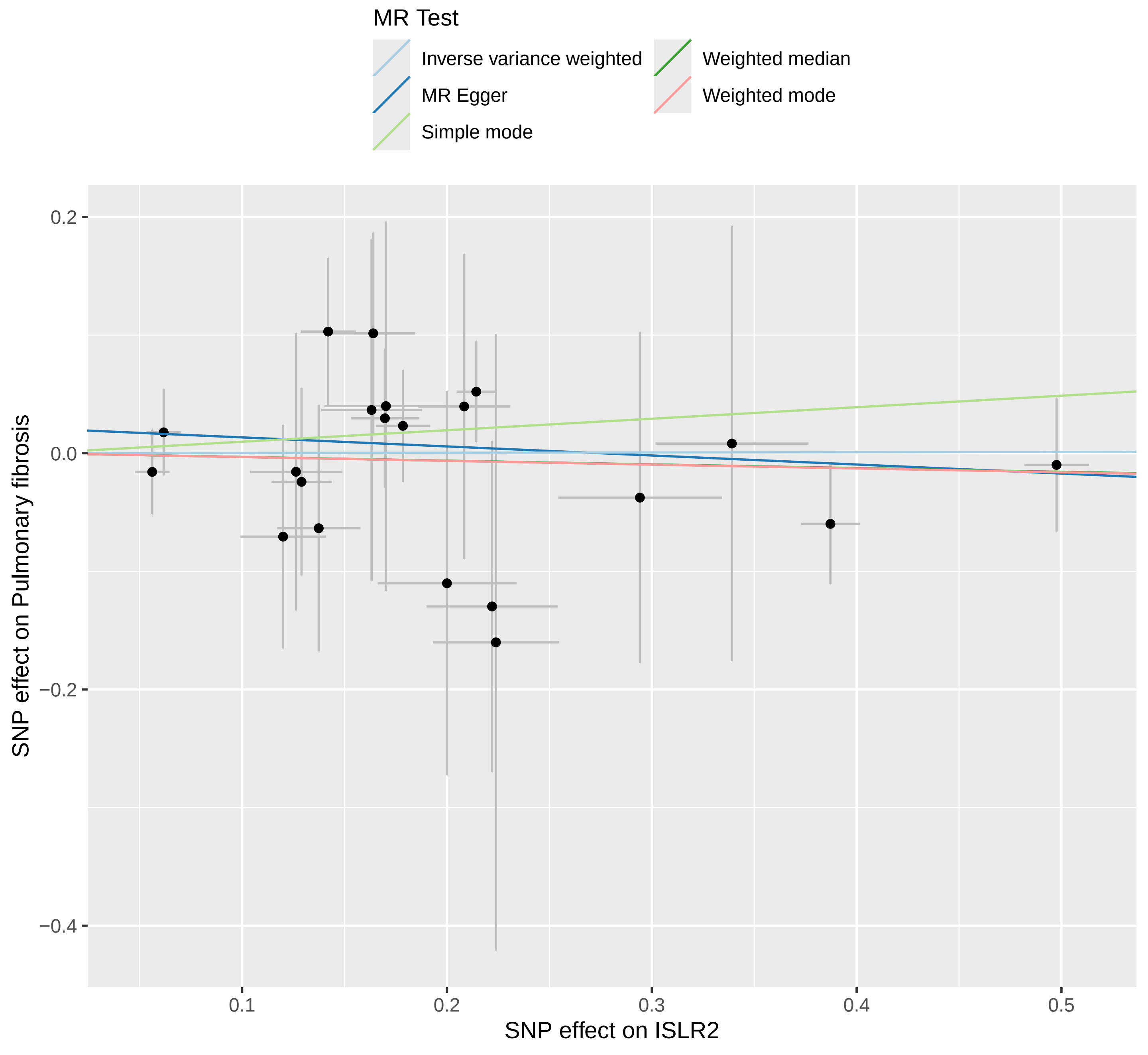

MR Test
Inverse variance weighted
MR Egger
Simple mode
Weighted median
Weighted mode
0.2
0.0
−0.2
−0.4
0.1
0.2
0.3
0.4
0.5
SNP effect on Pulmonary fibrosis
SNP effect on ISLR2

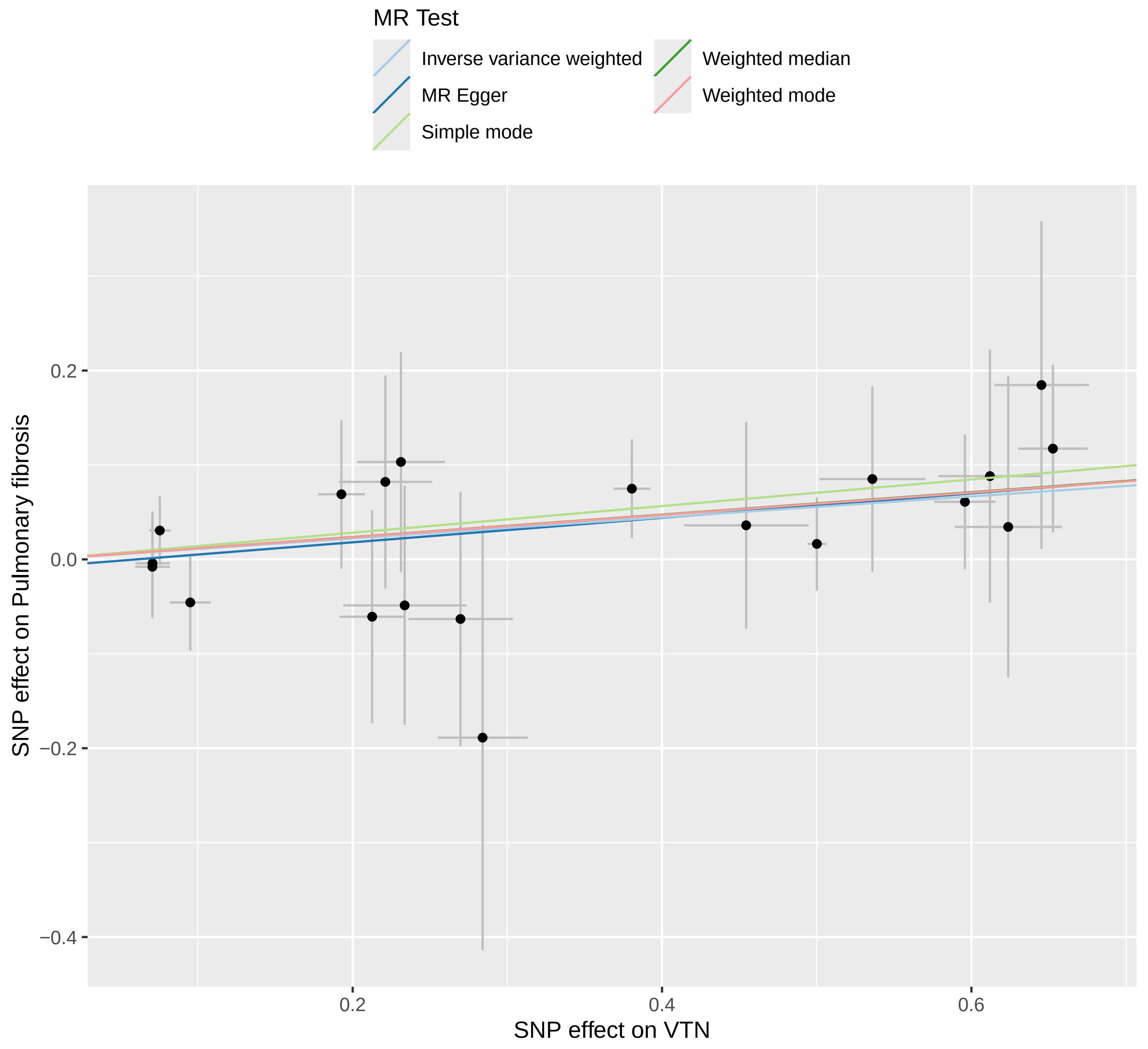

MR Test
Inverse variance weighted
MR Egger
Simple mode
Weighted median
Weighted mode
SNP effect on Pulmonary fibrosis
0.2
0.0
−0.2
−0.4
0.2
0.4
0.6
SNP effect on VTN

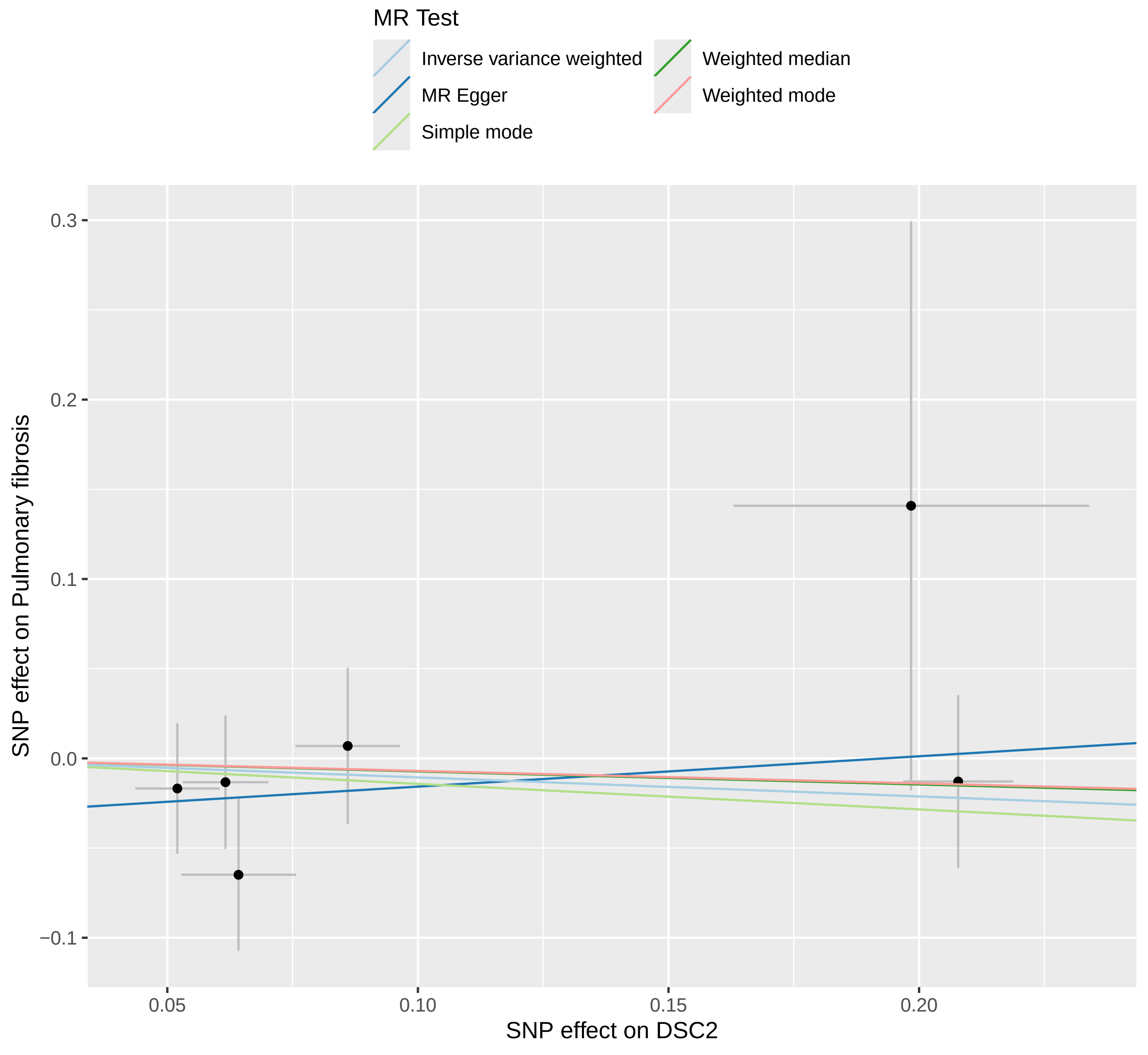

MR Test
Inverse variance weighted
MR Egger
Simple mode
Weighted median
Weighted mode
0.3
0.2
0.1
0.0
−0.1
0.05
0.10
0.15
0.20
SNP effect on Pulmonary fibrosis
SNP effect on DSC2

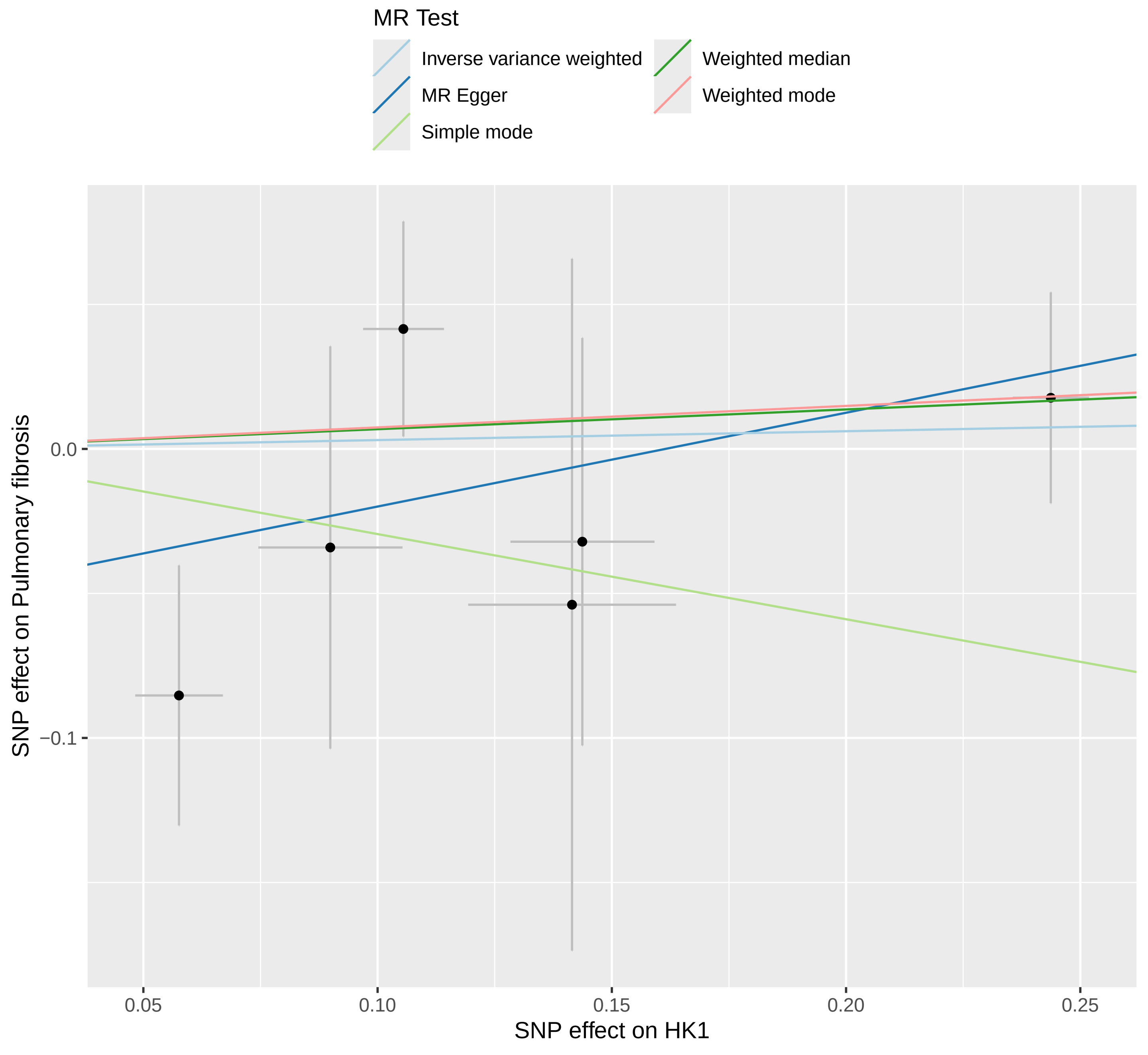
MR Test
Inverse variance weighted
MR Egger
Simple mode
Weighted median
Weighted mode
SNP effect on Pulmonary fibrosis
0.0
−0.1
0.05
0.10
0.15
0.20
0.25
SNP effect on HK1

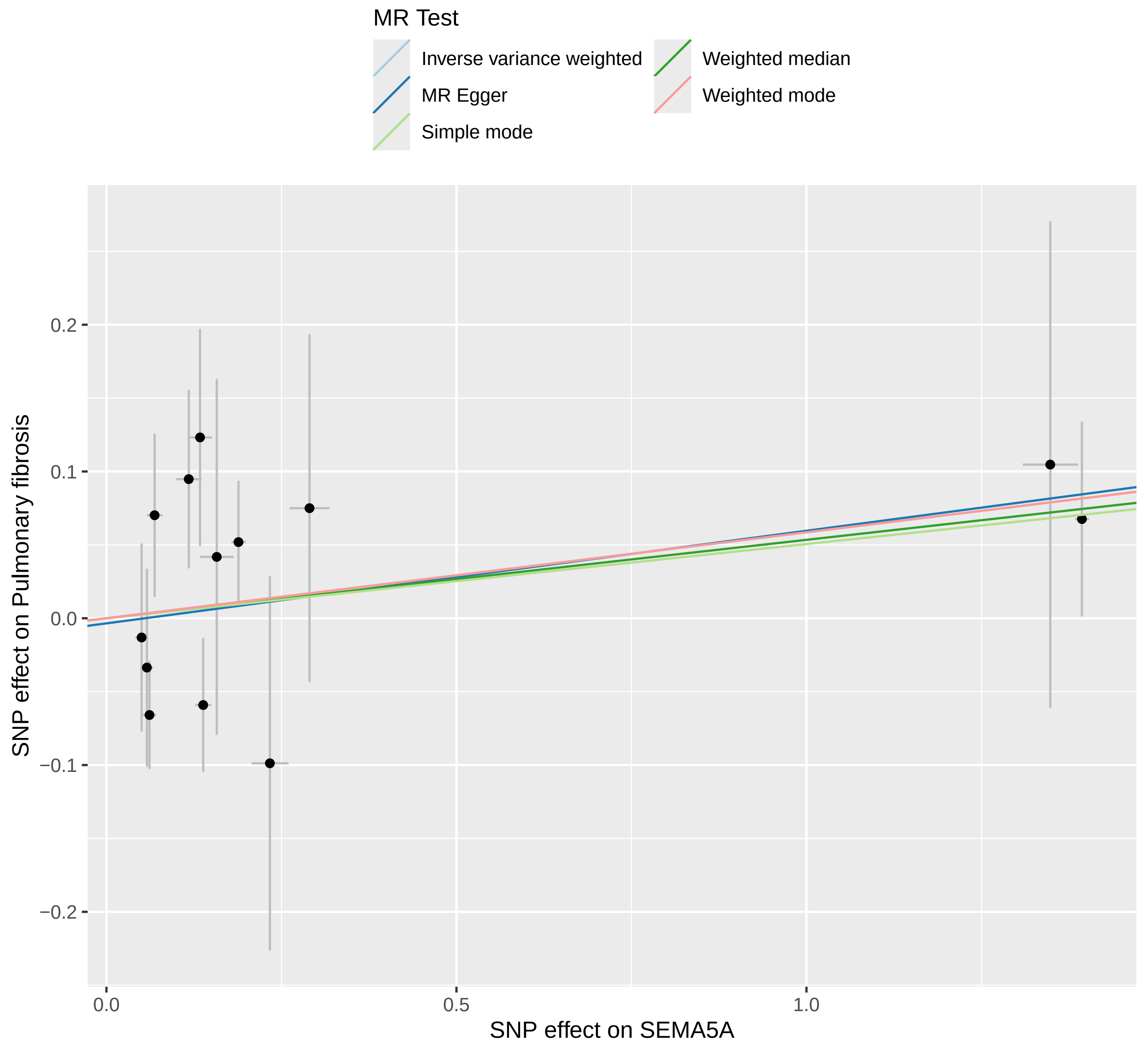
MR Test
Inverse variance weighted
MR Egger
Simple mode
Weighted median
Weighted mode
0.2
0.1
0.0
−0.1
−0.2
0.0
0.5
1.0
SNP effect on Pulmonary fibrosis
SNP effect on SEMA5A

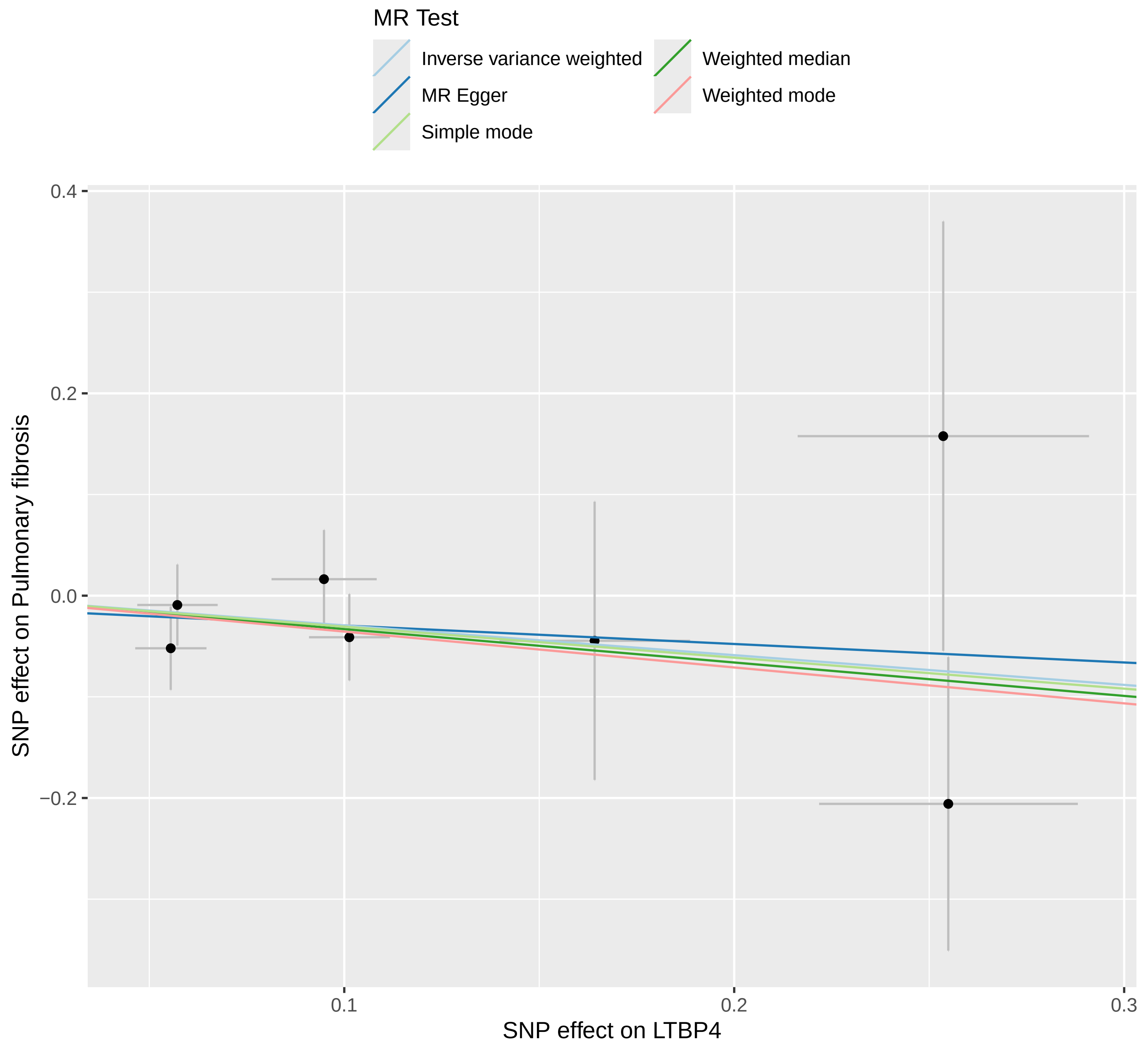
MR Test
Inverse variance weighted
MR Egger
Simple mode
Weighted median
Weighted mode
0.4
0.2
0.0
−0.2
0.1
0.2
0.3
SNP effect on Pulmonary fibrosis
SNP effect on LTBP4

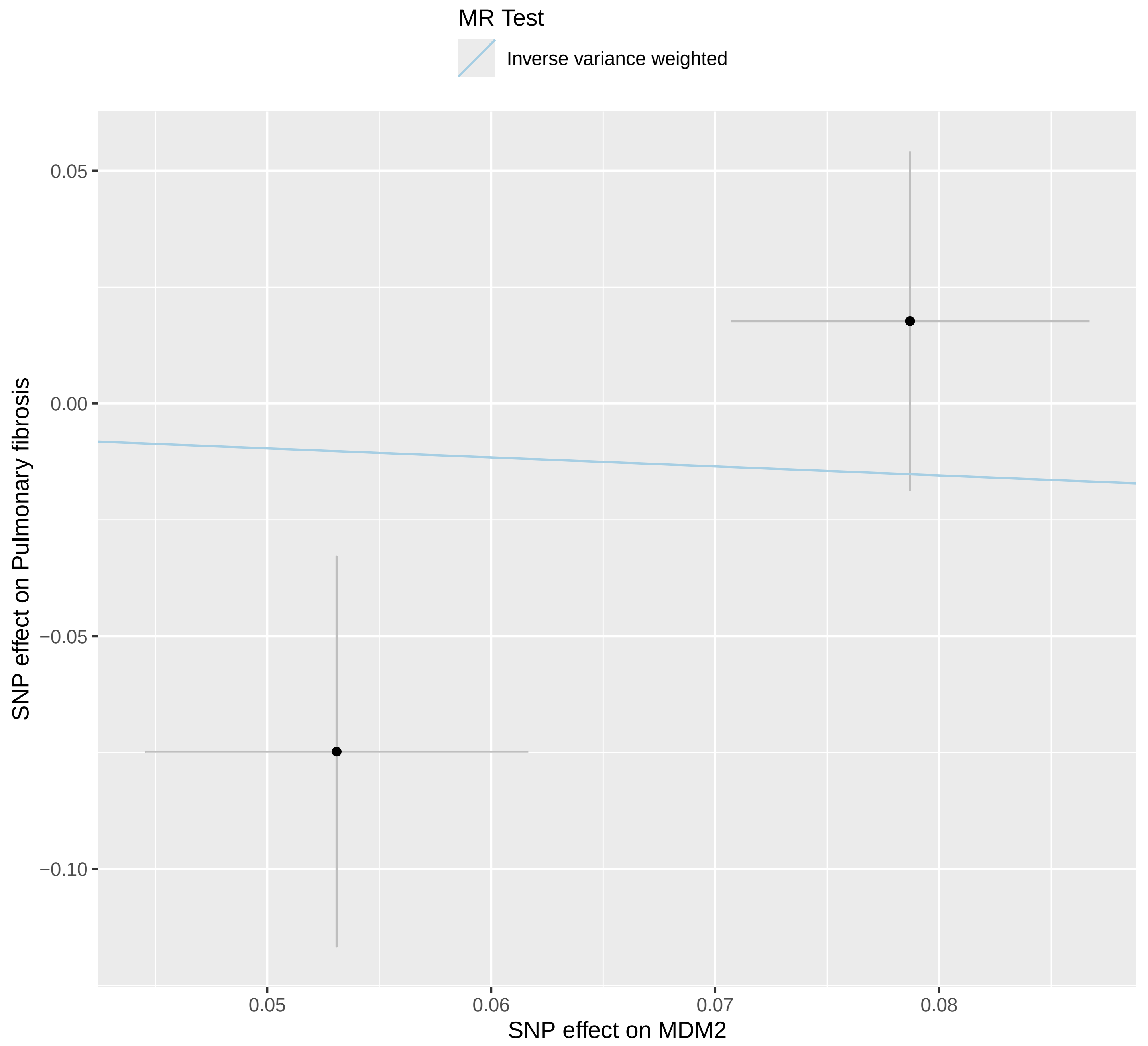

MR Test
Inverse variance weighted
0.05
0.00
−0.05
−0.10
0.05
0.06
0.07
0.08
SNP effect on Pulmonary fibrosis
SNP effect on MDM2

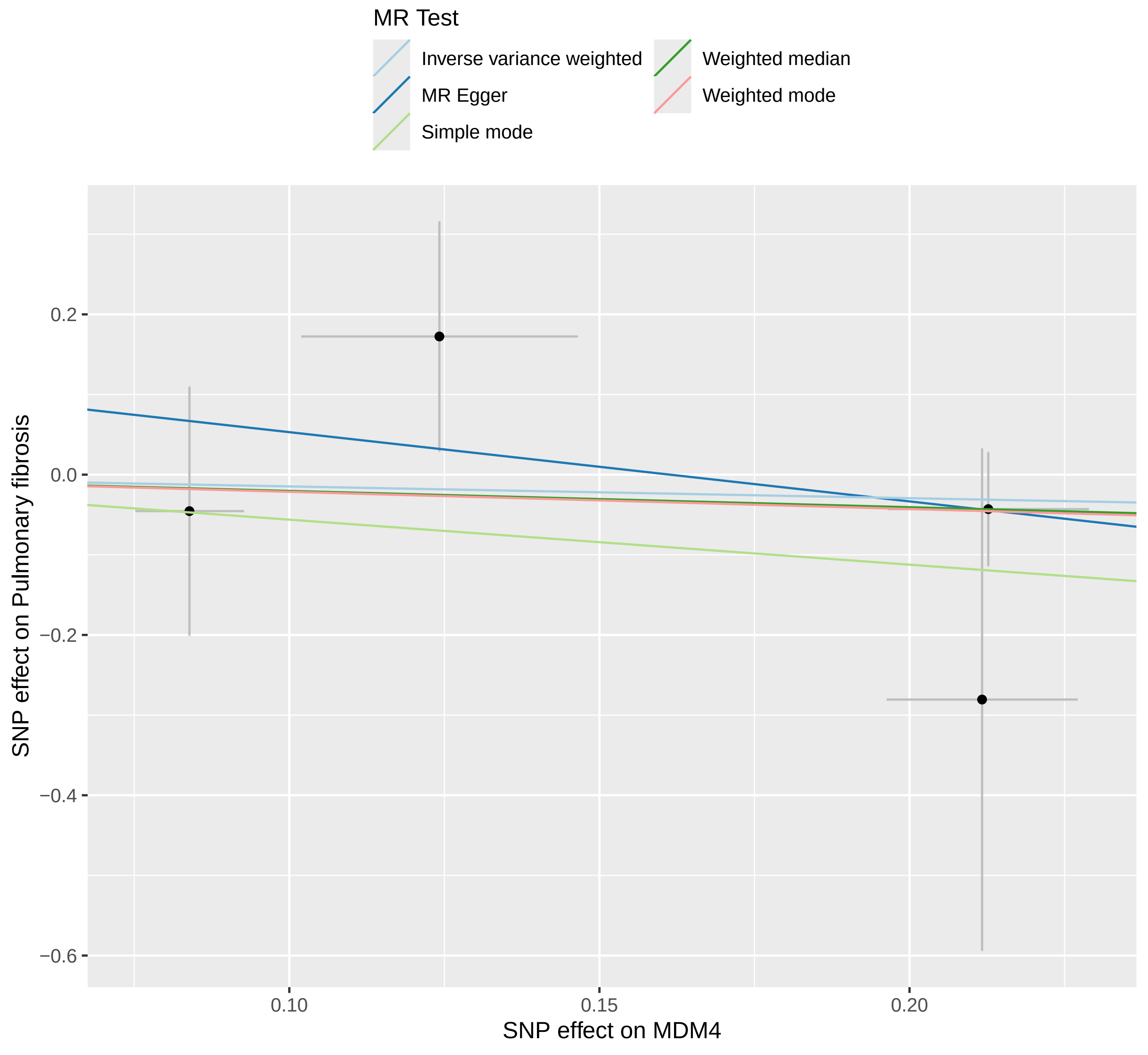
MR Test
Inverse variance weighted
MR Egger
Simple mode
Weighted median
Weighted mode
0.2
0.0
−0.2
−0.4
−0.6
SNP effect on Pulmonary fibrosis
0.10
0.15
0.20
SNP effect on MDM4

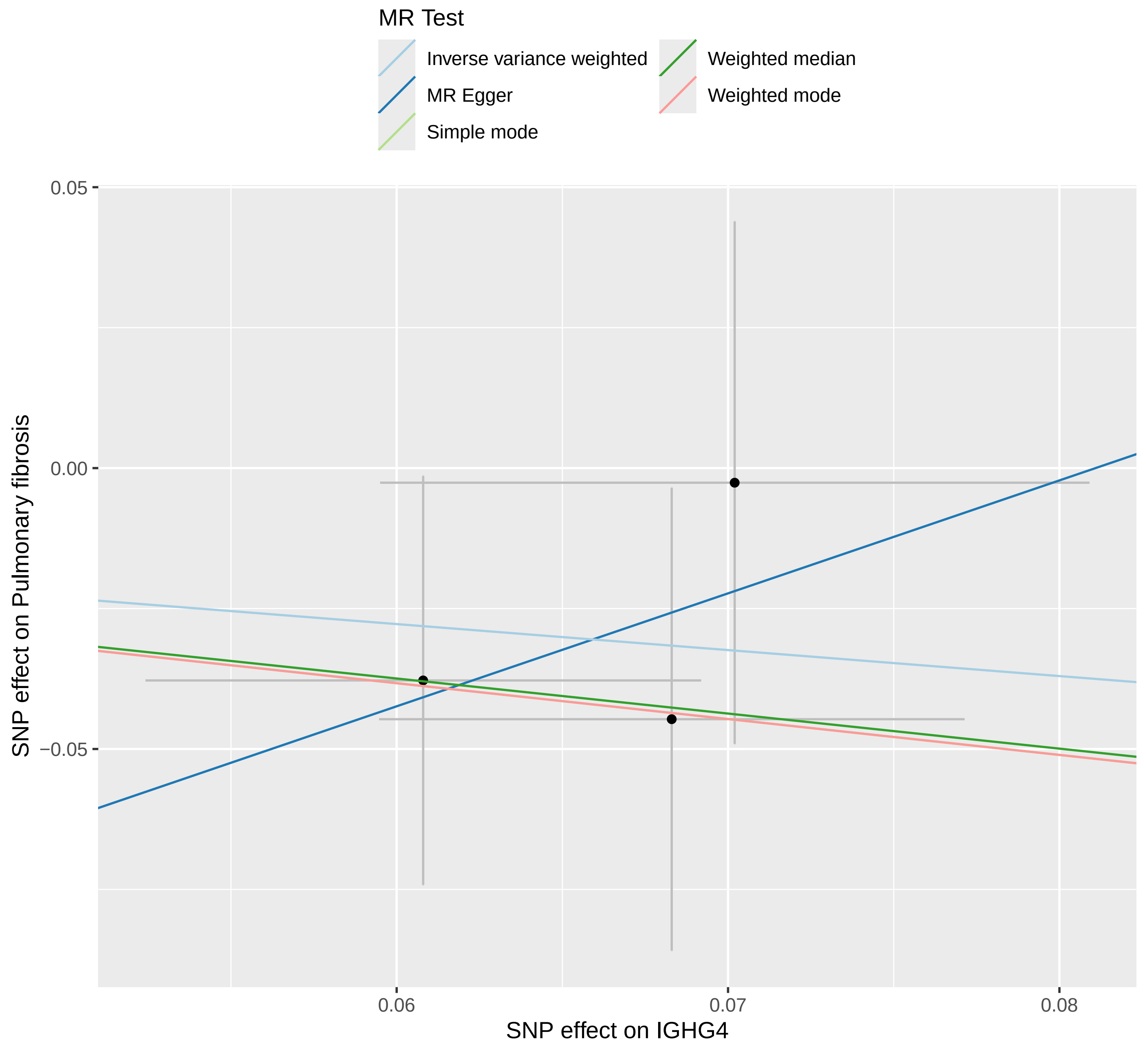

MR Test
Inverse variance weighted
MR Egger
Simple mode
Weighted median
Weighted mode
0.05
0.00
−0.05
0.06
0.07
0.08
SNP effect on Pulmonary fibrosis
SNP effect on IGHG4

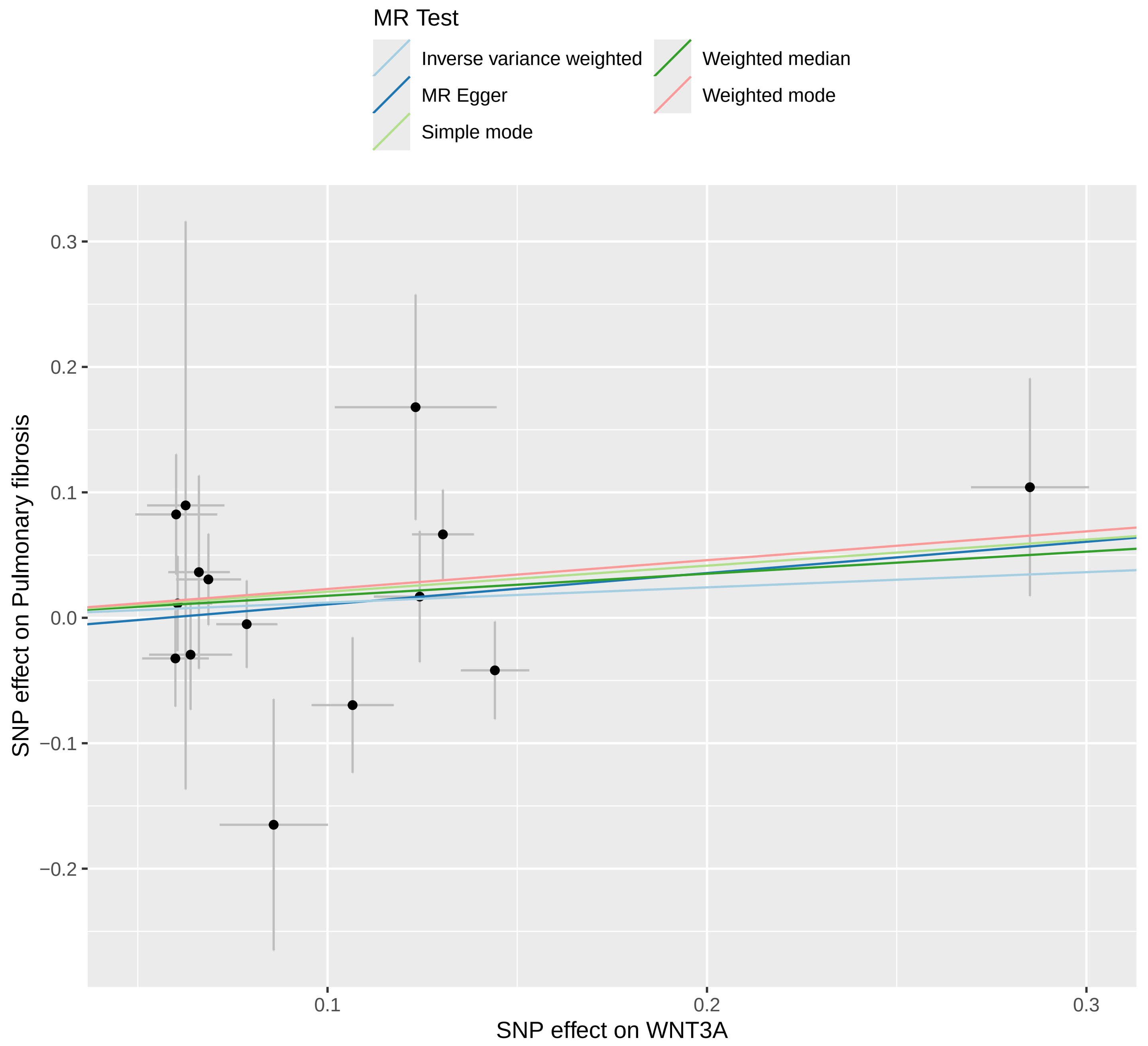
MR Test
Inverse variance weighted
MR Egger
Simple mode
Weighted median
Weighted mode
SNP effect on Pulmonary fibrosis
0.3
0.2
0.1
0.0
−0.1
−0.2
0.1
0.2
0.3
SNP effect on WNT3A

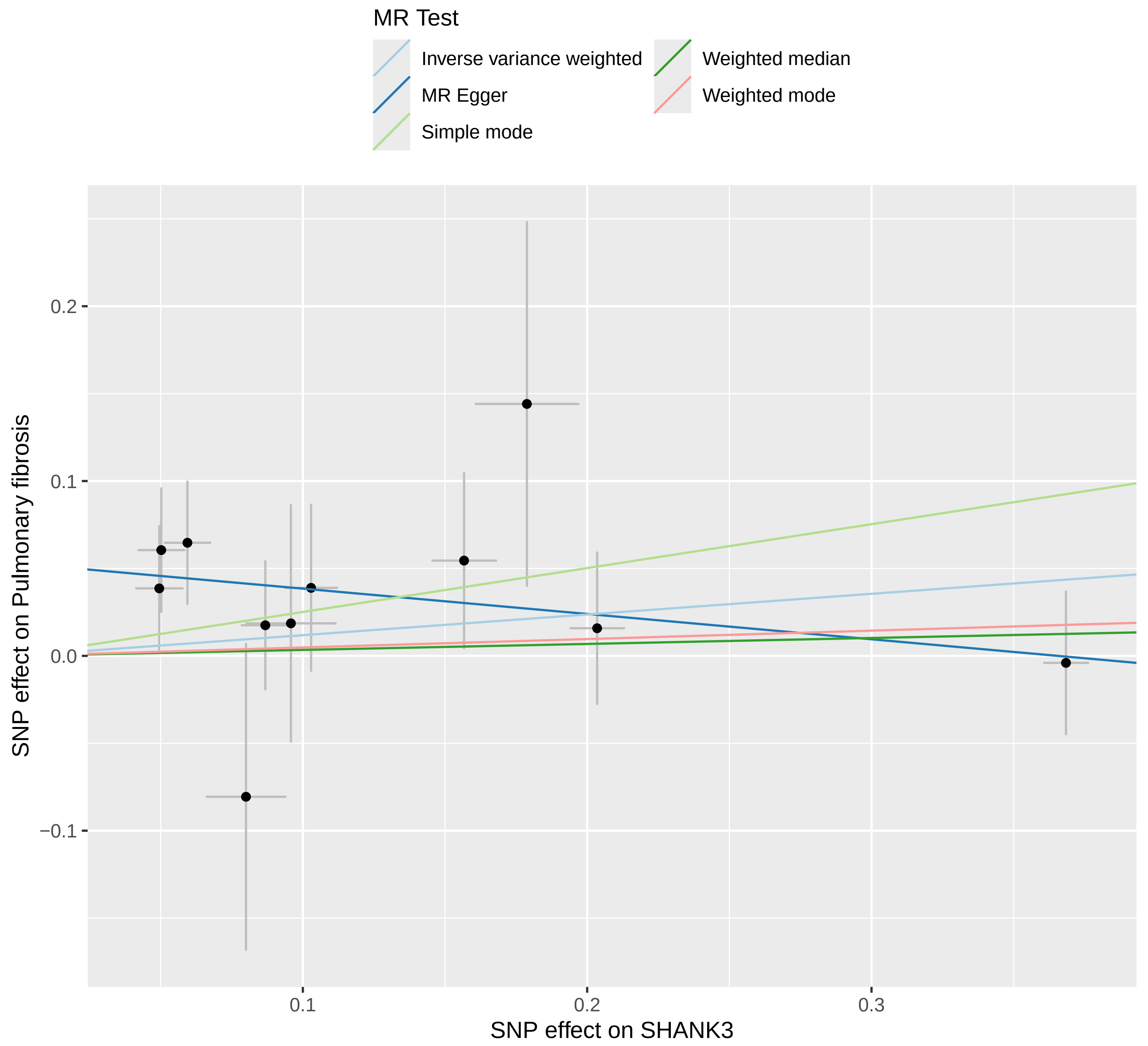

MR Test
Inverse variance weighted
Weighted median
MR Egger
Weighted mode
Simple mode
SNP effect on Pulmonary fibrosis
0.2
0.1
0.0
−0.1
0.1
0.2
0.3
SNP effect on SHANK3

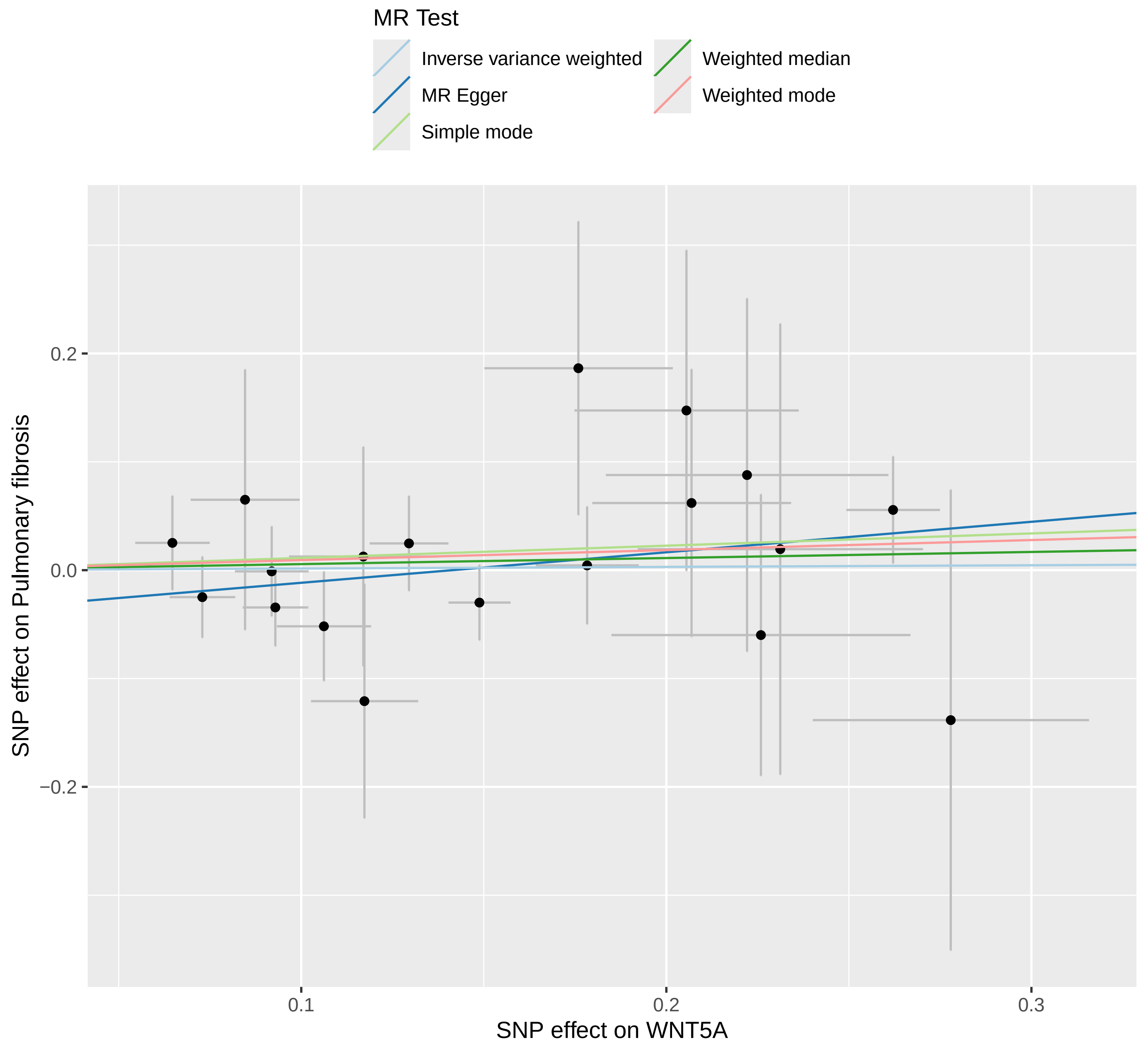
MR Test
Inverse variance weighted
MR Egger
Simple mode
Weighted median
Weighted mode
SNP effect on Pulmonary fibrosis
0.2
0.0
−0.2
0.1
0.2
0.3
SNP effect on WNT5A

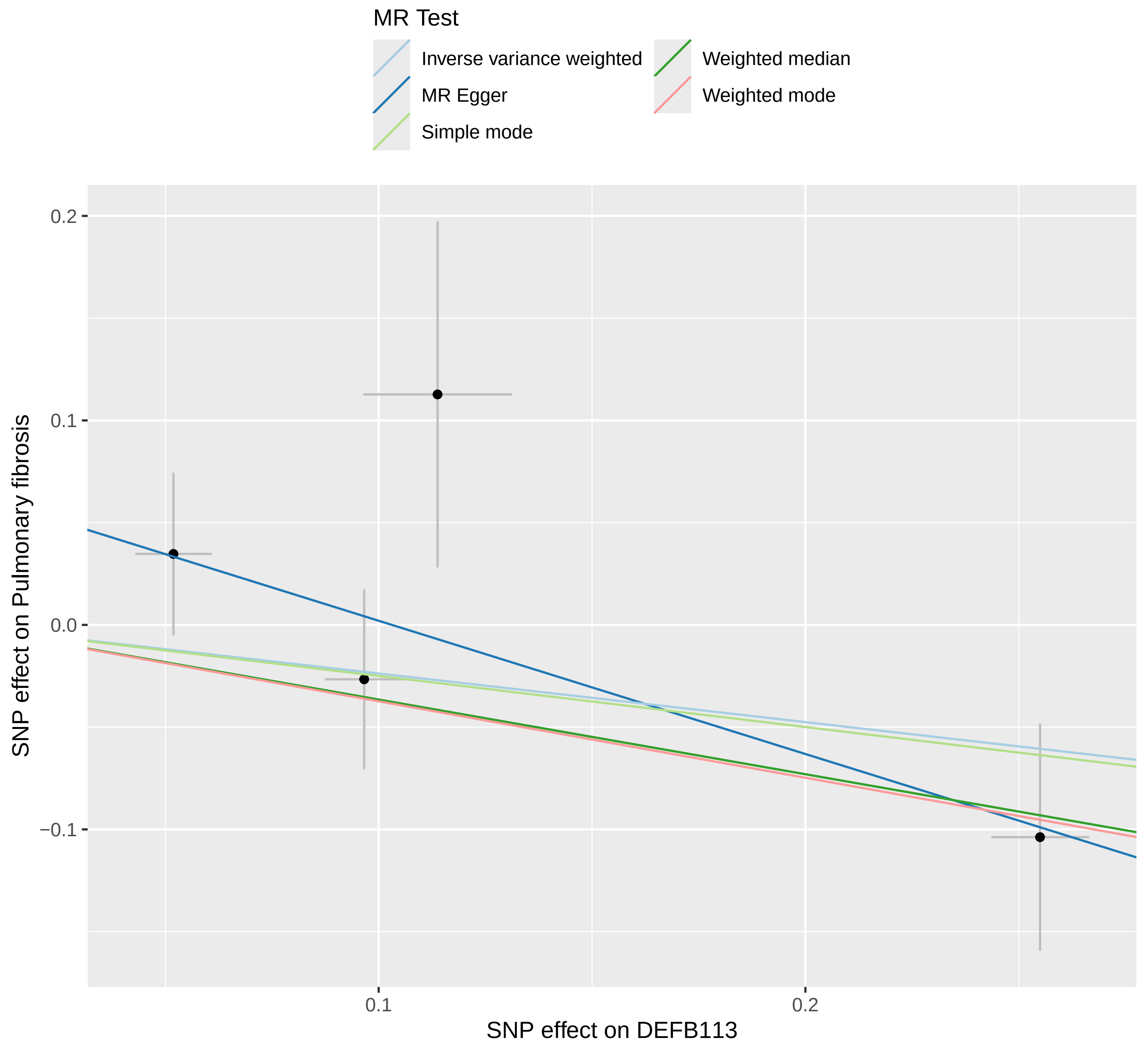
MR Test
Inverse variance weighted
MR Egger
Simple mode
Weighted median
Weighted mode
0.2
0.1
0.0
−0.1
0.1
0.2
SNP effect on Pulmonary fibrosis
SNP effect on DEFB113

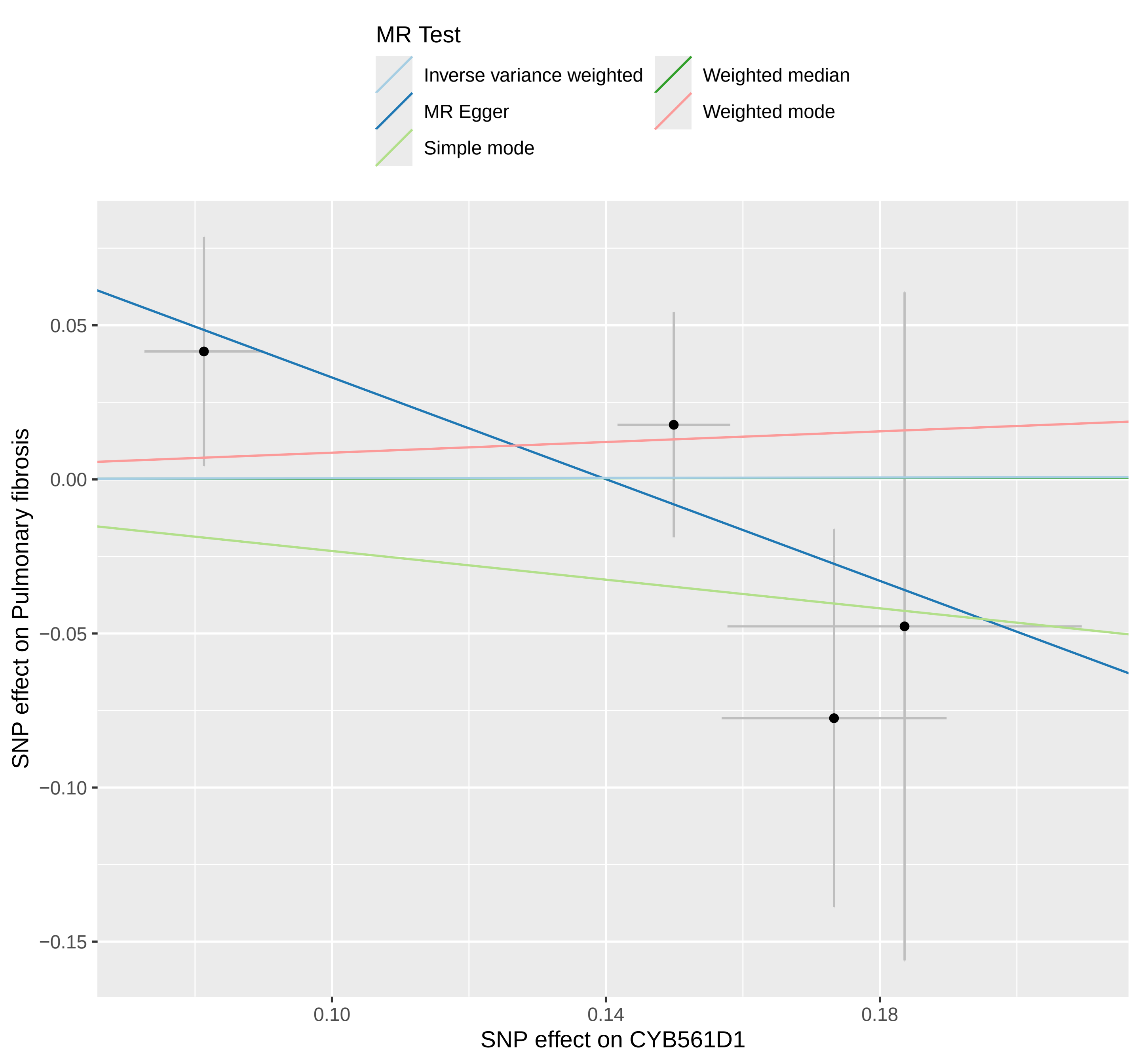
MR Test
Inverse variance weighted
MR Egger
Simple mode
Weighted median
Weighted mode
0.05
0.00
−0.05
−0.10
−0.15
0.10
0.14
0.18
SNP effect on Pulmonary fibrosis
SNP effect on CYB561D1

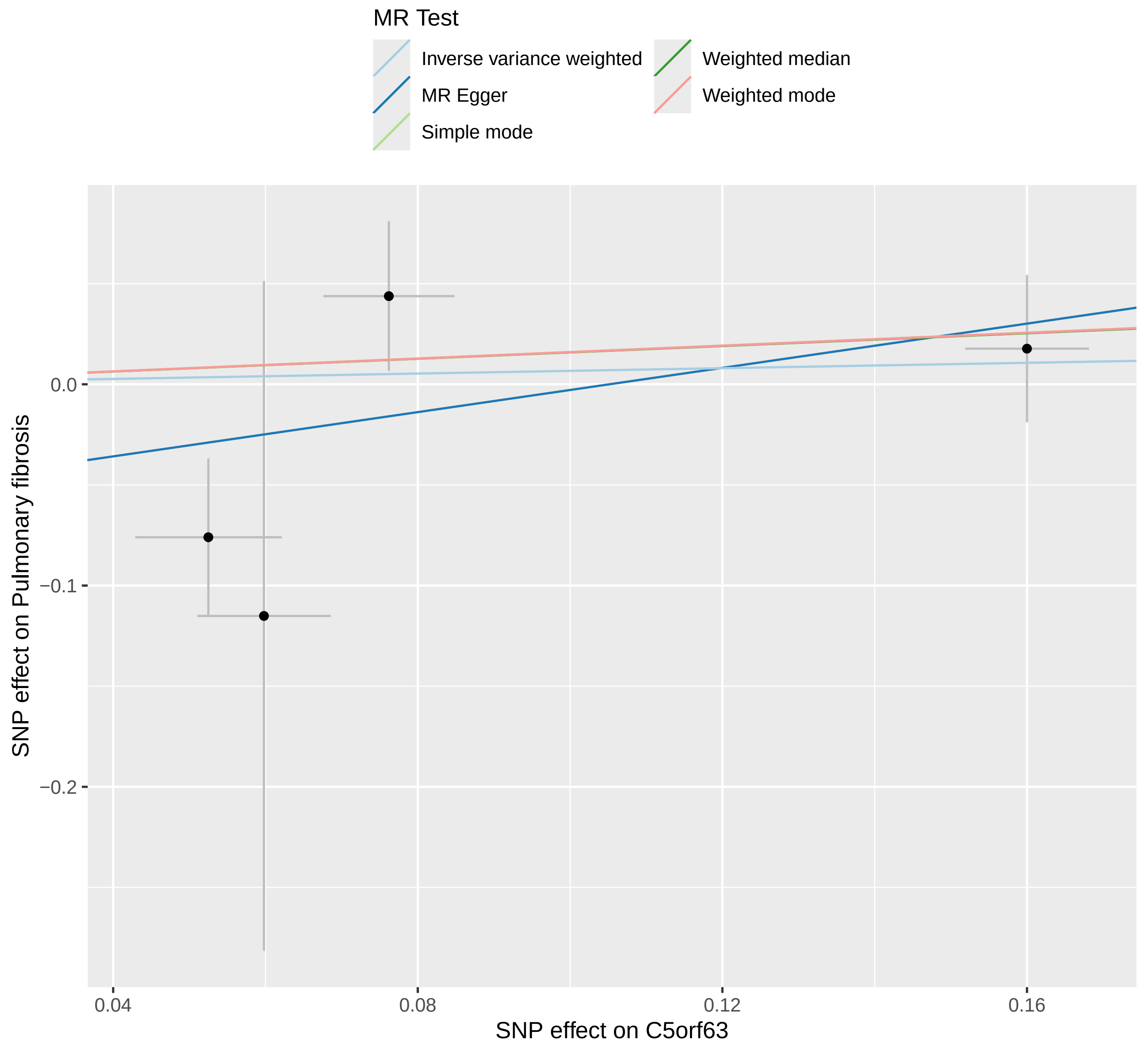
MR Test
Inverse variance weighted
MR Egger
Simple mode
Weighted median
Weighted mode
0.0
−0.1
−0.2
SNP effect on Pulmonary fibrosis
0.04
0.08
0.12
0.16
SNP effect on C5orf63

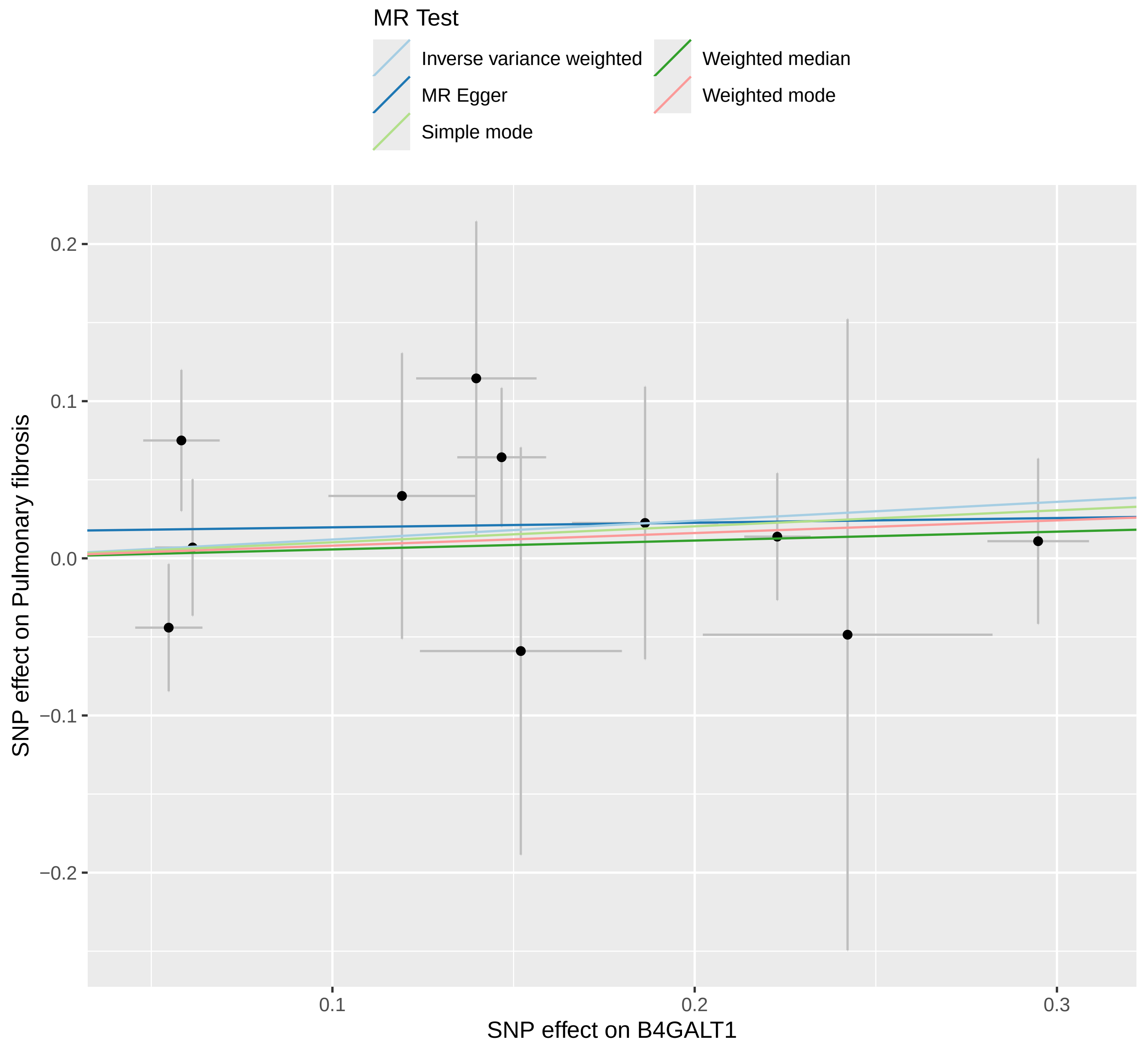

MR Test
Inverse variance weighted
MR Egger
Simple mode
Weighted median
Weighted mode
0.2
0.1
0.0
−0.1
−0.2
SNP effect on Pulmonary fibrosis
0.1
0.2
0.3
SNP effect on B4GALT1

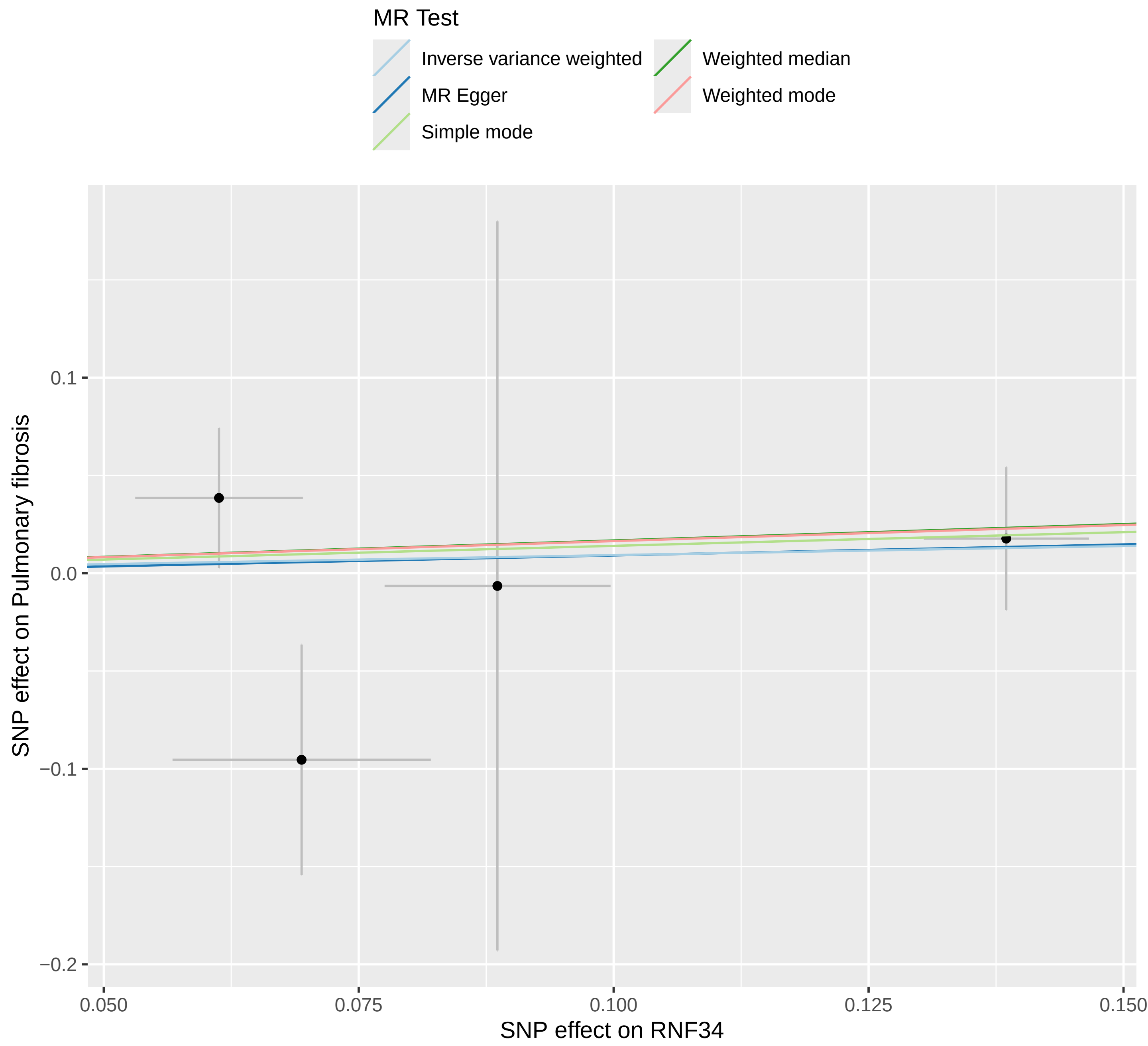
MR Test
Inverse variance weighted
MR Egger
Simple mode
Weighted median
Weighted mode
0.1
0.0
−0.1
−0.2
SNP effect on Pulmonary fibrosis
0.050
0.075
0.100
0.125
0.150
SNP effect on RNF34

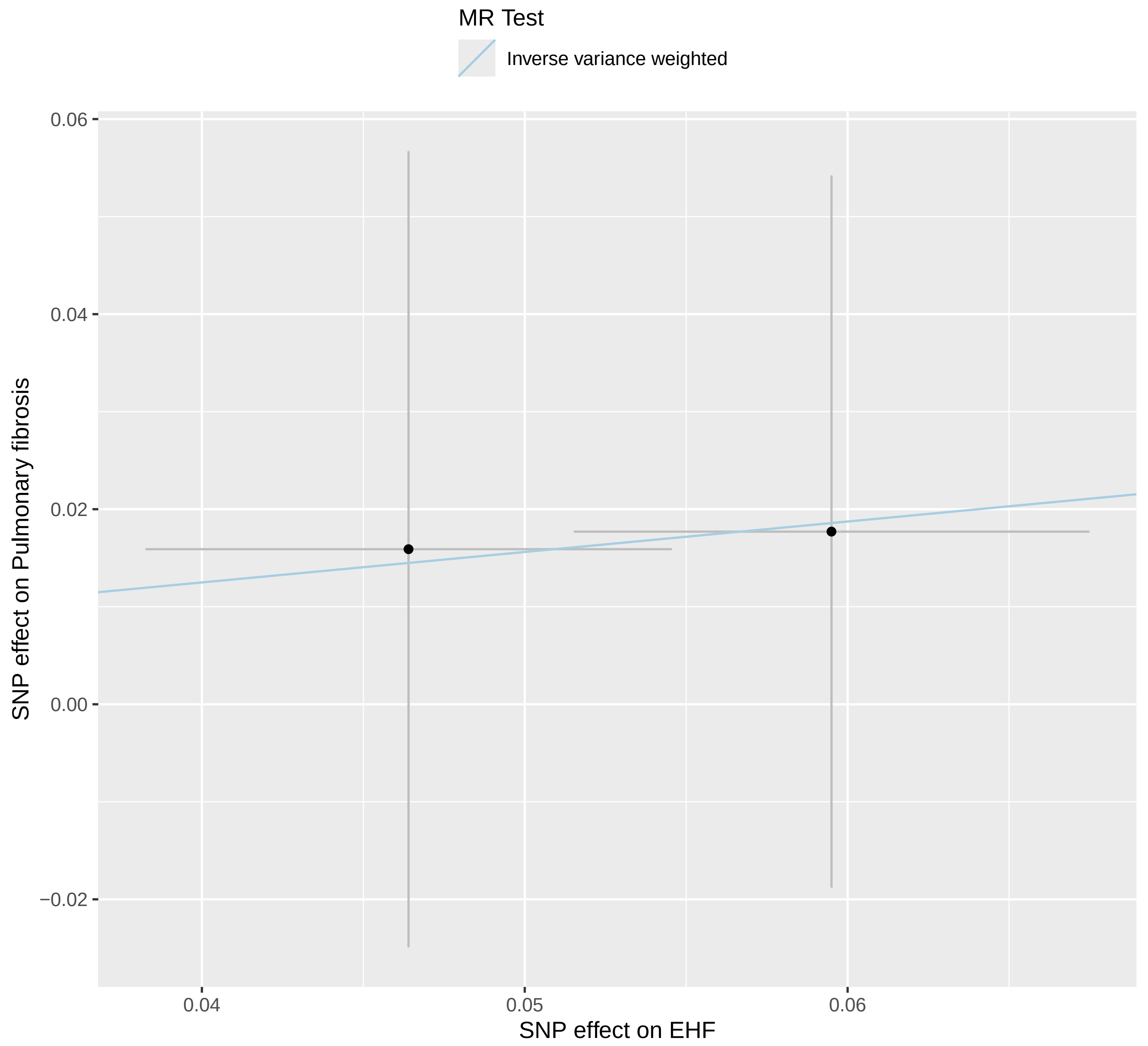
MR Test
Inverse variance weighted
0.06
0.04
0.02
0.00
−0.02
0.04
0.05
0.06
SNP effect on Pulmonary fibrosis
SNP effect on EHF

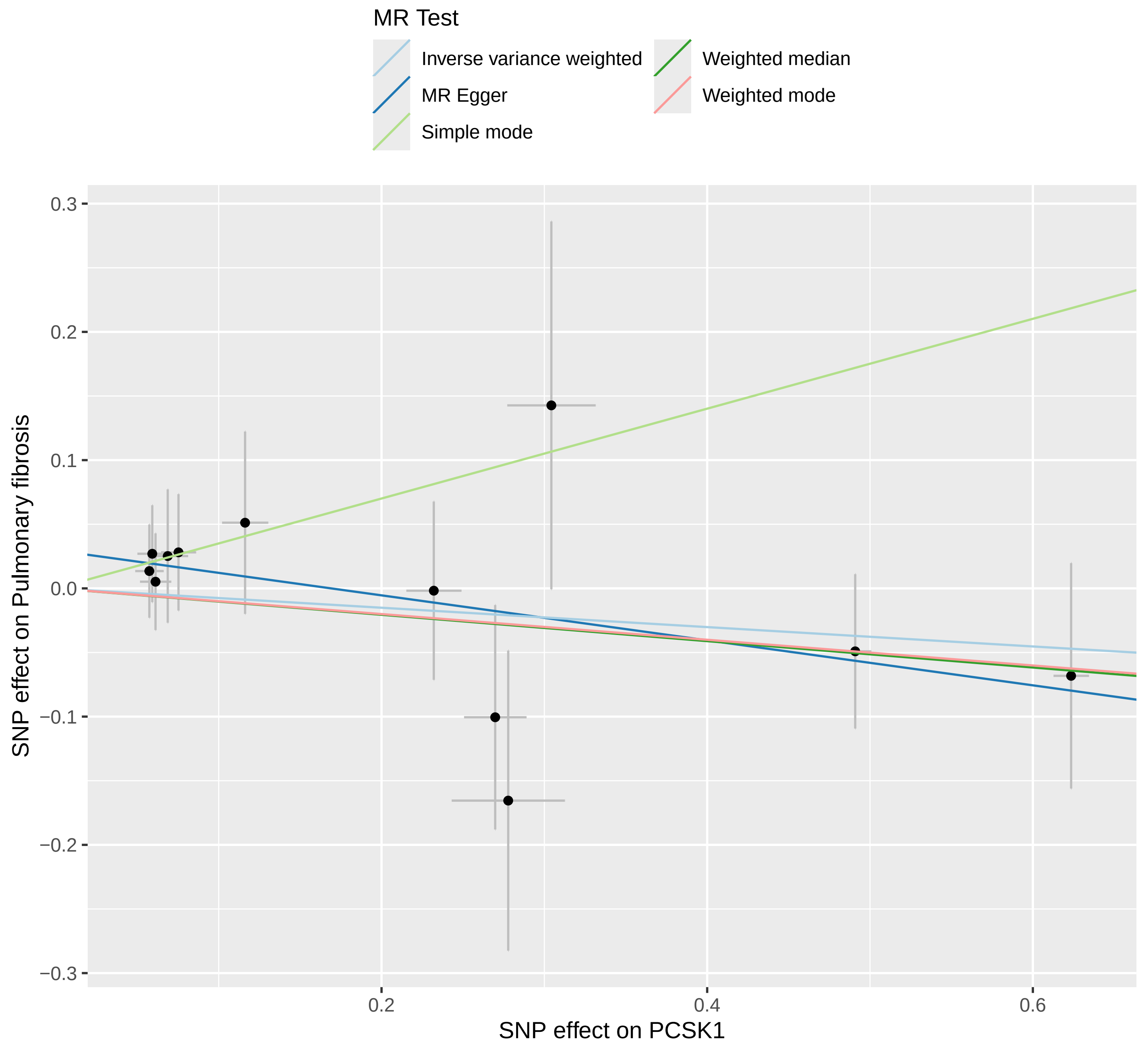
MR Test
Inverse variance weighted
MR Egger
Simple mode
Weighted median
Weighted mode
0.3
0.2
0.1
0.0
−0.1
−0.2
−0.3
0.2
0.4
0.6
SNP effect on Pulmonary fibrosis
SNP effect on PCSK1

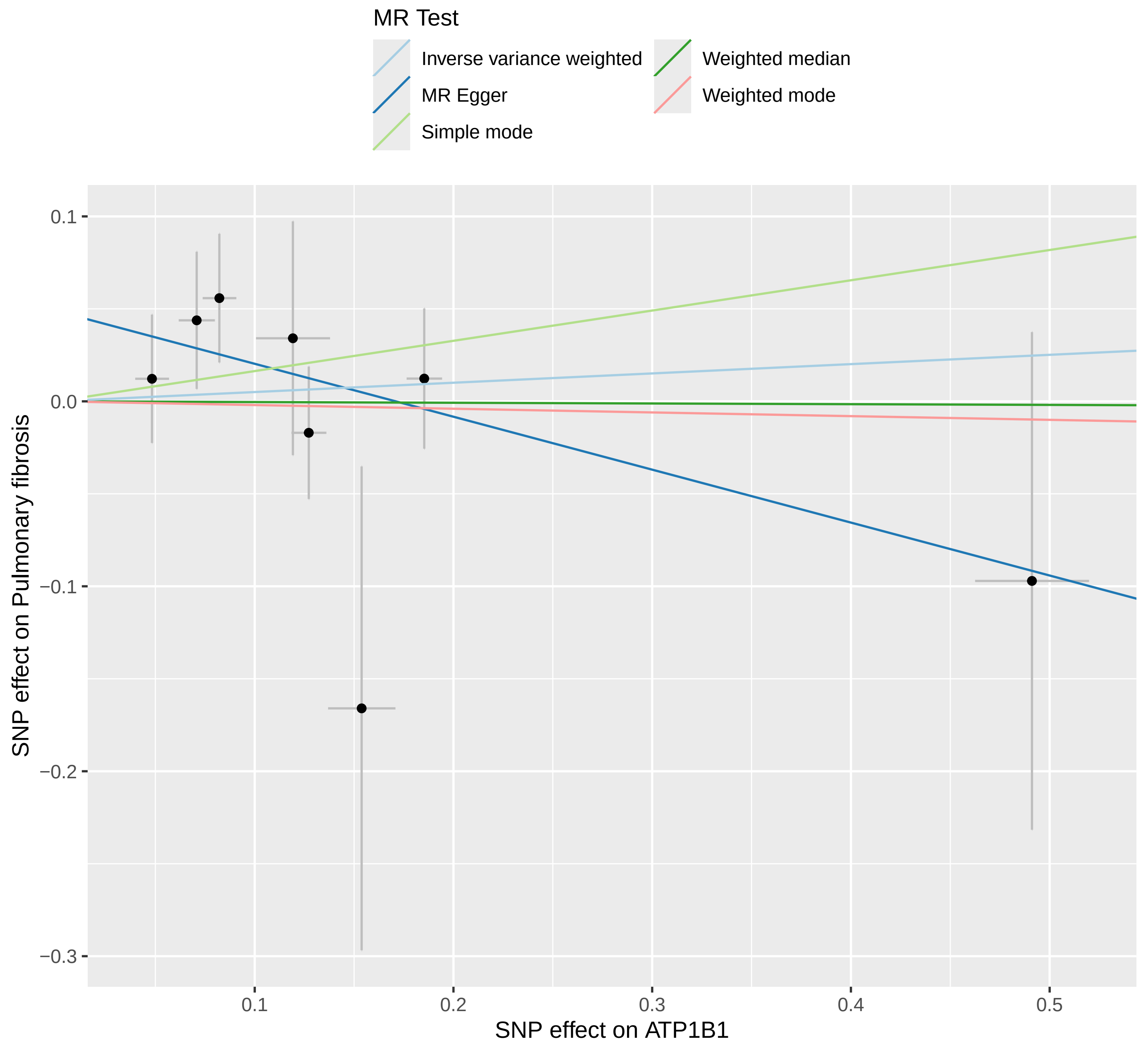
MR Test
Inverse variance weighted
MR Egger
Simple mode
Weighted median
Weighted mode
0.1
0.0
−0.1
−0.2
−0.3
0.1
0.2
0.3
0.4
0.5
SNP effect on Pulmonary fibrosis
SNP effect on ATP1B1

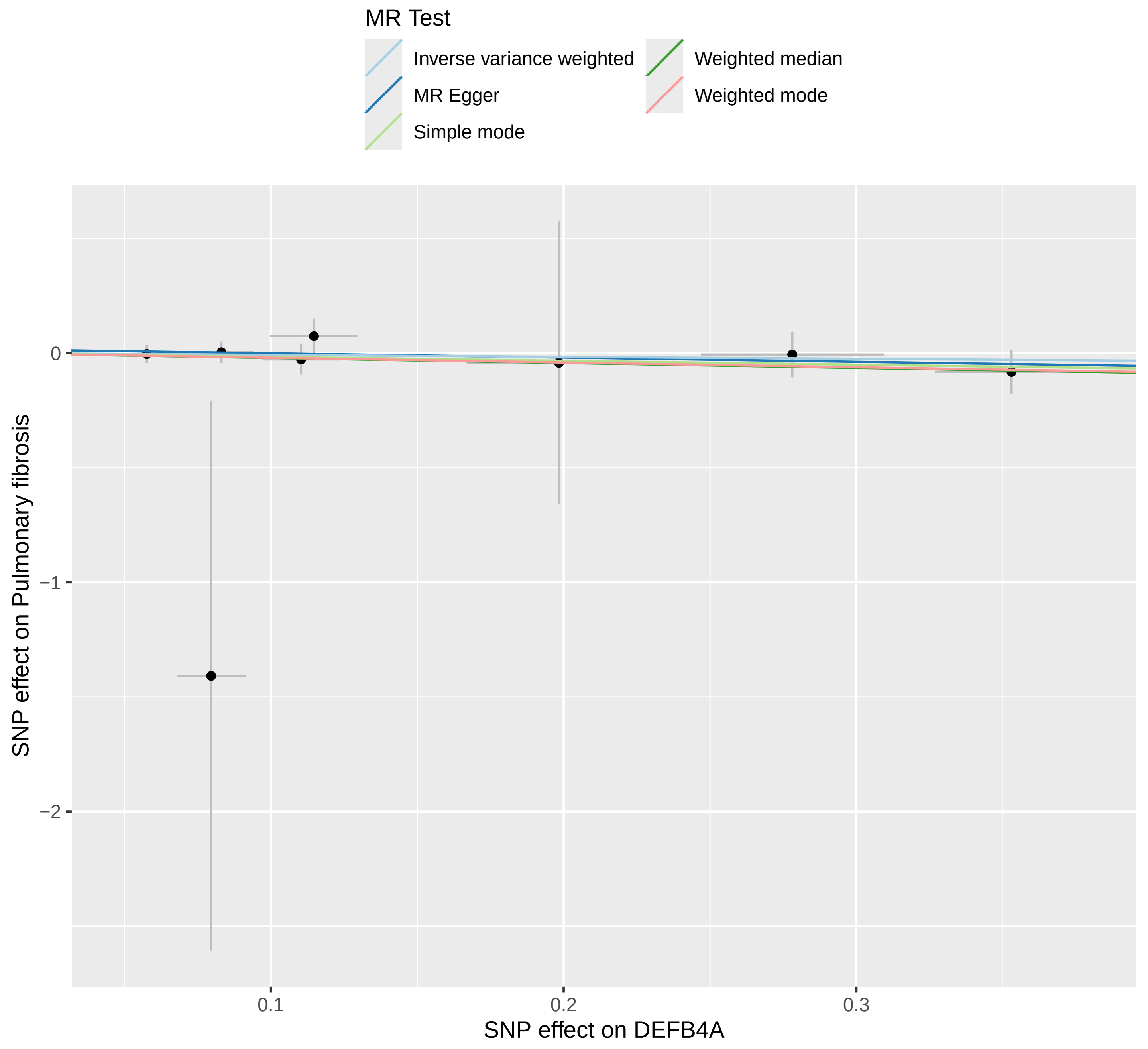
MR Test
Inverse variance weighted
MR Egger
Simple mode
Weighted median
Weighted mode
0
−1
−2
0.1
0.2
0.3
SNP effect on Pulmonary fibrosis
SNP effect on DEFB4A

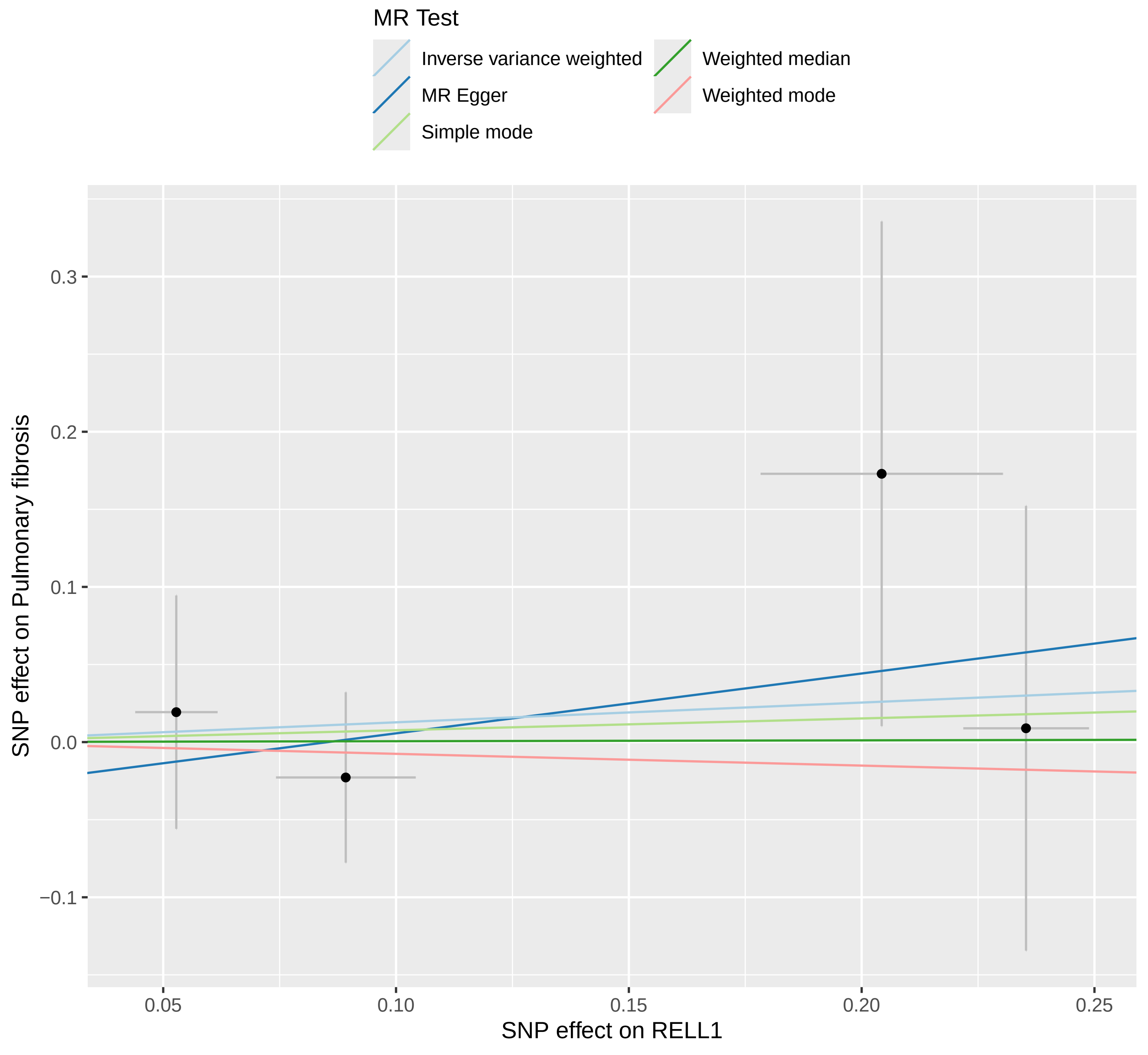
MR Test
Inverse variance weighted
MR Egger
Simple mode
Weighted median
Weighted mode
SNP effect on Pulmonary fibrosis
0.3
0.2
0.1
0.0
−0.1
0.05
0.10
0.15
0.20
0.25
SNP effect on RELL1

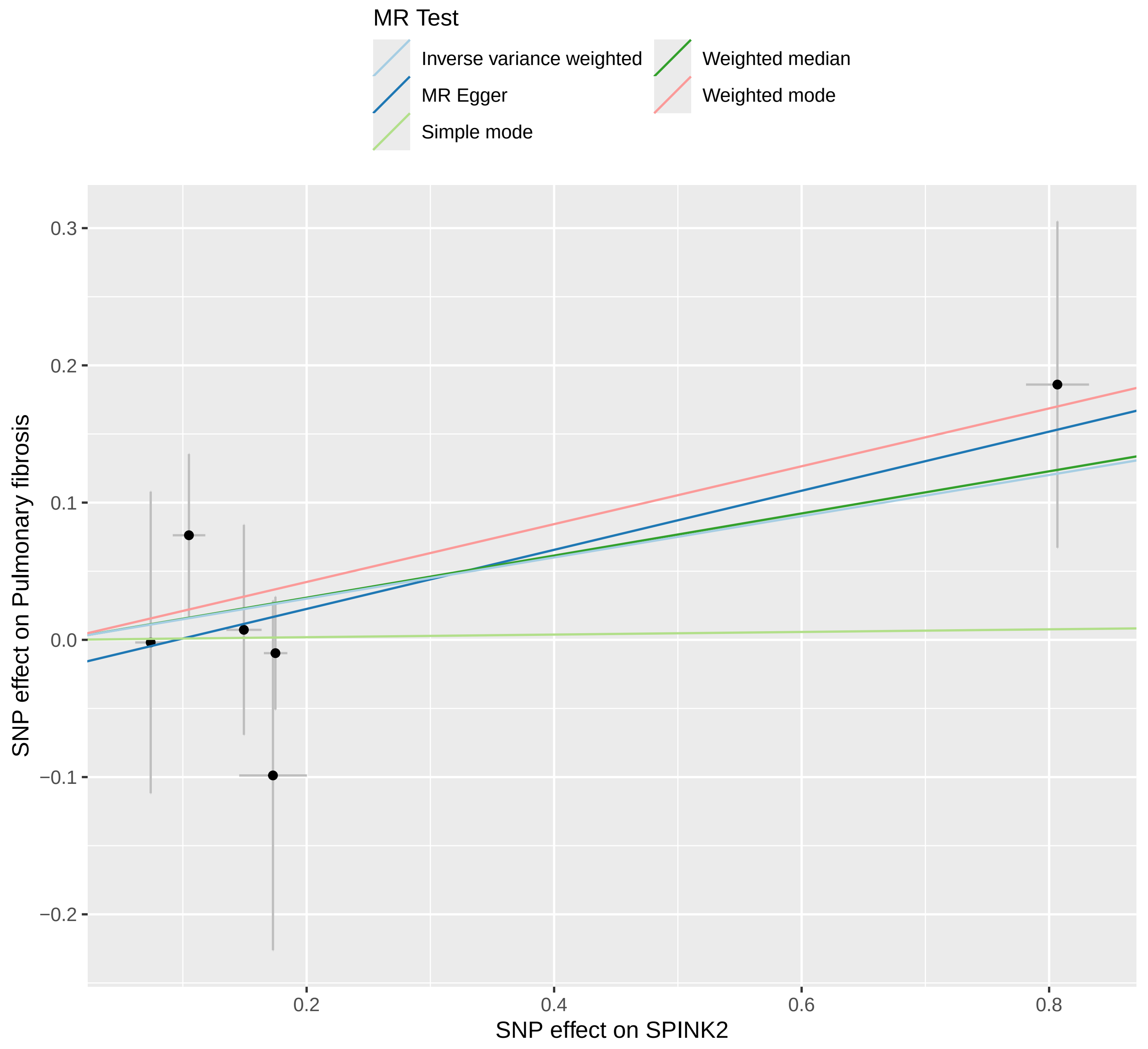
MR Test
Inverse variance weighted
MR Egger
Simple mode
Weighted median
Weighted mode
0.3
0.2
0.1
0.0
−0.1
−0.2
SNP effect on Pulmonary fibrosis
0.2
0.4
0.6
0.8
SNP effect on SPINK2

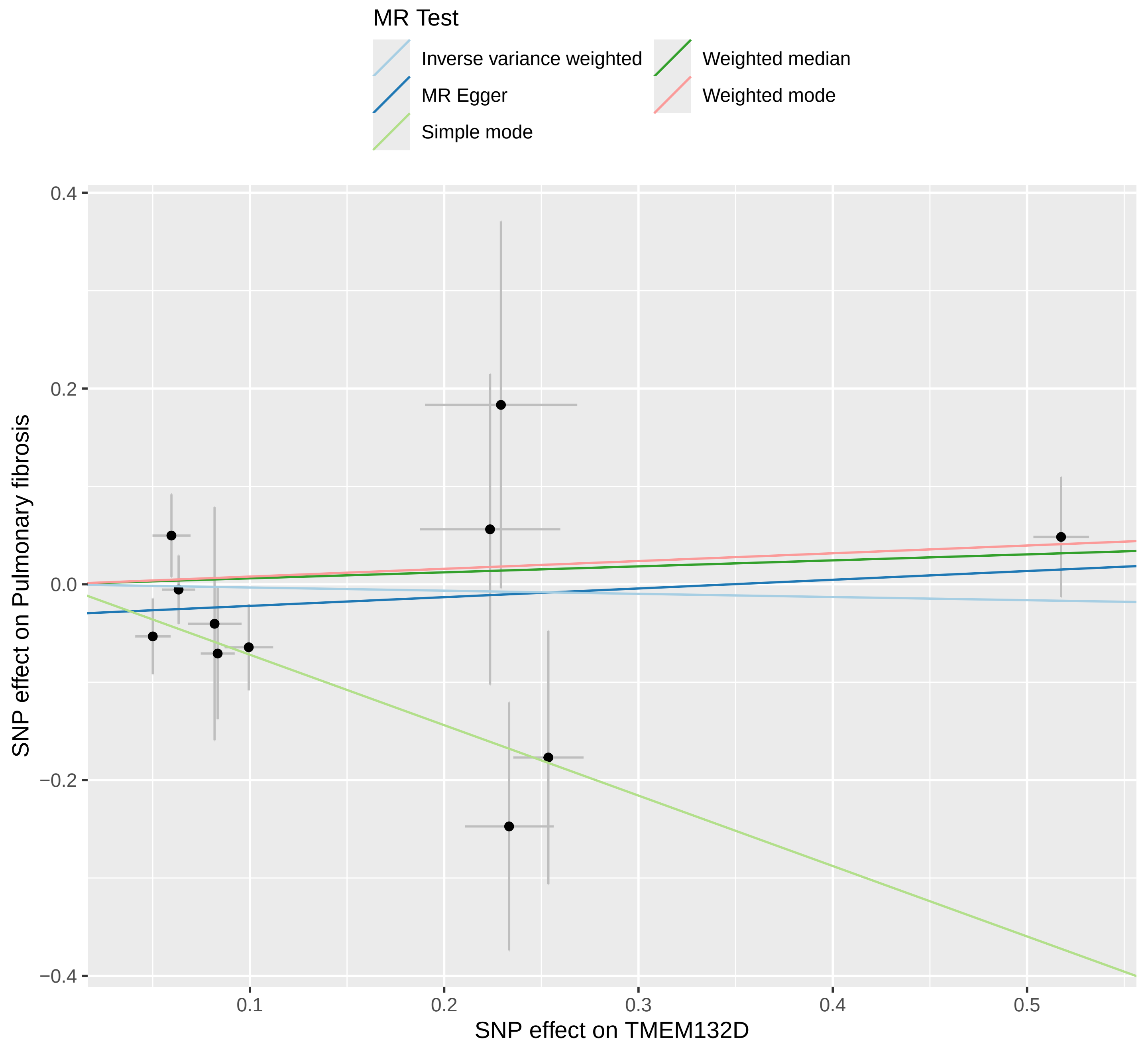
MR Test
Inverse variance weighted
MR Egger
Simple mode
Weighted median
Weighted mode
0.4
0.2
0.0
−0.2
−0.4
SNP effect on Pulmonary fibrosis
0.1
0.2
0.3
0.4
0.5
SNP effect on TMEM132D

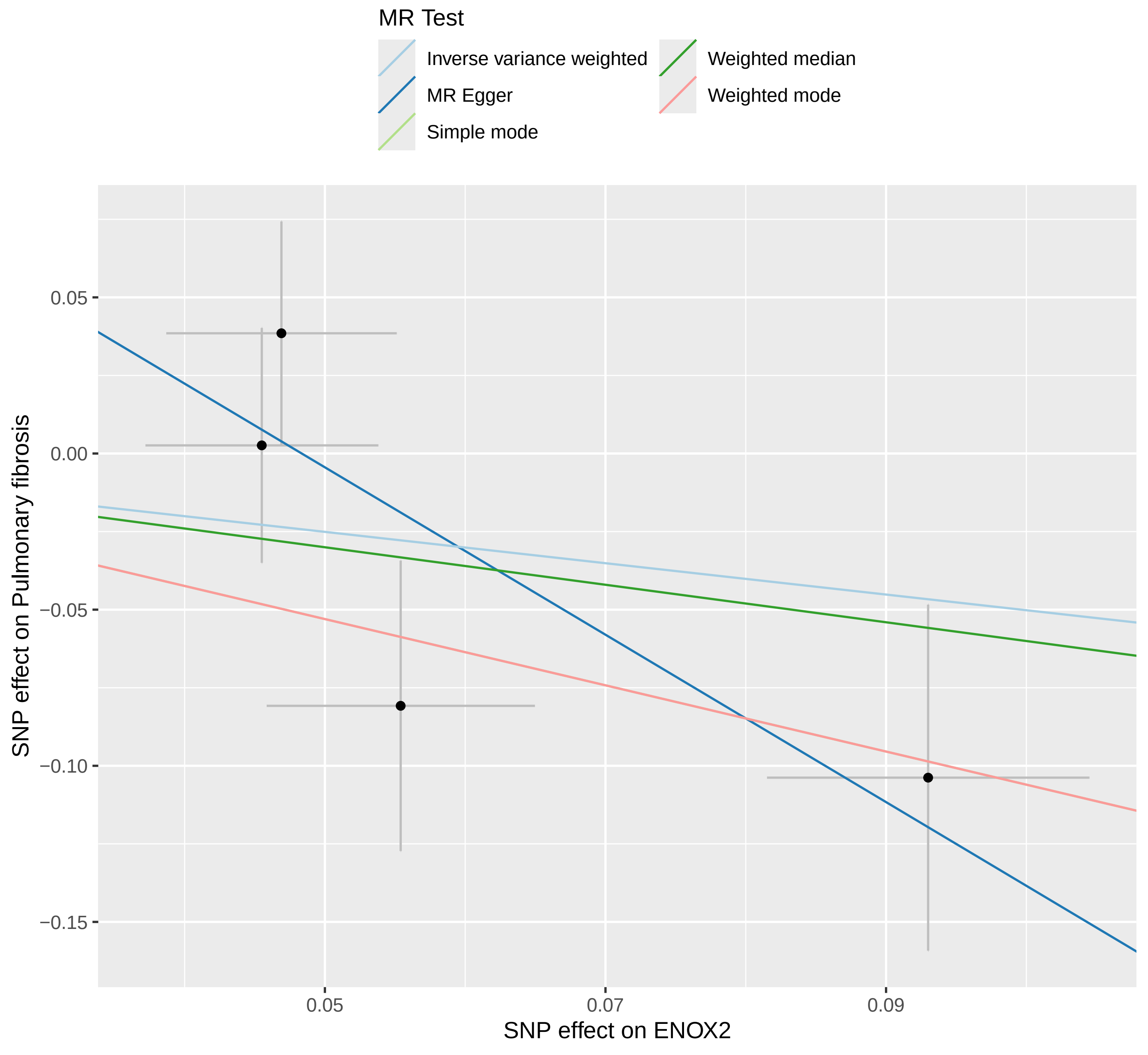

MR Test
Inverse variance weighted
MR Egger
Simple mode
Weighted median
Weighted mode
0.05
0.00
−0.05
−0.10
−0.15
0.05
0.07
0.09
SNP effect on Pulmonary fibrosis
SNP effect on ENOX2

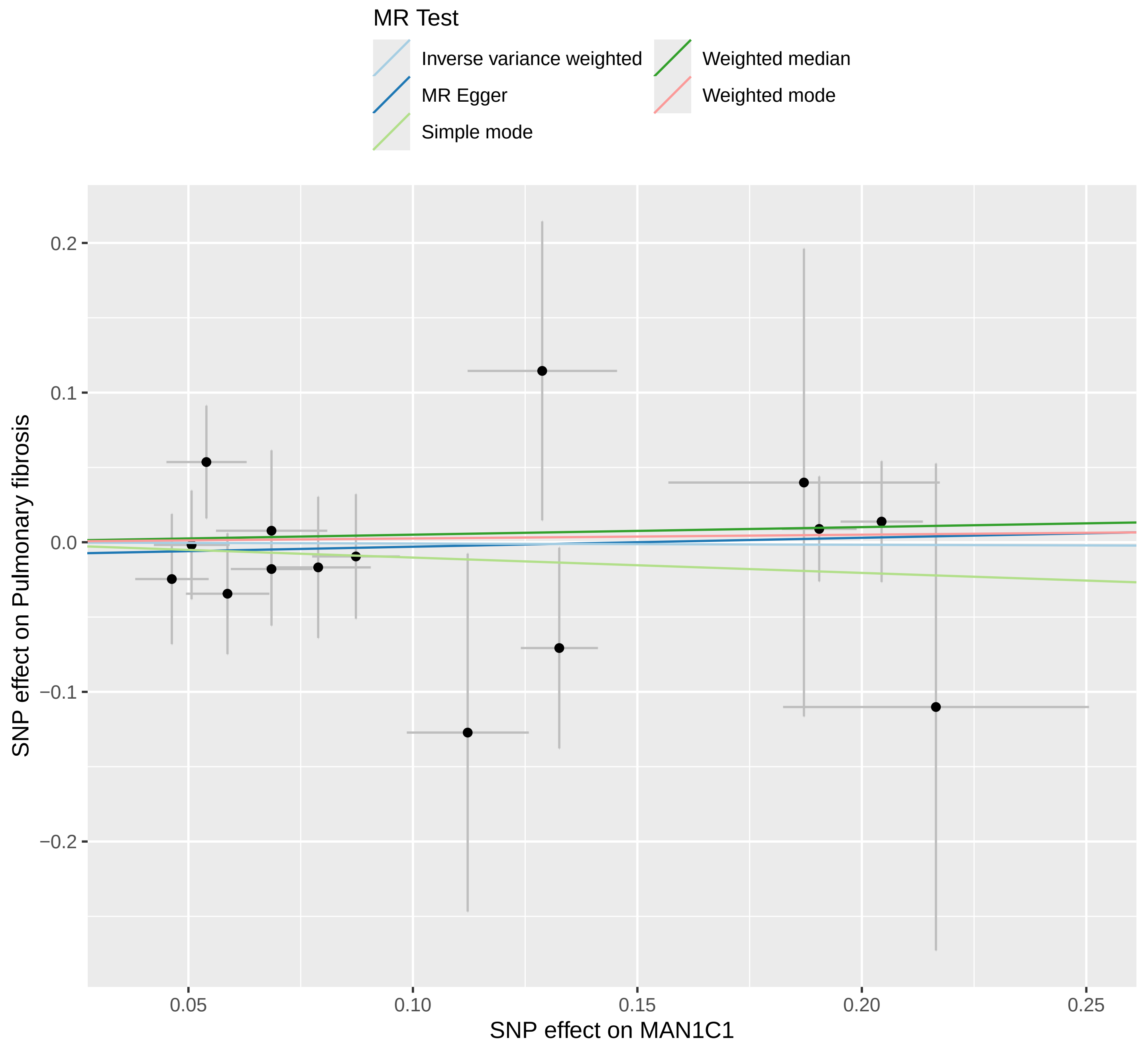
MR Test
Inverse variance weighted
MR Egger
Simple mode
Weighted median
Weighted mode
0.2
0.1
0.0
−0.1
−0.2
0.05
0.10
0.15
0.20
0.25
SNP effect on Pulmonary fibrosis
SNP effect on MAN1C1

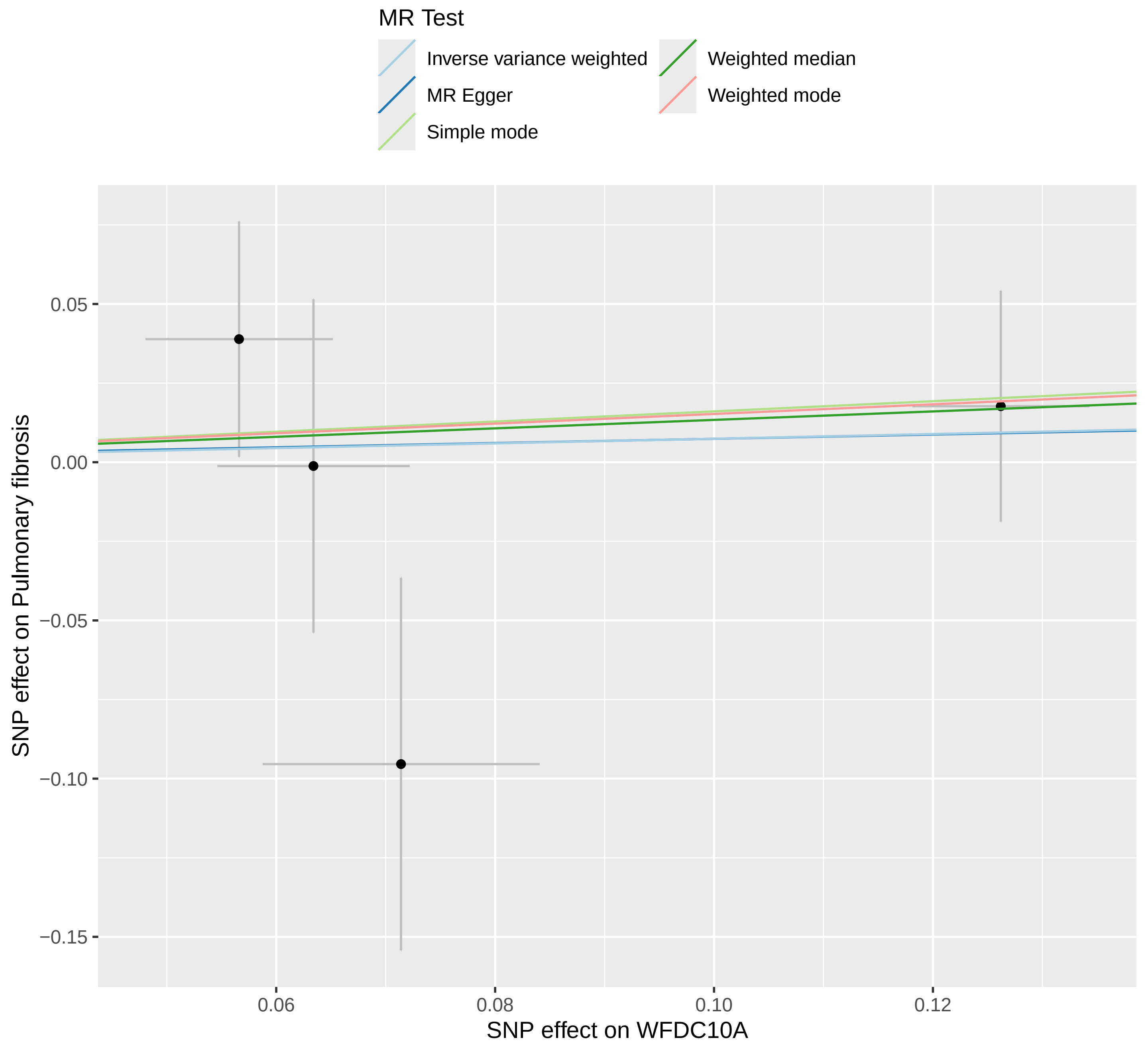
MR Test
Inverse variance weighted
MR Egger
Simple mode
Weighted median
Weighted mode
0.05
0.00
−0.05
−0.10
−0.15
0.06
0.08
0.10
0.12
SNP effect on WFDC10A
SNP effect on Pulmonary fibrosis

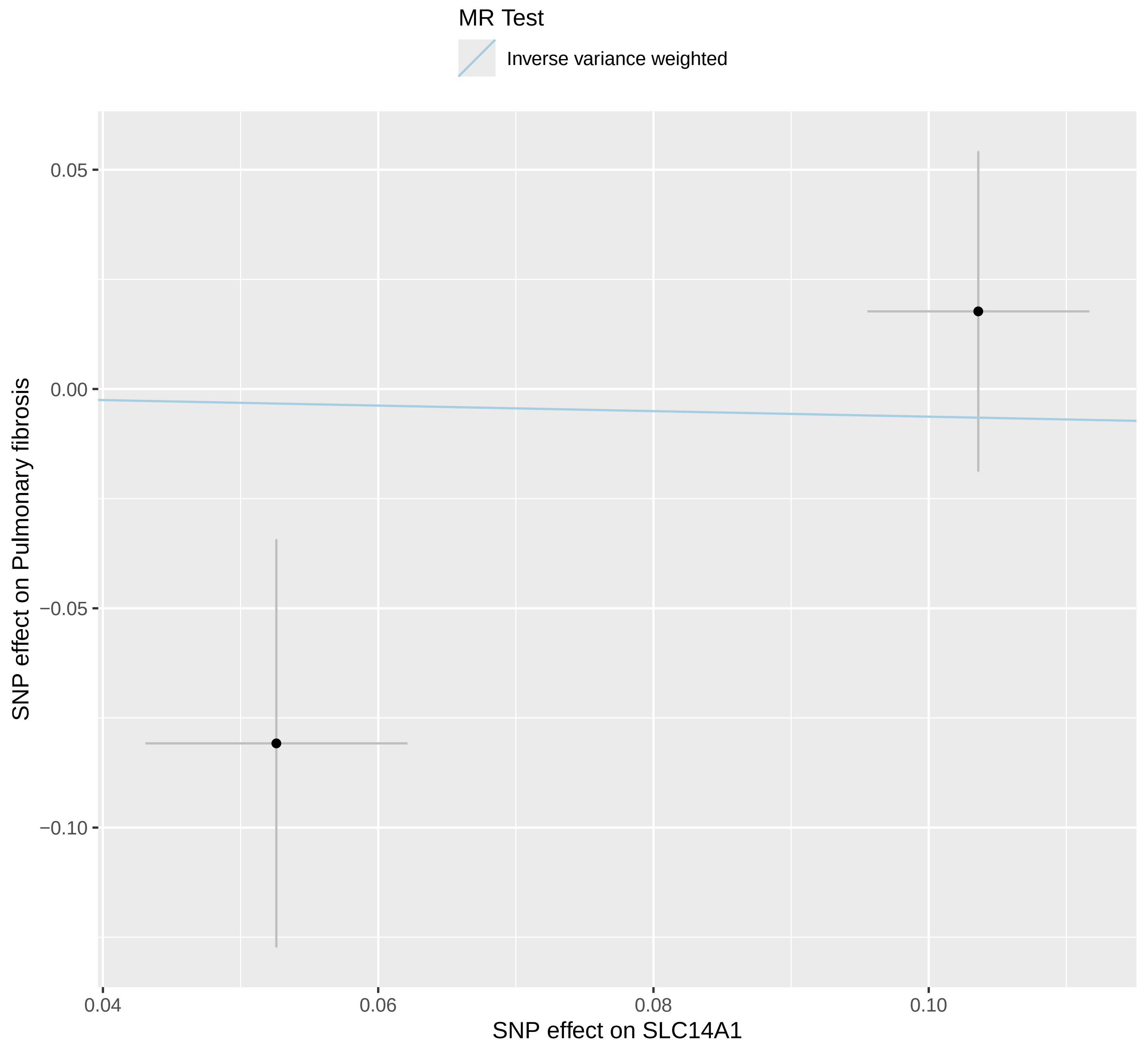
MR Test
Inverse variance weighted
0.05
0.00
−0.05
−0.10
SNP effect on Pulmonary fibrosis
0.04
0.06
0.08
0.10
SNP effect on SLC14A1

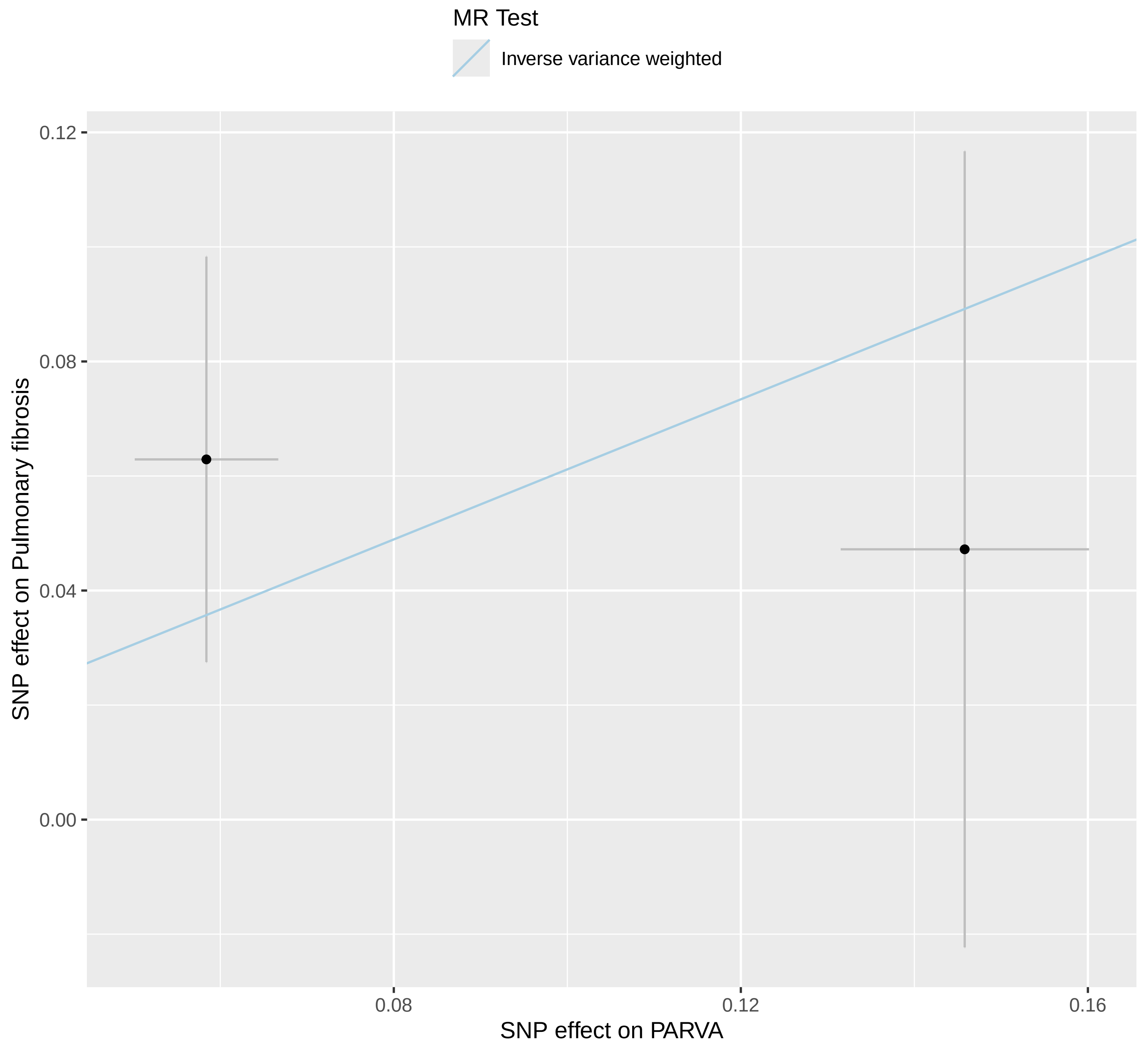
MR Test
Inverse variance weighted
0.12
0.08
0.04
0.00
SNP effect on Pulmonary fibrosis
0.08
0.12
0.16
SNP effect on PARVA

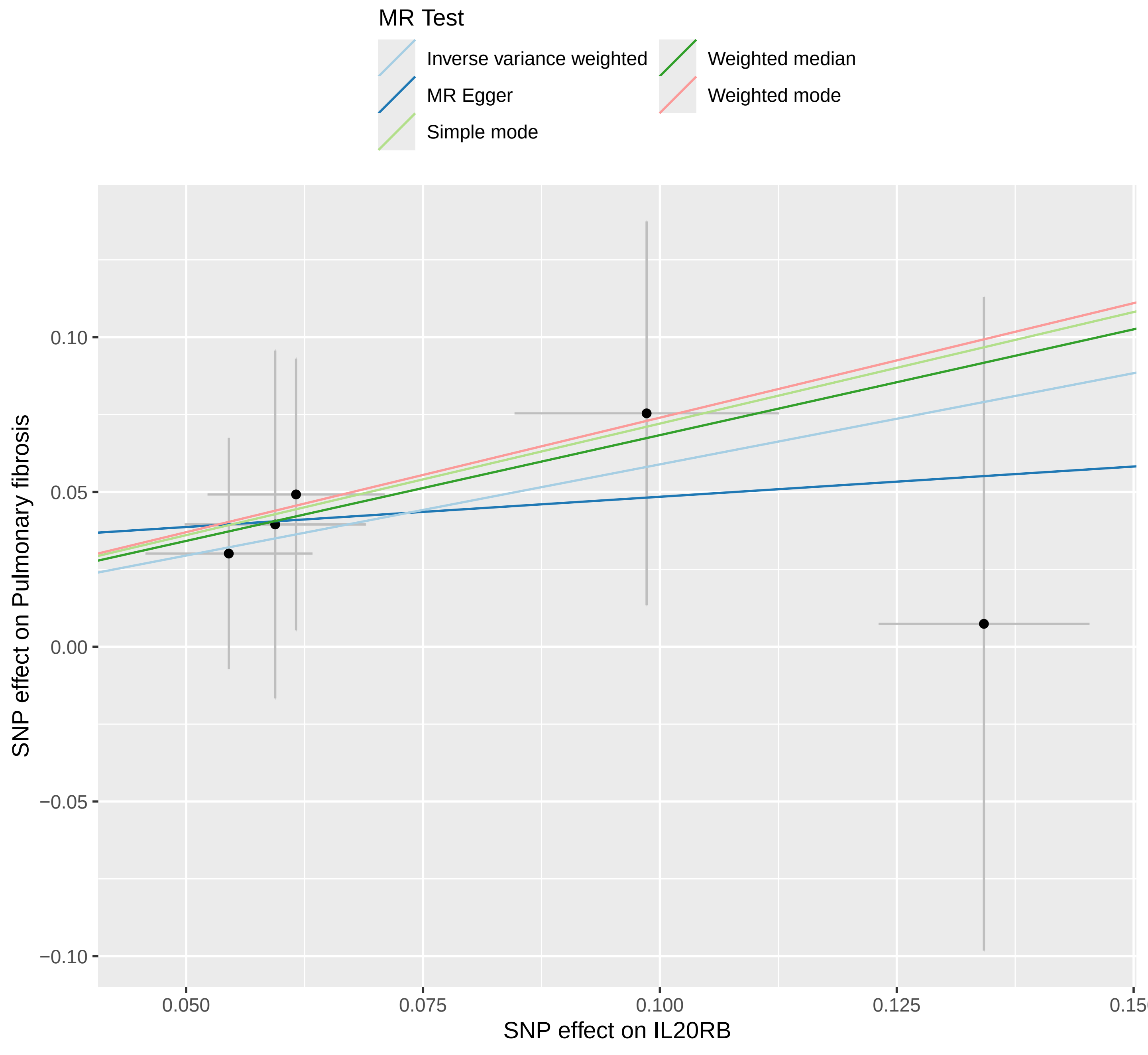
MR Test
Inverse variance weighted
MR Egger
Simple mode
Weighted median
Weighted mode
SNP effect on Pulmonary fibrosis
0.10
0.05
0.00
−0.05
−0.10
0.050
0.075
0.100
0.125
SNP effect on IL20RB

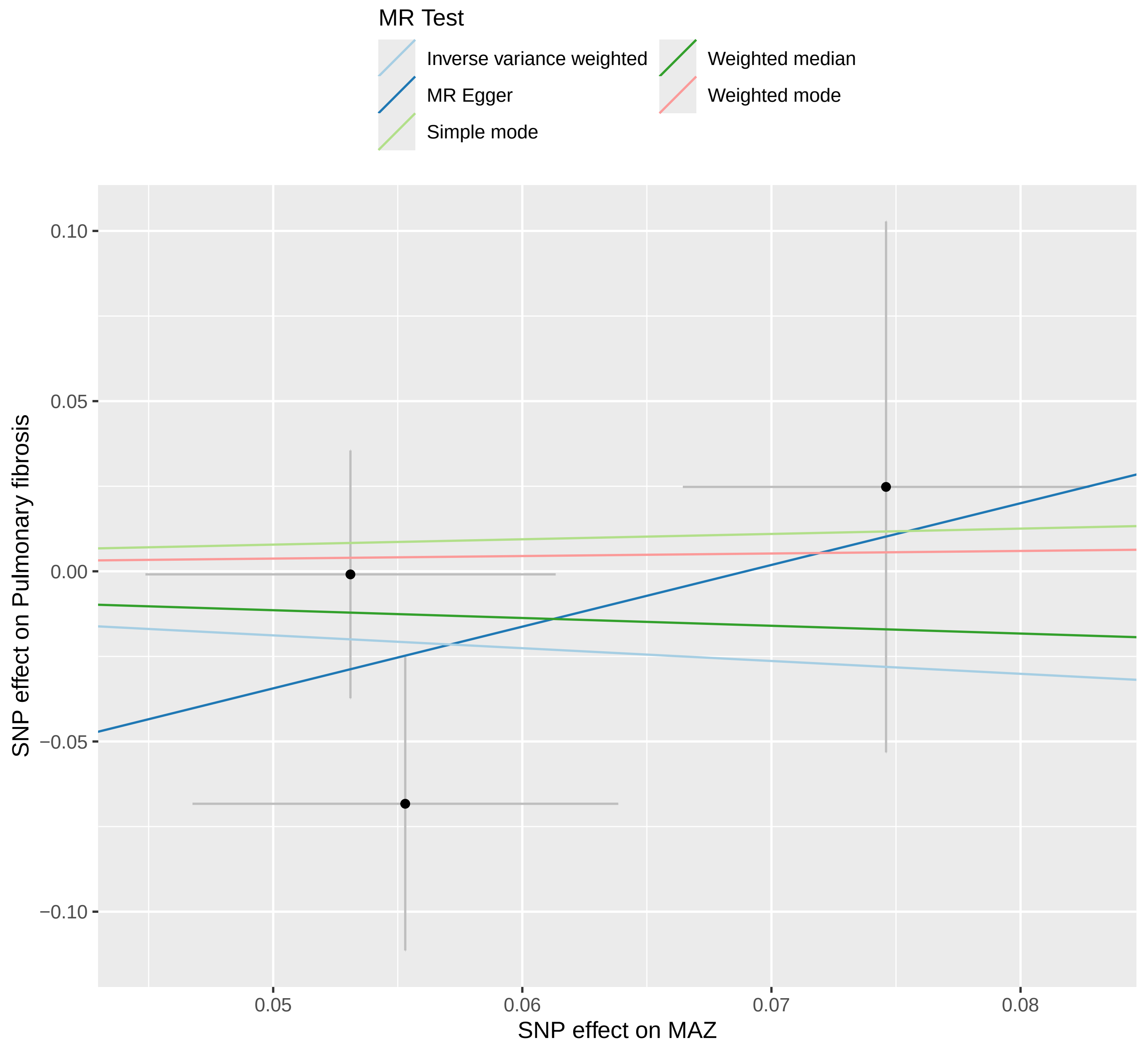

MR Test
Inverse variance weighted
MR Egger
Simple mode
Weighted median
Weighted mode
0.10
0.05
0.00
−0.05
−0.10
SNP effect on Pulmonary fibrosis
0.05
0.06
0.07
0.08
SNP effect on MAZ

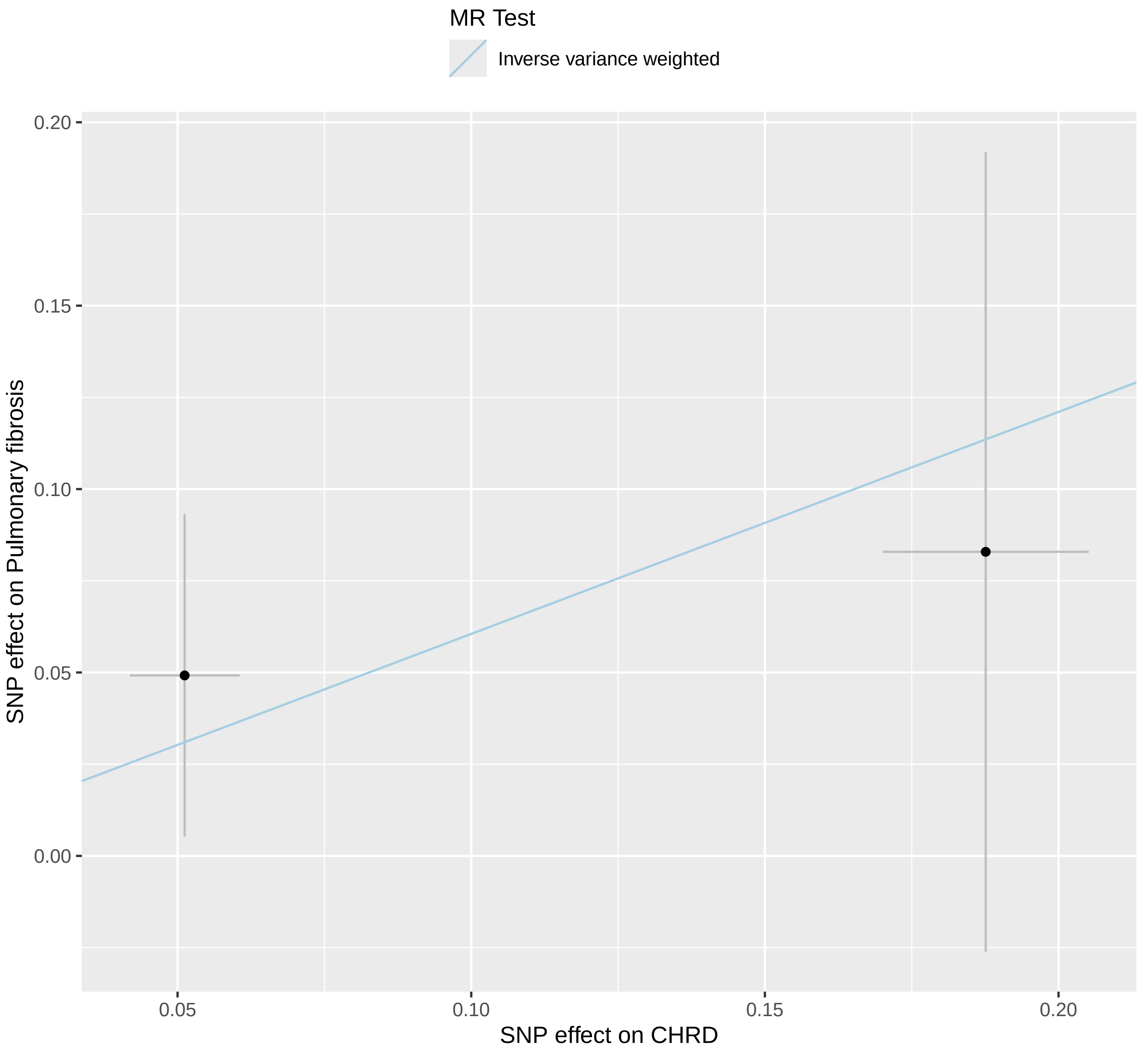
MR Test
Inverse variance weighted
SNP effect on Pulmonary fibrosis
0.20
0.15
0.10
0.05
0.00
0.05
0.10
0.15
0.20
SNP effect on CHRD

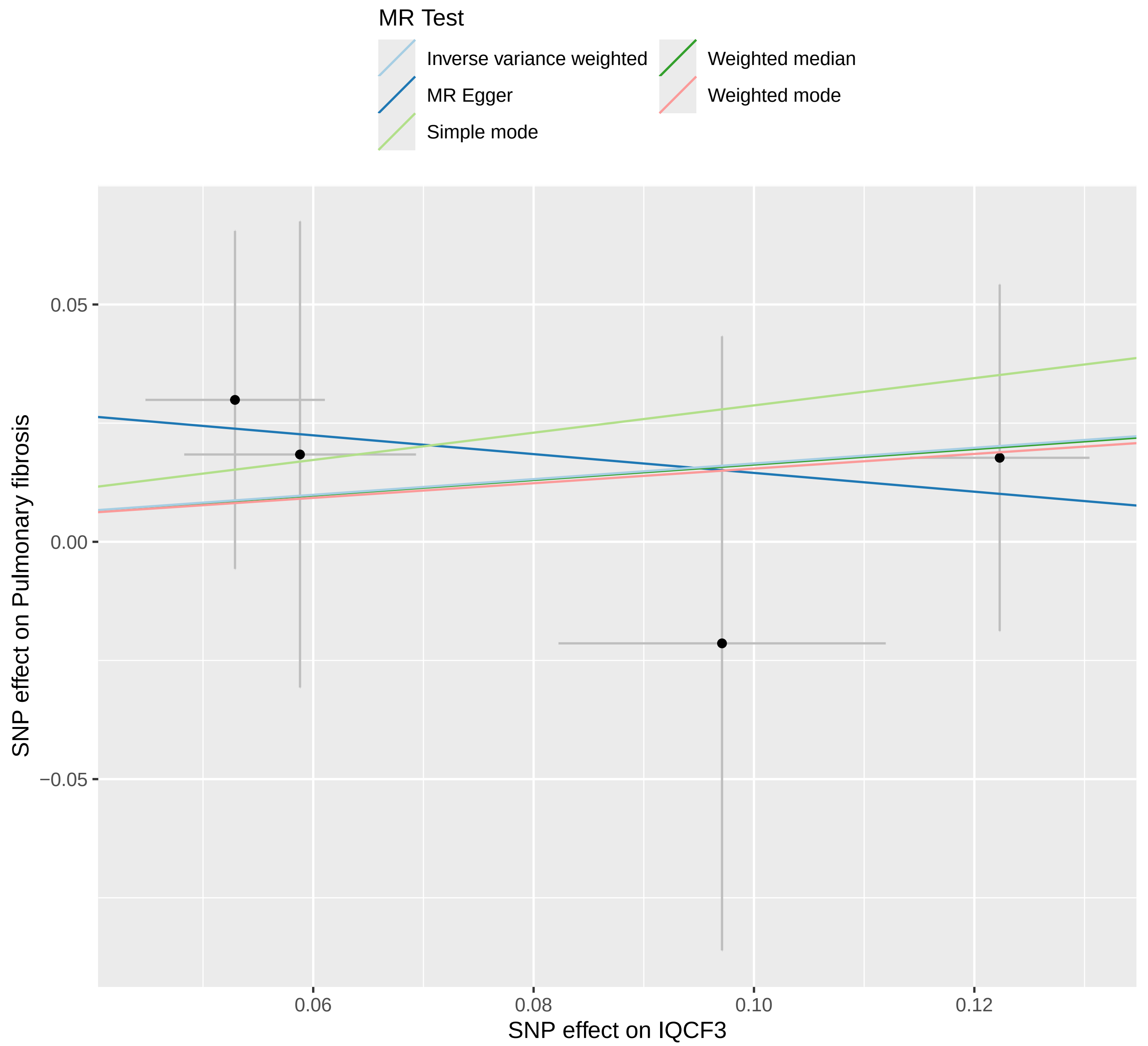
MR Test
Inverse variance weighted
MR Egger
Simple mode
Weighted median
Weighted mode
0.05
0.00
−0.05
0.06
0.08
0.10
0.12
SNP effect on IQCF3
SNP effect on Pulmonary fibrosis

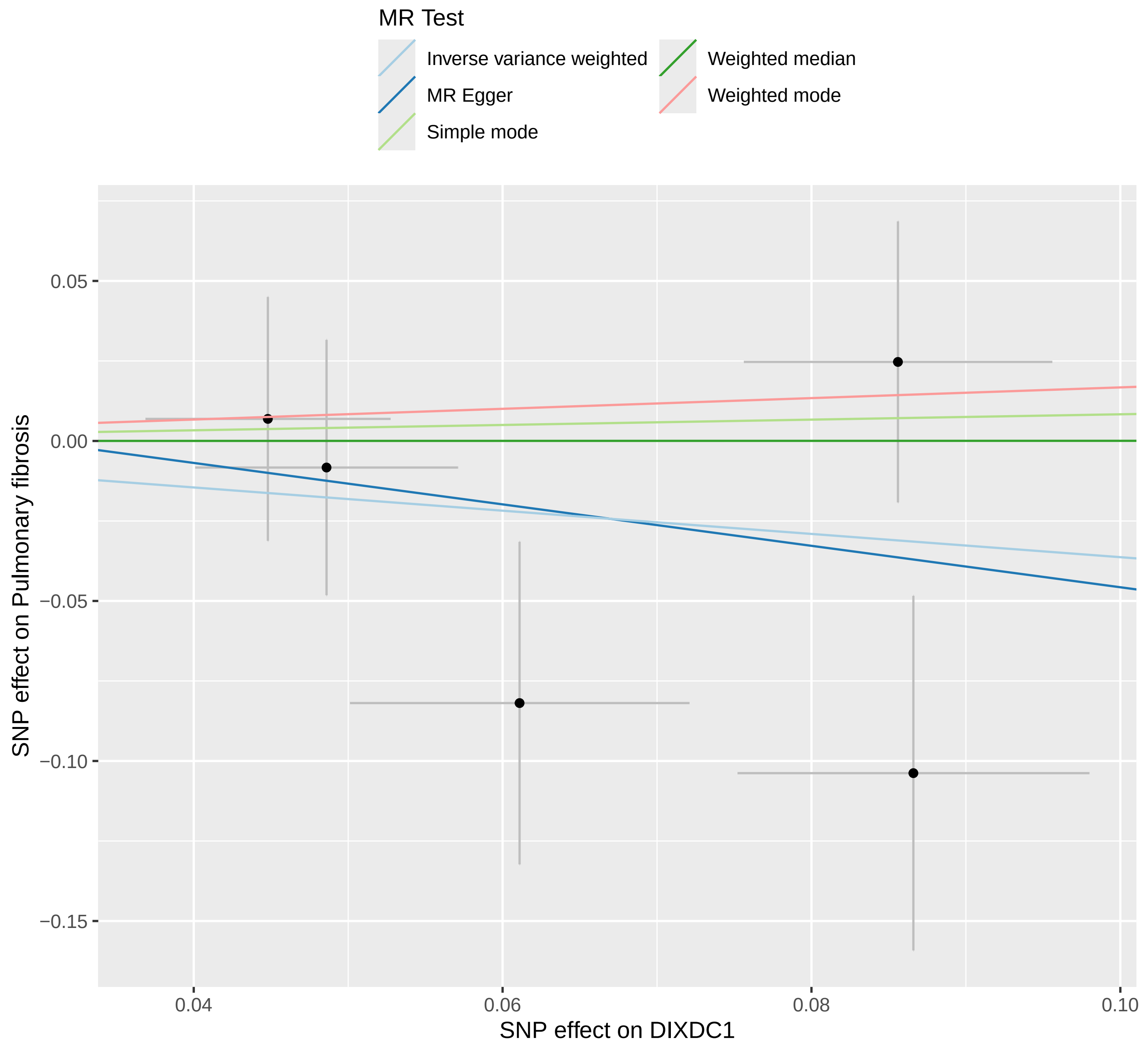
MR Test
Inverse variance weighted
MR Egger
Simple mode
Weighted median
Weighted mode
0.05
0.00
−0.05
−0.10
−0.15
SNP effect on Pulmonary fibrosis
0.04
0.06
0.08
0.10
SNP effect on DIXDC1

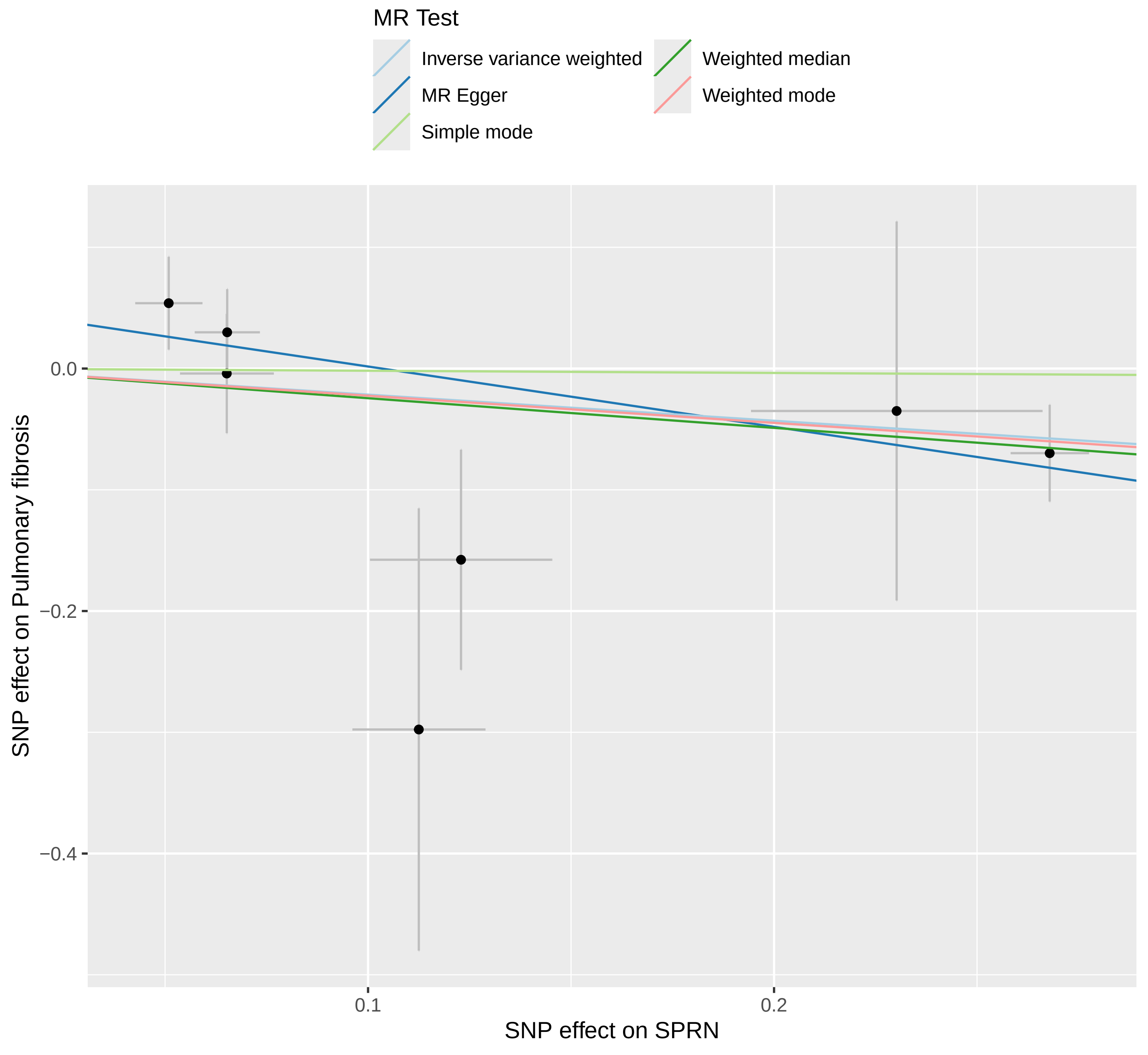
MR Test
Inverse variance weighted
MR Egger
Simple mode
Weighted median
Weighted mode
0.0
−0.2
−0.4
0.1
0.2
SNP effect on Pulmonary fibrosis
SNP effect on SPRN

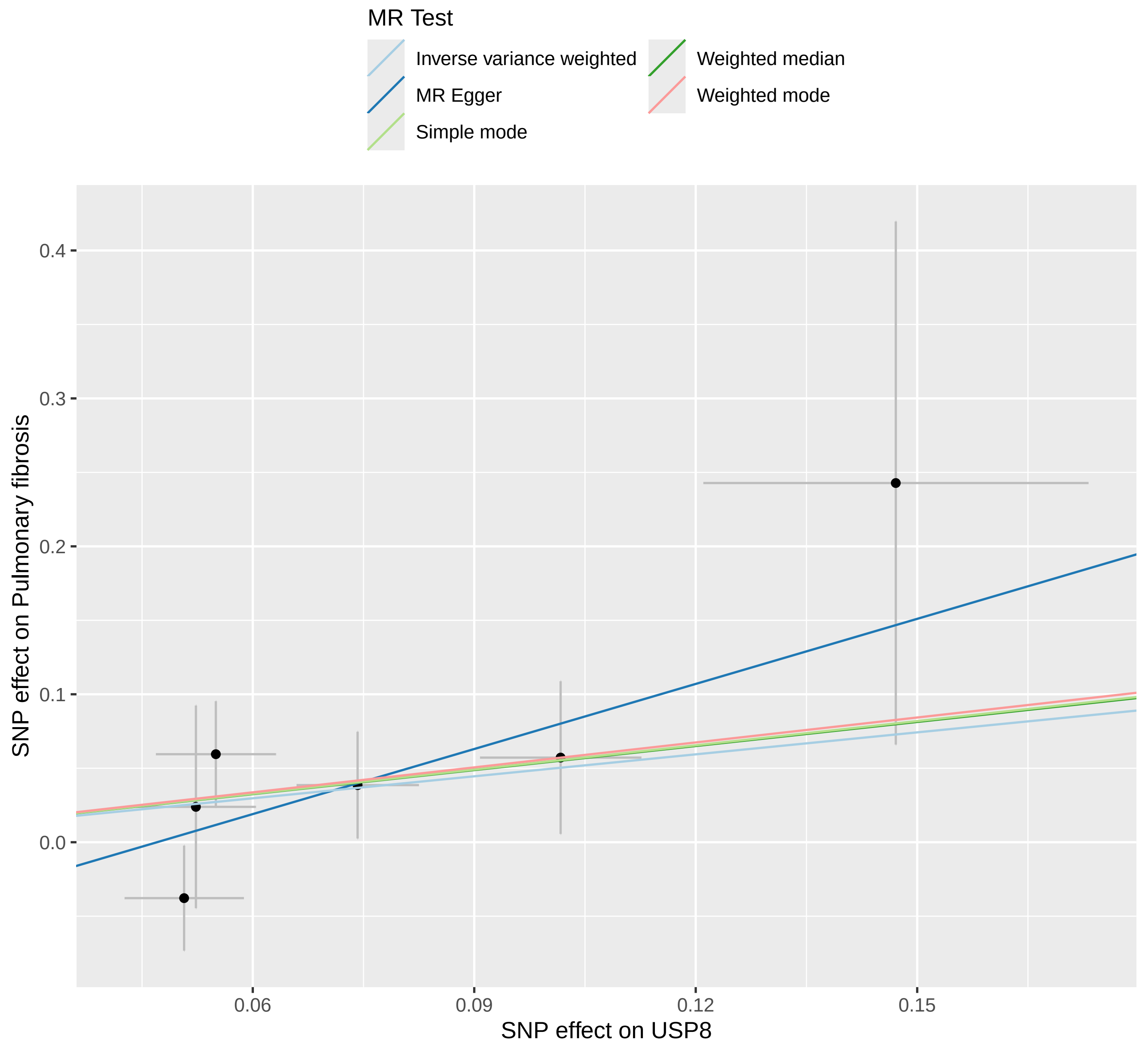
MR Test
Inverse variance weighted
MR Egger
Simple mode
Weighted median
Weighted mode
SNP effect on Pulmonary fibrosis
0.4
0.3
0.2
0.1
0.0
0.06
0.09
0.12
0.15
SNP effect on USP8

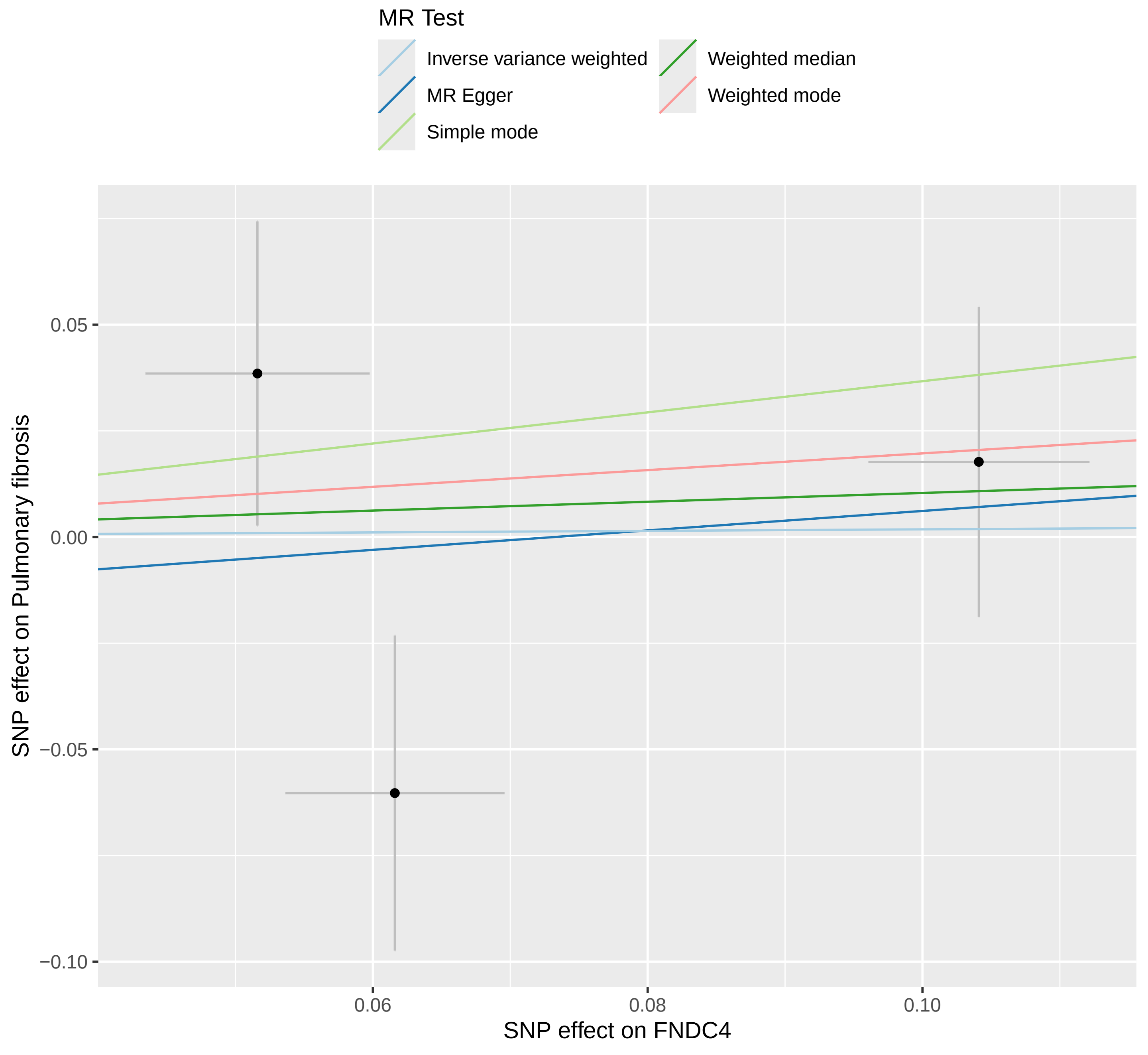
MR Test
Inverse variance weighted
MR Egger
Simple mode
Weighted median
Weighted mode
SNP effect on Pulmonary fibrosis
0.05
0.00
−0.05
−0.10
0.06
0.08
0.10
SNP effect on FNDC4

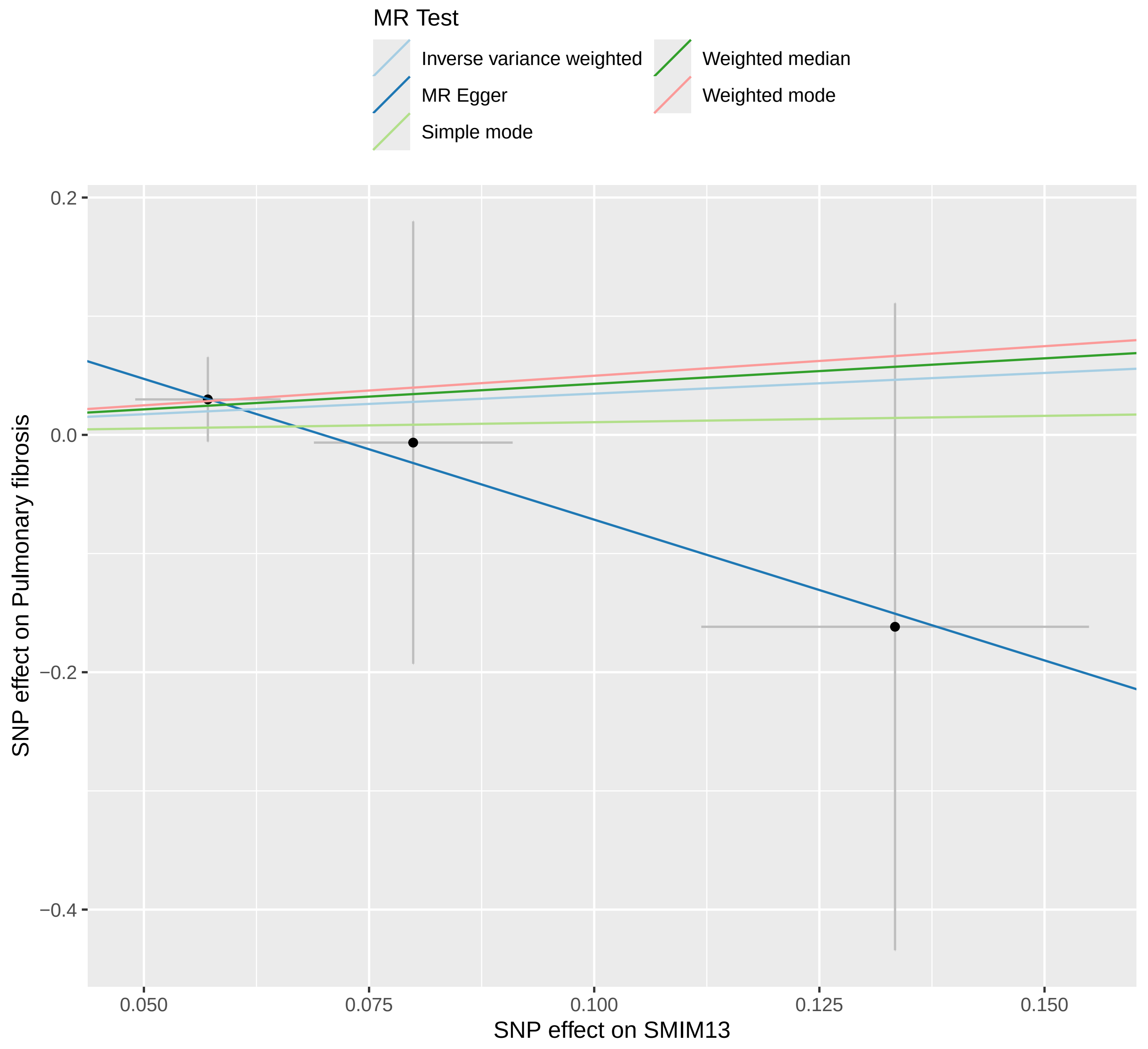

MR Test
Inverse variance weighted
MR Egger
Simple mode
Weighted median
Weighted mode
0.2
0.0
−0.2
−0.4
0.050
0.075
0.100
0.125
0.150
SNP effect on Pulmonary fibrosis
SNP effect on SMIM13

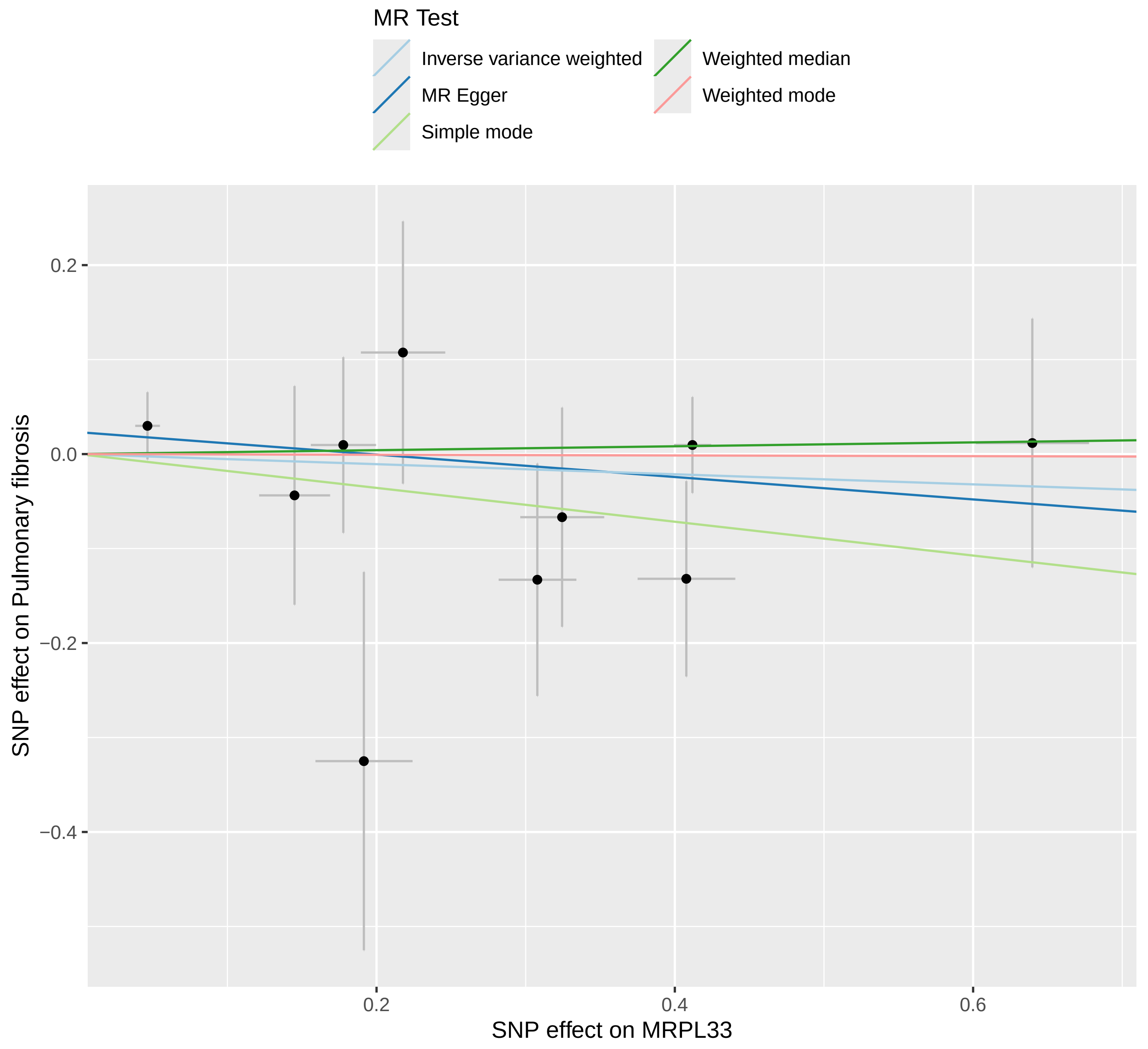
MR Test
Inverse variance weighted
MR Egger
Simple mode
Weighted median
Weighted mode
0.2
0.0
−0.2
−0.4
SNP effect on Pulmonary fibrosis
0.2
0.4
0.6
SNP effect on MRPL33

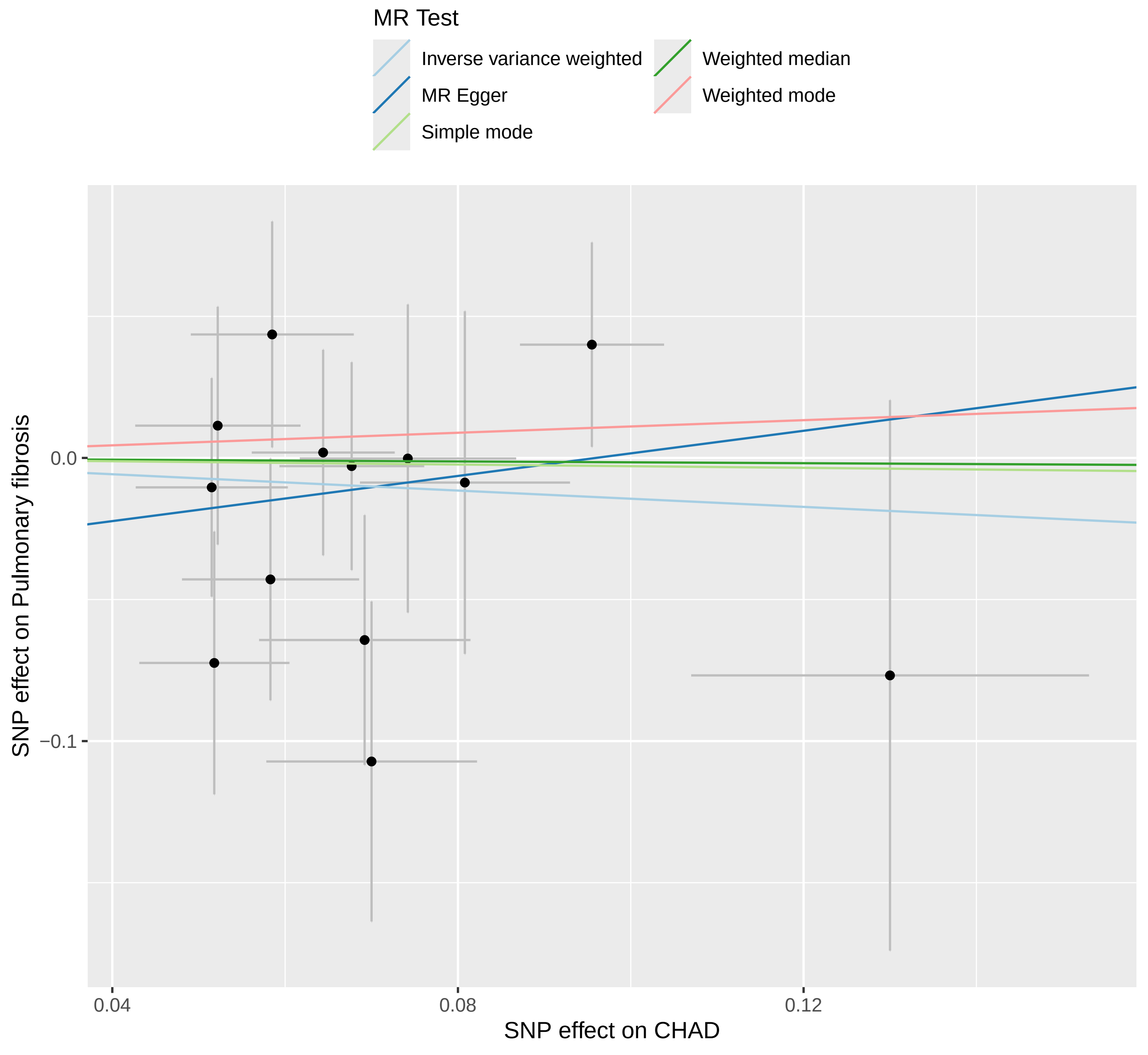
MR Test
Inverse variance weighted
MR Egger
Simple mode
Weighted median
Weighted mode
SNP effect on Pulmonary fibrosis
0.0
−0.1
0.04
0.08
0.12
SNP effect on CHAD

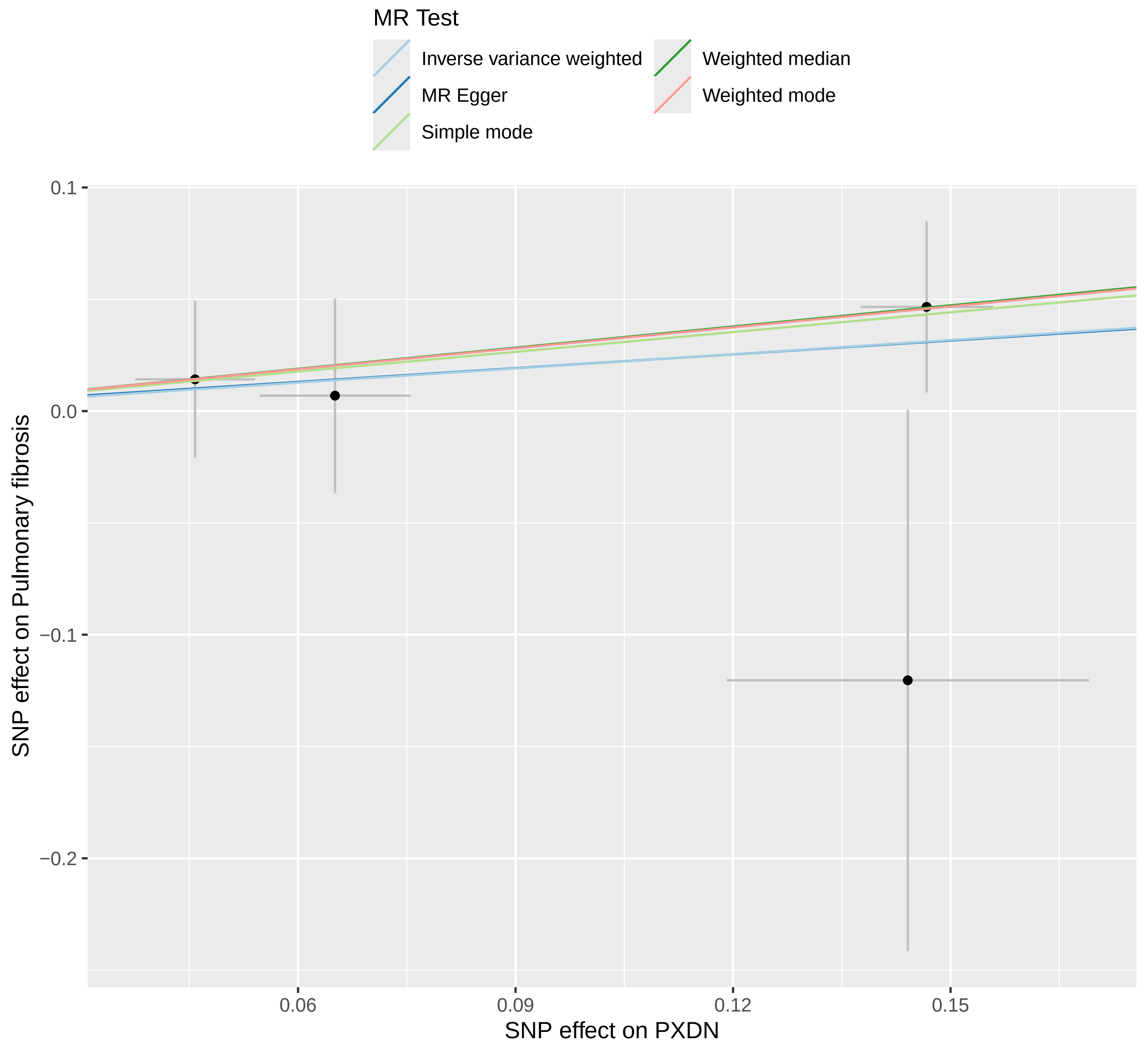
MR Test
Inverse variance weighted
MR Egger
Simple mode
Weighted median
Weighted mode
0.1
0.0
−0.1
−0.2
SNP effect on Pulmonary fibrosis
0.06
0.09
0.12
0.15
SNP effect on PXDN

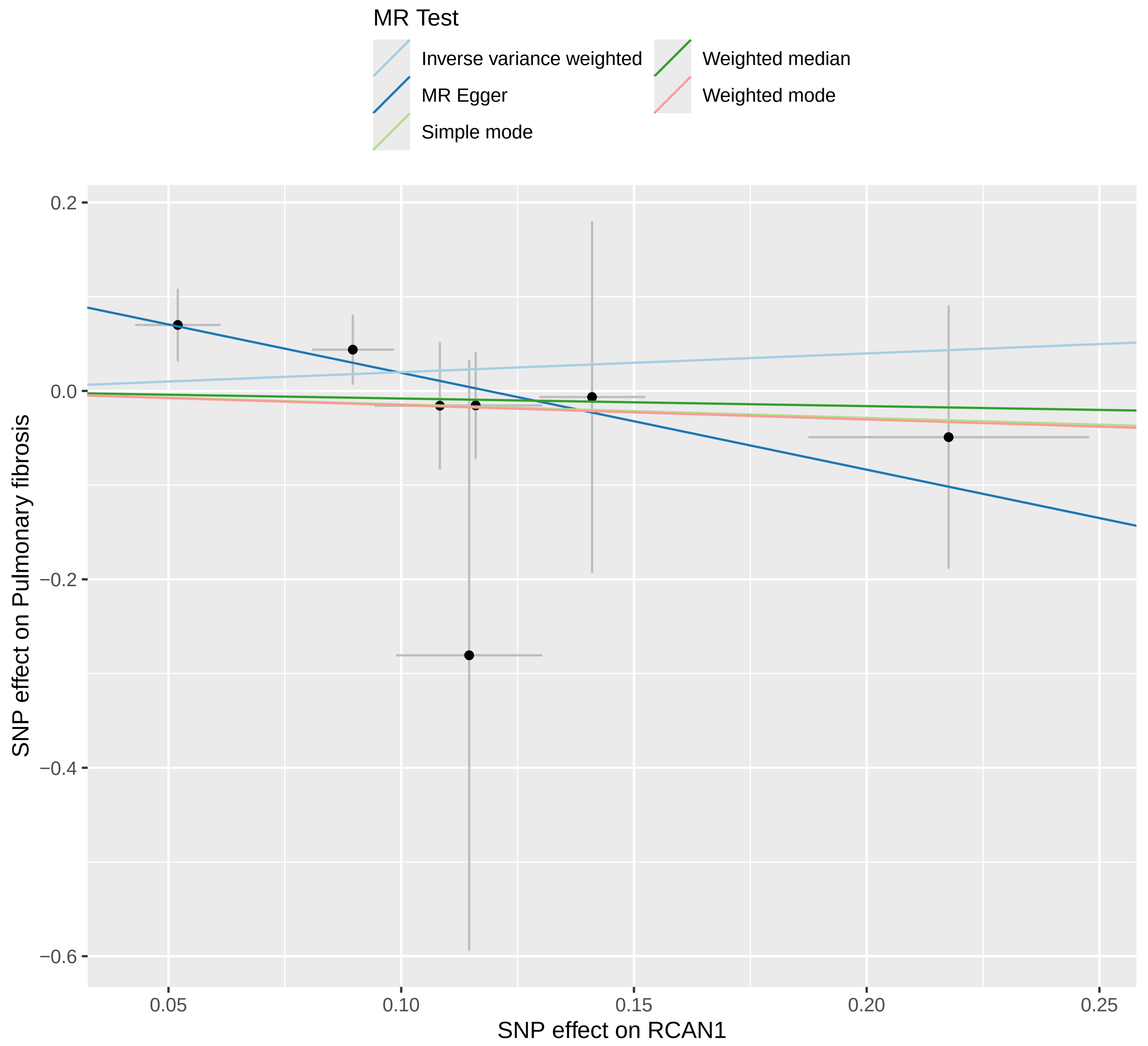
MR Test
Inverse variance weighted
MR Egger
Simple mode
Weighted median
Weighted mode
0.2
0.0
−0.2
−0.4
−0.6
SNP effect on Pulmonary fibrosis
0.05
0.10
0.15
0.20
0.25
SNP effect on RCAN1

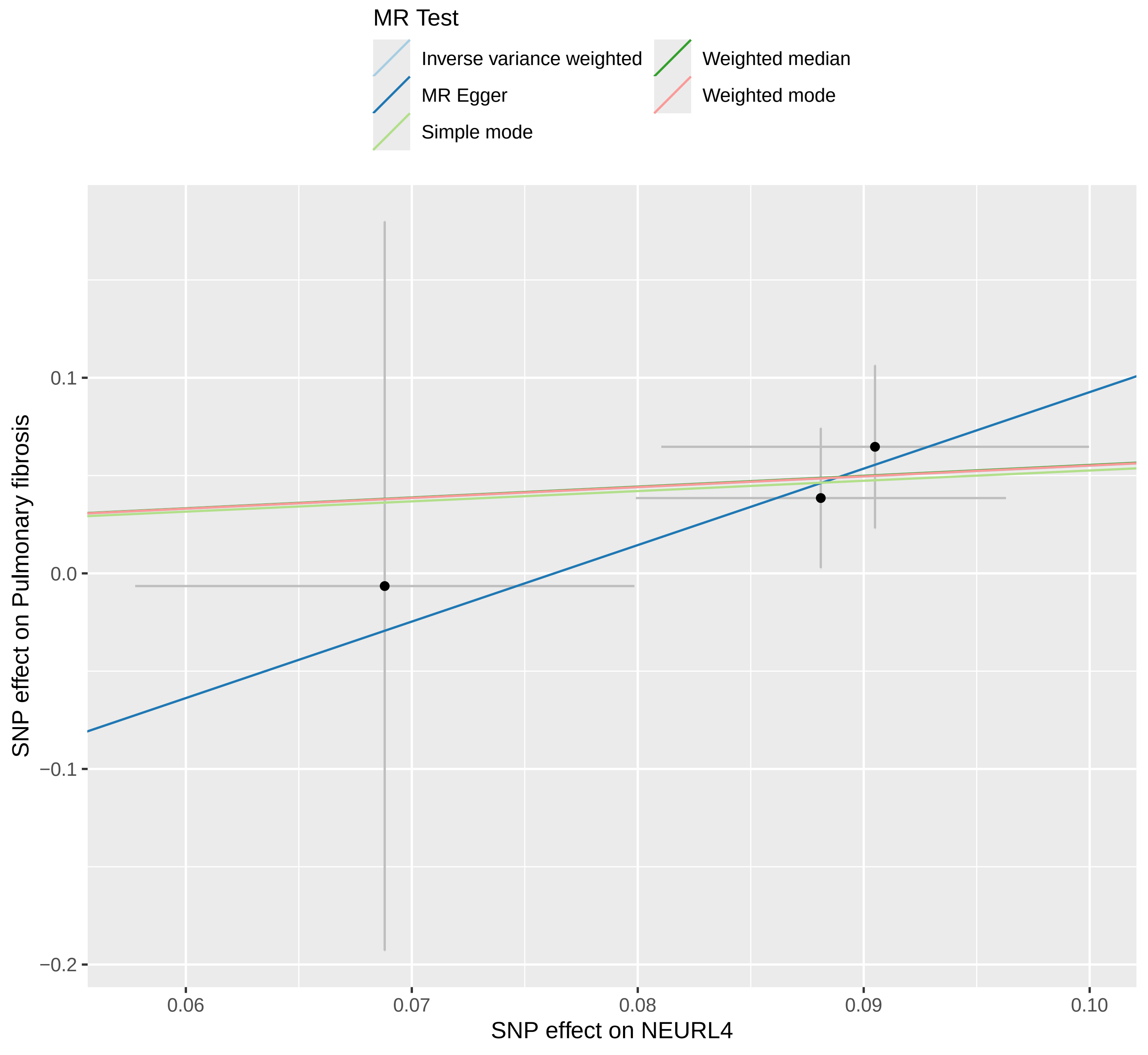
MR Test
Inverse variance weighted
MR Egger
Simple mode
Weighted median
Weighted mode
0.1
0.0
−0.1
−0.2
0.06
0.07
0.08
0.09
0.10
SNP effect on Pulmonary fibrosis
SNP effect on NEURL4

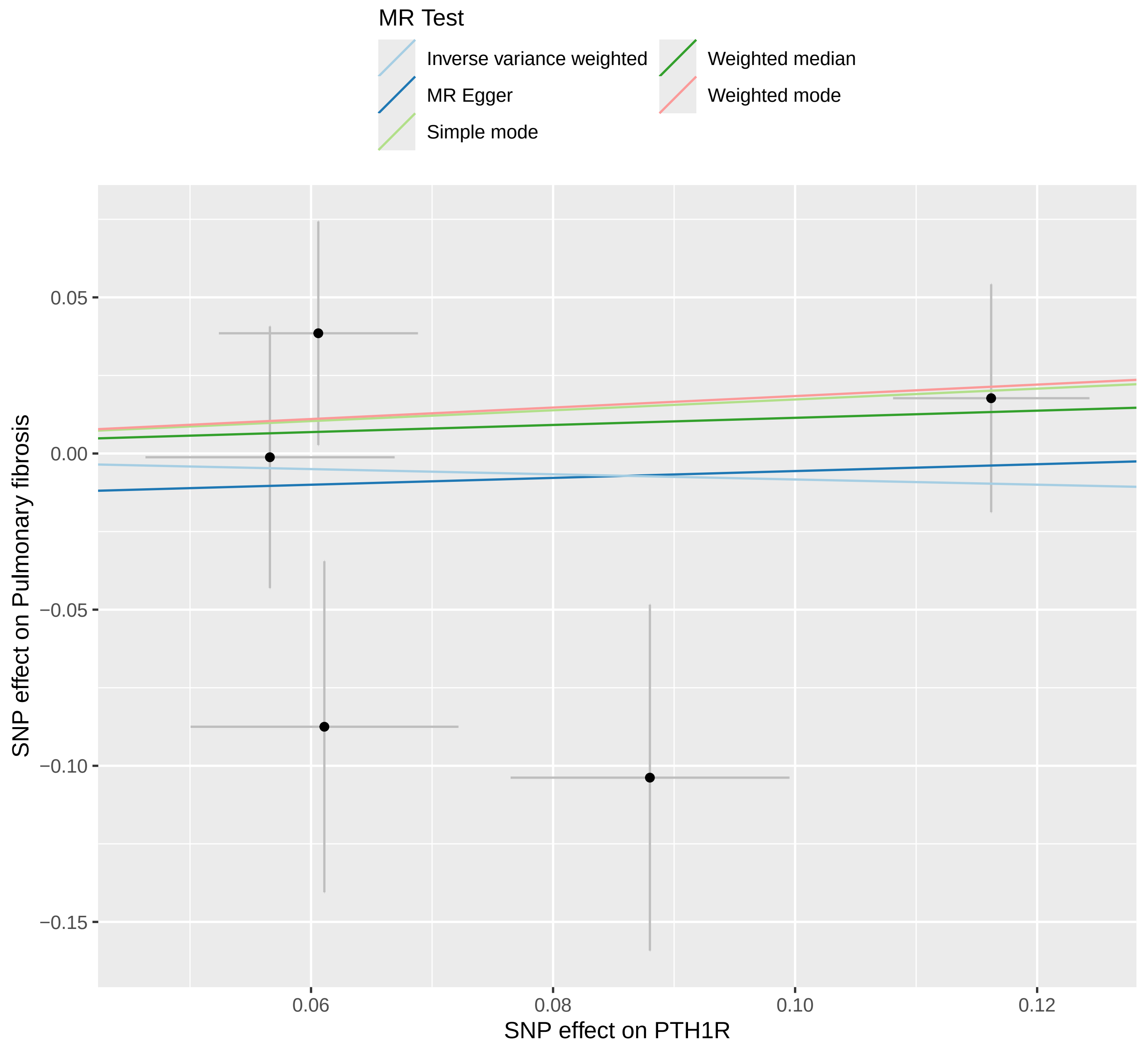
MR Test
Inverse variance weighted
MR Egger
Simple mode
Weighted median
Weighted mode
0.05
0.00
−0.05
−0.10
−0.15
0.06
0.08
0.10
0.12
SNP effect on Pulmonary fibrosis
SNP effect on PTH1R

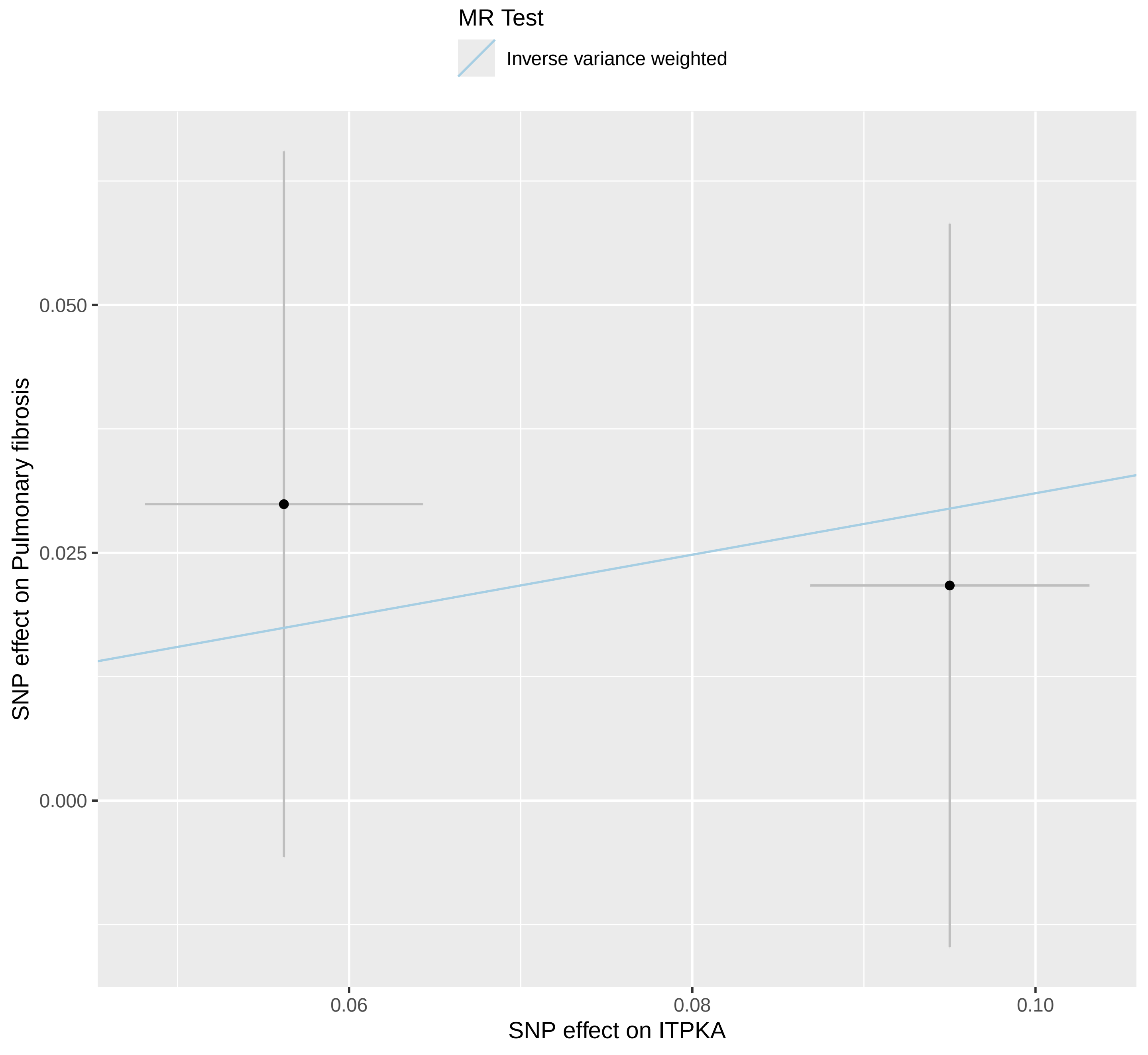

MR Test
Inverse variance weighted
SNP effect on Pulmonary fibrosis
0.050
0.025
0.000
0.06
0.08
0.10
SNP effect on ITPKA

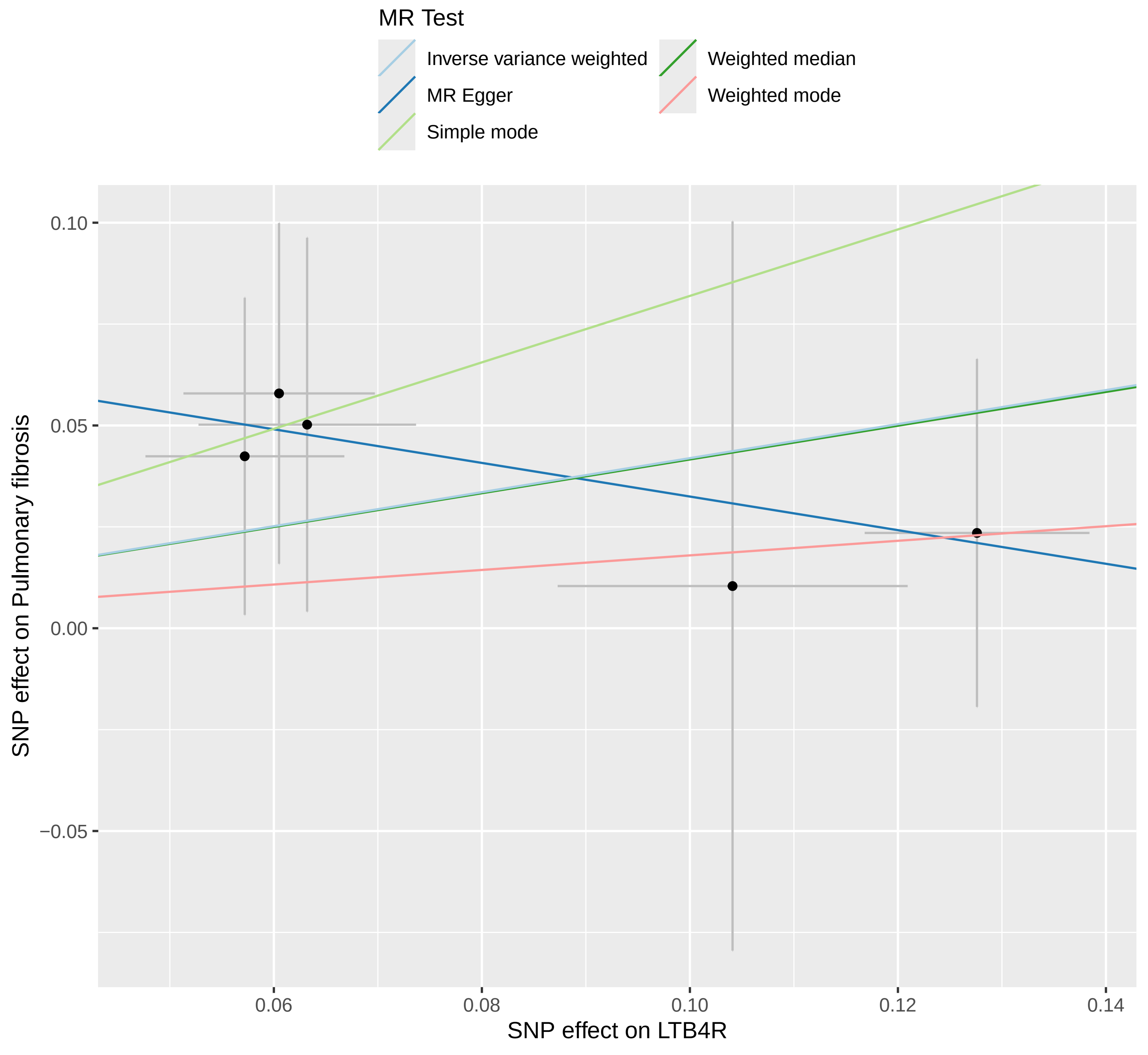

MR Test
Inverse variance weighted
MR Egger
Simple mode
Weighted median
Weighted mode
0.10
0.05
0.00
−0.05
0.06
0.08
0.10
0.12
0.14
SNP effect on LTB4R
SNP effect on Pulmonary fibrosis

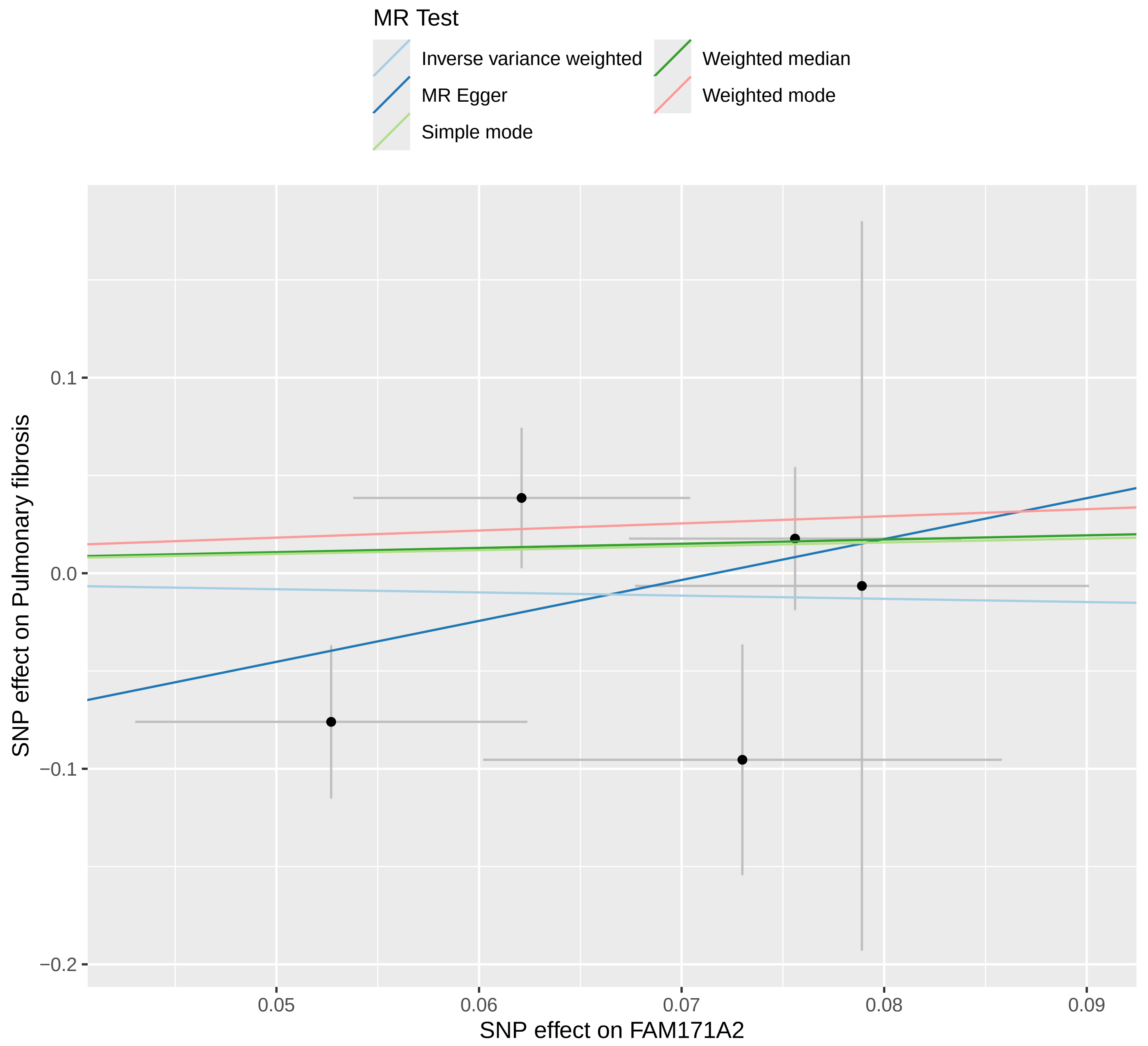
MR Test
Inverse variance weighted
MR Egger
Simple mode
Weighted median
Weighted mode
0.1
0.0
−0.1
−0.2
SNP effect on Pulmonary fibrosis
0.05
0.06
0.07
0.08
0.09
SNP effect on FAM171A2

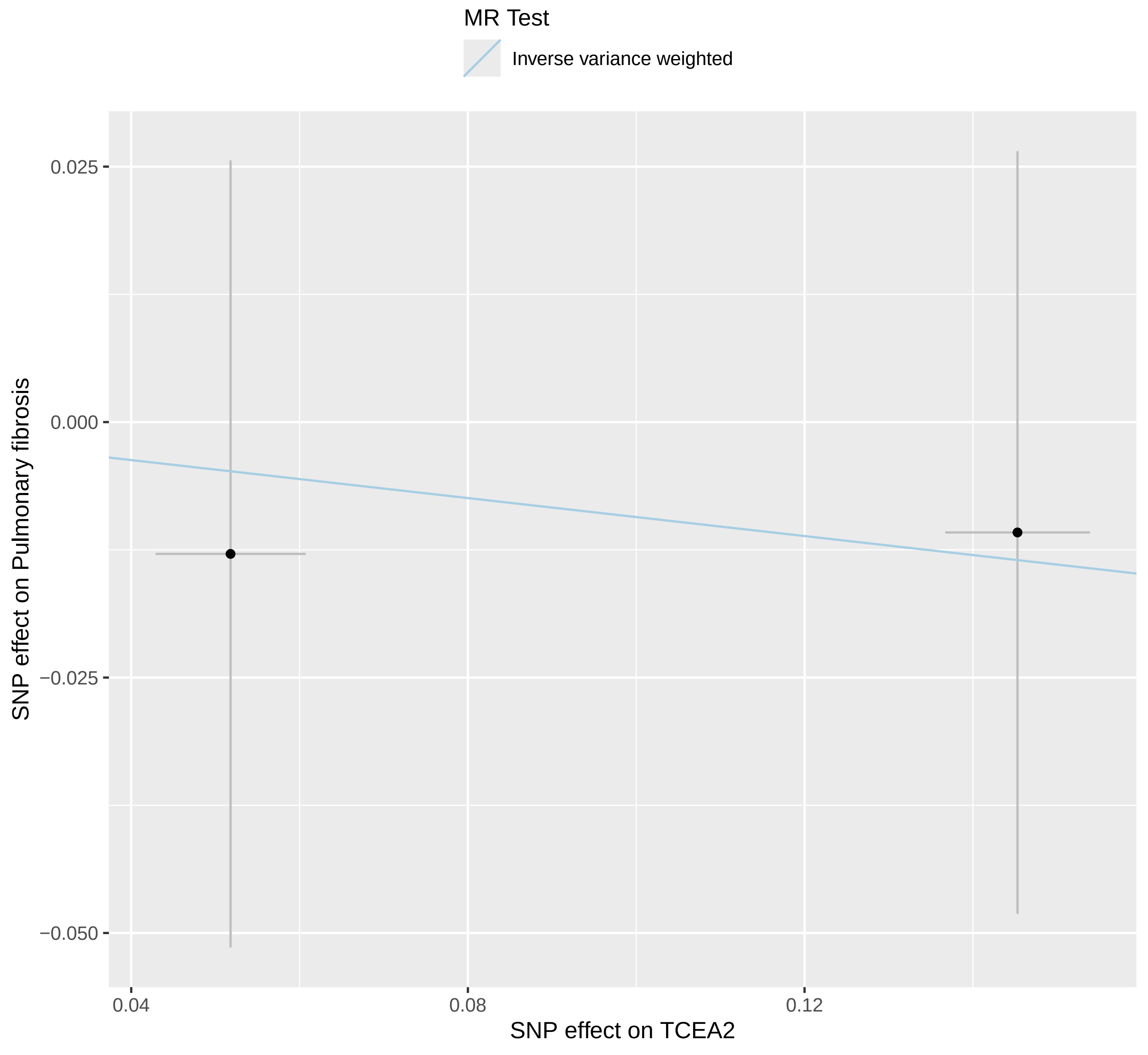

MR Test
Inverse variance weighted
0.025
0.000
−0.025
−0.050
SNP effect on Pulmonary fibrosis
0.04
0.08
0.12
SNP effect on TCEA2

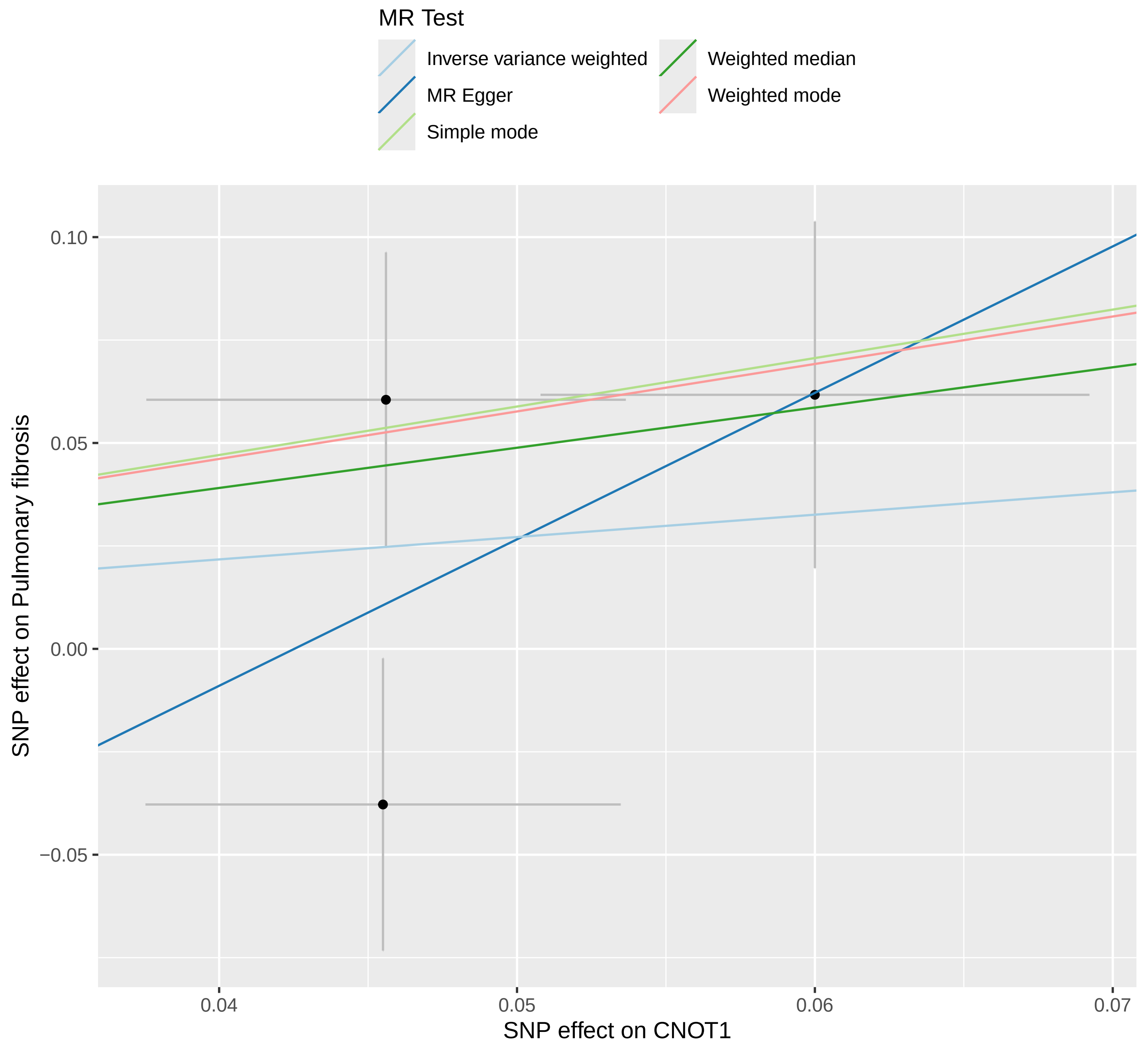

MR Test
Inverse variance weighted
MR Egger
Simple mode
Weighted median
Weighted mode
SNP effect on Pulmonary fibrosis
0.10
0.05
0.00
−0.05
0.04
0.05
0.06
0.07
SNP effect on CNOT1

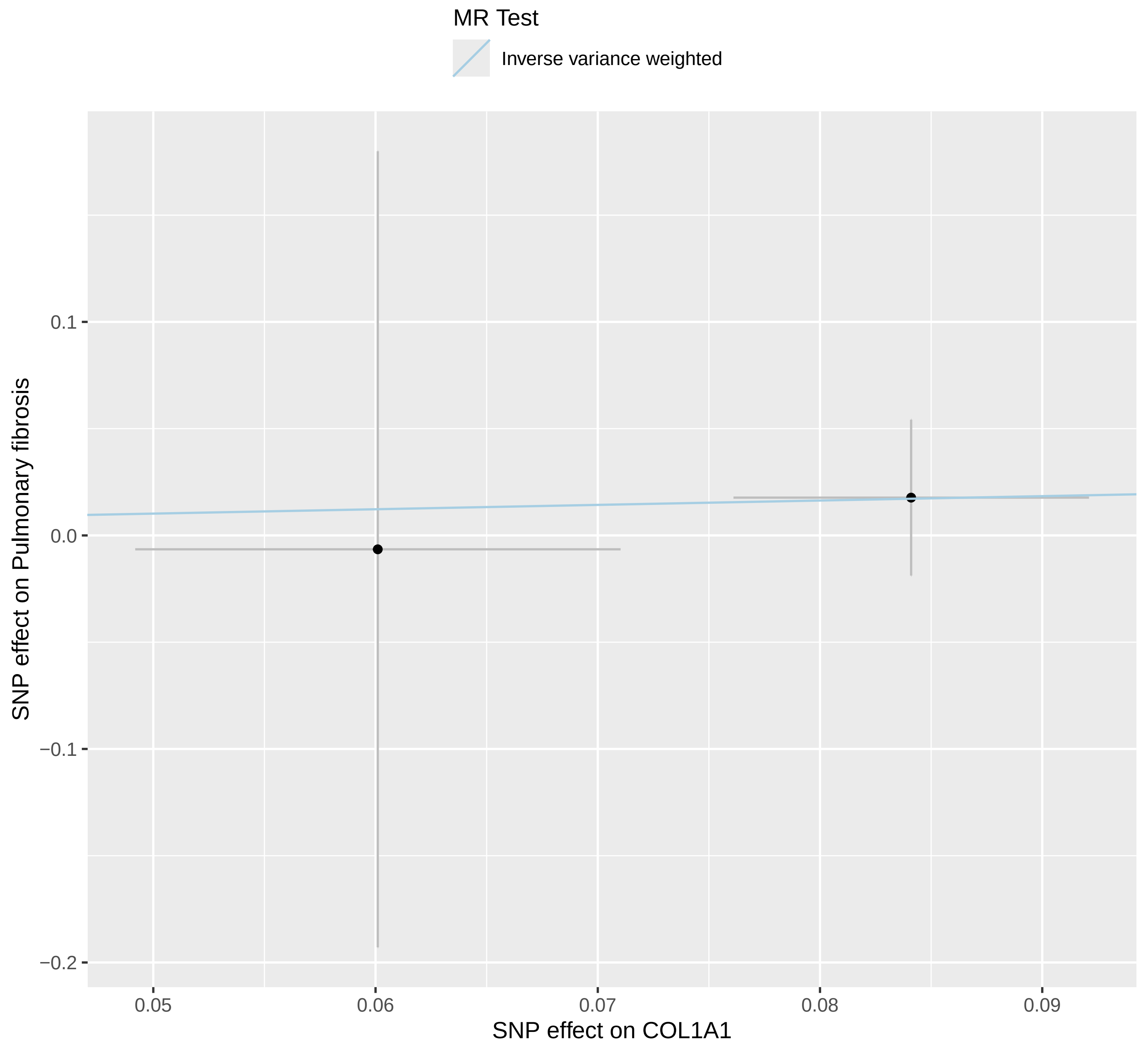
MR Test
Inverse variance weighted
0.1
0.0
−0.1
−0.2
0.05
0.06
0.07
0.08
0.09
SNP effect on Pulmonary fibrosis
SNP effect on COL1A1

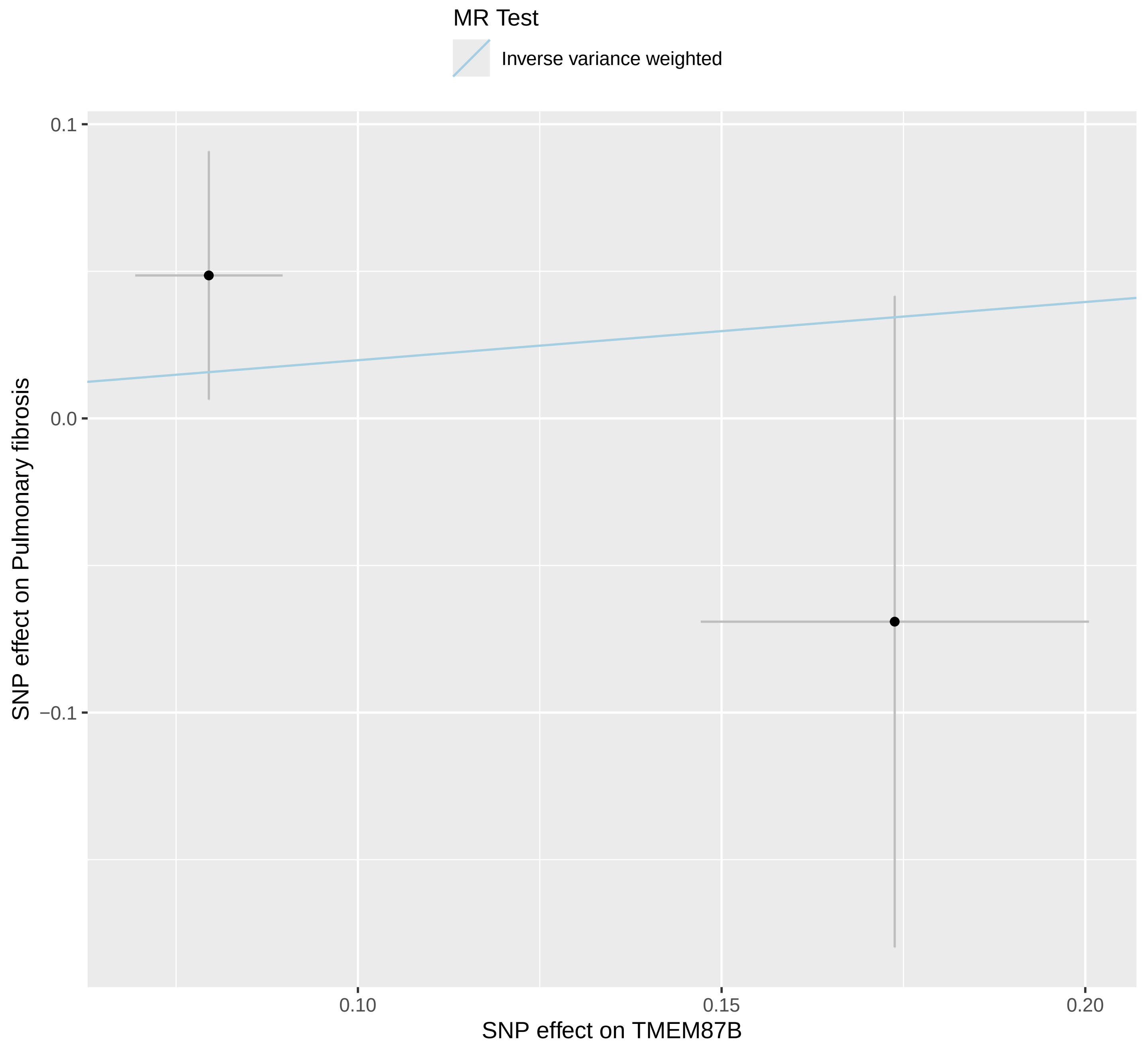

MR Test
Inverse variance weighted
0.1
0.0
−0.1
SNP effect on Pulmonary fibrosis
0.10
0.15
0.20
SNP effect on TMEM87B

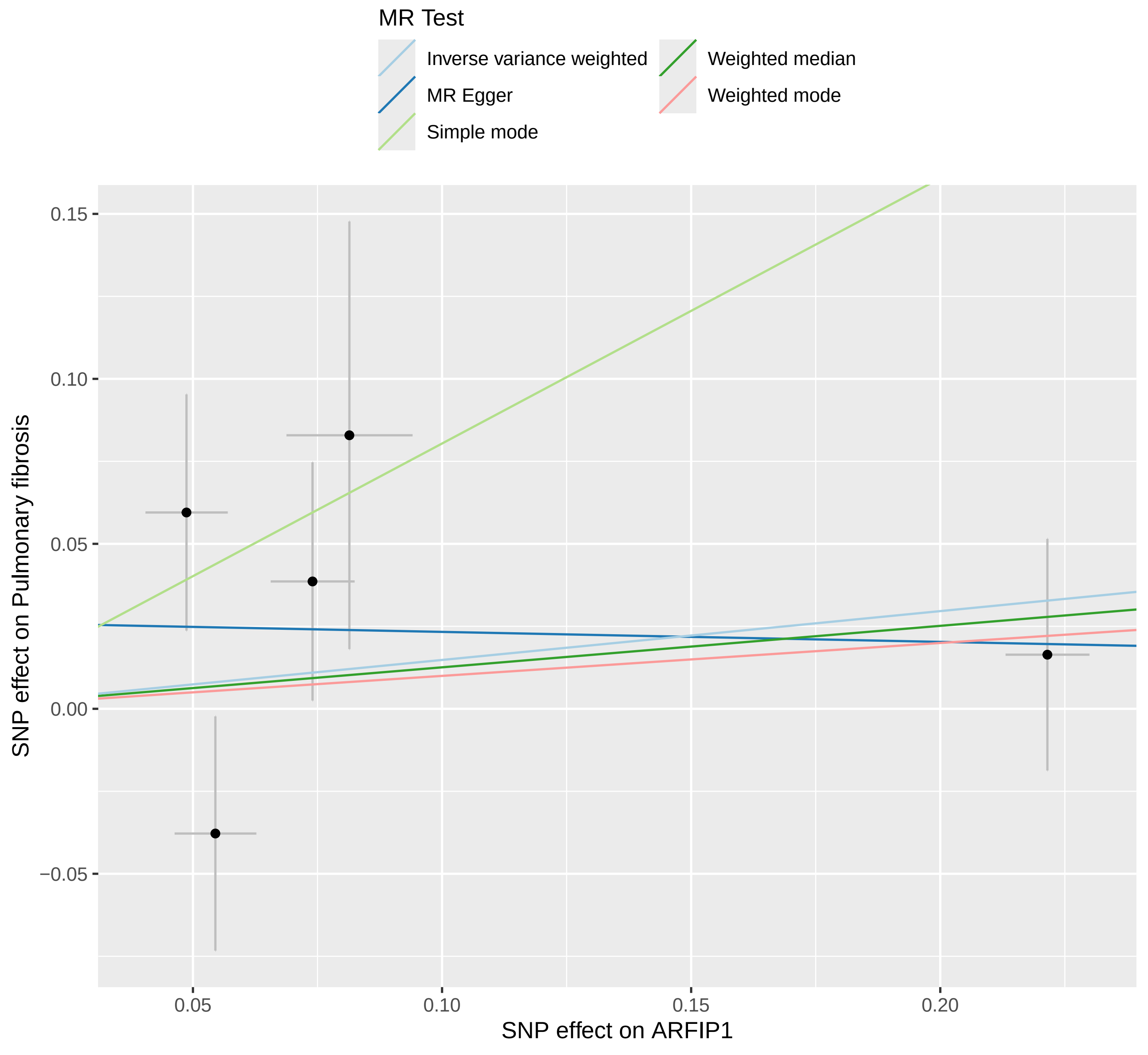

MR Test
Inverse variance weighted
MR Egger
Simple mode
Weighted median
Weighted mode
0.15
0.10
0.05
0.00
−0.05
0.05
0.10
0.15
0.20
SNP effect on Pulmonary fibrosis
SNP effect on ARFIP1

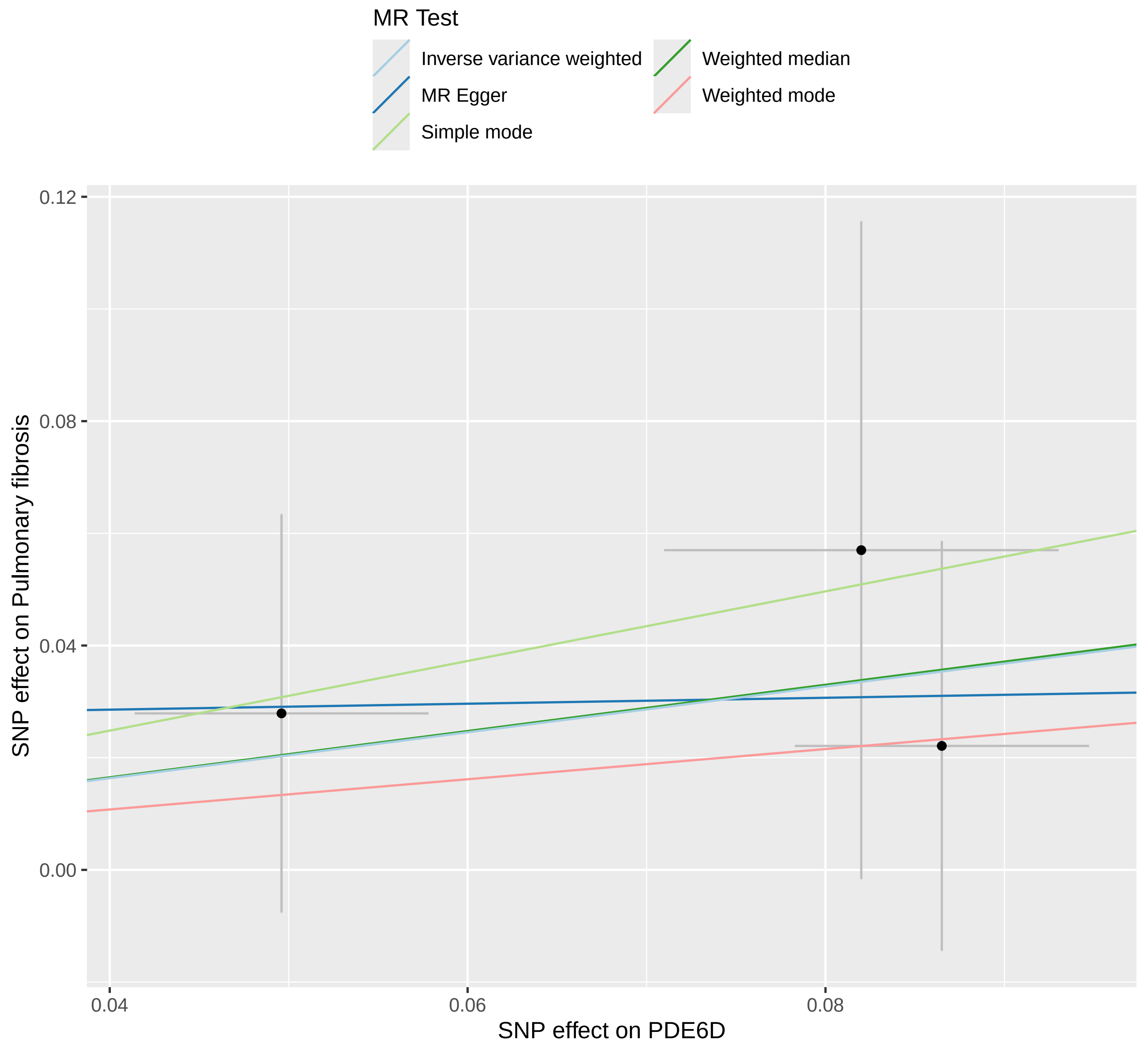
MR Test
Inverse variance weighted
MR Egger
Simple mode
Weighted median
Weighted mode
0.12
0.08
0.04
0.00
SNP effect on Pulmonary fibrosis
0.04
0.06
0.08
SNP effect on PDE6D

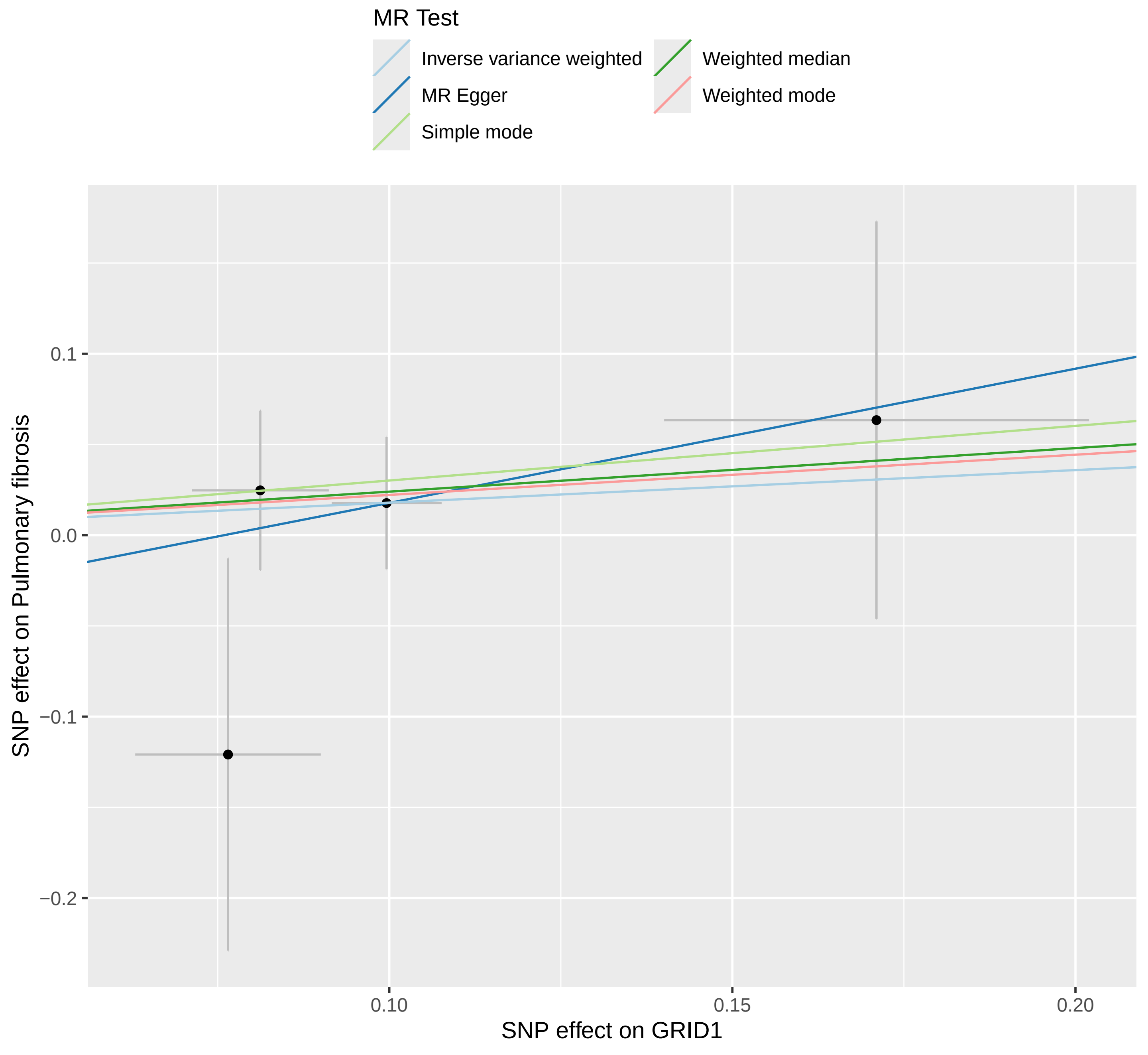
MR Test
Inverse variance weighted
MR Egger
Simple mode
Weighted median
Weighted mode
0.1
0.0
−0.1
−0.2
SNP effect on Pulmonary fibrosis
0.10
0.15
0.20
SNP effect on GRID1

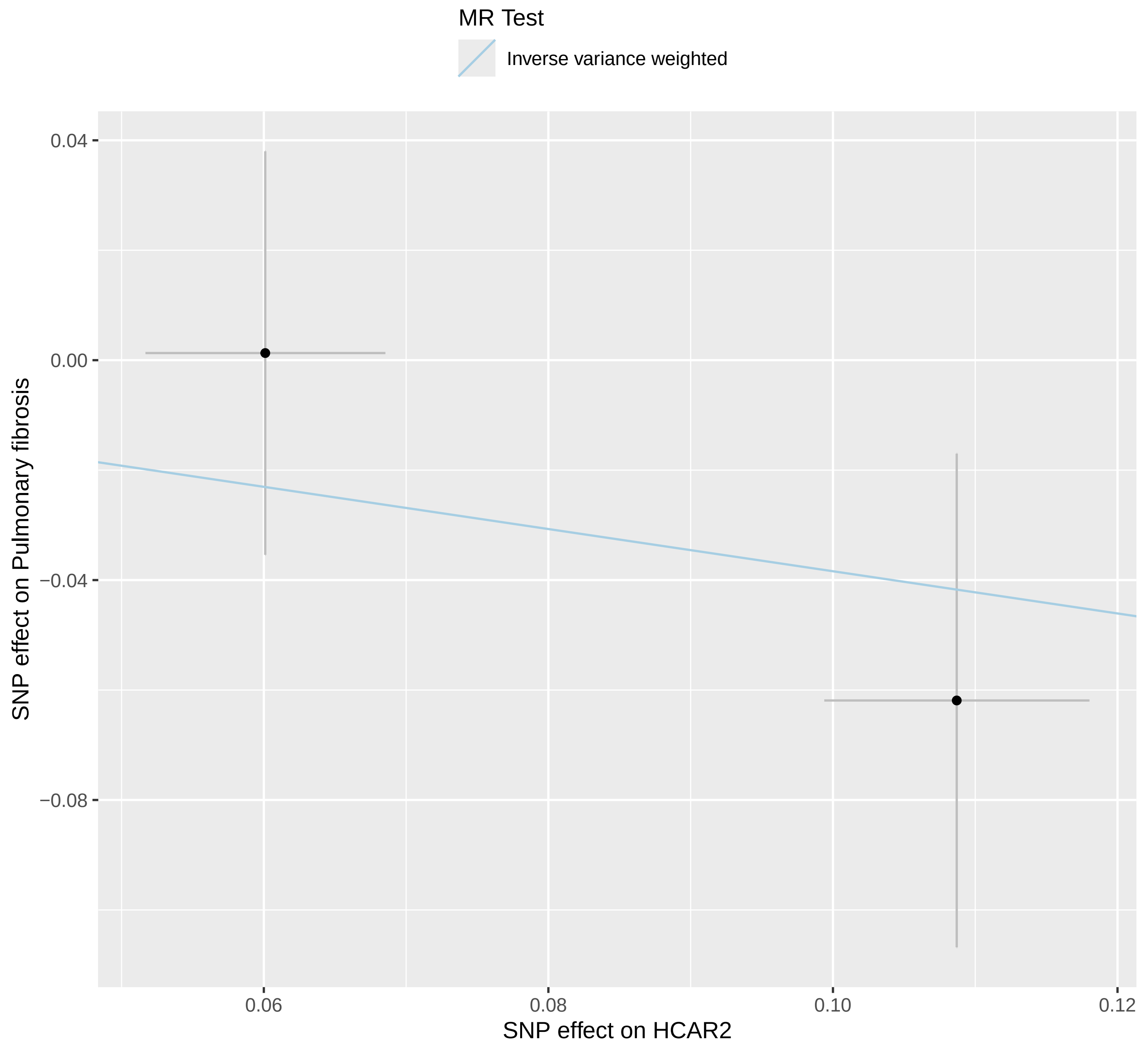

MR Test
Inverse variance weighted
0.04
0.00
−0.04
−0.08
0.06
0.08
0.10
0.12
SNP effect on HCAR2
SNP effect on Pulmonary fibrosis

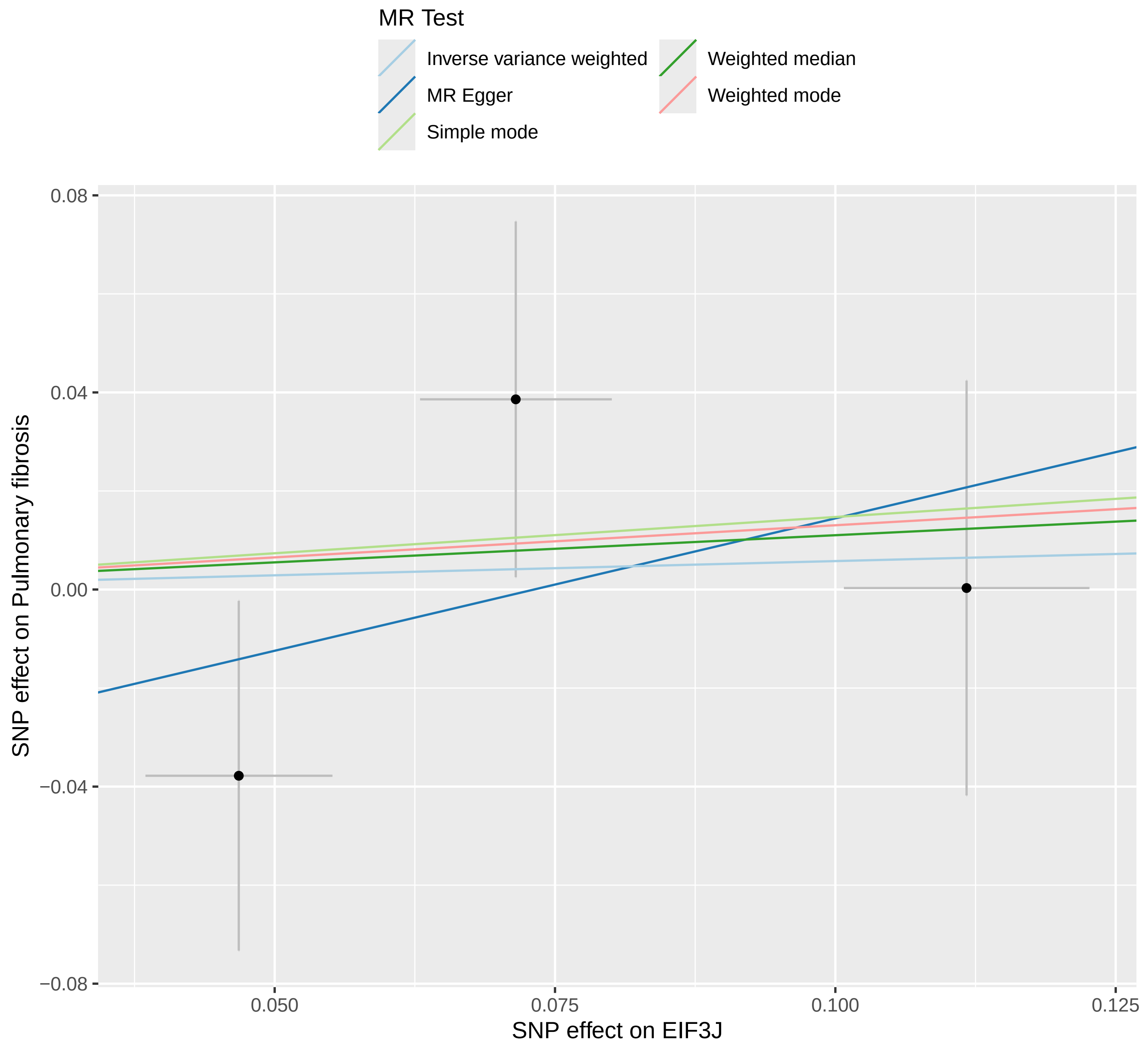

MR Test
Inverse variance weighted
MR Egger
Simple mode
Weighted median
Weighted mode
0.08
0.04
0.00
−0.04
−0.08
0.050
0.075
0.100
0.125
SNP effect on EIF3J
SNP effect on Pulmonary fibrosis

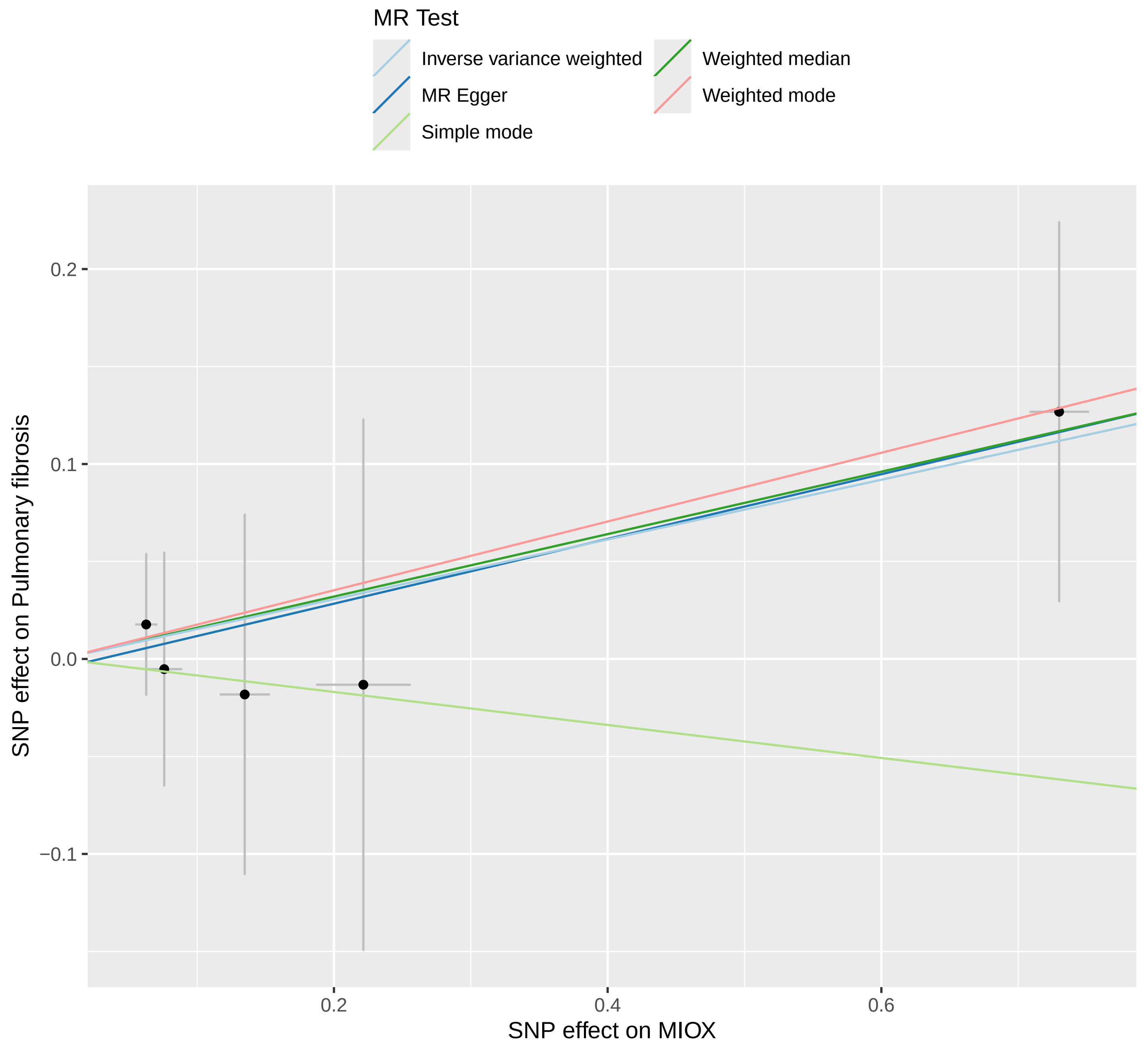
MR Test
Inverse variance weighted
MR Egger
Simple mode
Weighted median
Weighted mode
0.2
0.1
0.0
−0.1
0.2
0.4
0.6
SNP effect on MIOX
SNP effect on Pulmonary fibrosis

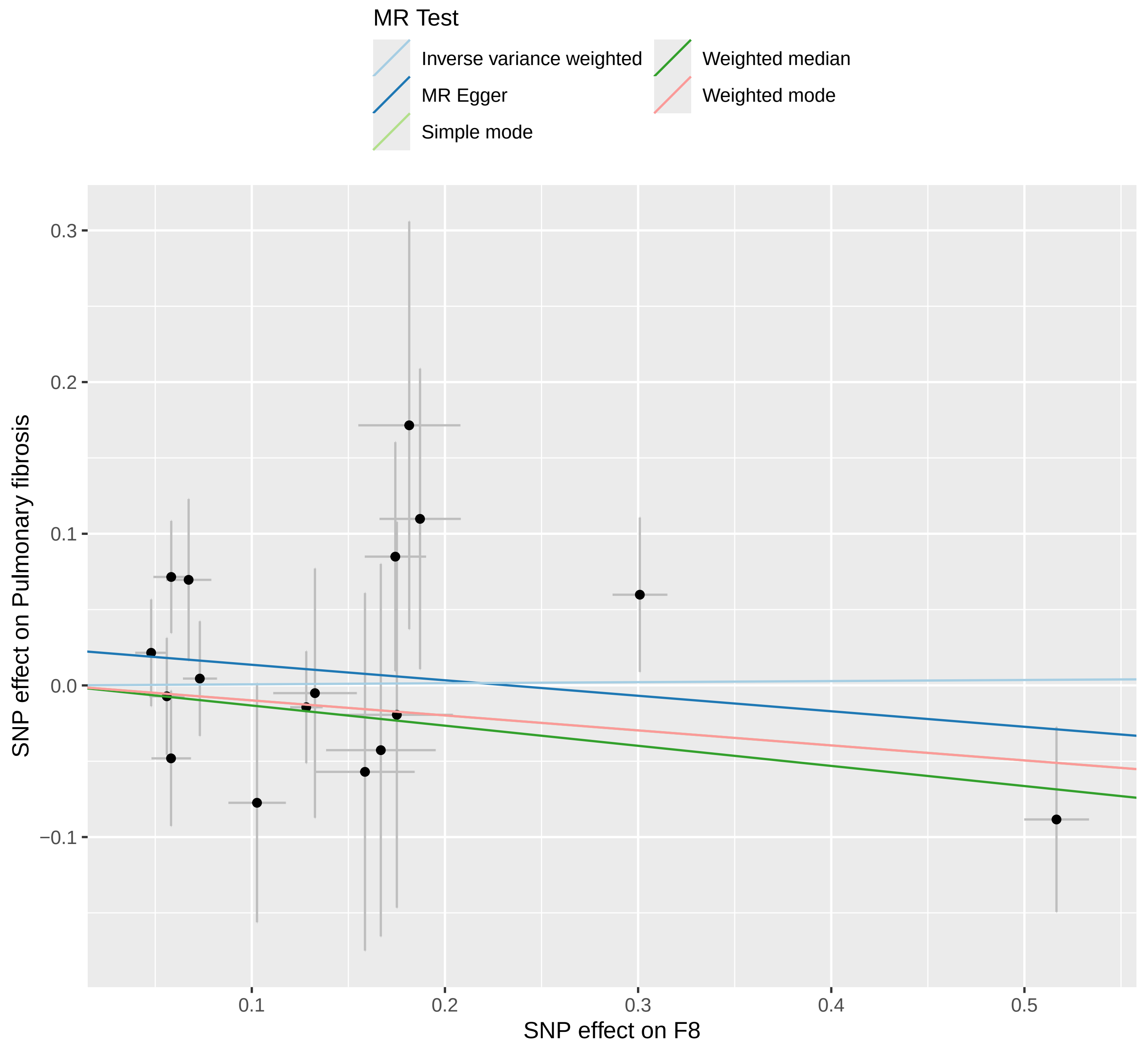

MR Test
Inverse variance weighted
MR Egger
Simple mode
Weighted median
Weighted mode
SNP effect on Pulmonary fibrosis
0.3
0.2
0.1
0.0
−0.1
0.1
0.2
0.3
0.4
0.5
SNP effect on F8

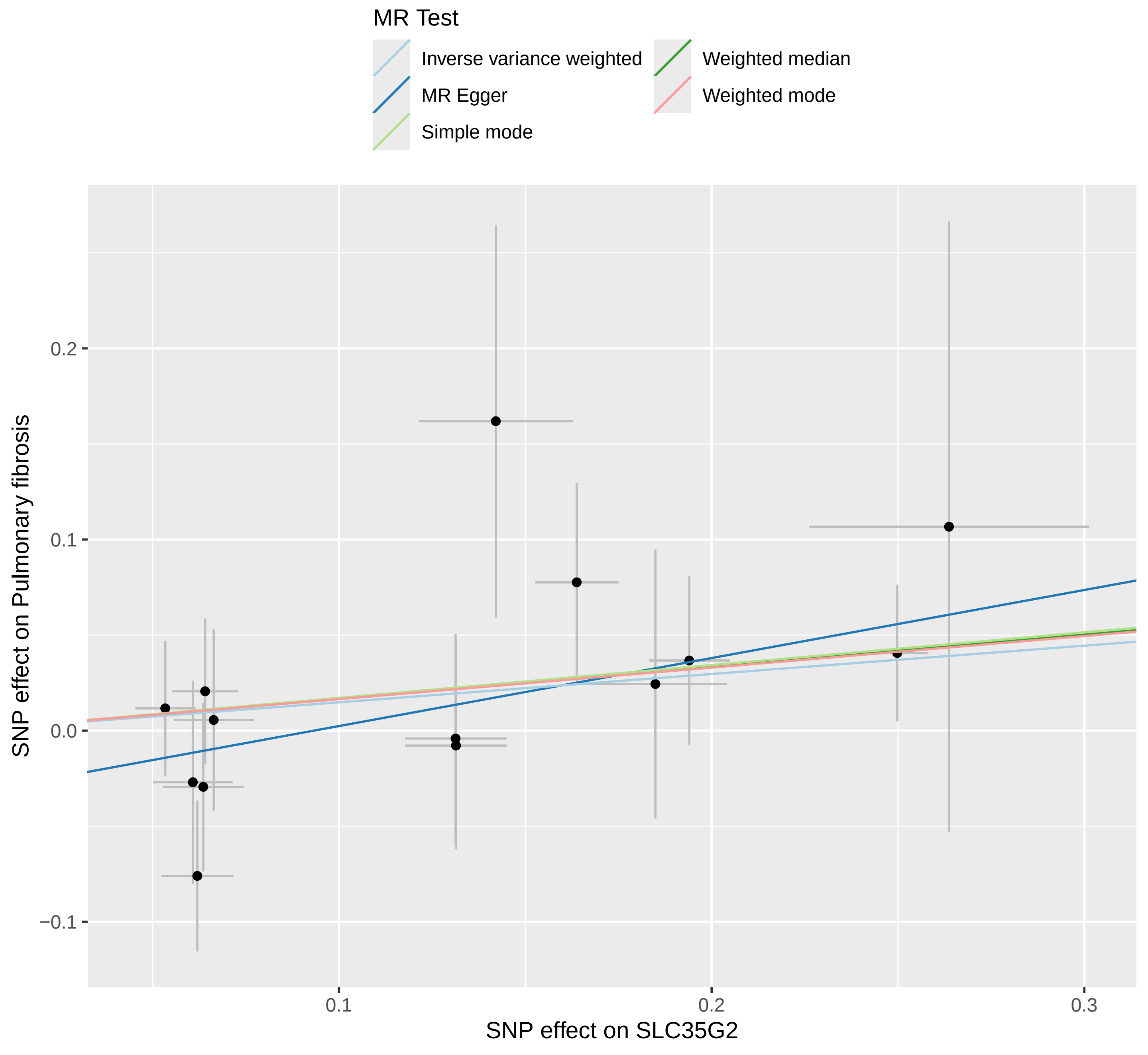
MR Test
Inverse variance weighted
MR Egger
Simple mode
Weighted median
Weighted mode
SNP effect on Pulmonary fibrosis
0.2
0.1
0.0
−0.1
0.1
0.2
0.3
SNP effect on SLC35G2

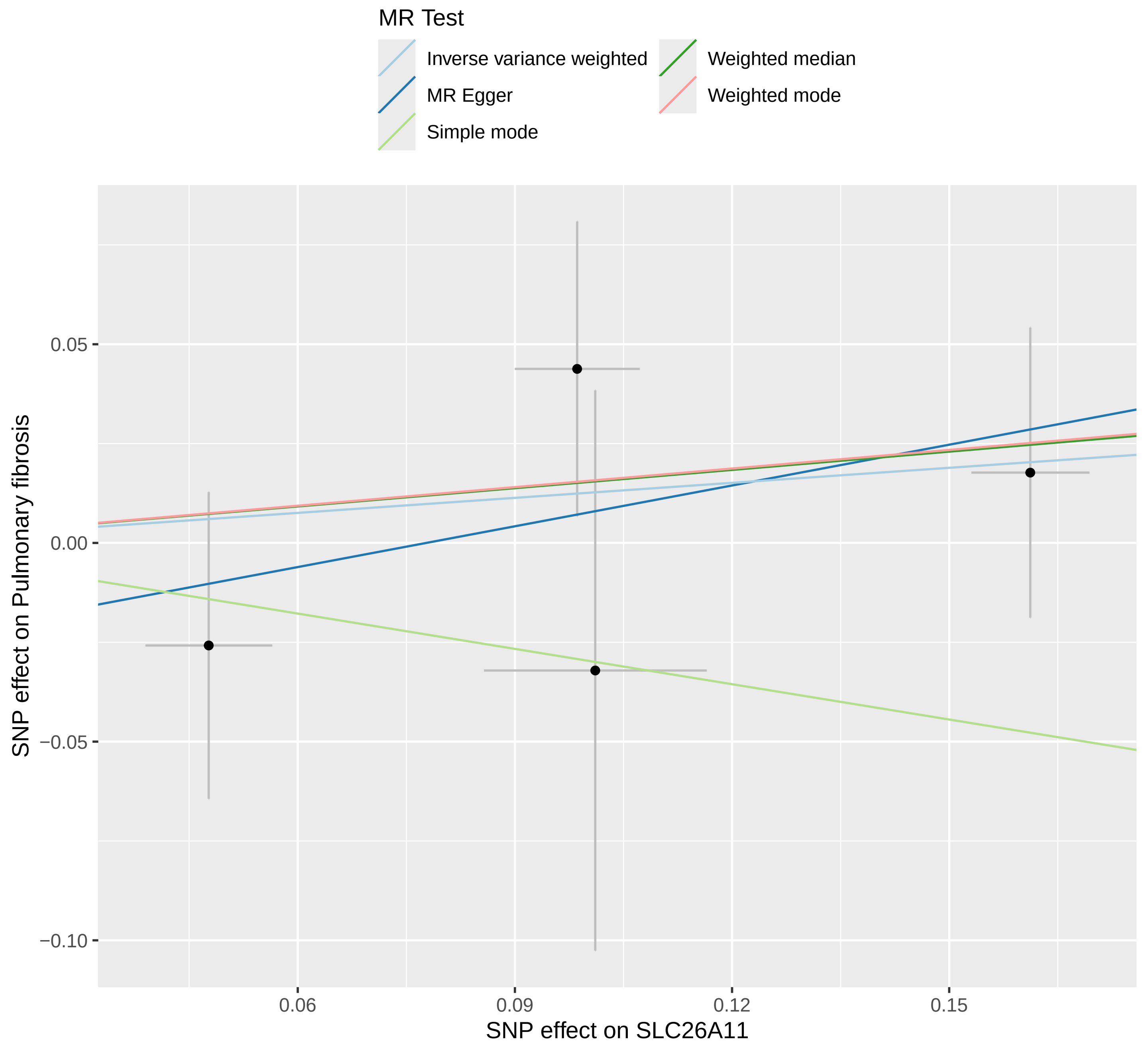
MR Test
Inverse variance weighted
MR Egger
Simple mode
Weighted median
Weighted mode
0.05
0.00
−0.05
−0.10
0.06
0.09
0.12
0.15
SNP effect on Pulmonary fibrosis
SNP effect on SLC26A11

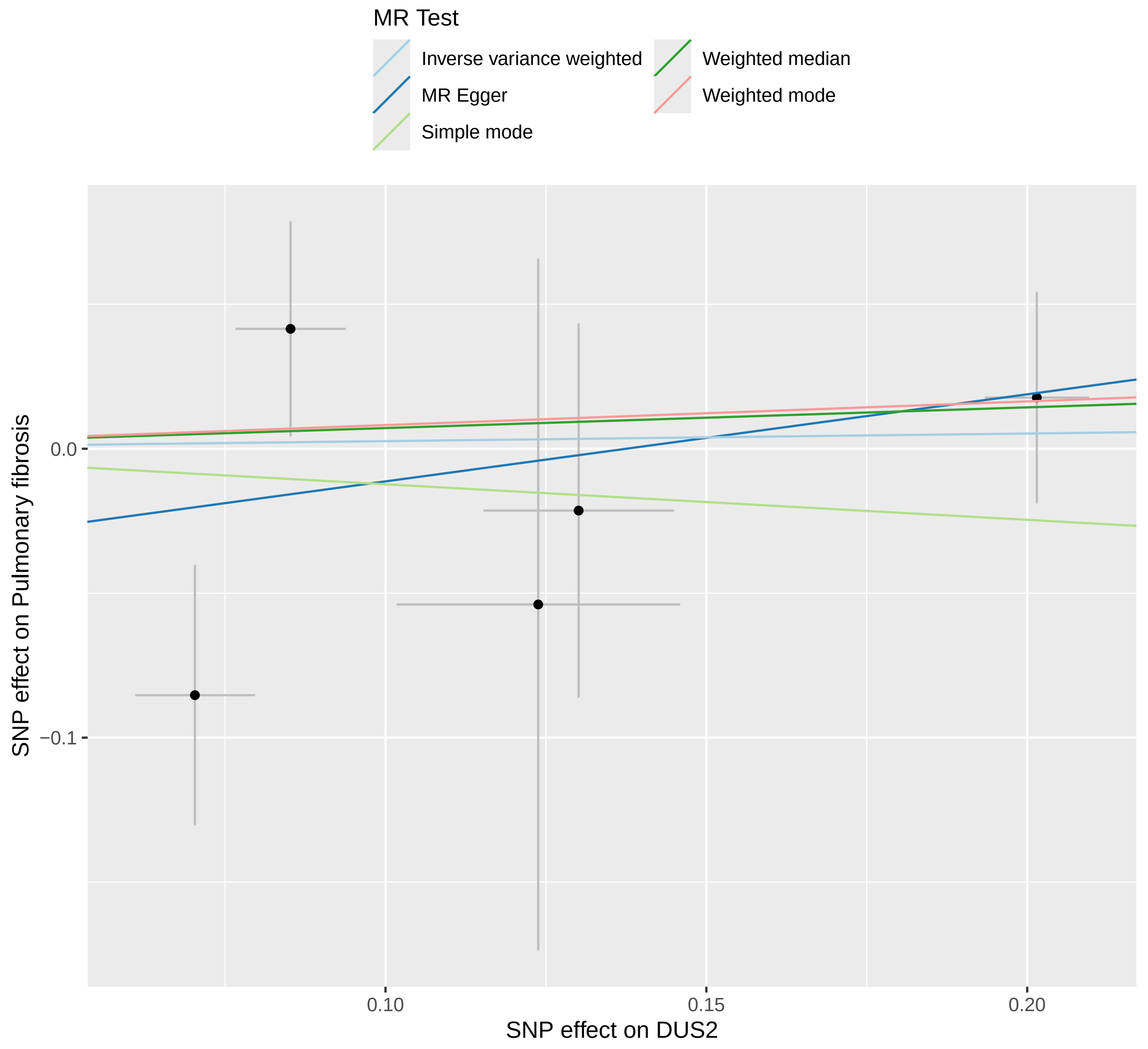
MR Test
Inverse variance weighted
MR Egger
Simple mode
Weighted median
Weighted mode
0.0
−0.1
0.10
0.15
0.20
SNP effect on Pulmonary fibrosis
SNP effect on DUS2

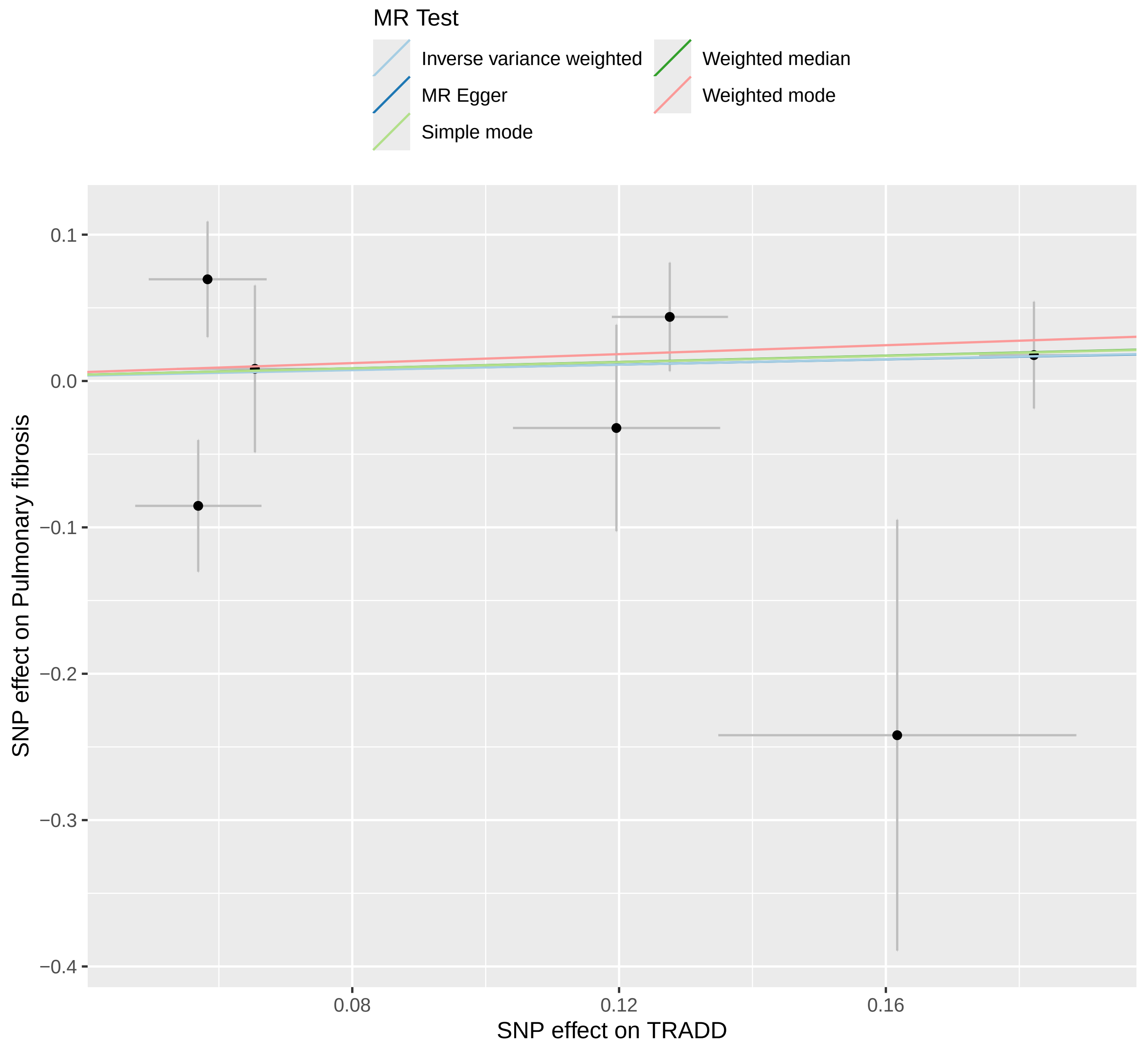

MR Test
Inverse variance weighted
MR Egger
Simple mode
Weighted median
Weighted mode
0.1
0.0
−0.1
−0.2
−0.3
−0.4
SNP effect on Pulmonary fibrosis
0.08
0.12
0.16
SNP effect on TRADD

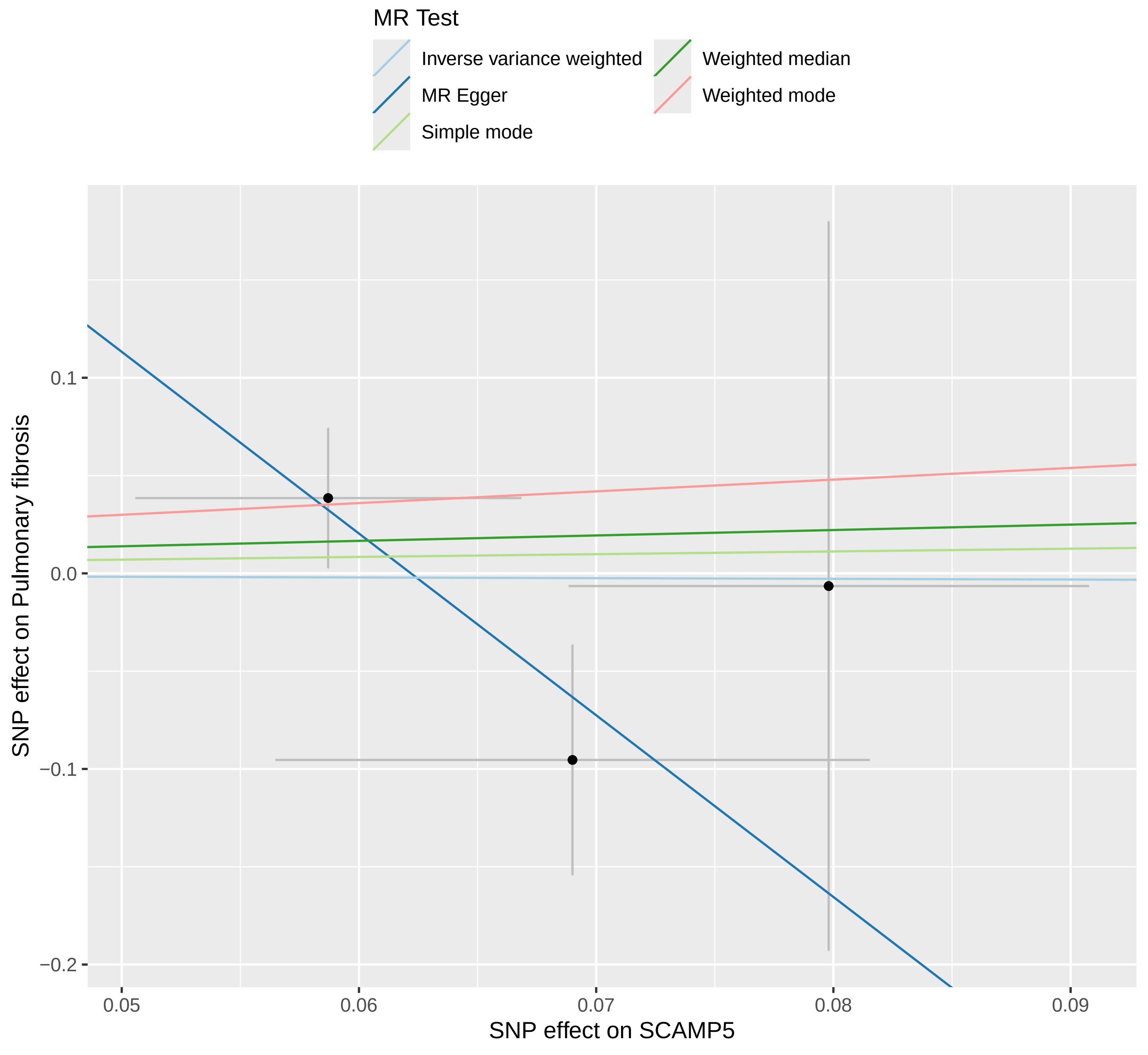
MR Test
Inverse variance weighted
MR Egger
Simple mode
Weighted median
Weighted mode
SNP effect on Pulmonary fibrosis
0.1
0.0
−0.1
−0.2
0.05
0.06
0.07
0.08
0.09
SNP effect on SCAMP5

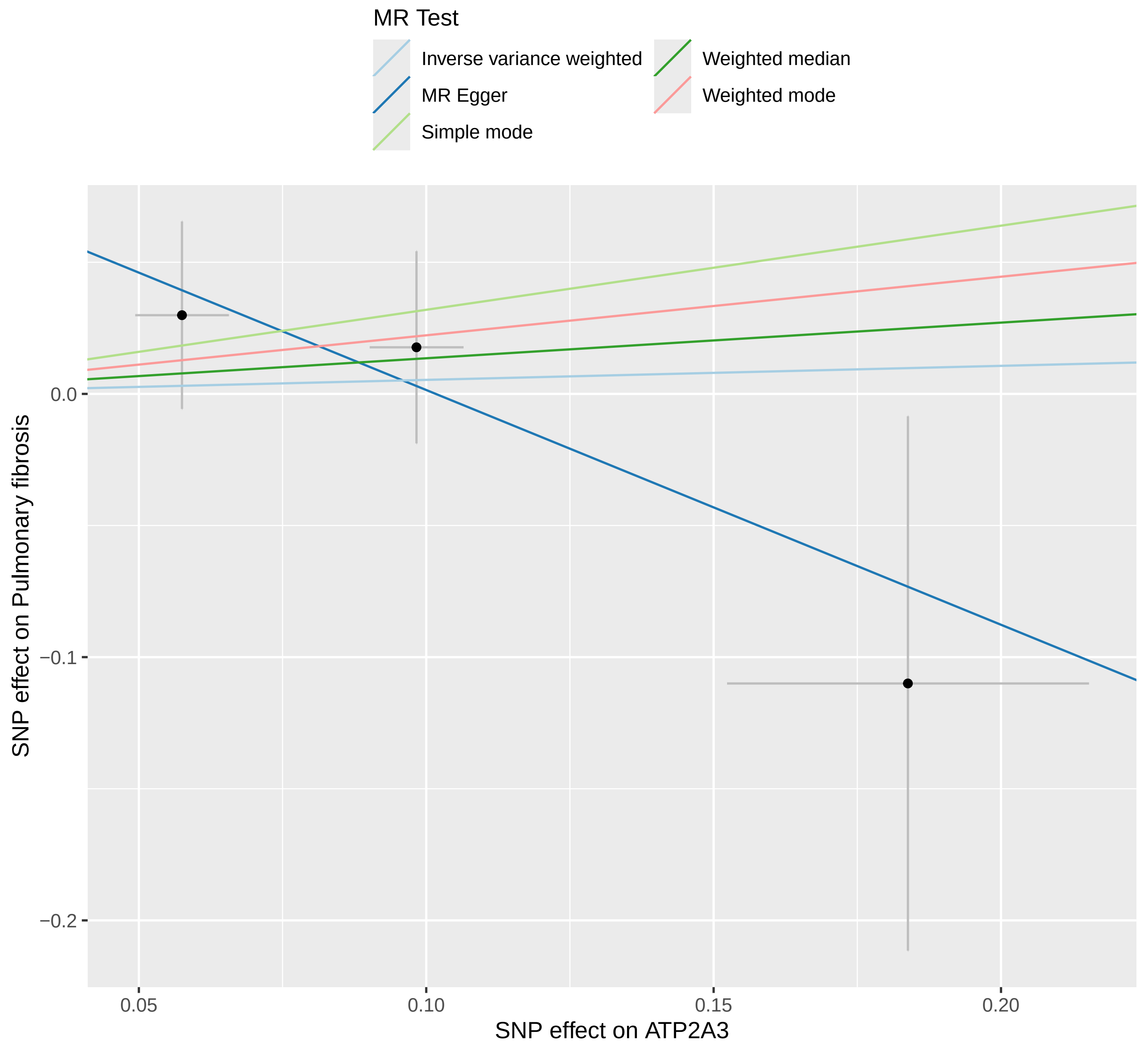

MR Test
Inverse variance weighted
MR Egger
Simple mode
Weighted median
Weighted mode
SNP effect on Pulmonary fibrosis
0.0
−0.1
−0.2
0.05
0.10
0.15
0.20
SNP effect on ATP2A3

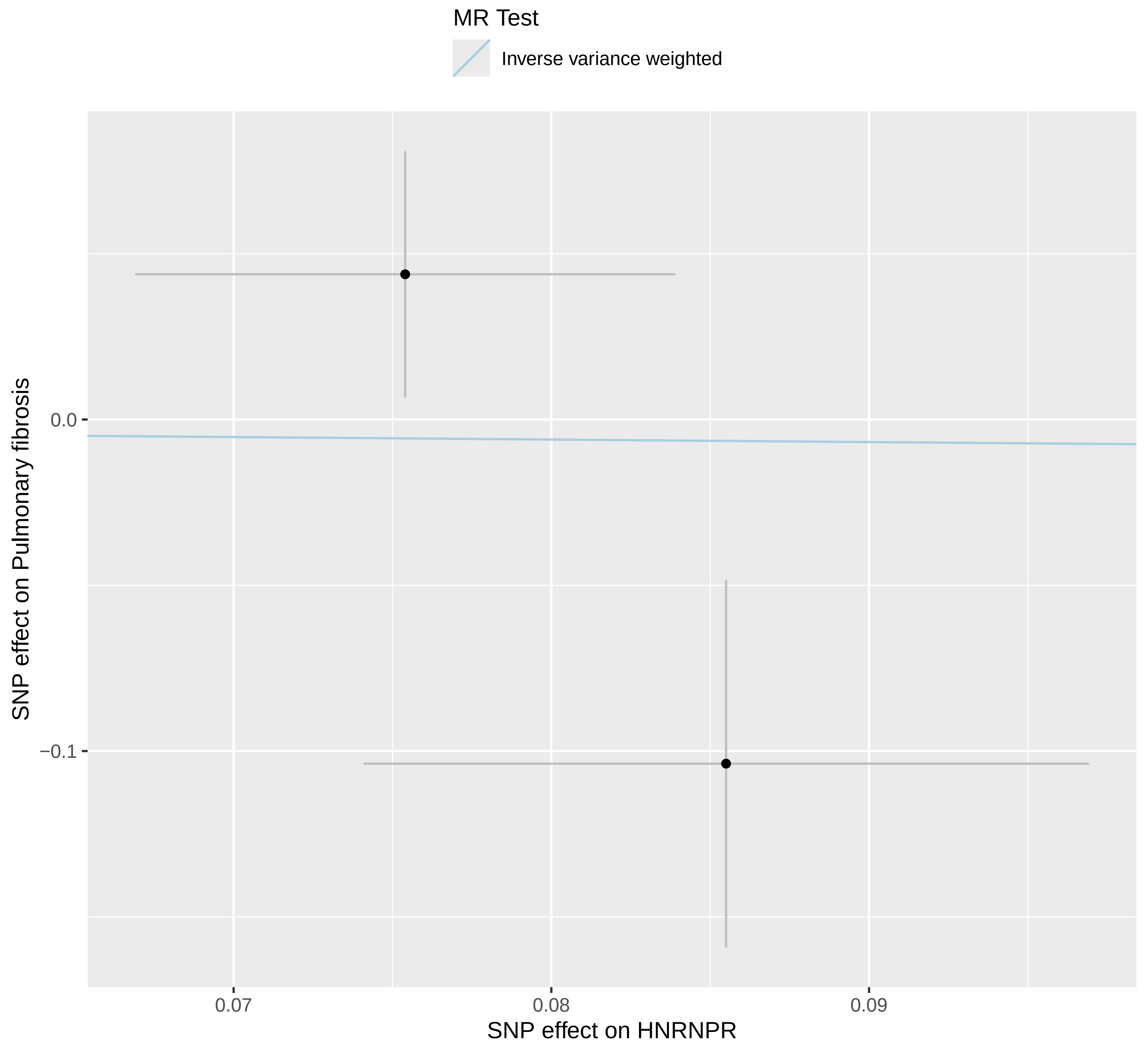
MR Test
Inverse variance weighted
SNP effect on Pulmonary fibrosis
0.0
−0.1
0.07
0.08
0.09
SNP effect on HNRNPR

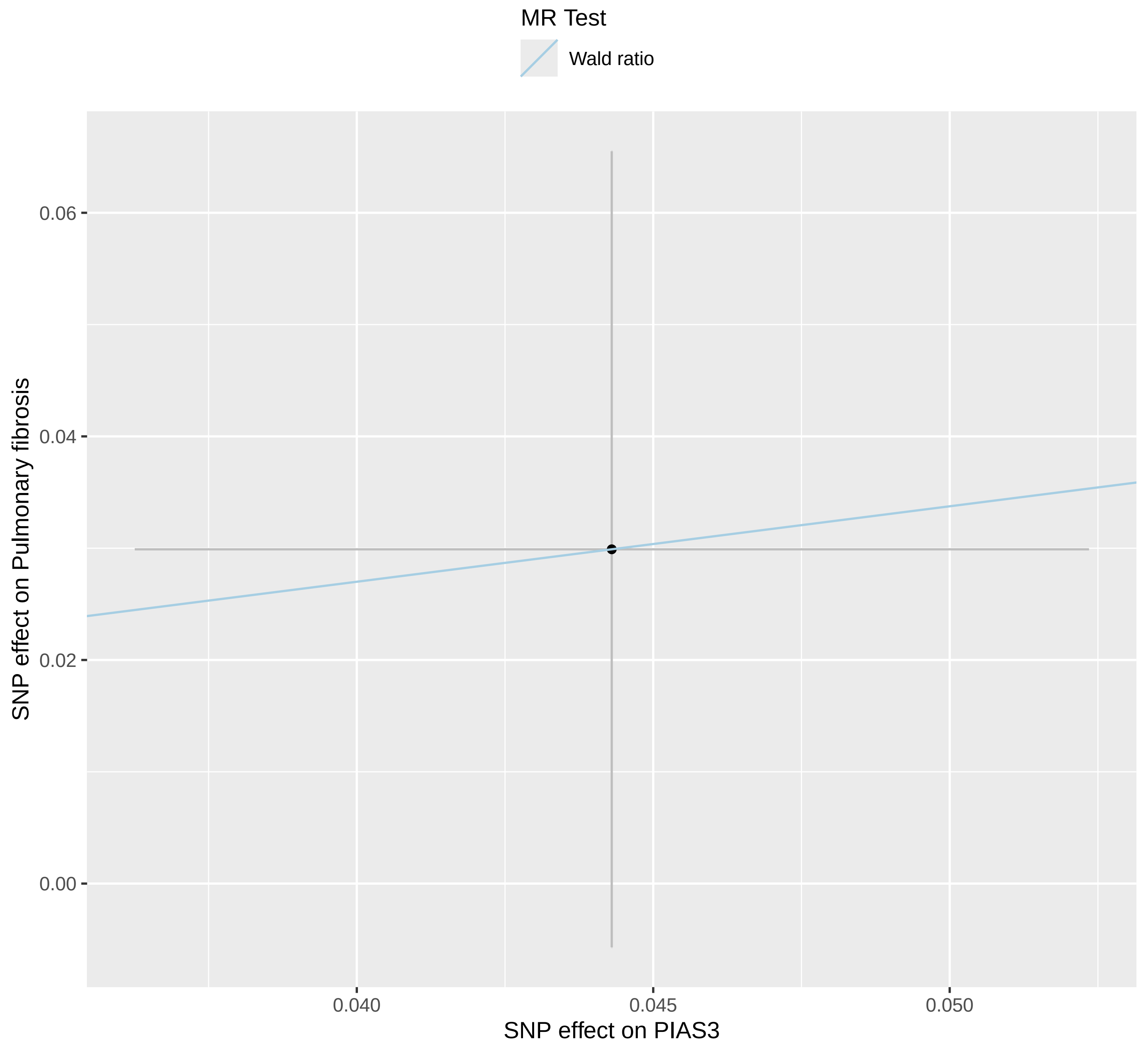

MR Test
Wald ratio
0.06
0.04
0.02
0.00
0.040
0.045
0.050
SNP effect on Pulmonary fibrosis
SNP effect on PIAS3

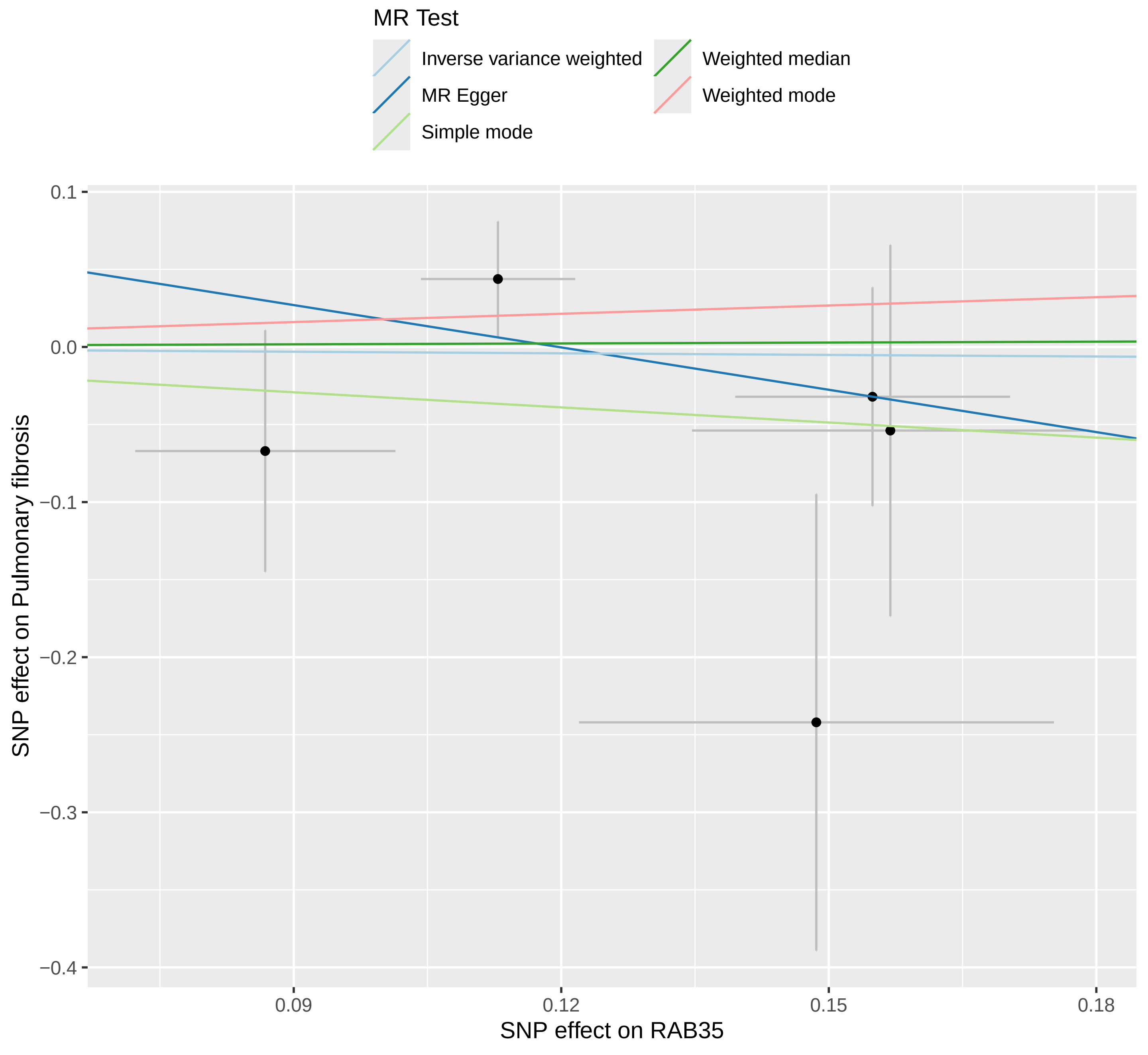
MR Test
Inverse variance weighted
Weighted median
MR Egger
Weighted mode
Simple mode
0.1
0.0
−0.1
−0.2
−0.3
−0.4
0.09
0.12
0.15
0.18
SNP effect on Pulmonary fibrosis
SNP effect on RAB35

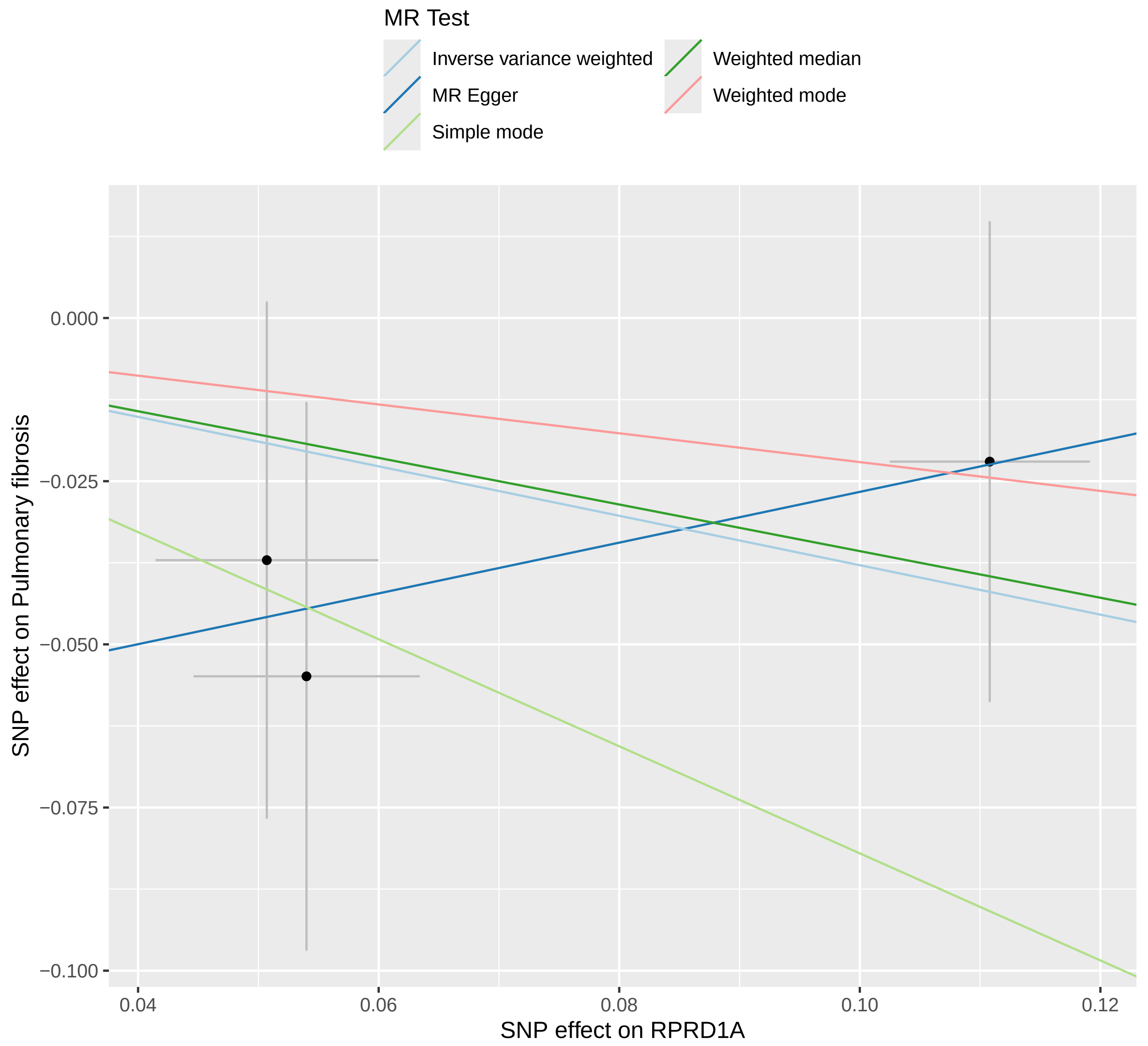
MR Test
Inverse variance weighted
MR Egger
Simple mode
Weighted median
Weighted mode
SNP effect on Pulmonary fibrosis
0.000
−0.025
−0.050
−0.075
−0.100
0.04
0.06
0.08
0.10
0.12
SNP effect on RPRD1A

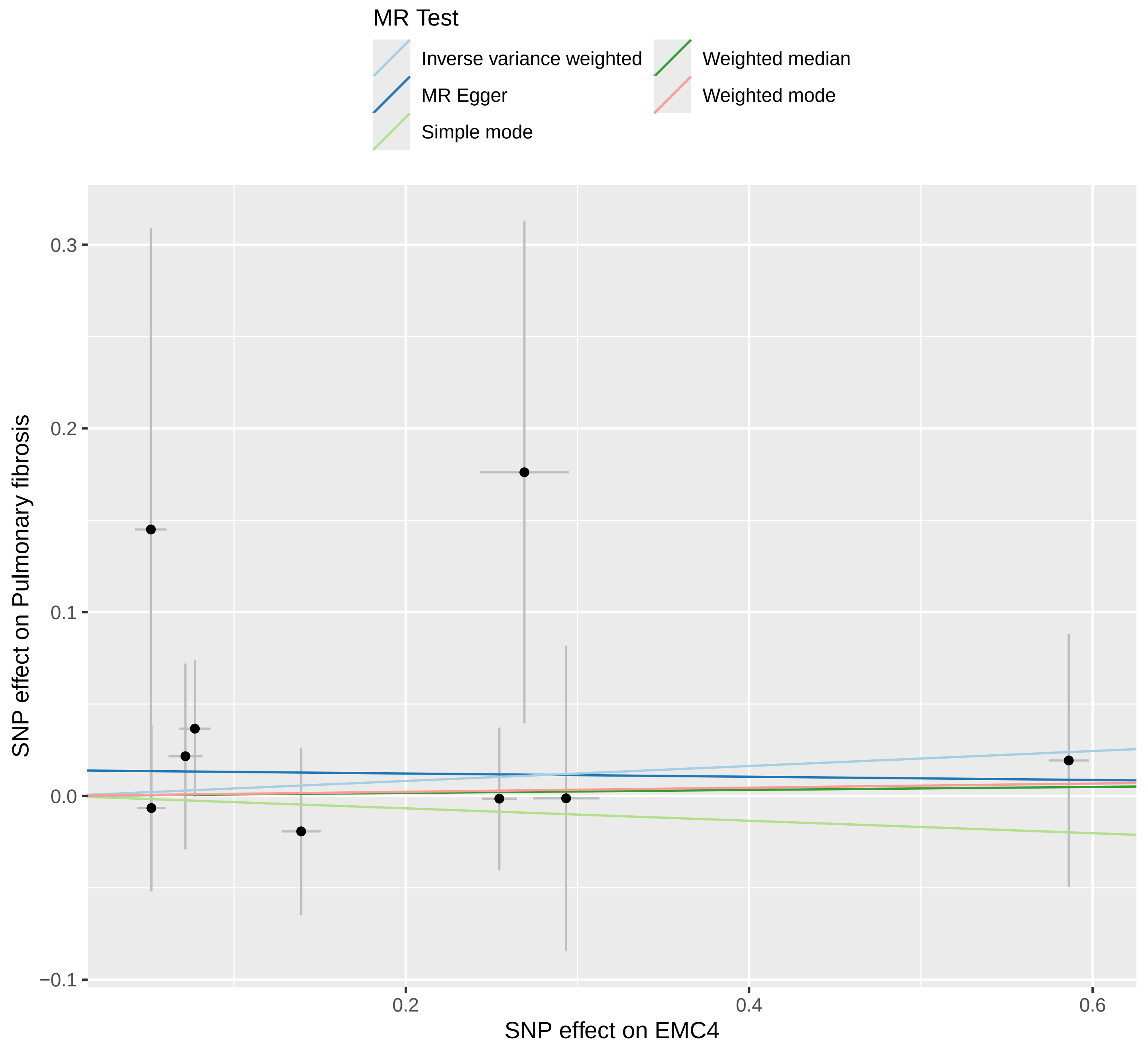
MR Test
Inverse variance weighted
MR Egger
Simple mode
Weighted median
Weighted mode
0.3
0.2
0.1
0.0
−0.1
0.2
0.4
0.6
SNP effect on Pulmonary fibrosis
SNP effect on EMC4

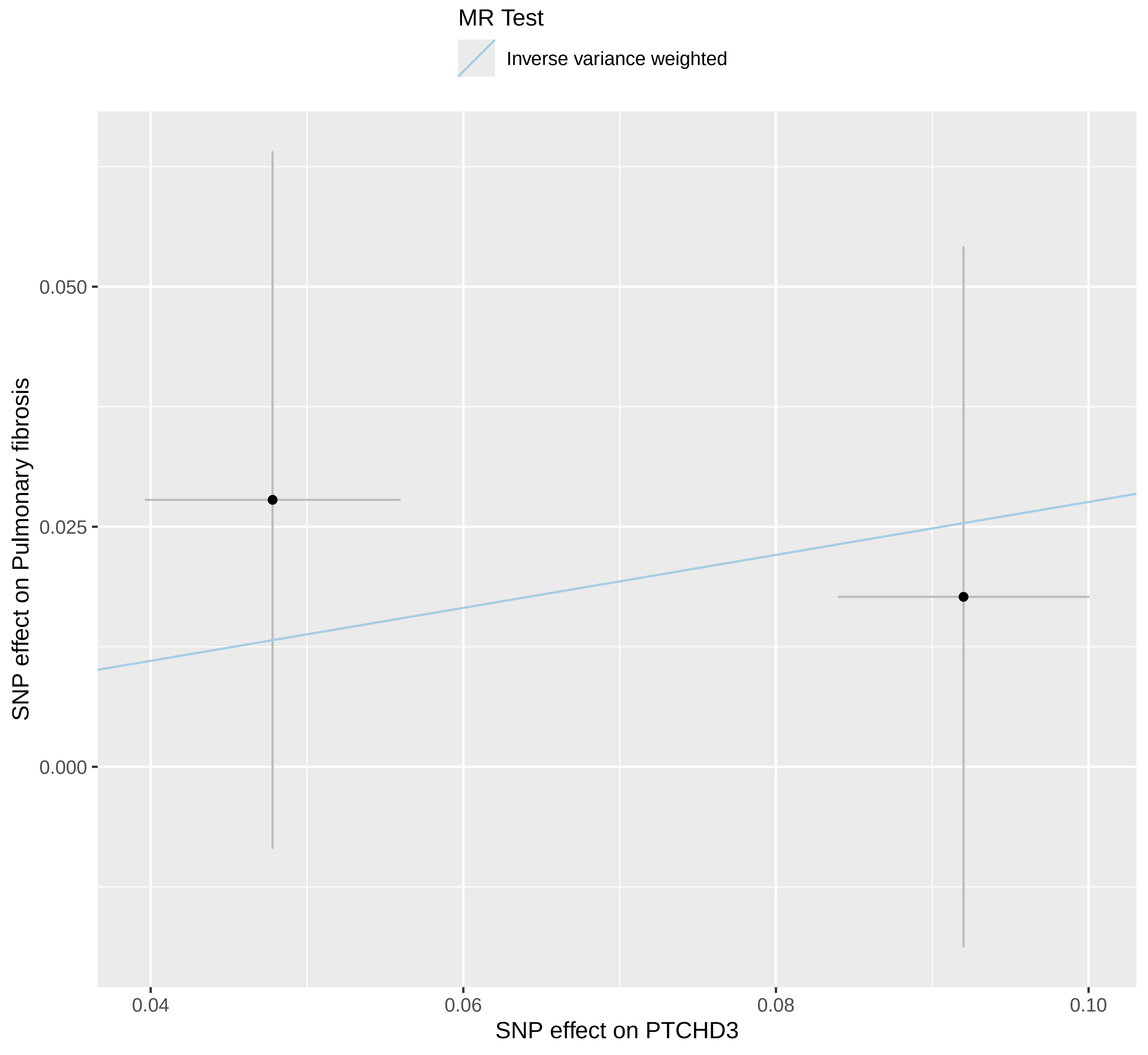

MR Test
Inverse variance weighted
0.050
0.025
0.000
0.04
0.06
0.08
0.10
SNP effect on PTCHD3
SNP effect on Pulmonary fibrosis

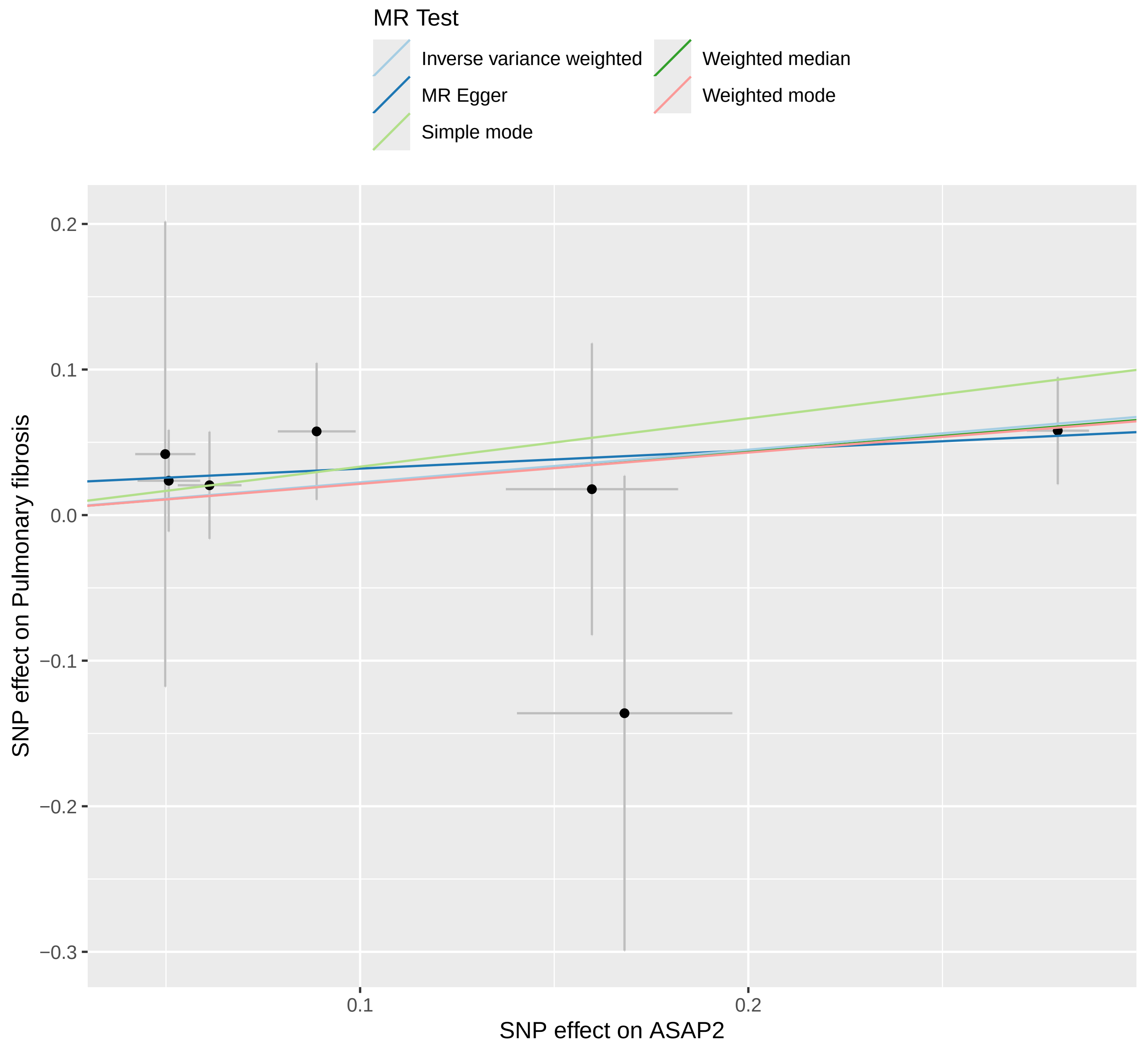
MR Test
Inverse variance weighted
MR Egger
Simple mode
Weighted median
Weighted mode
0.2
0.1
0.0
−0.1
−0.2
−0.3
0.1
0.2
SNP effect on ASAP2
SNP effect on Pulmonary fibrosis

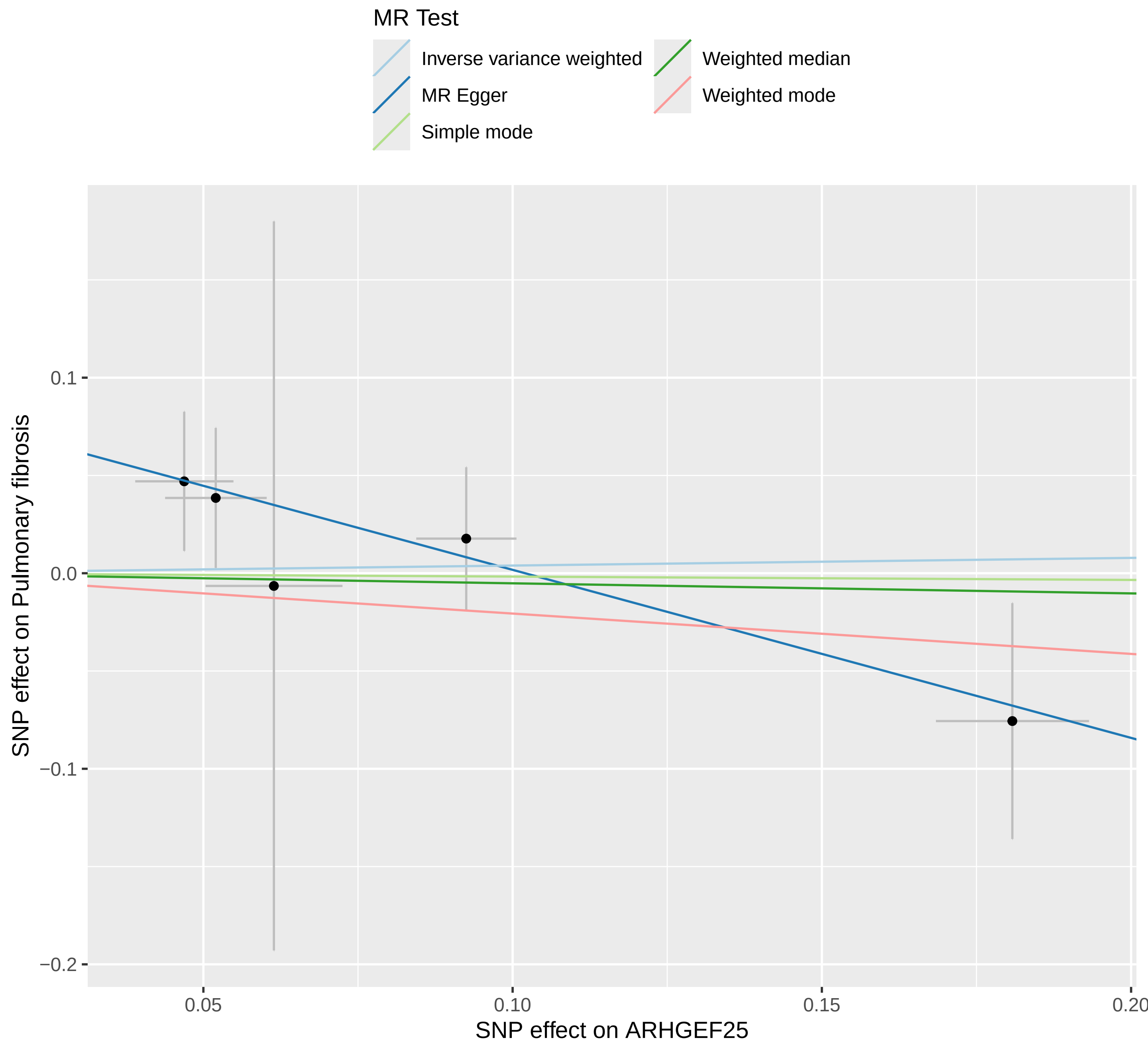

MR Test
Inverse variance weighted
MR Egger
Simple mode
Weighted median
Weighted mode
0.1
0.0
−0.1
−0.2
SNP effect on Pulmonary fibrosis
0.05
0.10
0.15
0.20
SNP effect on ARHGEF25

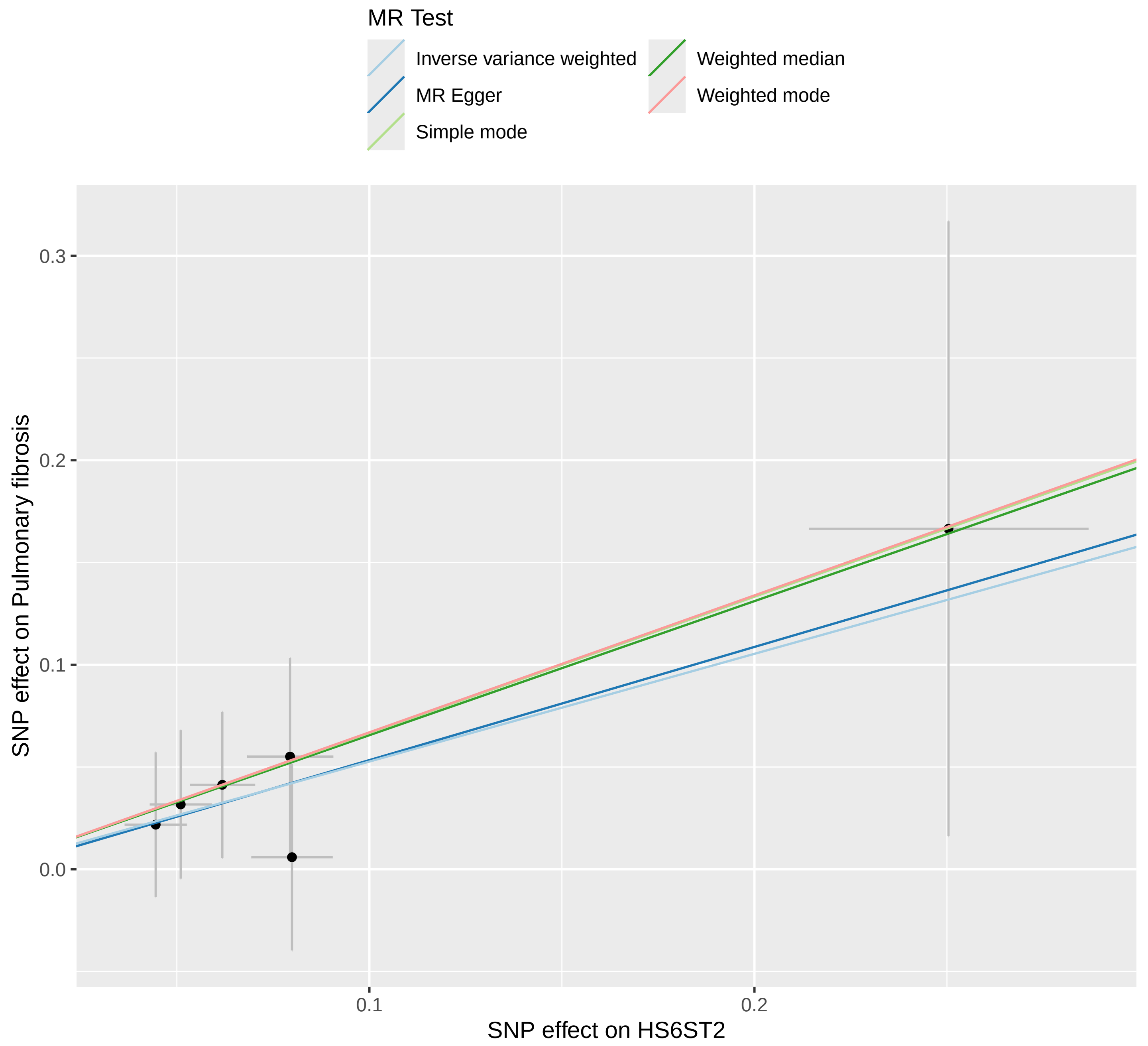
MR Test
Inverse variance weighted
MR Egger
Simple mode
Weighted median
Weighted mode
0.3
0.2
0.1
0.0
0.1
0.2
SNP effect on HS6ST2
SNP effect on Pulmonary fibrosis

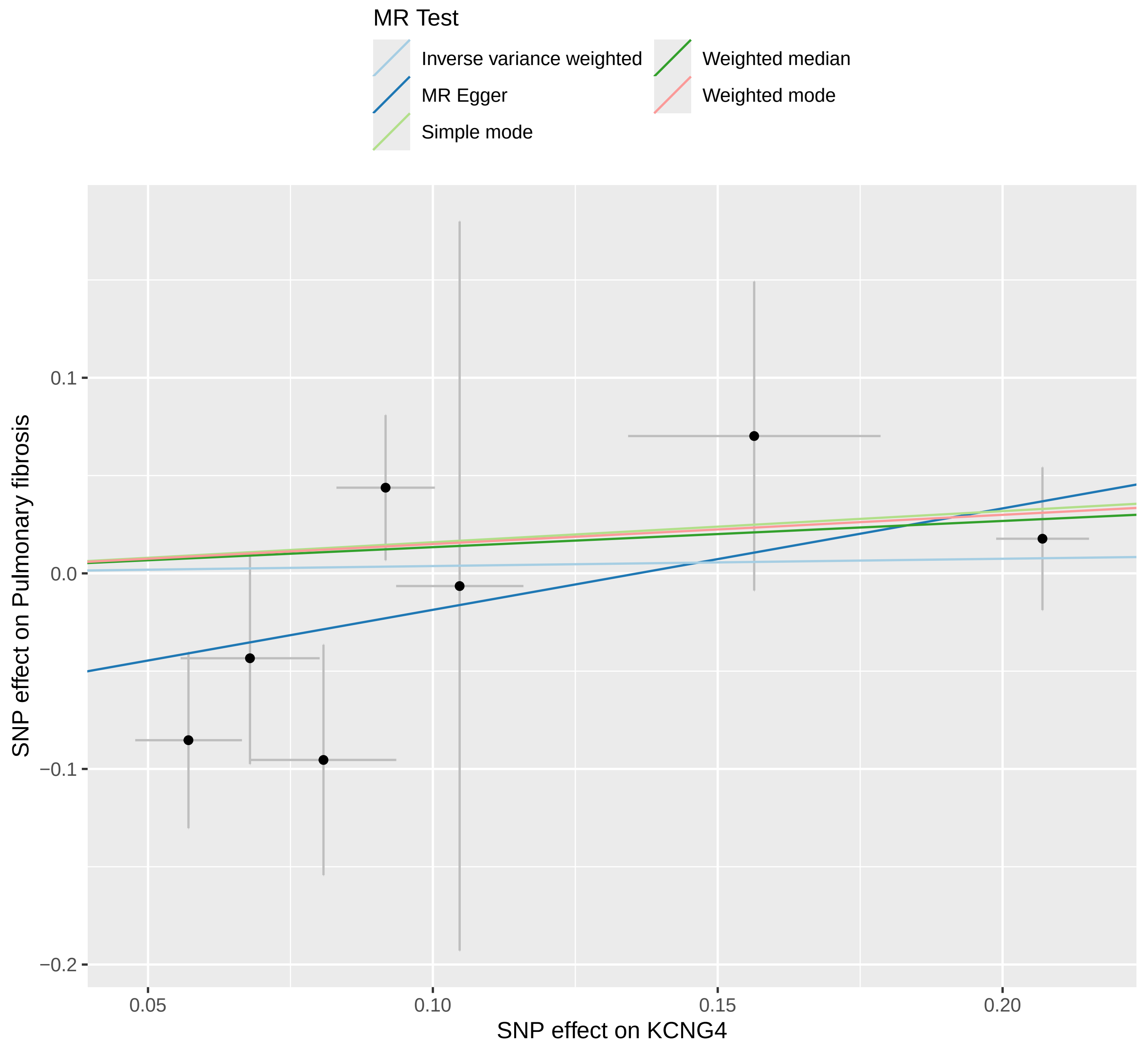
MR Test
Inverse variance weighted
MR Egger
Simple mode
Weighted median
Weighted mode
SNP effect on Pulmonary fibrosis
0.1
0.0
−0.1
−0.2
0.05
0.10
0.15
0.20
SNP effect on KCNG4

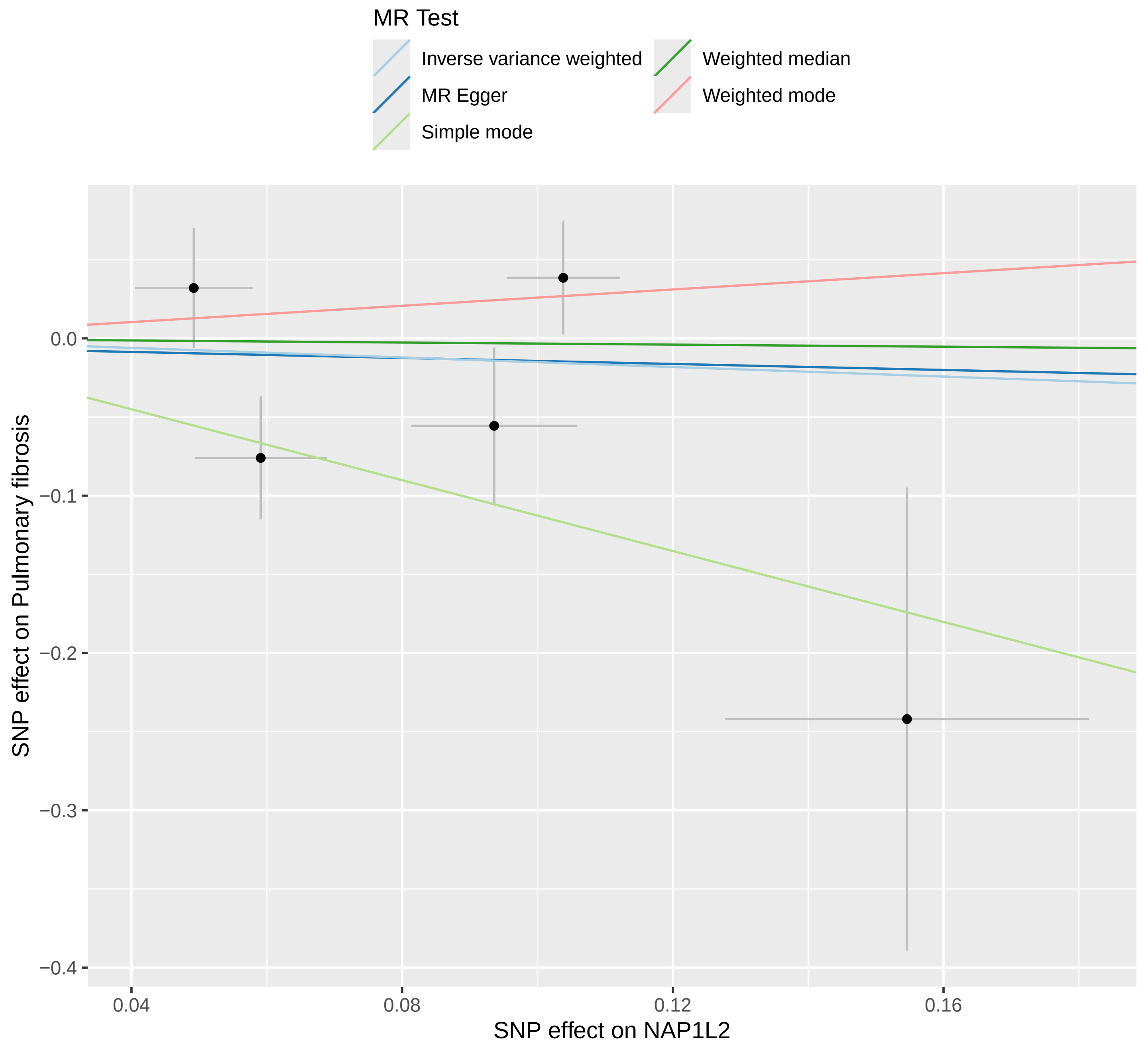
MR Test
Inverse variance weighted
MR Egger
Simple mode
Weighted median
Weighted mode
SNP effect on Pulmonary fibrosis
0.0
−0.1
−0.2
−0.3
−0.4
0.04
0.08
0.12
0.16
SNP effect on NAP1L2

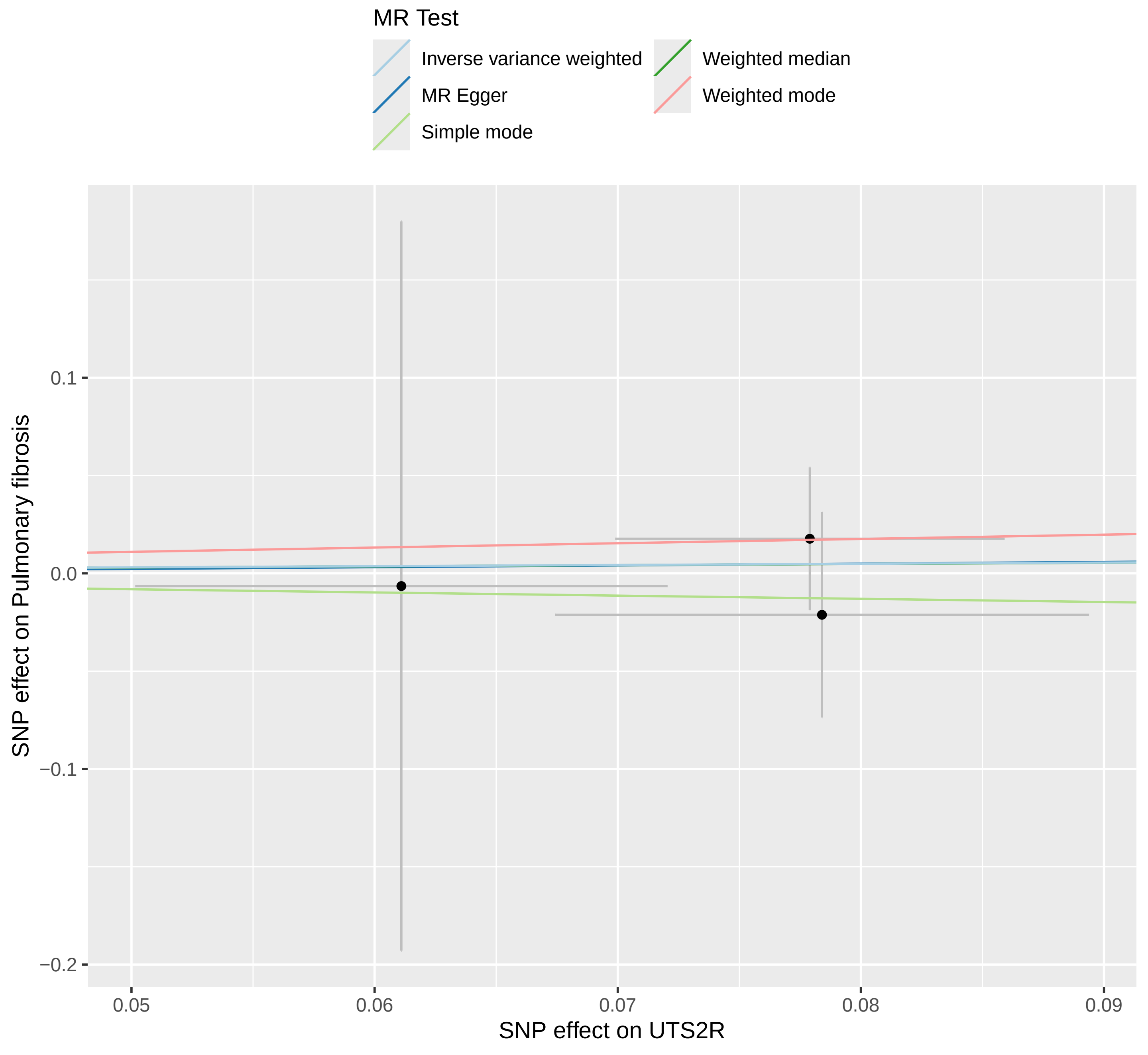

MR Test
Inverse variance weighted
MR Egger
Simple mode
Weighted median
Weighted mode
0.1
0.0
−0.1
−0.2
SNP effect on Pulmonary fibrosis
0.05
0.06
0.07
0.08
0.09
SNP effect on UTS2R

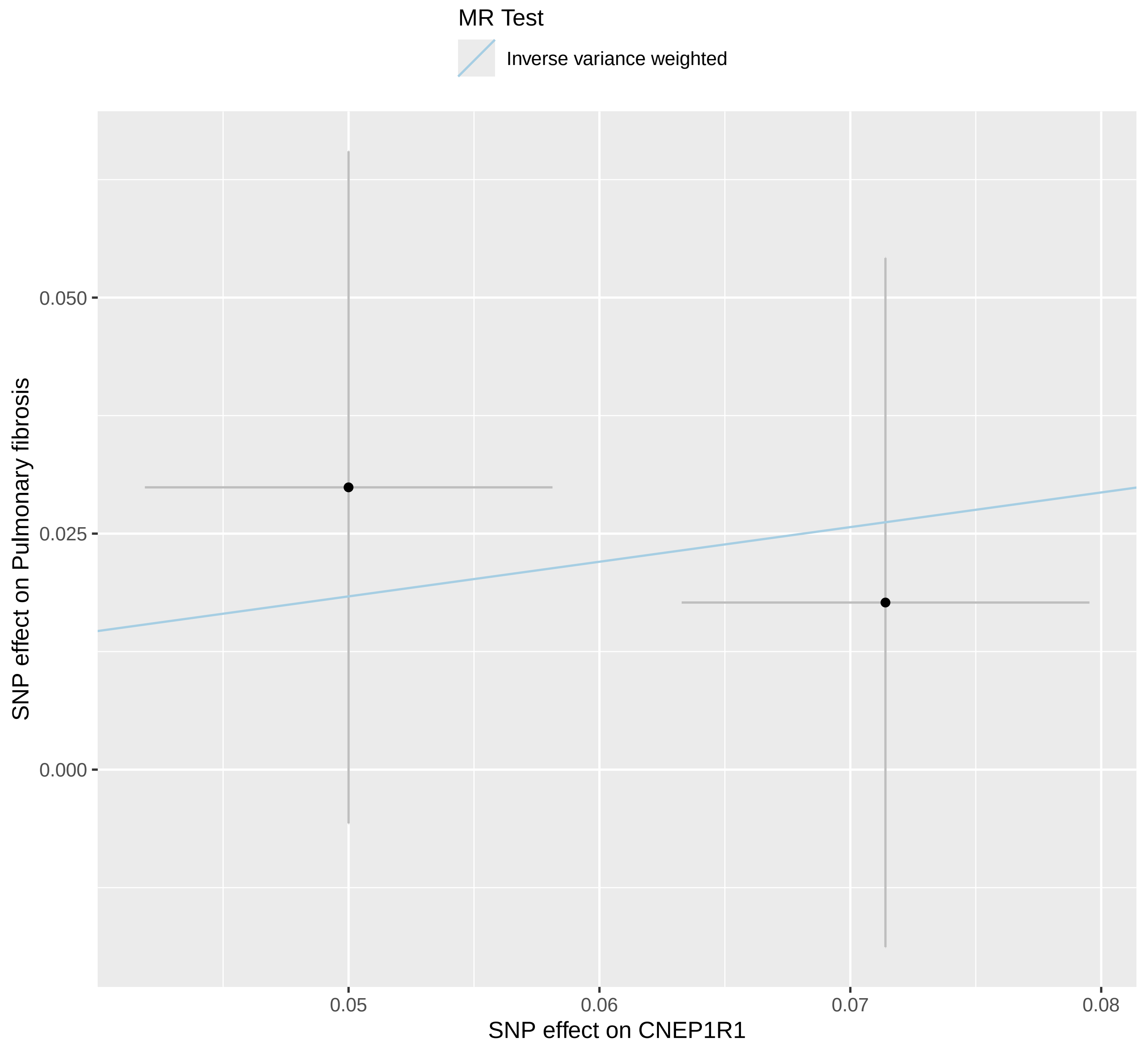
MR Test
Inverse variance weighted
0.050
0.025
0.000
0.05
0.06
0.07
0.08
SNP effect on CNEP1R1
SNP effect on Pulmonary fibrosis

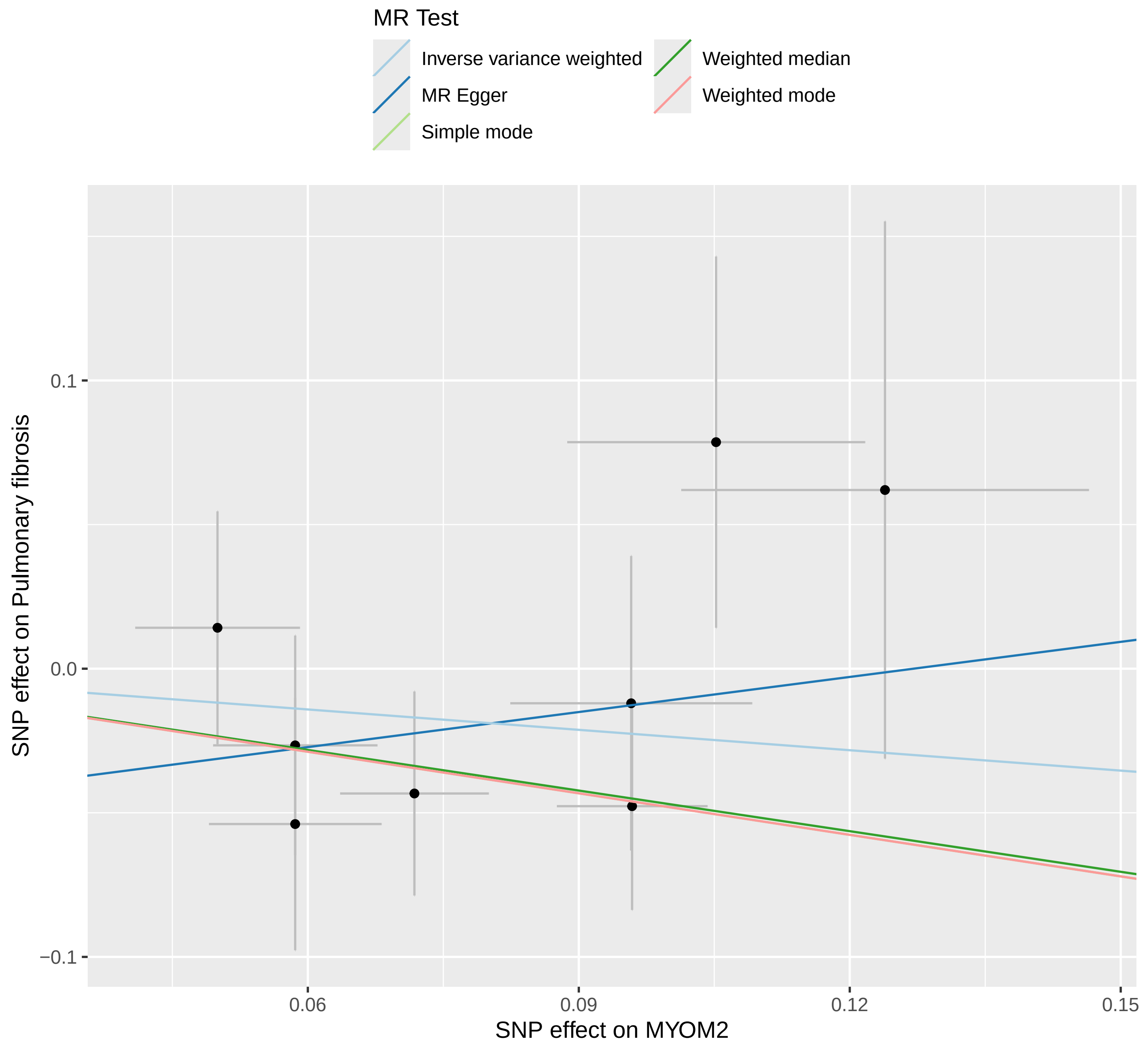
MR Test
Inverse variance weighted
MR Egger
Simple mode
Weighted median
Weighted mode
0.1
0.0
−0.1
0.06
0.09
0.12
0.15
SNP effect on Pulmonary fibrosis
SNP effect on MYOM2

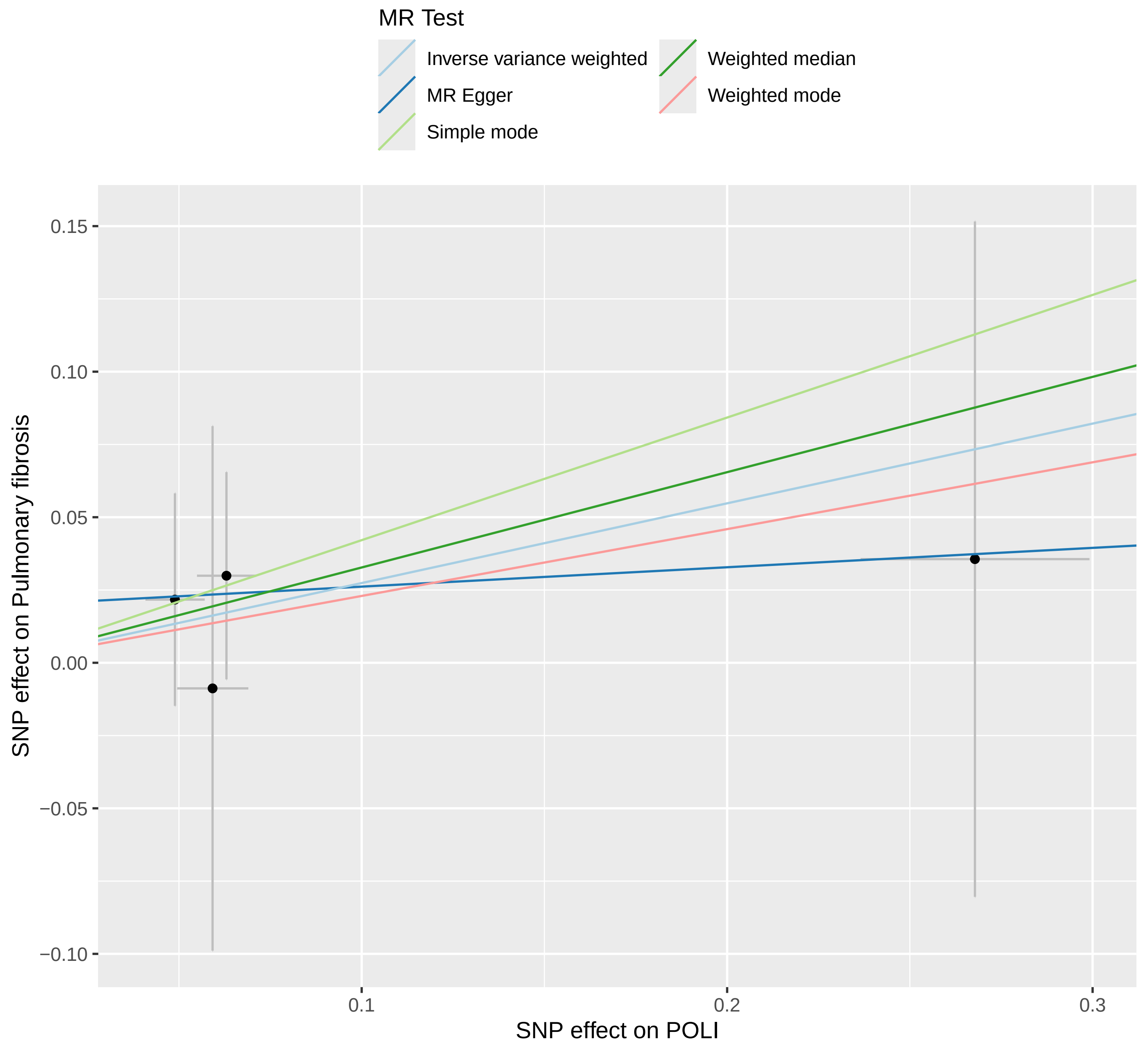
MR Test
Inverse variance weighted
MR Egger
Simple mode
Weighted median
Weighted mode
0.15
0.10
0.05
0.00
−0.05
−0.10
SNP effect on Pulmonary fibrosis
0.1
0.2
0.3
SNP effect on POLI

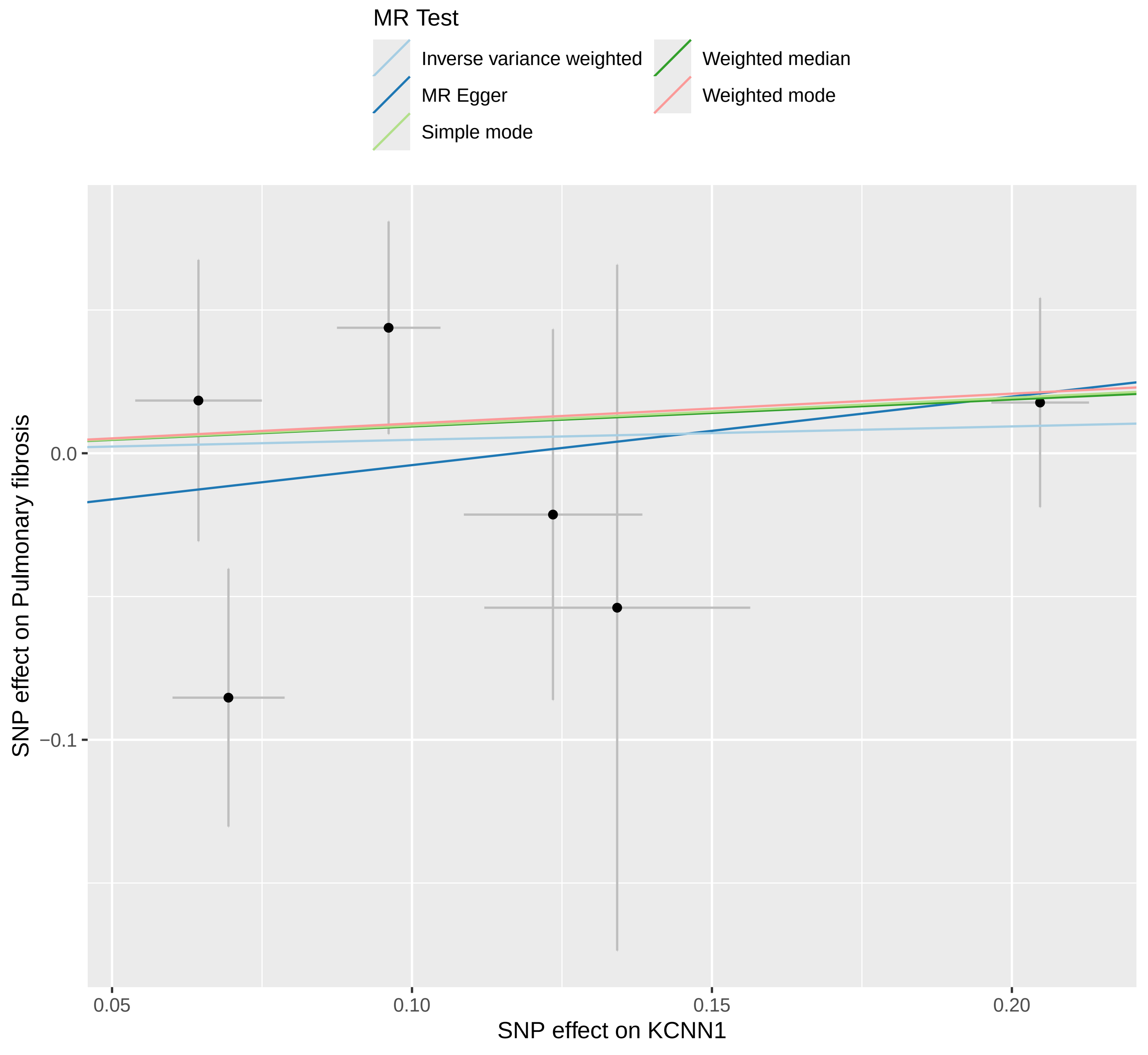

MR Test
Inverse variance weighted
MR Egger
Simple mode
Weighted median
Weighted mode
0.0
−0.1
0.05
0.10
0.15
0.20
SNP effect on KCNN1
SNP effect on Pulmonary fibrosis

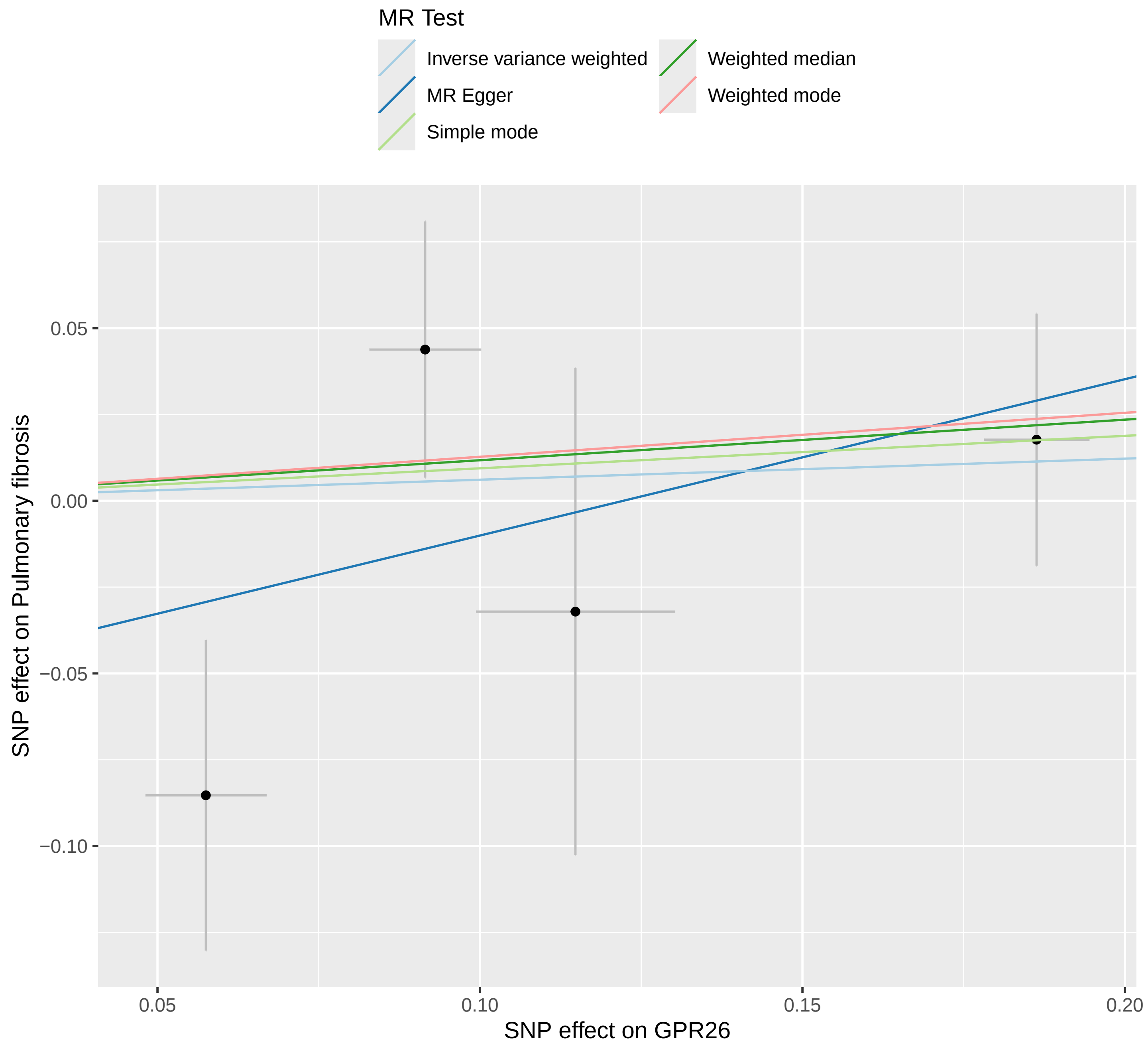
MR Test
Inverse variance weighted
MR Egger
Simple mode
Weighted median
Weighted mode
0.05
0.00
−0.05
−0.10
0.05
0.10
0.15
0.20
SNP effect on GPR26
SNP effect on Pulmonary fibrosis

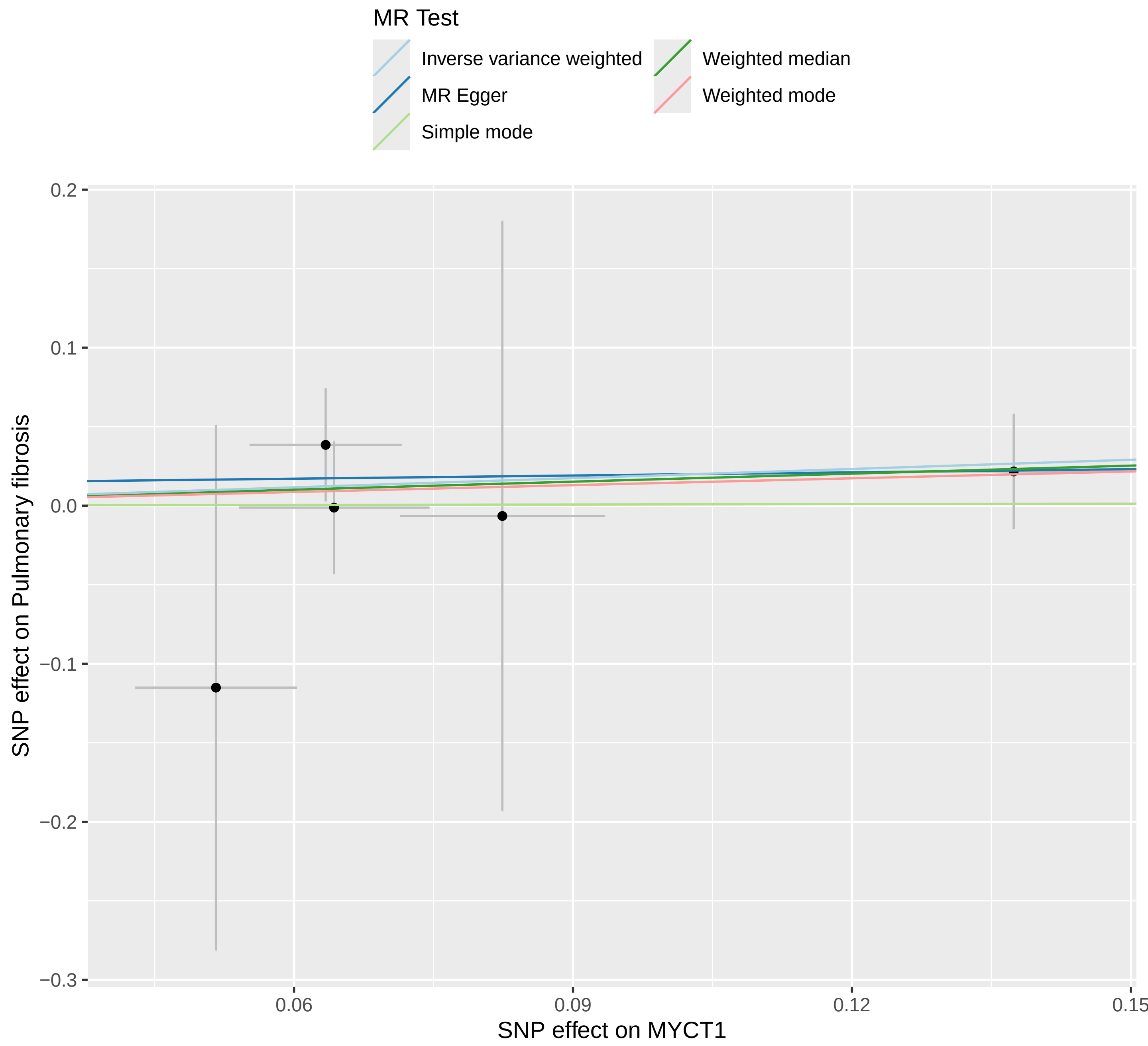
MR Test
Inverse variance weighted
MR Egger
Simple mode
Weighted median
Weighted mode
0.2
0.1
0.0
−0.1
−0.2
−0.3
SNP effect on Pulmonary fibrosis
0.06
0.09
0.12
0.15
SNP effect on MYCT1

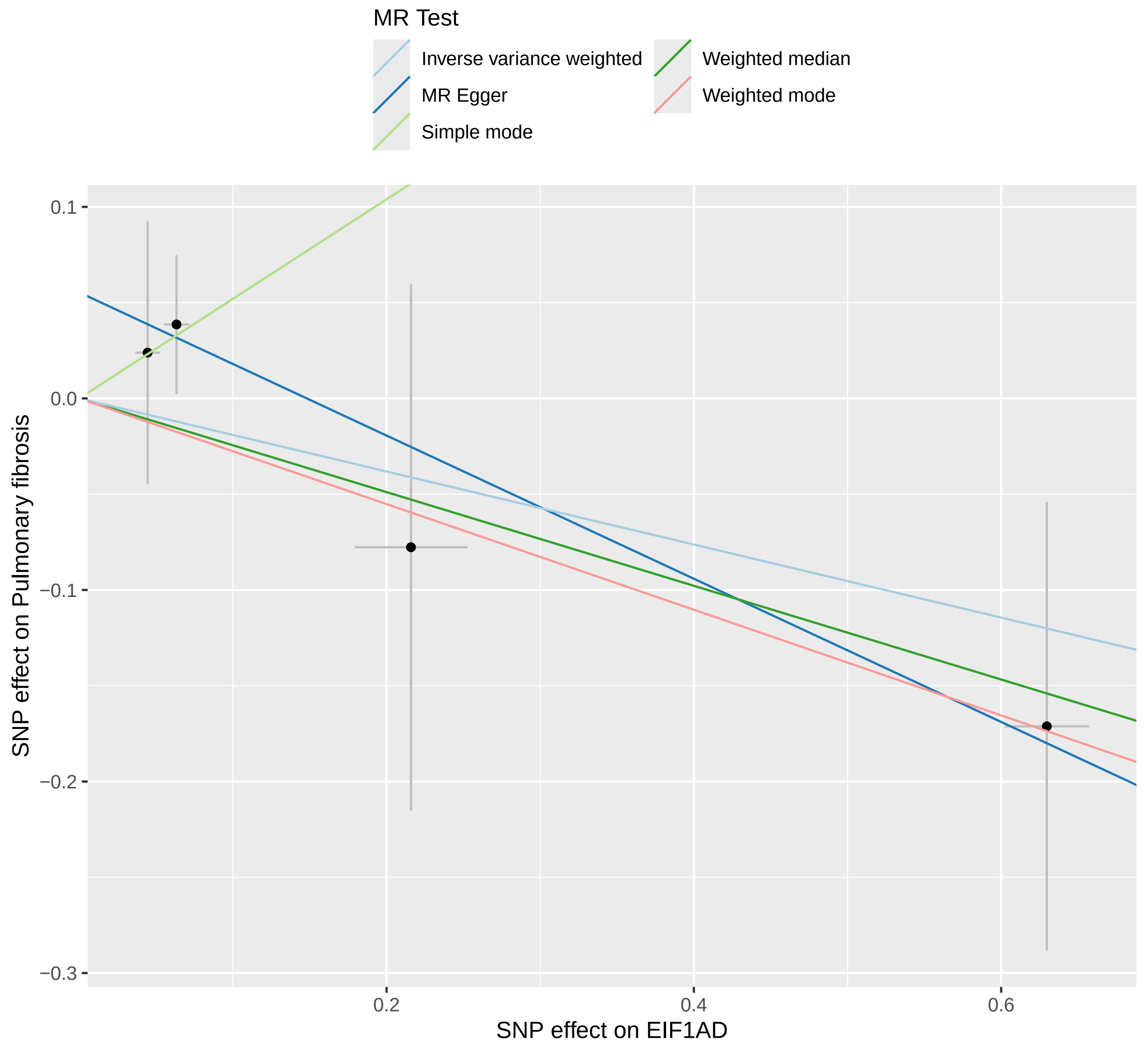

MR Test
Inverse variance weighted
MR Egger
Simple mode
Weighted median
Weighted mode
SNP effect on Pulmonary fibrosis
0.1
0.0
−0.1
−0.2
−0.3
0.2
0.4
0.6
SNP effect on EIF1AD

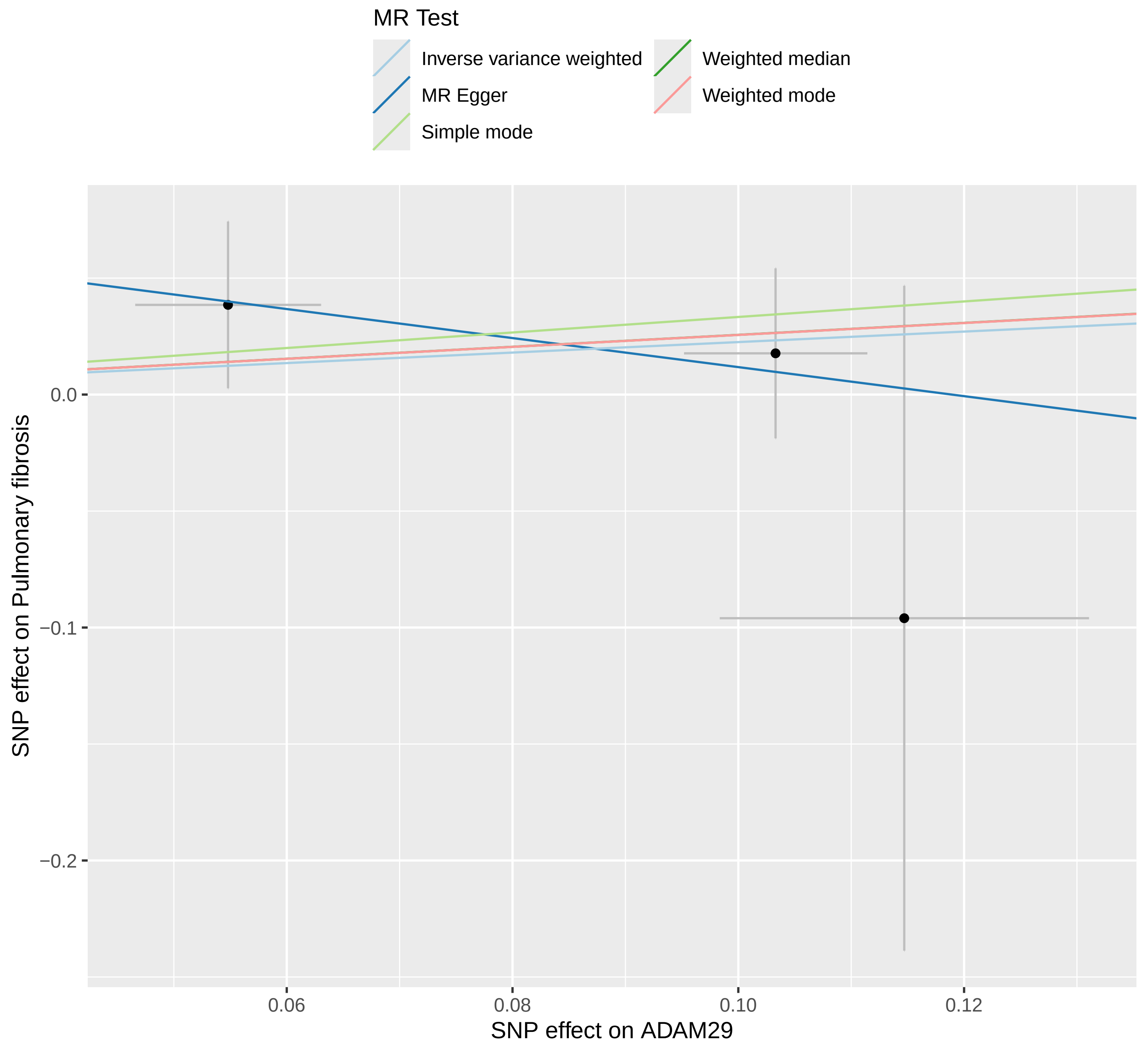
MR Test
Inverse variance weighted
MR Egger
Simple mode
Weighted median
Weighted mode
SNP effect on Pulmonary fibrosis
0.0
−0.1
−0.2
0.06
0.08
0.10
0.12
SNP effect on ADAM29

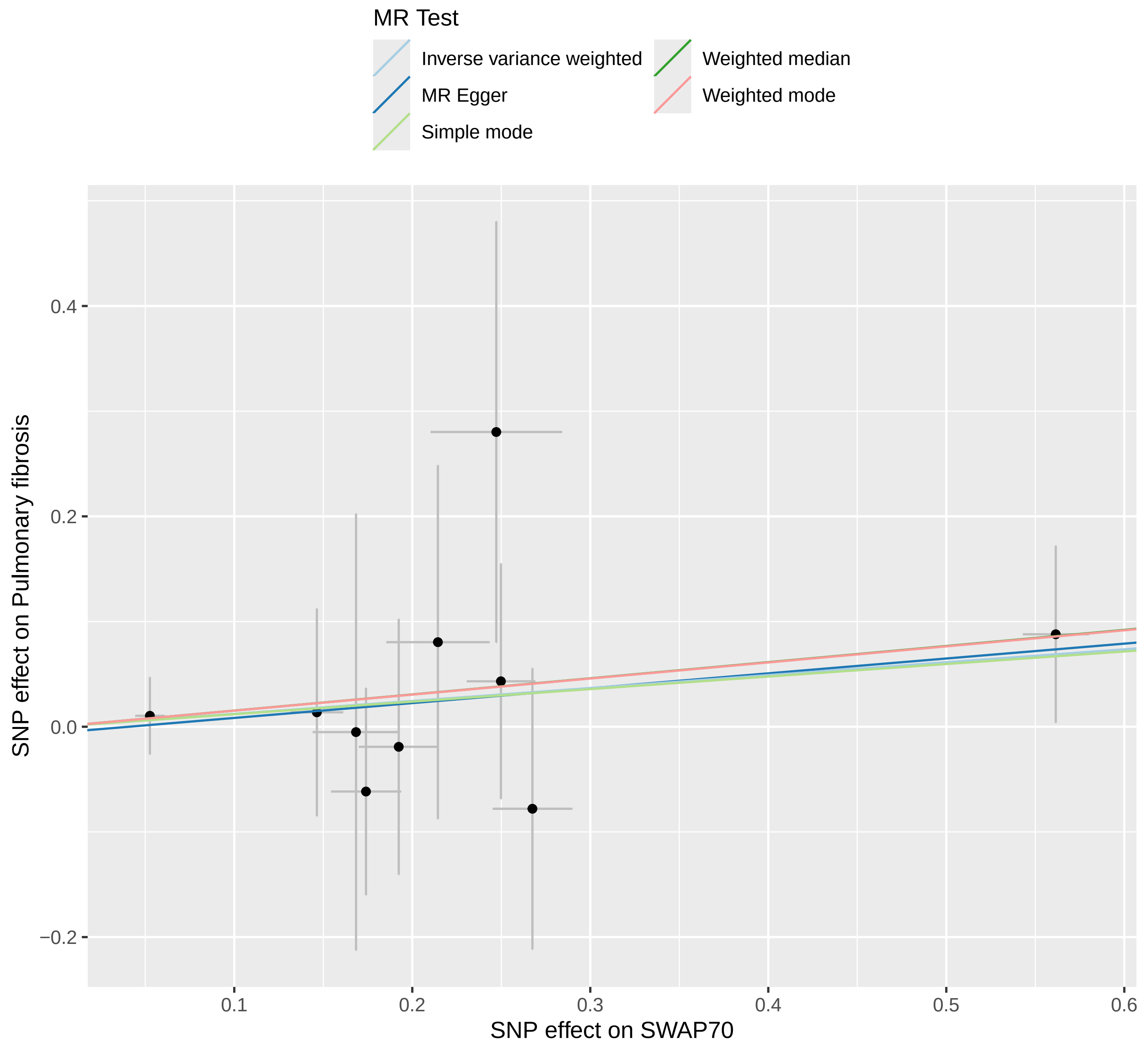
MR Test
Inverse variance weighted
MR Egger
Simple mode
Weighted median
Weighted mode
0.4
0.2
0.0
−0.2
0.1
0.2
0.3
0.4
0.5
0.6
SNP effect on SWAP70
SNP effect on Pulmonary fibrosis

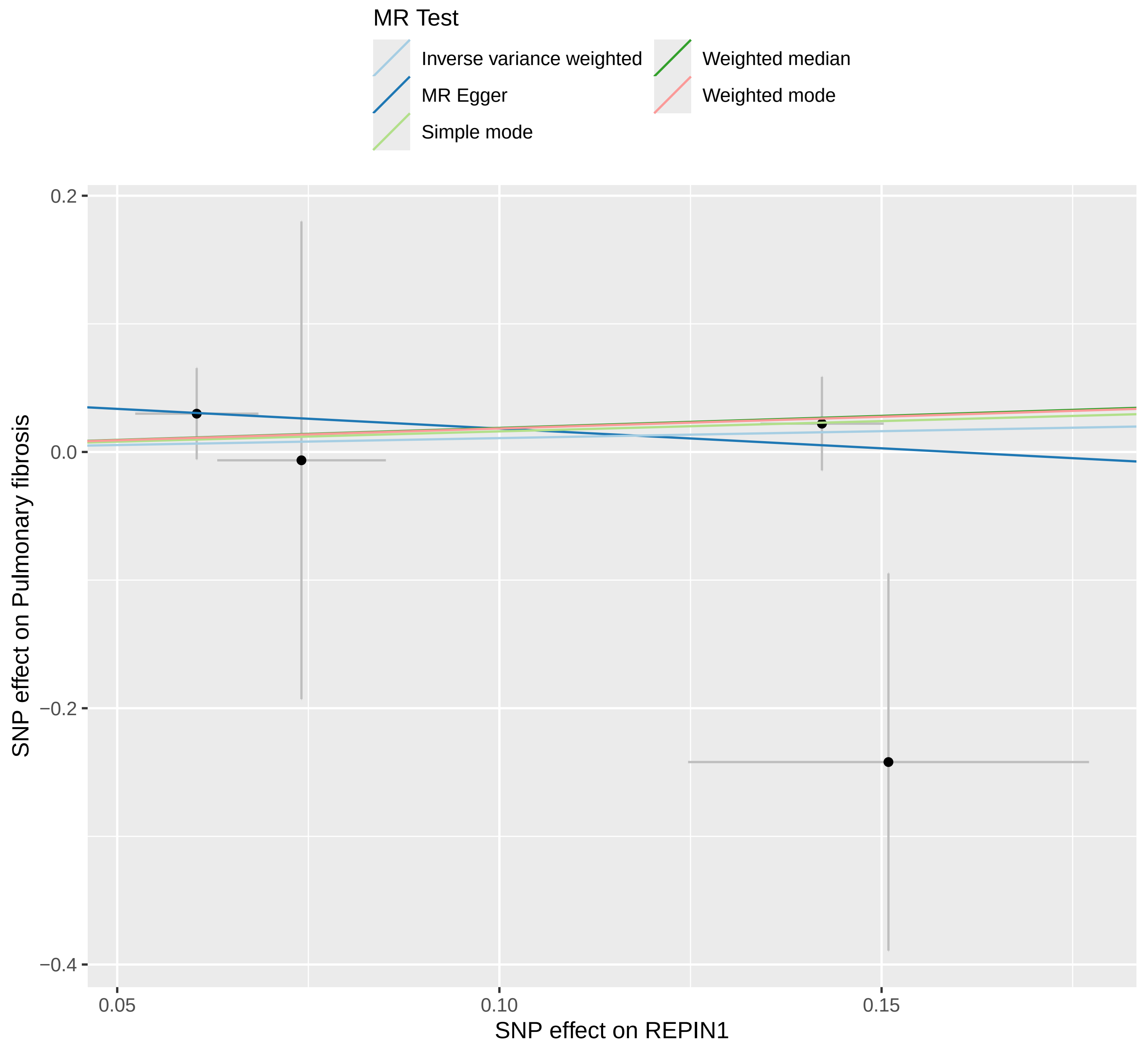

MR Test
Inverse variance weighted
MR Egger
Simple mode
Weighted median
Weighted mode
0.2
0.0
−0.2
−0.4
SNP effect on Pulmonary fibrosis
0.05
0.10
0.15
SNP effect on REPIN1

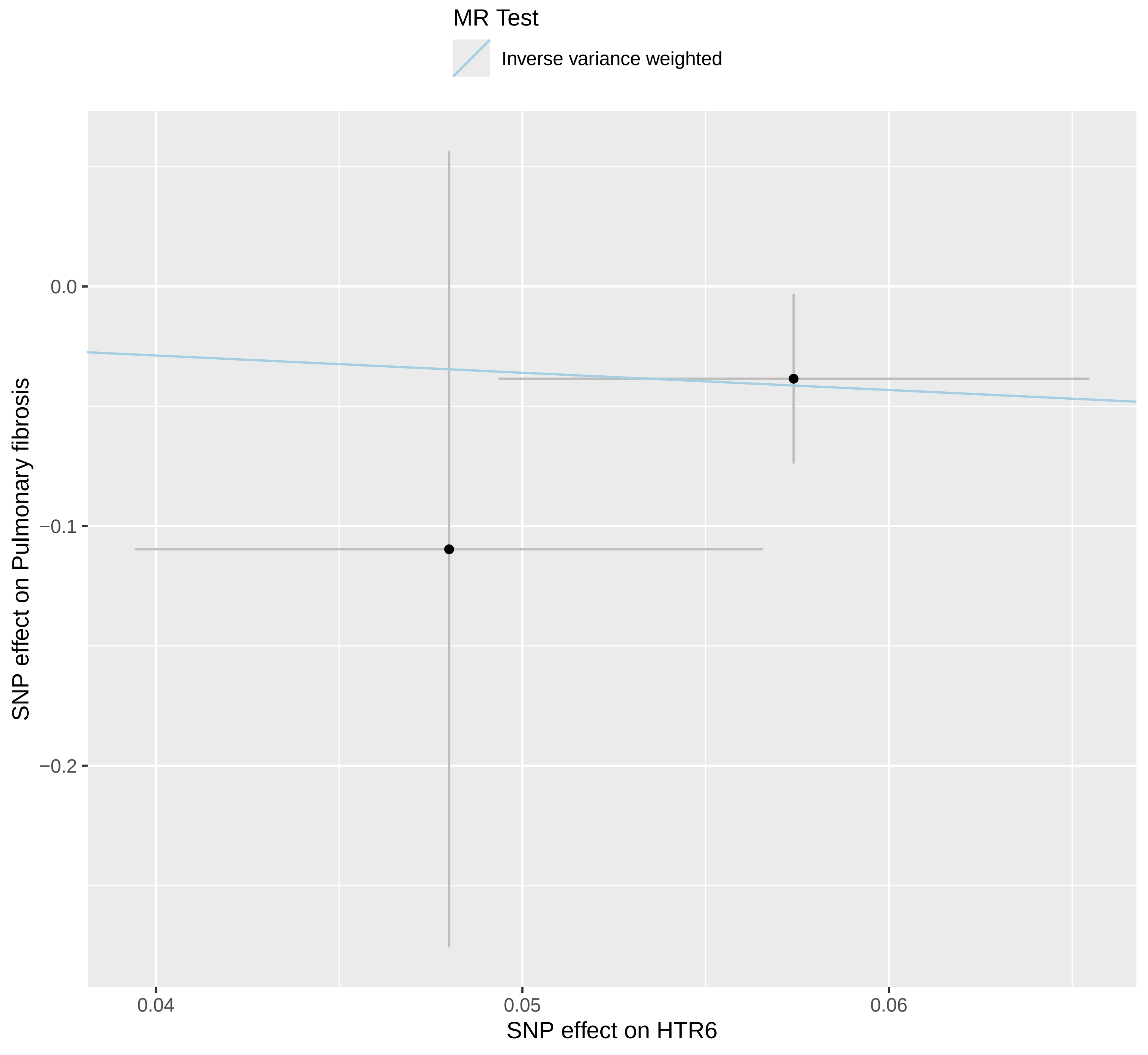
MR Test
Inverse variance weighted
0.0
−0.1
−0.2
SNP effect on Pulmonary fibrosis
0.04
0.05
0.06
SNP effect on HTR6

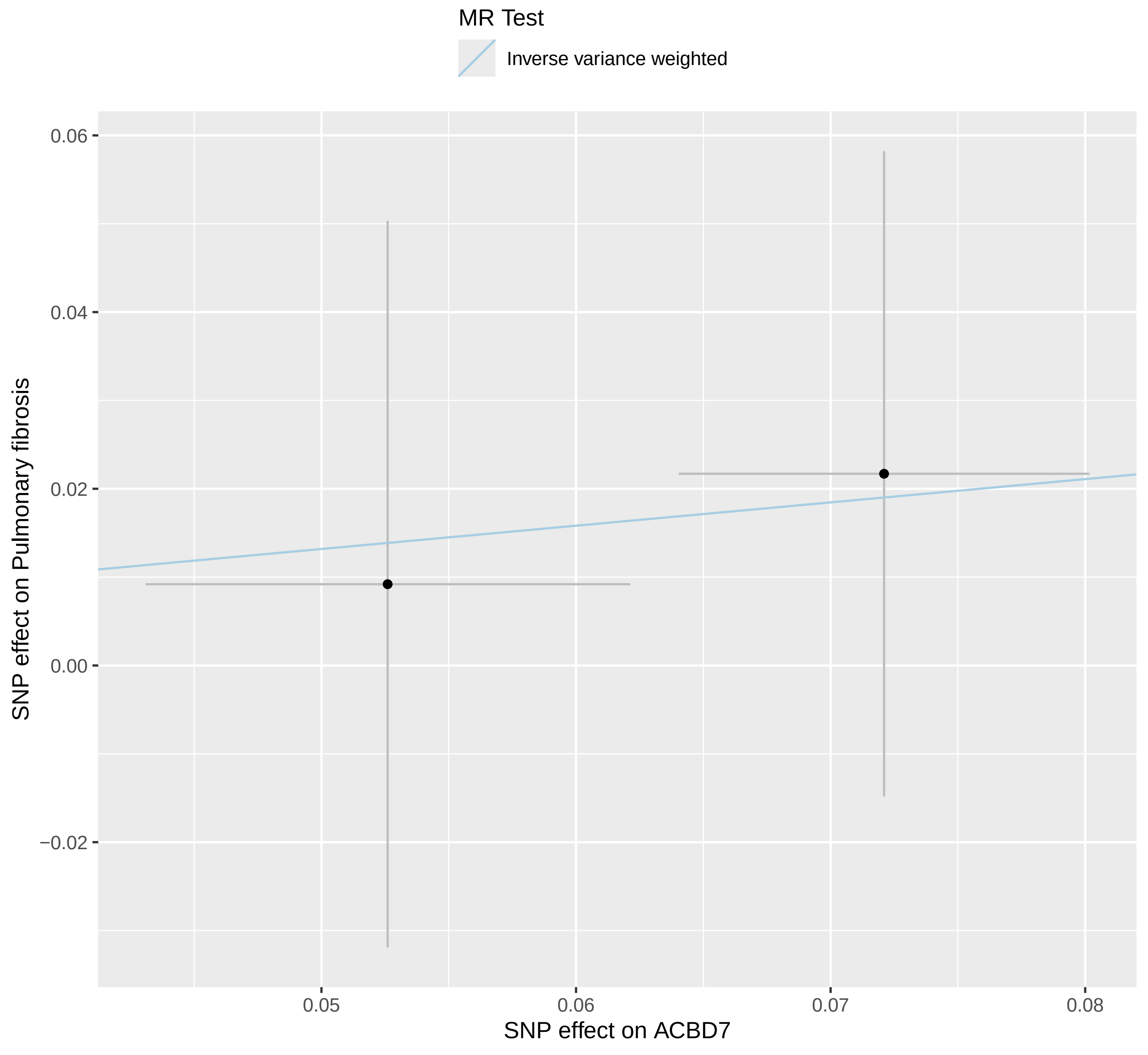
MR Test
Inverse variance weighted
SNP effect on Pulmonary fibrosis
0.06
0.04
0.02
0.00
−0.02
0.05
0.06
0.07
0.08
SNP effect on ACBD7

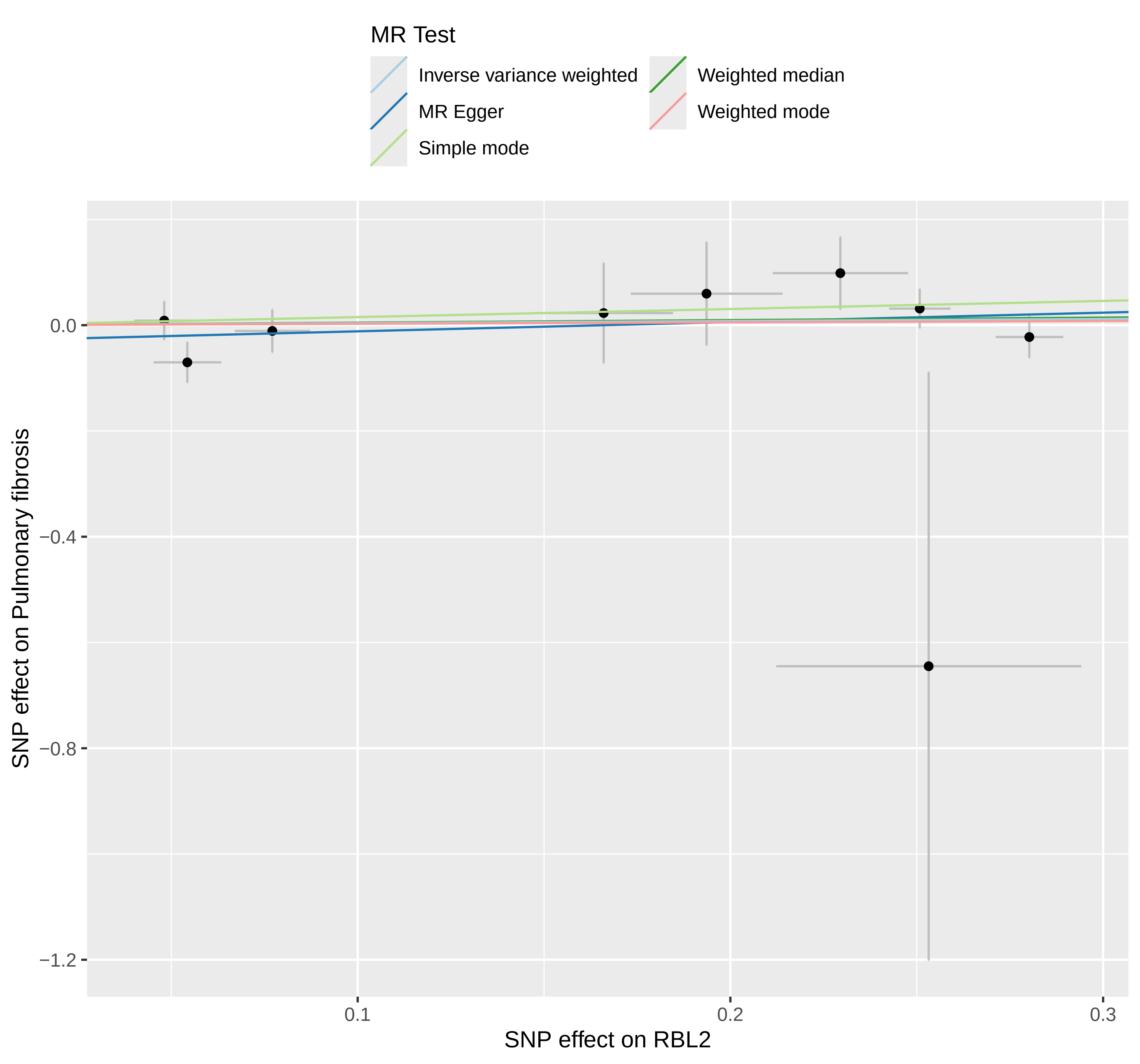
MR Test
Inverse variance weighted
MR Egger
Simple mode
Weighted median
Weighted mode
0.0
−0.4
−0.8
−1.2
0.1
0.2
0.3
SNP effect on Pulmonary fibrosis
SNP effect on RBL2

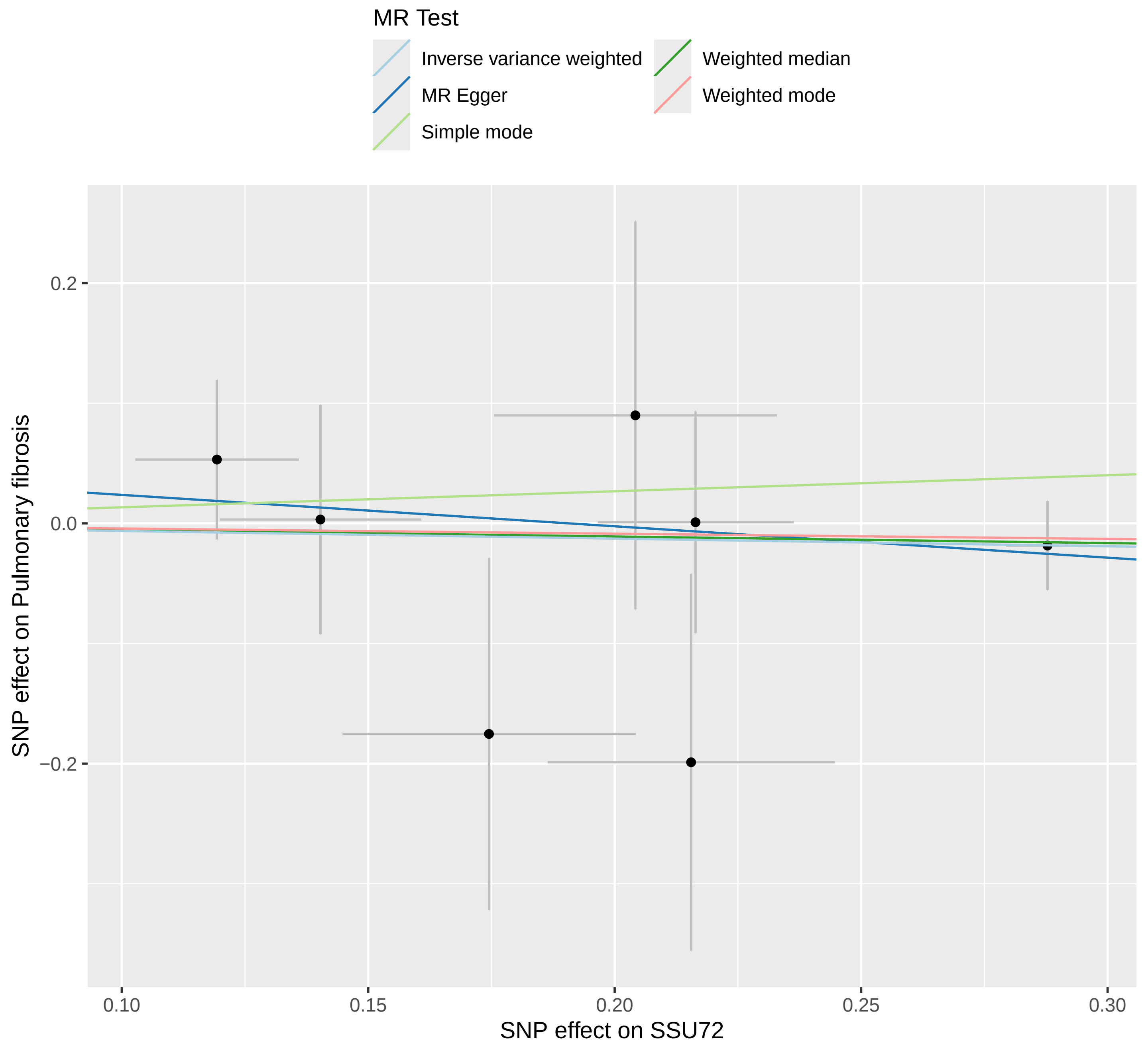
MR Test
Inverse variance weighted
MR Egger
Simple mode
Weighted median
Weighted mode
0.2
0.0
−0.2
0.10
0.15
0.20
0.25
0.30
SNP effect on Pulmonary fibrosis
SNP effect on SSU72

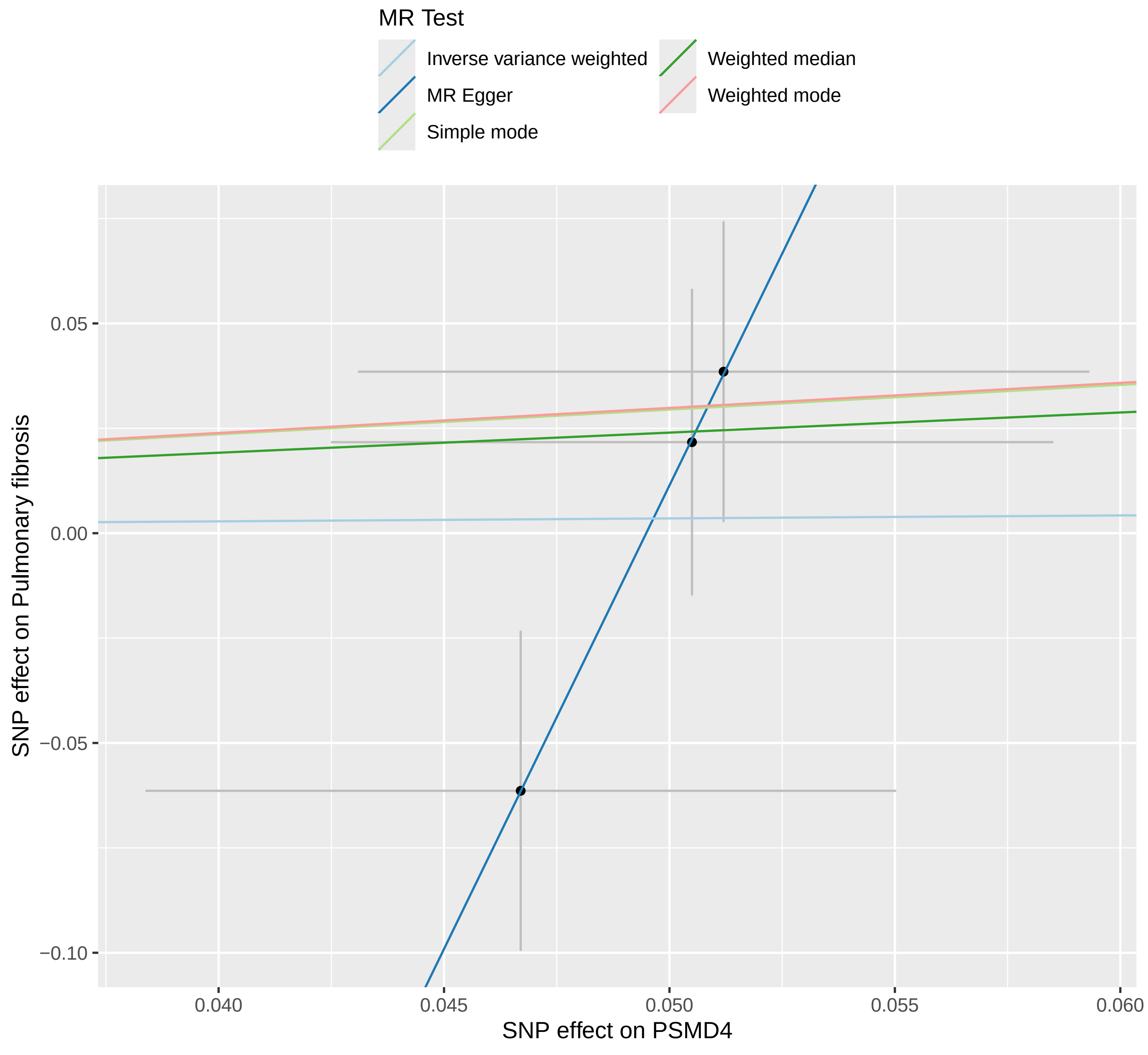

MR Test
Inverse variance weighted
MR Egger
Simple mode
Weighted median
Weighted mode
0.05
0.00
−0.05
−0.10
0.040
0.045
0.050
0.055
0.060
SNP effect on PSMD4
SNP effect on Pulmonary fibrosis

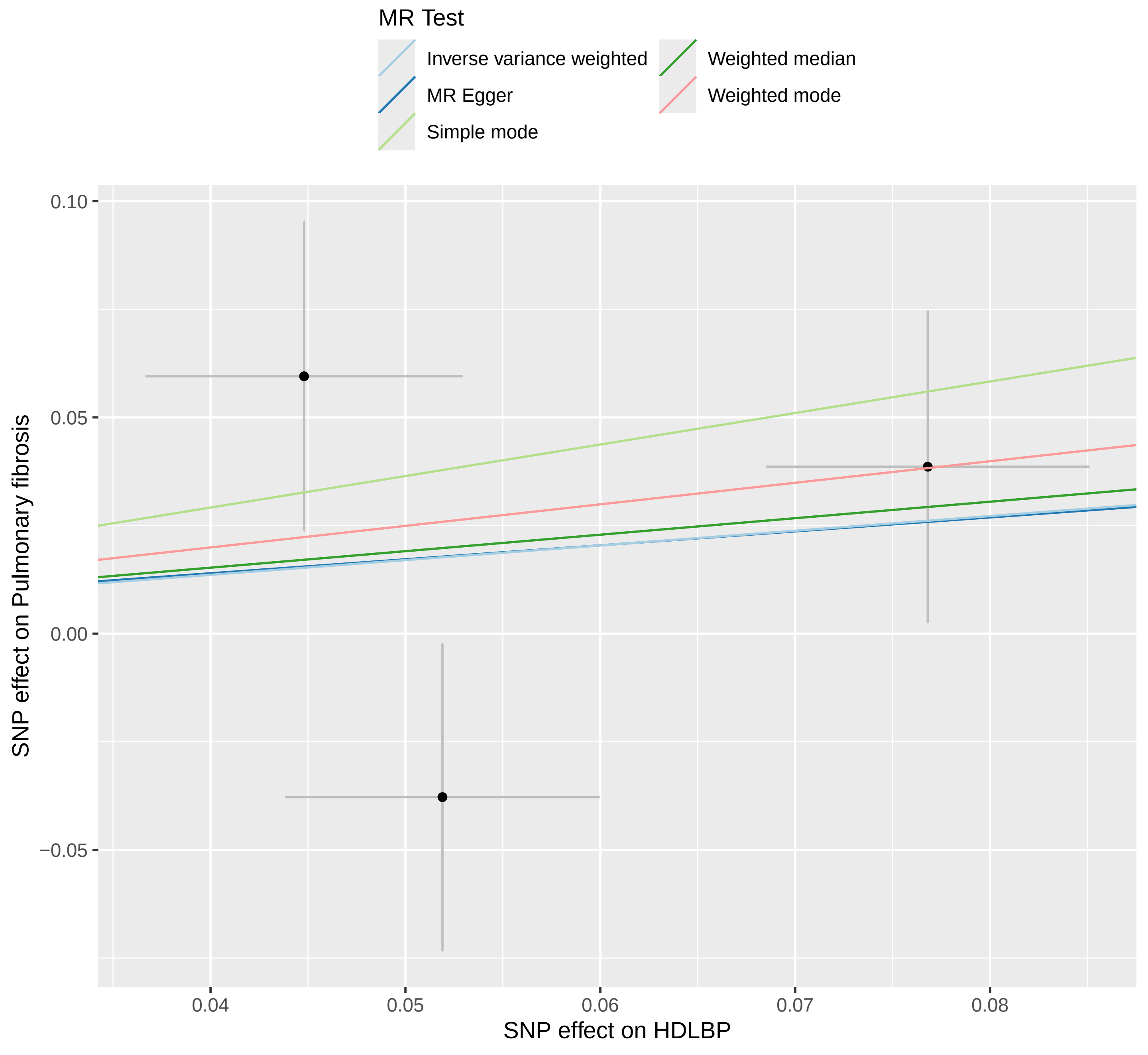
MR Test
Inverse variance weighted
MR Egger
Simple mode
Weighted median
Weighted mode
0.10
0.05
0.00
−0.05
0.04
0.05
0.06
0.07
0.08
SNP effect on Pulmonary fibrosis
SNP effect on HDLBP

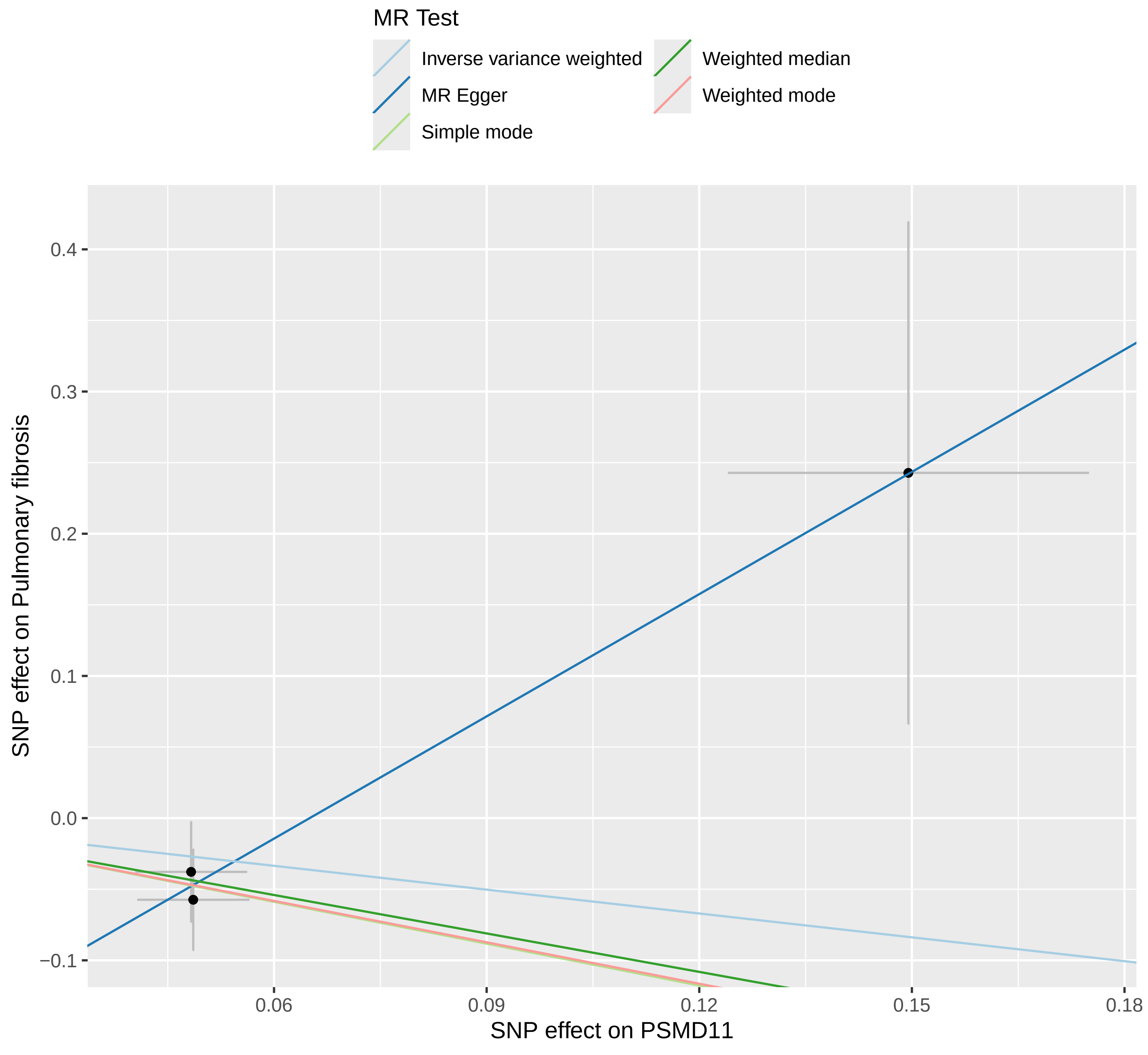

MR Test
Inverse variance weighted
MR Egger
Simple mode
Weighted median
Weighted mode
SNP effect on Pulmonary fibrosis
0.4
0.3
0.2
0.1
0.0
−0.1
0.06
0.09
0.12
0.15
0.18
SNP effect on PSMD11

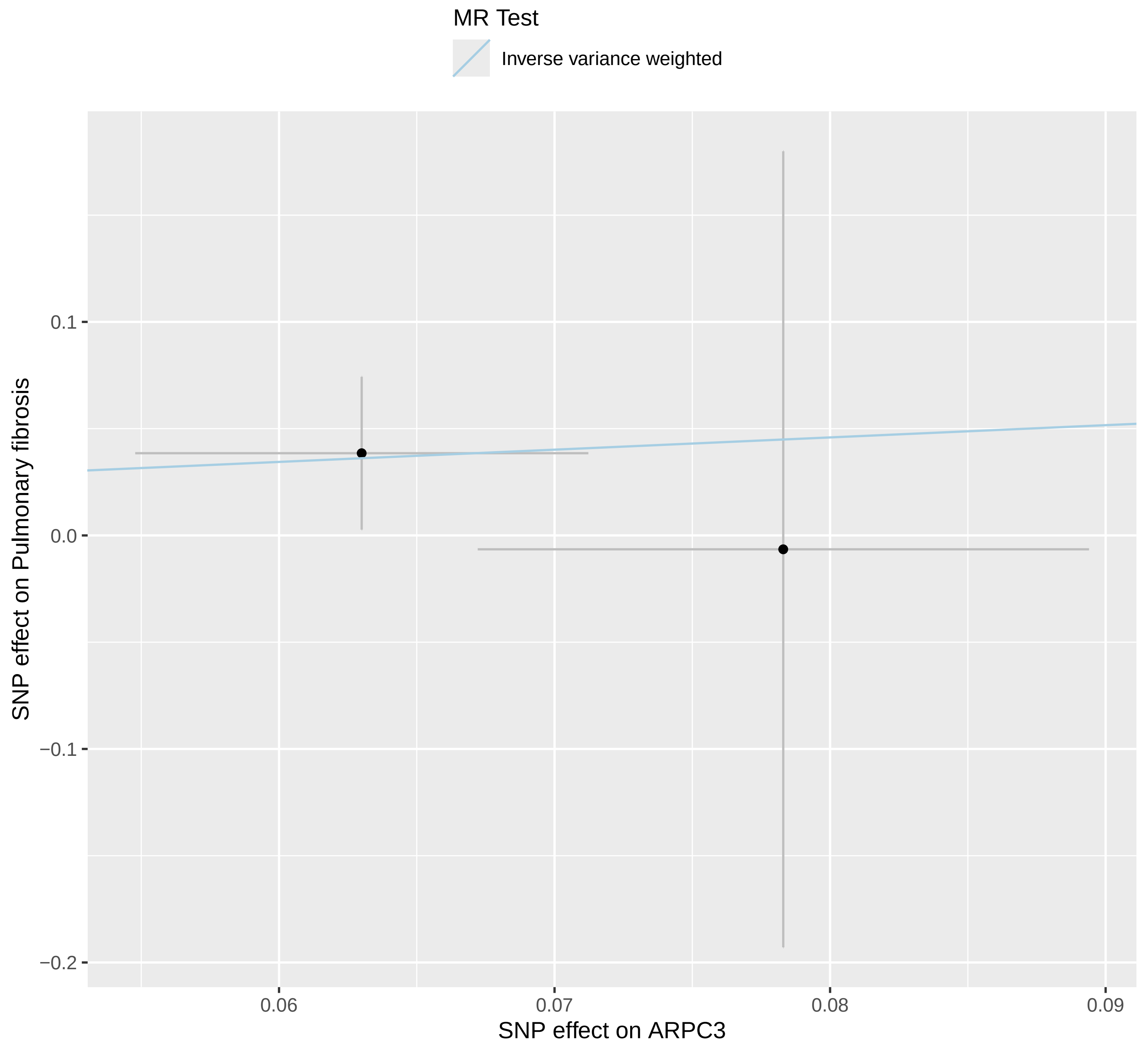
MR Test
Inverse variance weighted
0.1
0.0
−0.1
−0.2
0.06
0.07
0.08
0.09
SNP effect on ARPC3
SNP effect on Pulmonary fibrosis

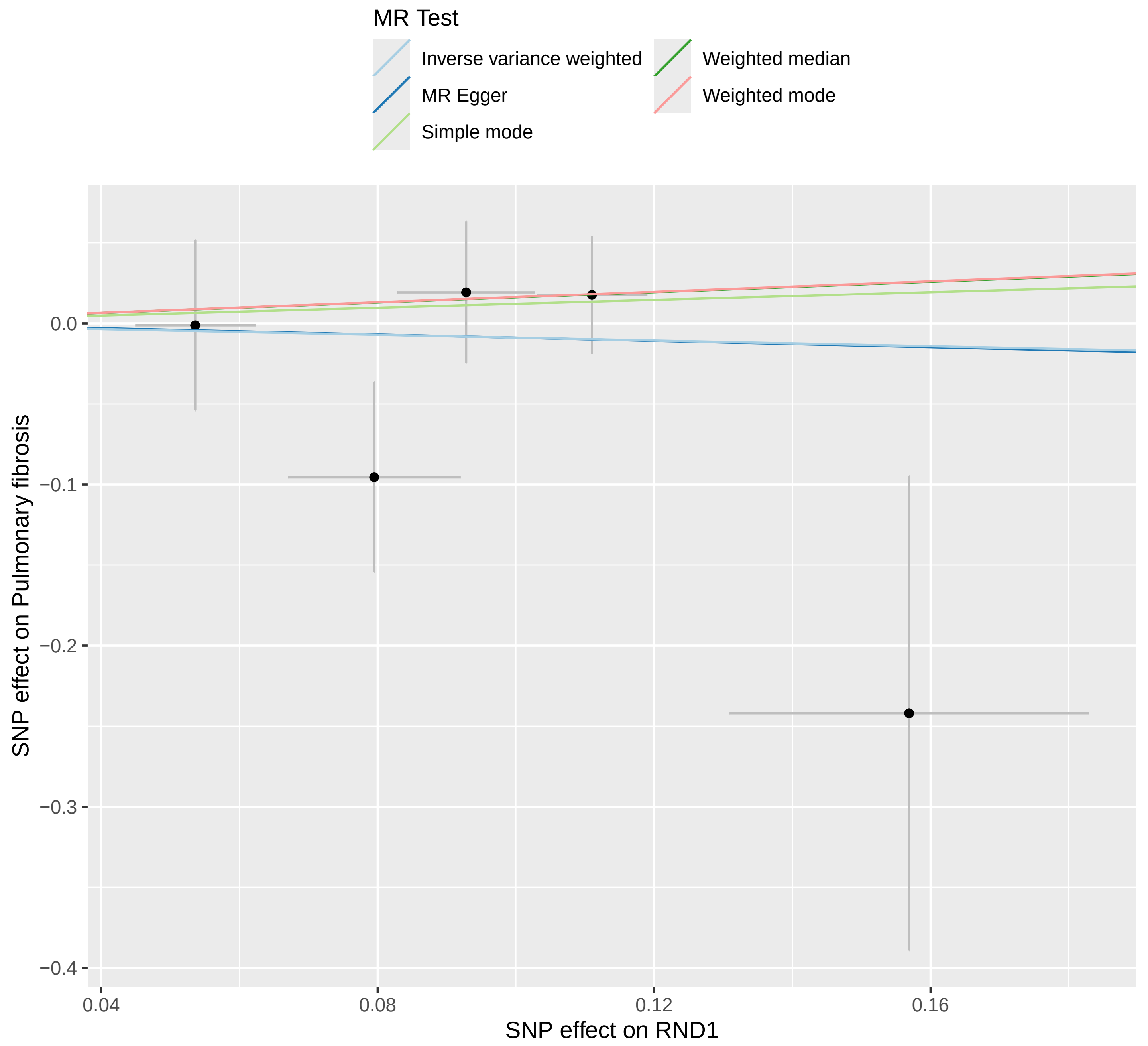
MR Test
Inverse variance weighted
MR Egger
Simple mode
Weighted median
Weighted mode
SNP effect on Pulmonary fibrosis
0.0
−0.1
−0.2
−0.3
−0.4
0.04
0.08
0.12
0.16
SNP effect on RND1

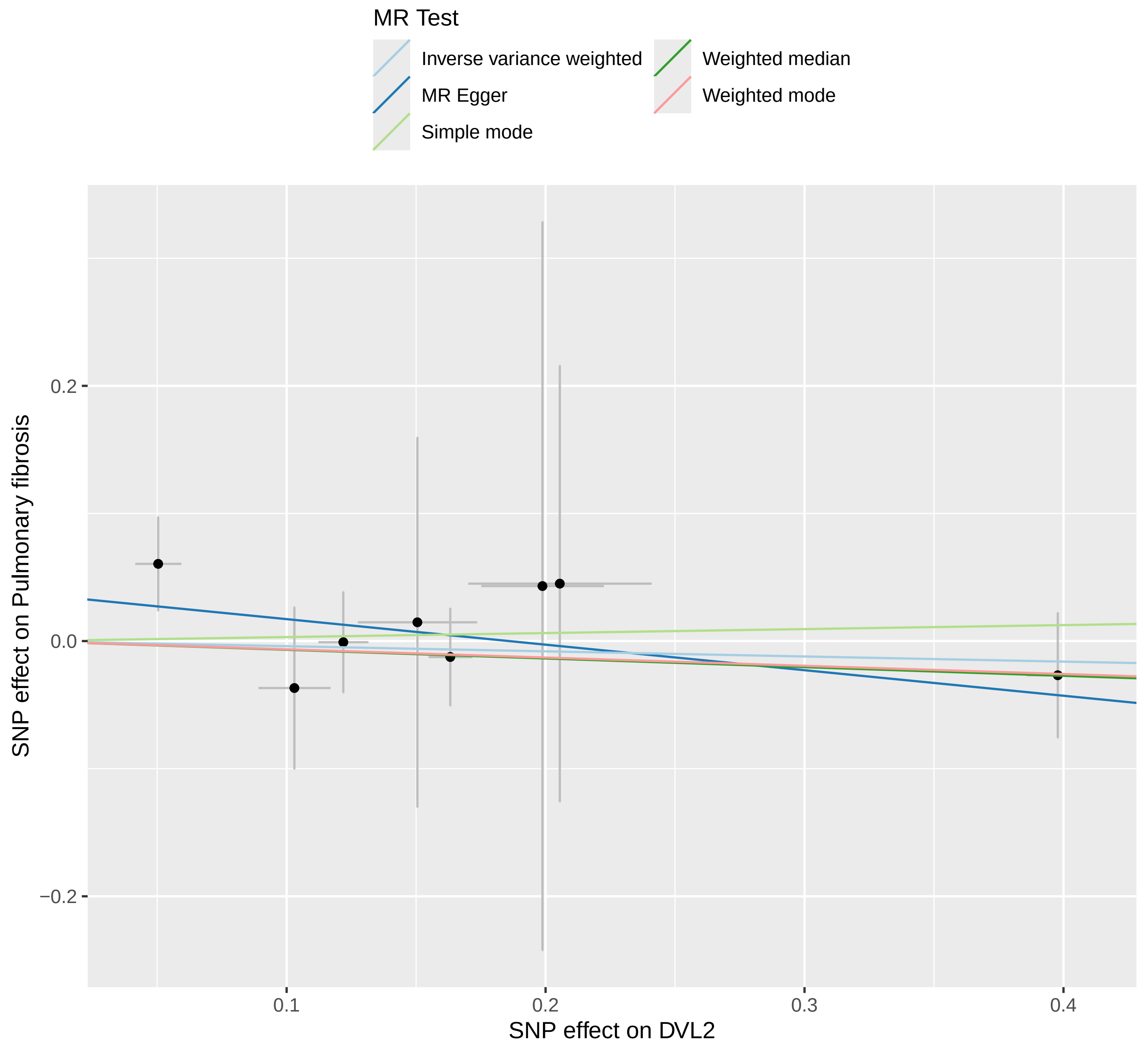
MR Test
Inverse variance weighted
MR Egger
Simple mode
Weighted median
Weighted mode
0.2
0.0
−0.2
0.1
0.2
0.3
0.4
SNP effect on Pulmonary fibrosis
SNP effect on DVL2

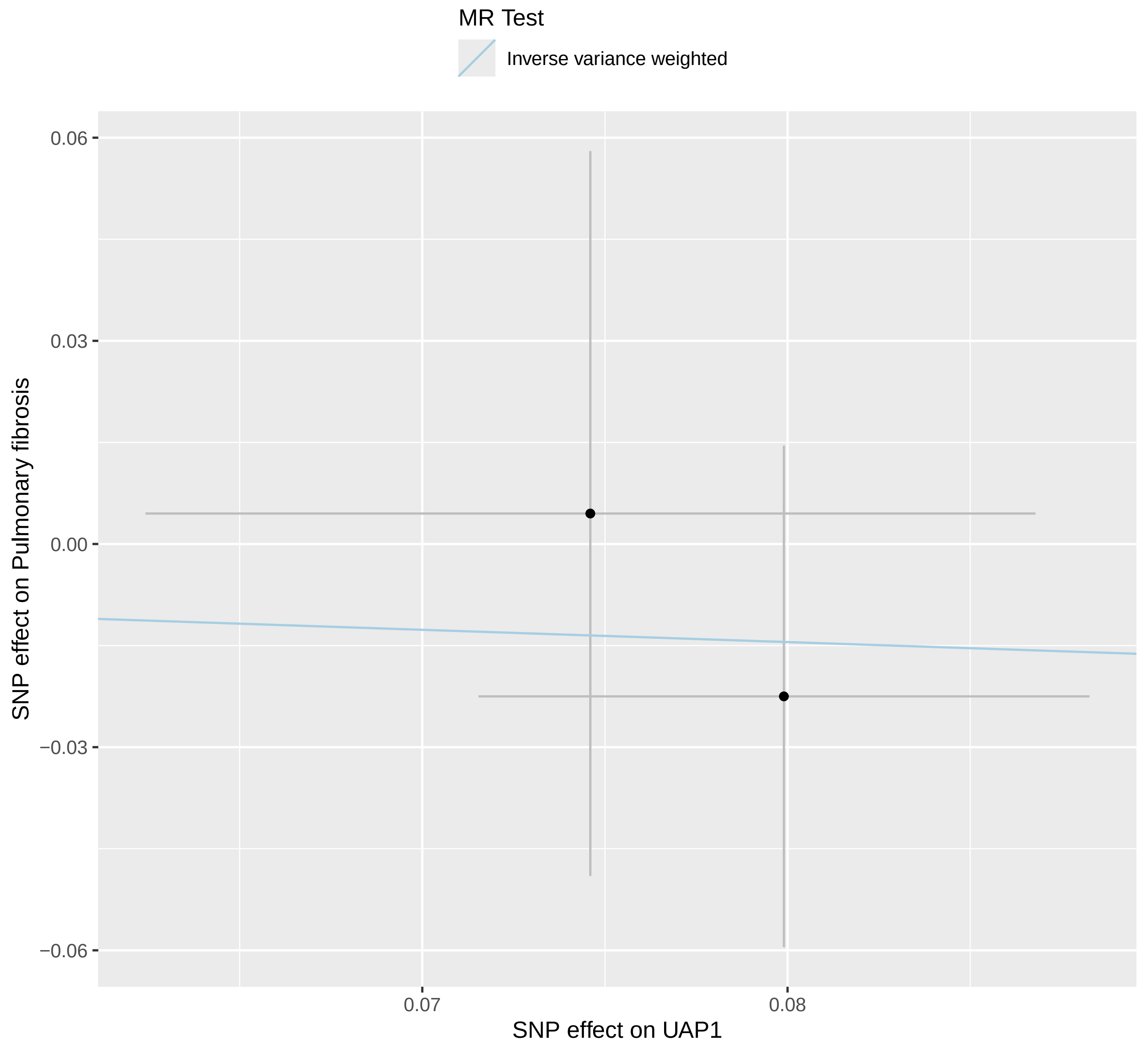

MR Test
Inverse variance weighted
0.06
0.03
0.00
−0.03
−0.06
SNP effect on Pulmonary fibrosis
0.07
0.08
SNP effect on UAP1

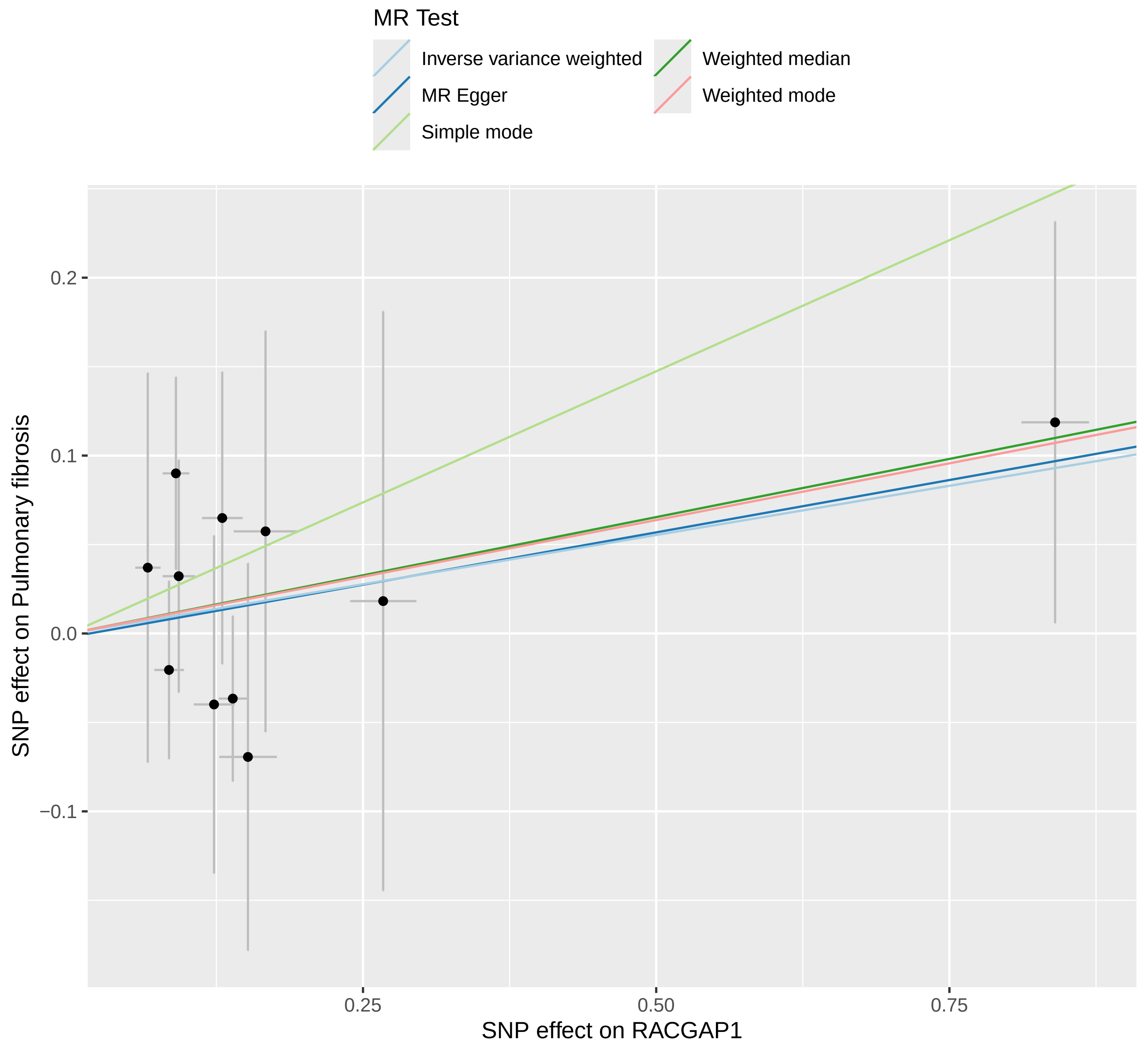

MR Test
Inverse variance weighted
MR Egger
Simple mode
Weighted median
Weighted mode
SNP effect on Pulmonary fibrosis
0.2
0.1
0.0
−0.1
0.25
0.50
0.75
SNP effect on RACGAP1

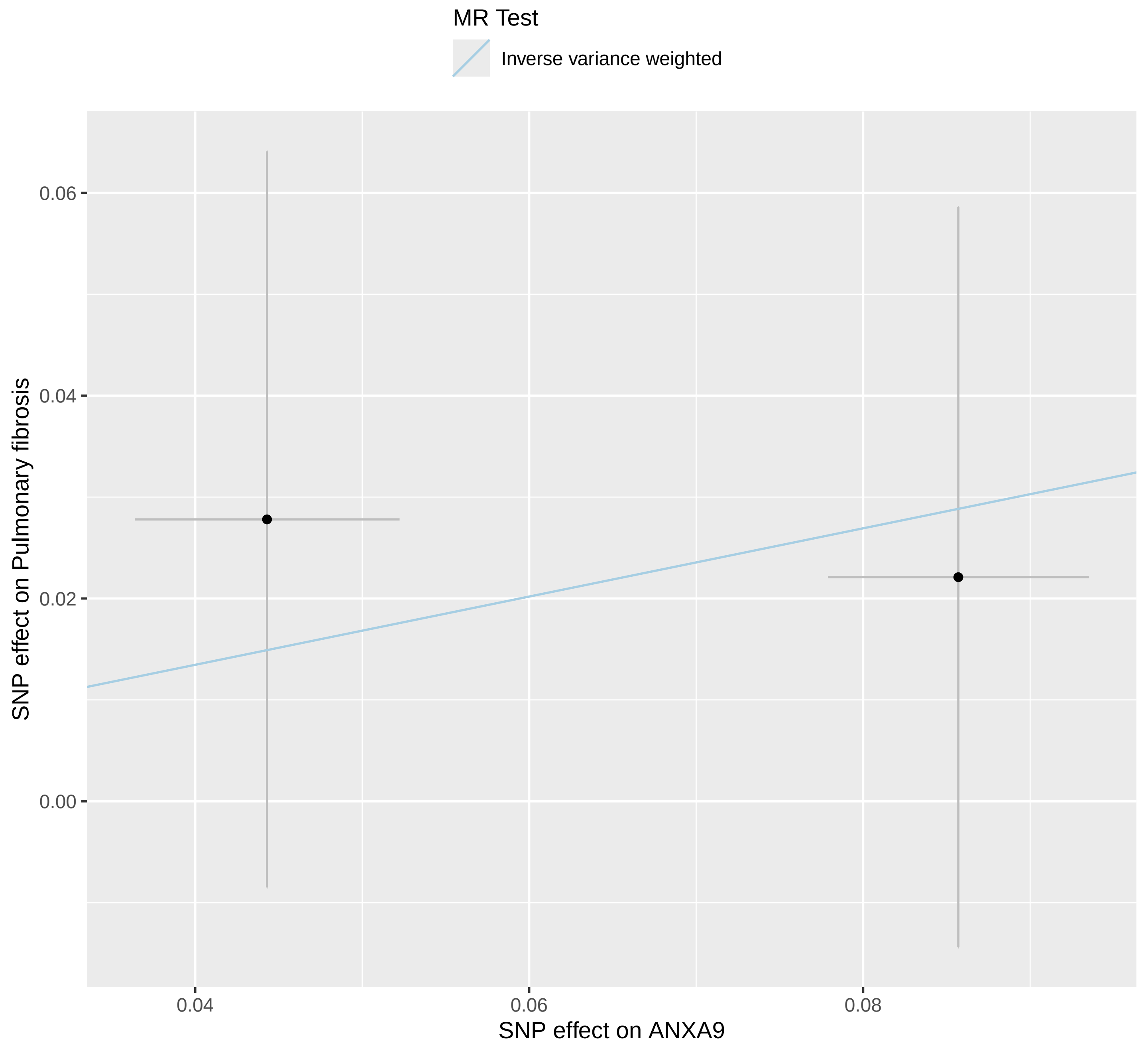

MR Test
Inverse variance weighted
SNP effect on Pulmonary fibrosis
0.06
0.04
0.02
0.00
0.04
0.06
0.08
SNP effect on ANXA9

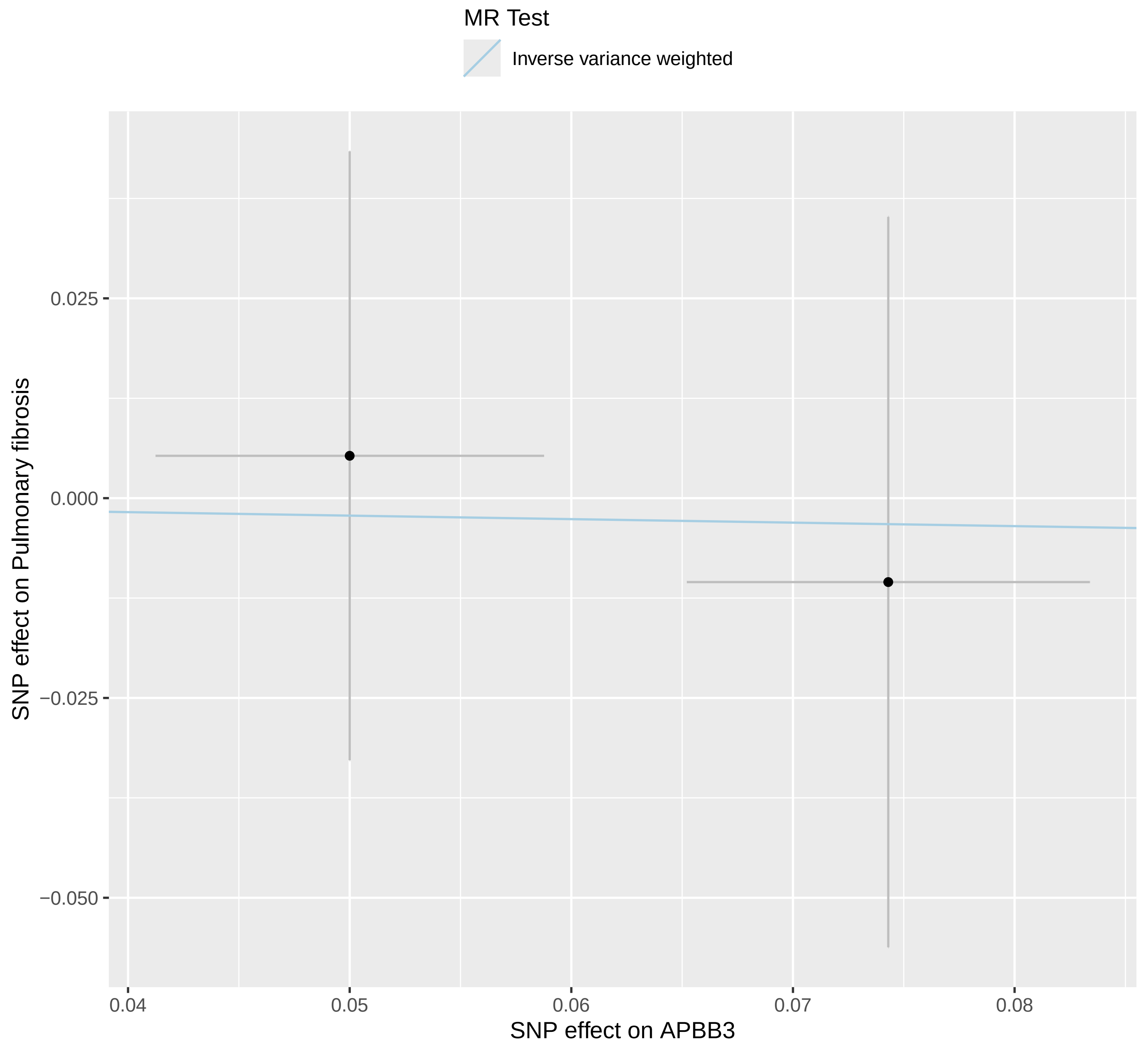
MR Test
Inverse variance weighted
0.025
0.000
−0.025
−0.050
0.04
0.05
0.06
0.07
0.08
SNP effect on Pulmonary fibrosis
SNP effect on APBB3

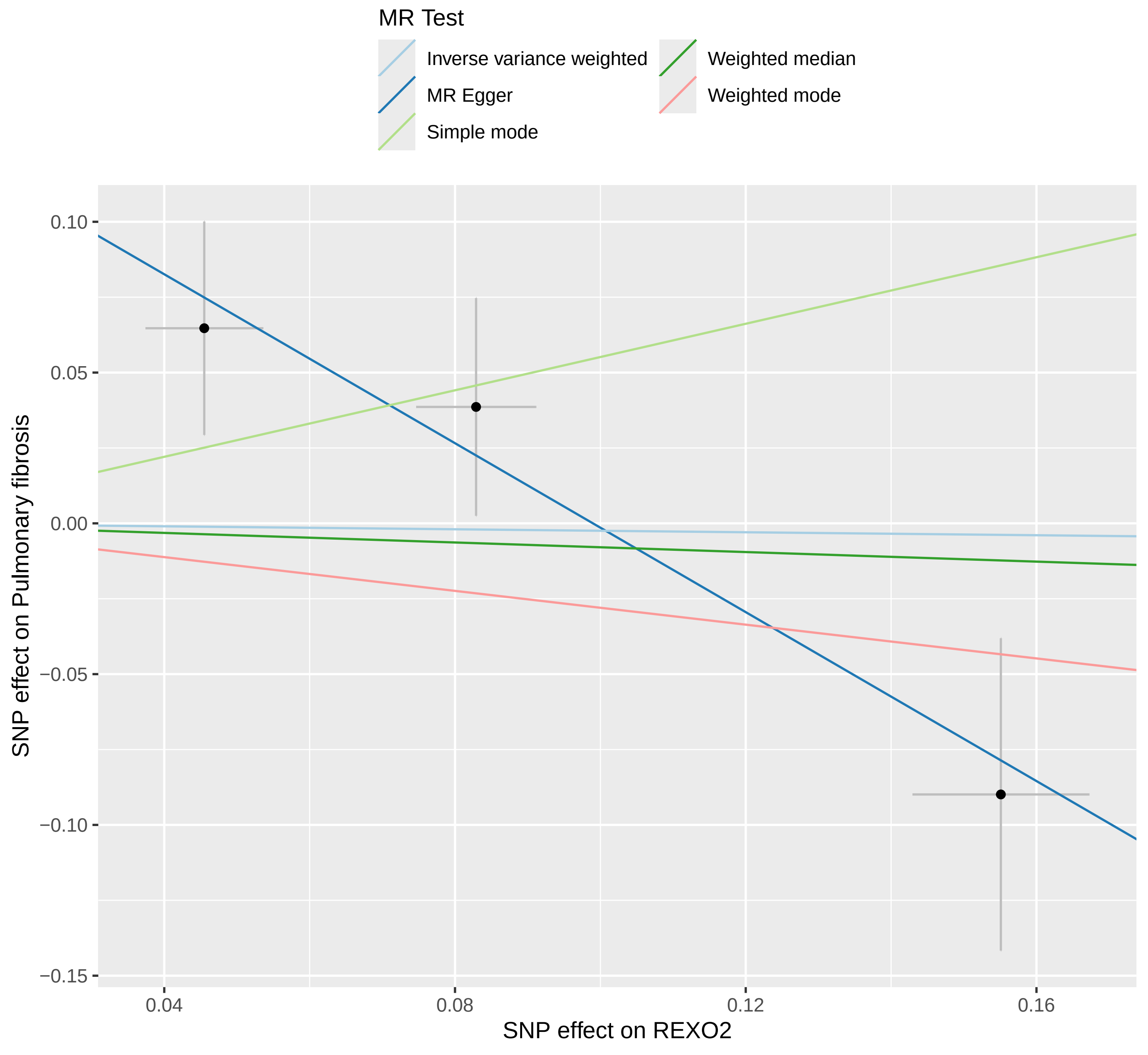
MR Test
Inverse variance weighted
MR Egger
Simple mode
Weighted median
Weighted mode
0.10
0.05
0.00
−0.05
−0.10
−0.15
SNP effect on Pulmonary fibrosis
0.04
0.08
0.12
0.16
SNP effect on REXO2

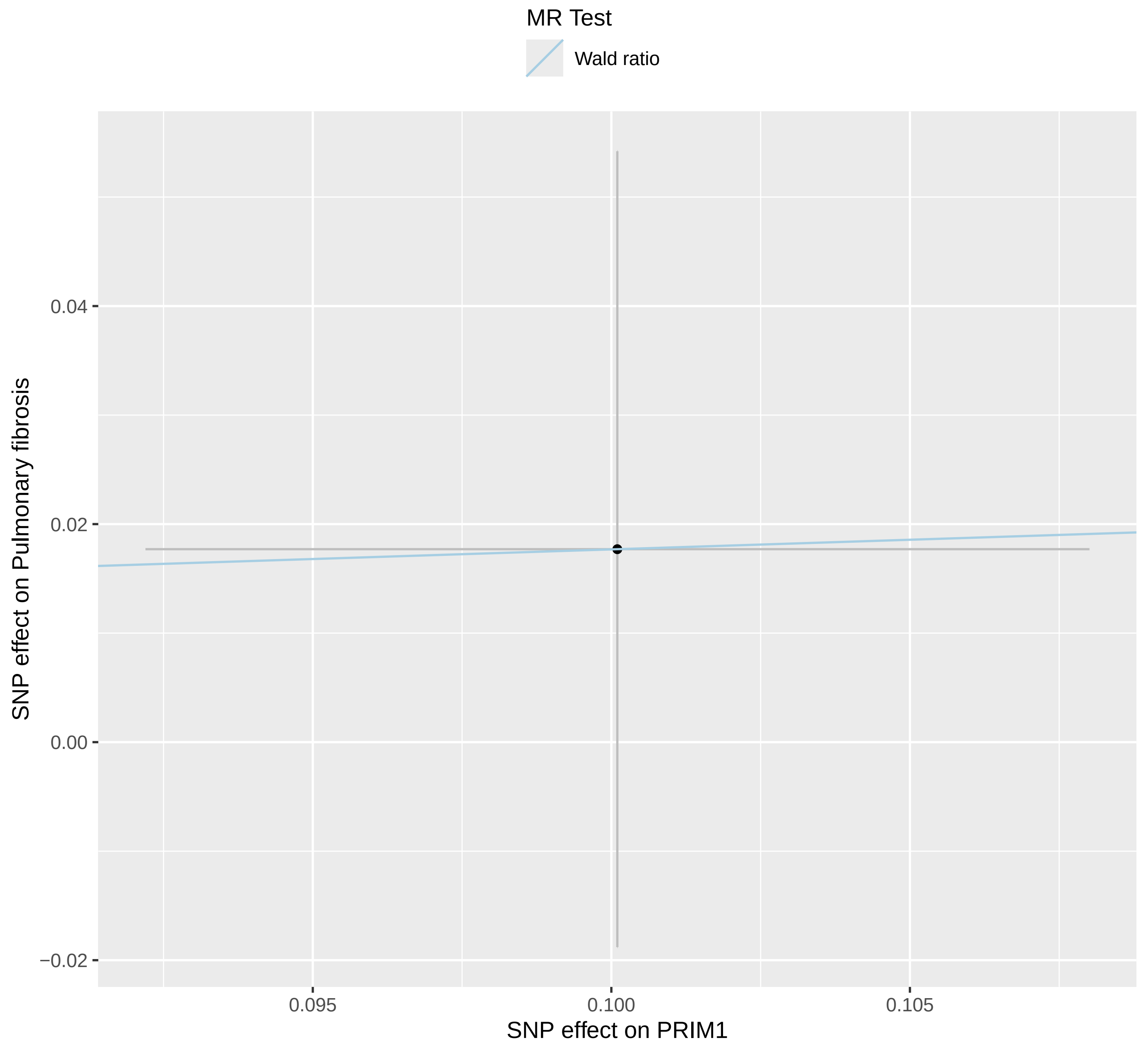

MR Test
Wald ratio
SNP effect on Pulmonary fibrosis
0.04
0.02
0.00
−0.02
0.095
0.100
0.105
SNP effect on PRIM1

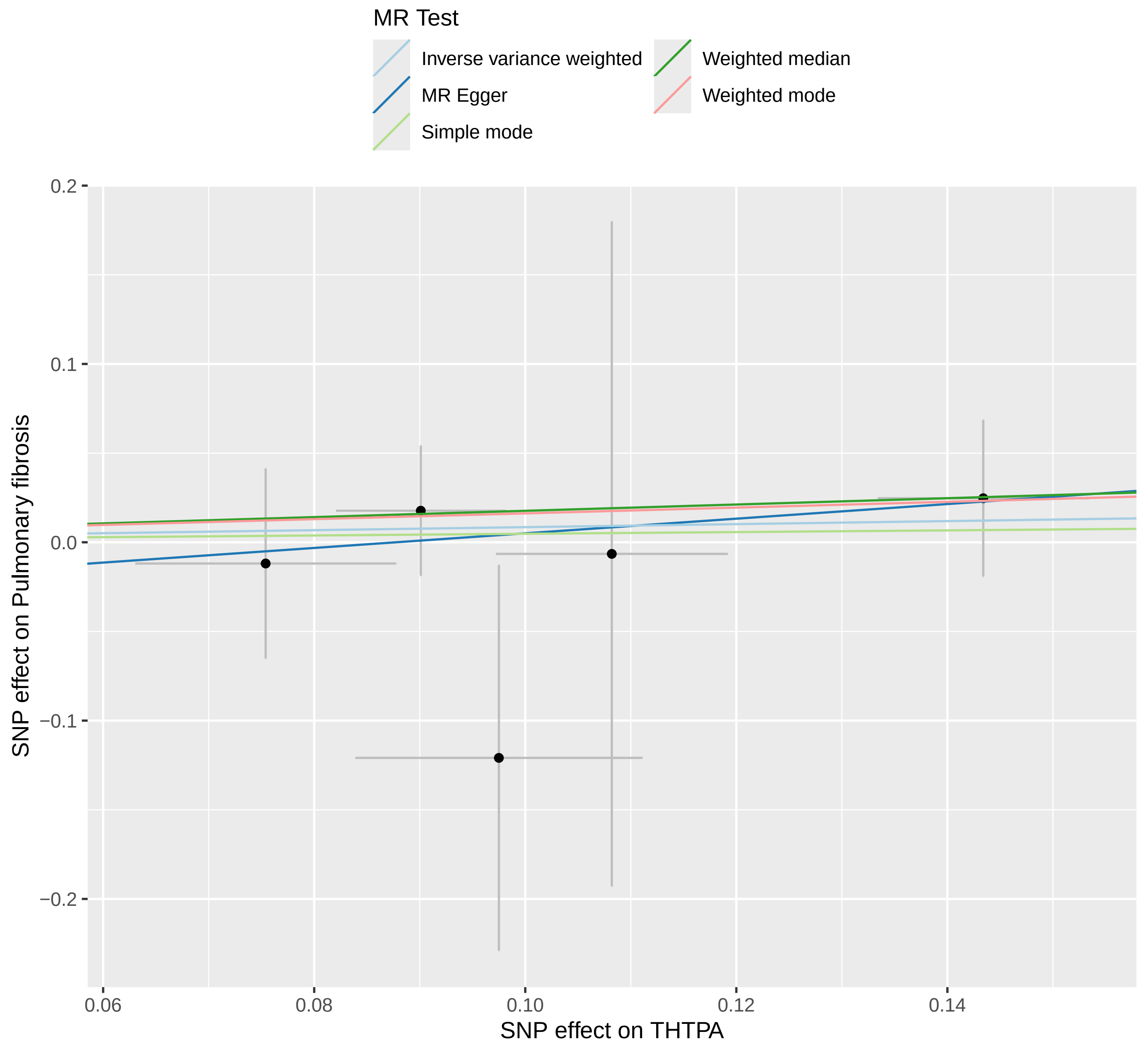
MR Test
Inverse variance weighted
MR Egger
Simple mode
Weighted median
Weighted mode
0.2
0.1
0.0
−0.1
−0.2
0.06
0.08
0.10
0.12
0.14
SNP effect on THTPA
SNP effect on Pulmonary fibrosis

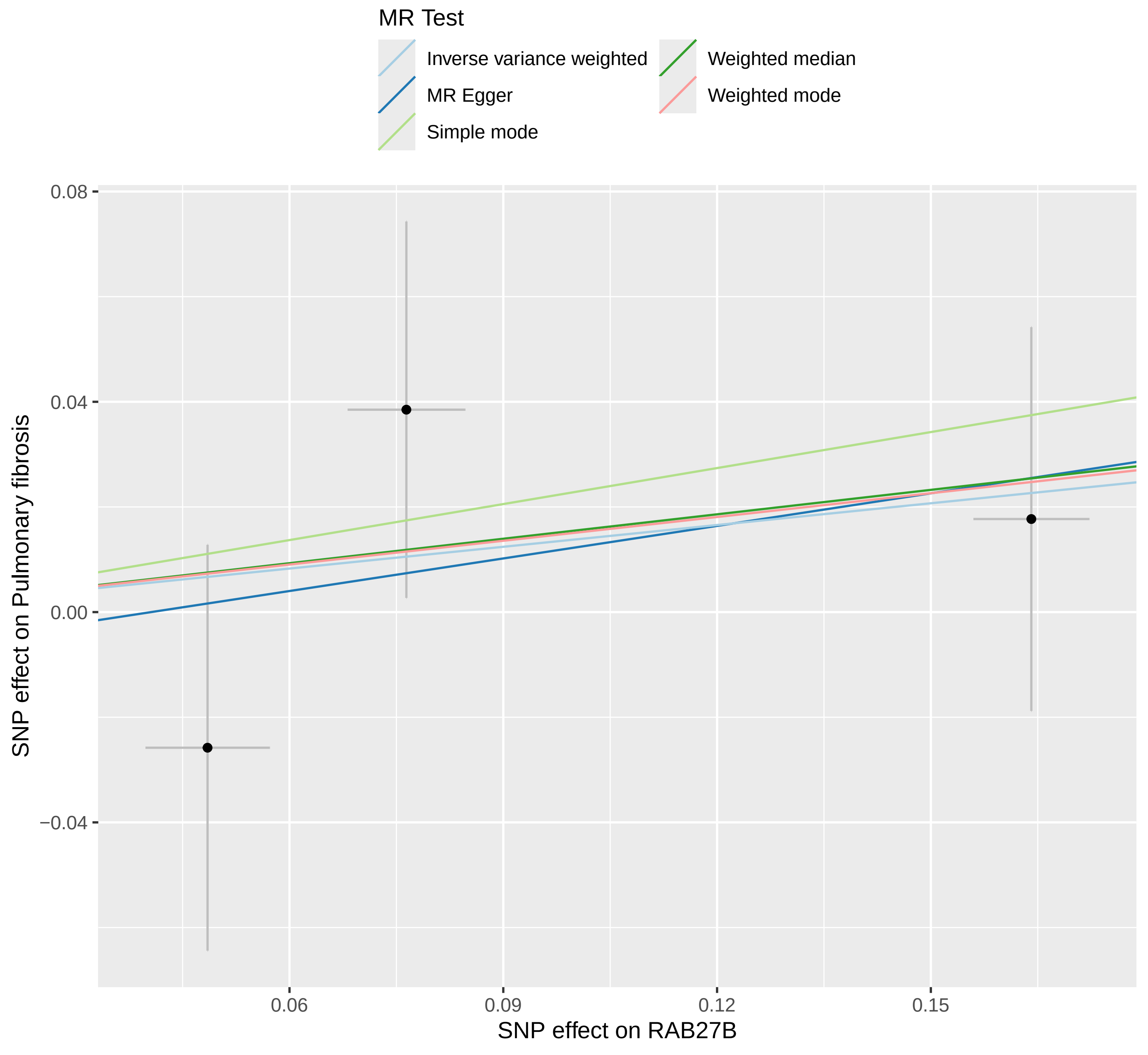
MR Test
Inverse variance weighted
MR Egger
Simple mode
Weighted median
Weighted mode
0.08
0.04
0.00
−0.04
0.06
0.09
0.12
0.15
SNP effect on Pulmonary fibrosis
SNP effect on RAB27B

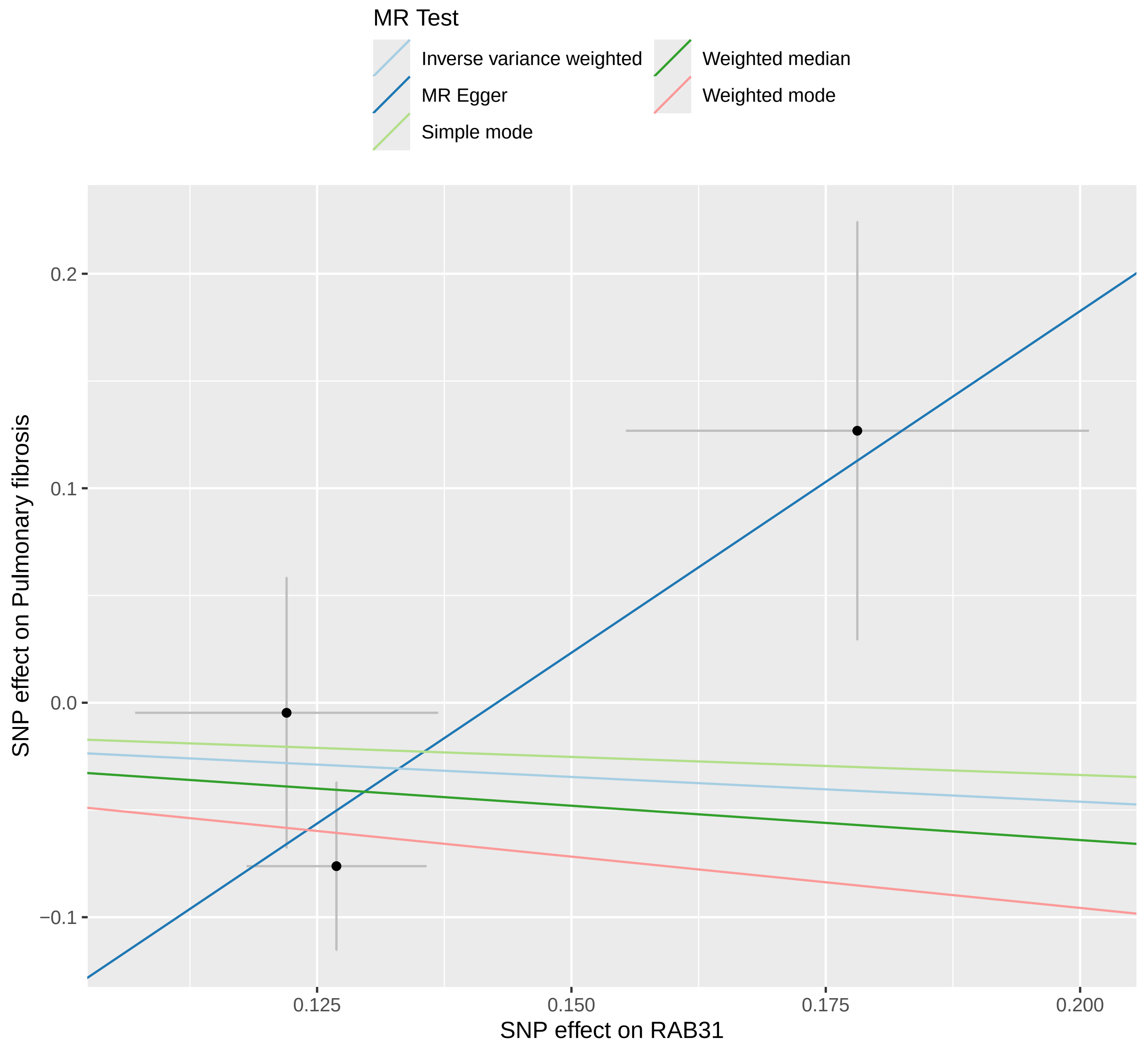
MR Test
Inverse variance weighted
MR Egger
Simple mode
Weighted median
Weighted mode
0.2
0.1
0.0
−0.1
0.125
0.150
0.175
0.200
SNP effect on Pulmonary fibrosis
SNP effect on RAB31

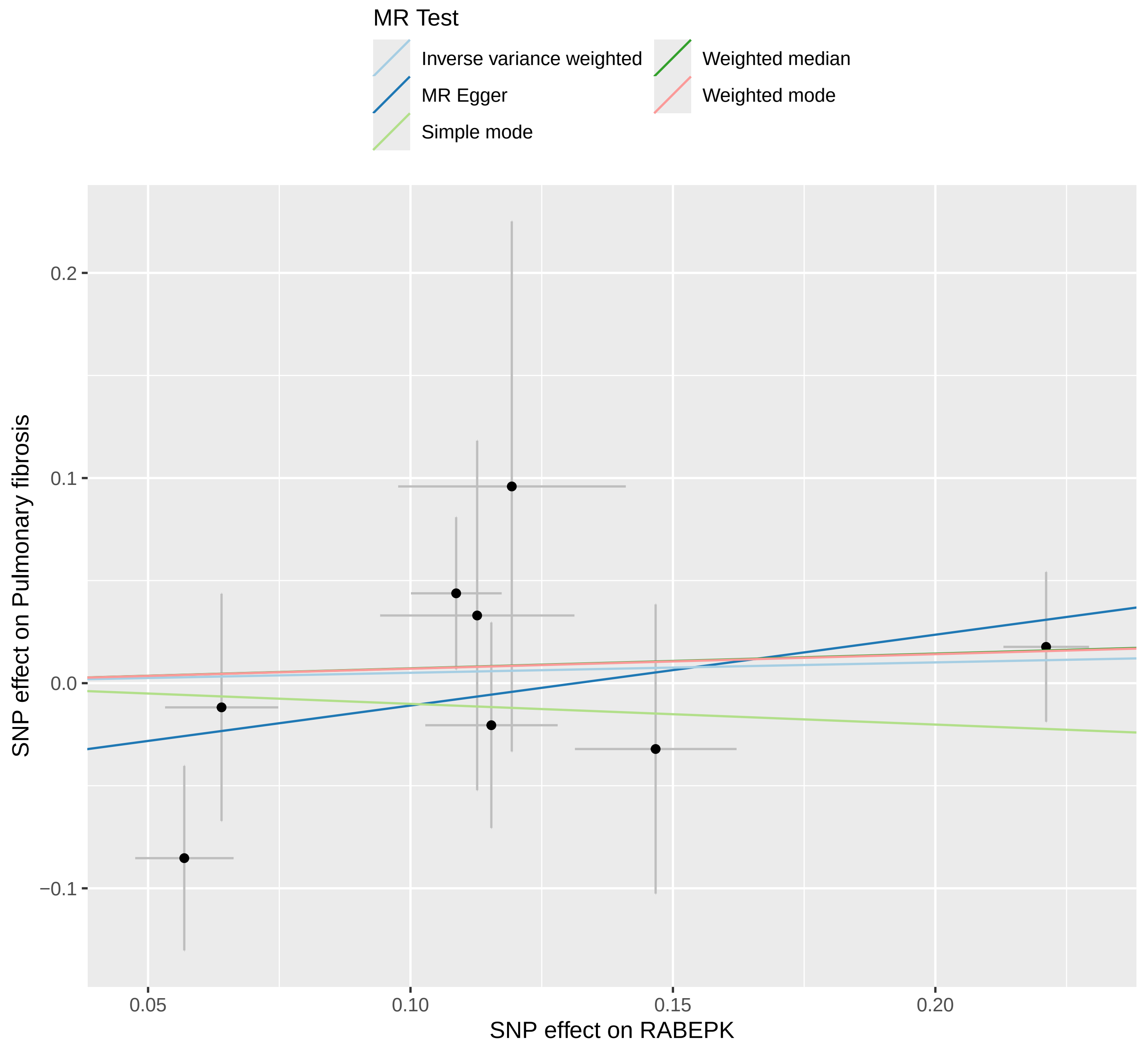
MR Test
Inverse variance weighted
MR Egger
Simple mode
Weighted median
Weighted mode
SNP effect on Pulmonary fibrosis
0.2
0.1
0.0
−0.1
0.05
0.10
0.15
0.20
SNP effect on RABEPK

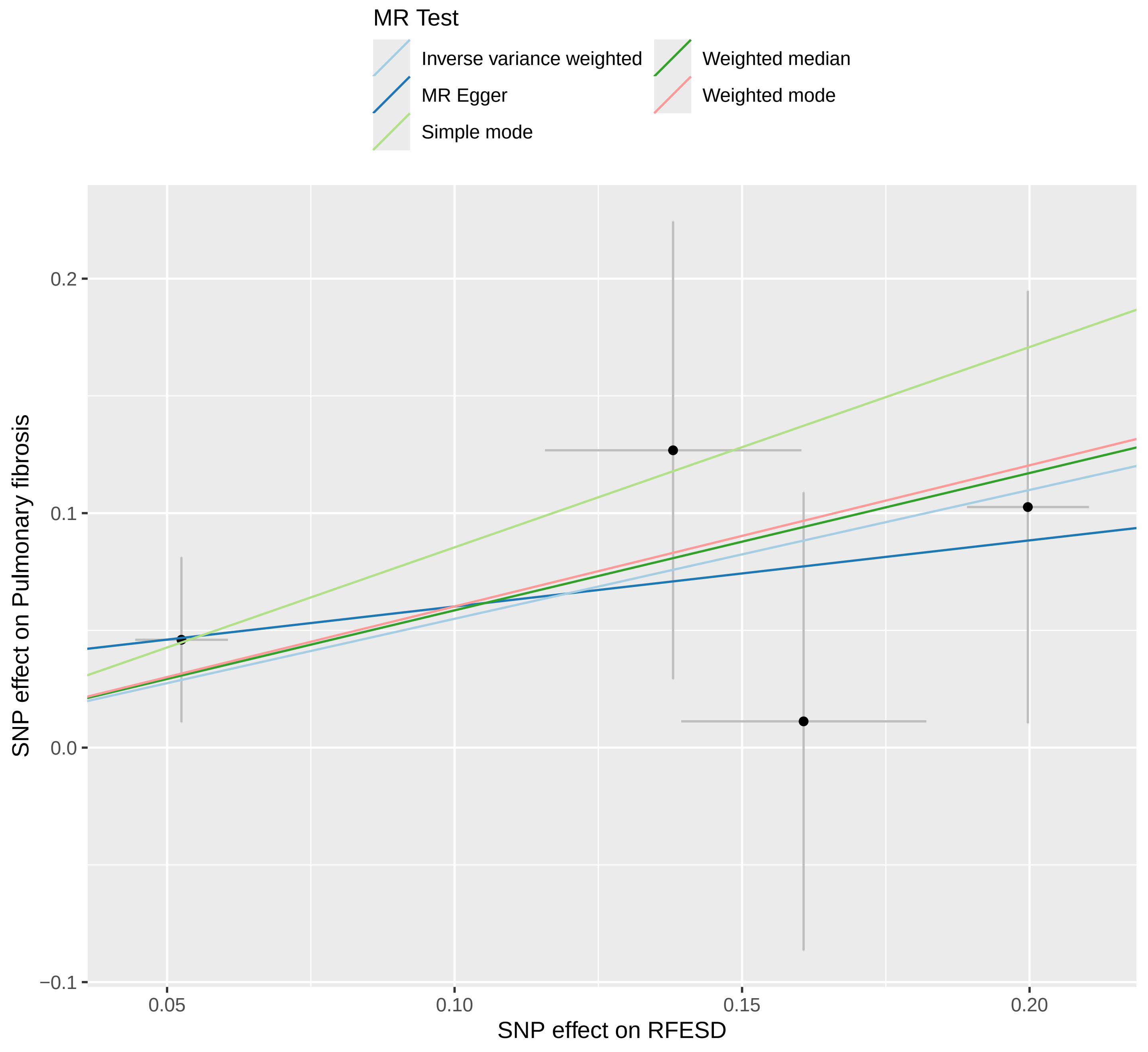
MR Test
Inverse variance weighted
MR Egger
Simple mode
Weighted median
Weighted mode
0.2
0.1
0.0
−0.1
0.05
0.10
0.15
0.20
SNP effect on RFESD
SNP effect on Pulmonary fibrosis

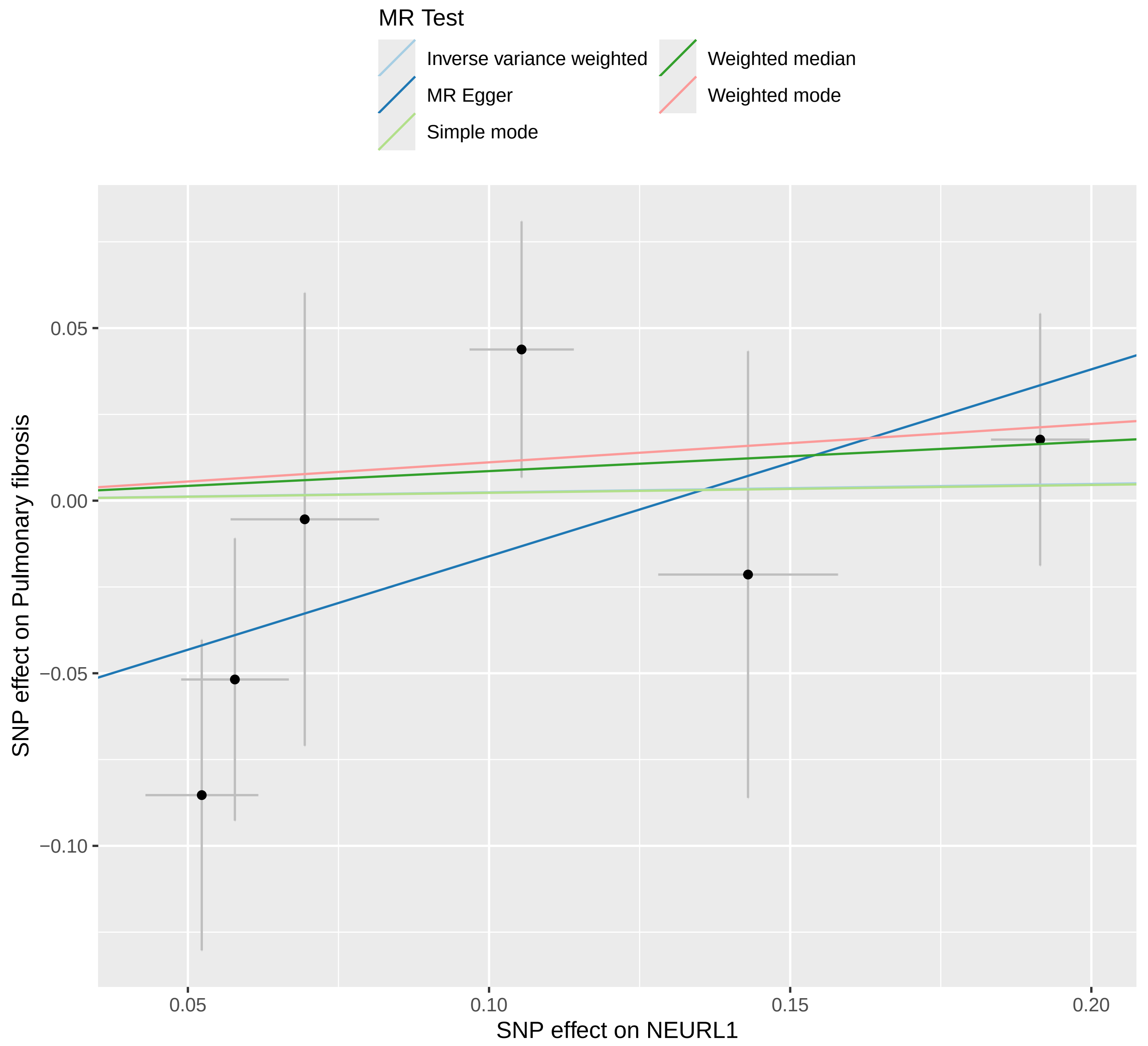
MR Test
Inverse variance weighted
MR Egger
Simple mode
Weighted median
Weighted mode
0.05
0.00
−0.05
−0.10
0.05
0.10
0.15
0.20
SNP effect on Pulmonary fibrosis
SNP effect on NEURL1

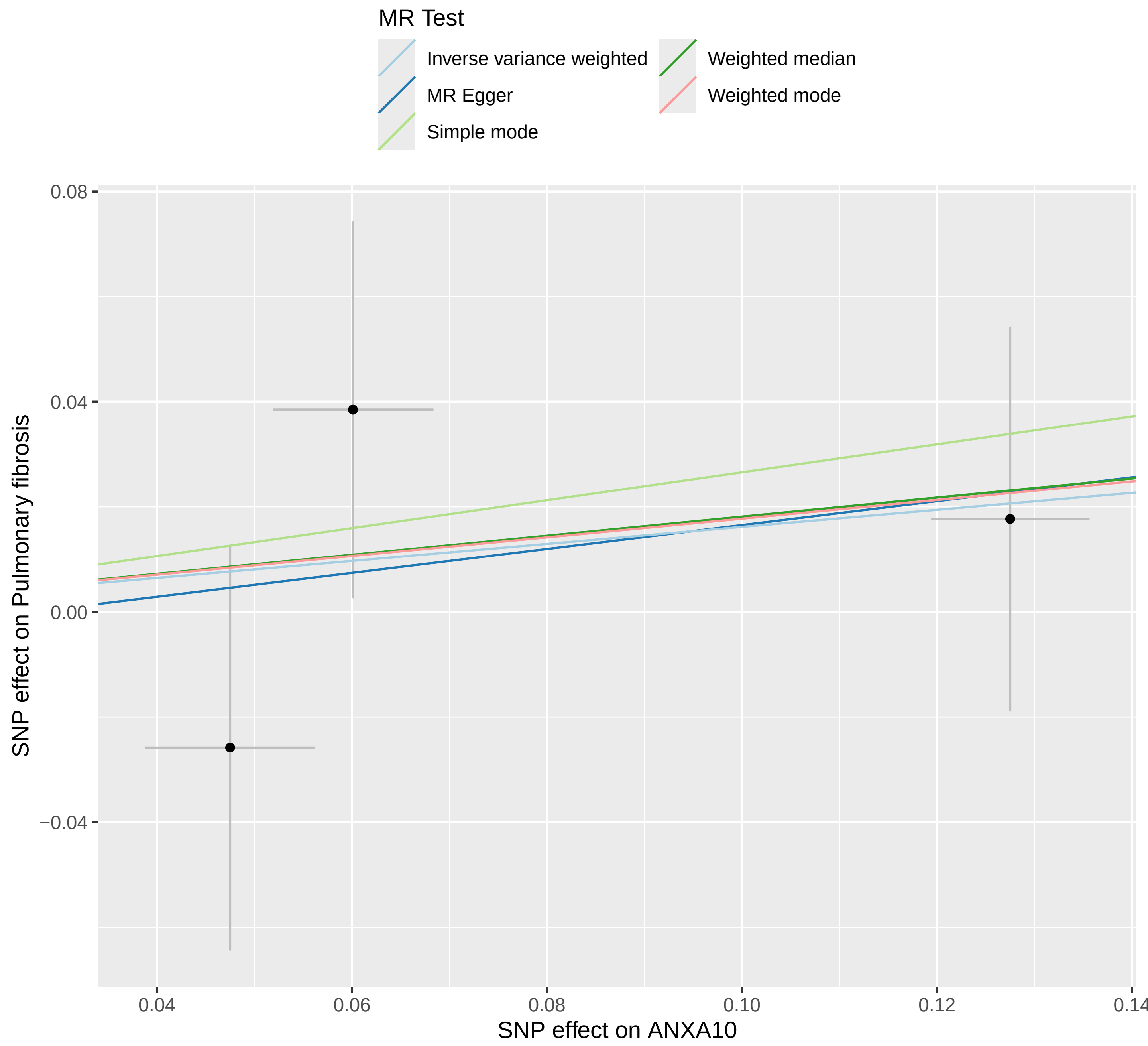

MR Test
Inverse variance weighted
MR Egger
Simple mode
Weighted median
Weighted mode
0.08
0.04
0.00
−0.04
SNP effect on Pulmonary fibrosis
0.04
0.06
0.08
0.10
0.12
0.14
SNP effect on ANXA10

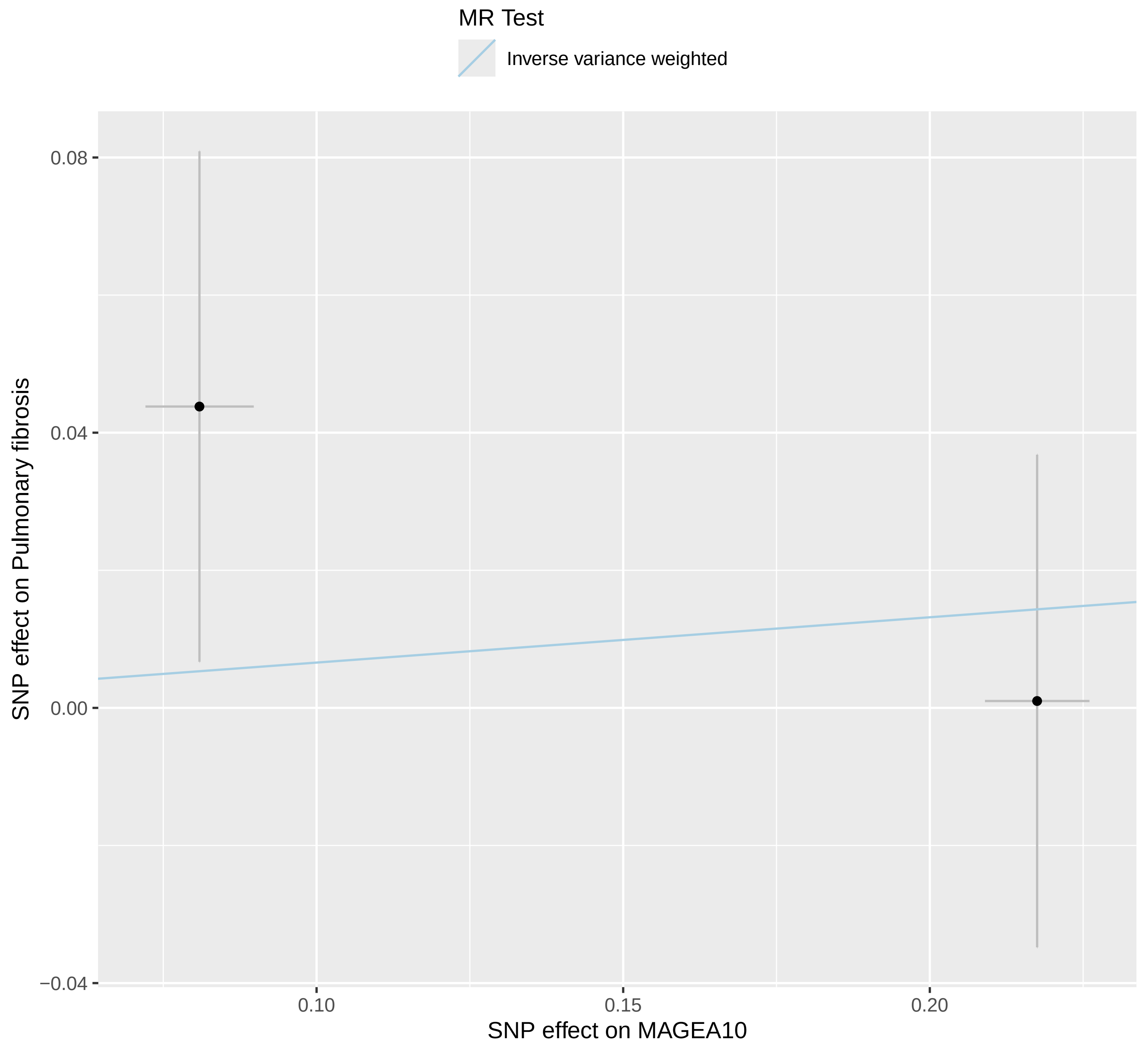
MR Test
Inverse variance weighted
0.08
0.04
0.00
−0.04
0.10
0.15
0.20
SNP effect on Pulmonary fibrosis
SNP effect on MAGEA10

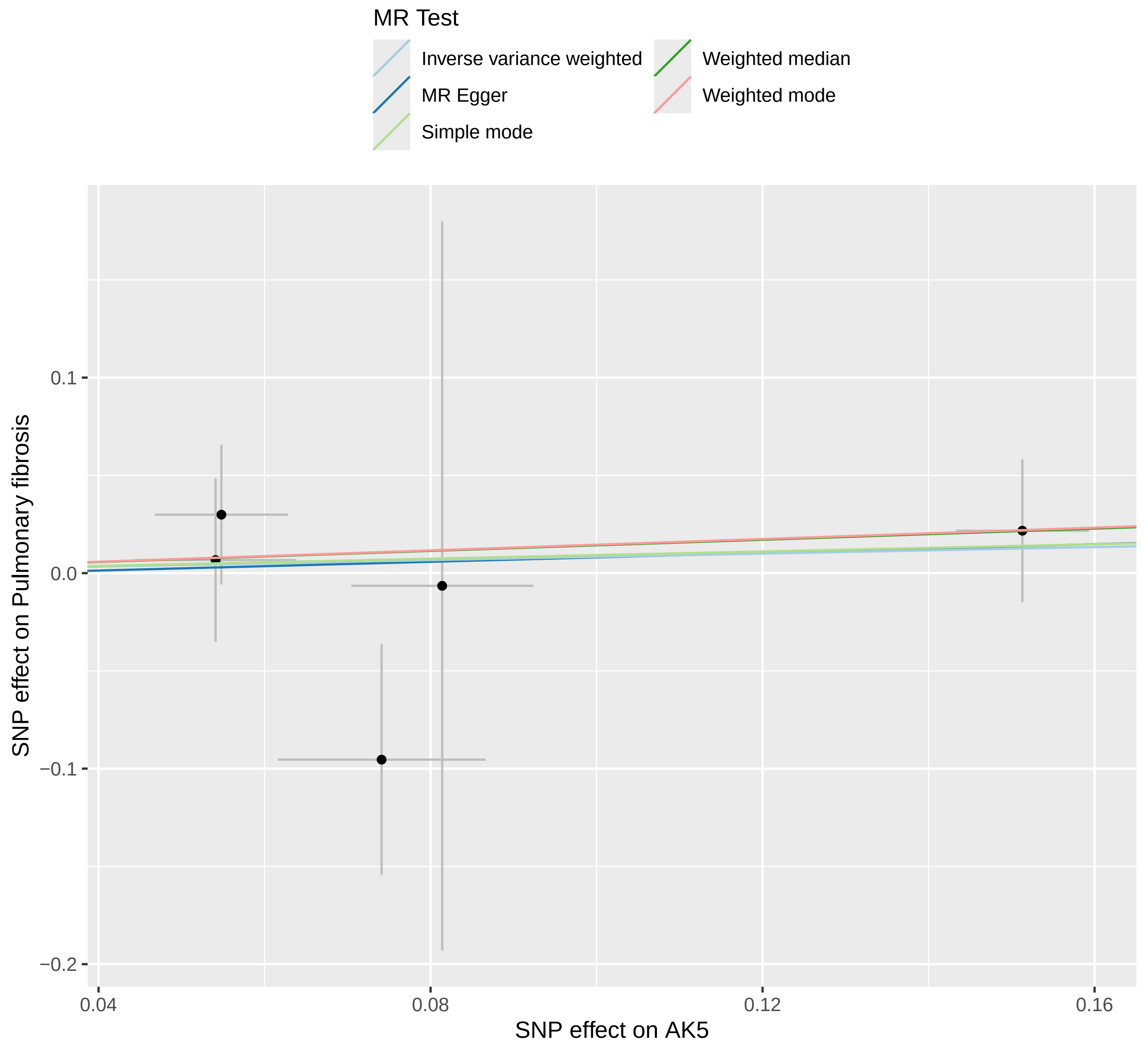
MR Test
Inverse variance weighted
MR Egger
Simple mode
Weighted median
Weighted mode
0.1
0.0
−0.1
−0.2
0.04
0.08
0.12
0.16
SNP effect on Pulmonary fibrosis
SNP effect on AK5

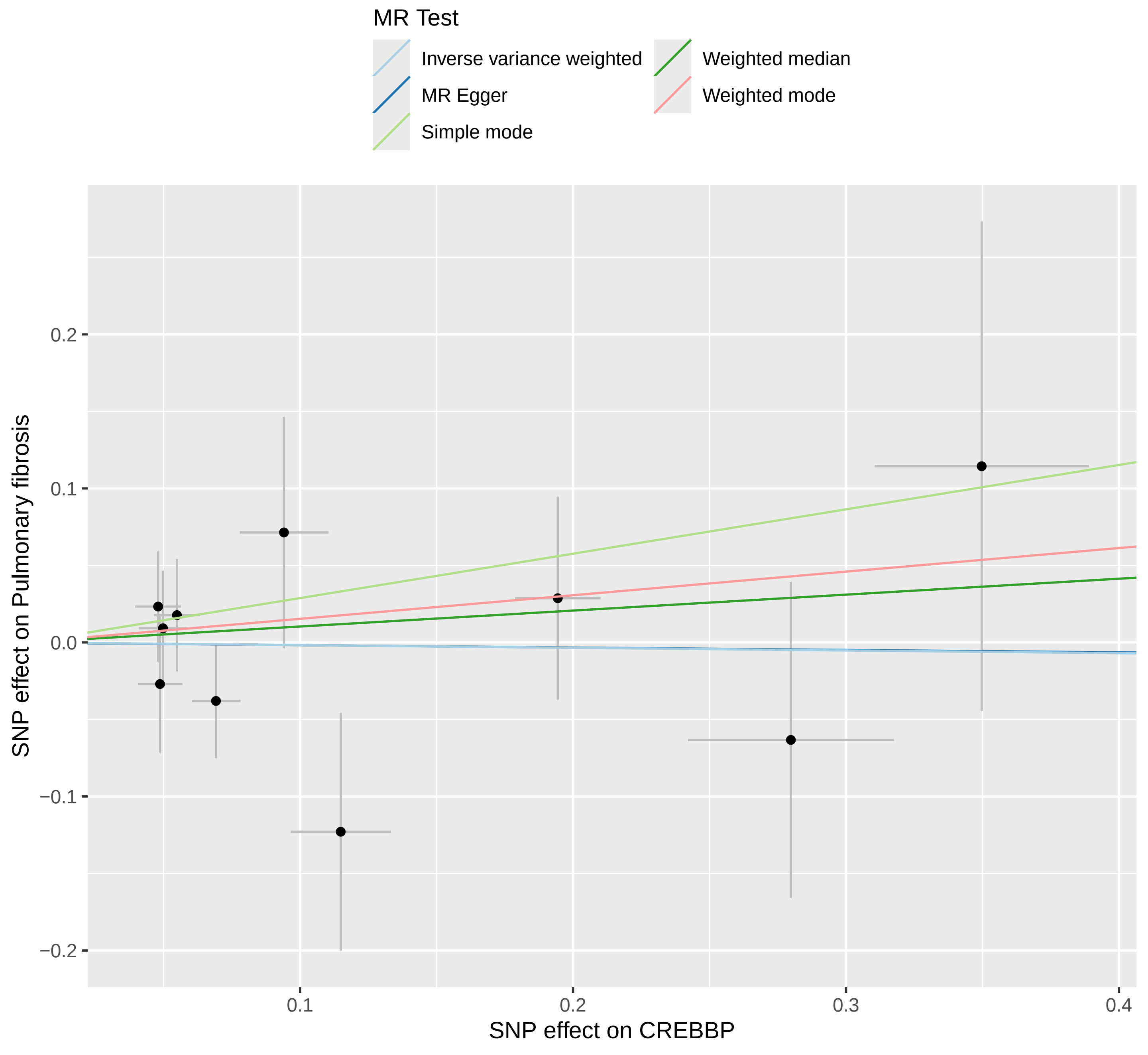
MR Test
Inverse variance weighted
MR Egger
Simple mode
Weighted median
Weighted mode
SNP effect on Pulmonary fibrosis
0.2
0.1
0.0
−0.1
−0.2
0.1
0.2
0.3
0.4
SNP effect on CREBBP

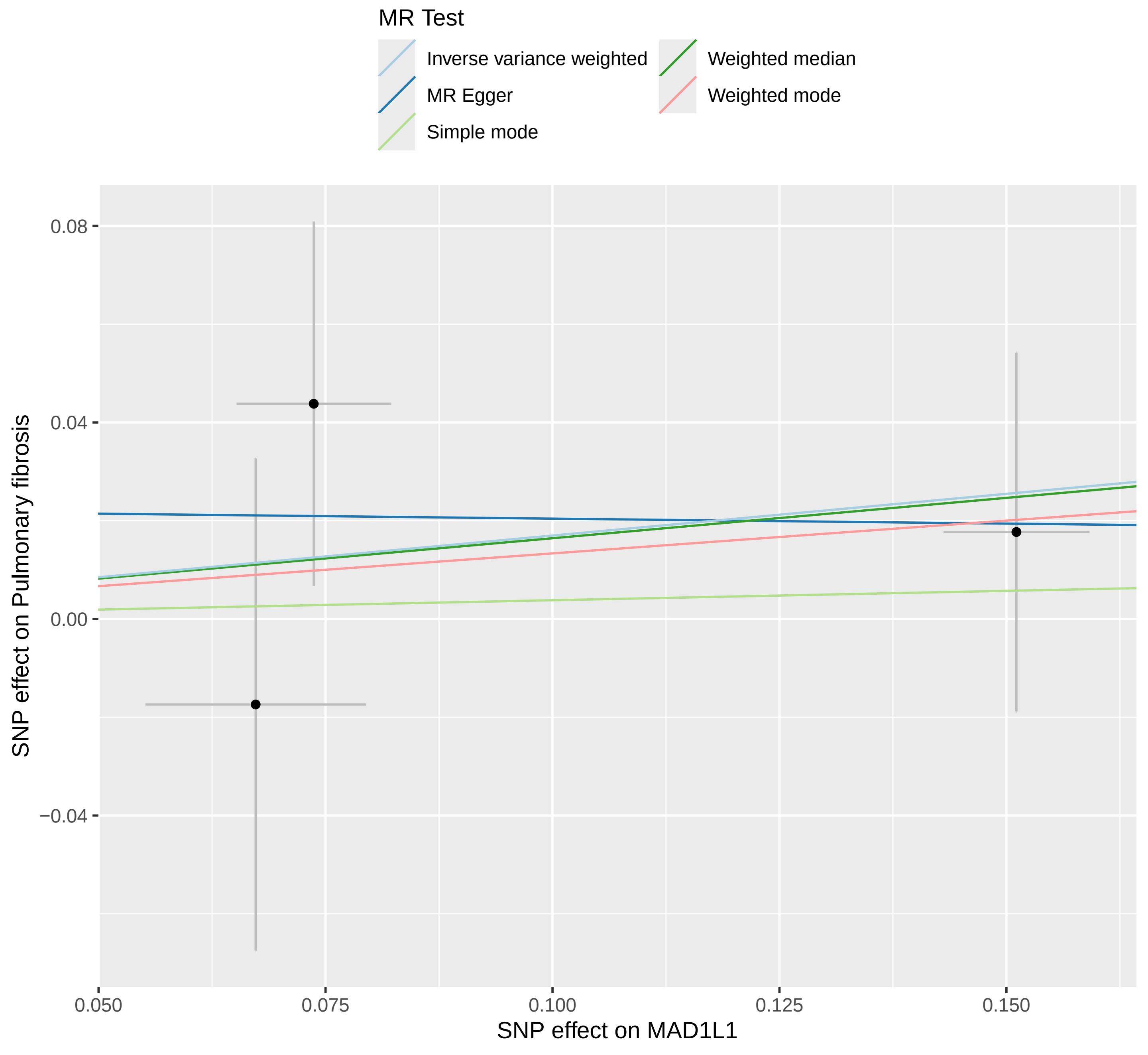
MR Test
Inverse variance weighted
MR Egger
Simple mode
Weighted median
Weighted mode
SNP effect on Pulmonary fibrosis
0.08
0.04
0.00
−0.04
0.050
0.075
0.100
0.125
0.150
SNP effect on MAD1L1

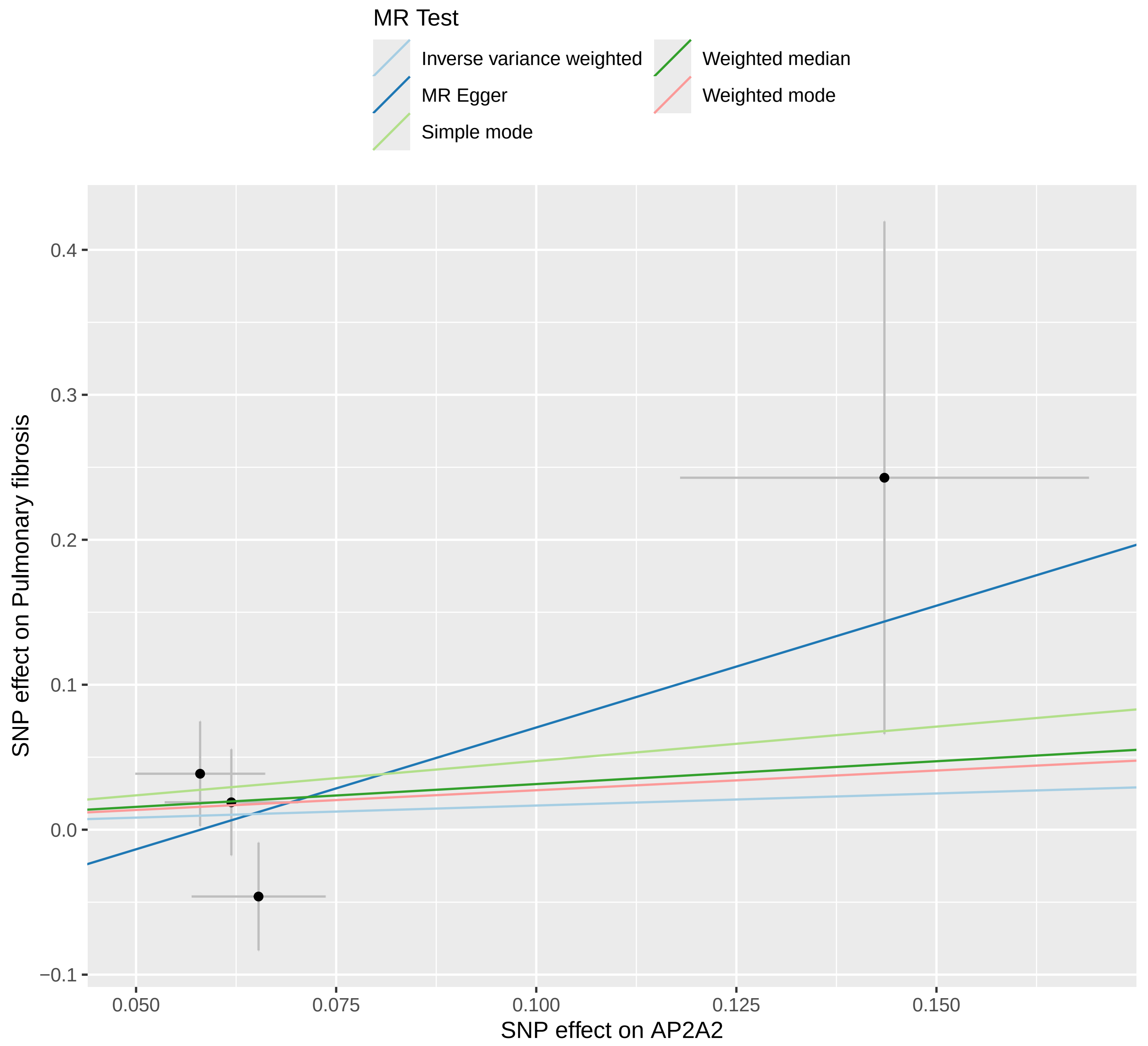
MR Test
Inverse variance weighted
MR Egger
Simple mode
Weighted median
Weighted mode
SNP effect on Pulmonary fibrosis
0.4
0.3
0.2
0.1
0.0
−0.1
0.050
0.075
0.100
0.125
0.150
SNP effect on AP2A2

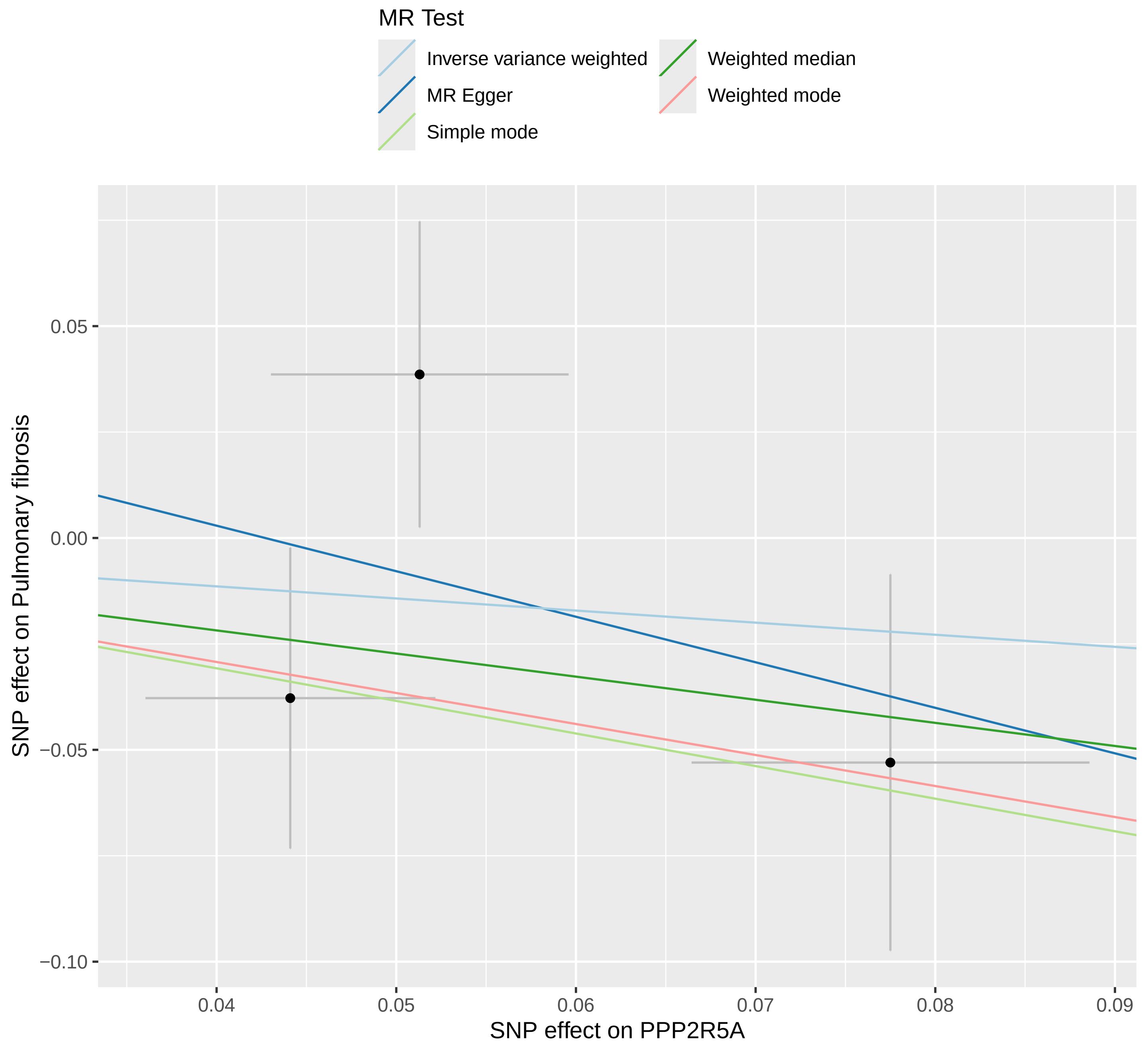
MR Test
Inverse variance weighted
MR Egger
Simple mode
Weighted median
Weighted mode
0.05
0.00
−0.05
−0.10
SNP effect on Pulmonary fibrosis
0.04
0.05
0.06
0.07
0.08
0.09
SNP effect on PPP2R5A

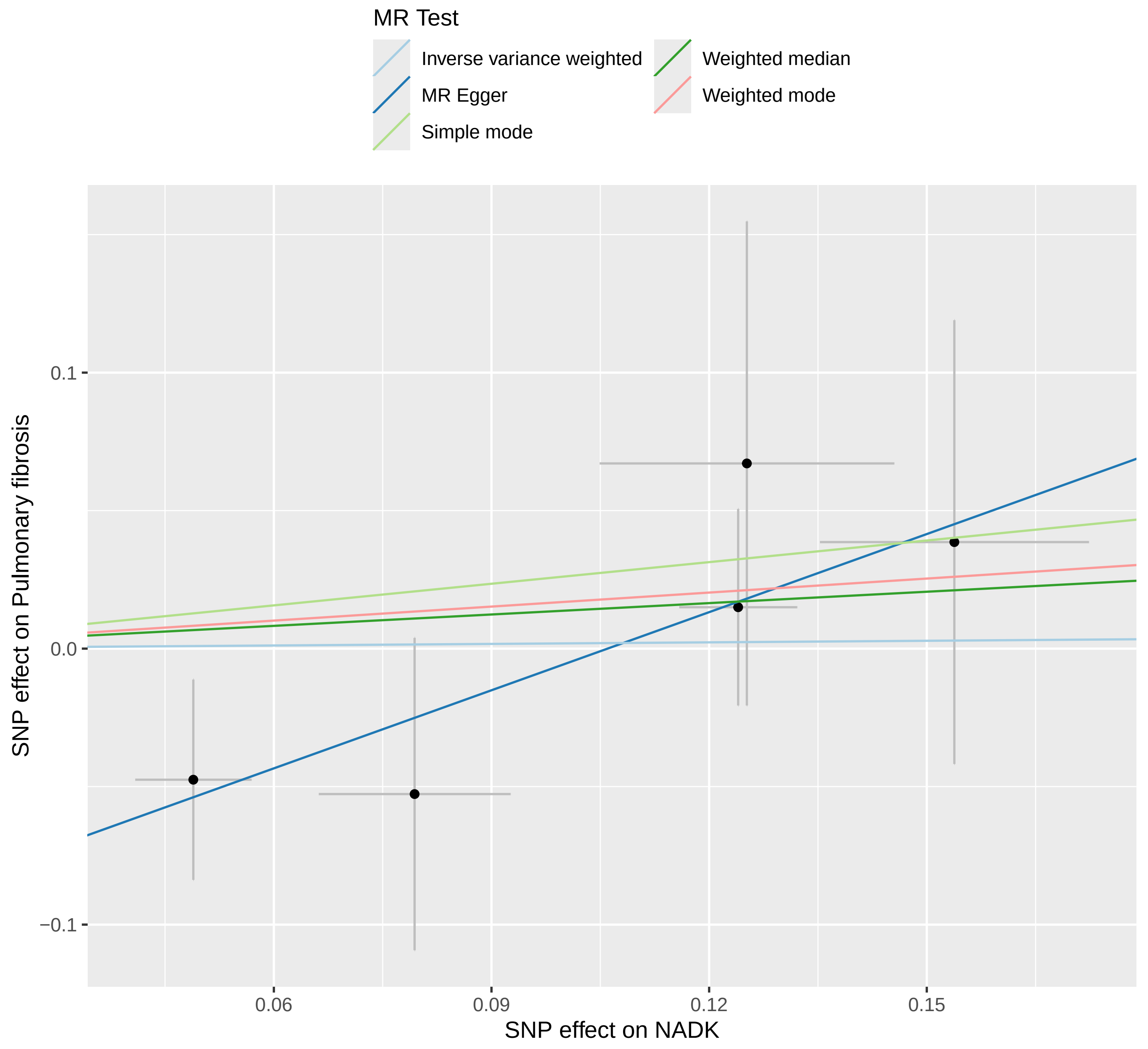
MR Test
Inverse variance weighted
MR Egger
Simple mode
Weighted median
Weighted mode
0.1
0.0
−0.1
0.06
0.09
0.12
0.15
SNP effect on Pulmonary fibrosis
SNP effect on NADK

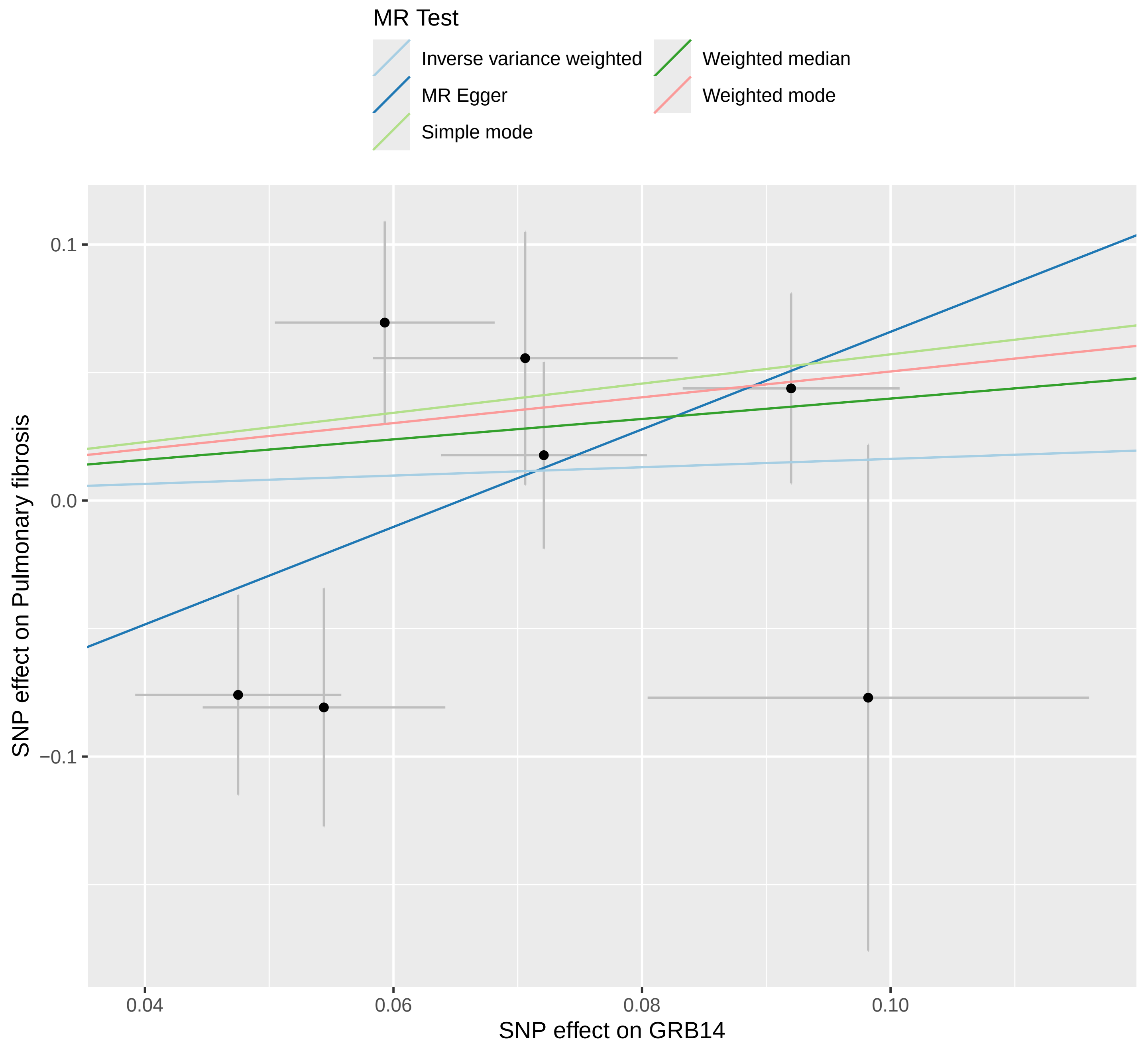

MR Test
Inverse variance weighted
MR Egger
Simple mode
Weighted median
Weighted mode
0.1
0.0
−0.1
SNP effect on Pulmonary fibrosis
0.04
0.06
0.08
0.10
SNP effect on GRB14

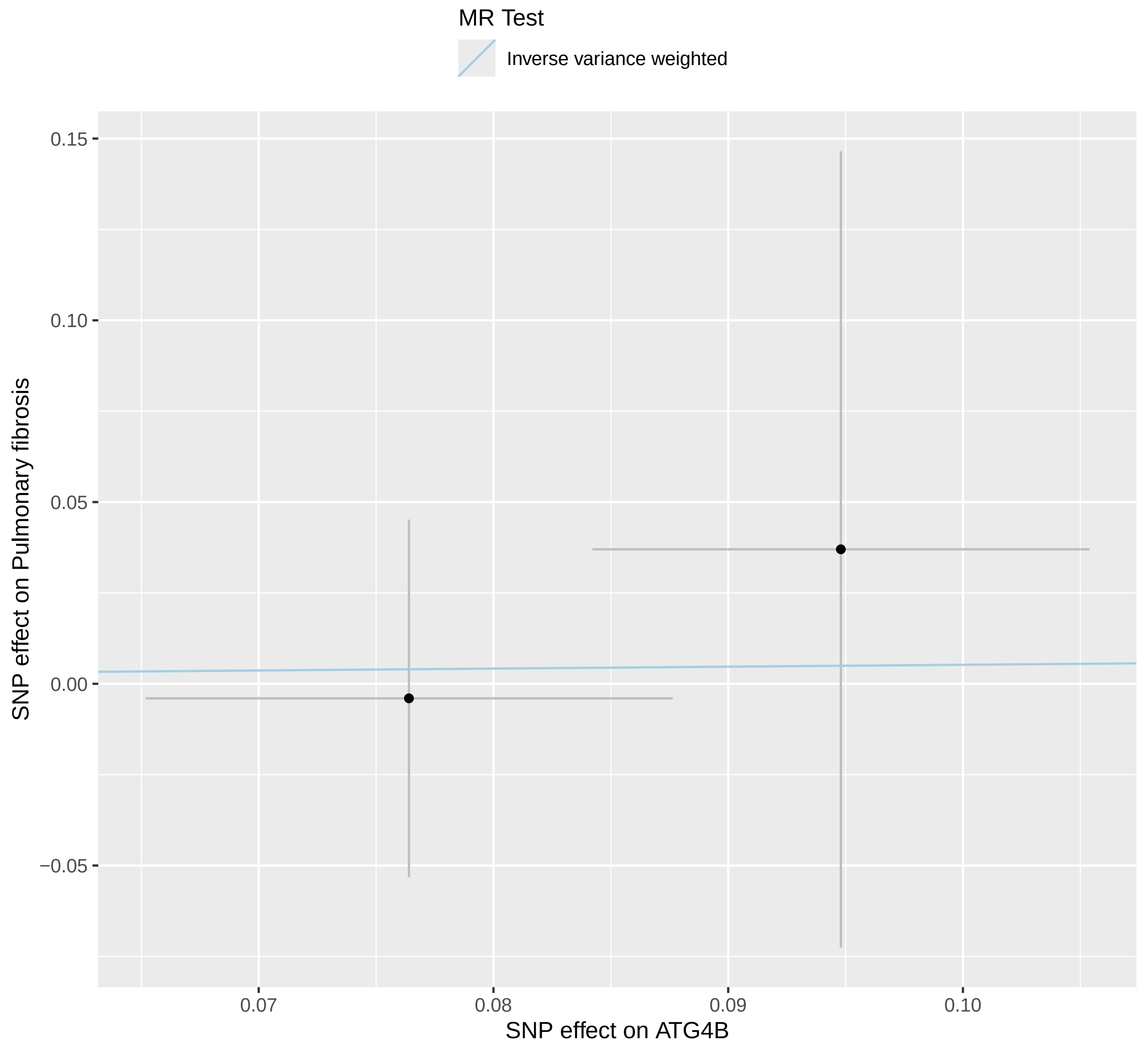

MR Test
Inverse variance weighted
0.15
0.10
0.05
0.00
−0.05
0.07
0.08
0.09
0.10
SNP effect on ATG4B
SNP effect on Pulmonary fibrosis

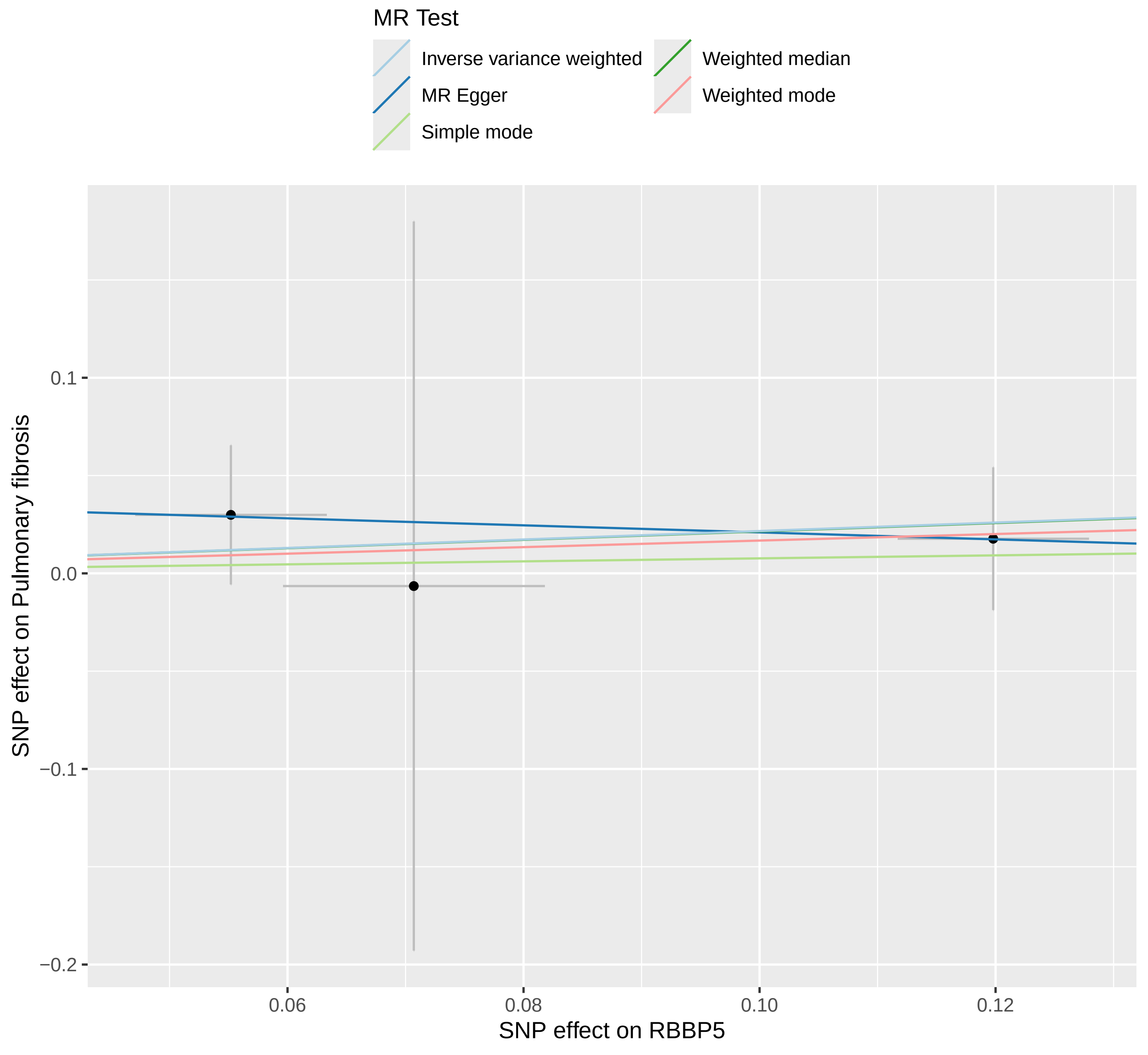
MR Test
Inverse variance weighted
MR Egger
Simple mode
Weighted median
Weighted mode
SNP effect on Pulmonary fibrosis
0.1
0.0
−0.1
−0.2
0.06
0.08
0.10
0.12
SNP effect on RBBP5

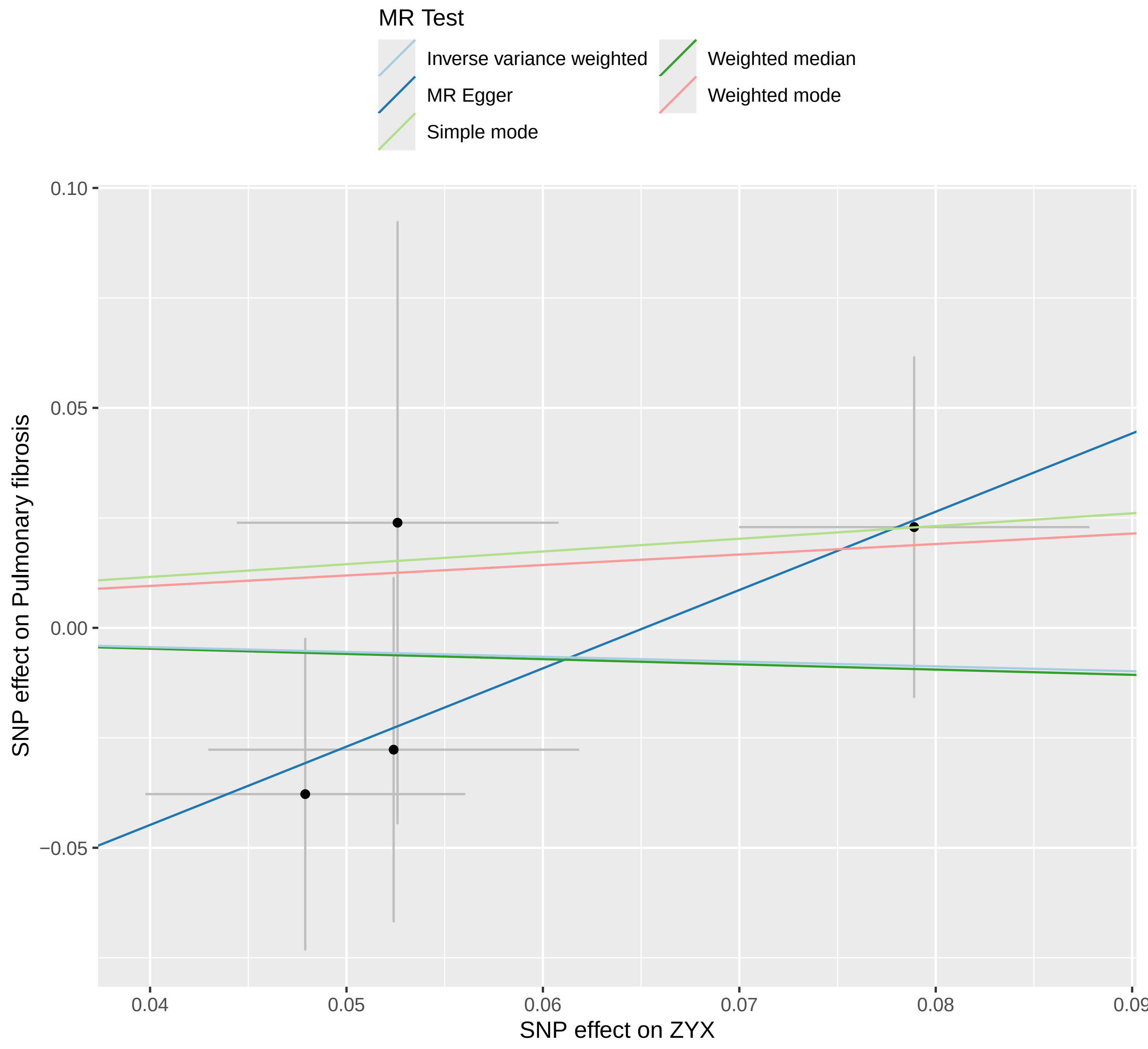

MR Test
Inverse variance weighted
MR Egger
Simple mode
Weighted median
Weighted mode
0.10
0.05
0.00
−0.05
SNP effect on Pulmonary fibrosis
0.04
0.05
0.06
0.07
0.08
SNP effect on ZYX

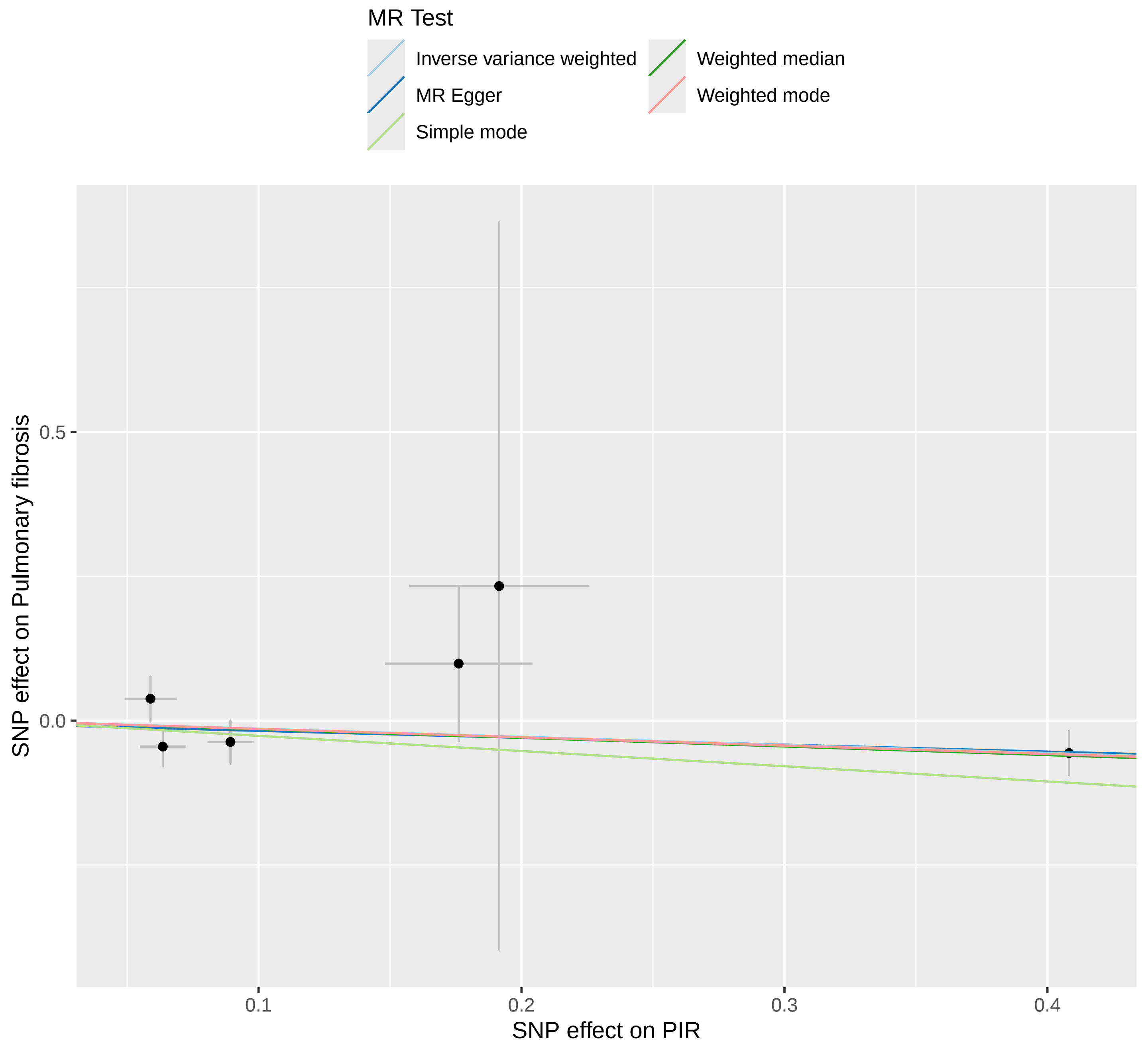
MR Test
Inverse variance weighted
MR Egger
Simple mode
Weighted median
Weighted mode
SNP effect on Pulmonary fibrosis
0.5
0.0
0.1
0.2
0.3
0.4
SNP effect on PIR

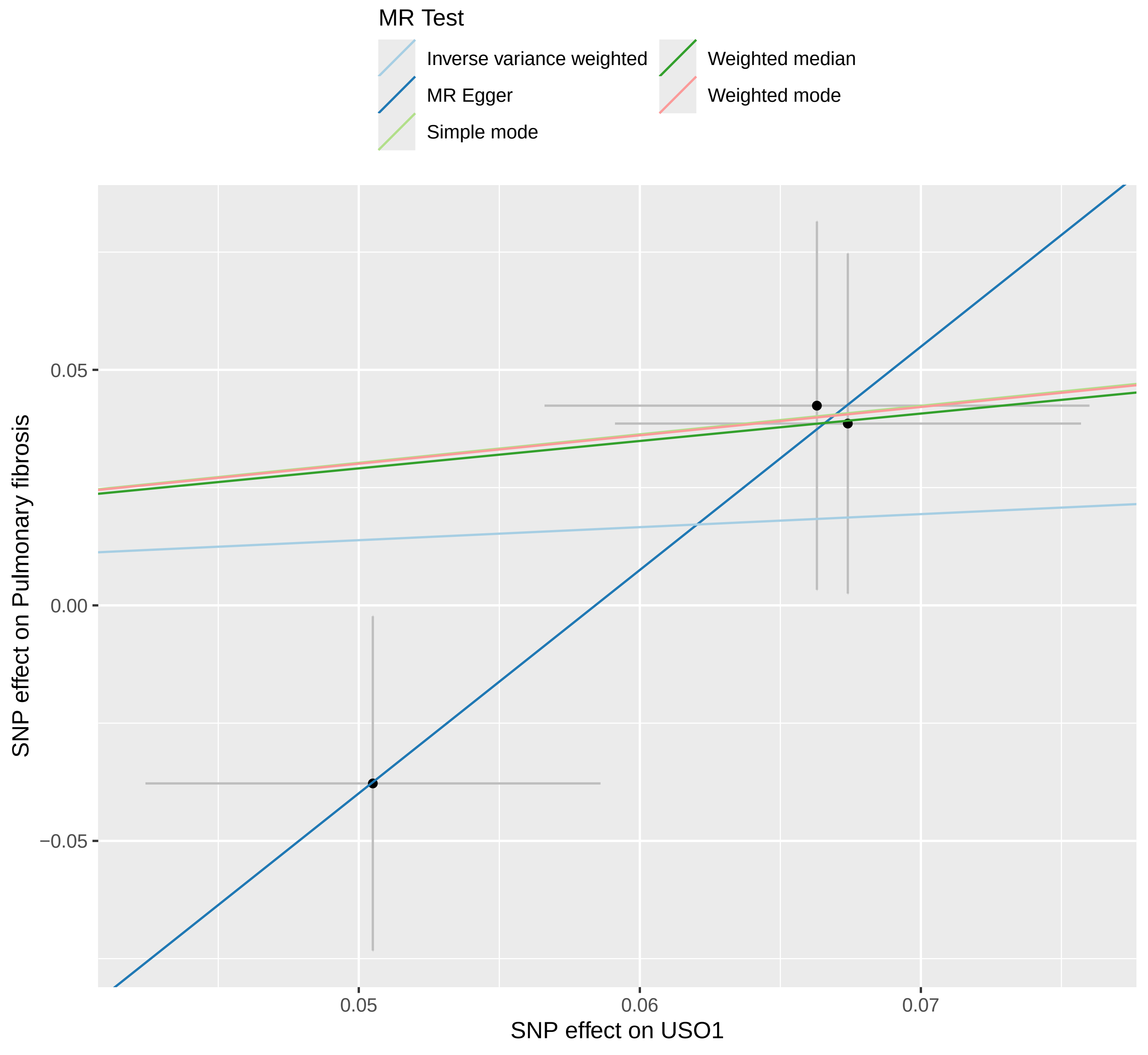
MR Test
Inverse variance weighted
MR Egger
Simple mode
Weighted median
Weighted mode
0.05
0.00
−0.05
SNP effect on Pulmonary fibrosis
0.05
0.06
0.07
SNP effect on USO1

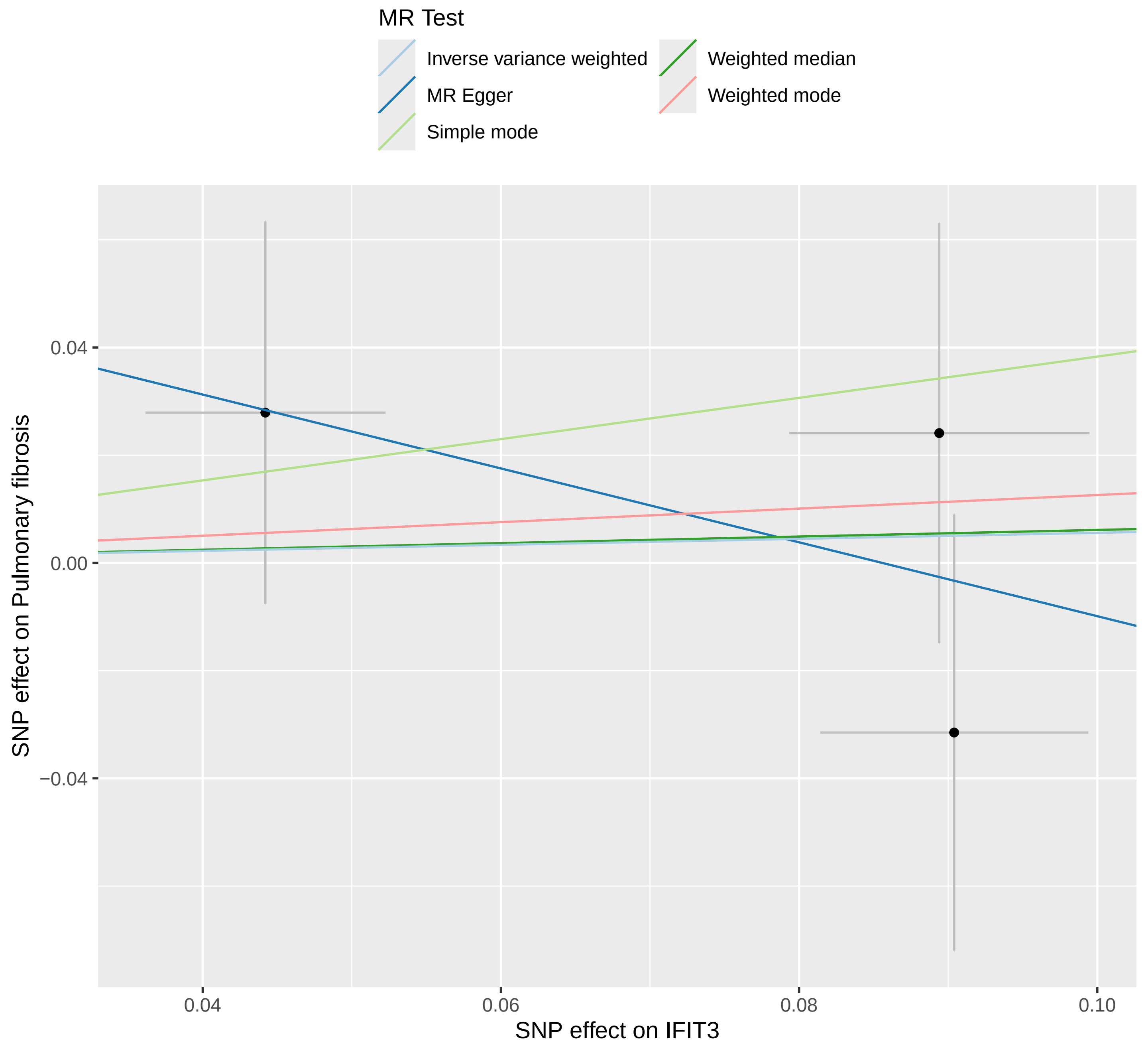

MR Test
Inverse variance weighted
MR Egger
Simple mode
Weighted median
Weighted mode
0.04
0.00
−0.04
0.04
0.06
0.08
0.10
SNP effect on Pulmonary fibrosis
SNP effect on IFIT3

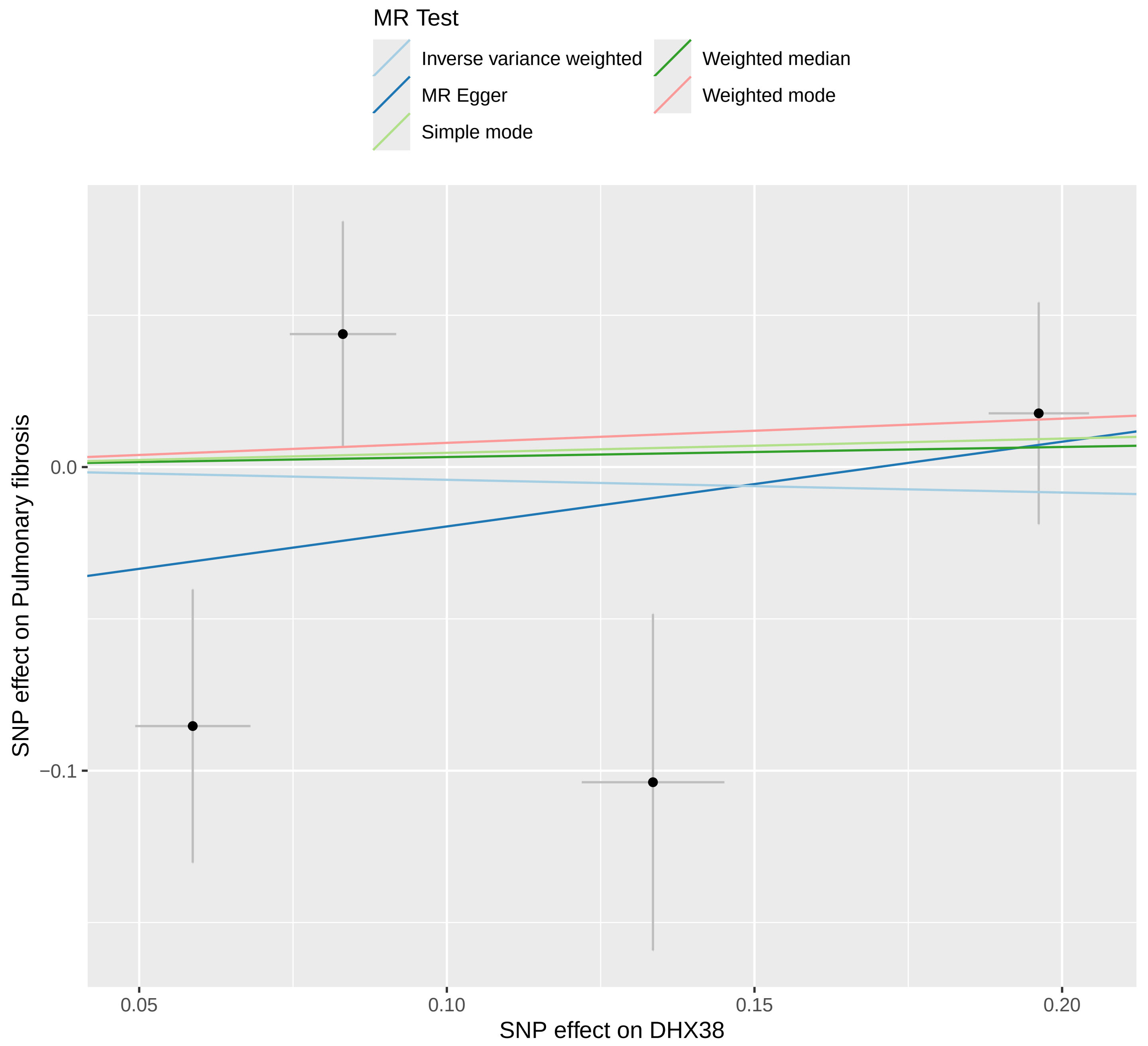
MR Test
Inverse variance weighted
MR Egger
Simple mode
Weighted median
Weighted mode
SNP effect on Pulmonary fibrosis
0.0
−0.1
0.05
0.10
0.15
0.20
SNP effect on DHX38

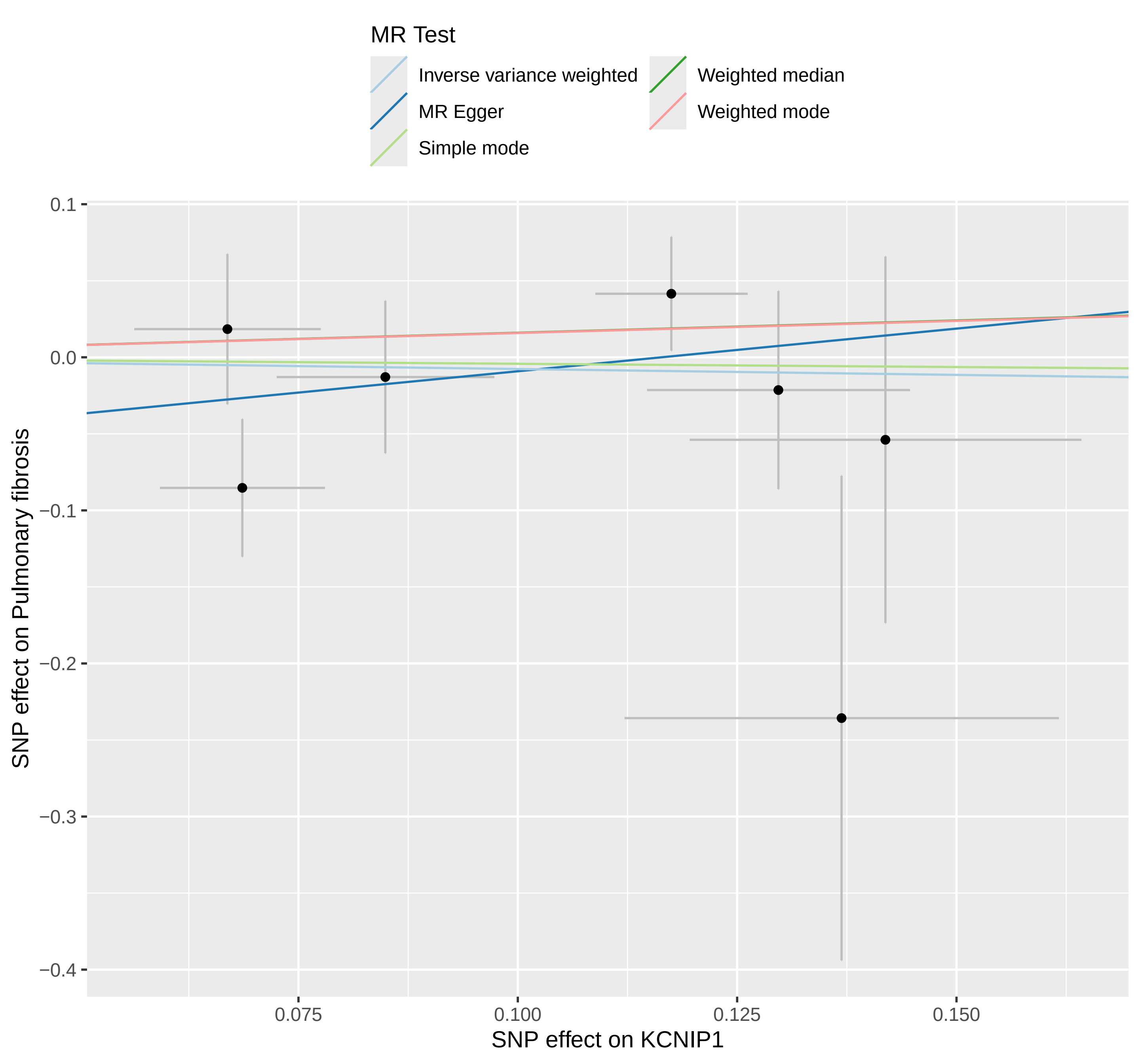
MR Test
Inverse variance weighted
MR Egger
Simple mode
Weighted median
Weighted mode
0.1
0.0
−0.1
−0.2
−0.3
−0.4
0.075
0.100
0.125
0.150
SNP effect on KCNIP1
SNP effect on Pulmonary fibrosis

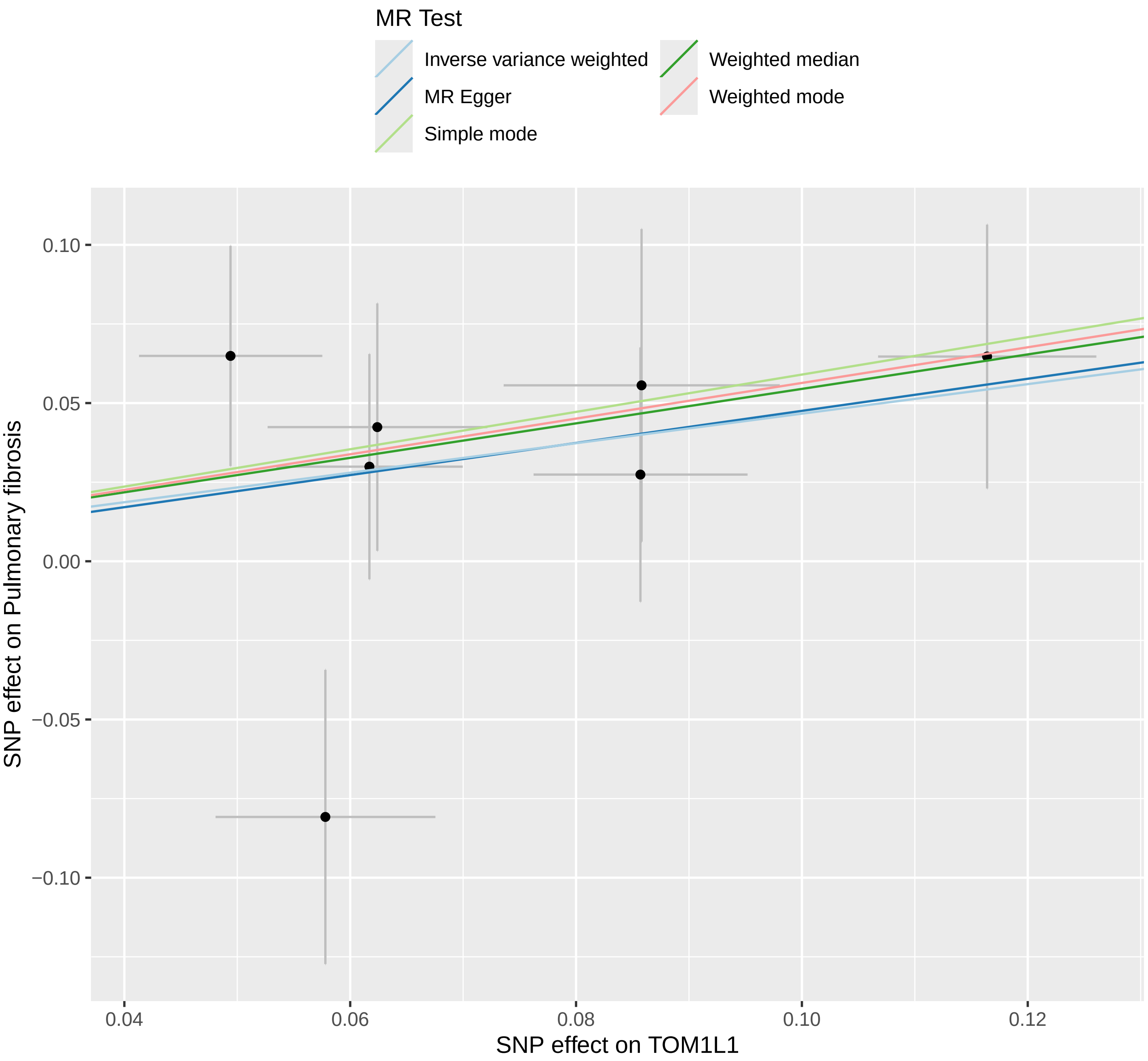
MR Test
Inverse variance weighted
MR Egger
Simple mode
Weighted median
Weighted mode
0.10
0.05
0.00
−0.05
−0.10
SNP effect on Pulmonary fibrosis
0.04
0.06
0.08
0.10
0.12
SNP effect on TOM1L1

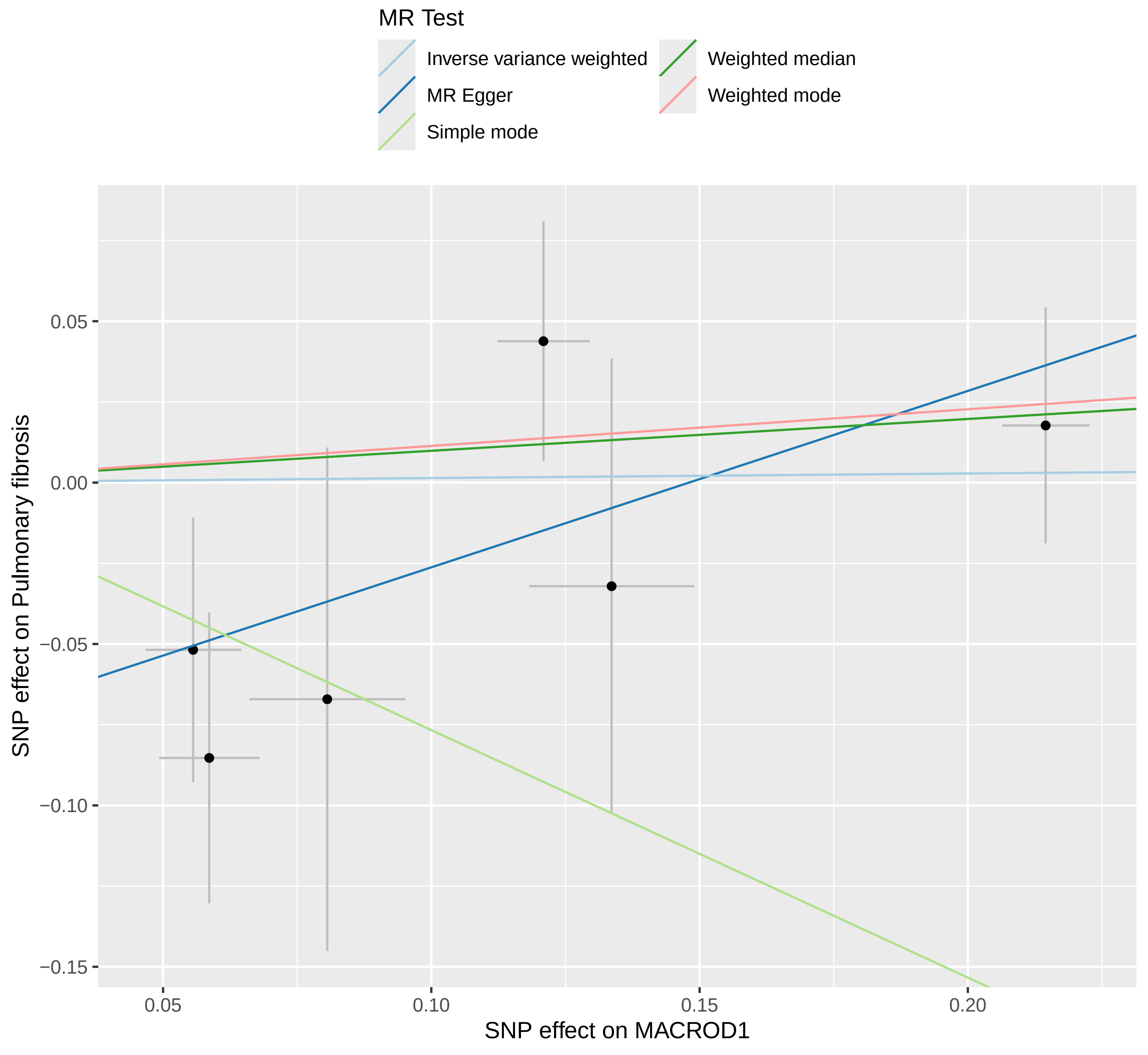
MR Test
Inverse variance weighted
MR Egger
Simple mode
Weighted median
Weighted mode
SNP effect on Pulmonary fibrosis
0.05
0.00
−0.05
−0.10
−0.15
0.05
0.10
0.15
0.20
SNP effect on MACROD1

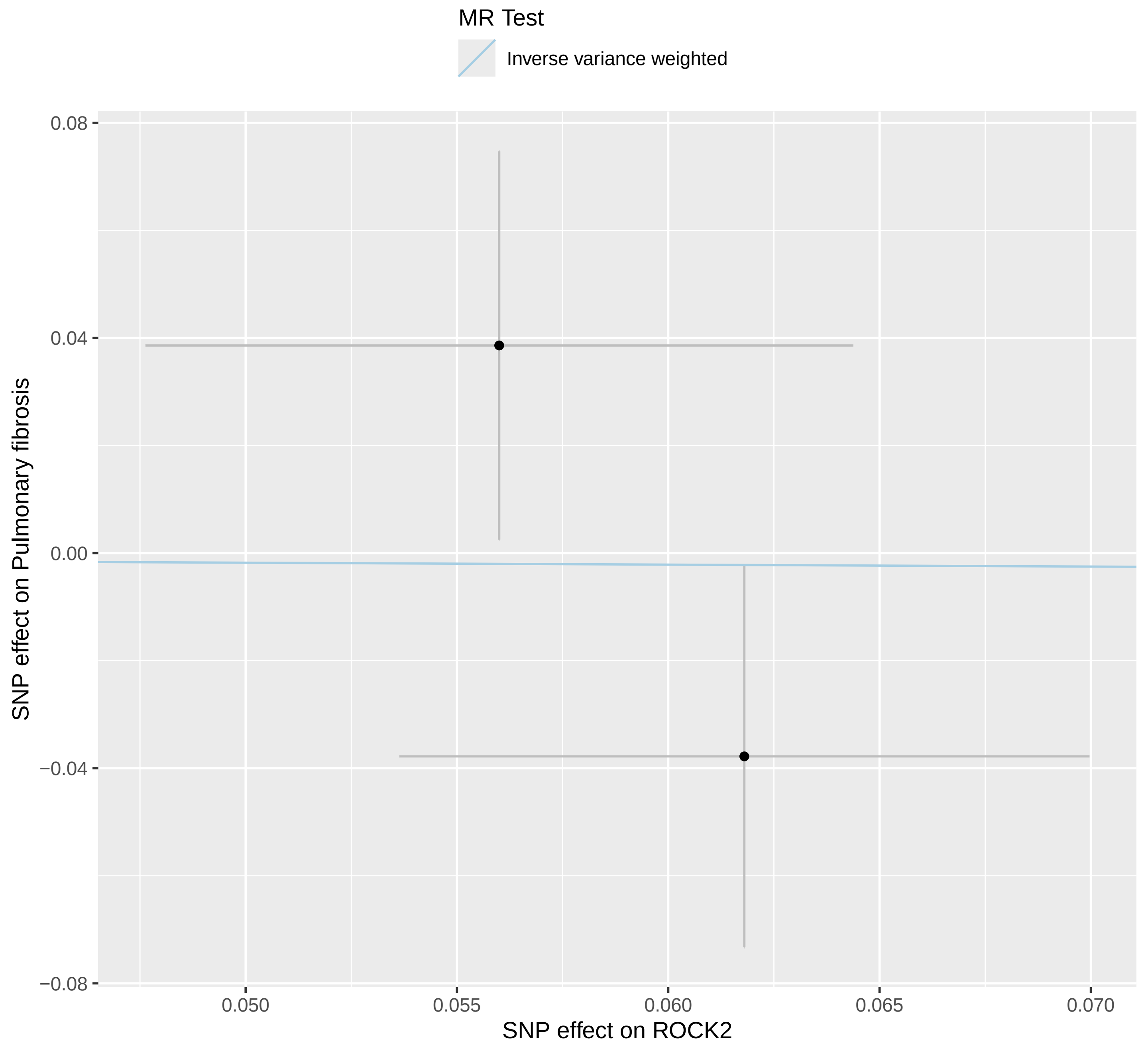
MR Test
Inverse variance weighted
0.08
0.04
0.00
−0.04
−0.08
SNP effect on Pulmonary fibrosis
0.050
0.055
0.060
0.065
0.070
SNP effect on ROCK2

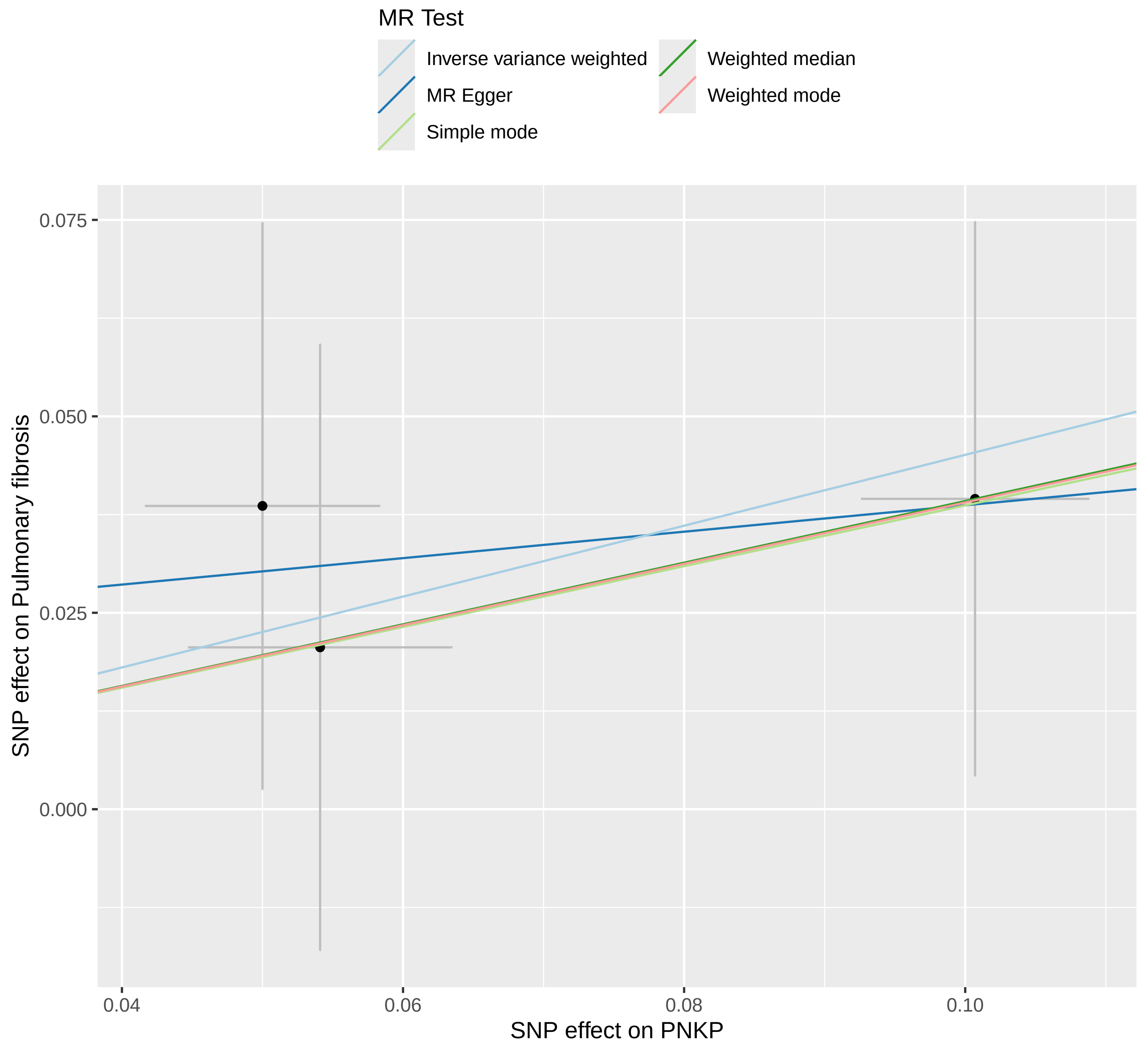

MR Test
Inverse variance weighted
MR Egger
Simple mode
Weighted median
Weighted mode
0.075
0.050
0.025
0.000
SNP effect on Pulmonary fibrosis
0.04
0.06
0.08
0.10
SNP effect on PNKP

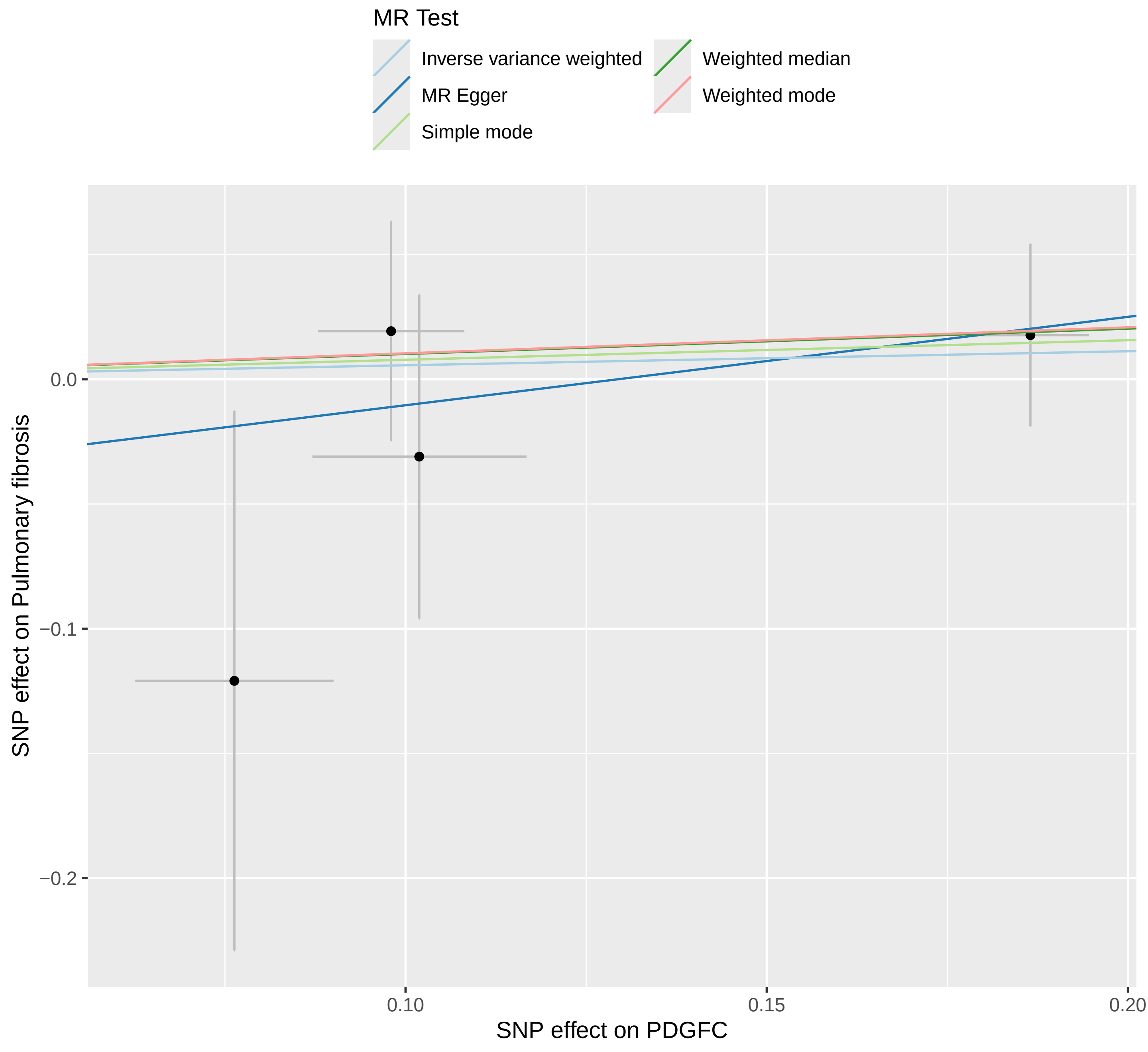
MR Test
Inverse variance weighted
MR Egger
Simple mode
Weighted median
Weighted mode
0.0
−0.1
−0.2
0.10
0.15
0.20
SNP effect on PDGFC
SNP effect on Pulmonary fibrosis

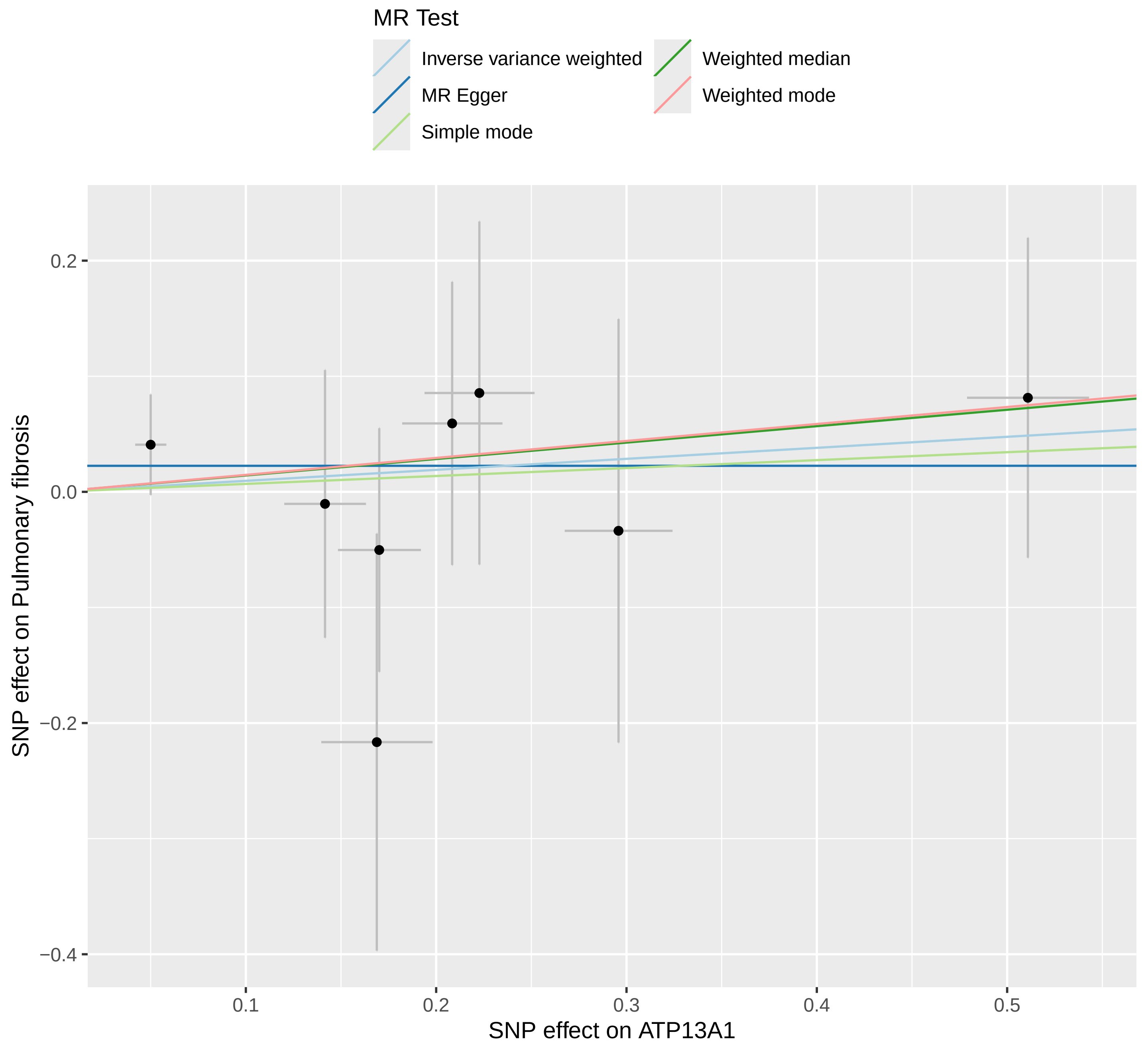
MR Test
Inverse variance weighted
MR Egger
Simple mode
Weighted median
Weighted mode
SNP effect on Pulmonary fibrosis
0.2
0.0
−0.2
−0.4
0.1
0.2
0.3
0.4
0.5
SNP effect on ATP13A1

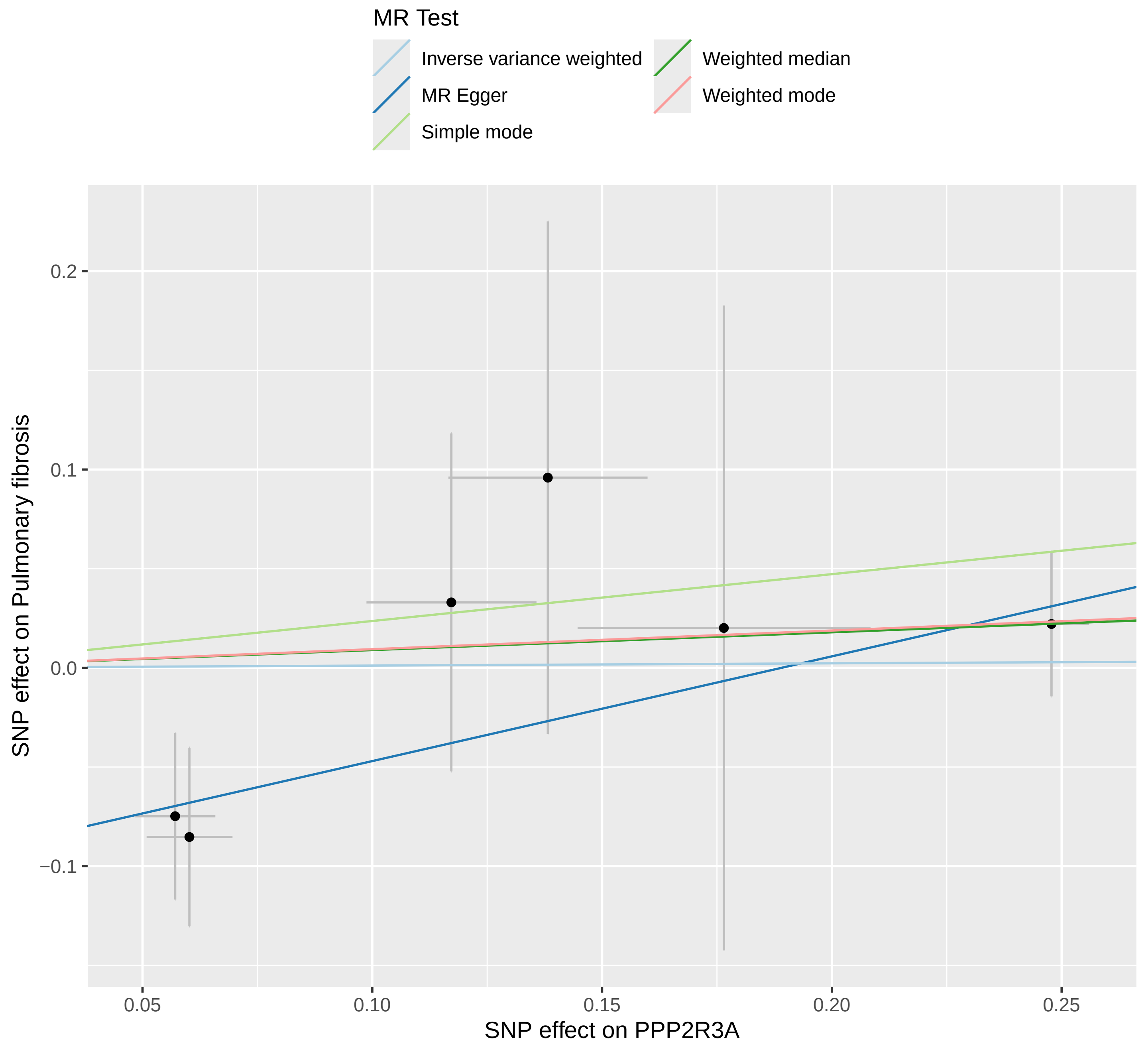
MR Test
Inverse variance weighted
MR Egger
Simple mode
Weighted median
Weighted mode
0.2
0.1
0.0
−0.1
SNP effect on Pulmonary fibrosis
0.05
0.10
0.15
0.20
0.25
SNP effect on PPP2R3A

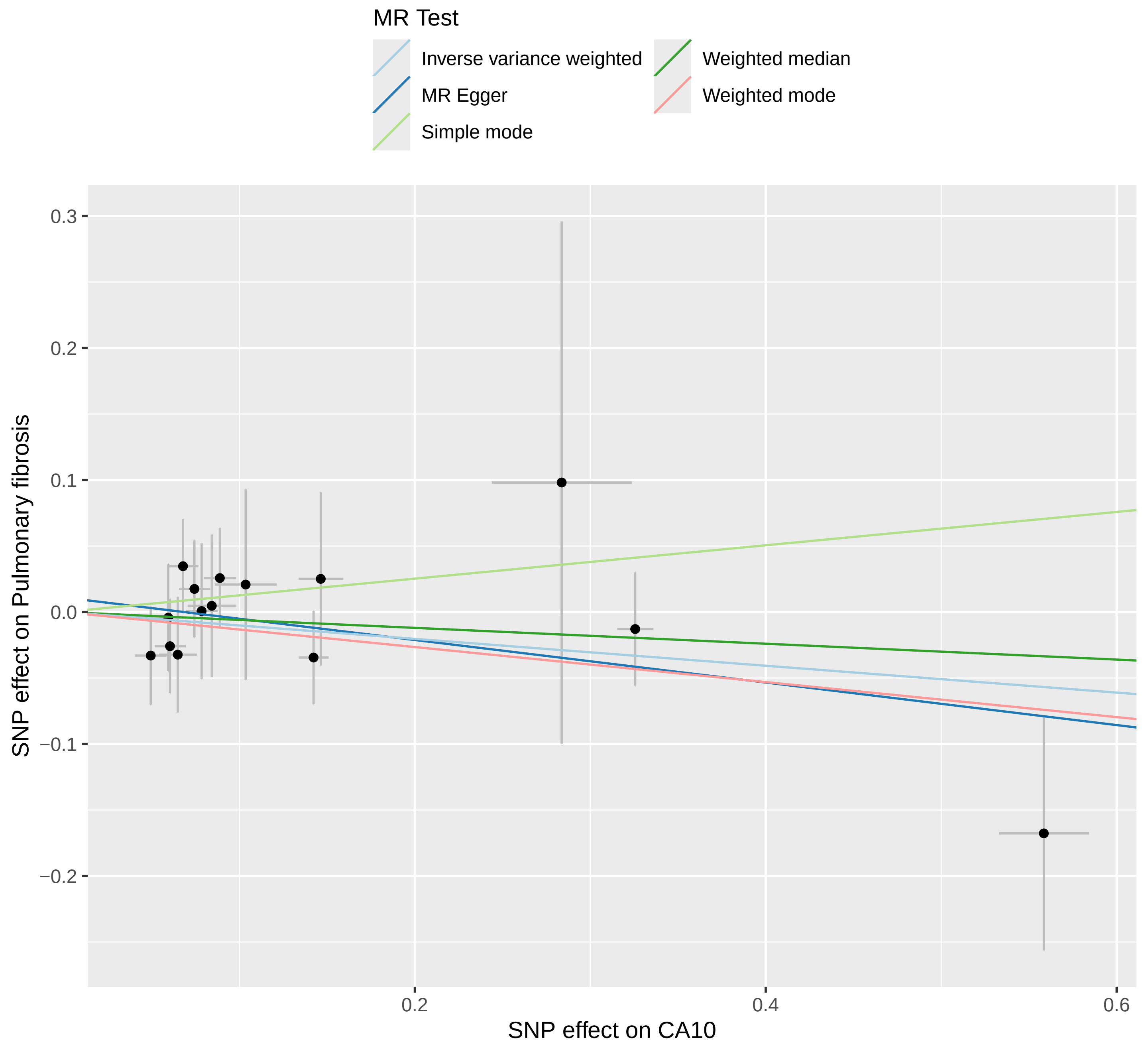
MR Test
Inverse variance weighted
MR Egger
Simple mode
Weighted median
Weighted mode
0.3
0.2
0.1
0.0
−0.1
−0.2
SNP effect on Pulmonary fibrosis
0.2
0.4
0.6
SNP effect on CA10

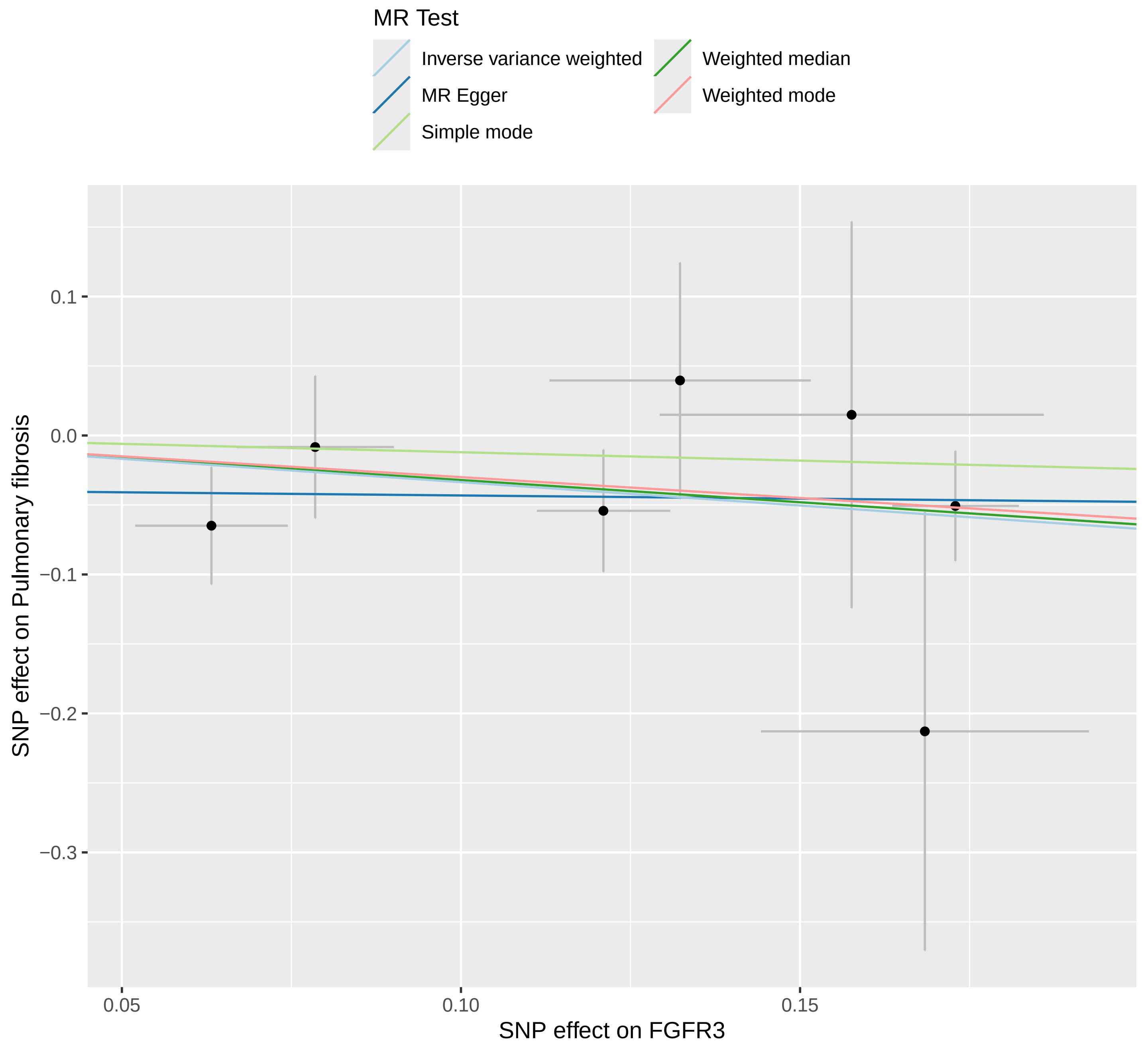
MR Test
Inverse variance weighted
MR Egger
Simple mode
Weighted median
Weighted mode
0.1
0.0
−0.1
−0.2
−0.3
0.05
0.10
0.15
SNP effect on FGFR3
SNP effect on Pulmonary fibrosis

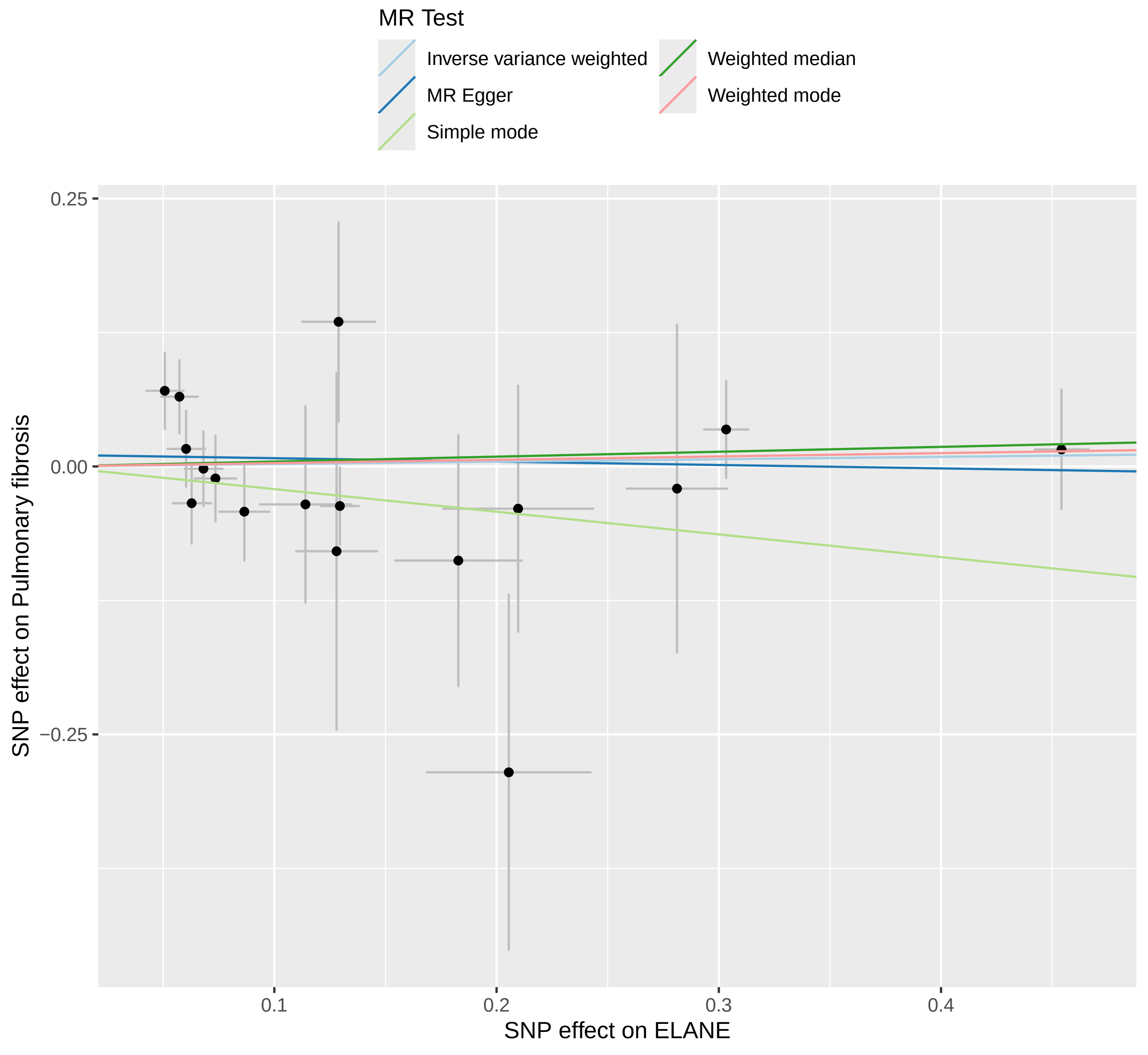
MR Test
Inverse variance weighted
MR Egger
Simple mode
Weighted median
Weighted mode
0.25
0.00
−0.25
SNP effect on Pulmonary fibrosis
0.1
0.2
0.3
0.4
SNP effect on ELANE

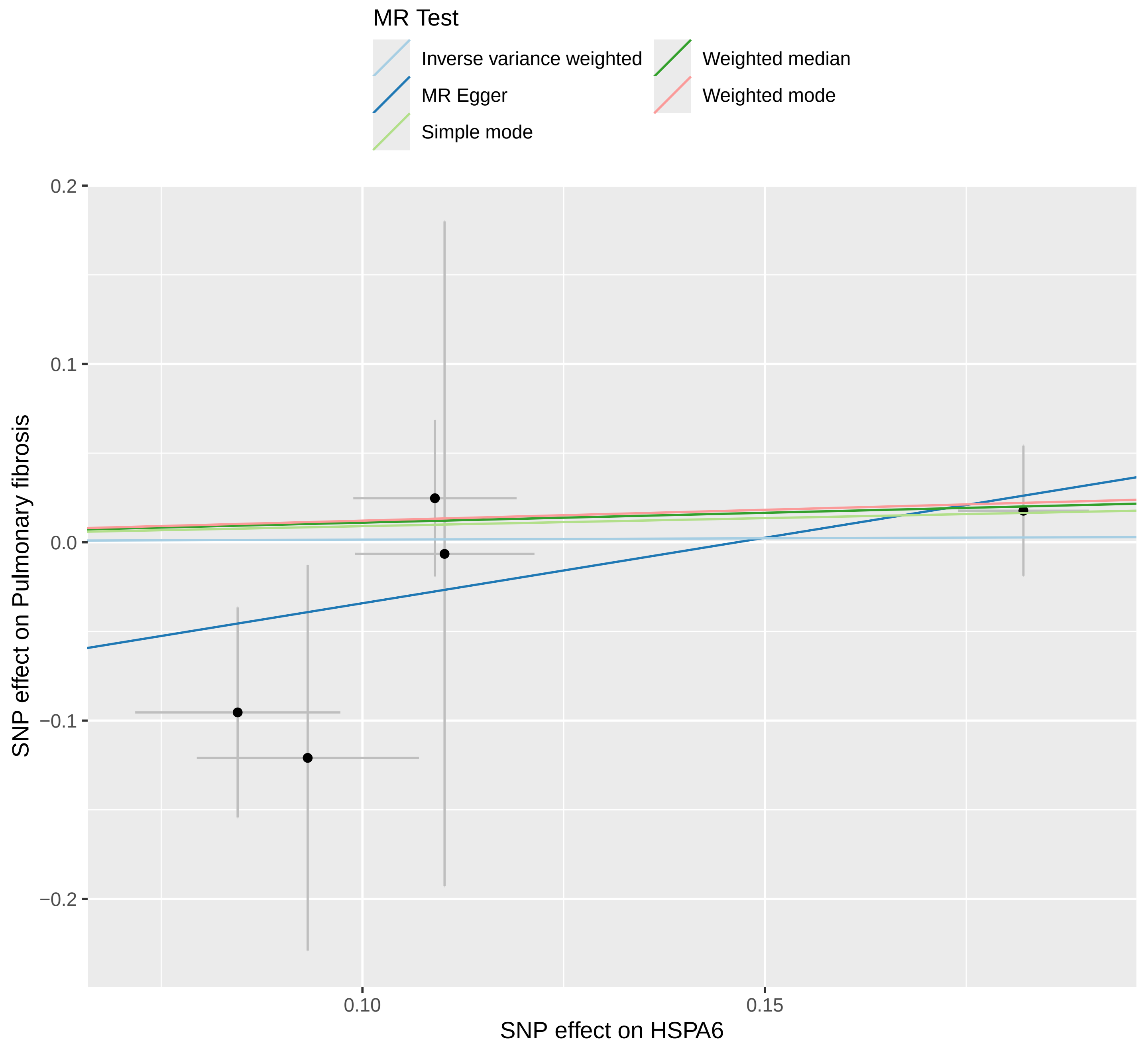
MR Test
Inverse variance weighted
MR Egger
Simple mode
Weighted median
Weighted mode
0.2
0.1
0.0
−0.1
−0.2
0.10
0.15
SNP effect on HSPA6
SNP effect on Pulmonary fibrosis

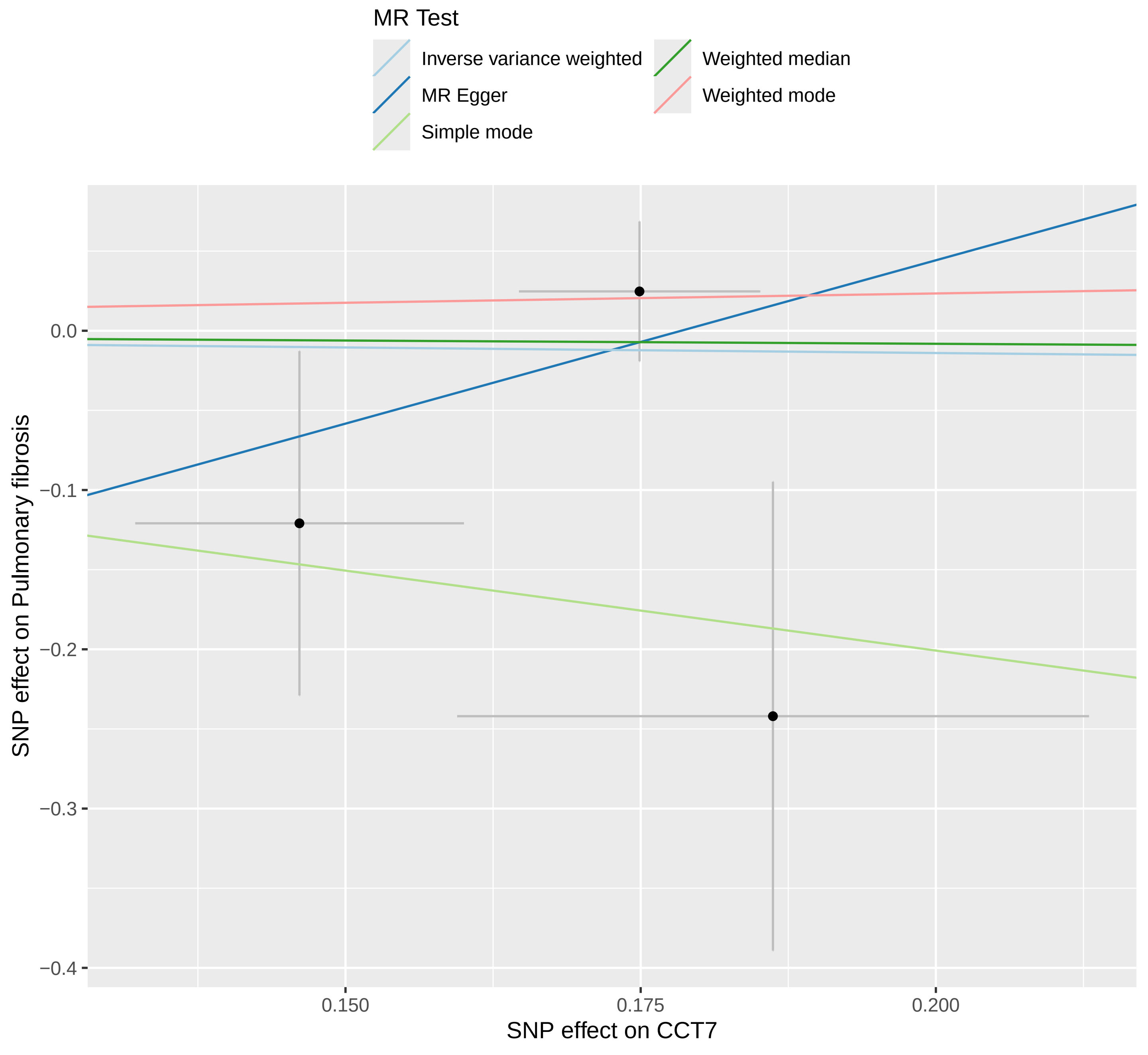

MR Test
Inverse variance weighted
MR Egger
Simple mode
Weighted median
Weighted mode
SNP effect on Pulmonary fibrosis
0.0
−0.1
−0.2
−0.3
−0.4
0.150
0.175
0.200
SNP effect on CCT7

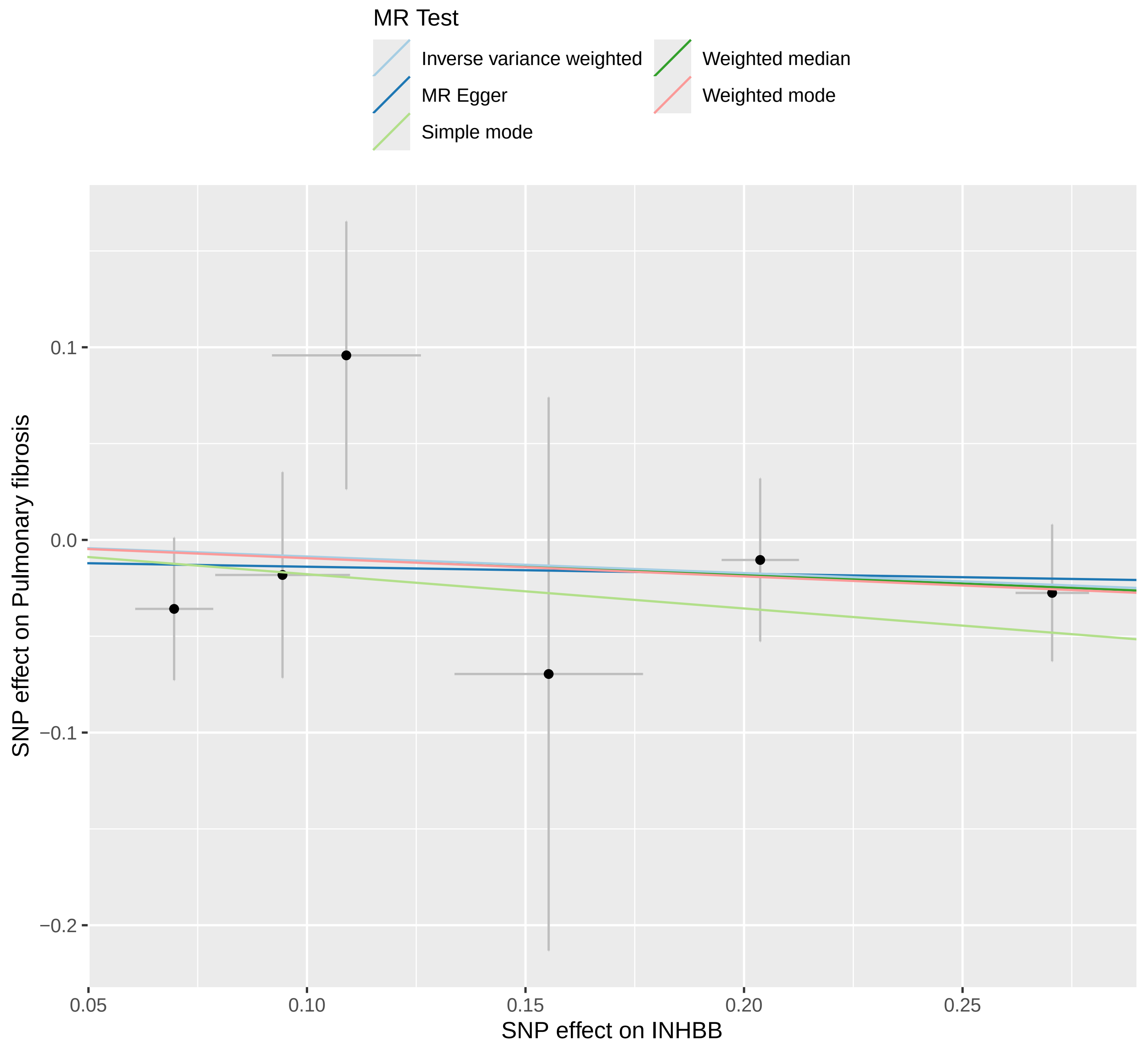
MR Test
Inverse variance weighted
MR Egger
Simple mode
Weighted median
Weighted mode
SNP effect on Pulmonary fibrosis
0.1
0.0
−0.1
−0.2
0.05
0.10
0.15
0.20
0.25
SNP effect on INHBB

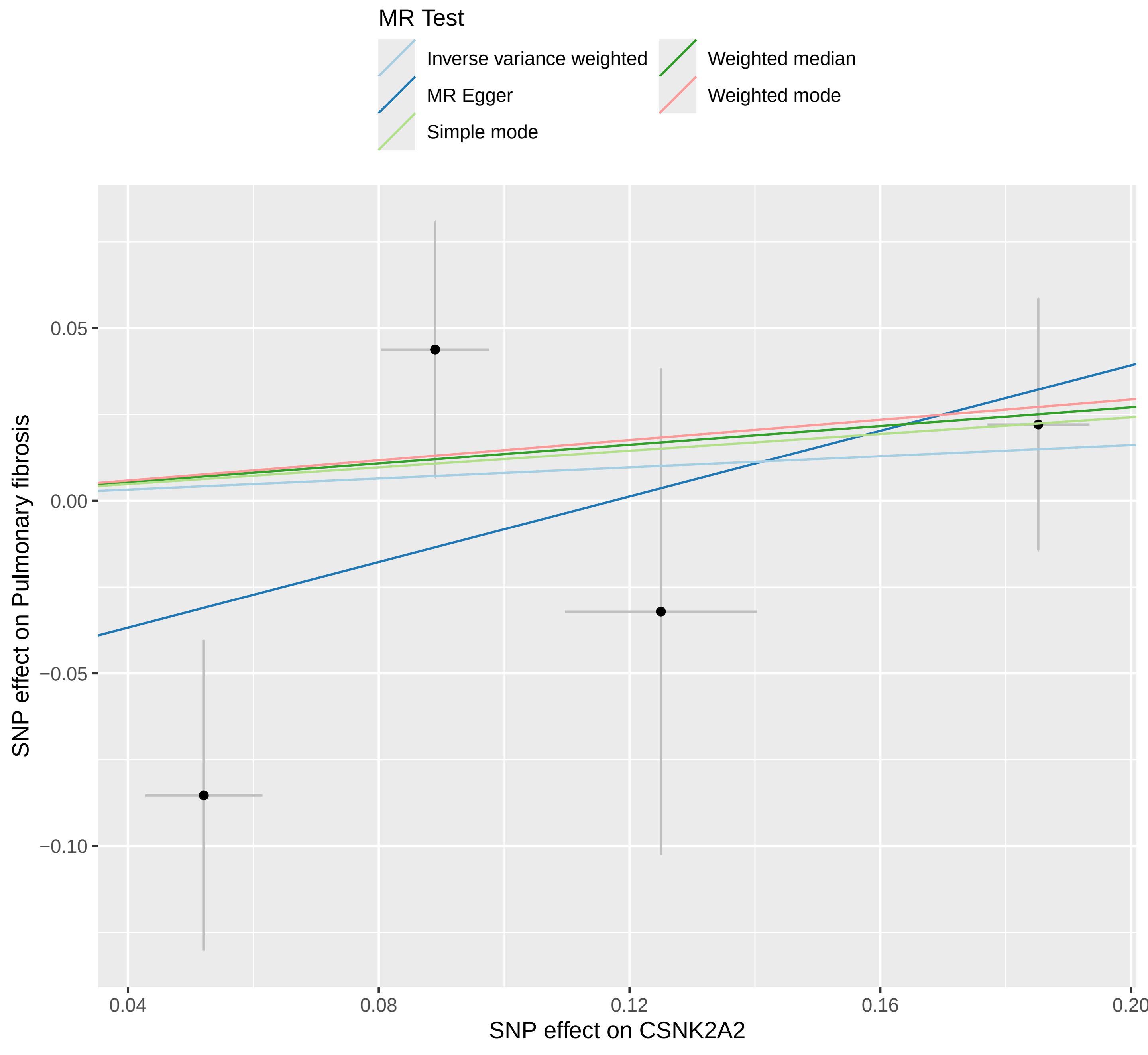
MR Test
Inverse variance weighted
MR Egger
Simple mode
Weighted median
Weighted mode
0.05
0.00
−0.05
−0.10
0.04
0.08
0.12
0.16
0.20
SNP effect on CSNK2A2
SNP effect on Pulmonary fibrosis

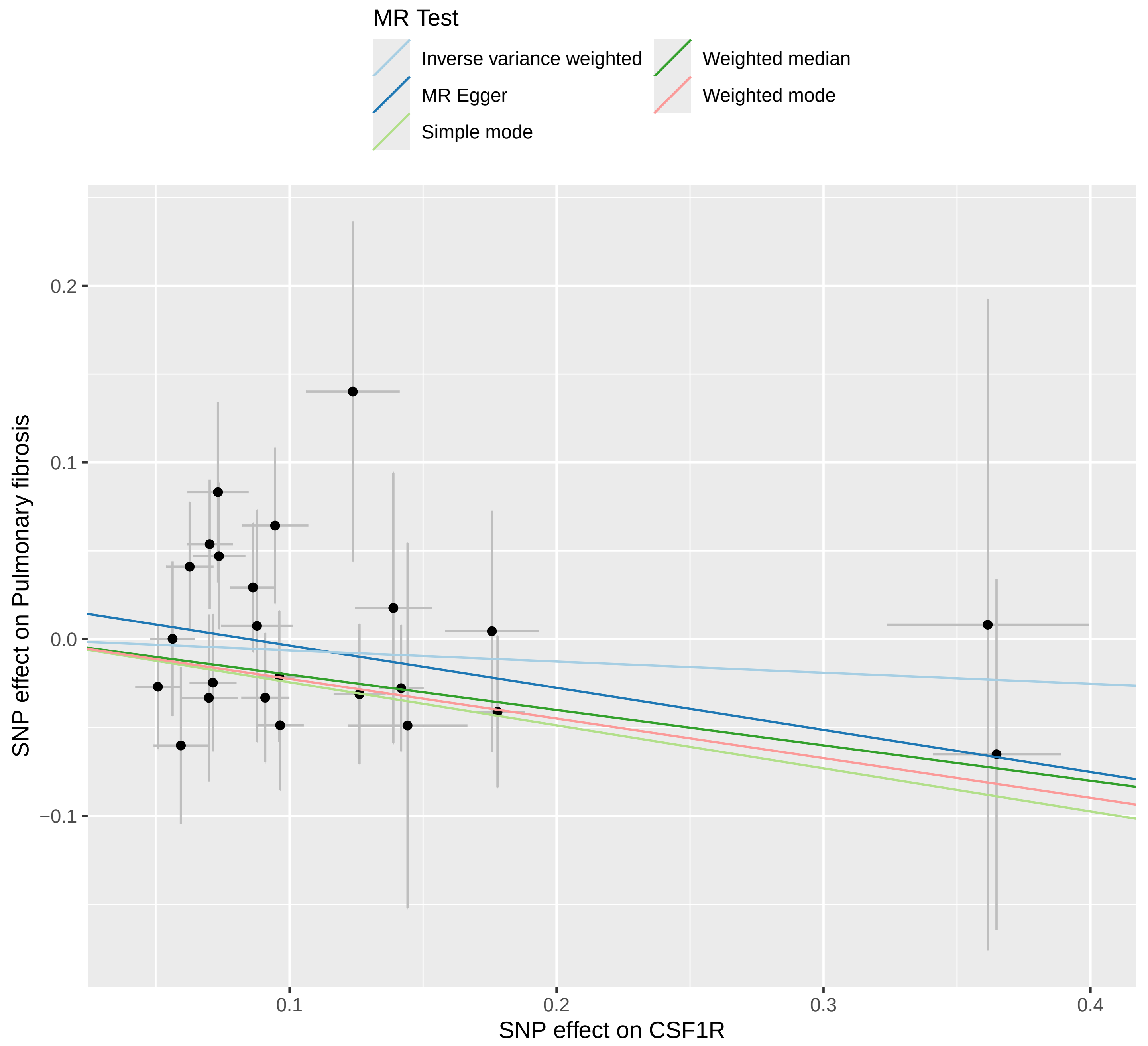
MR Test
Inverse variance weighted
MR Egger
Simple mode
Weighted median
Weighted mode
0.2
0.1
0.0
−0.1
0.1
0.2
0.3
0.4
SNP effect on CSF1R
SNP effect on Pulmonary fibrosis

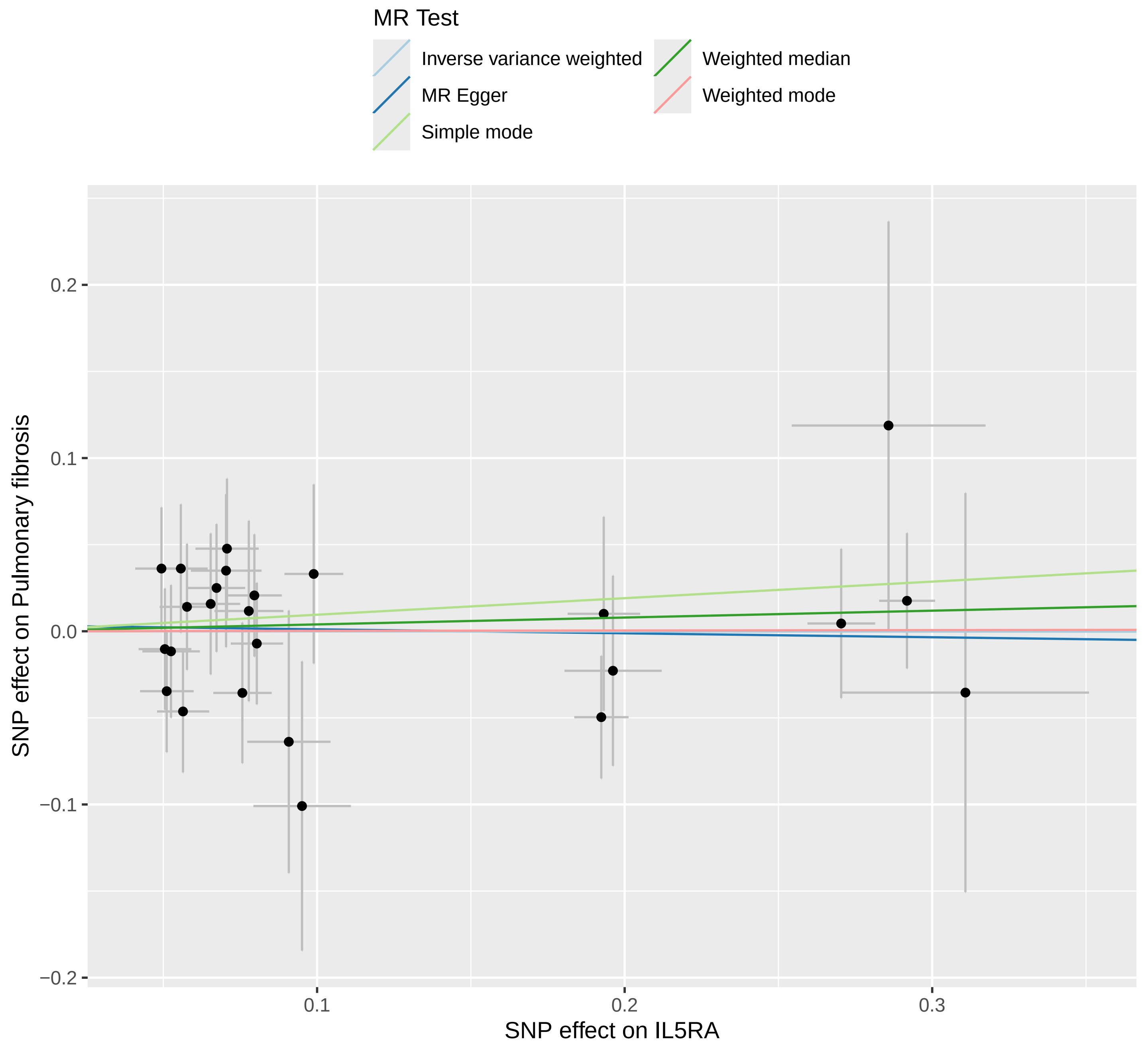
MR Test
Inverse variance weighted
MR Egger
Simple mode
Weighted median
Weighted mode
0.2
0.1
0.0
−0.1
−0.2
SNP effect on Pulmonary fibrosis
0.1
0.2
0.3
SNP effect on IL5RA

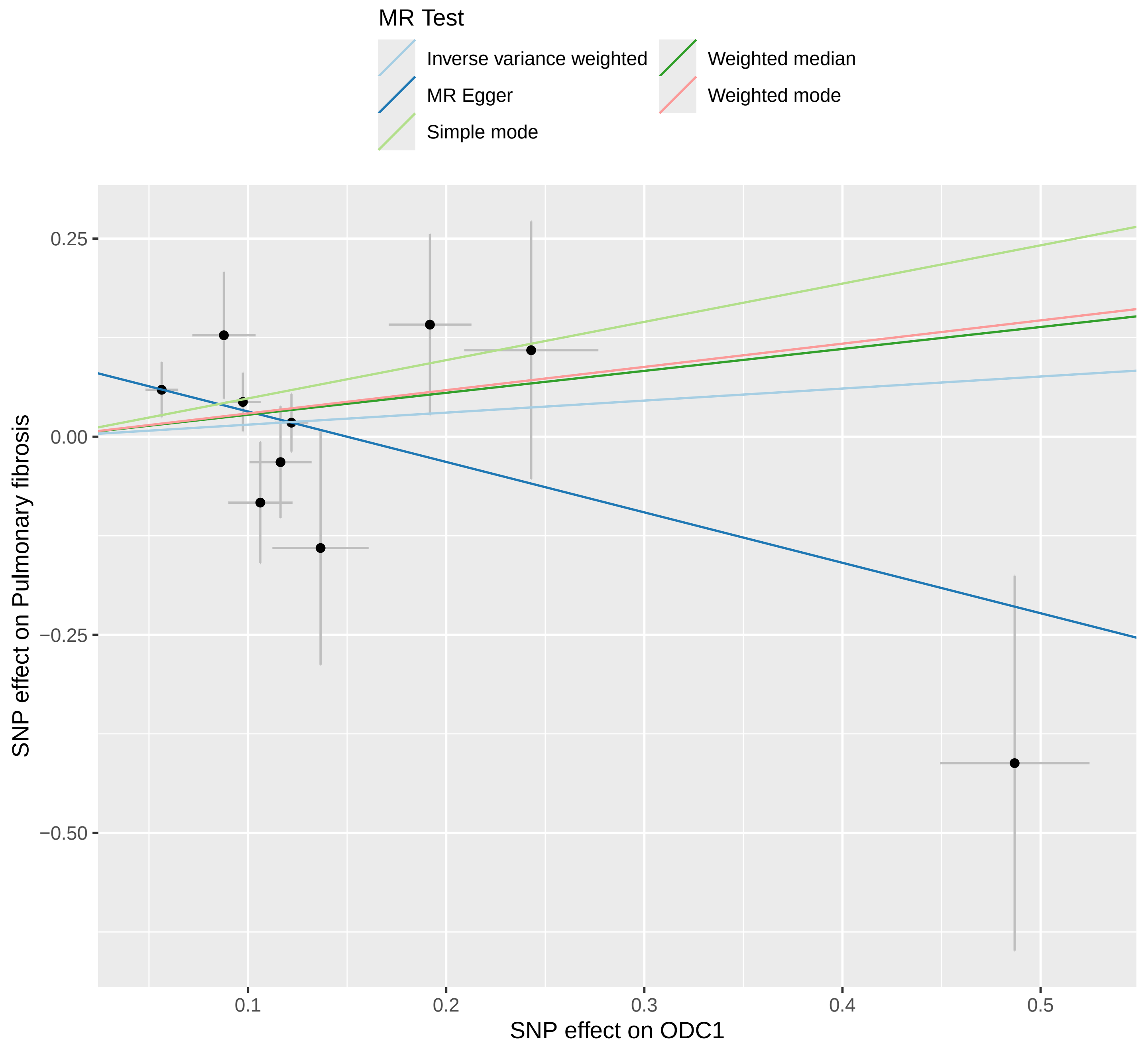

MR Test
Inverse variance weighted
MR Egger
Simple mode
Weighted median
Weighted mode
0.25
0.00
−0.25
−0.50
SNP effect on Pulmonary fibrosis
0.1
0.2
0.3
0.4
0.5
SNP effect on ODC1

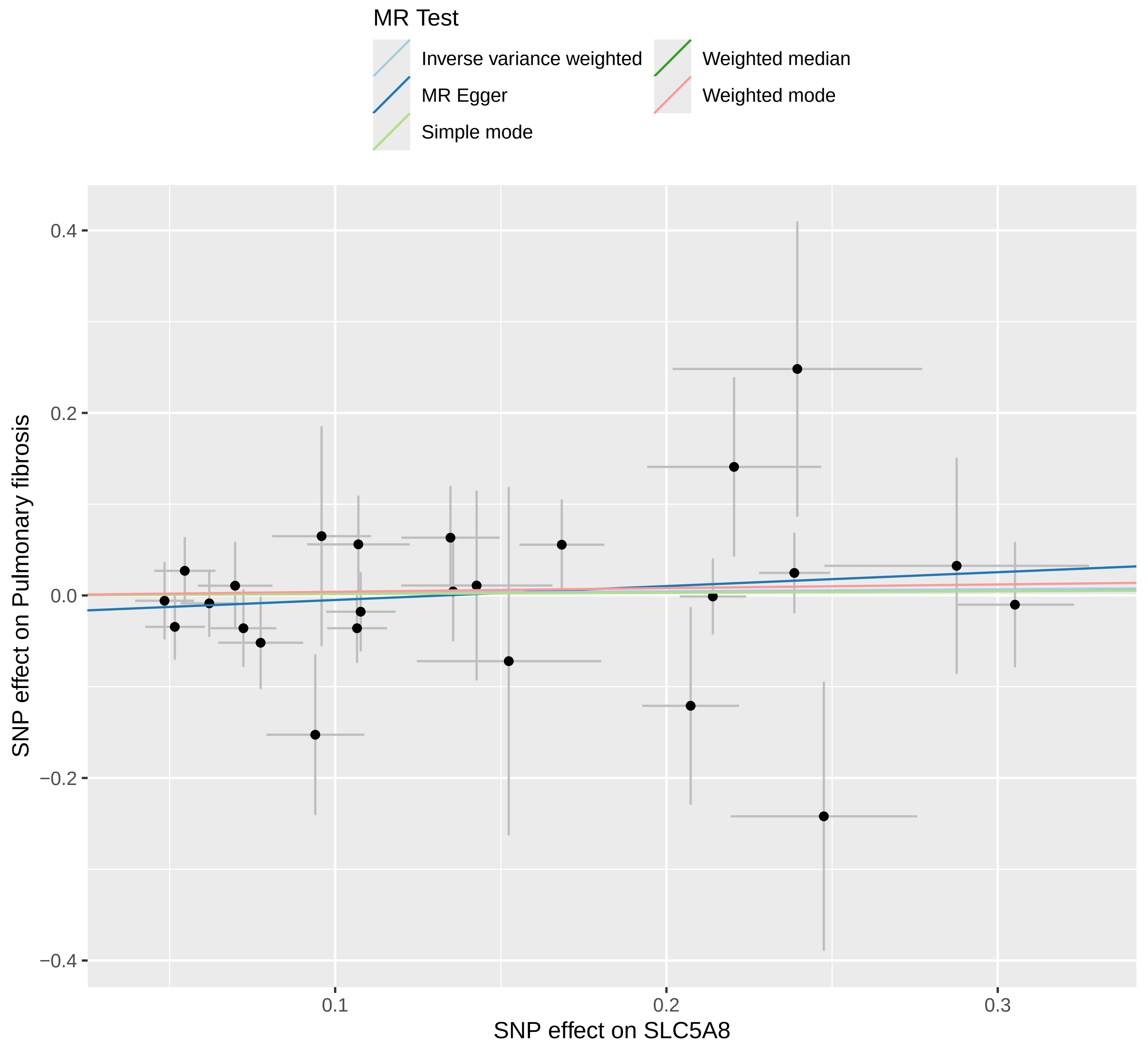

MR Test
Inverse variance weighted
MR Egger
Simple mode
Weighted median
Weighted mode
0.4
0.2
0.0
−0.2
−0.4
SNP effect on Pulmonary fibrosis
0.1
0.2
0.3
SNP effect on SLC5A8

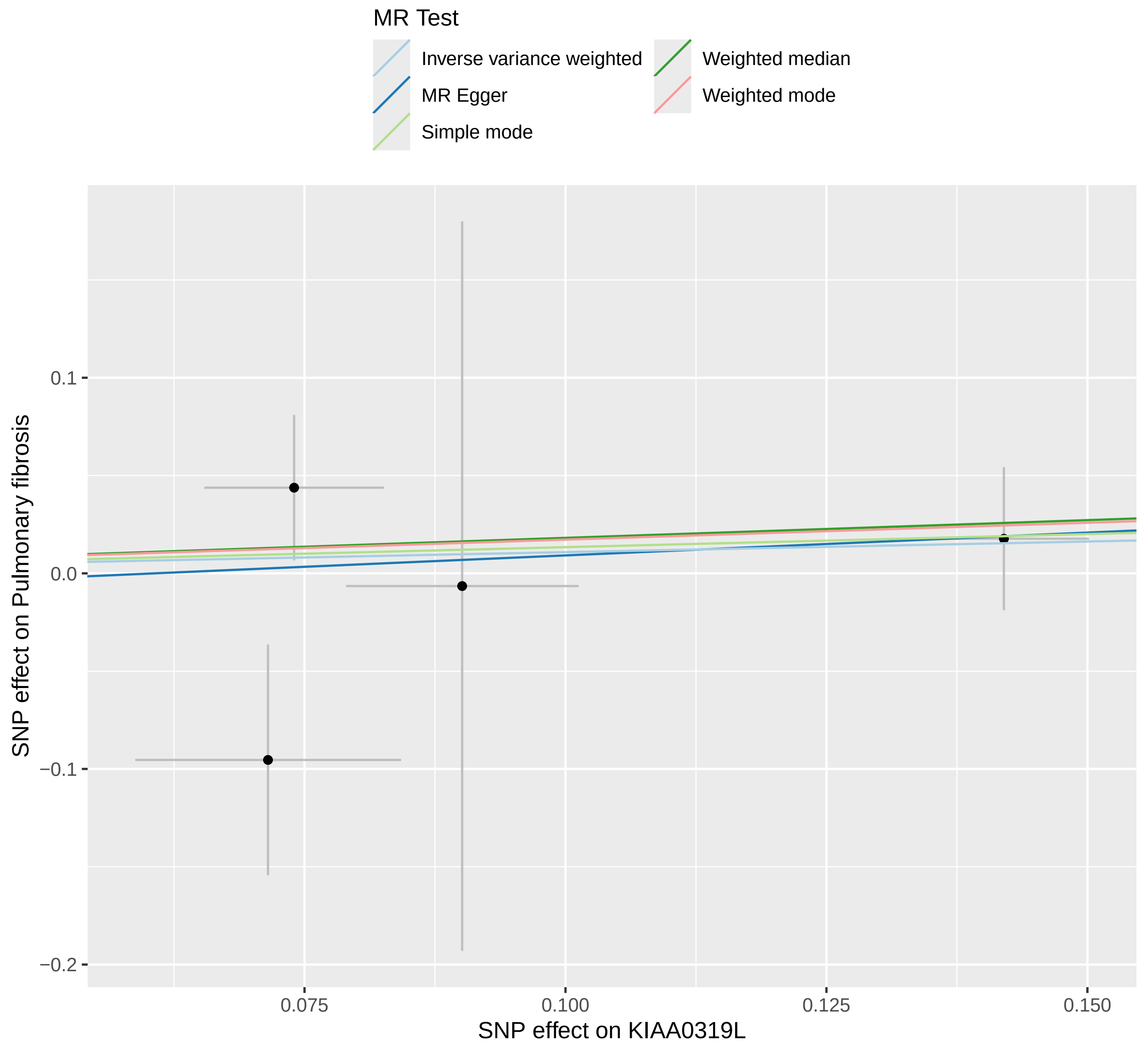
MR Test
Inverse variance weighted
MR Egger
Simple mode
Weighted median
Weighted mode
SNP effect on Pulmonary fibrosis
0.1
0.0
−0.1
−0.2
0.075
0.100
0.125
0.150
SNP effect on KIAA0319L

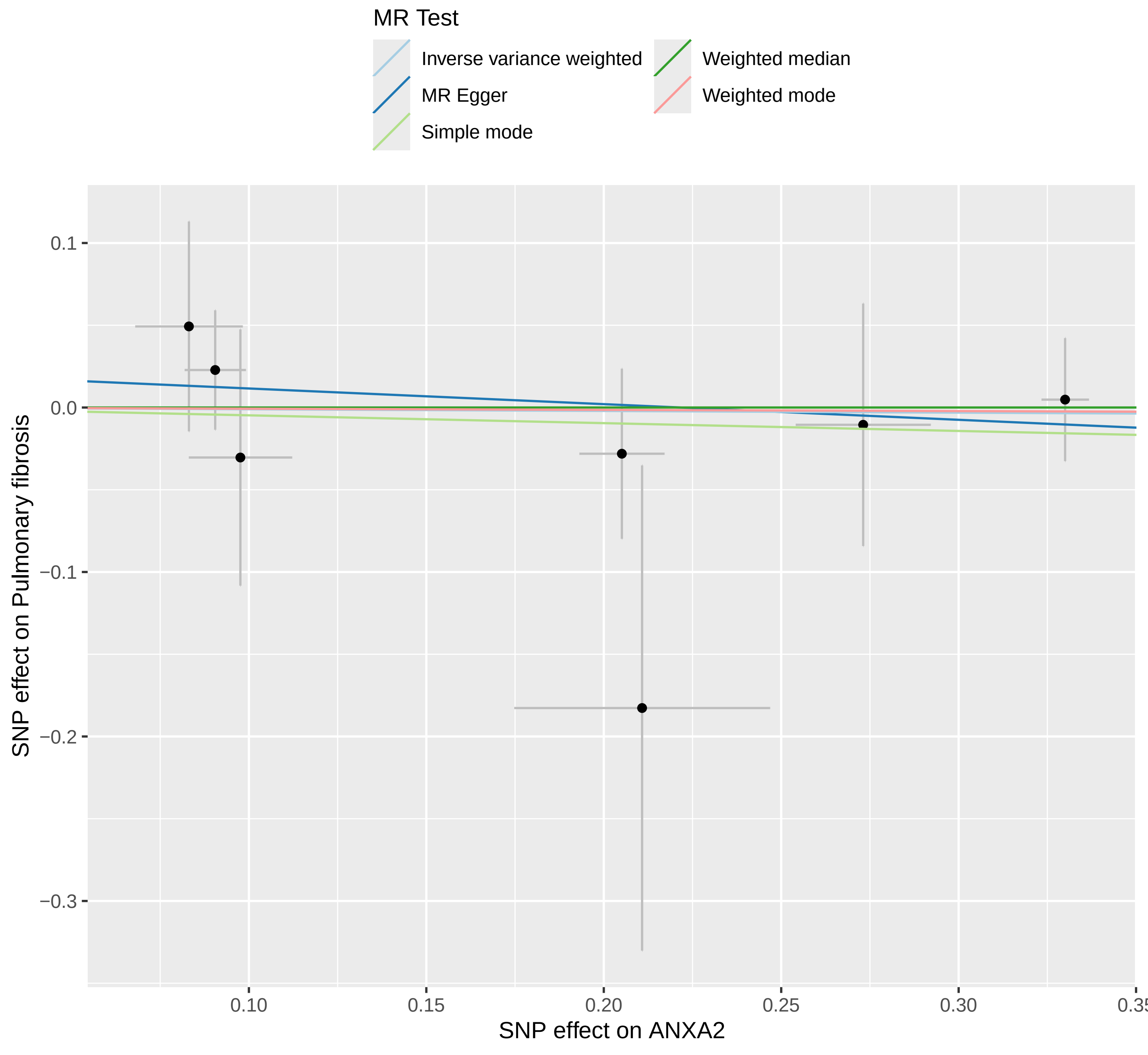

MR Test
Inverse variance weighted
MR Egger
Simple mode
Weighted median
Weighted mode
0.1
0.0
−0.1
−0.2
−0.3
0.10
0.15
0.20
0.25
0.30
SNP effect on ANXA2
SNP effect on Pulmonary fibrosis

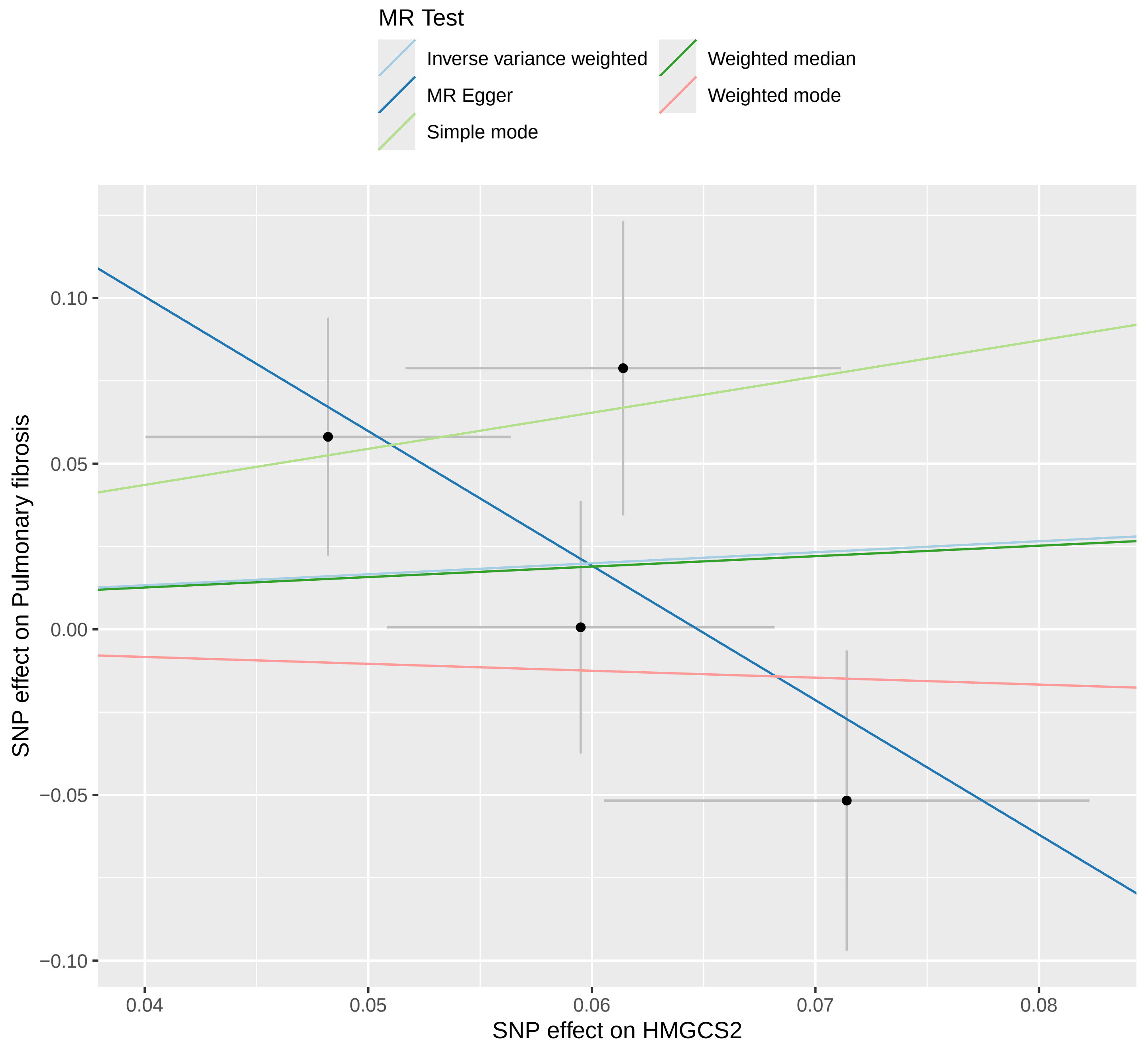

MR Test
Inverse variance weighted
MR Egger
Simple mode
Weighted median
Weighted mode
0.10
0.05
0.00
−0.05
−0.10
SNP effect on Pulmonary fibrosis
0.04
0.05
0.06
0.07
0.08
SNP effect on HMGCS2

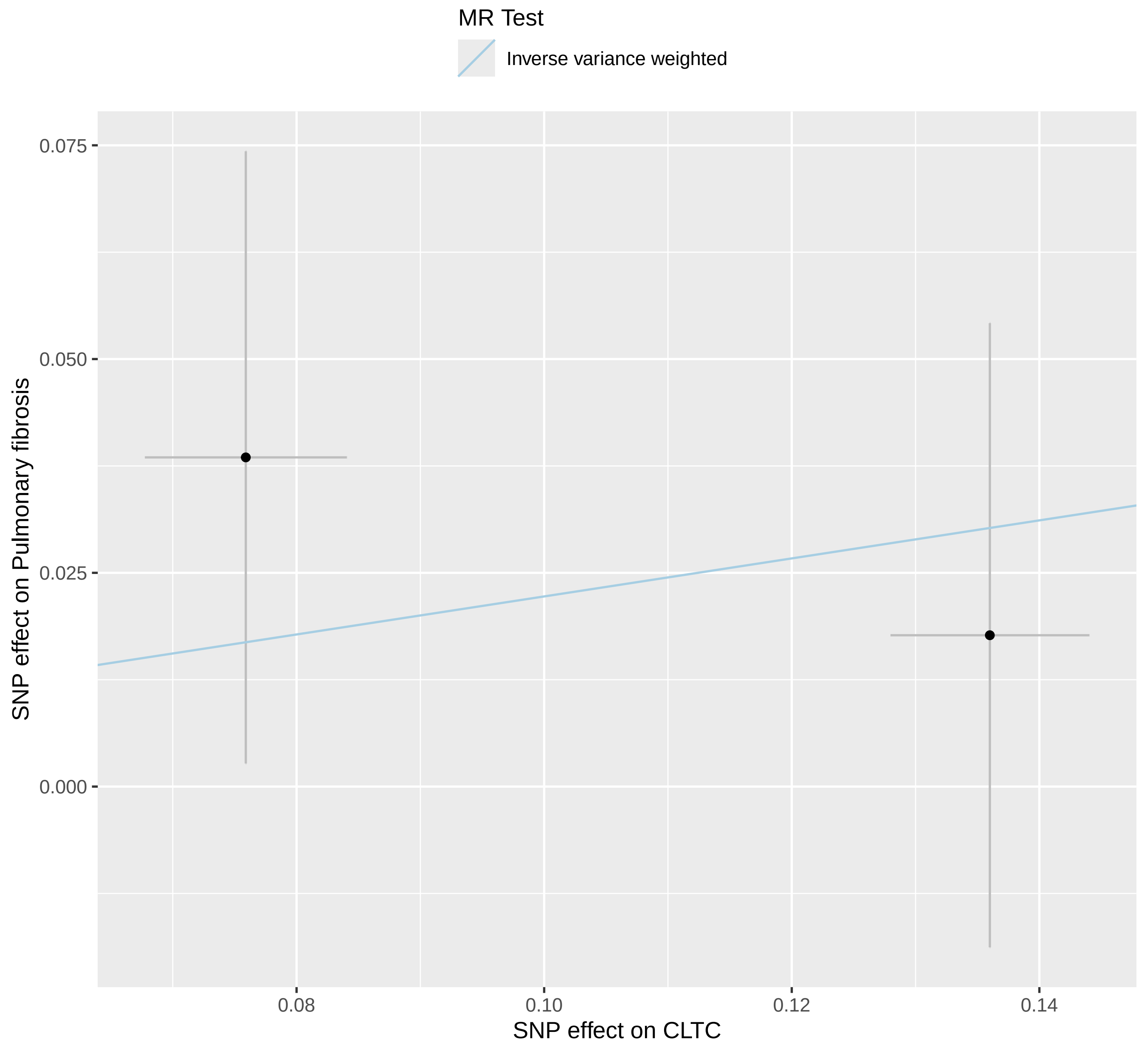
MR Test
Inverse variance weighted
0.075
0.050
0.025
0.000
SNP effect on Pulmonary fibrosis
0.08
0.10
0.12
0.14
SNP effect on CLTC

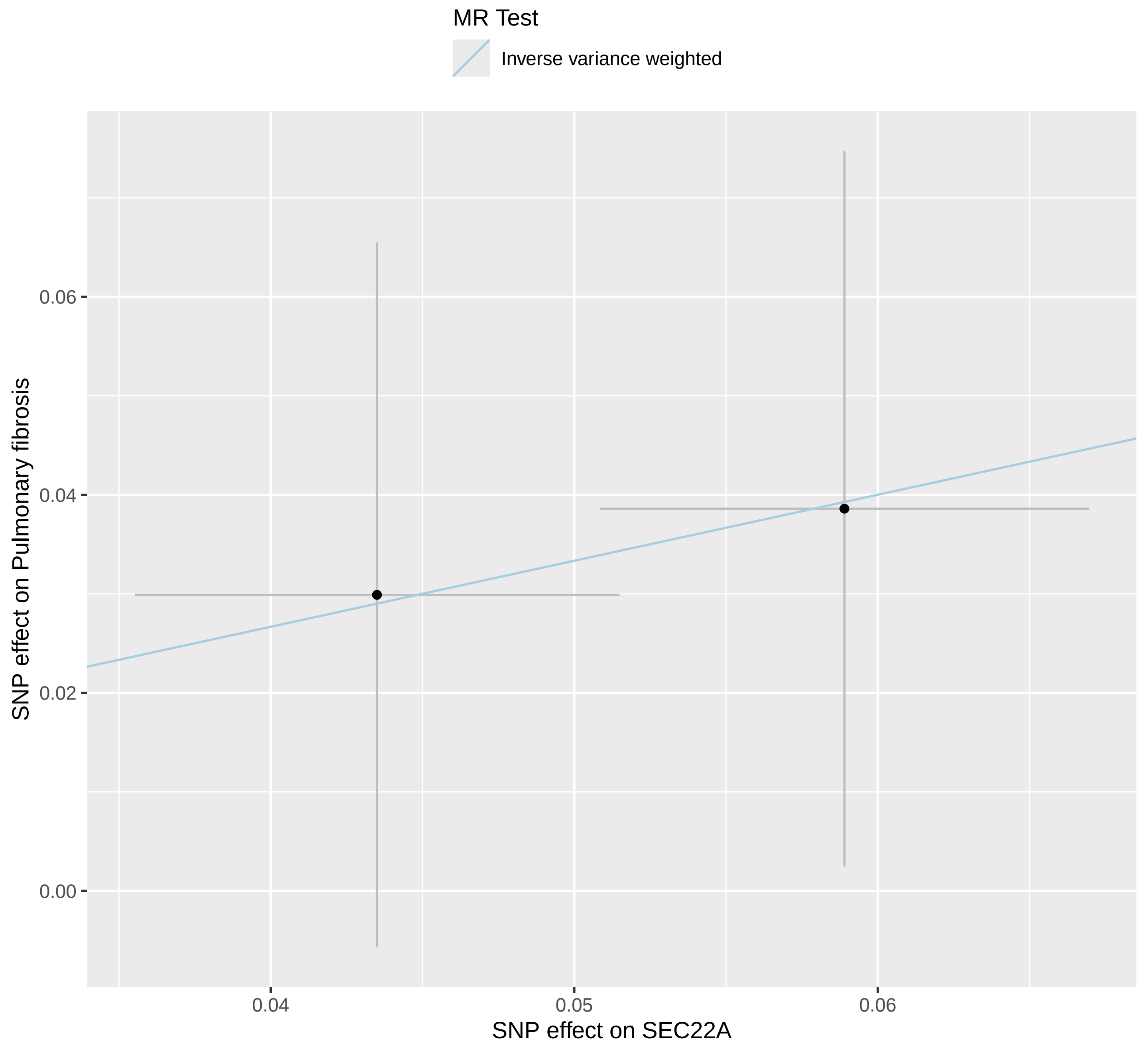
MR Test
Inverse variance weighted
0.06
0.04
0.02
0.00
0.04
0.05
0.06
SNP effect on Pulmonary fibrosis
SNP effect on SEC22A

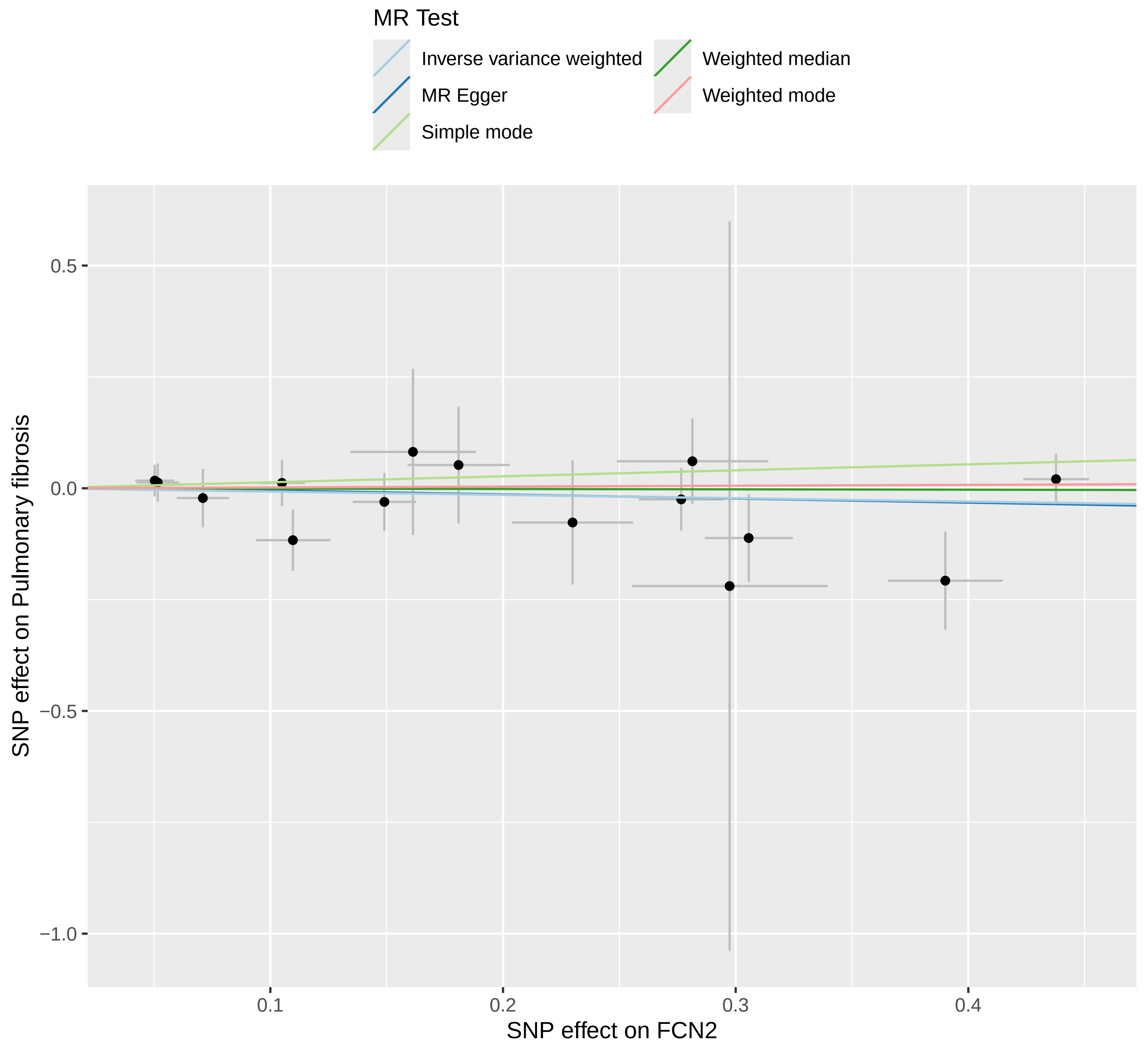

MR Test
Inverse variance weighted
MR Egger
Simple mode
Weighted median
Weighted mode
0.5
0.0
−0.5
−1.0
SNP effect on Pulmonary fibrosis
0.1
0.2
0.3
0.4
SNP effect on FCN2

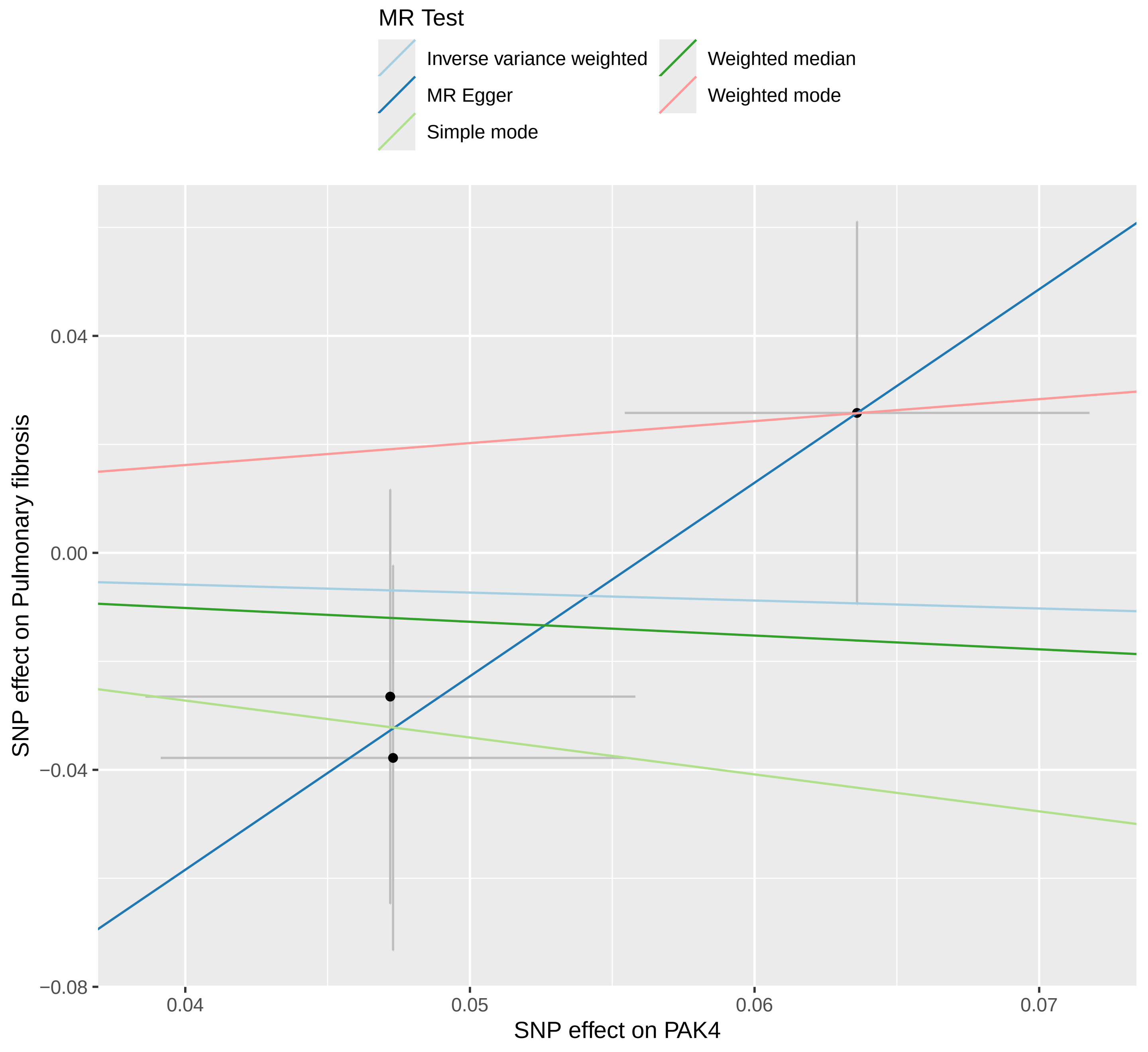

MR Test
Inverse variance weighted
MR Egger
Simple mode
Weighted median
Weighted mode
0.04
0.00
−0.04
−0.08
0.04
0.05
0.06
0.07
SNP effect on Pulmonary fibrosis
SNP effect on PAK4

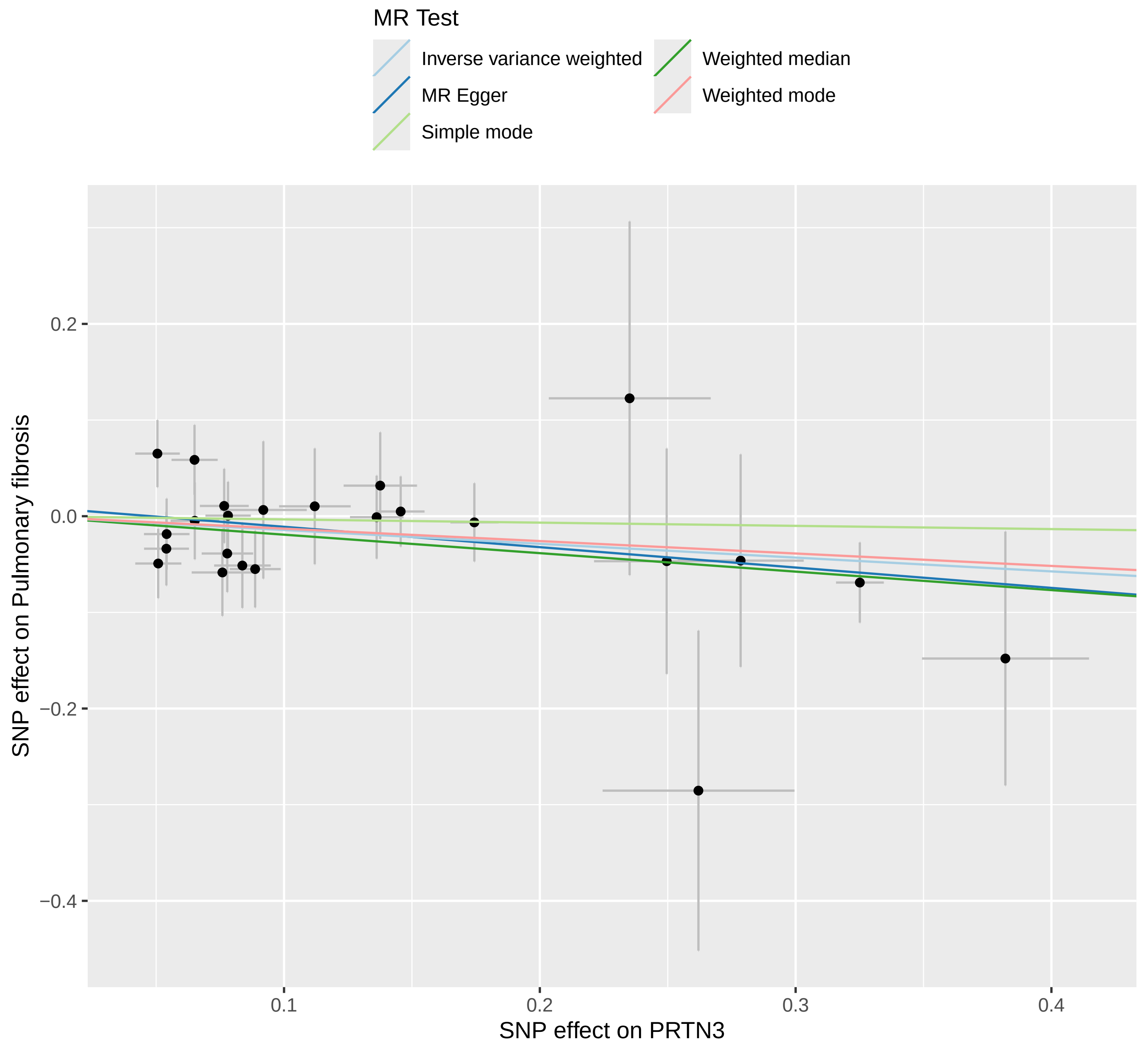
MR Test
Inverse variance weighted
Weighted median
MR Egger
Weighted mode
Simple mode
0.2
0.0
−0.2
−0.4
0.1
0.2
0.3
0.4
SNP effect on Pulmonary fibrosis
SNP effect on PRTN3

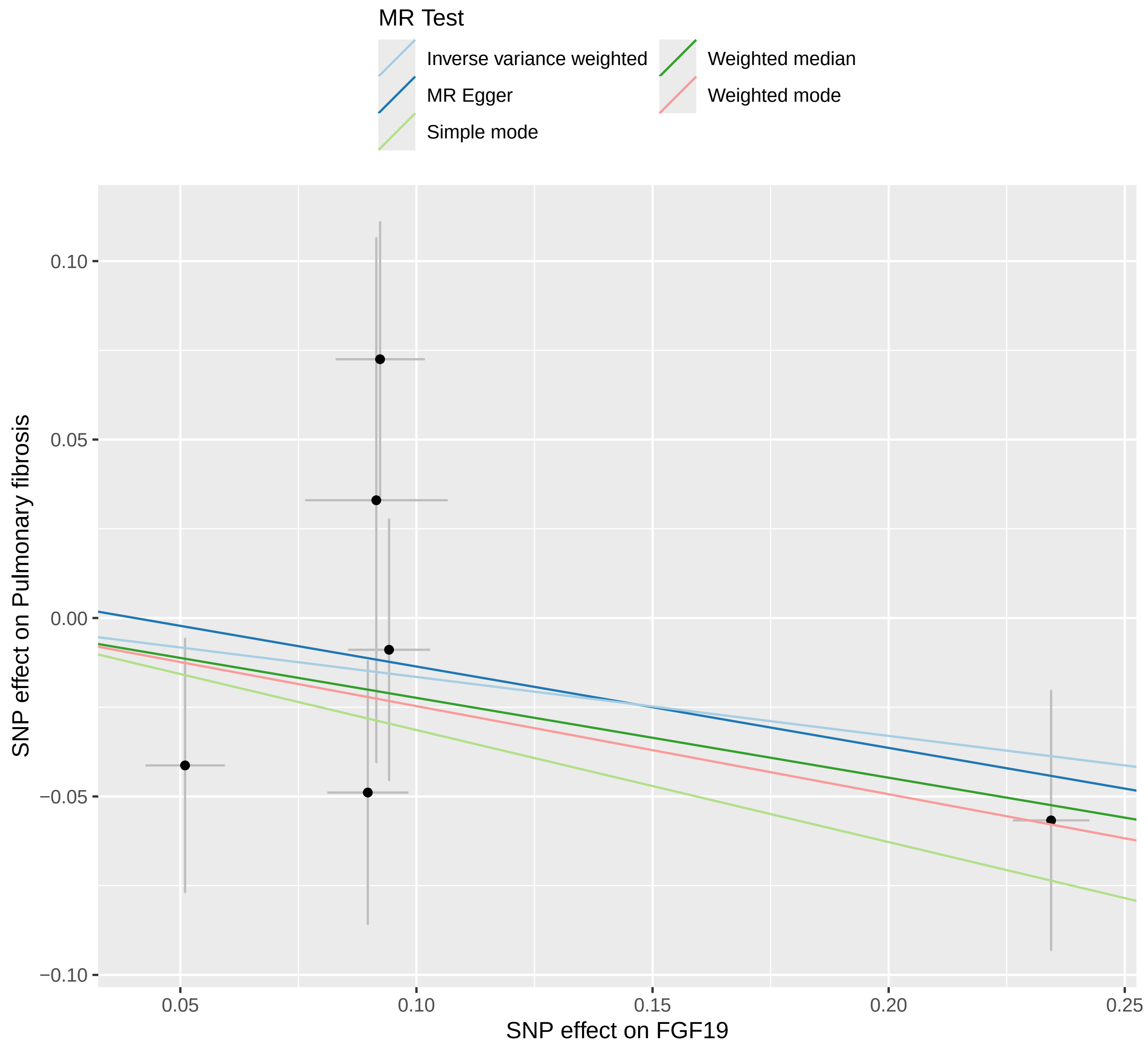

MR Test
Inverse variance weighted
MR Egger
Simple mode
Weighted median
Weighted mode
SNP effect on Pulmonary fibrosis
0.10
0.05
0.00
−0.05
−0.10
0.05
0.10
0.15
0.20
0.25
SNP effect on FGF19

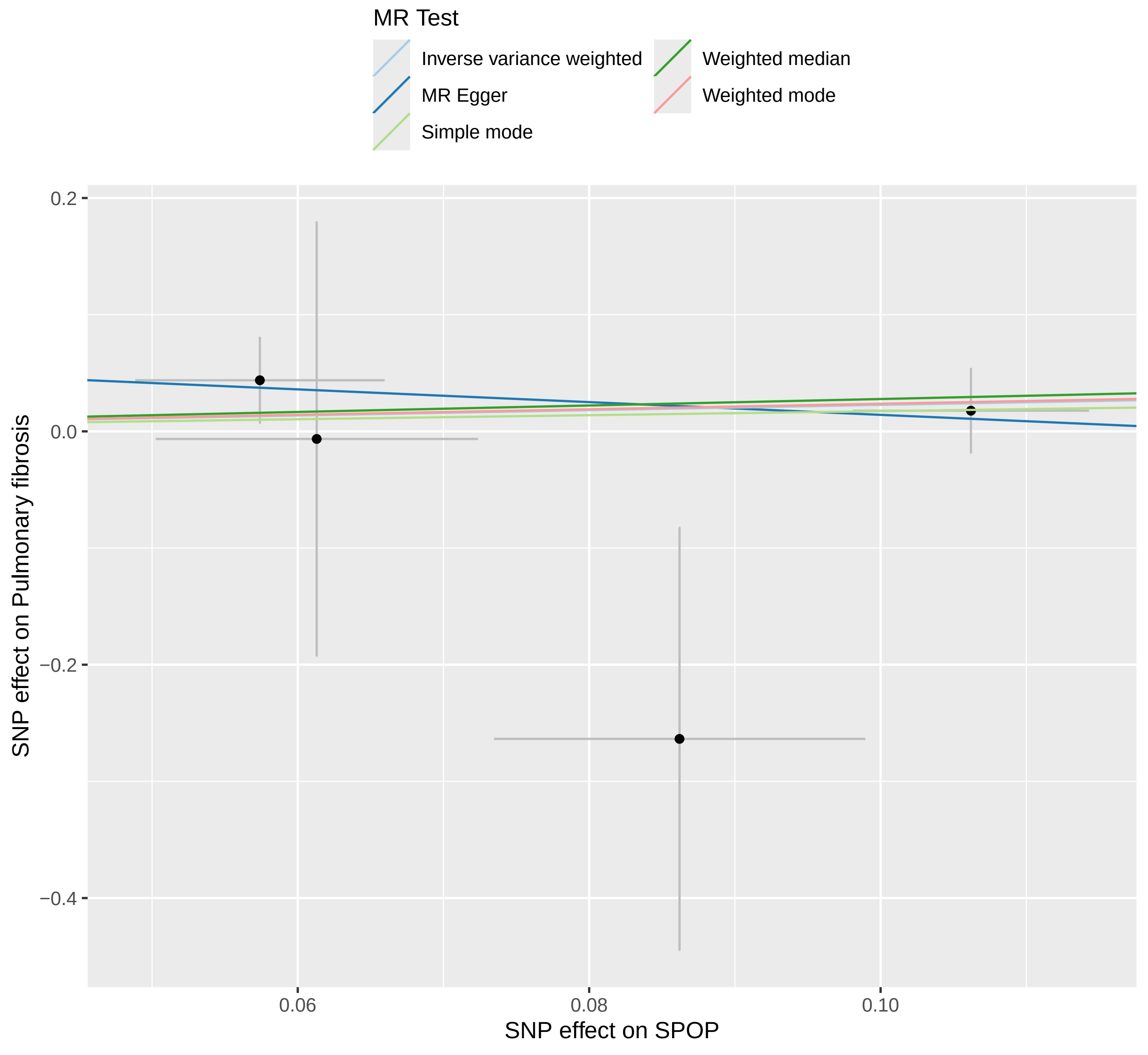
MR Test
Inverse variance weighted
MR Egger
Simple mode
Weighted median
Weighted mode
0.2
0.0
−0.2
−0.4
0.06
0.08
0.10
SNP effect on Pulmonary fibrosis
SNP effect on SPOP

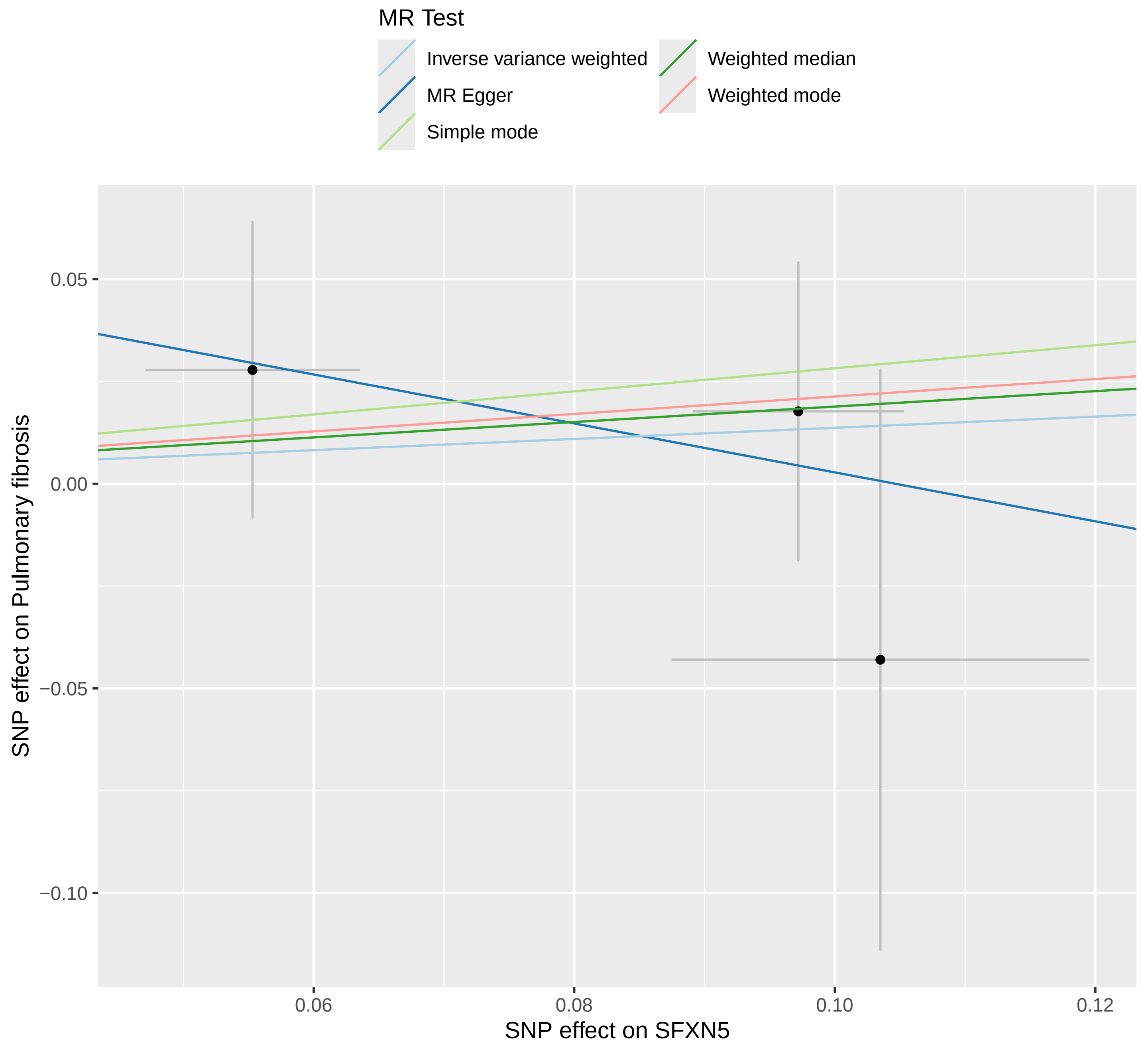
MR Test
Inverse variance weighted
MR Egger
Simple mode
Weighted median
Weighted mode
0.05
0.00
−0.05
−0.10
0.06
0.08
0.10
0.12
SNP effect on Pulmonary fibrosis
SNP effect on SFXN5

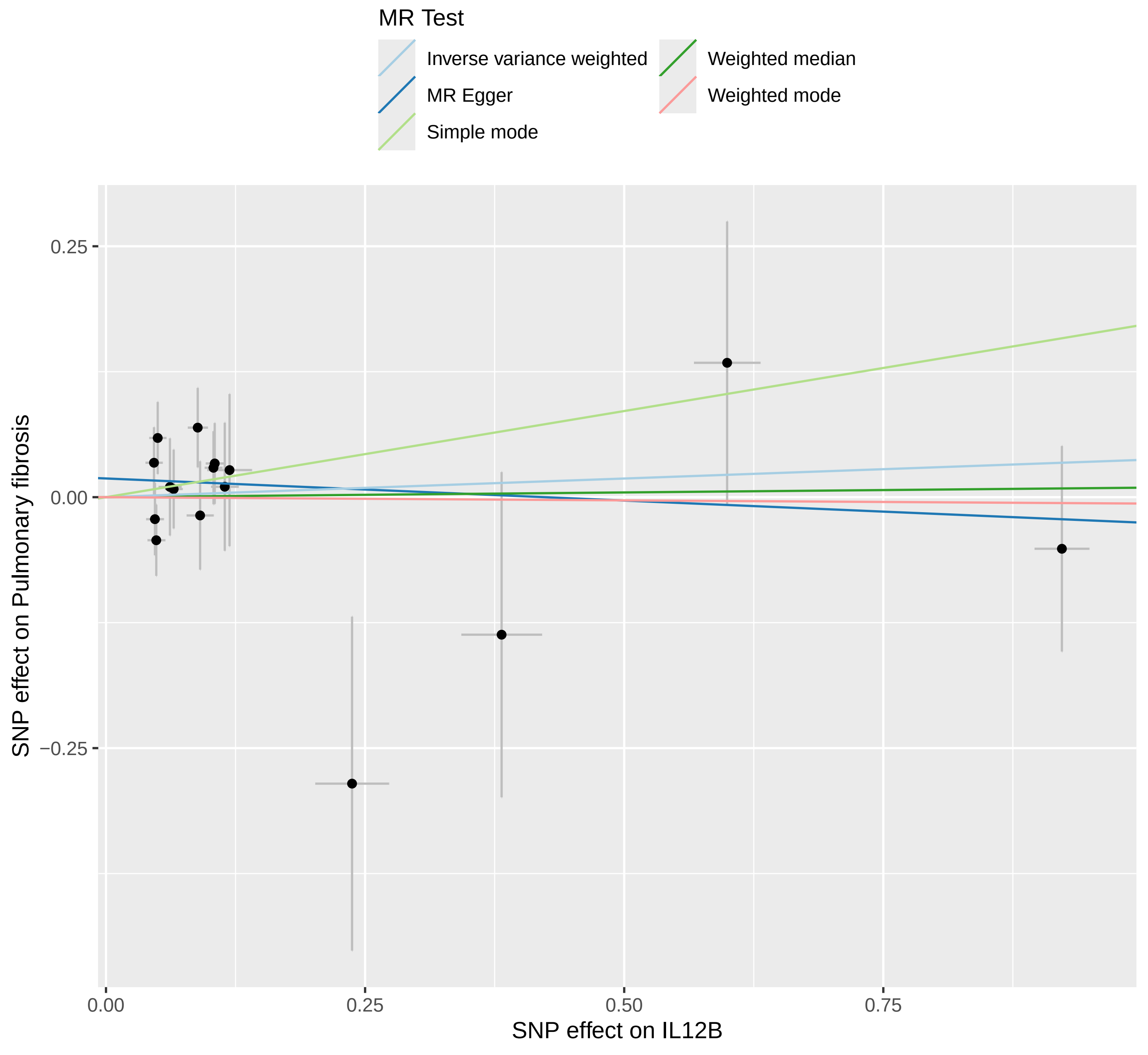
MR Test
Inverse variance weighted
MR Egger
Simple mode
Weighted median
Weighted mode
0.25
0.00
−0.25
SNP effect on Pulmonary fibrosis
0.00
0.25
0.50
0.75
SNP effect on IL12B

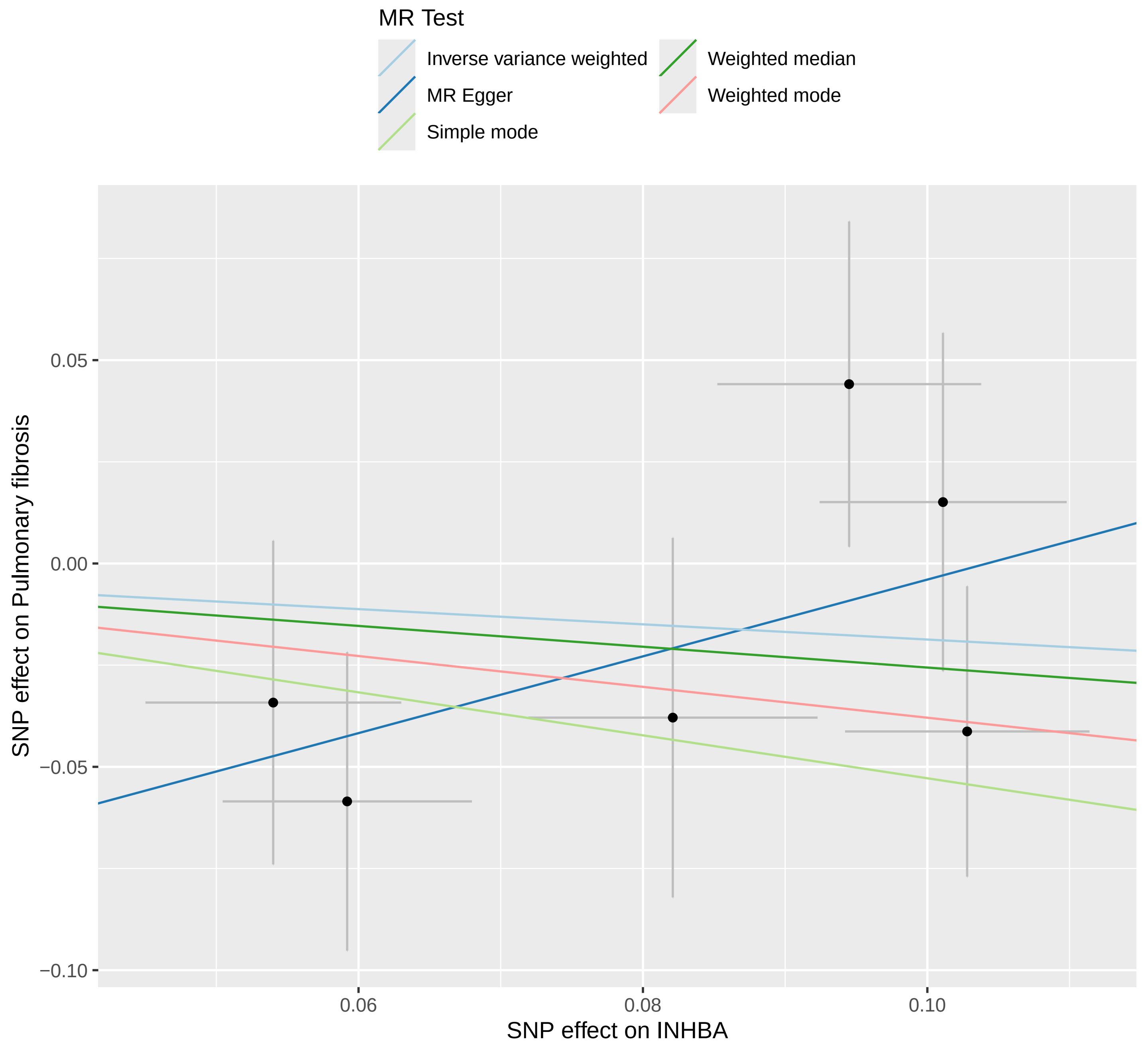

MR Test
Inverse variance weighted
MR Egger
Simple mode
Weighted median
Weighted mode
SNP effect on Pulmonary fibrosis
0.05
0.00
−0.05
−0.10
0.06
0.08
0.10
SNP effect on INHBA

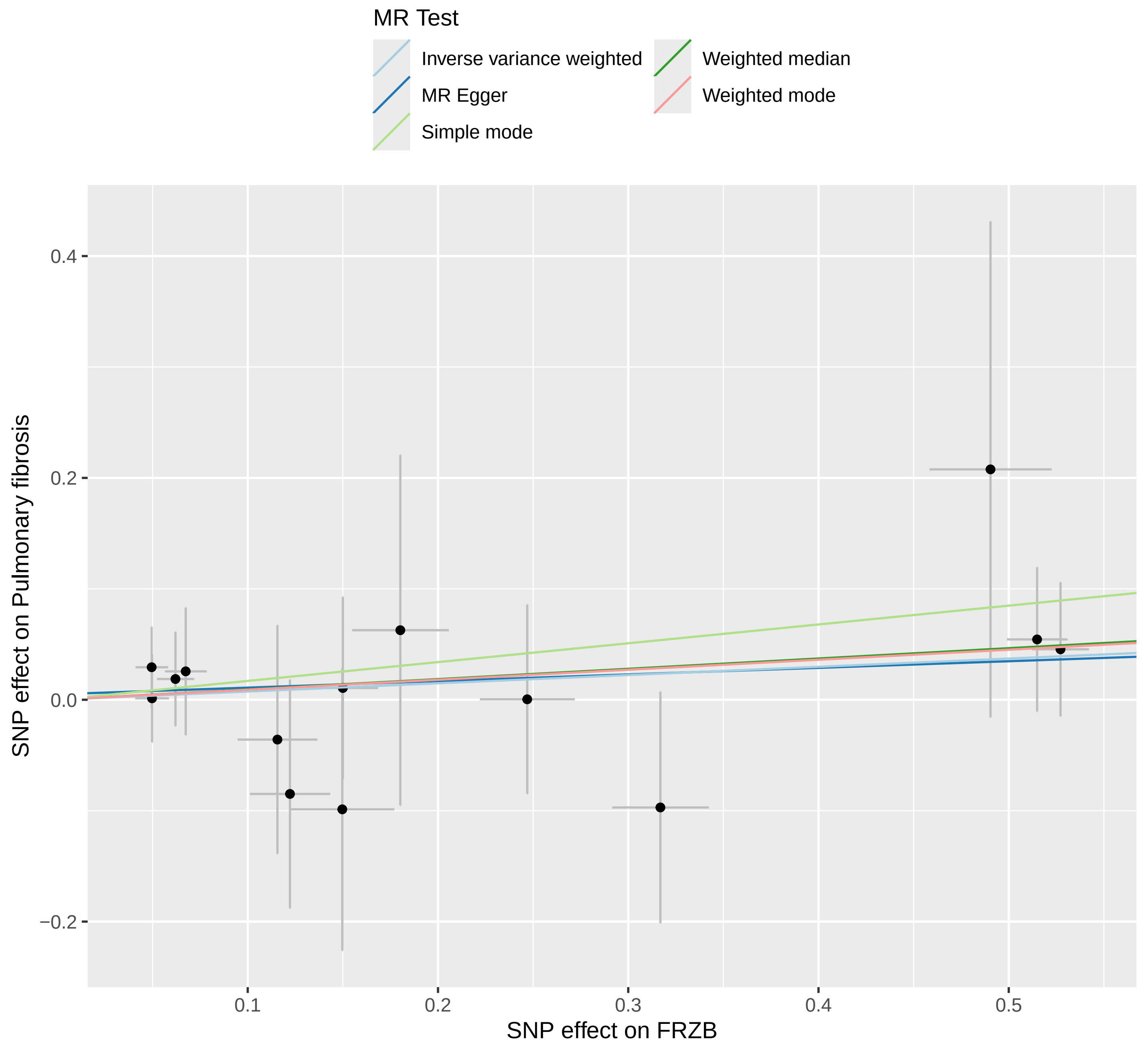
MR Test
Inverse variance weighted
MR Egger
Simple mode
Weighted median
Weighted mode
SNP effect on Pulmonary fibrosis
0.4
0.2
0.0
−0.2
0.1
0.2
0.3
0.4
0.5
SNP effect on FRZB

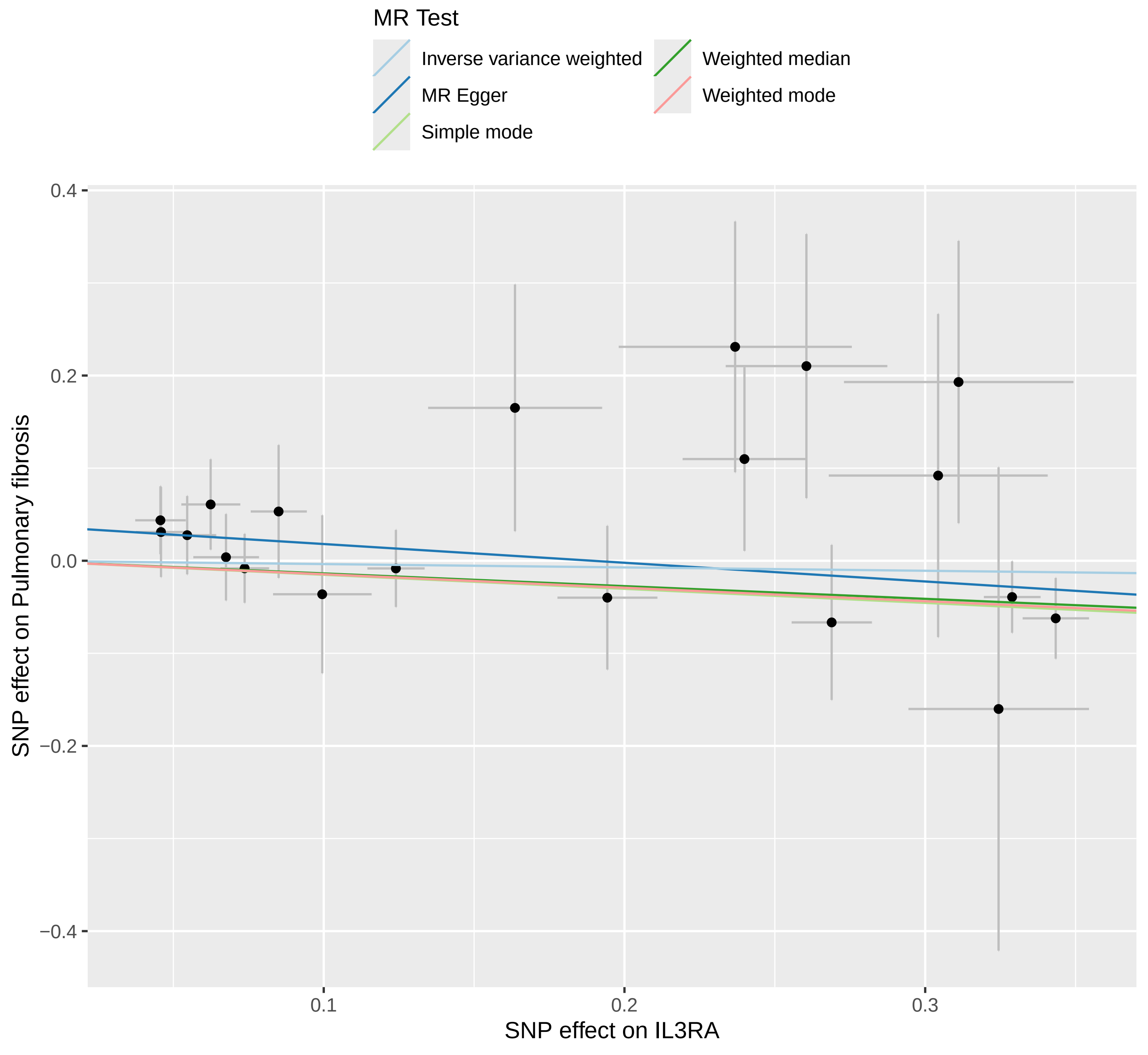
MR Test
Inverse variance weighted
MR Egger
Simple mode
Weighted median
Weighted mode
0.4
0.2
0.0
−0.2
−0.4
SNP effect on Pulmonary fibrosis
0.1
0.2
0.3
SNP effect on IL3RA

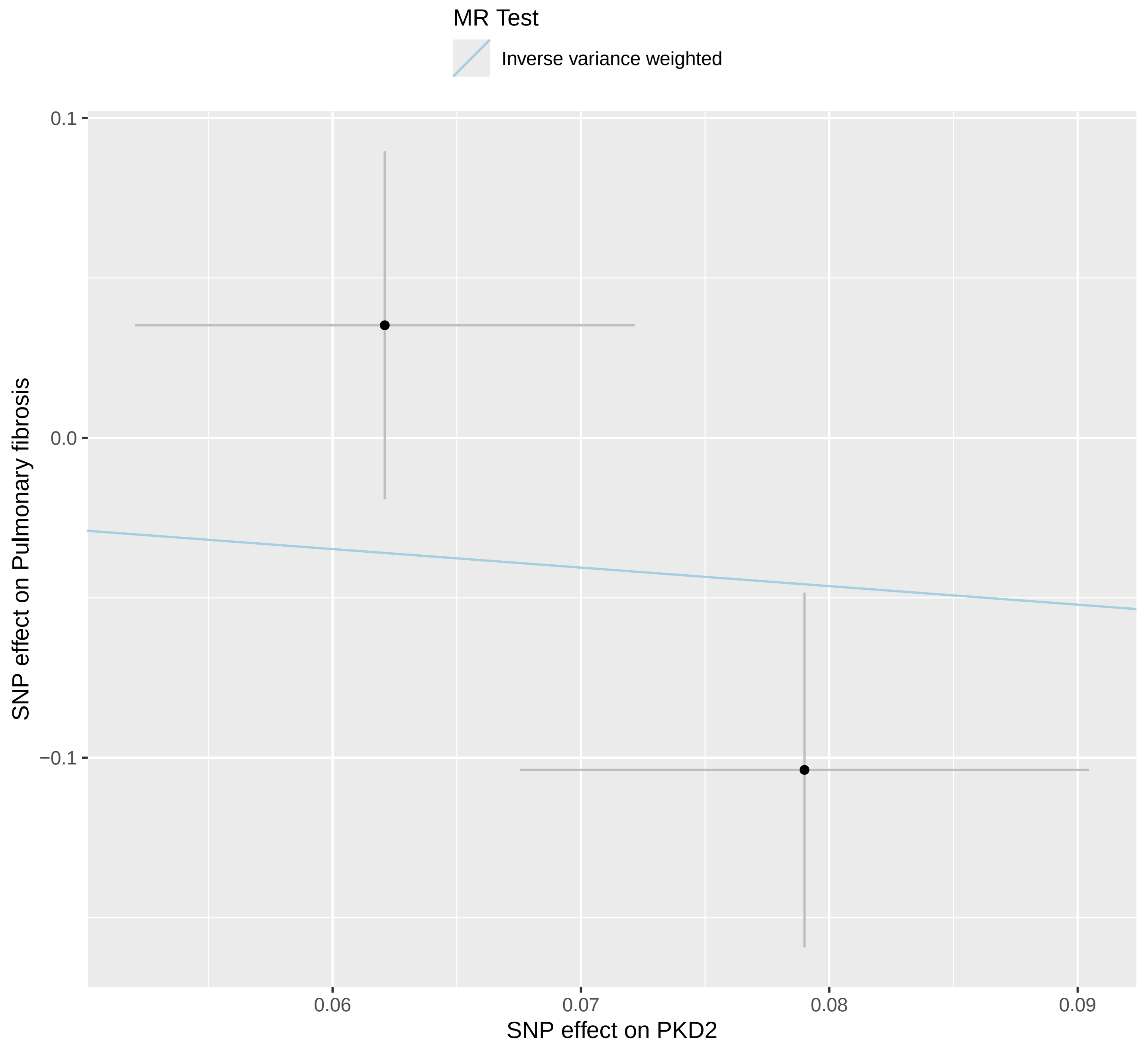
MR Test
Inverse variance weighted
0.1
0.0
−0.1
SNP effect on Pulmonary fibrosis
0.06
0.07
0.08
0.09
SNP effect on PKD2

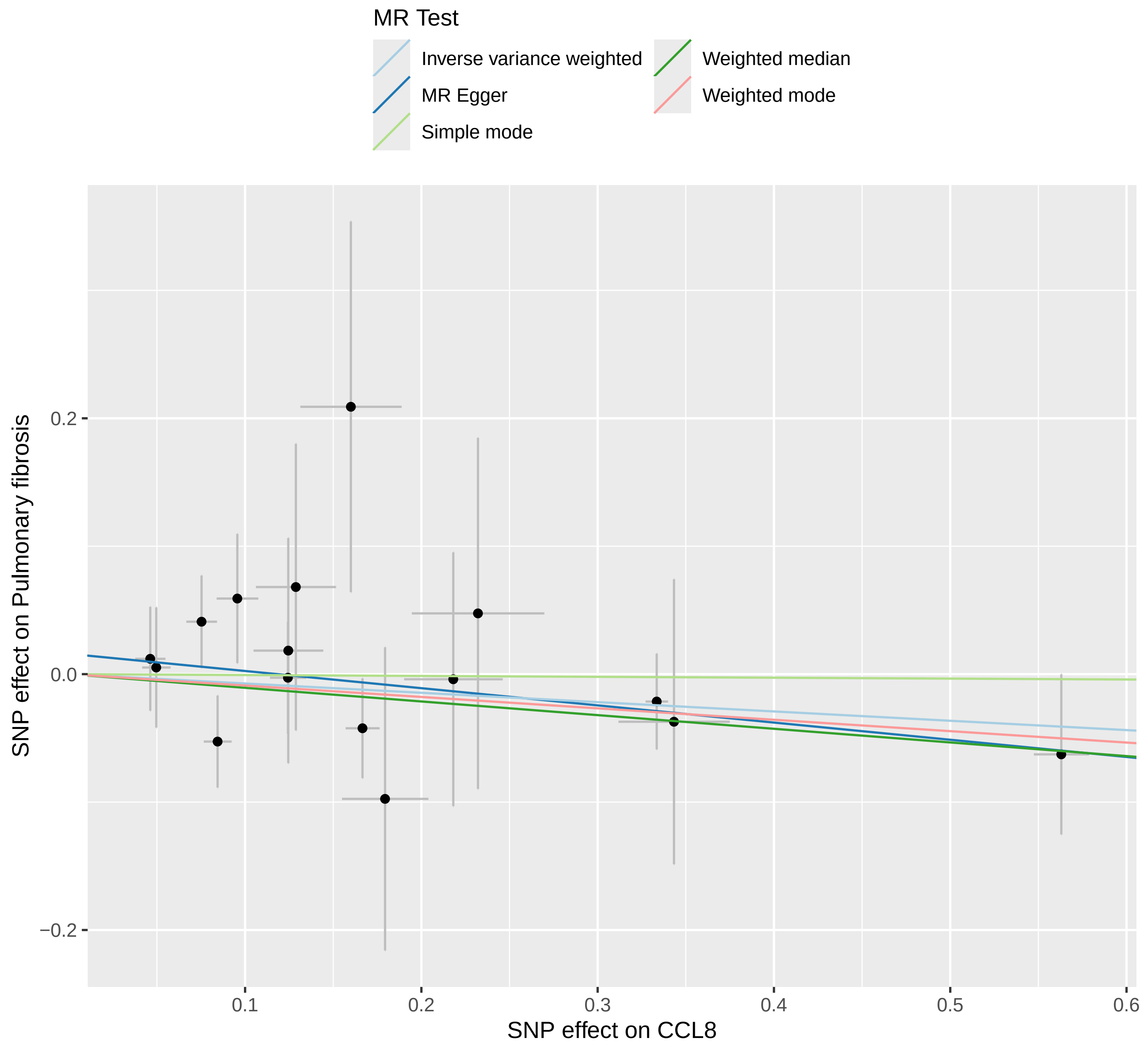
MR Test
Inverse variance weighted
MR Egger
Simple mode
Weighted median
Weighted mode
SNP effect on Pulmonary fibrosis
0.2
0.0
−0.2
0.1
0.2
0.3
0.4
0.5
0.6
SNP effect on CCL8

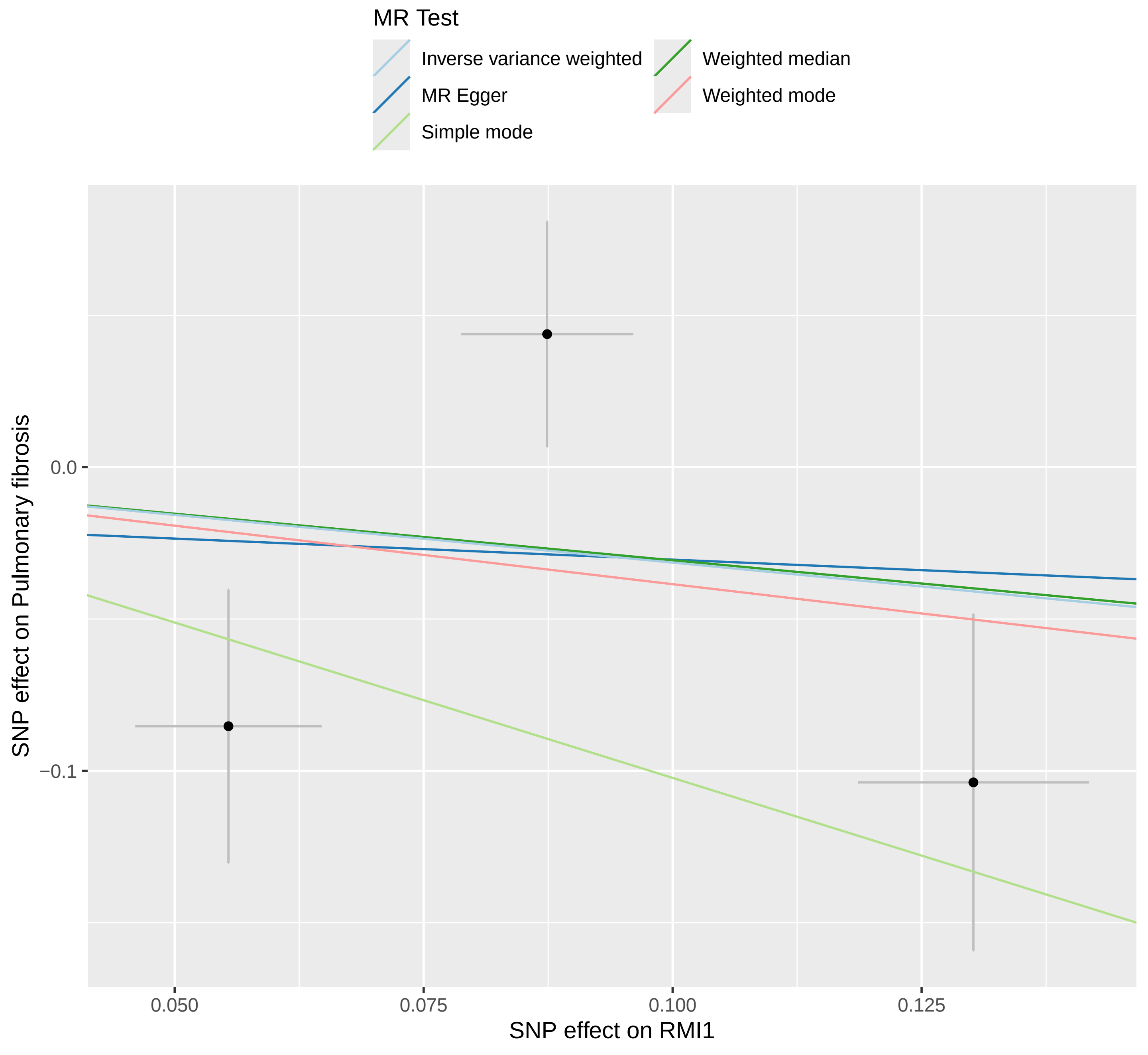
MR Test
Inverse variance weighted
MR Egger
Simple mode
Weighted median
Weighted mode
0.0
−0.1
0.050
0.075
0.100
0.125
SNP effect on Pulmonary fibrosis
SNP effect on RMI1

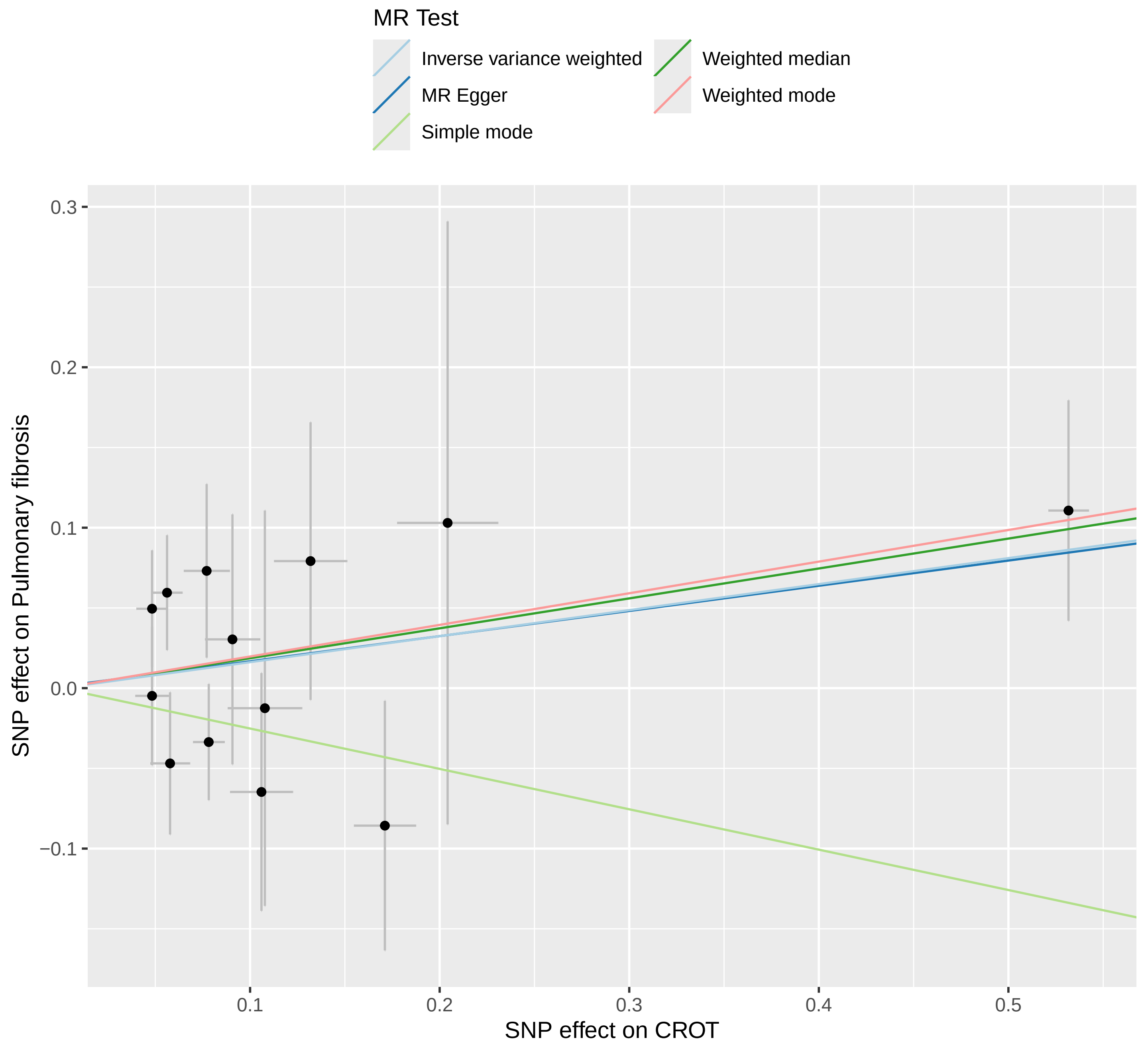
MR Test
Inverse variance weighted
MR Egger
Simple mode
Weighted median
Weighted mode
0.3
0.2
0.1
0.0
−0.1
0.1
0.2
0.3
0.4
0.5
SNP effect on Pulmonary fibrosis
SNP effect on CROT

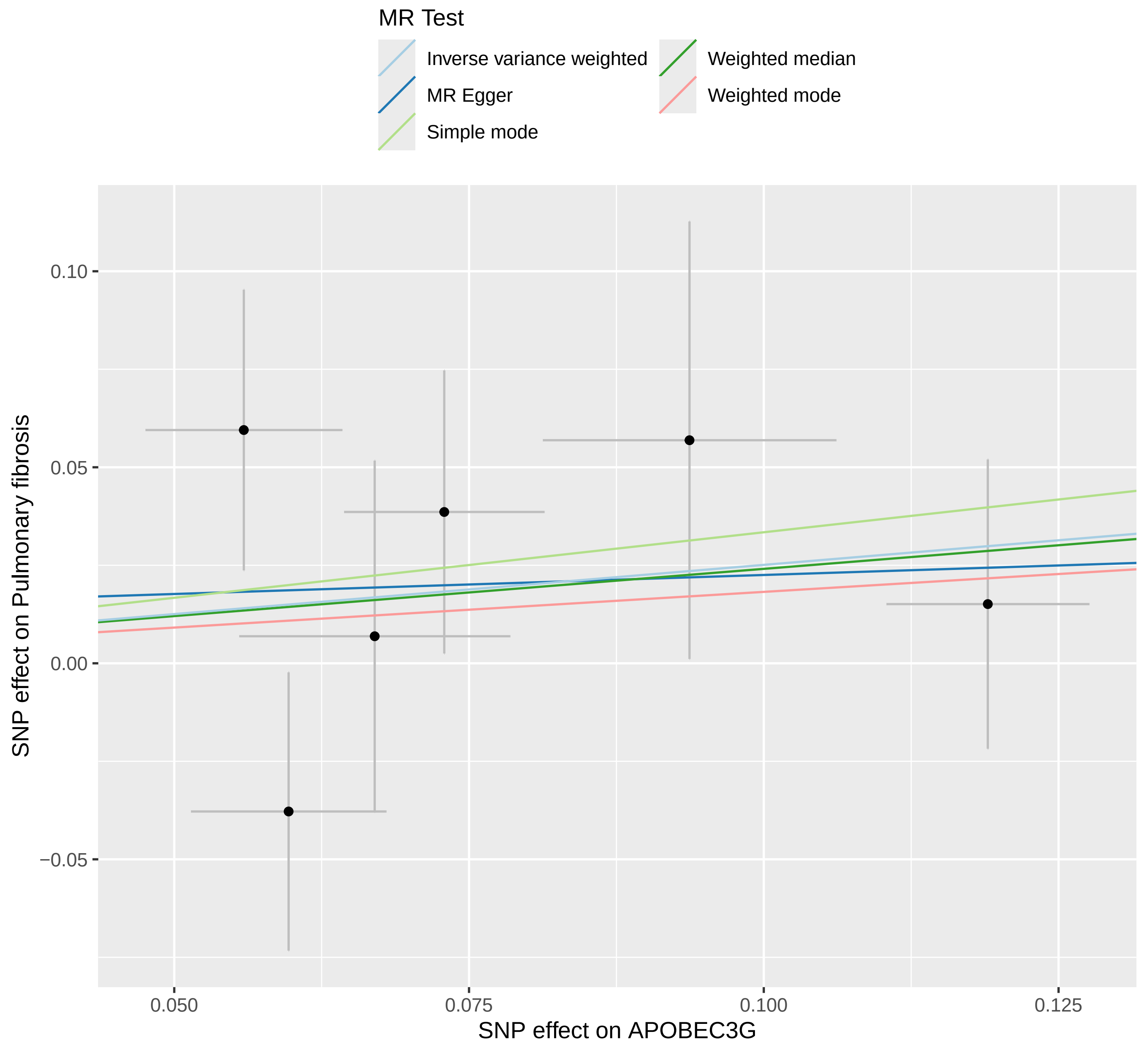
MR Test
Inverse variance weighted
MR Egger
Simple mode
Weighted median
Weighted mode
0.10
0.05
0.00
−0.05
SNP effect on Pulmonary fibrosis
0.050
0.075
0.100
0.125
SNP effect on APOBEC3G

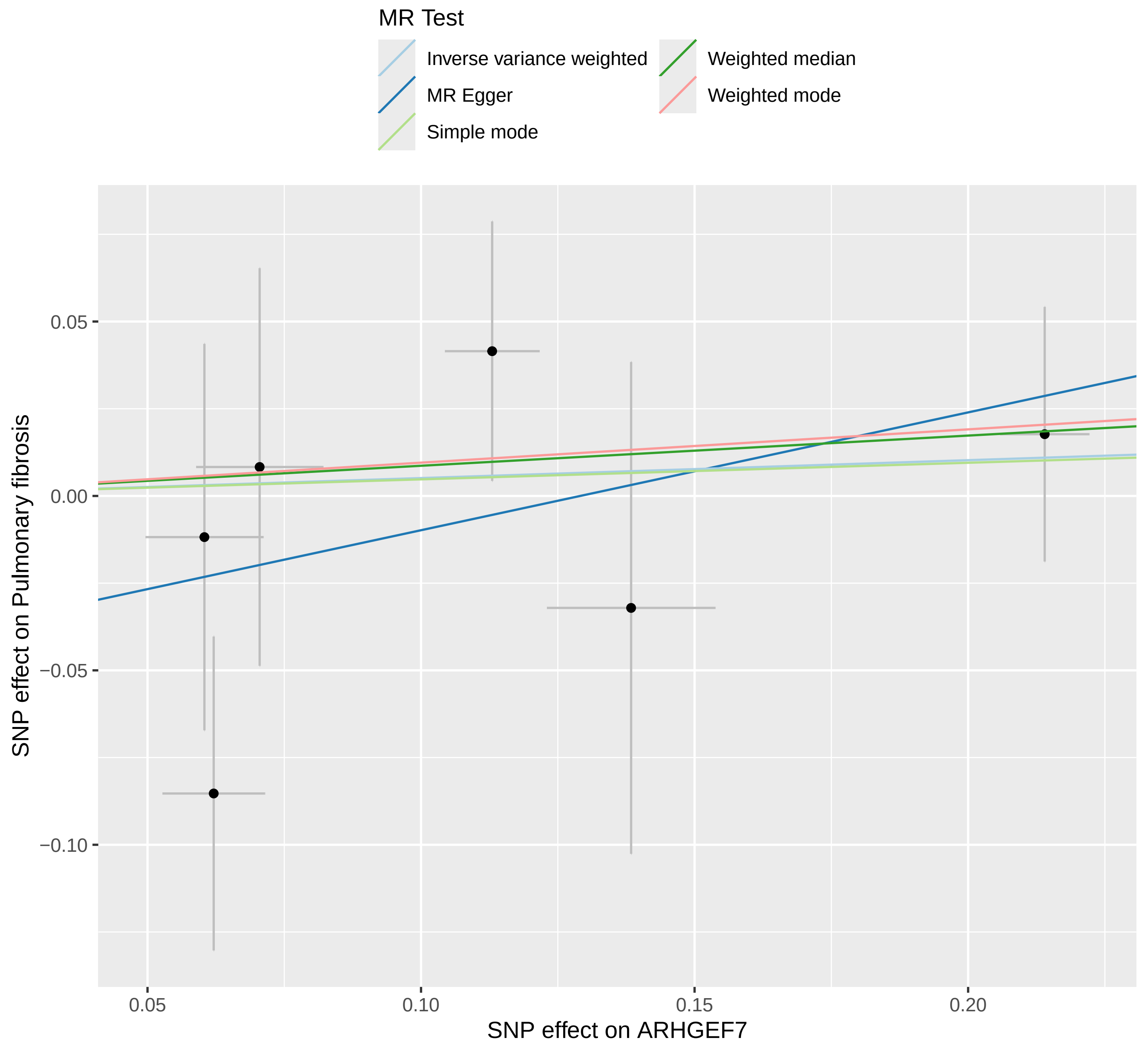

MR Test
Inverse variance weighted
MR Egger
Simple mode
Weighted median
Weighted mode
0.05
0.00
−0.05
−0.10
0.05
0.10
0.15
0.20
SNP effect on Pulmonary fibrosis
SNP effect on ARHGEF7

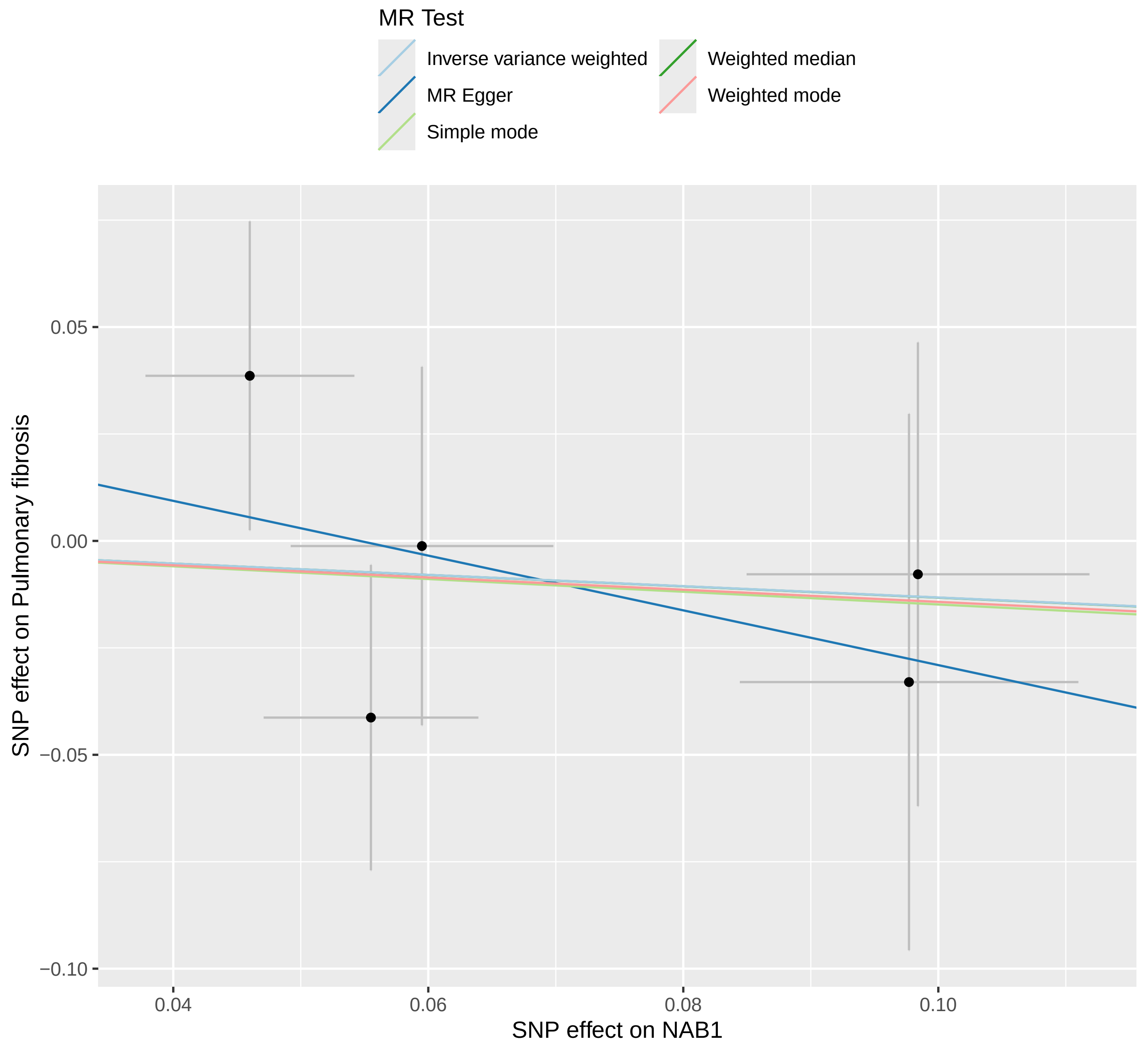
MR Test
Inverse variance weighted
MR Egger
Simple mode
Weighted median
Weighted mode
SNP effect on Pulmonary fibrosis
0.05
0.00
−0.05
−0.10
0.04
0.06
0.08
0.10
SNP effect on NAB1

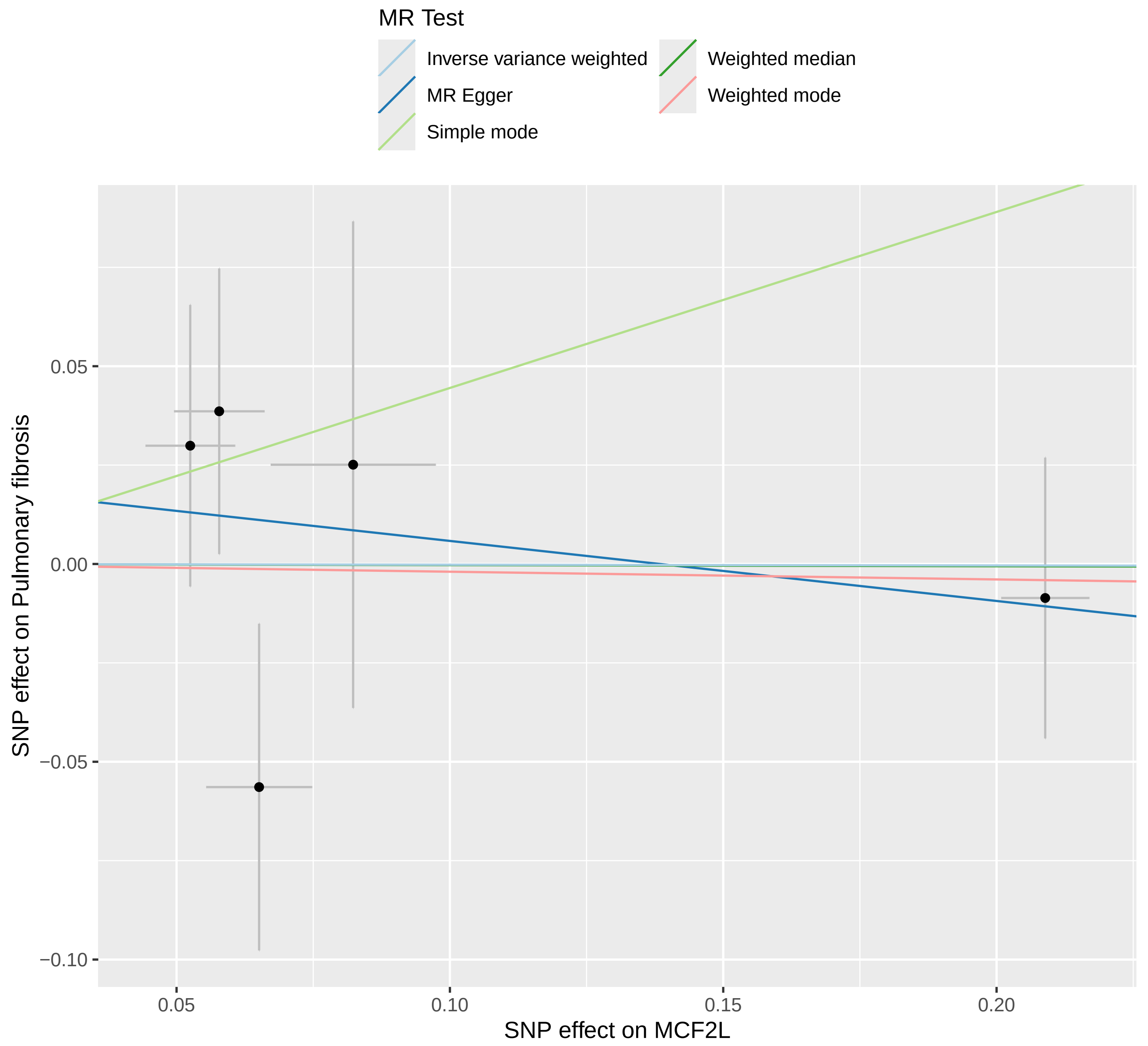

MR Test
Inverse variance weighted
MR Egger
Simple mode
Weighted median
Weighted mode
0.05
0.00
−0.05
−0.10
SNP effect on Pulmonary fibrosis
0.05
0.10
0.15
0.20
SNP effect on MCF2L

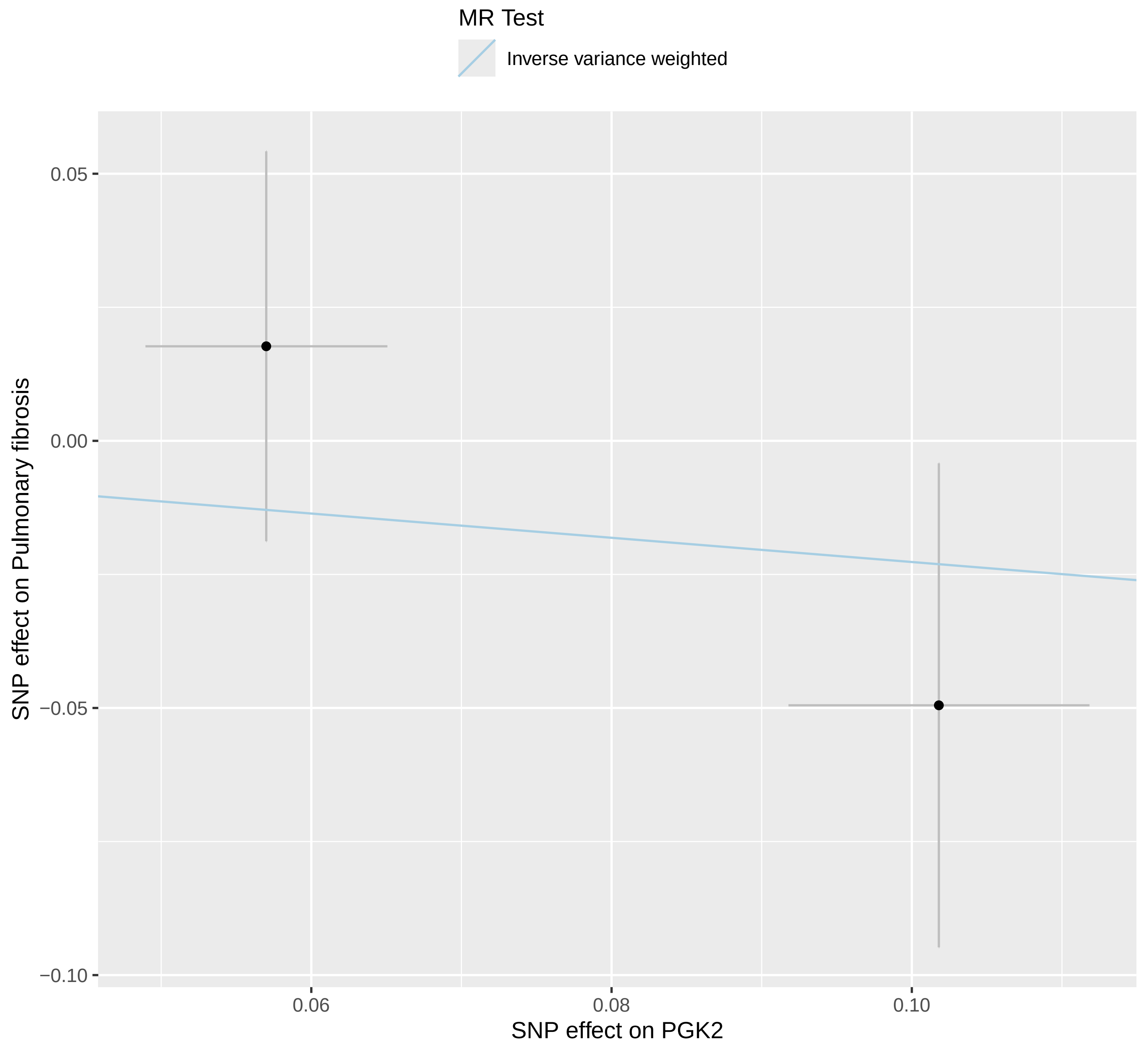
MR Test
Inverse variance weighted
0.05
0.00
−0.05
−0.10
0.06
0.08
0.10
SNP effect on Pulmonary fibrosis
SNP effect on PGK2

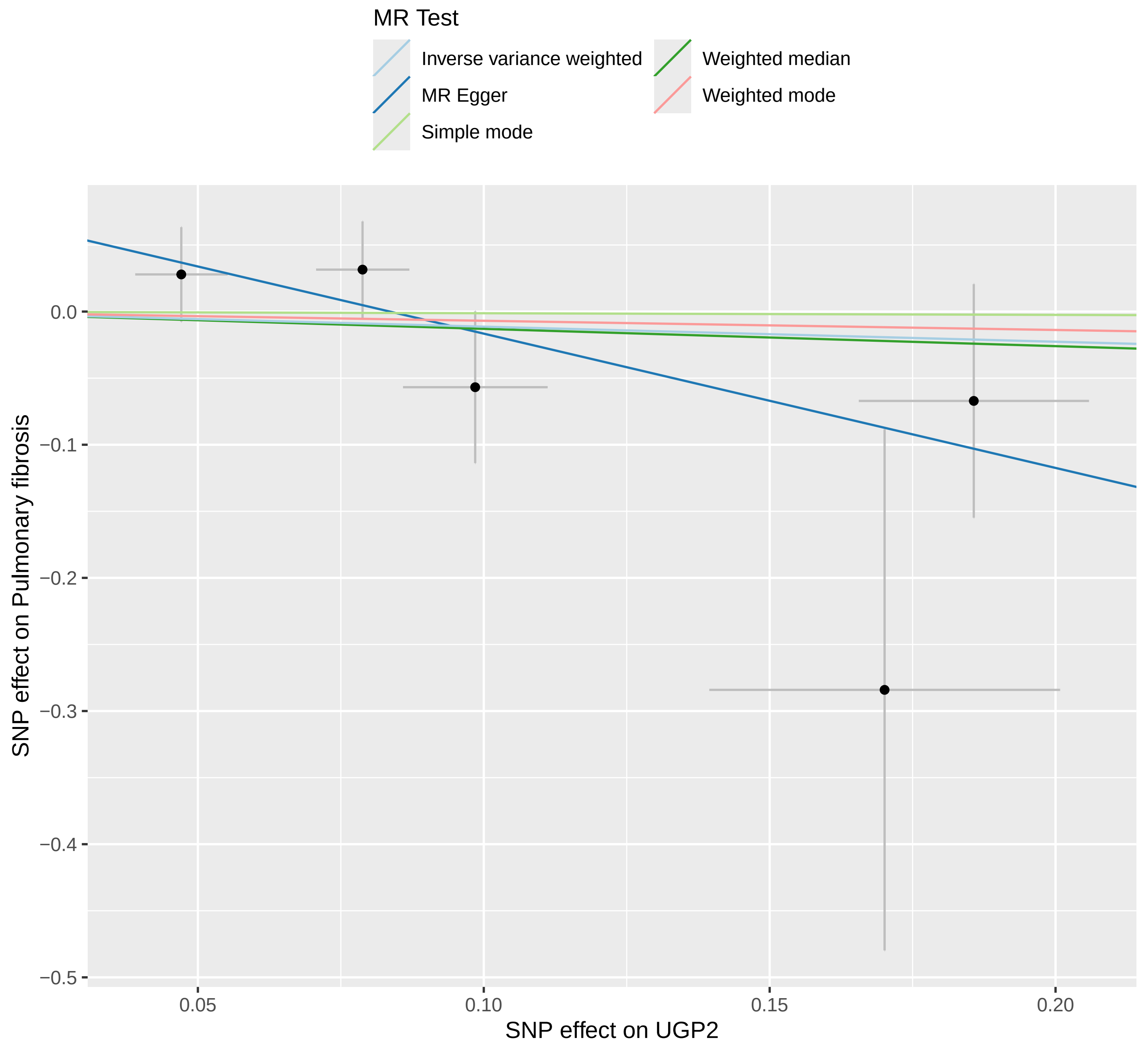
MR Test
Inverse variance weighted
MR Egger
Simple mode
Weighted median
Weighted mode
SNP effect on Pulmonary fibrosis
0.0
−0.1
−0.2
−0.3
−0.4
−0.5
0.05
0.10
0.15
0.20
SNP effect on UGP2

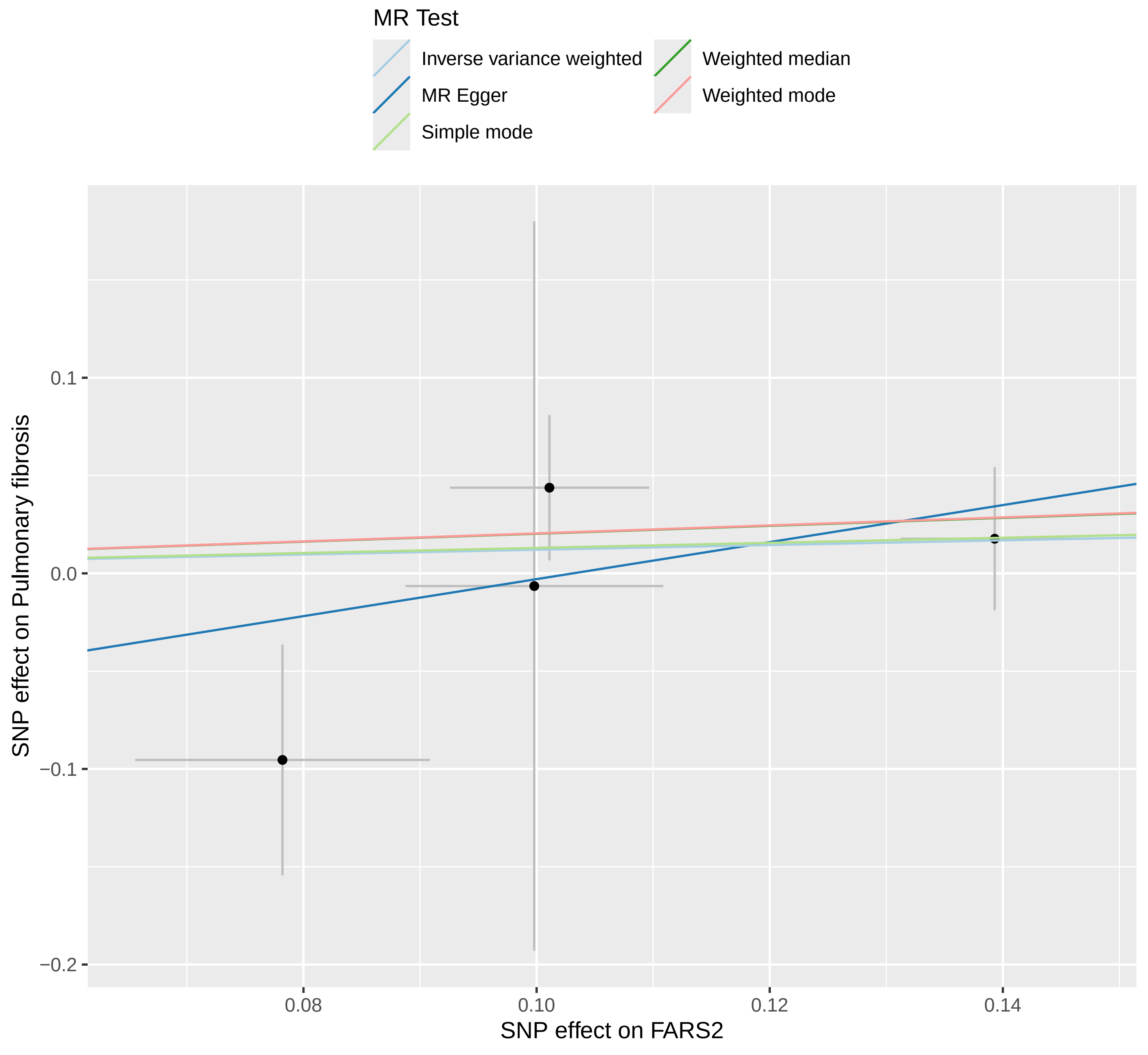

MR Test
Inverse variance weighted
MR Egger
Simple mode
Weighted median
Weighted mode
0.1
0.0
−0.1
−0.2
0.08
0.10
0.12
0.14
SNP effect on Pulmonary fibrosis
SNP effect on FARS2

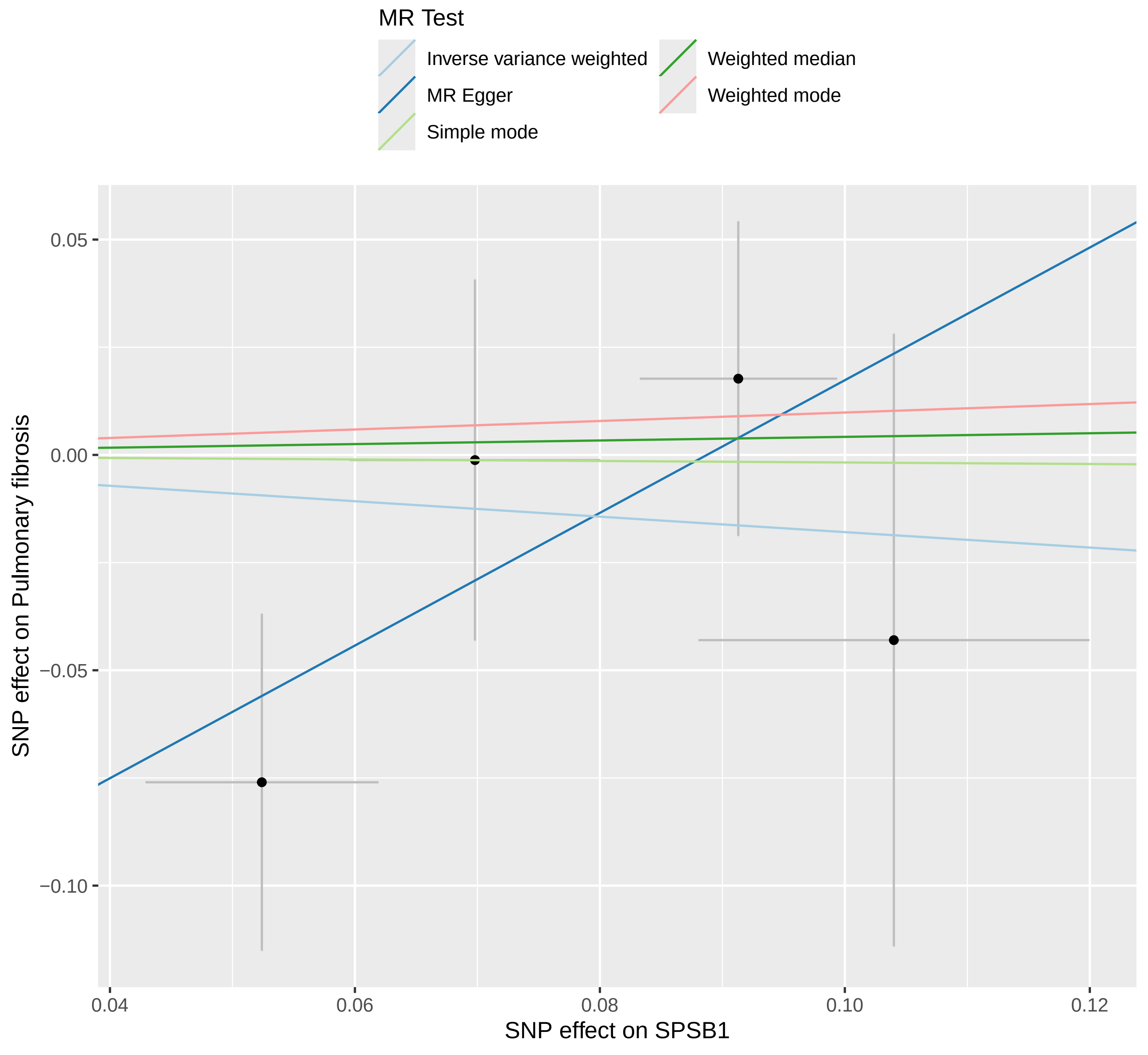
MR Test
Inverse variance weighted
MR Egger
Simple mode
Weighted median
Weighted mode
0.05
0.00
−0.05
−0.10
0.04
0.06
0.08
0.10
0.12
SNP effect on Pulmonary fibrosis
SNP effect on SPSB1

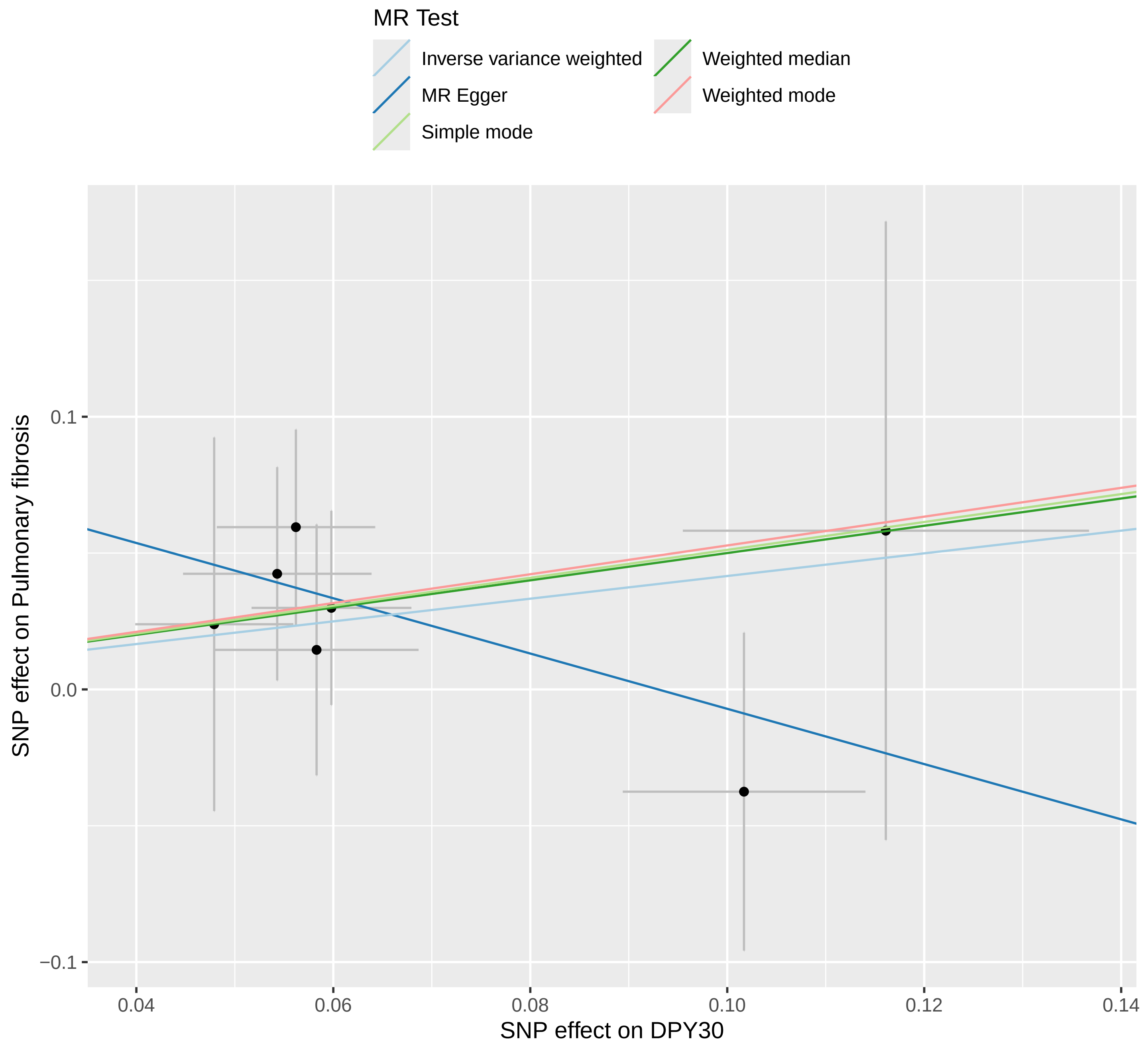
MR Test
Inverse variance weighted
MR Egger
Simple mode
Weighted median
Weighted mode
0.1
0.0
−0.1
0.04
0.06
0.08
0.10
0.12
0.14
SNP effect on DPY30
SNP effect on Pulmonary fibrosis

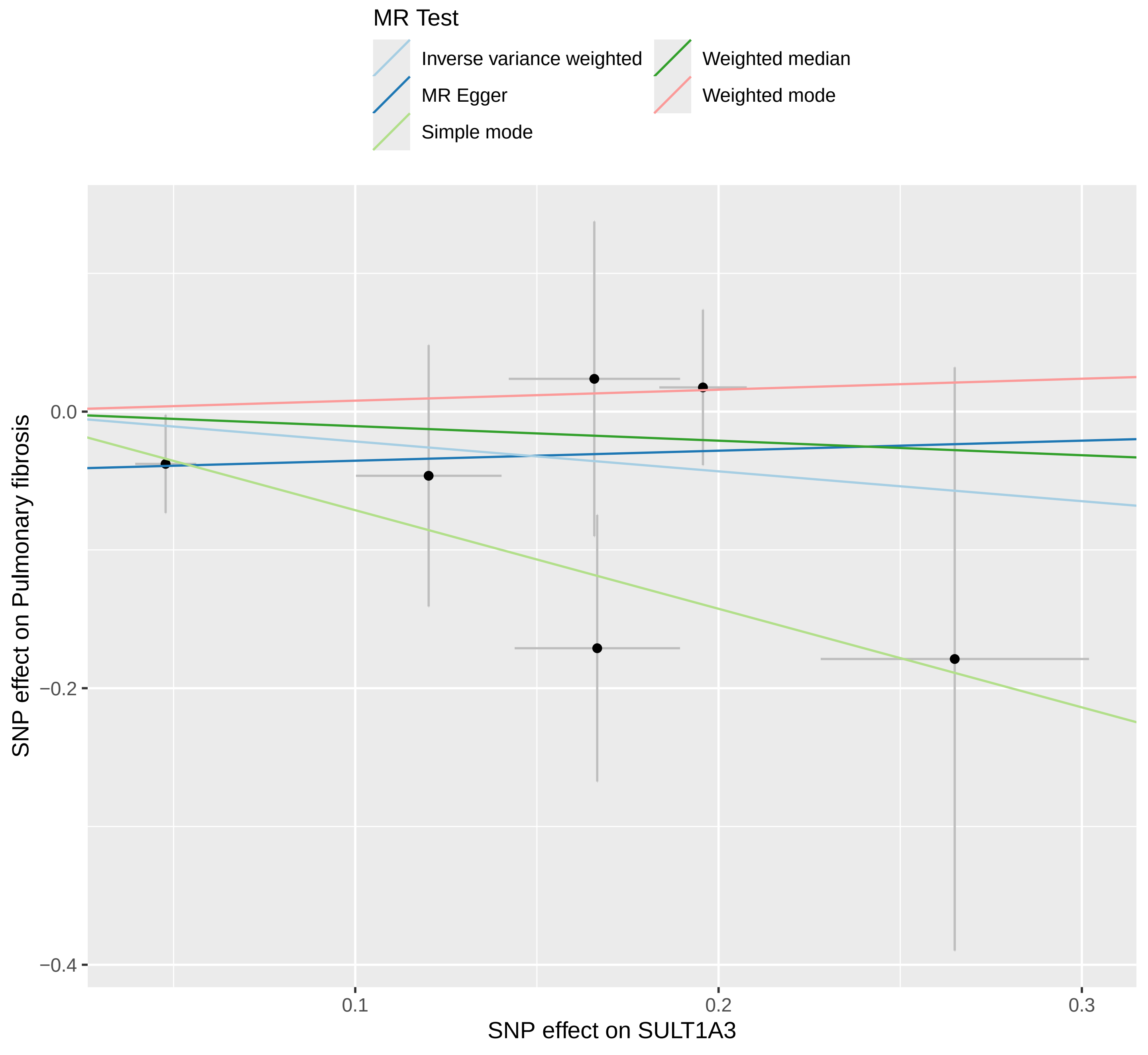
MR Test
Inverse variance weighted
MR Egger
Simple mode
Weighted median
Weighted mode
0.0
−0.2
−0.4
0.1
0.2
0.3
SNP effect on Pulmonary fibrosis
SNP effect on SULT1A3

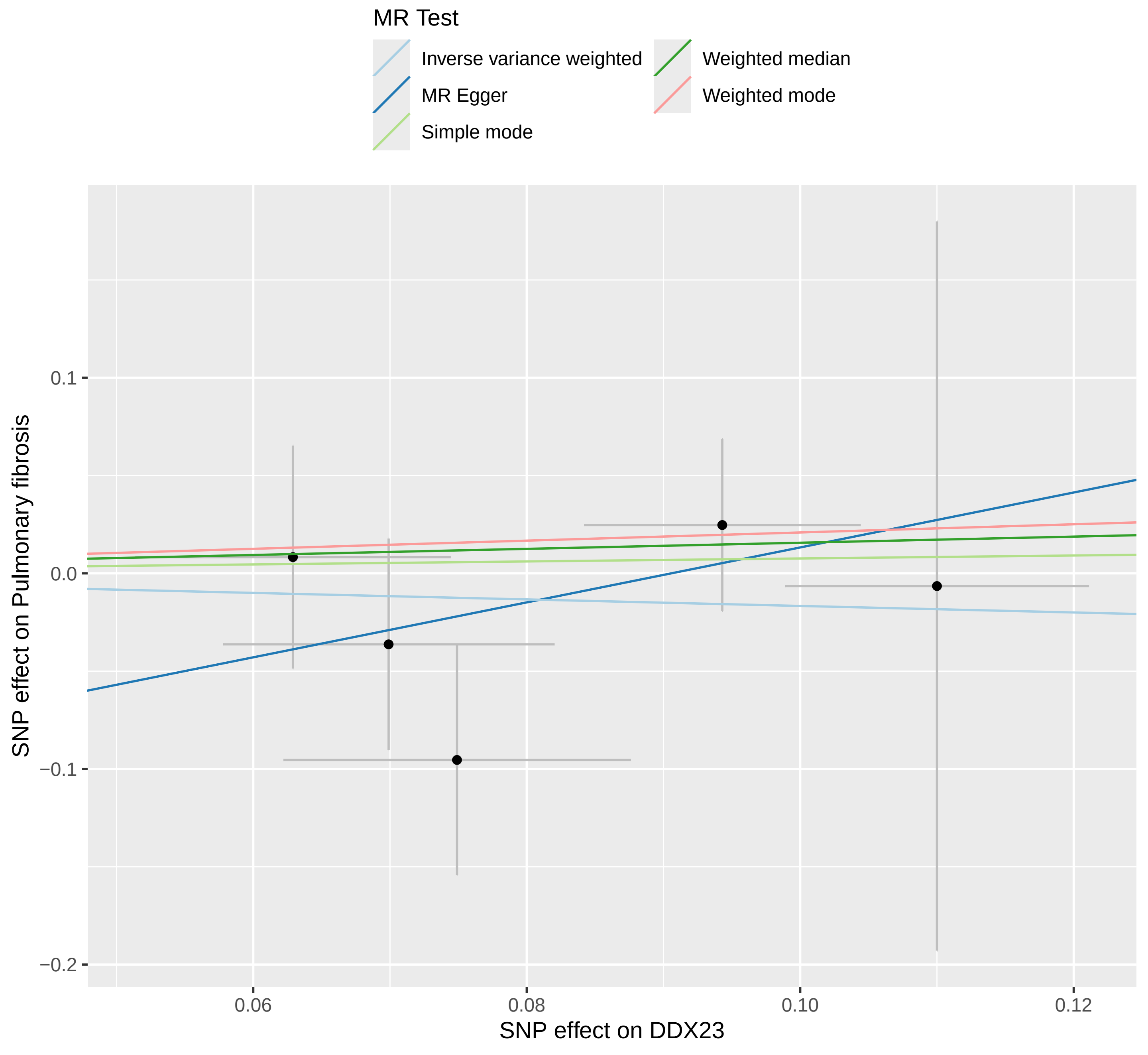
MR Test
Inverse variance weighted
MR Egger
Simple mode
Weighted median
Weighted mode
0.1
0.0
−0.1
−0.2
0.06
0.08
0.10
0.12
SNP effect on Pulmonary fibrosis
SNP effect on DDX23

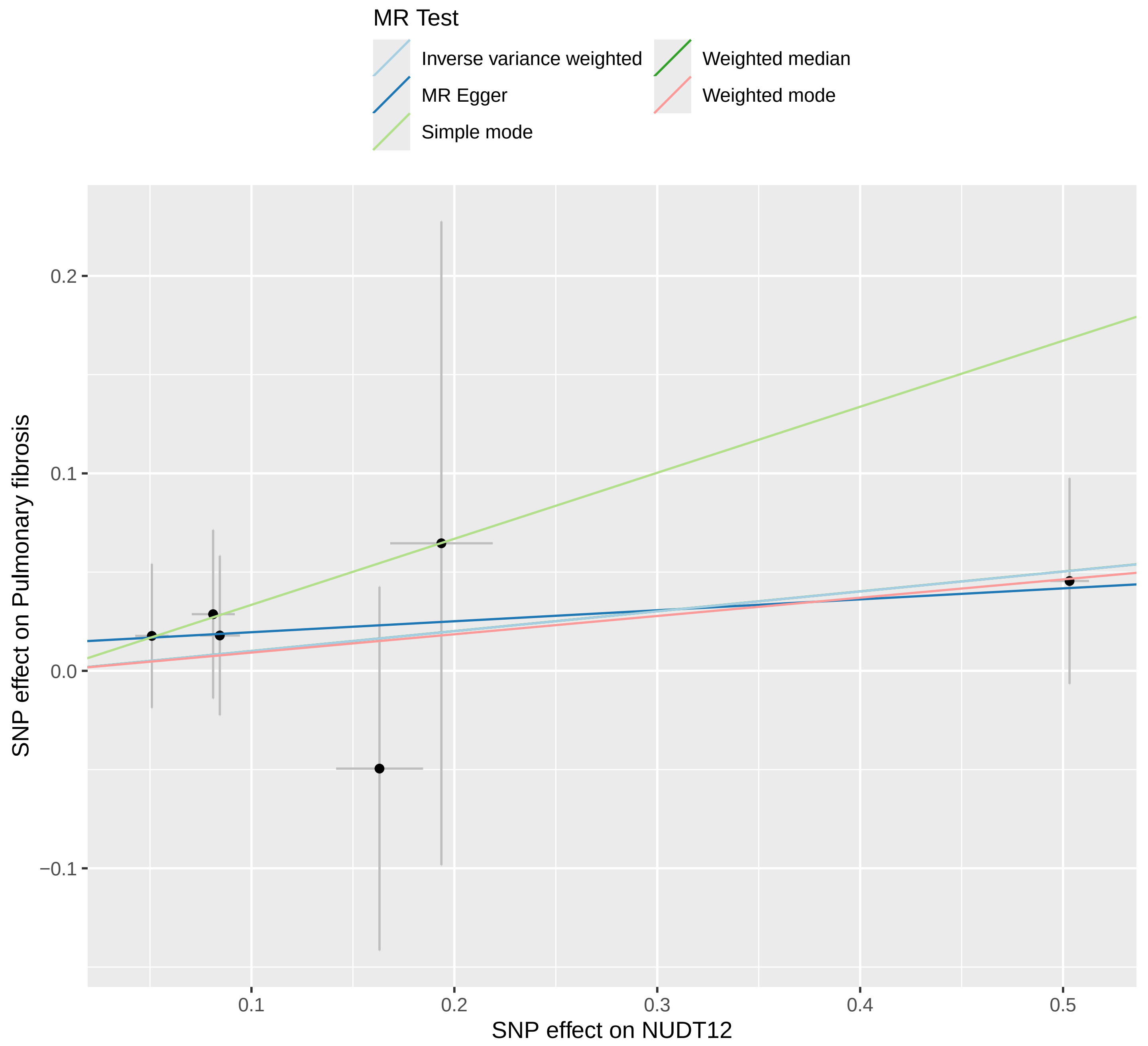

MR Test
Inverse variance weighted
MR Egger
Simple mode
Weighted median
Weighted mode
SNP effect on Pulmonary fibrosis
0.2
0.1
0.0
−0.1
0.1
0.2
0.3
0.4
0.5
SNP effect on NUDT12

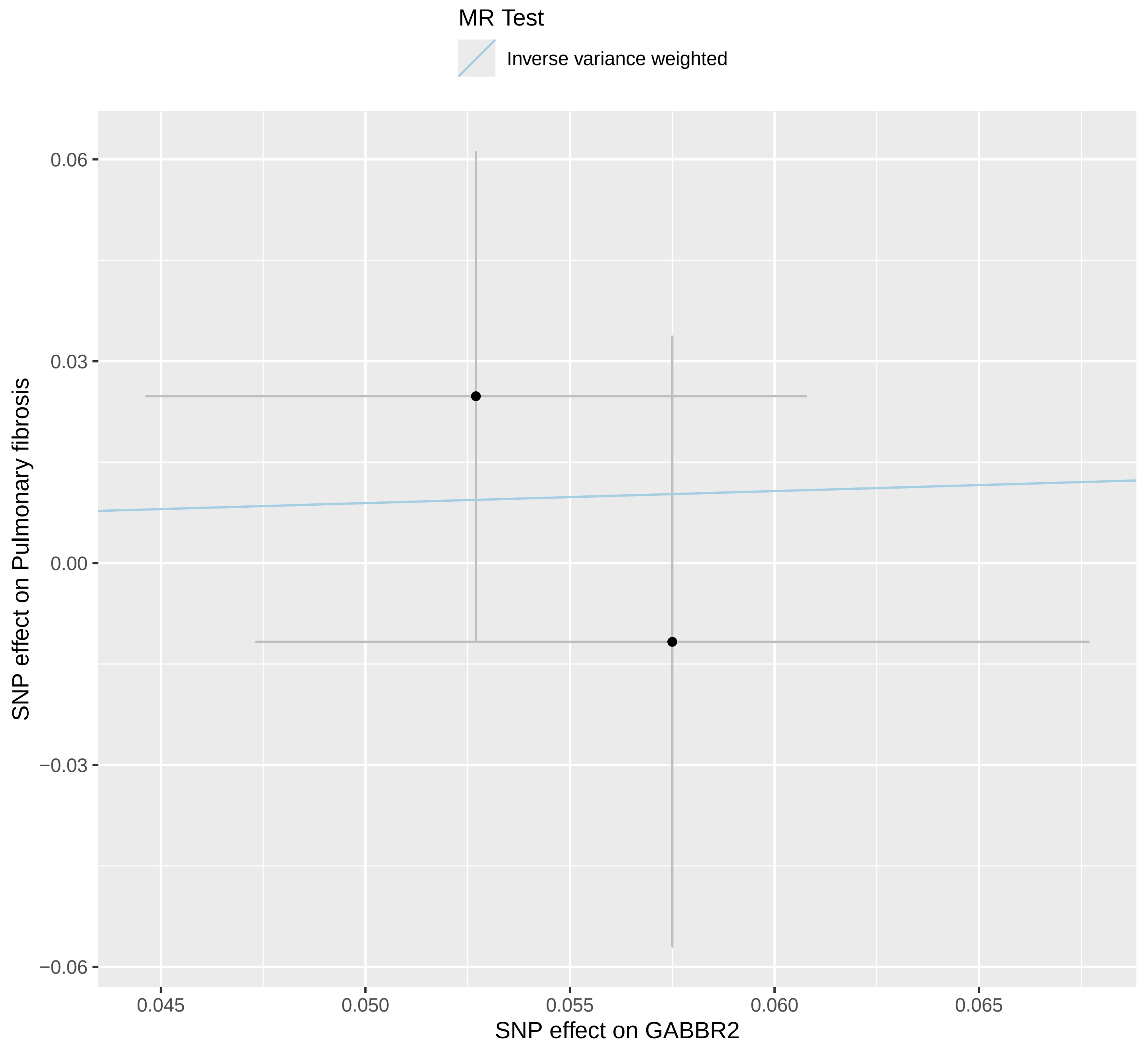
MR Test
Inverse variance weighted
0.06
0.03
0.00
−0.03
−0.06
SNP effect on Pulmonary fibrosis
0.045
0.050
0.055
0.060
0.065
SNP effect on GABBR2

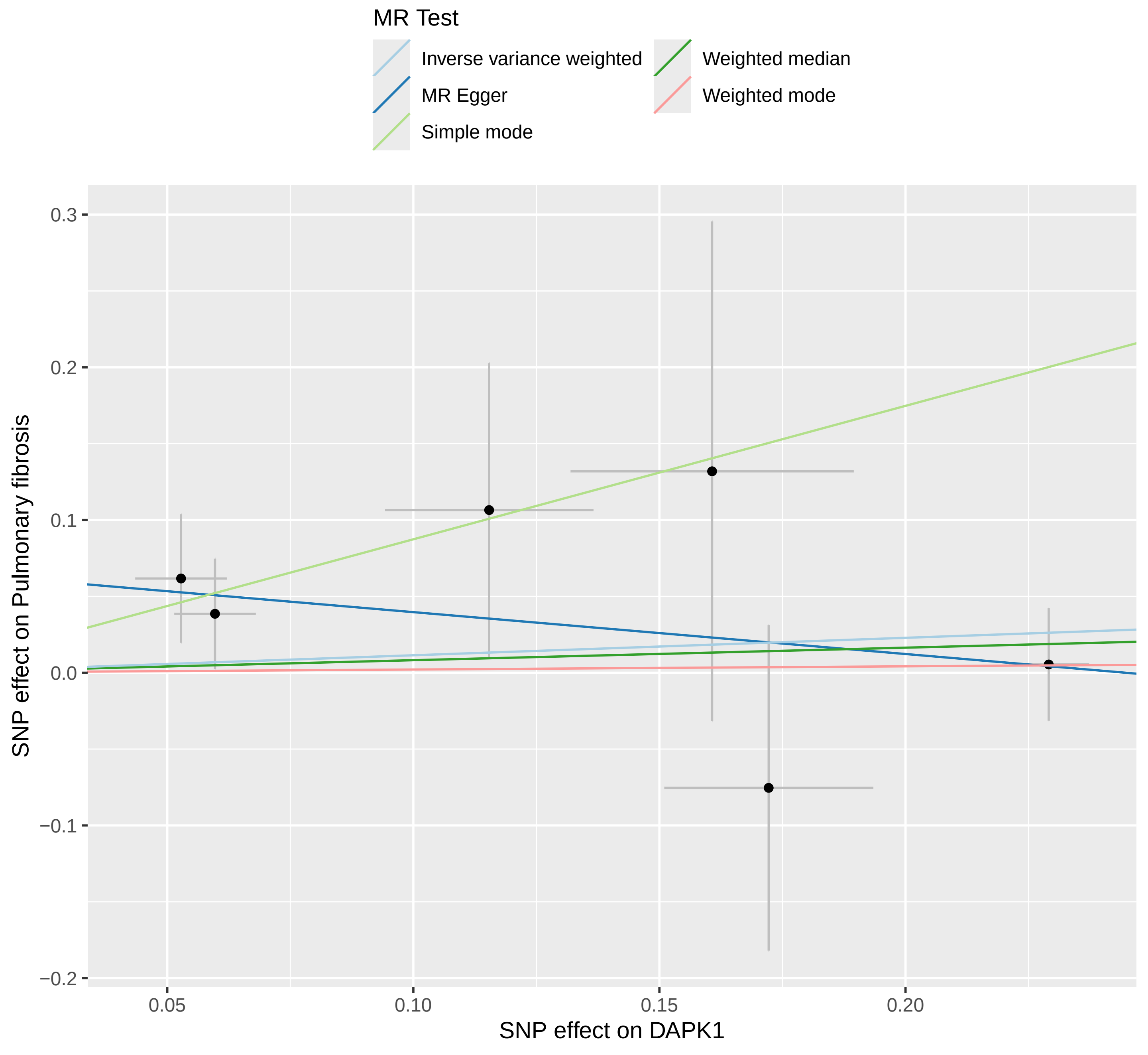
MR Test
Inverse variance weighted
MR Egger
Simple mode
Weighted median
Weighted mode
0.3
0.2
0.1
0.0
−0.1
−0.2
0.05
0.10
0.15
0.20
SNP effect on DAPK1
SNP effect on Pulmonary fibrosis

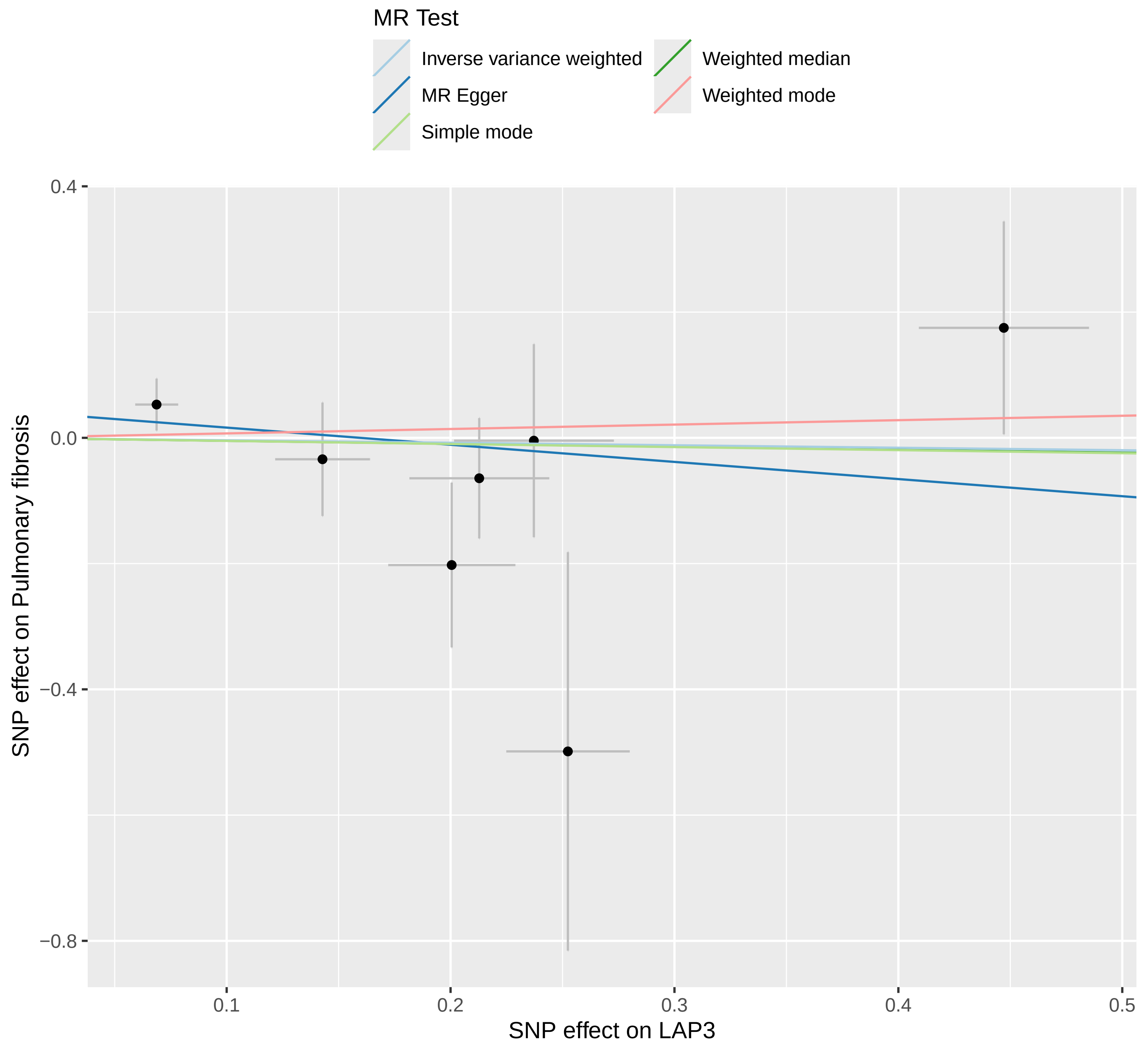
MR Test
Inverse variance weighted
MR Egger
Simple mode
Weighted median
Weighted mode
0.4
0.0
−0.4
−0.8
0.1
0.2
0.3
0.4
0.5
SNP effect on Pulmonary fibrosis
SNP effect on LAP3

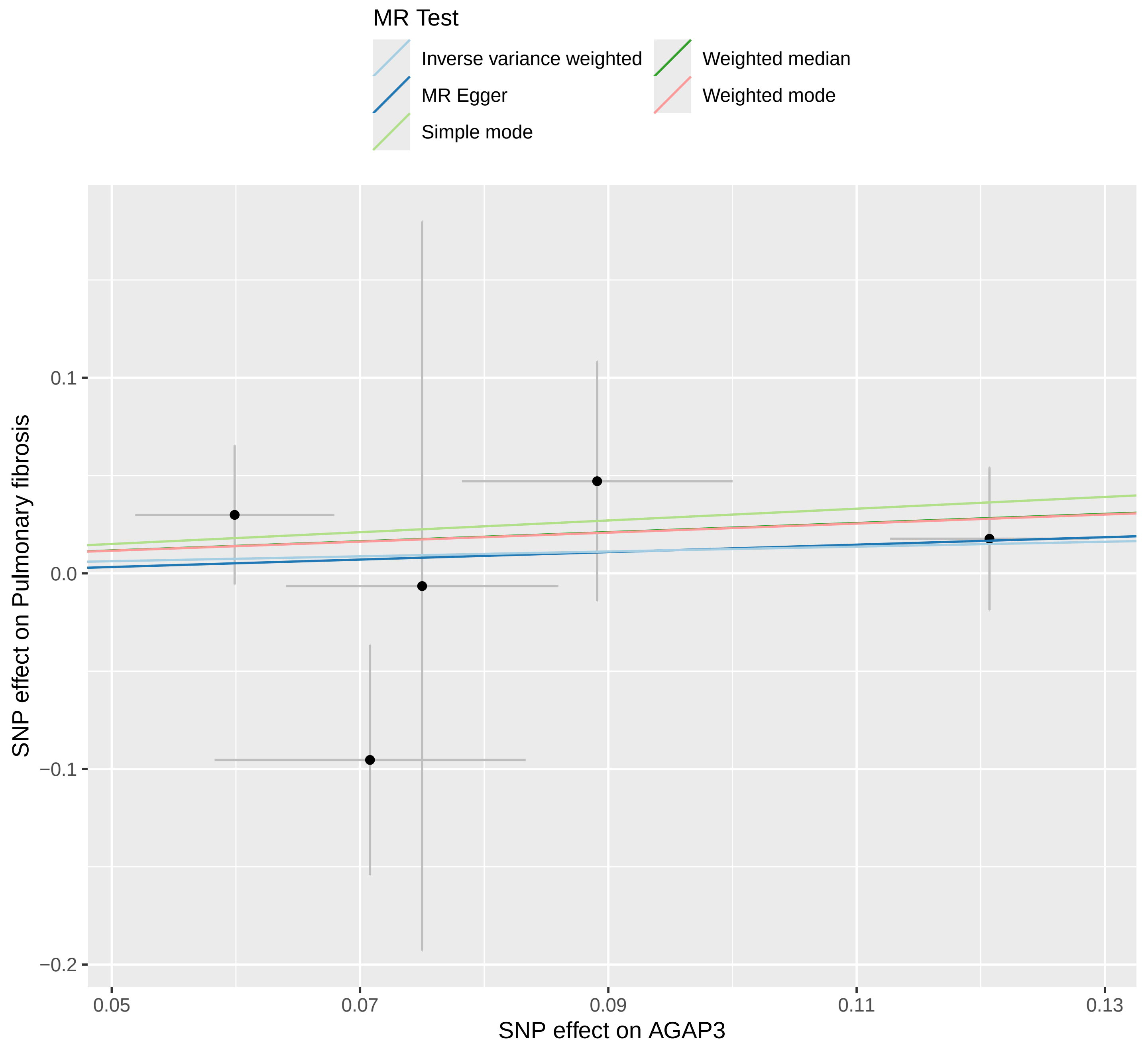
MR Test
Inverse variance weighted
MR Egger
Simple mode
Weighted median
Weighted mode
0.1
0.0
−0.1
−0.2
SNP effect on Pulmonary fibrosis
0.05
0.07
0.09
0.11
0.13
SNP effect on AGAP3

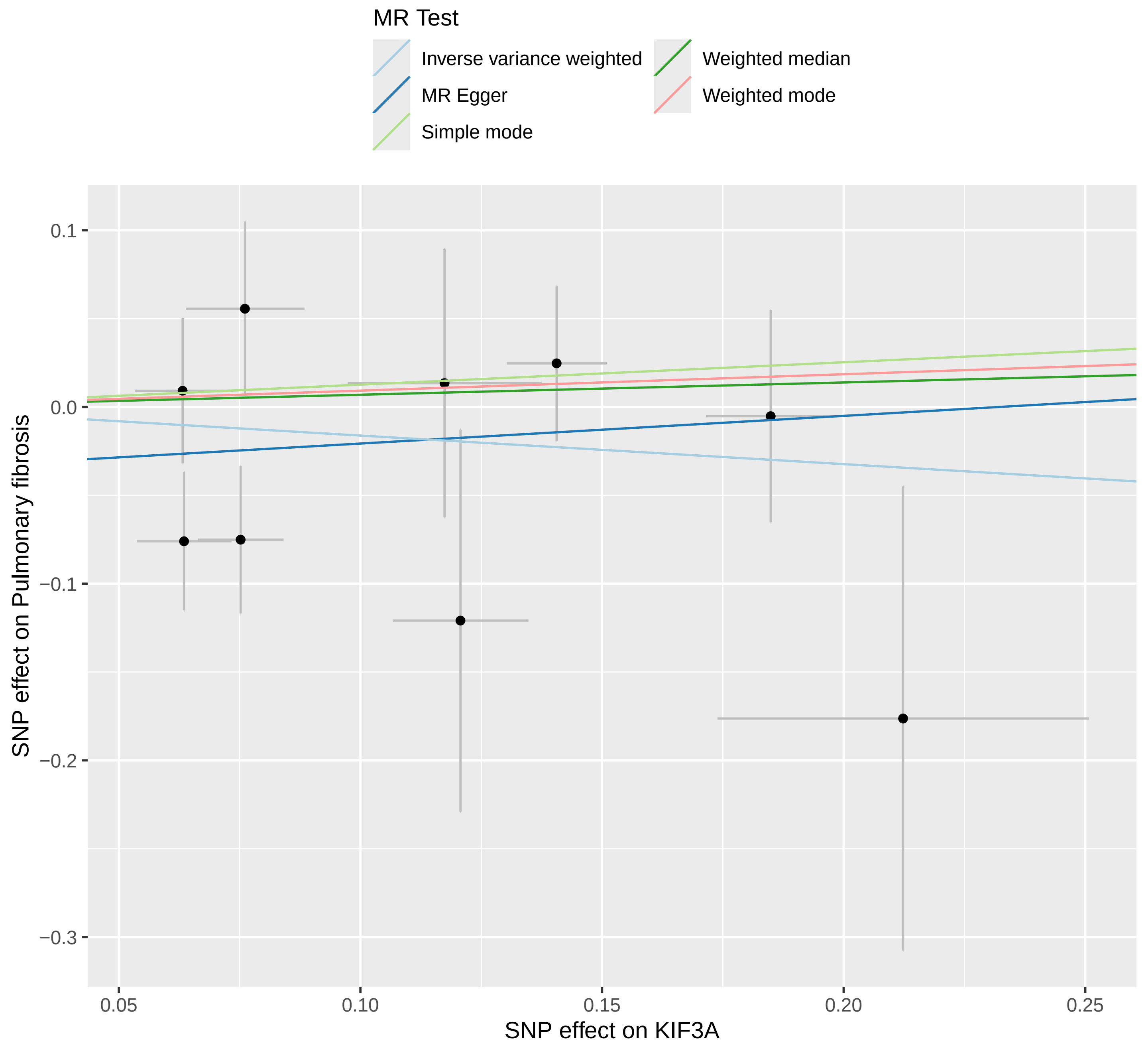
MR Test
Inverse variance weighted
MR Egger
Simple mode
Weighted median
Weighted mode
SNP effect on Pulmonary fibrosis
0.1
0.0
−0.1
−0.2
−0.3
0.05
0.10
0.15
0.20
0.25
SNP effect on KIF3A

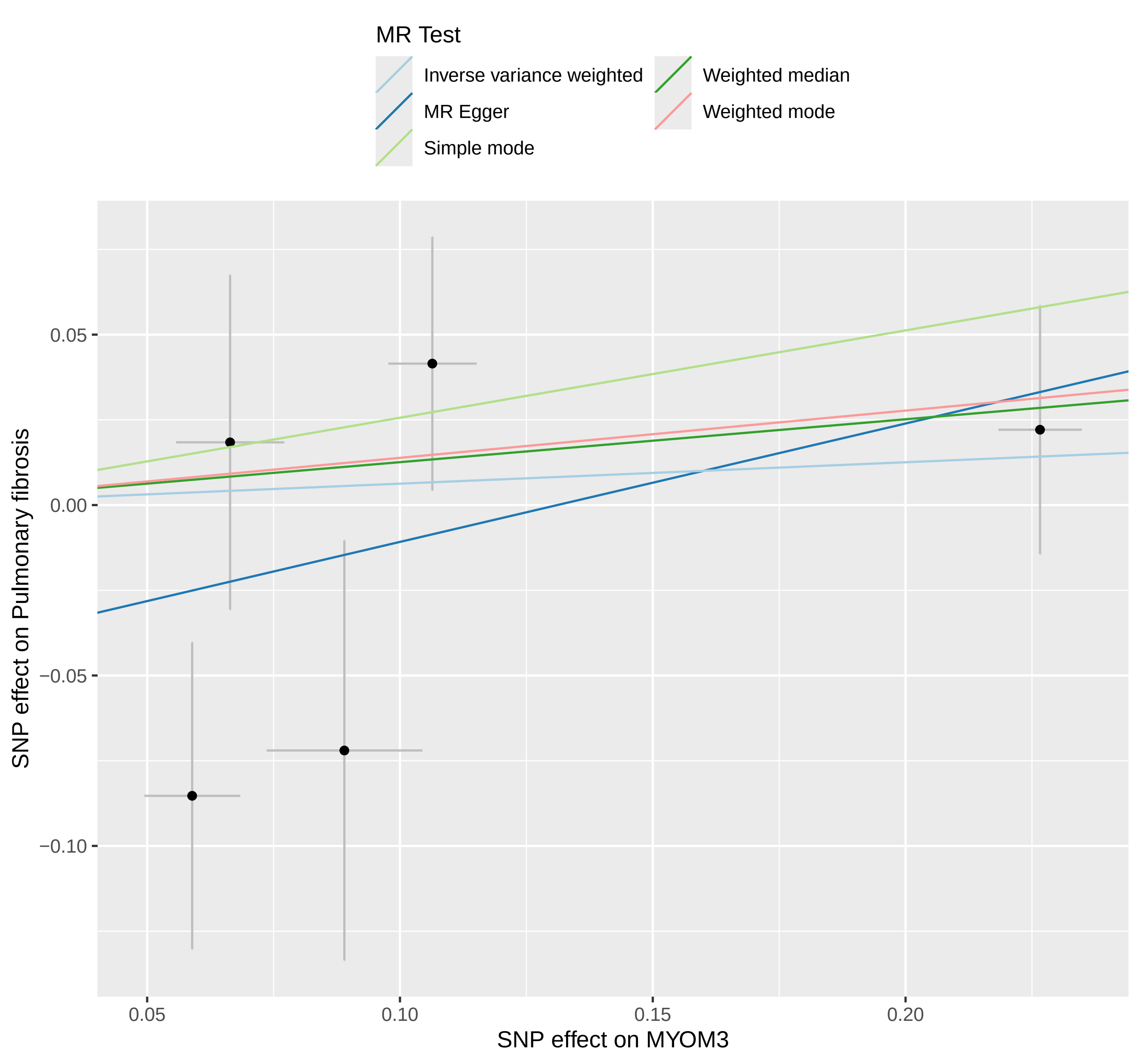
MR Test
Inverse variance weighted
MR Egger
Simple mode
Weighted median
Weighted mode
0.05
0.00
−0.05
−0.10
0.05
0.10
0.15
0.20
SNP effect on Pulmonary fibrosis
SNP effect on MYOM3

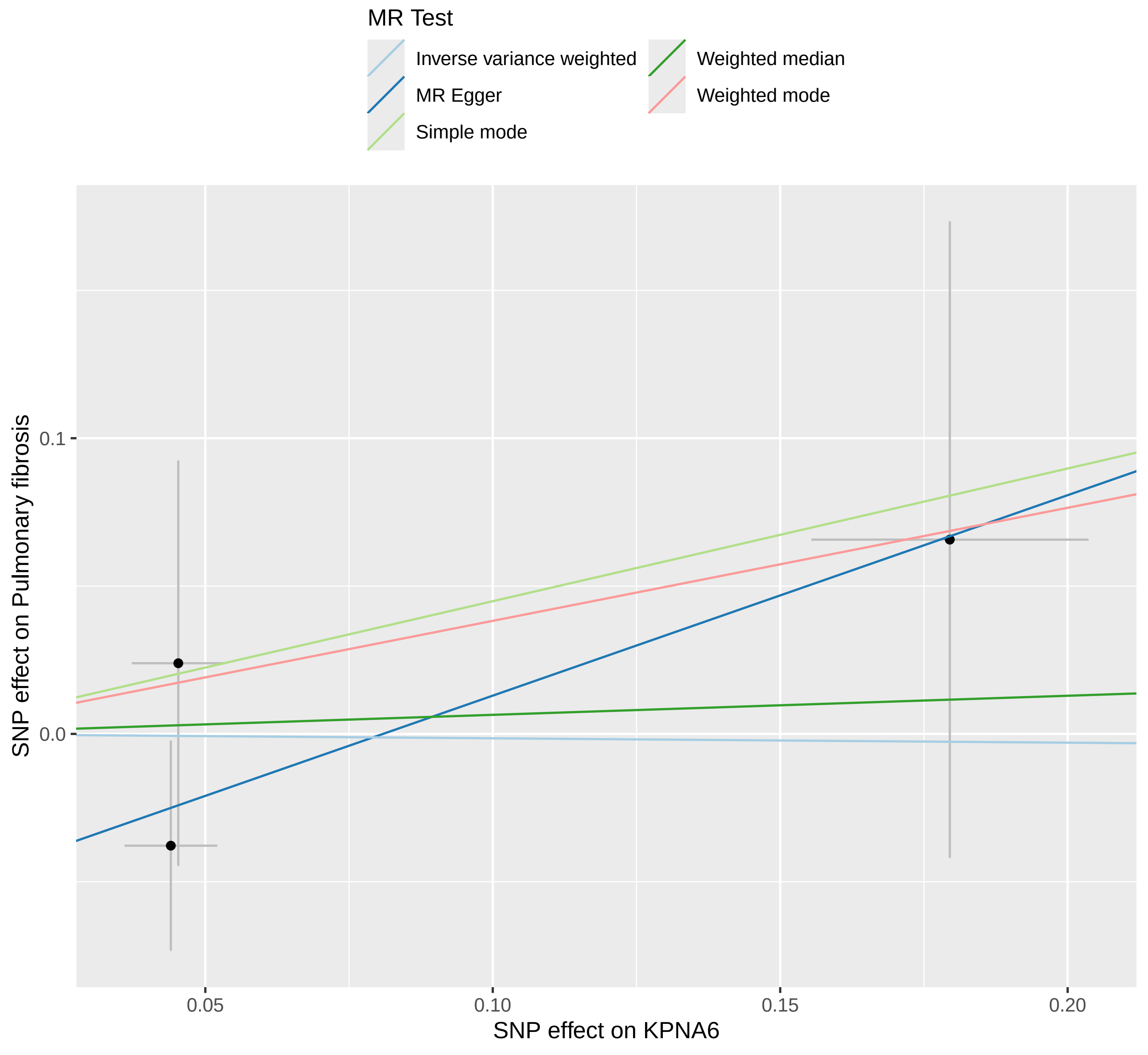
MR Test
Inverse variance weighted
MR Egger
Simple mode
Weighted median
Weighted mode
SNP effect on Pulmonary fibrosis
0.1
0.0
0.05
0.10
0.15
0.20
SNP effect on KPNA6

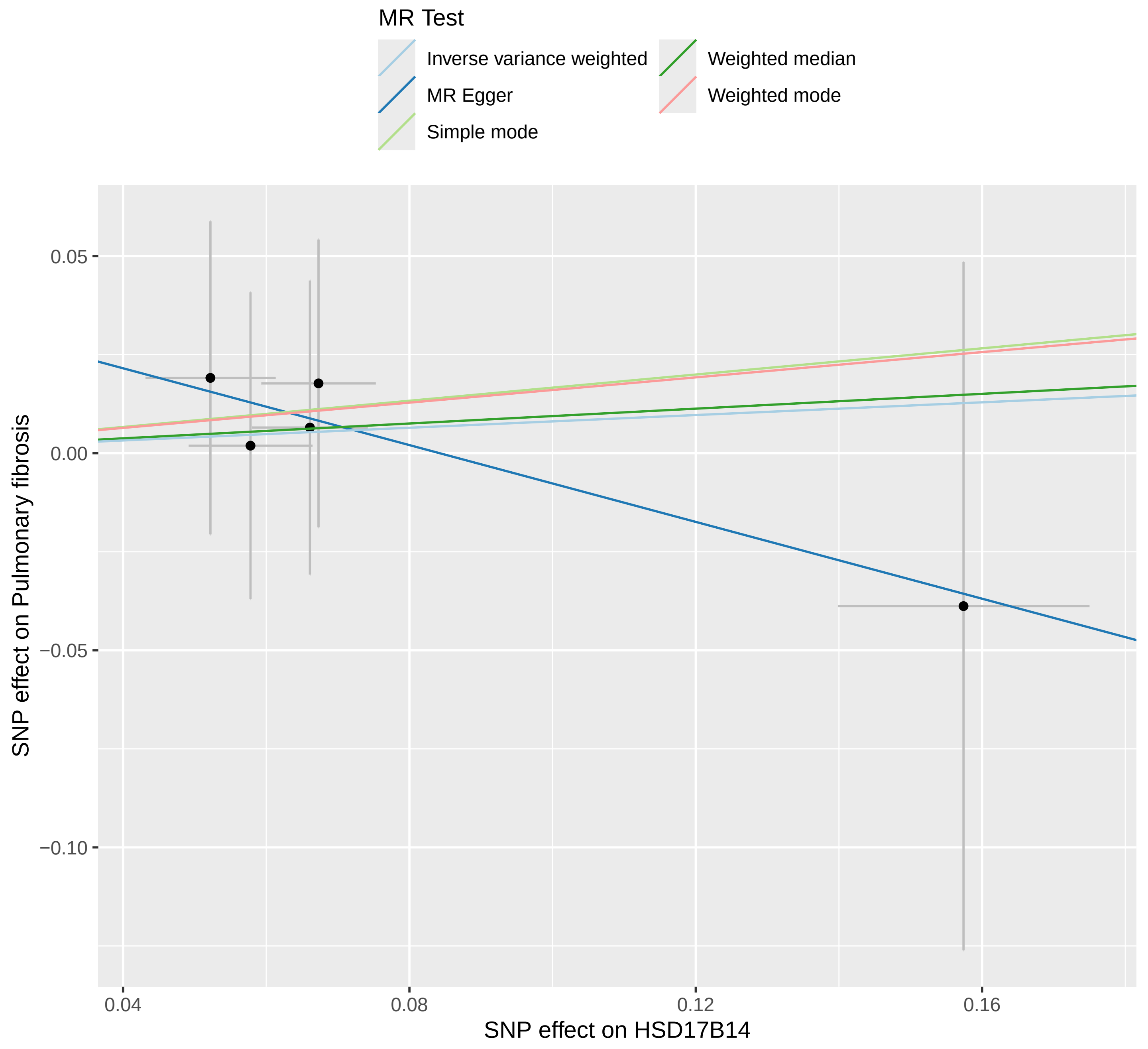
MR Test
Inverse variance weighted
MR Egger
Simple mode
Weighted median
Weighted mode
0.05
0.00
−0.05
−0.10
0.04
0.08
0.12
0.16
SNP effect on Pulmonary fibrosis
SNP effect on HSD17B14

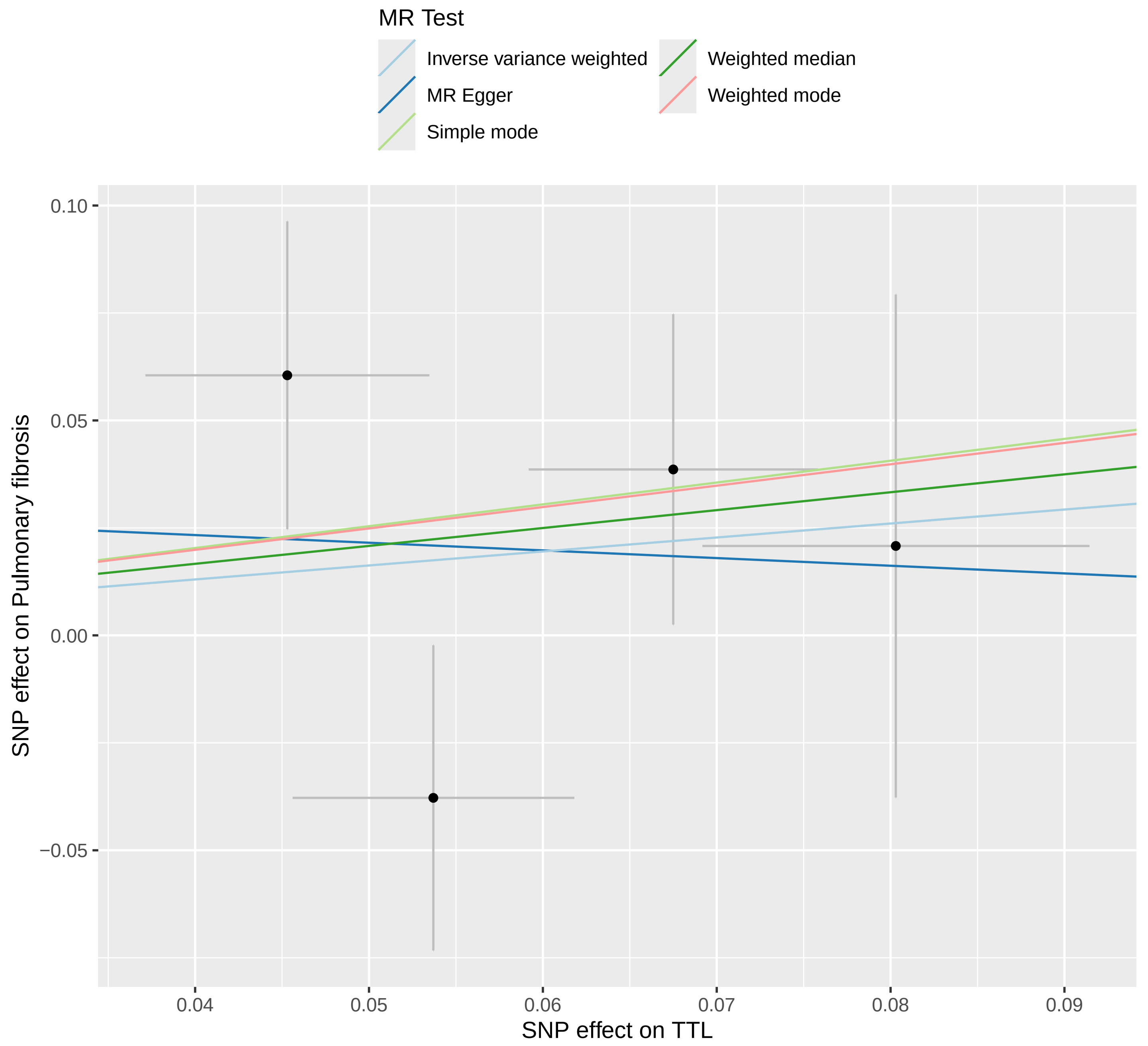
MR Test
Inverse variance weighted
MR Egger
Simple mode
Weighted median
Weighted mode
0.10
0.05
0.00
−0.05
0.04
0.05
0.06
0.07
0.08
0.09
SNP effect on Pulmonary fibrosis
SNP effect on TTL

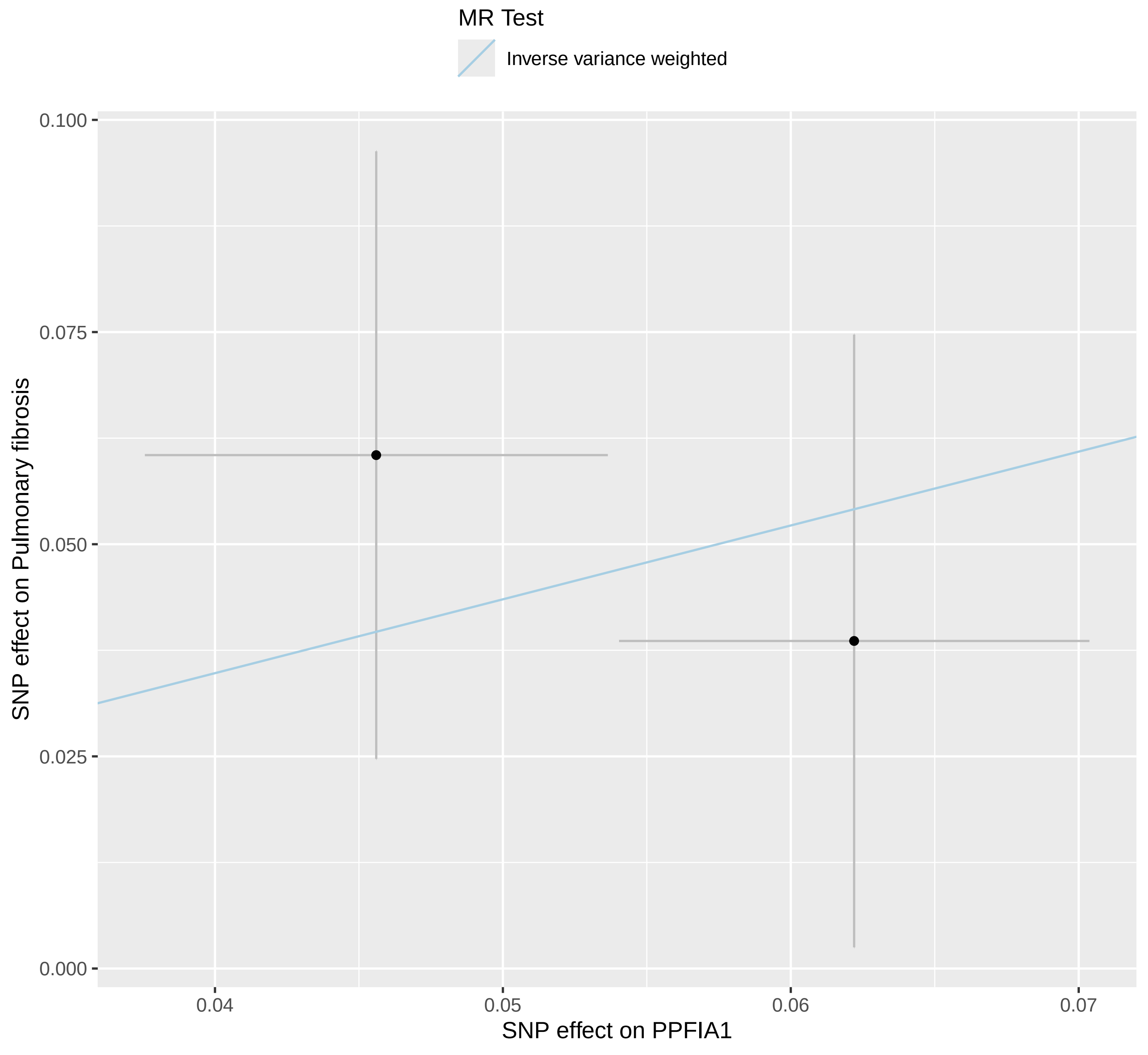
MR Test
Inverse variance weighted
0.100
0.075
0.050
0.025
0.000
0.04
0.05
0.06
0.07
SNP effect on Pulmonary fibrosis
SNP effect on PPFIA1

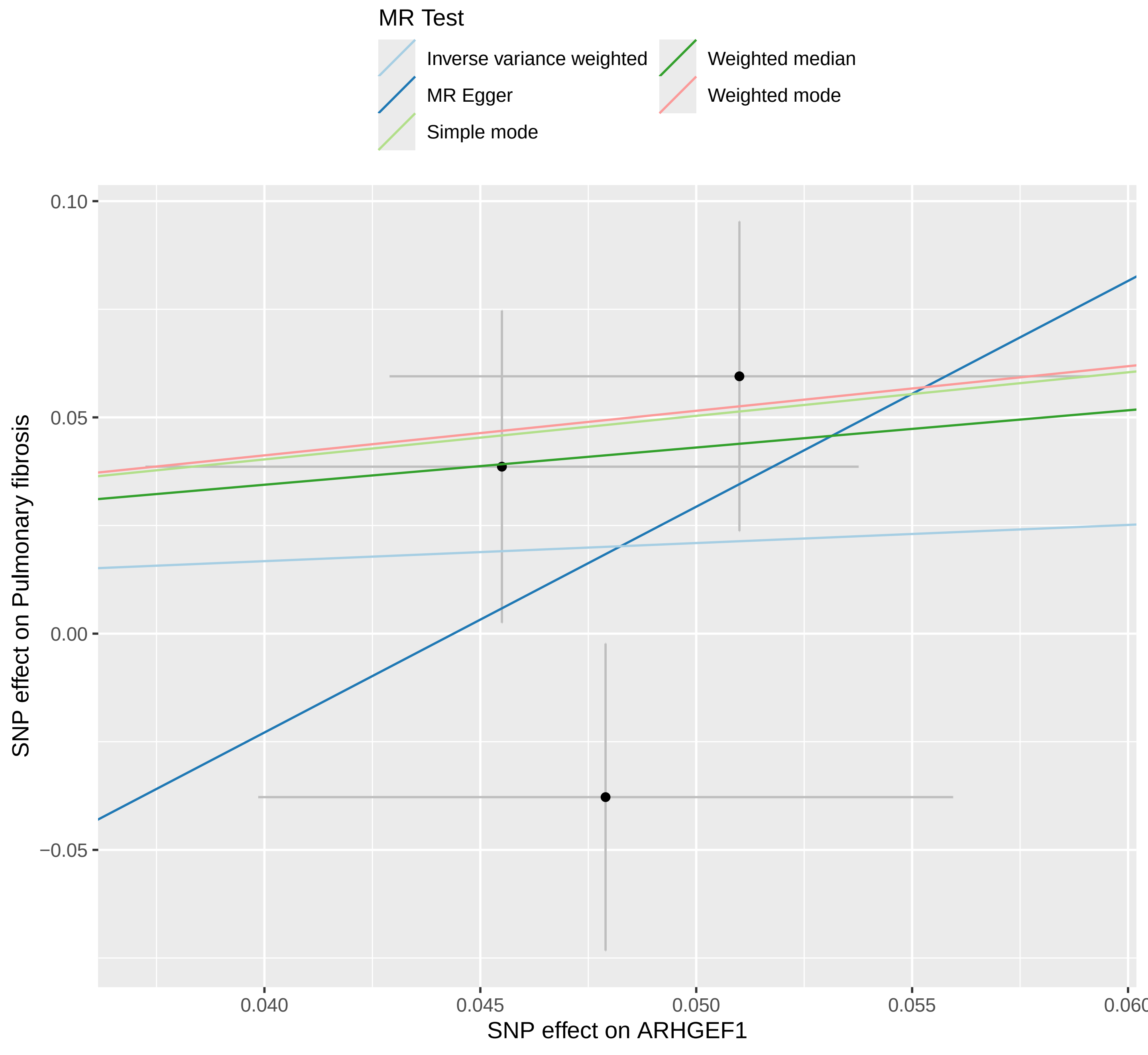

MR Test
Inverse variance weighted
MR Egger
Simple mode
Weighted median
Weighted mode
0.10
0.05
0.00
−0.05
SNP effect on Pulmonary fibrosis
0.040
0.045
0.050
0.055
0.060
SNP effect on ARHGEF1

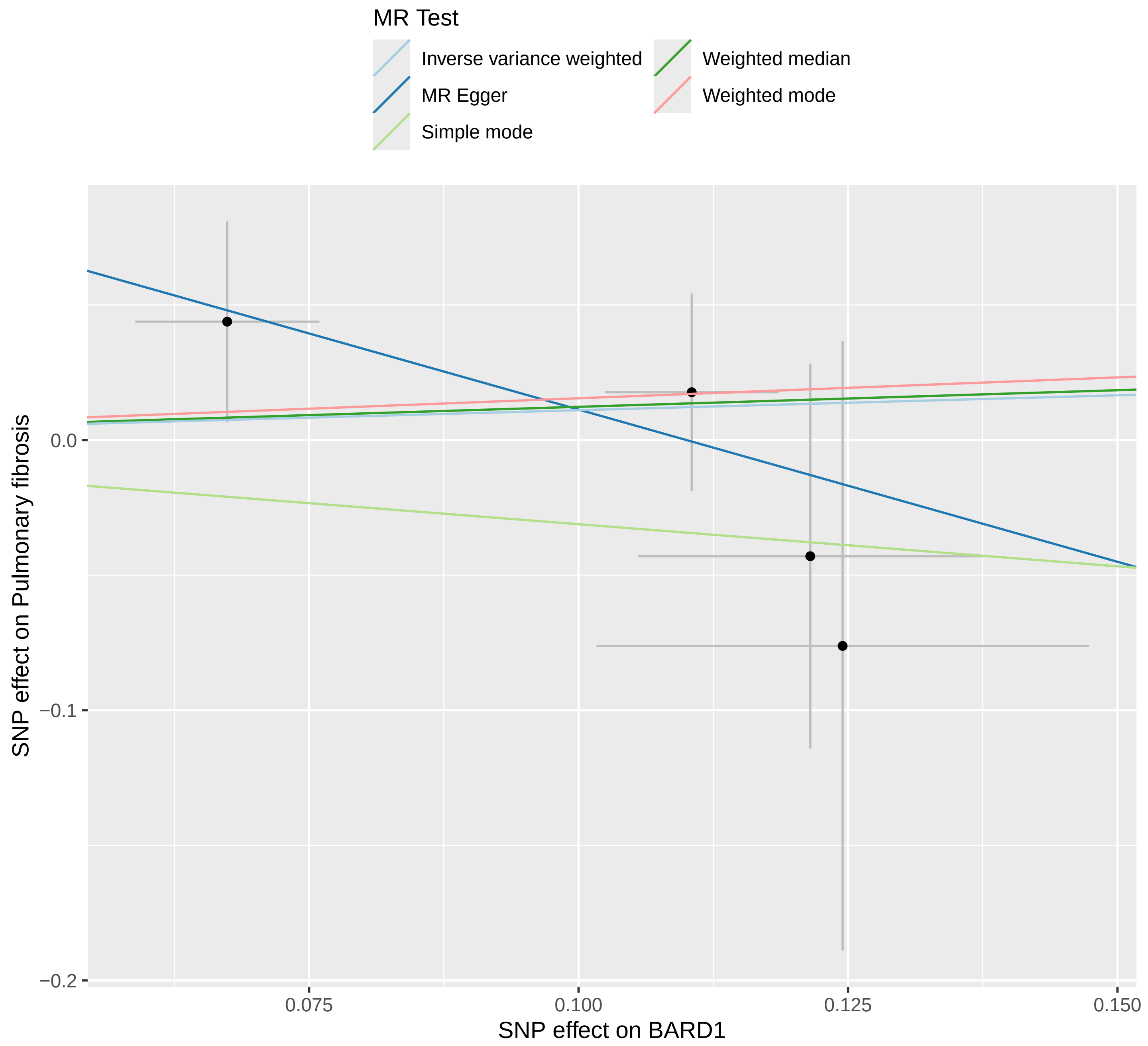

MR Test
Inverse variance weighted
MR Egger
Simple mode
Weighted median
Weighted mode
SNP effect on Pulmonary fibrosis
0.0
−0.1
−0.2
0.075
0.100
0.125
0.150
SNP effect on BARD1

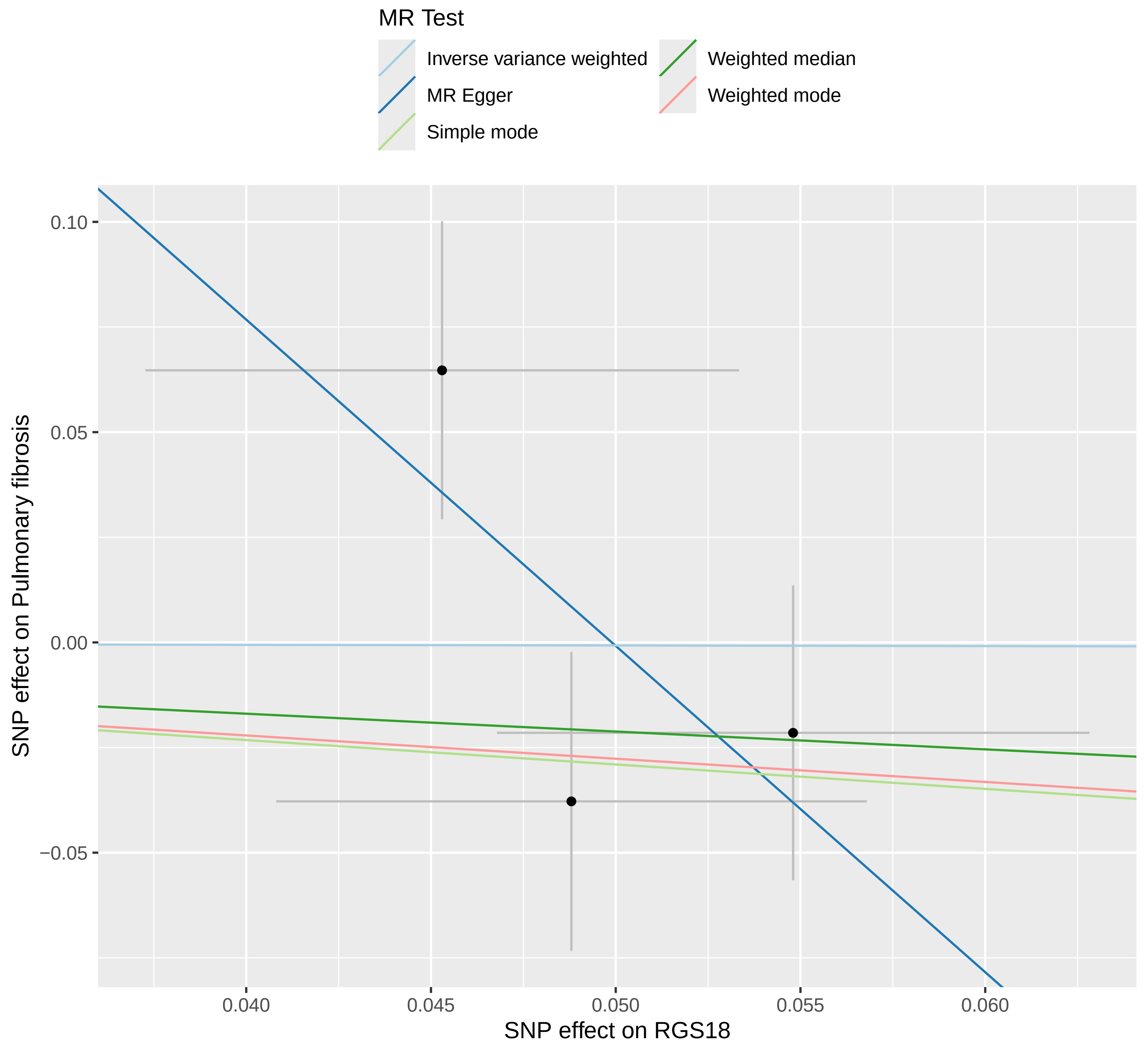
MR Test
Inverse variance weighted
MR Egger
Simple mode
Weighted median
Weighted mode
0.10
0.05
0.00
−0.05
SNP effect on Pulmonary fibrosis
0.040
0.045
0.050
0.055
0.060
SNP effect on RGS18

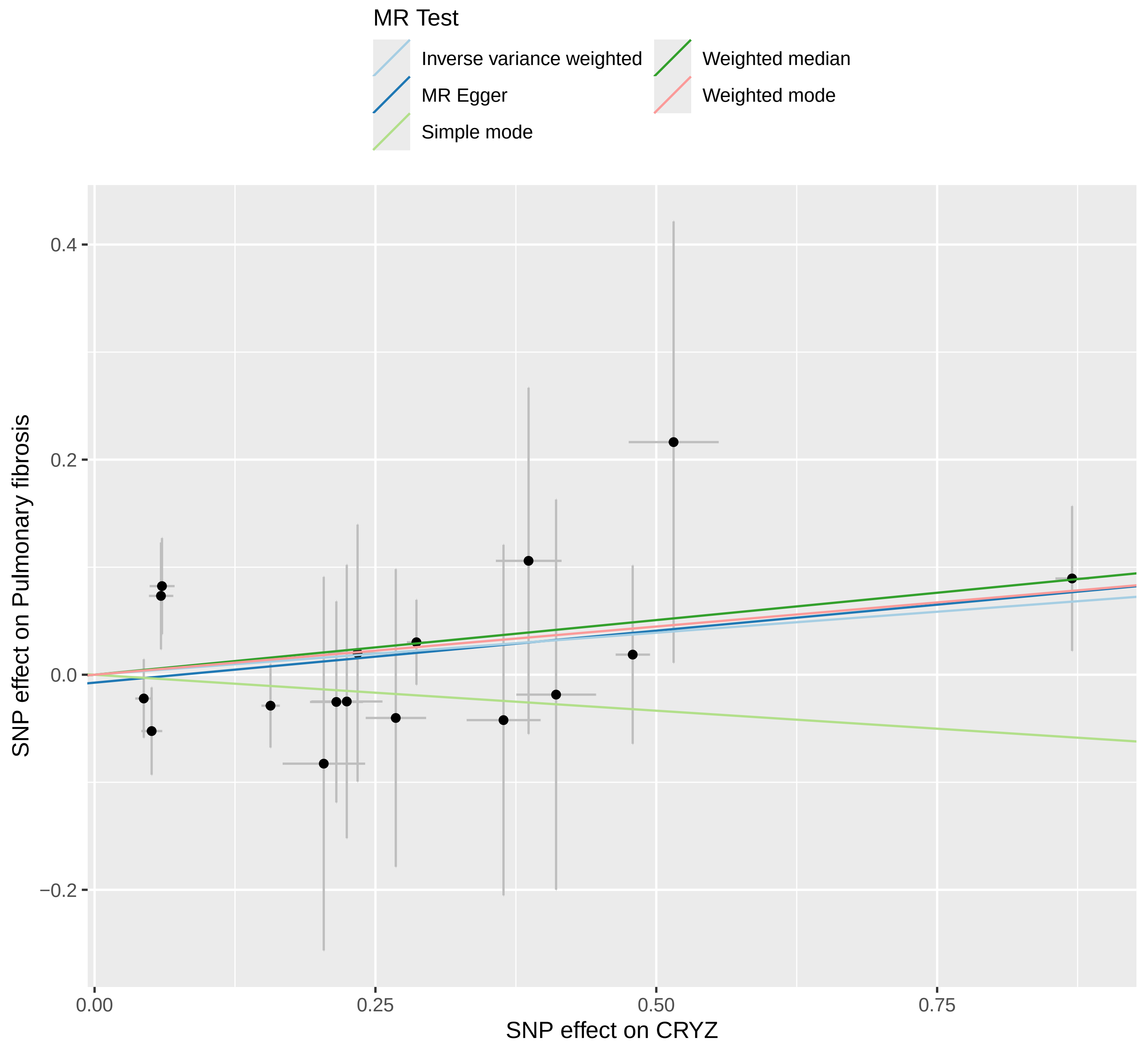
MR Test
Inverse variance weighted
MR Egger
Simple mode
Weighted median
Weighted mode
SNP effect on Pulmonary fibrosis
SNP effect on CRYZ
0.4
0.2
0.0
−0.2
0.00
0.25
0.50
0.75

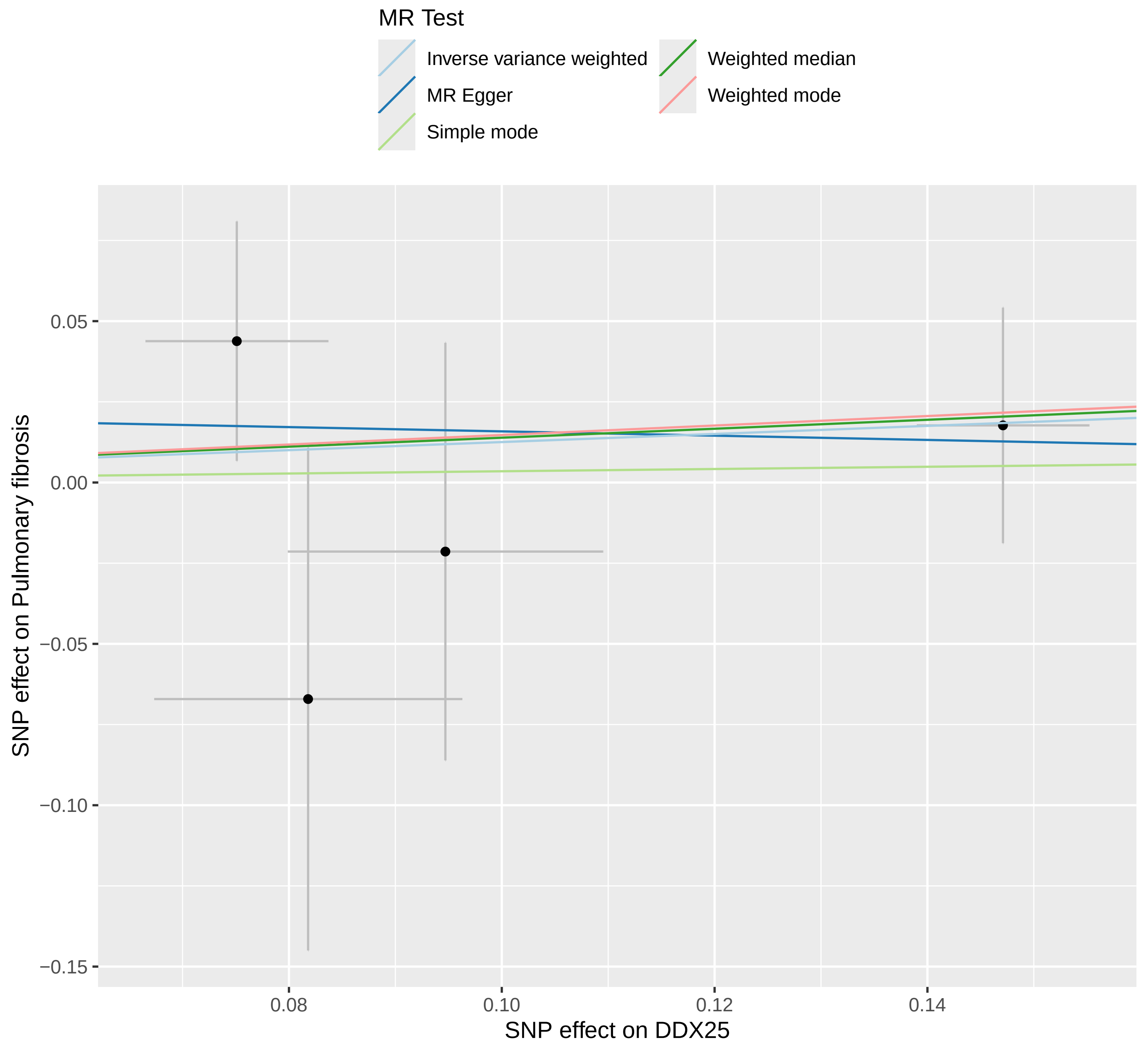
MR Test
Inverse variance weighted
MR Egger
Simple mode
Weighted median
Weighted mode
0.05
0.00
−0.05
−0.10
−0.15
SNP effect on Pulmonary fibrosis
0.08
0.10
0.12
0.14
SNP effect on DDX25

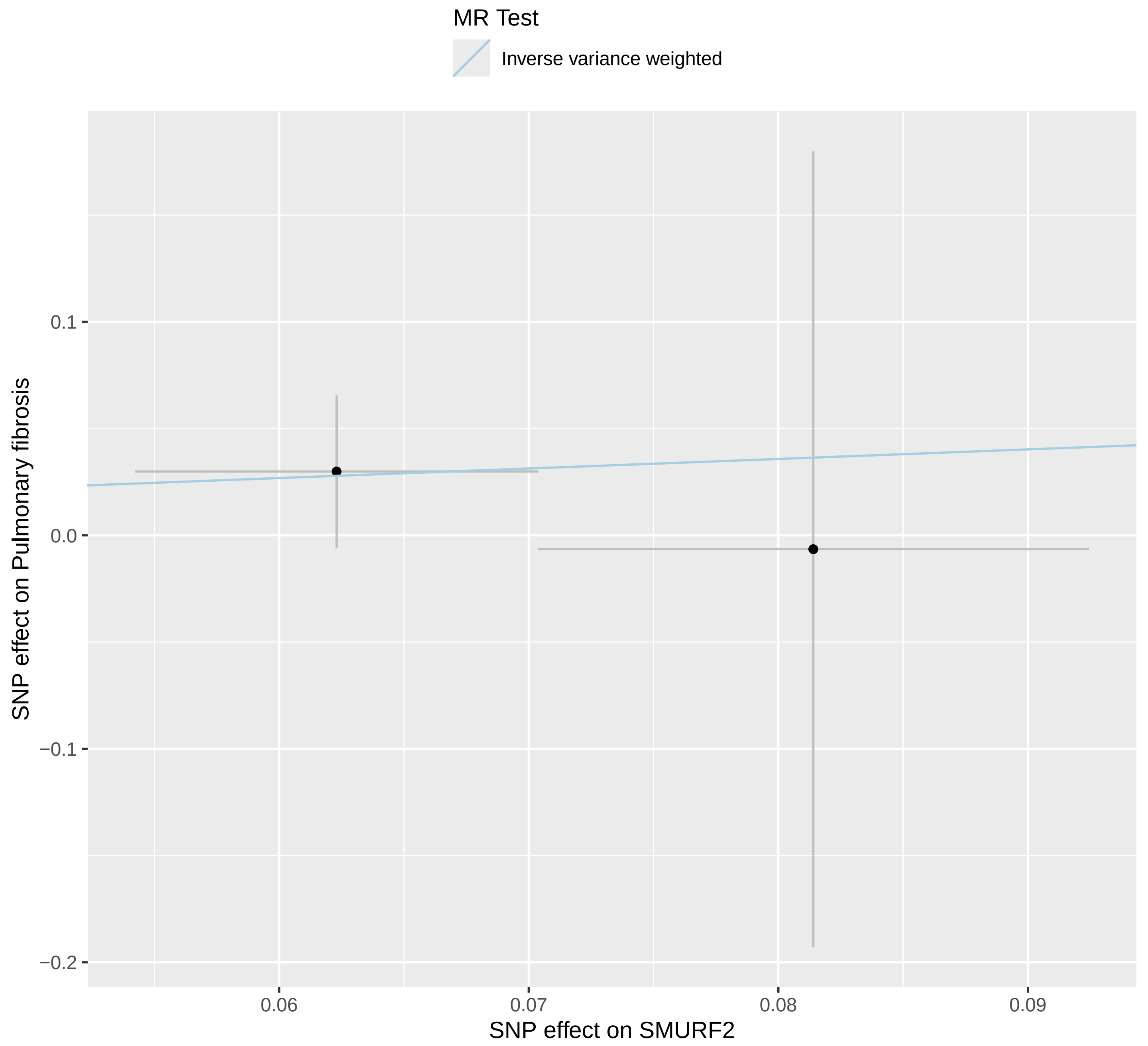

MR Test
Inverse variance weighted
0.1
0.0
−0.1
−0.2
0.06
0.07
0.08
0.09
SNP effect on Pulmonary fibrosis
SNP effect on SMURF2

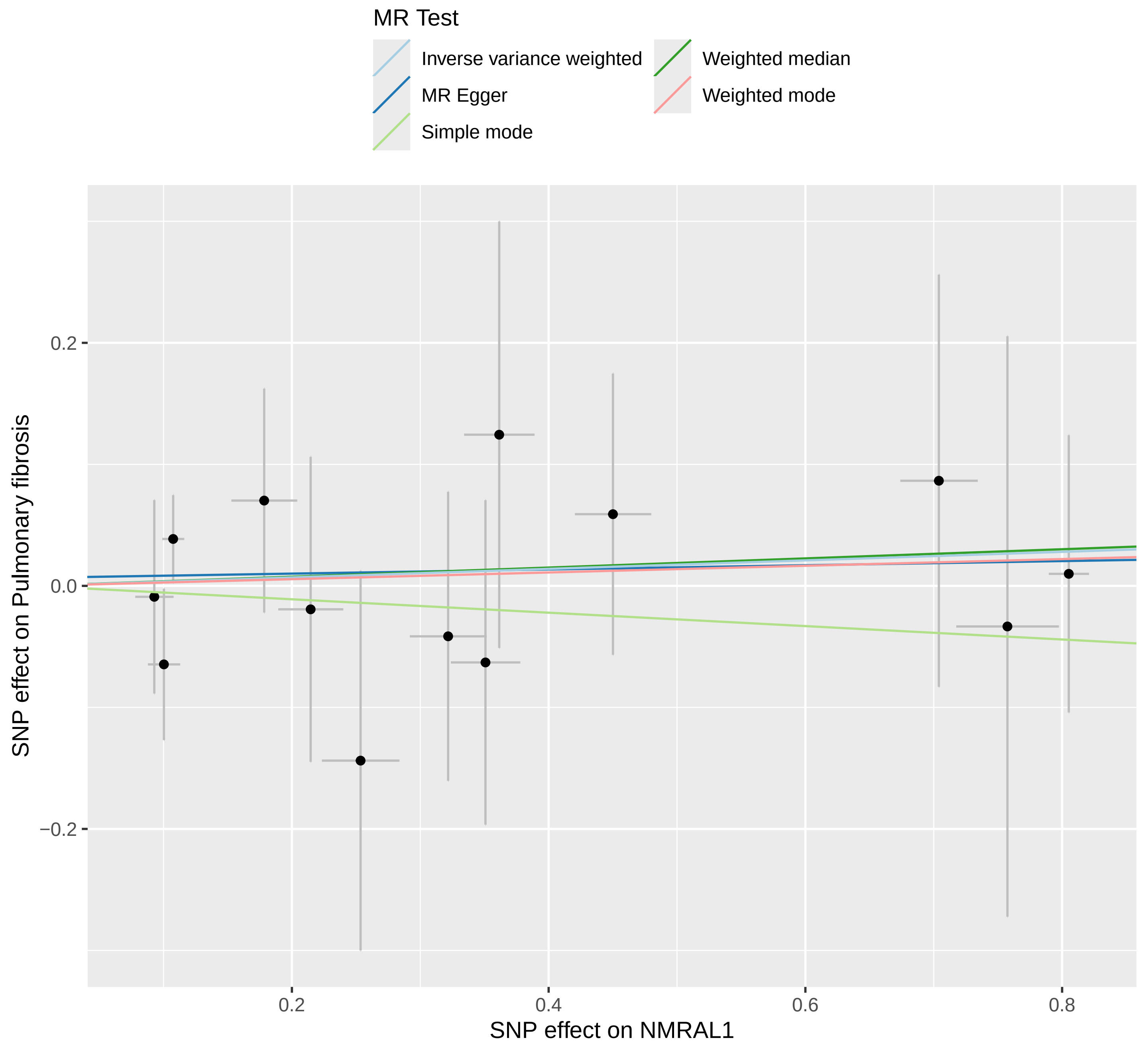
MR Test
Inverse variance weighted
MR Egger
Simple mode
Weighted median
Weighted mode
SNP effect on Pulmonary fibrosis
0.2
0.0
−0.2
0.2
0.4
0.6
0.8
SNP effect on NMRAL1

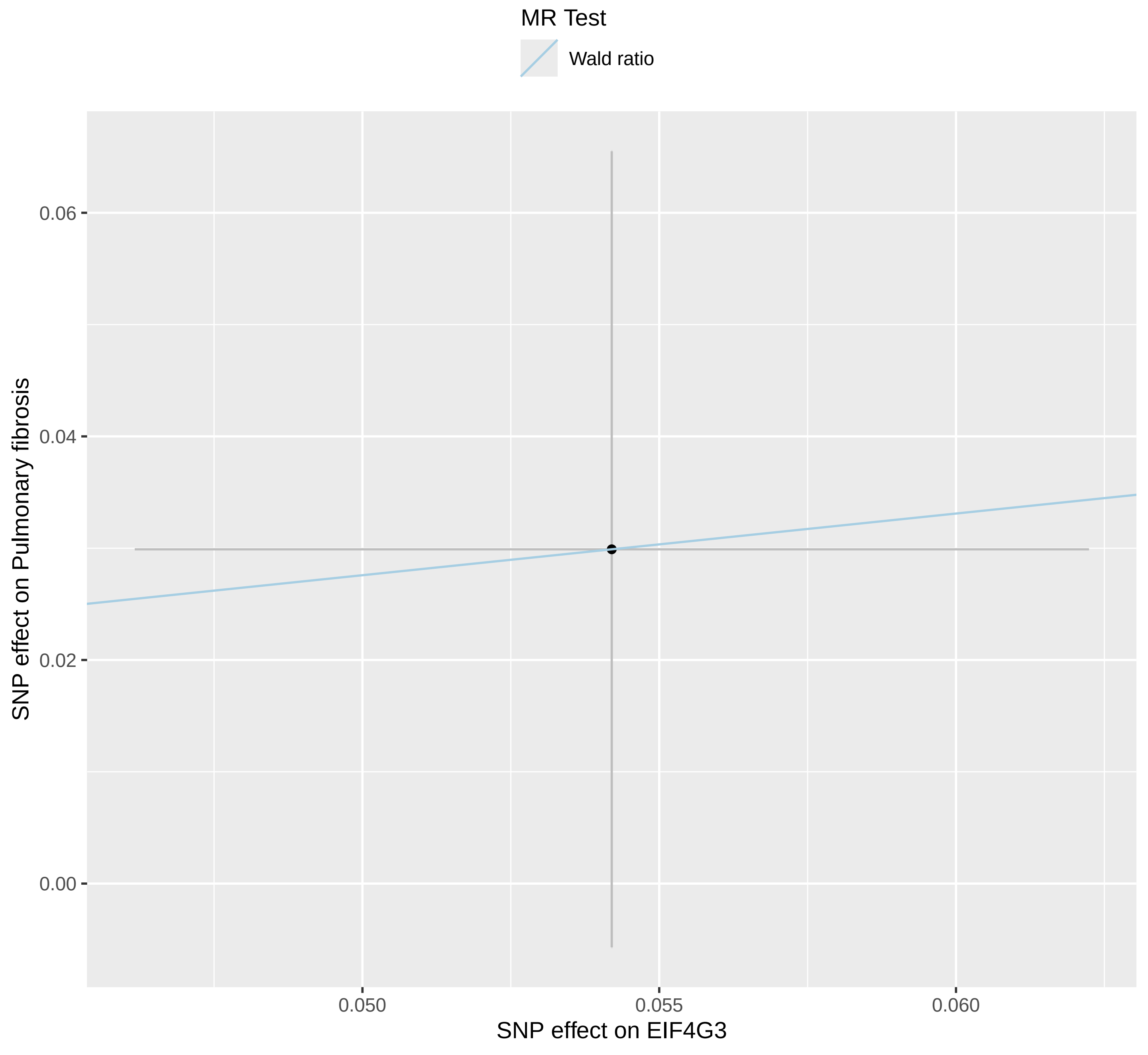

MR Test
Wald ratio
0.06
0.04
0.02
0.00
0.050
0.055
0.060
SNP effect on Pulmonary fibrosis
SNP effect on EIF4G3

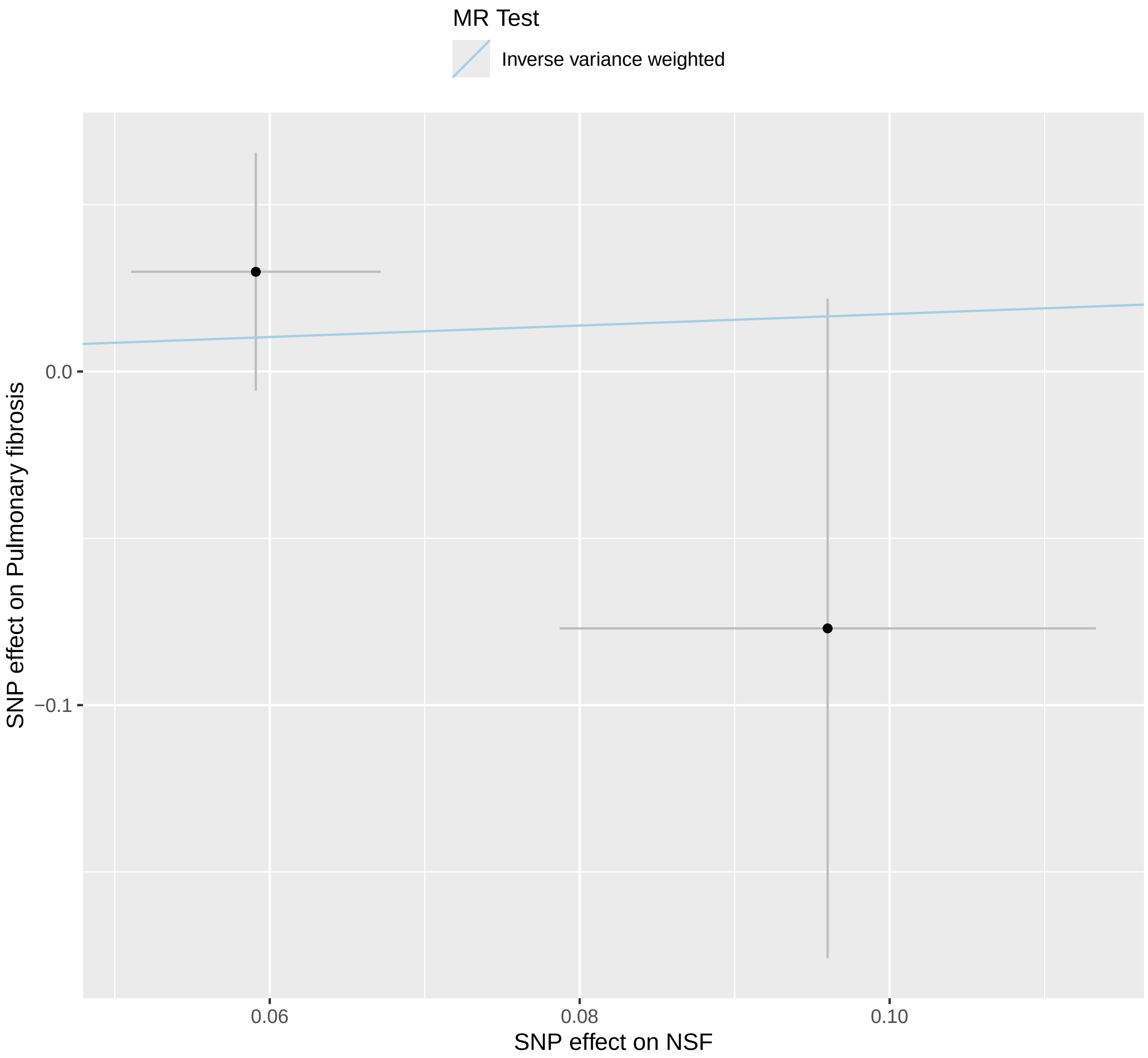

MR Test
Inverse variance weighted
0.0
−0.1
0.06
0.08
0.10
SNP effect on NSF
SNP effect on Pulmonary fibrosis

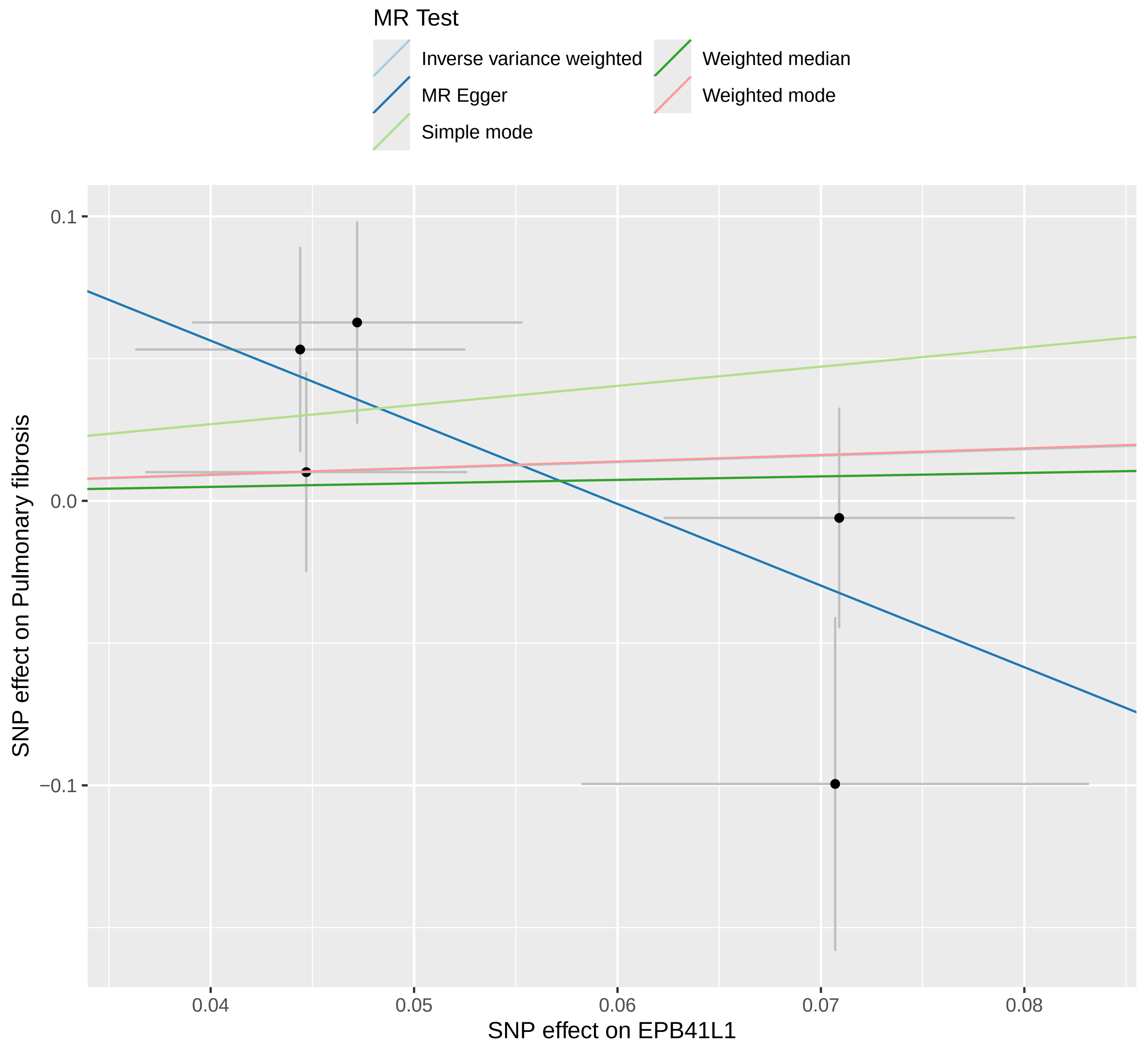
MR Test
Inverse variance weighted
MR Egger
Simple mode
Weighted median
Weighted mode
0.1
0.0
−0.1
0.04
0.05
0.06
0.07
0.08
SNP effect on EPB41L1
SNP effect on Pulmonary fibrosis

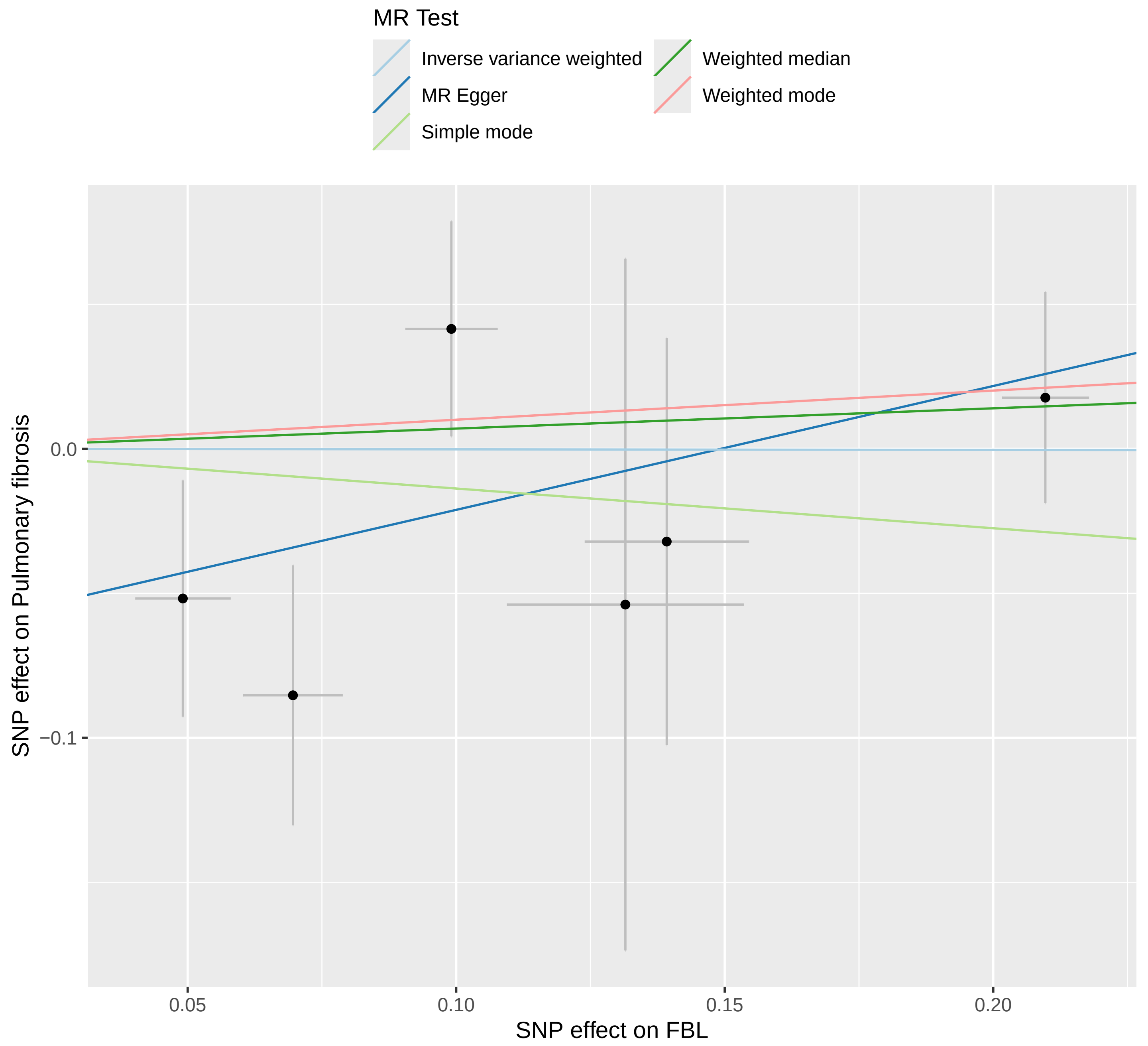
MR Test
Inverse variance weighted
MR Egger
Simple mode
Weighted median
Weighted mode
0.0
−0.1
0.05
0.10
0.15
0.20
SNP effect on FBL
SNP effect on Pulmonary fibrosis

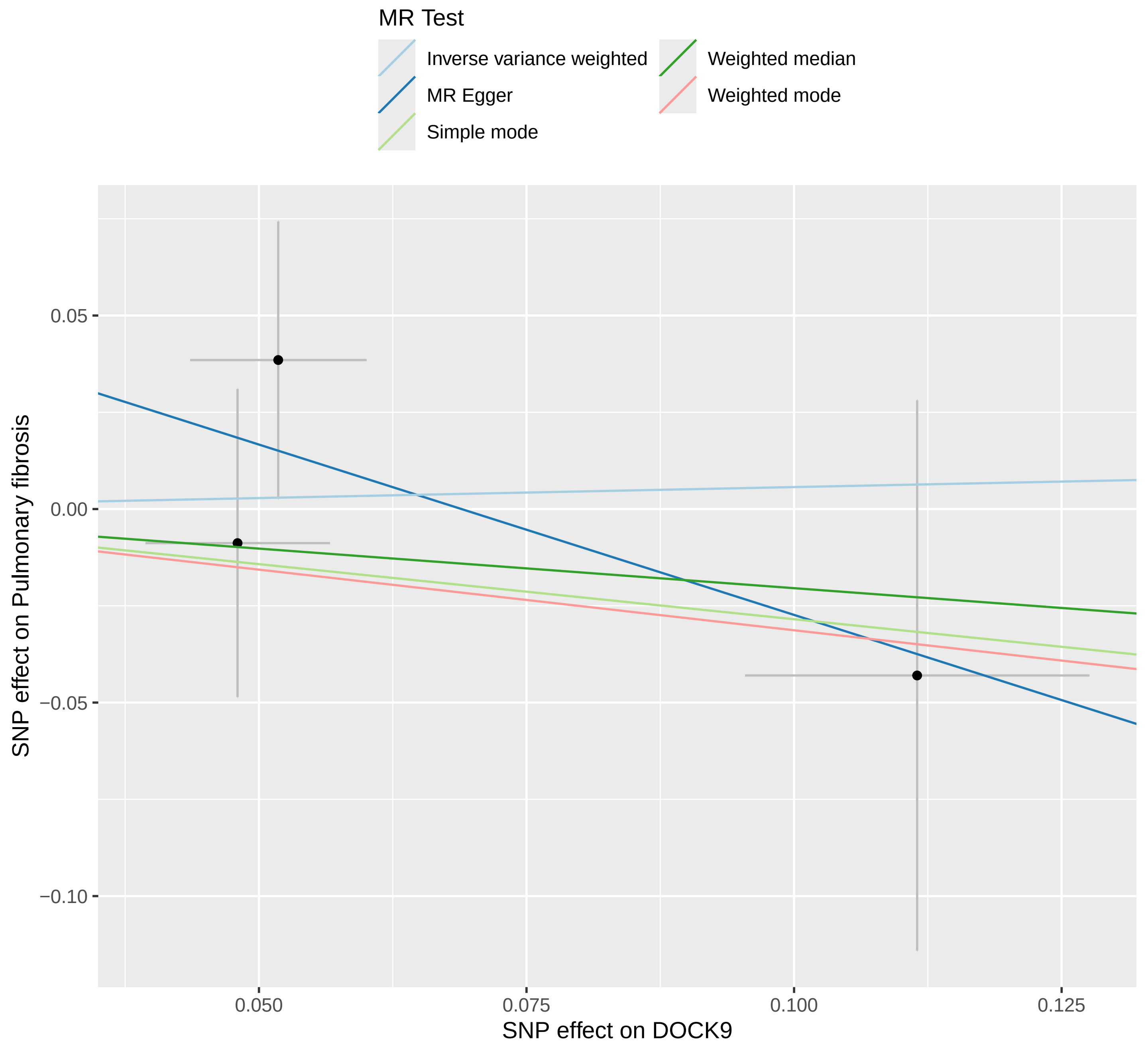
MR Test
Inverse variance weighted
MR Egger
Simple mode
Weighted median
Weighted mode
0.05
0.00
−0.05
−0.10
0.050
0.075
0.100
0.125
SNP effect on Pulmonary fibrosis
SNP effect on DOCK9

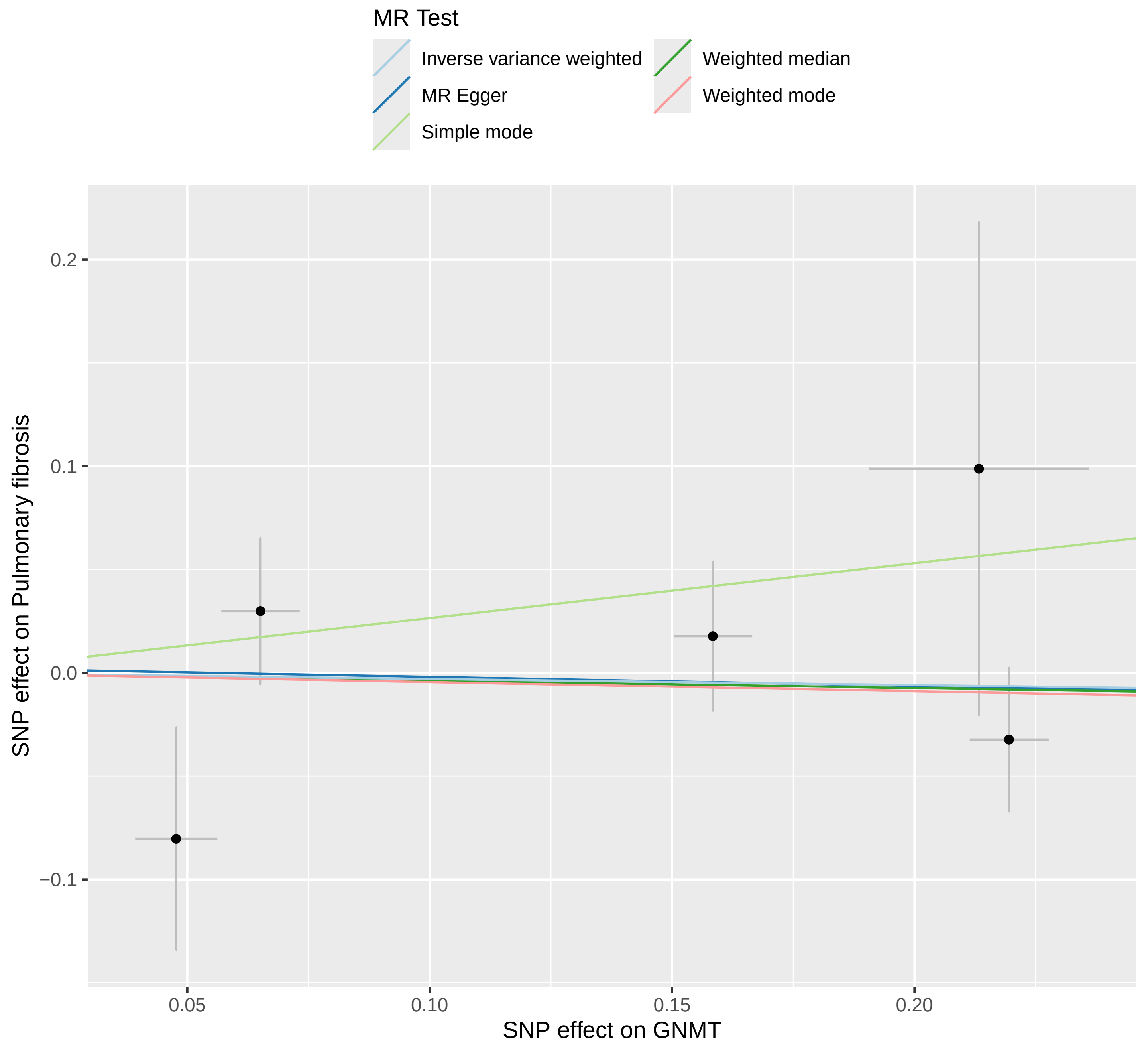
MR Test
Inverse variance weighted
MR Egger
Simple mode
Weighted median
Weighted mode
0.2
0.1
0.0
−0.1
SNP effect on Pulmonary fibrosis
0.05
0.10
0.15
0.20
SNP effect on GNMT

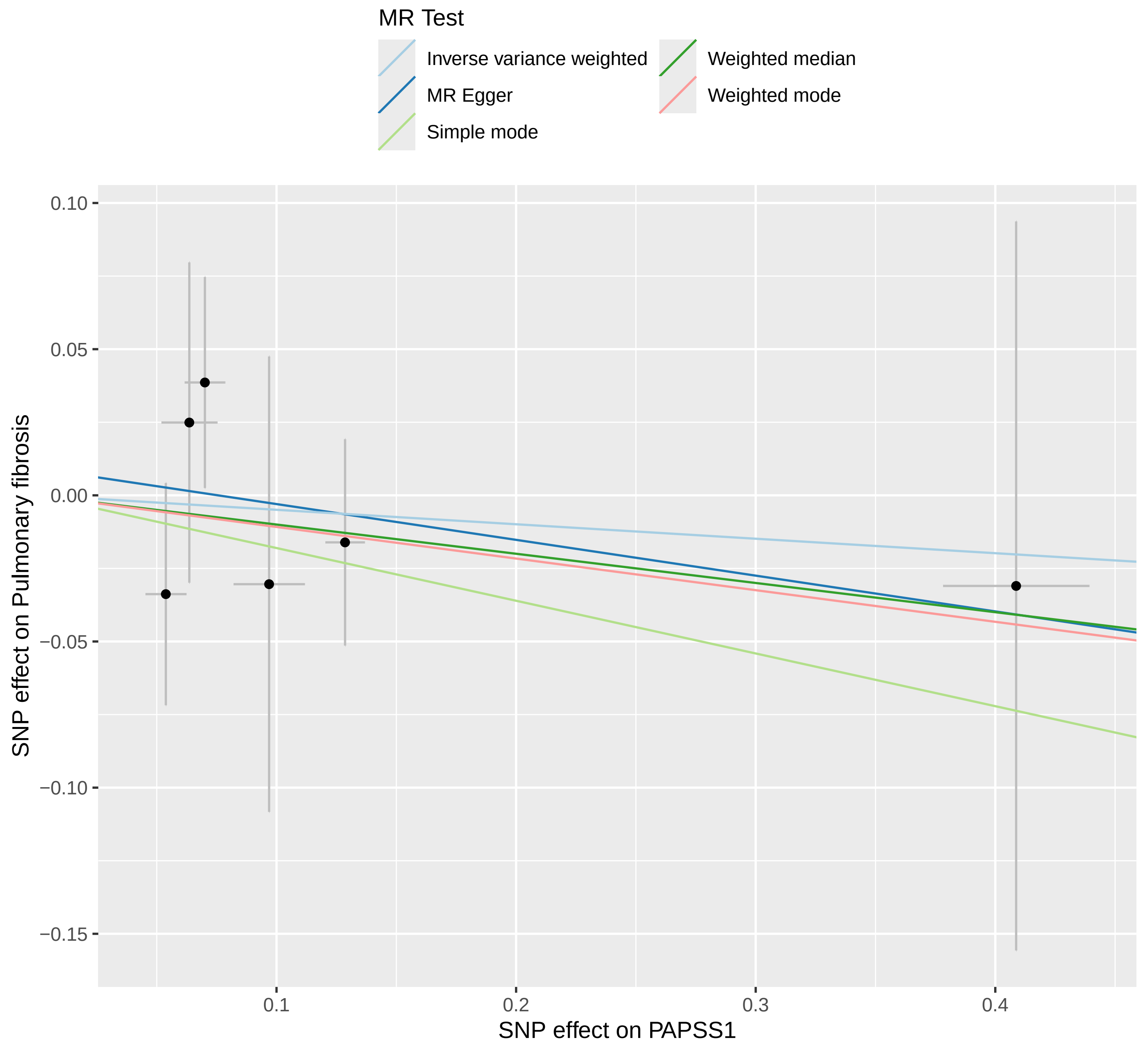

MR Test
Inverse variance weighted
MR Egger
Simple mode
Weighted median
Weighted mode
0.10
0.05
0.00
−0.05
−0.10
−0.15
0.1
0.2
0.3
0.4
SNP effect on Pulmonary fibrosis
SNP effect on PAPSS1

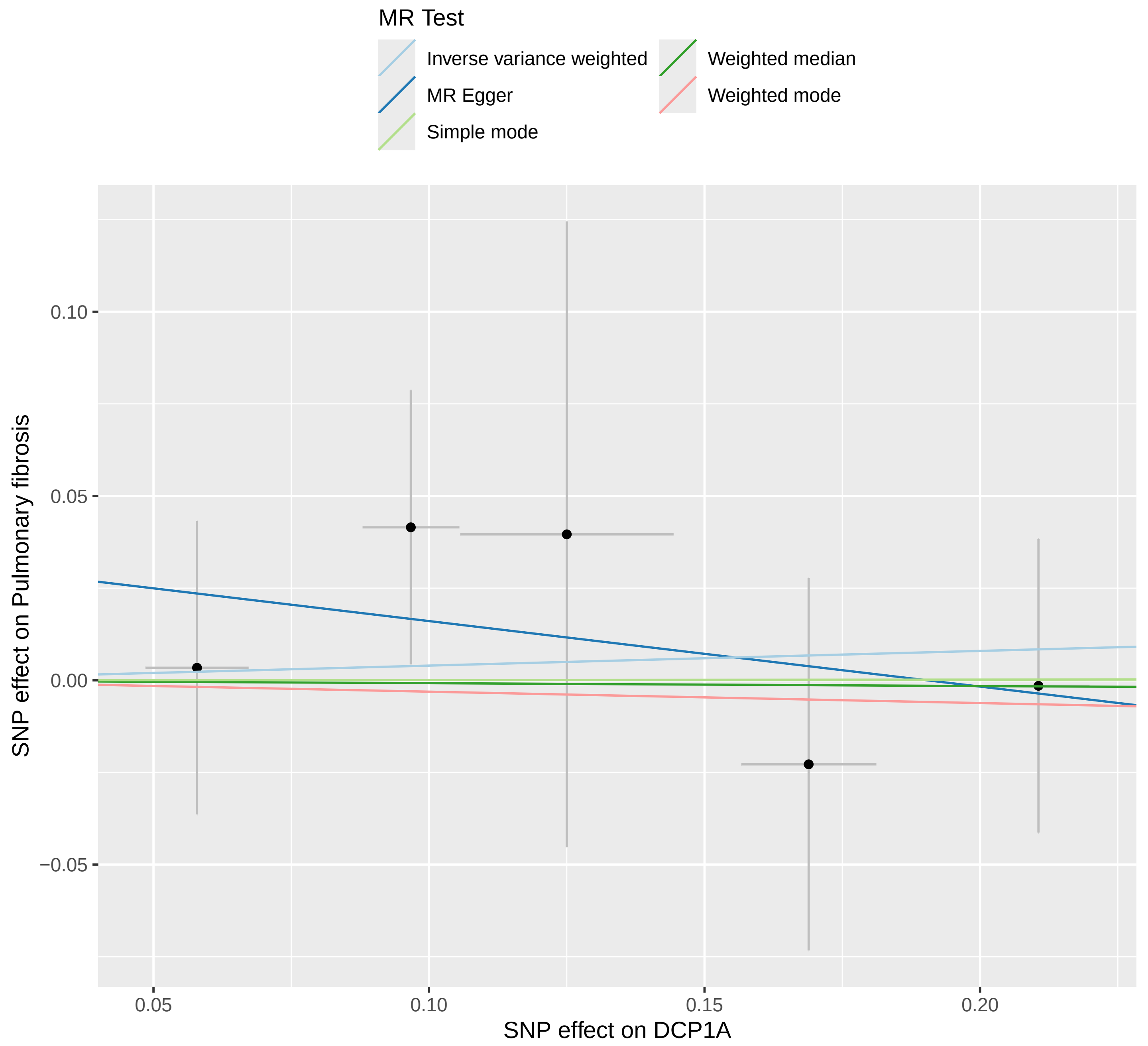
MR Test
Inverse variance weighted
MR Egger
Simple mode
Weighted median
Weighted mode
SNP effect on Pulmonary fibrosis
0.10
0.05
0.00
−0.05
0.05
0.10
0.15
0.20
SNP effect on DCP1A

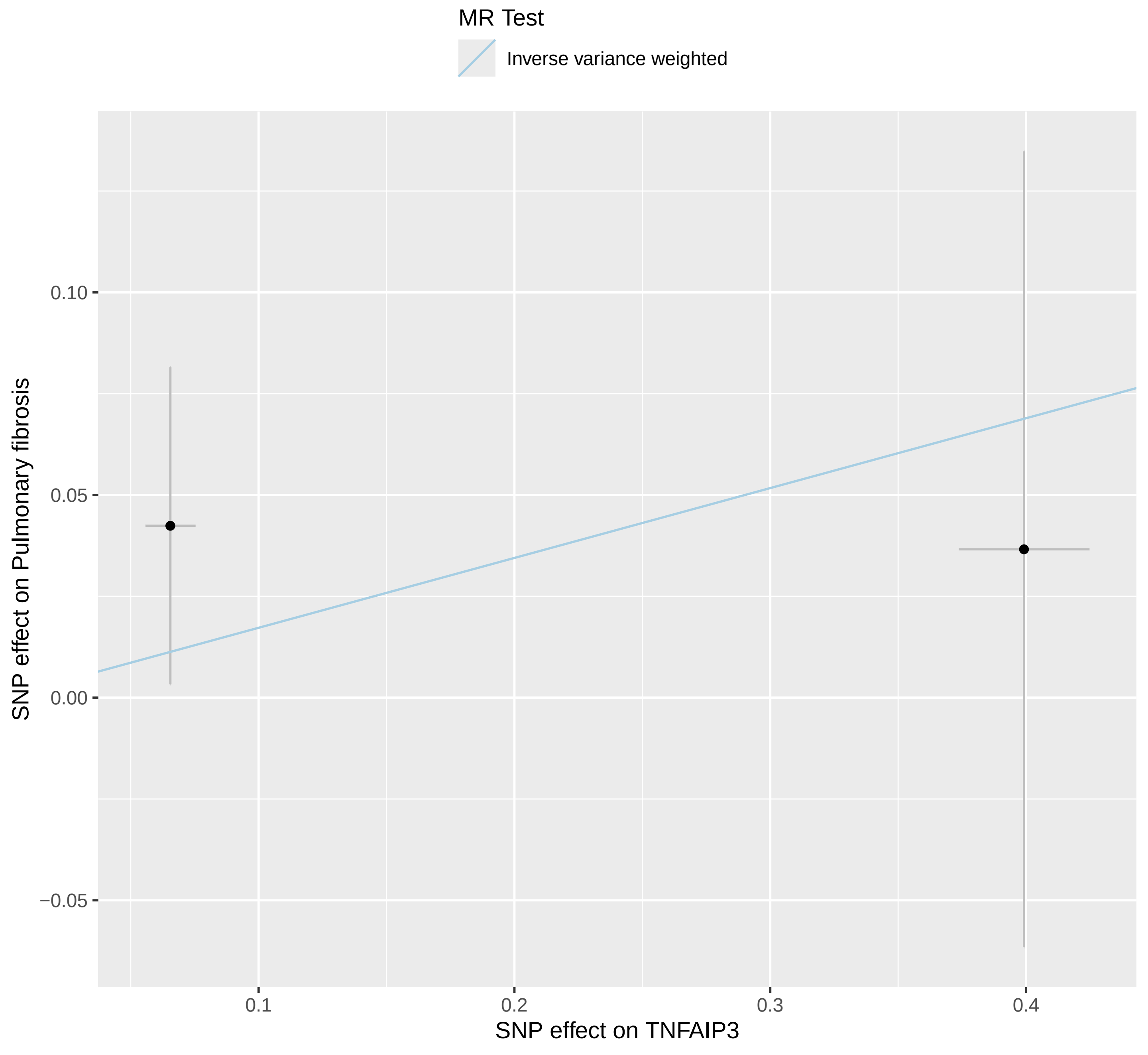

MR Test
Inverse variance weighted
0.10
0.05
0.00
−0.05
SNP effect on Pulmonary fibrosis
0.1
0.2
0.3
0.4
SNP effect on TNFAIP3

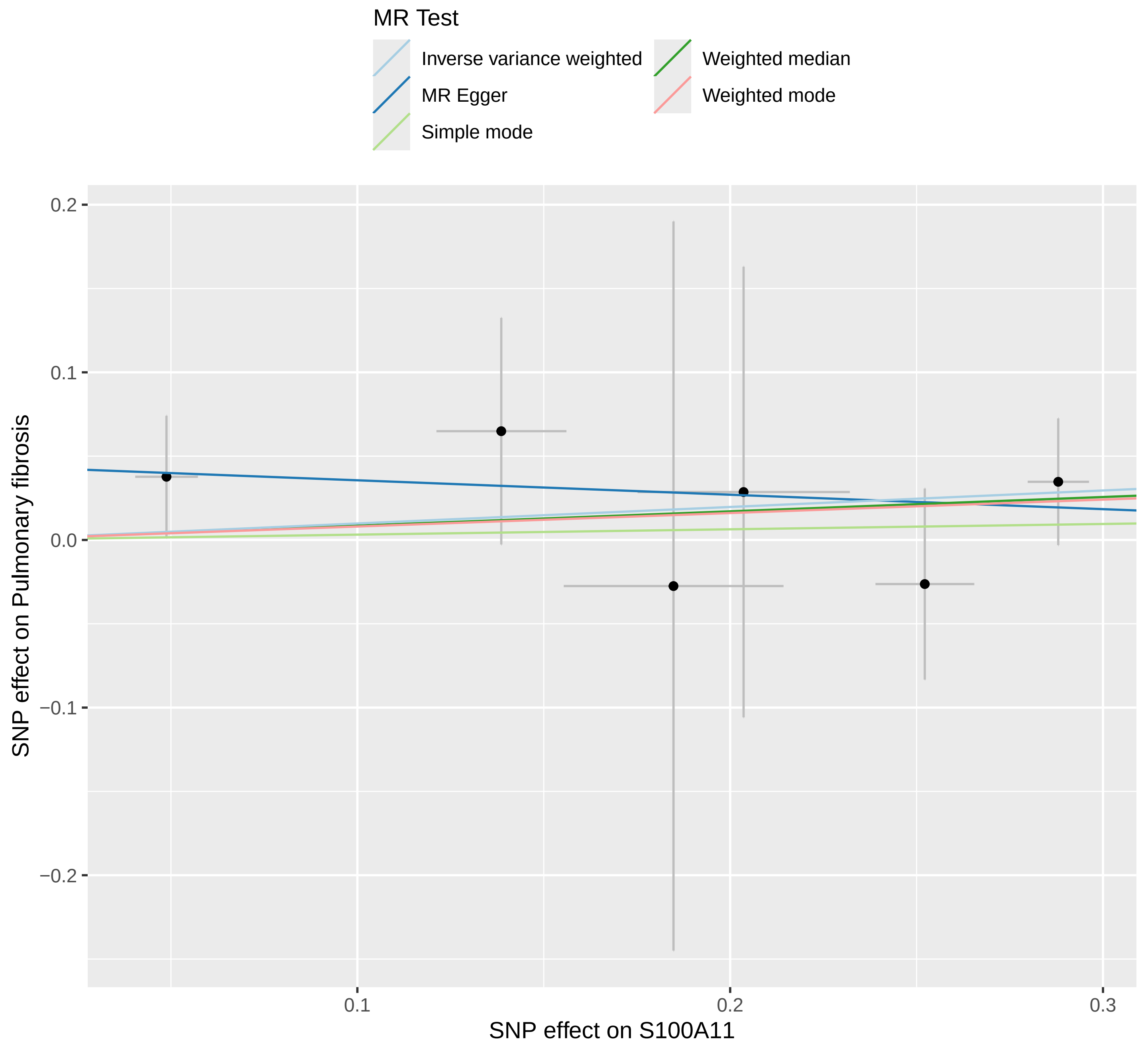
MR Test
Inverse variance weighted
MR Egger
Simple mode
Weighted median
Weighted mode
0.2
0.1
0.0
−0.1
−0.2
0.1
0.2
0.3
SNP effect on S100A11
SNP effect on Pulmonary fibrosis

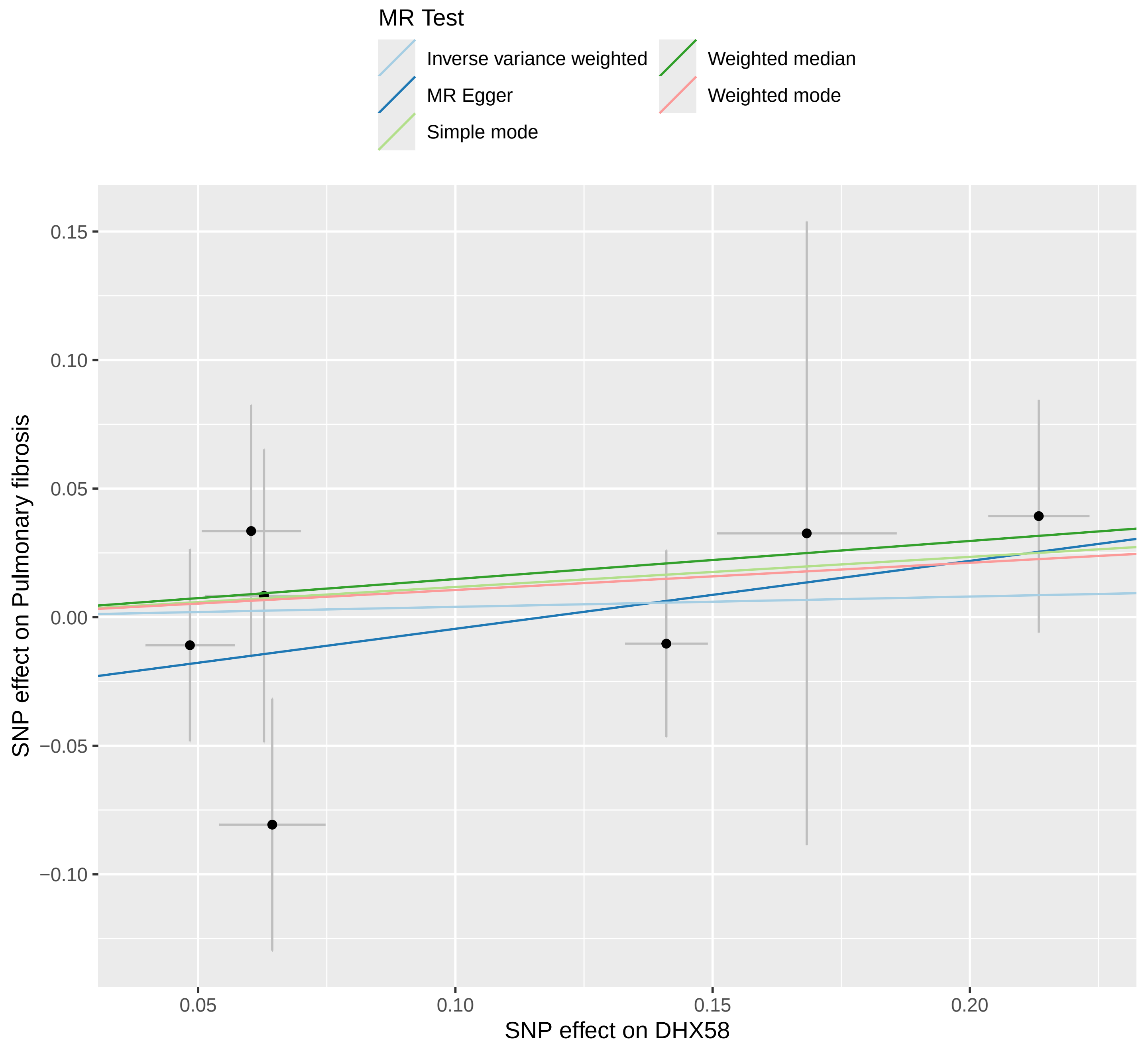

MR Test
Inverse variance weighted
MR Egger
Simple mode
Weighted median
Weighted mode
0.15
0.10
0.05
0.00
−0.05
−0.10
SNP effect on Pulmonary fibrosis
0.05
0.10
0.15
0.20
SNP effect on DHX58

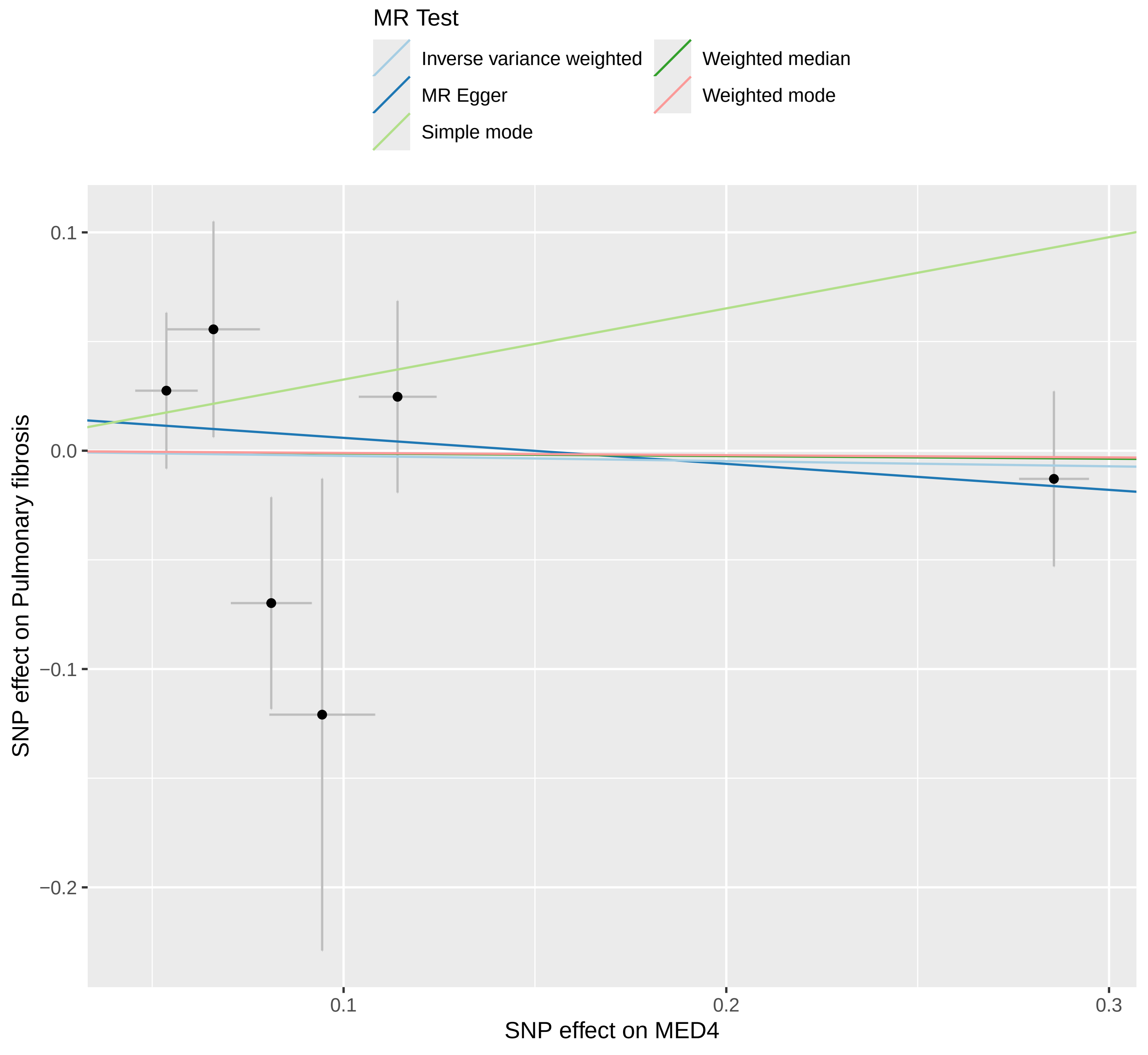
MR Test
Inverse variance weighted
MR Egger
Simple mode
Weighted median
Weighted mode
0.1
0.0
−0.1
−0.2
SNP effect on Pulmonary fibrosis
0.1
0.2
0.3
SNP effect on MED4

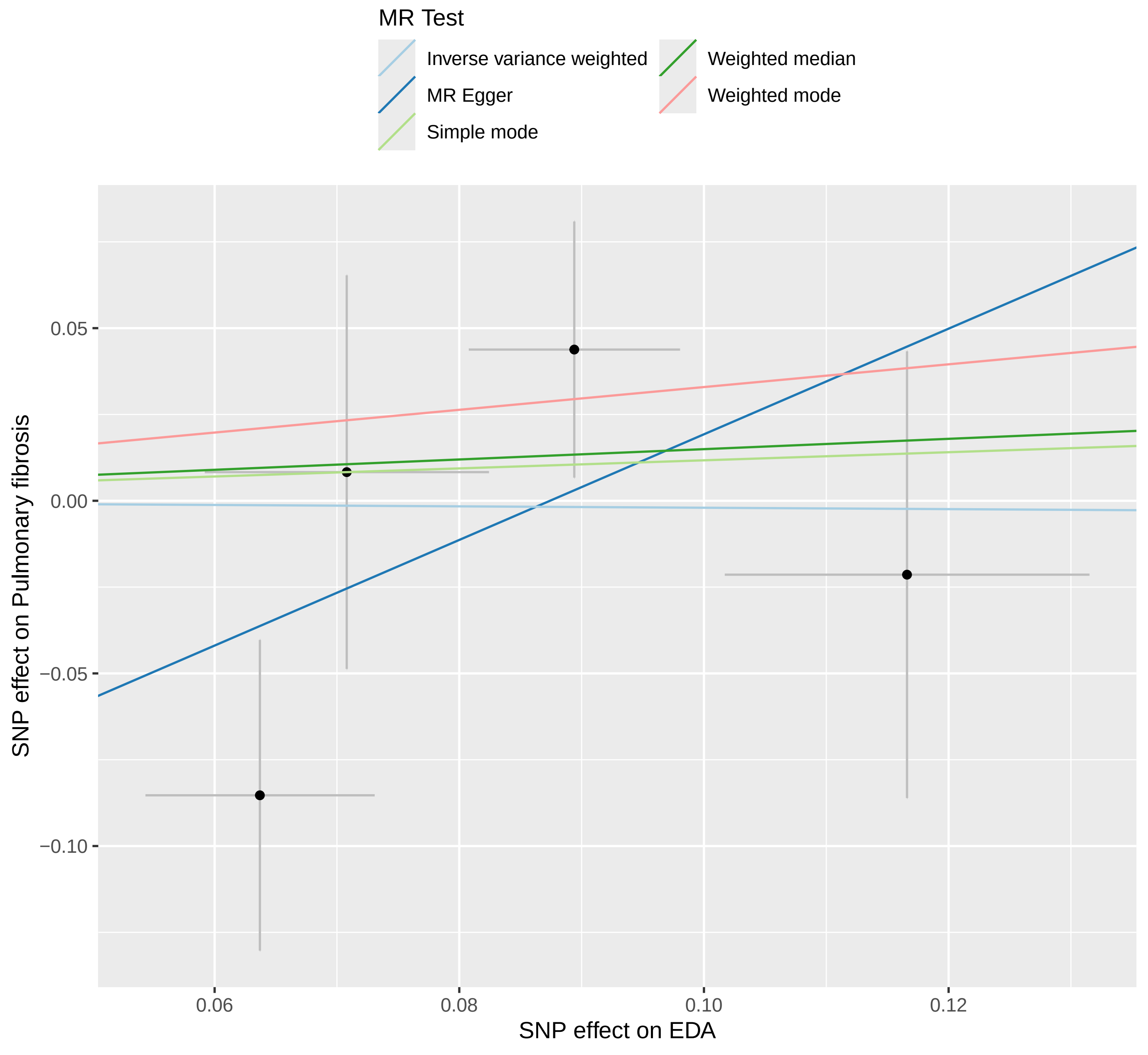
MR Test
Inverse variance weighted
MR Egger
Simple mode
Weighted median
Weighted mode
0.05
0.00
−0.05
−0.10
SNP effect on Pulmonary fibrosis
0.06
0.08
0.10
0.12
SNP effect on EDA

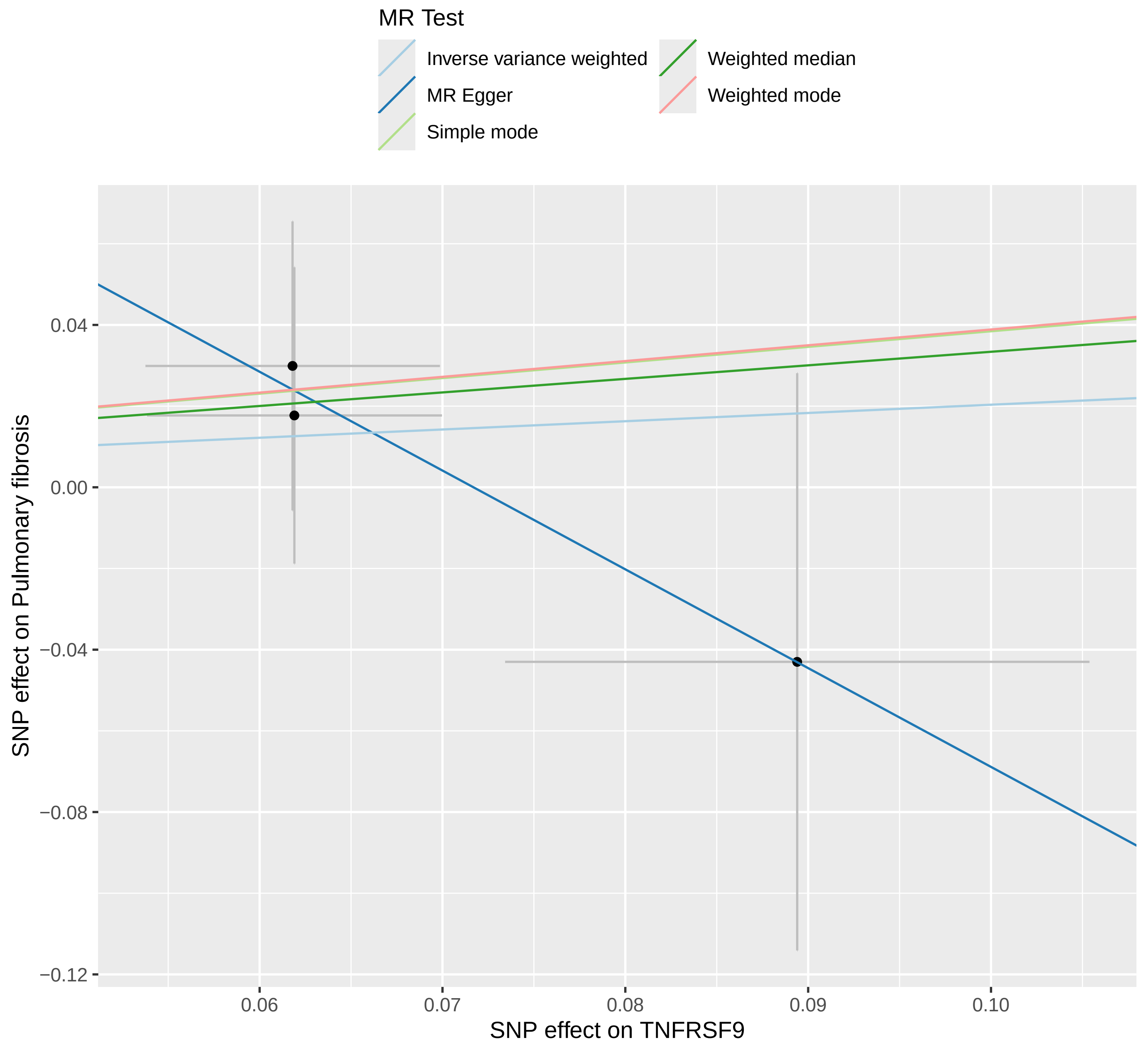
MR Test
Inverse variance weighted
MR Egger
Simple mode
Weighted median
Weighted mode
SNP effect on Pulmonary fibrosis
0.04
0.00
−0.04
−0.08
−0.12
0.06
0.07
0.08
0.09
0.10
SNP effect on TNFRSF9

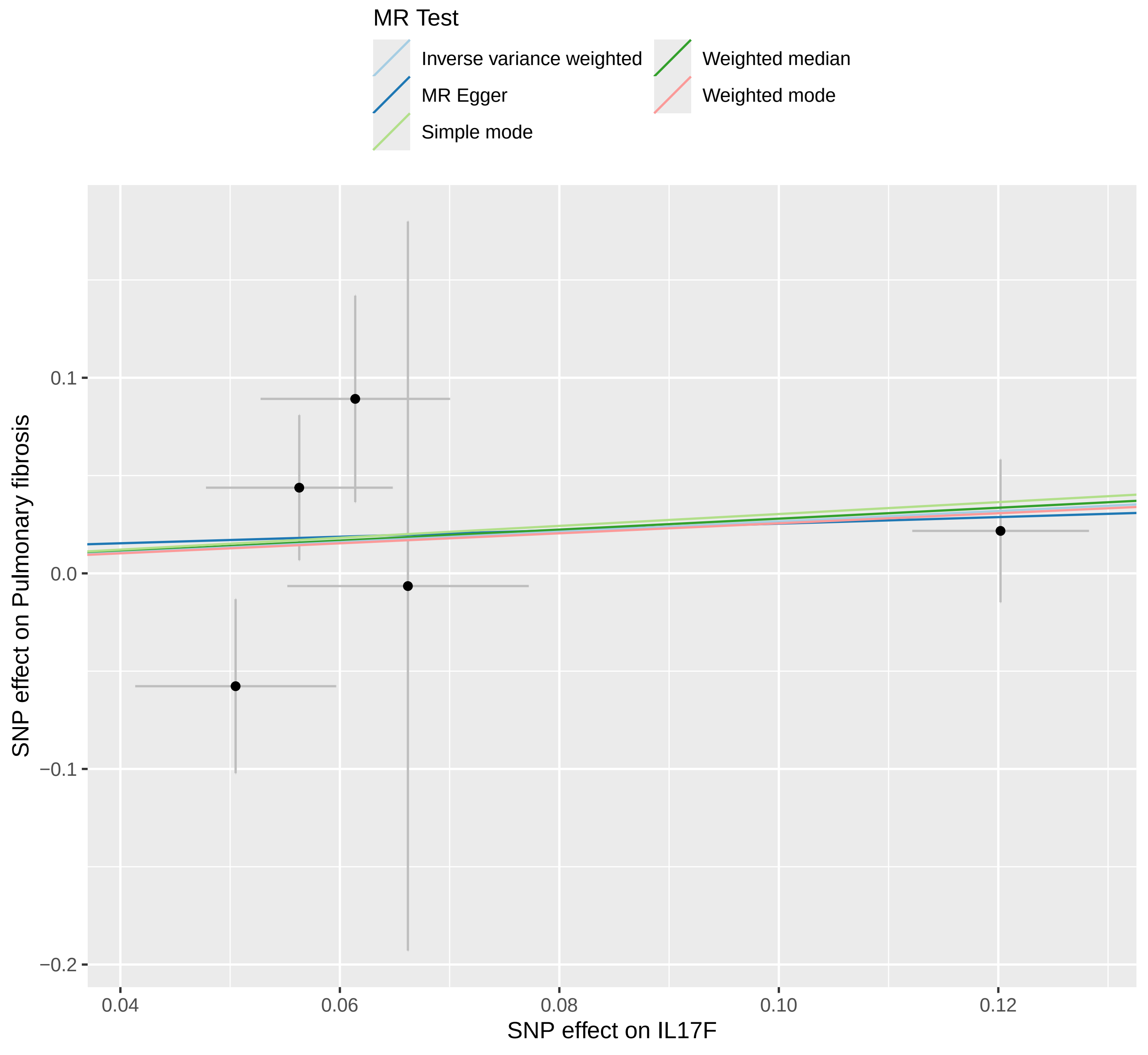
MR Test
Inverse variance weighted
MR Egger
Simple mode
Weighted median
Weighted mode
SNP effect on Pulmonary fibrosis
0.1
0.0
−0.1
−0.2
0.04
0.06
0.08
0.10
0.12
SNP effect on IL17F

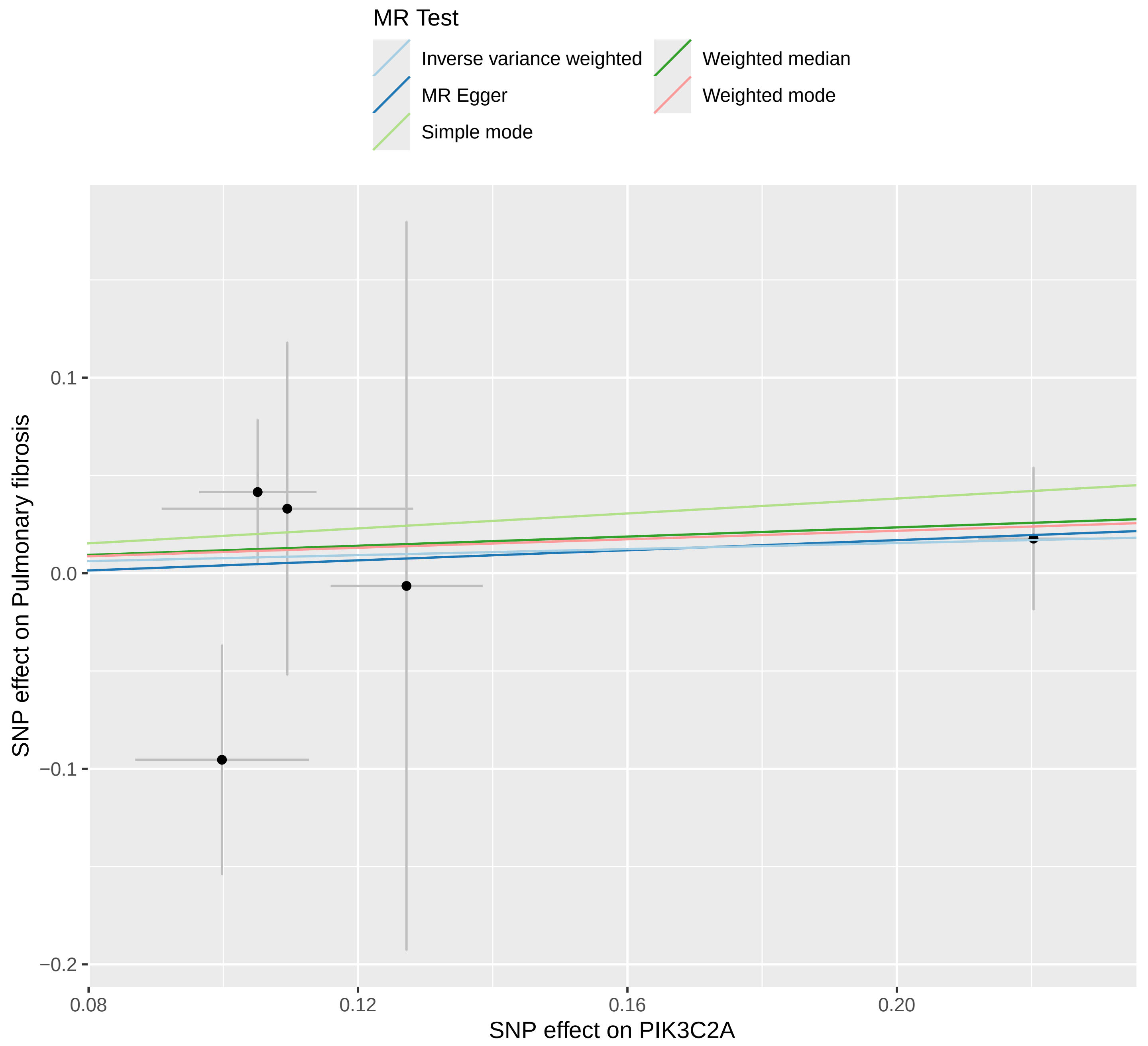
MR Test
Inverse variance weighted
MR Egger
Simple mode
Weighted median
Weighted mode
SNP effect on Pulmonary fibrosis
0.1
0.0
−0.1
−0.2
0.08
0.12
0.16
0.20
SNP effect on PIK3C2A

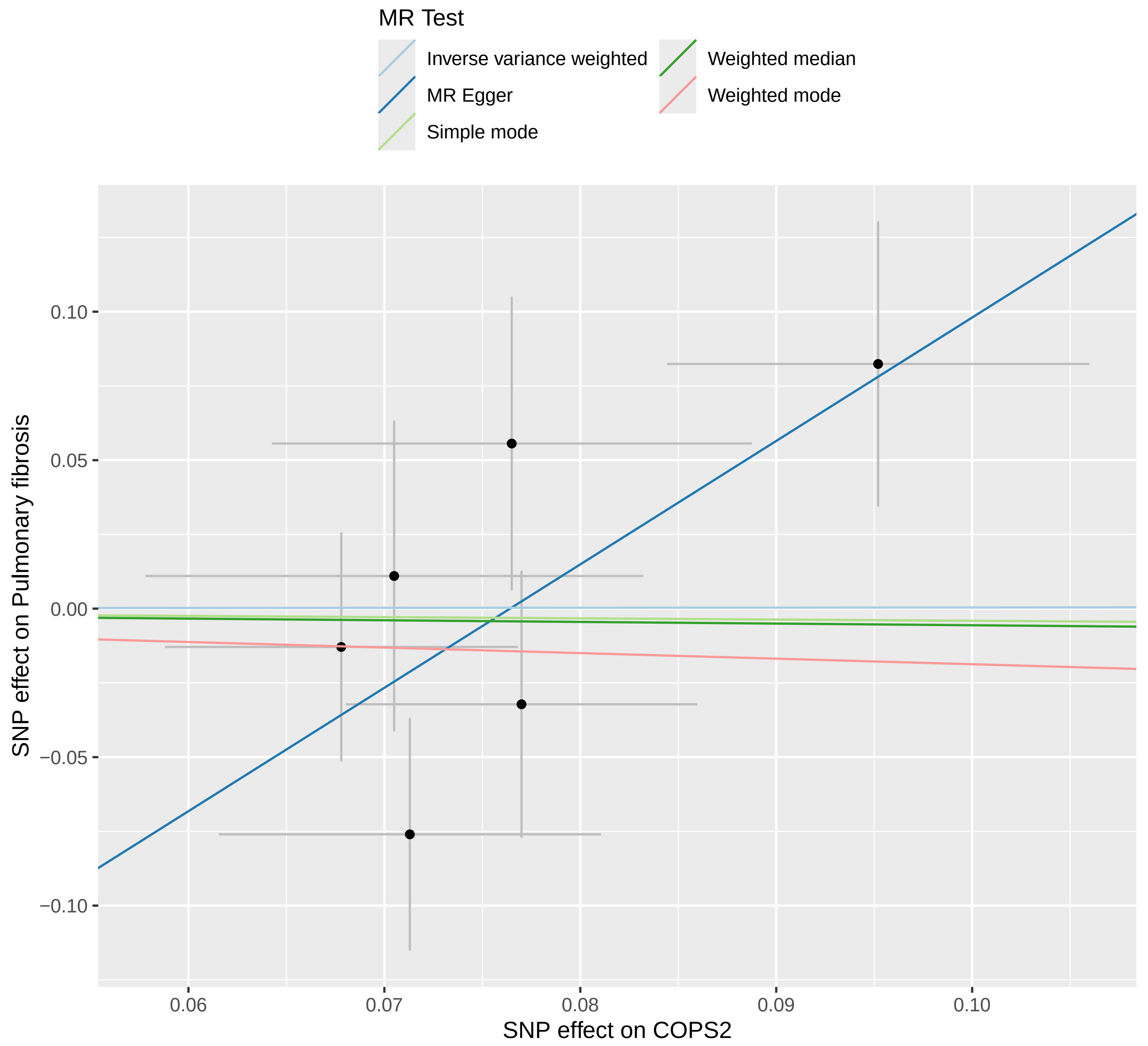

MR Test
Inverse variance weighted
MR Egger
Simple mode
Weighted median
Weighted mode
0.10
0.05
0.00
−0.05
−0.10
SNP effect on Pulmonary fibrosis
0.06
0.07
0.08
0.09
0.10
SNP effect on COPS2

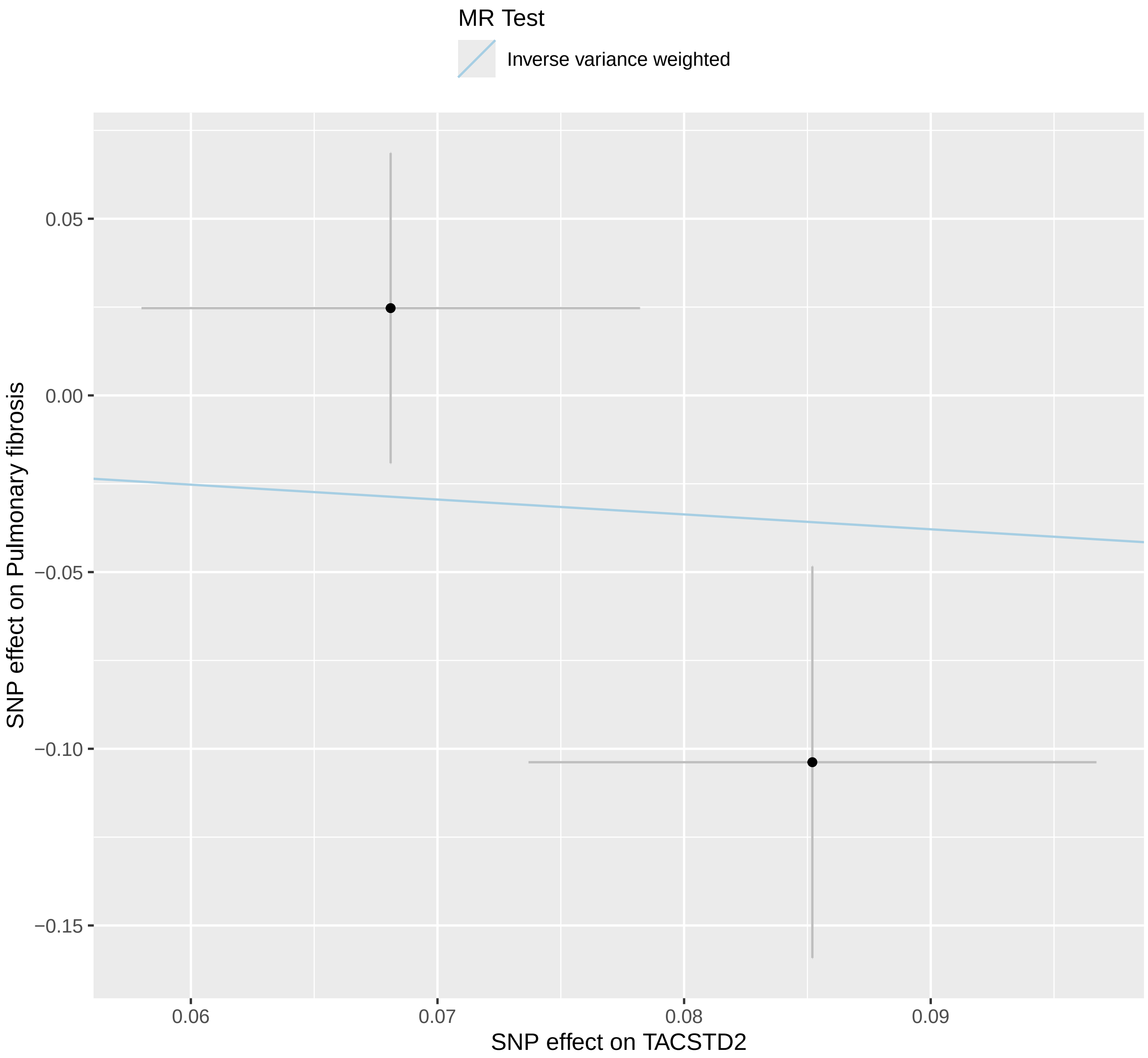

MR Test
Inverse variance weighted
0.05
0.00
−0.05
−0.10
−0.15
0.06
0.07
0.08
0.09
SNP effect on TACSTD2
SNP effect on Pulmonary fibrosis

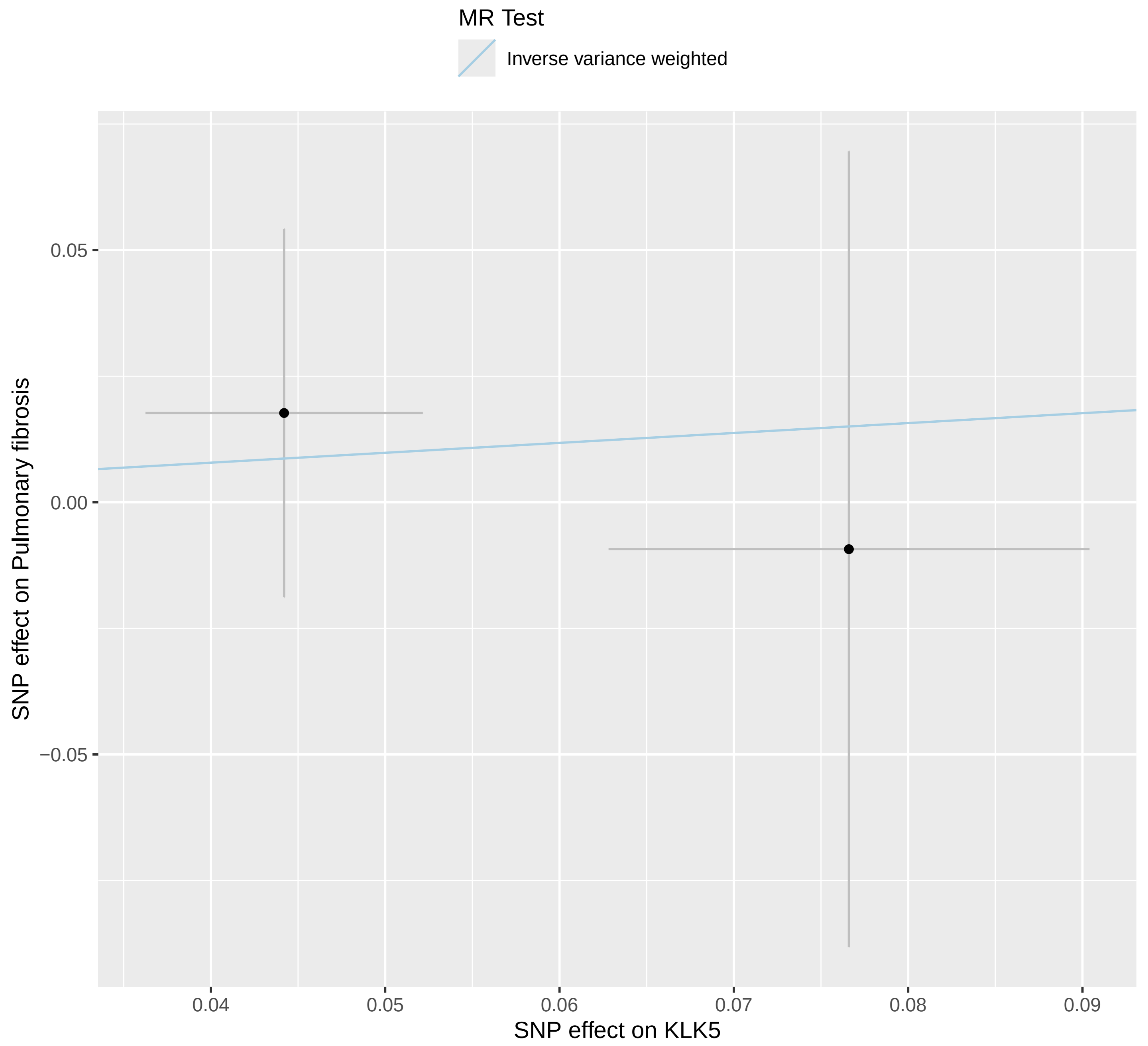

MR Test
Inverse variance weighted
0.05
0.00
−0.05
SNP effect on Pulmonary fibrosis
0.04
0.05
0.06
0.07
0.08
0.09
SNP effect on KLK5

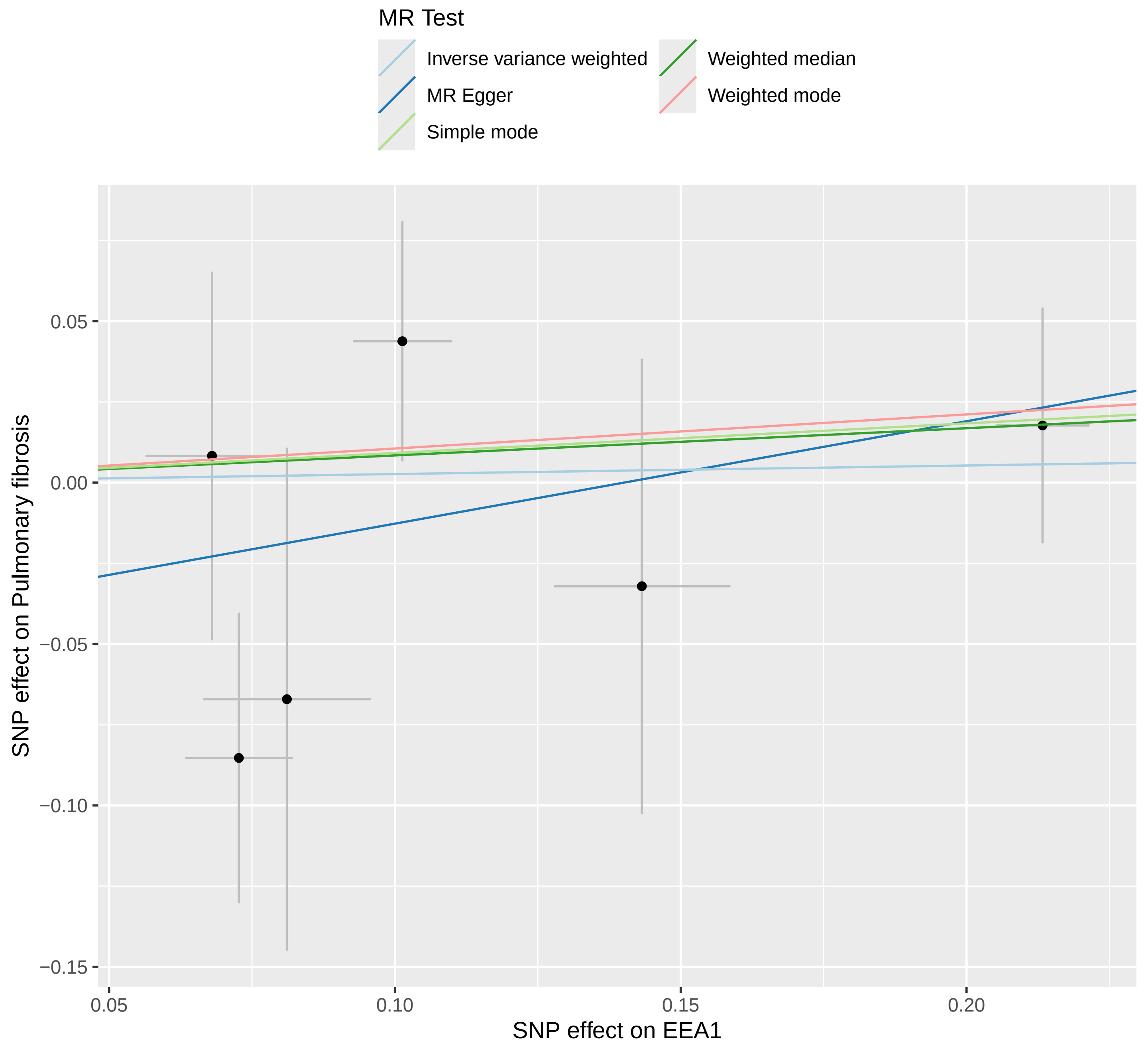
MR Test
Inverse variance weighted
MR Egger
Simple mode
Weighted median
Weighted mode
0.05
0.00
−0.05
−0.10
−0.15
0.05
0.10
0.15
0.20
SNP effect on Pulmonary fibrosis
SNP effect on EEA1

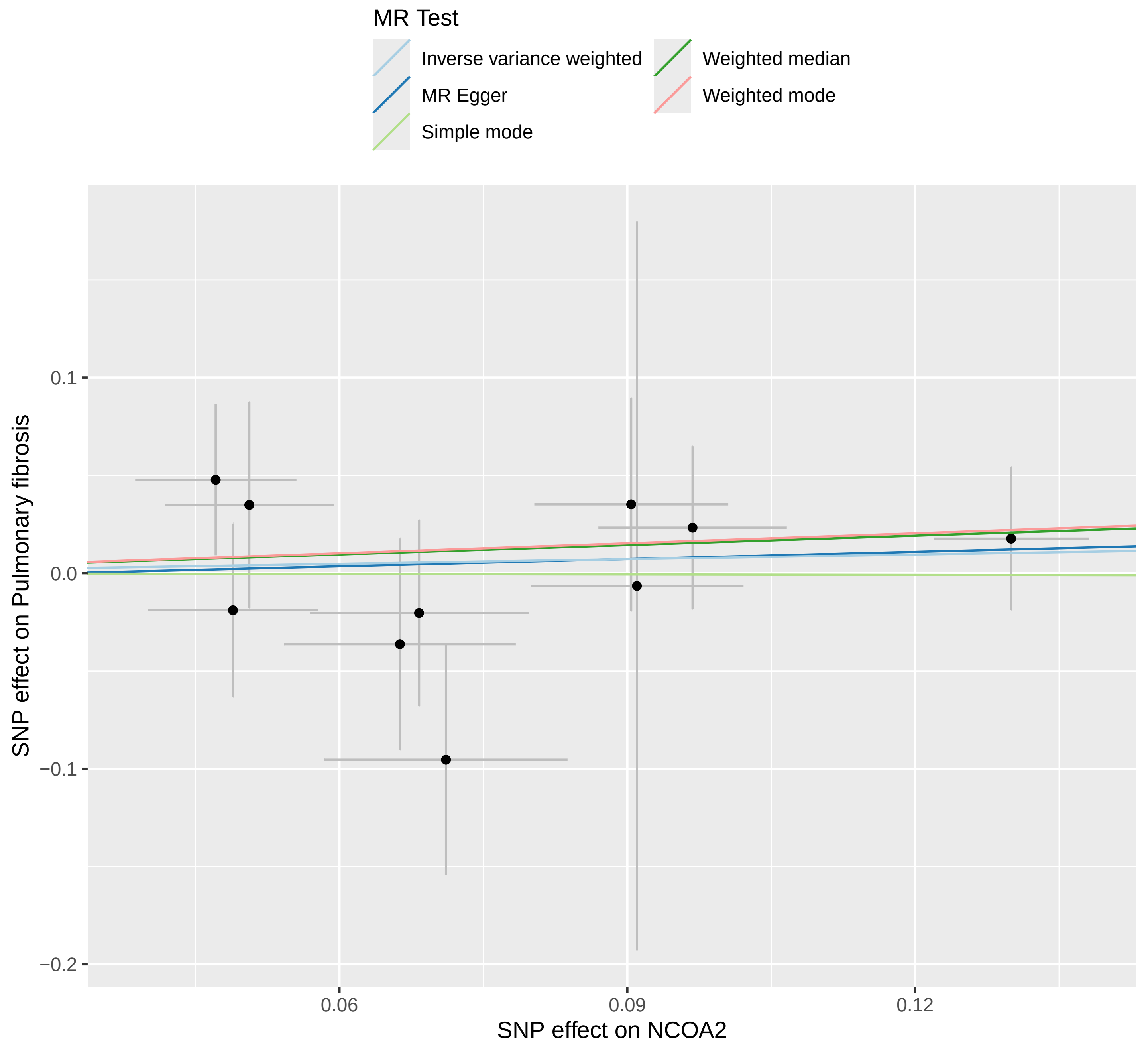

MR Test
Inverse variance weighted
MR Egger
Simple mode
Weighted median
Weighted mode
0.1
0.0
−0.1
−0.2
SNP effect on Pulmonary fibrosis
0.06
0.09
0.12
SNP effect on NCOA2

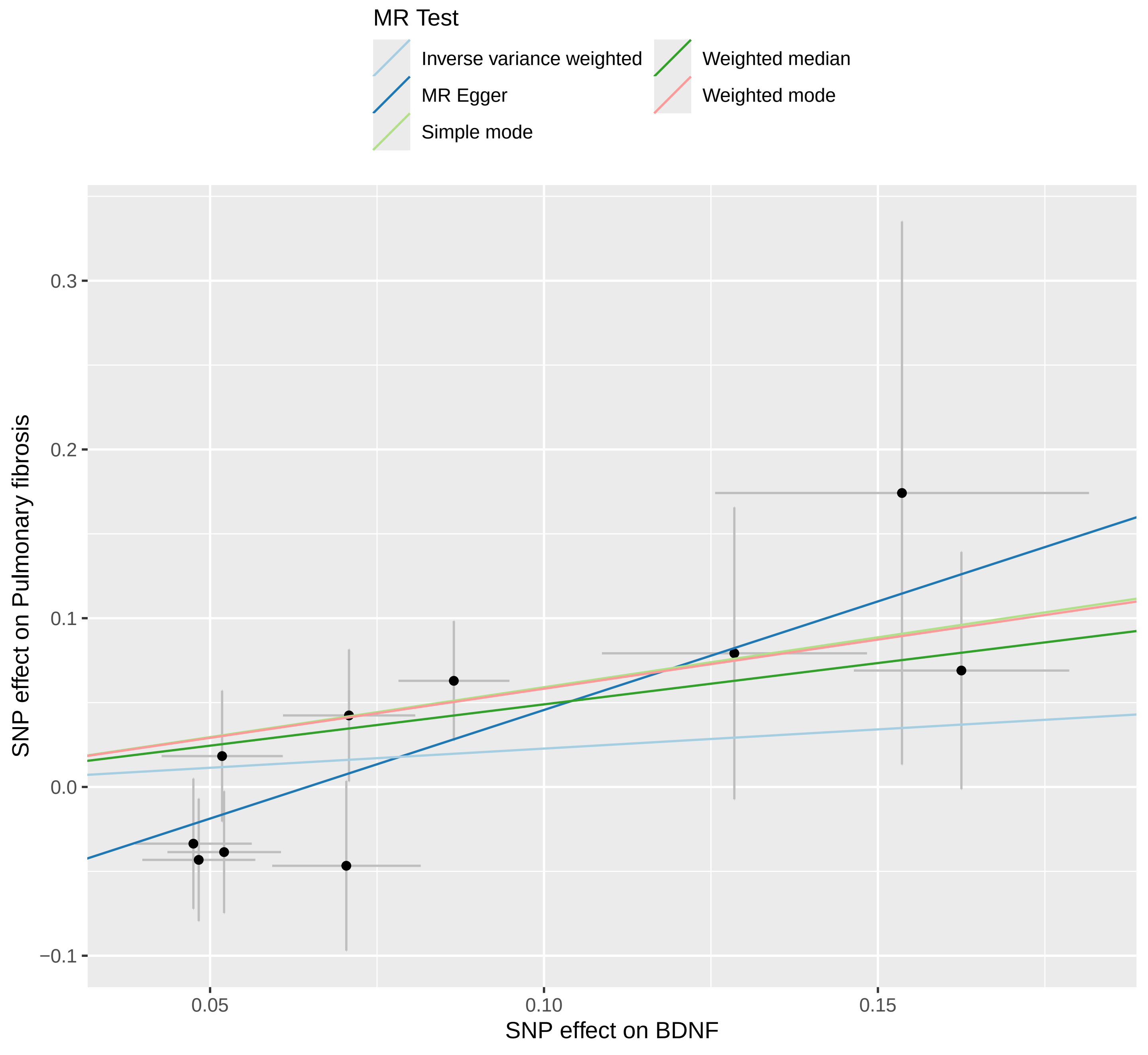

MR Test
Inverse variance weighted
MR Egger
Simple mode
Weighted median
Weighted mode
0.3
0.2
0.1
0.0
−0.1
0.05
0.10
0.15
SNP effect on Pulmonary fibrosis
SNP effect on BDNF

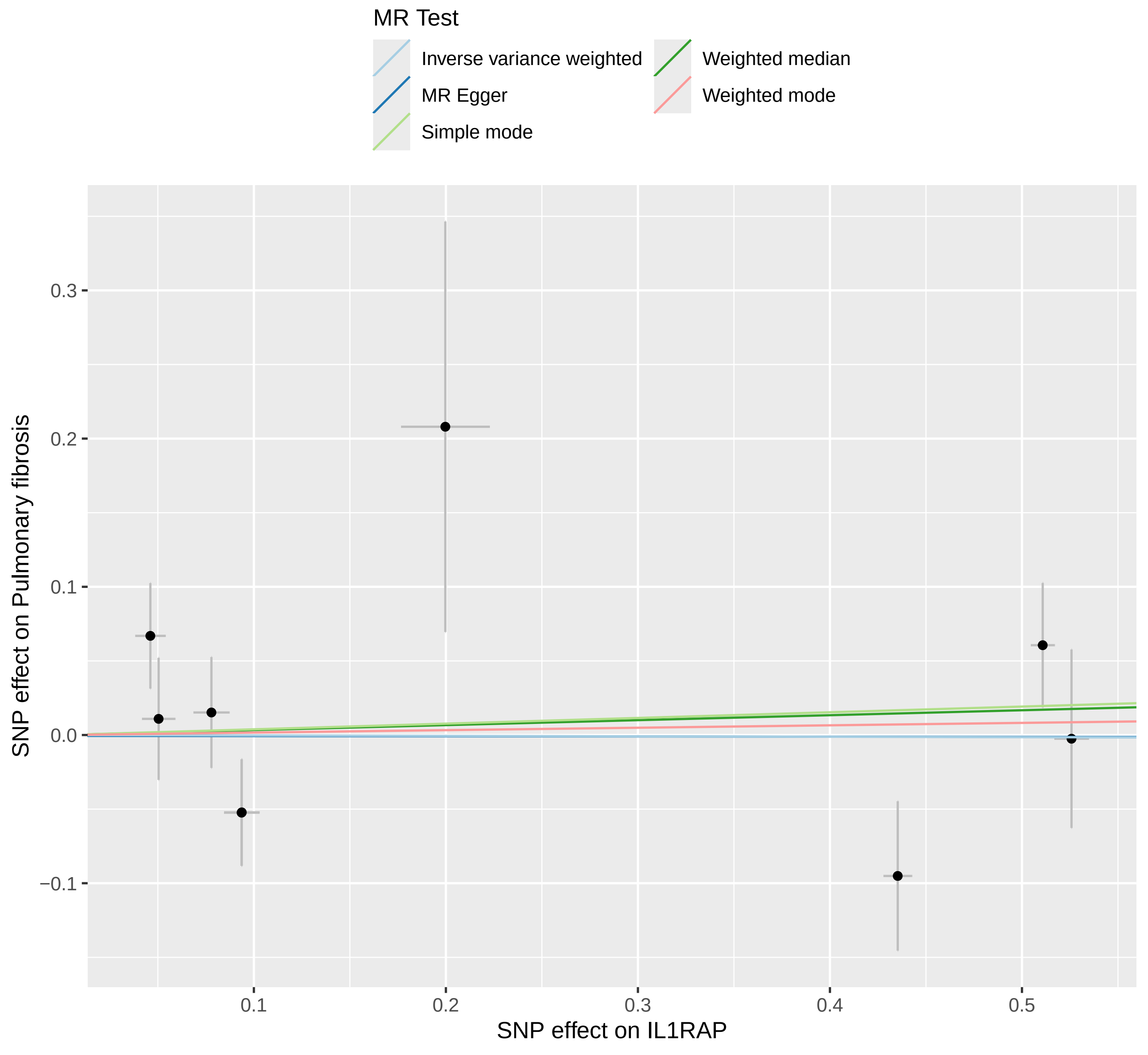

MR Test
Inverse variance weighted
MR Egger
Simple mode
Weighted median
Weighted mode
0.3
0.2
0.1
0.0
−0.1
0.1
0.2
0.3
0.4
0.5
SNP effect on Pulmonary fibrosis
SNP effect on IL1RAP

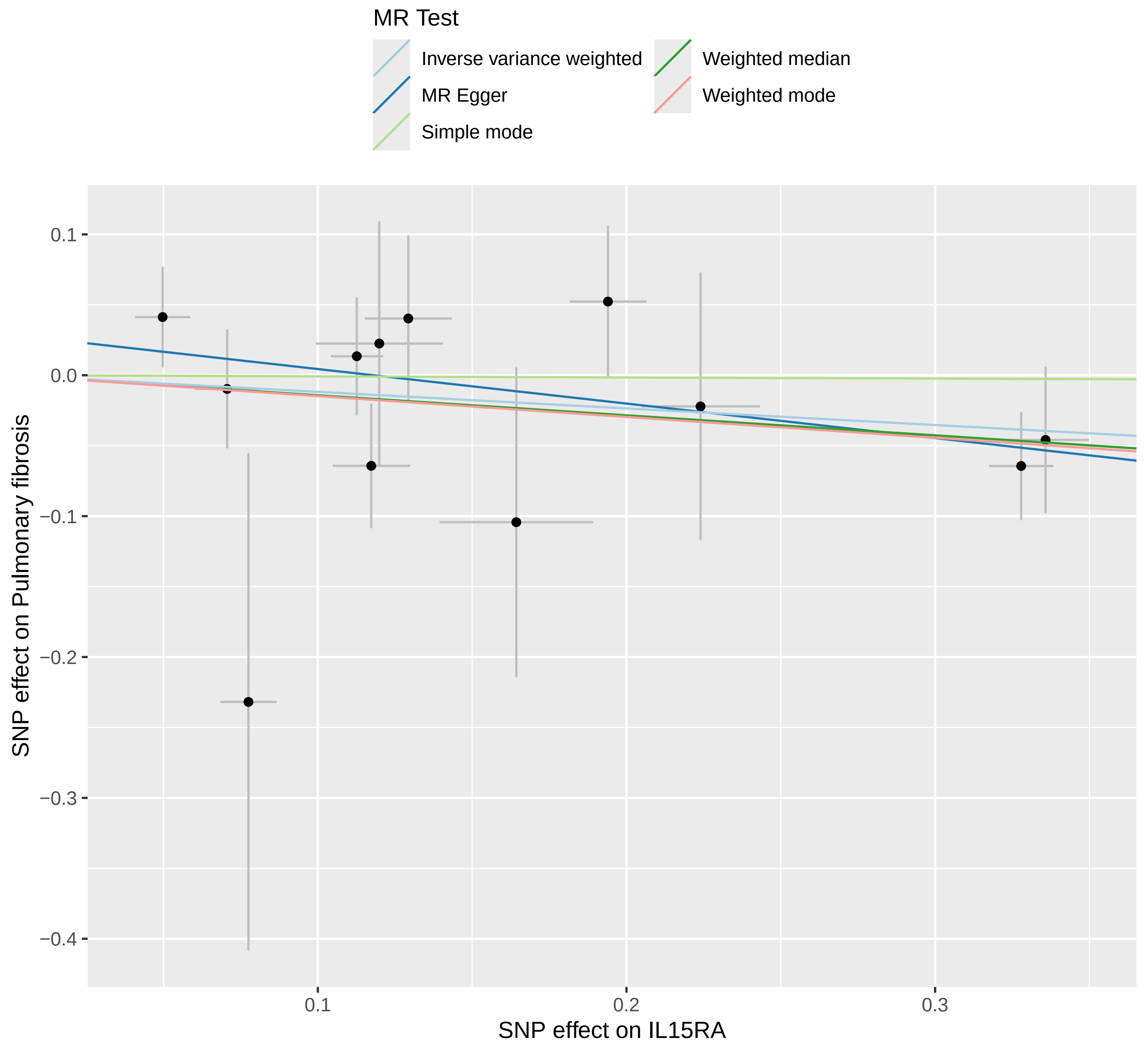

MR Test
Inverse variance weighted
MR Egger
Simple mode
Weighted median
Weighted mode
0.1
0.0
−0.1
−0.2
−0.3
−0.4
SNP effect on Pulmonary fibrosis
0.1
0.2
0.3
SNP effect on IL15RA

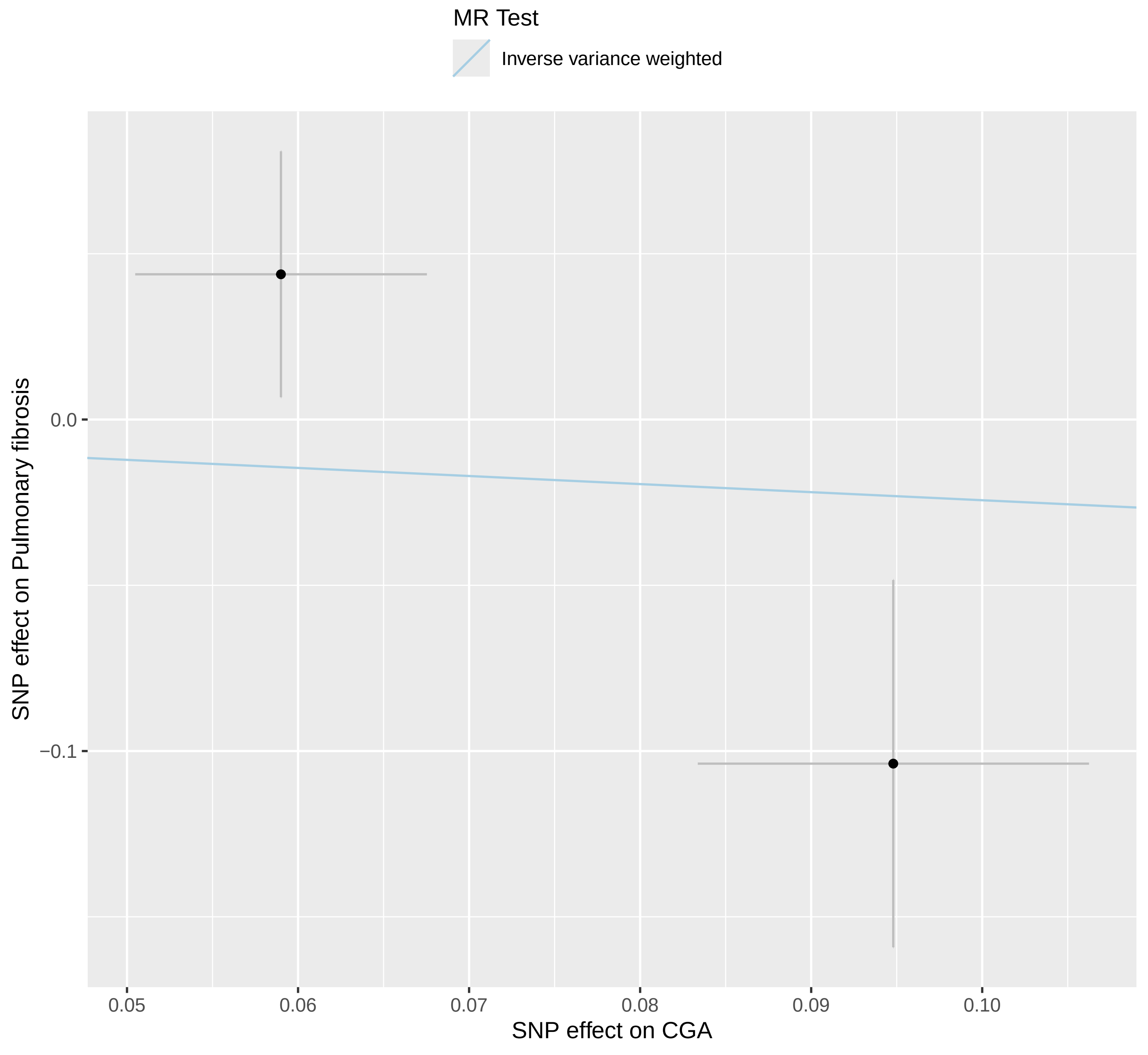
MR Test
Inverse variance weighted
SNP effect on Pulmonary fibrosis
0.0
−0.1
0.05
0.06
0.07
0.08
0.09
0.10
SNP effect on CGA

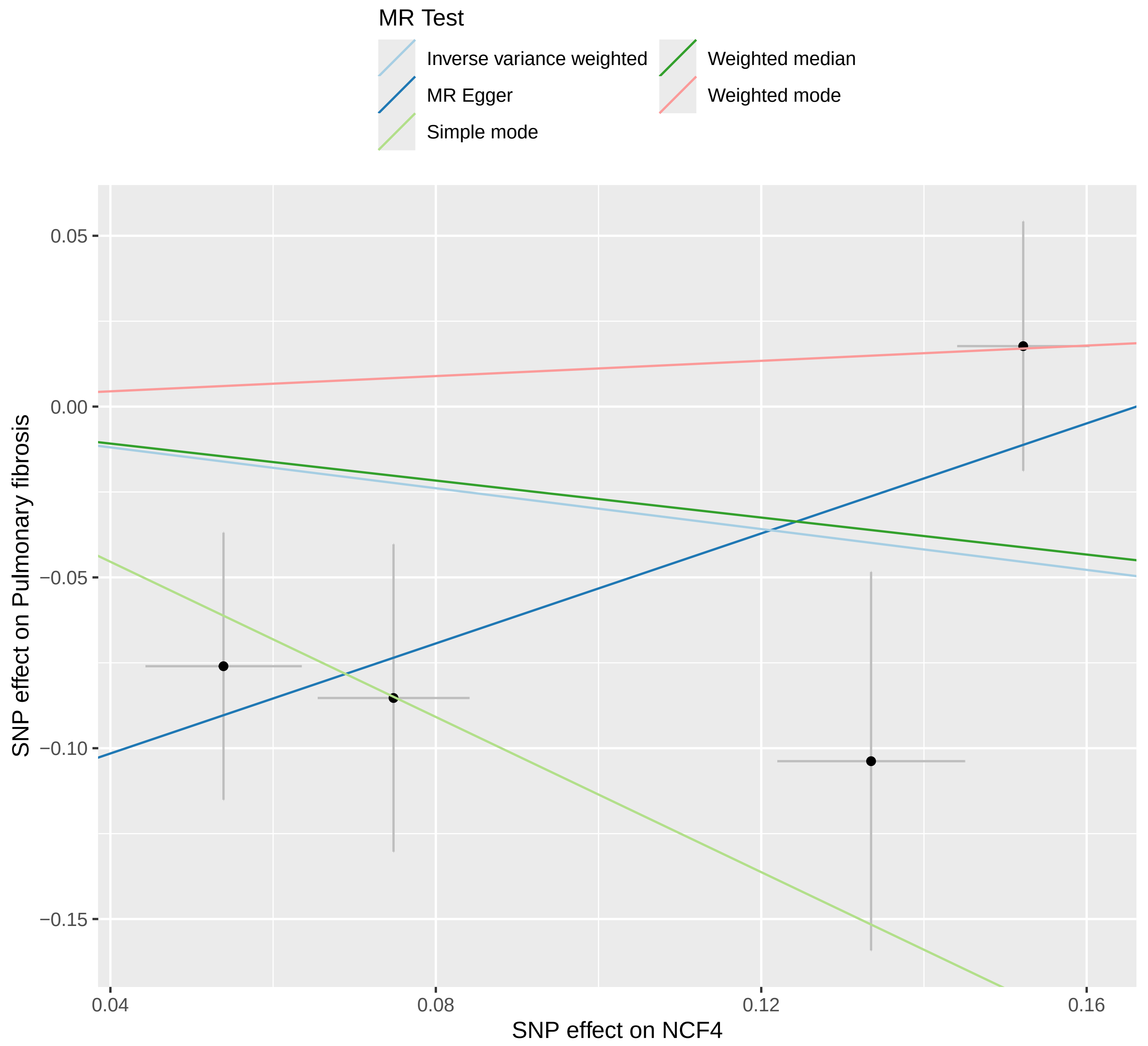
MR Test
Inverse variance weighted
MR Egger
Simple mode
Weighted median
Weighted mode
0.05
0.00
−0.05
−0.10
−0.15
0.04
0.08
0.12
0.16
SNP effect on Pulmonary fibrosis
SNP effect on NCF4

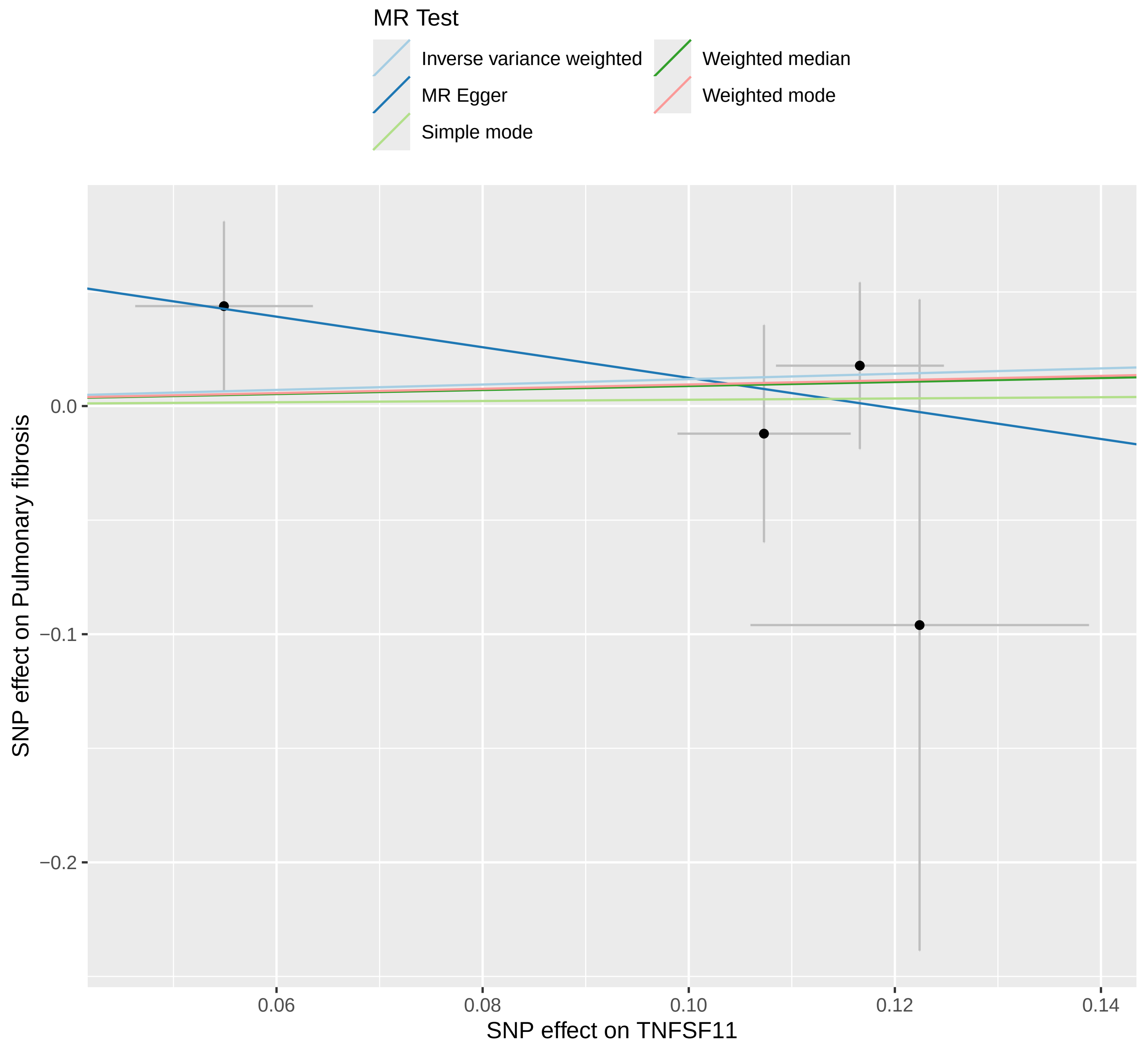

MR Test
Inverse variance weighted
MR Egger
Simple mode
Weighted median
Weighted mode
0.0
−0.1
−0.2
SNP effect on Pulmonary fibrosis
0.06
0.08
0.10
0.12
0.14
SNP effect on TNFSF11

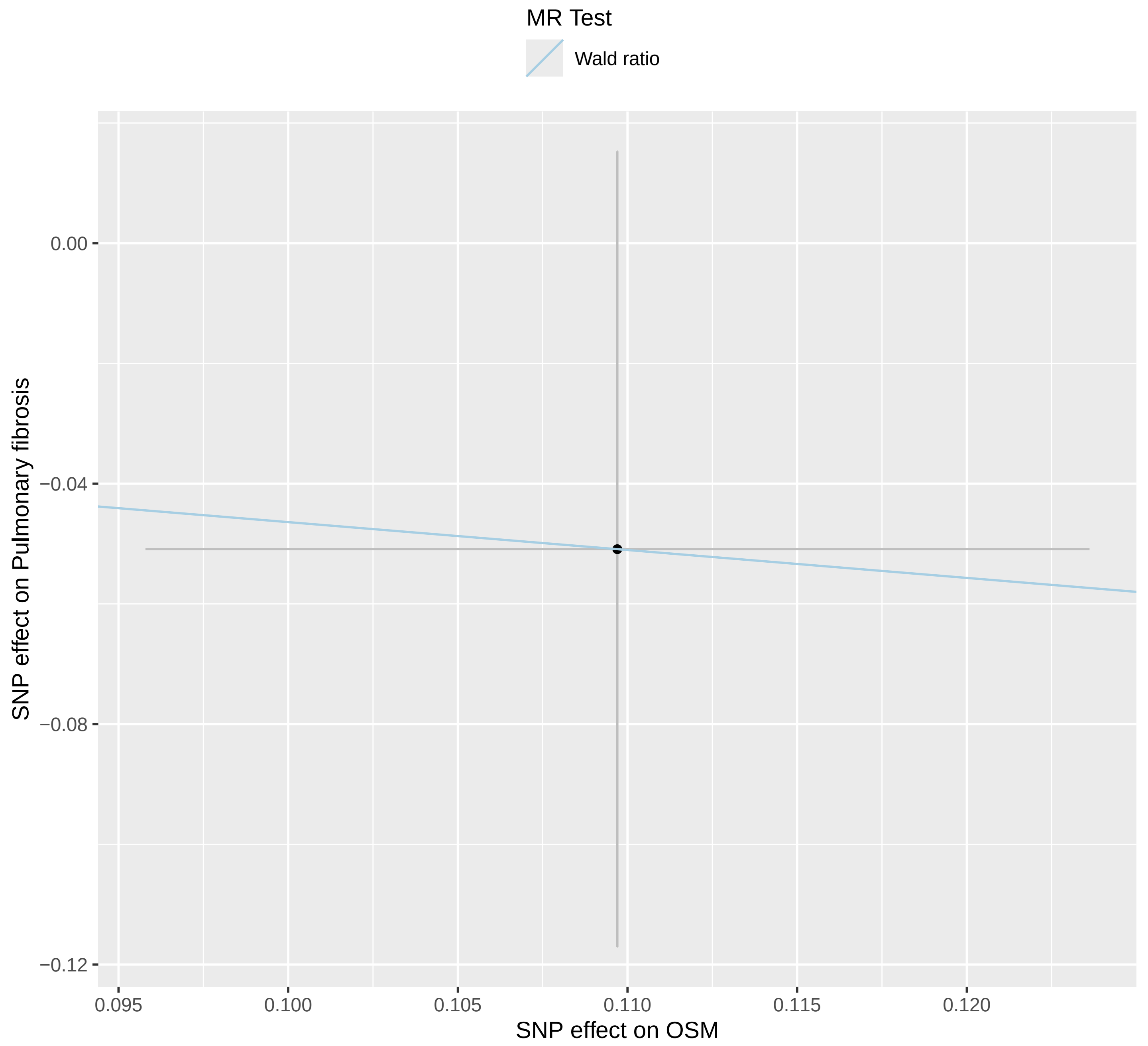

MR Test
Wald ratio
0.00
−0.04
−0.08
−0.12
SNP effect on Pulmonary fibrosis
0.095
0.100
0.105
0.110
0.115
0.120
SNP effect on OSM

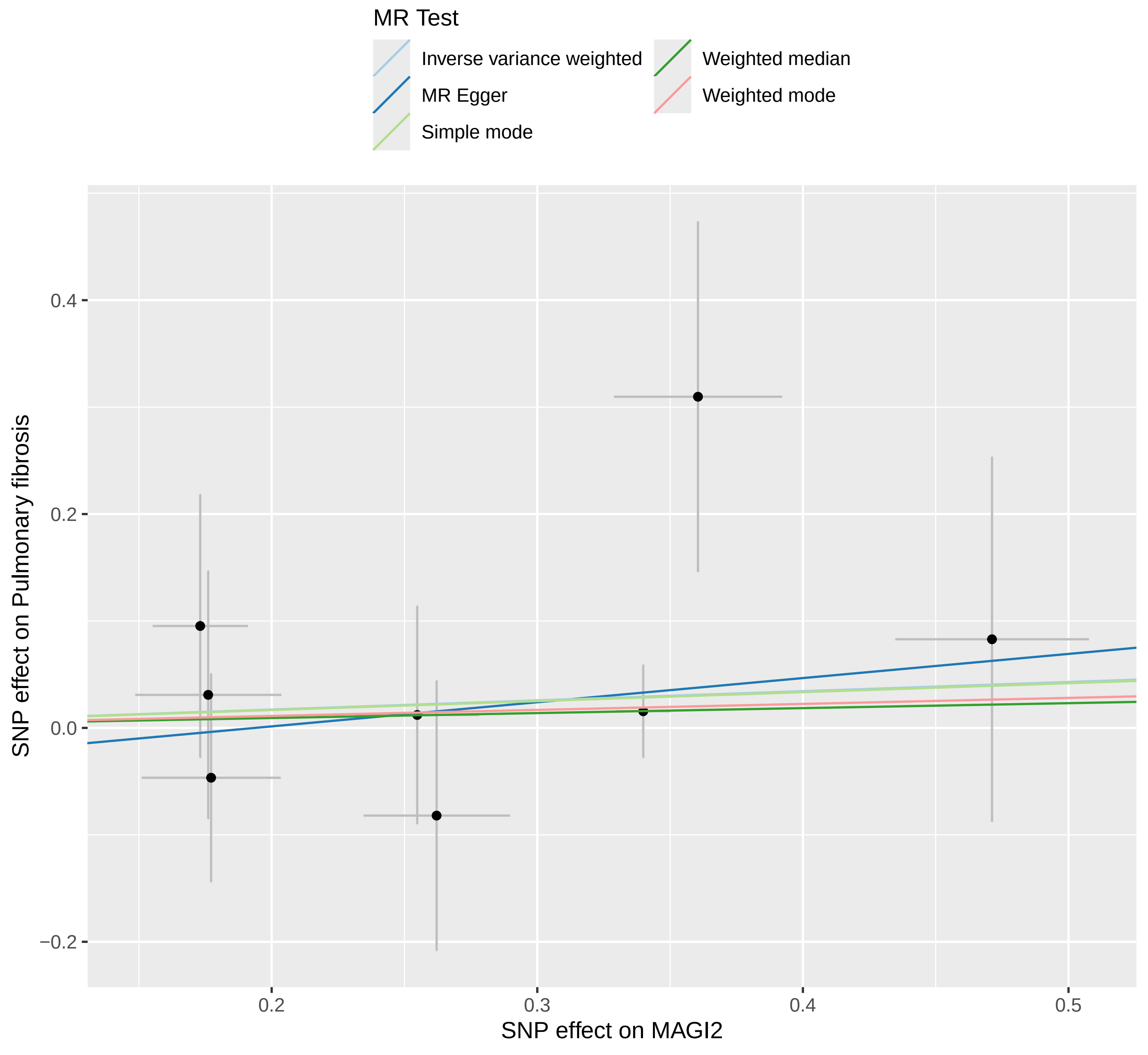
MR Test
Inverse variance weighted
MR Egger
Simple mode
Weighted median
Weighted mode
SNP effect on Pulmonary fibrosis
SNP effect on MAGI2
0.4
0.2
0.0
−0.2
0.2
0.3
0.4
0.5

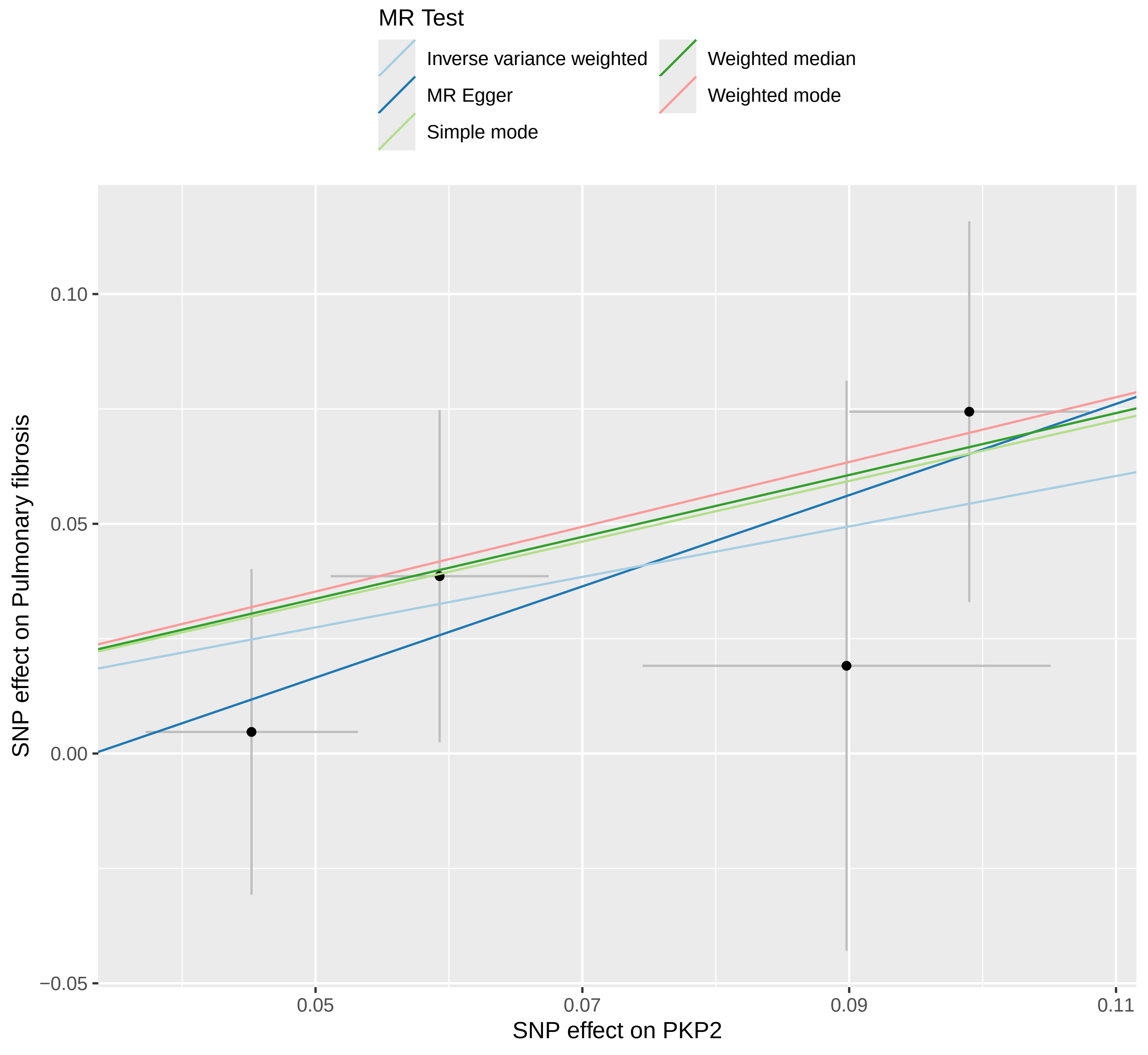
MR Test
Inverse variance weighted
MR Egger
Simple mode
Weighted median
Weighted mode
0.10
0.05
0.00
−0.05
0.05
0.07
0.09
0.11
SNP effect on Pulmonary fibrosis
SNP effect on PKP2

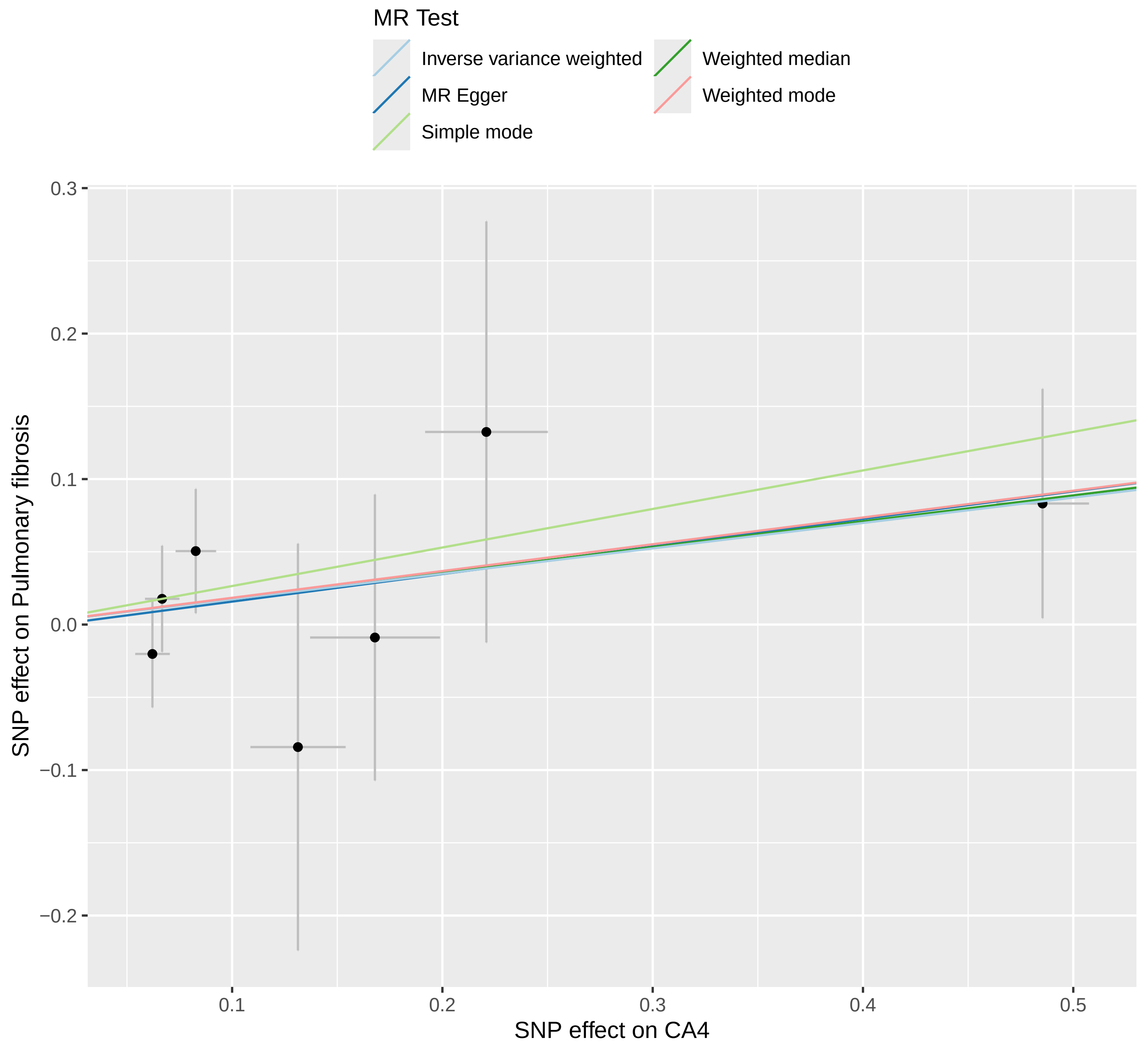
MR Test
Inverse variance weighted
MR Egger
Simple mode
Weighted median
Weighted mode
0.3
0.2
0.1
0.0
−0.1
−0.2
SNP effect on Pulmonary fibrosis
0.1
0.2
0.3
0.4
0.5
SNP effect on CA4

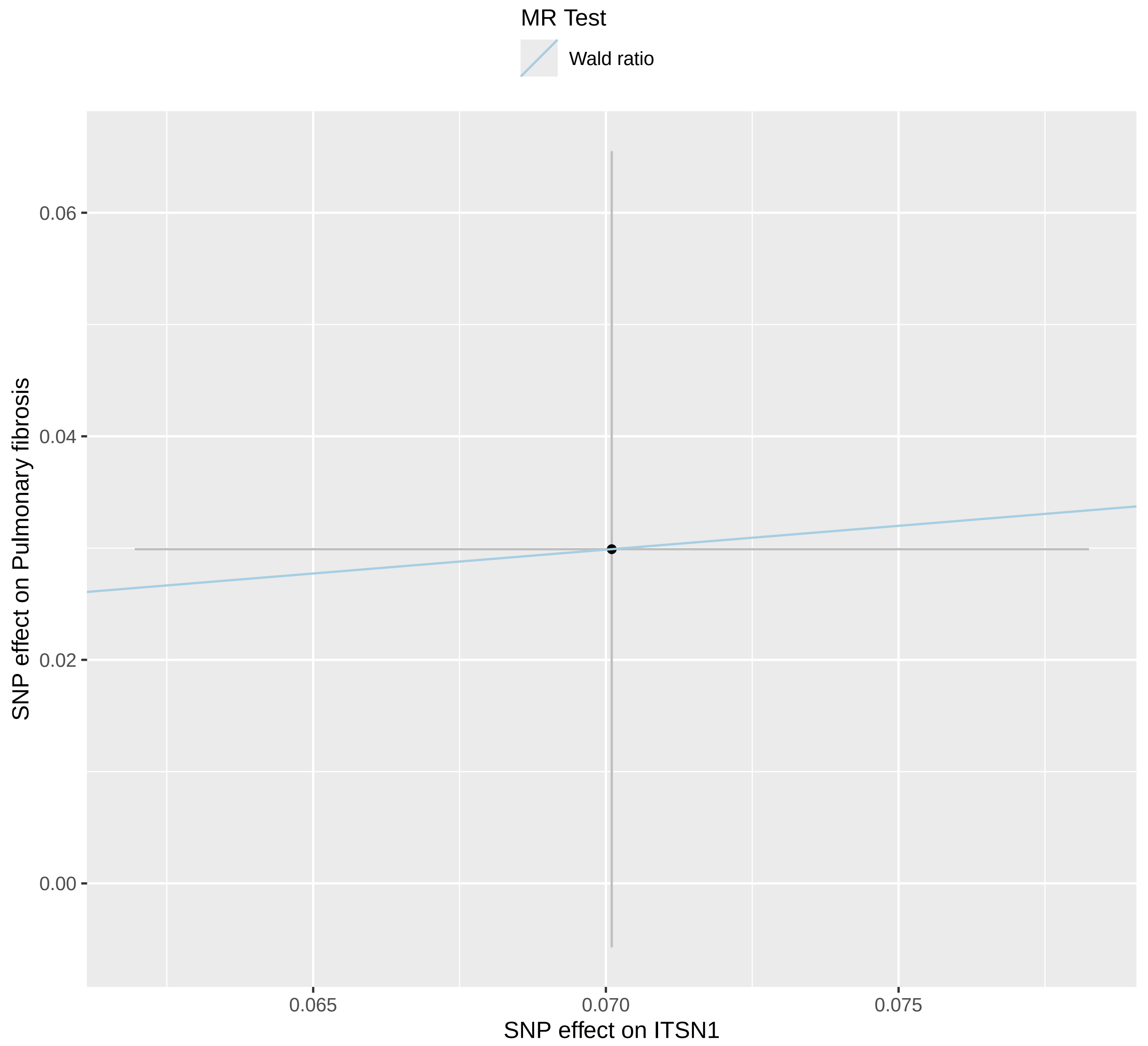
MR Test
Wald ratio
SNP effect on Pulmonary fibrosis
0.06
0.04
0.02
0.00
0.065
0.070
0.075
SNP effect on ITSN1

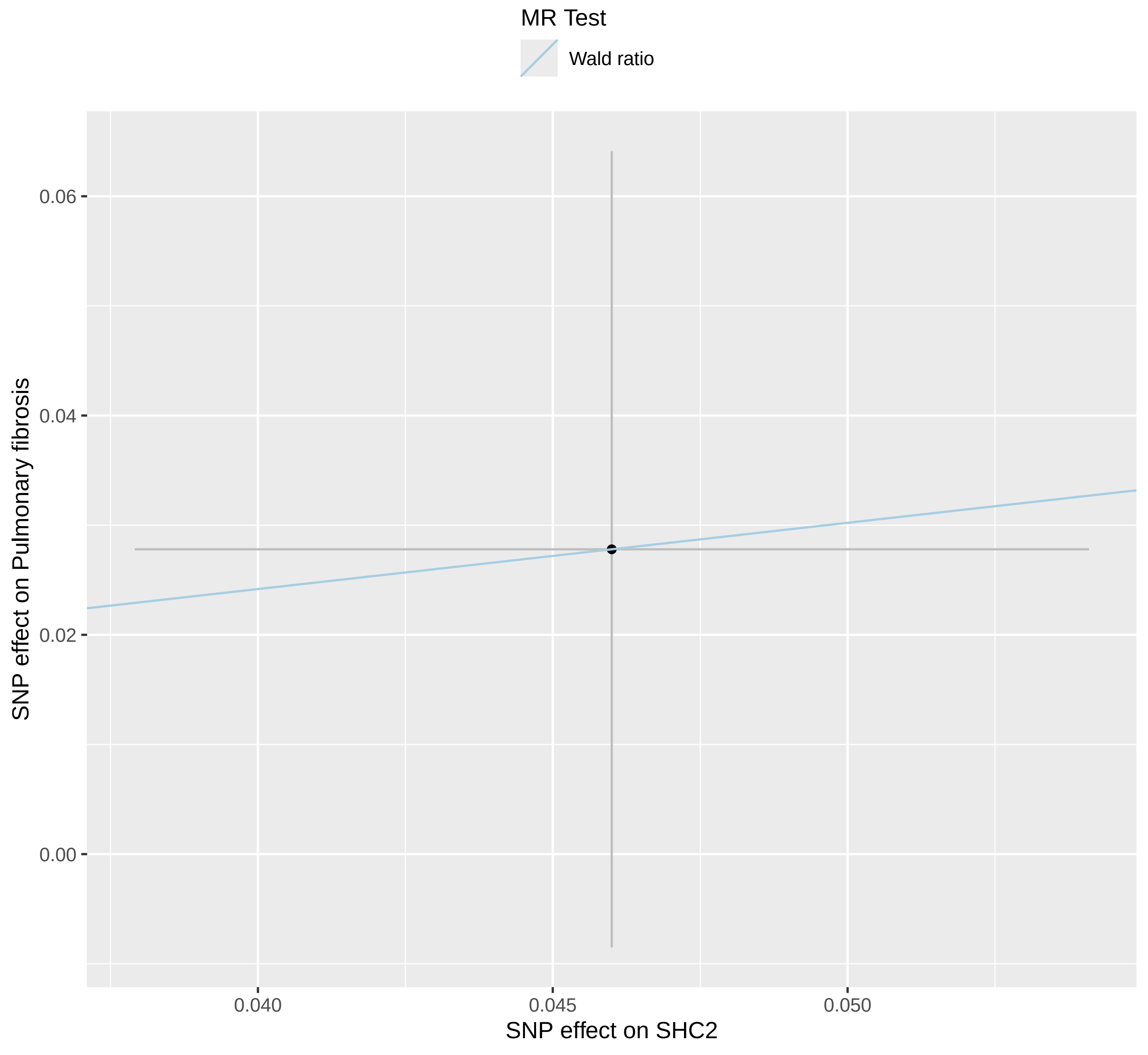
MR Test
Wald ratio
0.06
0.04
0.02
0.00
0.040
0.045
0.050
SNP effect on SHC2
SNP effect on Pulmonary fibrosis

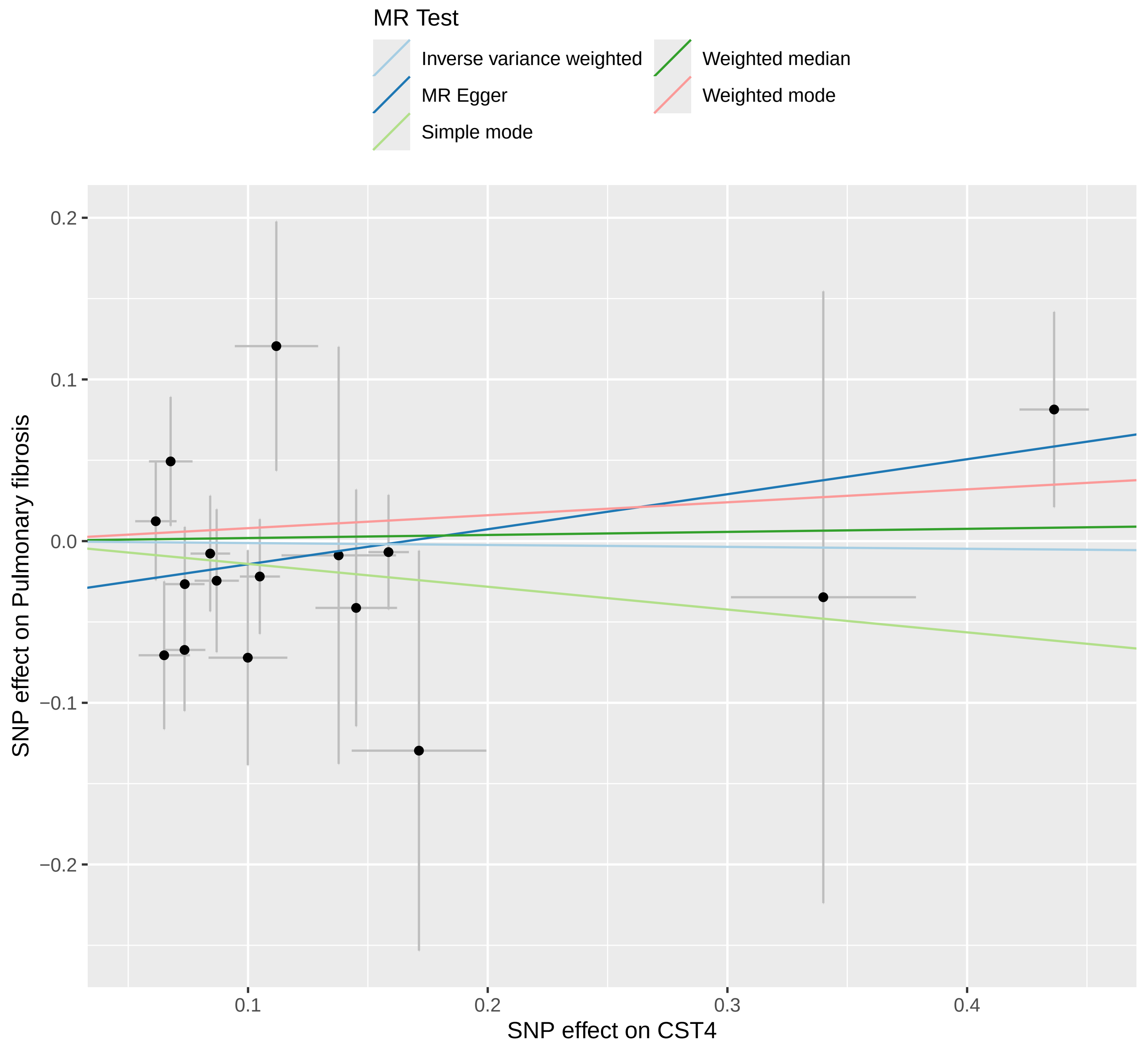
MR Test
Inverse variance weighted
MR Egger
Simple mode
Weighted median
Weighted mode
SNP effect on Pulmonary fibrosis
0.2
0.1
0.0
−0.1
−0.2
0.1
0.2
0.3
0.4
SNP effect on CST4

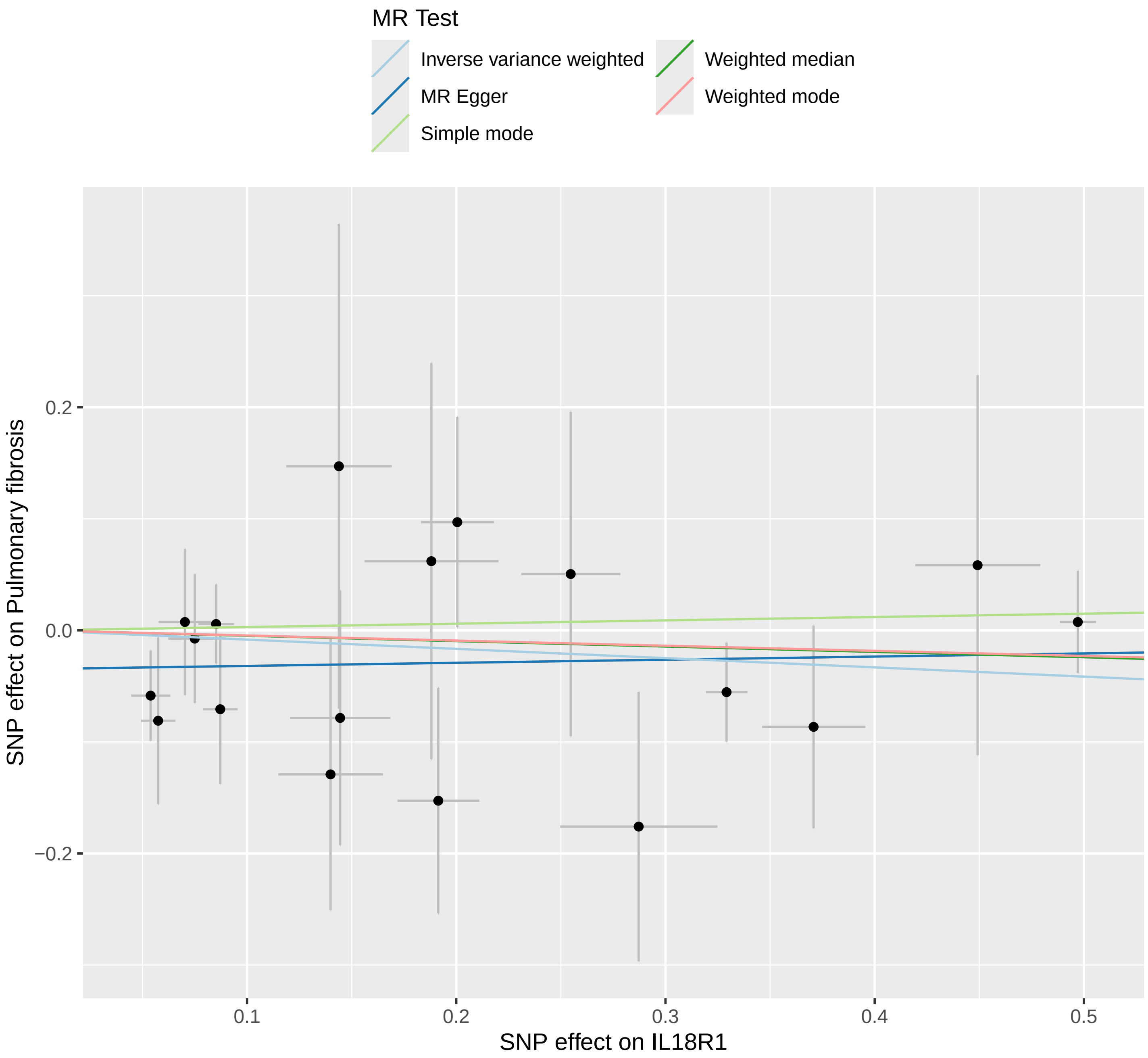
MR Test
Inverse variance weighted
MR Egger
Simple mode
Weighted median
Weighted mode
0.2
0.0
−0.2
0.1
0.2
0.3
0.4
0.5
SNP effect on Pulmonary fibrosis
SNP effect on IL18R1

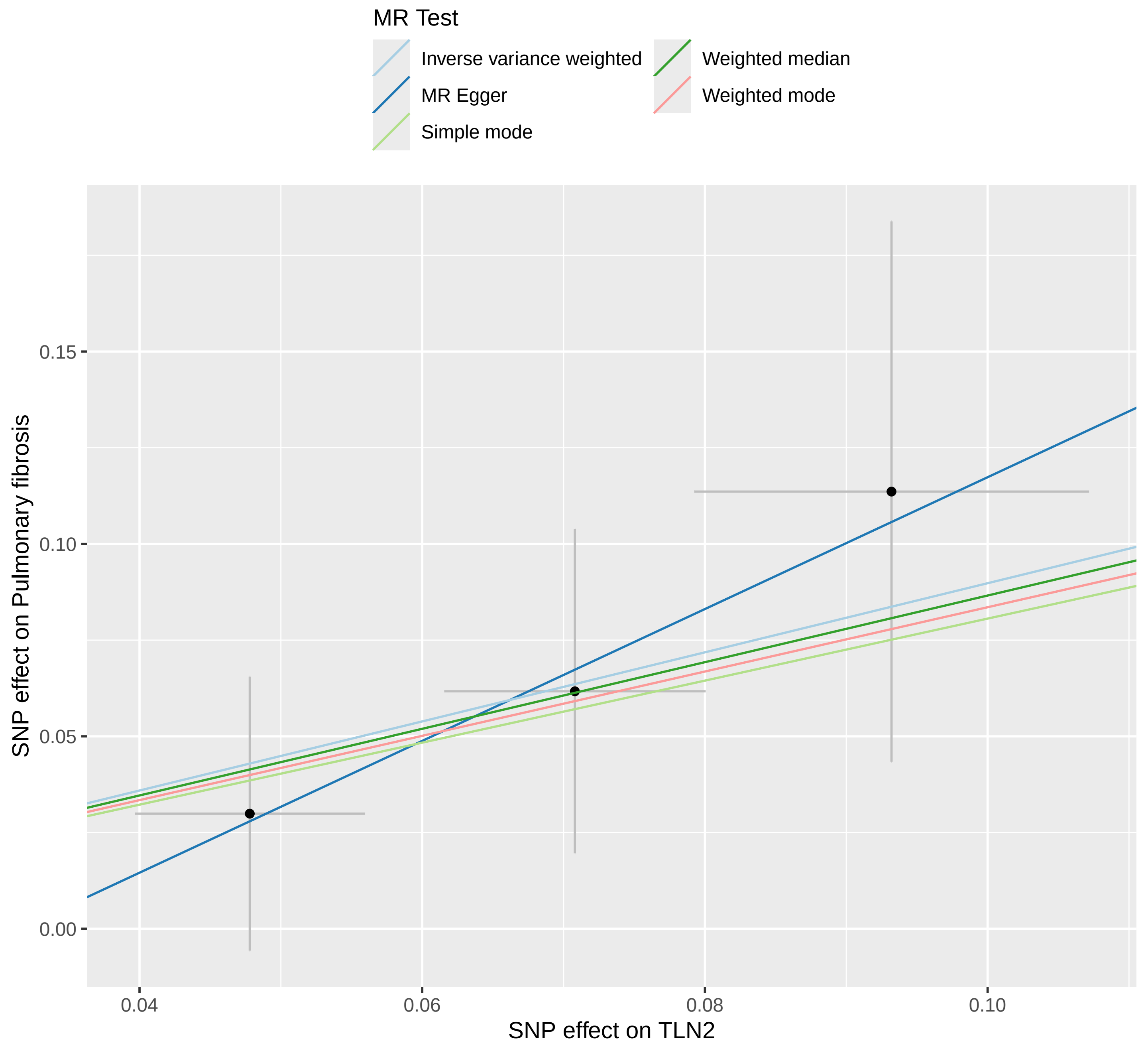

MR Test
Inverse variance weighted
MR Egger
Simple mode
Weighted median
Weighted mode
SNP effect on Pulmonary fibrosis
0.15
0.10
0.05
0.00
0.04
0.06
0.08
0.10
SNP effect on TLN2

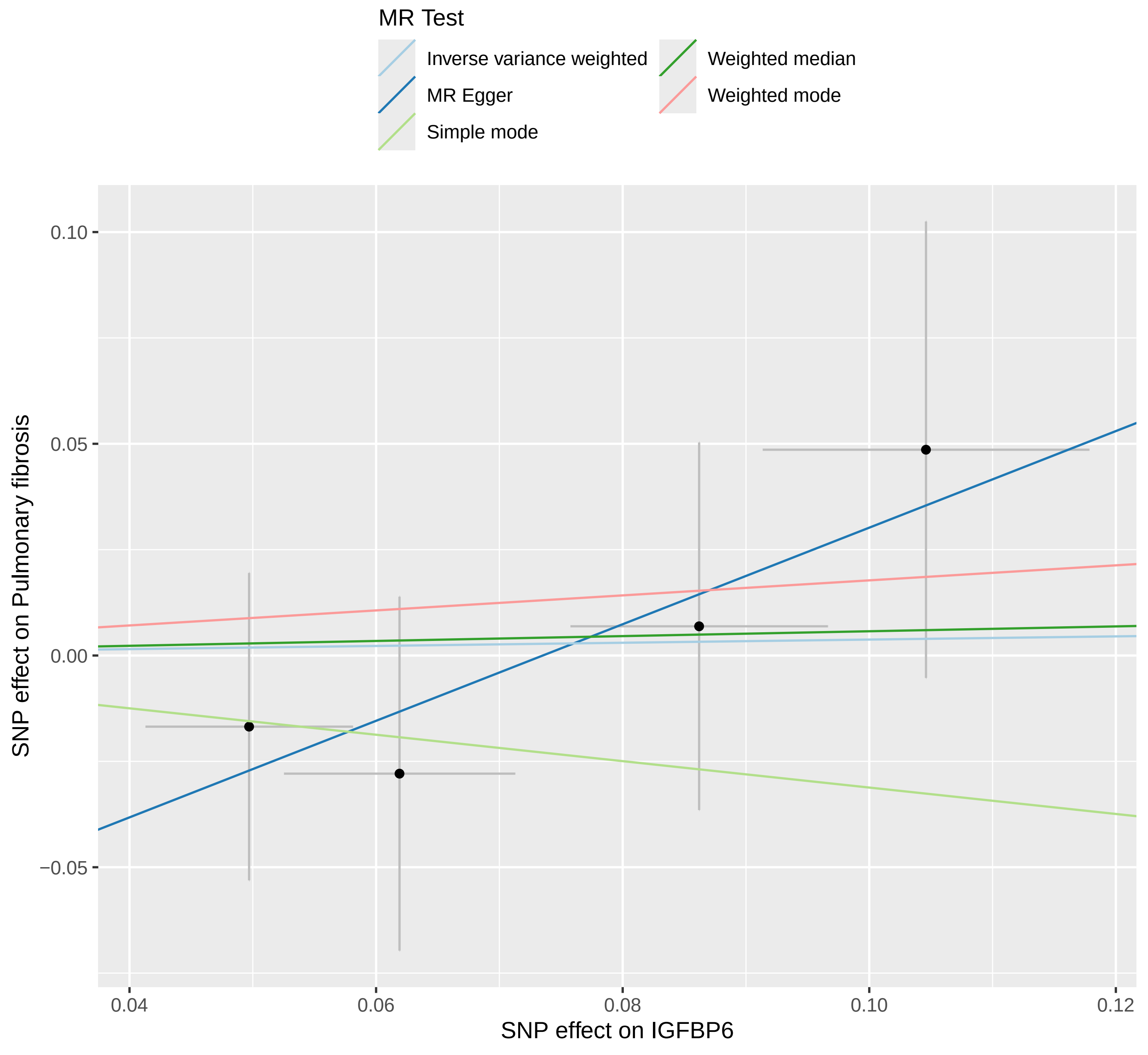
MR Test
Inverse variance weighted
MR Egger
Simple mode
Weighted median
Weighted mode
0.10
0.05
0.00
−0.05
SNP effect on Pulmonary fibrosis
0.04
0.06
0.08
0.10
0.12
SNP effect on IGFBP6

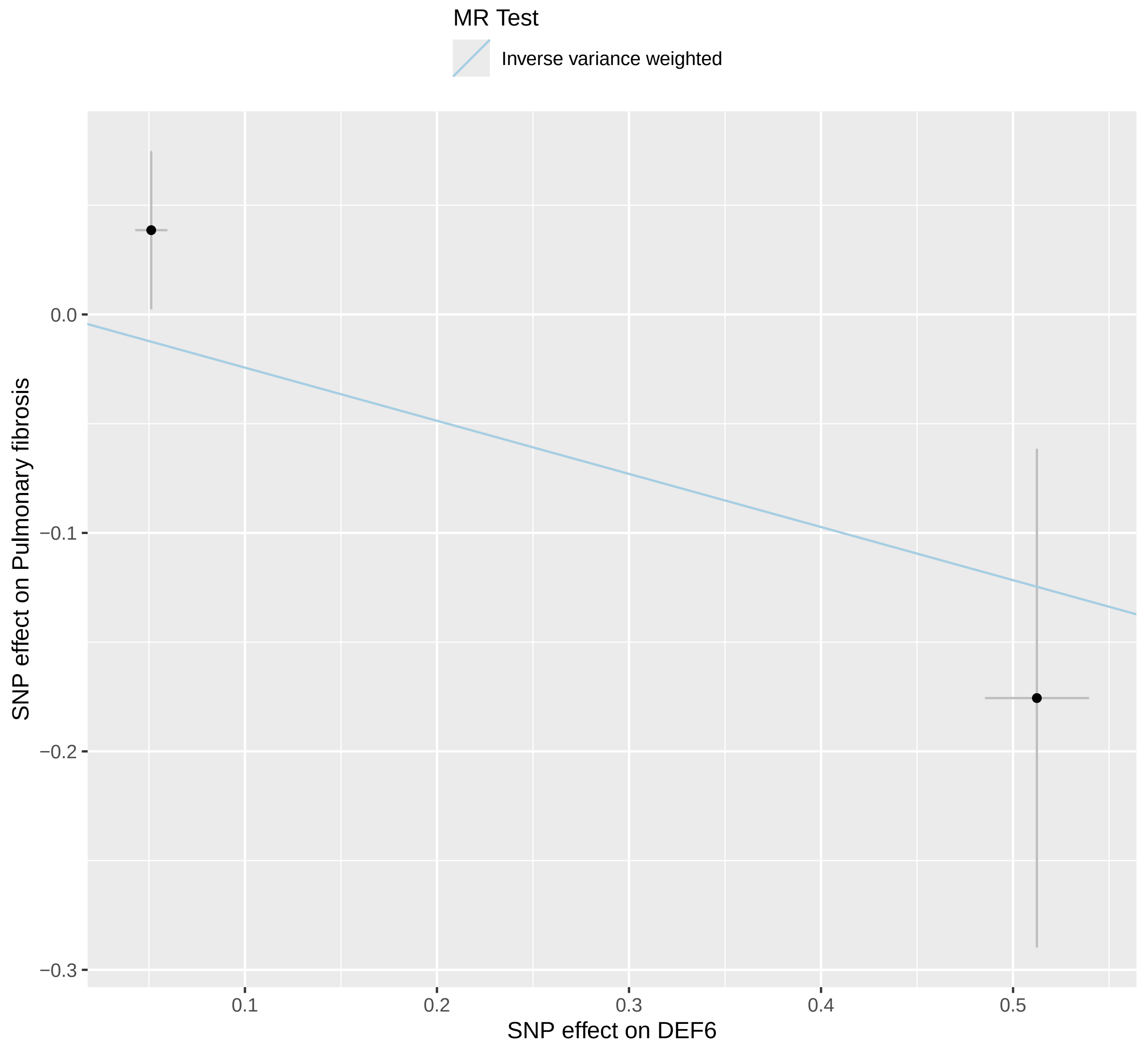
MR Test
Inverse variance weighted
0.0
−0.1
−0.2
−0.3
SNP effect on Pulmonary fibrosis
0.1
0.2
0.3
0.4
0.5
SNP effect on DEF6

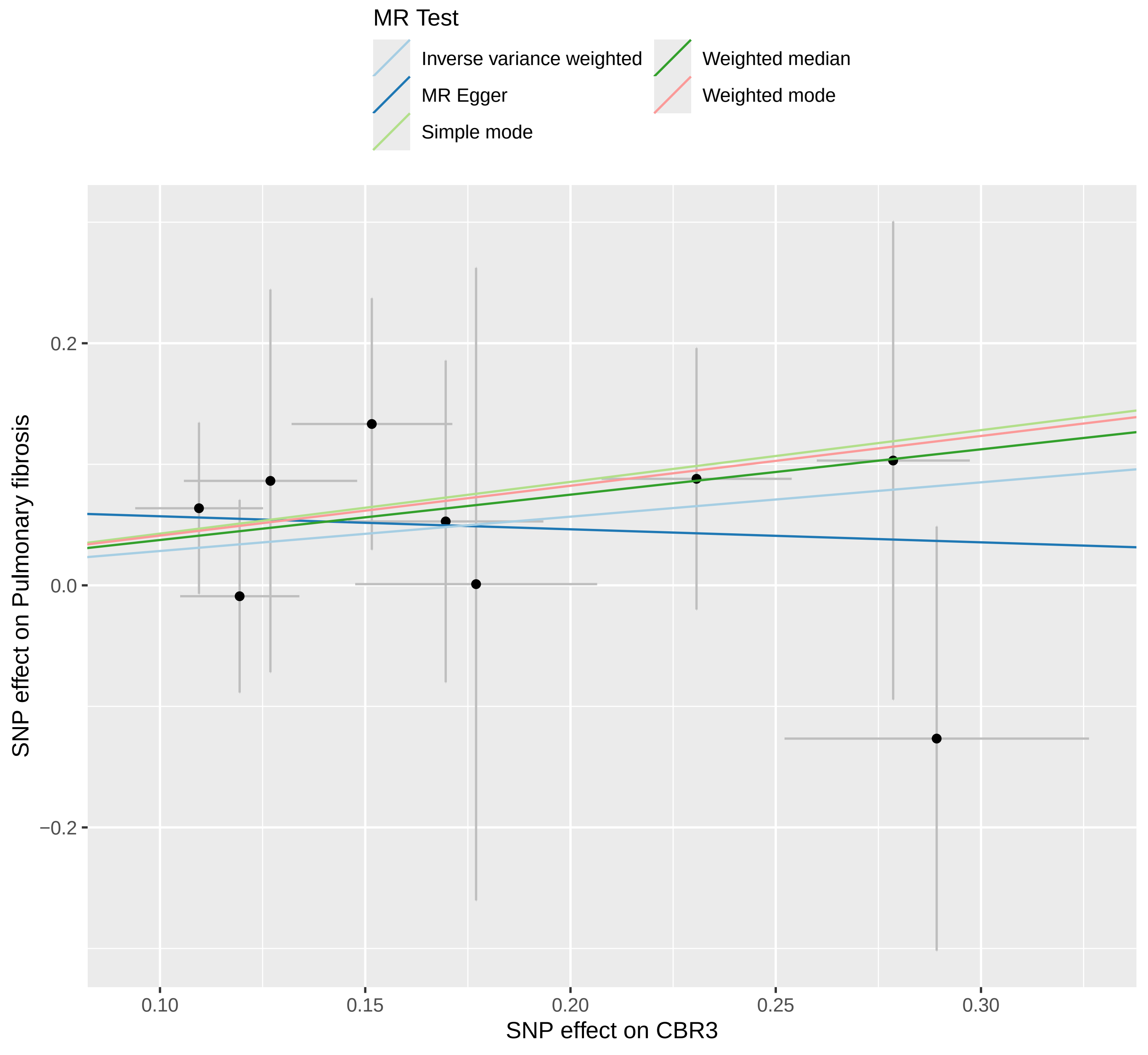

MR Test
Inverse variance weighted
MR Egger
Simple mode
Weighted median
Weighted mode
0.2
0.0
−0.2
0.10
0.15
0.20
0.25
0.30
SNP effect on CBR3
SNP effect on Pulmonary fibrosis

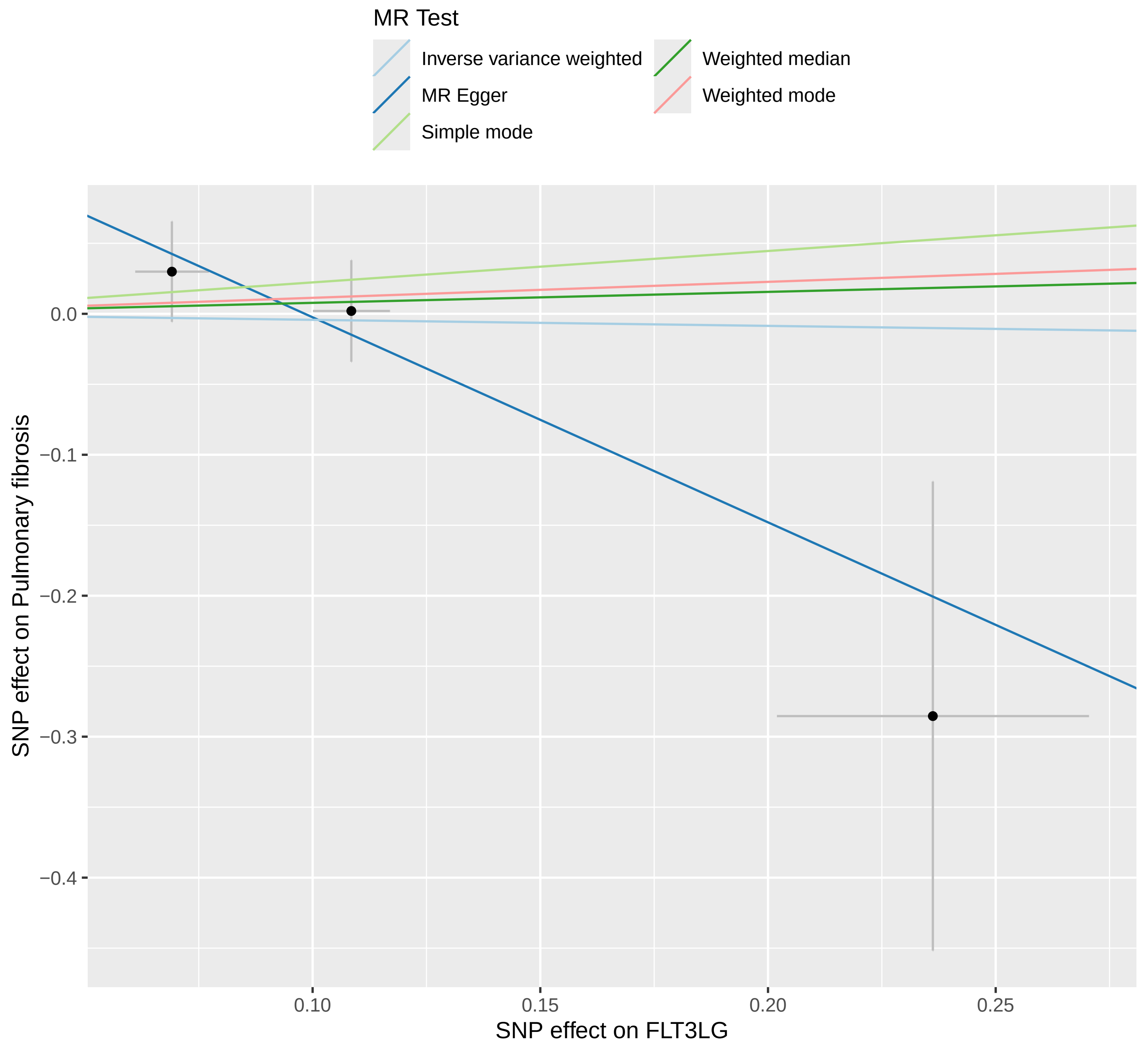

MR Test
Inverse variance weighted
MR Egger
Simple mode
Weighted median
Weighted mode
0.0
−0.1
−0.2
−0.3
−0.4
SNP effect on Pulmonary fibrosis
0.10
0.15
0.20
0.25
SNP effect on FLT3LG

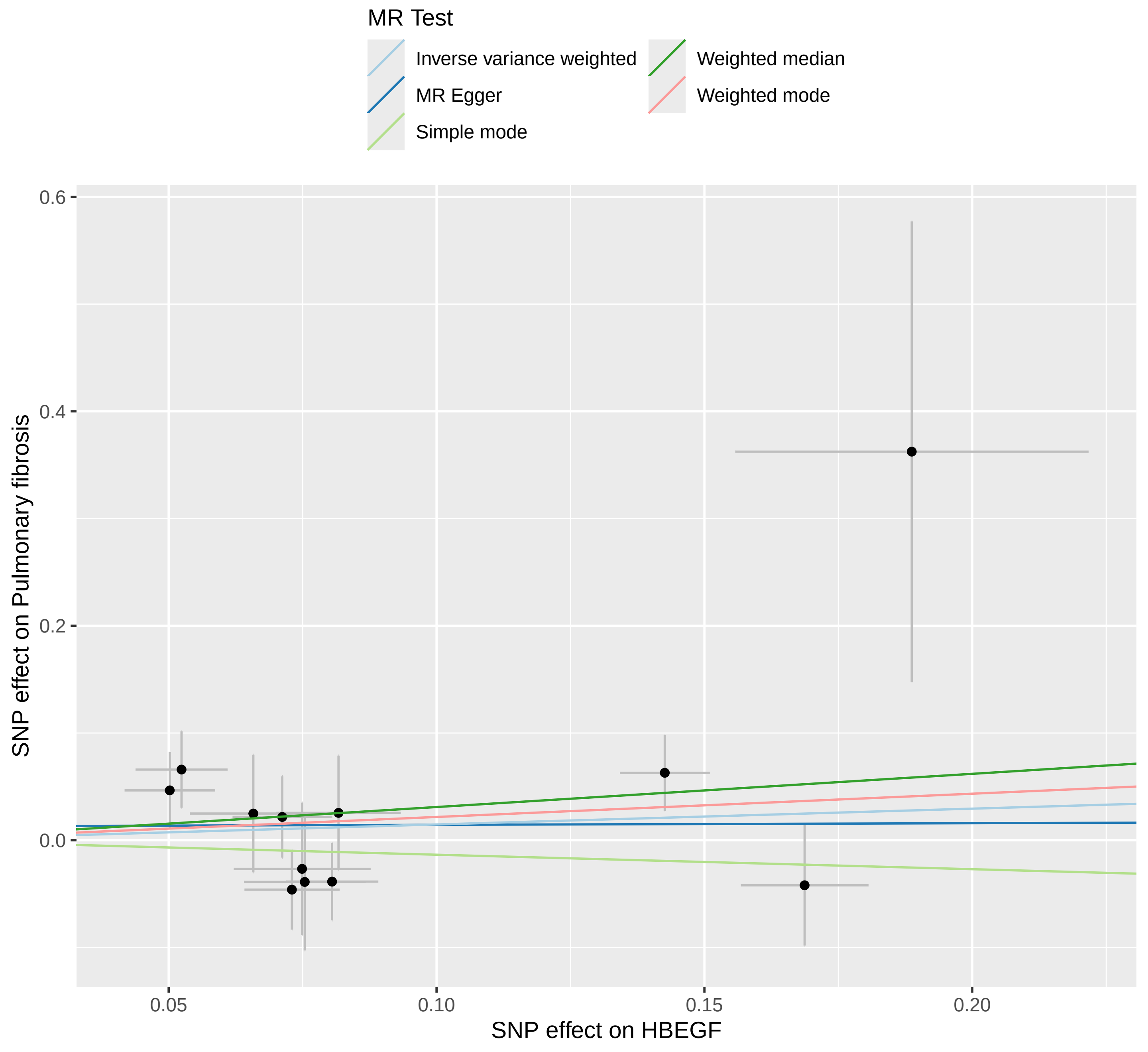
MR Test
Inverse variance weighted
MR Egger
Simple mode
Weighted median
Weighted mode
0.6
0.4
0.2
0.0
0.05
0.10
0.15
0.20
SNP effect on Pulmonary fibrosis
SNP effect on HBEGF

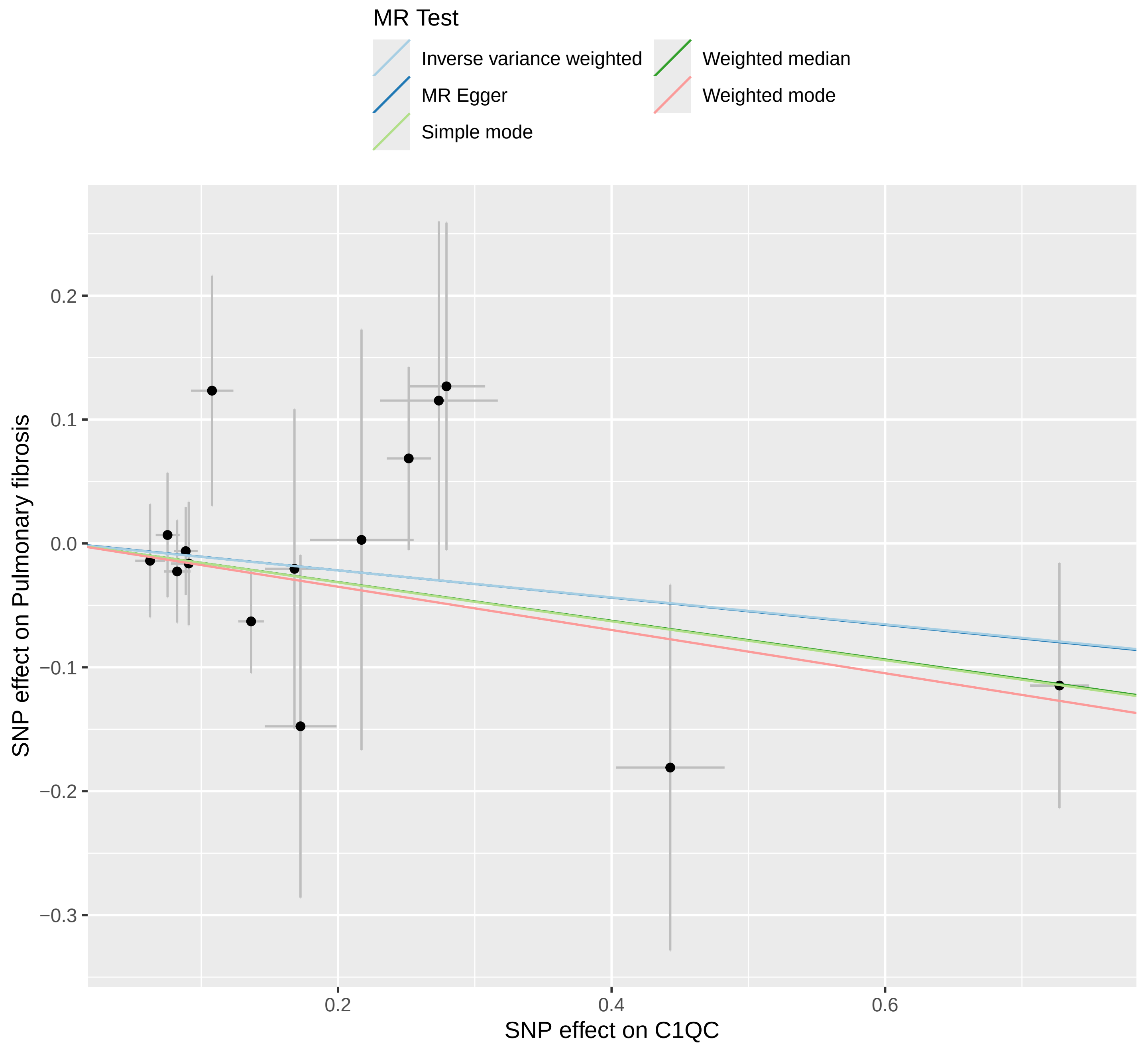
MR Test
Inverse variance weighted
MR Egger
Simple mode
Weighted median
Weighted mode
SNP effect on Pulmonary fibrosis
0.2
0.1
0.0
−0.1
−0.2
−0.3
0.2
0.4
0.6
SNP effect on C1QC

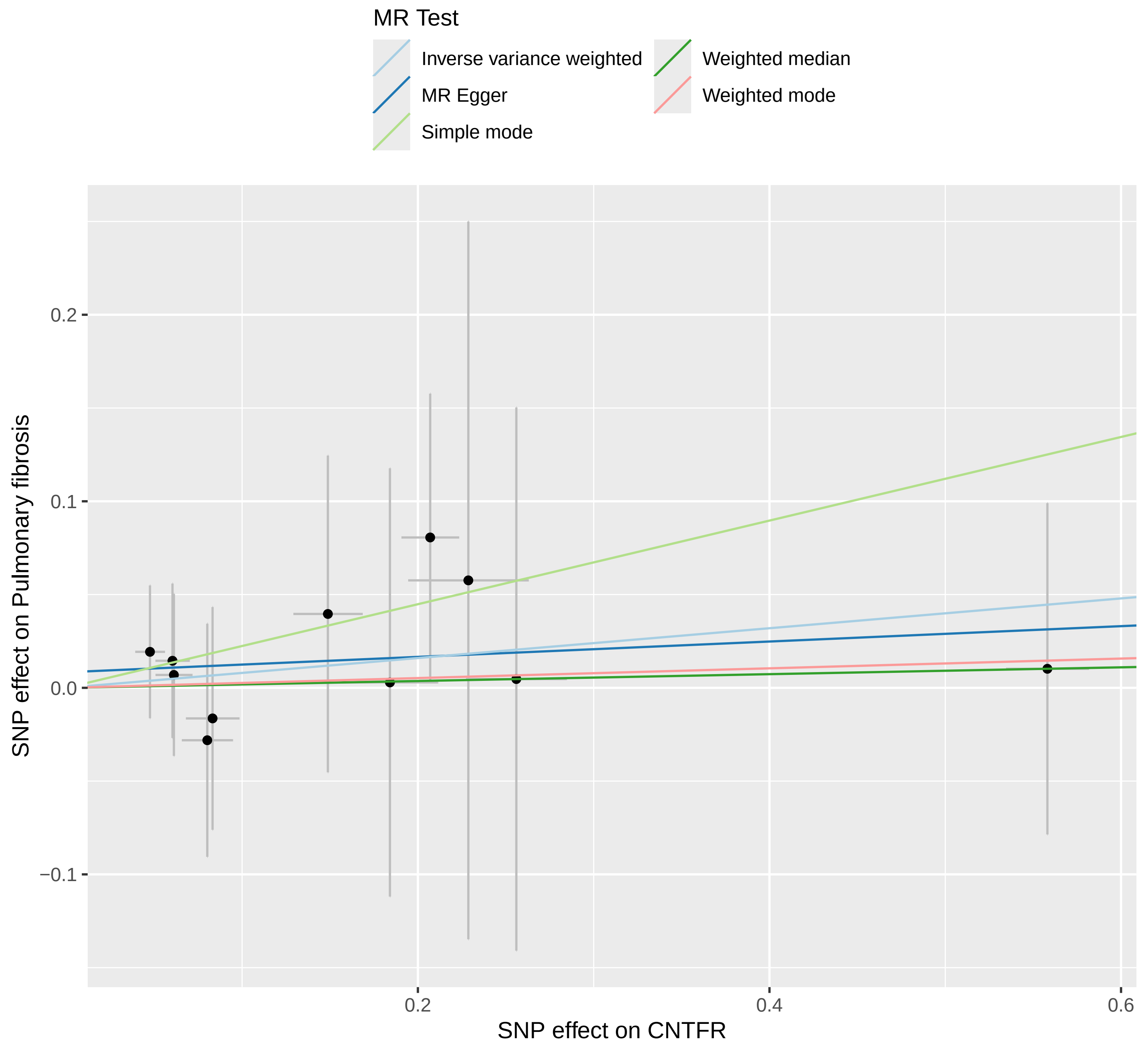

MR Test
Inverse variance weighted
MR Egger
Simple mode
Weighted median
Weighted mode
0.2
0.1
0.0
−0.1
SNP effect on Pulmonary fibrosis
0.2
0.4
0.6
SNP effect on CNTFR

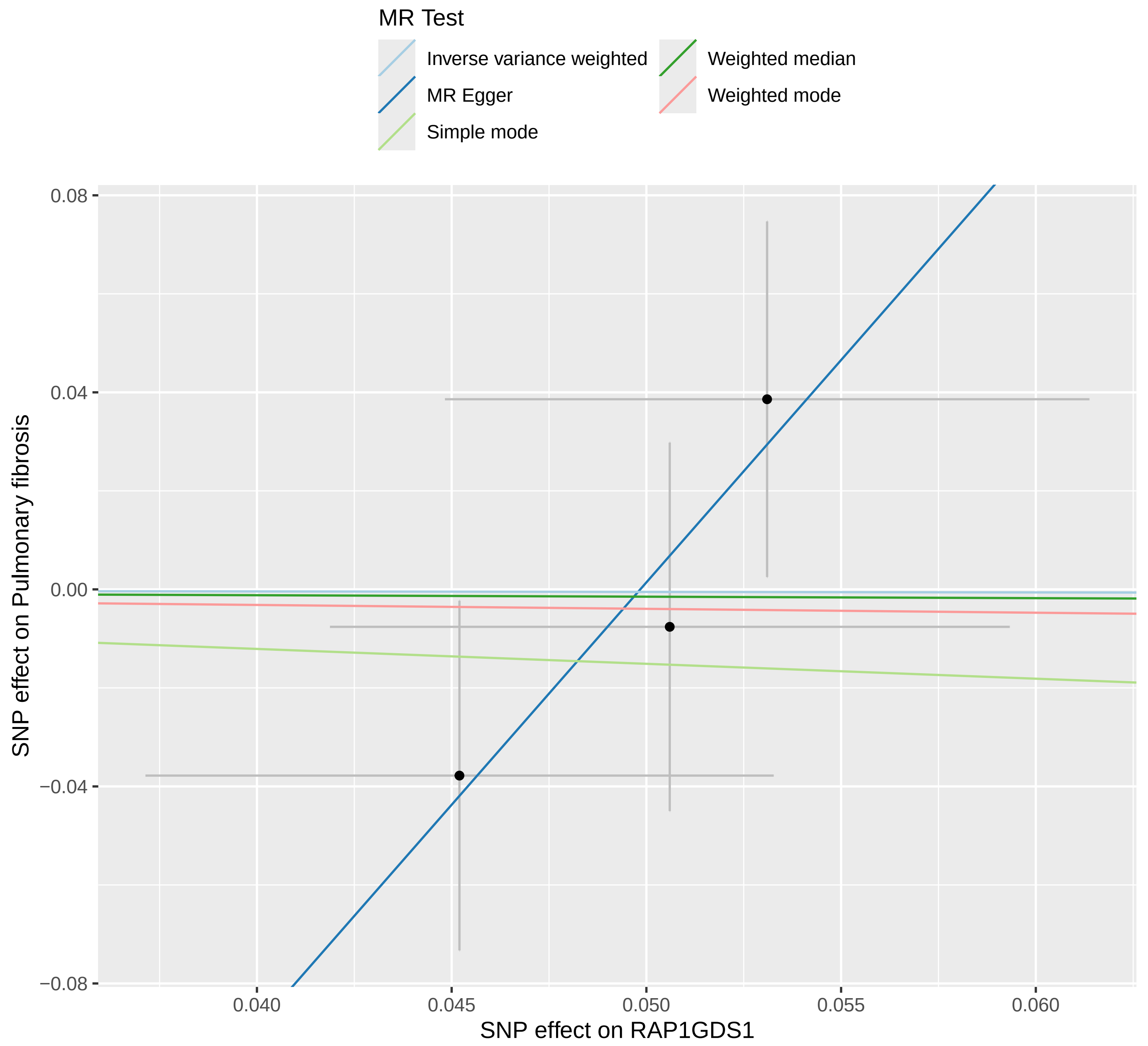
MR Test
Inverse variance weighted
MR Egger
Simple mode
Weighted median
Weighted mode
0.08
0.04
0.00
−0.04
−0.08
SNP effect on Pulmonary fibrosis
0.040
0.045
0.050
0.055
0.060
SNP effect on RAP1GDS1

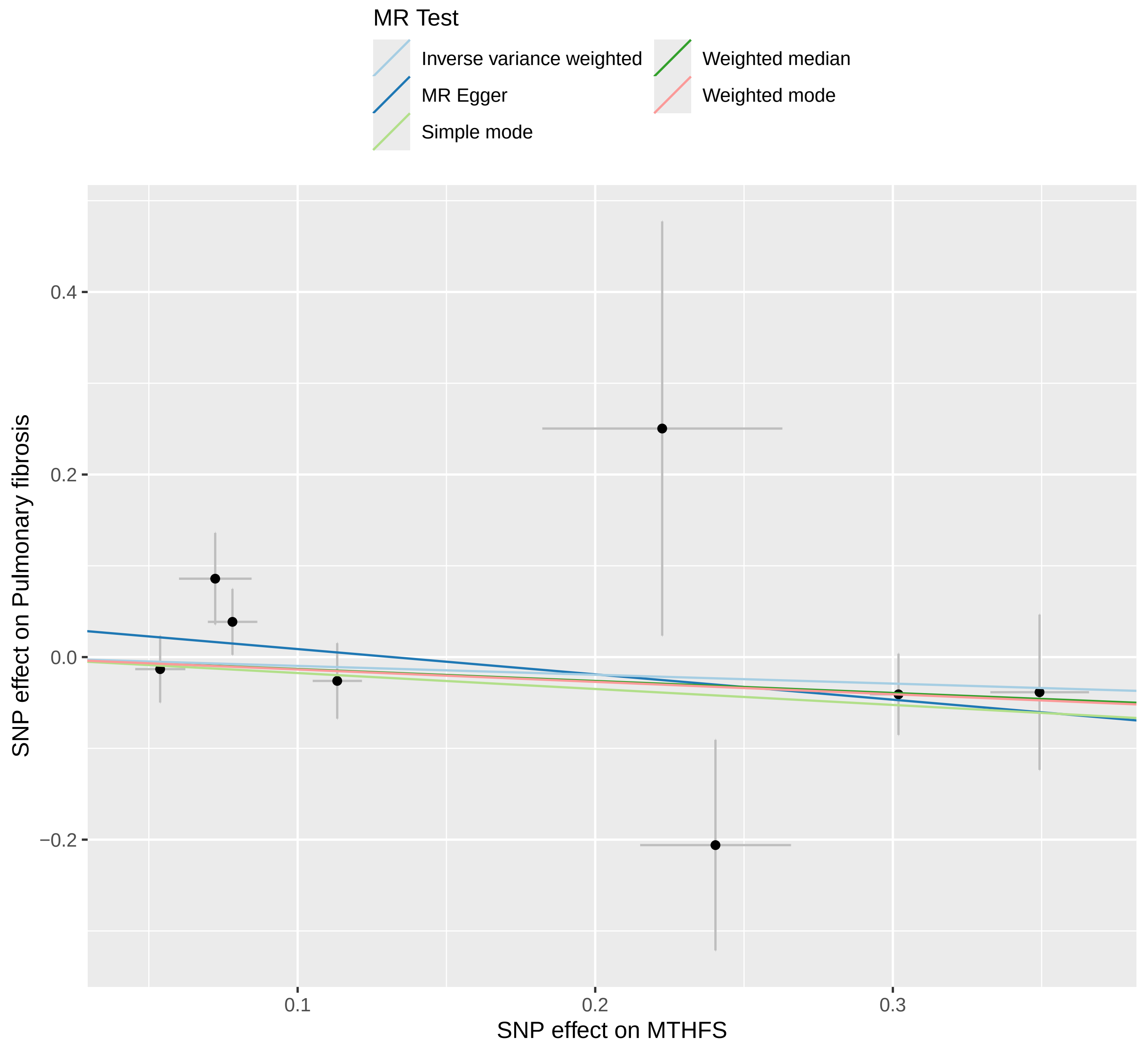

MR Test
Inverse variance weighted
MR Egger
Simple mode
Weighted median
Weighted mode
SNP effect on Pulmonary fibrosis
0.4
0.2
0.0
−0.2
0.1
0.2
0.3
SNP effect on MTHFS

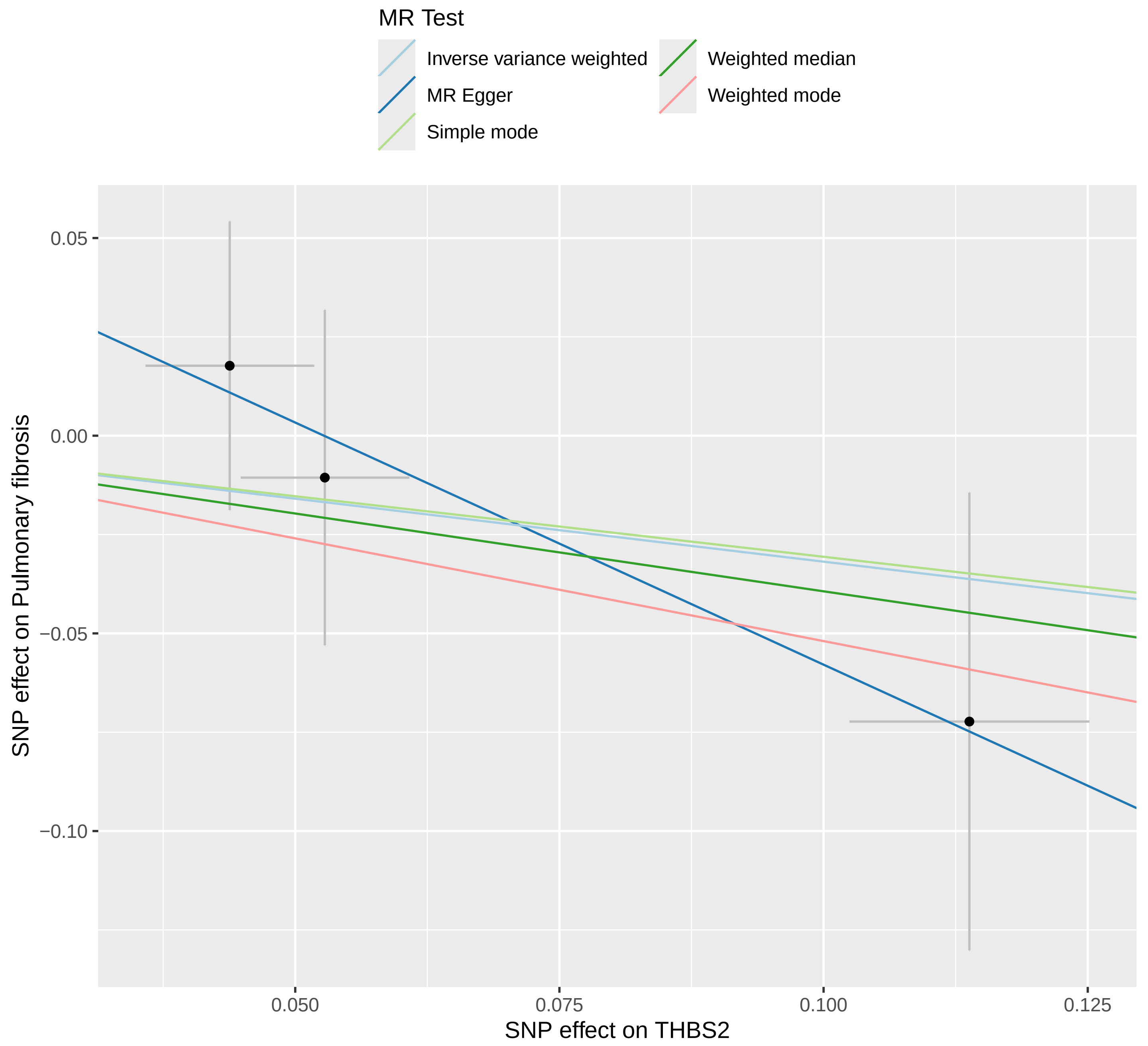
MR Test
Inverse variance weighted
MR Egger
Simple mode
Weighted median
Weighted mode
0.05
0.00
−0.05
−0.10
0.050
0.075
0.100
0.125
SNP effect on Pulmonary fibrosis
SNP effect on THBS2

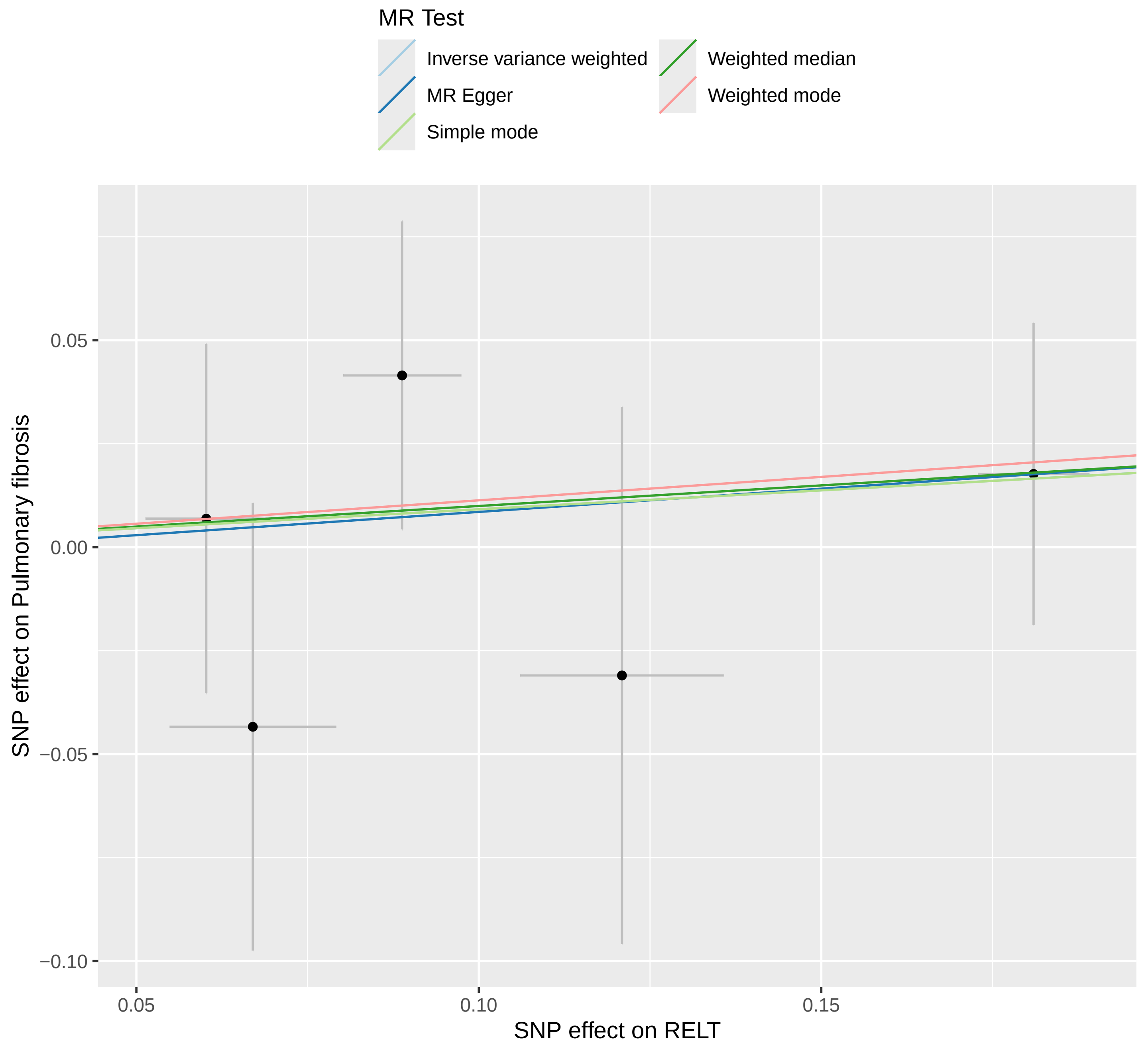

MR Test
Inverse variance weighted
MR Egger
Simple mode
Weighted median
Weighted mode
0.05
0.00
−0.05
−0.10
SNP effect on Pulmonary fibrosis
0.05
0.10
0.15
SNP effect on RELT

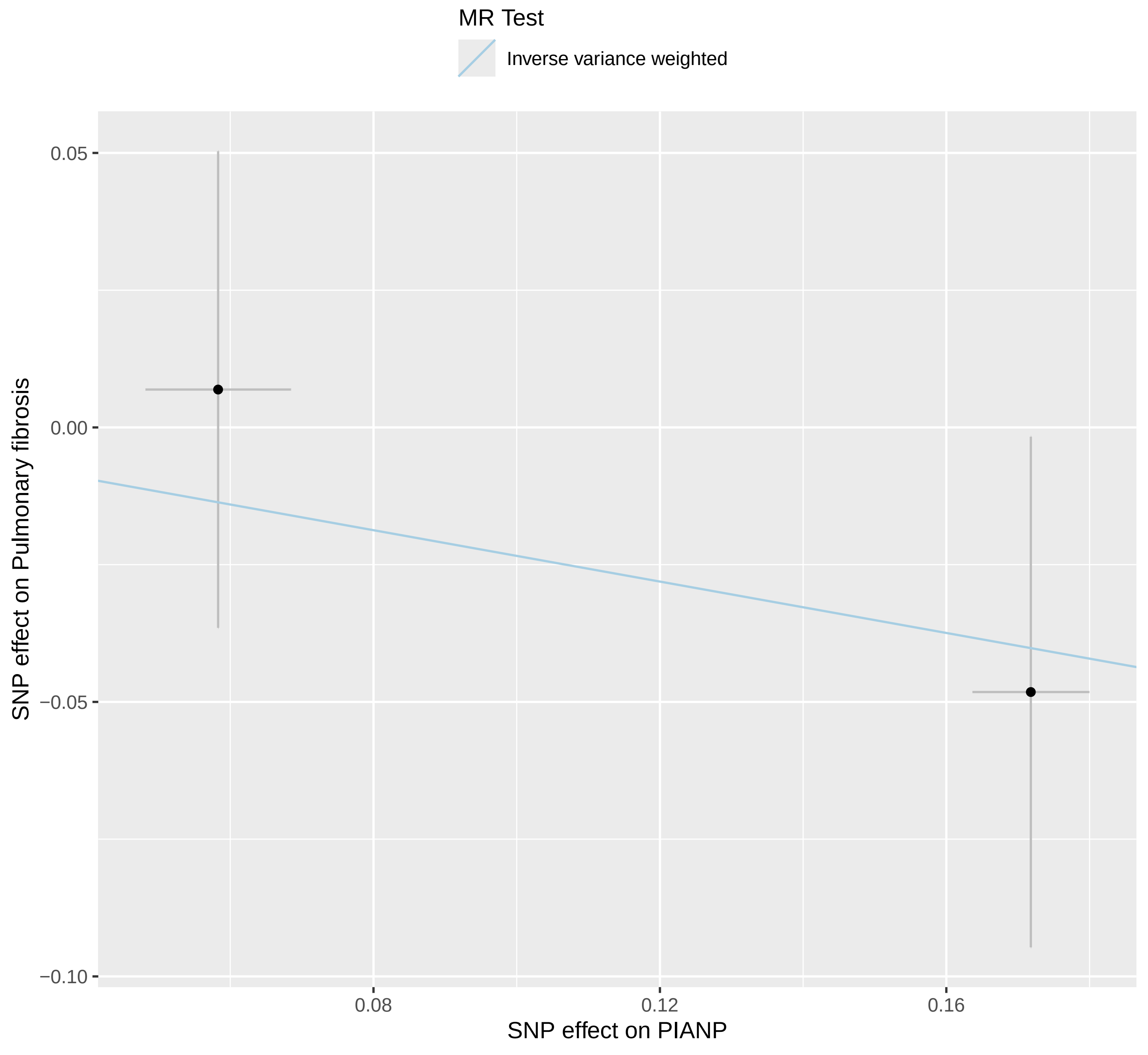
MR Test
Inverse variance weighted
0.05
0.00
−0.05
−0.10
0.08
0.12
0.16
SNP effect on PIANP
SNP effect on Pulmonary fibrosis

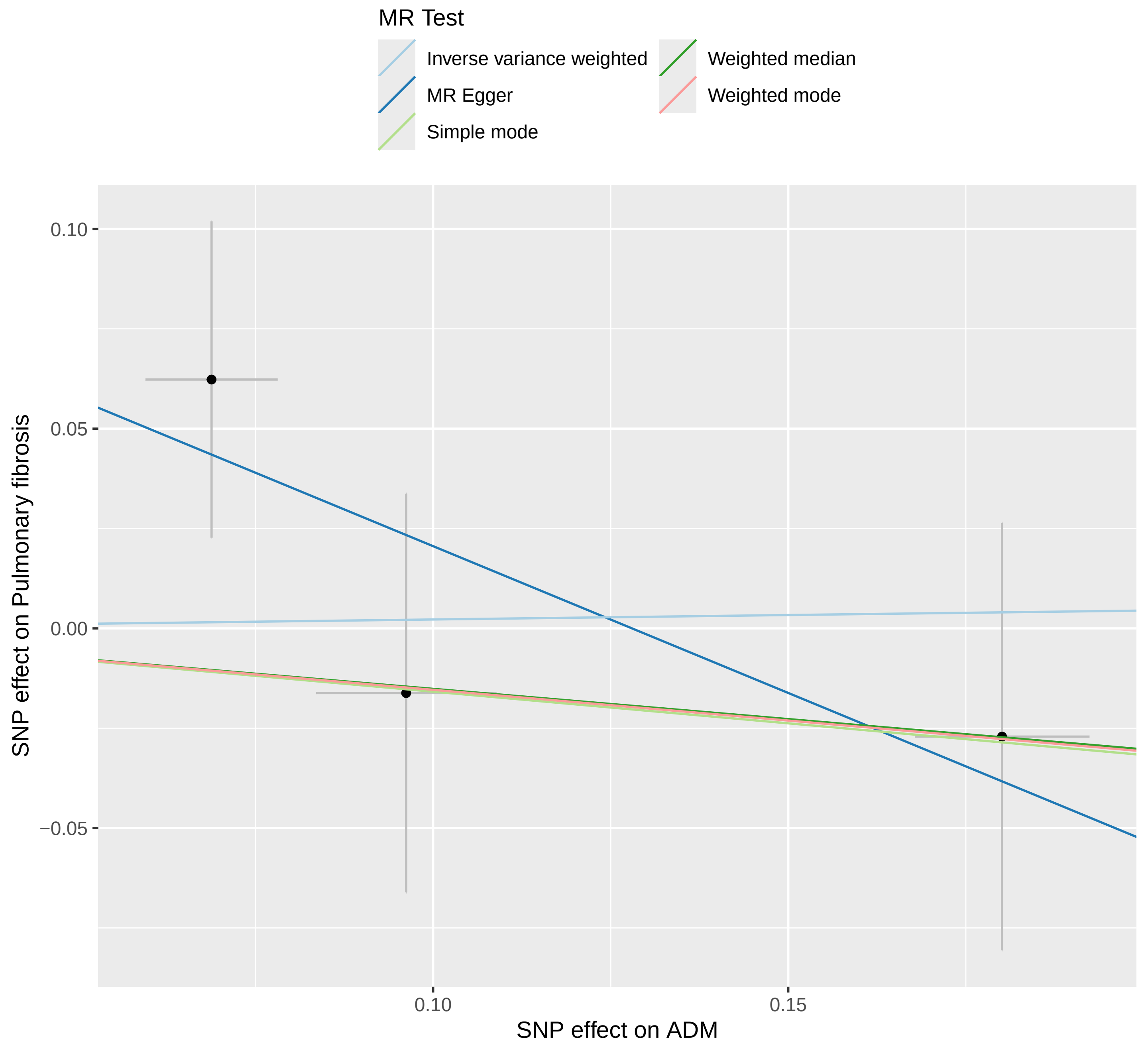
MR Test
Inverse variance weighted
MR Egger
Simple mode
Weighted median
Weighted mode
0.10
0.05
0.00
−0.05
SNP effect on Pulmonary fibrosis
0.10
0.15
SNP effect on ADM

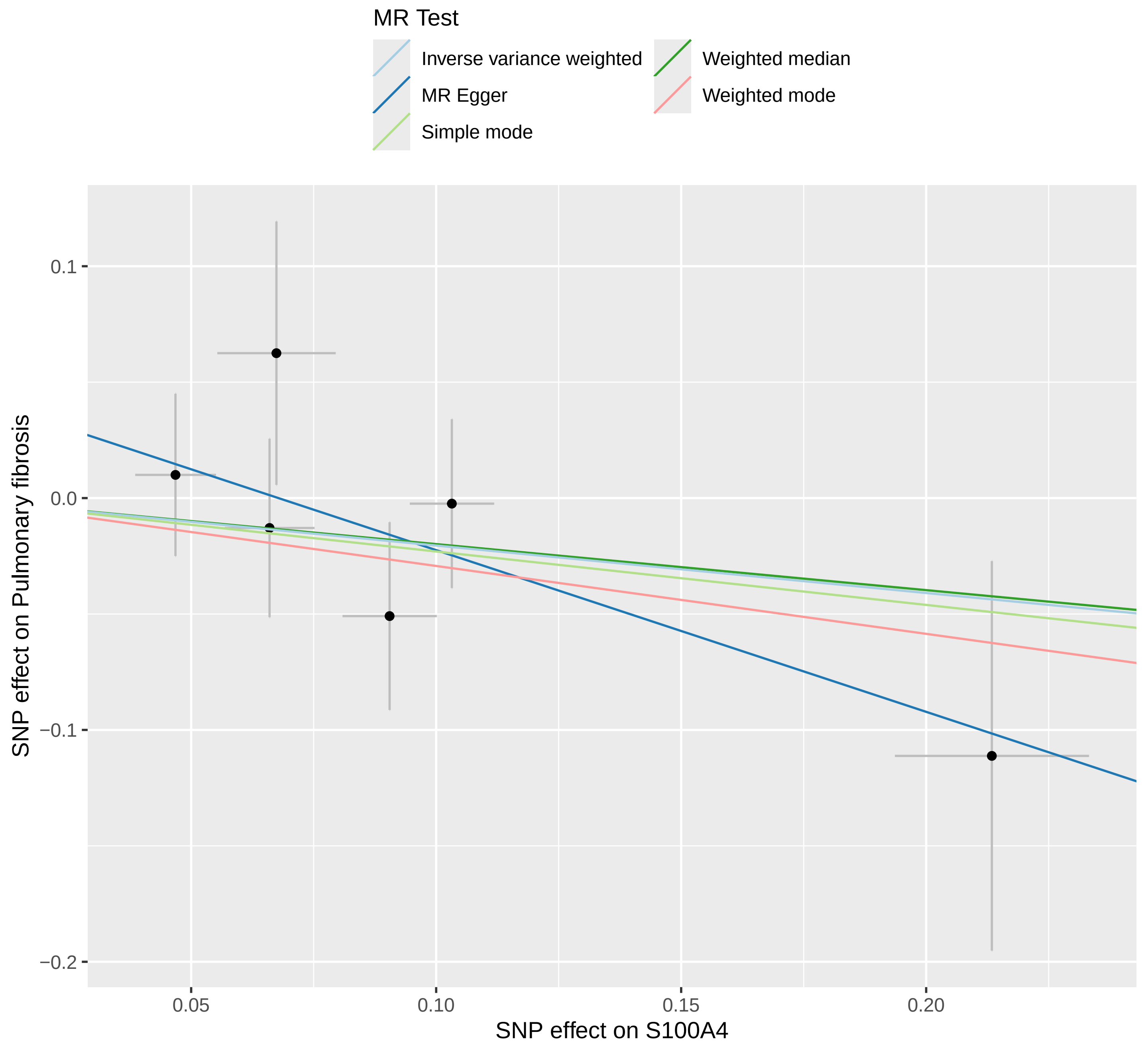
MR Test
Inverse variance weighted
MR Egger
Simple mode
Weighted median
Weighted mode
SNP effect on Pulmonary fibrosis
0.1
0.0
−0.1
−0.2
0.05
0.10
0.15
0.20
SNP effect on S100A4

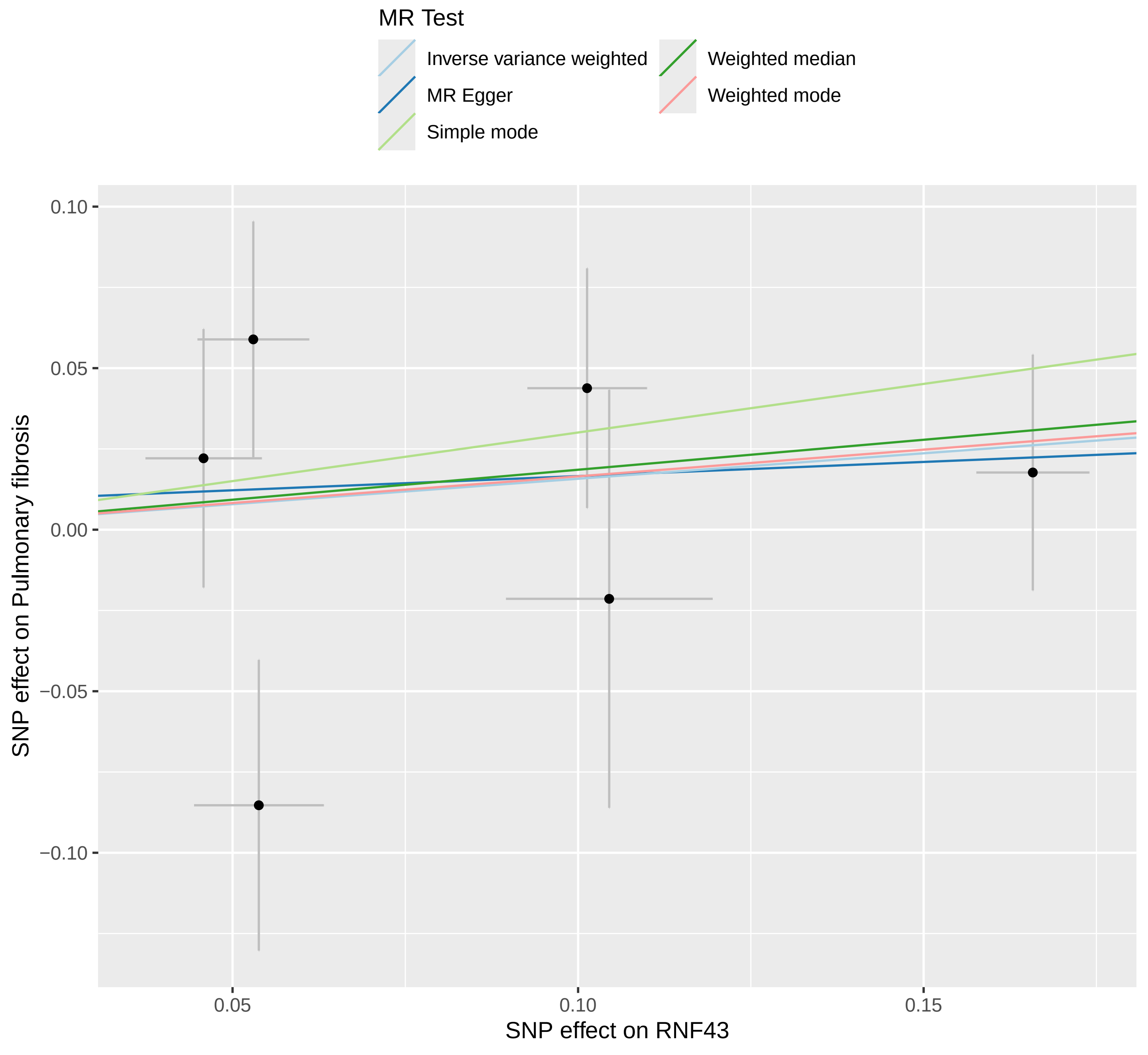
MR Test
Inverse variance weighted
MR Egger
Simple mode
Weighted median
Weighted mode
0.10
0.05
0.00
−0.05
−0.10
SNP effect on Pulmonary fibrosis
0.05
0.10
0.15
SNP effect on RNF43

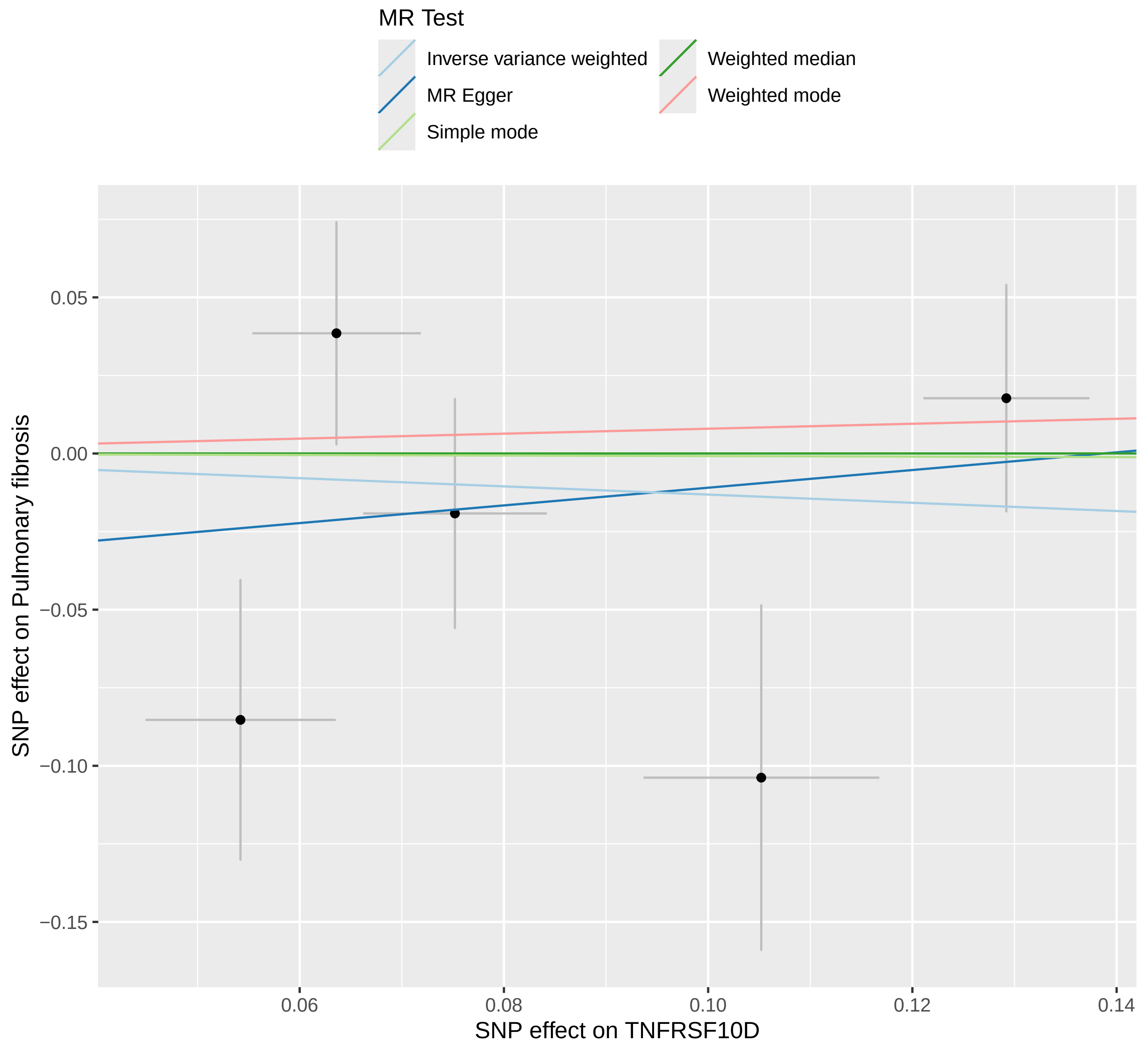
MR Test
Inverse variance weighted
MR Egger
Simple mode
Weighted median
Weighted mode
0.05
0.00
−0.05
−0.10
−0.15
SNP effect on Pulmonary fibrosis
0.06
0.08
0.10
0.12
0.14
SNP effect on TNFRSF10D

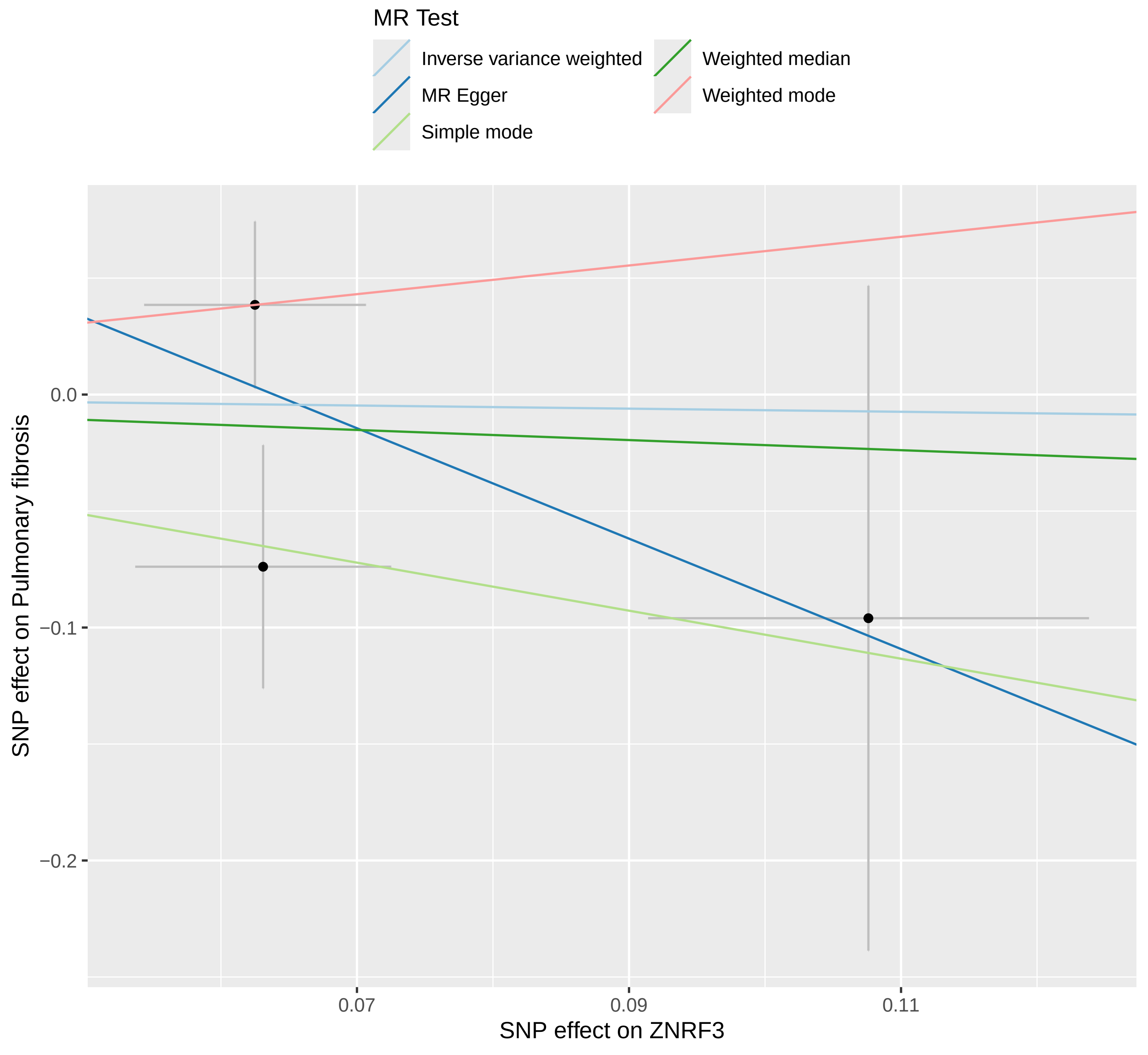
MR Test
Inverse variance weighted
MR Egger
Simple mode
Weighted median
Weighted mode
SNP effect on Pulmonary fibrosis
0.0
−0.1
−0.2
0.07
0.09
0.11
SNP effect on ZNRF3

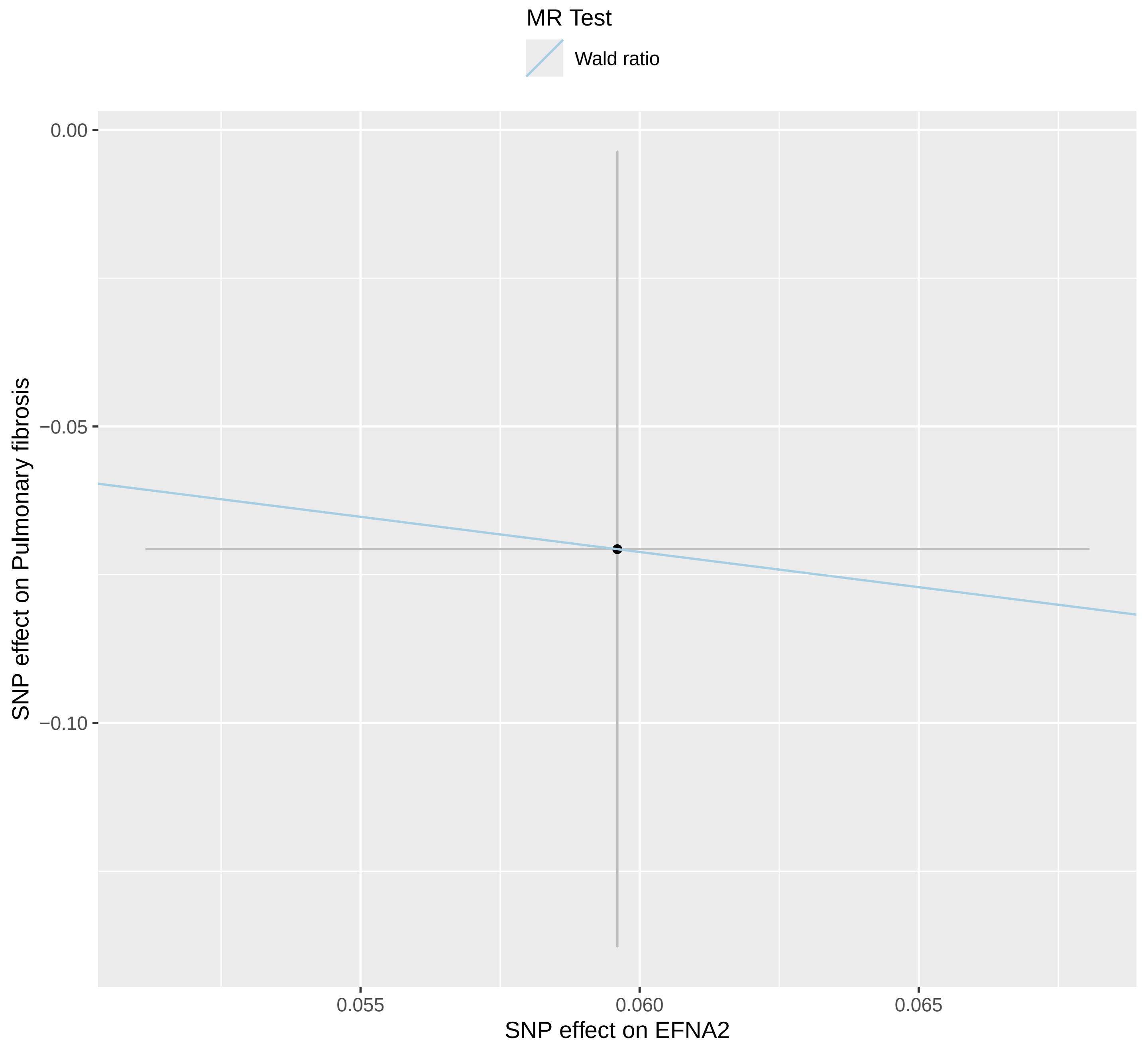
MR Test
Wald ratio
0.00
−0.05
−0.10
0.055
0.060
0.065
SNP effect on Pulmonary fibrosis
SNP effect on EFNA2

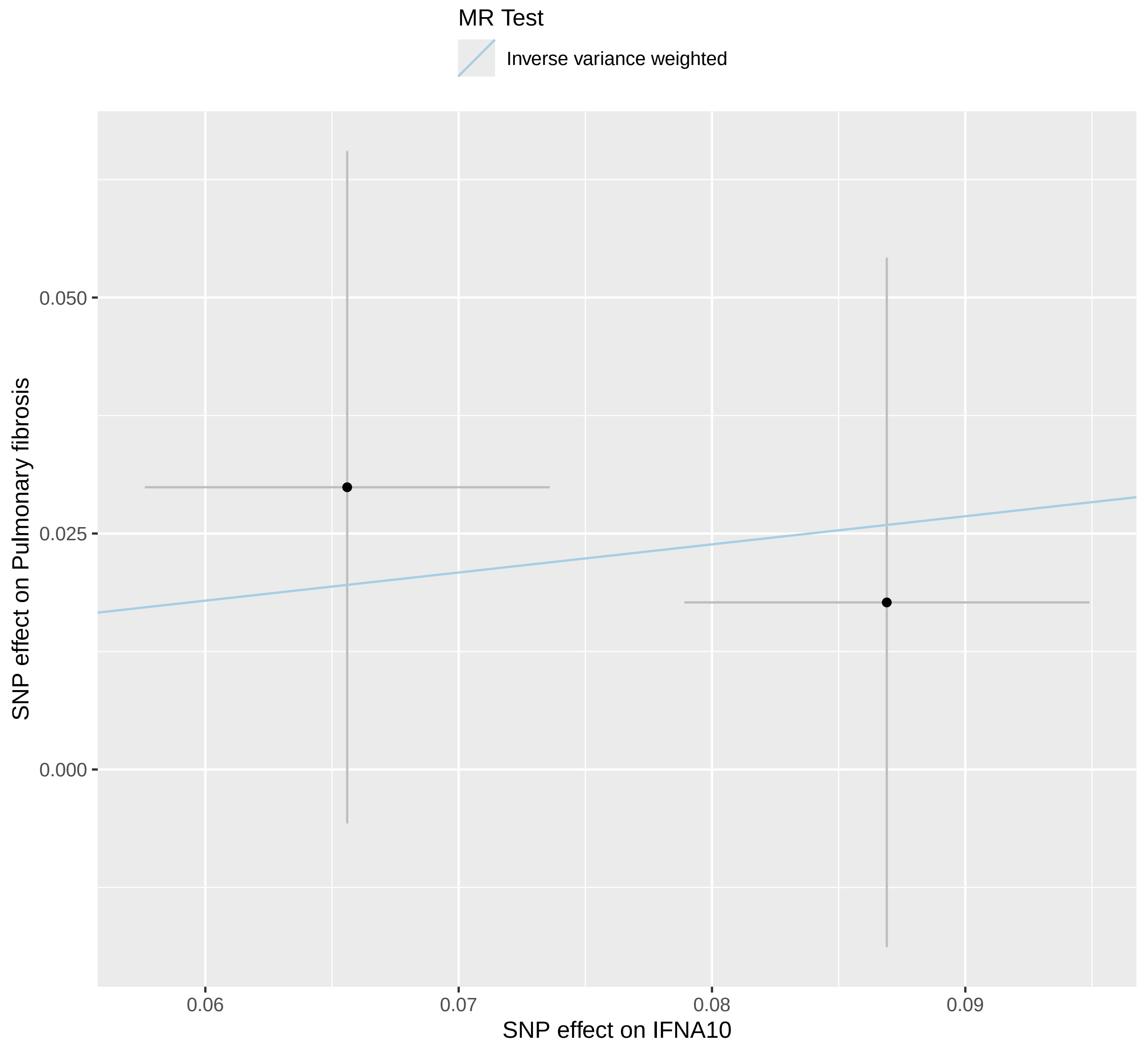

MR Test
Inverse variance weighted
0.050
0.025
0.000
0.06
0.07
0.08
0.09
SNP effect on Pulmonary fibrosis
SNP effect on IFNA10

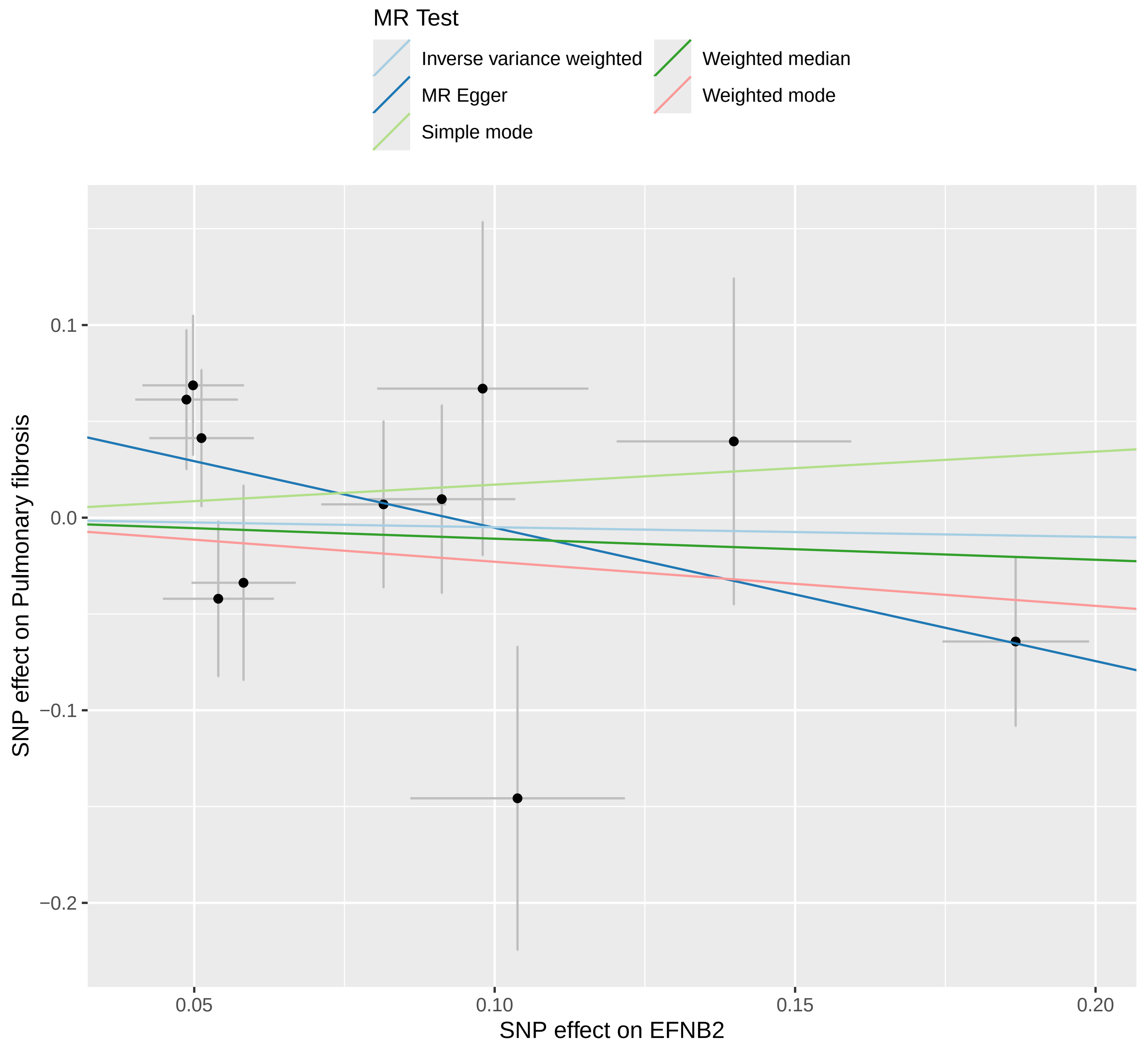
MR Test
Inverse variance weighted
MR Egger
Simple mode
Weighted median
Weighted mode
0.1
0.0
−0.1
−0.2
SNP effect on Pulmonary fibrosis
0.05
0.10
0.15
0.20
SNP effect on EFNB2

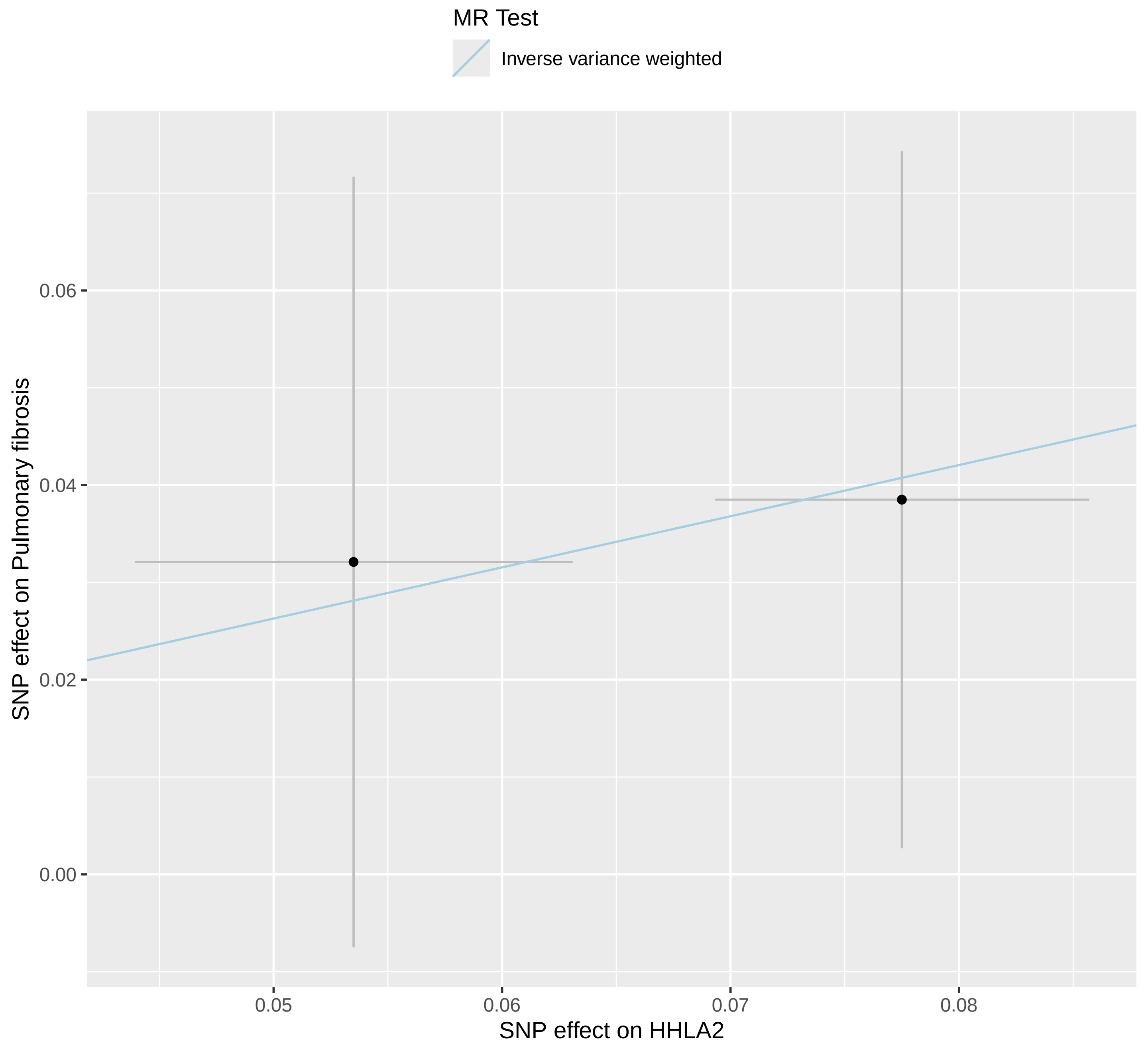
MR Test
Inverse variance weighted
0.06
0.04
0.02
0.00
SNP effect on Pulmonary fibrosis
0.05
0.06
0.07
0.08
SNP effect on HHLA2

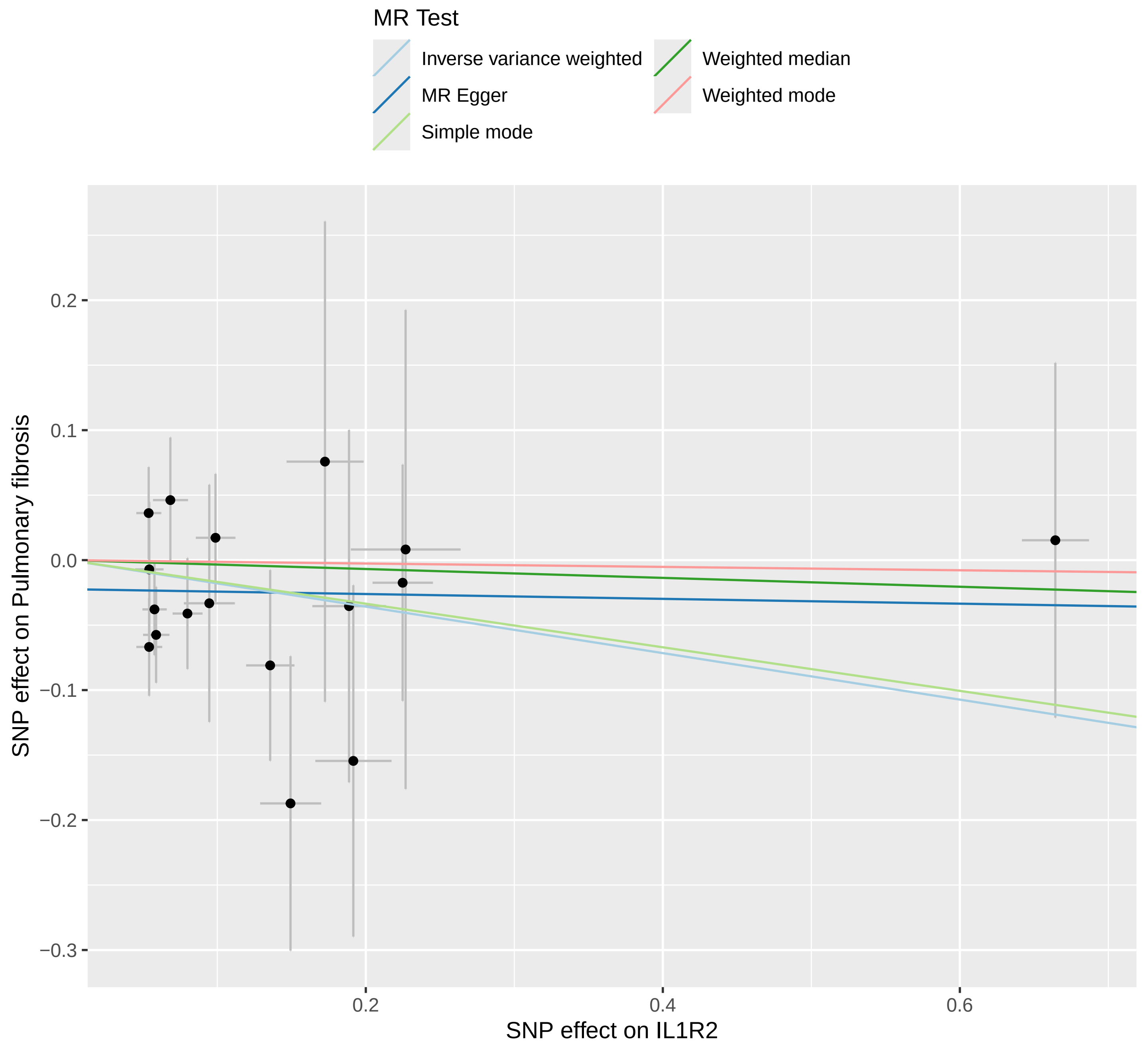

MR Test
Inverse variance weighted
MR Egger
Simple mode
Weighted median
Weighted mode
0.2
0.1
0.0
−0.1
−0.2
−0.3
SNP effect on Pulmonary fibrosis
0.2
0.4
0.6
SNP effect on IL1R2

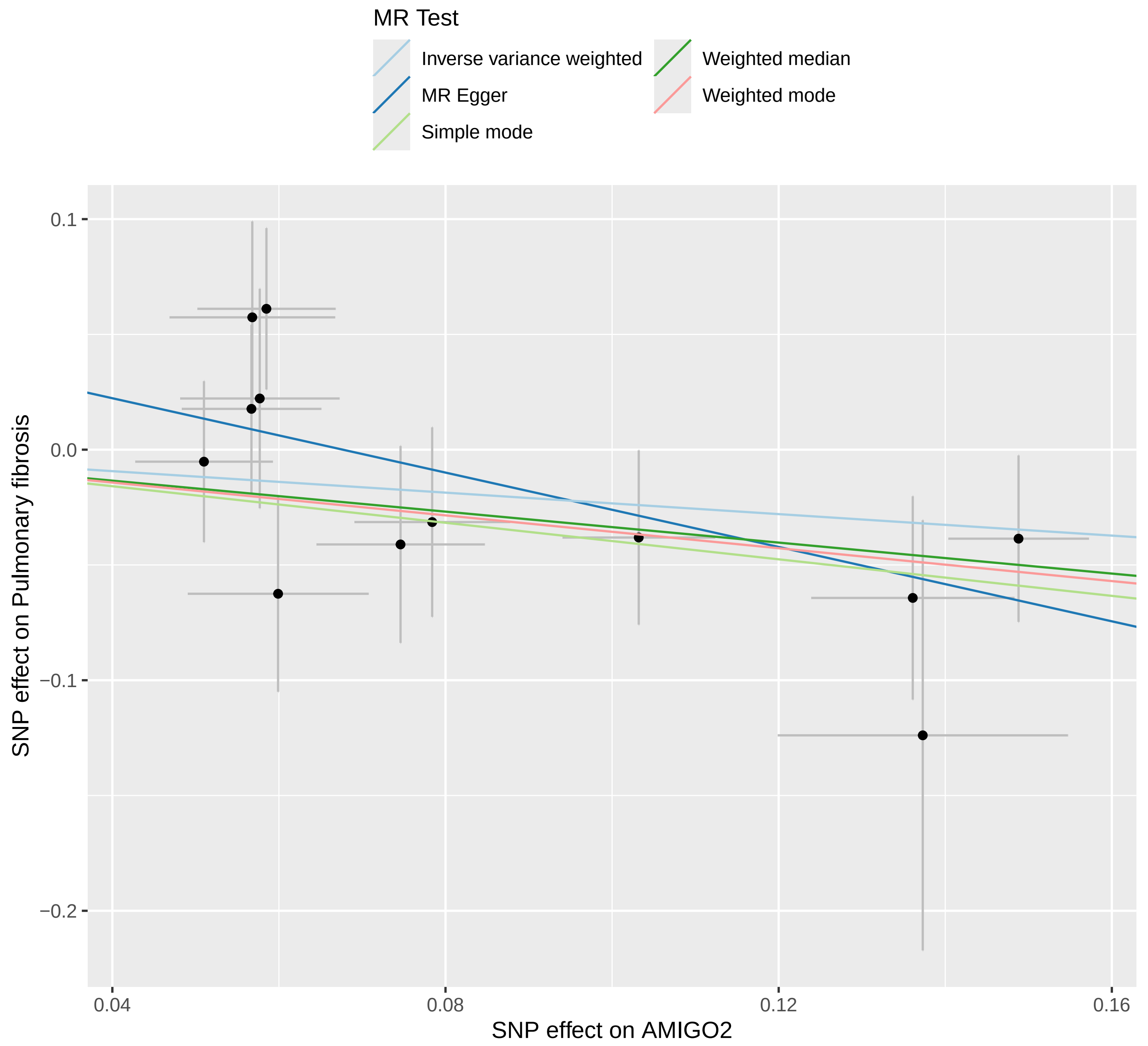

MR Test
Inverse variance weighted
MR Egger
Simple mode
Weighted median
Weighted mode
0.1
0.0
−0.1
−0.2
0.04
0.08
0.12
0.16
SNP effect on AMIGO2
SNP effect on Pulmonary fibrosis

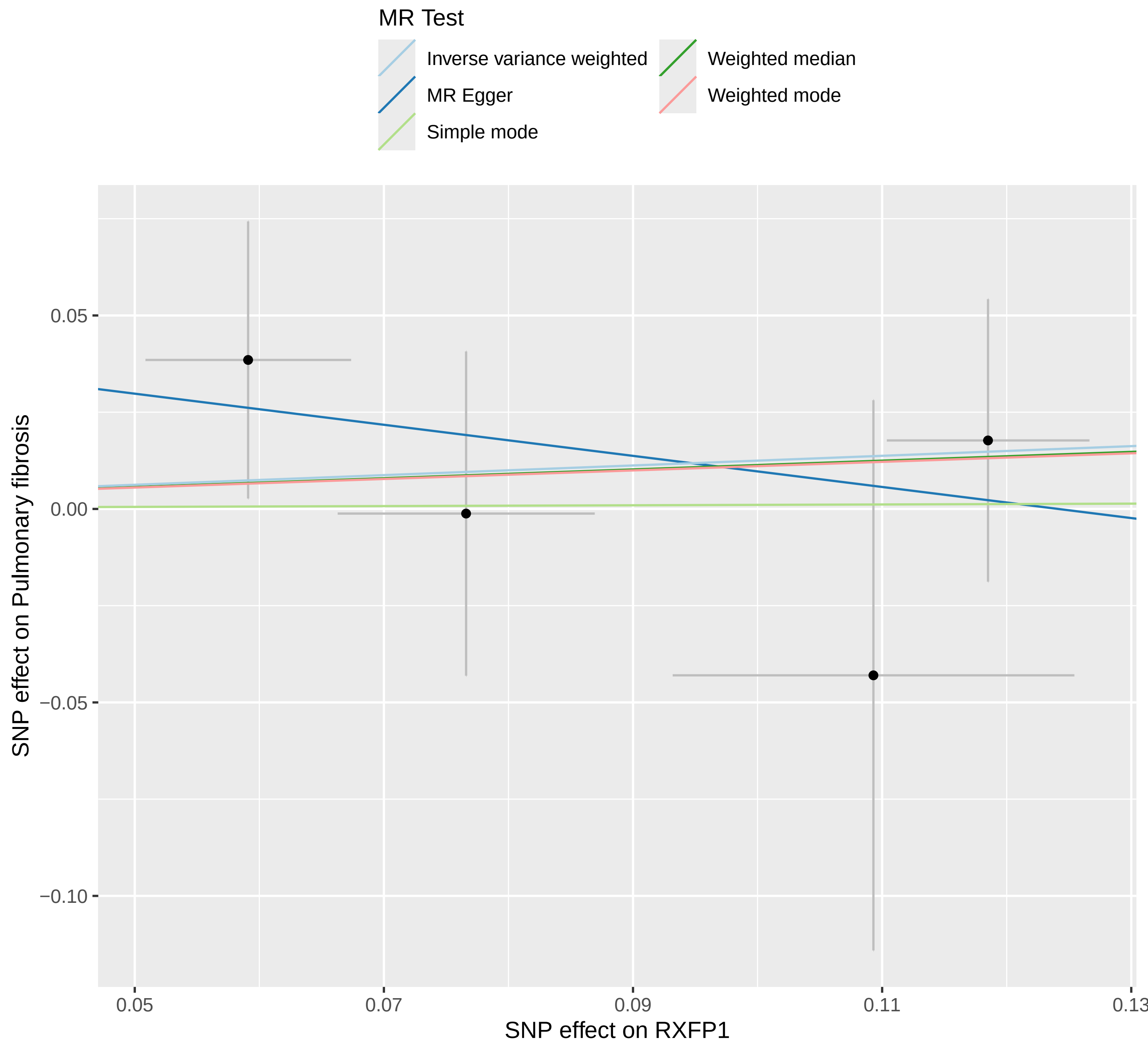
MR Test
Inverse variance weighted
MR Egger
Simple mode
Weighted median
Weighted mode
0.05
0.00
−0.05
−0.10
SNP effect on Pulmonary fibrosis
0.05
0.07
0.09
0.11
0.13
SNP effect on RXFP1

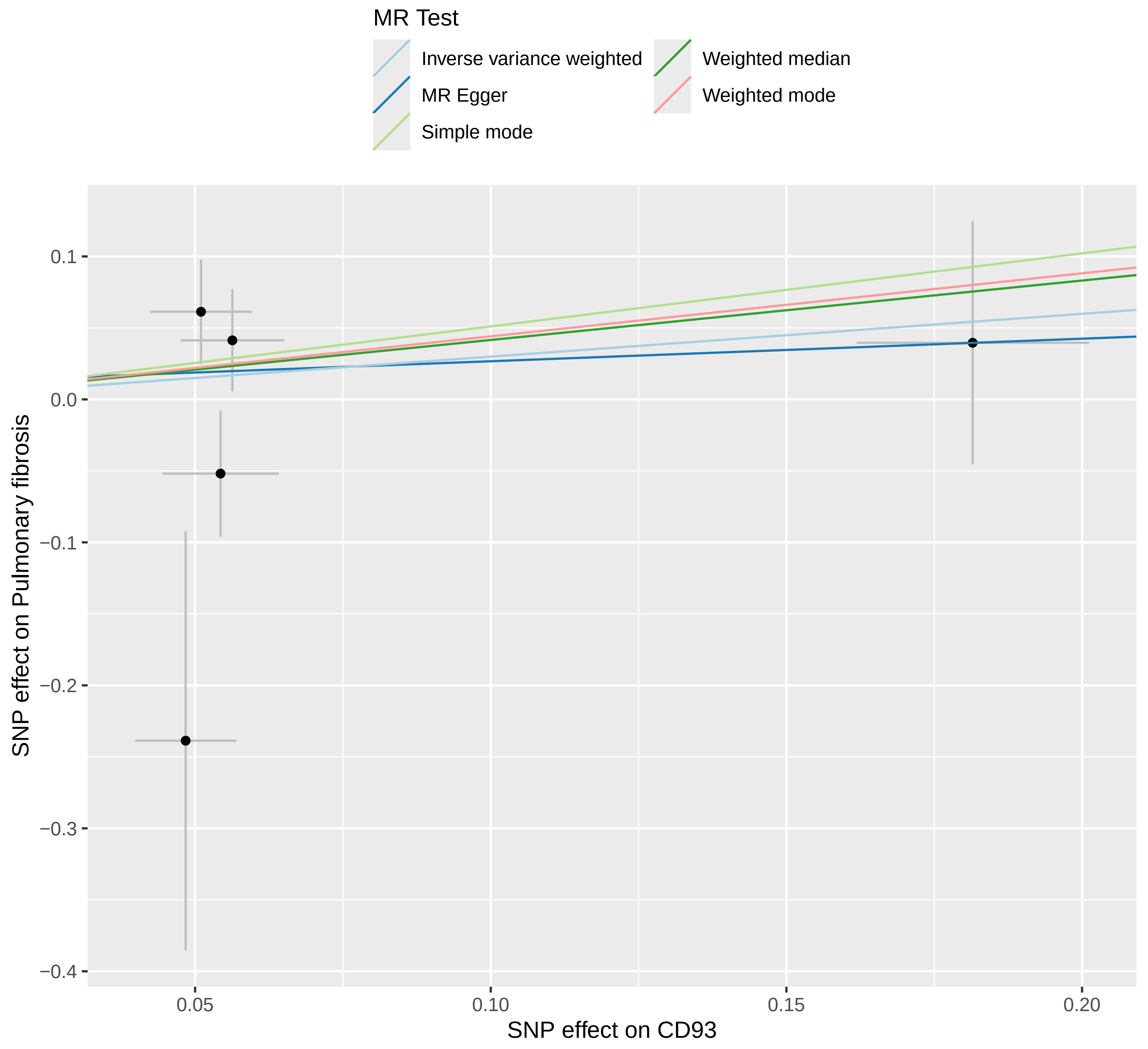
MR Test
Inverse variance weighted
MR Egger
Simple mode
Weighted median
Weighted mode
0.1
0.0
−0.1
−0.2
−0.3
−0.4
SNP effect on Pulmonary fibrosis
0.05
0.10
0.15
0.20
SNP effect on CD93

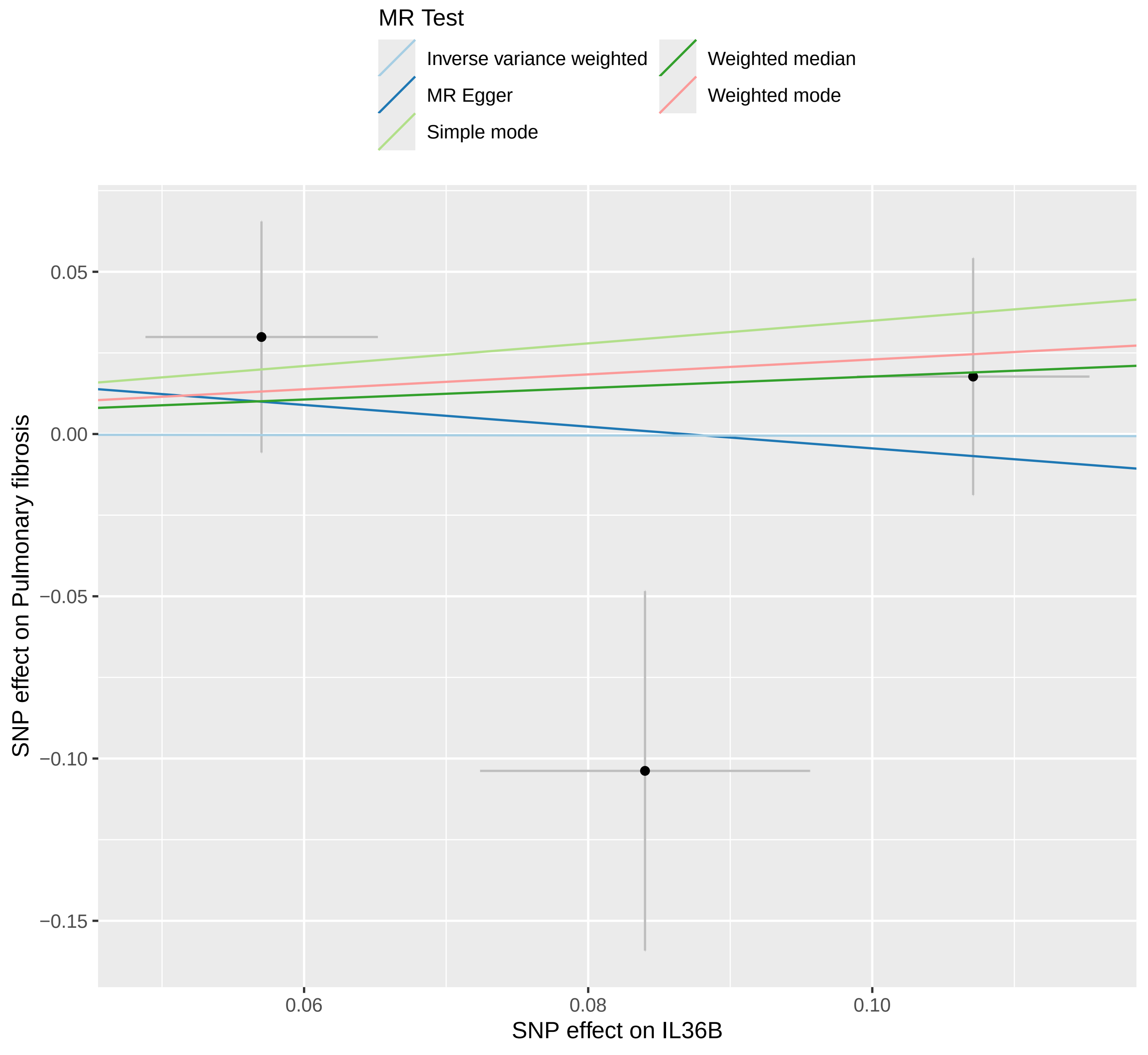

MR Test
Inverse variance weighted
MR Egger
Simple mode
Weighted median
Weighted mode
0.05
0.00
−0.05
−0.10
−0.15
SNP effect on Pulmonary fibrosis
0.06
0.08
0.10
SNP effect on IL36B

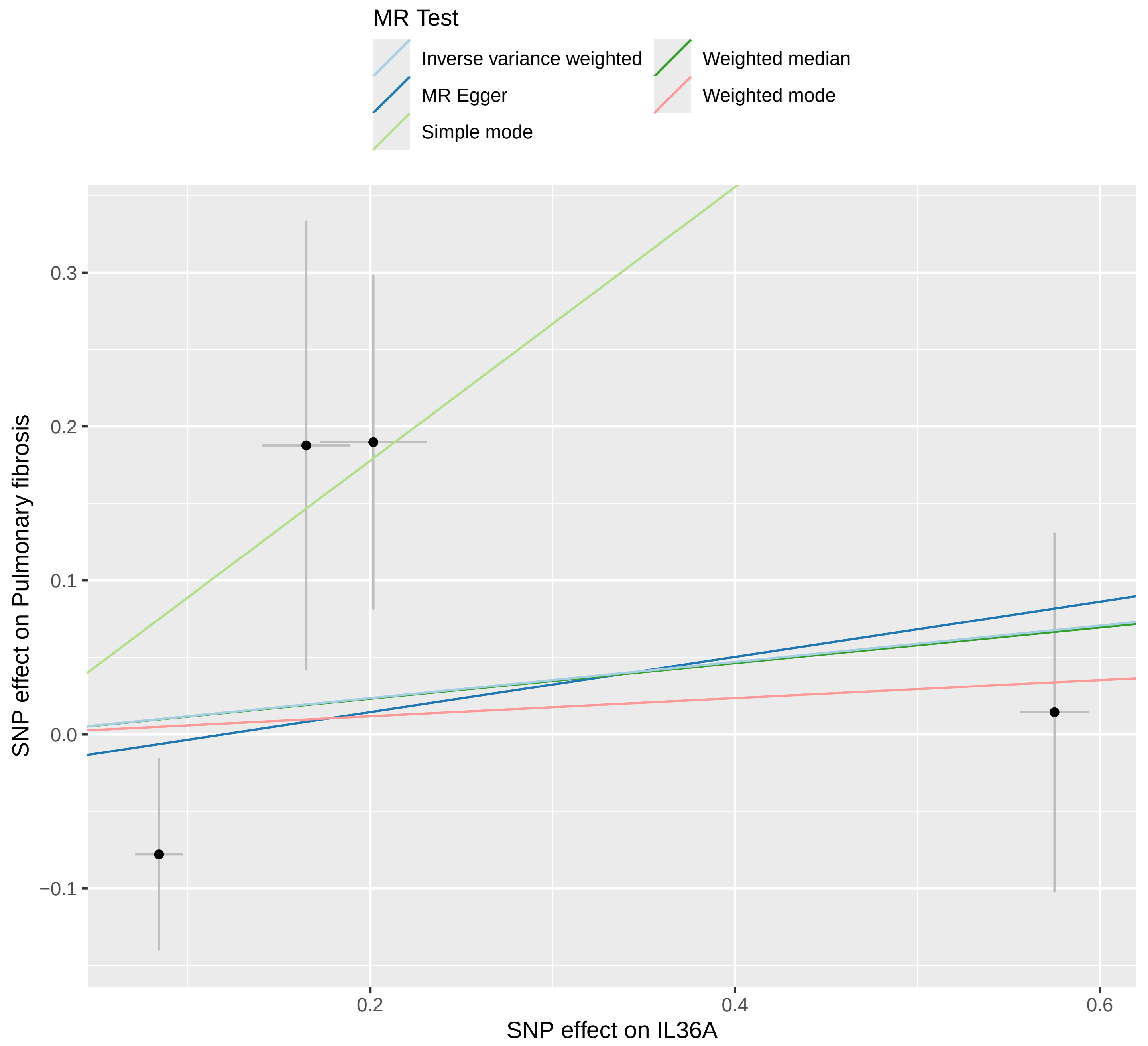
MR Test
Inverse variance weighted
MR Egger
Simple mode
Weighted median
Weighted mode
0.3
0.2
0.1
0.0
−0.1
0.2
0.4
0.6
SNP effect on Pulmonary fibrosis
SNP effect on IL36A

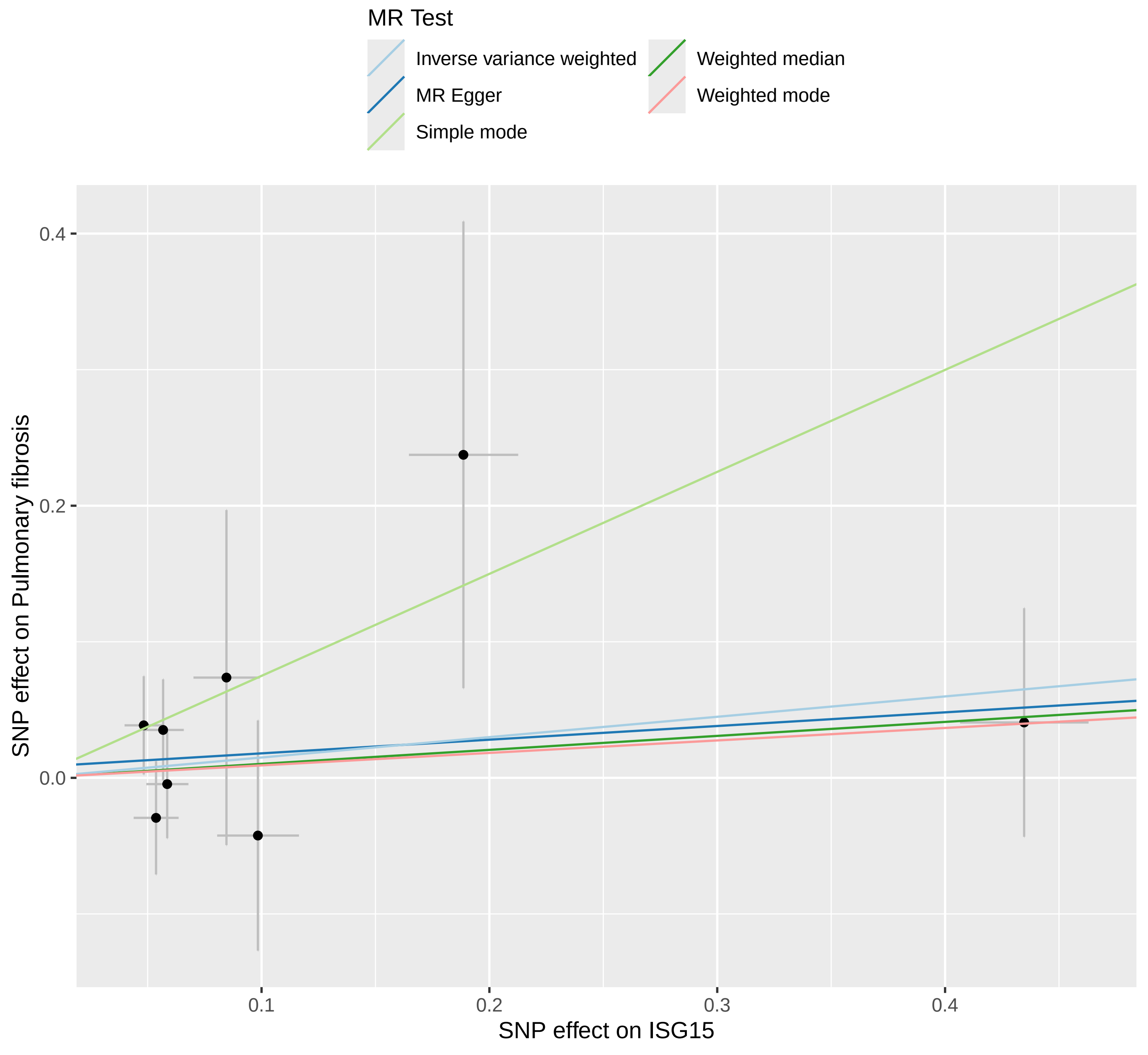
MR Test
Inverse variance weighted
MR Egger
Simple mode
Weighted median
Weighted mode
0.4
0.2
0.0
0.1
0.2
0.3
0.4
SNP effect on Pulmonary fibrosis
SNP effect on ISG15

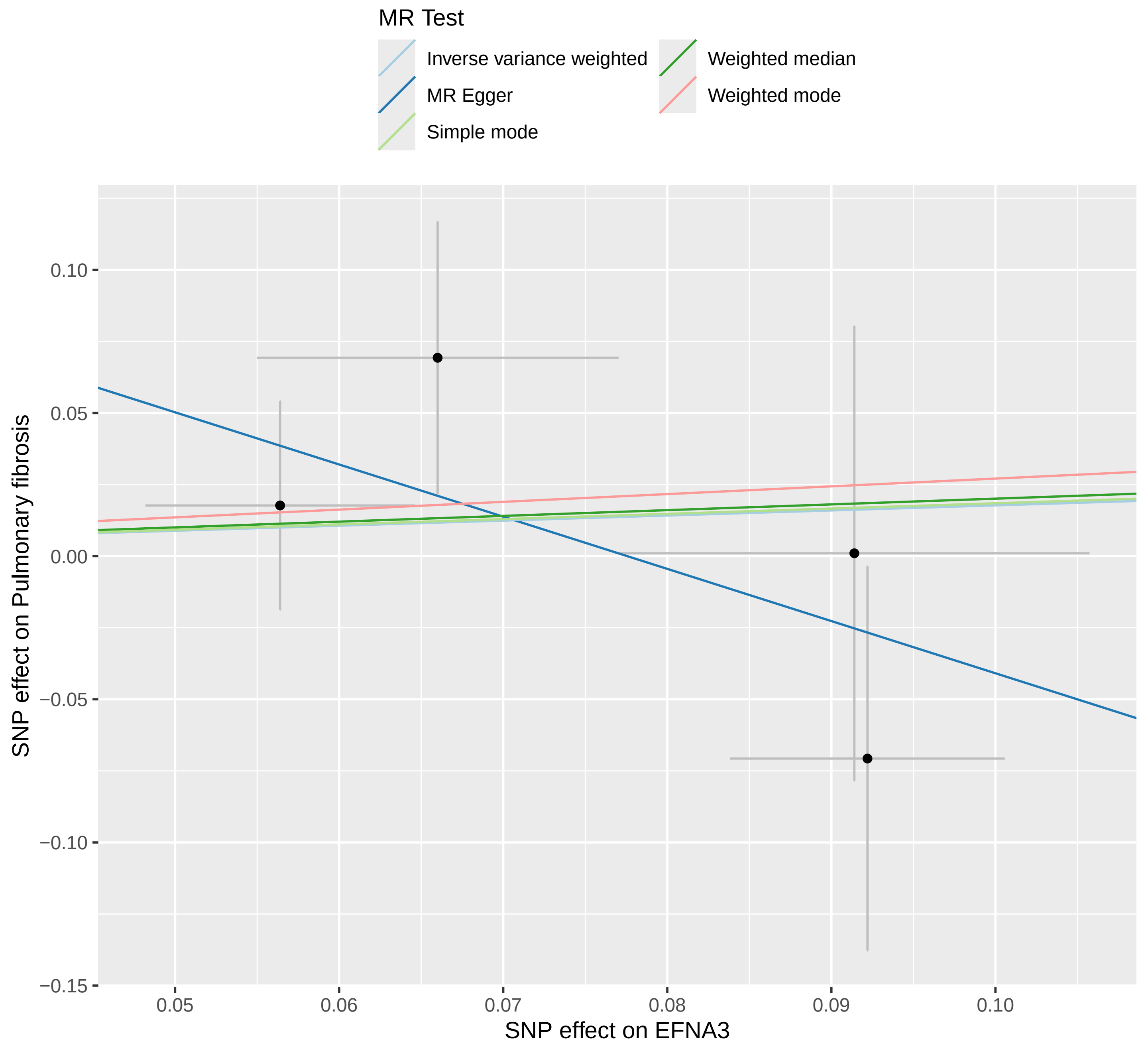

MR Test
Inverse variance weighted
MR Egger
Simple mode
Weighted median
Weighted mode
SNP effect on Pulmonary fibrosis
0.10
0.05
0.00
−0.05
−0.10
−0.15
0.05
0.06
0.07
0.08
0.09
0.10
SNP effect on EFNA3

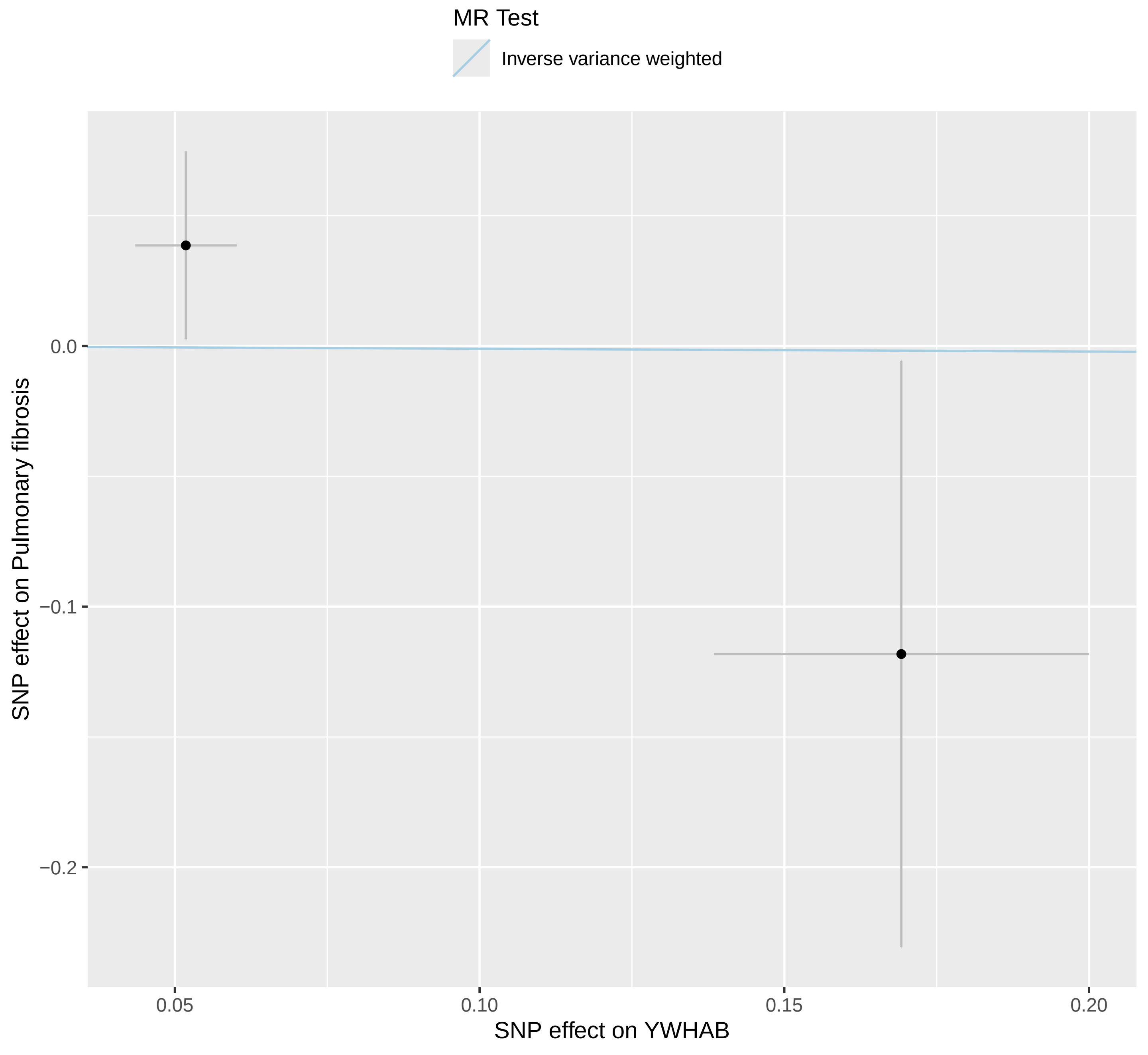
MR Test
Inverse variance weighted
0.0
−0.1
−0.2
SNP effect on Pulmonary fibrosis
0.05
0.10
0.15
0.20
SNP effect on YWHAB

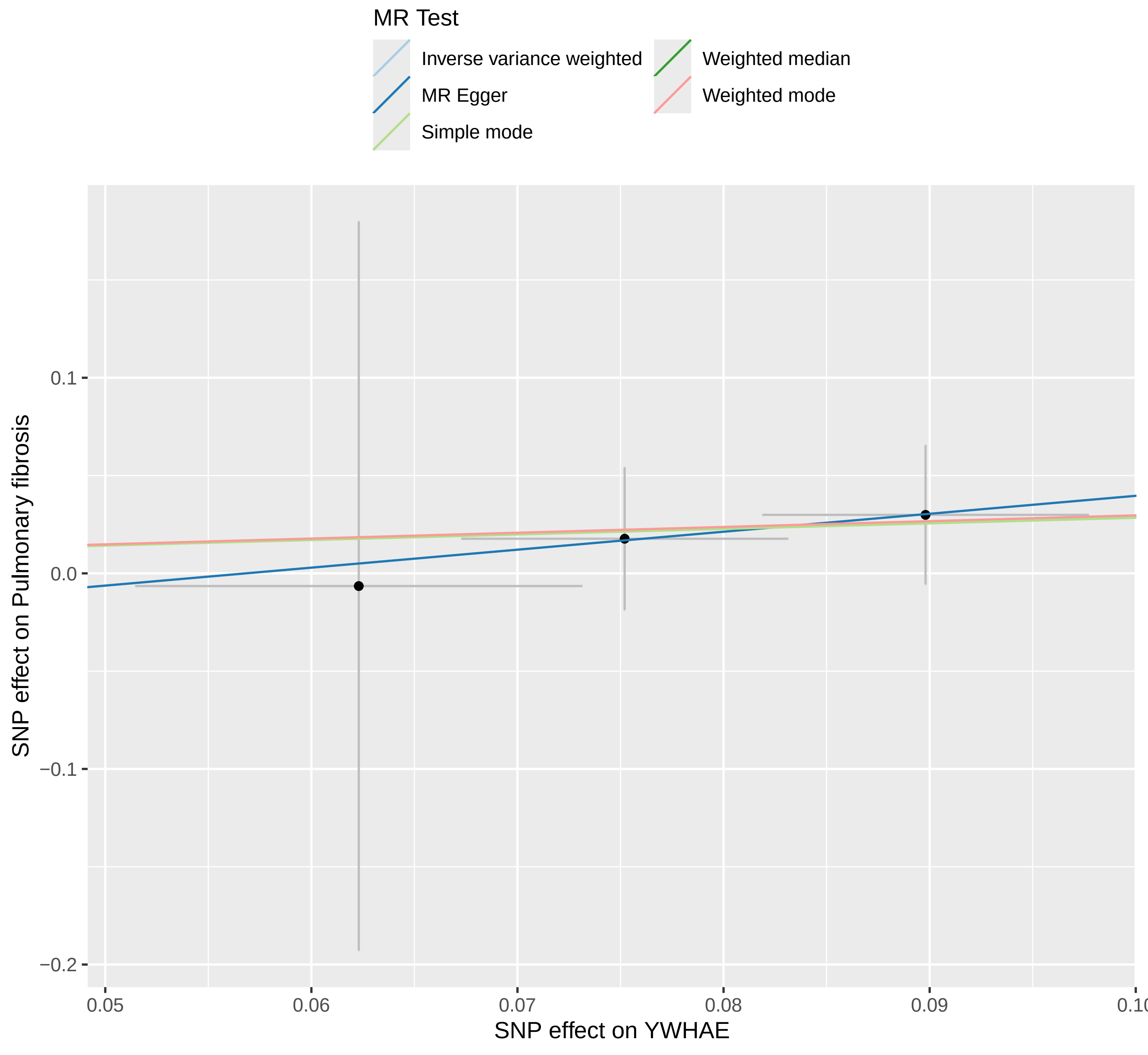
MR Test
Inverse variance weighted
MR Egger
Simple mode
Weighted median
Weighted mode
0.1
0.0
−0.1
−0.2
0.05
0.06
0.07
0.08
0.09
SNP effect on Pulmonary fibrosis
SNP effect on YWHAE

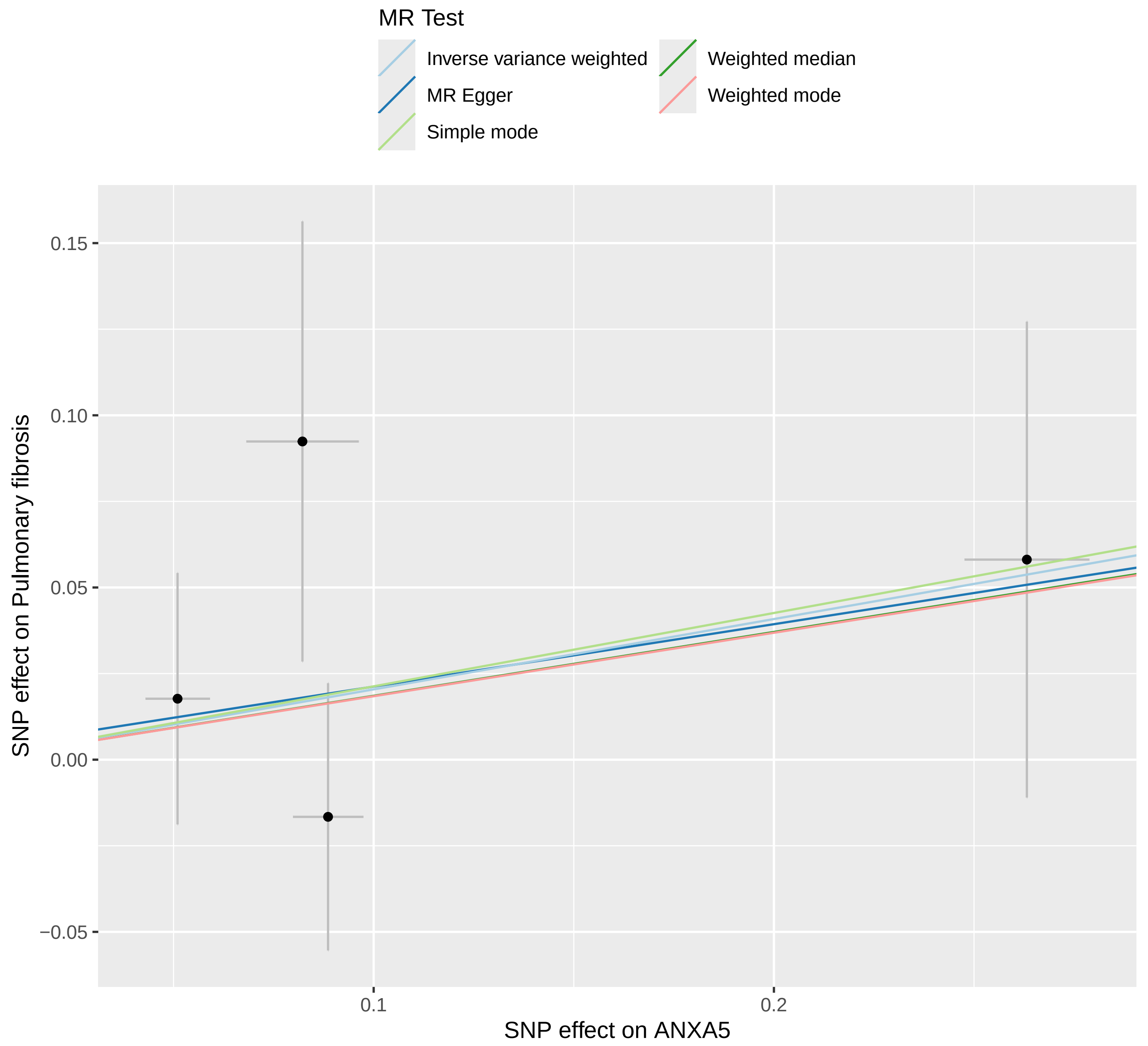

MR Test
Inverse variance weighted
MR Egger
Simple mode
Weighted median
Weighted mode
0.15
0.10
0.05
0.00
−0.05
0.1
0.2
SNP effect on Pulmonary fibrosis
SNP effect on ANXA5

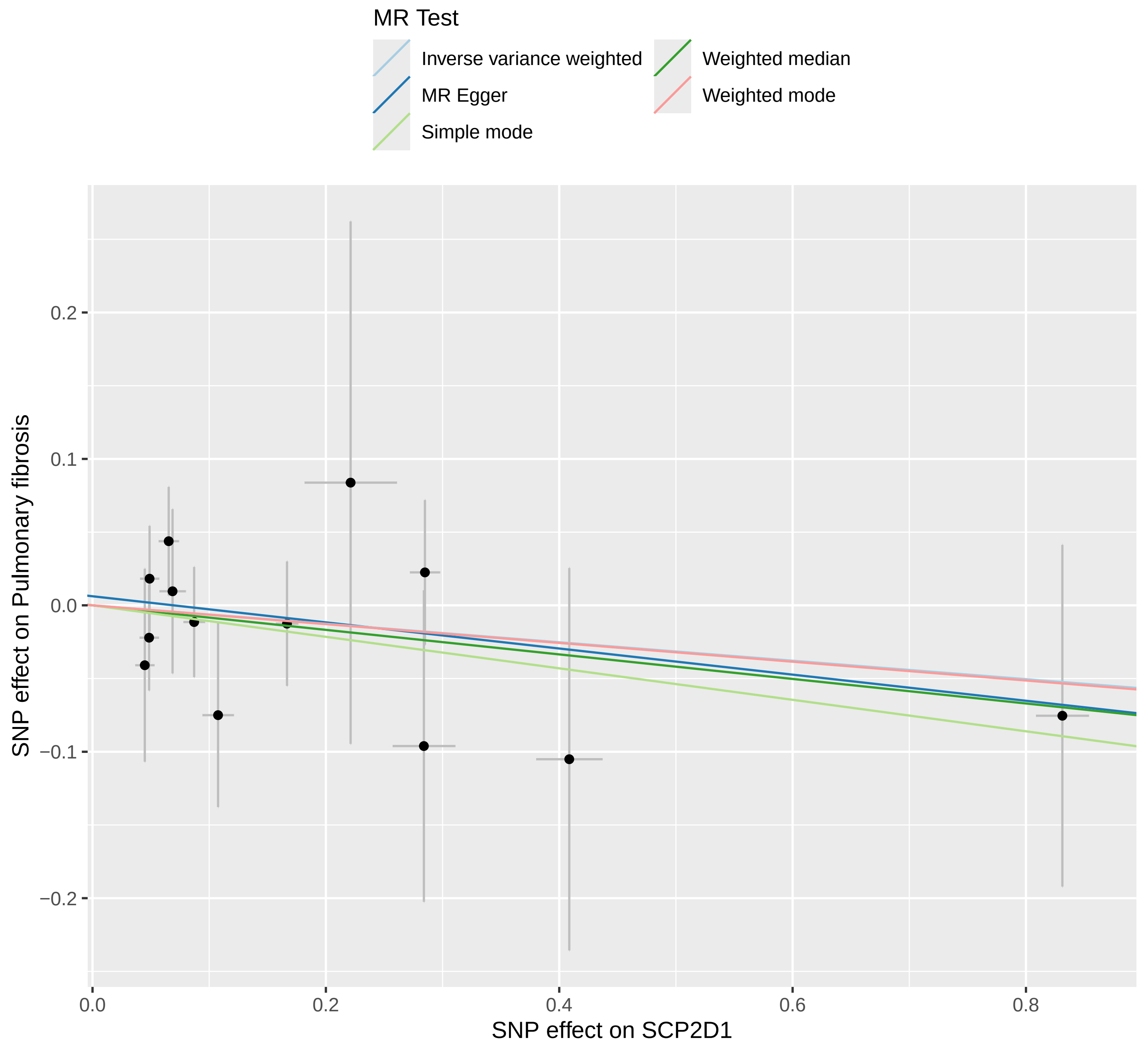
MR Test
Inverse variance weighted
MR Egger
Simple mode
Weighted median
Weighted mode
SNP effect on Pulmonary fibrosis
0.2
0.1
0.0
−0.1
−0.2
0.0
0.2
0.4
0.6
0.8
SNP effect on SCP2D1

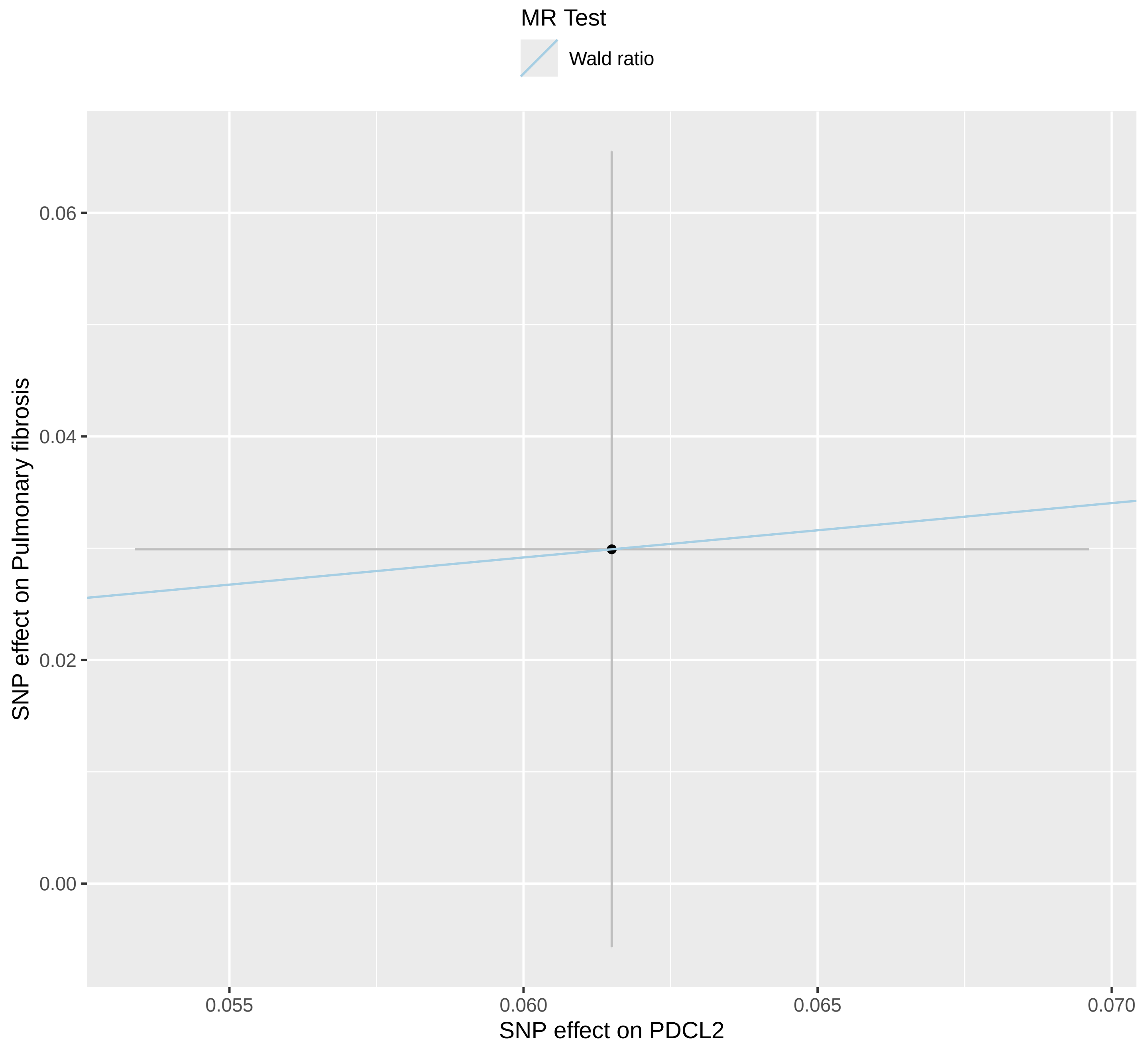

MR Test
Wald ratio
SNP effect on Pulmonary fibrosis
0.06
0.04
0.02
0.00
0.055
0.060
0.065
0.070
SNP effect on PDCL2

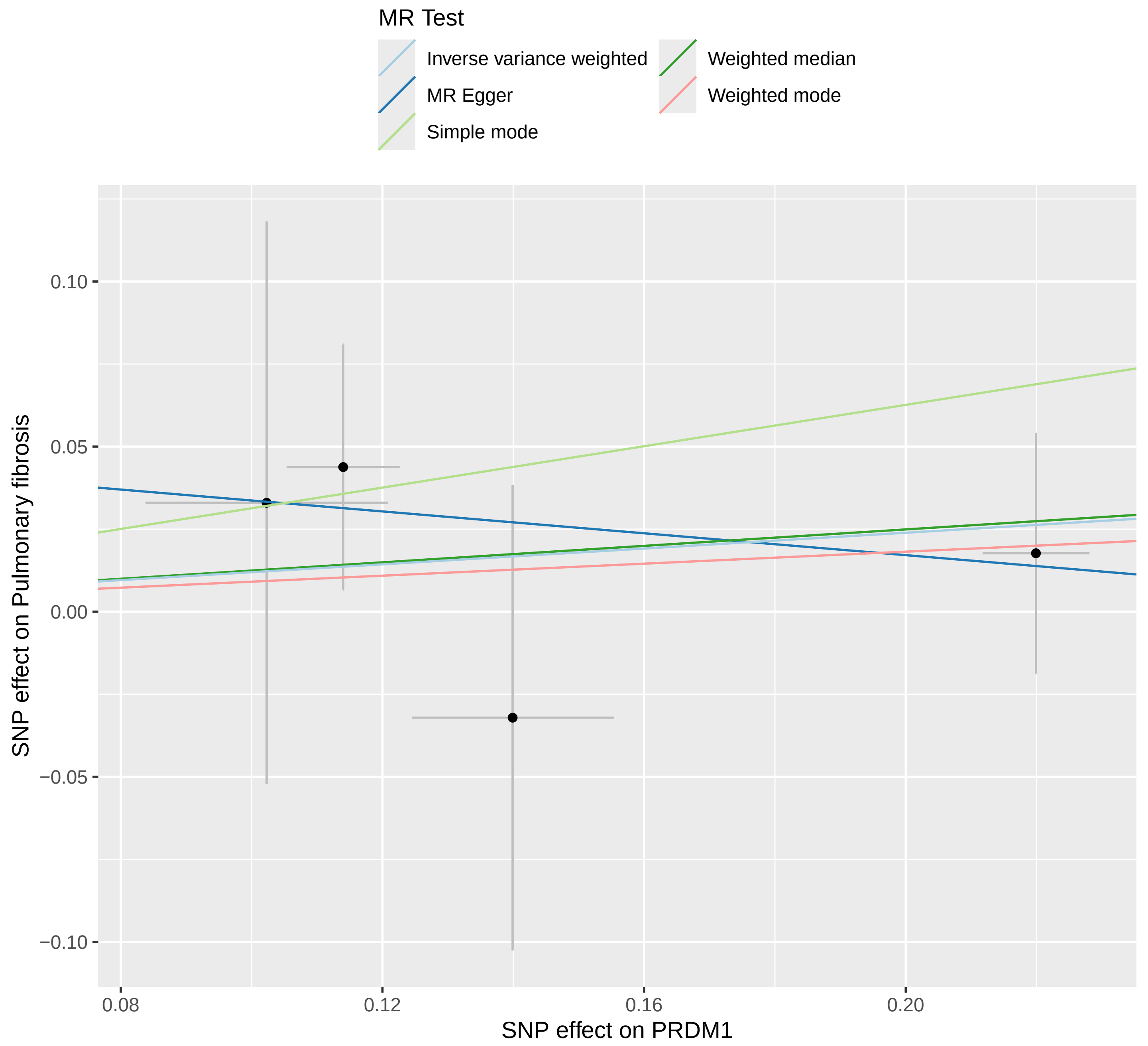
MR Test
Inverse variance weighted
MR Egger
Simple mode
Weighted median
Weighted mode
0.10
0.05
0.00
−0.05
−0.10
SNP effect on Pulmonary fibrosis
0.08
0.12
0.16
0.20
SNP effect on PRDM1

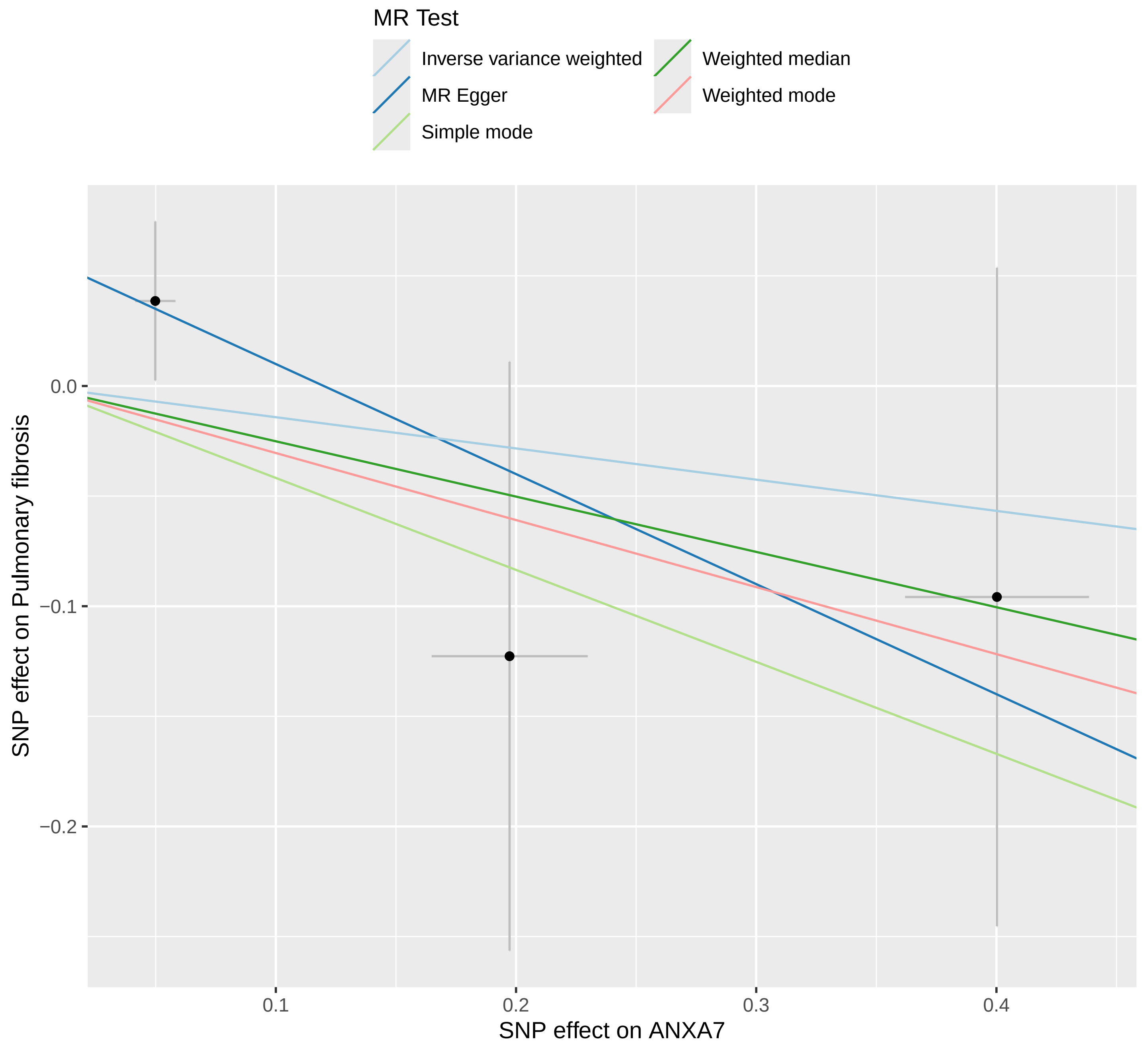
MR Test
Inverse variance weighted
MR Egger
Simple mode
Weighted median
Weighted mode
SNP effect on Pulmonary fibrosis
0.0
−0.1
−0.2
0.1
0.2
0.3
0.4
SNP effect on ANXA7

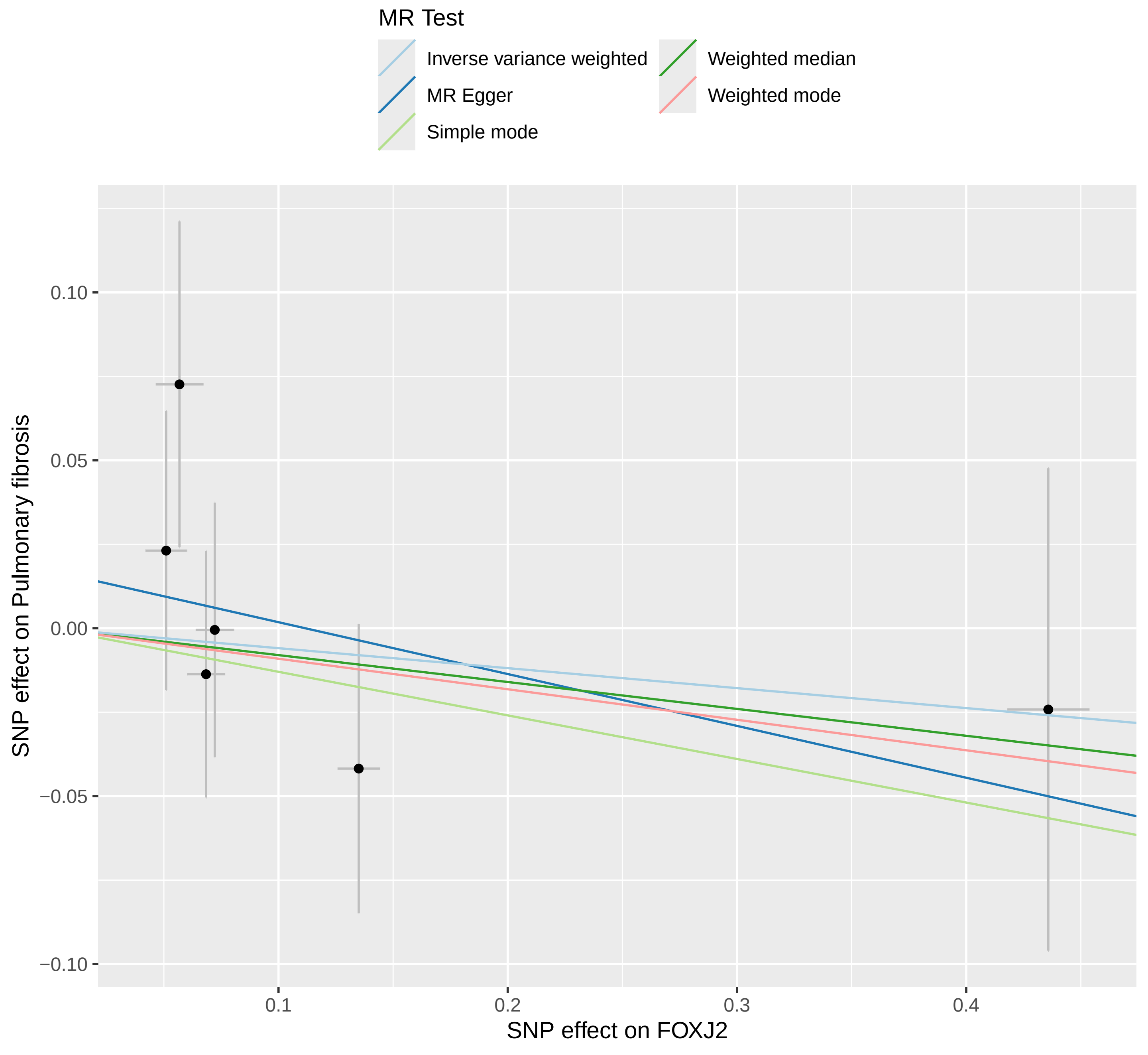
MR Test
Inverse variance weighted
MR Egger
Simple mode
Weighted median
Weighted mode
SNP effect on Pulmonary fibrosis
0.10
0.05
0.00
−0.05
−0.10
0.1
0.2
0.3
0.4
SNP effect on FOXJ2

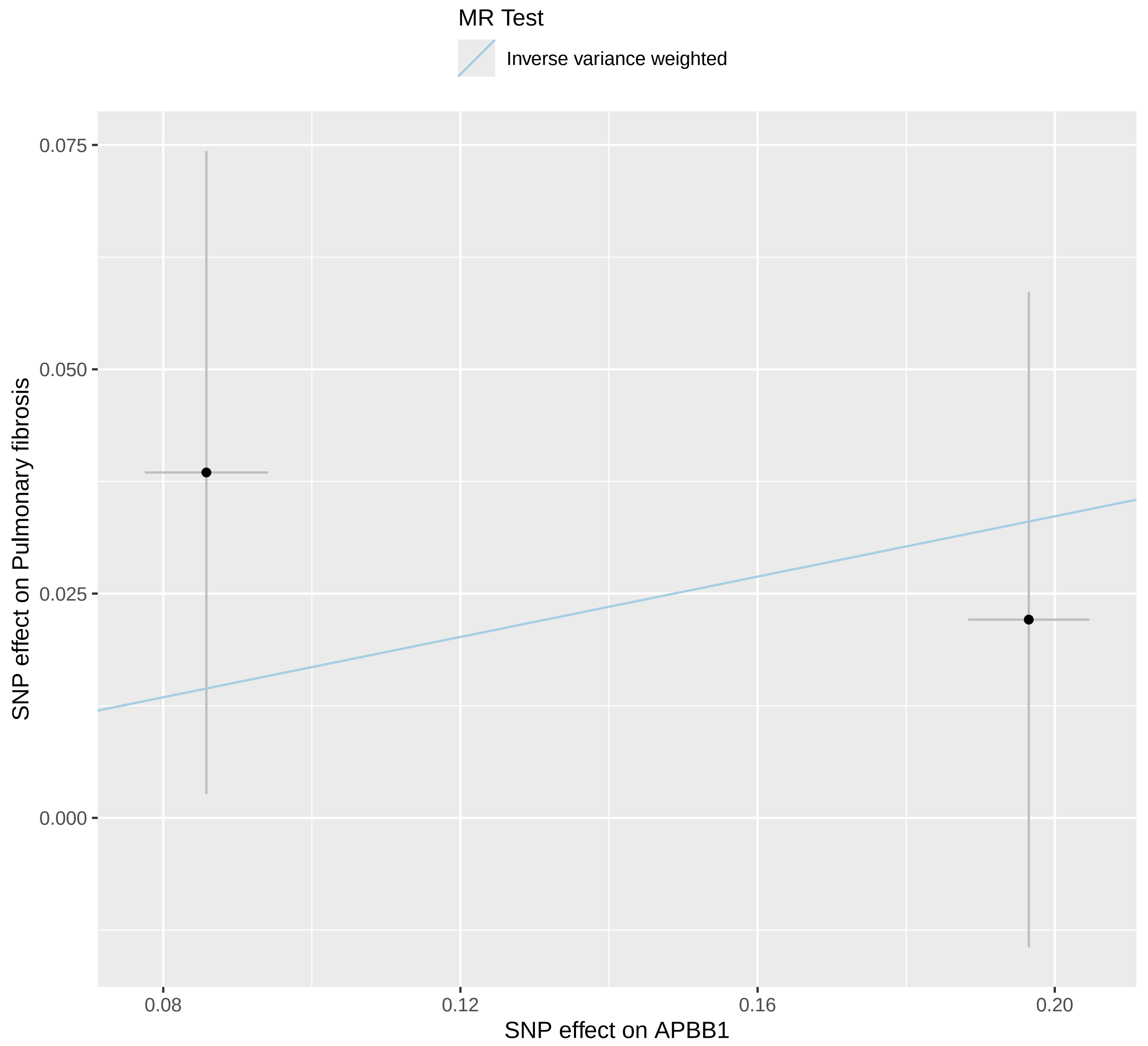

MR Test
Inverse variance weighted
0.075
0.050
0.025
0.000
0.08
0.12
0.16
0.20
SNP effect on Pulmonary fibrosis
SNP effect on APBB1

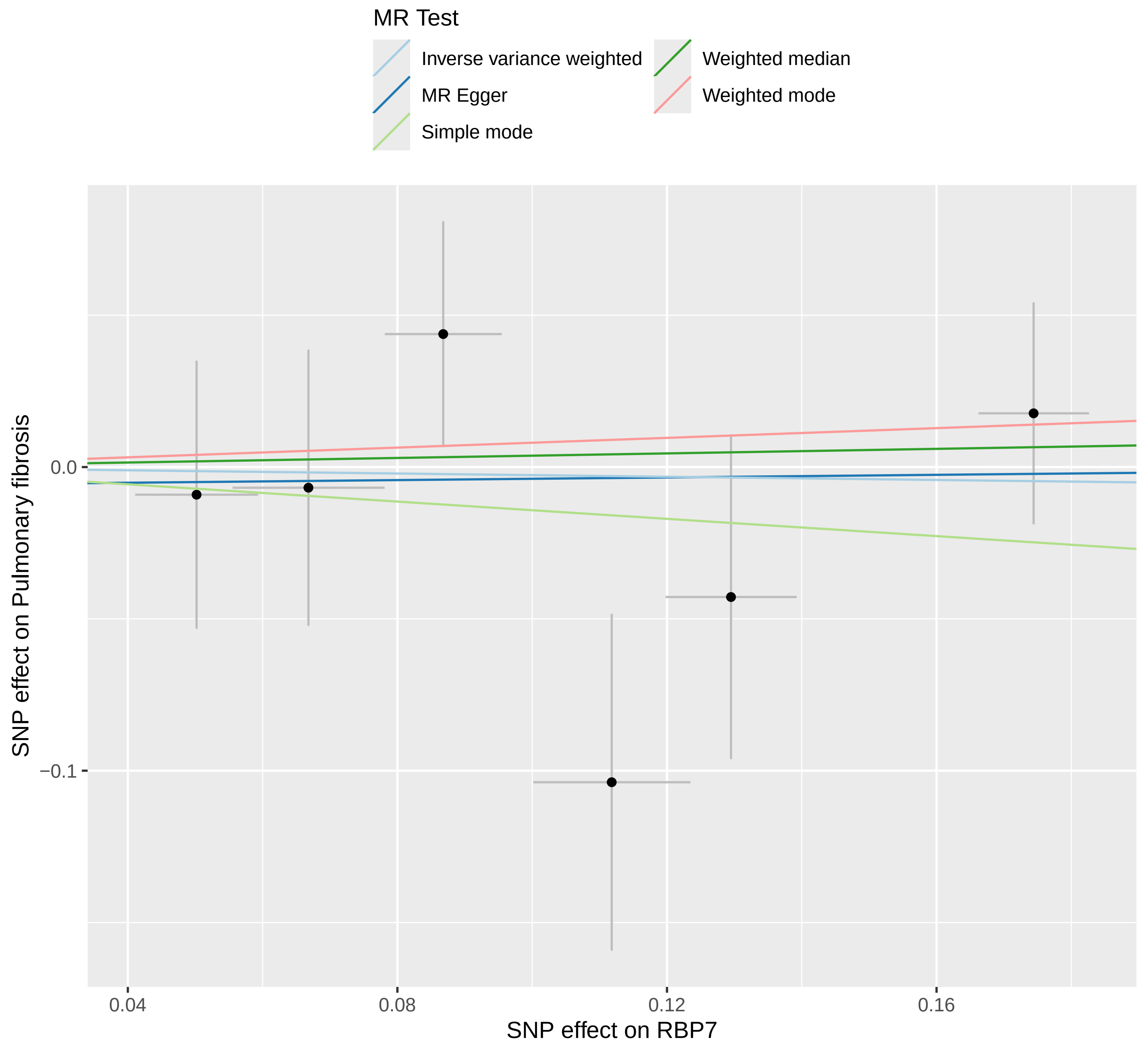
MR Test
Inverse variance weighted
MR Egger
Simple mode
Weighted median
Weighted mode
0.0
−0.1
SNP effect on Pulmonary fibrosis
0.04
0.08
0.12
0.16
SNP effect on RBP7

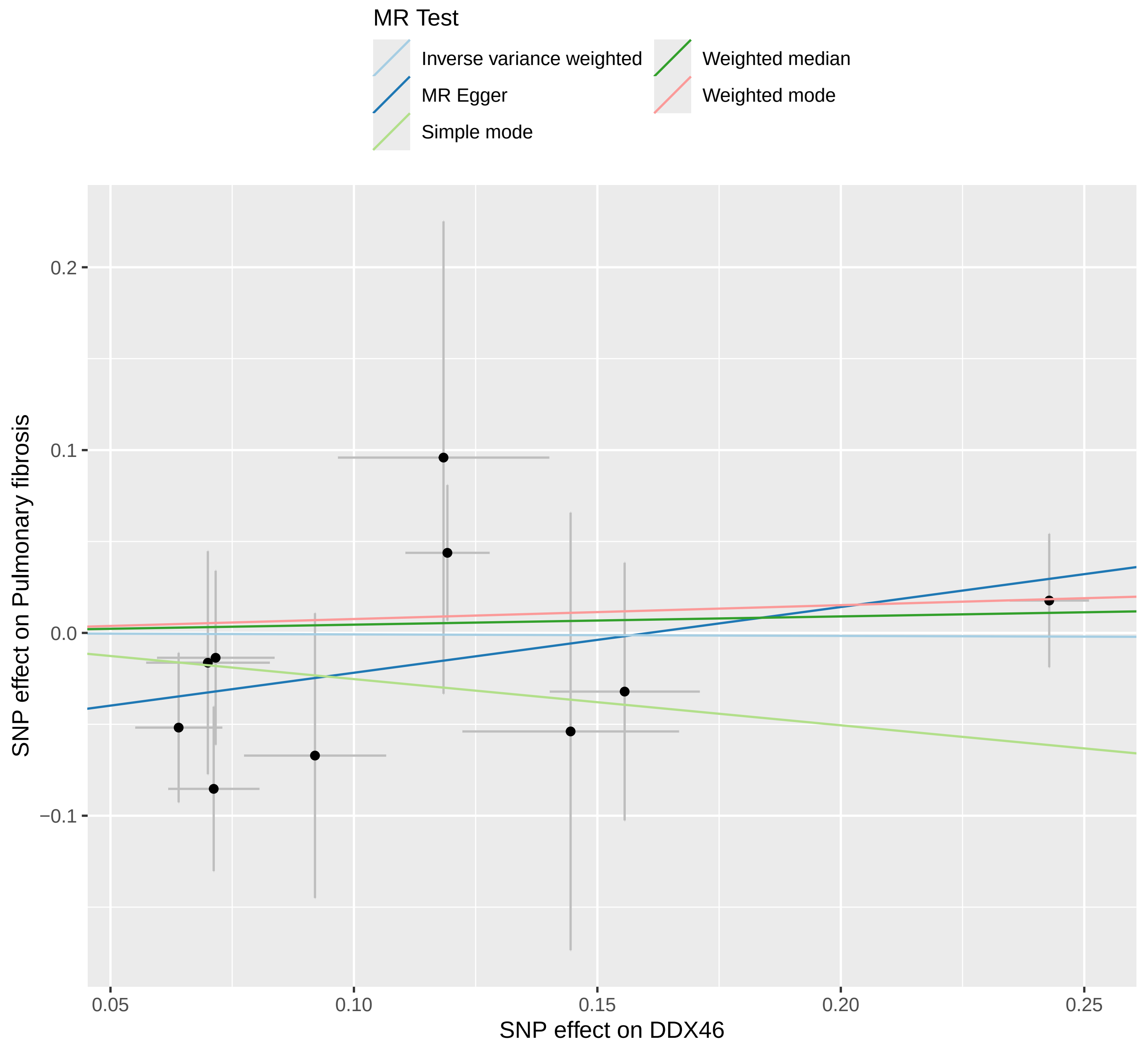
MR Test
Inverse variance weighted
MR Egger
Simple mode
Weighted median
Weighted mode
SNP effect on Pulmonary fibrosis
0.2
0.1
0.0
−0.1
0.05
0.10
0.15
0.20
0.25
SNP effect on DDX46

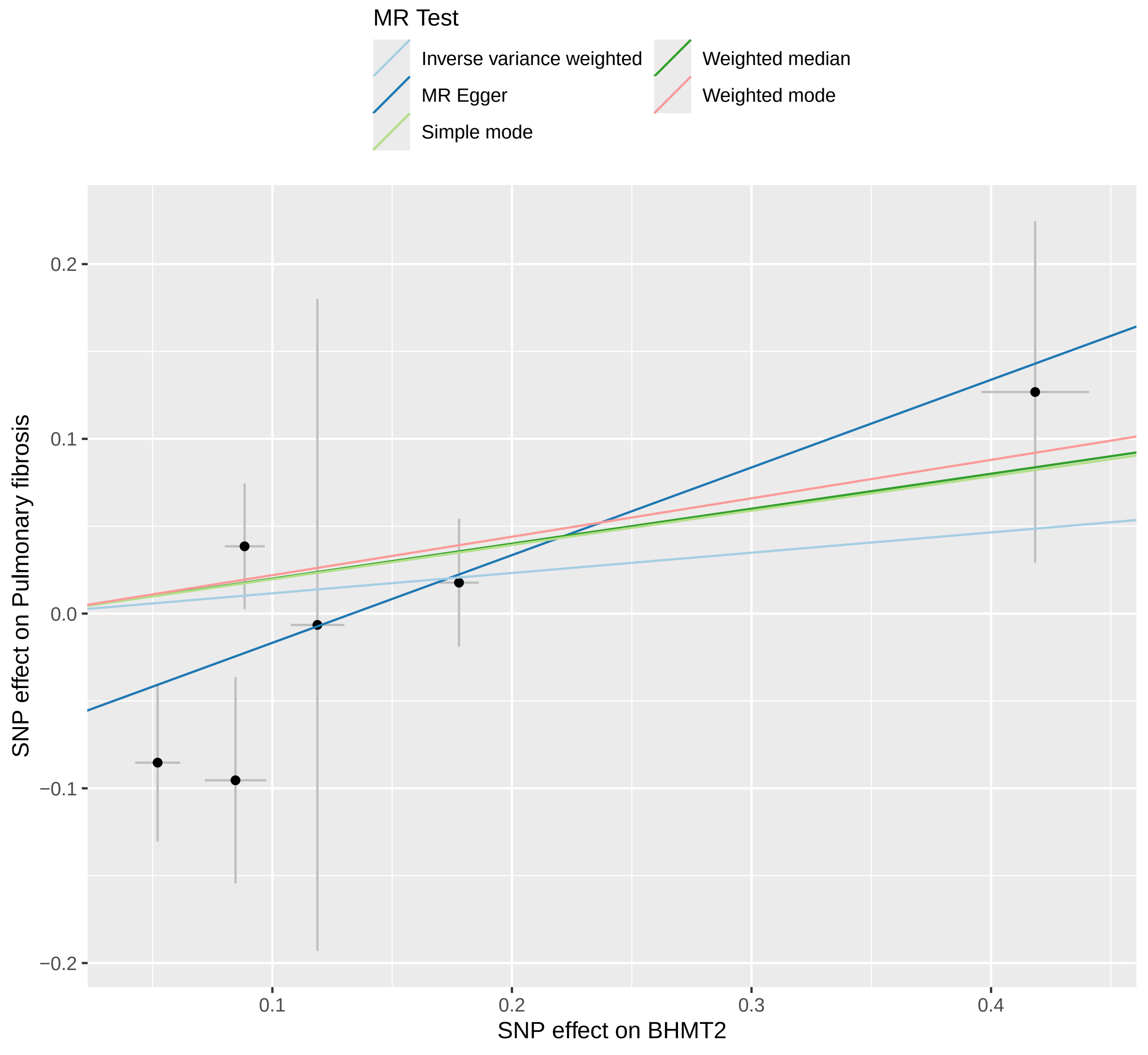
MR Test
Inverse variance weighted
MR Egger
Simple mode
Weighted median
Weighted mode
SNP effect on Pulmonary fibrosis
0.2
0.1
0.0
−0.1
−0.2
0.1
0.2
0.3
0.4
SNP effect on BHMT2

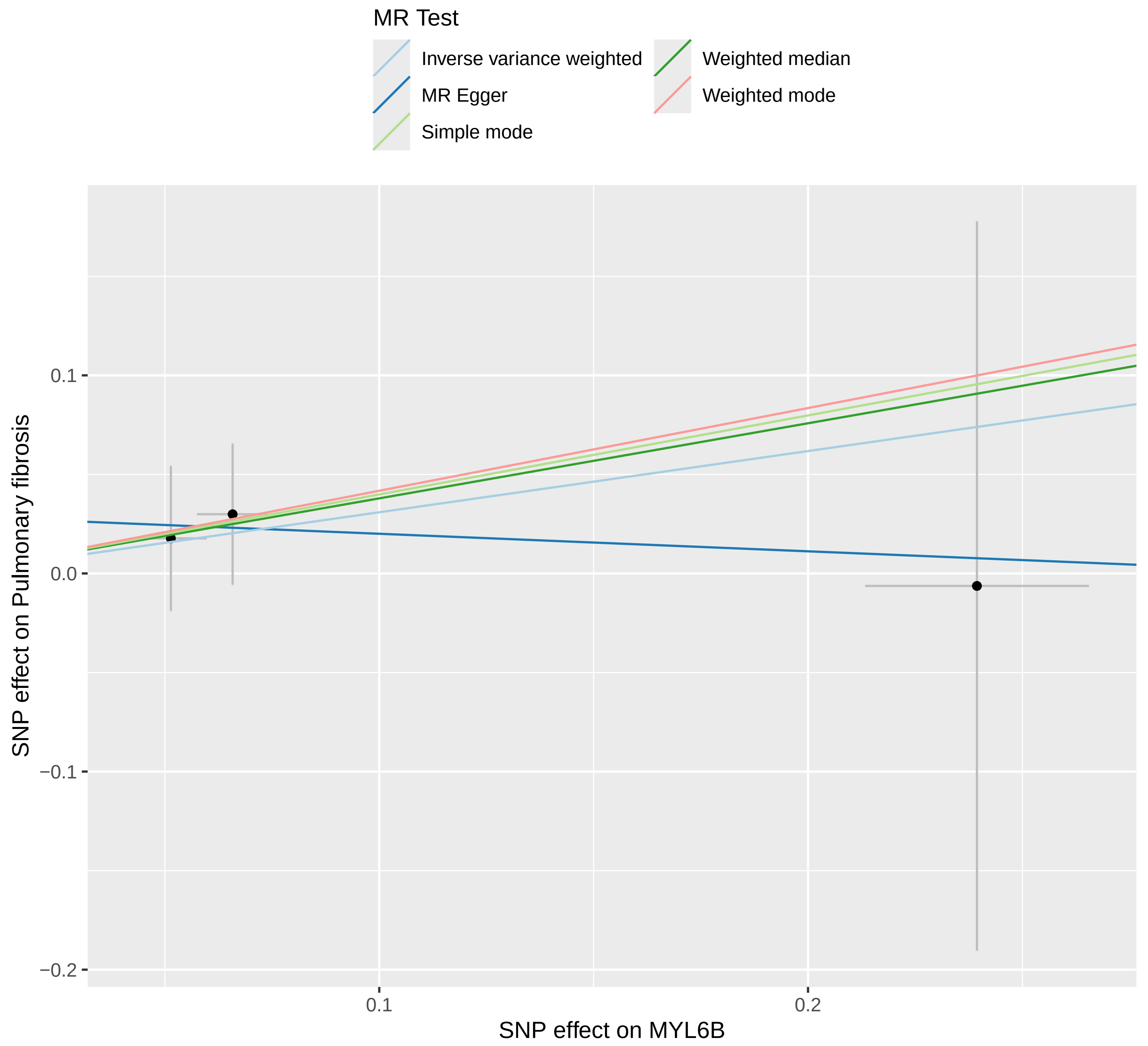
MR Test
Inverse variance weighted
MR Egger
Simple mode
Weighted median
Weighted mode
0.1
0.0
−0.1
−0.2
0.1
0.2
SNP effect on Pulmonary fibrosis
SNP effect on MYL6B

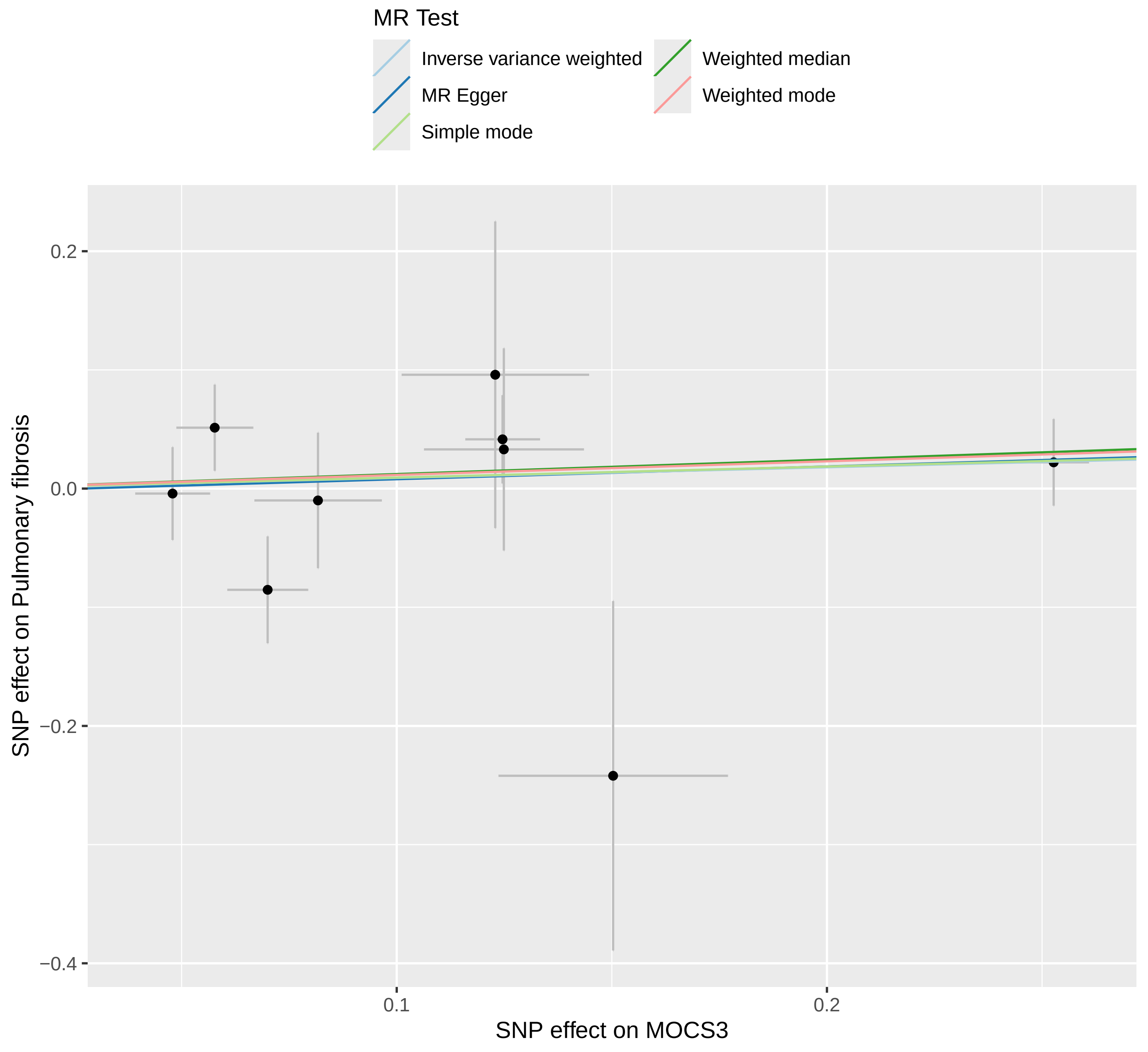

MR Test
Inverse variance weighted
MR Egger
Simple mode
Weighted median
Weighted mode
0.2
0.0
−0.2
−0.4
0.1
0.2
SNP effect on Pulmonary fibrosis
SNP effect on MOCS3

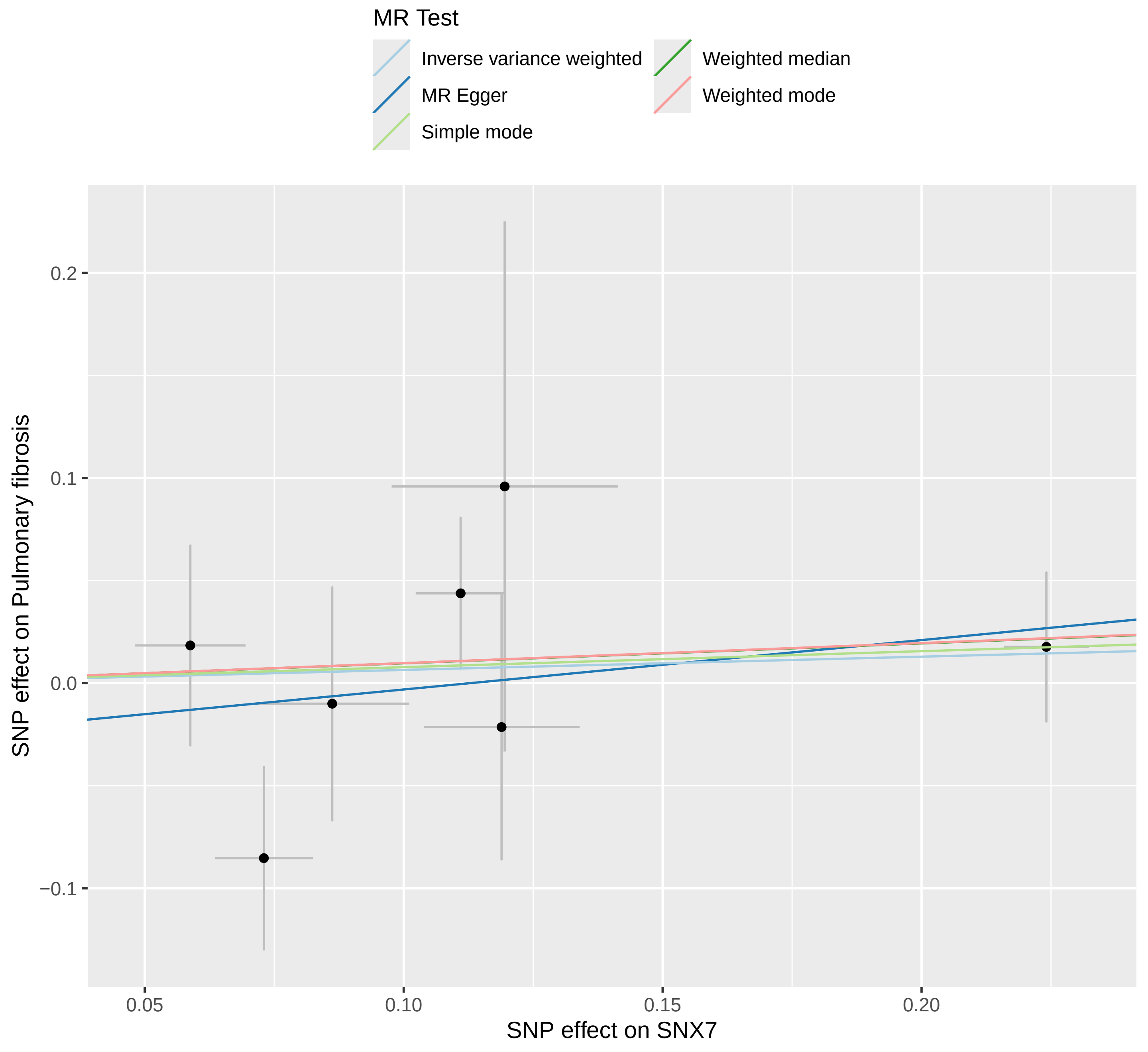
MR Test
Inverse variance weighted
MR Egger
Simple mode
Weighted median
Weighted mode
0.2
0.1
0.0
−0.1
SNP effect on Pulmonary fibrosis
0.05
0.10
0.15
0.20
SNP effect on SNX7

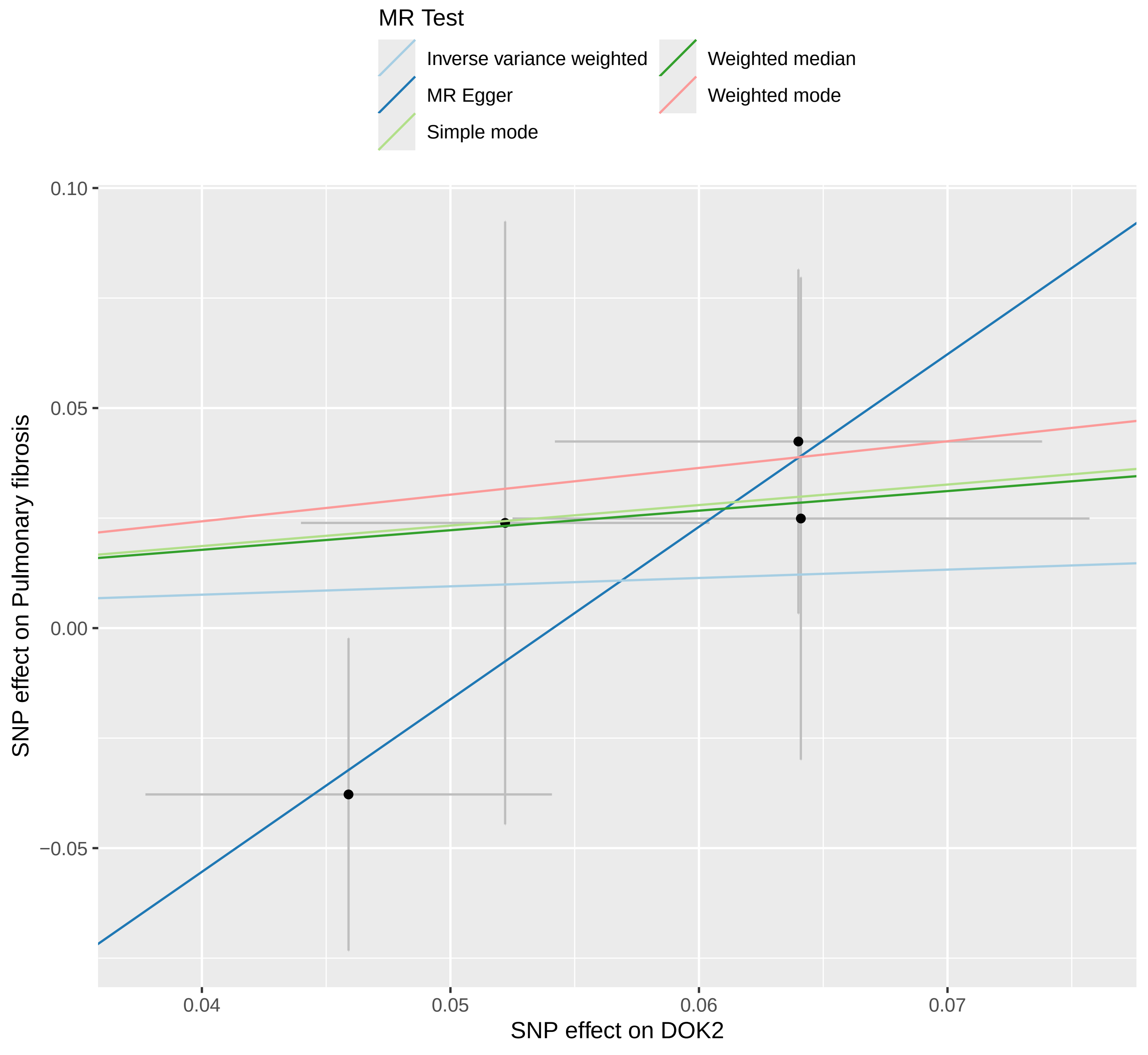

MR Test
Inverse variance weighted
MR Egger
Simple mode
Weighted median
Weighted mode
SNP effect on Pulmonary fibrosis
0.10
0.05
0.00
−0.05
0.04
0.05
0.06
0.07
SNP effect on DOK2

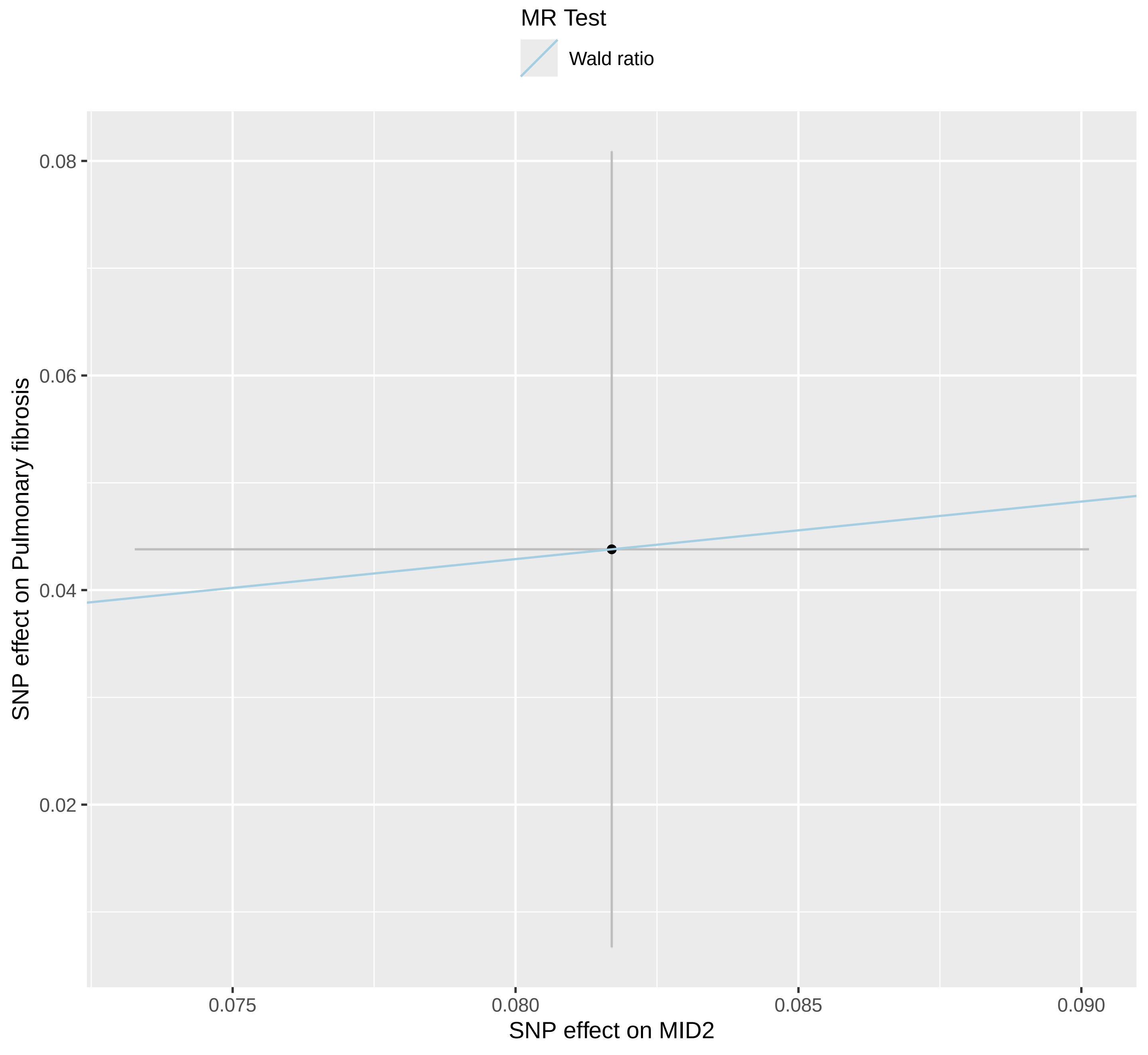
MR Test
Wald ratio
0.08
0.06
0.04
0.02
SNP effect on Pulmonary fibrosis
0.075
0.080
0.085
0.090
SNP effect on MID2

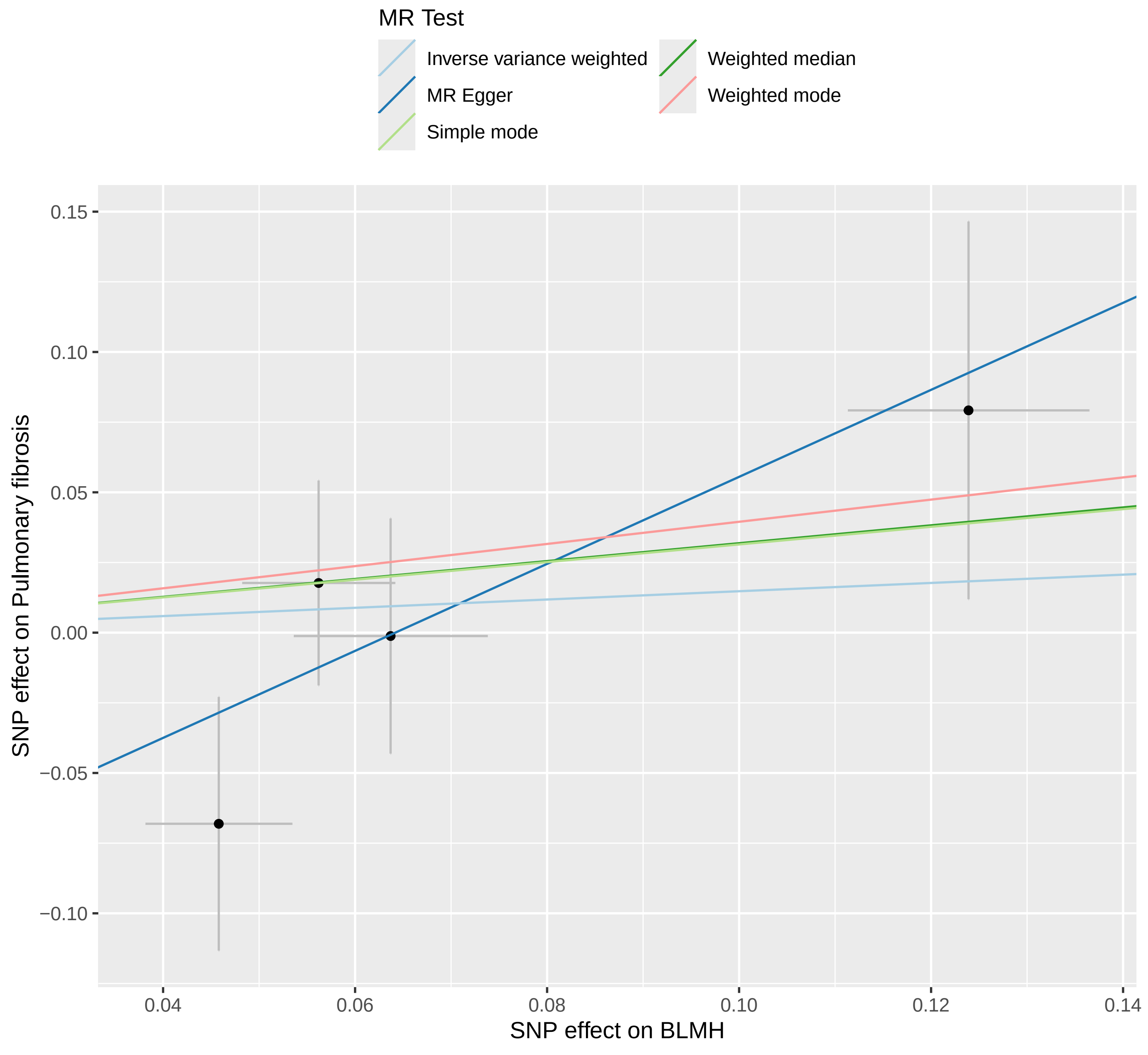

MR Test
Inverse variance weighted
MR Egger
Simple mode
Weighted median
Weighted mode
0.15
0.10
0.05
0.00
−0.05
−0.10
SNP effect on Pulmonary fibrosis
0.04
0.06
0.08
0.10
0.12
0.14
SNP effect on BLMH

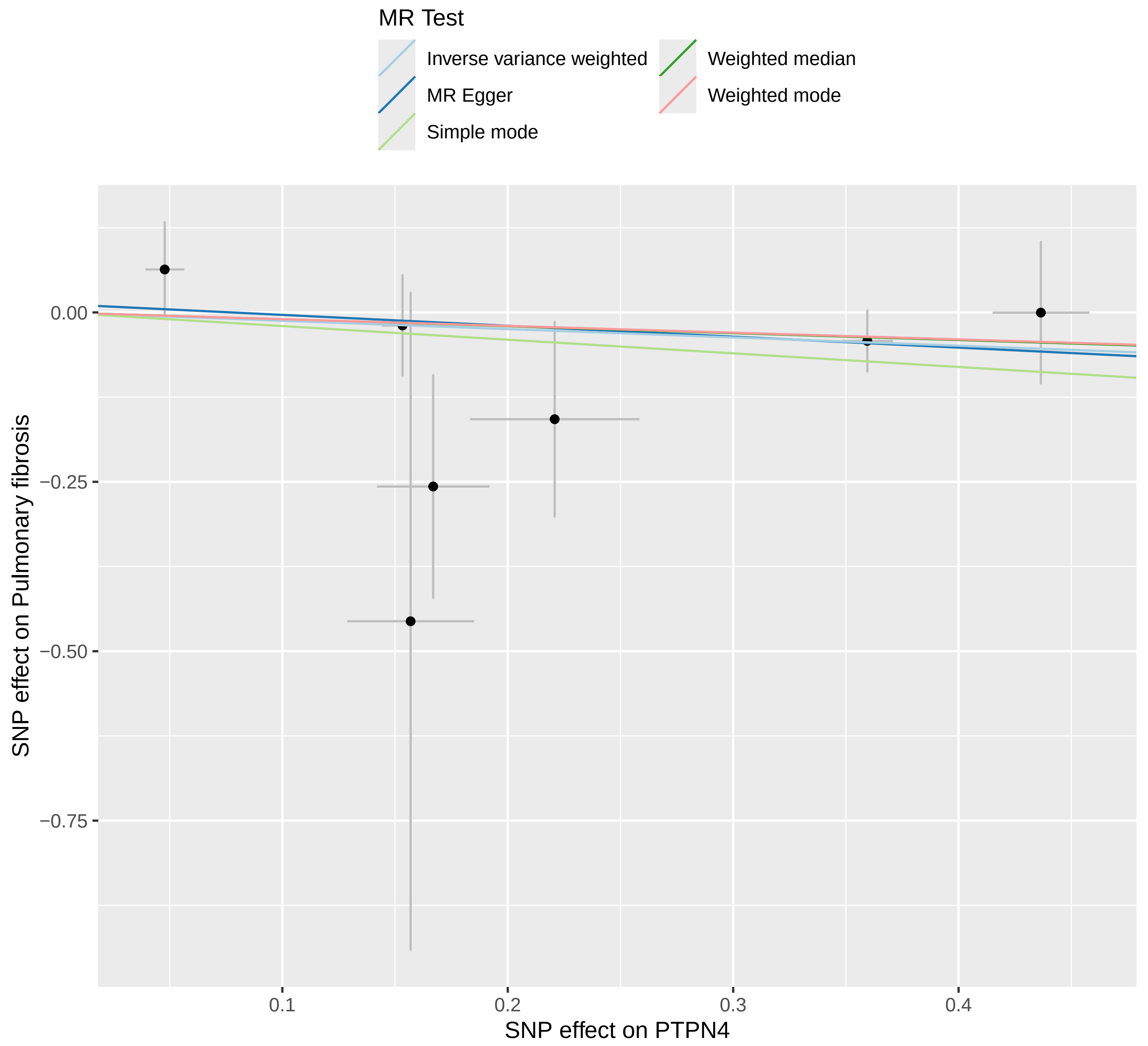

MR Test
Inverse variance weighted
MR Egger
Simple mode
Weighted median
Weighted mode
SNP effect on Pulmonary fibrosis
0.00
−0.25
−0.50
−0.75
SNP effect on PTPN4
0.1
0.2
0.3
0.4

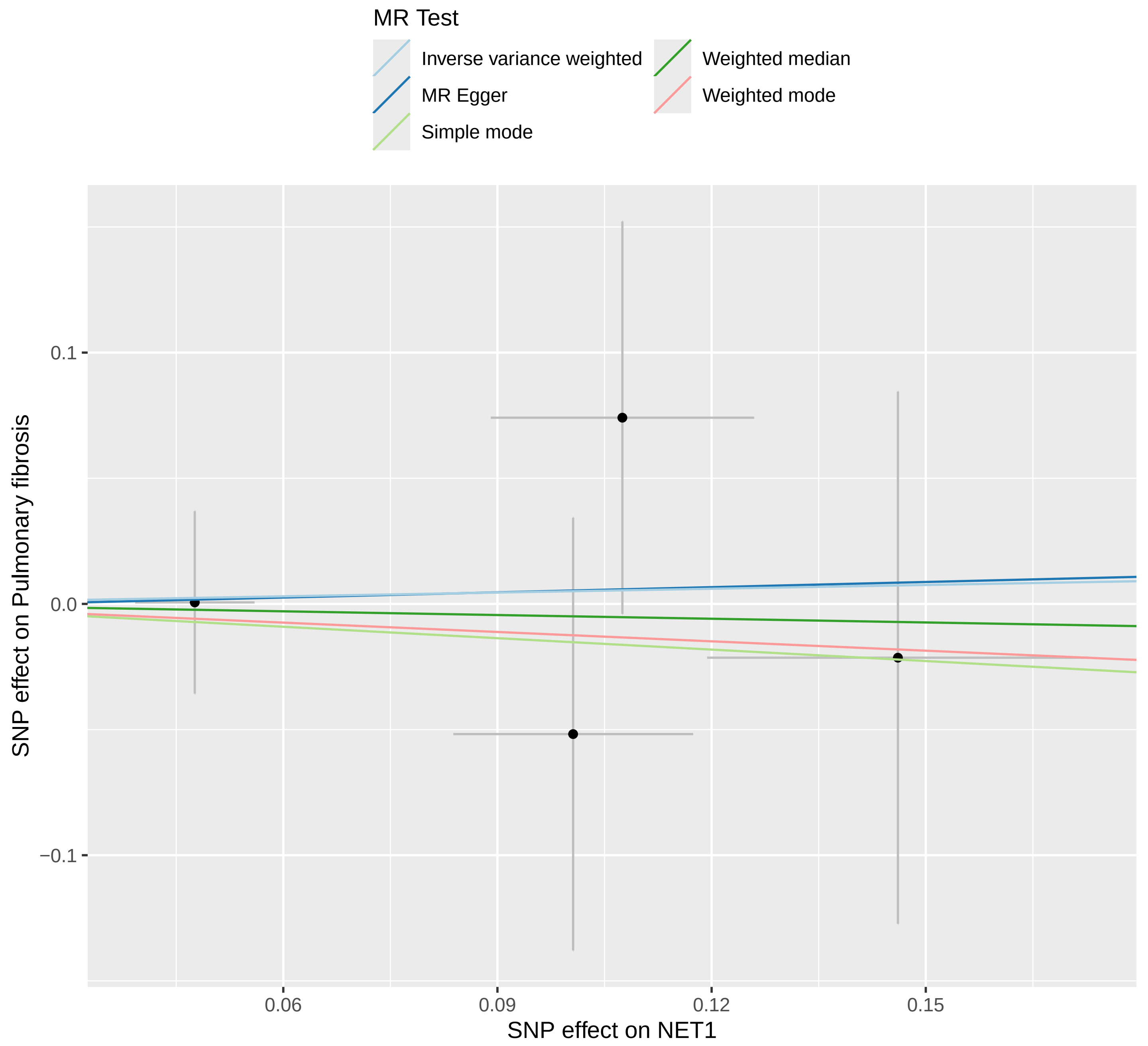
MR Test
Inverse variance weighted
MR Egger
Simple mode
Weighted median
Weighted mode
0.1
0.0
−0.1
0.06
0.09
0.12
0.15
SNP effect on NET1
SNP effect on Pulmonary fibrosis

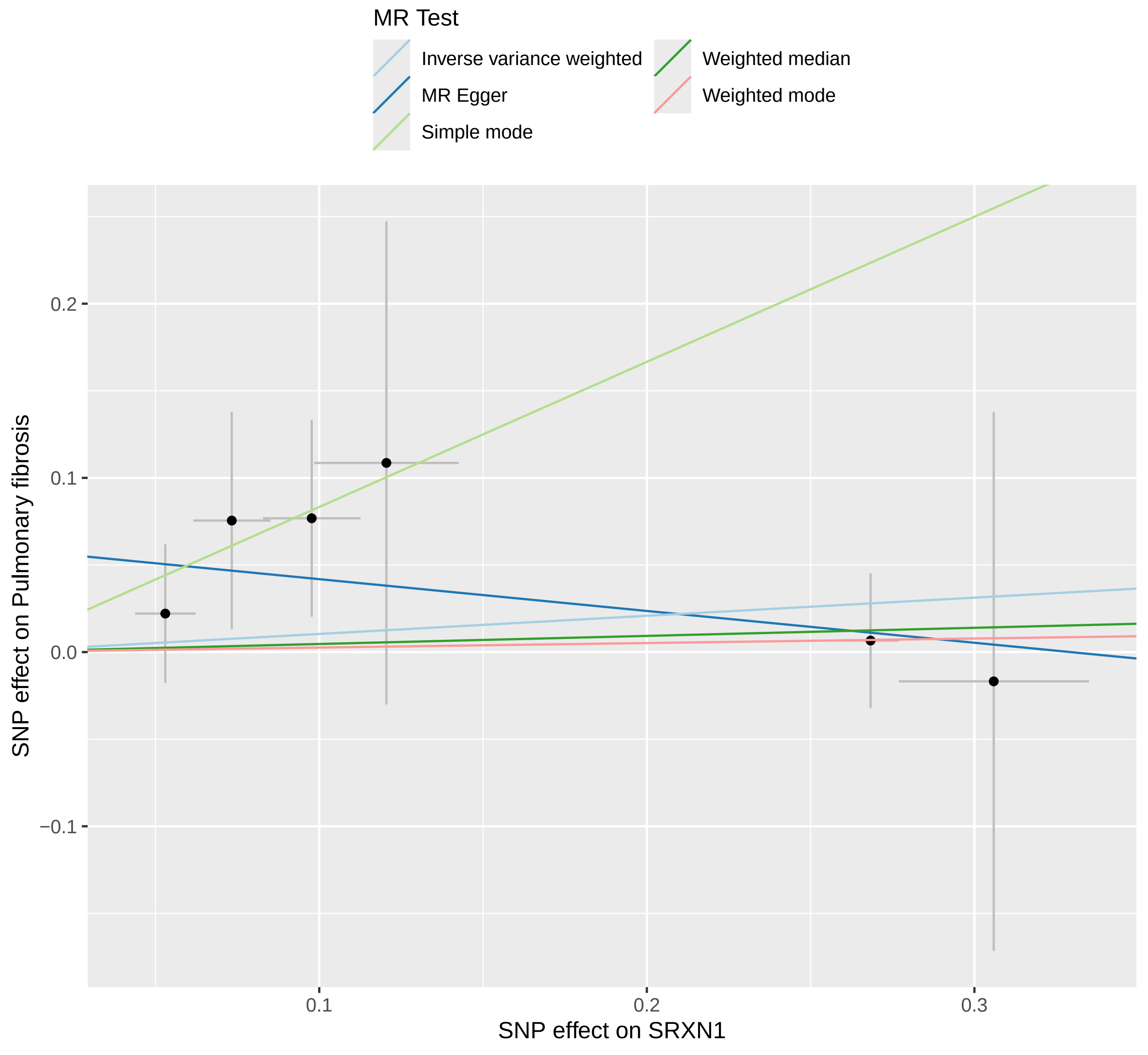
MR Test
Inverse variance weighted
MR Egger
Simple mode
Weighted median
Weighted mode
0.2
0.1
0.0
−0.1
0.1
0.2
0.3
SNP effect on Pulmonary fibrosis
SNP effect on SRXN1

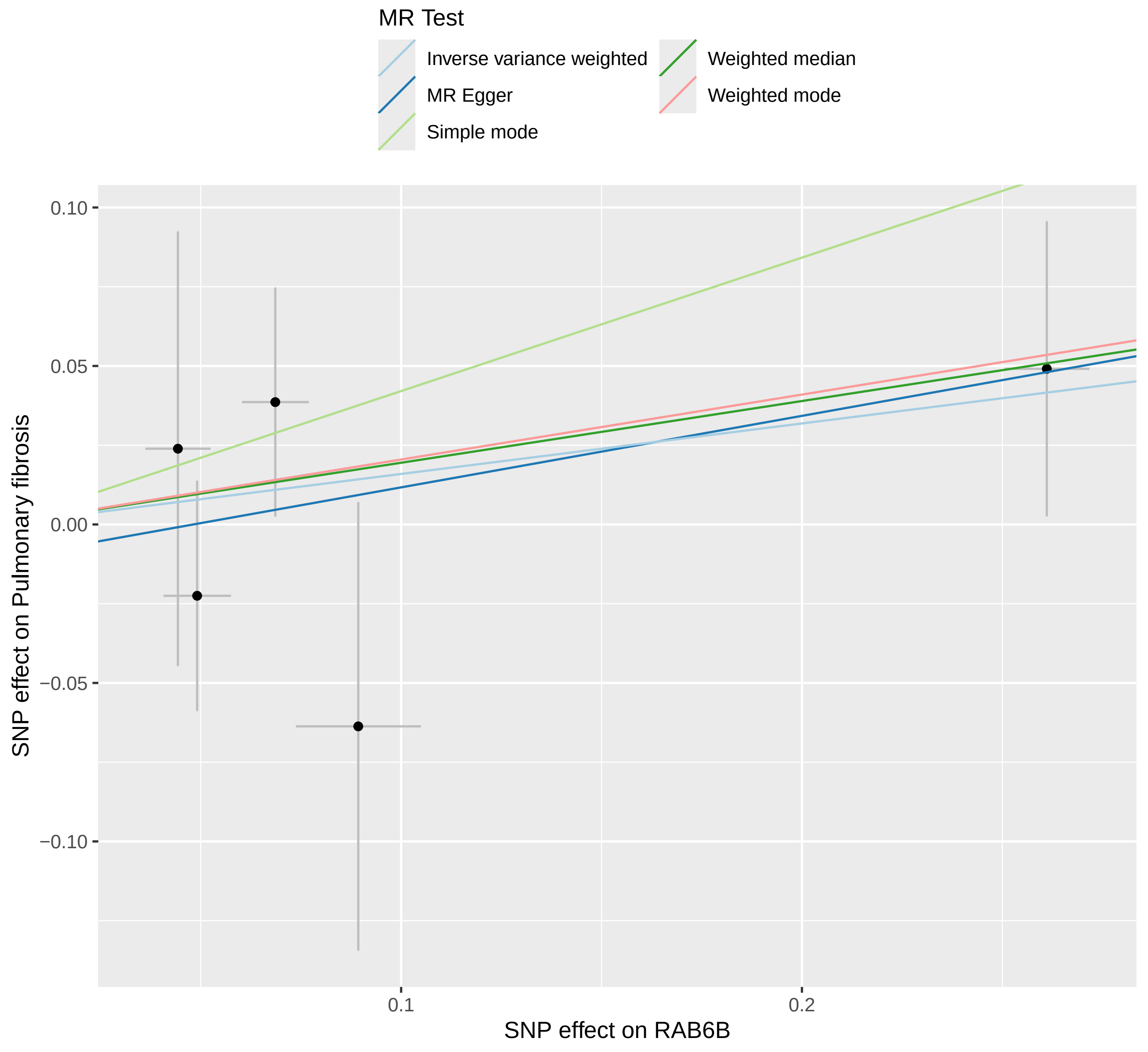

MR Test
Inverse variance weighted
MR Egger
Simple mode
Weighted median
Weighted mode
0.10
0.05
0.00
−0.05
−0.10
SNP effect on Pulmonary fibrosis
0.1
0.2
SNP effect on RAB6B

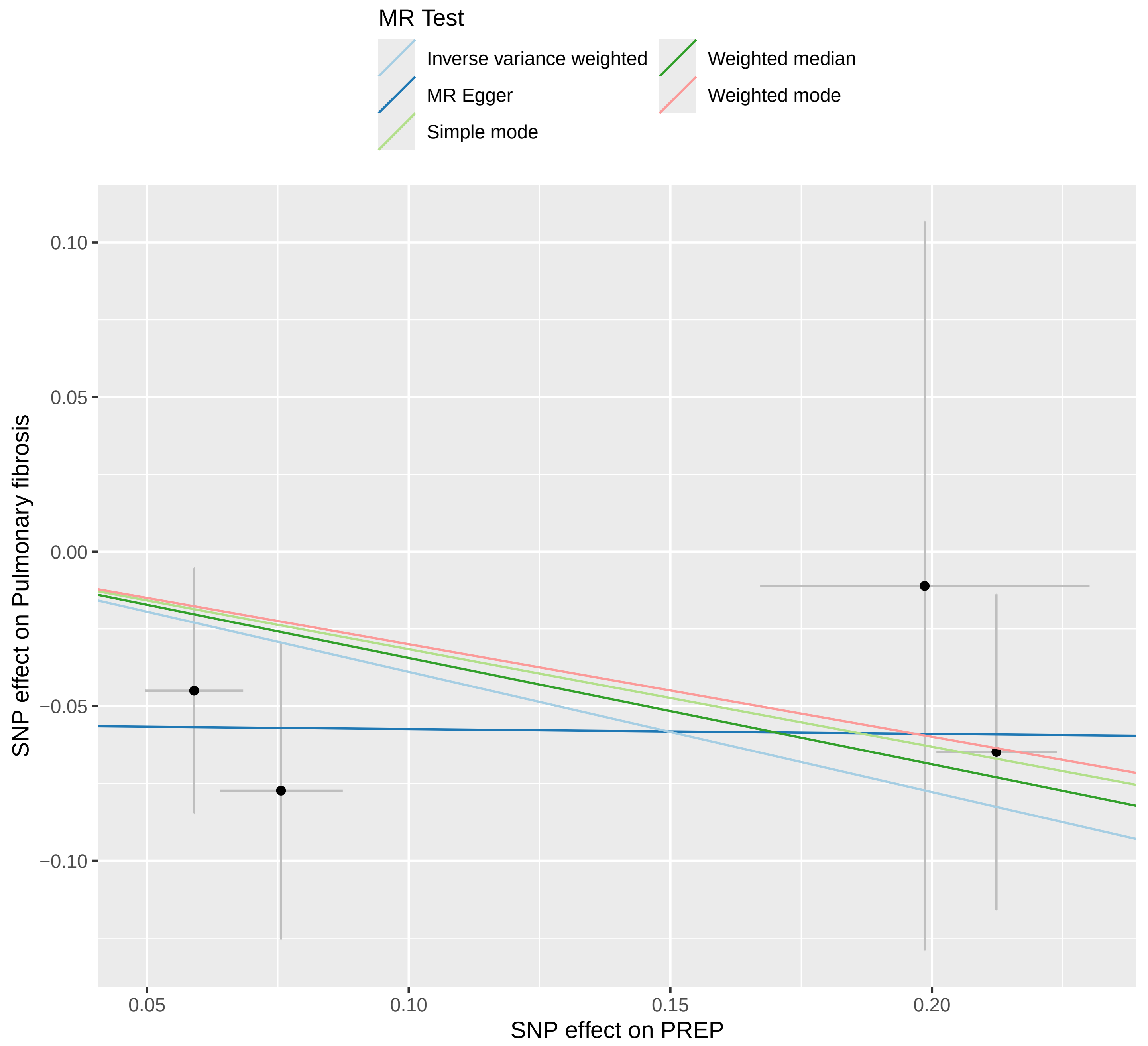
MR Test
Inverse variance weighted
MR Egger
Simple mode
Weighted median
Weighted mode
0.10
0.05
0.00
−0.05
−0.10
SNP effect on Pulmonary fibrosis
0.05
0.10
0.15
0.20
SNP effect on PREP

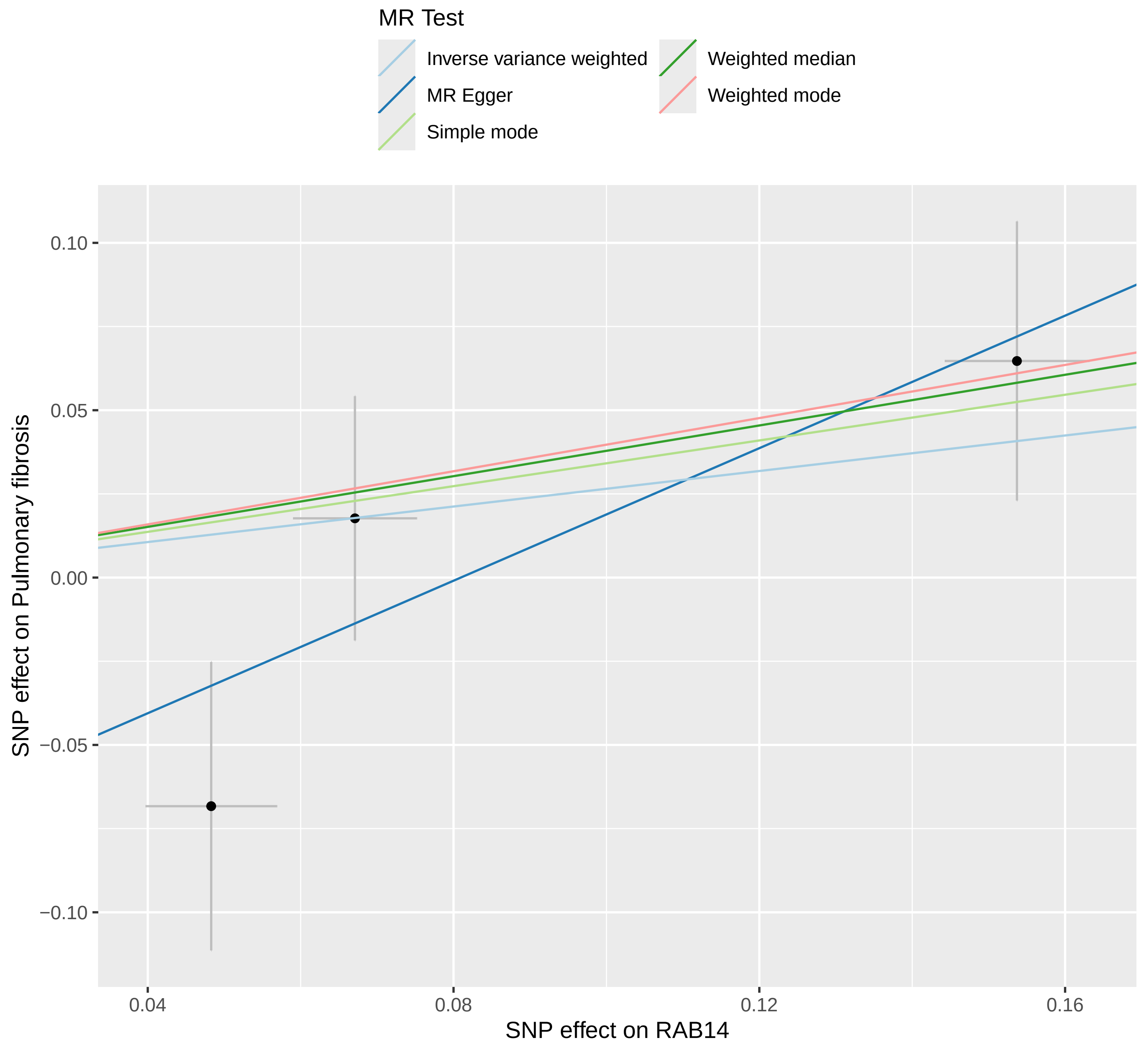
MR Test
Inverse variance weighted
MR Egger
Simple mode
Weighted median
Weighted mode
0.10
0.05
0.00
−0.05
−0.10
0.04
0.08
0.12
0.16
SNP effect on Pulmonary fibrosis
SNP effect on RAB14

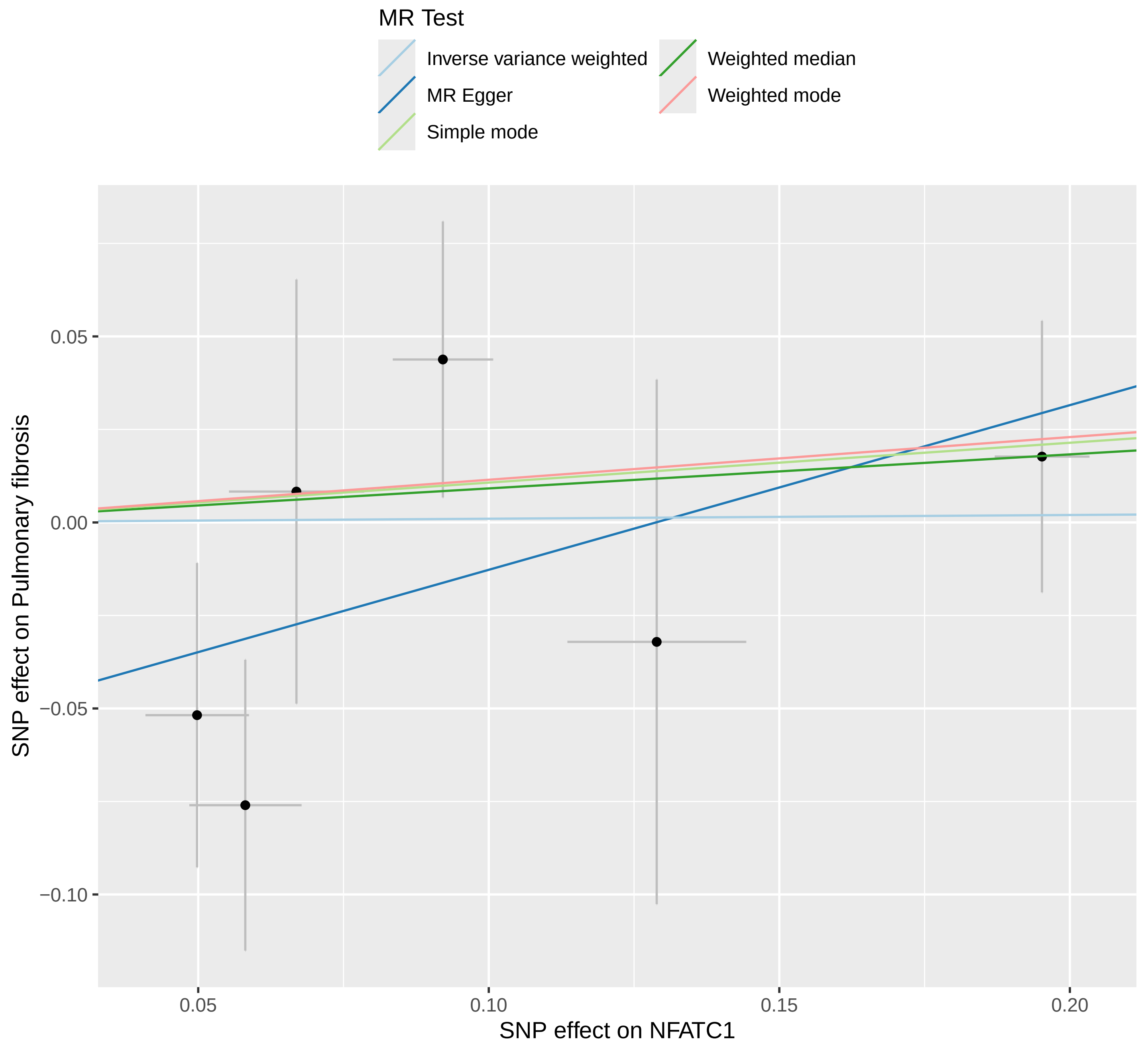
MR Test
Inverse variance weighted
MR Egger
Simple mode
Weighted median
Weighted mode
0.05
0.00
−0.05
−0.10
0.05
0.10
0.15
0.20
SNP effect on NFATC1
SNP effect on Pulmonary fibrosis

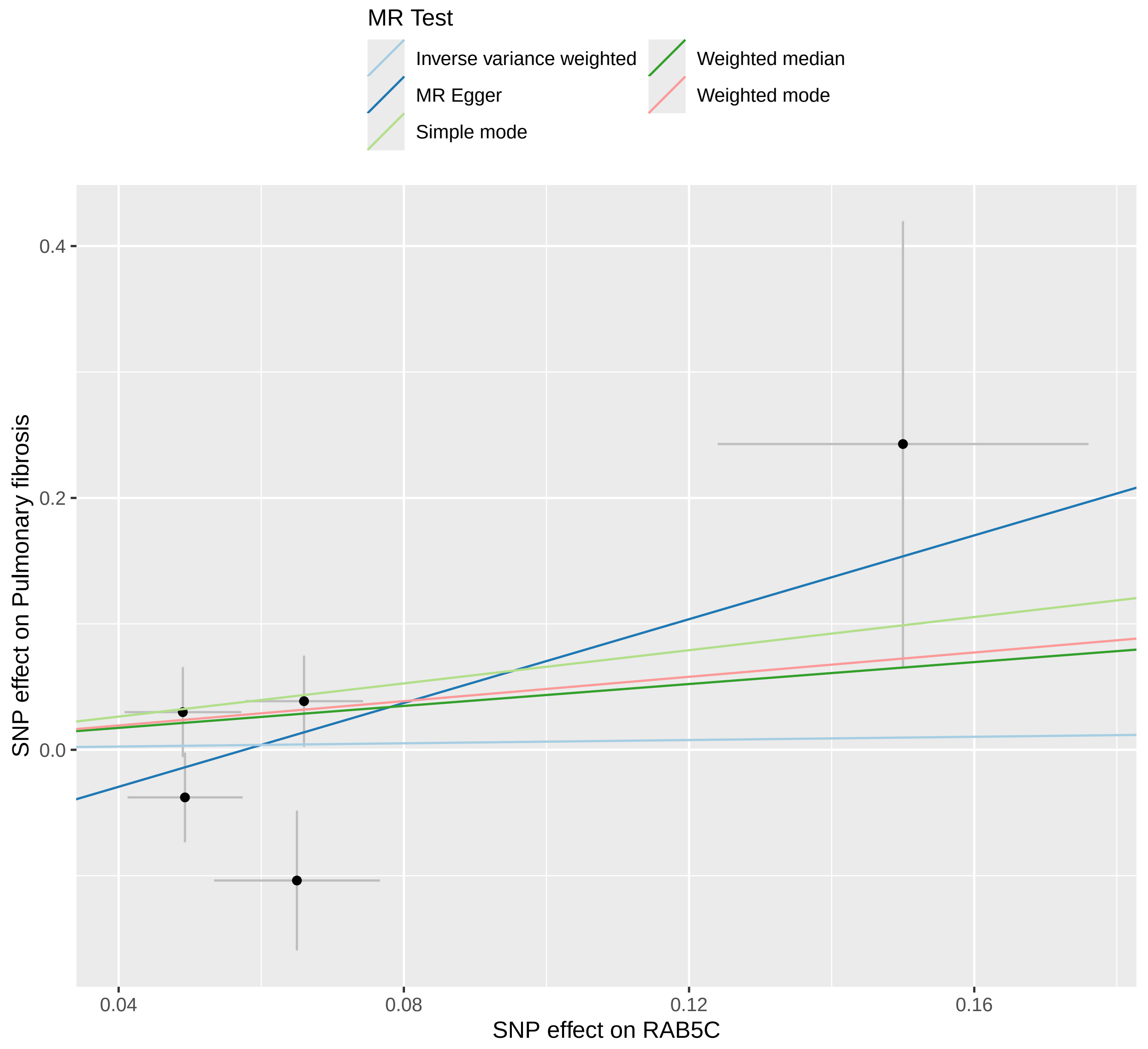
MR Test
Inverse variance weighted
MR Egger
Simple mode
Weighted median
Weighted mode
SNP effect on Pulmonary fibrosis
0.4
0.2
0.0
0.04
0.08
0.12
0.16
SNP effect on RAB5C

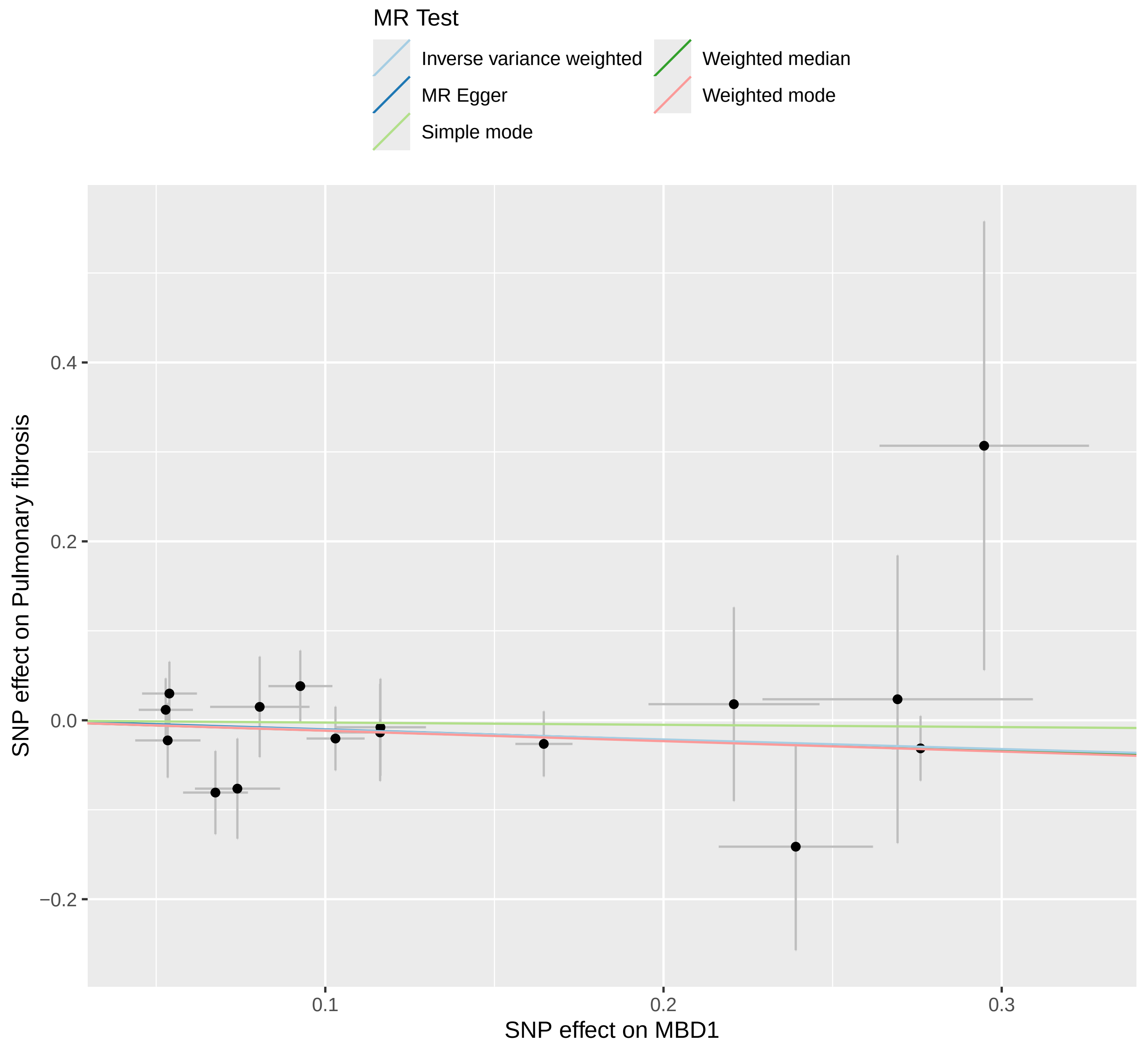

MR Test
Inverse variance weighted
MR Egger
Simple mode
Weighted median
Weighted mode
0.4
0.2
0.0
−0.2
SNP effect on Pulmonary fibrosis
0.1
0.2
0.3
SNP effect on MBD1

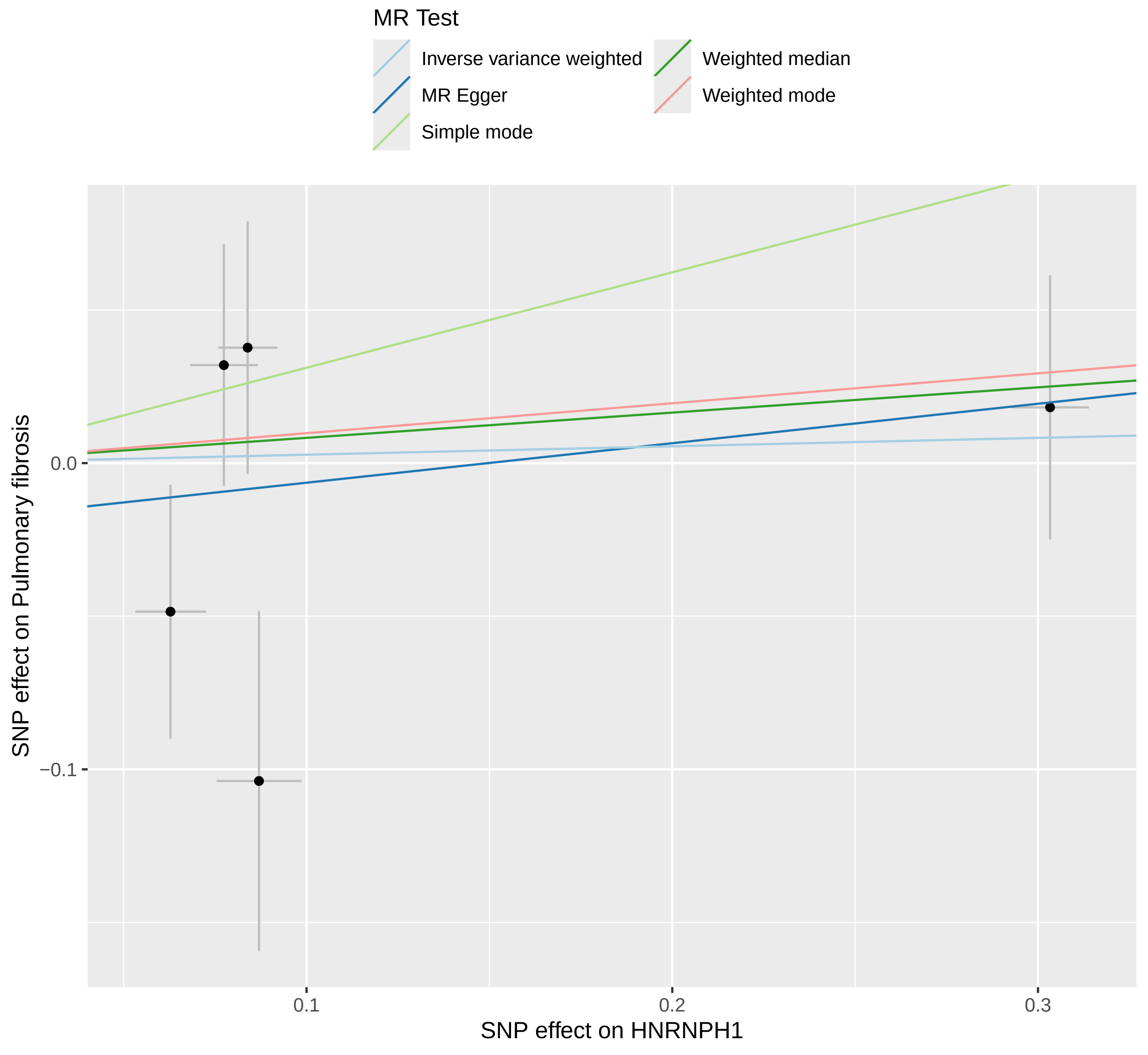
MR Test
Inverse variance weighted
MR Egger
Simple mode
Weighted median
Weighted mode
SNP effect on Pulmonary fibrosis
0.0
−0.1
0.1
0.2
0.3
SNP effect on HNRNPH1

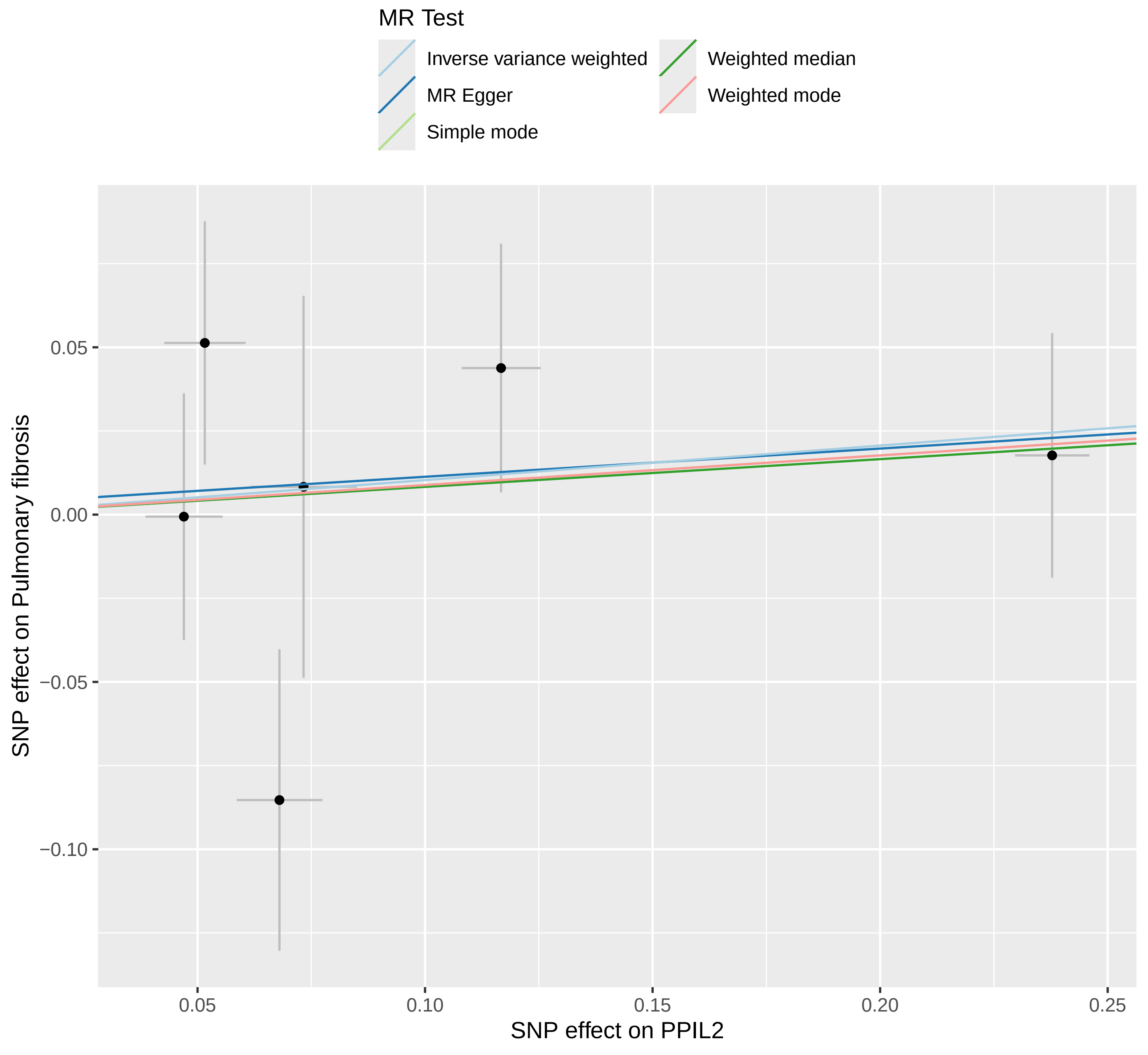
MR Test
Inverse variance weighted
MR Egger
Simple mode
Weighted median
Weighted mode
0.05
0.00
−0.05
−0.10
SNP effect on Pulmonary fibrosis
0.05
0.10
0.15
0.20
0.25
SNP effect on PPIL2

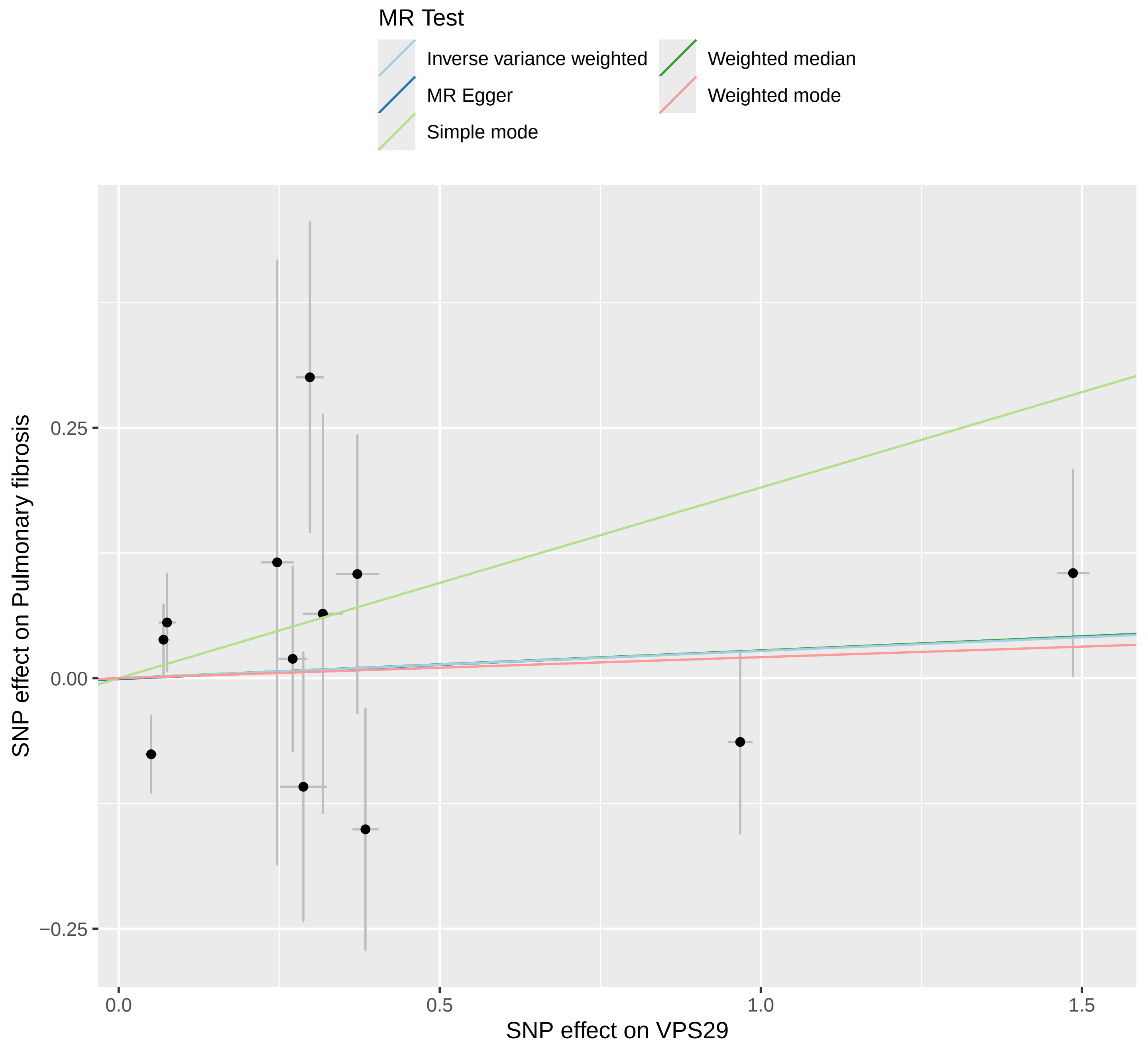
MR Test
Inverse variance weighted
MR Egger
Simple mode
Weighted median
Weighted mode
0.25
0.00
−0.25
0.0
0.5
1.0
1.5
SNP effect on Pulmonary fibrosis
SNP effect on VPS29

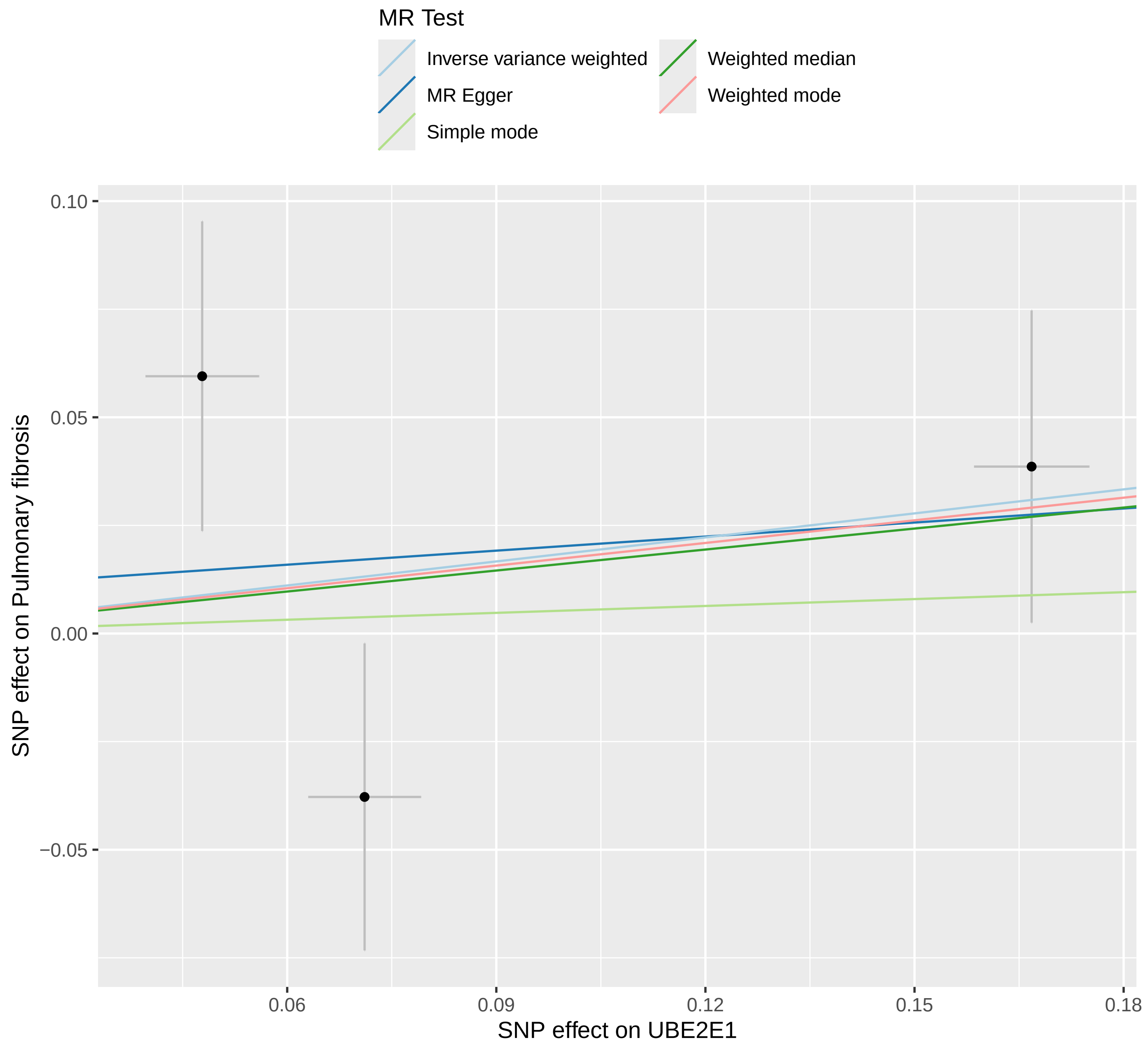
MR Test
Inverse variance weighted
MR Egger
Simple mode
Weighted median
Weighted mode
0.10
0.05
0.00
−0.05
SNP effect on Pulmonary fibrosis
0.06
0.09
0.12
0.15
0.18
SNP effect on UBE2E1

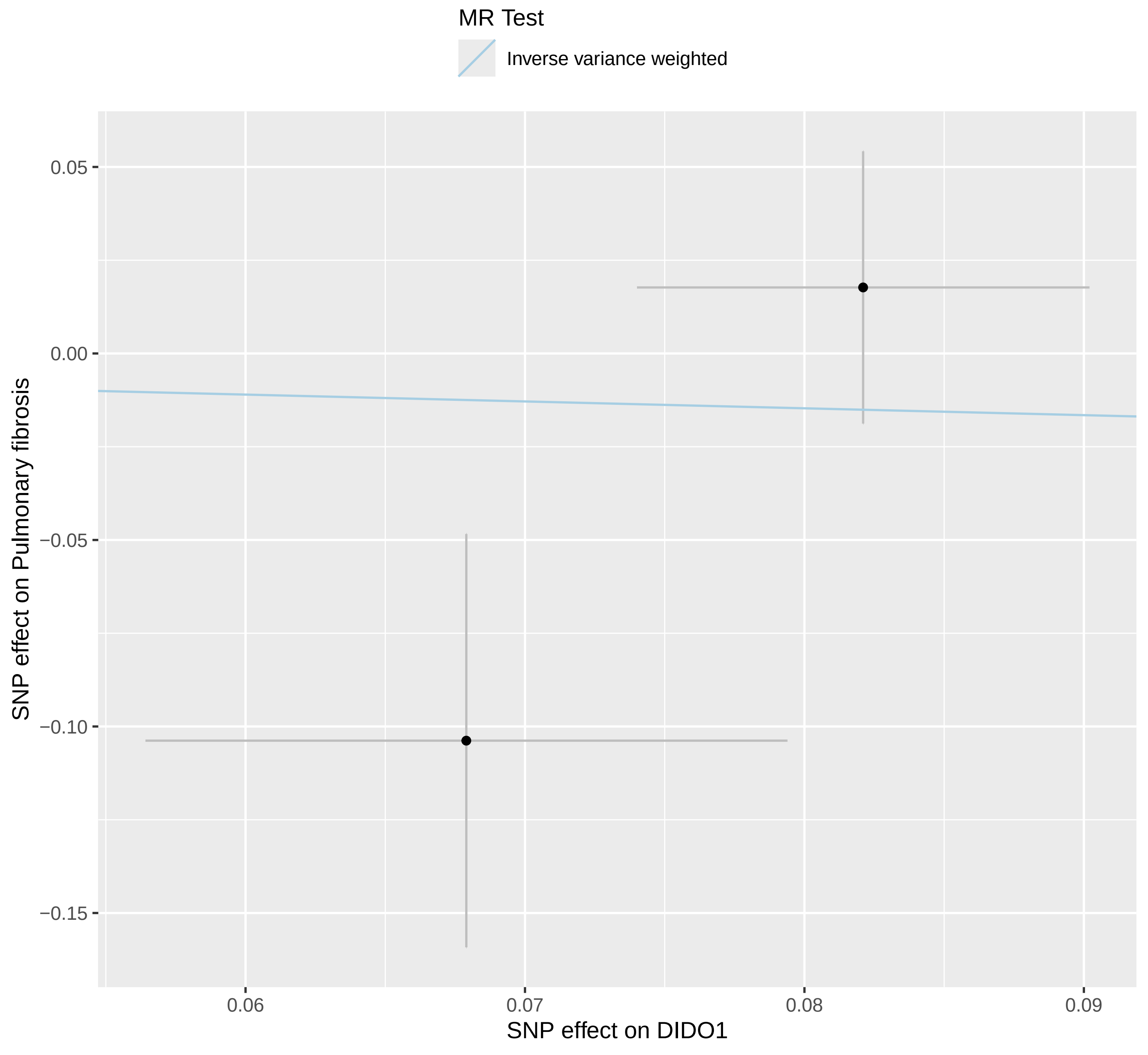

MR Test
Inverse variance weighted
0.05
0.00
−0.05
−0.10
−0.15
SNP effect on Pulmonary fibrosis
0.06
0.07
0.08
0.09
SNP effect on DIDO1

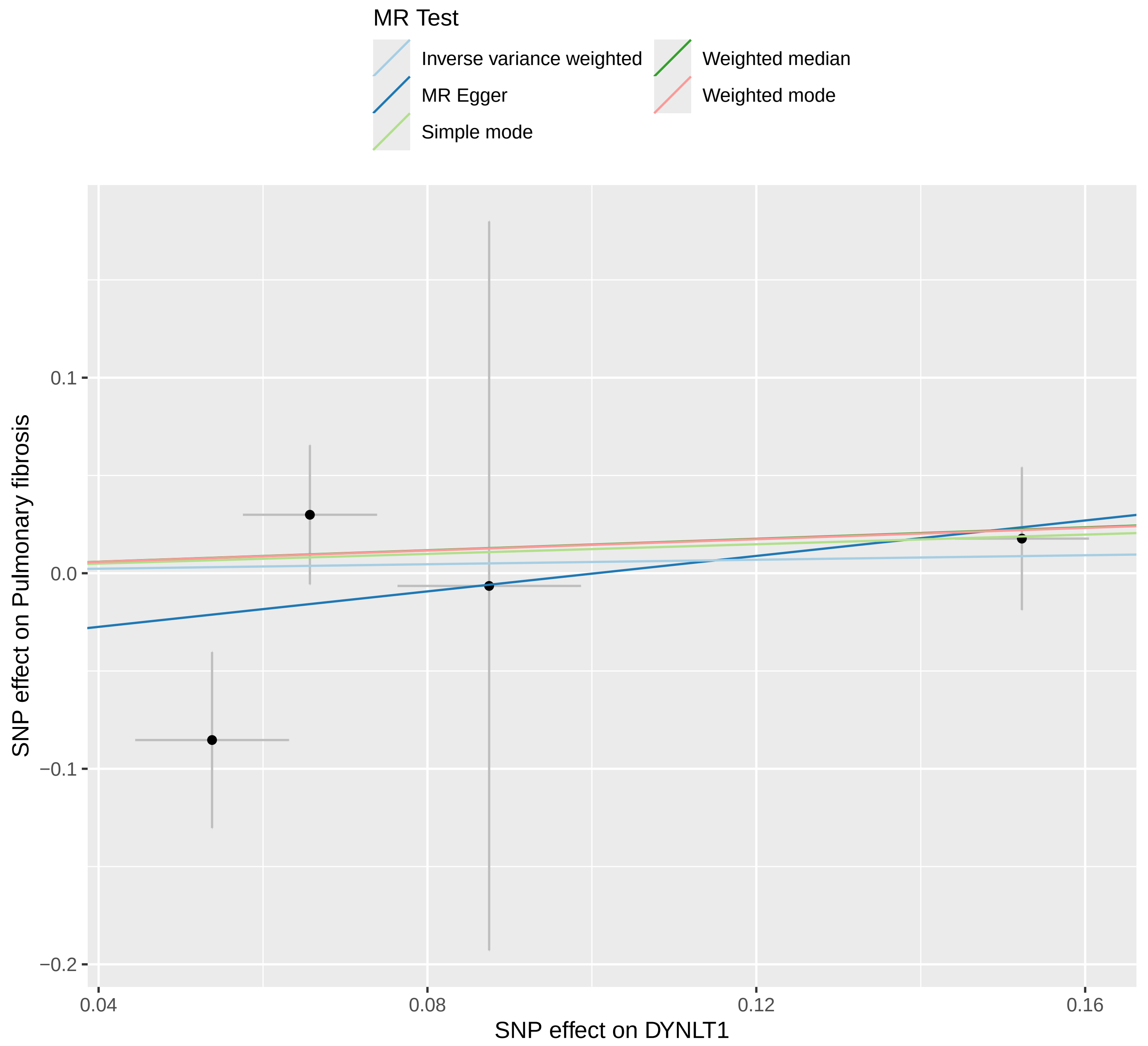
MR Test
Inverse variance weighted
MR Egger
Simple mode
Weighted median
Weighted mode
0.1
0.0
−0.1
−0.2
SNP effect on Pulmonary fibrosis
0.04
0.08
0.12
0.16
SNP effect on DYNLT1

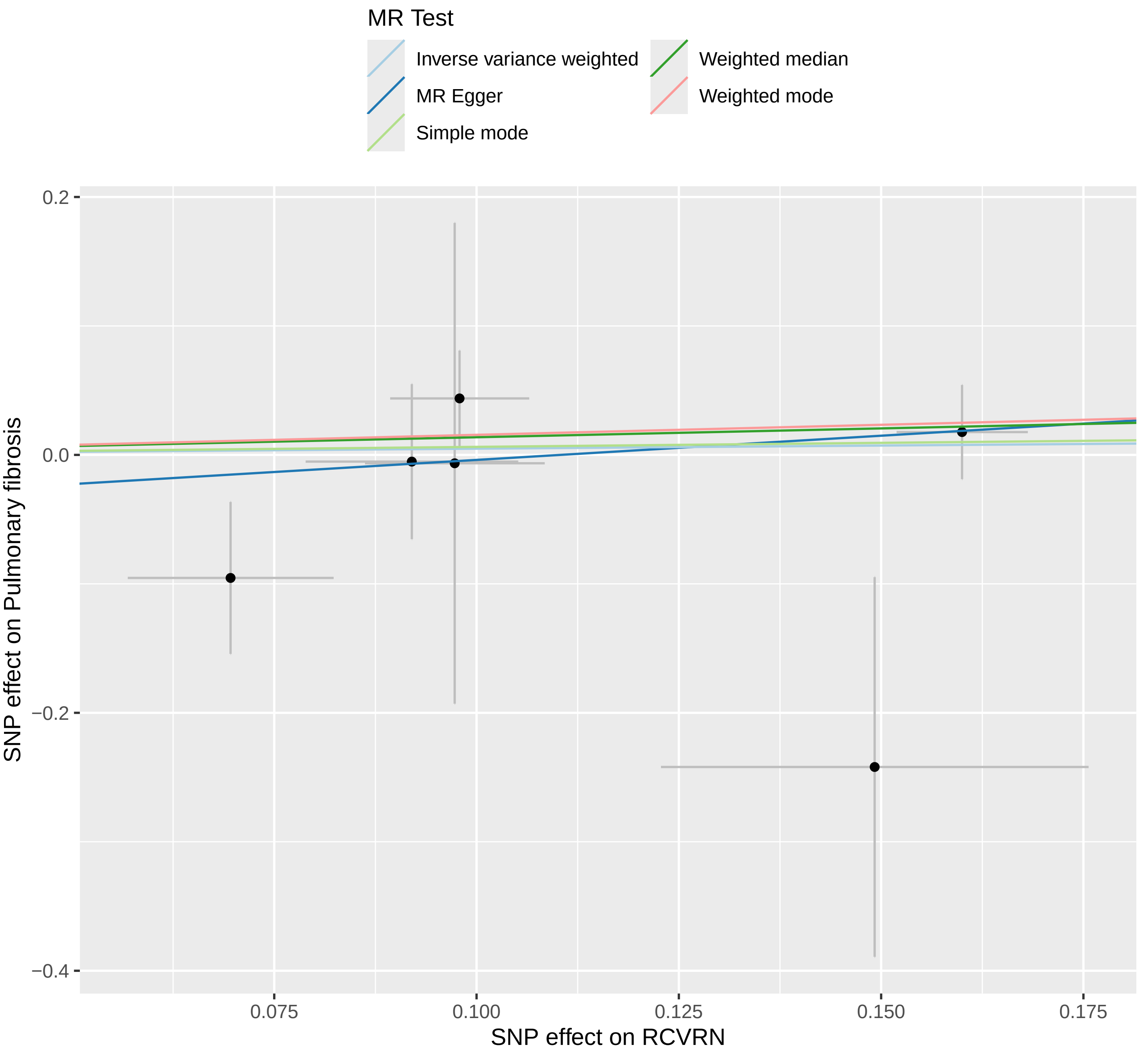
MR Test
Inverse variance weighted
MR Egger
Simple mode
Weighted median
Weighted mode
0.2
0.0
−0.2
−0.4
0.075
0.100
0.125
0.150
0.175
SNP effect on Pulmonary fibrosis
SNP effect on RCVRN

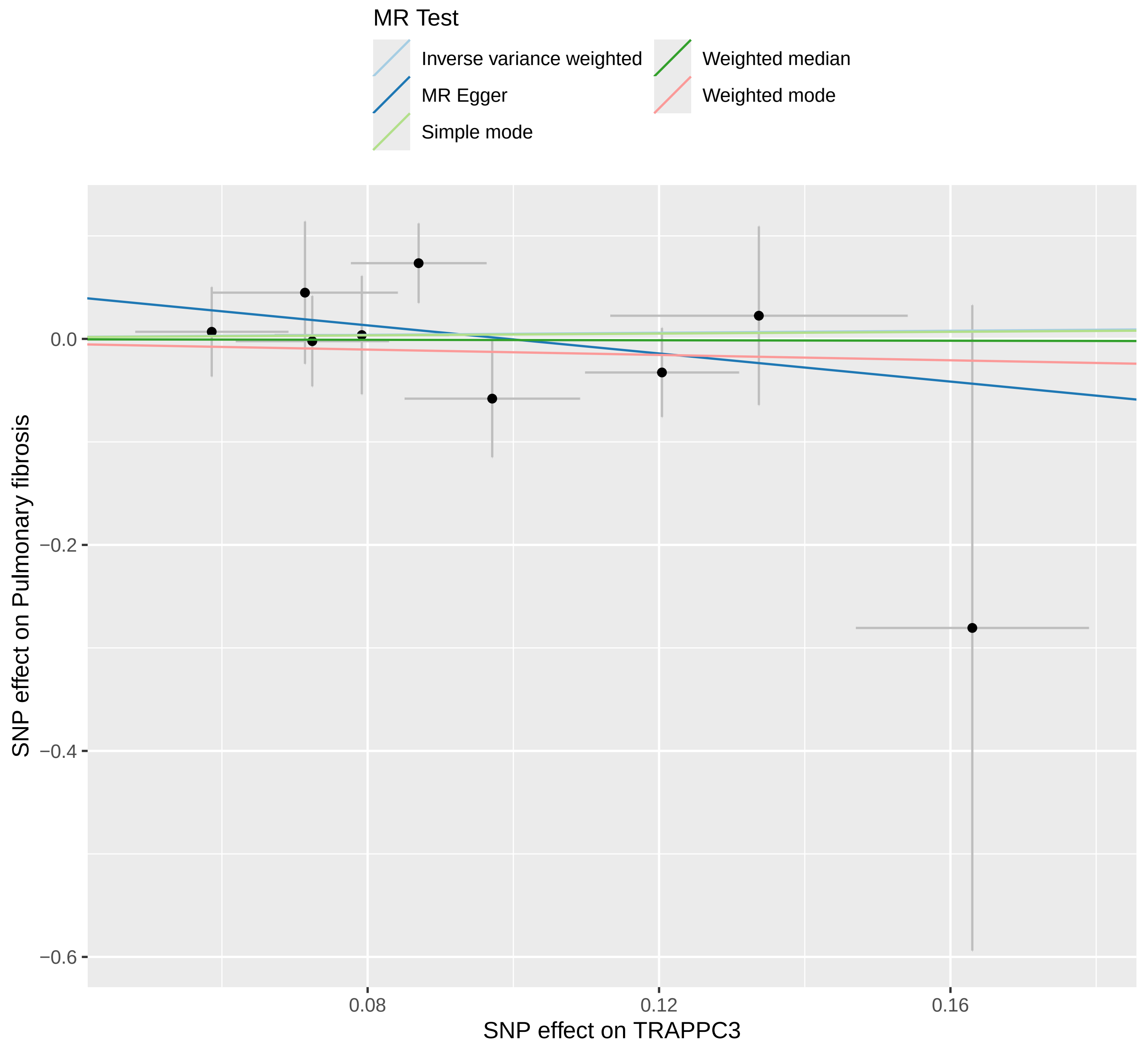
MR Test
Inverse variance weighted
MR Egger
Simple mode
Weighted median
Weighted mode
0.0
−0.2
−0.4
−0.6
0.08
0.12
0.16
SNP effect on TRAPPC3
SNP effect on Pulmonary fibrosis

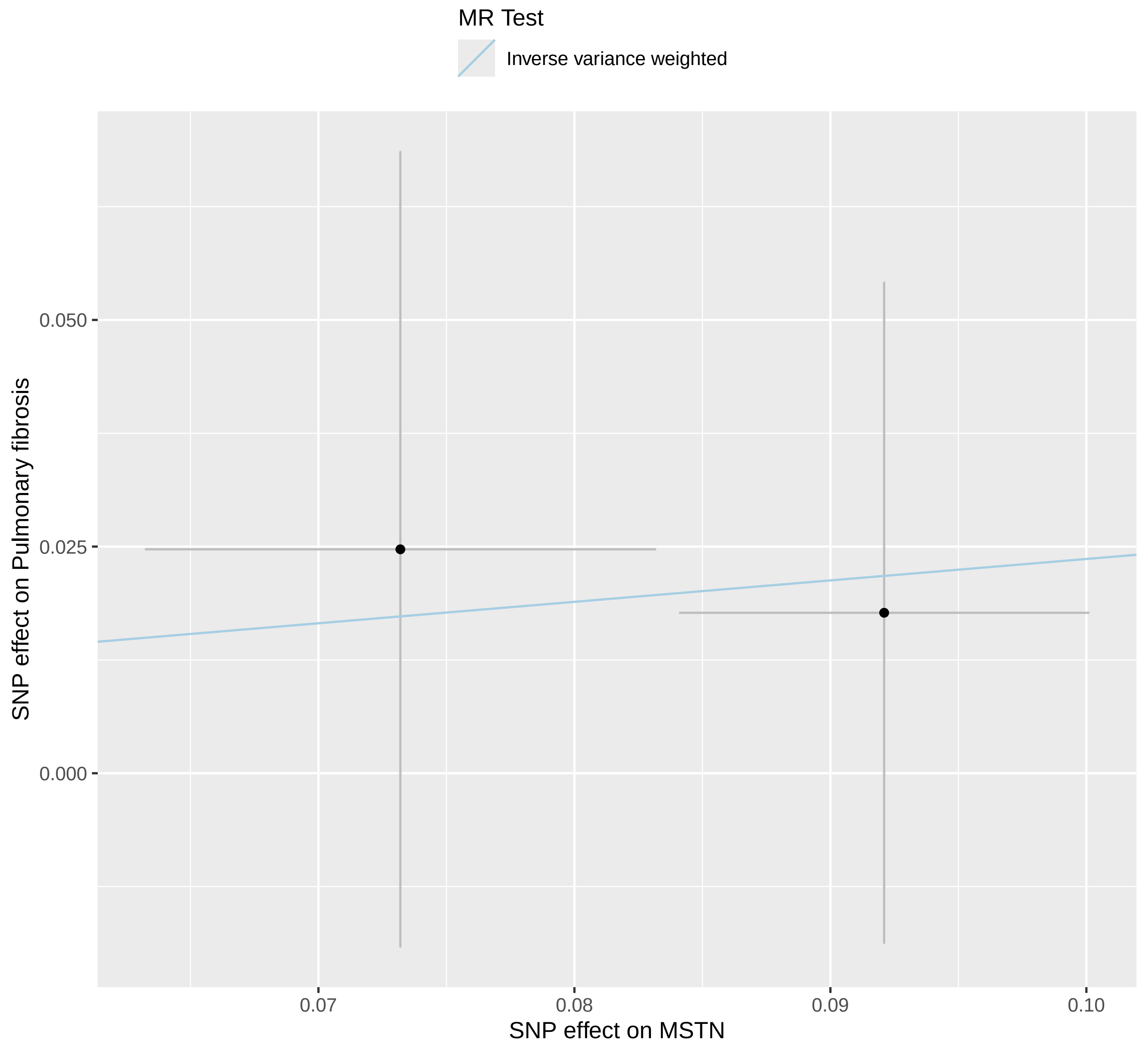

MR Test
Inverse variance weighted
0.050
0.025
0.000
0.07
0.08
0.09
0.10
SNP effect on MSTN
SNP effect on Pulmonary fibrosis

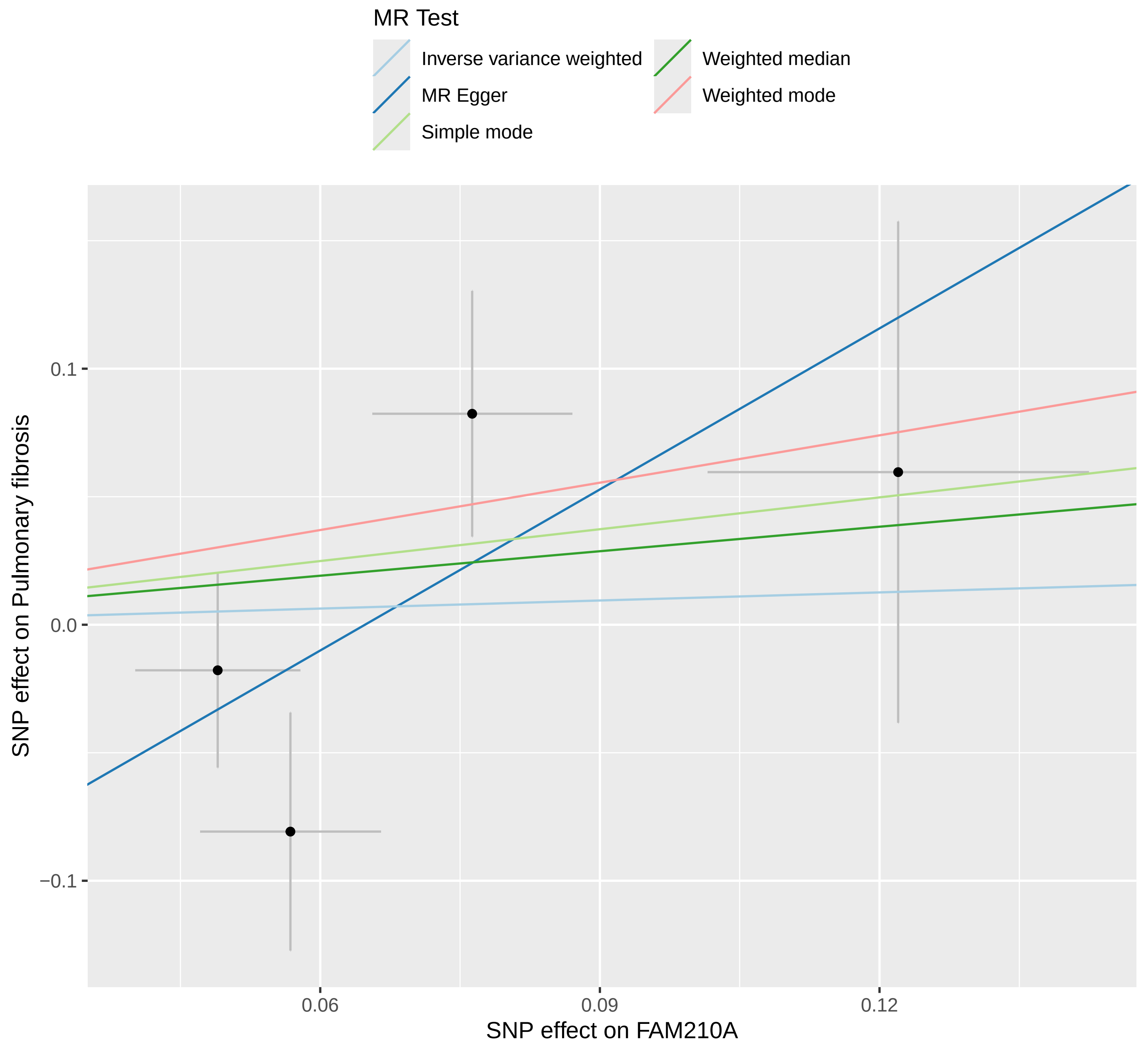
MR Test
Inverse variance weighted
MR Egger
Simple mode
Weighted median
Weighted mode
0.1
0.0
−0.1
0.06
0.09
0.12
SNP effect on Pulmonary fibrosis
SNP effect on FAM210A

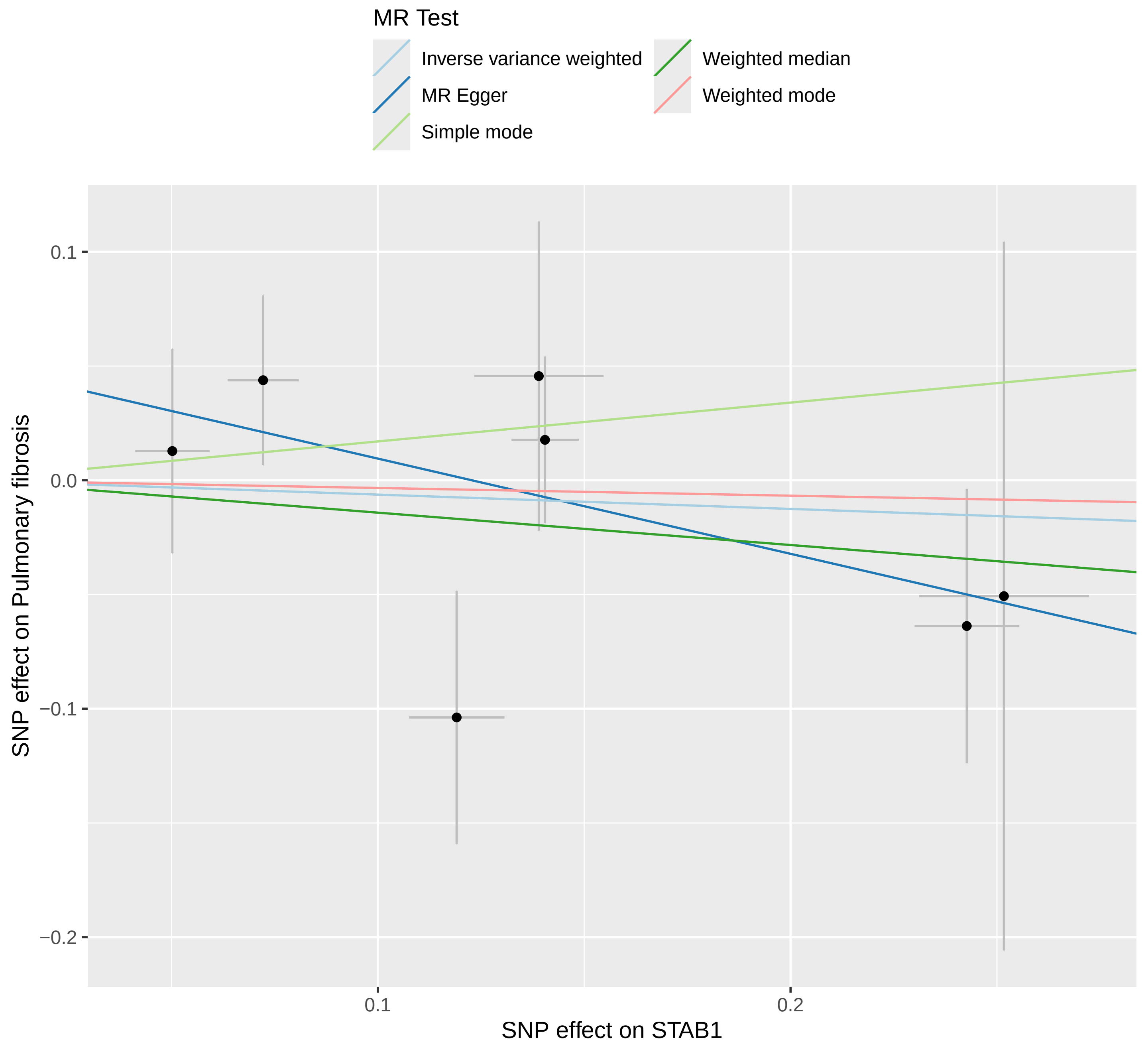
MR Test
Inverse variance weighted
MR Egger
Simple mode
Weighted median
Weighted mode
SNP effect on Pulmonary fibrosis
0.1
0.0
−0.1
−0.2
0.1
0.2
SNP effect on STAB1

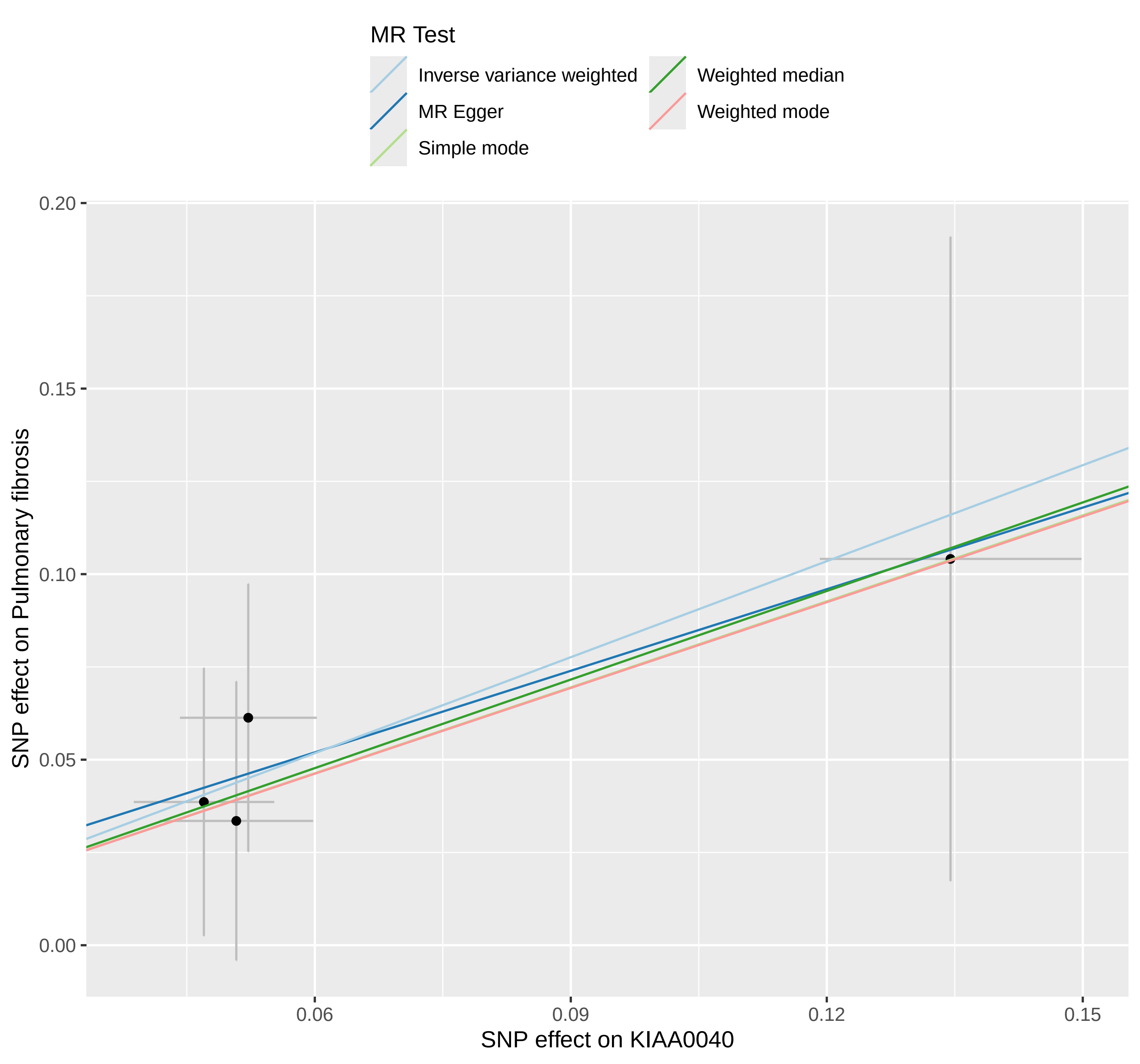
MR Test
Inverse variance weighted
MR Egger
Simple mode
Weighted median
Weighted mode
0.20
0.15
0.10
0.05
0.00
SNP effect on Pulmonary fibrosis
0.06
0.09
0.12
0.15
SNP effect on KIAA0040

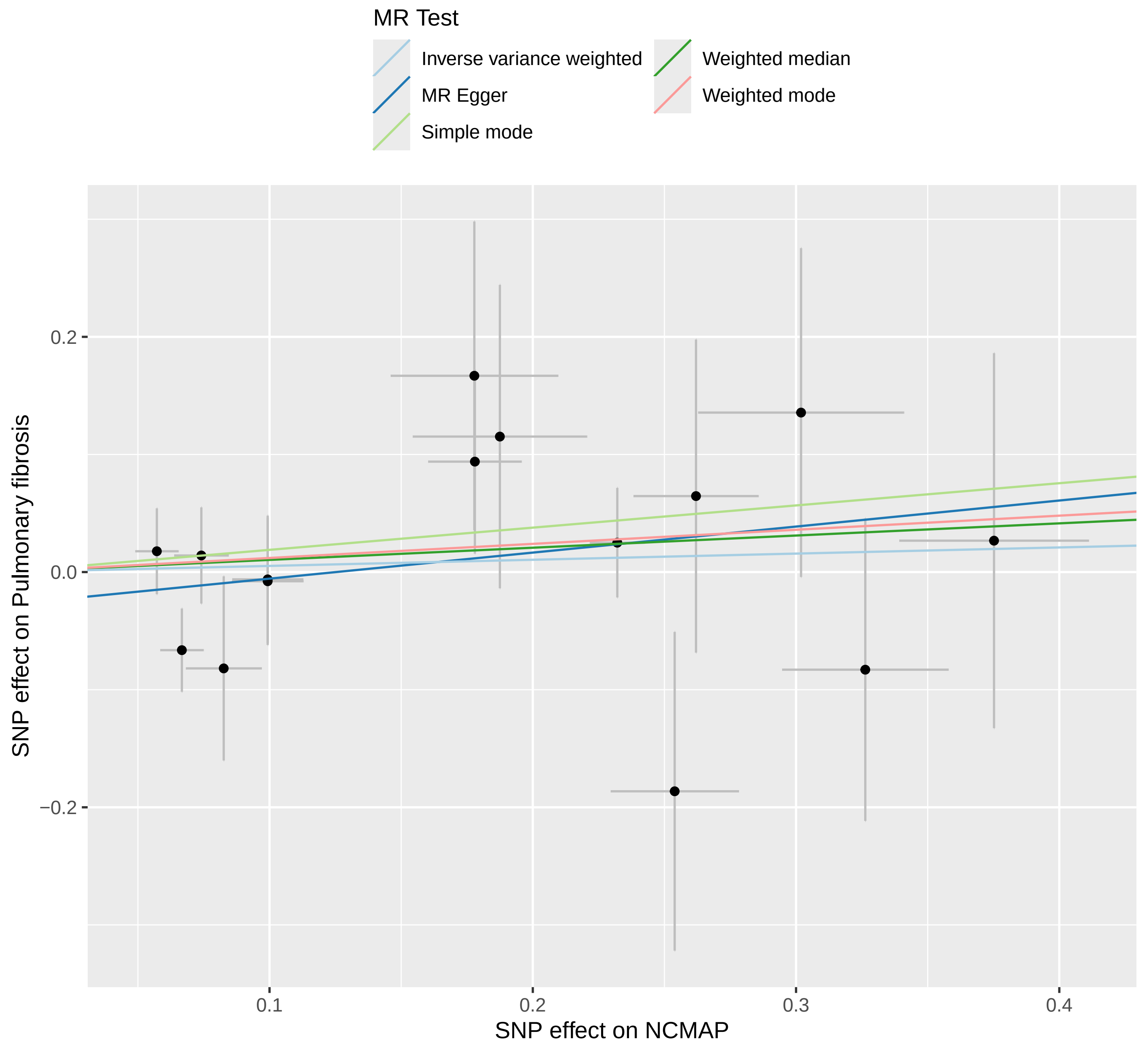
MR Test
Inverse variance weighted
MR Egger
Simple mode
Weighted median
Weighted mode
SNP effect on Pulmonary fibrosis
0.2
0.0
−0.2
0.1
0.2
0.3
0.4
SNP effect on NCMAP

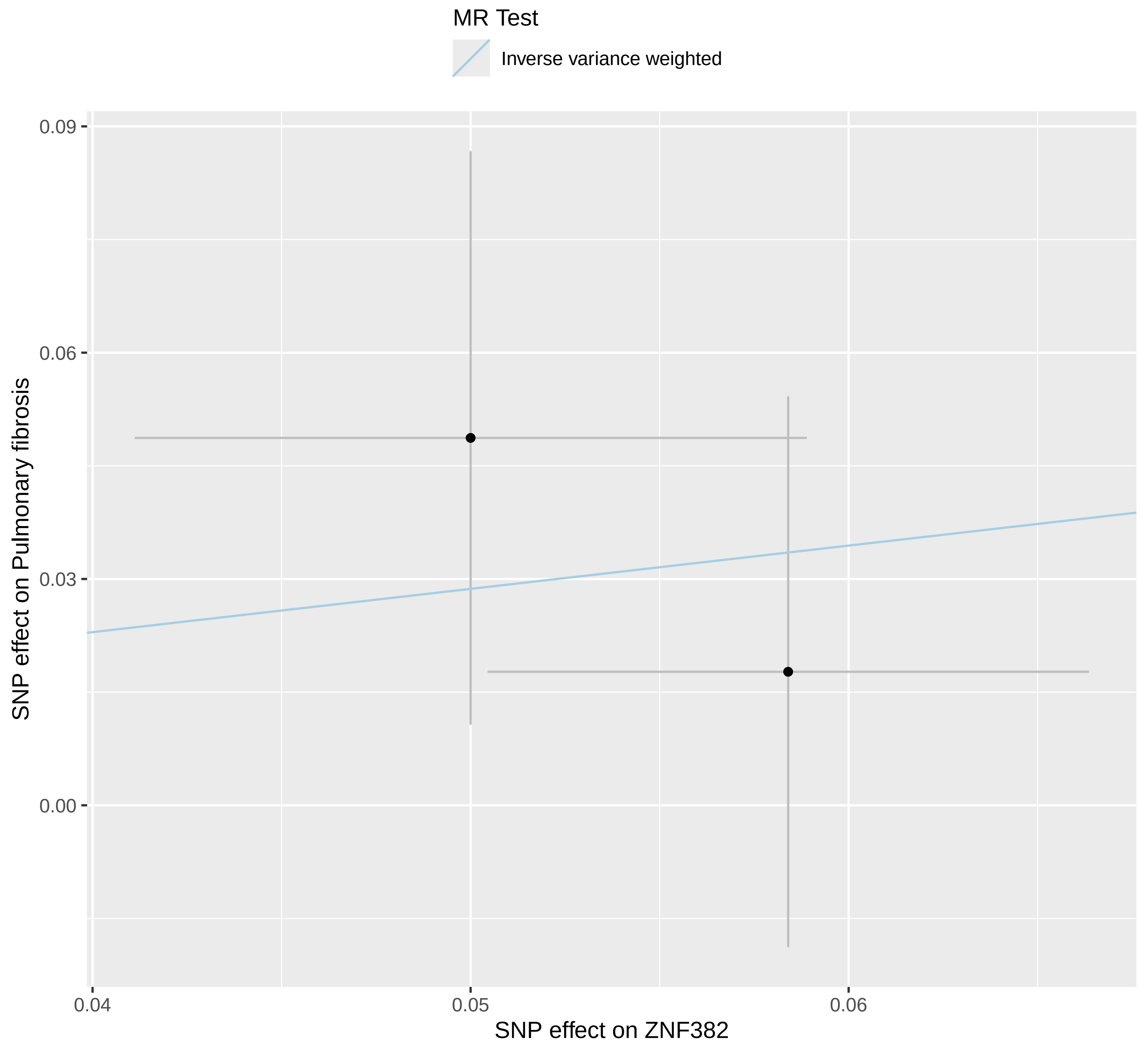

MR Test
Inverse variance weighted
0.09
0.06
0.03
0.00
SNP effect on Pulmonary fibrosis
0.04
0.05
0.06
SNP effect on ZNF382

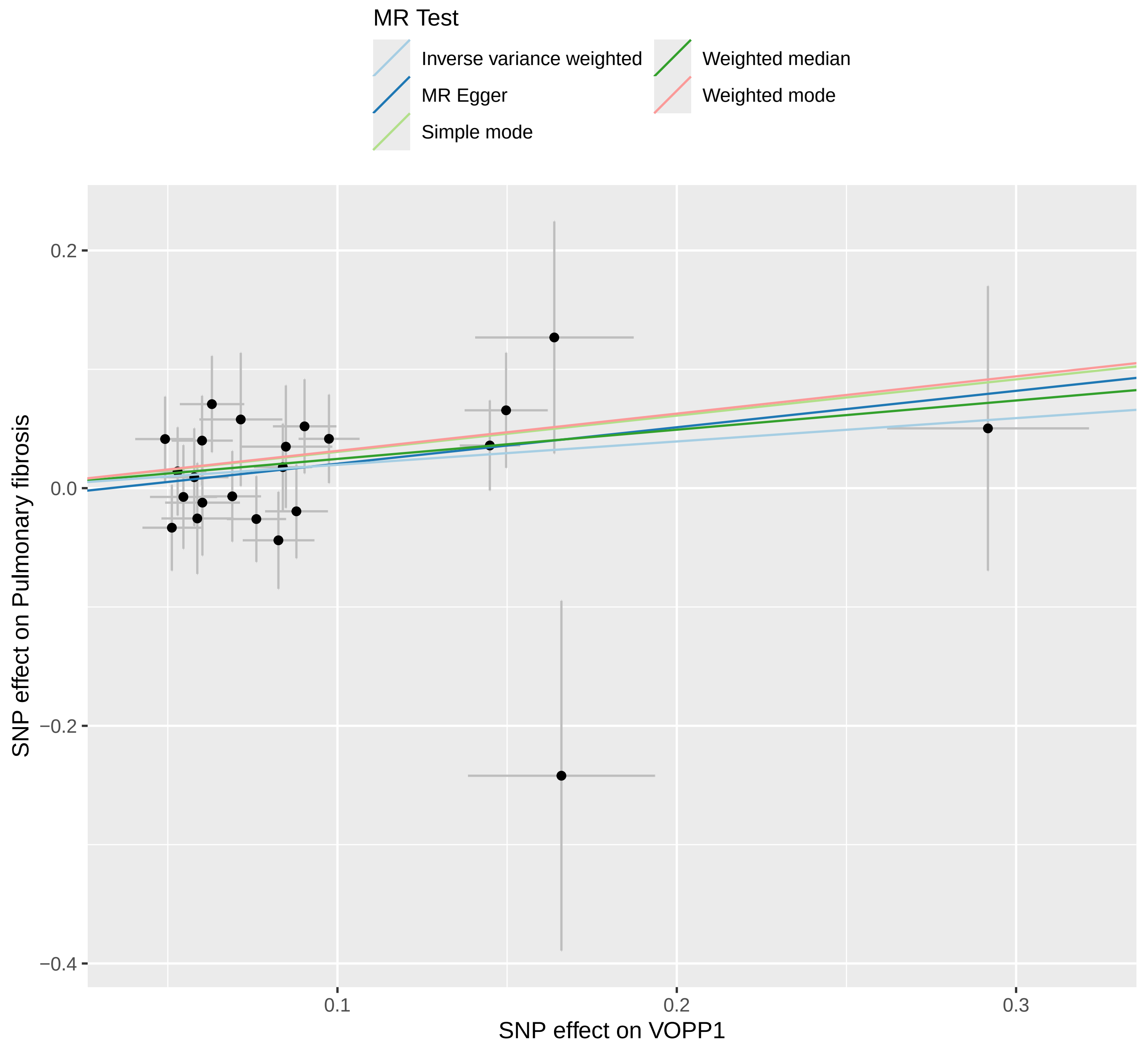
MR Test
Inverse variance weighted
MR Egger
Simple mode
Weighted median
Weighted mode
0.2
0.0
−0.2
−0.4
0.1
0.2
0.3
SNP effect on Pulmonary fibrosis
SNP effect on VOPP1

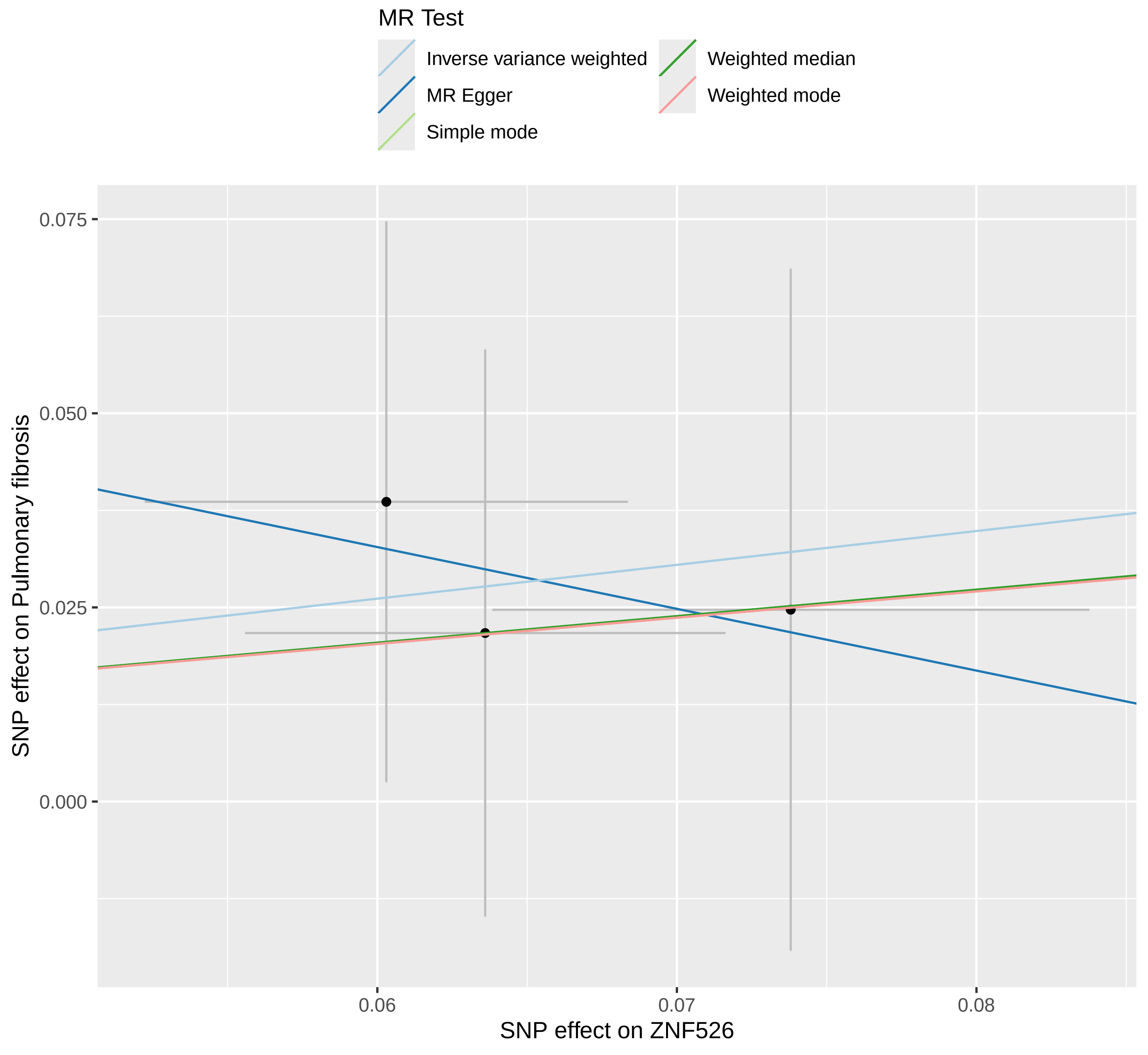

MR Test
Inverse variance weighted
MR Egger
Simple mode
Weighted median
Weighted mode
0.075
0.050
0.025
0.000
SNP effect on Pulmonary fibrosis
0.06
0.07
0.08
SNP effect on ZNF526

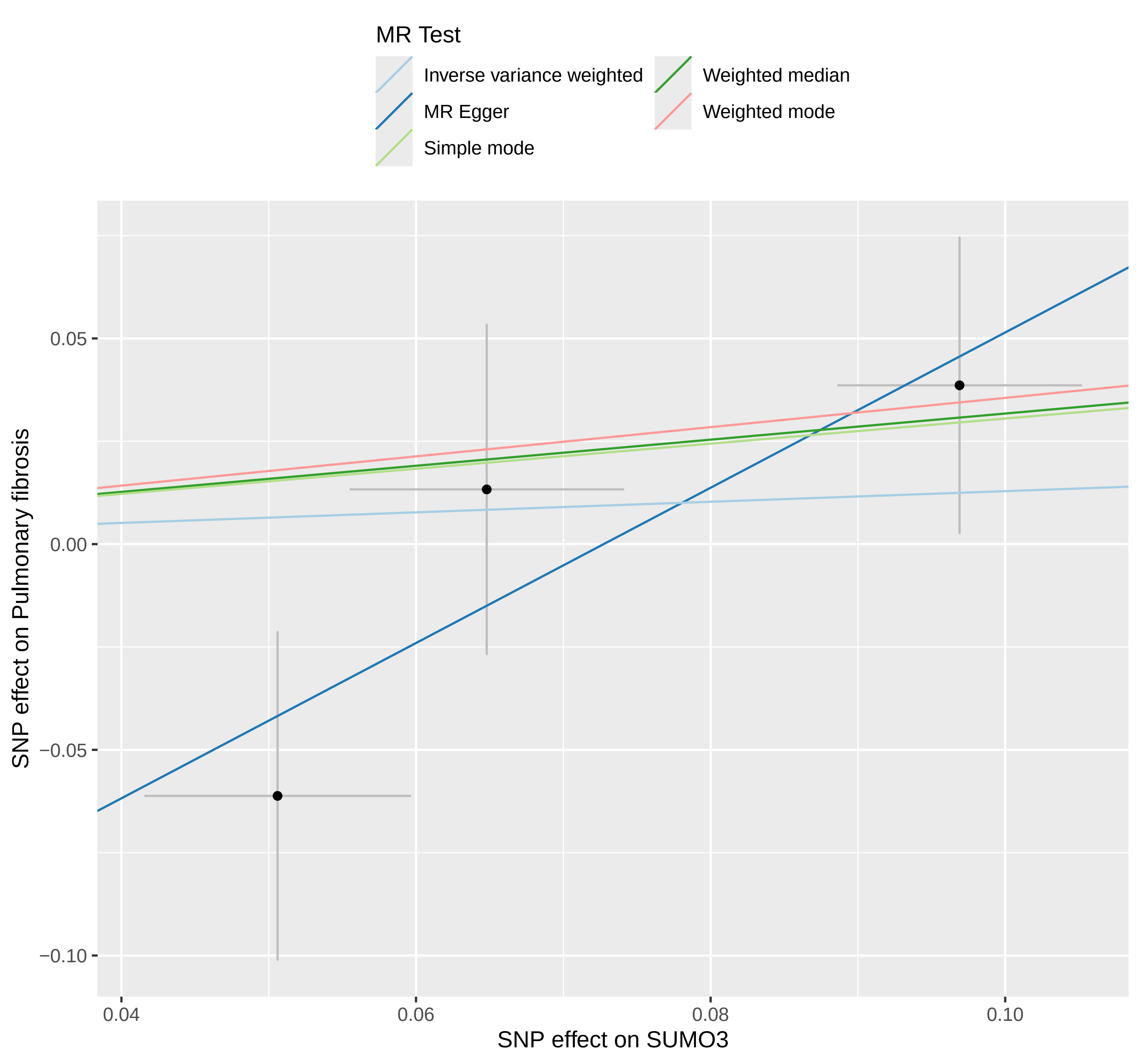

MR Test
Inverse variance weighted
MR Egger
Simple mode
Weighted median
Weighted mode
0.05
0.00
−0.05
−0.10
0.04
0.06
0.08
0.10
SNP effect on Pulmonary fibrosis
SNP effect on SUMO3

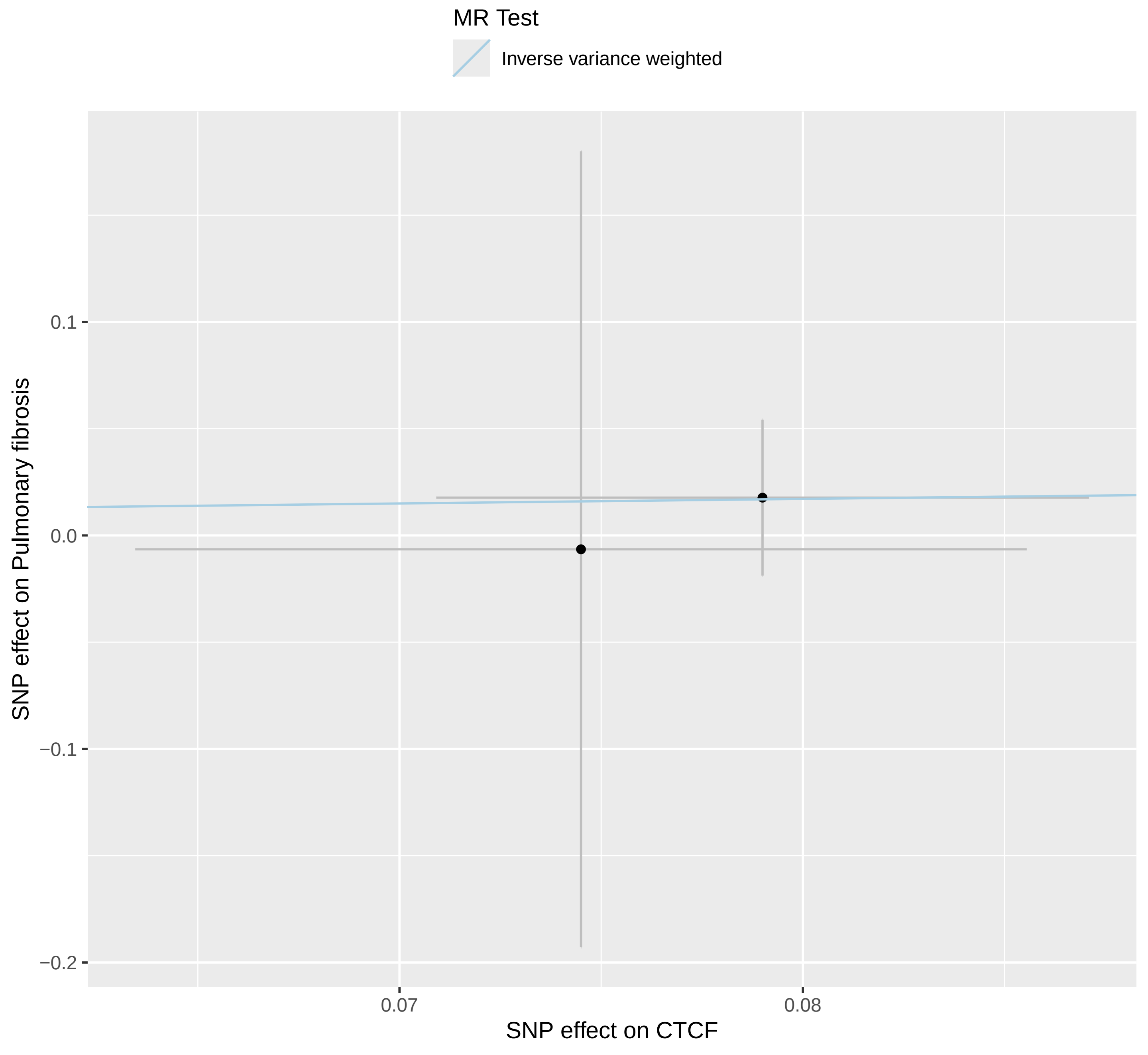

MR Test
Inverse variance weighted
SNP effect on Pulmonary fibrosis
0.1
0.0
−0.1
−0.2
0.07
0.08
SNP effect on CTCF

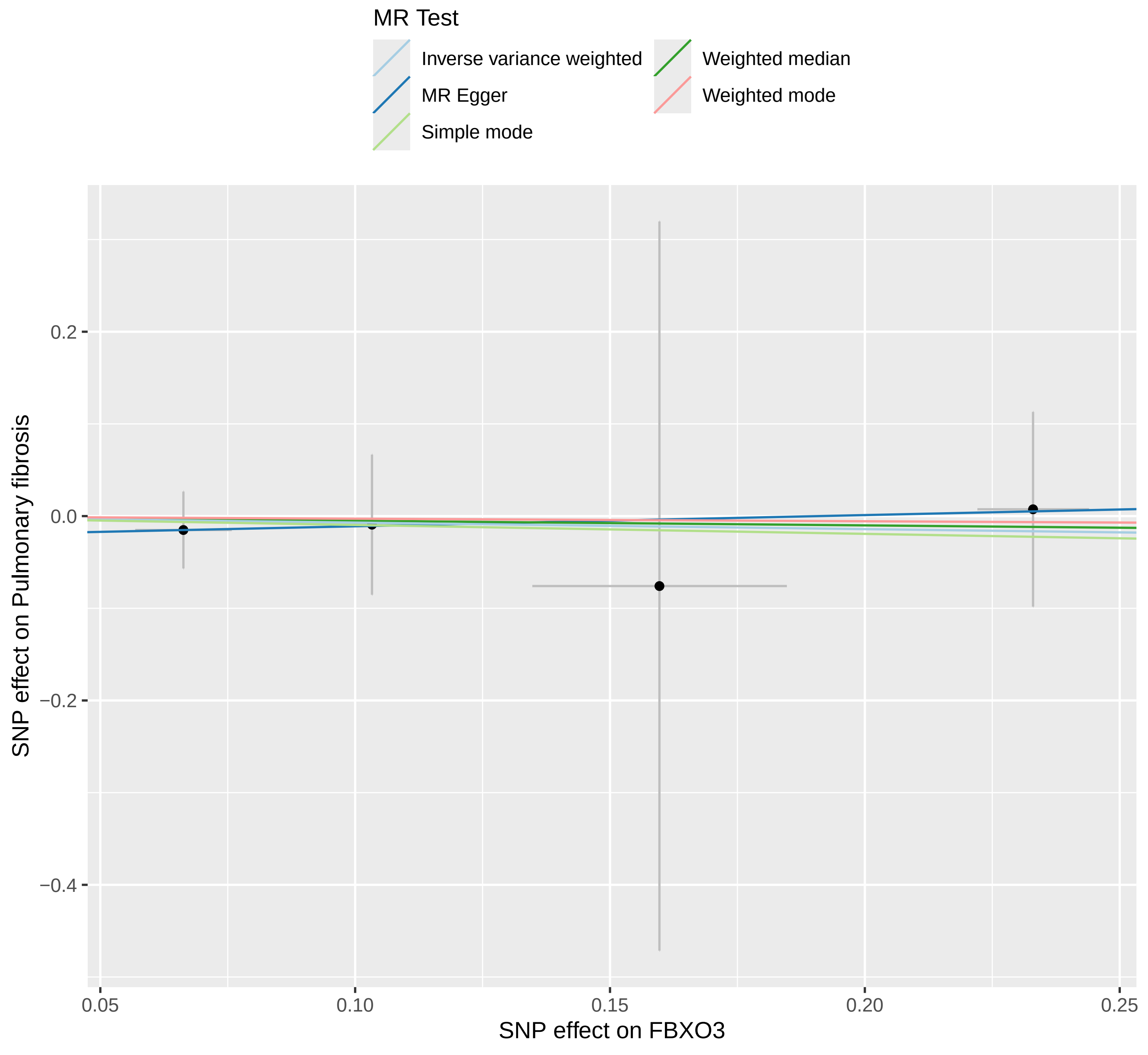

MR Test
Inverse variance weighted
MR Egger
Simple mode
Weighted median
Weighted mode
0.2
0.0
−0.2
−0.4
0.05
0.10
0.15
0.20
0.25
SNP effect on Pulmonary fibrosis
SNP effect on FBXO3

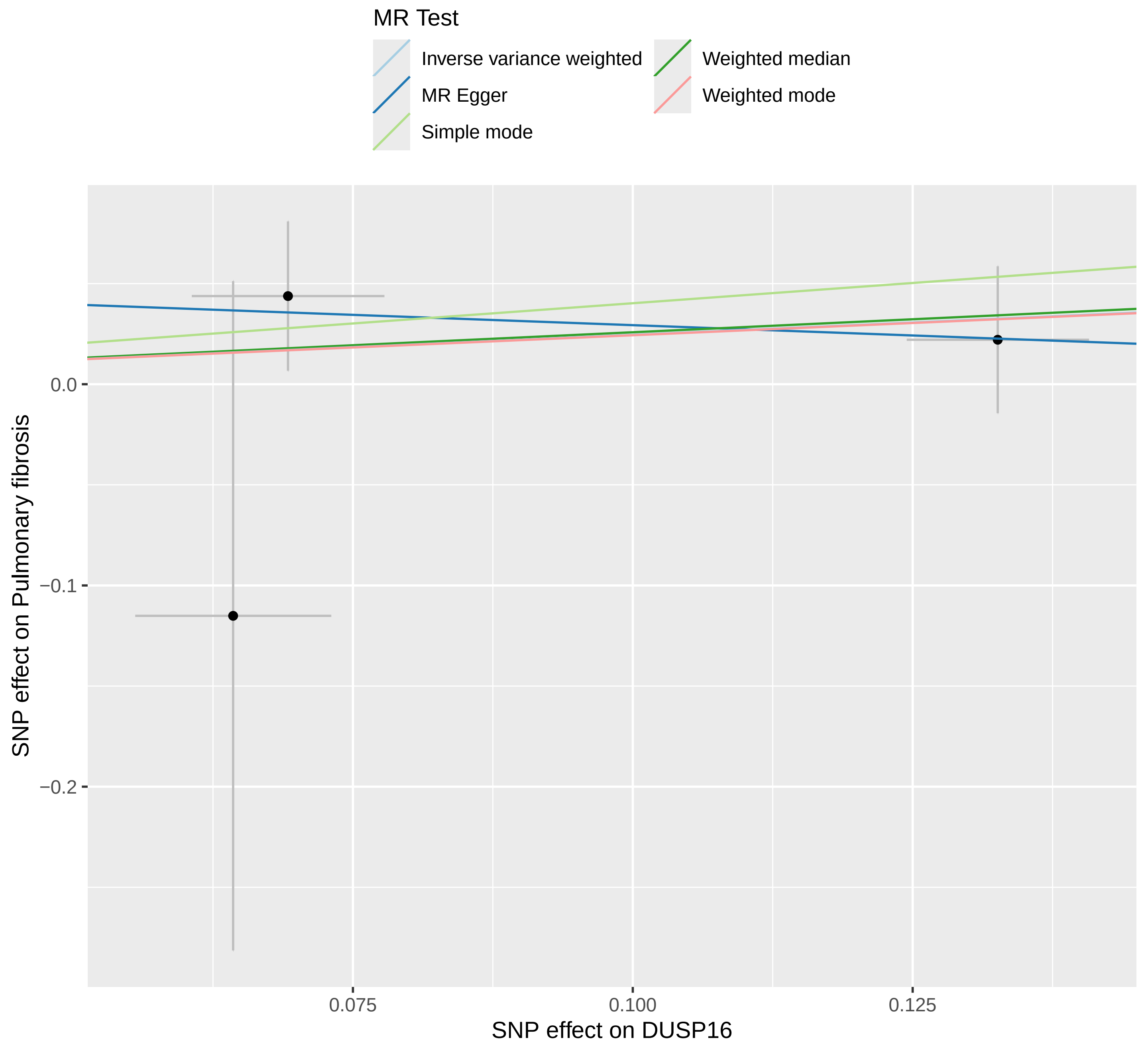
MR Test
Inverse variance weighted
MR Egger
Simple mode
Weighted median
Weighted mode
0.0
−0.1
−0.2
SNP effect on Pulmonary fibrosis
0.075
0.100
0.125
SNP effect on DUSP16

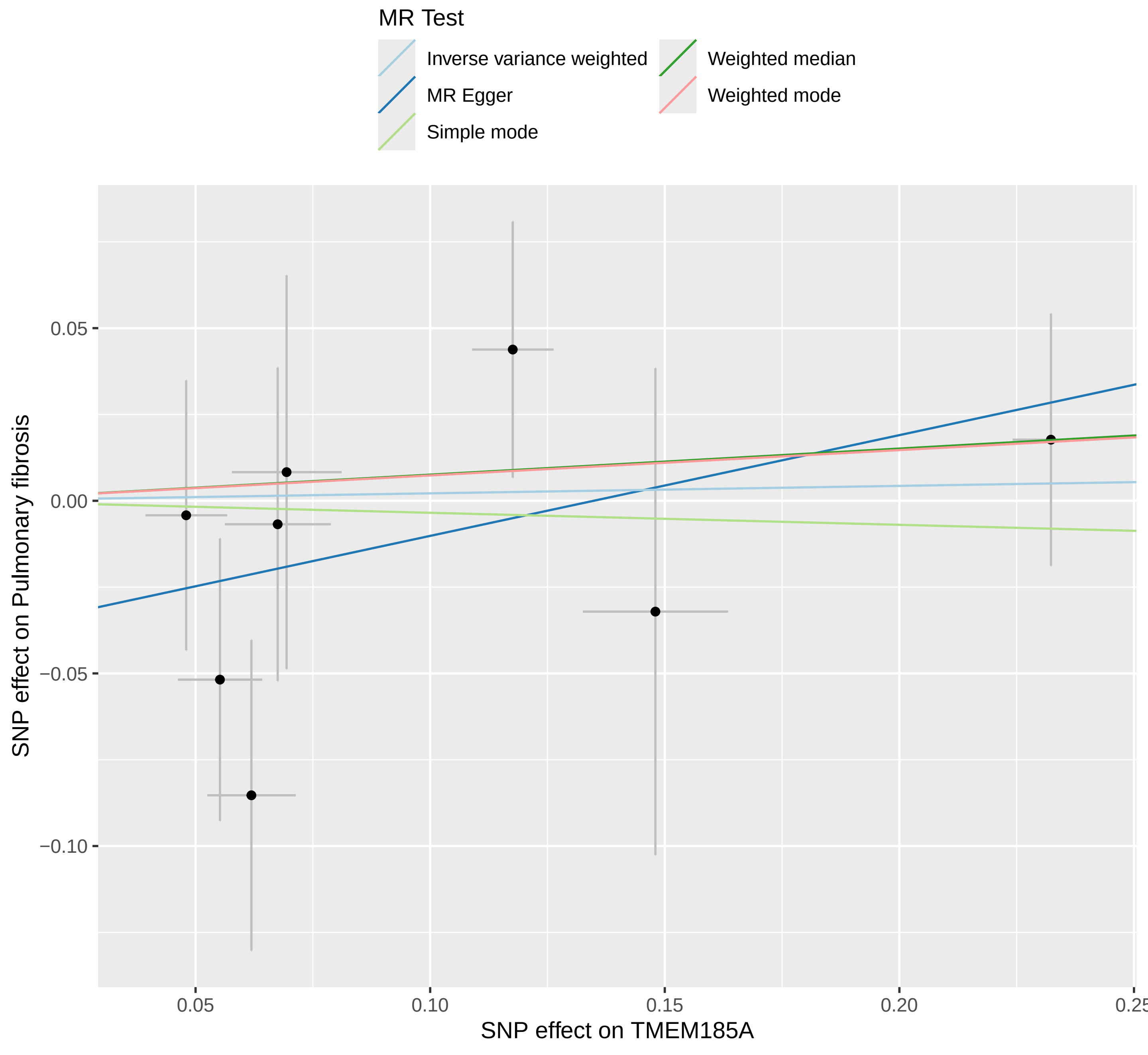

MR Test
Inverse variance weighted
MR Egger
Simple mode
Weighted median
Weighted mode
0.05
0.00
−0.05
−0.10
SNP effect on Pulmonary fibrosis
0.05
0.10
0.15
0.20
0.25
SNP effect on TMEM185A

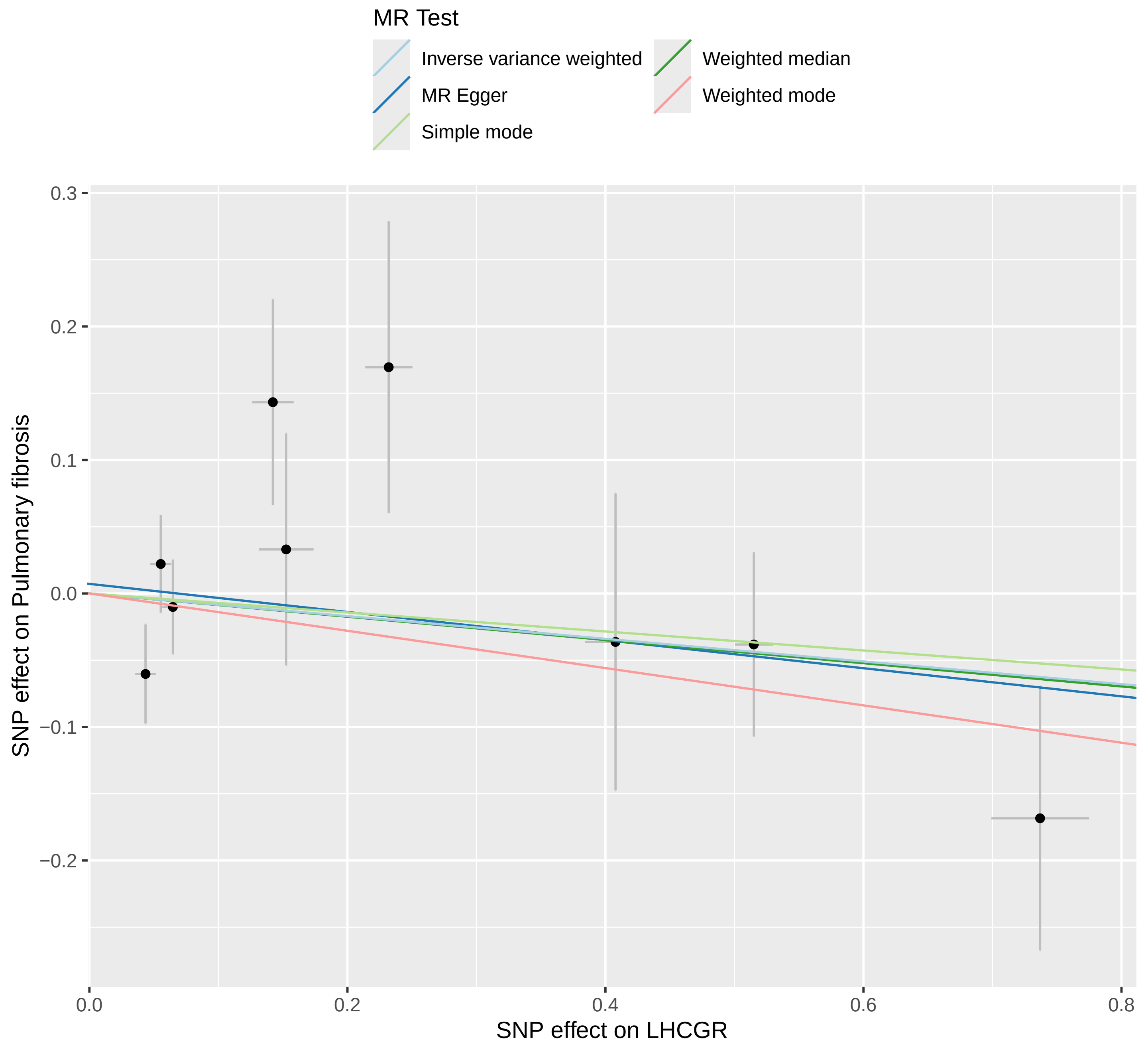
MR Test
Inverse variance weighted
MR Egger
Simple mode
Weighted median
Weighted mode
0.3
0.2
0.1
0.0
−0.1
−0.2
0.0
0.2
0.4
0.6
0.8
SNP effect on Pulmonary fibrosis
SNP effect on LHCGR

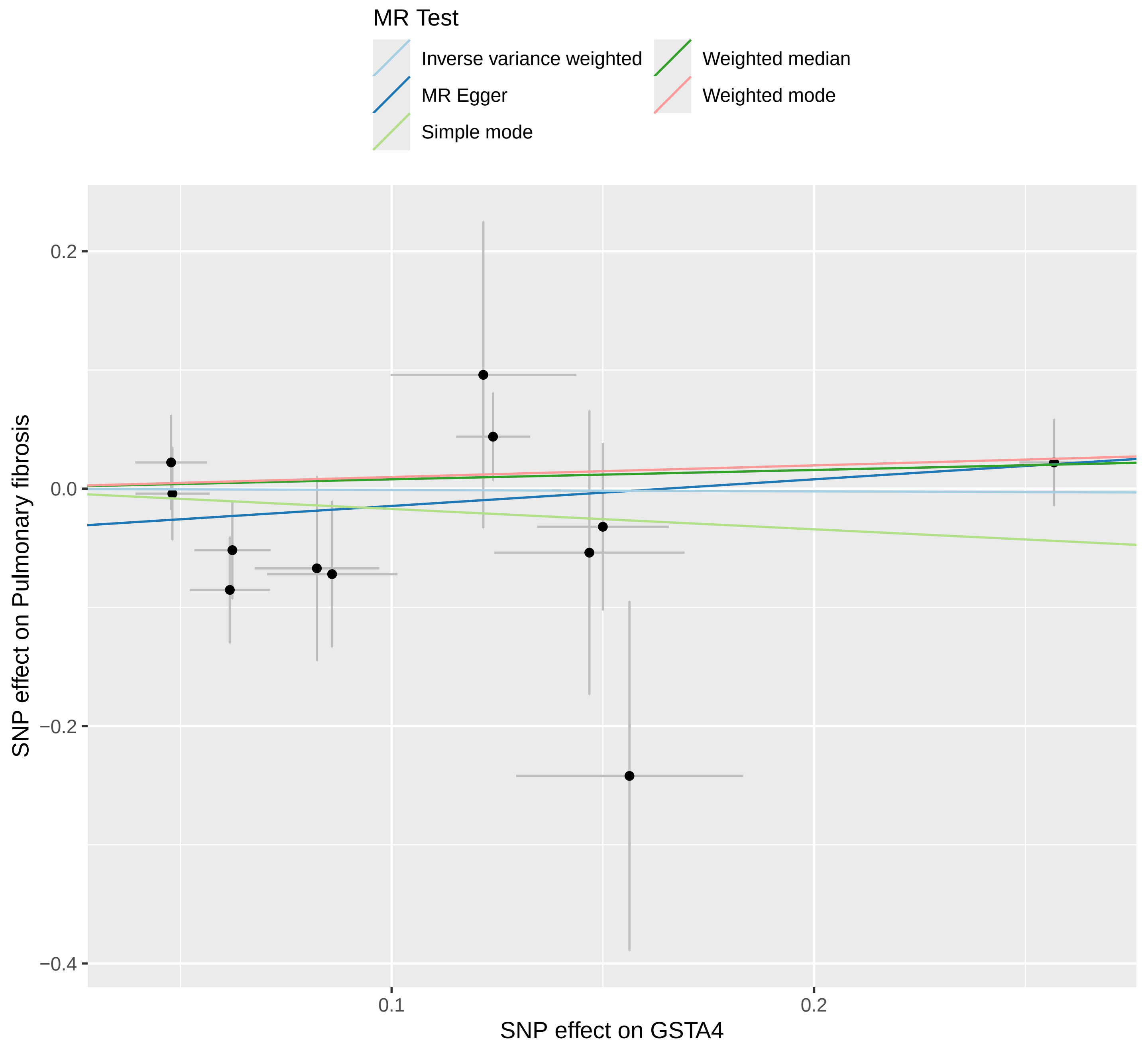

MR Test
Inverse variance weighted
MR Egger
Simple mode
Weighted median
Weighted mode
0.2
0.0
−0.2
−0.4
0.1
0.2
SNP effect on Pulmonary fibrosis
SNP effect on GSTA4

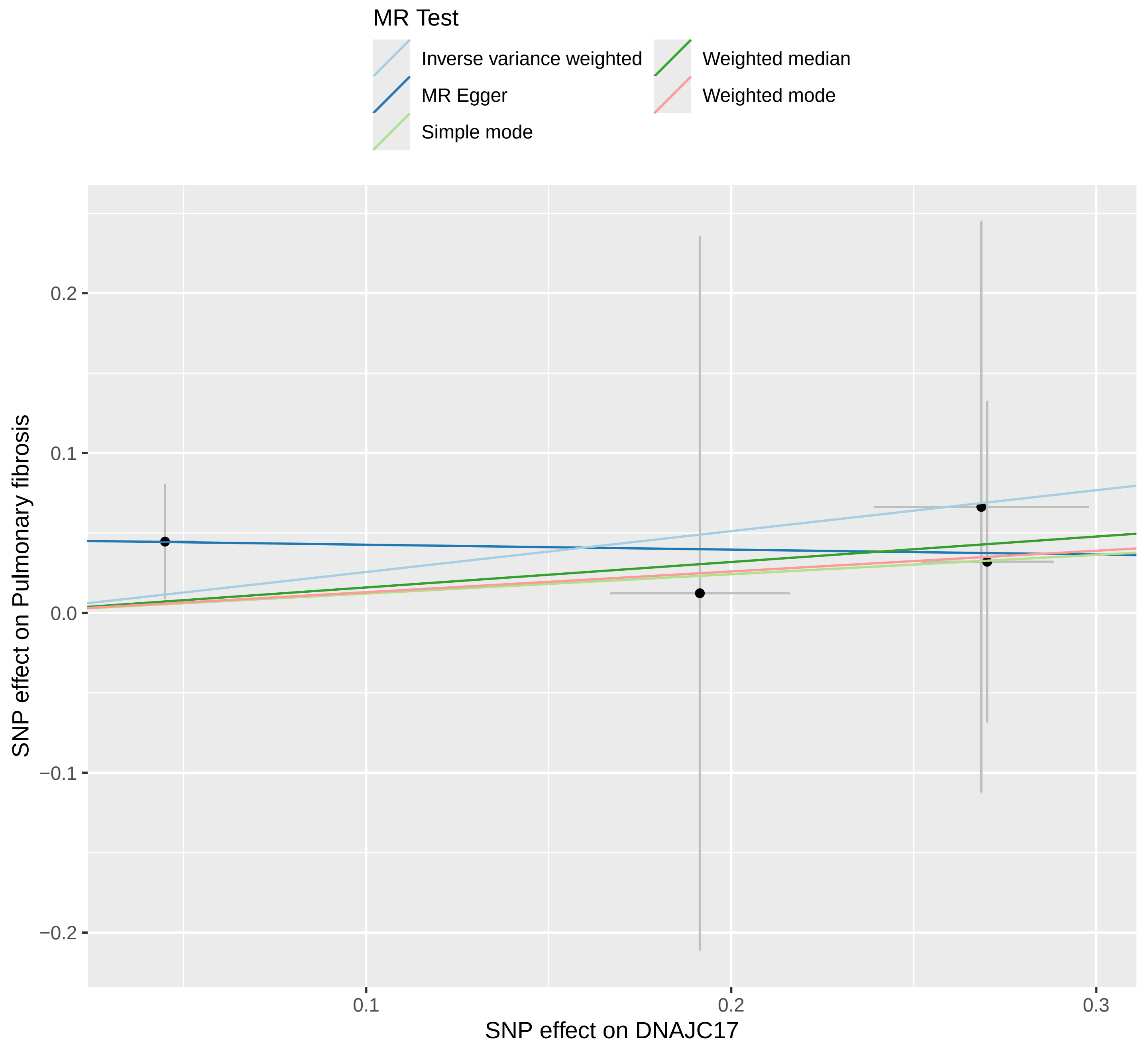
MR Test
Inverse variance weighted
MR Egger
Simple mode
Weighted median
Weighted mode
0.2
0.1
0.0
−0.1
−0.2
SNP effect on Pulmonary fibrosis
0.1
0.2
0.3
SNP effect on DNAJC17

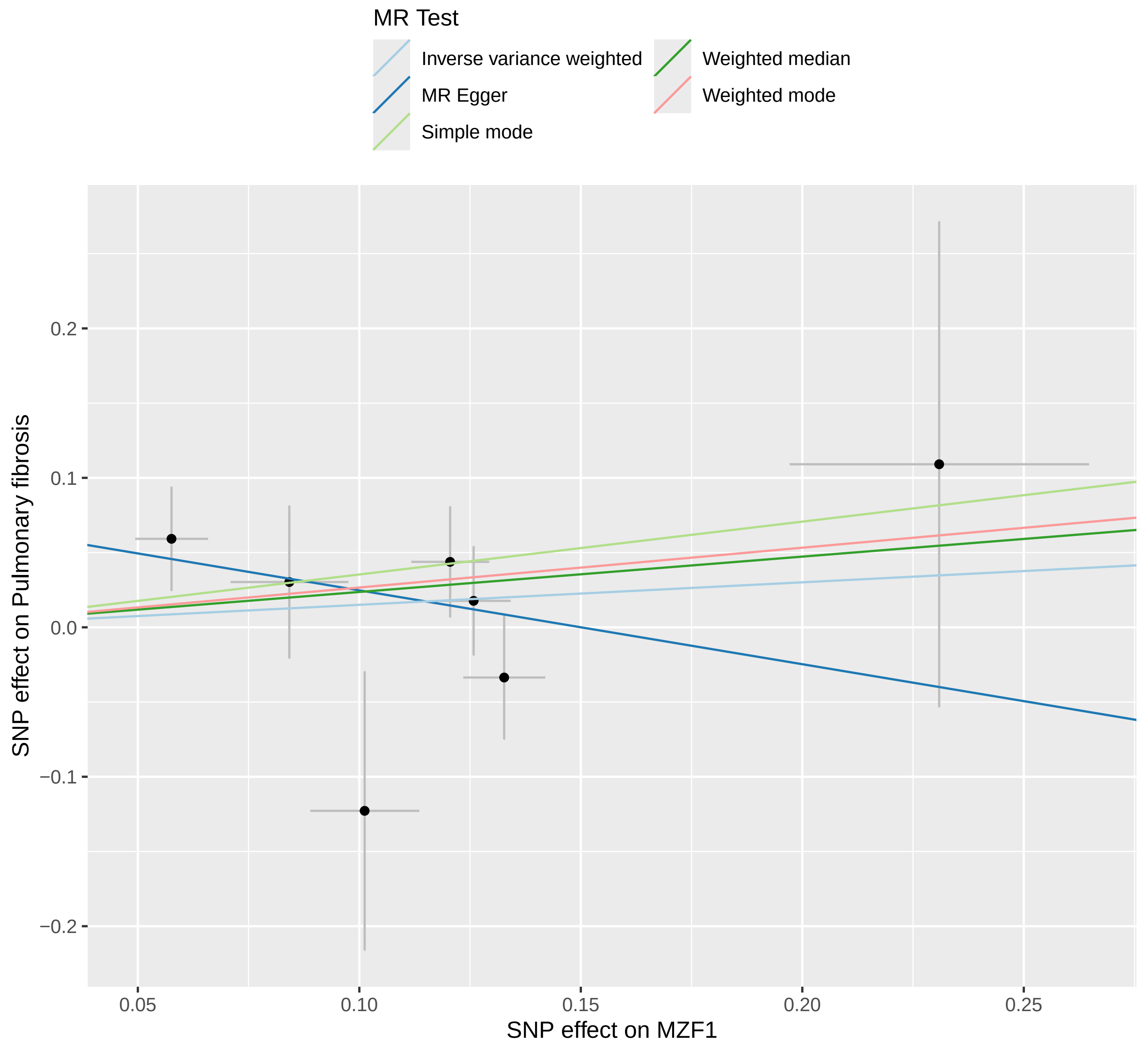

MR Test
Inverse variance weighted
MR Egger
Simple mode
Weighted median
Weighted mode
0.2
0.1
0.0
−0.1
−0.2
SNP effect on Pulmonary fibrosis
0.05
0.10
0.15
0.20
0.25
SNP effect on MZF1

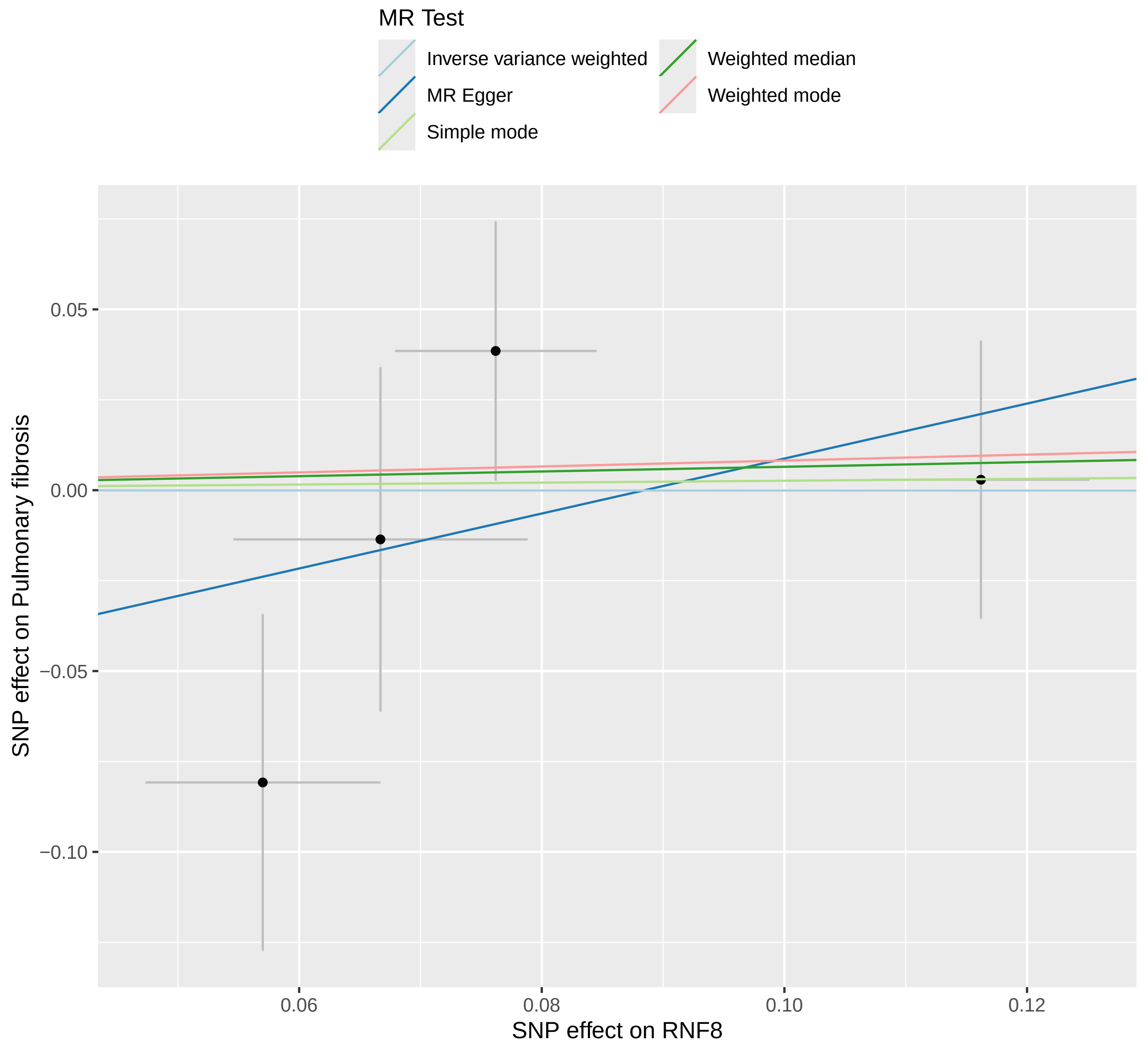
MR Test
Inverse variance weighted
MR Egger
Simple mode
Weighted median
Weighted mode
0.05
0.00
−0.05
−0.10
SNP effect on Pulmonary fibrosis
0.06
0.08
0.10
0.12
SNP effect on RNF8

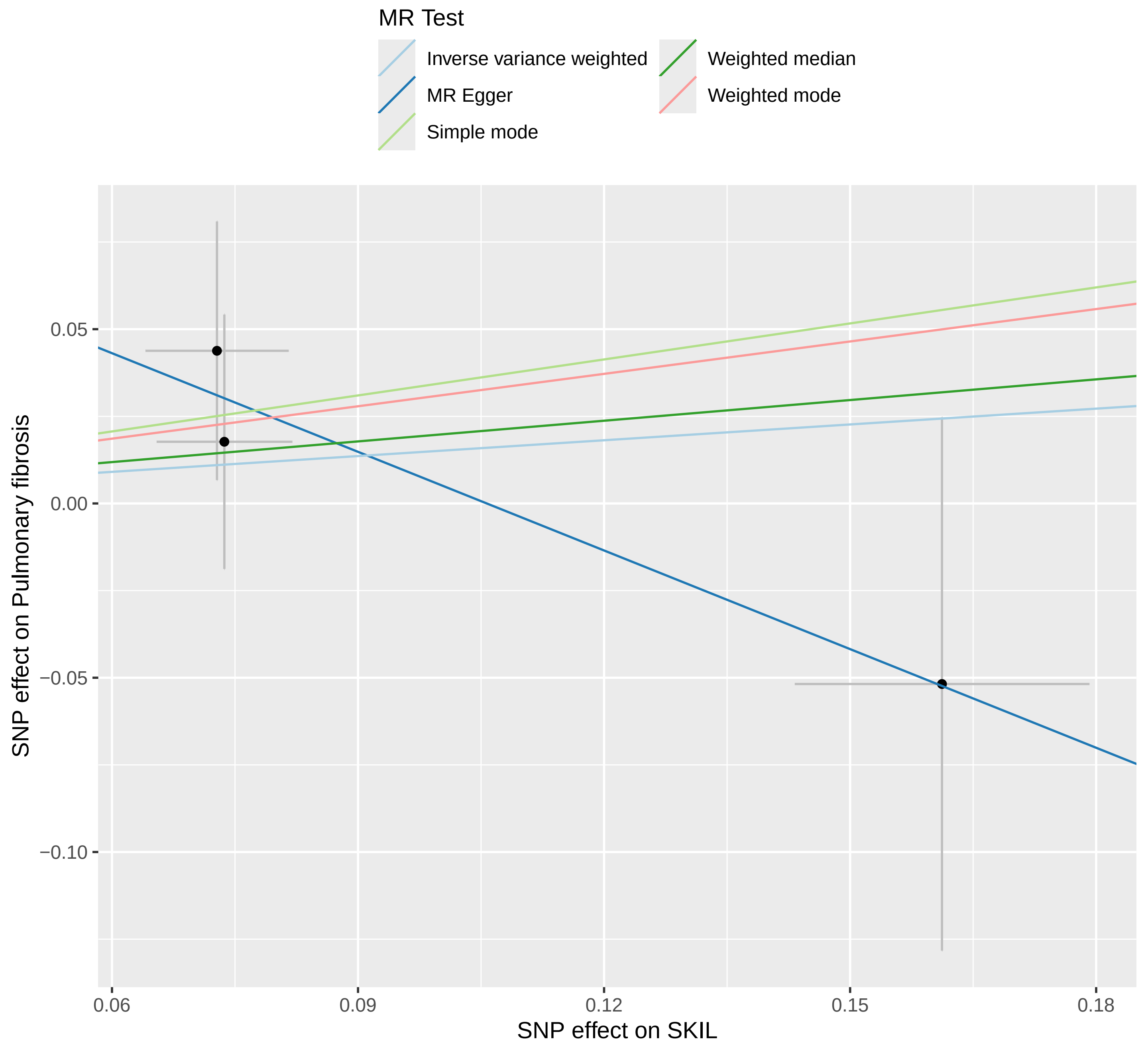

MR Test
Inverse variance weighted
MR Egger
Simple mode
Weighted median
Weighted mode
0.05
0.00
−0.05
−0.10
SNP effect on Pulmonary fibrosis
0.06
0.09
0.12
0.15
0.18
SNP effect on SKIL

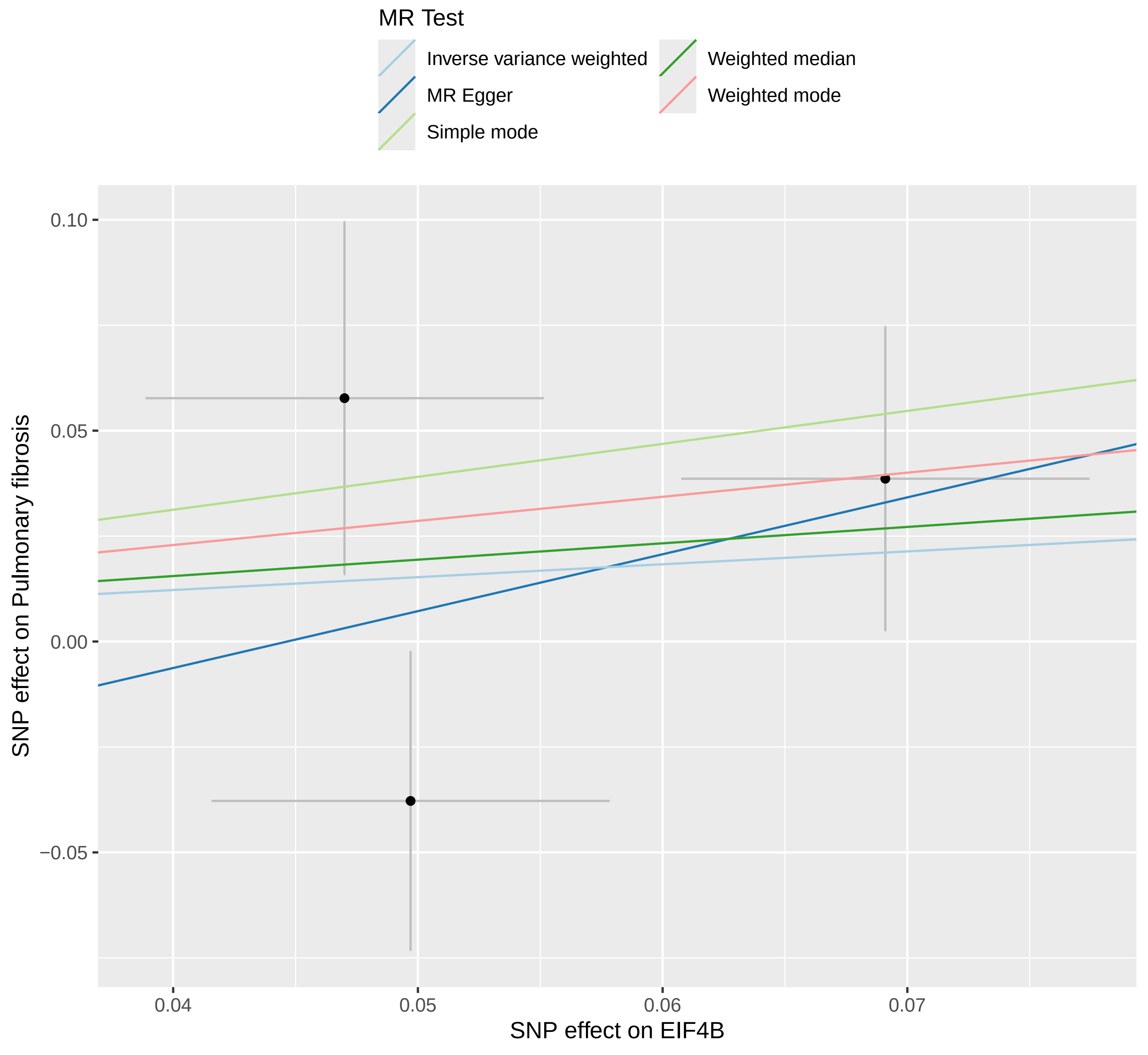
MR Test
Inverse variance weighted
MR Egger
Simple mode
Weighted median
Weighted mode
0.10
0.05
0.00
−0.05
0.04
0.05
0.06
0.07
SNP effect on Pulmonary fibrosis
SNP effect on EIF4B

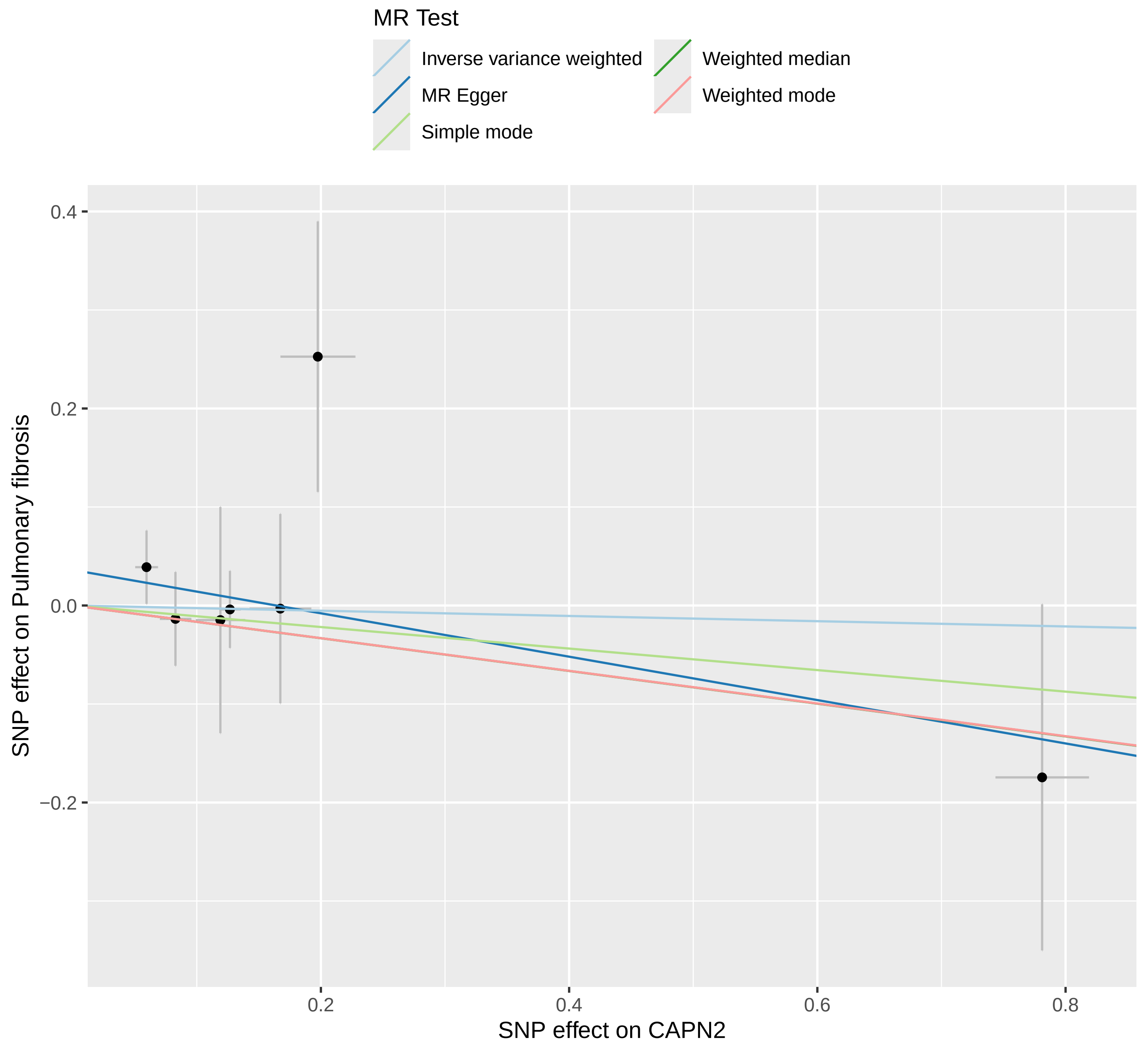

MR Test
Inverse variance weighted
MR Egger
Simple mode
Weighted median
Weighted mode
SNP effect on Pulmonary fibrosis
0.4
0.2
0.0
−0.2
SNP effect on CAPN2
0.2
0.4
0.6
0.8

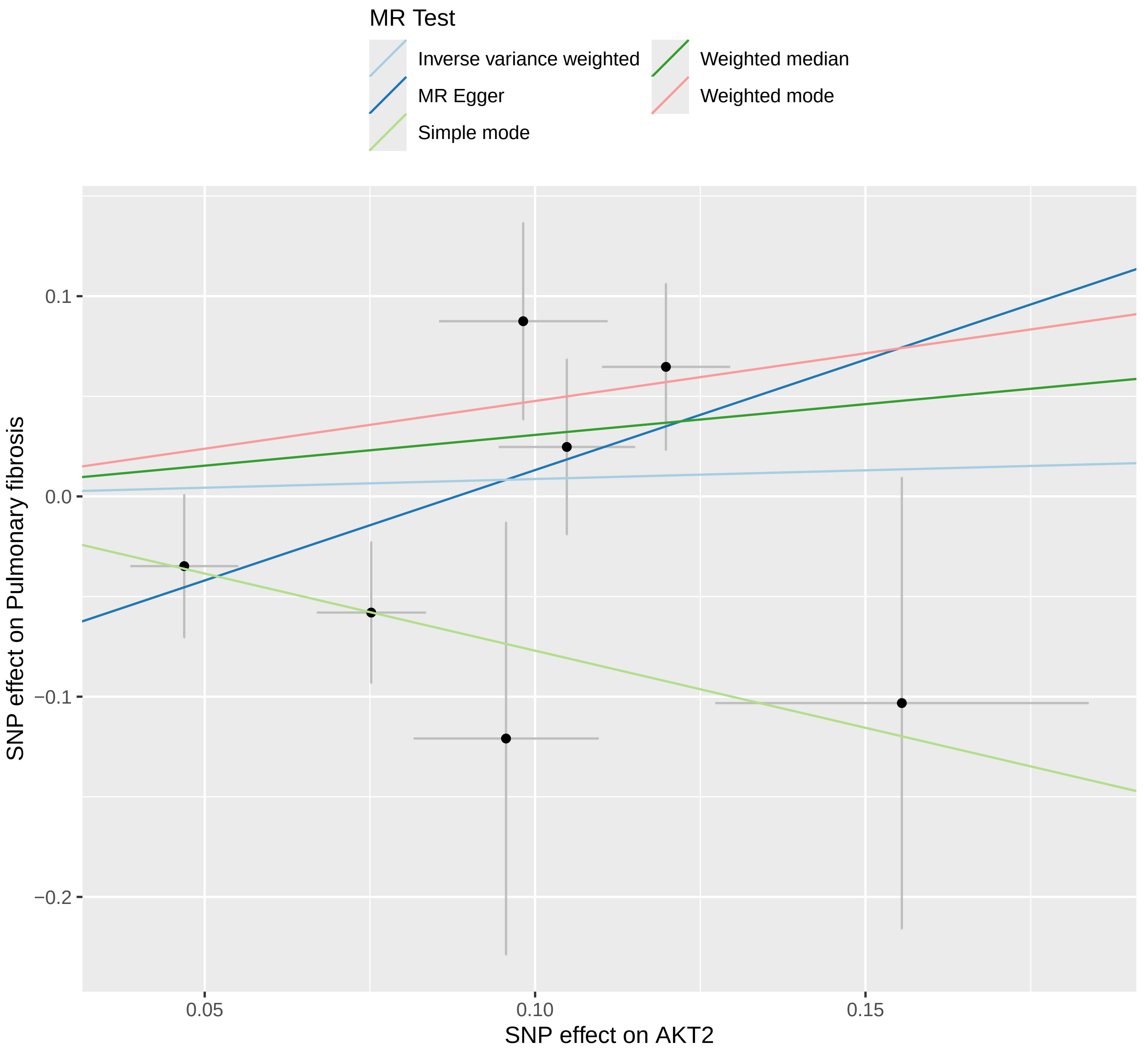

MR Test
Inverse variance weighted
MR Egger
Simple mode
Weighted median
Weighted mode
0.1
0.0
−0.1
−0.2
0.05
0.10
0.15
SNP effect on Pulmonary fibrosis
SNP effect on AKT2

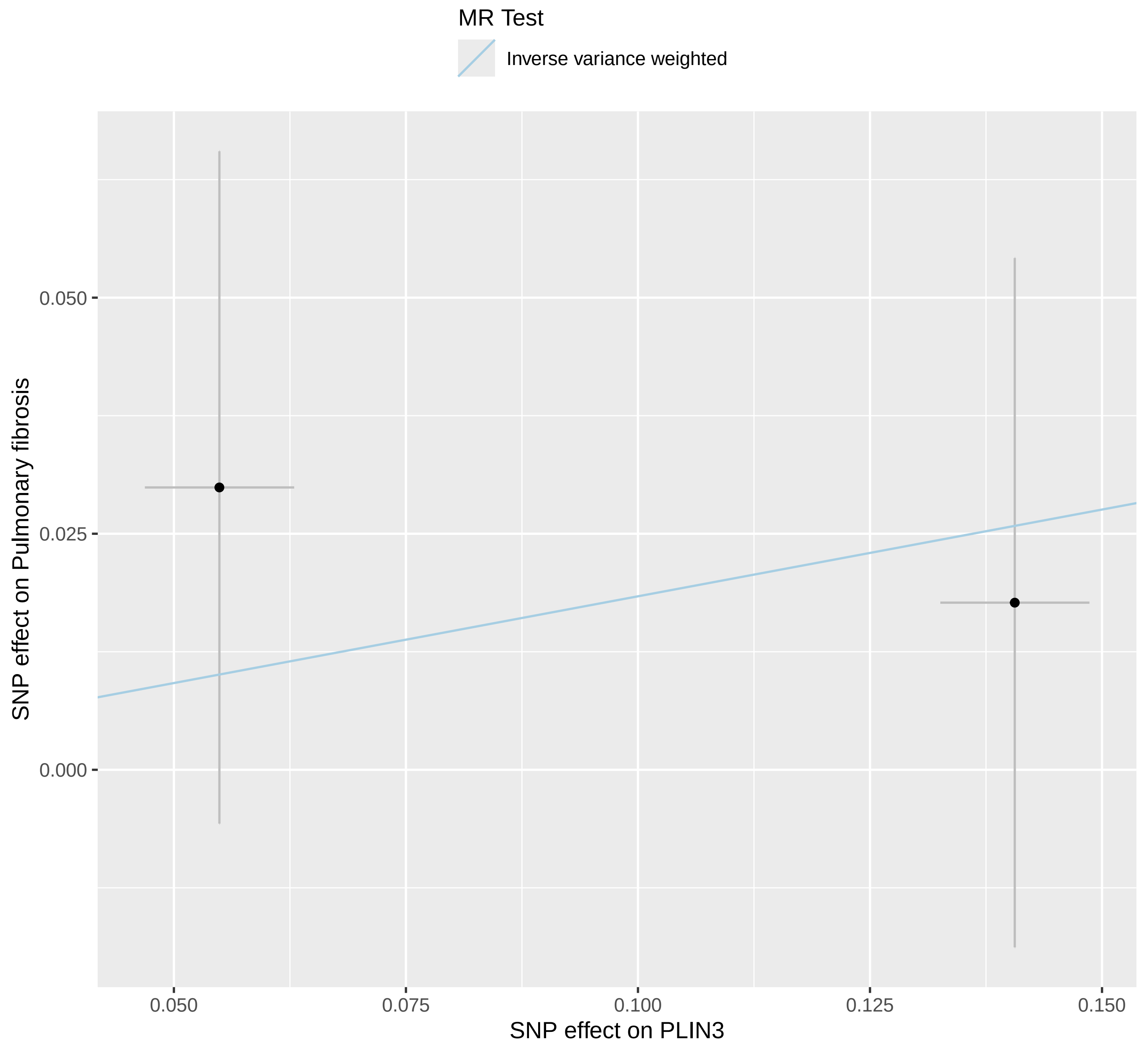

MR Test
Inverse variance weighted
0.050
0.025
0.000
SNP effect on Pulmonary fibrosis
0.050
0.075
0.100
0.125
0.150
SNP effect on PLIN3

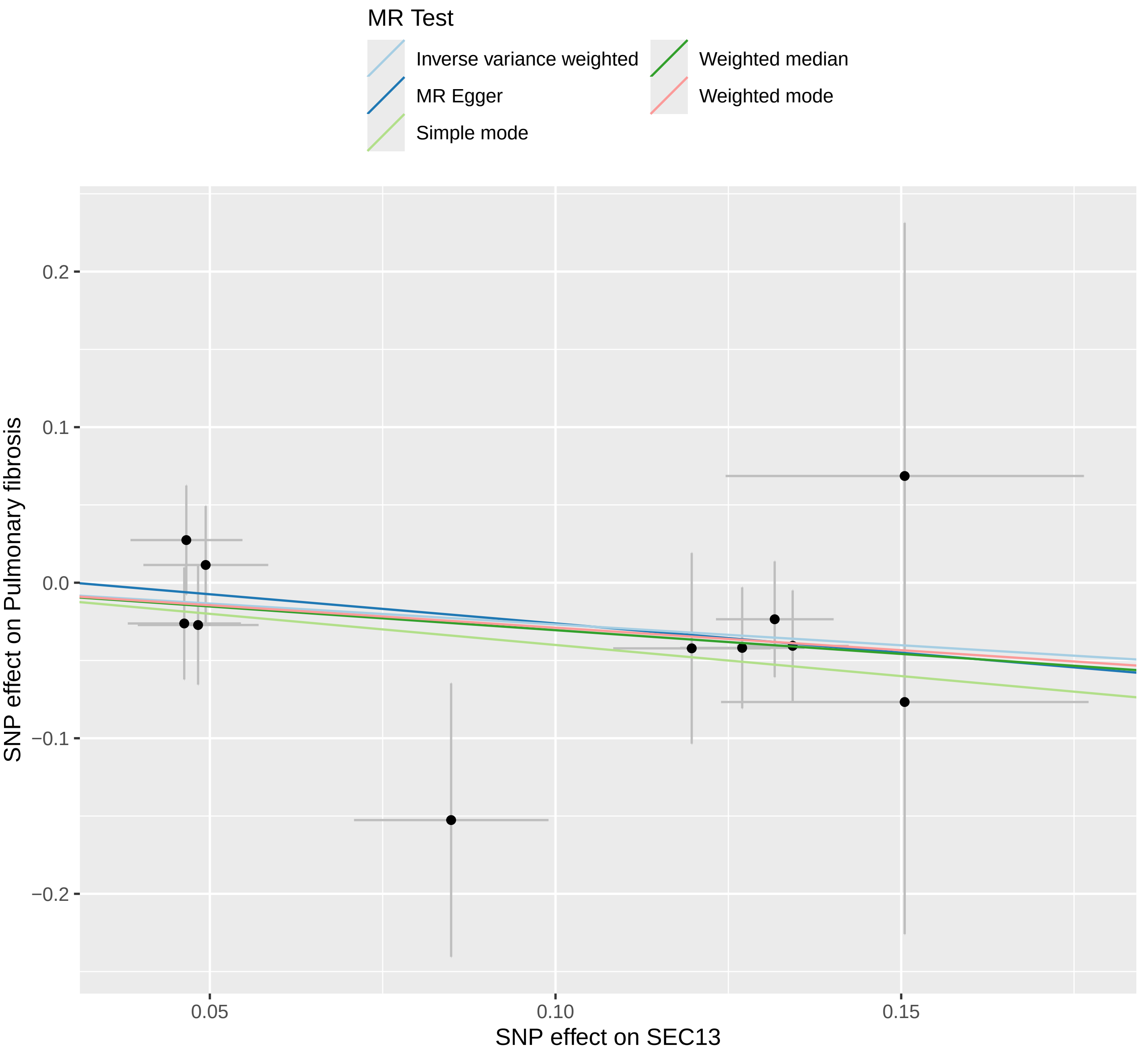

MR Test
Inverse variance weighted
MR Egger
Simple mode
Weighted median
Weighted mode
0.2
0.1
0.0
−0.1
−0.2
SNP effect on Pulmonary fibrosis
0.05
0.10
0.15
SNP effect on SEC13

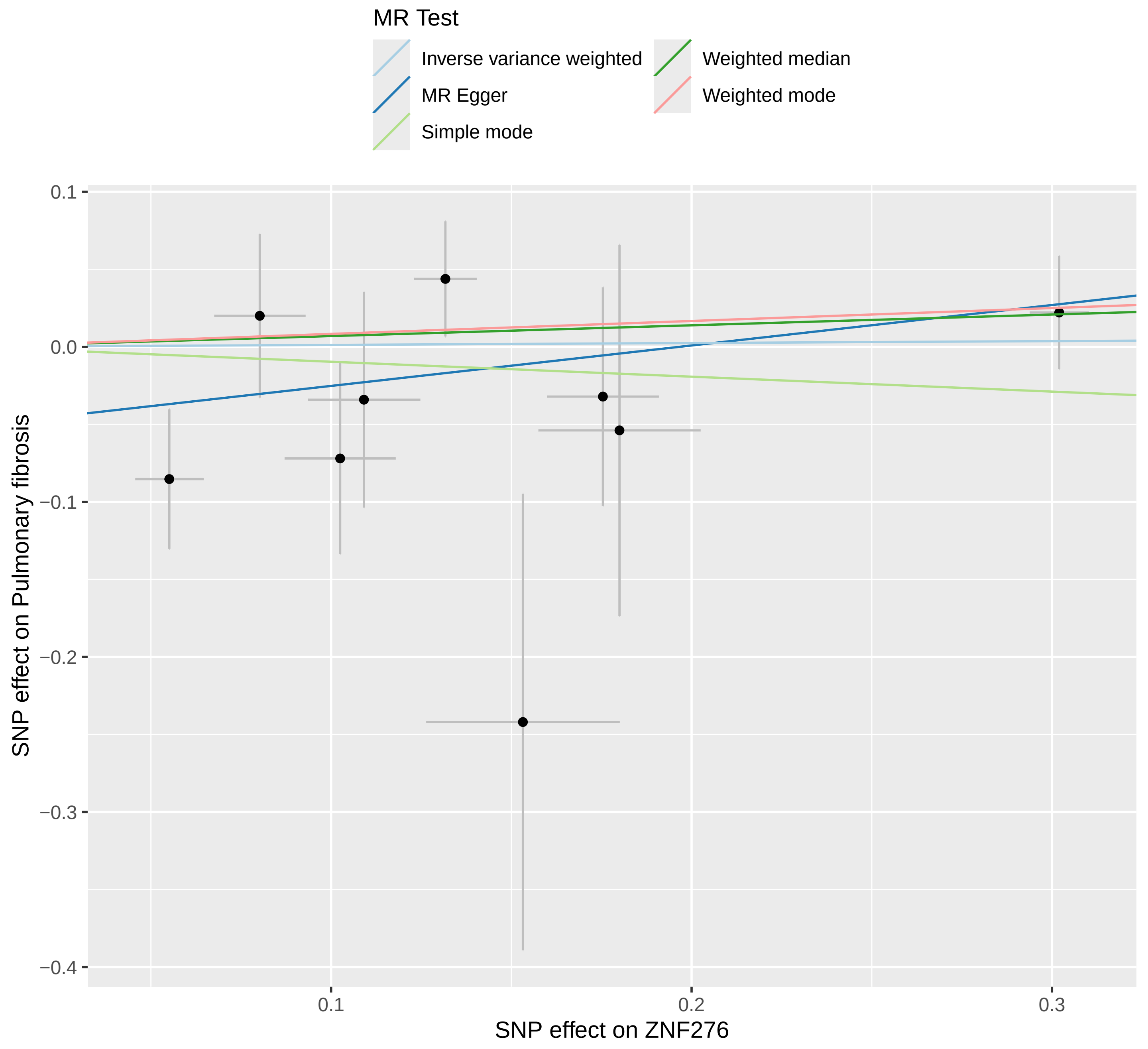
MR Test
Inverse variance weighted
MR Egger
Simple mode
Weighted median
Weighted mode
0.1
0.0
−0.1
−0.2
−0.3
−0.4
SNP effect on Pulmonary fibrosis
0.1
0.2
0.3
SNP effect on ZNF276

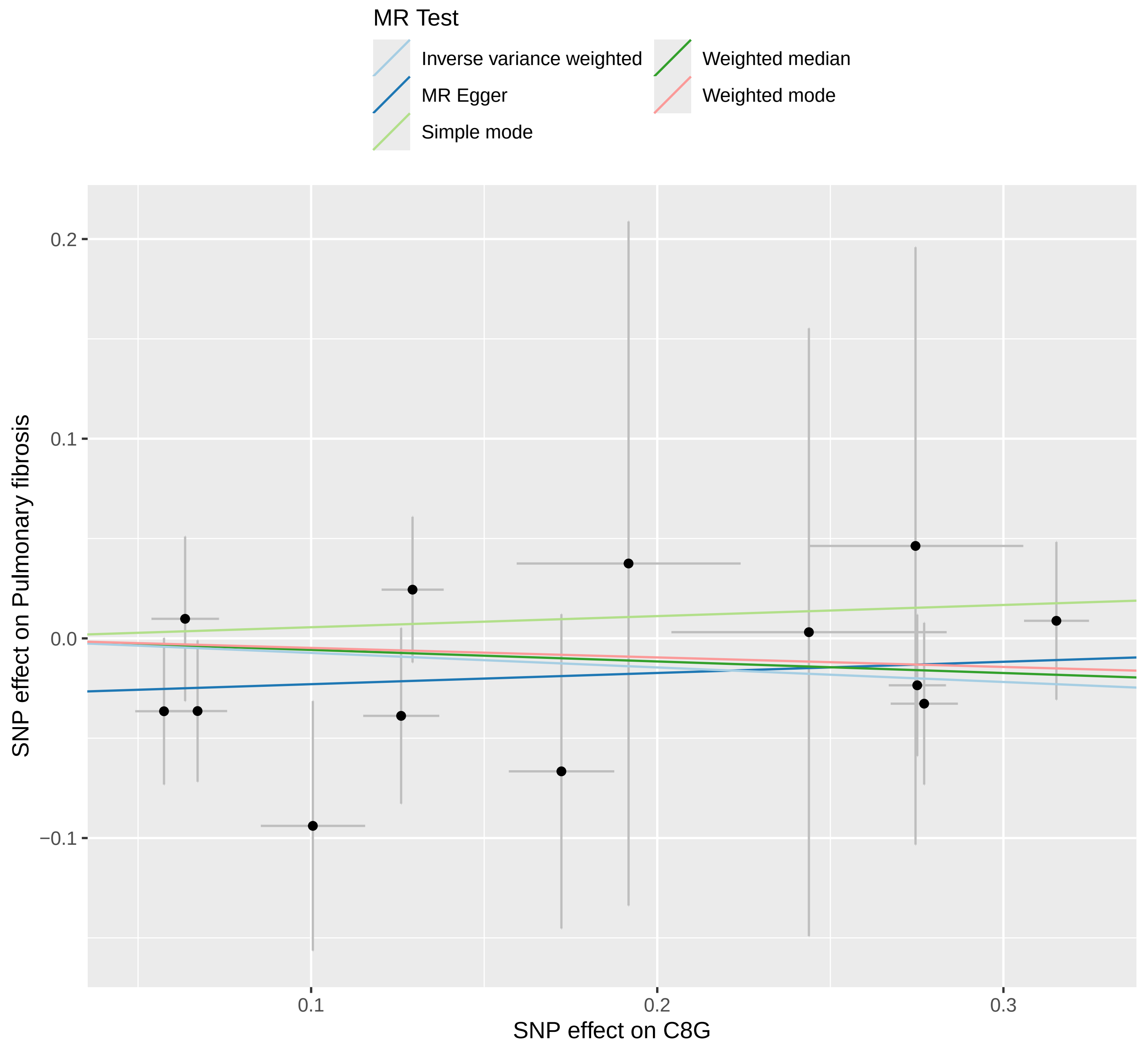
MR Test
Inverse variance weighted
MR Egger
Simple mode
Weighted median
Weighted mode
SNP effect on Pulmonary fibrosis
0.2
0.1
0.0
−0.1
0.1
0.2
0.3
SNP effect on C8G

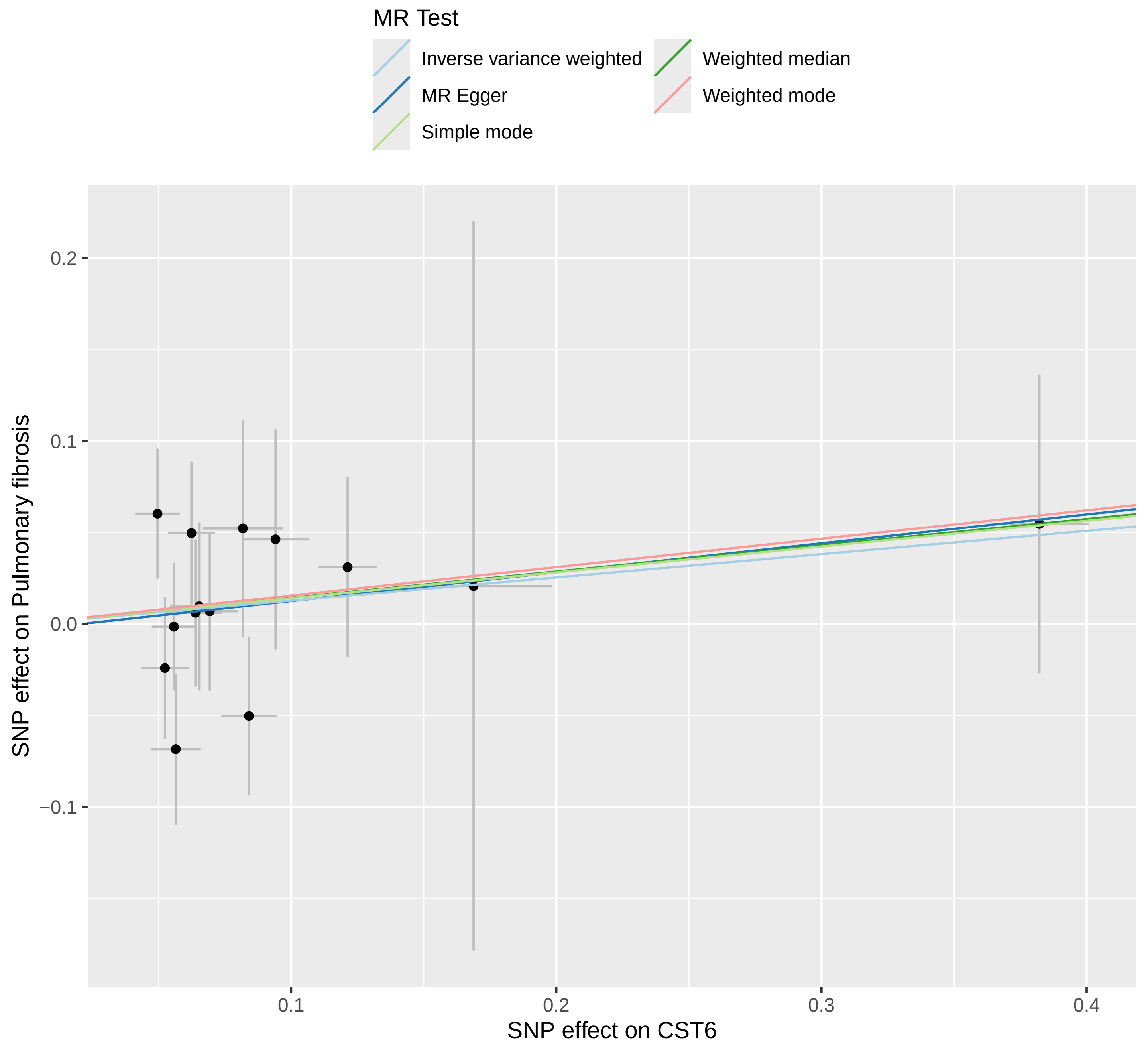
MR Test
Inverse variance weighted
MR Egger
Simple mode
Weighted median
Weighted mode
SNP effect on Pulmonary fibrosis
0.2
0.1
0.0
−0.1
0.1
0.2
0.3
0.4
SNP effect on CST6

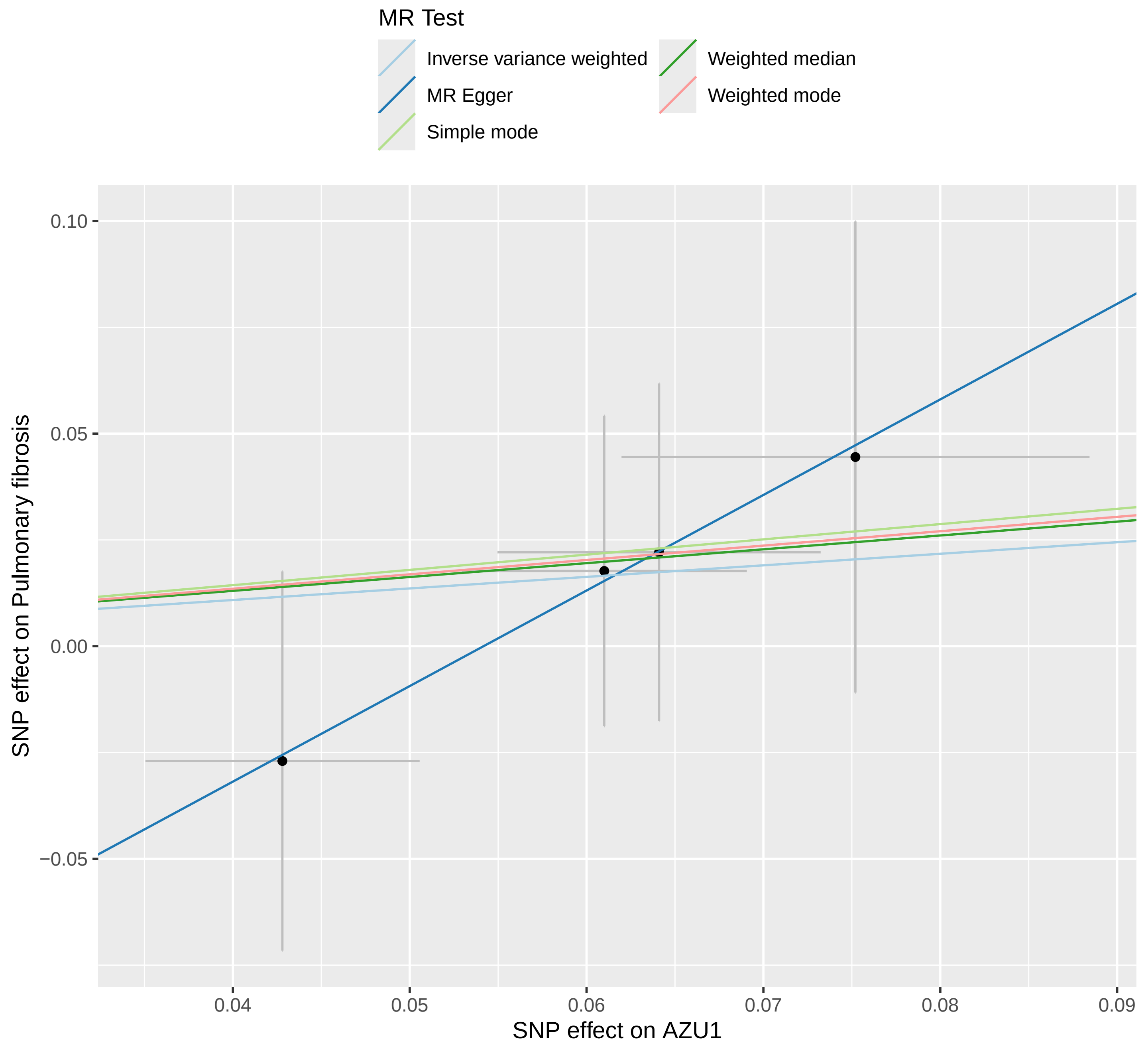

MR Test
Inverse variance weighted
MR Egger
Simple mode
Weighted median
Weighted mode
0.10
0.05
0.00
−0.05
SNP effect on Pulmonary fibrosis
0.04
0.05
0.06
0.07
0.08
0.09
SNP effect on AZU1

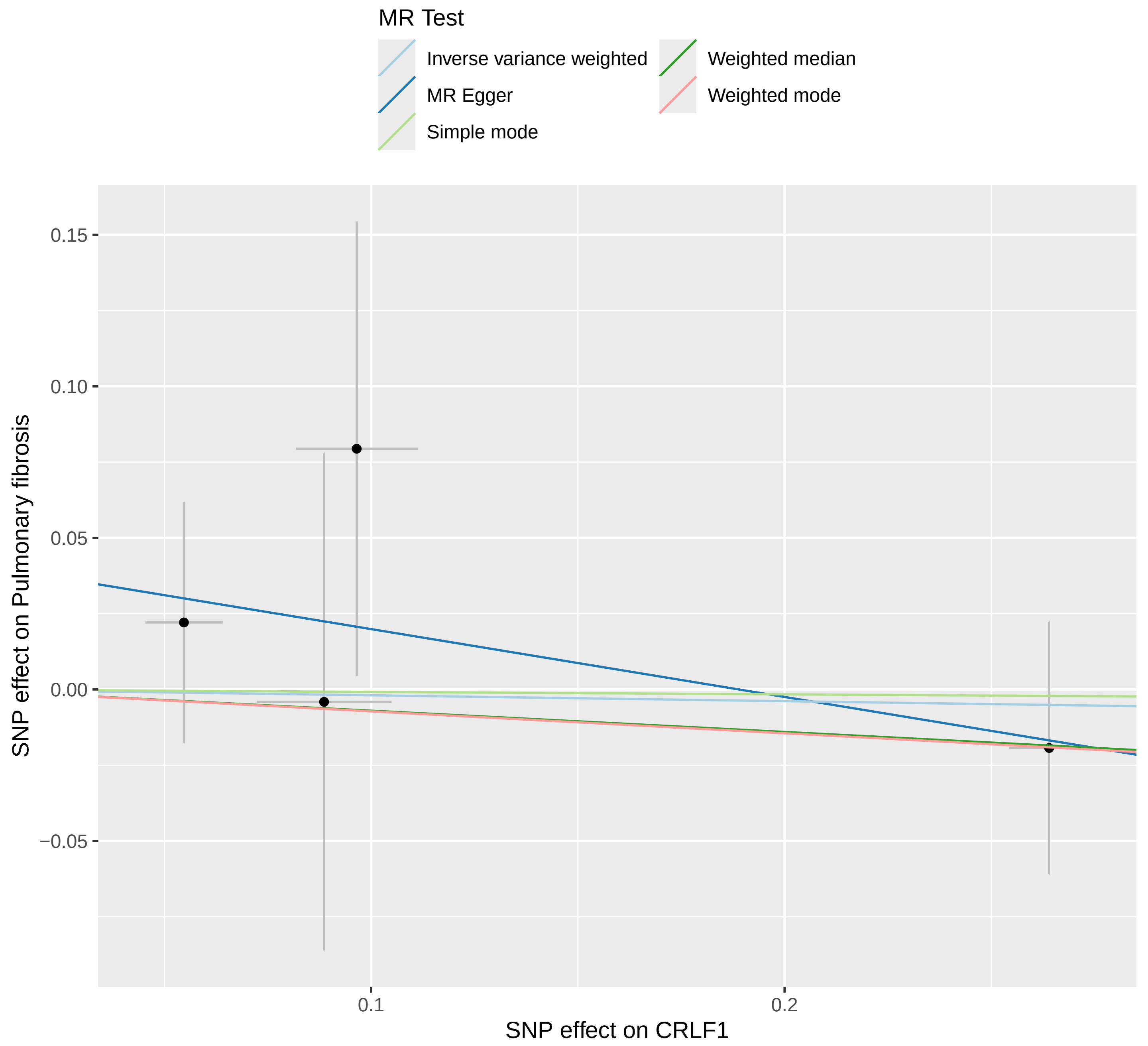

MR Test
Inverse variance weighted
MR Egger
Simple mode
Weighted median
Weighted mode
0.15
0.10
0.05
0.00
−0.05
SNP effect on Pulmonary fibrosis
0.1
0.2
SNP effect on CRLF1

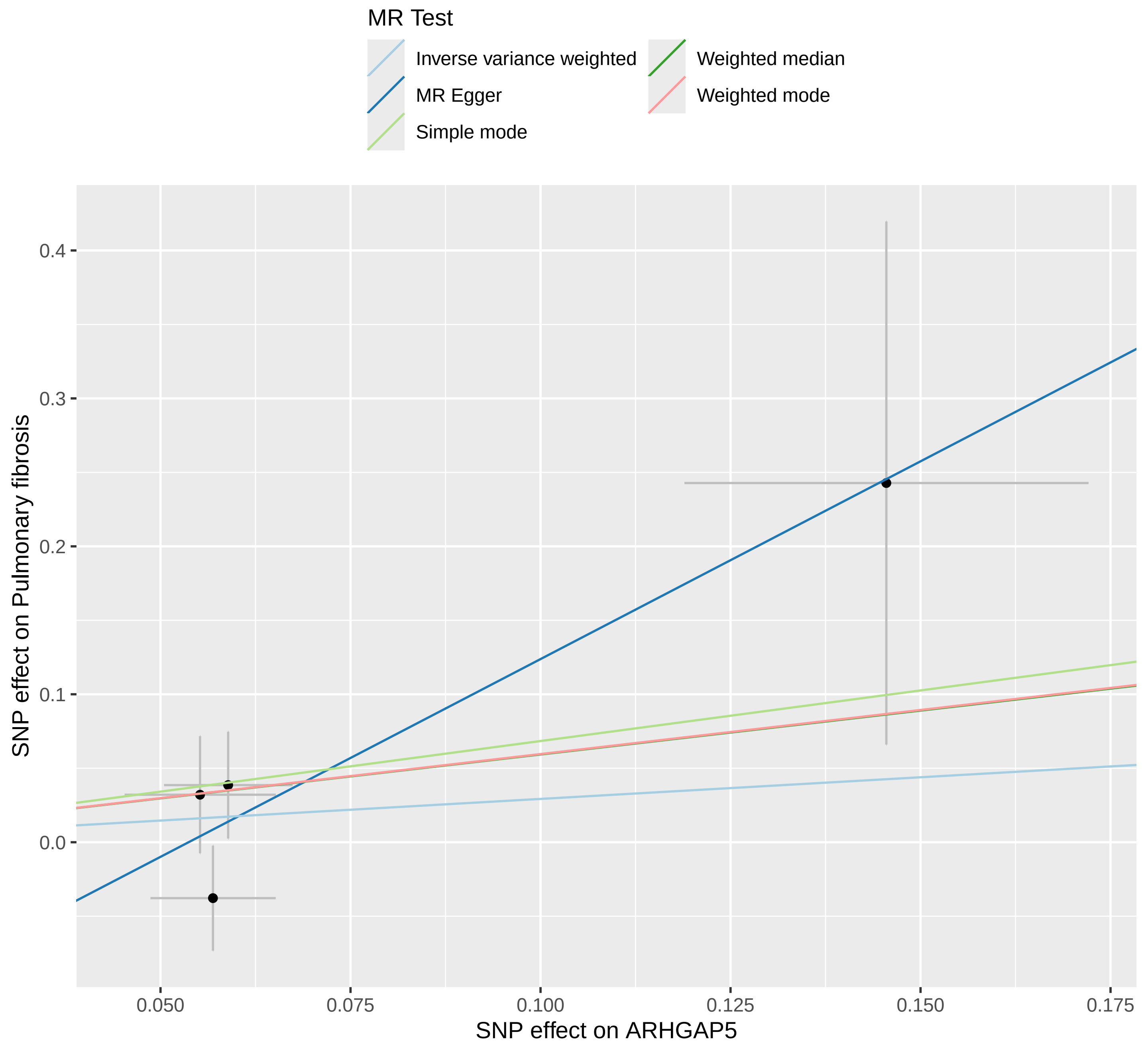

MR Test
Inverse variance weighted
MR Egger
Simple mode
Weighted median
Weighted mode
SNP effect on Pulmonary fibrosis
0.4
0.3
0.2
0.1
0.0
0.050
0.075
0.100
0.125
0.150
0.175
SNP effect on ARHGAP5

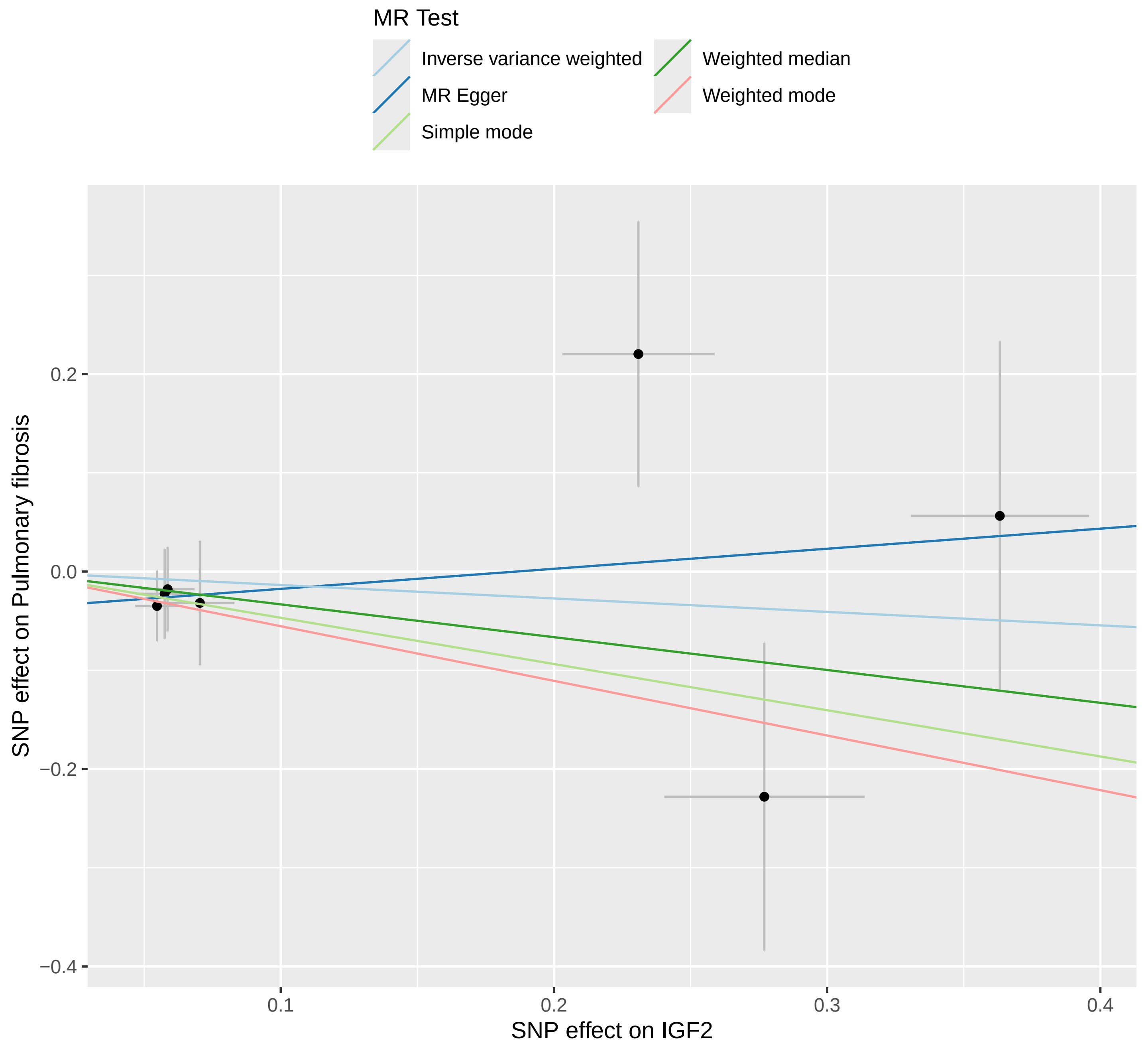
MR Test
Inverse variance weighted
MR Egger
Simple mode
Weighted median
Weighted mode
SNP effect on Pulmonary fibrosis
0.2
0.0
−0.2
−0.4
0.1
0.2
0.3
0.4
SNP effect on IGF2

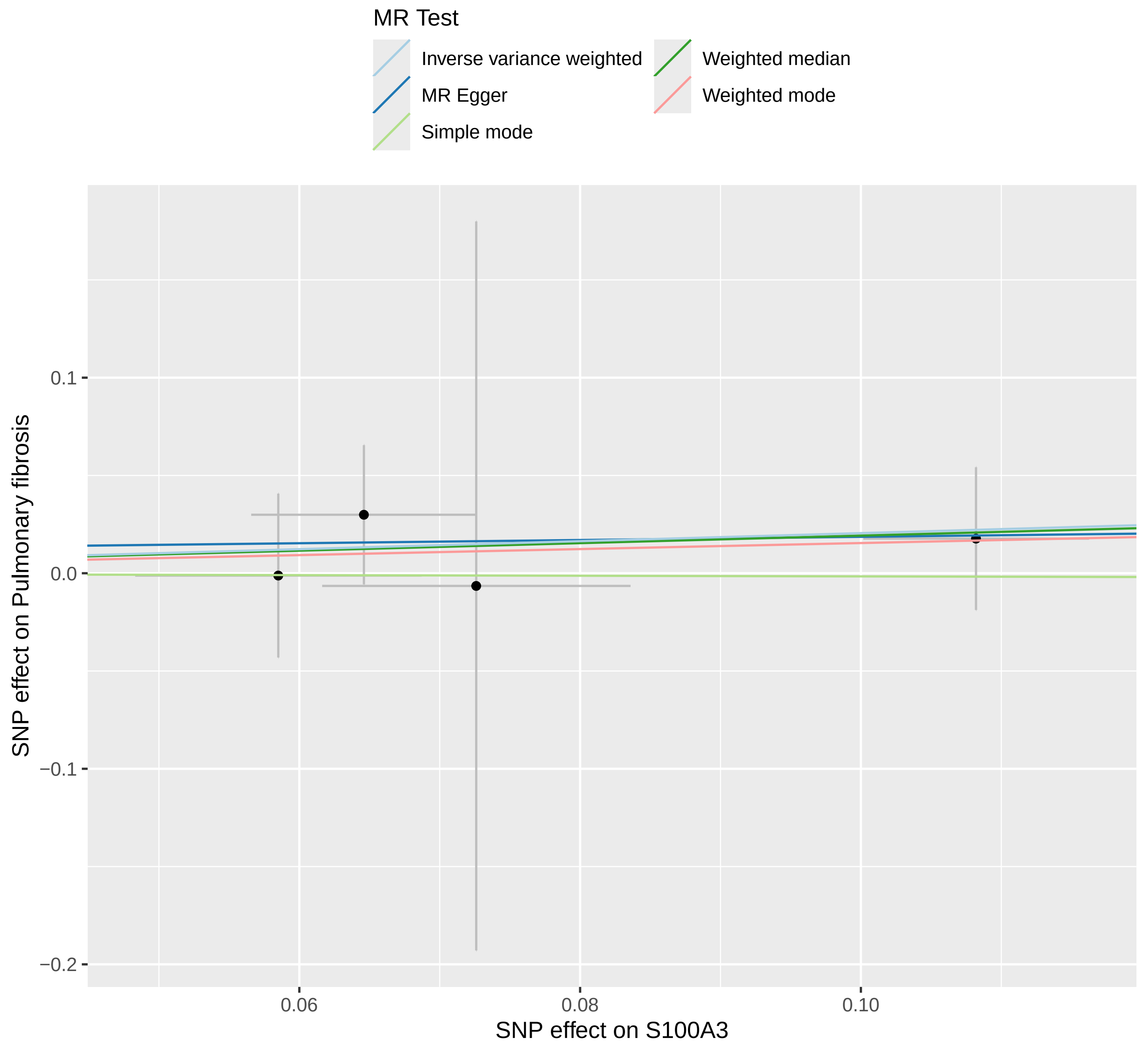
MR Test
Inverse variance weighted
MR Egger
Simple mode
Weighted median
Weighted mode
0.1
0.0
−0.1
−0.2
SNP effect on Pulmonary fibrosis
0.06
0.08
0.10
SNP effect on S100A3

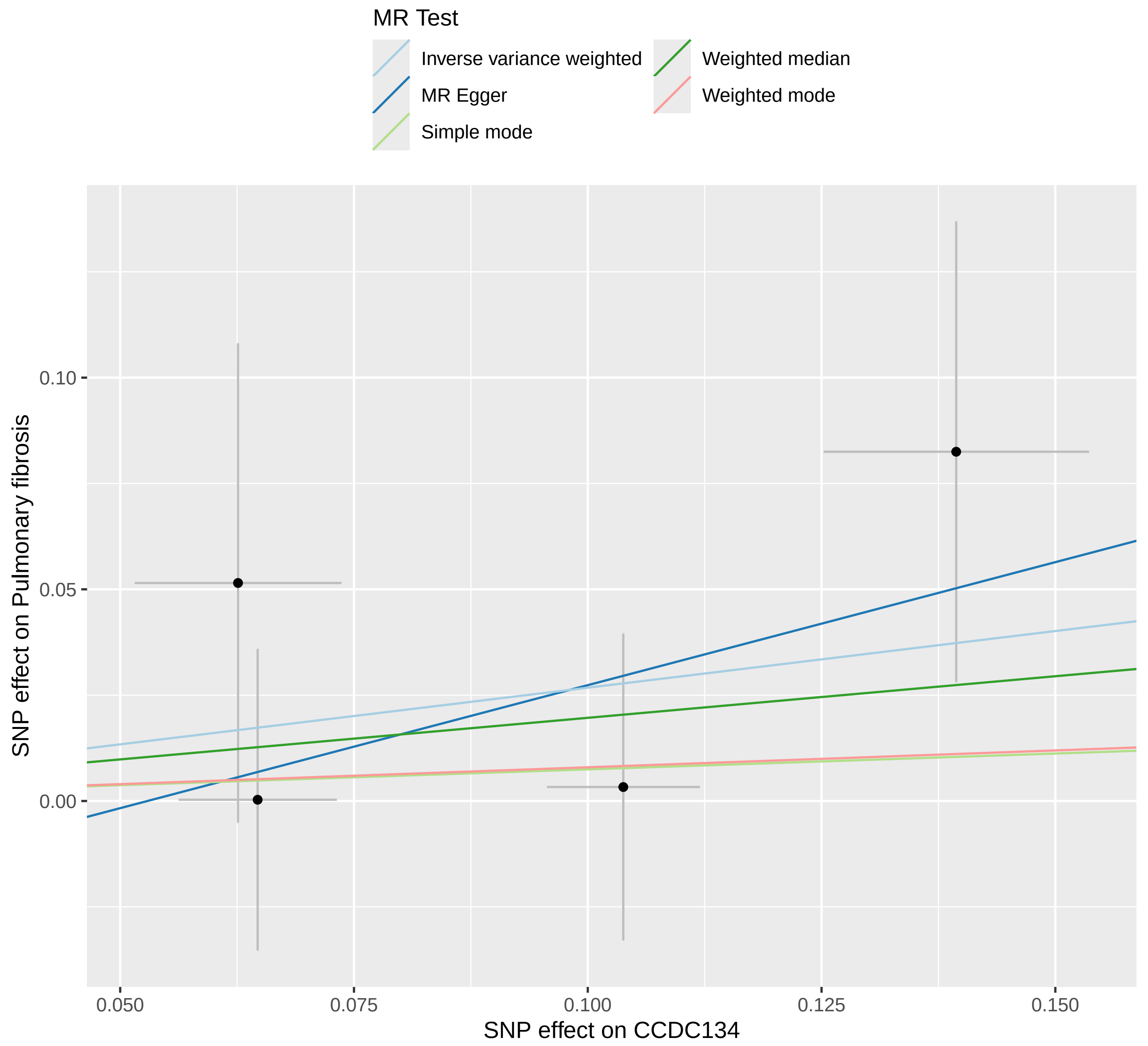
MR Test
Inverse variance weighted
MR Egger
Simple mode
Weighted median
Weighted mode
SNP effect on Pulmonary fibrosis
0.10
0.05
0.00
0.050
0.075
0.100
0.125
0.150
SNP effect on CCDC134

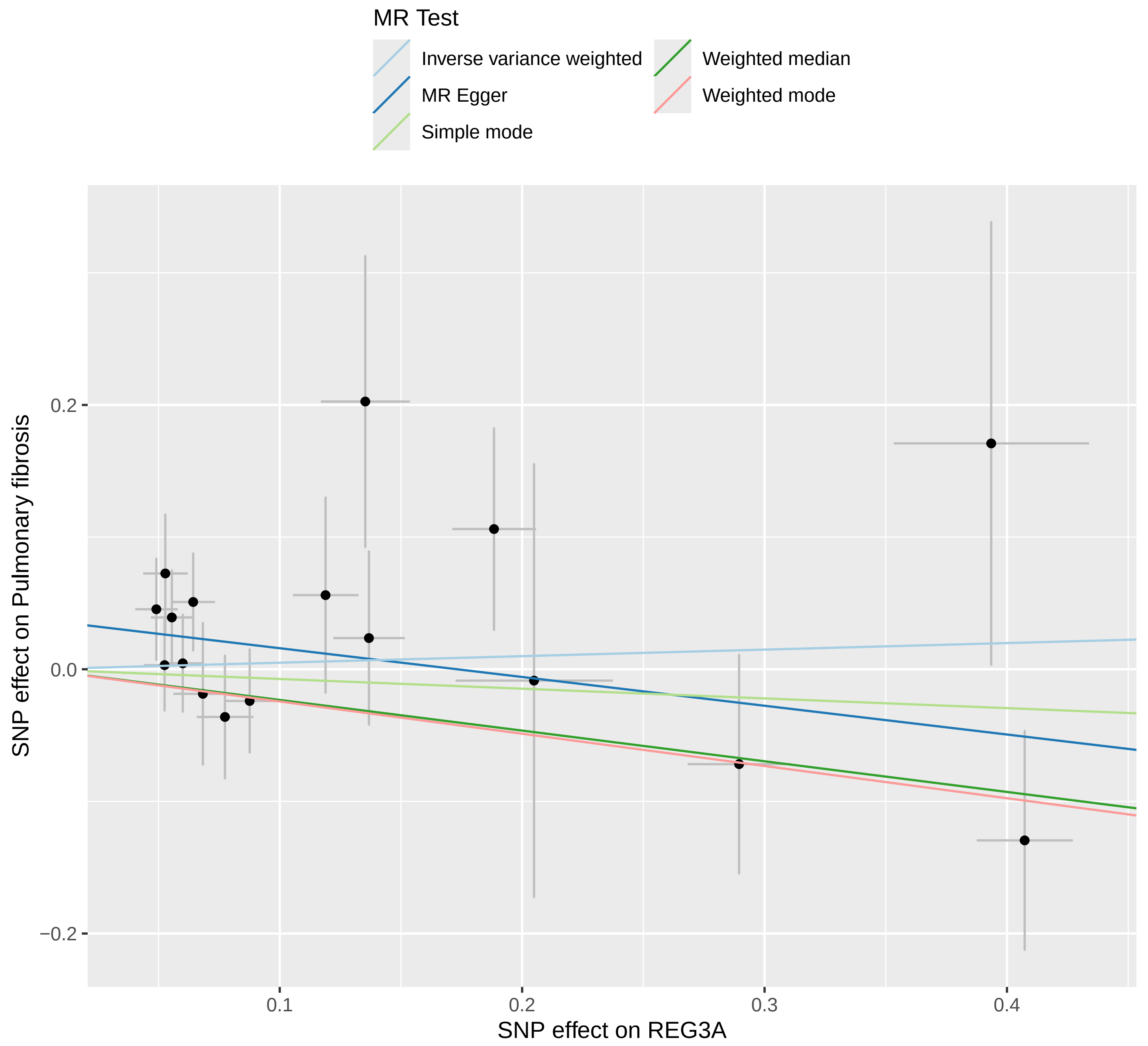

MR Test
Inverse variance weighted
MR Egger
Simple mode
Weighted median
Weighted mode
0.2
0.0
−0.2
SNP effect on Pulmonary fibrosis
0.1
0.2
0.3
0.4
SNP effect on REG3A

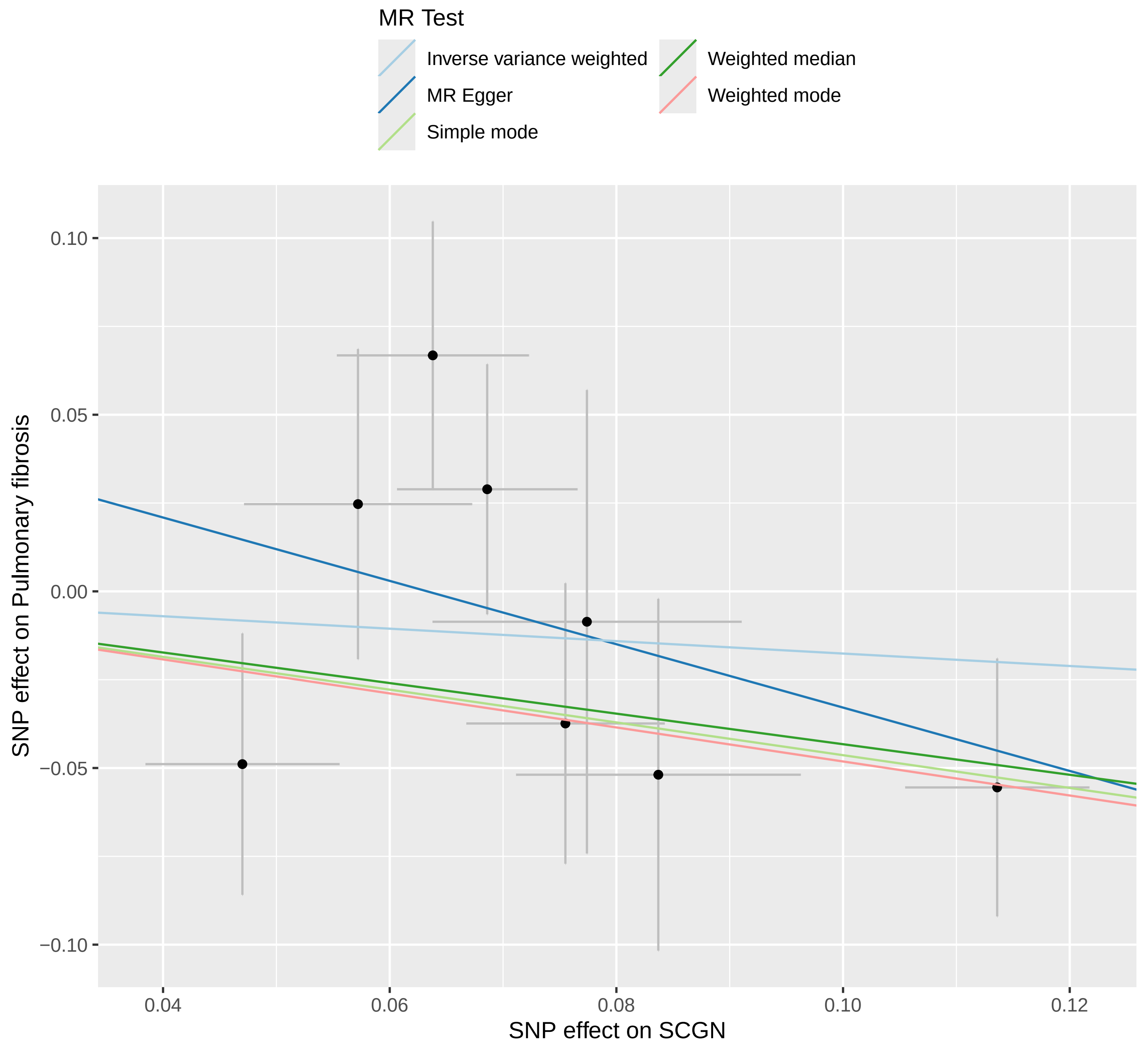

MR Test
Inverse variance weighted
MR Egger
Simple mode
Weighted median
Weighted mode
0.10
0.05
0.00
−0.05
−0.10
0.04
0.06
0.08
0.10
0.12
SNP effect on Pulmonary fibrosis
SNP effect on SCGN

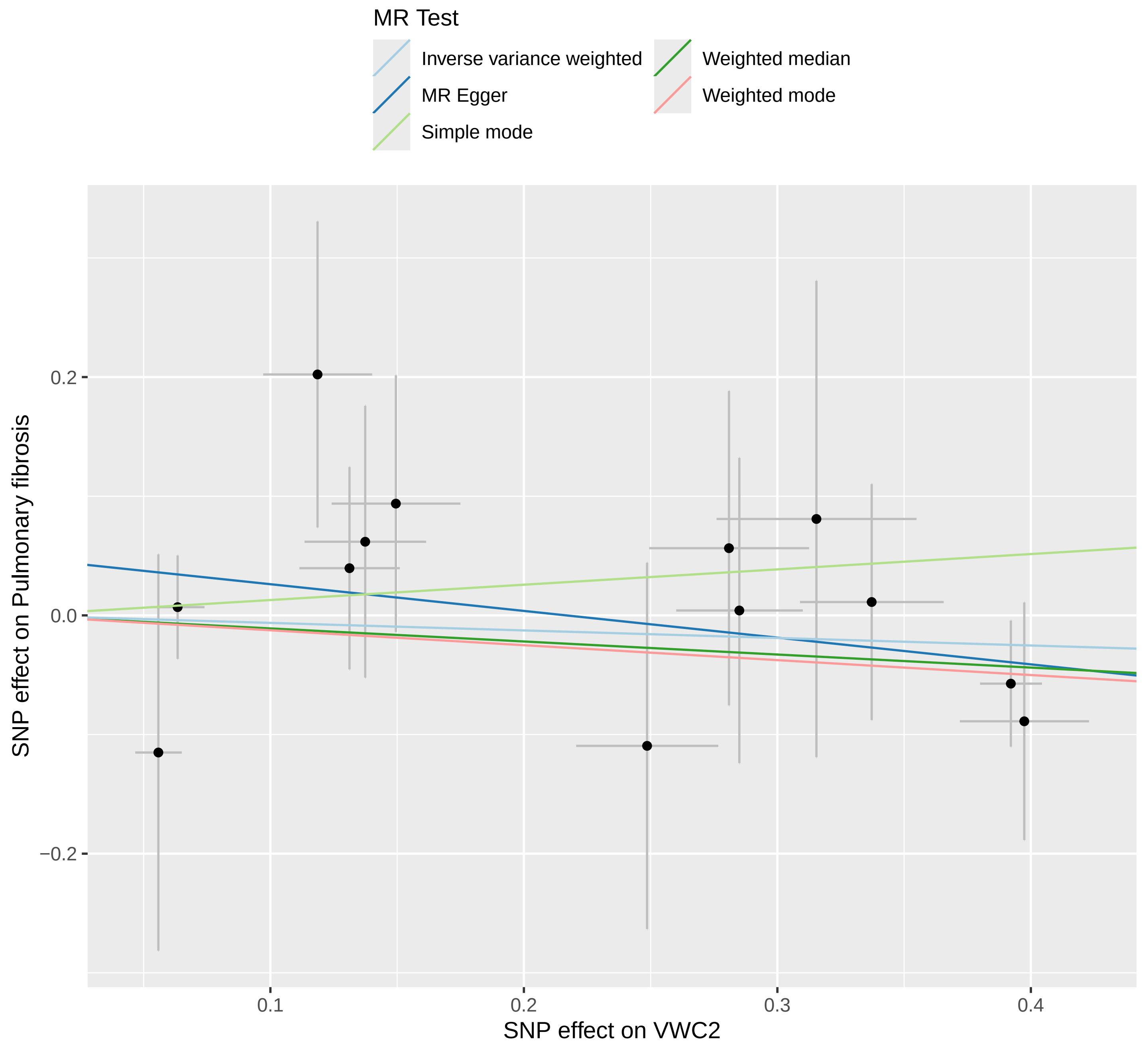
MR Test
Inverse variance weighted
MR Egger
Simple mode
Weighted median
Weighted mode
SNP effect on Pulmonary fibrosis
0.2
0.0
−0.2
0.1
0.2
0.3
0.4
SNP effect on VWC2

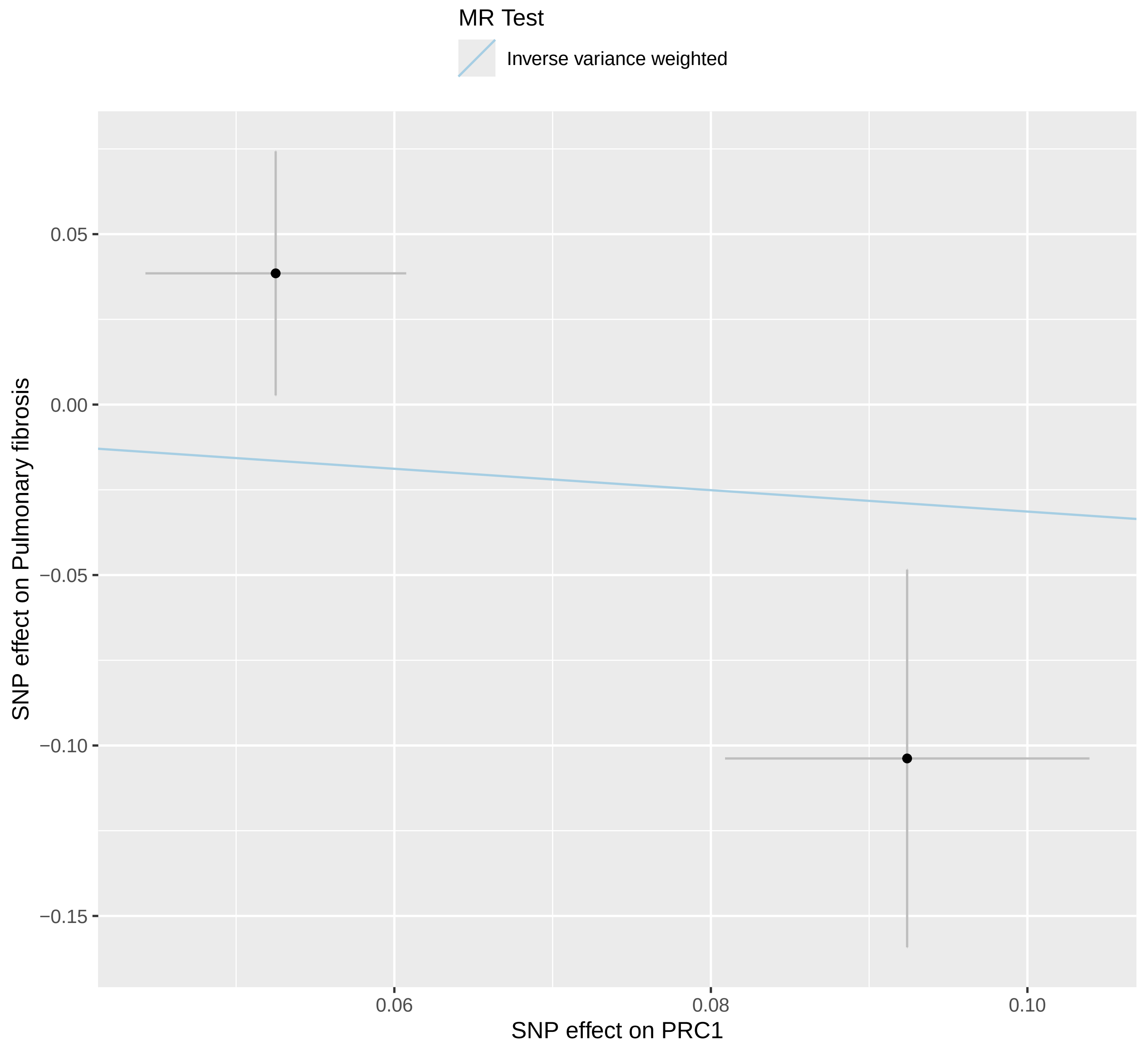
MR Test
Inverse variance weighted
SNP effect on Pulmonary fibrosis
0.05
0.00
−0.05
−0.10
−0.15
0.06
0.08
0.10
SNP effect on PRC1

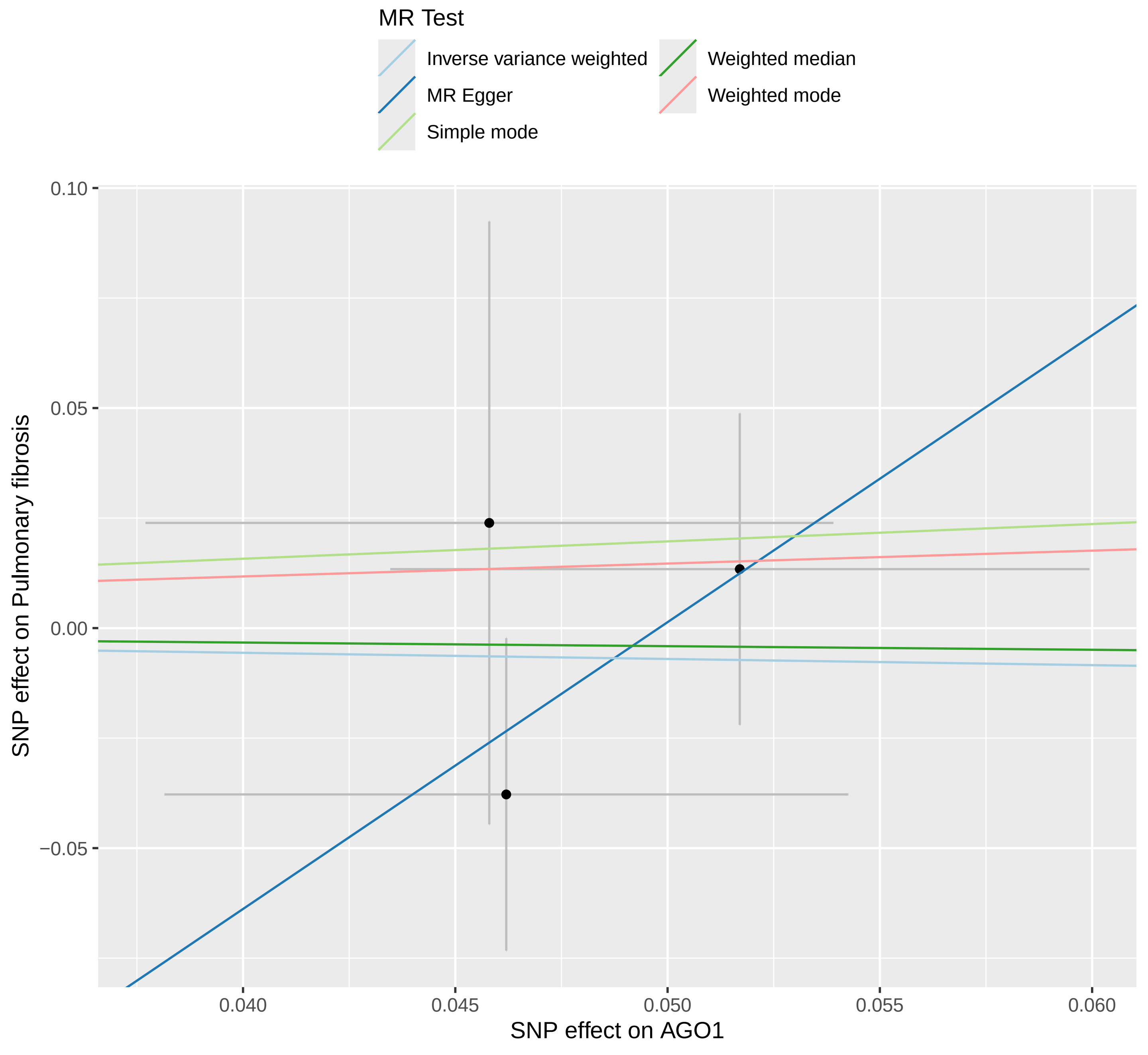

MR Test
Inverse variance weighted
MR Egger
Simple mode
Weighted median
Weighted mode
0.10
0.05
0.00
−0.05
0.040
0.045
0.050
0.055
0.060
SNP effect on Pulmonary fibrosis
SNP effect on AGO1

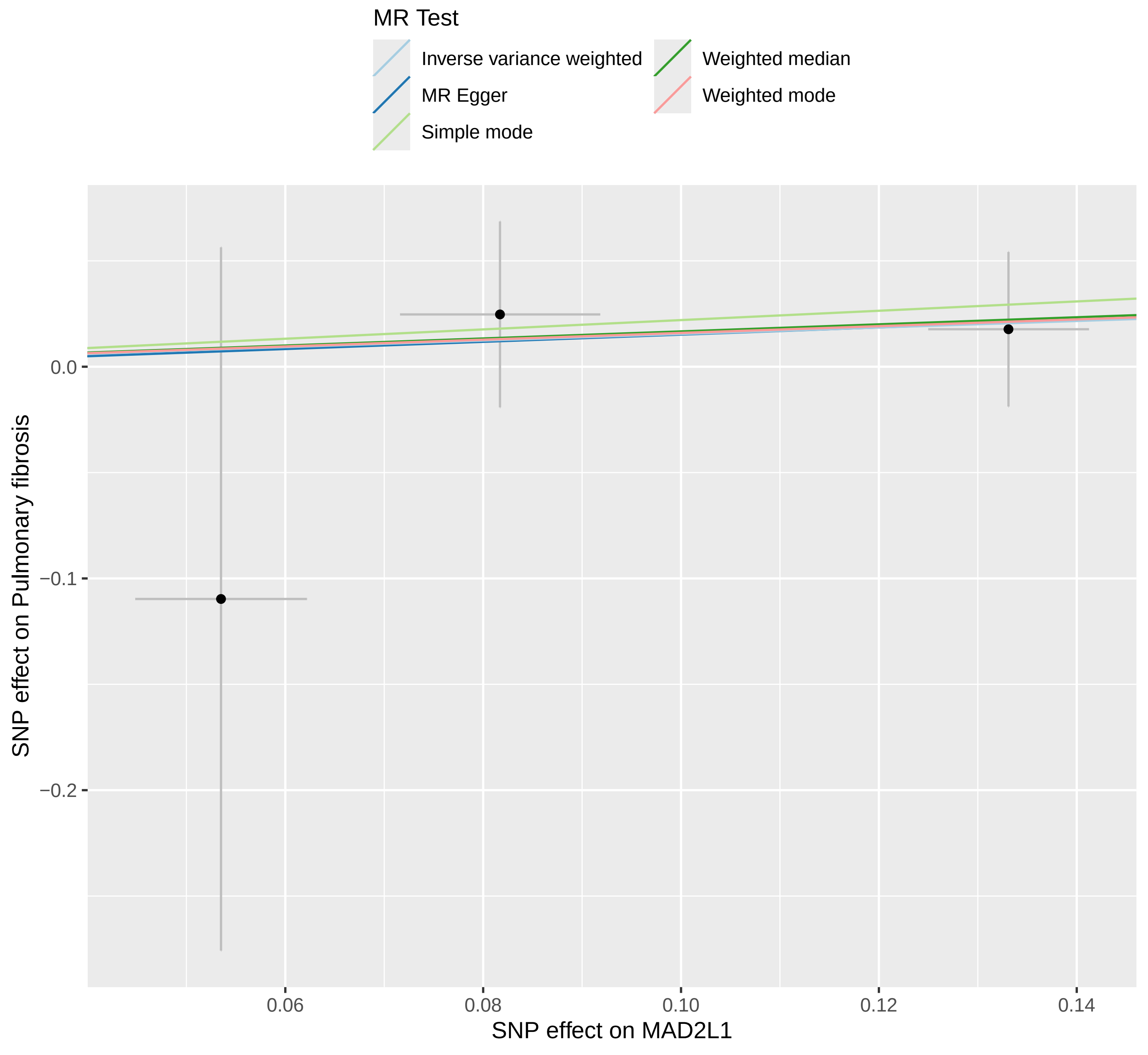
MR Test
Inverse variance weighted
MR Egger
Simple mode
Weighted median
Weighted mode
0.0
−0.1
−0.2
0.06
0.08
0.10
0.12
0.14
SNP effect on MAD2L1
SNP effect on Pulmonary fibrosis

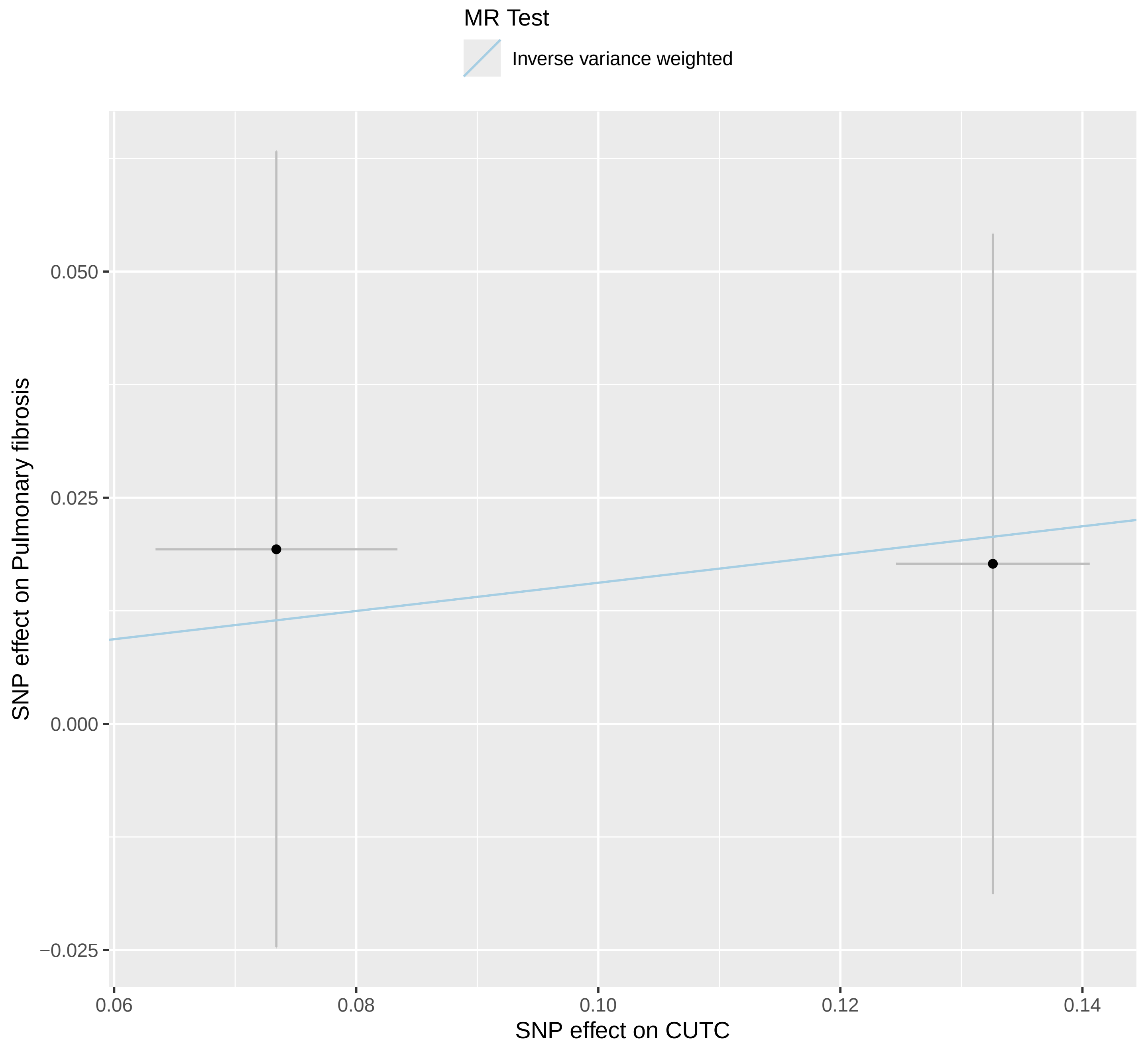

MR Test
Inverse variance weighted
SNP effect on Pulmonary fibrosis
0.050
0.025
0.000
−0.025
0.06
0.08
0.10
0.12
0.14
SNP effect on CUTC

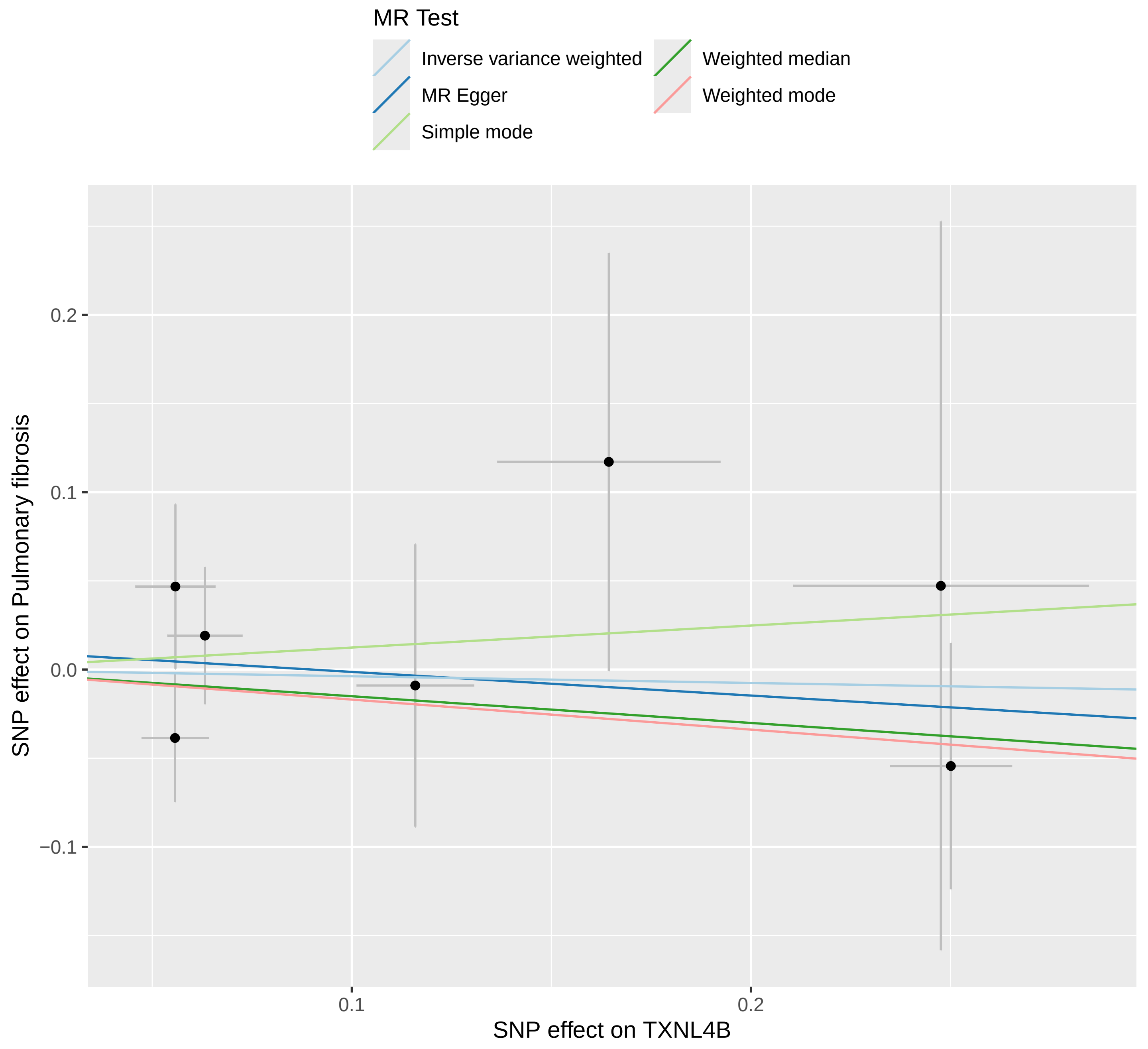
MR Test
Inverse variance weighted
MR Egger
Simple mode
Weighted median
Weighted mode
0.2
0.1
0.0
−0.1
0.1
0.2
SNP effect on TXNL4B
SNP effect on Pulmonary fibrosis

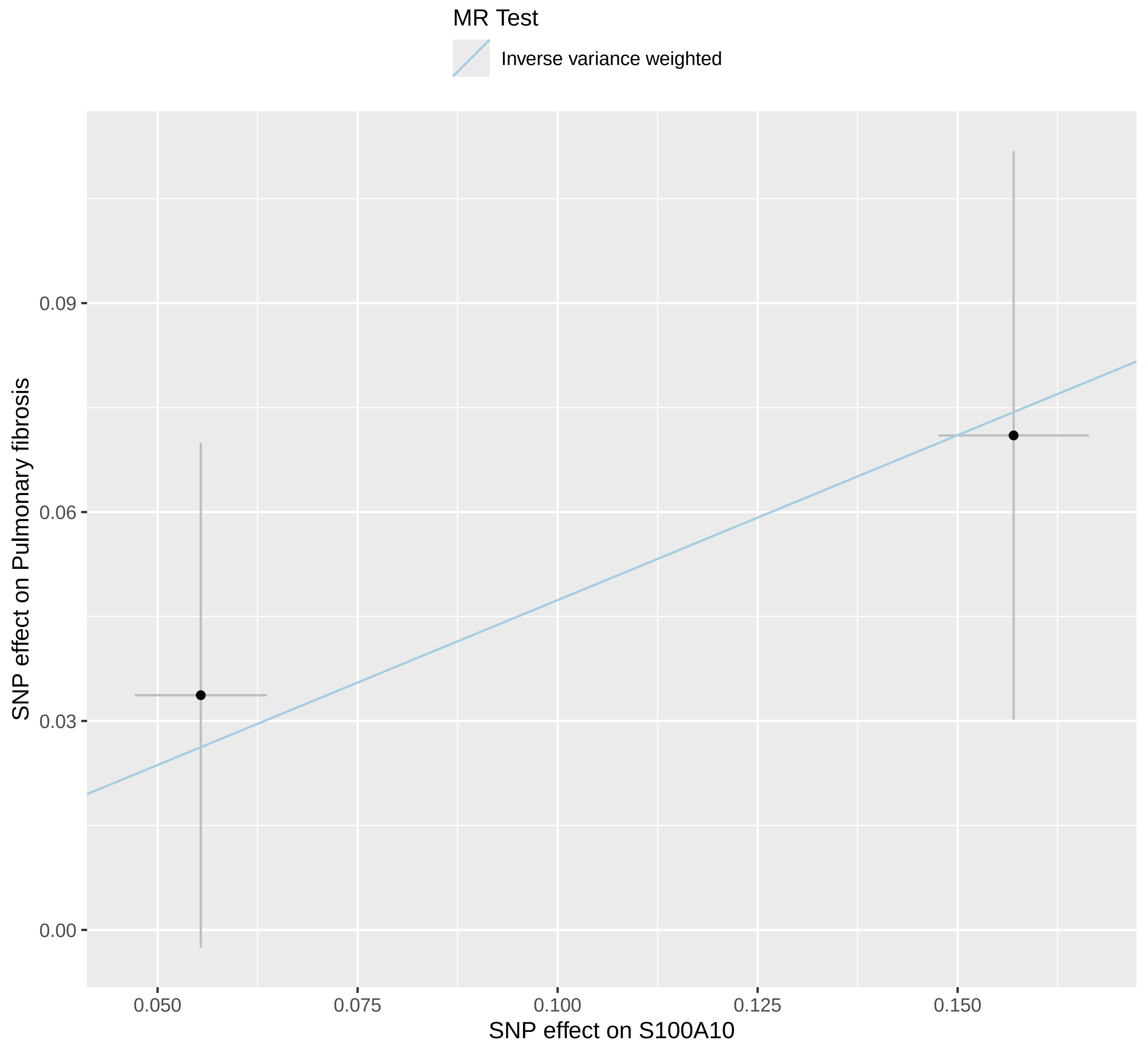

MR Test
Inverse variance weighted
0.09
0.06
0.03
0.00
SNP effect on Pulmonary fibrosis
0.050
0.075
0.100
0.125
0.150
SNP effect on S100A10

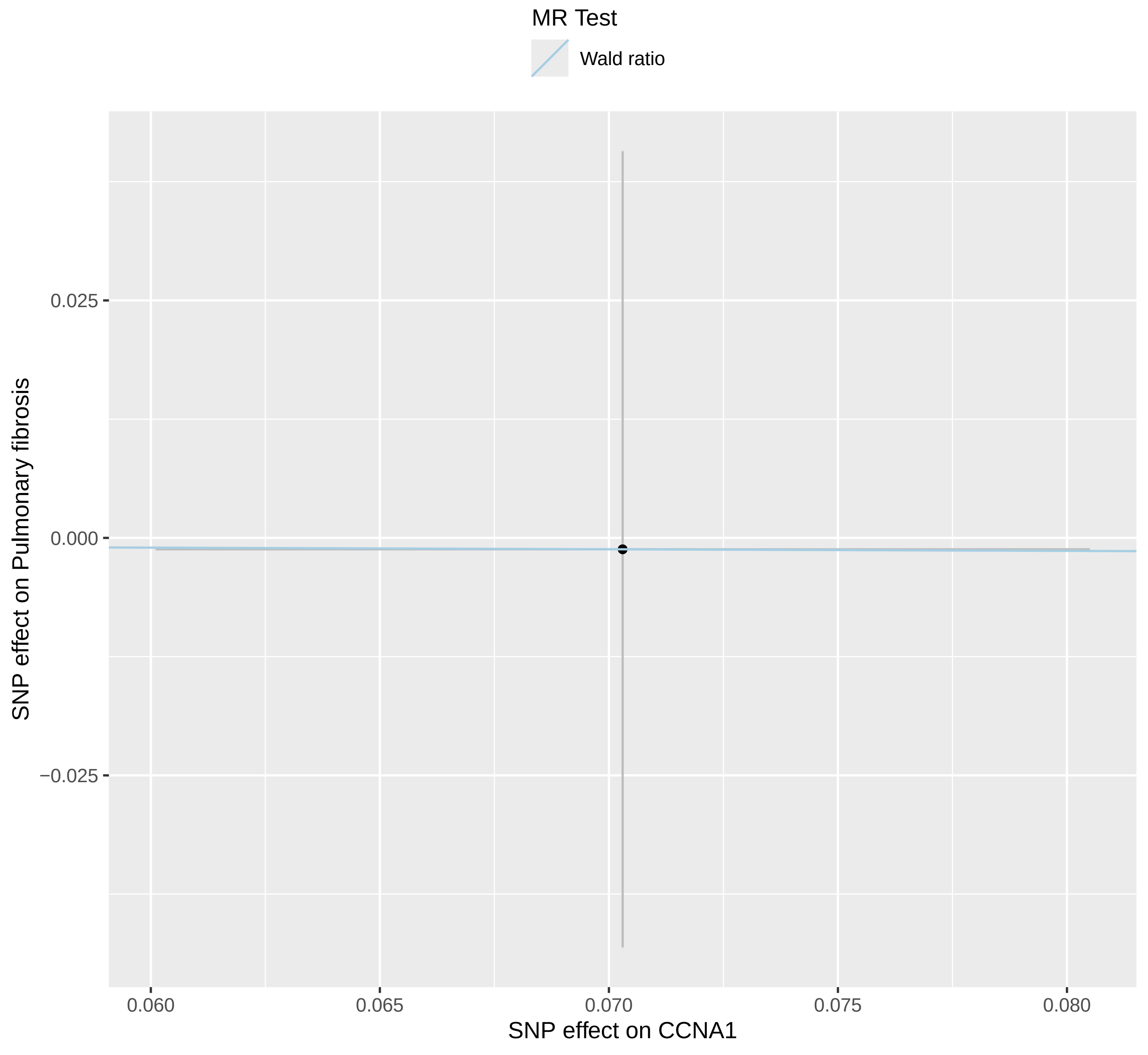

MR Test
Wald ratio
0.025
0.000
−0.025
0.060
0.065
0.070
0.075
0.080
SNP effect on Pulmonary fibrosis
SNP effect on CCNA1

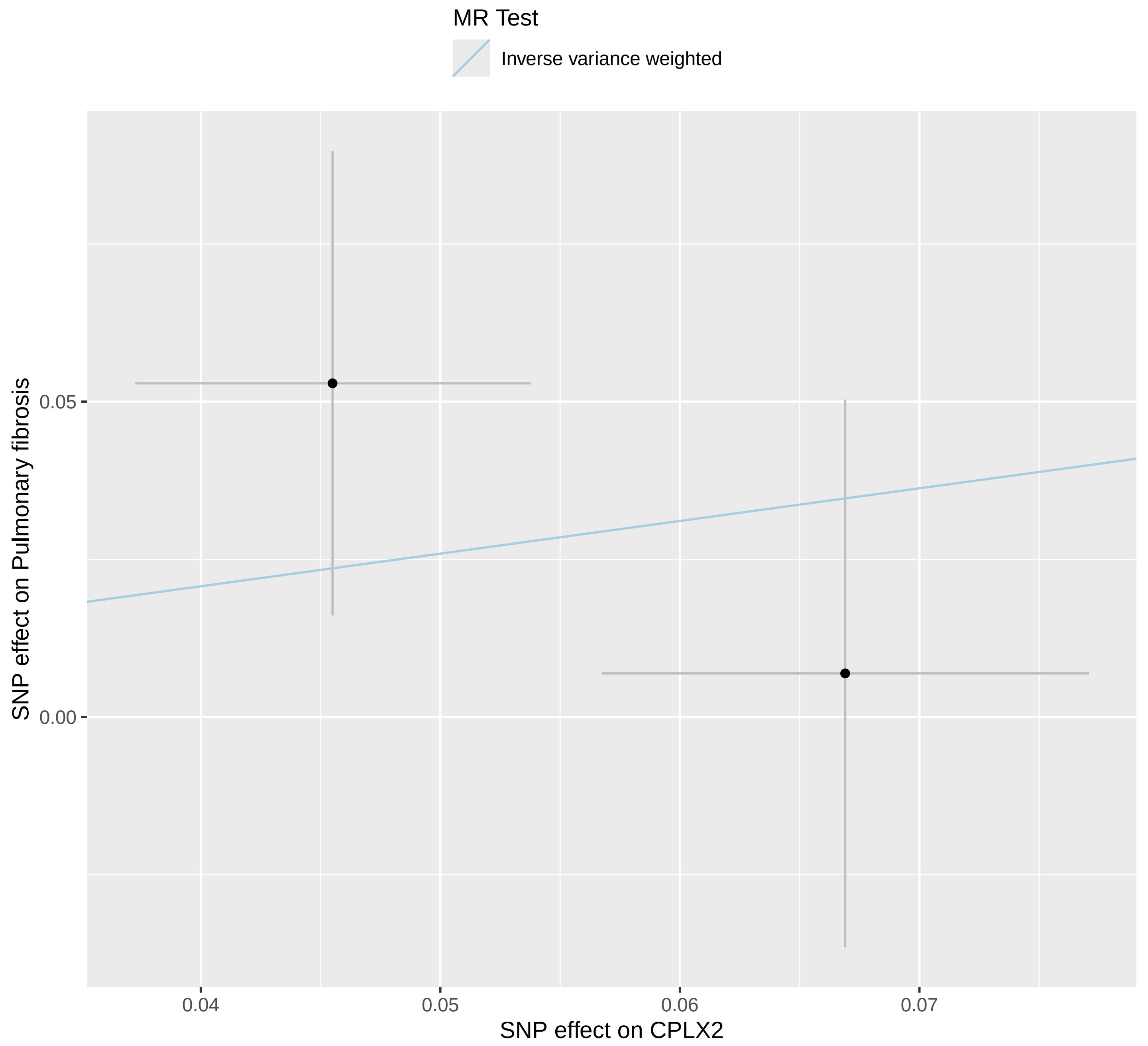

MR Test
Inverse variance weighted
SNP effect on Pulmonary fibrosis
0.05
0.00
0.04
0.05
0.06
0.07
SNP effect on CPLX2

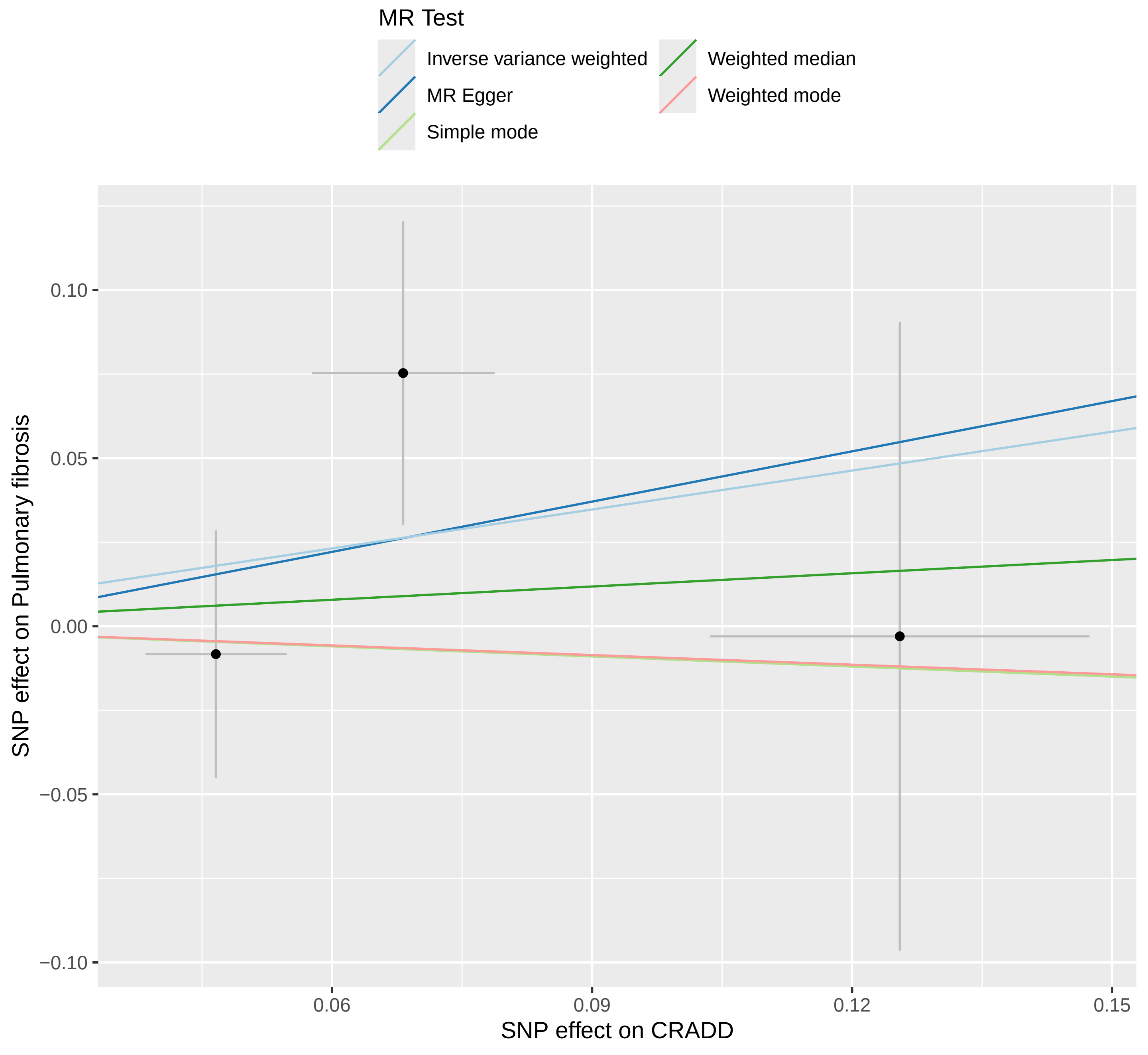

MR Test
Inverse variance weighted
MR Egger
Simple mode
Weighted median
Weighted mode
0.10
0.05
0.00
−0.05
−0.10
0.06
0.09
0.12
0.15
SNP effect on Pulmonary fibrosis
SNP effect on CRADD

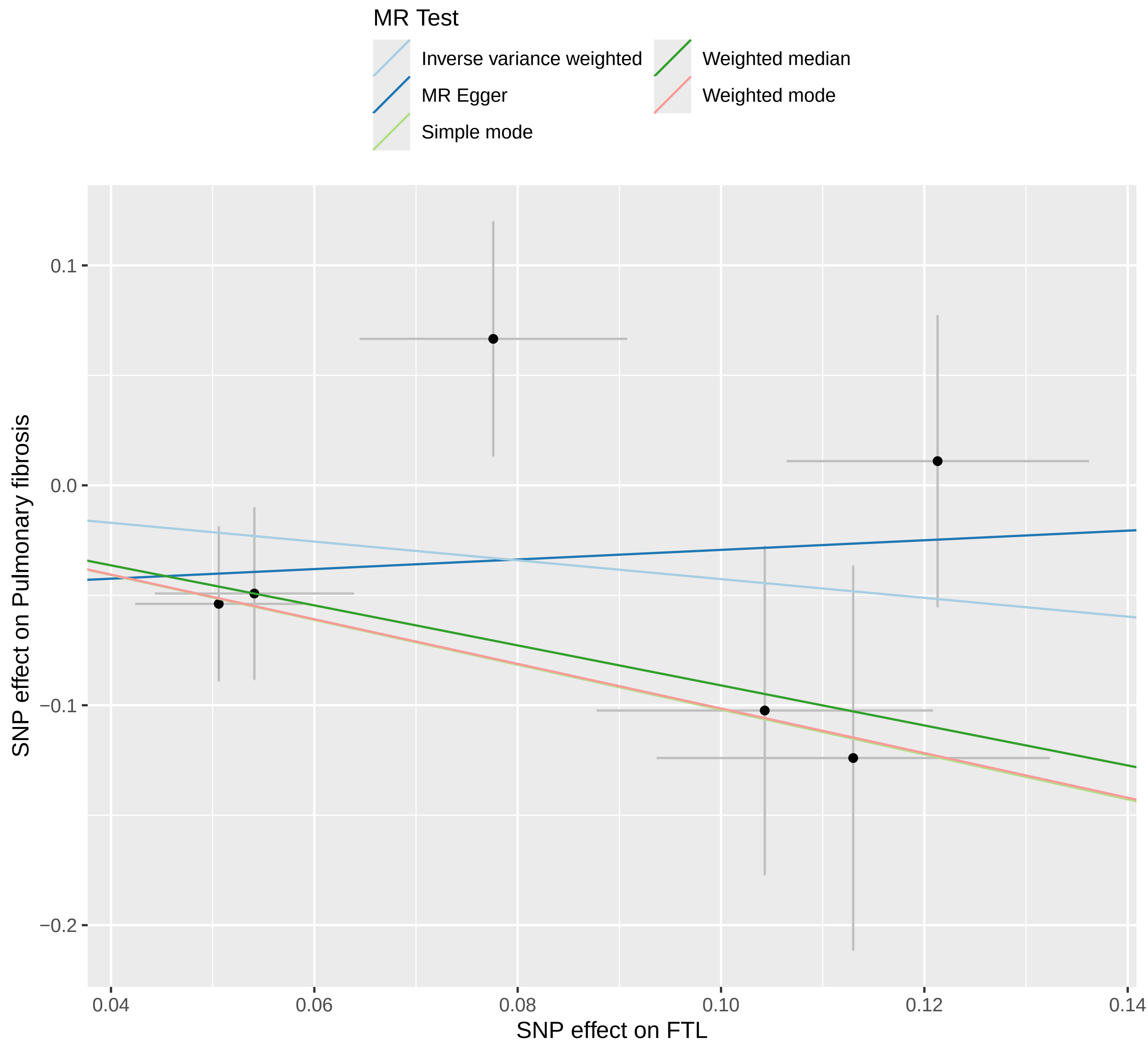
MR Test
Inverse variance weighted
MR Egger
Simple mode
Weighted median
Weighted mode
0.1
0.0
−0.1
−0.2
SNP effect on Pulmonary fibrosis
0.04
0.06
0.08
0.10
0.12
0.14
SNP effect on FTL

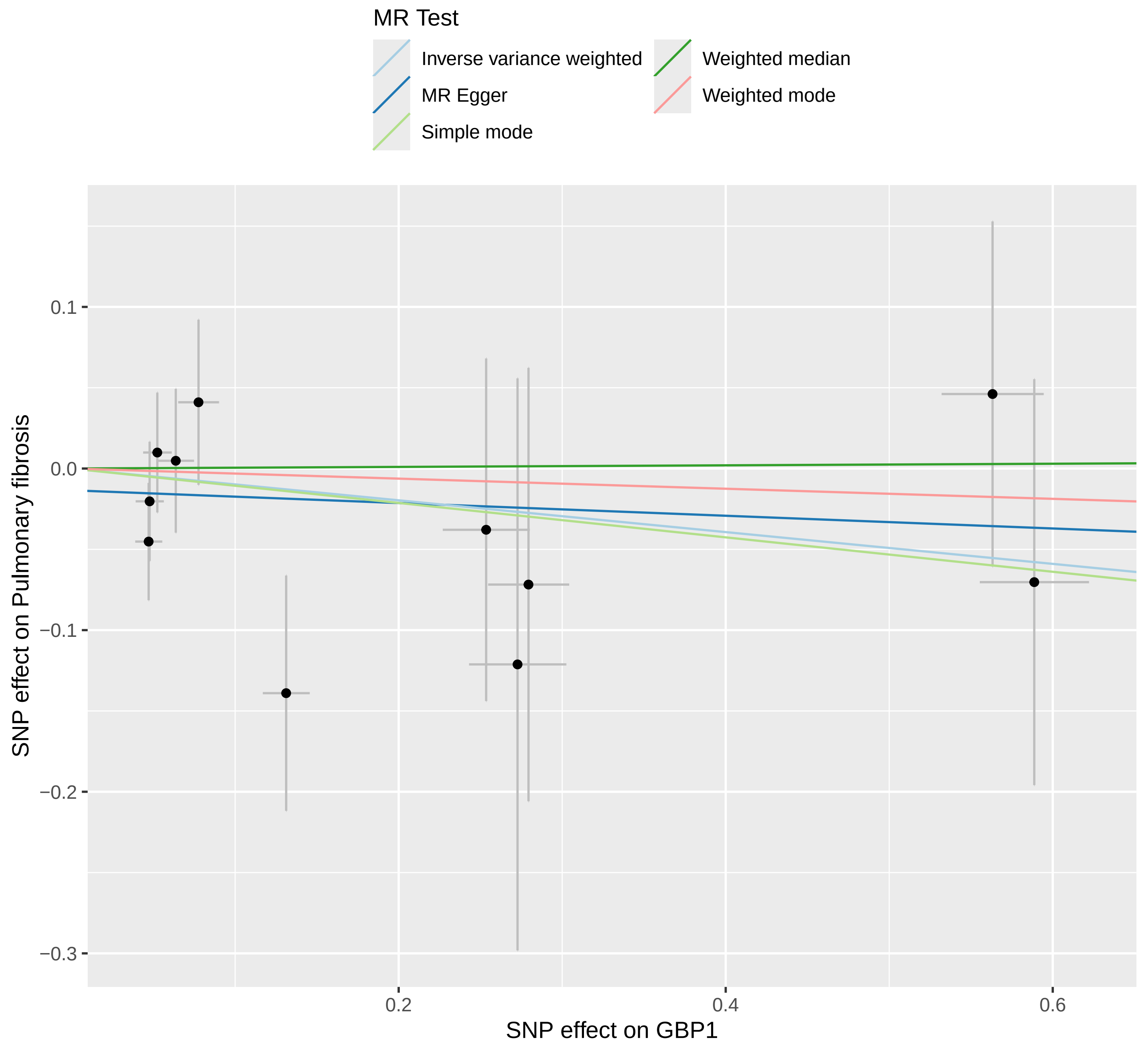

MR Test
Inverse variance weighted
MR Egger
Simple mode
Weighted median
Weighted mode
0.1
0.0
−0.1
−0.2
−0.3
SNP effect on Pulmonary fibrosis
0.2
0.4
0.6
SNP effect on GBP1

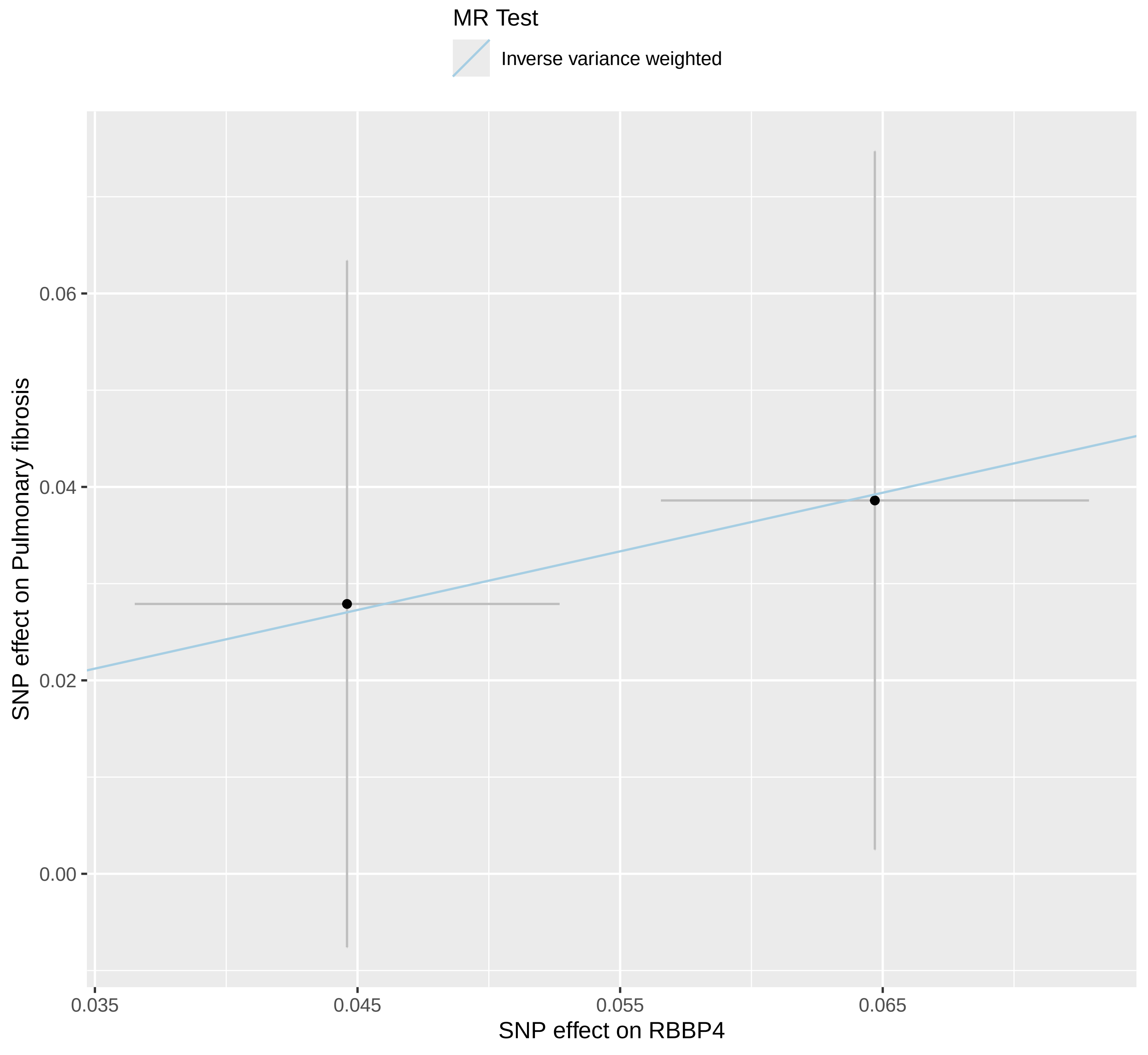

MR Test
Inverse variance weighted
0.06
0.04
0.02
0.00
SNP effect on Pulmonary fibrosis
0.035
0.045
0.055
0.065
SNP effect on RBBP4

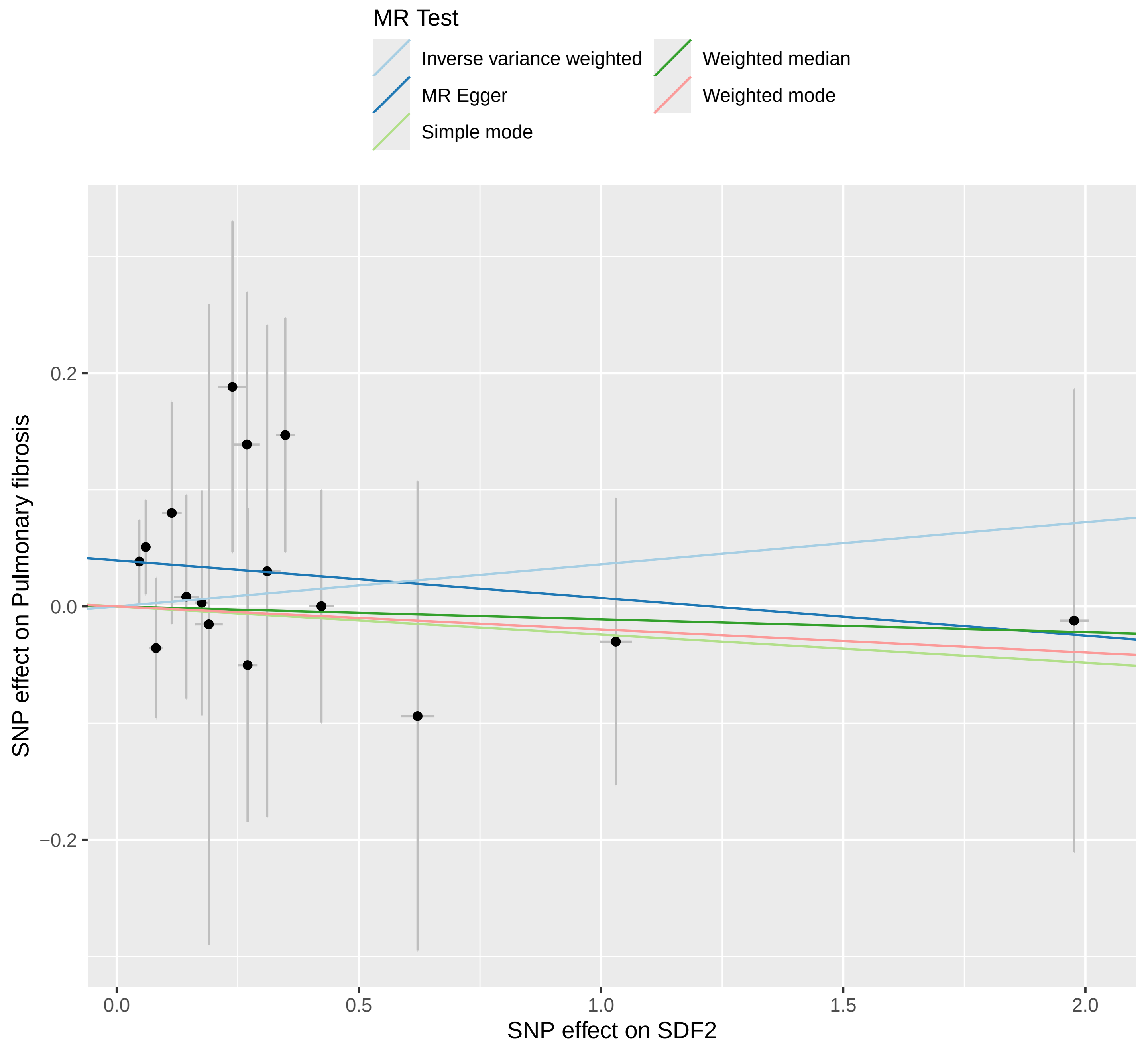

MR Test
Inverse variance weighted
MR Egger
Simple mode
Weighted median
Weighted mode
SNP effect on Pulmonary fibrosis
0.2
0.0
−0.2
0.0
0.5
1.0
1.5
2.0
SNP effect on SDF2

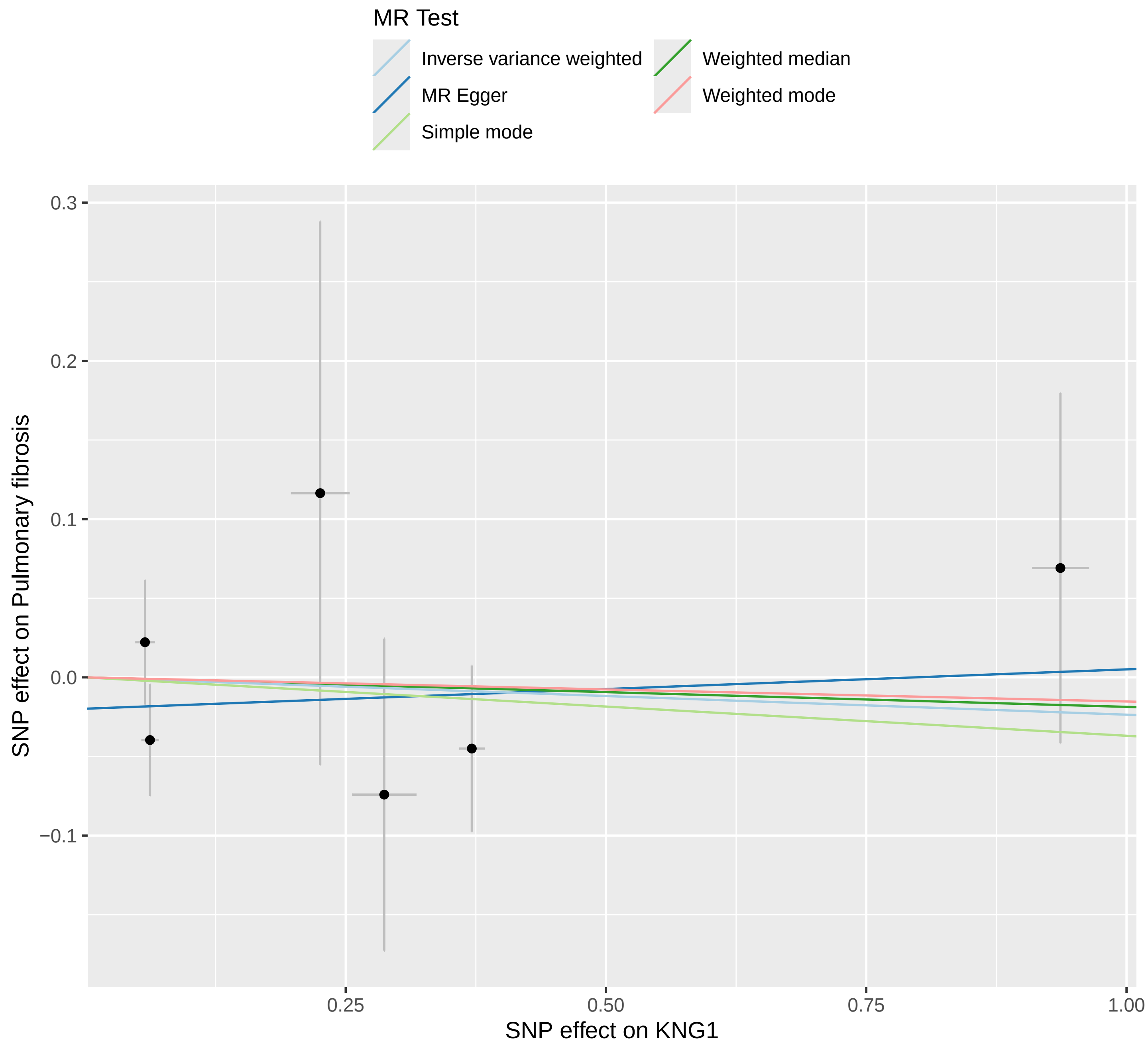

MR Test
Inverse variance weighted
MR Egger
Simple mode
Weighted median
Weighted mode
0.3
0.2
0.1
0.0
−0.1
0.25
0.50
0.75
1.00
SNP effect on Pulmonary fibrosis
SNP effect on KNG1

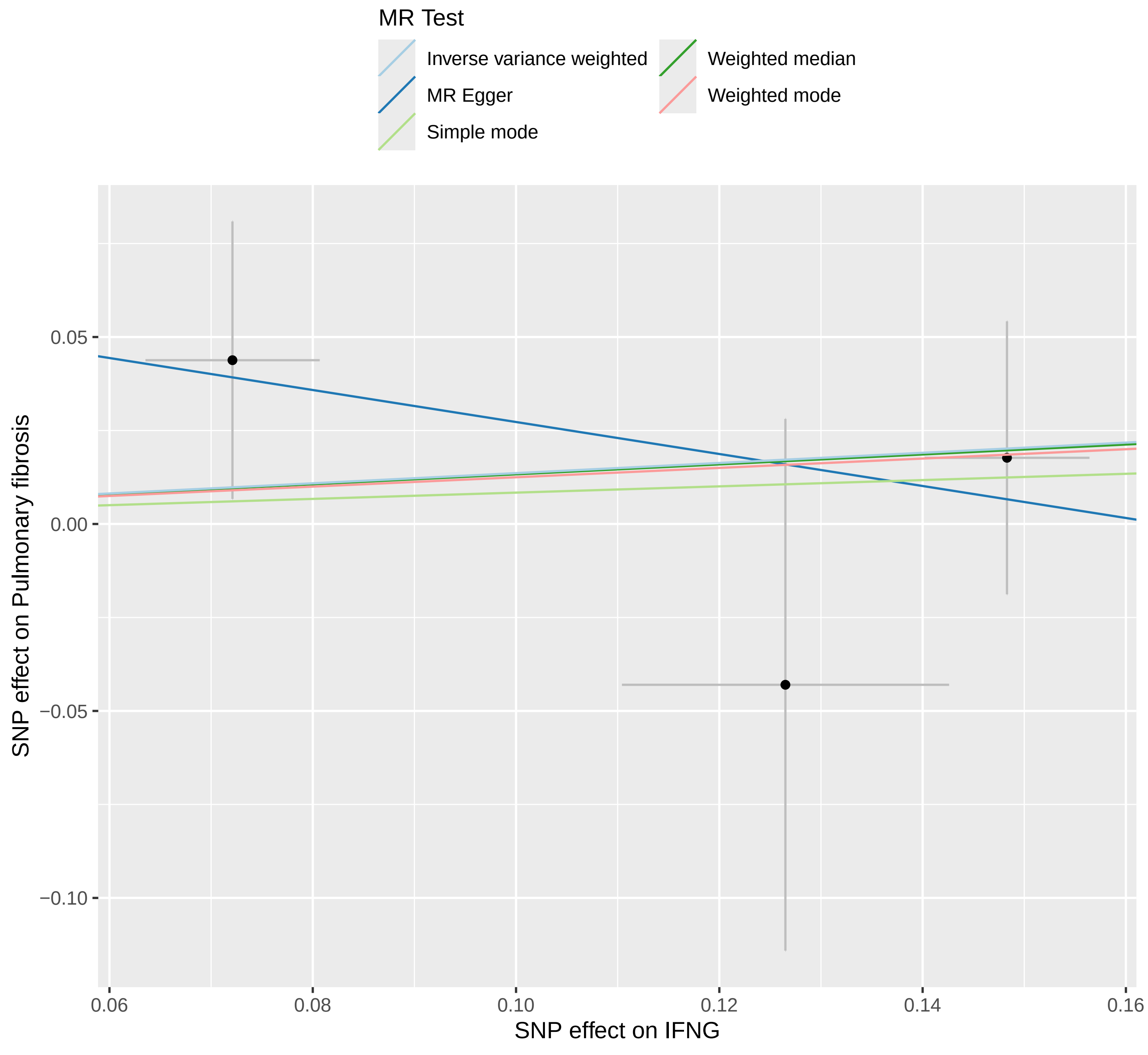

MR Test
Inverse variance weighted
MR Egger
Simple mode
Weighted median
Weighted mode
0.05
0.00
−0.05
−0.10
0.06
0.08
0.10
0.12
0.14
0.16
SNP effect on IFNG
SNP effect on Pulmonary fibrosis

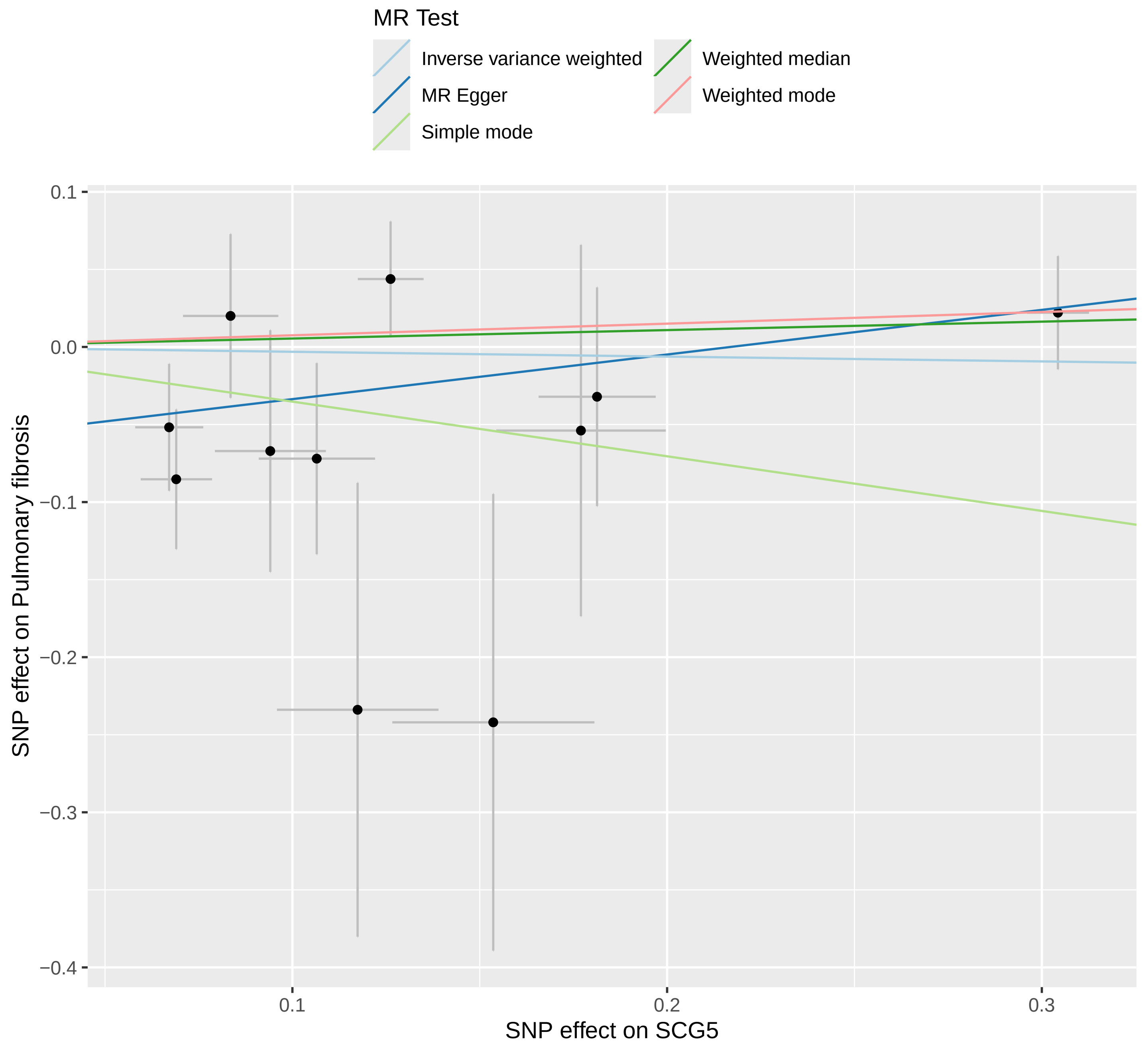

MR Test
Inverse variance weighted
MR Egger
Simple mode
Weighted median
Weighted mode
0.1
0.0
−0.1
−0.2
−0.3
−0.4
SNP effect on Pulmonary fibrosis
0.1
0.2
0.3
SNP effect on SCG5

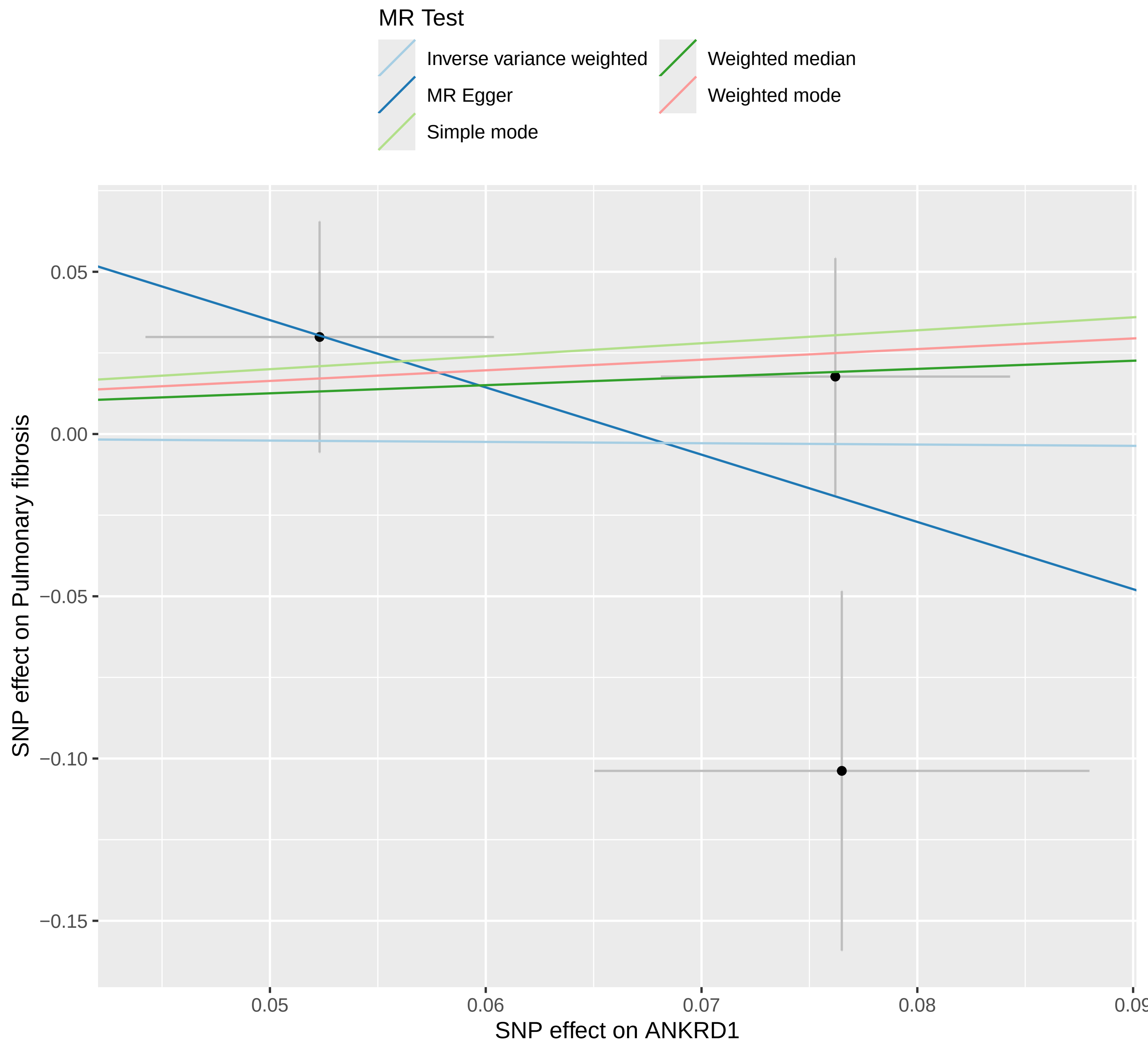

MR Test
Inverse variance weighted
MR Egger
Simple mode
Weighted median
Weighted mode
0.05
0.00
−0.05
−0.10
−0.15
0.05
0.06
0.07
0.08
SNP effect on Pulmonary fibrosis
SNP effect on ANKRD1

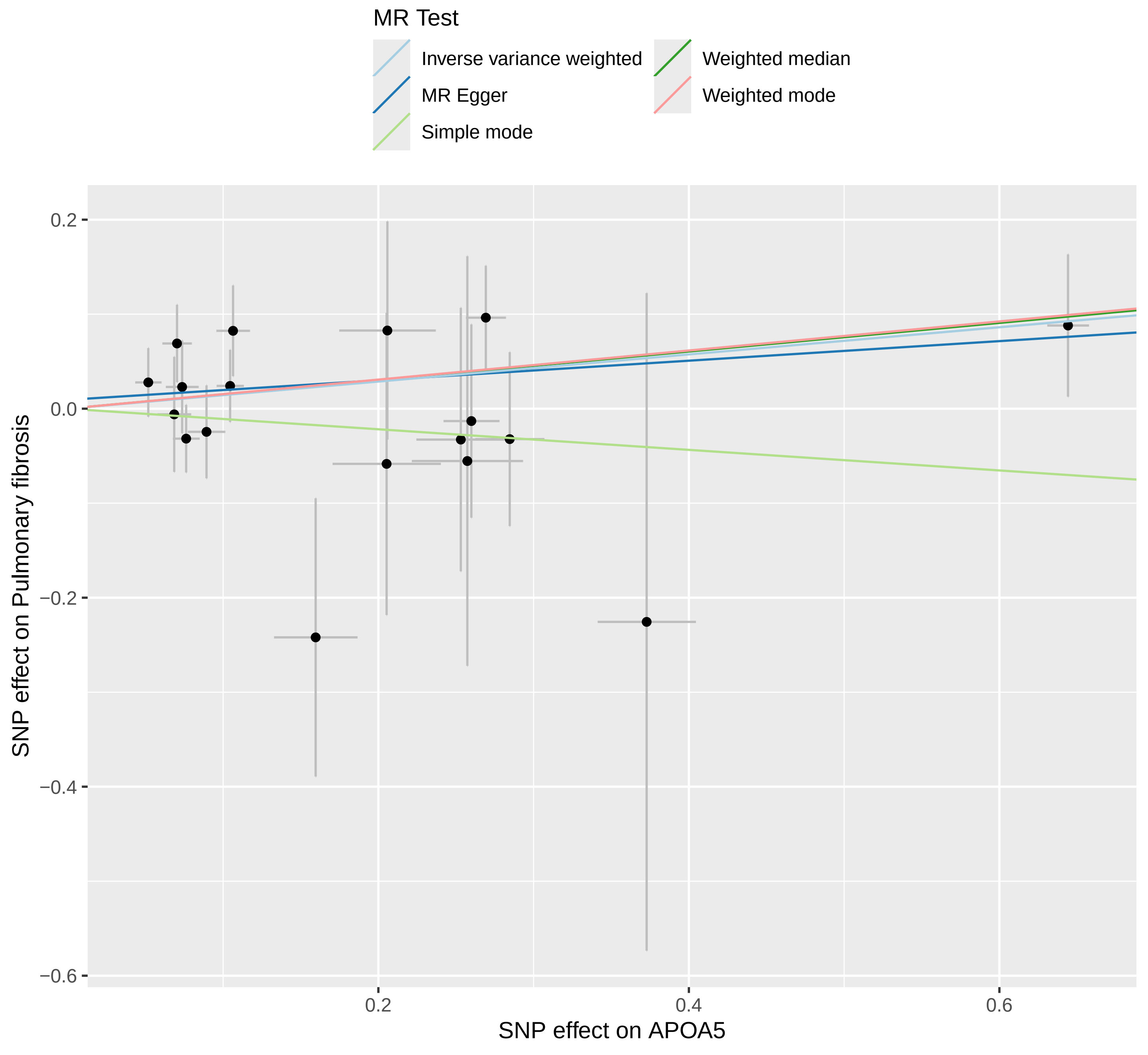
MR Test
Inverse variance weighted
MR Egger
Simple mode
Weighted median
Weighted mode
0.2
0.0
−0.2
−0.4
−0.6
SNP effect on Pulmonary fibrosis
0.2
0.4
0.6
SNP effect on APOA5

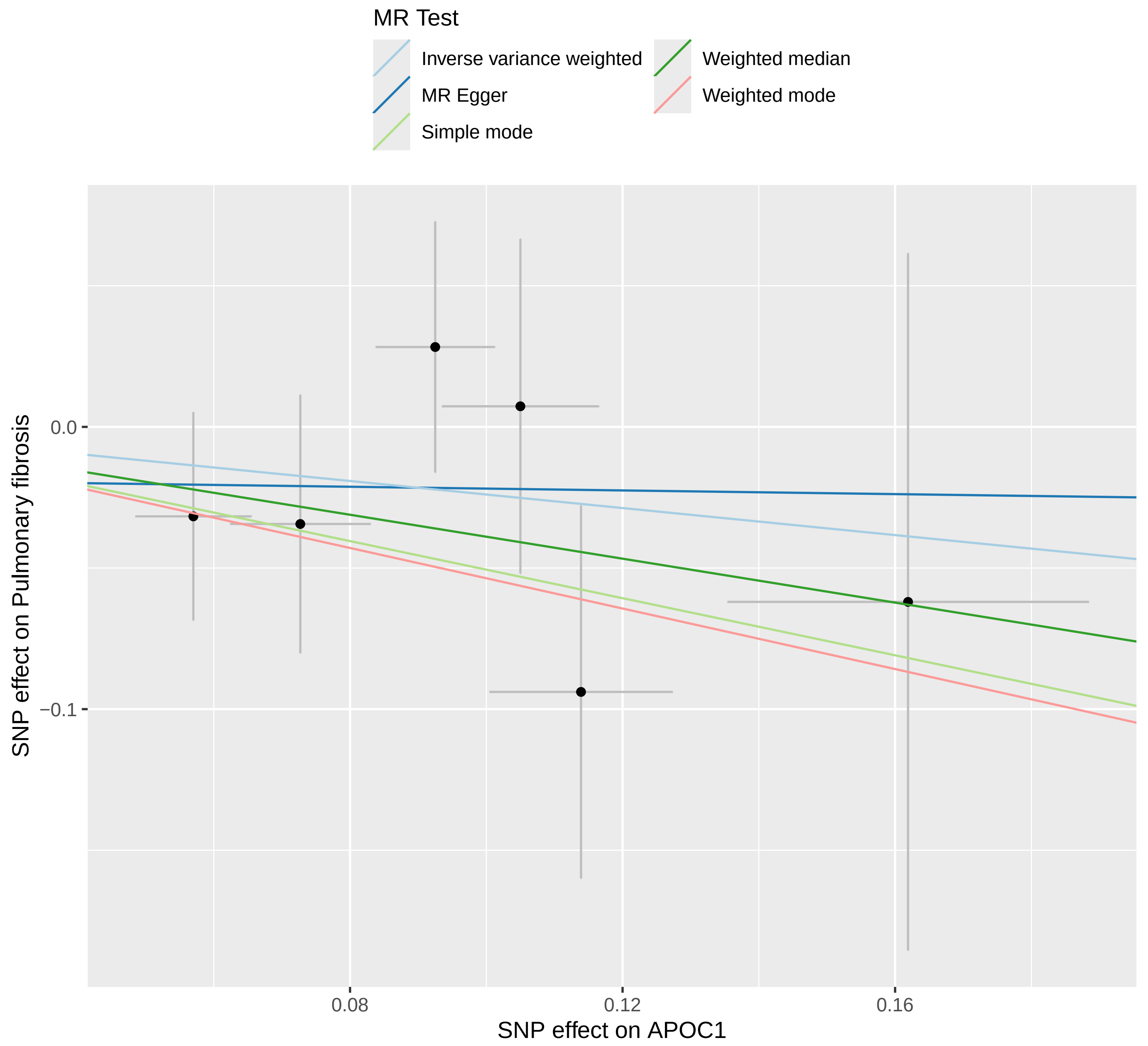
MR Test
Inverse variance weighted
MR Egger
Simple mode
Weighted median
Weighted mode
0.0
−0.1
SNP effect on Pulmonary fibrosis
0.08
0.12
0.16
SNP effect on APOC1

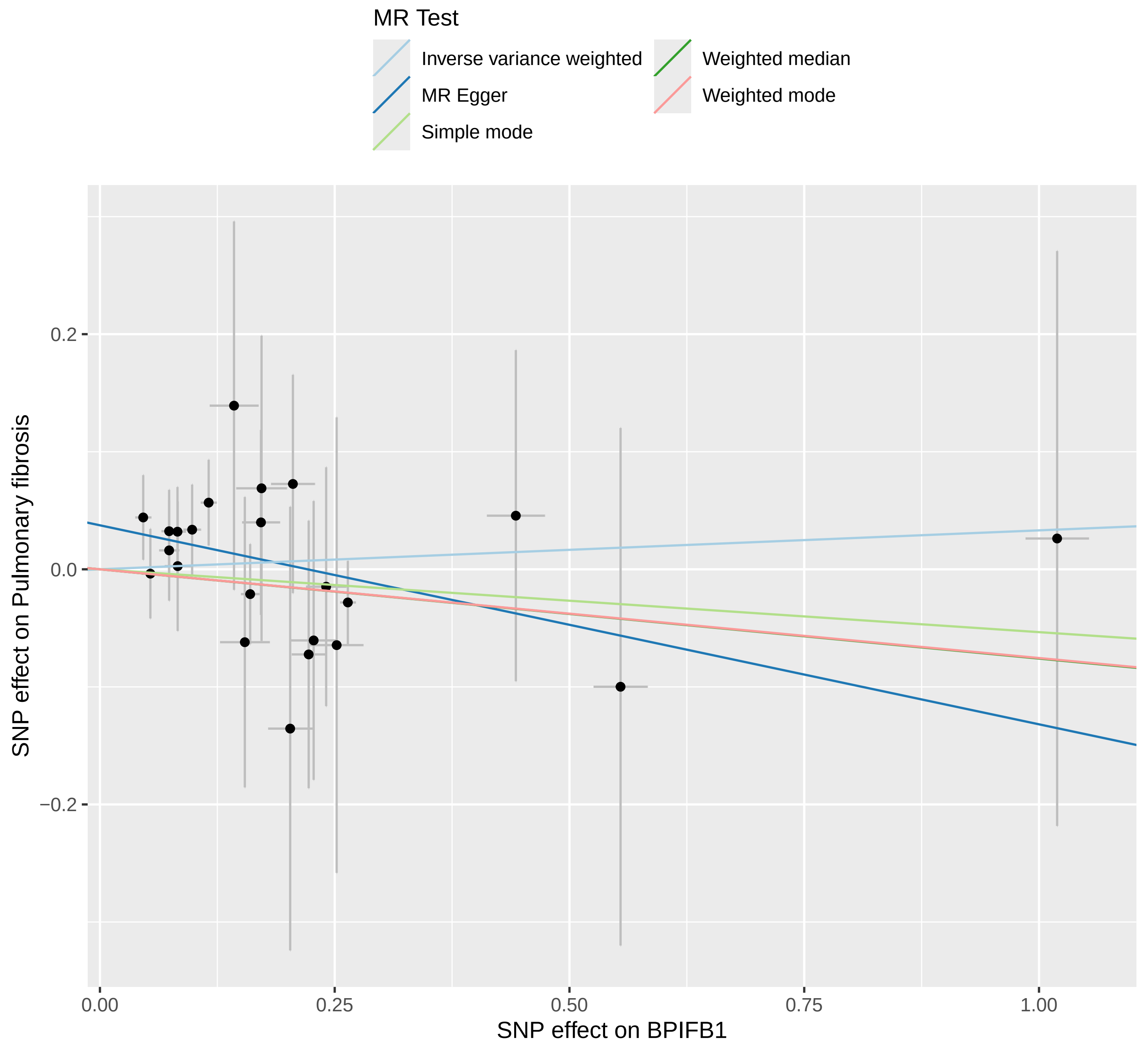
MR Test
Inverse variance weighted
MR Egger
Simple mode
Weighted median
Weighted mode
SNP effect on Pulmonary fibrosis
0.2
0.0
−0.2
0.00
0.25
0.50
0.75
1.00
SNP effect on BPIFB1

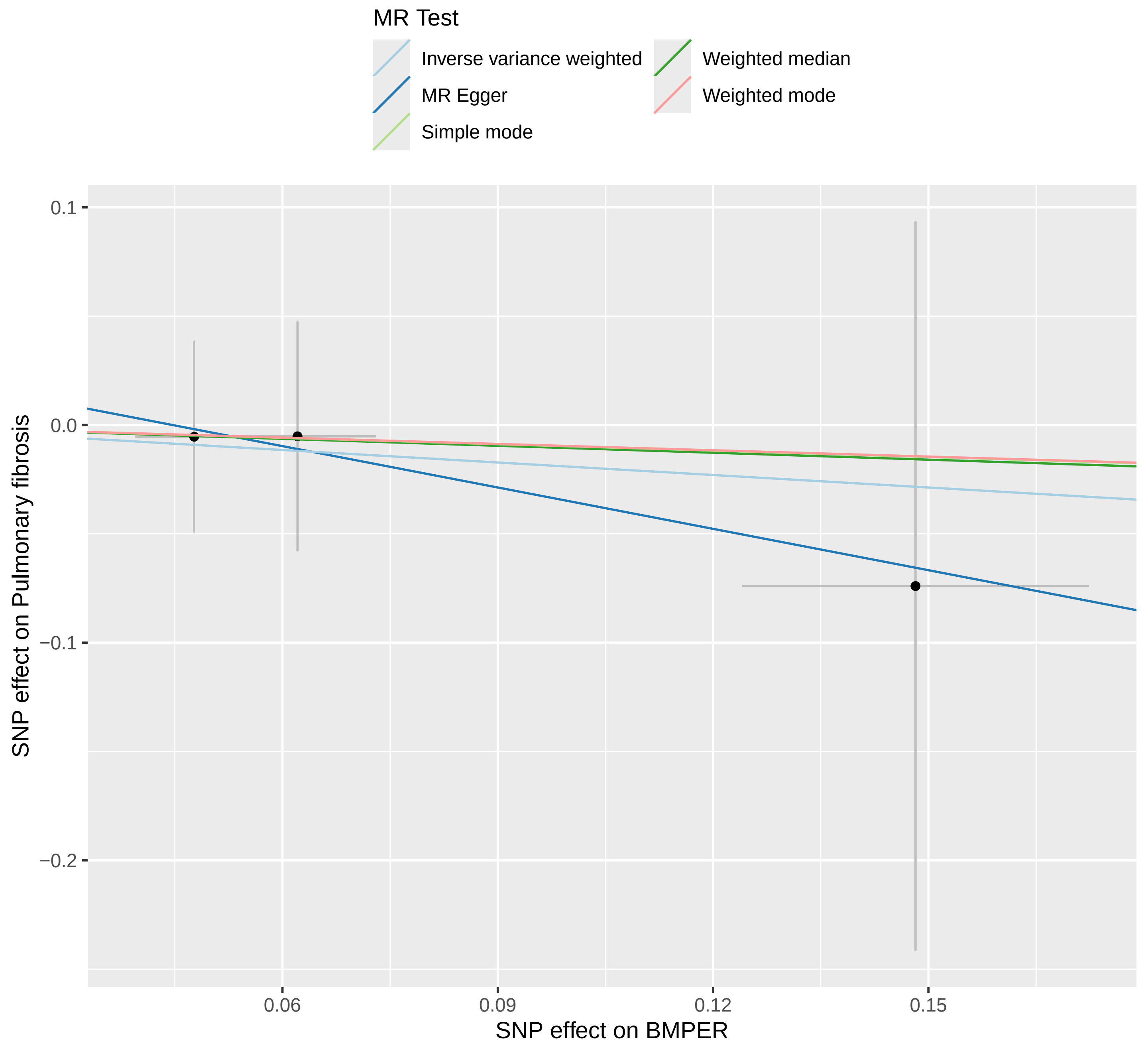
MR Test
Inverse variance weighted
MR Egger
Simple mode
Weighted median
Weighted mode
0.1
0.0
−0.1
−0.2
0.06
0.09
0.12
0.15
SNP effect on BMPER
SNP effect on Pulmonary fibrosis

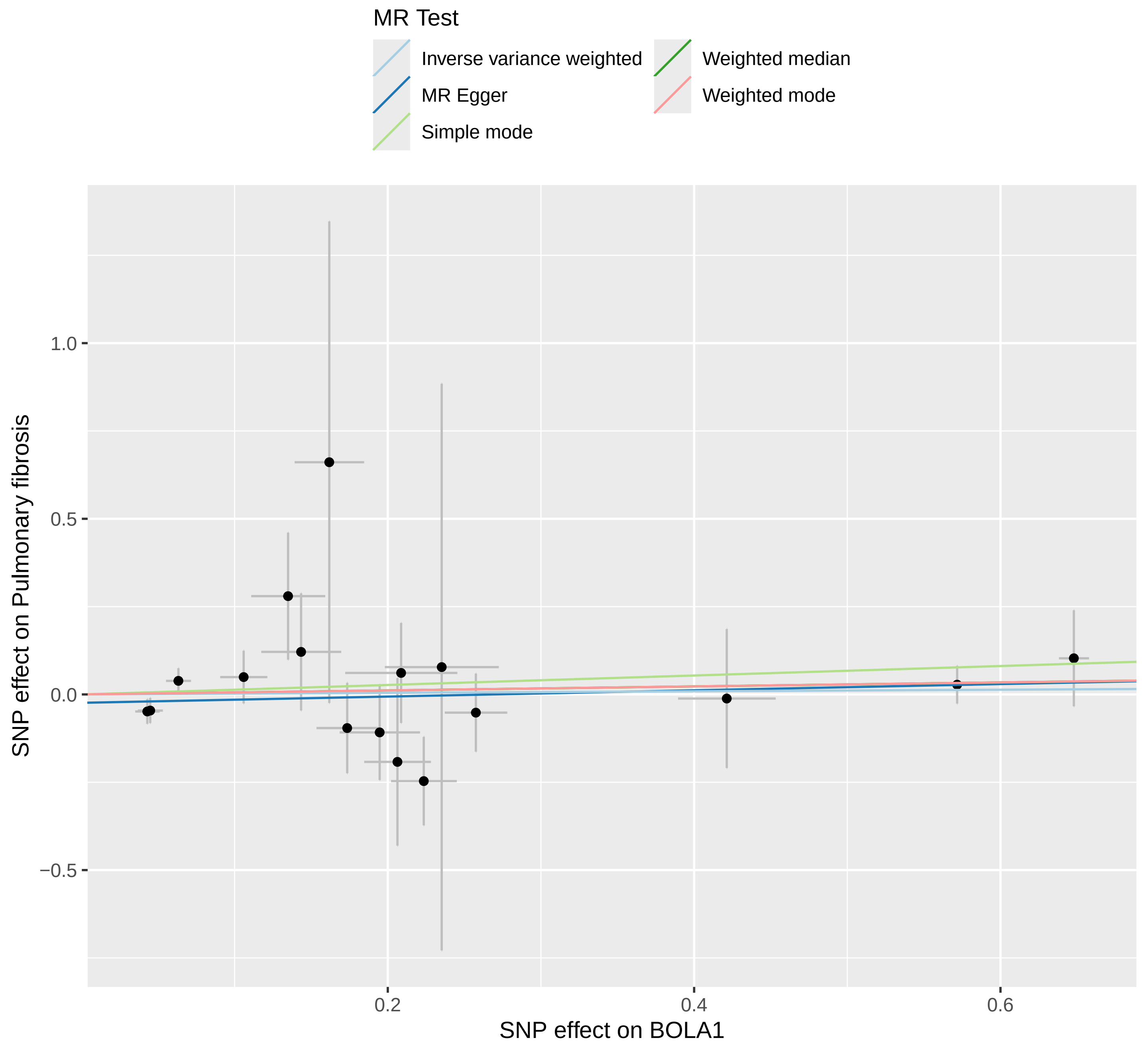
MR Test
Inverse variance weighted
MR Egger
Simple mode
Weighted median
Weighted mode
1.0
0.5
0.0
−0.5
0.2
0.4
0.6
SNP effect on Pulmonary fibrosis
SNP effect on BOLA1

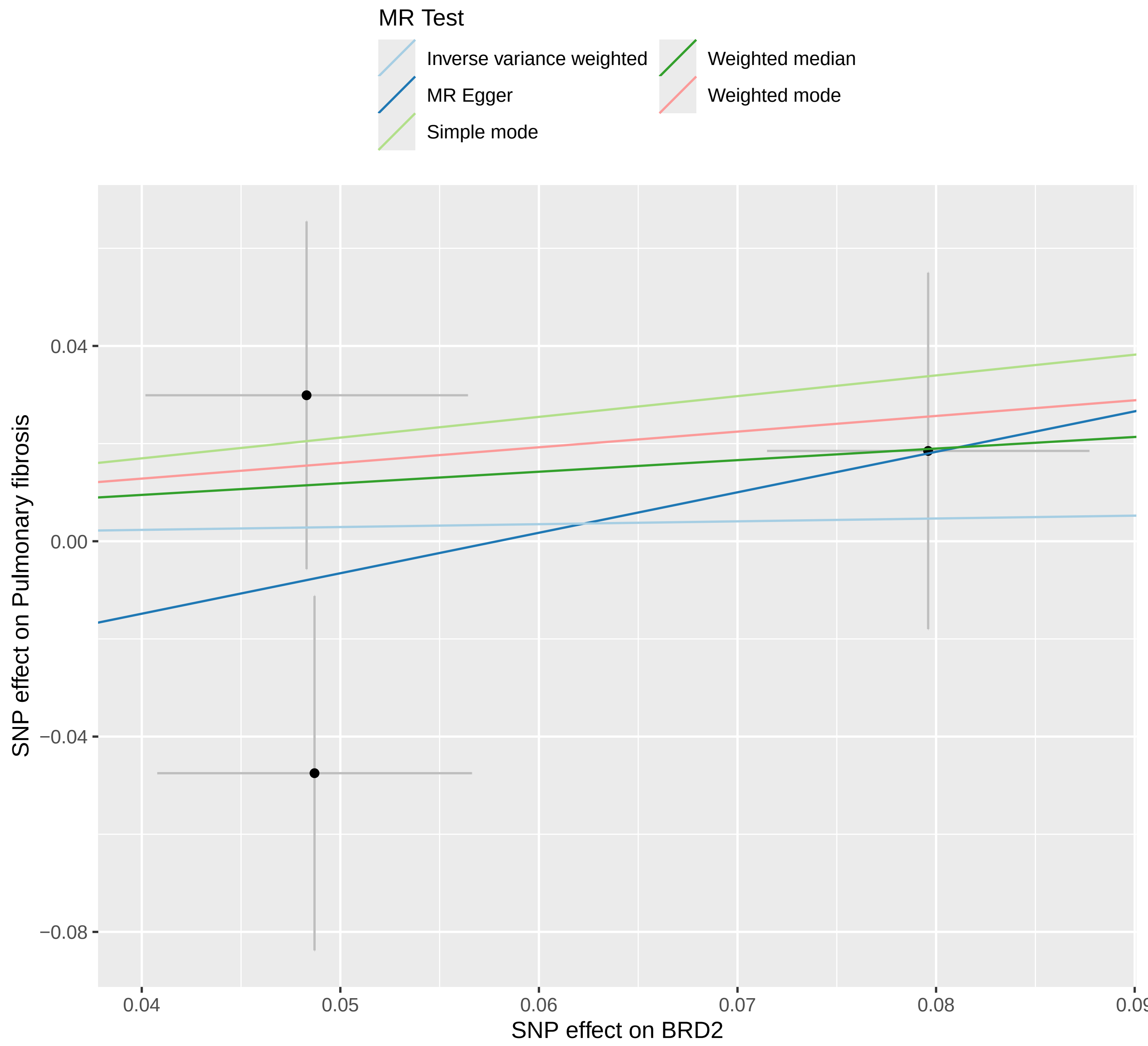
MR Test
Inverse variance weighted
MR Egger
Simple mode
Weighted median
Weighted mode
0.04
0.00
−0.04
−0.08
SNP effect on Pulmonary fibrosis
0.04
0.05
0.06
0.07
0.08
SNP effect on BRD2

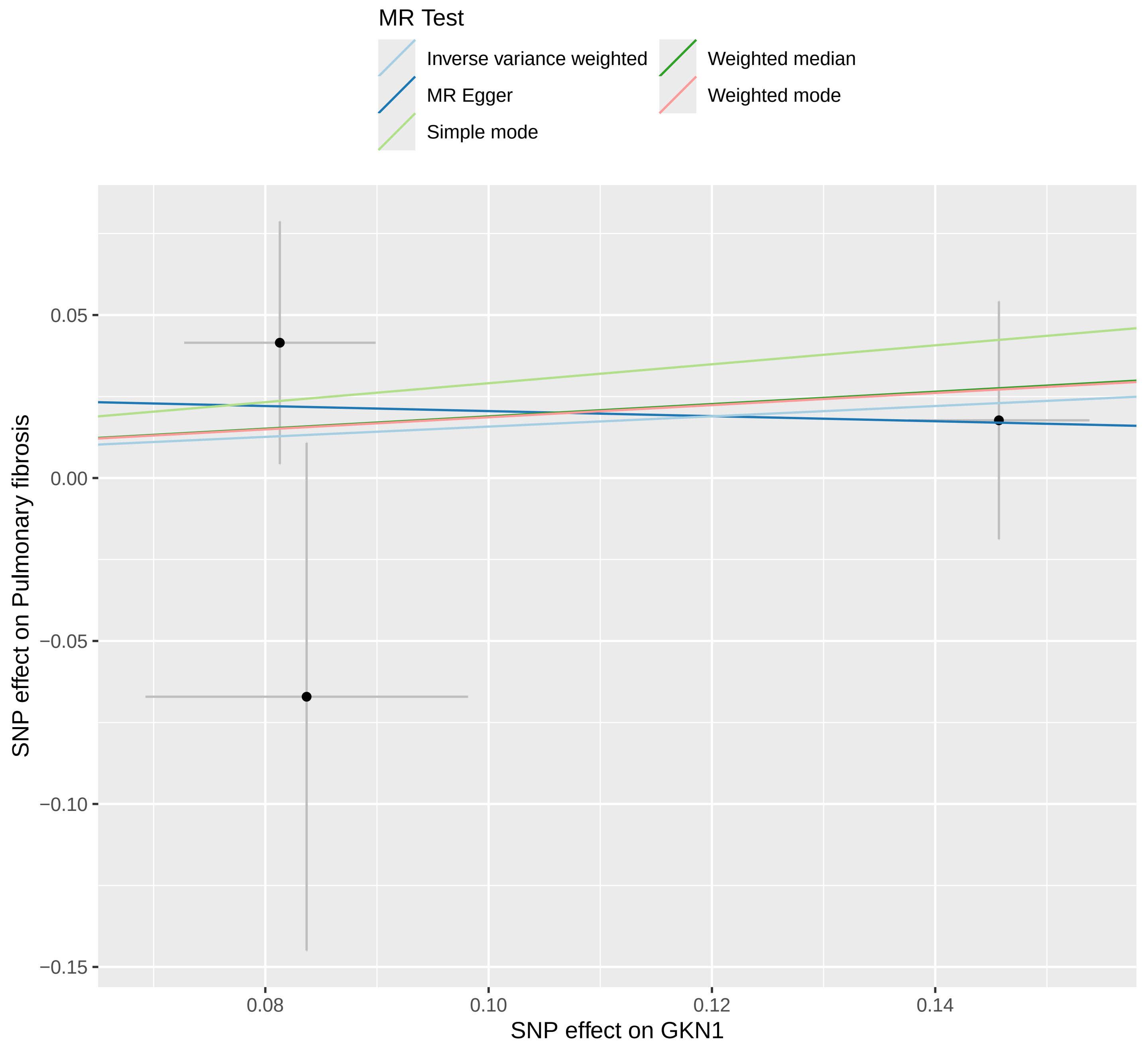

MR Test
Inverse variance weighted
MR Egger
Simple mode
Weighted median
Weighted mode
0.05
0.00
−0.05
−0.10
−0.15
SNP effect on Pulmonary fibrosis
0.08
0.10
0.12
0.14
SNP effect on GKN1

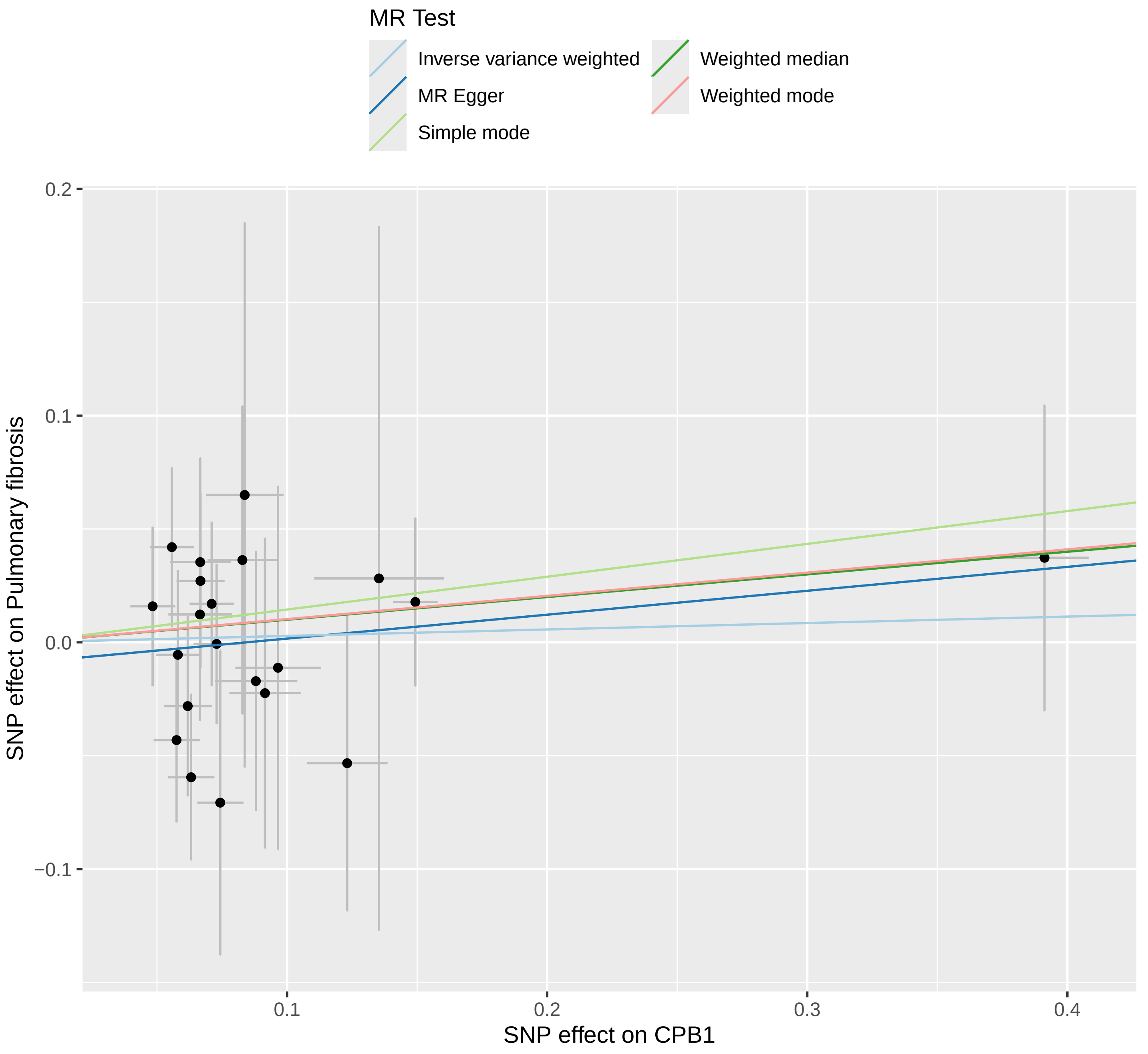

MR Test
Inverse variance weighted
MR Egger
Simple mode
Weighted median
Weighted mode
0.2
0.1
0.0
−0.1
0.1
0.2
0.3
0.4
SNP effect on Pulmonary fibrosis
SNP effect on CPB1

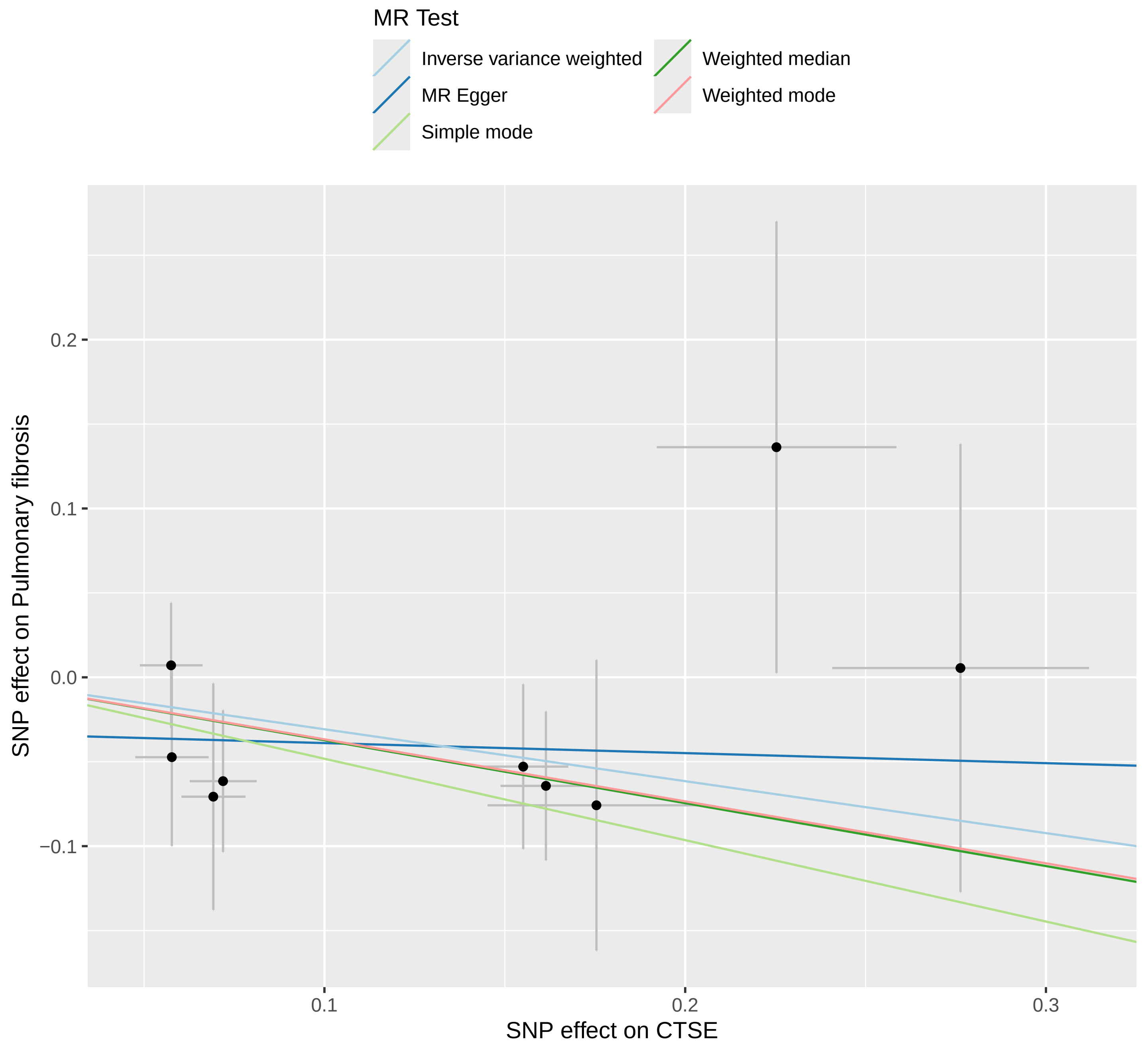
MR Test
Inverse variance weighted
MR Egger
Simple mode
Weighted median
Weighted mode
SNP effect on Pulmonary fibrosis
0.2
0.1
0.0
−0.1
0.1
0.2
0.3
SNP effect on CTSE

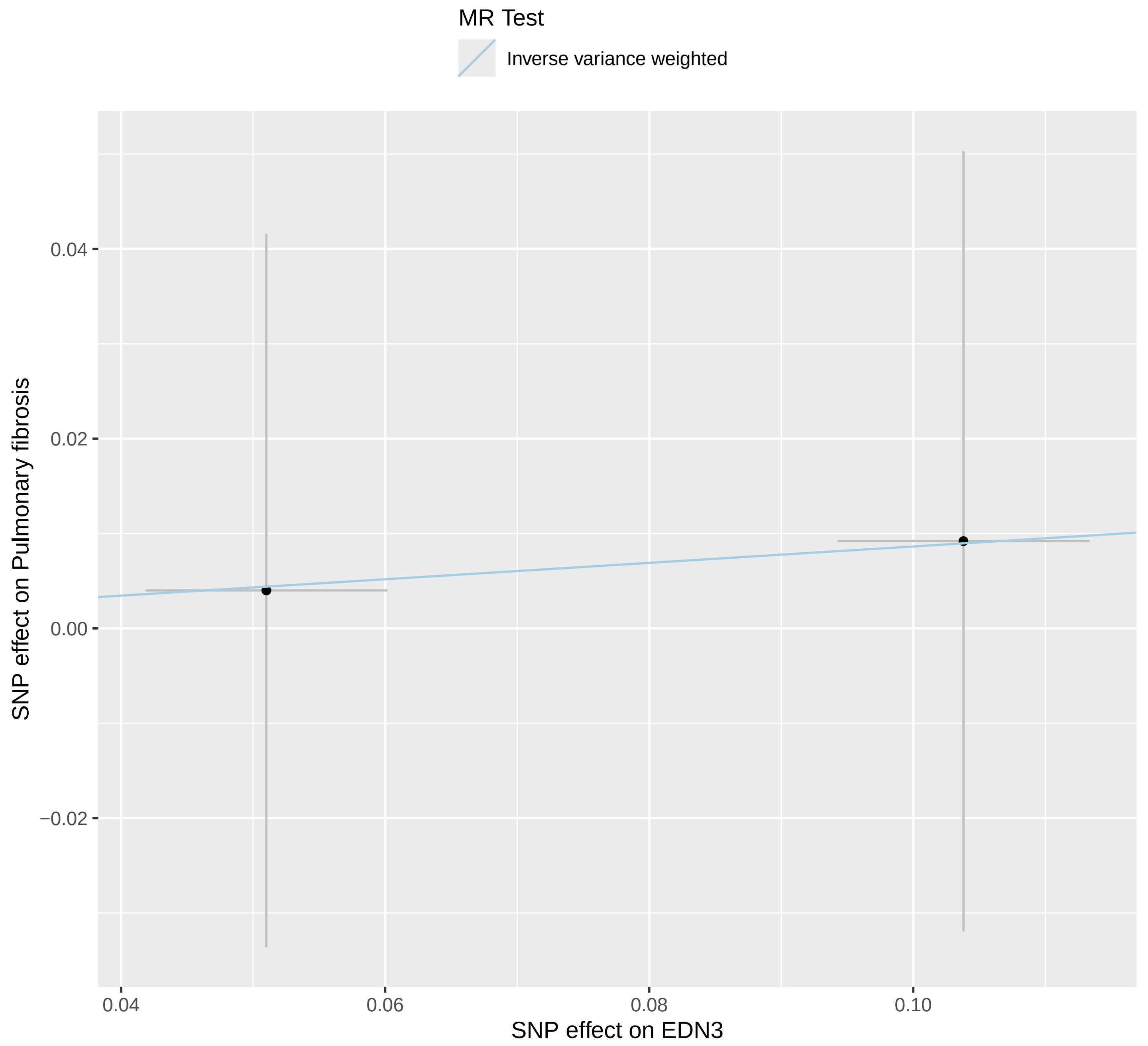
MR Test
Inverse variance weighted
SNP effect on Pulmonary fibrosis
0.04
0.02
0.00
−0.02
0.04
0.06
0.08
0.10
SNP effect on EDN3

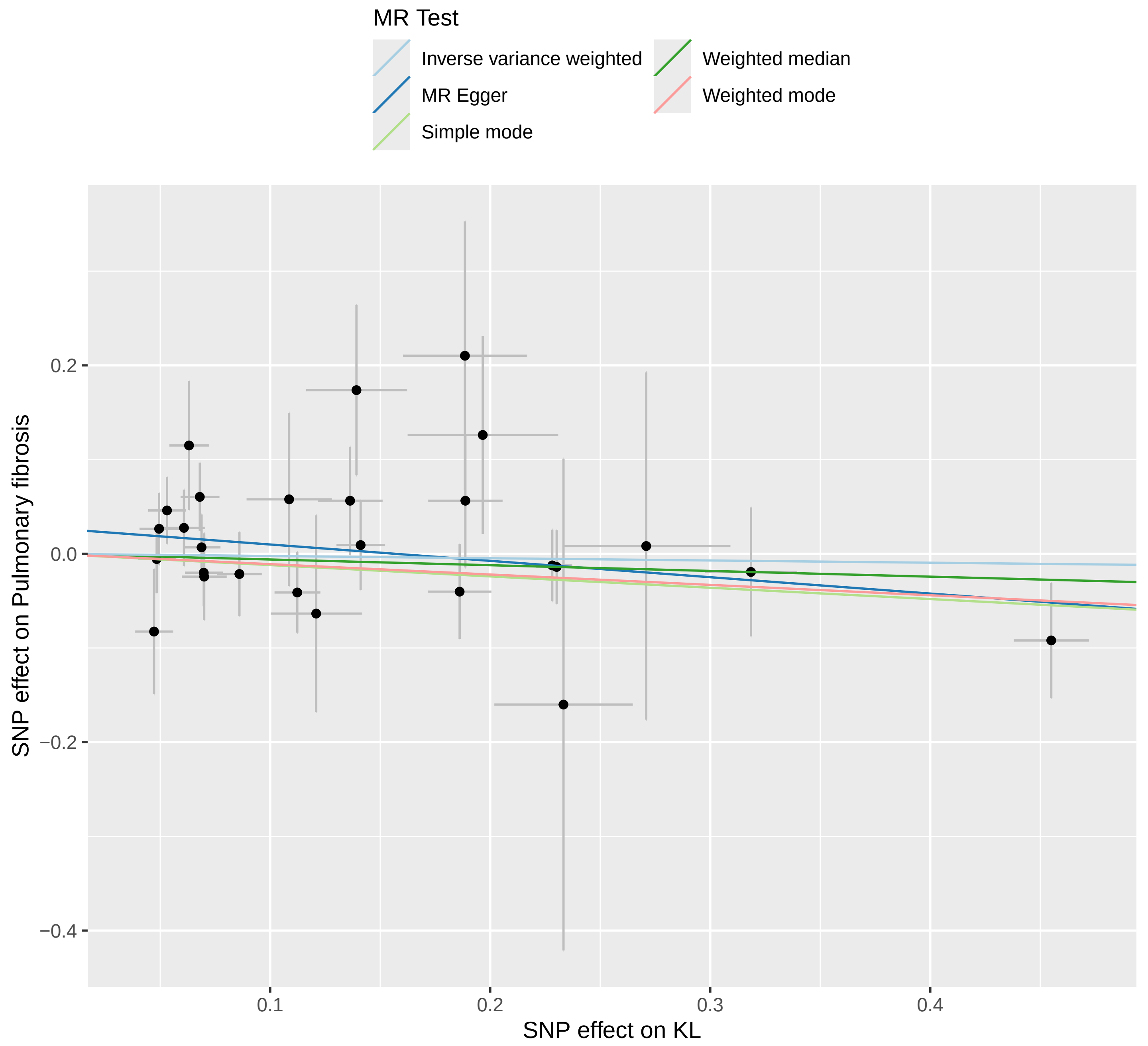

MR Test
Inverse variance weighted
MR Egger
Simple mode
Weighted median
Weighted mode
0.2
0.0
−0.2
−0.4
0.1
0.2
0.3
0.4
SNP effect on Pulmonary fibrosis
SNP effect on KL

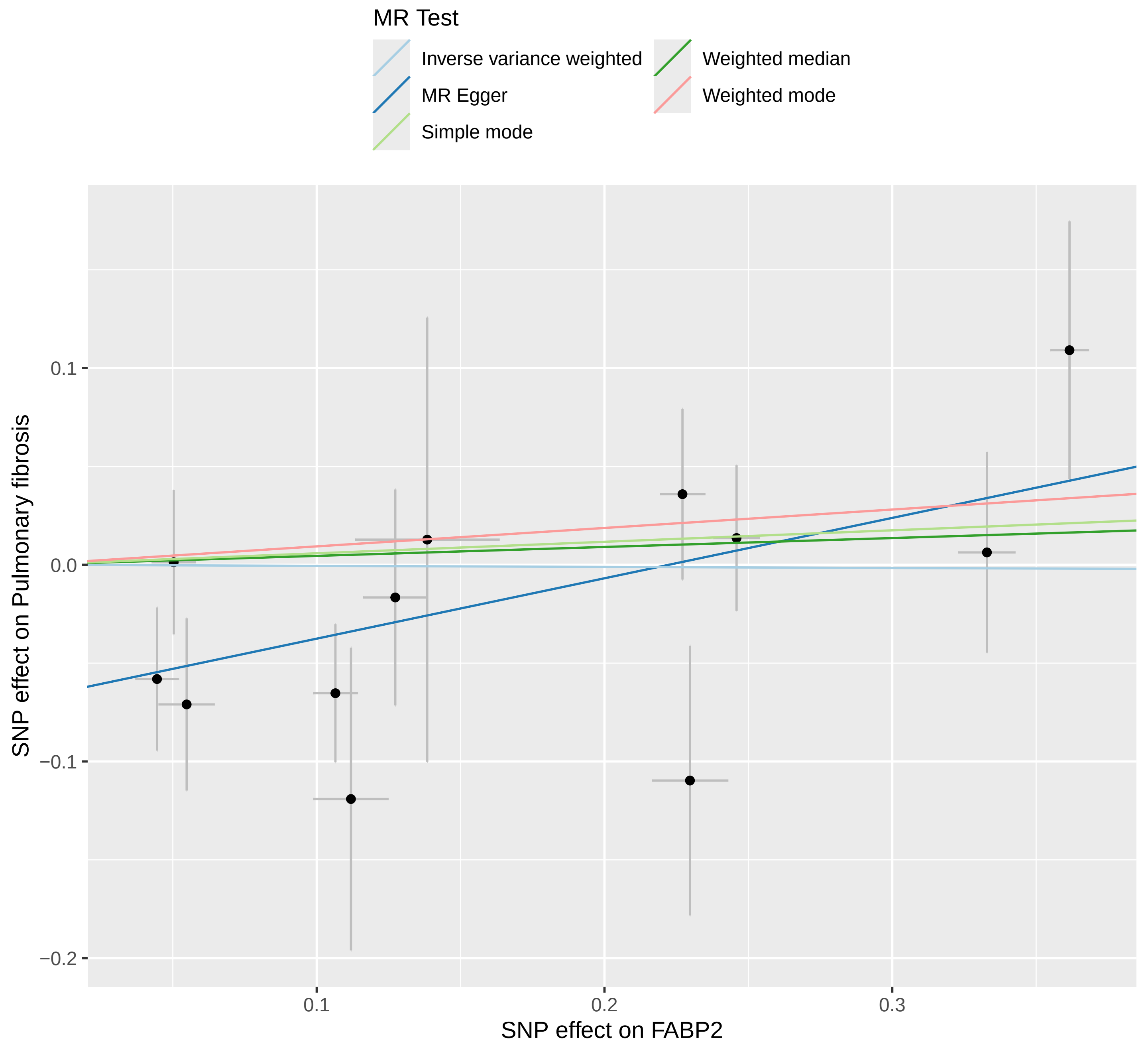
MR Test
Inverse variance weighted
MR Egger
Simple mode
Weighted median
Weighted mode
SNP effect on Pulmonary fibrosis
0.1
0.0
−0.1
−0.2
0.1
0.2
0.3
SNP effect on FABP2

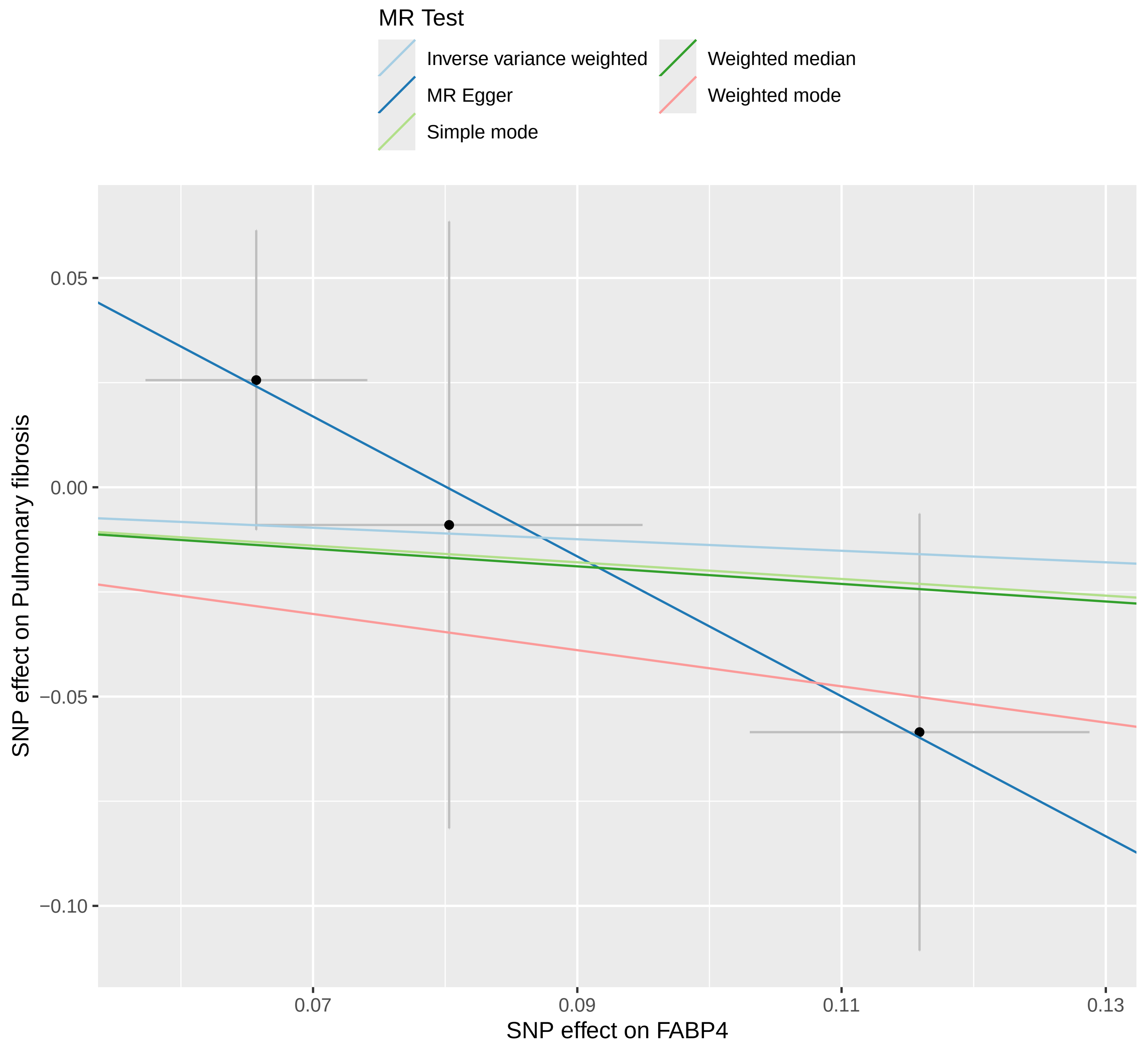
MR Test
Inverse variance weighted
MR Egger
Simple mode
Weighted median
Weighted mode
0.05
0.00
−0.05
−0.10
0.07
0.09
0.11
0.13
SNP effect on FABP4
SNP effect on Pulmonary fibrosis

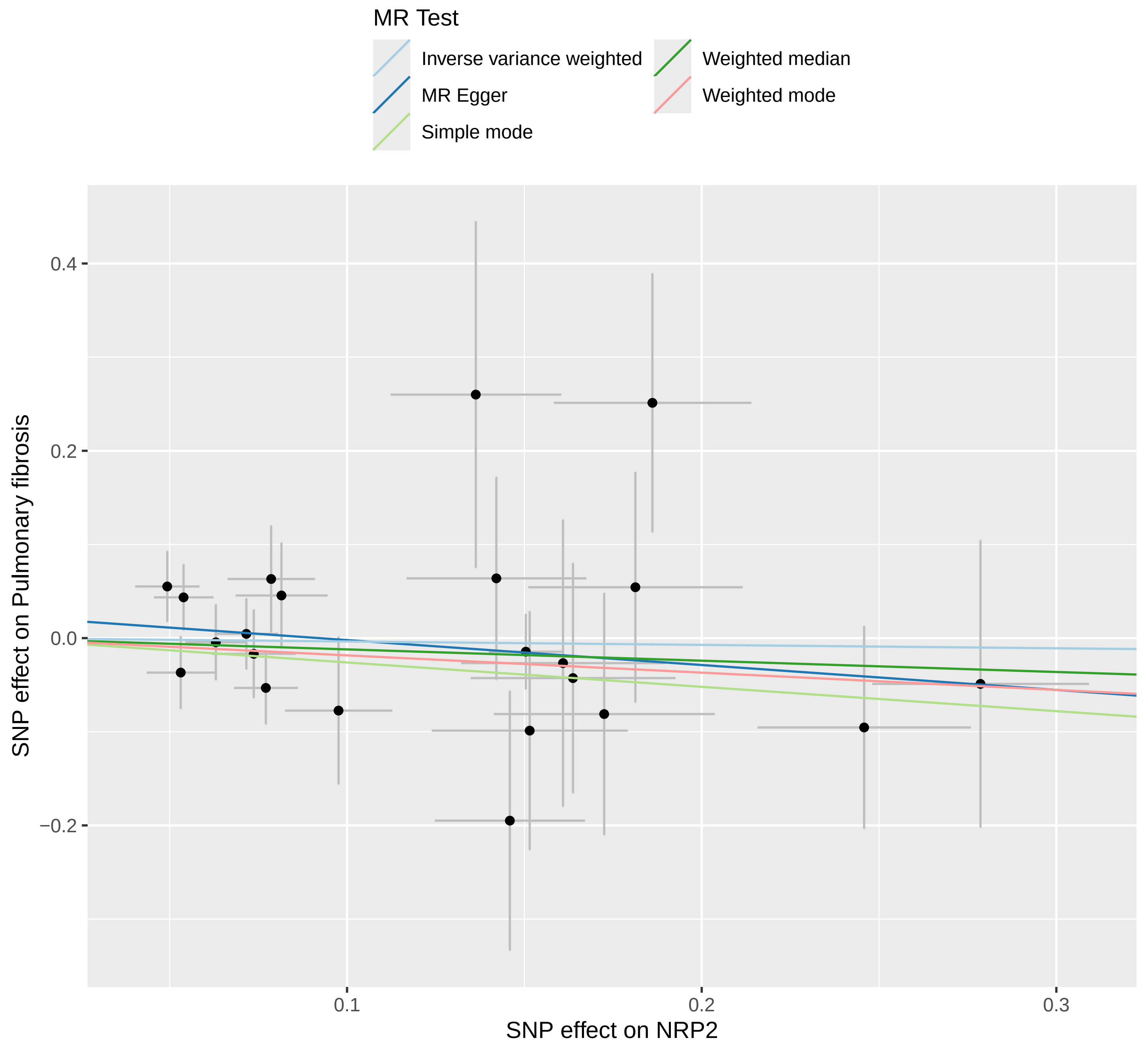

MR Test
Inverse variance weighted
MR Egger
Simple mode
Weighted median
Weighted mode
0.4
0.2
0.0
−0.2
0.1
0.2
0.3
SNP effect on Pulmonary fibrosis
SNP effect on NRP2

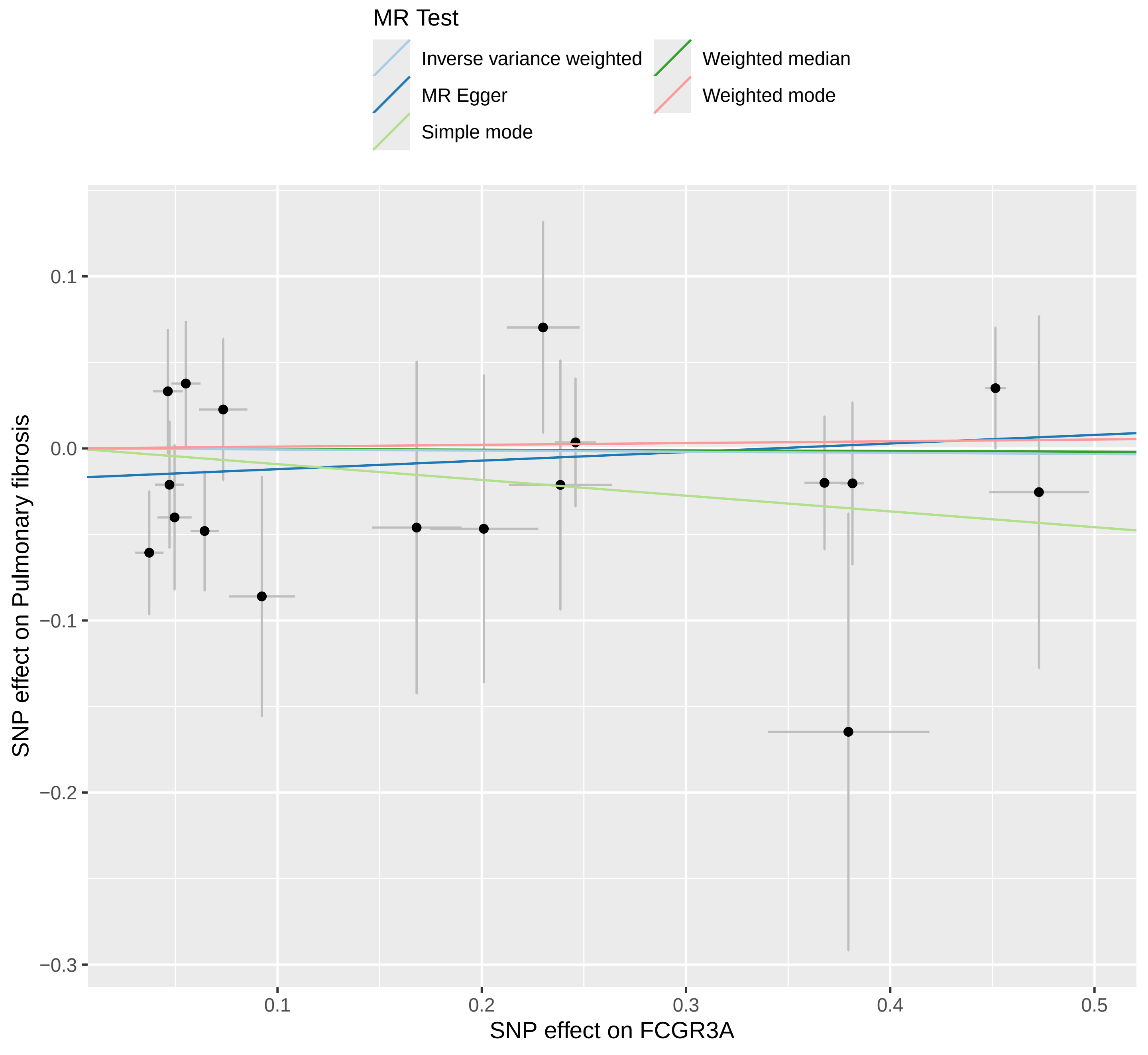
MR Test
Inverse variance weighted
MR Egger
Simple mode
Weighted median
Weighted mode
0.1
0.0
−0.1
−0.2
−0.3
SNP effect on Pulmonary fibrosis
0.1
0.2
0.3
0.4
0.5
SNP effect on FCGR3A

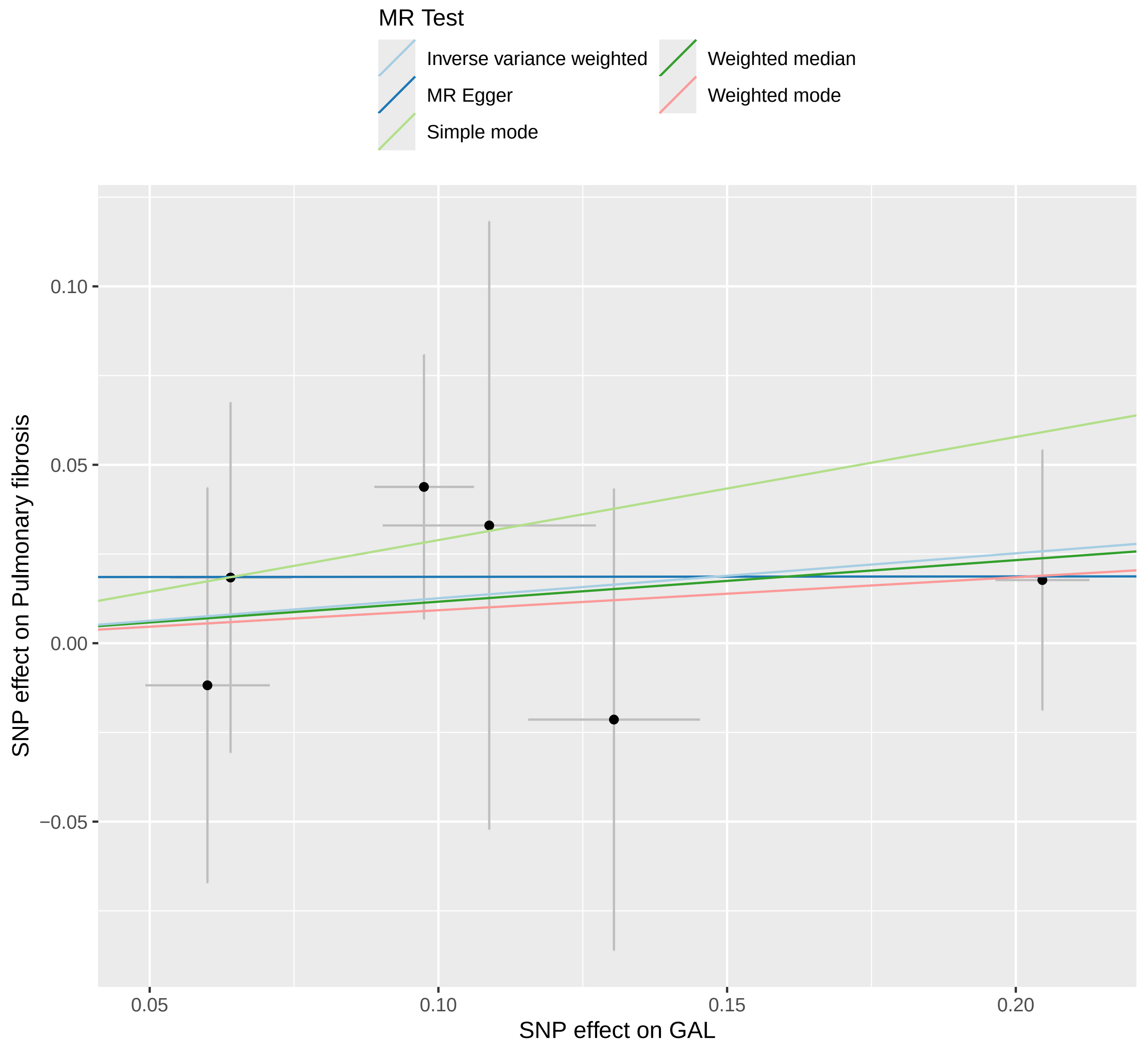

MR Test
Inverse variance weighted
MR Egger
Simple mode
Weighted median
Weighted mode
SNP effect on Pulmonary fibrosis
0.10
0.05
0.00
−0.05
SNP effect on GAL
0.05
0.10
0.15
0.20

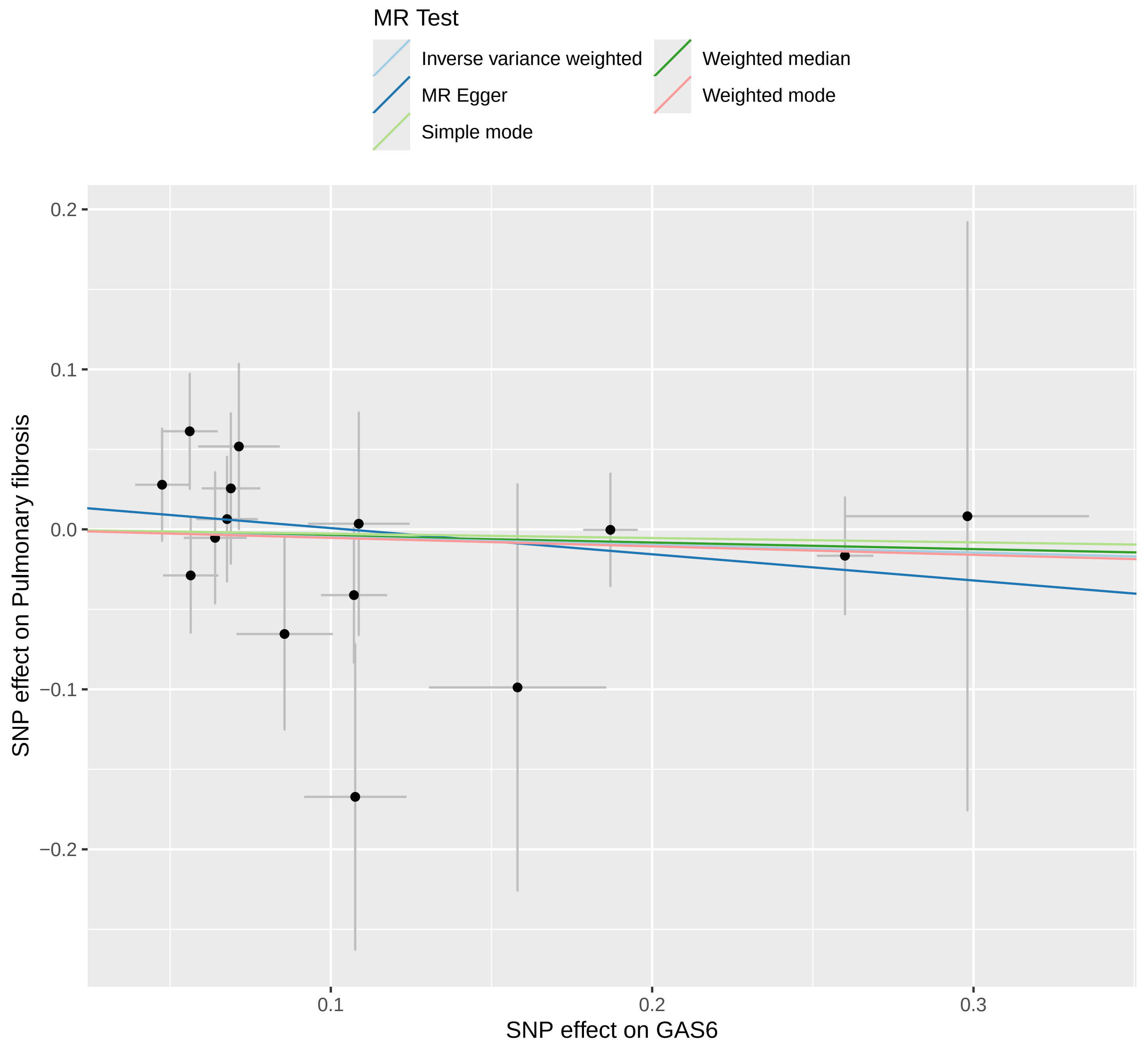

MR Test
Inverse variance weighted
MR Egger
Simple mode
Weighted median
Weighted mode
0.2
0.1
0.0
−0.1
−0.2
SNP effect on Pulmonary fibrosis
0.1
0.2
0.3
SNP effect on GAS6

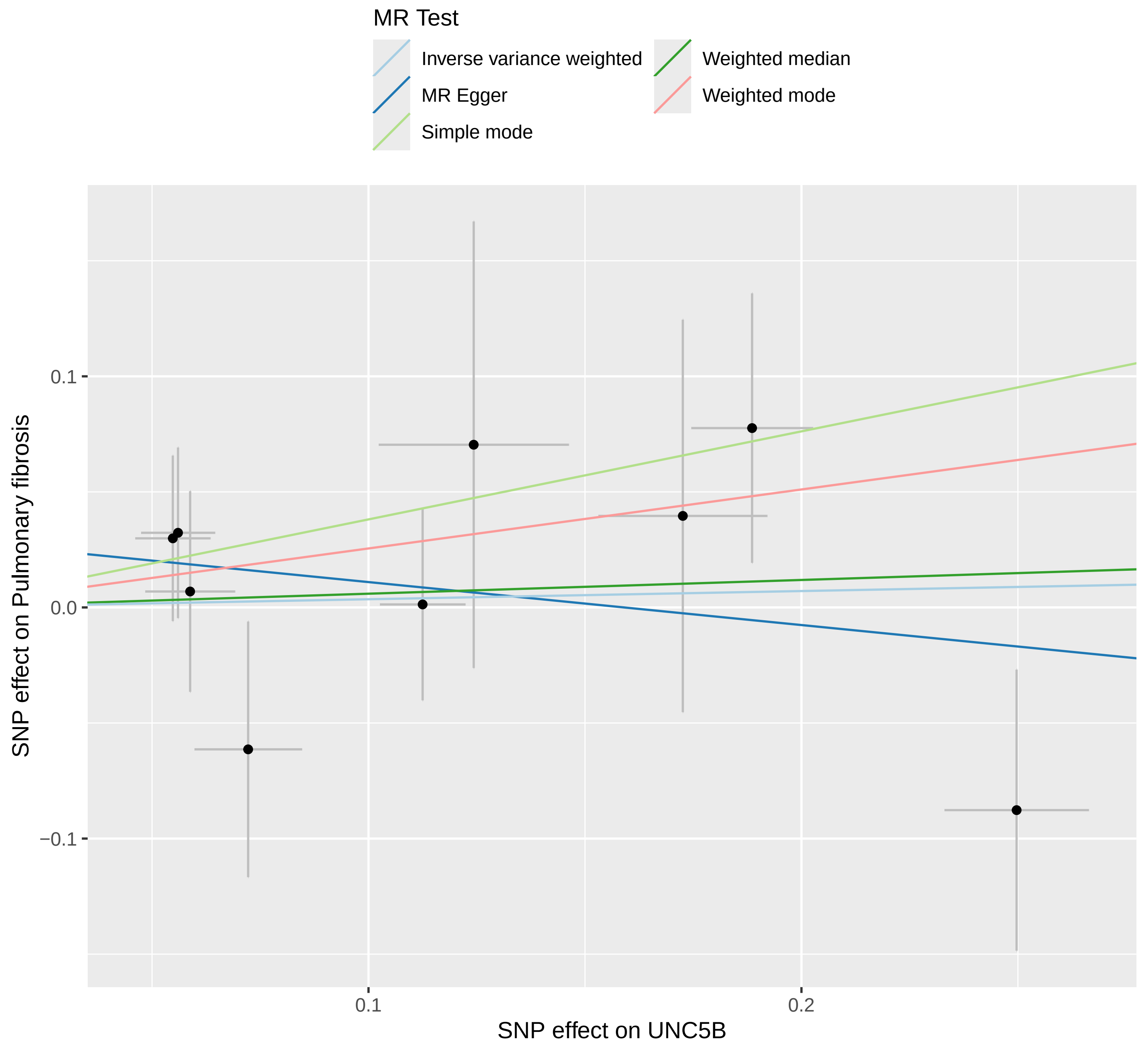

MR Test
Inverse variance weighted
MR Egger
Simple mode
Weighted median
Weighted mode
0.1
0.0
−0.1
SNP effect on Pulmonary fibrosis
0.1
0.2
SNP effect on UNC5B

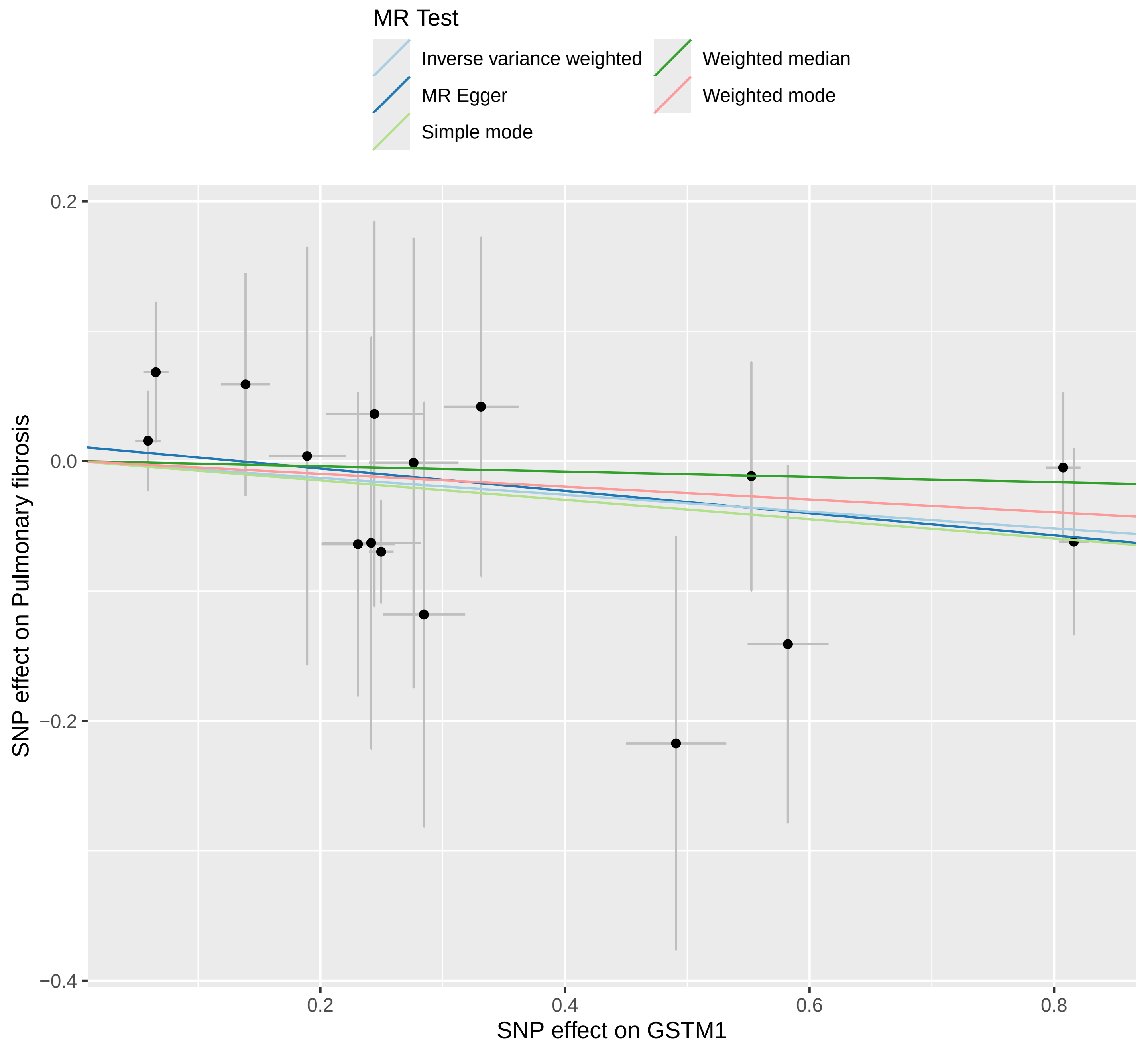
MR Test
Inverse variance weighted
MR Egger
Simple mode
Weighted median
Weighted mode
0.2
0.0
−0.2
−0.4
0.2
0.4
0.6
0.8
SNP effect on Pulmonary fibrosis
SNP effect on GSTM1

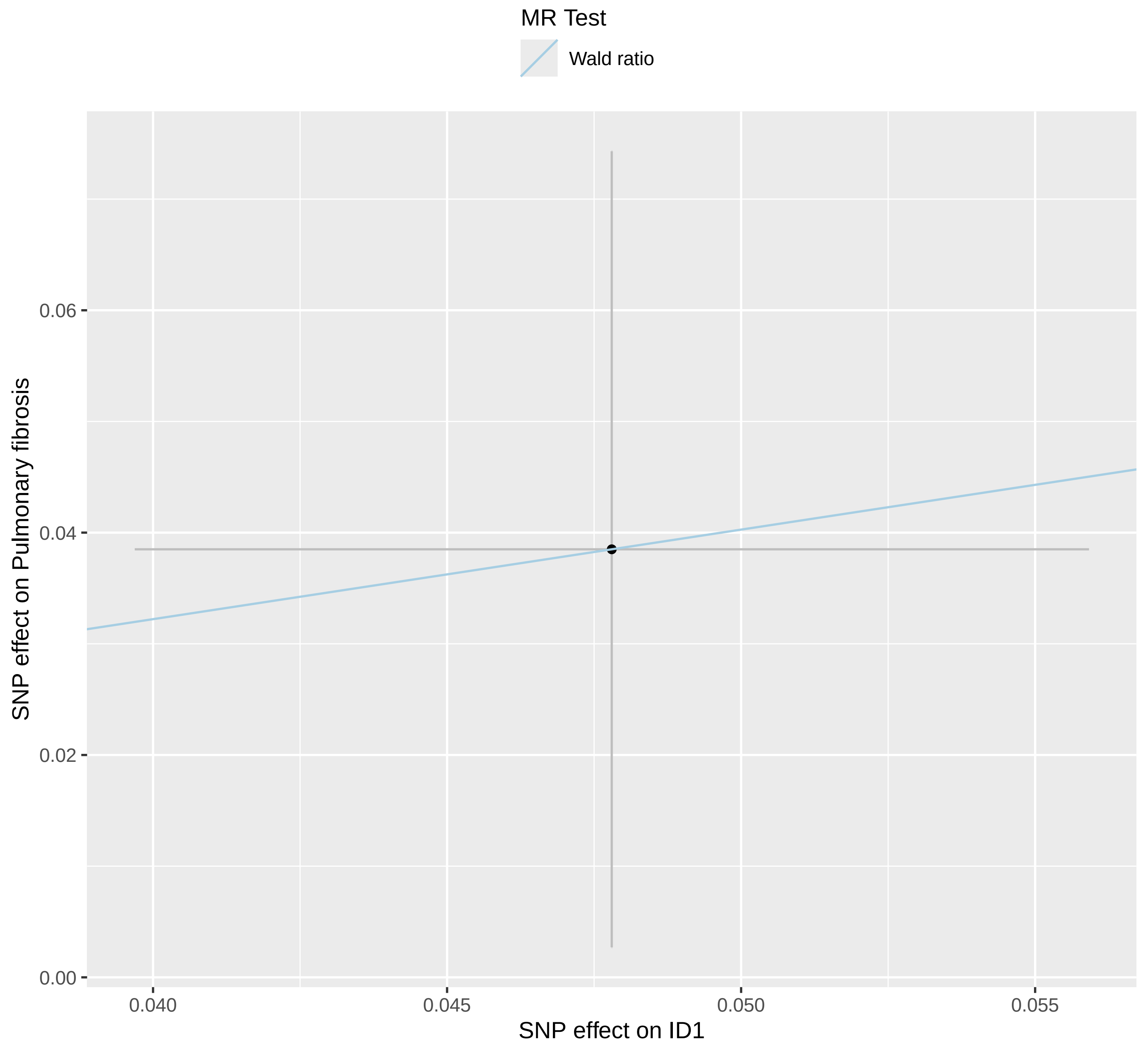

MR Test
Wald ratio
SNP effect on Pulmonary fibrosis
0.06
0.04
0.02
0.00
0.040
0.045
0.050
0.055
SNP effect on ID1

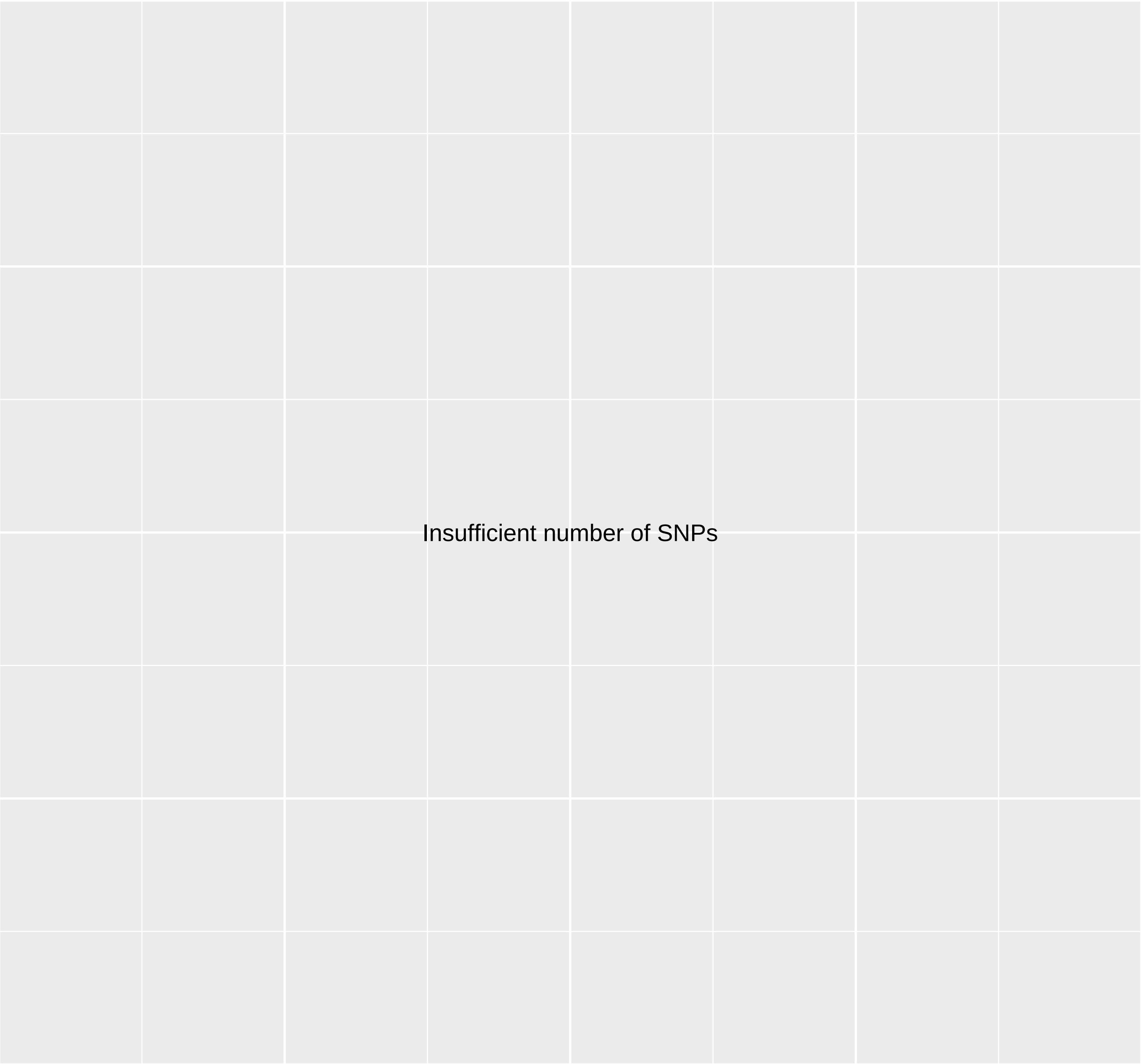
Insufficient number of SNPs

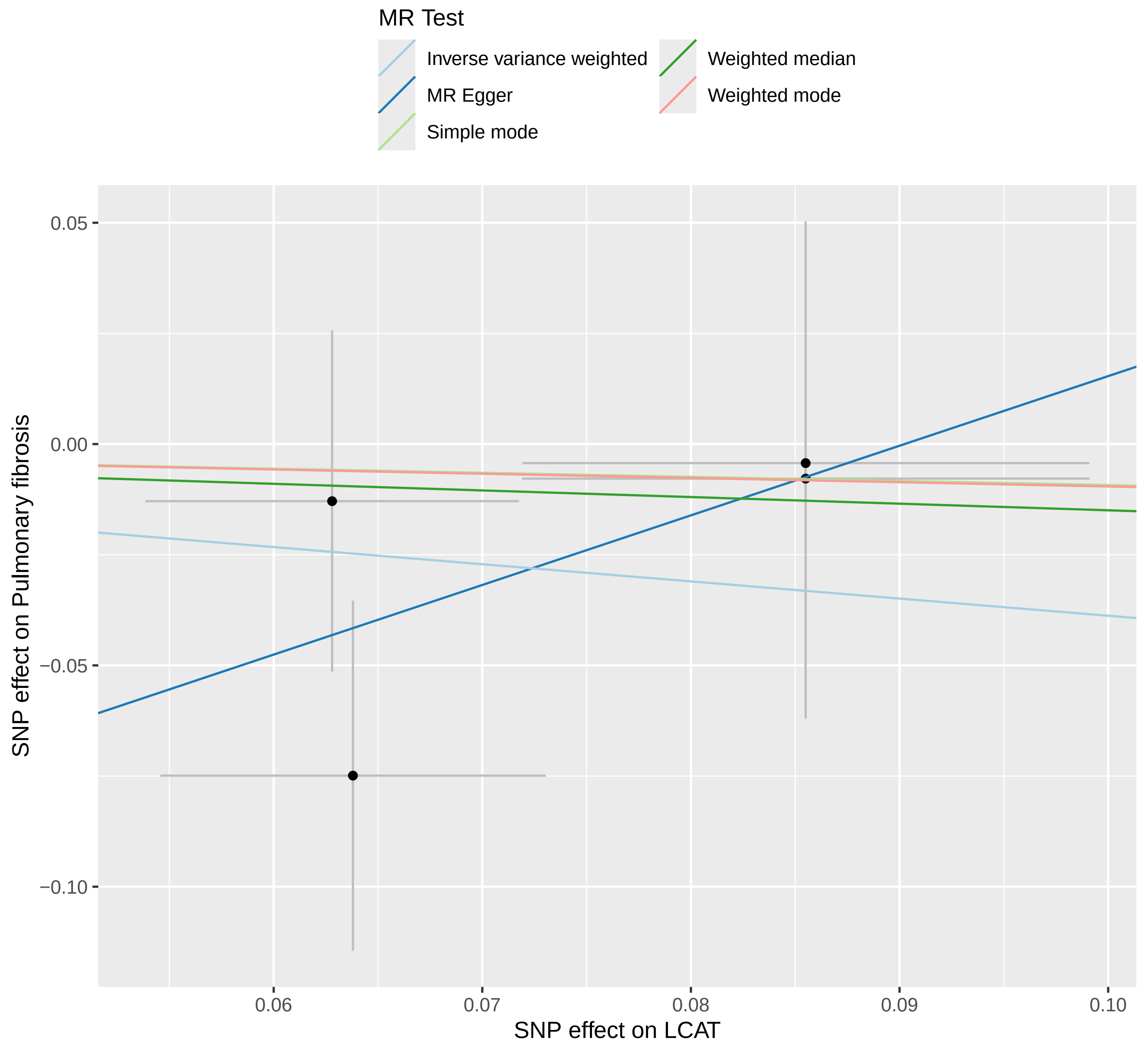
MR Test
Inverse variance weighted
MR Egger
Simple mode
Weighted median
Weighted mode
0.05
0.00
−0.05
−0.10
0.06
0.07
0.08
0.09
0.10
SNP effect on LCAT
SNP effect on Pulmonary fibrosis

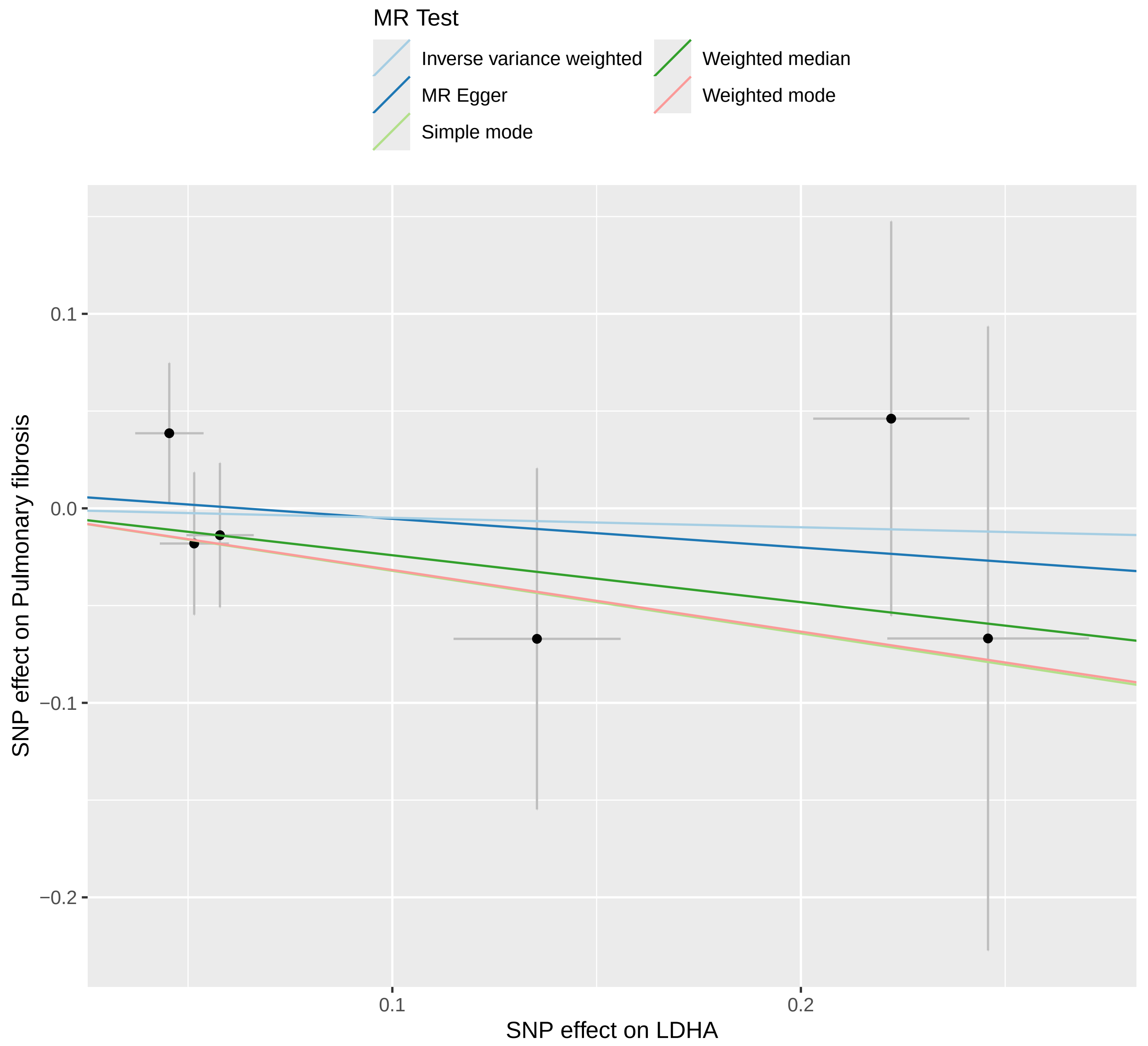

MR Test
Inverse variance weighted
MR Egger
Simple mode
Weighted median
Weighted mode
0.1
0.0
−0.1
−0.2
SNP effect on Pulmonary fibrosis
0.1
0.2
SNP effect on LDHA

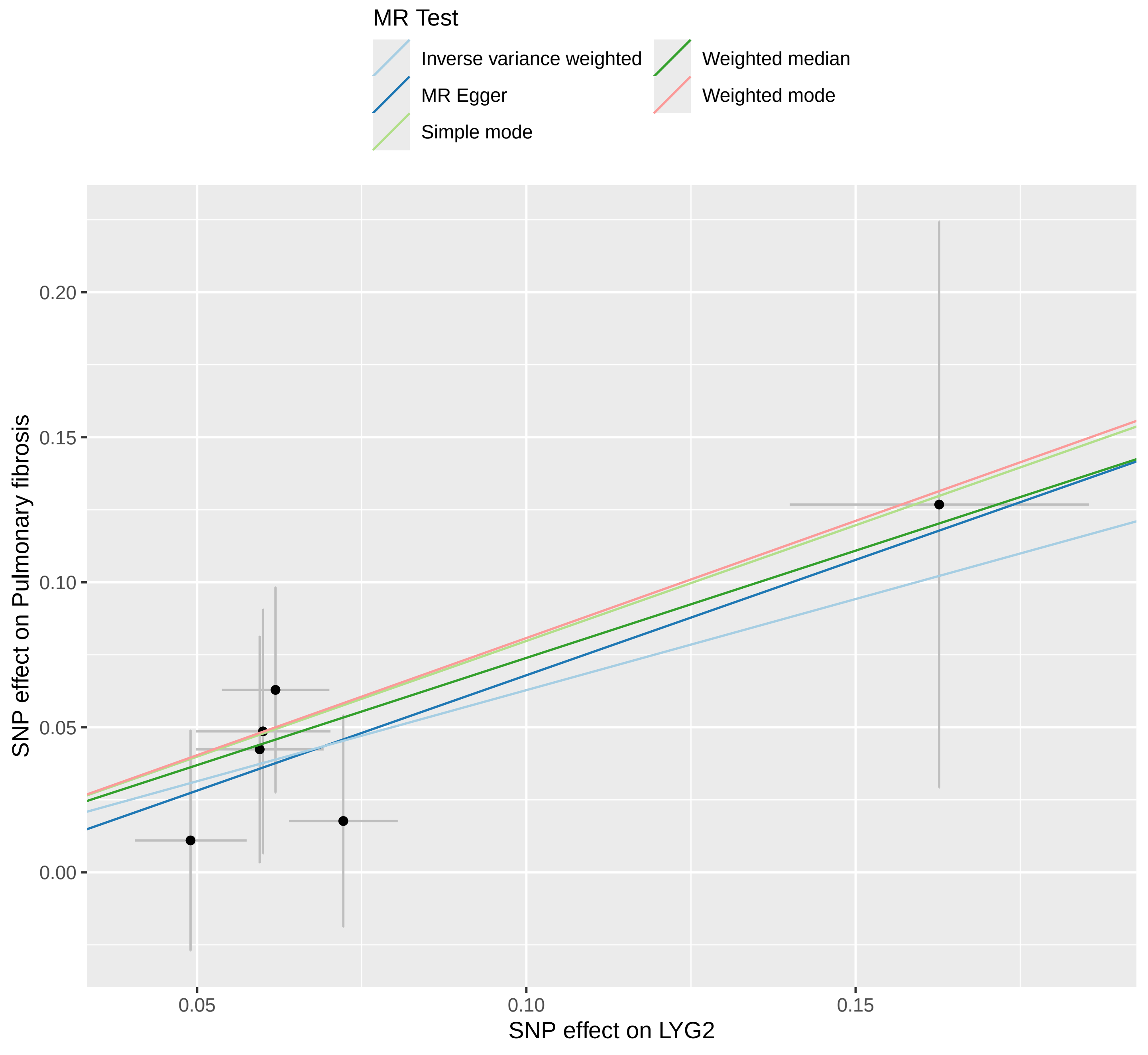
MR Test
Inverse variance weighted
MR Egger
Simple mode
Weighted median
Weighted mode
SNP effect on Pulmonary fibrosis
0.20
0.15
0.10
0.05
0.00
0.05
0.10
0.15
SNP effect on LYG2

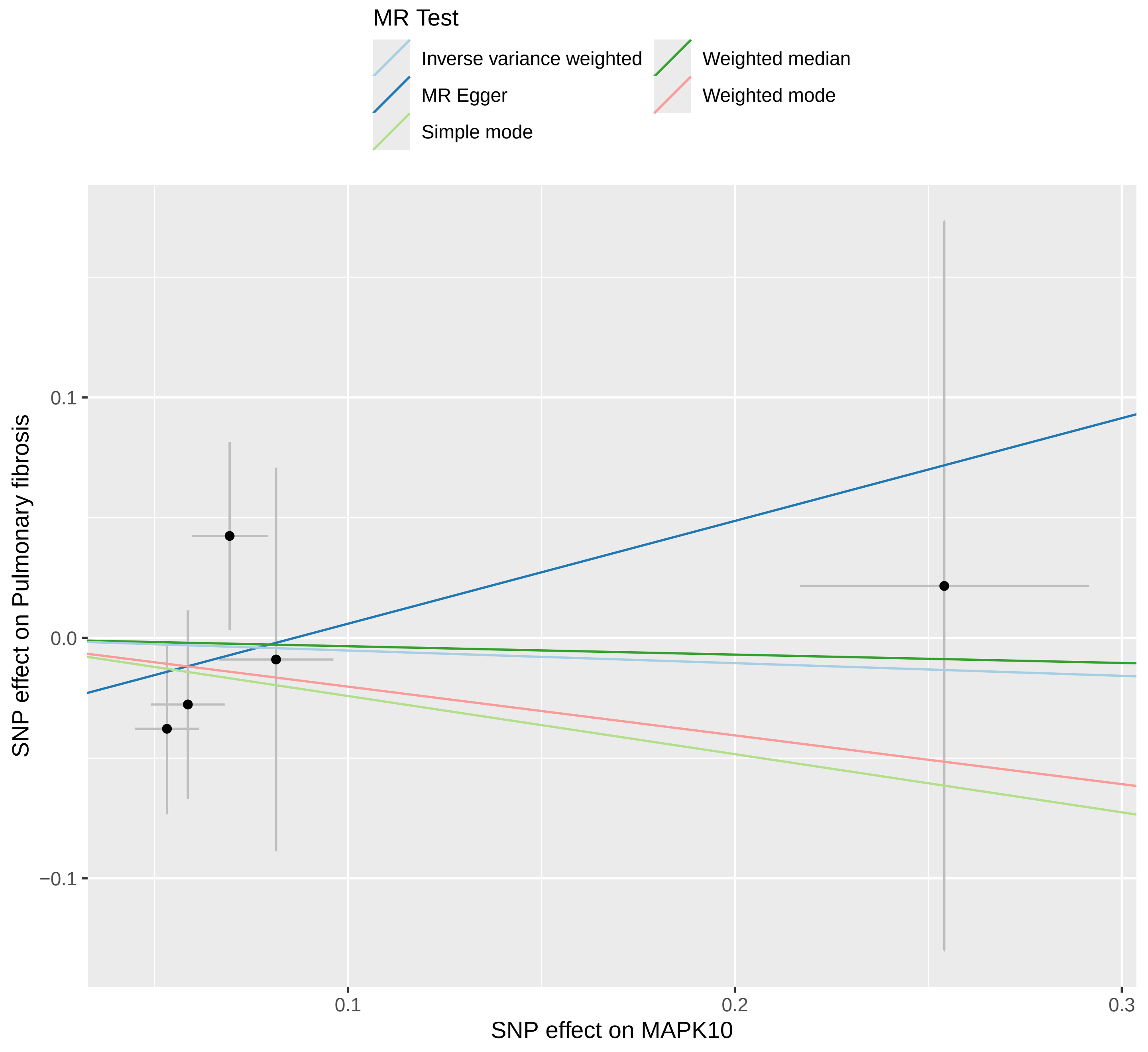

MR Test
Inverse variance weighted
MR Egger
Simple mode
Weighted median
Weighted mode
0.1
0.0
−0.1
0.1
0.2
0.3
SNP effect on Pulmonary fibrosis
SNP effect on MAPK10

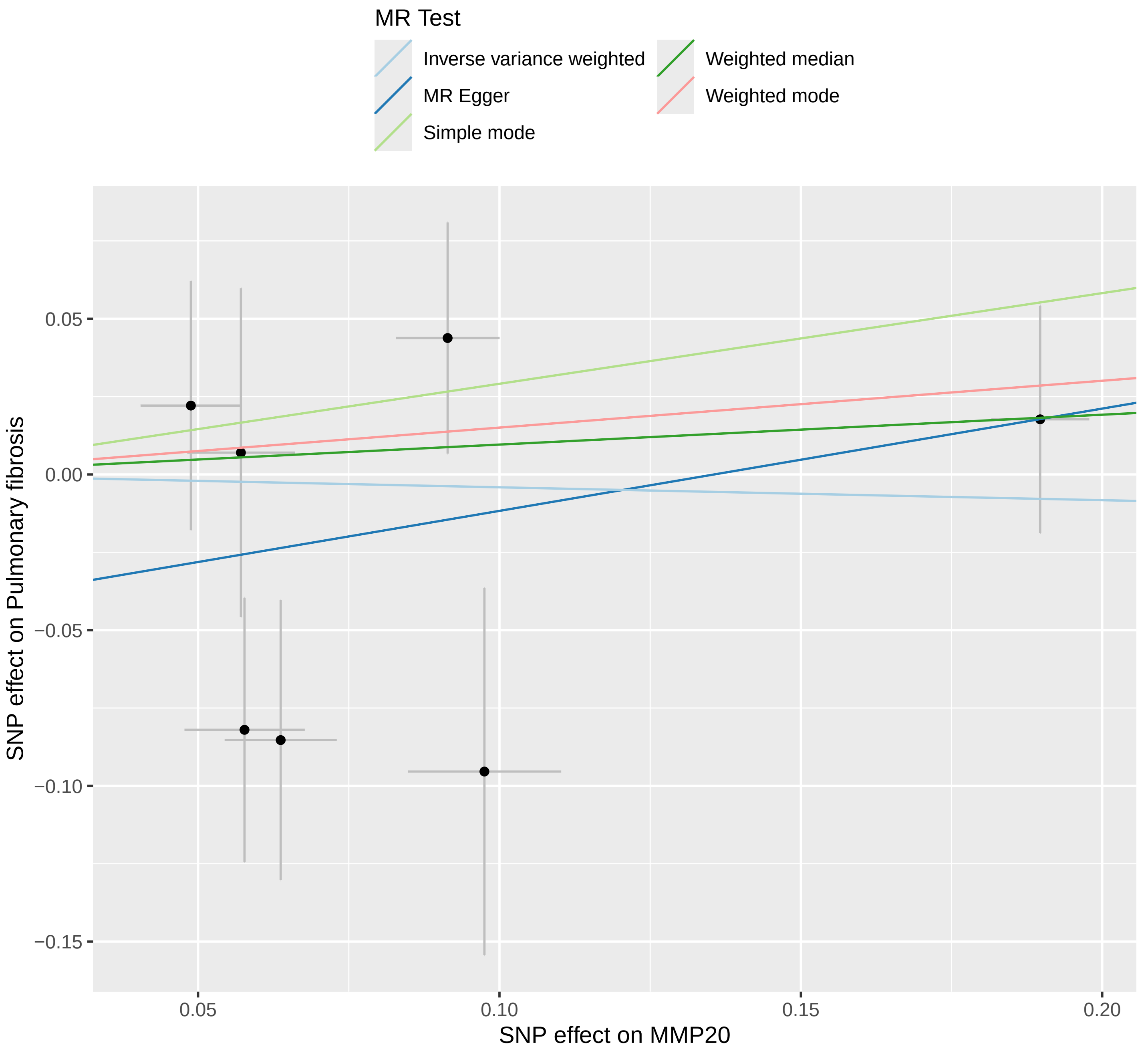
MR Test
Inverse variance weighted
MR Egger
Simple mode
Weighted median
Weighted mode
SNP effect on Pulmonary fibrosis
0.05
0.00
−0.05
−0.10
−0.15
0.05
0.10
0.15
0.20
SNP effect on MMP20

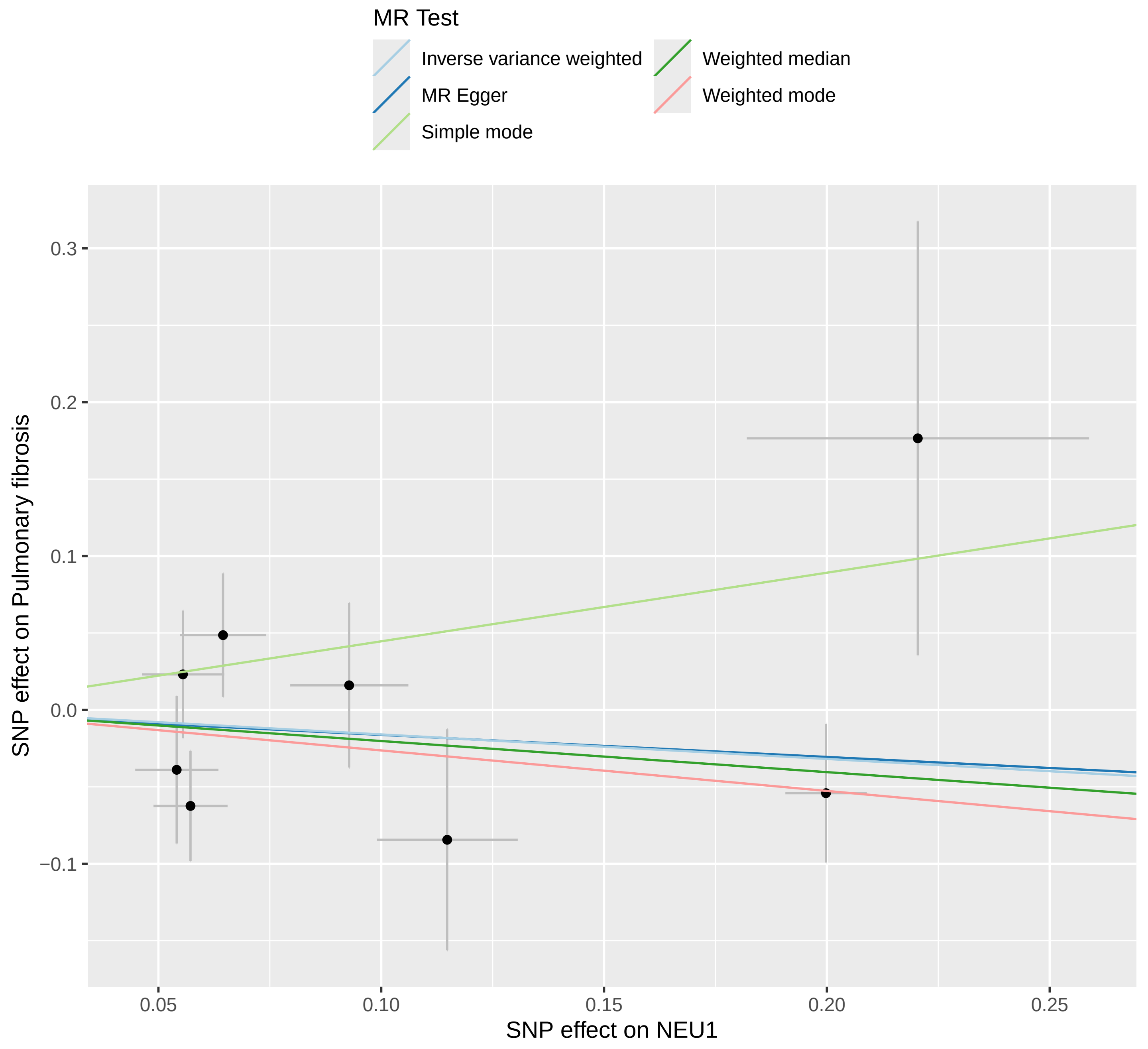
MR Test
Inverse variance weighted
MR Egger
Simple mode
Weighted median
Weighted mode
SNP effect on Pulmonary fibrosis
0.3
0.2
0.1
0.0
−0.1
0.05
0.10
0.15
0.20
0.25
SNP effect on NEU1

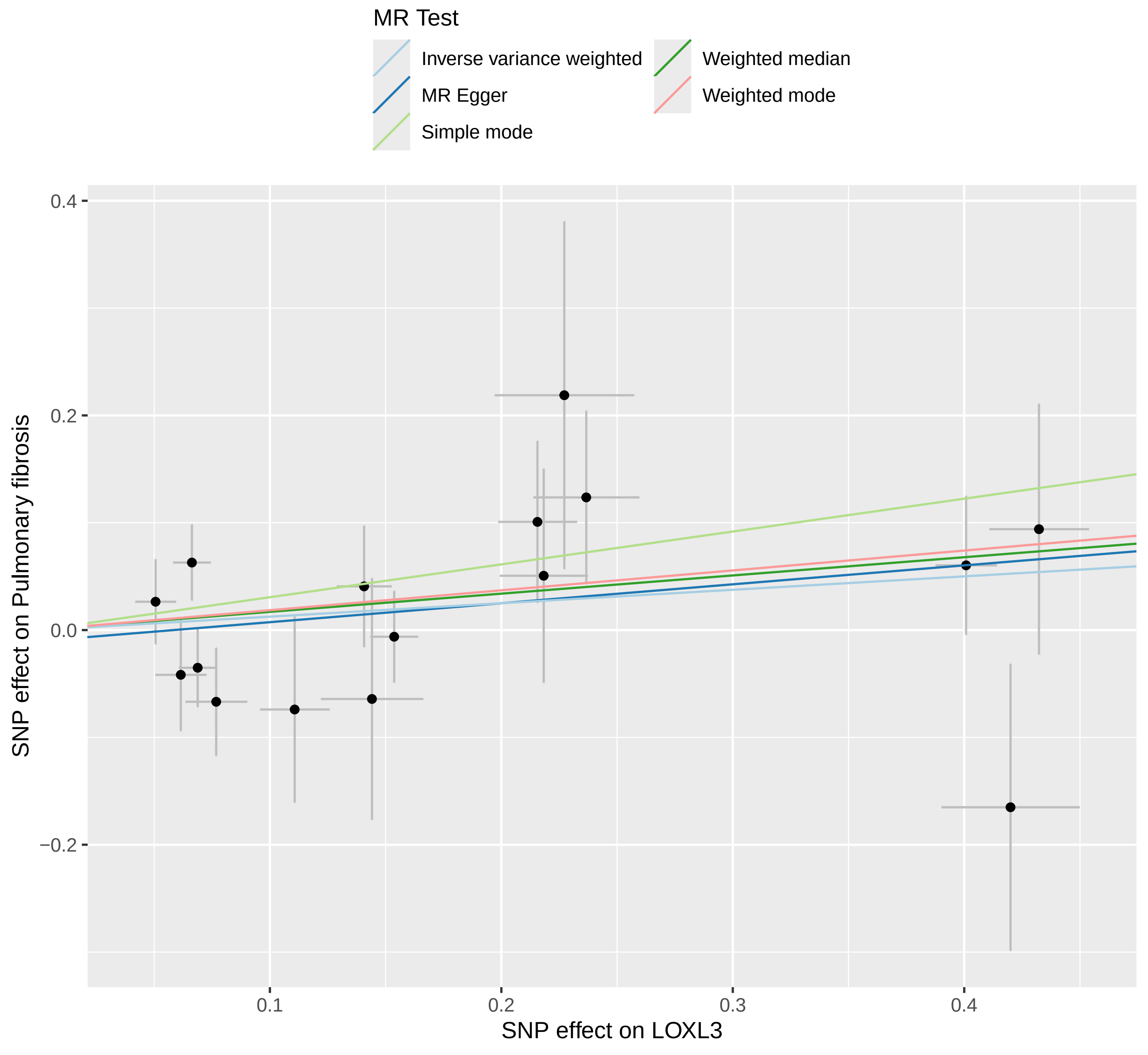

MR Test
Inverse variance weighted
MR Egger
Simple mode
Weighted median
Weighted mode
SNP effect on Pulmonary fibrosis
SNP effect on LOXL3
0.4
0.2
0.0
−0.2
0.1
0.2
0.3
0.4

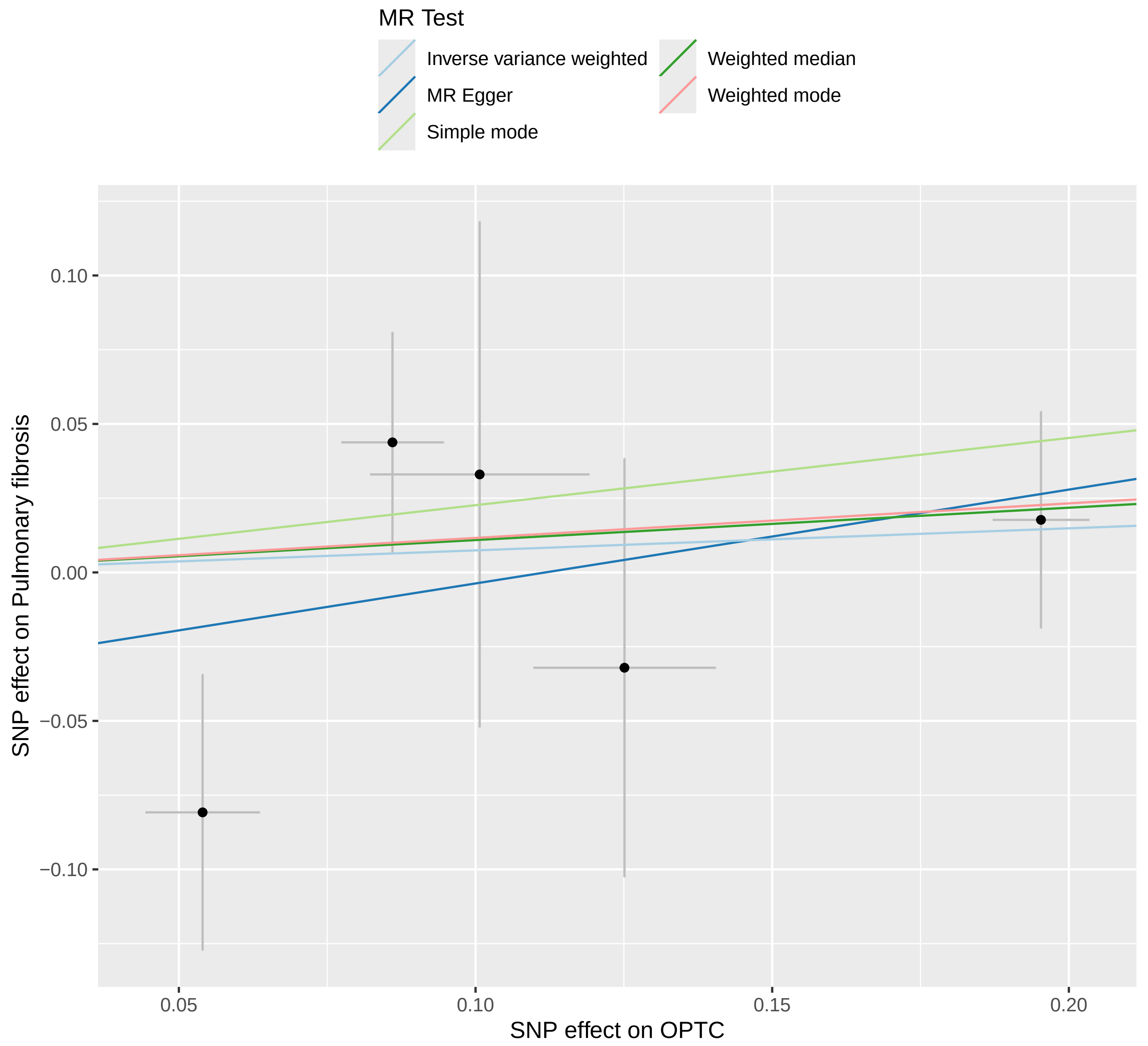

MR Test
Inverse variance weighted
MR Egger
Simple mode
Weighted median
Weighted mode
0.10
0.05
0.00
−0.05
−0.10
SNP effect on Pulmonary fibrosis
0.05
0.10
0.15
0.20
SNP effect on OPTC

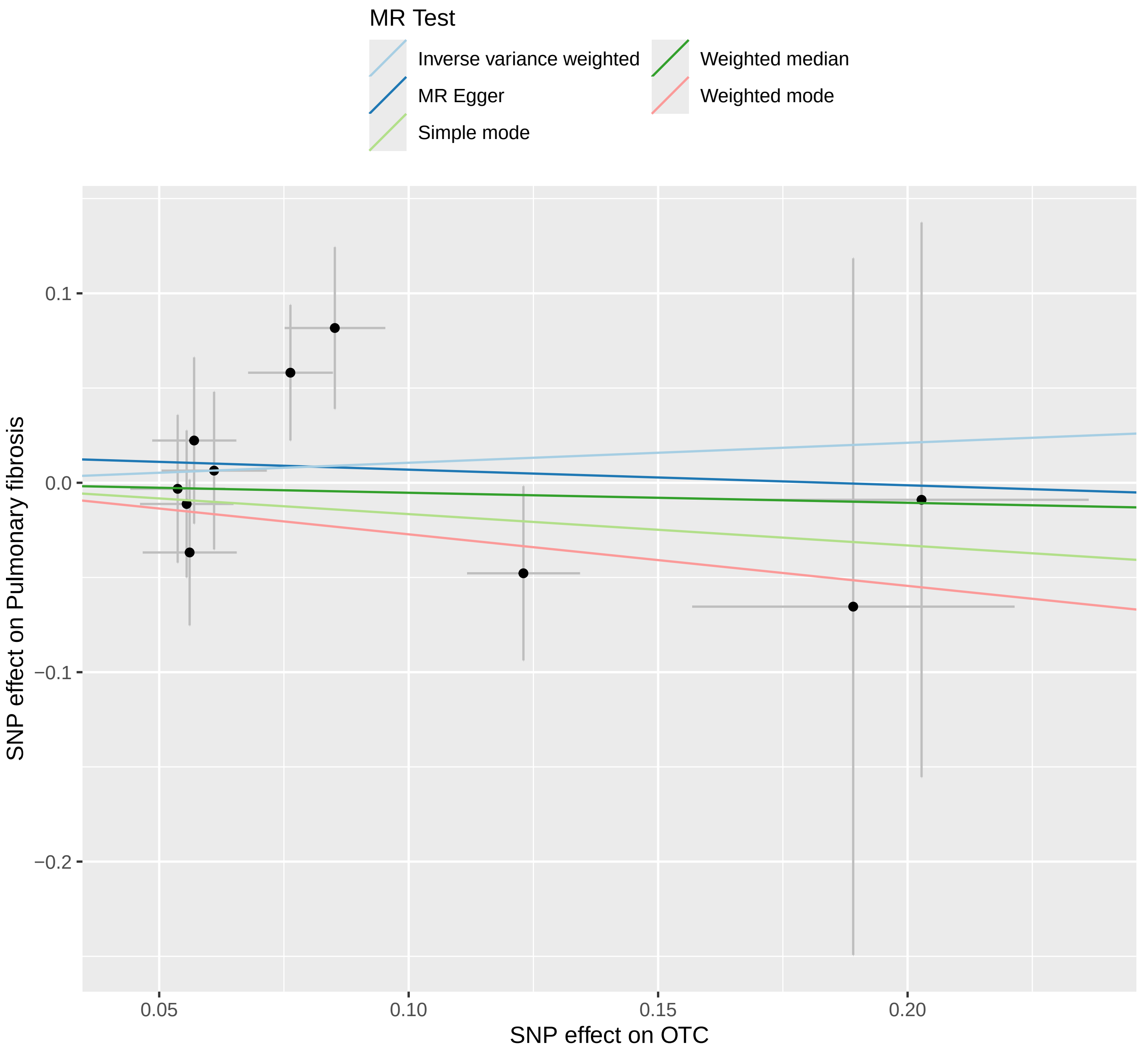

MR Test
Inverse variance weighted
MR Egger
Simple mode
Weighted median
Weighted mode
0.1
0.0
−0.1
−0.2
0.05
0.10
0.15
0.20
SNP effect on Pulmonary fibrosis
SNP effect on OTC

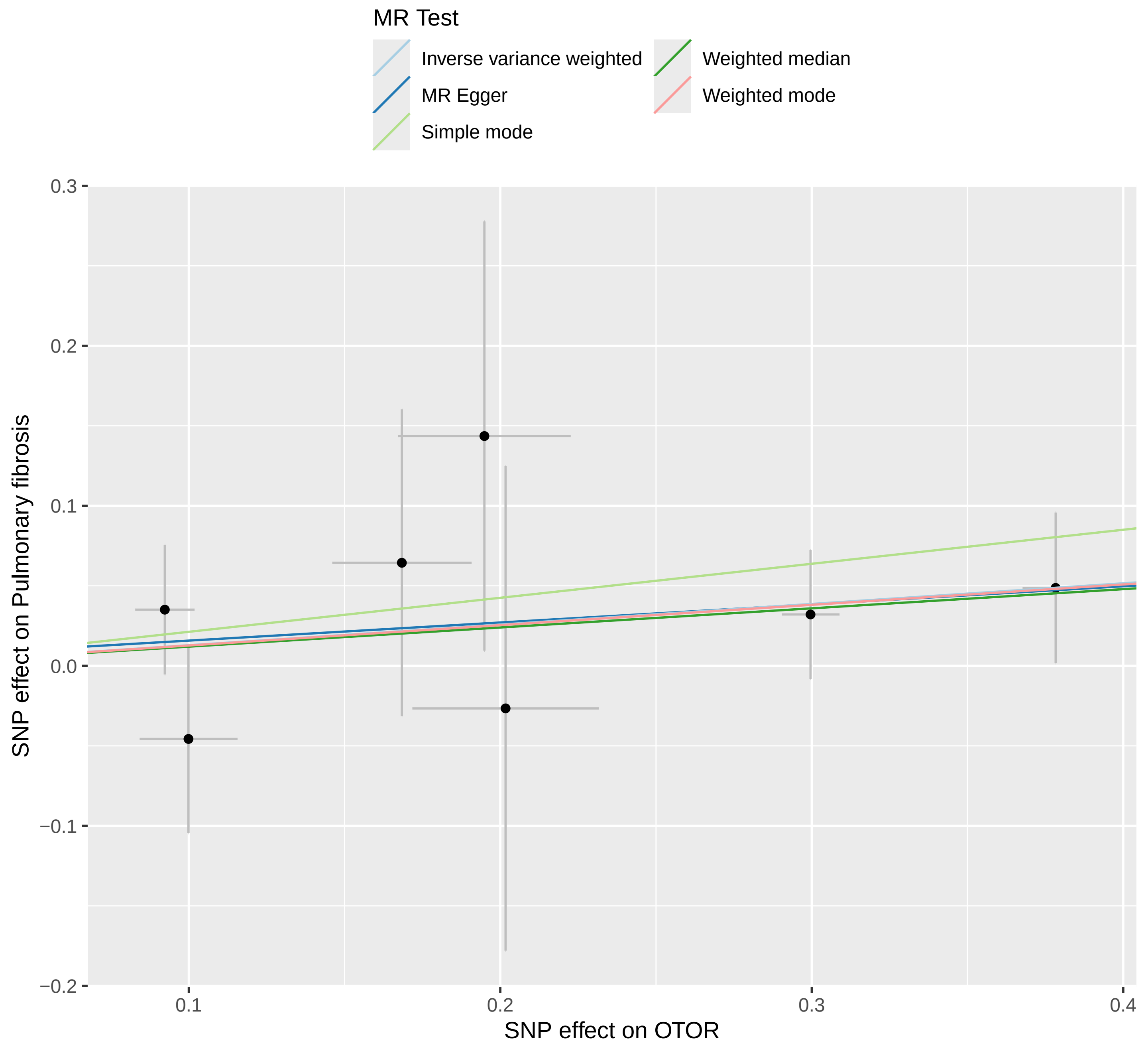

MR Test
Inverse variance weighted
MR Egger
Simple mode
Weighted median
Weighted mode
0.3
0.2
0.1
0.0
−0.1
−0.2
0.1
0.2
0.3
0.4
SNP effect on Pulmonary fibrosis
SNP effect on OTOR

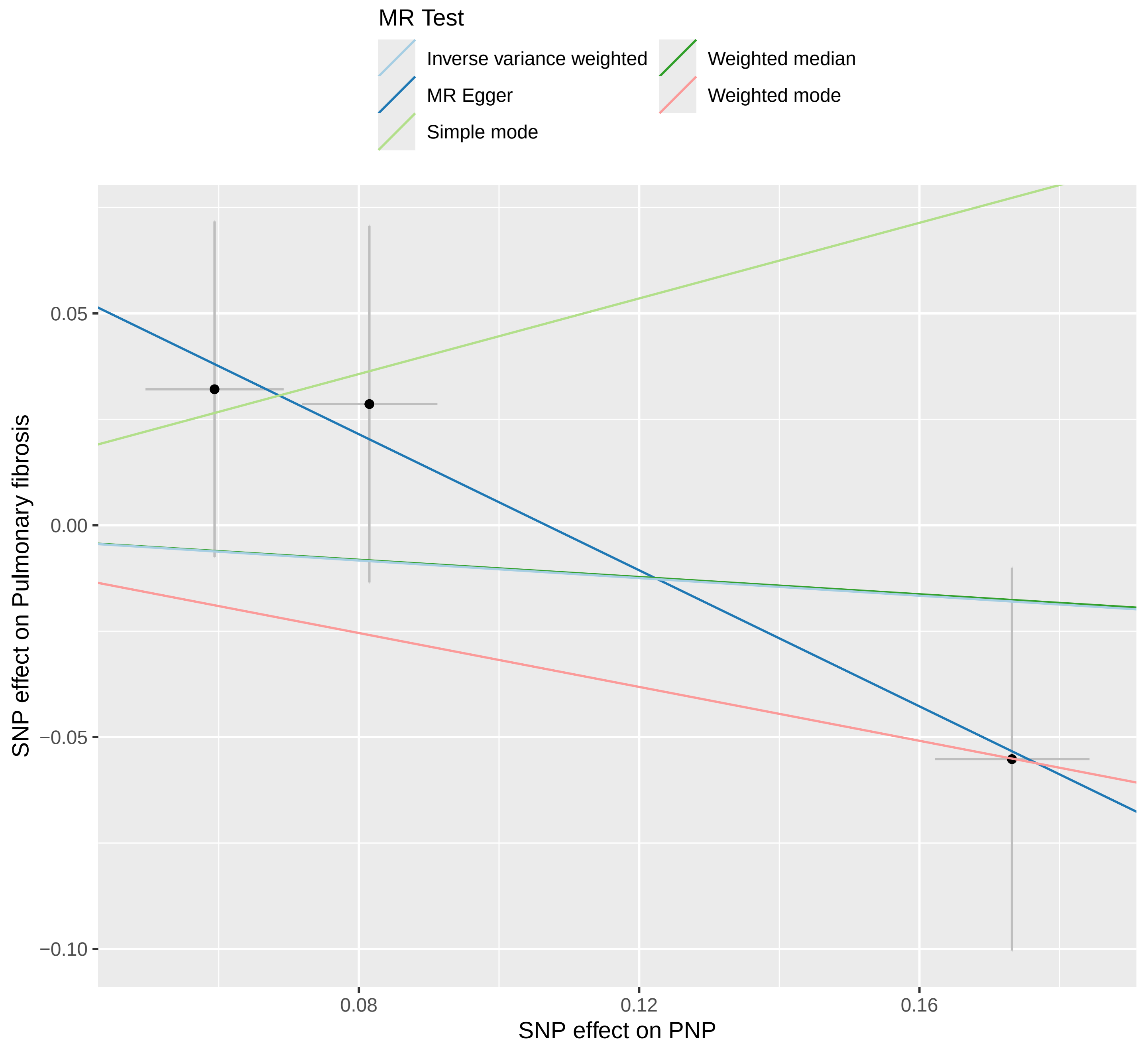
MR Test
Inverse variance weighted
MR Egger
Simple mode
Weighted median
Weighted mode
0.05
0.00
−0.05
−0.10
SNP effect on Pulmonary fibrosis
0.08
0.12
0.16
SNP effect on PNP

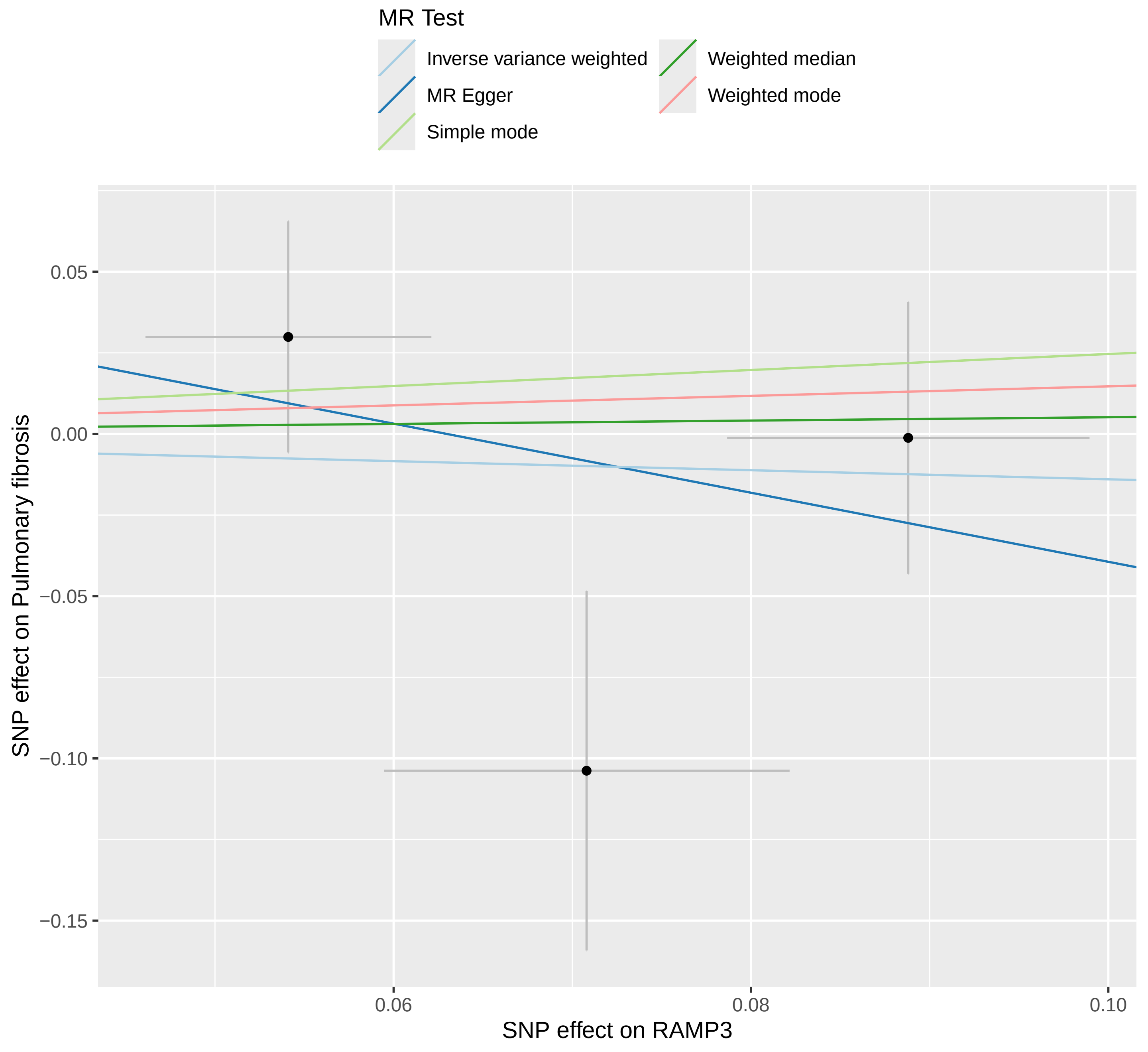

MR Test
Inverse variance weighted
MR Egger
Simple mode
Weighted median
Weighted mode
0.05
0.00
−0.05
−0.10
−0.15
SNP effect on Pulmonary fibrosis
0.06
0.08
0.10
SNP effect on RAMP3

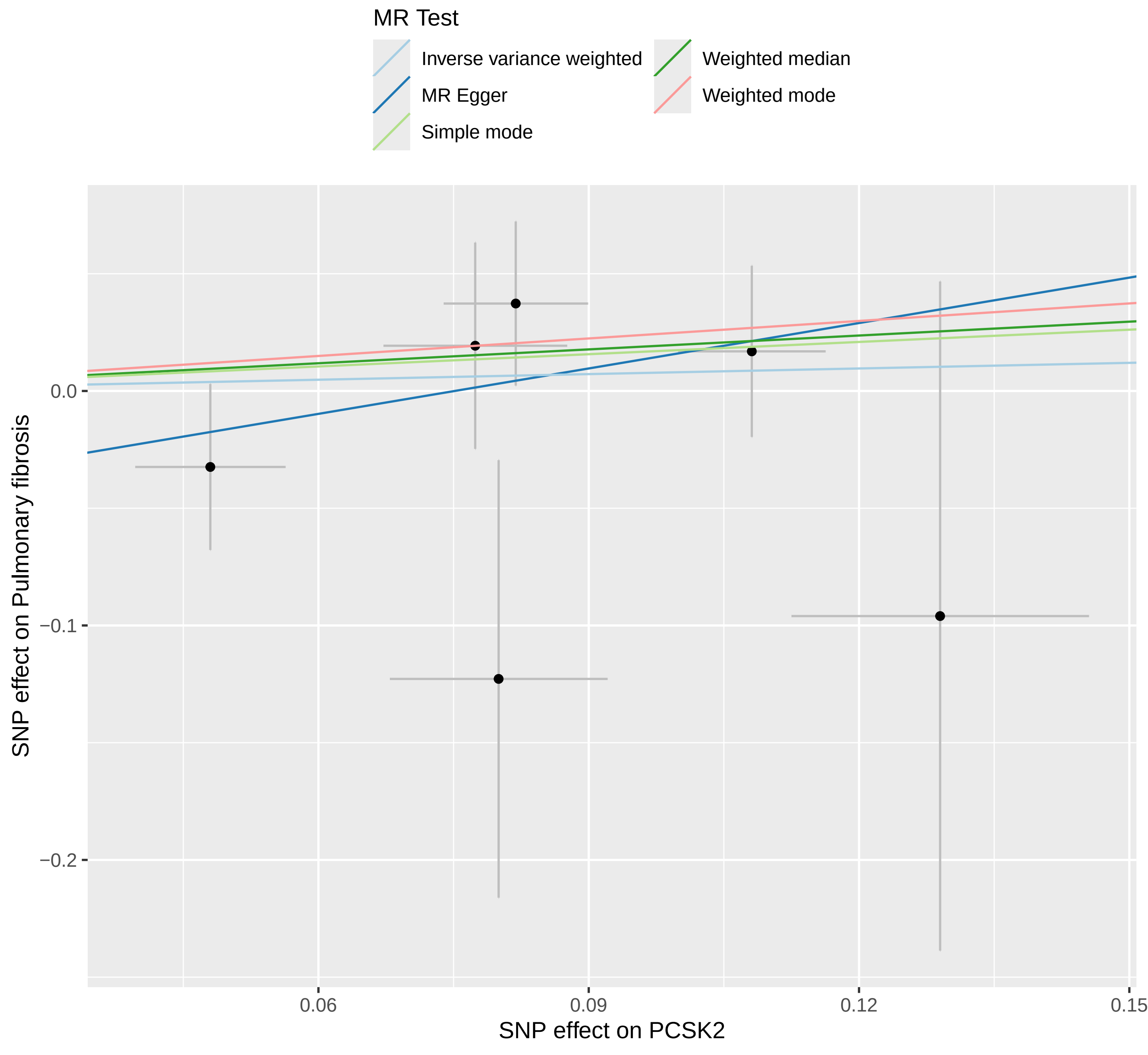
MR Test
Inverse variance weighted
MR Egger
Simple mode
Weighted median
Weighted mode
0.0
−0.1
−0.2
0.06
0.09
0.12
0.15
SNP effect on Pulmonary fibrosis
SNP effect on PCSK2

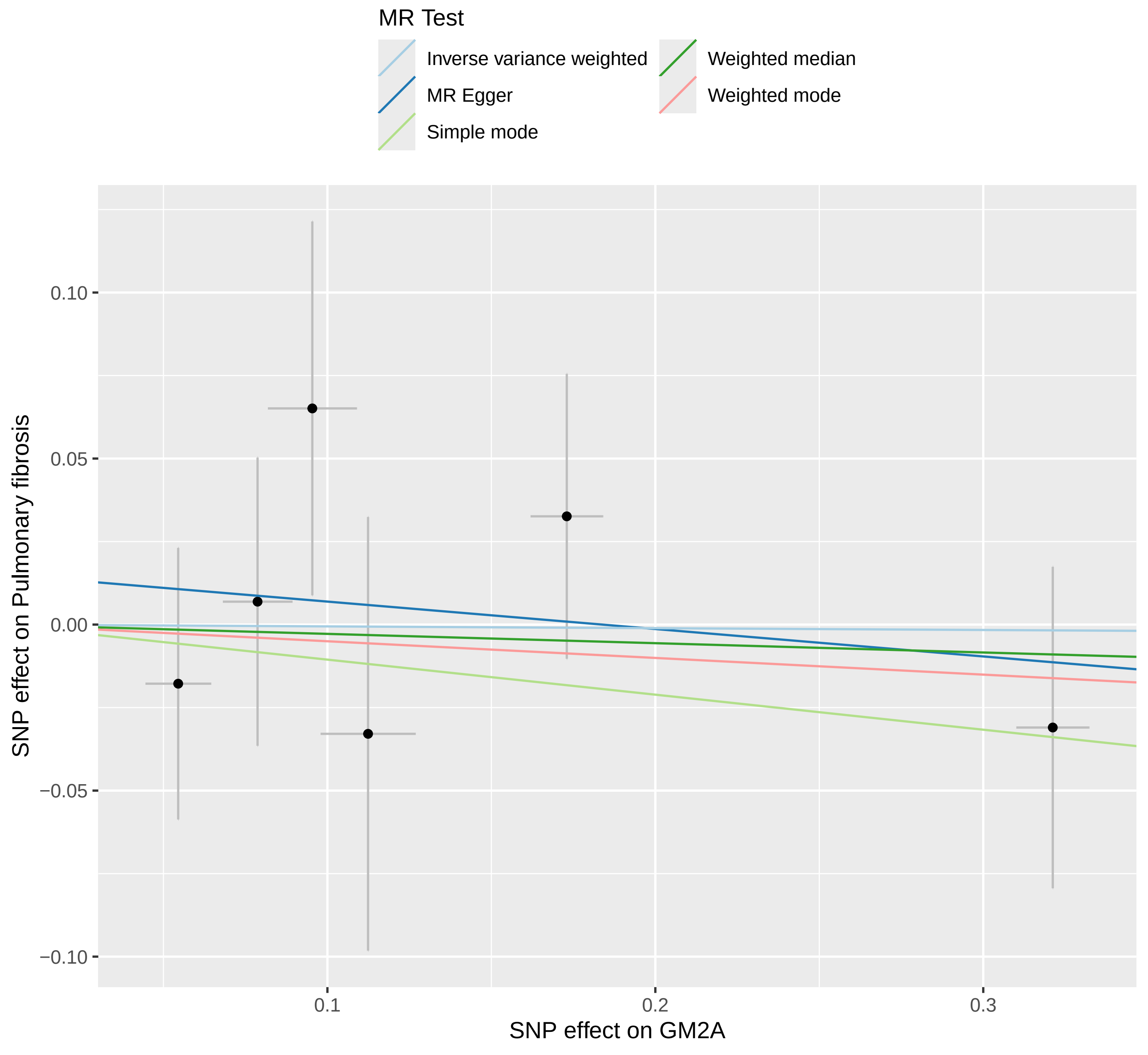

MR Test
Inverse variance weighted
MR Egger
Simple mode
Weighted median
Weighted mode
0.10
0.05
0.00
−0.05
−0.10
SNP effect on Pulmonary fibrosis
0.1
0.2
0.3
SNP effect on GM2A

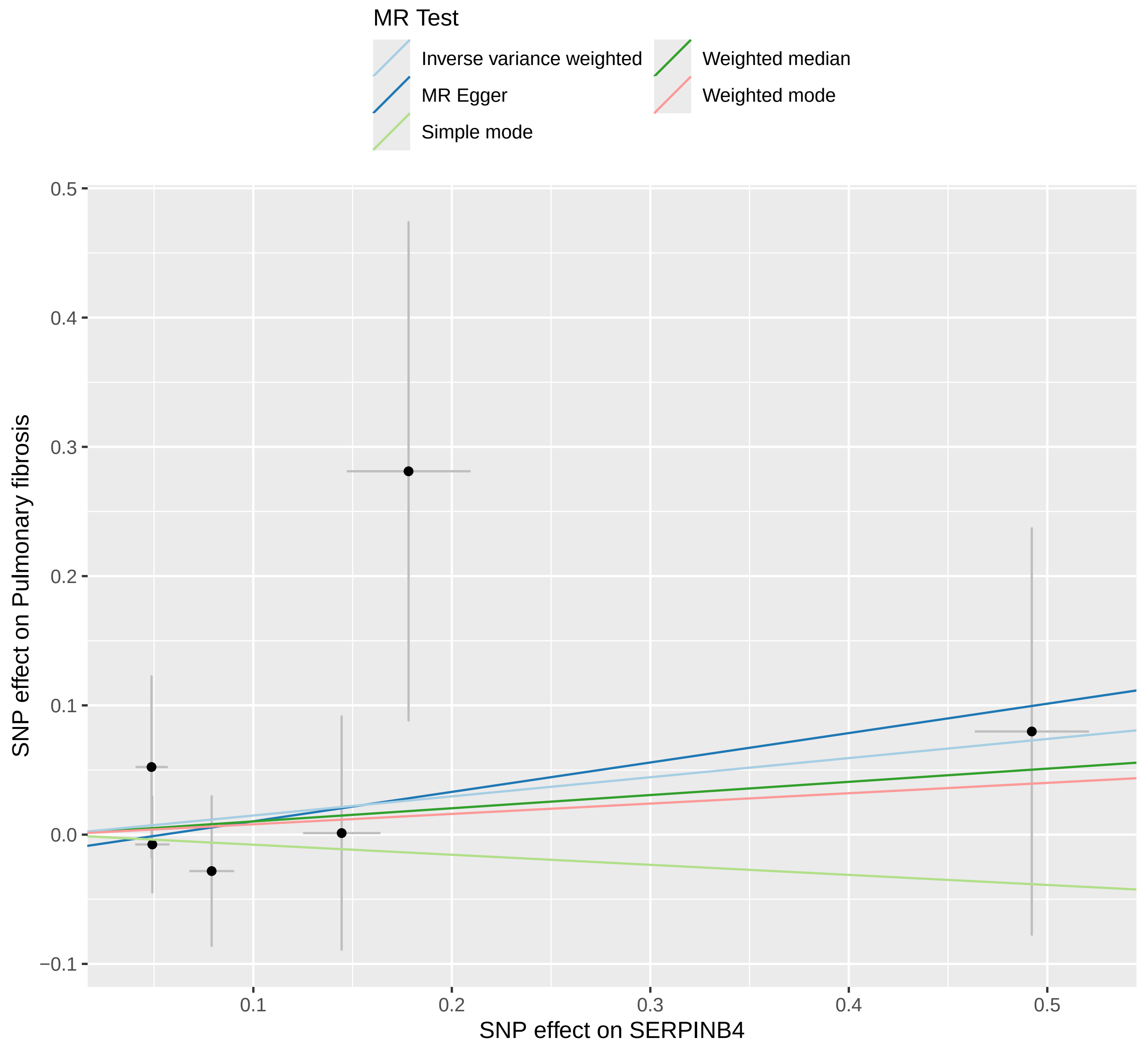

MR Test
Inverse variance weighted
MR Egger
Simple mode
Weighted median
Weighted mode
SNP effect on Pulmonary fibrosis
SNP effect on SERPINB4
0.5
0.4
0.3
0.2
0.1
0.0
−0.1
0.1
0.2
0.3
0.4
0.5

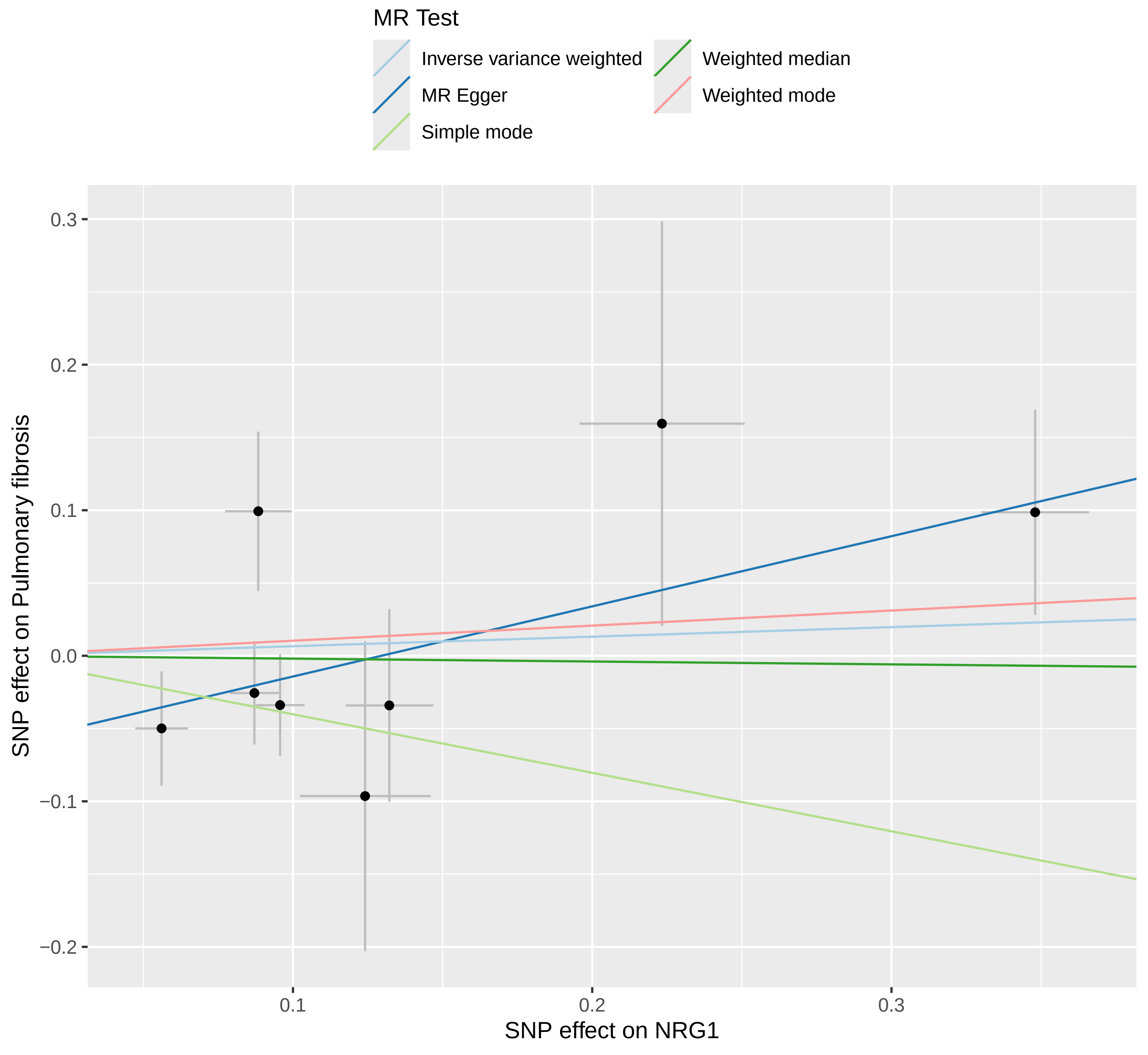
MR Test
Inverse variance weighted
MR Egger
Simple mode
Weighted median
Weighted mode
0.3
0.2
0.1
0.0
−0.1
−0.2
0.1
0.2
0.3
SNP effect on Pulmonary fibrosis
SNP effect on NRG1

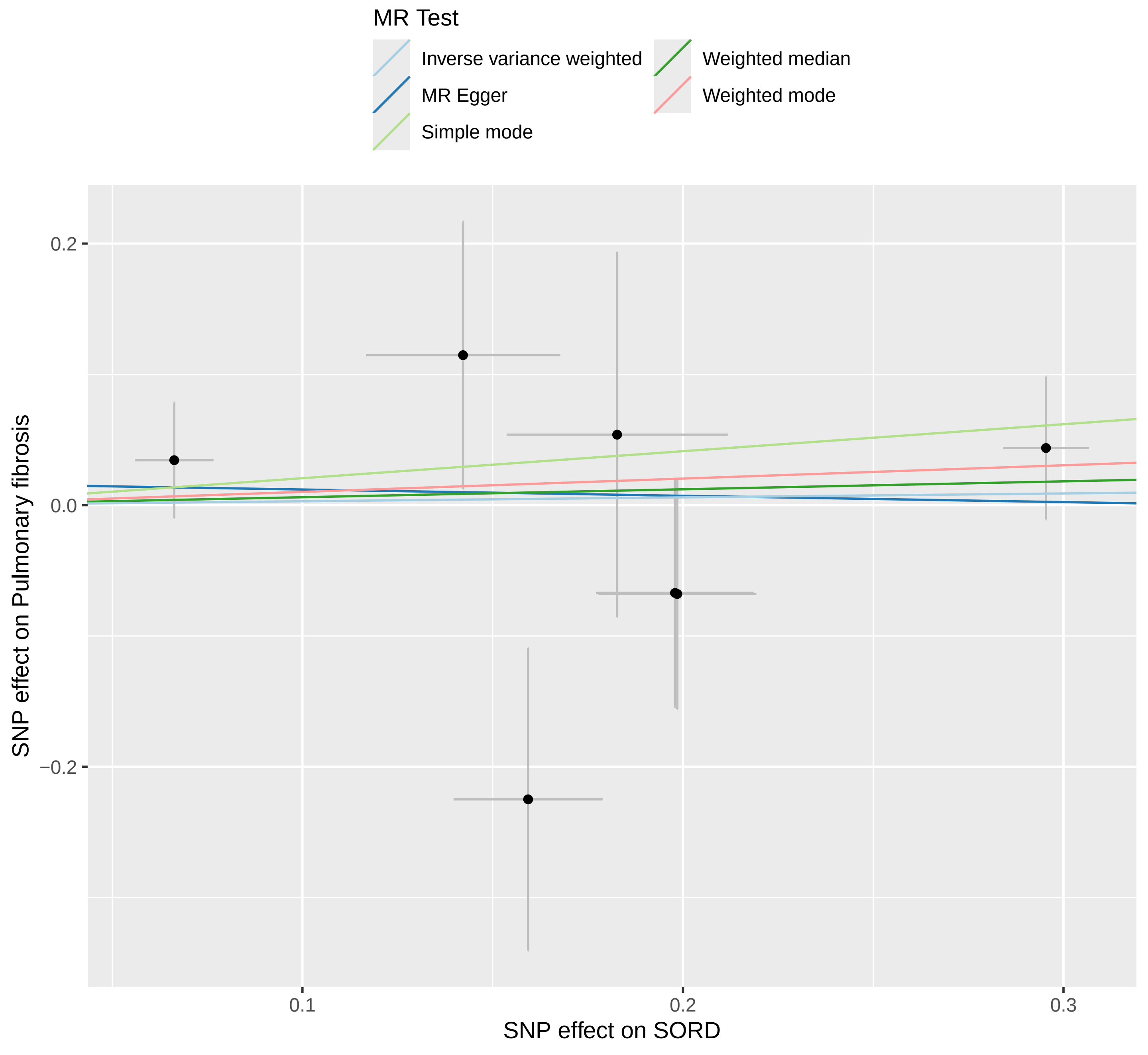
MR Test
Inverse variance weighted
MR Egger
Simple mode
Weighted median
Weighted mode
0.2
0.0
−0.2
0.1
0.2
0.3
SNP effect on Pulmonary fibrosis
SNP effect on SORD

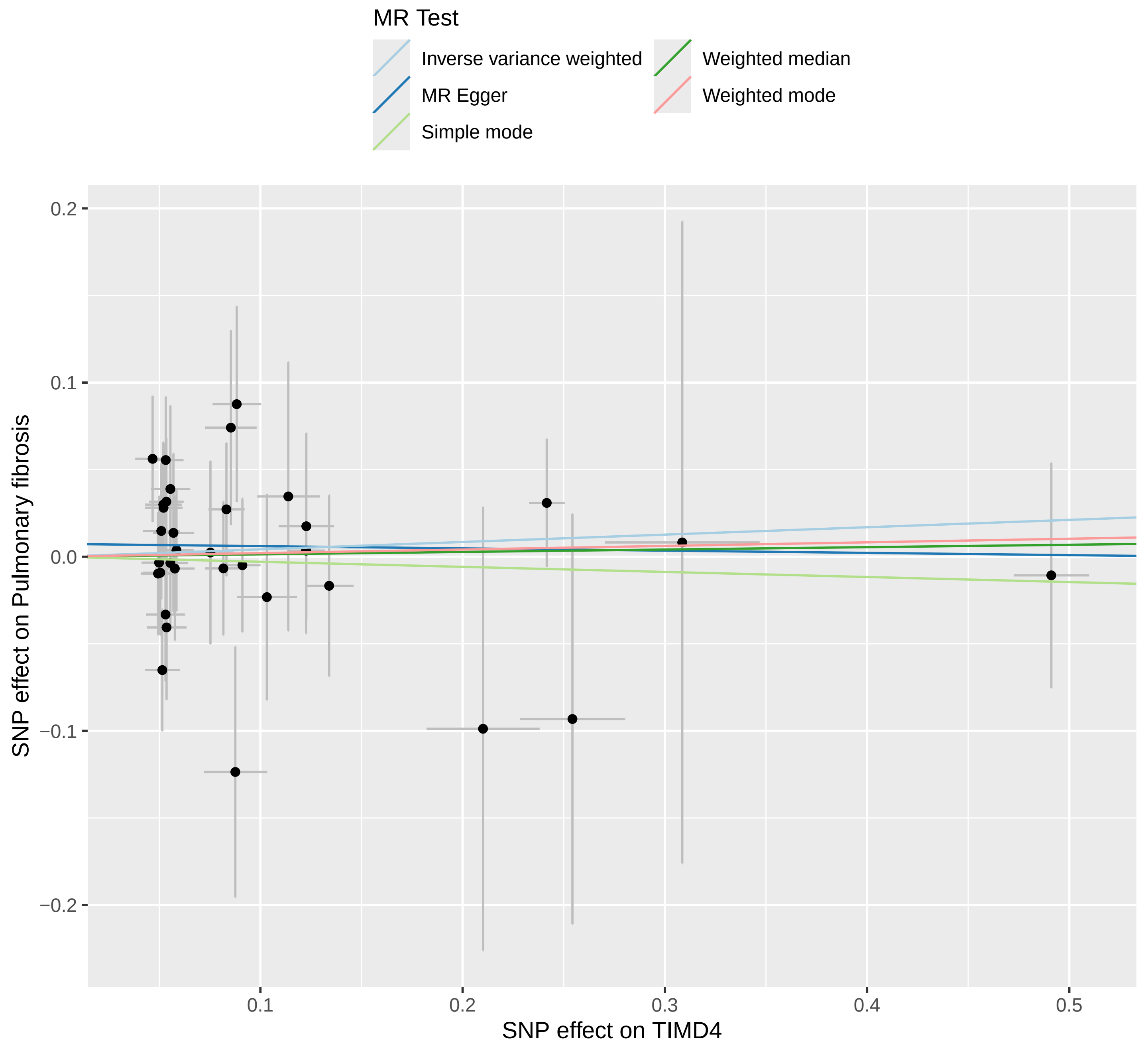
MR Test
Inverse variance weighted
MR Egger
Simple mode
Weighted median
Weighted mode
0.2
0.1
0.0
−0.1
−0.2
0.1
0.2
0.3
0.4
0.5
SNP effect on TIMD4
SNP effect on Pulmonary fibrosis

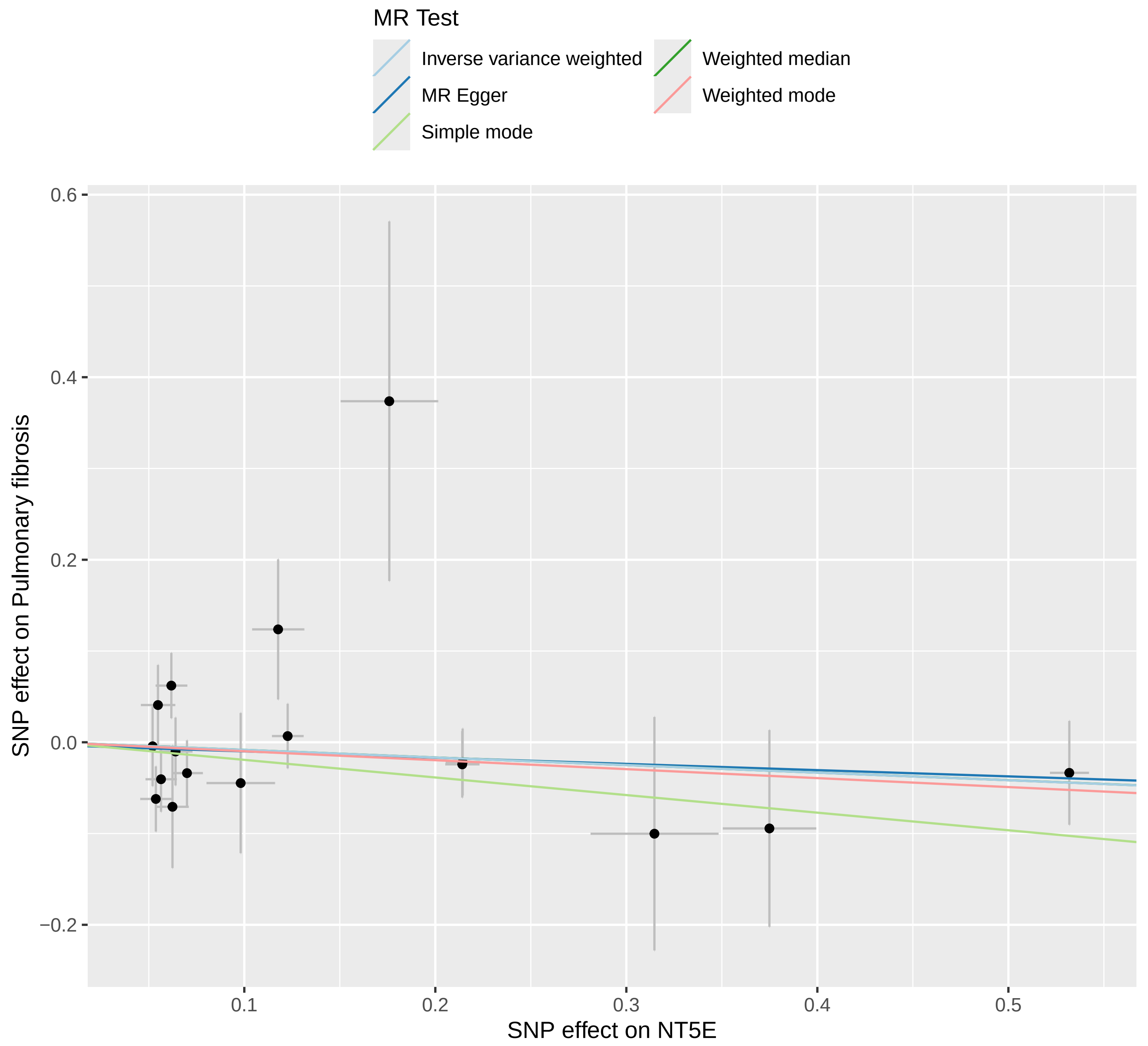
MR Test
Inverse variance weighted
MR Egger
Simple mode
Weighted median
Weighted mode
SNP effect on Pulmonary fibrosis
0.6
0.4
0.2
0.0
−0.2
0.1
0.2
0.3
0.4
0.5
SNP effect on NT5E

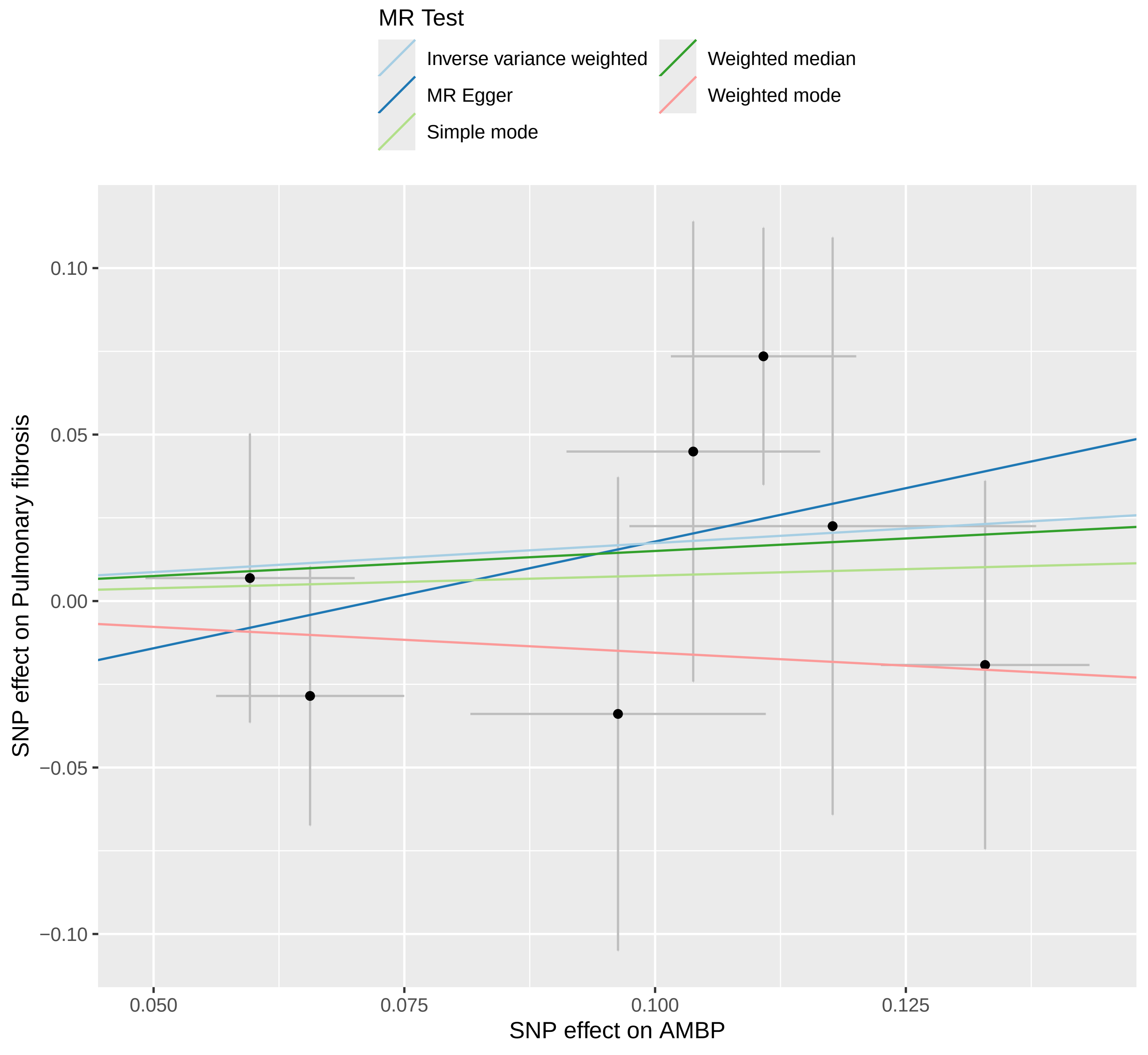
MR Test
Inverse variance weighted
MR Egger
Simple mode
Weighted median
Weighted mode
0.10
0.05
0.00
−0.05
−0.10
SNP effect on Pulmonary fibrosis
0.050
0.075
0.100
0.125
SNP effect on AMBP

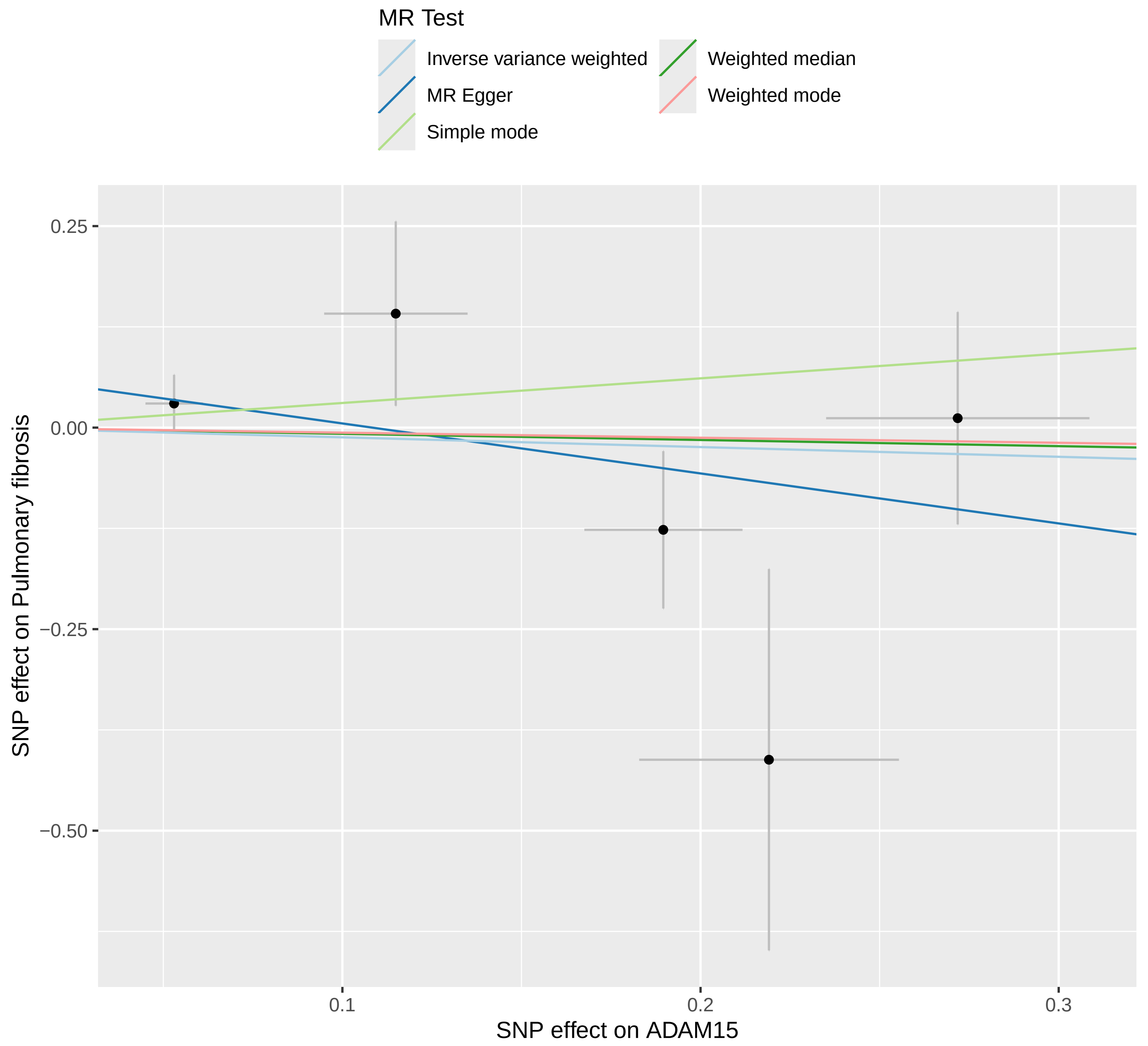
MR Test
Inverse variance weighted
MR Egger
Simple mode
Weighted median
Weighted mode
0.25
0.00
−0.25
−0.50
0.1
0.2
0.3
SNP effect on Pulmonary fibrosis
SNP effect on ADAM15

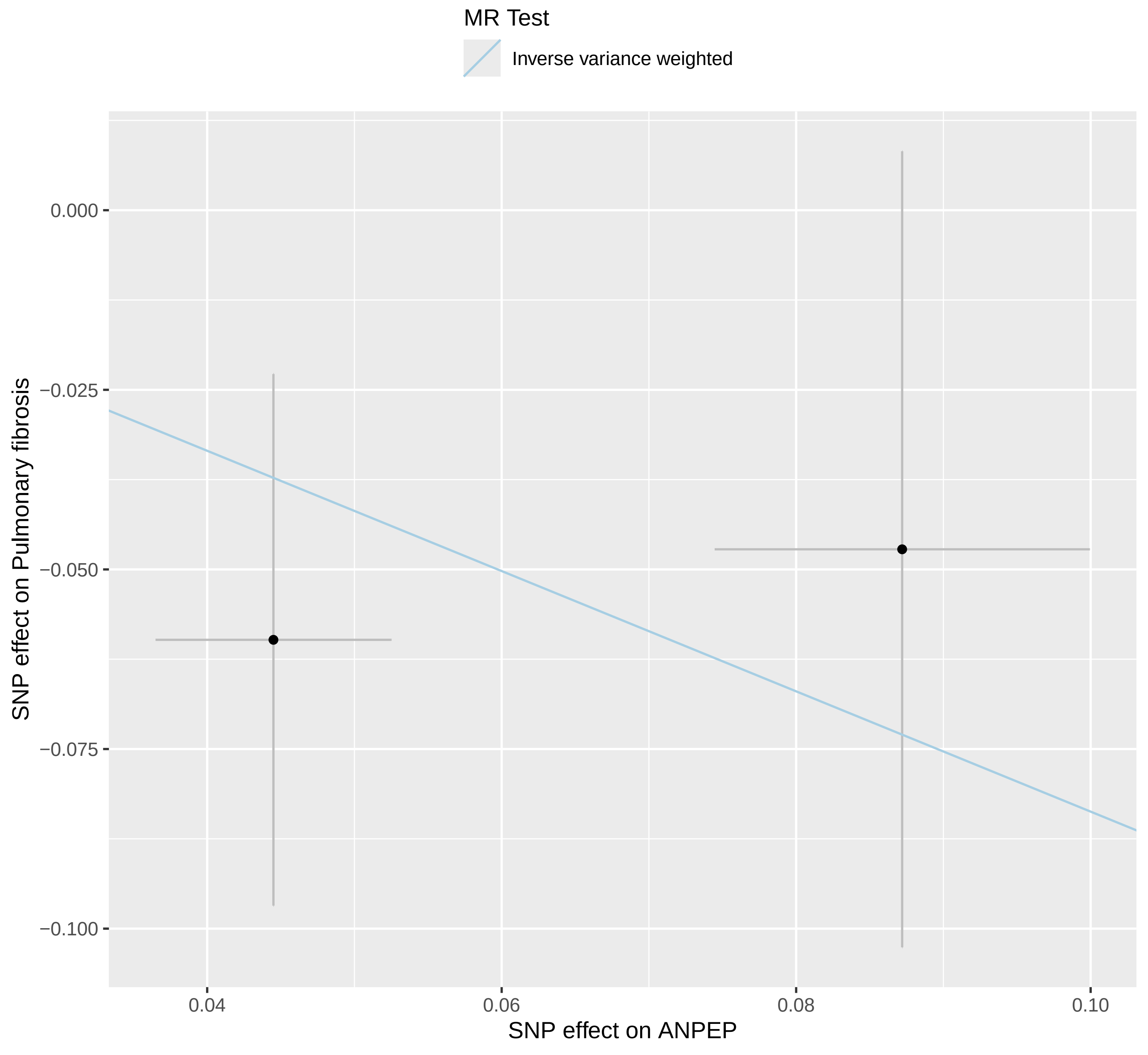

MR Test
Inverse variance weighted
SNP effect on Pulmonary fibrosis
0.000
−0.025
−0.050
−0.075
−0.100
0.04
0.06
0.08
0.10
SNP effect on ANPEP

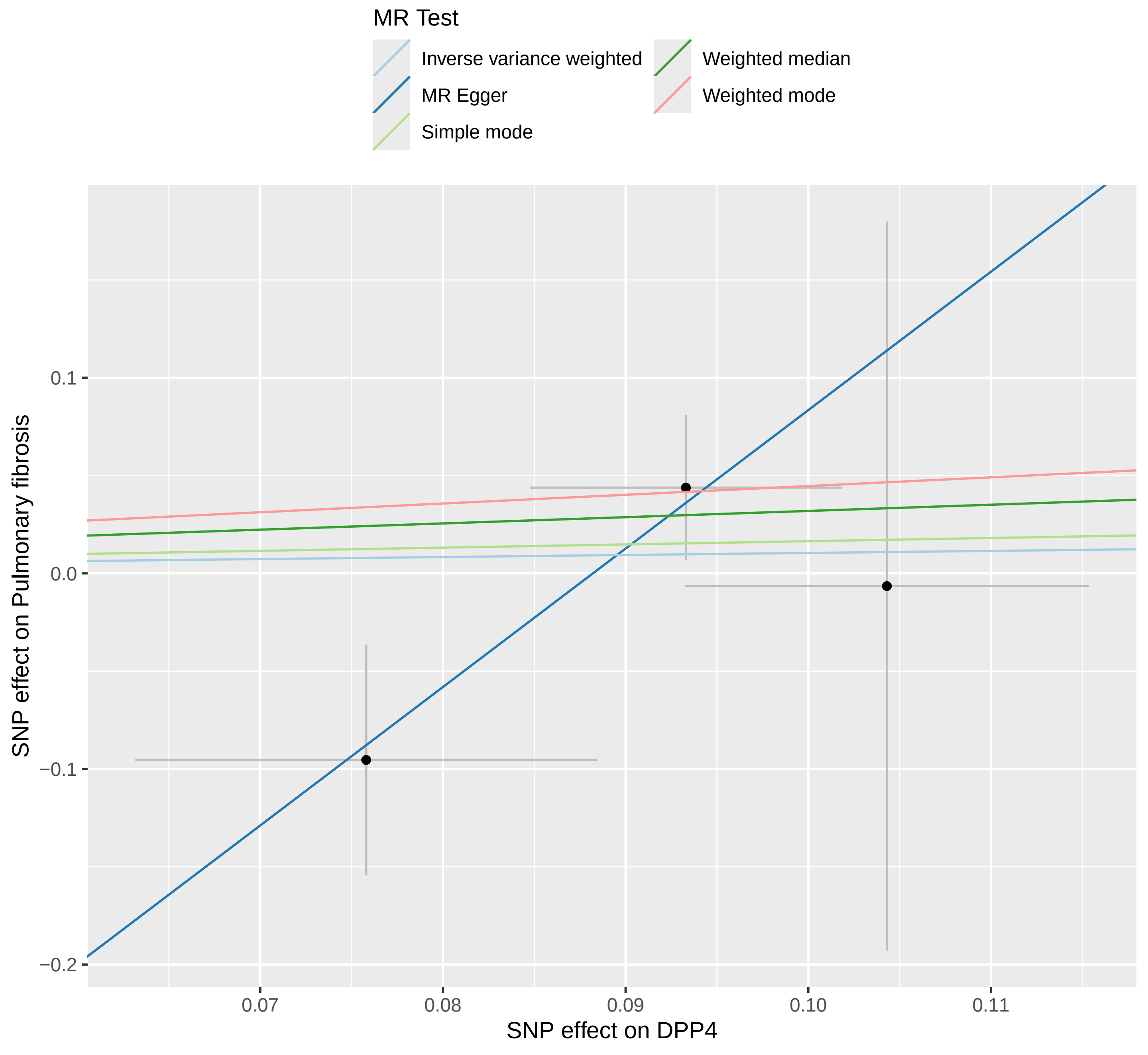

MR Test
Inverse variance weighted
MR Egger
Simple mode
Weighted median
Weighted mode
0.1
0.0
−0.1
−0.2
0.07
0.08
0.09
0.10
0.11
SNP effect on Pulmonary fibrosis
SNP effect on DPP4

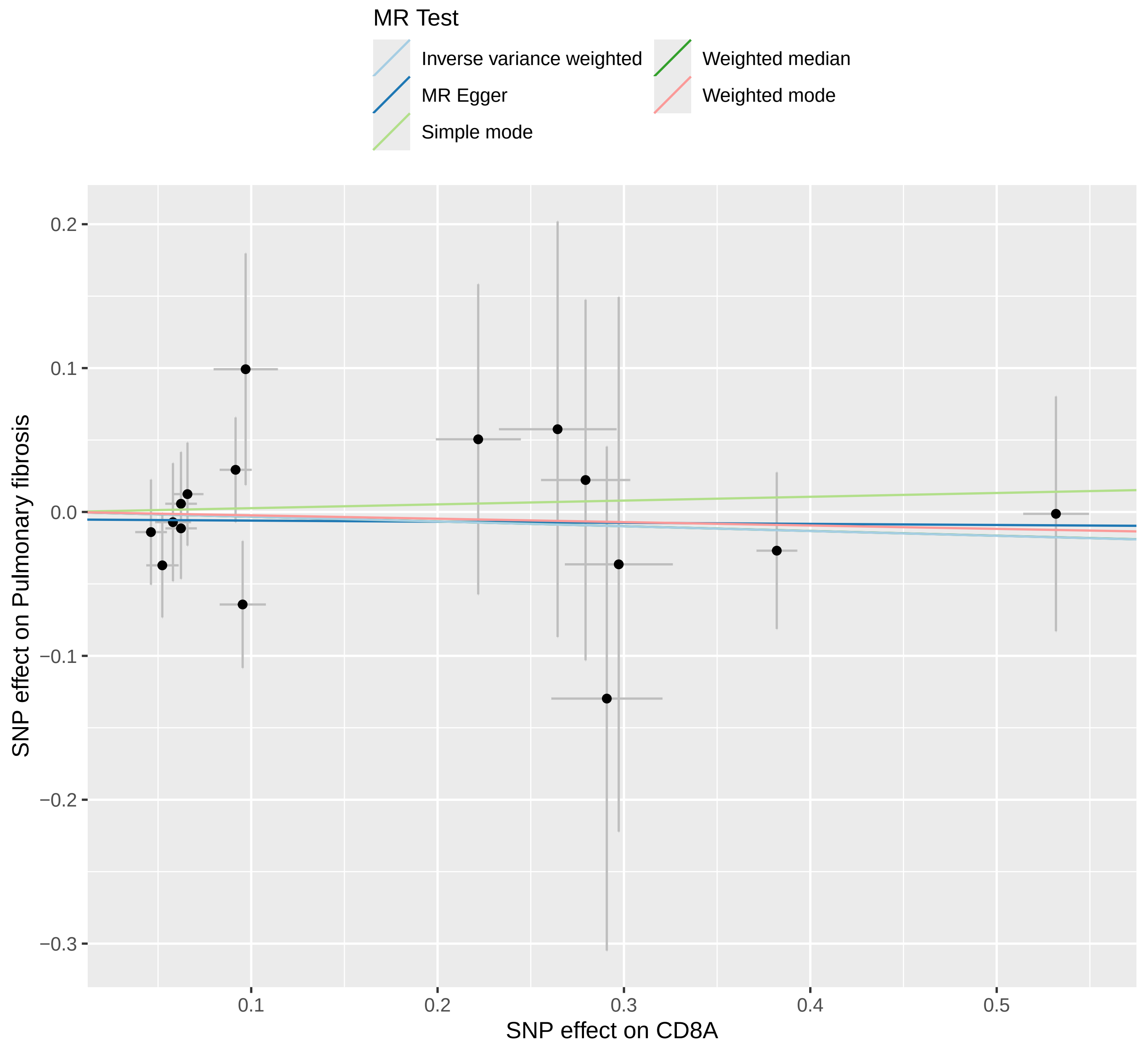
MR Test
Inverse variance weighted
Weighted median
MR Egger
Weighted mode
Simple mode
0.2
0.1
0.0
−0.1
−0.2
−0.3
0.1
0.2
0.3
0.4
0.5
SNP effect on Pulmonary fibrosis
SNP effect on CD8A

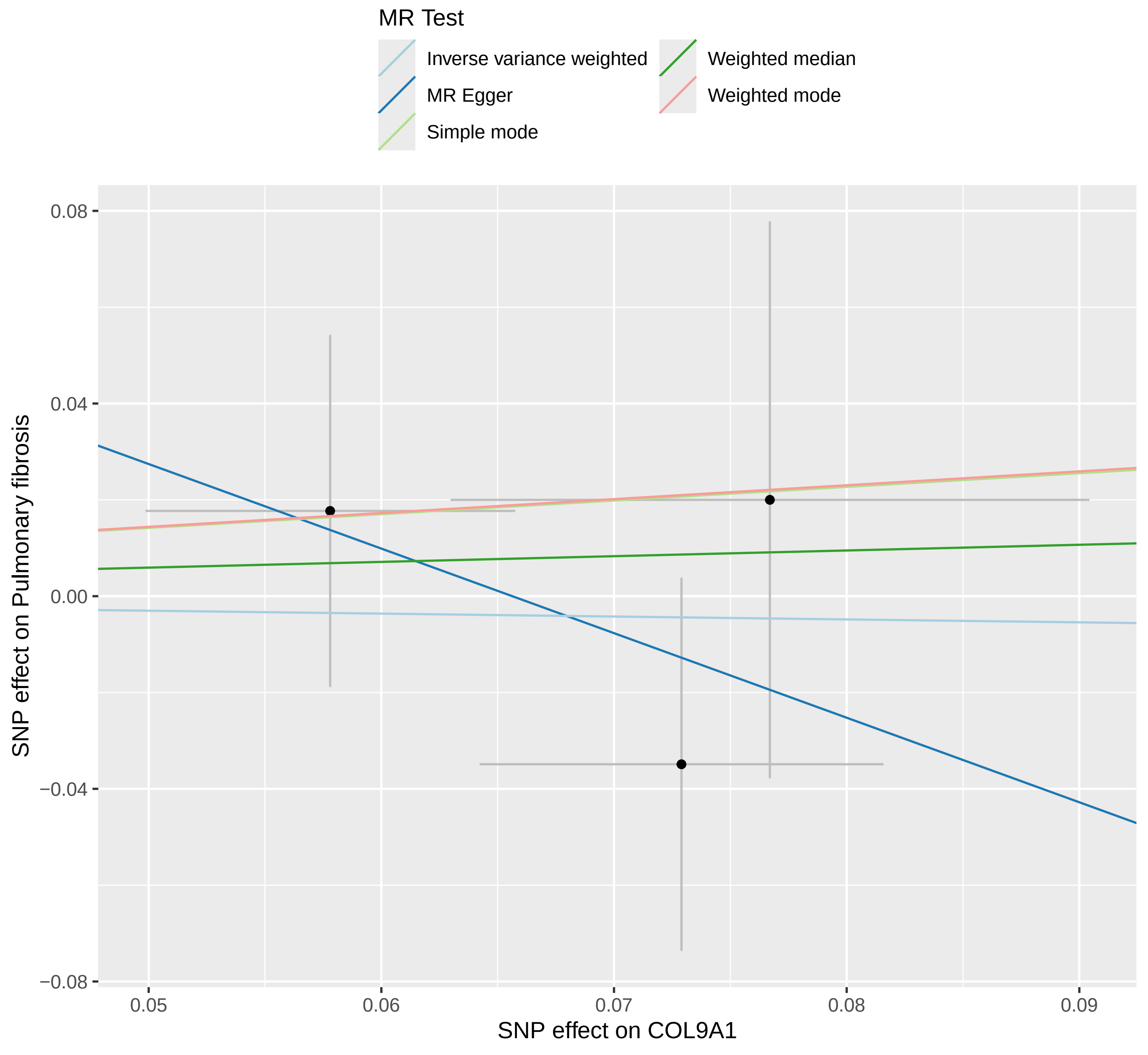
MR Test
Inverse variance weighted
MR Egger
Simple mode
Weighted median
Weighted mode
0.08
0.04
0.00
−0.04
−0.08
0.05
0.06
0.07
0.08
0.09
SNP effect on Pulmonary fibrosis
SNP effect on COL9A1

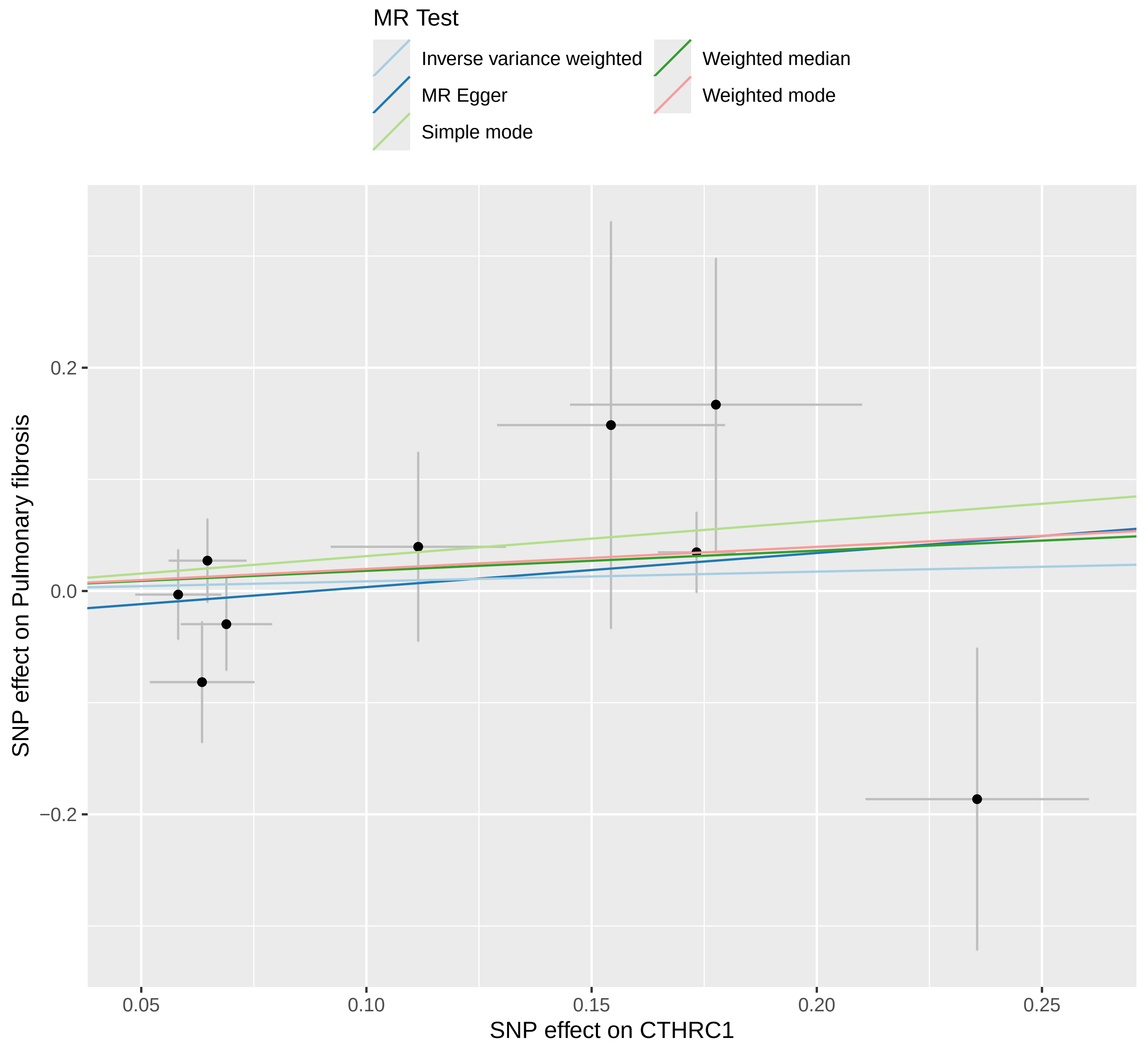

MR Test
Inverse variance weighted
MR Egger
Simple mode
Weighted median
Weighted mode
SNP effect on Pulmonary fibrosis
0.2
0.0
−0.2
0.05
0.10
0.15
0.20
0.25
SNP effect on CTHRC1

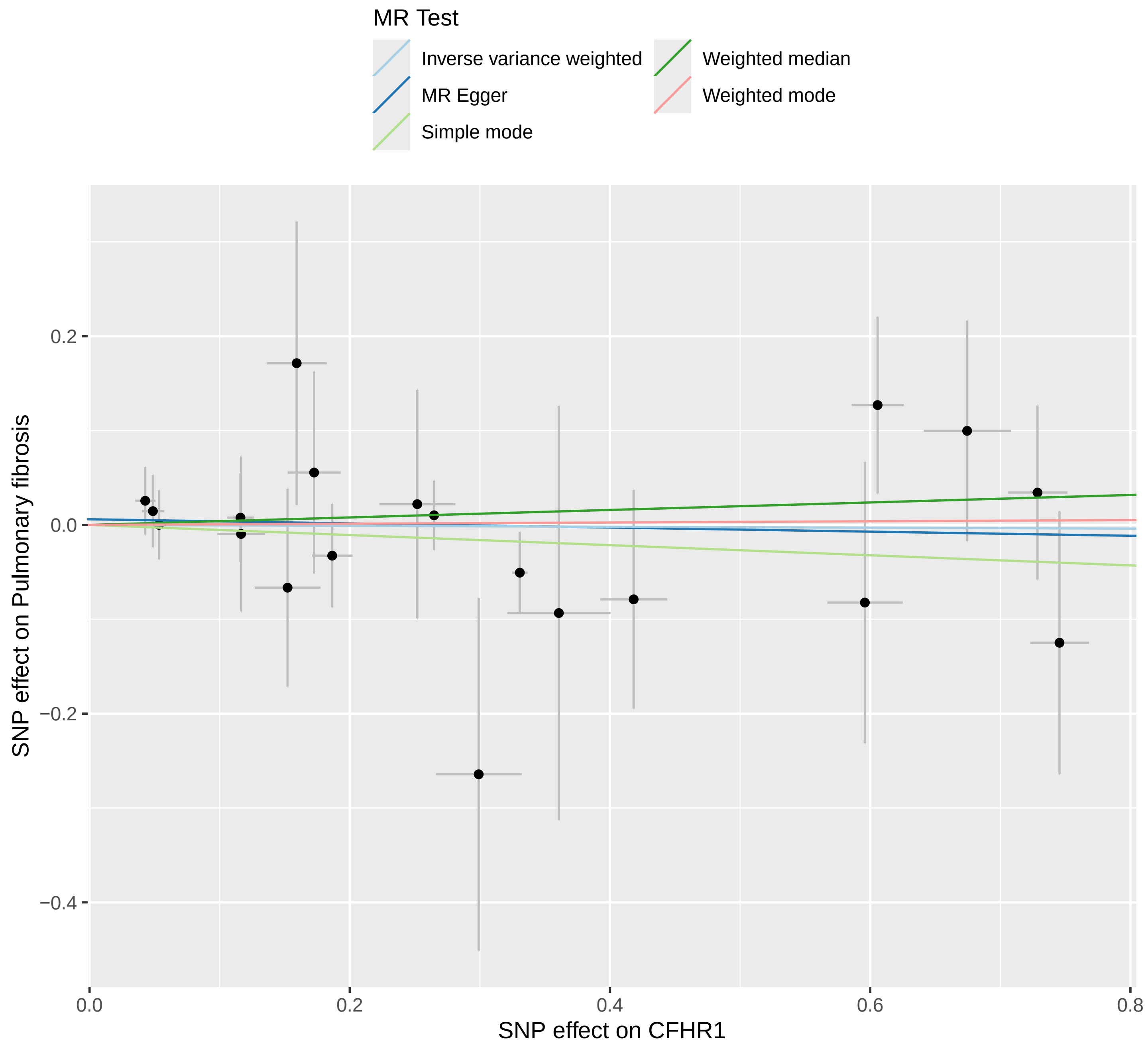
MR Test
Inverse variance weighted
MR Egger
Simple mode
Weighted median
Weighted mode
SNP effect on Pulmonary fibrosis
0.2
0.0
−0.2
−0.4
0.0
0.2
0.4
0.6
0.8
SNP effect on CFHR1

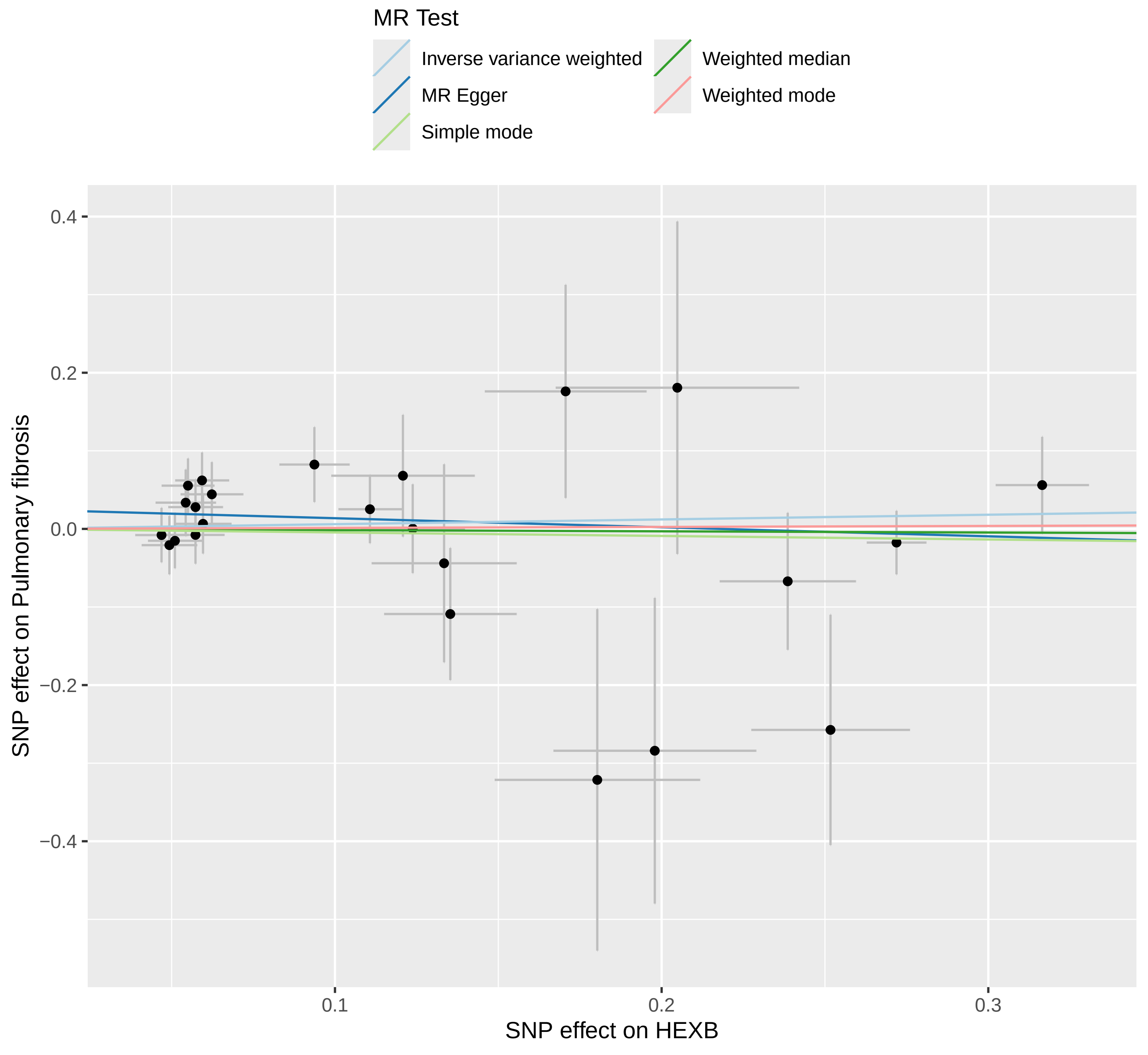

MR Test
Inverse variance weighted
MR Egger
Simple mode
Weighted median
Weighted mode
0.4
0.2
0.0
−0.2
−0.4
0.1
0.2
0.3
SNP effect on Pulmonary fibrosis
SNP effect on HEXB

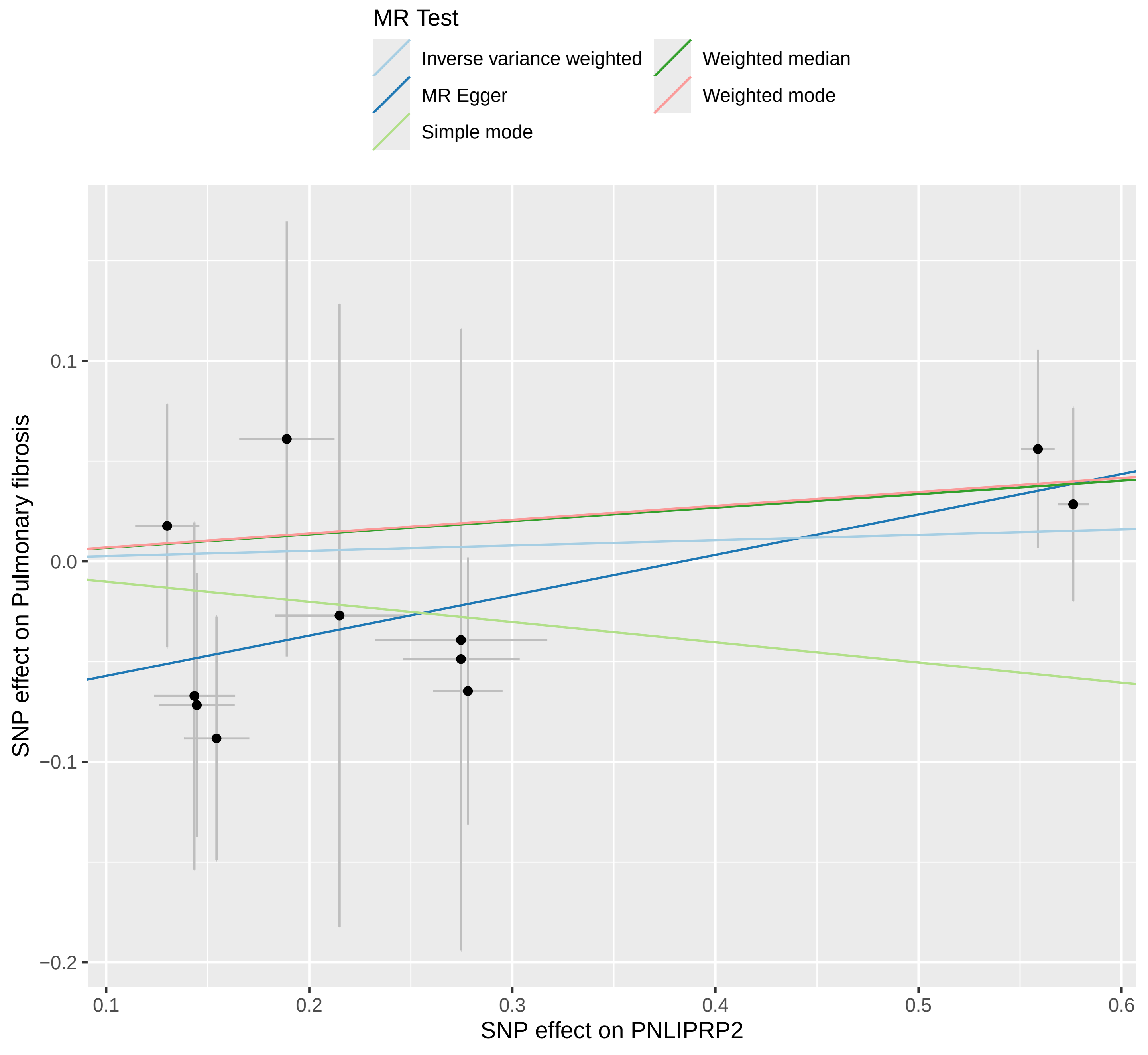
MR Test
Inverse variance weighted
MR Egger
Simple mode
Weighted median
Weighted mode
SNP effect on Pulmonary fibrosis
0.1
0.0
−0.1
−0.2
0.1
0.2
0.3
0.4
0.5
0.6
SNP effect on PNLIPRP2

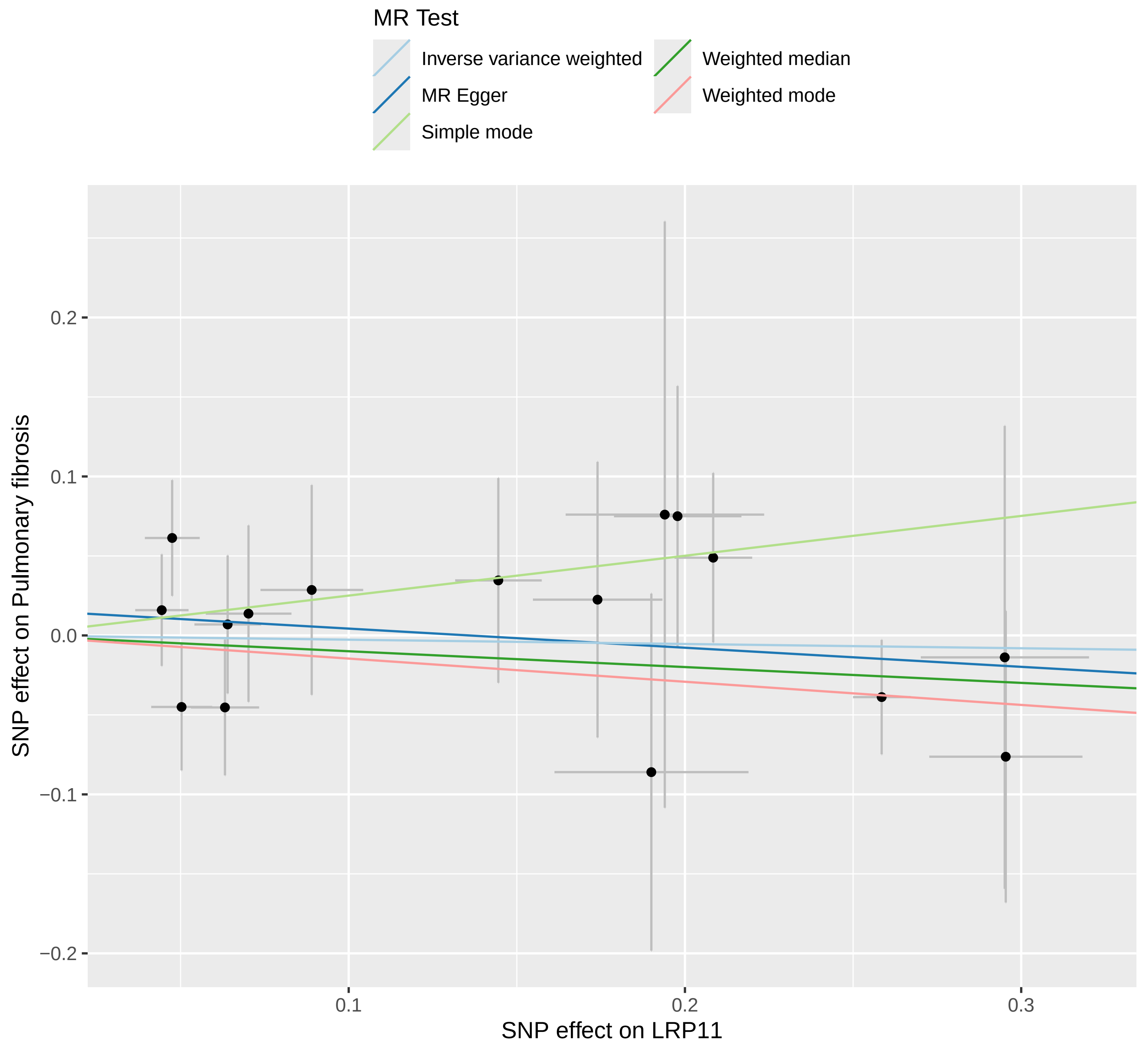
MR Test
Inverse variance weighted
MR Egger
Simple mode
Weighted median
Weighted mode
SNP effect on Pulmonary fibrosis
0.2
0.1
0.0
−0.1
−0.2
0.1
0.2
0.3
SNP effect on LRP11

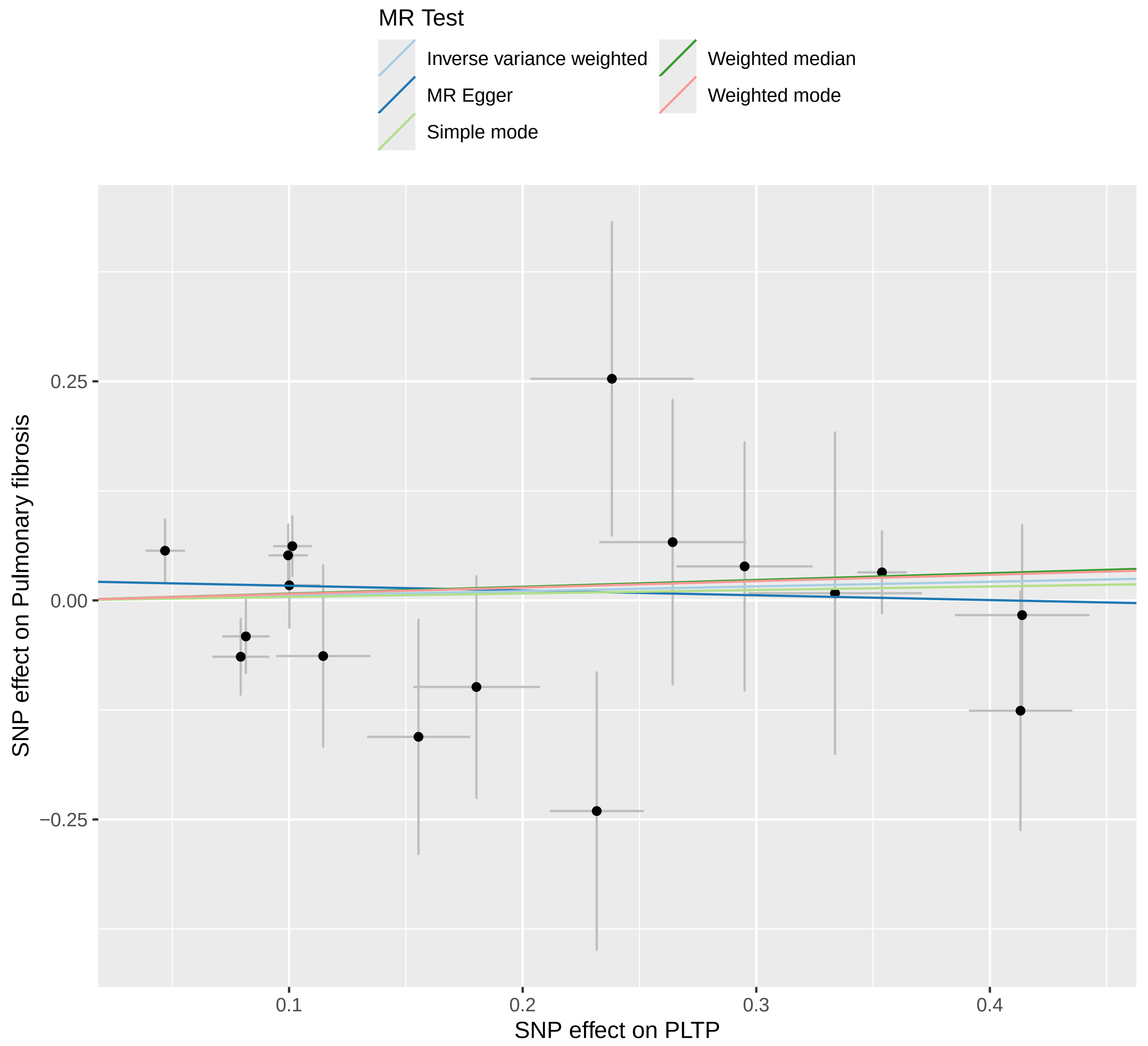
MR Test
Inverse variance weighted
MR Egger
Simple mode
Weighted median
Weighted mode
0.25
0.00
−0.25
SNP effect on Pulmonary fibrosis
0.1
0.2
0.3
0.4
SNP effect on PLTP

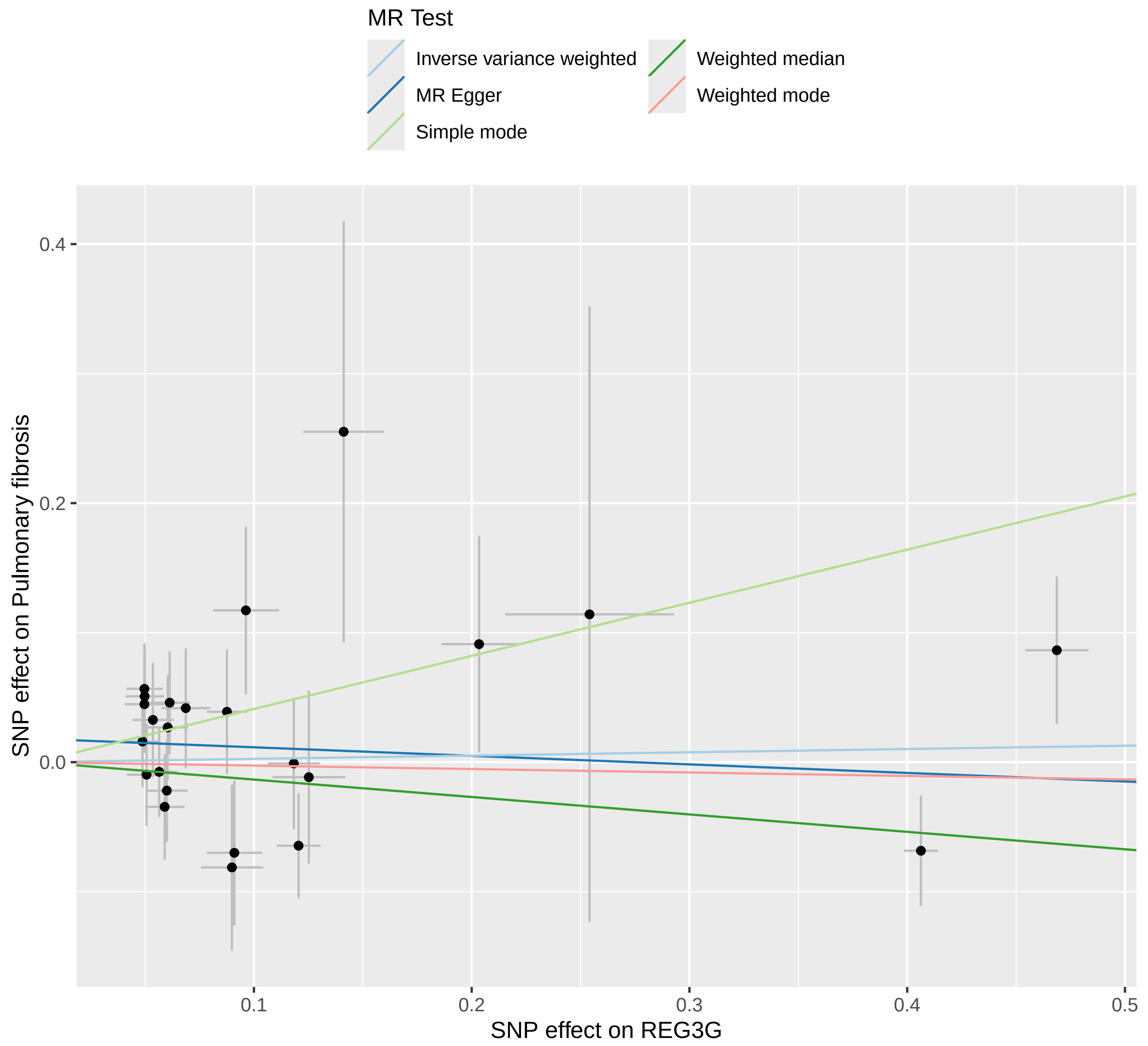
MR Test
Inverse variance weighted
MR Egger
Simple mode
Weighted median
Weighted mode
SNP effect on Pulmonary fibrosis
0.4
0.2
0.0
0.1
0.2
0.3
0.4
0.5
SNP effect on REG3G

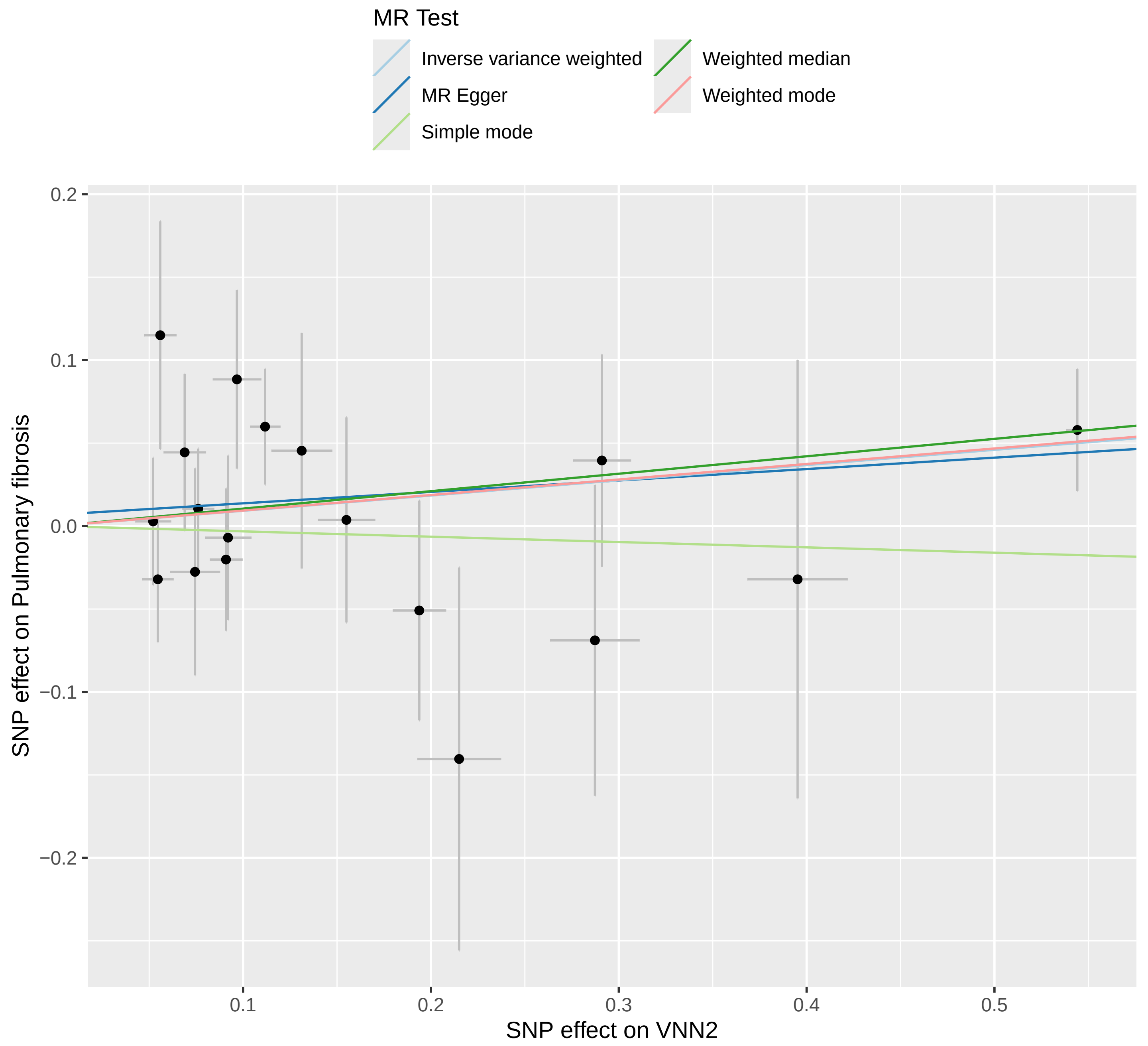

MR Test
Inverse variance weighted
MR Egger
Simple mode
Weighted median
Weighted mode
0.2
0.1
0.0
−0.1
−0.2
SNP effect on Pulmonary fibrosis
0.1
0.2
0.3
0.4
0.5
SNP effect on VNN2

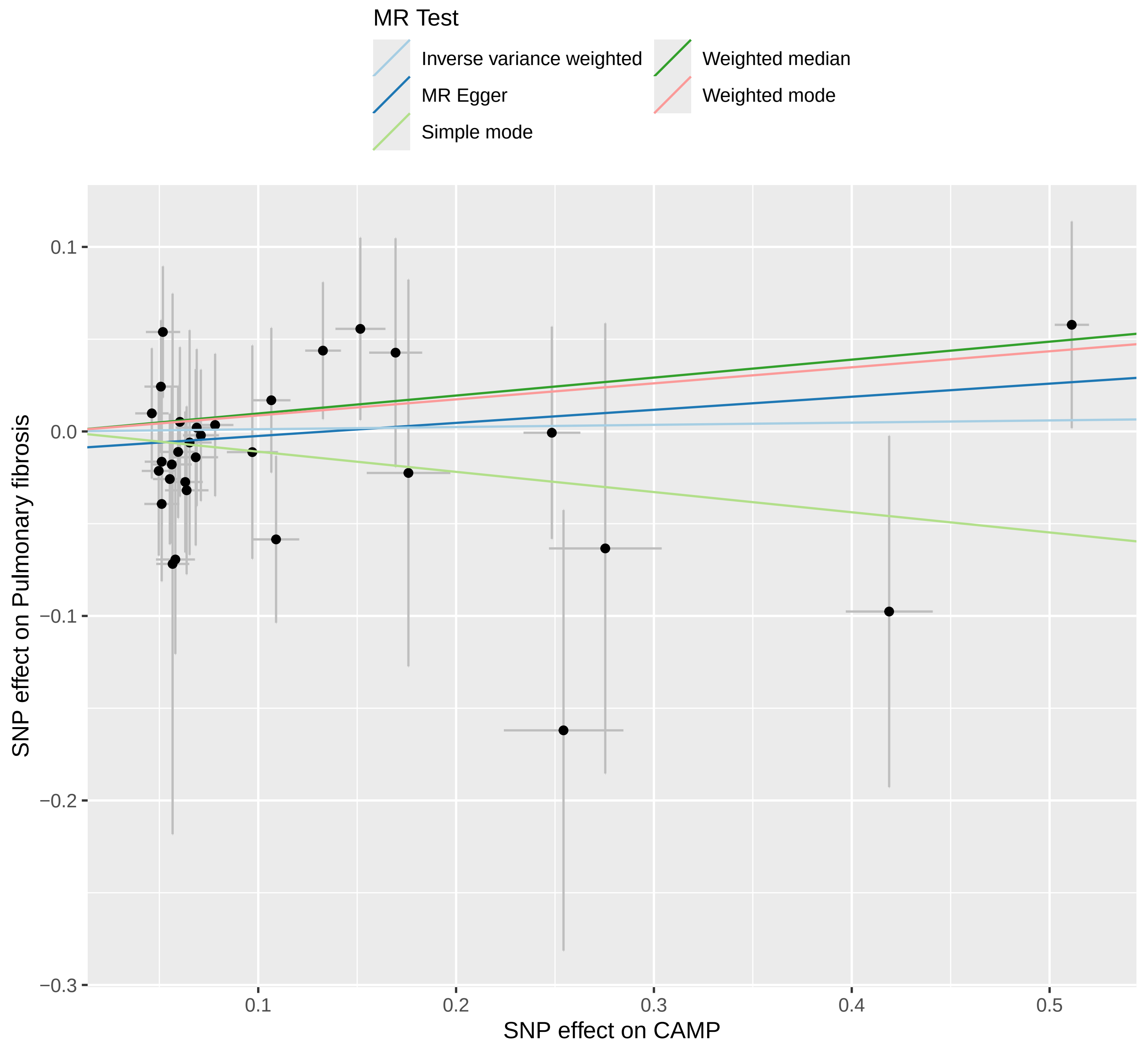
MR Test
Inverse variance weighted
MR Egger
Simple mode
Weighted median
Weighted mode
0.1
0.0
−0.1
−0.2
−0.3
SNP effect on Pulmonary fibrosis
0.1
0.2
0.3
0.4
0.5
SNP effect on CAMP

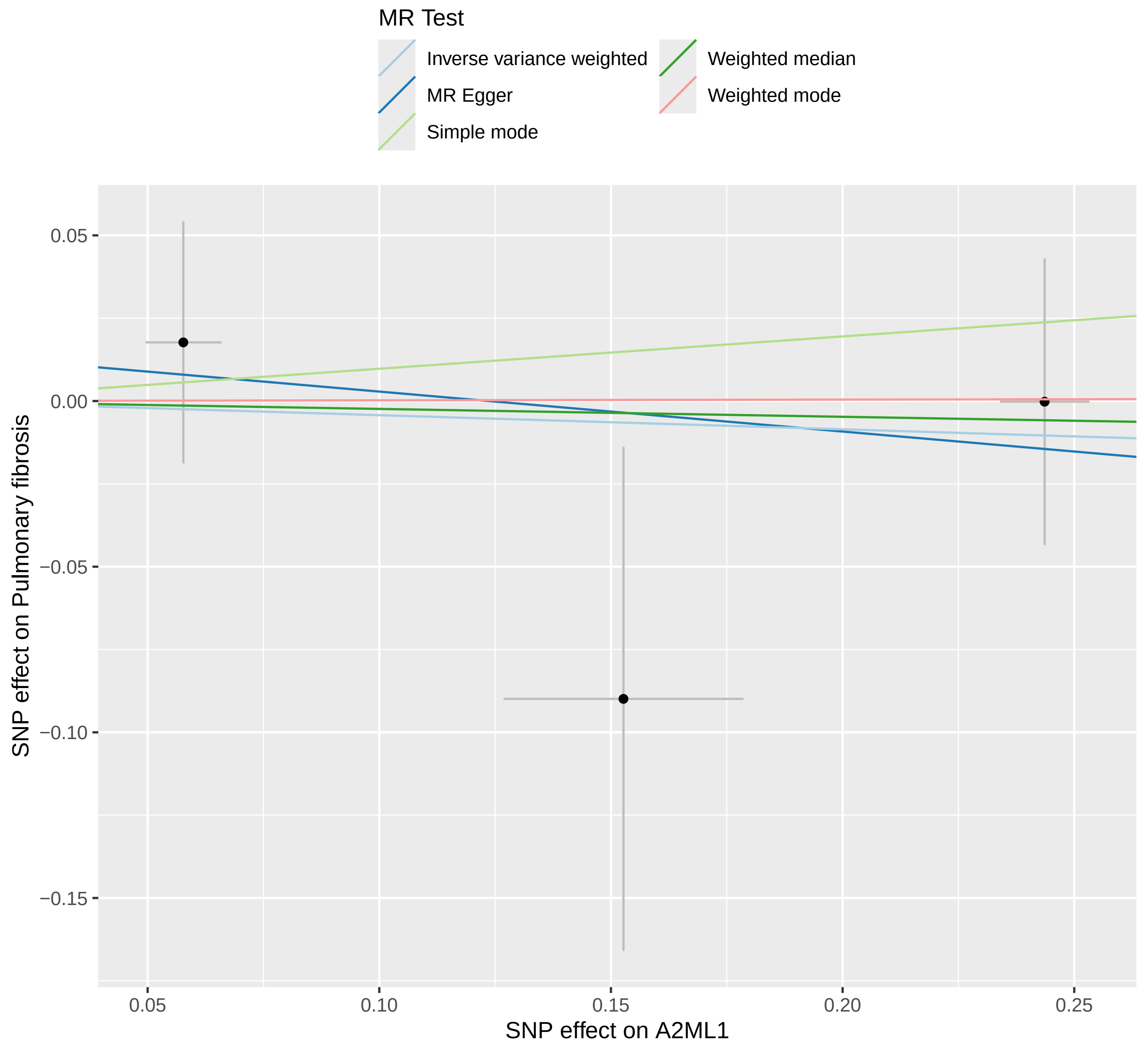

MR Test
Inverse variance weighted
MR Egger
Simple mode
Weighted median
Weighted mode
0.05
0.00
−0.05
−0.10
−0.15
SNP effect on Pulmonary fibrosis
0.05
0.10
0.15
0.20
0.25
SNP effect on A2ML1

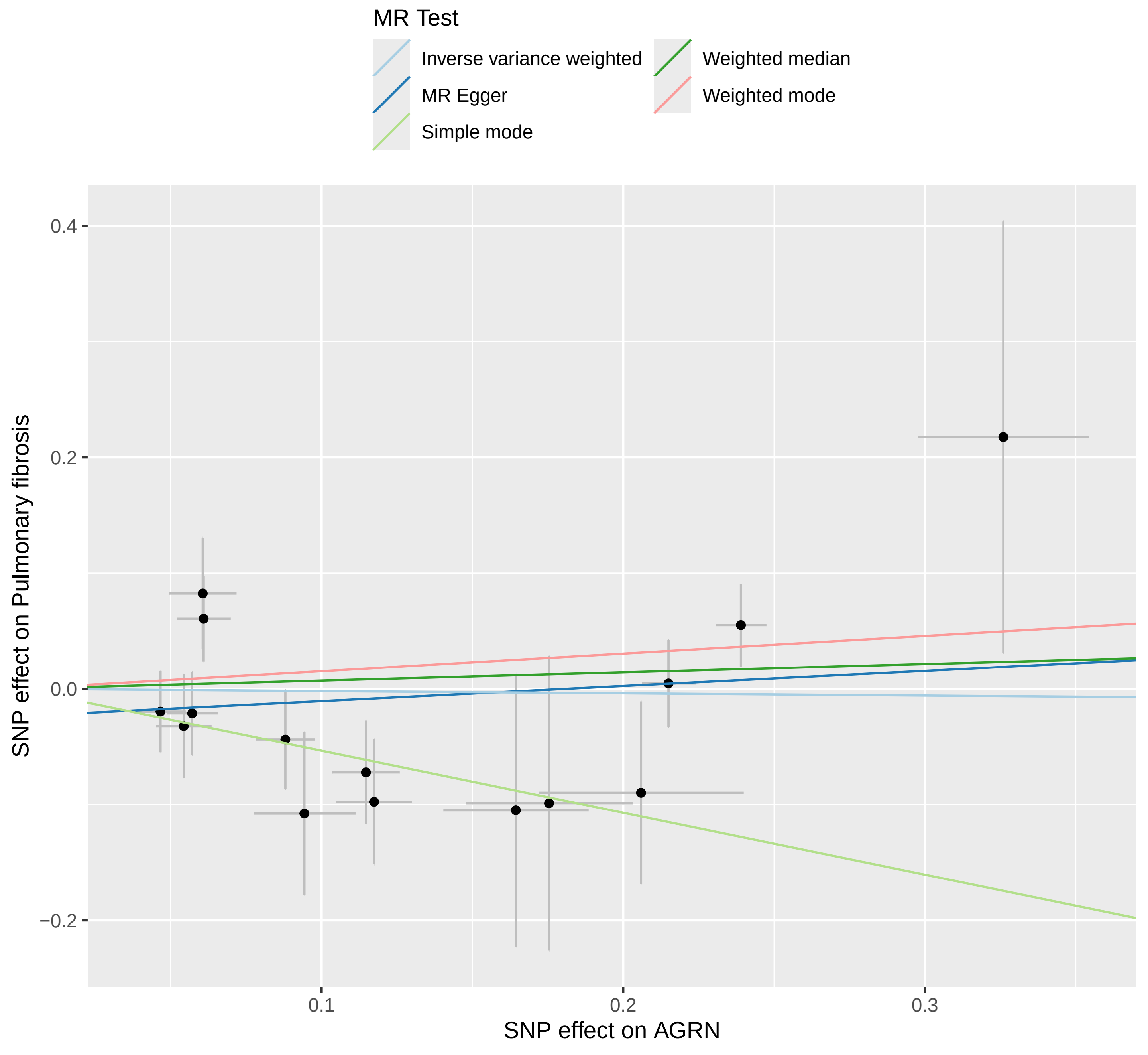

MR Test
Inverse variance weighted
MR Egger
Simple mode
Weighted median
Weighted mode
SNP effect on Pulmonary fibrosis
0.4
0.2
0.0
−0.2
0.1
0.2
0.3
SNP effect on AGRN

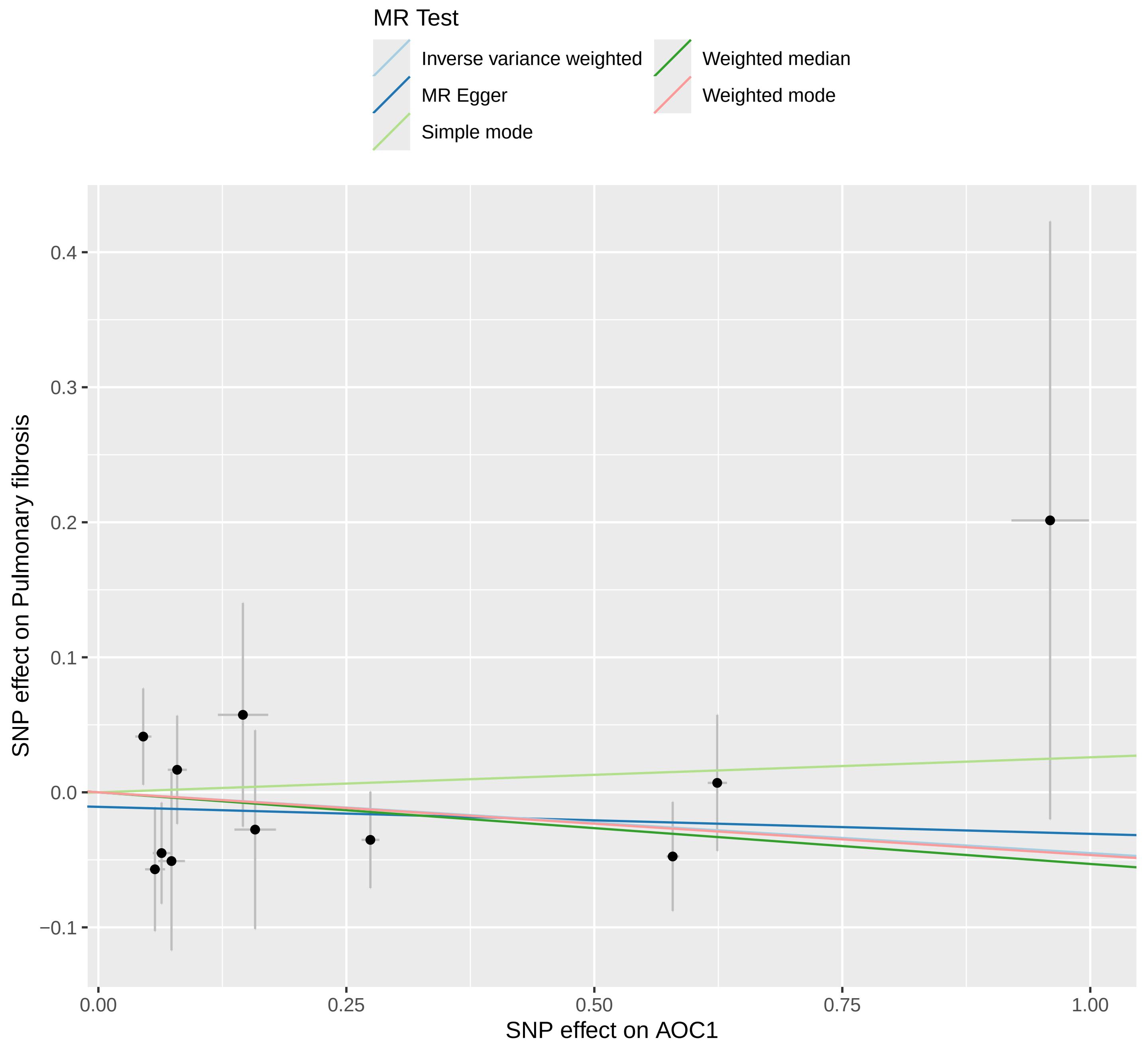
MR Test
Inverse variance weighted
MR Egger
Simple mode
Weighted median
Weighted mode
0.4
0.3
0.2
0.1
0.0
−0.1
SNP effect on Pulmonary fibrosis
0.00
0.25
0.50
0.75
1.00
SNP effect on AOC1

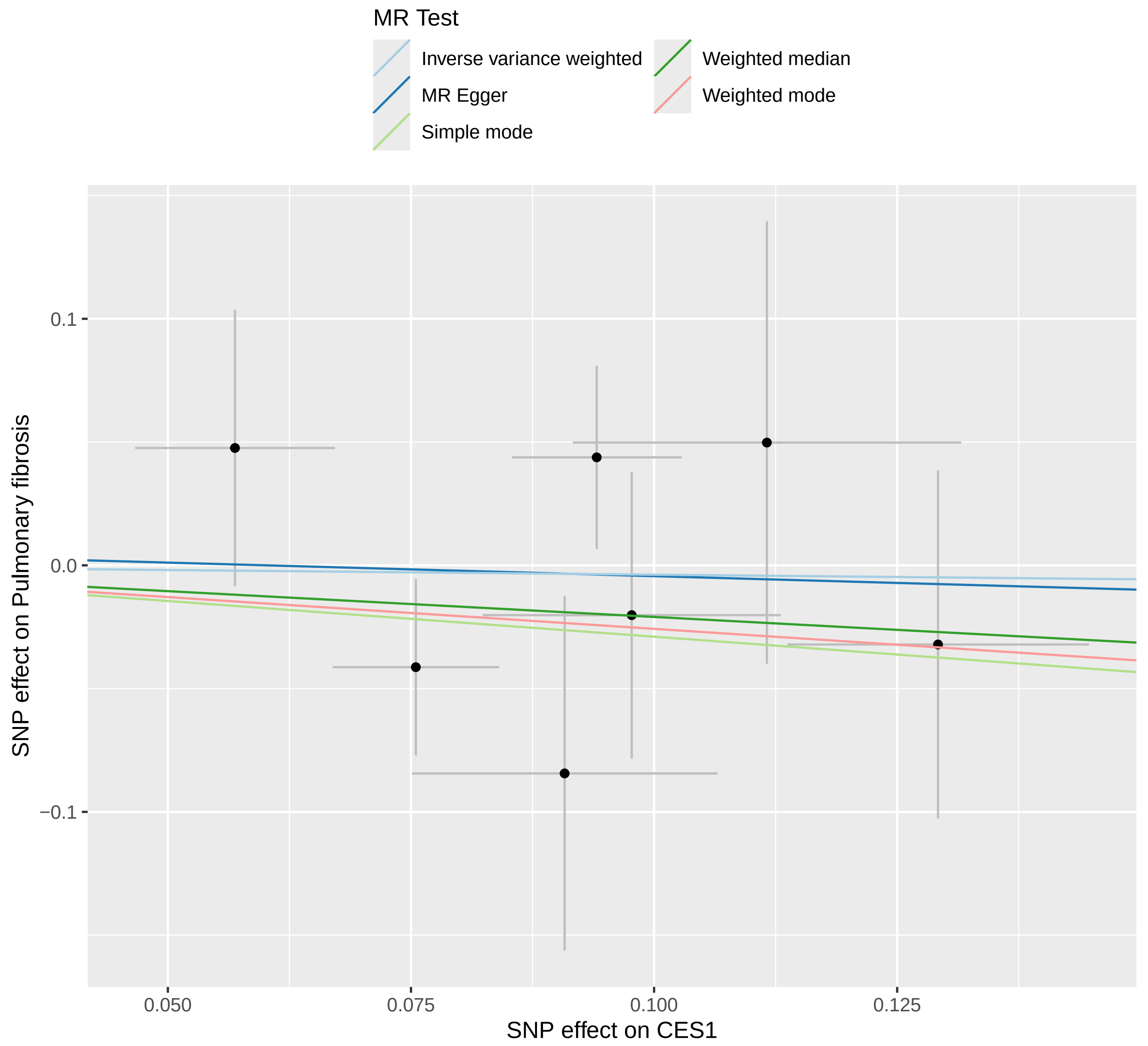

MR Test
Inverse variance weighted
MR Egger
Simple mode
Weighted median
Weighted mode
0.1
0.0
−0.1
SNP effect on Pulmonary fibrosis
0.050
0.075
0.100
0.125
SNP effect on CES1

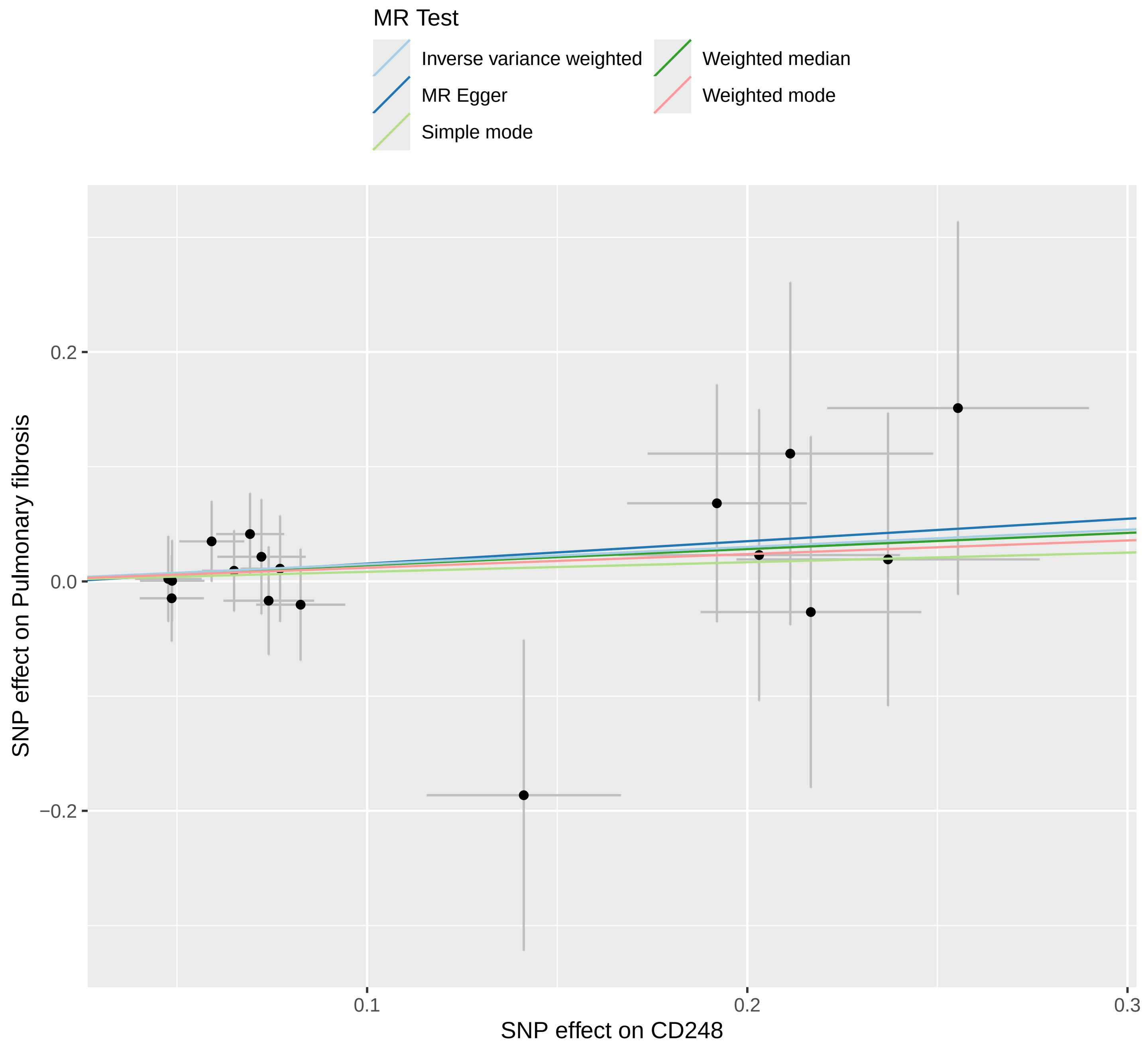
MR Test
Inverse variance weighted
MR Egger
Simple mode
Weighted median
Weighted mode
0.2
0.0
−0.2
SNP effect on Pulmonary fibrosis
0.1
0.2
0.3
SNP effect on CD248

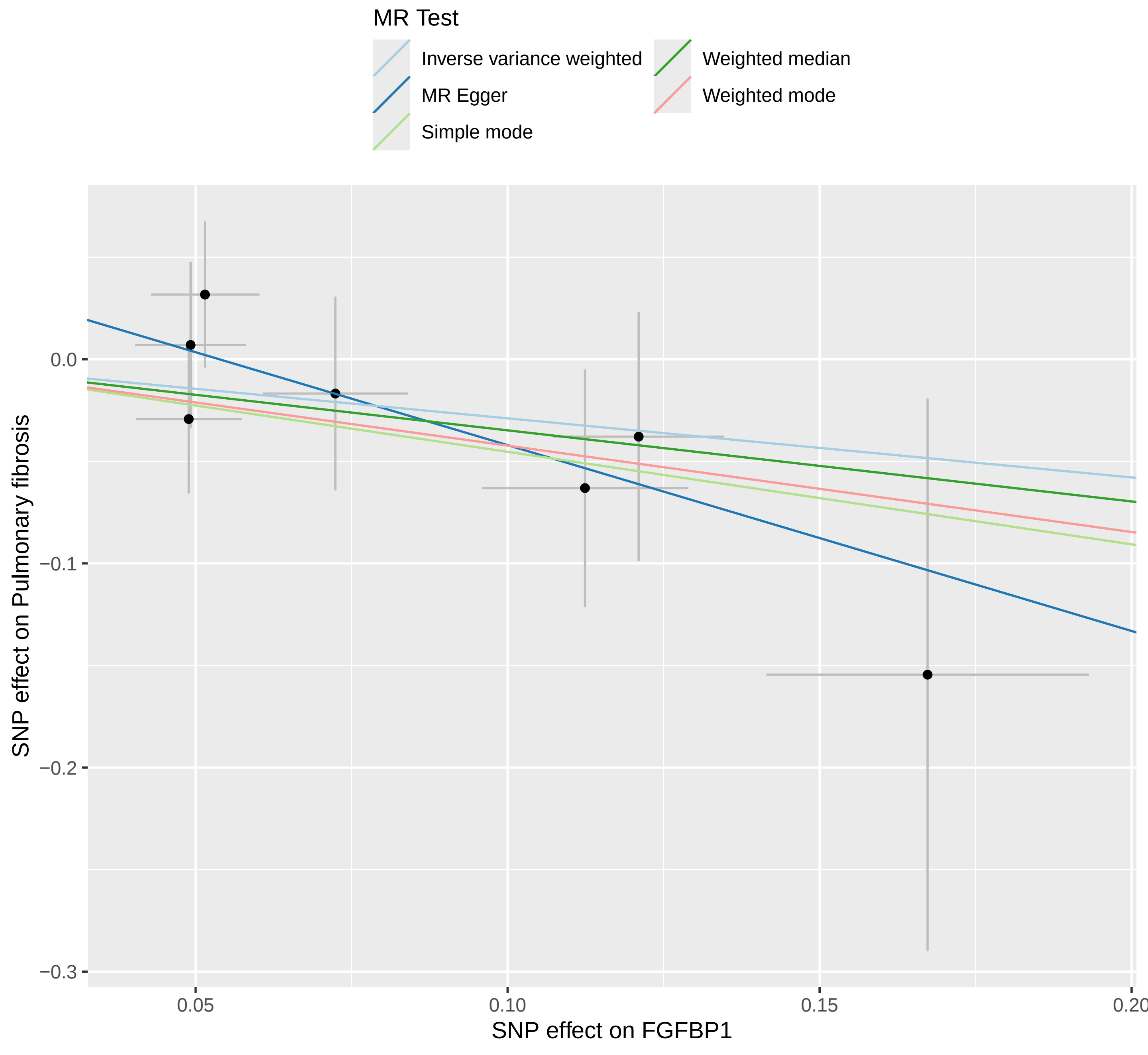
MR Test
Inverse variance weighted
MR Egger
Simple mode
Weighted median
Weighted mode
0.0
−0.1
−0.2
−0.3
0.05
0.10
0.15
0.20
SNP effect on Pulmonary fibrosis
SNP effect on FGFBP1

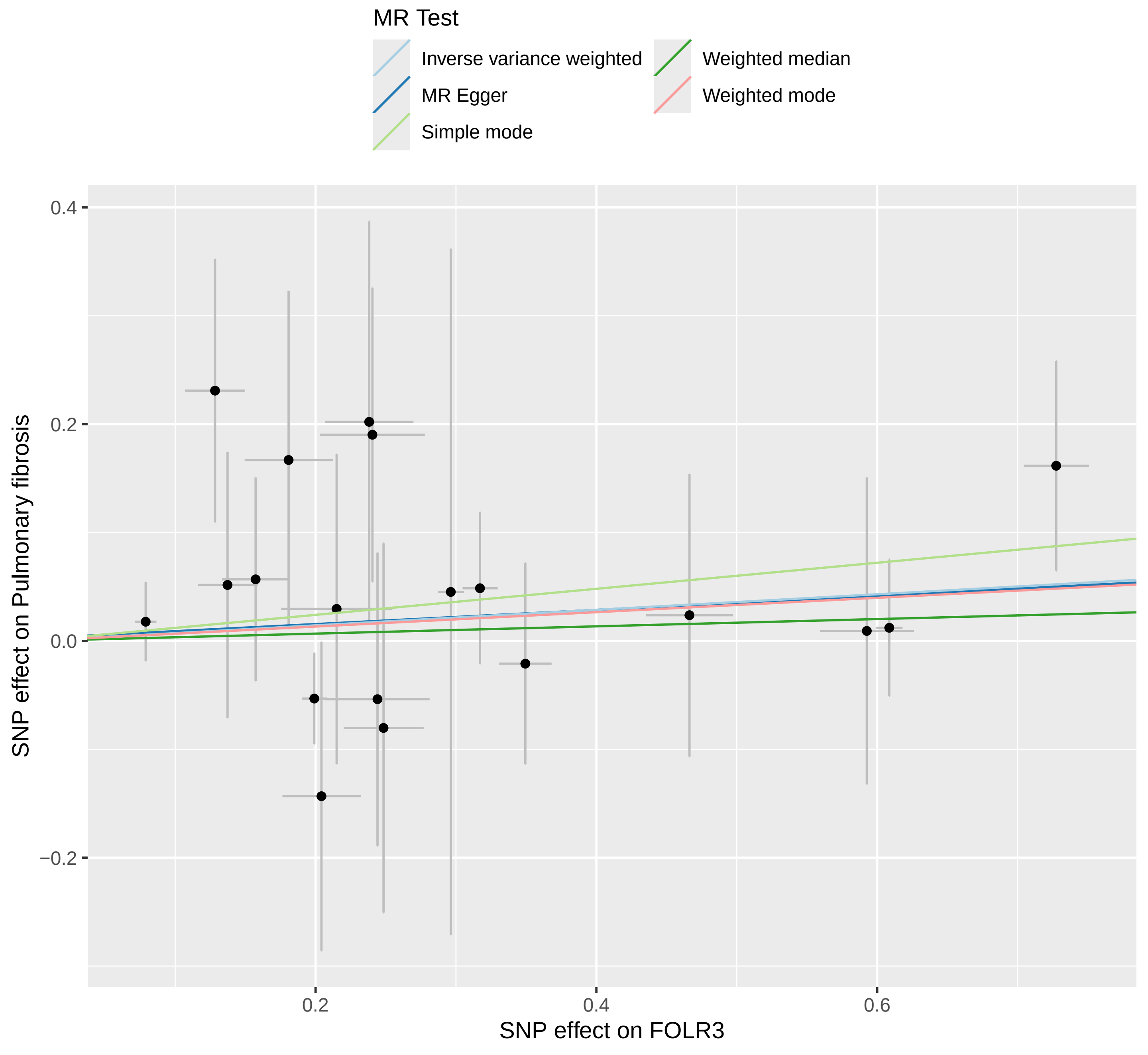
MR Test
Inverse variance weighted
MR Egger
Simple mode
Weighted median
Weighted mode
0.4
0.2
0.0
−0.2
0.2
0.4
0.6
SNP effect on Pulmonary fibrosis
SNP effect on FOLR3

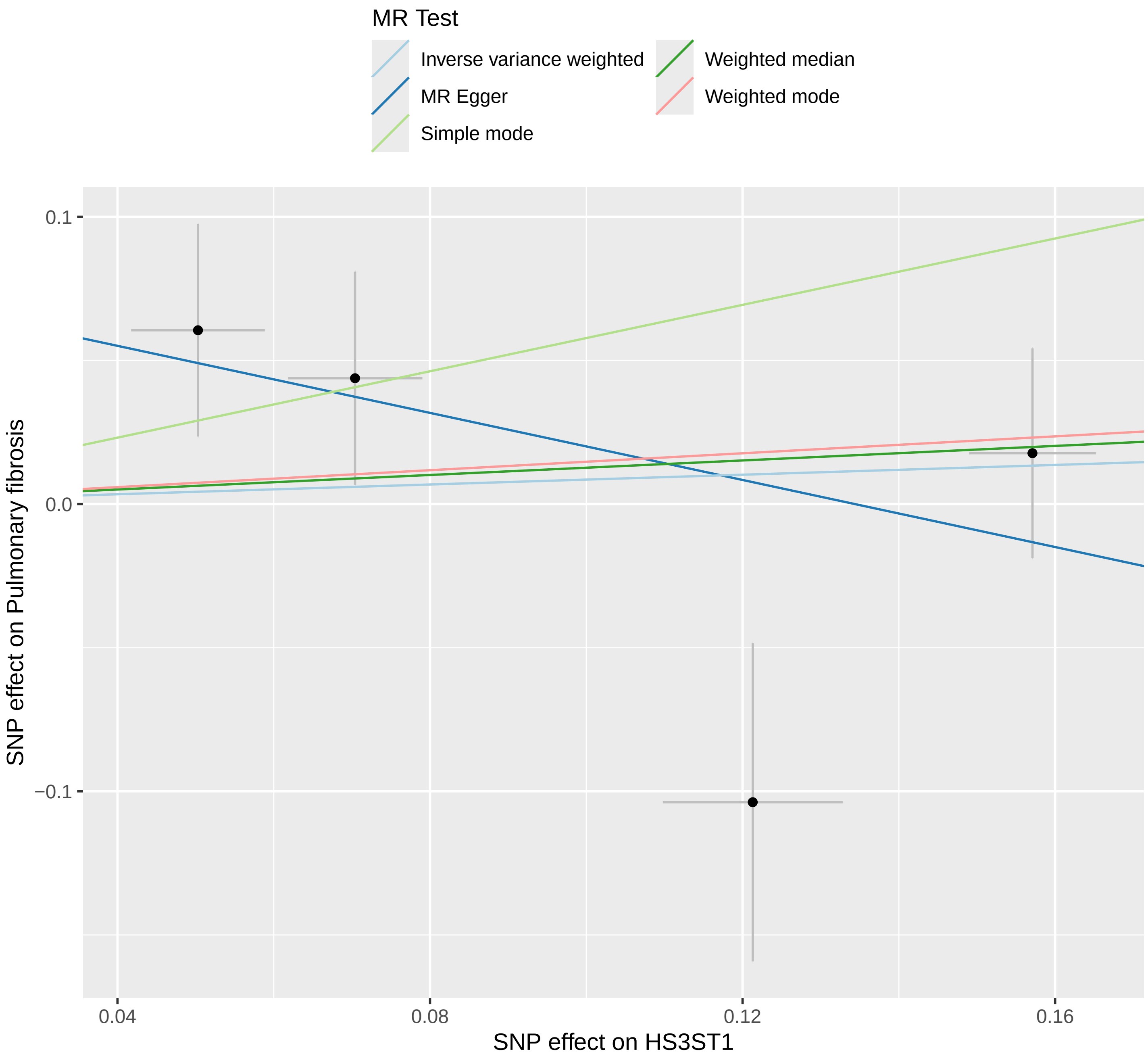
MR Test
Inverse variance weighted
MR Egger
Simple mode
Weighted median
Weighted mode
0.1
0.0
−0.1
SNP effect on Pulmonary fibrosis
0.04
0.08
0.12
0.16
SNP effect on HS3ST1

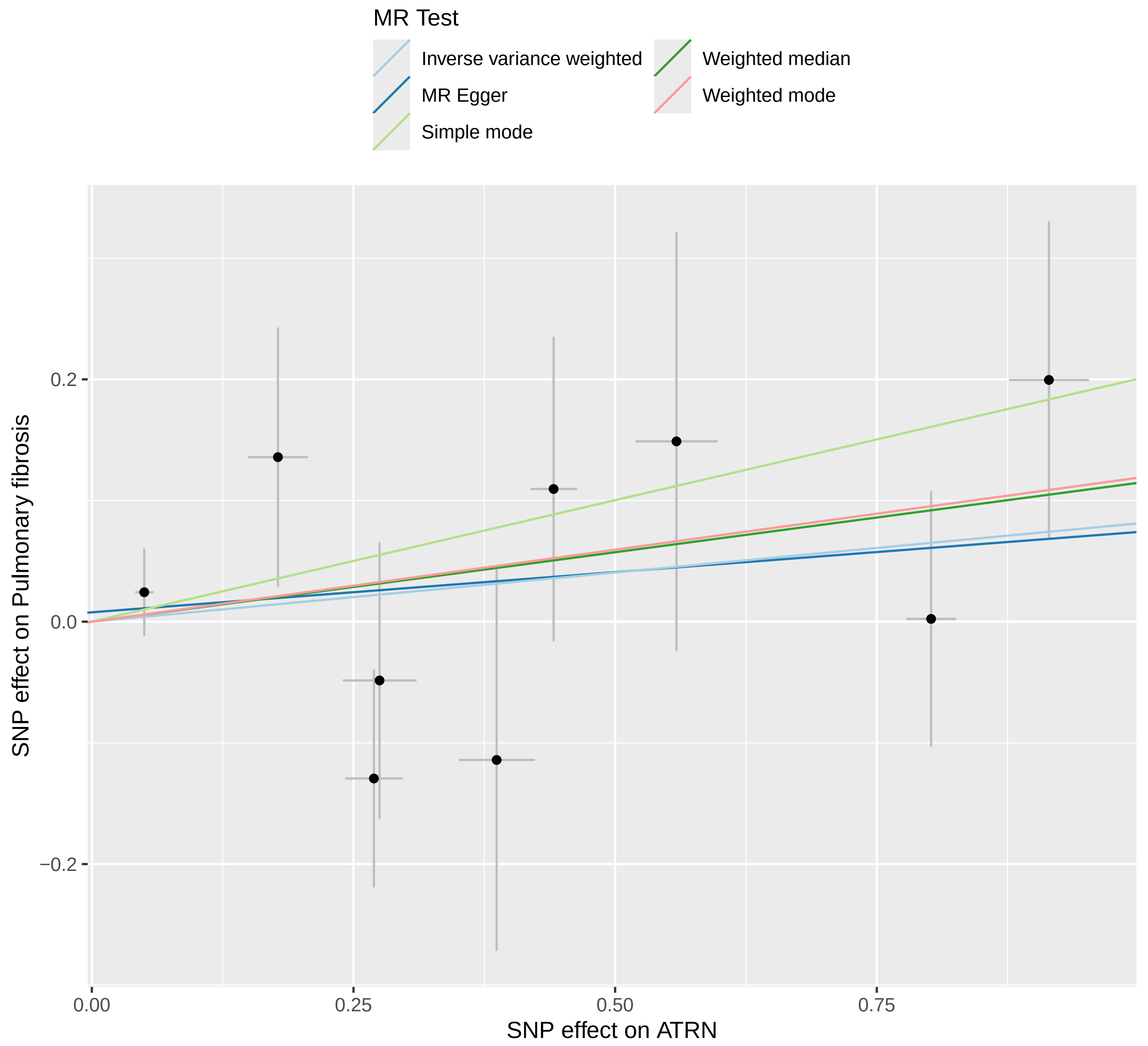

MR Test
Inverse variance weighted
MR Egger
Simple mode
Weighted median
Weighted mode
0.2
0.0
−0.2
0.00
0.25
0.50
0.75
SNP effect on ATRN
SNP effect on Pulmonary fibrosis

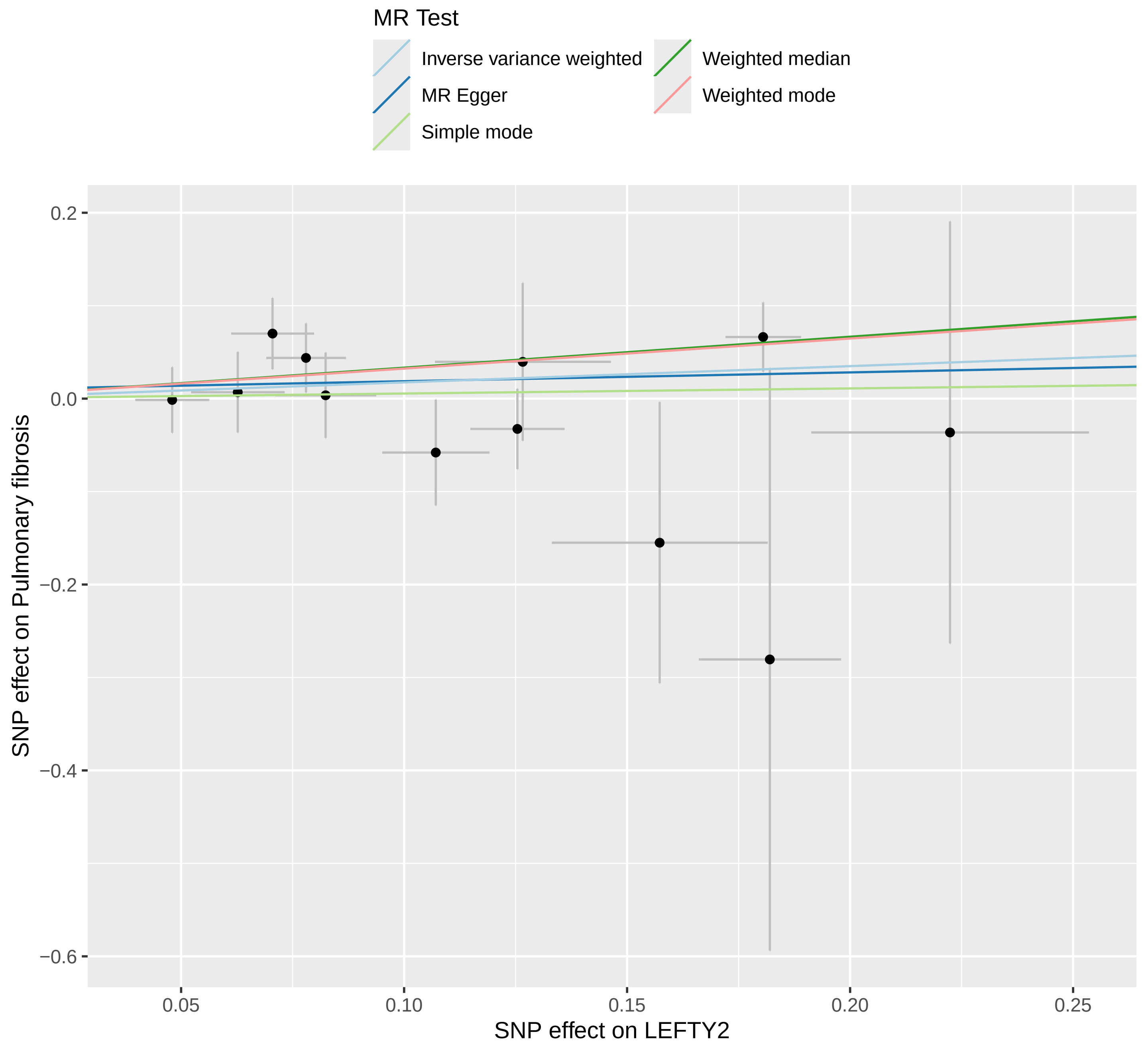
MR Test
Inverse variance weighted
MR Egger
Simple mode
Weighted median
Weighted mode
0.2
0.0
−0.2
−0.4
−0.6
SNP effect on Pulmonary fibrosis
0.05
0.10
0.15
0.20
0.25
SNP effect on LEFTY2

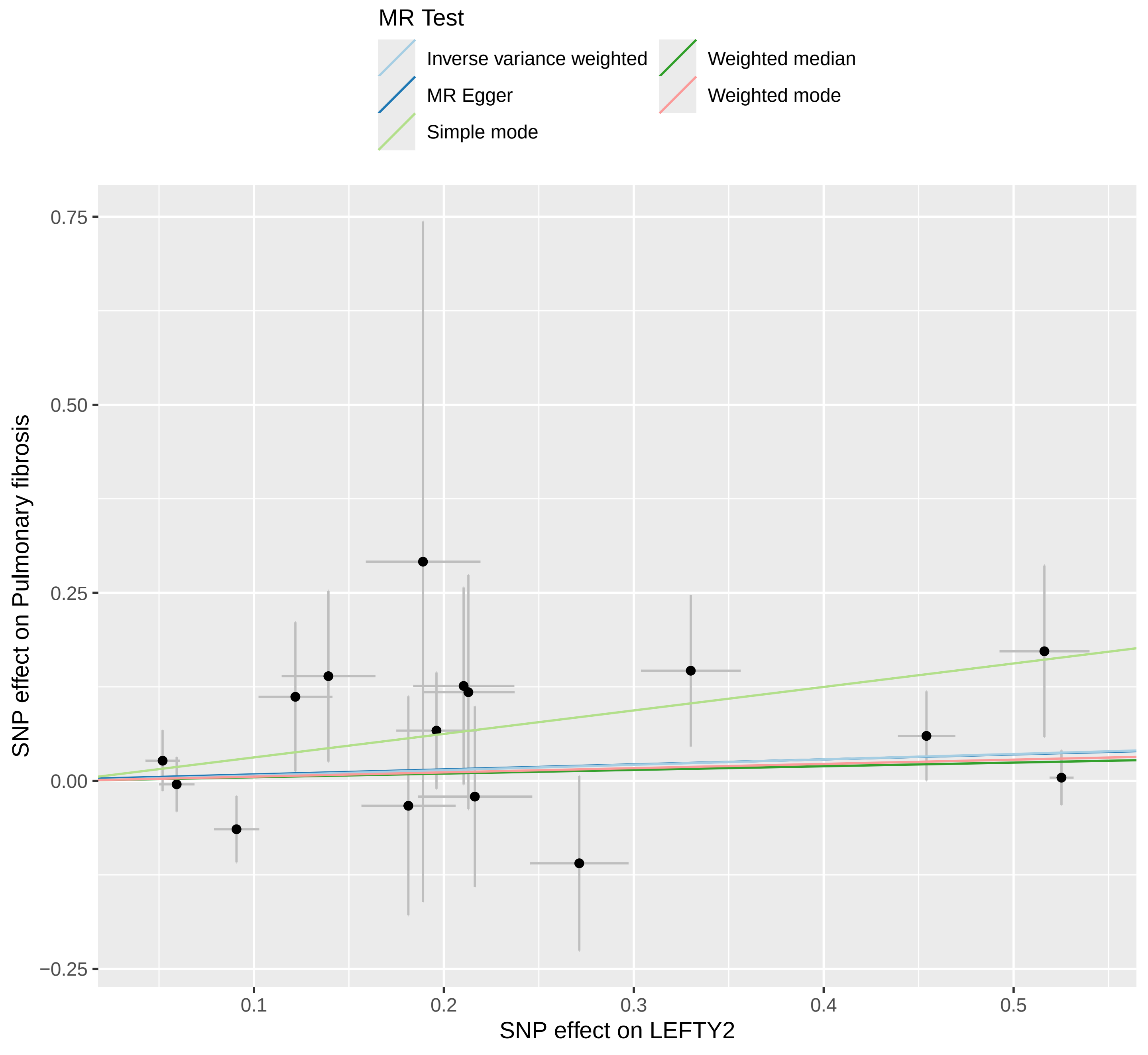

MR Test
Inverse variance weighted
MR Egger
Simple mode
Weighted median
Weighted mode
0.75
0.50
0.25
0.00
−0.25
0.1
0.2
0.3
0.4
0.5
SNP effect on LEFTY2
SNP effect on Pulmonary fibrosis

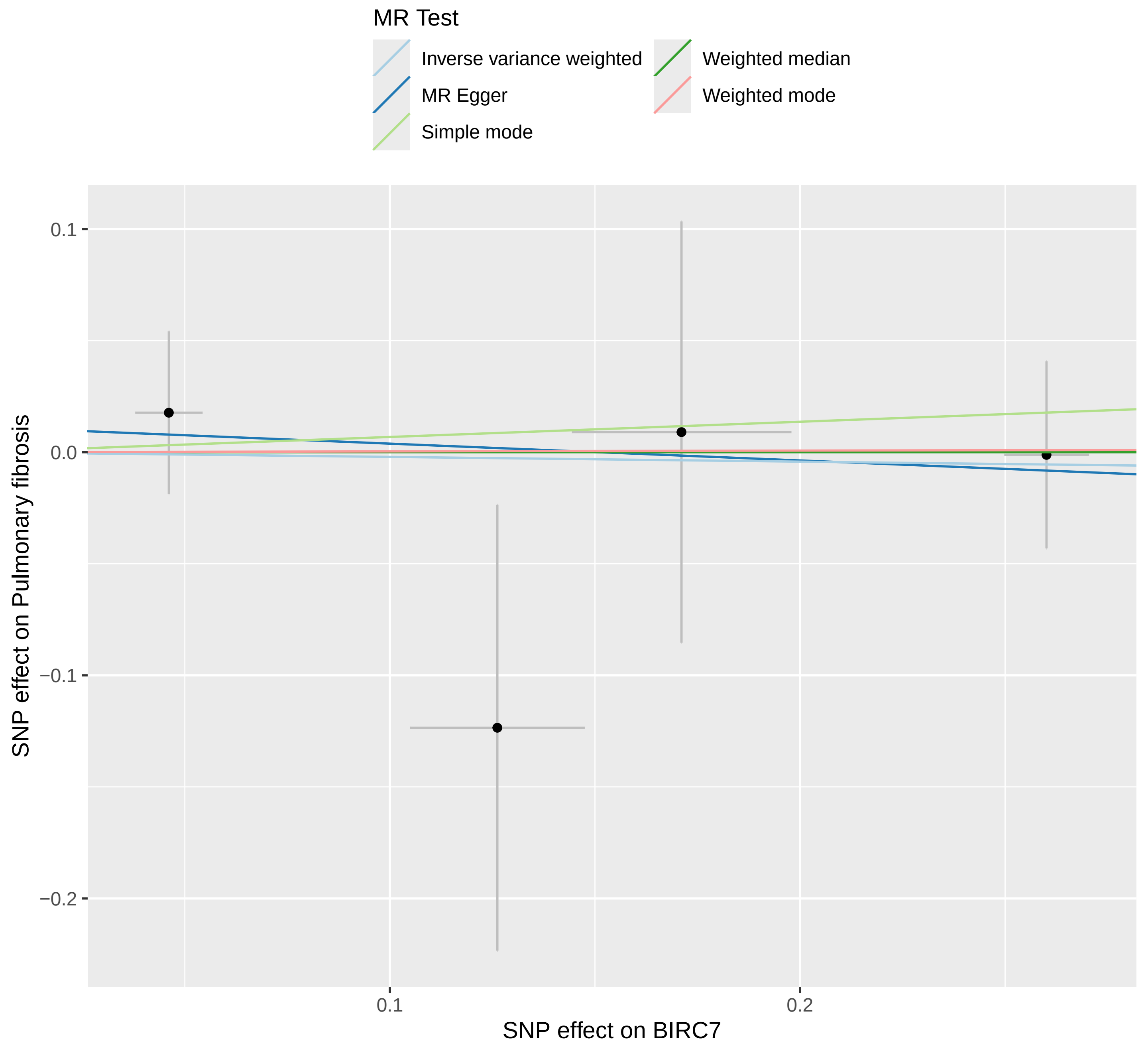
MR Test
Inverse variance weighted
MR Egger
Simple mode
Weighted median
Weighted mode
0.1
0.0
−0.1
−0.2
SNP effect on Pulmonary fibrosis
0.1
0.2
SNP effect on BIRC7

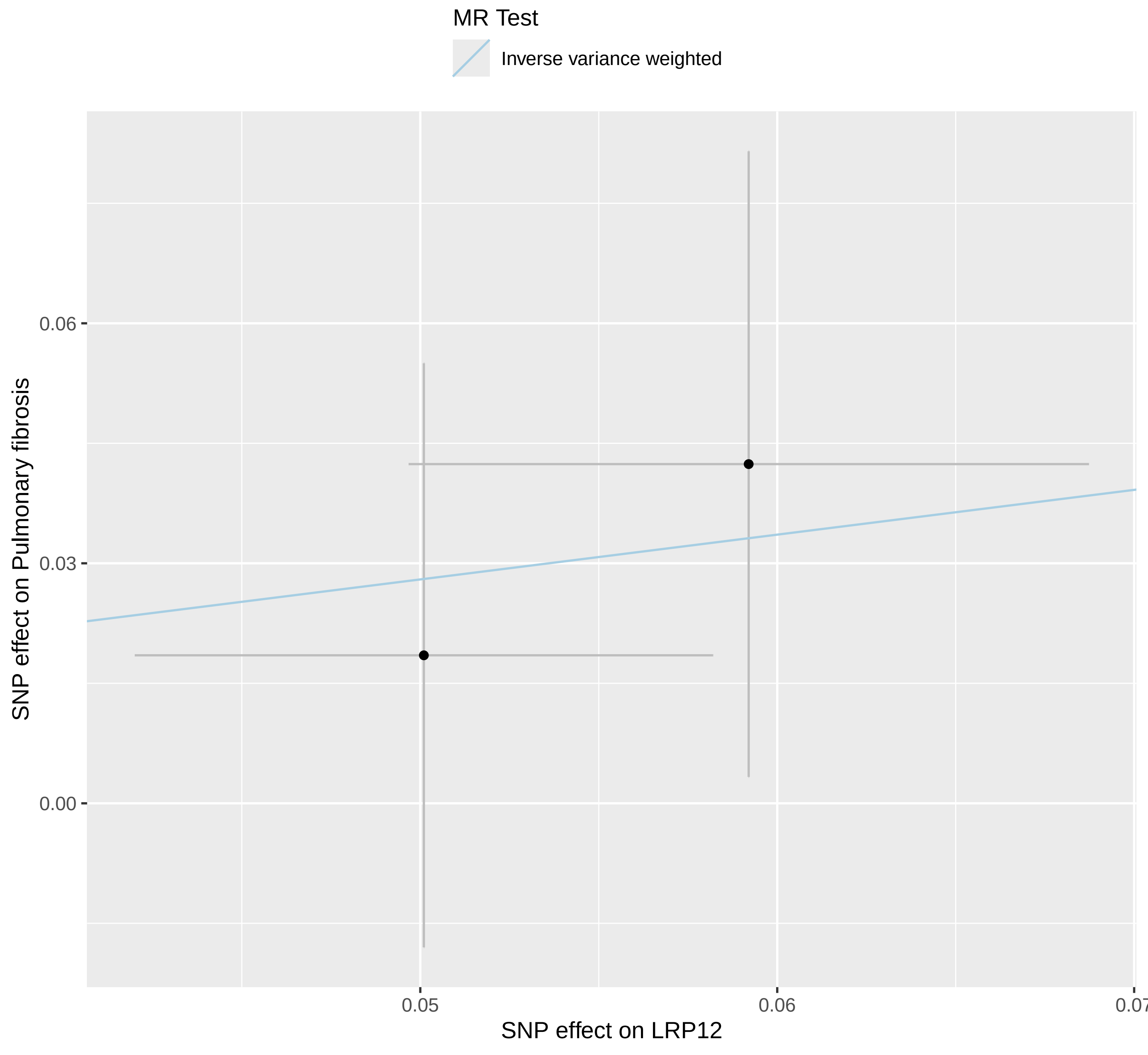
MR Test
Inverse variance weighted
SNP effect on Pulmonary fibrosis
0.06
0.03
0.00
0.05
0.06
0.07
SNP effect on LRP12

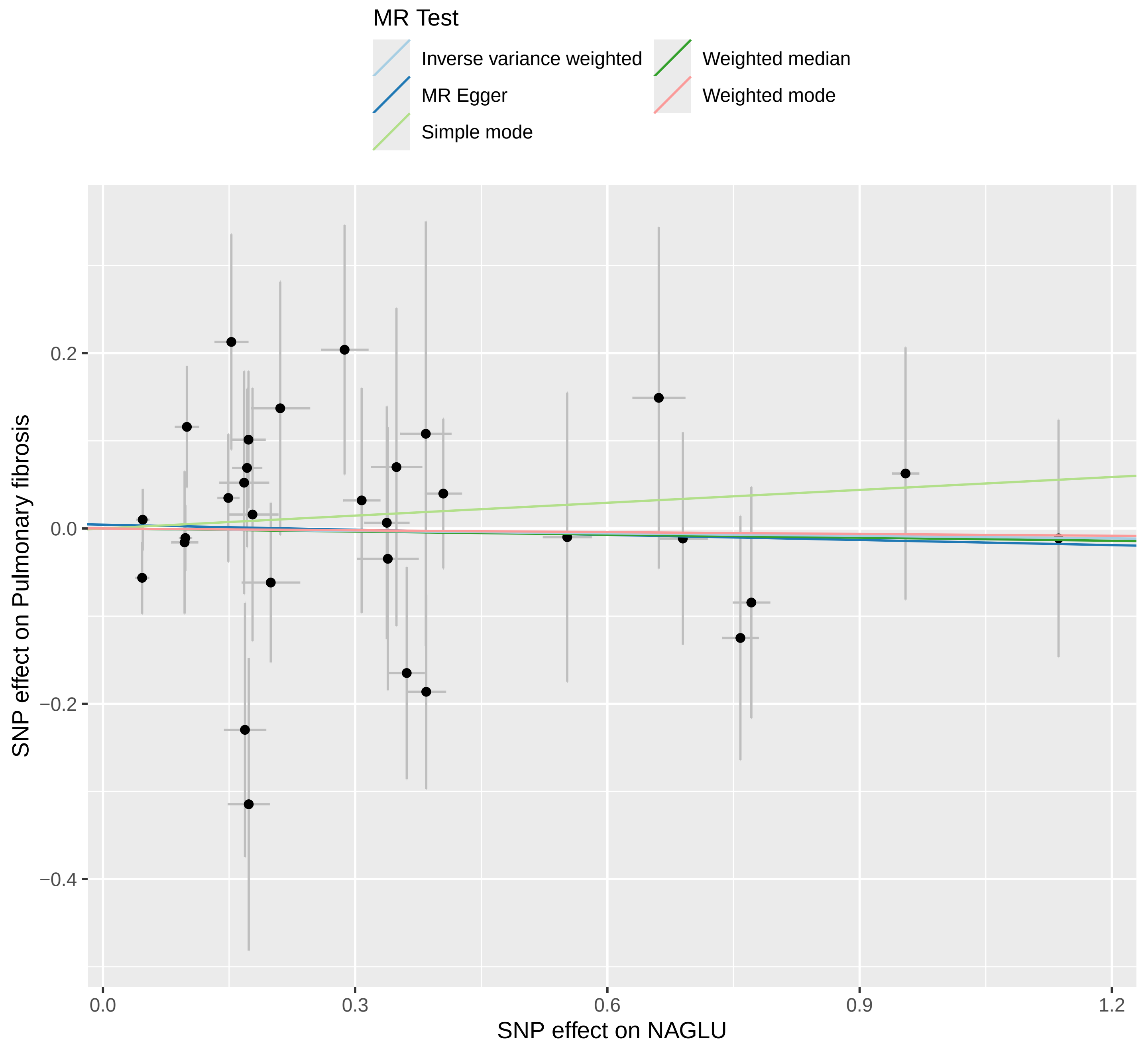
MR Test
Inverse variance weighted
MR Egger
Simple mode
Weighted median
Weighted mode
SNP effect on Pulmonary fibrosis
0.2
0.0
−0.2
−0.4
0.0
0.3
0.6
0.9
1.2
SNP effect on NAGLU

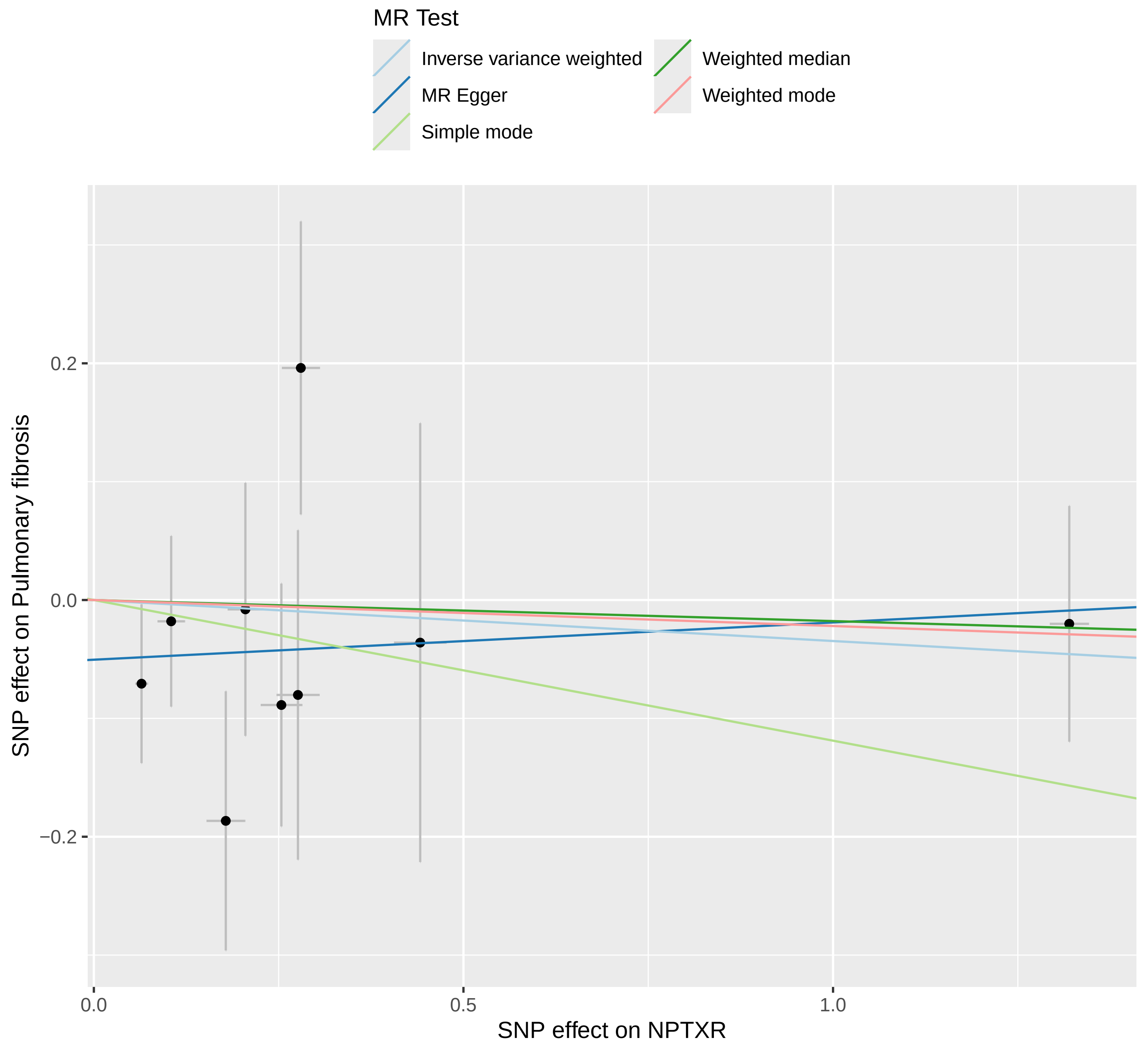

MR Test
Inverse variance weighted
MR Egger
Simple mode
Weighted median
Weighted mode
0.2
0.0
−0.2
SNP effect on Pulmonary fibrosis
0.0
0.5
1.0
SNP effect on NPTXR

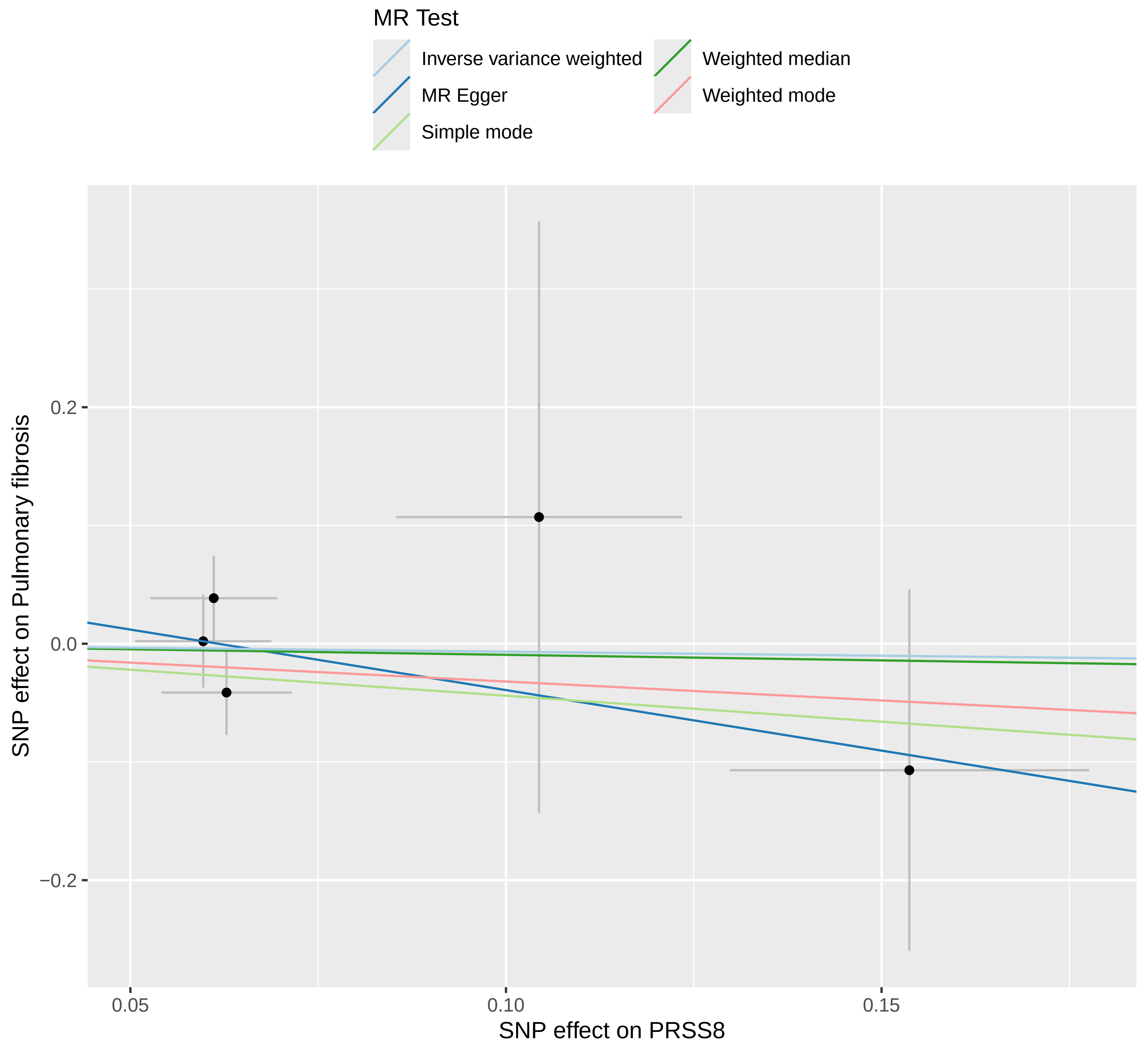

MR Test
Inverse variance weighted
MR Egger
Simple mode
Weighted median
Weighted mode
SNP effect on Pulmonary fibrosis
0.2
0.0
−0.2
0.05
0.10
0.15
SNP effect on PRSS8

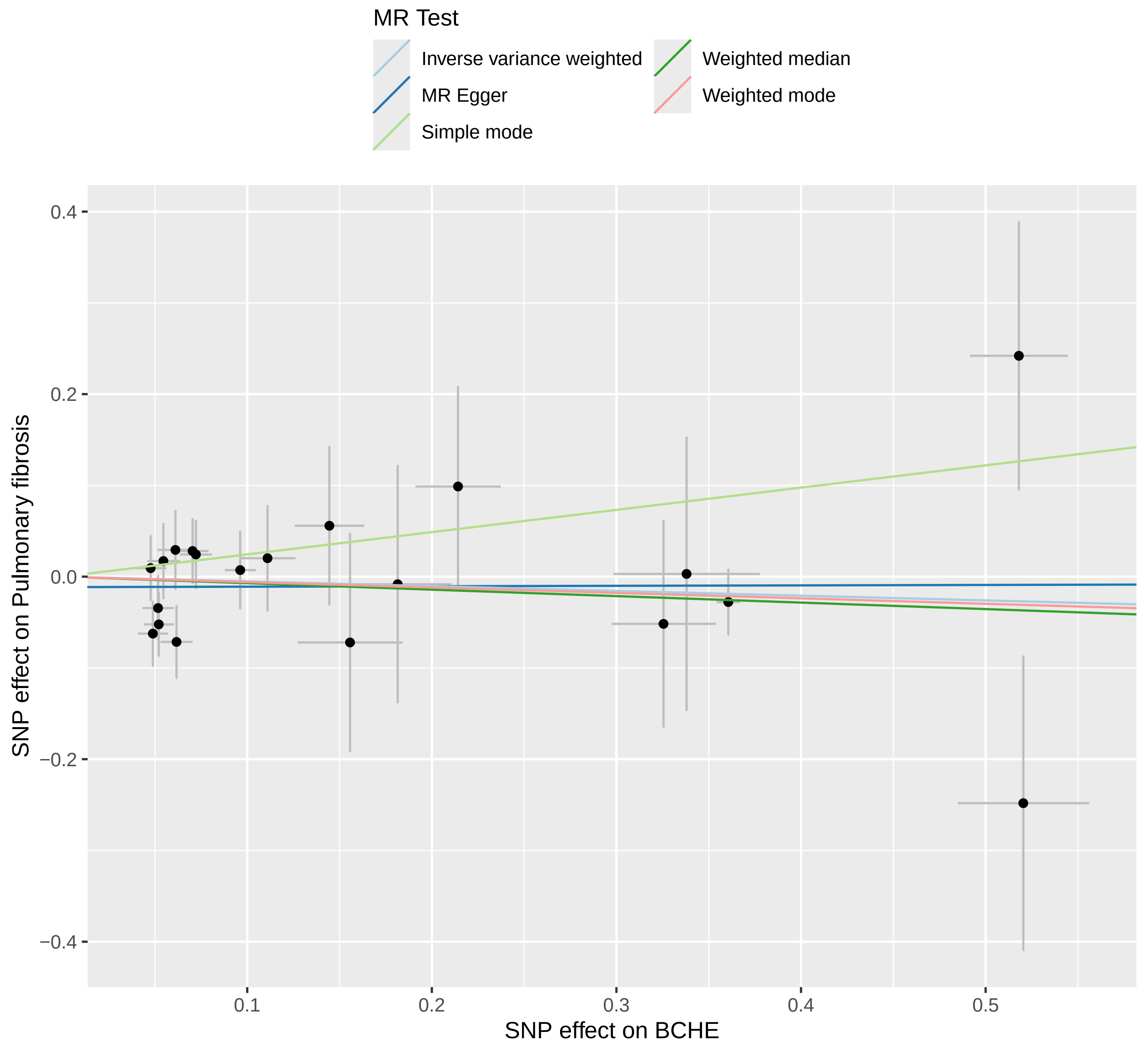

MR Test
Inverse variance weighted
MR Egger
Simple mode
Weighted median
Weighted mode
0.4
0.2
0.0
−0.2
−0.4
SNP effect on Pulmonary fibrosis
0.1
0.2
0.3
0.4
0.5
SNP effect on BCHE

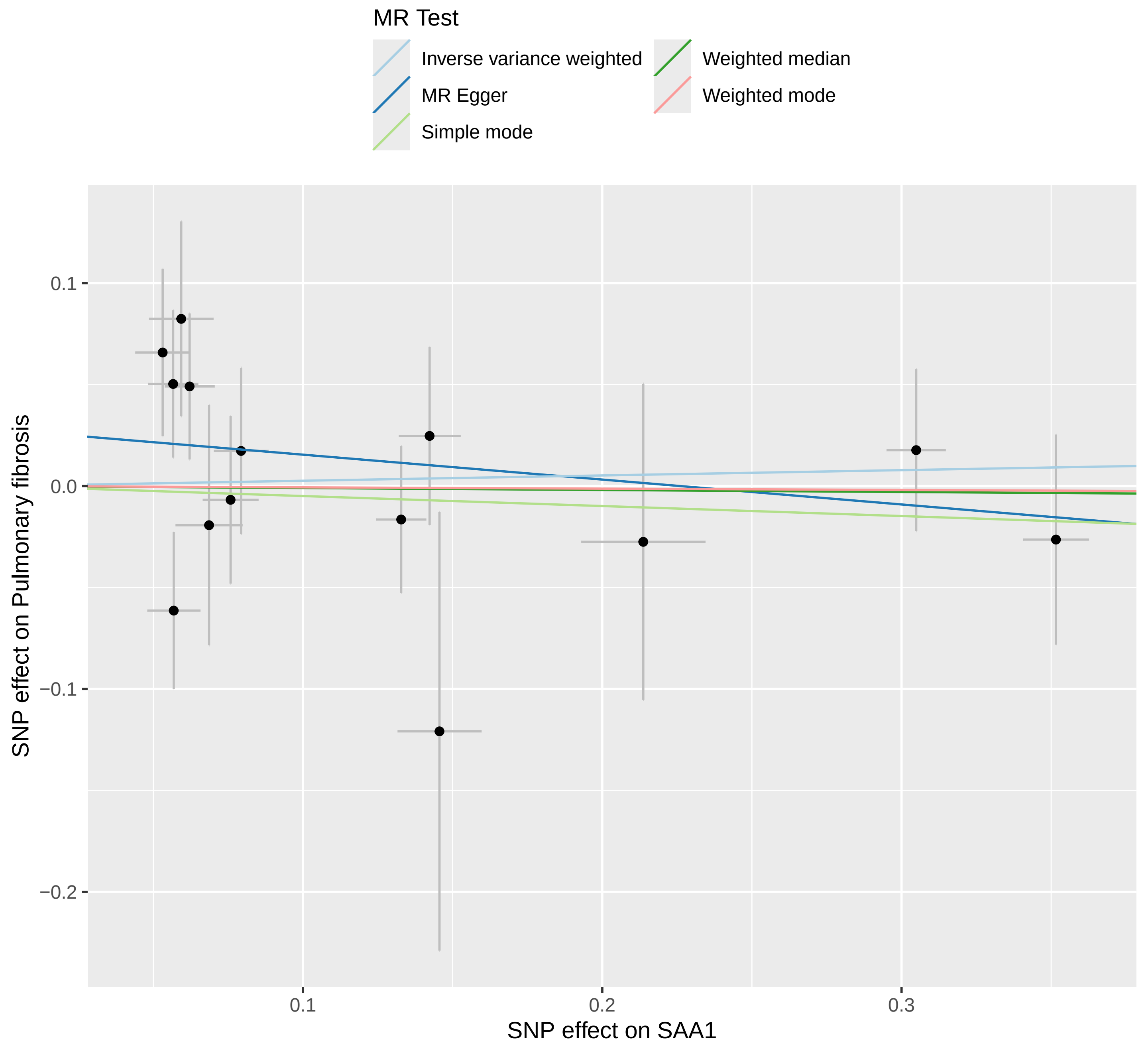

MR Test
Inverse variance weighted
MR Egger
Simple mode
Weighted median
Weighted mode
0.1
0.0
−0.1
−0.2
0.1
0.2
0.3
SNP effect on Pulmonary fibrosis
SNP effect on SAA1

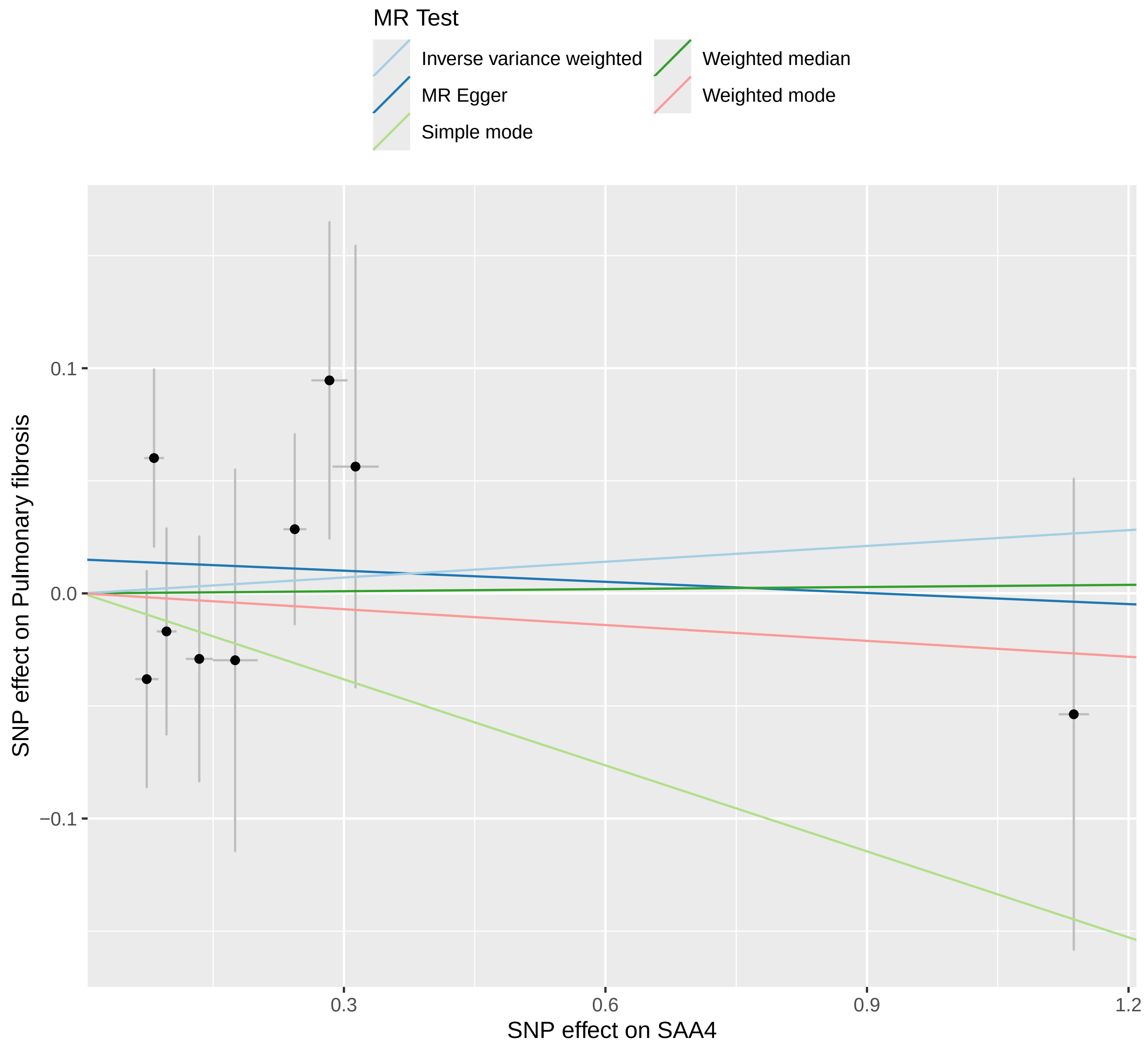
MR Test
Inverse variance weighted
MR Egger
Simple mode
Weighted median
Weighted mode
0.1
0.0
−0.1
0.3
0.6
0.9
1.2
SNP effect on SAA4
SNP effect on Pulmonary fibrosis

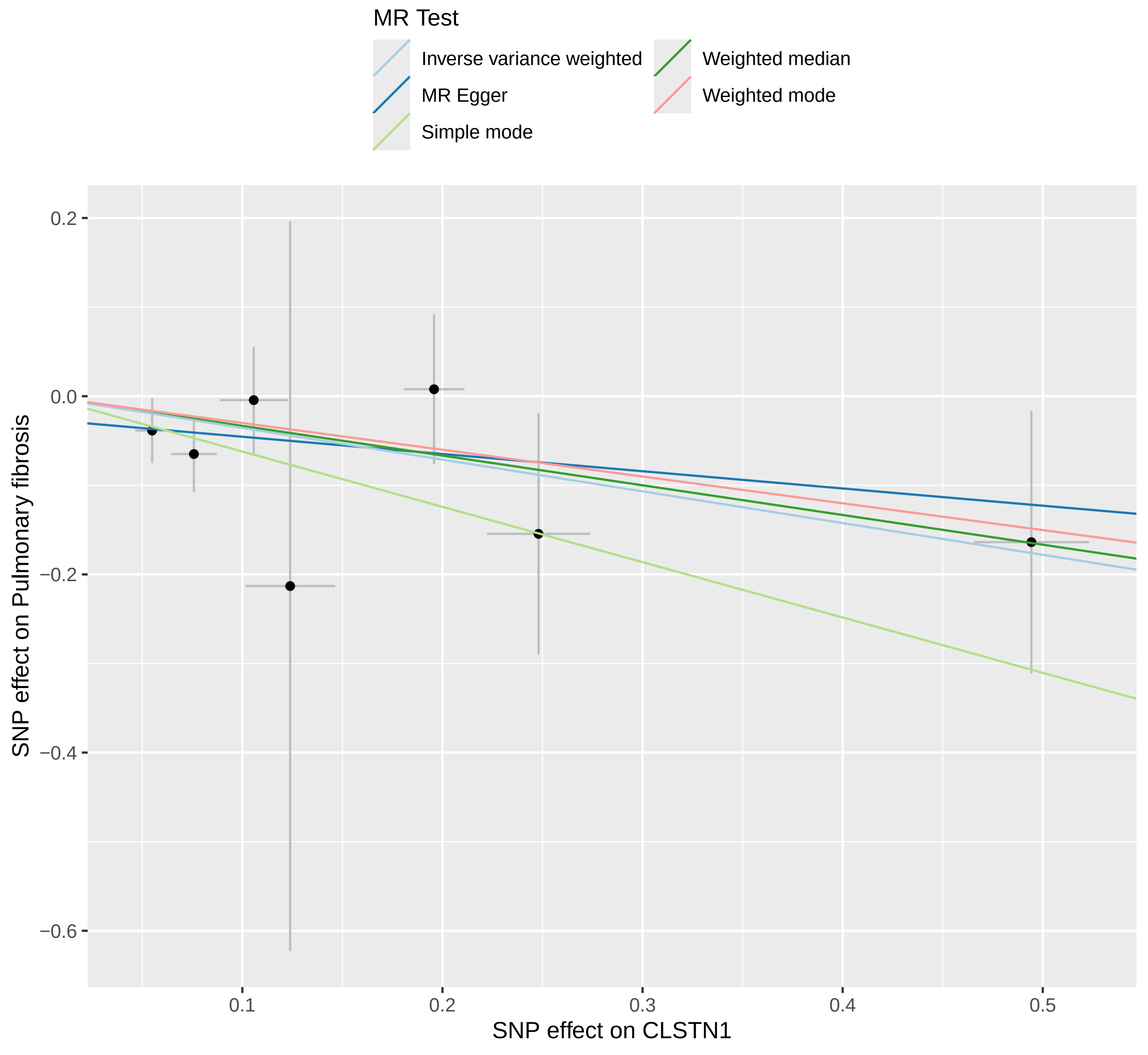
MR Test
Inverse variance weighted
MR Egger
Simple mode
Weighted median
Weighted mode
0.2
0.0
−0.2
−0.4
−0.6
SNP effect on Pulmonary fibrosis
0.1
0.2
0.3
0.4
0.5
SNP effect on CLSTN1

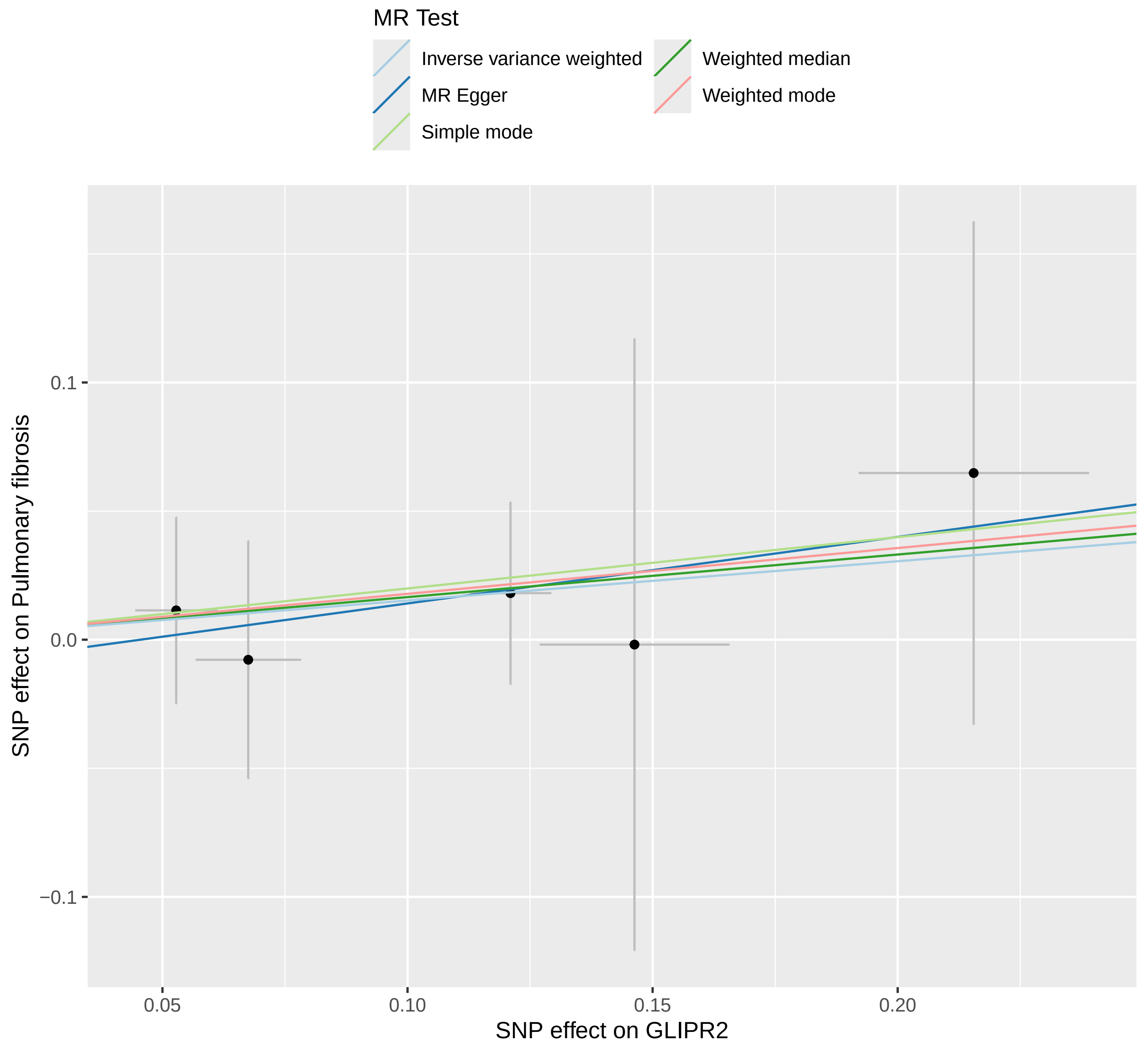
MR Test
Inverse variance weighted
MR Egger
Simple mode
Weighted median
Weighted mode
0.1
0.0
−0.1
0.05
0.10
0.15
0.20
SNP effect on Pulmonary fibrosis
SNP effect on GLIPR2

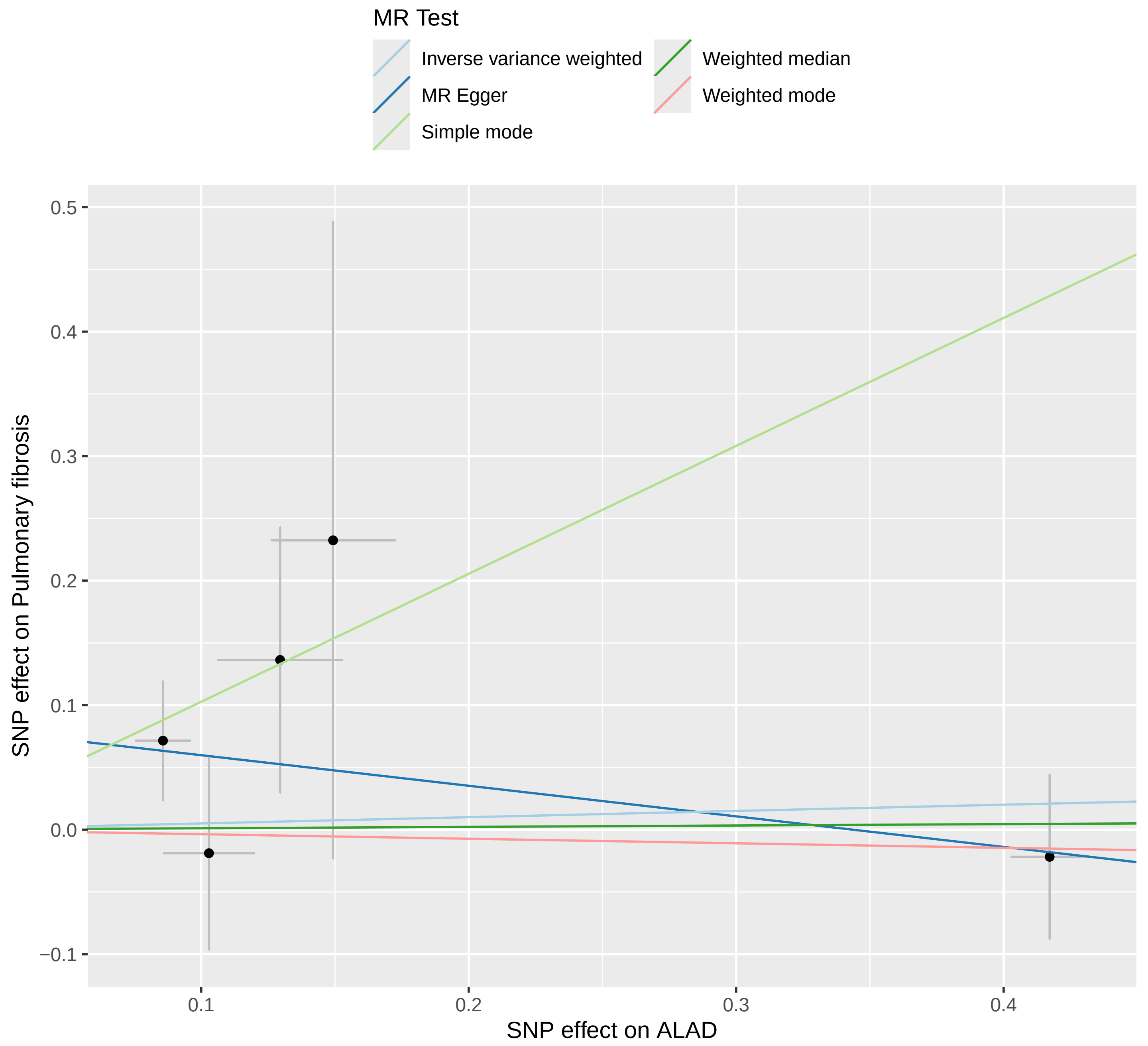
MR Test
Inverse variance weighted
MR Egger
Simple mode
Weighted median
Weighted mode
SNP effect on Pulmonary fibrosis
0.5
0.4
0.3
0.2
0.1
0.0
−0.1
0.1
0.2
0.3
0.4
SNP effect on ALAD

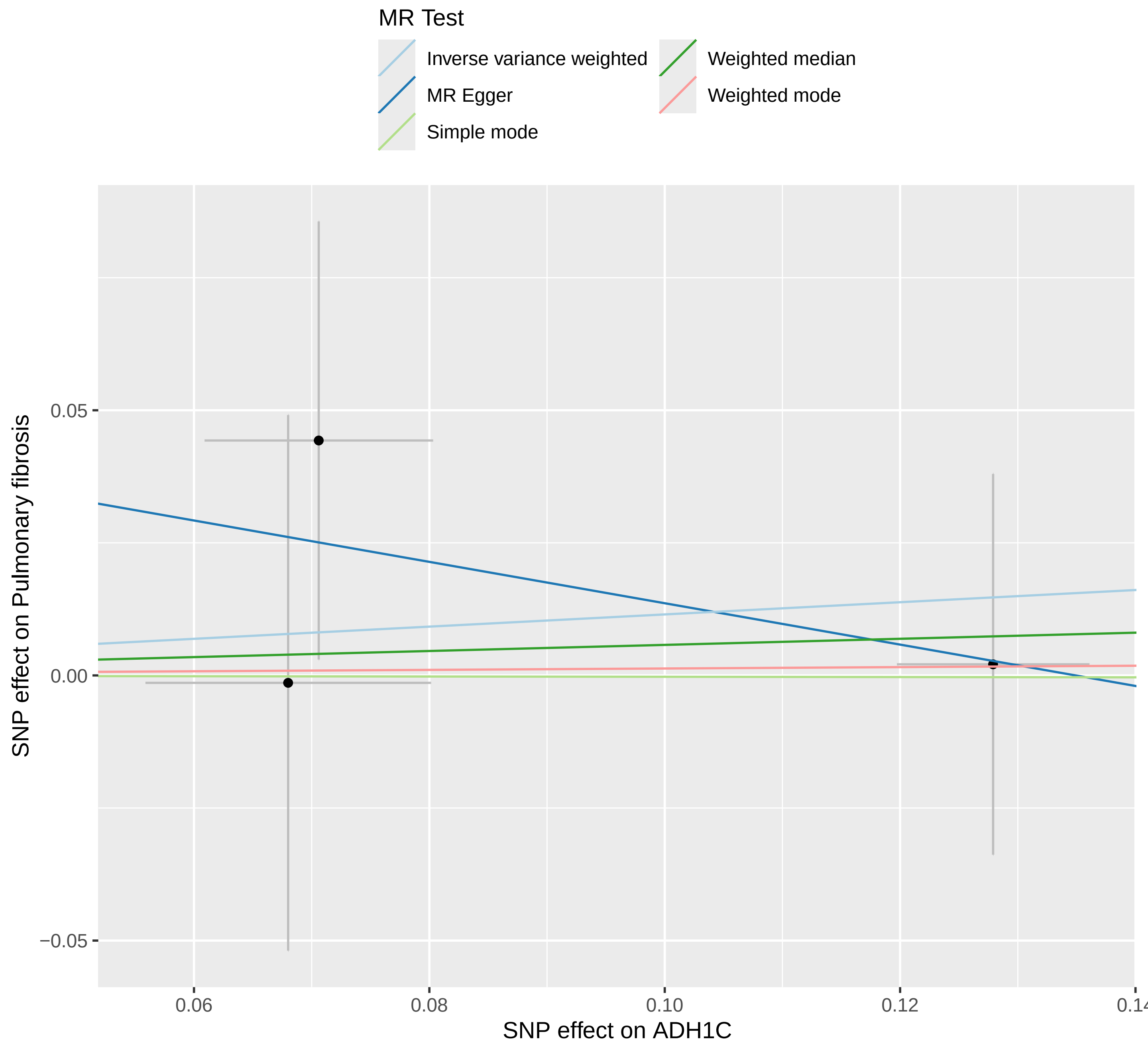

MR Test
Inverse variance weighted
MR Egger
Simple mode
Weighted median
Weighted mode
0.05
0.00
−0.05
0.06
0.08
0.10
0.12
0.14
SNP effect on ADH1C
SNP effect on Pulmonary fibrosis

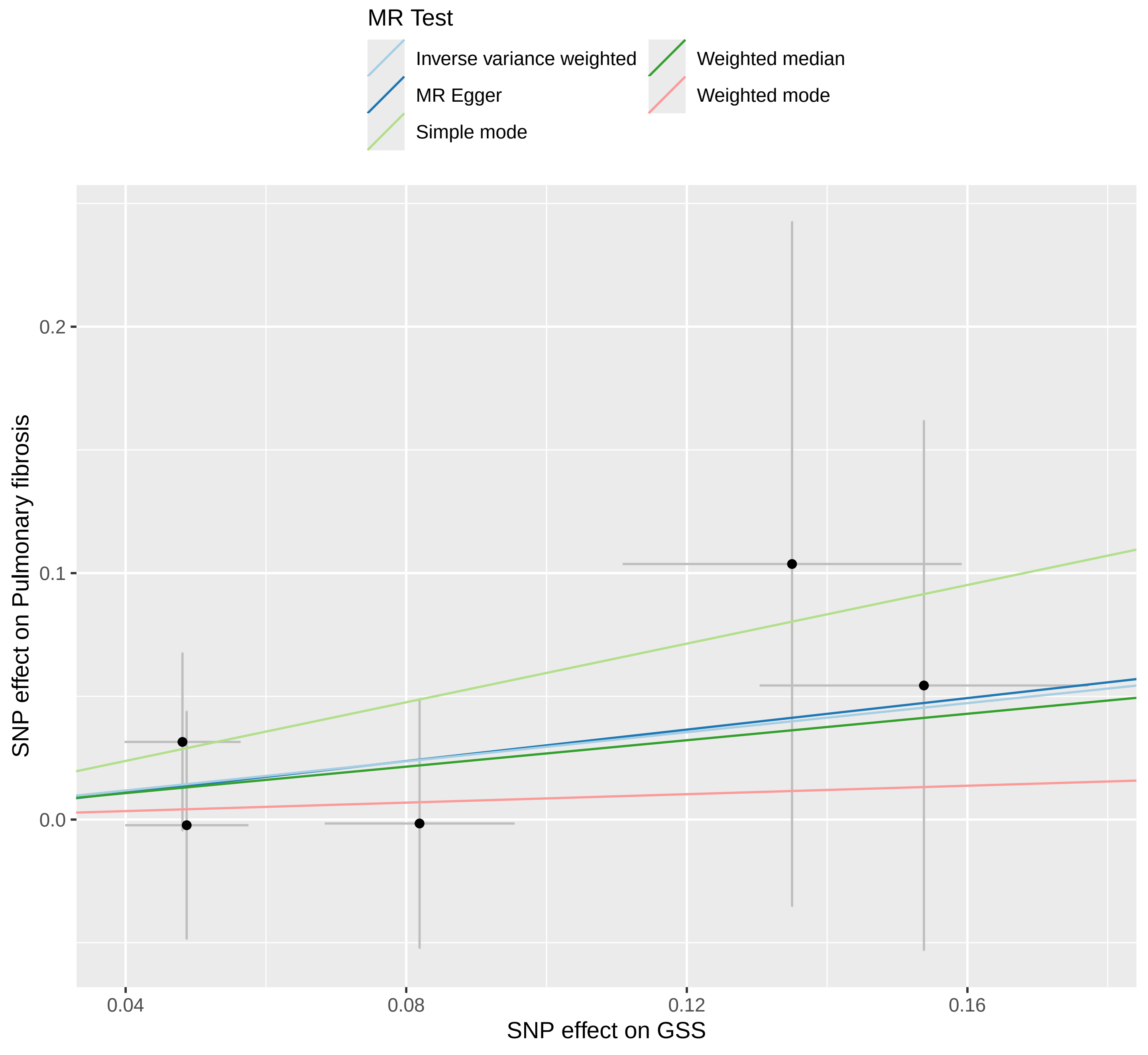

MR Test
Inverse variance weighted
MR Egger
Simple mode
Weighted median
Weighted mode
0.2
0.1
0.0
SNP effect on Pulmonary fibrosis
0.04
0.08
0.12
0.16
SNP effect on GSS

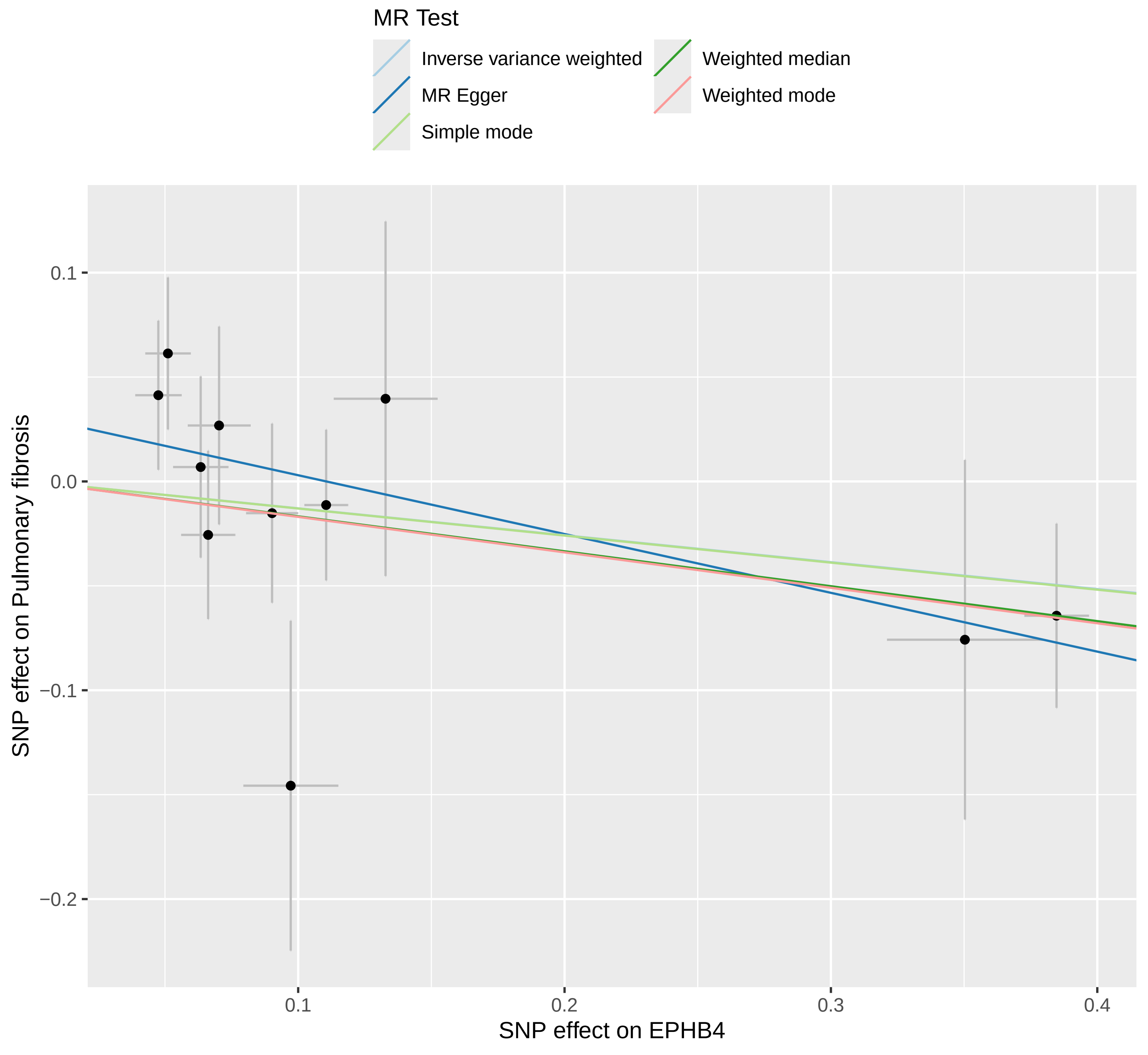
MR Test
Inverse variance weighted
MR Egger
Simple mode
Weighted median
Weighted mode
SNP effect on Pulmonary fibrosis
0.1
0.0
−0.1
−0.2
0.1
0.2
0.3
0.4
SNP effect on EPHB4

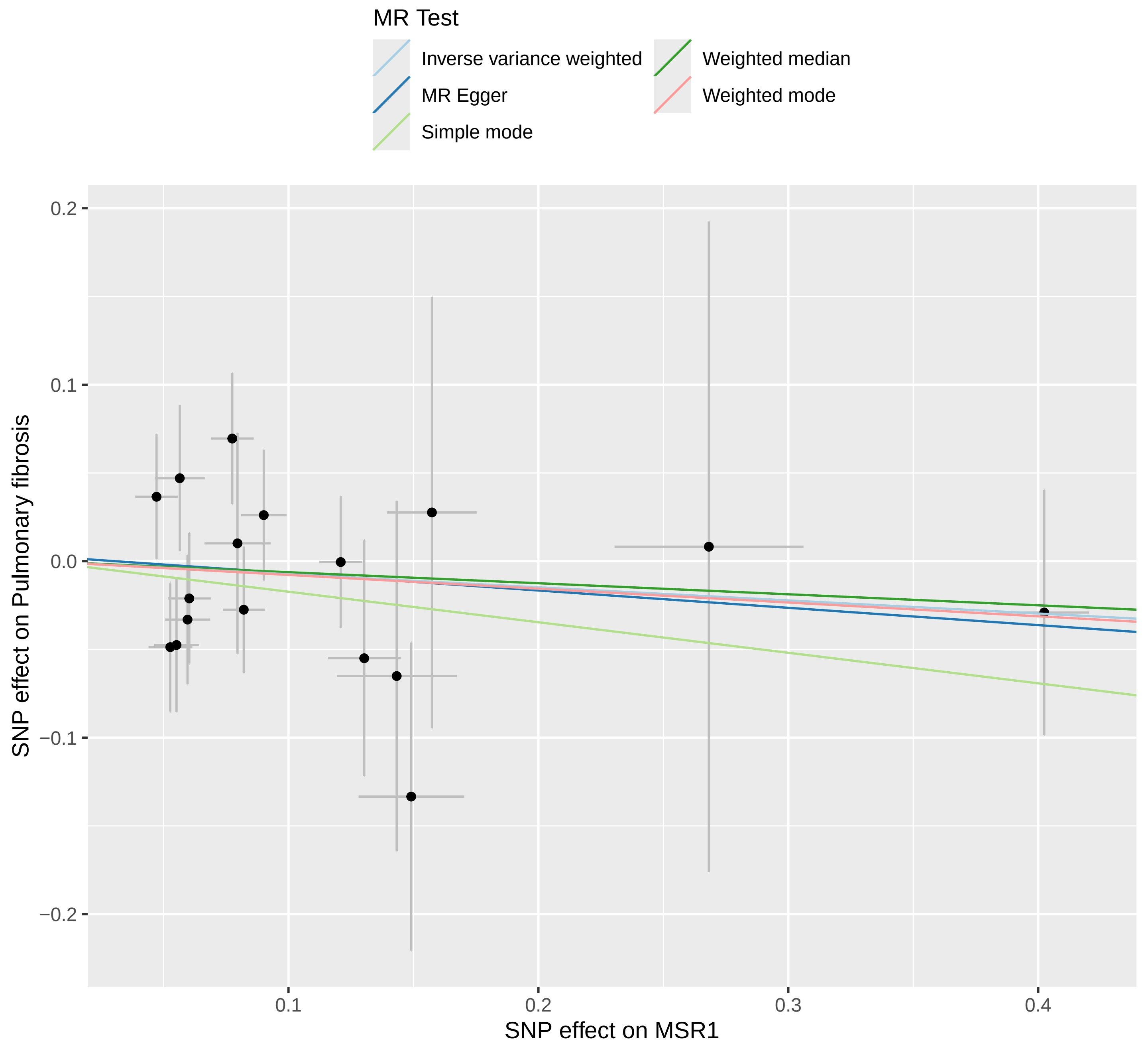
MR Test
Inverse variance weighted
MR Egger
Simple mode
Weighted median
Weighted mode
0.2
0.1
0.0
−0.1
−0.2
0.1
0.2
0.3
0.4
SNP effect on MSR1
SNP effect on Pulmonary fibrosis

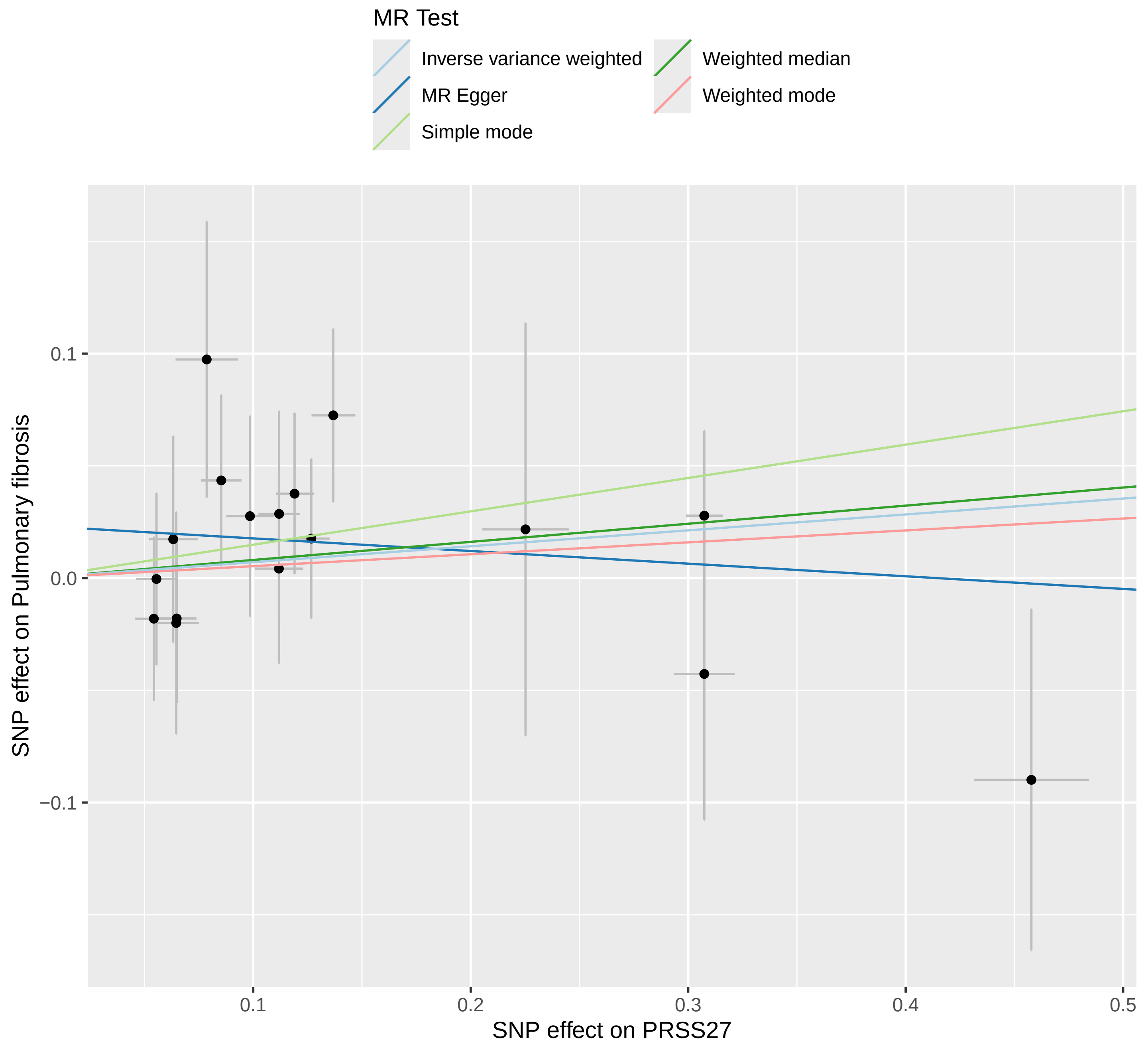

MR Test
Inverse variance weighted
MR Egger
Simple mode
Weighted median
Weighted mode
SNP effect on Pulmonary fibrosis
0.1
0.0
−0.1
0.1
0.2
0.3
0.4
0.5
SNP effect on PRSS27

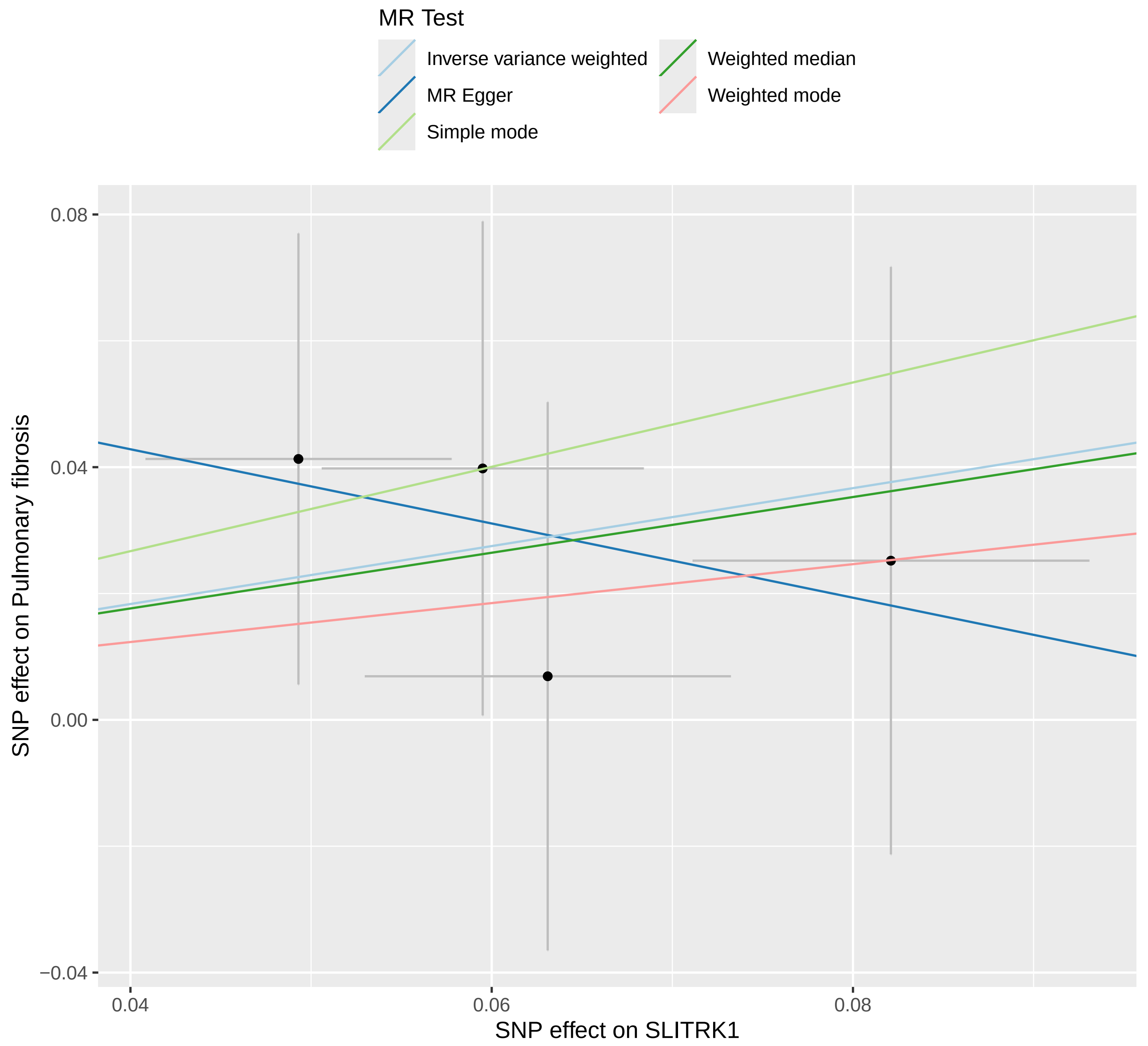
MR Test
Inverse variance weighted
MR Egger
Simple mode
Weighted median
Weighted mode
0.08
0.04
0.00
−0.04
SNP effect on Pulmonary fibrosis
0.04
0.06
0.08
SNP effect on SLITRK1

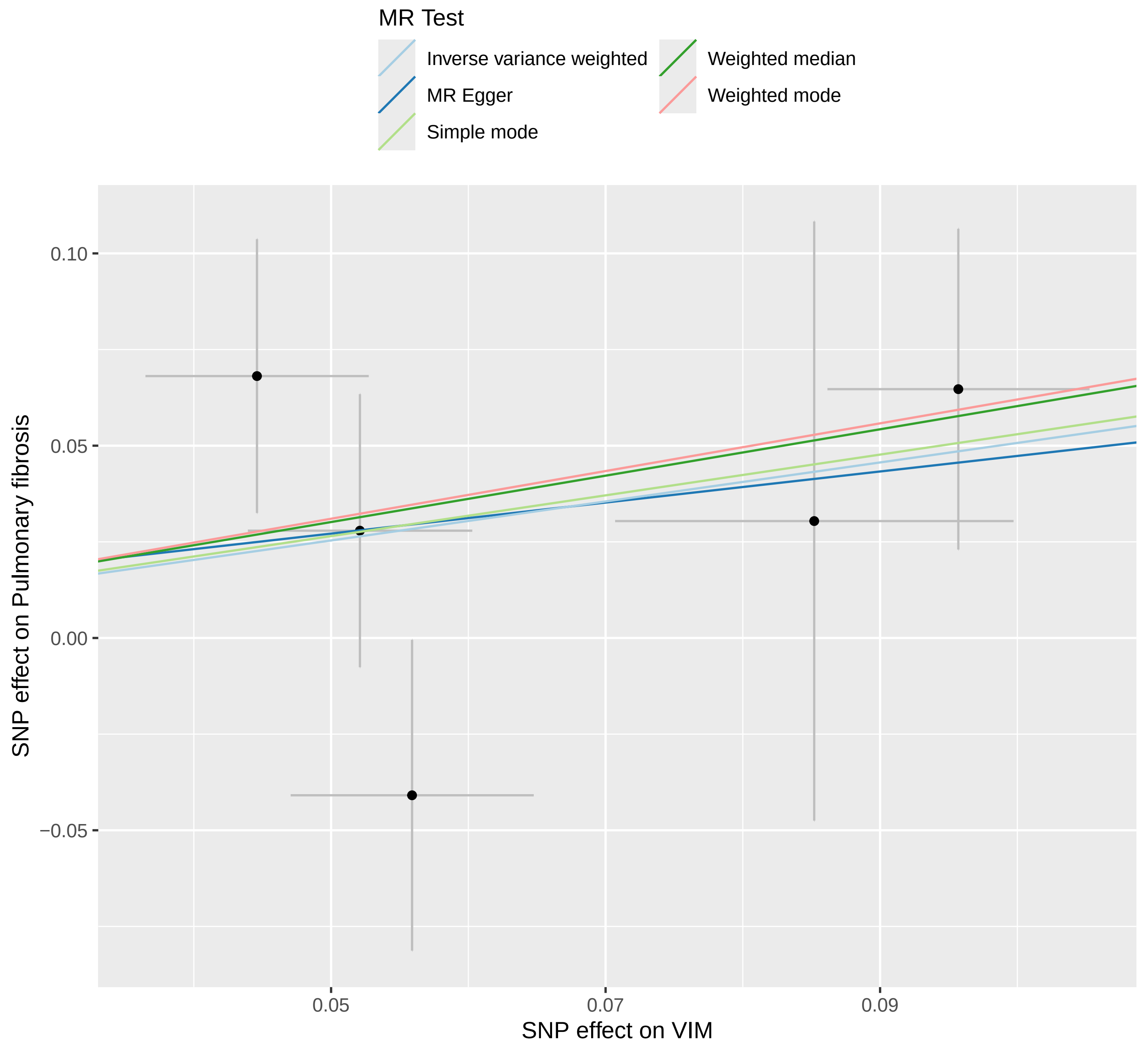
MR Test
Inverse variance weighted
MR Egger
Simple mode
Weighted median
Weighted mode
0.10
0.05
0.00
−0.05
SNP effect on Pulmonary fibrosis
0.05
0.07
0.09
SNP effect on VIM

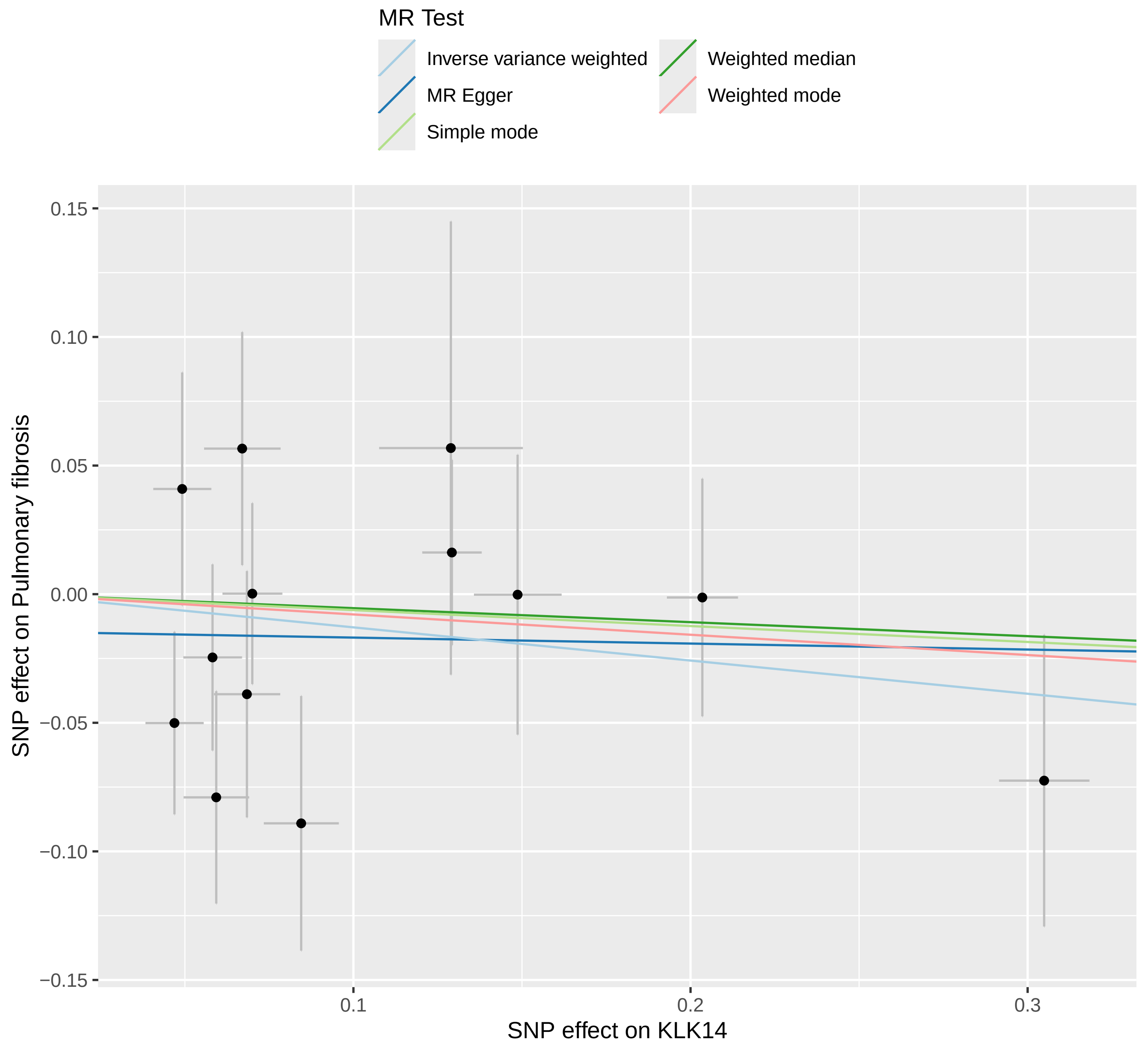

MR Test
Inverse variance weighted
MR Egger
Simple mode
Weighted median
Weighted mode
0.15
0.10
0.05
0.00
−0.05
−0.10
−0.15
SNP effect on Pulmonary fibrosis
0.1
0.2
0.3
SNP effect on KLK14

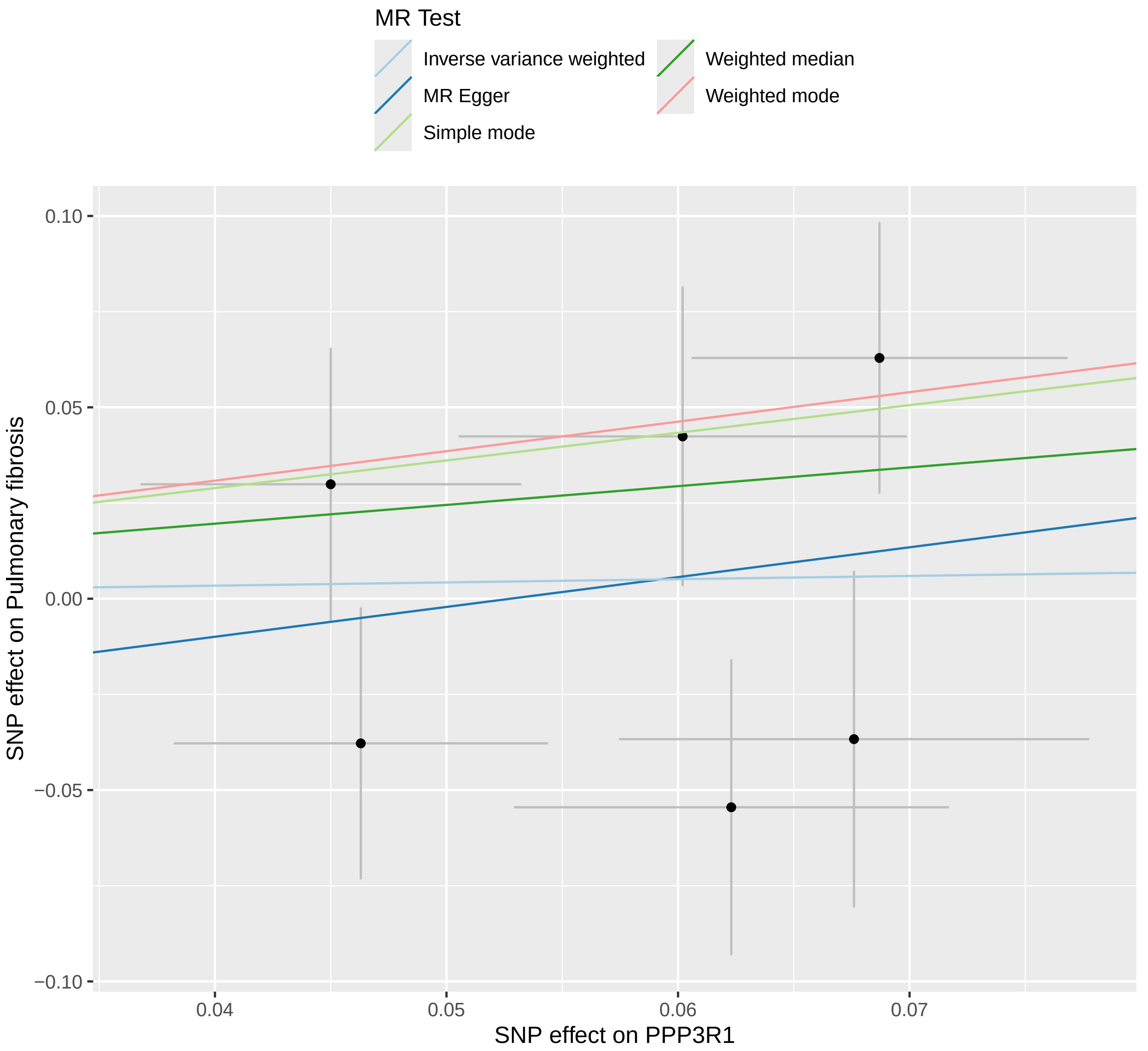

MR Test
Inverse variance weighted
MR Egger
Simple mode
Weighted median
Weighted mode
SNP effect on Pulmonary fibrosis
0.10
0.05
0.00
−0.05
−0.10
0.04
0.05
0.06
0.07
SNP effect on PPP3R1

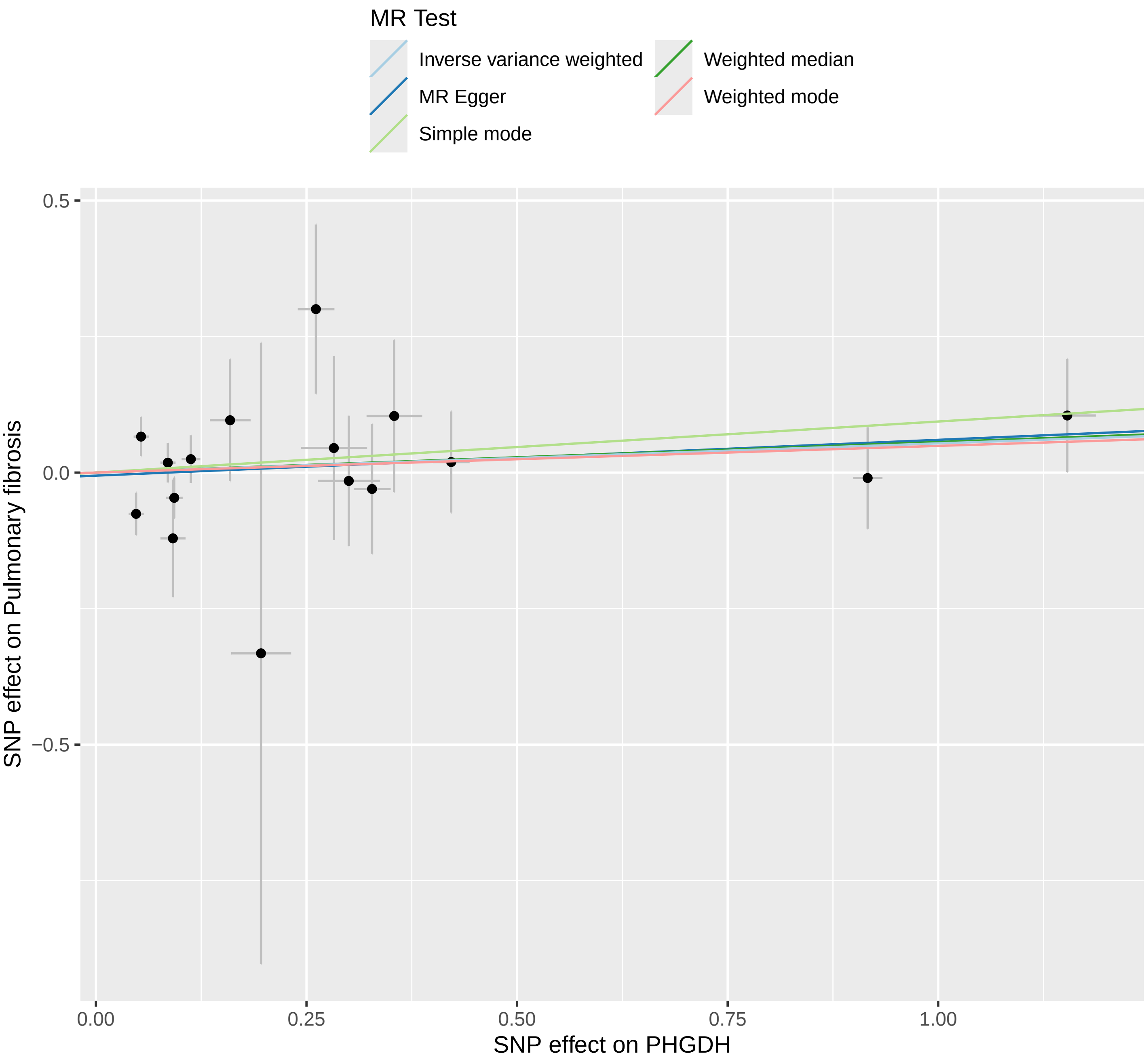
MR Test
Inverse variance weighted
MR Egger
Simple mode
Weighted median
Weighted mode
0.5
0.0
−0.5
SNP effect on Pulmonary fibrosis
0.00
0.25
0.50
0.75
1.00
SNP effect on PHGDH

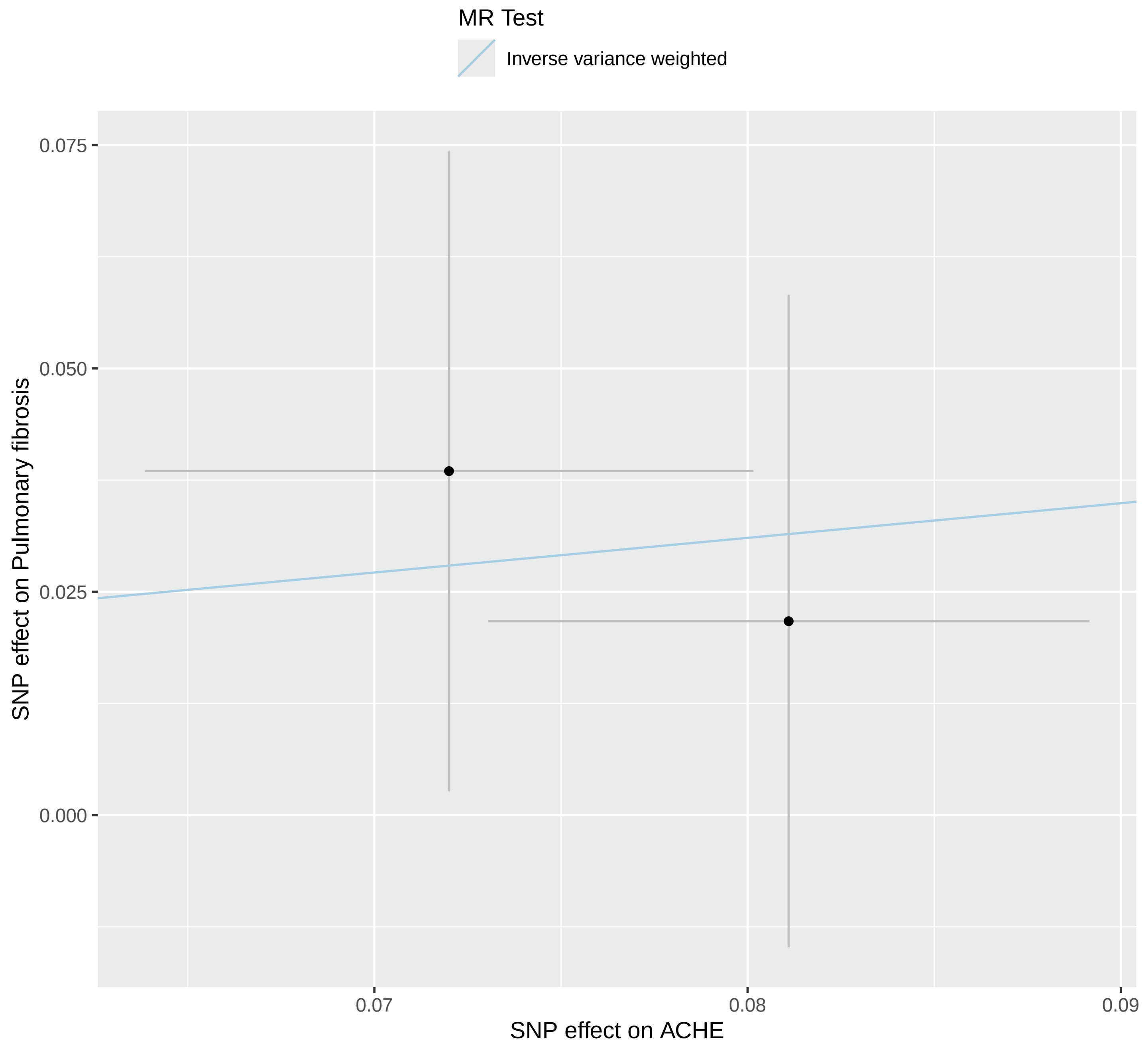

MR Test
Inverse variance weighted
0.075
0.050
0.025
0.000
SNP effect on Pulmonary fibrosis
0.07
0.08
0.09
SNP effect on ACHE

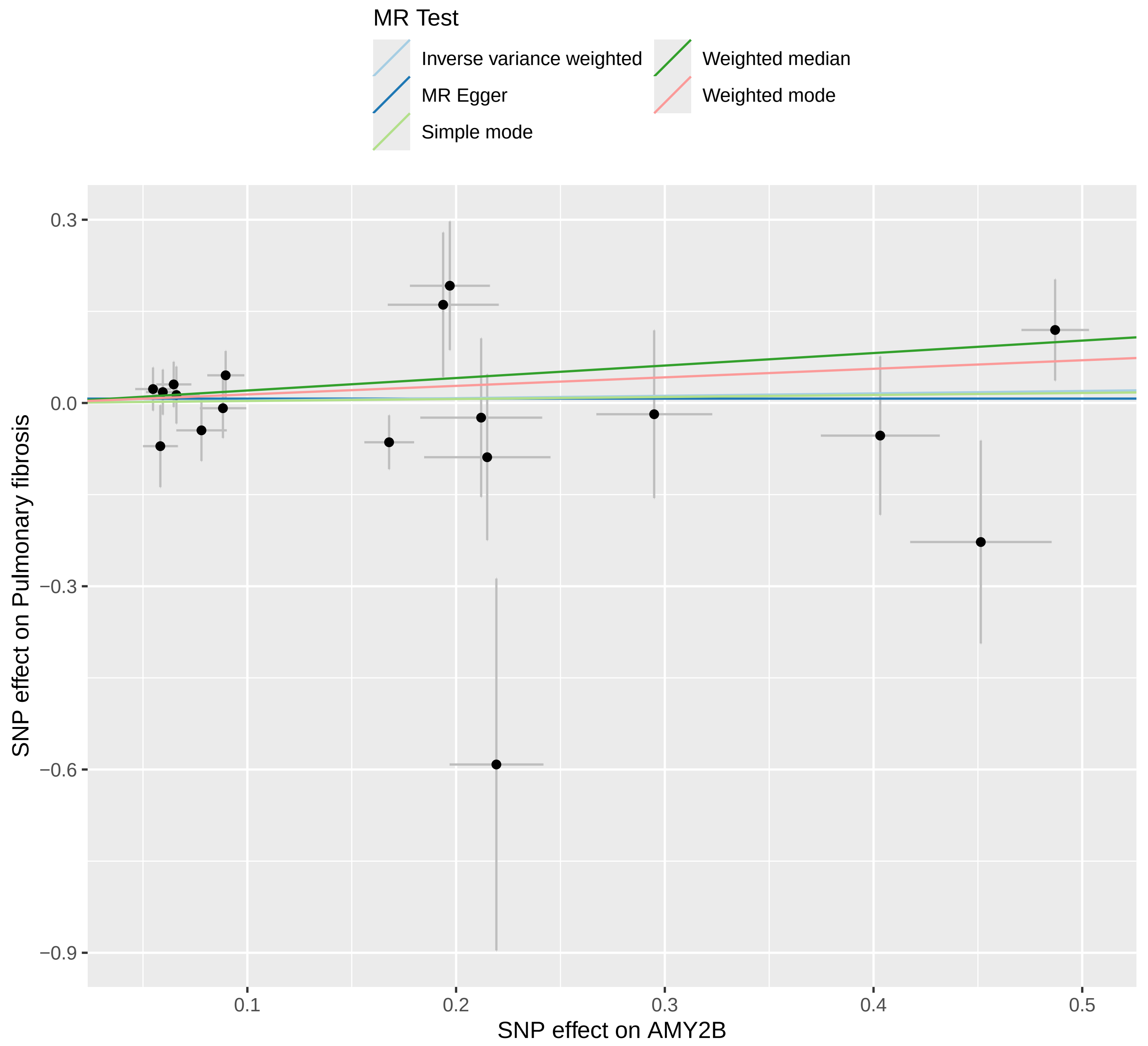

MR Test
Inverse variance weighted
MR Egger
Simple mode
Weighted median
Weighted mode
0.3
0.0
−0.3
−0.6
−0.9
0.1
0.2
0.3
0.4
0.5
SNP effect on AMY2B
SNP effect on Pulmonary fibrosis

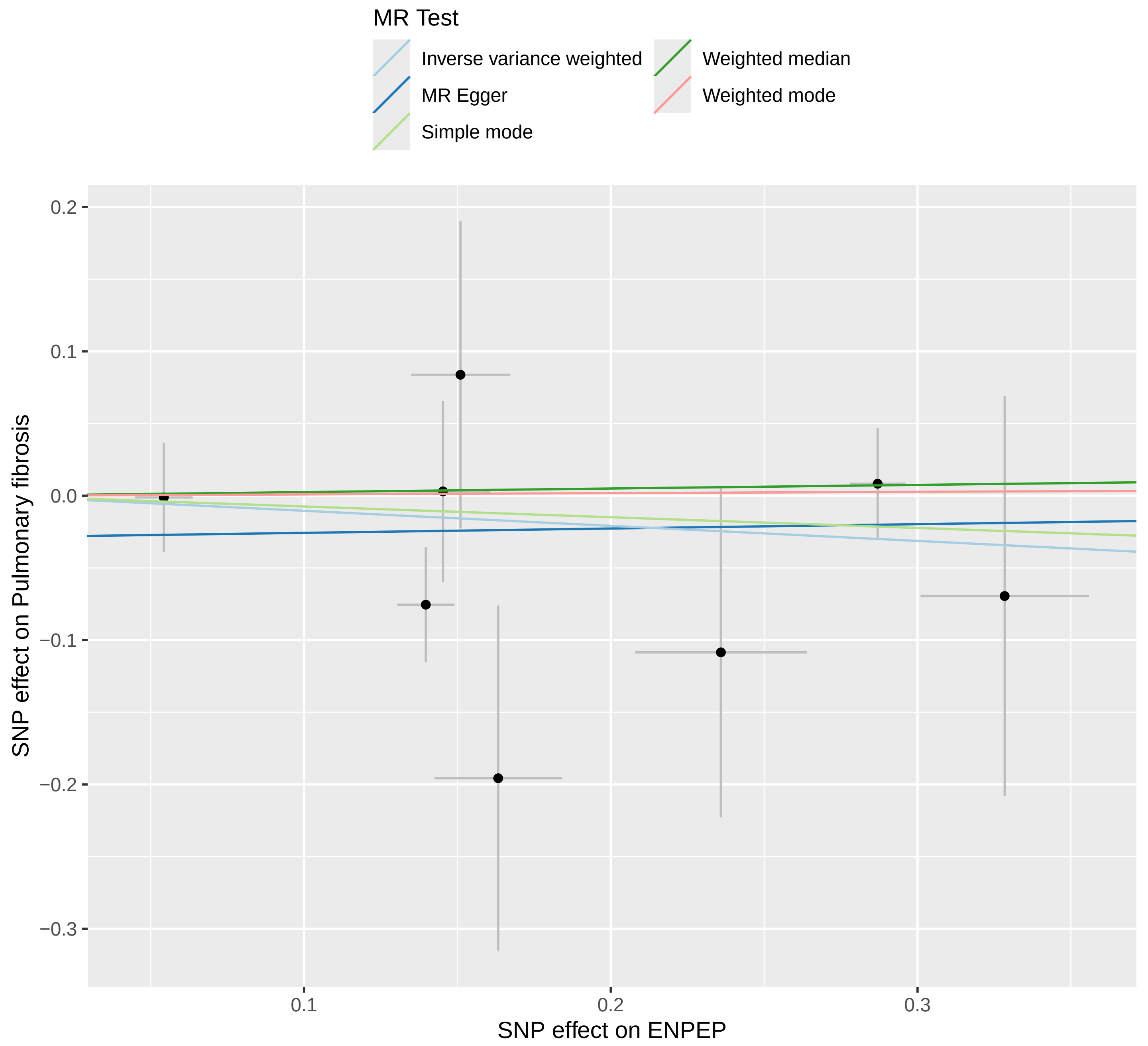

MR Test
Inverse variance weighted
MR Egger
Simple mode
Weighted median
Weighted mode
SNP effect on Pulmonary fibrosis
0.2
0.1
0.0
−0.1
−0.2
−0.3
0.1
0.2
0.3
SNP effect on ENPEP

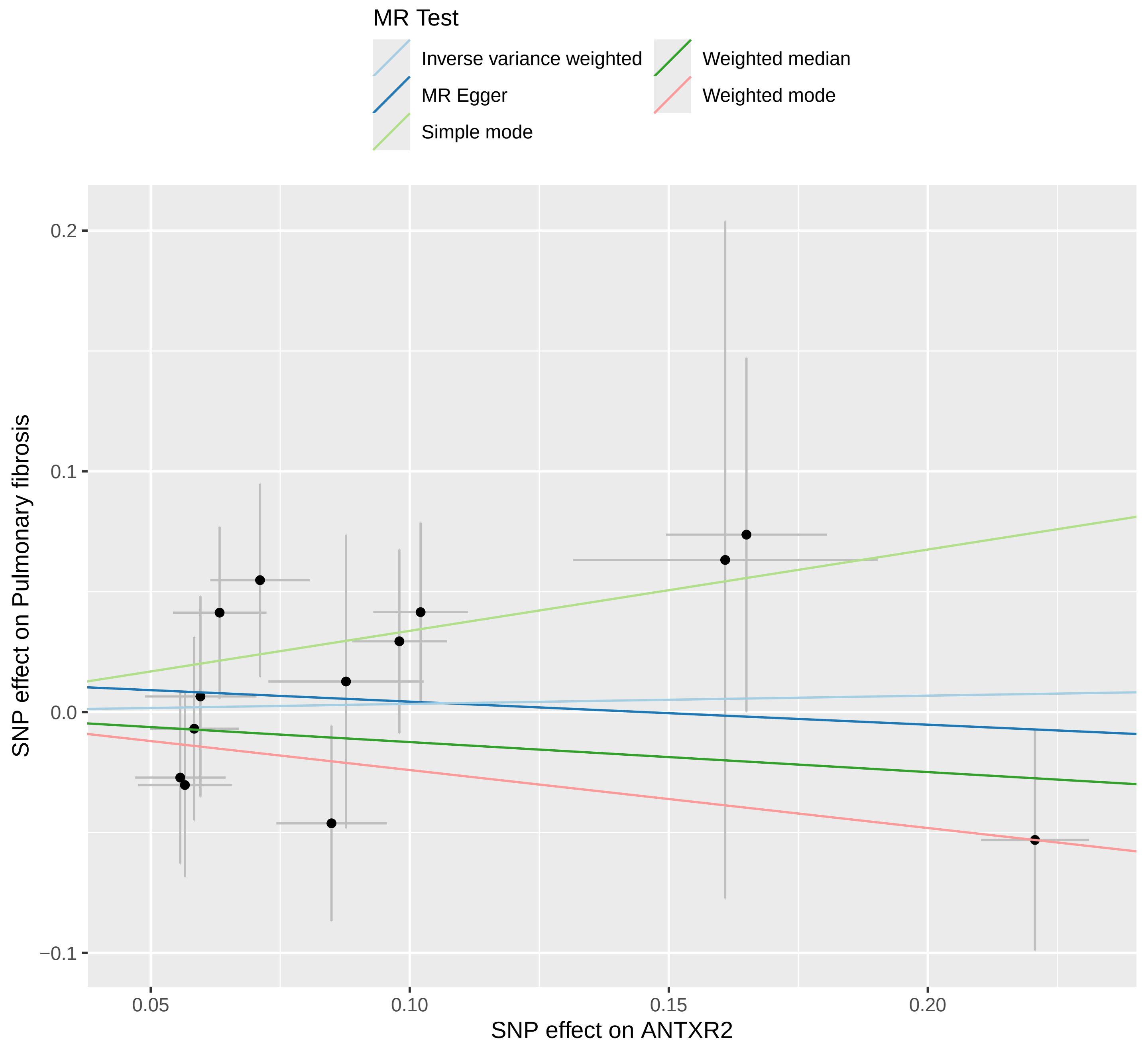

MR Test
Inverse variance weighted
MR Egger
Simple mode
Weighted median
Weighted mode
SNP effect on Pulmonary fibrosis
0.2
0.1
0.0
−0.1
0.05
0.10
0.15
0.20
SNP effect on ANTXR2

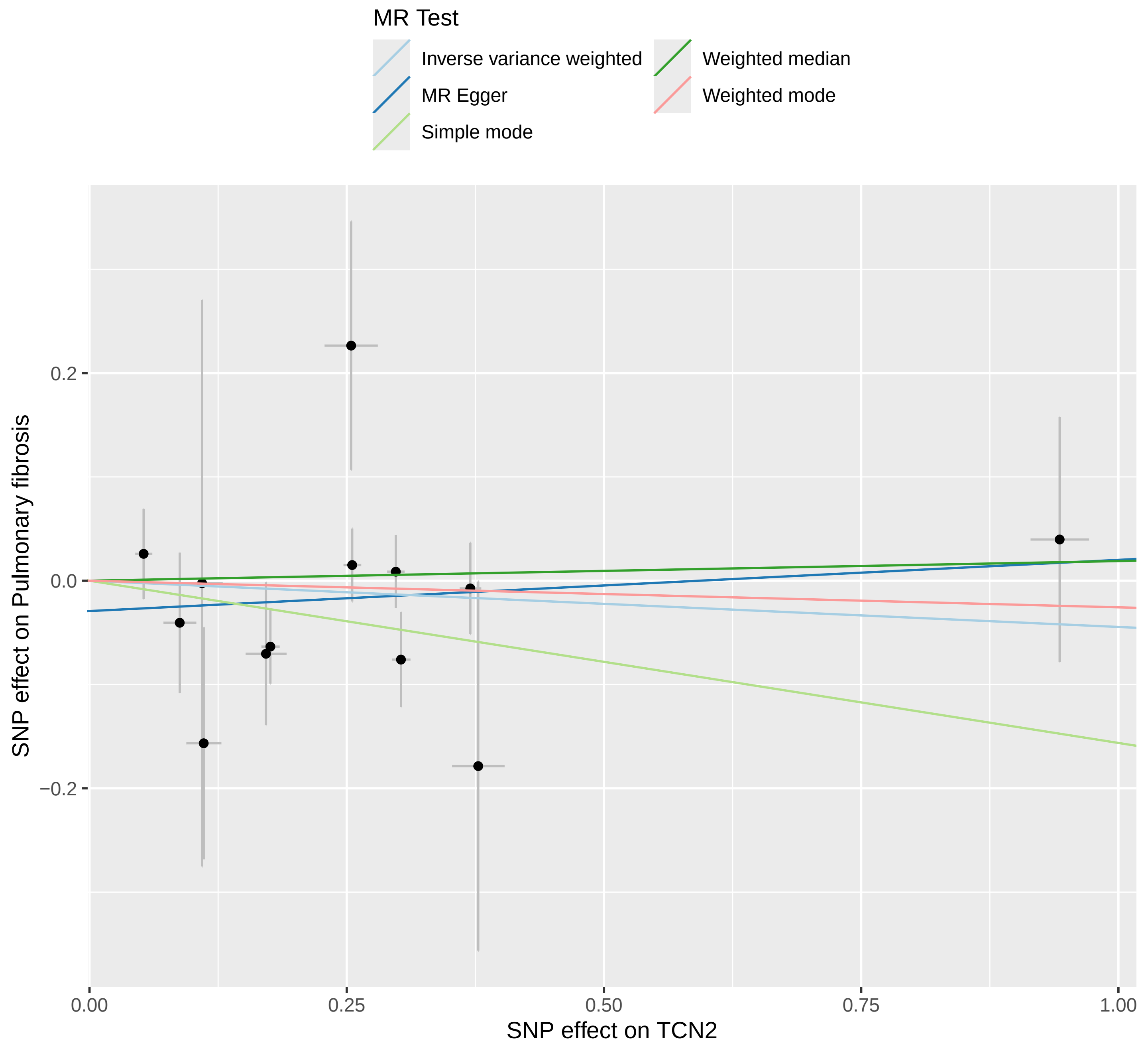
MR Test
Inverse variance weighted
MR Egger
Simple mode
Weighted median
Weighted mode
0.2
0.0
−0.2
SNP effect on Pulmonary fibrosis
0.00
0.25
0.50
0.75
1.00
SNP effect on TCN2

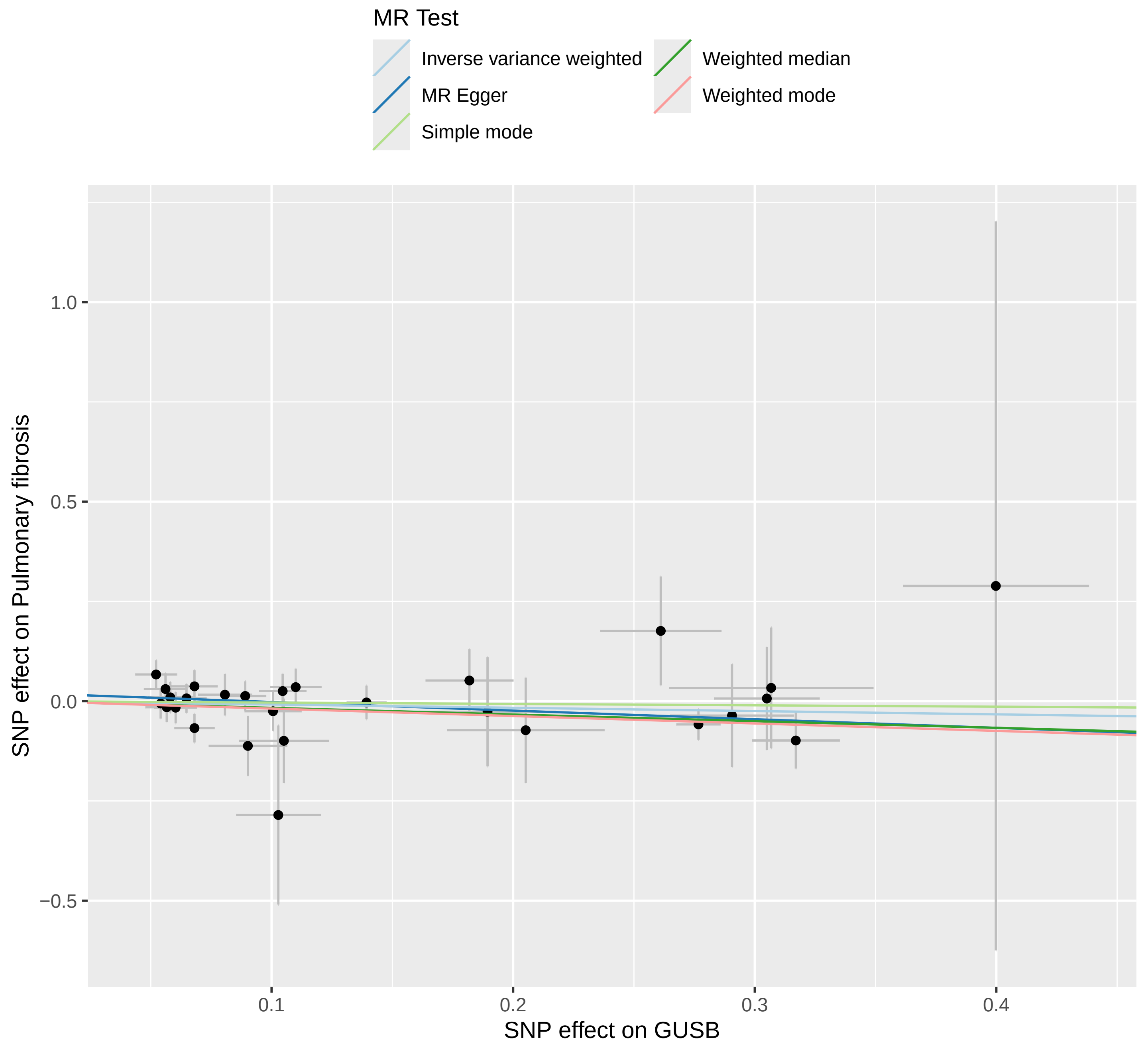

MR Test
Inverse variance weighted
MR Egger
Simple mode
Weighted median
Weighted mode
1.0
0.5
0.0
−0.5
0.1
0.2
0.3
0.4
SNP effect on Pulmonary fibrosis
SNP effect on GUSB

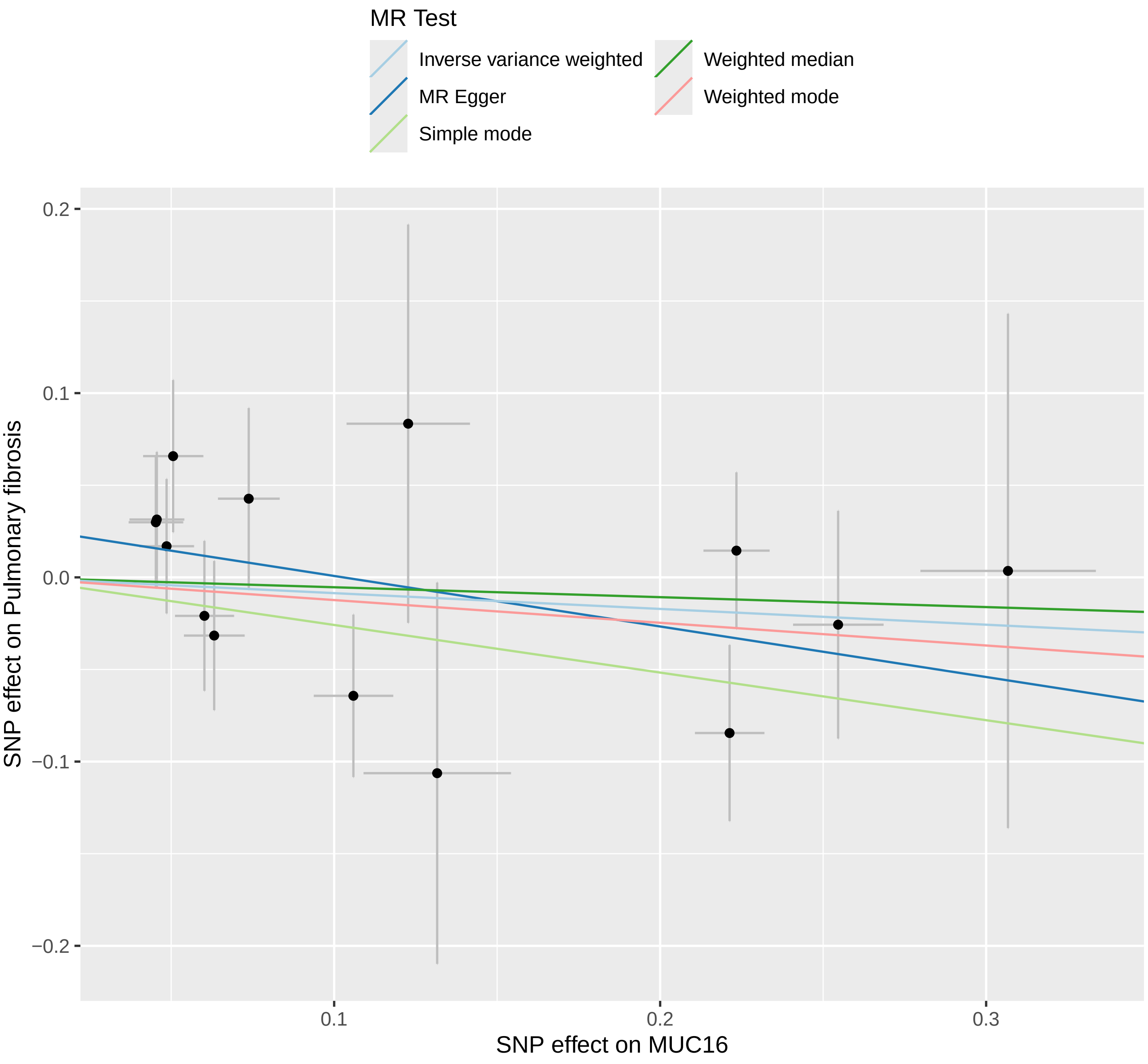
MR Test
Inverse variance weighted
MR Egger
Simple mode
Weighted median
Weighted mode
0.2
0.1
0.0
−0.1
−0.2
SNP effect on Pulmonary fibrosis
0.1
0.2
0.3
SNP effect on MUC16

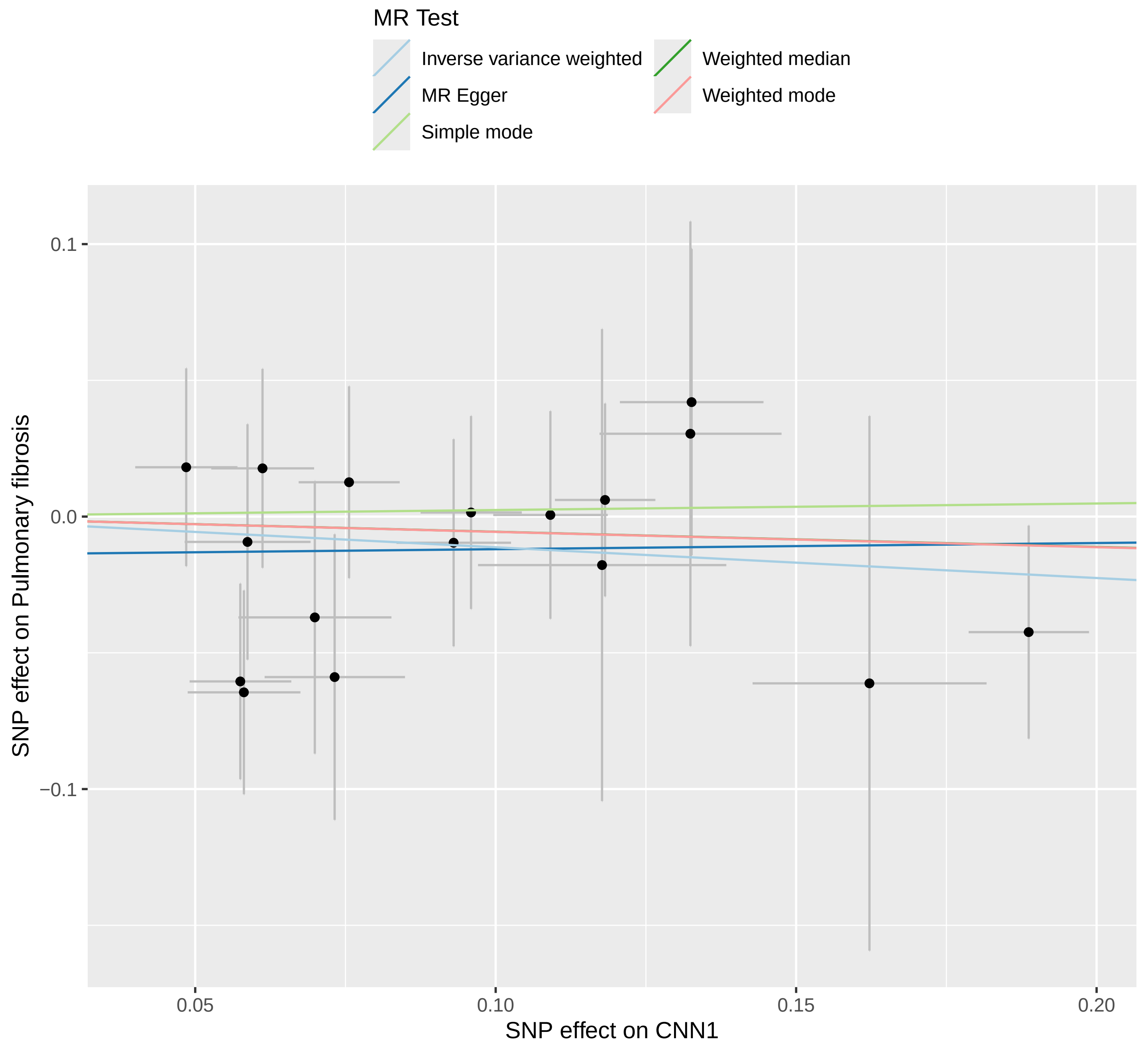

MR Test
Inverse variance weighted
MR Egger
Simple mode
Weighted median
Weighted mode
SNP effect on Pulmonary fibrosis
0.1
0.0
−0.1
0.05
0.10
0.15
0.20
SNP effect on CNN1

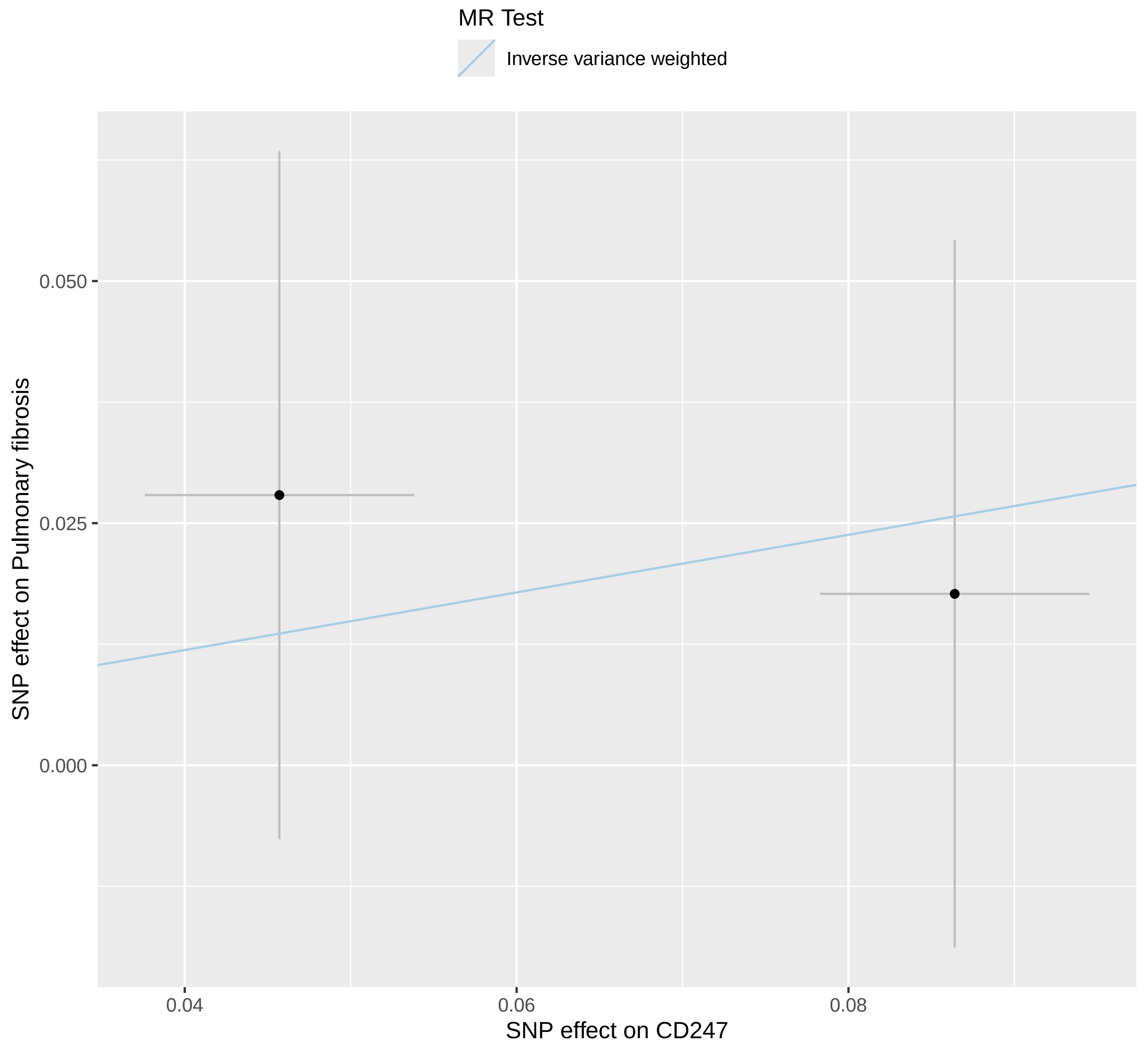
MR Test
Inverse variance weighted
SNP effect on Pulmonary fibrosis
0.050
0.025
0.000
0.04
0.06
0.08
SNP effect on CD247

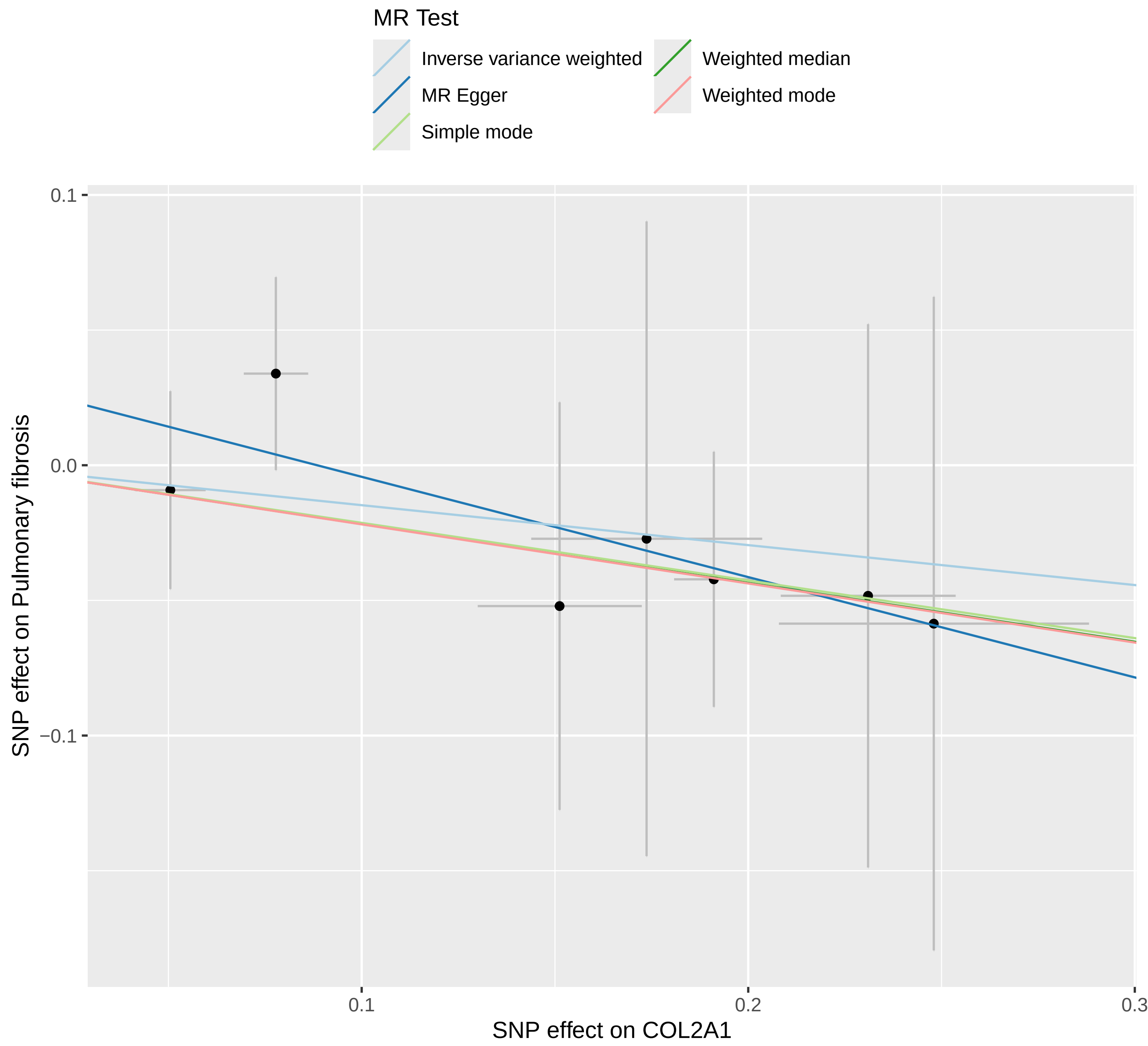
MR Test
Inverse variance weighted
MR Egger
Simple mode
Weighted median
Weighted mode
0.1
0.0
−0.1
SNP effect on Pulmonary fibrosis
0.1
0.2
0.3
SNP effect on COL2A1

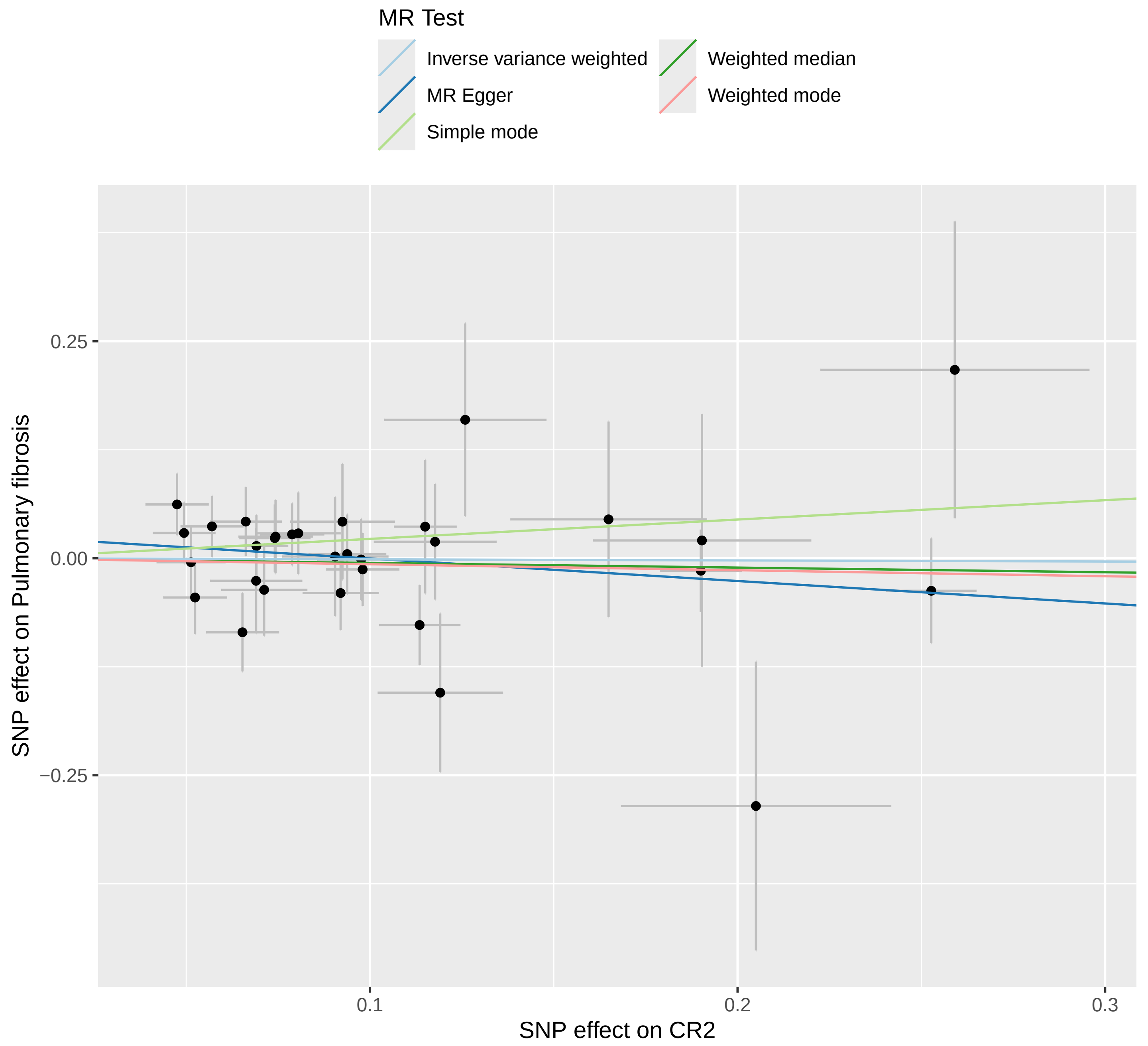

MR Test
Inverse variance weighted
MR Egger
Simple mode
Weighted median
Weighted mode
0.25
0.00
−0.25
0.1
0.2
0.3
SNP effect on Pulmonary fibrosis
SNP effect on CR2

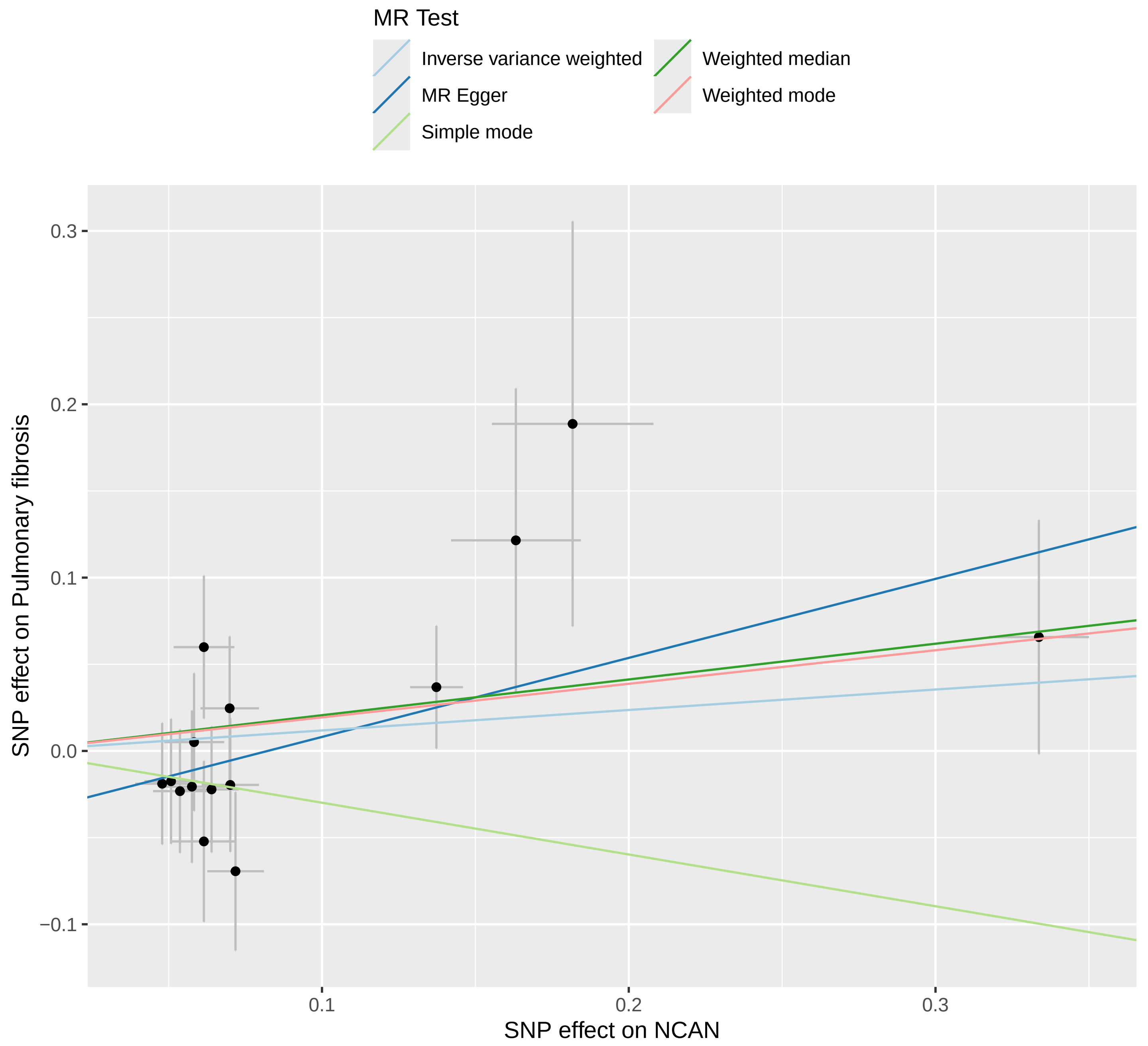

MR Test
Inverse variance weighted
MR Egger
Simple mode
Weighted median
Weighted mode
SNP effect on Pulmonary fibrosis
0.3
0.2
0.1
0.0
−0.1
0.1
0.2
0.3
SNP effect on NCAN

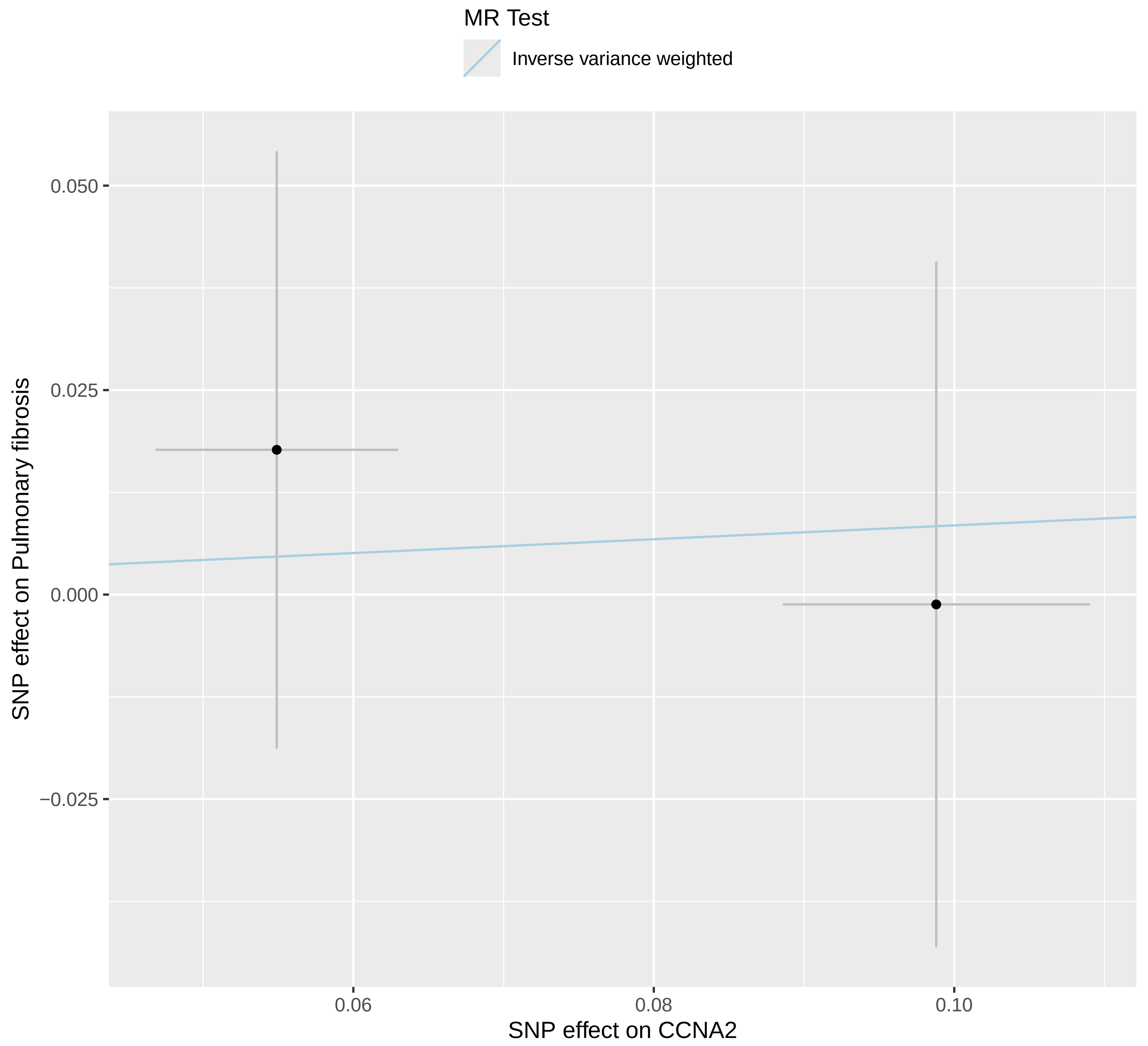

MR Test
Inverse variance weighted
0.050
0.025
0.000
−0.025
SNP effect on Pulmonary fibrosis
0.06
0.08
0.10
SNP effect on CCNA2

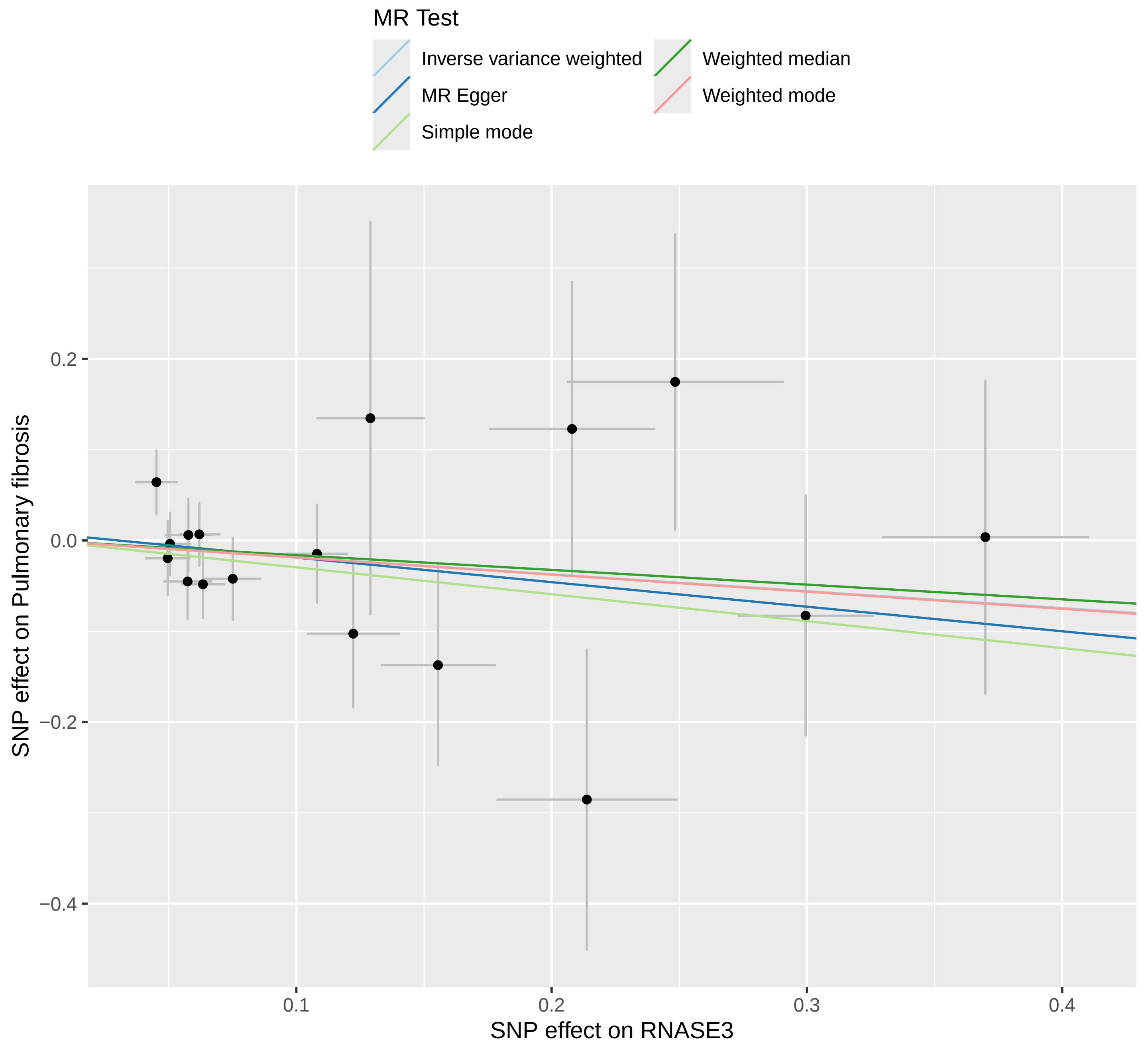
MR Test
Inverse variance weighted
MR Egger
Simple mode
Weighted median
Weighted mode
SNP effect on Pulmonary fibrosis
0.2
0.0
−0.2
−0.4
0.1
0.2
0.3
0.4
SNP effect on RNASE3

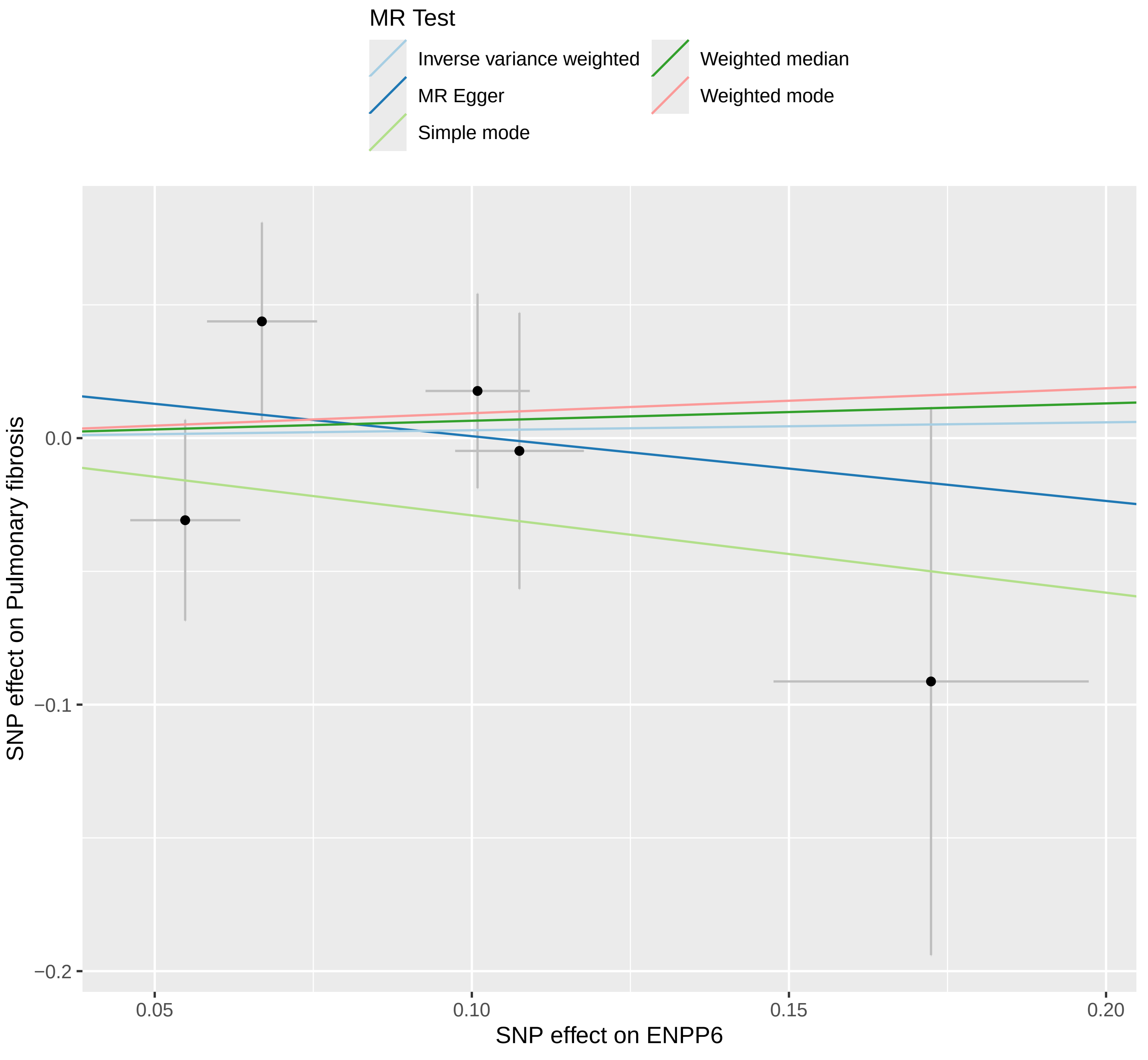
MR Test
Inverse variance weighted
MR Egger
Simple mode
Weighted median
Weighted mode
0.0
−0.1
−0.2
0.05
0.10
0.15
0.20
SNP effect on Pulmonary fibrosis
SNP effect on ENPP6

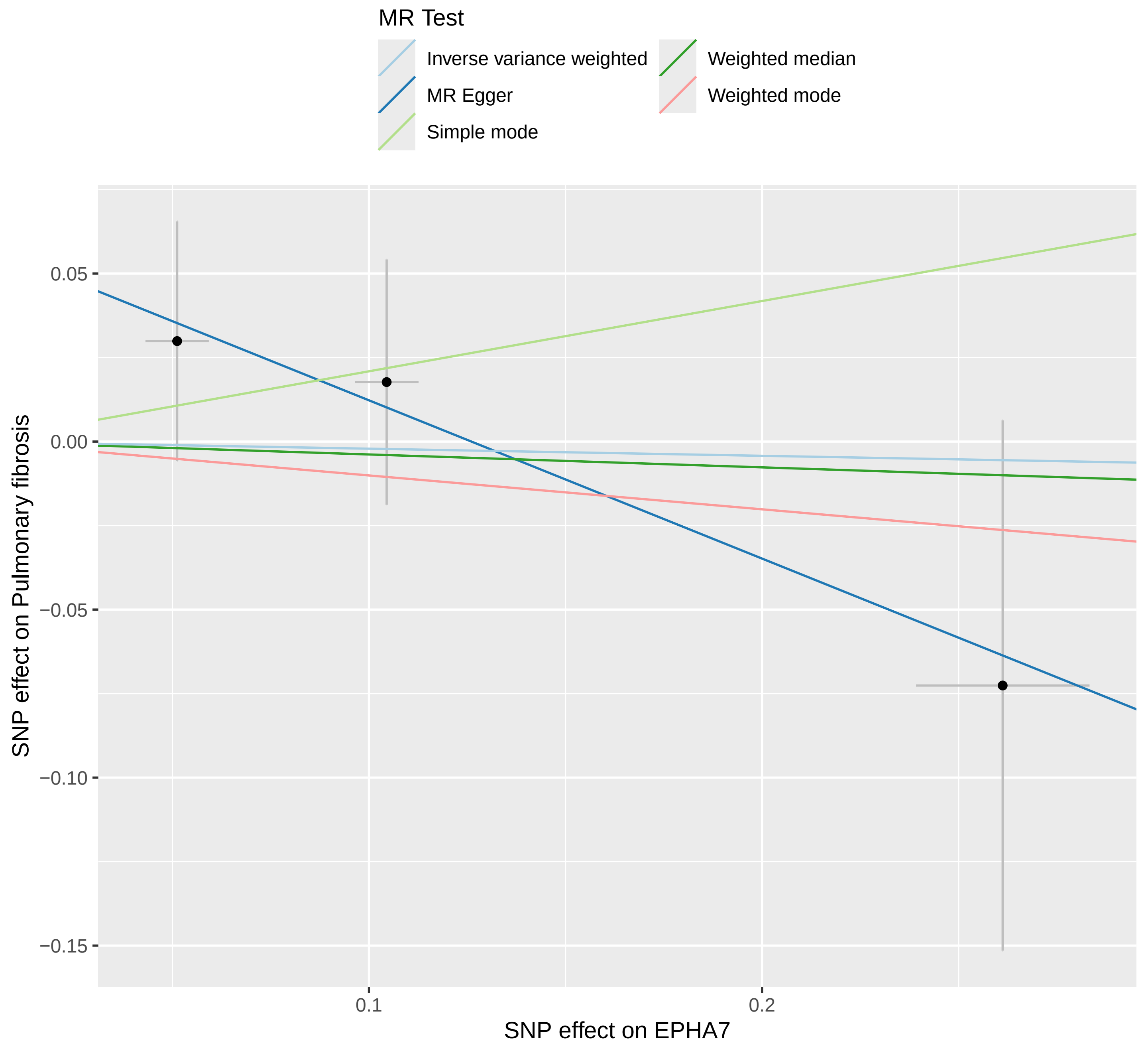

MR Test
Inverse variance weighted
MR Egger
Simple mode
Weighted median
Weighted mode
0.05
0.00
−0.05
−0.10
−0.15
SNP effect on Pulmonary fibrosis
0.1
0.2
SNP effect on EPHA7

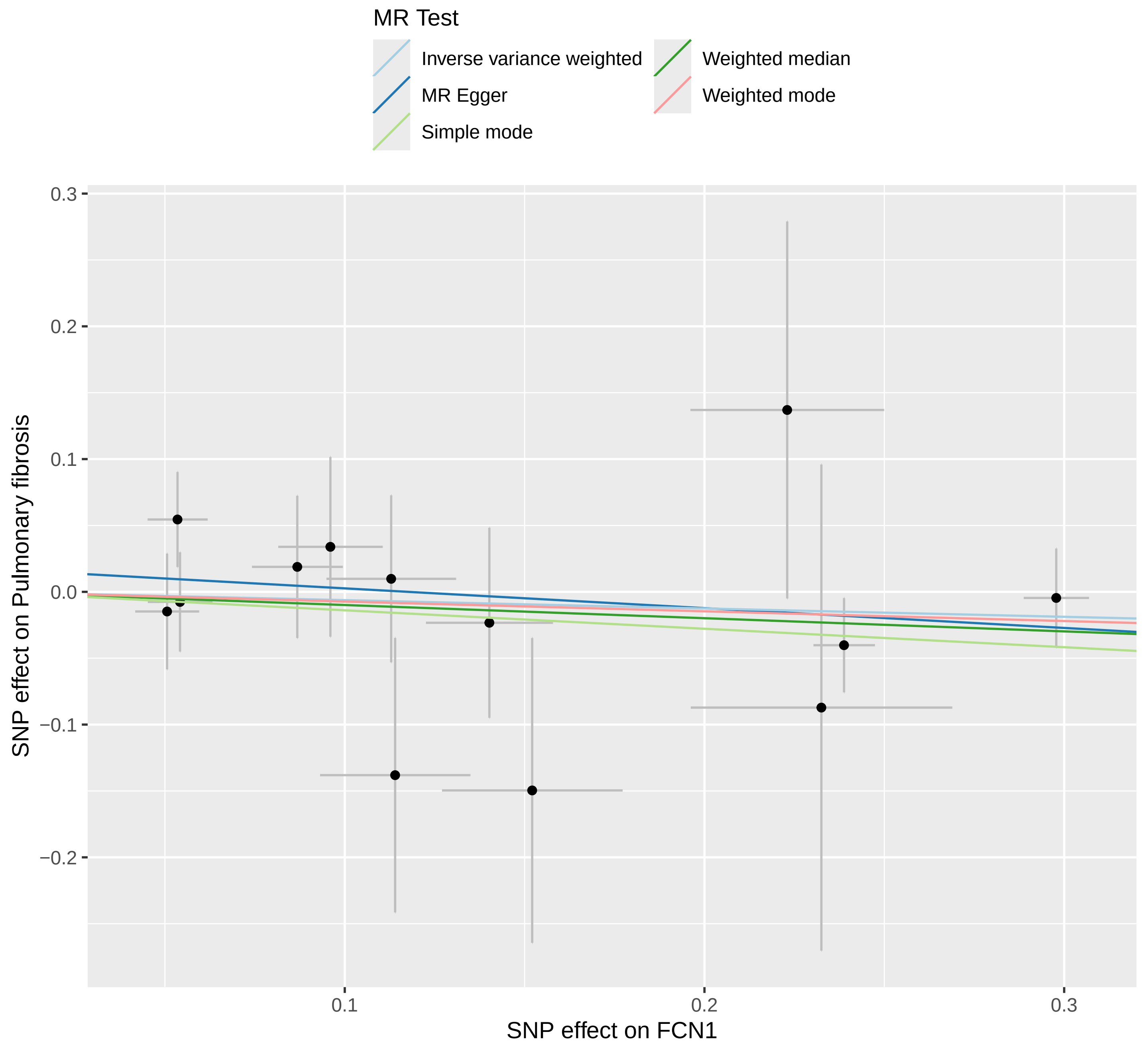
MR Test
Inverse variance weighted
MR Egger
Simple mode
Weighted median
Weighted mode
SNP effect on Pulmonary fibrosis
0.3
0.2
0.1
0.0
−0.1
−0.2
0.1
0.2
0.3
SNP effect on FCN1

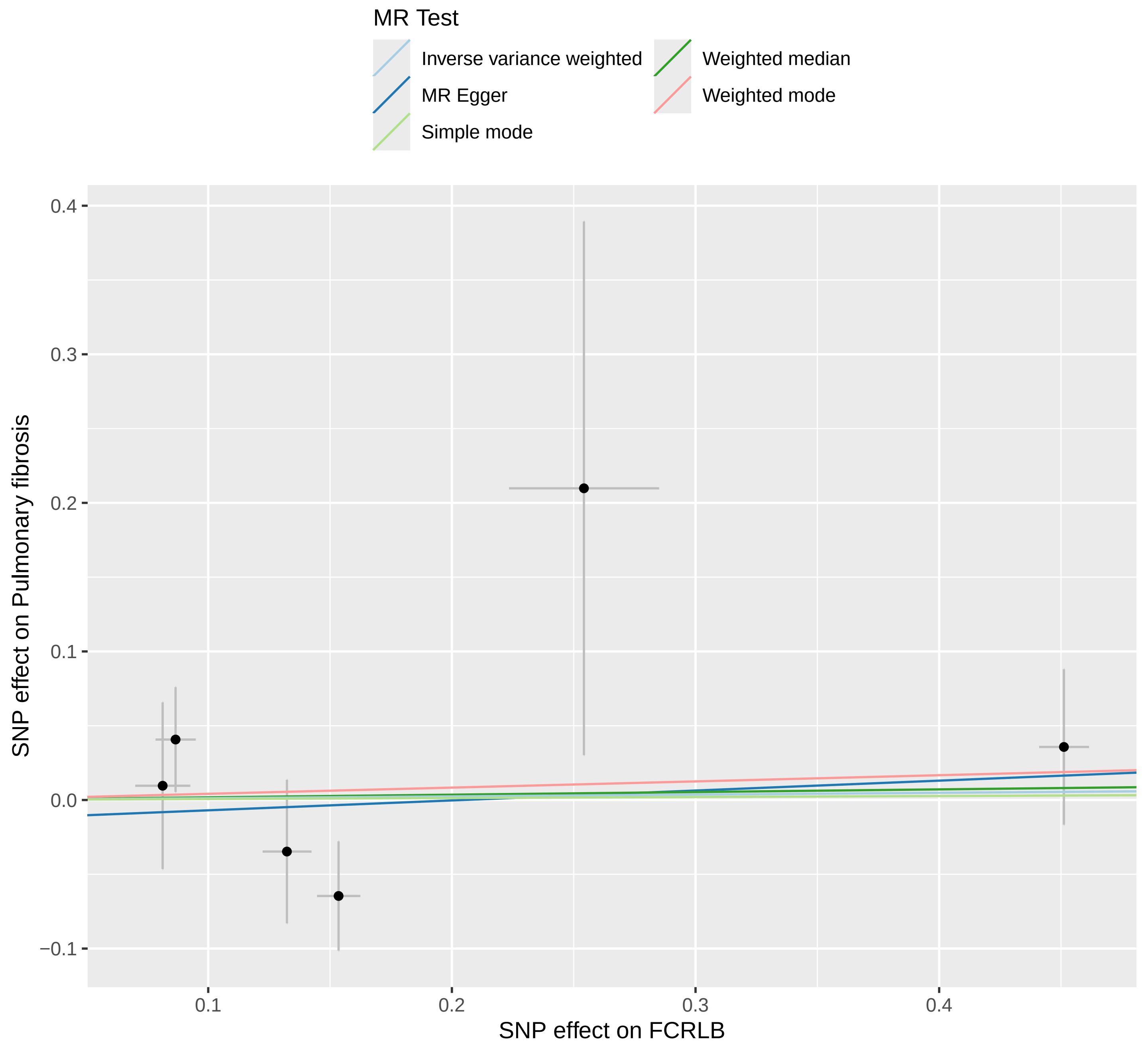
MR Test
Inverse variance weighted
MR Egger
Simple mode
Weighted median
Weighted mode
SNP effect on Pulmonary fibrosis
SNP effect on FCRLB
0.4
0.3
0.2
0.1
0.0
−0.1
0.1
0.2
0.3
0.4

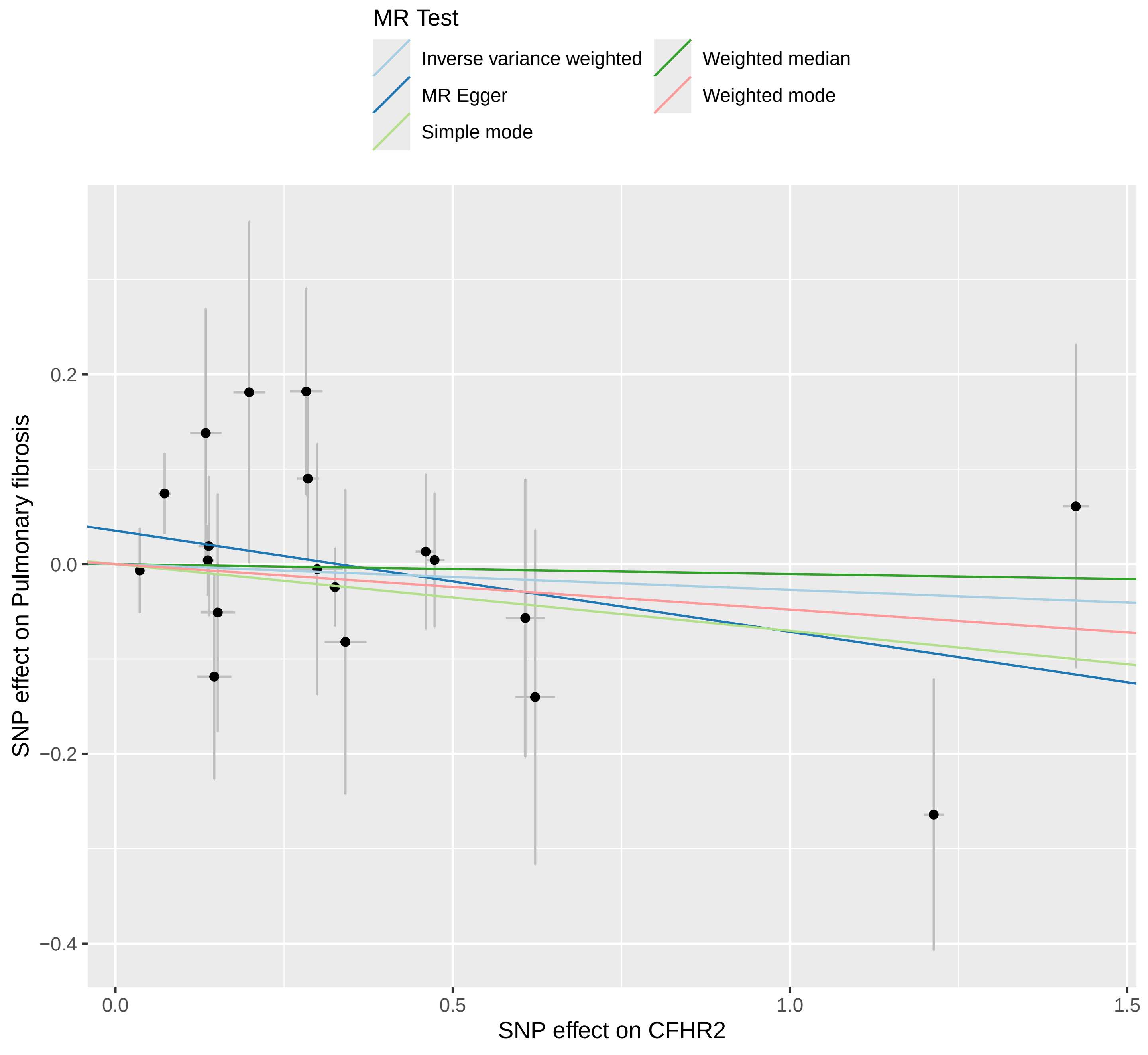
MR Test
Inverse variance weighted
MR Egger
Simple mode
Weighted median
Weighted mode
SNP effect on Pulmonary fibrosis
0.2
0.0
−0.2
−0.4
0.0
0.5
1.0
1.5
SNP effect on CFHR2

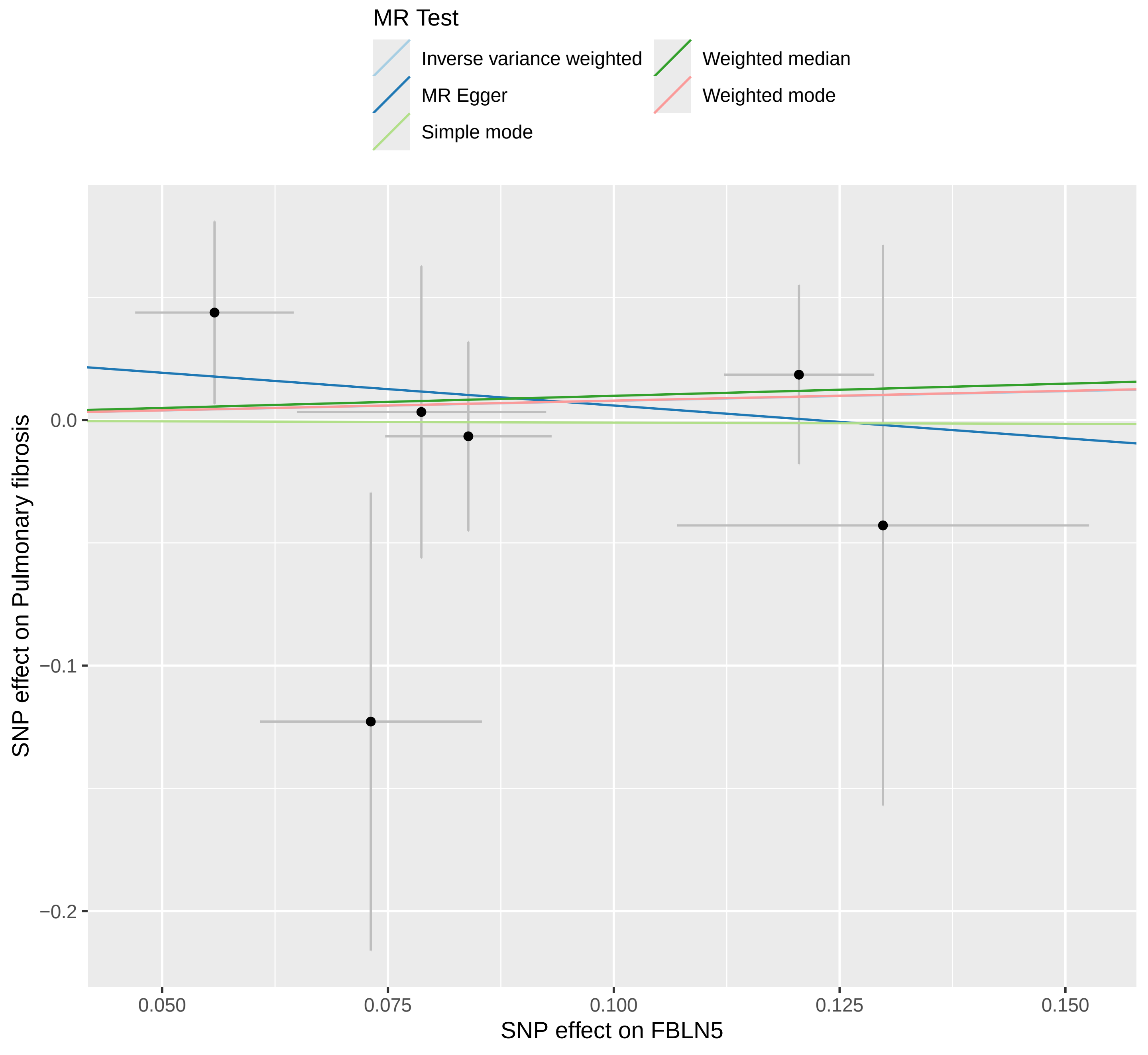
MR Test
Inverse variance weighted
MR Egger
Simple mode
Weighted median
Weighted mode
SNP effect on Pulmonary fibrosis
0.0
−0.1
−0.2
0.050
0.075
0.100
0.125
0.150
SNP effect on FBLN5

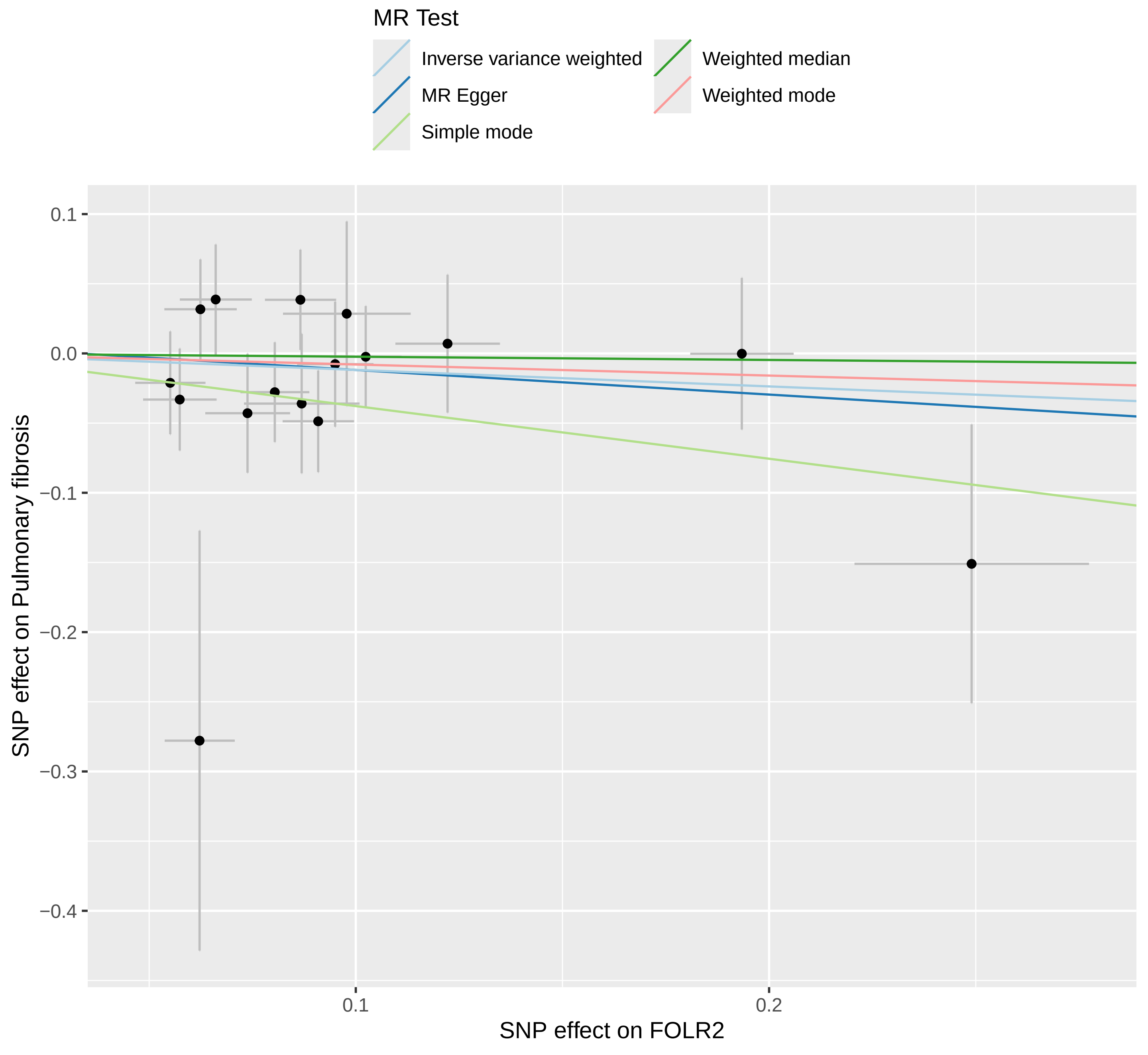

MR Test
Inverse variance weighted
MR Egger
Simple mode
Weighted median
Weighted mode
0.1
0.0
−0.1
−0.2
−0.3
−0.4
SNP effect on Pulmonary fibrosis
0.1
0.2
SNP effect on FOLR2

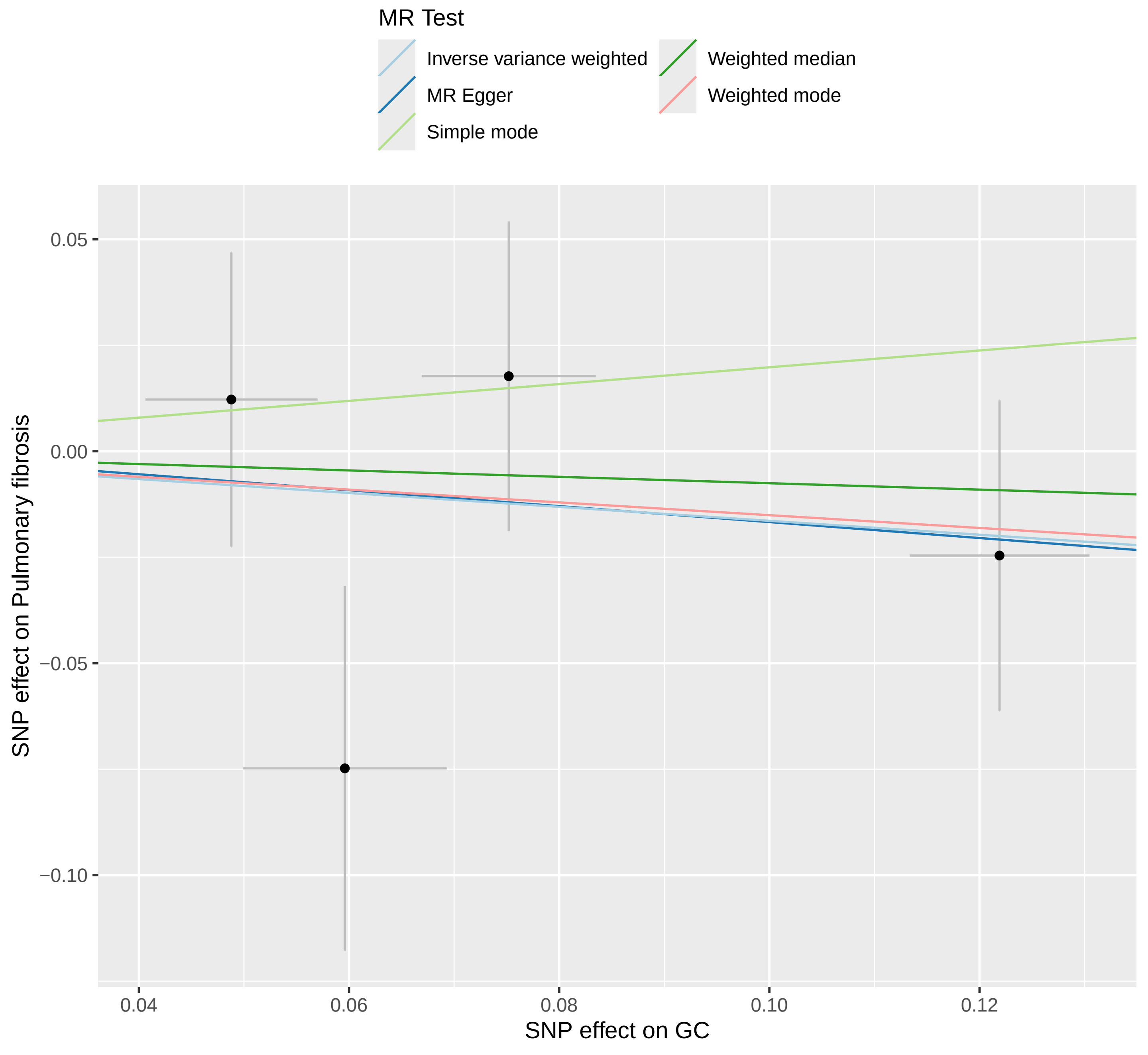
MR Test
Inverse variance weighted
MR Egger
Simple mode
Weighted median
Weighted mode
0.05
0.00
−0.05
−0.10
SNP effect on Pulmonary fibrosis
0.04
0.06
0.08
0.10
0.12
SNP effect on GC

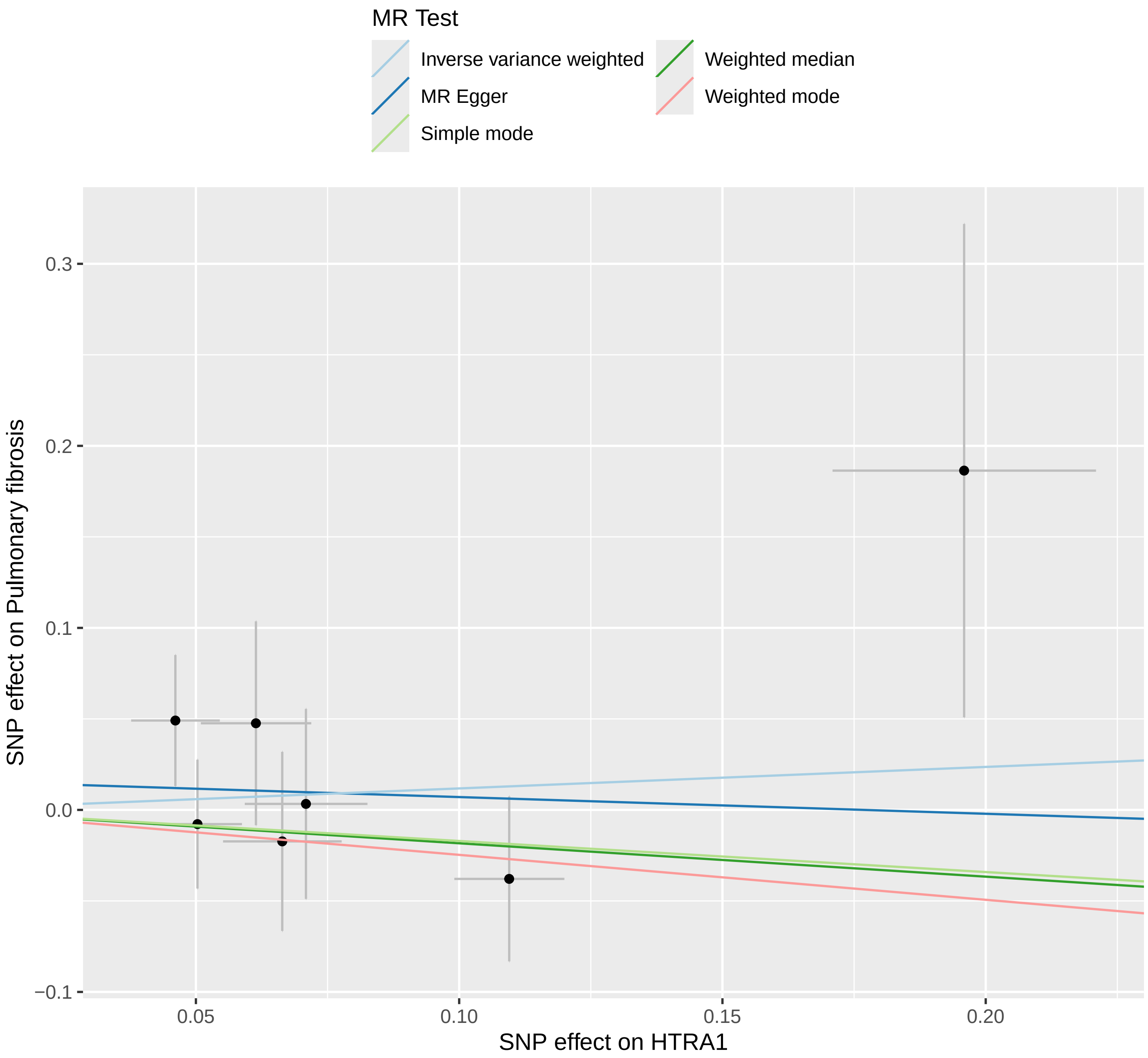
MR Test
Inverse variance weighted
MR Egger
Simple mode
Weighted median
Weighted mode
0.3
0.2
0.1
0.0
−0.1
0.05
0.10
0.15
0.20
SNP effect on Pulmonary fibrosis
SNP effect on HTRA1

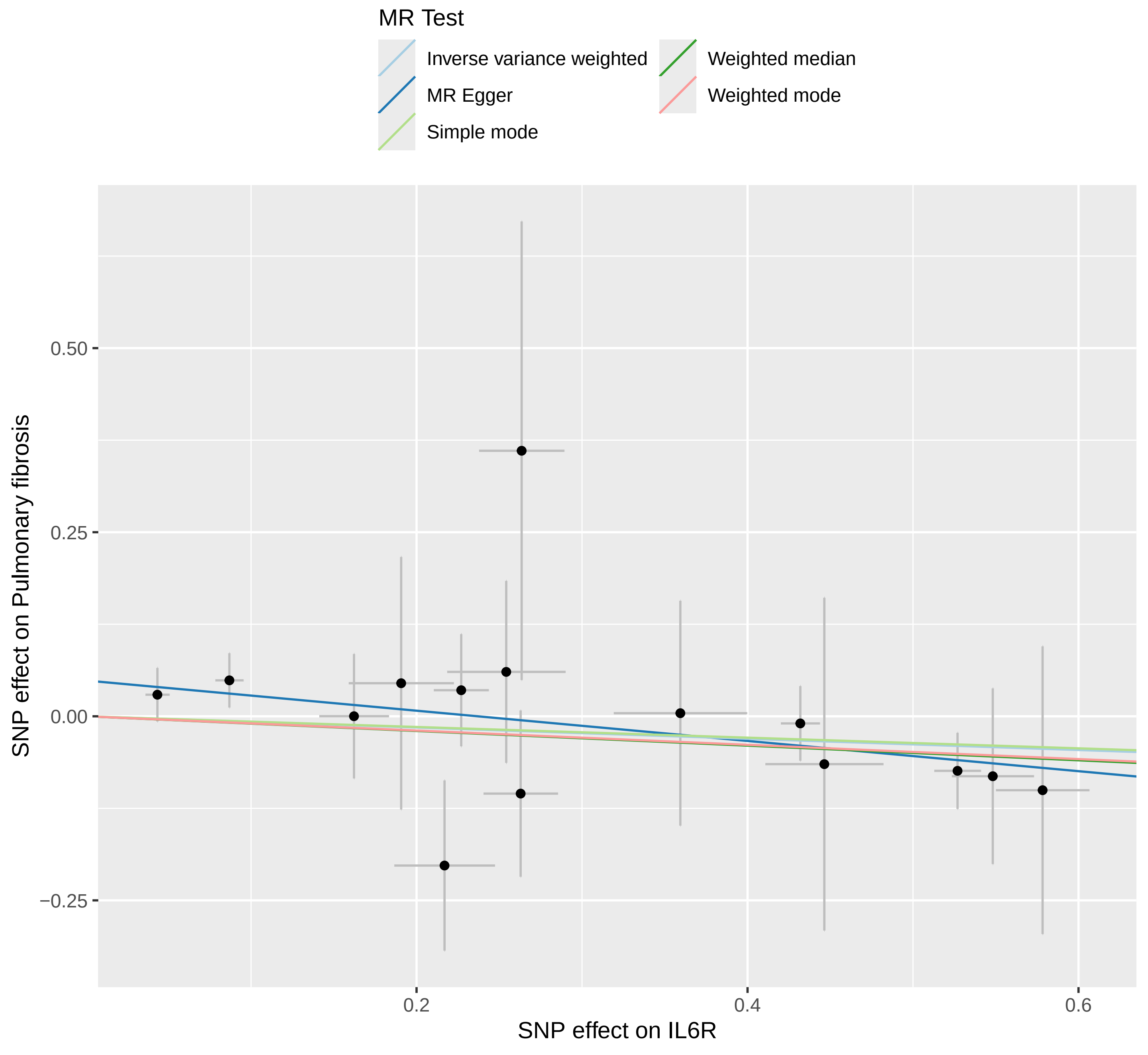
MR Test
Inverse variance weighted
MR Egger
Simple mode
Weighted median
Weighted mode
SNP effect on Pulmonary fibrosis
0.50
0.25
0.00
−0.25
0.2
0.4
0.6
SNP effect on IL6R

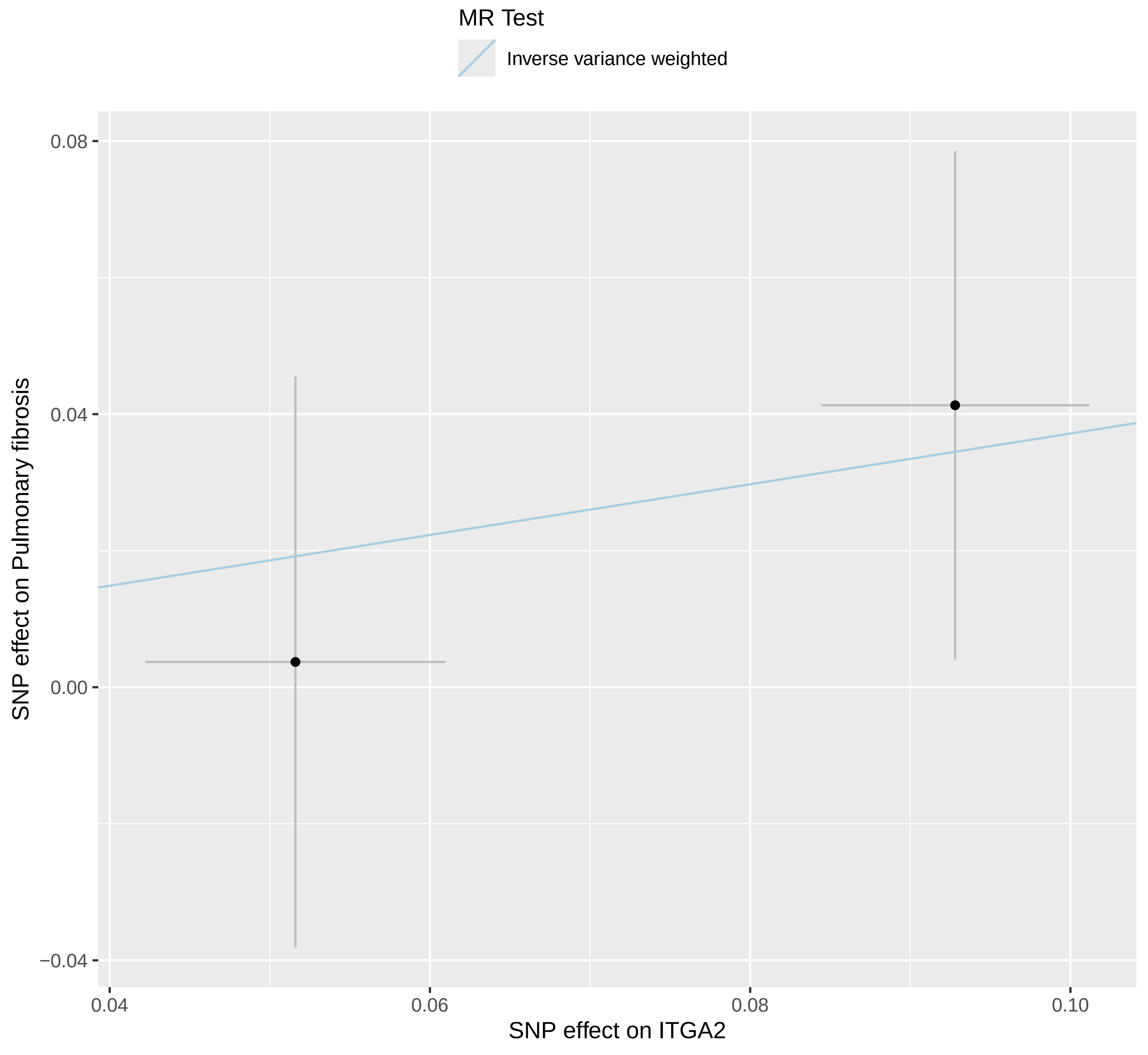

MR Test
Inverse variance weighted
0.08
0.04
0.00
−0.04
SNP effect on Pulmonary fibrosis
0.04
0.06
0.08
0.10
SNP effect on ITGA2

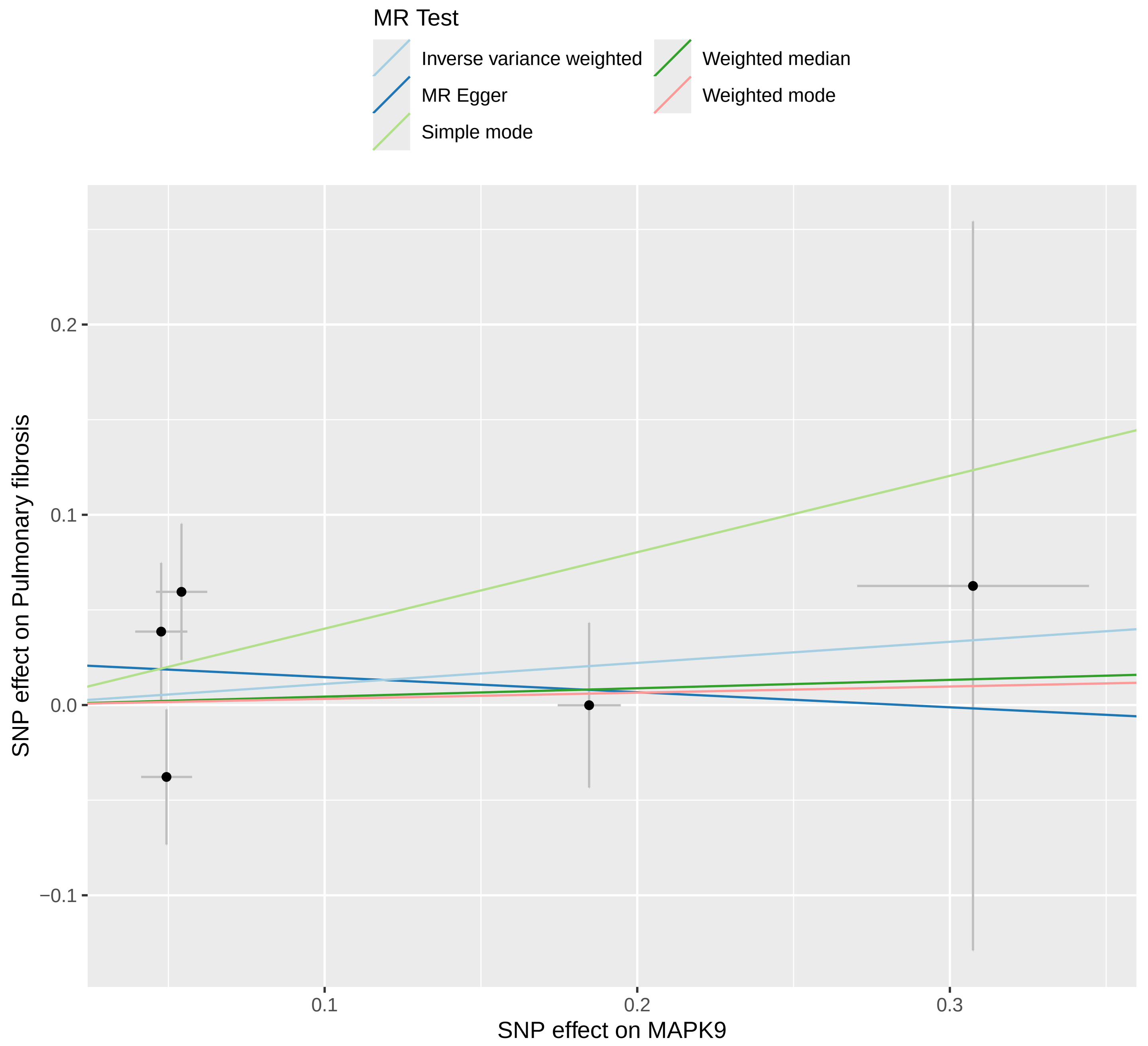

MR Test
Inverse variance weighted
MR Egger
Simple mode
Weighted median
Weighted mode
0.2
0.1
0.0
−0.1
0.1
0.2
0.3
SNP effect on Pulmonary fibrosis
SNP effect on MAPK9

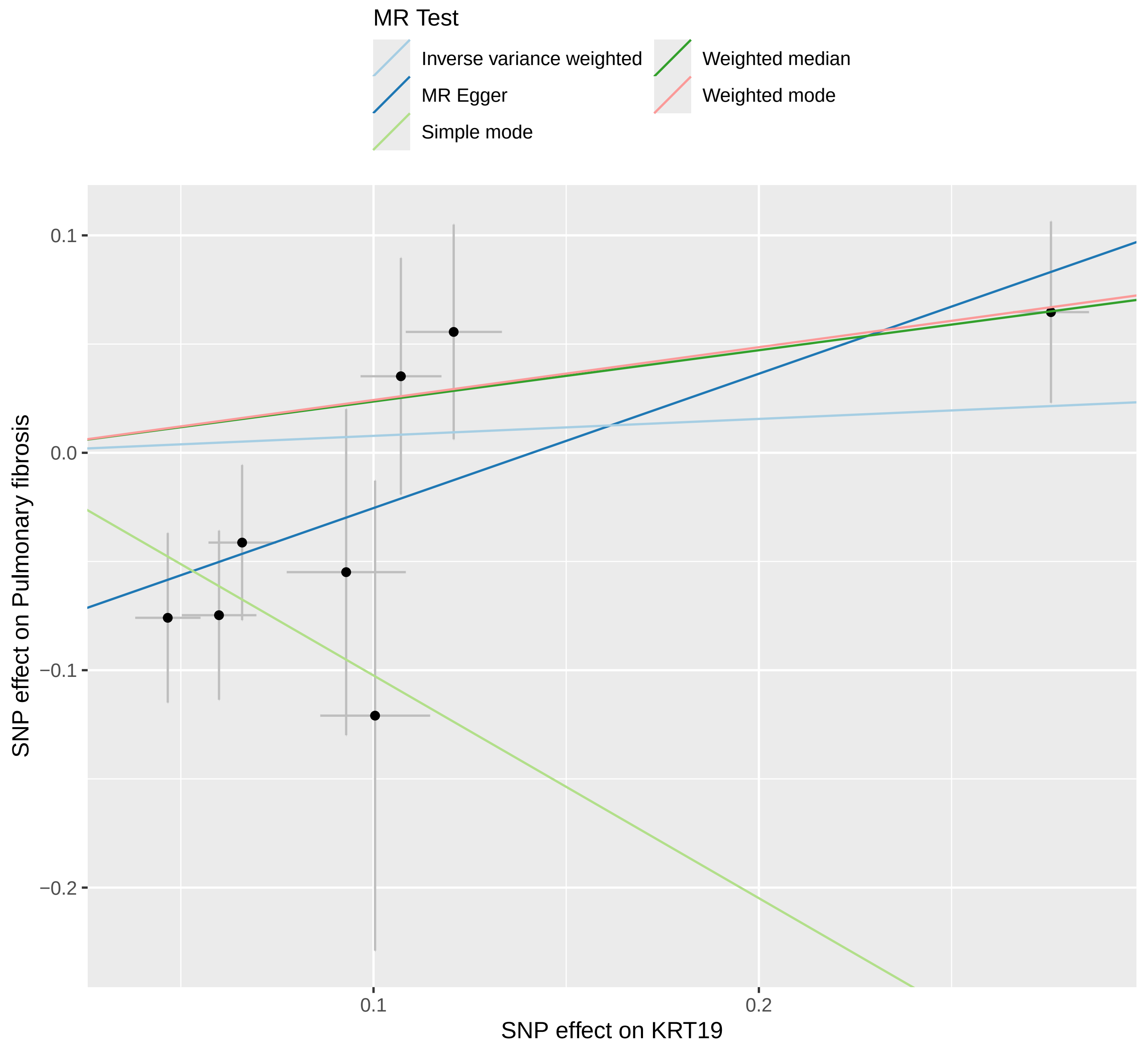

MR Test
Inverse variance weighted
MR Egger
Simple mode
Weighted median
Weighted mode
0.1
0.0
−0.1
−0.2
SNP effect on Pulmonary fibrosis
0.1
0.2
SNP effect on KRT19

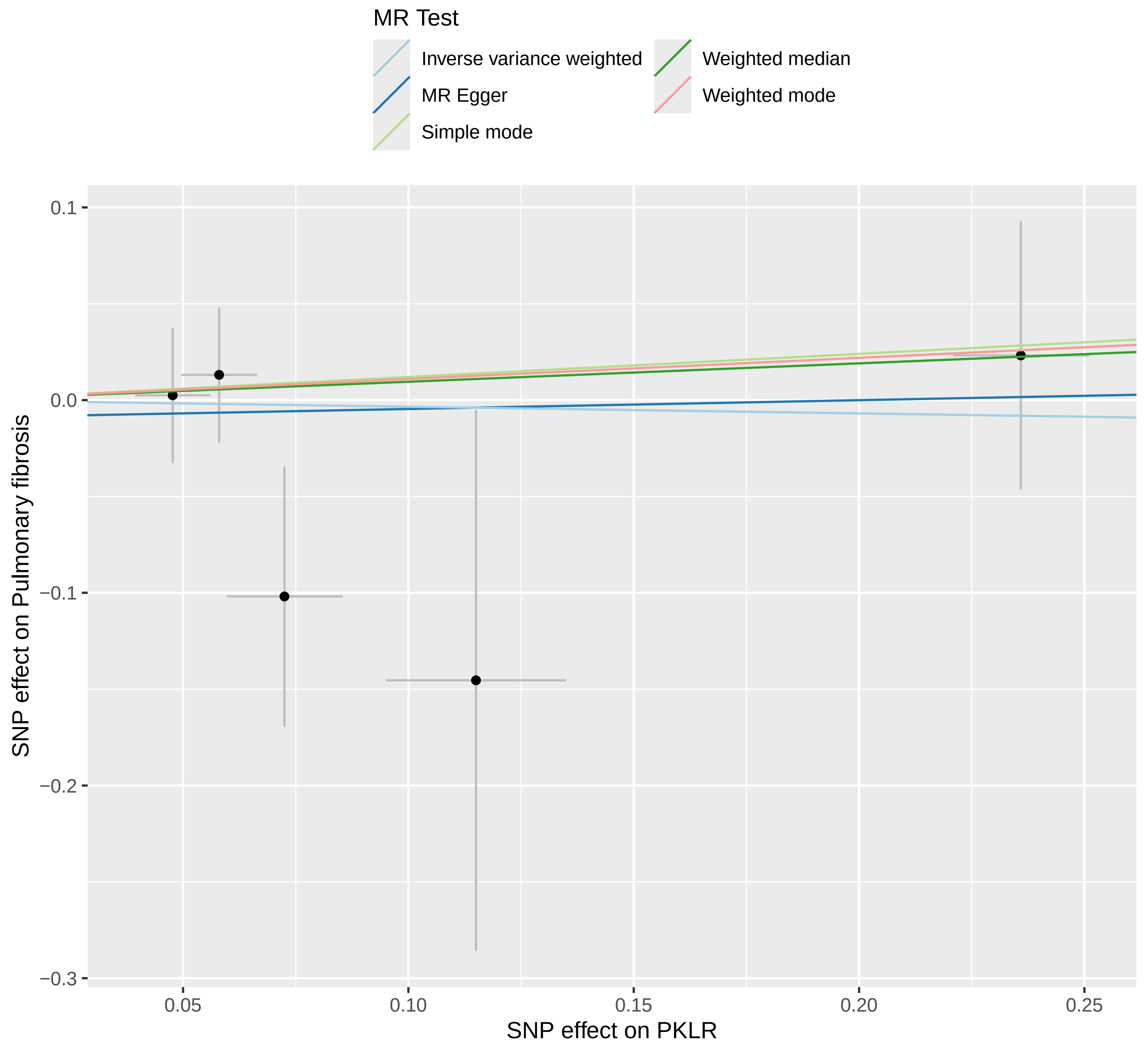

MR Test
Inverse variance weighted
MR Egger
Simple mode
Weighted median
Weighted mode
0.1
0.0
−0.1
−0.2
−0.3
SNP effect on Pulmonary fibrosis
0.05
0.10
0.15
0.20
0.25
SNP effect on PKLR

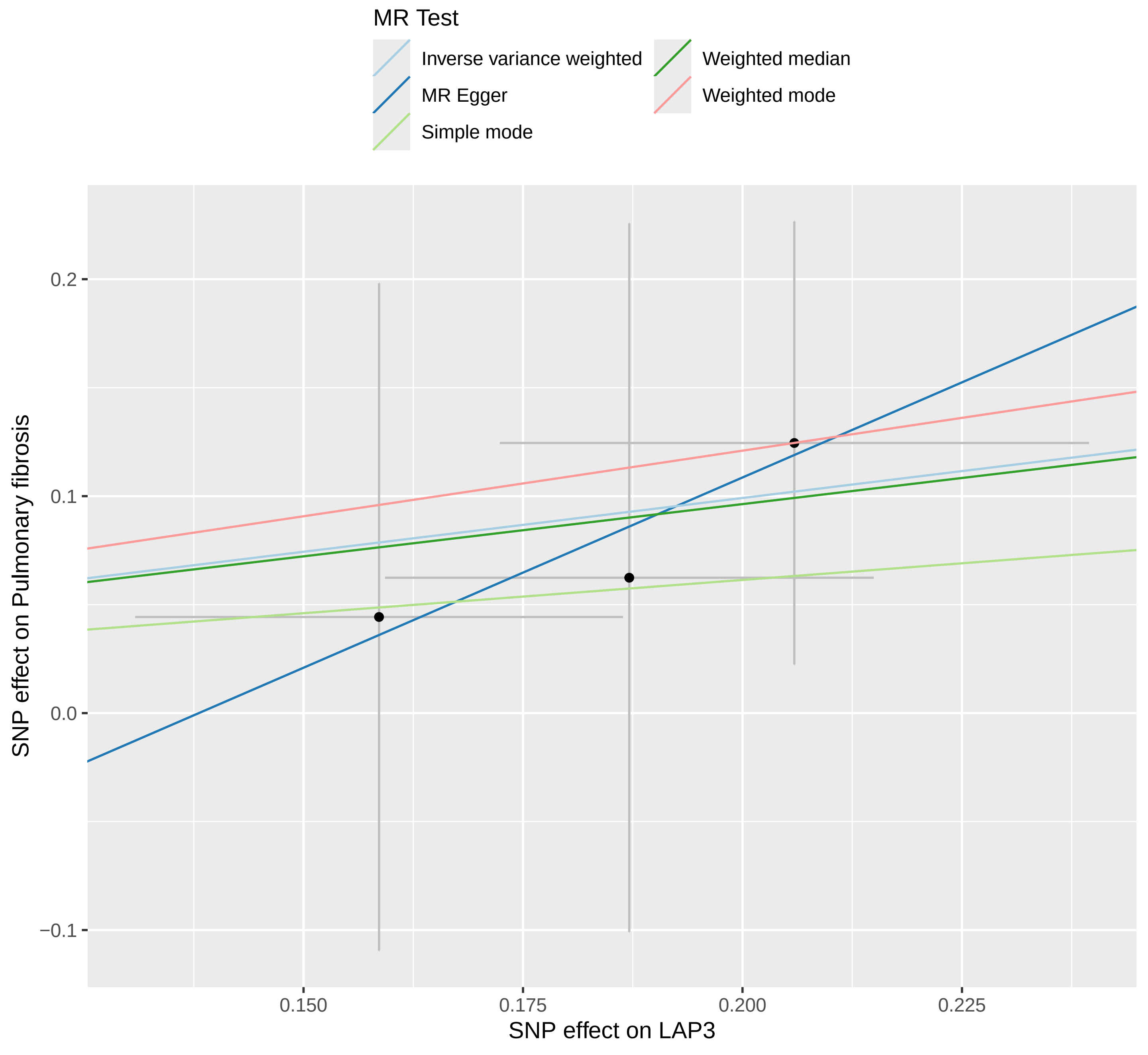
MR Test
Inverse variance weighted
MR Egger
Simple mode
Weighted median
Weighted mode
0.2
0.1
0.0
−0.1
SNP effect on Pulmonary fibrosis
0.150
0.175
0.200
0.225
SNP effect on LAP3

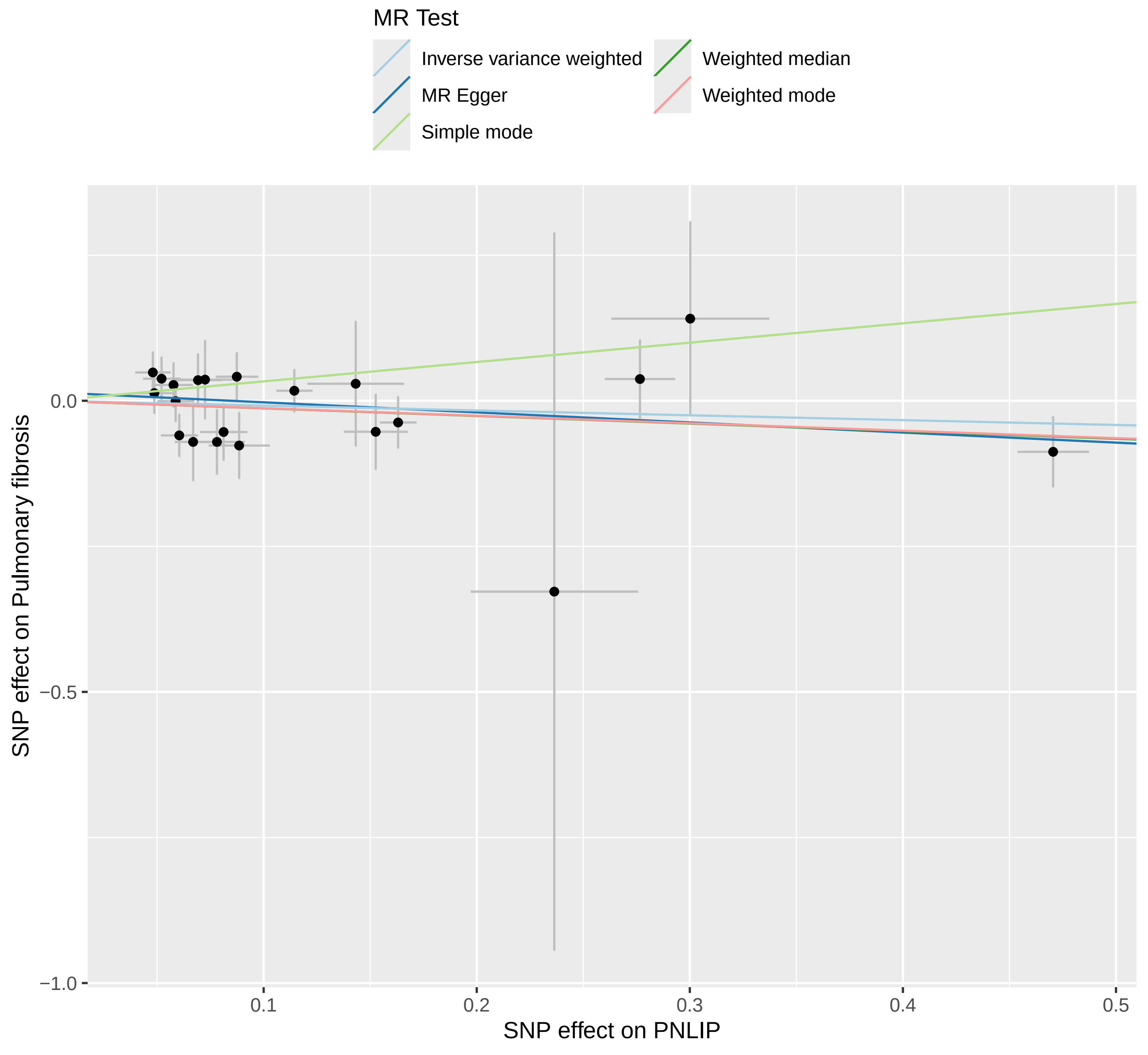

MR Test
Inverse variance weighted
MR Egger
Simple mode
Weighted median
Weighted mode
0.0
−0.5
−1.0
0.1
0.2
0.3
0.4
0.5
SNP effect on PNLIP
SNP effect on Pulmonary fibrosis

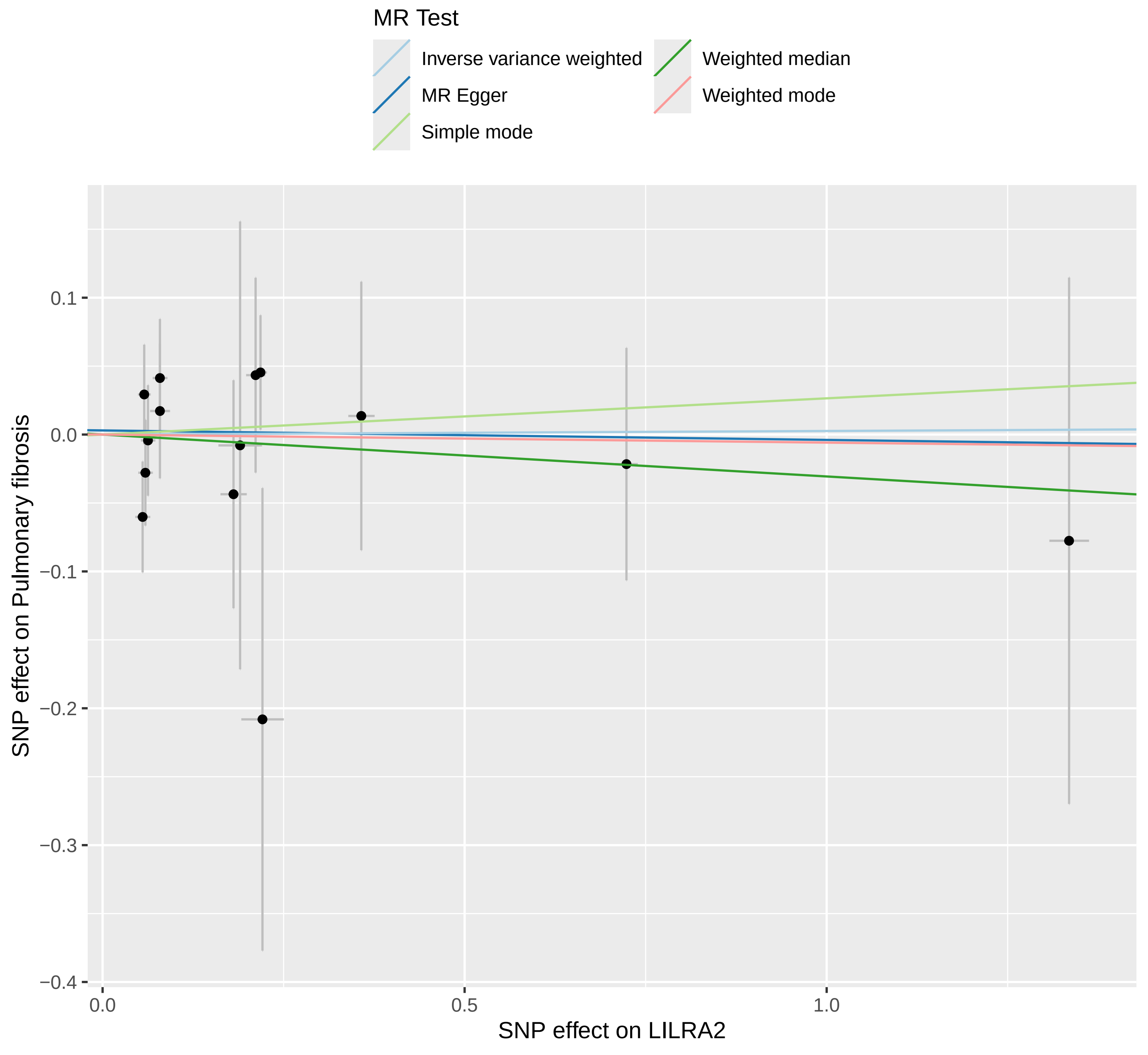
MR Test
Inverse variance weighted
MR Egger
Simple mode
Weighted median
Weighted mode
SNP effect on Pulmonary fibrosis
0.1
0.0
−0.1
−0.2
−0.3
−0.4
0.0
0.5
1.0
SNP effect on LILRA2

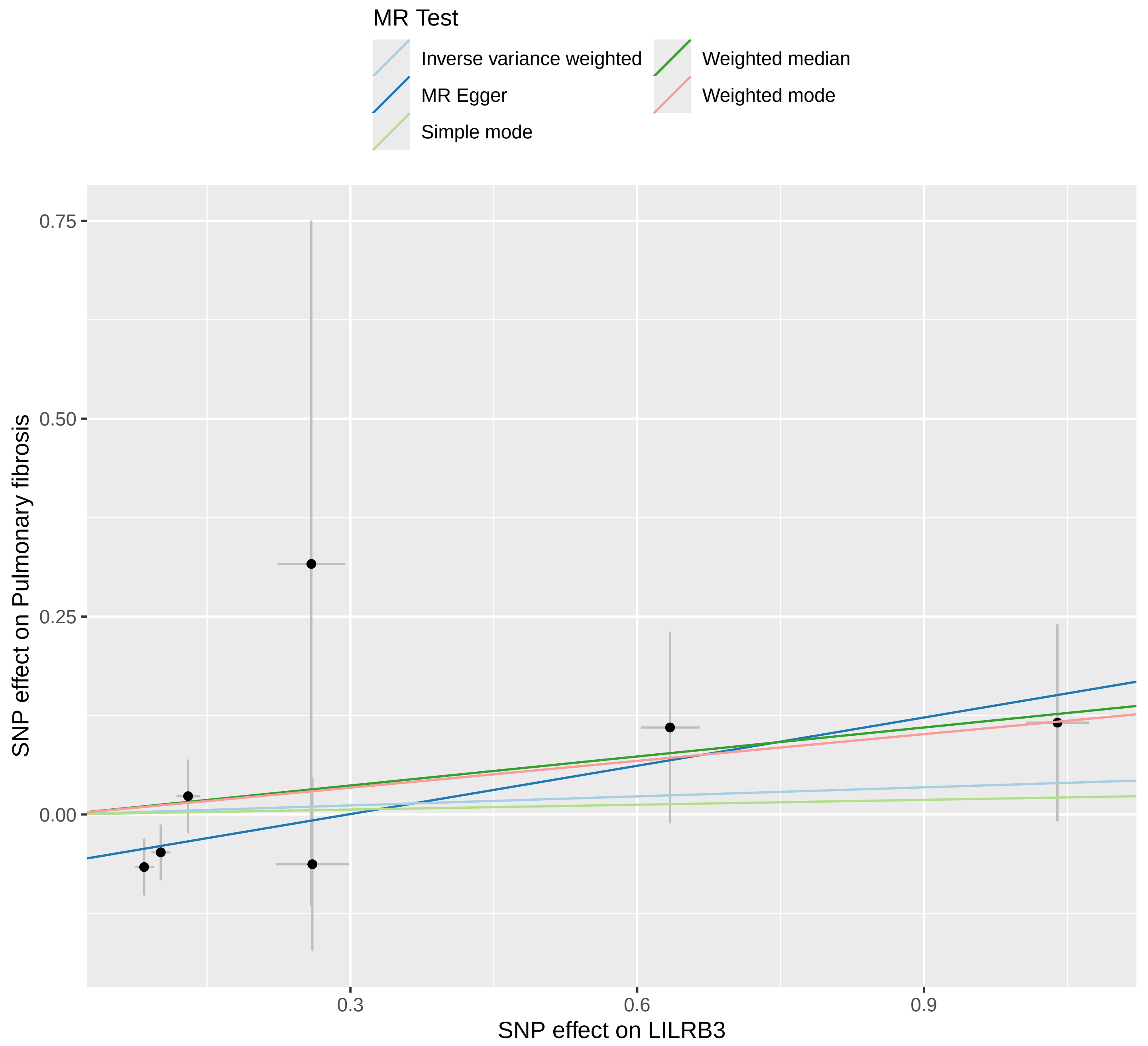

MR Test
Inverse variance weighted
MR Egger
Simple mode
Weighted median
Weighted mode
0.75
0.50
0.25
0.00
0.3
0.6
0.9
SNP effect on Pulmonary fibrosis
SNP effect on LILRB3

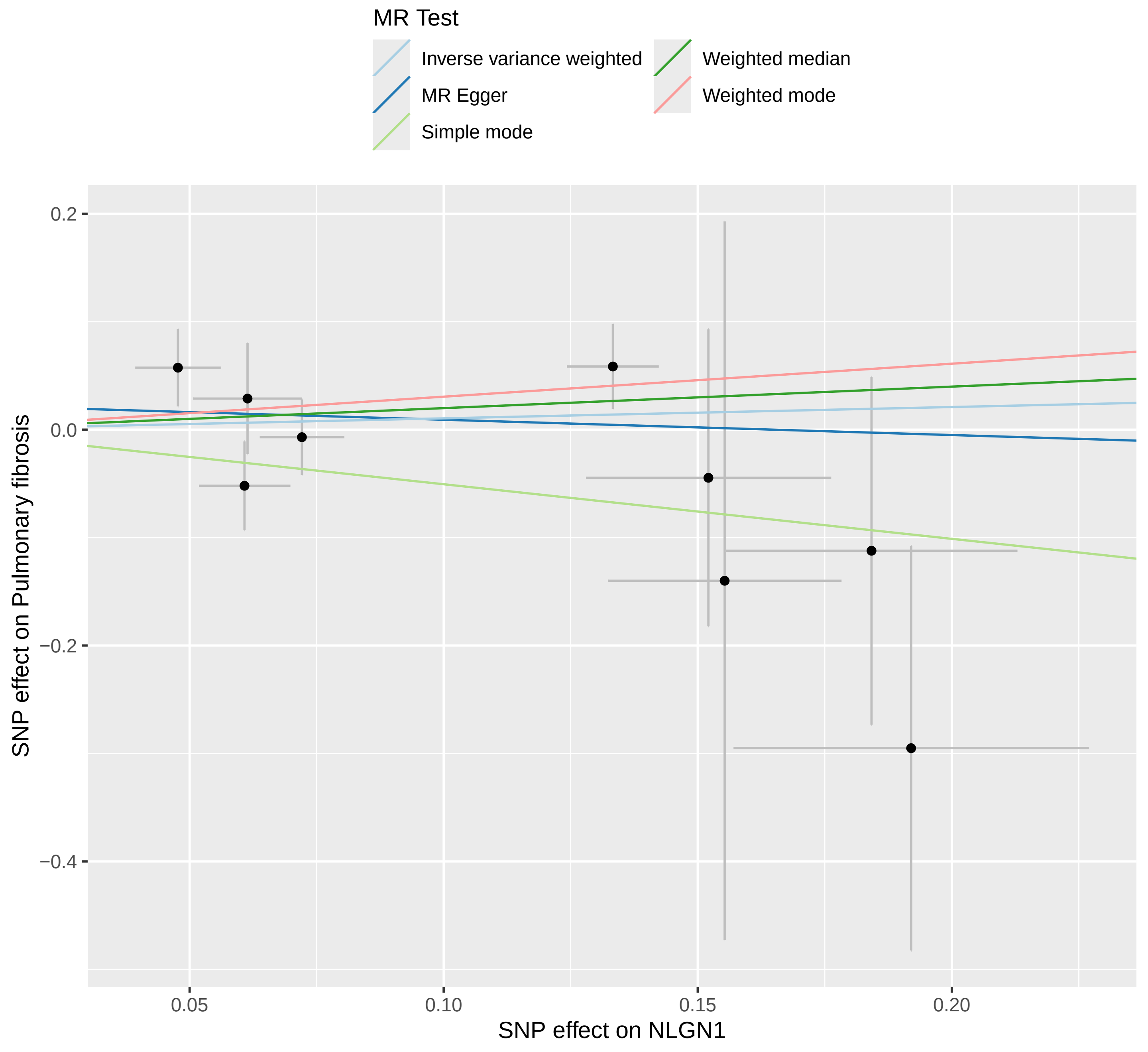
MR Test
Inverse variance weighted
MR Egger
Simple mode
Weighted median
Weighted mode
0.2
0.0
−0.2
−0.4
SNP effect on Pulmonary fibrosis
0.05
0.10
0.15
0.20
SNP effect on NLGN1

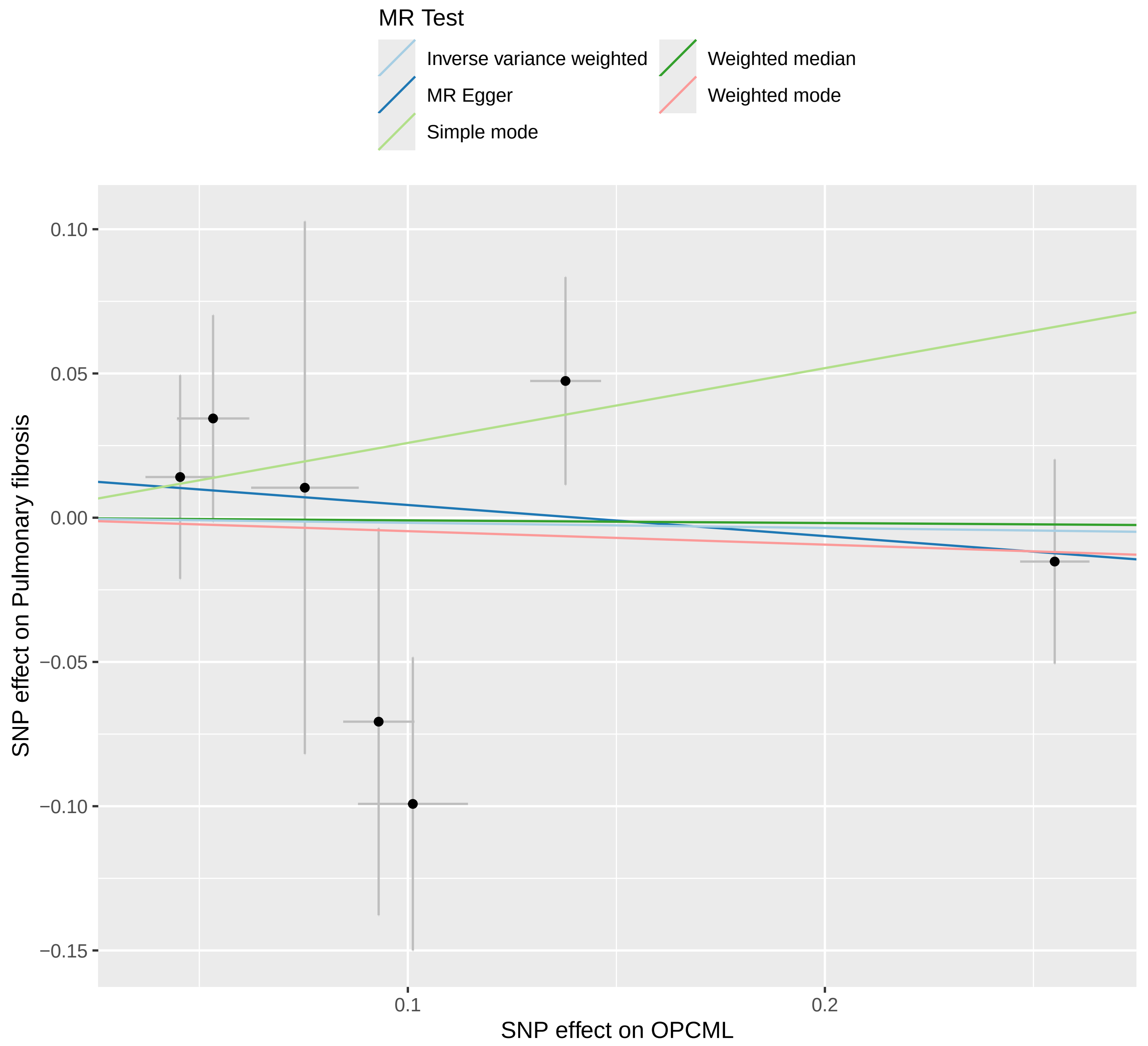
MR Test
Inverse variance weighted
MR Egger
Simple mode
Weighted median
Weighted mode
0.10
0.05
0.00
−0.05
−0.10
−0.15
SNP effect on Pulmonary fibrosis
0.1
0.2
SNP effect on OPCML

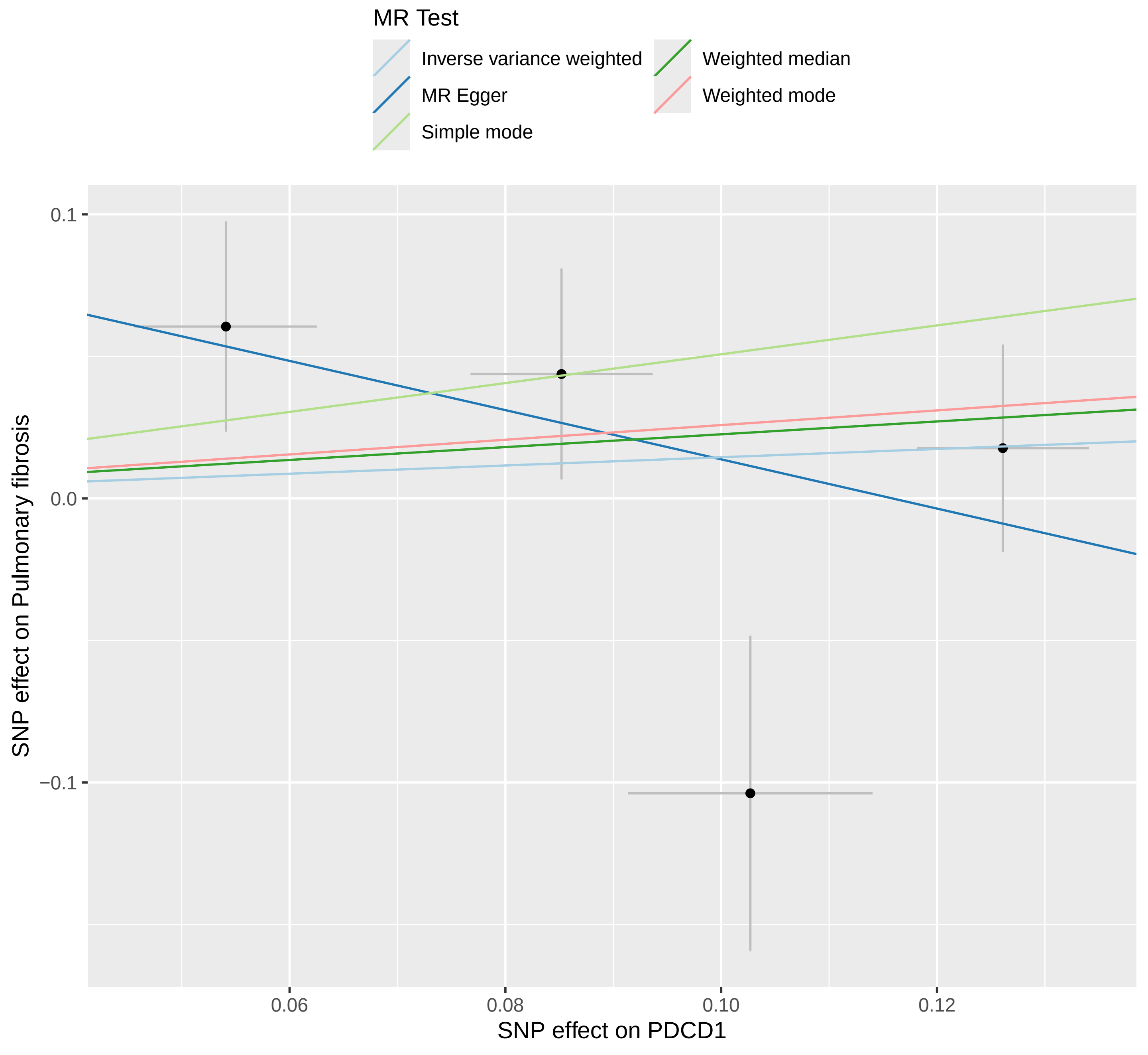

MR Test
Inverse variance weighted
MR Egger
Simple mode
Weighted median
Weighted mode
0.1
0.0
−0.1
SNP effect on Pulmonary fibrosis
0.06
0.08
0.10
0.12
SNP effect on PDCD1

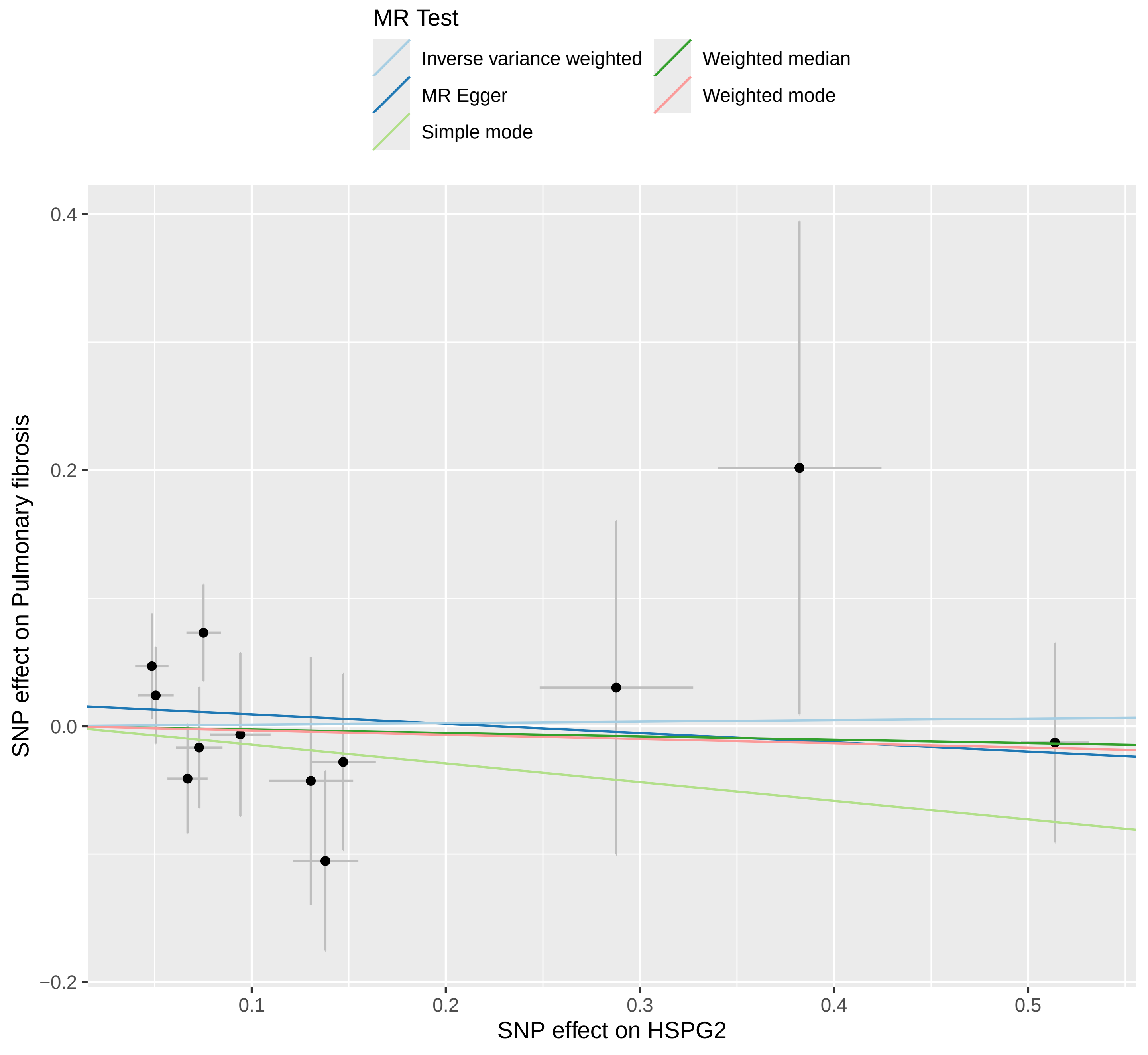
MR Test
Inverse variance weighted
MR Egger
Simple mode
Weighted median
Weighted mode
0.4
0.2
0.0
−0.2
SNP effect on Pulmonary fibrosis
0.1
0.2
0.3
0.4
0.5
SNP effect on HSPG2

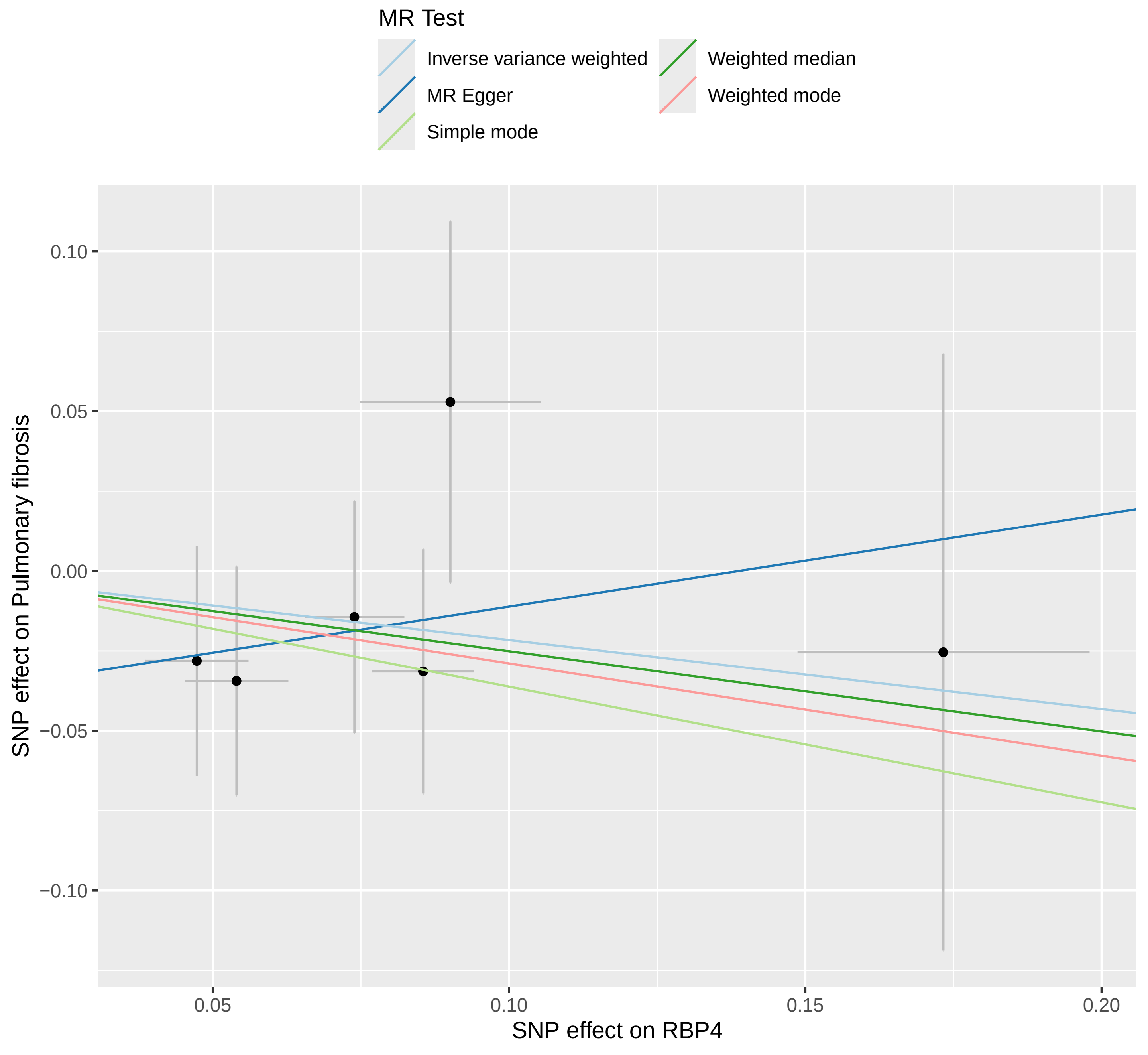

MR Test
Inverse variance weighted
MR Egger
Simple mode
Weighted median
Weighted mode
0.10
0.05
0.00
−0.05
−0.10
SNP effect on Pulmonary fibrosis
0.05
0.10
0.15
0.20
SNP effect on RBP4

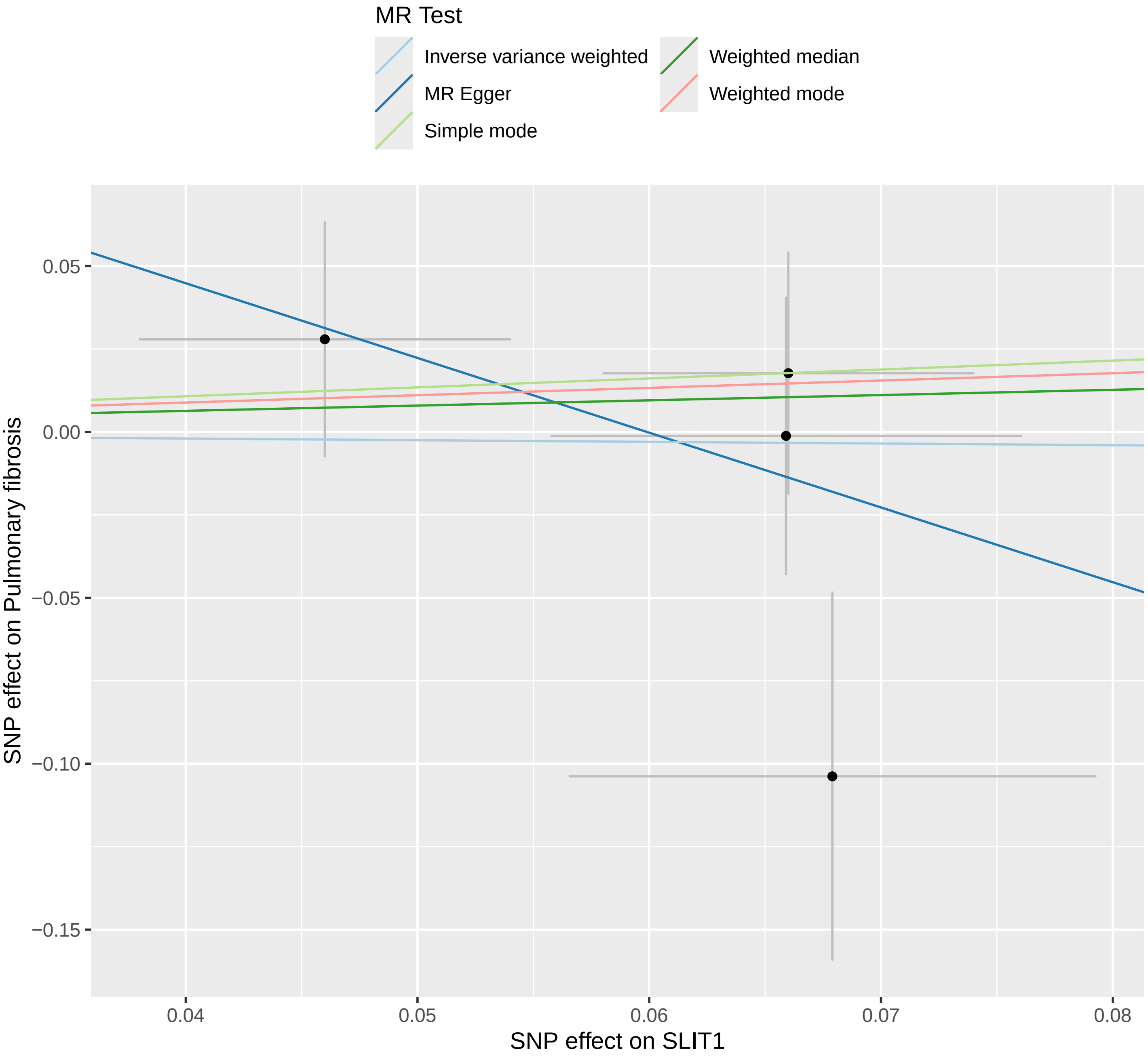
MR Test
Inverse variance weighted
MR Egger
Simple mode
Weighted median
Weighted mode
0.05
0.00
−0.05
−0.10
−0.15
0.04
0.05
0.06
0.07
0.08
SNP effect on Pulmonary fibrosis
SNP effect on SLIT1

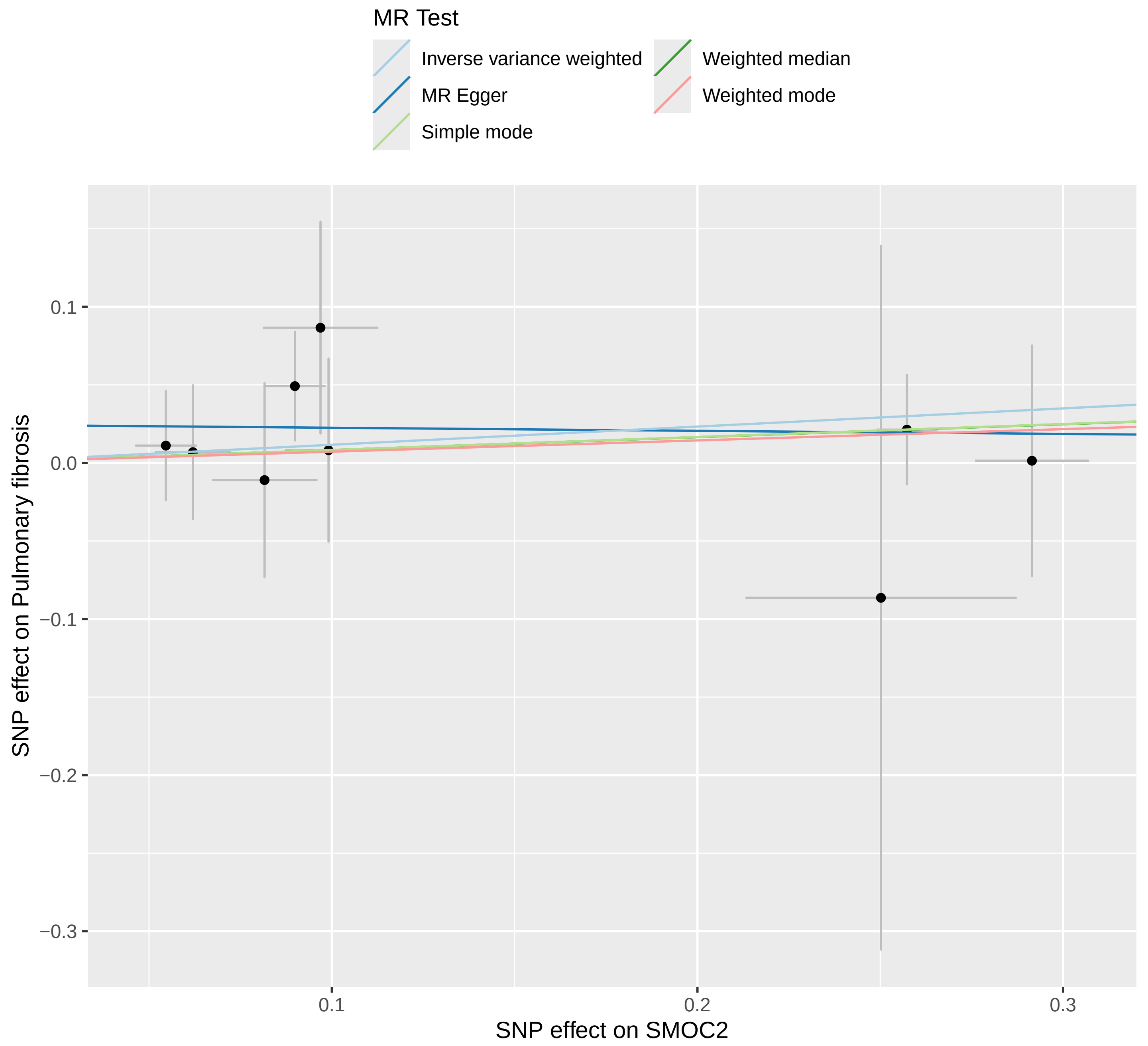

MR Test
Inverse variance weighted
MR Egger
Simple mode
Weighted median
Weighted mode
SNP effect on Pulmonary fibrosis
0.1
0.0
−0.1
−0.2
−0.3
0.1
0.2
0.3
SNP effect on SMOC2

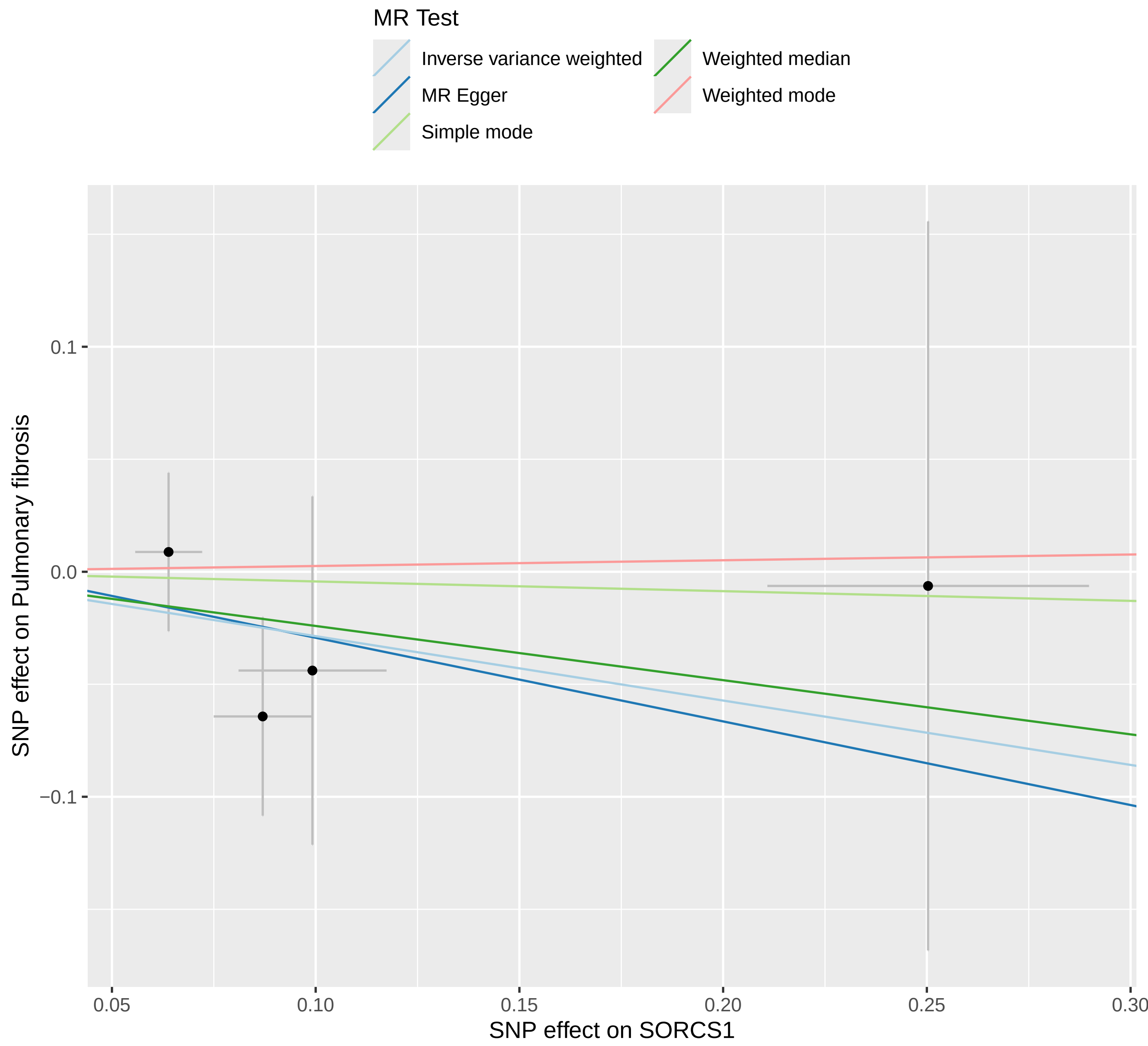
MR Test
Inverse variance weighted
MR Egger
Simple mode
Weighted median
Weighted mode
0.1
0.0
−0.1
0.05
0.10
0.15
0.20
0.25
0.30
SNP effect on SORCS1
SNP effect on Pulmonary fibrosis

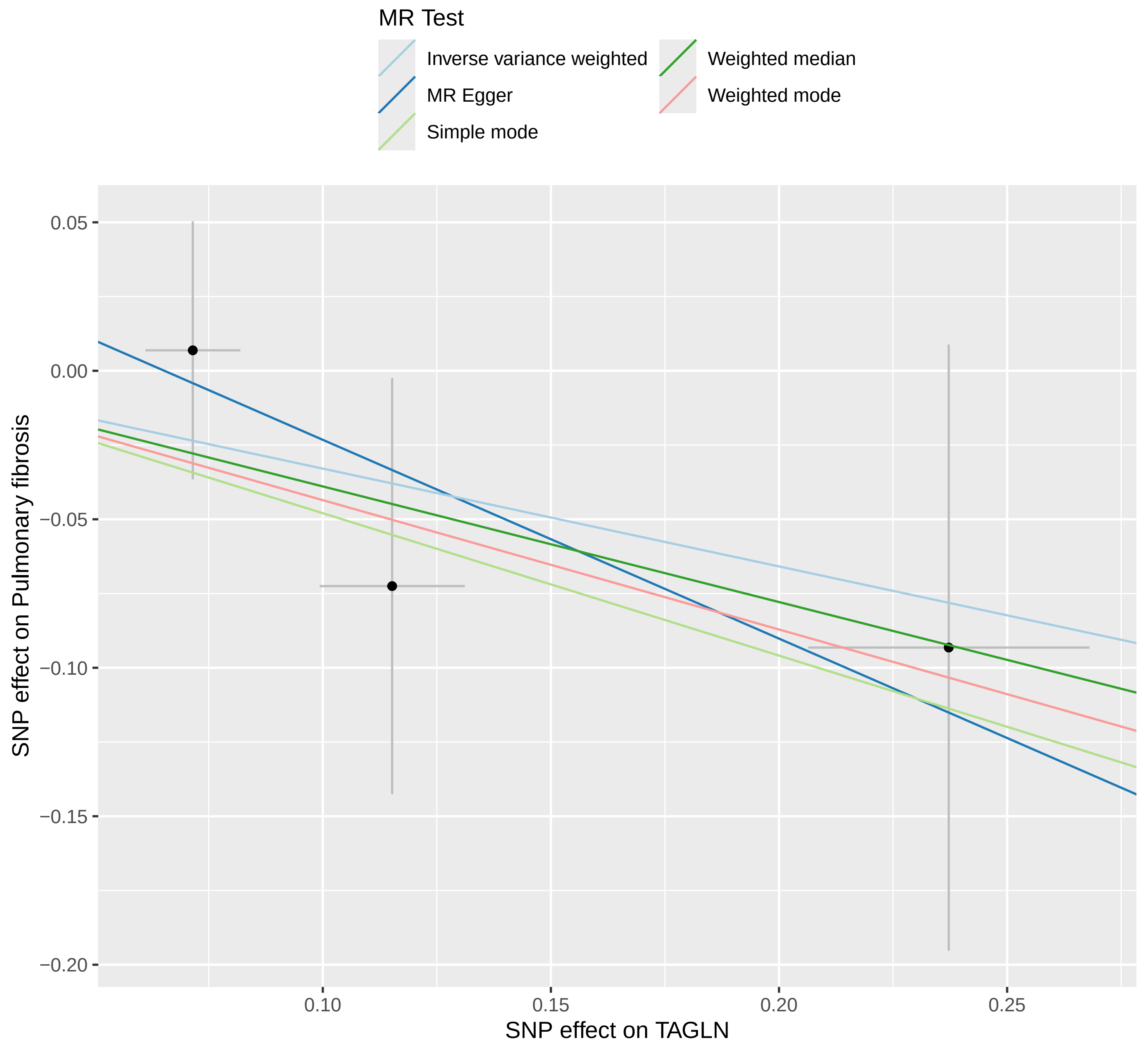

MR Test
Inverse variance weighted
MR Egger
Simple mode
Weighted median
Weighted mode
SNP effect on Pulmonary fibrosis
0.05
0.00
−0.05
−0.10
−0.15
−0.20
0.10
0.15
0.20
0.25
SNP effect on TAGLN

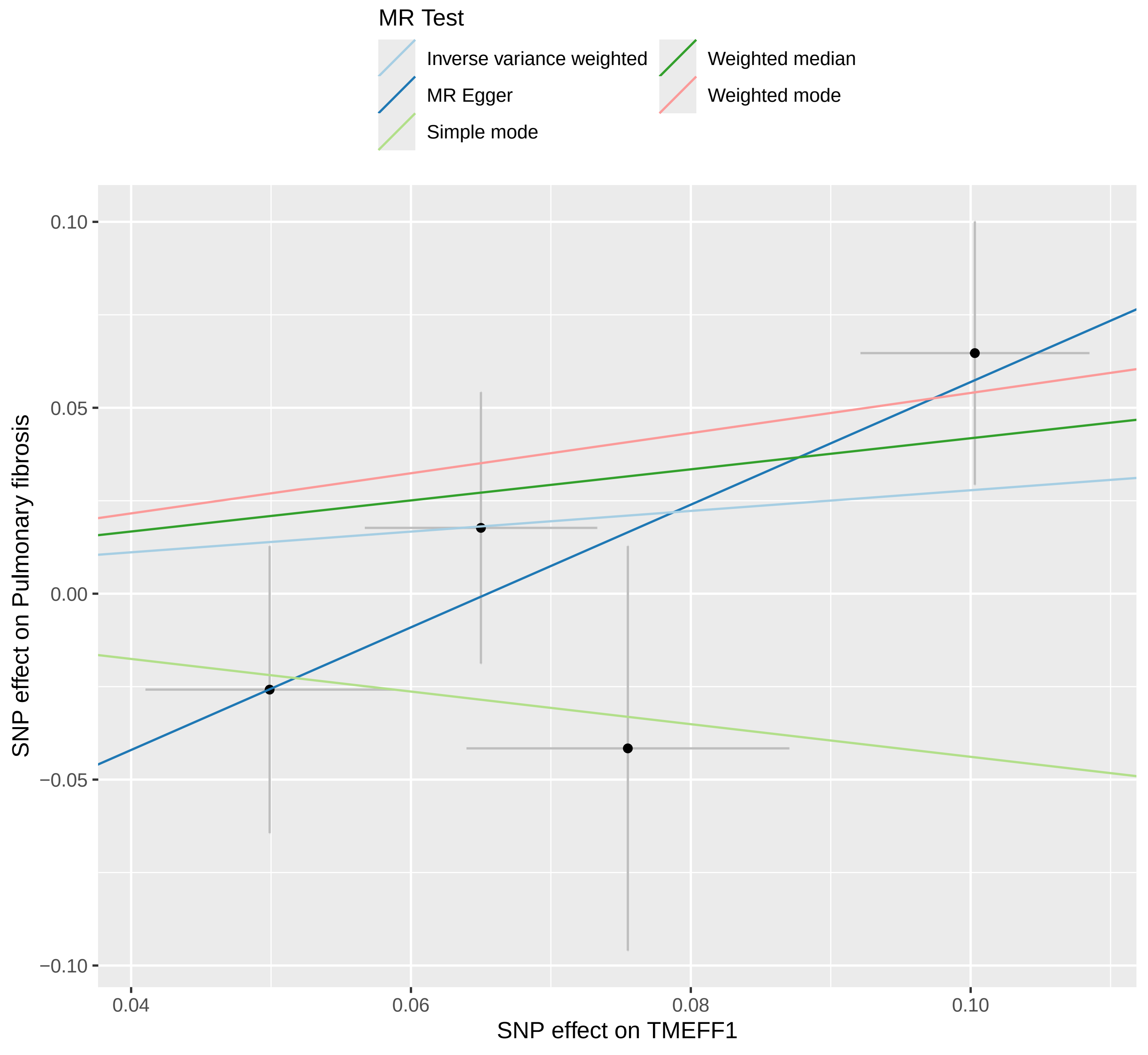

MR Test
Inverse variance weighted
MR Egger
Simple mode
Weighted median
Weighted mode
0.10
0.05
0.00
−0.05
−0.10
SNP effect on Pulmonary fibrosis
0.04
0.06
0.08
0.10
SNP effect on TMEFF1

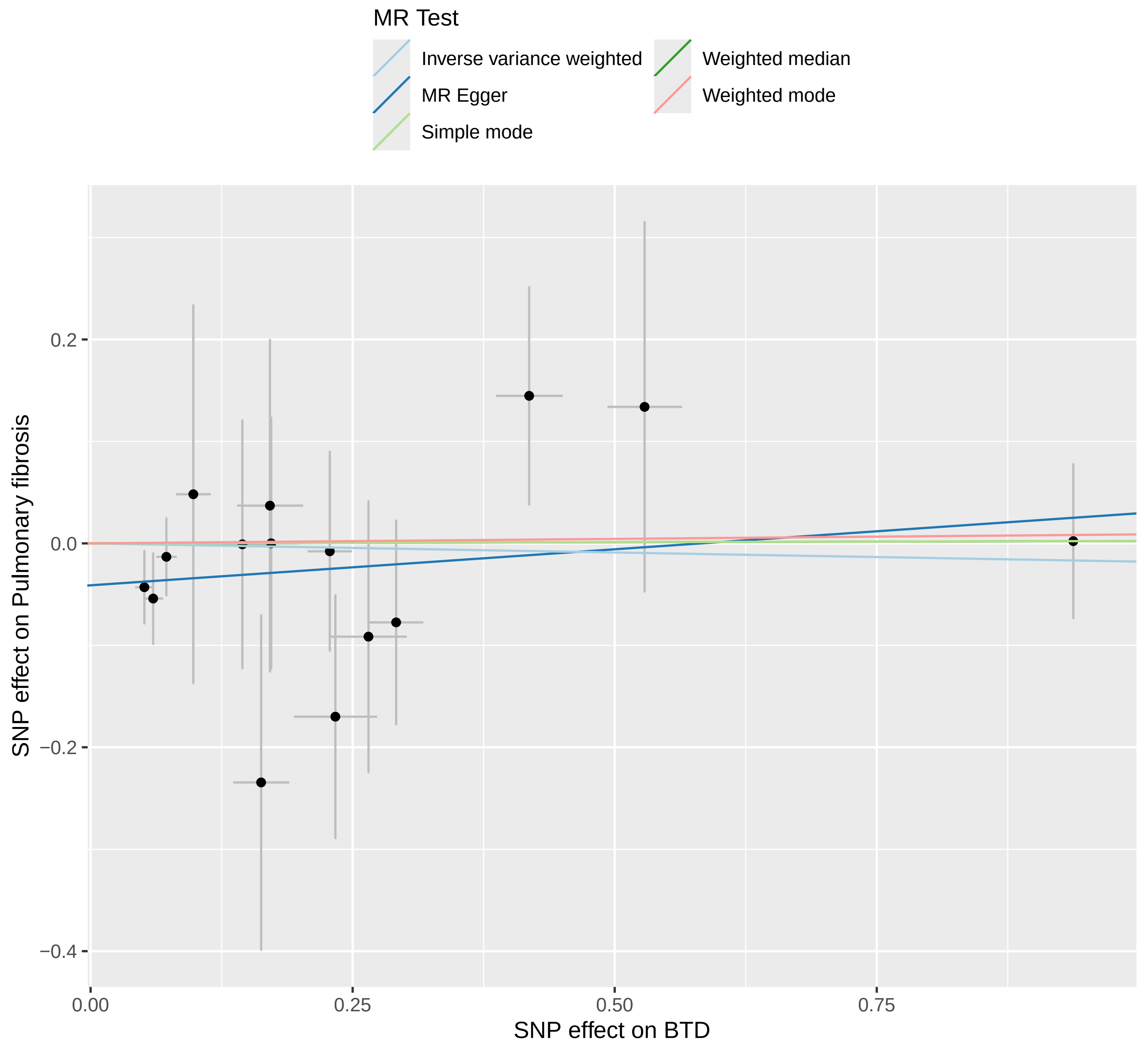
MR Test
Inverse variance weighted
MR Egger
Simple mode
Weighted median
Weighted mode
0.2
0.0
−0.2
−0.4
0.00
0.25
0.50
0.75
SNP effect on BTD
SNP effect on Pulmonary fibrosis

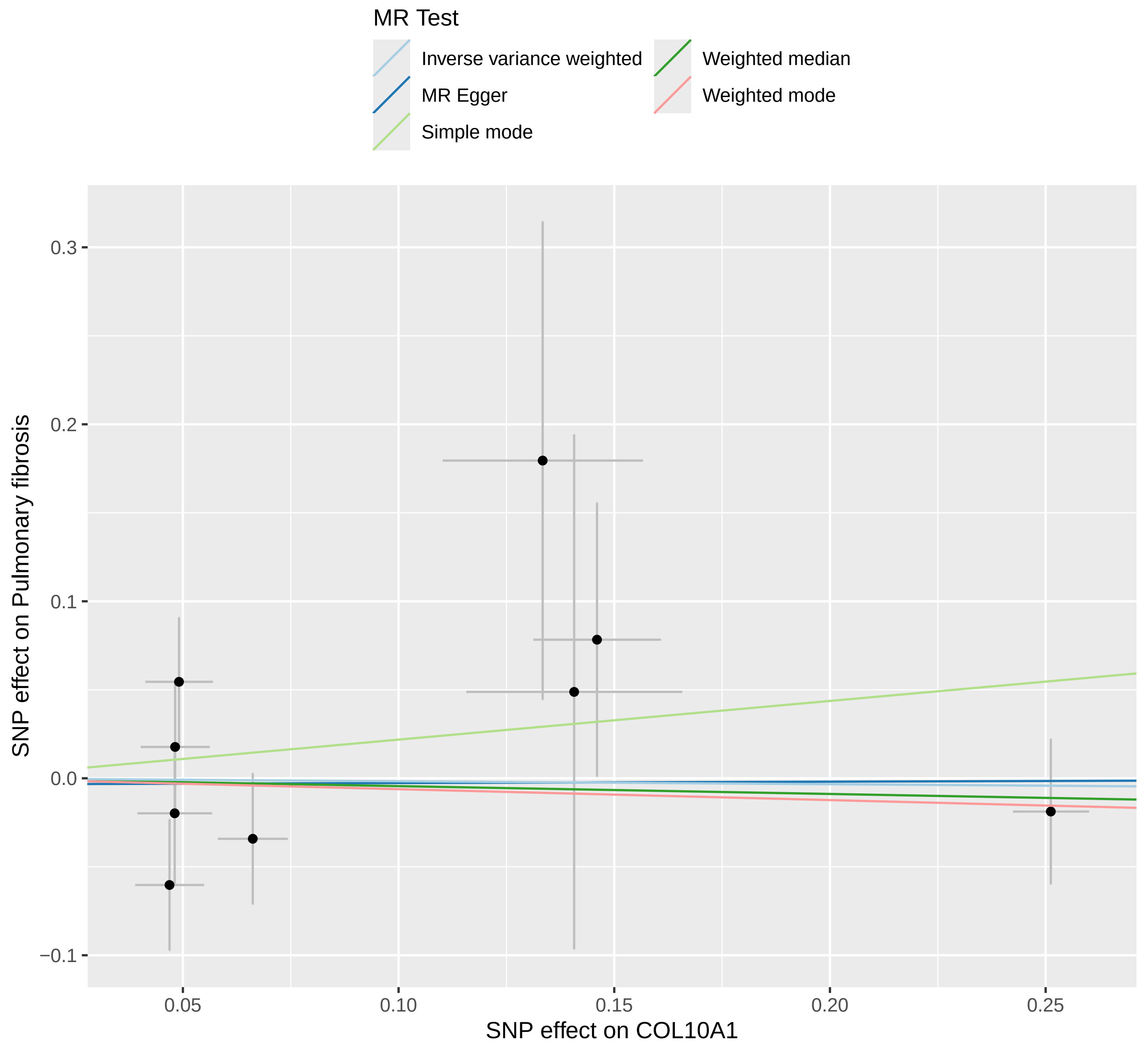

MR Test
Inverse variance weighted
MR Egger
Simple mode
Weighted median
Weighted mode
0.3
0.2
0.1
0.0
−0.1
SNP effect on Pulmonary fibrosis
0.05
0.10
0.15
0.20
0.25
SNP effect on COL10A1

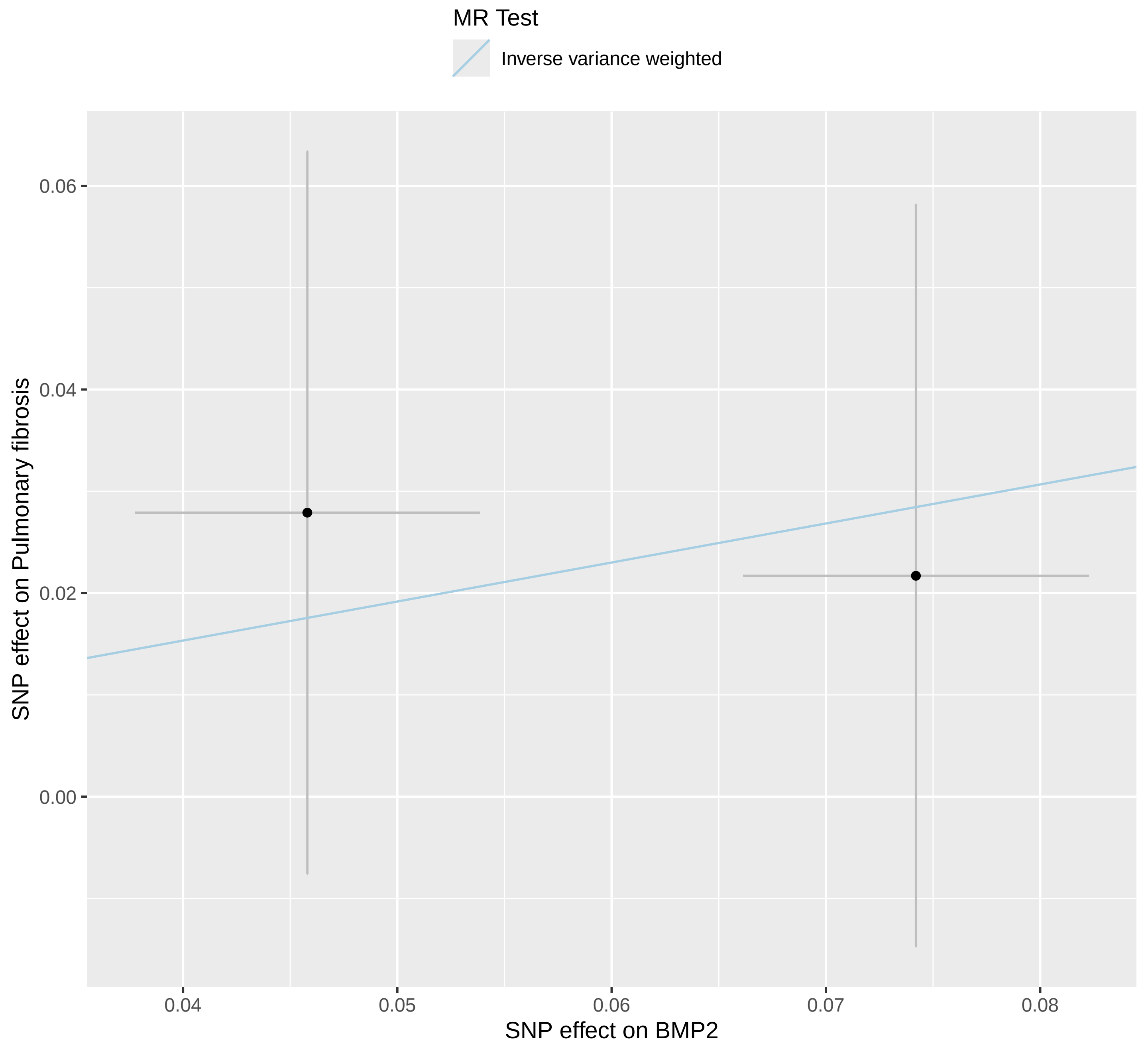
MR Test
Inverse variance weighted
SNP effect on Pulmonary fibrosis
0.06
0.04
0.02
0.00
0.04
0.05
0.06
0.07
0.08
SNP effect on BMP2

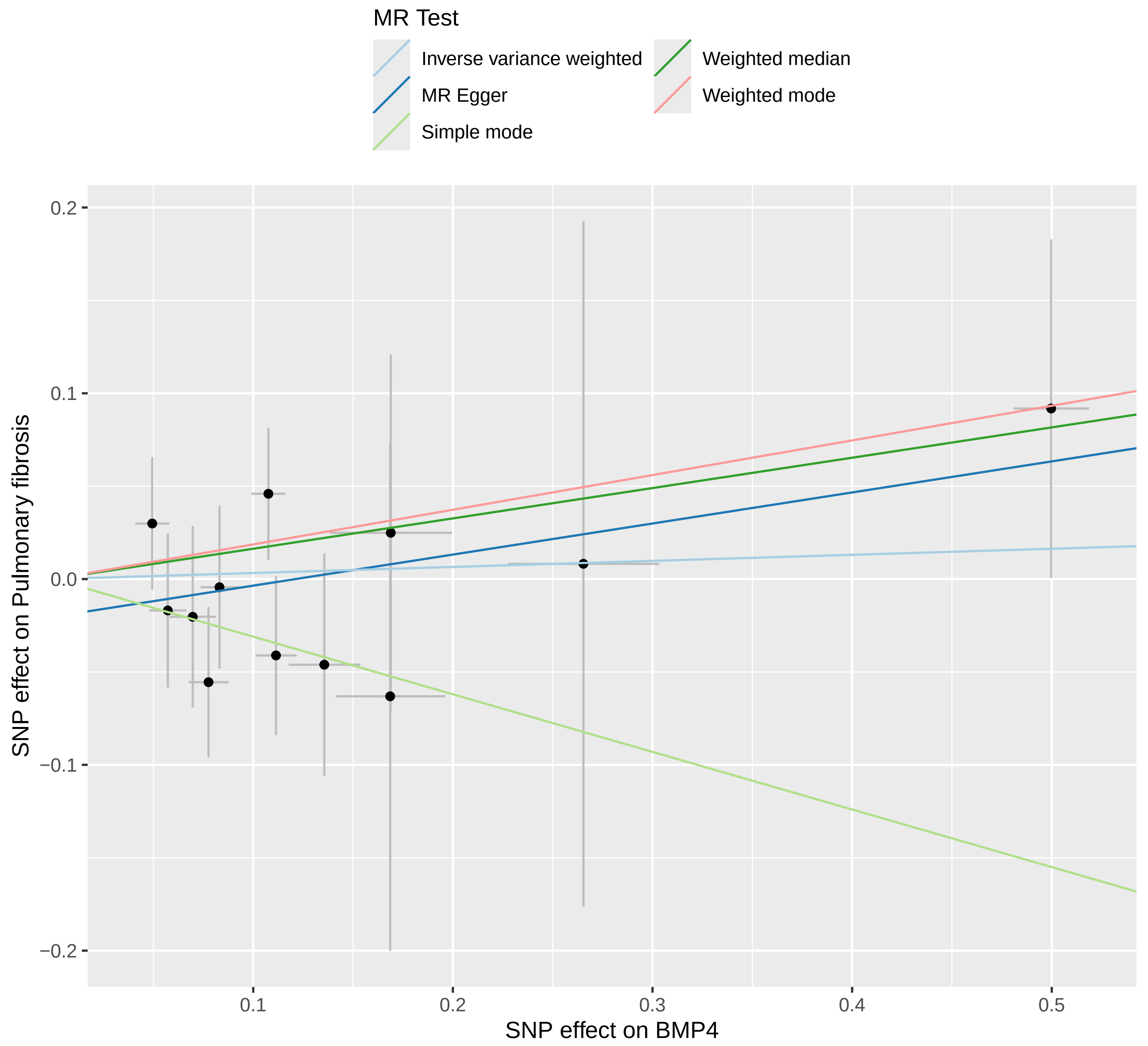

MR Test
Inverse variance weighted
MR Egger
Simple mode
Weighted median
Weighted mode
0.2
0.1
0.0
−0.1
−0.2
SNP effect on Pulmonary fibrosis
0.1
0.2
0.3
0.4
0.5
SNP effect on BMP4

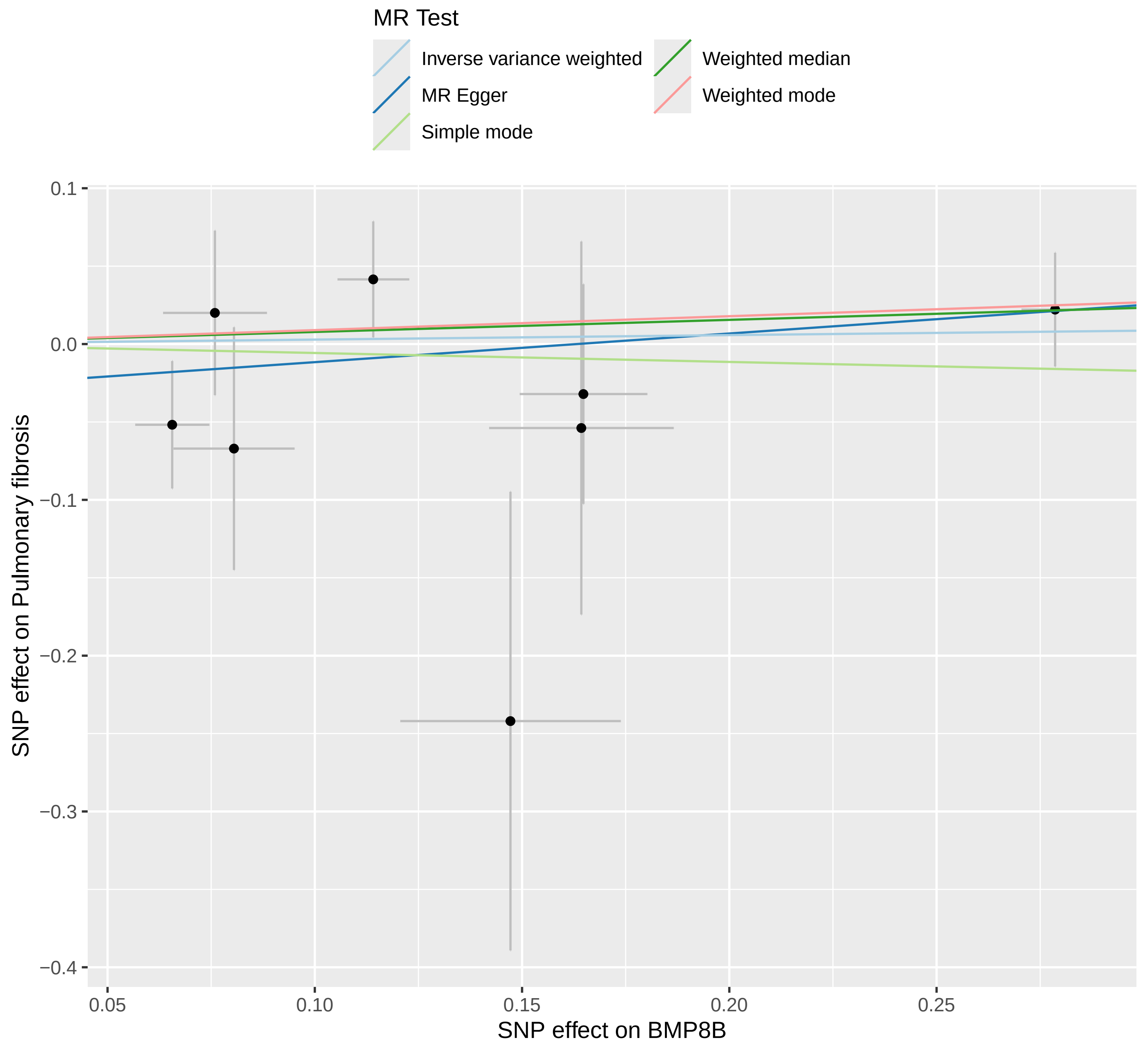
MR Test
Inverse variance weighted
MR Egger
Simple mode
Weighted median
Weighted mode
0.1
0.0
−0.1
−0.2
−0.3
−0.4
SNP effect on Pulmonary fibrosis
0.05
0.10
0.15
0.20
0.25
SNP effect on BMP8B

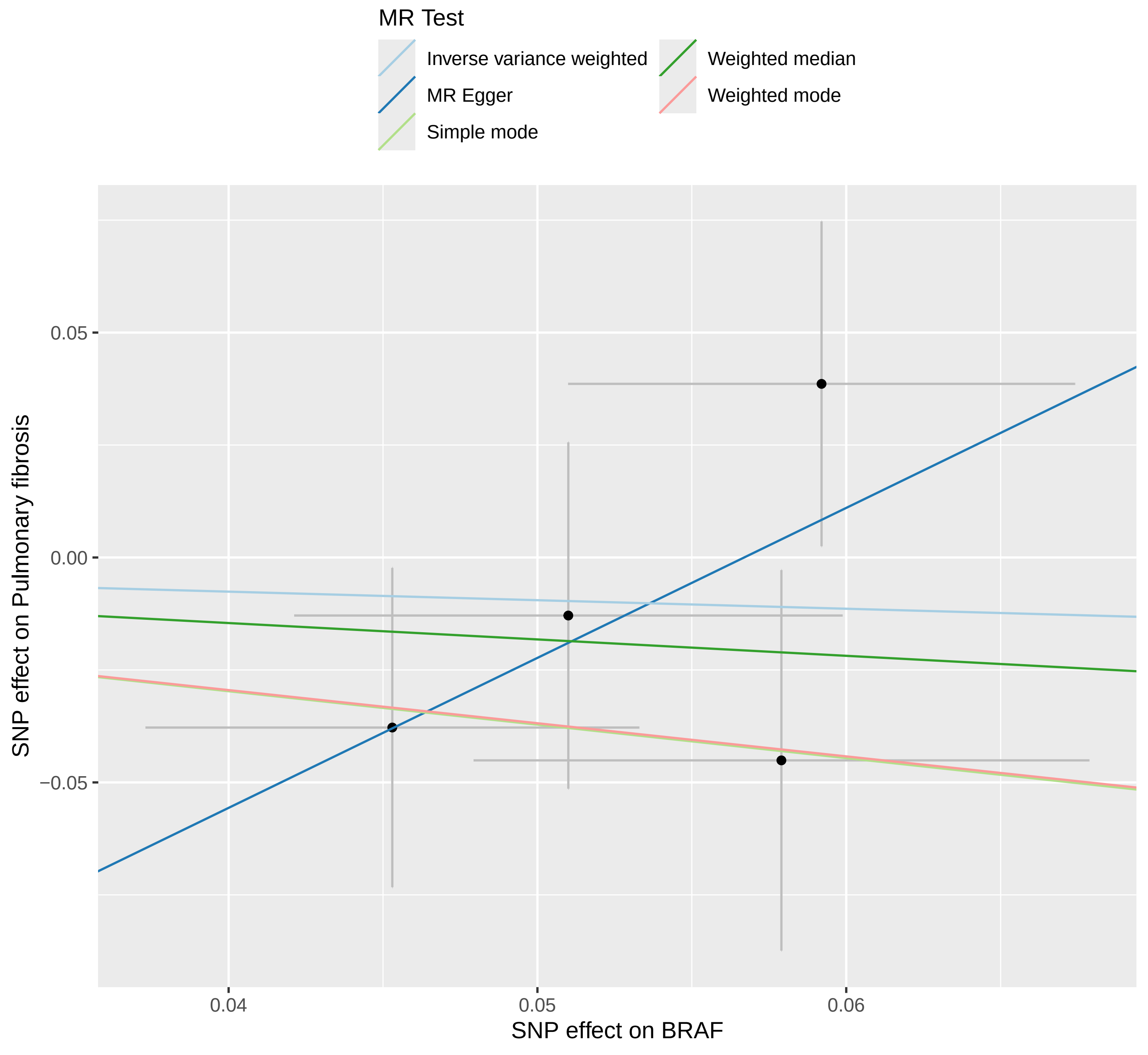

MR Test
Inverse variance weighted
MR Egger
Simple mode
Weighted median
Weighted mode
0.05
0.00
−0.05
0.04
0.05
0.06
SNP effect on Pulmonary fibrosis
SNP effect on BRAF

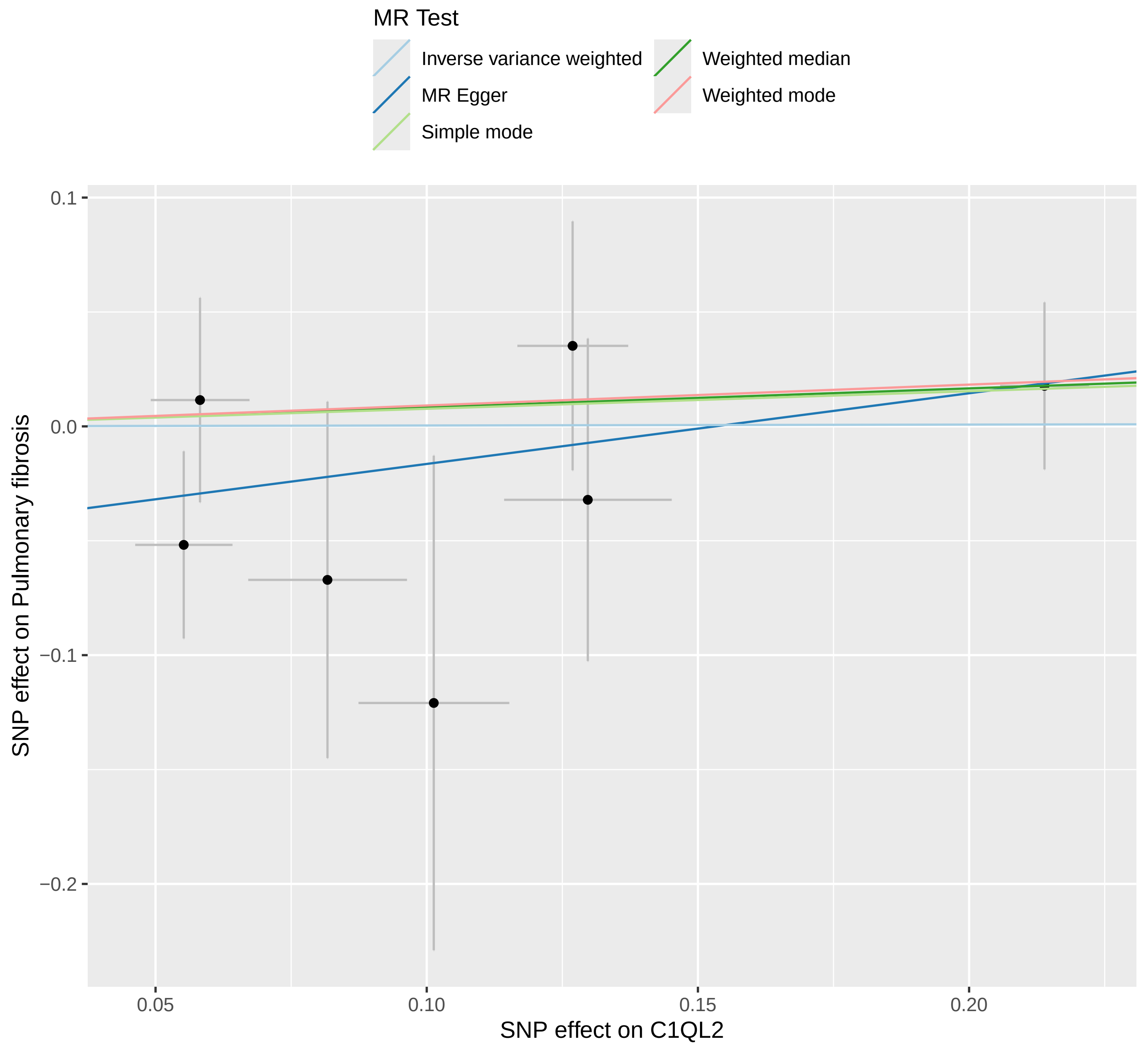

MR Test
Inverse variance weighted
MR Egger
Simple mode
Weighted median
Weighted mode
0.1
0.0
−0.1
−0.2
0.05
0.10
0.15
0.20
SNP effect on C1QL2
SNP effect on Pulmonary fibrosis

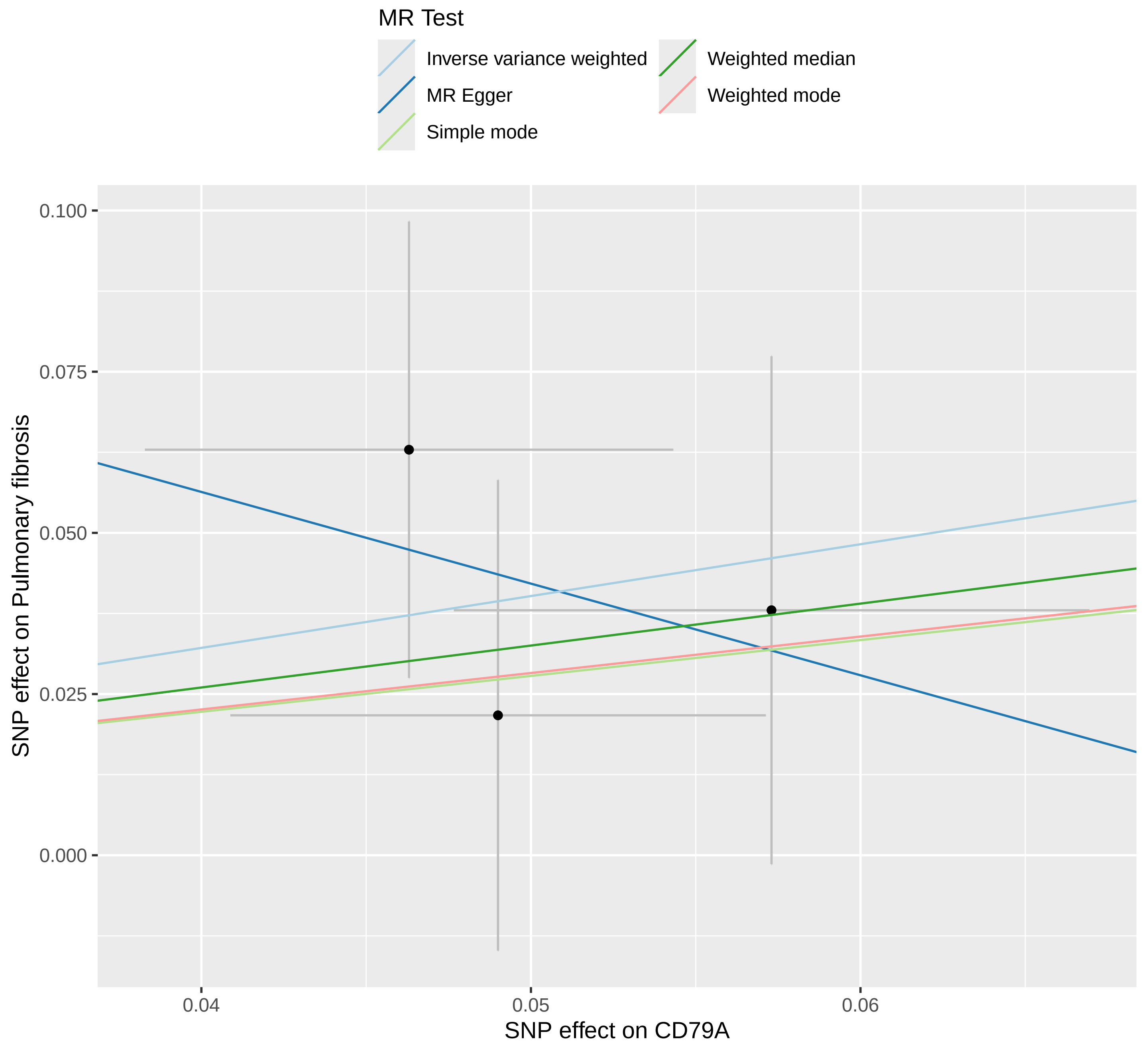
MR Test
Inverse variance weighted
MR Egger
Simple mode
Weighted median
Weighted mode
0.100
0.075
0.050
0.025
0.000
SNP effect on Pulmonary fibrosis
0.04
0.05
0.06
SNP effect on CD79A

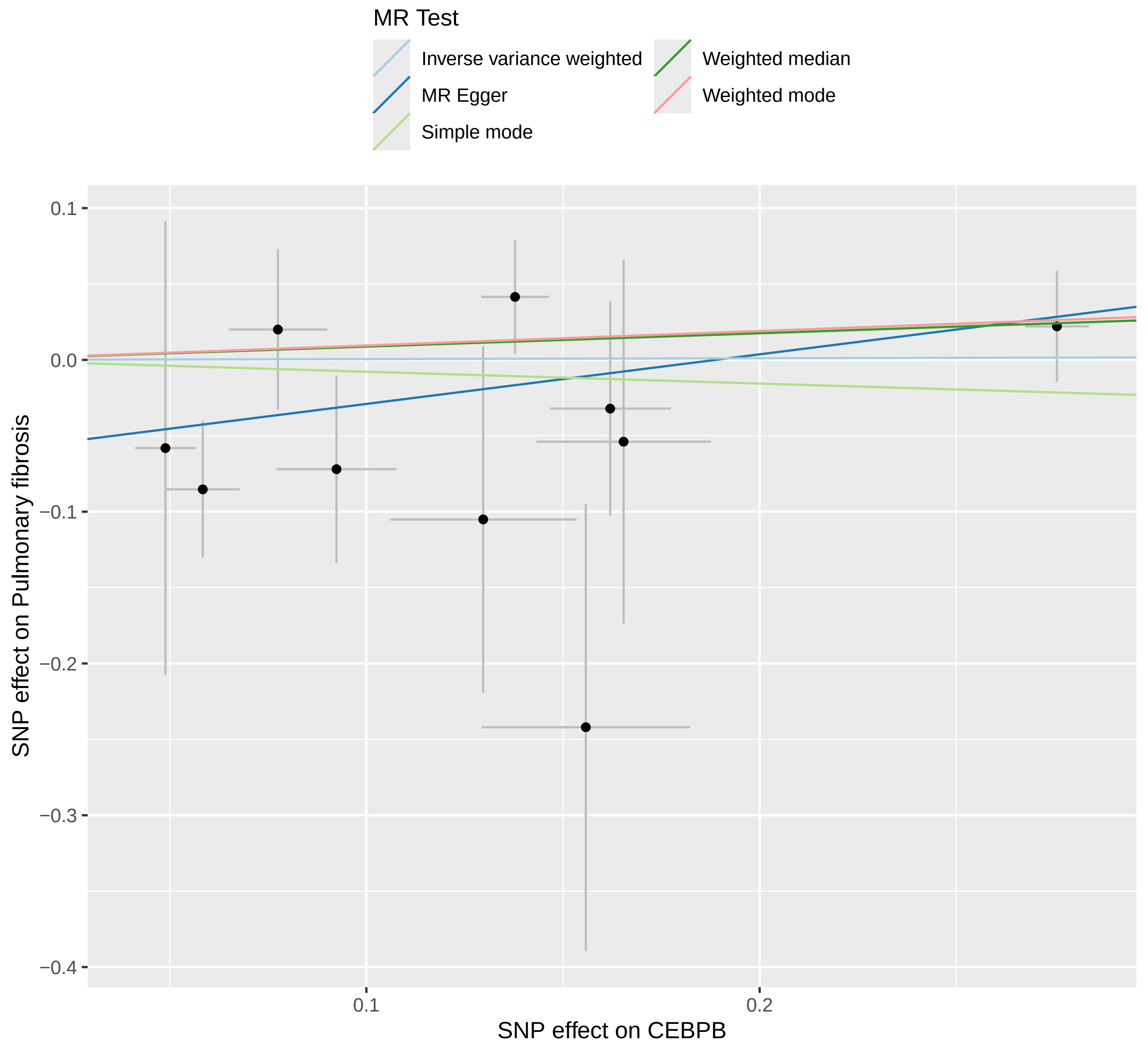
MR Test
Inverse variance weighted
MR Egger
Simple mode
Weighted median
Weighted mode
0.1
0.0
−0.1
−0.2
−0.3
−0.4
SNP effect on Pulmonary fibrosis
0.1
0.2
SNP effect on CEBPB

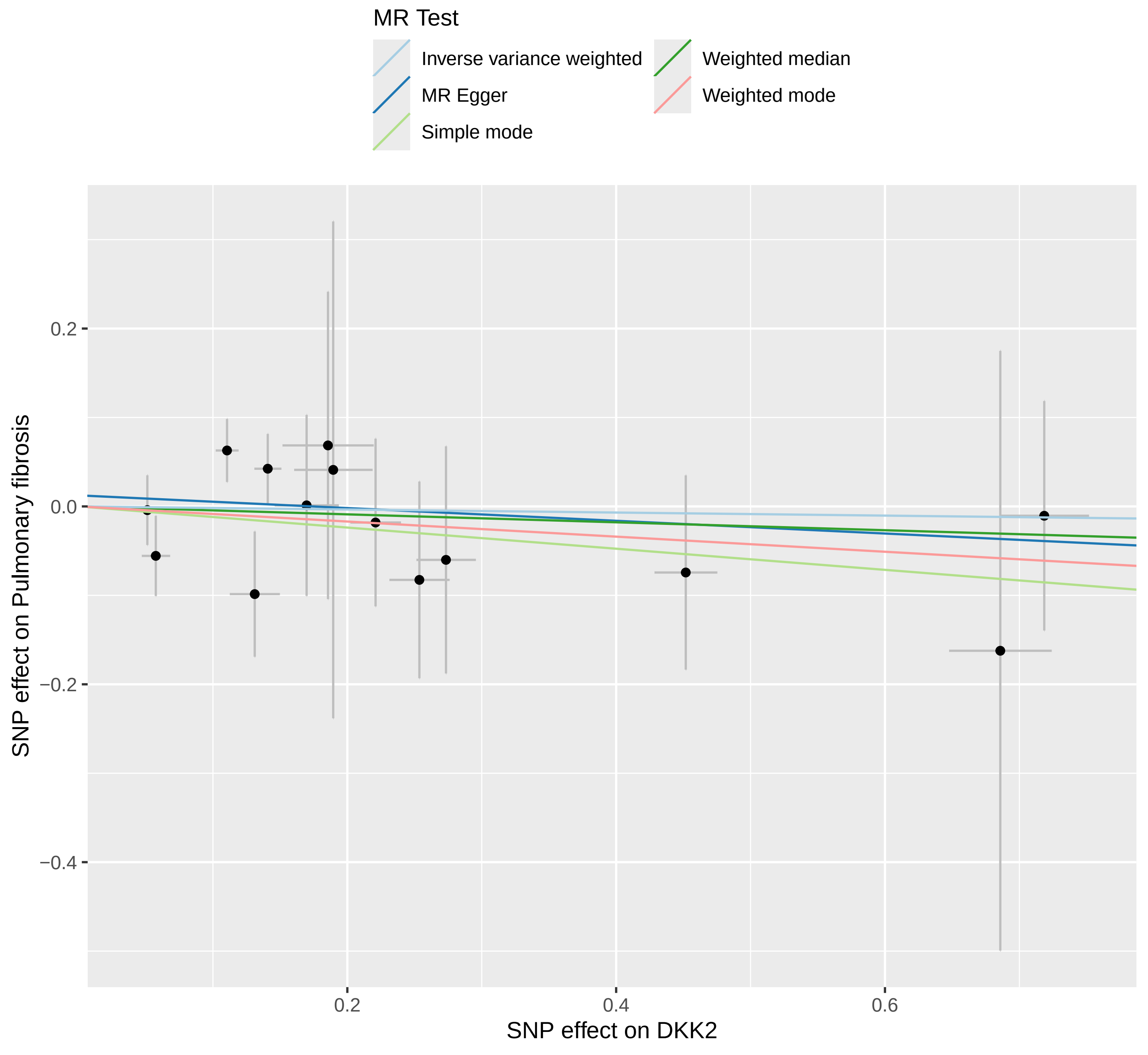

MR Test
Inverse variance weighted
MR Egger
Simple mode
Weighted median
Weighted mode
0.2
0.0
−0.2
−0.4
SNP effect on Pulmonary fibrosis
0.2
0.4
0.6
SNP effect on DKK2

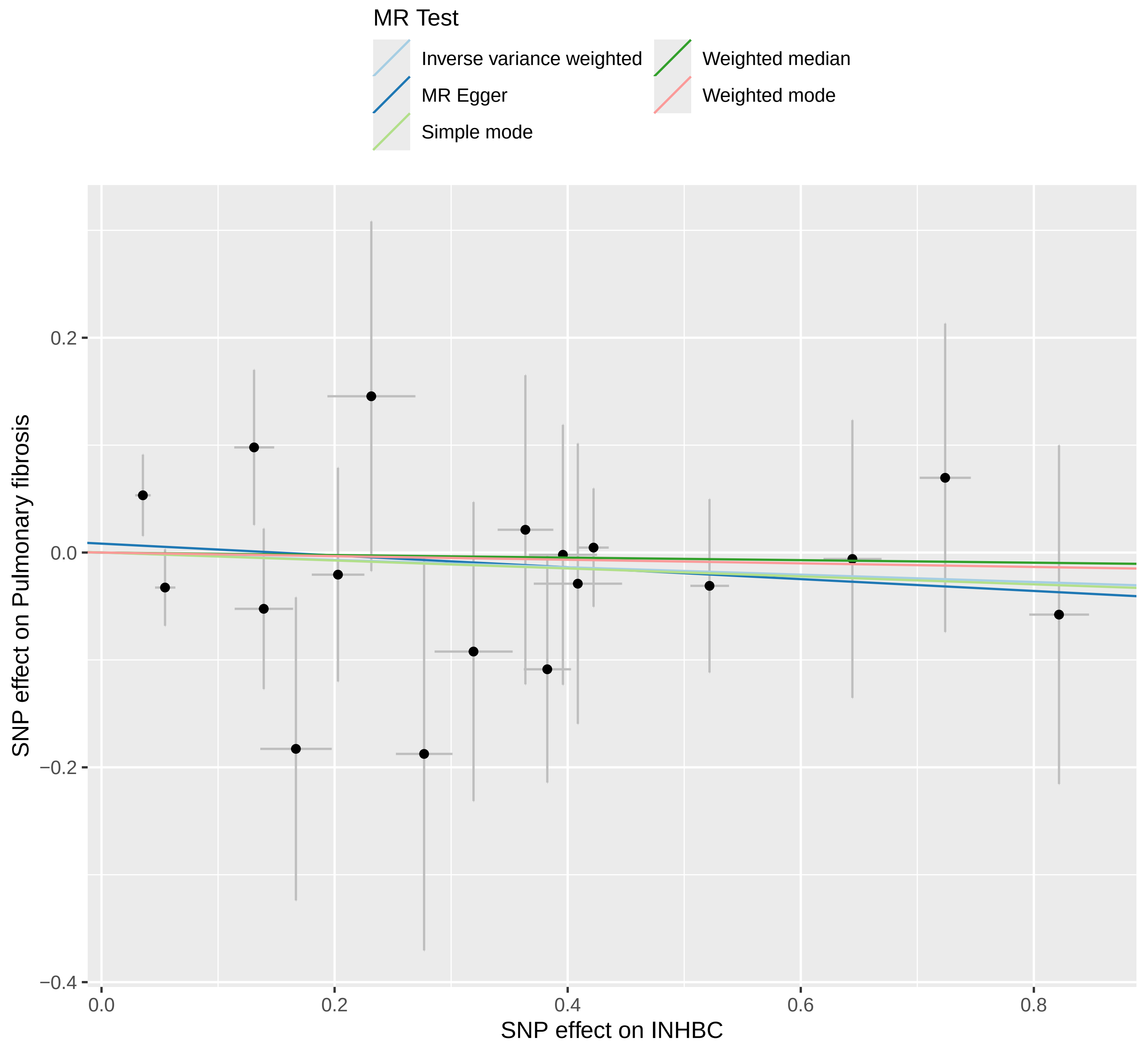
MR Test
Inverse variance weighted
MR Egger
Simple mode
Weighted median
Weighted mode
0.2
0.0
−0.2
−0.4
0.0
0.2
0.4
0.6
0.8
SNP effect on Pulmonary fibrosis
SNP effect on INHBC

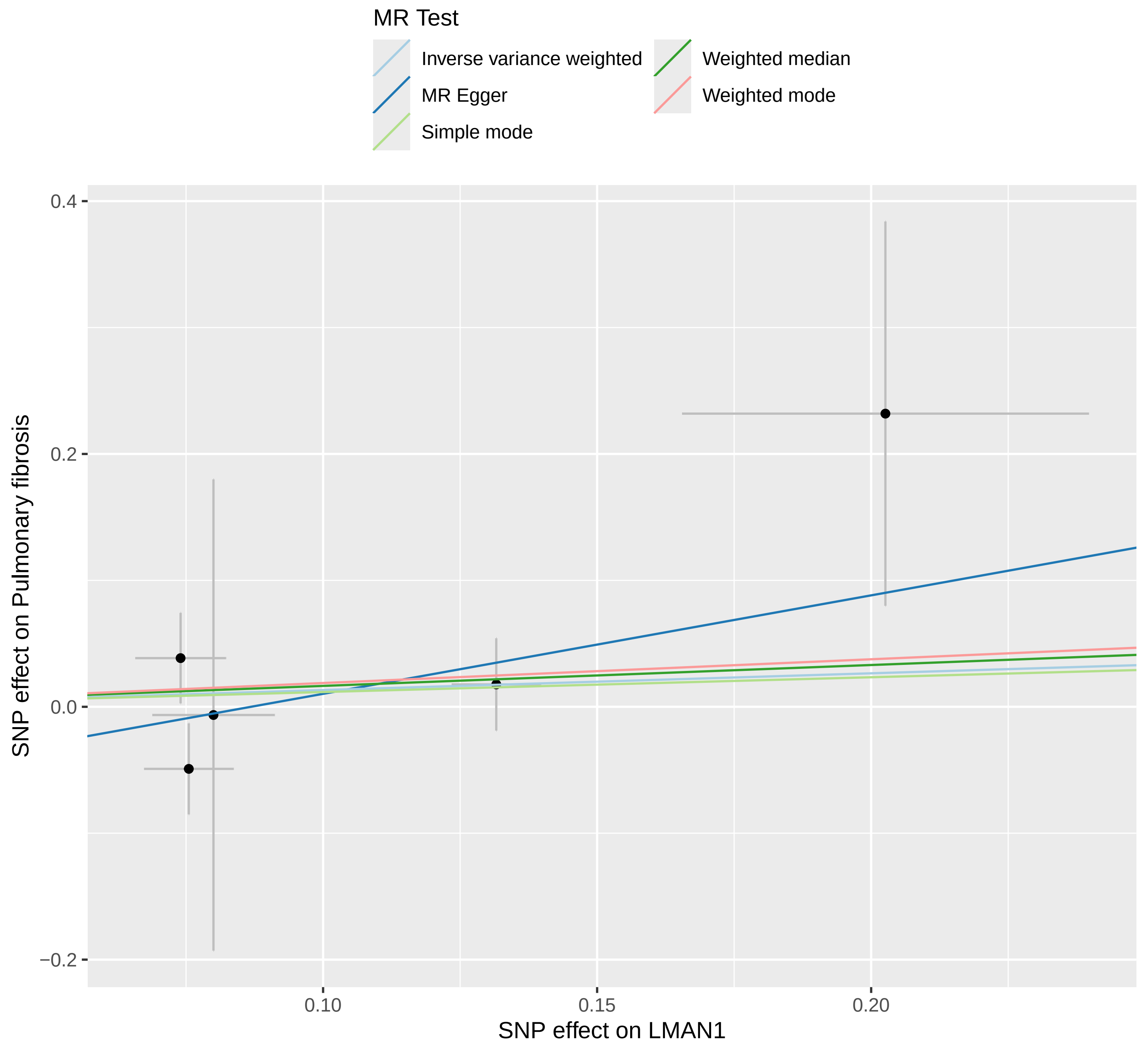

MR Test
Inverse variance weighted
MR Egger
Simple mode
Weighted median
Weighted mode
0.4
0.2
0.0
−0.2
0.10
0.15
0.20
SNP effect on Pulmonary fibrosis
SNP effect on LMAN1

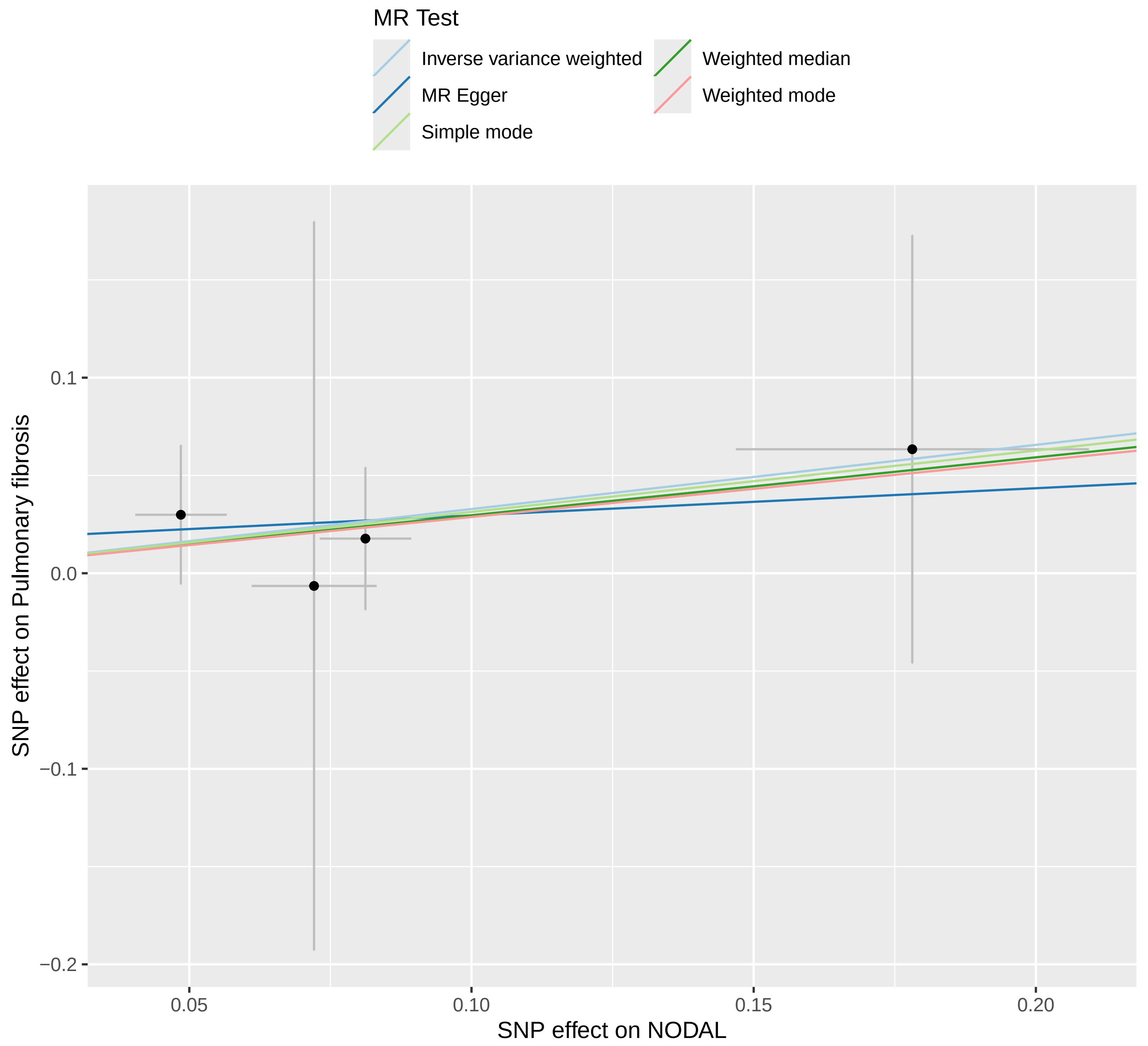

MR Test
Inverse variance weighted
MR Egger
Simple mode
Weighted median
Weighted mode
0.1
0.0
−0.1
−0.2
SNP effect on Pulmonary fibrosis
0.05
0.10
0.15
0.20
SNP effect on NODAL

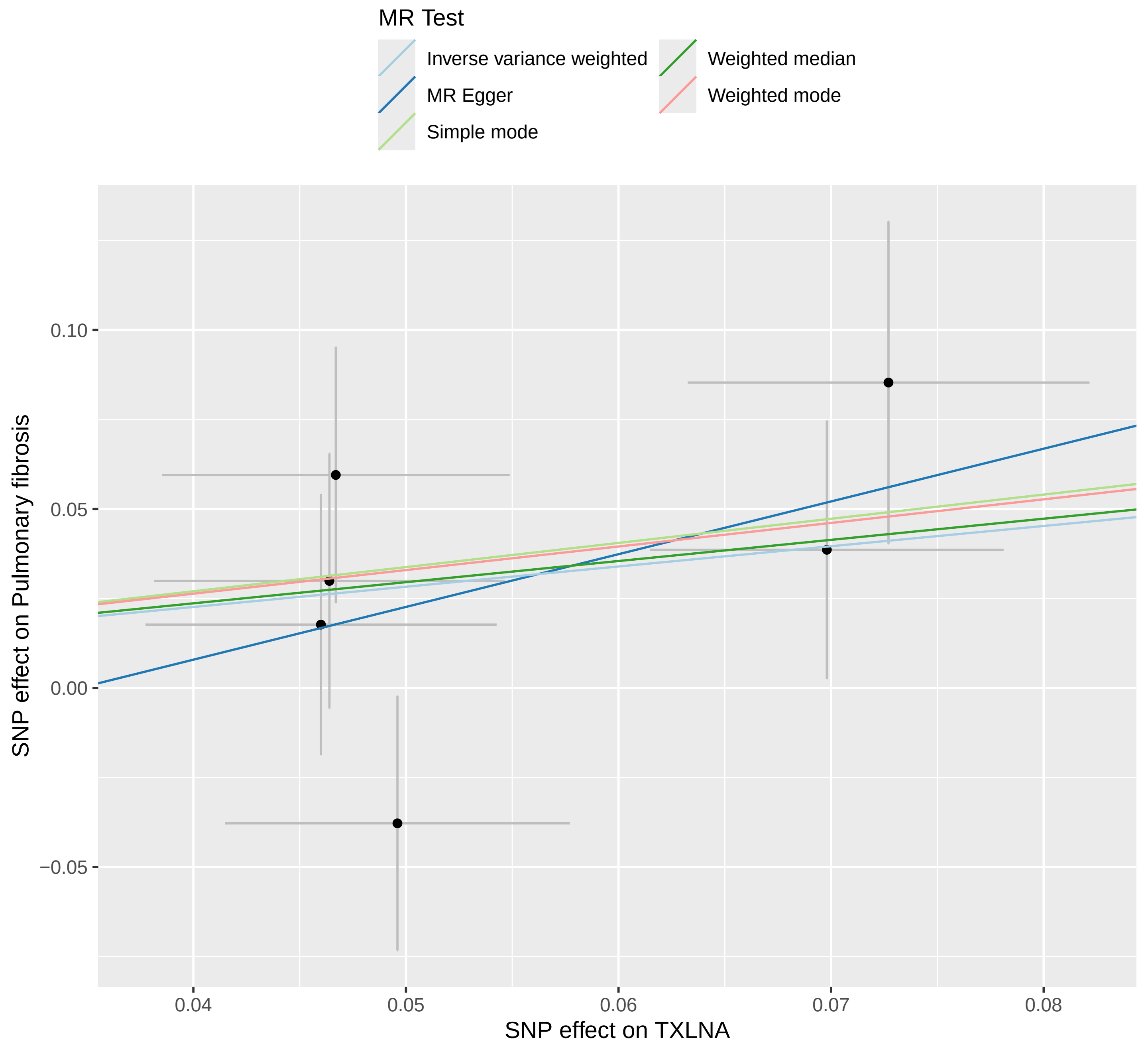

MR Test
Inverse variance weighted
MR Egger
Simple mode
Weighted median
Weighted mode
SNP effect on Pulmonary fibrosis
0.10
0.05
0.00
−0.05
0.04
0.05
0.06
0.07
0.08
SNP effect on TXLNA

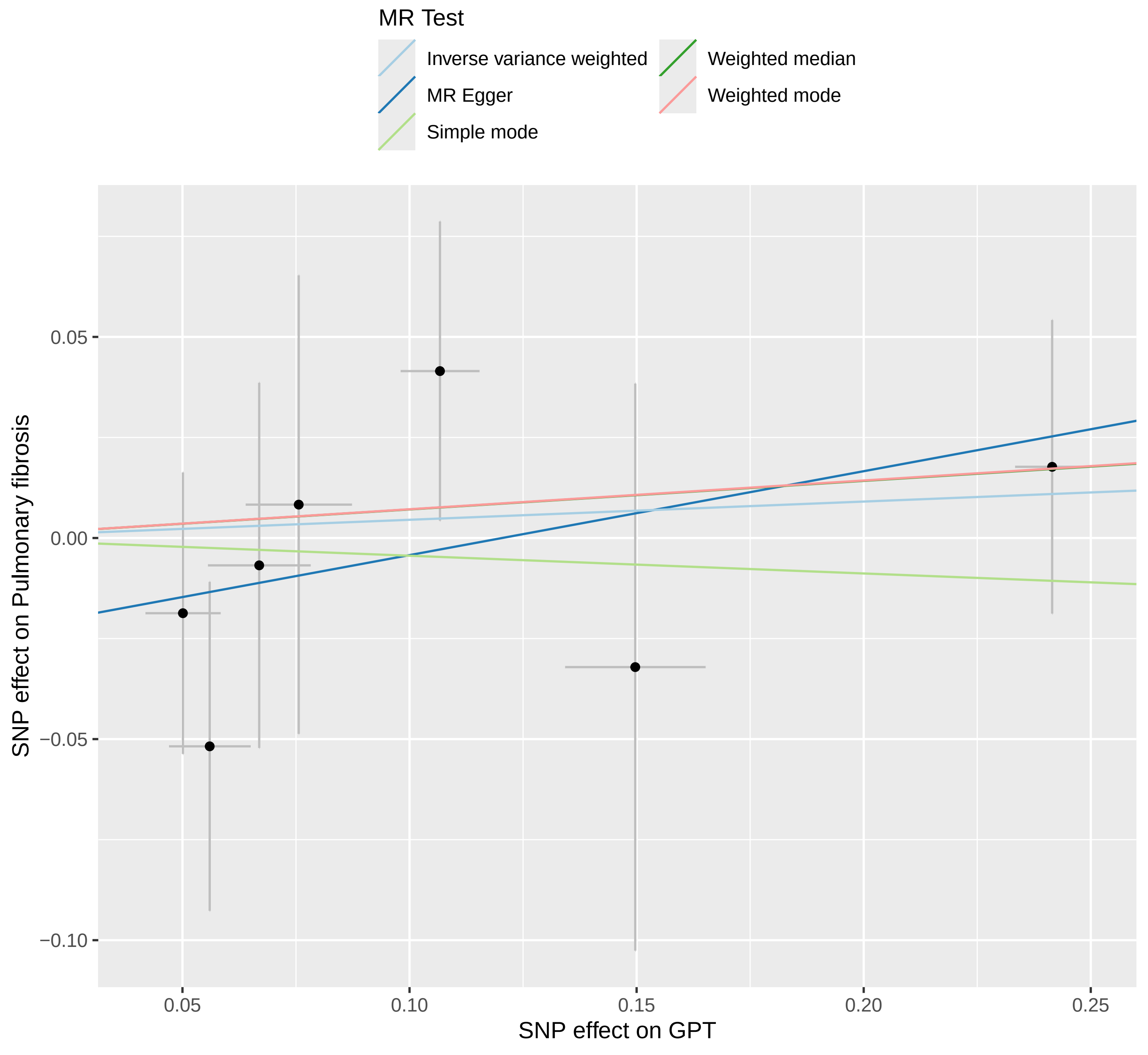
MR Test
Inverse variance weighted
MR Egger
Simple mode
Weighted median
Weighted mode
SNP effect on Pulmonary fibrosis
0.05
0.00
−0.05
−0.10
0.05
0.10
0.15
0.20
0.25
SNP effect on GPT

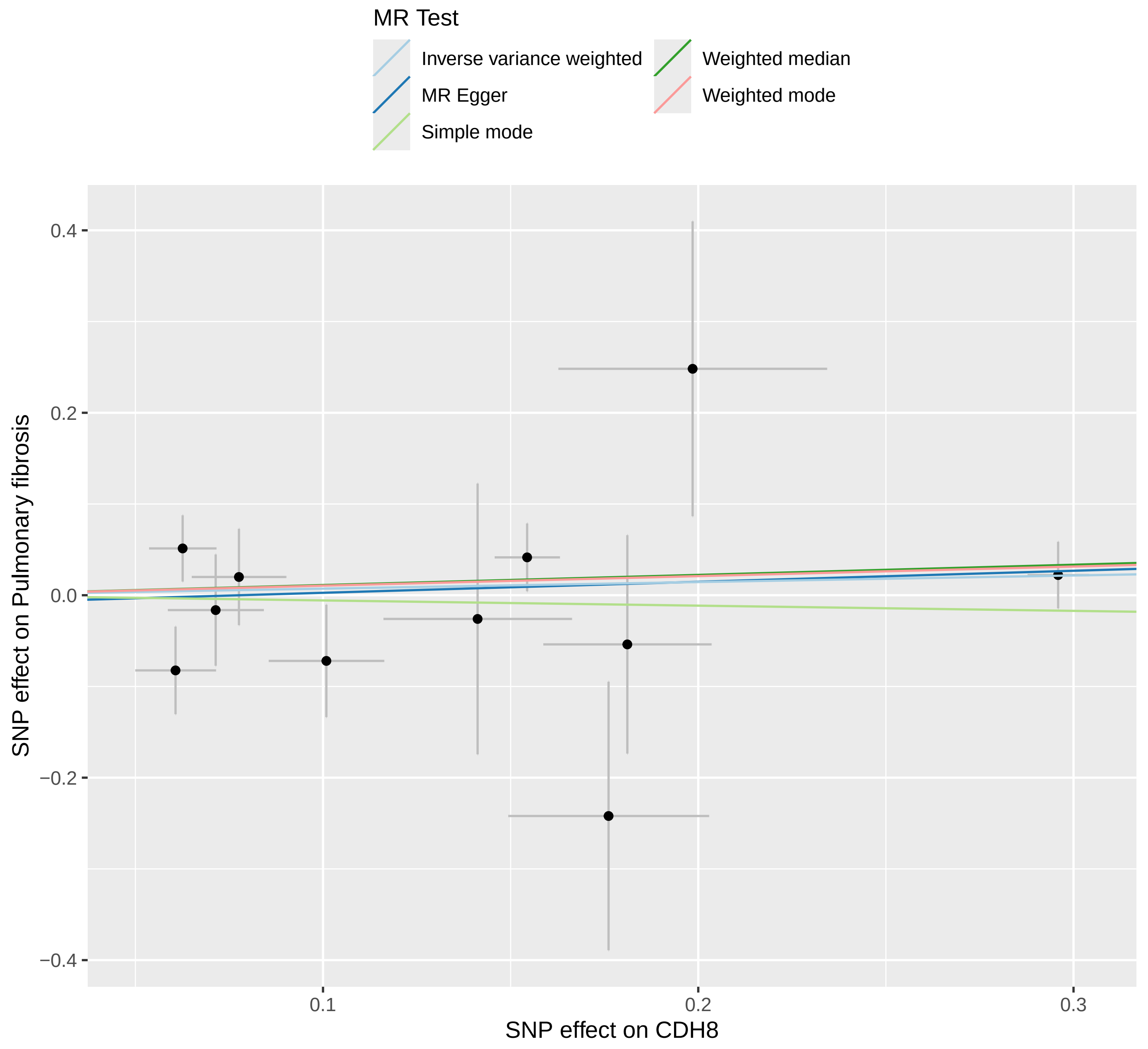

MR Test
Inverse variance weighted
MR Egger
Simple mode
Weighted median
Weighted mode
0.4
0.2
0.0
−0.2
−0.4
0.1
0.2
0.3
SNP effect on Pulmonary fibrosis
SNP effect on CDH8

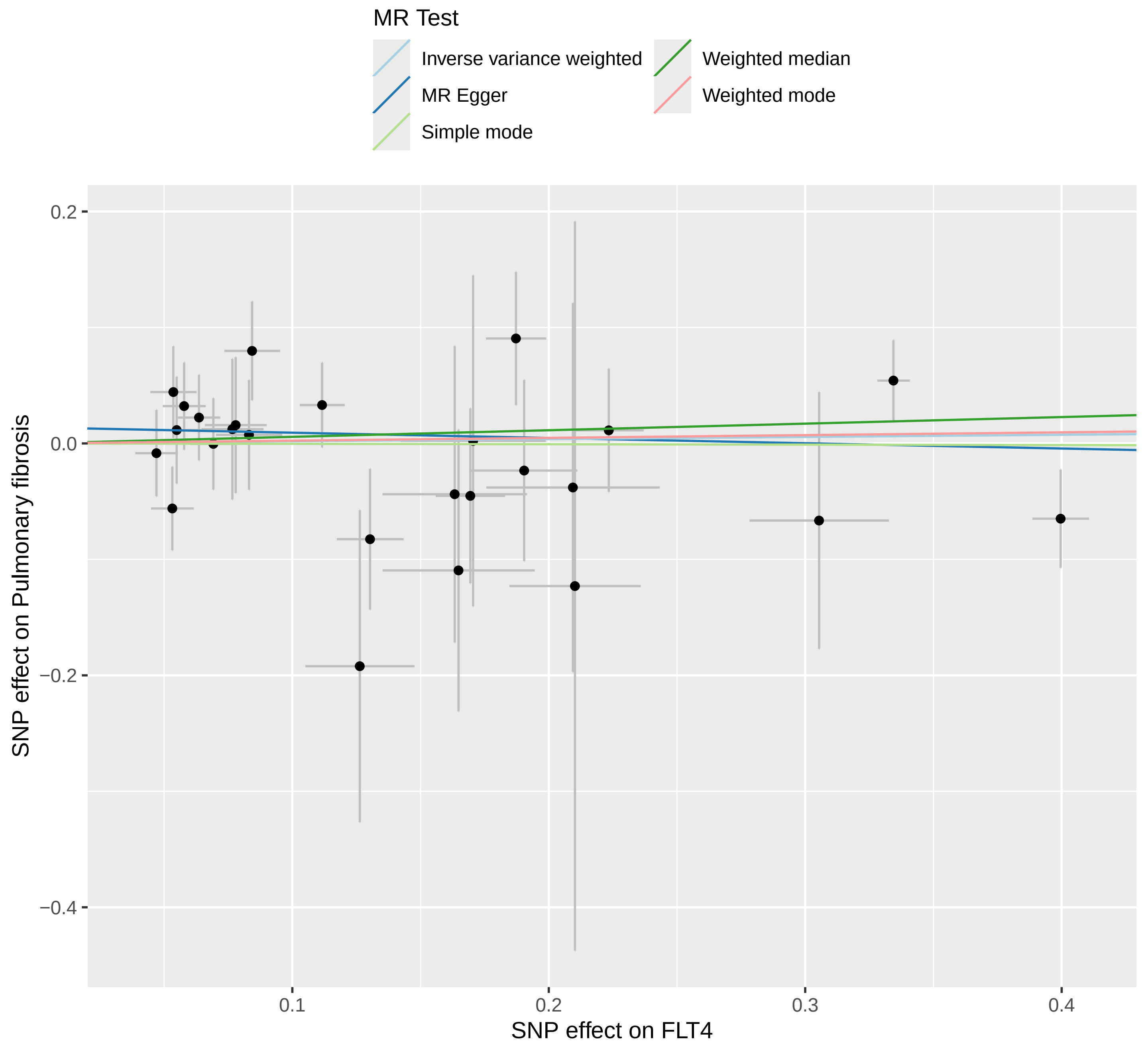
MR Test
Inverse variance weighted
MR Egger
Simple mode
Weighted median
Weighted mode
0.2
0.0
−0.2
−0.4
0.1
0.2
0.3
0.4
SNP effect on Pulmonary fibrosis
SNP effect on FLT4

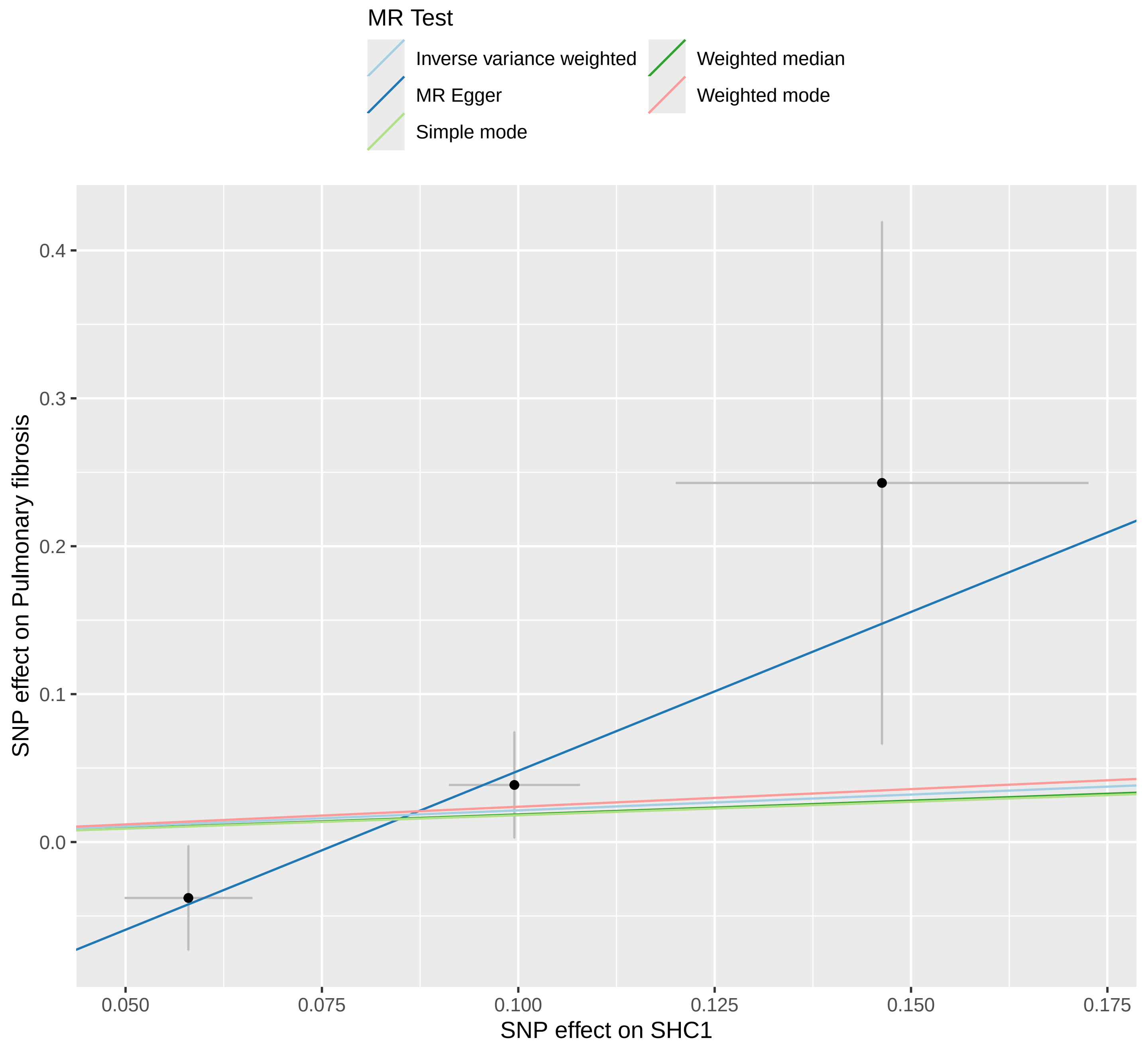

MR Test
Inverse variance weighted
MR Egger
Simple mode
Weighted median
Weighted mode
0.4
0.3
0.2
0.1
0.0
SNP effect on Pulmonary fibrosis
0.050
0.075
0.100
0.125
0.150
0.175
SNP effect on SHC1

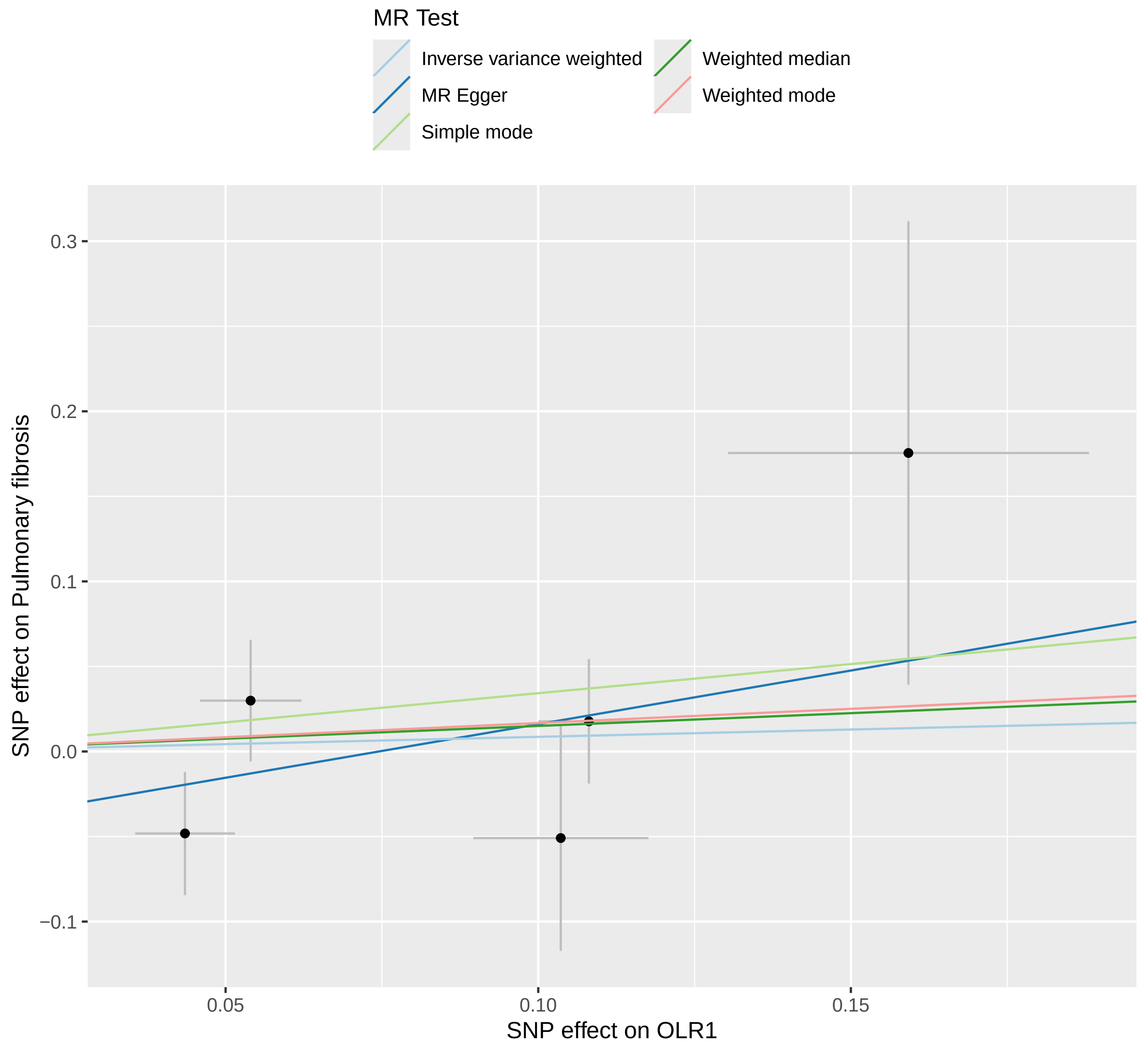

MR Test
Inverse variance weighted
MR Egger
Simple mode
Weighted median
Weighted mode
0.3
0.2
0.1
0.0
−0.1
0.05
0.10
0.15
SNP effect on Pulmonary fibrosis
SNP effect on OLR1

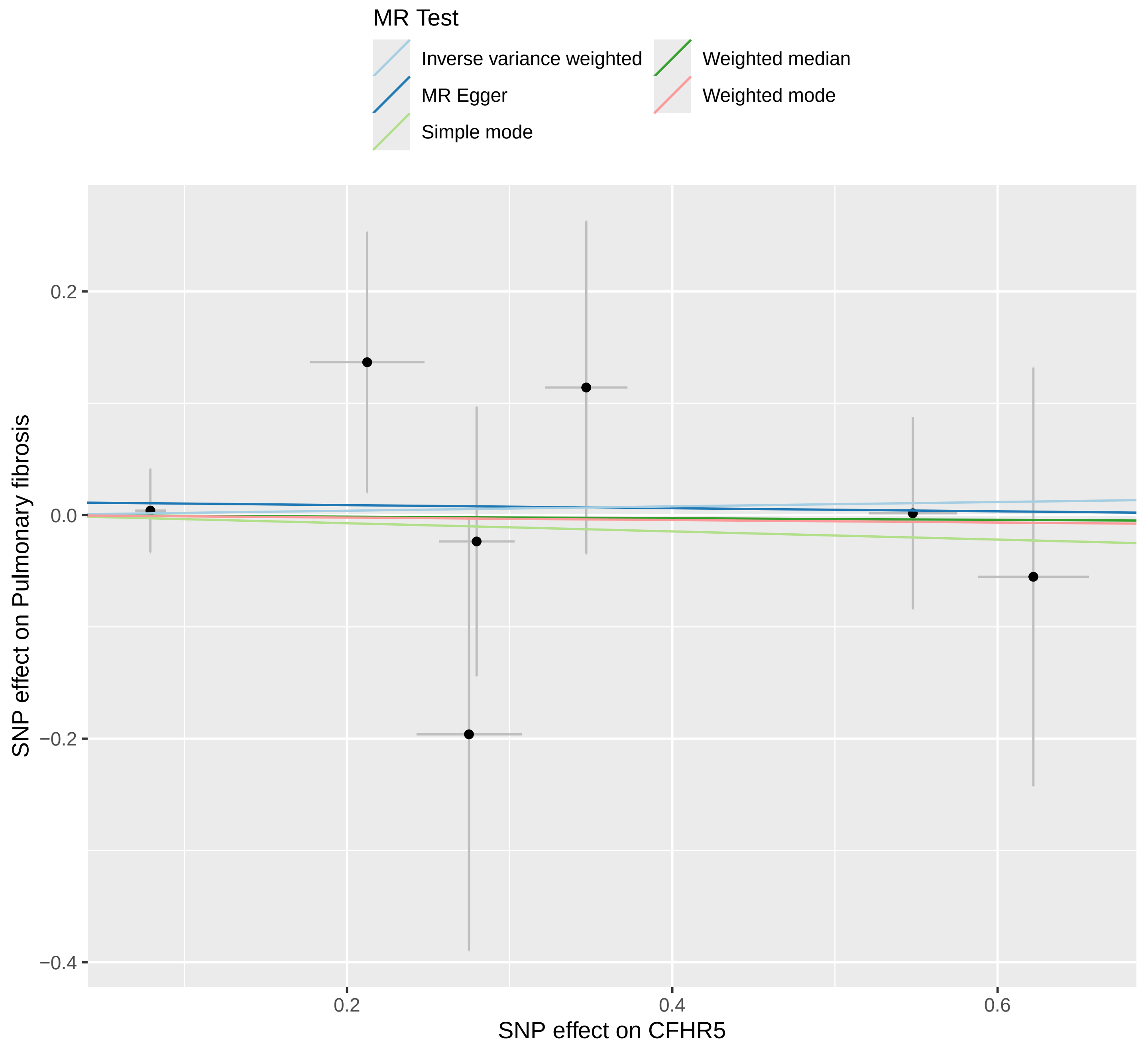
MR Test
Inverse variance weighted
MR Egger
Simple mode
Weighted median
Weighted mode
SNP effect on Pulmonary fibrosis
0.2
0.0
−0.2
−0.4
0.2
0.4
0.6
SNP effect on CFHR5

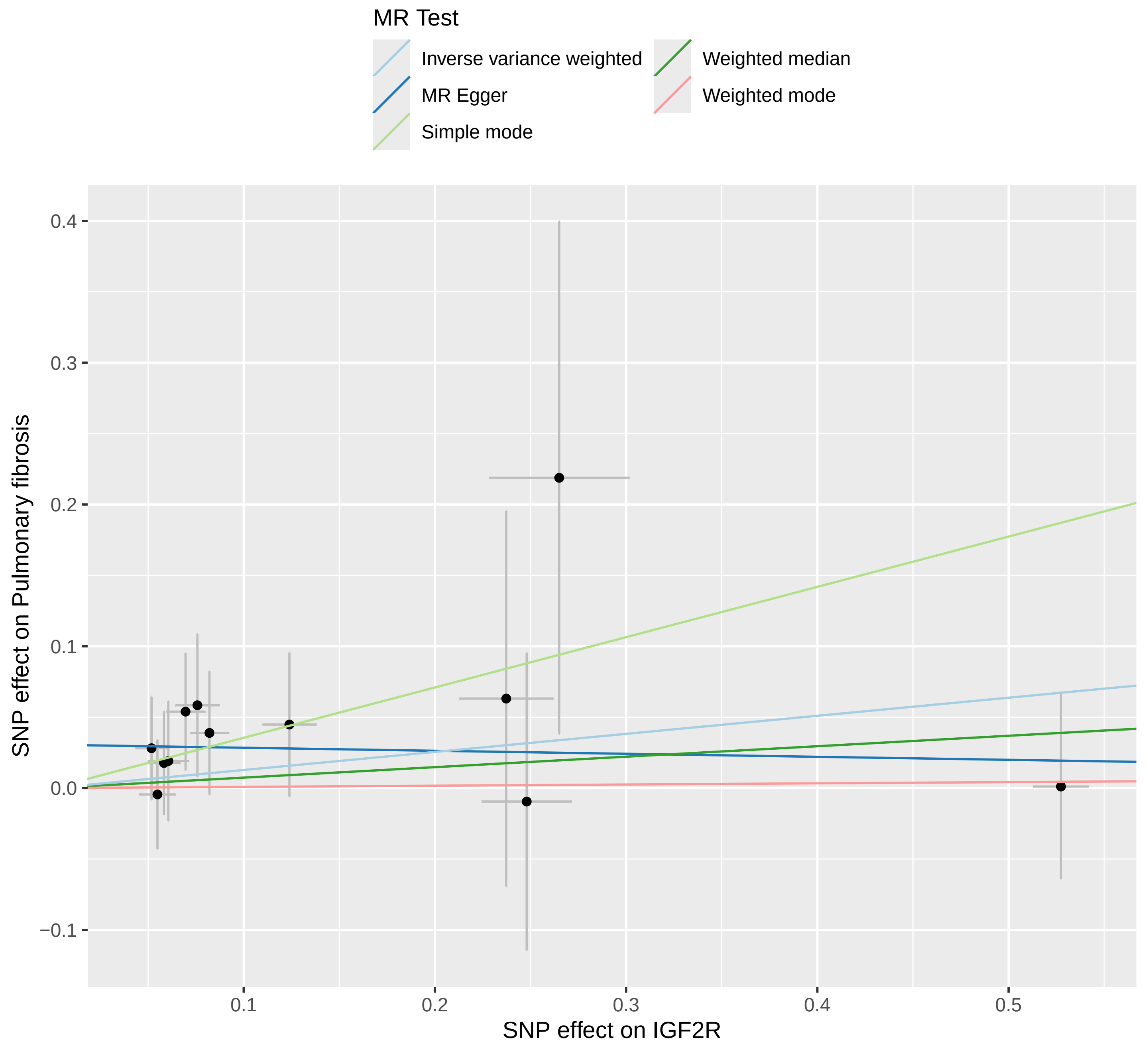
MR Test
Inverse variance weighted
MR Egger
Simple mode
Weighted median
Weighted mode
SNP effect on Pulmonary fibrosis
0.4
0.3
0.2
0.1
0.0
−0.1
0.1
0.2
0.3
0.4
0.5
SNP effect on IGF2R

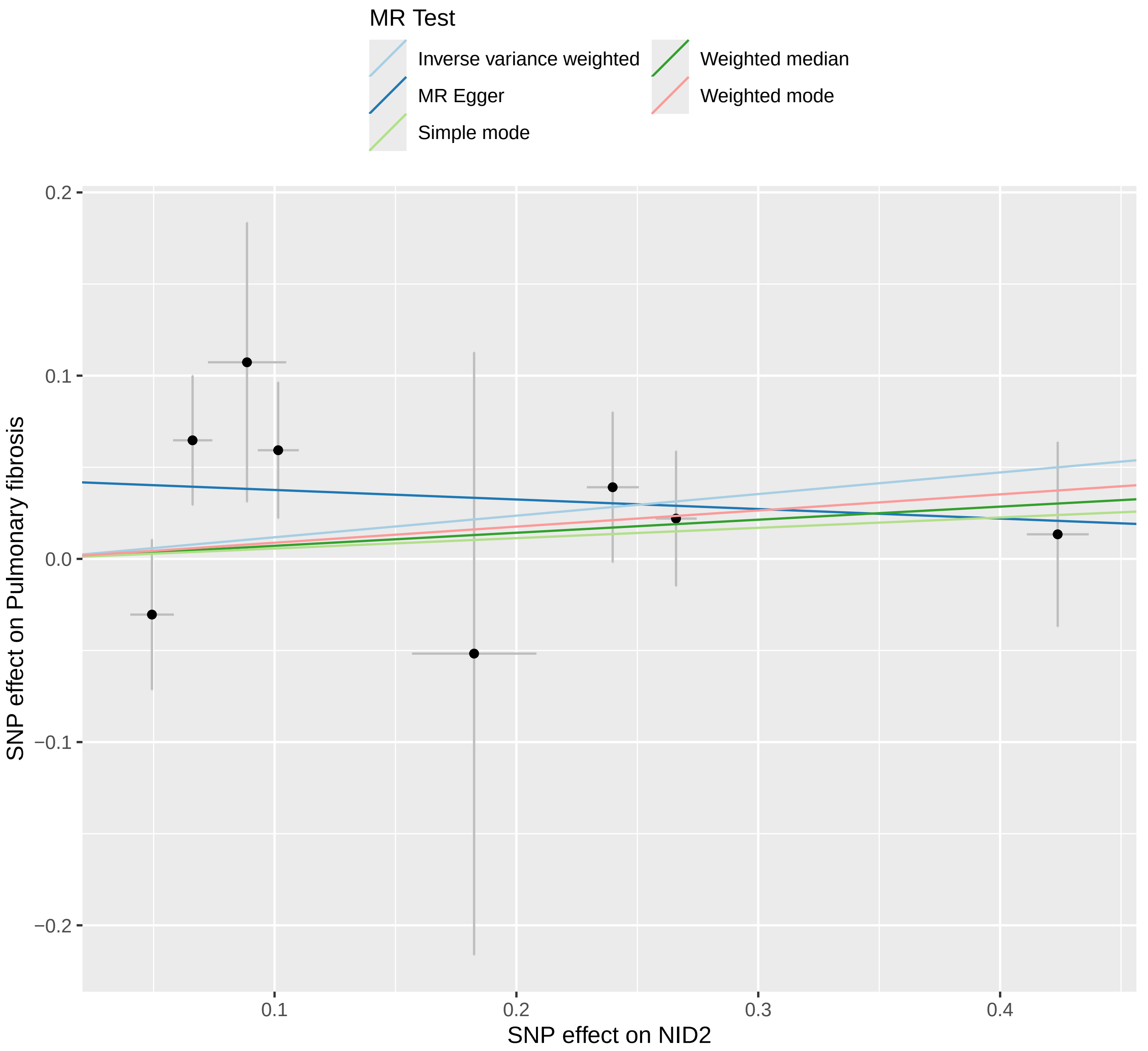

MR Test
Inverse variance weighted
MR Egger
Simple mode
Weighted median
Weighted mode
0.2
0.1
0.0
−0.1
−0.2
SNP effect on Pulmonary fibrosis
0.1
0.2
0.3
0.4
SNP effect on NID2

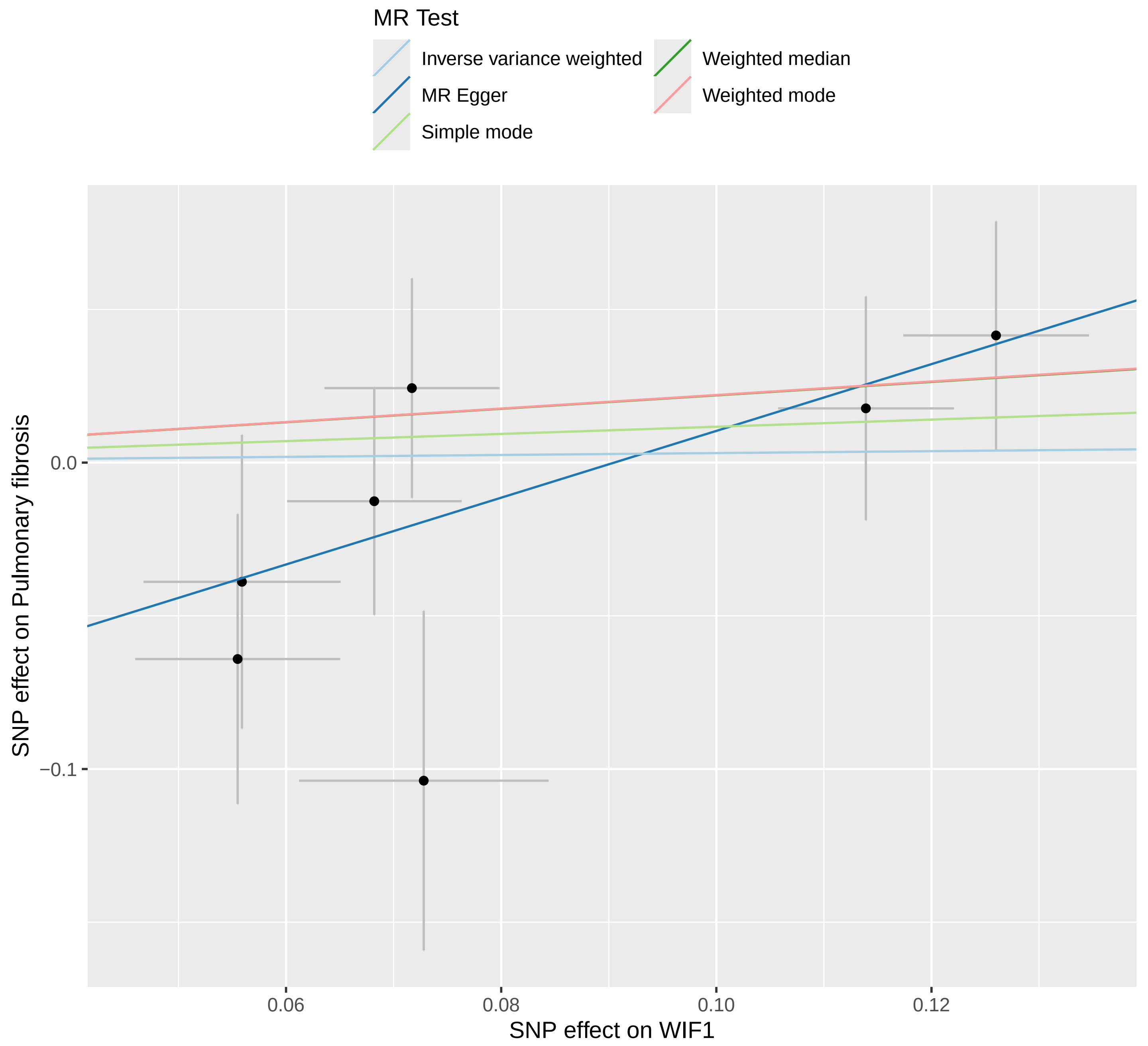
MR Test
Inverse variance weighted
MR Egger
Simple mode
Weighted median
Weighted mode
0.0
−0.1
0.06
0.08
0.10
0.12
SNP effect on Pulmonary fibrosis
SNP effect on WIF1

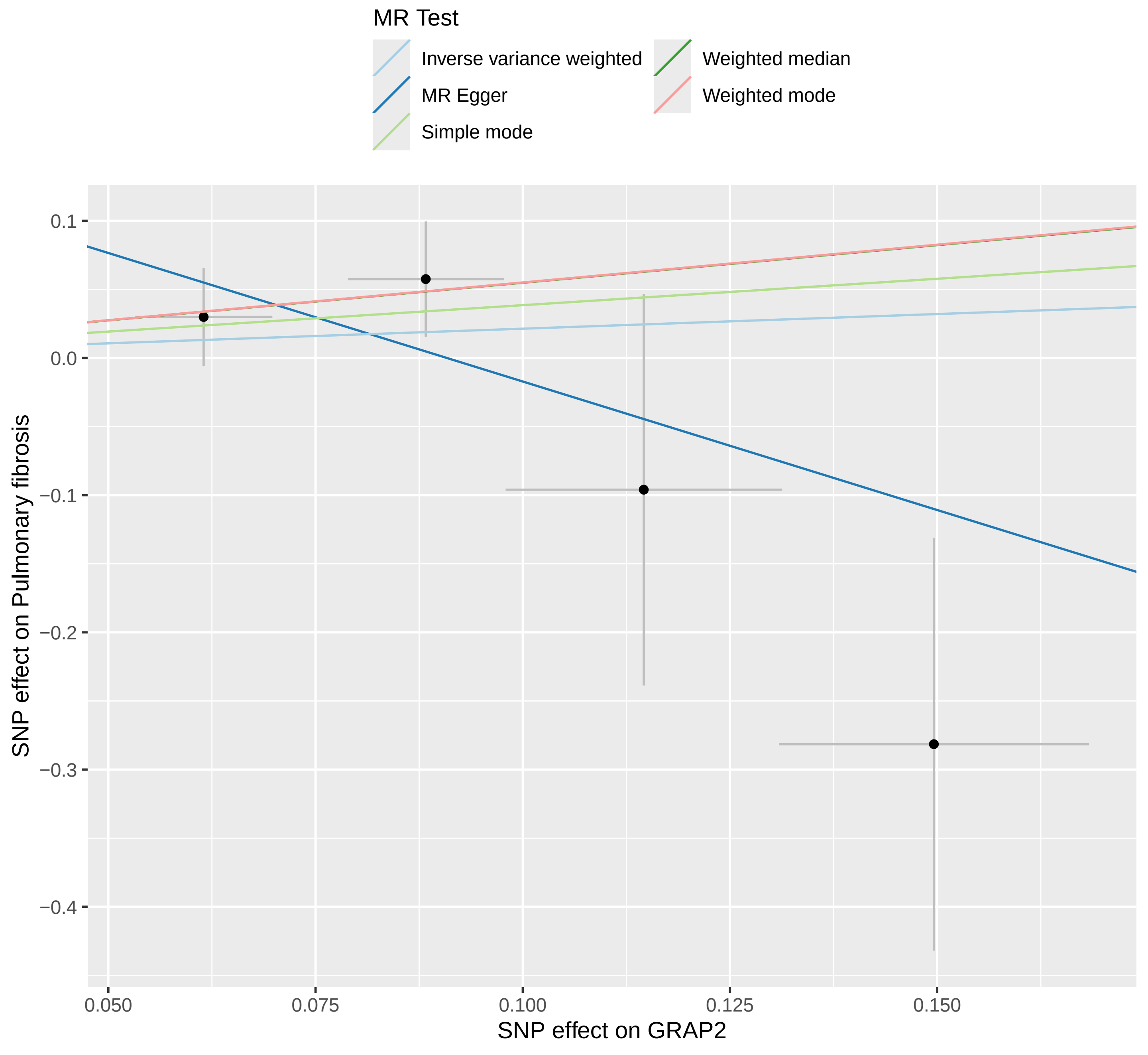

MR Test
Inverse variance weighted
MR Egger
Simple mode
Weighted median
Weighted mode
SNP effect on Pulmonary fibrosis
0.1
0.0
−0.1
−0.2
−0.3
−0.4
0.050
0.075
0.100
0.125
0.150
SNP effect on GRAP2

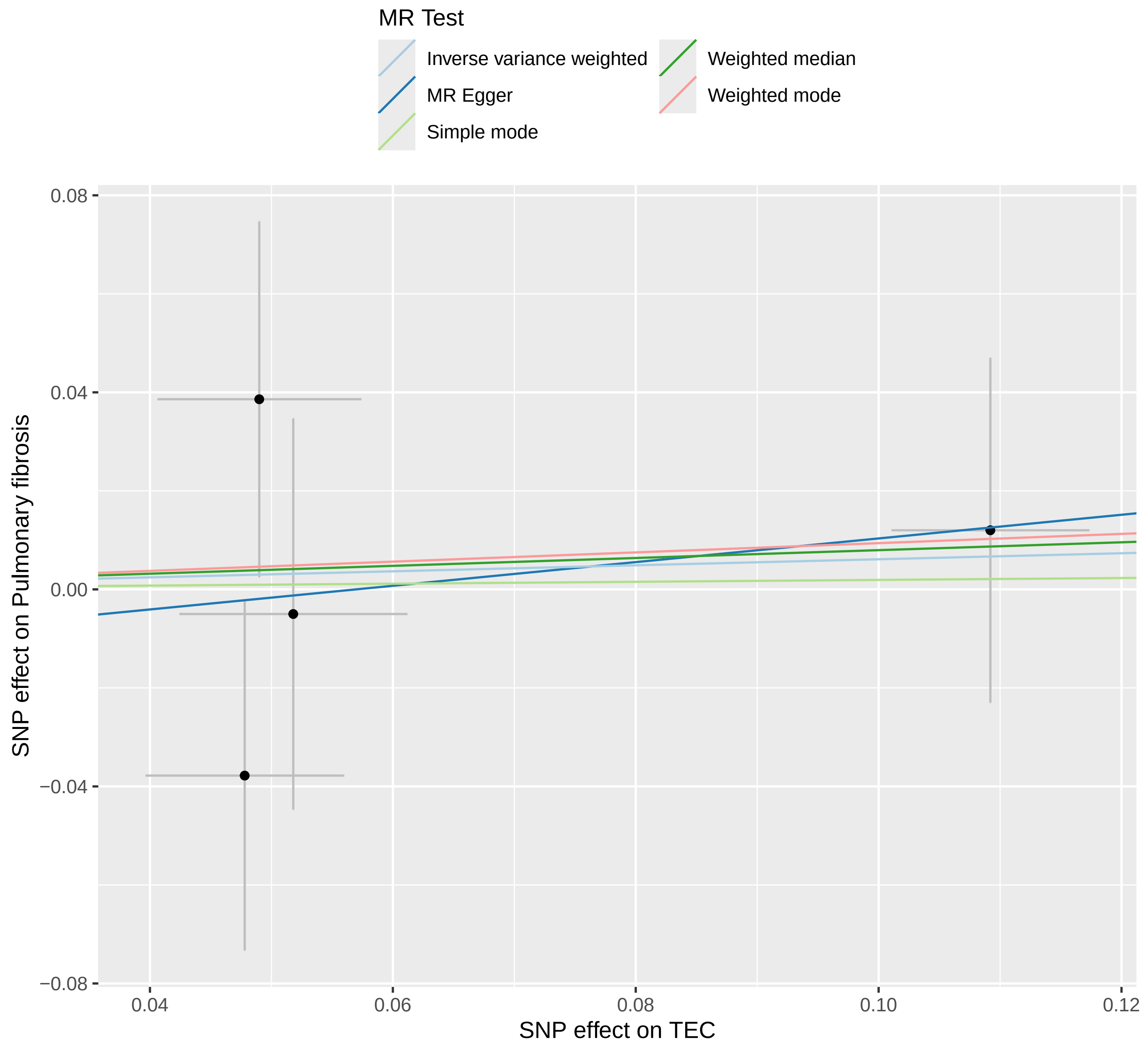
MR Test
Inverse variance weighted
MR Egger
Simple mode
Weighted median
Weighted mode
0.08
0.04
0.00
−0.04
−0.08
SNP effect on Pulmonary fibrosis
0.04
0.06
0.08
0.10
0.12
SNP effect on TEC

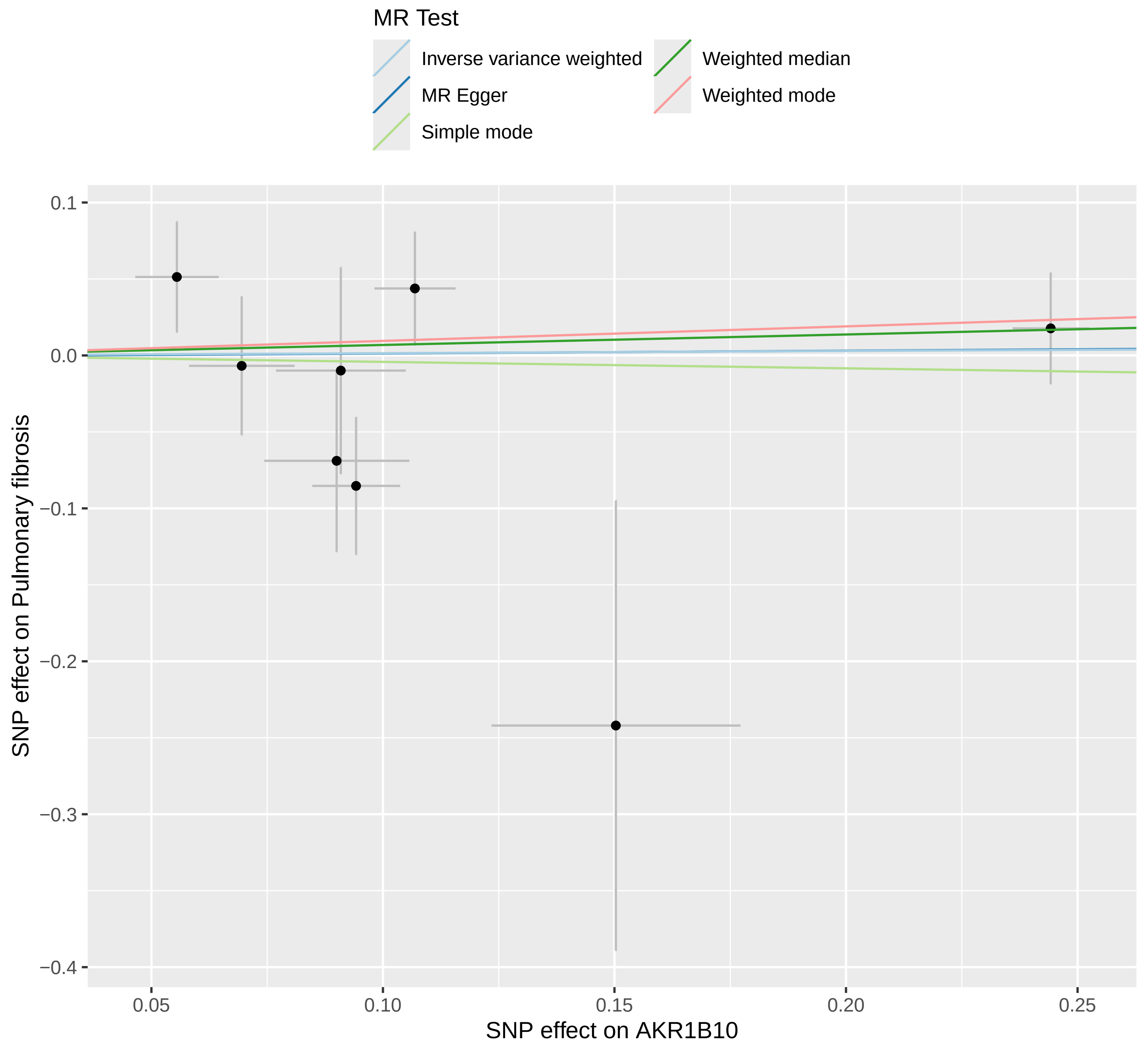
MR Test
Inverse variance weighted
MR Egger
Simple mode
Weighted median
Weighted mode
0.1
0.0
−0.1
−0.2
−0.3
−0.4
SNP effect on Pulmonary fibrosis
0.05
0.10
0.15
0.20
0.25
SNP effect on AKR1B10

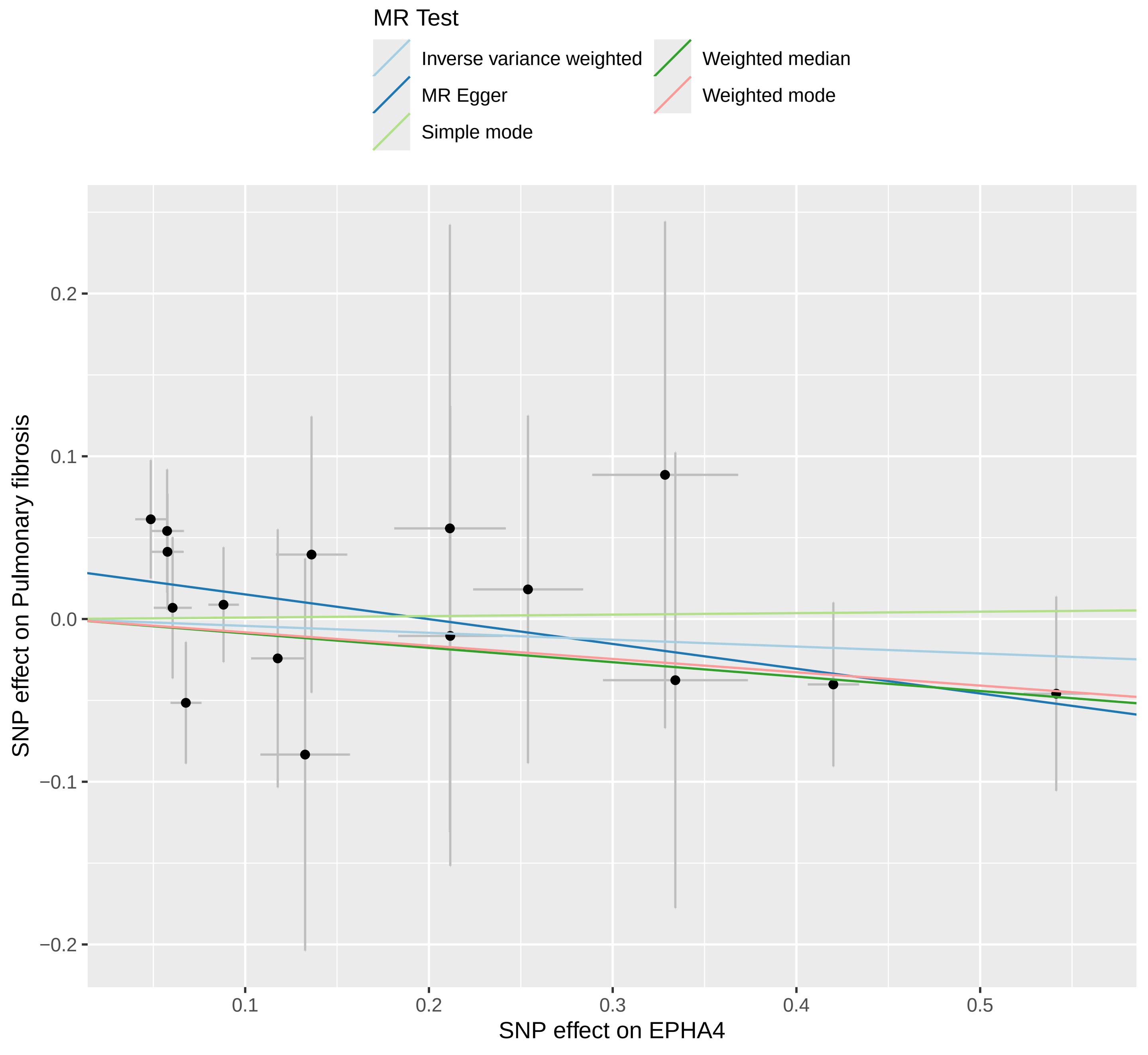

MR Test
Inverse variance weighted
MR Egger
Simple mode
Weighted median
Weighted mode
SNP effect on Pulmonary fibrosis
0.2
0.1
0.0
−0.1
−0.2
0.1
0.2
0.3
0.4
0.5
SNP effect on EPHA4

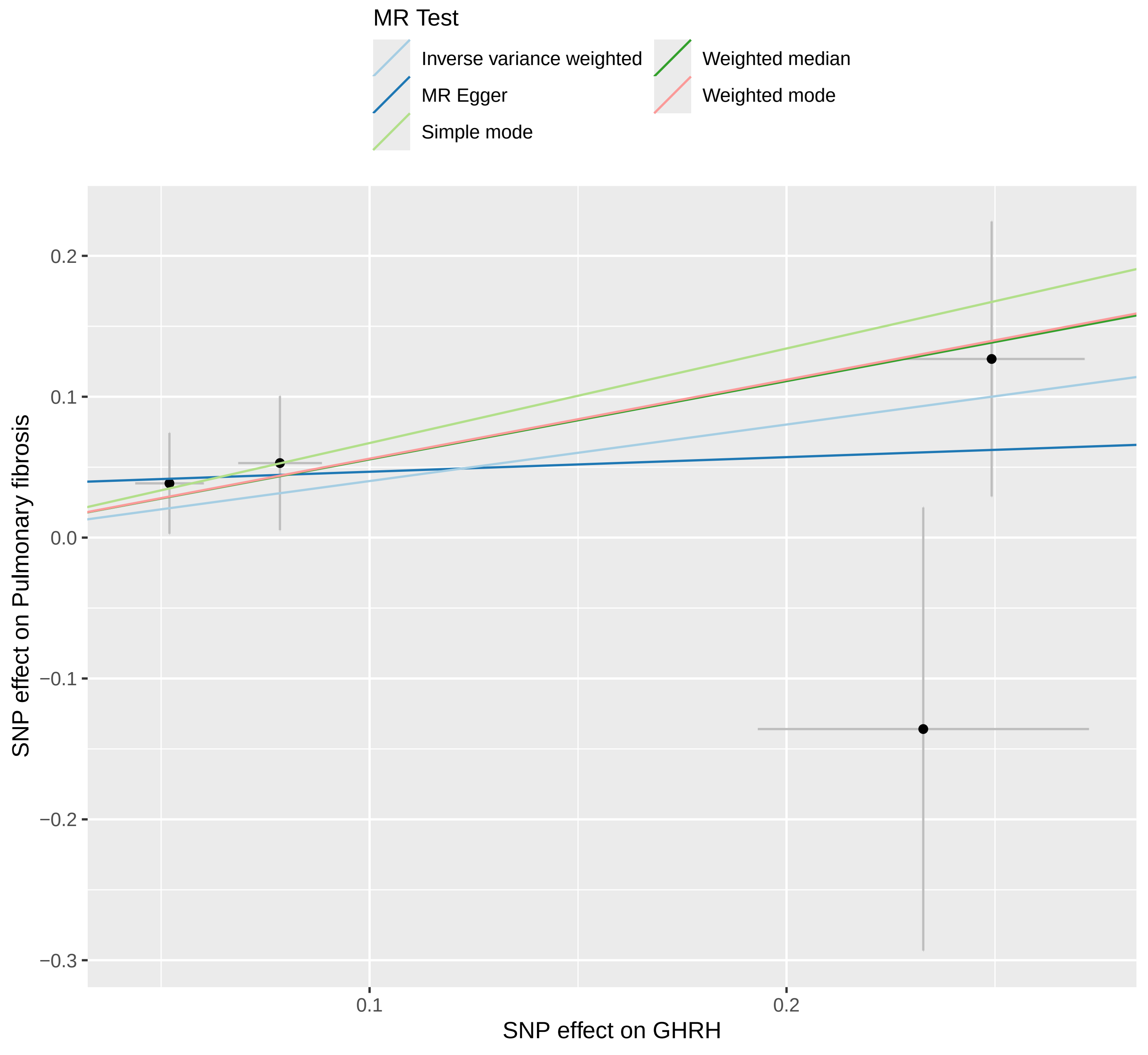
MR Test
Inverse variance weighted
MR Egger
Simple mode
Weighted median
Weighted mode
0.2
0.1
0.0
−0.1
−0.2
−0.3
SNP effect on Pulmonary fibrosis
0.1
0.2
SNP effect on GHRH

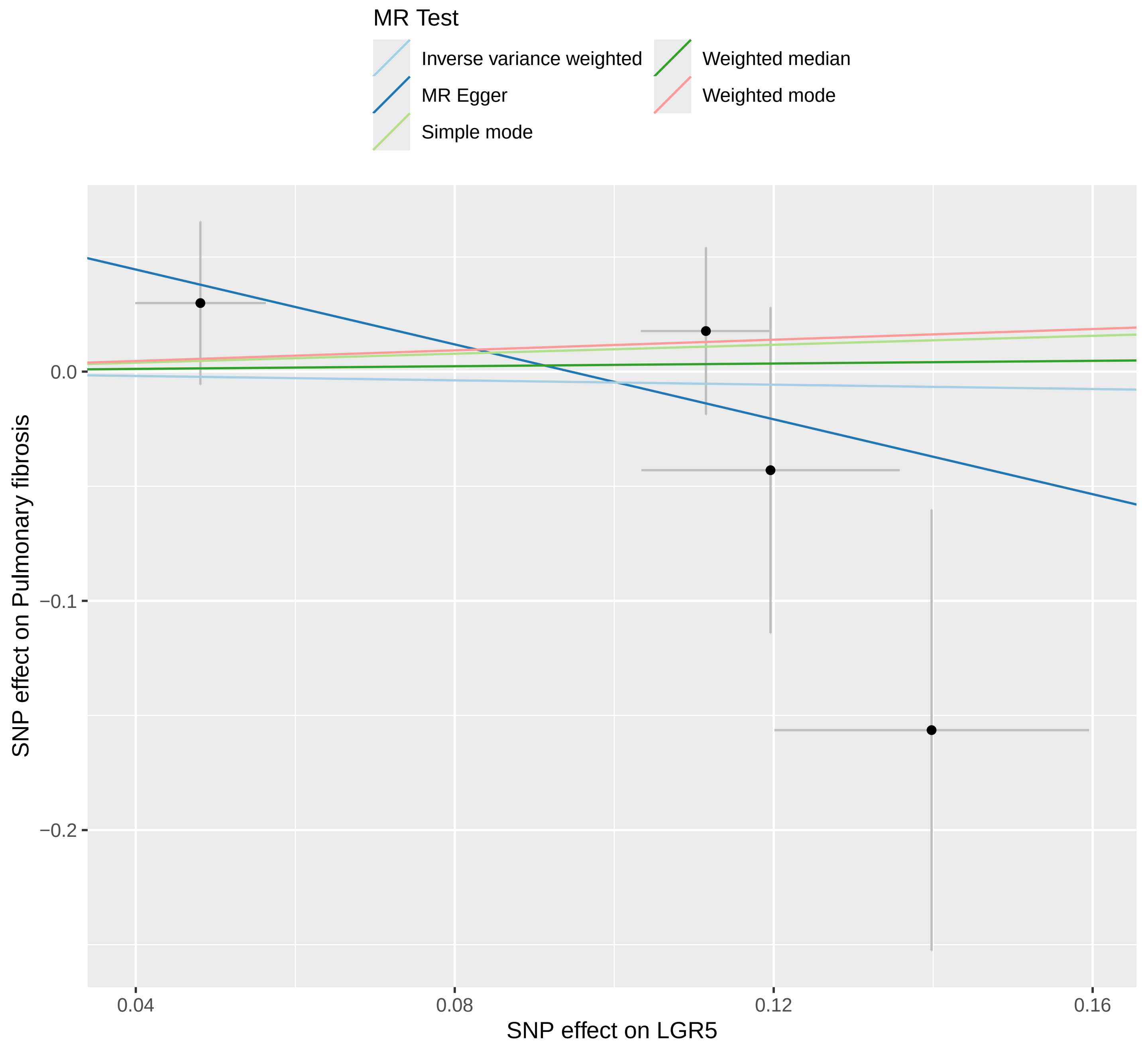

MR Test
Inverse variance weighted
MR Egger
Simple mode
Weighted median
Weighted mode
0.0
−0.1
−0.2
SNP effect on Pulmonary fibrosis
0.04
0.08
0.12
0.16
SNP effect on LGR5

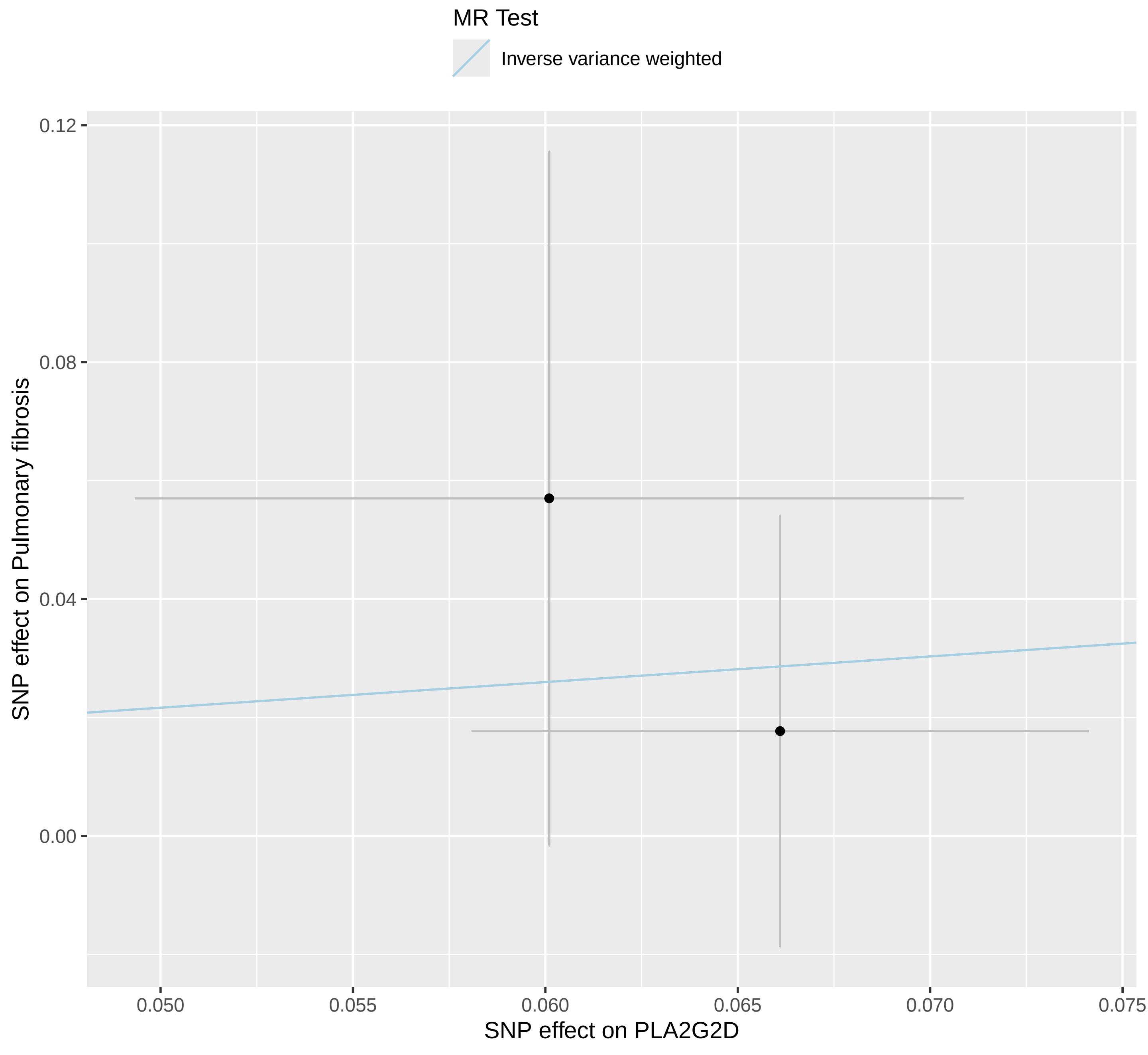
MR Test
Inverse variance weighted
0.12
0.08
0.04
0.00
SNP effect on Pulmonary fibrosis
0.050
0.055
0.060
0.065
0.070
0.075
SNP effect on PLA2G2D

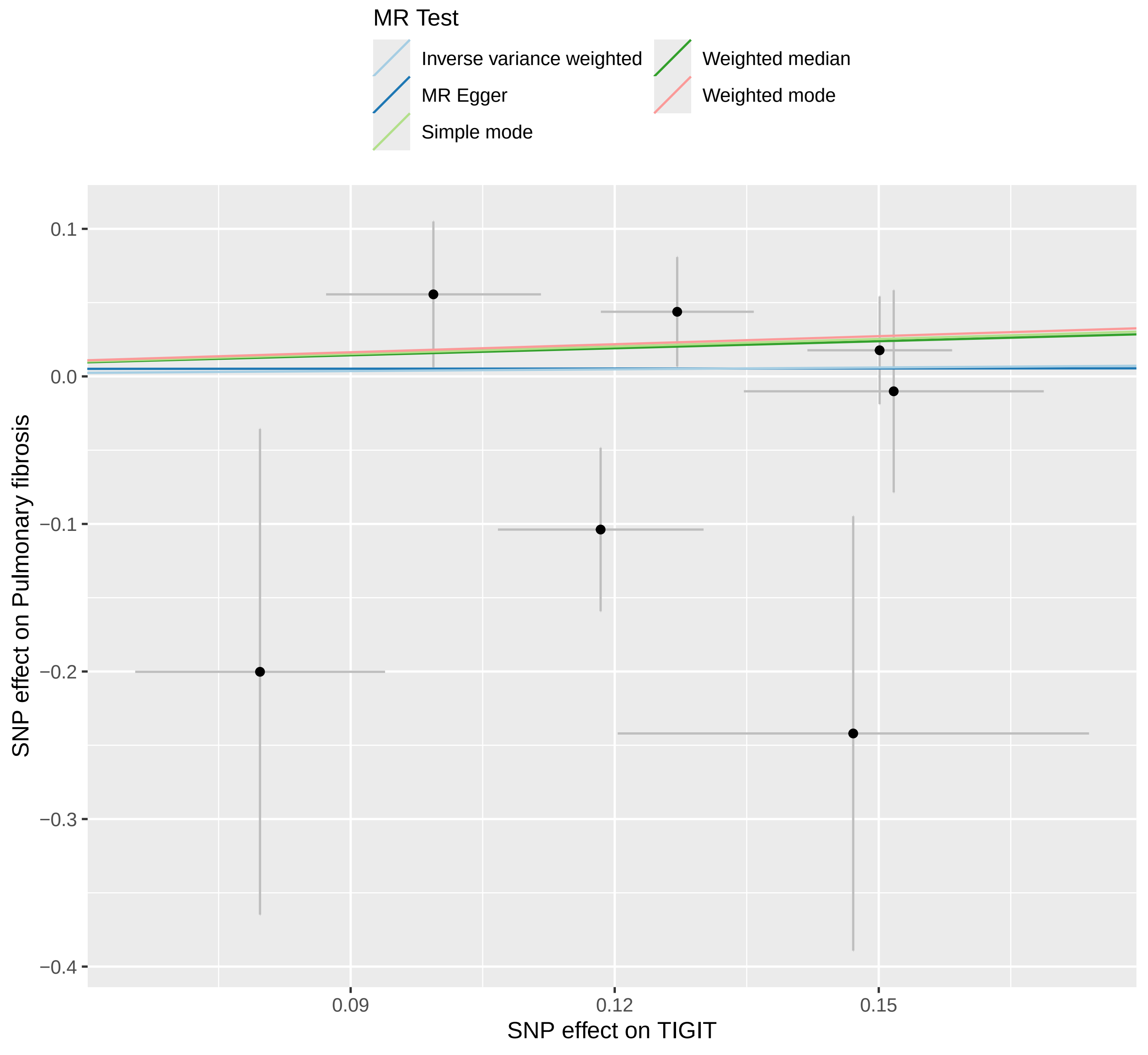
MR Test
Inverse variance weighted
MR Egger
Simple mode
Weighted median
Weighted mode
0.1
0.0
−0.1
−0.2
−0.3
−0.4
SNP effect on Pulmonary fibrosis
0.09
0.12
0.15
SNP effect on TIGIT

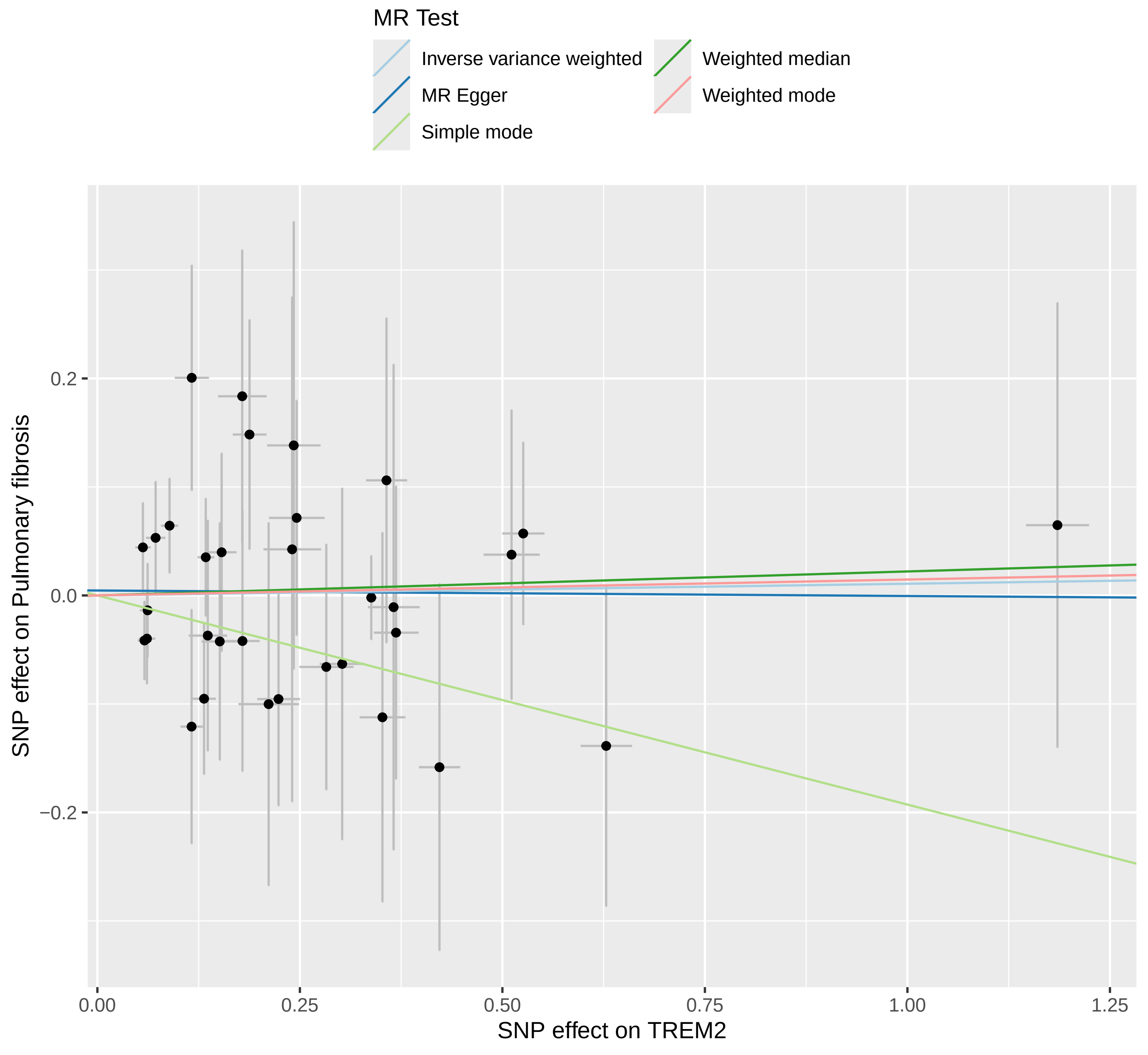

MR Test
Inverse variance weighted
MR Egger
Simple mode
Weighted median
Weighted mode
0.2
0.0
−0.2
SNP effect on Pulmonary fibrosis
0.00
0.25
0.50
0.75
1.00
1.25
SNP effect on TREM2

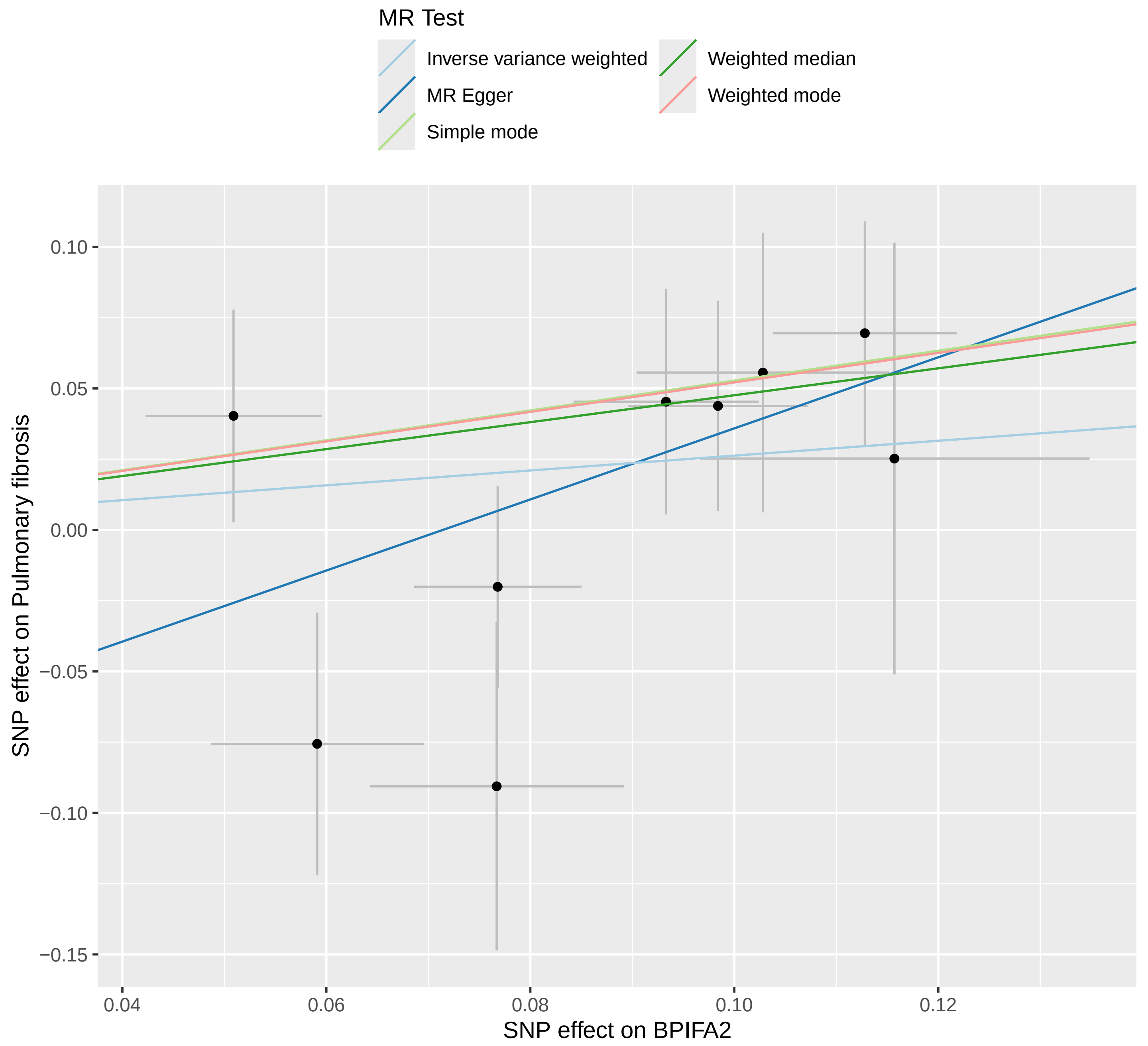

MR Test
Inverse variance weighted
MR Egger
Simple mode
Weighted median
Weighted mode
0.10
0.05
0.00
−0.05
−0.10
−0.15
SNP effect on Pulmonary fibrosis
0.04
0.06
0.08
0.10
0.12
SNP effect on BPIFA2

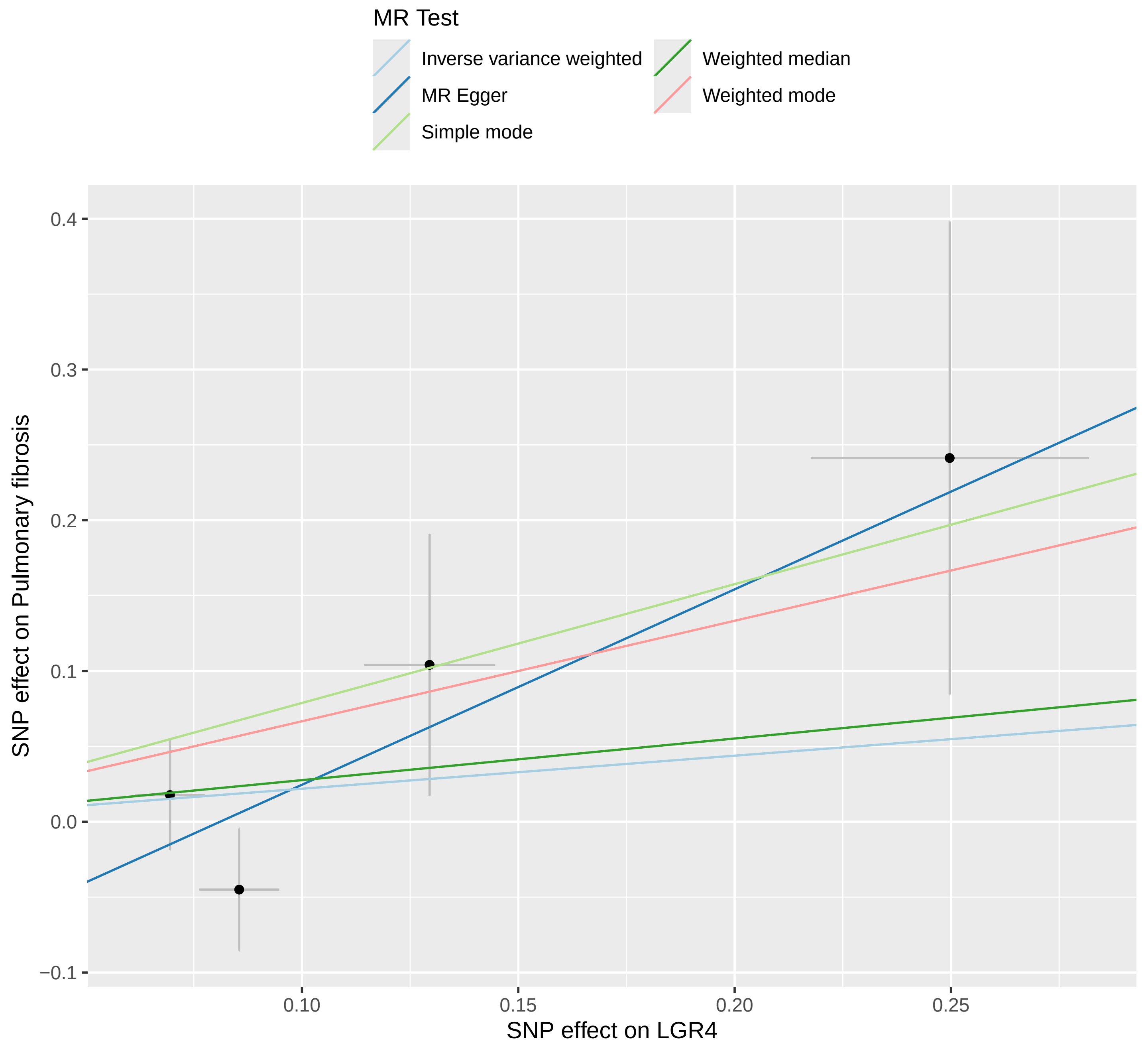

MR Test
Inverse variance weighted
MR Egger
Simple mode
Weighted median
Weighted mode
SNP effect on Pulmonary fibrosis
0.4
0.3
0.2
0.1
0.0
−0.1
0.10
0.15
0.20
0.25
SNP effect on LGR4

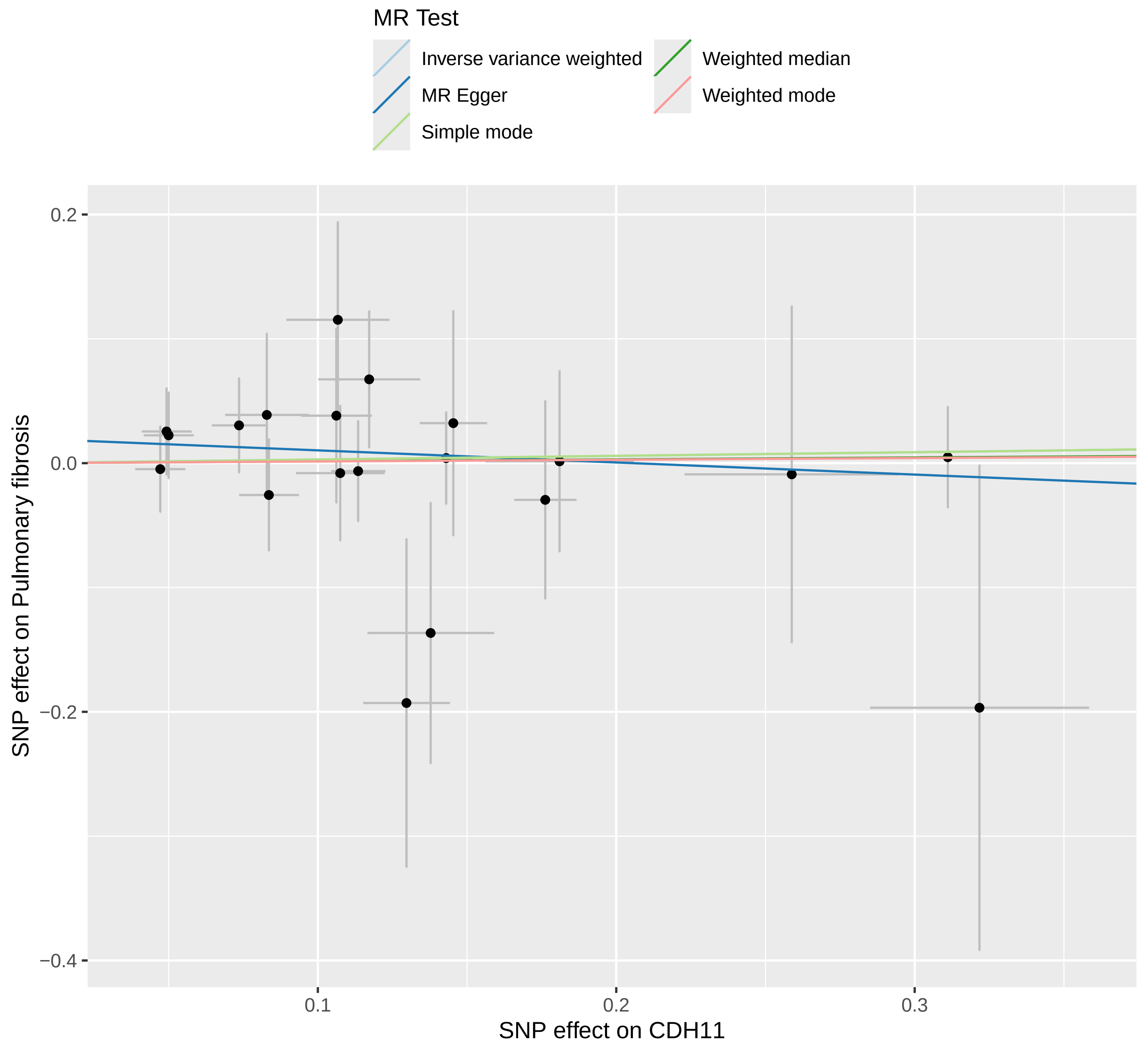

MR Test
Inverse variance weighted
MR Egger
Simple mode
Weighted median
Weighted mode
0.2
0.0
−0.2
−0.4
0.1
0.2
0.3
SNP effect on Pulmonary fibrosis
SNP effect on CDH11

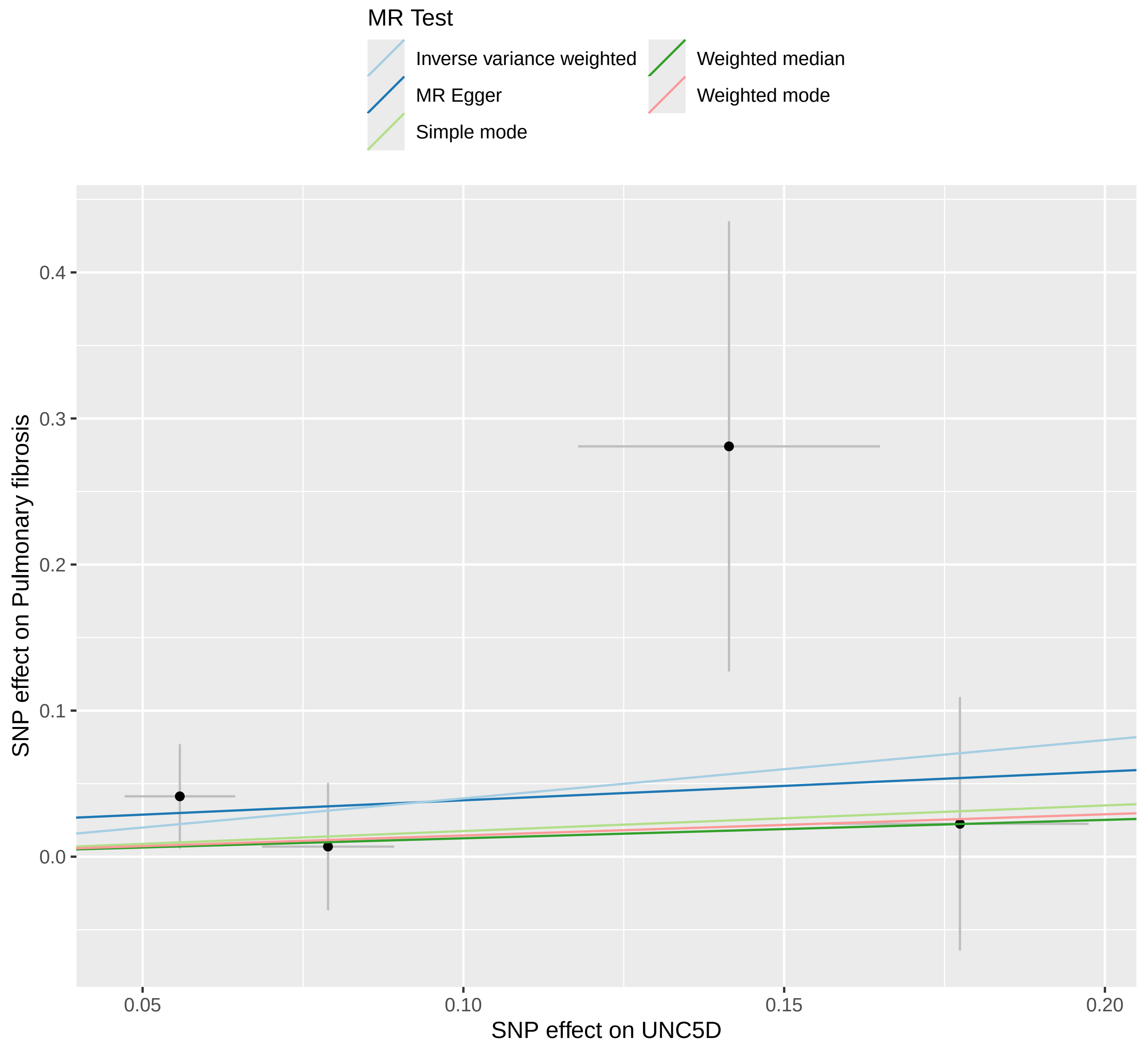
MR Test
Inverse variance weighted
MR Egger
Simple mode
Weighted median
Weighted mode
SNP effect on Pulmonary fibrosis
0.4
0.3
0.2
0.1
0.0
0.05
0.10
0.15
0.20
SNP effect on UNC5D

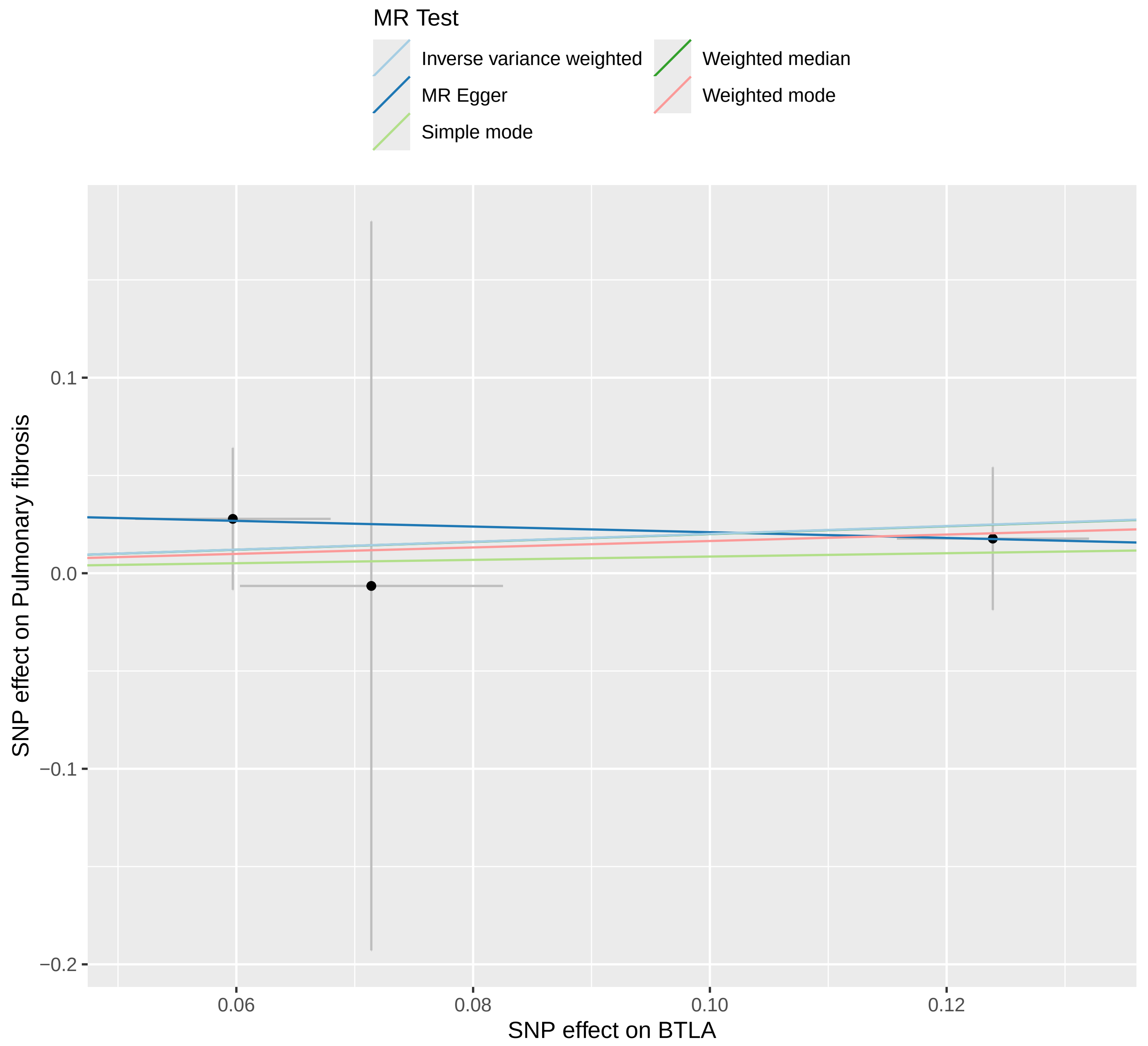
MR Test
Inverse variance weighted
MR Egger
Simple mode
Weighted median
Weighted mode
SNP effect on Pulmonary fibrosis
0.1
0.0
−0.1
−0.2
0.06
0.08
0.10
0.12
SNP effect on BTLA

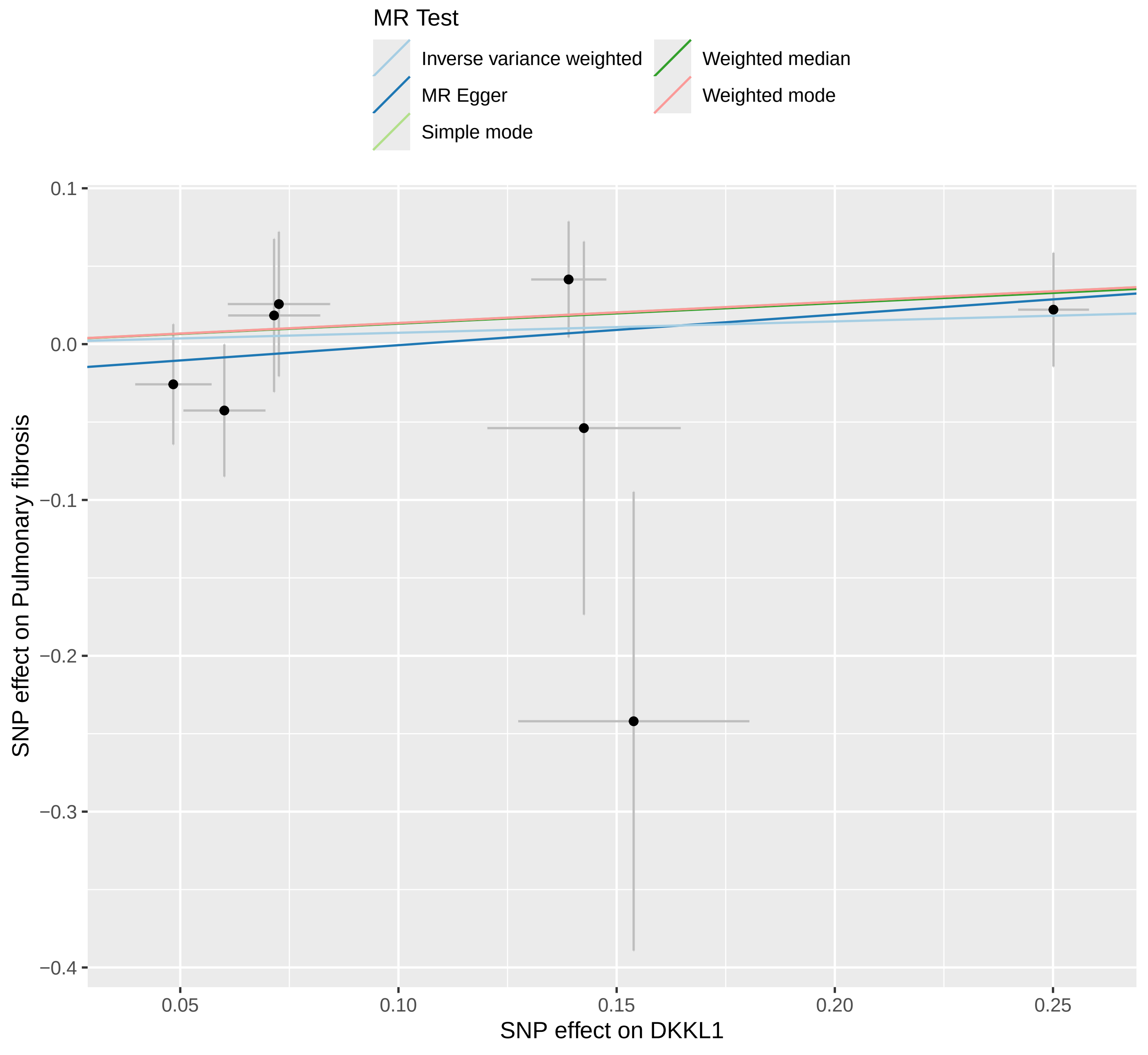

MR Test
Inverse variance weighted
MR Egger
Simple mode
Weighted median
Weighted mode
0.1
0.0
−0.1
−0.2
−0.3
−0.4
SNP effect on Pulmonary fibrosis
0.05
0.10
0.15
0.20
0.25
SNP effect on DKKL1

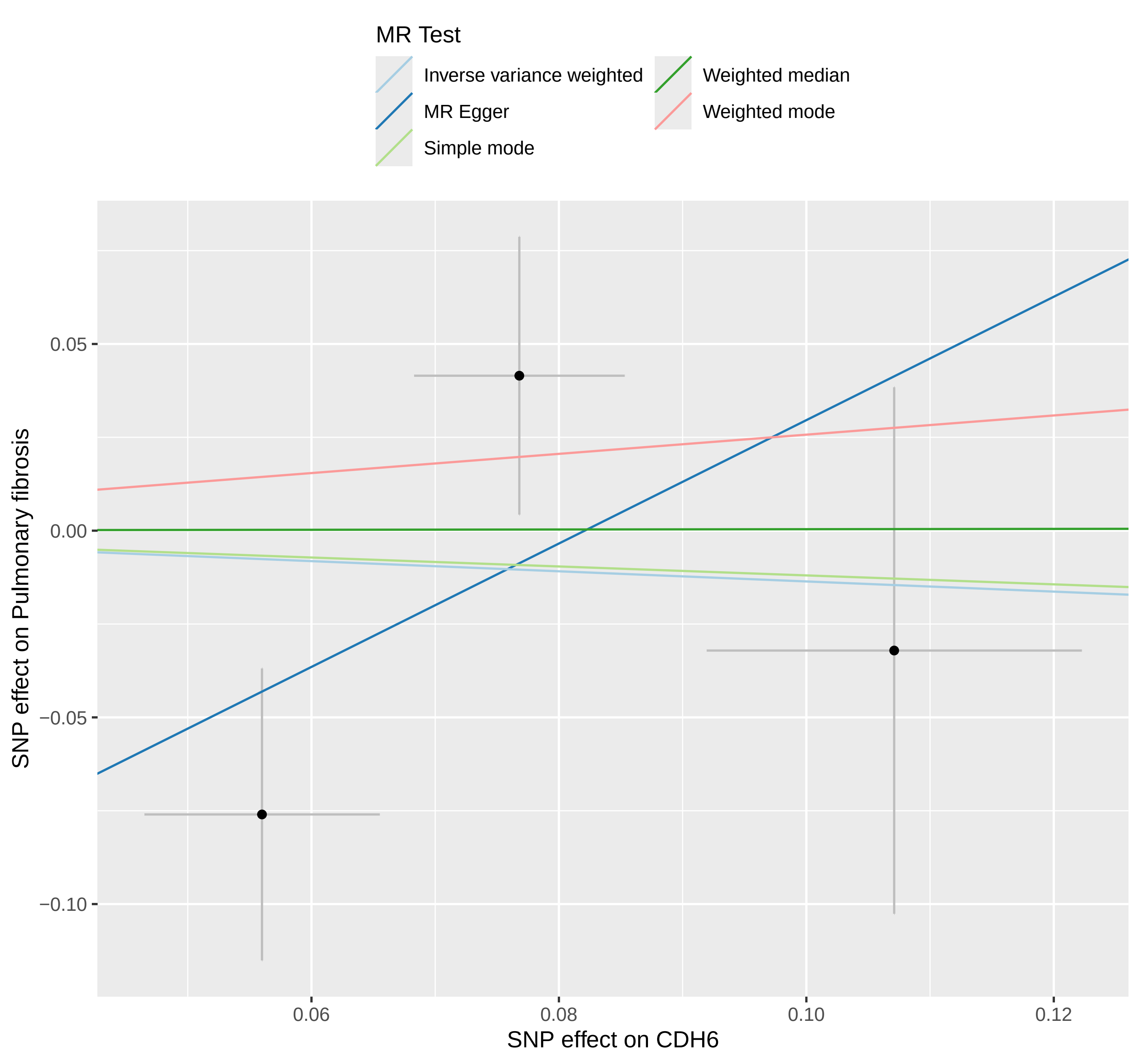

MR Test
Inverse variance weighted
MR Egger
Simple mode
Weighted median
Weighted mode
0.05
0.00
−0.05
−0.10
SNP effect on Pulmonary fibrosis
0.06
0.08
0.10
0.12
SNP effect on CDH6

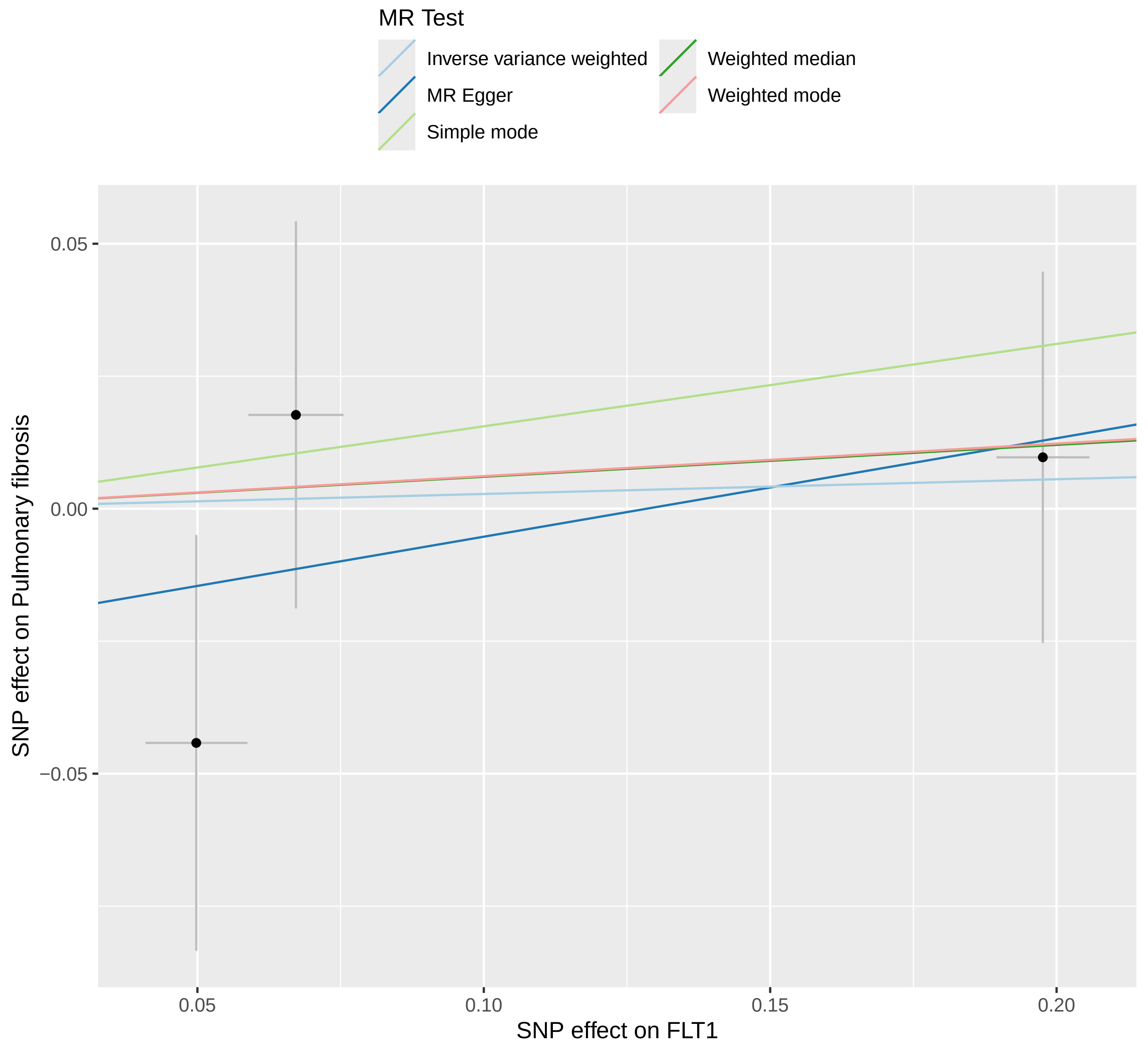
MR Test
Inverse variance weighted
MR Egger
Simple mode
Weighted median
Weighted mode
0.05
0.00
−0.05
0.05
0.10
0.15
0.20
SNP effect on Pulmonary fibrosis
SNP effect on FLT1

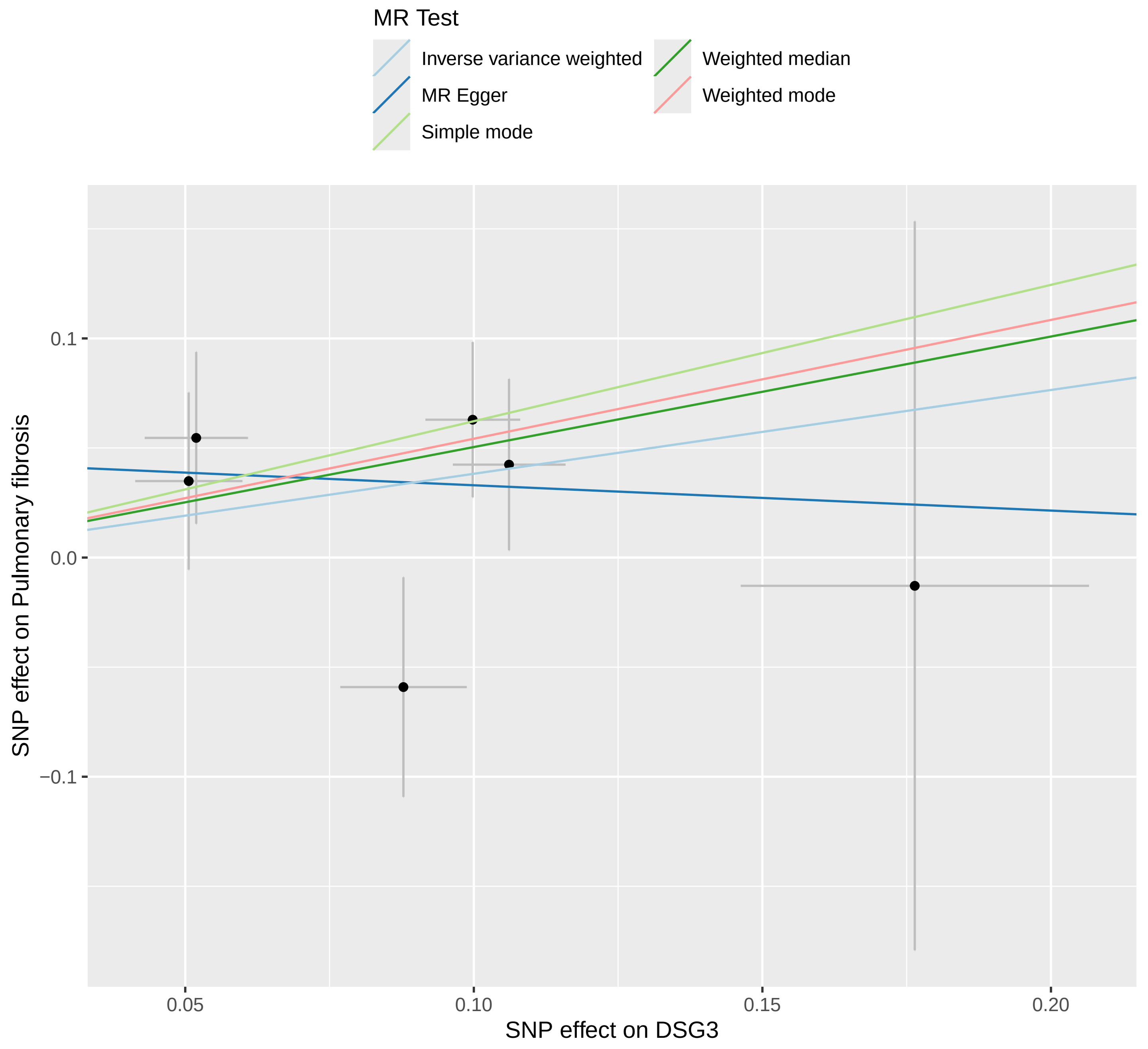

MR Test
Inverse variance weighted
MR Egger
Simple mode
Weighted median
Weighted mode
SNP effect on Pulmonary fibrosis
0.1
0.0
−0.1
0.05
0.10
0.15
0.20
SNP effect on DSG3

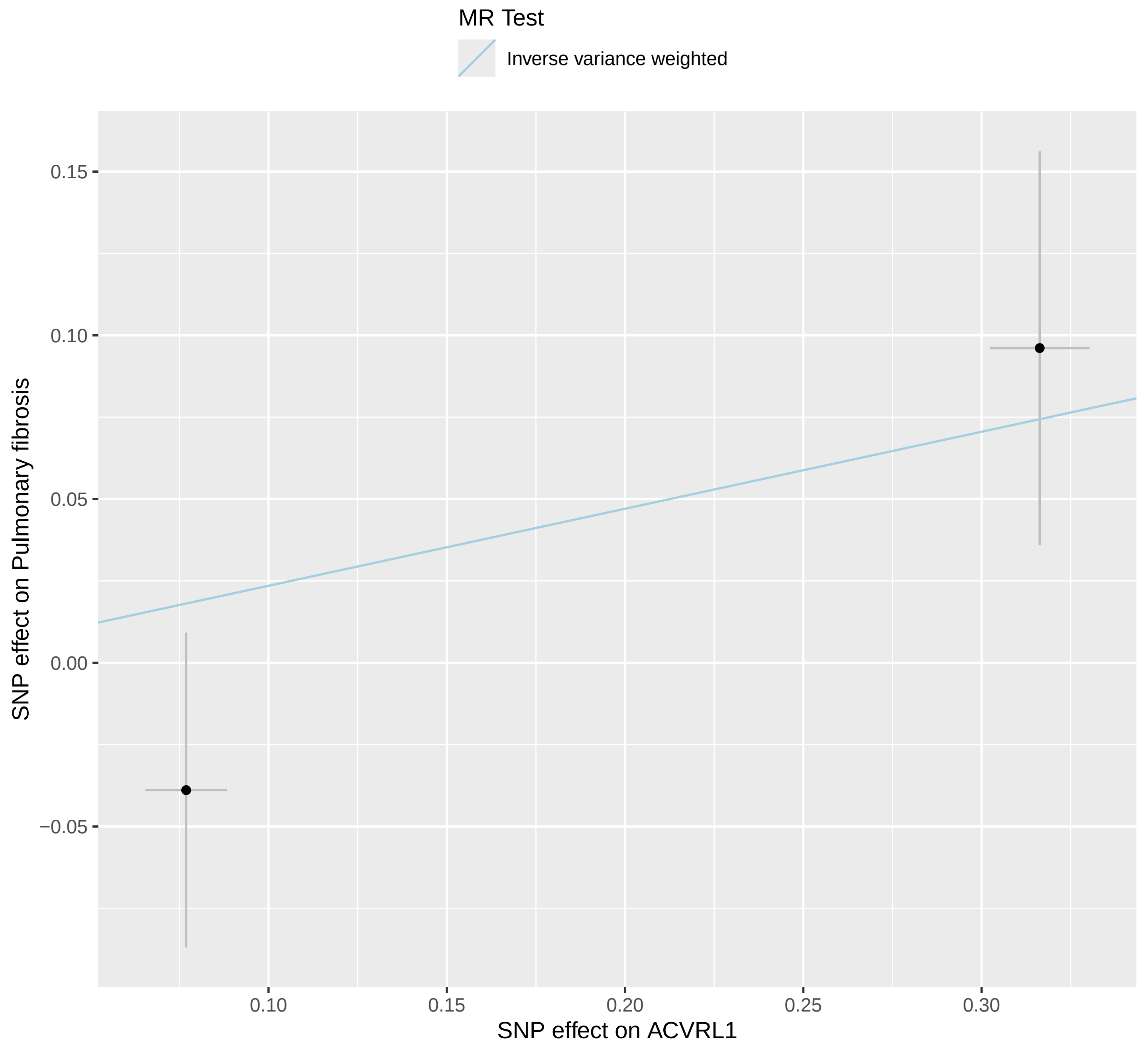
MR Test
Inverse variance weighted
0.15
0.10
0.05
0.00
−0.05
0.10
0.15
0.20
0.25
0.30
SNP effect on Pulmonary fibrosis
SNP effect on ACVRL1

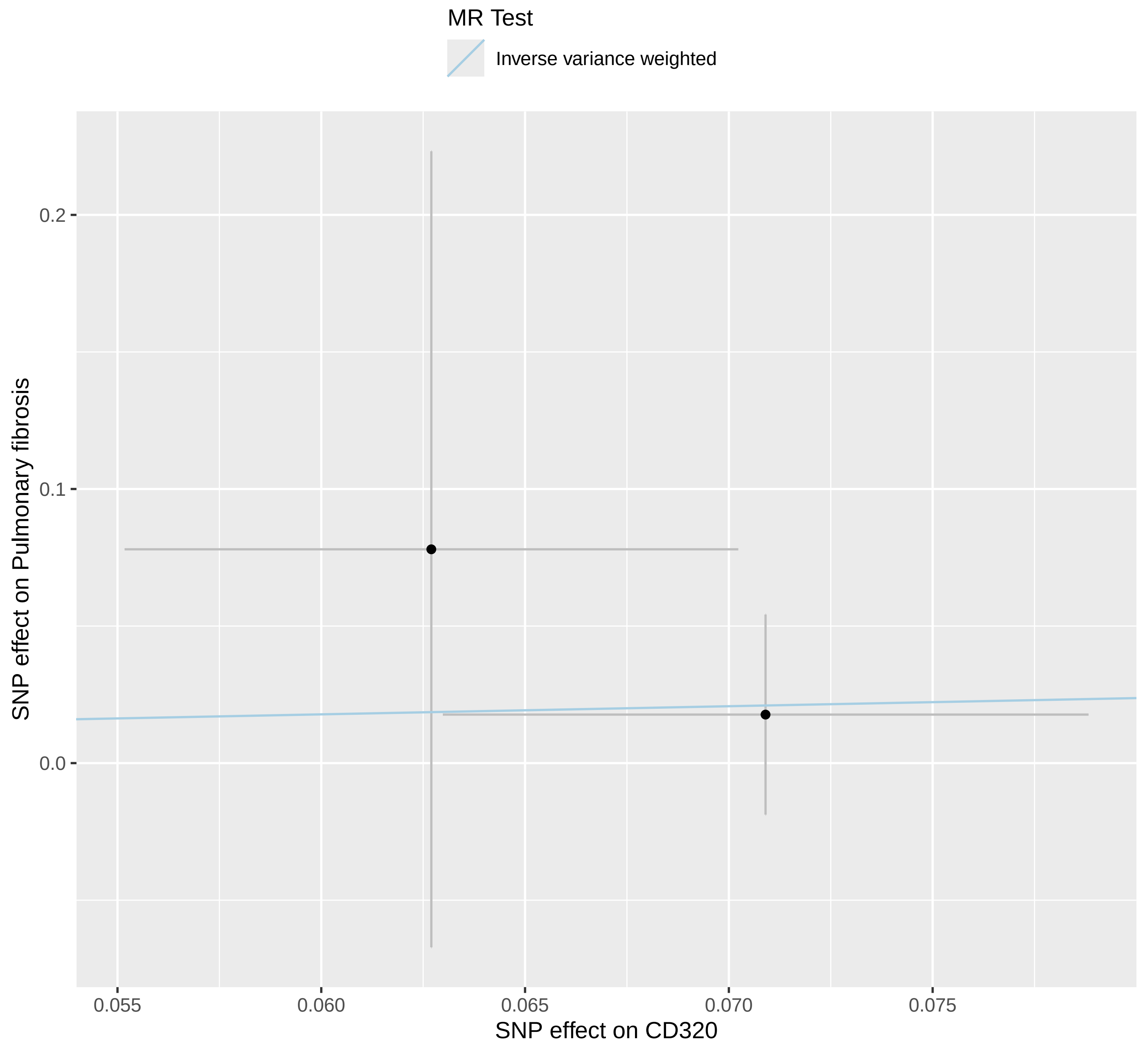
MR Test
Inverse variance weighted
0.2
0.1
0.0
SNP effect on Pulmonary fibrosis
0.055
0.060
0.065
0.070
0.075
SNP effect on CD320

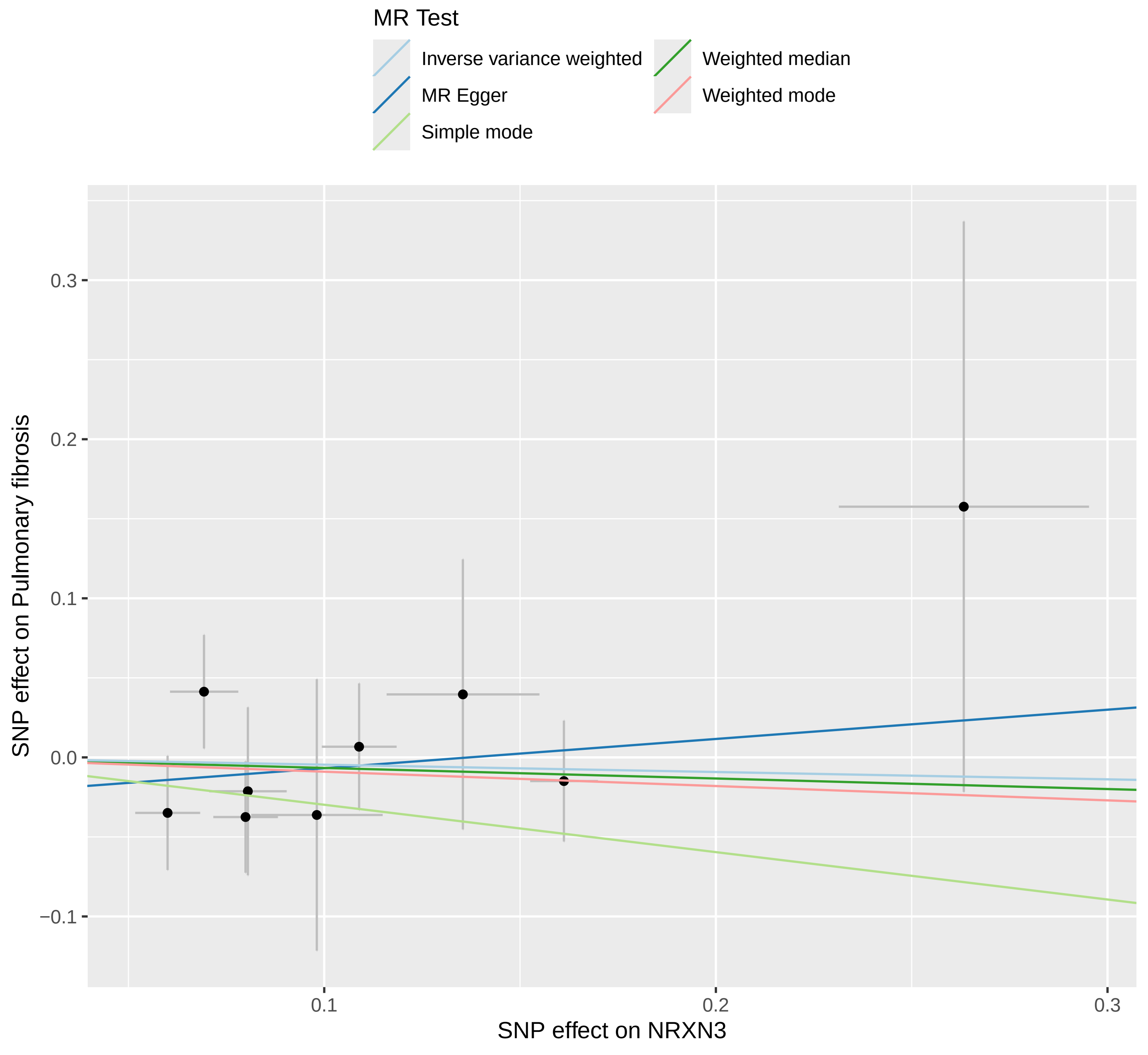

MR Test
Inverse variance weighted
MR Egger
Simple mode
Weighted median
Weighted mode
SNP effect on Pulmonary fibrosis
0.3
0.2
0.1
0.0
−0.1
0.1
0.2
0.3
SNP effect on NRXN3

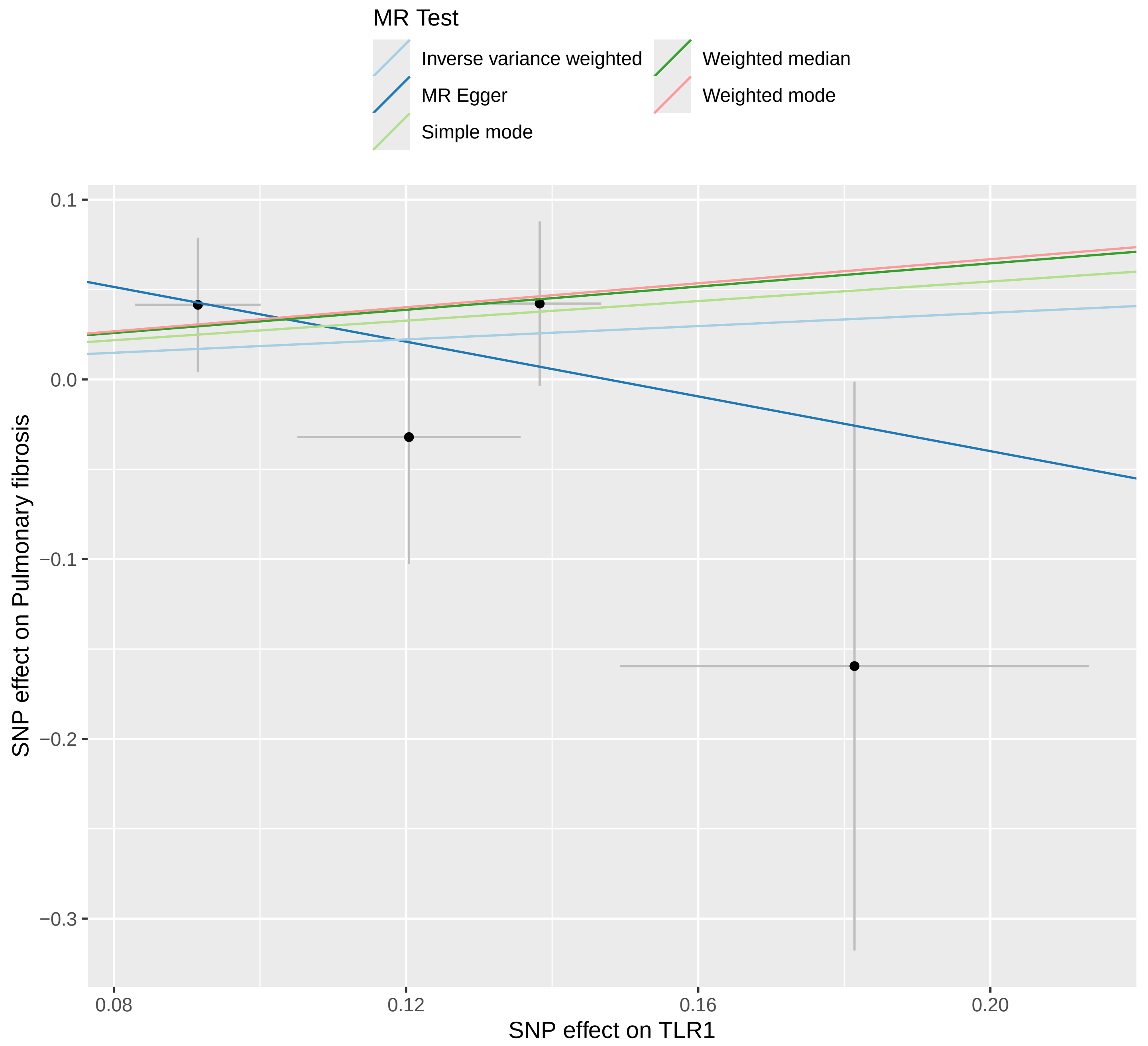
MR Test
Inverse variance weighted
MR Egger
Simple mode
Weighted median
Weighted mode
0.1
0.0
−0.1
−0.2
−0.3
SNP effect on Pulmonary fibrosis
0.08
0.12
0.16
0.20
SNP effect on TLR1

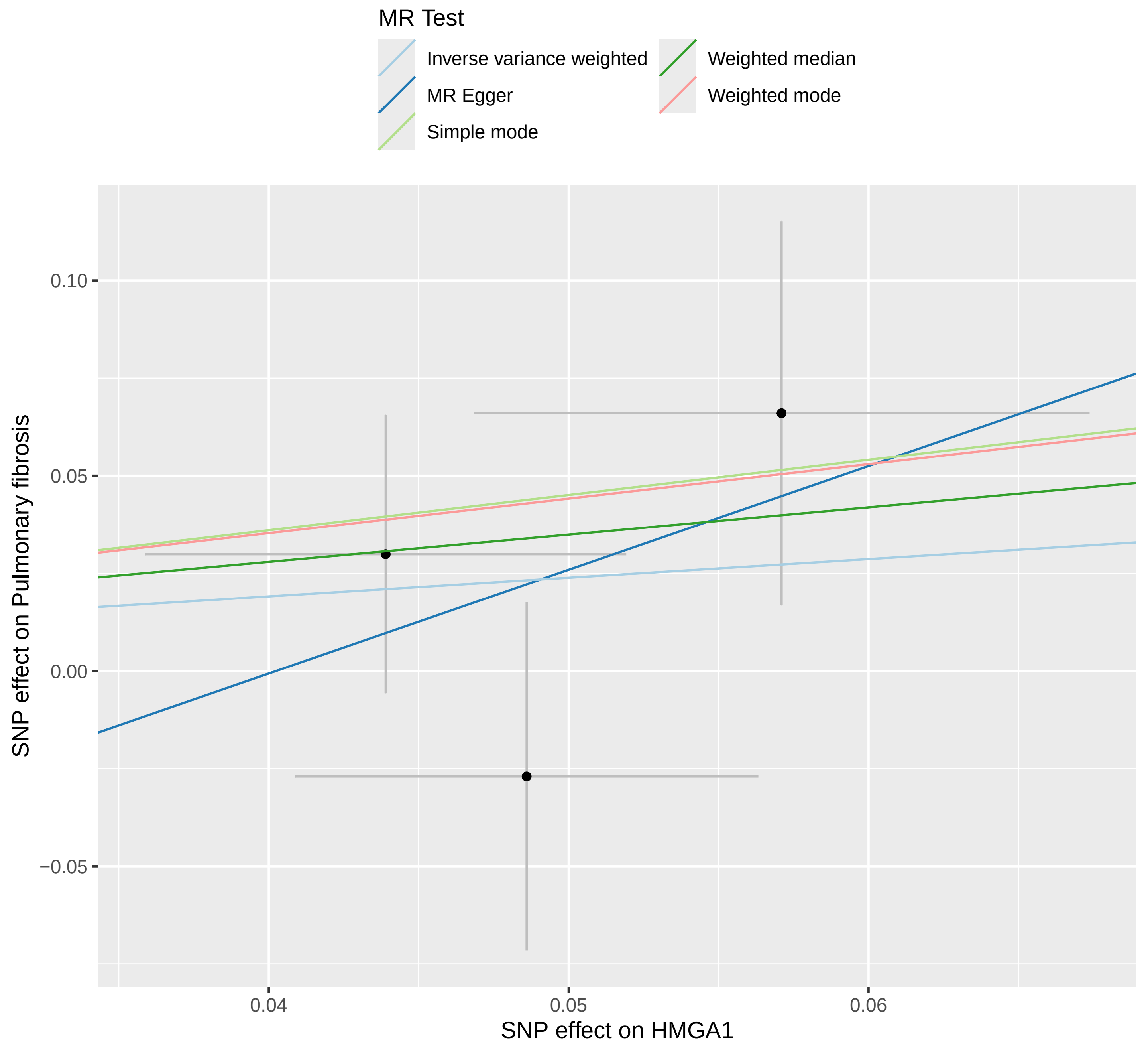

MR Test
Inverse variance weighted
MR Egger
Simple mode
Weighted median
Weighted mode
0.10
0.05
0.00
−0.05
0.04
0.05
0.06
SNP effect on Pulmonary fibrosis
SNP effect on HMGA1

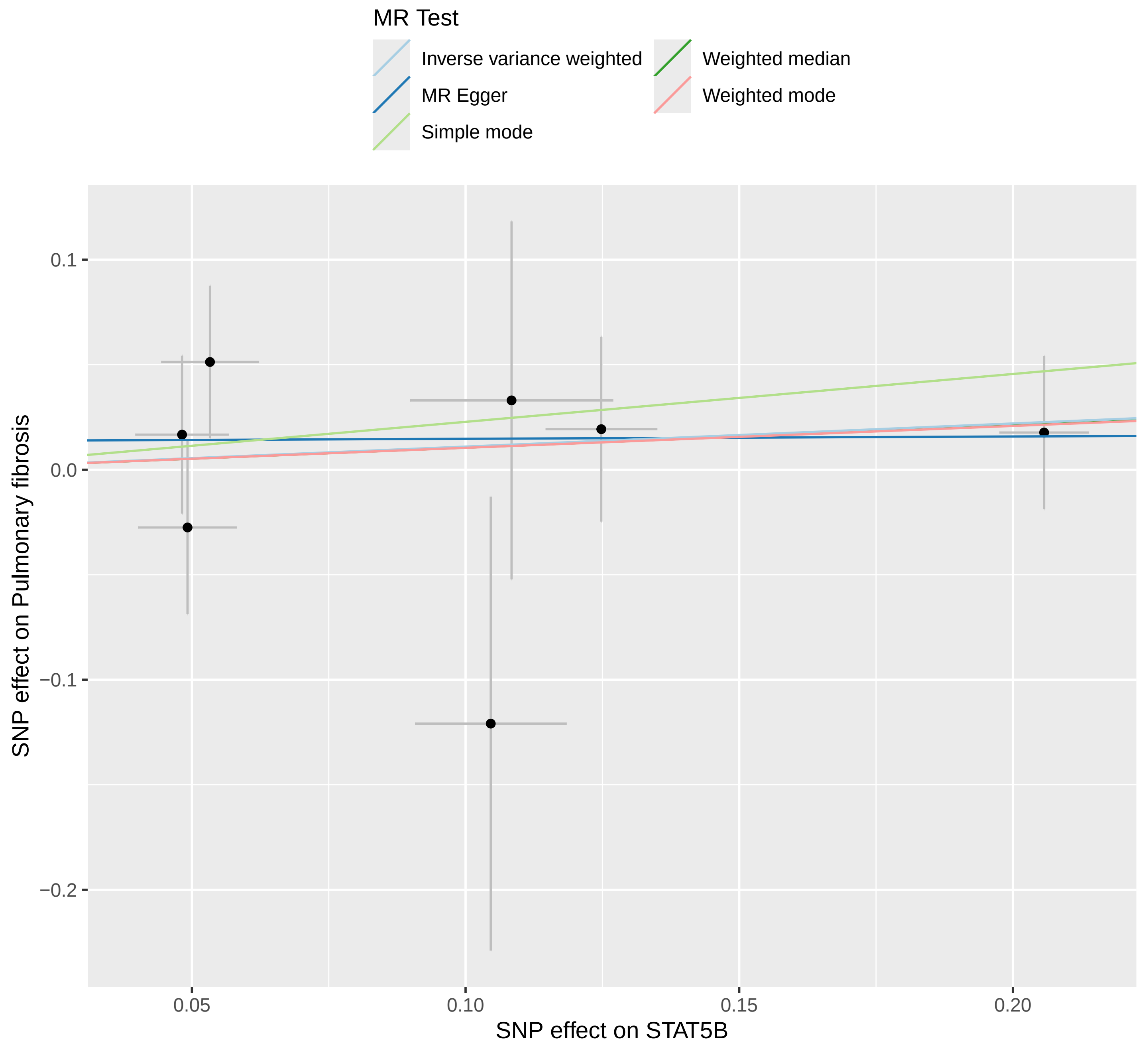
MR Test
Inverse variance weighted
MR Egger
Simple mode
Weighted median
Weighted mode
0.1
0.0
−0.1
−0.2
0.05
0.10
0.15
0.20
SNP effect on STAT5B
SNP effect on Pulmonary fibrosis

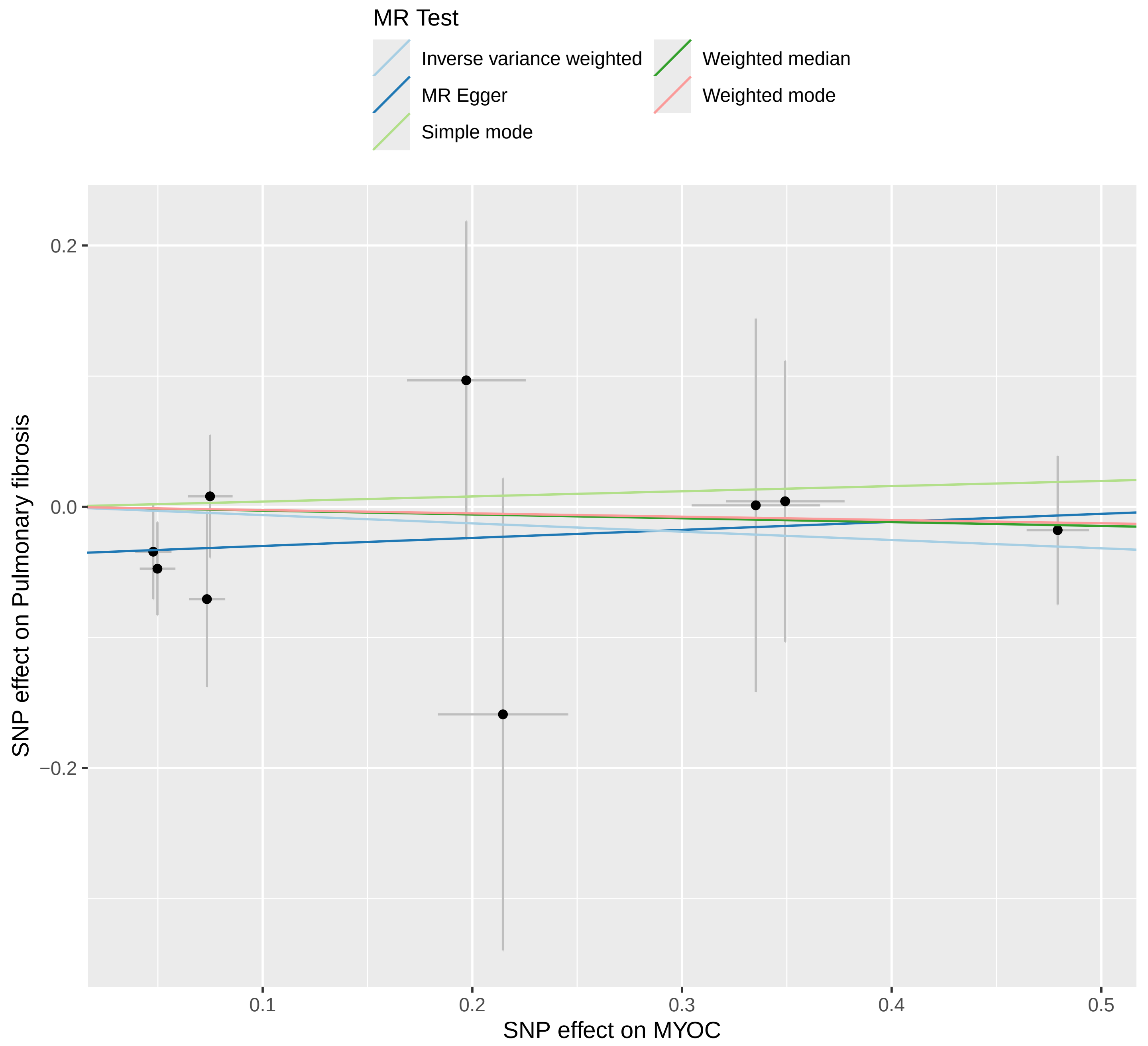

MR Test
Inverse variance weighted
MR Egger
Simple mode
Weighted median
Weighted mode
0.2
0.0
−0.2
SNP effect on Pulmonary fibrosis
0.1
0.2
0.3
0.4
0.5
SNP effect on MYOC

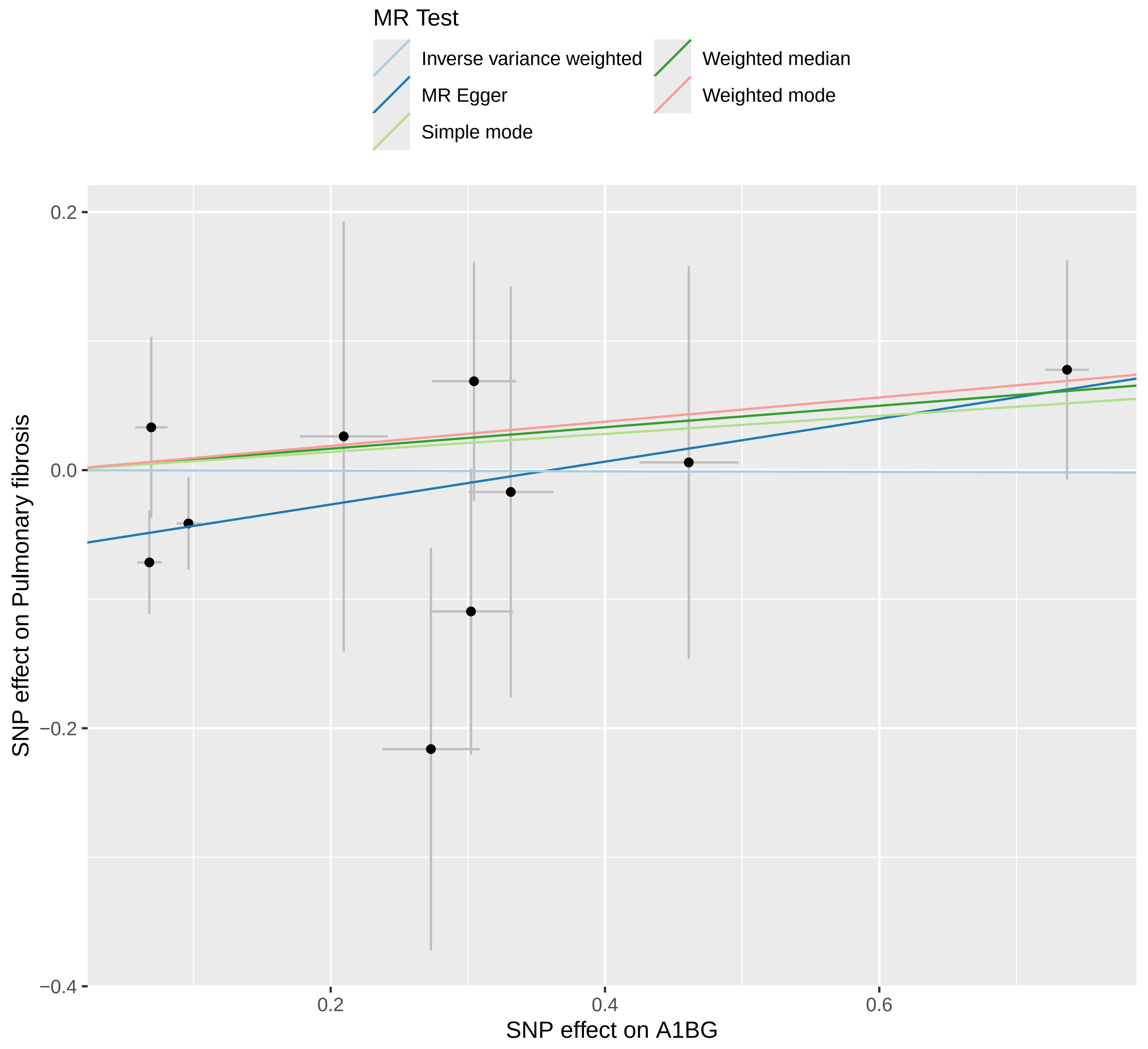
MR Test
Inverse variance weighted
MR Egger
Simple mode
Weighted median
Weighted mode
0.2
0.0
−0.2
−0.4
SNP effect on Pulmonary fibrosis
0.2
0.4
0.6
SNP effect on A1BG

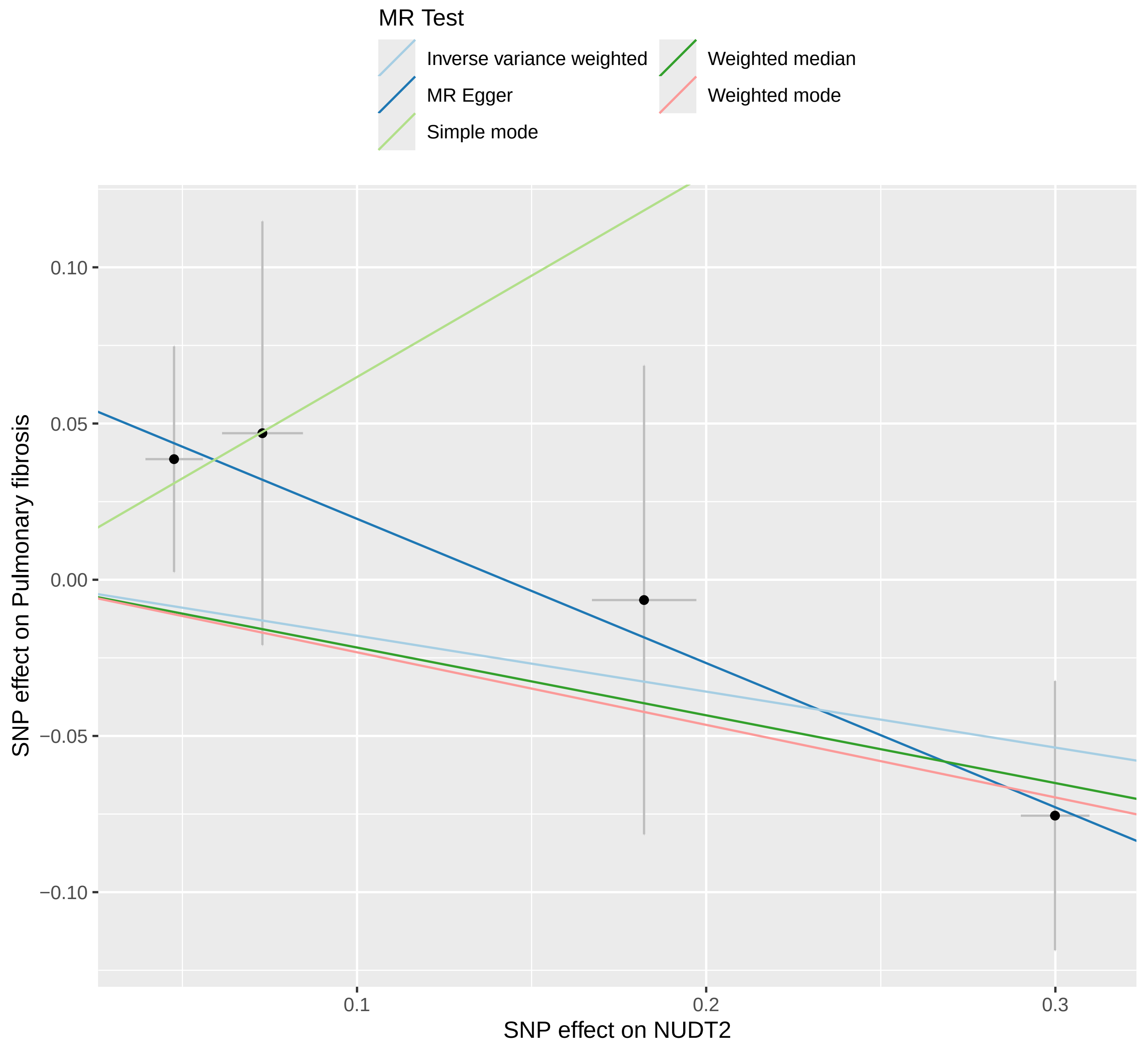

MR Test
Inverse variance weighted
MR Egger
Simple mode
Weighted median
Weighted mode
0.10
0.05
0.00
−0.05
−0.10
0.1
0.2
0.3
SNP effect on Pulmonary fibrosis
SNP effect on NUDT2

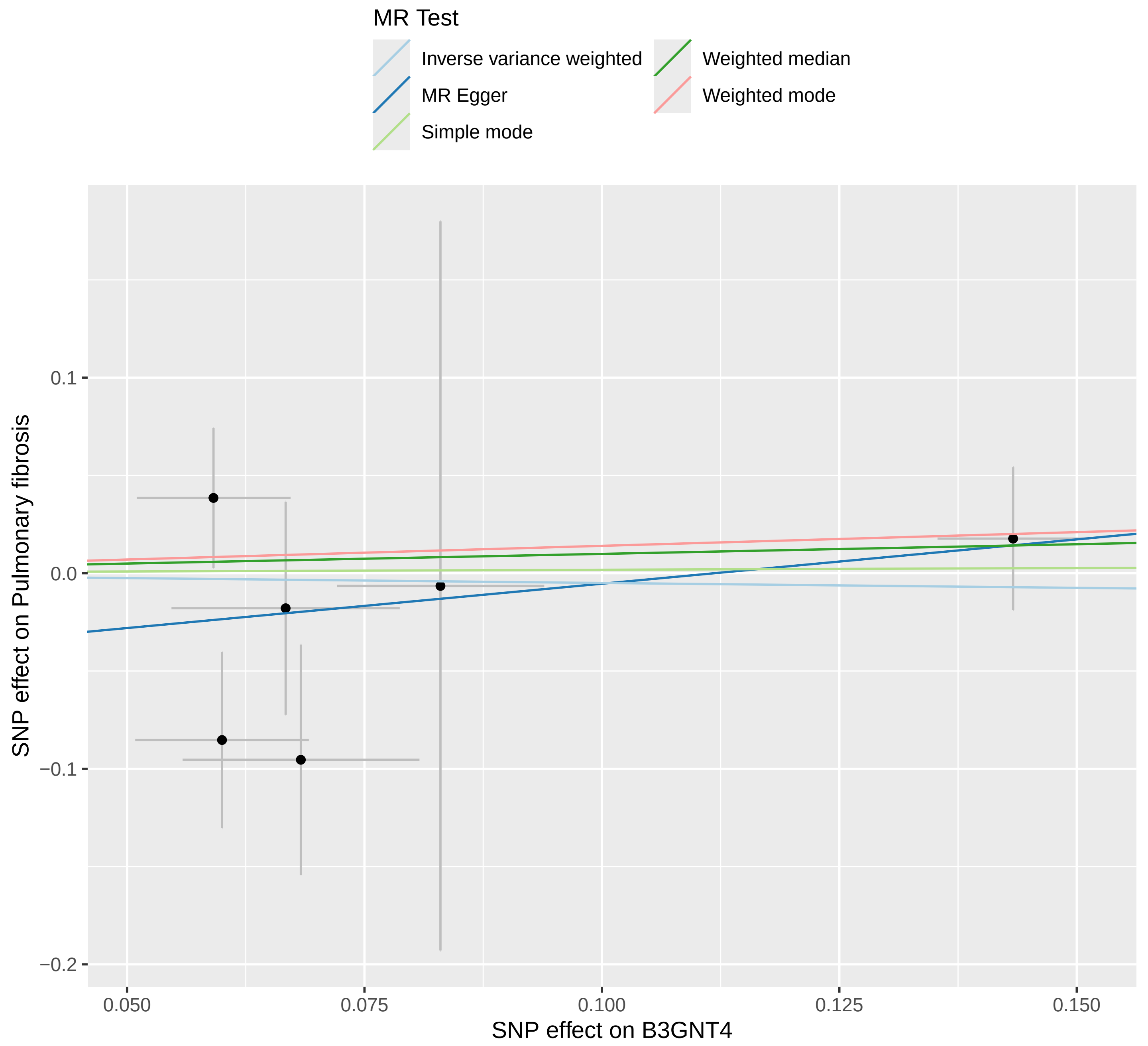

MR Test
Inverse variance weighted
MR Egger
Simple mode
Weighted median
Weighted mode
0.1
0.0
−0.1
−0.2
SNP effect on Pulmonary fibrosis
0.050
0.075
0.100
0.125
0.150
SNP effect on B3GNT4

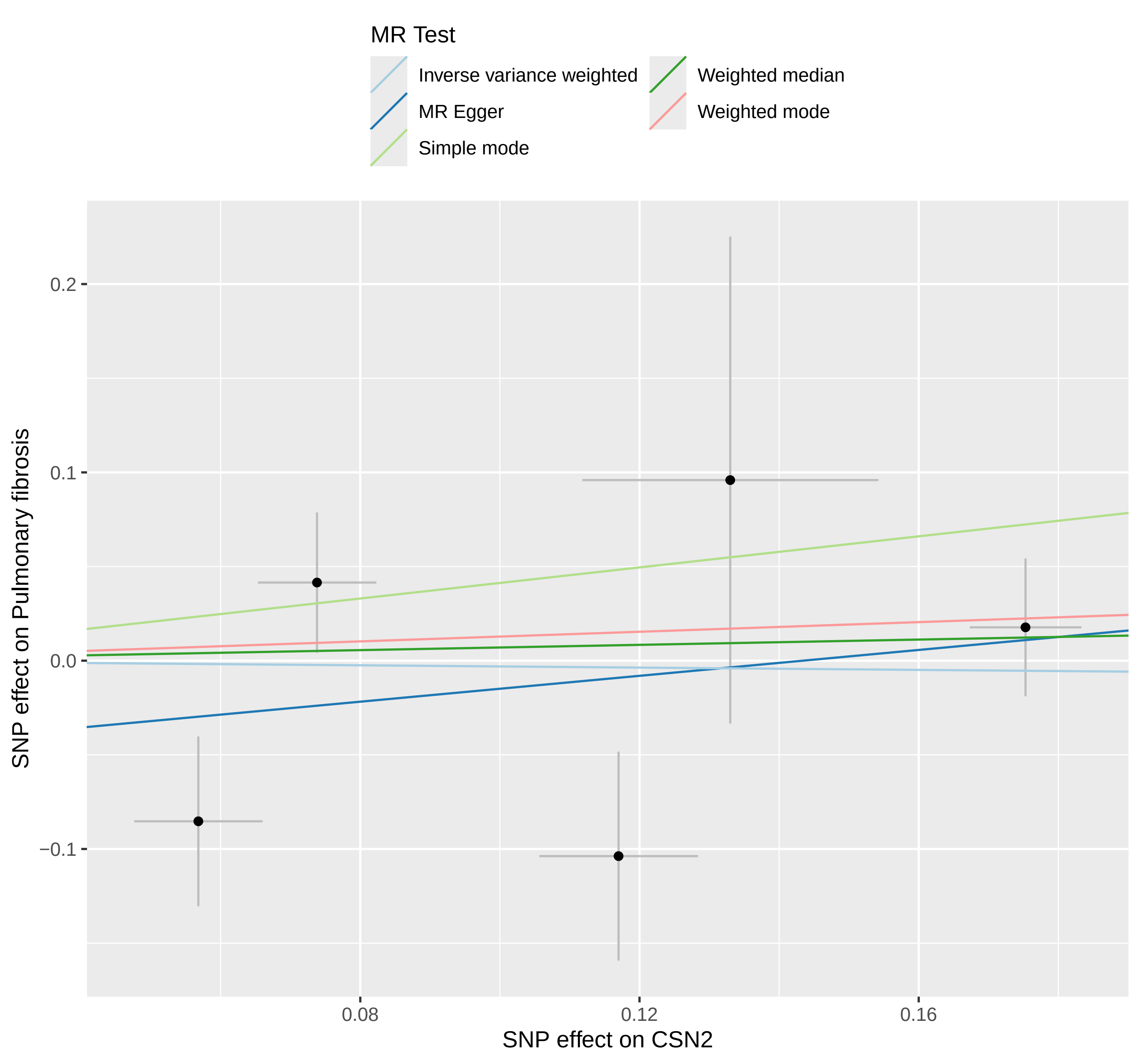
MR Test
Inverse variance weighted
MR Egger
Simple mode
Weighted median
Weighted mode
SNP effect on Pulmonary fibrosis
0.2
0.1
0.0
−0.1
0.08
0.12
0.16
SNP effect on CSN2

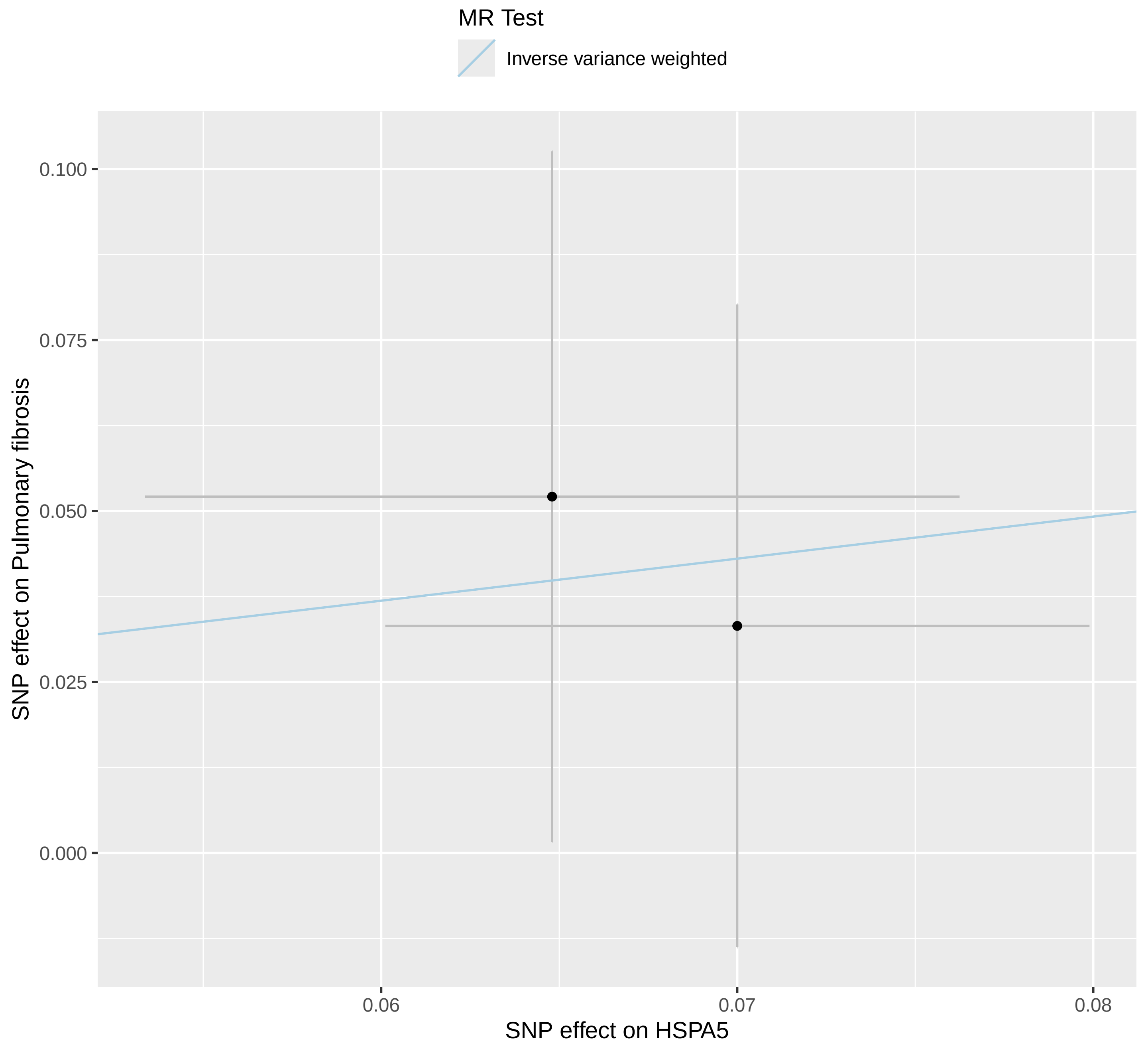

MR Test
Inverse variance weighted
0.100
0.075
0.050
0.025
0.000
SNP effect on Pulmonary fibrosis
0.06
0.07
0.08
SNP effect on HSPA5

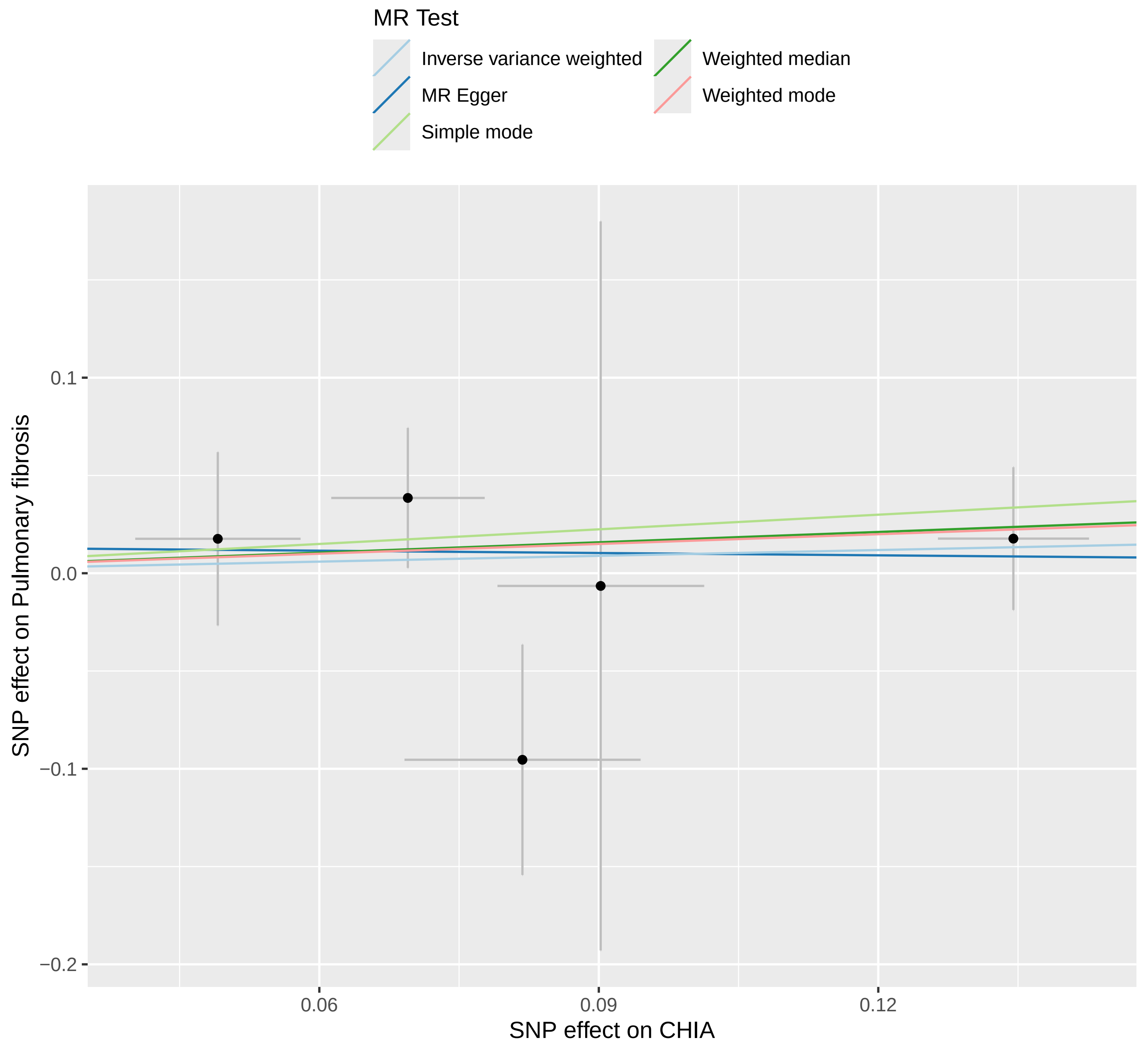
MR Test
Inverse variance weighted
MR Egger
Simple mode
Weighted median
Weighted mode
0.1
0.0
−0.1
−0.2
0.06
0.09
0.12
SNP effect on Pulmonary fibrosis
SNP effect on CHIA

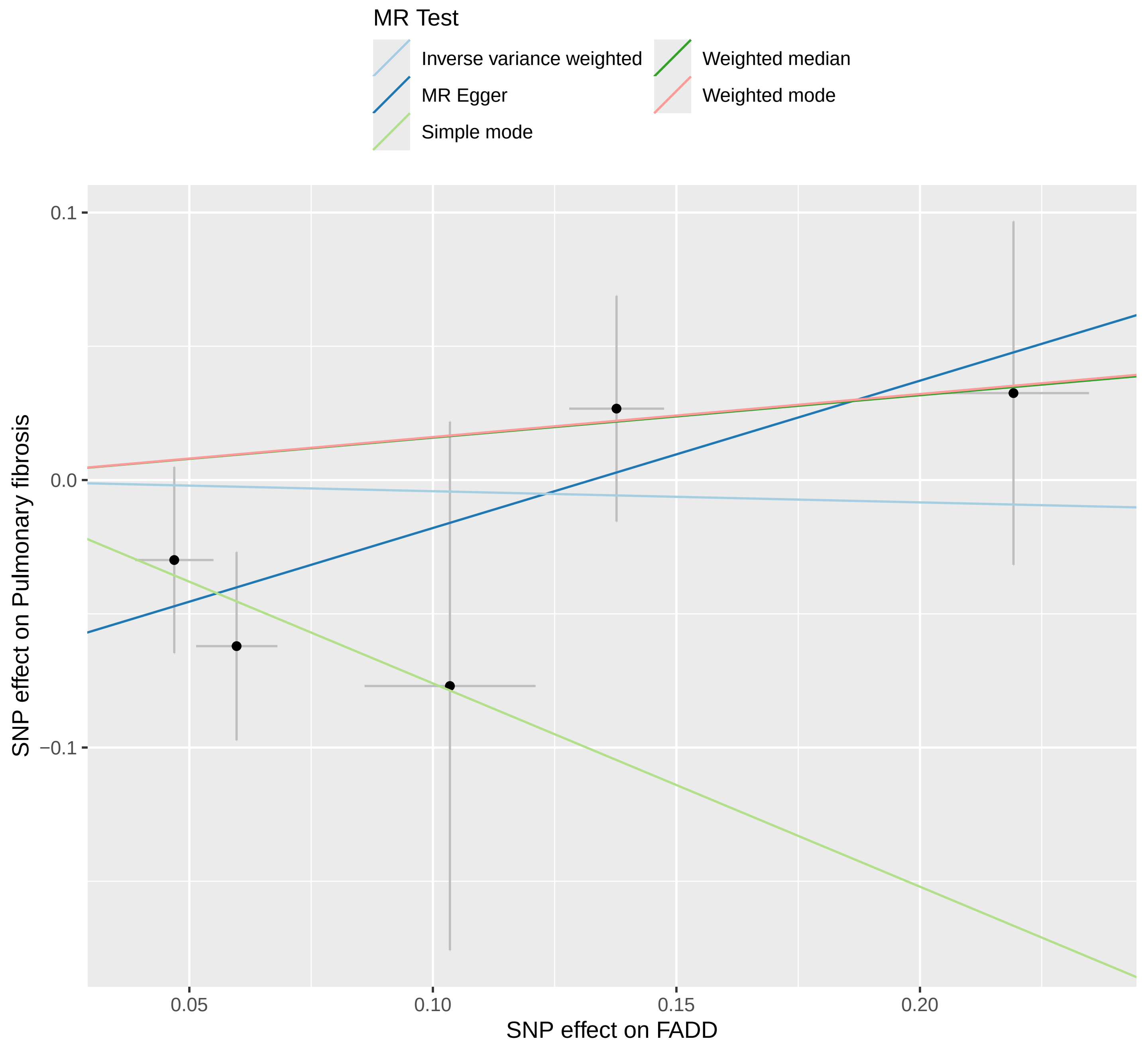
MR Test
Inverse variance weighted
MR Egger
Simple mode
Weighted median
Weighted mode
0.1
0.0
−0.1
0.05
0.10
0.15
0.20
SNP effect on Pulmonary fibrosis
SNP effect on FADD

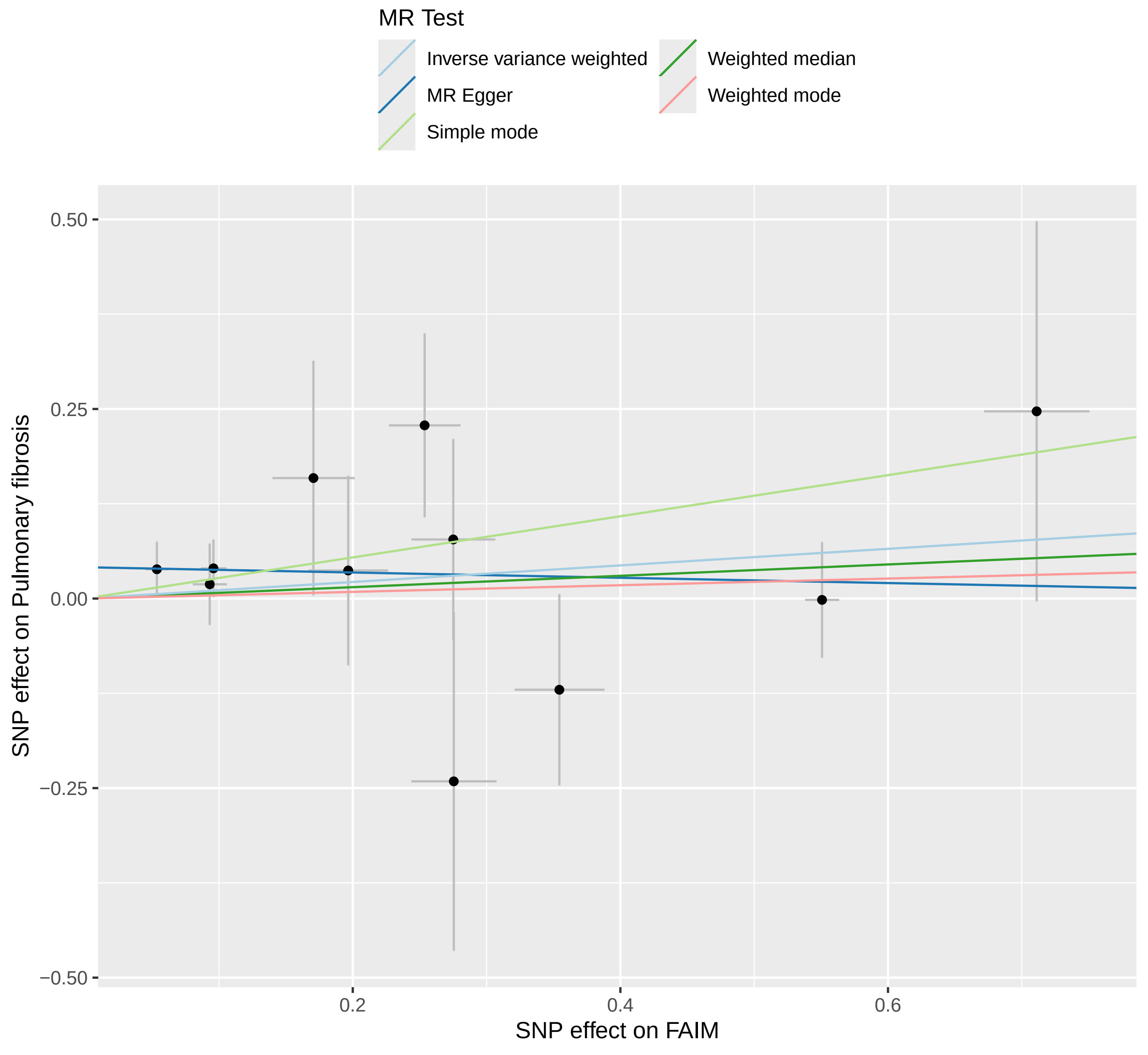
MR Test
Inverse variance weighted
MR Egger
Simple mode
Weighted median
Weighted mode
0.50
0.25
0.00
−0.25
−0.50
SNP effect on Pulmonary fibrosis
0.2
0.4
0.6
SNP effect on FAIM

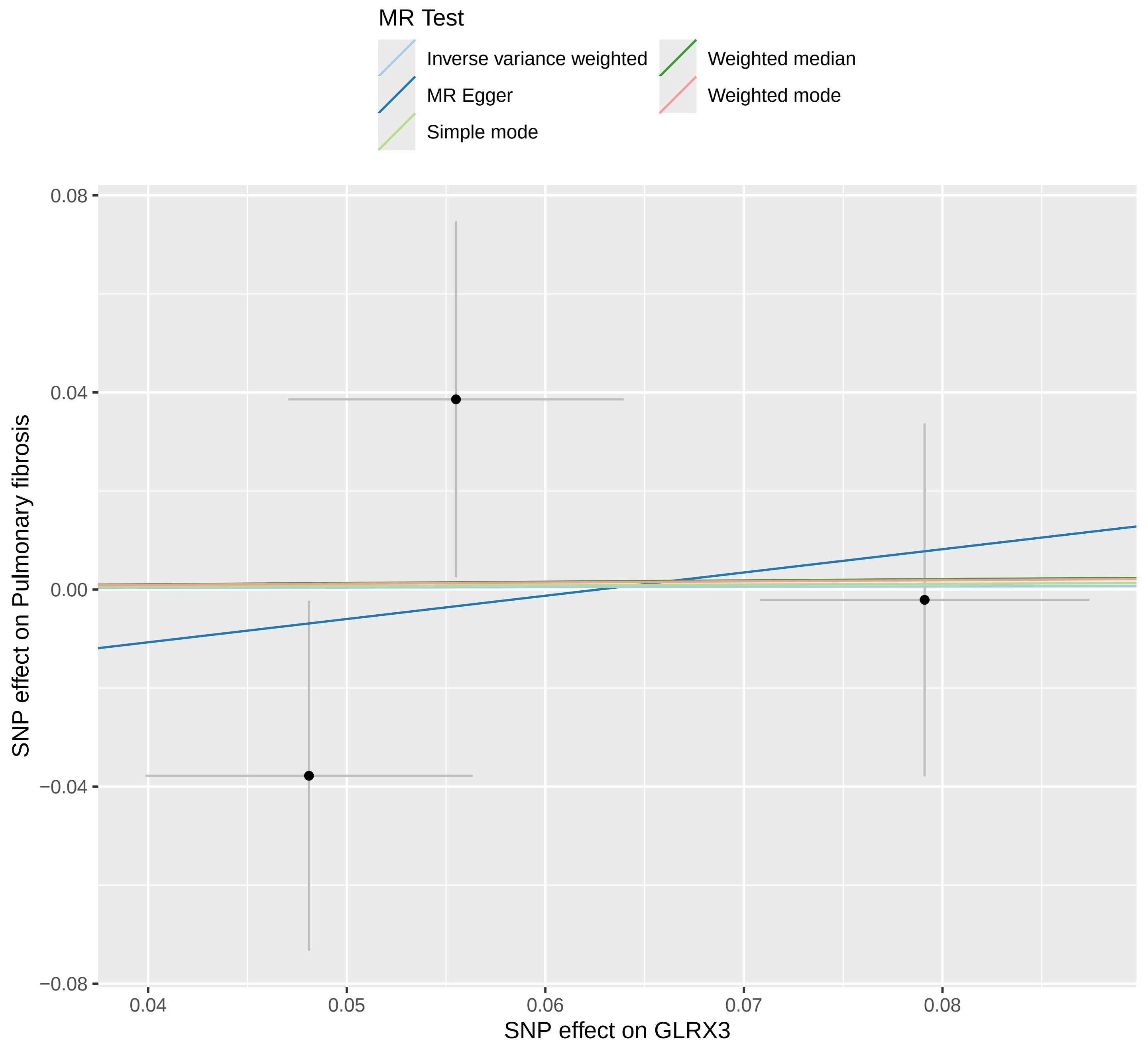
MR Test
Inverse variance weighted
MR Egger
Simple mode
Weighted median
Weighted mode
0.08
0.04
0.00
−0.04
−0.08
SNP effect on Pulmonary fibrosis
0.04
0.05
0.06
0.07
0.08
SNP effect on GLRX3

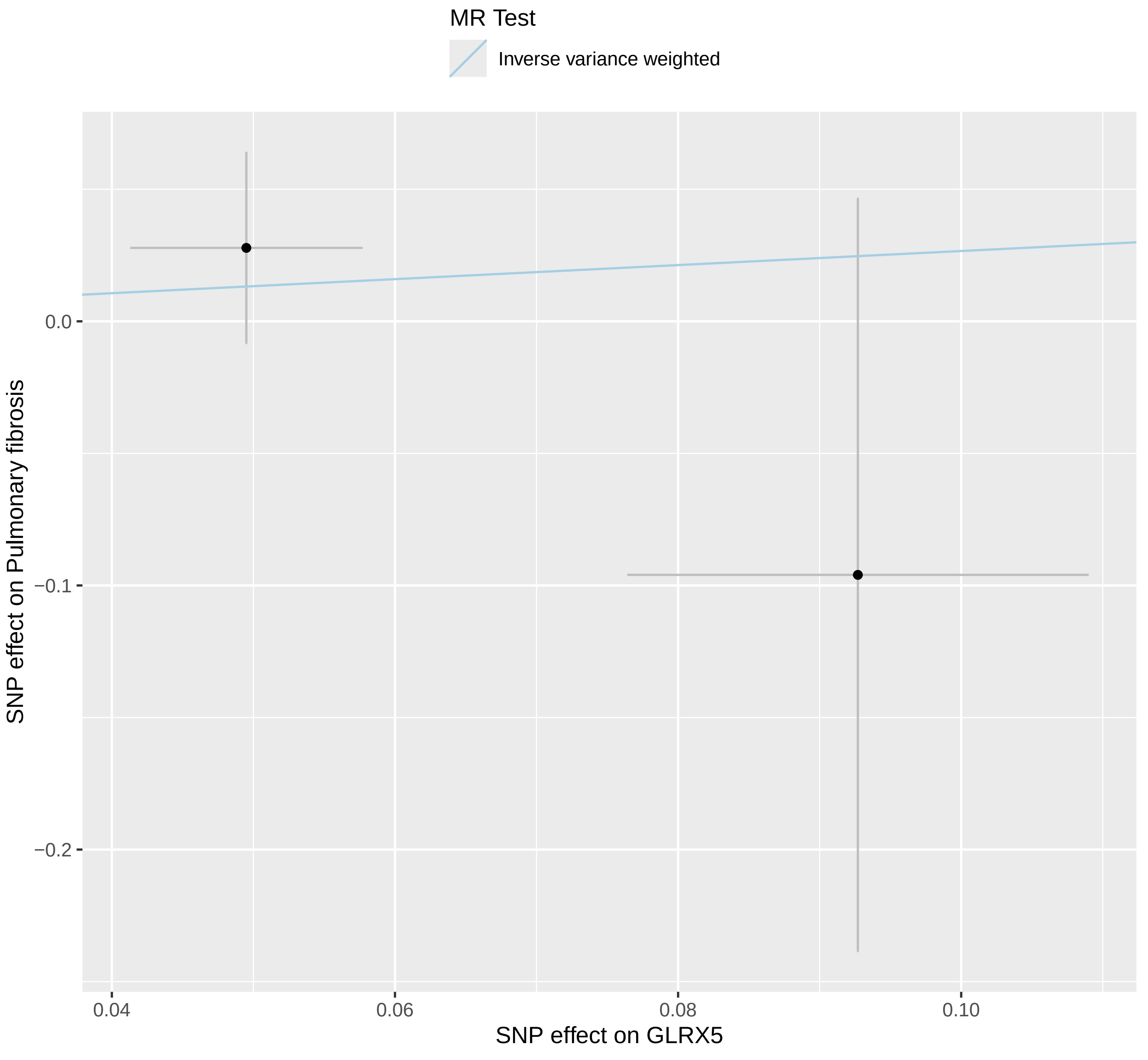

MR Test
Inverse variance weighted
SNP effect on Pulmonary fibrosis
0.0
−0.1
−0.2
0.04
0.06
0.08
0.10
SNP effect on GLRX5

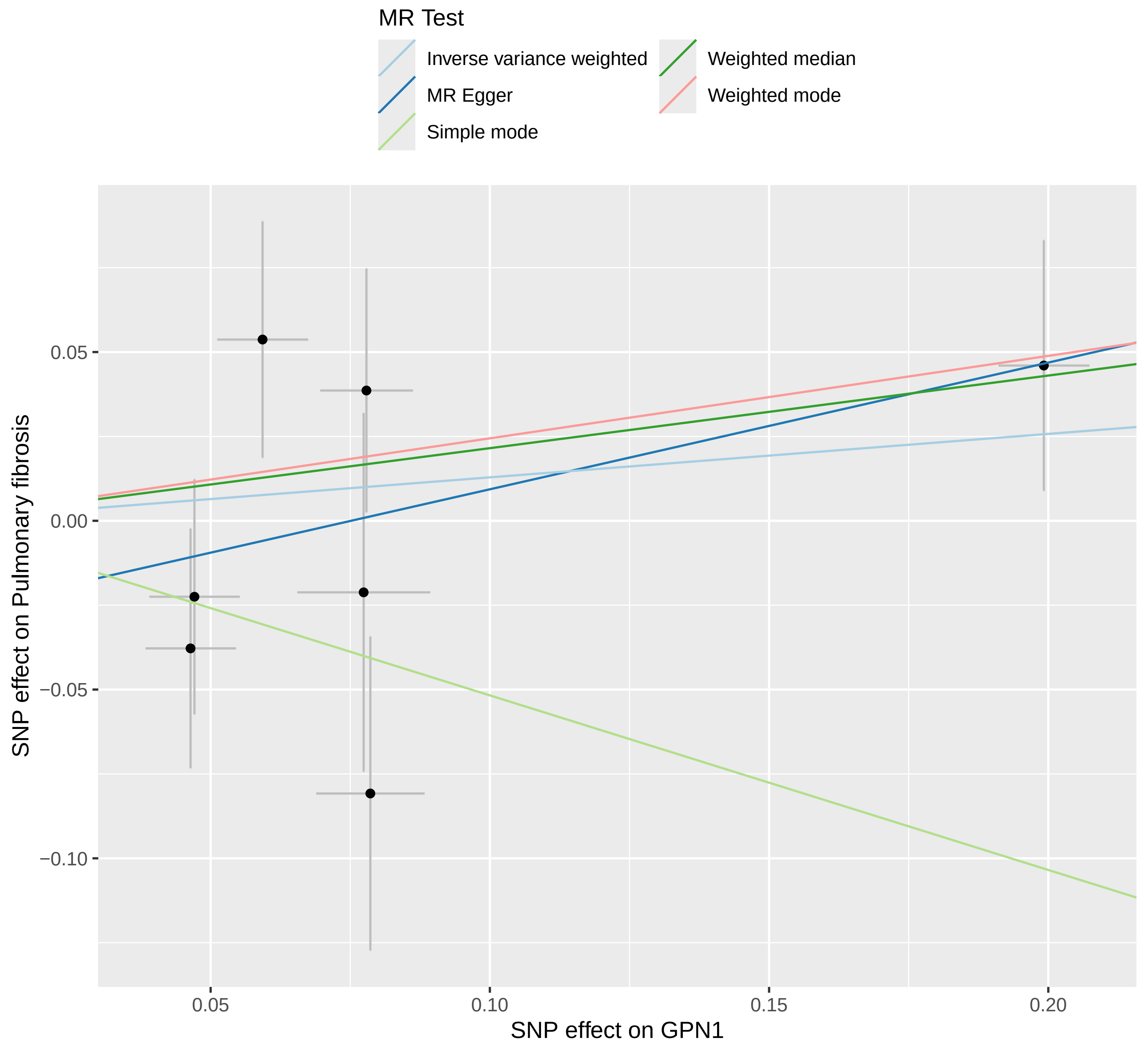

MR Test
Inverse variance weighted
MR Egger
Simple mode
Weighted median
Weighted mode
SNP effect on Pulmonary fibrosis
0.05
0.00
−0.05
−0.10
0.05
0.10
0.15
0.20
SNP effect on GPN1

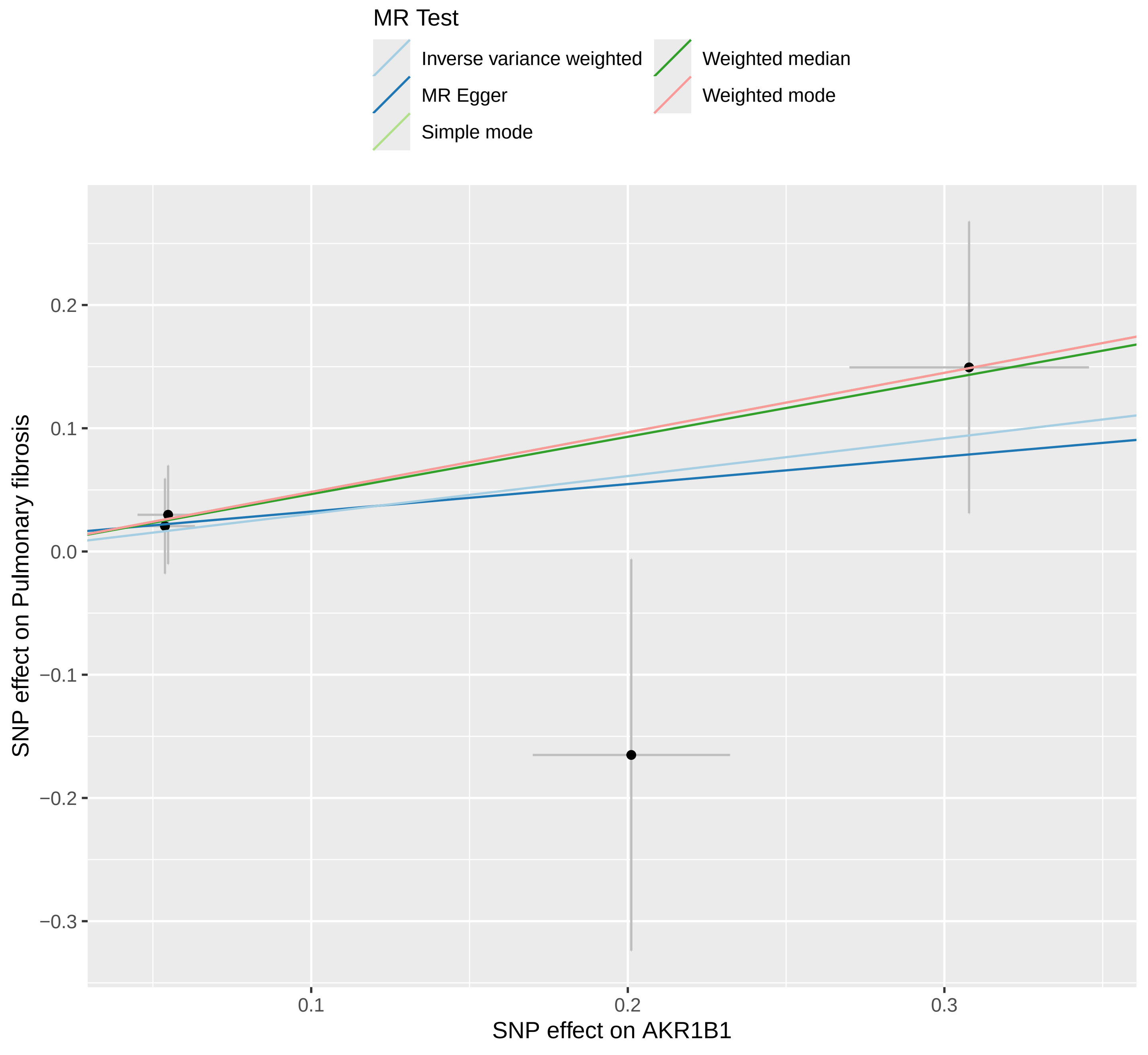
MR Test
Inverse variance weighted
MR Egger
Simple mode
Weighted median
Weighted mode
0.2
0.1
0.0
−0.1
−0.2
−0.3
SNP effect on Pulmonary fibrosis
0.1
0.2
0.3
SNP effect on AKR1B1

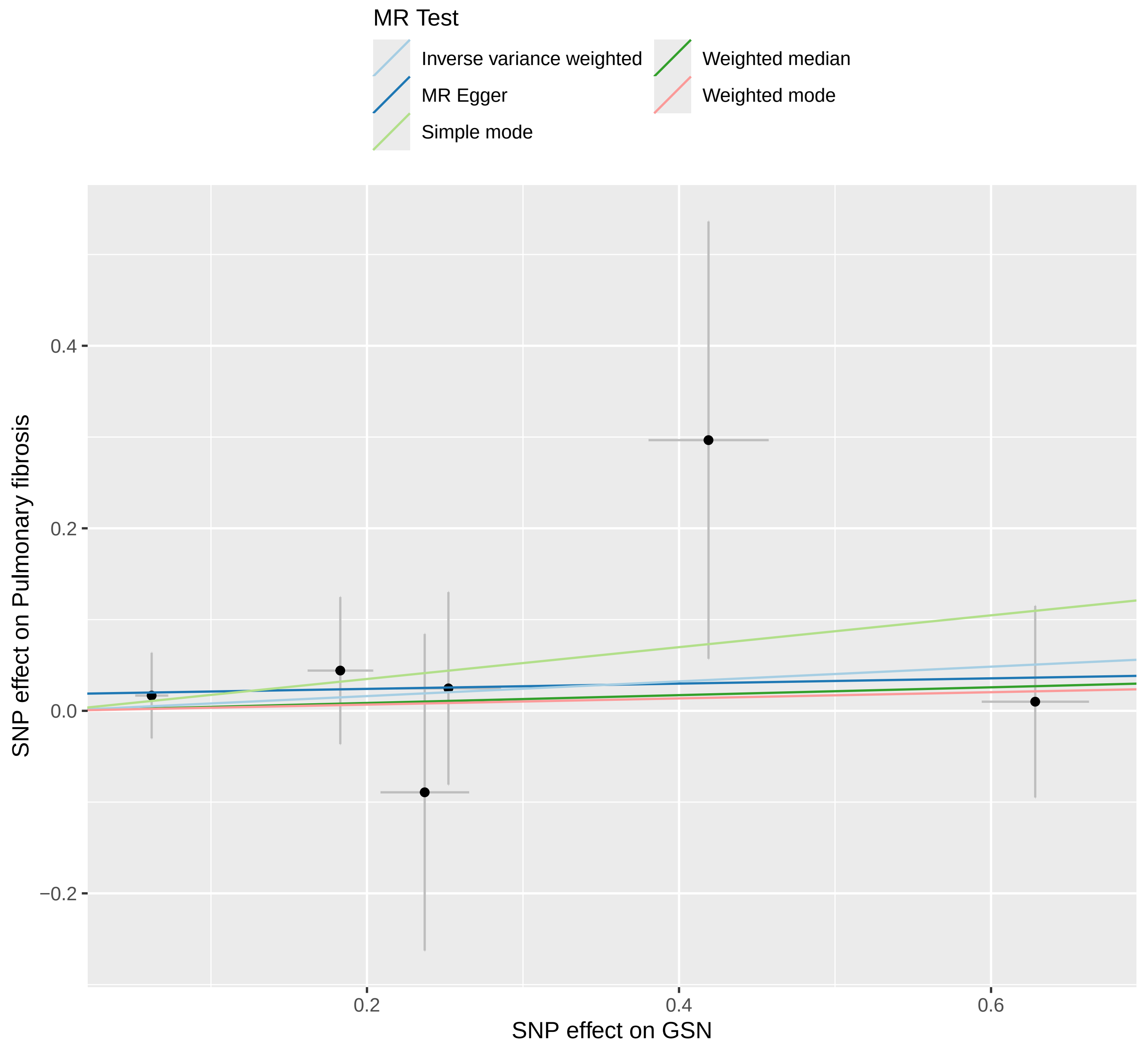
MR Test
Inverse variance weighted
MR Egger
Simple mode
Weighted median
Weighted mode
SNP effect on Pulmonary fibrosis
0.4
0.2
0.0
−0.2
0.2
0.4
0.6
SNP effect on GSN

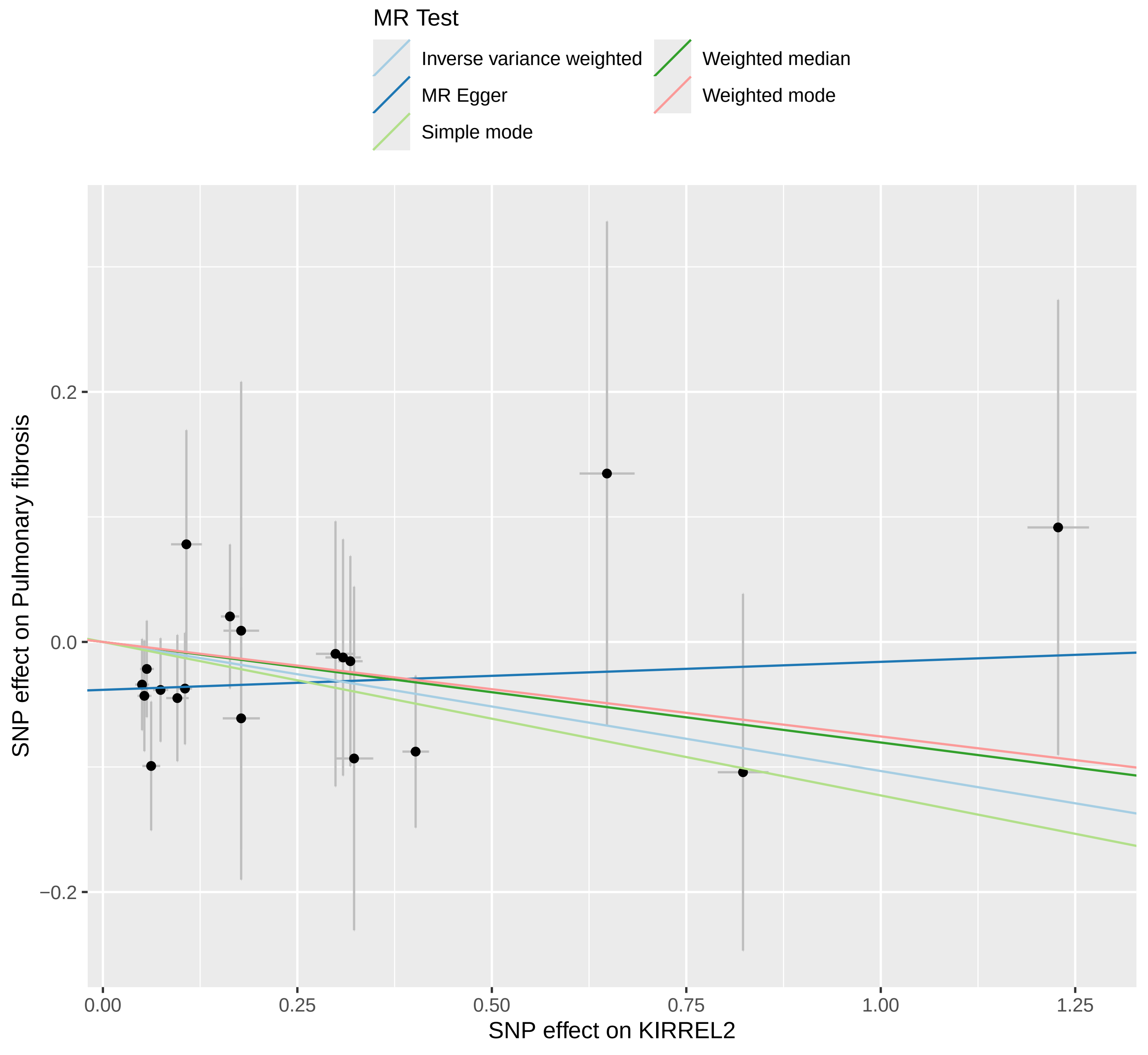

MR Test
Inverse variance weighted
MR Egger
Simple mode
Weighted median
Weighted mode
0.2
0.0
−0.2
SNP effect on Pulmonary fibrosis
0.00
0.25
0.50
0.75
1.00
1.25
SNP effect on KIRREL2

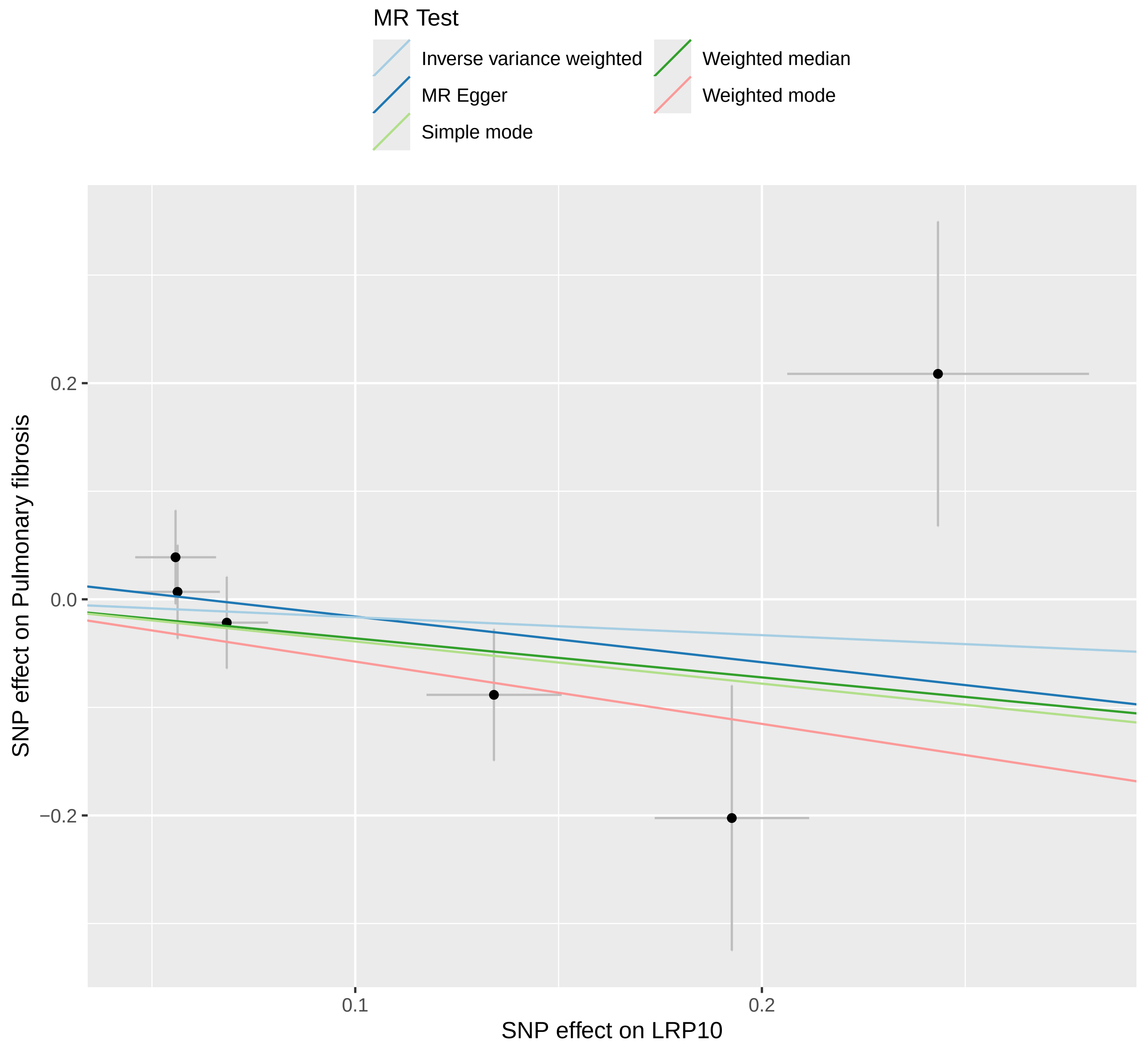

MR Test
Inverse variance weighted
MR Egger
Simple mode
Weighted median
Weighted mode
0.2
0.0
−0.2
SNP effect on Pulmonary fibrosis
0.1
0.2
SNP effect on LRP10

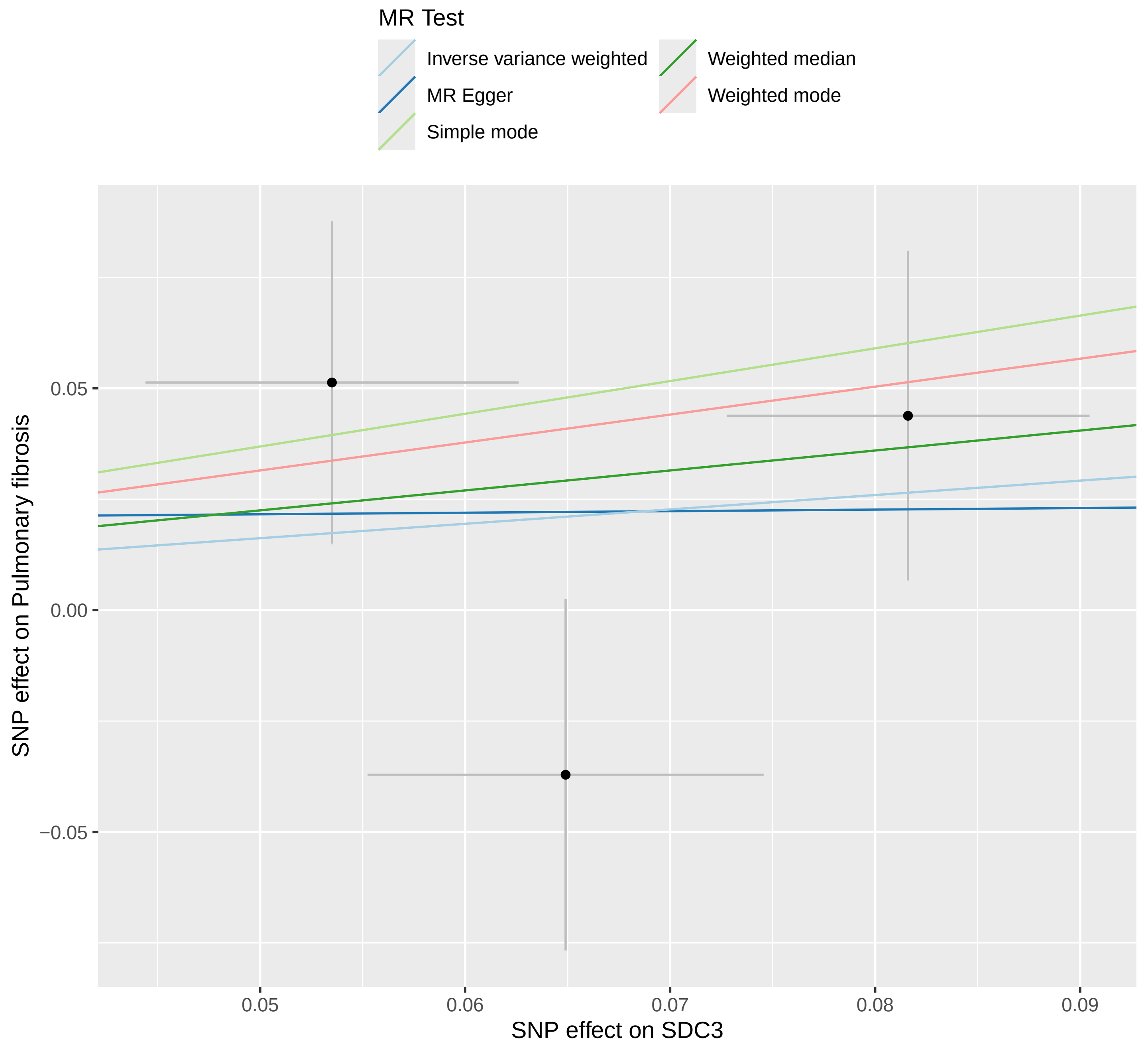

MR Test
Inverse variance weighted
MR Egger
Simple mode
Weighted median
Weighted mode
0.05
0.00
−0.05
SNP effect on Pulmonary fibrosis
0.05
0.06
0.07
0.08
0.09
SNP effect on SDC3

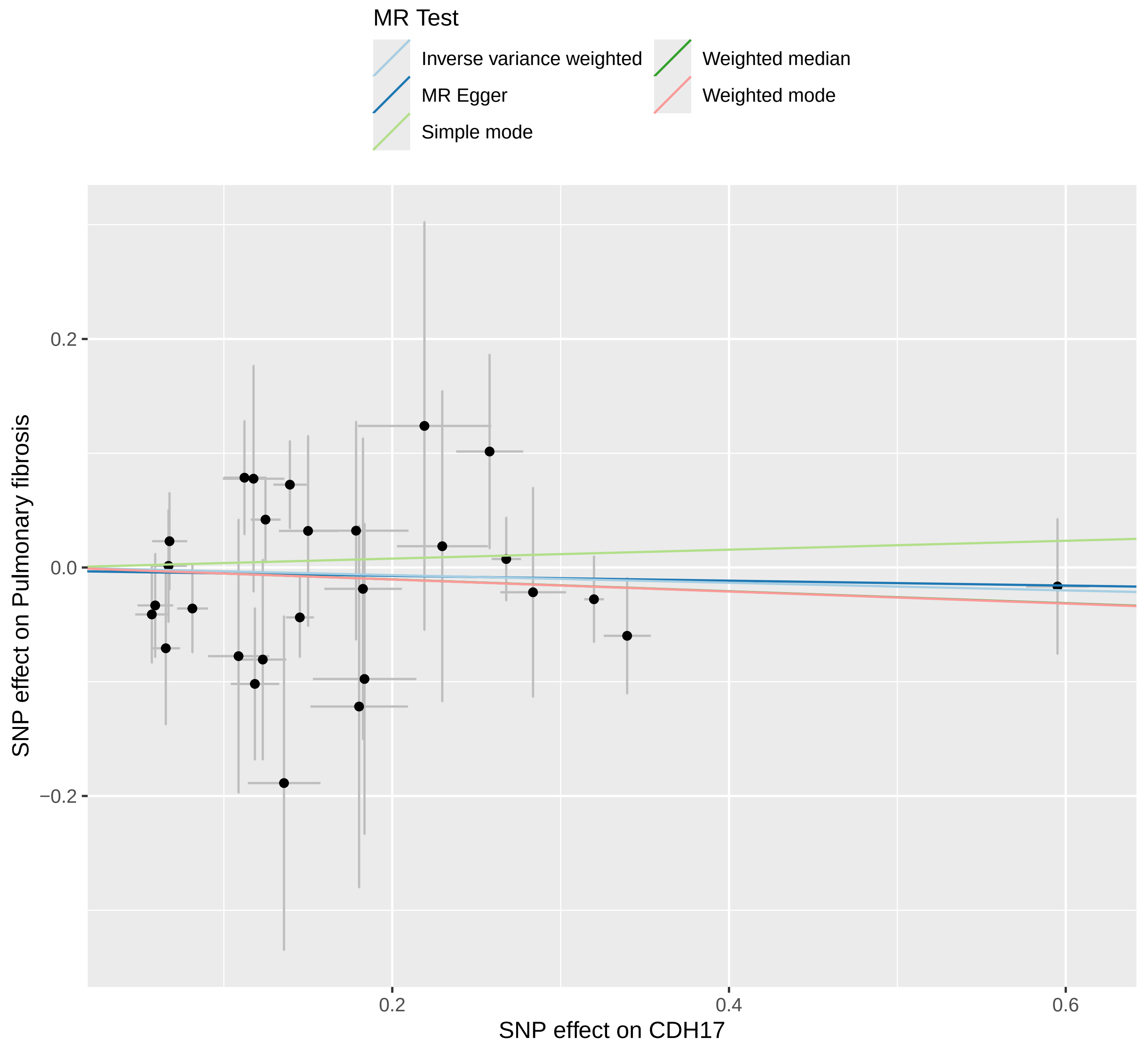

MR Test
Inverse variance weighted
MR Egger
Simple mode
Weighted median
Weighted mode
0.2
0.0
−0.2
SNP effect on Pulmonary fibrosis
0.2
0.4
0.6
SNP effect on CDH17

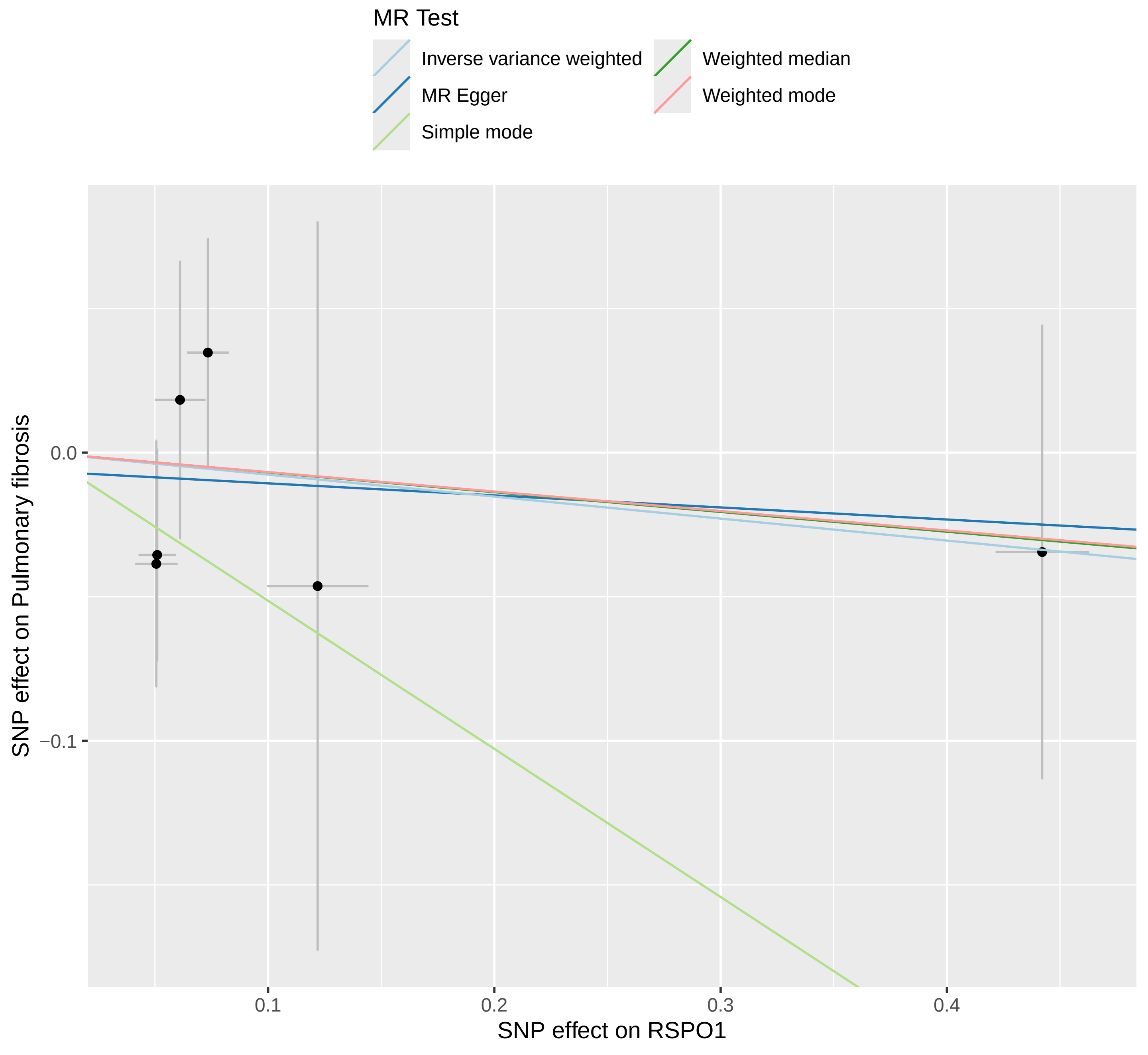
MR Test
Inverse variance weighted
MR Egger
Simple mode
Weighted median
Weighted mode
0.0
−0.1
0.1
0.2
0.3
0.4
SNP effect on RSPO1
SNP effect on Pulmonary fibrosis

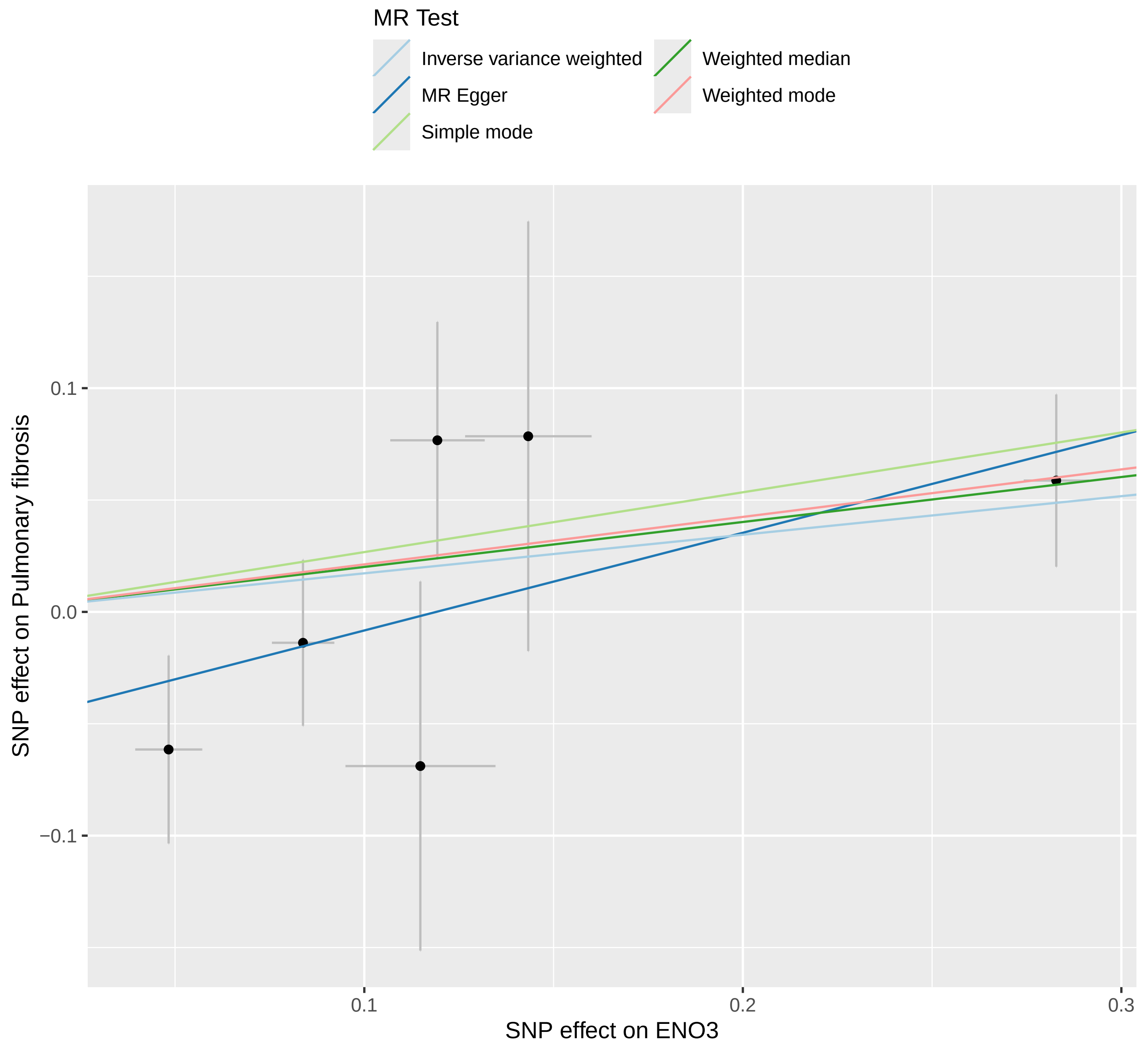

MR Test
Inverse variance weighted
MR Egger
Simple mode
Weighted median
Weighted mode
0.1
0.0
−0.1
SNP effect on Pulmonary fibrosis
0.1
0.2
0.3
SNP effect on ENO3

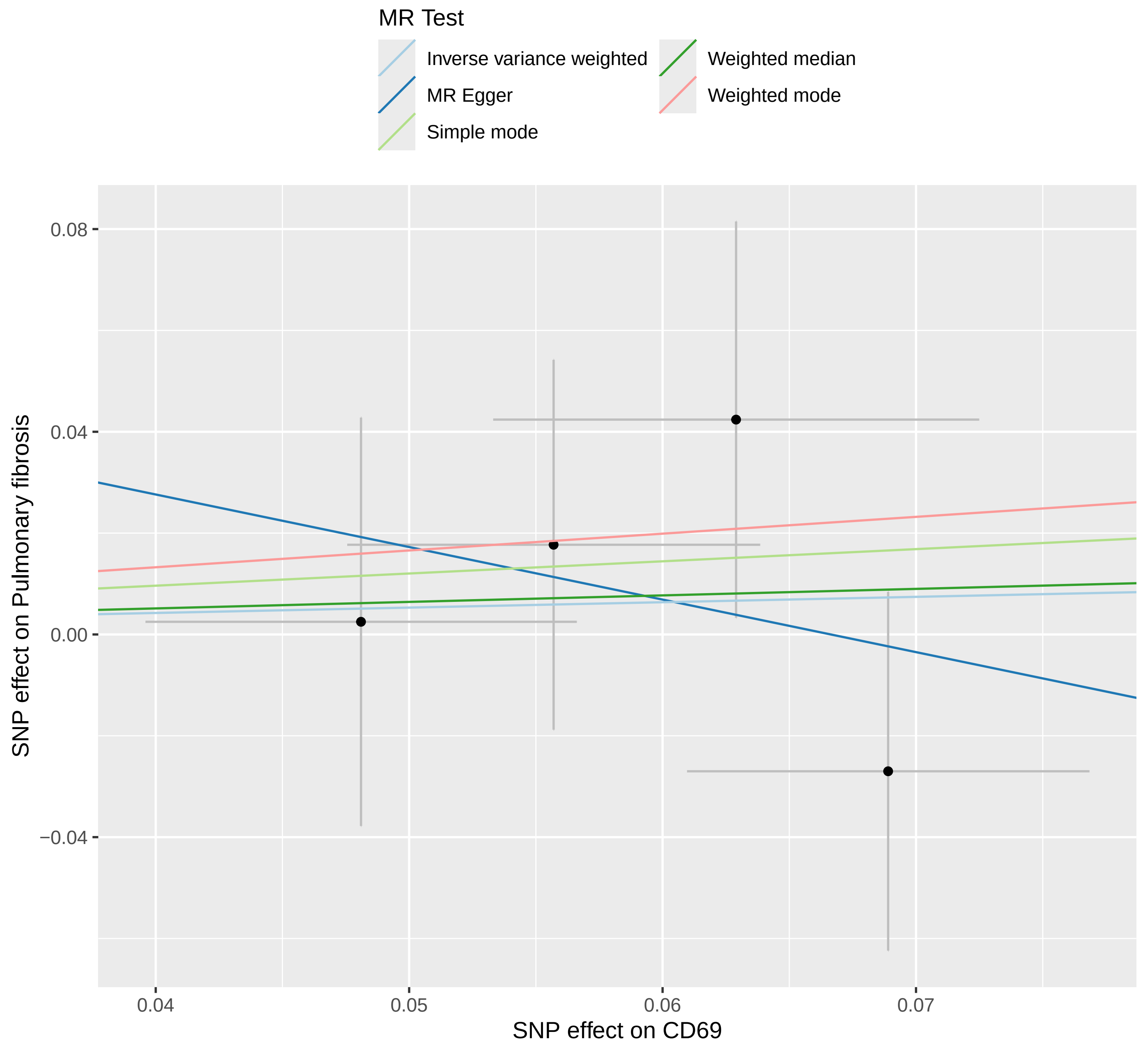

MR Test
Inverse variance weighted
MR Egger
Simple mode
Weighted median
Weighted mode
0.08
0.04
0.00
−0.04
SNP effect on Pulmonary fibrosis
0.04
0.05
0.06
0.07
SNP effect on CD69

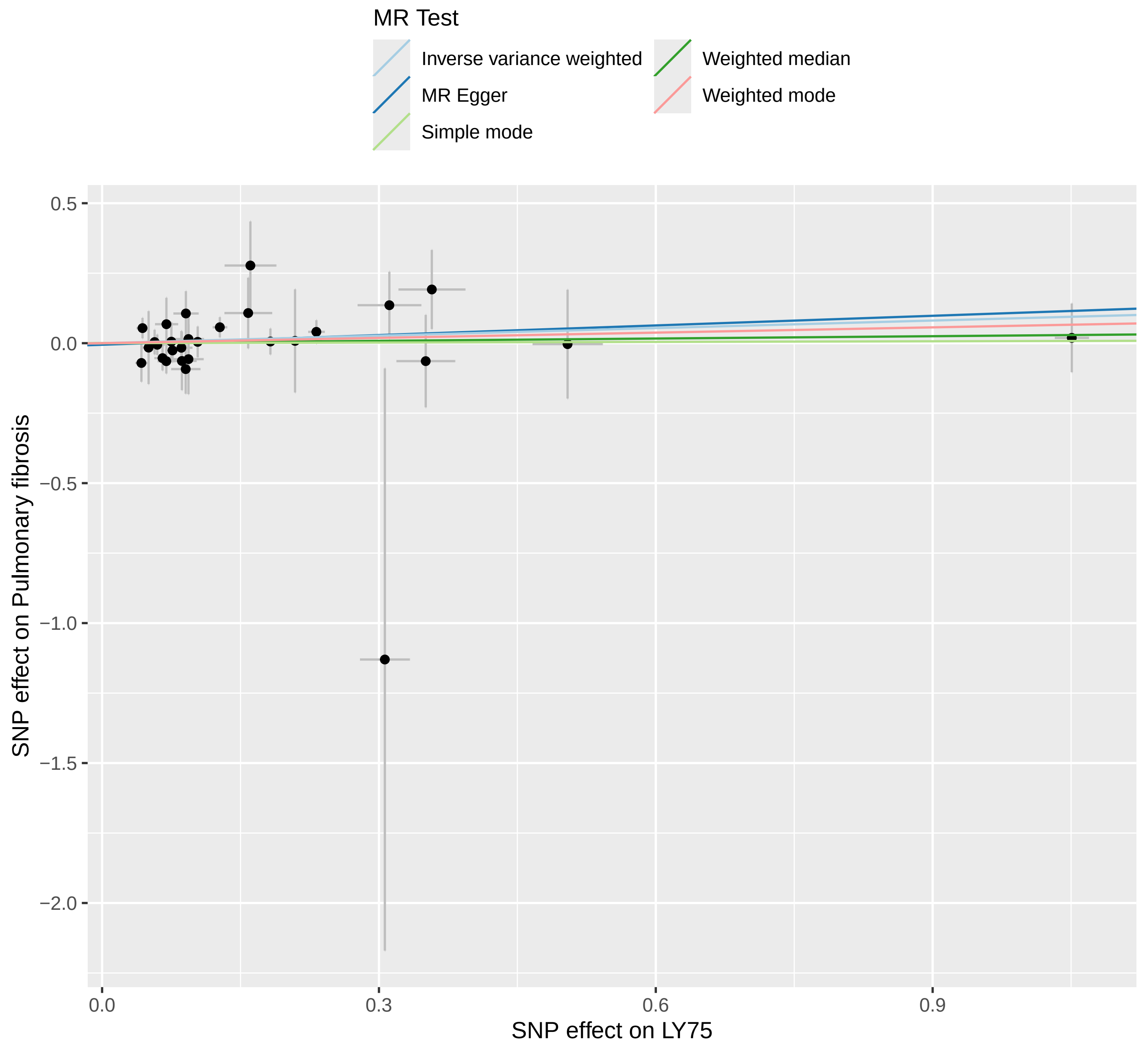

MR Test
Inverse variance weighted
MR Egger
Simple mode
Weighted median
Weighted mode
0.5
0.0
−0.5
−1.0
−1.5
−2.0
SNP effect on Pulmonary fibrosis
0.0
0.3
0.6
0.9
SNP effect on LY75

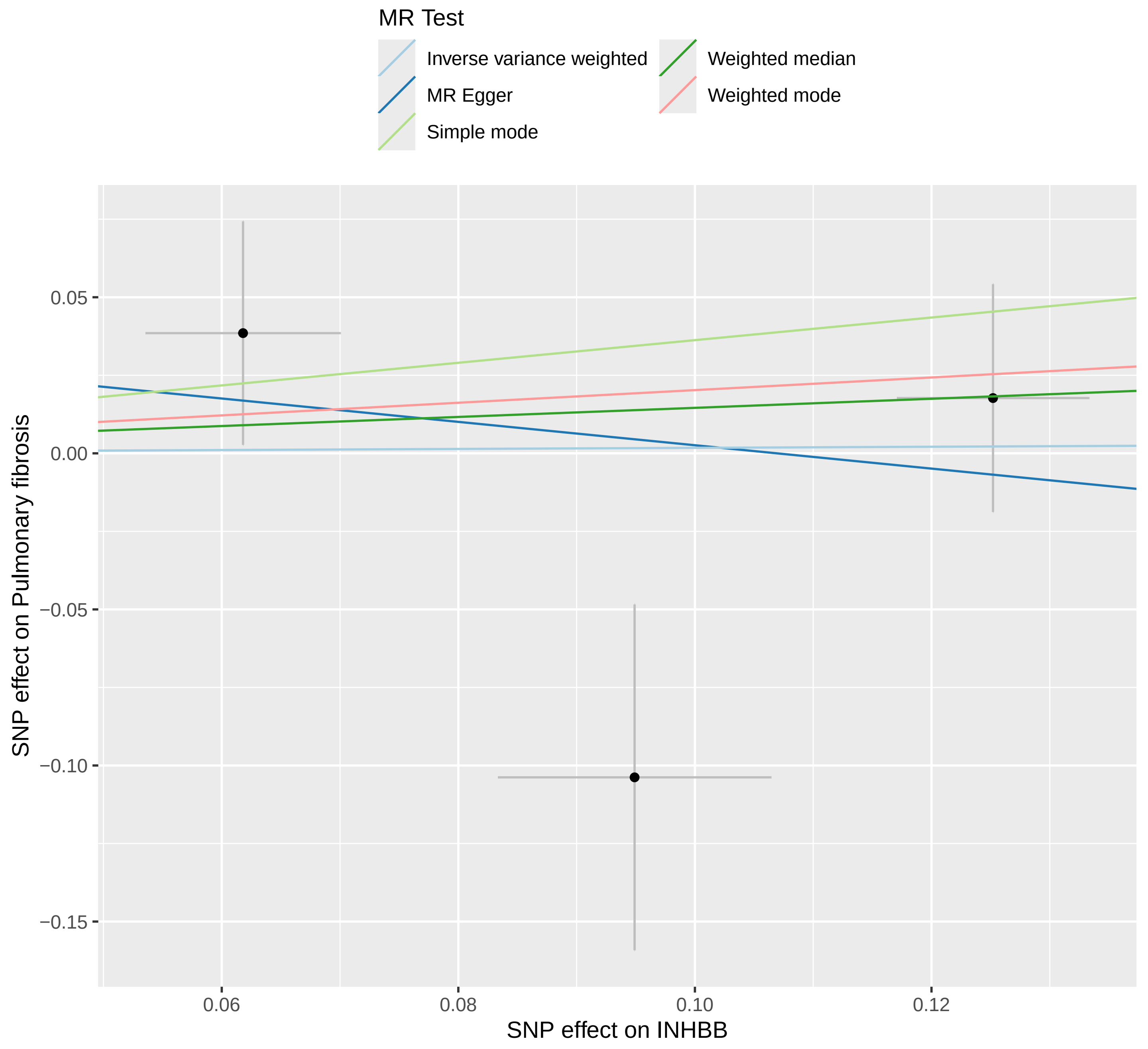

MR Test
Inverse variance weighted
MR Egger
Simple mode
Weighted median
Weighted mode
0.05
0.00
−0.05
−0.10
−0.15
SNP effect on Pulmonary fibrosis
0.06
0.08
0.10
0.12
SNP effect on INHBB

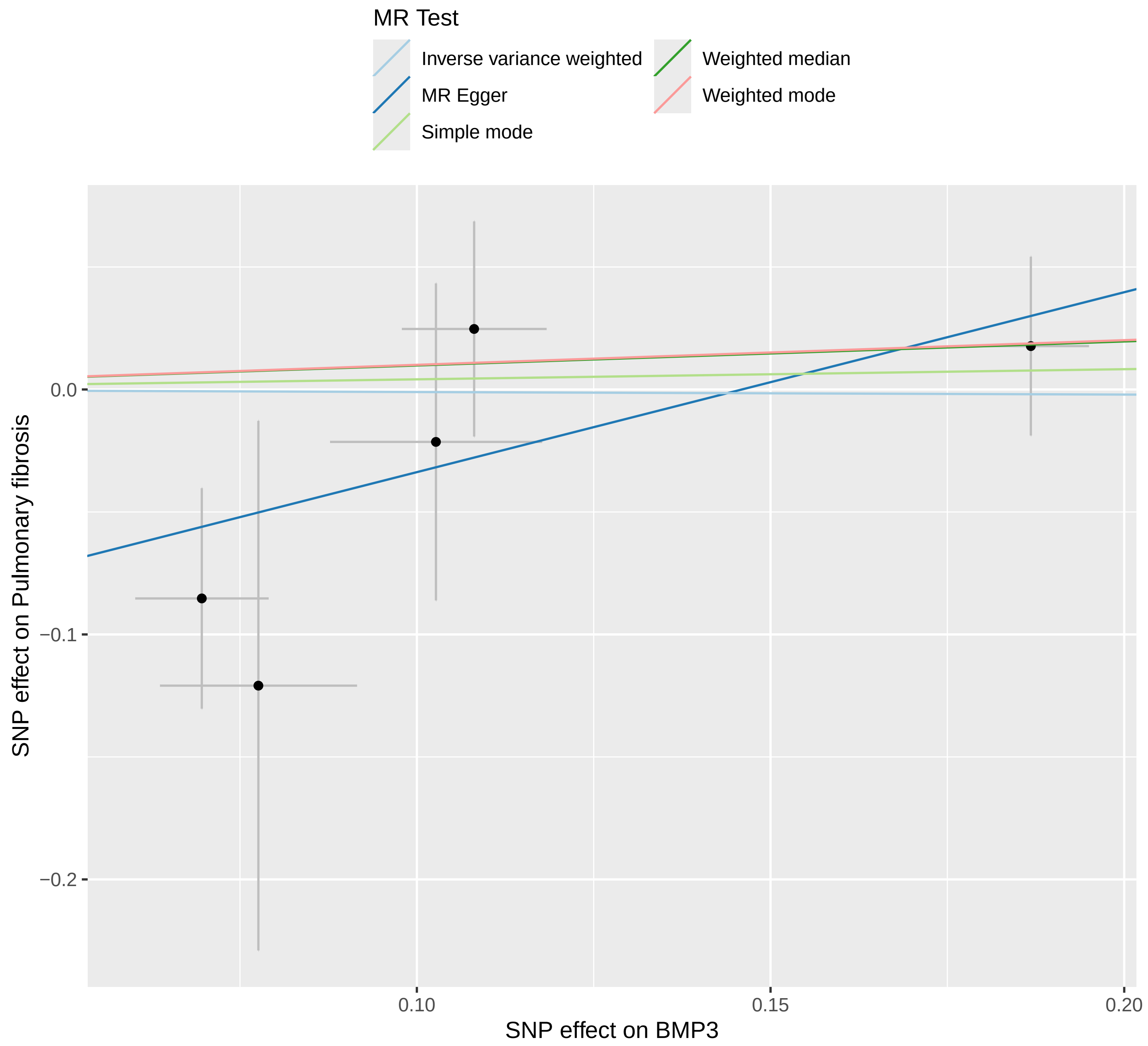

MR Test
Inverse variance weighted
MR Egger
Simple mode
Weighted median
Weighted mode
0.0
−0.1
−0.2
SNP effect on Pulmonary fibrosis
0.10
0.15
0.20
SNP effect on BMP3

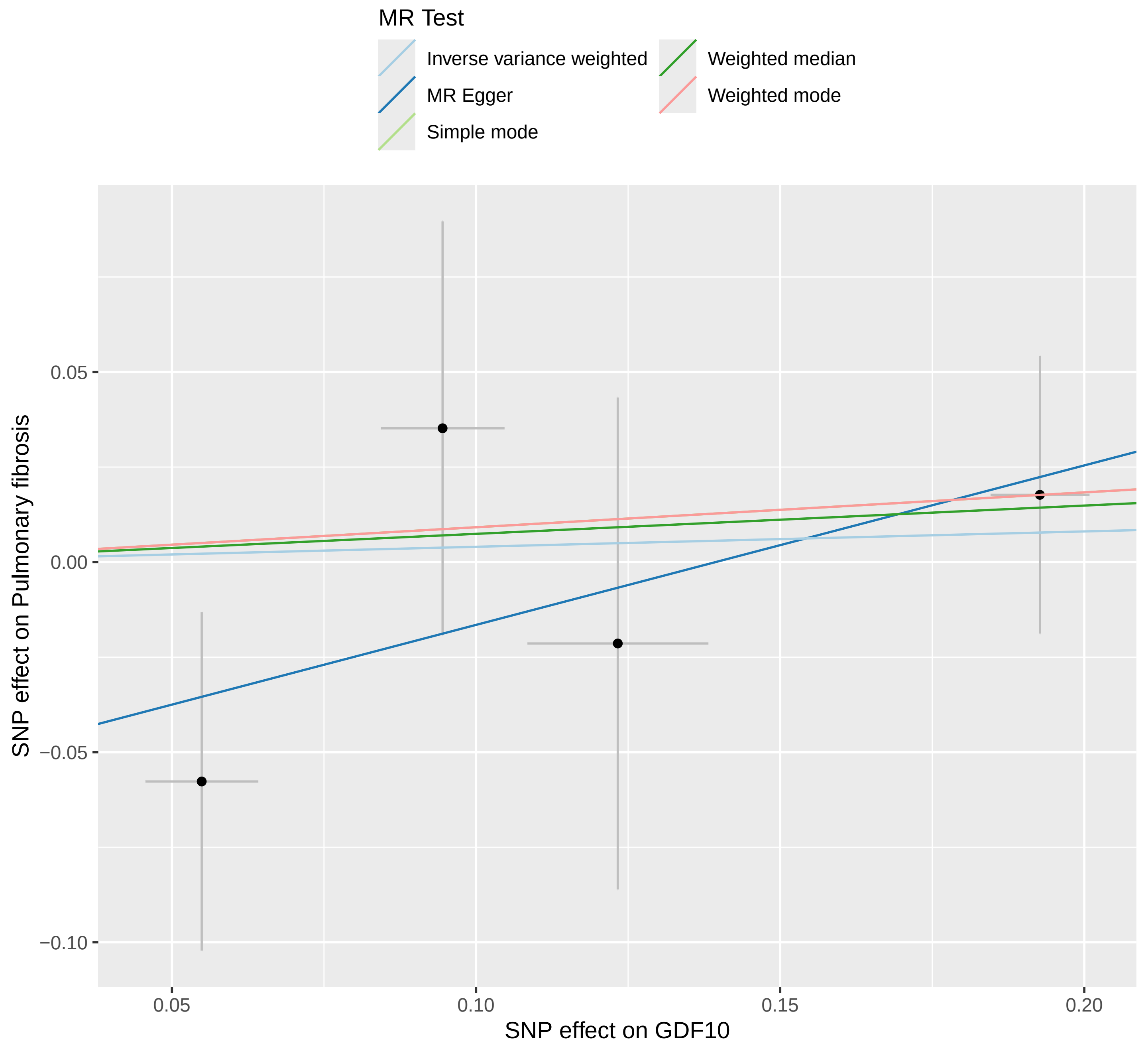

MR Test
Inverse variance weighted
MR Egger
Simple mode
Weighted median
Weighted mode
0.05
0.00
−0.05
−0.10
SNP effect on Pulmonary fibrosis
0.05
0.10
0.15
0.20
SNP effect on GDF10

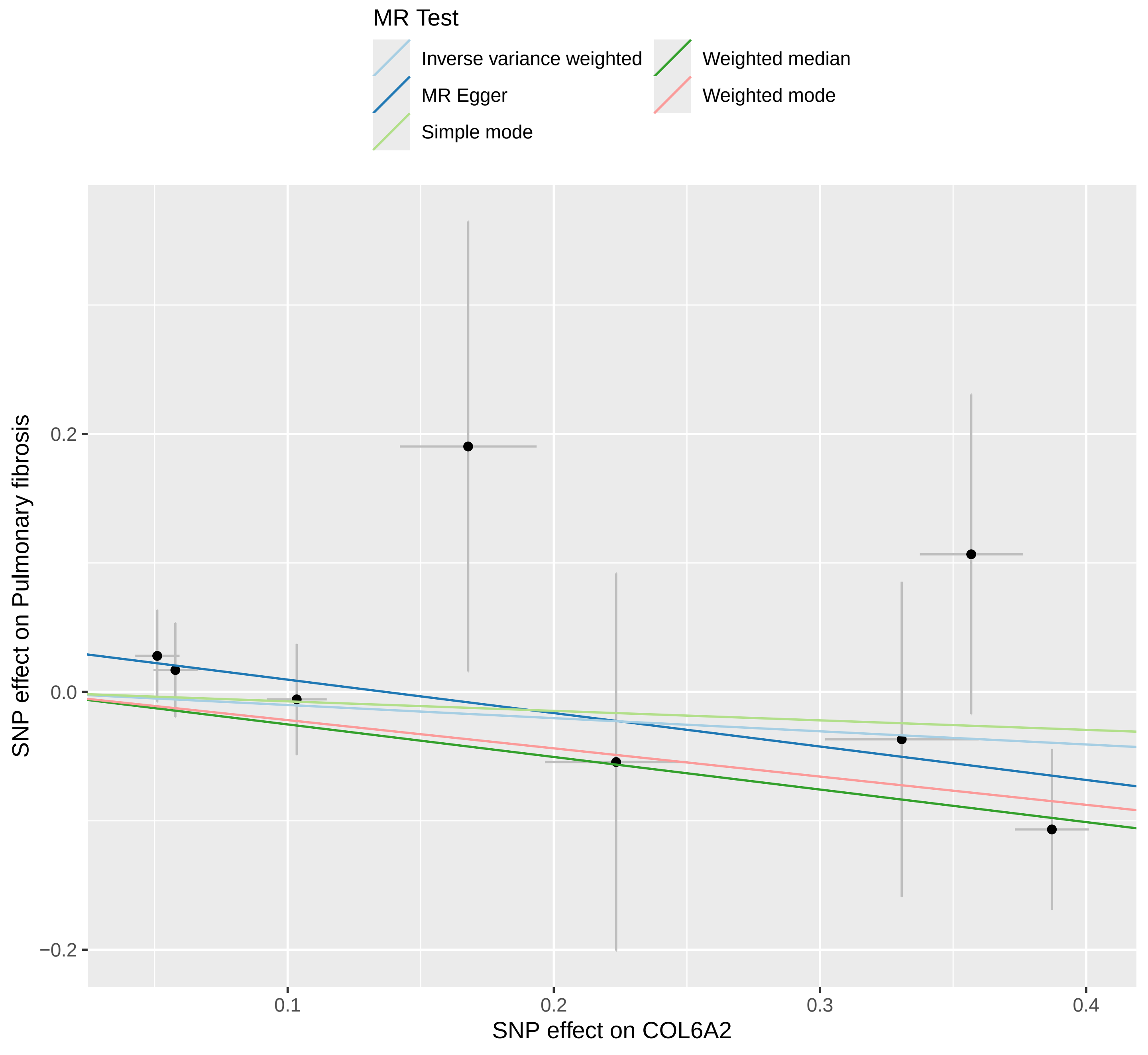

MR Test
Inverse variance weighted
MR Egger
Simple mode
Weighted median
Weighted mode
0.2
0.0
−0.2
0.1
0.2
0.3
0.4
SNP effect on Pulmonary fibrosis
SNP effect on COL6A2

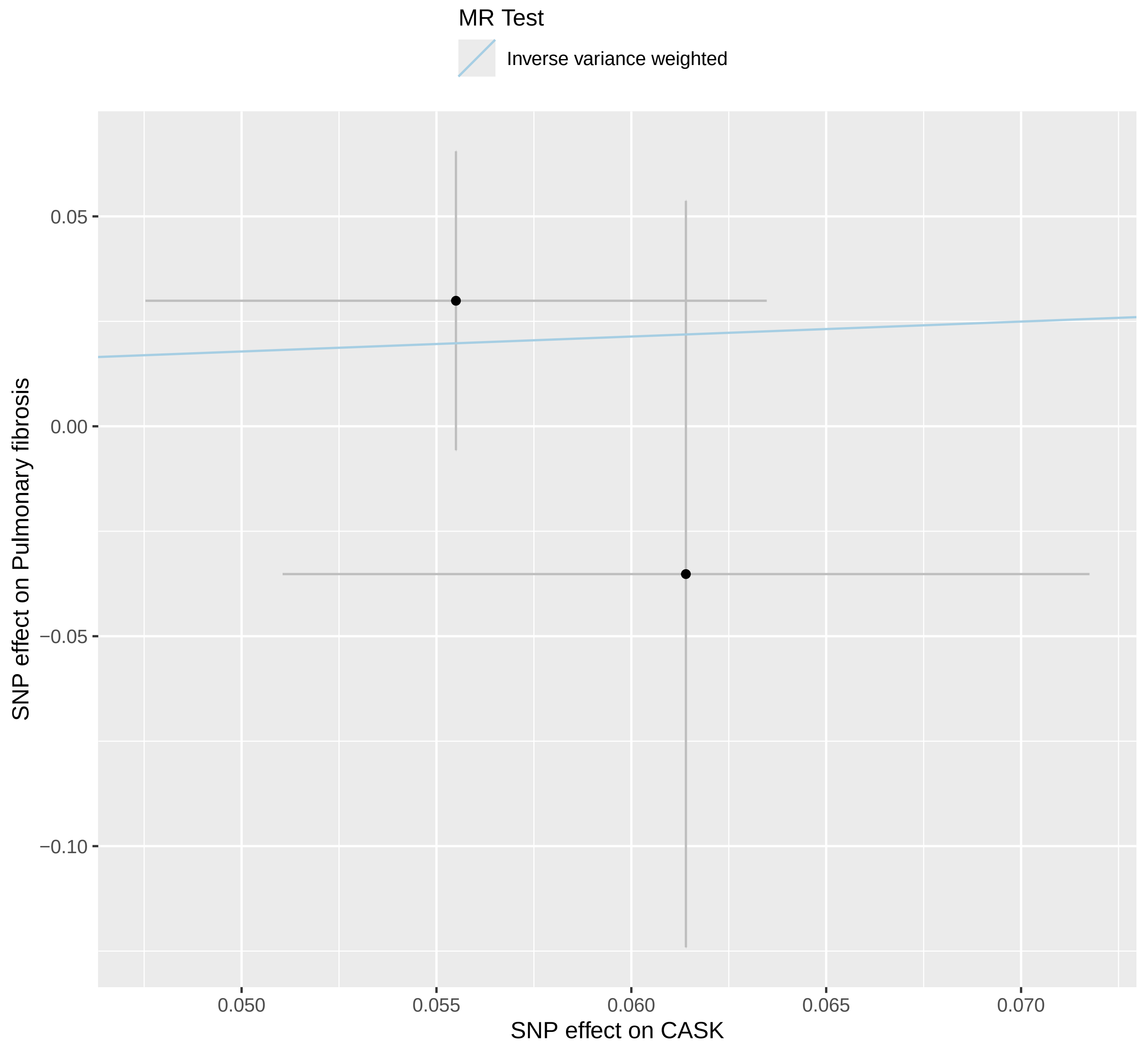
MR Test
Inverse variance weighted
SNP effect on Pulmonary fibrosis
0.05
0.00
−0.05
−0.10
0.050
0.055
0.060
0.065
0.070
SNP effect on CASK

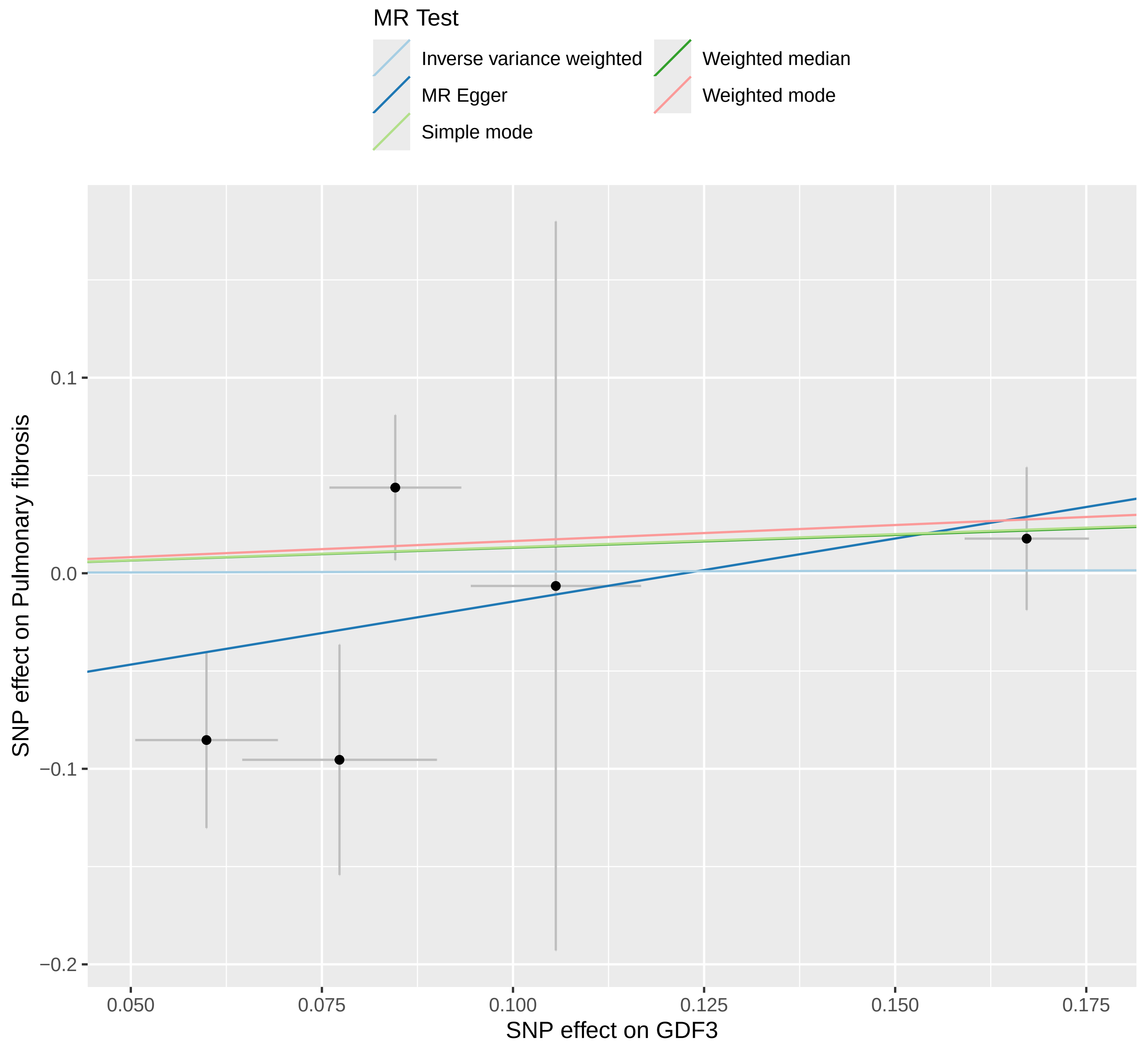

MR Test
Inverse variance weighted
MR Egger
Simple mode
Weighted median
Weighted mode
0.1
0.0
−0.1
−0.2
SNP effect on Pulmonary fibrosis
0.050
0.075
0.100
0.125
0.150
0.175
SNP effect on GDF3

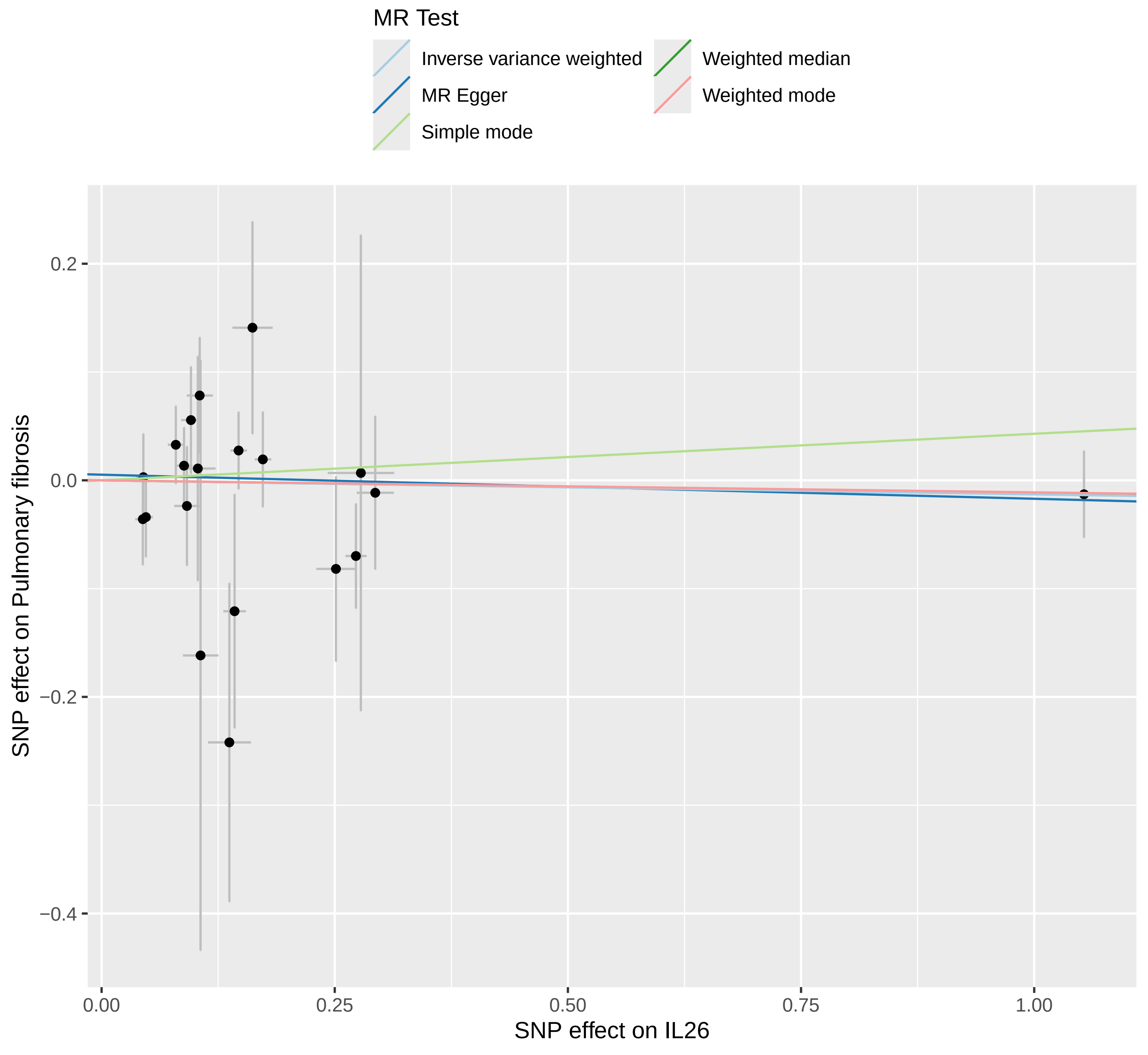

MR Test
Inverse variance weighted
MR Egger
Simple mode
Weighted median
Weighted mode
0.2
0.0
−0.2
−0.4
SNP effect on Pulmonary fibrosis
0.00
0.25
0.50
0.75
1.00
SNP effect on IL26

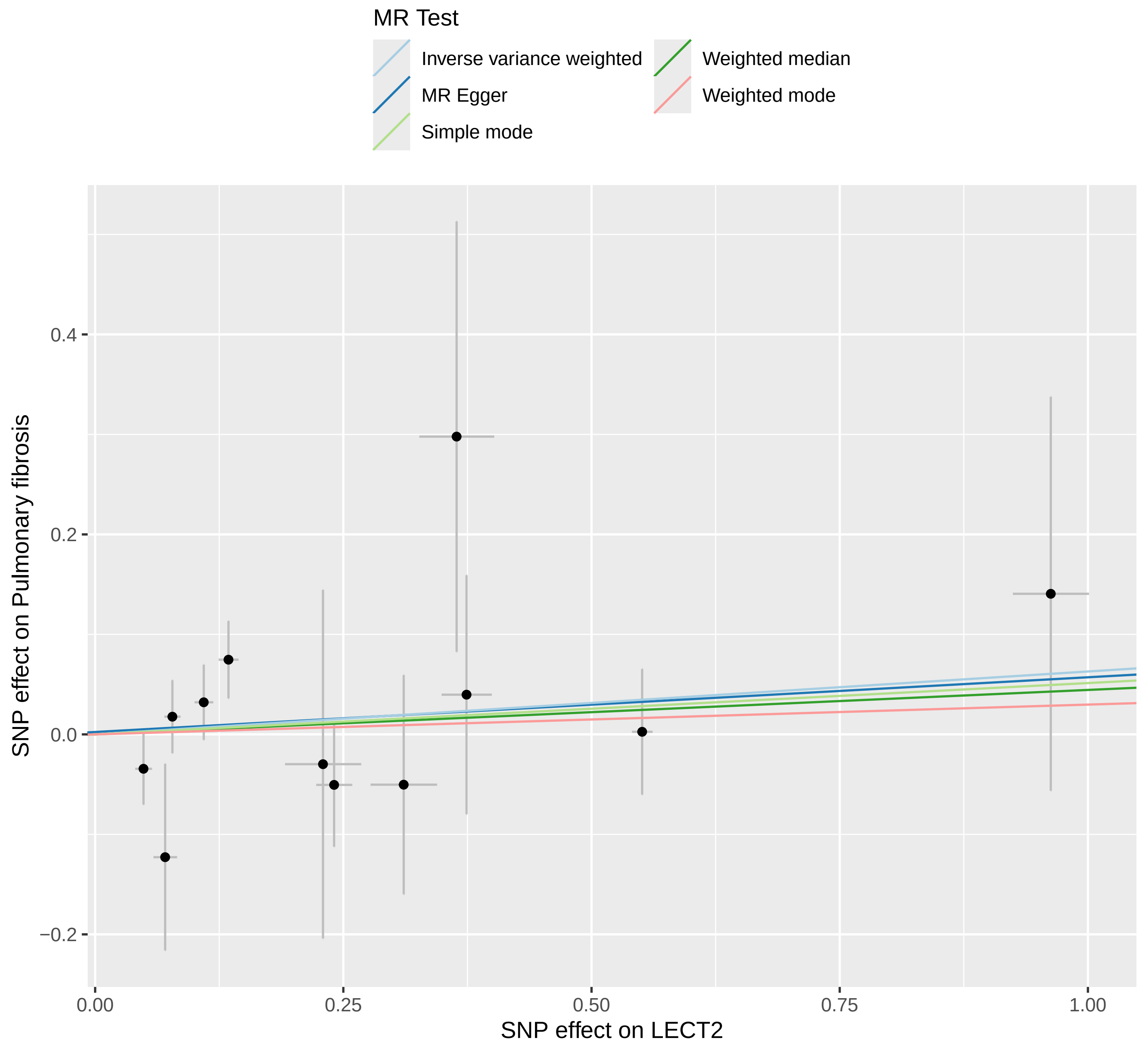
MR Test
Inverse variance weighted
MR Egger
Simple mode
Weighted median
Weighted mode
SNP effect on Pulmonary fibrosis
0.4
0.2
0.0
−0.2
0.00
0.25
0.50
0.75
1.00
SNP effect on LECT2

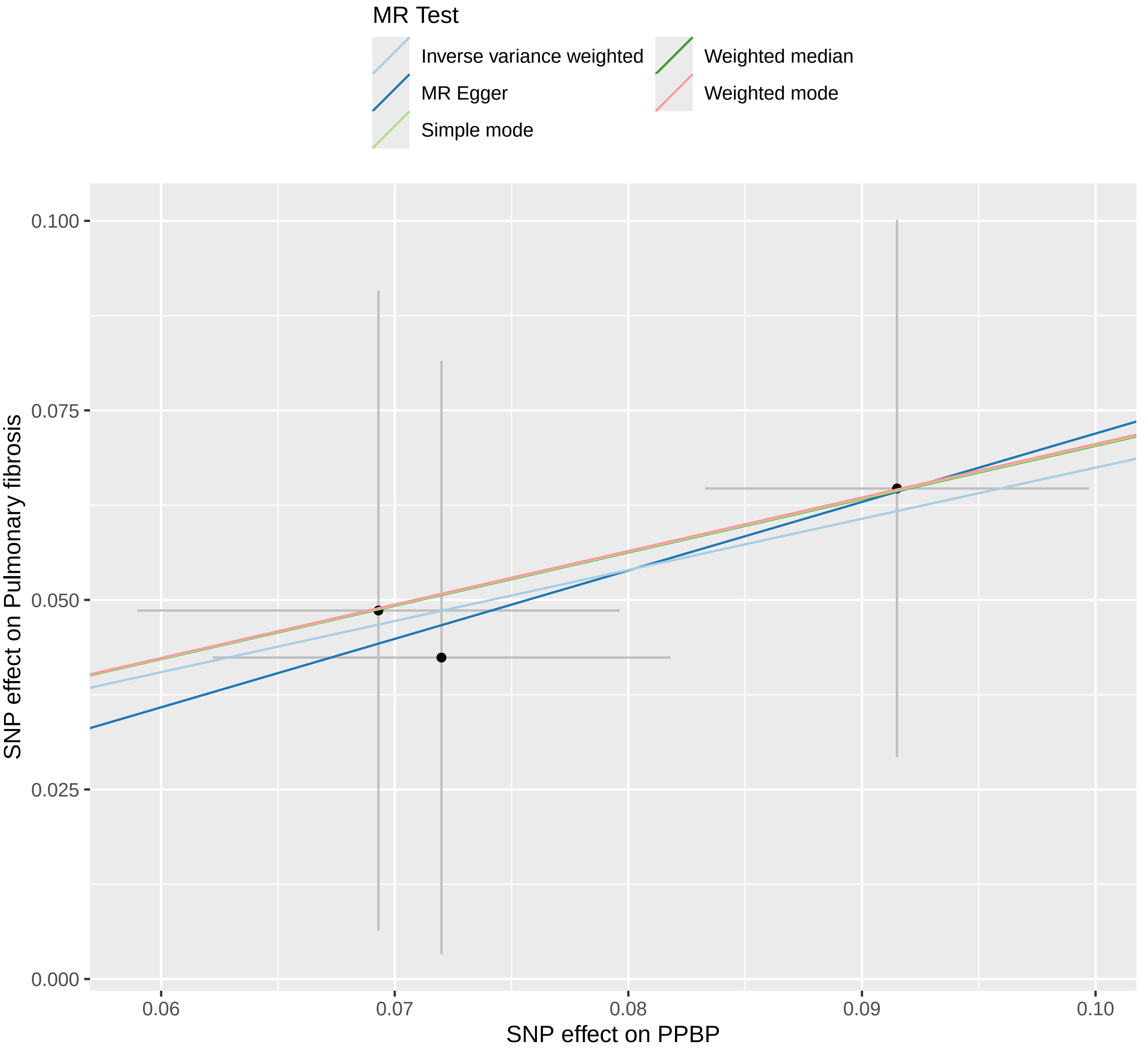

MR Test
Inverse variance weighted
MR Egger
Simple mode
Weighted median
Weighted mode
0.100
0.075
0.050
0.025
0.000
0.06
0.07
0.08
0.09
0.10
SNP effect on Pulmonary fibrosis
SNP effect on PPBP

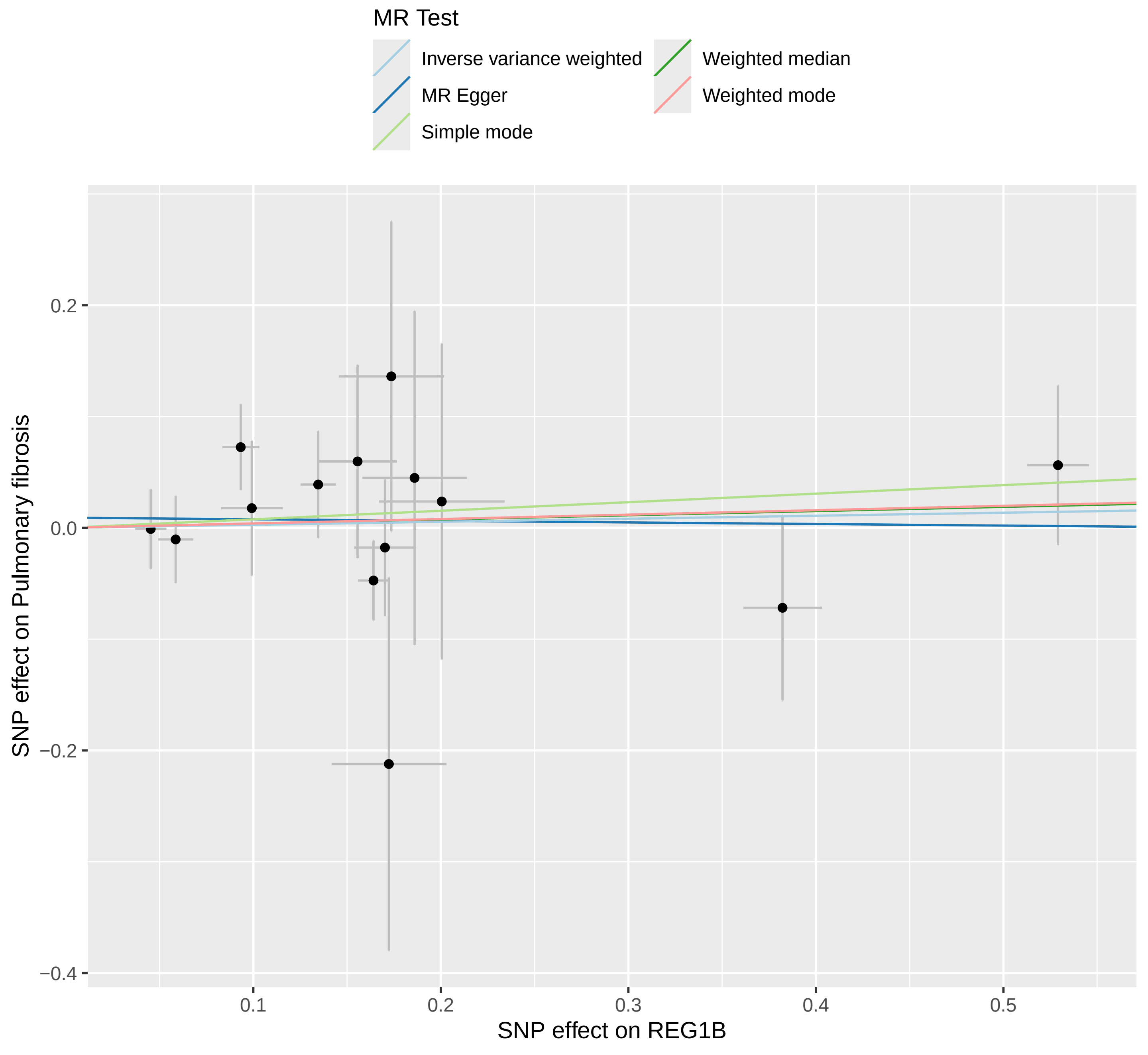
MR Test
Inverse variance weighted
MR Egger
Simple mode
Weighted median
Weighted mode
0.2
0.0
−0.2
−0.4
0.1
0.2
0.3
0.4
0.5
SNP effect on REG1B
SNP effect on Pulmonary fibrosis

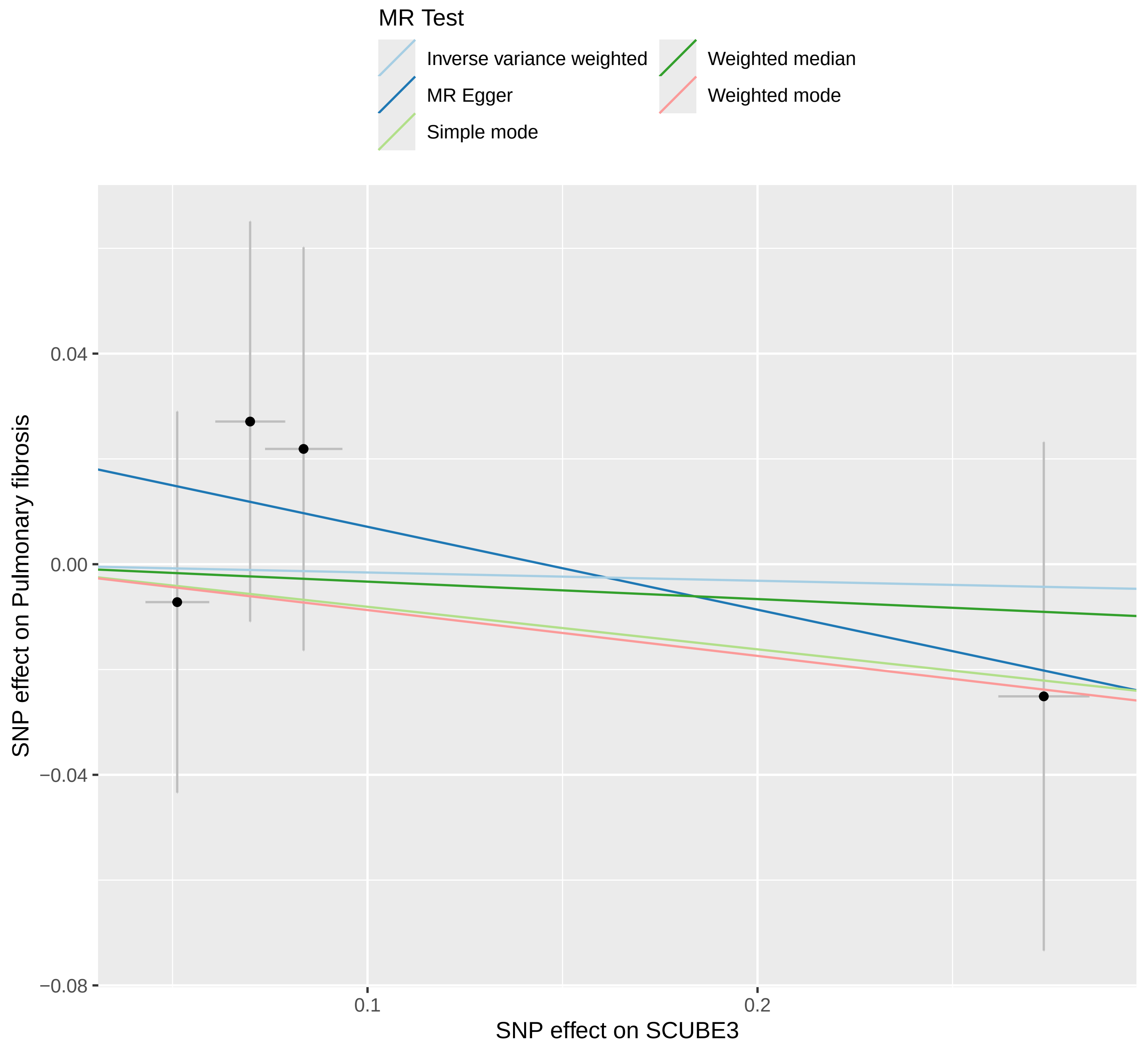

MR Test
Inverse variance weighted
MR Egger
Simple mode
Weighted median
Weighted mode
0.04
0.00
−0.04
−0.08
0.1
0.2
SNP effect on Pulmonary fibrosis
SNP effect on SCUBE3

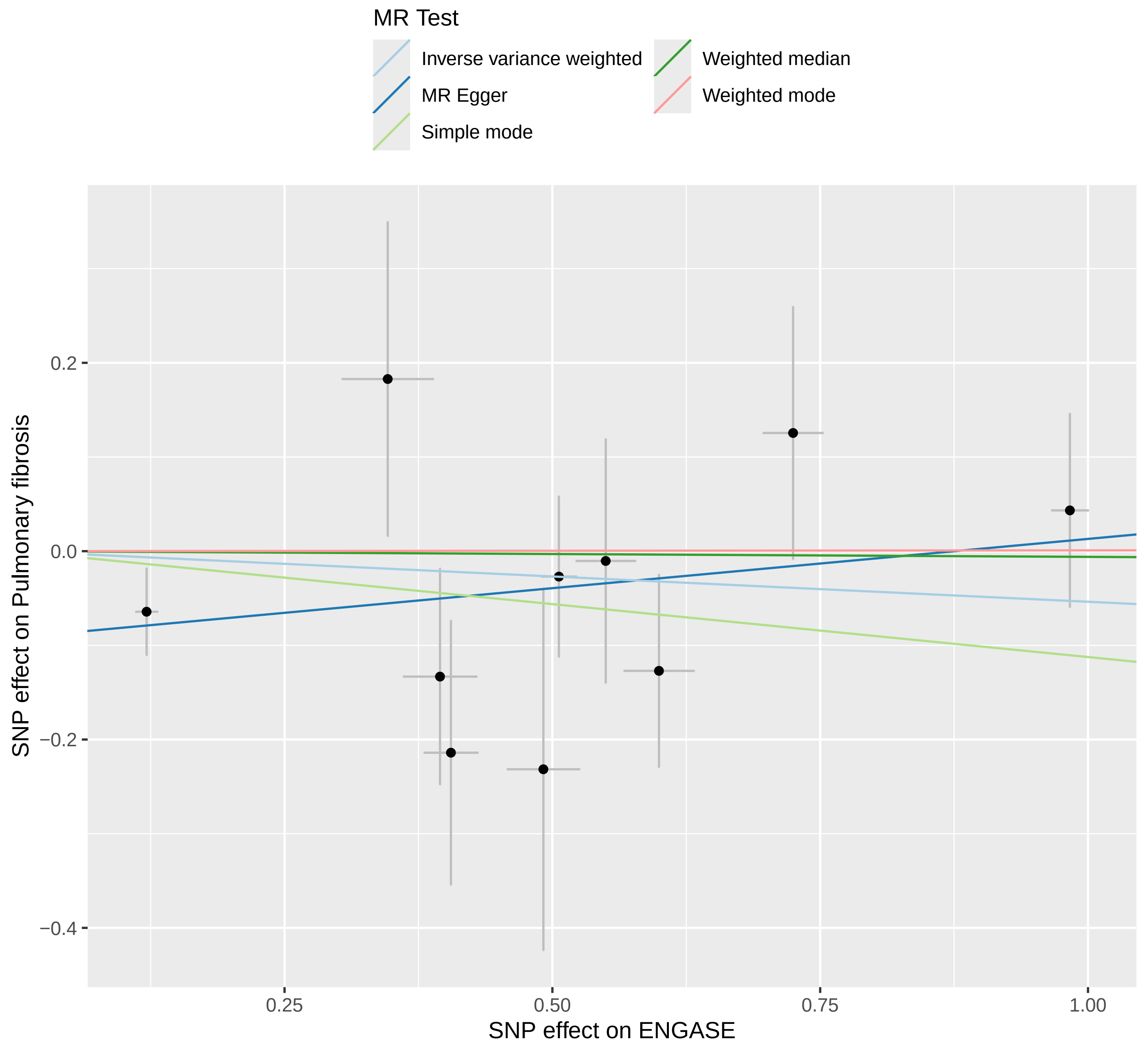
MR Test
Inverse variance weighted
MR Egger
Simple mode
Weighted median
Weighted mode
0.2
0.0
−0.2
−0.4
0.25
0.50
0.75
1.00
SNP effect on Pulmonary fibrosis
SNP effect on ENGASE

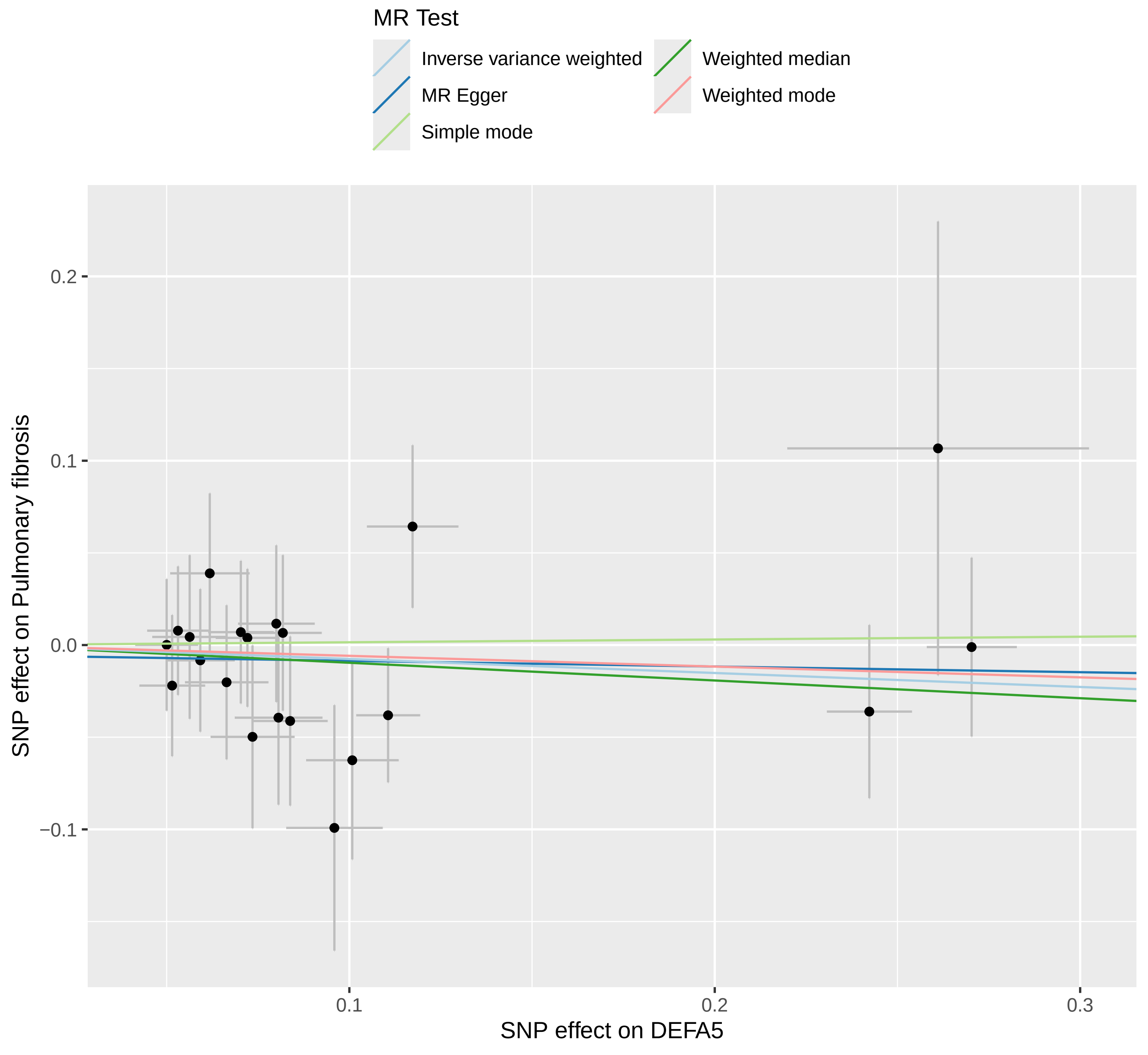

MR Test
Inverse variance weighted
MR Egger
Simple mode
Weighted median
Weighted mode
0.2
0.1
0.0
−0.1
SNP effect on Pulmonary fibrosis
0.1
0.2
0.3
SNP effect on DEFA5

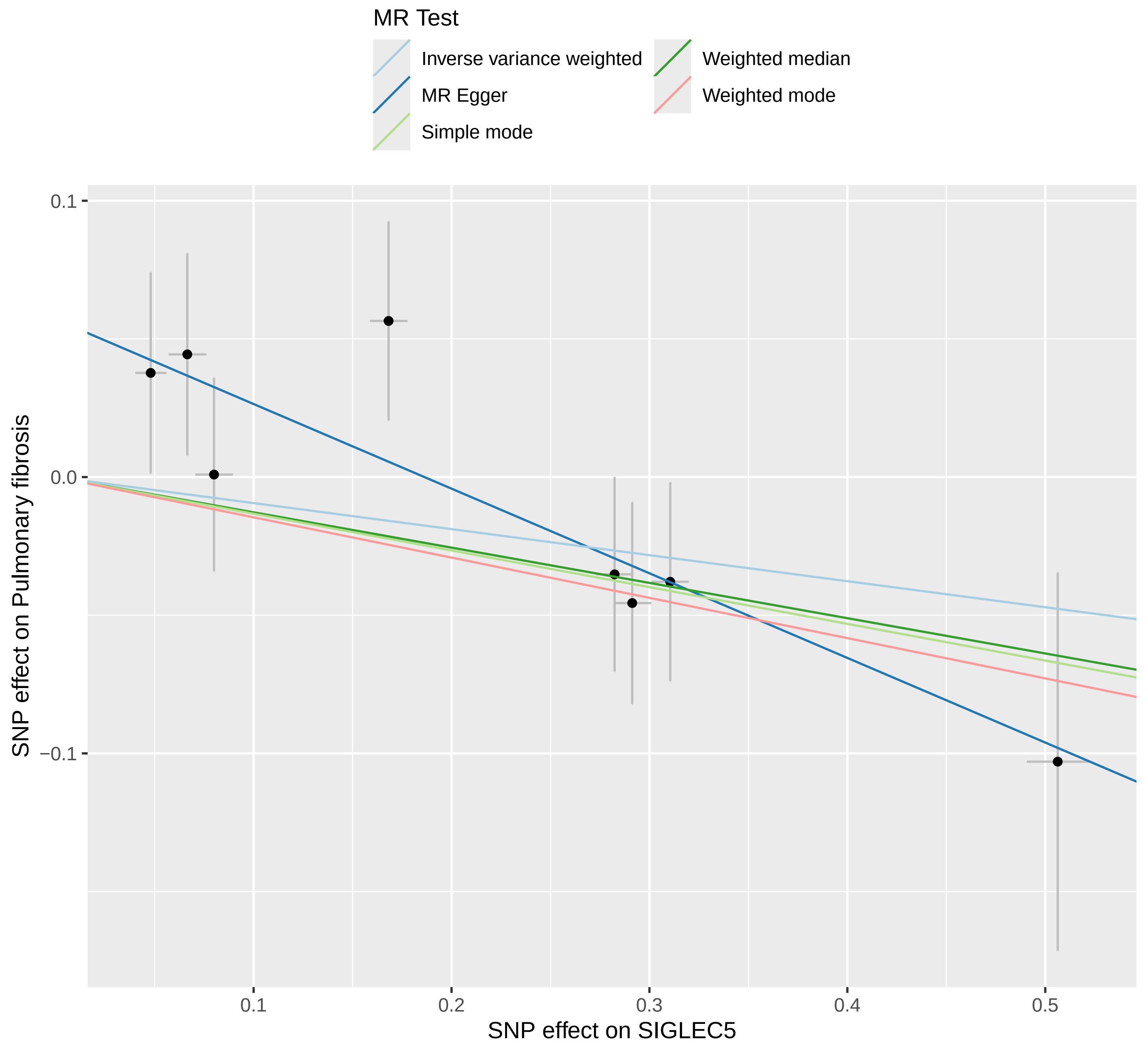
MR Test
Inverse variance weighted
MR Egger
Simple mode
Weighted median
Weighted mode
0.1
0.0
−0.1
0.1
0.2
0.3
0.4
0.5
SNP effect on Pulmonary fibrosis
SNP effect on SIGLEC5

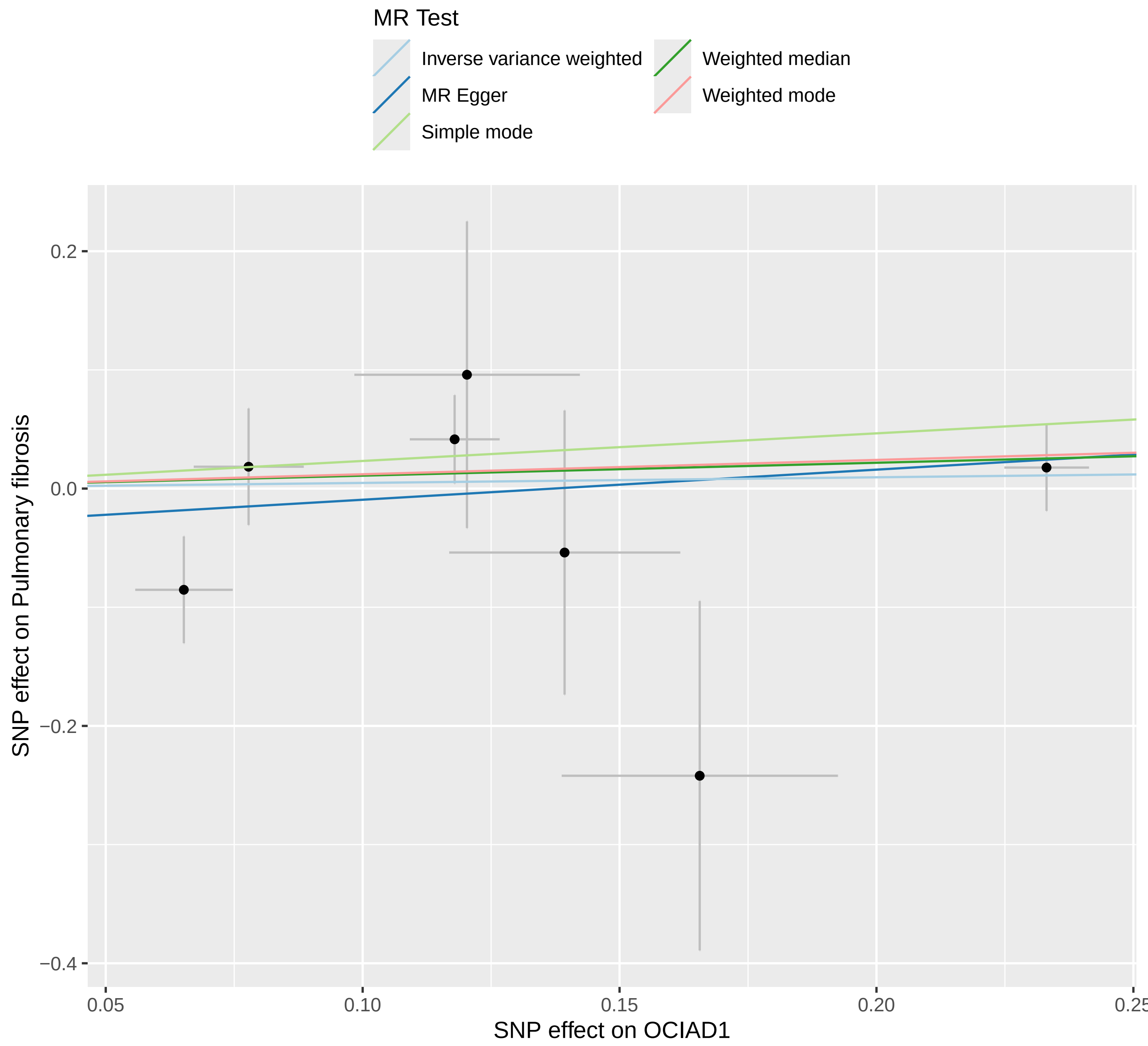

MR Test
Inverse variance weighted
MR Egger
Simple mode
Weighted median
Weighted mode
0.2
0.0
−0.2
−0.4
0.05
0.10
0.15
0.20
0.25
SNP effect on OCIAD1
SNP effect on Pulmonary fibrosis

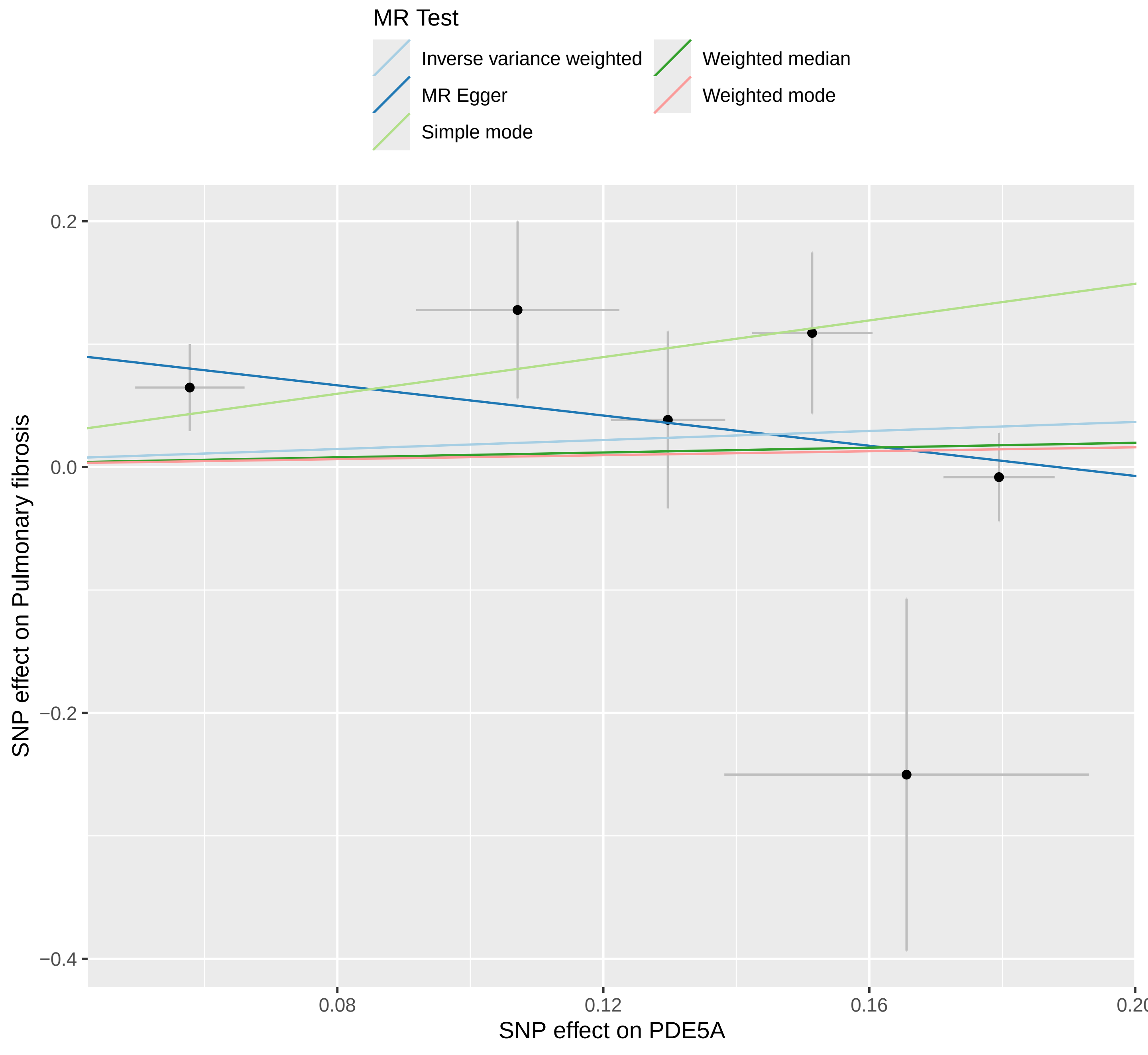
MR Test
Inverse variance weighted
MR Egger
Simple mode
Weighted median
Weighted mode
0.2
0.0
−0.2
−0.4
SNP effect on Pulmonary fibrosis
0.08
0.12
0.16
SNP effect on PDE5A

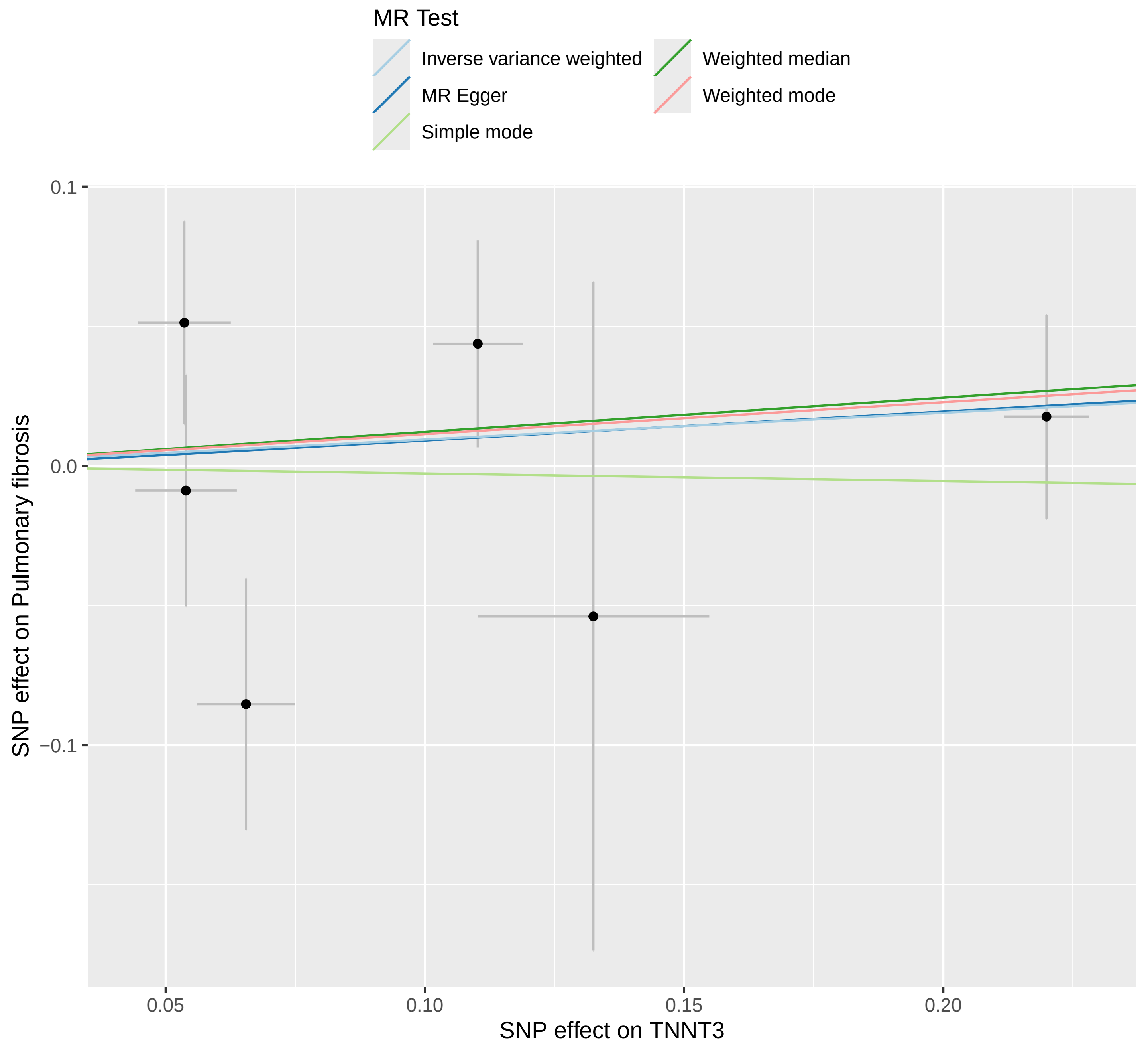

MR Test
Inverse variance weighted
MR Egger
Simple mode
Weighted median
Weighted mode
0.1
0.0
−0.1
0.05
0.10
0.15
0.20
SNP effect on Pulmonary fibrosis
SNP effect on TNNT3

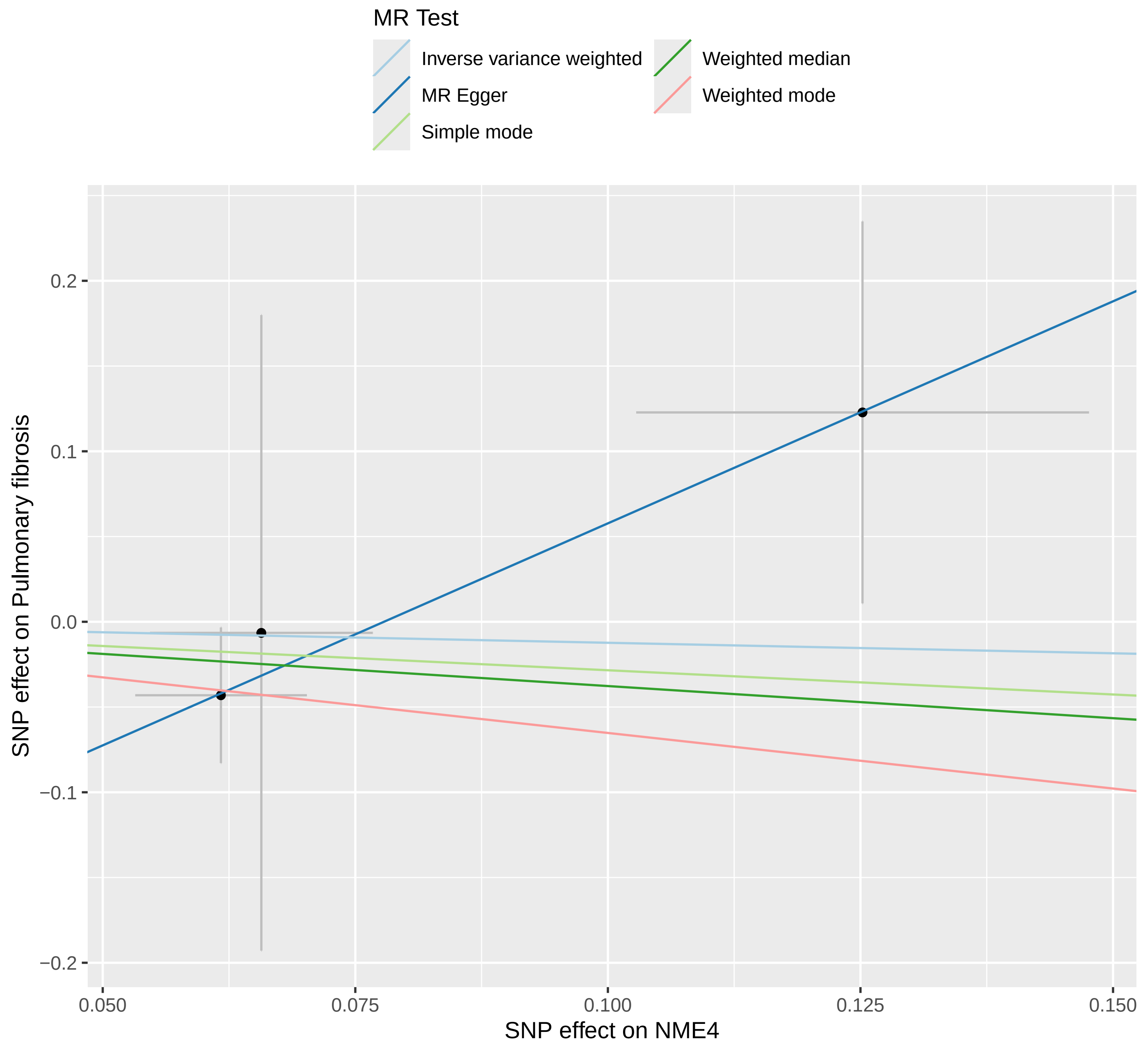

MR Test
Inverse variance weighted
MR Egger
Simple mode
Weighted median
Weighted mode
0.2
0.1
0.0
−0.1
−0.2
SNP effect on Pulmonary fibrosis
0.050
0.075
0.100
0.125
0.150
SNP effect on NME4

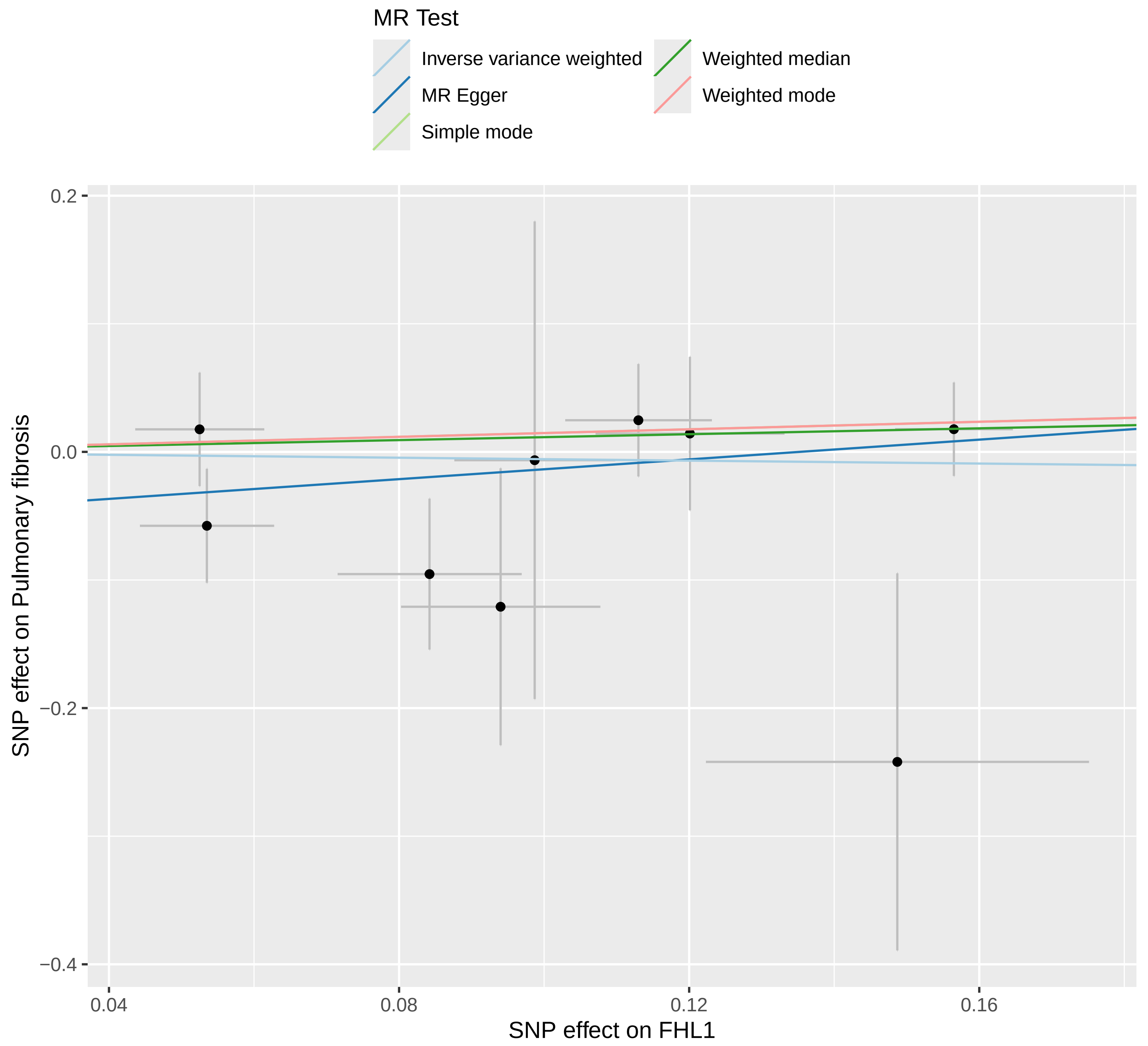

MR Test
Inverse variance weighted
MR Egger
Simple mode
Weighted median
Weighted mode
0.2
0.0
−0.2
−0.4
0.04
0.08
0.12
0.16
SNP effect on FHL1
SNP effect on Pulmonary fibrosis

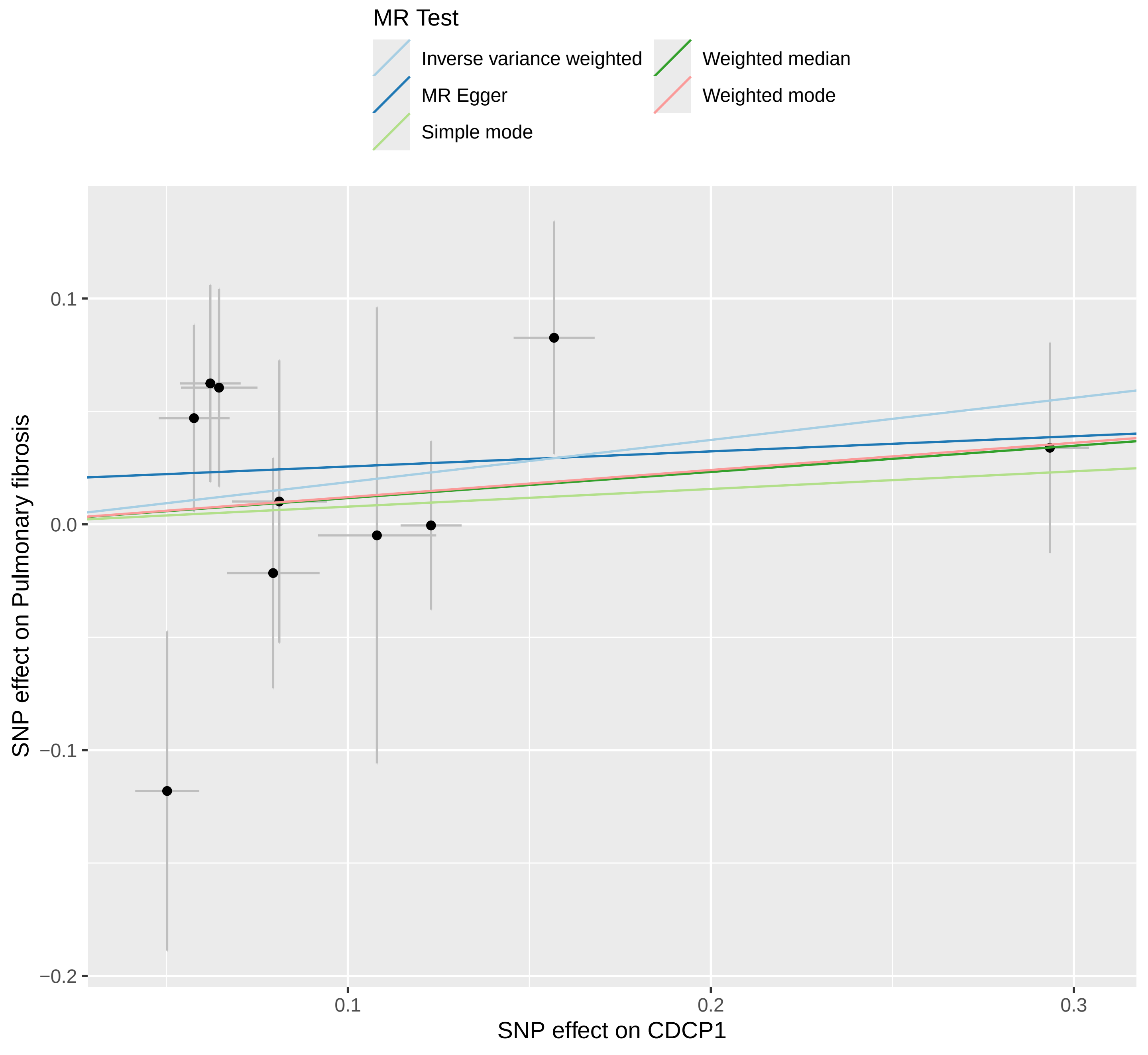

MR Test
Inverse variance weighted
MR Egger
Simple mode
Weighted median
Weighted mode
0.1
0.0
−0.1
−0.2
0.1
0.2
0.3
SNP effect on Pulmonary fibrosis
SNP effect on CDCP1

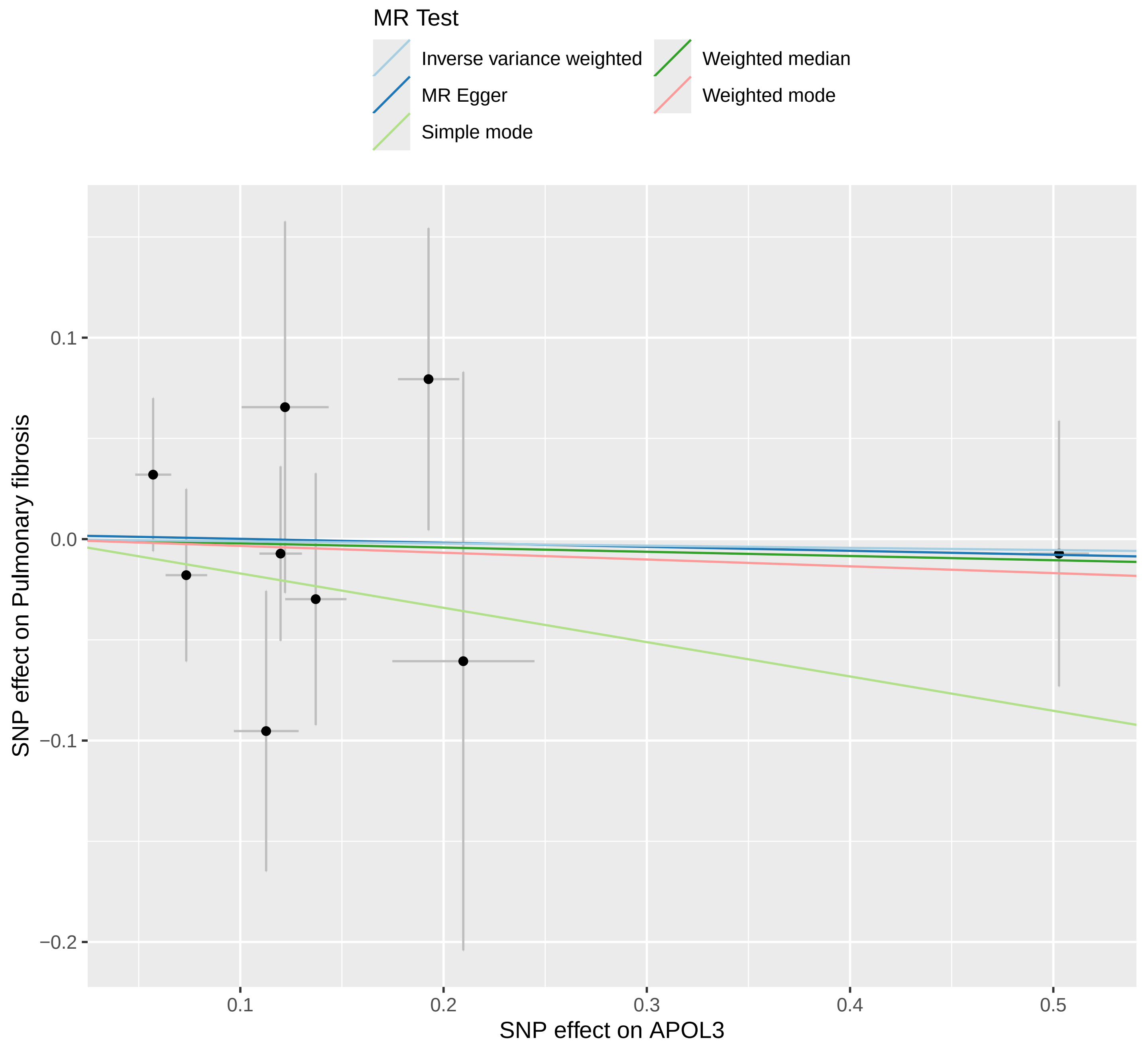

MR Test
Inverse variance weighted
MR Egger
Simple mode
Weighted median
Weighted mode
0.1
0.0
−0.1
−0.2
SNP effect on Pulmonary fibrosis
0.1
0.2
0.3
0.4
0.5
SNP effect on APOL3

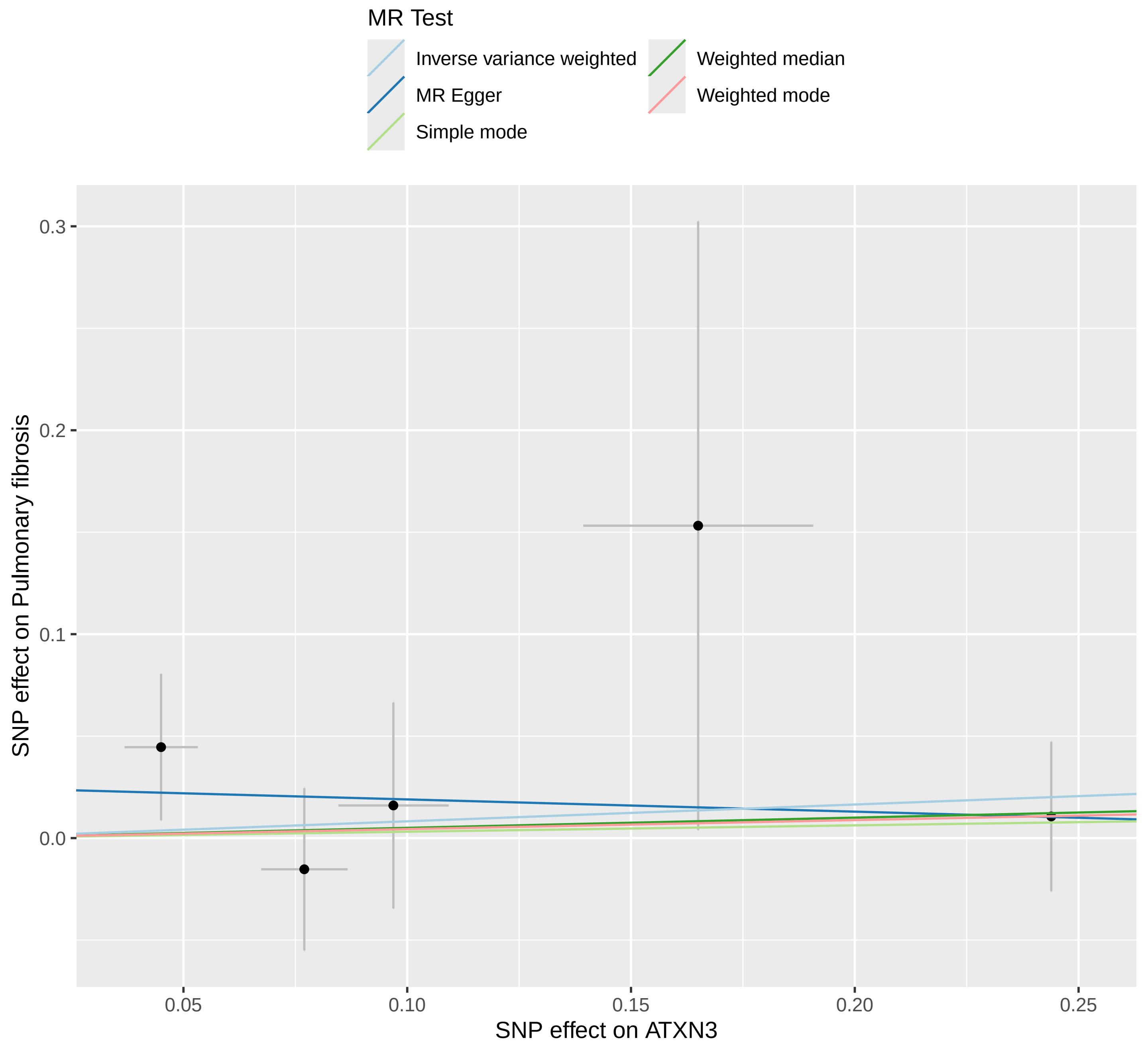

MR Test
Inverse variance weighted
MR Egger
Simple mode
Weighted median
Weighted mode
SNP effect on Pulmonary fibrosis
0.3
0.2
0.1
0.0
0.05
0.10
0.15
0.20
0.25
SNP effect on ATXN3

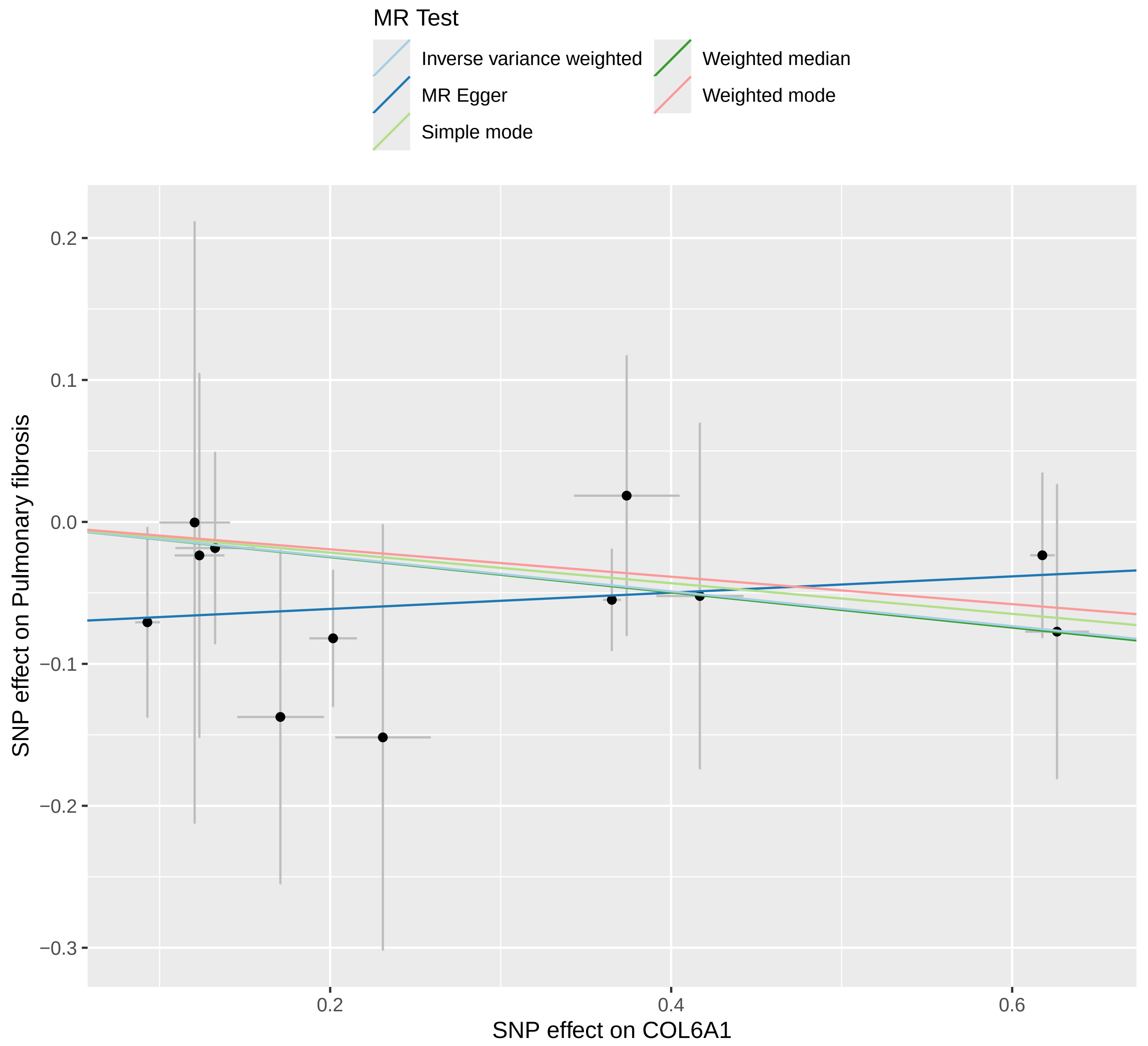
MR Test
Inverse variance weighted
MR Egger
Simple mode
Weighted median
Weighted mode
SNP effect on Pulmonary fibrosis
0.2
0.1
0.0
−0.1
−0.2
−0.3
0.2
0.4
0.6
SNP effect on COL6A1

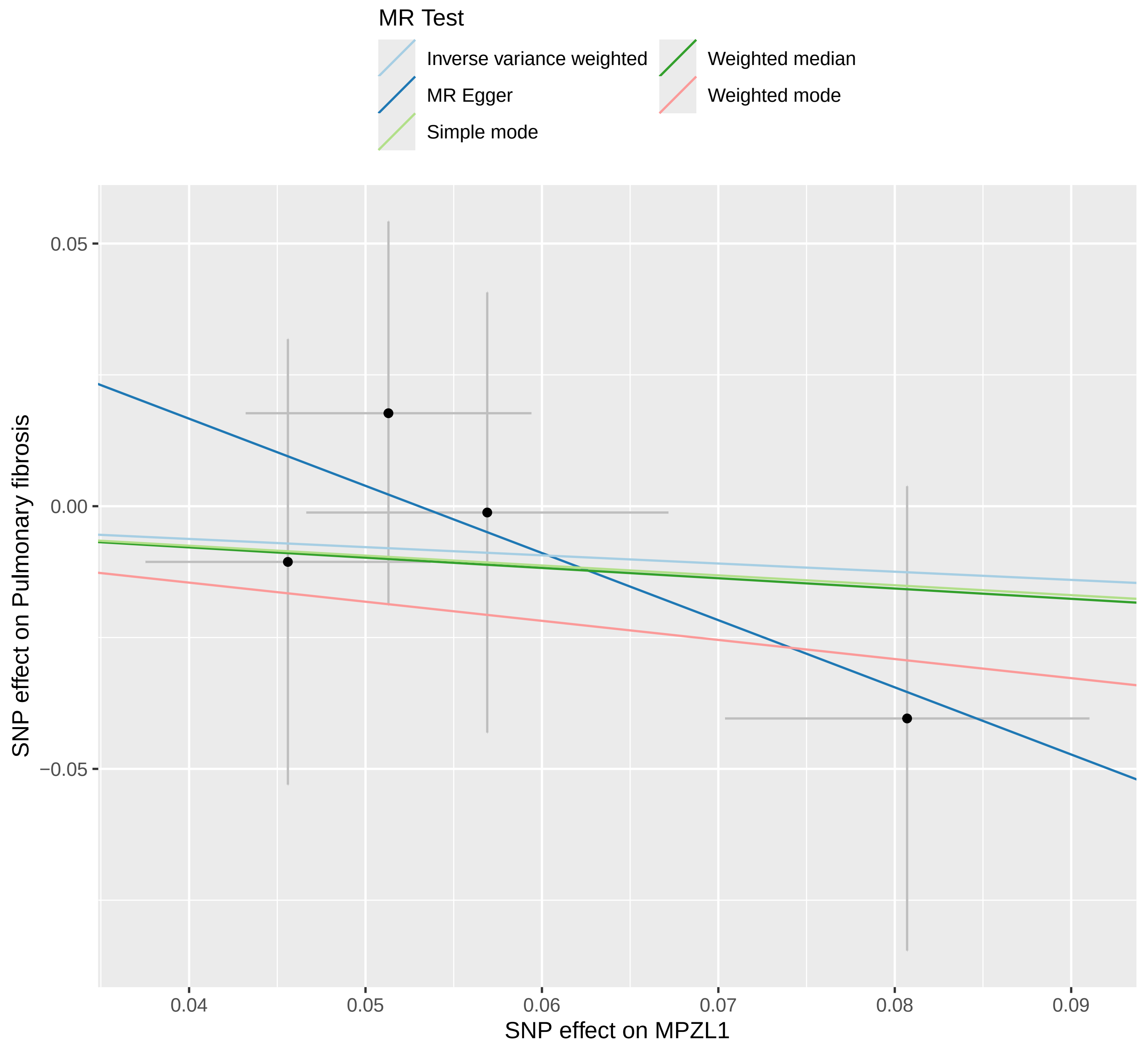

MR Test
Inverse variance weighted
MR Egger
Simple mode
Weighted median
Weighted mode
0.05
0.00
−0.05
SNP effect on Pulmonary fibrosis
0.04
0.05
0.06
0.07
0.08
0.09
SNP effect on MPZL1

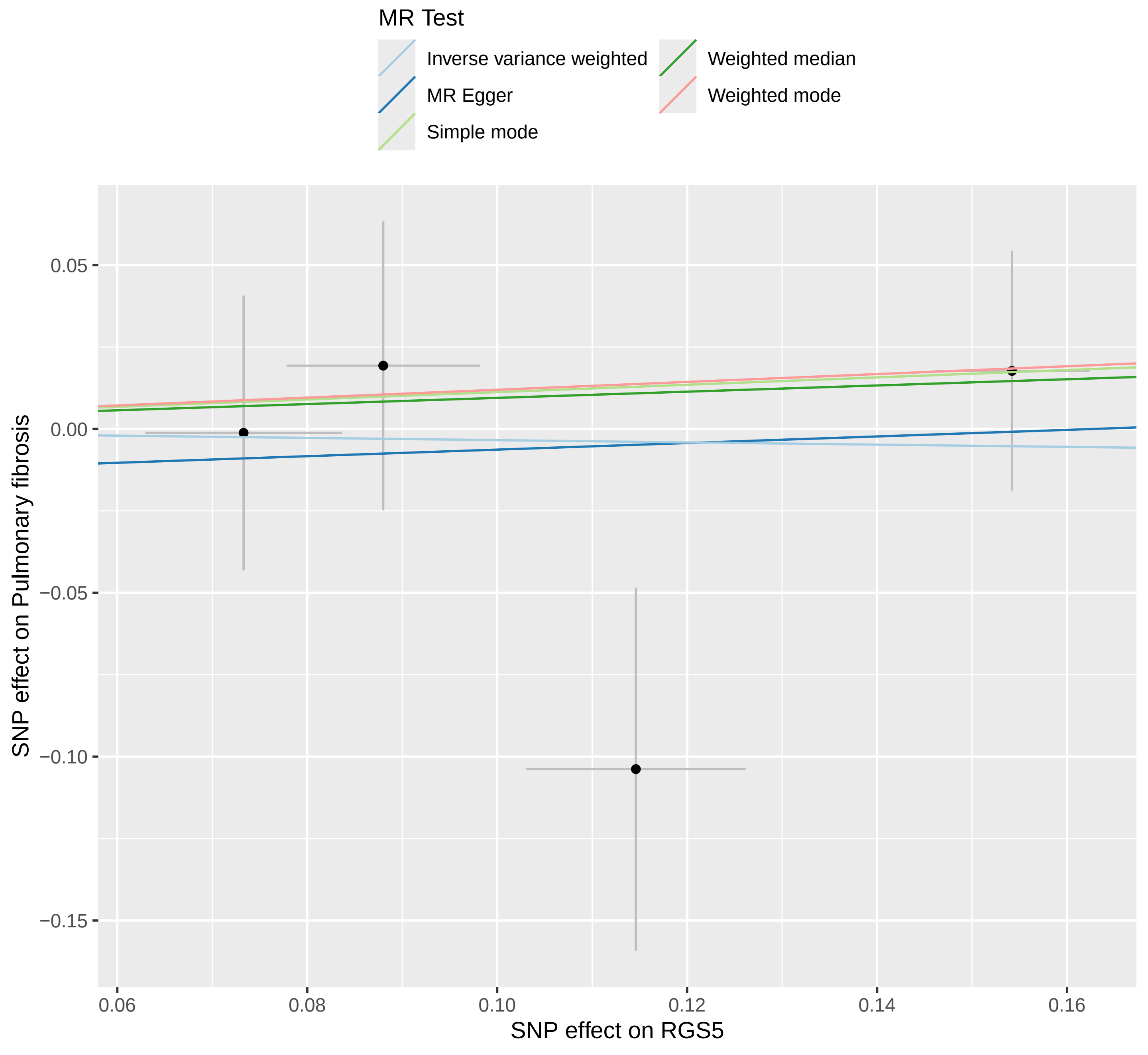

MR Test
Inverse variance weighted
MR Egger
Simple mode
Weighted median
Weighted mode
SNP effect on Pulmonary fibrosis
0.05
0.00
−0.05
−0.10
−0.15
0.06
0.08
0.10
0.12
0.14
0.16
SNP effect on RGS5

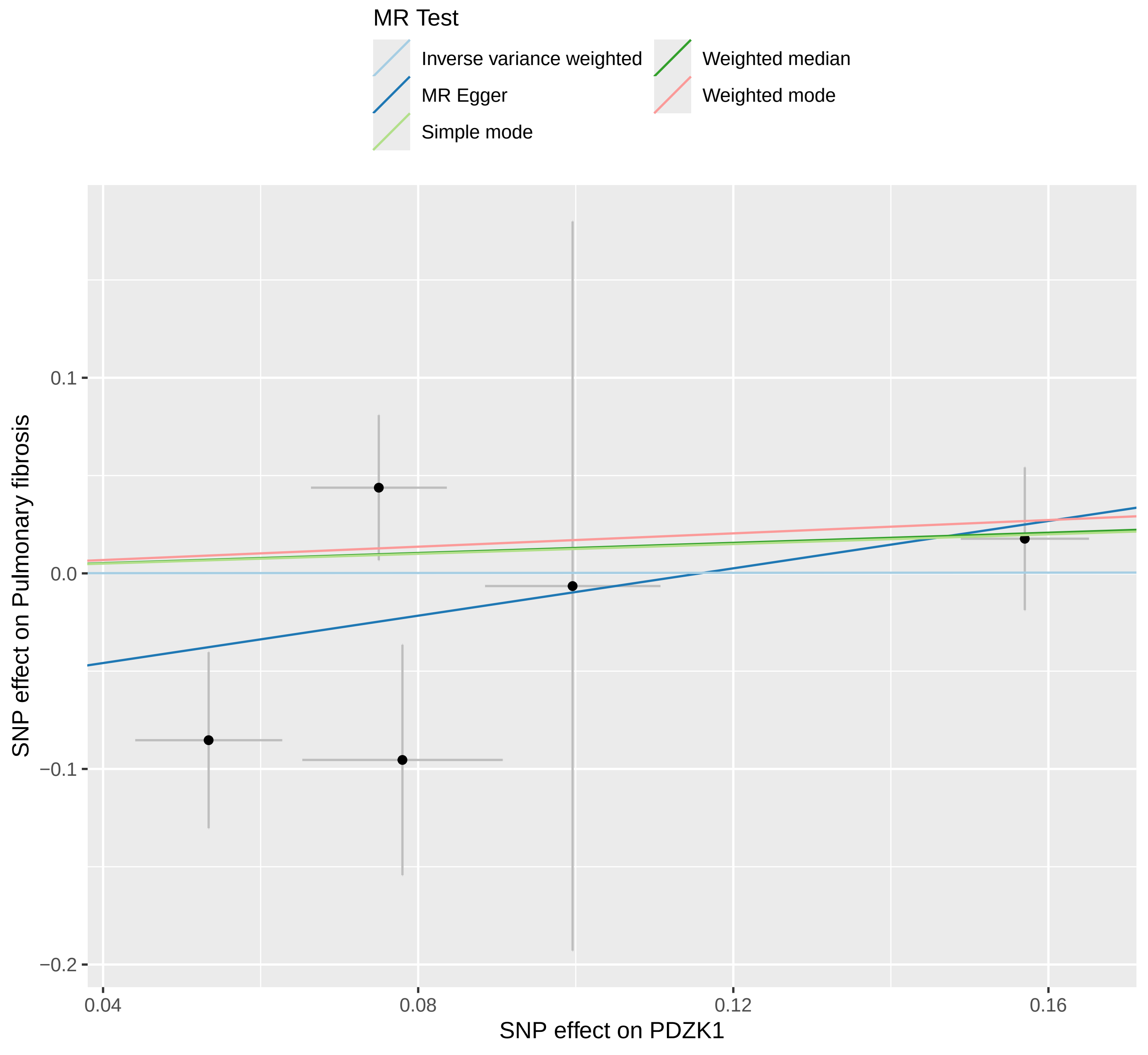
MR Test
Inverse variance weighted
MR Egger
Simple mode
Weighted median
Weighted mode
0.1
0.0
−0.1
−0.2
SNP effect on Pulmonary fibrosis
0.04
0.08
0.12
0.16
SNP effect on PDZK1

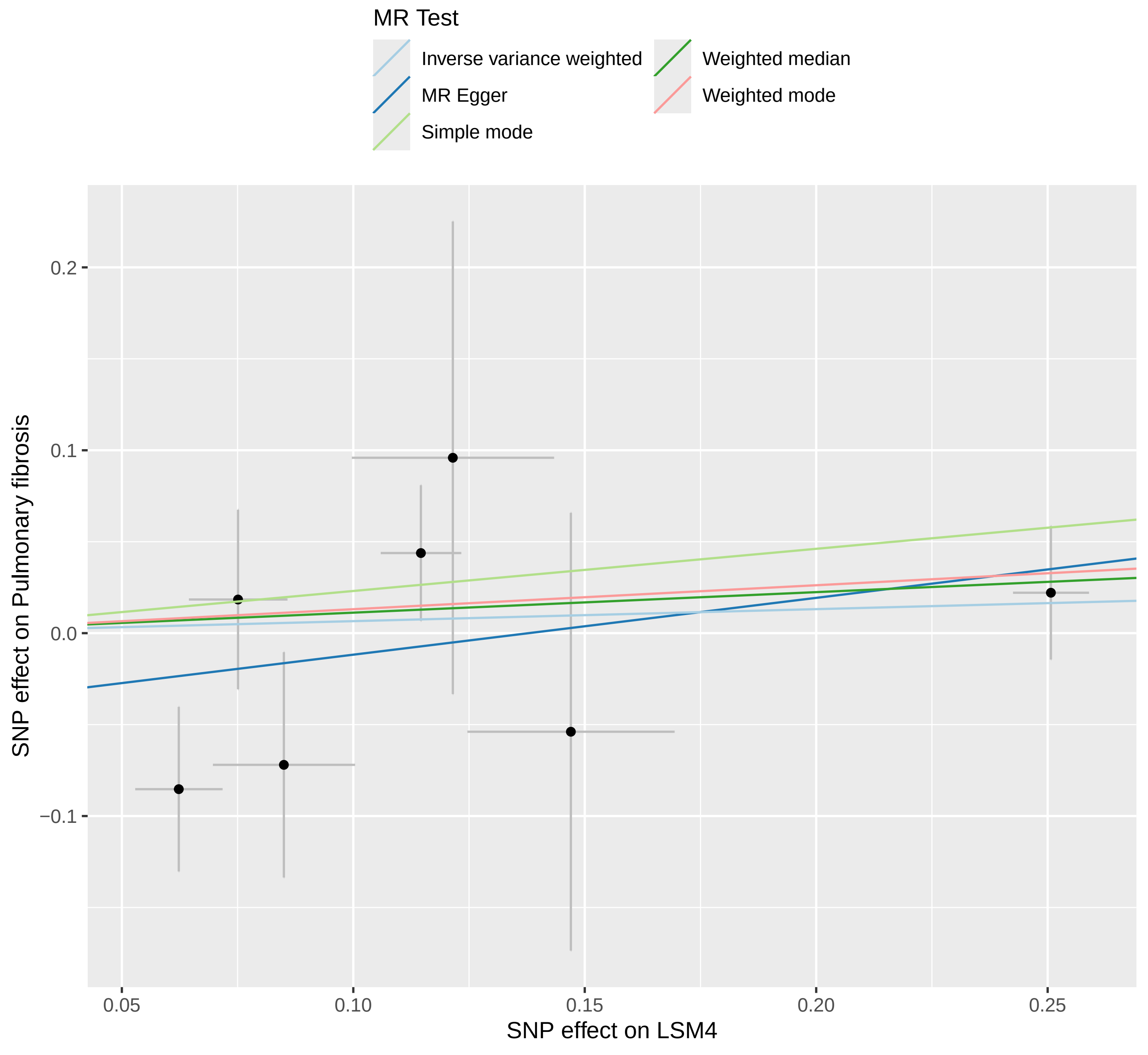

MR Test
Inverse variance weighted
MR Egger
Simple mode
Weighted median
Weighted mode
0.2
0.1
0.0
−0.1
0.05
0.10
0.15
0.20
0.25
SNP effect on LSM4
SNP effect on Pulmonary fibrosis

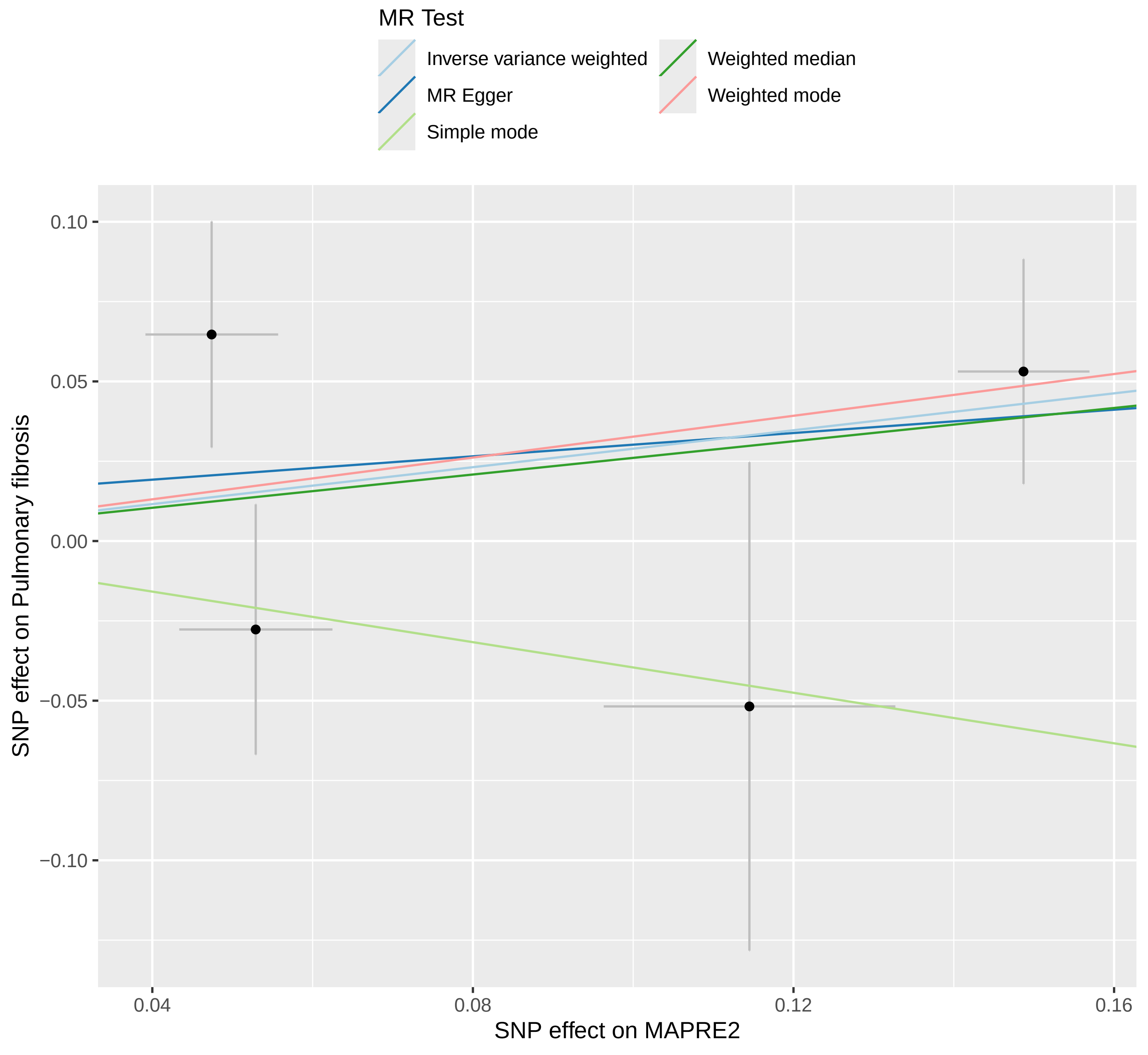
MR Test
Inverse variance weighted
MR Egger
Simple mode
Weighted median
Weighted mode
0.10
0.05
0.00
−0.05
−0.10
0.04
0.08
0.12
0.16
SNP effect on MAPRE2
SNP effect on Pulmonary fibrosis

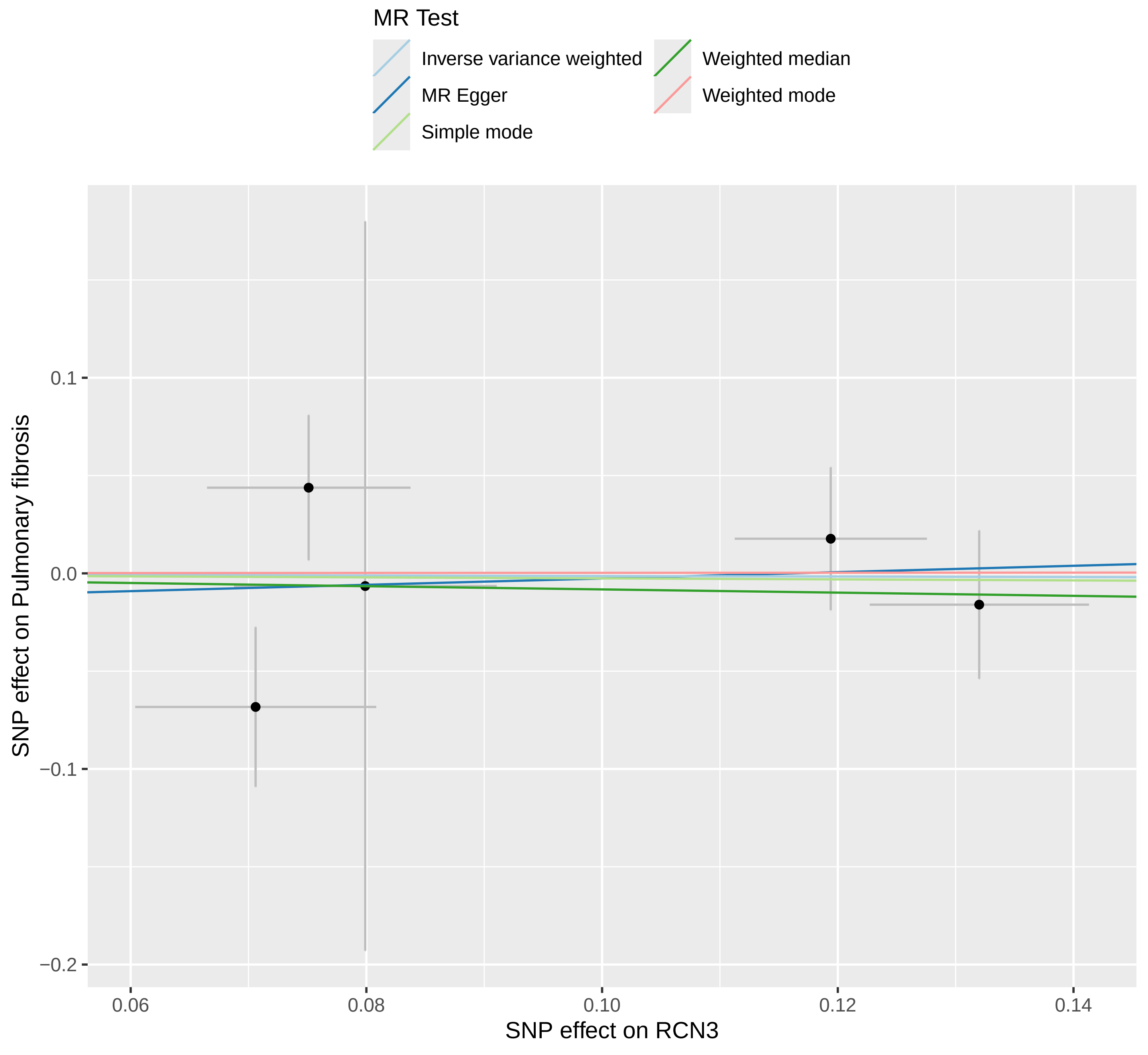

MR Test
Inverse variance weighted
MR Egger
Simple mode
Weighted median
Weighted mode
0.1
0.0
−0.1
−0.2
SNP effect on Pulmonary fibrosis
0.06
0.08
0.10
0.12
0.14
SNP effect on RCN3

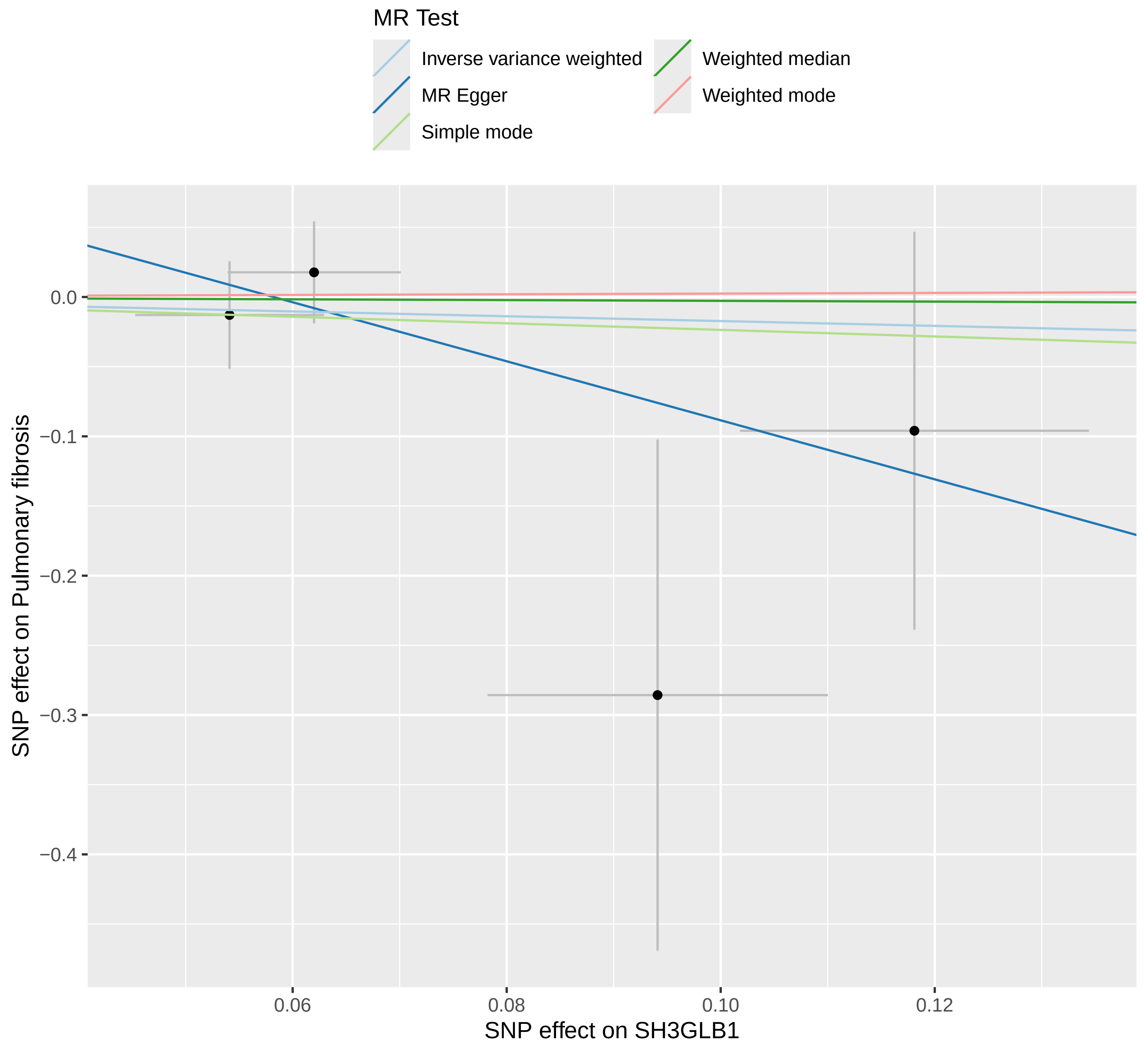

MR Test
Inverse variance weighted
MR Egger
Simple mode
Weighted median
Weighted mode
0.0
−0.1
−0.2
−0.3
−0.4
SNP effect on Pulmonary fibrosis
0.06
0.08
0.10
0.12
SNP effect on SH3GLB1

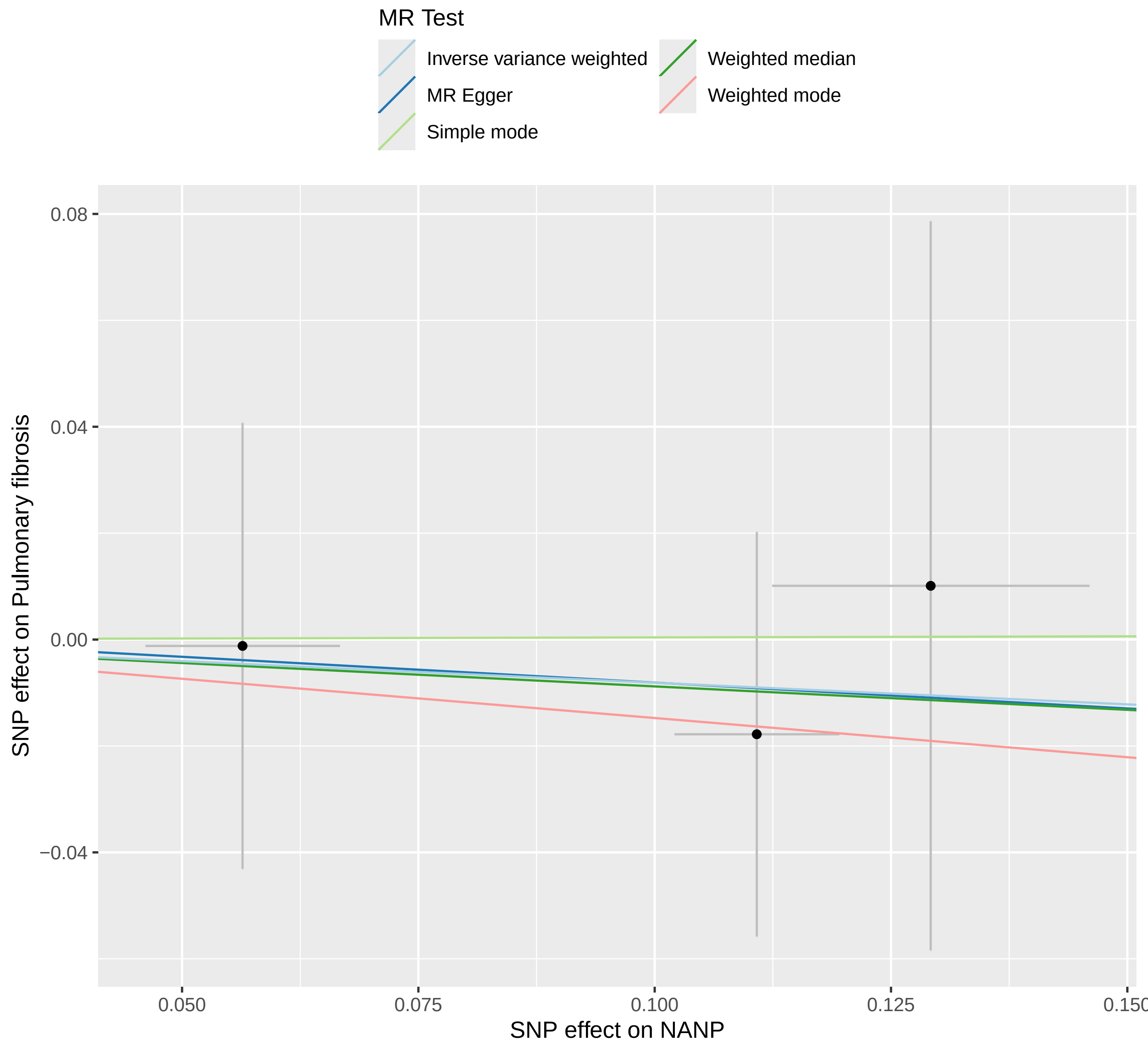

MR Test
Inverse variance weighted
MR Egger
Simple mode
Weighted median
Weighted mode
0.08
0.04
0.00
−0.04
SNP effect on Pulmonary fibrosis
0.050
0.075
0.100
0.125
0.150
SNP effect on NANP

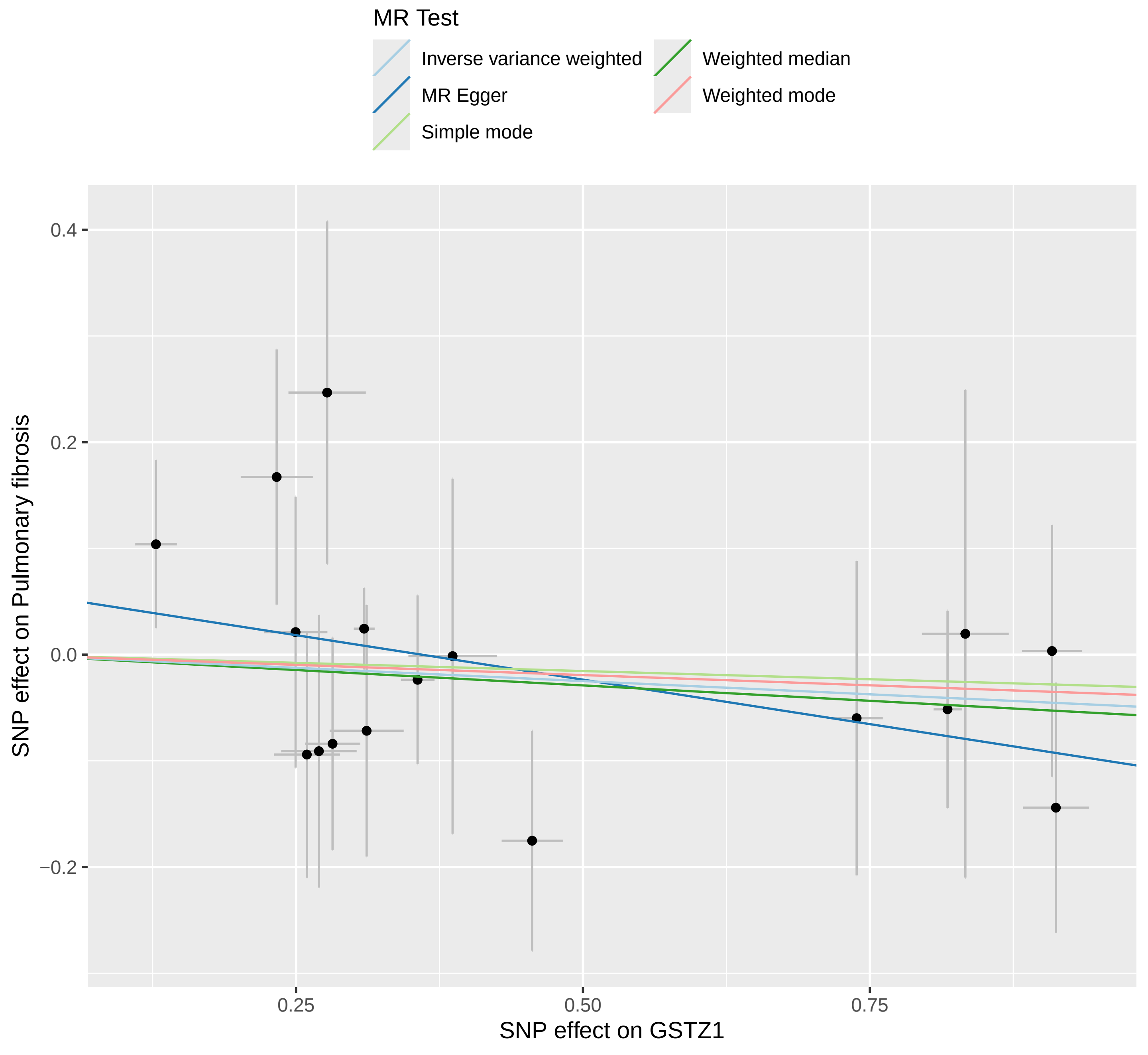

MR Test
Inverse variance weighted
MR Egger
Simple mode
Weighted median
Weighted mode
0.4
0.2
0.0
−0.2
0.25
0.50
0.75
SNP effect on Pulmonary fibrosis
SNP effect on GSTZ1

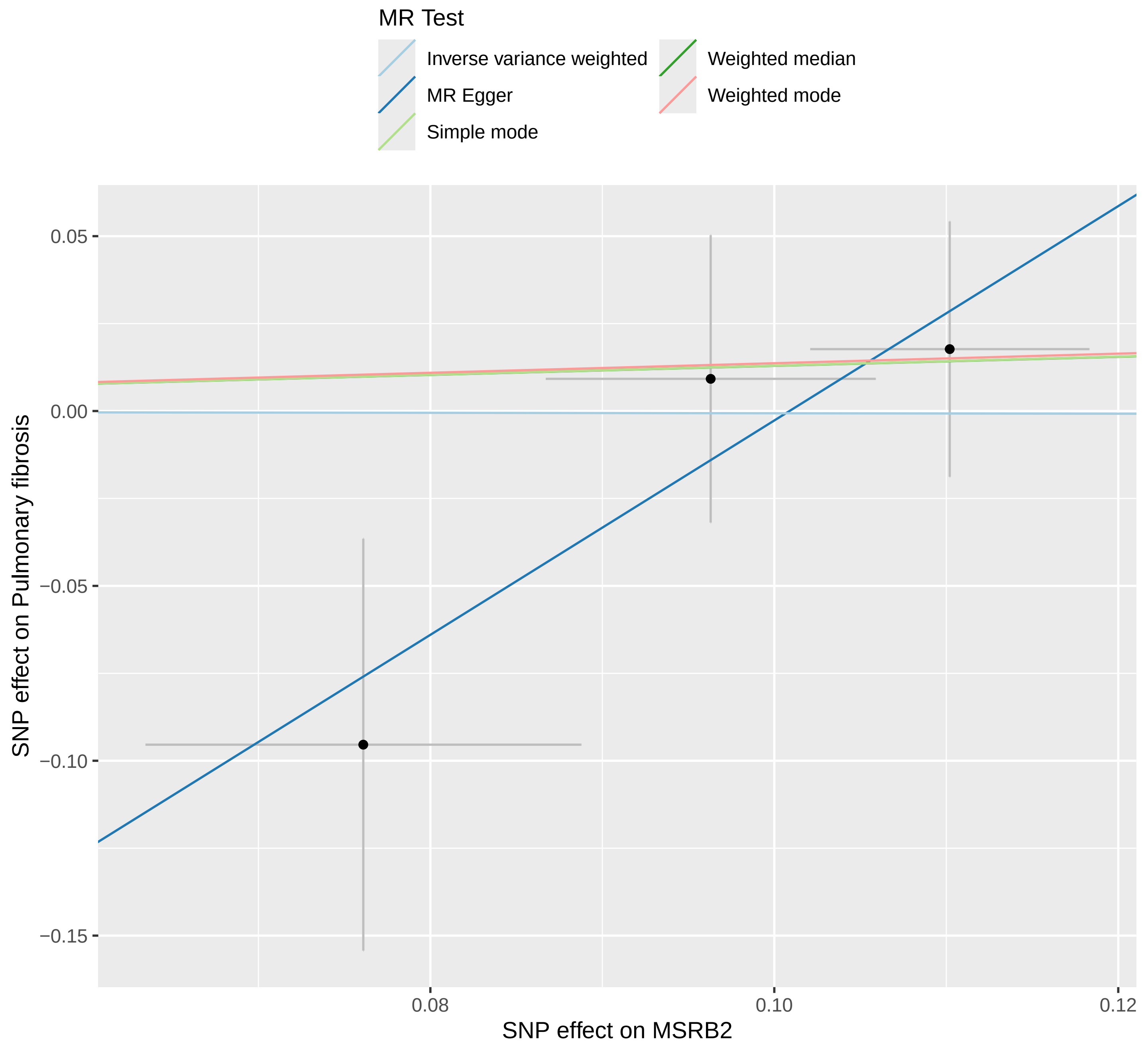
MR Test
Inverse variance weighted
MR Egger
Simple mode
Weighted median
Weighted mode
0.05
0.00
−0.05
−0.10
−0.15
0.08
0.10
0.12
SNP effect on Pulmonary fibrosis
SNP effect on MSRB2

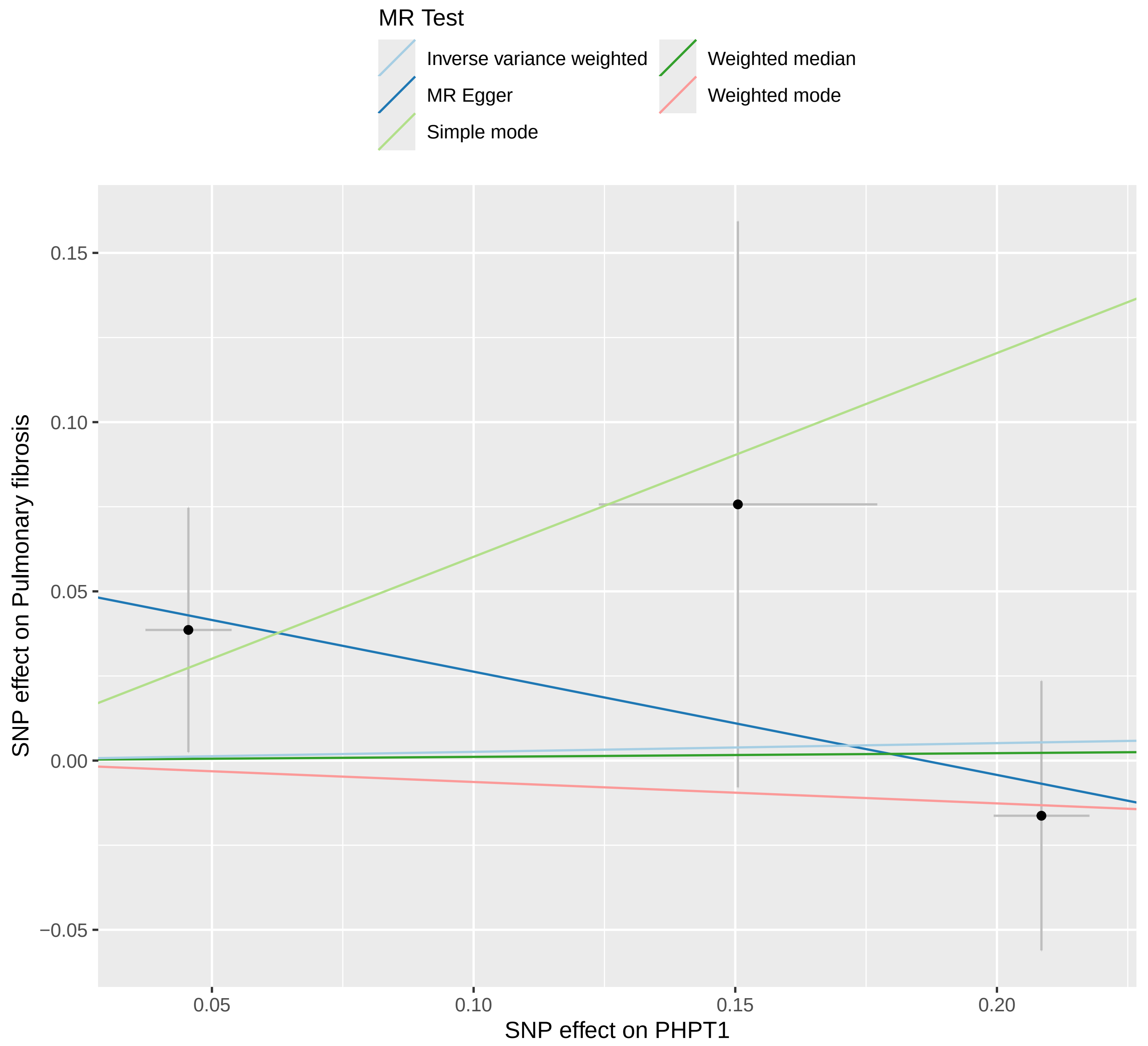
MR Test
Inverse variance weighted
MR Egger
Simple mode
Weighted median
Weighted mode
0.15
0.10
0.05
0.00
−0.05
SNP effect on Pulmonary fibrosis
0.05
0.10
0.15
0.20
SNP effect on PHPT1

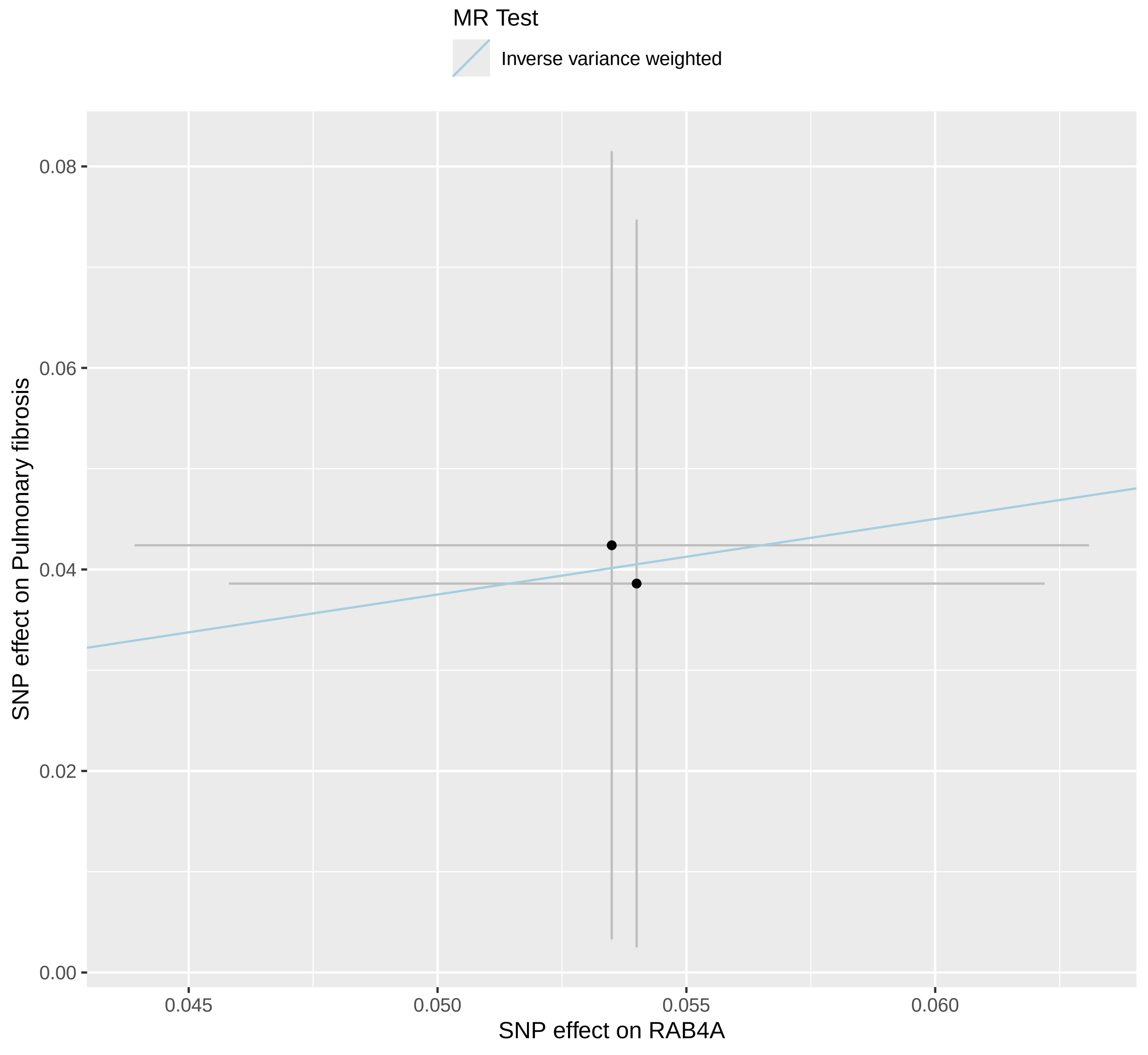

MR Test
Inverse variance weighted
0.08
0.06
0.04
0.02
0.00
0.045
0.050
0.055
0.060
SNP effect on Pulmonary fibrosis
SNP effect on RAB4A

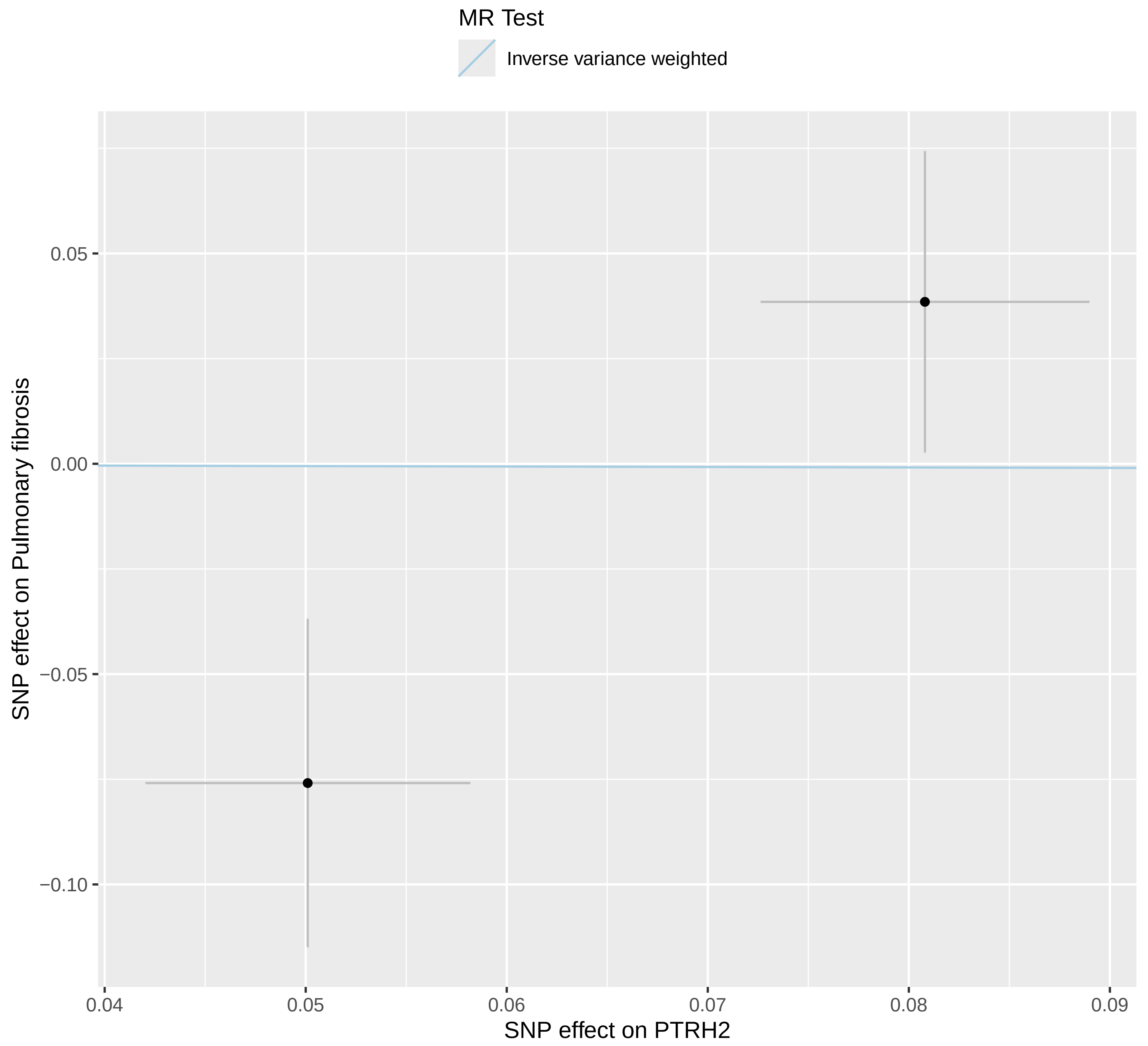
MR Test
Inverse variance weighted
0.05
0.00
−0.05
−0.10
0.04
0.05
0.06
0.07
0.08
0.09
SNP effect on Pulmonary fibrosis
SNP effect on PTRH2

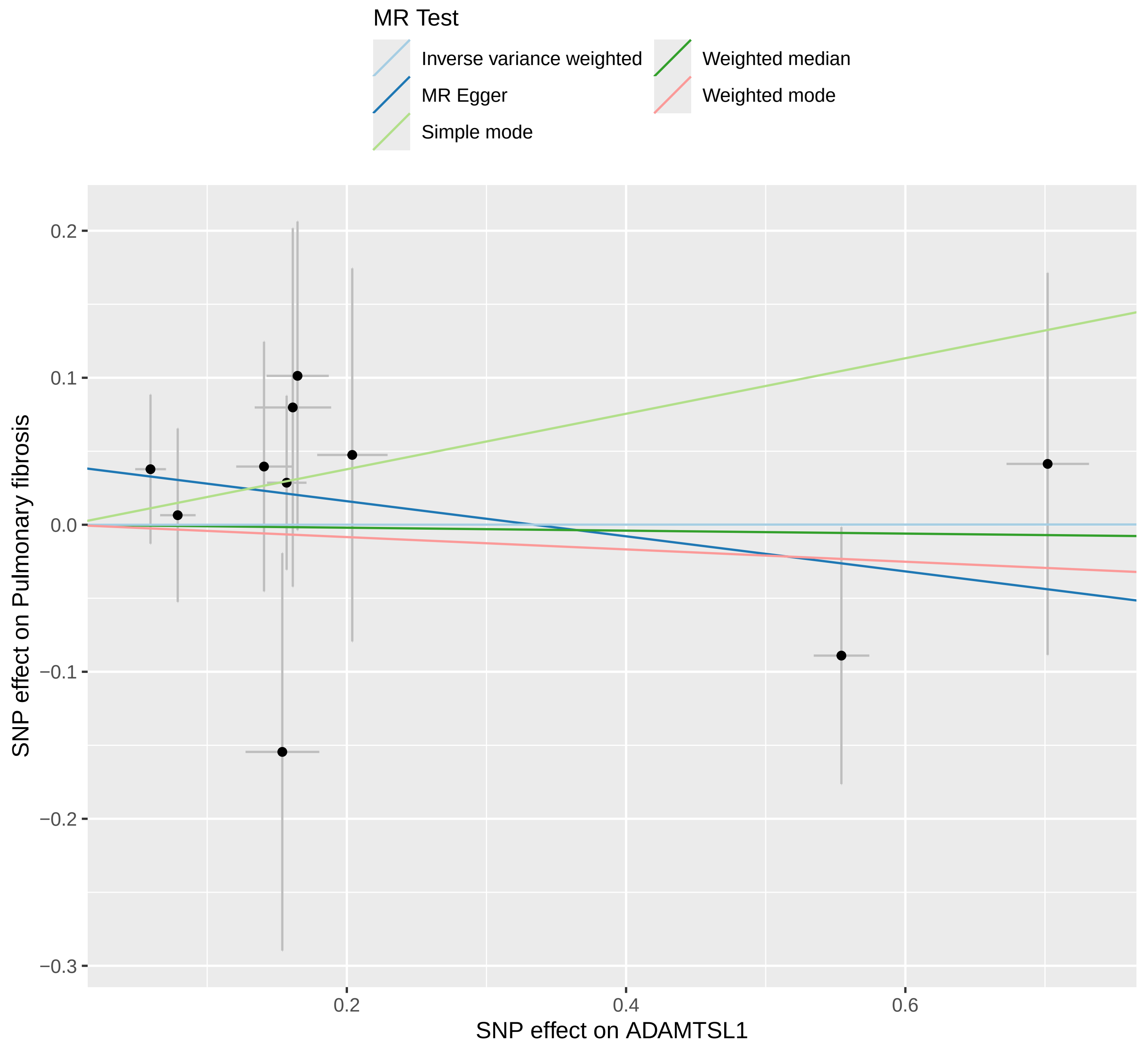

MR Test
Inverse variance weighted
MR Egger
Simple mode
Weighted median
Weighted mode
0.2
0.1
0.0
−0.1
−0.2
−0.3
SNP effect on Pulmonary fibrosis
0.2
0.4
0.6
SNP effect on ADAMTSL1

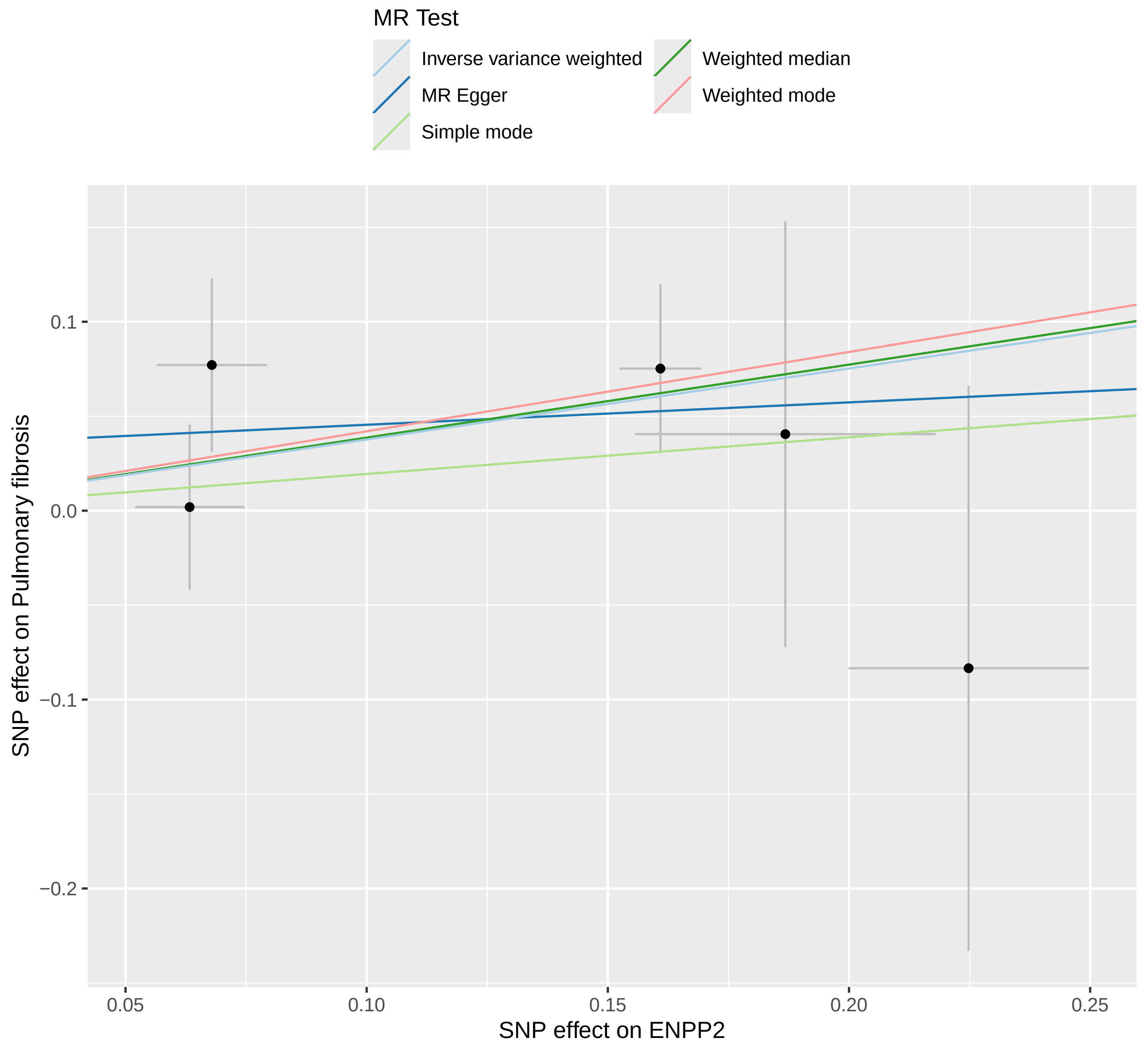
MR Test
Inverse variance weighted
MR Egger
Simple mode
Weighted median
Weighted mode
SNP effect on Pulmonary fibrosis
0.1
0.0
−0.1
−0.2
0.05
0.10
0.15
0.20
0.25
SNP effect on ENPP2

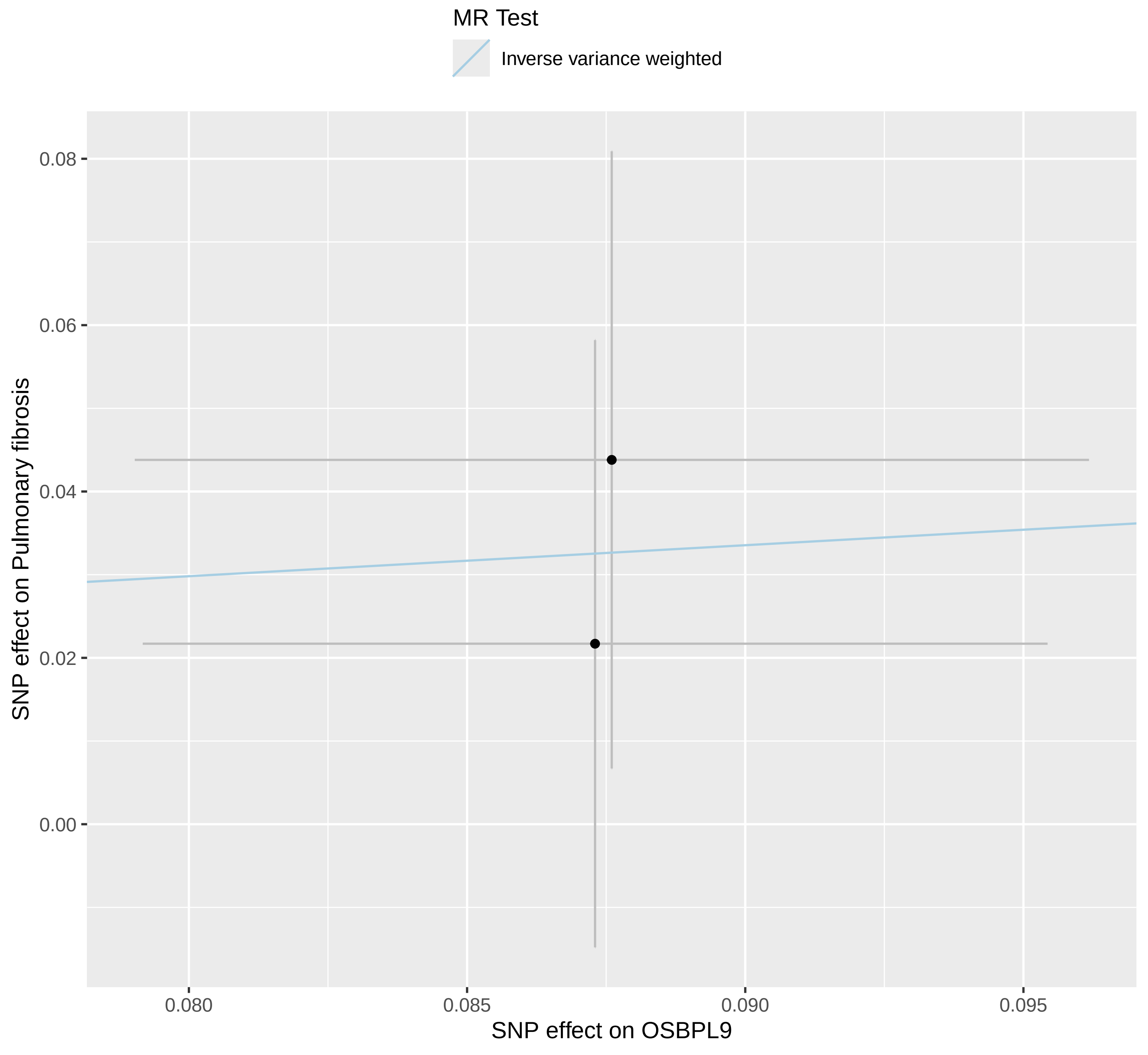

MR Test
Inverse variance weighted
0.08
0.06
0.04
0.02
0.00
SNP effect on Pulmonary fibrosis
0.080
0.085
0.090
0.095
SNP effect on OSBPL9

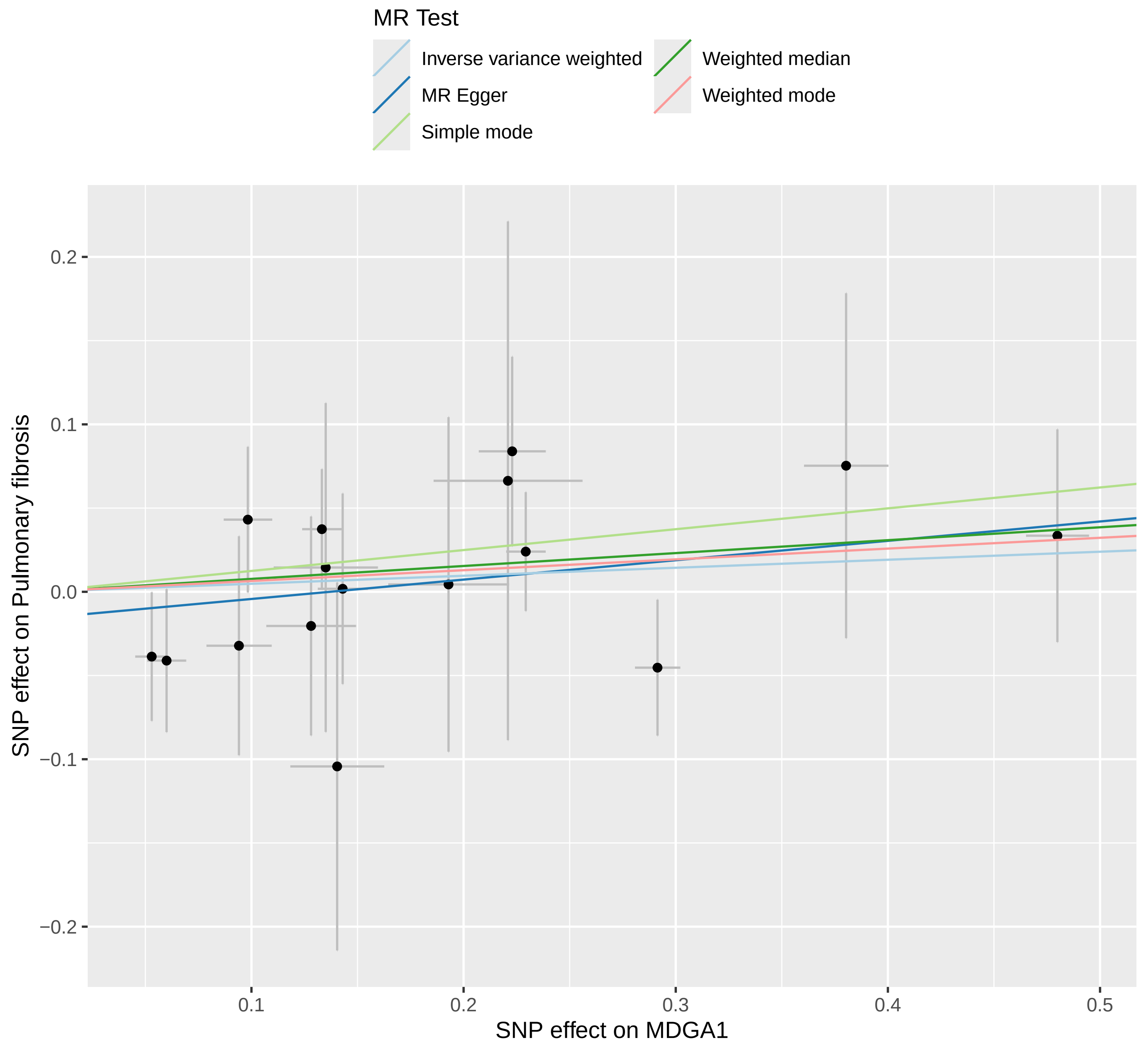

MR Test
Inverse variance weighted
MR Egger
Simple mode
Weighted median
Weighted mode
0.2
0.1
0.0
−0.1
−0.2
SNP effect on Pulmonary fibrosis
0.1
0.2
0.3
0.4
0.5
SNP effect on MDGA1

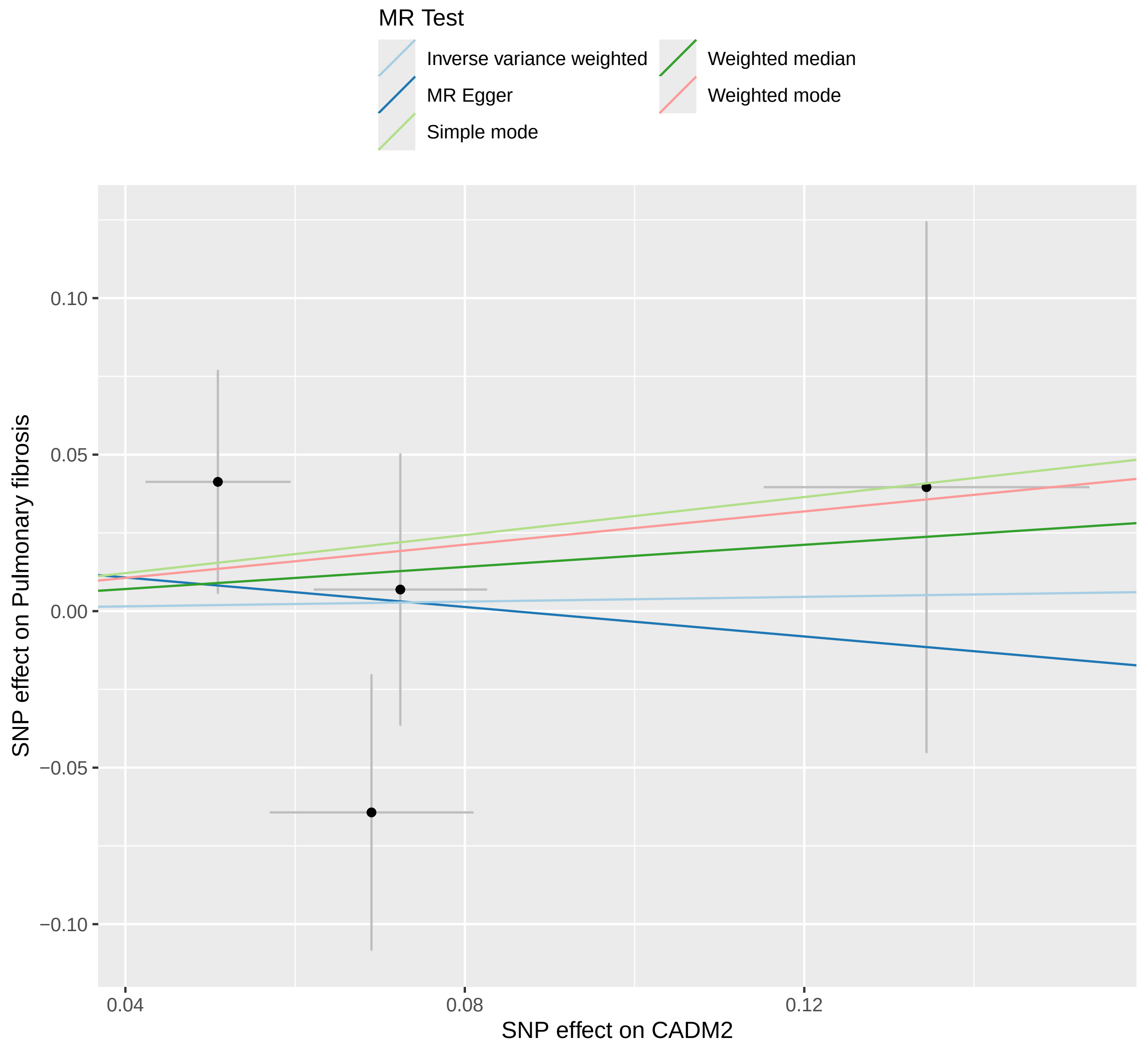

MR Test
Inverse variance weighted
MR Egger
Simple mode
Weighted median
Weighted mode
0.10
0.05
0.00
−0.05
−0.10
SNP effect on Pulmonary fibrosis
0.04
0.08
0.12
SNP effect on CADM2

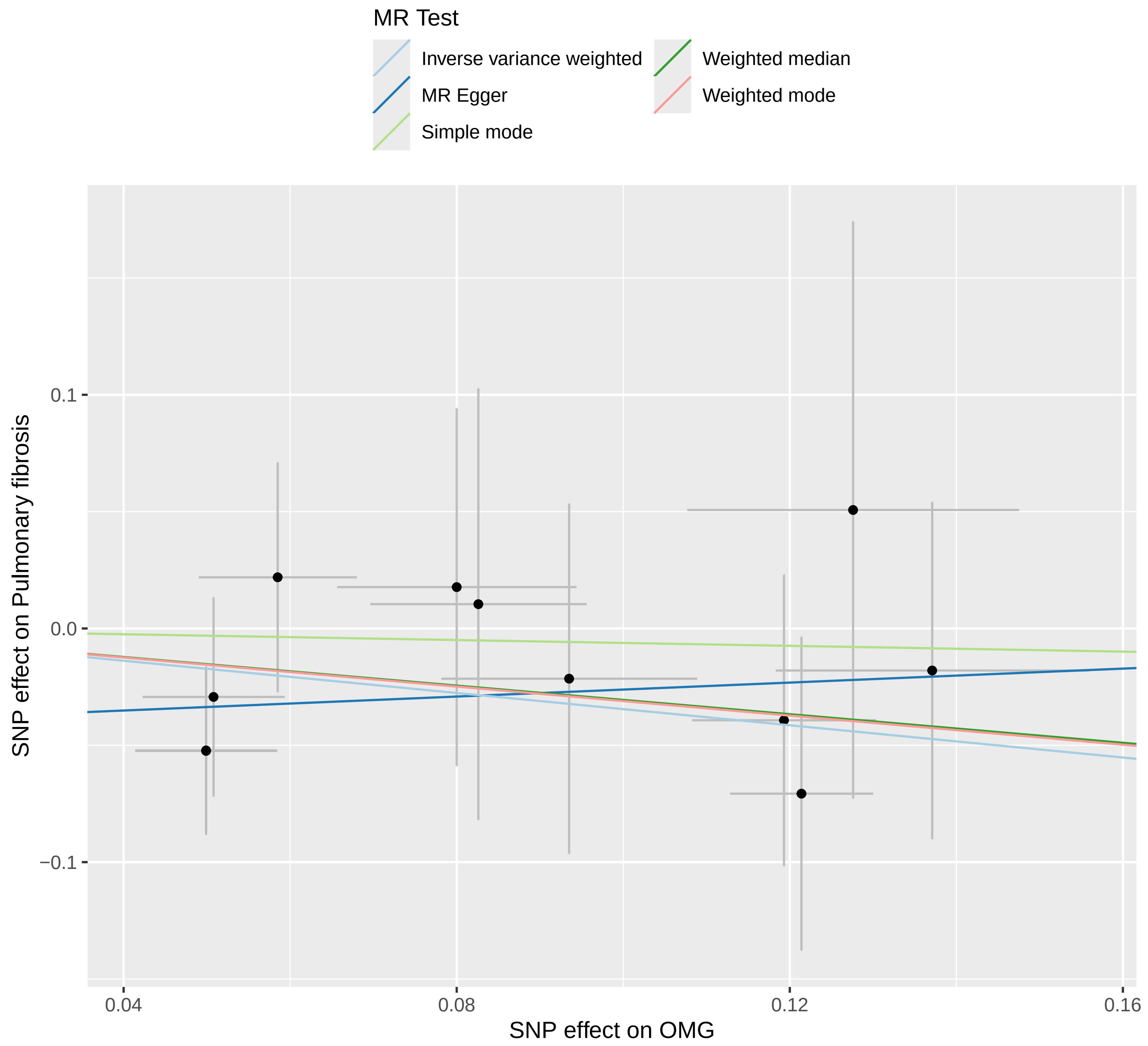
MR Test
Inverse variance weighted
MR Egger
Simple mode
Weighted median
Weighted mode
0.1
0.0
−0.1
0.04
0.08
0.12
0.16
SNP effect on Pulmonary fibrosis
SNP effect on OMG

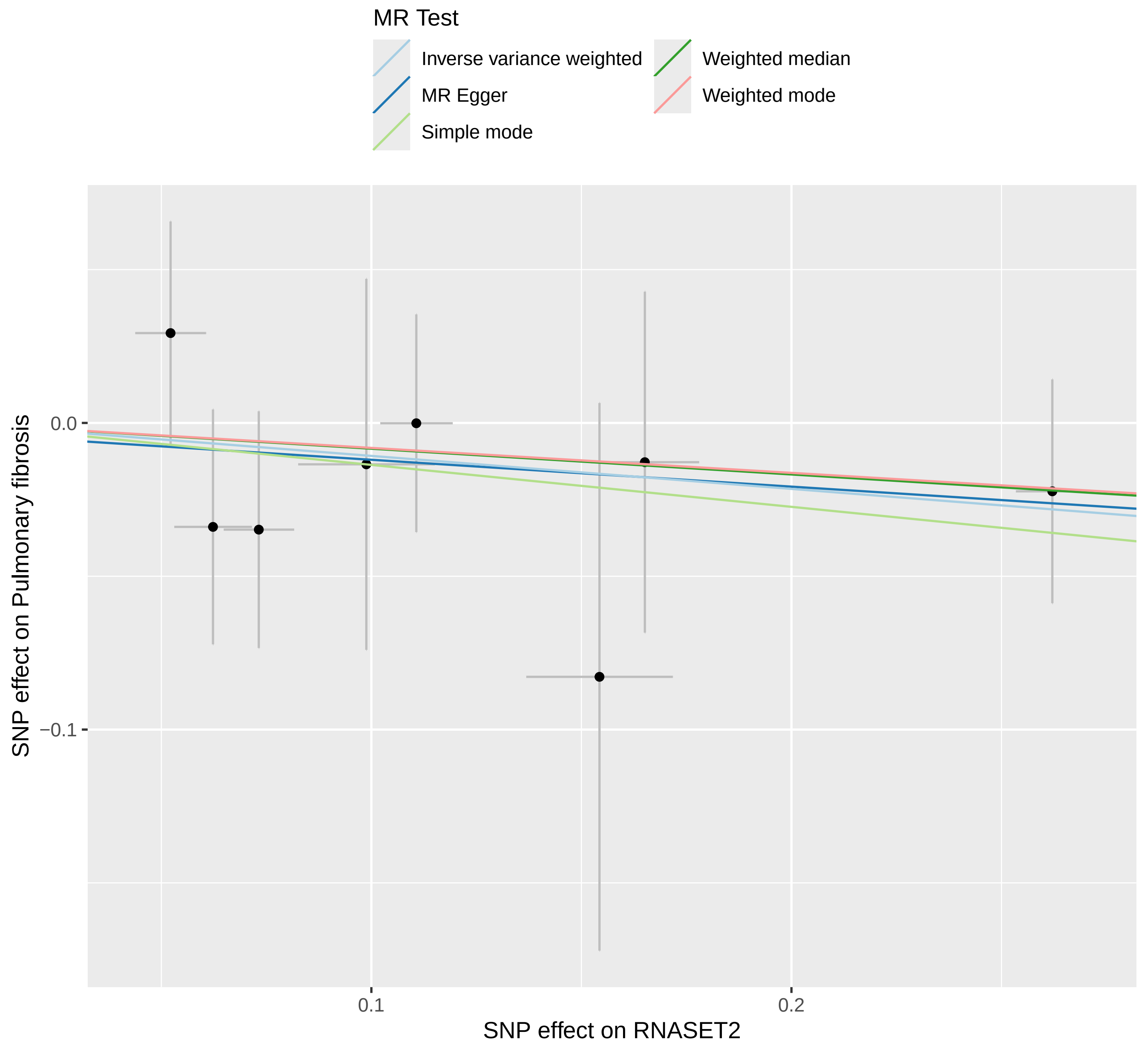
MR Test
Inverse variance weighted
MR Egger
Simple mode
Weighted median
Weighted mode
SNP effect on Pulmonary fibrosis
0.0
−0.1
0.1
0.2
SNP effect on RNASET2

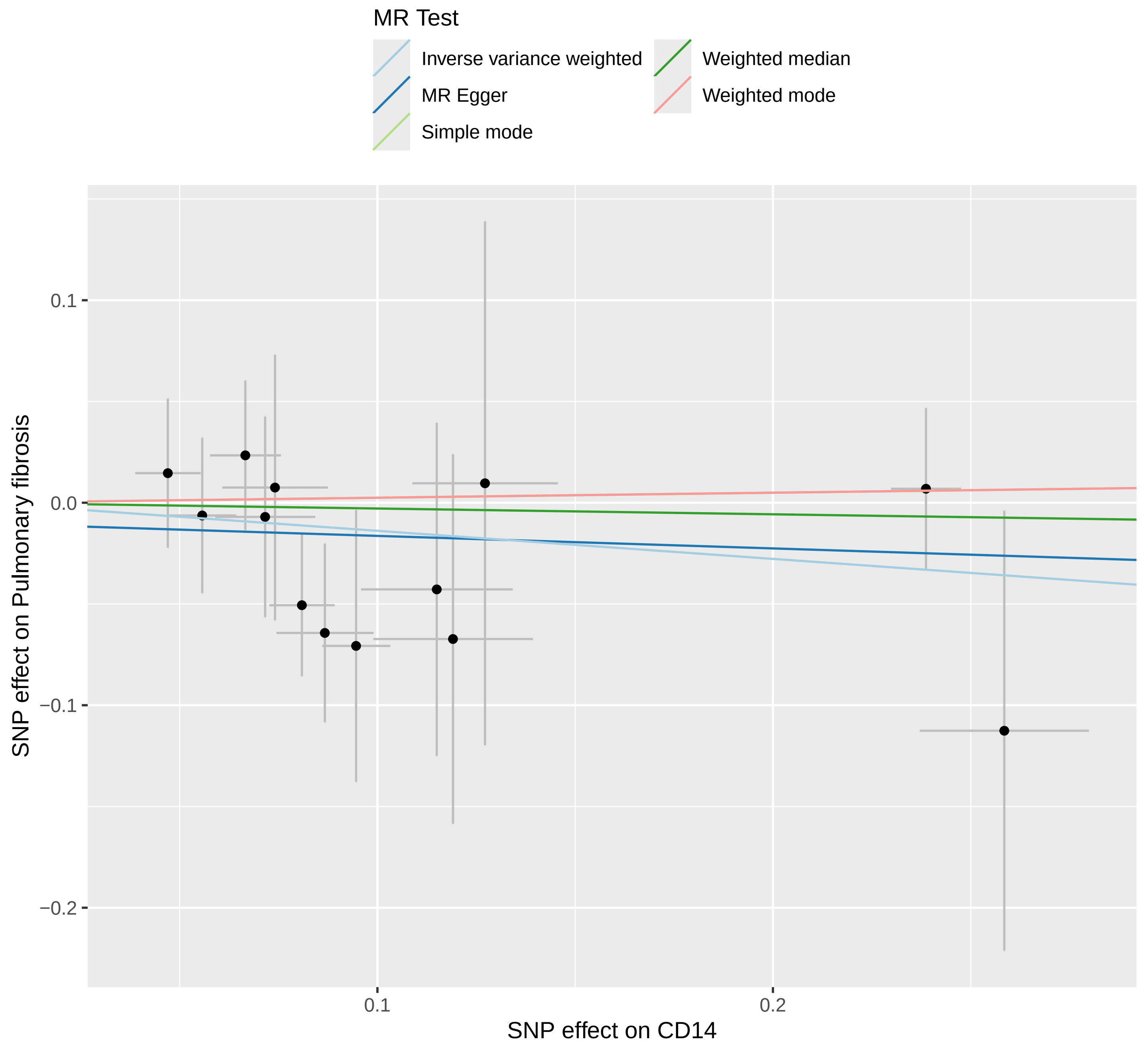

MR Test
Inverse variance weighted
MR Egger
Simple mode
Weighted median
Weighted mode
0.1
0.0
−0.1
−0.2
SNP effect on Pulmonary fibrosis
0.1
0.2
SNP effect on CD14

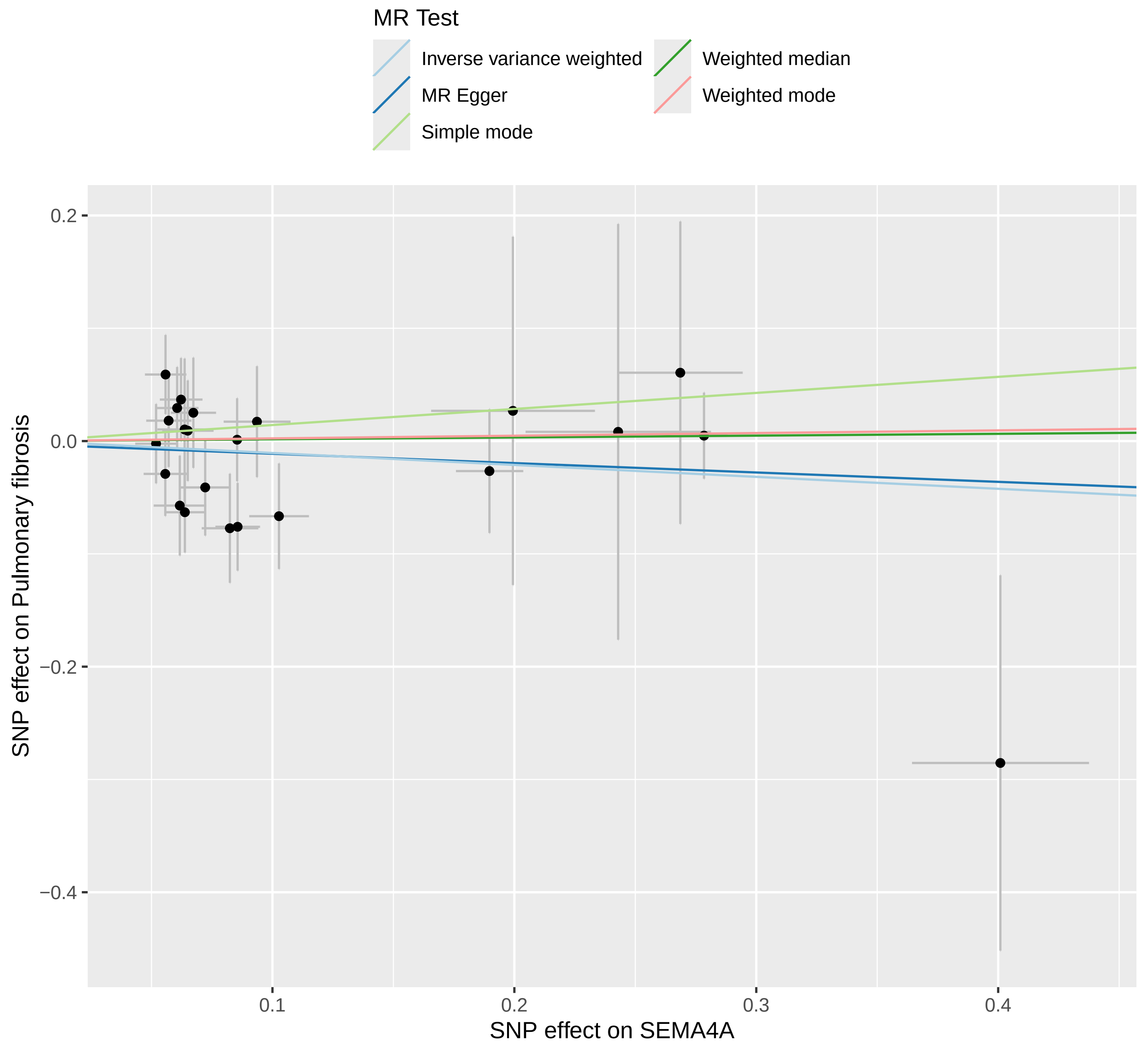
MR Test
Inverse variance weighted
MR Egger
Simple mode
Weighted median
Weighted mode
0.2
0.0
−0.2
−0.4
SNP effect on Pulmonary fibrosis
0.1
0.2
0.3
0.4
SNP effect on SEMA4A

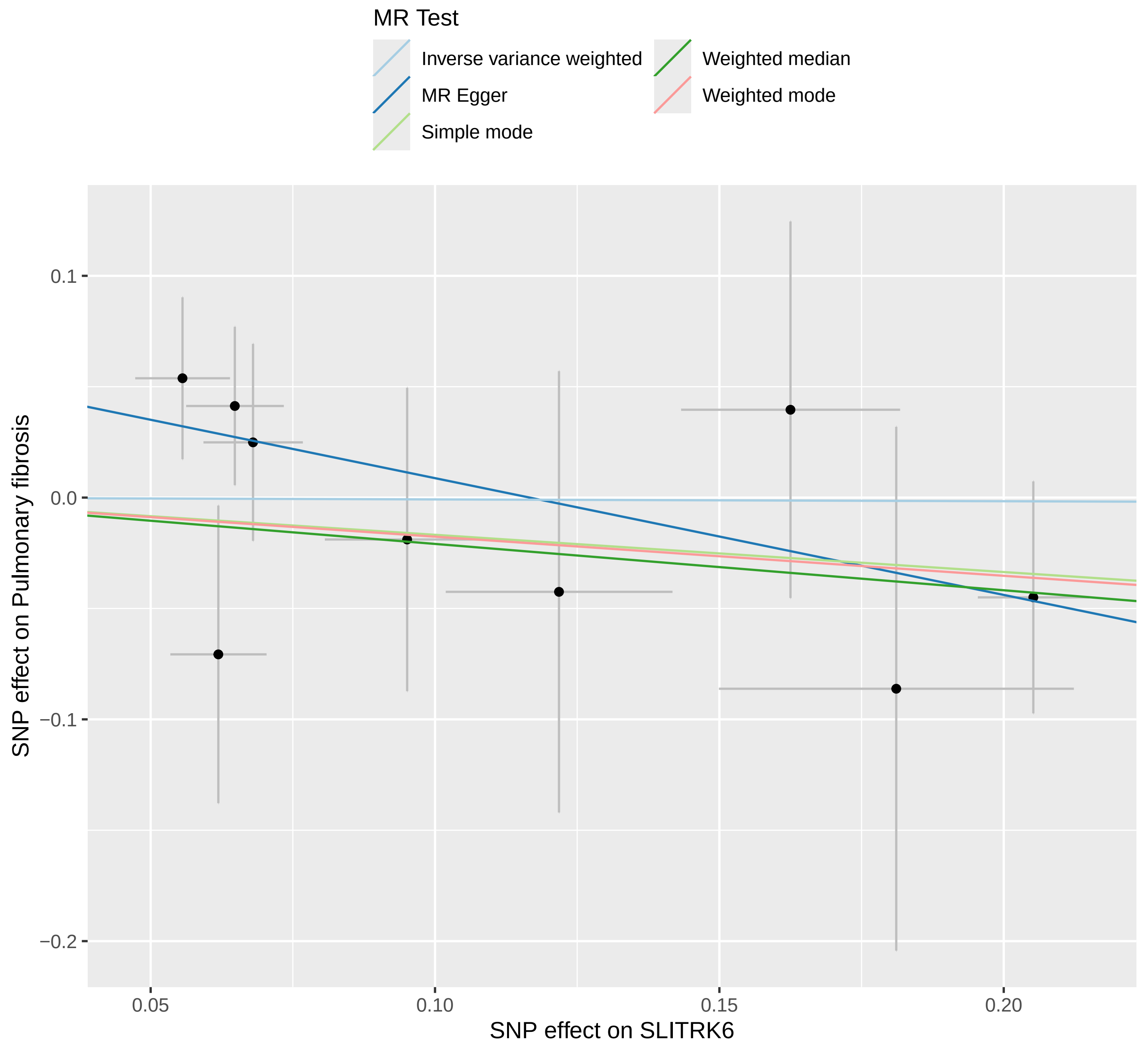
MR Test
Inverse variance weighted
MR Egger
Simple mode
Weighted median
Weighted mode
SNP effect on Pulmonary fibrosis
0.1
0.0
−0.1
−0.2
0.05
0.10
0.15
0.20
SNP effect on SLITRK6

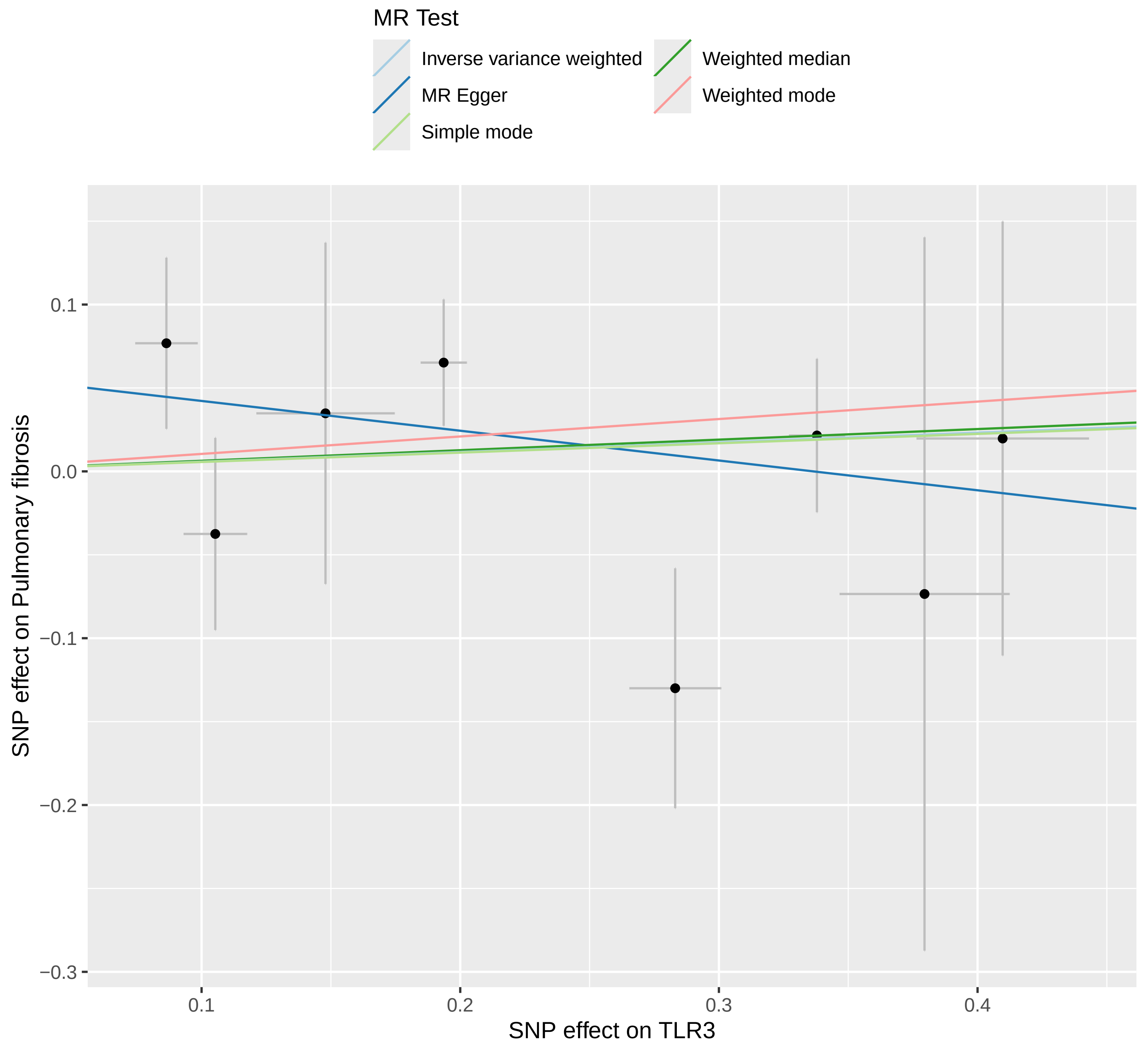
MR Test
Inverse variance weighted
MR Egger
Simple mode
Weighted median
Weighted mode
0.1
0.0
−0.1
−0.2
−0.3
0.1
0.2
0.3
0.4
SNP effect on Pulmonary fibrosis
SNP effect on TLR3

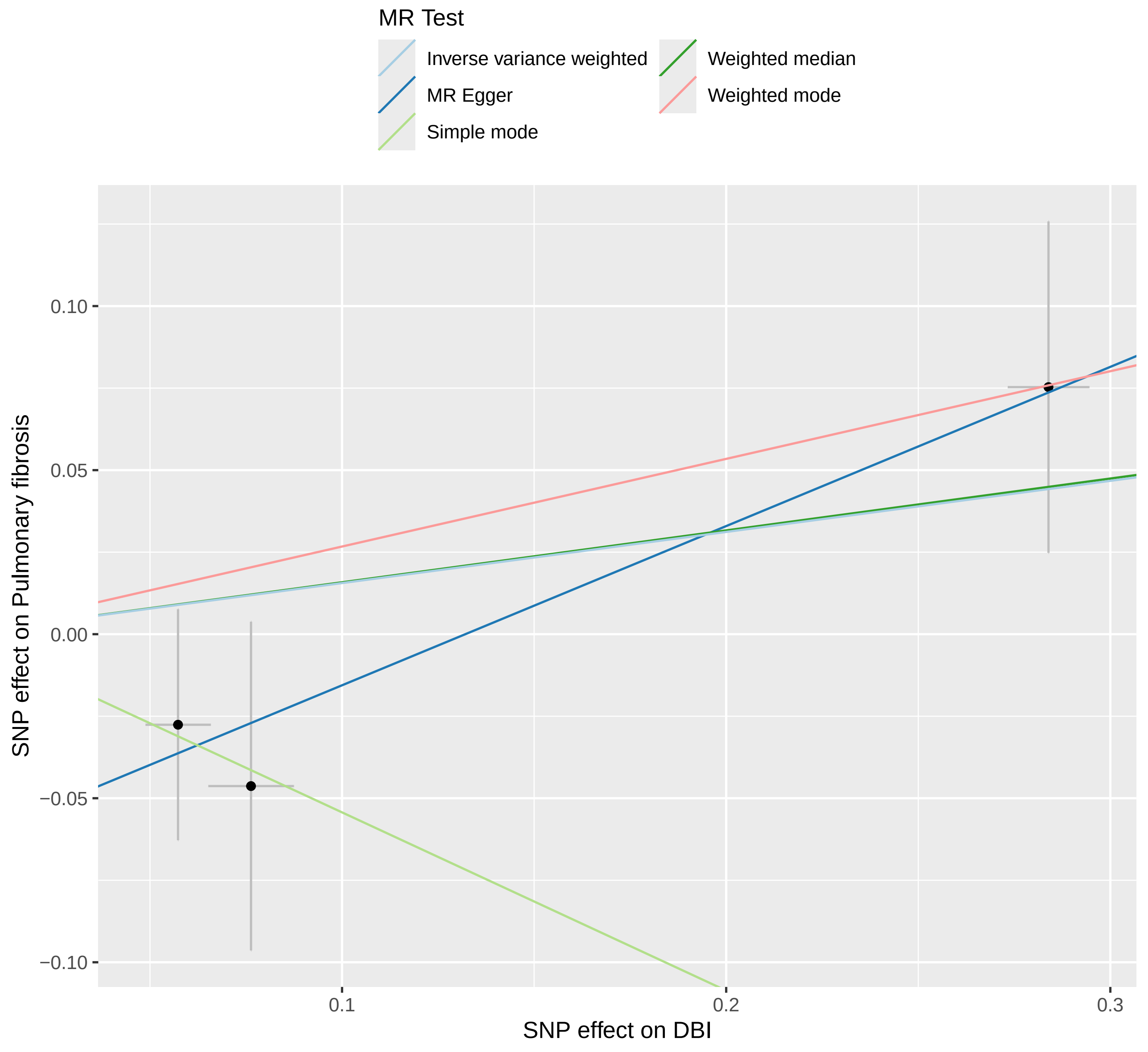
MR Test
Inverse variance weighted
MR Egger
Simple mode
Weighted median
Weighted mode
0.10
0.05
0.00
−0.05
−0.10
0.1
0.2
0.3
SNP effect on Pulmonary fibrosis
SNP effect on DBI

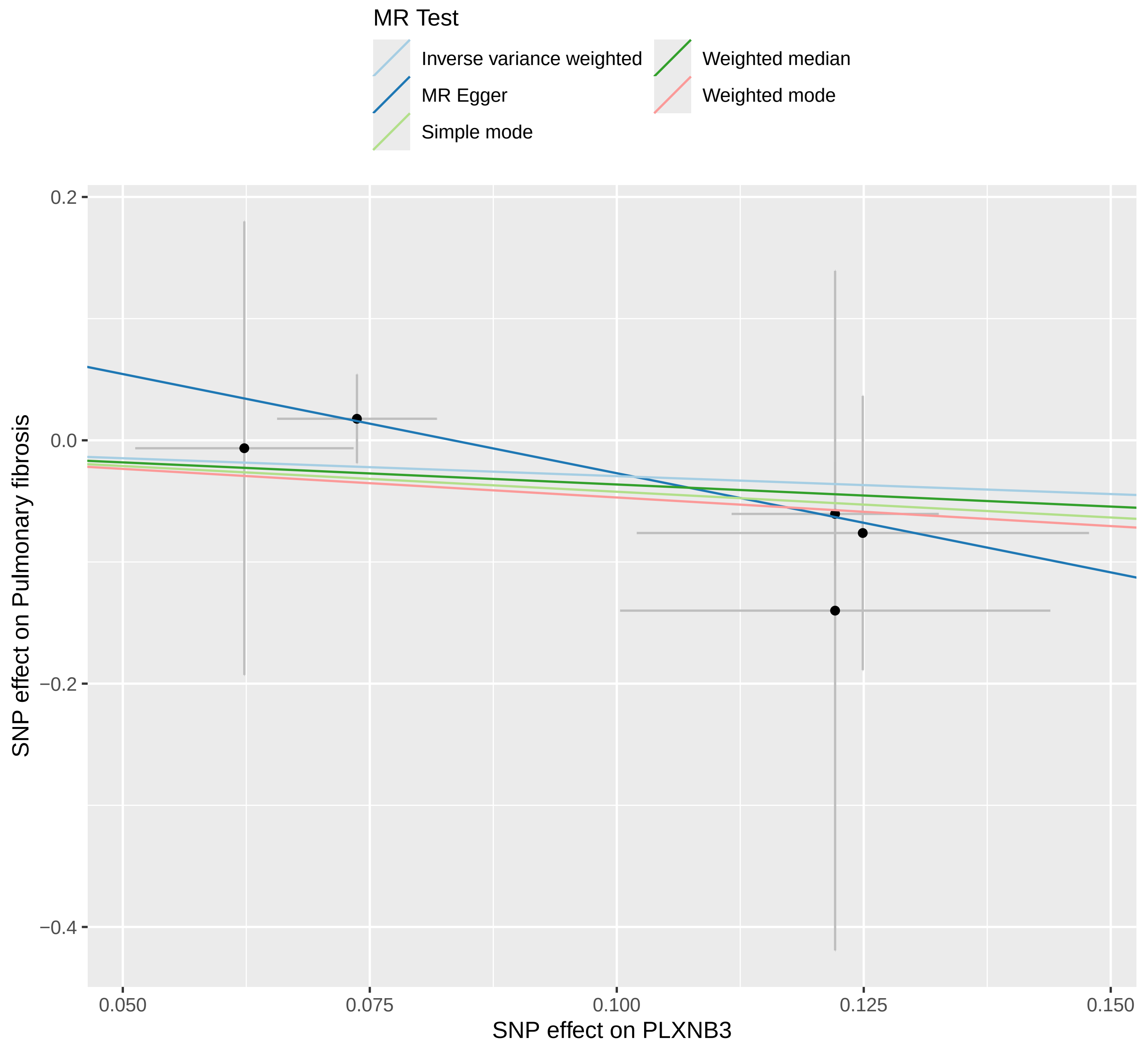

MR Test
Inverse variance weighted
MR Egger
Simple mode
Weighted median
Weighted mode
0.2
0.0
−0.2
−0.4
0.050
0.075
0.100
0.125
0.150
SNP effect on Pulmonary fibrosis
SNP effect on PLXNB3

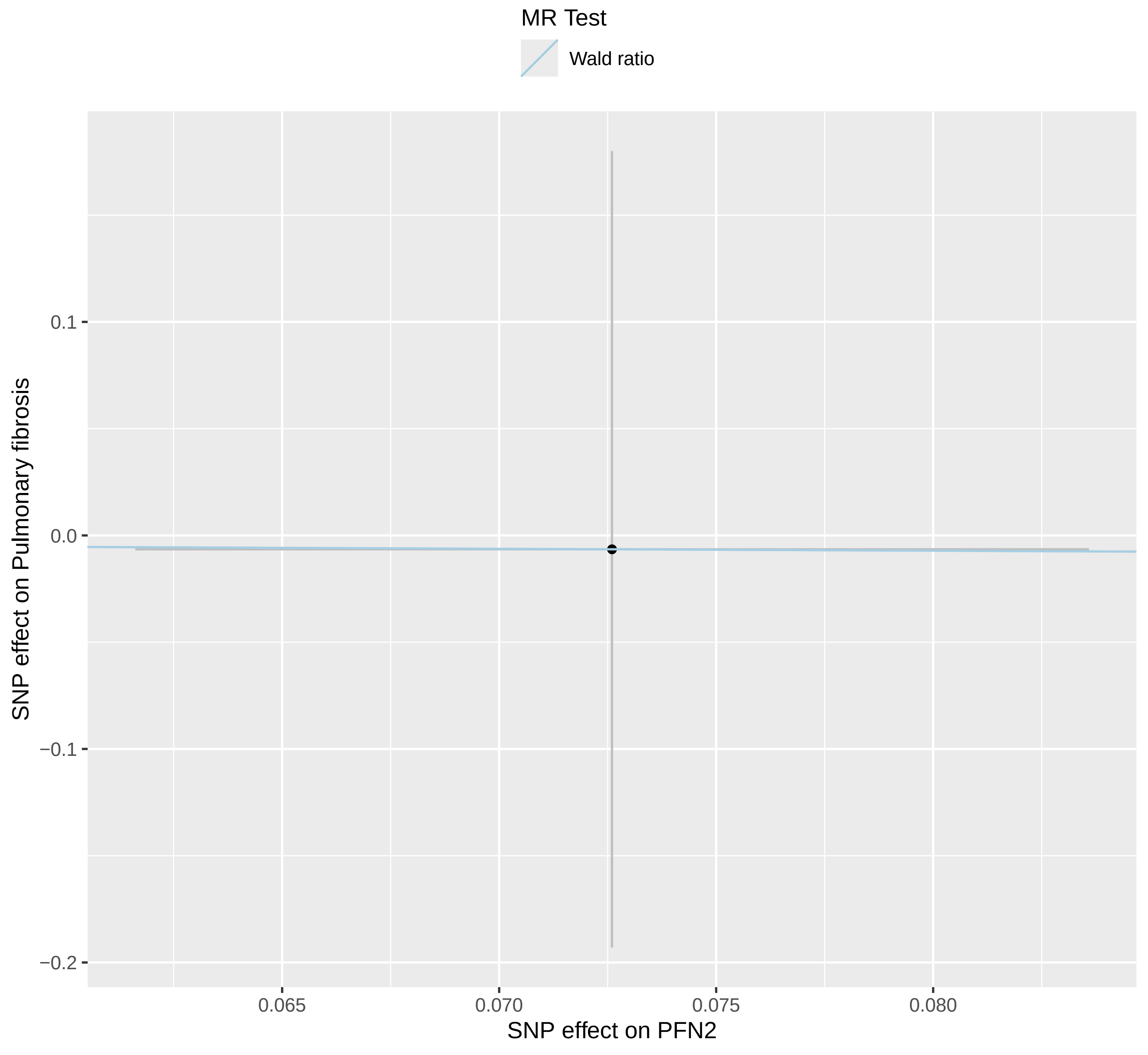

MR Test
Wald ratio
0.1
0.0
−0.1
−0.2
SNP effect on Pulmonary fibrosis
0.065
0.070
0.075
0.080
SNP effect on PFN2

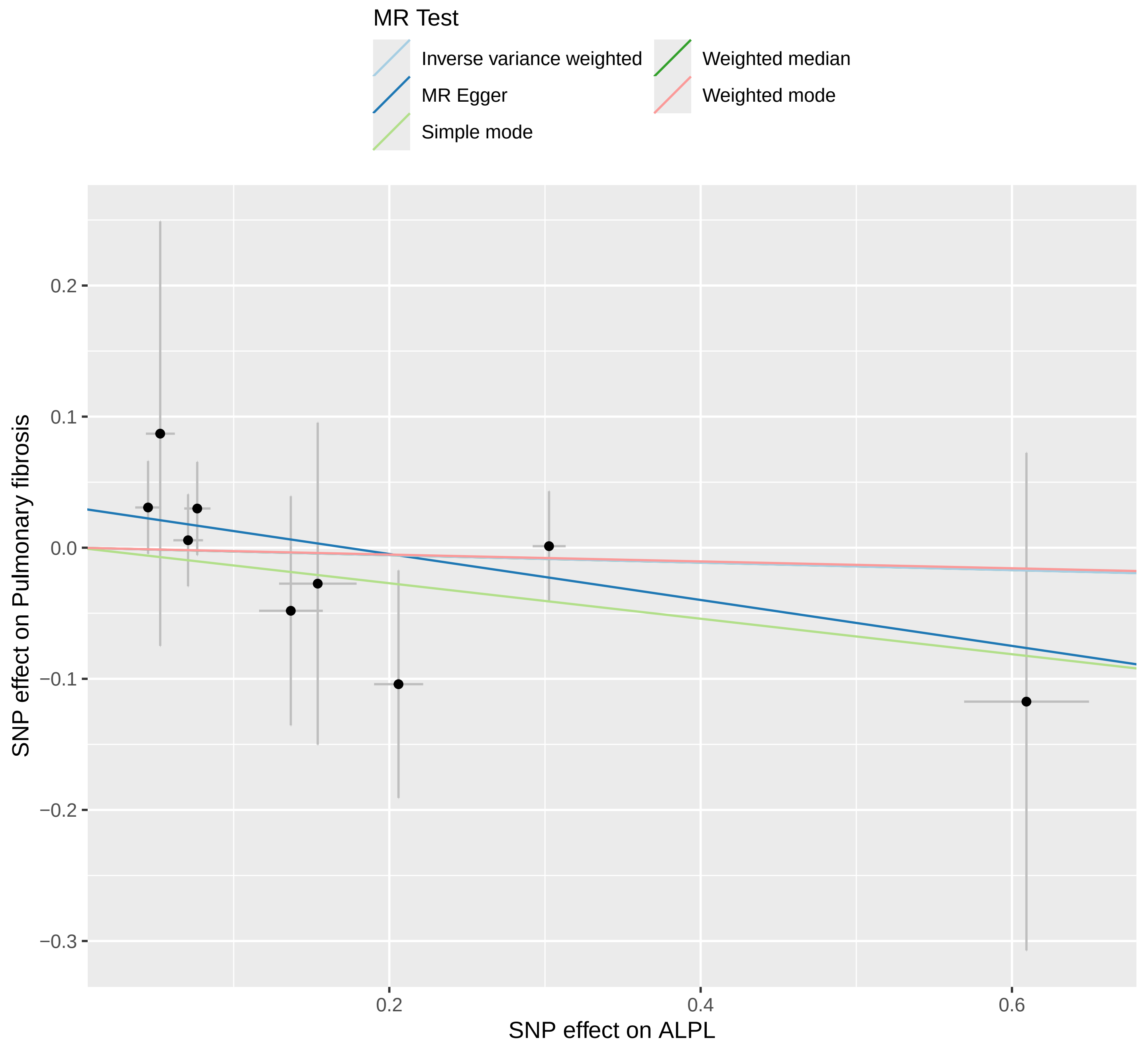

MR Test
Inverse variance weighted
MR Egger
Simple mode
Weighted median
Weighted mode
0.2
0.1
0.0
−0.1
−0.2
−0.3
SNP effect on Pulmonary fibrosis
0.2
0.4
0.6
SNP effect on ALPL

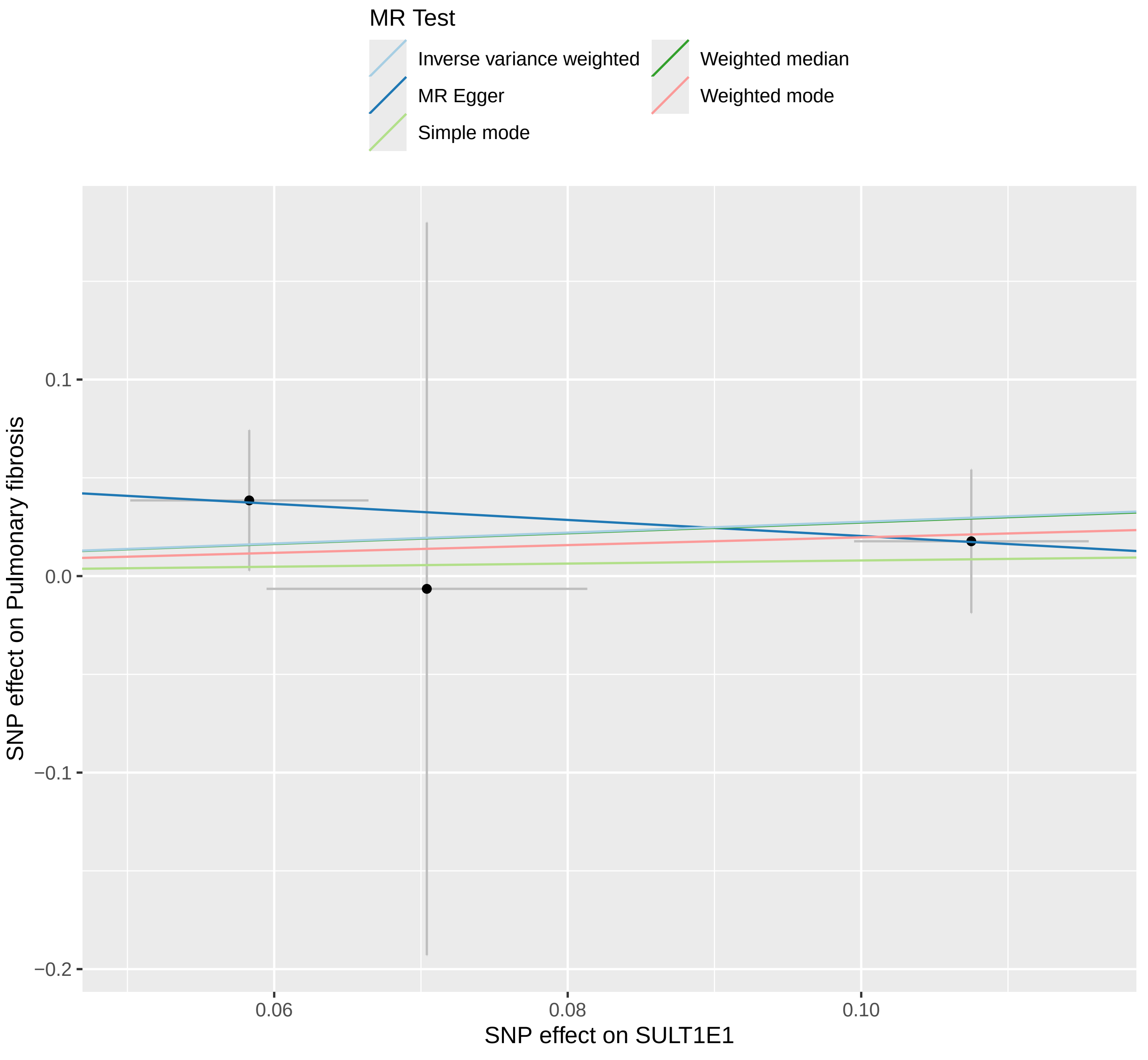

MR Test
Inverse variance weighted
MR Egger
Simple mode
Weighted median
Weighted mode
0.1
0.0
−0.1
−0.2
SNP effect on Pulmonary fibrosis
0.06
0.08
0.10
SNP effect on SULT1E1

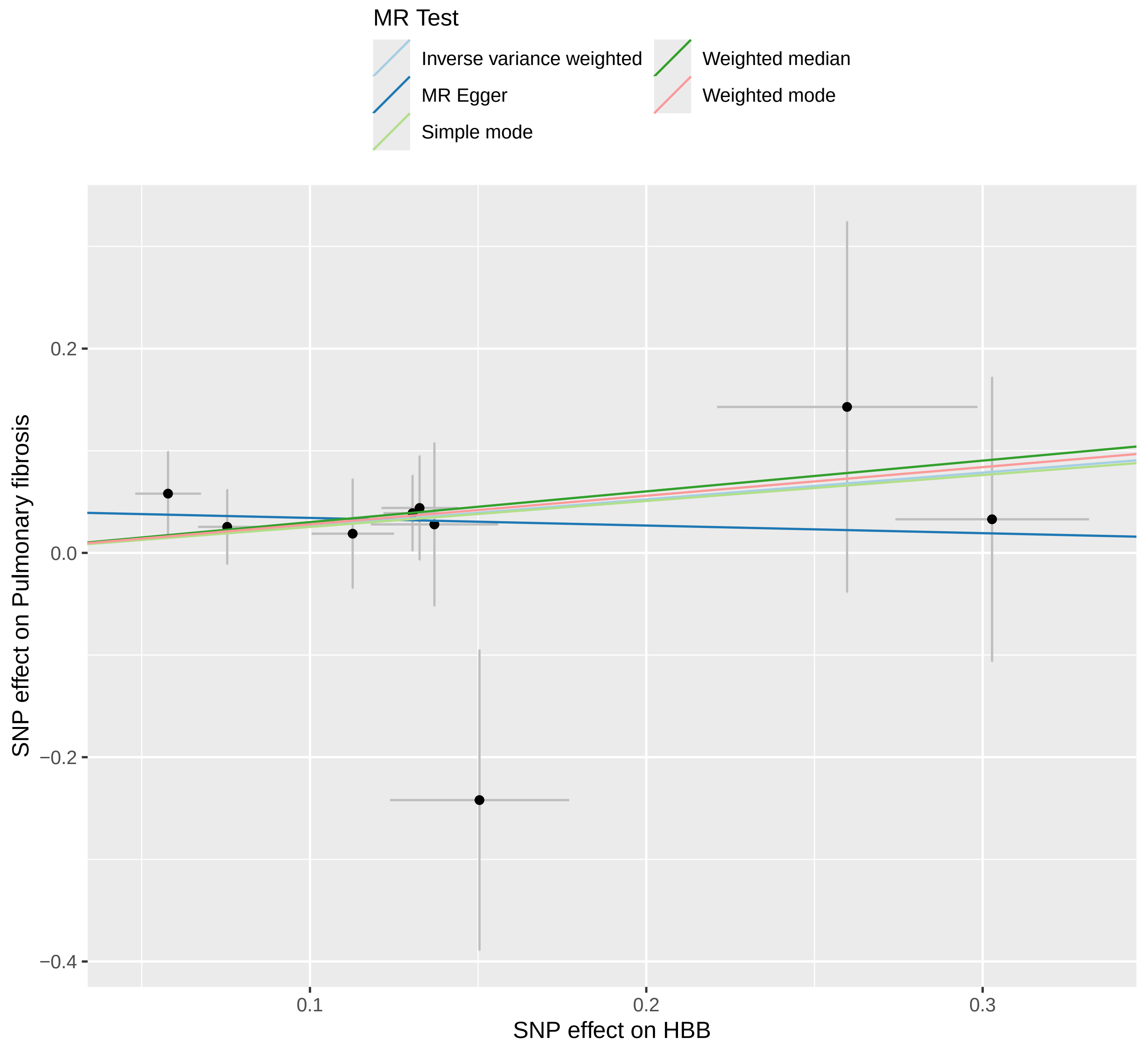
MR Test
Inverse variance weighted
MR Egger
Simple mode
Weighted median
Weighted mode
SNP effect on Pulmonary fibrosis
0.2
0.0
−0.2
−0.4
0.1
0.2
0.3
SNP effect on HBB

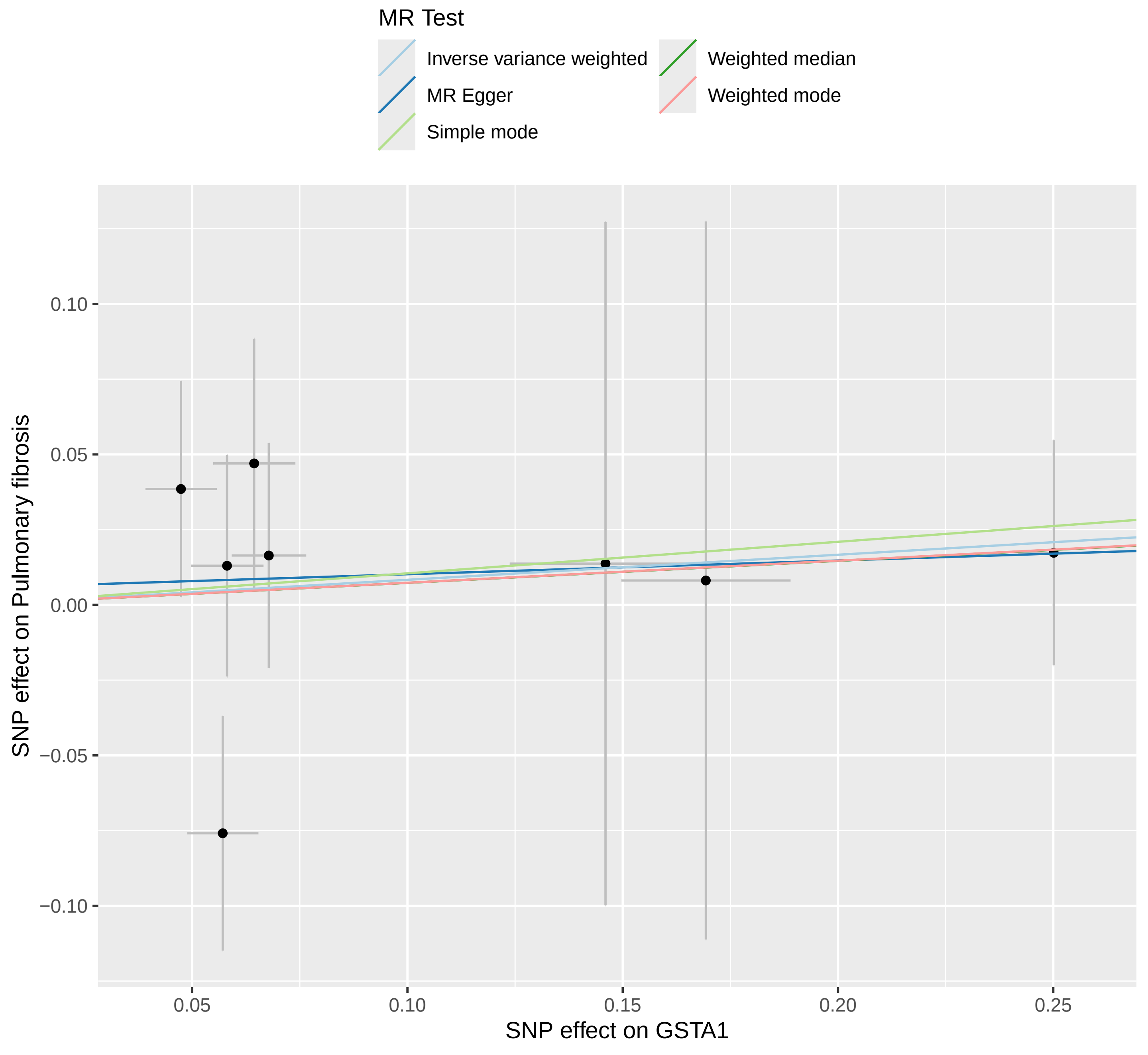

MR Test
Inverse variance weighted
MR Egger
Simple mode
Weighted median
Weighted mode
SNP effect on Pulmonary fibrosis
0.10
0.05
0.00
−0.05
−0.10
0.05
0.10
0.15
0.20
0.25
SNP effect on GSTA1

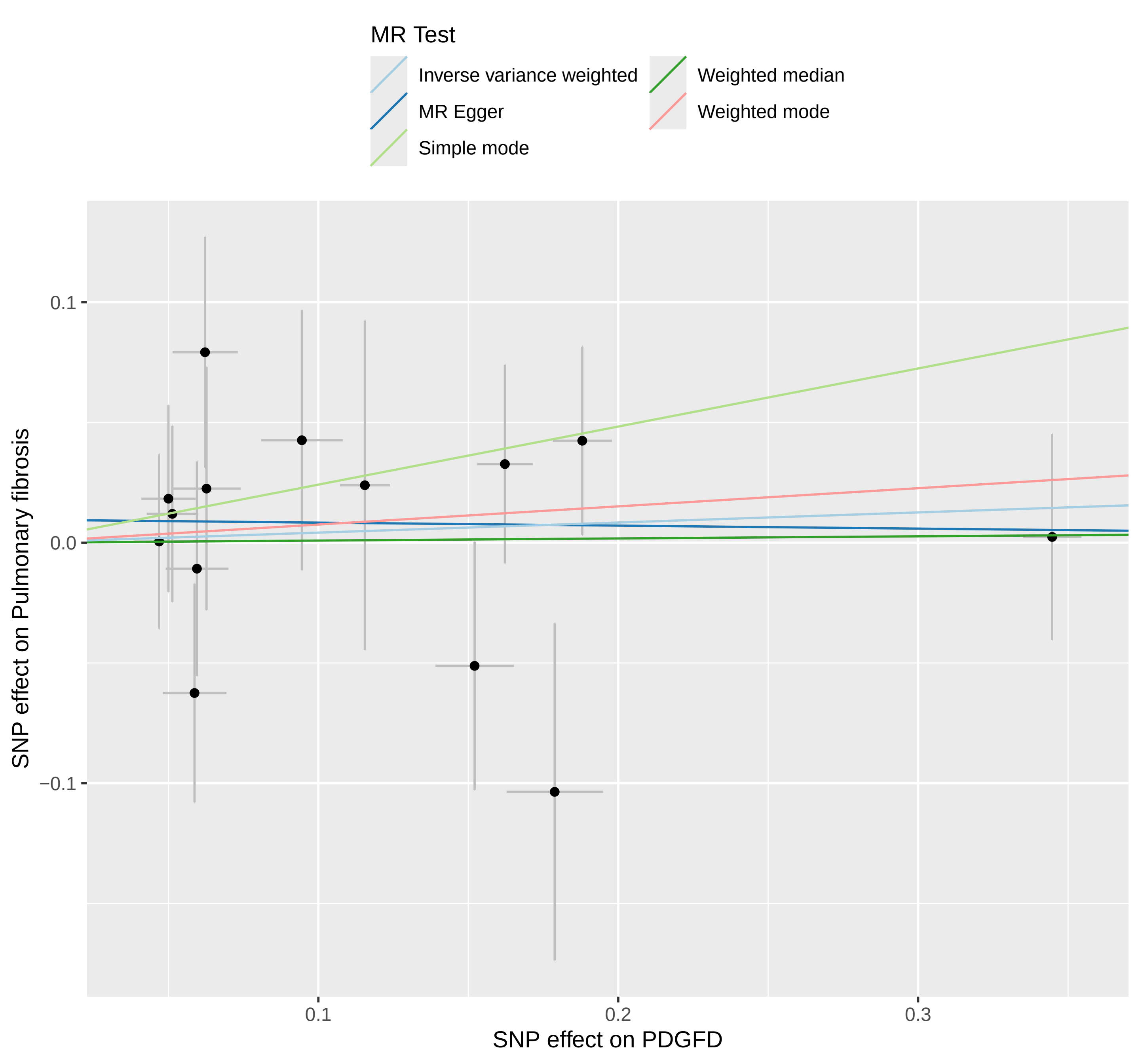

MR Test
Inverse variance weighted
MR Egger
Simple mode
Weighted median
Weighted mode
SNP effect on Pulmonary fibrosis
0.1
0.0
−0.1
0.1
0.2
0.3
SNP effect on PDGFD

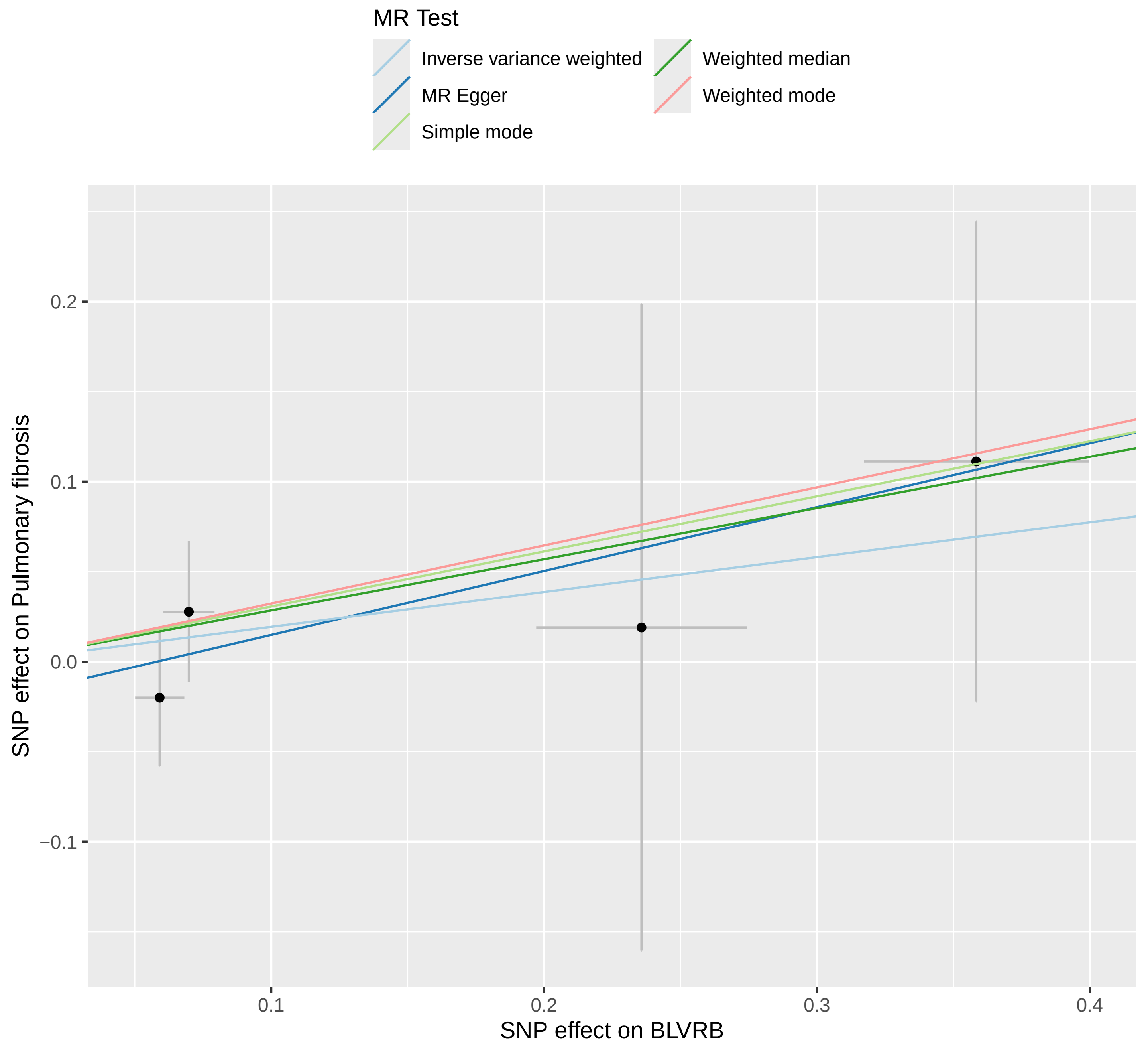

MR Test
Inverse variance weighted
MR Egger
Simple mode
Weighted median
Weighted mode
0.2
0.1
0.0
−0.1
0.1
0.2
0.3
0.4
SNP effect on Pulmonary fibrosis
SNP effect on BLVRB

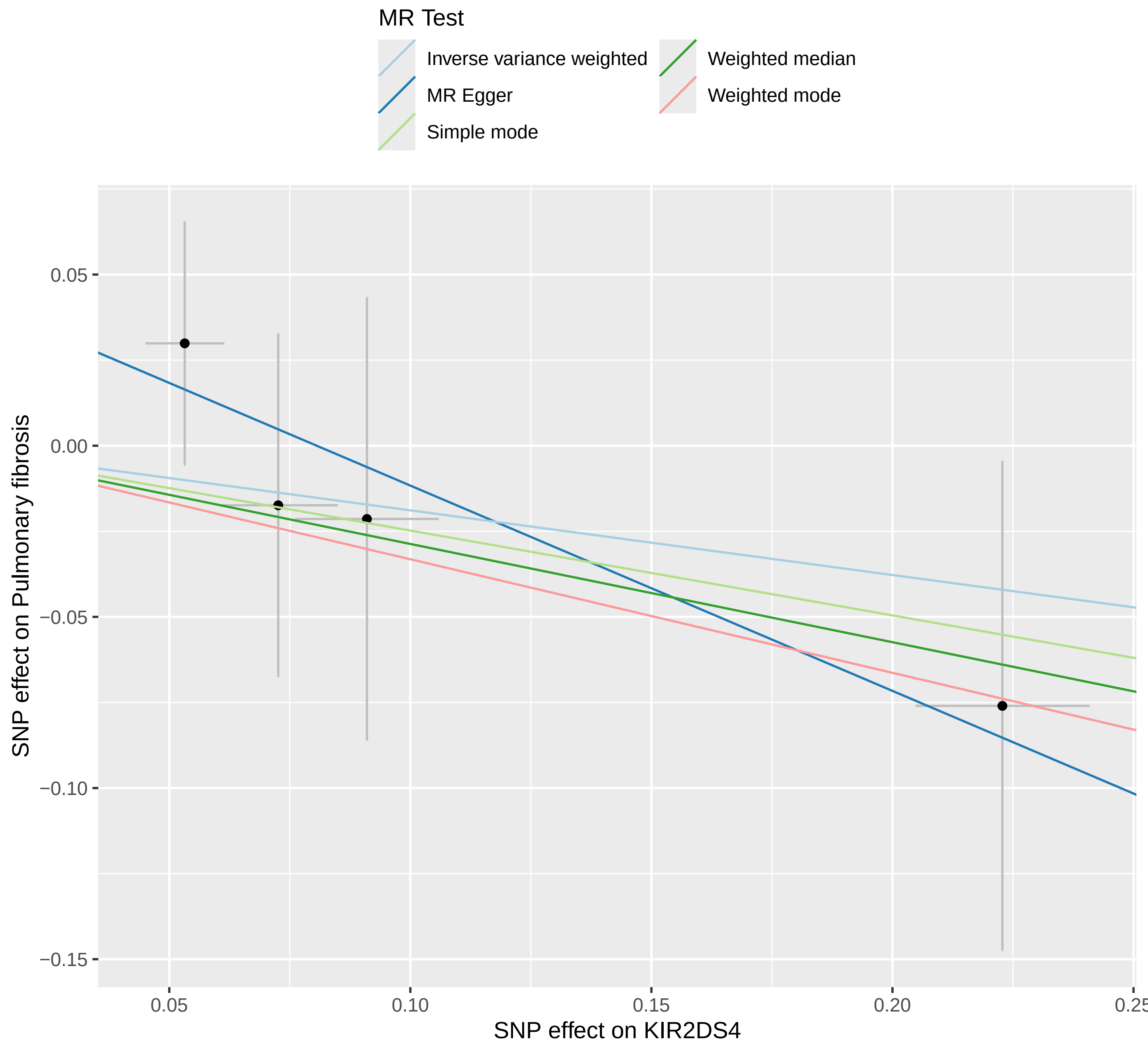
MR Test
Inverse variance weighted
MR Egger
Simple mode
Weighted median
Weighted mode
SNP effect on Pulmonary fibrosis
0.05
0.00
−0.05
−0.10
−0.15
0.05
0.10
0.15
0.20
0.25
SNP effect on KIR2DS4

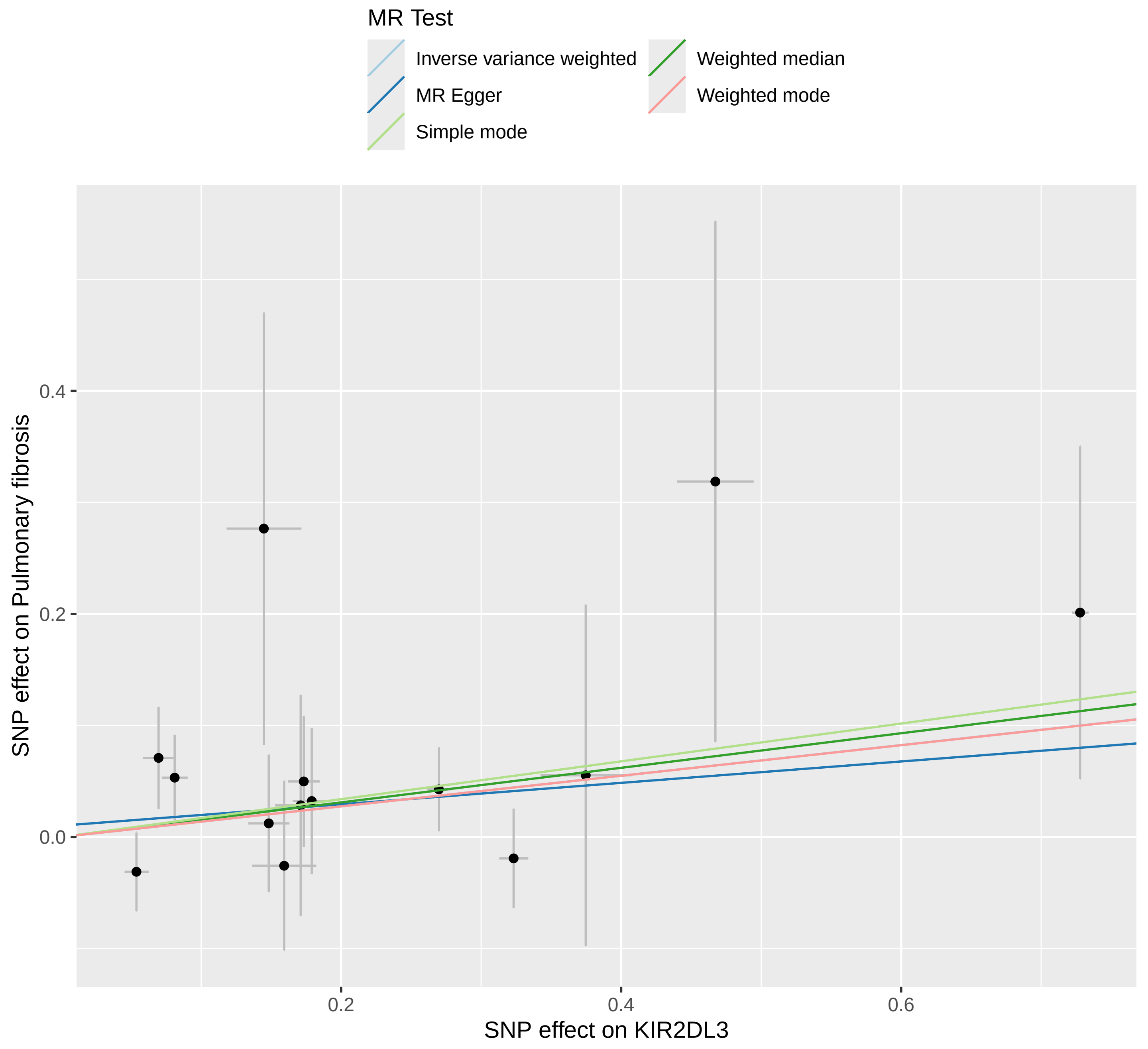

MR Test
Inverse variance weighted
MR Egger
Simple mode
Weighted median
Weighted mode
0.4
0.2
0.0
SNP effect on Pulmonary fibrosis
0.2
0.4
0.6
SNP effect on KIR2DL3

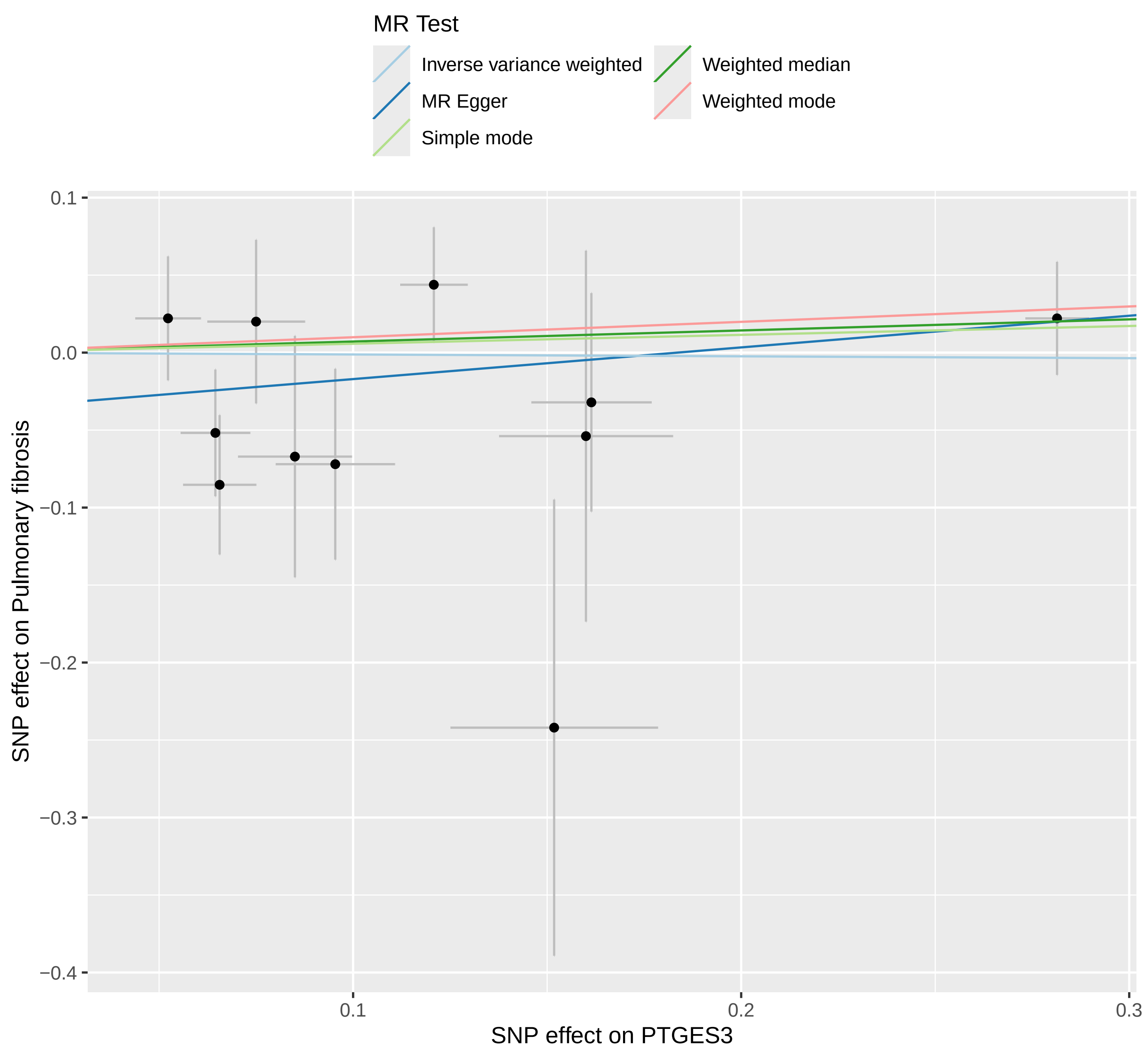

MR Test
Inverse variance weighted
MR Egger
Simple mode
Weighted median
Weighted mode
0.1
0.0
−0.1
−0.2
−0.3
−0.4
SNP effect on Pulmonary fibrosis
0.1
0.2
0.3
SNP effect on PTGES3

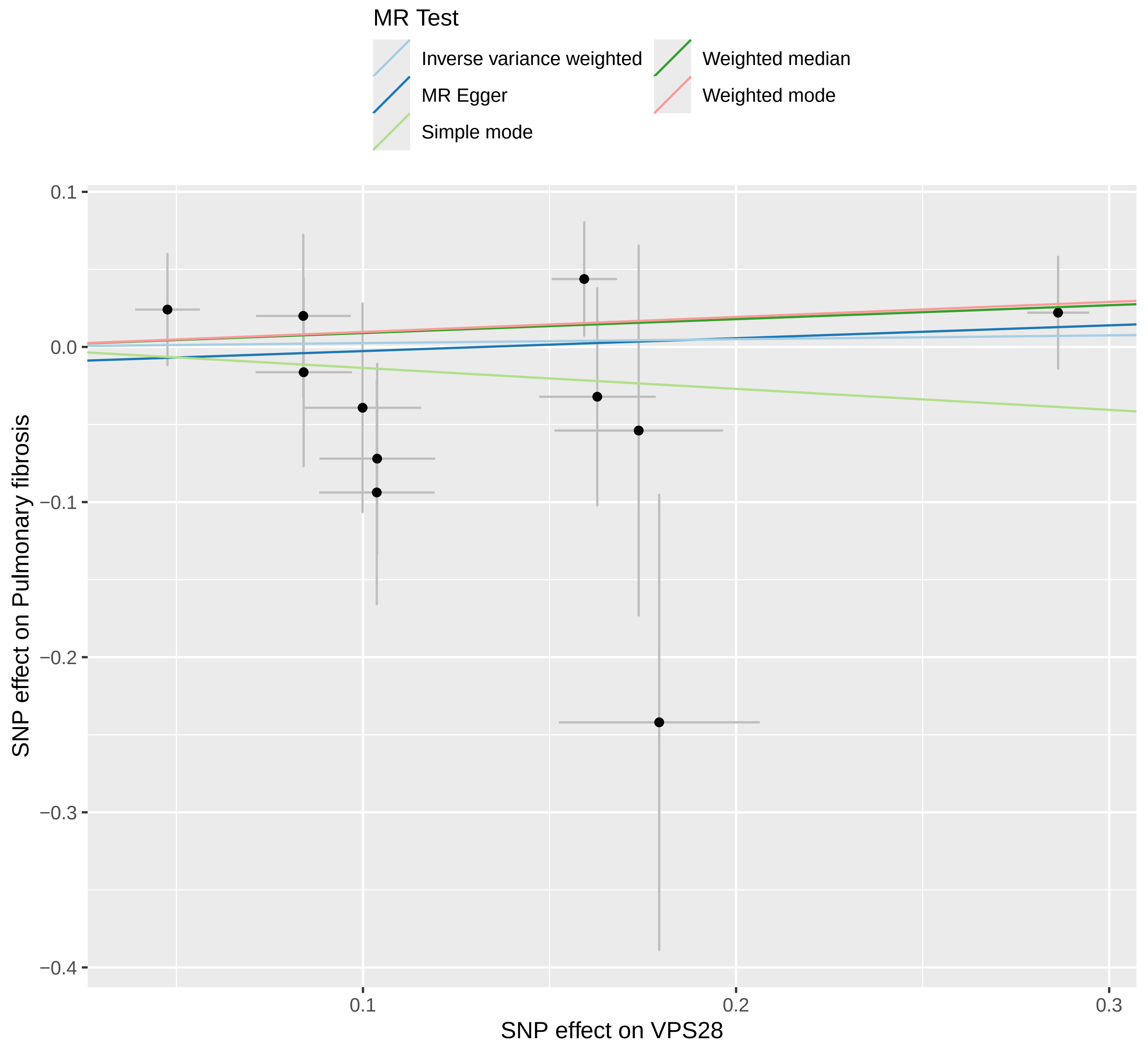

MR Test
Inverse variance weighted
MR Egger
Simple mode
Weighted median
Weighted mode
0.1
0.0
−0.1
−0.2
−0.3
−0.4
SNP effect on Pulmonary fibrosis
0.1
0.2
0.3
SNP effect on VPS28

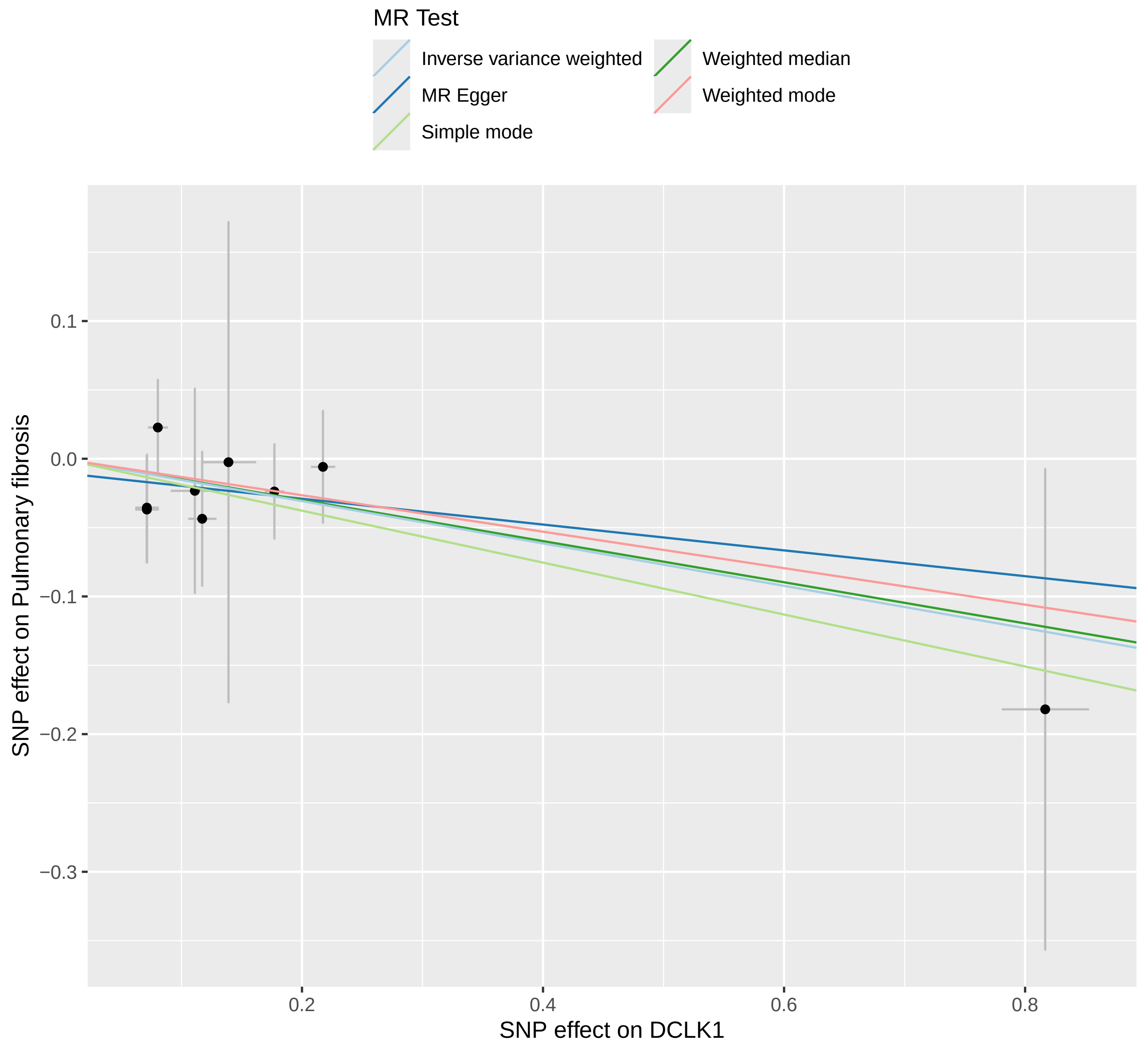

MR Test
Inverse variance weighted
MR Egger
Simple mode
Weighted median
Weighted mode
0.1
0.0
−0.1
−0.2
−0.3
SNP effect on Pulmonary fibrosis
0.2
0.4
0.6
0.8
SNP effect on DCLK1

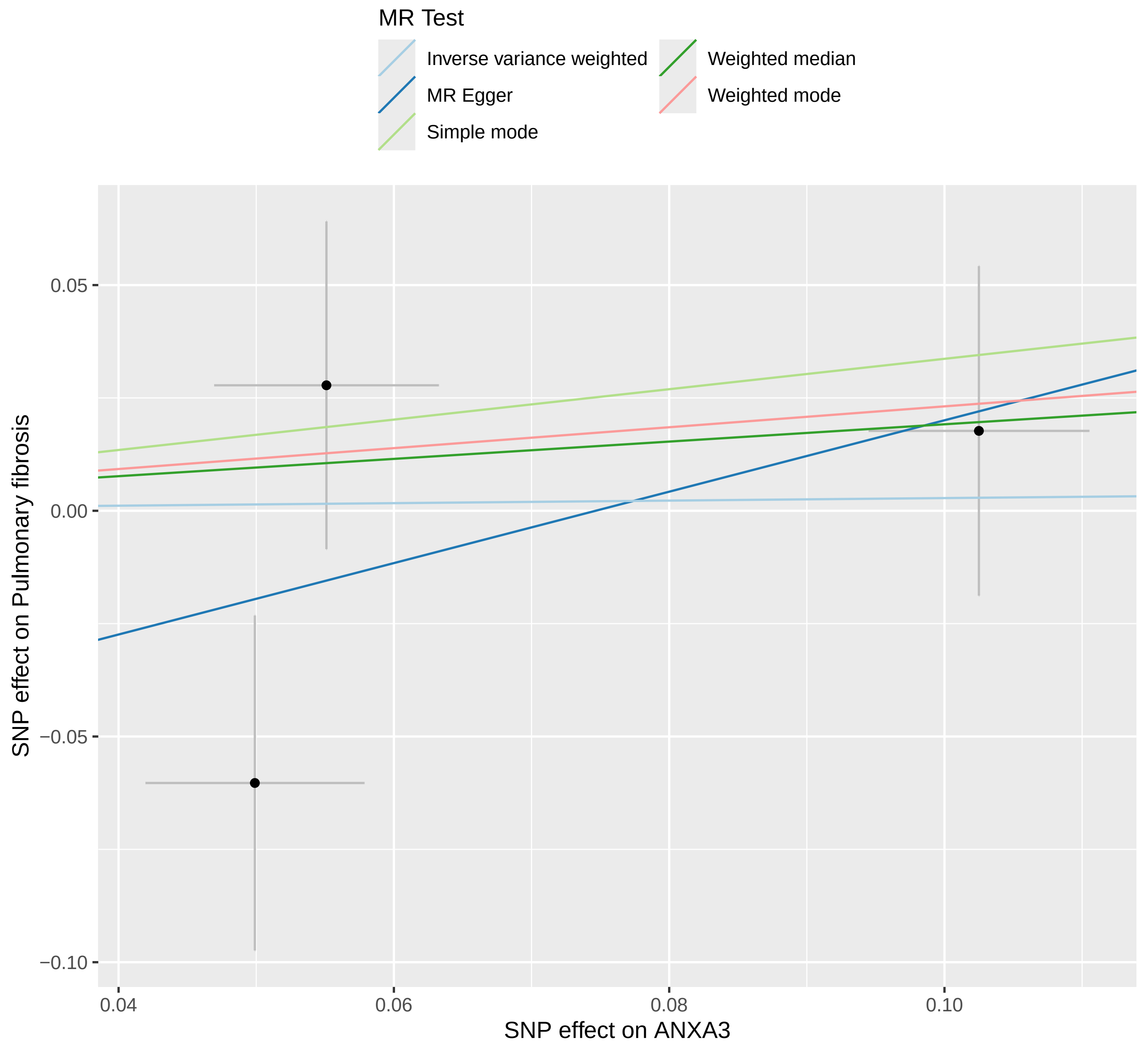
MR Test
Inverse variance weighted
MR Egger
Simple mode
Weighted median
Weighted mode
0.05
0.00
−0.05
−0.10
SNP effect on Pulmonary fibrosis
0.04
0.06
0.08
0.10
SNP effect on ANXA3

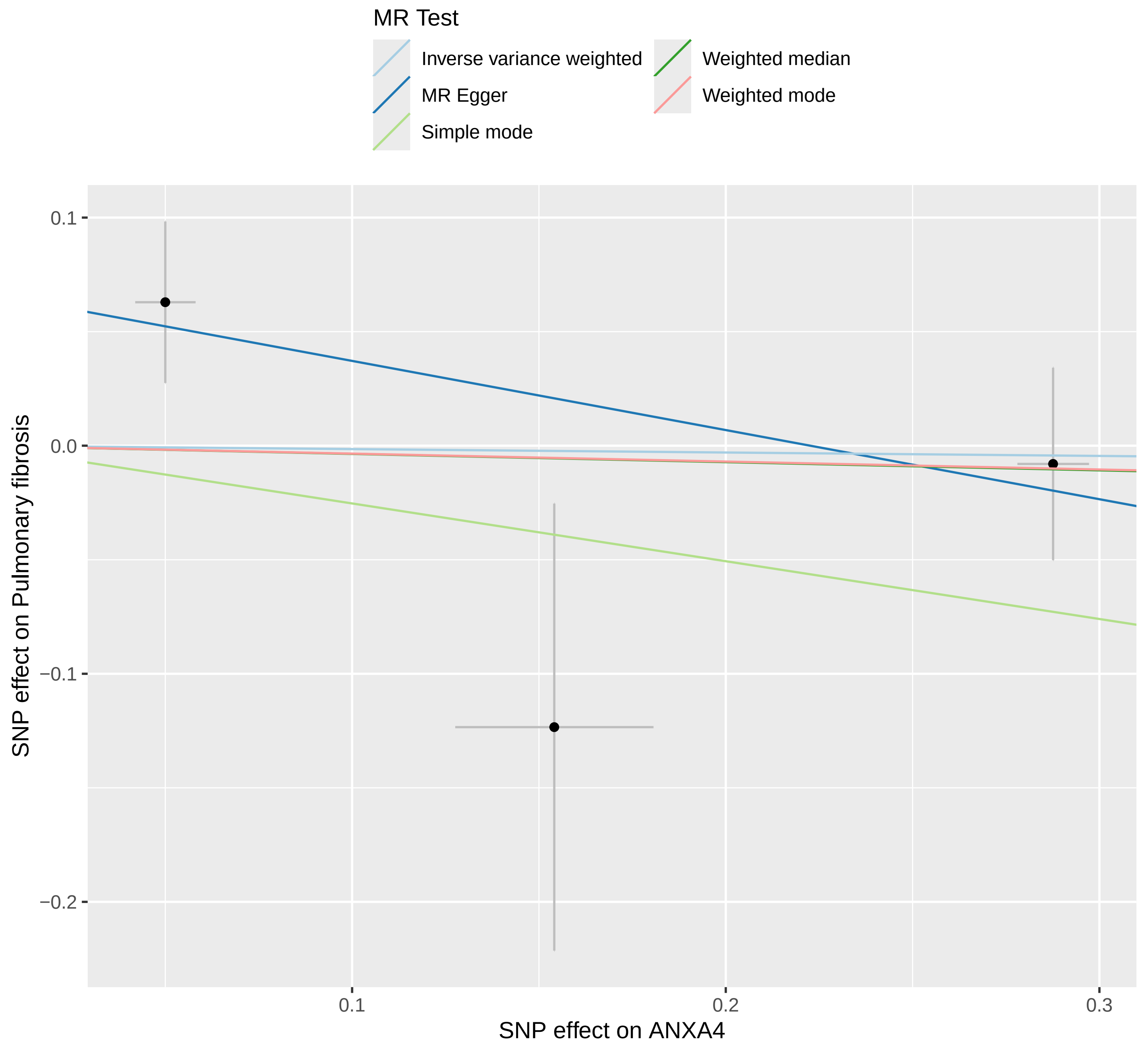

MR Test
Inverse variance weighted
MR Egger
Simple mode
Weighted median
Weighted mode
0.1
0.0
−0.1
−0.2
SNP effect on Pulmonary fibrosis
0.1
0.2
0.3
SNP effect on ANXA4

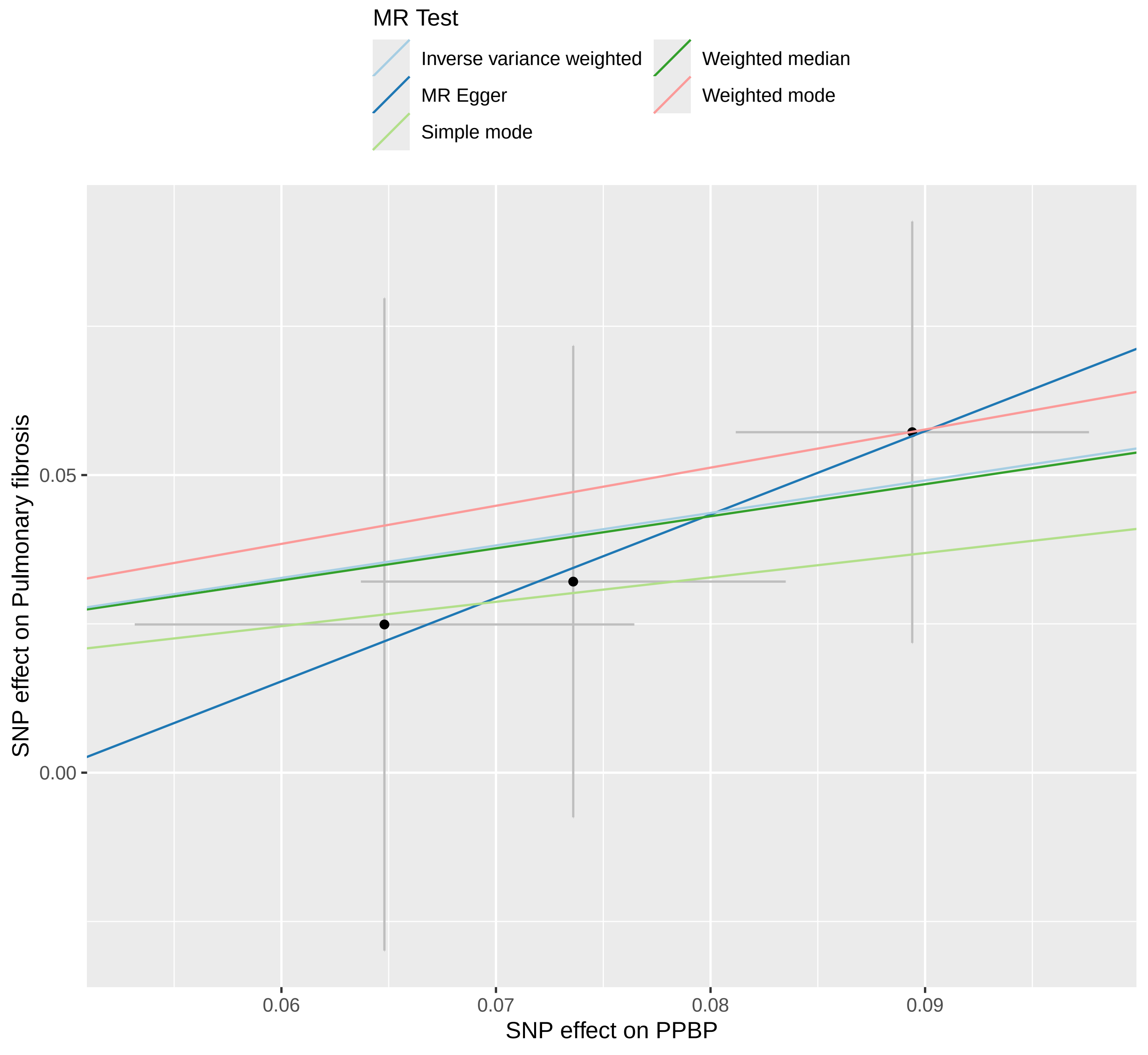
MR Test
Inverse variance weighted
MR Egger
Simple mode
Weighted median
Weighted mode
SNP effect on Pulmonary fibrosis
0.05
0.00
0.06
0.07
0.08
0.09
SNP effect on PPBP

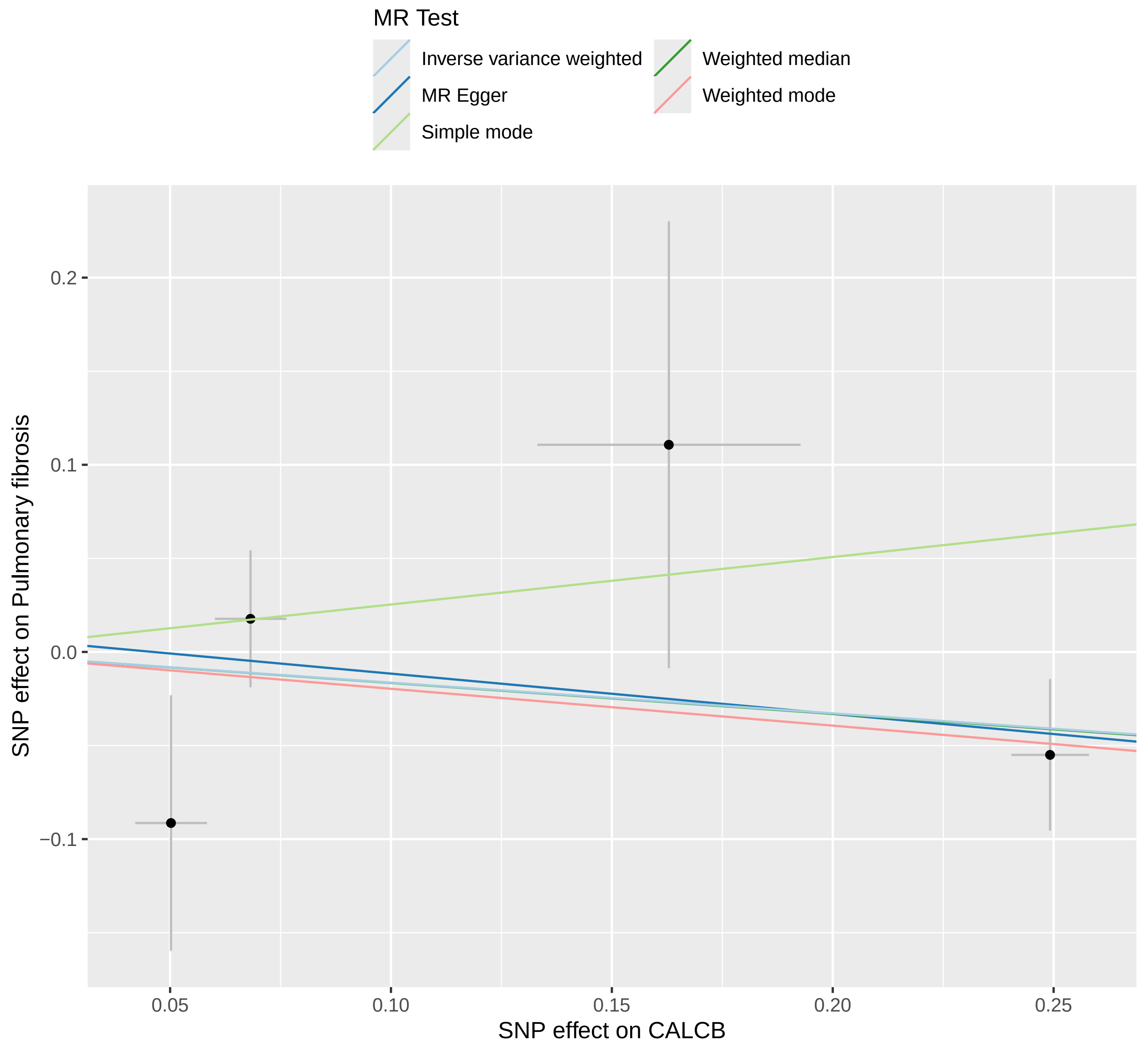

MR Test
Inverse variance weighted
MR Egger
Simple mode
Weighted median
Weighted mode
0.2
0.1
0.0
−0.1
0.05
0.10
0.15
0.20
0.25
SNP effect on Pulmonary fibrosis
SNP effect on CALCB

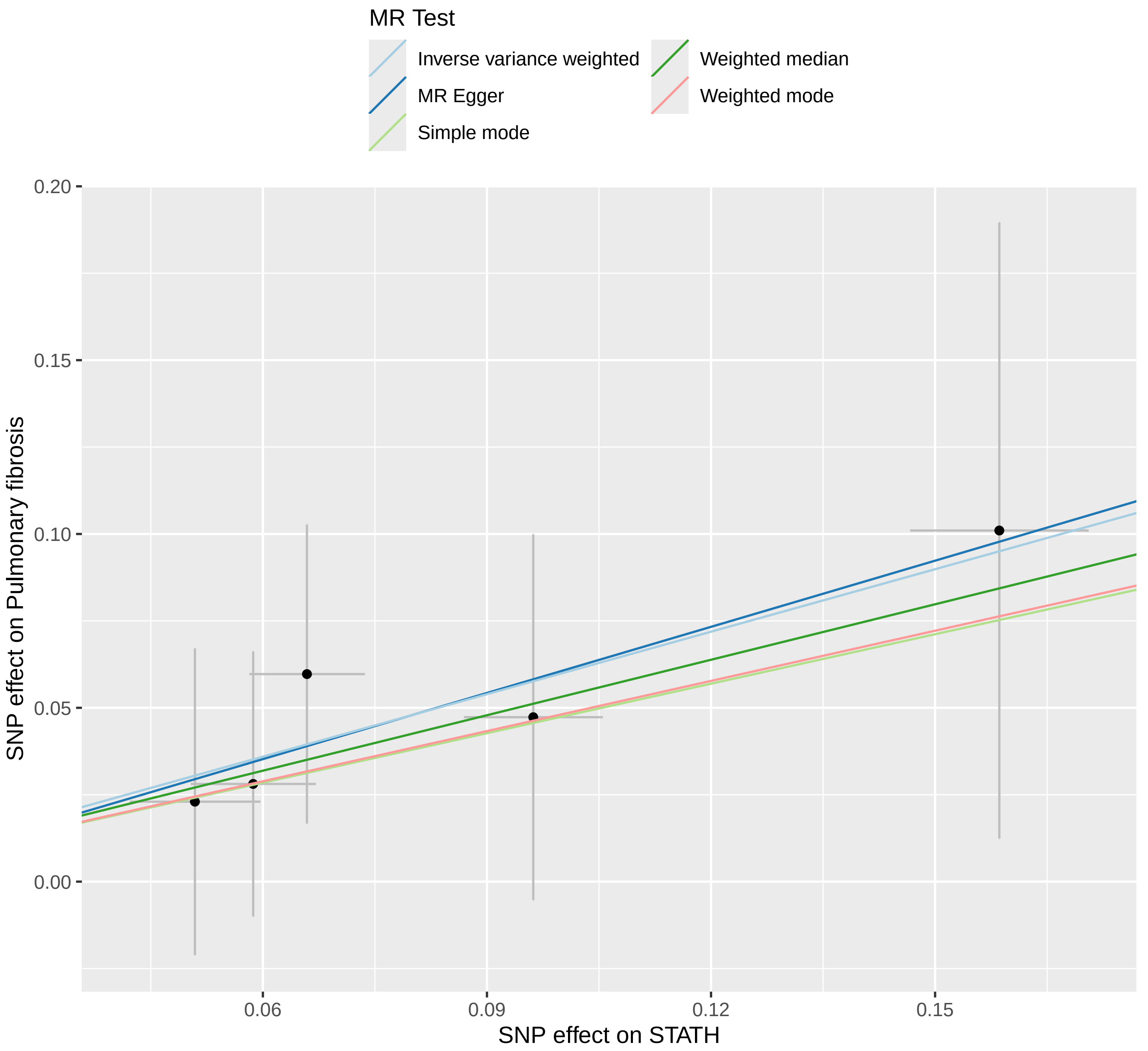
MR Test
Inverse variance weighted
MR Egger
Simple mode
Weighted median
Weighted mode
0.20
0.15
0.10
0.05
0.00
SNP effect on Pulmonary fibrosis
0.06
0.09
0.12
0.15
SNP effect on STATH

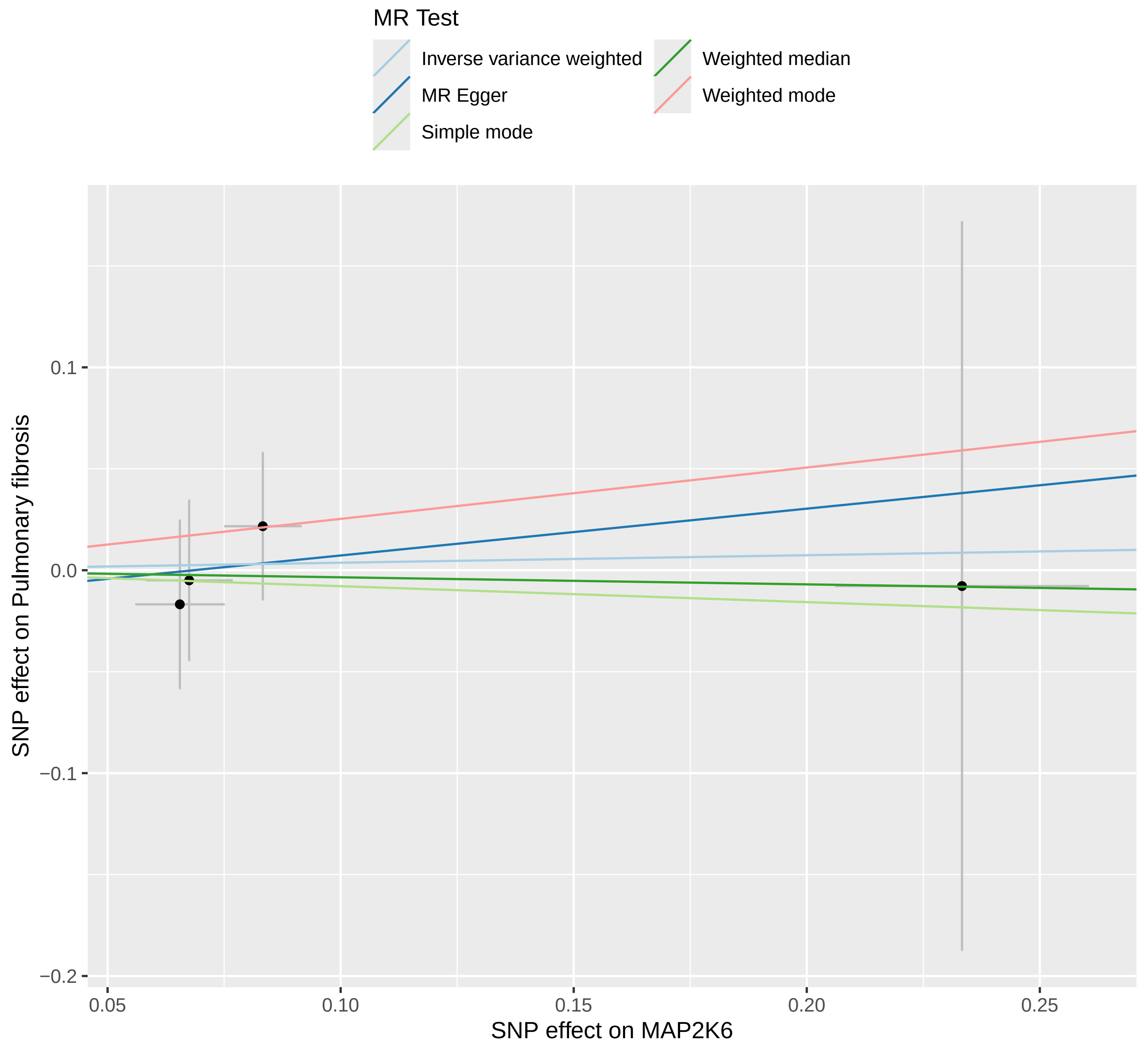

MR Test
Inverse variance weighted
MR Egger
Simple mode
Weighted median
Weighted mode
0.1
0.0
−0.1
−0.2
0.05
0.10
0.15
0.20
0.25
SNP effect on Pulmonary fibrosis
SNP effect on MAP2K6

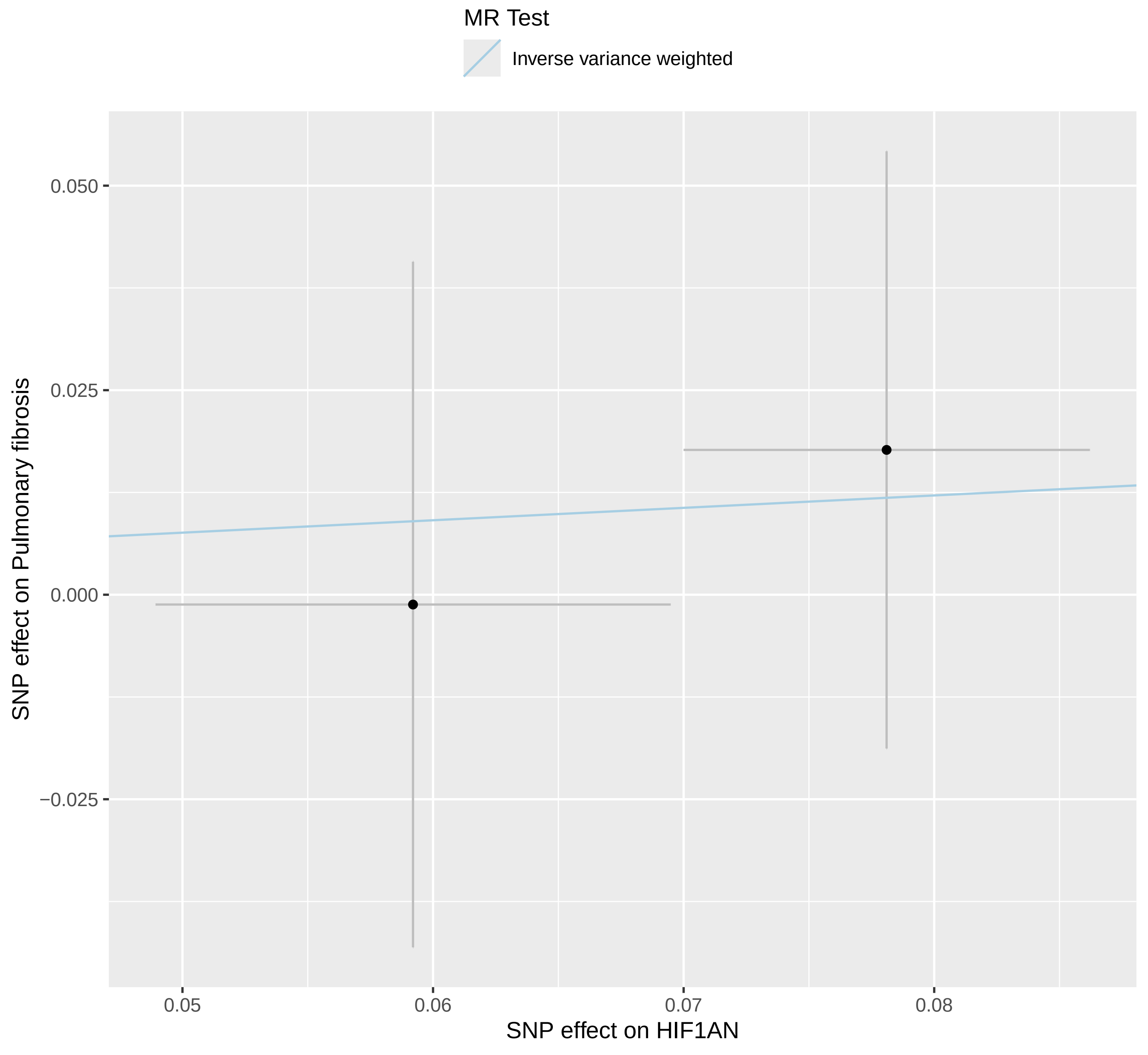

MR Test
Inverse variance weighted
0.050
0.025
0.000
−0.025
0.05
0.06
0.07
0.08
SNP effect on HIF1AN
SNP effect on Pulmonary fibrosis

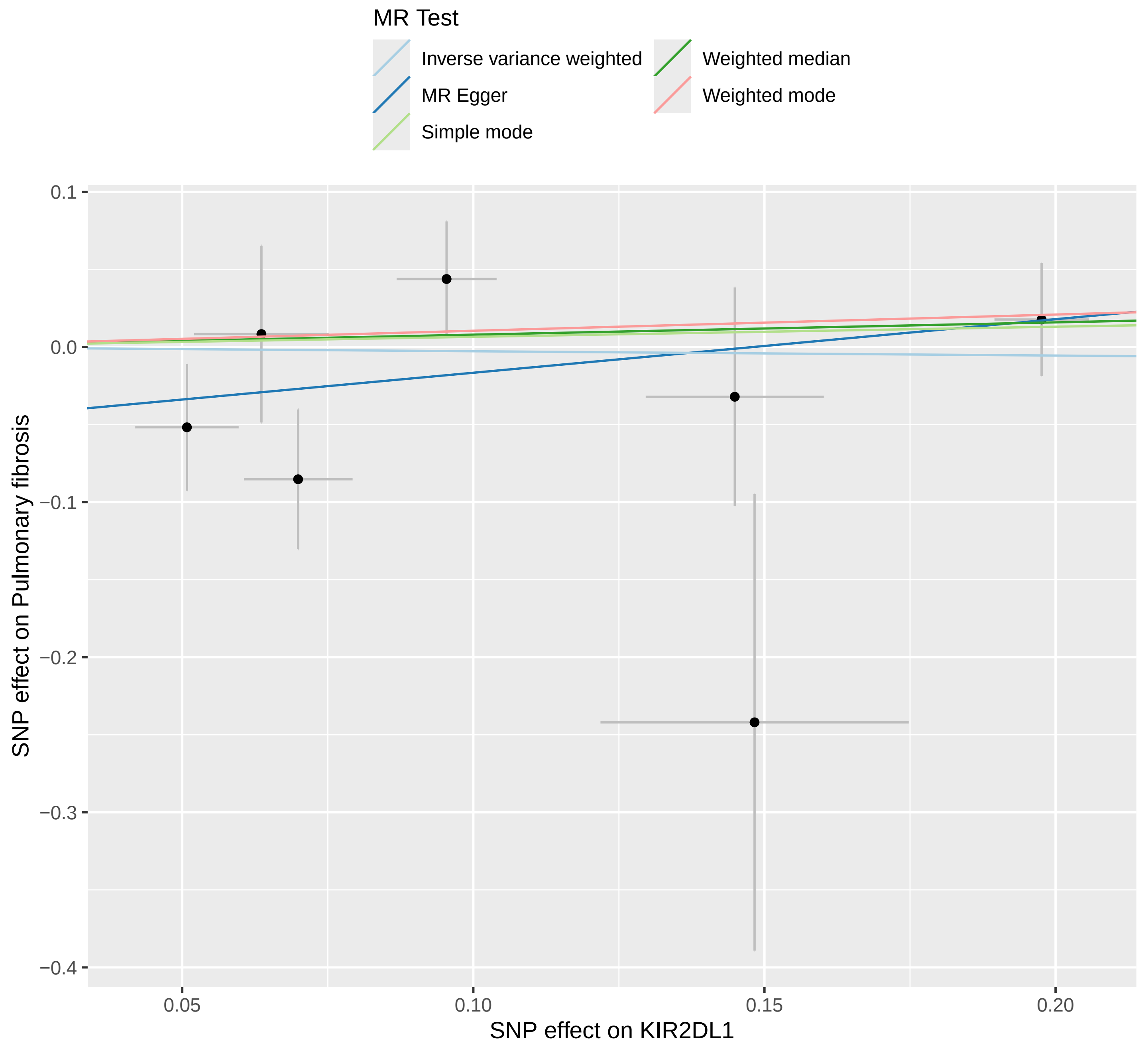

MR Test
Inverse variance weighted
MR Egger
Simple mode
Weighted median
Weighted mode
0.1
0.0
−0.1
−0.2
−0.3
−0.4
0.05
0.10
0.15
0.20
SNP effect on KIR2DL1
SNP effect on Pulmonary fibrosis

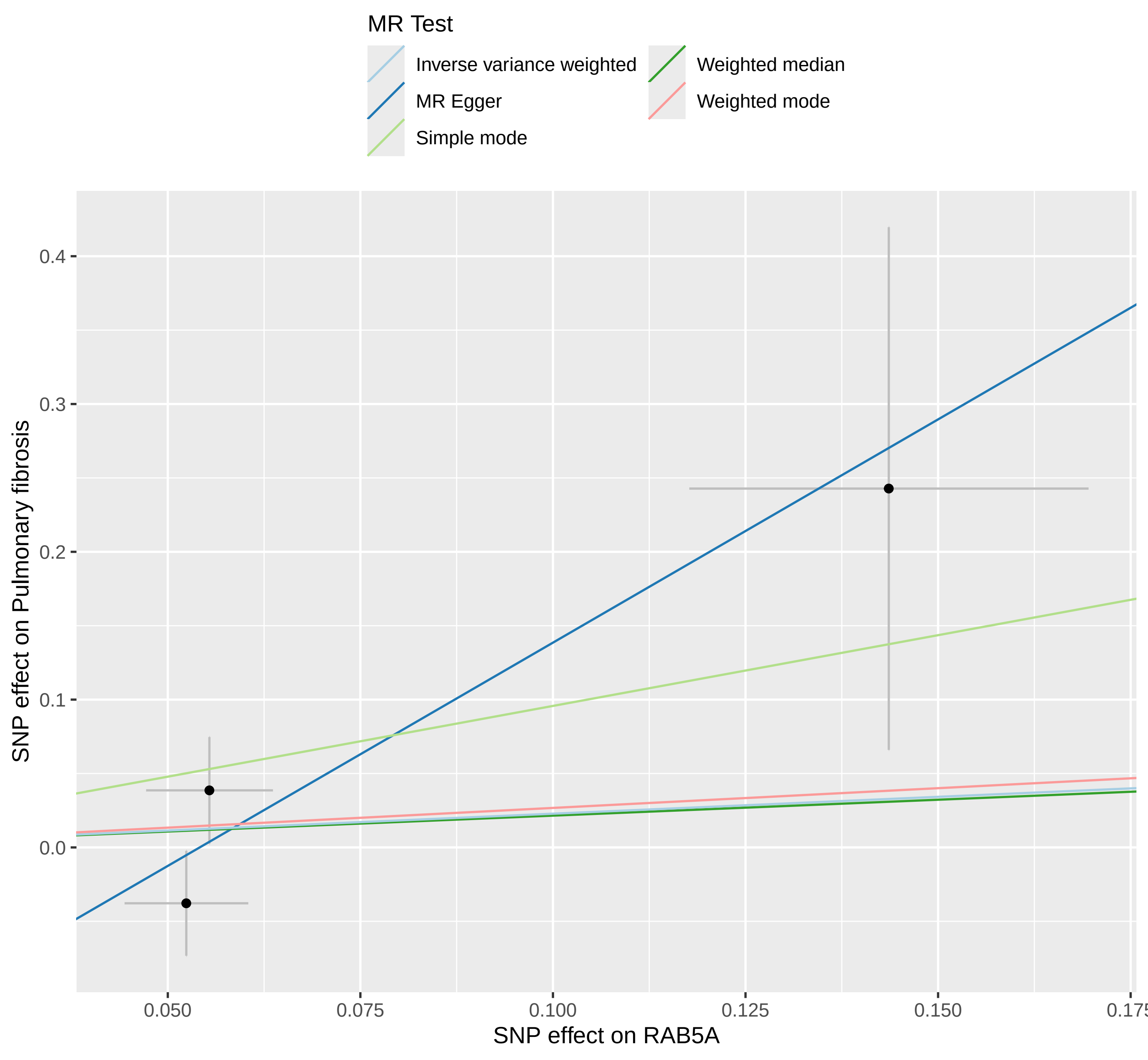

MR Test
Inverse variance weighted
MR Egger
Simple mode
Weighted median
Weighted mode
0.4
0.3
0.2
0.1
0.0
SNP effect on Pulmonary fibrosis
0.050
0.075
0.100
0.125
0.150
0.175
SNP effect on RAB5A

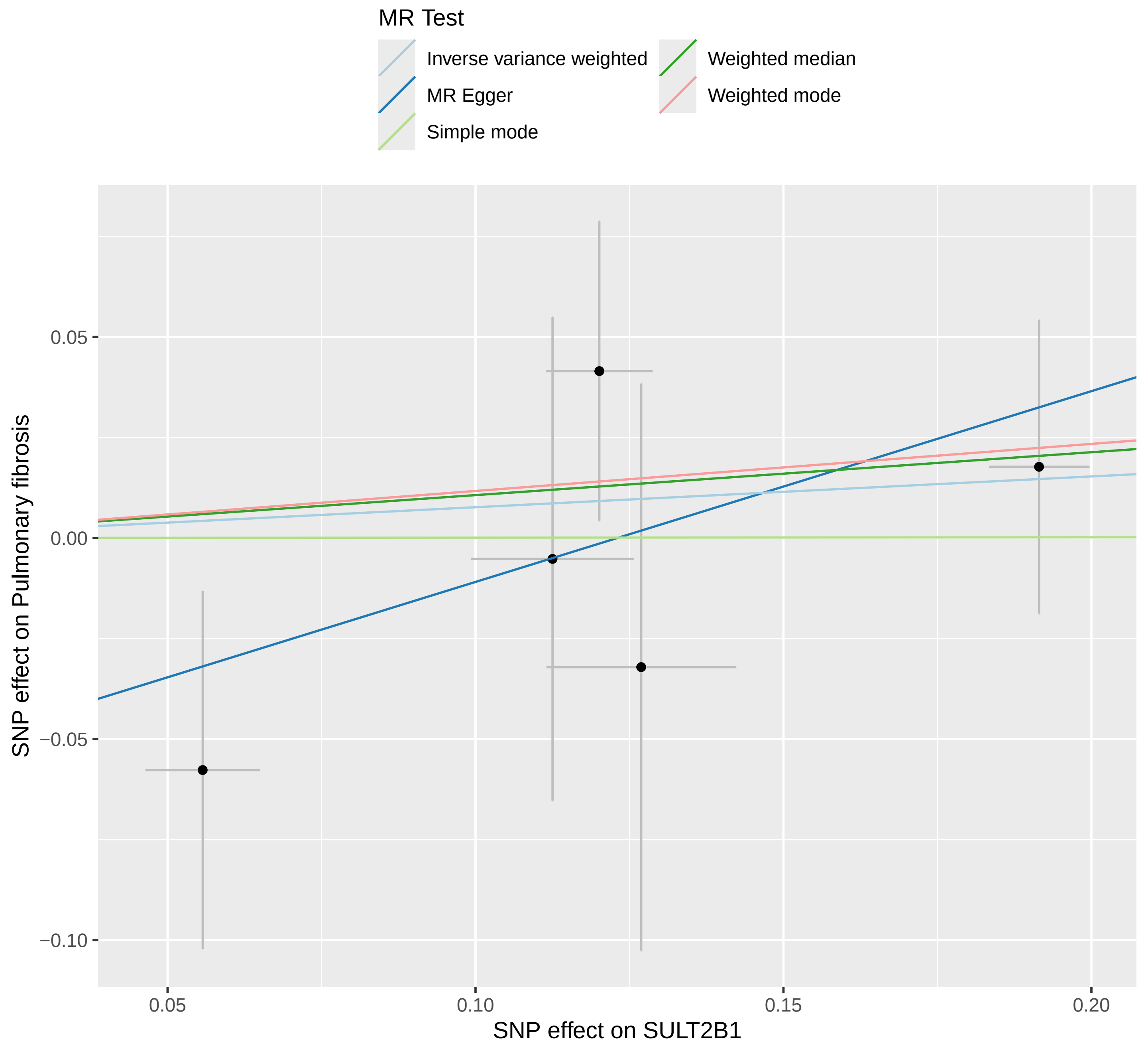

MR Test
Inverse variance weighted
MR Egger
Simple mode
Weighted median
Weighted mode
0.05
0.00
−0.05
−0.10
0.05
0.10
0.15
0.20
SNP effect on Pulmonary fibrosis
SNP effect on SULT2B1

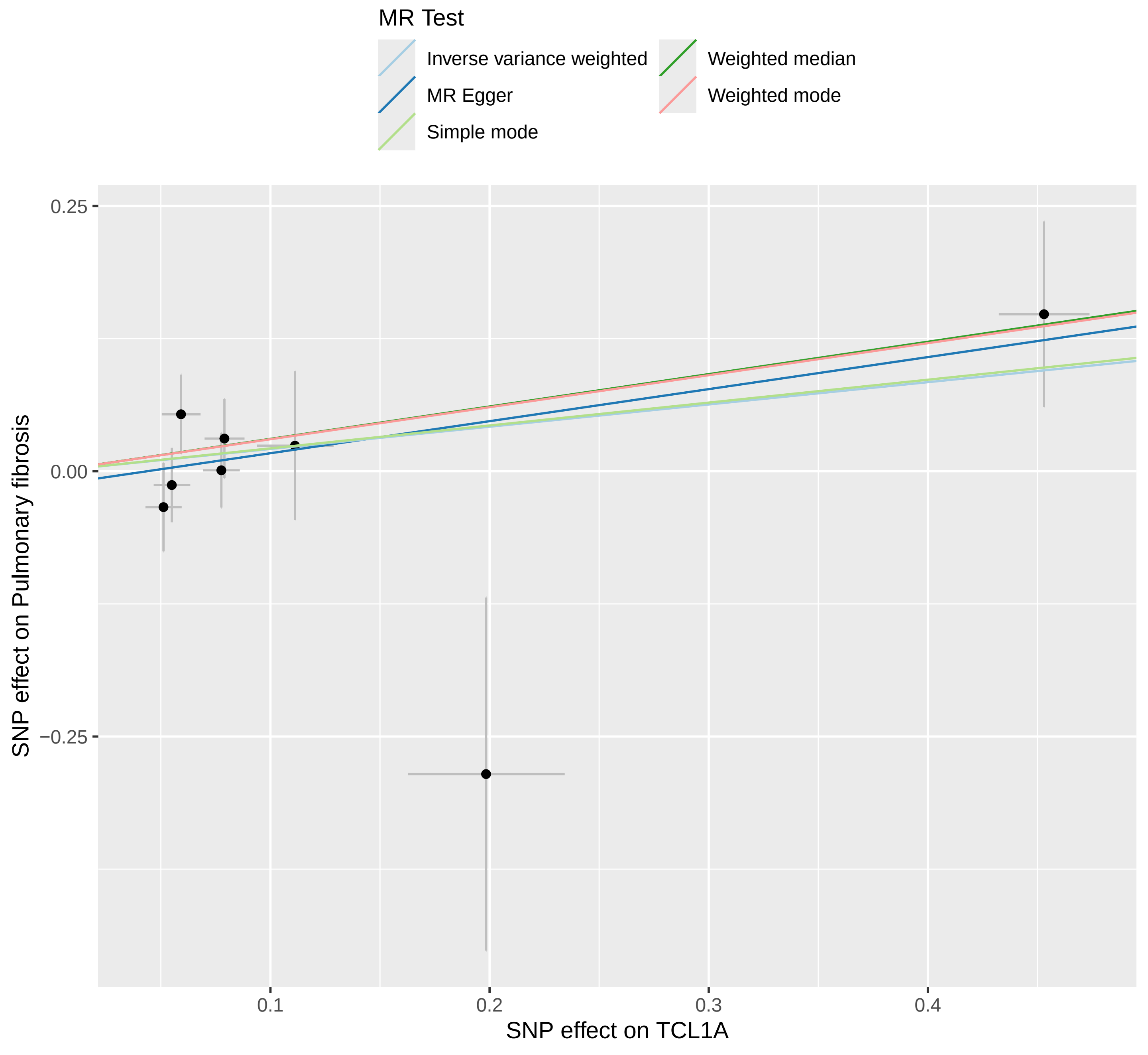

MR Test
Inverse variance weighted
MR Egger
Simple mode
Weighted median
Weighted mode
0.25
0.00
−0.25
SNP effect on Pulmonary fibrosis
0.1
0.2
0.3
0.4
SNP effect on TCL1A

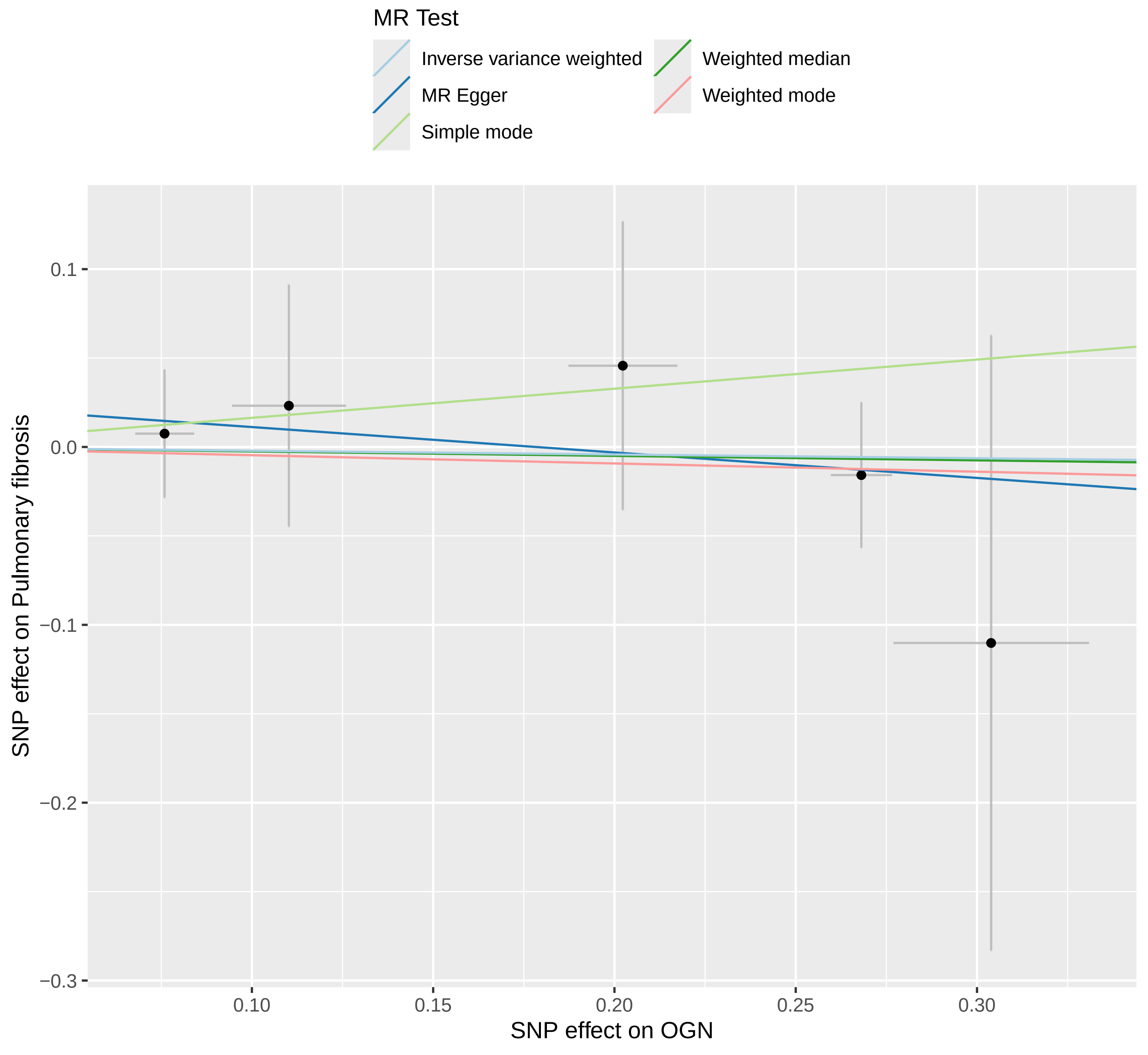

MR Test
Inverse variance weighted
MR Egger
Simple mode
Weighted median
Weighted mode
0.1
0.0
−0.1
−0.2
−0.3
0.10
0.15
0.20
0.25
0.30
SNP effect on Pulmonary fibrosis
SNP effect on OGN

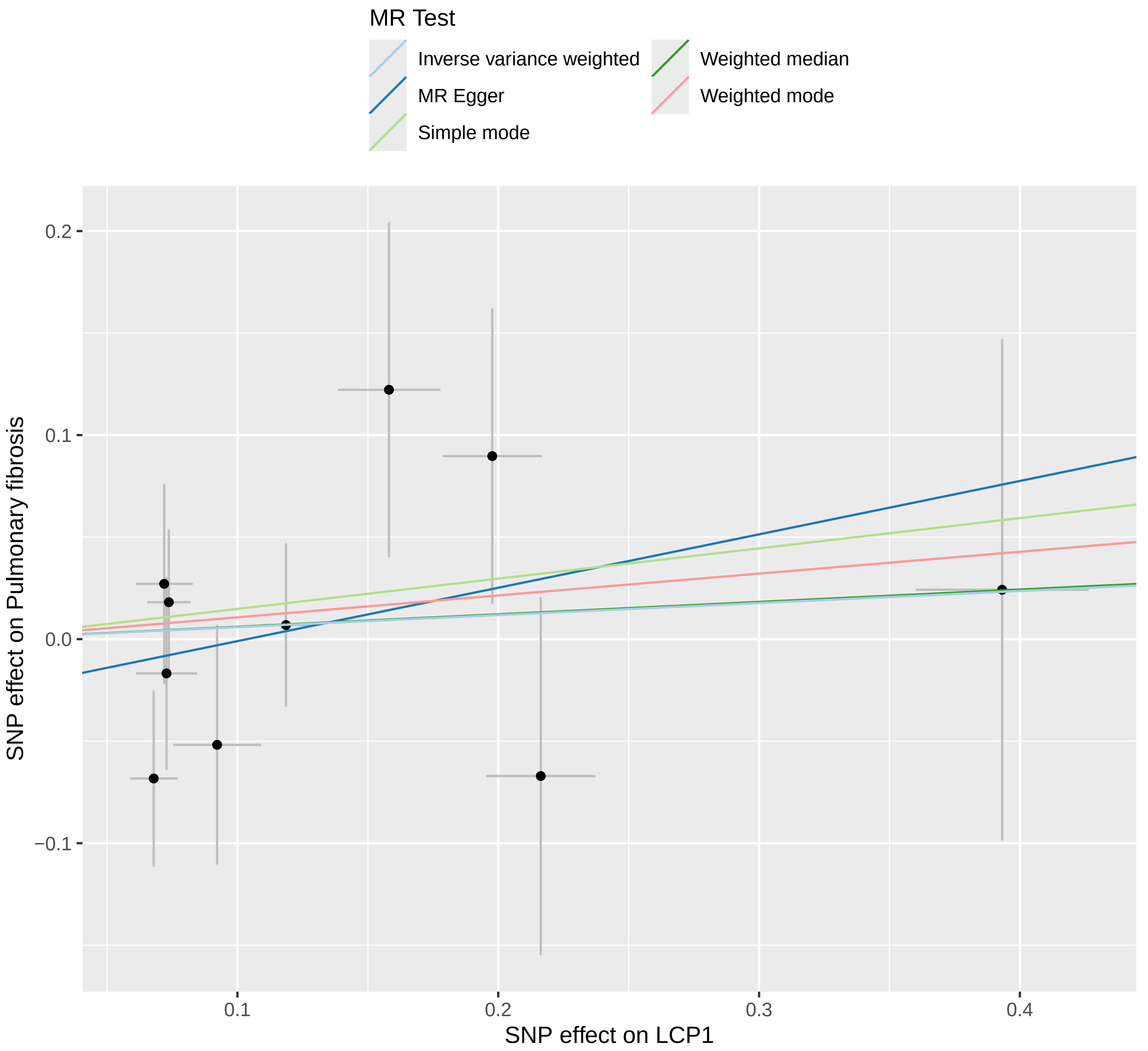

MR Test
Inverse variance weighted
MR Egger
Simple mode
Weighted median
Weighted mode
0.2
0.1
0.0
−0.1
SNP effect on Pulmonary fibrosis
0.1
0.2
0.3
0.4
SNP effect on LCP1

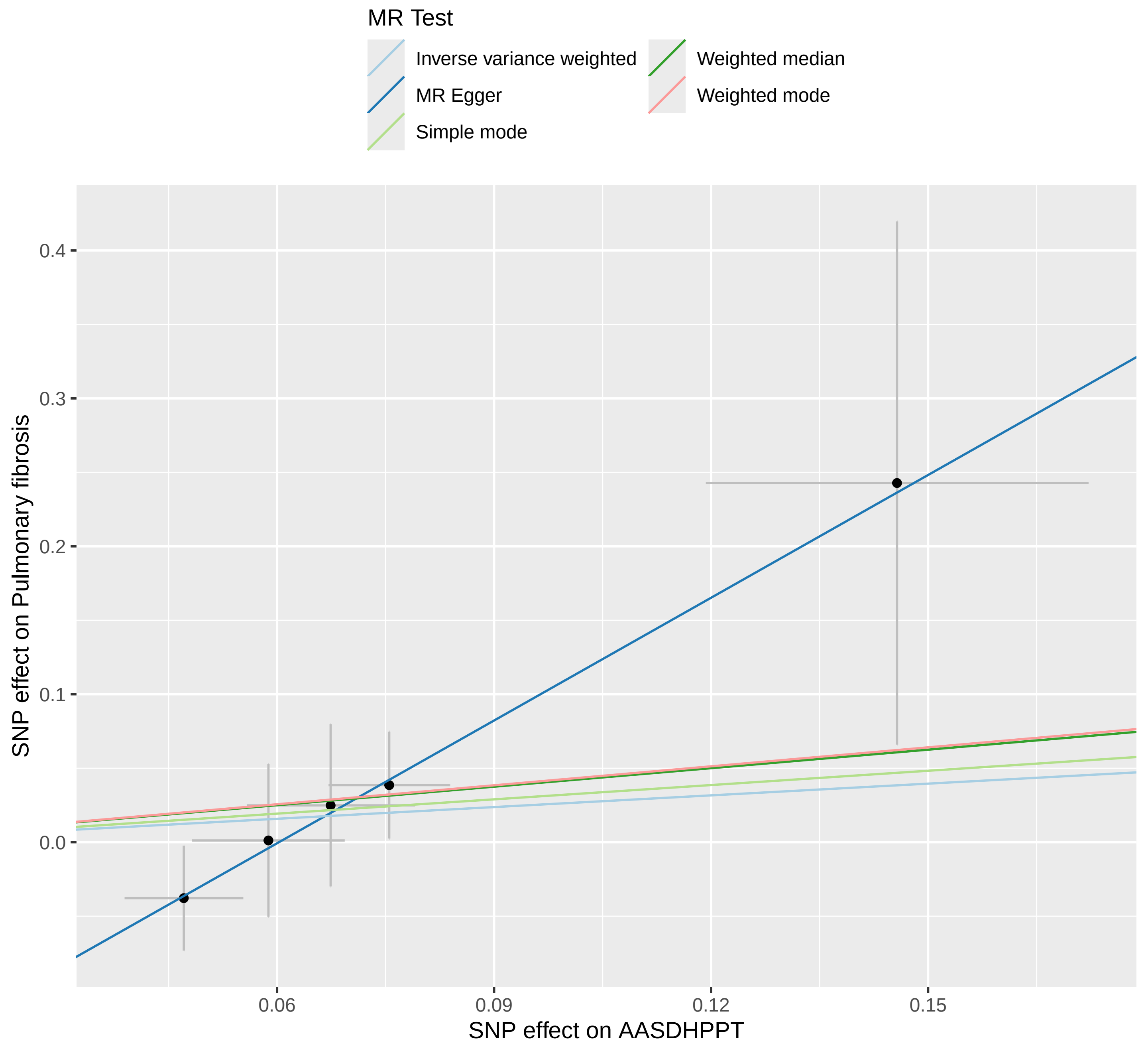

MR Test
Inverse variance weighted
MR Egger
Simple mode
Weighted median
Weighted mode
0.4
0.3
0.2
0.1
0.0
SNP effect on Pulmonary fibrosis
0.06
0.09
0.12
0.15
SNP effect on AASDHPPT

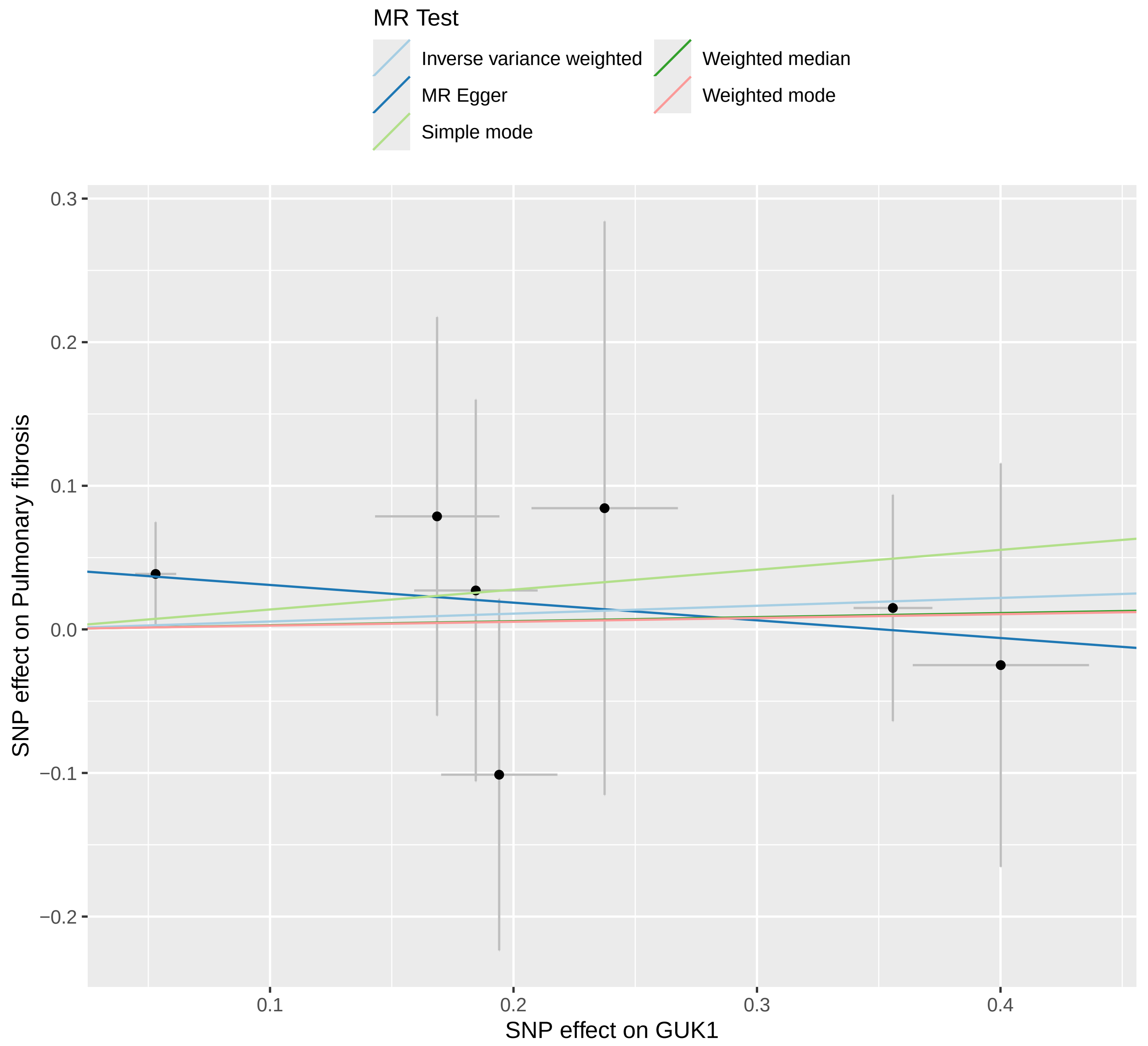

MR Test
Inverse variance weighted
MR Egger
Simple mode
Weighted median
Weighted mode
0.3
0.2
0.1
0.0
−0.1
−0.2
0.1
0.2
0.3
0.4
SNP effect on GUK1
SNP effect on Pulmonary fibrosis

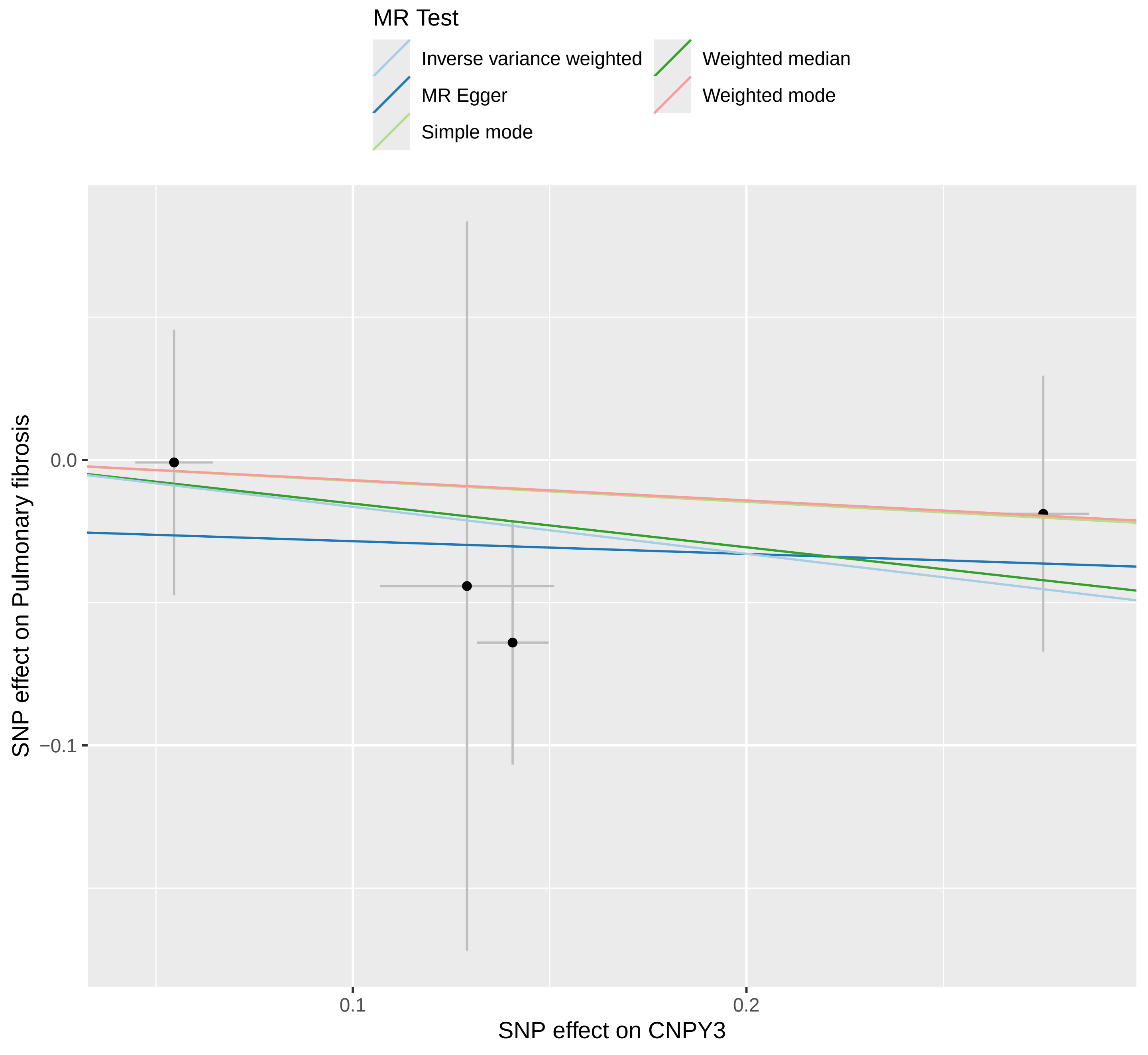

MR Test
Inverse variance weighted
MR Egger
Simple mode
Weighted median
Weighted mode
0.0
−0.1
0.1
0.2
SNP effect on Pulmonary fibrosis
SNP effect on CNPY3

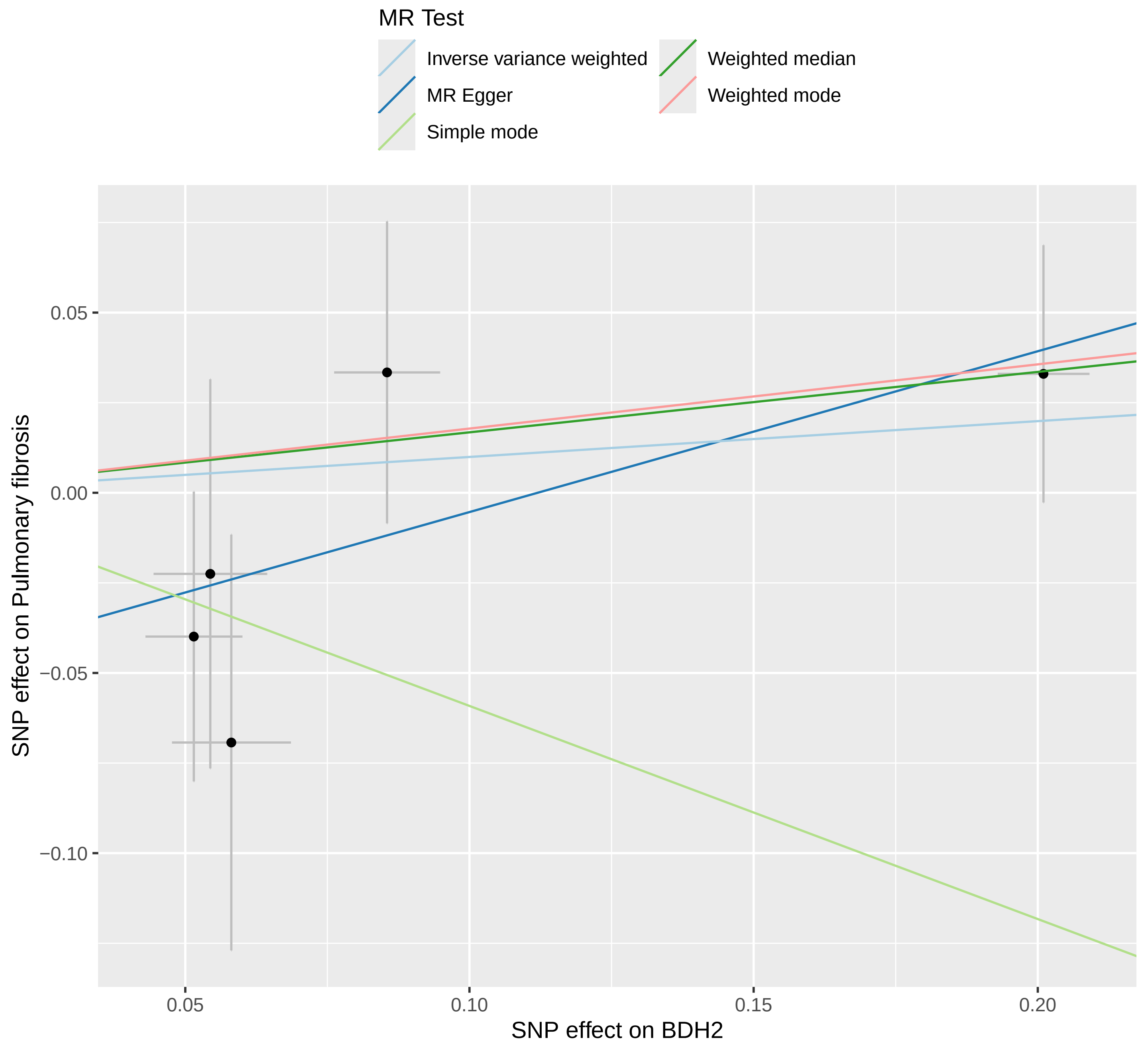

MR Test
Inverse variance weighted
MR Egger
Simple mode
Weighted median
Weighted mode
0.05
0.00
−0.05
−0.10
0.05
0.10
0.15
0.20
SNP effect on BDH2
SNP effect on Pulmonary fibrosis

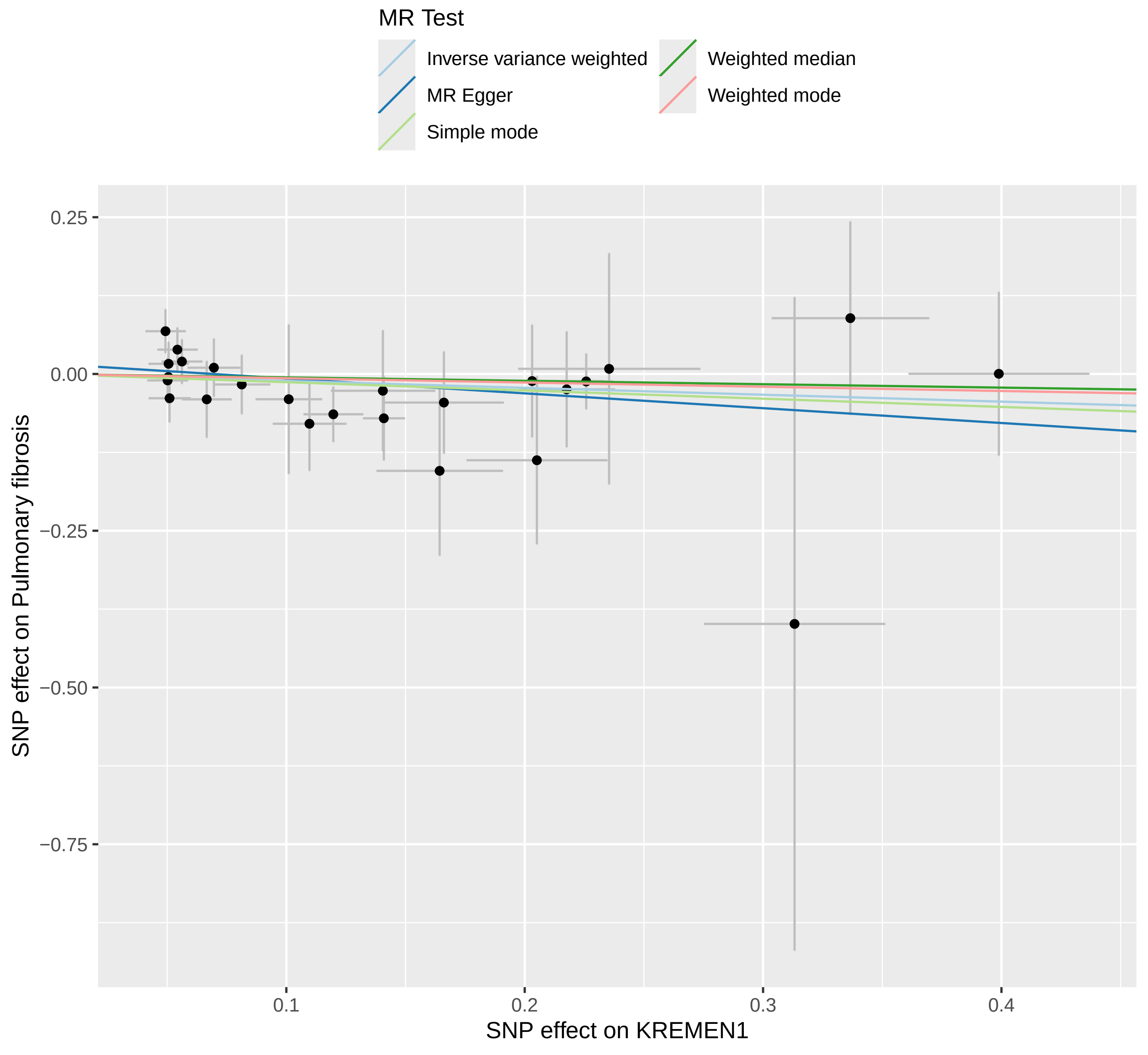

MR Test
Inverse variance weighted
MR Egger
Simple mode
Weighted median
Weighted mode
0.25
0.00
−0.25
−0.50
−0.75
0.1
0.2
0.3
0.4
SNP effect on Pulmonary fibrosis
SNP effect on KREMEN1

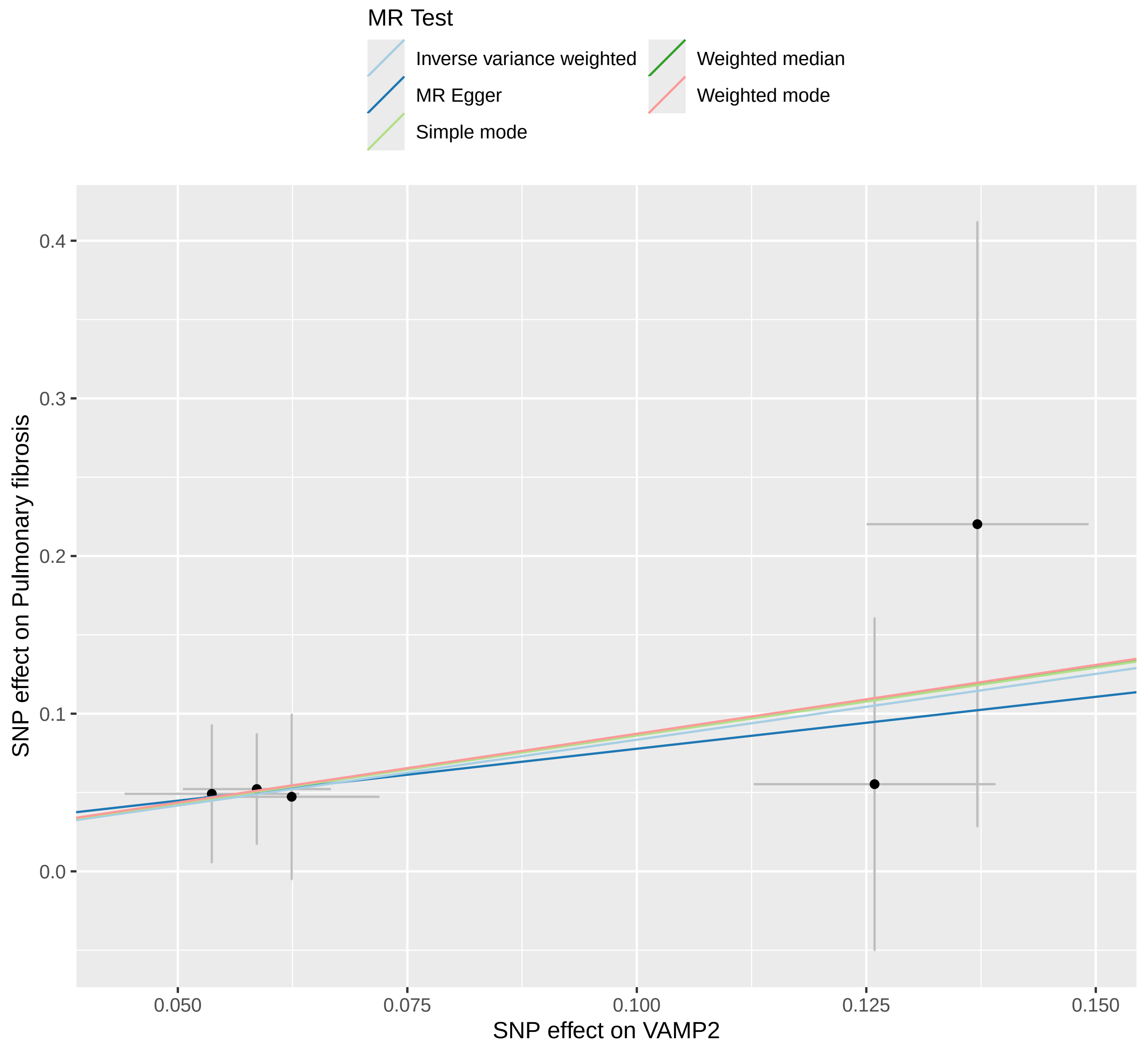

MR Test
Inverse variance weighted
MR Egger
Simple mode
Weighted median
Weighted mode
SNP effect on Pulmonary fibrosis
0.4
0.3
0.2
0.1
0.0
0.050
0.075
0.100
0.125
0.150
SNP effect on VAMP2

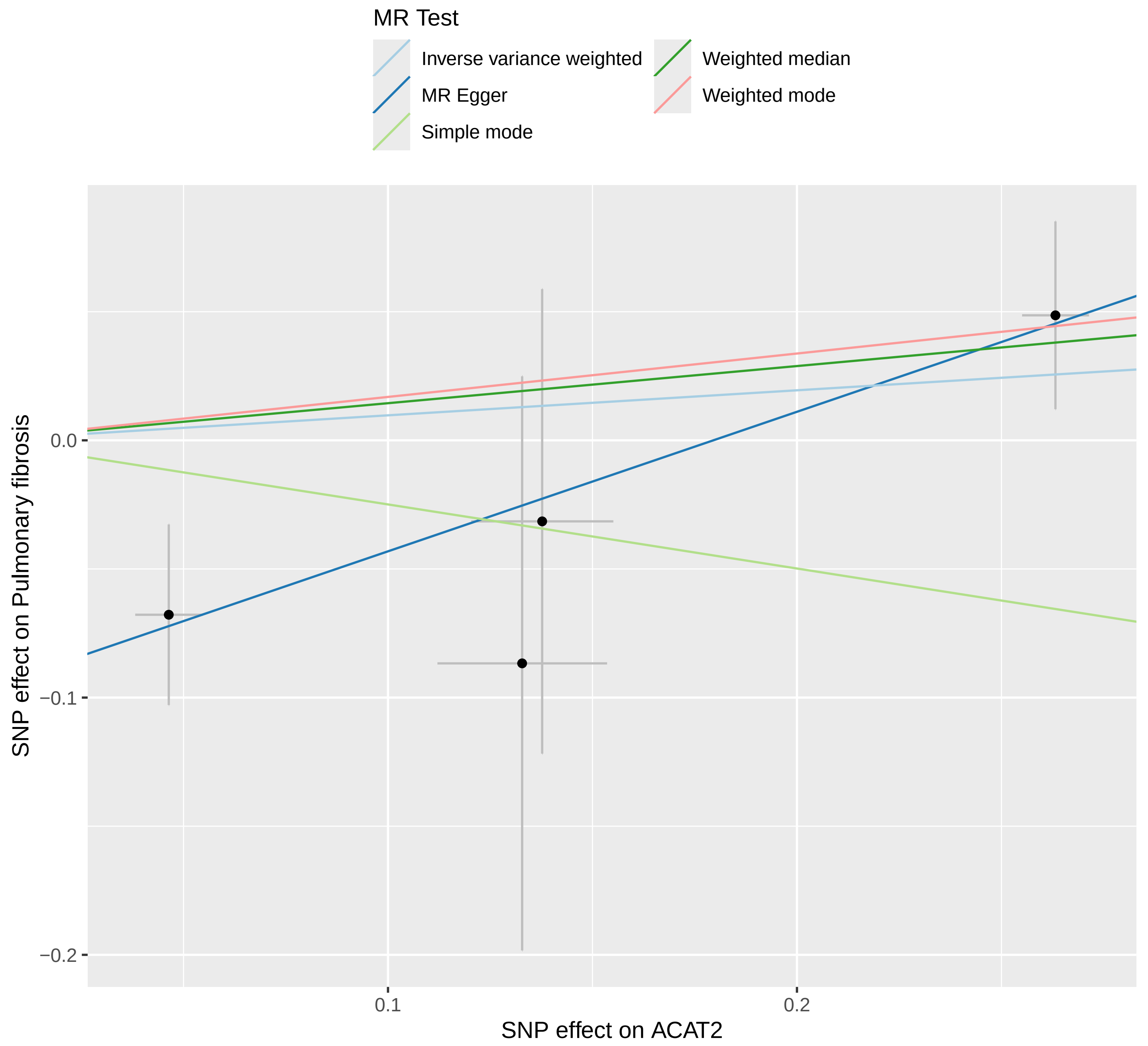
MR Test
Inverse variance weighted
MR Egger
Simple mode
Weighted median
Weighted mode
SNP effect on Pulmonary fibrosis
0.0
−0.1
−0.2
0.1
0.2
SNP effect on ACAT2

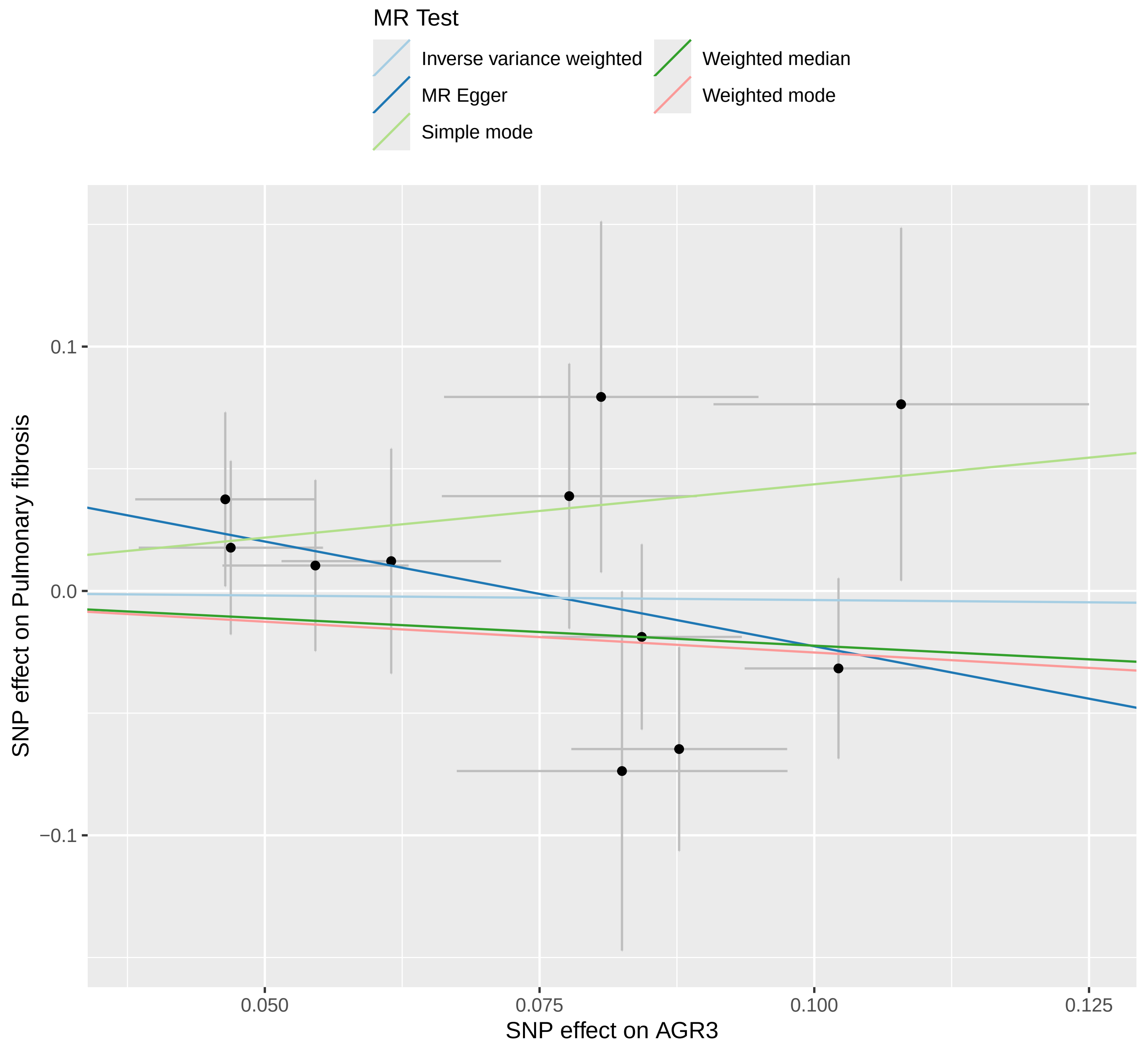

MR Test
Inverse variance weighted
MR Egger
Simple mode
Weighted median
Weighted mode
0.1
0.0
−0.1
0.050
0.075
0.100
0.125
SNP effect on Pulmonary fibrosis
SNP effect on AGR3

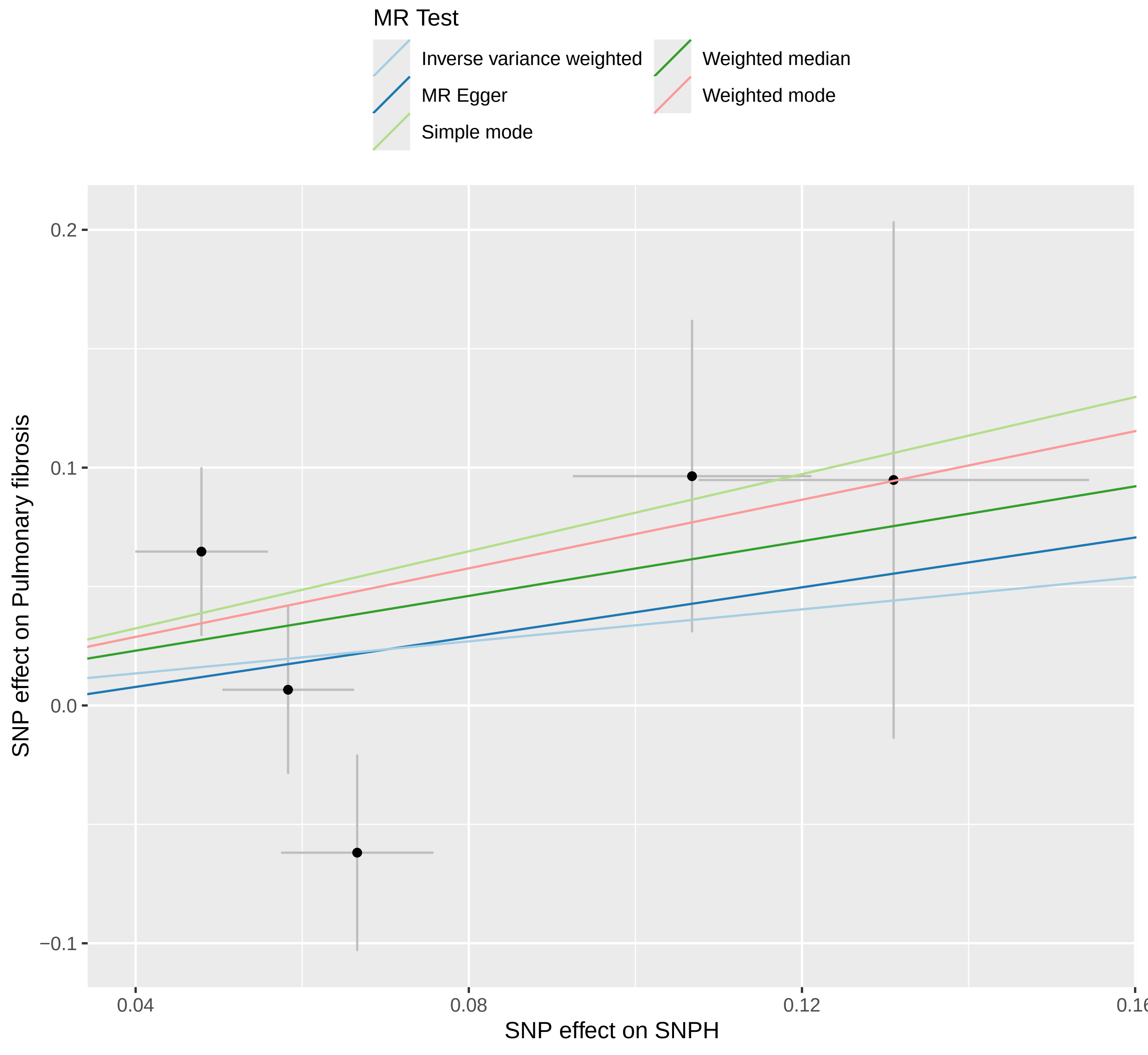

MR Test
Inverse variance weighted
MR Egger
Simple mode
Weighted median
Weighted mode
SNP effect on Pulmonary fibrosis
0.2
0.1
0.0
−0.1
0.04
0.08
0.12
0.16
SNP effect on SNPH

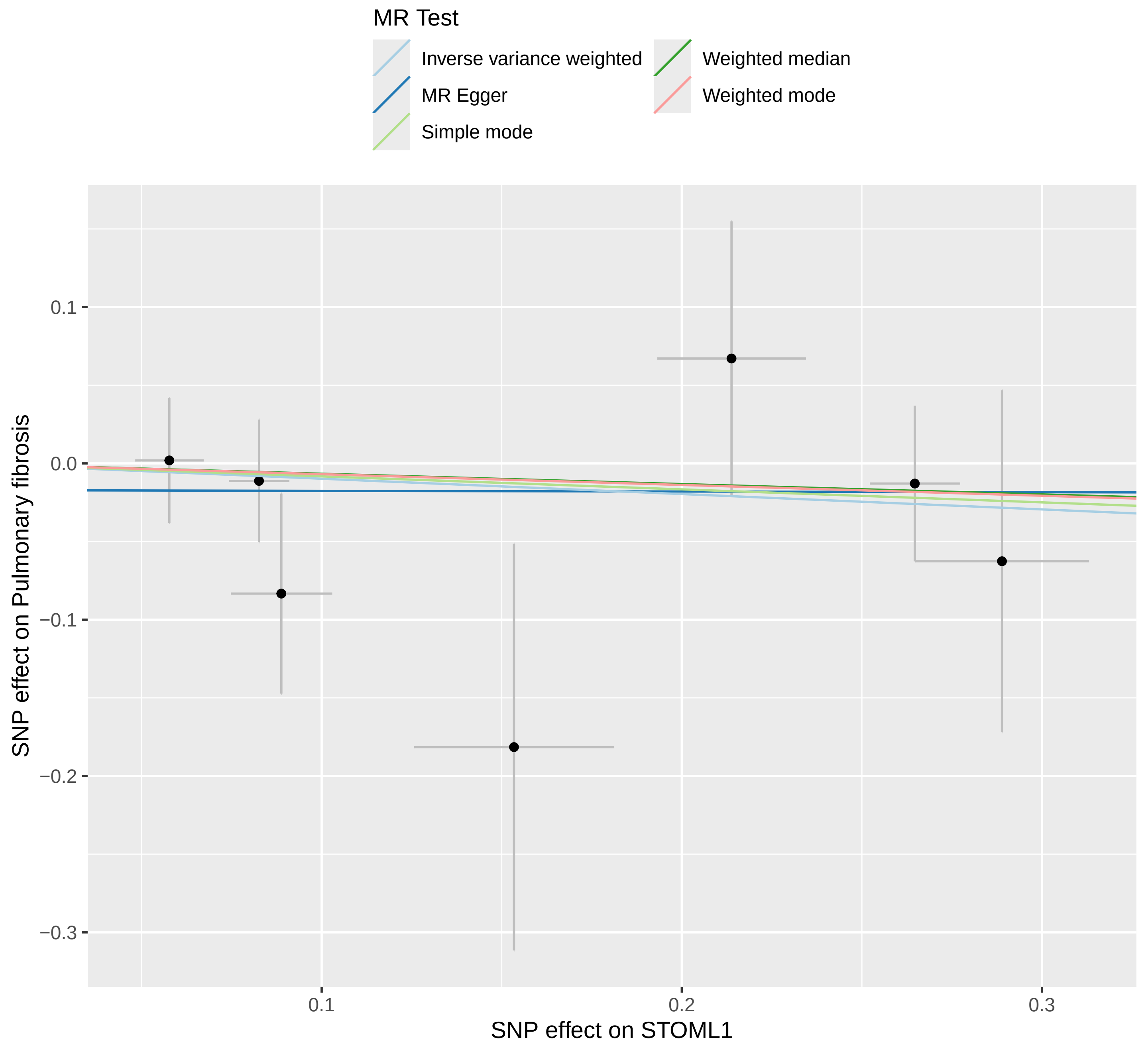

MR Test
Inverse variance weighted
MR Egger
Simple mode
Weighted median
Weighted mode
0.1
0.0
−0.1
−0.2
−0.3
SNP effect on Pulmonary fibrosis
0.1
0.2
0.3
SNP effect on STOML1

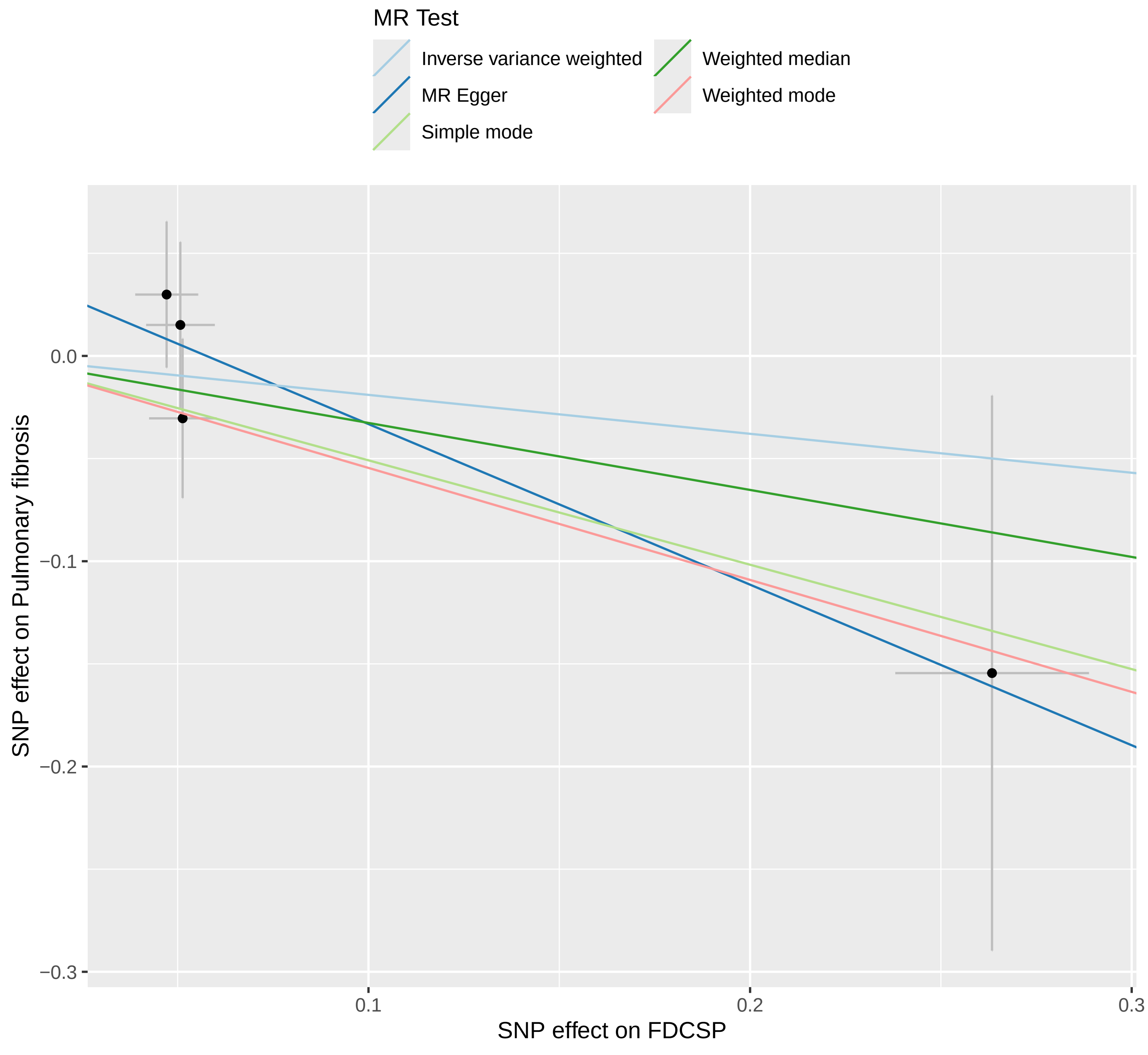
MR Test
Inverse variance weighted
MR Egger
Simple mode
Weighted median
Weighted mode
0.0
−0.1
−0.2
−0.3
SNP effect on Pulmonary fibrosis
0.1
0.2
0.3
SNP effect on FDCSP

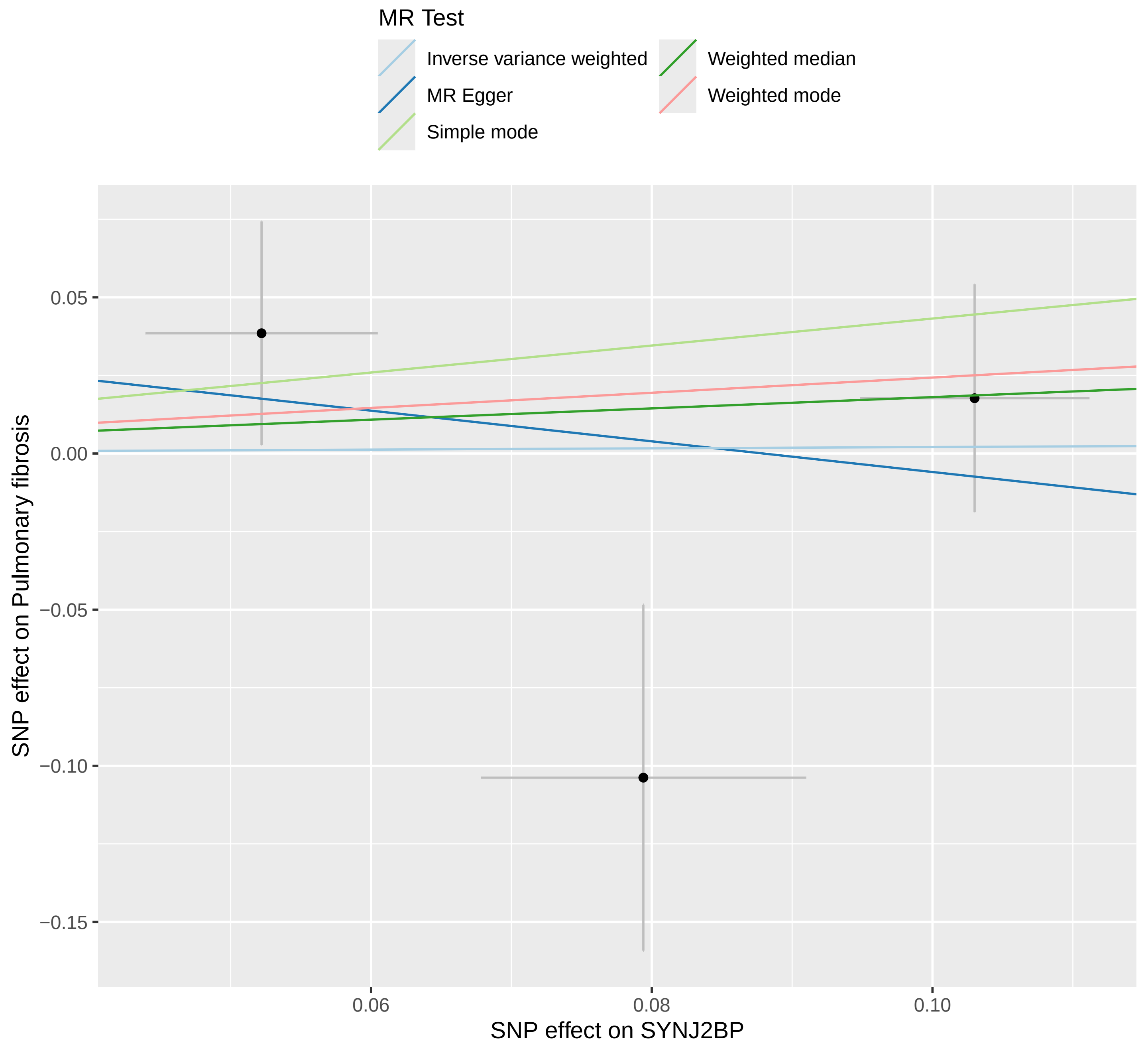

MR Test
Inverse variance weighted
MR Egger
Simple mode
Weighted median
Weighted mode
0.05
0.00
−0.05
−0.10
−0.15
0.06
0.08
0.10
SNP effect on Pulmonary fibrosis
SNP effect on SYNJ2BP

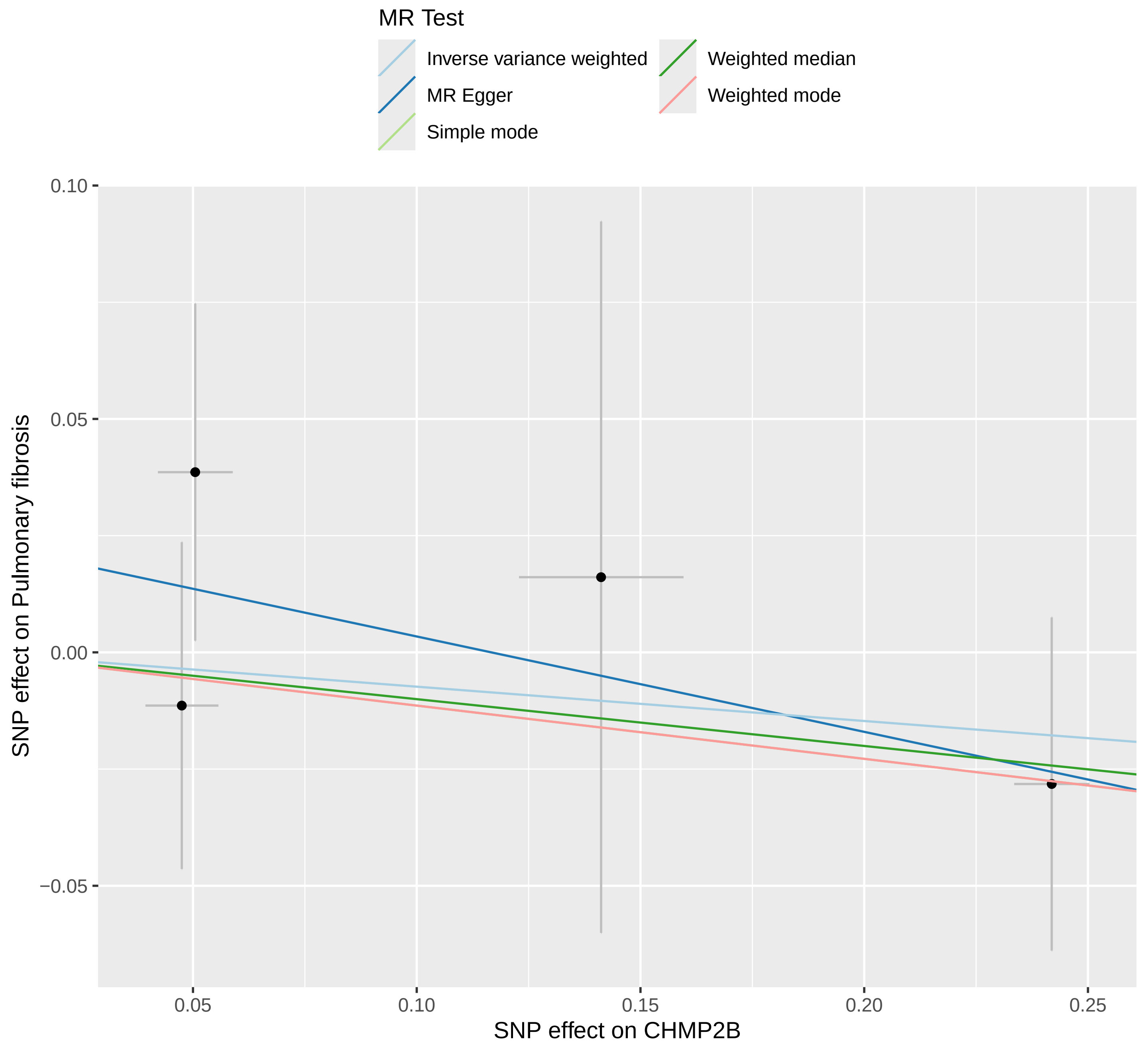
MR Test
Inverse variance weighted
MR Egger
Simple mode
Weighted median
Weighted mode
0.10
0.05
0.00
−0.05
SNP effect on Pulmonary fibrosis
0.05
0.10
0.15
0.20
0.25
SNP effect on CHMP2B

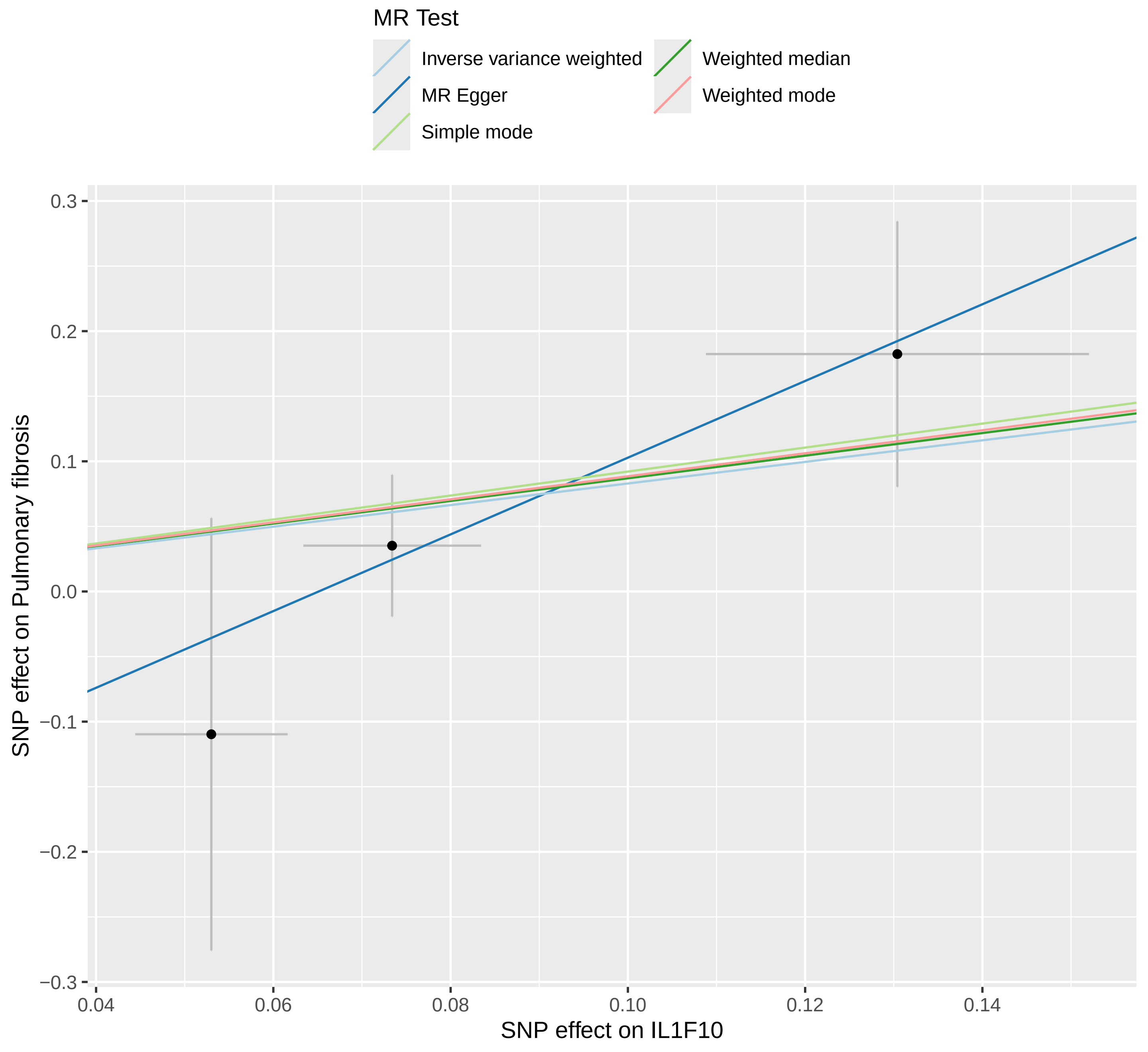
MR Test
Inverse variance weighted
MR Egger
Simple mode
Weighted median
Weighted mode
0.3
0.2
0.1
0.0
−0.1
−0.2
−0.3
SNP effect on Pulmonary fibrosis
0.04
0.06
0.08
0.10
0.12
0.14
SNP effect on IL1F10

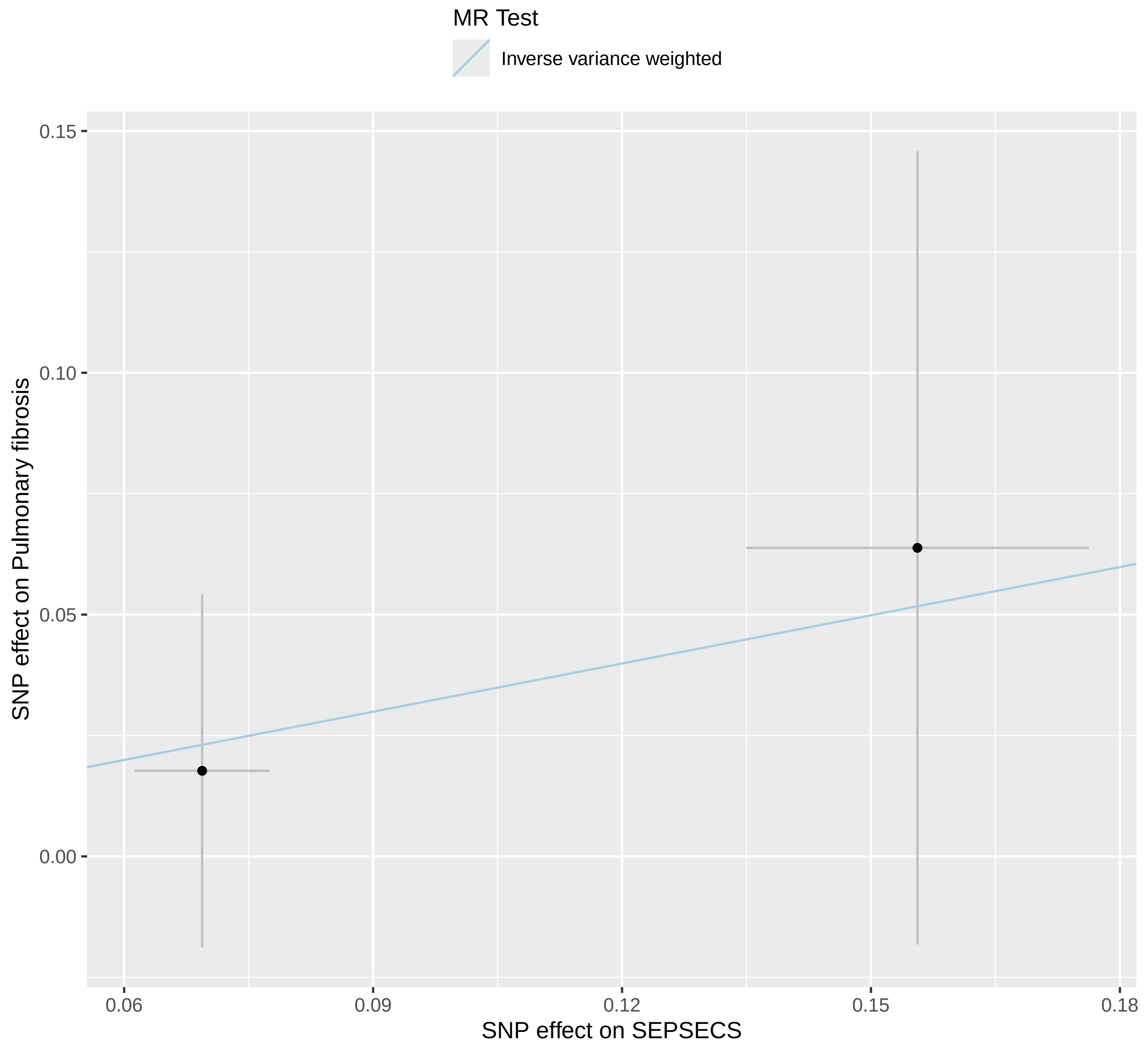

MR Test
Inverse variance weighted
0.15
0.10
0.05
0.00
SNP effect on Pulmonary fibrosis
0.06
0.09
0.12
0.15
0.18
SNP effect on SEPSECS

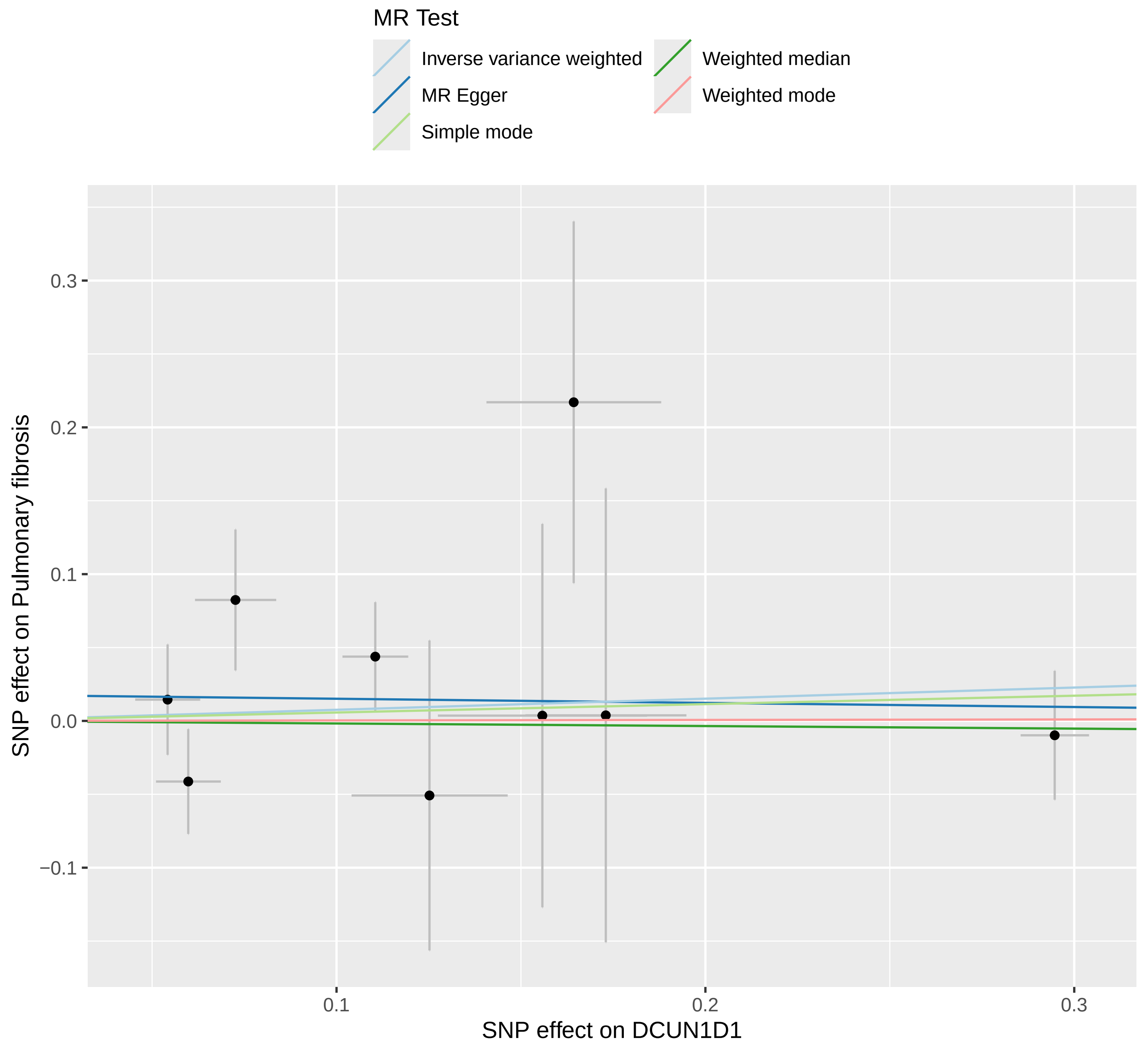

MR Test
Inverse variance weighted
MR Egger
Simple mode
Weighted median
Weighted mode
0.3
0.2
0.1
0.0
−0.1
0.1
0.2
0.3
SNP effect on Pulmonary fibrosis
SNP effect on DCUN1D1

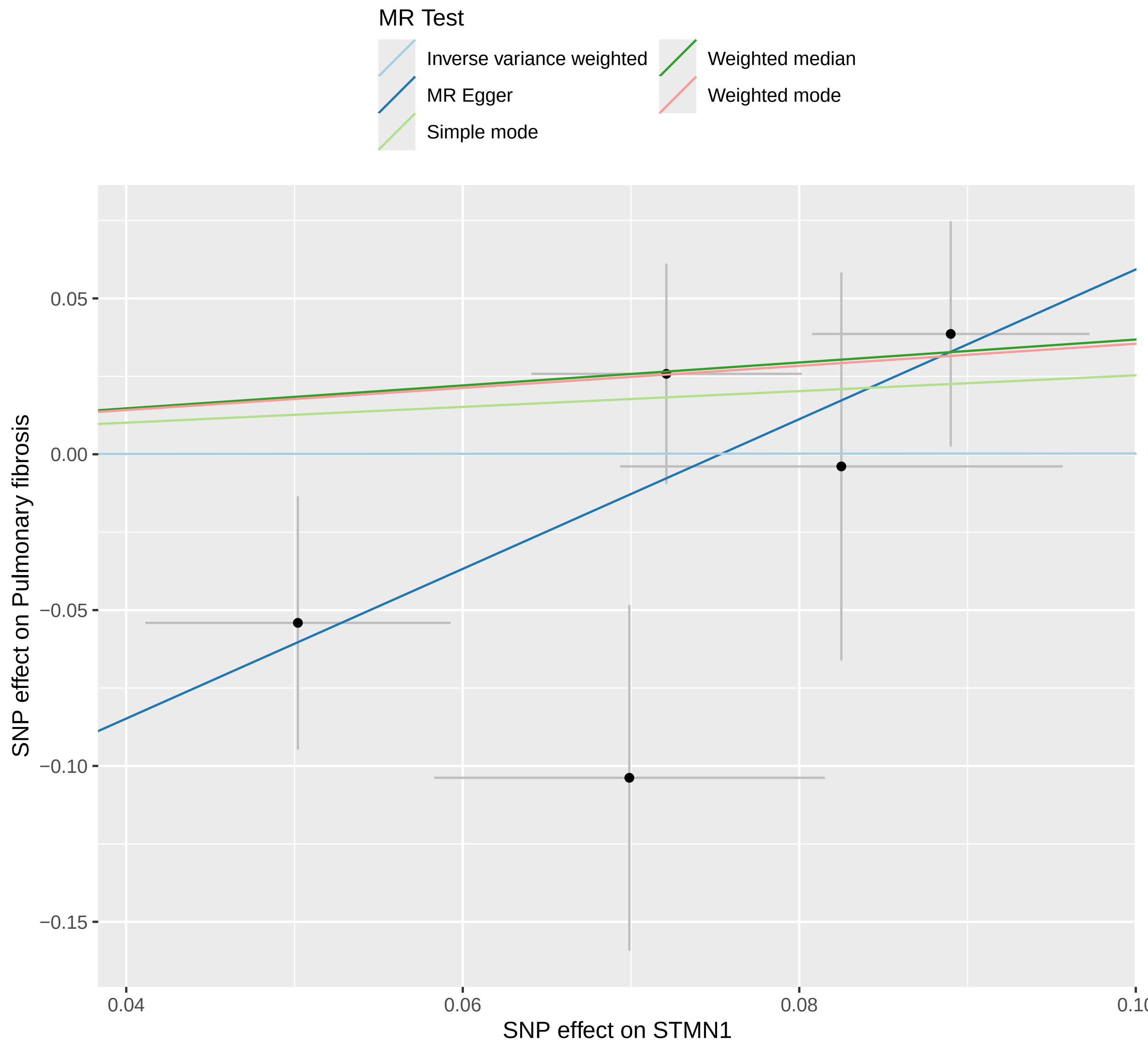
MR Test
Inverse variance weighted
MR Egger
Simple mode
Weighted median
Weighted mode
0.05
0.00
−0.05
−0.10
−0.15
SNP effect on Pulmonary fibrosis
0.04
0.06
0.08
0.10
SNP effect on STMN1

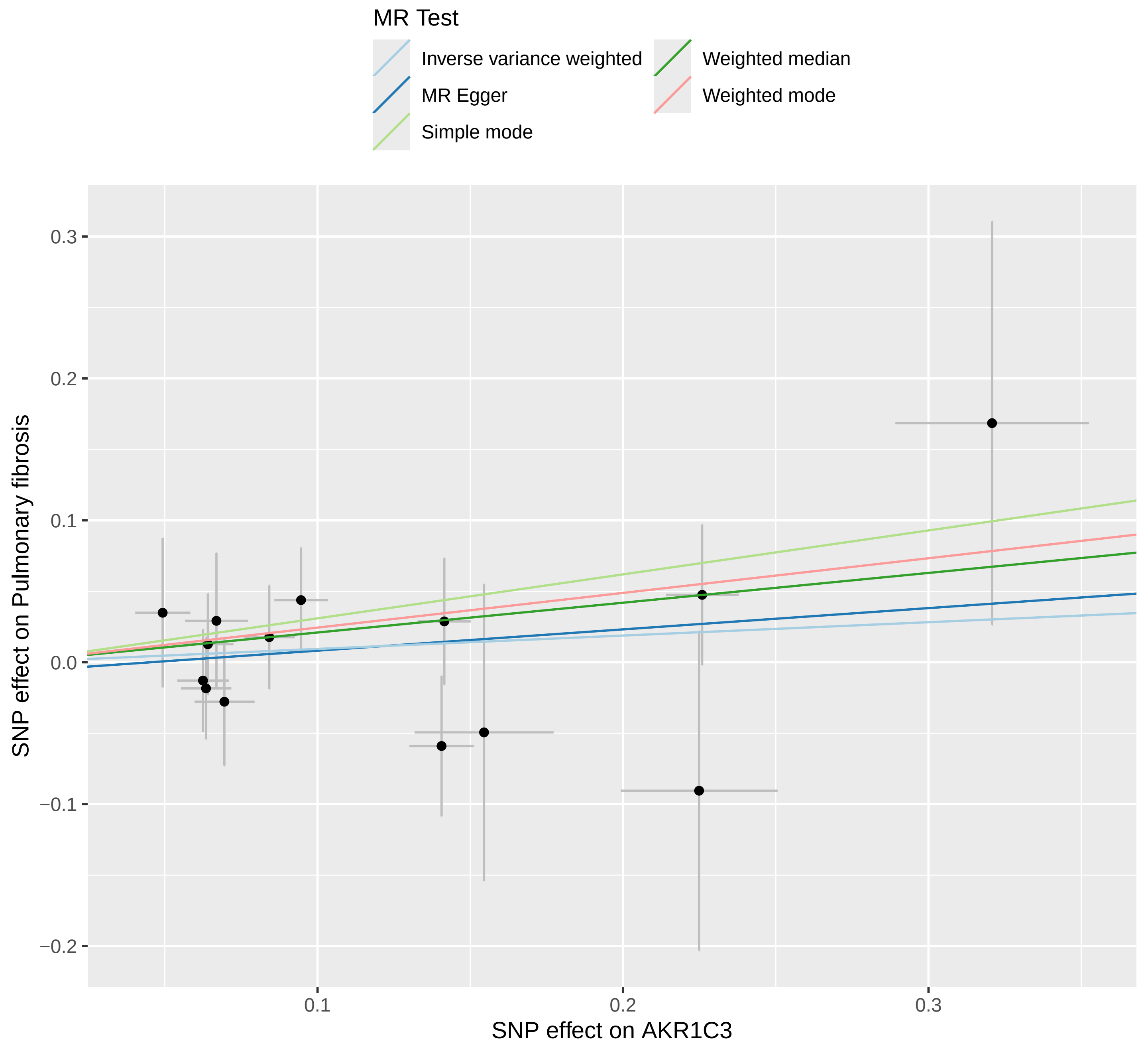

MR Test
Inverse variance weighted
MR Egger
Simple mode
Weighted median
Weighted mode
0.3
0.2
0.1
0.0
−0.1
−0.2
SNP effect on Pulmonary fibrosis
0.1
0.2
0.3
SNP effect on AKR1C3

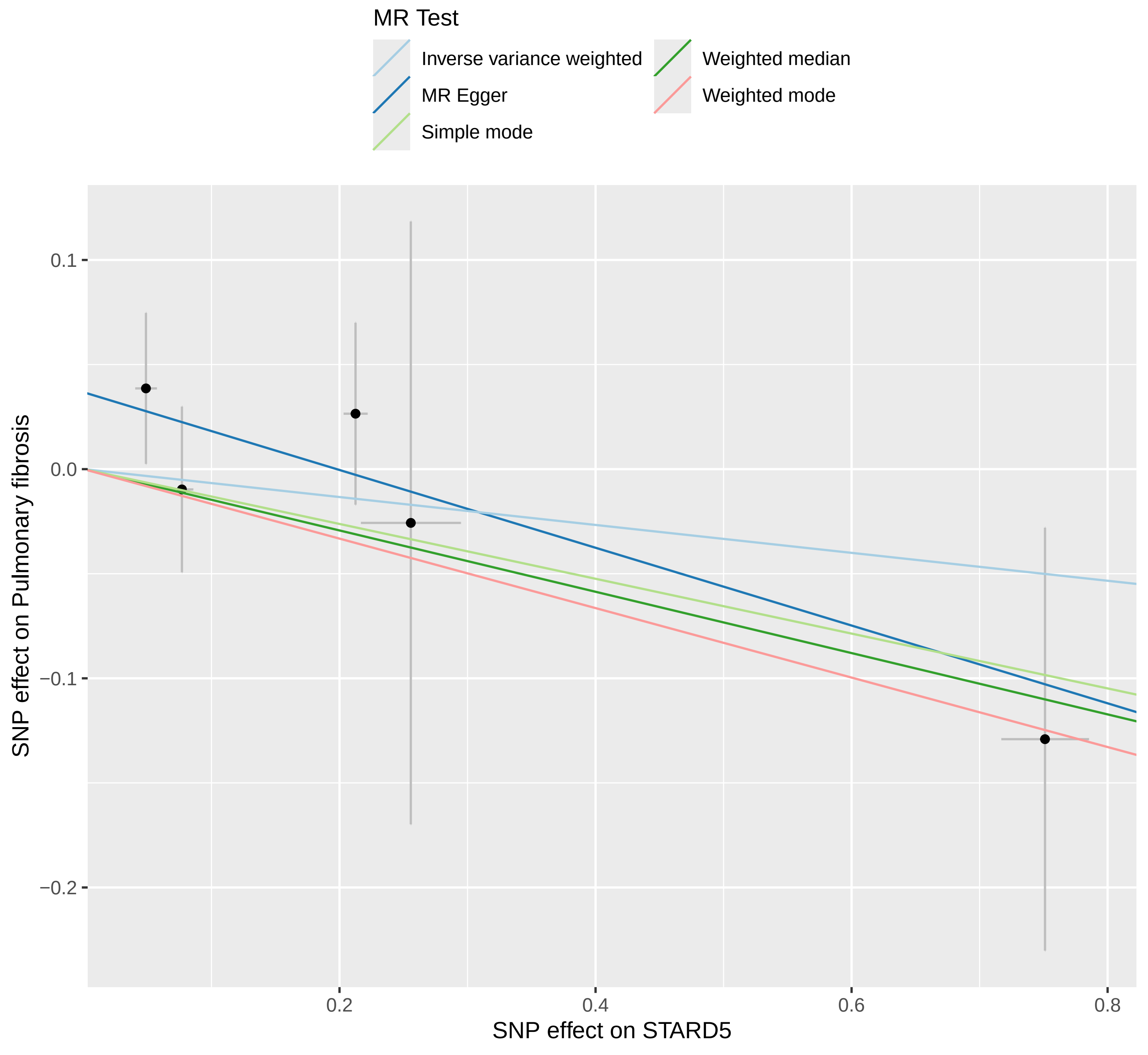
MR Test
Inverse variance weighted
MR Egger
Simple mode
Weighted median
Weighted mode
0.1
0.0
−0.1
−0.2
0.2
0.4
0.6
0.8
SNP effect on Pulmonary fibrosis
SNP effect on STARD5

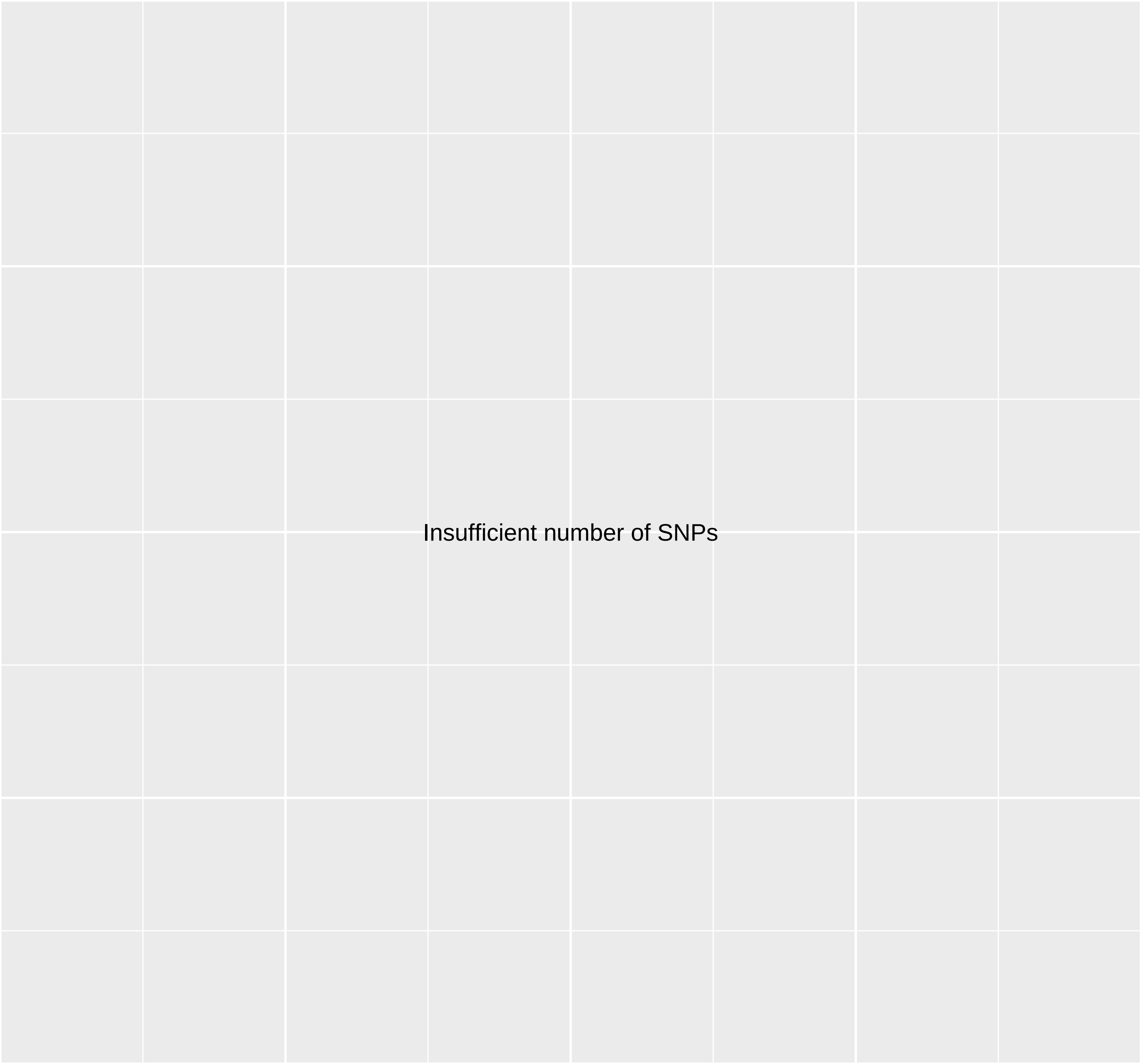
Insufficient number of SNPs

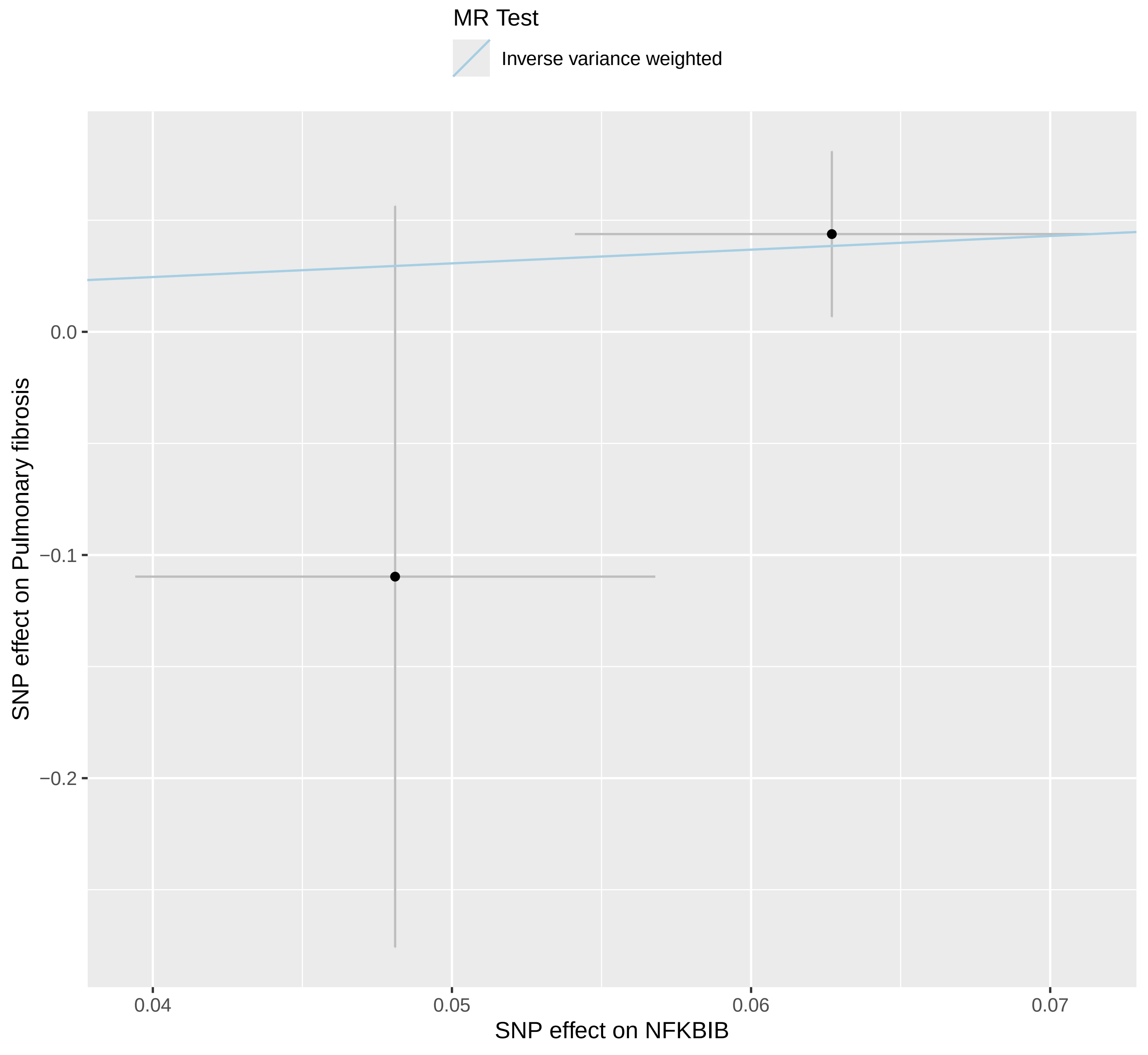
MR Test
Inverse variance weighted
0.0
−0.1
−0.2
0.04
0.05
0.06
0.07
SNP effect on Pulmonary fibrosis
SNP effect on NFKBIB

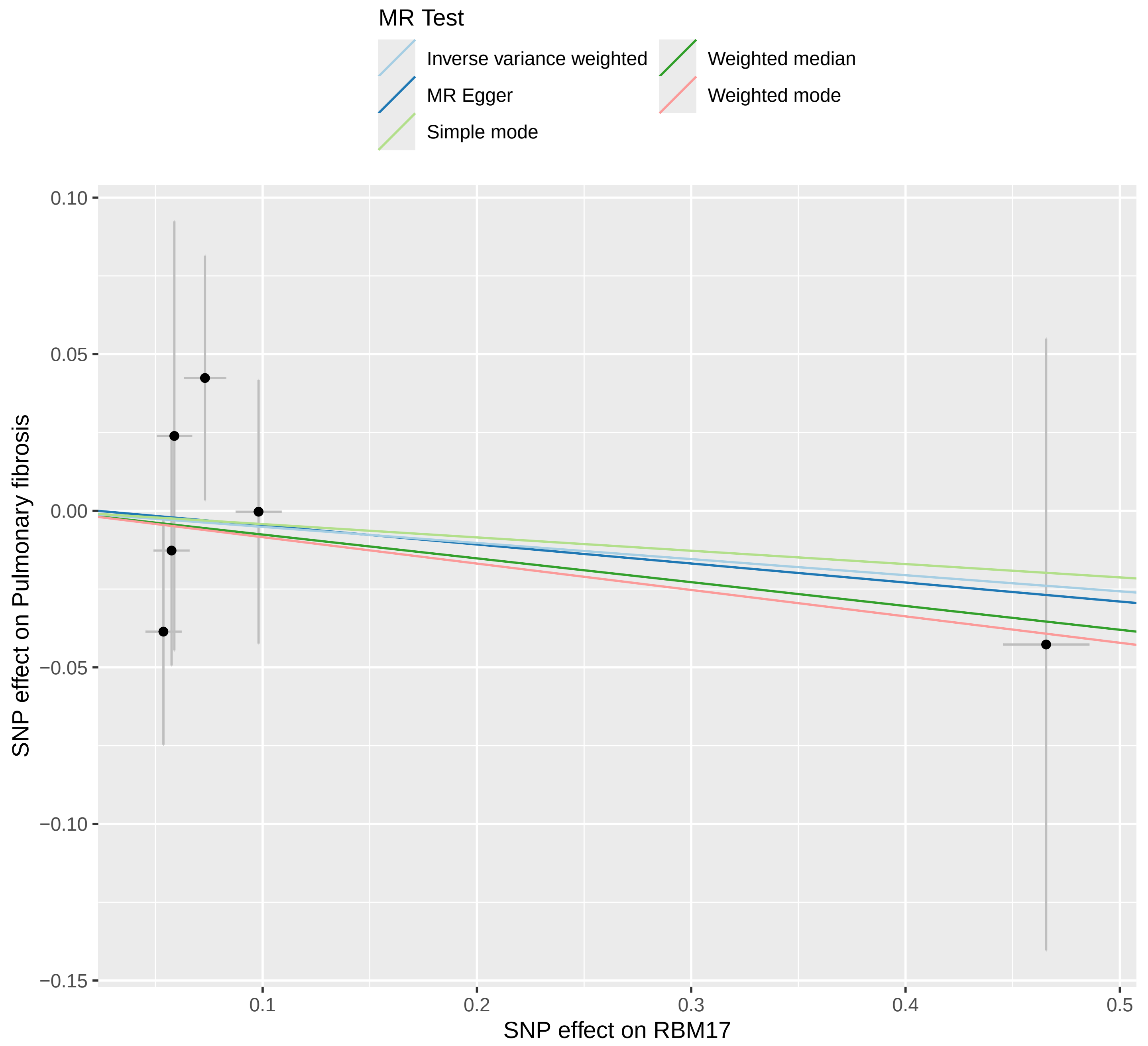
MR Test
Inverse variance weighted
MR Egger
Simple mode
Weighted median
Weighted mode
0.10
0.05
0.00
−0.05
−0.10
−0.15
0.1
0.2
0.3
0.4
0.5
SNP effect on RBM17
SNP effect on Pulmonary fibrosis

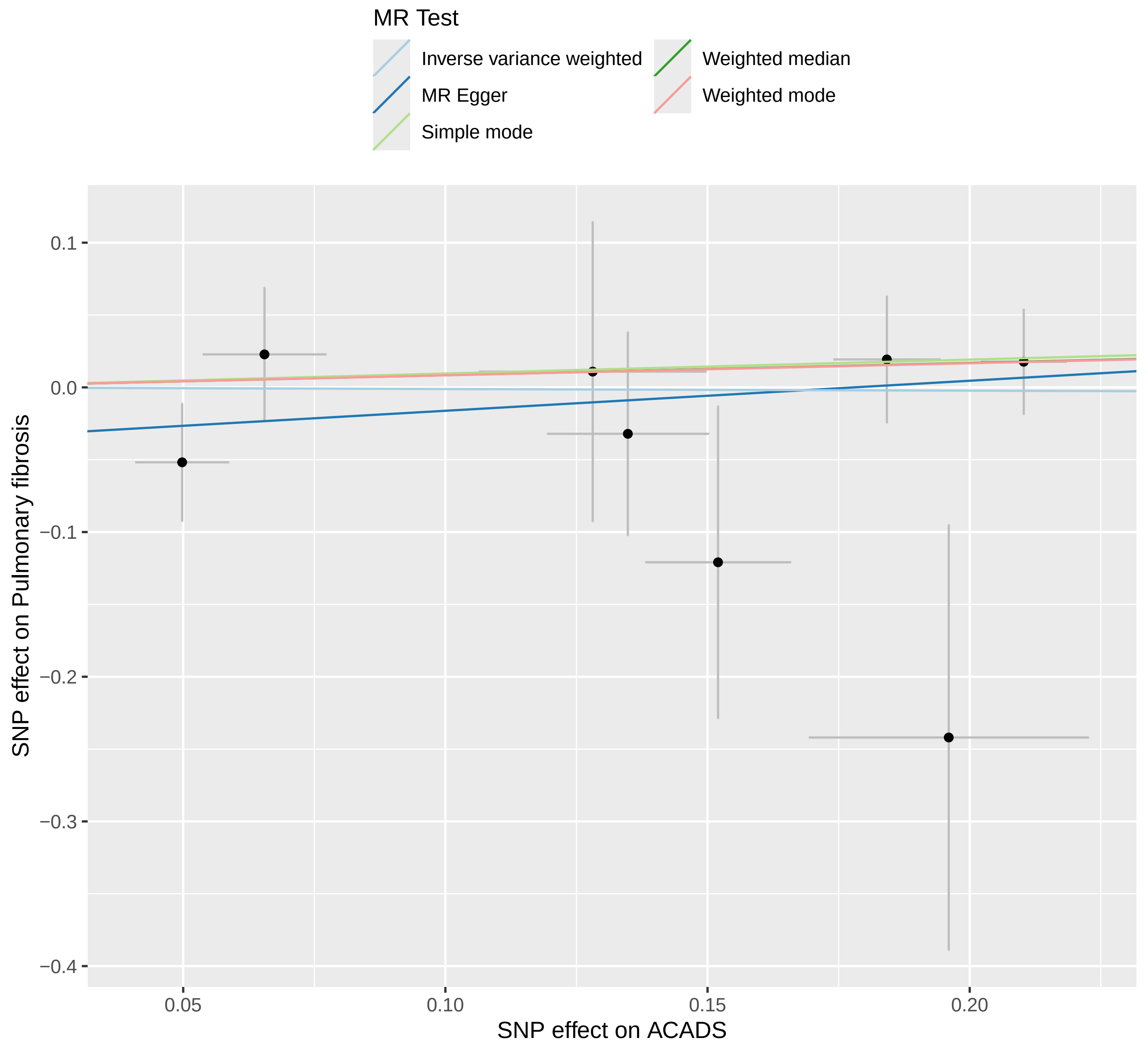

MR Test
Inverse variance weighted
MR Egger
Simple mode
Weighted median
Weighted mode
SNP effect on Pulmonary fibrosis
0.1
0.0
−0.1
−0.2
−0.3
−0.4
0.05
0.10
0.15
0.20
SNP effect on ACADS

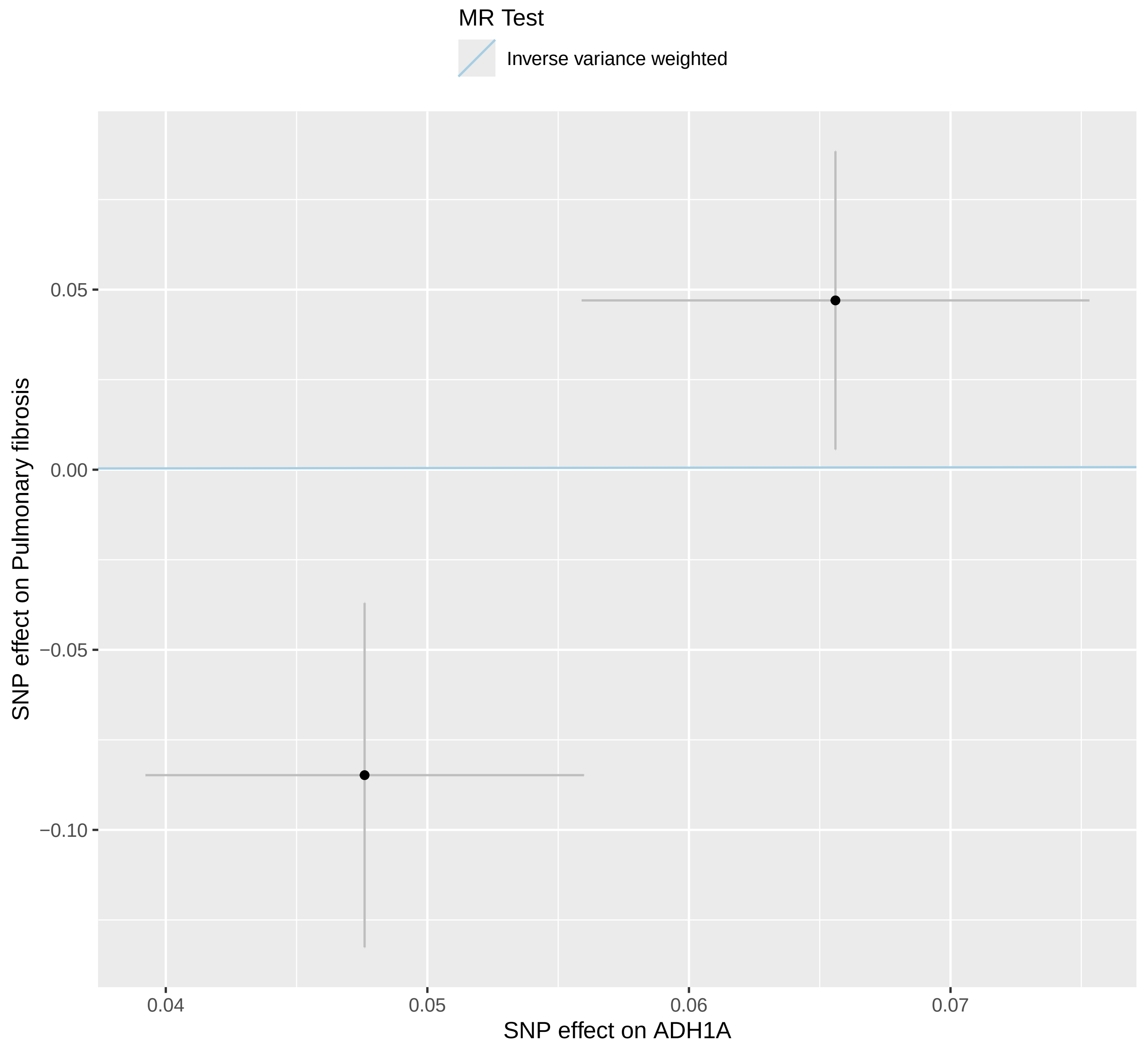

MR Test
Inverse variance weighted
SNP effect on Pulmonary fibrosis
0.05
0.00
−0.05
−0.10
0.04
0.05
0.06
0.07
SNP effect on ADH1A

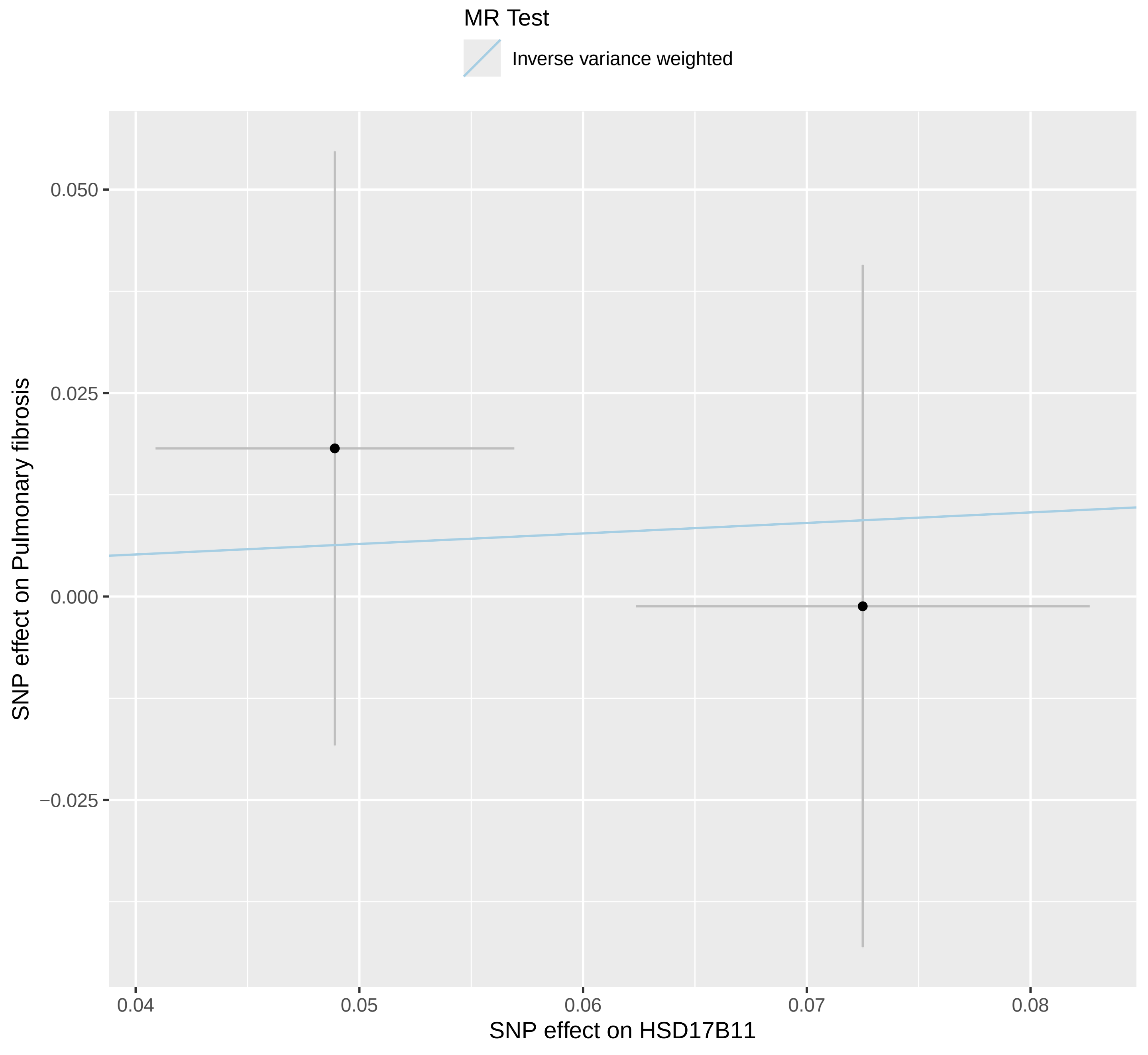

MR Test
Inverse variance weighted
0.050
0.025
0.000
−0.025
0.04
0.05
0.06
0.07
0.08
SNP effect on HSD17B11
SNP effect on Pulmonary fibrosis

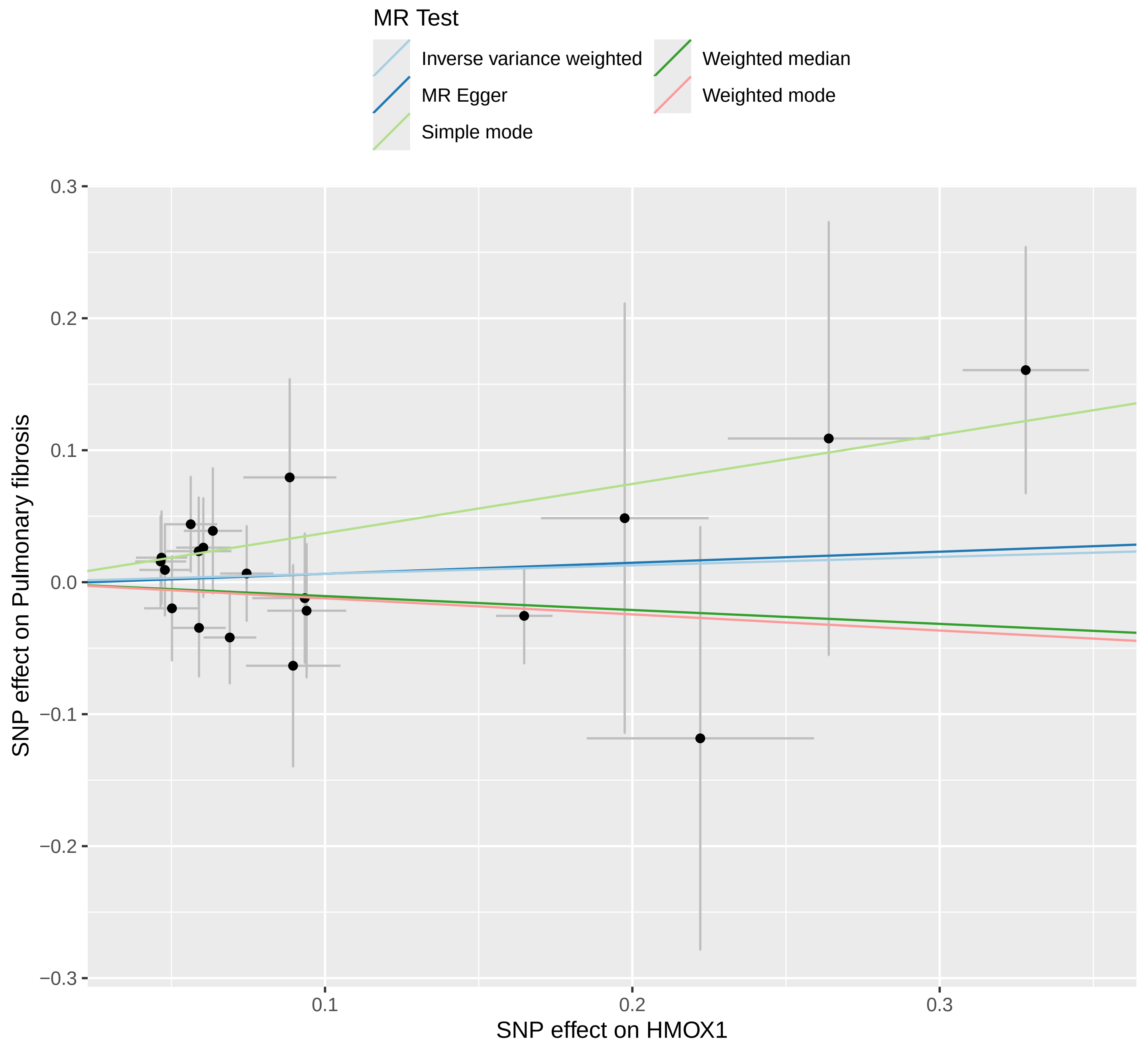

MR Test
Inverse variance weighted
MR Egger
Simple mode
Weighted median
Weighted mode
0.3
0.2
0.1
0.0
−0.1
−0.2
−0.3
SNP effect on Pulmonary fibrosis
0.1
0.2
0.3
SNP effect on HMOX1

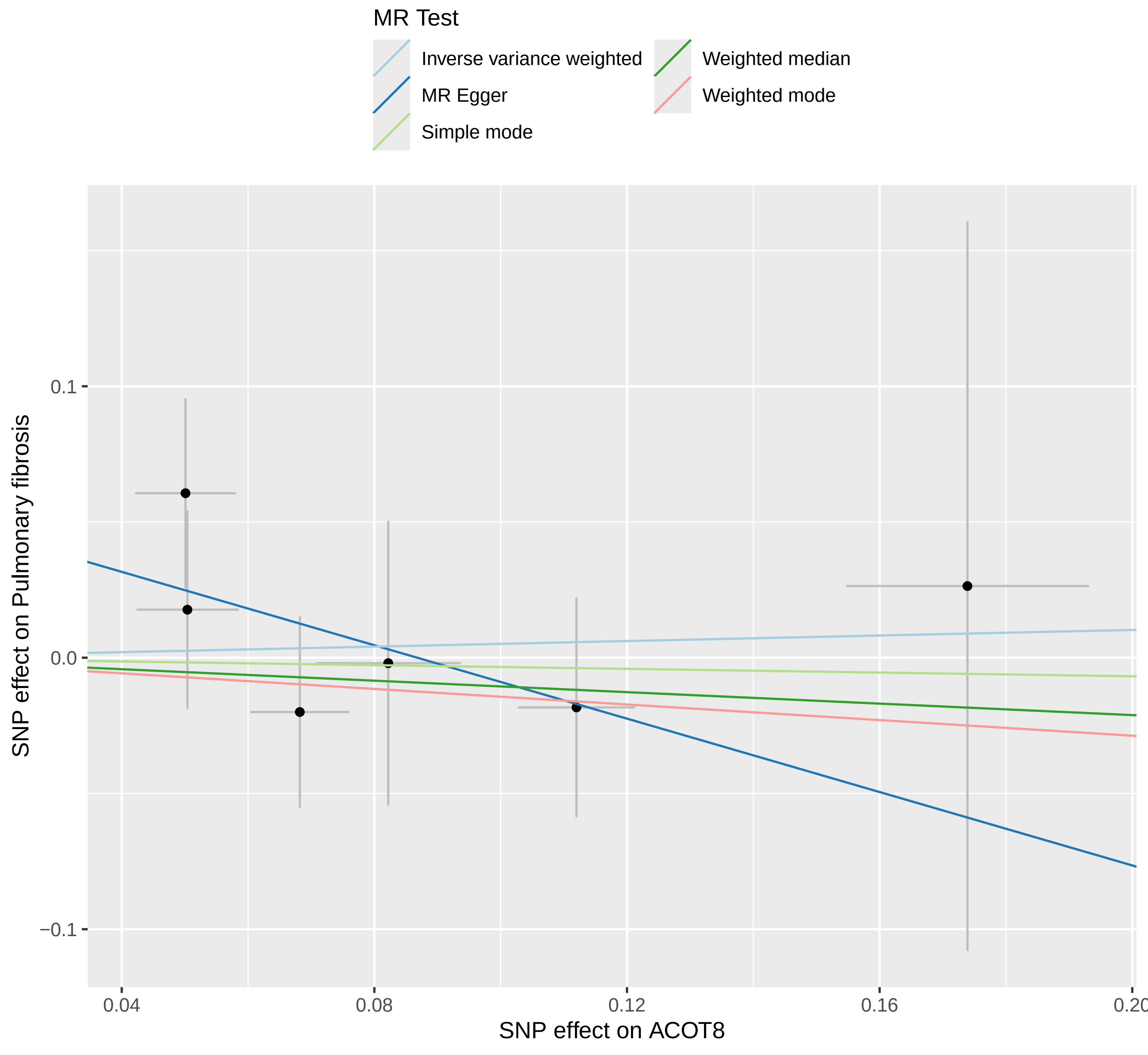

MR Test
Inverse variance weighted
MR Egger
Simple mode
Weighted median
Weighted mode
0.1
0.0
−0.1
SNP effect on Pulmonary fibrosis
0.04
0.08
0.12
0.16
0.20
SNP effect on ACOT8

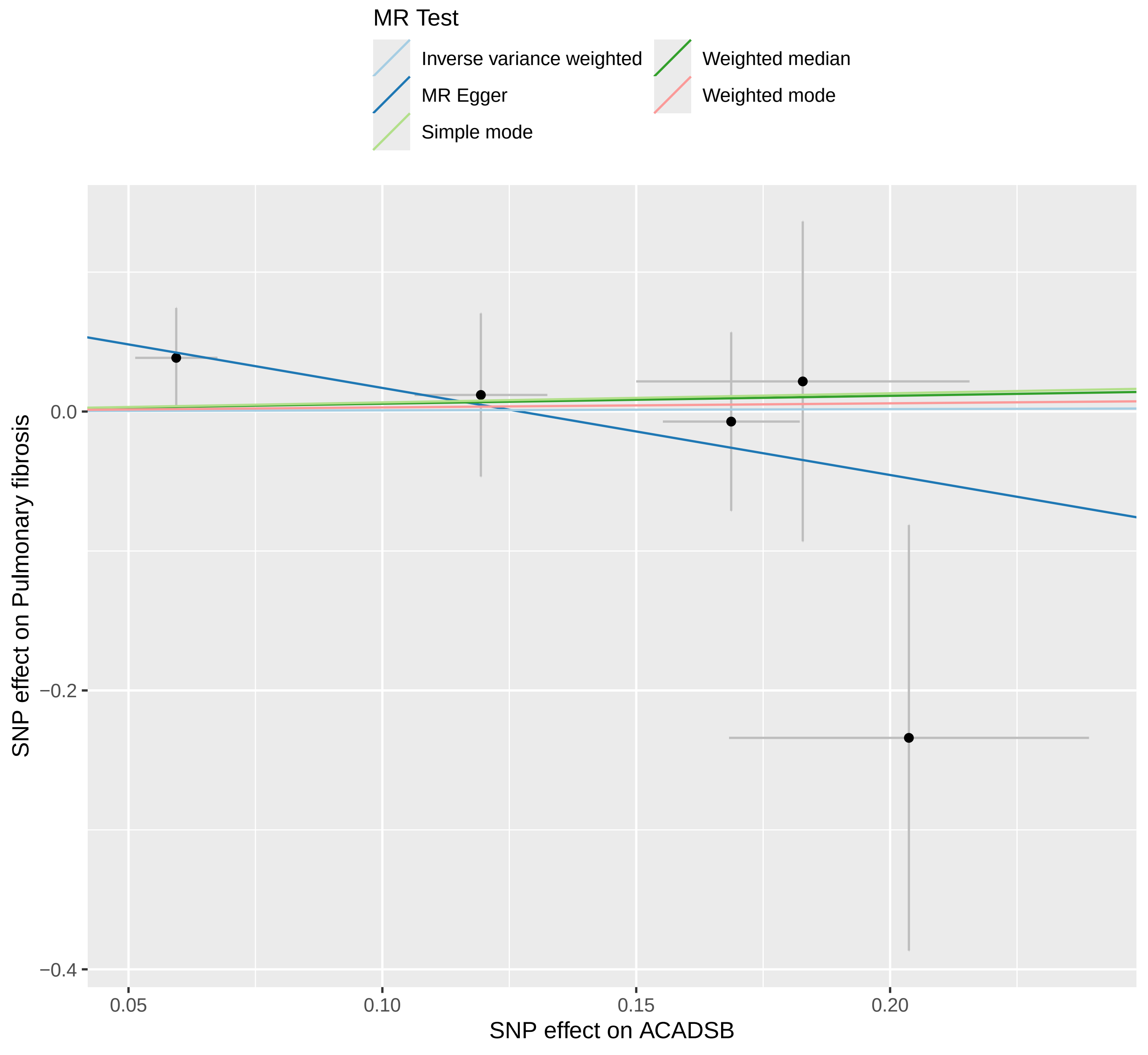
MR Test
Inverse variance weighted
MR Egger
Simple mode
Weighted median
Weighted mode
SNP effect on Pulmonary fibrosis
0.0
−0.2
−0.4
0.05
0.10
0.15
0.20
SNP effect on ACADSB

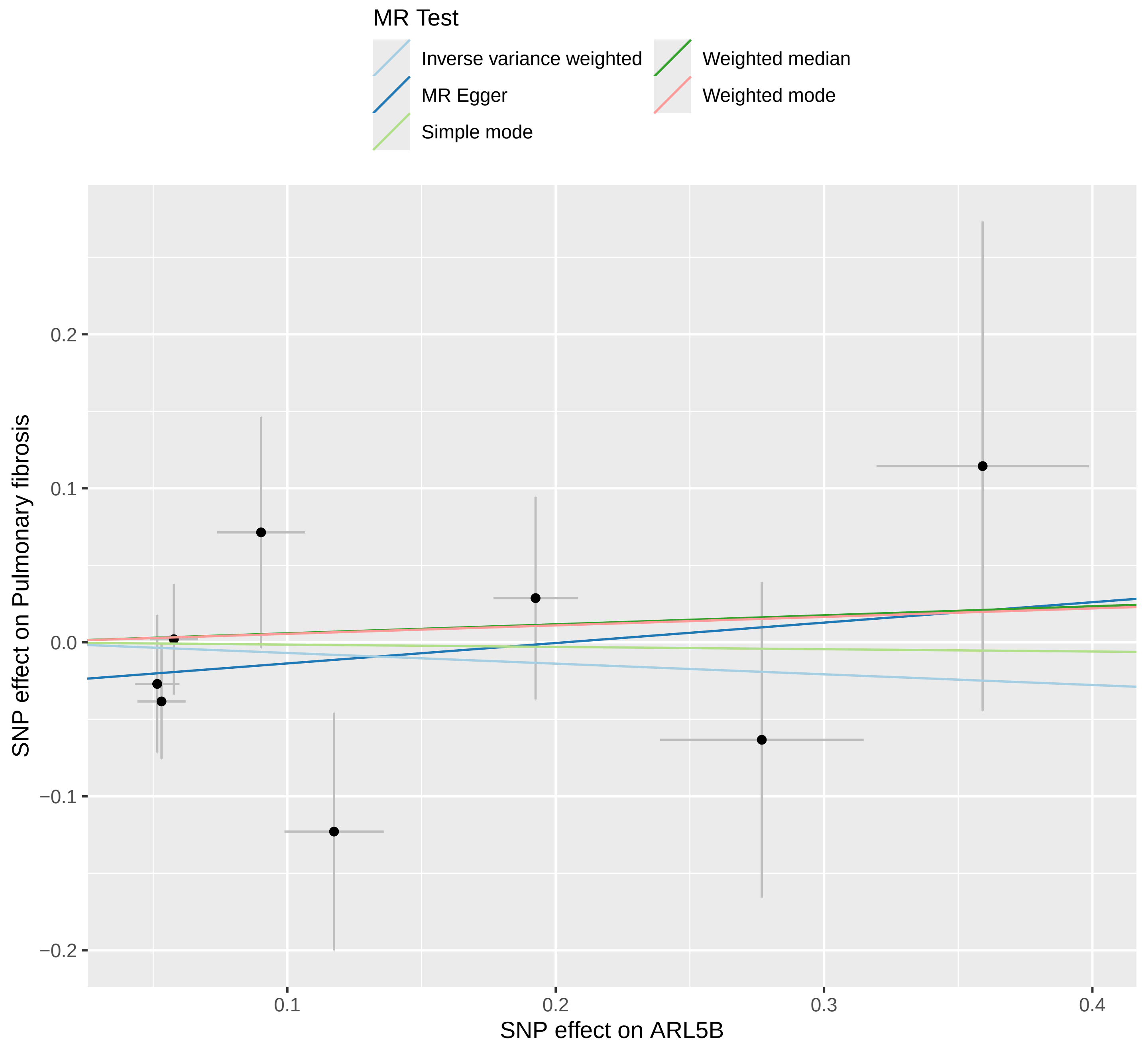

MR Test
Inverse variance weighted
MR Egger
Simple mode
Weighted median
Weighted mode
0.2
0.1
0.0
−0.1
−0.2
SNP effect on Pulmonary fibrosis
0.1
0.2
0.3
0.4
SNP effect on ARL5B

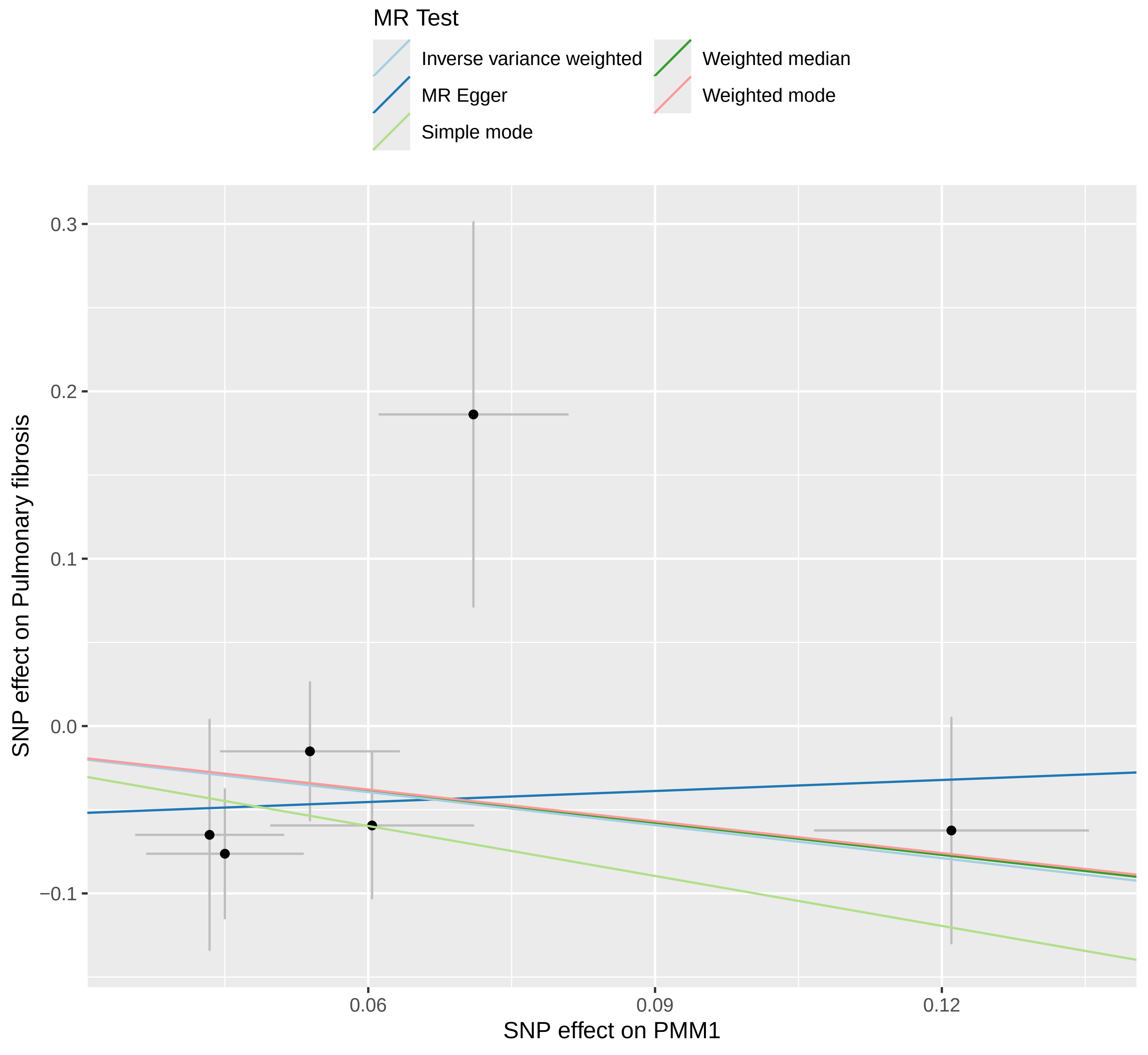
MR Test
Inverse variance weighted
MR Egger
Simple mode
Weighted median
Weighted mode
0.3
0.2
0.1
0.0
−0.1
0.06
0.09
0.12
SNP effect on Pulmonary fibrosis
SNP effect on PMM1

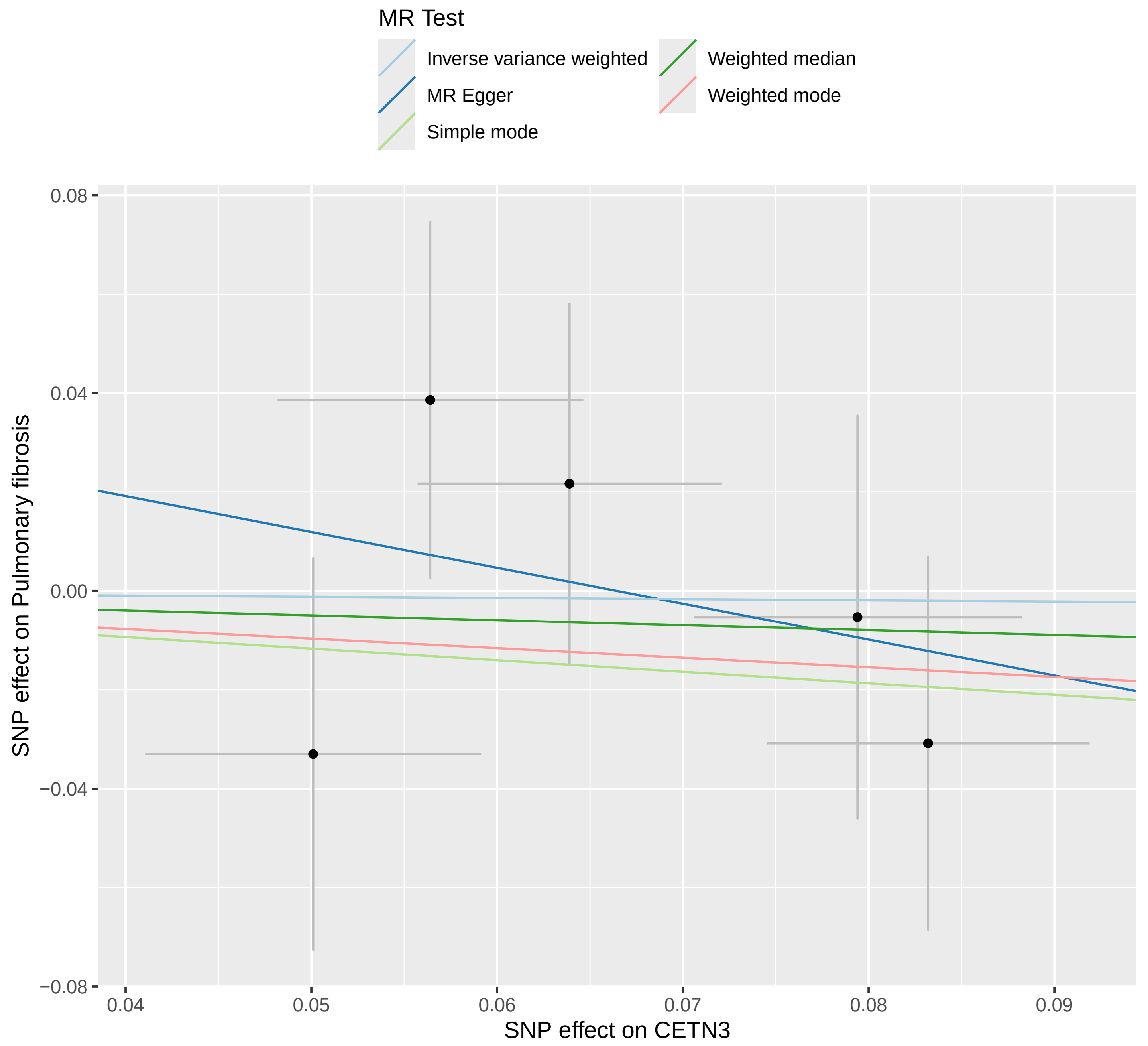
MR Test
Inverse variance weighted
MR Egger
Simple mode
Weighted median
Weighted mode
0.08
0.04
0.00
−0.04
−0.08
0.04
0.05
0.06
0.07
0.08
0.09
SNP effect on Pulmonary fibrosis
SNP effect on CETN3

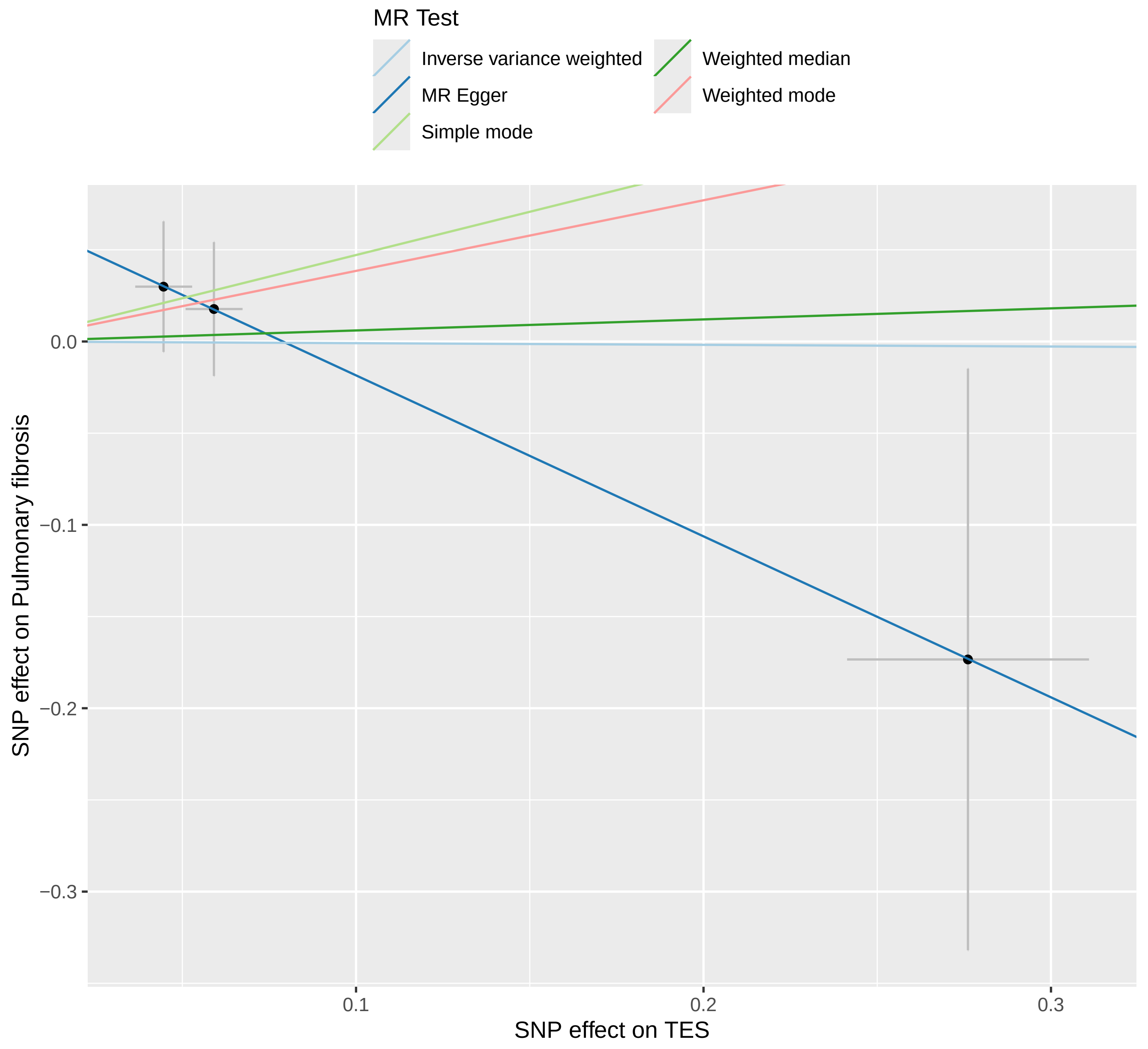
MR Test
Inverse variance weighted
MR Egger
Simple mode
Weighted median
Weighted mode
SNP effect on Pulmonary fibrosis
0.0
−0.1
−0.2
−0.3
0.1
0.2
0.3
SNP effect on TES

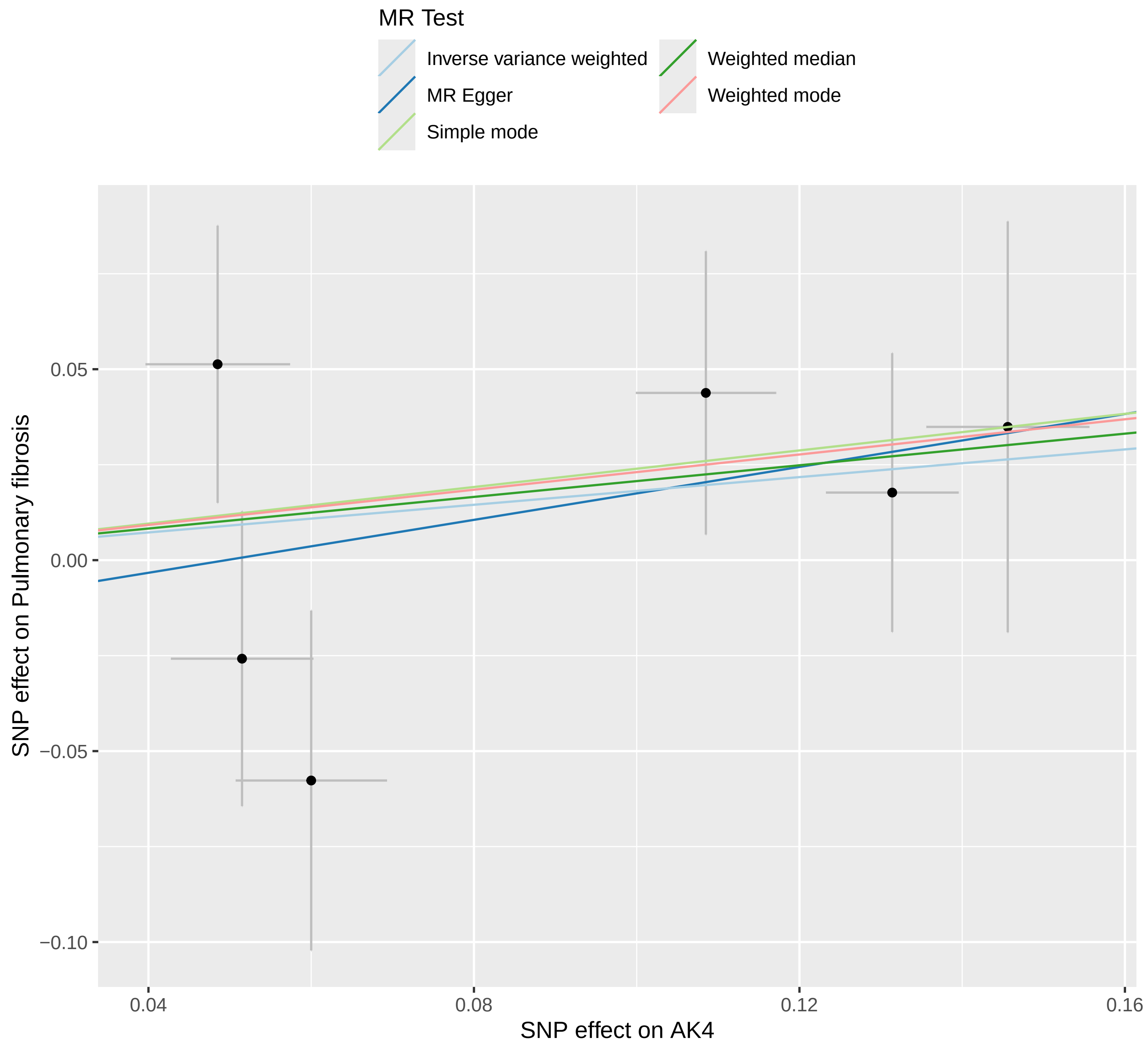

MR Test
Inverse variance weighted
MR Egger
Simple mode
Weighted median
Weighted mode
0.05
0.00
−0.05
−0.10
SNP effect on Pulmonary fibrosis
0.04
0.08
0.12
0.16
SNP effect on AK4

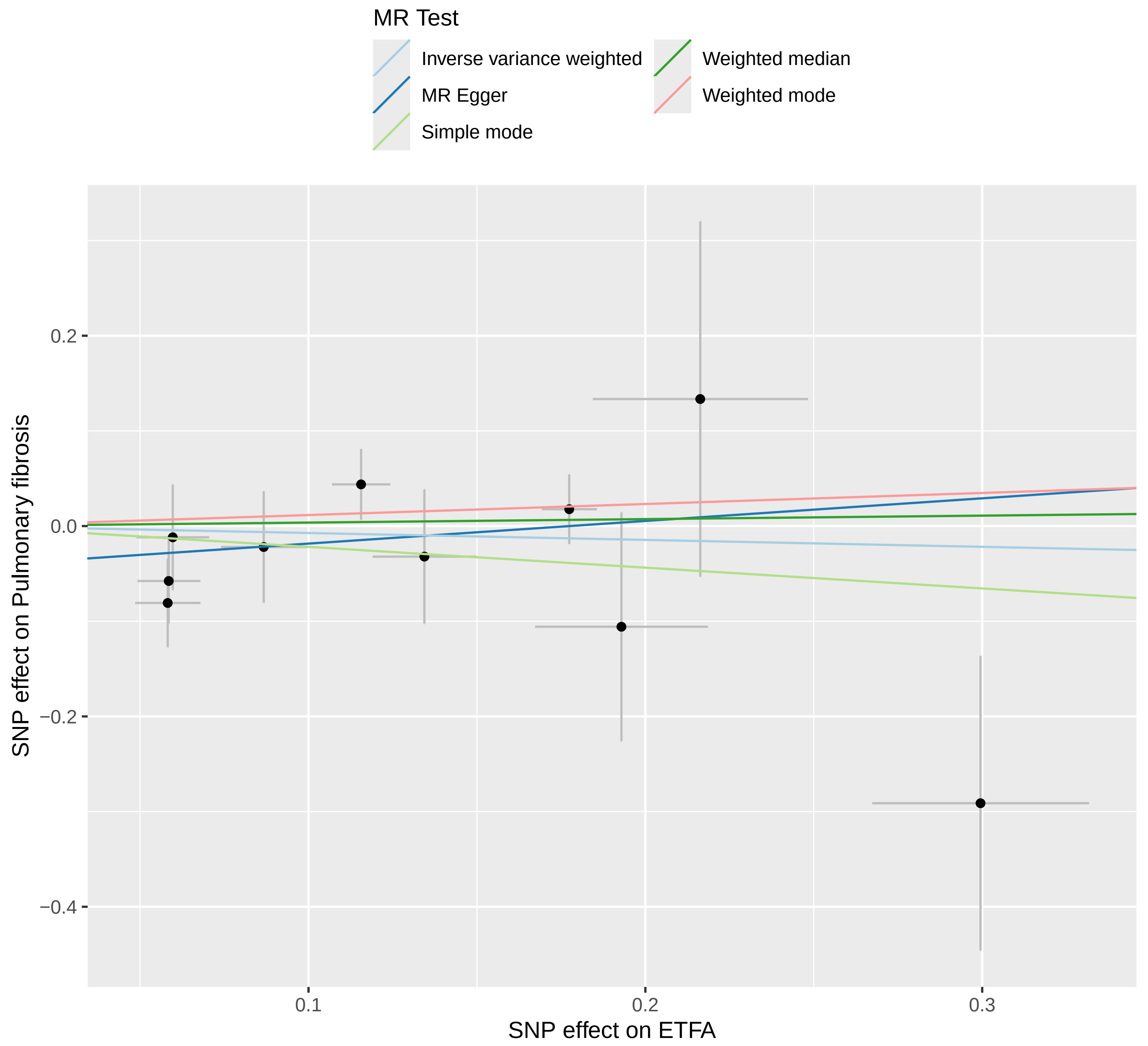
MR Test
Inverse variance weighted
MR Egger
Simple mode
Weighted median
Weighted mode
0.2
0.0
−0.2
−0.4
0.1
0.2
0.3
SNP effect on Pulmonary fibrosis
SNP effect on ETFA

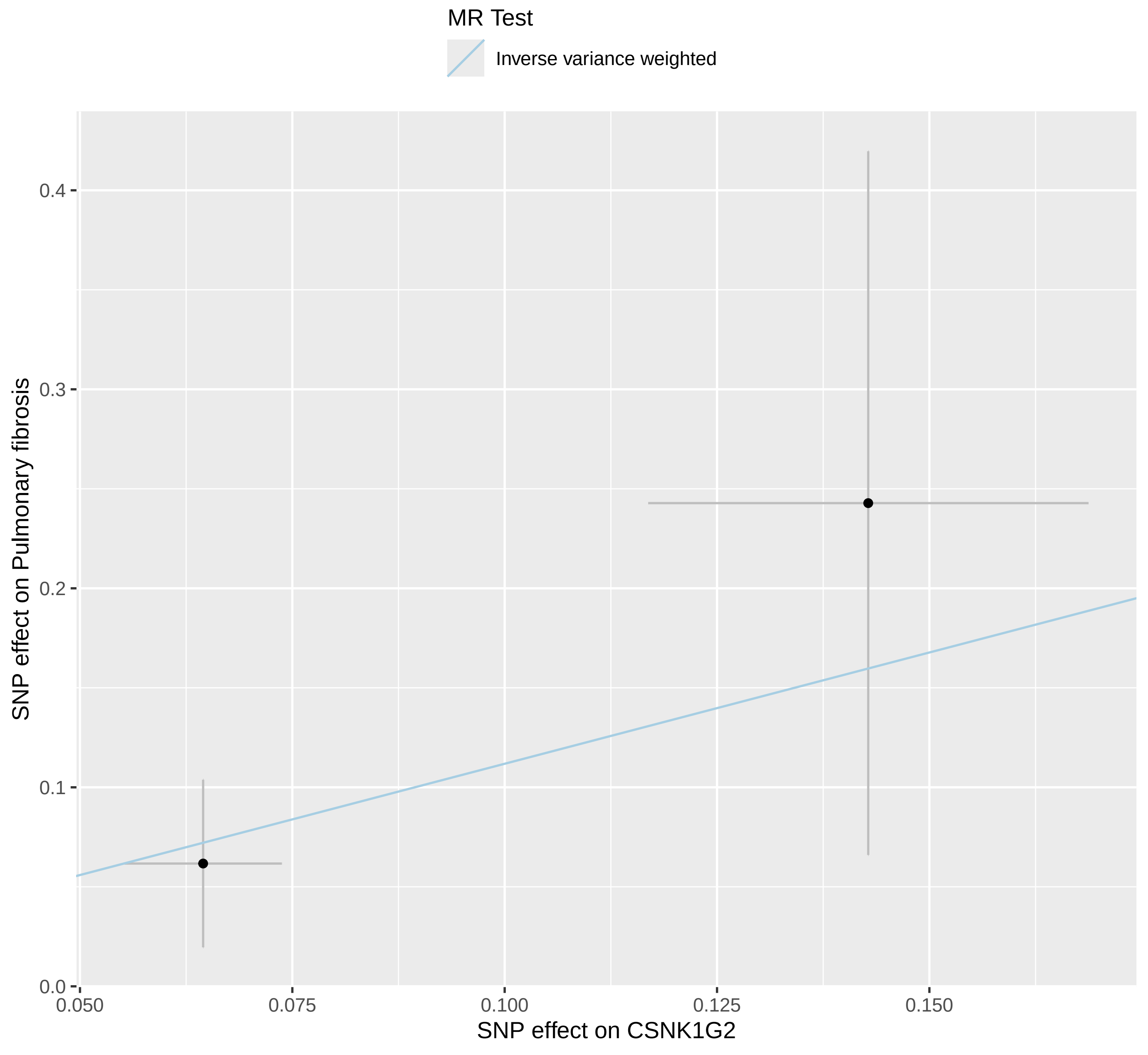
MR Test
Inverse variance weighted
0.4
0.3
0.2
0.1
0.0
SNP effect on Pulmonary fibrosis
0.050
0.075
0.100
0.125
0.150
SNP effect on CSNK1G2

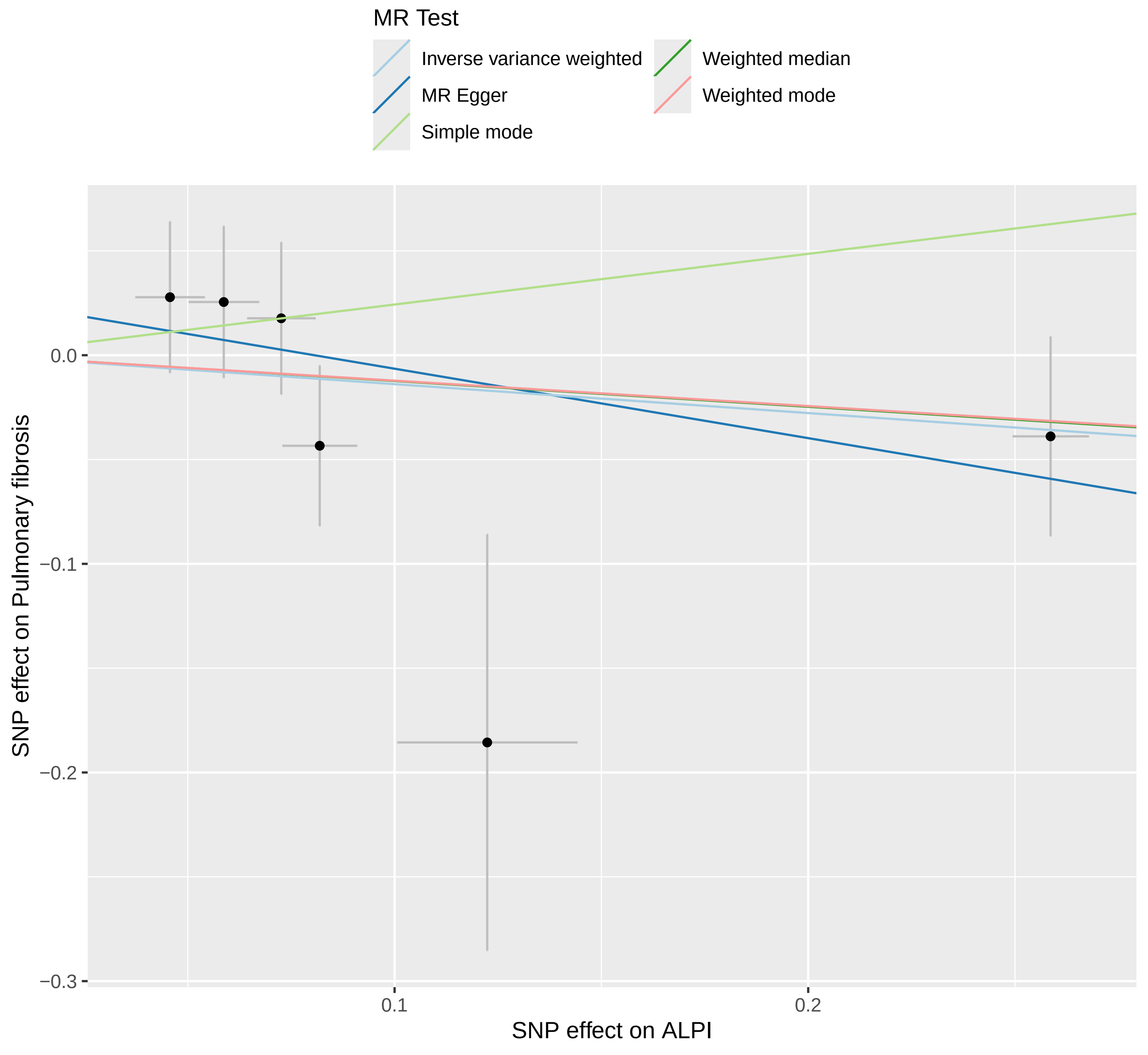
MR Test
Inverse variance weighted
MR Egger
Simple mode
Weighted median
Weighted mode
0.0
−0.1
−0.2
−0.3
0.1
0.2
SNP effect on Pulmonary fibrosis
SNP effect on ALPI

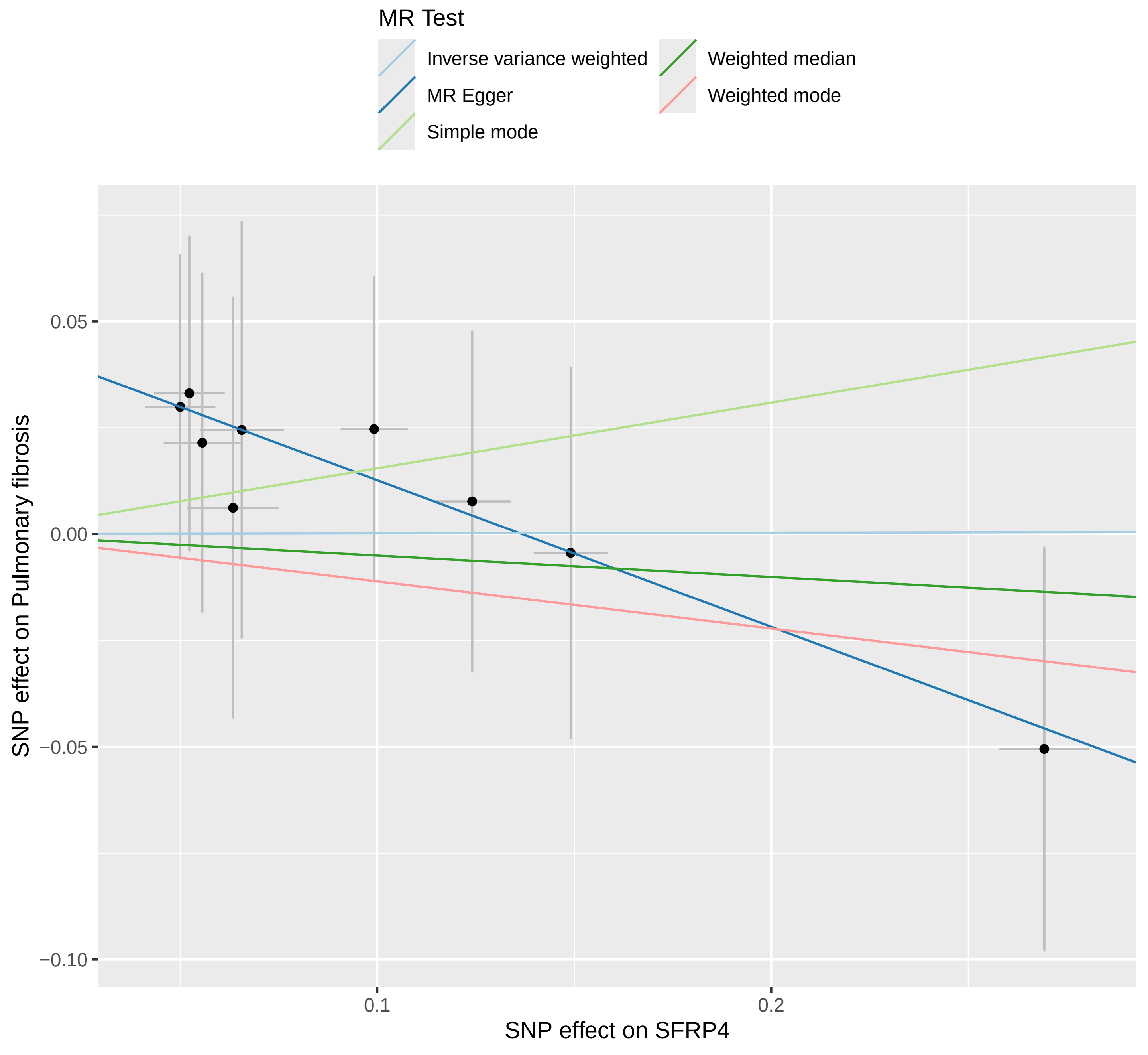
MR Test
Inverse variance weighted
MR Egger
Simple mode
Weighted median
Weighted mode
0.05
0.00
−0.05
−0.10
0.1
0.2
SNP effect on Pulmonary fibrosis
SNP effect on SFRP4

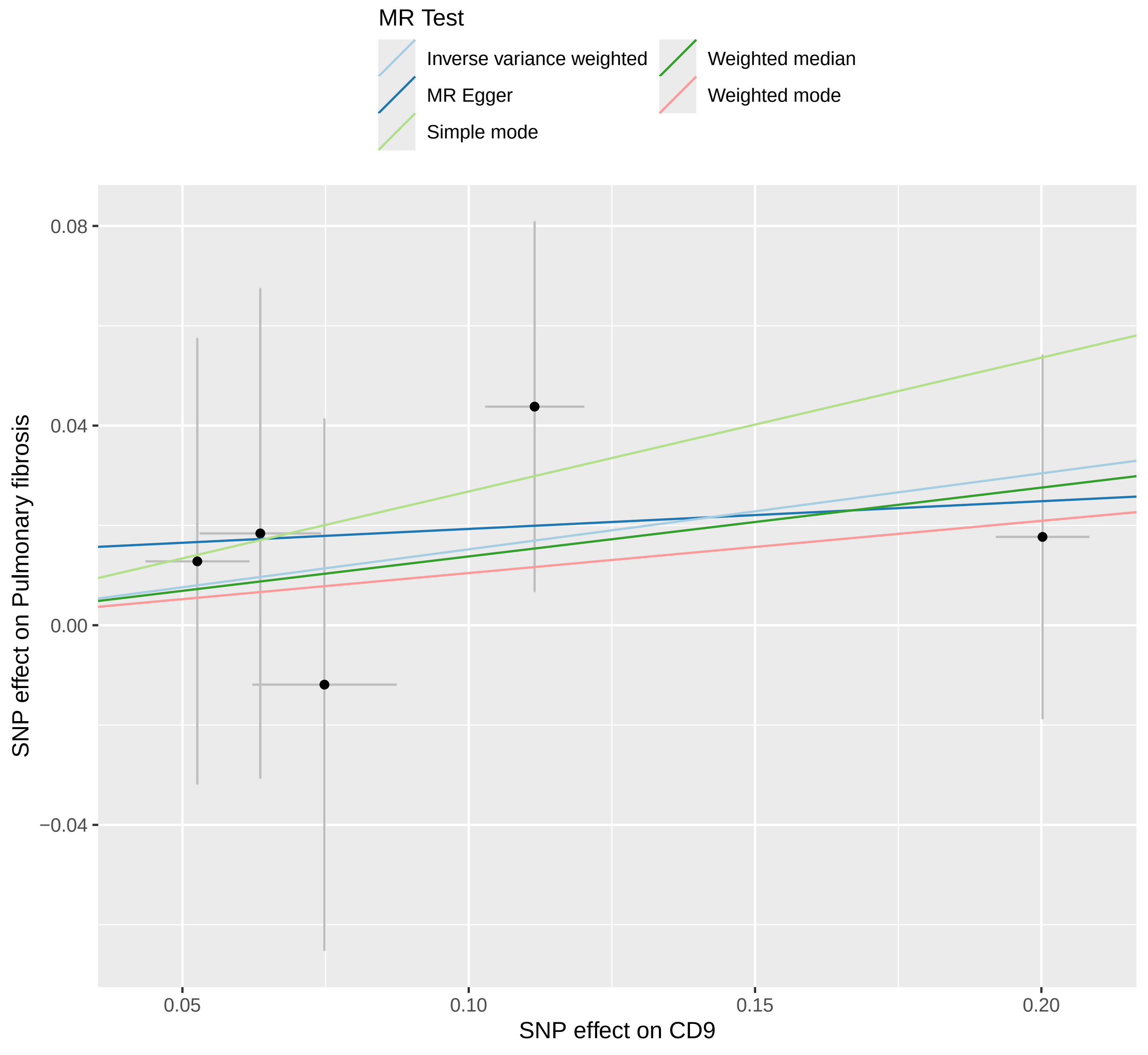

MR Test
Inverse variance weighted
MR Egger
Simple mode
Weighted median
Weighted mode
0.08
0.04
0.00
−0.04
0.05
0.10
0.15
0.20
SNP effect on Pulmonary fibrosis
SNP effect on CD9

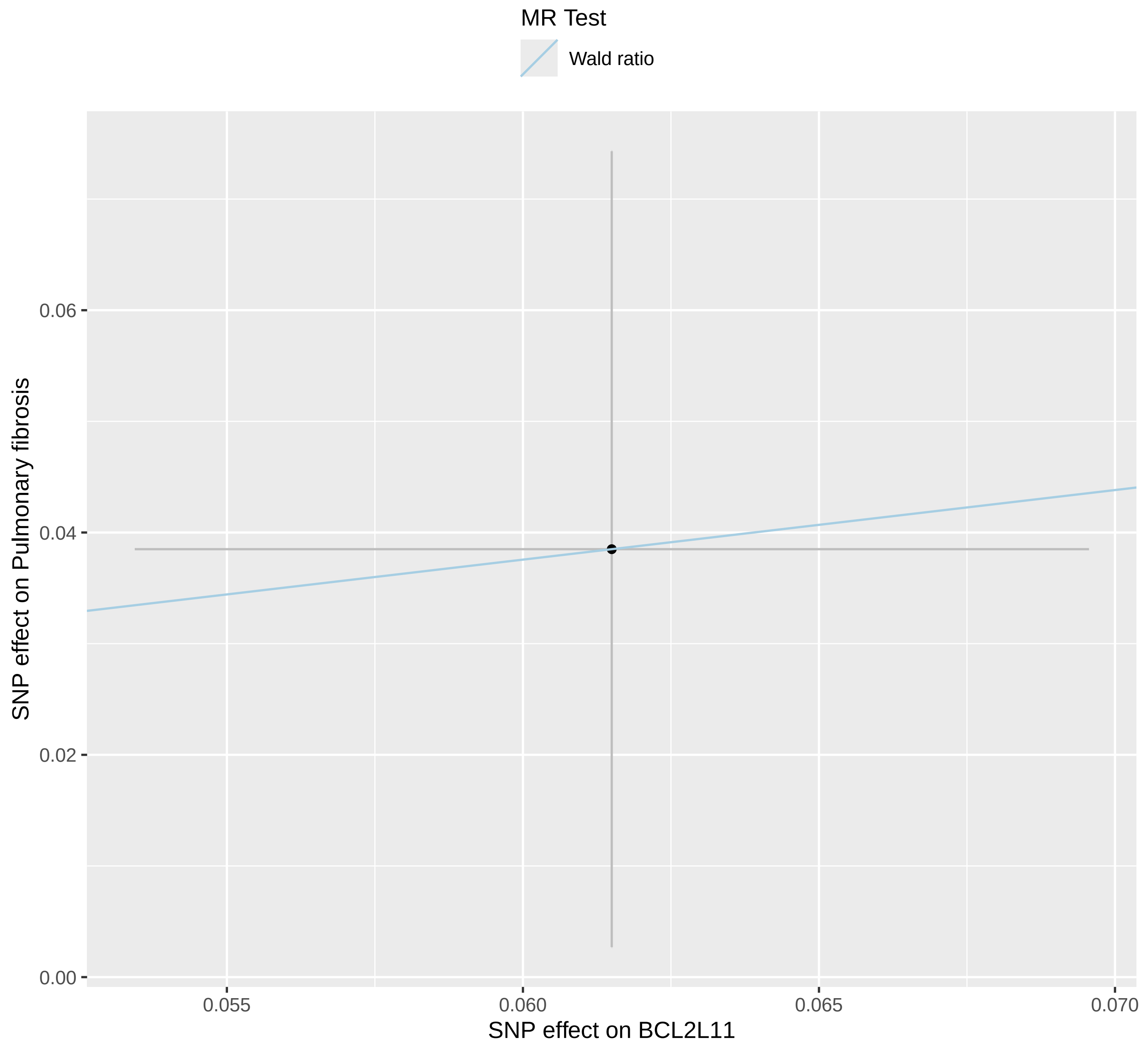

MR Test
Wald ratio
0.06
0.04
0.02
0.00
SNP effect on Pulmonary fibrosis
0.055
0.060
0.065
0.070
SNP effect on BCL2L11

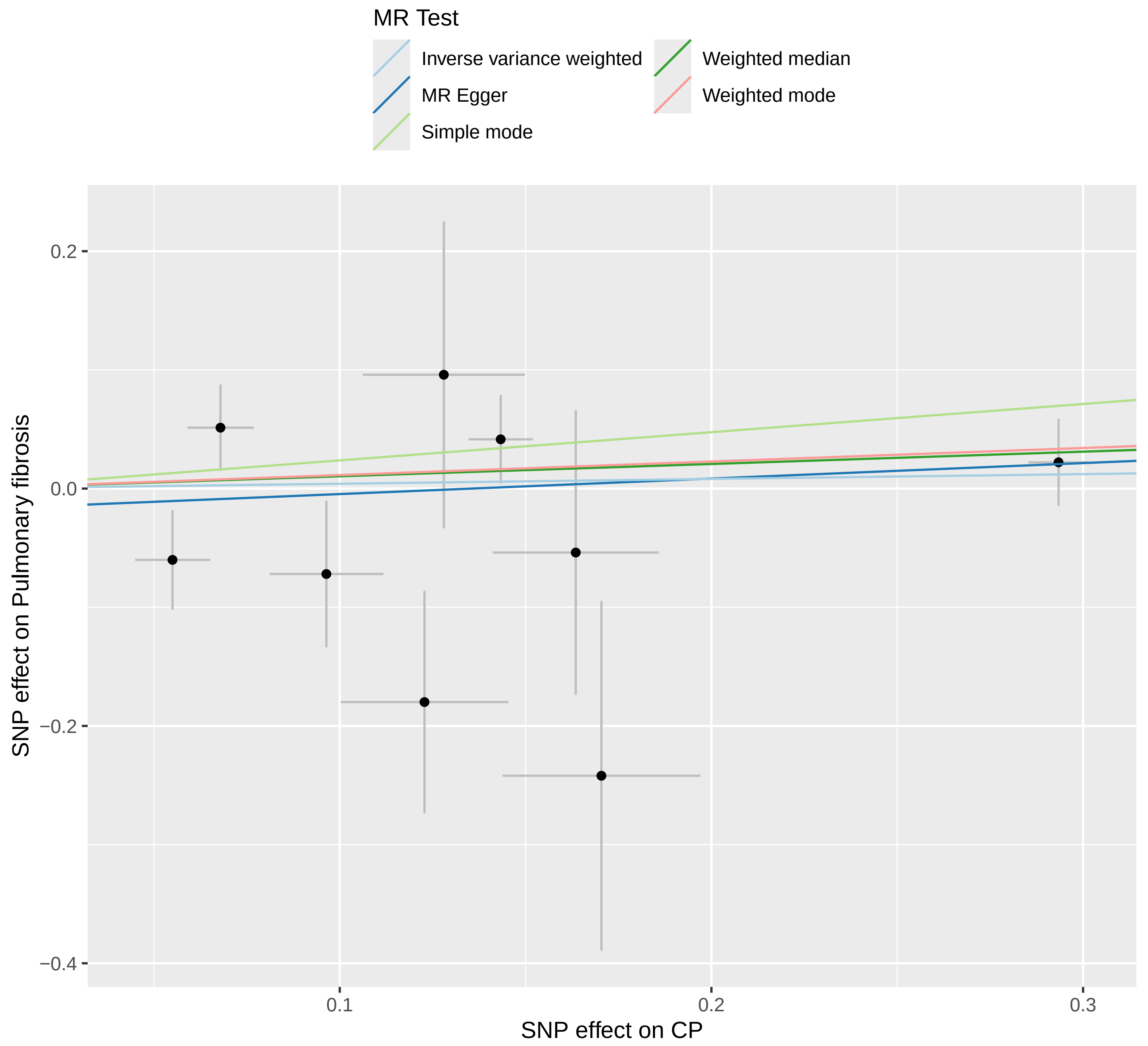
MR Test
Inverse variance weighted
MR Egger
Simple mode
Weighted median
Weighted mode
0.2
0.0
−0.2
−0.4
0.1
0.2
0.3
SNP effect on Pulmonary fibrosis
SNP effect on CP

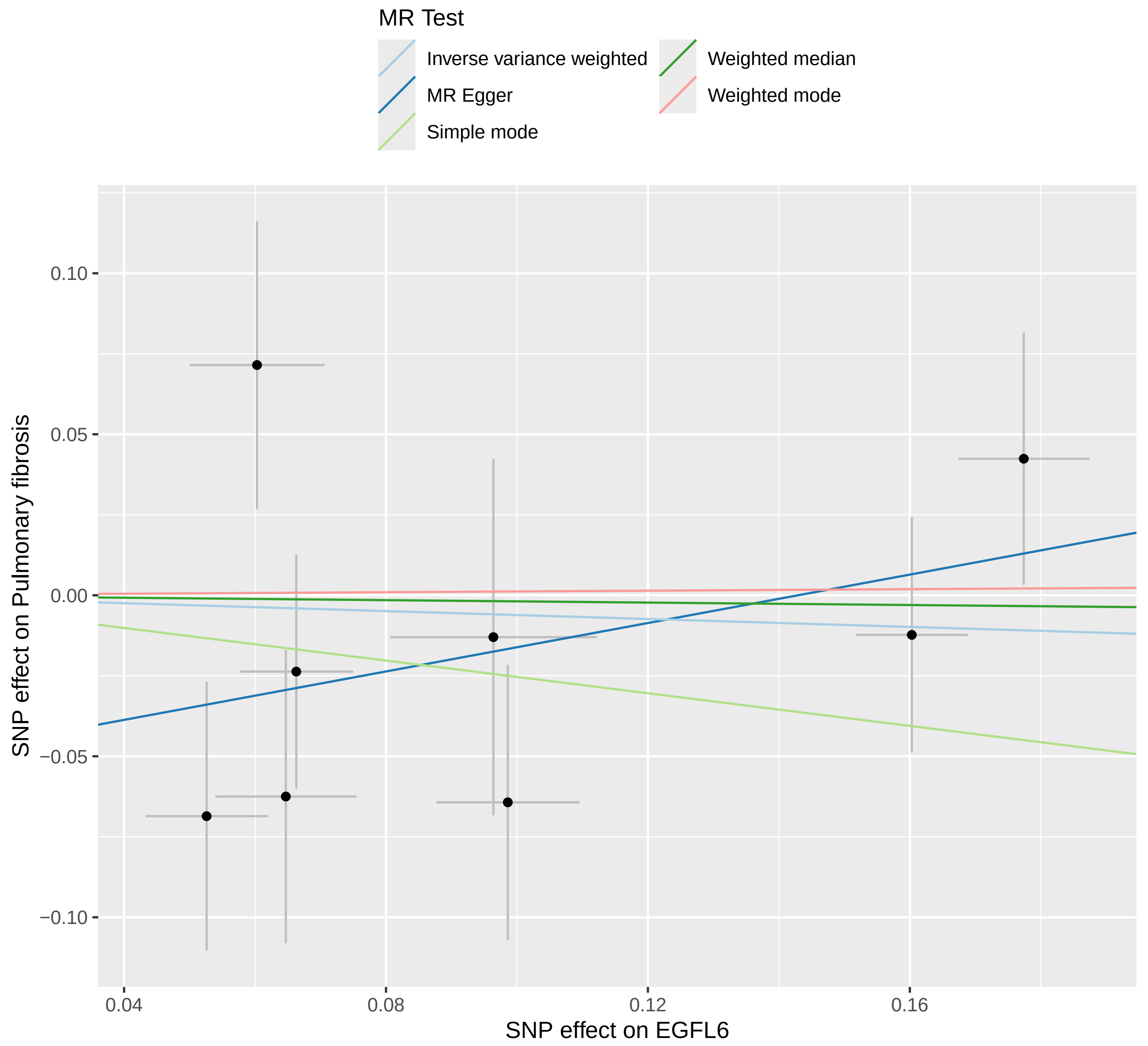

MR Test
Inverse variance weighted
MR Egger
Simple mode
Weighted median
Weighted mode
0.10
0.05
0.00
−0.05
−0.10
SNP effect on Pulmonary fibrosis
0.04
0.08
0.12
0.16
SNP effect on EGFL6

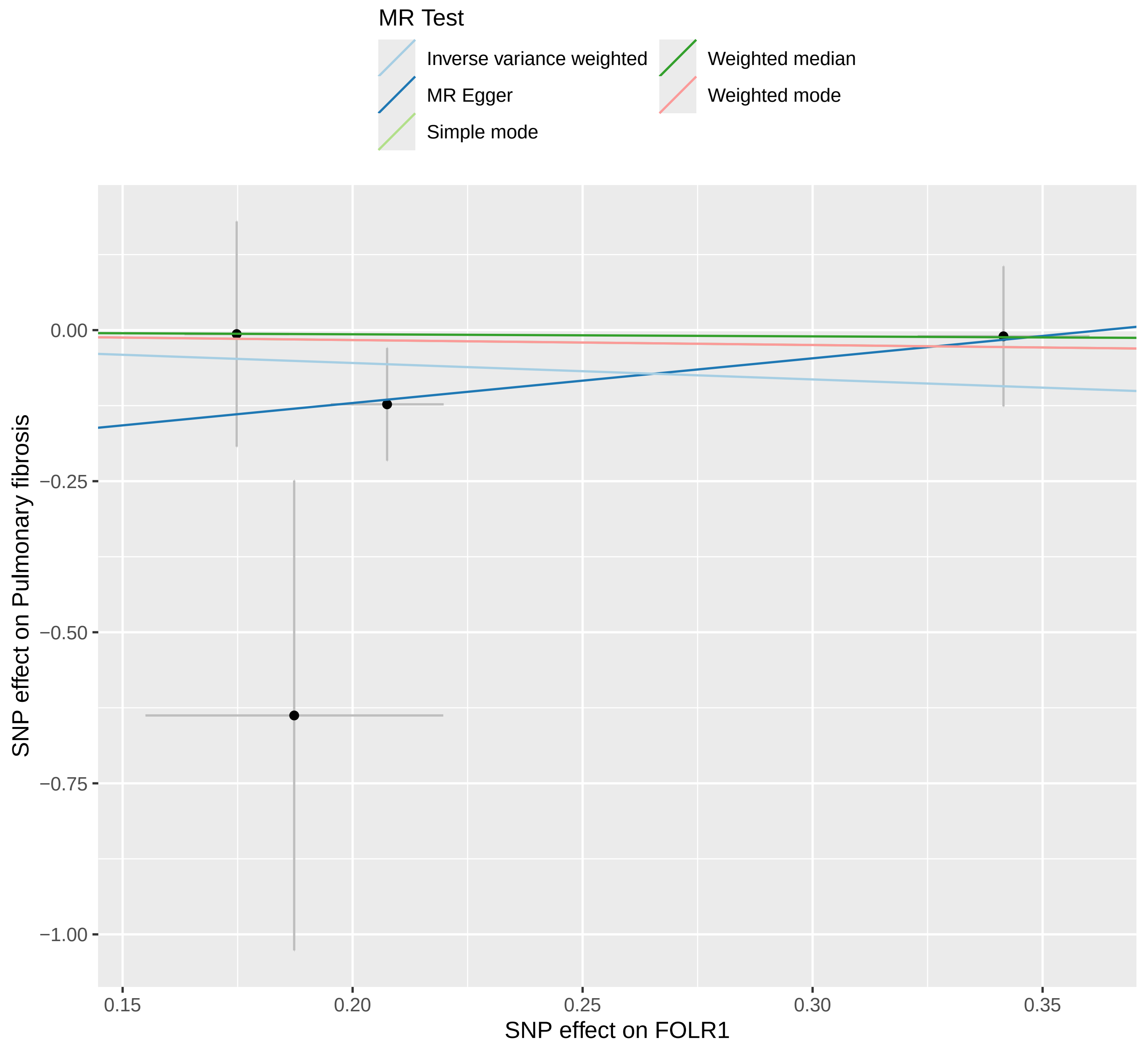

MR Test
Inverse variance weighted
MR Egger
Simple mode
Weighted median
Weighted mode
0.00
−0.25
−0.50
−0.75
−1.00
0.15
0.20
0.25
0.30
0.35
SNP effect on Pulmonary fibrosis
SNP effect on FOLR1

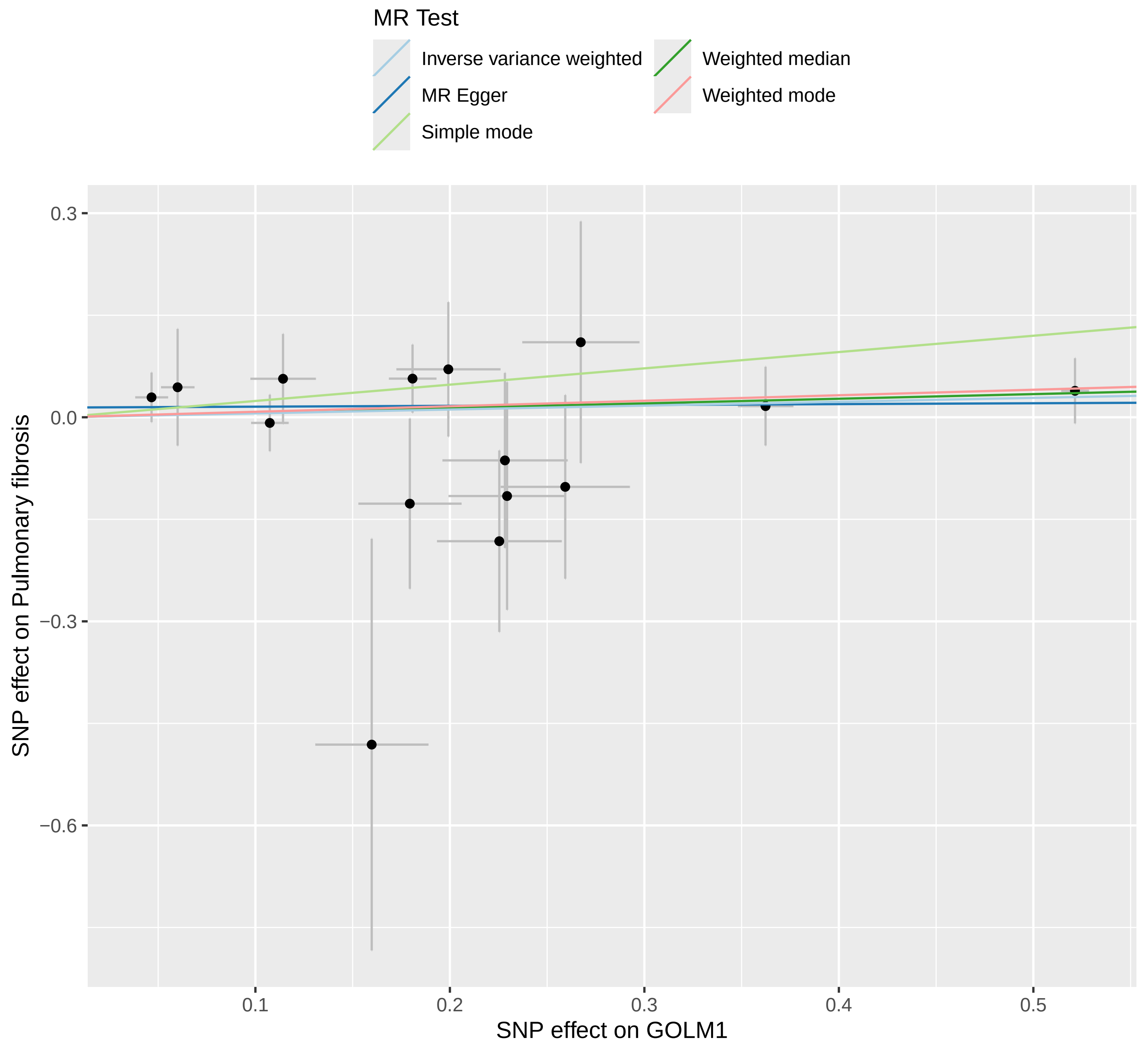

MR Test
Inverse variance weighted
MR Egger
Simple mode
Weighted median
Weighted mode
0.3
0.0
−0.3
−0.6
0.1
0.2
0.3
0.4
0.5
SNP effect on Pulmonary fibrosis
SNP effect on GOLM1

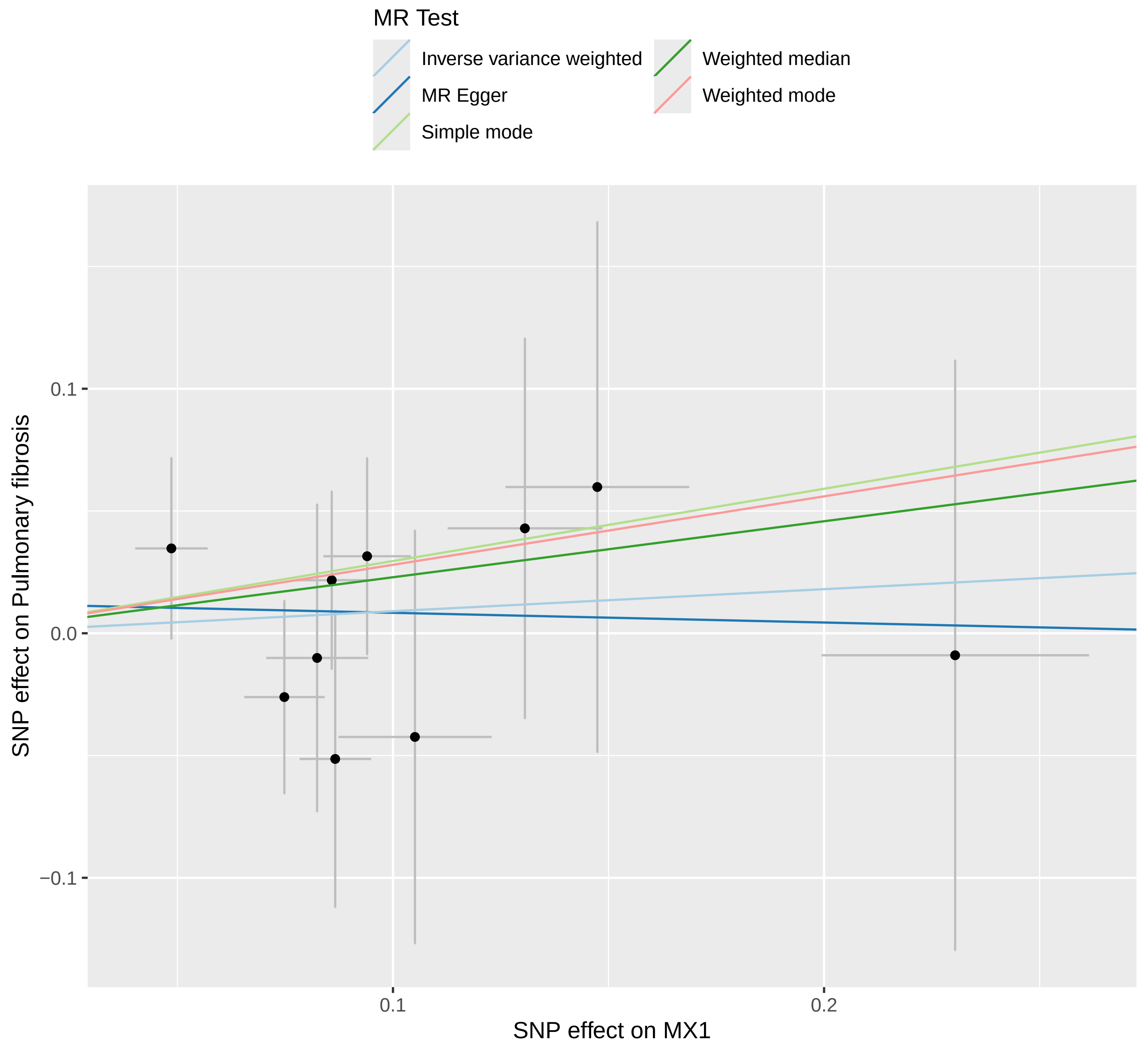

MR Test
Inverse variance weighted
MR Egger
Simple mode
Weighted median
Weighted mode
0.1
0.0
−0.1
SNP effect on Pulmonary fibrosis
0.1
0.2
SNP effect on MX1

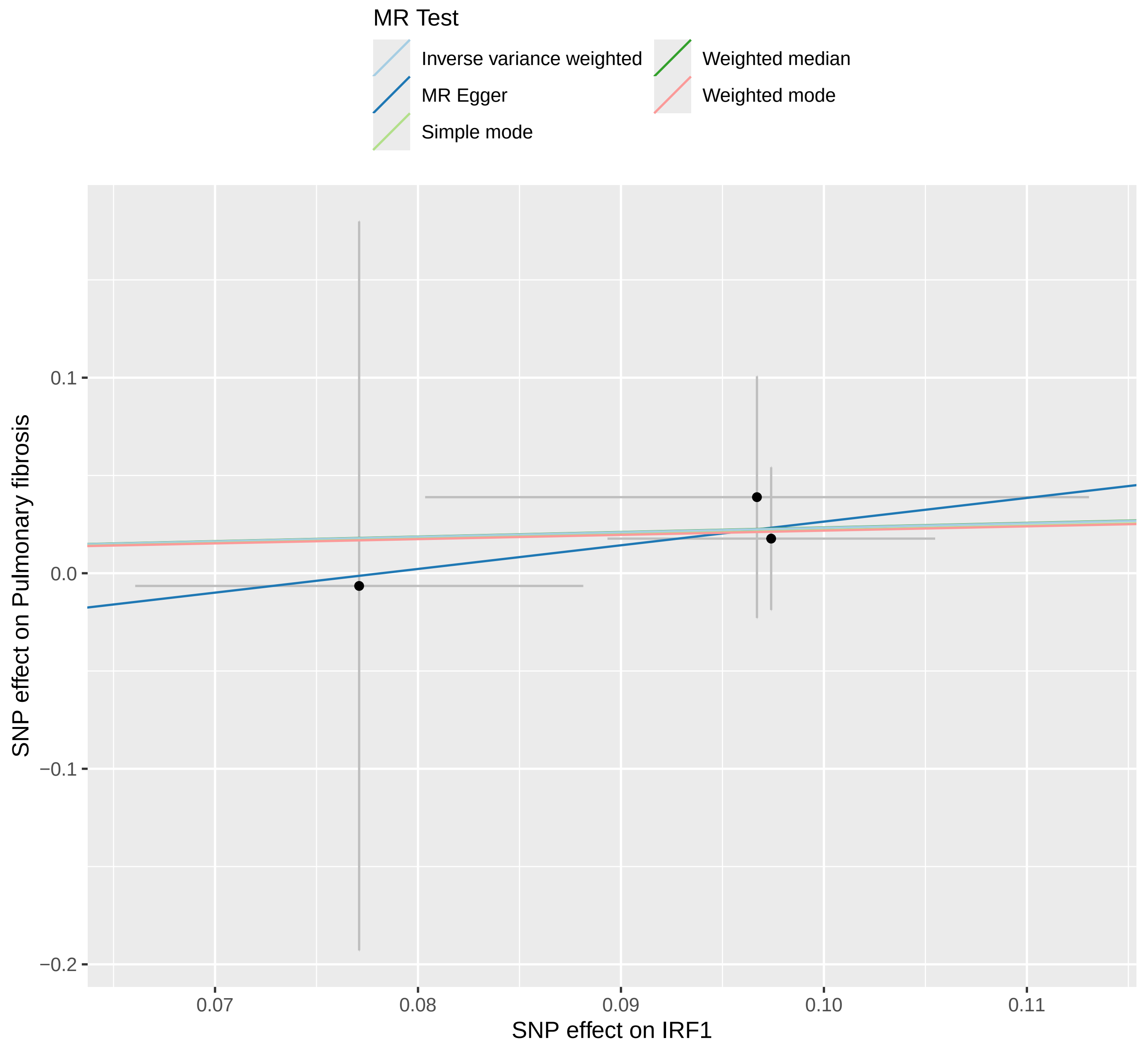

MR Test
Inverse variance weighted
MR Egger
Simple mode
Weighted median
Weighted mode
0.1
0.0
−0.1
−0.2
SNP effect on Pulmonary fibrosis
0.07
0.08
0.09
0.10
0.11
SNP effect on IRF1

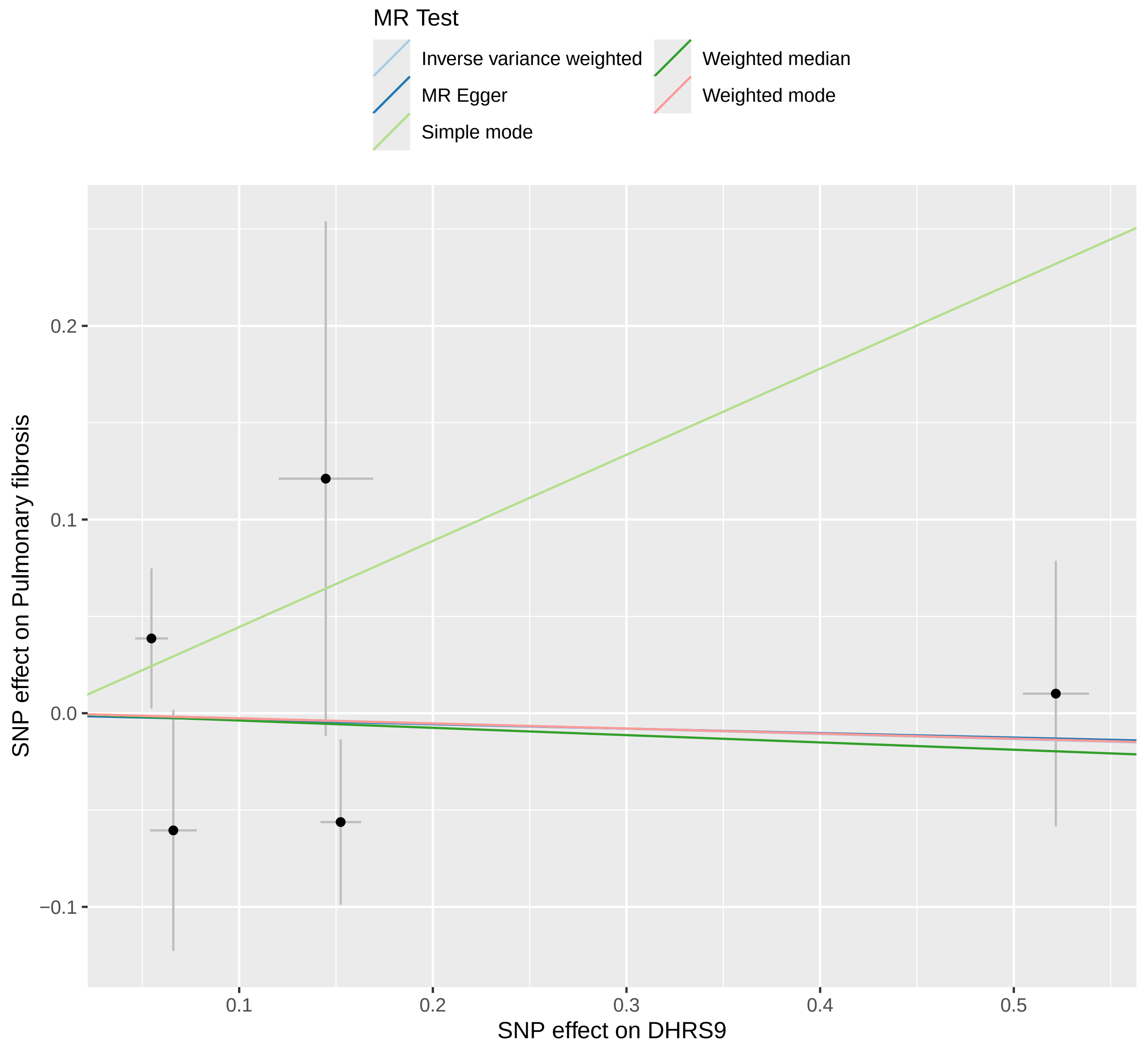

MR Test
Inverse variance weighted
MR Egger
Simple mode
Weighted median
Weighted mode
0.2
0.1
0.0
−0.1
0.1
0.2
0.3
0.4
0.5
SNP effect on Pulmonary fibrosis
SNP effect on DHRS9

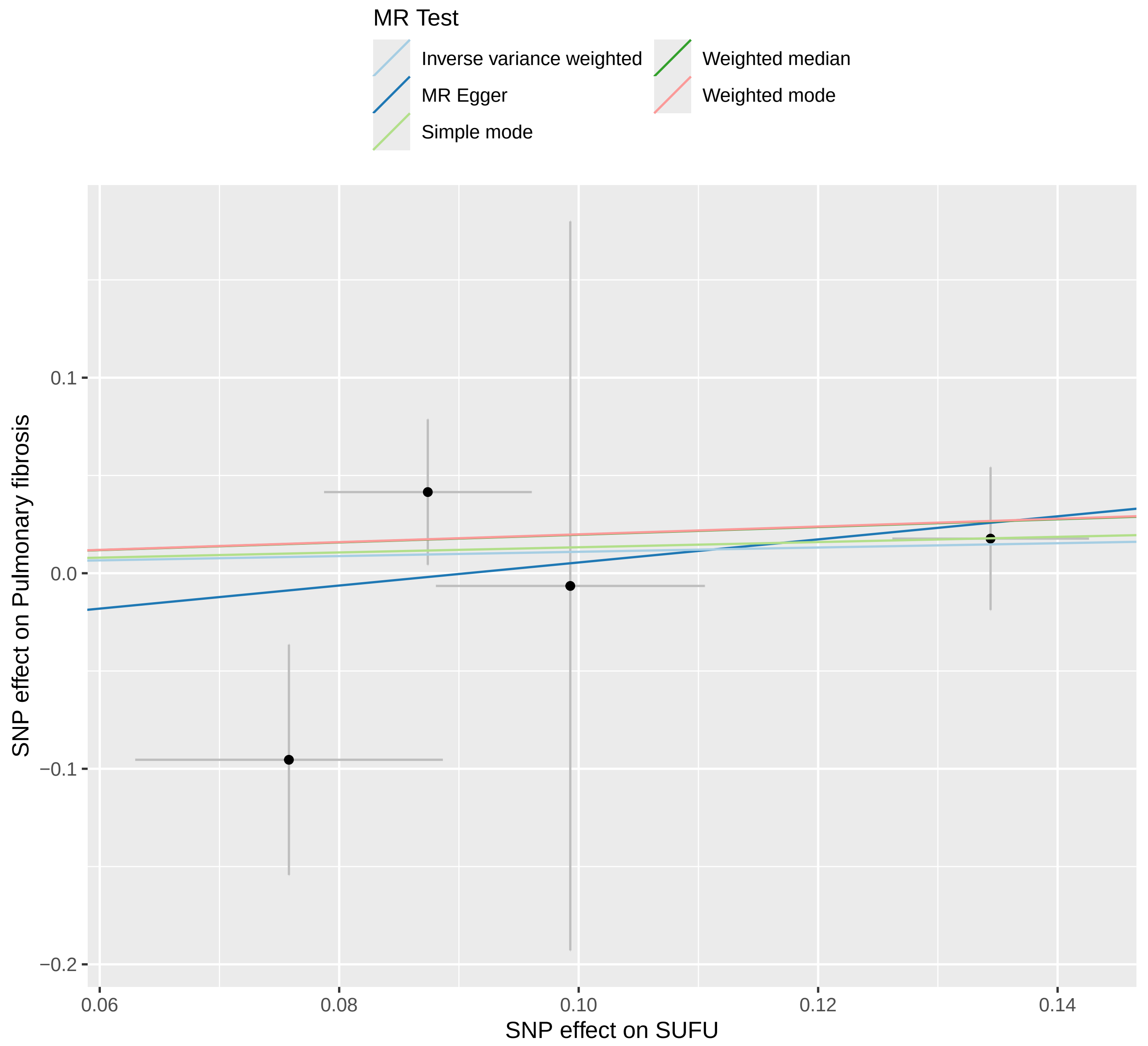

MR Test
Inverse variance weighted
MR Egger
Simple mode
Weighted median
Weighted mode
0.1
0.0
−0.1
−0.2
SNP effect on Pulmonary fibrosis
0.06
0.08
0.10
0.12
0.14
SNP effect on SUFU

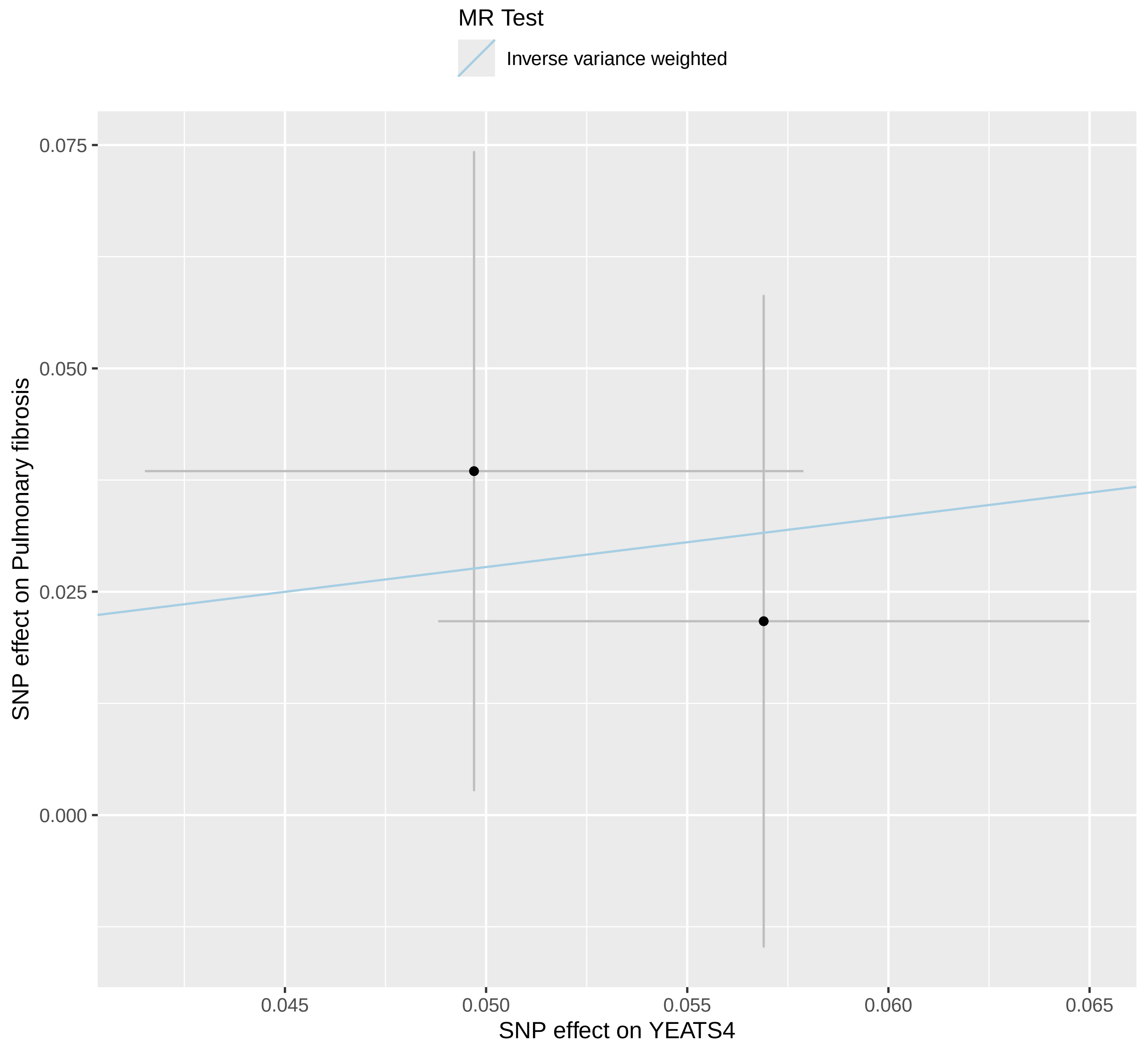
MR Test
Inverse variance weighted
0.075
0.050
0.025
0.000
SNP effect on Pulmonary fibrosis
0.045
0.050
0.055
0.060
0.065
SNP effect on YEATS4

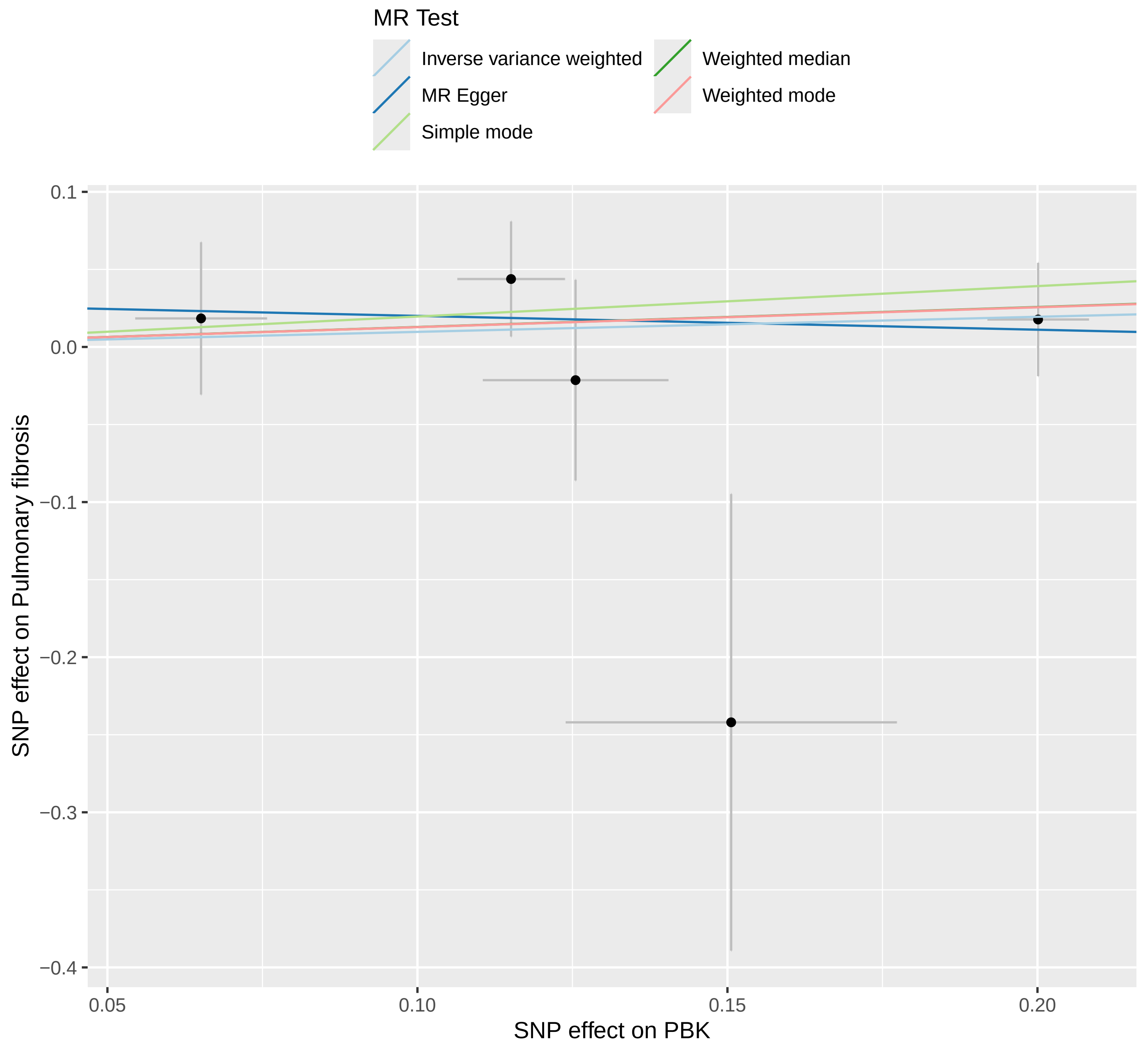
MR Test
Inverse variance weighted
MR Egger
Simple mode
Weighted median
Weighted mode
0.1
0.0
−0.1
−0.2
−0.3
−0.4
SNP effect on Pulmonary fibrosis
0.05
0.10
0.15
0.20
SNP effect on PBK

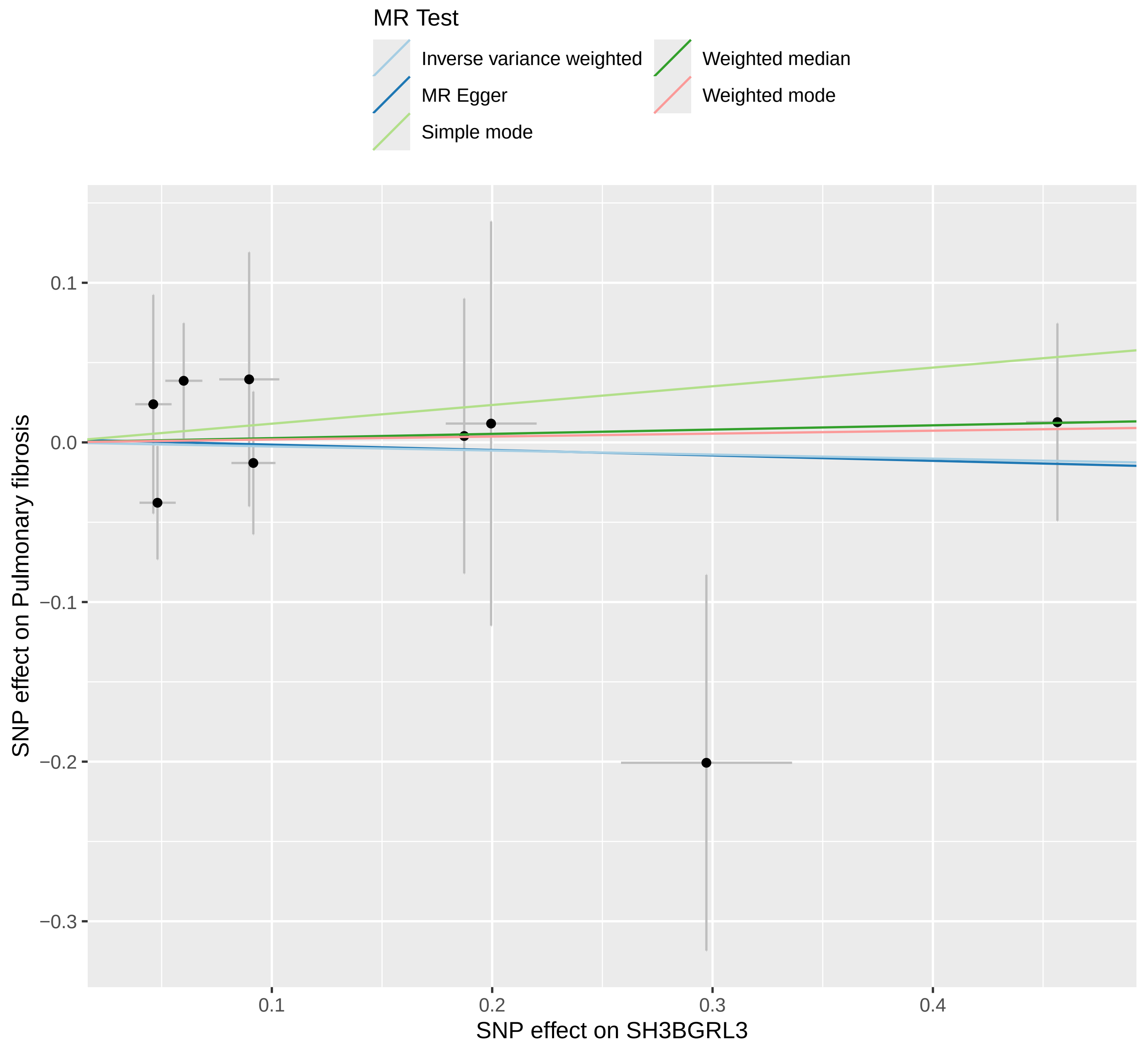

MR Test
Inverse variance weighted
MR Egger
Simple mode
Weighted median
Weighted mode
0.1
0.0
−0.1
−0.2
−0.3
0.1
0.2
0.3
0.4
SNP effect on SH3BGRL3
SNP effect on Pulmonary fibrosis

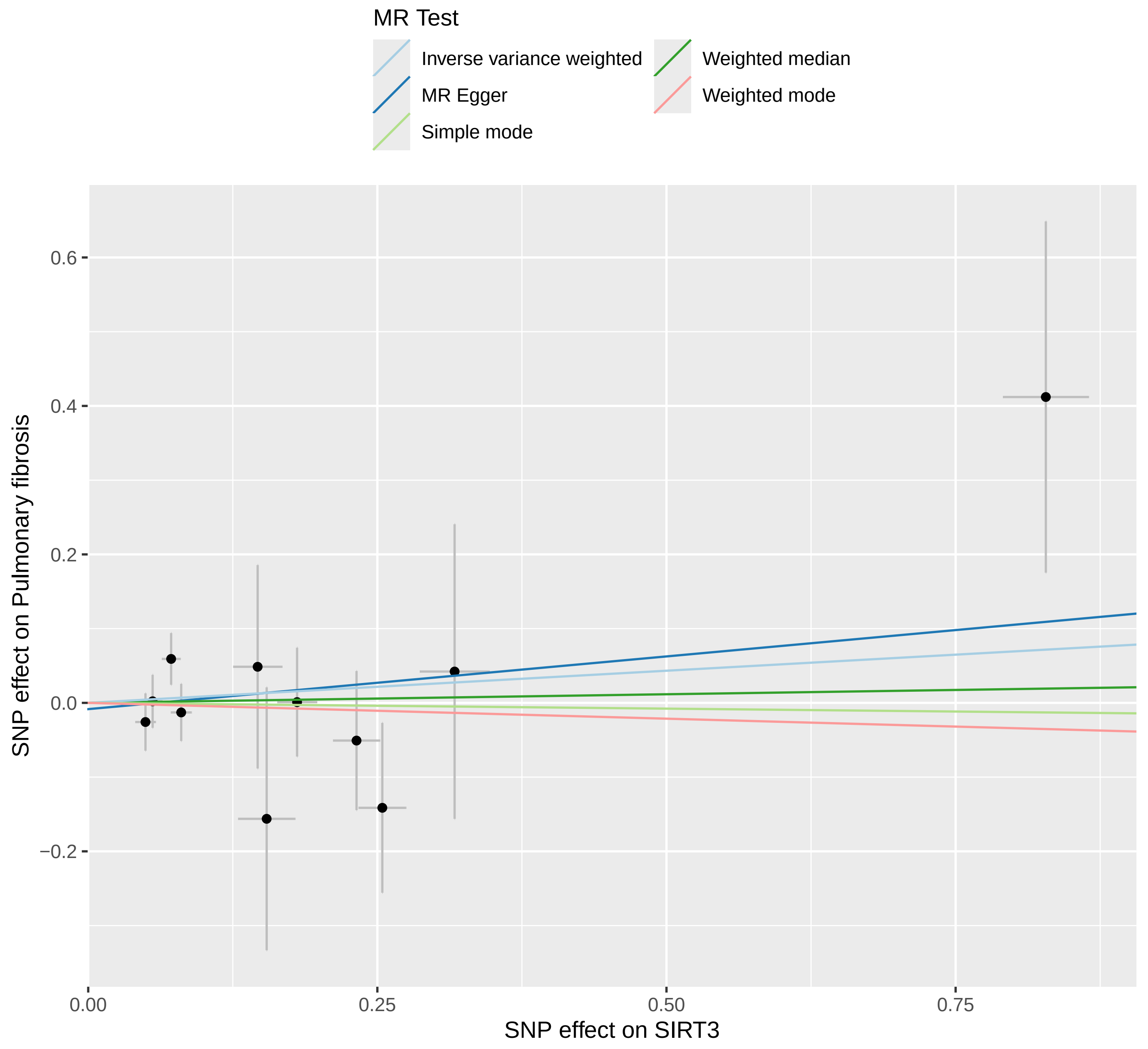

MR Test
Inverse variance weighted
MR Egger
Simple mode
Weighted median
Weighted mode
SNP effect on Pulmonary fibrosis
0.6
0.4
0.2
0.0
−0.2
0.00
0.25
0.50
0.75
SNP effect on SIRT3

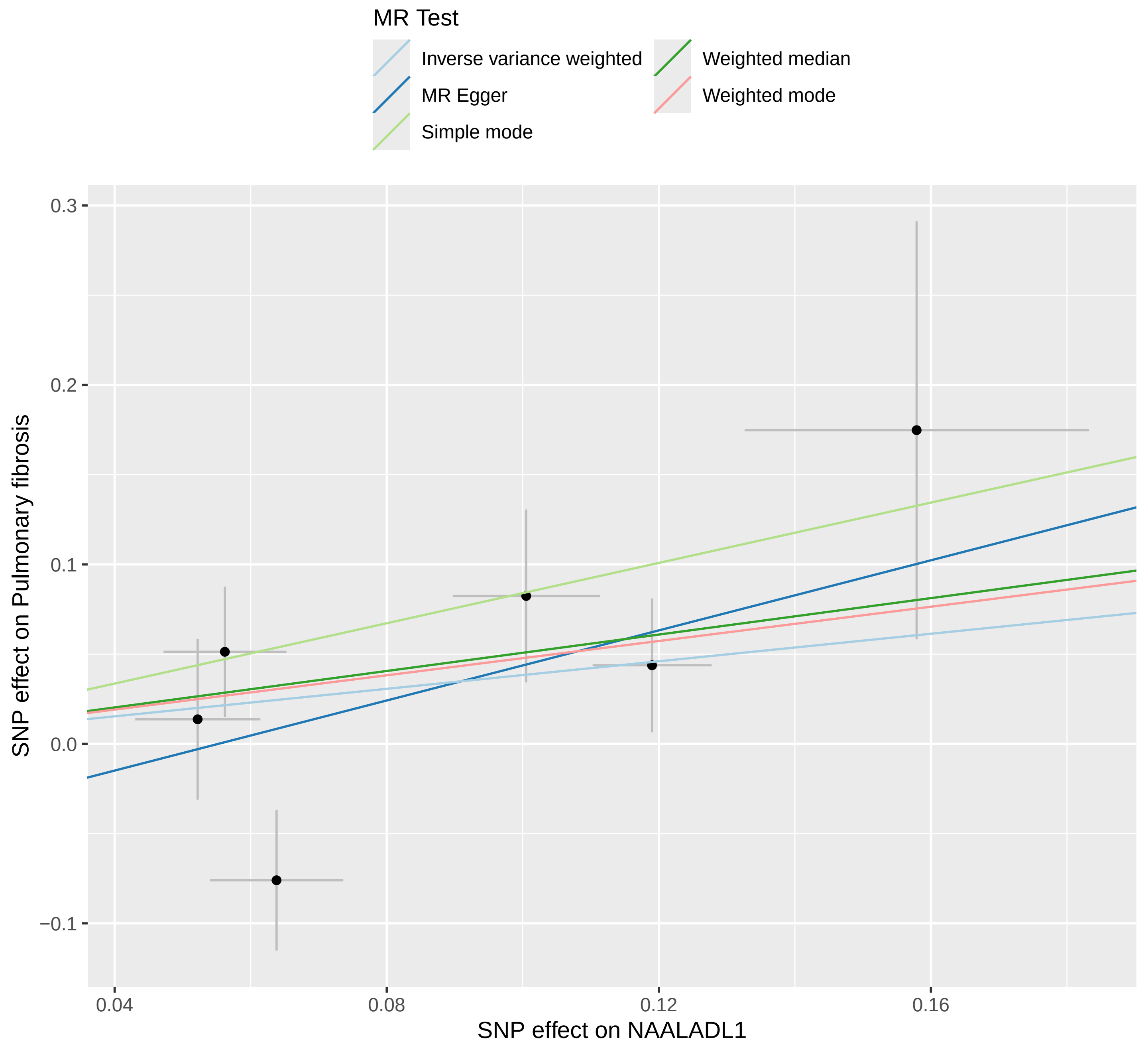

MR Test
Inverse variance weighted
MR Egger
Simple mode
Weighted median
Weighted mode
0.3
0.2
0.1
0.0
−0.1
0.04
0.08
0.12
0.16
SNP effect on Pulmonary fibrosis
SNP effect on NAALADL1

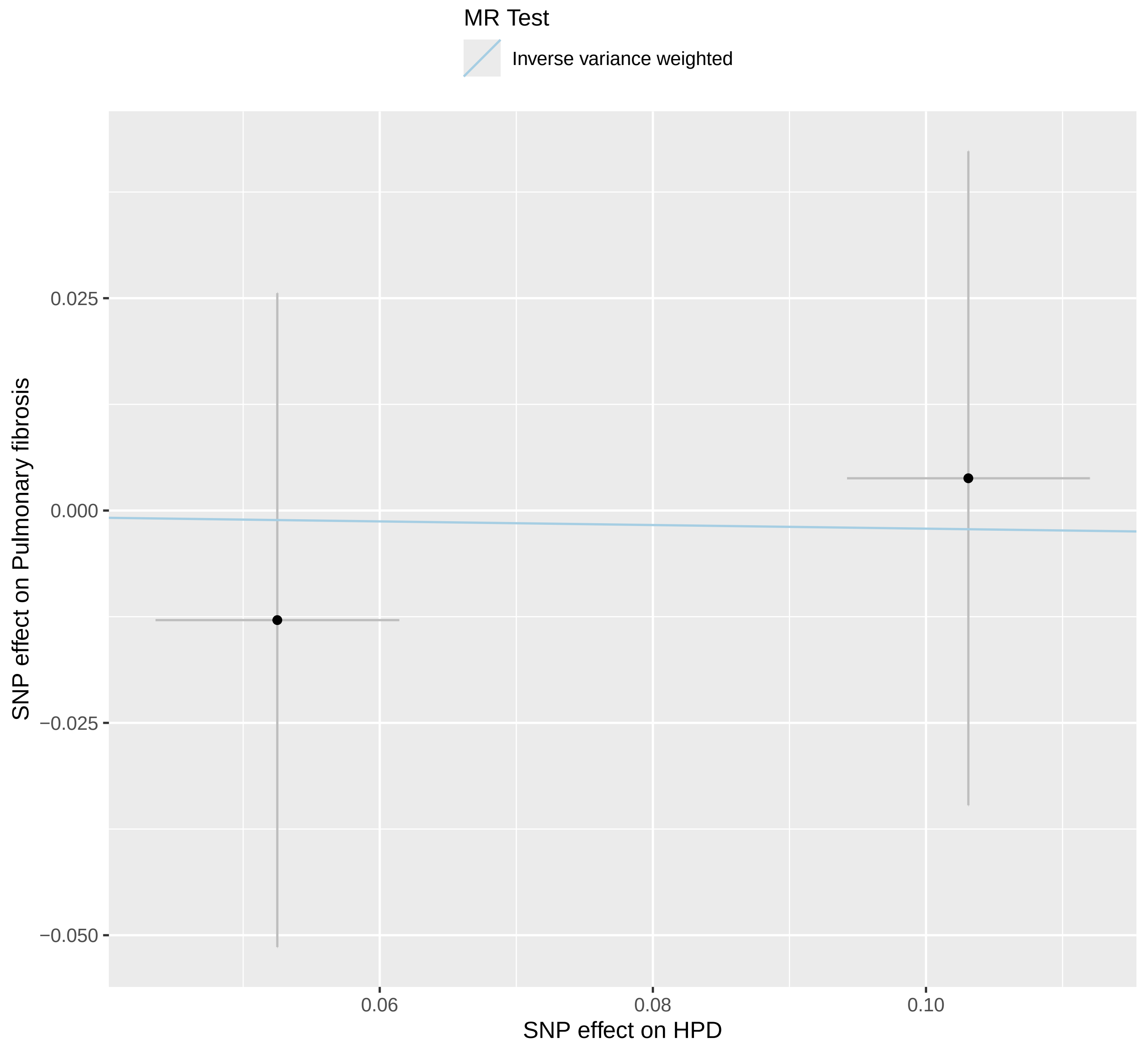

MR Test
Inverse variance weighted
0.025
0.000
−0.025
−0.050
SNP effect on Pulmonary fibrosis
0.06
0.08
0.10
SNP effect on HPD

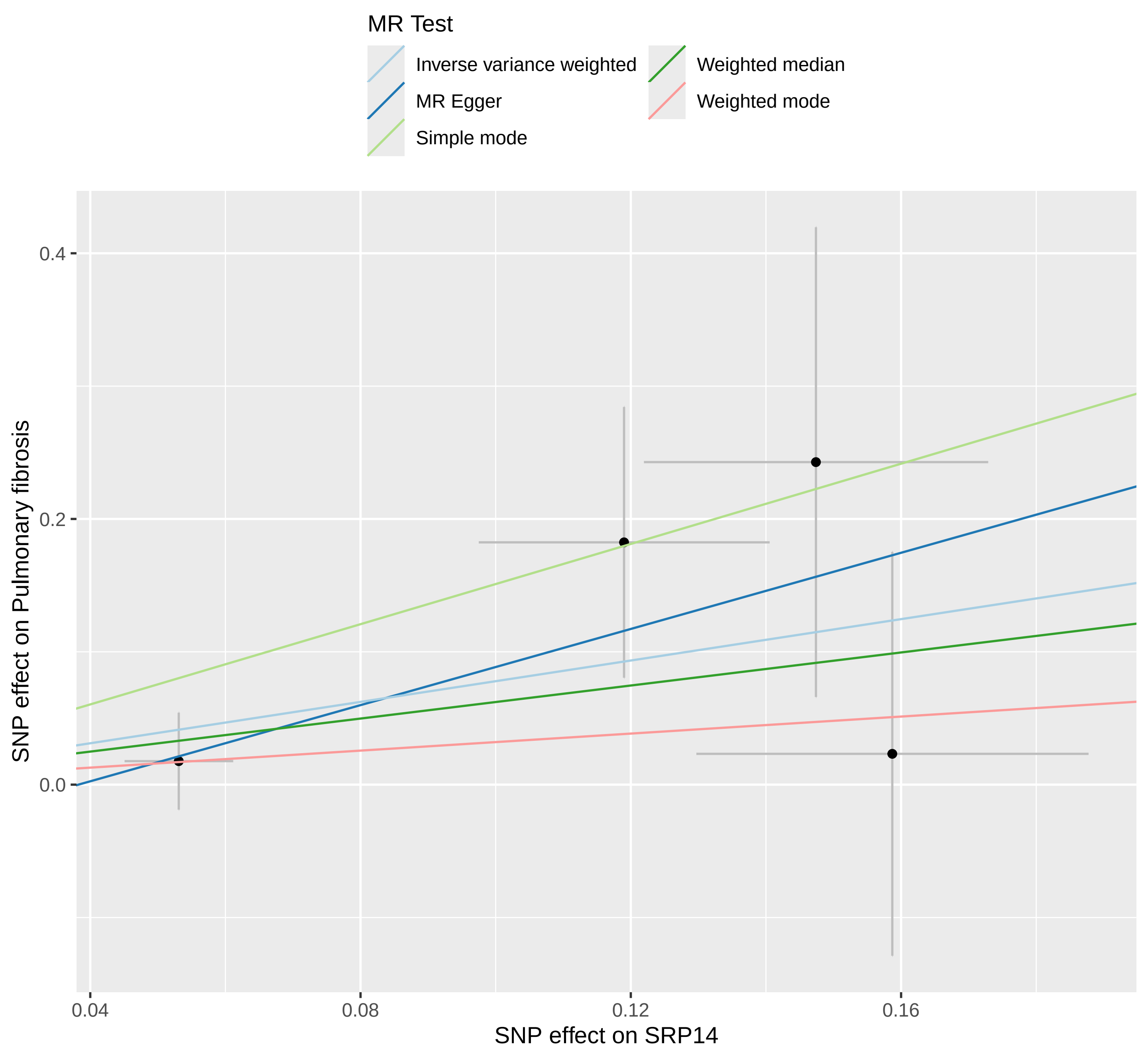
MR Test
Inverse variance weighted
MR Egger
Simple mode
Weighted median
Weighted mode
0.4
0.2
0.0
0.04
0.08
0.12
0.16
SNP effect on SRP14
SNP effect on Pulmonary fibrosis

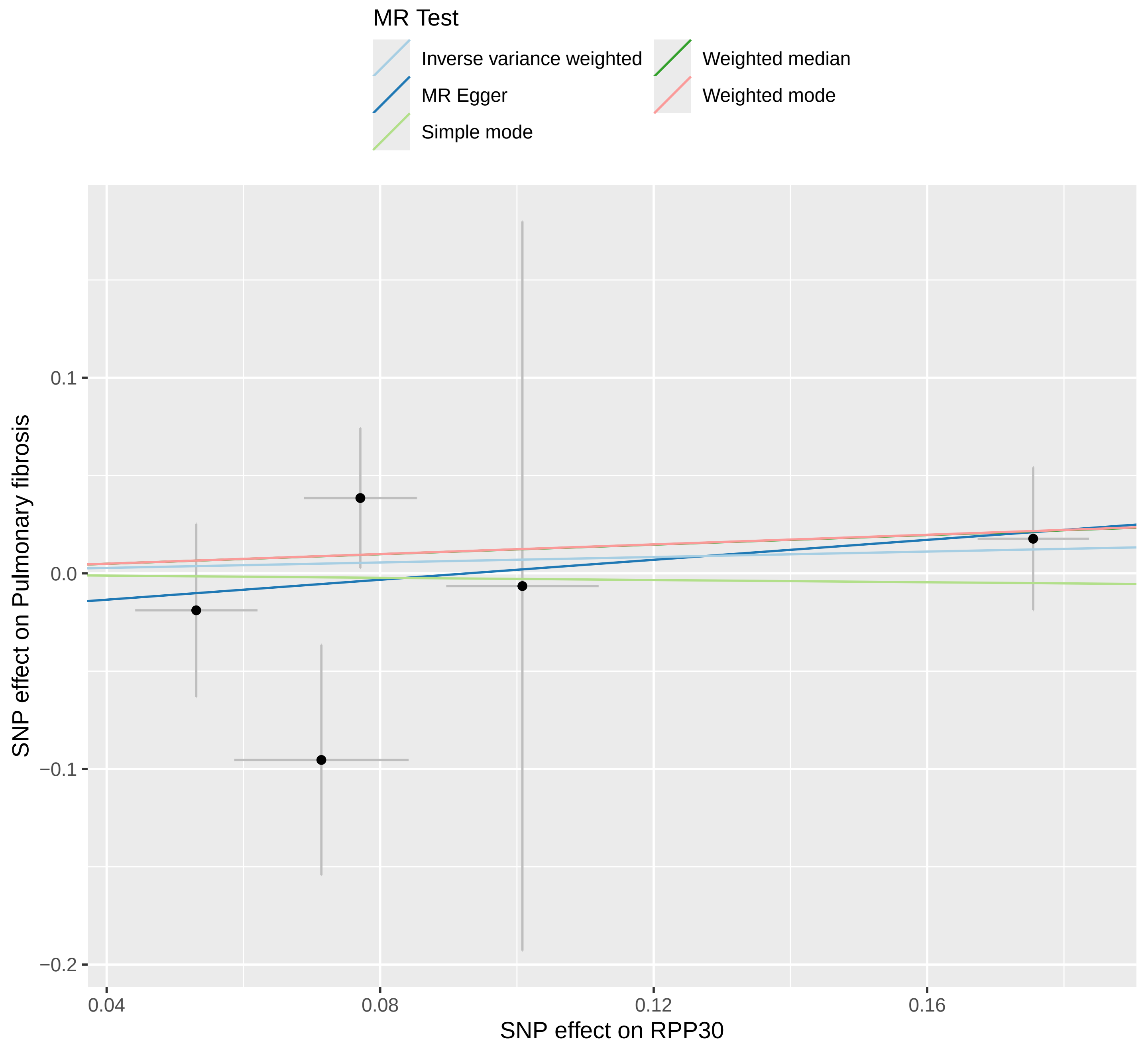
MR Test
Inverse variance weighted
MR Egger
Simple mode
Weighted median
Weighted mode
0.1
0.0
−0.1
−0.2
SNP effect on Pulmonary fibrosis
0.04
0.08
0.12
0.16
SNP effect on RPP30

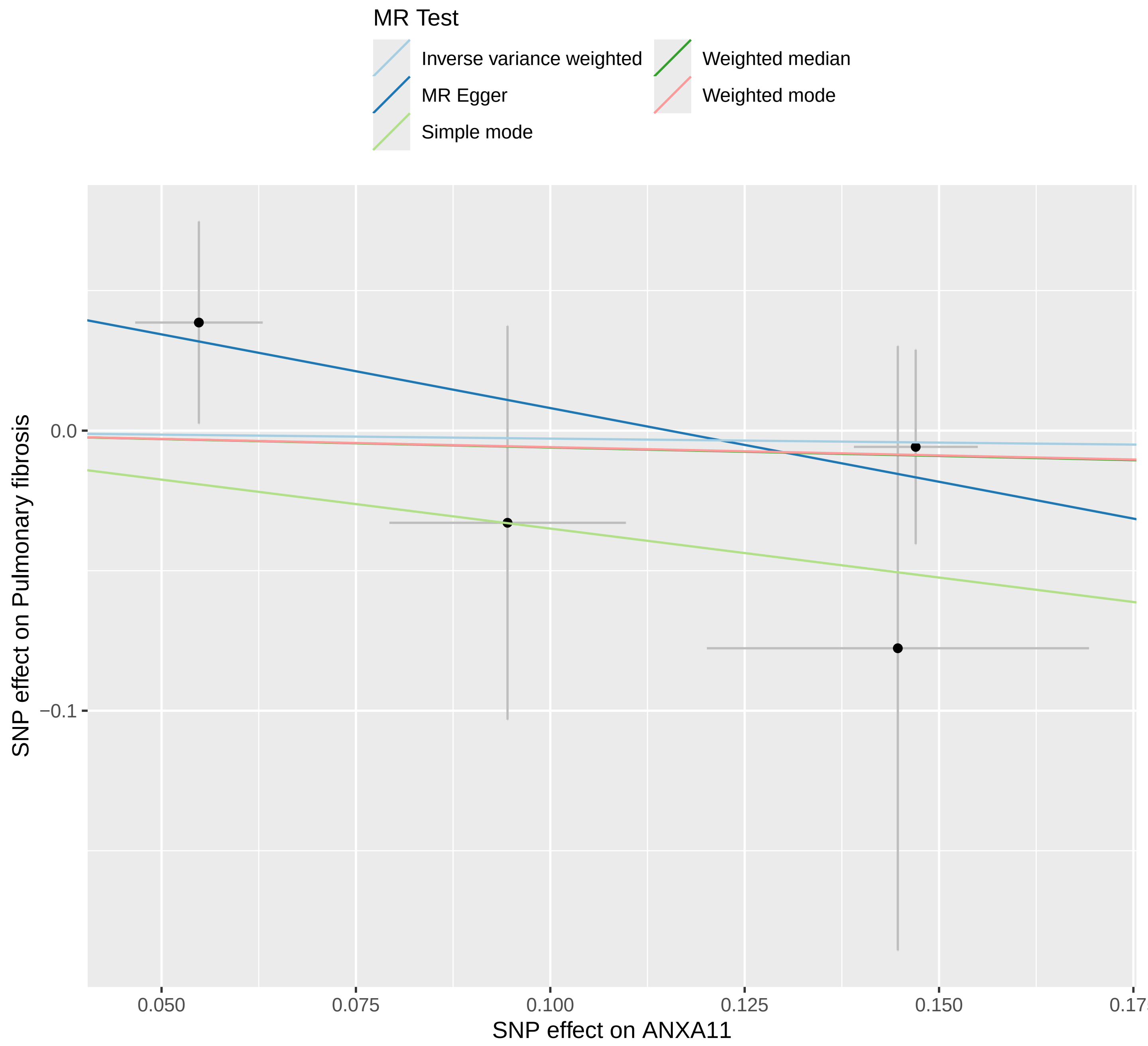
MR Test
Inverse variance weighted
MR Egger
Simple mode
Weighted median
Weighted mode
0.0
−0.1
SNP effect on Pulmonary fibrosis
0.050
0.075
0.100
0.125
0.150
SNP effect on ANXA11

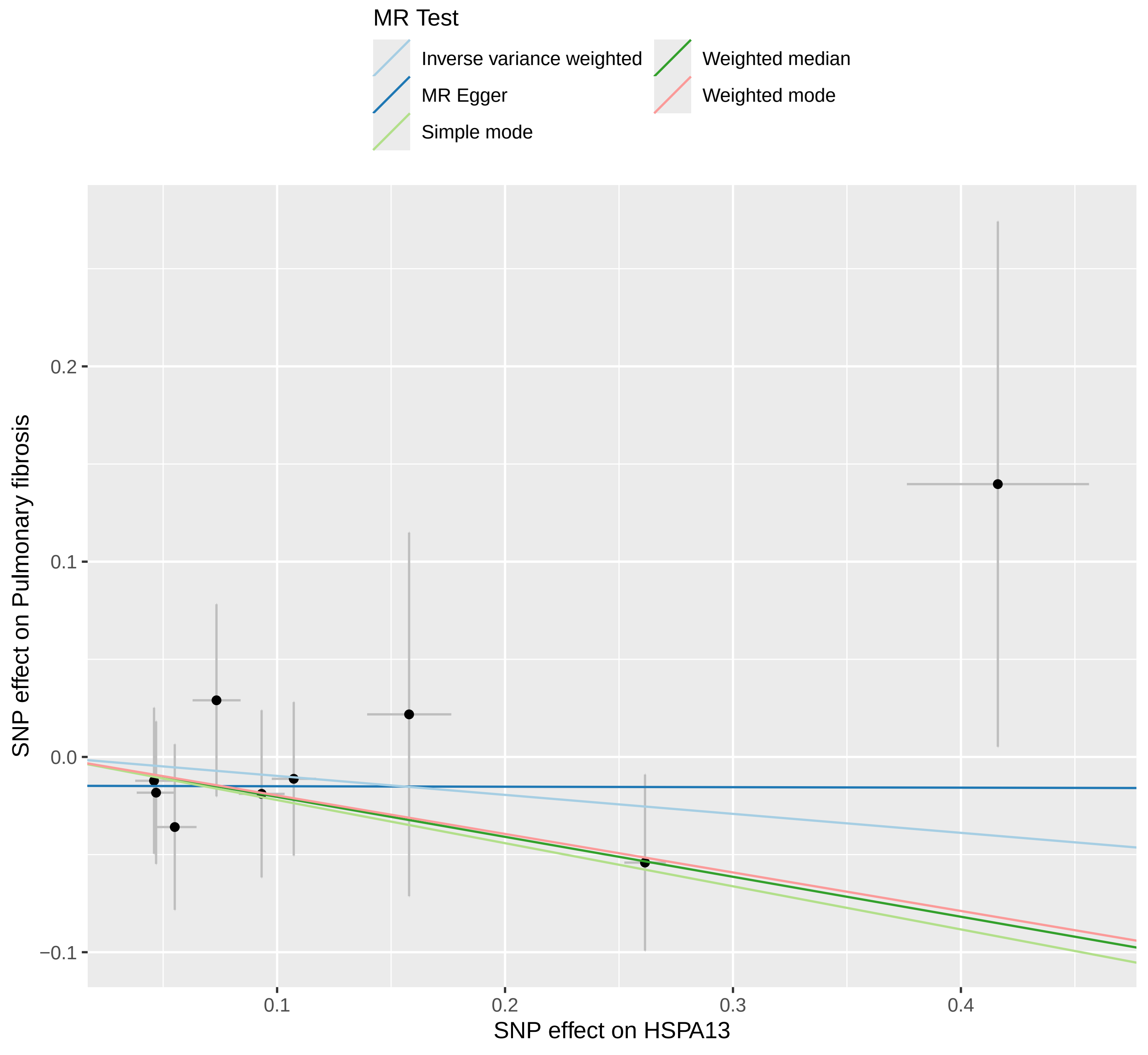
MR Test
Inverse variance weighted
MR Egger
Simple mode
Weighted median
Weighted mode
0.2
0.1
0.0
−0.1
0.1
0.2
0.3
0.4
SNP effect on Pulmonary fibrosis
SNP effect on HSPA13

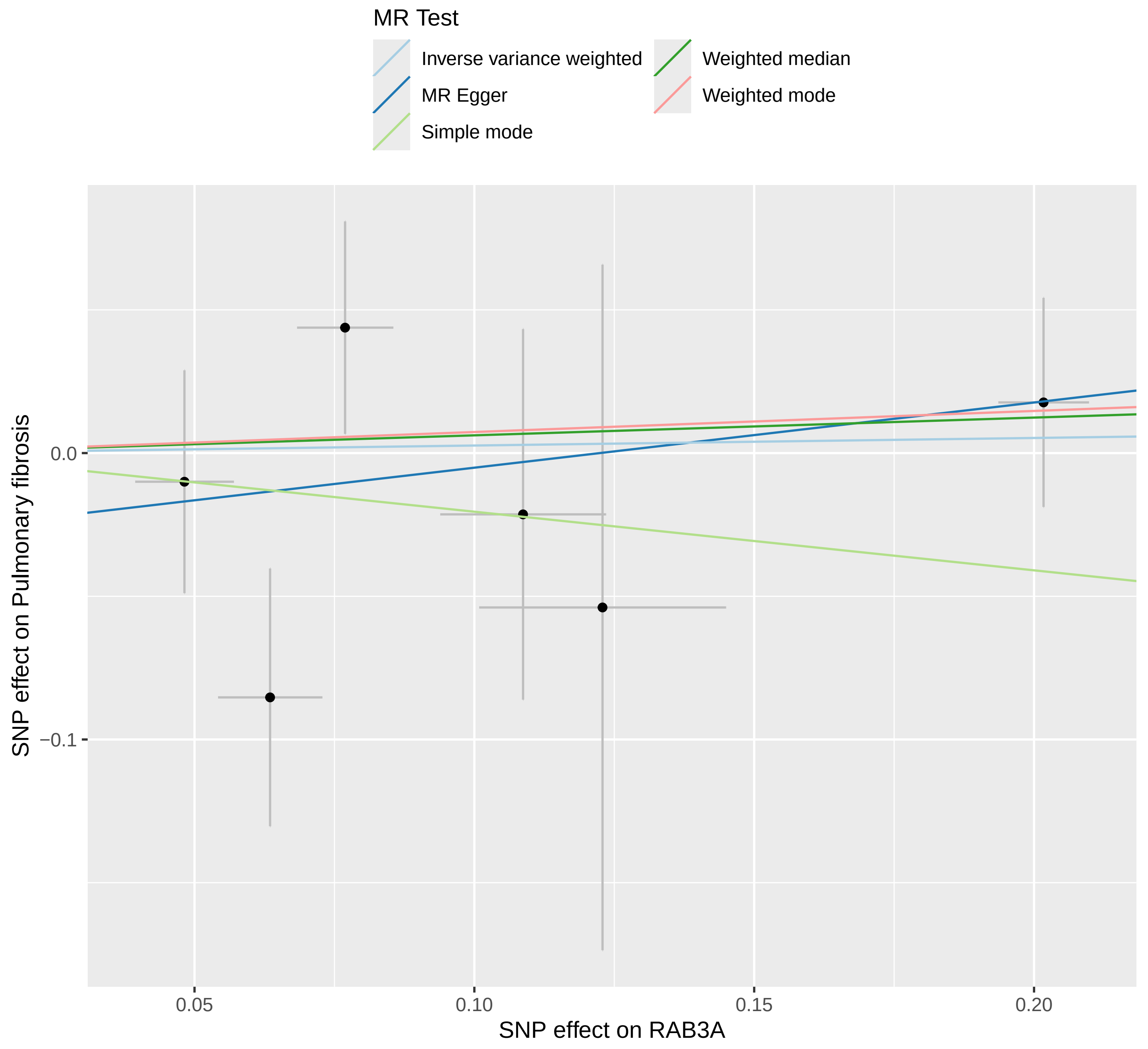

MR Test
Inverse variance weighted
MR Egger
Simple mode
Weighted median
Weighted mode
SNP effect on Pulmonary fibrosis
0.0
−0.1
0.05
0.10
0.15
0.20
SNP effect on RAB3A

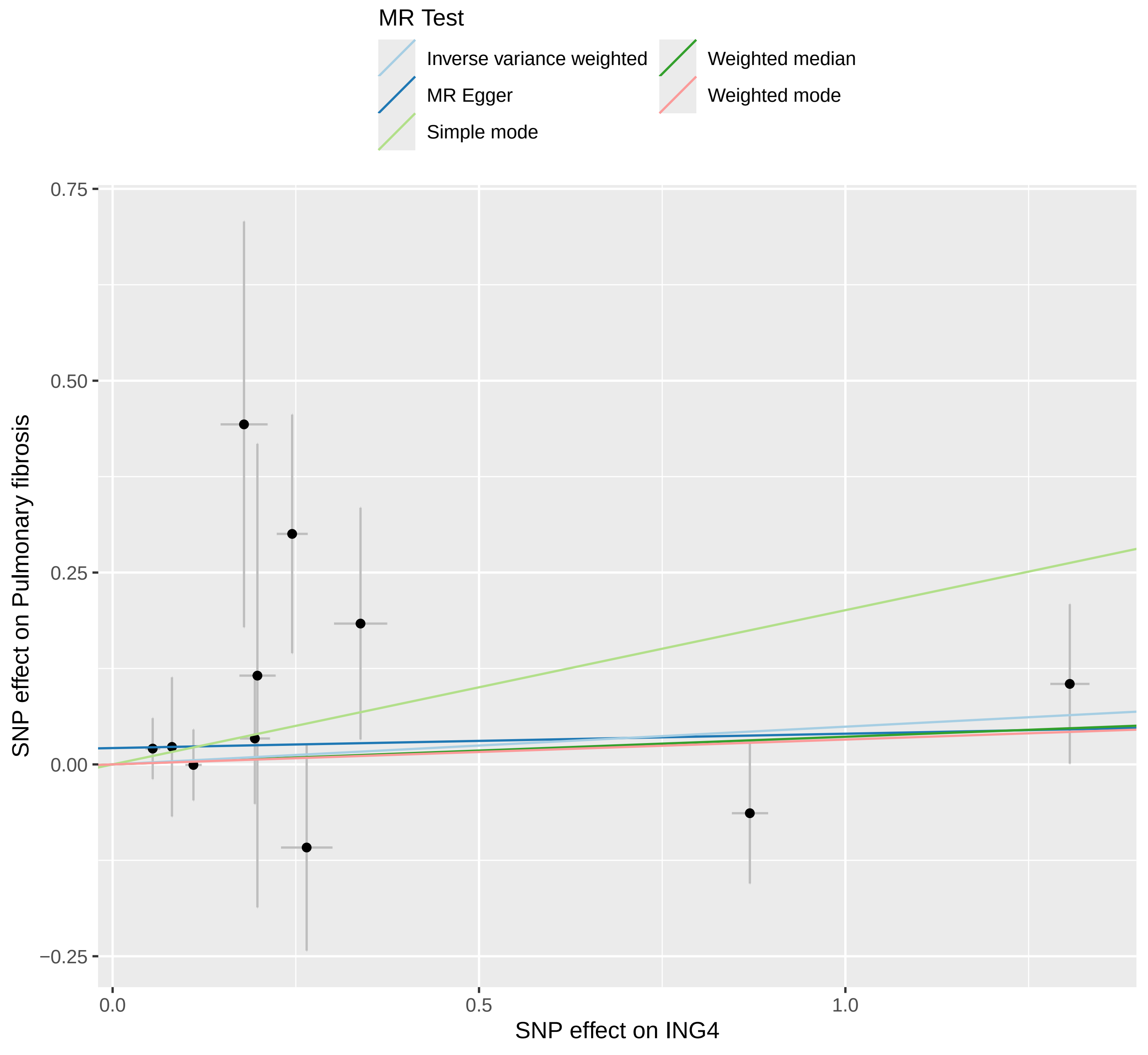
MR Test
Inverse variance weighted
MR Egger
Simple mode
Weighted median
Weighted mode
0.75
0.50
0.25
0.00
−0.25
0.0
0.5
1.0
SNP effect on Pulmonary fibrosis
SNP effect on ING4

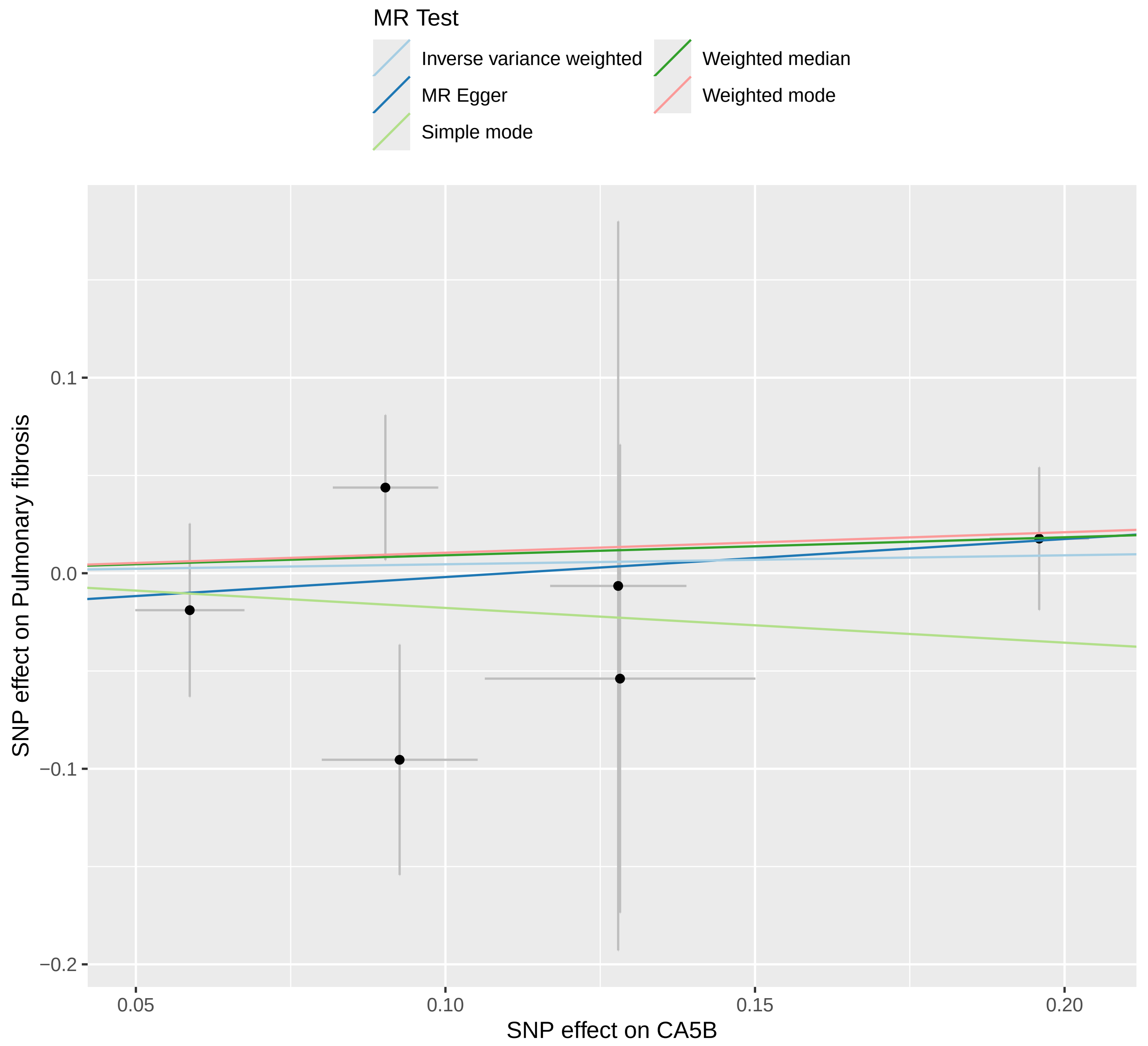

MR Test
Inverse variance weighted
MR Egger
Simple mode
Weighted median
Weighted mode
0.1
0.0
−0.1
−0.2
SNP effect on Pulmonary fibrosis
0.05
0.10
0.15
0.20
SNP effect on CA5B

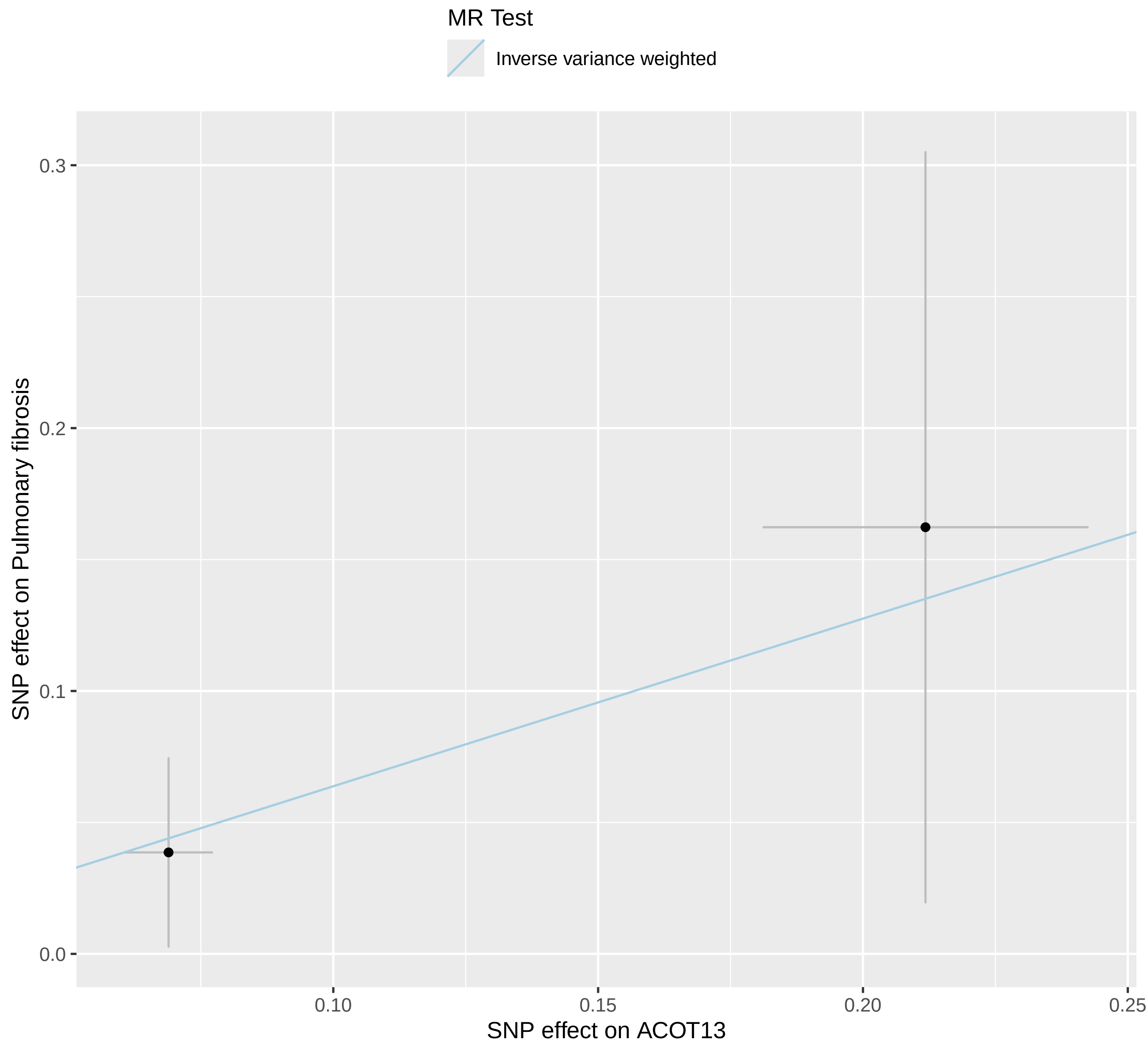

MR Test
Inverse variance weighted
0.3
0.2
0.1
0.0
SNP effect on Pulmonary fibrosis
0.10
0.15
0.20
0.25
SNP effect on ACOT13

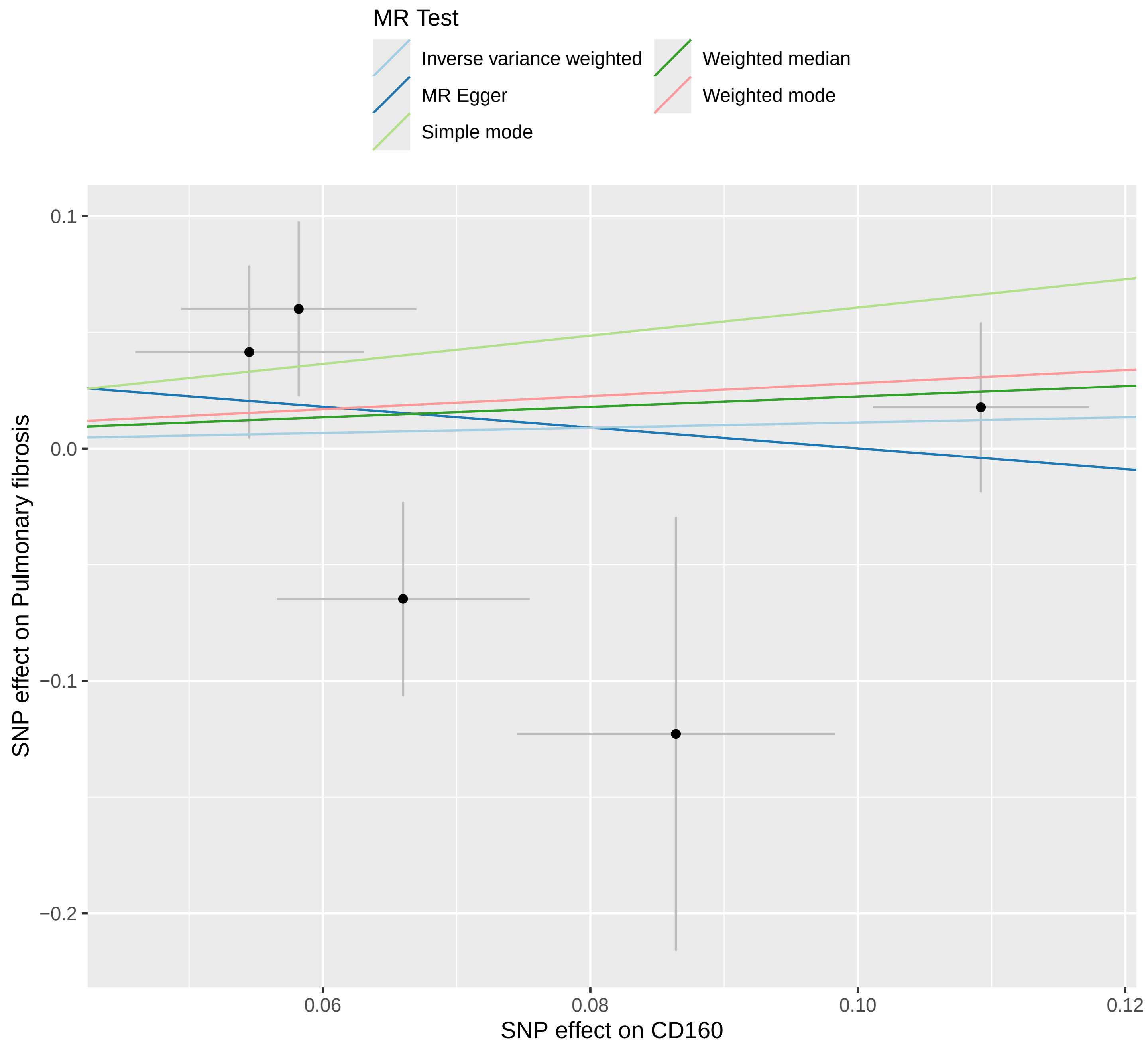
MR Test
Inverse variance weighted
MR Egger
Simple mode
Weighted median
Weighted mode
0.1
0.0
−0.1
−0.2
SNP effect on Pulmonary fibrosis
0.06
0.08
0.10
0.12
SNP effect on CD160

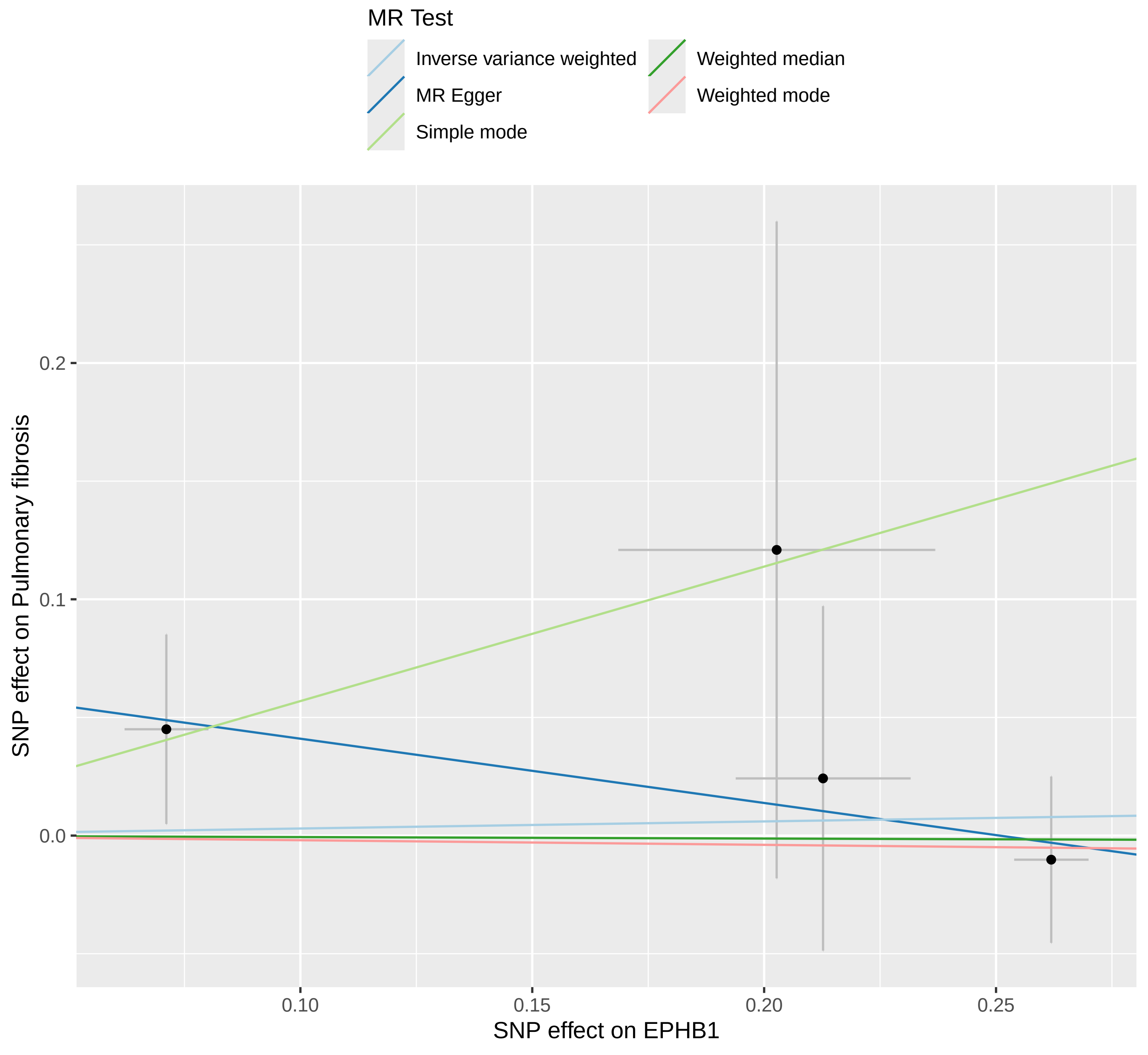

MR Test
Inverse variance weighted
MR Egger
Simple mode
Weighted median
Weighted mode
0.2
0.1
0.0
SNP effect on Pulmonary fibrosis
0.10
0.15
0.20
0.25
SNP effect on EPHB1

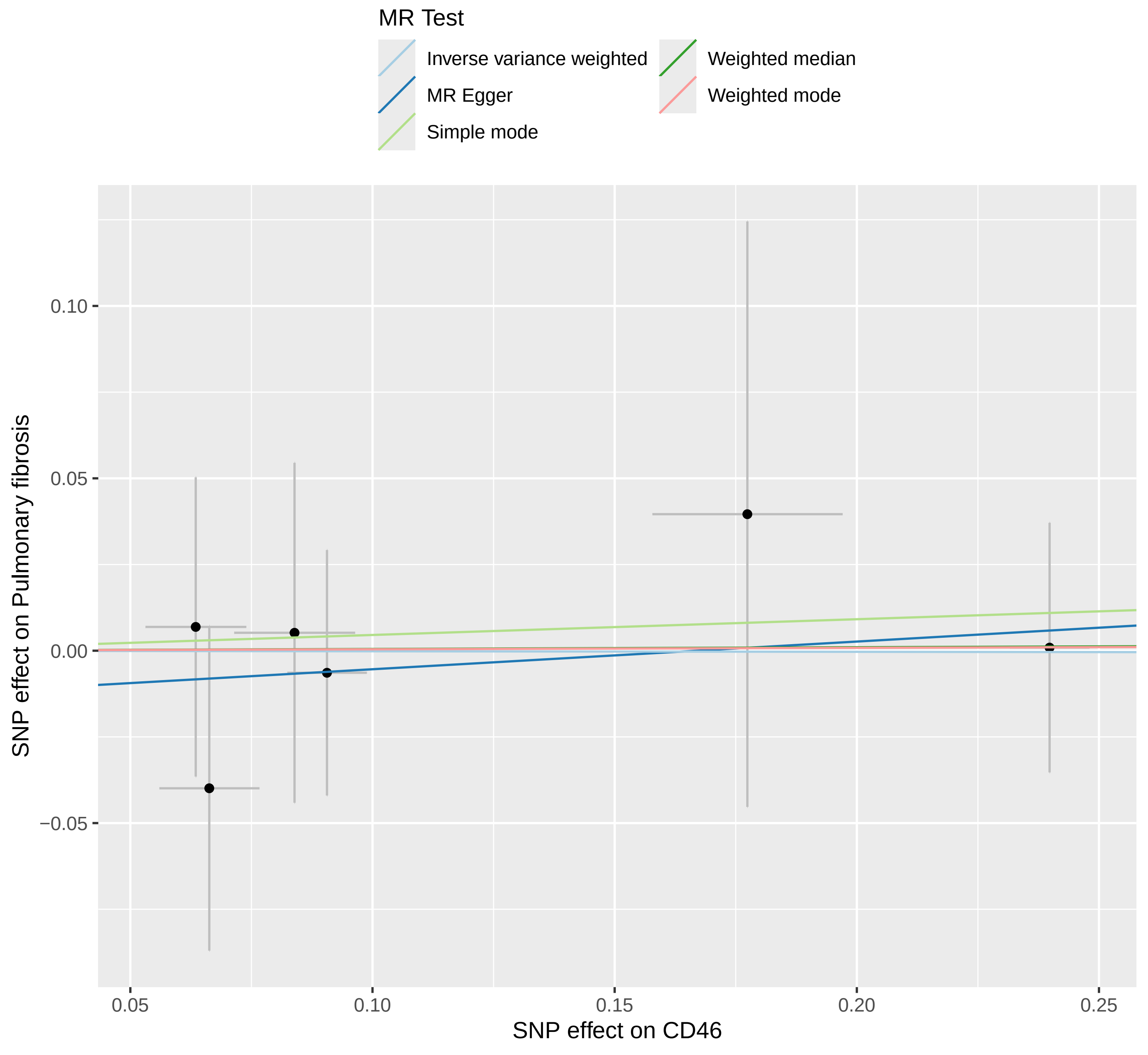
MR Test
Inverse variance weighted
MR Egger
Simple mode
Weighted median
Weighted mode
0.10
0.05
0.00
−0.05
SNP effect on Pulmonary fibrosis
0.05
0.10
0.15
0.20
0.25
SNP effect on CD46

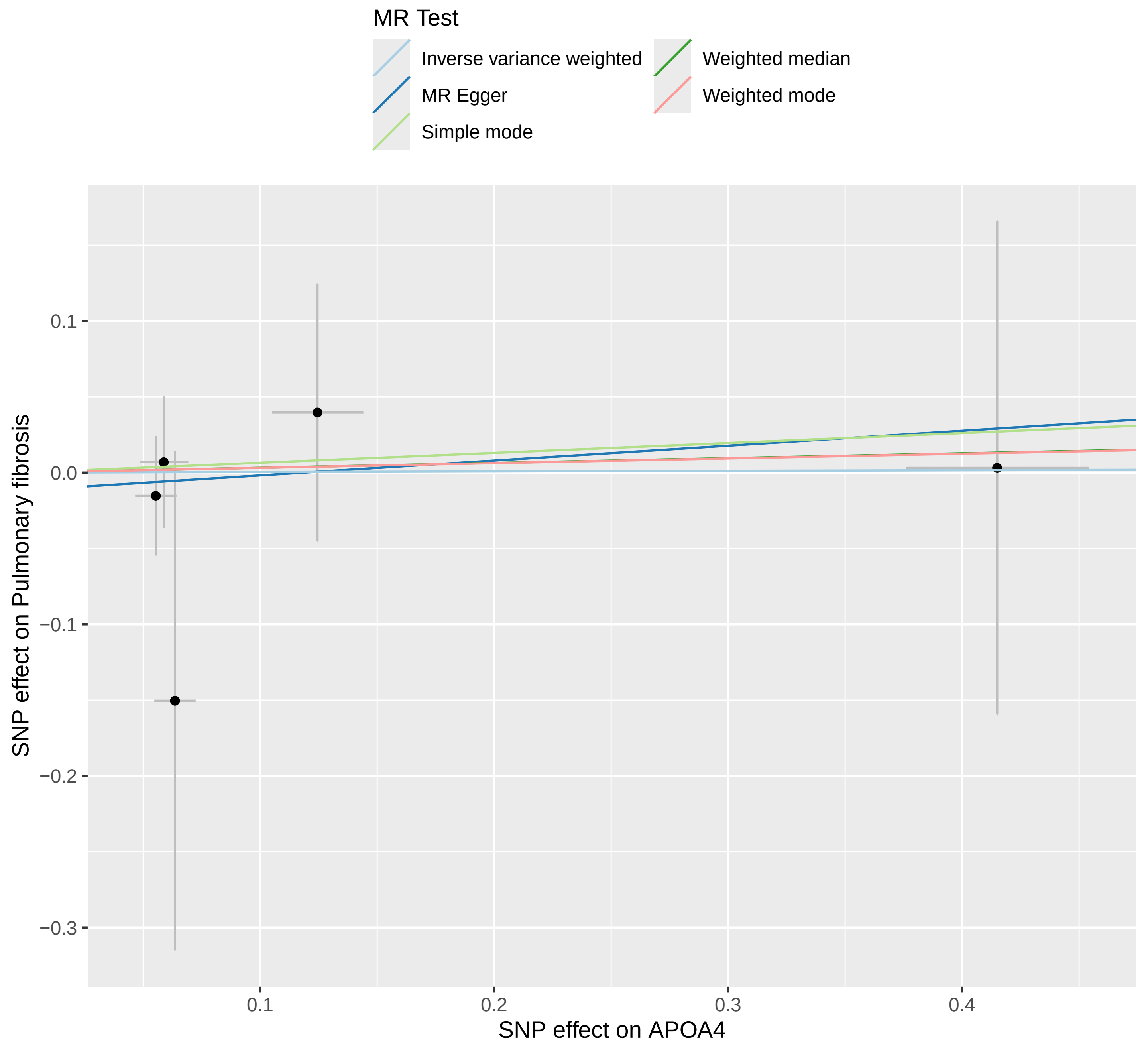

MR Test
Inverse variance weighted
MR Egger
Simple mode
Weighted median
Weighted mode
0.1
0.0
−0.1
−0.2
−0.3
SNP effect on Pulmonary fibrosis
0.1
0.2
0.3
0.4
SNP effect on APOA4

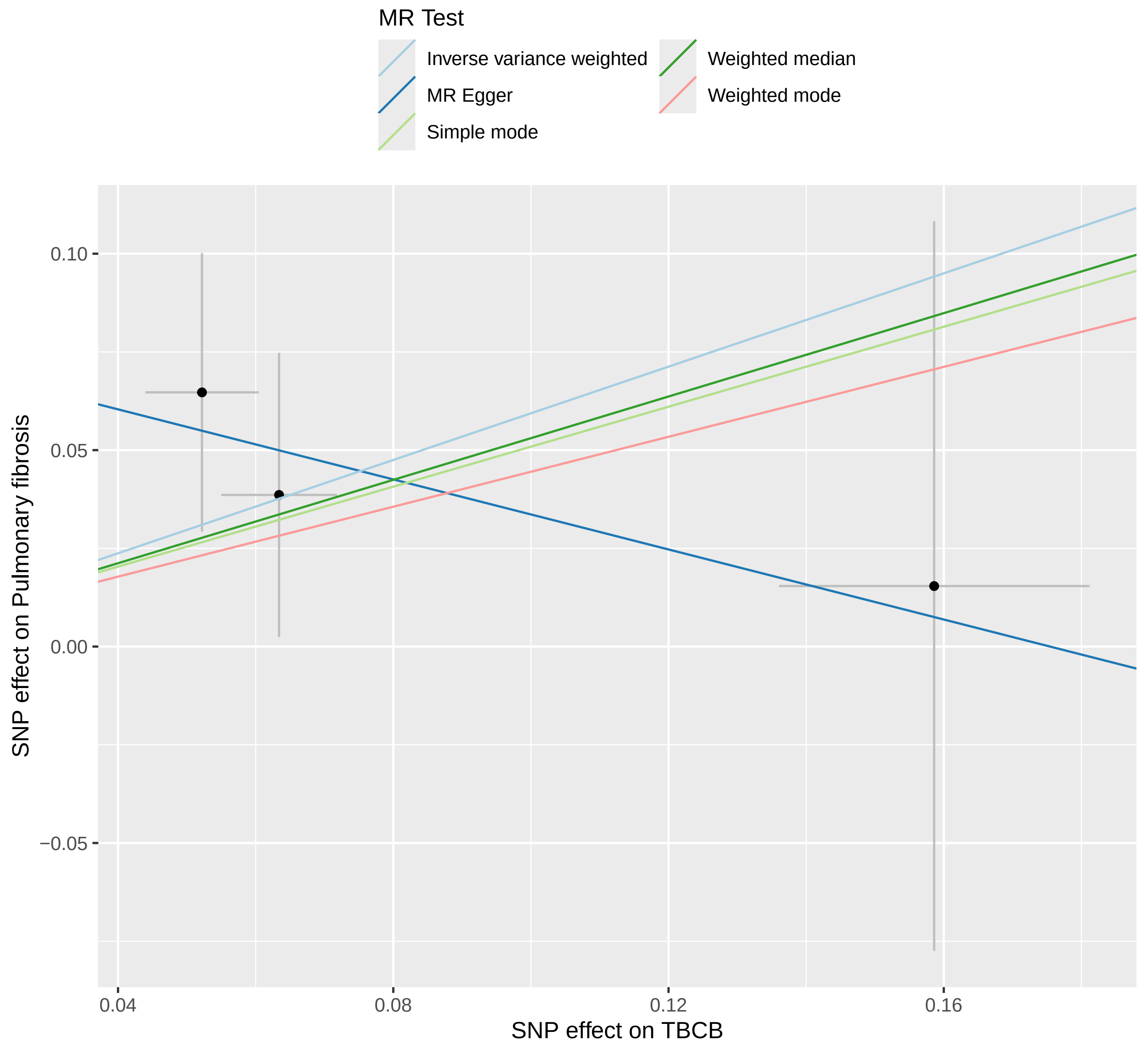

MR Test
Inverse variance weighted
MR Egger
Simple mode
Weighted median
Weighted mode
SNP effect on Pulmonary fibrosis
0.10
0.05
0.00
−0.05
0.04
0.08
0.12
0.16
SNP effect on TBCB

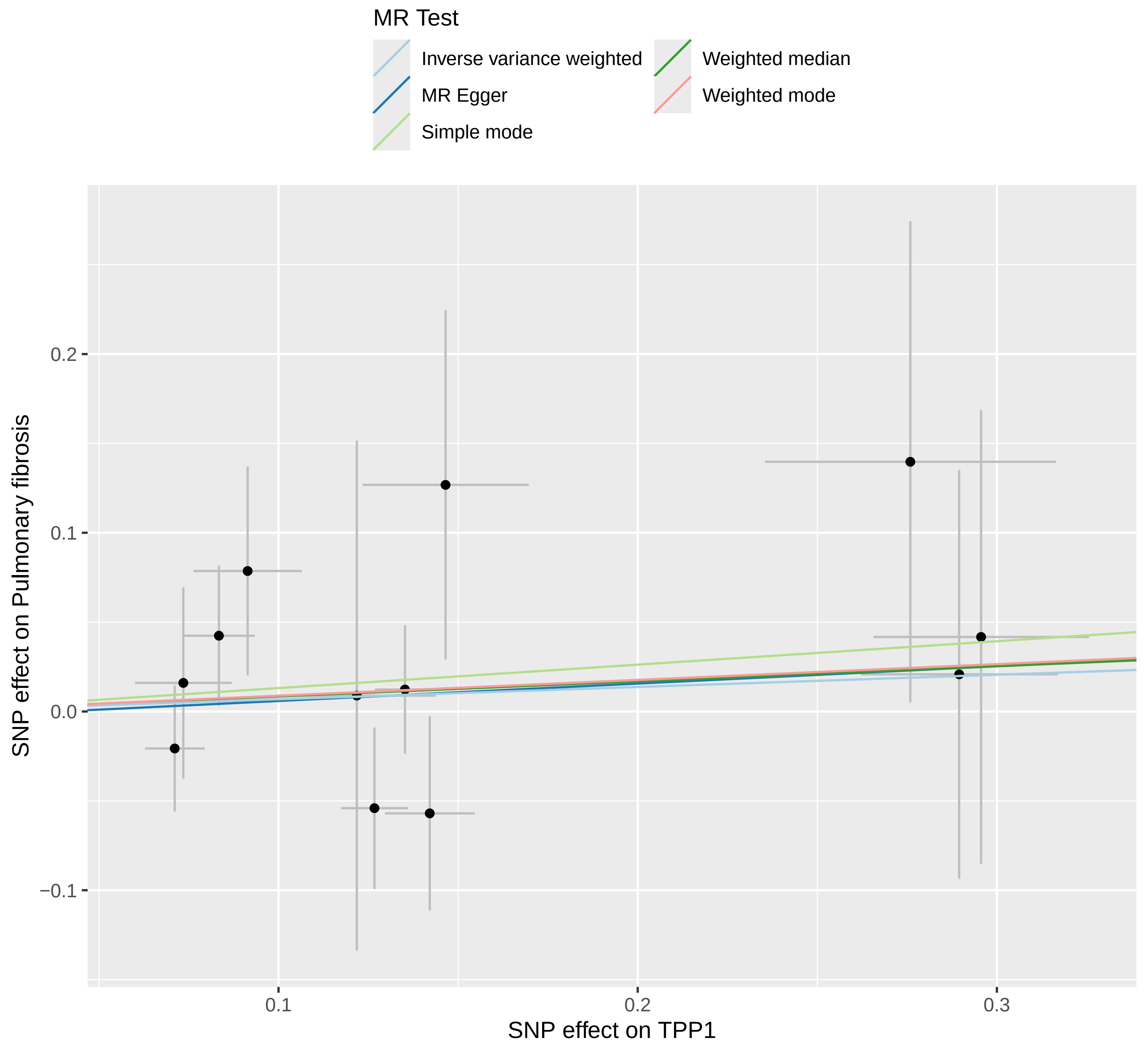

MR Test
Inverse variance weighted
MR Egger
Simple mode
Weighted median
Weighted mode
0.2
0.1
0.0
−0.1
SNP effect on Pulmonary fibrosis
0.1
0.2
0.3
SNP effect on TPP1

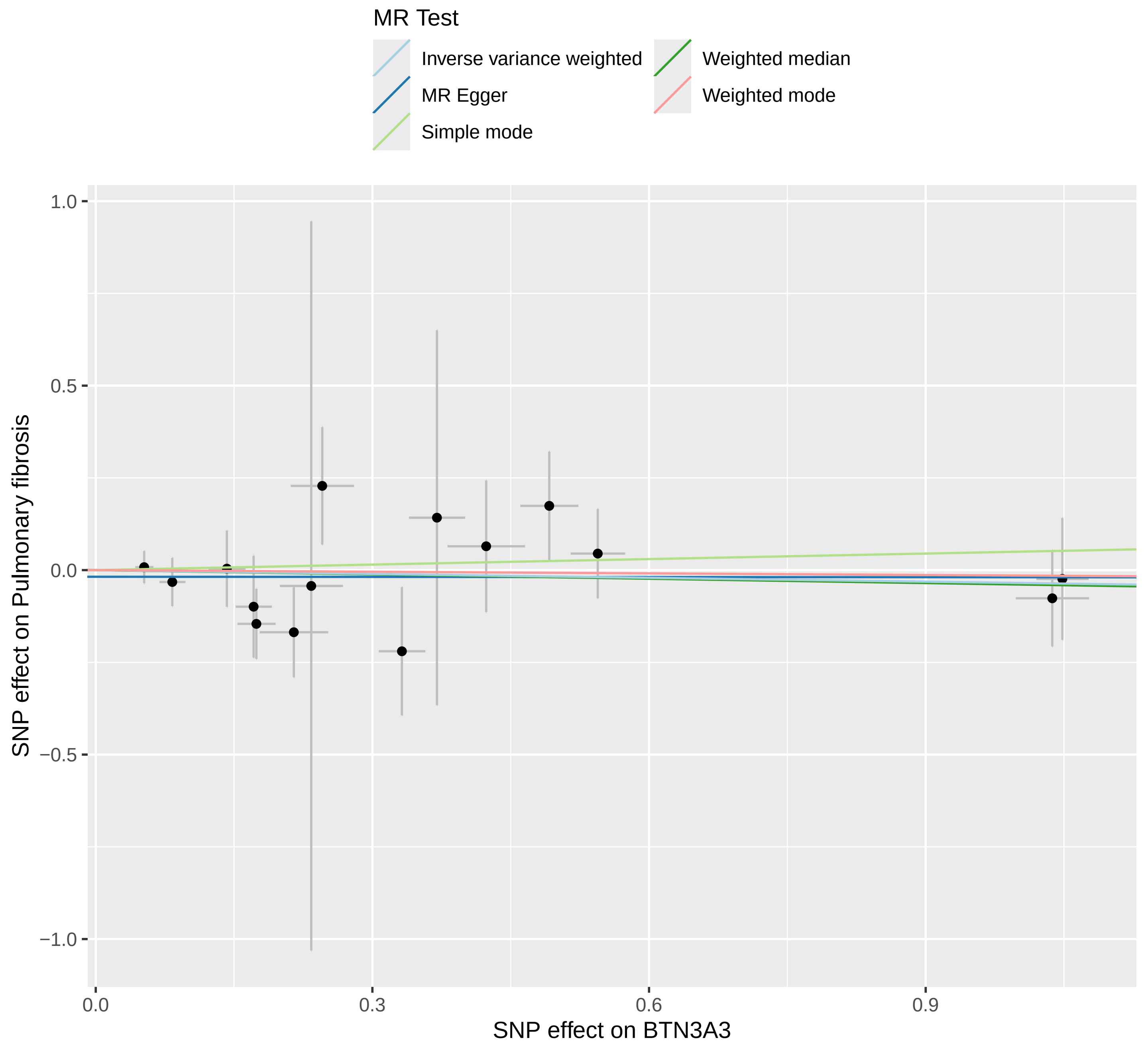

MR Test
Inverse variance weighted
MR Egger
Simple mode
Weighted median
Weighted mode
1.0
0.5
0.0
−0.5
−1.0
SNP effect on Pulmonary fibrosis
0.0
0.3
0.6
0.9
SNP effect on BTN3A3

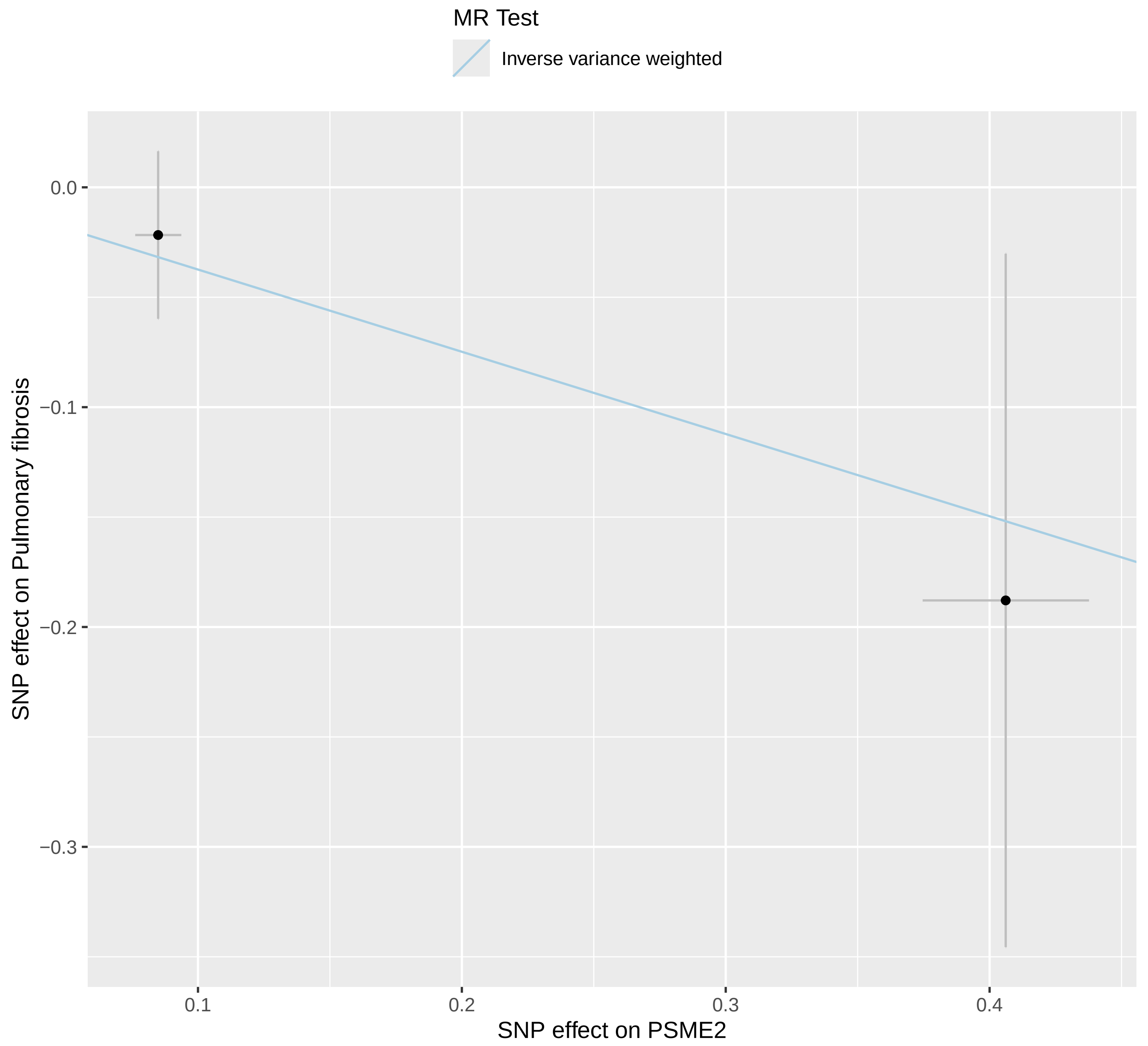

MR Test
Inverse variance weighted
0.0
−0.1
−0.2
−0.3
SNP effect on Pulmonary fibrosis
0.1
0.2
0.3
0.4
SNP effect on PSME2

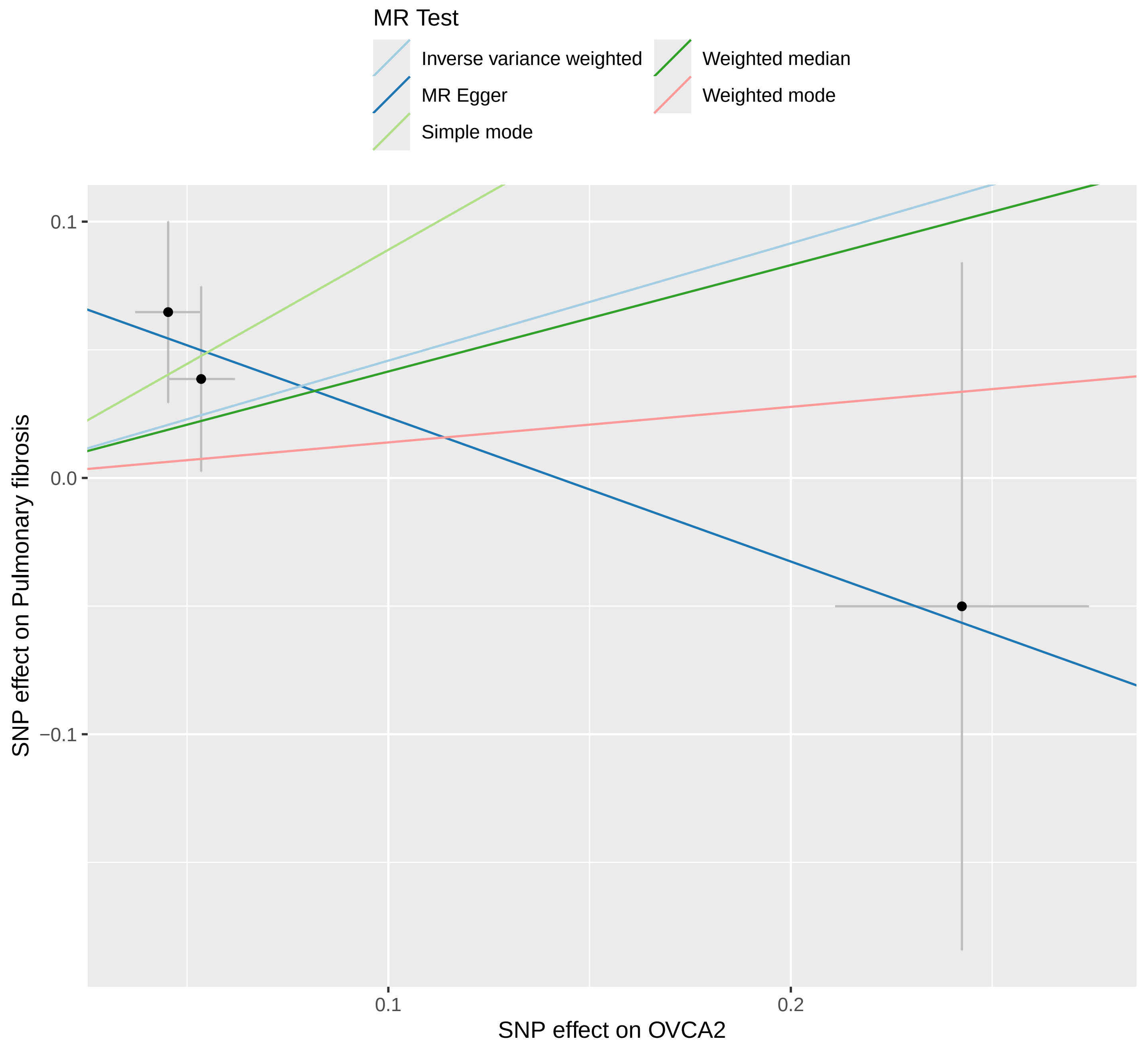
MR Test
Inverse variance weighted
MR Egger
Simple mode
Weighted median
Weighted mode
0.1
0.0
−0.1
SNP effect on Pulmonary fibrosis
0.1
0.2
SNP effect on OVCA2

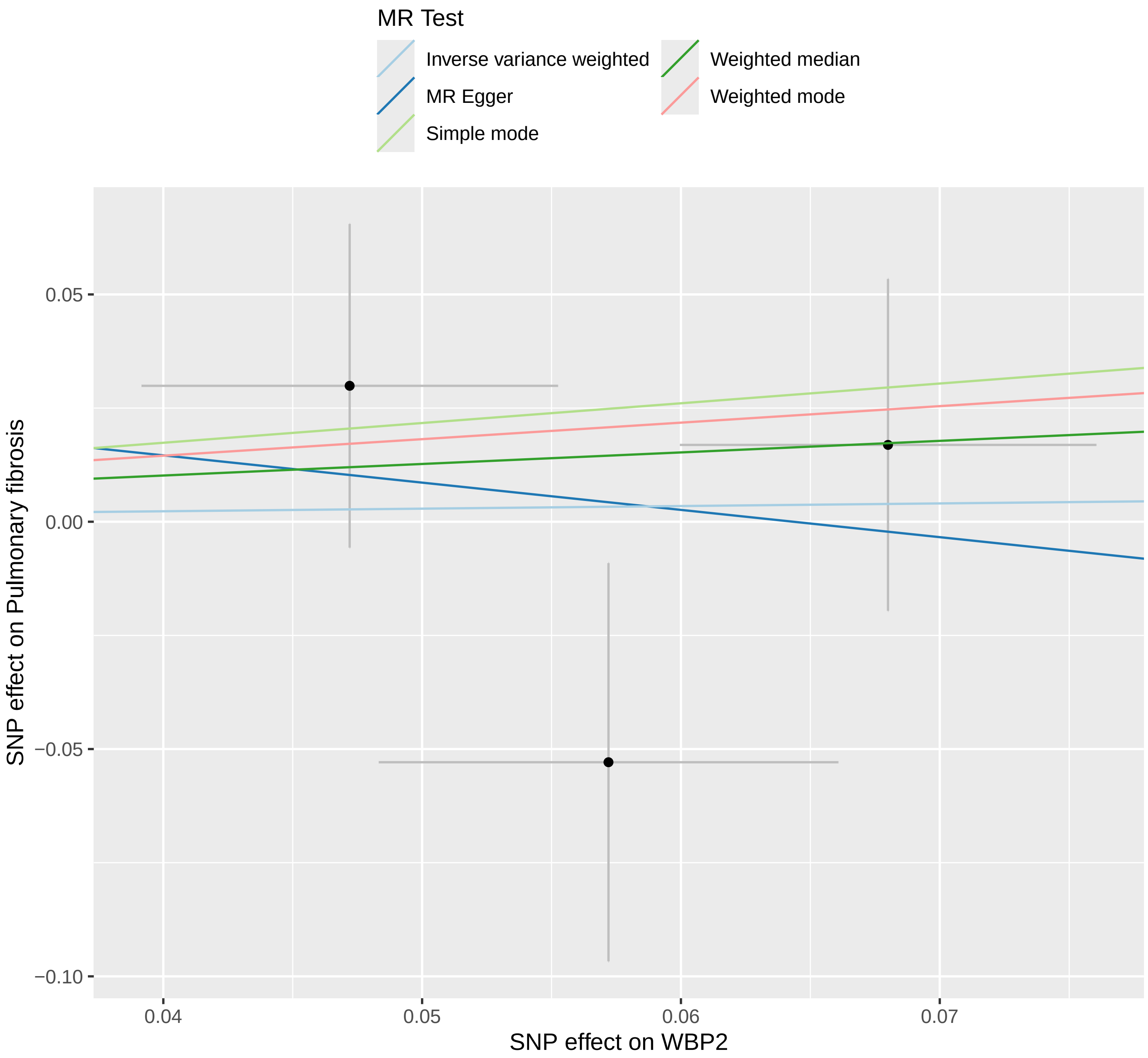
MR Test
Inverse variance weighted
MR Egger
Simple mode
Weighted median
Weighted mode
0.05
0.00
−0.05
−0.10
SNP effect on Pulmonary fibrosis
0.04
0.05
0.06
0.07
SNP effect on WBP2

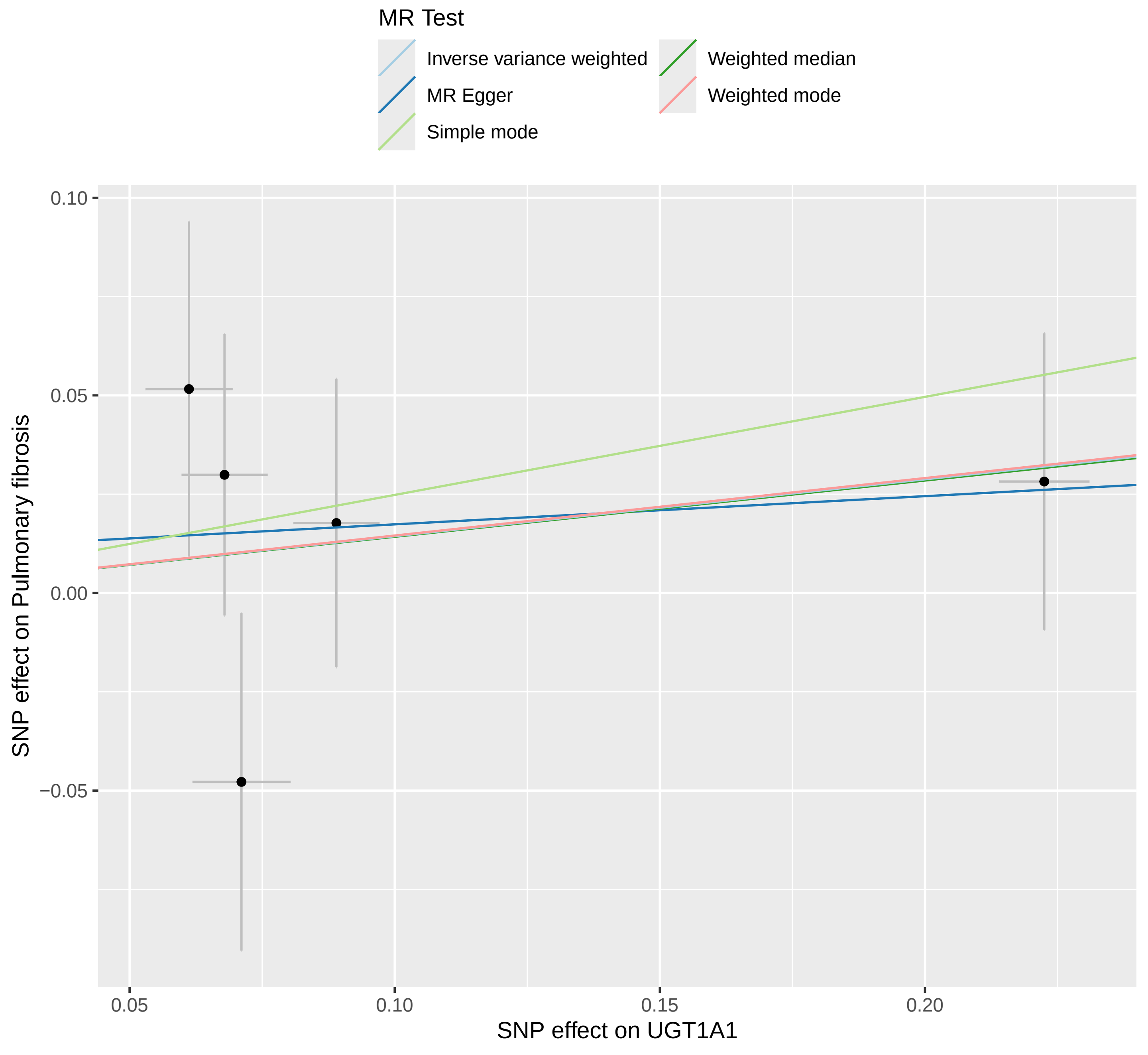
MR Test
Inverse variance weighted
MR Egger
Simple mode
Weighted median
Weighted mode
0.10
0.05
0.00
−0.05
SNP effect on Pulmonary fibrosis
0.05
0.10
0.15
0.20
SNP effect on UGT1A1

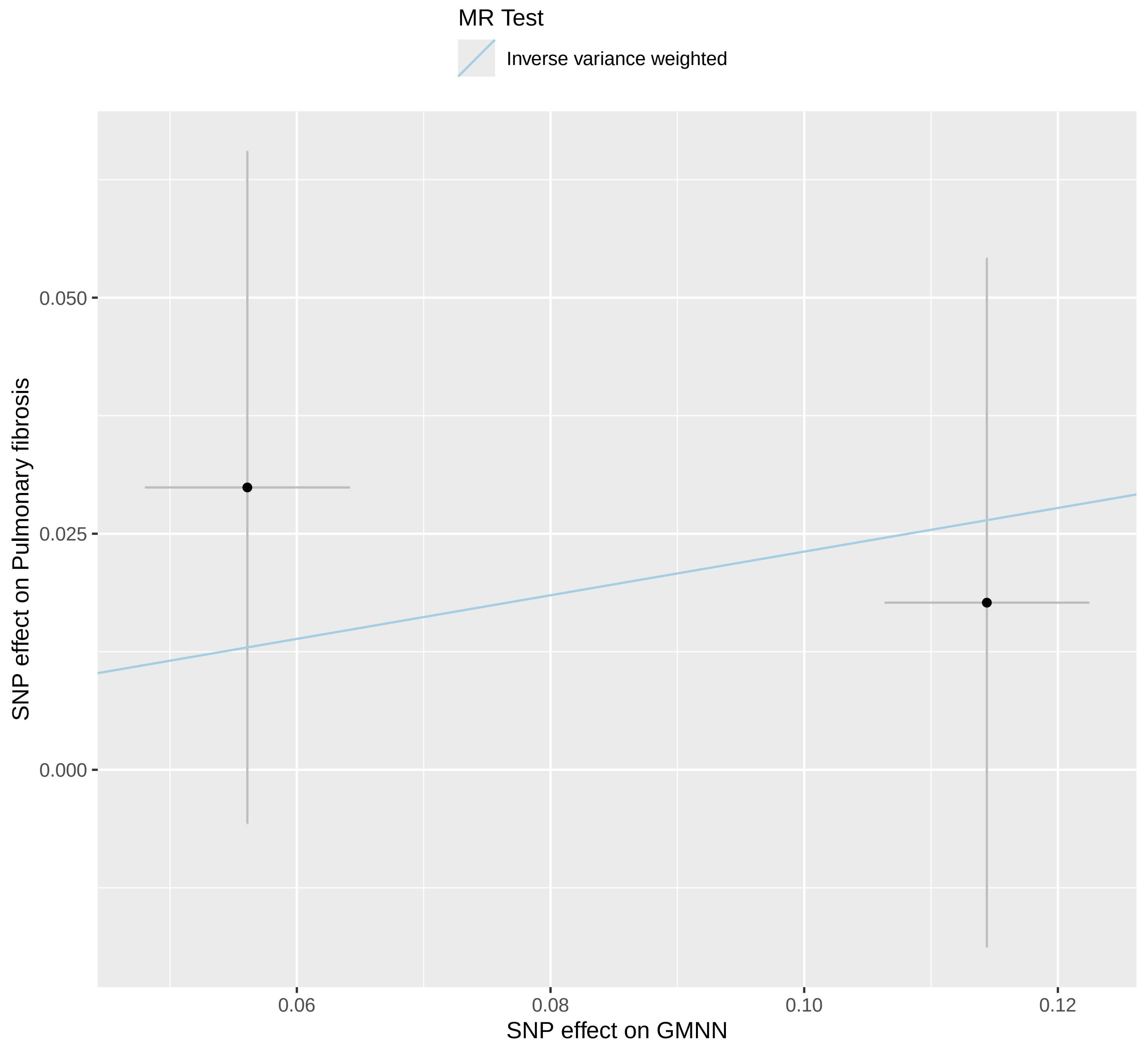

MR Test
Inverse variance weighted
0.050
0.025
0.000
0.06
0.08
0.10
0.12
SNP effect on GMNN
SNP effect on Pulmonary fibrosis

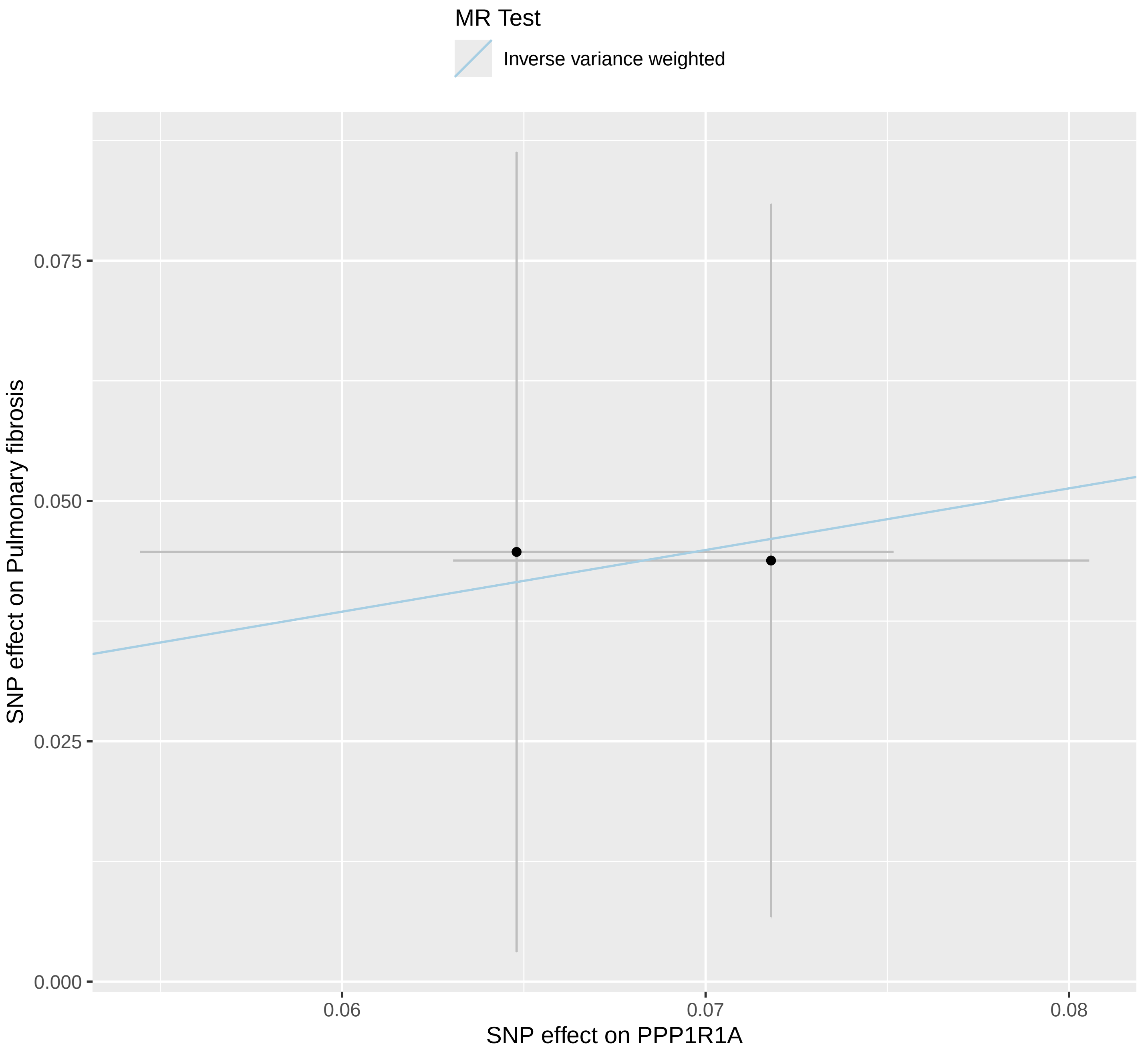

MR Test
Inverse variance weighted
0.075
0.050
0.025
0.000
0.06
0.07
0.08
SNP effect on Pulmonary fibrosis
SNP effect on PPP1R1A

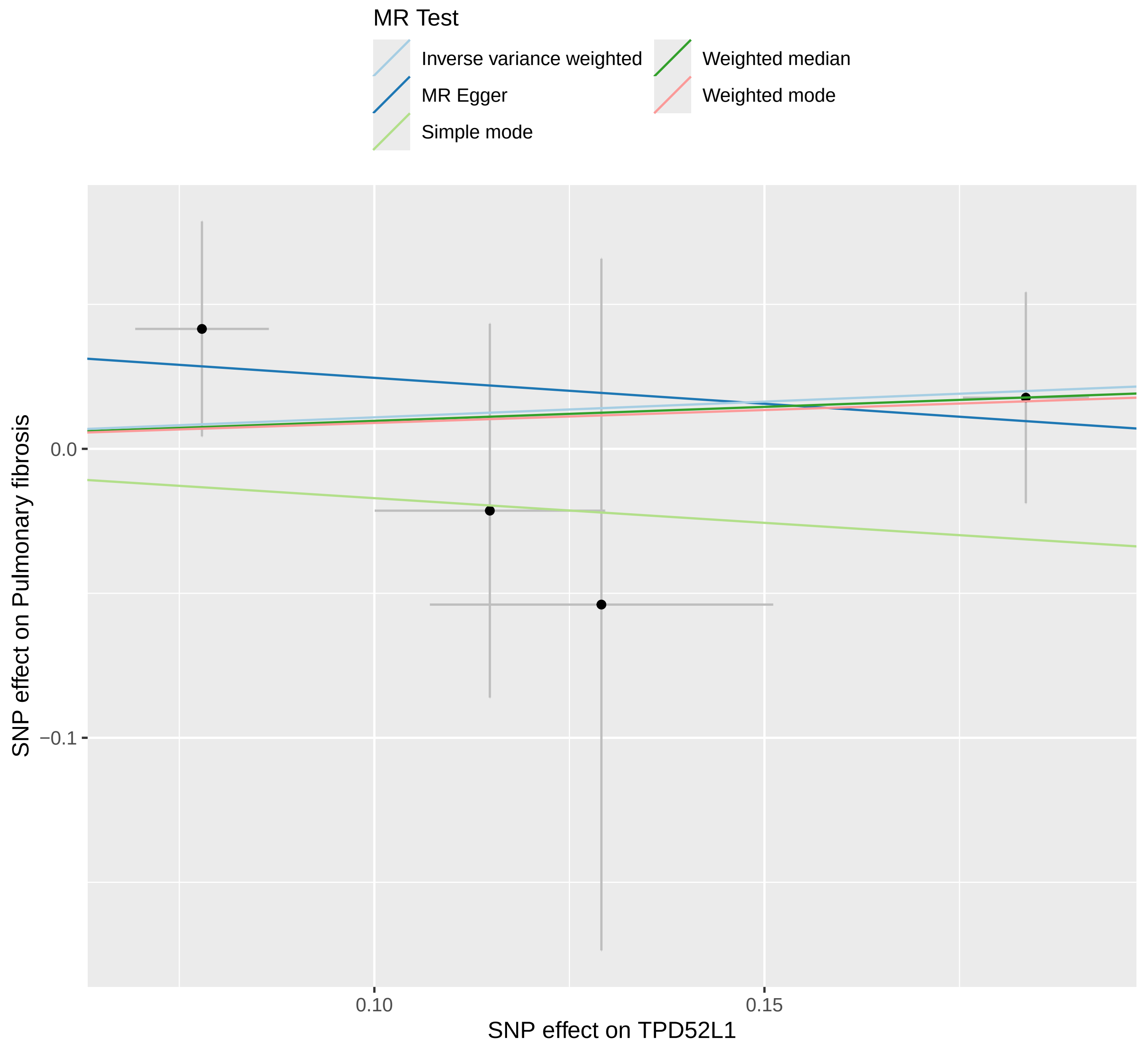

MR Test
Inverse variance weighted
MR Egger
Simple mode
Weighted median
Weighted mode
SNP effect on Pulmonary fibrosis
0.0
−0.1
0.10
0.15
SNP effect on TPD52L1

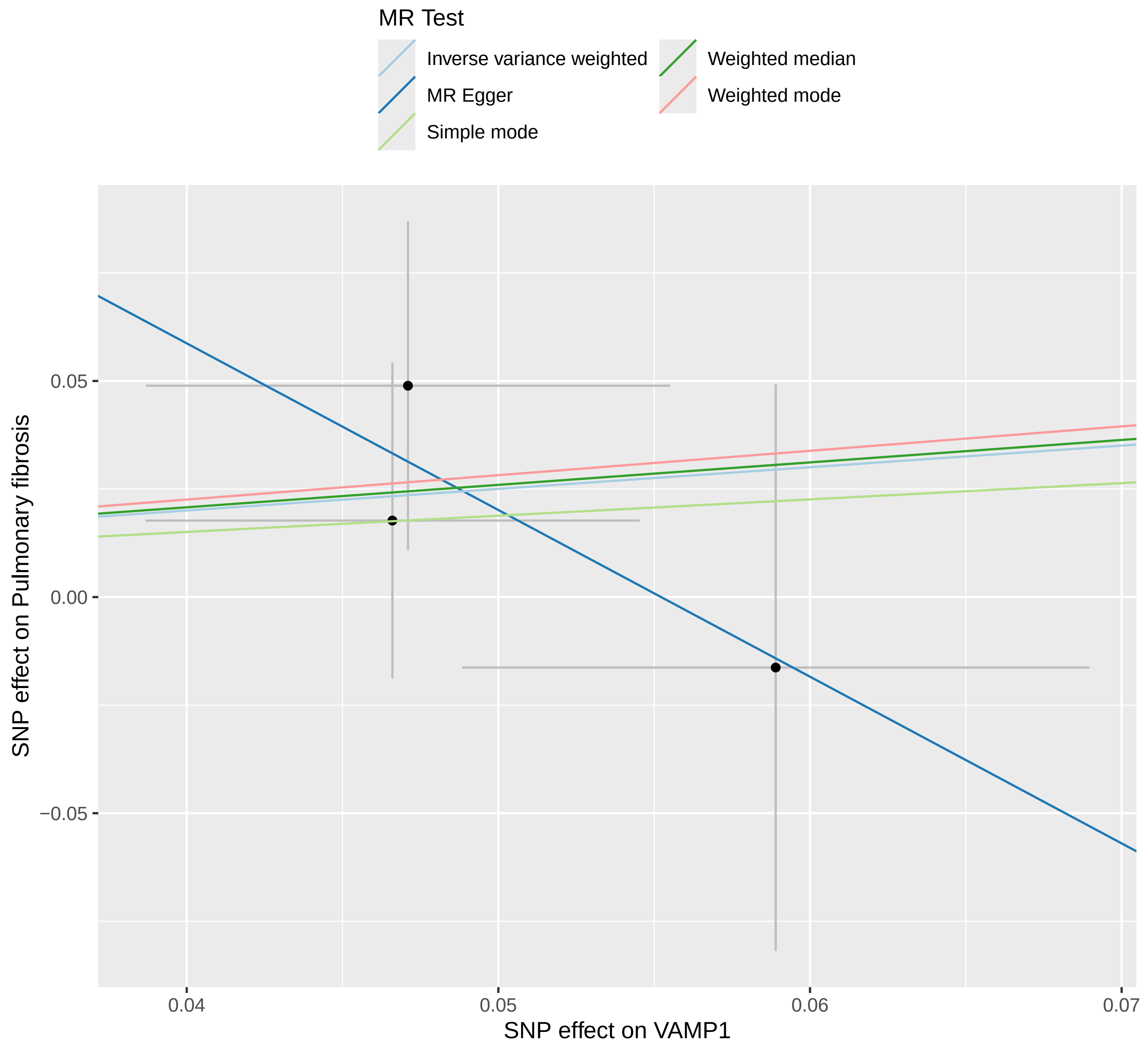
MR Test
Inverse variance weighted
MR Egger
Simple mode
Weighted median
Weighted mode
0.05
0.00
−0.05
SNP effect on Pulmonary fibrosis
0.04
0.05
0.06
0.07
SNP effect on VAMP1

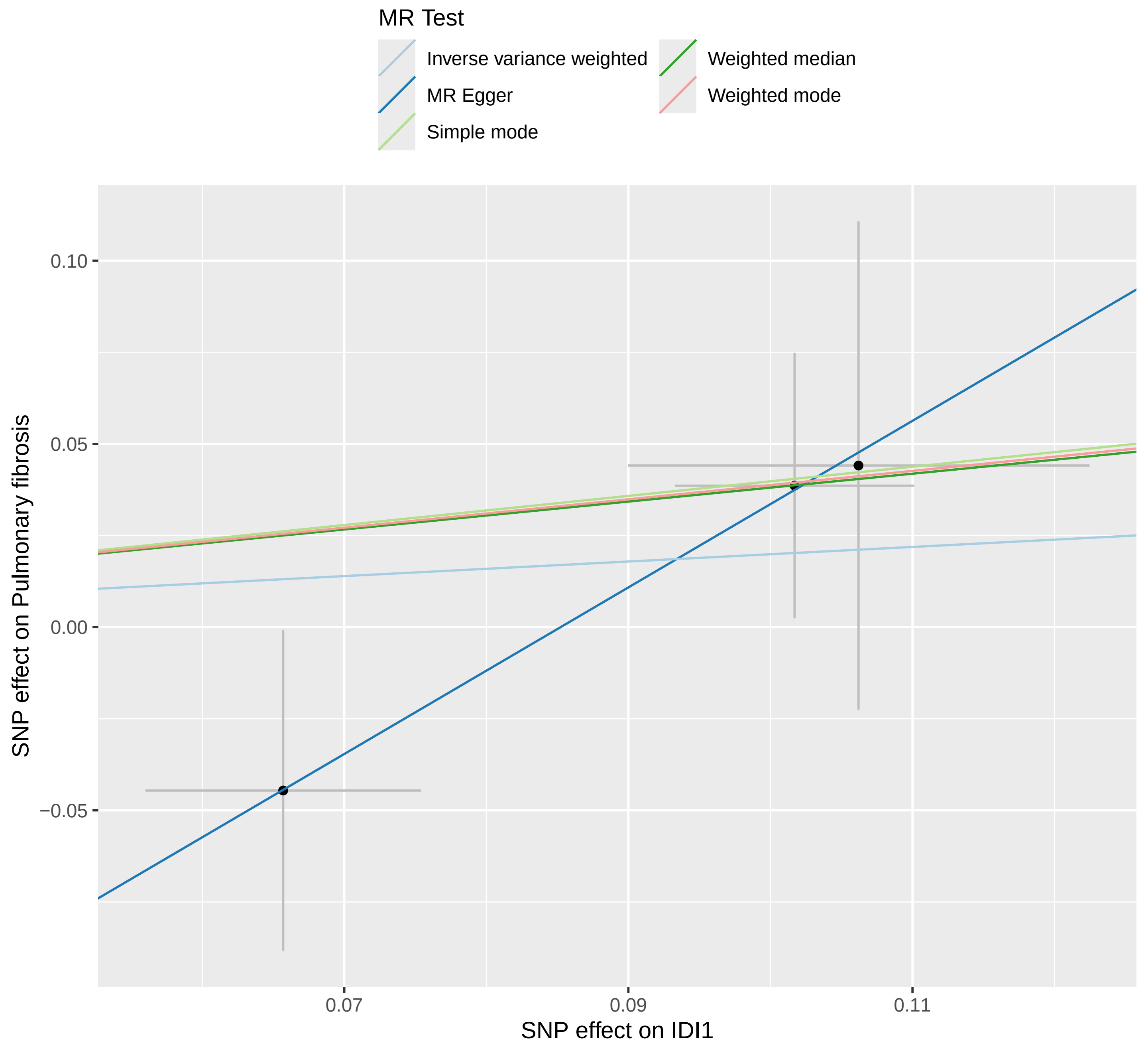
MR Test
Inverse variance weighted
MR Egger
Simple mode
Weighted median
Weighted mode
SNP effect on Pulmonary fibrosis
0.10
0.05
0.00
−0.05
0.07
0.09
0.11
SNP effect on IDI1

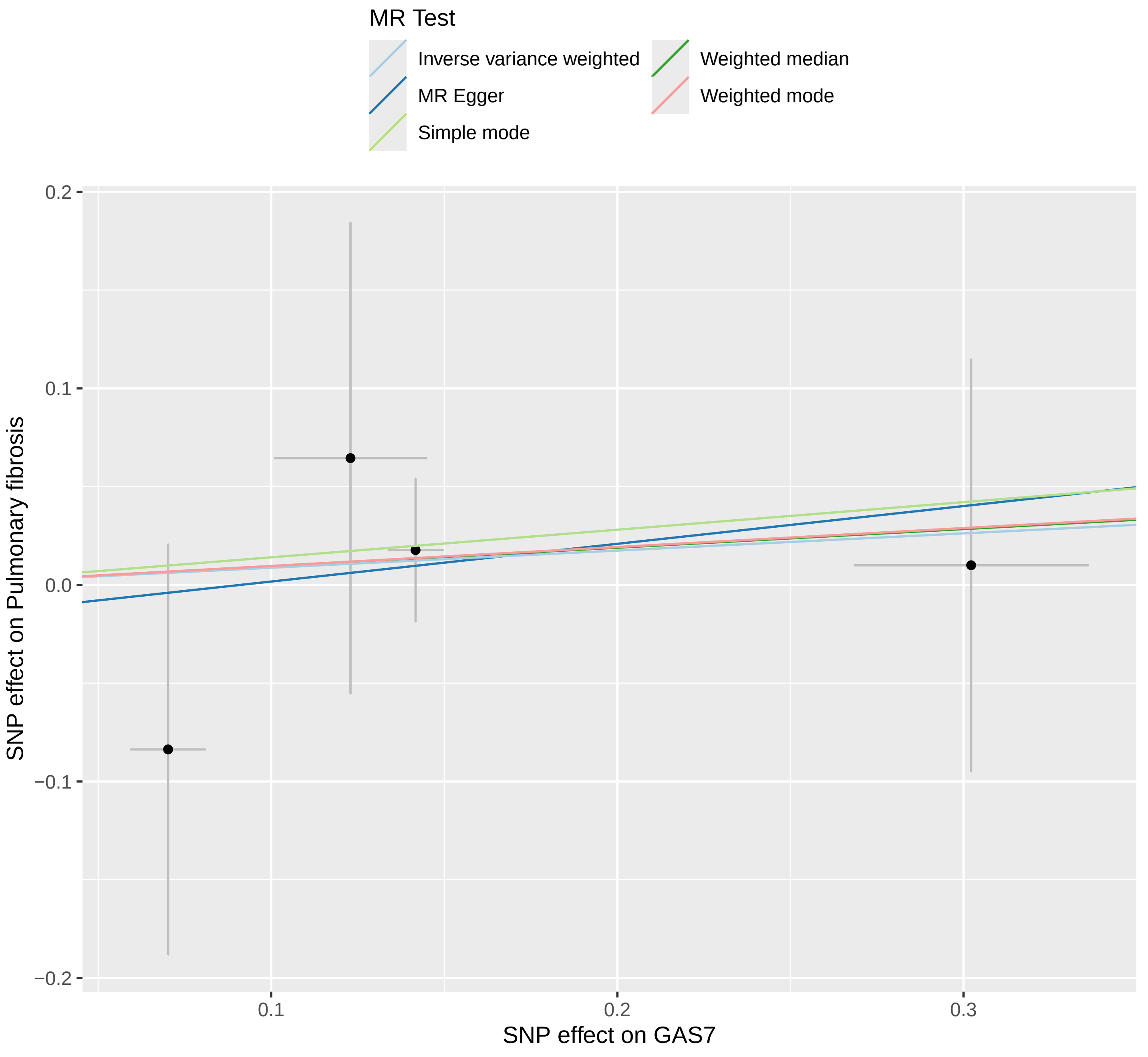
MR Test
Inverse variance weighted
MR Egger
Simple mode
Weighted median
Weighted mode
0.2
0.1
0.0
−0.1
−0.2
0.1
0.2
0.3
SNP effect on Pulmonary fibrosis
SNP effect on GAS7

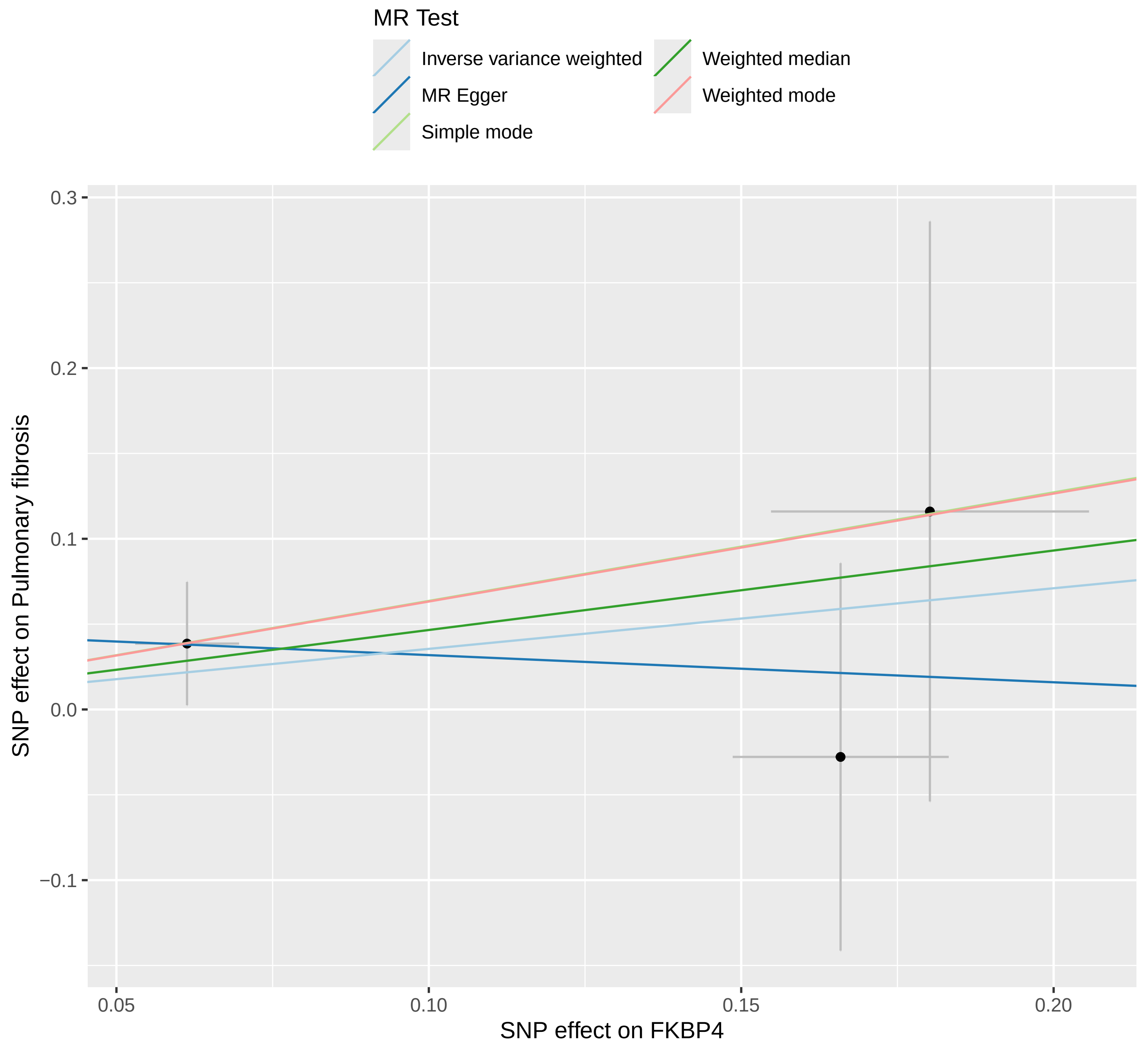

MR Test
Inverse variance weighted
MR Egger
Simple mode
Weighted median
Weighted mode
SNP effect on Pulmonary fibrosis
SNP effect on FKBP4
0.3
0.2
0.1
0.0
−0.1
0.05
0.10
0.15
0.20

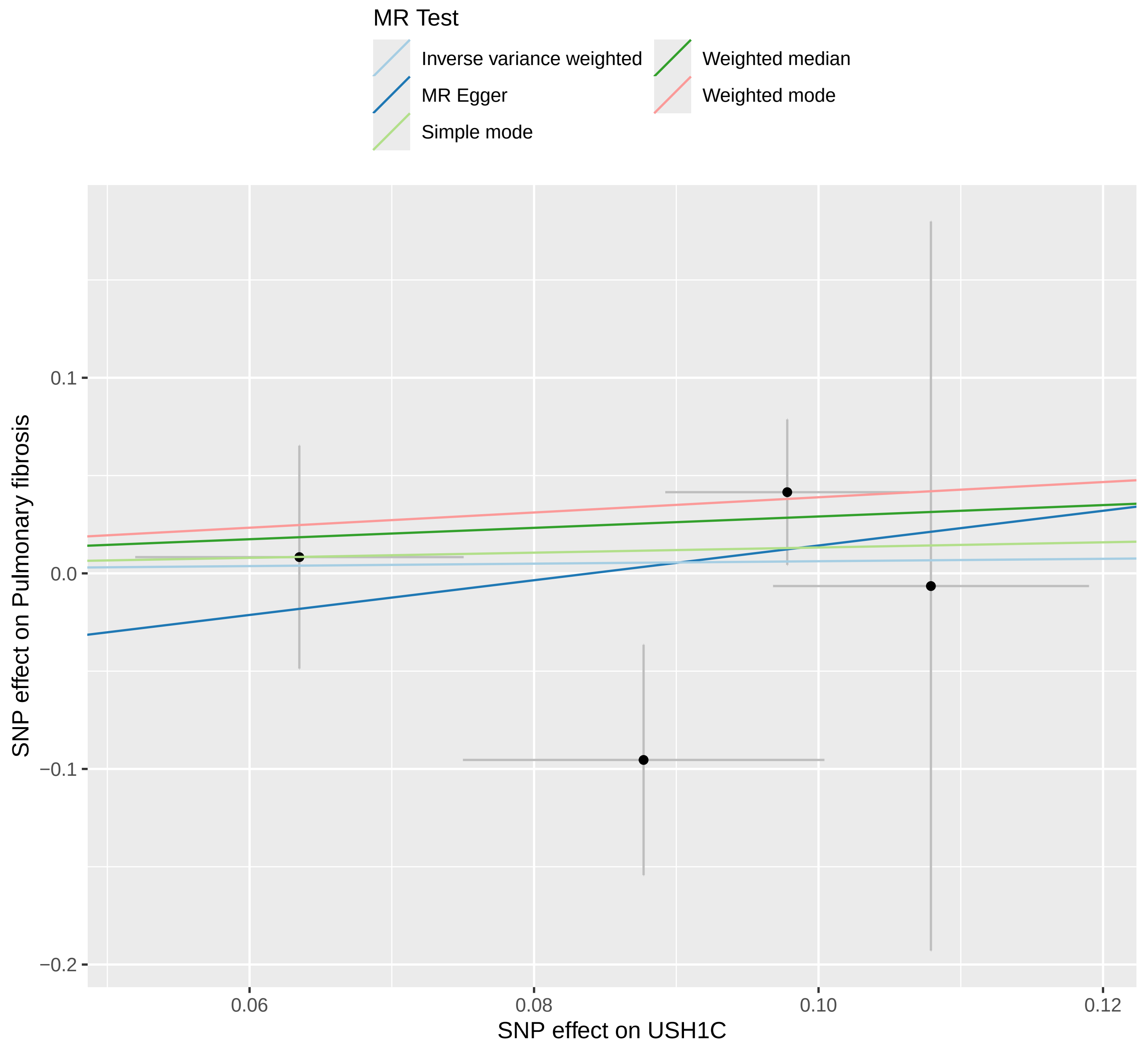

MR Test
Inverse variance weighted
MR Egger
Simple mode
Weighted median
Weighted mode
0.1
0.0
−0.1
−0.2
SNP effect on Pulmonary fibrosis
0.06
0.08
0.10
0.12
SNP effect on USH1C

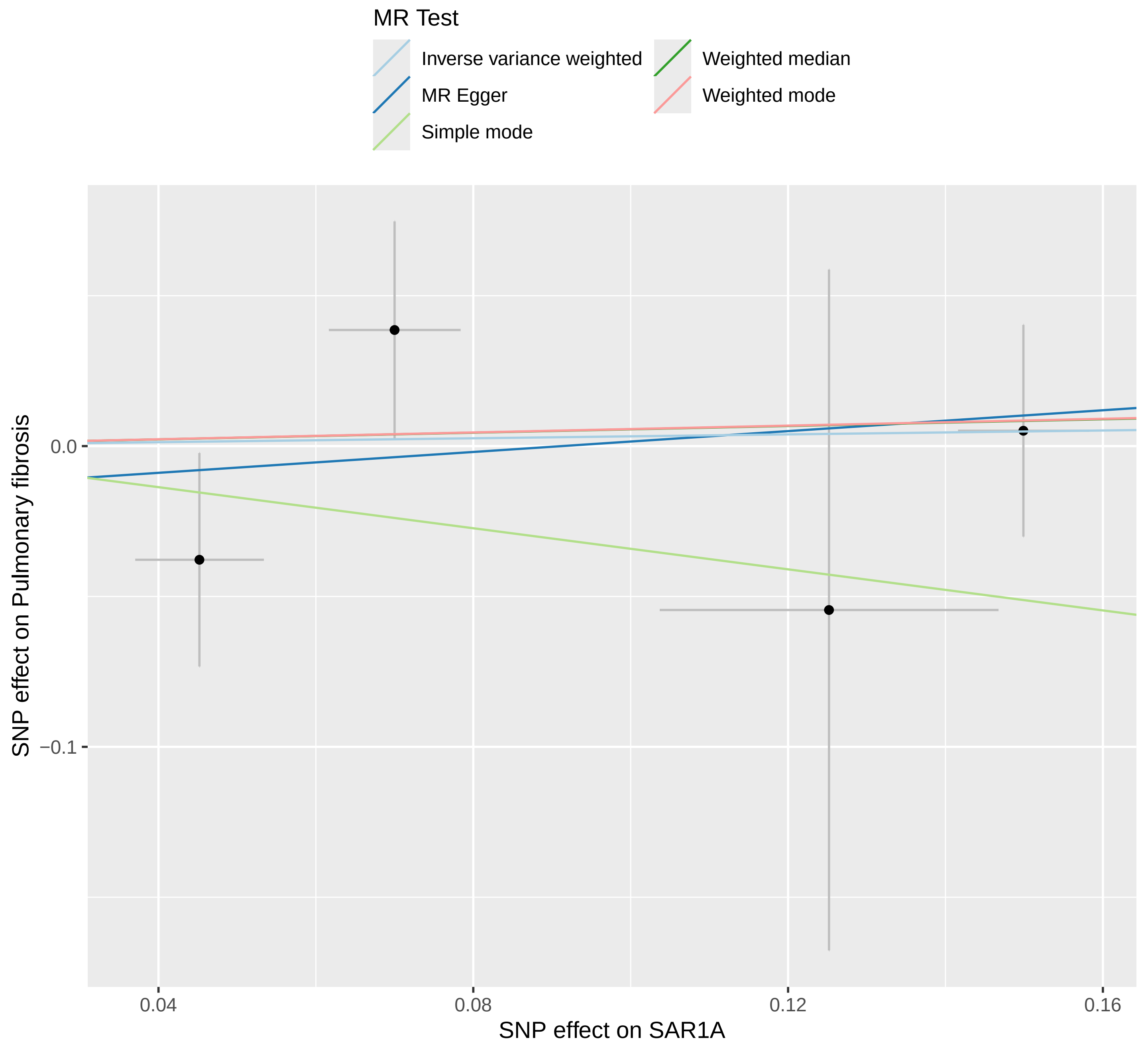
MR Test
Inverse variance weighted
MR Egger
Simple mode
Weighted median
Weighted mode
SNP effect on Pulmonary fibrosis
0.0
−0.1
0.04
0.08
0.12
0.16
SNP effect on SAR1A

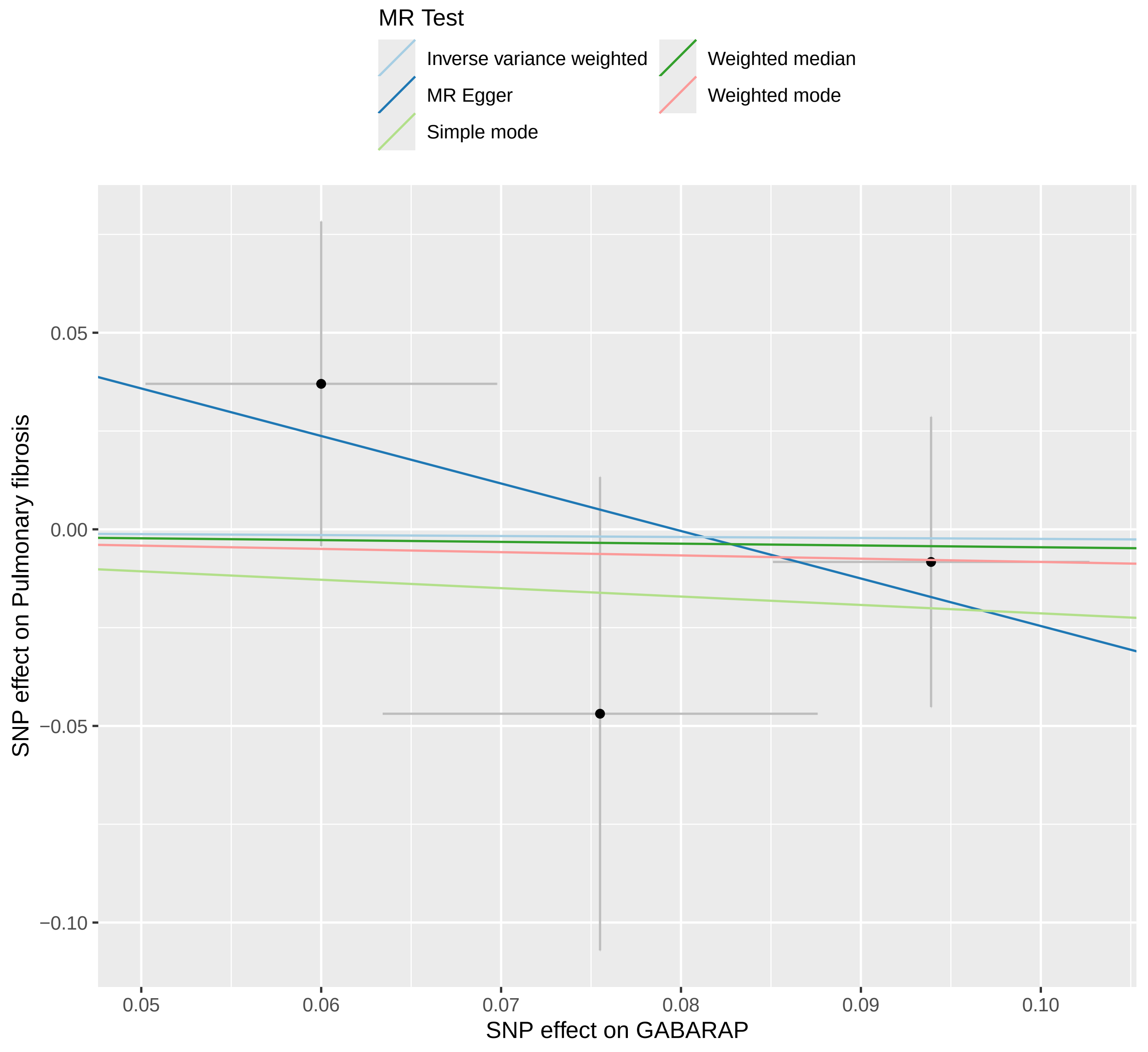
MR Test
Inverse variance weighted
MR Egger
Simple mode
Weighted median
Weighted mode
SNP effect on Pulmonary fibrosis
0.05
0.00
−0.05
−0.10
0.05
0.06
0.07
0.08
0.09
0.10
SNP effect on GABARAP

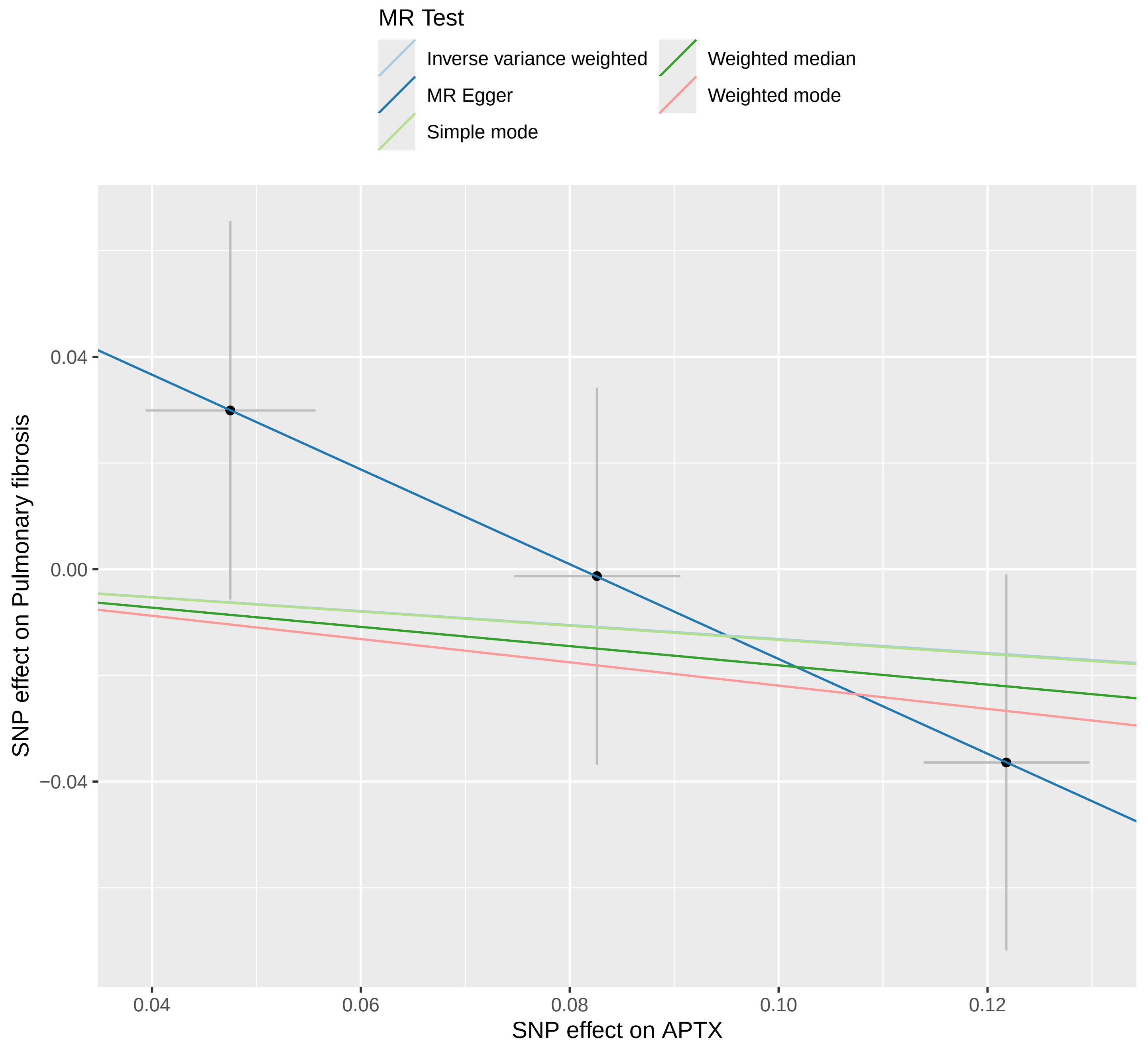
MR Test
Inverse variance weighted
MR Egger
Simple mode
Weighted median
Weighted mode
0.04
0.00
−0.04
SNP effect on Pulmonary fibrosis
0.04
0.06
0.08
0.10
0.12
SNP effect on APTX

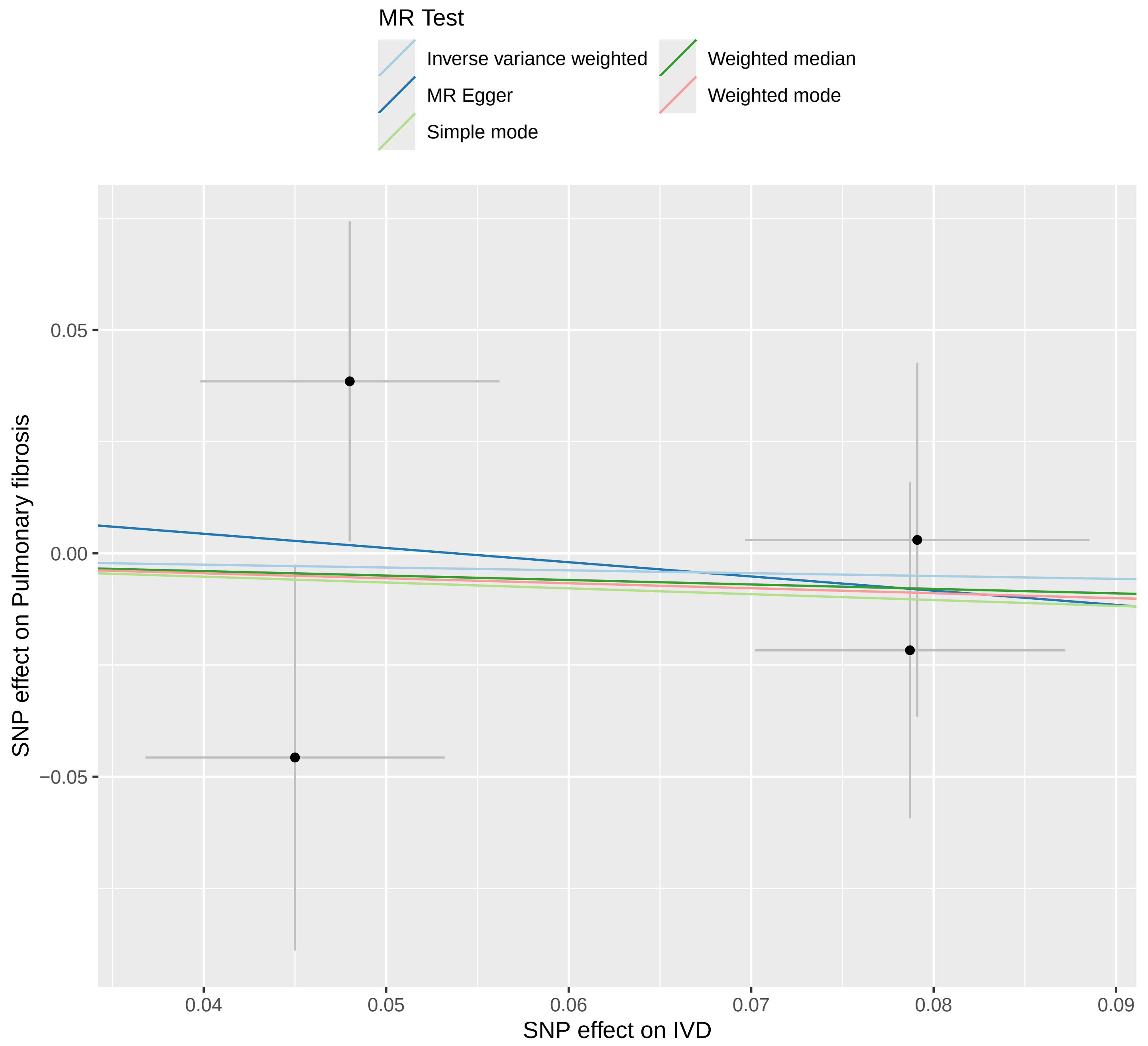

MR Test
Inverse variance weighted
MR Egger
Simple mode
Weighted median
Weighted mode
0.05
0.00
−0.05
0.04
0.05
0.06
0.07
0.08
0.09
SNP effect on Pulmonary fibrosis
SNP effect on IVD

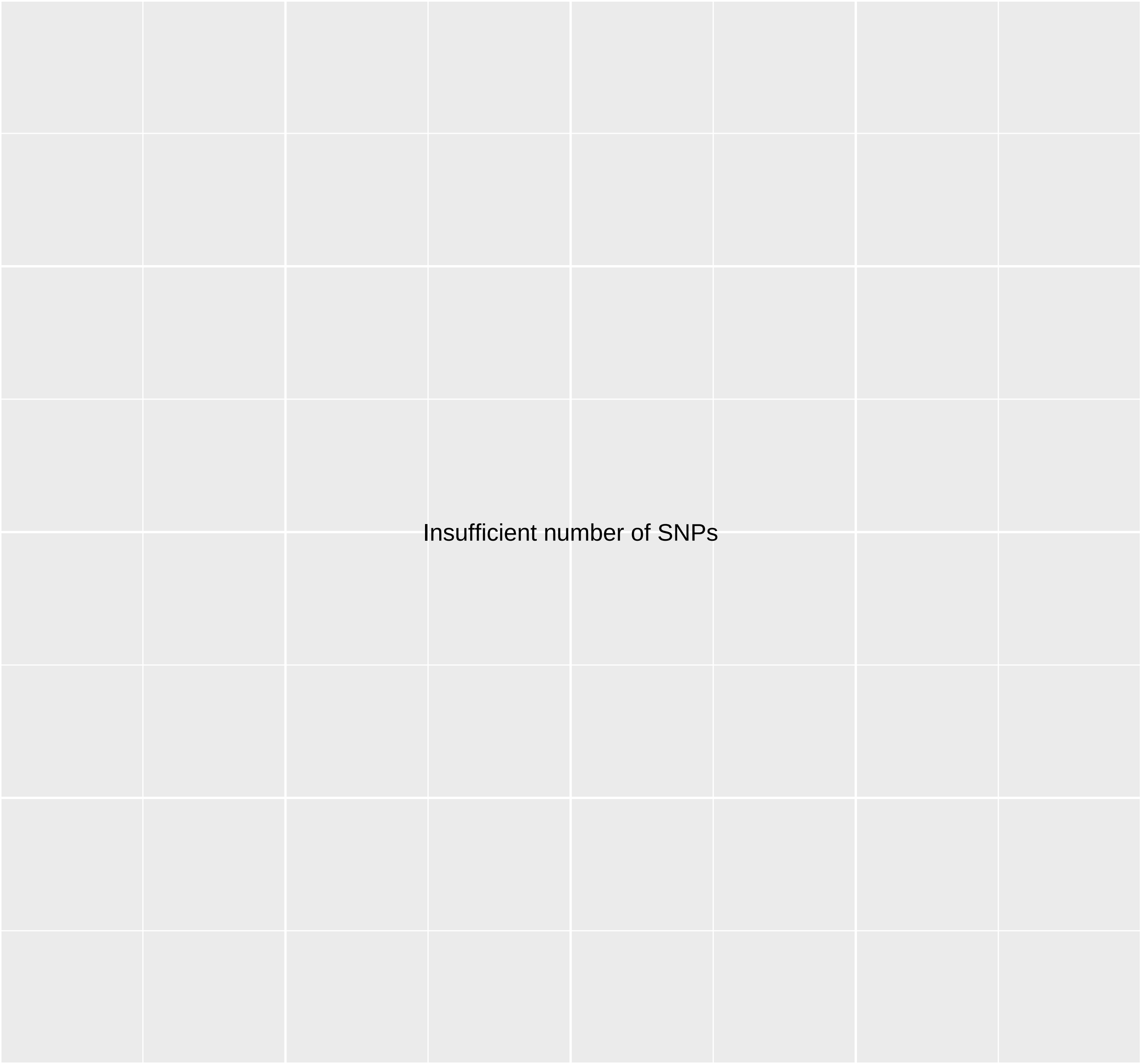
Insufficient number of SNPs

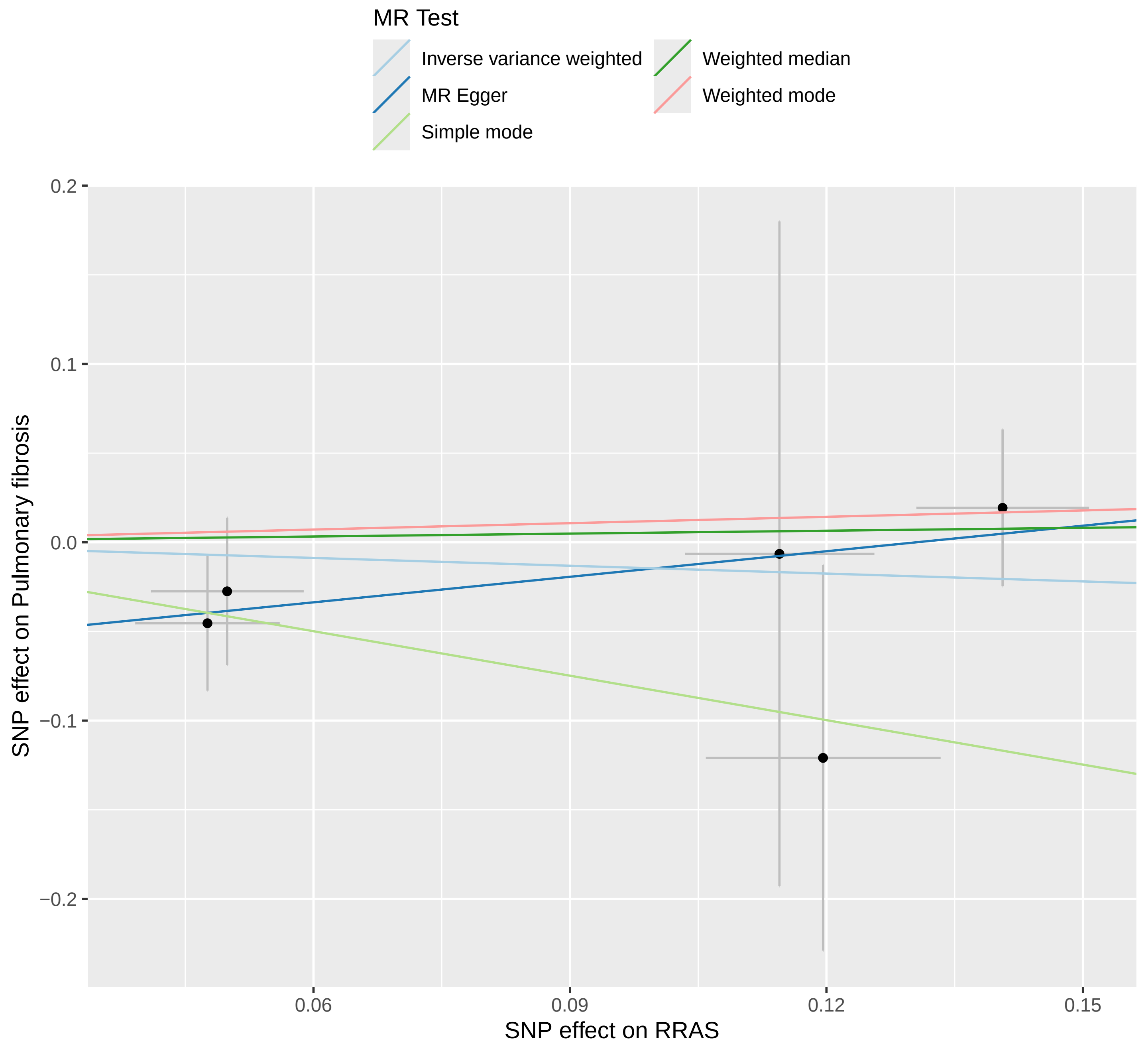

MR Test
Inverse variance weighted
MR Egger
Simple mode
Weighted median
Weighted mode
0.2
0.1
0.0
−0.1
−0.2
SNP effect on Pulmonary fibrosis
0.06
0.09
0.12
0.15
SNP effect on RRAS

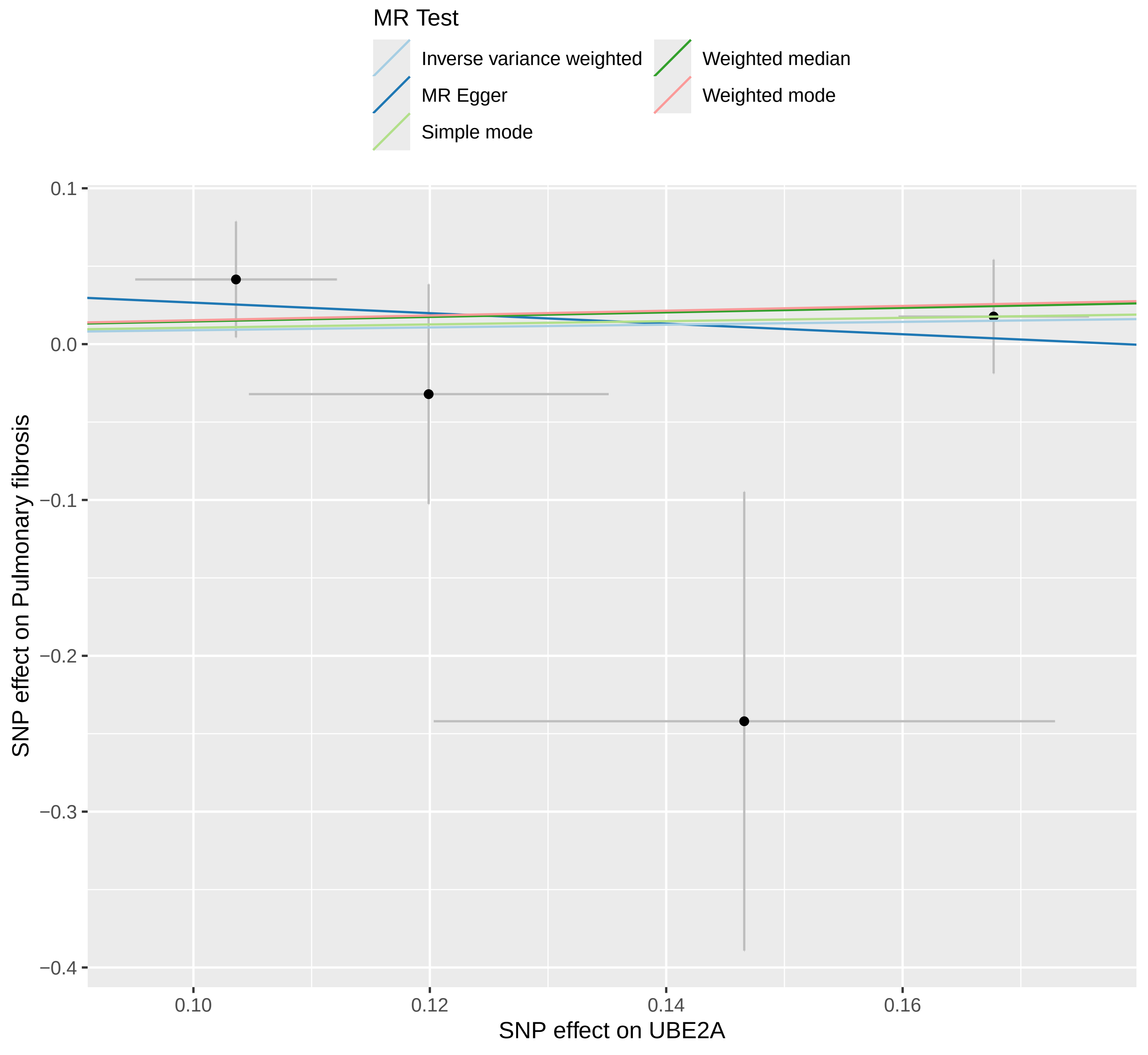
MR Test
Inverse variance weighted
MR Egger
Simple mode
Weighted median
Weighted mode
0.1
0.0
−0.1
−0.2
−0.3
−0.4
0.10
0.12
0.14
0.16
SNP effect on UBE2A
SNP effect on Pulmonary fibrosis

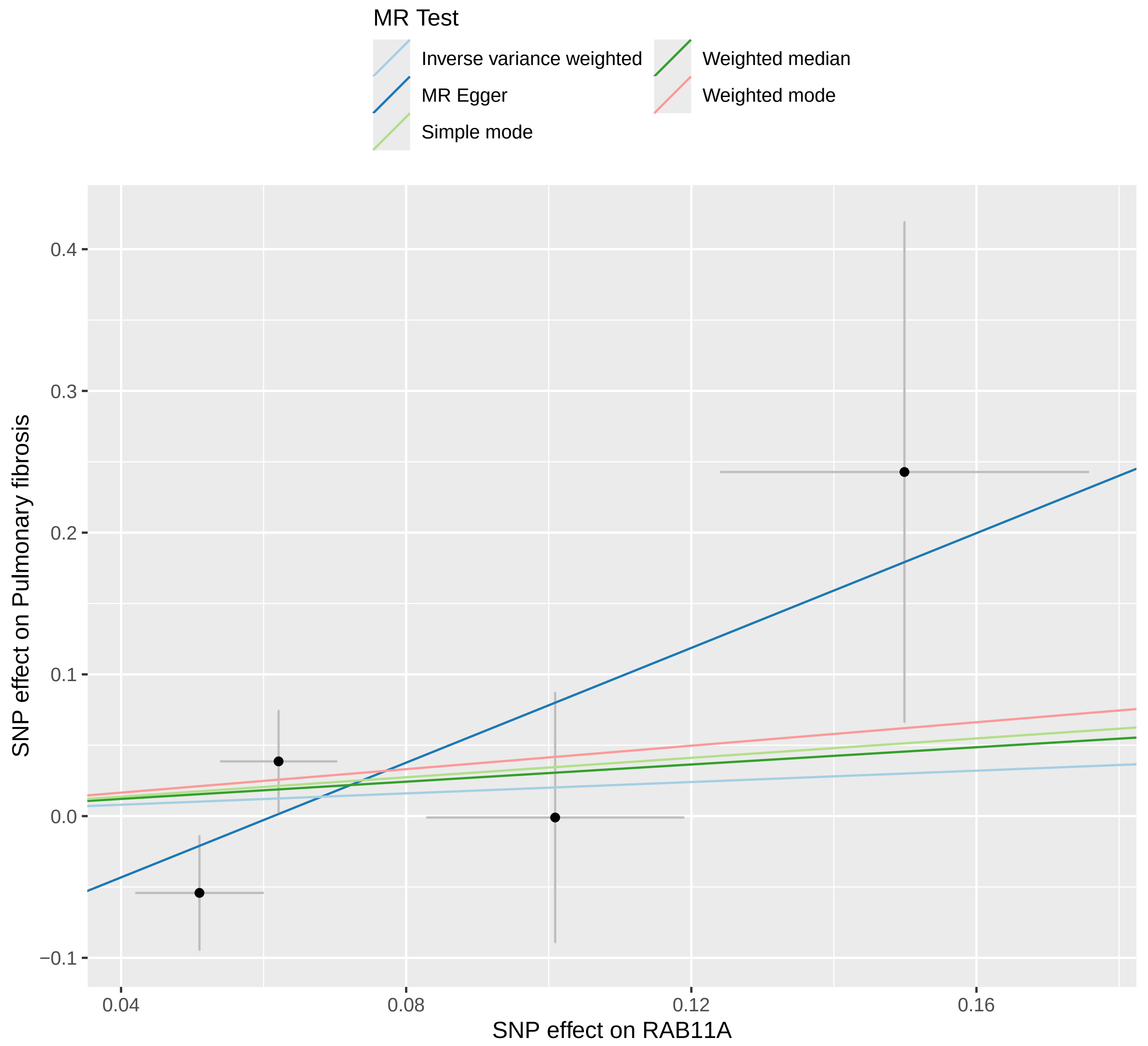

MR Test
Inverse variance weighted
MR Egger
Simple mode
Weighted median
Weighted mode
SNP effect on Pulmonary fibrosis
0.4
0.3
0.2
0.1
0.0
−0.1
0.04
0.08
0.12
0.16
SNP effect on RAB11A

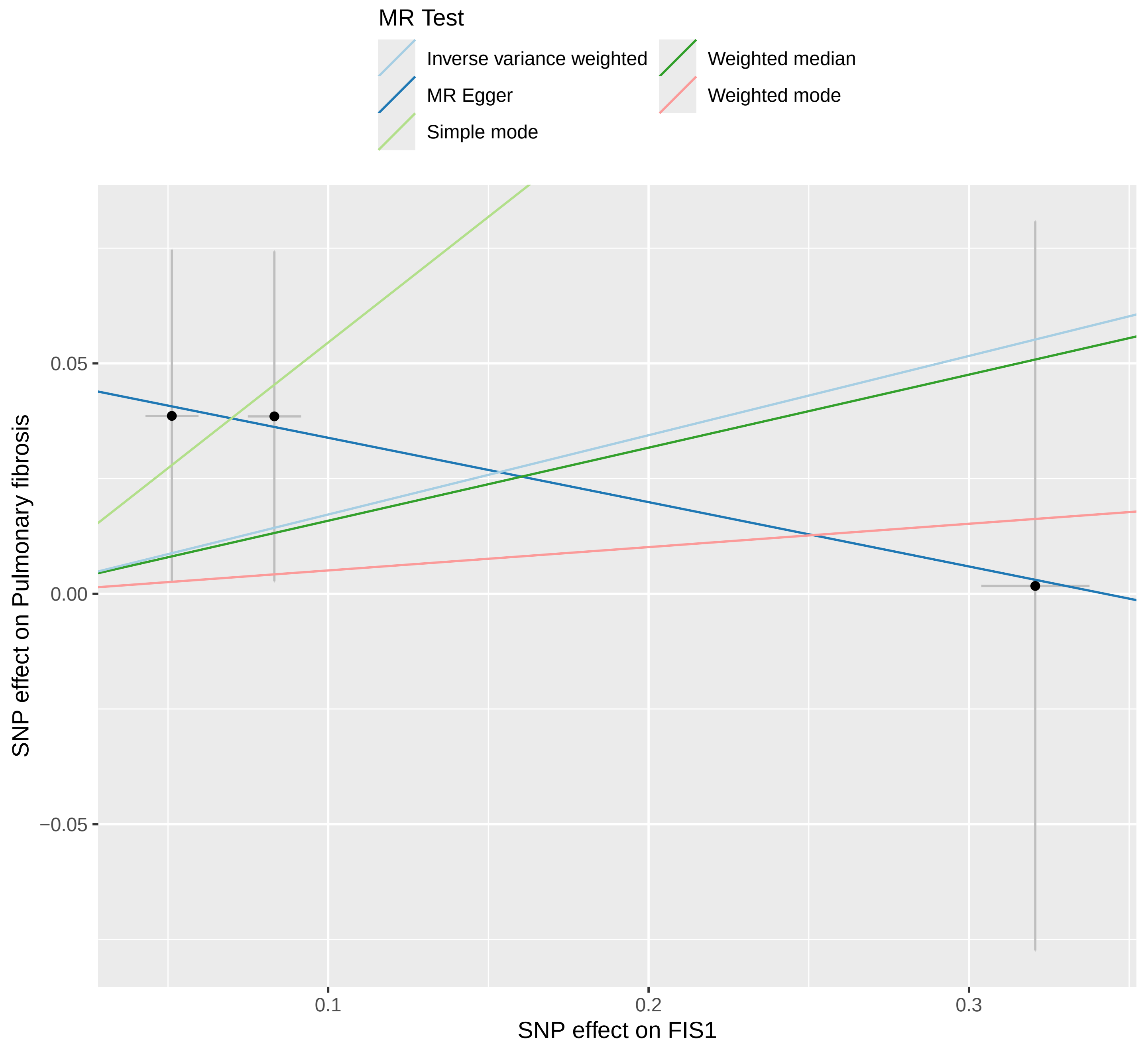

MR Test
Inverse variance weighted
MR Egger
Simple mode
Weighted median
Weighted mode
SNP effect on Pulmonary fibrosis
0.05
0.00
−0.05
0.1
0.2
0.3
SNP effect on FIS1

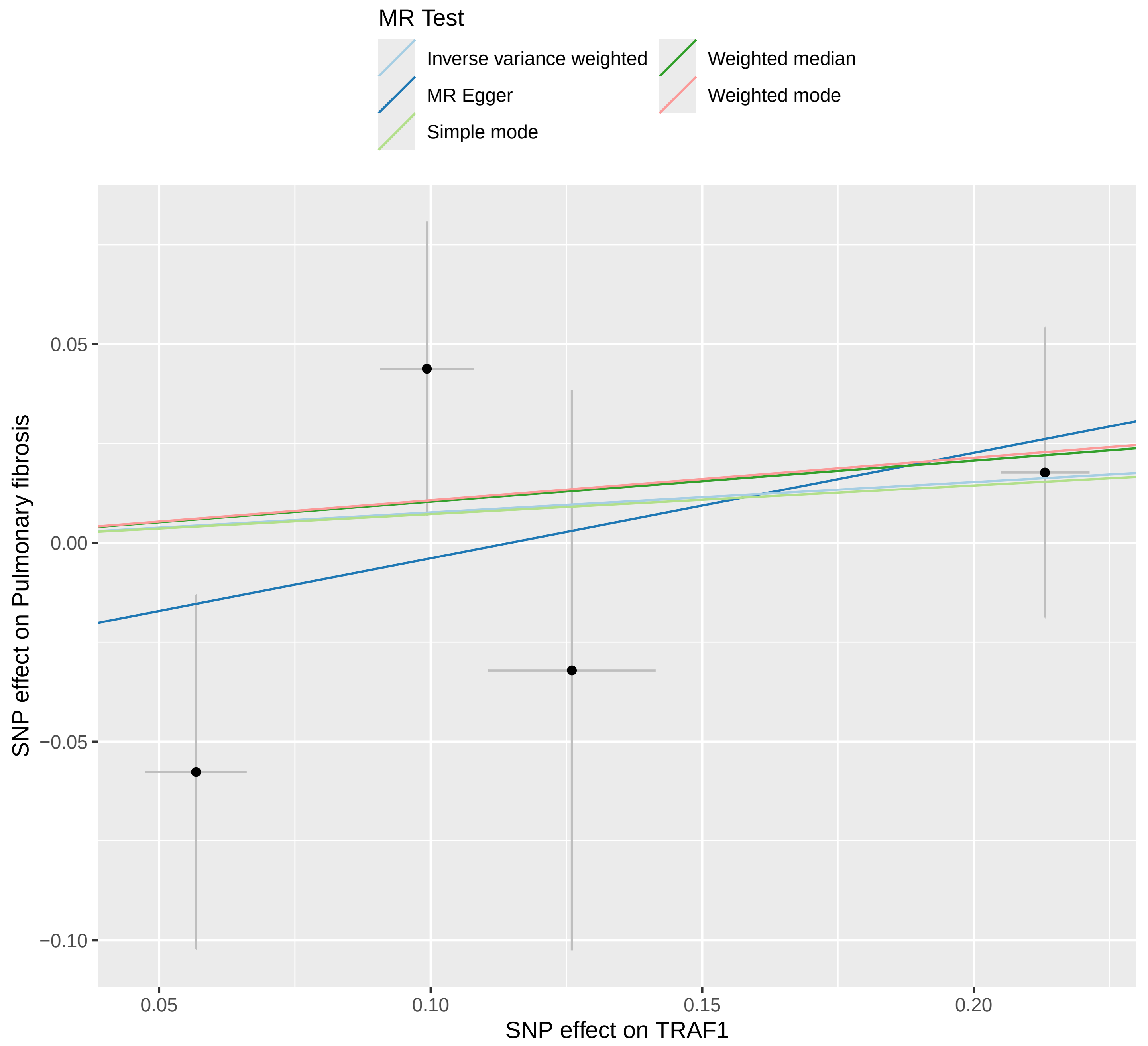

MR Test
Inverse variance weighted
MR Egger
Simple mode
Weighted median
Weighted mode
0.05
0.00
−0.05
−0.10
SNP effect on Pulmonary fibrosis
0.05
0.10
0.15
0.20
SNP effect on TRAF1

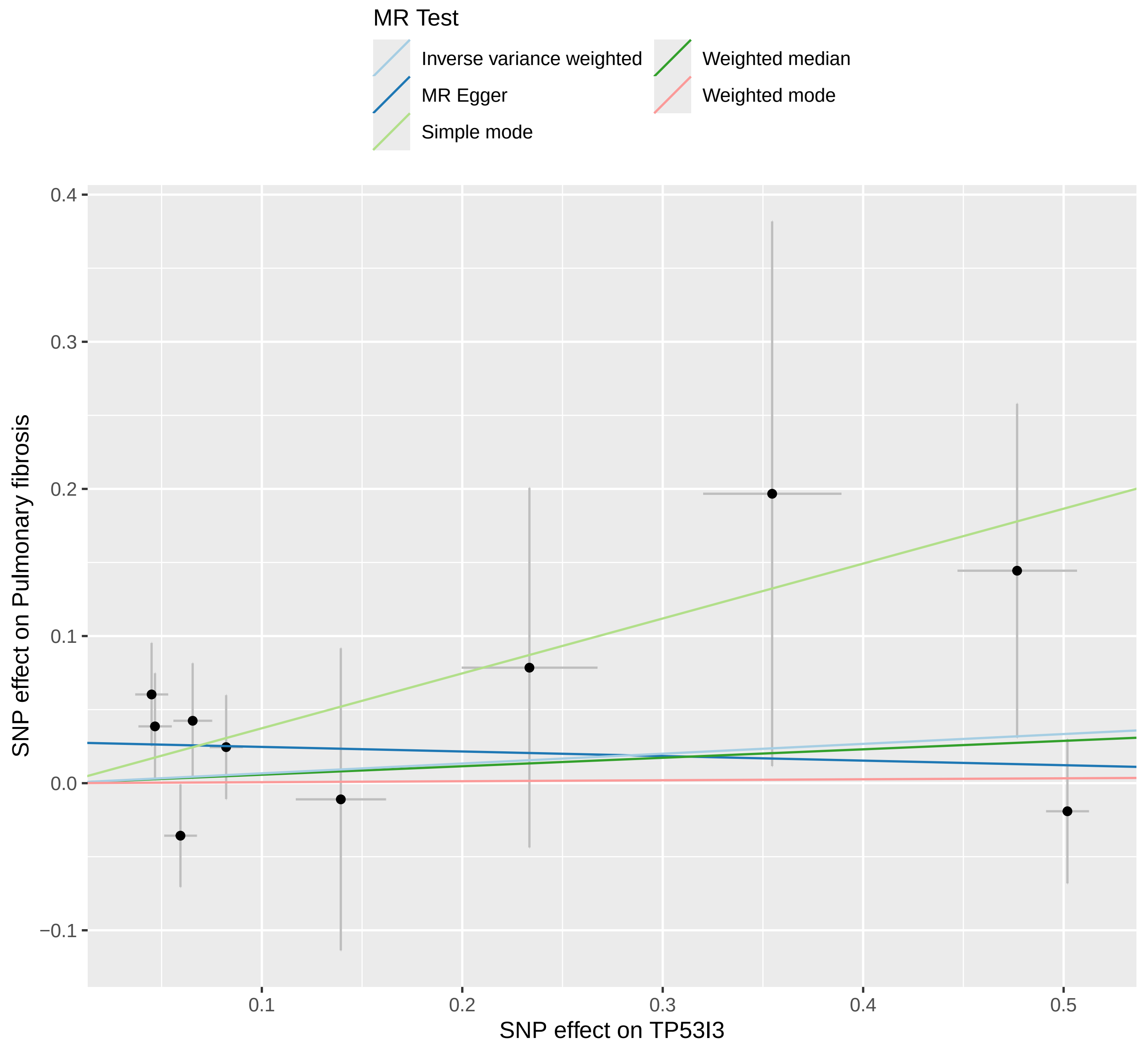
MR Test
Inverse variance weighted
MR Egger
Simple mode
Weighted median
Weighted mode
0.4
0.3
0.2
0.1
0.0
−0.1
SNP effect on Pulmonary fibrosis
0.1
0.2
0.3
0.4
0.5
SNP effect on TP53I3

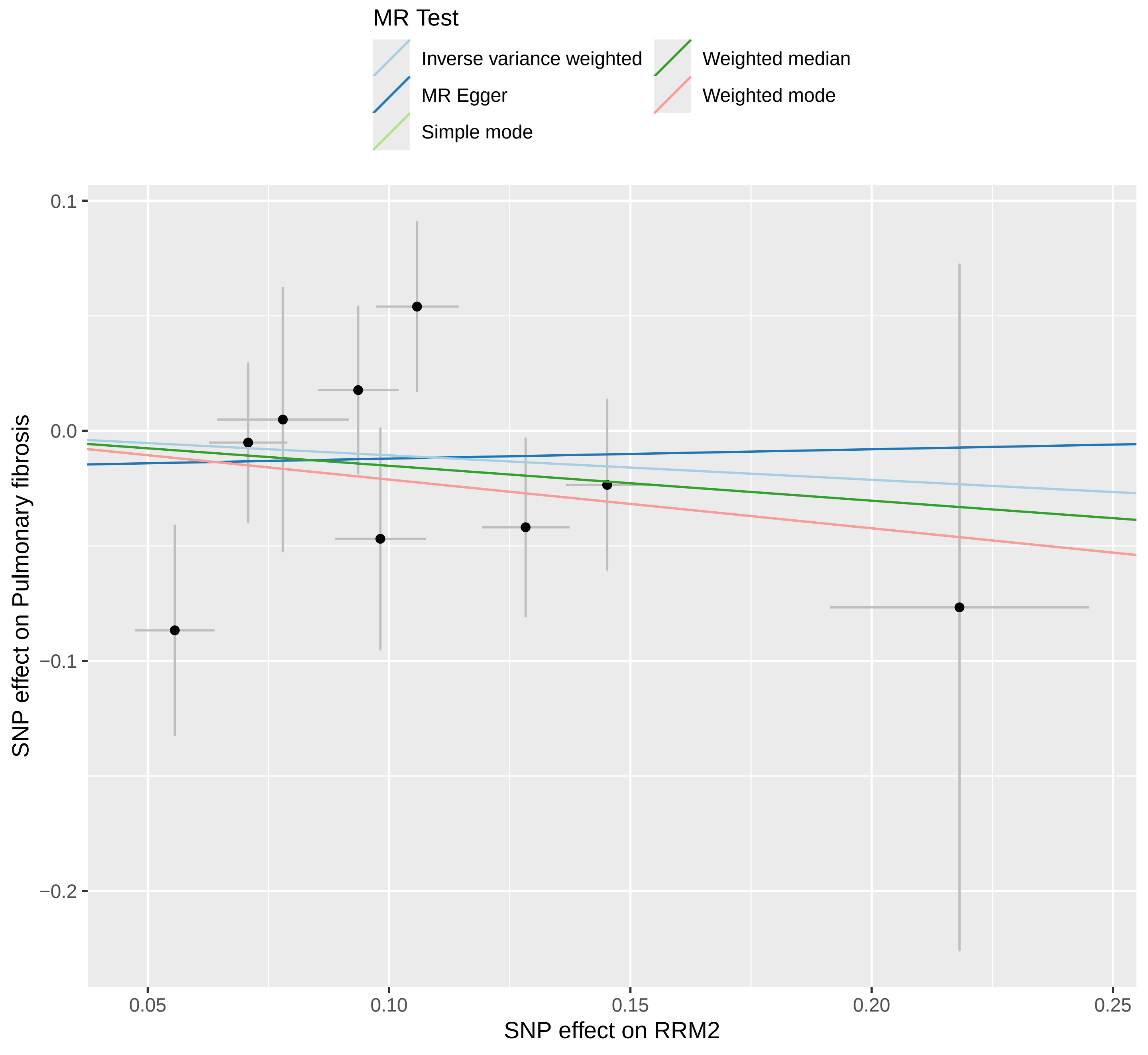
MR Test
Inverse variance weighted
MR Egger
Simple mode
Weighted median
Weighted mode
0.1
0.0
−0.1
−0.2
SNP effect on Pulmonary fibrosis
0.05
0.10
0.15
0.20
0.25
SNP effect on RRM2

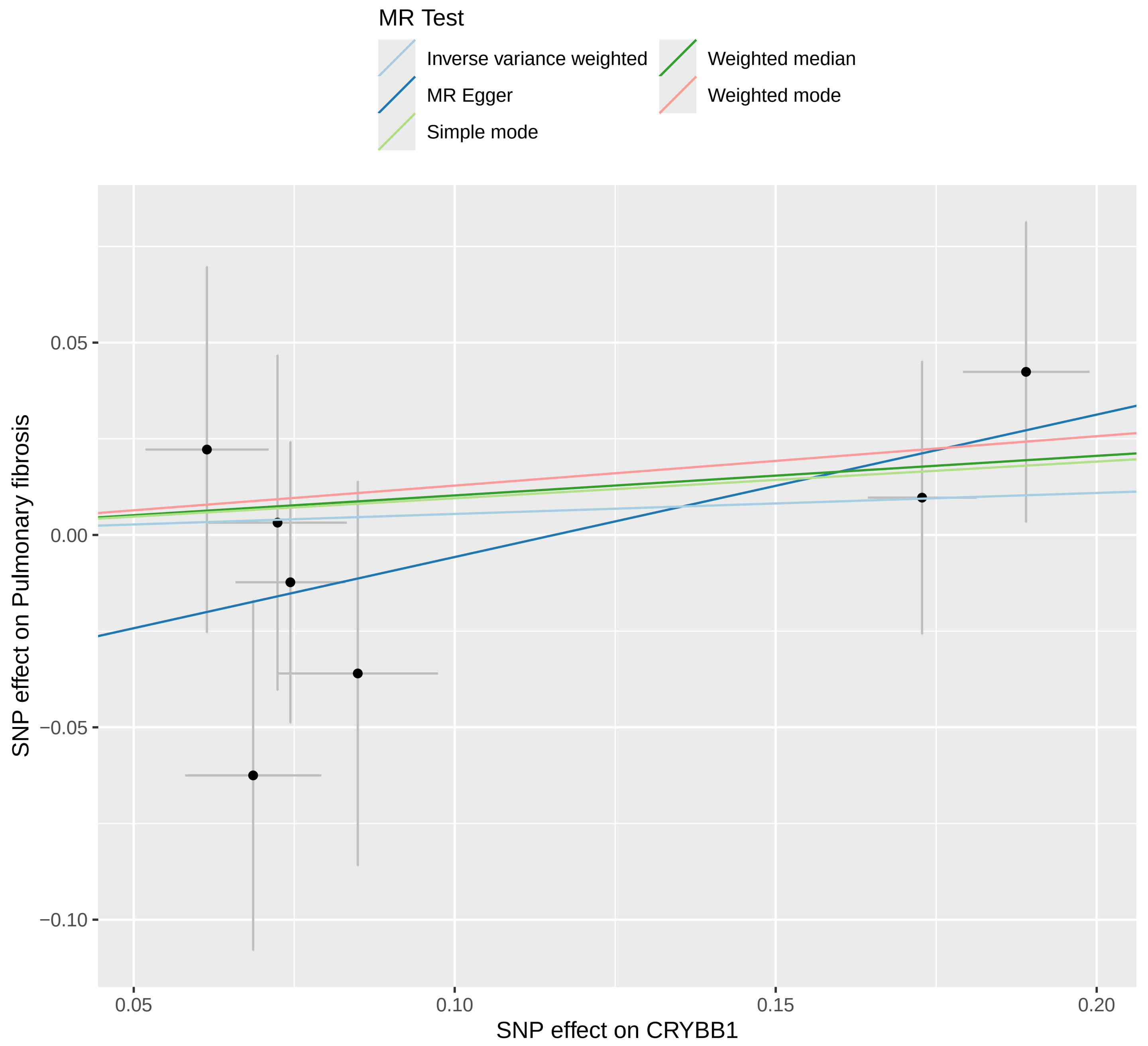

MR Test
Inverse variance weighted
MR Egger
Simple mode
Weighted median
Weighted mode
0.05
0.00
−0.05
−0.10
SNP effect on Pulmonary fibrosis
0.05
0.10
0.15
0.20
SNP effect on CRYBB1

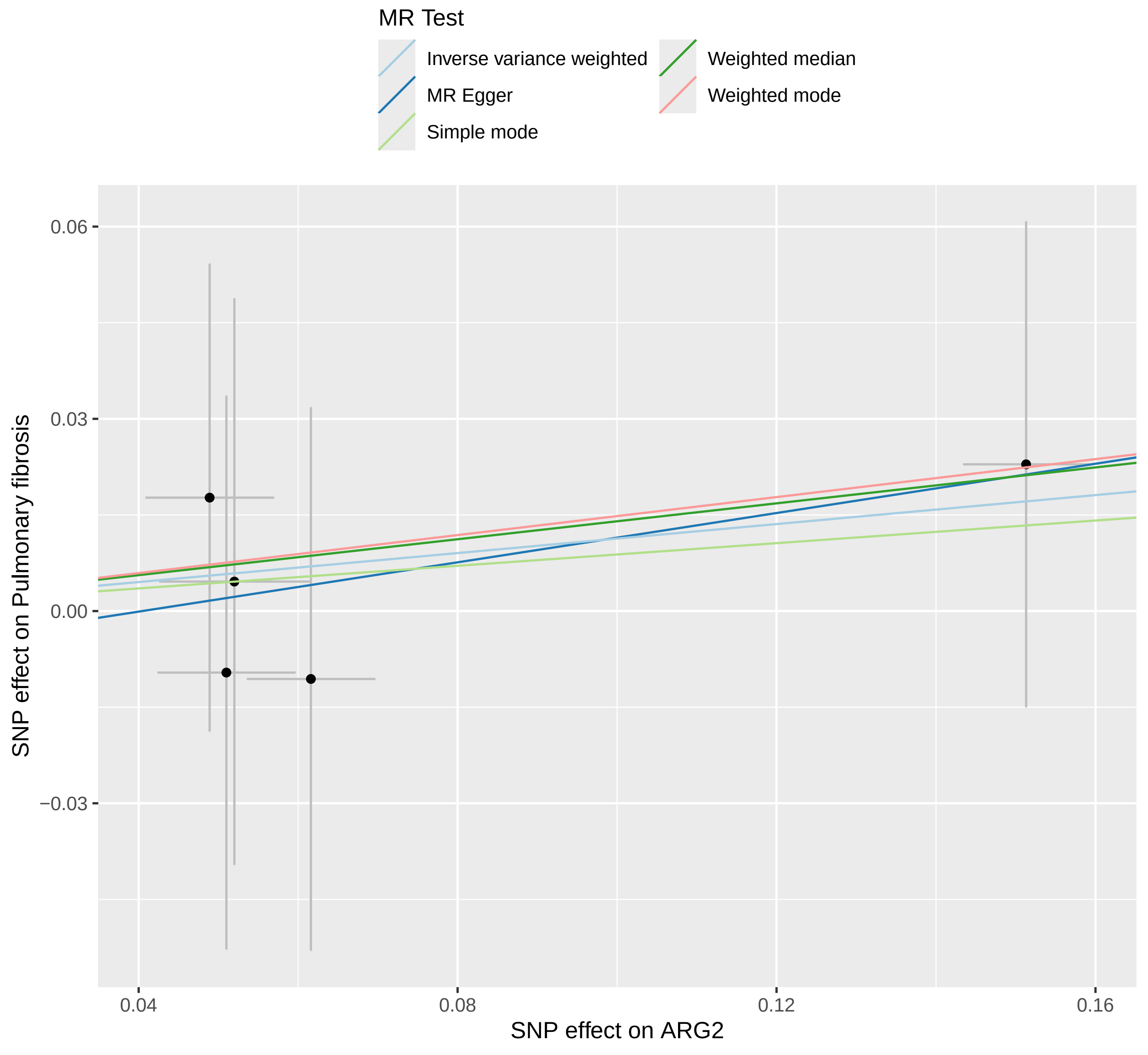
MR Test
Inverse variance weighted
MR Egger
Simple mode
Weighted median
Weighted mode
0.06
0.03
0.00
−0.03
0.04
0.08
0.12
0.16
SNP effect on Pulmonary fibrosis
SNP effect on ARG2

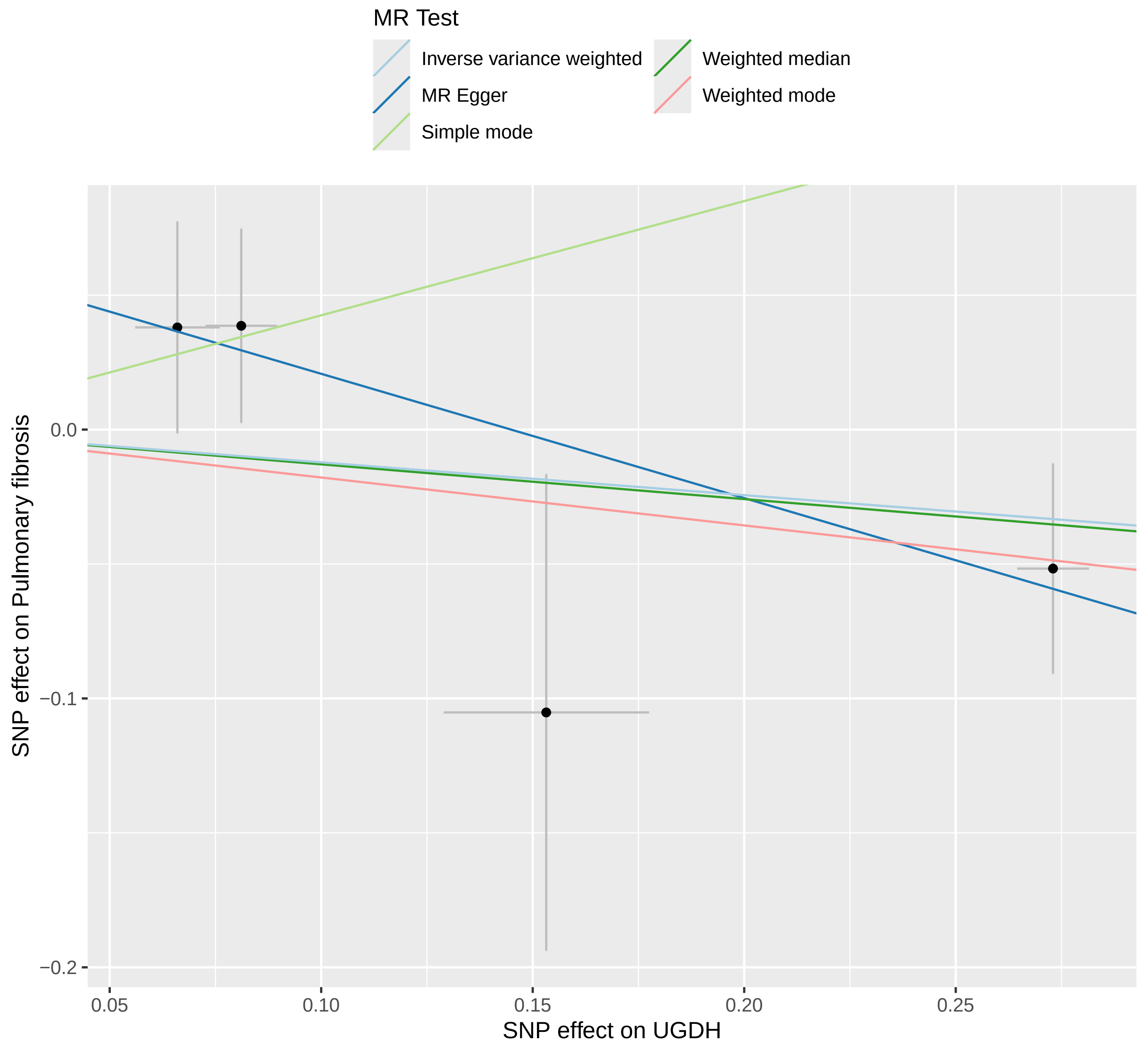

MR Test
Inverse variance weighted
MR Egger
Simple mode
Weighted median
Weighted mode
SNP effect on Pulmonary fibrosis
0.0
−0.1
−0.2
0.05
0.10
0.15
0.20
0.25
SNP effect on UGDH

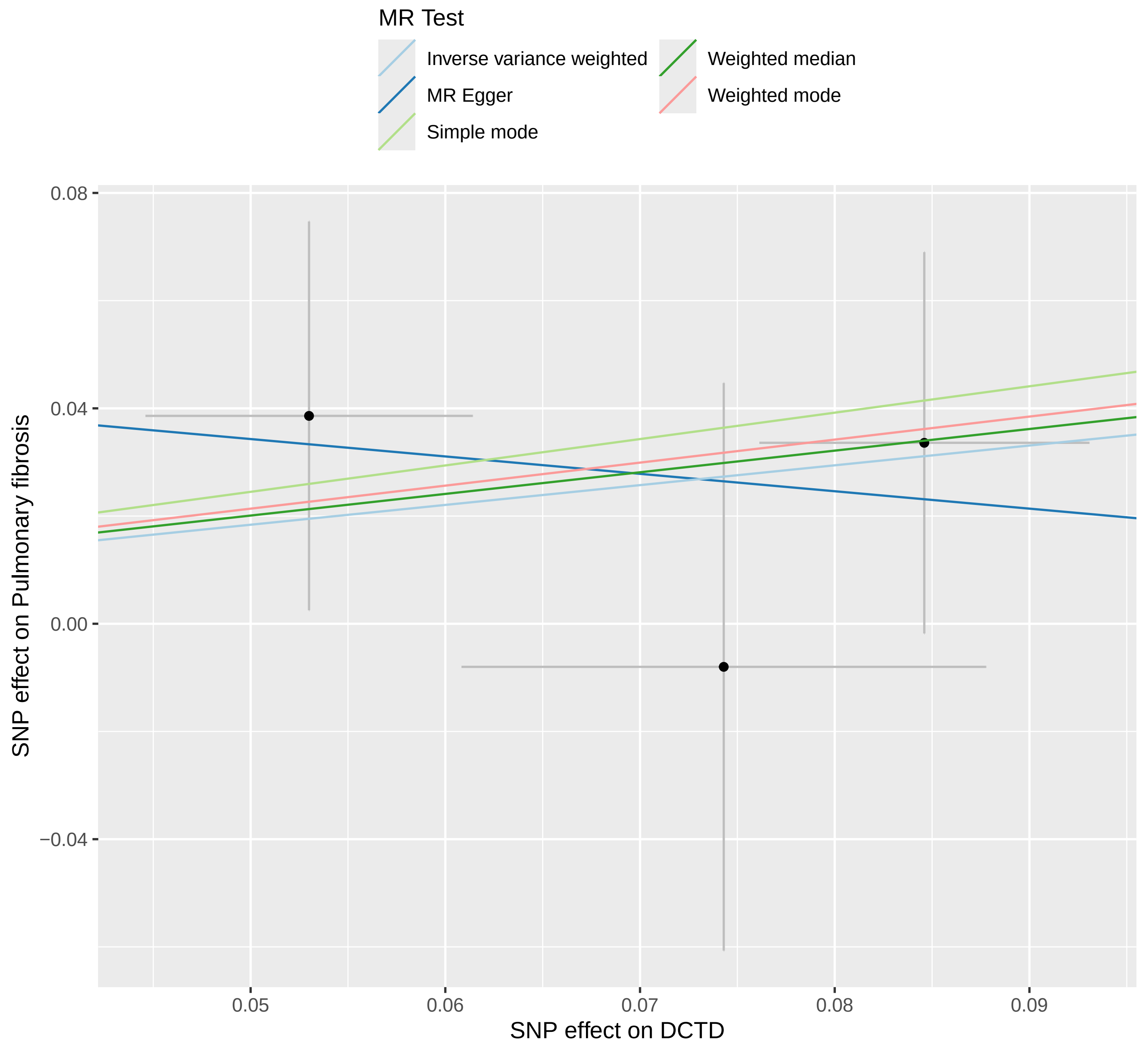
MR Test
Inverse variance weighted
MR Egger
Simple mode
Weighted median
Weighted mode
0.08
0.04
0.00
−0.04
SNP effect on Pulmonary fibrosis
0.05
0.06
0.07
0.08
0.09
SNP effect on DCTD

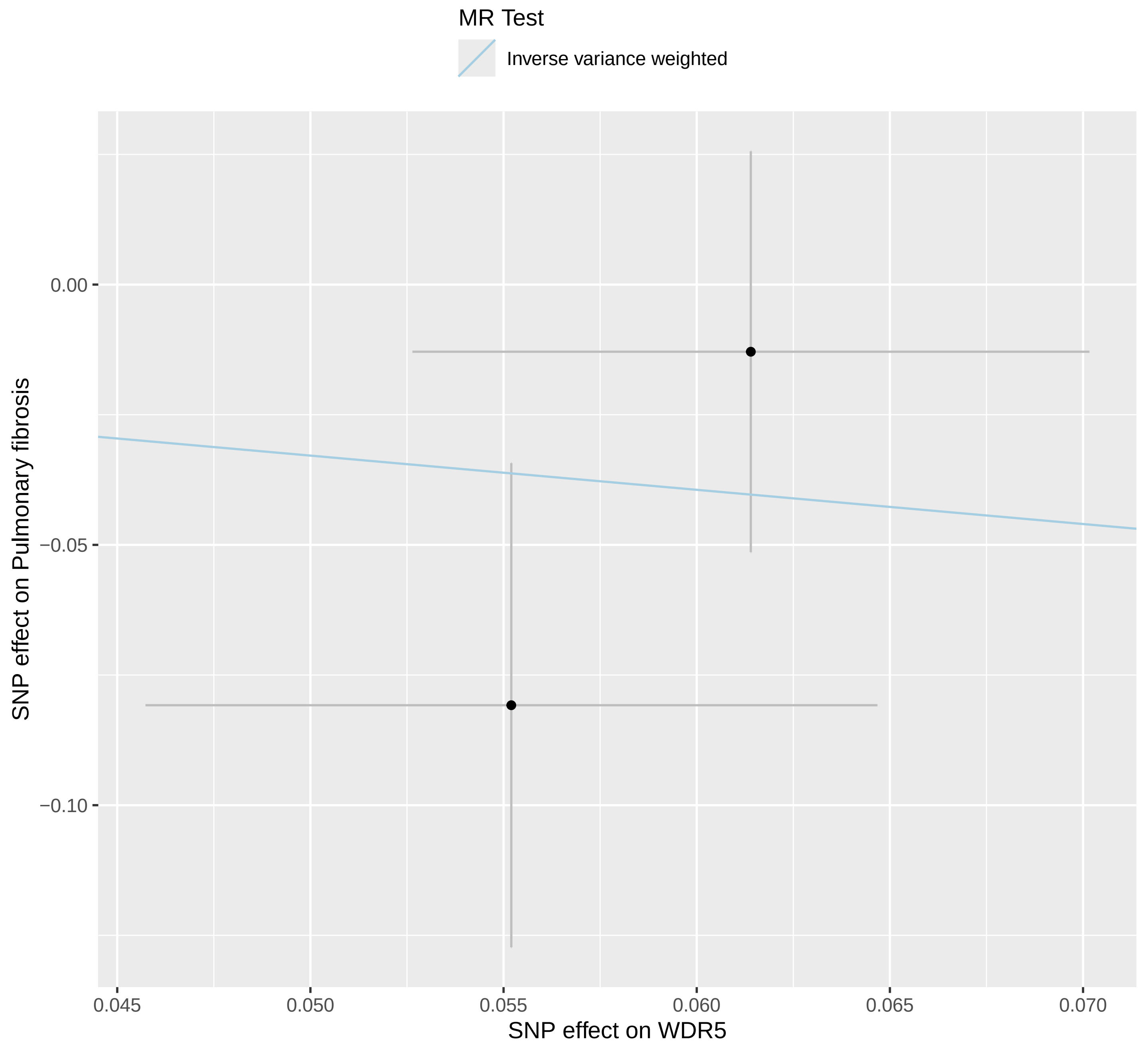
MR Test
Inverse variance weighted
0.00
−0.05
−0.10
SNP effect on Pulmonary fibrosis
0.045
0.050
0.055
0.060
0.065
0.070
SNP effect on WDR5

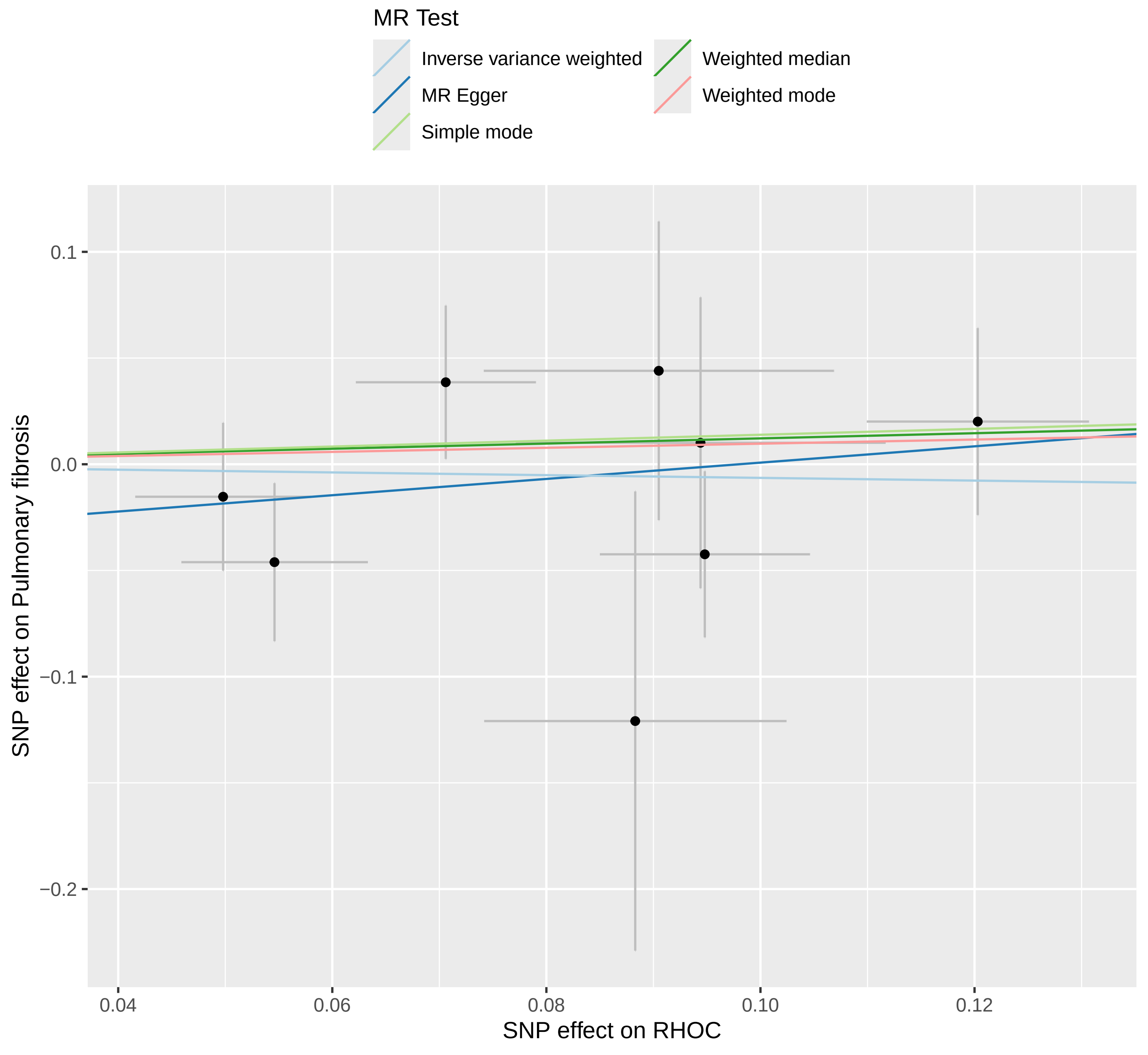

MR Test
Inverse variance weighted
MR Egger
Simple mode
Weighted median
Weighted mode
0.1
0.0
−0.1
−0.2
SNP effect on Pulmonary fibrosis
0.04
0.06
0.08
0.10
0.12
SNP effect on RHOC

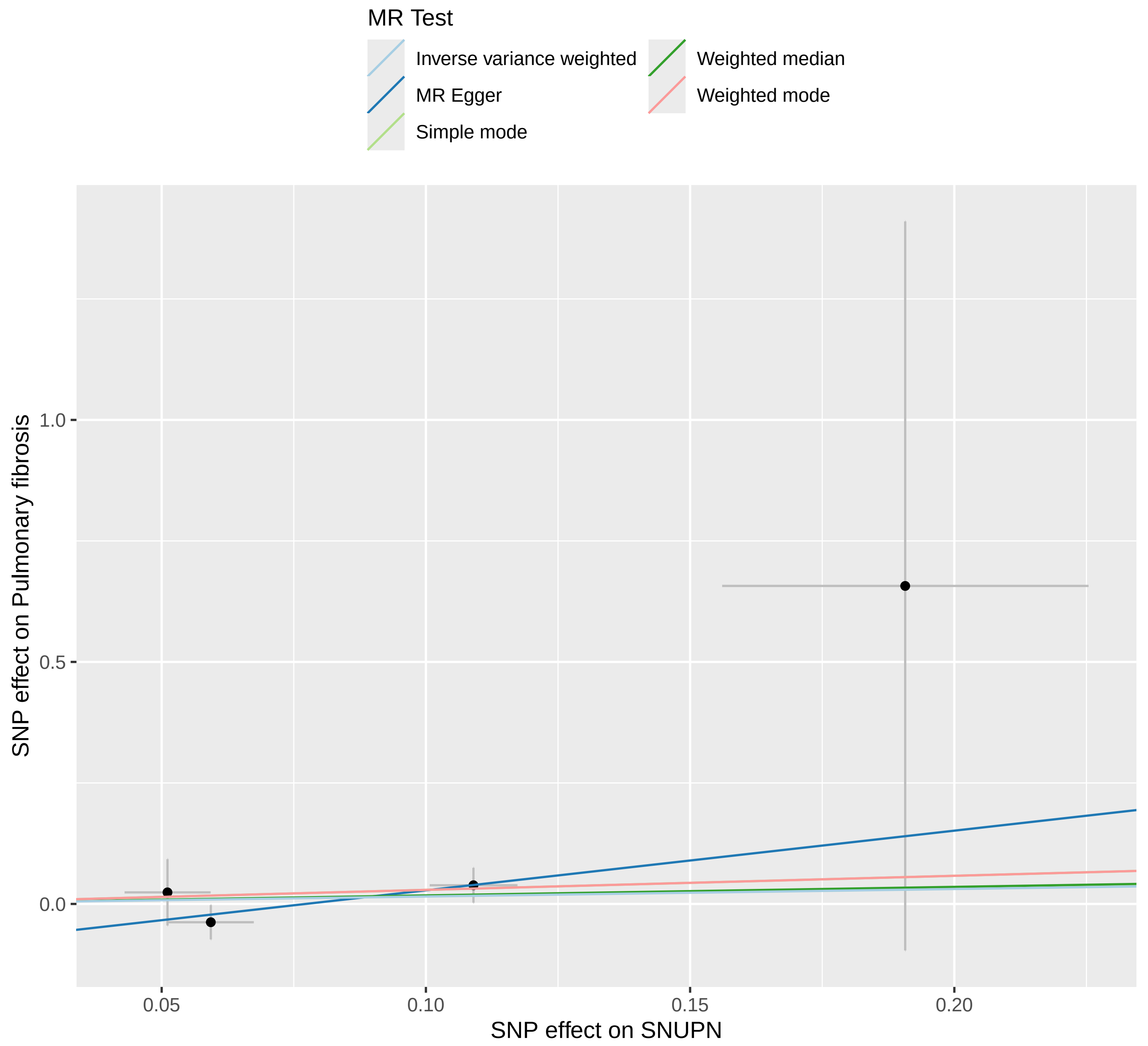
MR Test
Inverse variance weighted
MR Egger
Simple mode
Weighted median
Weighted mode
SNP effect on Pulmonary fibrosis
1.0
0.5
0.0
0.05
0.10
0.15
0.20
SNP effect on SNUPN

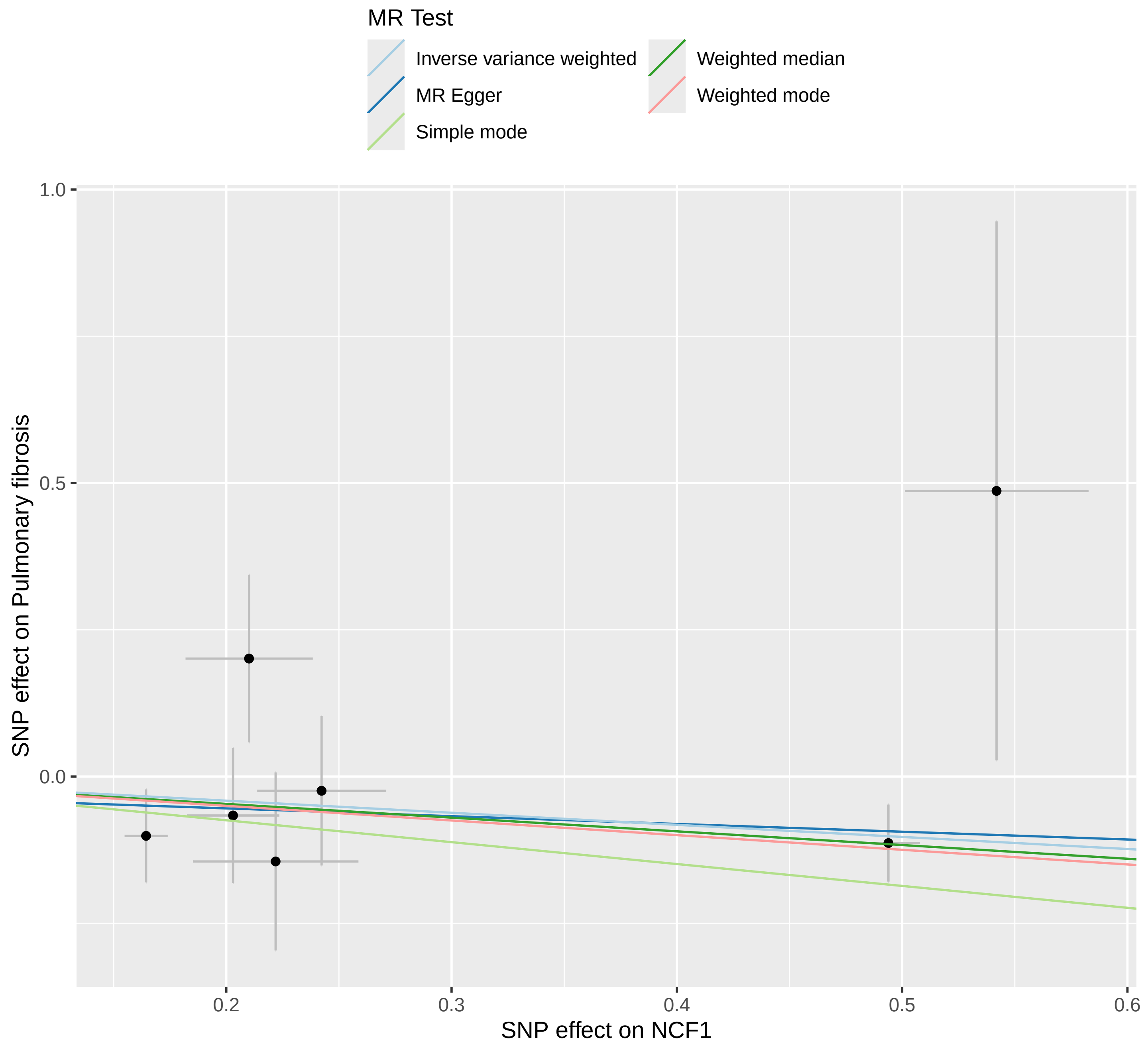

MR Test
Inverse variance weighted
MR Egger
Simple mode
Weighted median
Weighted mode
1.0
0.5
0.0
SNP effect on Pulmonary fibrosis
0.2
0.3
0.4
0.5
0.6
SNP effect on NCF1

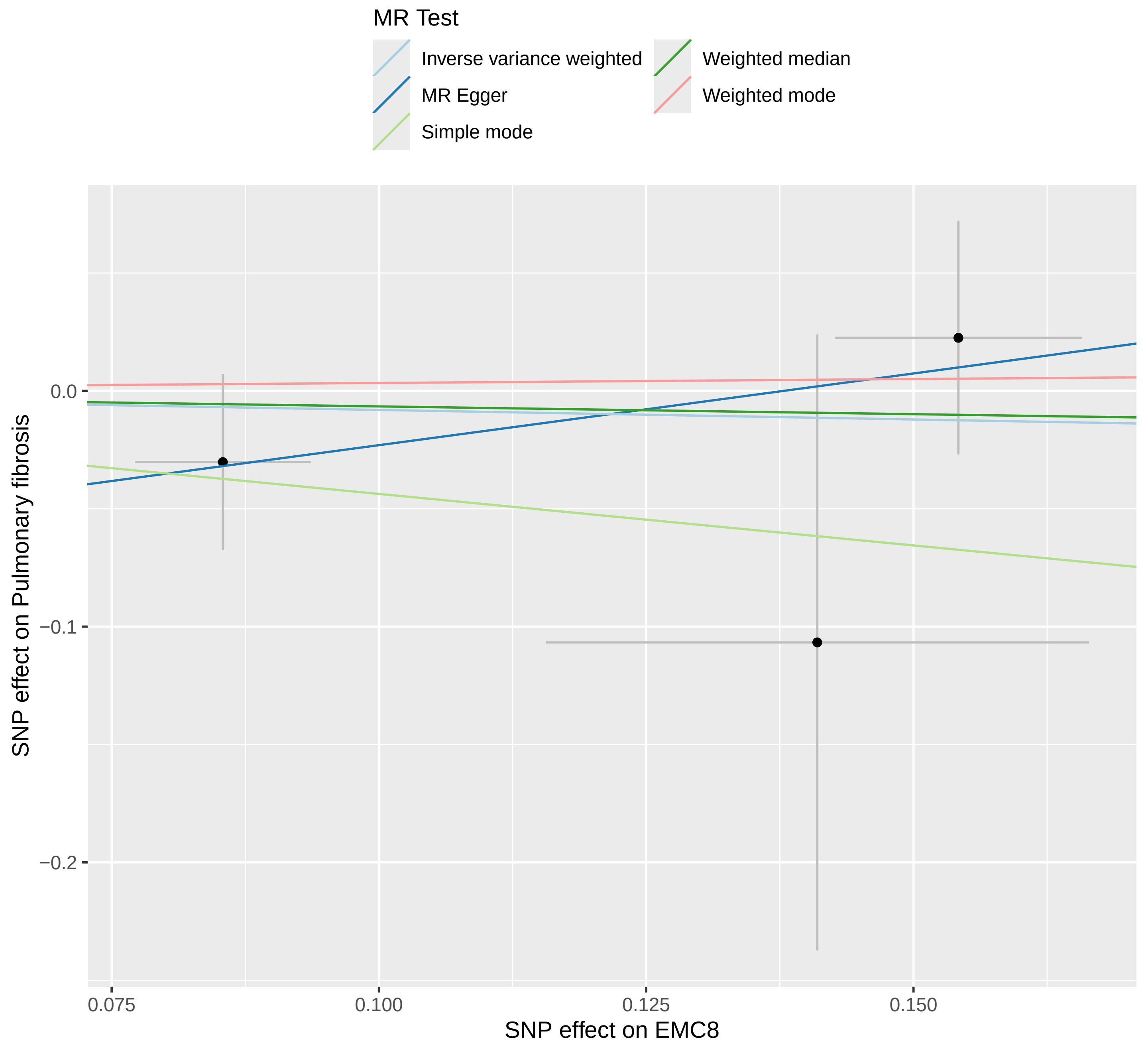
MR Test
Inverse variance weighted
MR Egger
Simple mode
Weighted median
Weighted mode
0.0
−0.1
−0.2
SNP effect on Pulmonary fibrosis
0.075
0.100
0.125
0.150
SNP effect on EMC8

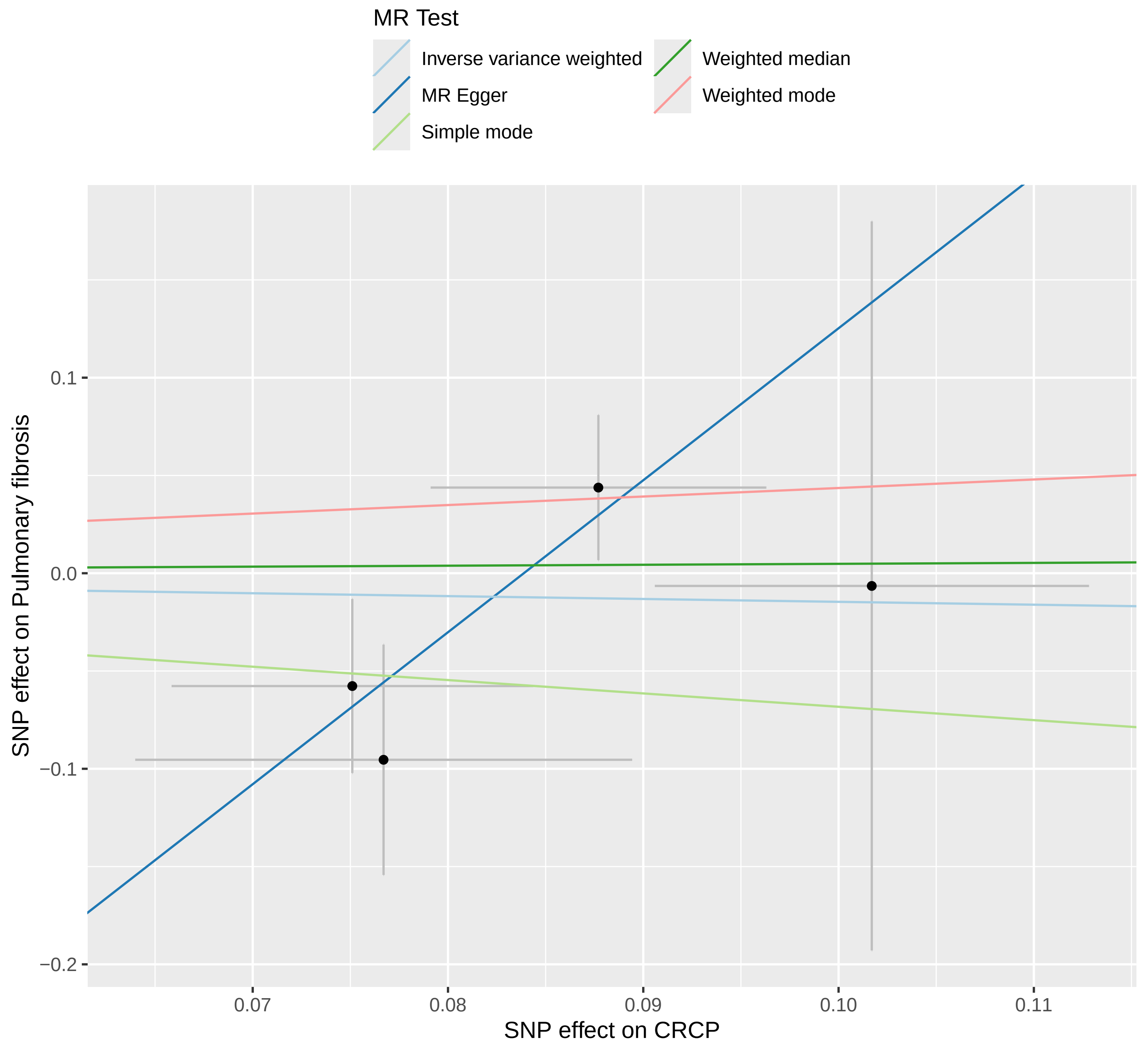

MR Test
Inverse variance weighted
MR Egger
Simple mode
Weighted median
Weighted mode
0.1
0.0
−0.1
−0.2
SNP effect on Pulmonary fibrosis
0.07
0.08
0.09
0.10
0.11
SNP effect on CRCP

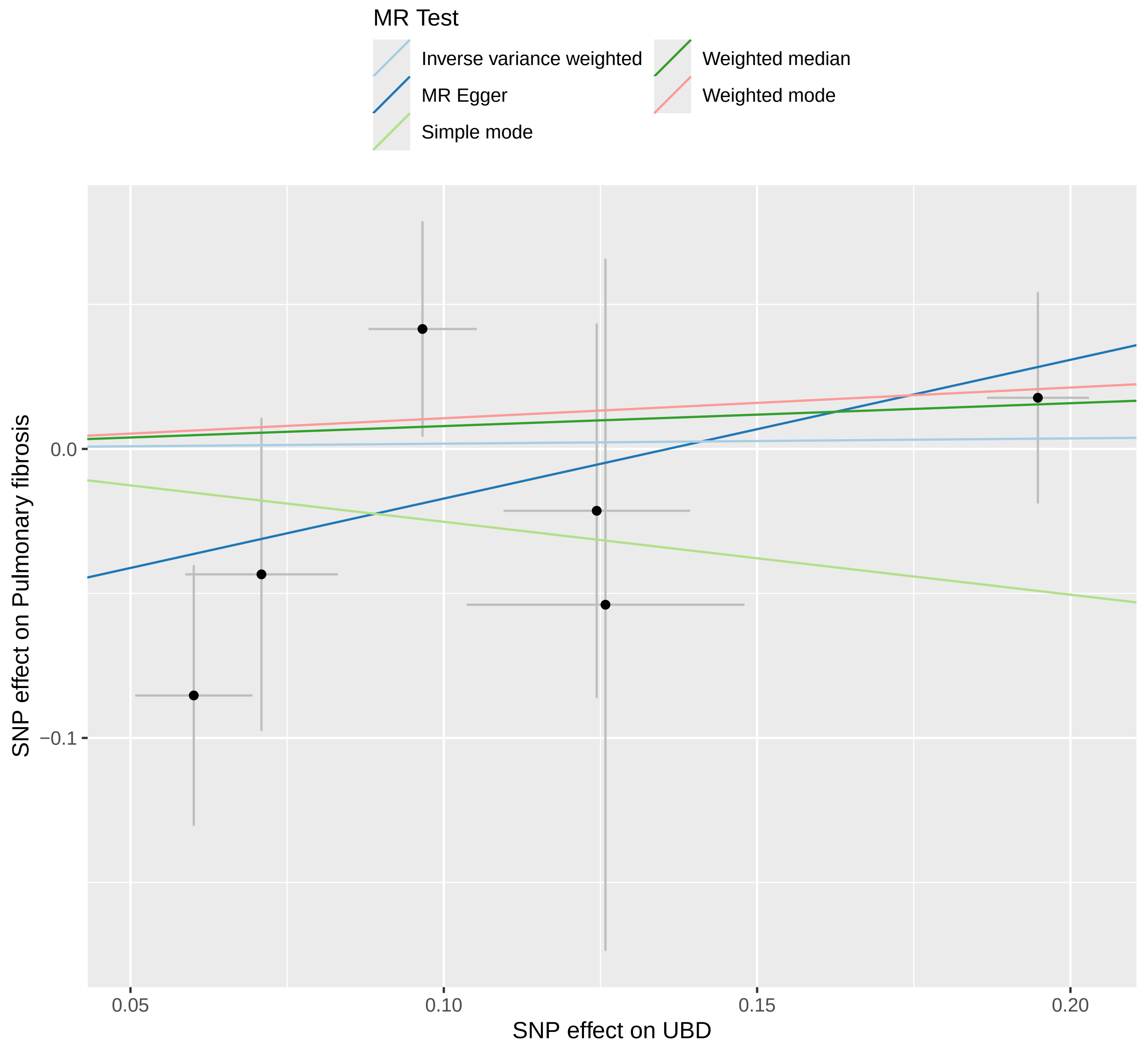
MR Test
Inverse variance weighted
MR Egger
Simple mode
Weighted median
Weighted mode
SNP effect on Pulmonary fibrosis
0.0
−0.1
0.05
0.10
0.15
0.20
SNP effect on UBD

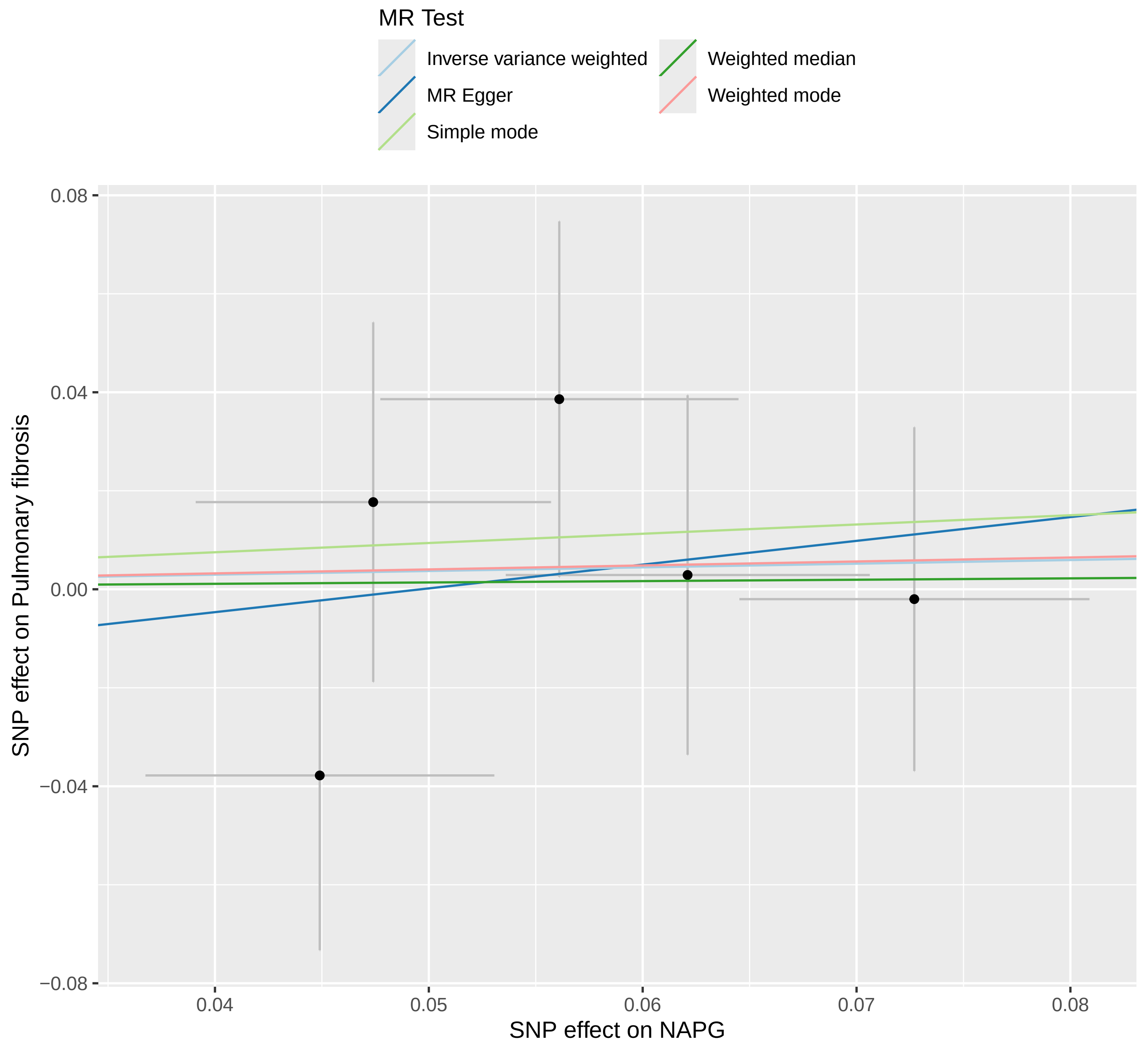

MR Test
Inverse variance weighted
MR Egger
Simple mode
Weighted median
Weighted mode
0.08
0.04
0.00
−0.04
−0.08
SNP effect on Pulmonary fibrosis
0.04
0.05
0.06
0.07
0.08
SNP effect on NAPG

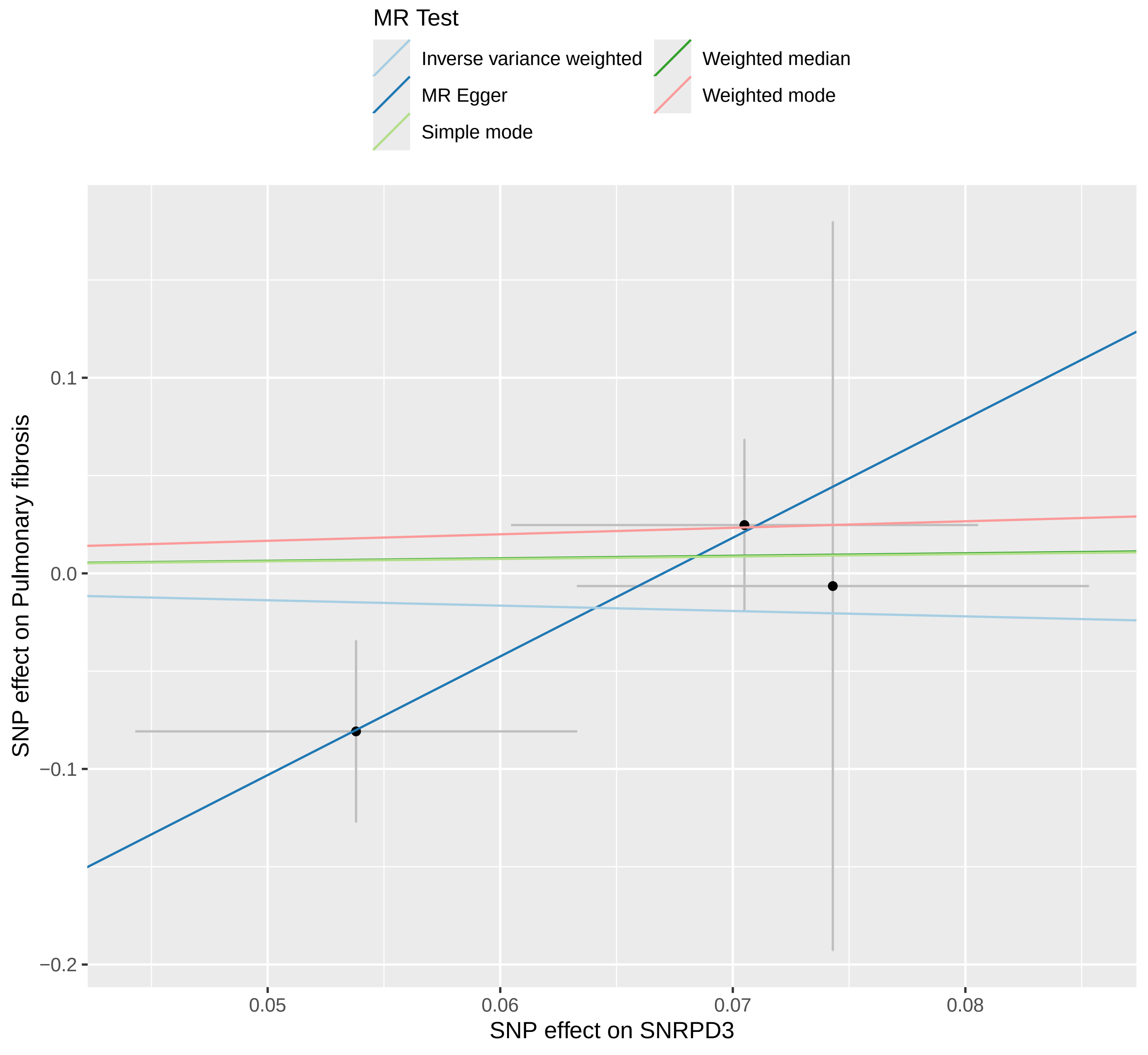

MR Test
Inverse variance weighted
MR Egger
Simple mode
Weighted median
Weighted mode
0.1
0.0
−0.1
−0.2
SNP effect on Pulmonary fibrosis
0.05
0.06
0.07
0.08
SNP effect on SNRPD3

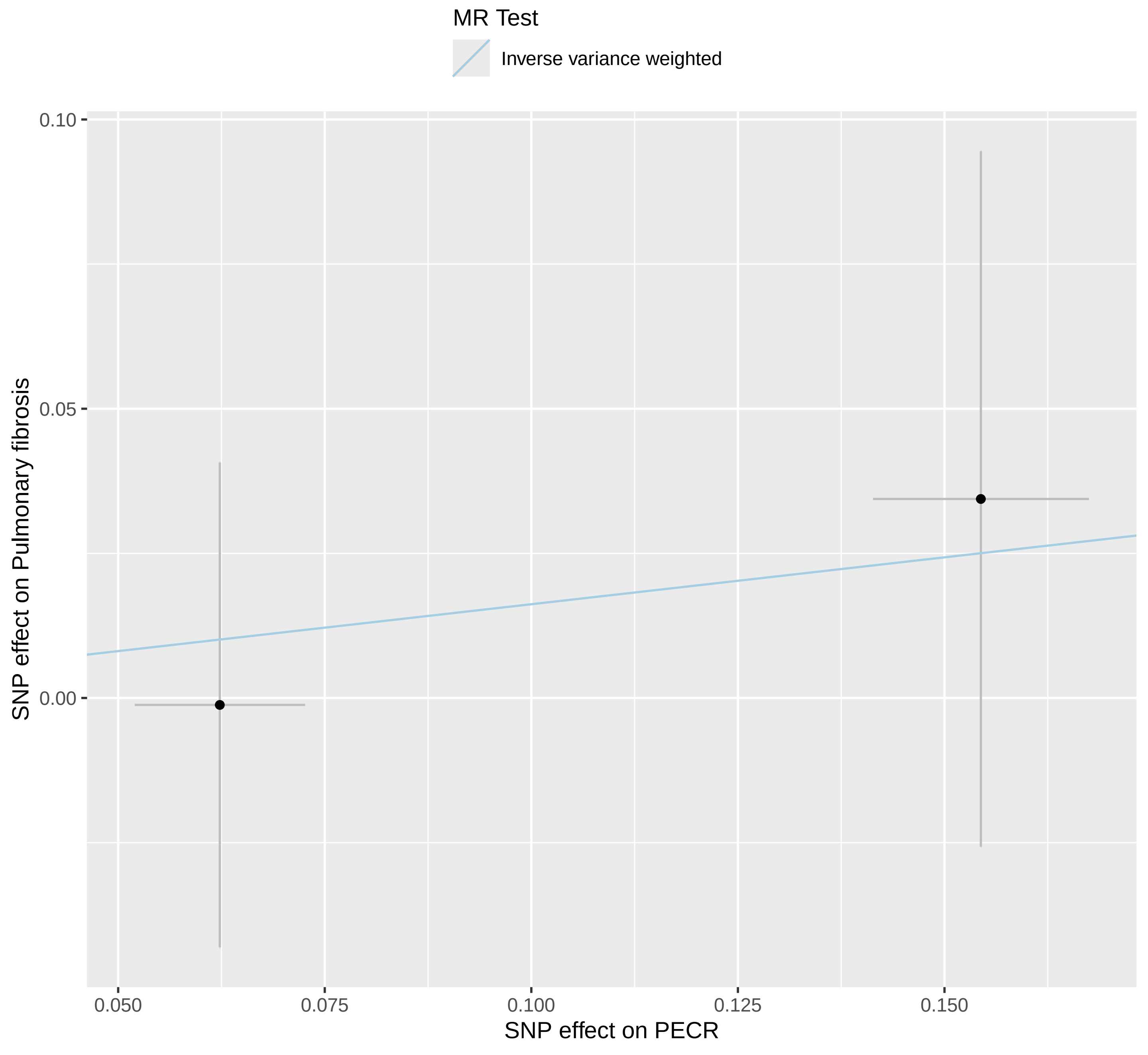

MR Test
Inverse variance weighted
0.10
0.05
0.00
SNP effect on Pulmonary fibrosis
0.050
0.075
0.100
0.125
0.150
SNP effect on PECR

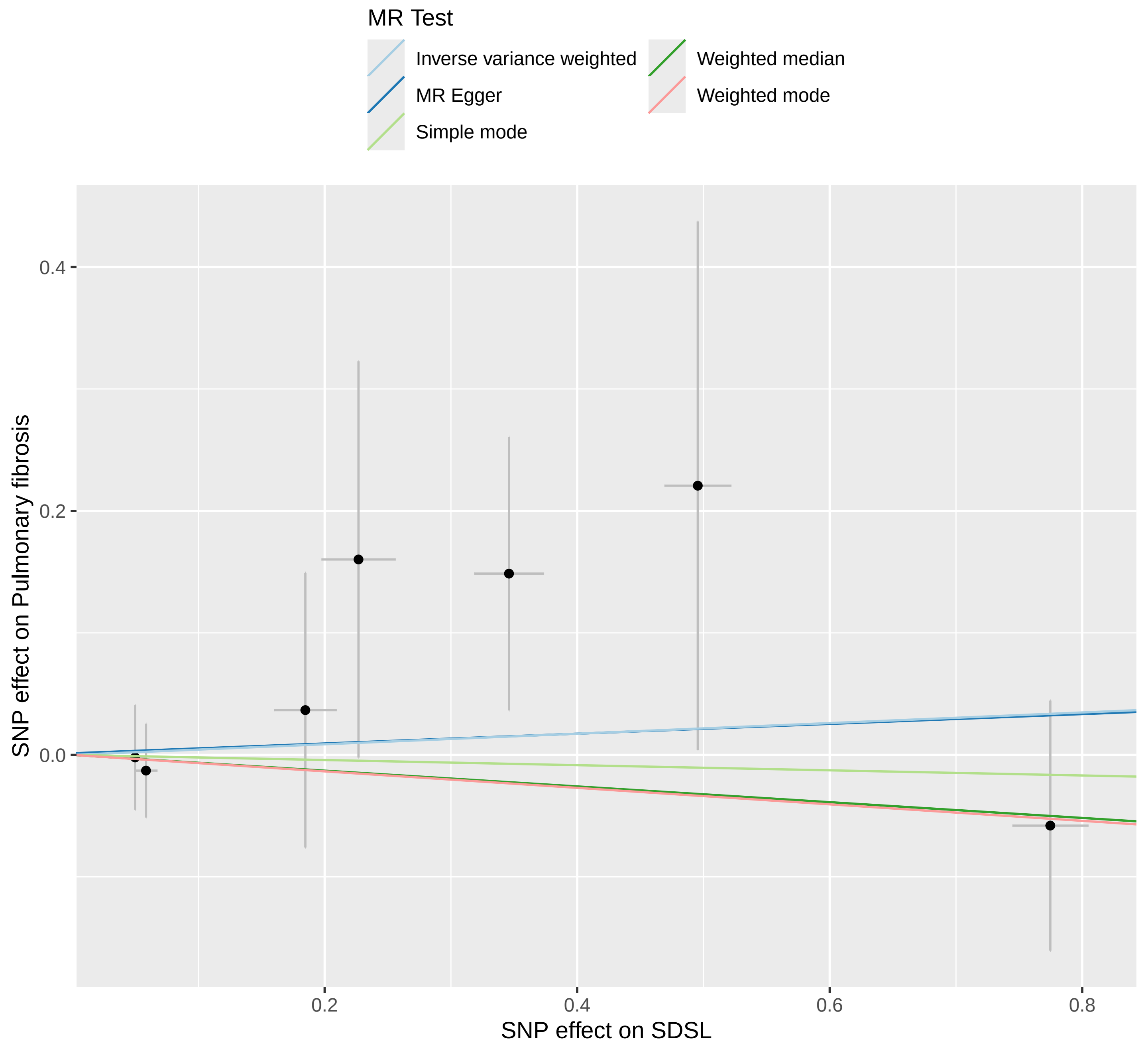

MR Test
Inverse variance weighted
MR Egger
Simple mode
Weighted median
Weighted mode
0.4
0.2
0.0
0.2
0.4
0.6
0.8
SNP effect on Pulmonary fibrosis
SNP effect on SDSL

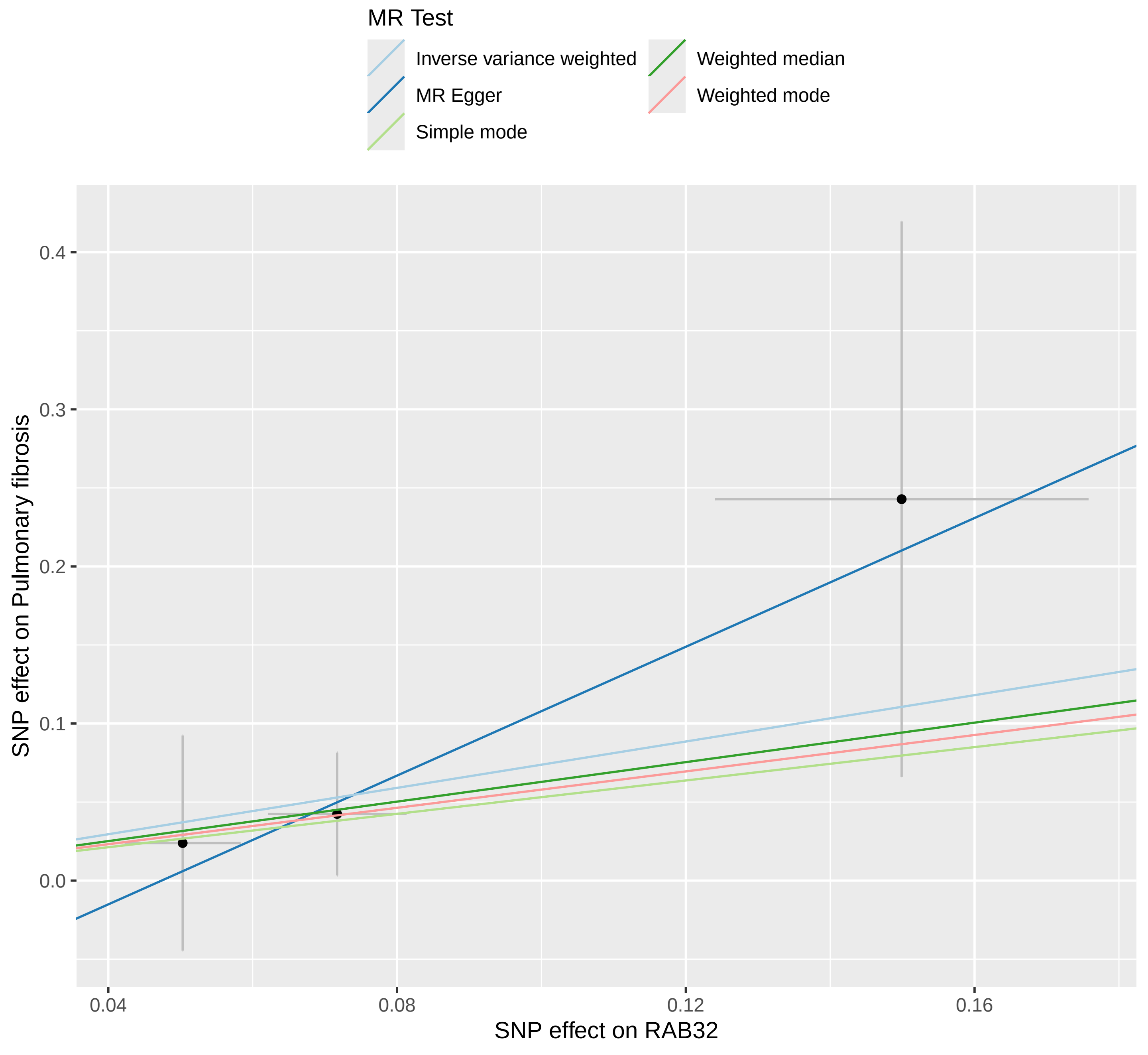
MR Test
Inverse variance weighted
MR Egger
Simple mode
Weighted median
Weighted mode
SNP effect on Pulmonary fibrosis
0.4
0.3
0.2
0.1
0.0
0.04
0.08
0.12
0.16
SNP effect on RAB32

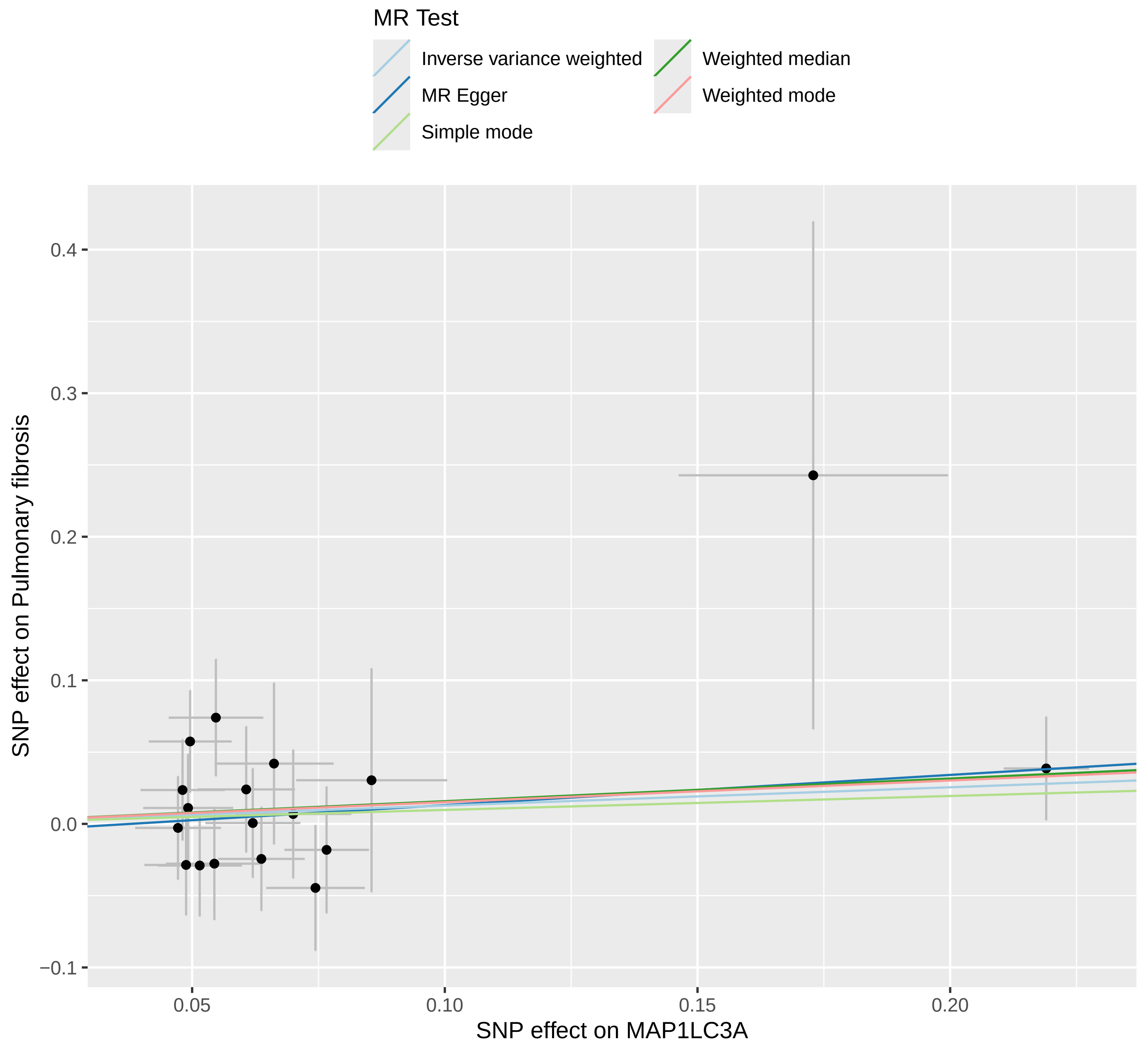

MR Test
Inverse variance weighted
MR Egger
Simple mode
Weighted median
Weighted mode
SNP effect on Pulmonary fibrosis
SNP effect on MAP1LC3A
0.4
0.3
0.2
0.1
0.0
−0.1
0.05
0.10
0.15
0.20

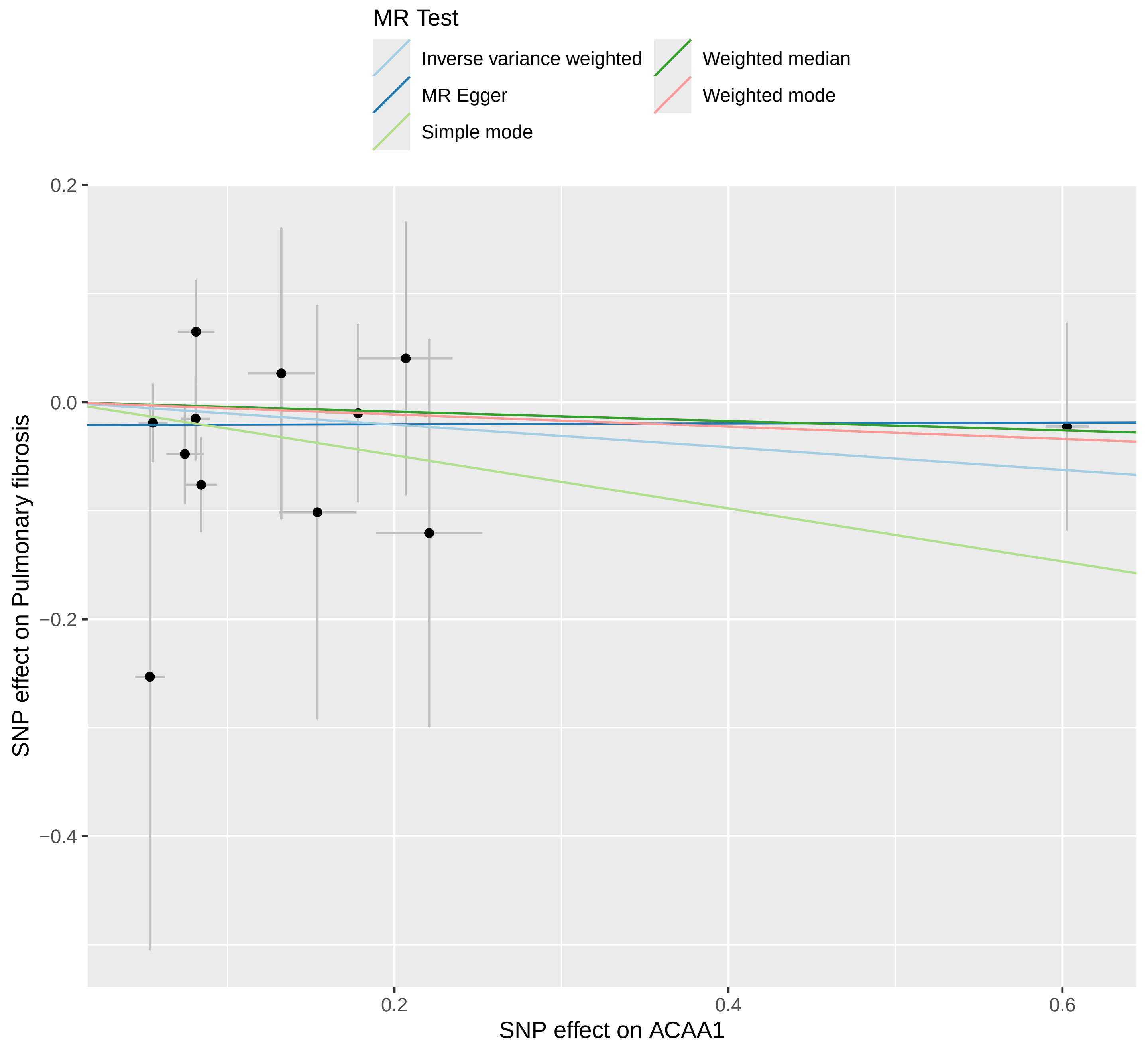
MR Test
Inverse variance weighted
MR Egger
Simple mode
Weighted median
Weighted mode
0.2
0.0
−0.2
−0.4
SNP effect on Pulmonary fibrosis
0.2
0.4
0.6
SNP effect on ACAA1

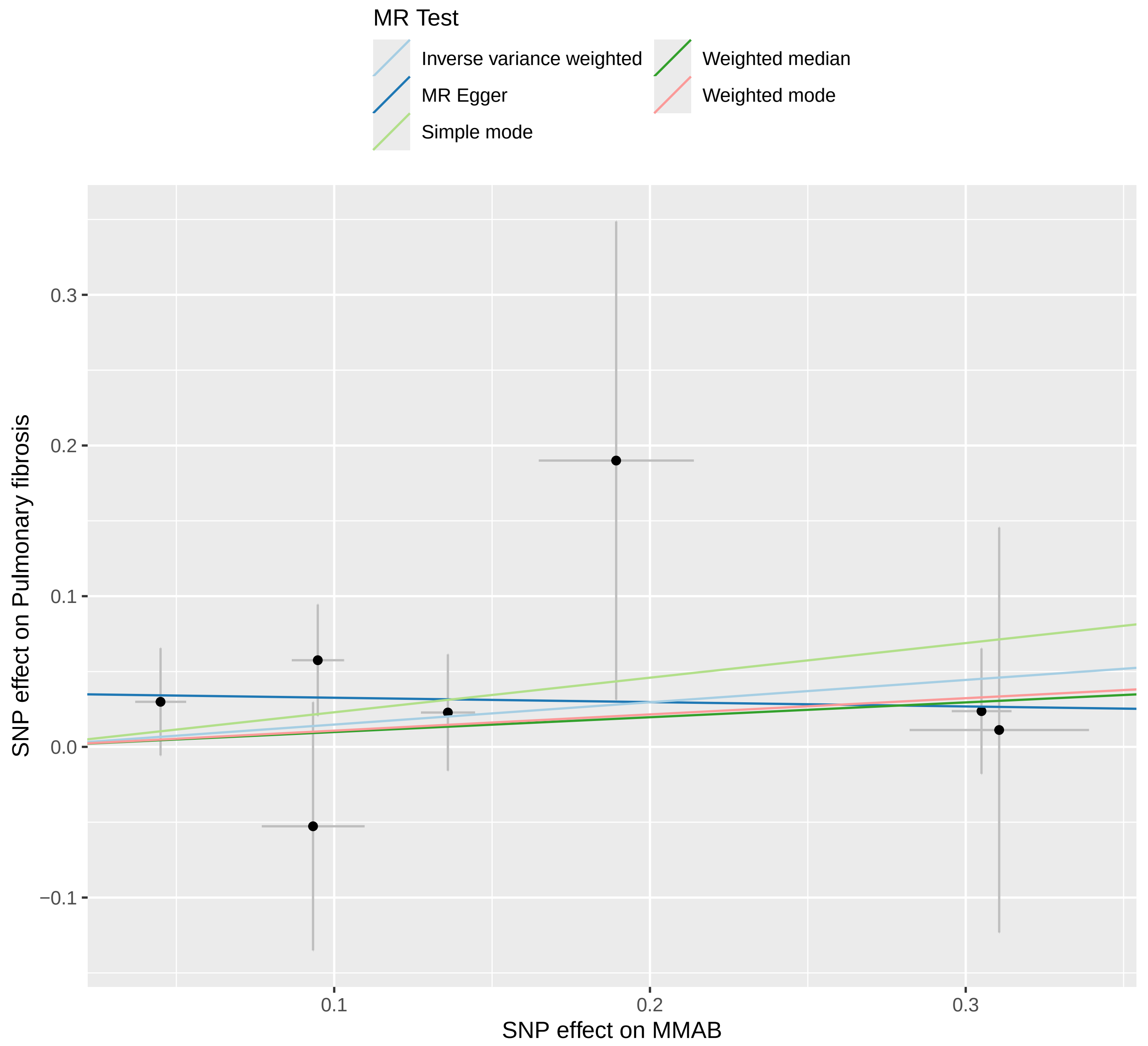
MR Test
Inverse variance weighted
MR Egger
Simple mode
Weighted median
Weighted mode
SNP effect on Pulmonary fibrosis
0.3
0.2
0.1
0.0
−0.1
0.1
0.2
0.3
SNP effect on MMAB

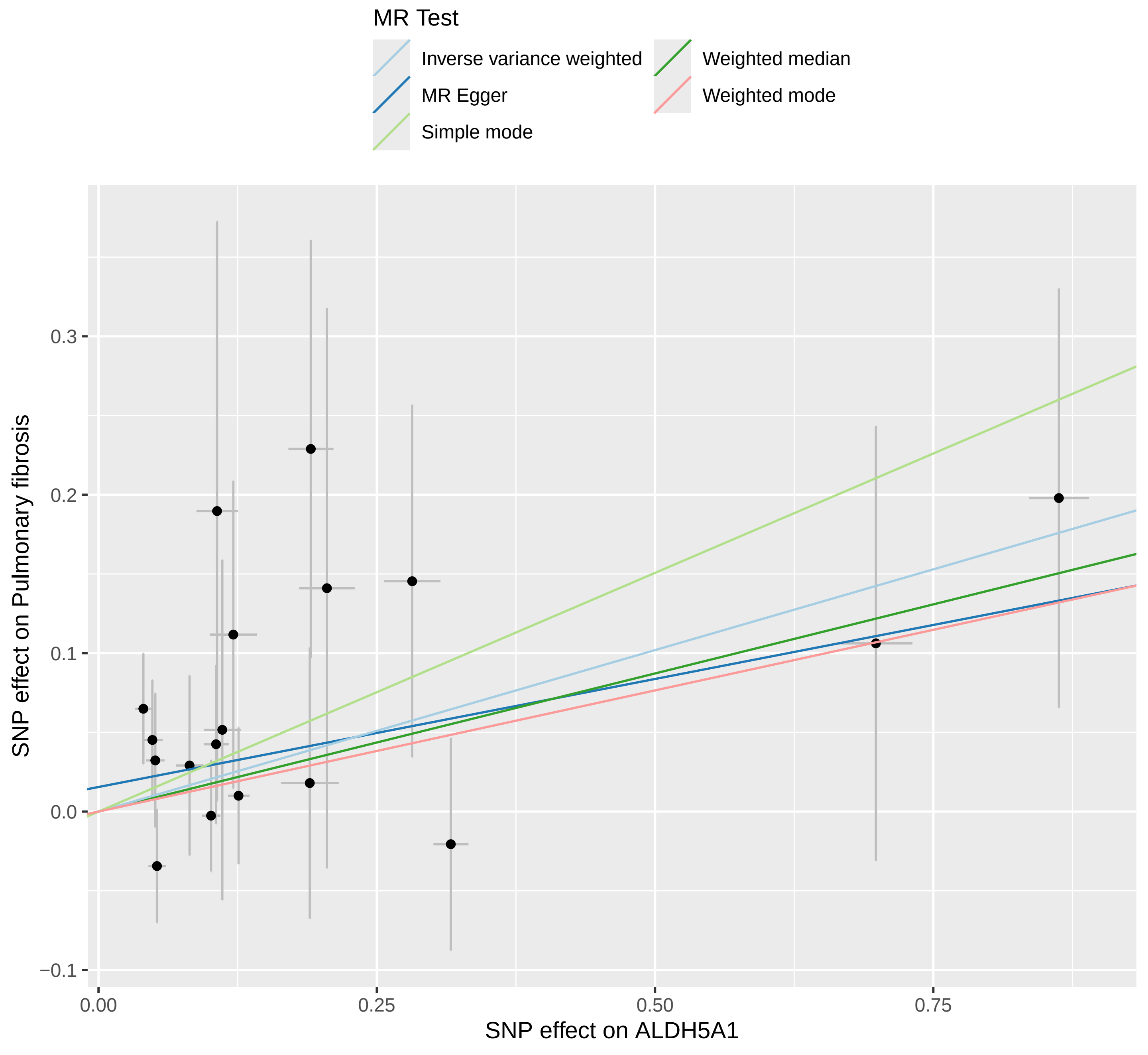

MR Test
Inverse variance weighted
MR Egger
Simple mode
Weighted median
Weighted mode
SNP effect on Pulmonary fibrosis
SNP effect on ALDH5A1
0.3
0.2
0.1
0.0
−0.1
0.00
0.25
0.50
0.75

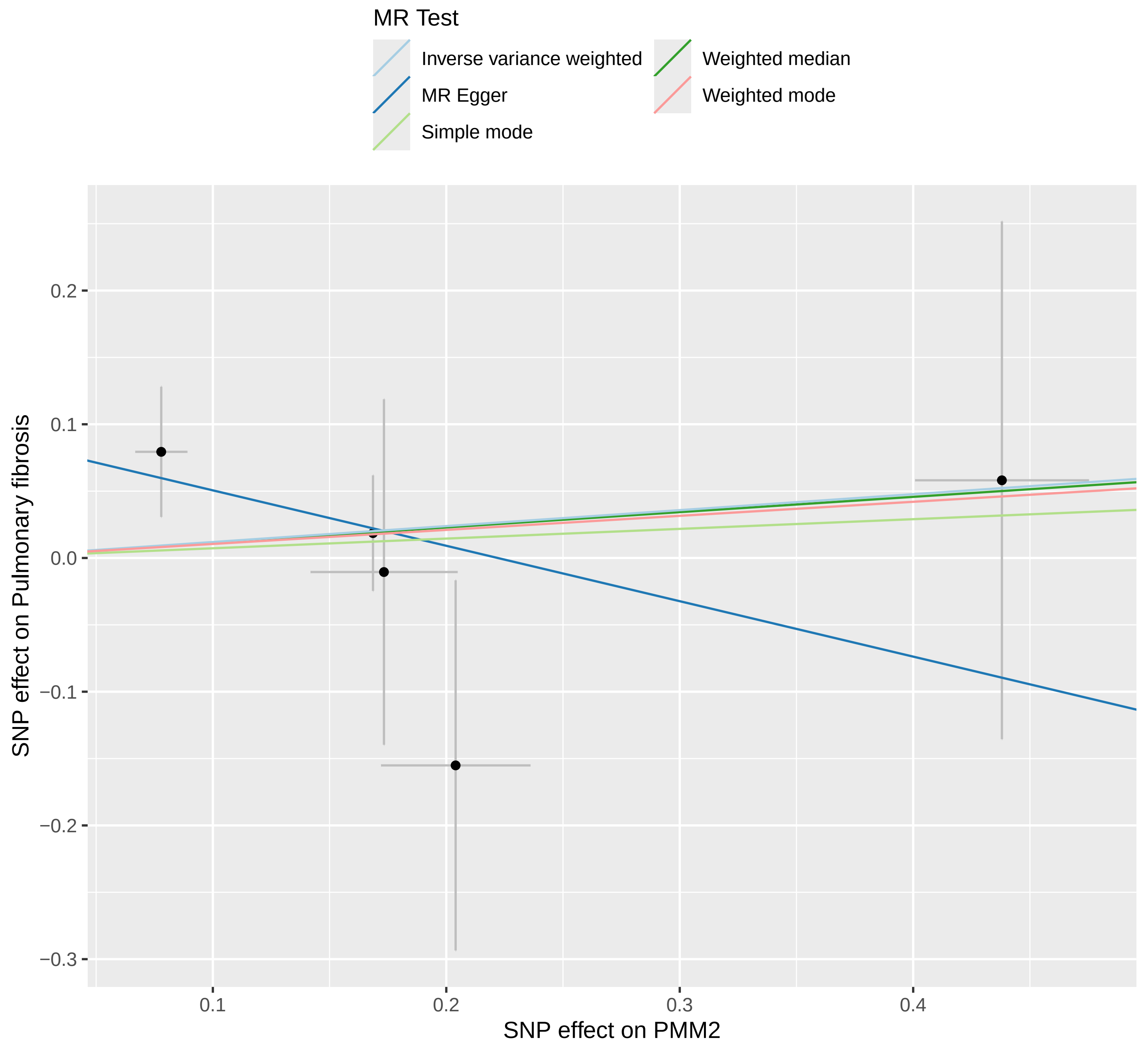

MR Test
Inverse variance weighted
MR Egger
Simple mode
Weighted median
Weighted mode
0.2
0.1
0.0
−0.1
−0.2
−0.3
SNP effect on Pulmonary fibrosis
0.1
0.2
0.3
0.4
SNP effect on PMM2

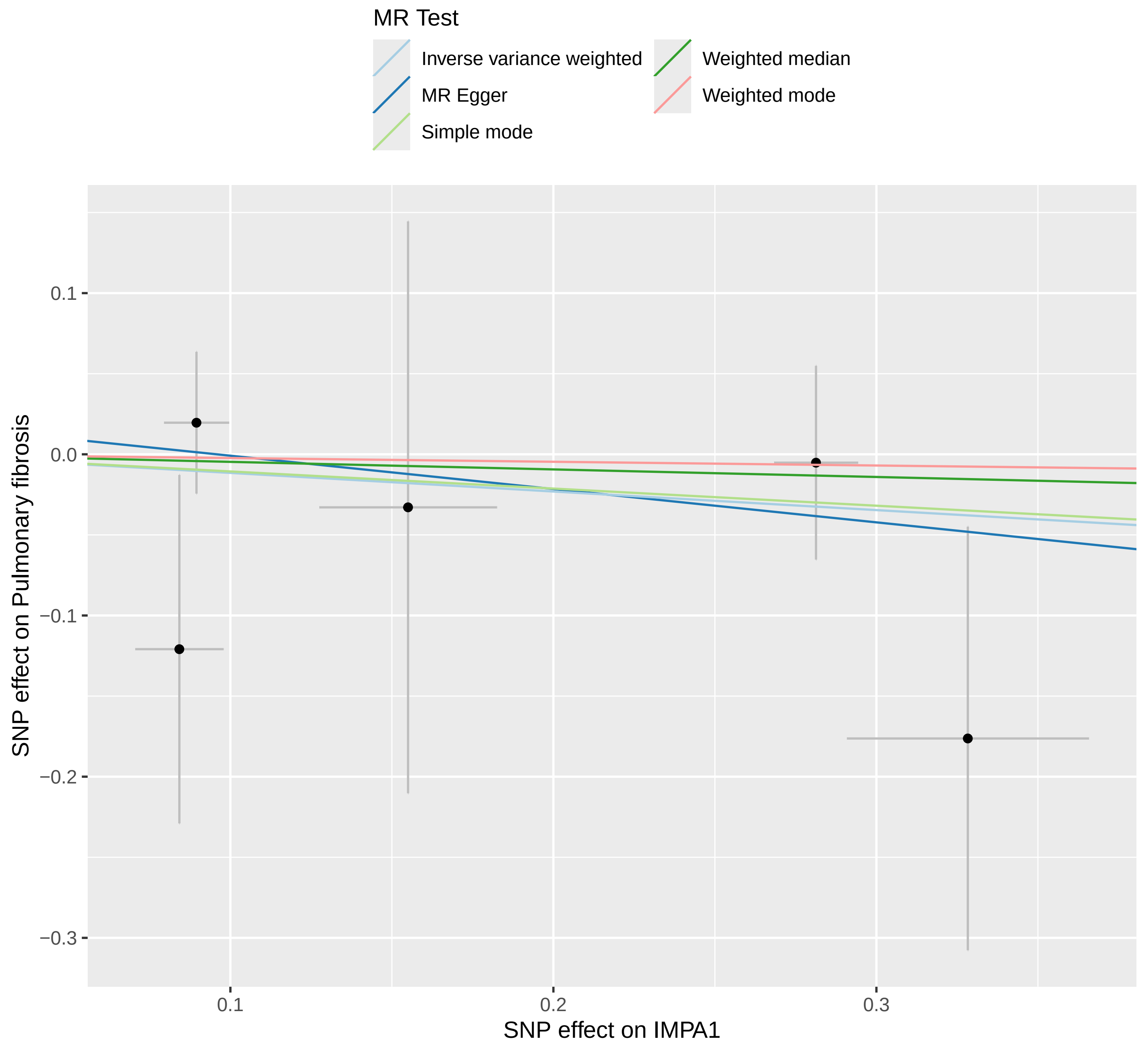
MR Test
Inverse variance weighted
MR Egger
Simple mode
Weighted median
Weighted mode
0.1
0.0
−0.1
−0.2
−0.3
SNP effect on Pulmonary fibrosis
0.1
0.2
0.3
SNP effect on IMPA1

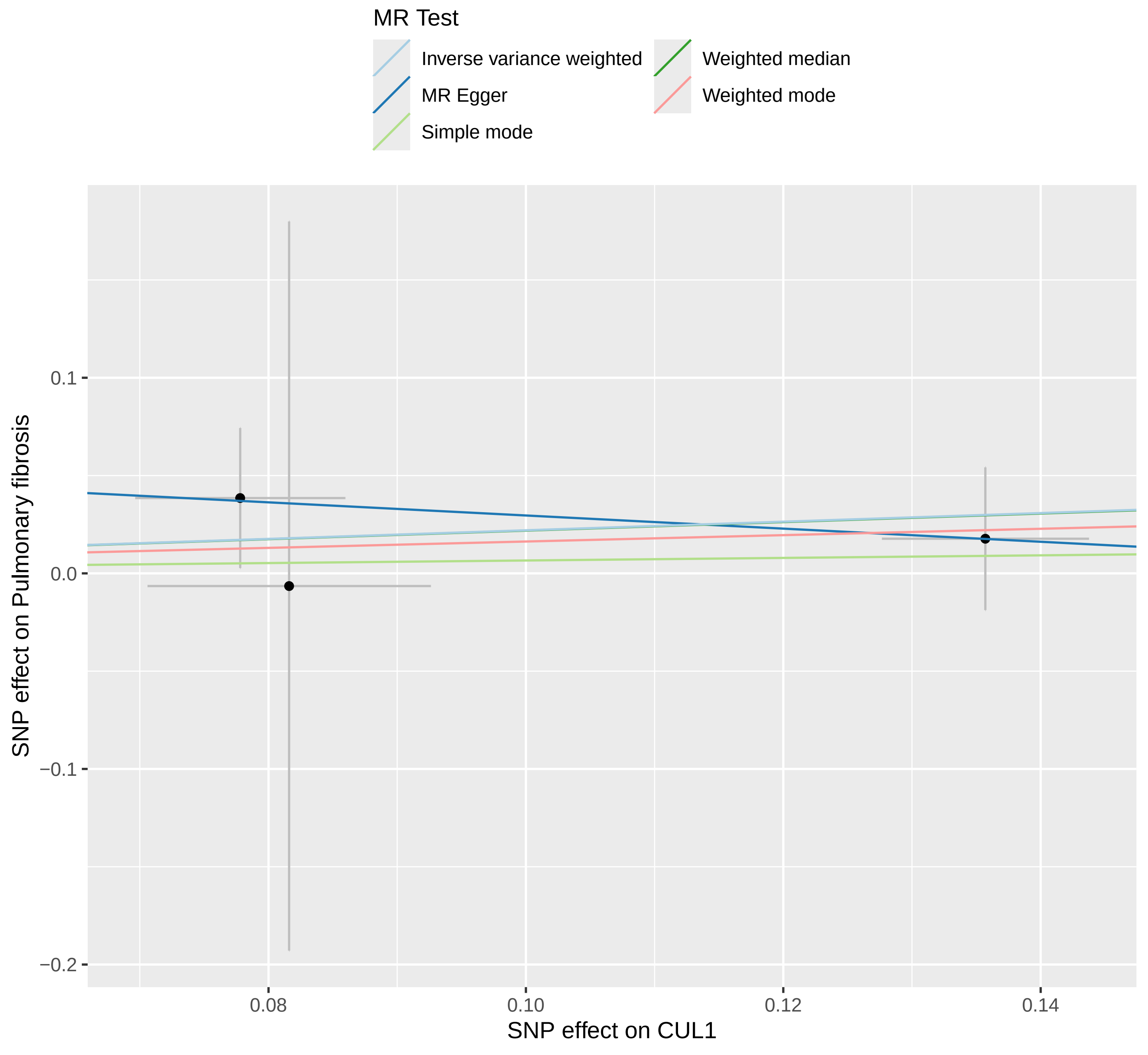

MR Test
Inverse variance weighted
MR Egger
Simple mode
Weighted median
Weighted mode
0.1
0.0
−0.1
−0.2
SNP effect on Pulmonary fibrosis
0.08
0.10
0.12
0.14
SNP effect on CUL1

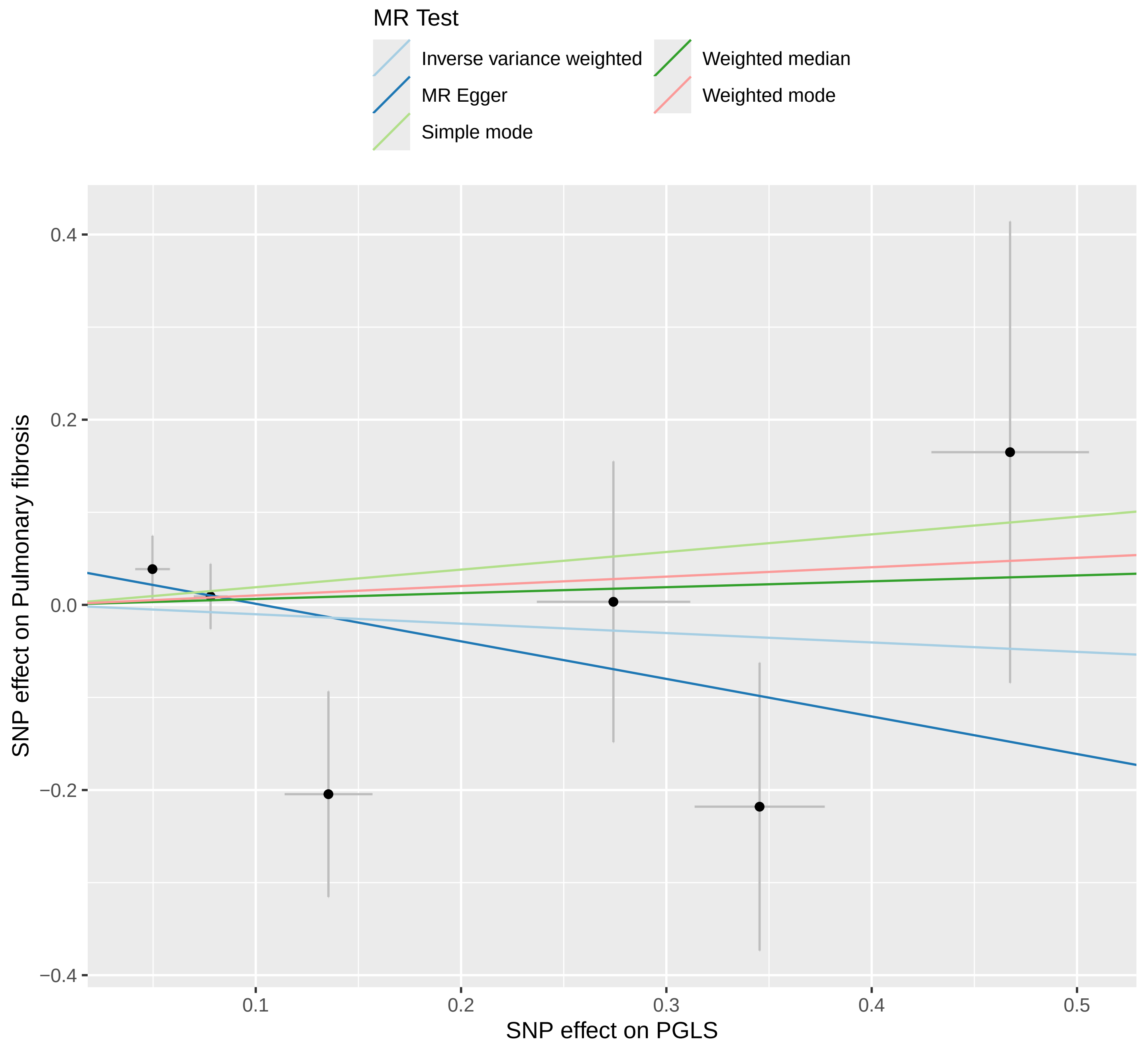

MR Test
Inverse variance weighted
MR Egger
Simple mode
Weighted median
Weighted mode
0.4
0.2
0.0
−0.2
−0.4
0.1
0.2
0.3
0.4
0.5
SNP effect on Pulmonary fibrosis
SNP effect on PGLS

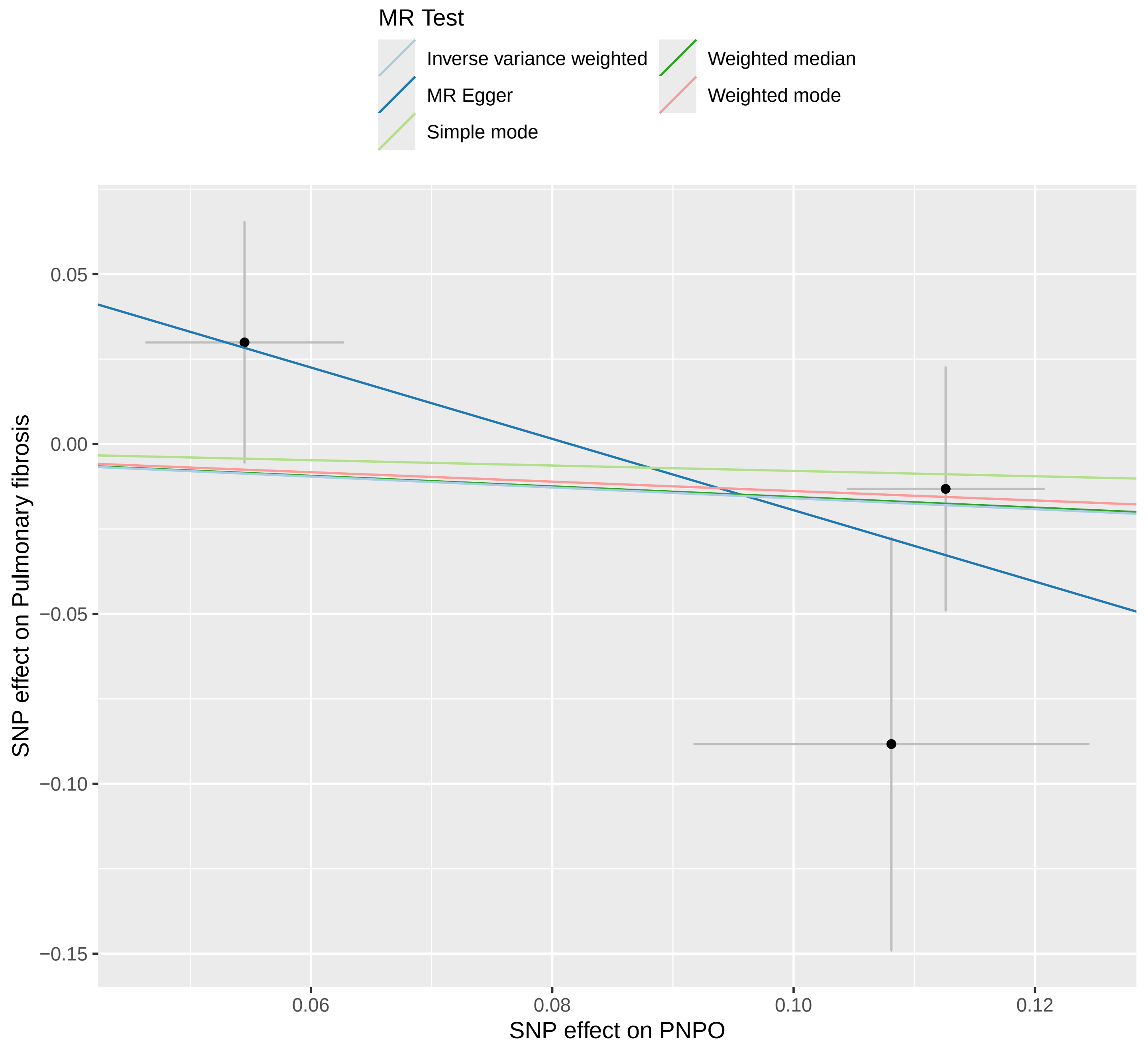

MR Test
Inverse variance weighted
MR Egger
Simple mode
Weighted median
Weighted mode
0.05
0.00
−0.05
−0.10
−0.15
SNP effect on Pulmonary fibrosis
0.06
0.08
0.10
0.12
SNP effect on PNPO

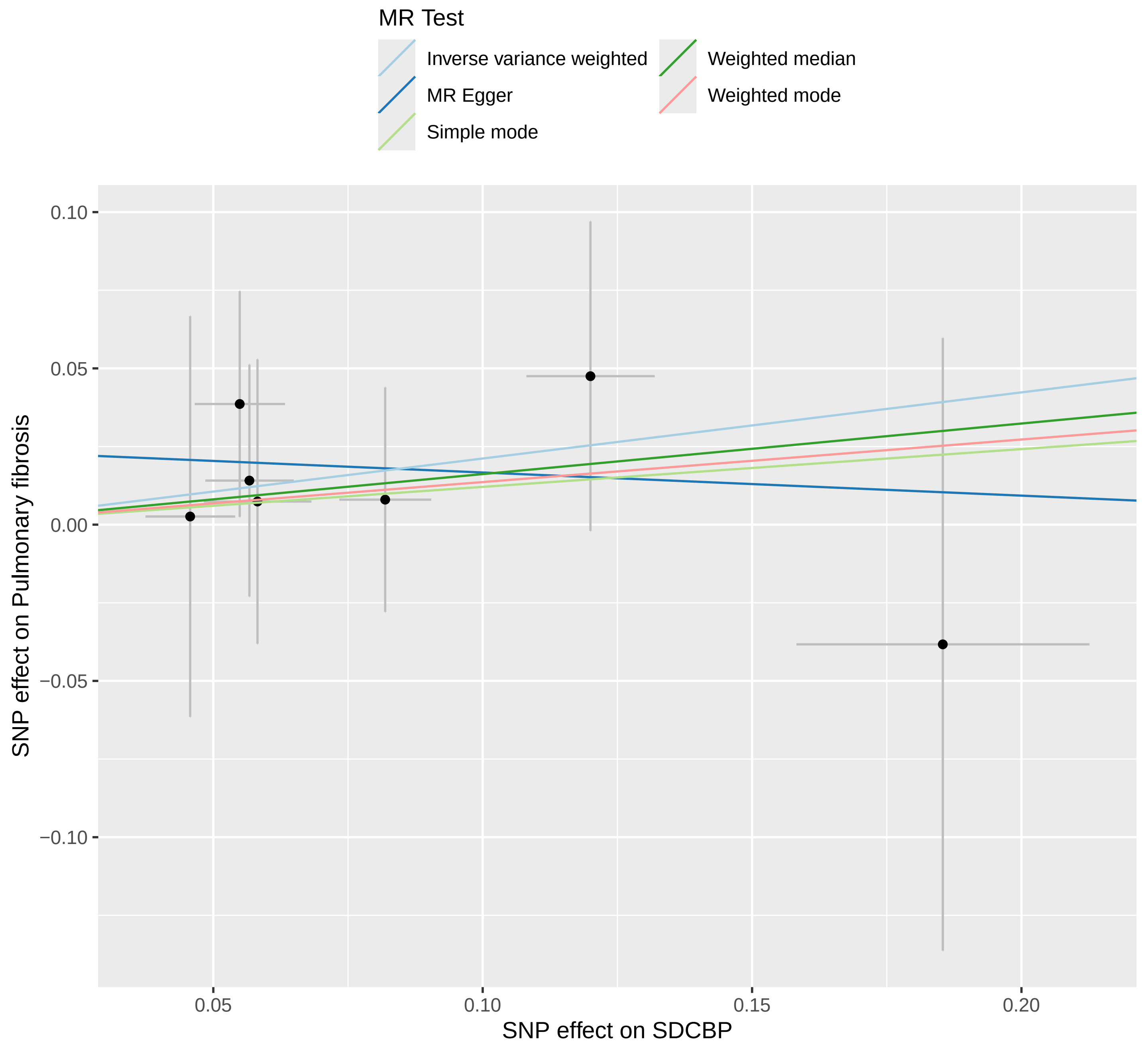

MR Test
Inverse variance weighted
MR Egger
Simple mode
Weighted median
Weighted mode
0.10
0.05
0.00
−0.05
−0.10
SNP effect on Pulmonary fibrosis
0.05
0.10
0.15
0.20
SNP effect on SDCBP

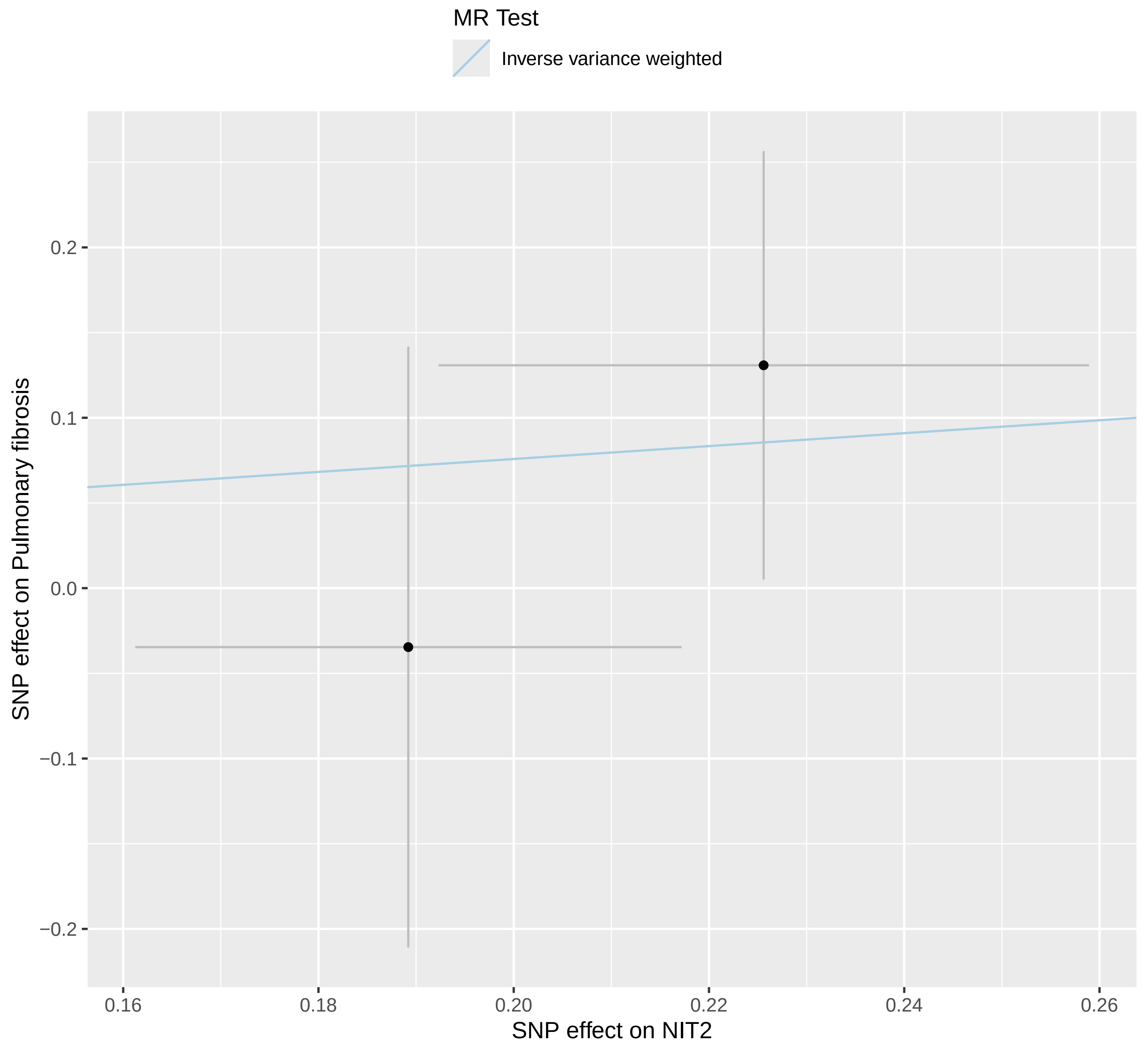
MR Test
Inverse variance weighted
SNP effect on Pulmonary fibrosis
SNP effect on NIT2
0.2
0.1
0.0
−0.1
−0.2
0.16
0.18
0.20
0.22
0.24
0.26

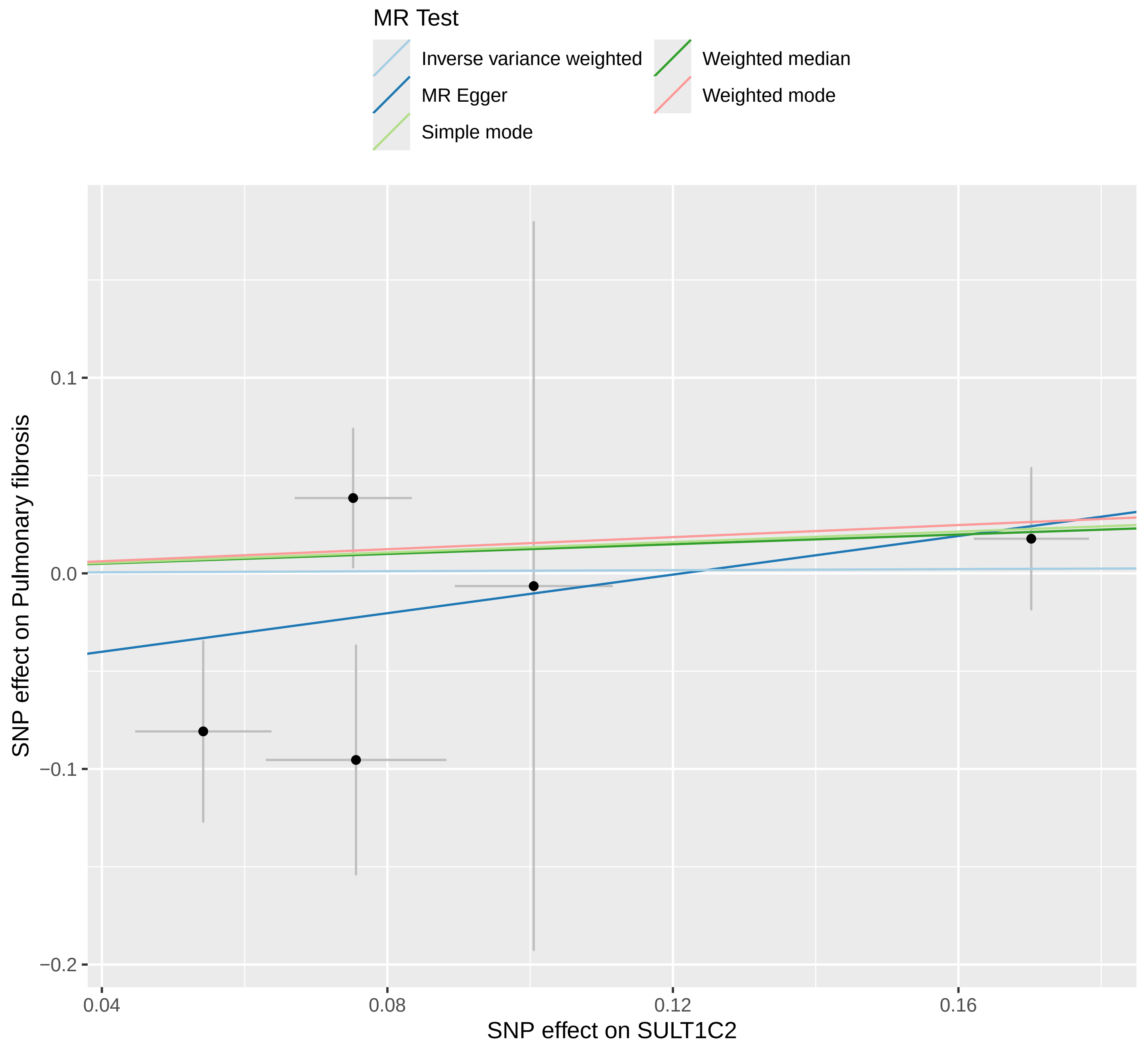
MR Test
Inverse variance weighted
MR Egger
Simple mode
Weighted median
Weighted mode
SNP effect on Pulmonary fibrosis
0.1
0.0
−0.1
−0.2
0.04
0.08
0.12
0.16
SNP effect on SULT1C2

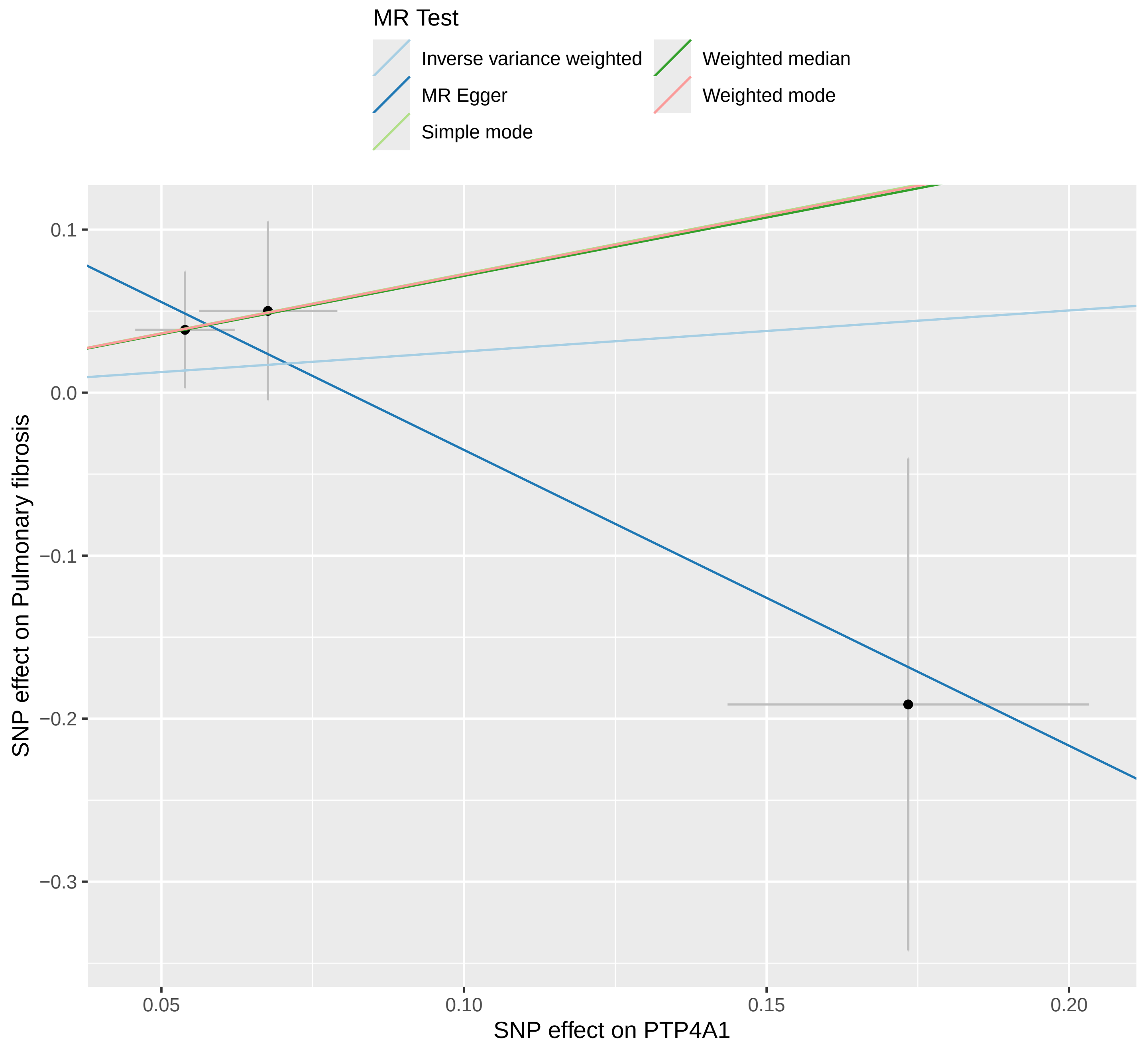

MR Test
Inverse variance weighted
MR Egger
Simple mode
Weighted median
Weighted mode
0.1
0.0
−0.1
−0.2
−0.3
SNP effect on Pulmonary fibrosis
0.05
0.10
0.15
0.20
SNP effect on PTP4A1

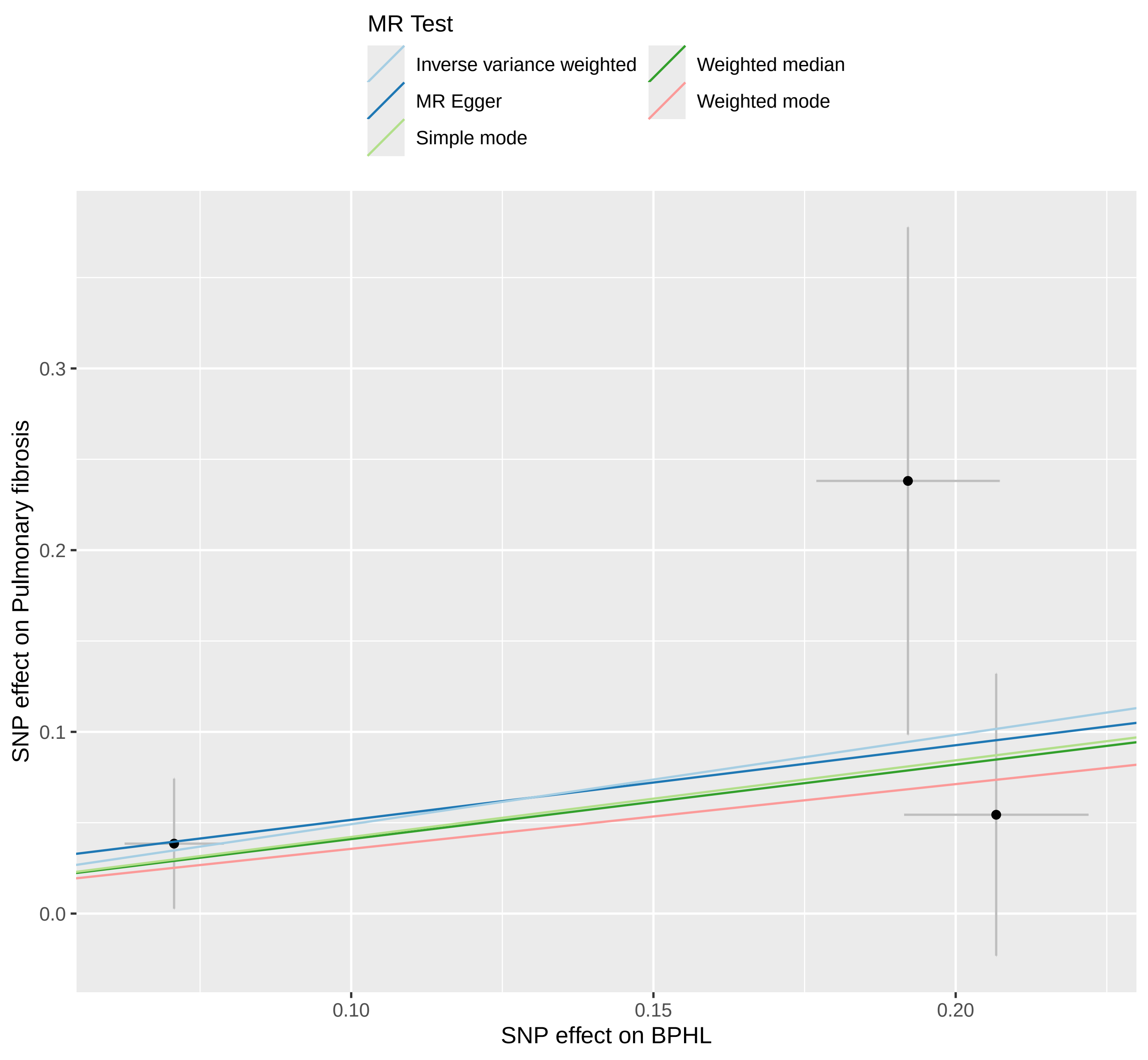
MR Test
Inverse variance weighted
MR Egger
Simple mode
Weighted median
Weighted mode
SNP effect on Pulmonary fibrosis
0.0
0.1
0.2
0.3
0.10
0.15
0.20
SNP effect on BPHL

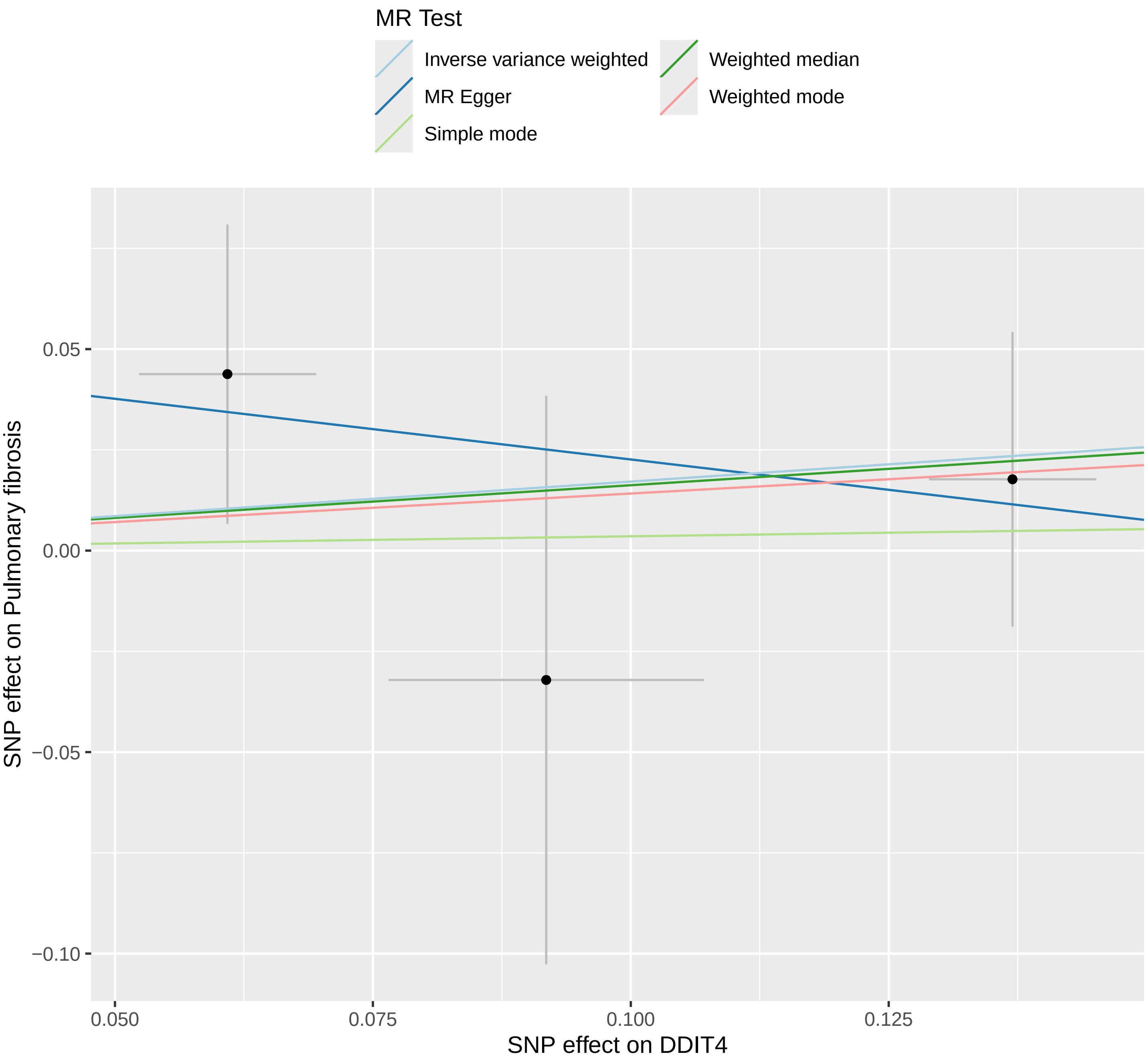
MR Test
Inverse variance weighted
MR Egger
Simple mode
Weighted median
Weighted mode
0.05
0.00
−0.05
−0.10
0.050
0.075
0.100
0.125
SNP effect on DDIT4
SNP effect on Pulmonary fibrosis

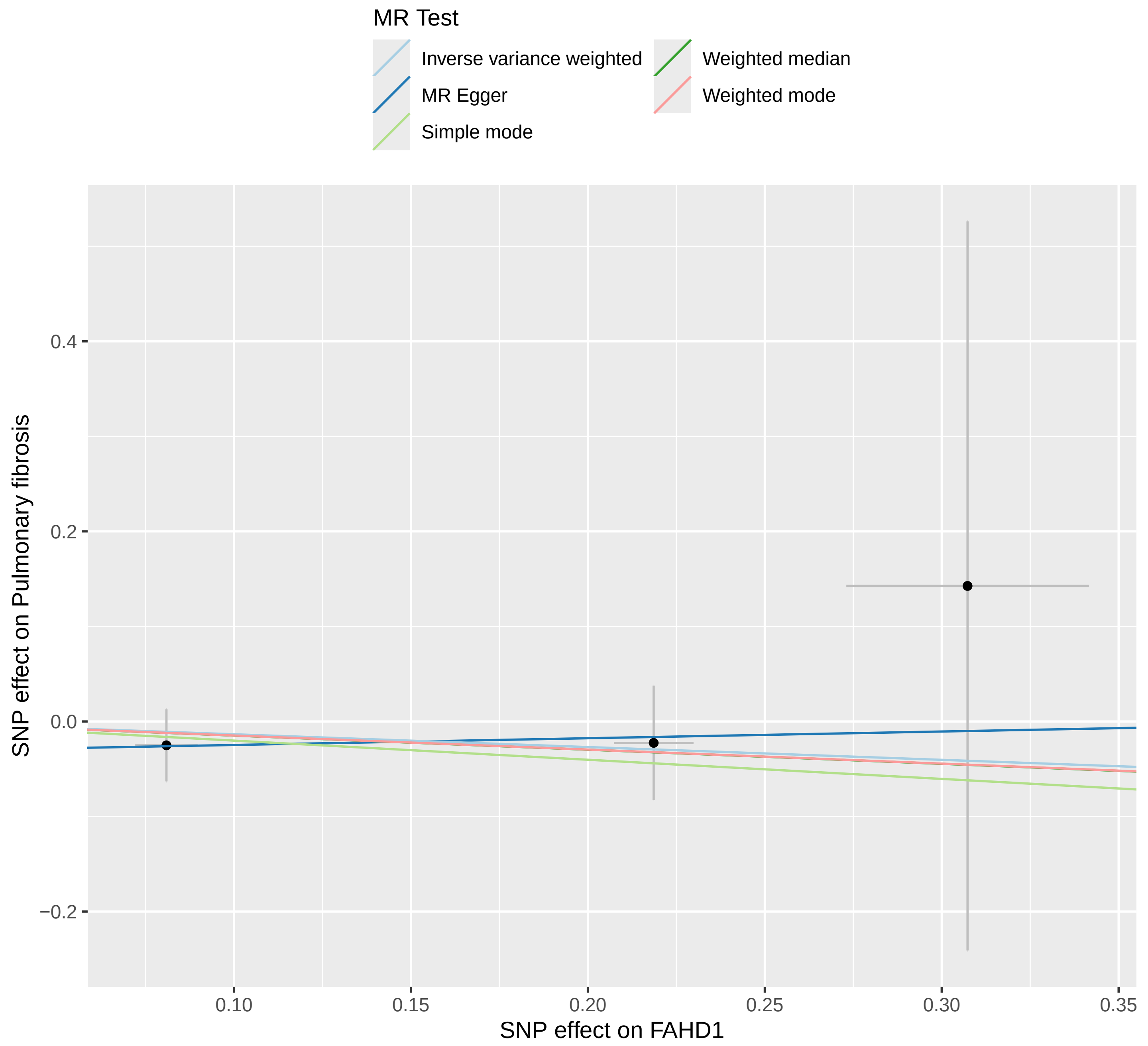
MR Test
Inverse variance weighted
MR Egger
Simple mode
Weighted median
Weighted mode
SNP effect on Pulmonary fibrosis
0.4
0.2
0.0
−0.2
0.10
0.15
0.20
0.25
0.30
0.35
SNP effect on FAHD1

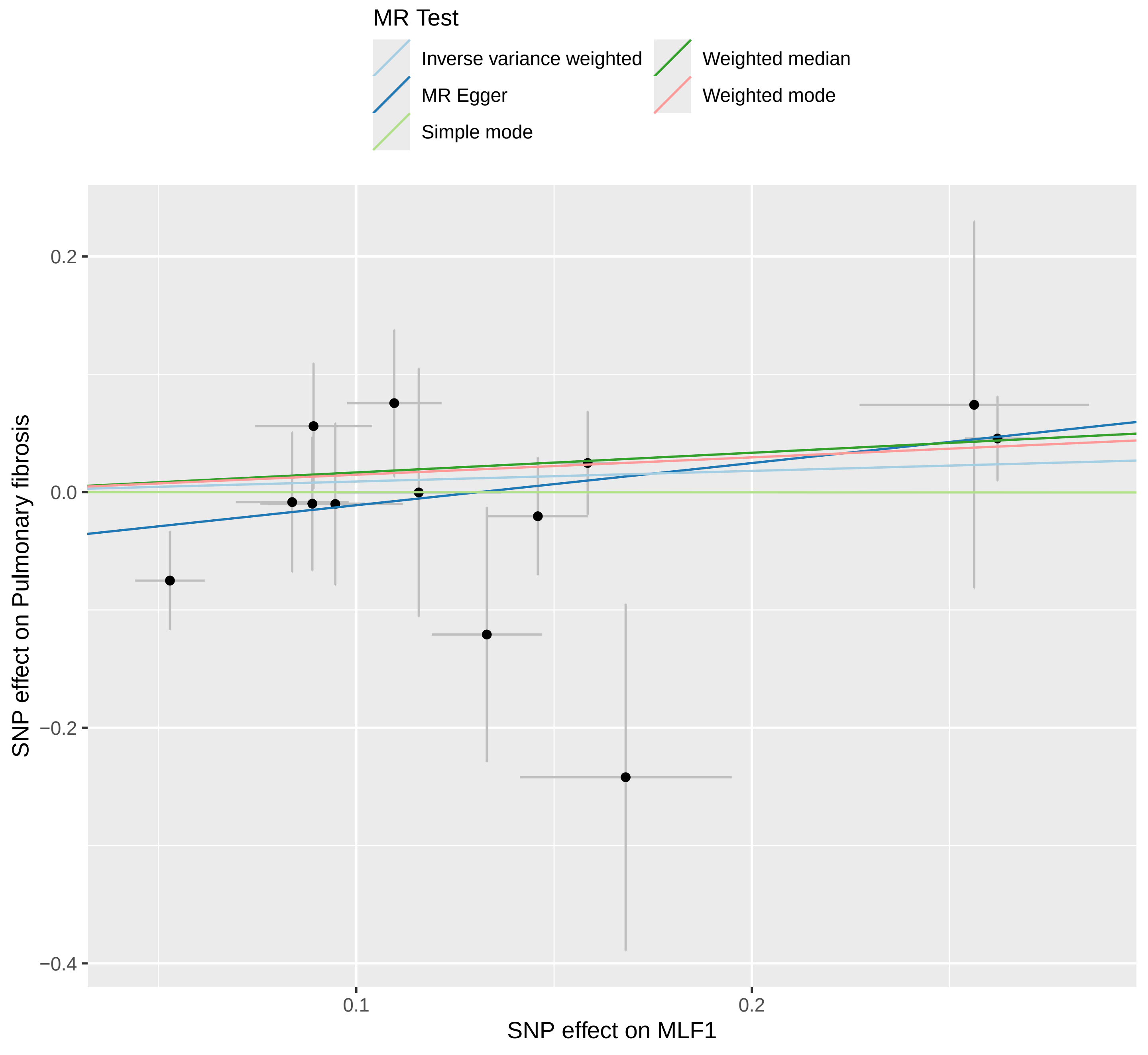
MR Test
Inverse variance weighted
MR Egger
Simple mode
Weighted median
Weighted mode
0.2
0.0
−0.2
−0.4
SNP effect on Pulmonary fibrosis
0.1
0.2
SNP effect on MLF1

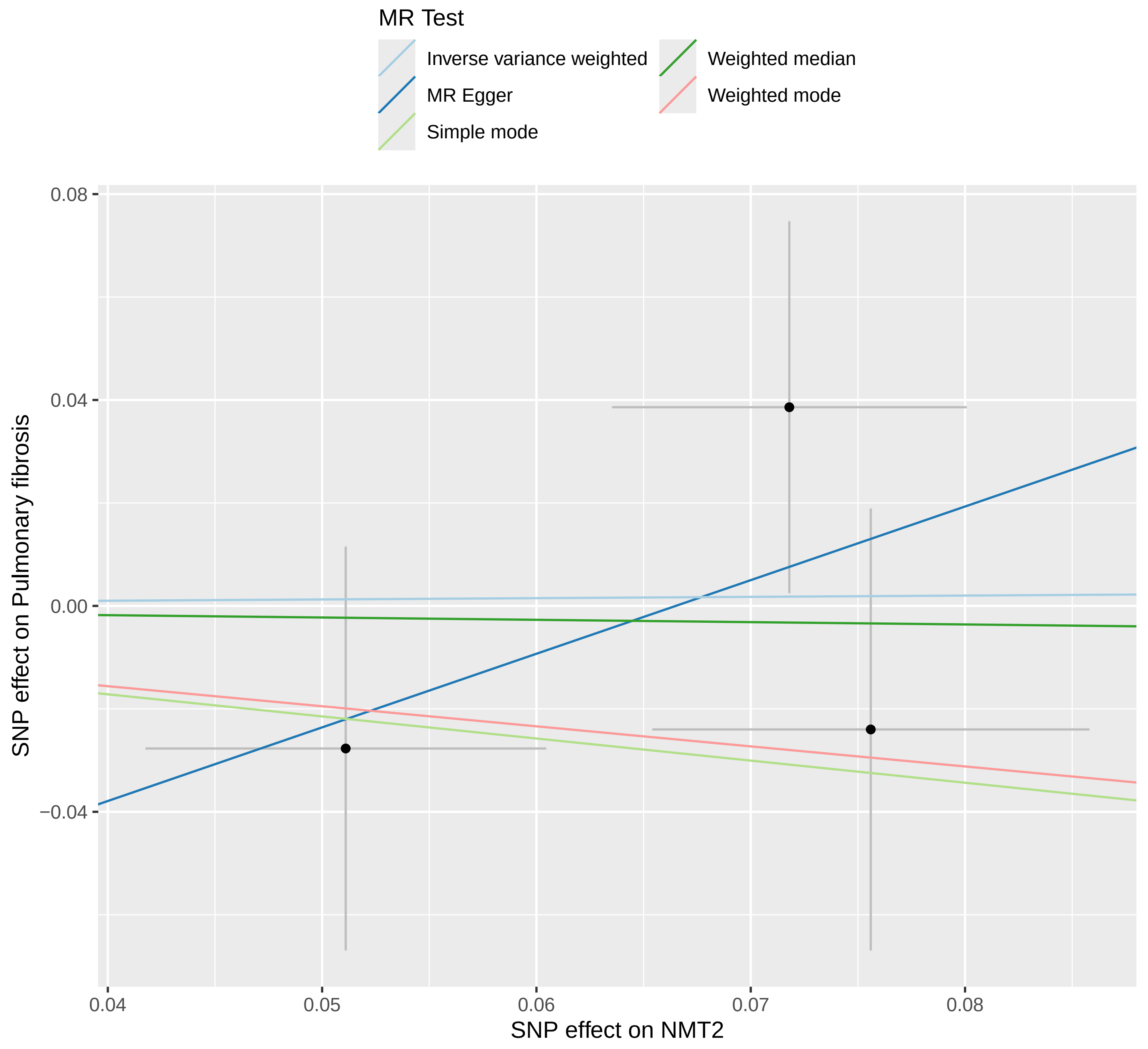

MR Test
Inverse variance weighted
MR Egger
Simple mode
Weighted median
Weighted mode
0.08
0.04
0.00
−0.04
0.04
0.05
0.06
0.07
0.08
SNP effect on Pulmonary fibrosis
SNP effect on NMT2

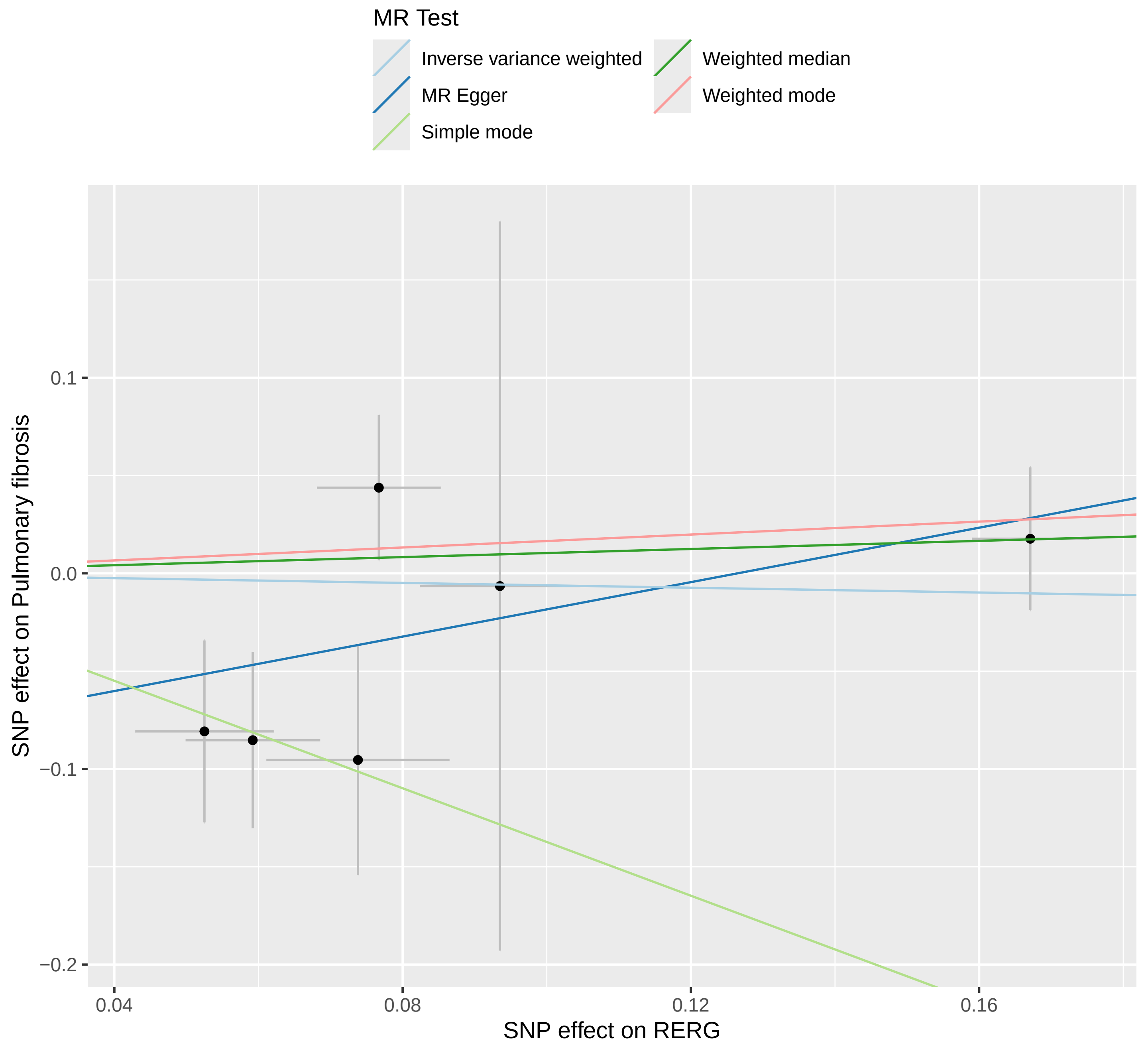

MR Test
Inverse variance weighted
MR Egger
Simple mode
Weighted median
Weighted mode
SNP effect on Pulmonary fibrosis
0.1
0.0
−0.1
−0.2
0.04
0.08
0.12
0.16
SNP effect on RERG

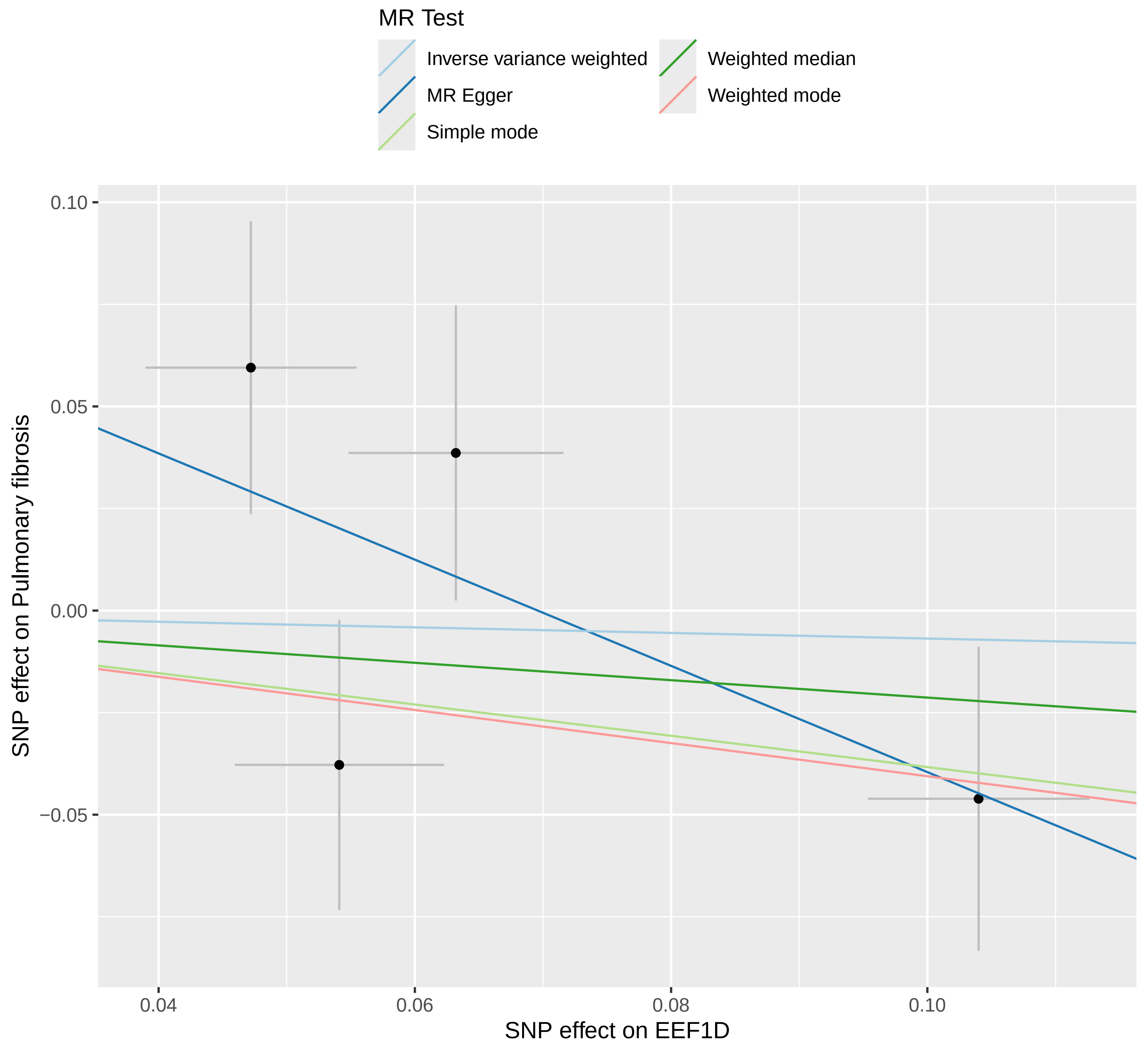
MR Test
Inverse variance weighted
MR Egger
Simple mode
Weighted median
Weighted mode
0.10
0.05
0.00
−0.05
SNP effect on Pulmonary fibrosis
0.04
0.06
0.08
0.10
SNP effect on EEF1D

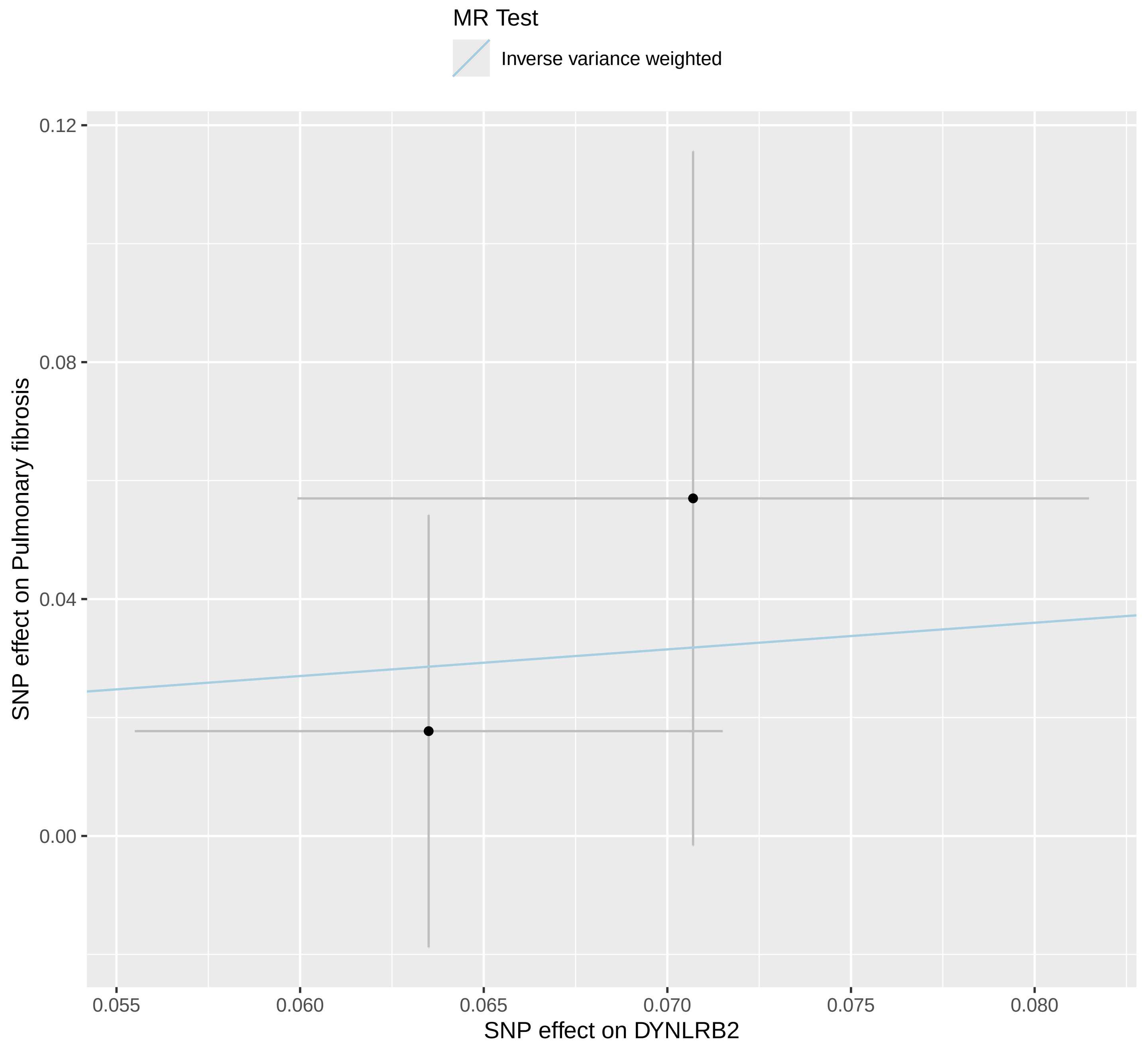

MR Test
Inverse variance weighted
0.12
0.08
0.04
0.00
SNP effect on Pulmonary fibrosis
0.055
0.060
0.065
0.070
0.075
0.080
SNP effect on DYNLRB2

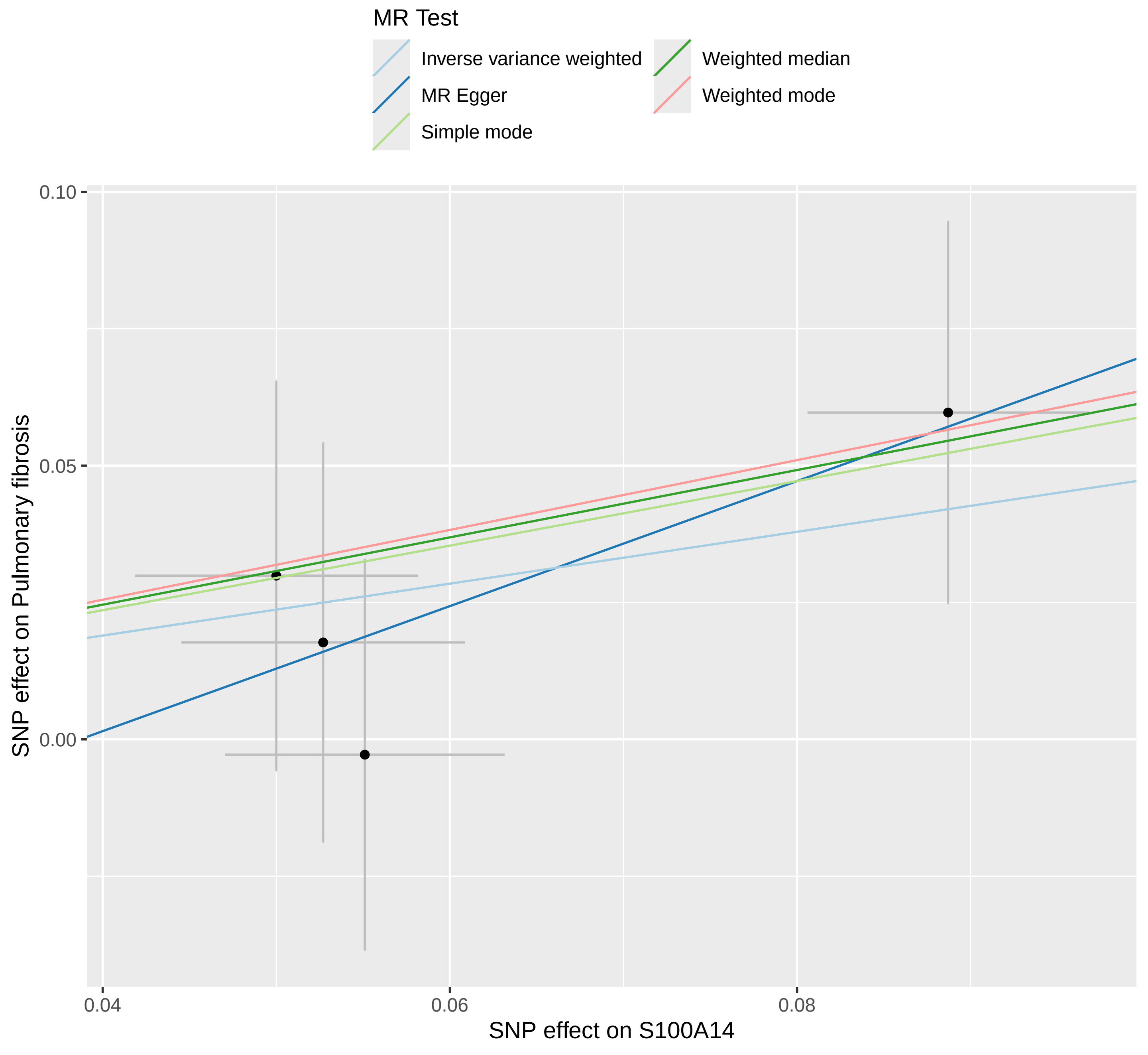
MR Test
Inverse variance weighted
MR Egger
Simple mode
Weighted median
Weighted mode
0.10
0.05
0.00
0.04
0.06
0.08
SNP effect on S100A14
SNP effect on Pulmonary fibrosis

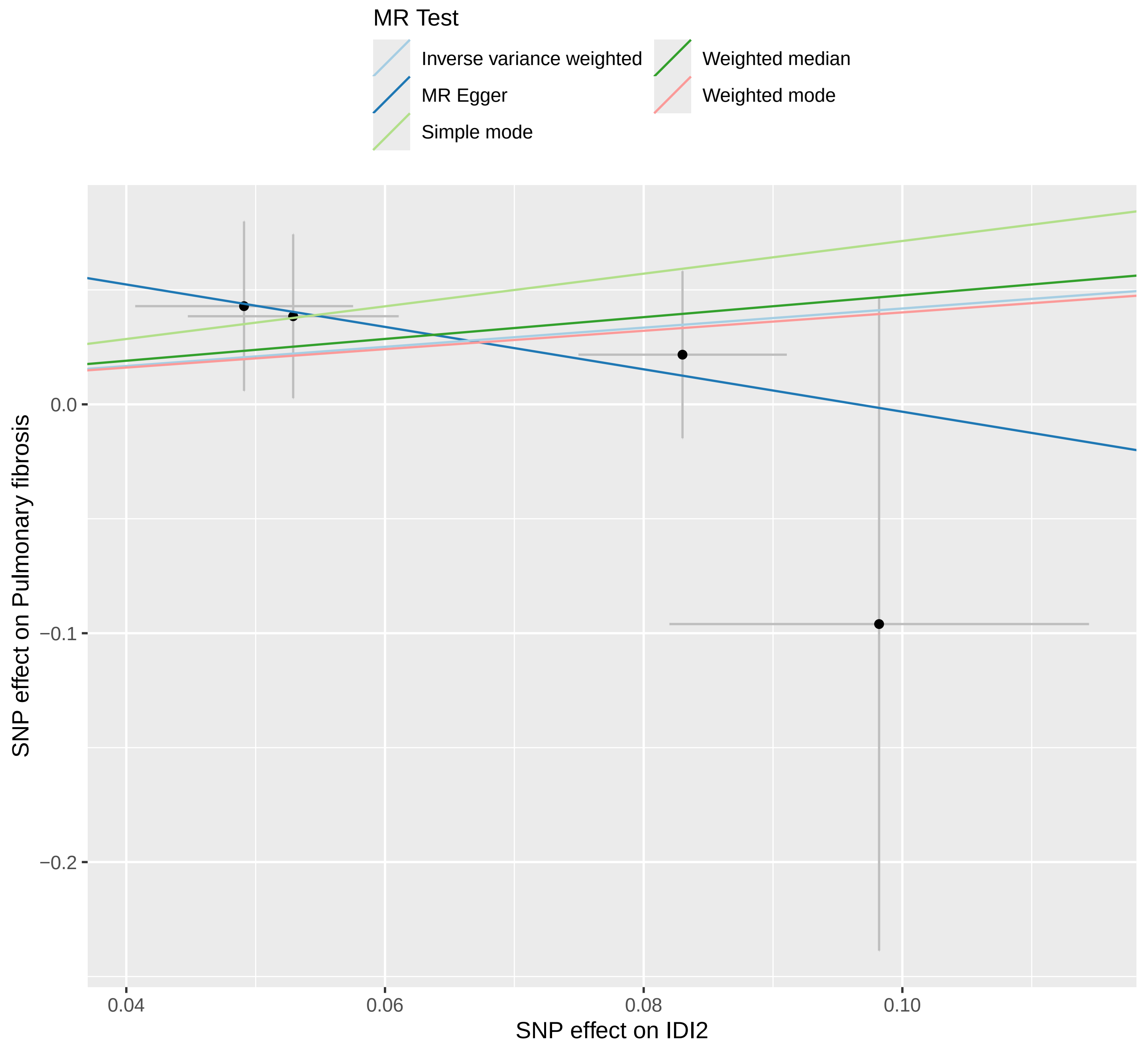

MR Test
Inverse variance weighted
MR Egger
Simple mode
Weighted median
Weighted mode
0.0
−0.1
−0.2
SNP effect on Pulmonary fibrosis
0.04
0.06
0.08
0.10
SNP effect on IDI2

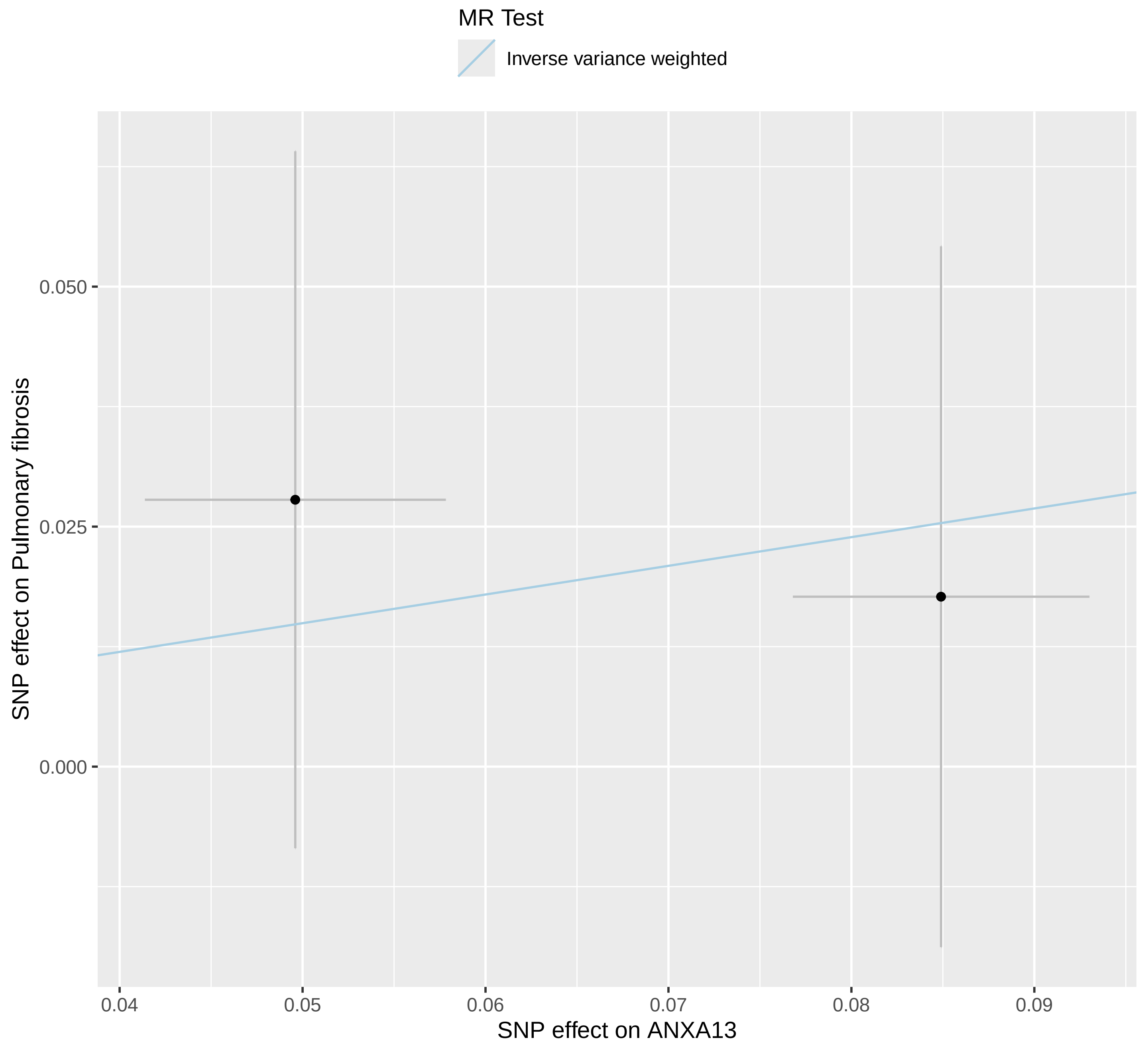

MR Test
Inverse variance weighted
SNP effect on Pulmonary fibrosis
0.050
0.025
0.000
0.04
0.05
0.06
0.07
0.08
0.09
SNP effect on ANXA13

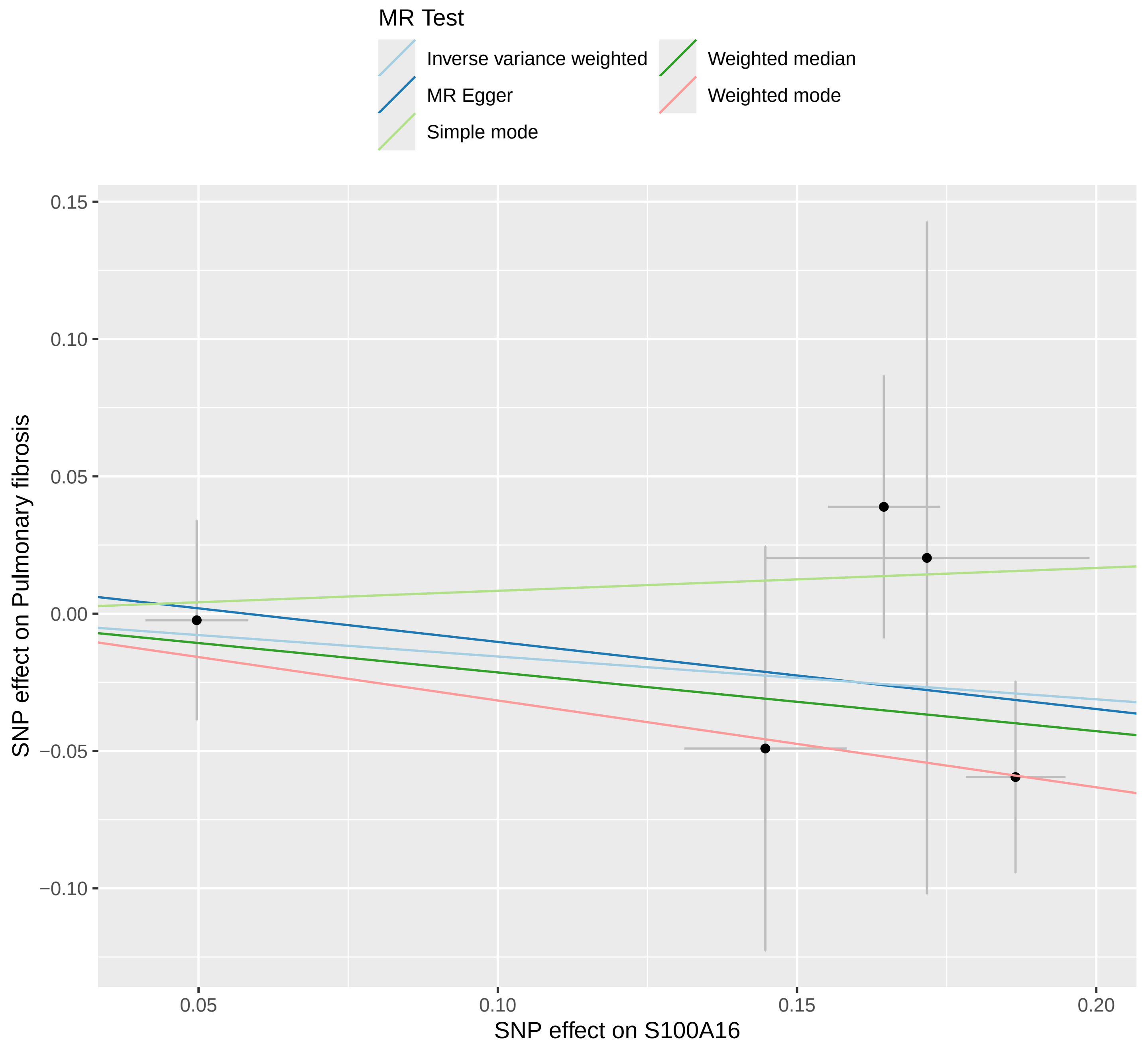
MR Test
Inverse variance weighted
MR Egger
Simple mode
Weighted median
Weighted mode
0.15
0.10
0.05
0.00
−0.05
−0.10
SNP effect on Pulmonary fibrosis
0.05
0.10
0.15
0.20
SNP effect on S100A16

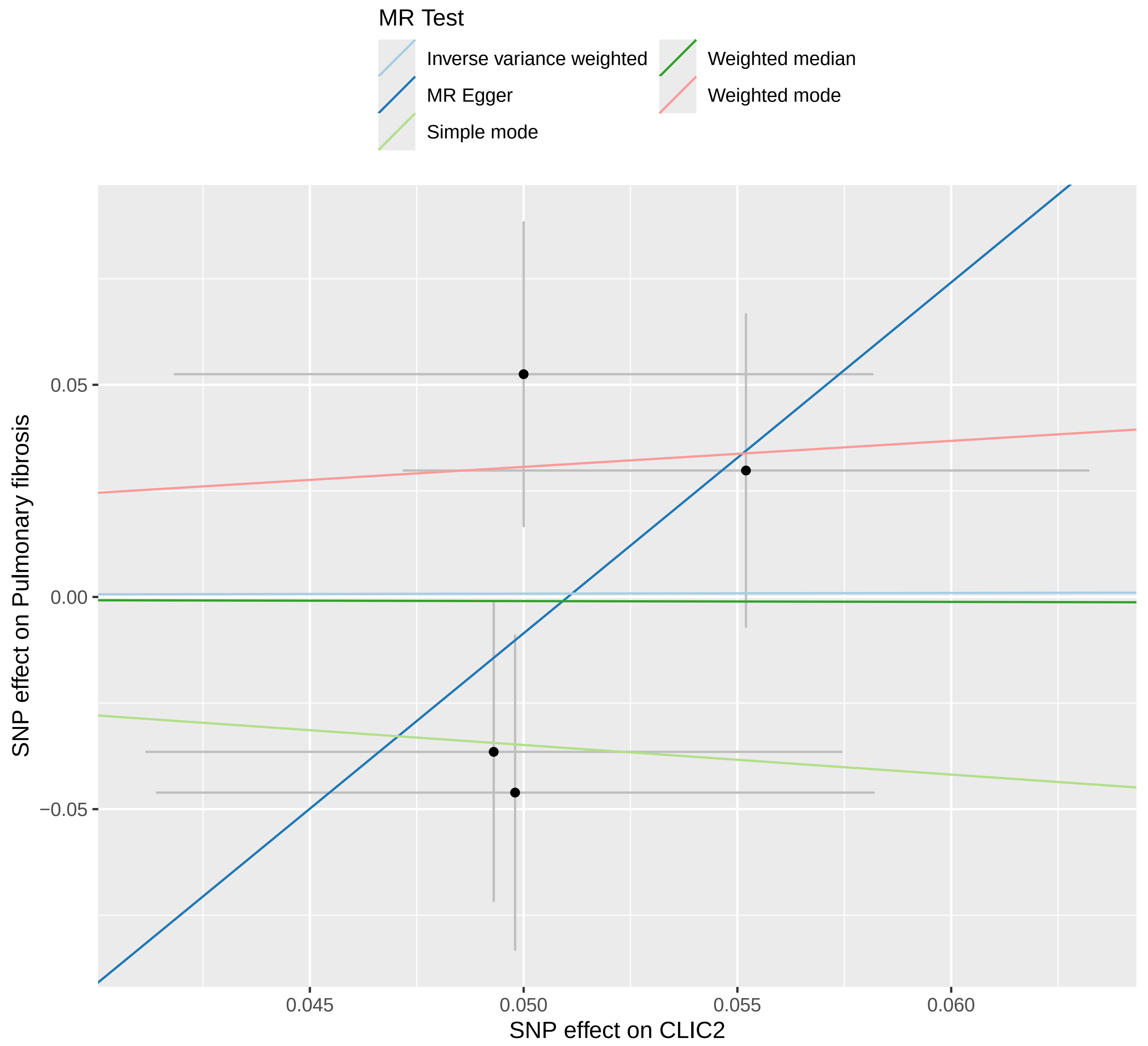

MR Test
Inverse variance weighted
MR Egger
Simple mode
Weighted median
Weighted mode
0.05
0.00
−0.05
SNP effect on Pulmonary fibrosis
0.045
0.050
0.055
0.060
SNP effect on CLIC2

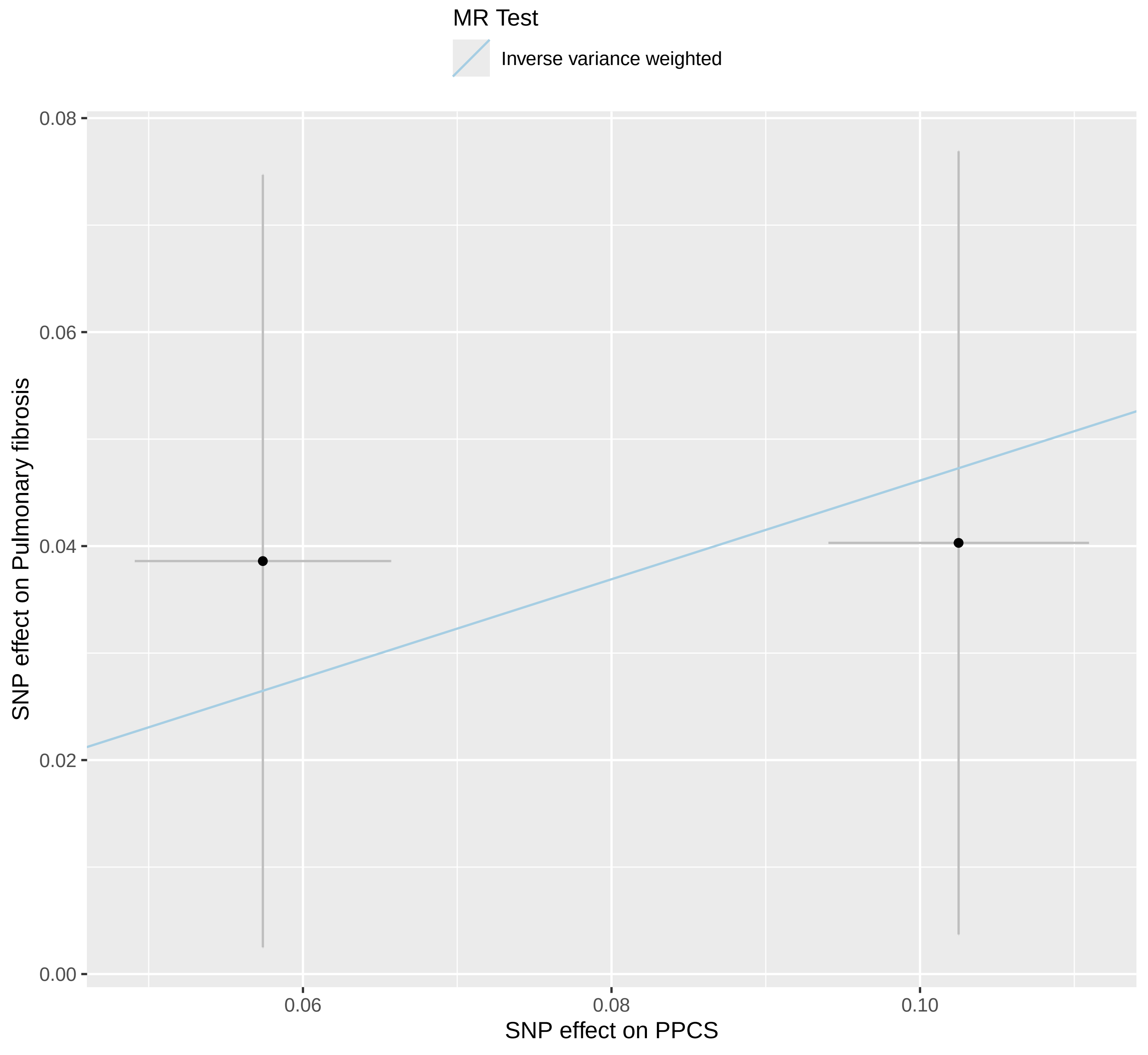
MR Test
Inverse variance weighted
0.08
0.06
0.04
0.02
0.00
0.06
0.08
0.10
SNP effect on Pulmonary fibrosis
SNP effect on PPCS

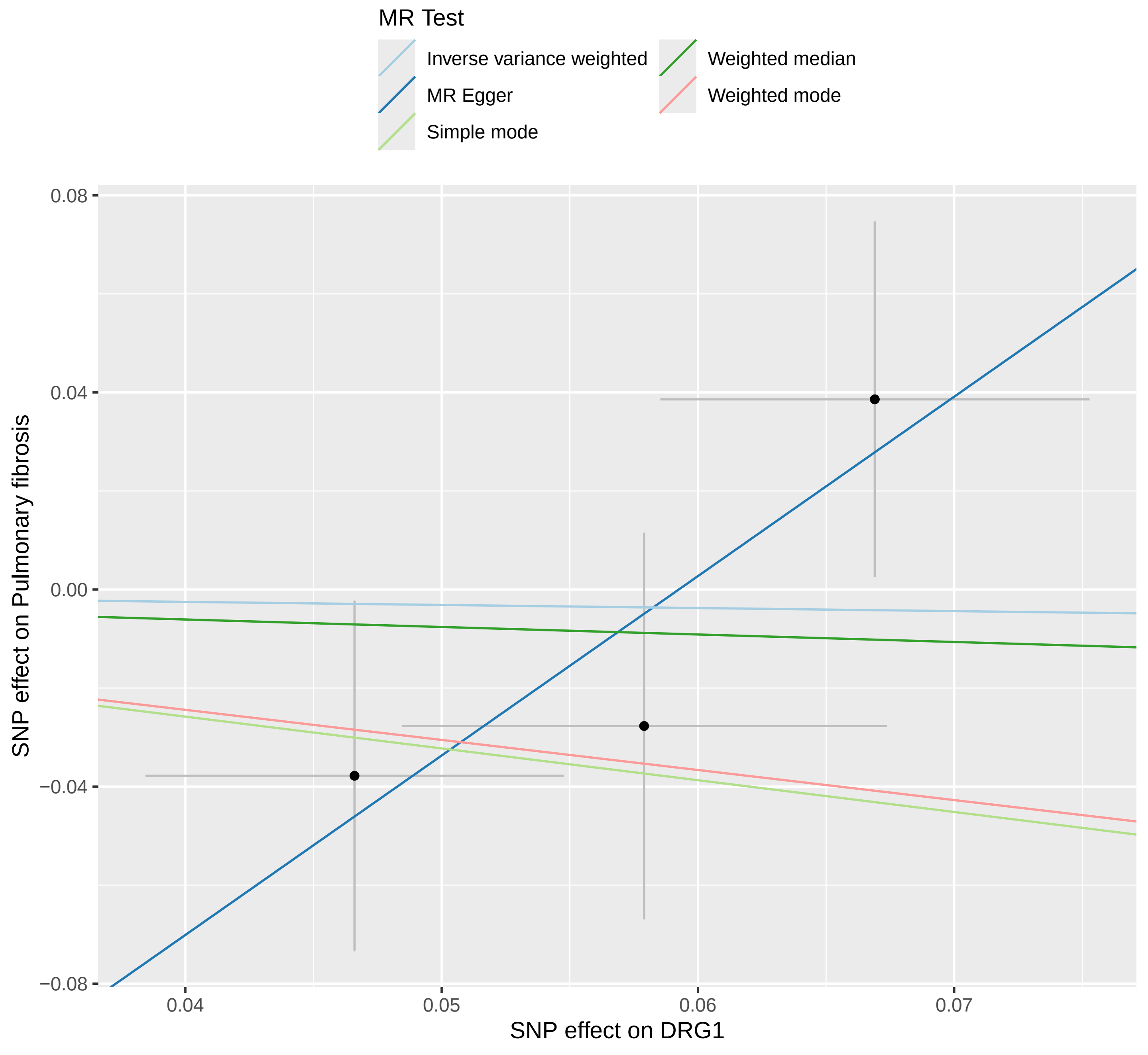

MR Test
Inverse variance weighted
MR Egger
Simple mode
Weighted median
Weighted mode
0.08
0.04
0.00
−0.04
−0.08
0.04
0.05
0.06
0.07
SNP effect on DRG1
SNP effect on Pulmonary fibrosis

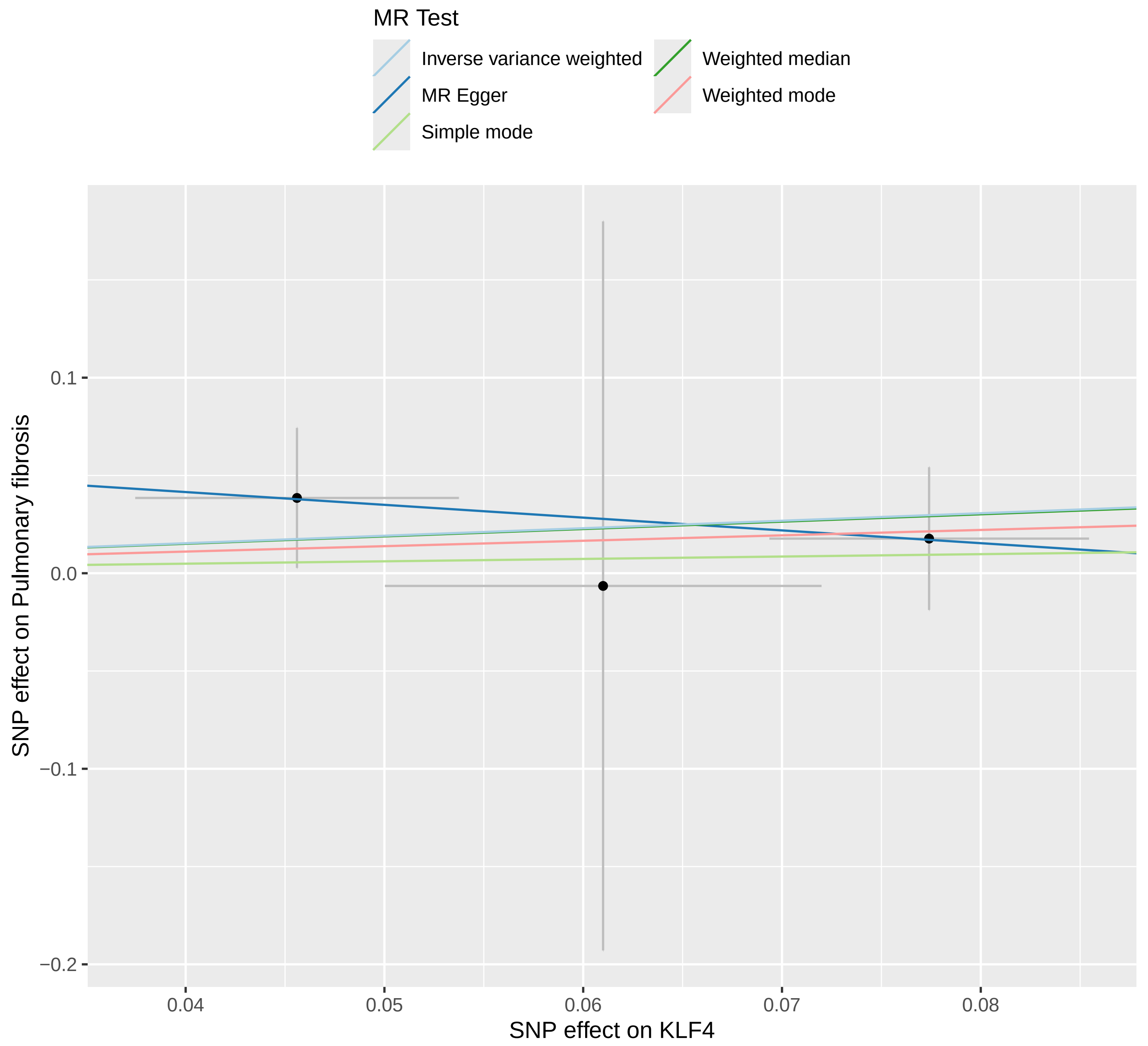
MR Test
Inverse variance weighted
MR Egger
Simple mode
Weighted median
Weighted mode
SNP effect on Pulmonary fibrosis
0.1
0.0
−0.1
−0.2
0.04
0.05
0.06
0.07
0.08
SNP effect on KLF4

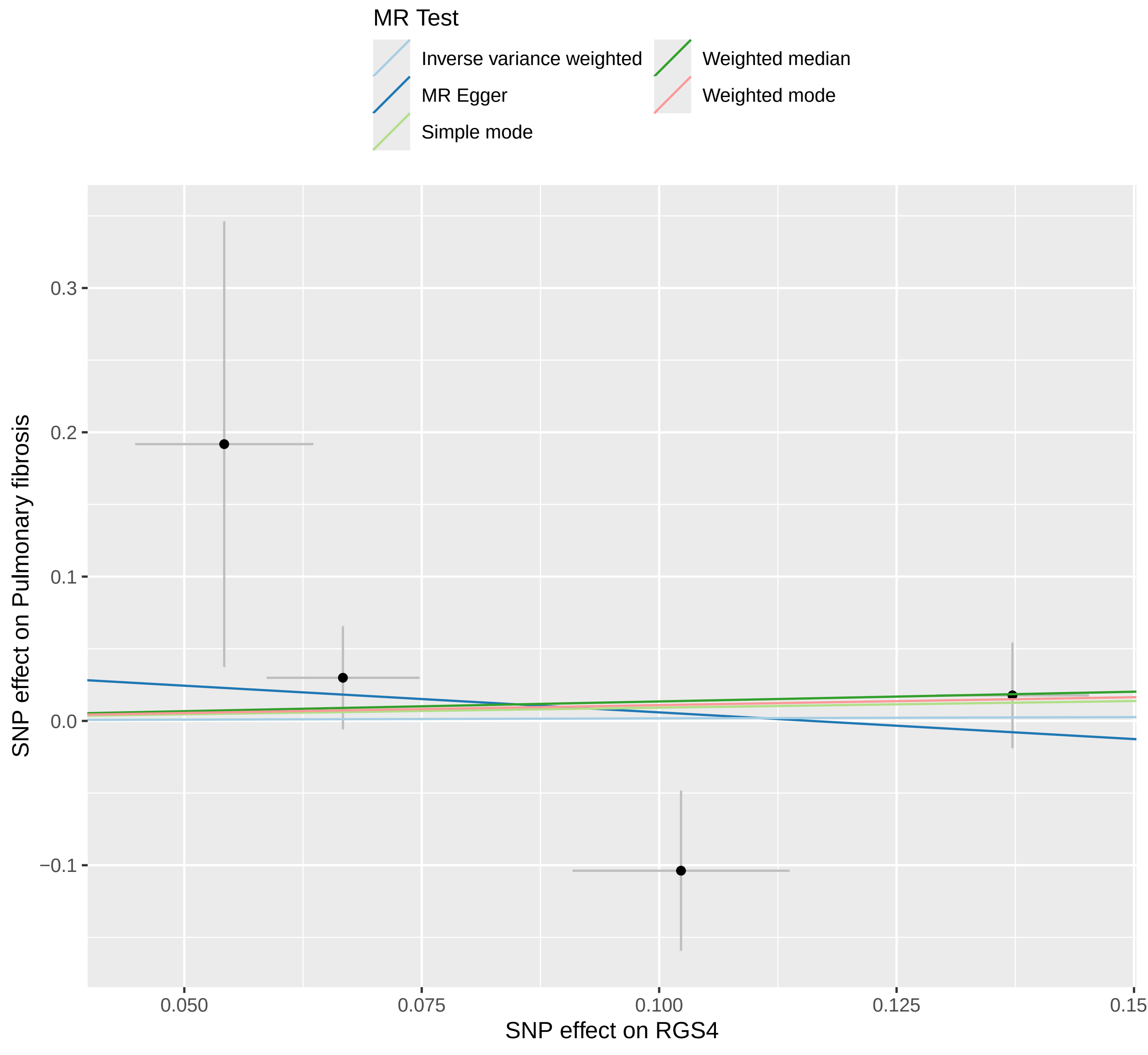
MR Test
Inverse variance weighted
MR Egger
Simple mode
Weighted median
Weighted mode
SNP effect on Pulmonary fibrosis
0.3
0.2
0.1
0.0
−0.1
0.050
0.075
0.100
0.125
0.15
SNP effect on RGS4

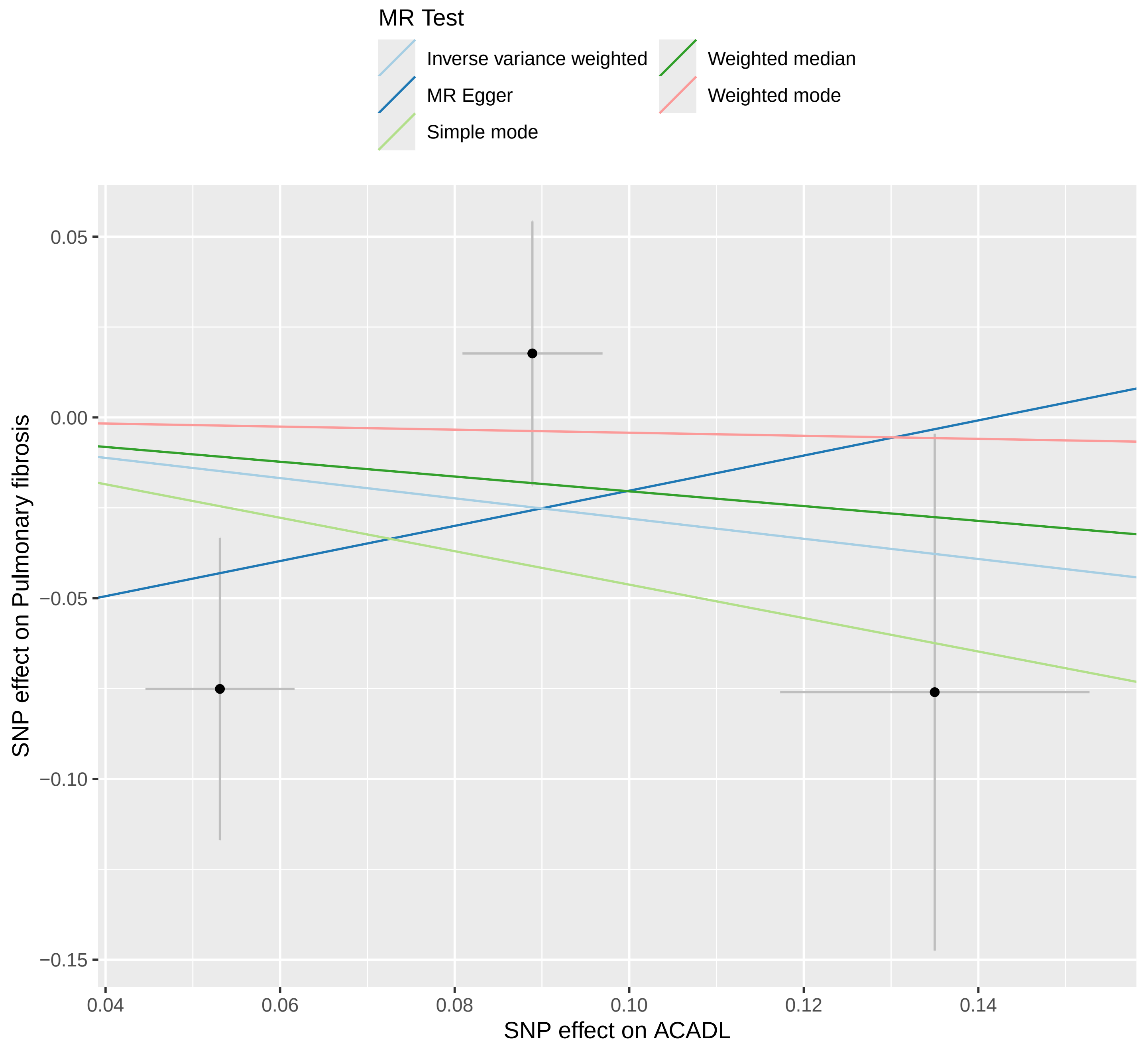
MR Test
Inverse variance weighted
MR Egger
Simple mode
Weighted median
Weighted mode
0.05
0.00
−0.05
−0.10
−0.15
0.04
0.06
0.08
0.10
0.12
0.14
SNP effect on ACADL
SNP effect on Pulmonary fibrosis

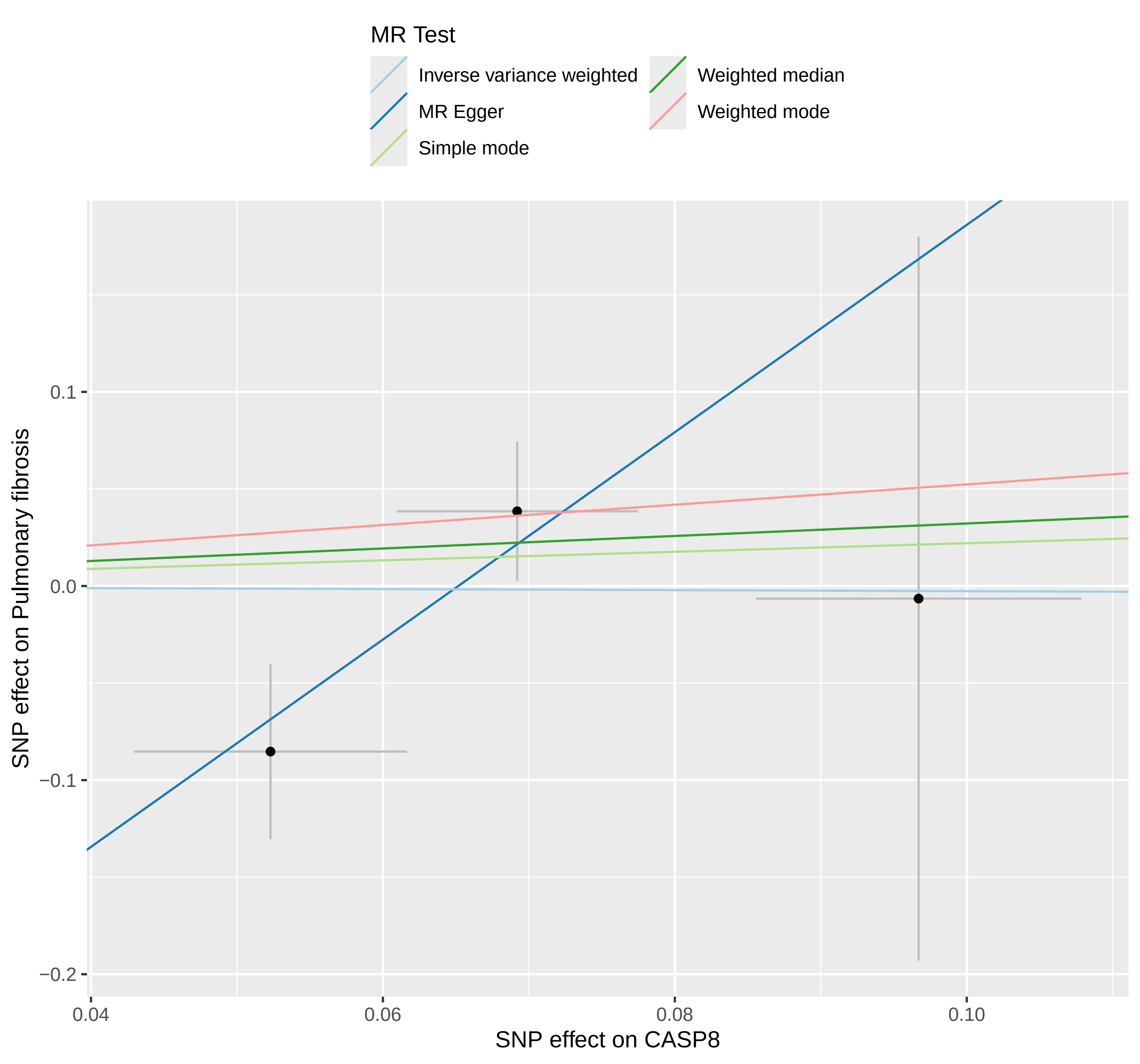
MR Test
Inverse variance weighted
MR Egger
Simple mode
Weighted median
Weighted mode
SNP effect on Pulmonary fibrosis
0.1
0.0
−0.1
−0.2
0.04
0.06
0.08
0.10
SNP effect on CASP8

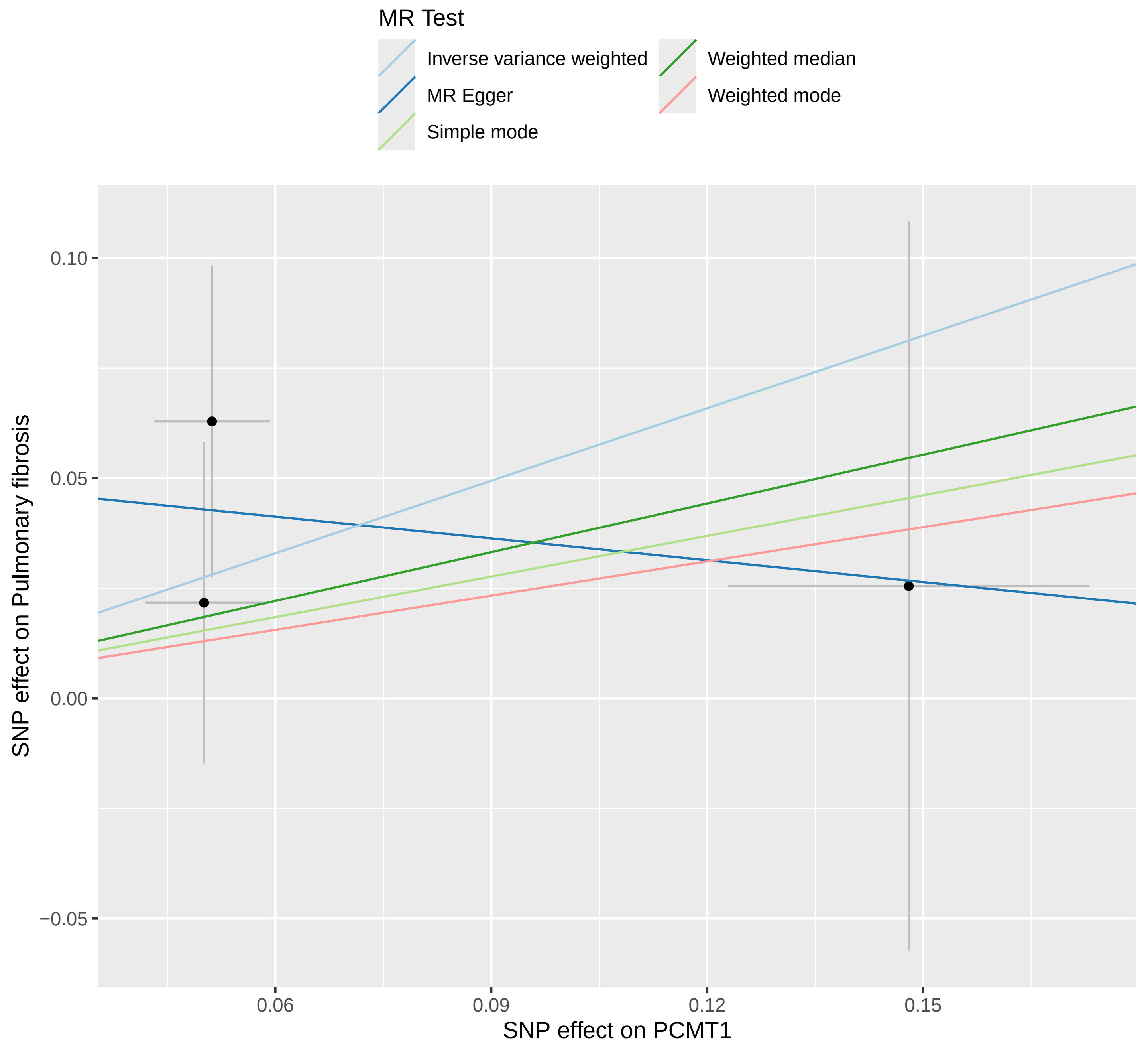

MR Test
Inverse variance weighted
MR Egger
Simple mode
Weighted median
Weighted mode
0.10
0.05
0.00
−0.05
0.06
0.09
0.12
0.15
SNP effect on PCMT1
SNP effect on Pulmonary fibrosis

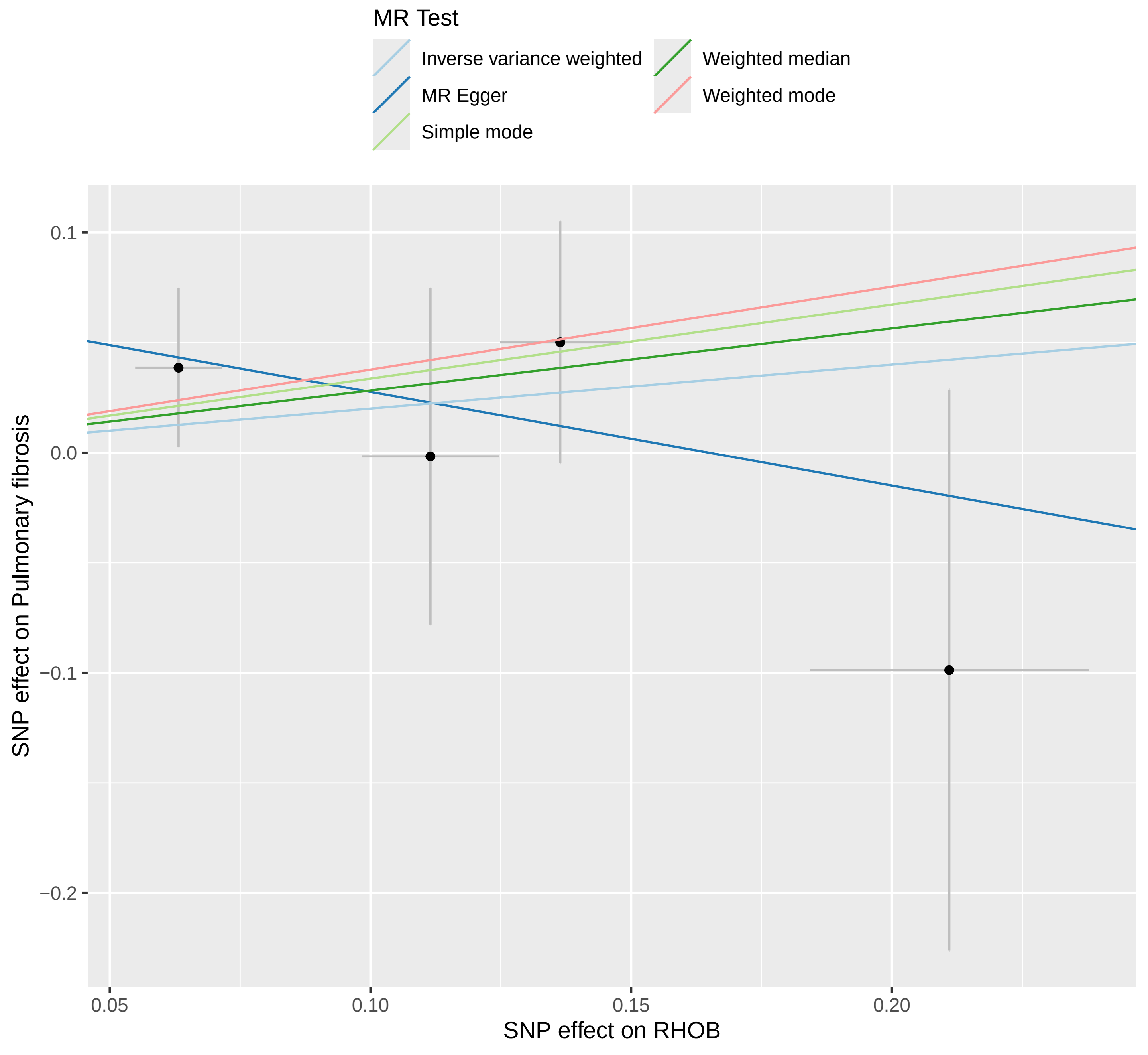

MR Test
Inverse variance weighted
MR Egger
Simple mode
Weighted median
Weighted mode
0.1
0.0
−0.1
−0.2
0.05
0.10
0.15
0.20
SNP effect on RHOB
SNP effect on Pulmonary fibrosis

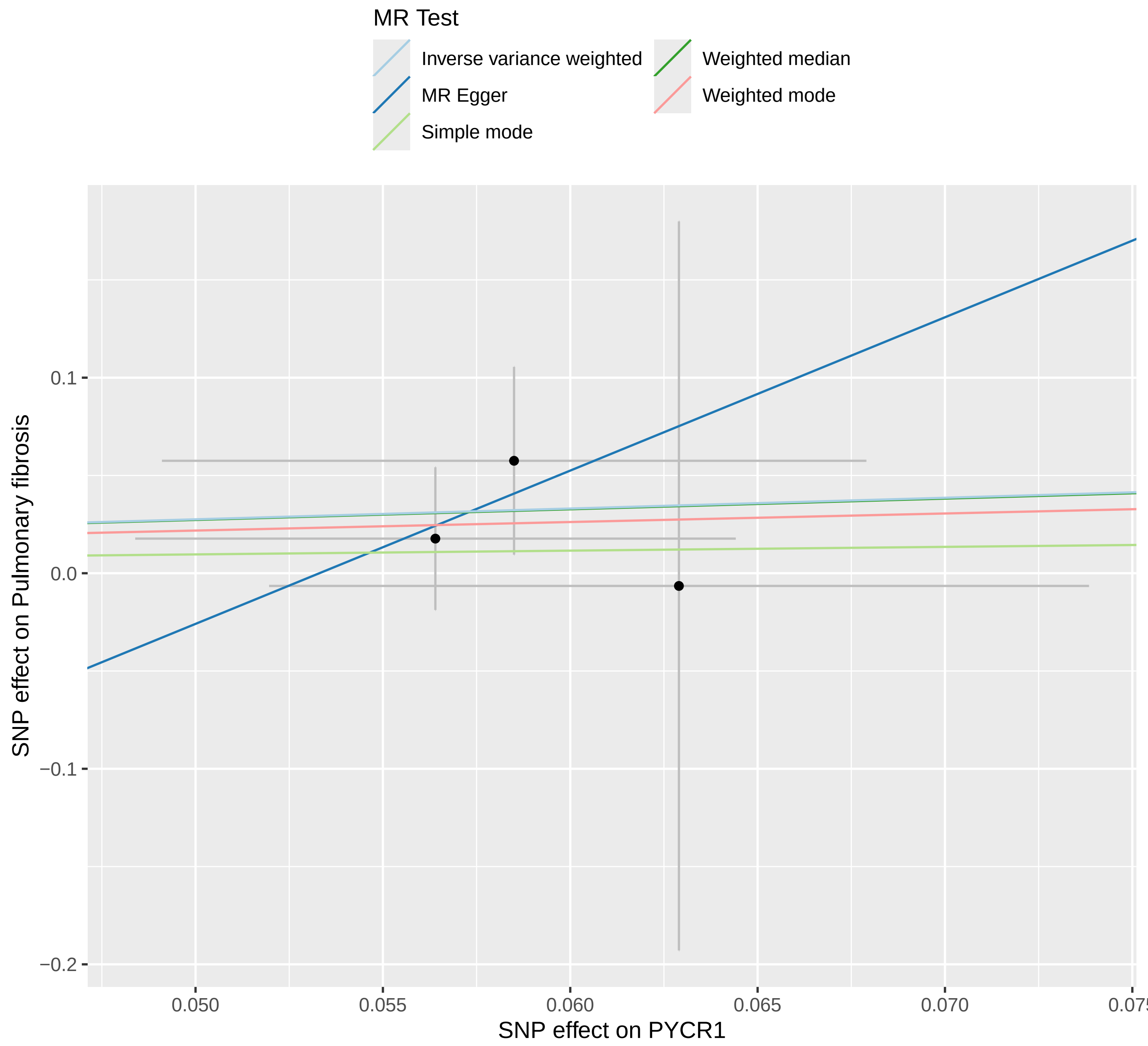

MR Test
Inverse variance weighted
MR Egger
Simple mode
Weighted median
Weighted mode
0.1
0.0
−0.1
−0.2
SNP effect on Pulmonary fibrosis
0.050
0.055
0.060
0.065
0.070
SNP effect on PYCR1

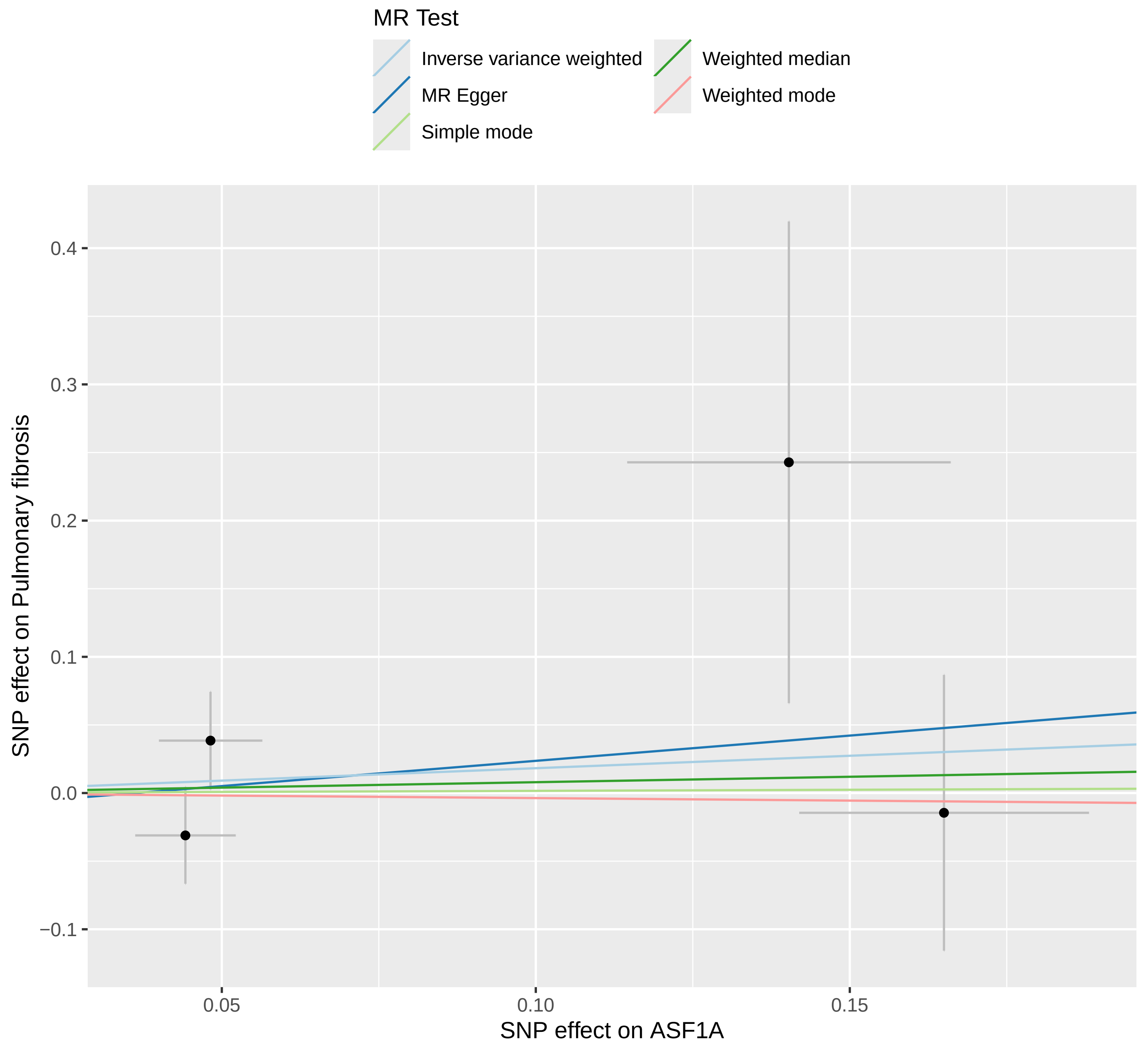

MR Test
Inverse variance weighted
MR Egger
Simple mode
Weighted median
Weighted mode
0.4
0.3
0.2
0.1
0.0
−0.1
0.05
0.10
0.15
SNP effect on Pulmonary fibrosis
SNP effect on ASF1A

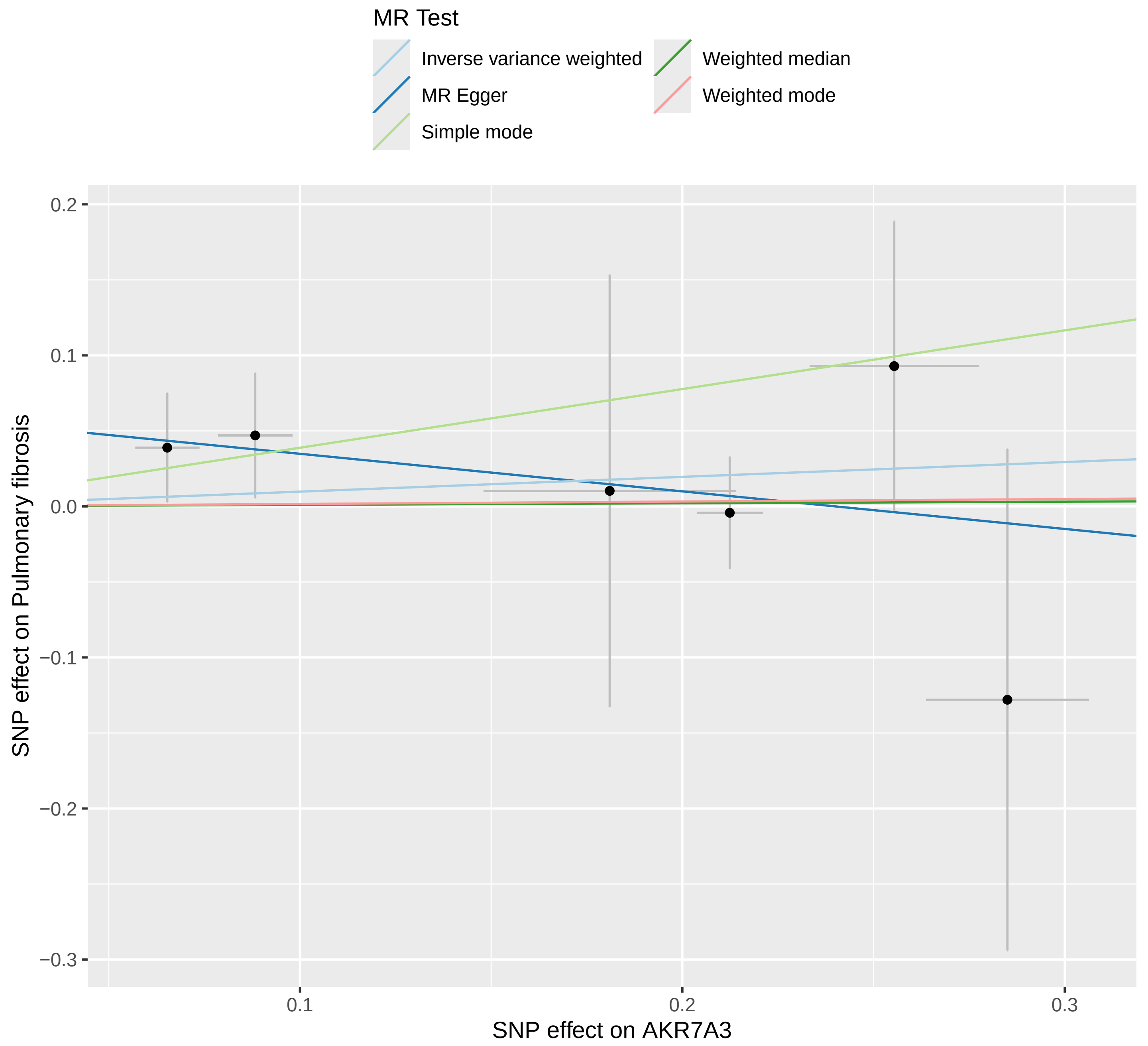
MR Test
Inverse variance weighted
MR Egger
Simple mode
Weighted median
Weighted mode
0.2
0.1
0.0
−0.1
−0.2
−0.3
SNP effect on Pulmonary fibrosis
0.1
0.2
0.3
SNP effect on AKR7A3

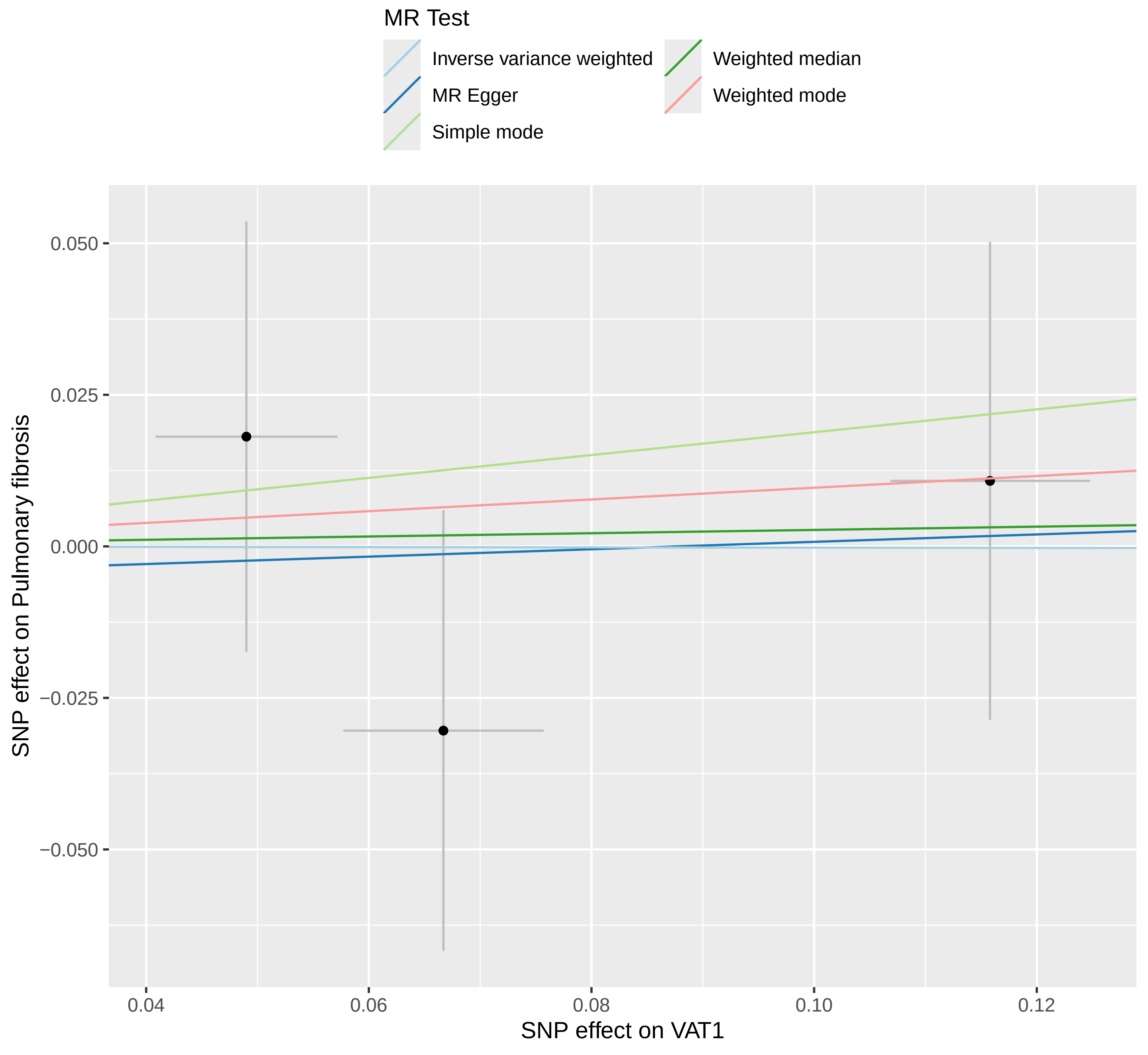

MR Test
Inverse variance weighted
MR Egger
Simple mode
Weighted median
Weighted mode
0.050
0.025
0.000
−0.025
−0.050
SNP effect on Pulmonary fibrosis
0.04
0.06
0.08
0.10
0.12
SNP effect on VAT1

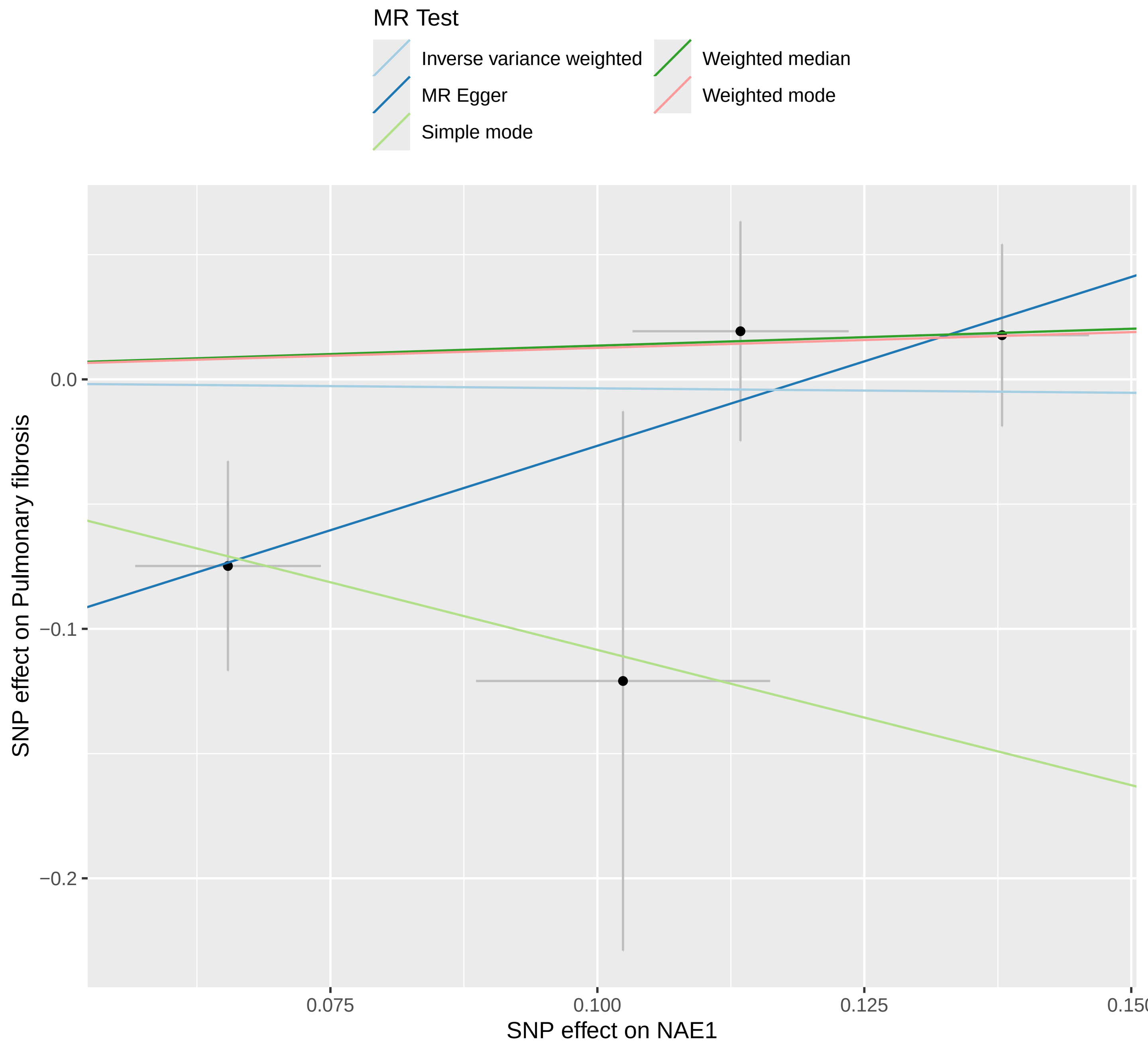

MR Test
Inverse variance weighted
MR Egger
Simple mode
Weighted median
Weighted mode
0.0
−0.1
−0.2
SNP effect on Pulmonary fibrosis
0.075
0.100
0.125
0.150
SNP effect on NAE1

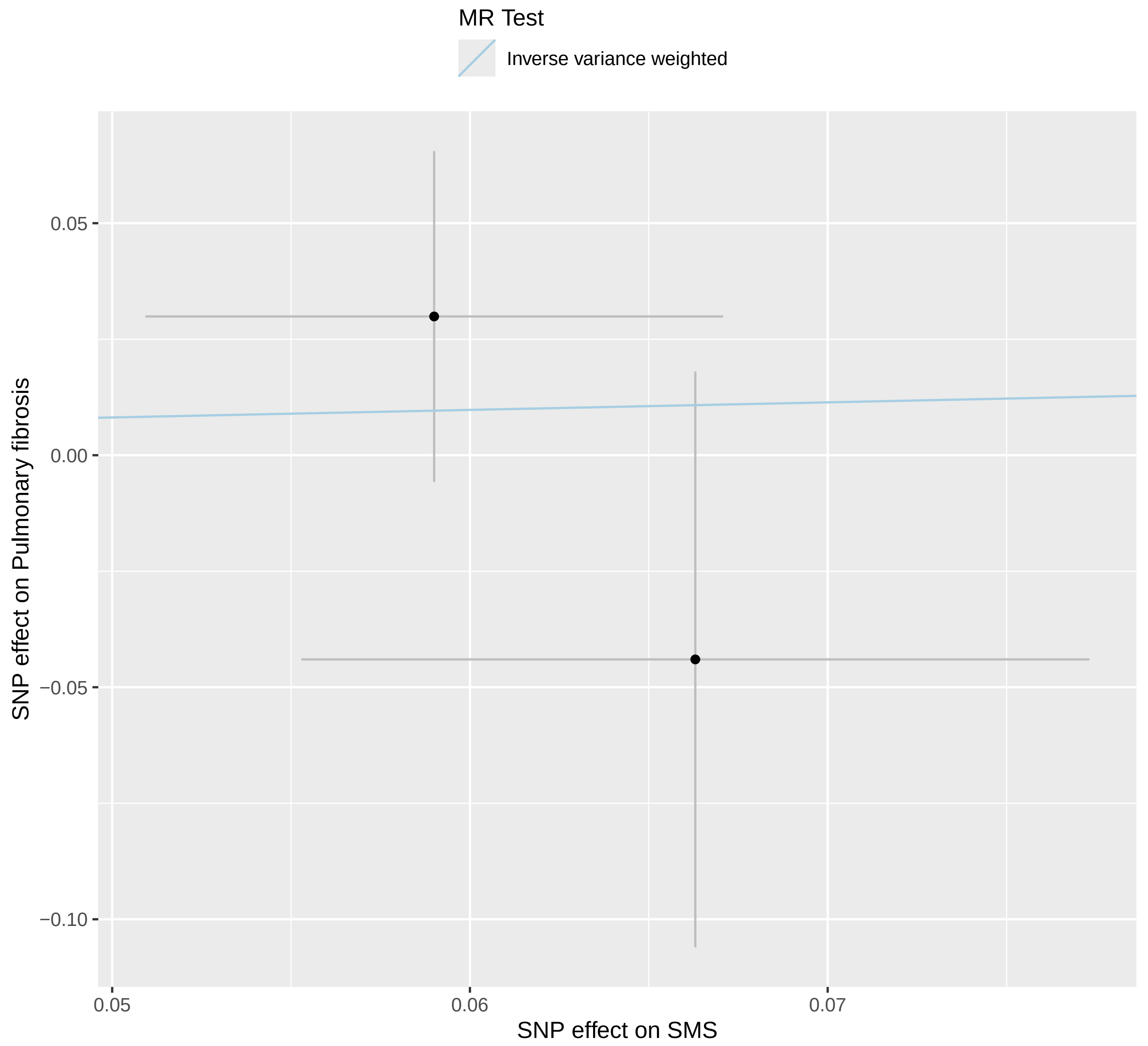
MR Test
Inverse variance weighted
0.05
0.00
−0.05
−0.10
SNP effect on Pulmonary fibrosis
0.05
0.06
0.07
SNP effect on SMS

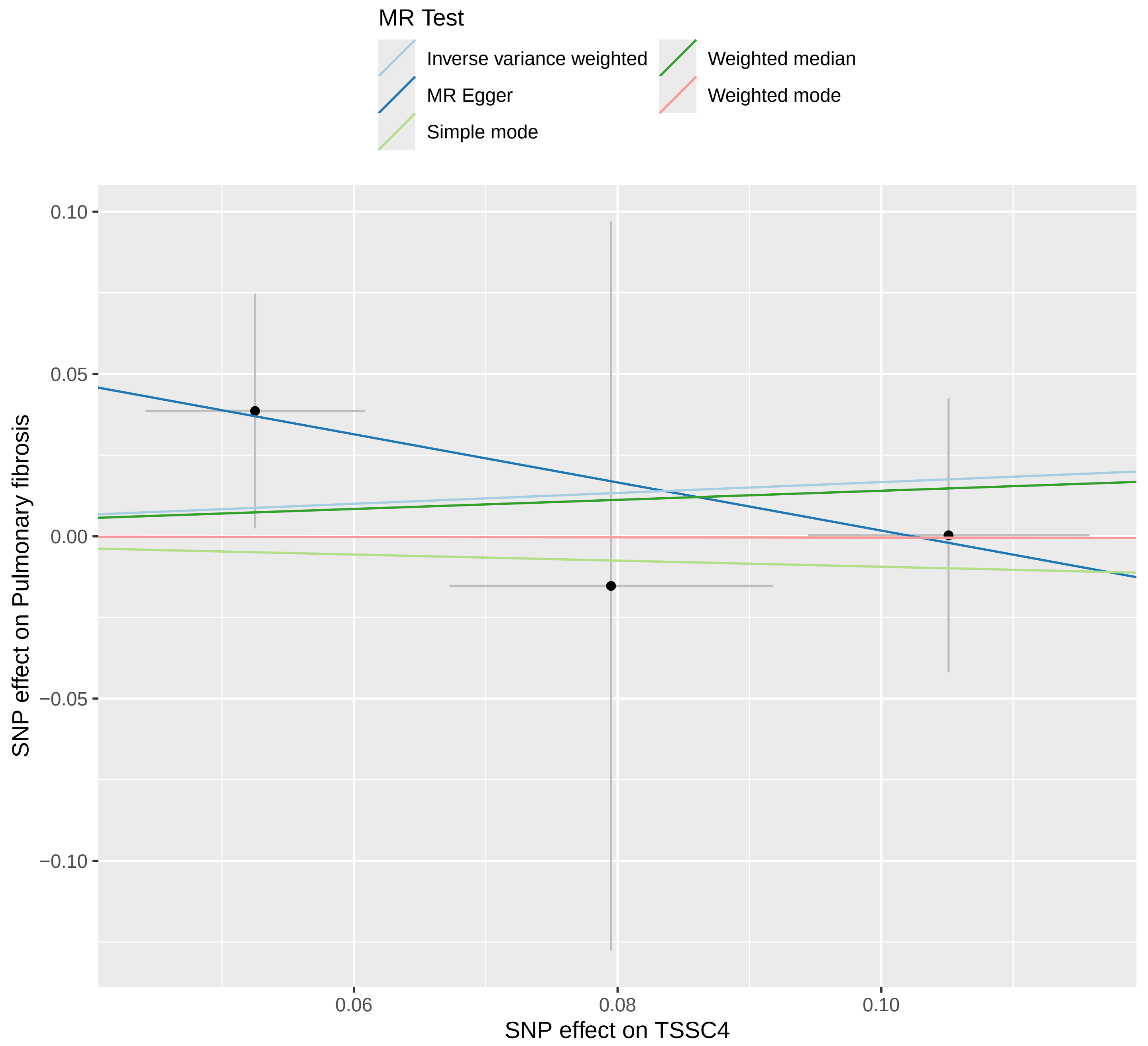
MR Test
Inverse variance weighted
MR Egger
Simple mode
Weighted median
Weighted mode
0.10
0.05
0.00
−0.05
−0.10
SNP effect on Pulmonary fibrosis
0.06
0.08
0.10
SNP effect on TSSC4

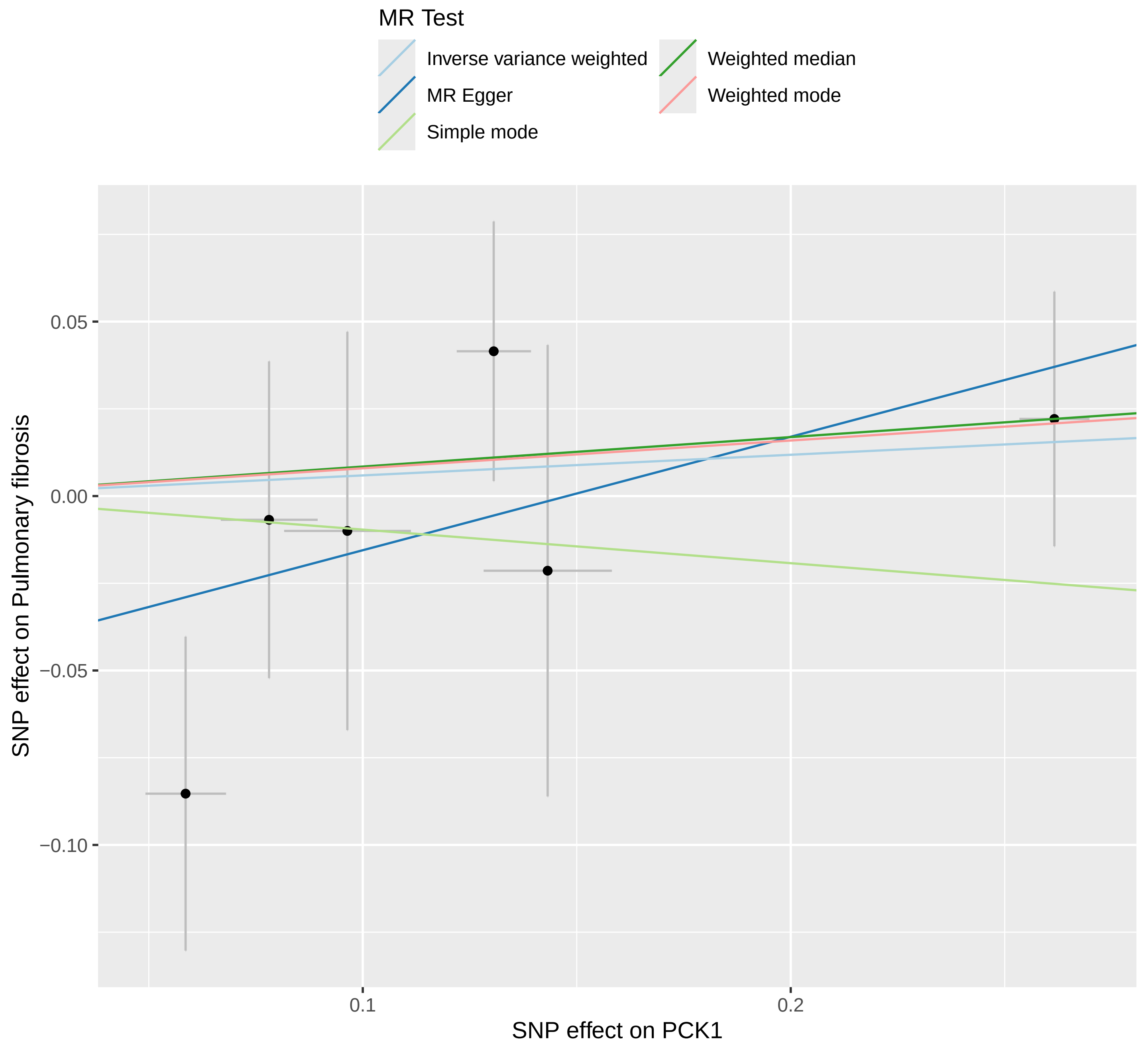

MR Test
Inverse variance weighted
MR Egger
Simple mode
Weighted median
Weighted mode
0.05
0.00
−0.05
−0.10
0.1
0.2
SNP effect on Pulmonary fibrosis
SNP effect on PCK1

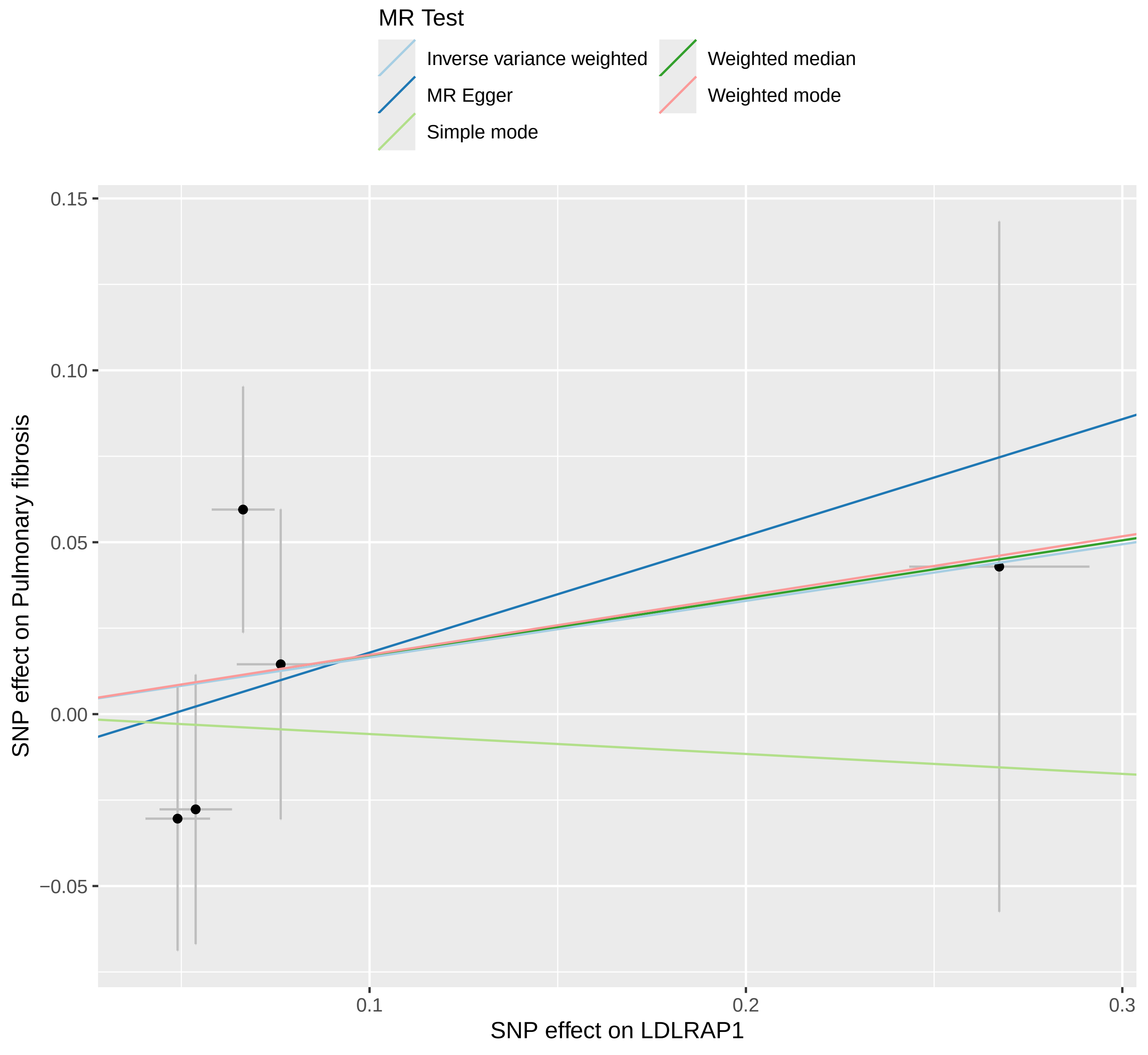

MR Test
Inverse variance weighted
MR Egger
Simple mode
Weighted median
Weighted mode
0.15
0.10
0.05
0.00
−0.05
0.1
0.2
0.3
SNP effect on Pulmonary fibrosis
SNP effect on LDLRAP1

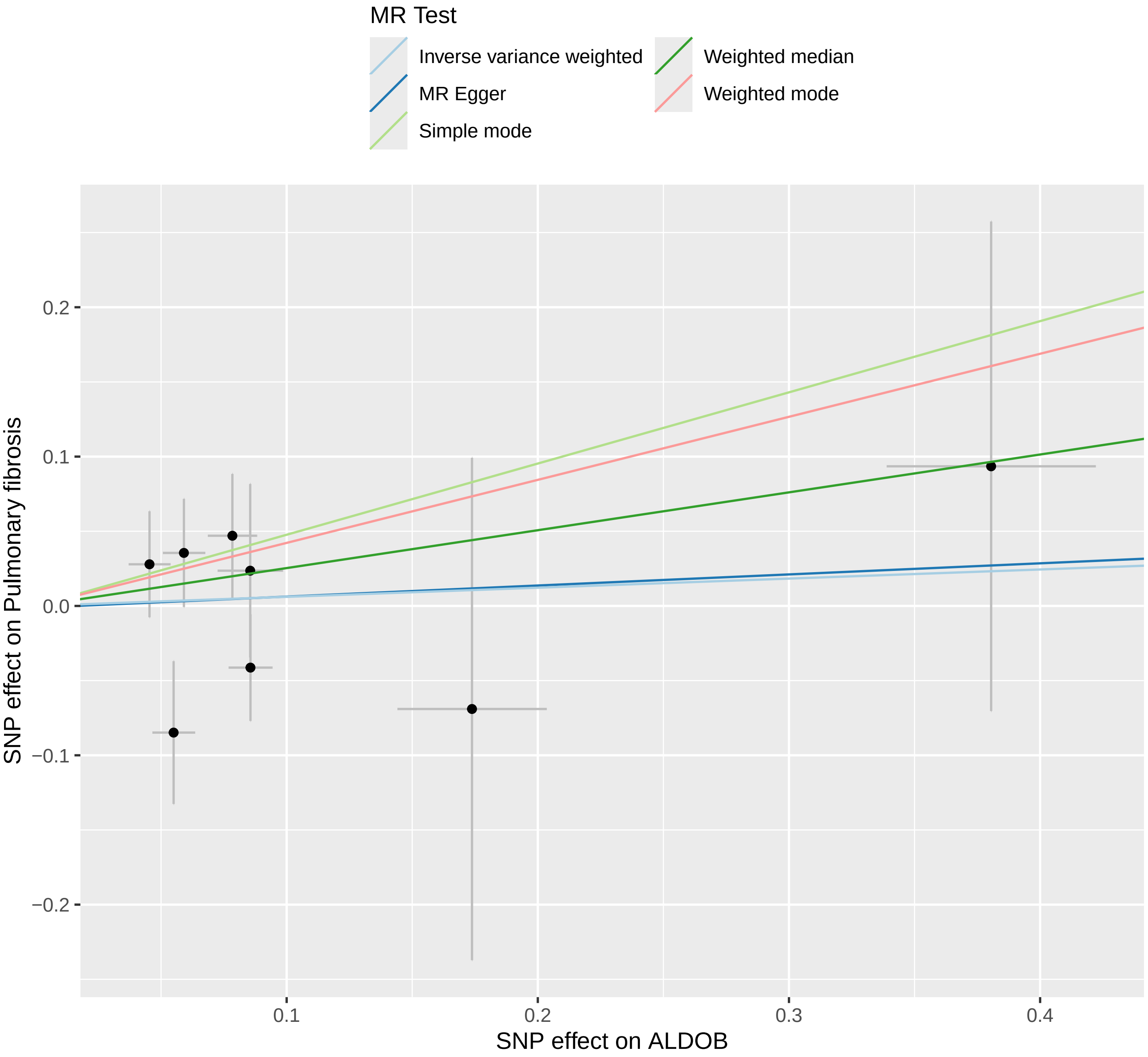
MR Test
Inverse variance weighted
MR Egger
Simple mode
Weighted median
Weighted mode
0.2
0.1
0.0
−0.1
−0.2
SNP effect on Pulmonary fibrosis
0.1
0.2
0.3
0.4
SNP effect on ALDOB

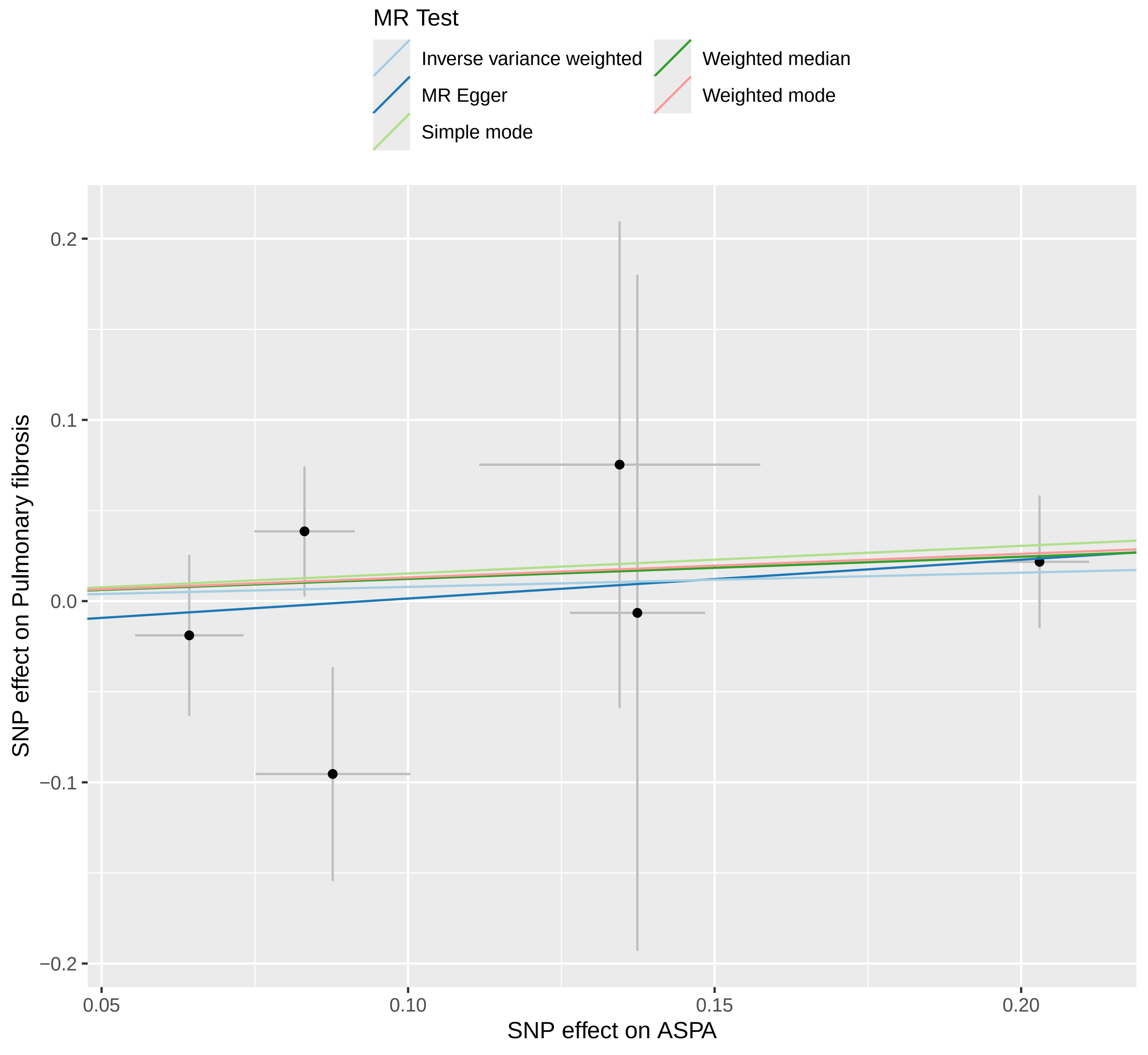
MR Test
Inverse variance weighted
MR Egger
Simple mode
Weighted median
Weighted mode
SNP effect on Pulmonary fibrosis
0.2
0.1
0.0
−0.1
−0.2
0.05
0.10
0.15
0.20
SNP effect on ASPA

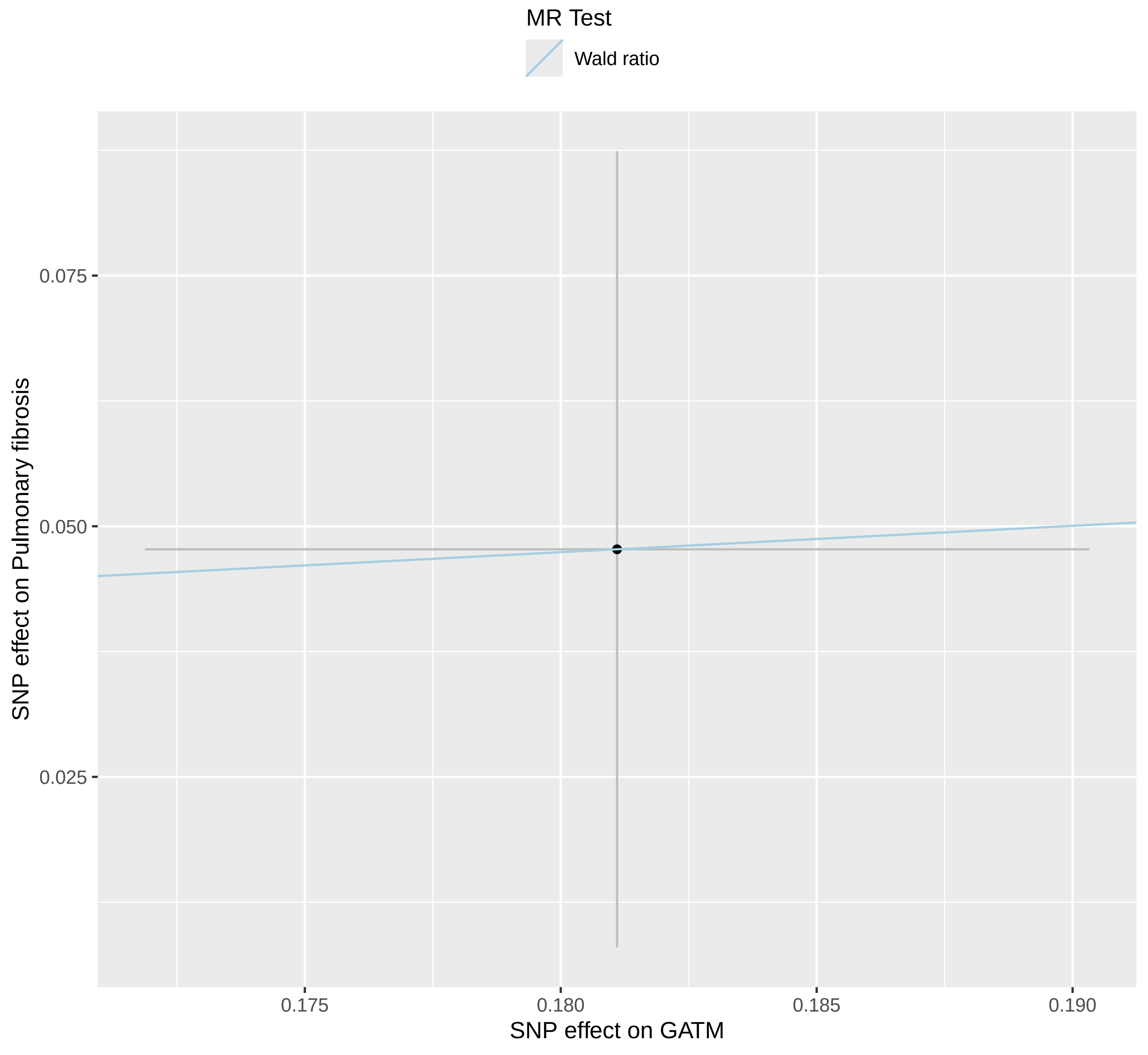
MR Test
Wald ratio
0.075
0.050
0.025
SNP effect on Pulmonary fibrosis
0.175
0.180
0.185
0.190
SNP effect on GATM

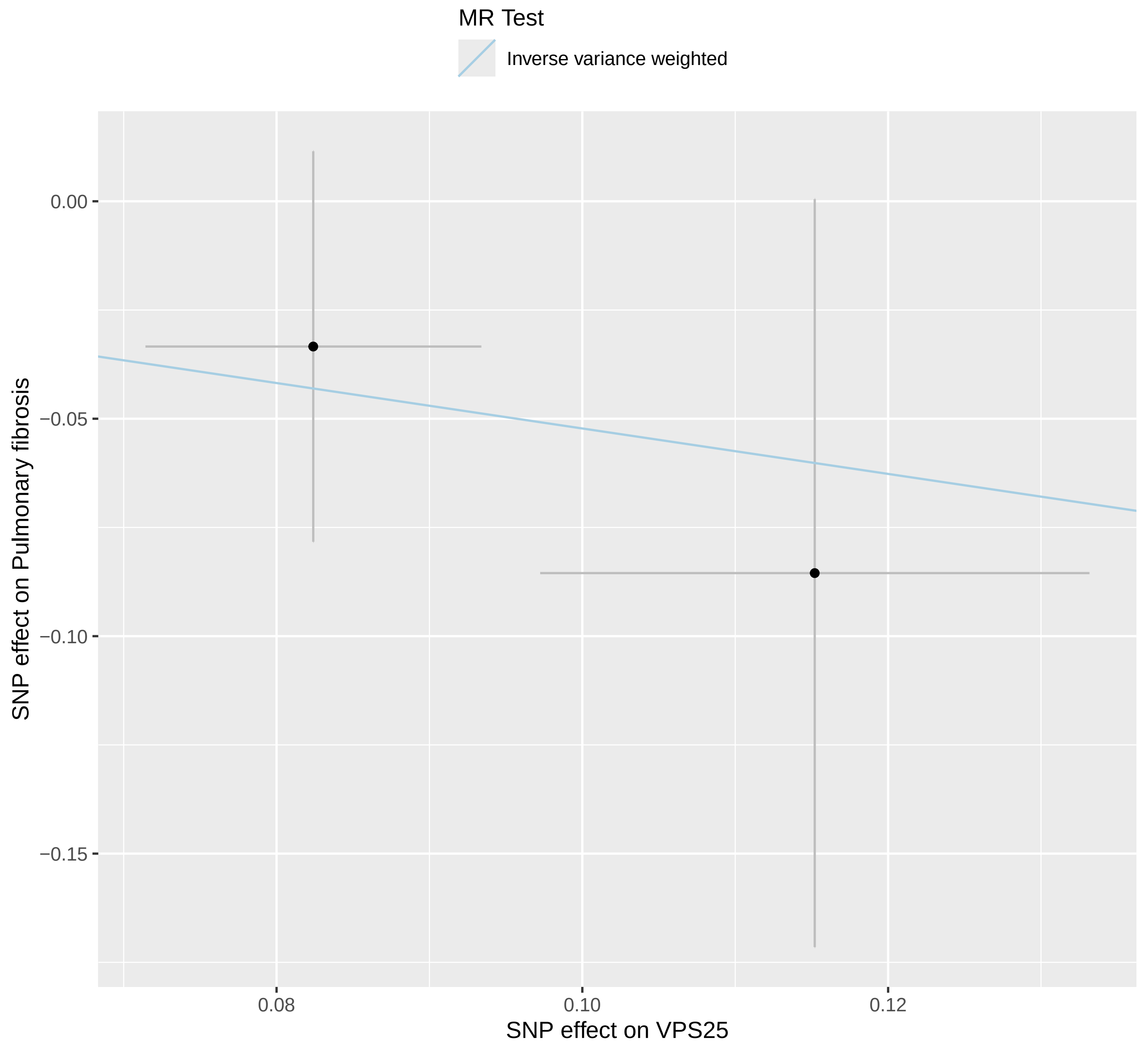

MR Test
Inverse variance weighted
SNP effect on Pulmonary fibrosis
0.00
−0.05
−0.10
−0.15
0.08
0.10
0.12
SNP effect on VPS25

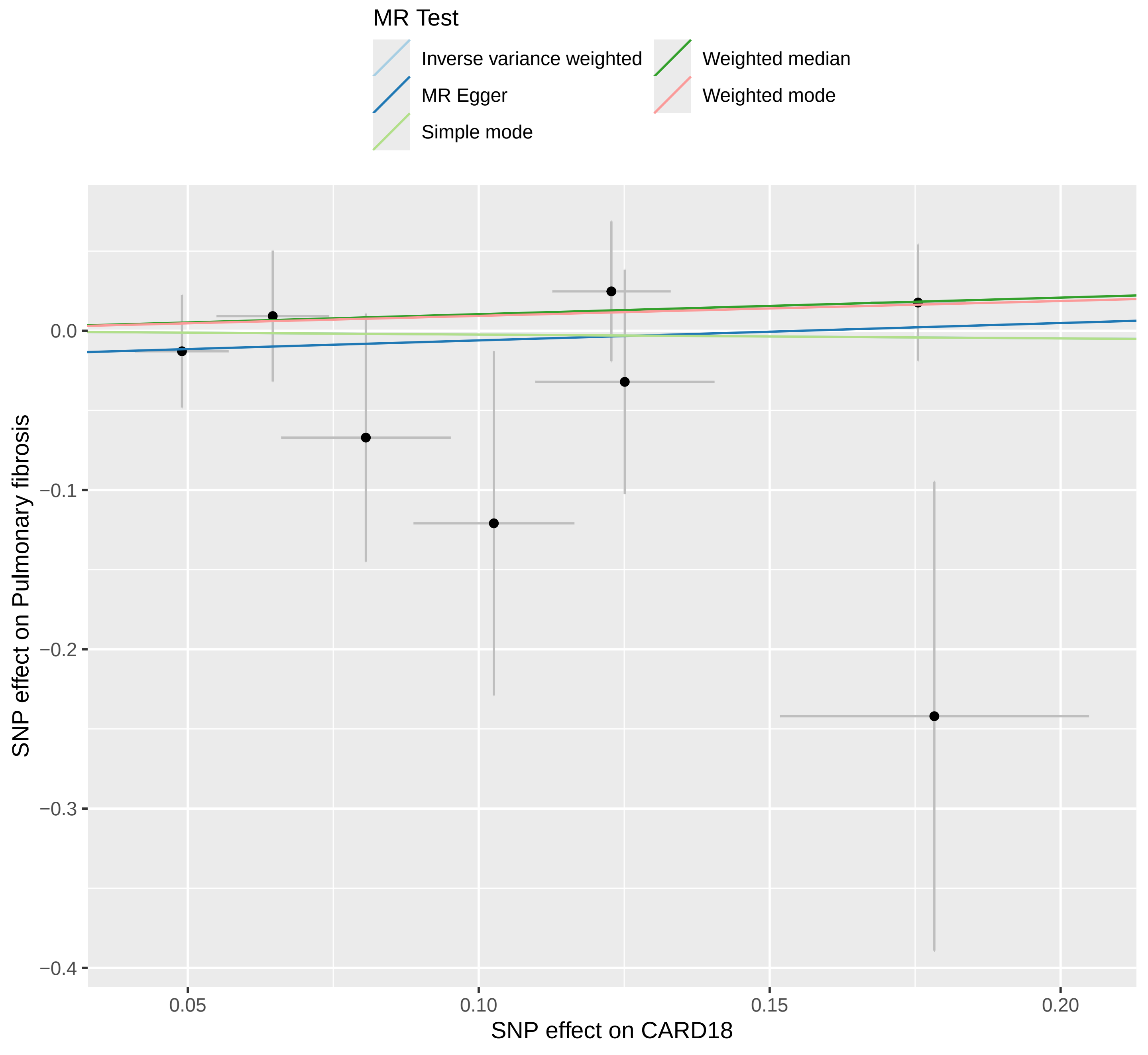

MR Test
Inverse variance weighted
MR Egger
Simple mode
Weighted median
Weighted mode
0.0
−0.1
−0.2
−0.3
−0.4
0.05
0.10
0.15
0.20
SNP effect on Pulmonary fibrosis
SNP effect on CARD18

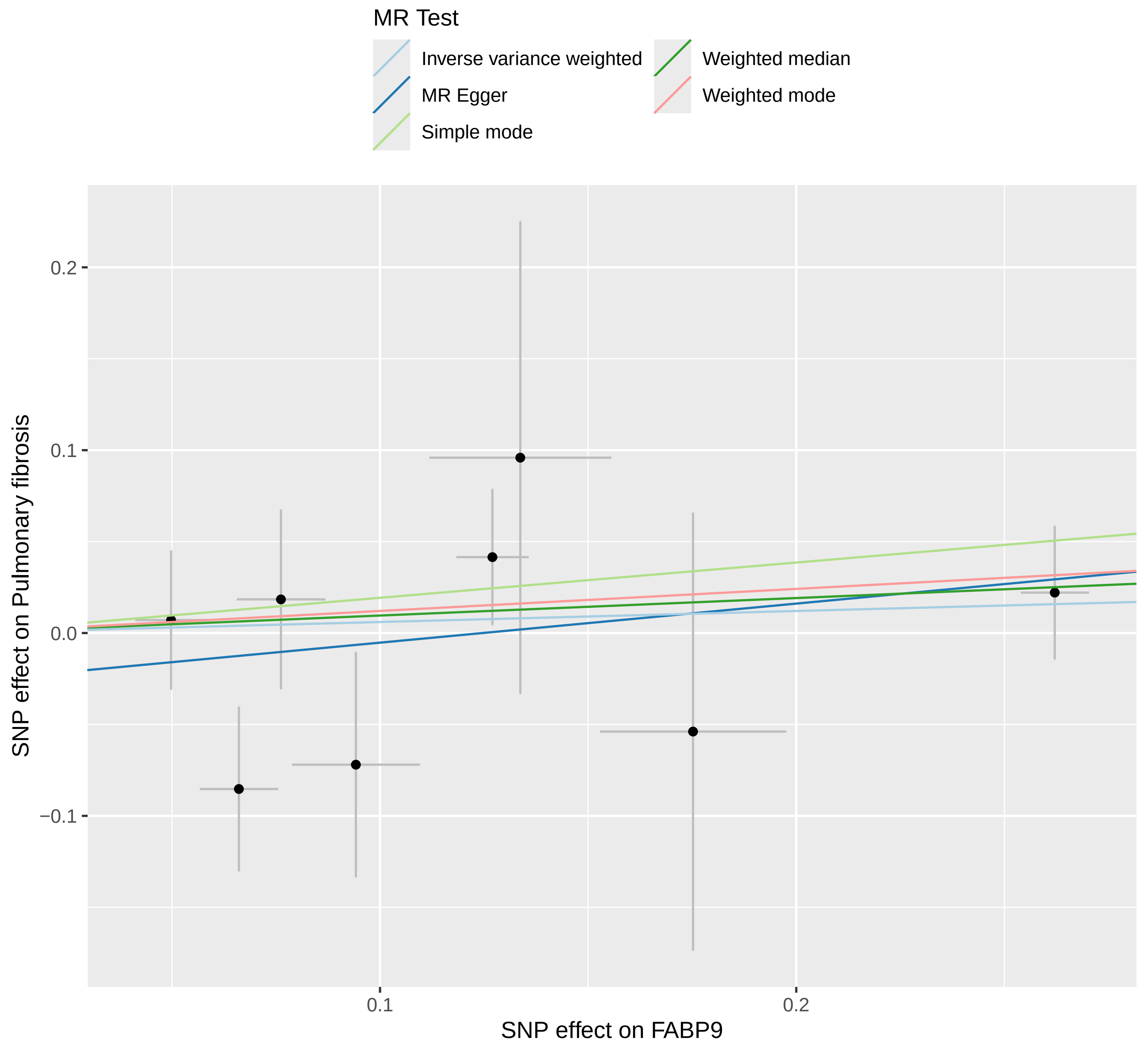
MR Test
Inverse variance weighted
MR Egger
Simple mode
Weighted median
Weighted mode
0.2
0.1
0.0
−0.1
SNP effect on Pulmonary fibrosis
0.1
0.2
SNP effect on FABP9

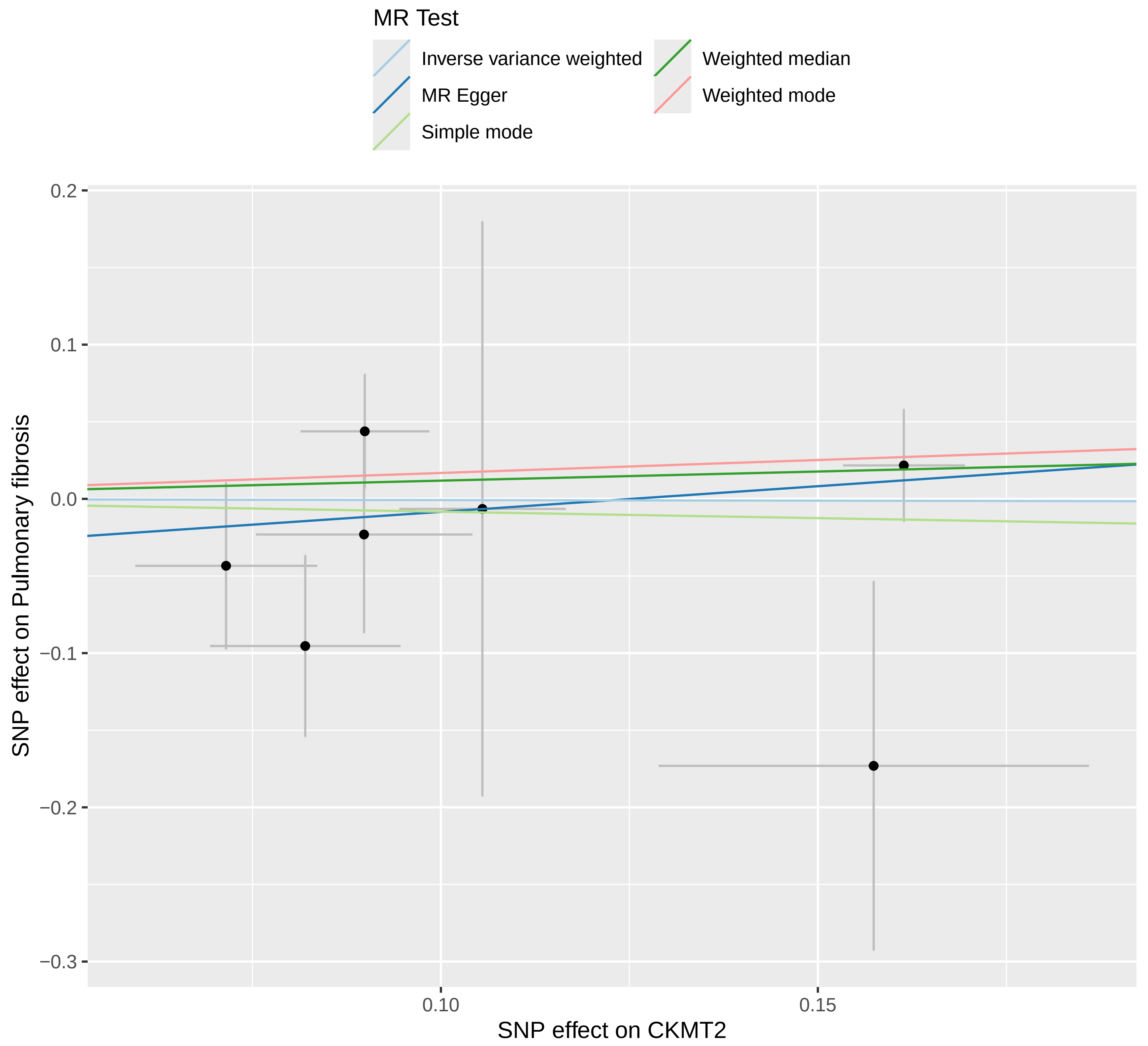

MR Test
Inverse variance weighted
MR Egger
Simple mode
Weighted median
Weighted mode
0.2
0.1
0.0
−0.1
−0.2
−0.3
SNP effect on Pulmonary fibrosis
0.10
0.15
SNP effect on CKMT2

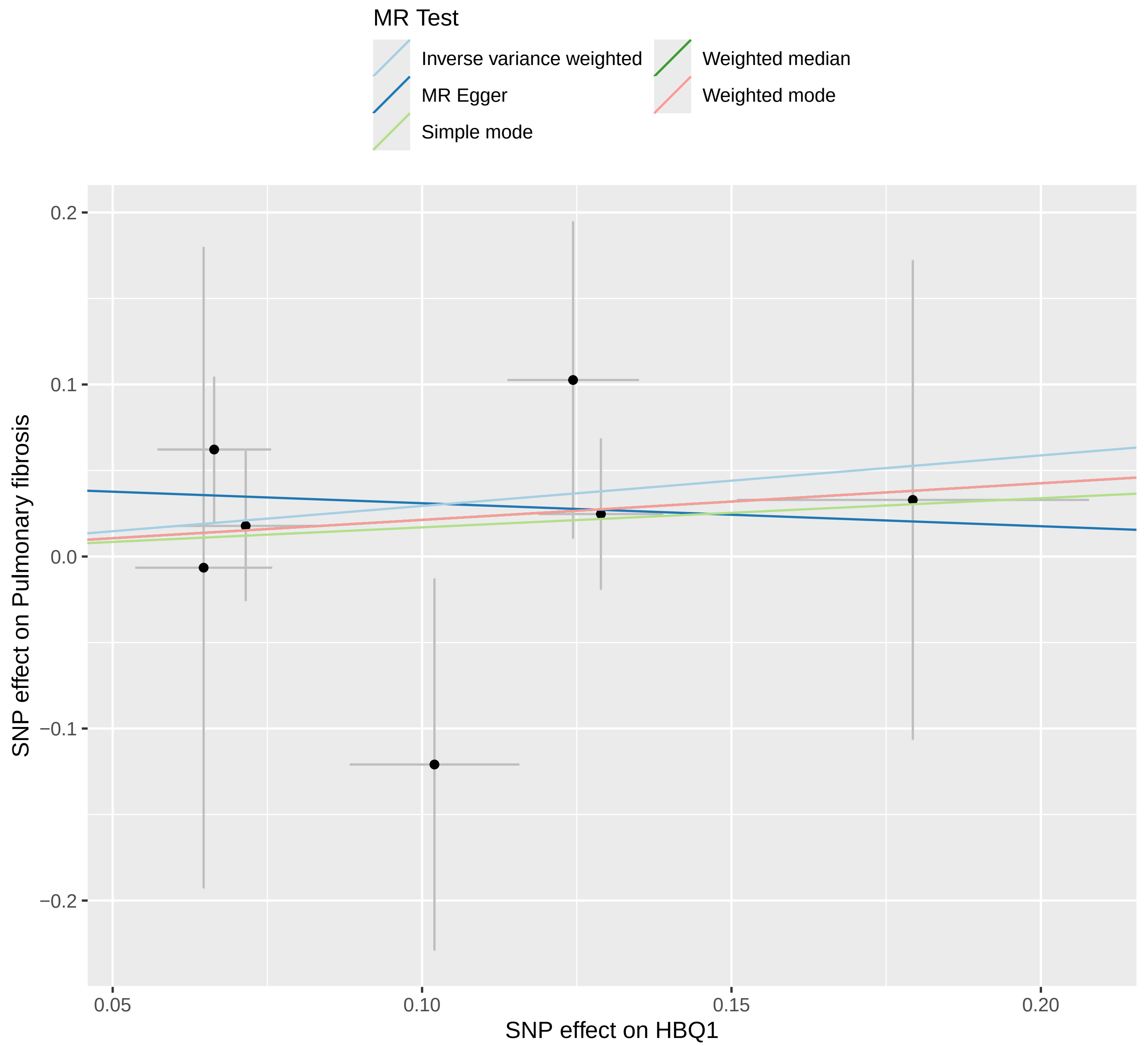
MR Test
Inverse variance weighted
MR Egger
Simple mode
Weighted median
Weighted mode
0.2
0.1
0.0
−0.1
−0.2
SNP effect on Pulmonary fibrosis
0.05
0.10
0.15
0.20
SNP effect on HBQ1

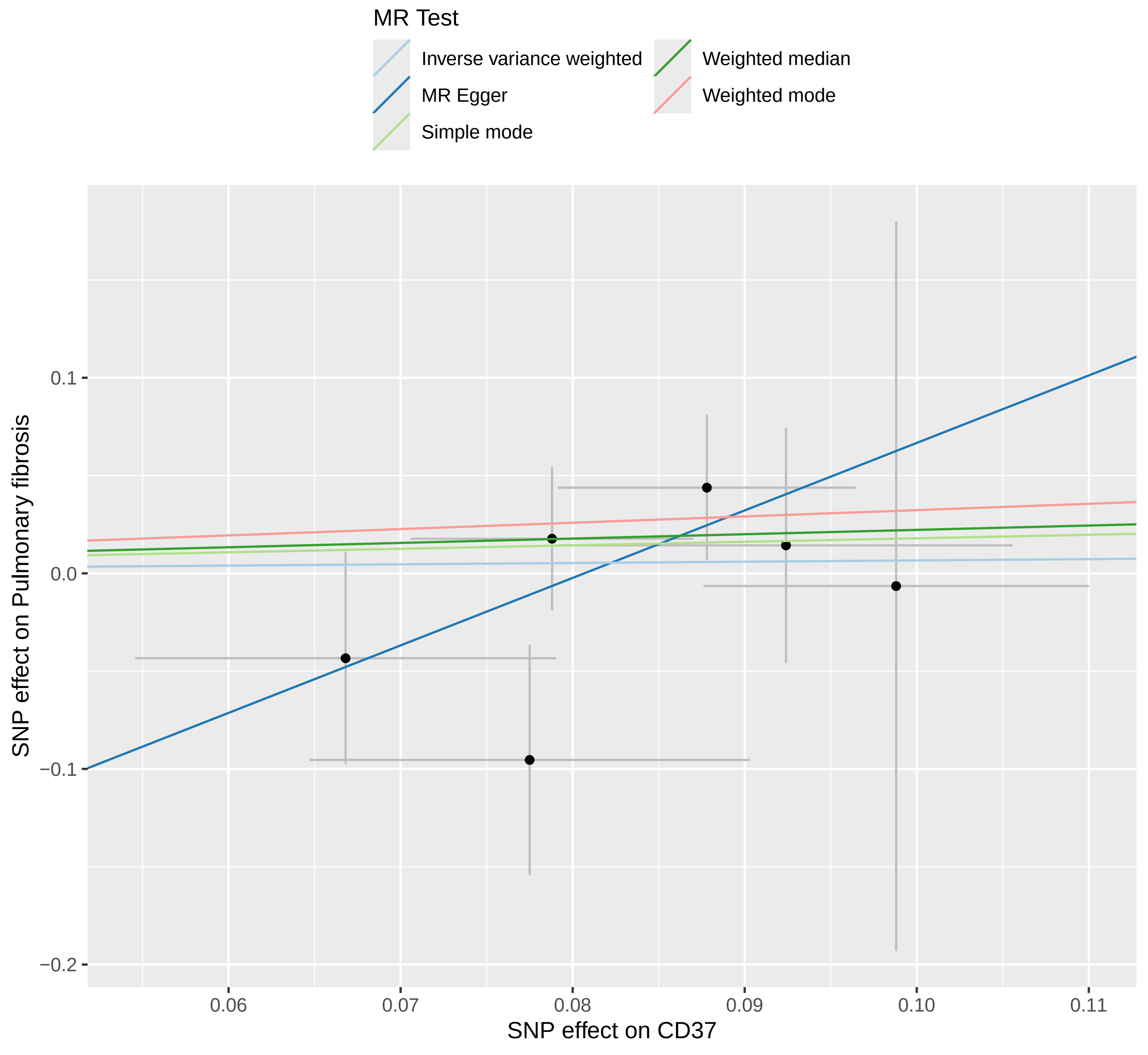

MR Test
Inverse variance weighted
MR Egger
Simple mode
Weighted median
Weighted mode
0.1
0.0
−0.1
−0.2
SNP effect on Pulmonary fibrosis
0.06
0.07
0.08
0.09
0.10
0.11
SNP effect on CD37

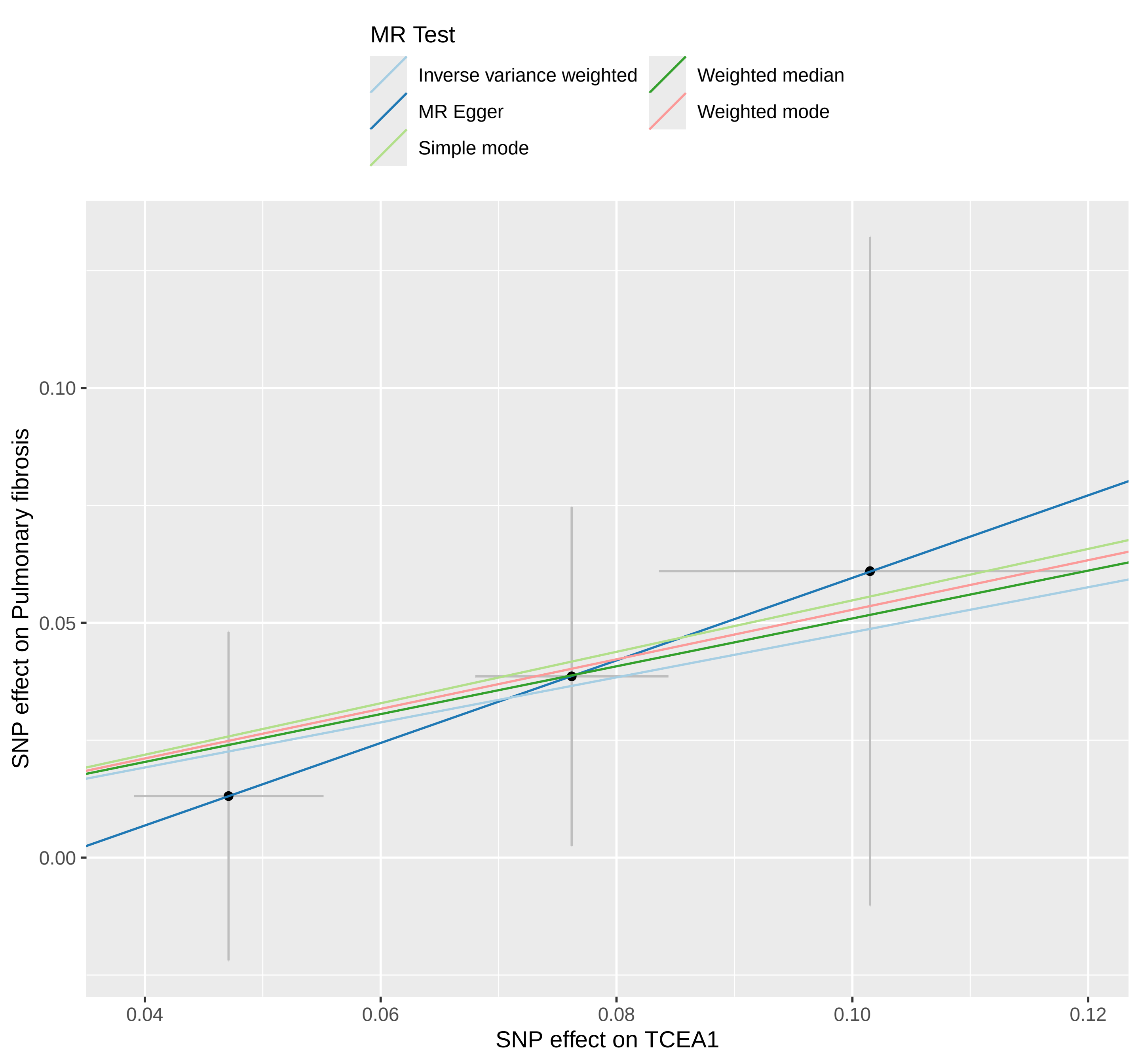

MR Test
Inverse variance weighted
MR Egger
Simple mode
Weighted median
Weighted mode
0.10
0.05
0.00
0.04
0.06
0.08
0.10
0.12
SNP effect on Pulmonary fibrosis
SNP effect on TCEA1

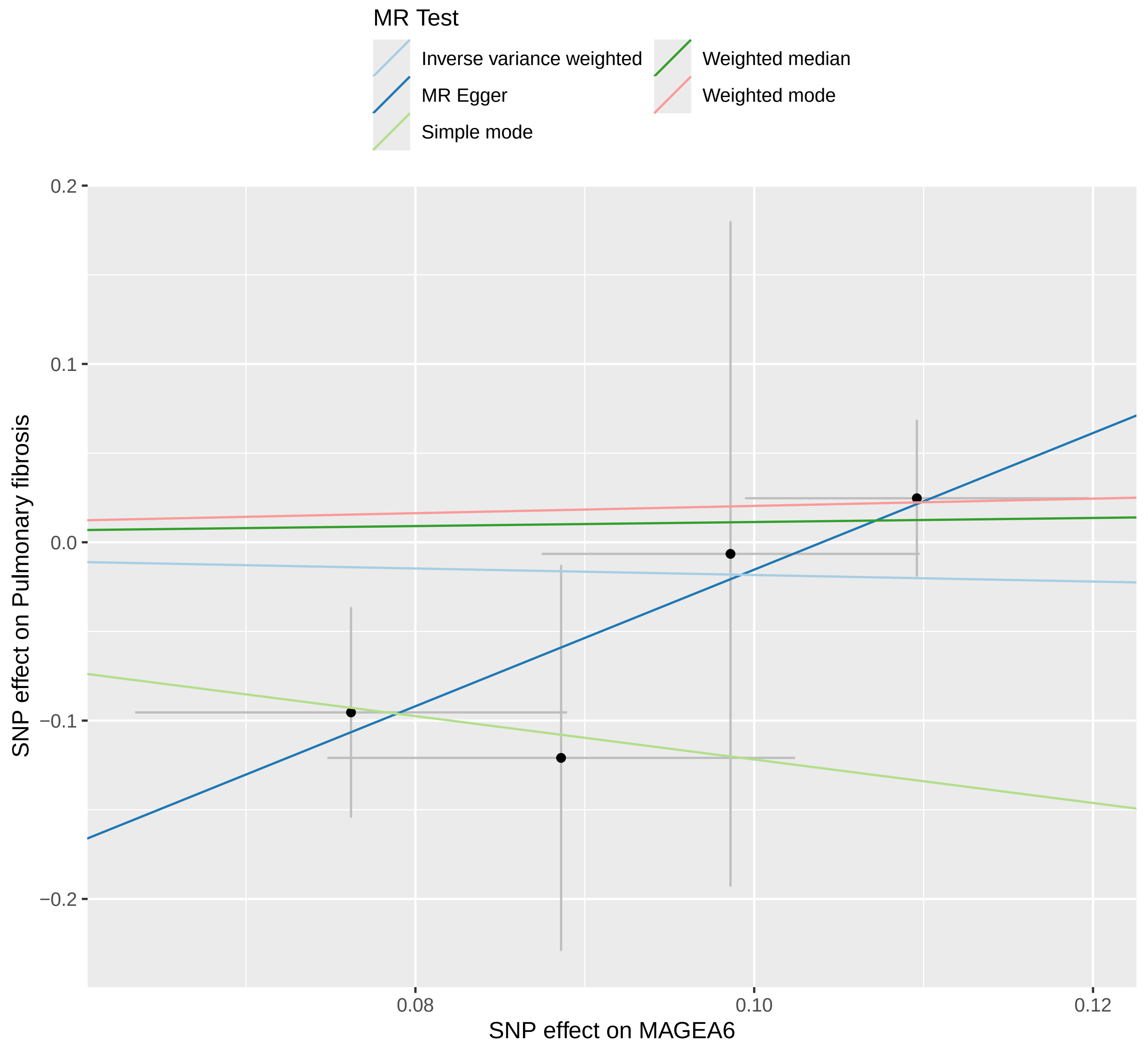
MR Test
Inverse variance weighted
MR Egger
Simple mode
Weighted median
Weighted mode
0.2
0.1
0.0
−0.1
−0.2
0.08
0.10
0.12
SNP effect on MAGEA6
SNP effect on Pulmonary fibrosis

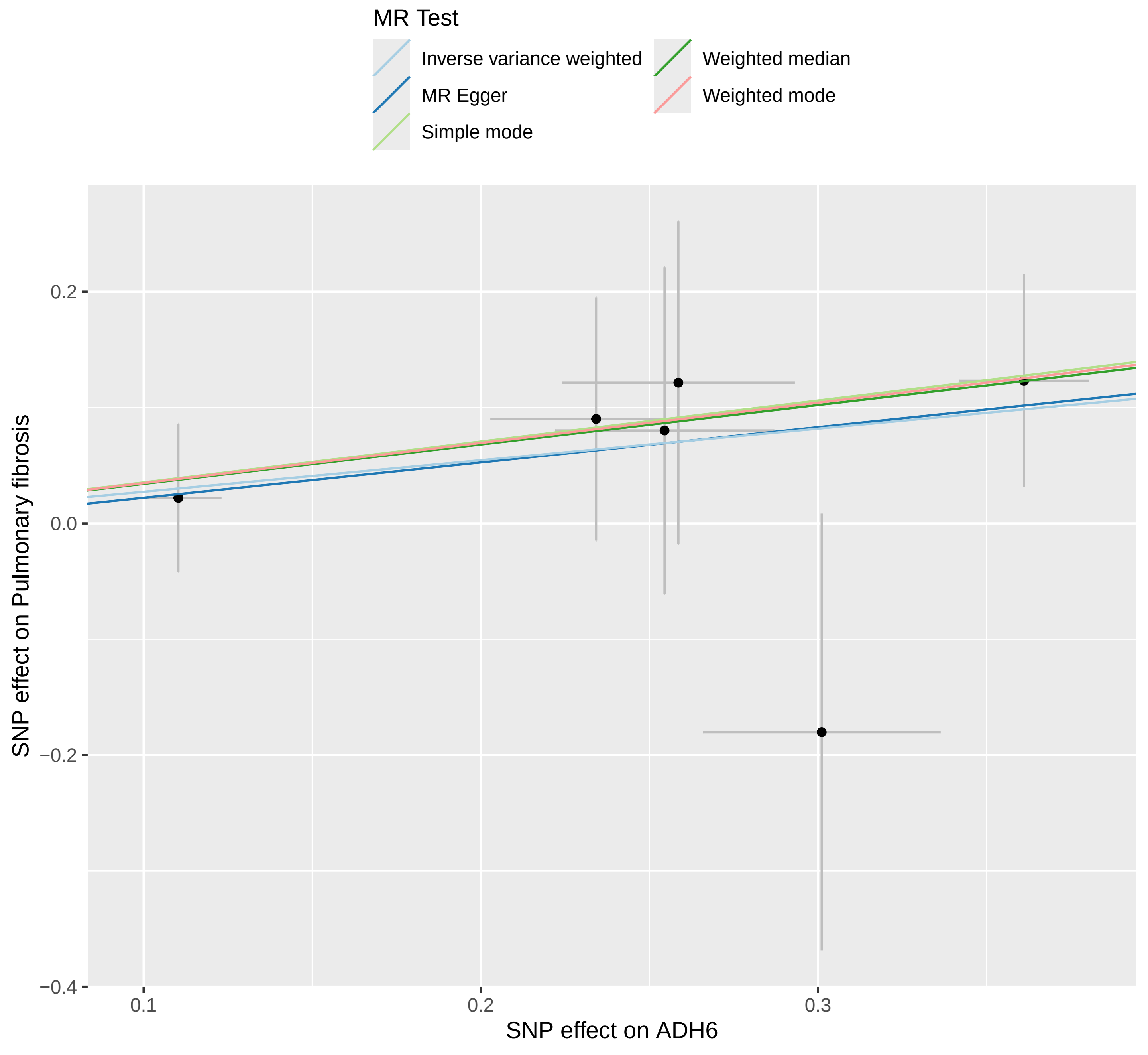
MR Test
Inverse variance weighted
MR Egger
Simple mode
Weighted median
Weighted mode
SNP effect on Pulmonary fibrosis
0.2
0.0
−0.2
−0.4
0.1
0.2
0.3
SNP effect on ADH6

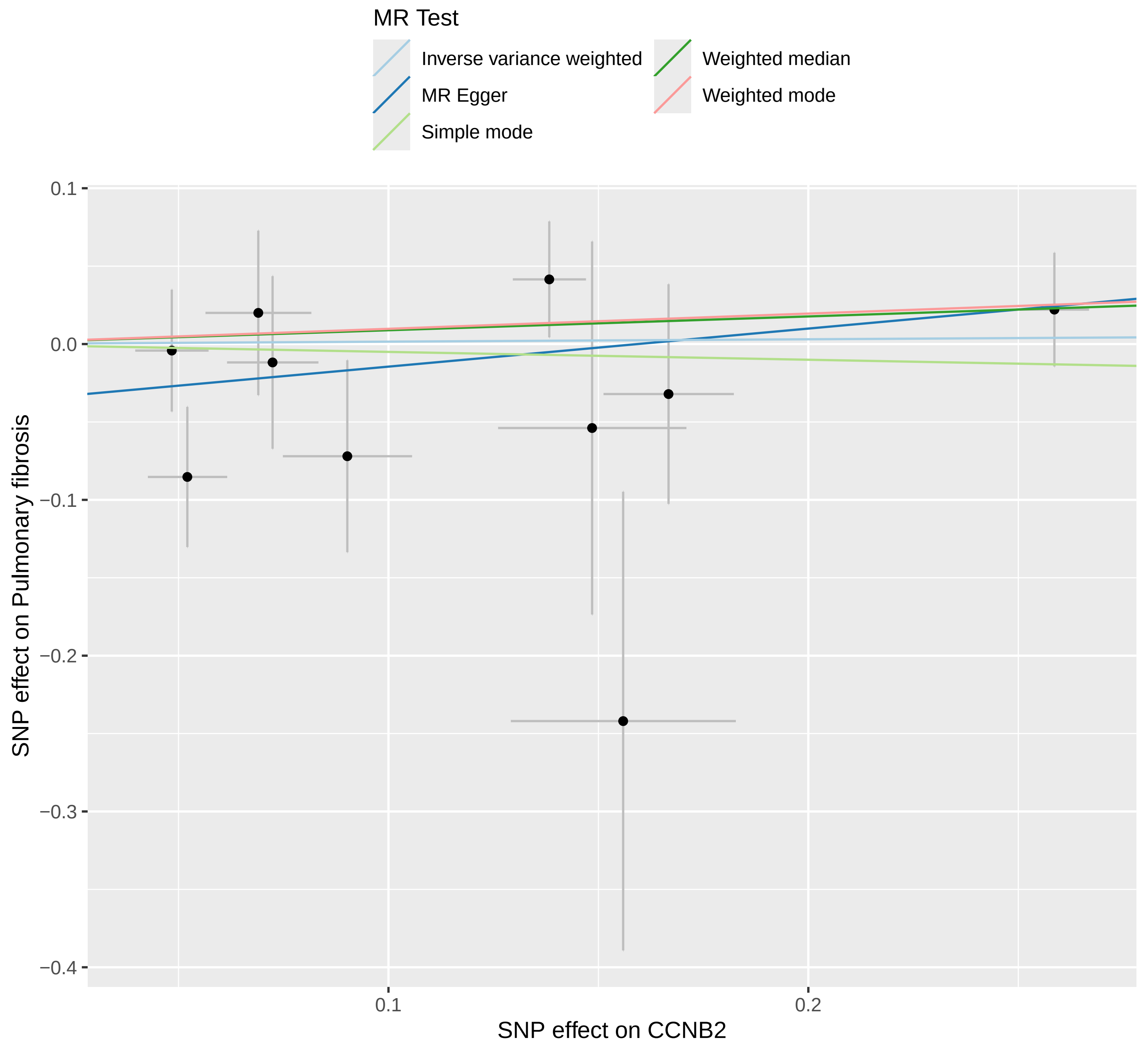

MR Test
Inverse variance weighted
MR Egger
Simple mode
Weighted median
Weighted mode
0.1
0.0
−0.1
−0.2
−0.3
−0.4
SNP effect on Pulmonary fibrosis
0.1
0.2
SNP effect on CCNB2

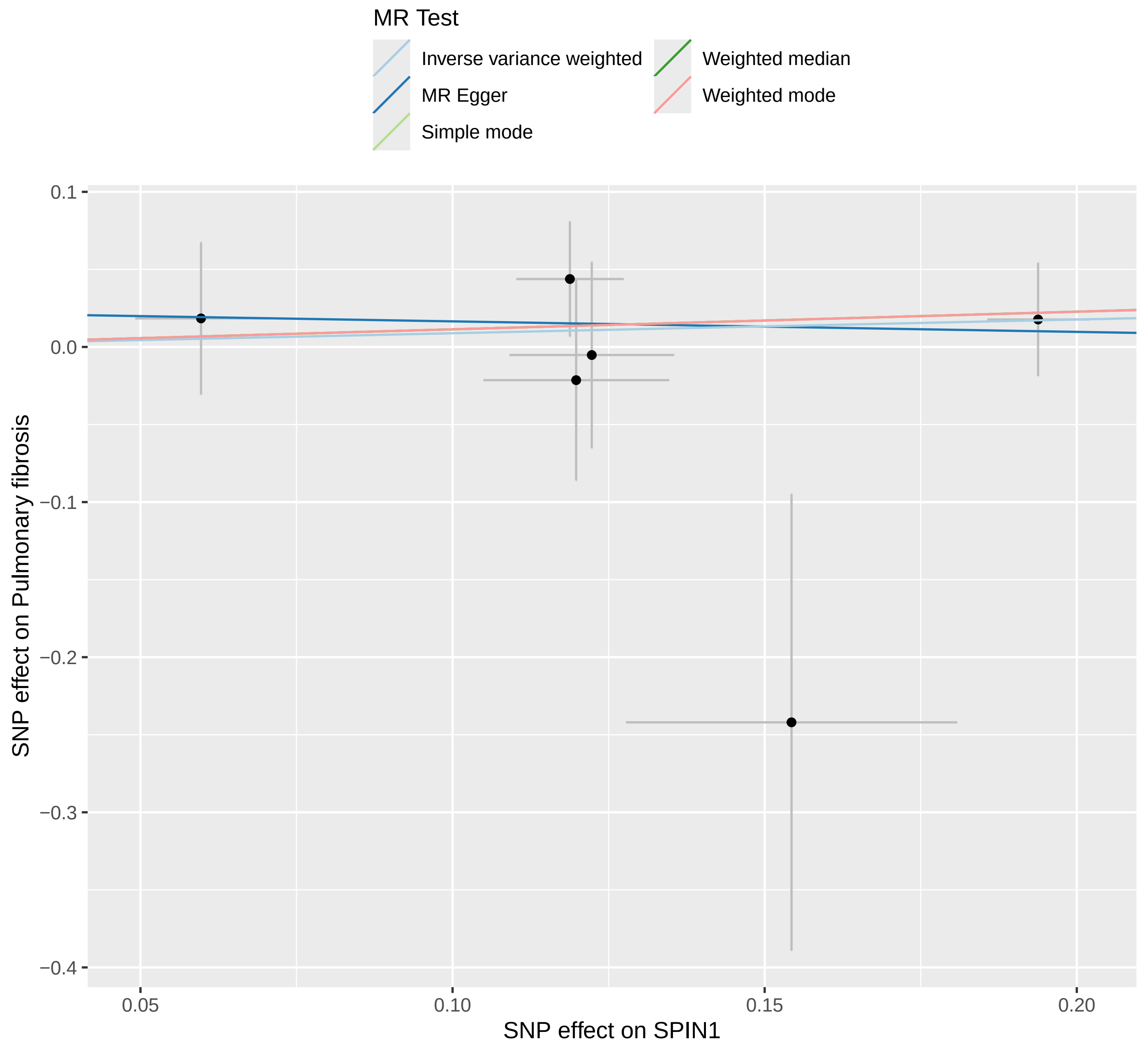
MR Test
Inverse variance weighted
MR Egger
Simple mode
Weighted median
Weighted mode
0.1
0.0
−0.1
−0.2
−0.3
−0.4
0.05
0.10
0.15
0.20
SNP effect on SPIN1
SNP effect on Pulmonary fibrosis

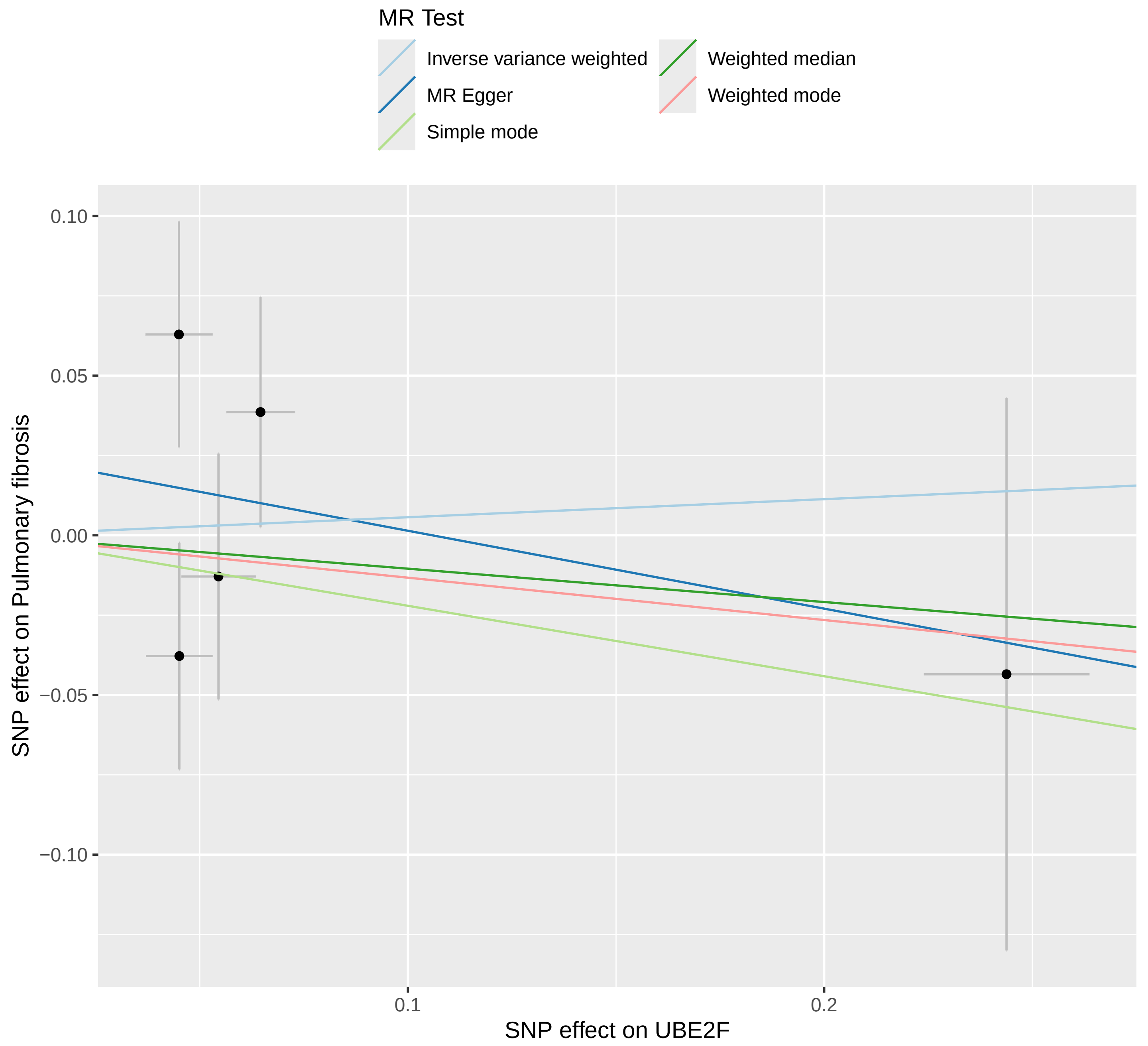

MR Test
Inverse variance weighted
MR Egger
Simple mode
Weighted median
Weighted mode
0.10
0.05
0.00
−0.05
−0.10
SNP effect on Pulmonary fibrosis
0.1
0.2
SNP effect on UBE2F

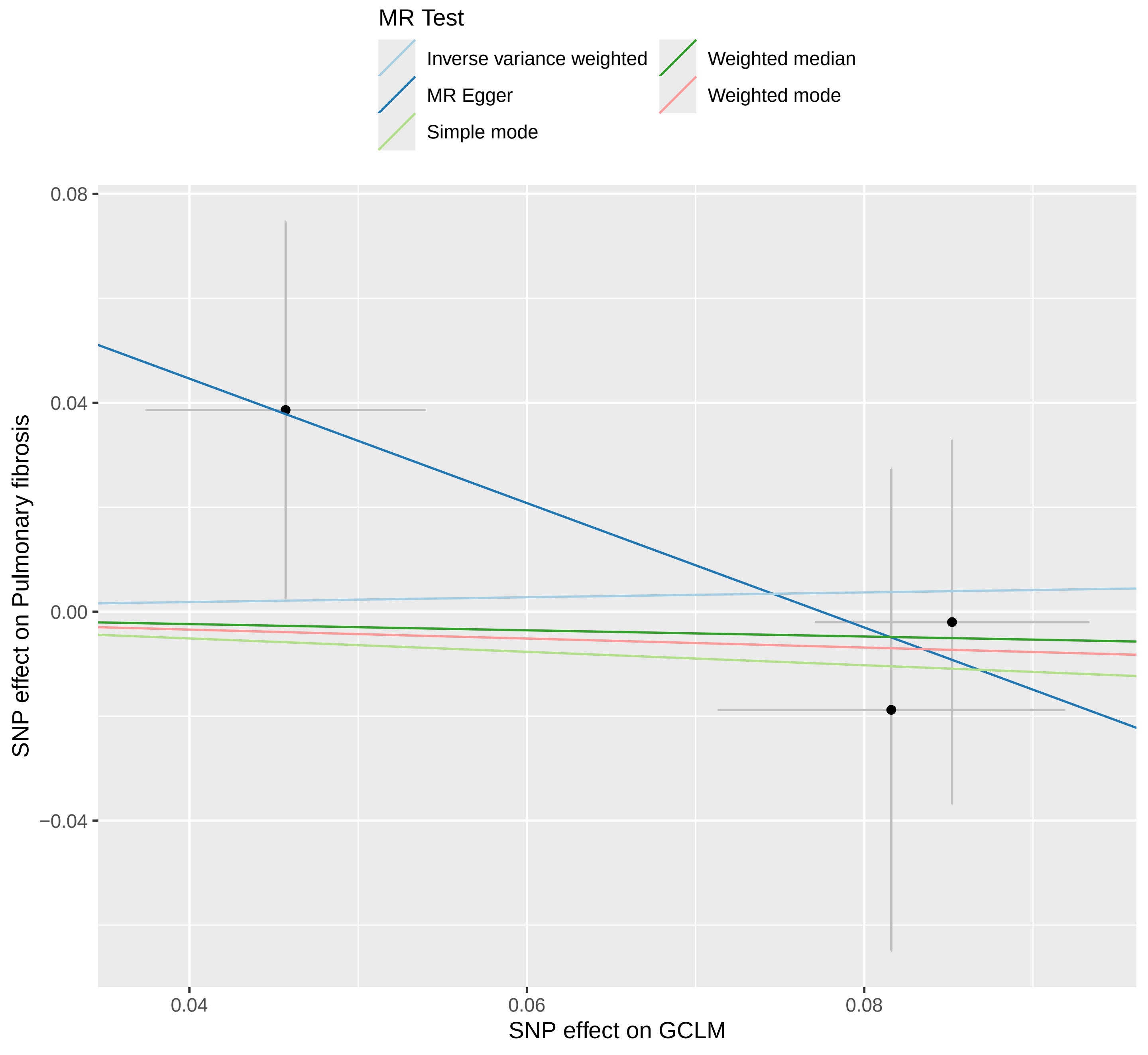
MR Test
Inverse variance weighted
MR Egger
Simple mode
Weighted median
Weighted mode
0.08
0.04
0.00
−0.04
SNP effect on Pulmonary fibrosis
0.04
0.06
0.08
SNP effect on GCLM

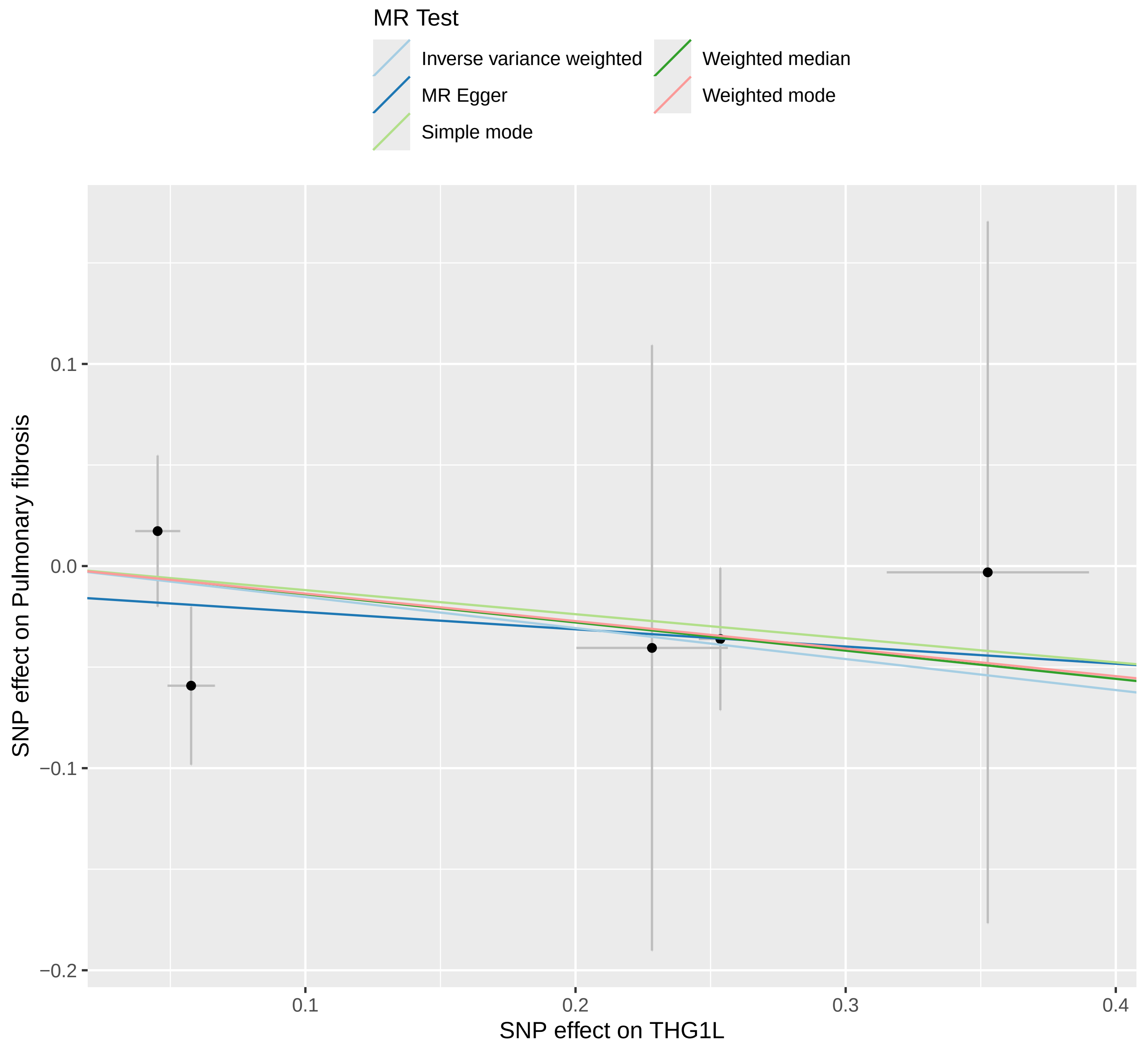

MR Test
Inverse variance weighted
MR Egger
Simple mode
Weighted median
Weighted mode
0.1
0.0
−0.1
−0.2
0.1
0.2
0.3
0.4
SNP effect on Pulmonary fibrosis
SNP effect on THG1L

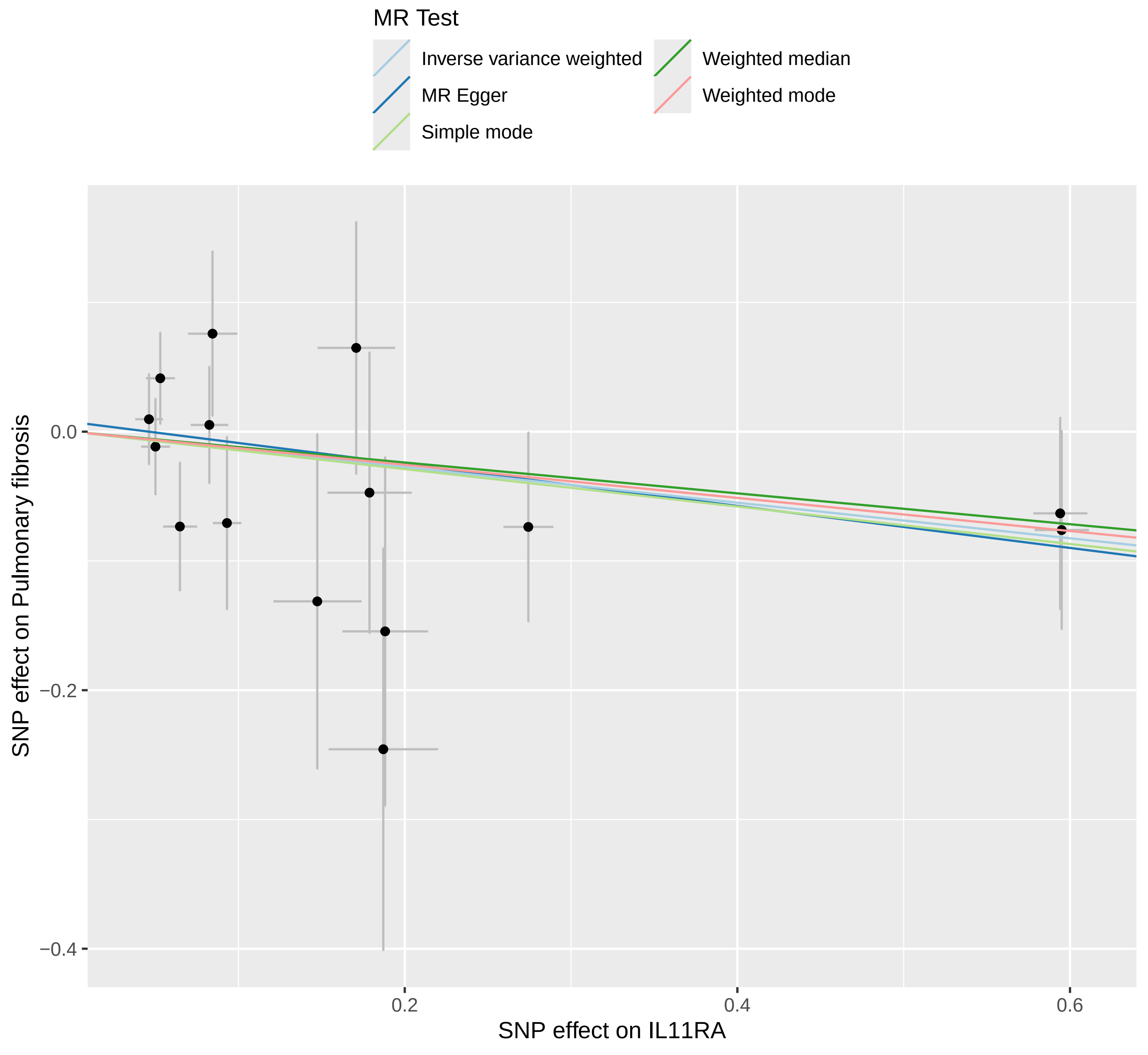
MR Test
Inverse variance weighted
MR Egger
Simple mode
Weighted median
Weighted mode
0.0
−0.2
−0.4
0.2
0.4
0.6
SNP effect on Pulmonary fibrosis
SNP effect on IL11RA

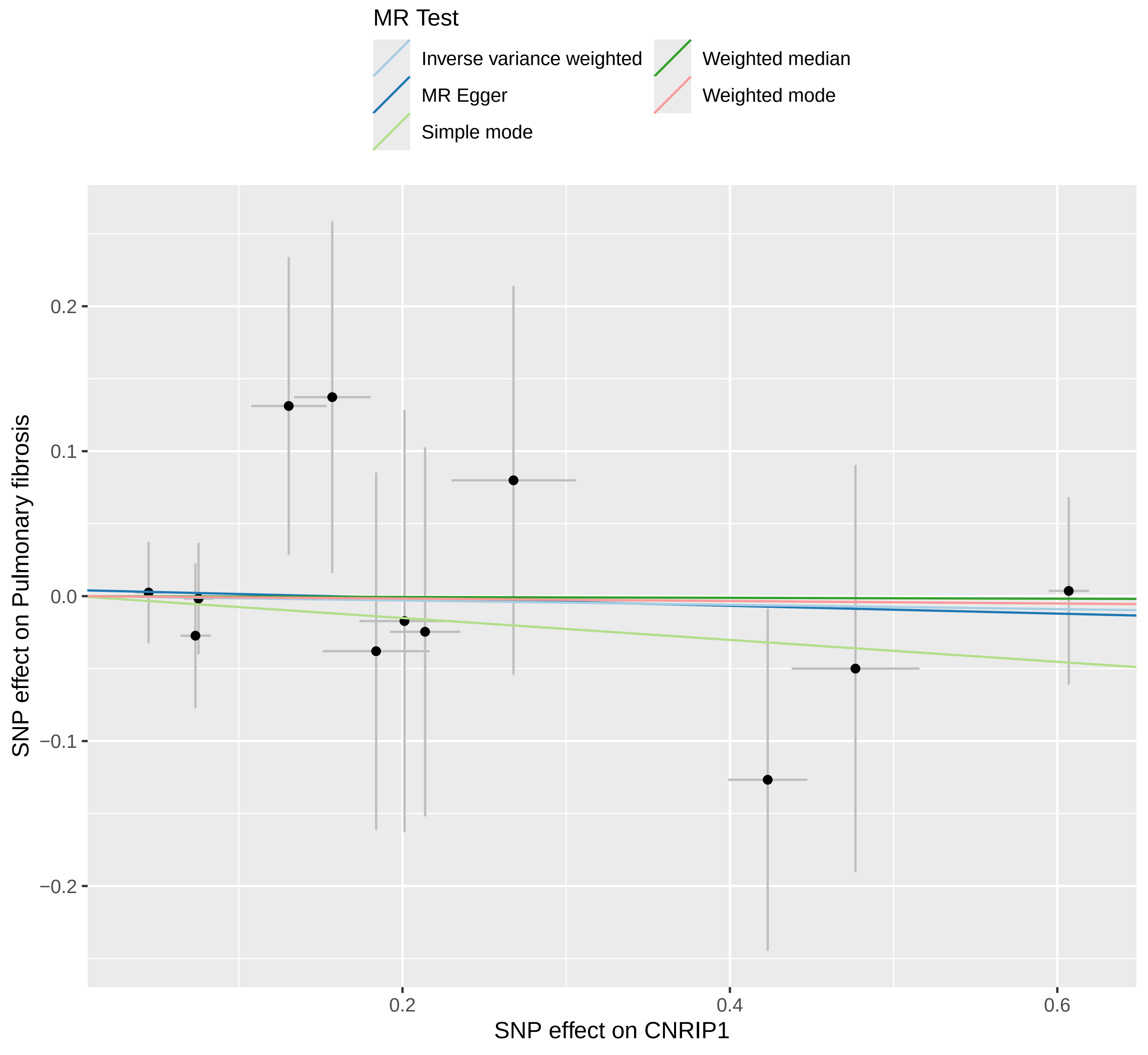

MR Test
Inverse variance weighted
MR Egger
Simple mode
Weighted median
Weighted mode
0.2
0.1
0.0
−0.1
−0.2
SNP effect on Pulmonary fibrosis
0.2
0.4
0.6
SNP effect on CNRIP1

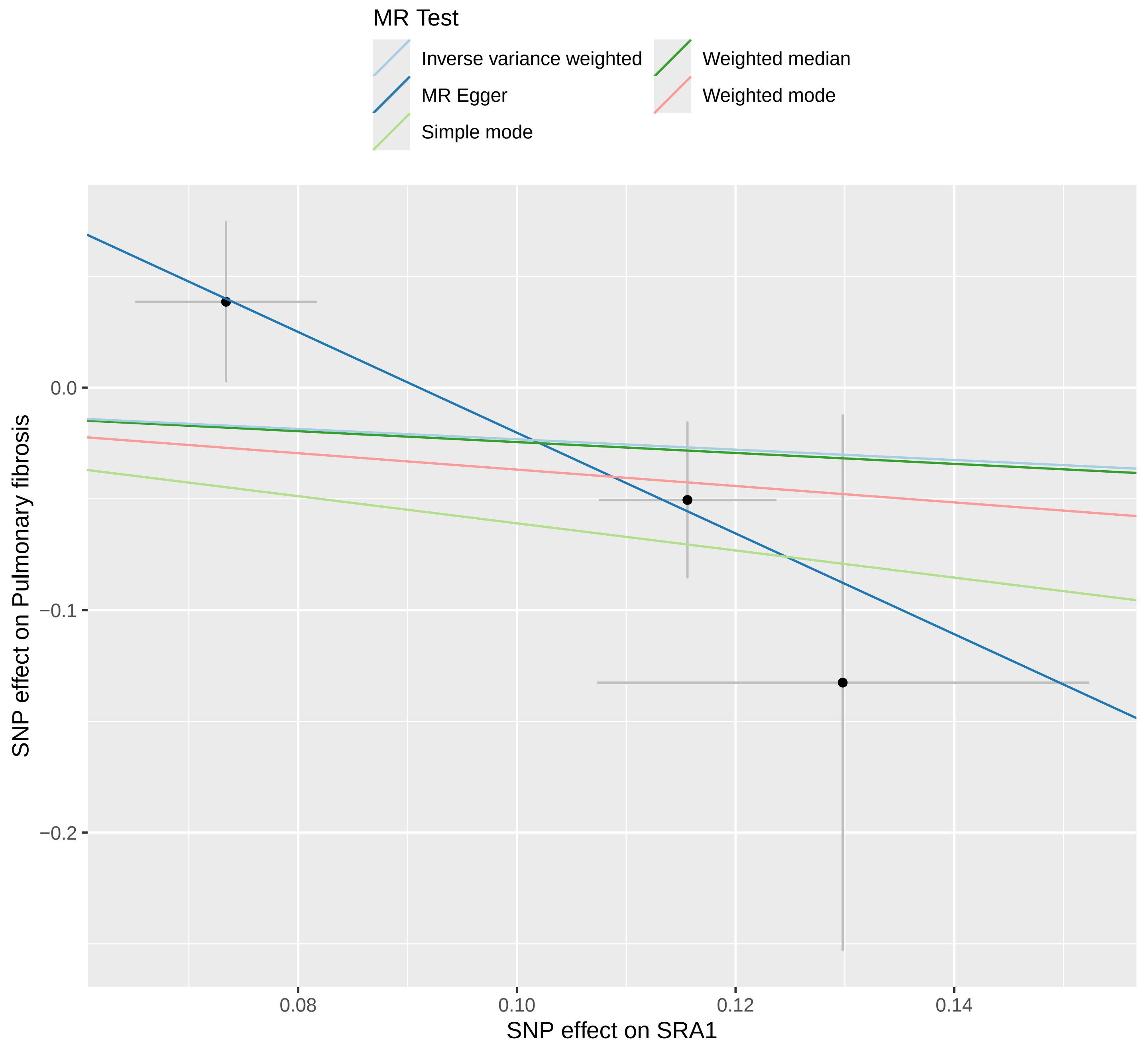

MR Test
Inverse variance weighted
MR Egger
Simple mode
Weighted median
Weighted mode
0.0
−0.1
−0.2
SNP effect on Pulmonary fibrosis
0.08
0.10
0.12
0.14
SNP effect on SRA1

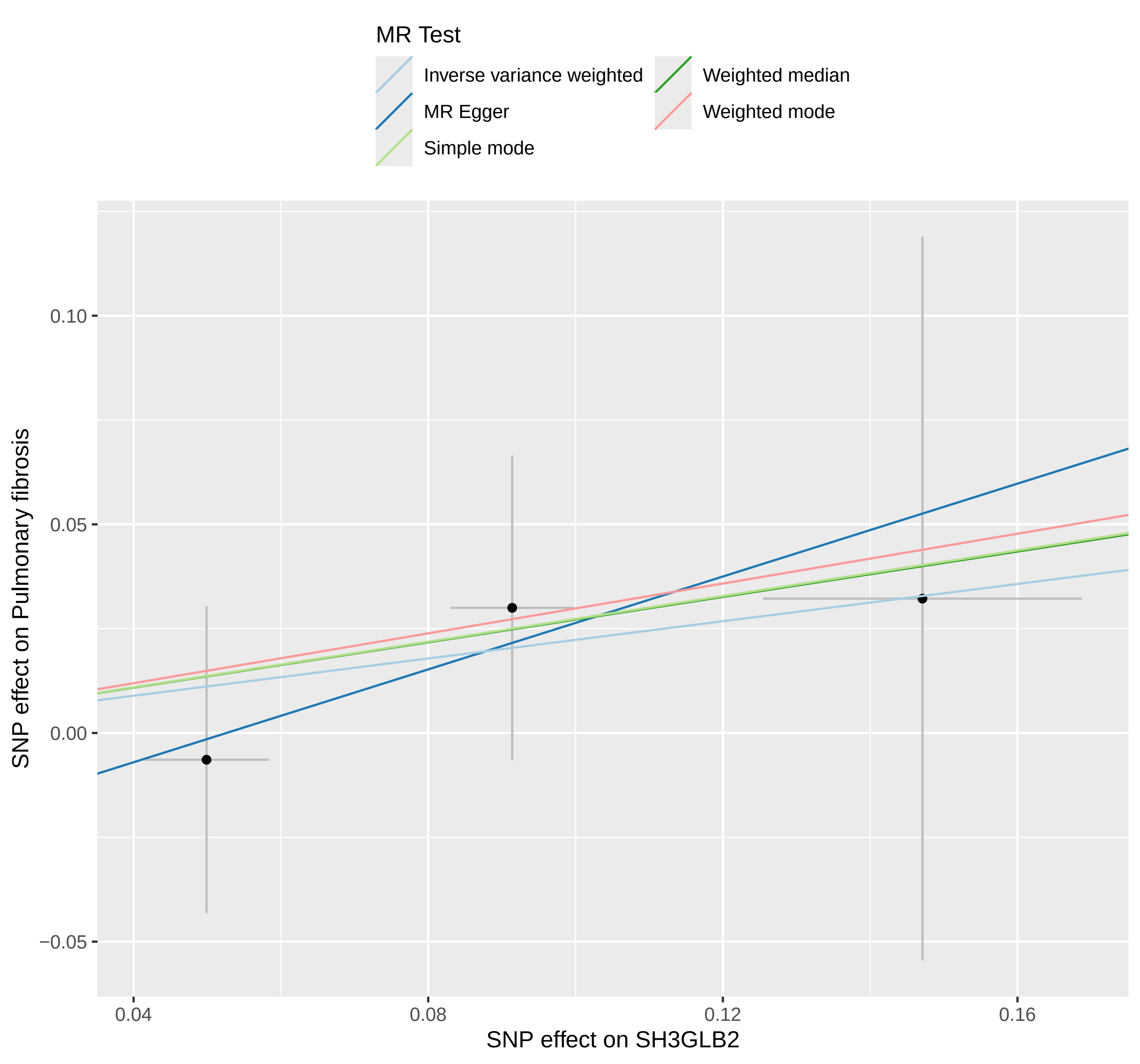

MR Test
Inverse variance weighted
MR Egger
Simple mode
Weighted median
Weighted mode
0.10
0.05
0.00
−0.05
SNP effect on Pulmonary fibrosis
0.04
0.08
0.12
0.16
SNP effect on SH3GLB2

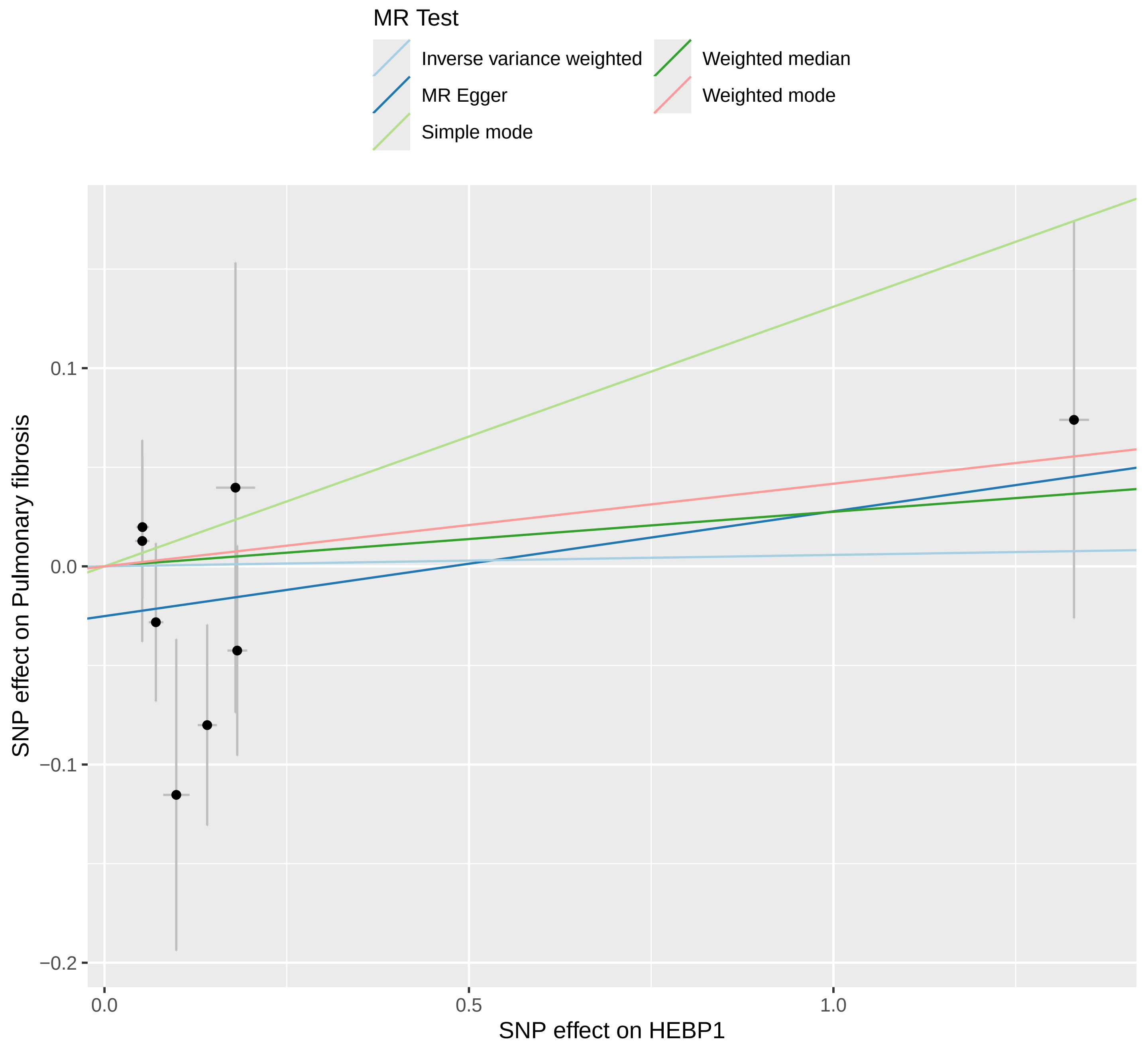

MR Test
Inverse variance weighted
MR Egger
Simple mode
Weighted median
Weighted mode
0.1
0.0
−0.1
−0.2
SNP effect on Pulmonary fibrosis
0.0
0.5
1.0
SNP effect on HEBP1

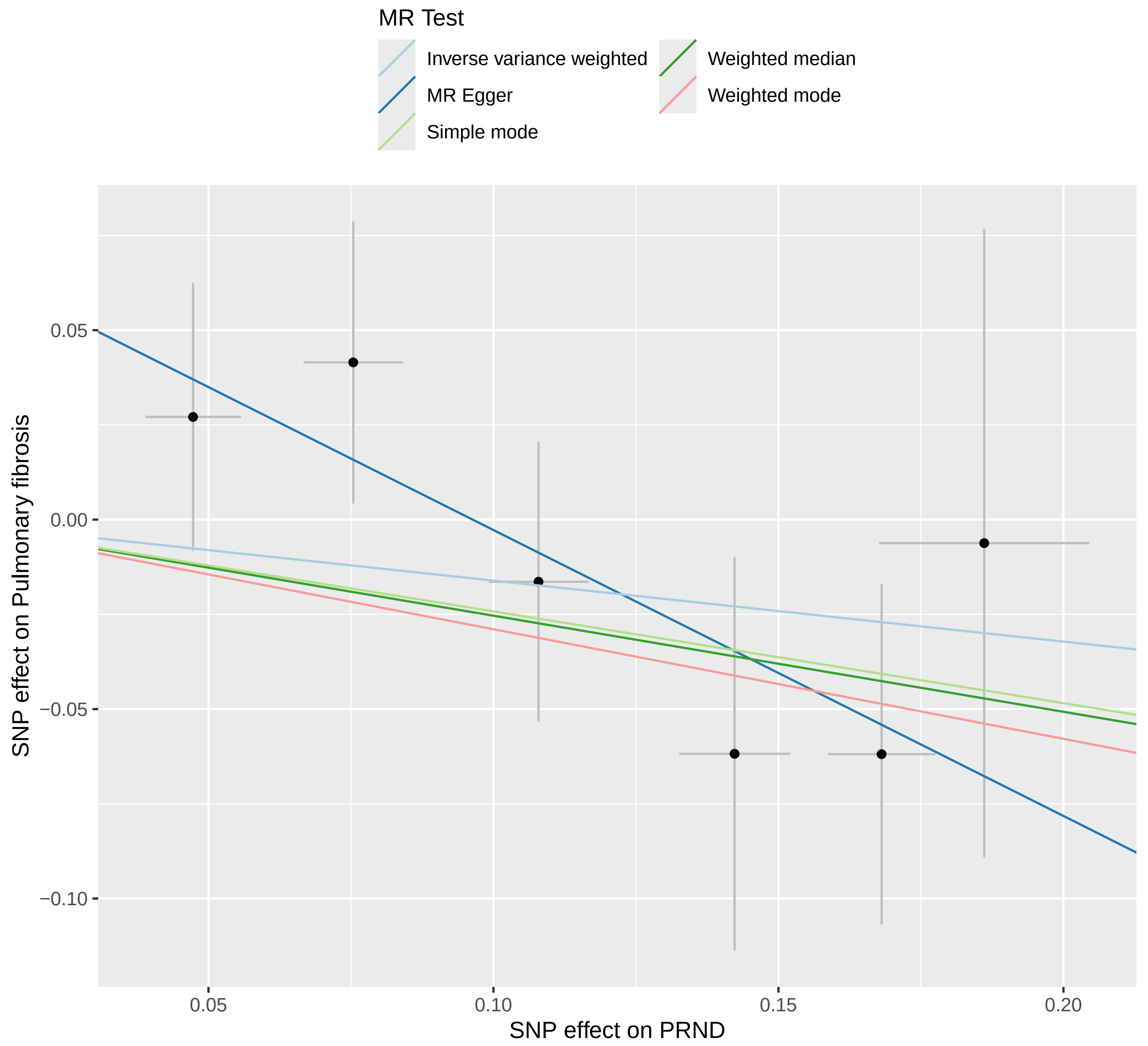
MR Test
Inverse variance weighted
MR Egger
Simple mode
Weighted median
Weighted mode
0.05
0.00
−0.05
−0.10
SNP effect on Pulmonary fibrosis
0.05
0.10
0.15
0.20
SNP effect on PRND

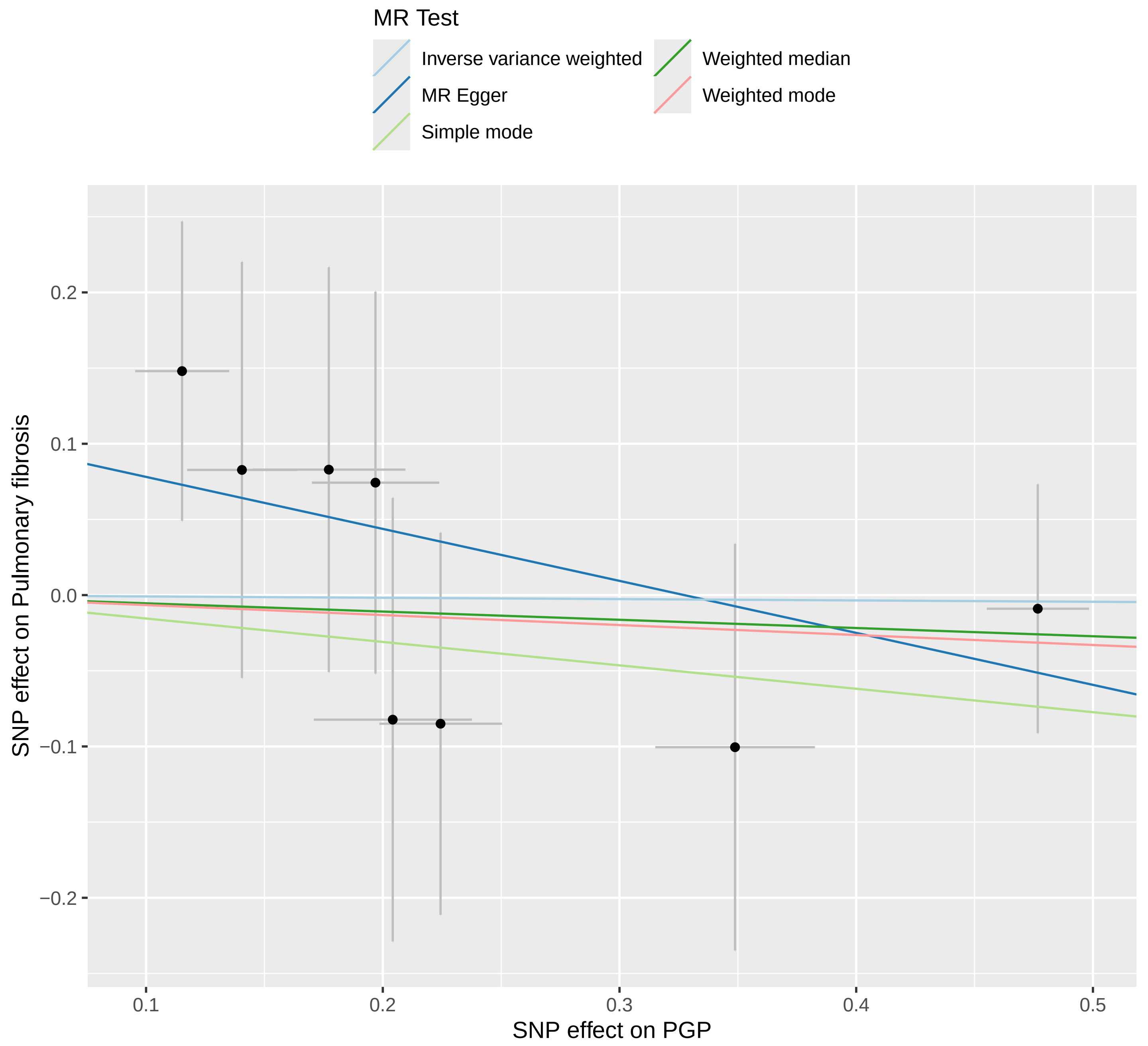

MR Test
Inverse variance weighted
MR Egger
Simple mode
Weighted median
Weighted mode
SNP effect on Pulmonary fibrosis
0.2
0.1
0.0
−0.1
−0.2
0.1
0.2
0.3
0.4
0.5
SNP effect on PGP

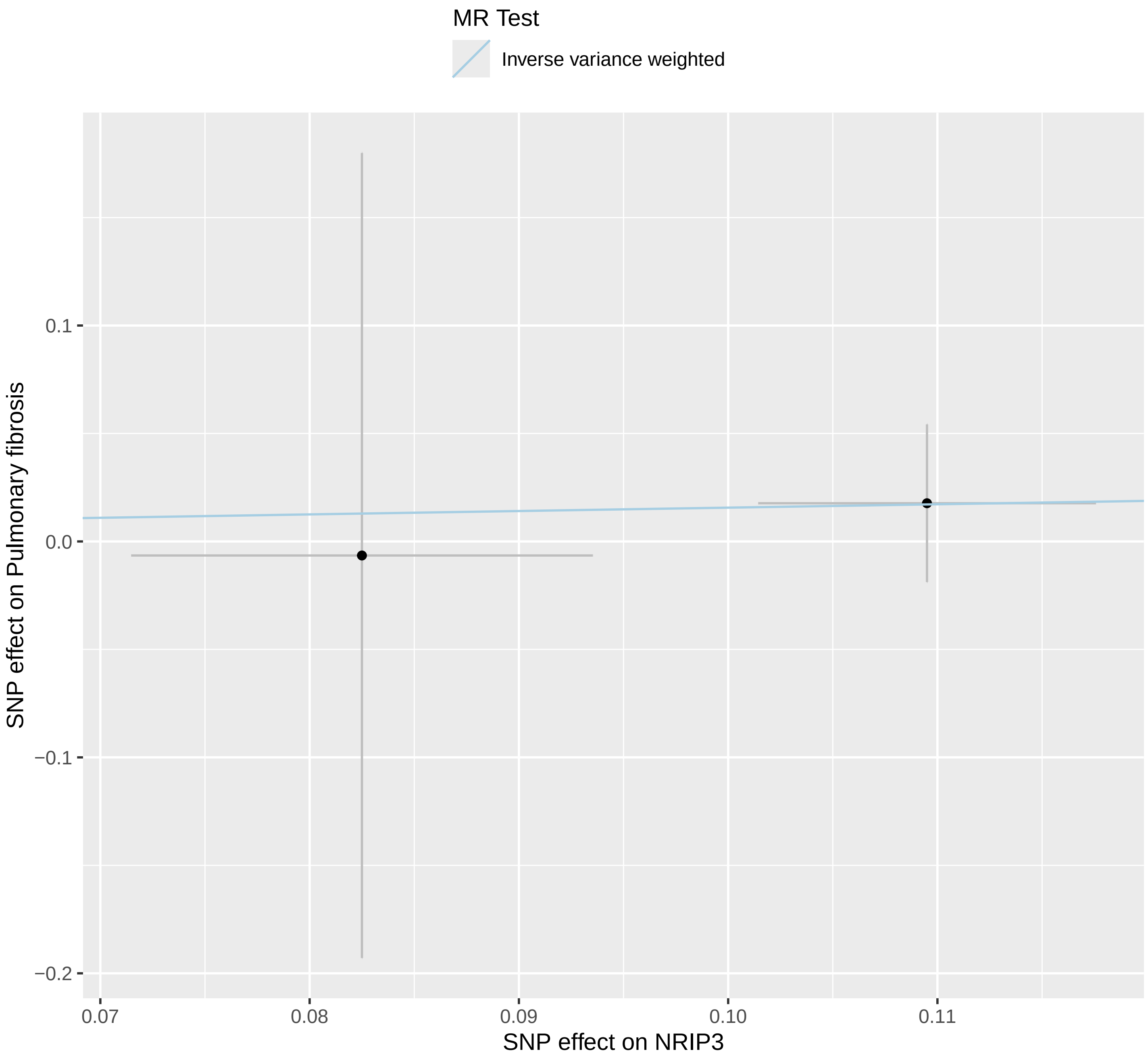
MR Test
Inverse variance weighted
SNP effect on Pulmonary fibrosis
0.1
0.0
−0.1
−0.2
0.07
0.08
0.09
0.10
0.11
SNP effect on NRIP3

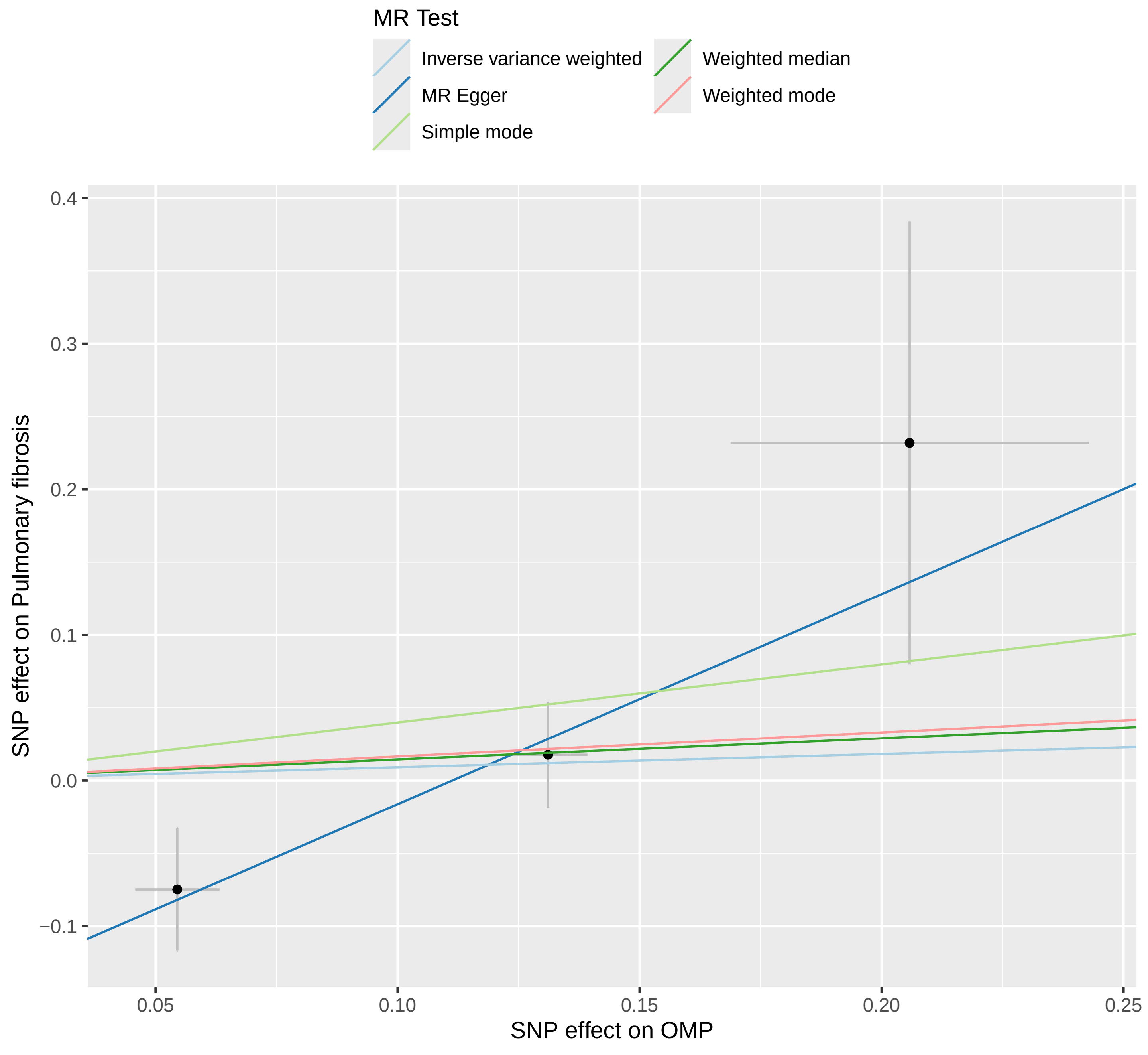

MR Test
Inverse variance weighted
MR Egger
Simple mode
Weighted median
Weighted mode
SNP effect on Pulmonary fibrosis
0.4
0.3
0.2
0.1
0.0
−0.1
0.05
0.10
0.15
0.20
0.25
SNP effect on OMP

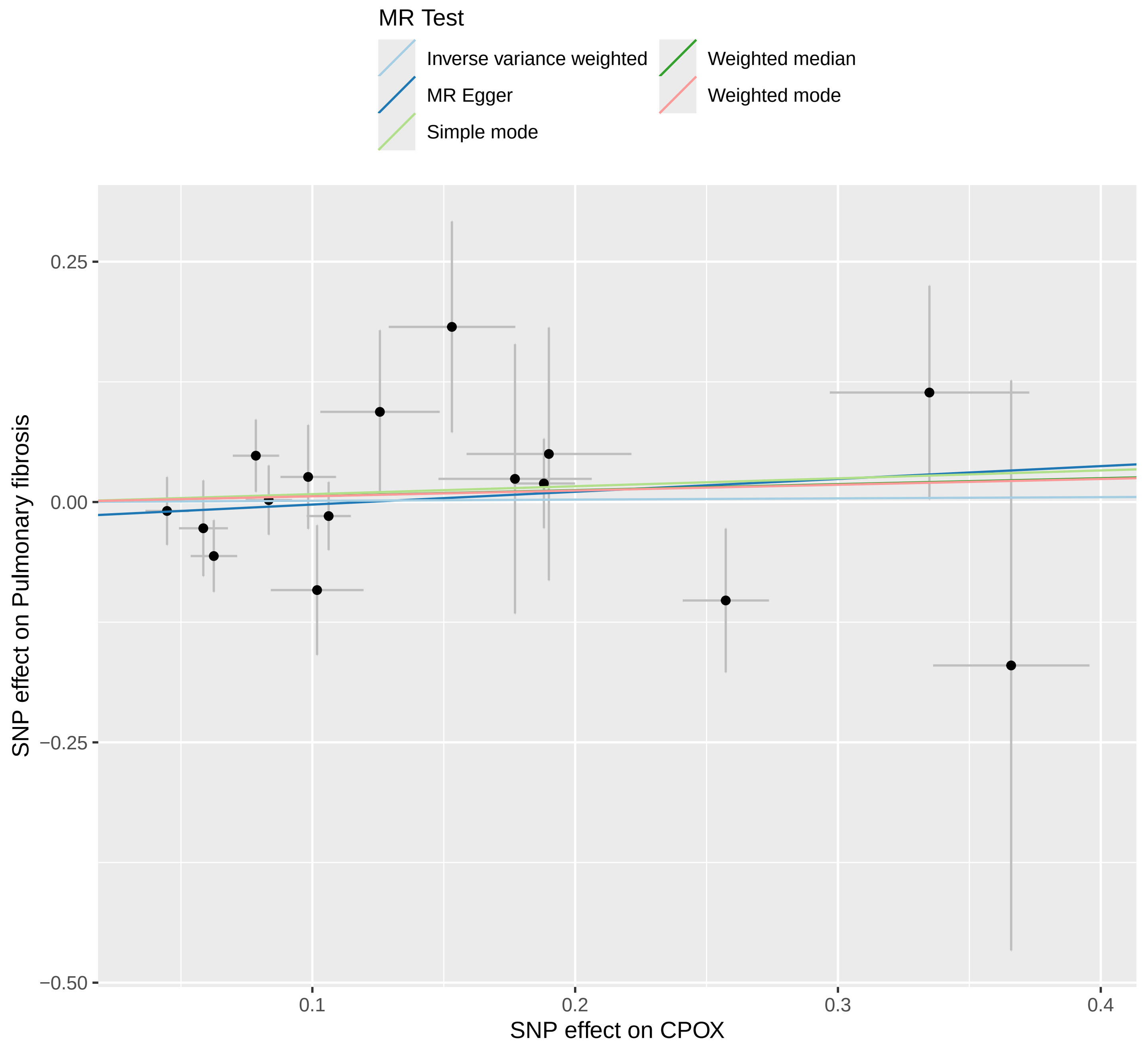
MR Test
Inverse variance weighted
MR Egger
Simple mode
Weighted median
Weighted mode
SNP effect on Pulmonary fibrosis
0.25
0.00
−0.25
−0.50
0.1
0.2
0.3
0.4
SNP effect on CPOX

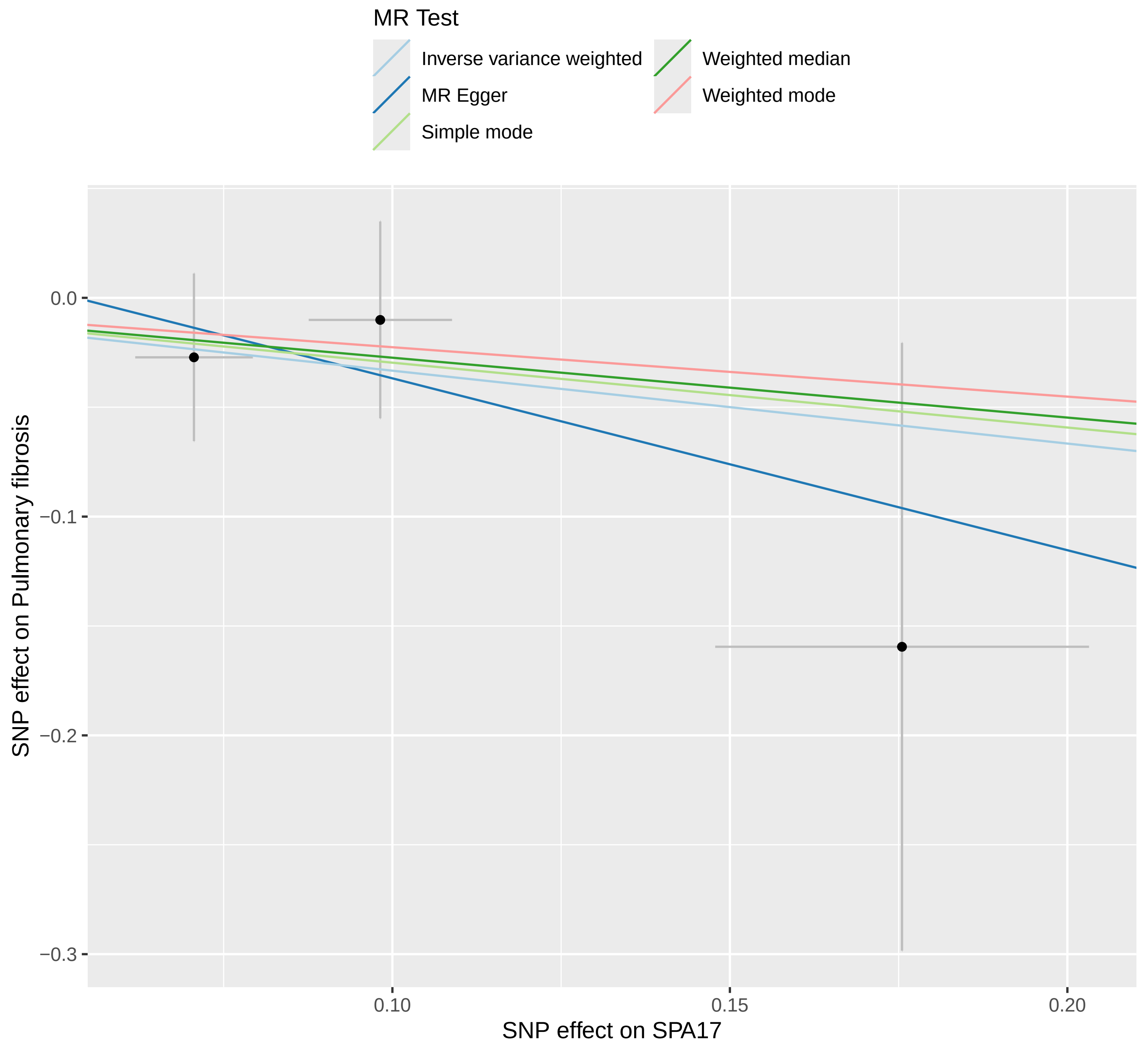
MR Test
Inverse variance weighted
MR Egger
Simple mode
Weighted median
Weighted mode
0.0
−0.1
−0.2
−0.3
SNP effect on Pulmonary fibrosis
0.10
0.15
0.20
SNP effect on SPA17

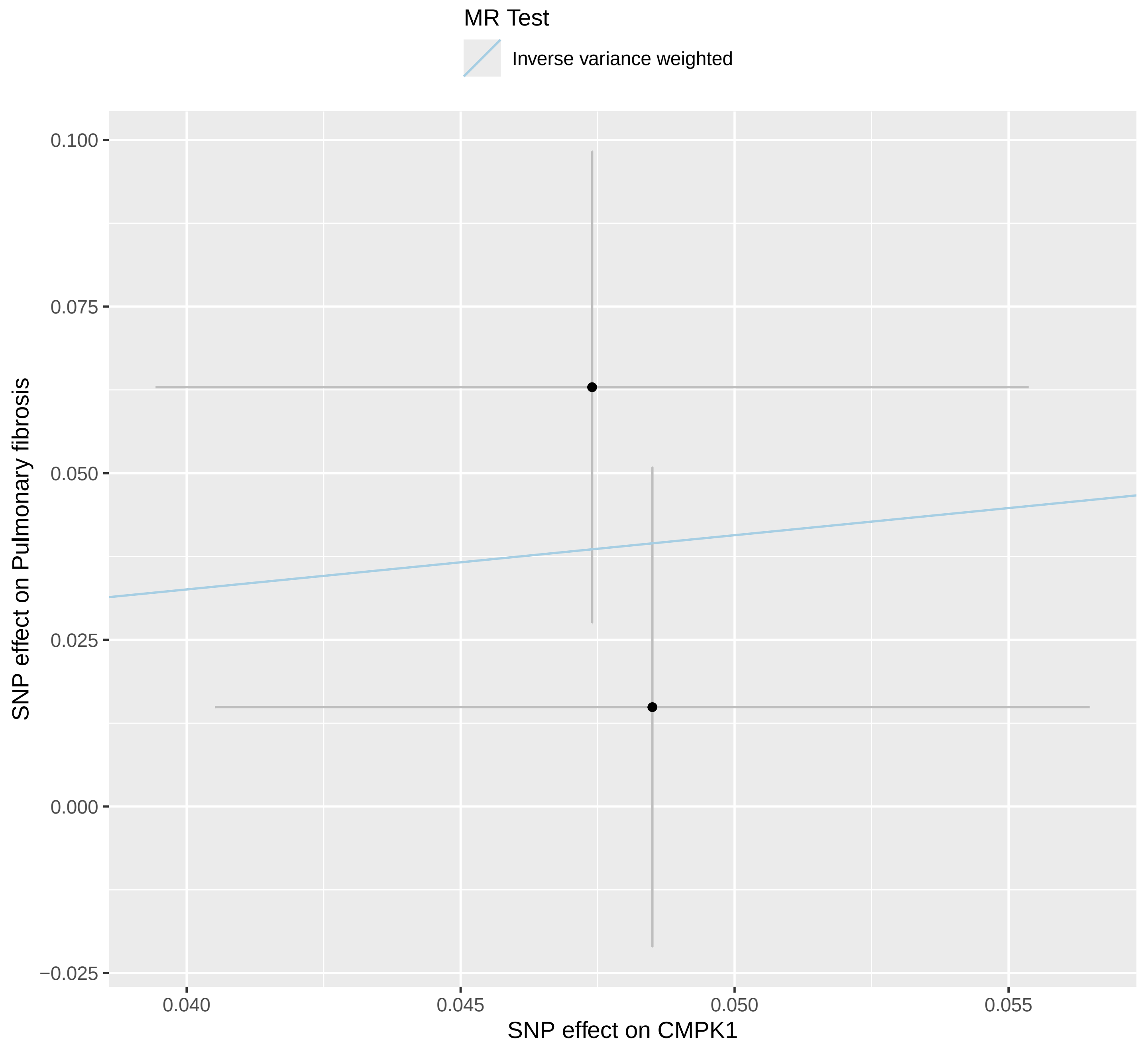

MR Test
Inverse variance weighted
SNP effect on Pulmonary fibrosis
0.100
0.075
0.050
0.025
0.000
−0.025
0.040
0.045
0.050
0.055
SNP effect on CMPK1

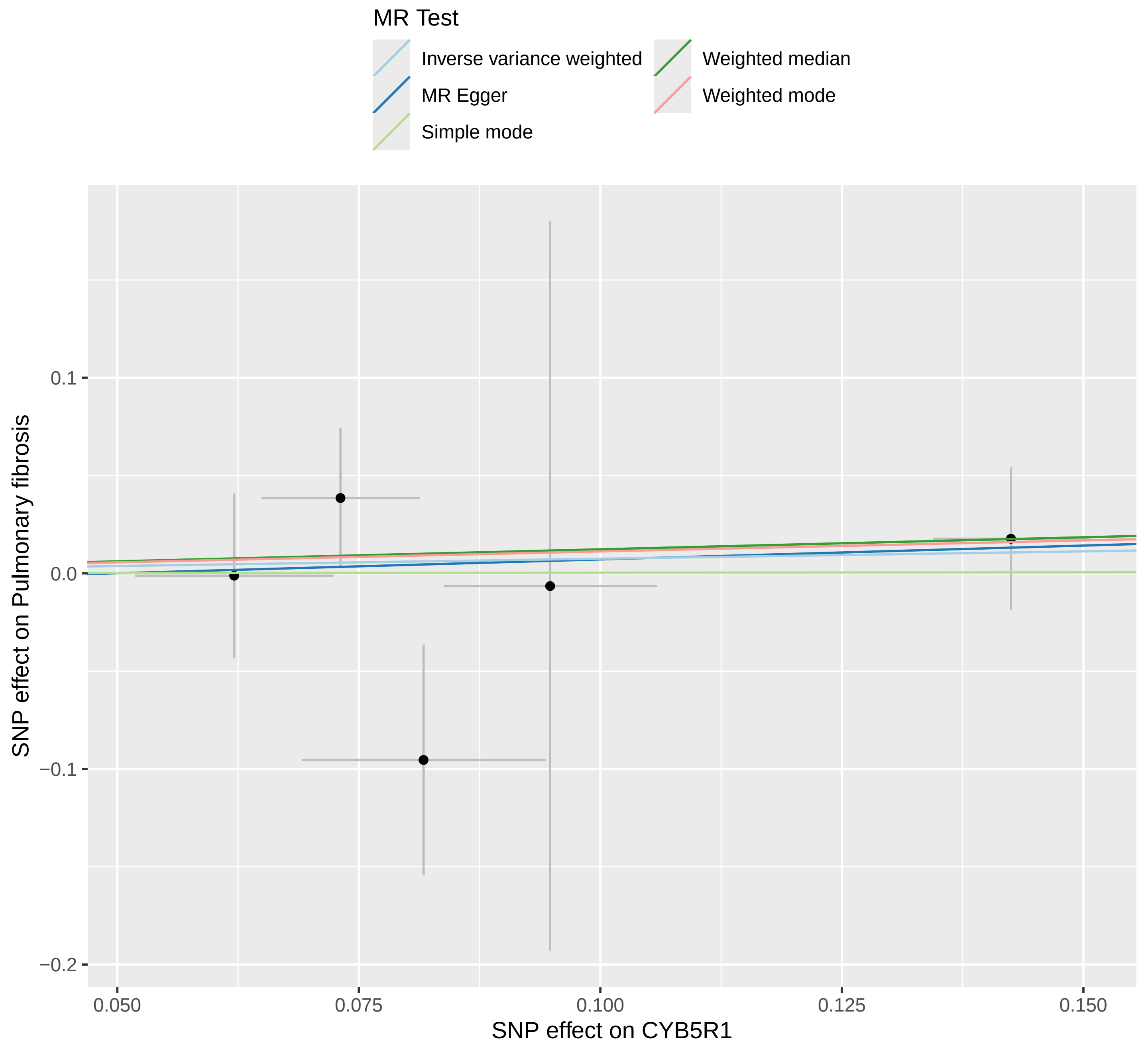

MR Test
Inverse variance weighted
MR Egger
Simple mode
Weighted median
Weighted mode
SNP effect on Pulmonary fibrosis
0.1
0.0
−0.1
−0.2
0.050
0.075
0.100
0.125
0.150
SNP effect on CYB5R1

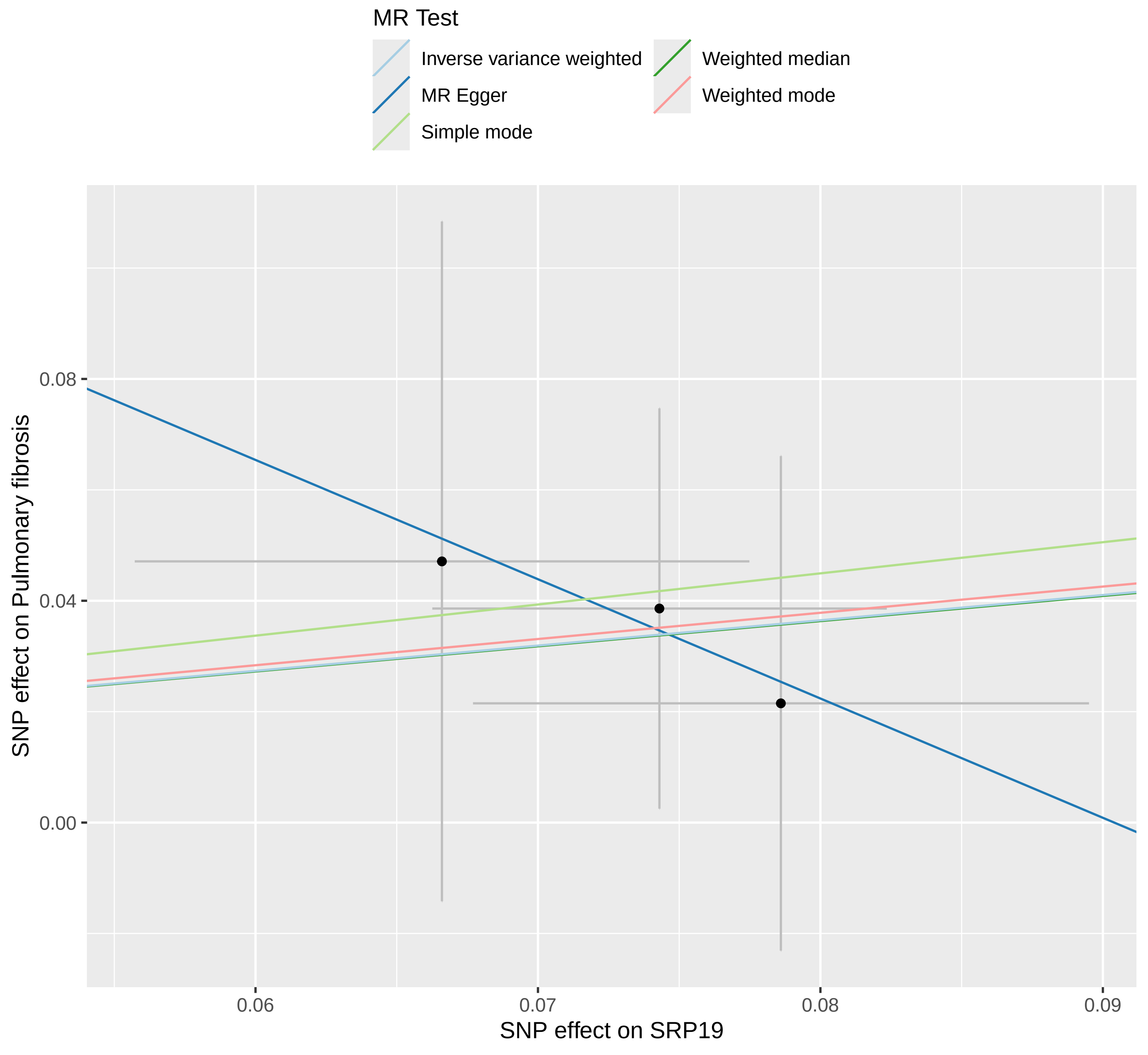

MR Test
Inverse variance weighted
MR Egger
Simple mode
Weighted median
Weighted mode
0.08
0.04
0.00
SNP effect on Pulmonary fibrosis
0.06
0.07
0.08
0.09
SNP effect on SRP19

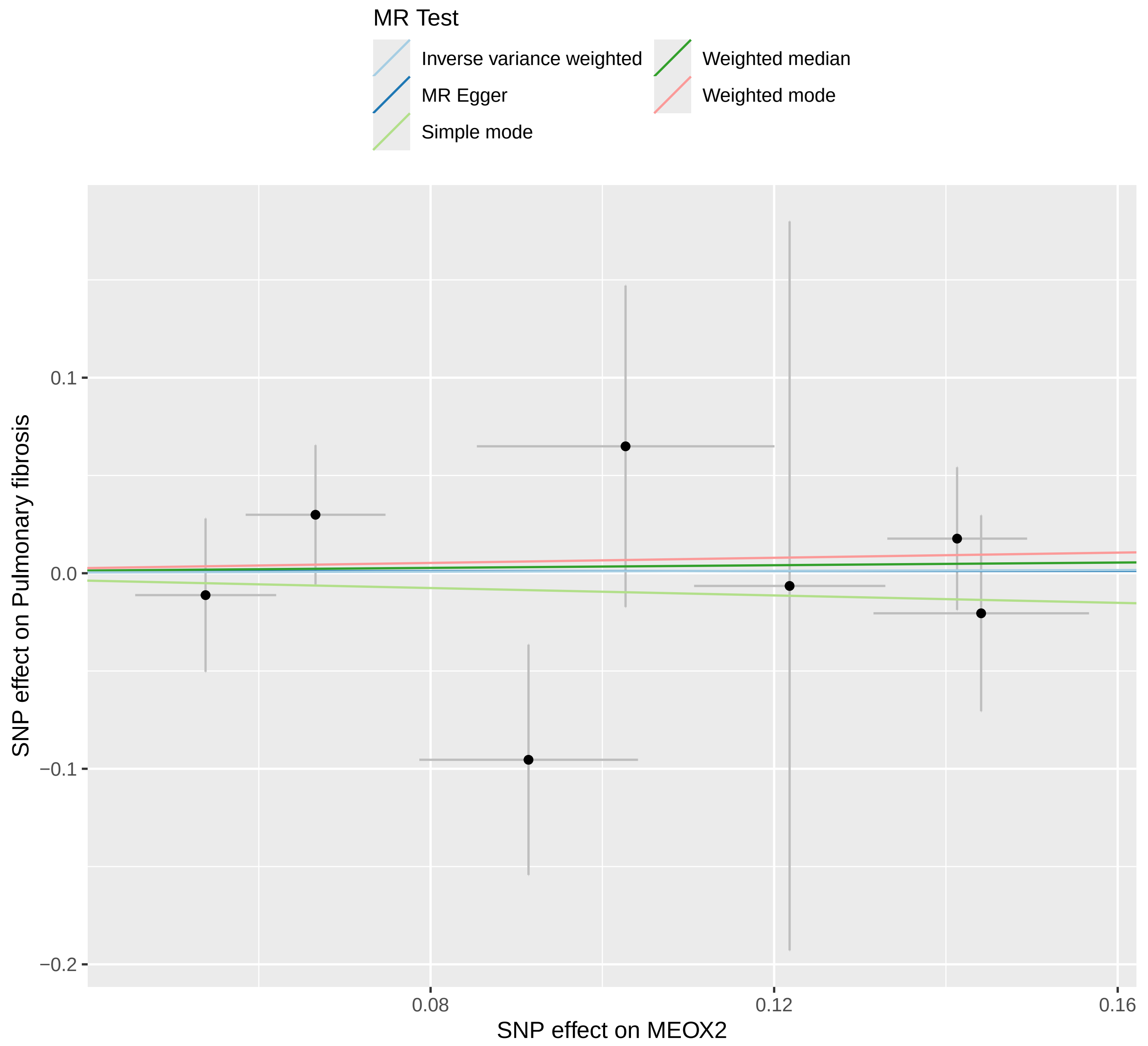
MR Test
Inverse variance weighted
MR Egger
Simple mode
Weighted median
Weighted mode
0.1
0.0
−0.1
−0.2
SNP effect on Pulmonary fibrosis
0.08
0.12
0.16
SNP effect on MEOX2

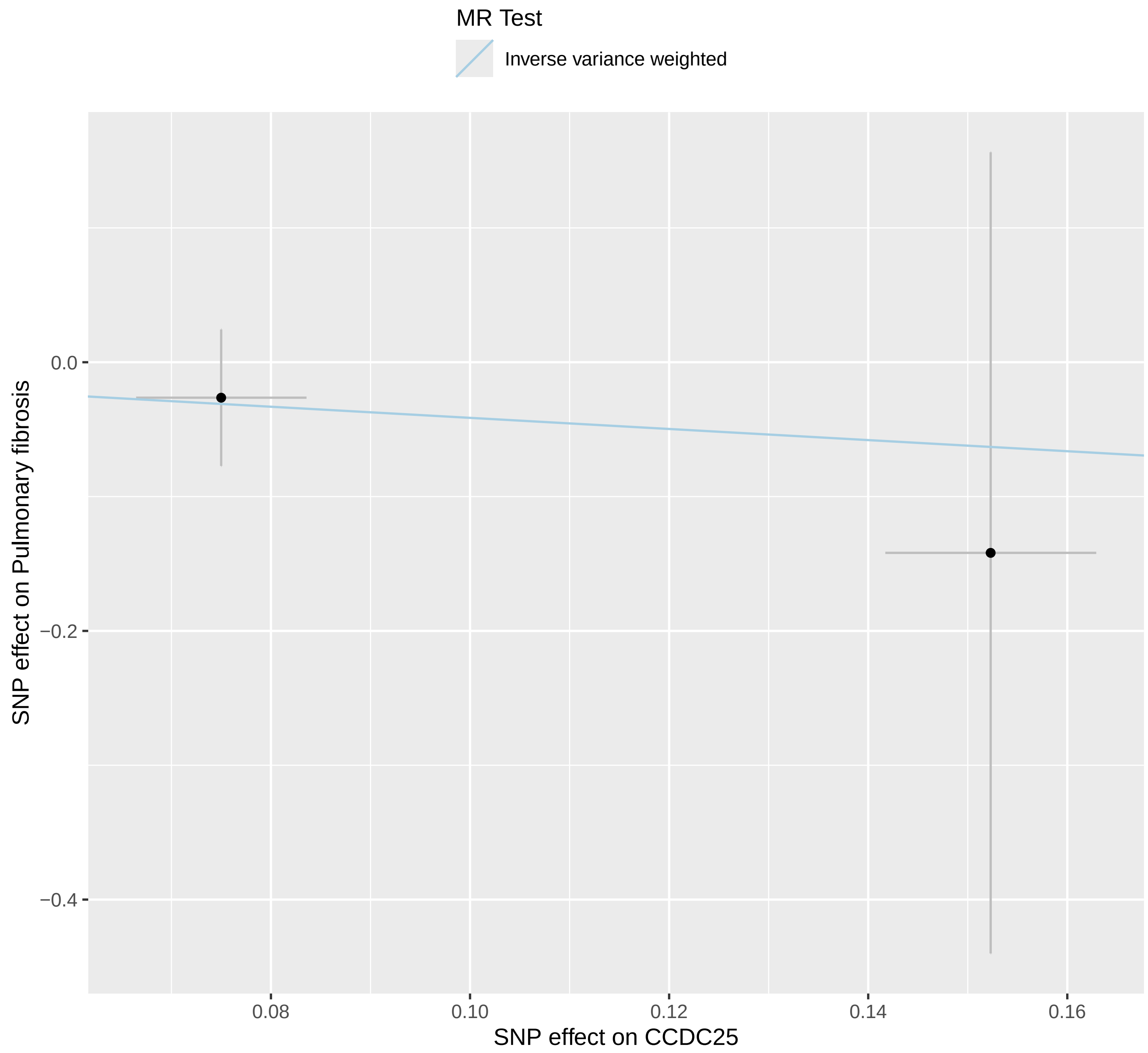

MR Test
Inverse variance weighted
SNP effect on Pulmonary fibrosis
0.0
−0.2
−0.4
0.08
0.10
0.12
0.14
0.16
SNP effect on CCDC25

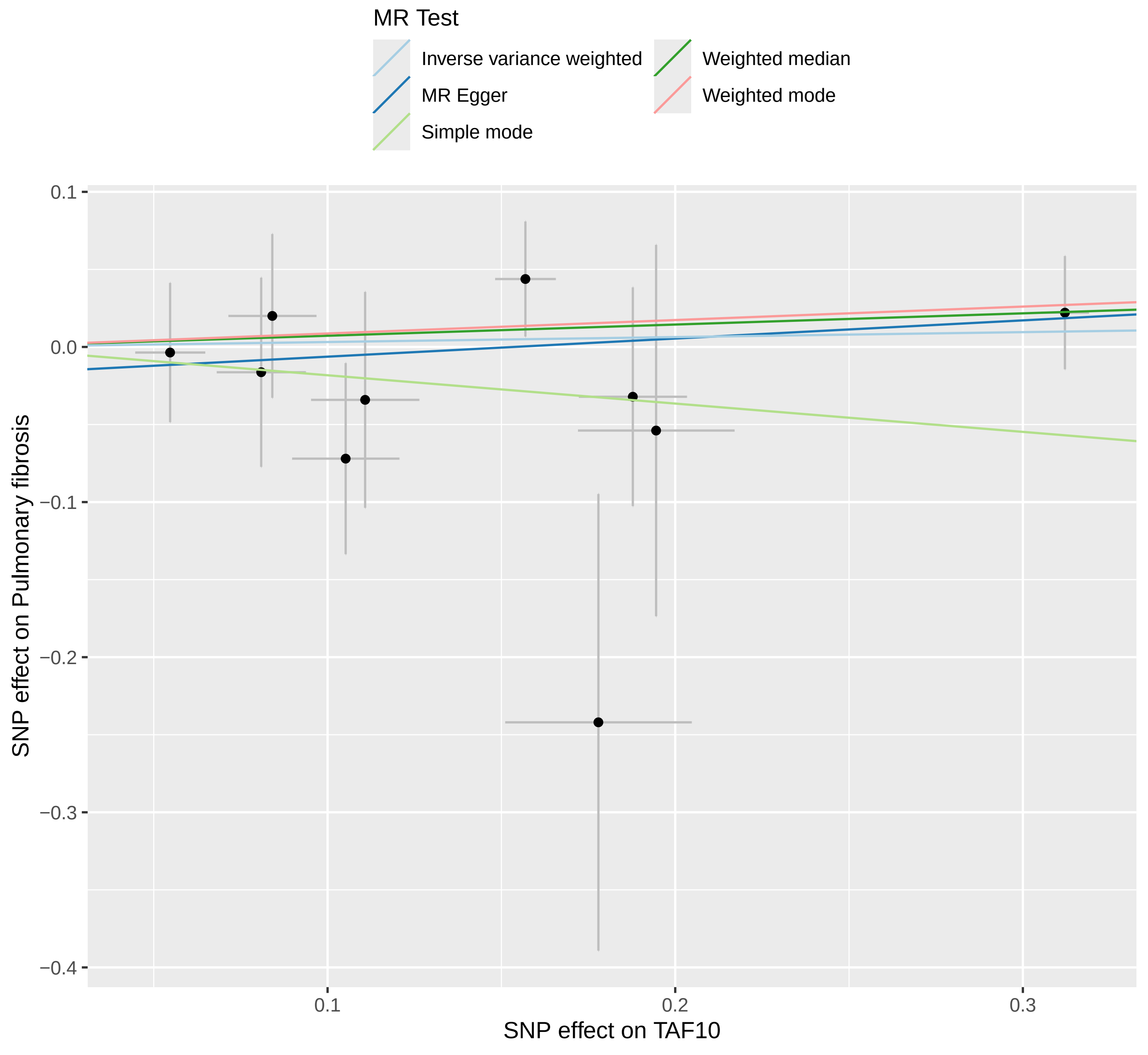

MR Test
Inverse variance weighted
MR Egger
Simple mode
Weighted median
Weighted mode
0.1
0.0
−0.1
−0.2
−0.3
−0.4
SNP effect on Pulmonary fibrosis
0.1
0.2
0.3
SNP effect on TAF10

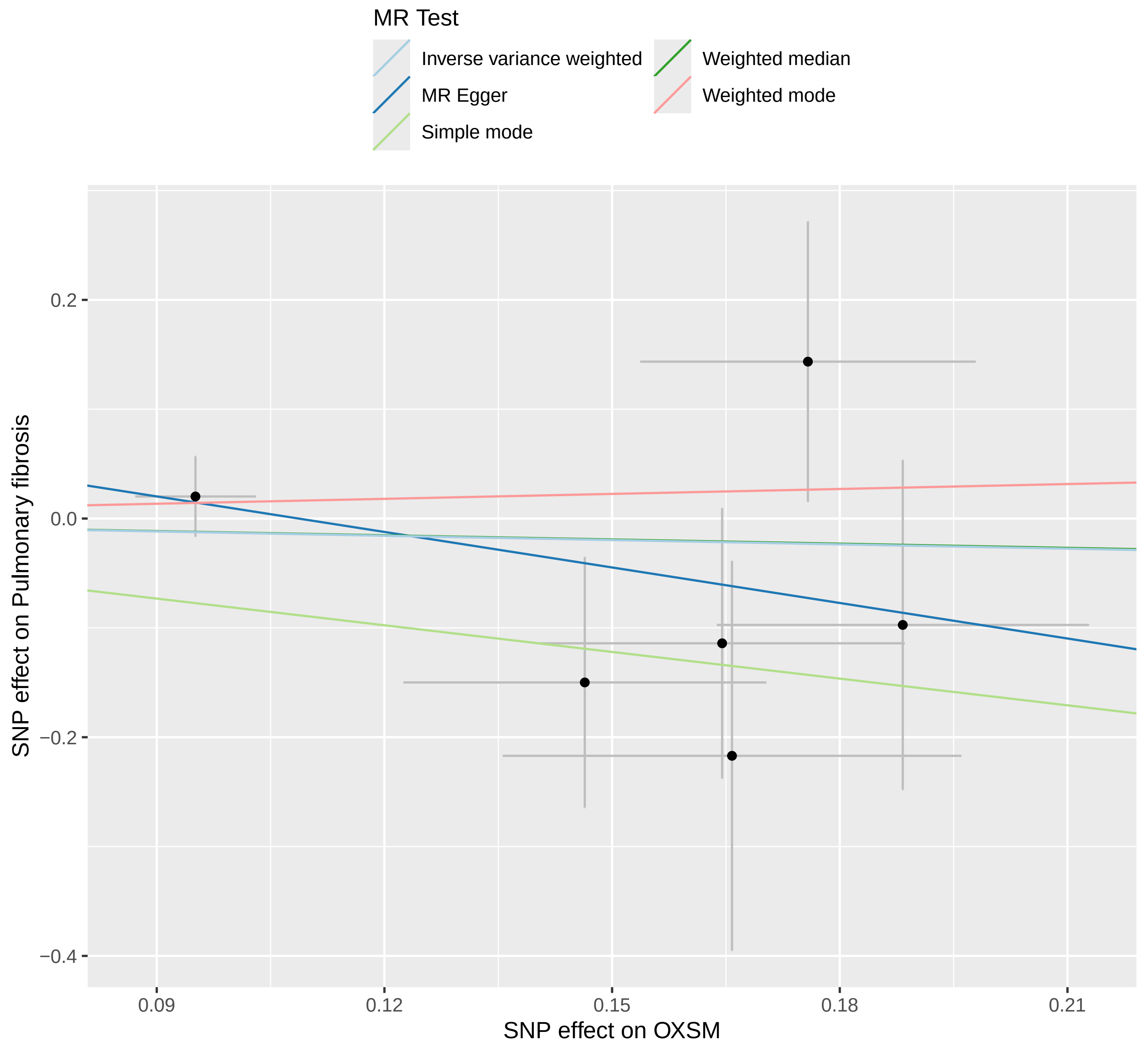

MR Test
Inverse variance weighted
MR Egger
Simple mode
Weighted median
Weighted mode
0.2
0.0
−0.2
−0.4
0.09
0.12
0.15
0.18
0.21
SNP effect on OXSM
SNP effect on Pulmonary fibrosis

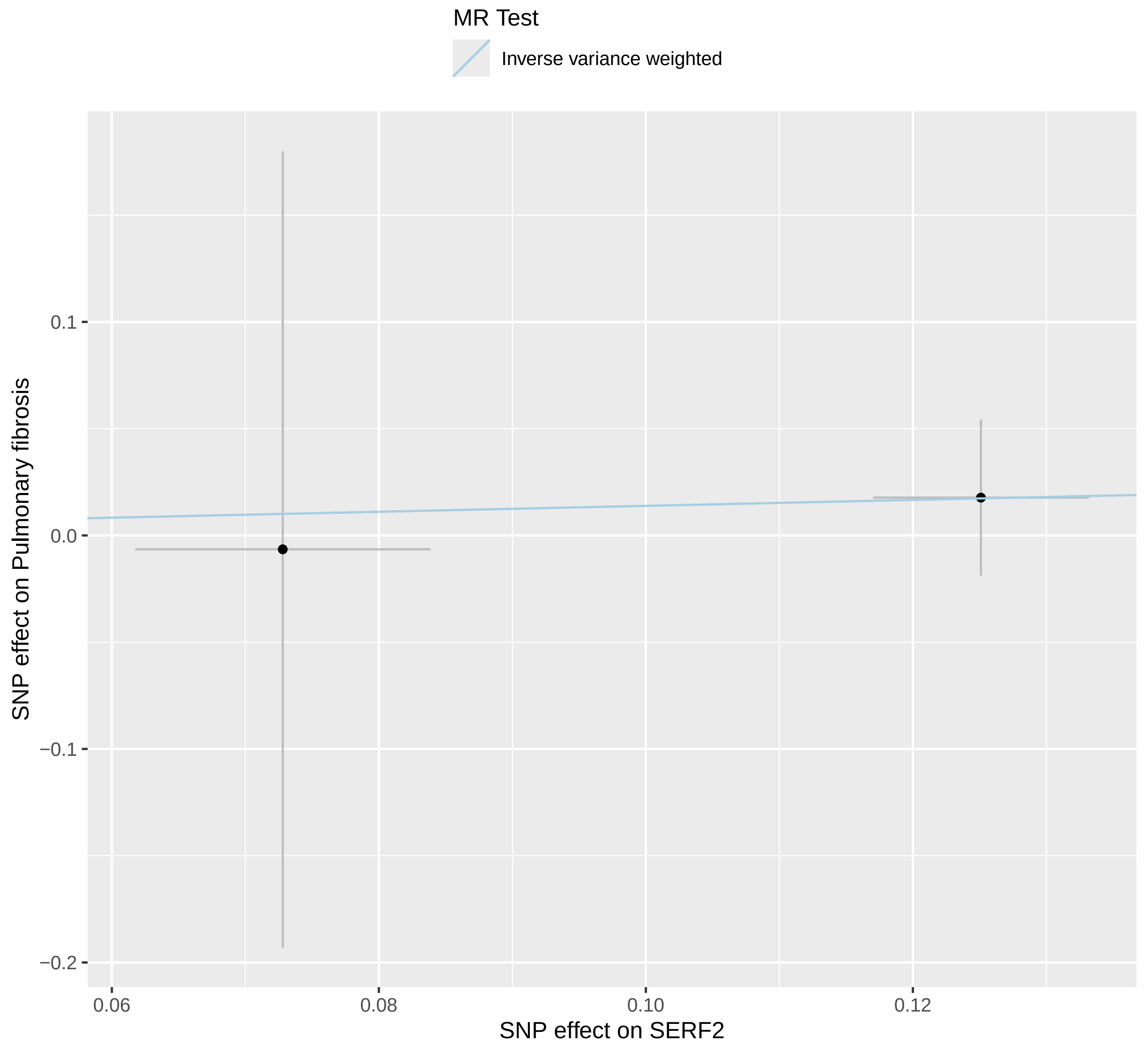

MR Test
Inverse variance weighted
0.1
0.0
−0.1
−0.2
SNP effect on Pulmonary fibrosis
0.06
0.08
0.10
0.12
SNP effect on SERF2

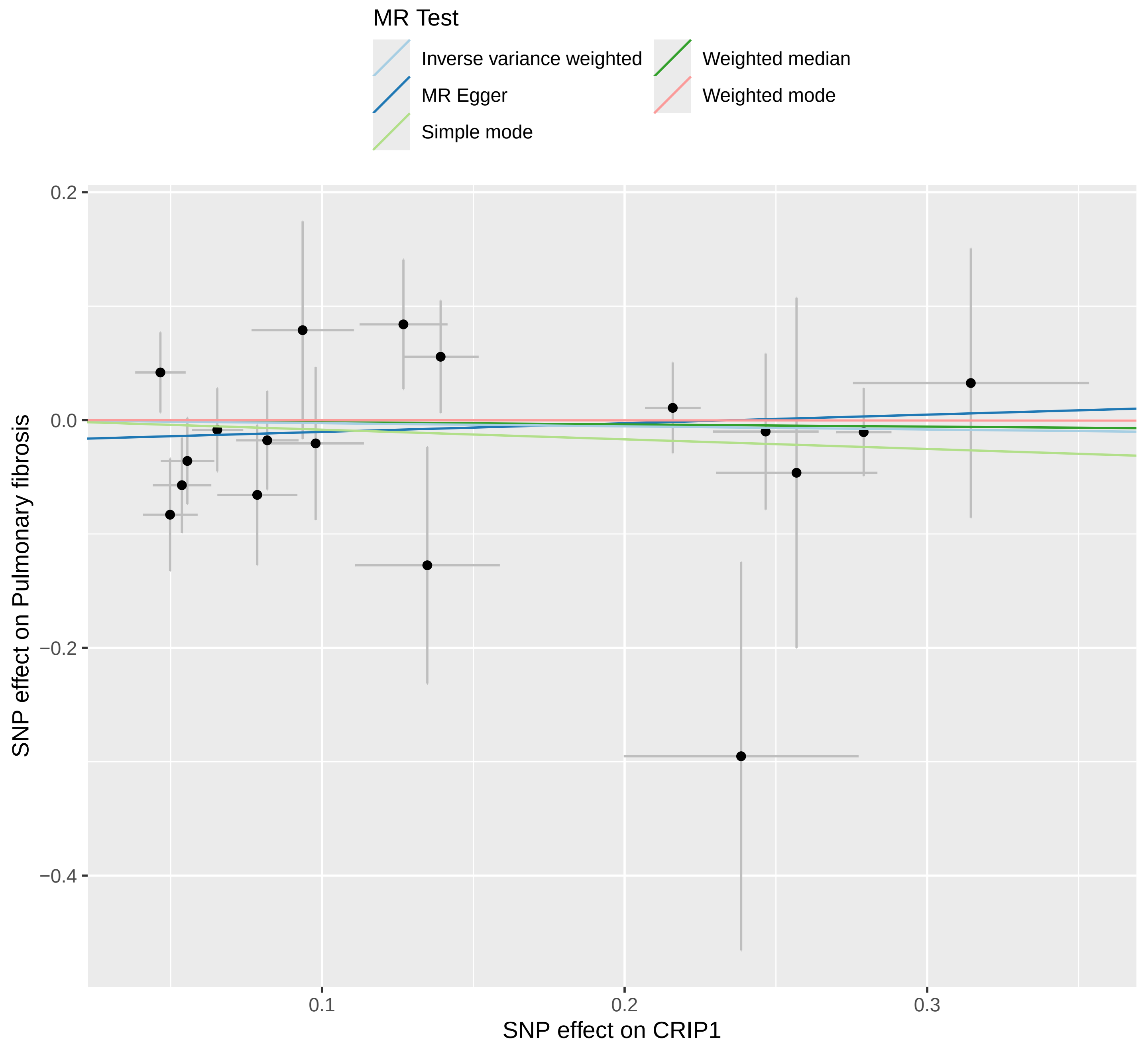

MR Test
Inverse variance weighted
MR Egger
Simple mode
Weighted median
Weighted mode
0.2
0.0
−0.2
−0.4
SNP effect on Pulmonary fibrosis
0.1
0.2
0.3
SNP effect on CRIP1

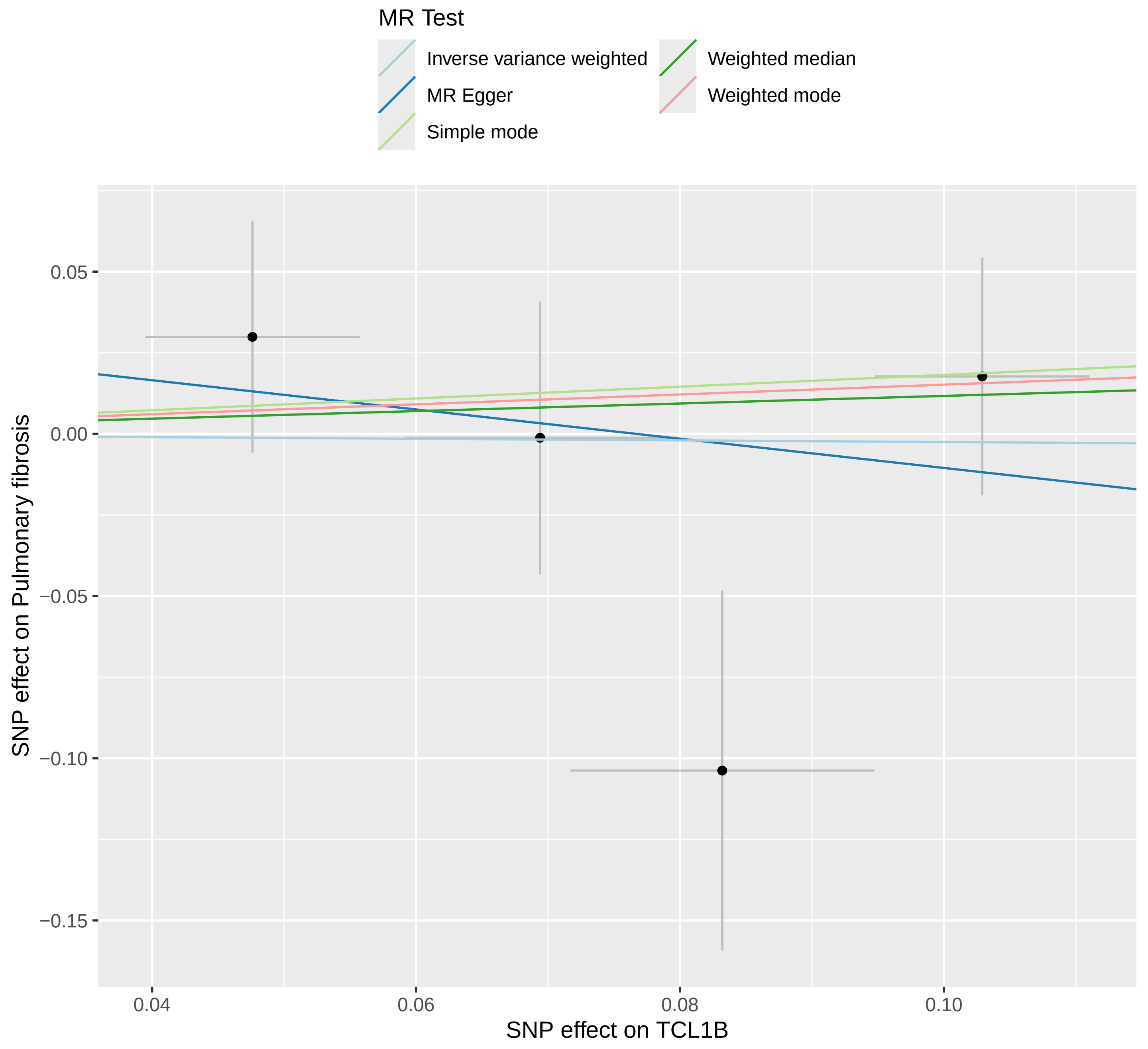
MR Test
Inverse variance weighted
MR Egger
Simple mode
Weighted median
Weighted mode
0.05
0.00
−0.05
−0.10
−0.15
0.04
0.06
0.08
0.10
SNP effect on TCL1B
SNP effect on Pulmonary fibrosis

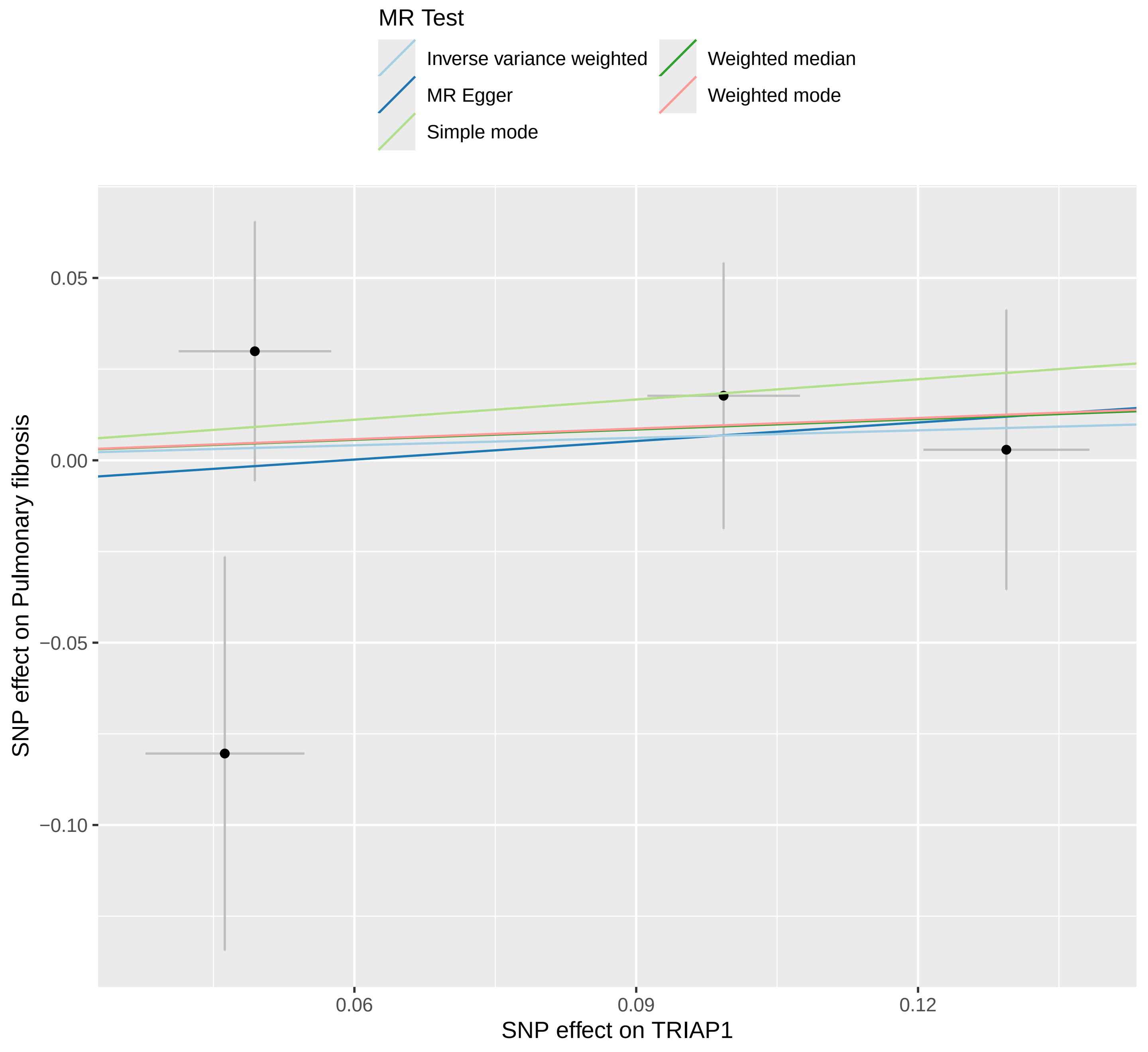

MR Test
Inverse variance weighted
MR Egger
Simple mode
Weighted median
Weighted mode
0.05
0.00
−0.05
−0.10
0.06
0.09
0.12
SNP effect on Pulmonary fibrosis
SNP effect on TRIAP1

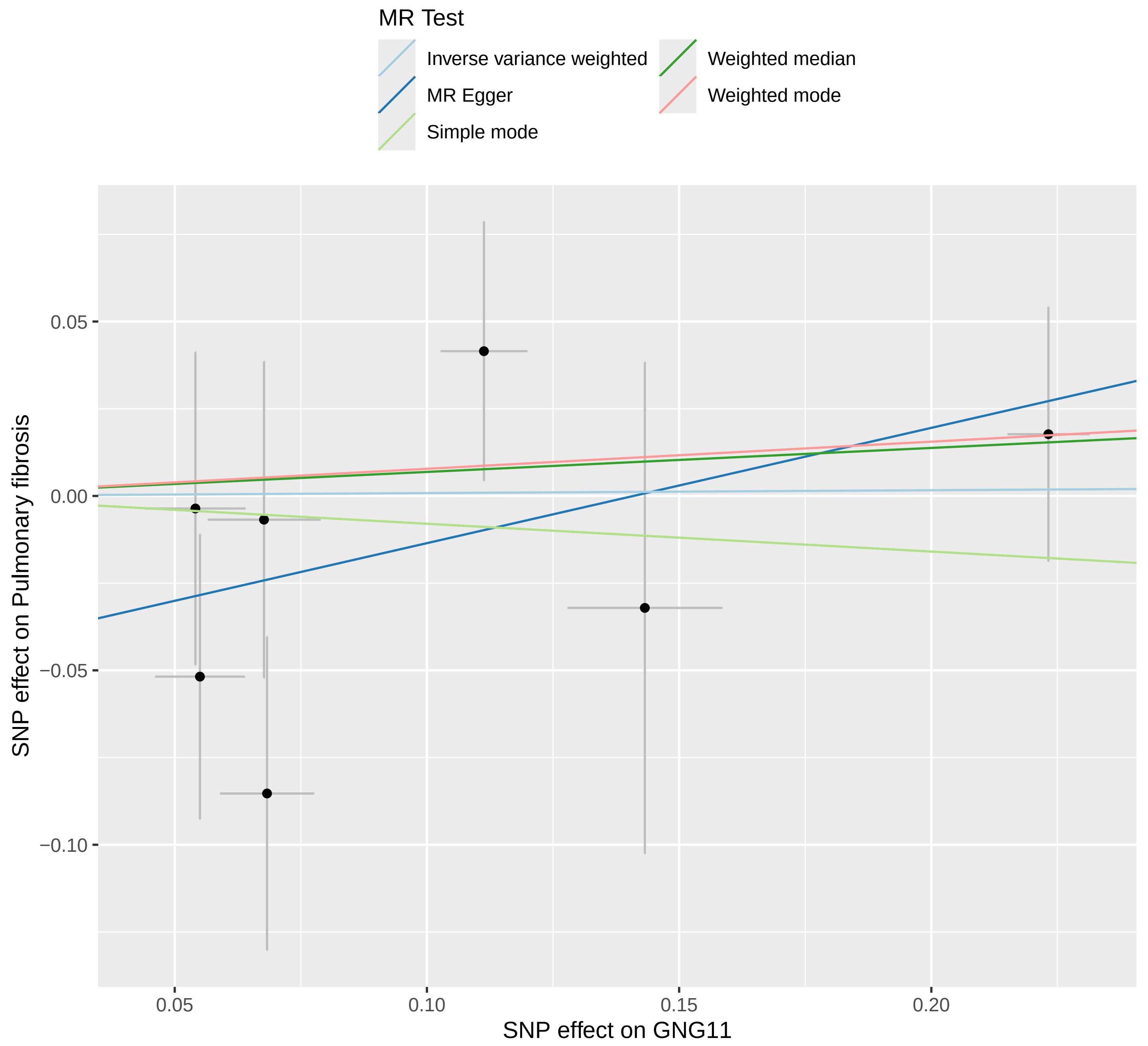

MR Test
Inverse variance weighted
MR Egger
Simple mode
Weighted median
Weighted mode
0.05
0.00
−0.05
−0.10
0.05
0.10
0.15
0.20
SNP effect on Pulmonary fibrosis
SNP effect on GNG11

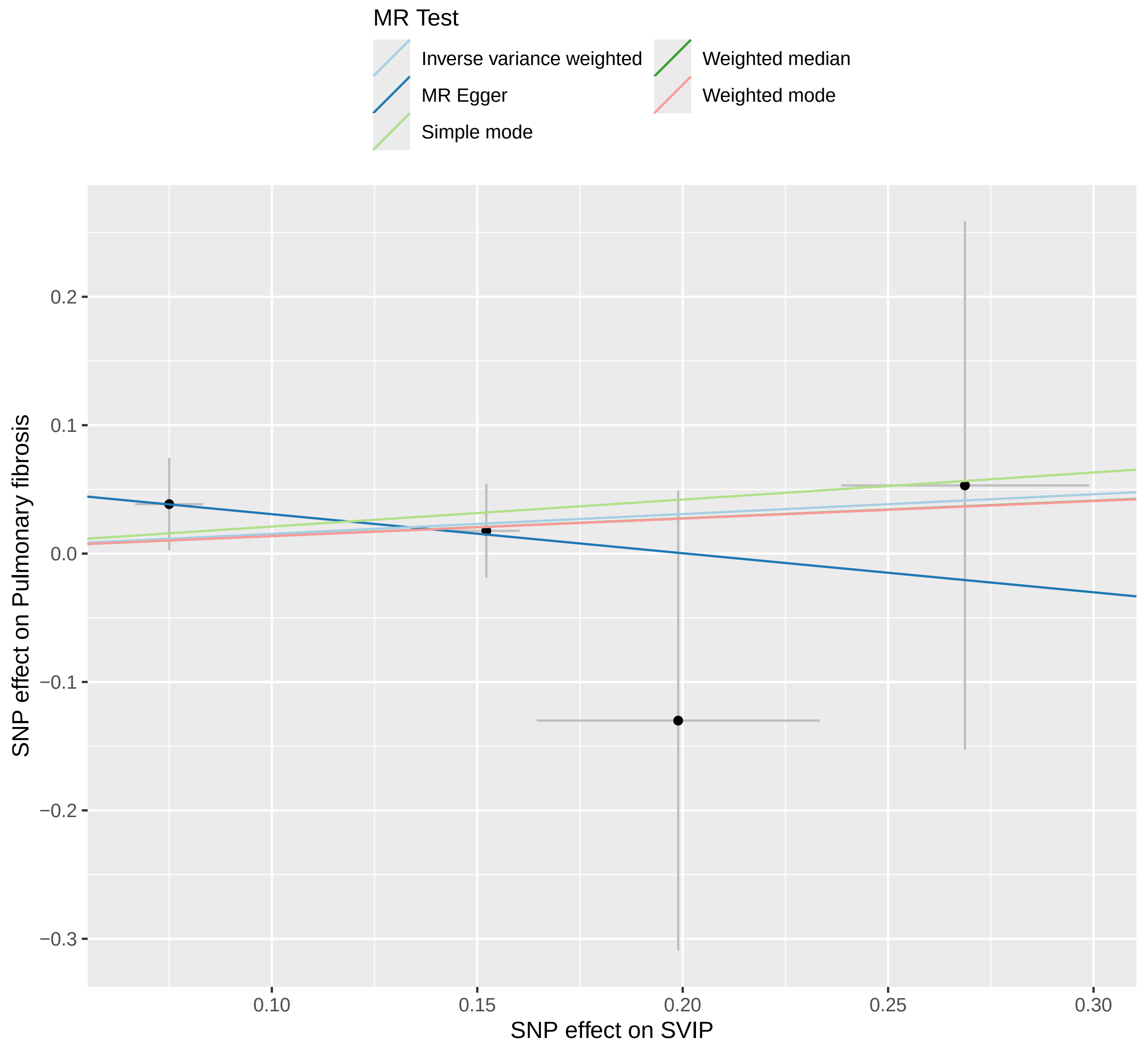

MR Test
Inverse variance weighted
MR Egger
Simple mode
Weighted median
Weighted mode
SNP effect on Pulmonary fibrosis
0.2
0.1
0.0
−0.1
−0.2
−0.3
0.10
0.15
0.20
0.25
0.30
SNP effect on SVIP

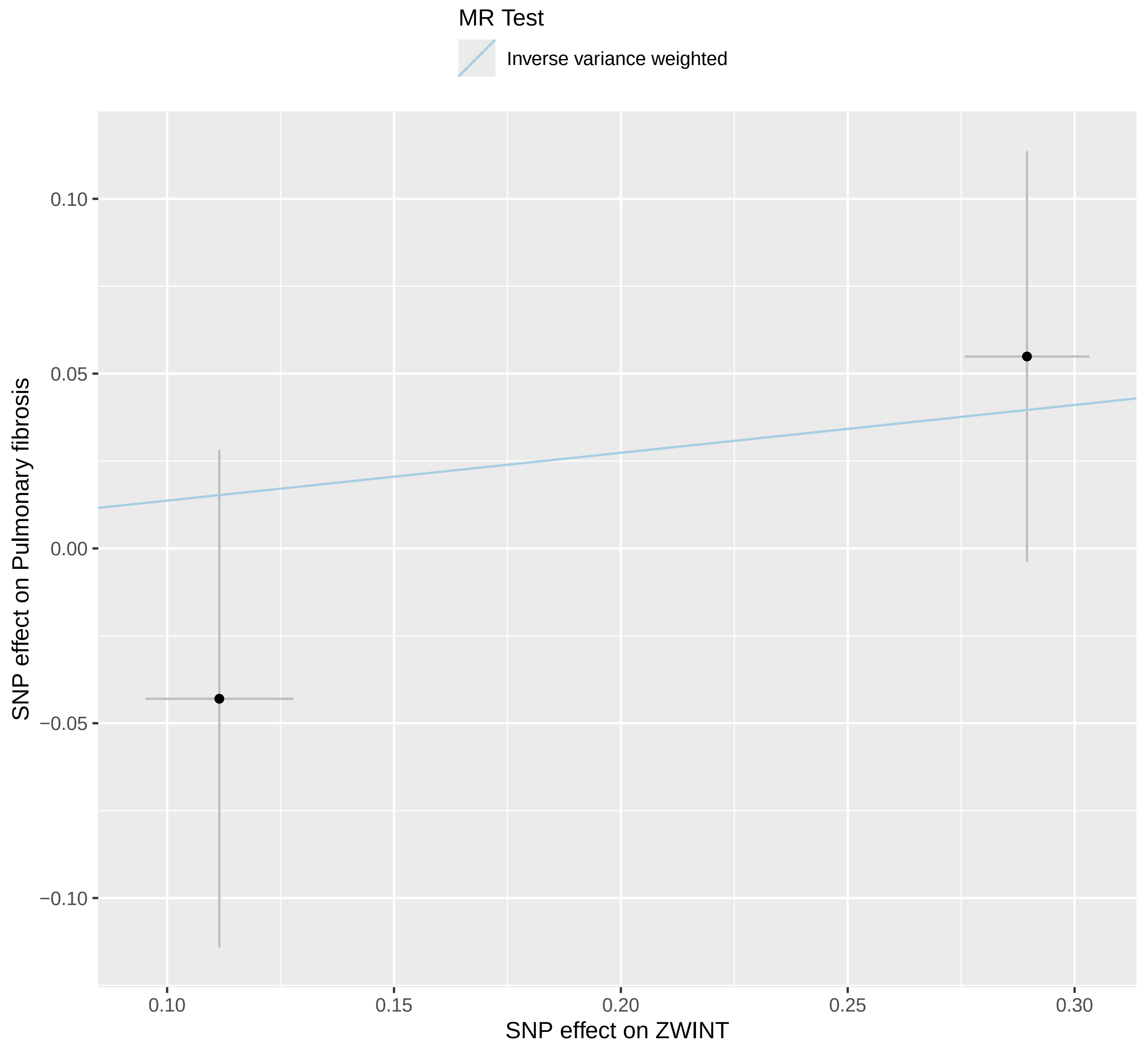
MR Test
Inverse variance weighted
SNP effect on Pulmonary fibrosis
SNP effect on ZWINT
0.10
0.05
0.00
−0.05
−0.10
0.10
0.15
0.20
0.25
0.30

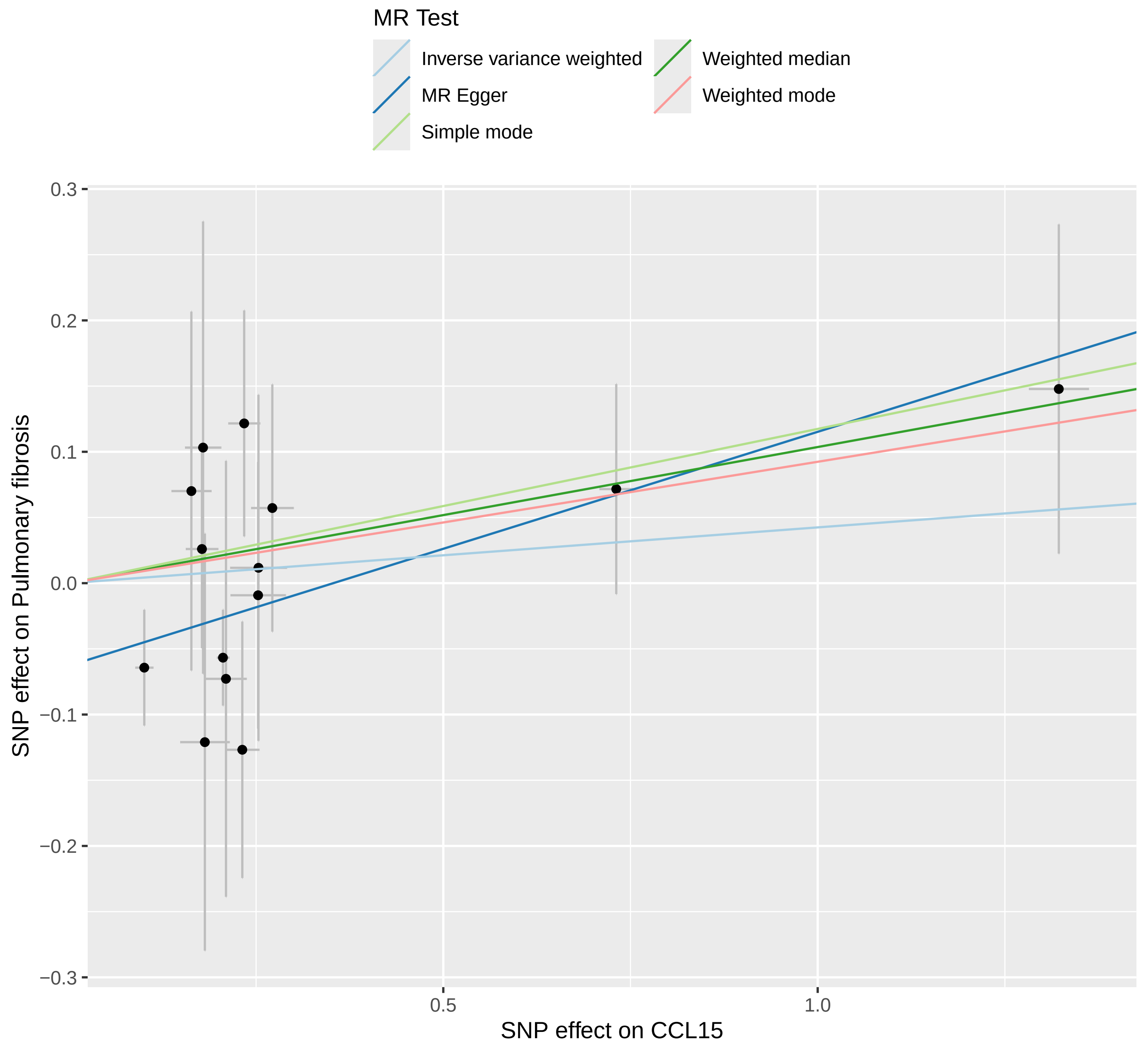

MR Test
Inverse variance weighted
MR Egger
Simple mode
Weighted median
Weighted mode
0.3
0.2
0.1
0.0
−0.1
−0.2
−0.3
SNP effect on Pulmonary fibrosis
0.5
1.0
SNP effect on CCL15

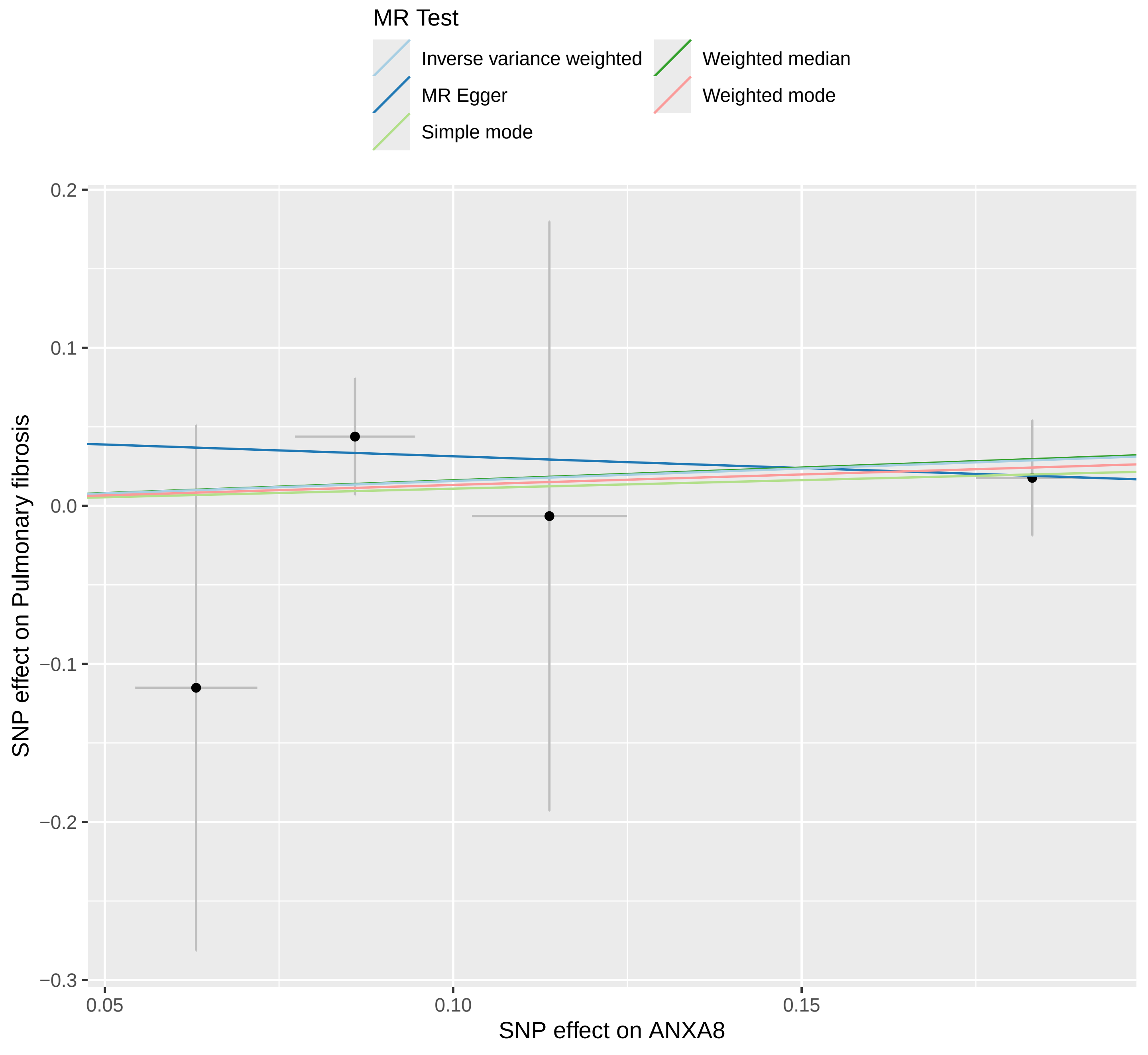
MR Test
Inverse variance weighted
MR Egger
Simple mode
Weighted median
Weighted mode
0.2
0.1
0.0
−0.1
−0.2
−0.3
0.05
0.10
0.15
SNP effect on Pulmonary fibrosis
SNP effect on ANXA8

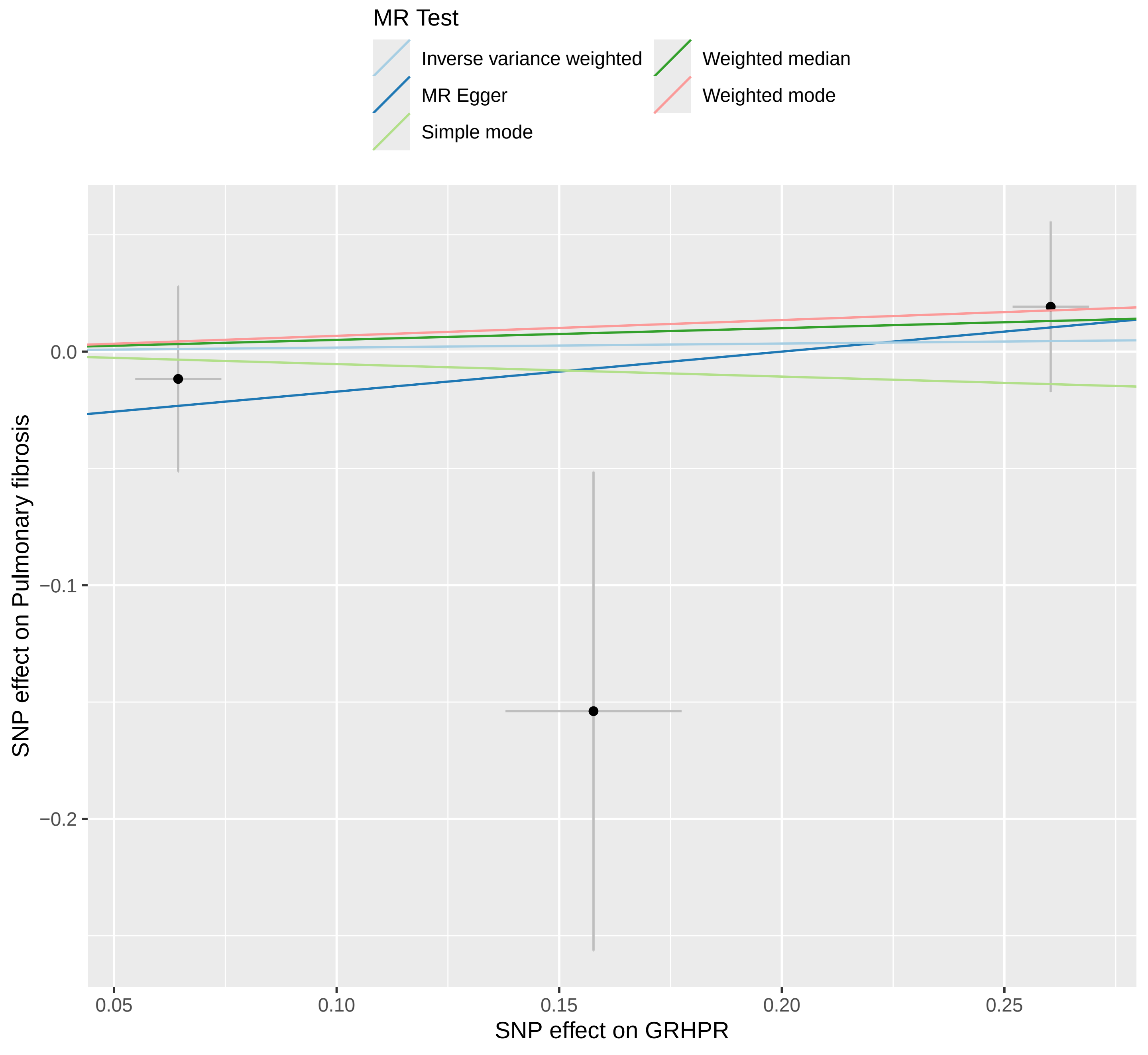
MR Test
Inverse variance weighted
MR Egger
Simple mode
Weighted median
Weighted mode
0.0
−0.1
−0.2
0.05
0.10
0.15
0.20
0.25
SNP effect on Pulmonary fibrosis
SNP effect on GRHPR

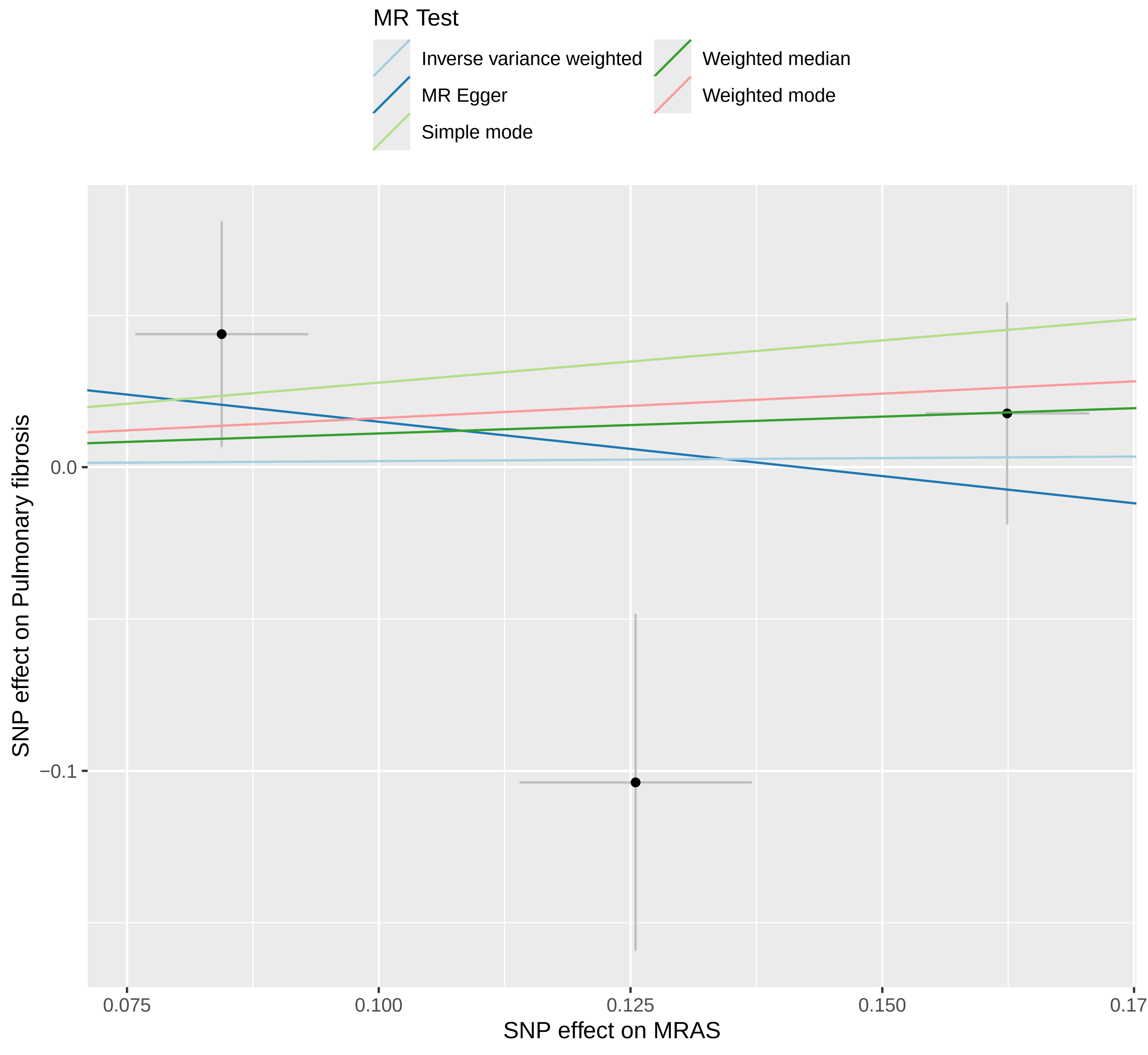
MR Test
Inverse variance weighted
MR Egger
Simple mode
Weighted median
Weighted mode
SNP effect on Pulmonary fibrosis
0.0
−0.1
0.075
0.100
0.125
0.150
0.17
SNP effect on MRAS

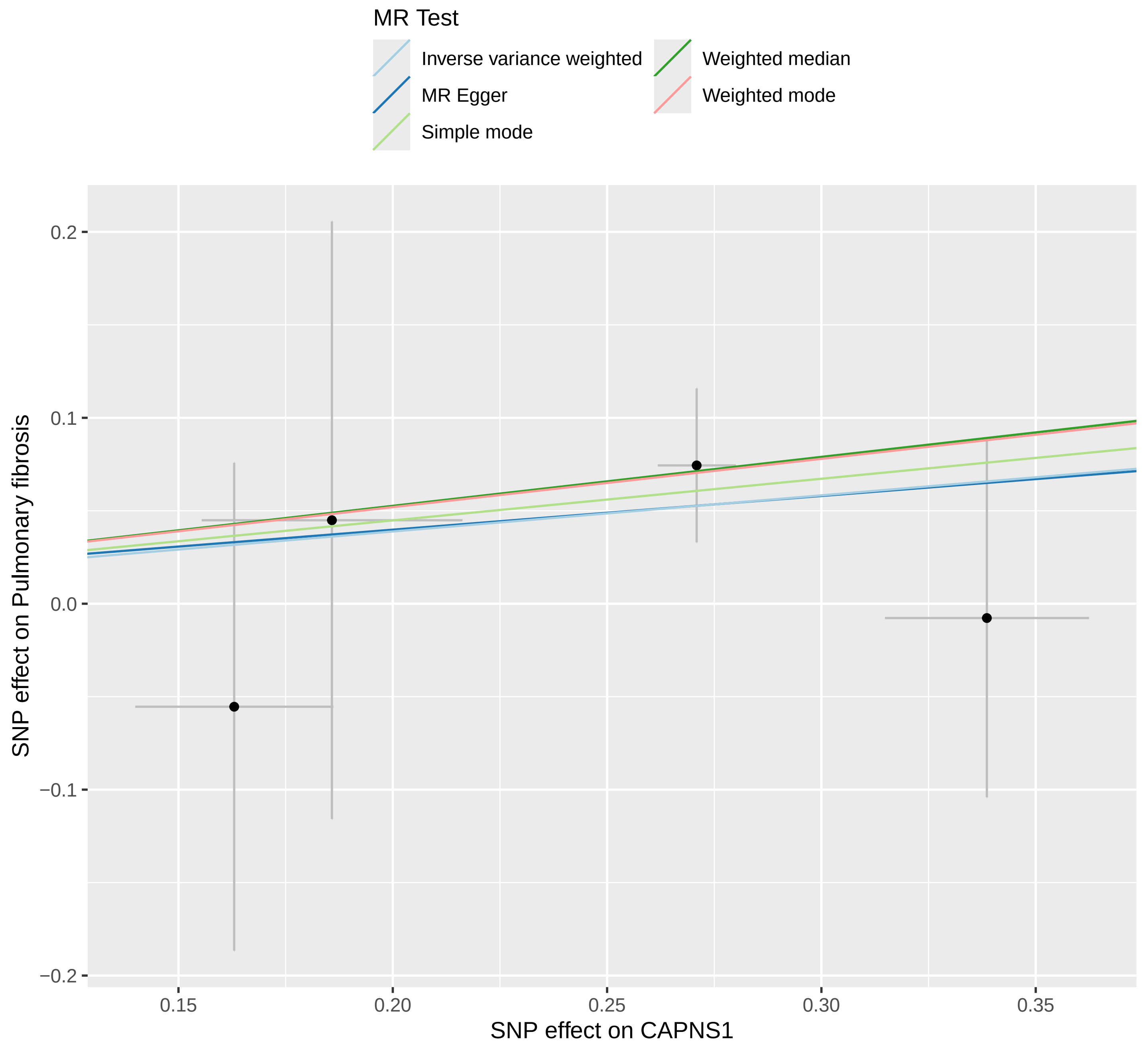
MR Test
Inverse variance weighted
MR Egger
Simple mode
Weighted median
Weighted mode
0.2
0.1
0.0
−0.1
−0.2
SNP effect on Pulmonary fibrosis
0.15
0.20
0.25
0.30
0.35
SNP effect on CAPNS1

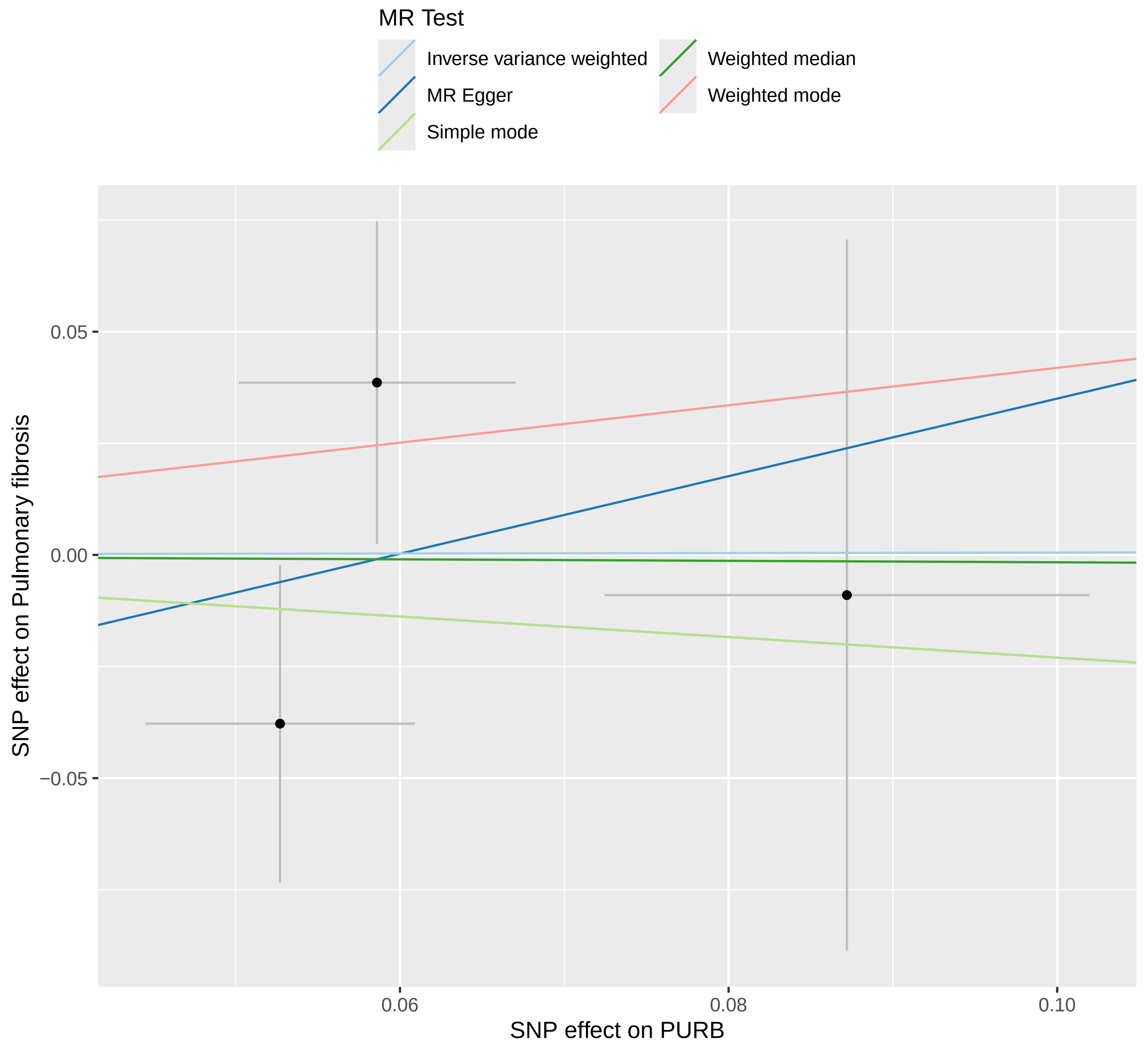

MR Test
Inverse variance weighted
MR Egger
Simple mode
Weighted median
Weighted mode
SNP effect on Pulmonary fibrosis
0.05
0.00
−0.05
0.06
0.08
0.10
SNP effect on PURB

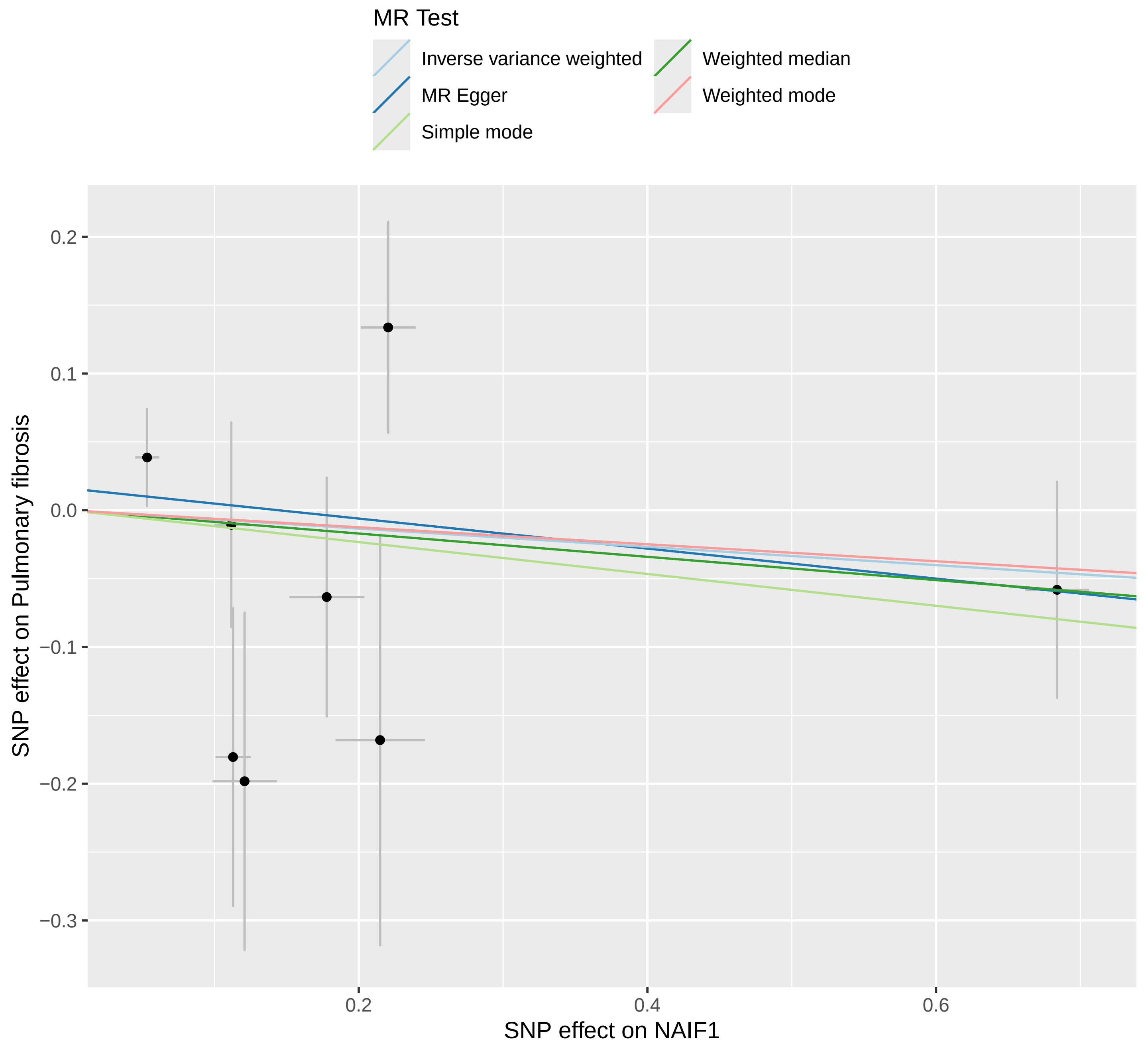

MR Test
Inverse variance weighted
MR Egger
Simple mode
Weighted median
Weighted mode
0.2
0.1
0.0
−0.1
−0.2
−0.3
SNP effect on Pulmonary fibrosis
0.2
0.4
0.6
SNP effect on NAIF1

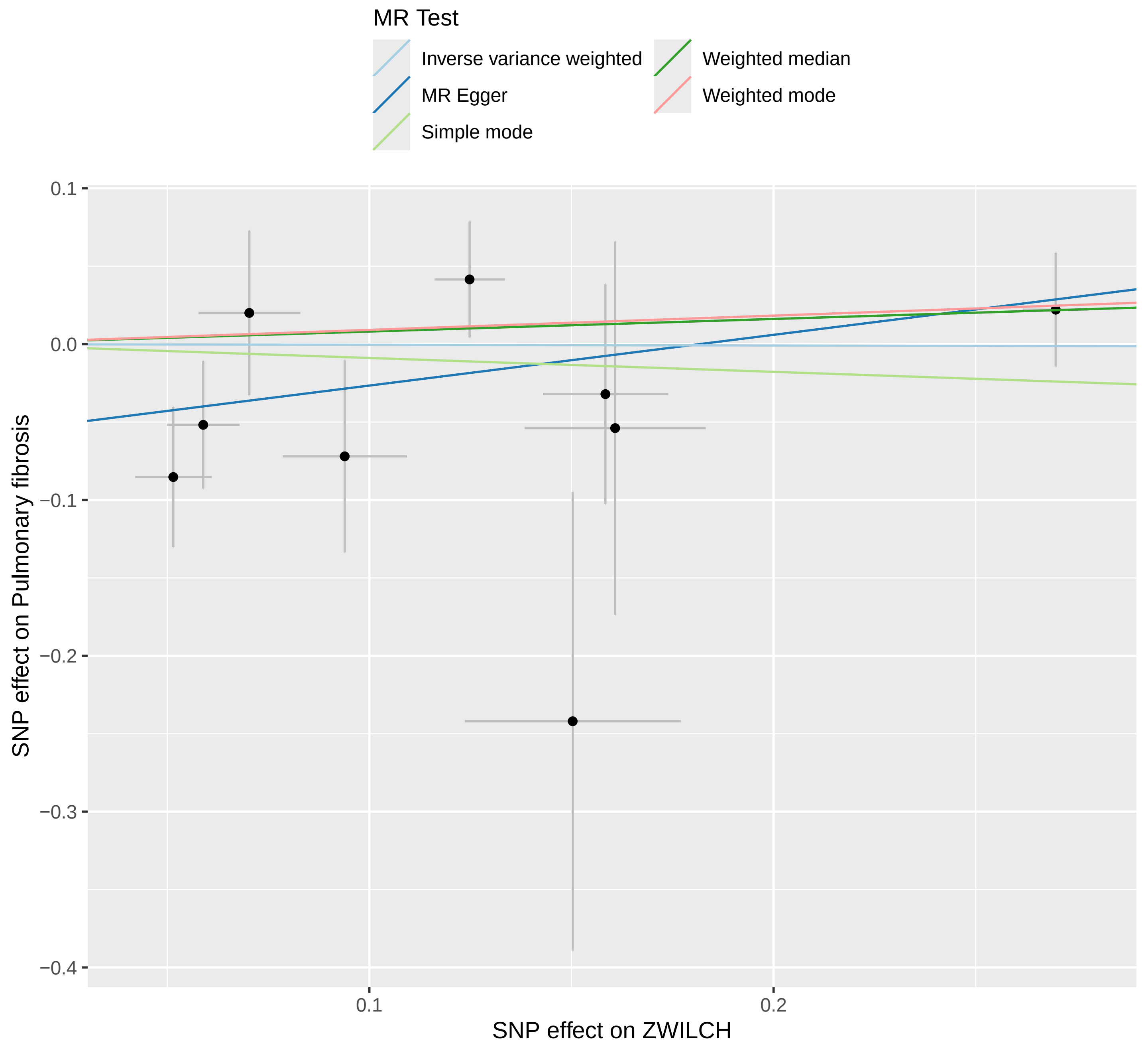
MR Test
Inverse variance weighted
MR Egger
Simple mode
Weighted median
Weighted mode
0.1
0.0
−0.1
−0.2
−0.3
−0.4
0.1
0.2
SNP effect on Pulmonary fibrosis
SNP effect on ZWILCH

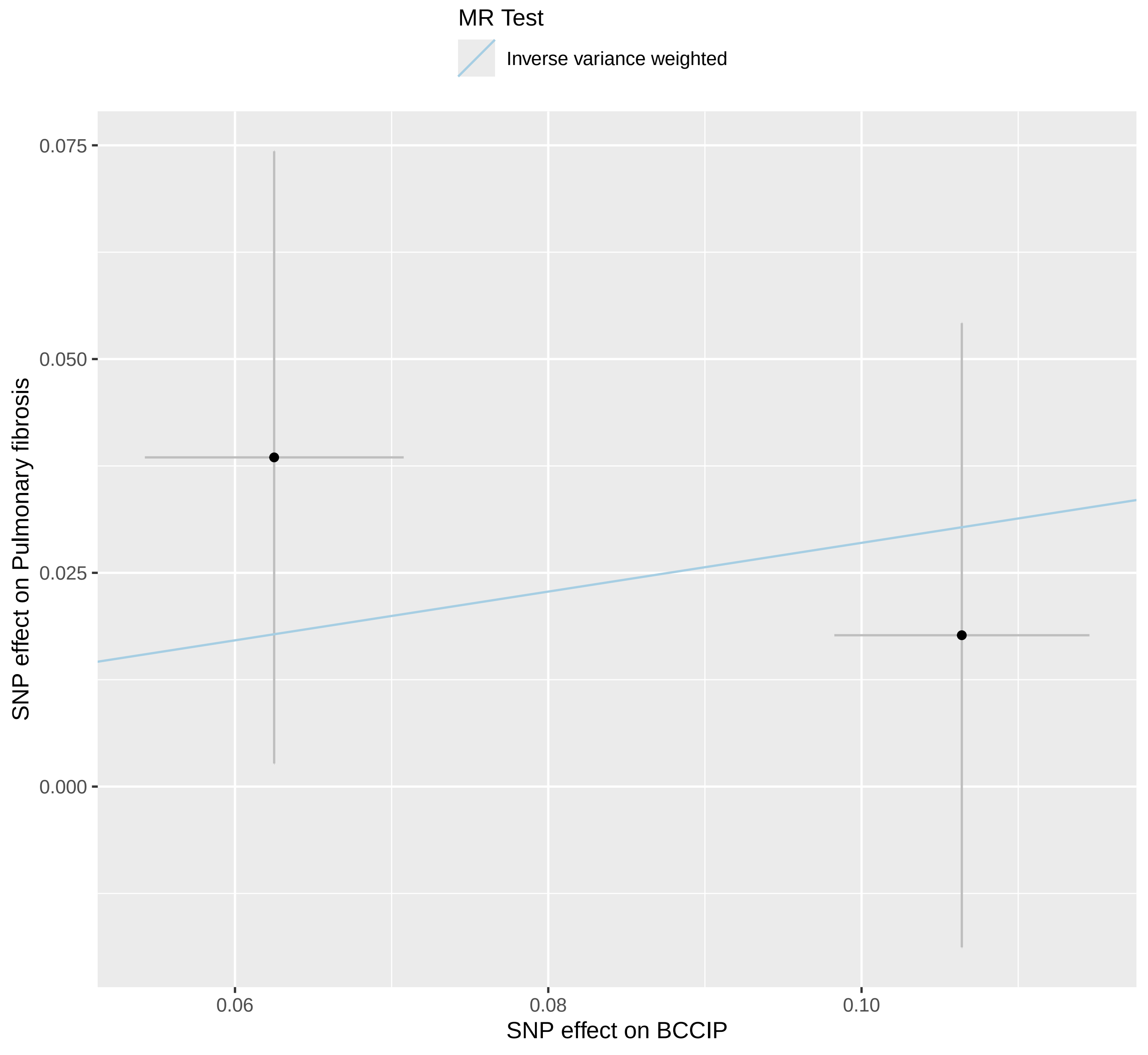

MR Test
Inverse variance weighted
0.075
0.050
0.025
0.000
SNP effect on Pulmonary fibrosis
0.06
0.08
0.10
SNP effect on BCCIP

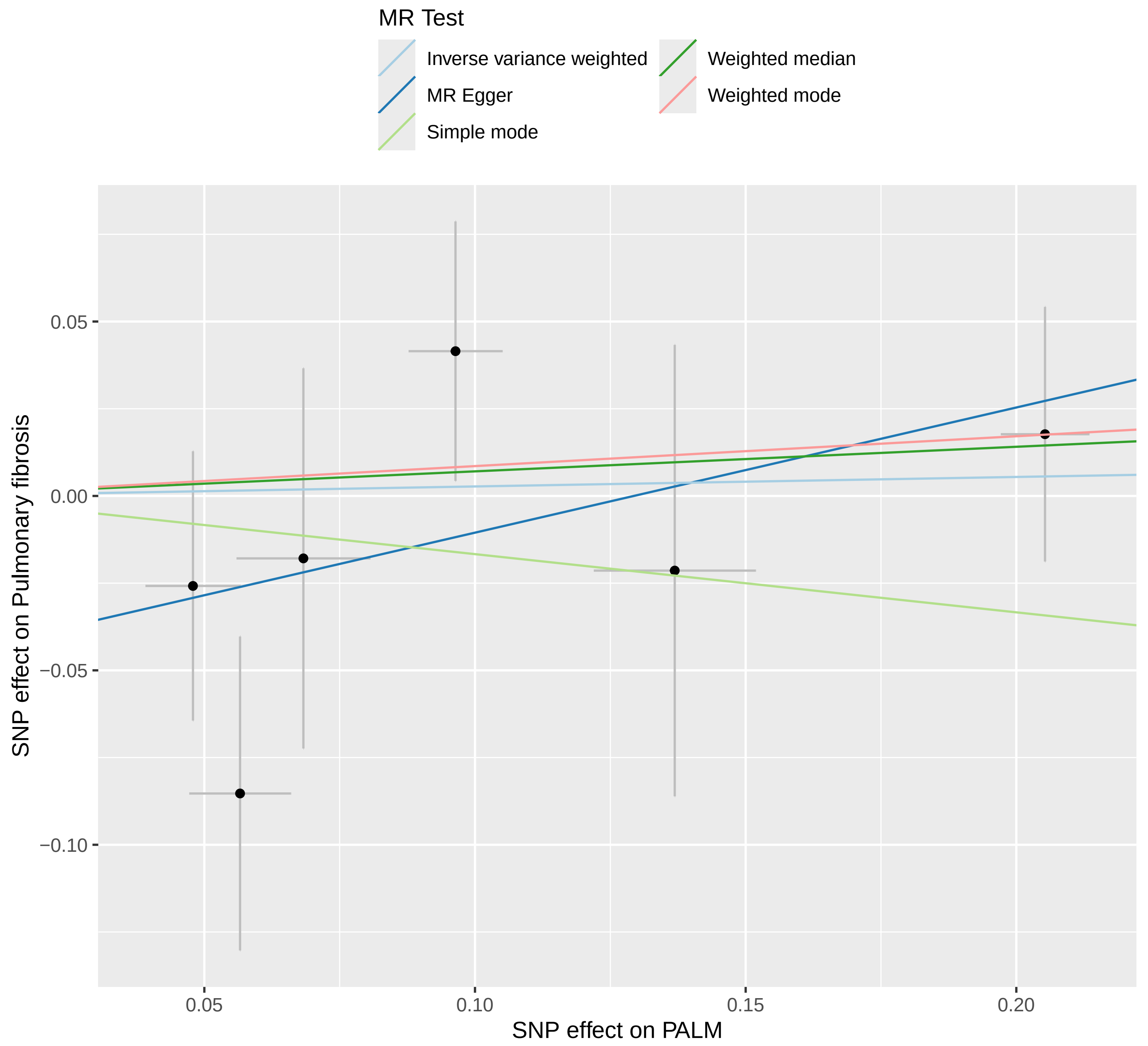

MR Test
Inverse variance weighted
MR Egger
Simple mode
Weighted median
Weighted mode
0.05
0.00
−0.05
−0.10
0.05
0.10
0.15
0.20
SNP effect on Pulmonary fibrosis
SNP effect on PALM

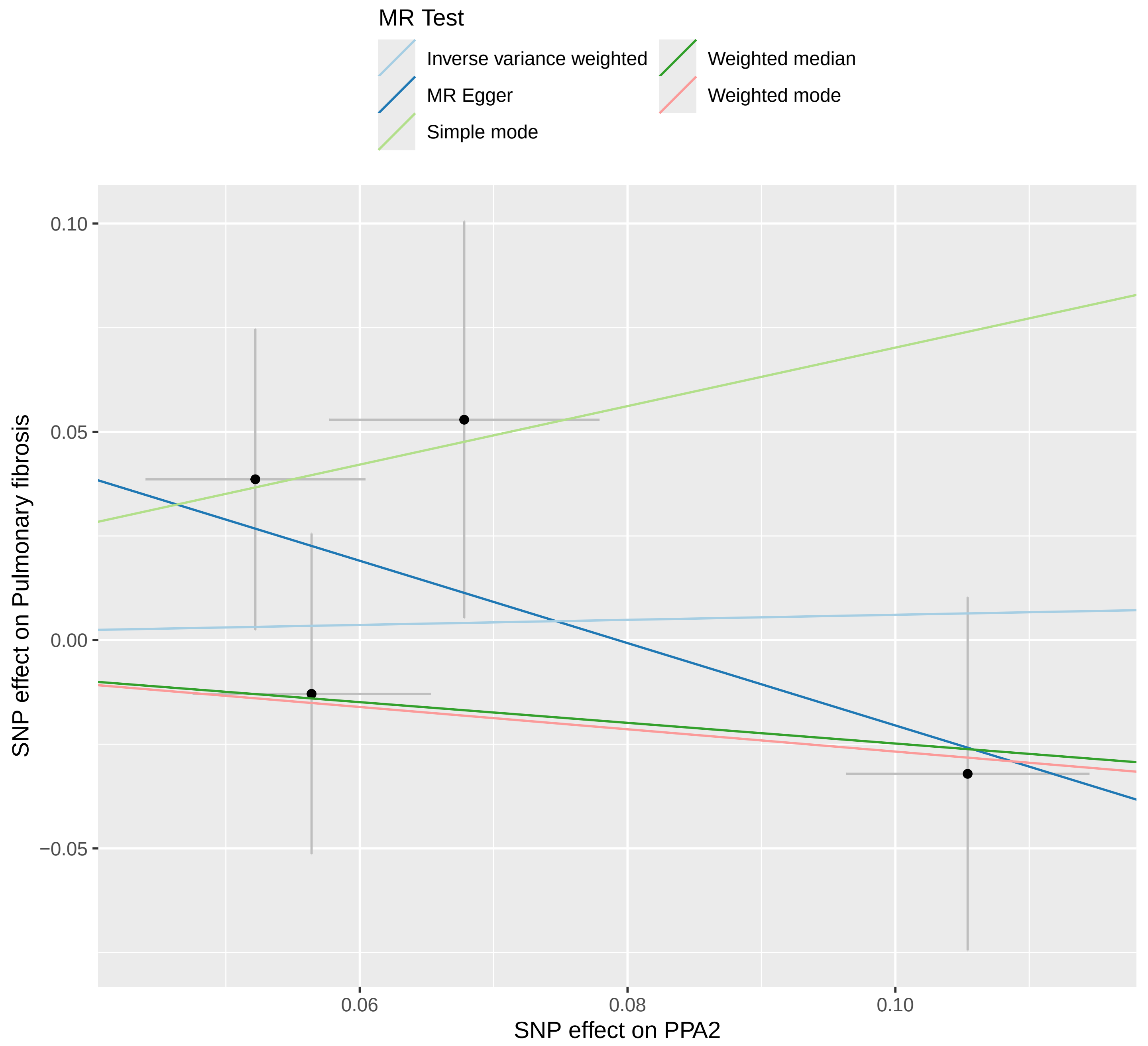
MR Test
Inverse variance weighted
MR Egger
Simple mode
Weighted median
Weighted mode
0.10
0.05
0.00
−0.05
0.06
0.08
0.10
SNP effect on Pulmonary fibrosis
SNP effect on PPA2

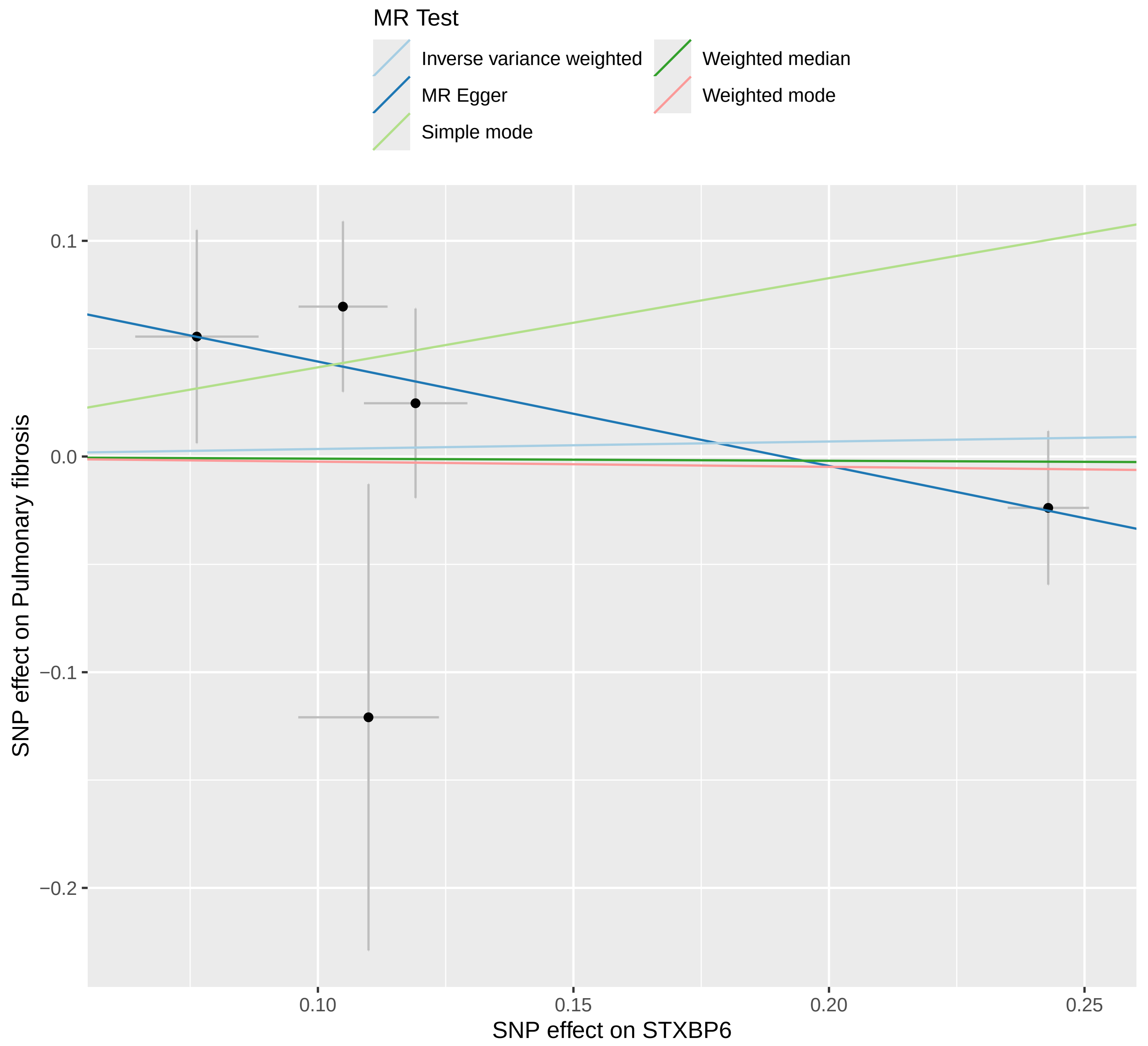

MR Test
Inverse variance weighted
MR Egger
Simple mode
Weighted median
Weighted mode
0.1
0.0
−0.1
−0.2
0.10
0.15
0.20
0.25
SNP effect on STXBP6
SNP effect on Pulmonary fibrosis

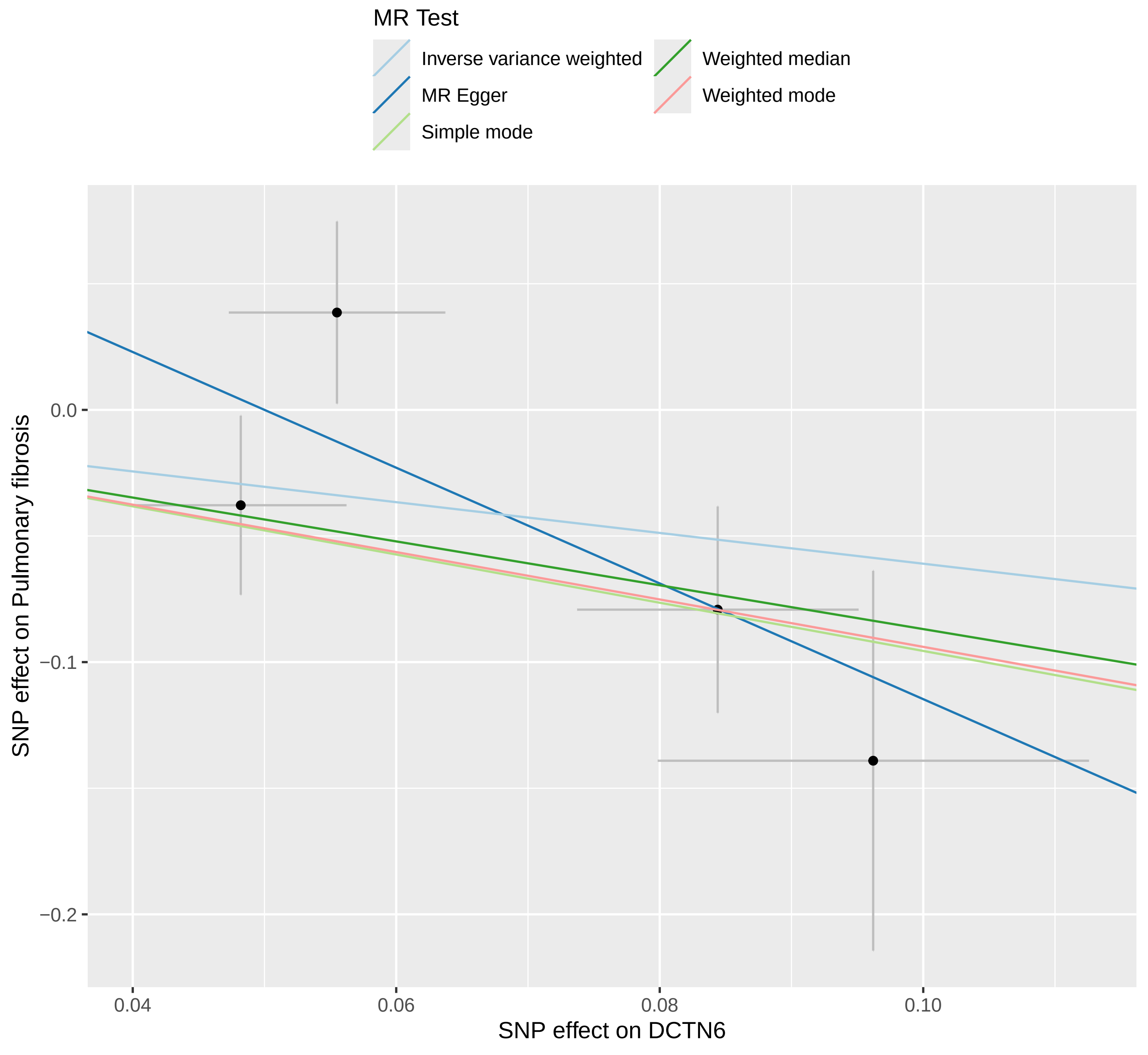
MR Test
Inverse variance weighted
MR Egger
Simple mode
Weighted median
Weighted mode
0.0
−0.1
−0.2
SNP effect on Pulmonary fibrosis
0.04
0.06
0.08
0.10
SNP effect on DCTN6

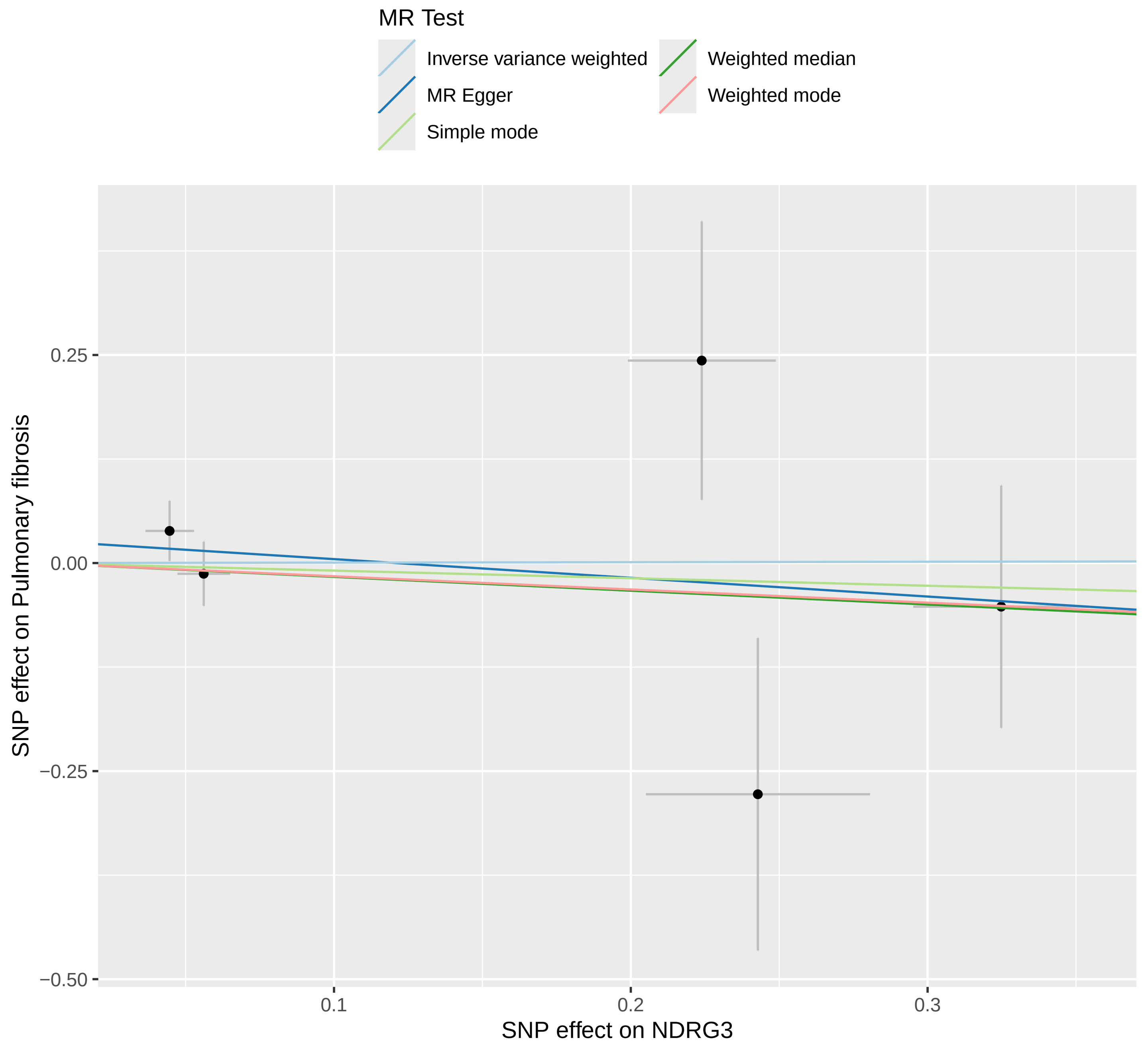
MR Test
Inverse variance weighted
MR Egger
Simple mode
Weighted median
Weighted mode
SNP effect on Pulmonary fibrosis
0.25
0.00
−0.25
−0.50
0.1
0.2
0.3
SNP effect on NDRG3

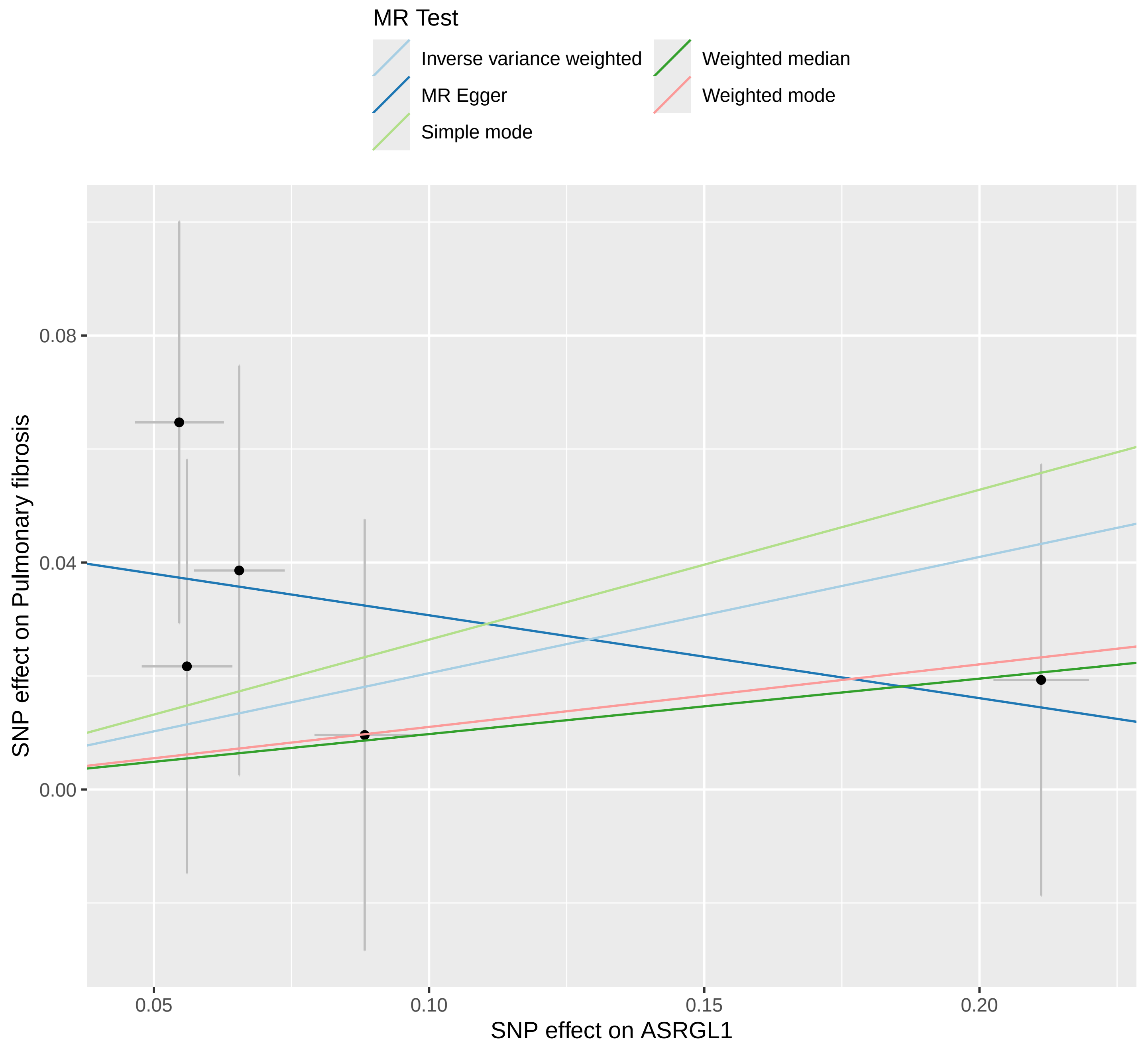
MR Test
Inverse variance weighted
MR Egger
Simple mode
Weighted median
Weighted mode
0.08
0.04
0.00
SNP effect on Pulmonary fibrosis
0.05
0.10
0.15
0.20
SNP effect on ASRGL1

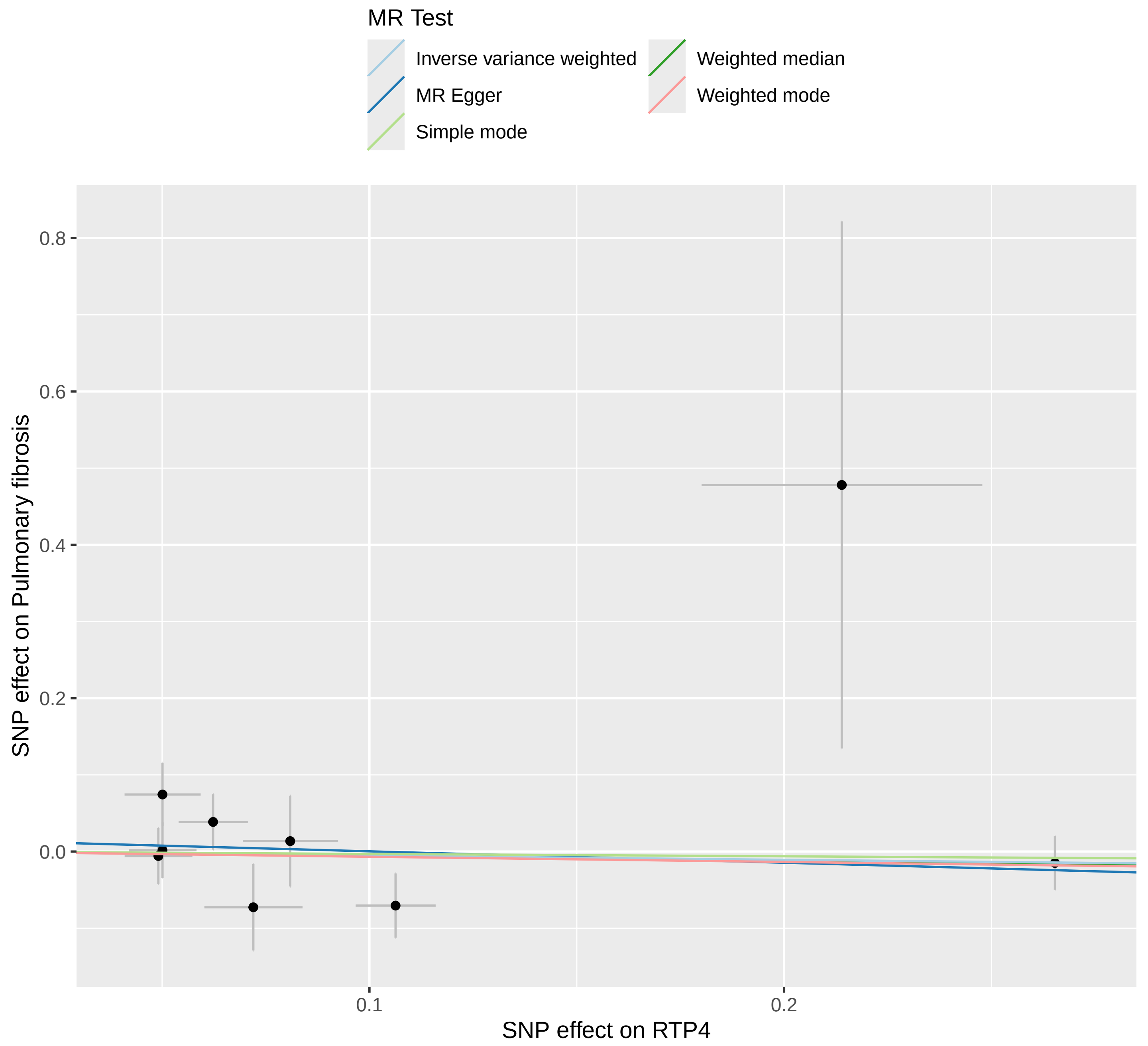
MR Test
Inverse variance weighted
MR Egger
Simple mode
Weighted median
Weighted mode
0.8
0.6
0.4
0.2
0.0
SNP effect on Pulmonary fibrosis
0.1
0.2
SNP effect on RTP4

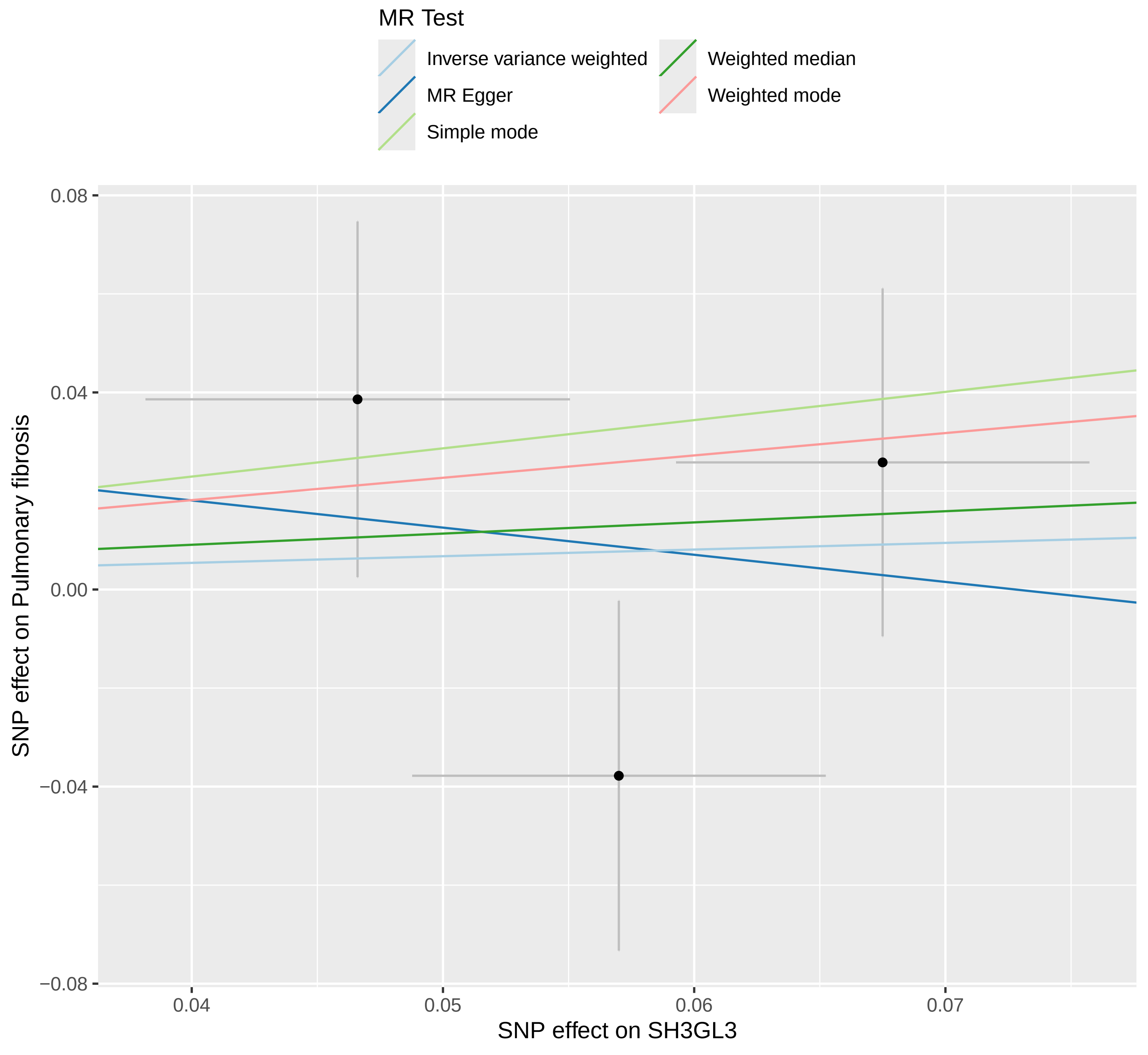

MR Test
Inverse variance weighted
MR Egger
Simple mode
Weighted median
Weighted mode
0.08
0.04
0.00
−0.04
−0.08
SNP effect on Pulmonary fibrosis
0.04
0.05
0.06
0.07
SNP effect on SH3GL3

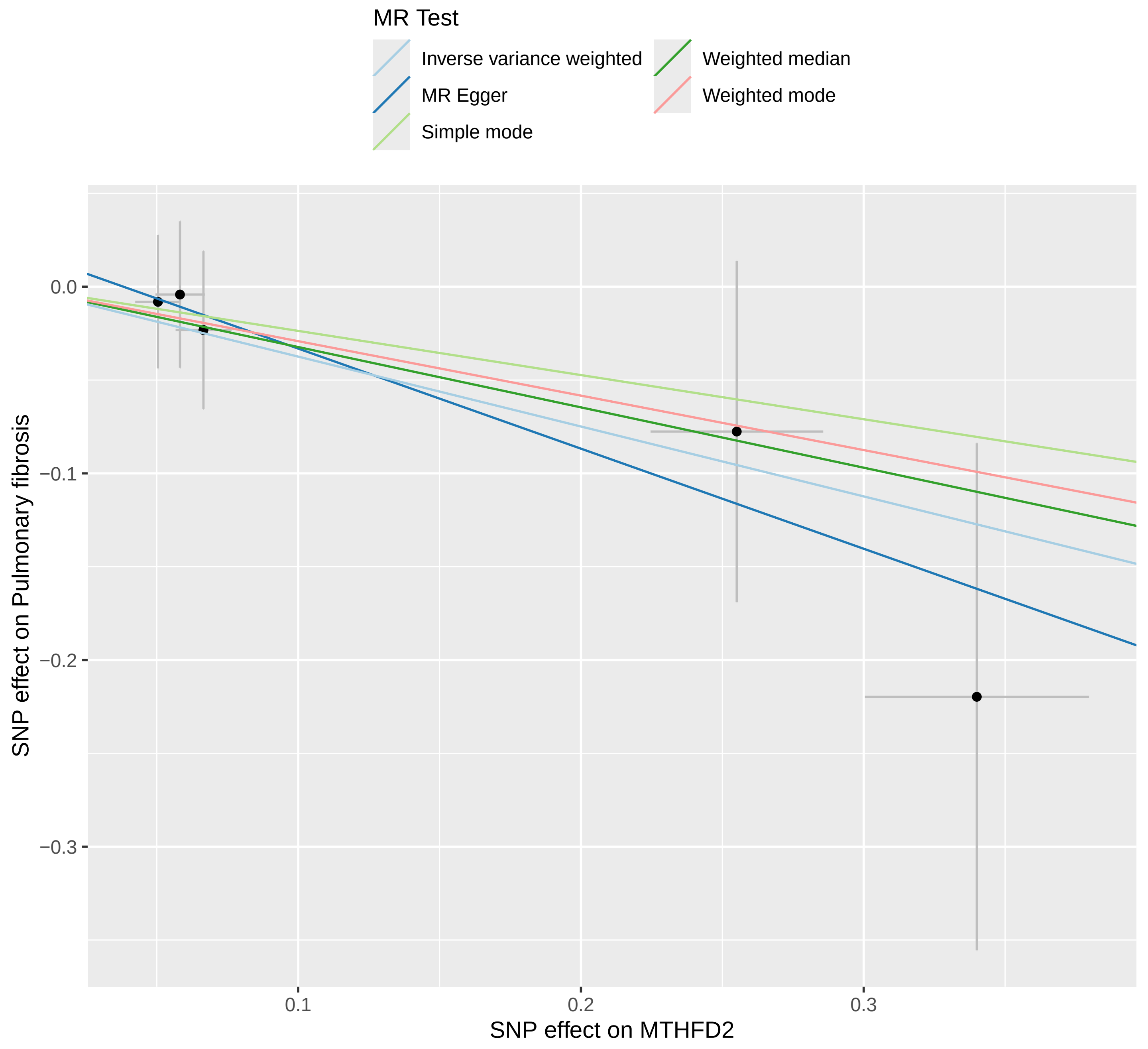

MR Test
Inverse variance weighted
MR Egger
Simple mode
Weighted median
Weighted mode
0.0
−0.1
−0.2
−0.3
0.1
0.2
0.3
SNP effect on Pulmonary fibrosis
SNP effect on MTHFD2

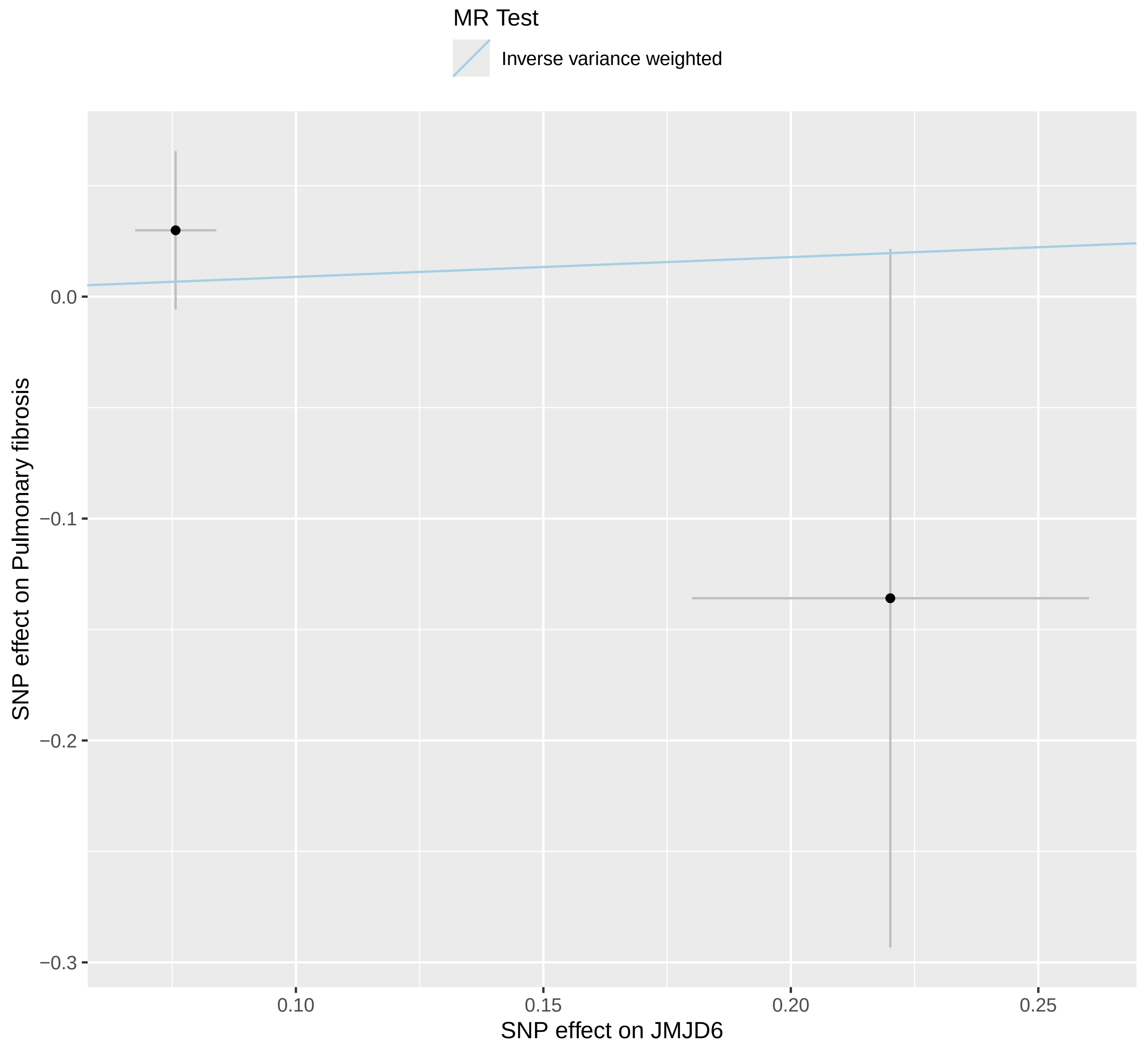

MR Test
Inverse variance weighted
0.0
−0.1
−0.2
−0.3
0.10
0.15
0.20
0.25
SNP effect on Pulmonary fibrosis
SNP effect on JMJD6

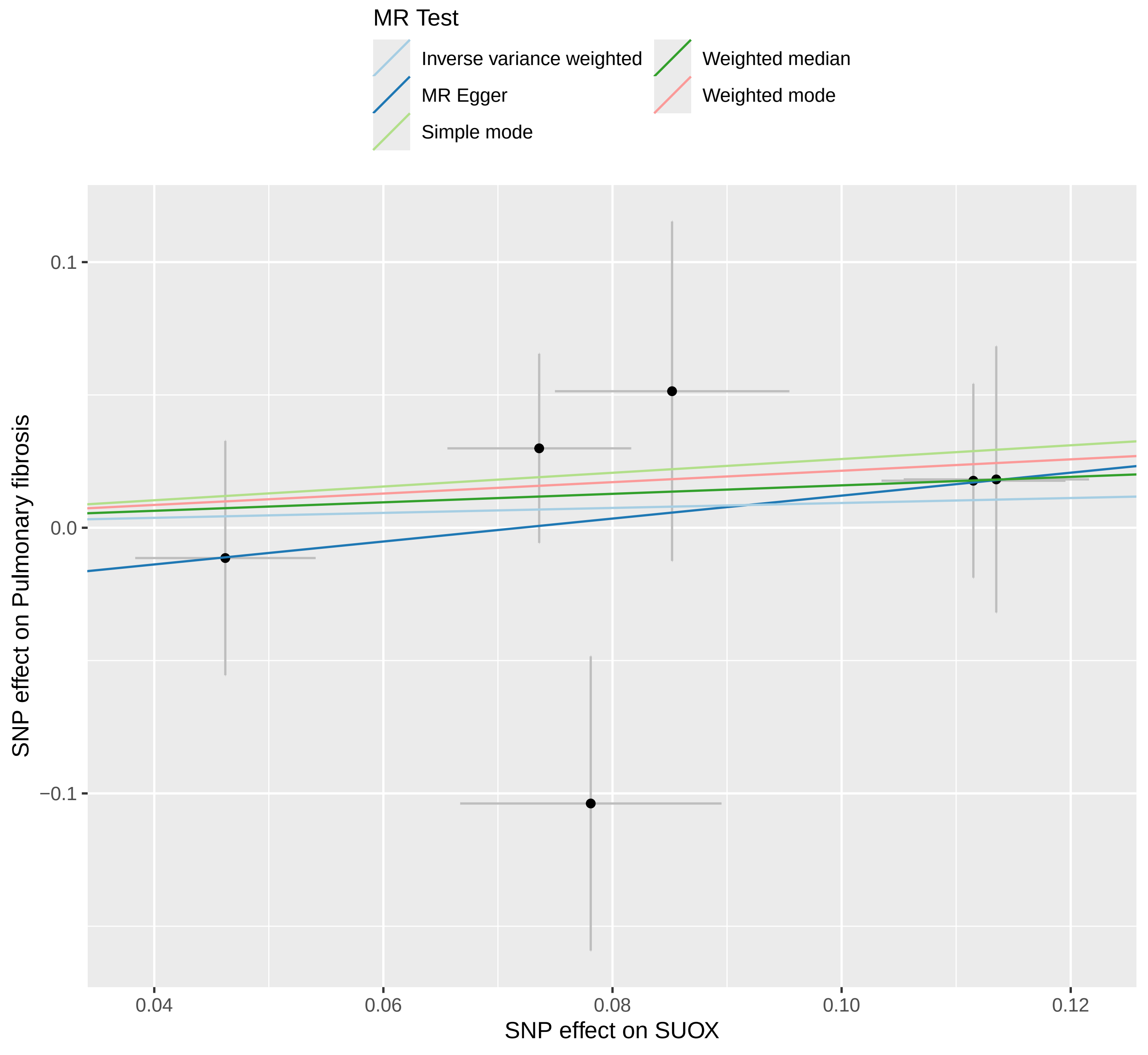
MR Test
Inverse variance weighted
MR Egger
Simple mode
Weighted median
Weighted mode
0.1
0.0
−0.1
SNP effect on Pulmonary fibrosis
0.04
0.06
0.08
0.10
0.12
SNP effect on SUOX

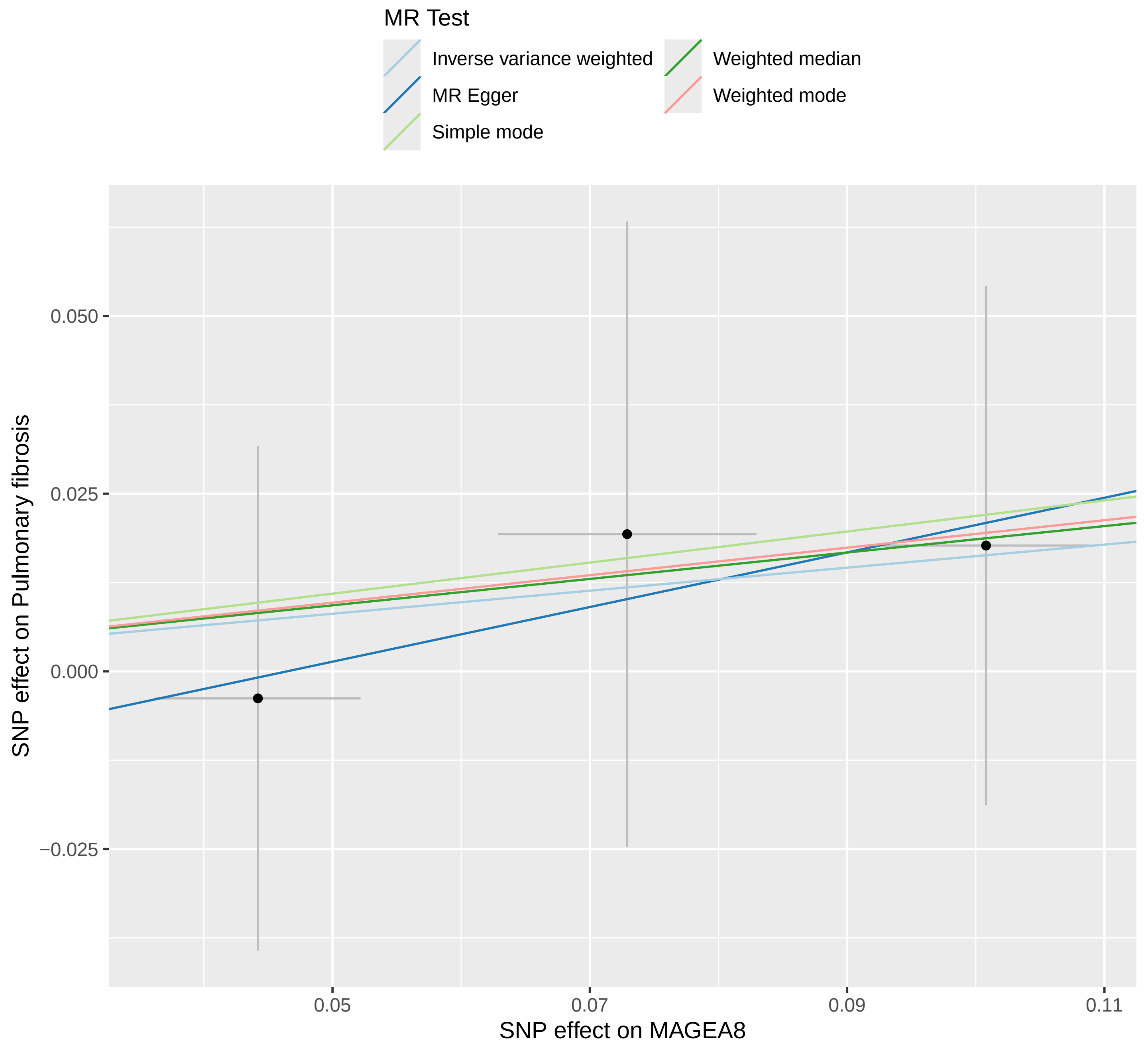

MR Test
Inverse variance weighted
MR Egger
Simple mode
Weighted median
Weighted mode
0.050
0.025
0.000
−0.025
0.05
0.07
0.09
0.11
SNP effect on MAGEA8
SNP effect on Pulmonary fibrosis

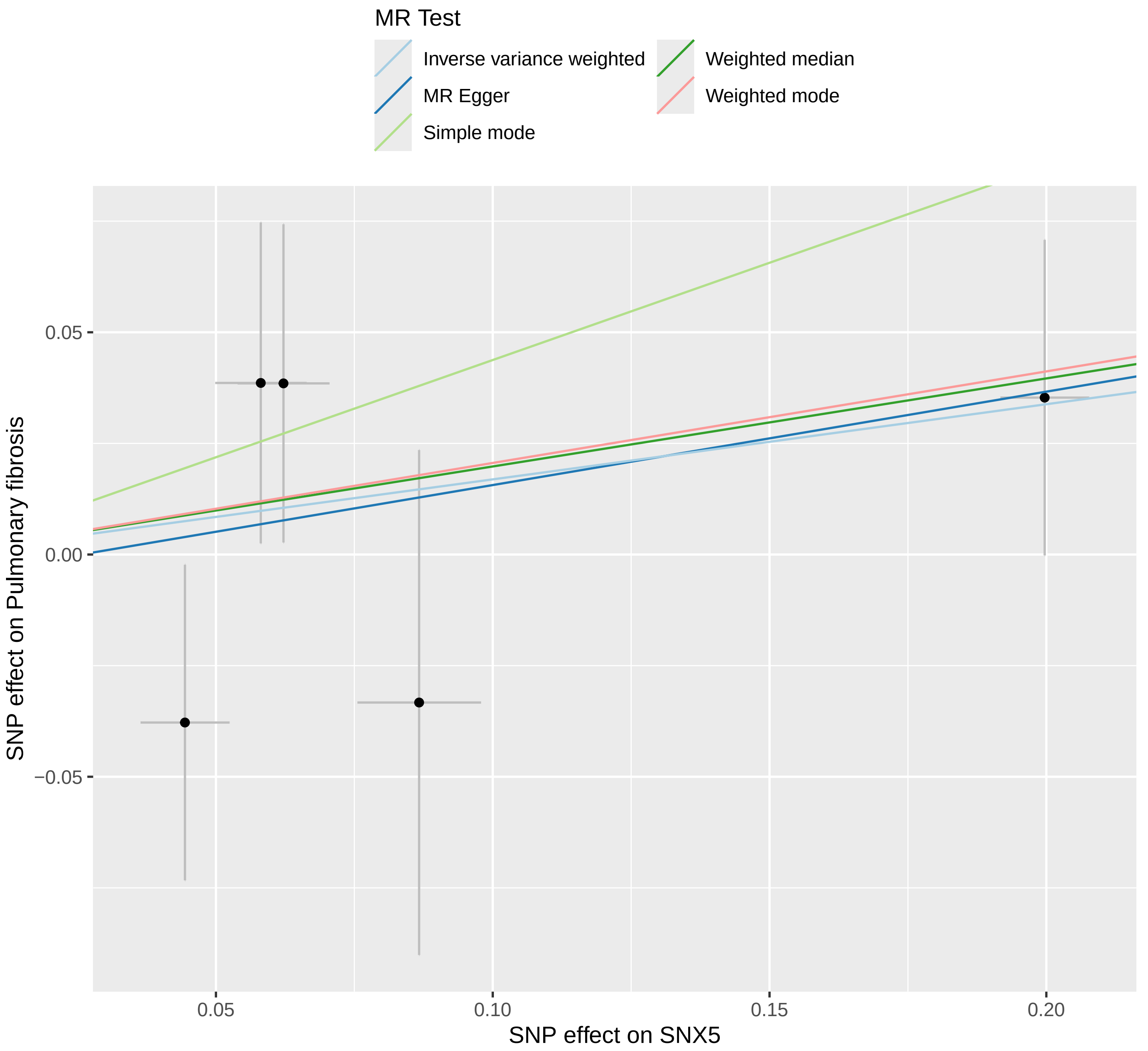

MR Test
Inverse variance weighted
MR Egger
Simple mode
Weighted median
Weighted mode
0.05
0.00
−0.05
SNP effect on Pulmonary fibrosis
0.05
0.10
0.15
0.20
SNP effect on SNX5

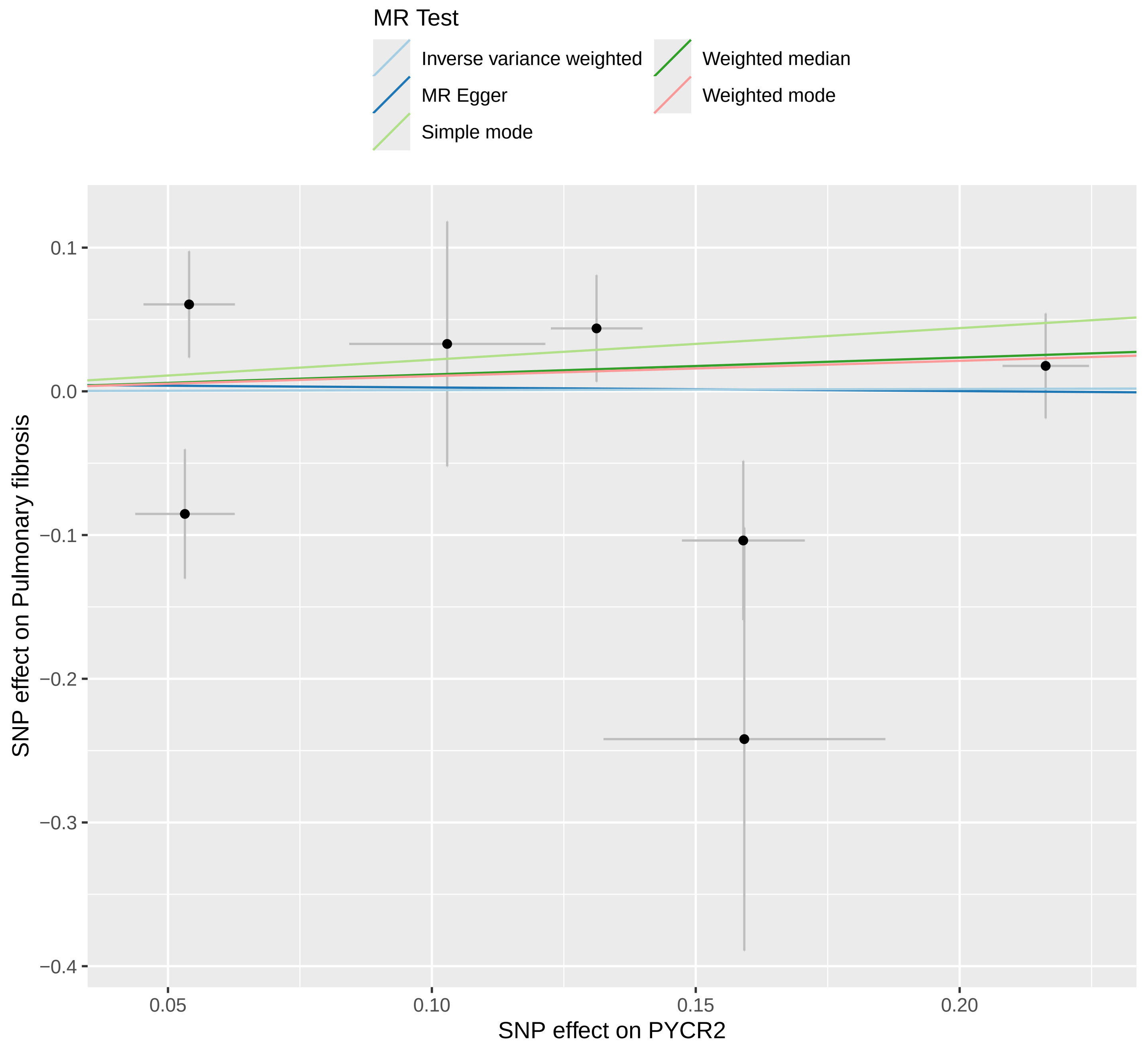

MR Test
Inverse variance weighted
MR Egger
Simple mode
Weighted median
Weighted mode
0.1
0.0
−0.1
−0.2
−0.3
−0.4
0.05
0.10
0.15
0.20
SNP effect on PYCR2
SNP effect on Pulmonary fibrosis

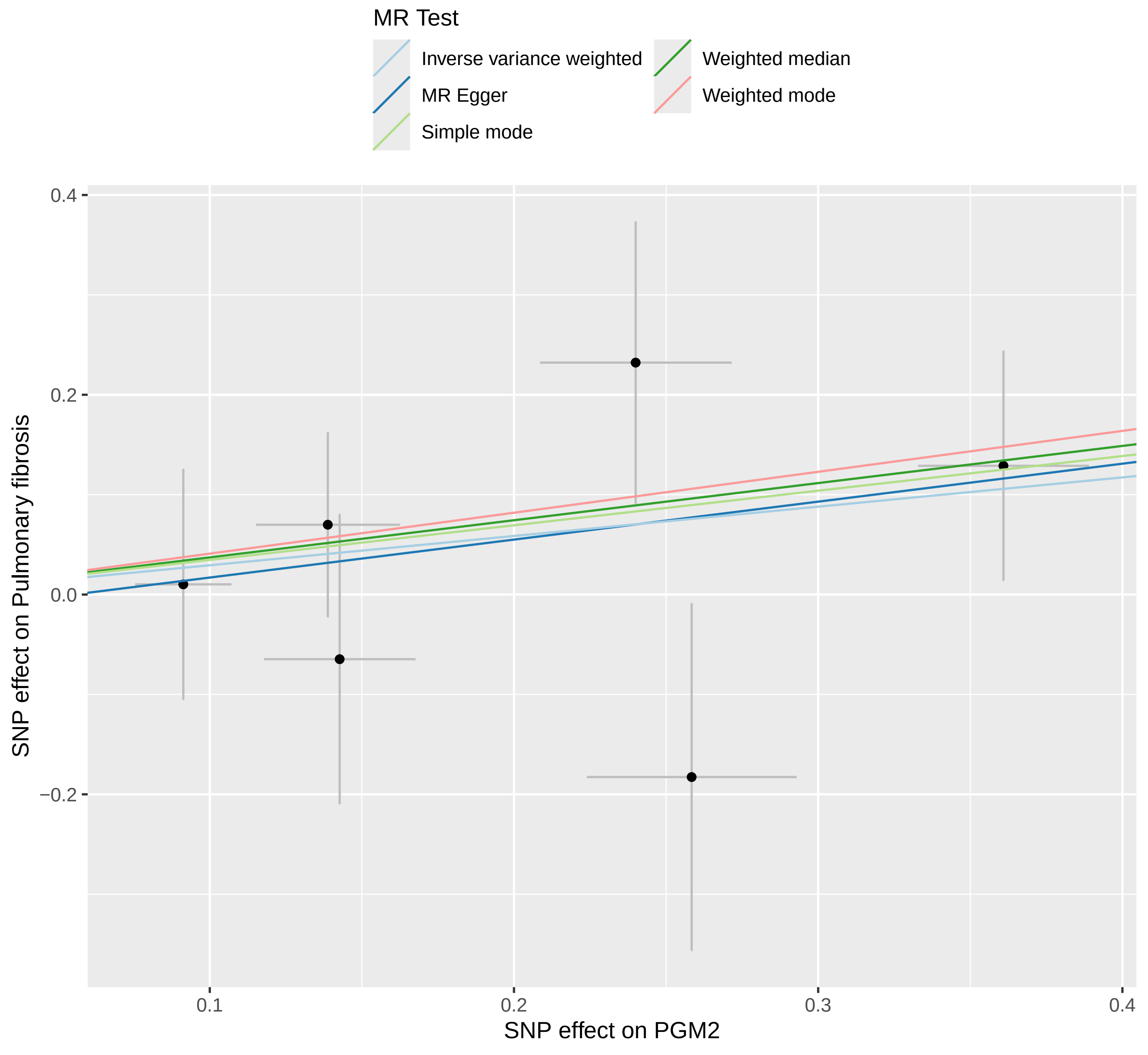

MR Test
Inverse variance weighted
MR Egger
Simple mode
Weighted median
Weighted mode
0.4
0.2
0.0
−0.2
0.1
0.2
0.3
0.4
SNP effect on PGM2
SNP effect on Pulmonary fibrosis

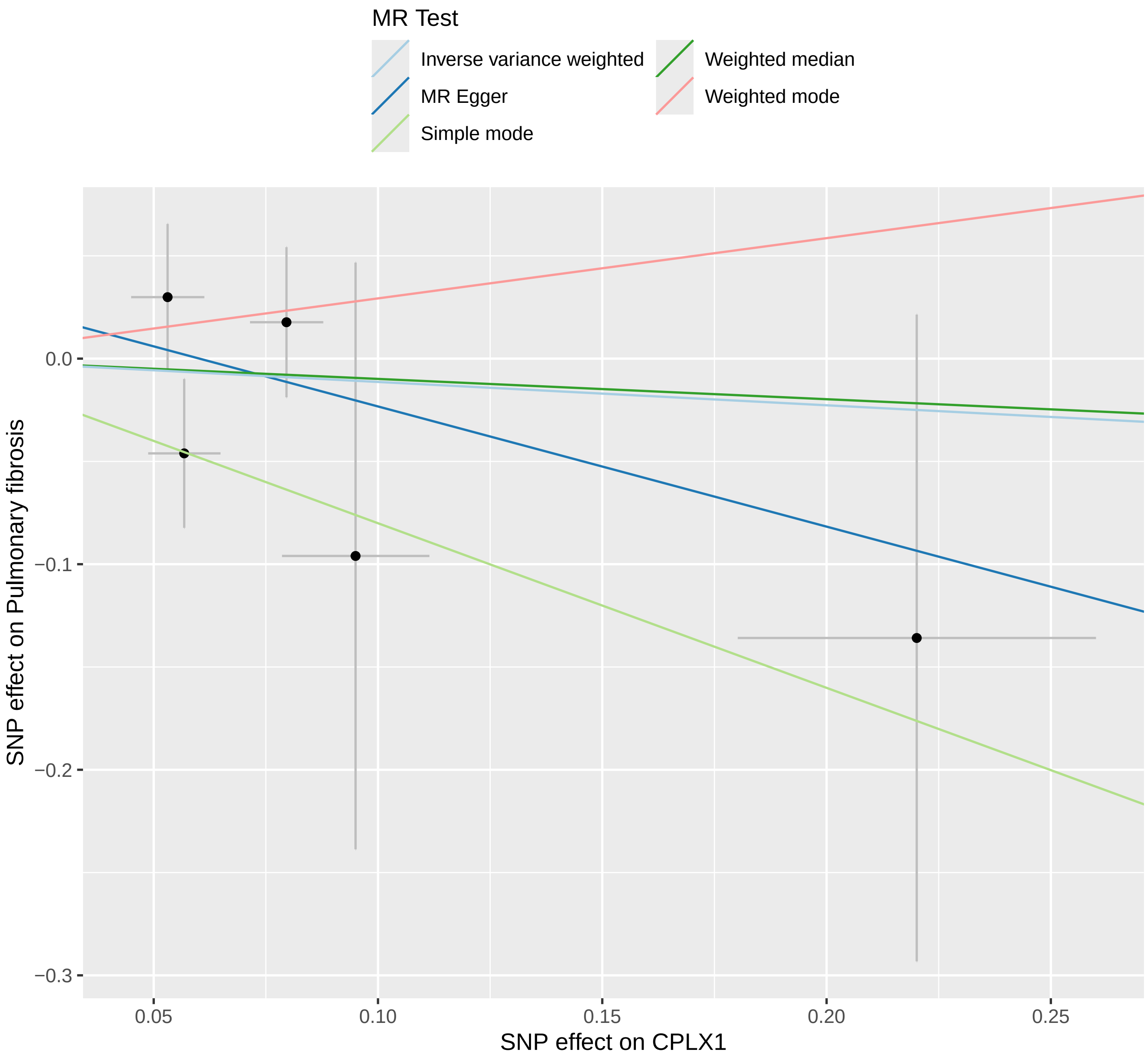
MR Test
Inverse variance weighted
MR Egger
Simple mode
Weighted median
Weighted mode
0.0
−0.1
−0.2
−0.3
0.05
0.10
0.15
0.20
0.25
SNP effect on Pulmonary fibrosis
SNP effect on CPLX1

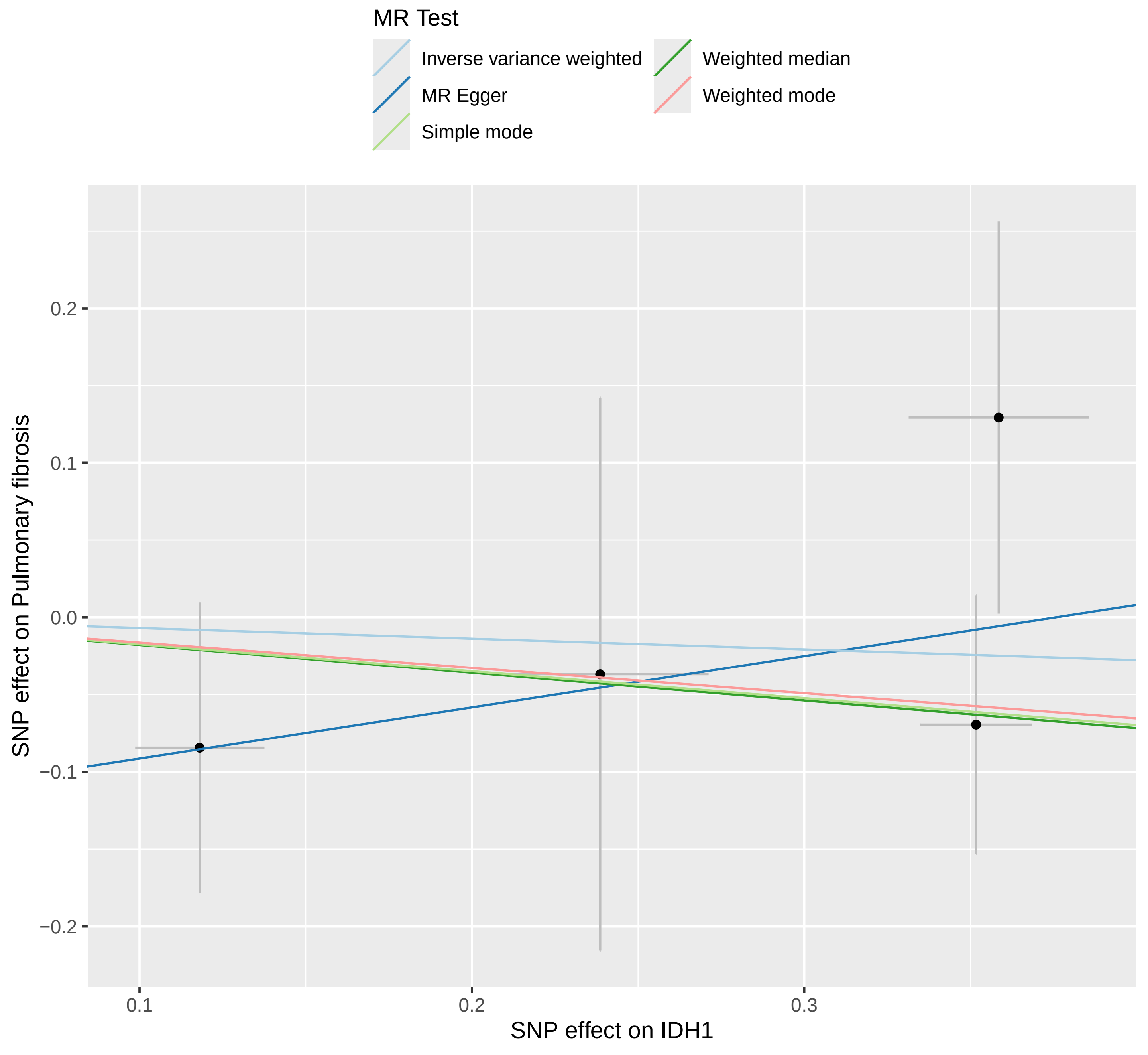

MR Test
Inverse variance weighted
MR Egger
Simple mode
Weighted median
Weighted mode
0.2
0.1
0.0
−0.1
−0.2
SNP effect on Pulmonary fibrosis
0.1
0.2
0.3
SNP effect on IDH1

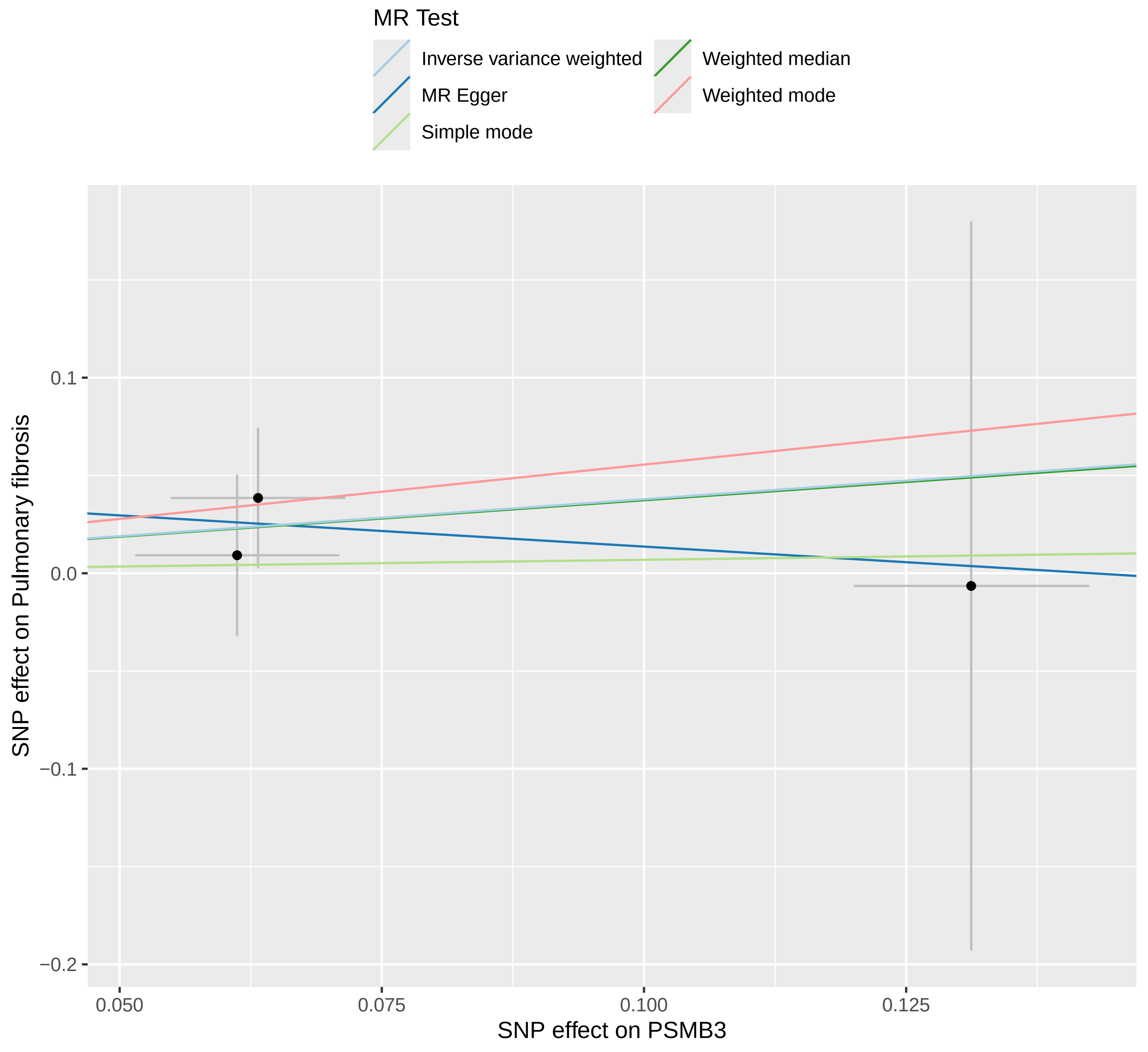

MR Test
Inverse variance weighted
MR Egger
Simple mode
Weighted median
Weighted mode
0.1
0.0
−0.1
−0.2
0.050
0.075
0.100
0.125
SNP effect on Pulmonary fibrosis
SNP effect on PSMB3

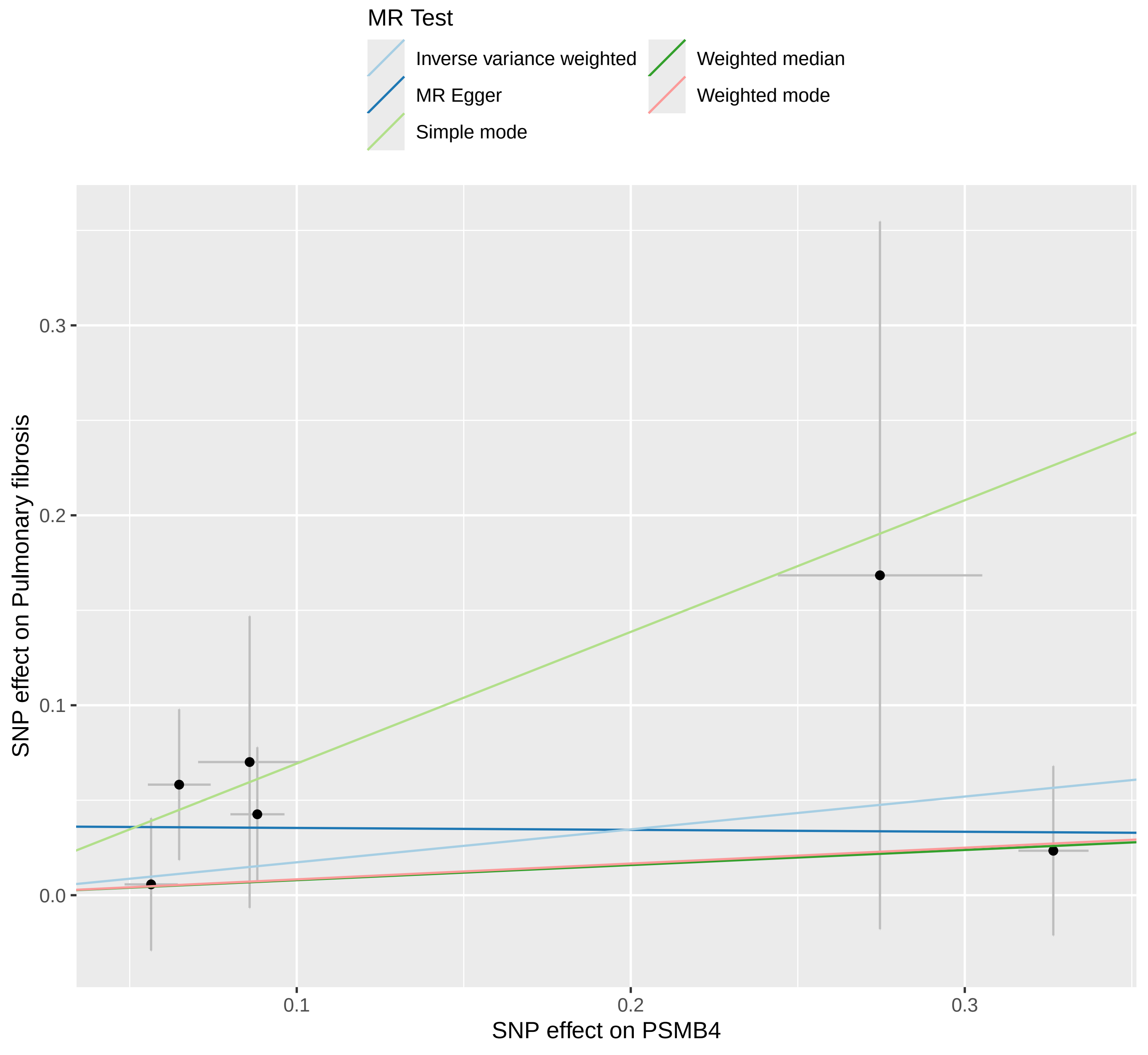
MR Test
Inverse variance weighted
MR Egger
Simple mode
Weighted median
Weighted mode
0.3
0.2
0.1
0.0
SNP effect on Pulmonary fibrosis
0.1
0.2
0.3
SNP effect on PSMB4

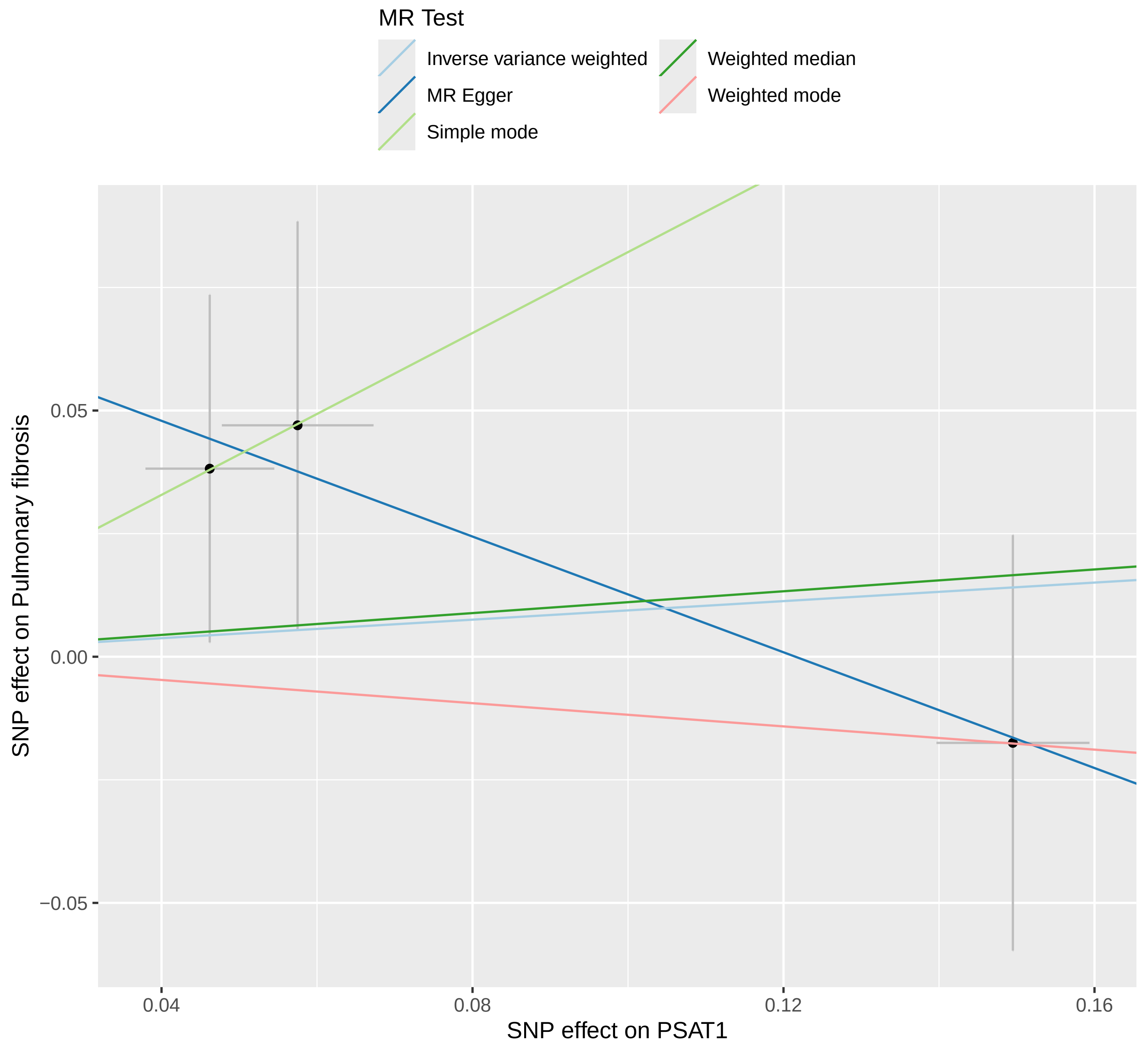

MR Test
Inverse variance weighted
MR Egger
Simple mode
Weighted median
Weighted mode
0.05
0.00
−0.05
SNP effect on Pulmonary fibrosis
0.04
0.08
0.12
0.16
SNP effect on PSAT1

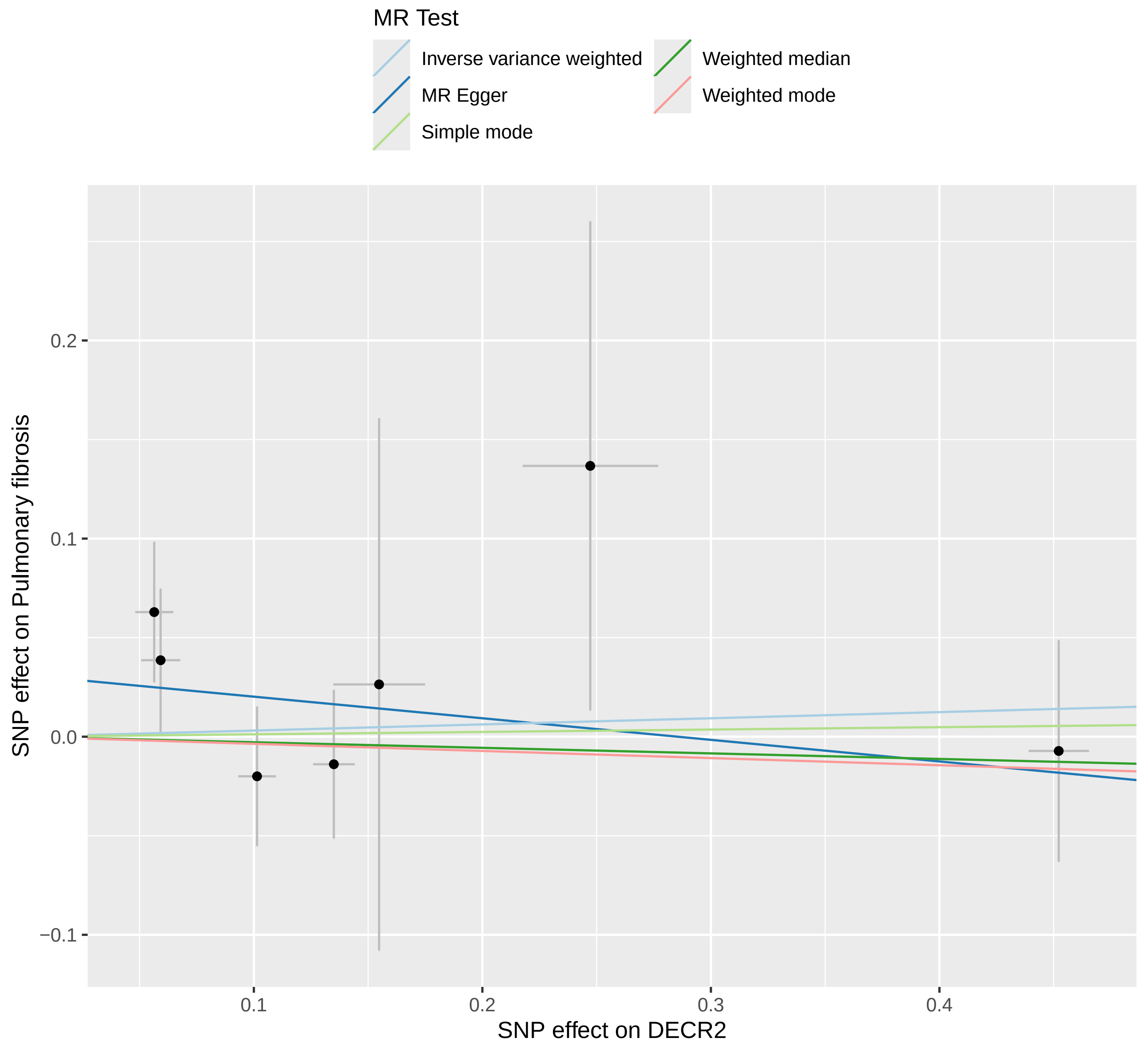
MR Test
Inverse variance weighted
MR Egger
Simple mode
Weighted median
Weighted mode
SNP effect on Pulmonary fibrosis
0.2
0.1
0.0
−0.1
0.1
0.2
0.3
0.4
SNP effect on DECR2

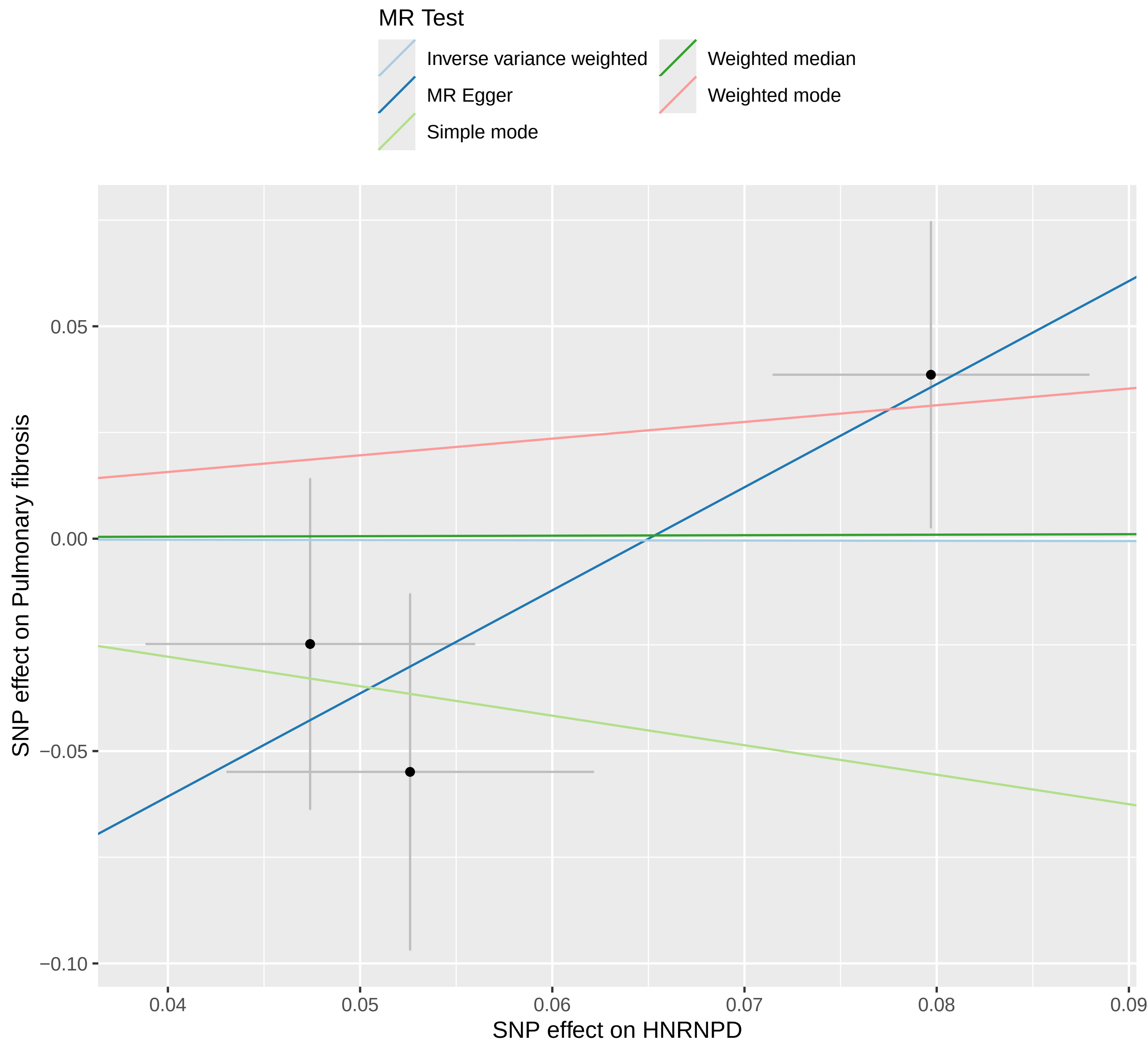

MR Test
Inverse variance weighted
MR Egger
Simple mode
Weighted median
Weighted mode
0.05
0.00
−0.05
−0.10
SNP effect on Pulmonary fibrosis
0.04
0.05
0.06
0.07
0.08
0.09
SNP effect on HNRNPD

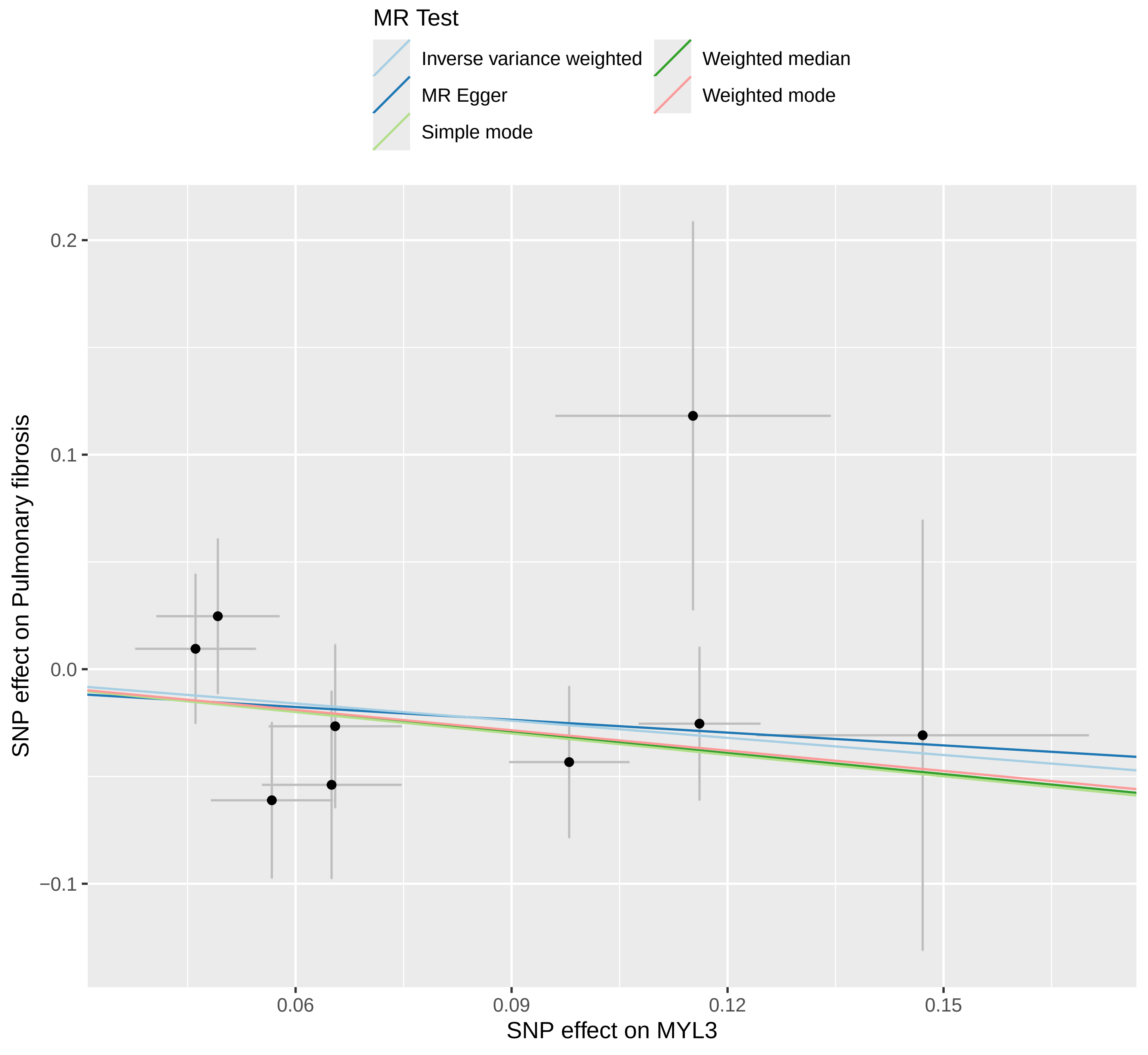

MR Test
Inverse variance weighted
MR Egger
Simple mode
Weighted median
Weighted mode
0.2
0.1
0.0
−0.1
0.06
0.09
0.12
0.15
SNP effect on Pulmonary fibrosis
SNP effect on MYL3

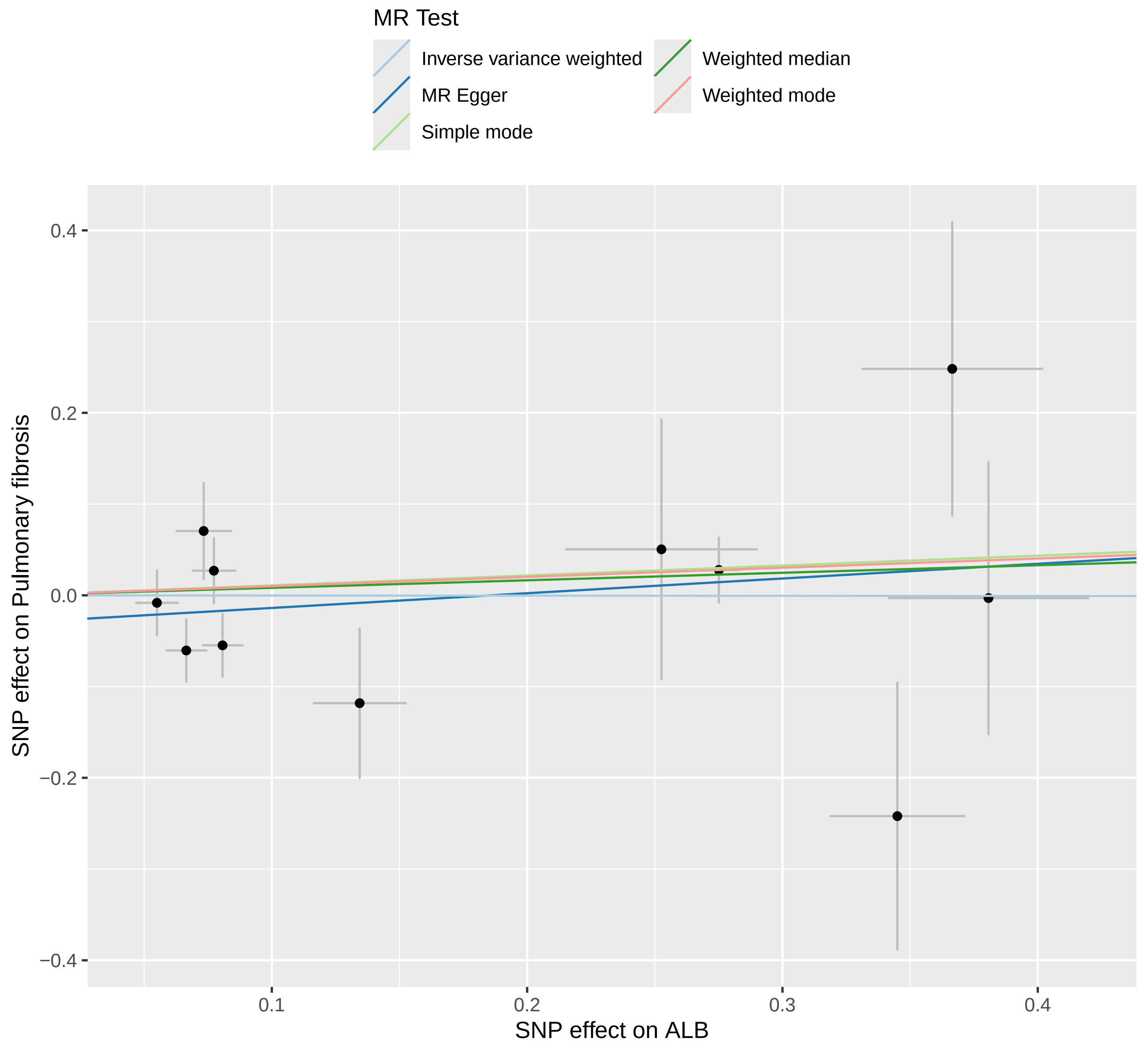

MR Test
Inverse variance weighted
MR Egger
Simple mode
Weighted median
Weighted mode
SNP effect on Pulmonary fibrosis
0.4
0.2
0.0
−0.2
−0.4
0.1
0.2
0.3
0.4
SNP effect on ALB

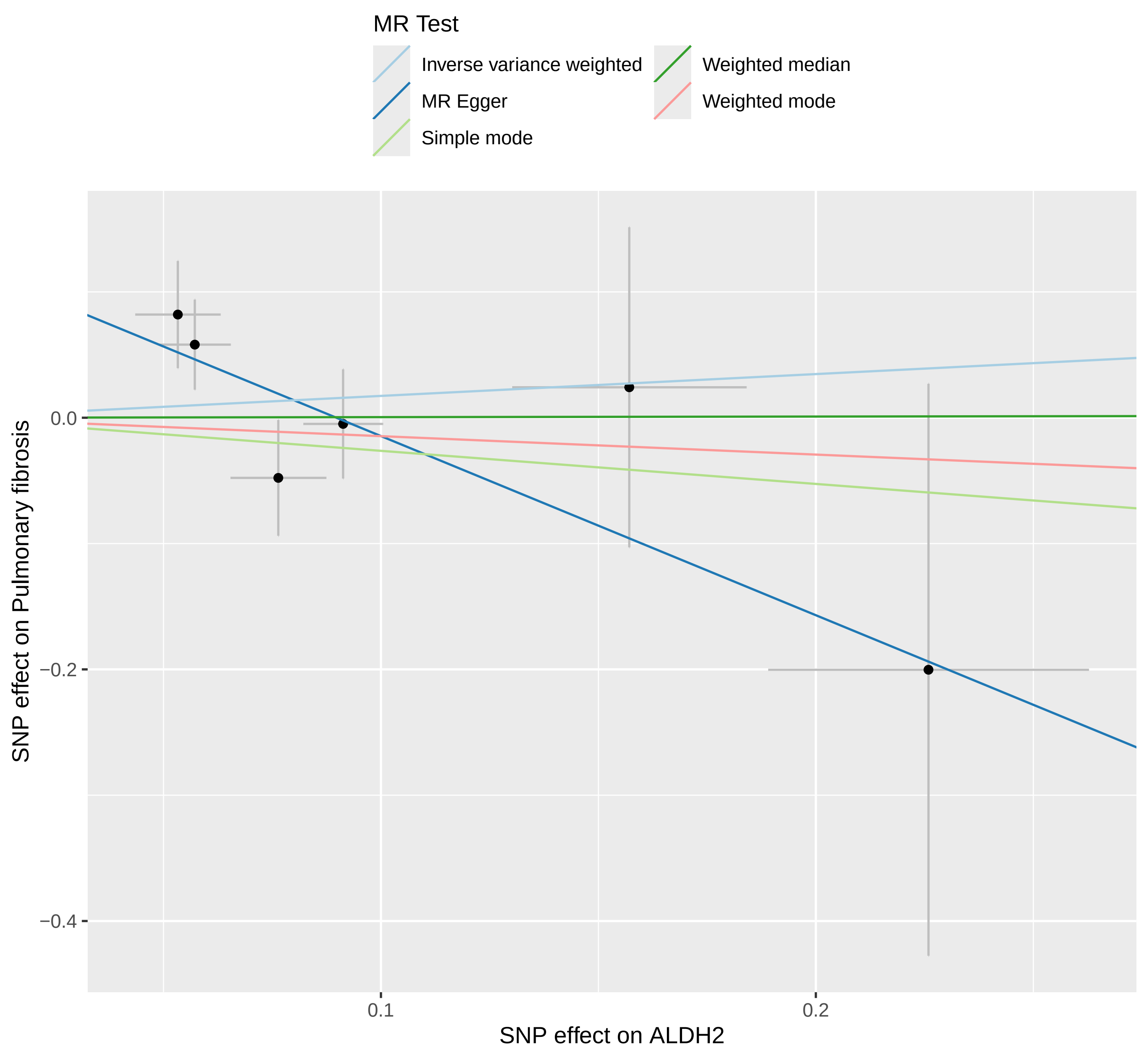
MR Test
Inverse variance weighted
MR Egger
Simple mode
Weighted median
Weighted mode
0.0
−0.2
−0.4
0.1
0.2
SNP effect on Pulmonary fibrosis
SNP effect on ALDH2

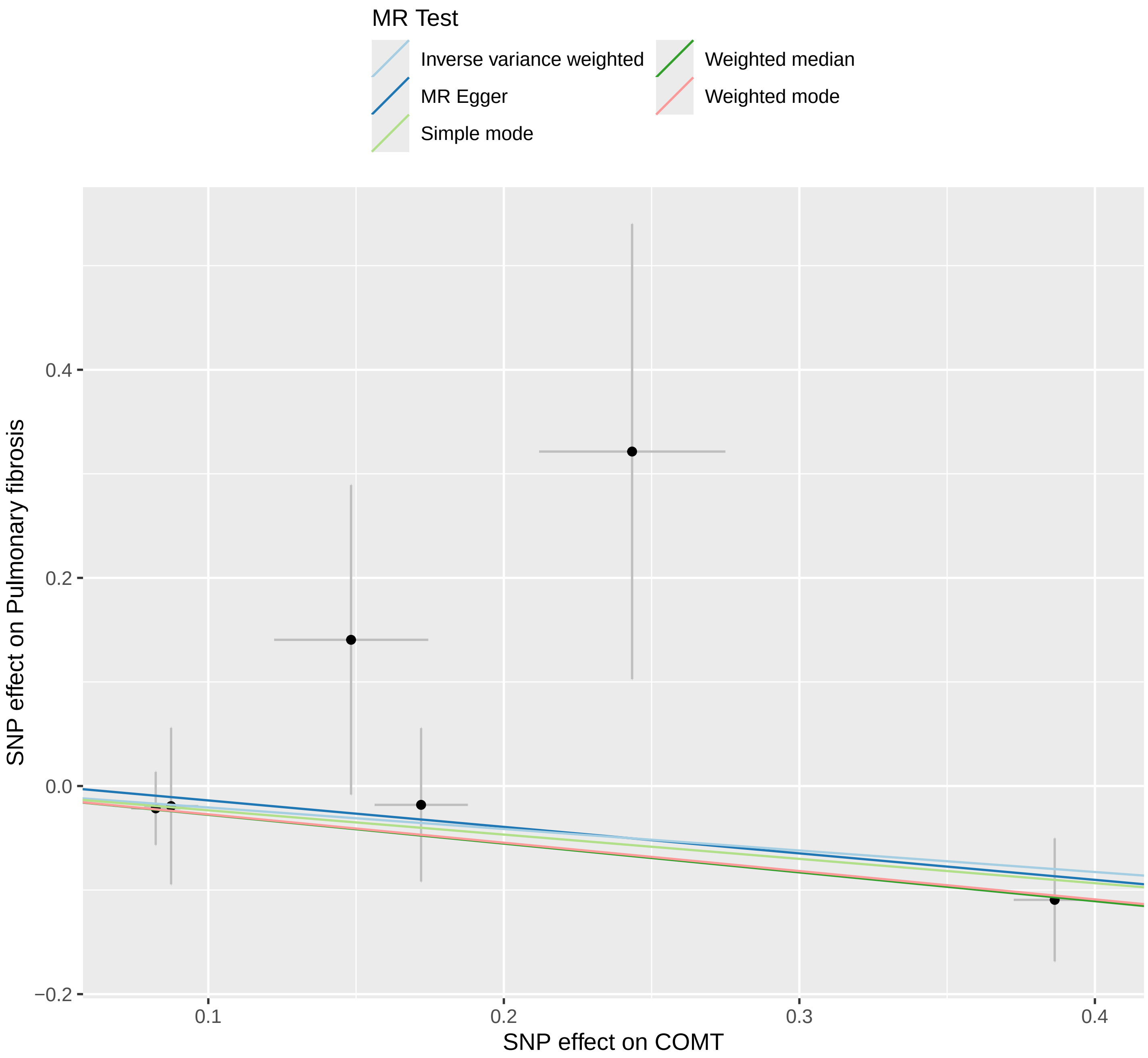
MR Test
Inverse variance weighted
MR Egger
Simple mode
Weighted median
Weighted mode
SNP effect on Pulmonary fibrosis
0.4
0.2
0.0
−0.2
0.1
0.2
0.3
0.4
SNP effect on COMT

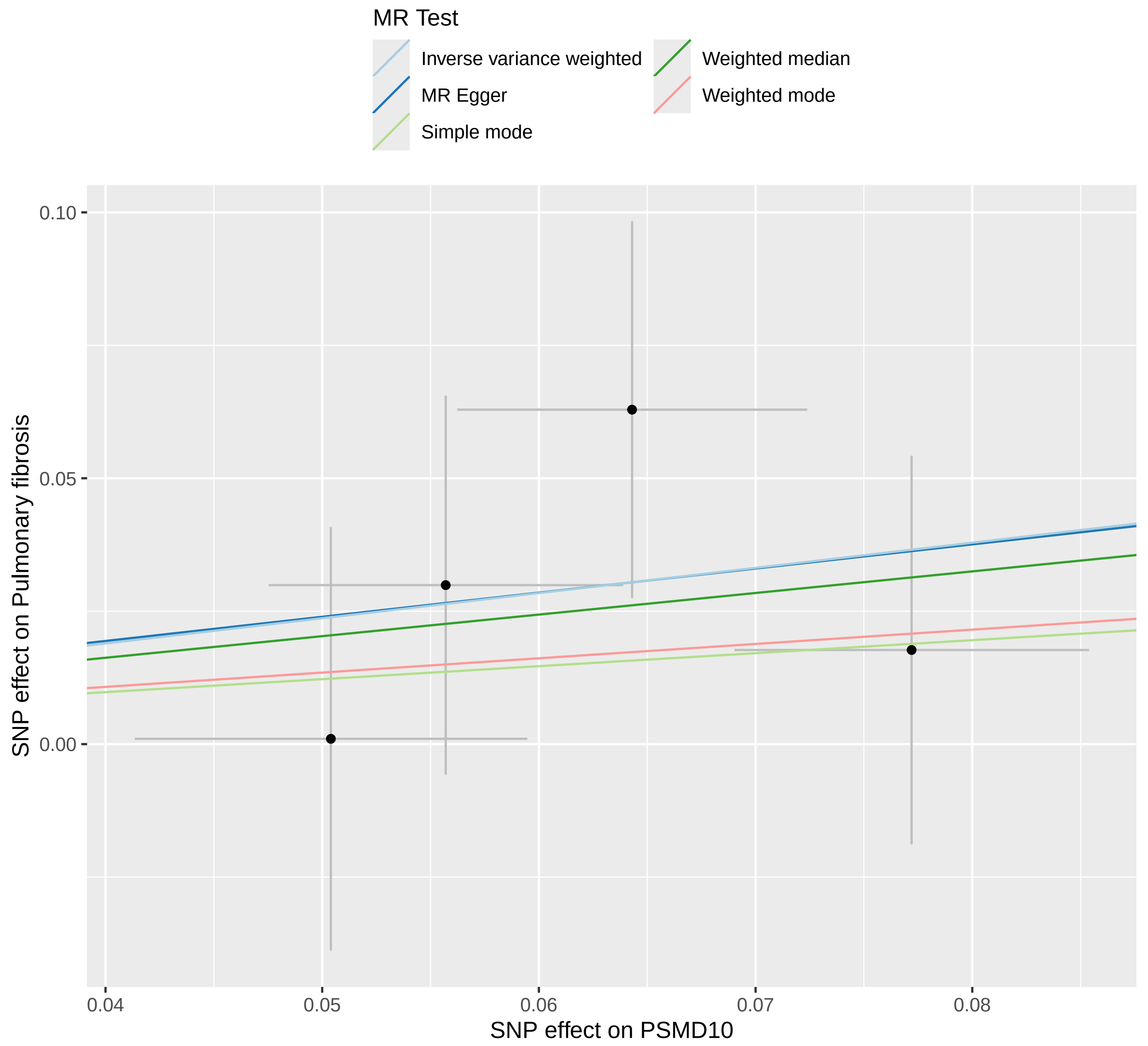

MR Test
Inverse variance weighted
MR Egger
Simple mode
Weighted median
Weighted mode
0.10
0.05
0.00
SNP effect on Pulmonary fibrosis
0.04
0.05
0.06
0.07
0.08
SNP effect on PSMD10

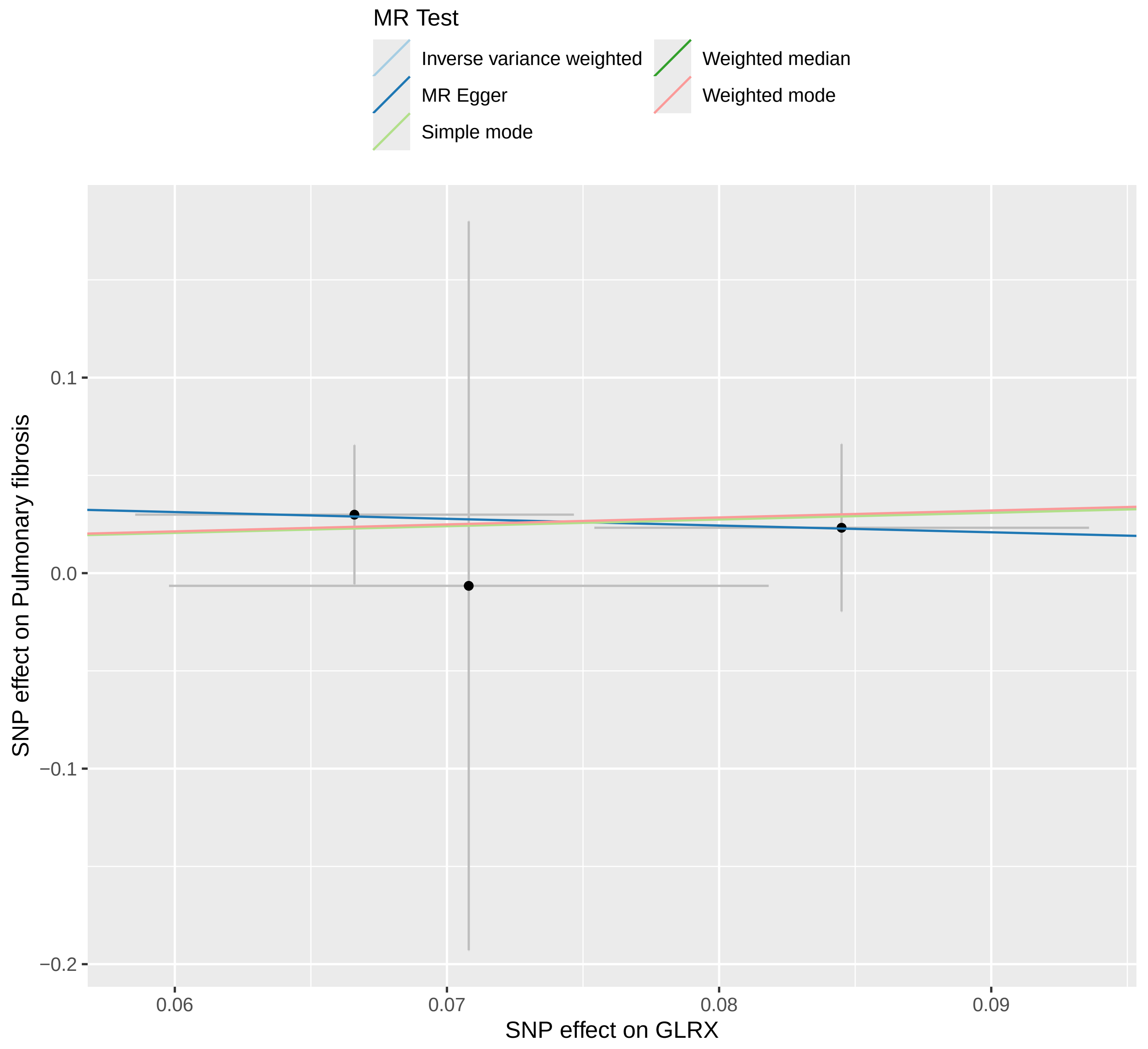

MR Test
Inverse variance weighted
MR Egger
Simple mode
Weighted median
Weighted mode
SNP effect on Pulmonary fibrosis
0.1
0.0
−0.1
−0.2
0.06
0.07
0.08
0.09
SNP effect on GLRX

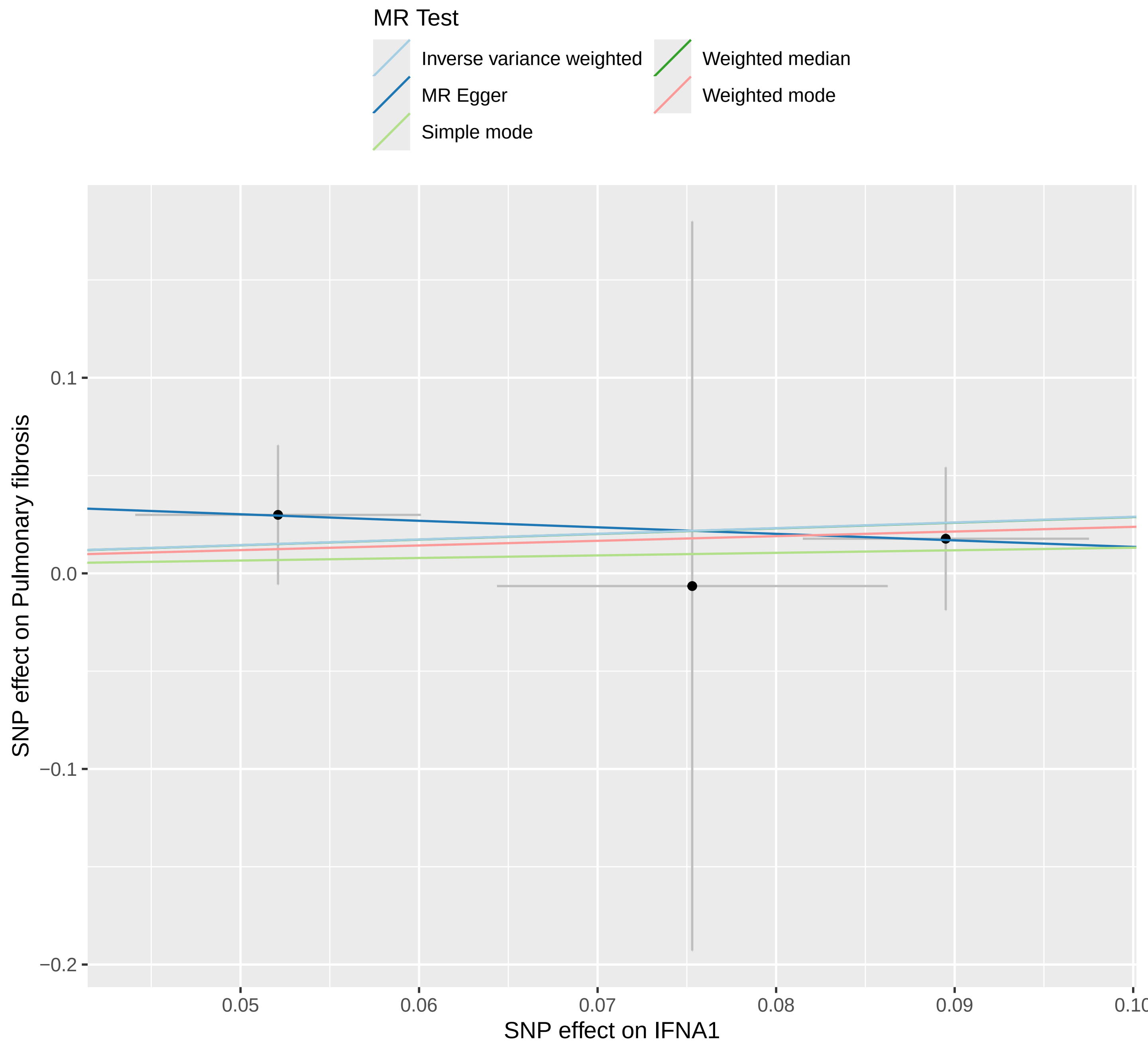
MR Test
Inverse variance weighted
MR Egger
Simple mode
Weighted median
Weighted mode
SNP effect on Pulmonary fibrosis
0.1
0.0
−0.1
−0.2
0.05
0.06
0.07
0.08
0.09
SNP effect on IFNA1

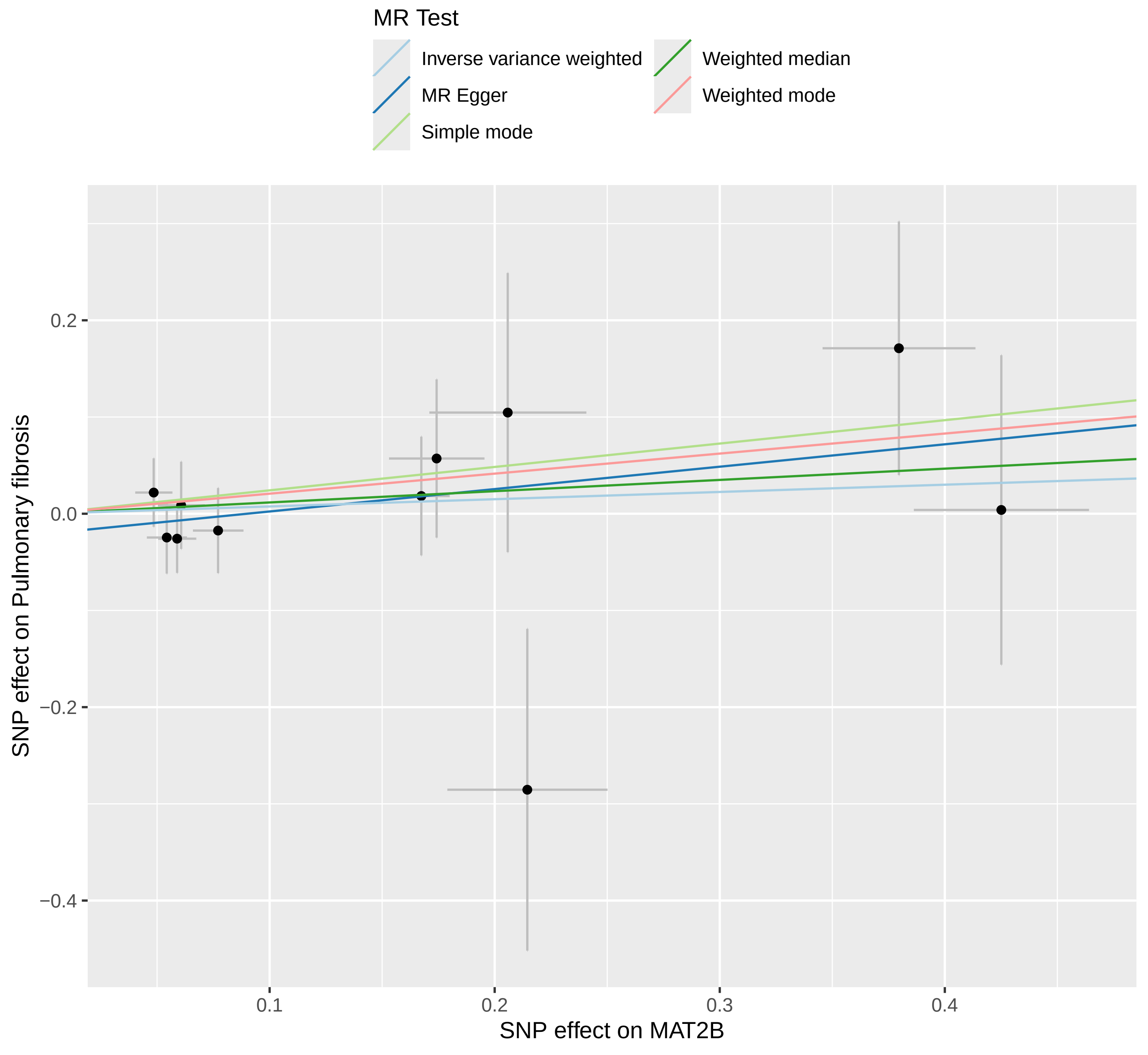
MR Test
Inverse variance weighted
MR Egger
Simple mode
Weighted median
Weighted mode
SNP effect on Pulmonary fibrosis
0.2
0.0
−0.2
−0.4
0.1
0.2
0.3
0.4
SNP effect on MAT2B

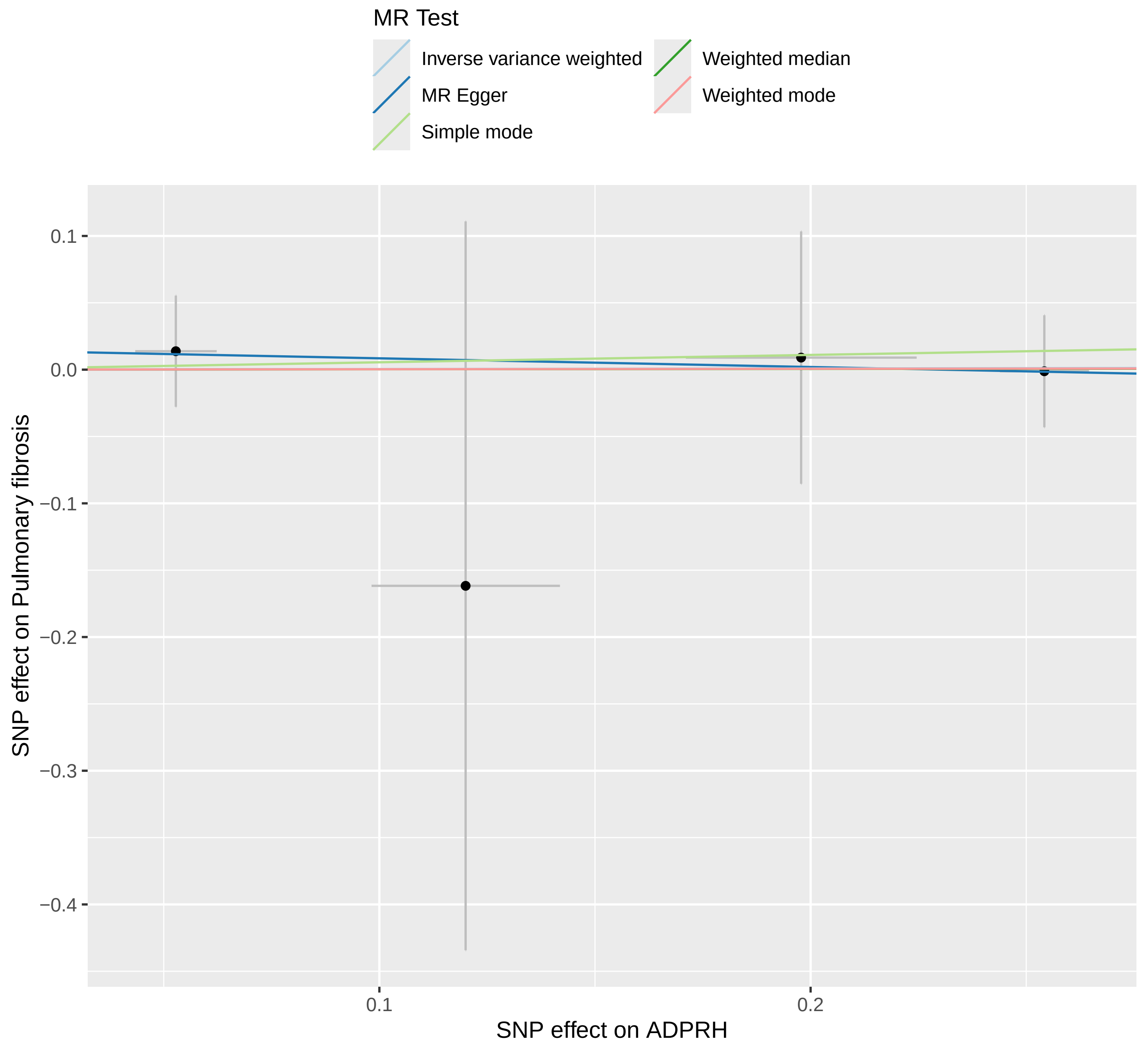
MR Test
Inverse variance weighted
MR Egger
Simple mode
Weighted median
Weighted mode
0.1
0.0
−0.1
−0.2
−0.3
−0.4
SNP effect on Pulmonary fibrosis
0.1
0.2
SNP effect on ADPRH

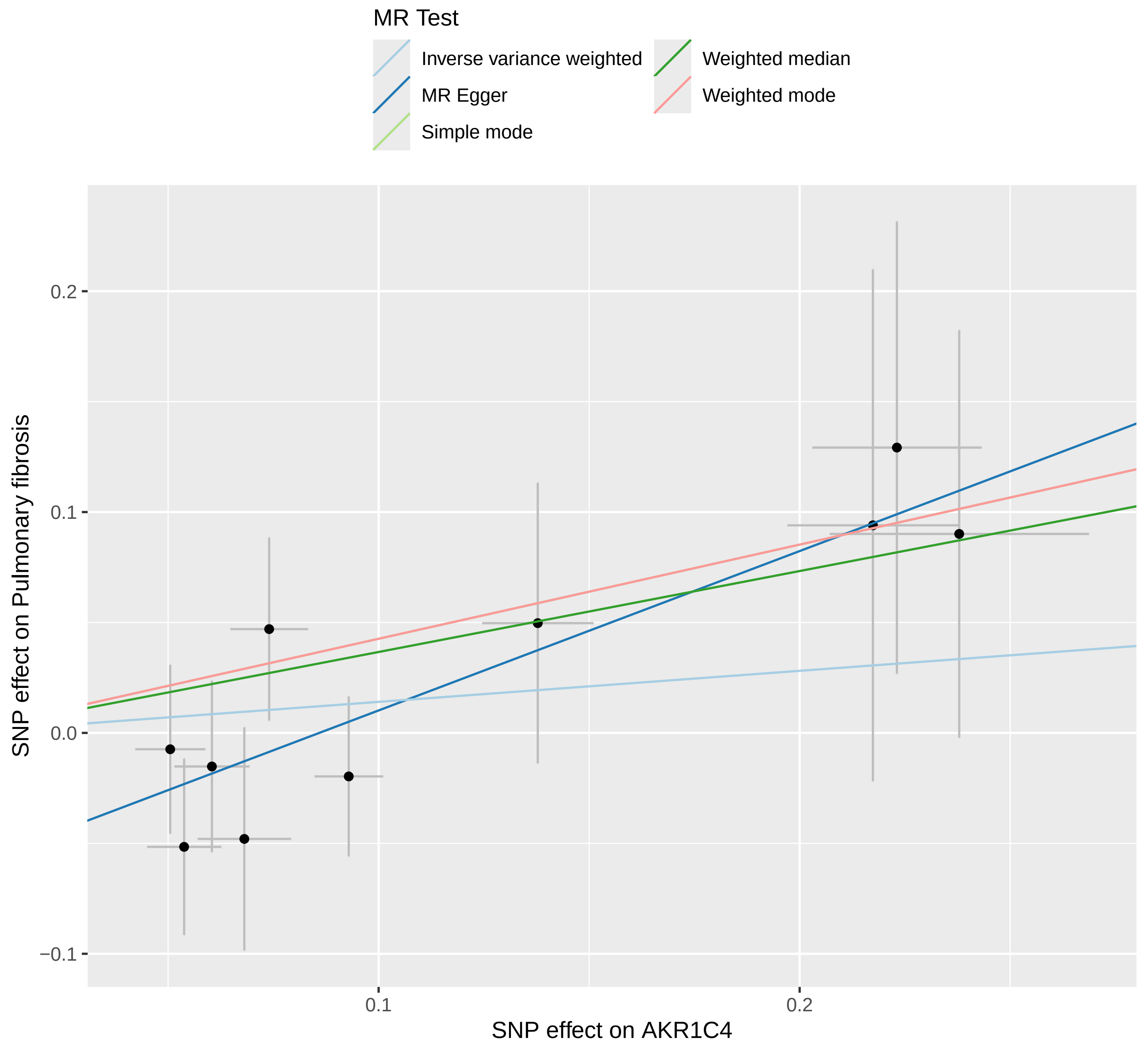

MR Test
Inverse variance weighted
MR Egger
Simple mode
Weighted median
Weighted mode
0.2
0.1
0.0
−0.1
SNP effect on Pulmonary fibrosis
0.1
0.2
SNP effect on AKR1C4

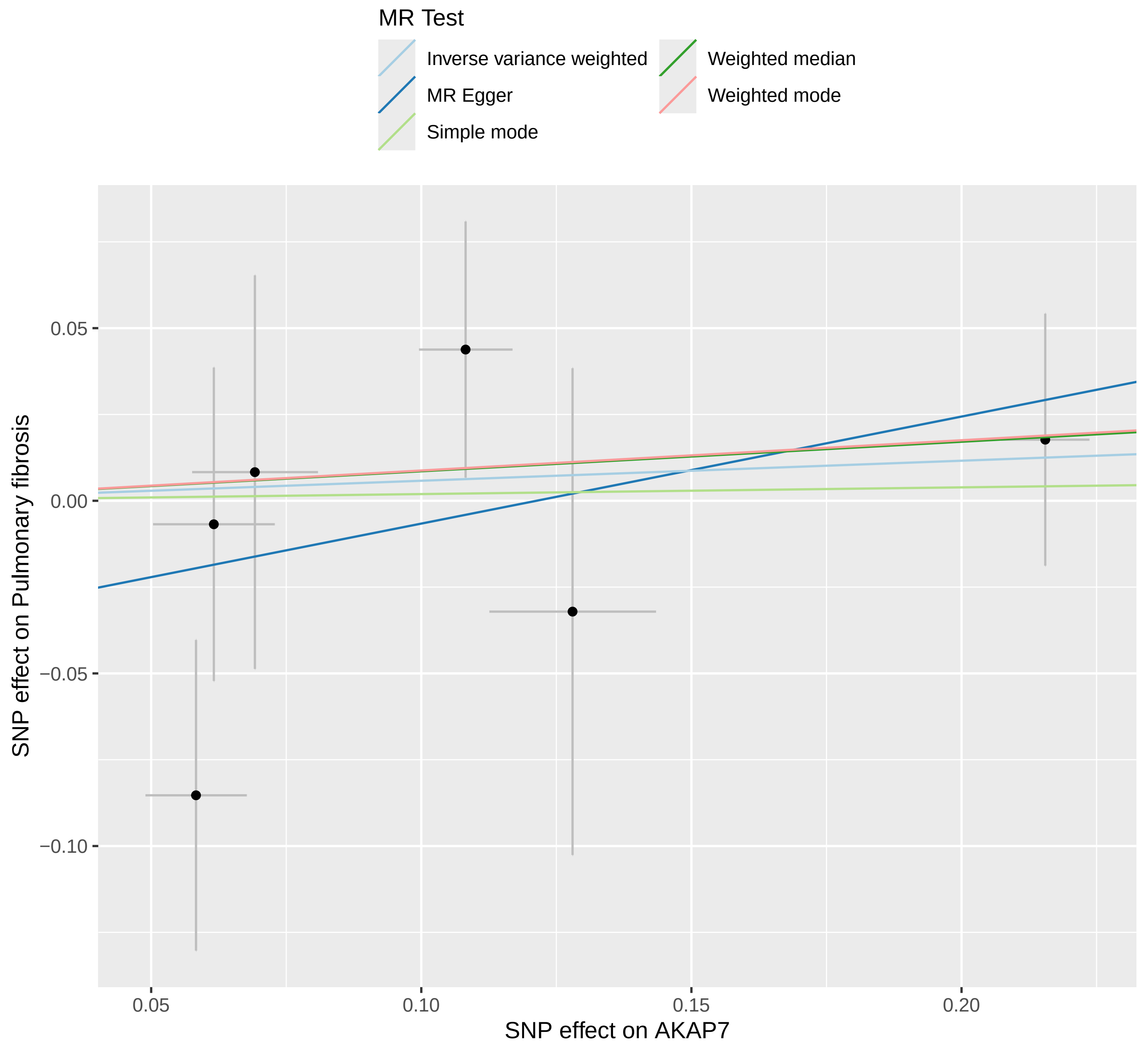

MR Test
Inverse variance weighted
MR Egger
Simple mode
Weighted median
Weighted mode
0.05
0.00
−0.05
−0.10
0.05
0.10
0.15
0.20
SNP effect on Pulmonary fibrosis
SNP effect on AKAP7

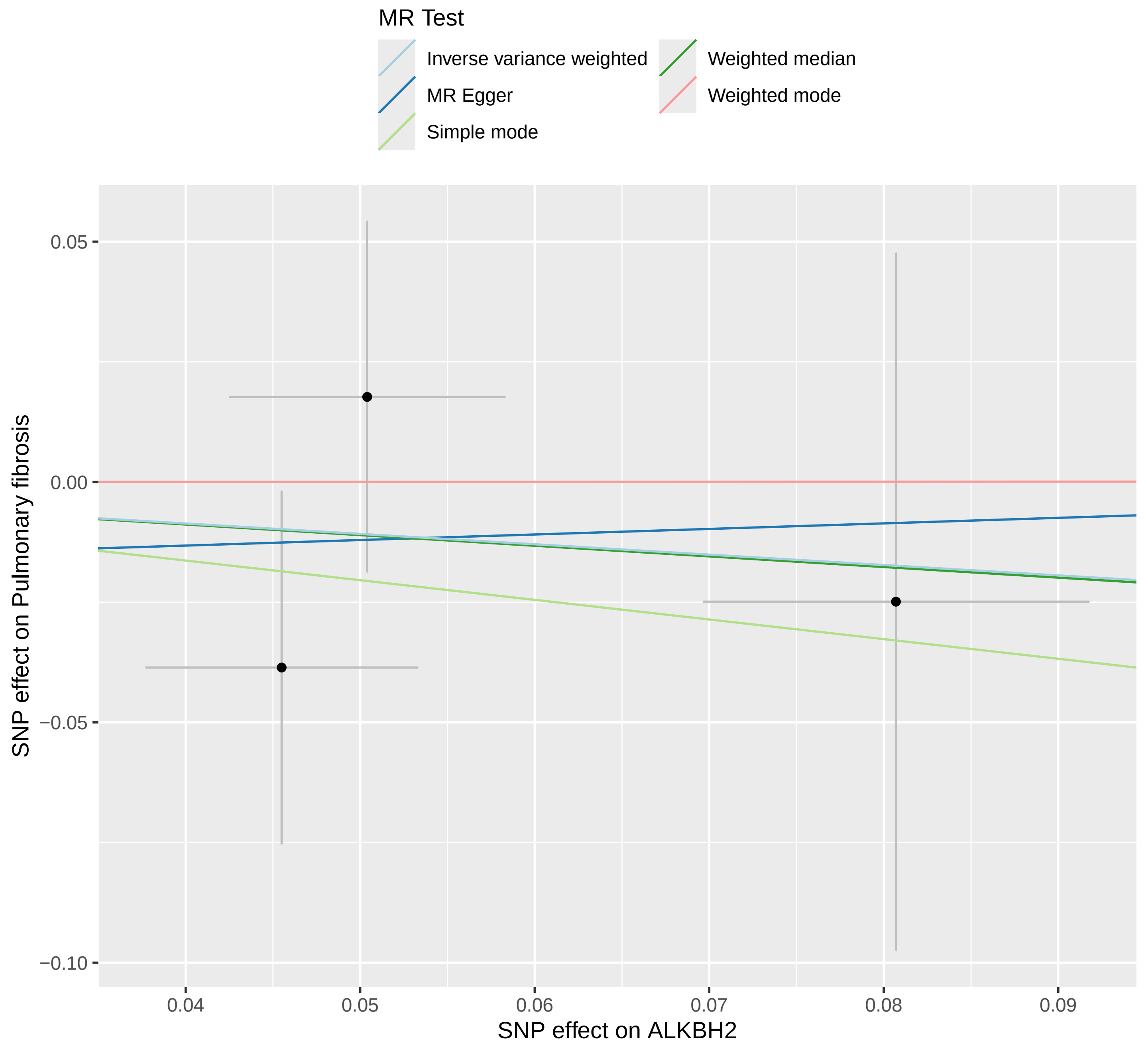
MR Test
Inverse variance weighted
MR Egger
Simple mode
Weighted median
Weighted mode
0.05
0.00
−0.05
−0.10
0.04
0.05
0.06
0.07
0.08
0.09
SNP effect on ALKBH2
SNP effect on Pulmonary fibrosis

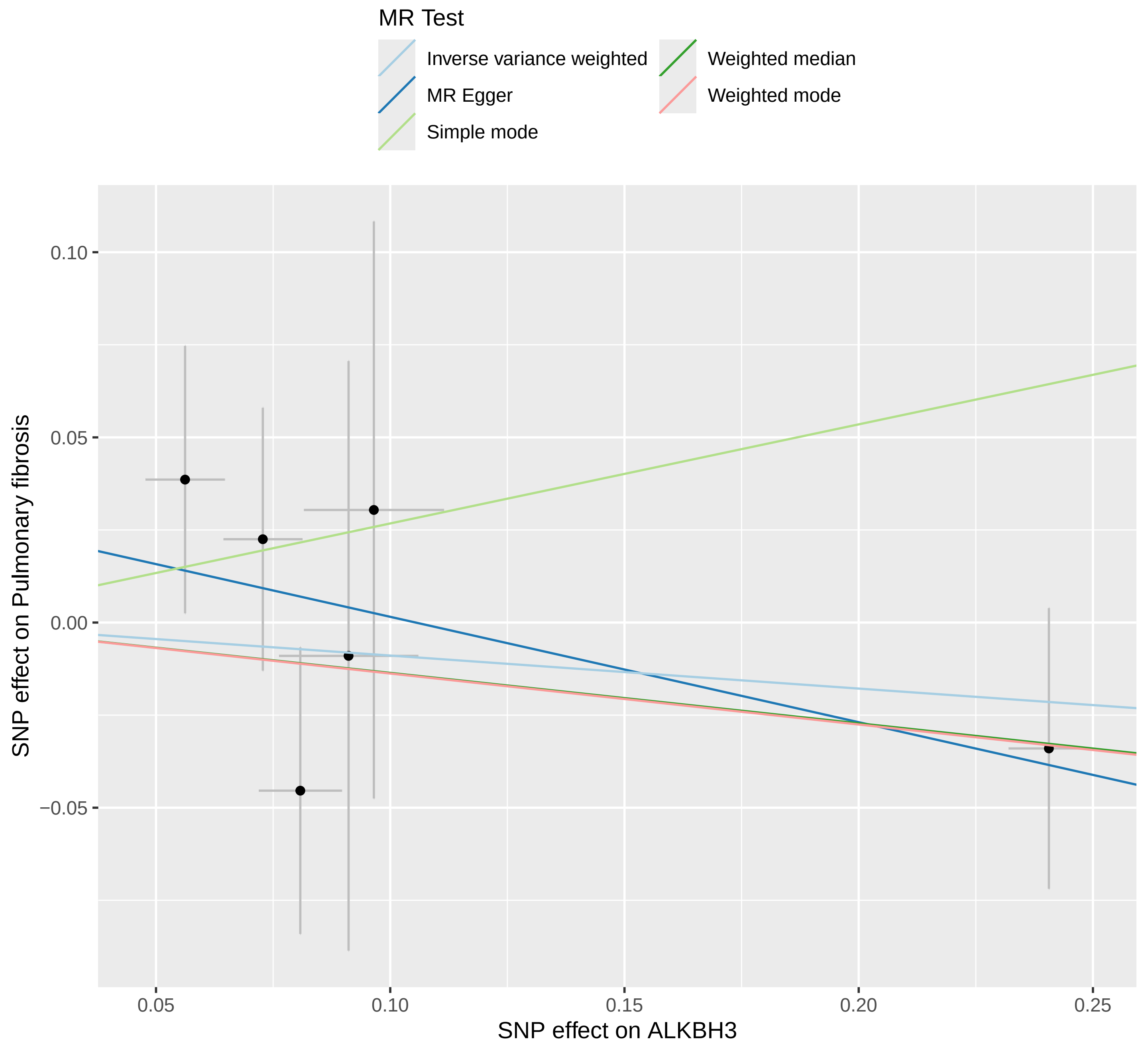

MR Test
Inverse variance weighted
MR Egger
Simple mode
Weighted median
Weighted mode
0.10
0.05
0.00
−0.05
0.05
0.10
0.15
0.20
0.25
SNP effect on Pulmonary fibrosis
SNP effect on ALKBH3

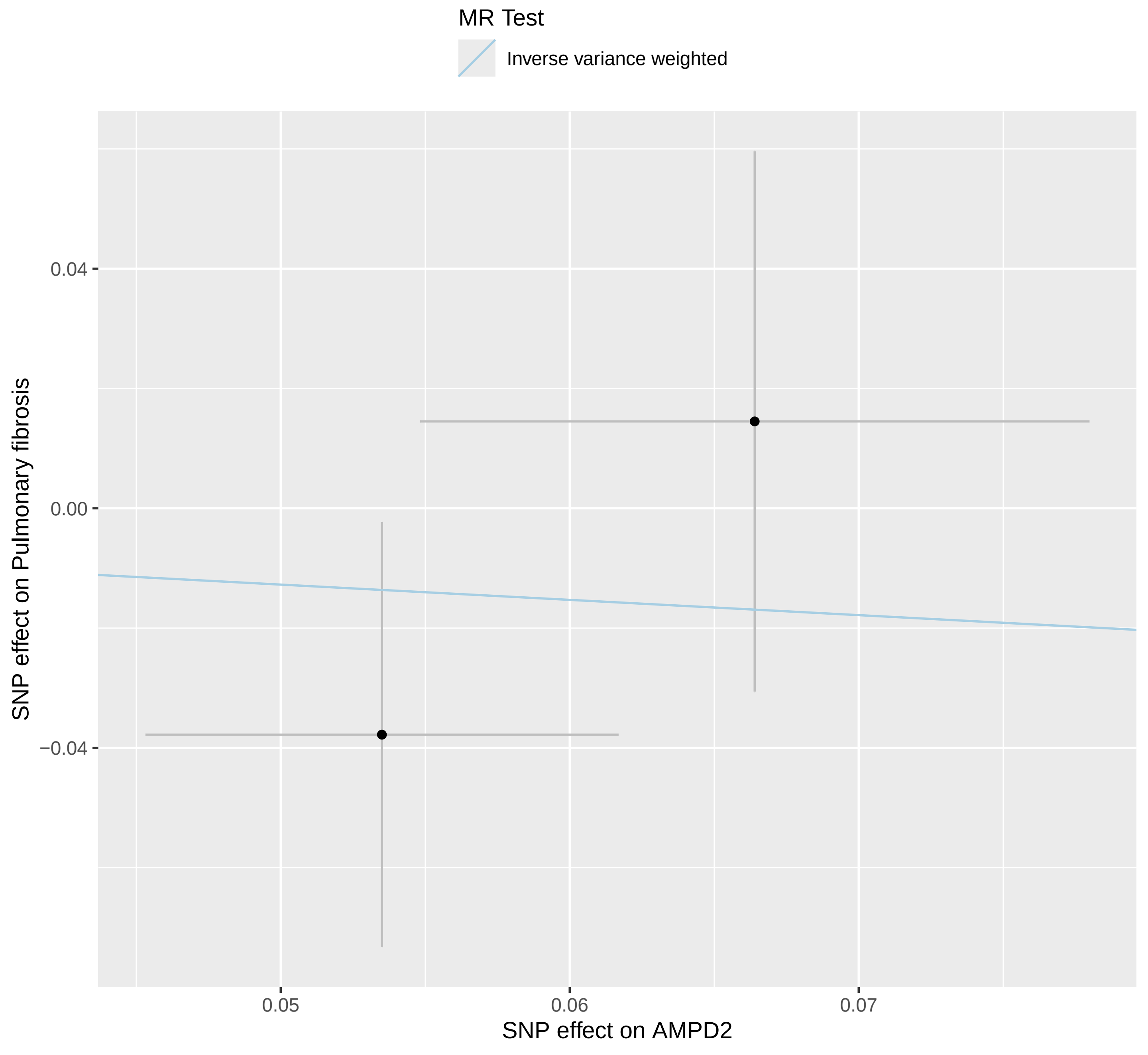
MR Test
Inverse variance weighted
0.04
0.00
−0.04
SNP effect on Pulmonary fibrosis
0.05
0.06
0.07
SNP effect on AMPD2

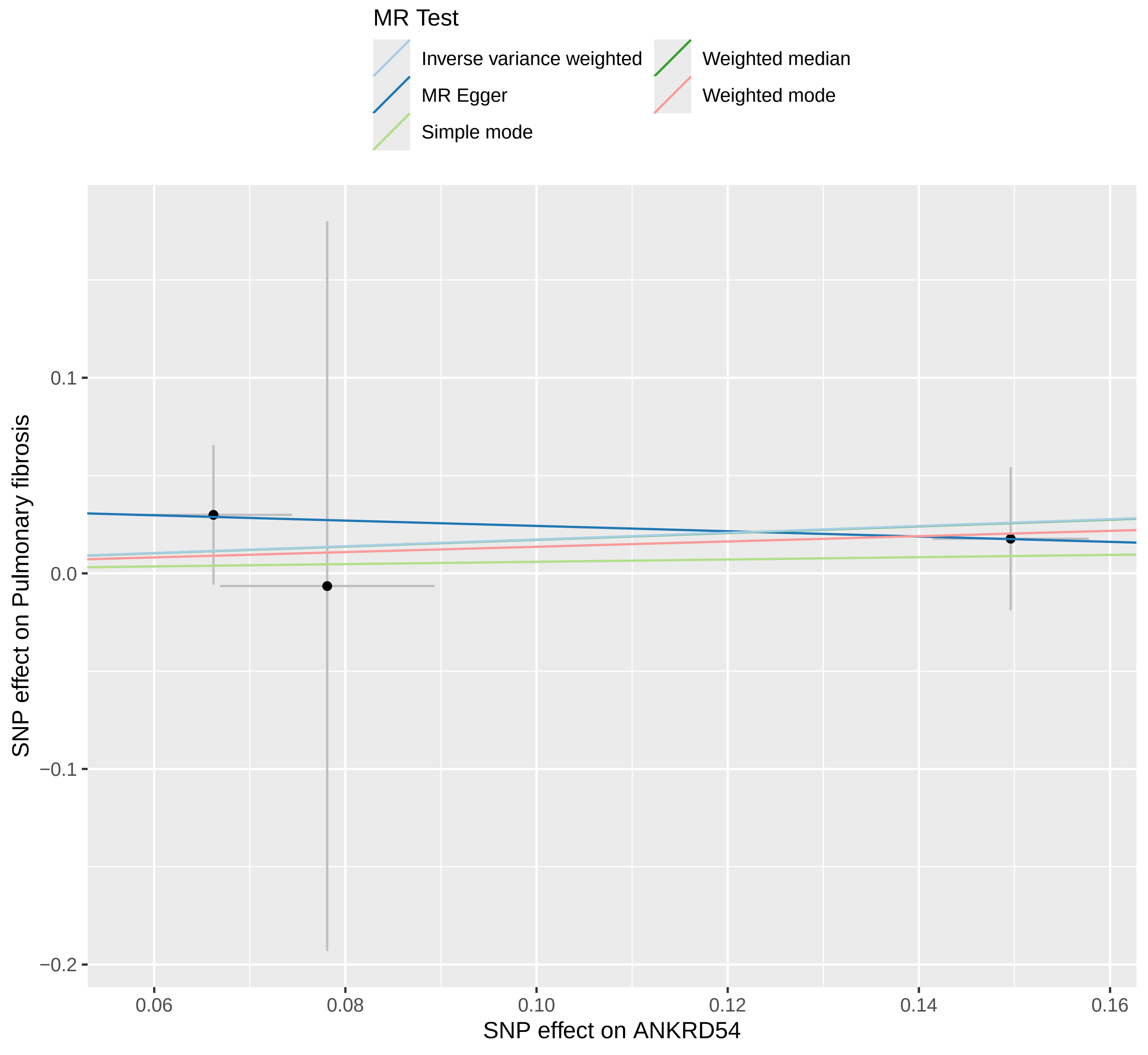
MR Test
Inverse variance weighted
MR Egger
Simple mode
Weighted median
Weighted mode
0.1
0.0
−0.1
−0.2
SNP effect on Pulmonary fibrosis
0.06
0.08
0.10
0.12
0.14
0.16
SNP effect on ANKRD54

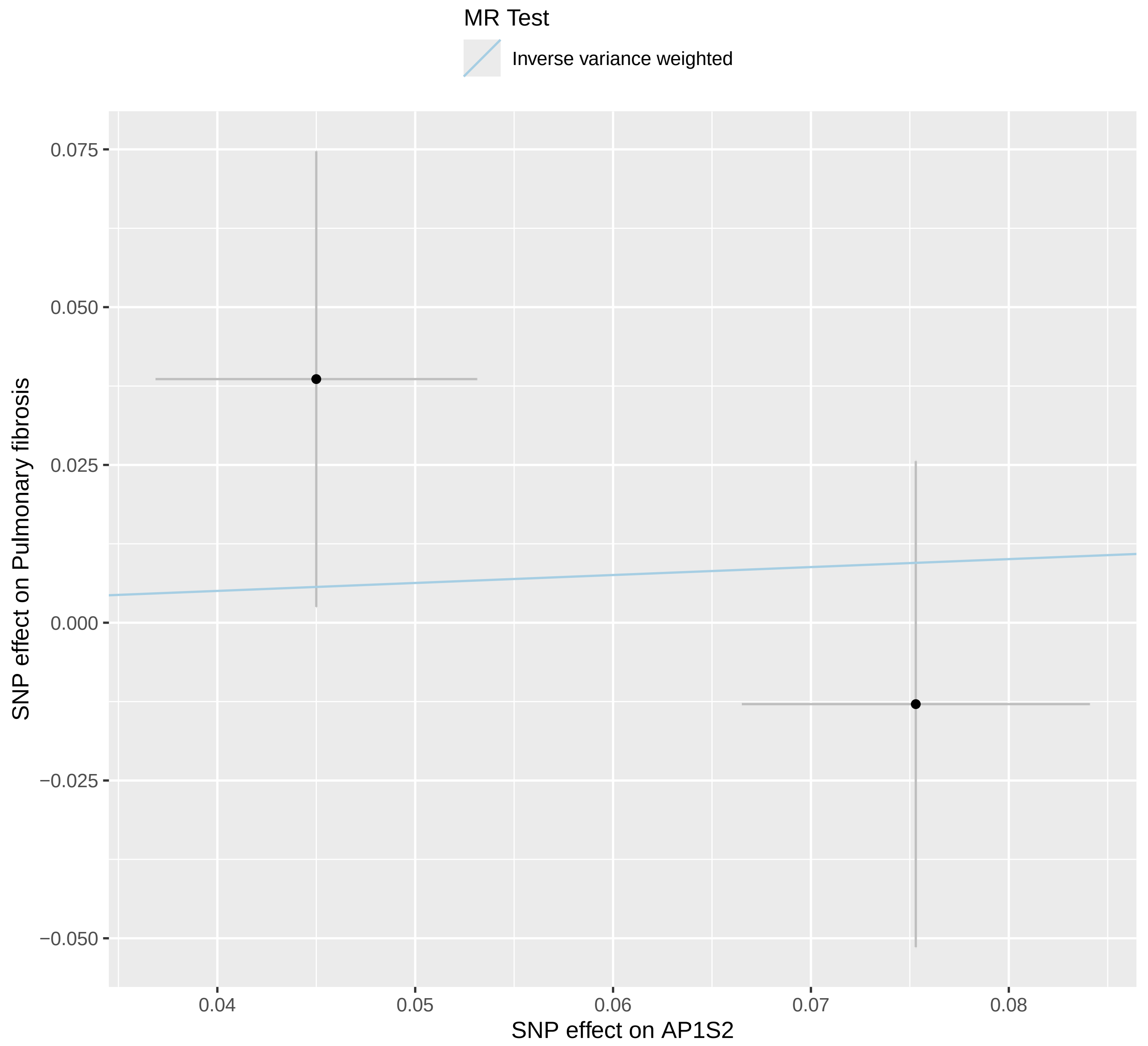

MR Test
Inverse variance weighted
0.075
0.050
0.025
0.000
−0.025
−0.050
SNP effect on Pulmonary fibrosis
0.04
0.05
0.06
0.07
0.08
SNP effect on AP1S2

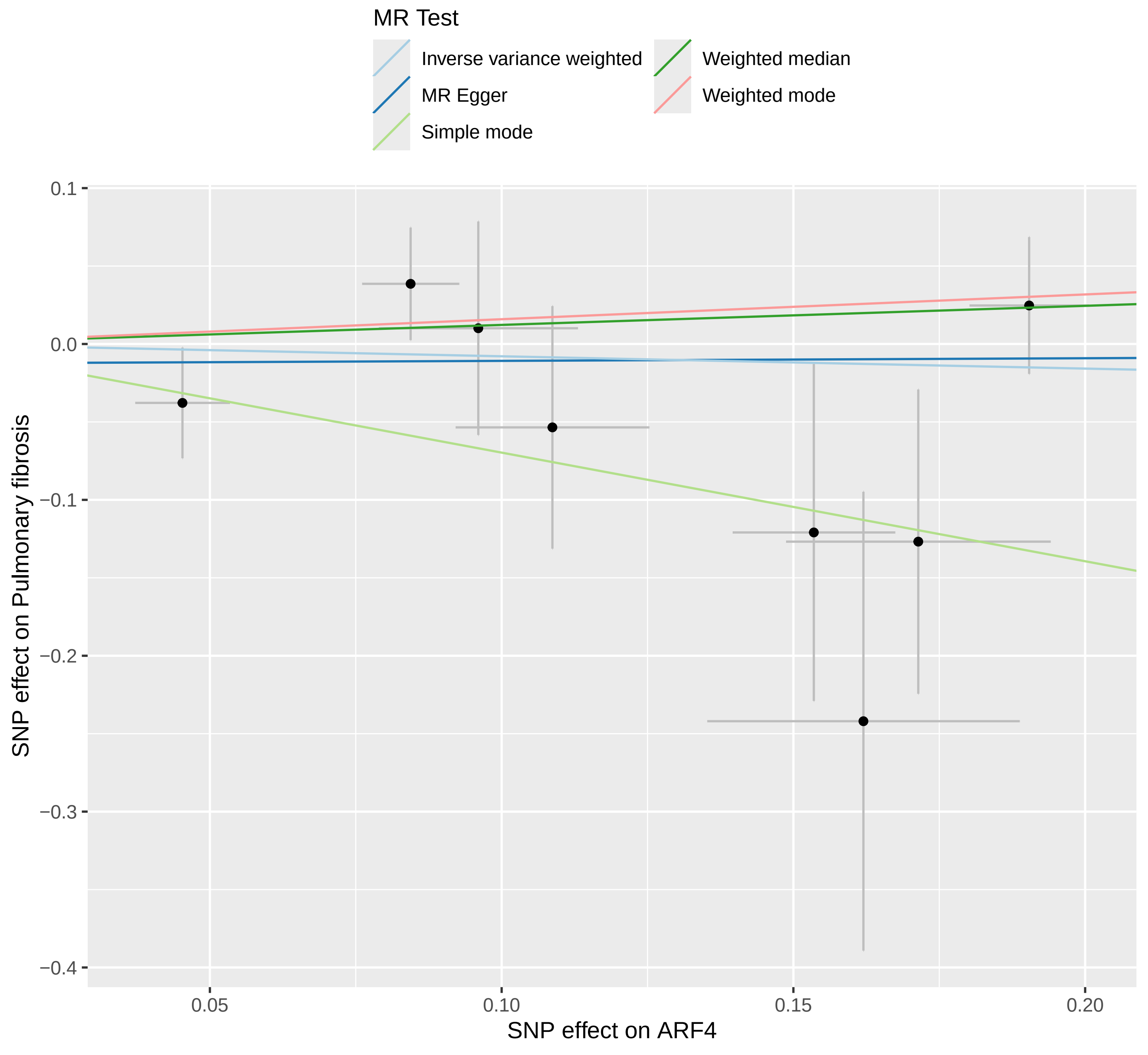
MR Test
Inverse variance weighted
MR Egger
Simple mode
Weighted median
Weighted mode
0.1
0.0
−0.1
−0.2
−0.3
−0.4
SNP effect on Pulmonary fibrosis
0.05
0.10
0.15
0.20
SNP effect on ARF4

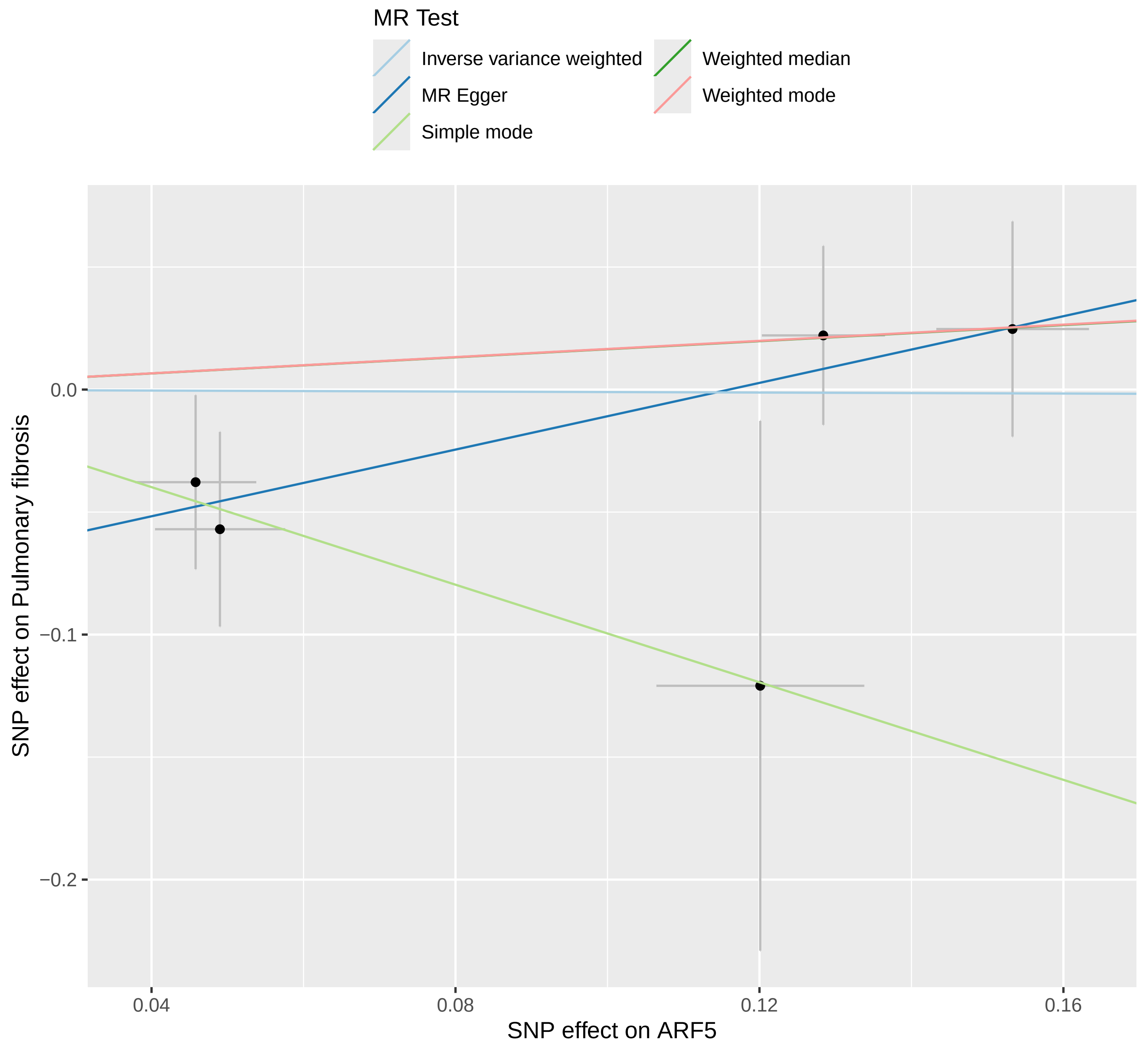

MR Test
Inverse variance weighted
MR Egger
Simple mode
Weighted median
Weighted mode
SNP effect on Pulmonary fibrosis
0.0
−0.1
−0.2
0.04
0.08
0.12
0.16
SNP effect on ARF5

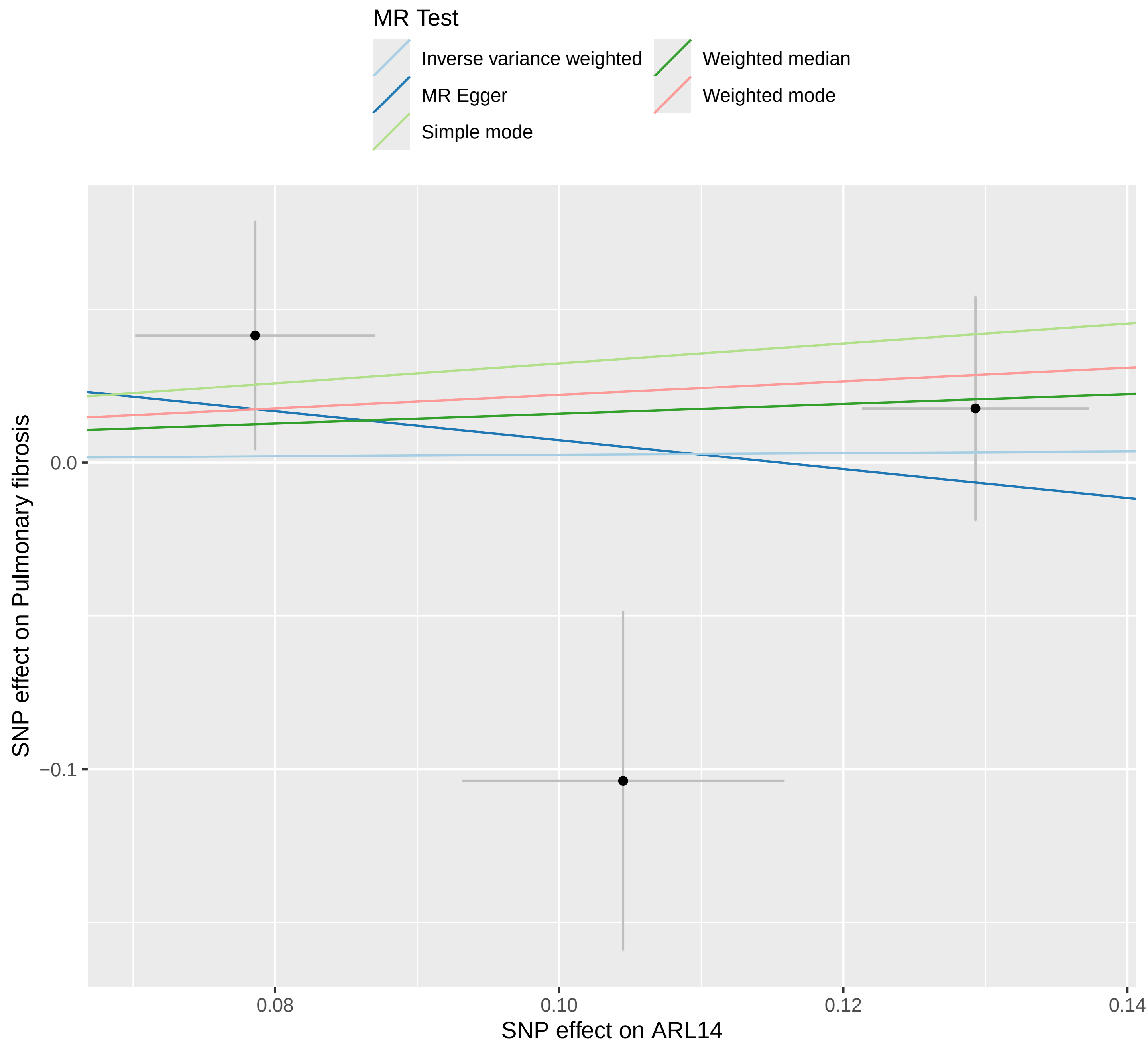

MR Test
Inverse variance weighted
MR Egger
Simple mode
Weighted median
Weighted mode
0.0
−0.1
SNP effect on Pulmonary fibrosis
0.08
0.10
0.12
0.14
SNP effect on ARL14

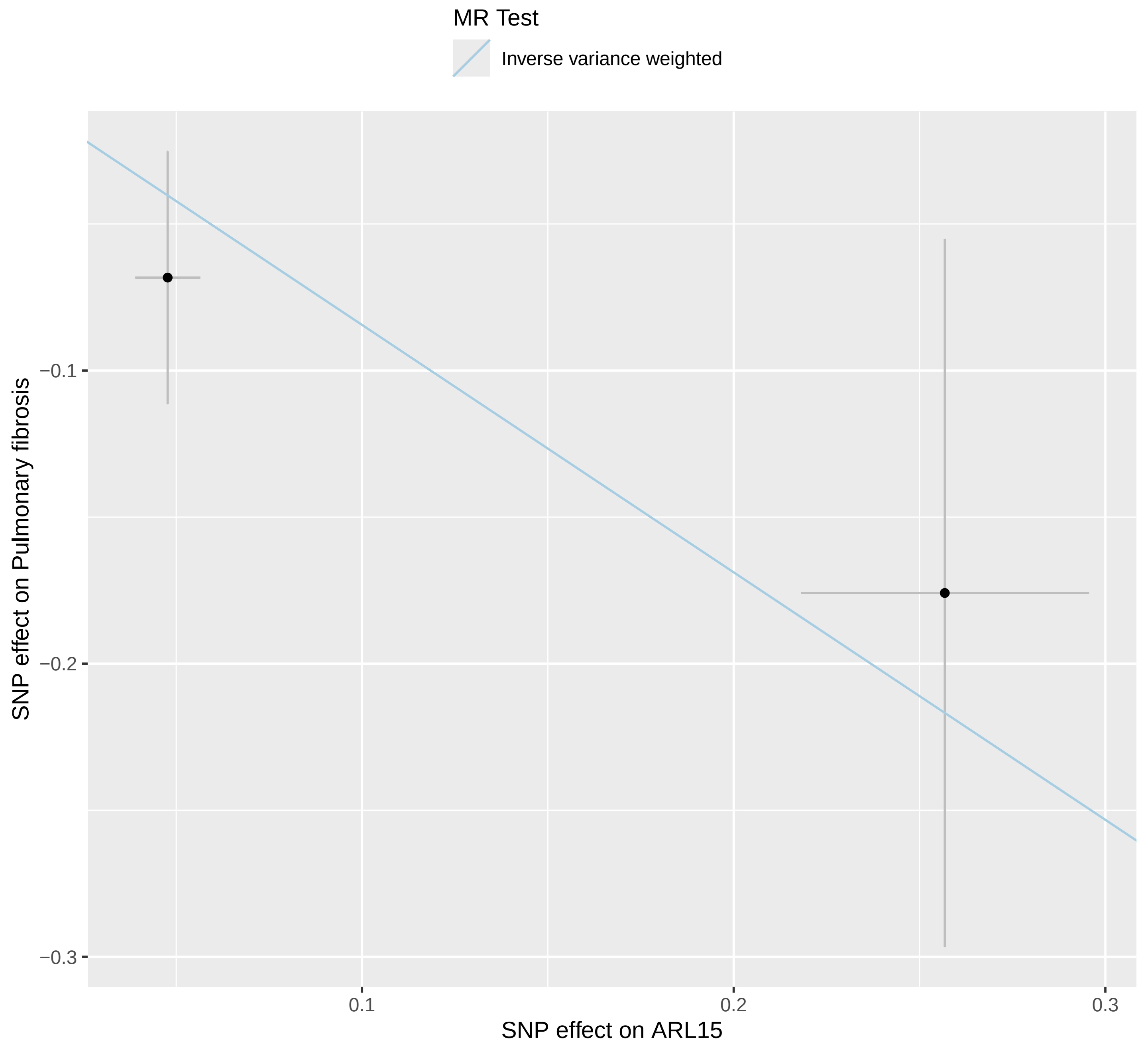
MR Test
Inverse variance weighted
−0.1
−0.2
−0.3
0.1
0.2
0.3
SNP effect on Pulmonary fibrosis
SNP effect on ARL15

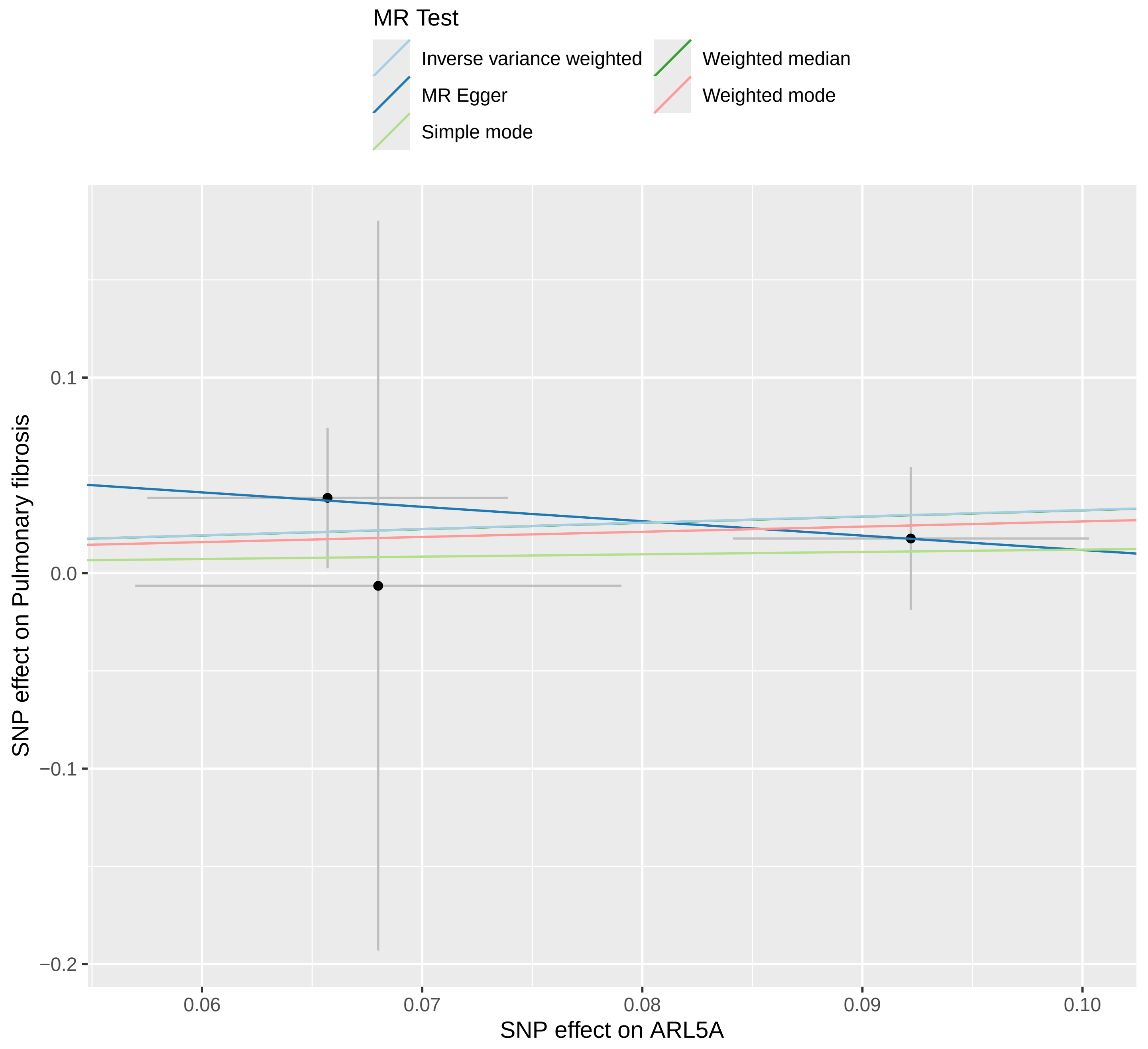

MR Test
Inverse variance weighted
MR Egger
Simple mode
Weighted median
Weighted mode
0.1
0.0
−0.1
−0.2
SNP effect on Pulmonary fibrosis
0.06
0.07
0.08
0.09
0.10
SNP effect on ARL5A

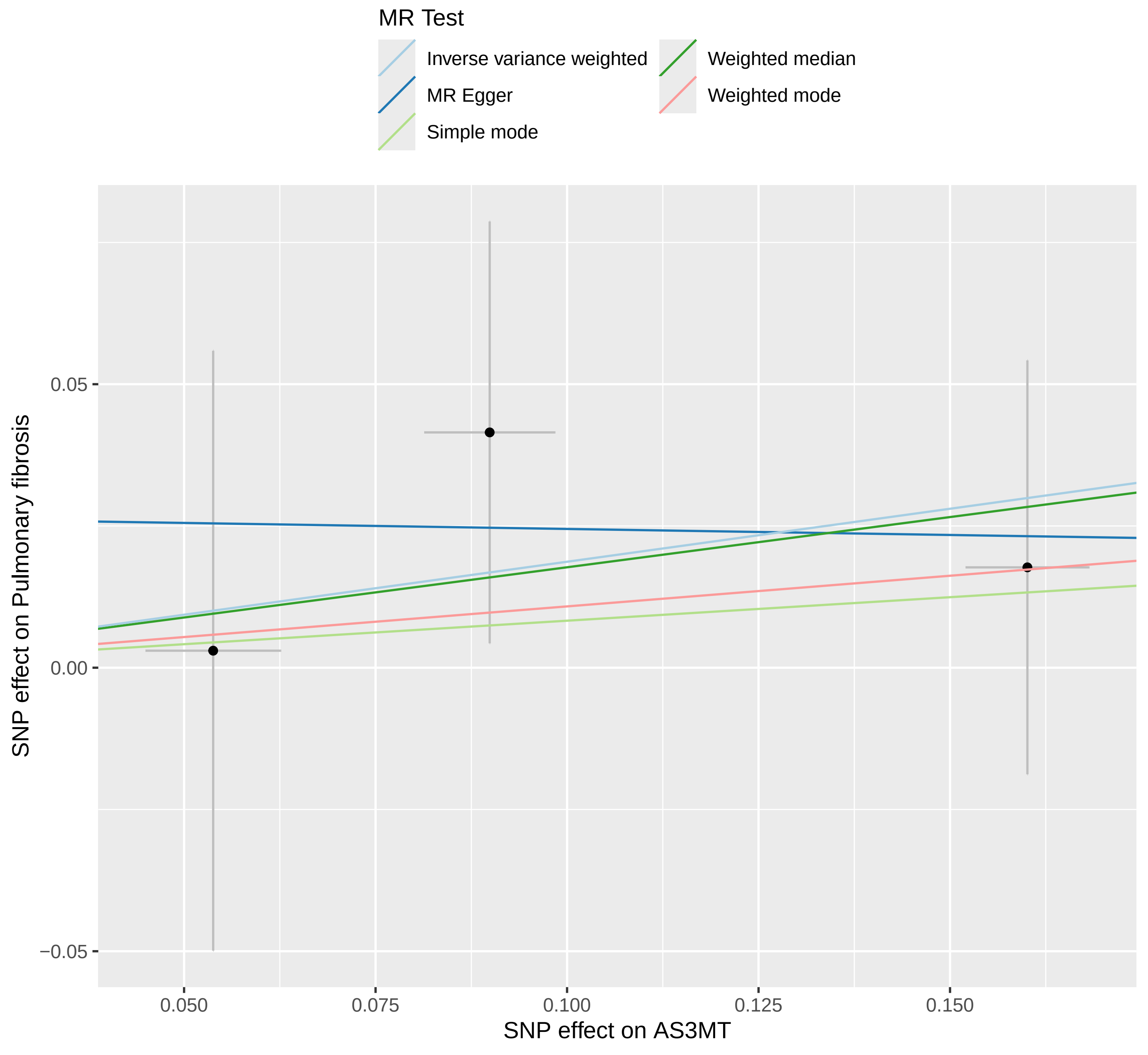
MR Test
Inverse variance weighted
MR Egger
Simple mode
Weighted median
Weighted mode
0.05
0.00
−0.05
0.050
0.075
0.100
0.125
0.150
SNP effect on AS3MT
SNP effect on Pulmonary fibrosis

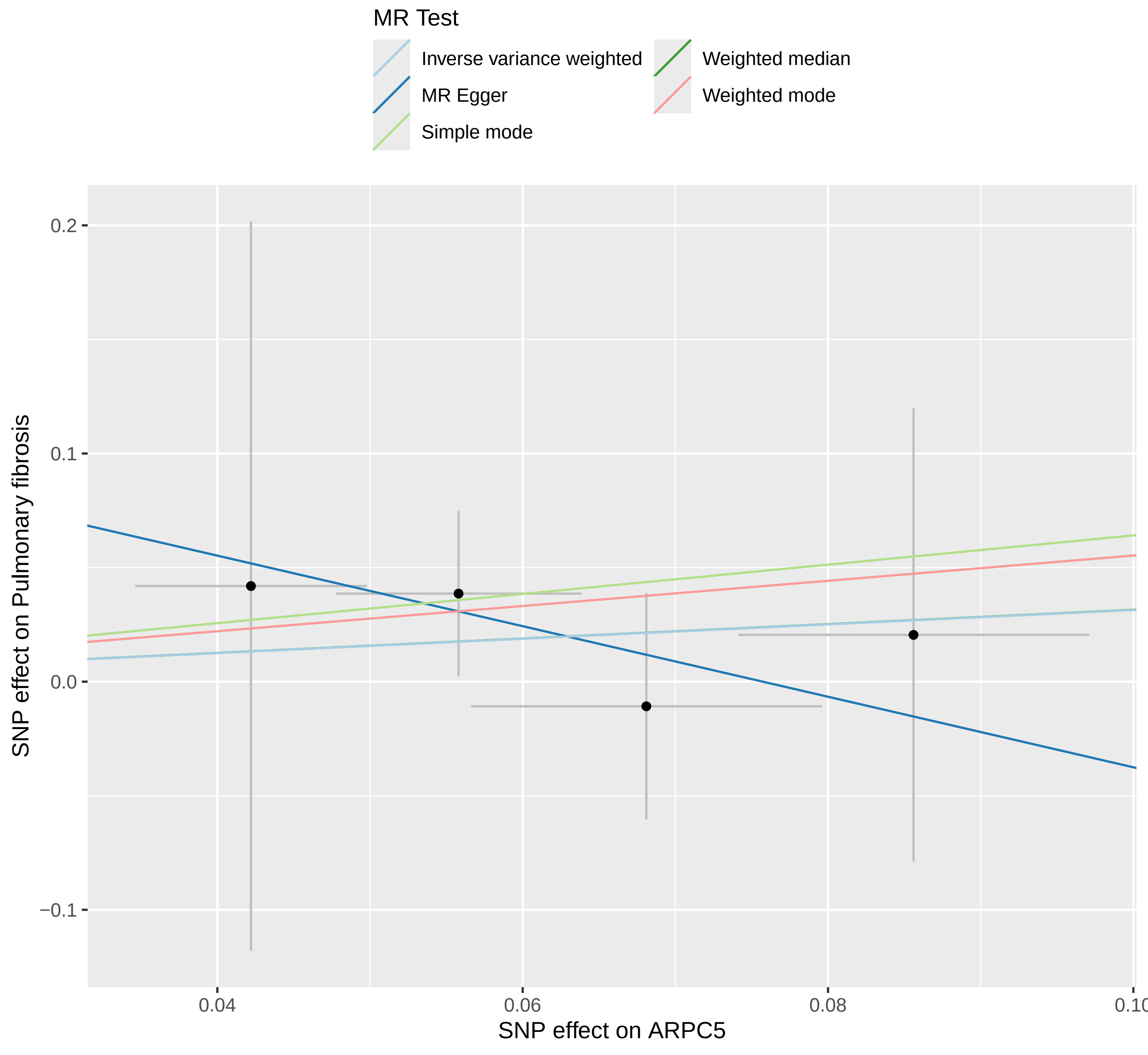
MR Test
Inverse variance weighted
MR Egger
Simple mode
Weighted median
Weighted mode
0.2
0.1
0.0
−0.1
0.04
0.06
0.08
0.10
SNP effect on Pulmonary fibrosis
SNP effect on ARPC5

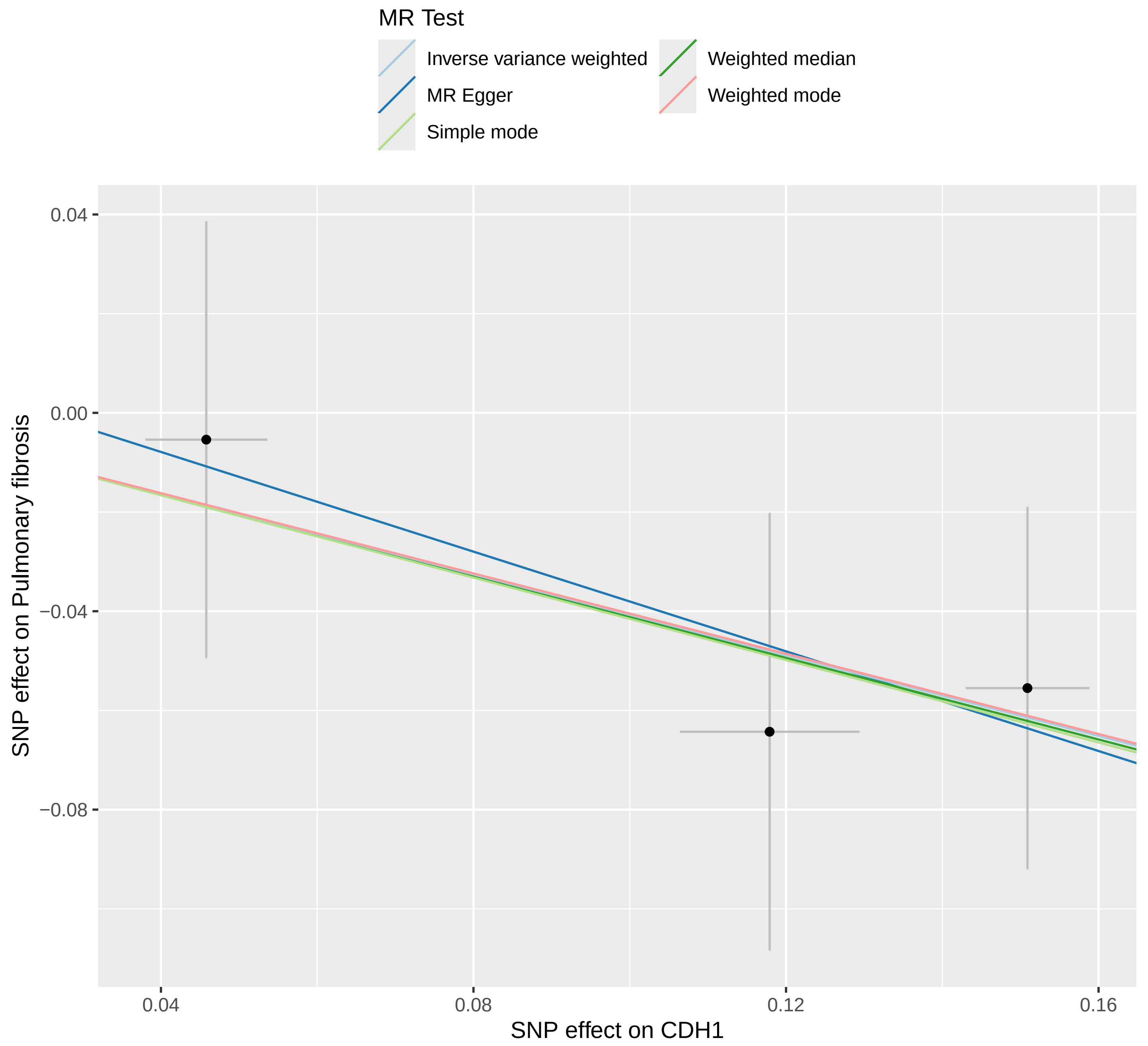

MR Test
Inverse variance weighted
MR Egger
Simple mode
Weighted median
Weighted mode
0.04
0.00
−0.04
−0.08
SNP effect on Pulmonary fibrosis
0.04
0.08
0.12
0.16
SNP effect on CDH1

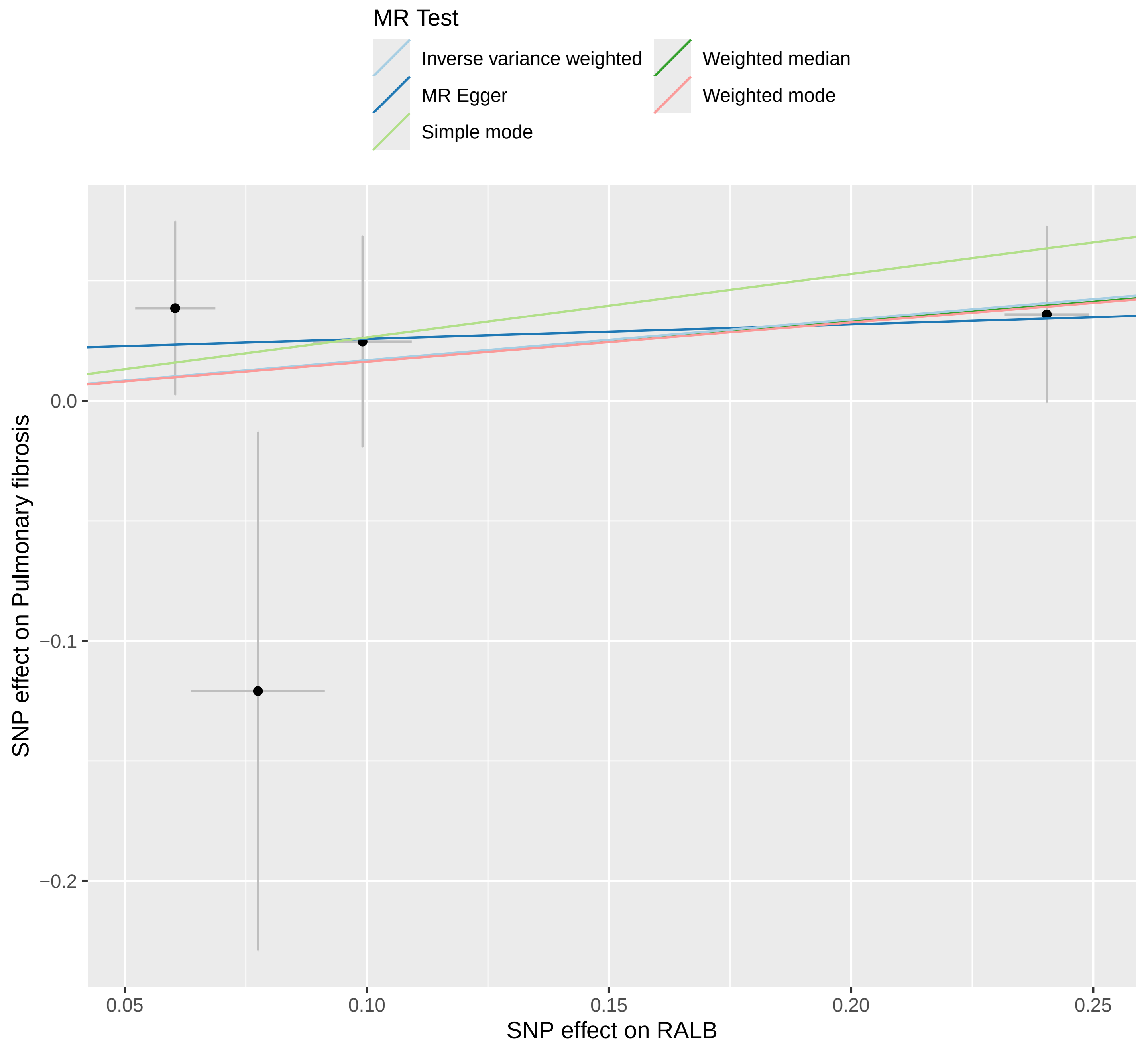
MR Test
Inverse variance weighted
MR Egger
Simple mode
Weighted median
Weighted mode
0.0
−0.1
−0.2
SNP effect on Pulmonary fibrosis
0.05
0.10
0.15
0.20
0.25
SNP effect on RALB

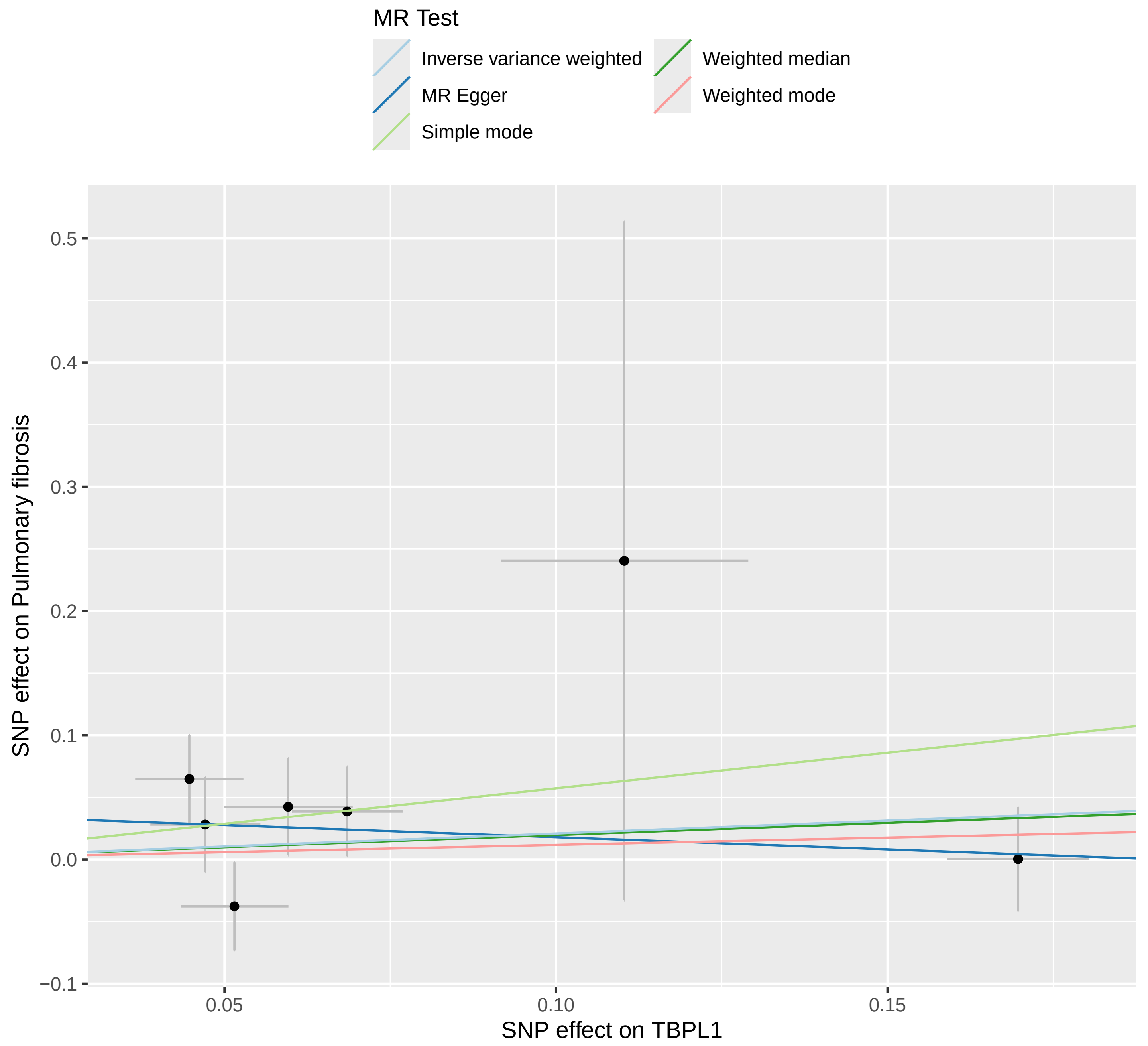
MR Test
Inverse variance weighted
MR Egger
Simple mode
Weighted median
Weighted mode
SNP effect on Pulmonary fibrosis
0.5
0.4
0.3
0.2
0.1
0.0
−0.1
0.05
0.10
0.15
SNP effect on TBPL1

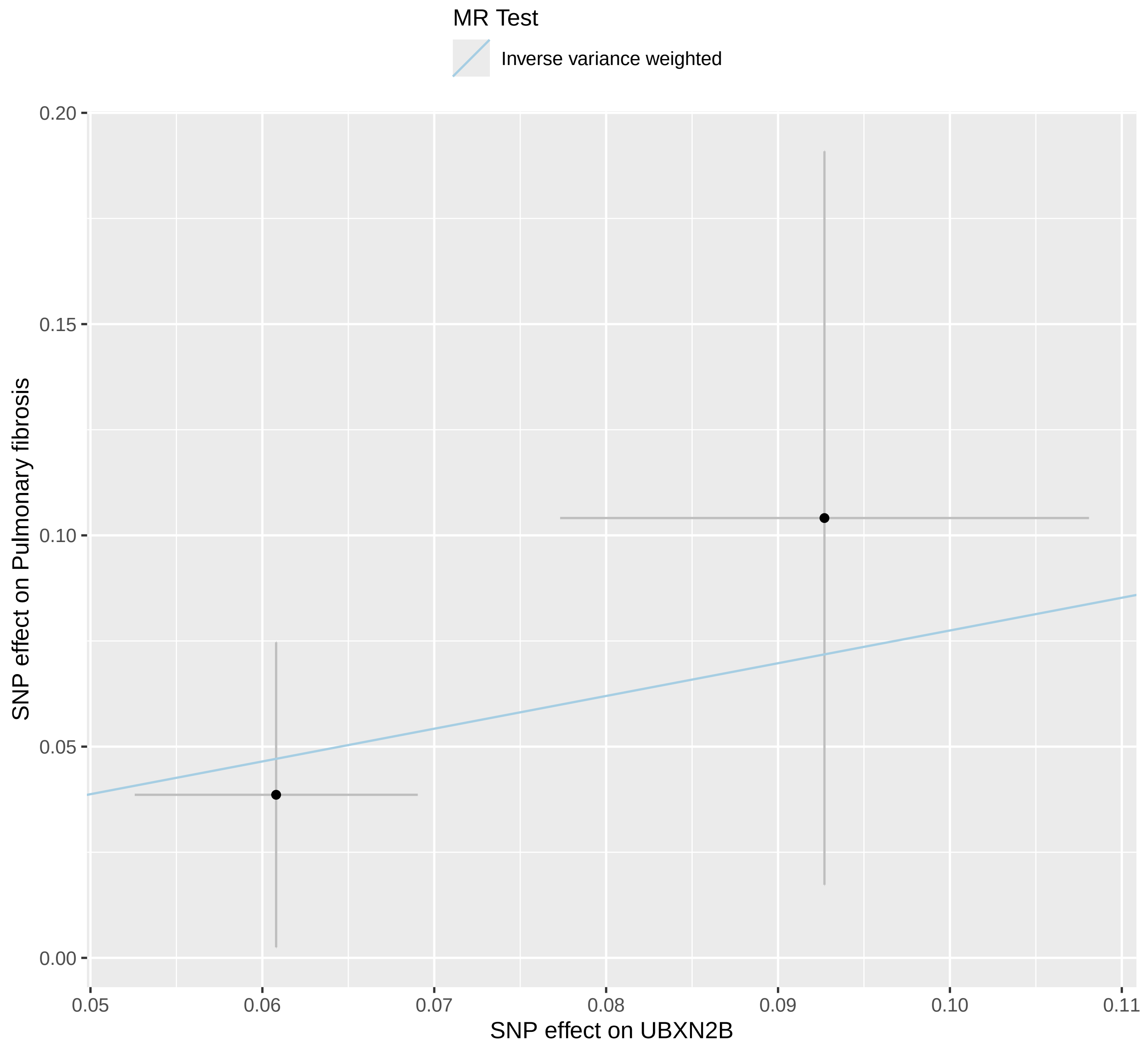

MR Test
Inverse variance weighted
0.20
0.15
0.10
0.05
0.00
SNP effect on Pulmonary fibrosis
0.05
0.06
0.07
0.08
0.09
0.10
0.11
SNP effect on UBXN2B

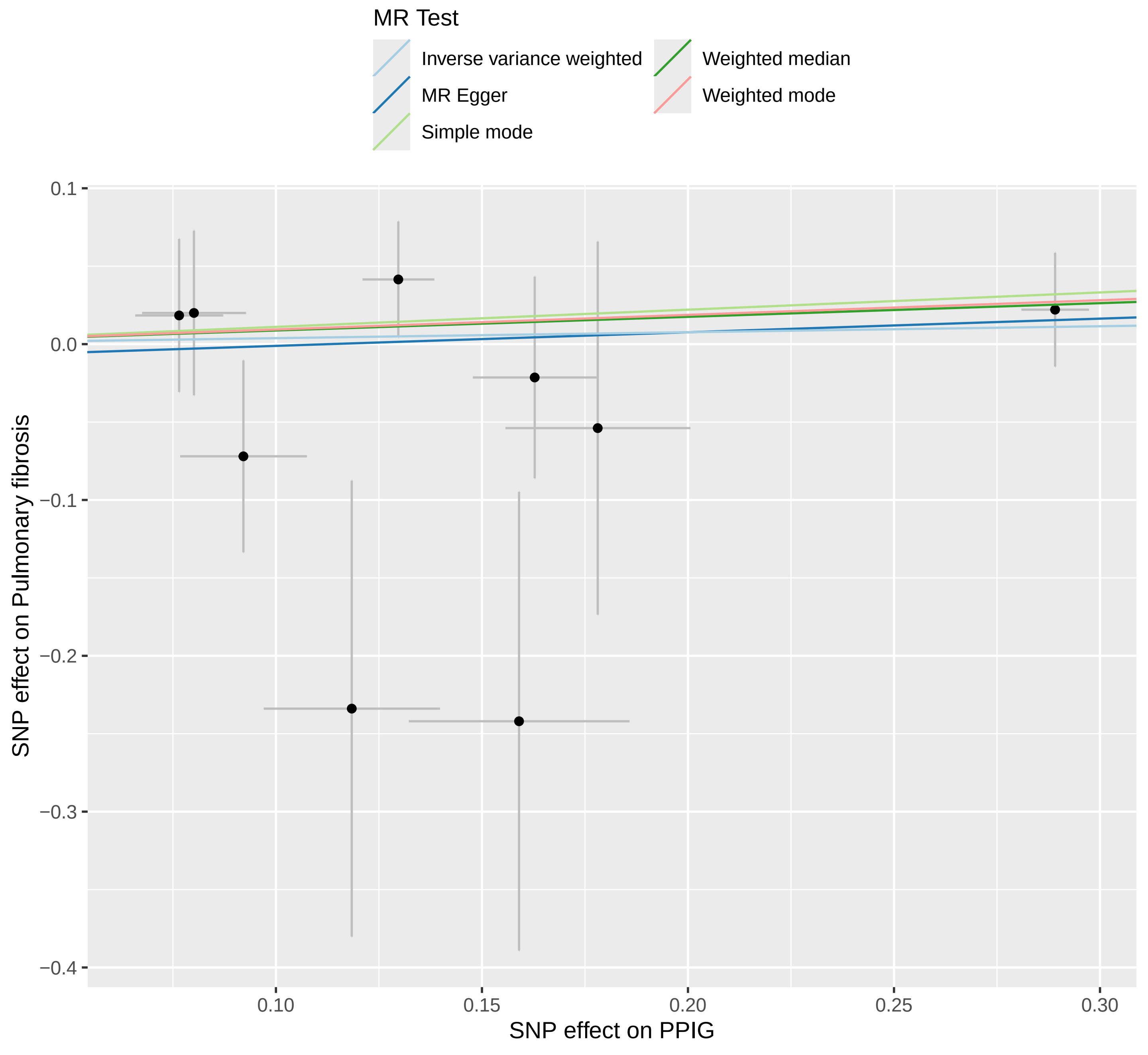

MR Test
Inverse variance weighted
MR Egger
Simple mode
Weighted median
Weighted mode
0.1
0.0
−0.1
−0.2
−0.3
−0.4
0.10
0.15
0.20
0.25
0.30
SNP effect on Pulmonary fibrosis
SNP effect on PPIG

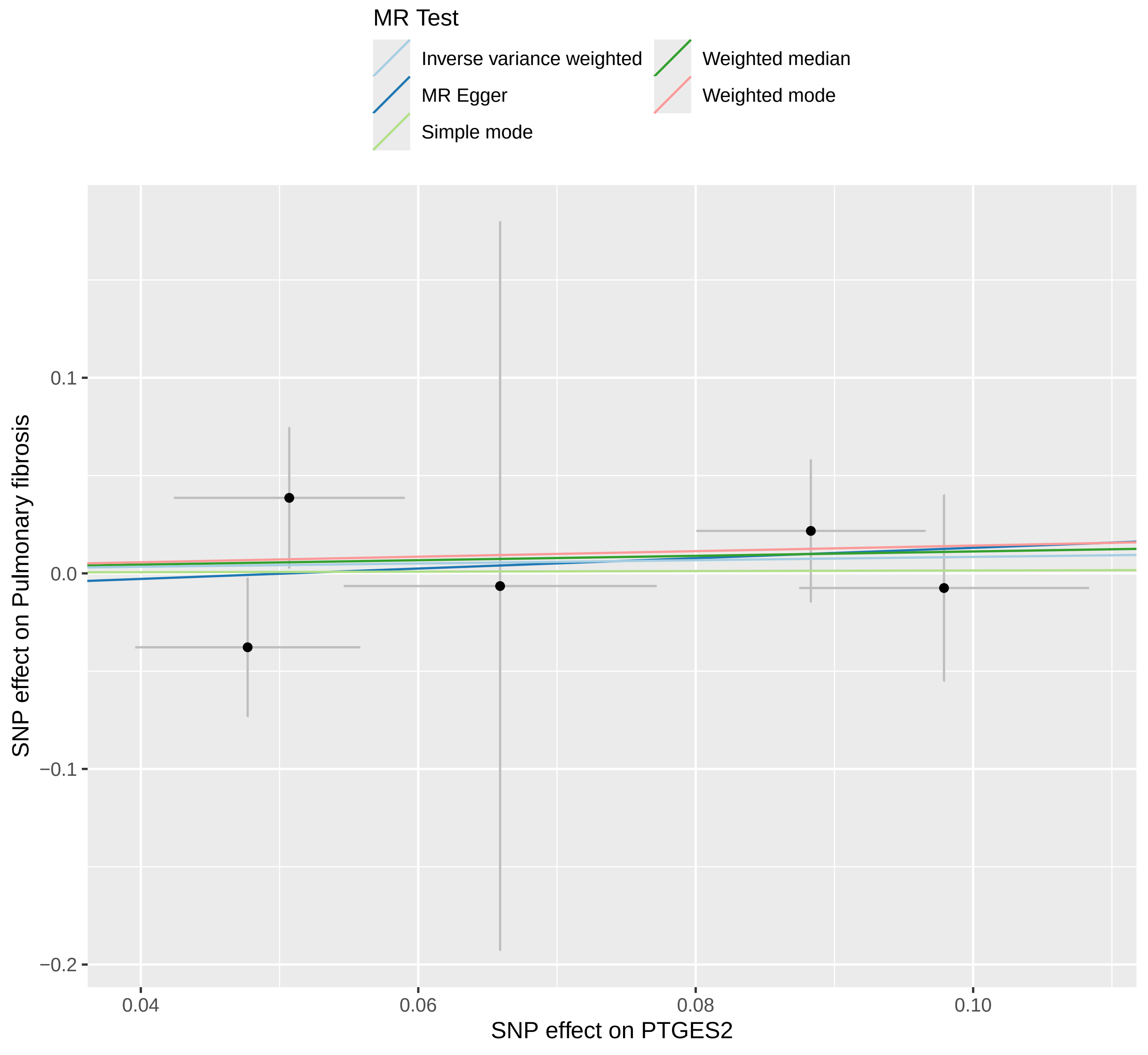
MR Test
Inverse variance weighted
MR Egger
Simple mode
Weighted median
Weighted mode
0.1
0.0
−0.1
−0.2
SNP effect on Pulmonary fibrosis
0.04
0.06
0.08
0.10
SNP effect on PTGES2

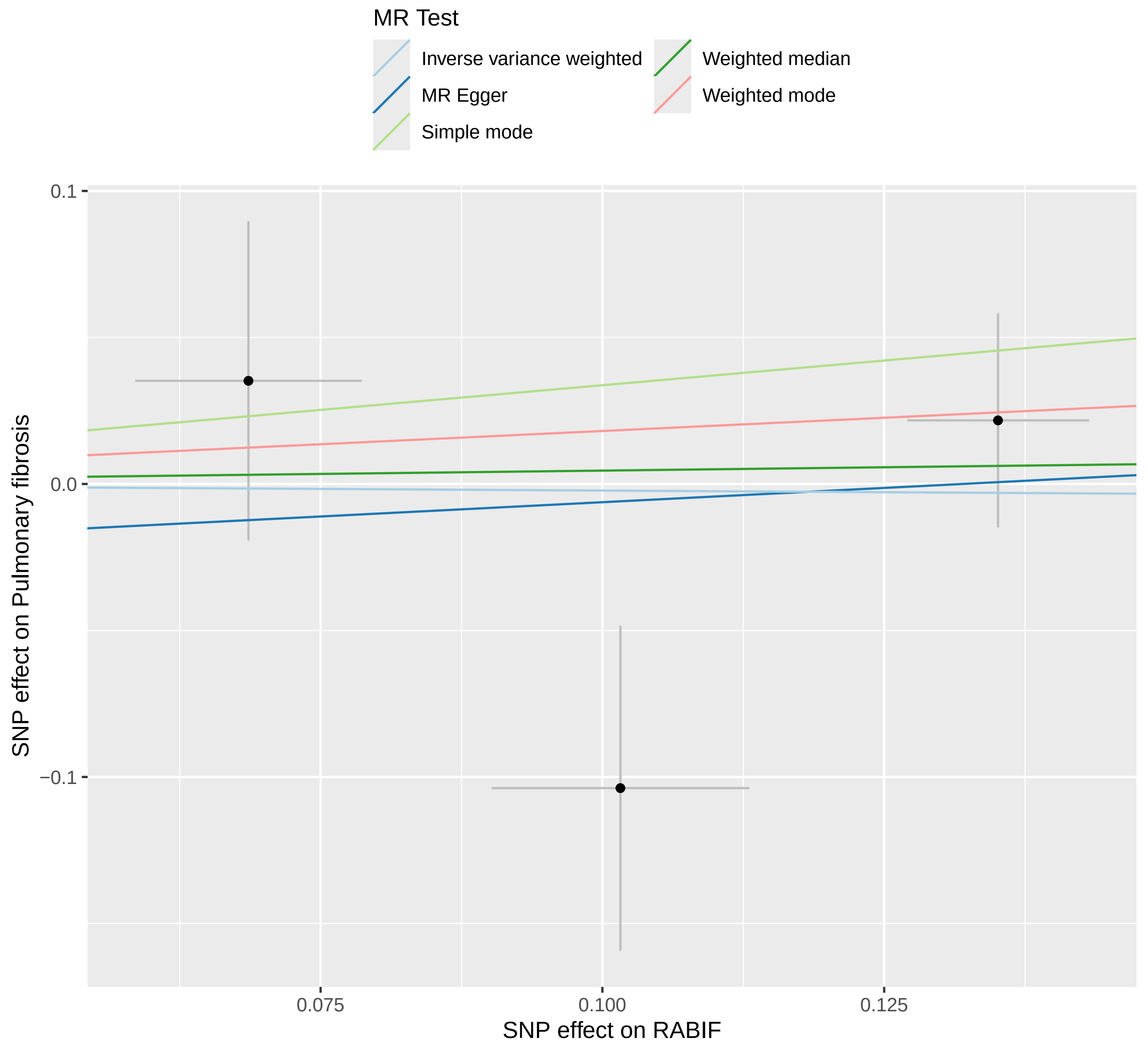
MR Test
Inverse variance weighted
MR Egger
Simple mode
Weighted median
Weighted mode
0.1
0.0
−0.1
0.075
0.100
0.125
SNP effect on Pulmonary fibrosis
SNP effect on RABIF

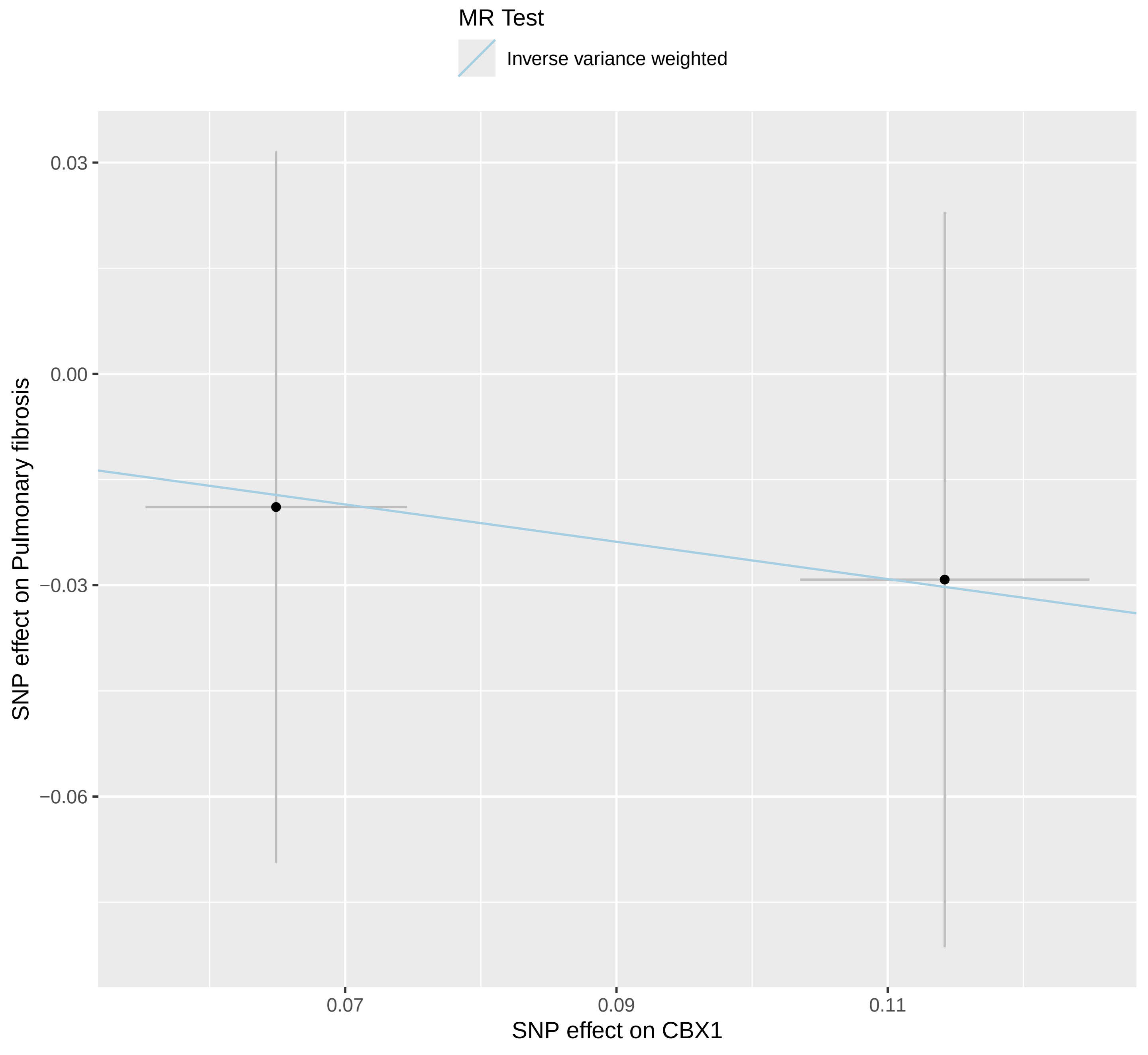

MR Test
Inverse variance weighted
0.03
0.00
−0.03
−0.06
SNP effect on Pulmonary fibrosis
0.07
0.09
0.11
SNP effect on CBX1

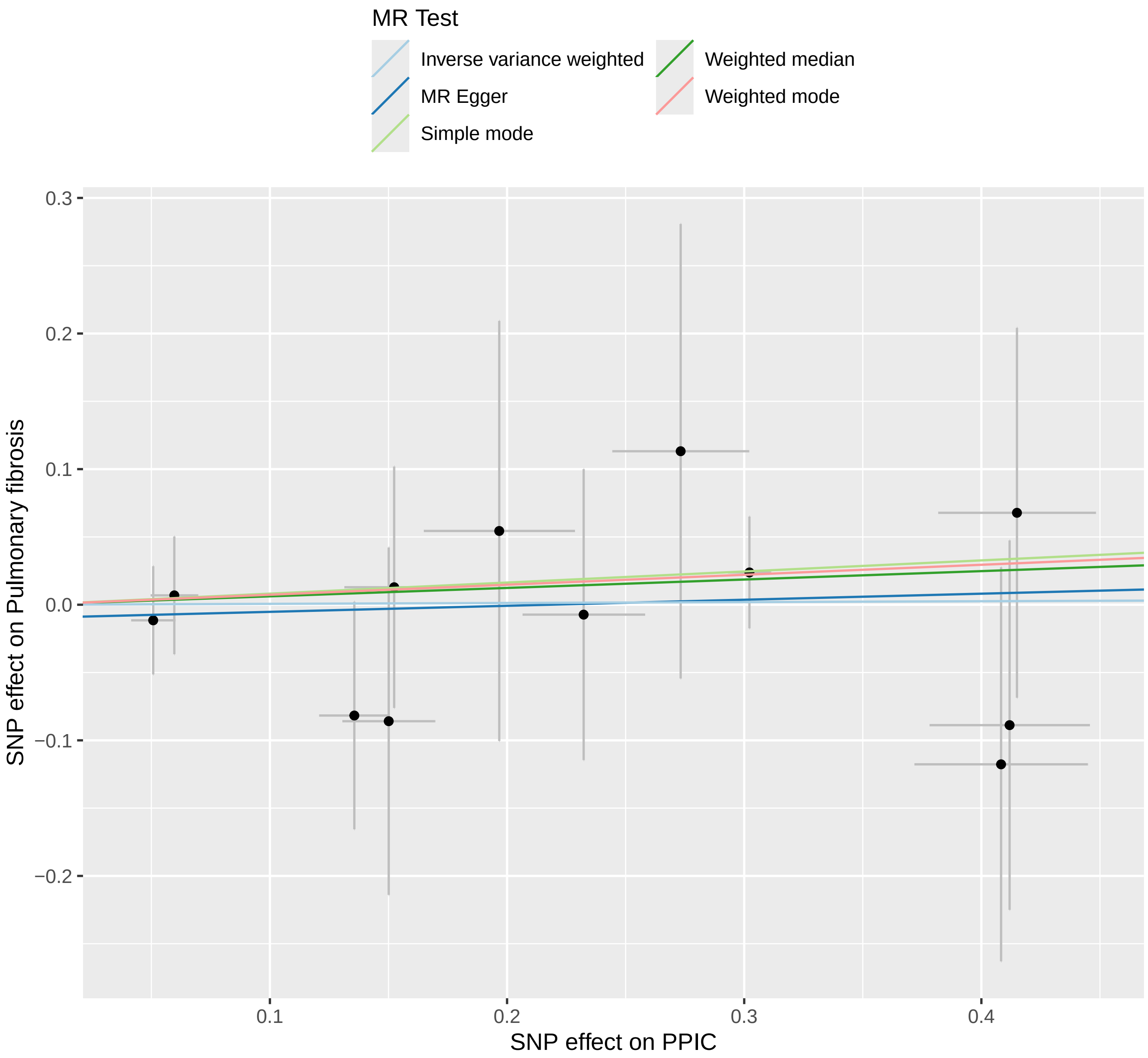

MR Test
Inverse variance weighted
MR Egger
Simple mode
Weighted median
Weighted mode
0.3
0.2
0.1
0.0
−0.1
−0.2
SNP effect on Pulmonary fibrosis
0.1
0.2
0.3
0.4
SNP effect on PPIC

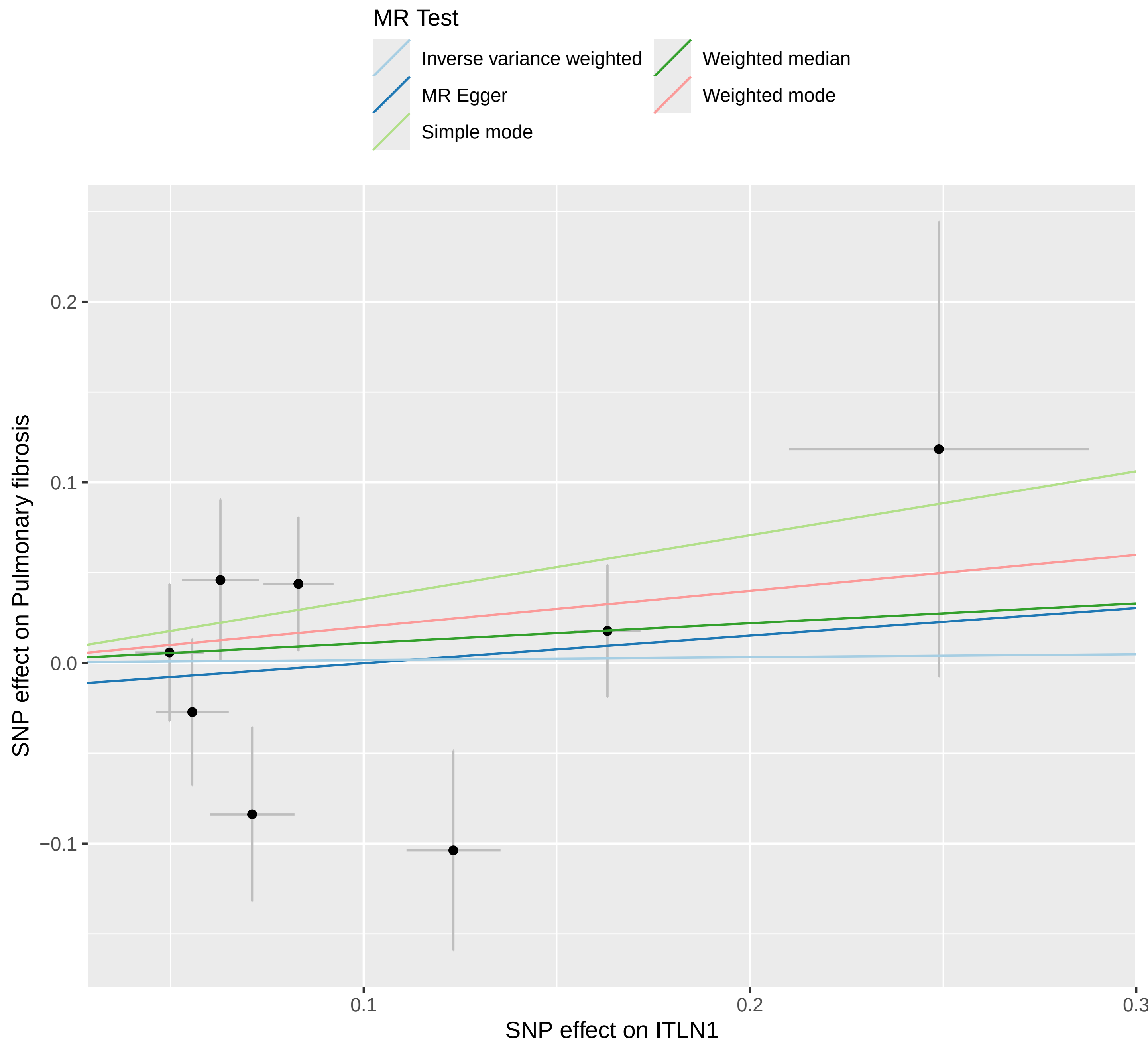
MR Test
Inverse variance weighted
MR Egger
Simple mode
Weighted median
Weighted mode
0.2
0.1
0.0
−0.1
0.1
0.2
0.3
SNP effect on Pulmonary fibrosis
SNP effect on ITLN1

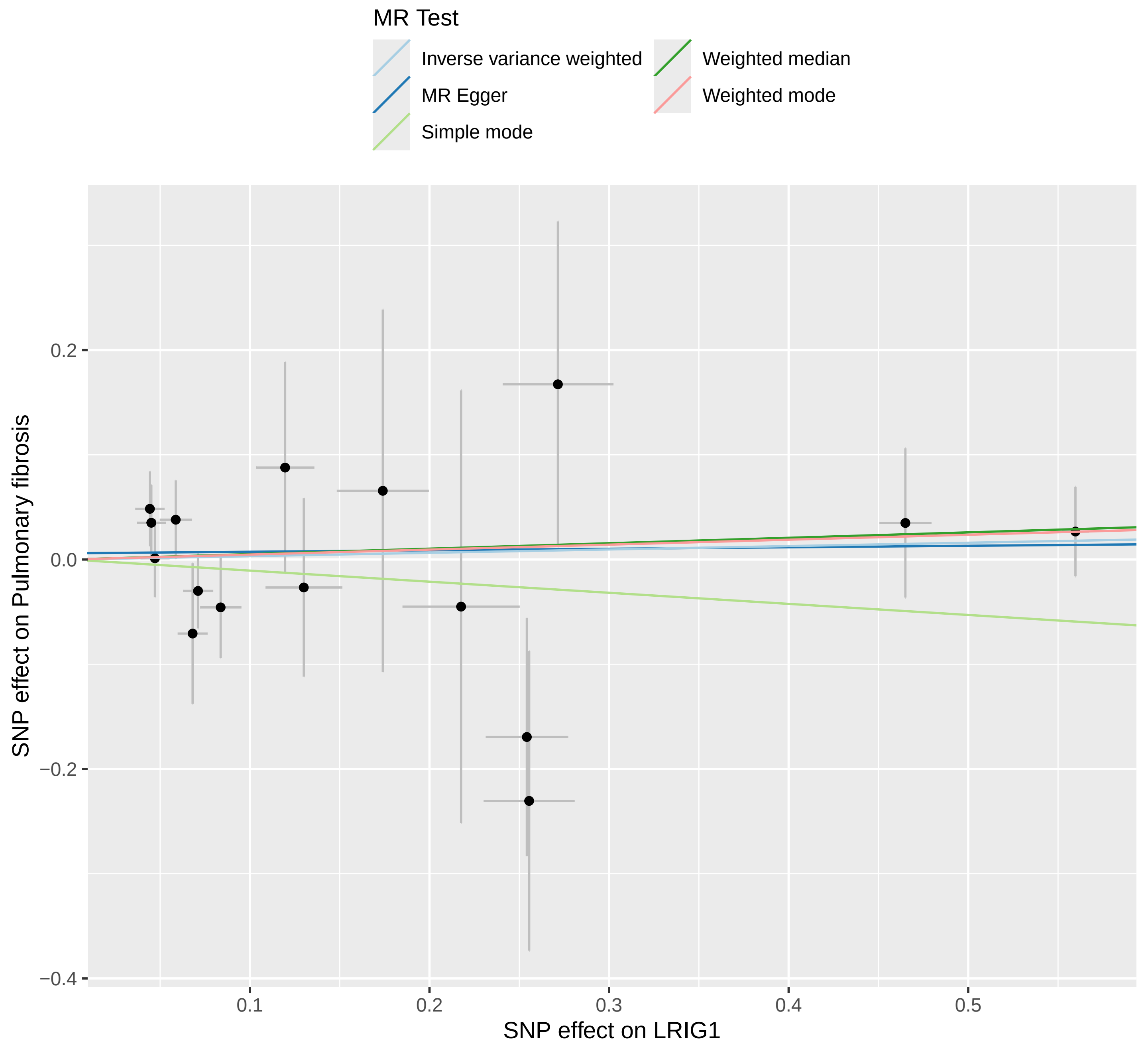
MR Test
Inverse variance weighted
MR Egger
Simple mode
Weighted median
Weighted mode
0.2
0.0
−0.2
−0.4
0.1
0.2
0.3
0.4
0.5
SNP effect on Pulmonary fibrosis
SNP effect on LRIG1

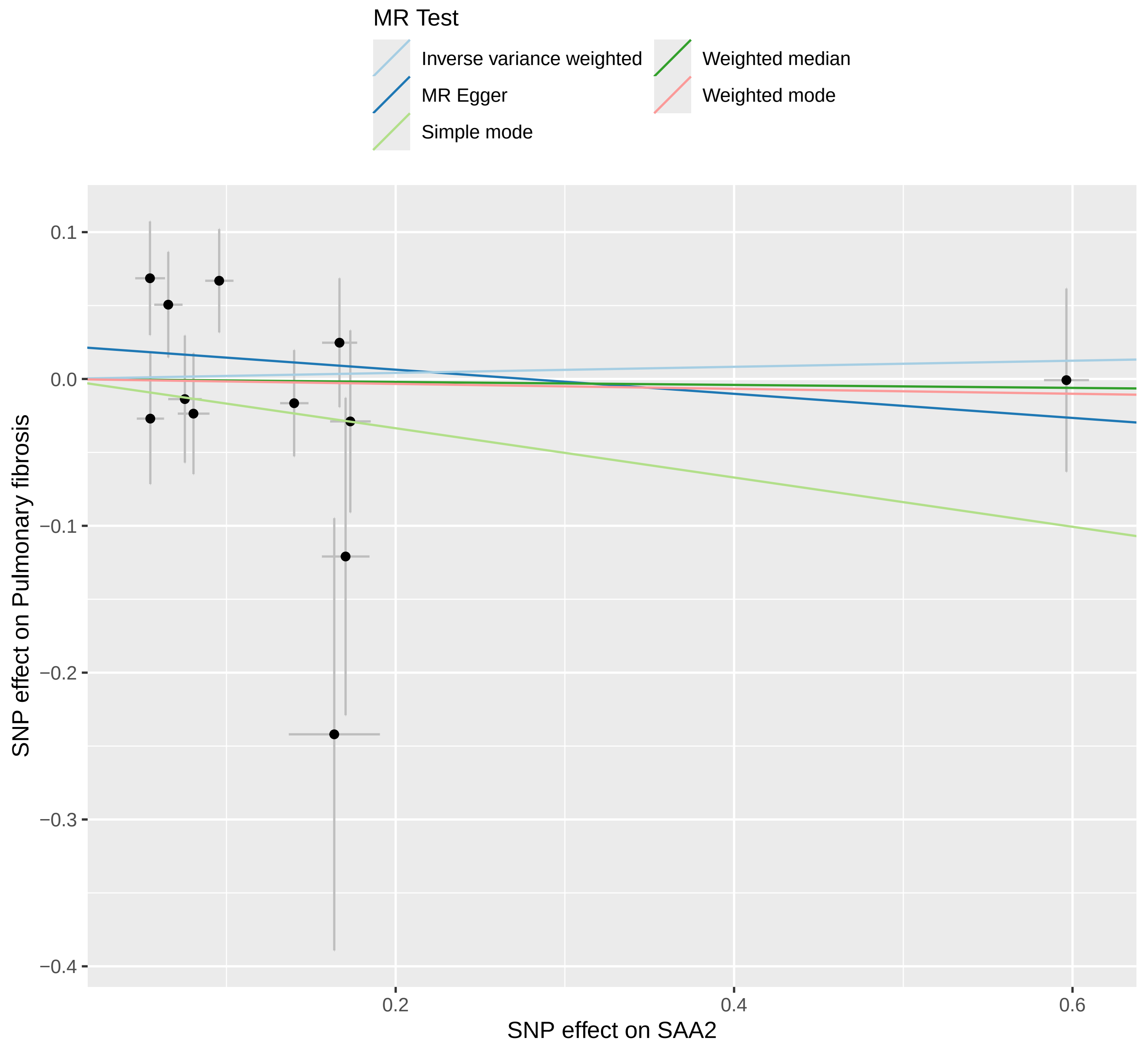
MR Test
Inverse variance weighted
MR Egger
Simple mode
Weighted median
Weighted mode
SNP effect on Pulmonary fibrosis
0.1
0.0
−0.1
−0.2
−0.3
−0.4
0.2
0.4
0.6
SNP effect on SAA2

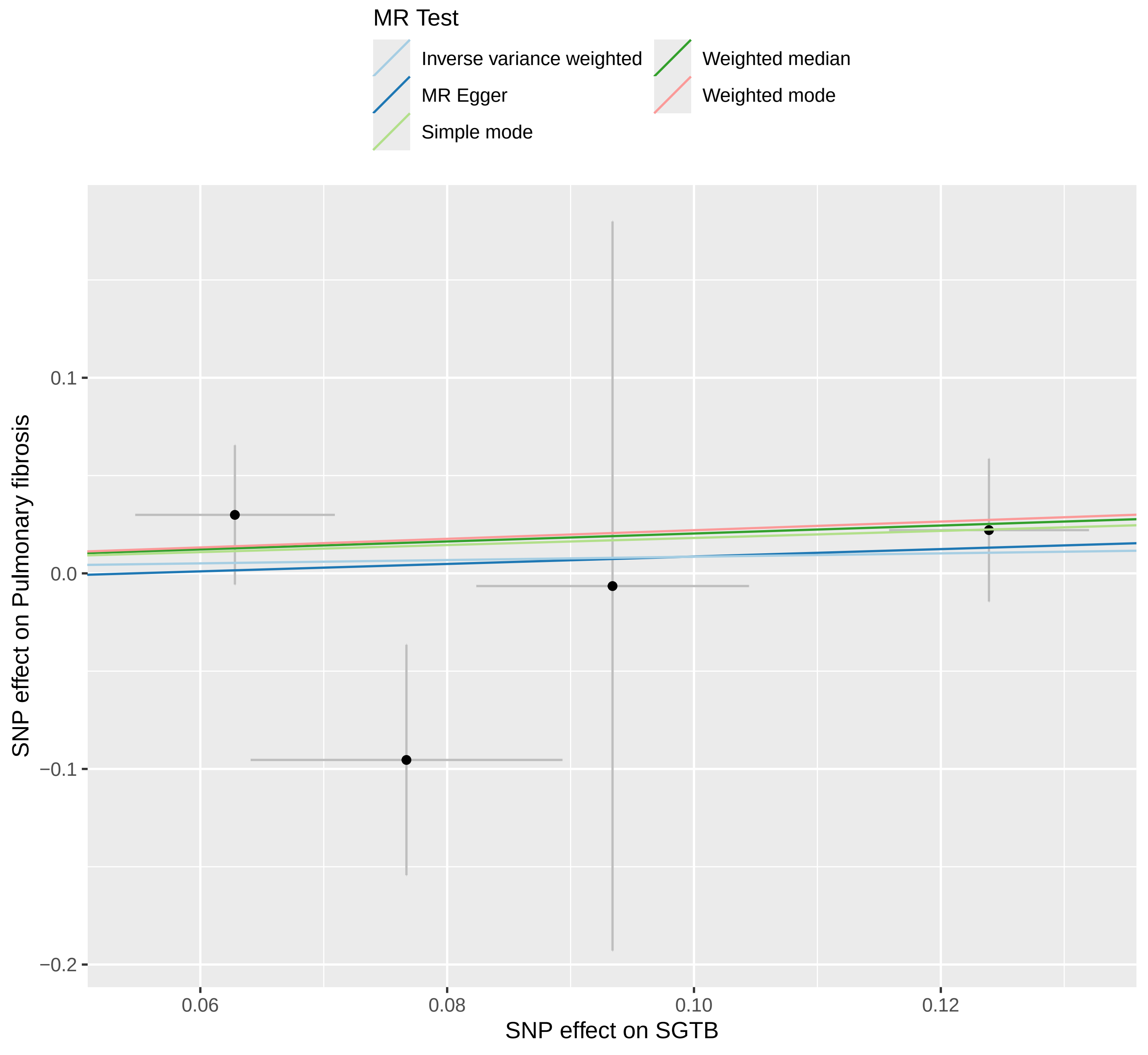

MR Test
Inverse variance weighted
MR Egger
Simple mode
Weighted median
Weighted mode
SNP effect on Pulmonary fibrosis
0.1
0.0
−0.1
−0.2
0.06
0.08
0.10
0.12
SNP effect on SGTB

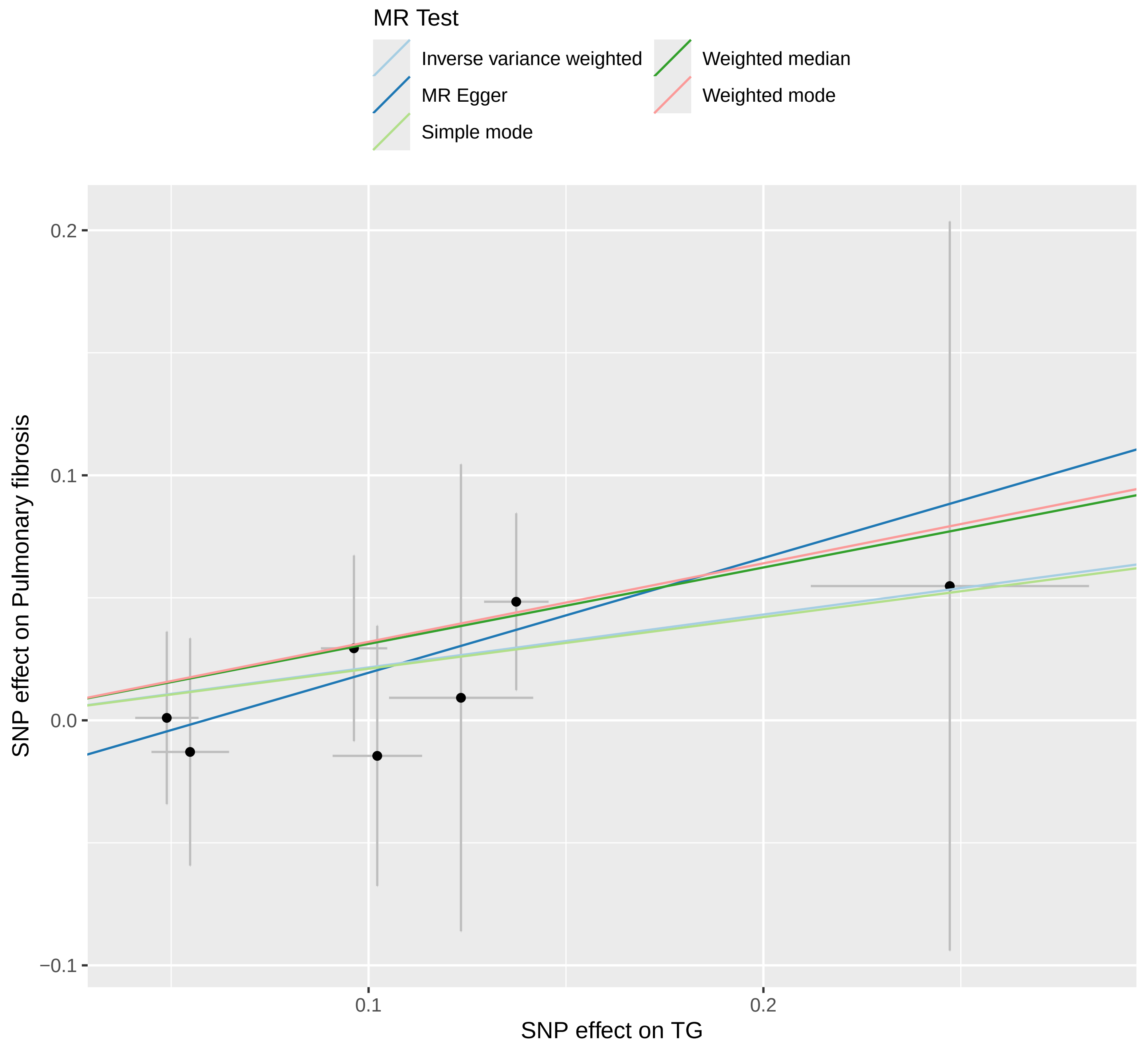

MR Test
Inverse variance weighted
MR Egger
Simple mode
Weighted median
Weighted mode
0.2
0.1
0.0
−0.1
0.1
0.2
SNP effect on Pulmonary fibrosis
SNP effect on TG

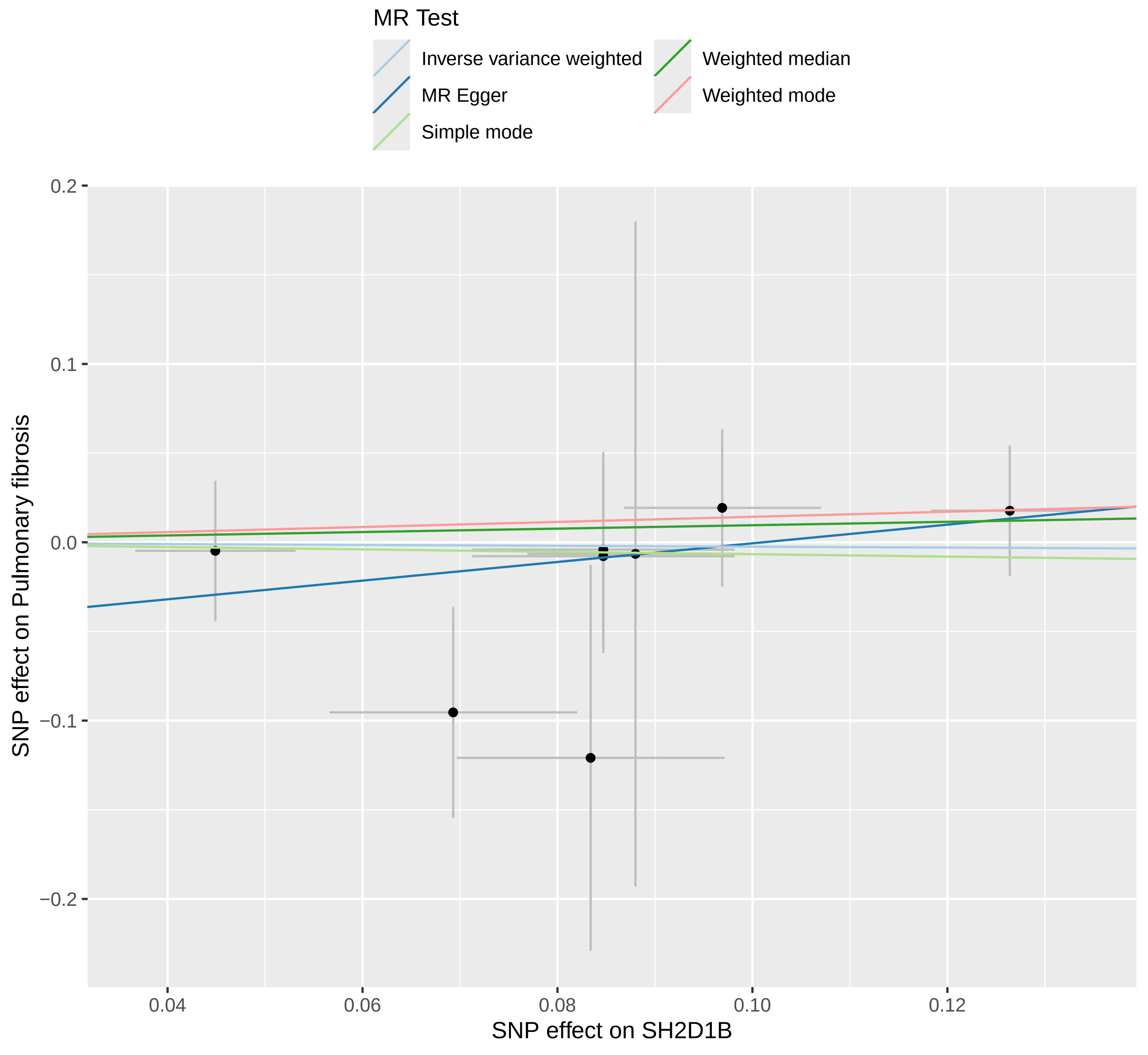

MR Test
Inverse variance weighted
MR Egger
Simple mode
Weighted median
Weighted mode
0.2
0.1
0.0
−0.1
−0.2
0.04
0.06
0.08
0.10
0.12
SNP effect on SH2D1B
SNP effect on Pulmonary fibrosis

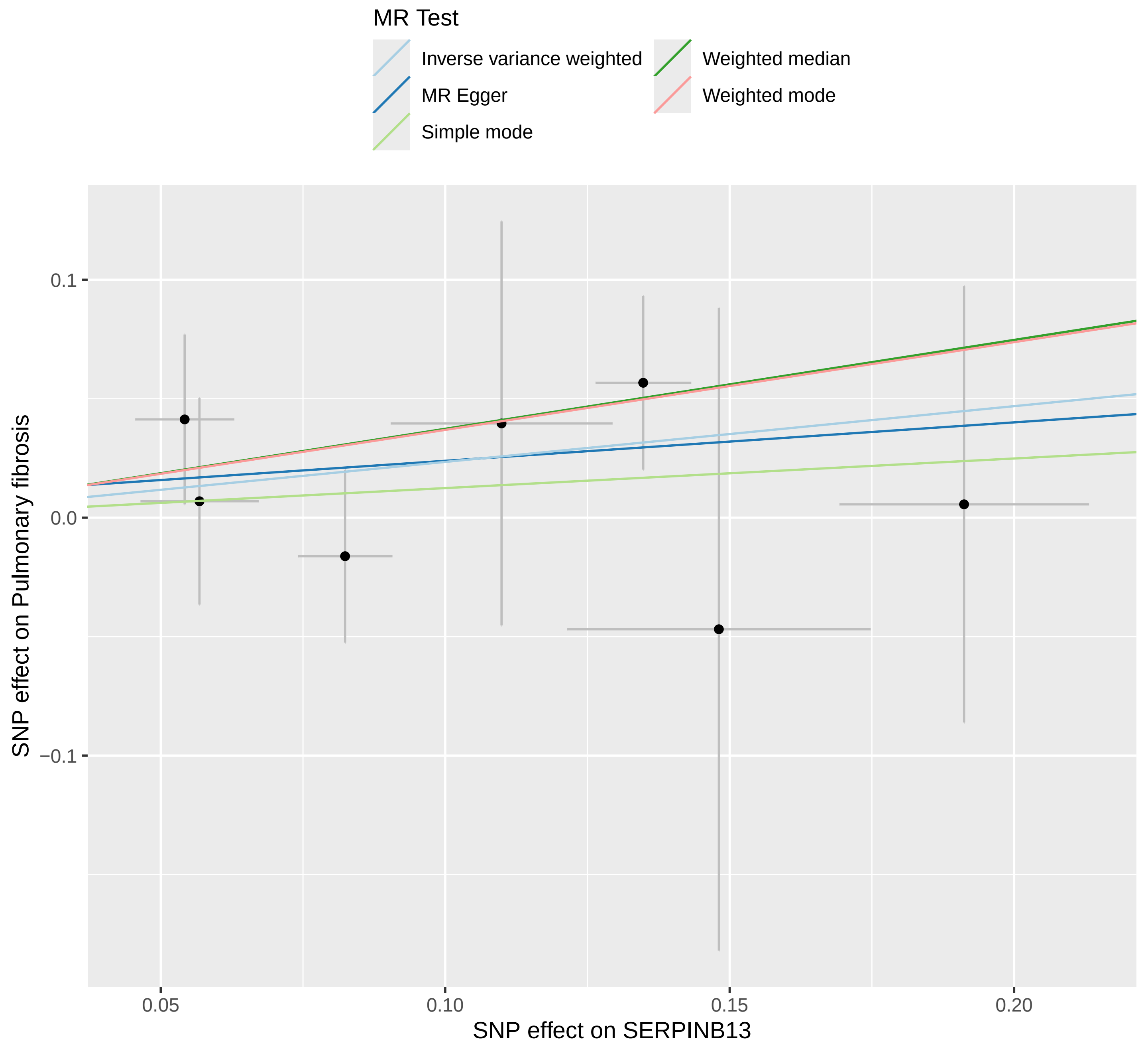

MR Test
Inverse variance weighted
MR Egger
Simple mode
Weighted median
Weighted mode
0.1
0.0
−0.1
0.05
0.10
0.15
0.20
SNP effect on Pulmonary fibrosis
SNP effect on SERPINB13

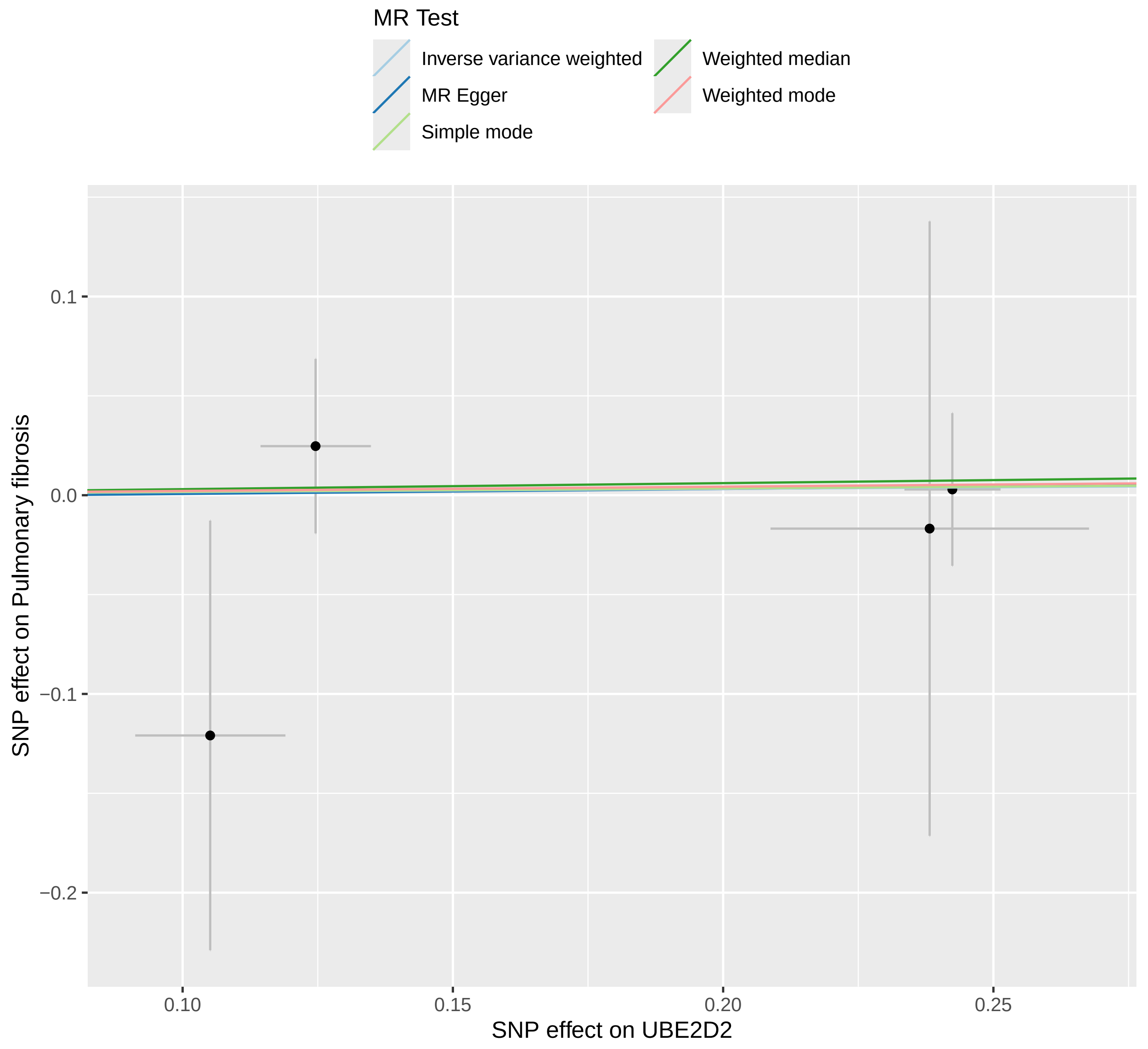
MR Test
Inverse variance weighted
MR Egger
Simple mode
Weighted median
Weighted mode
0.1
0.0
−0.1
−0.2
0.10
0.15
0.20
0.25
SNP effect on UBE2D2
SNP effect on Pulmonary fibrosis

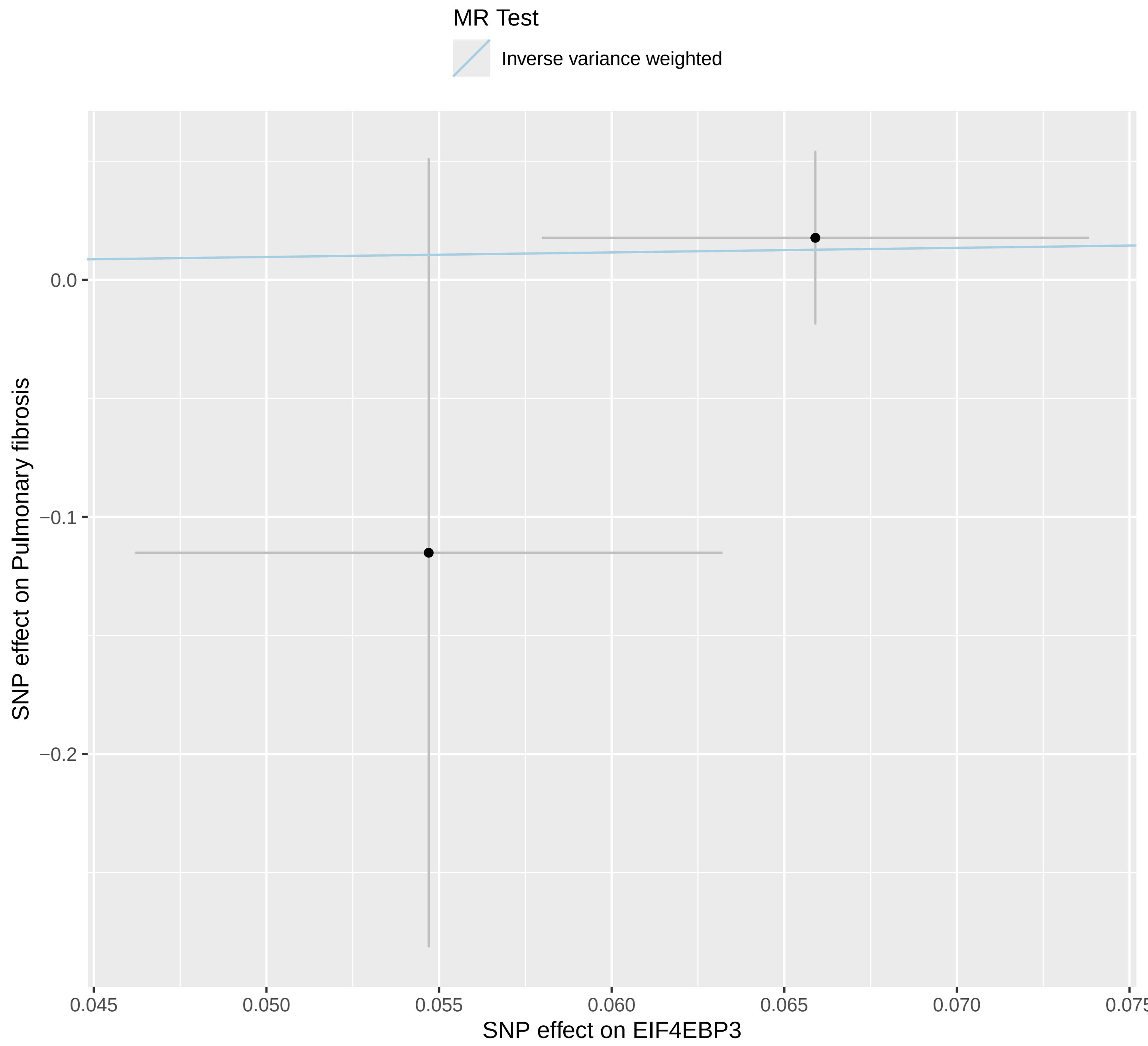
MR Test
Inverse variance weighted
0.0
−0.1
−0.2
SNP effect on Pulmonary fibrosis
0.045
0.050
0.055
0.060
0.065
0.070
0.075
SNP effect on EIF4EBP3

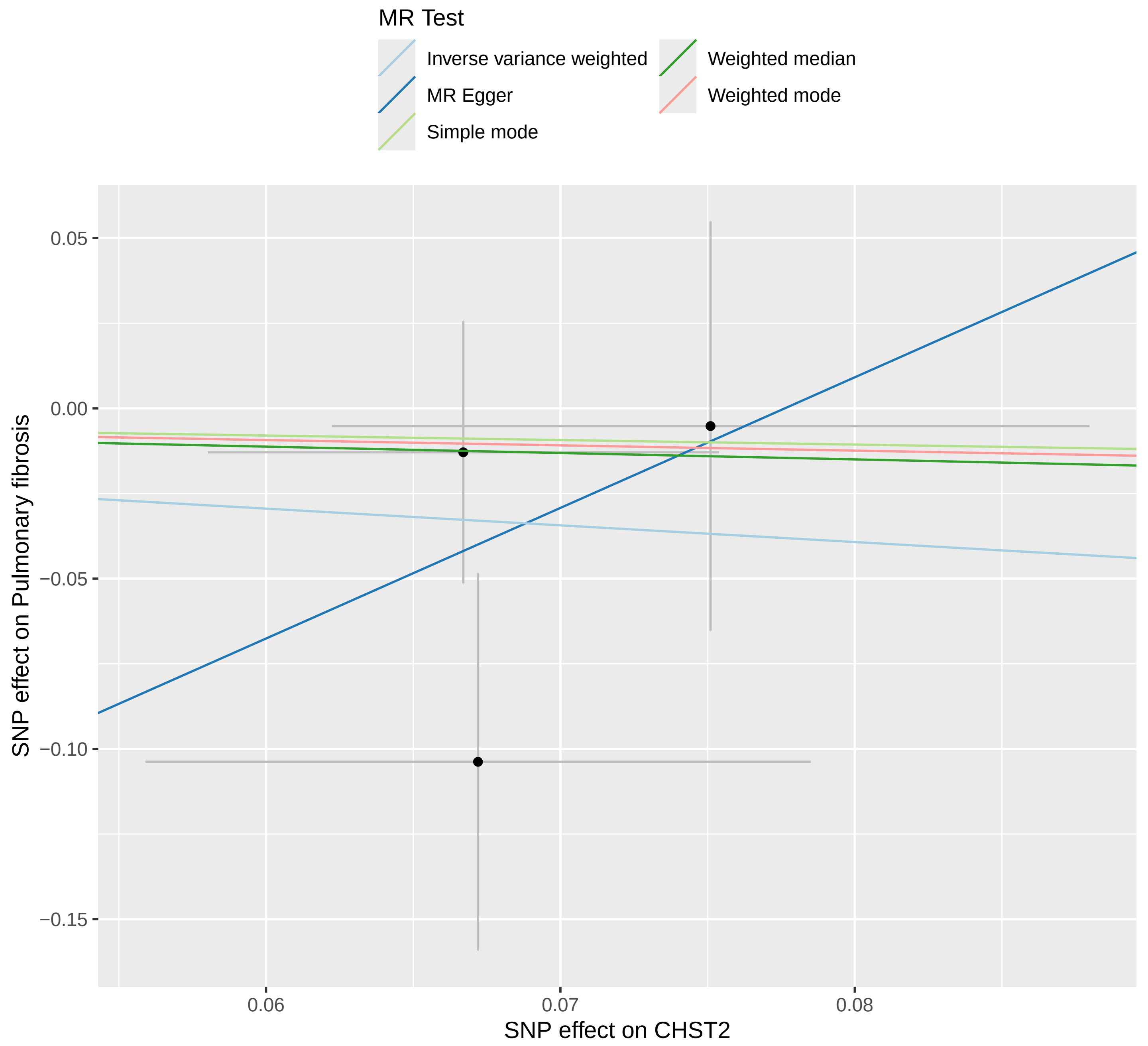

MR Test
Inverse variance weighted
MR Egger
Simple mode
Weighted median
Weighted mode
0.05
0.00
−0.05
−0.10
−0.15
0.06
0.07
0.08
SNP effect on Pulmonary fibrosis
SNP effect on CHST2

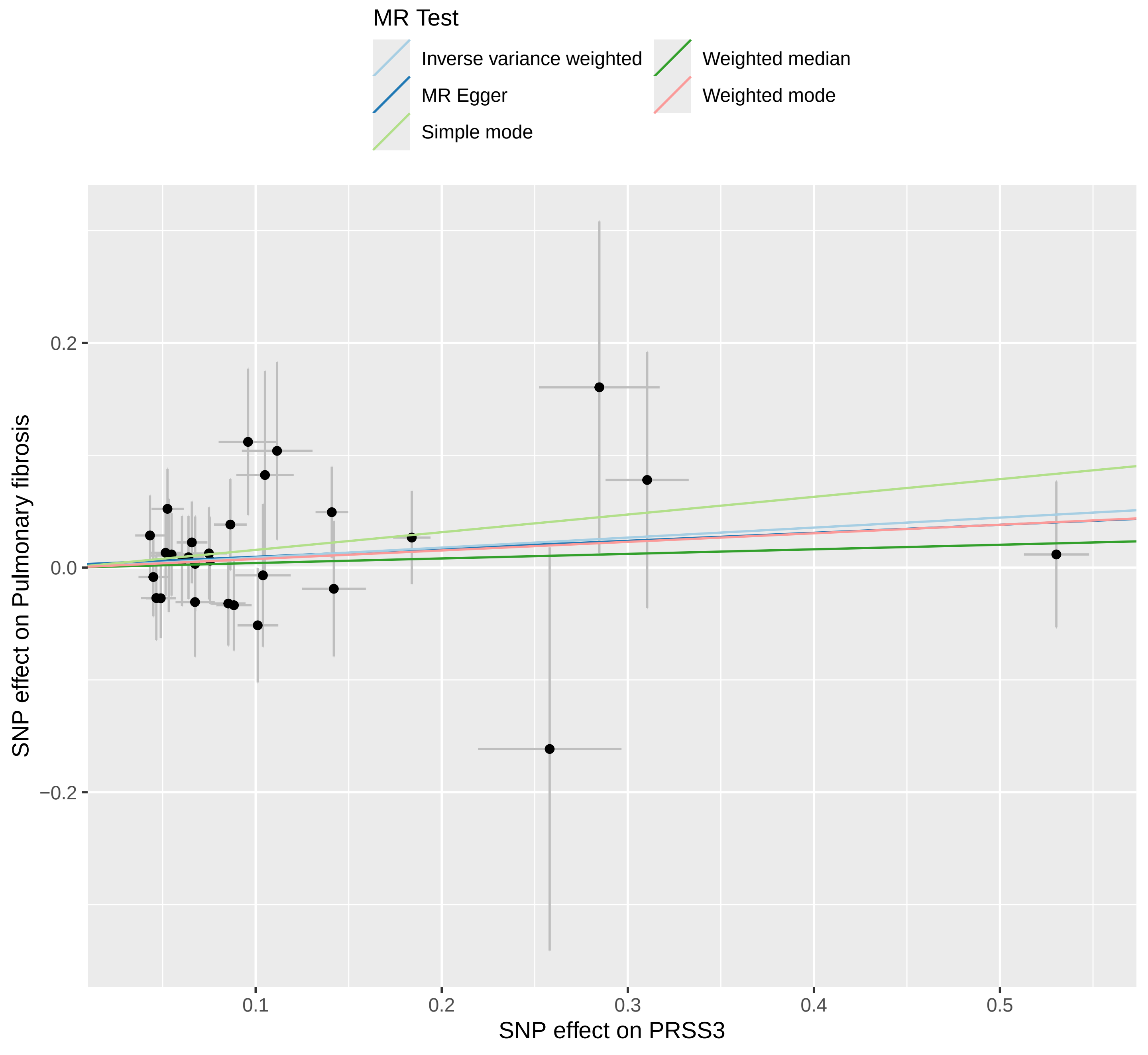

MR Test
Inverse variance weighted
MR Egger
Simple mode
Weighted median
Weighted mode
0.2
0.0
−0.2
SNP effect on Pulmonary fibrosis
0.1
0.2
0.3
0.4
0.5
SNP effect on PRSS3

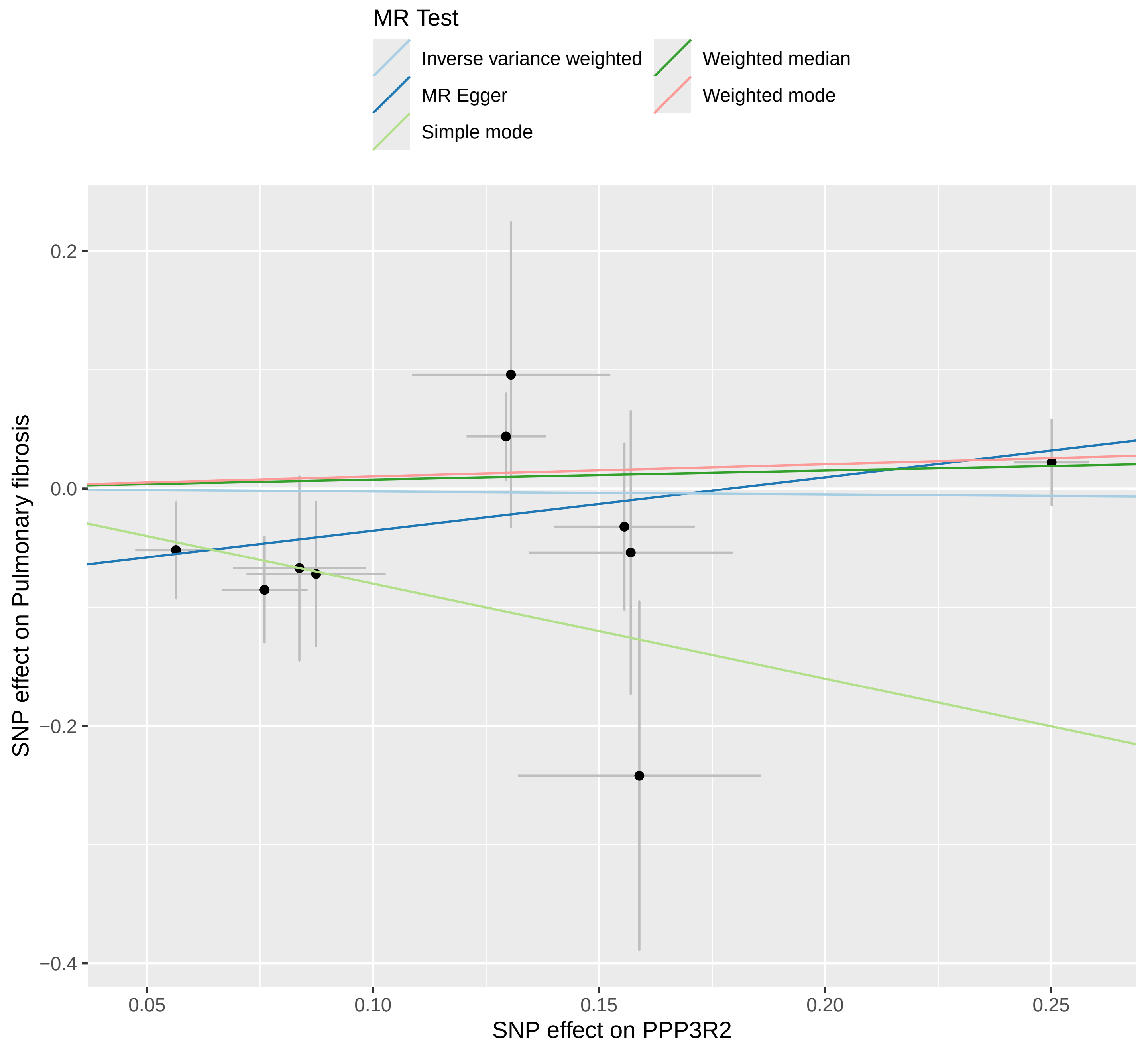

MR Test
Inverse variance weighted
MR Egger
Simple mode
Weighted median
Weighted mode
0.2
0.0
−0.2
−0.4
SNP effect on Pulmonary fibrosis
0.05
0.10
0.15
0.20
0.25
SNP effect on PPP3R2

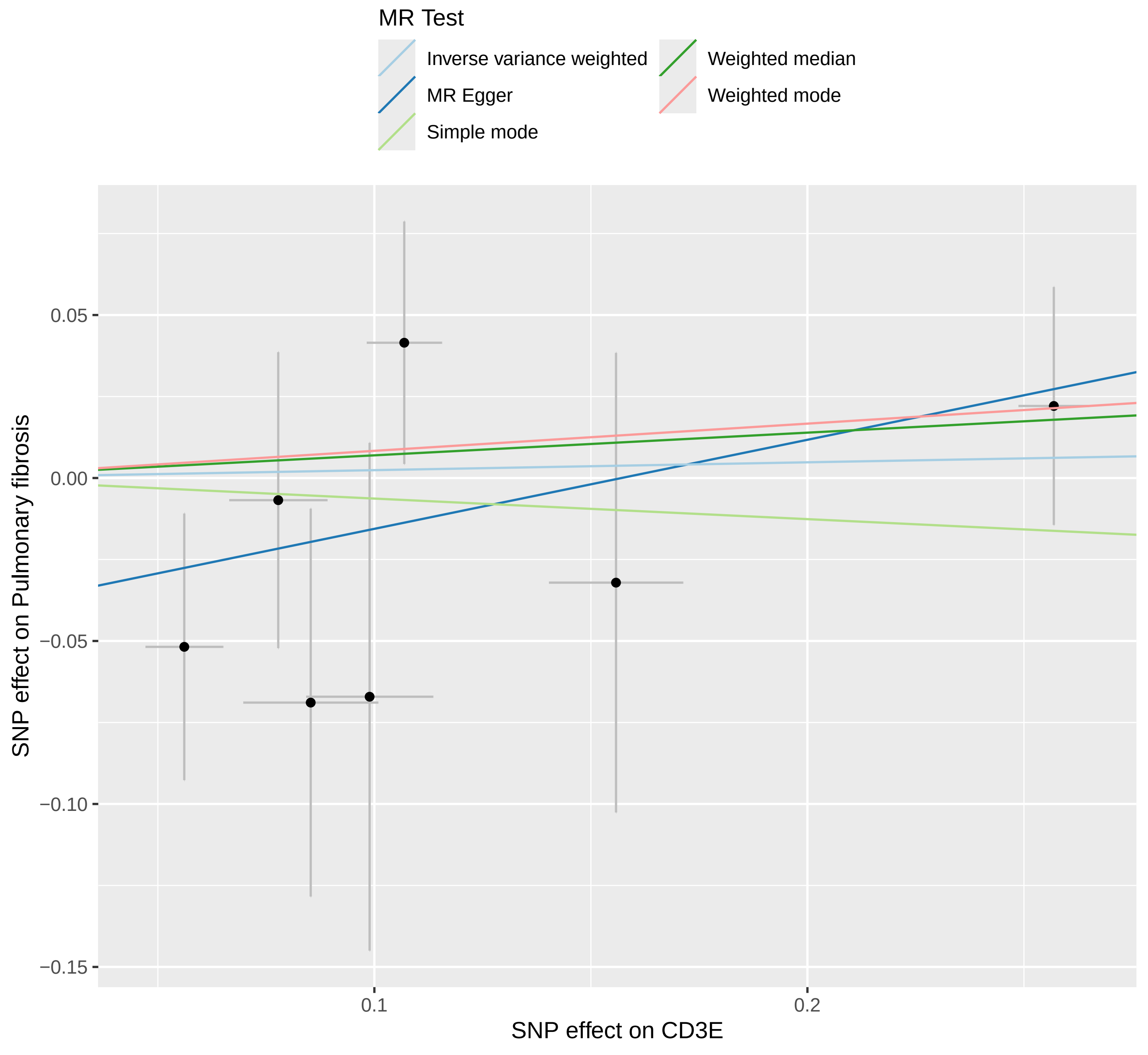

MR Test
Inverse variance weighted
MR Egger
Simple mode
Weighted median
Weighted mode
0.05
0.00
−0.05
−0.10
−0.15
0.1
0.2
SNP effect on Pulmonary fibrosis
SNP effect on CD3E

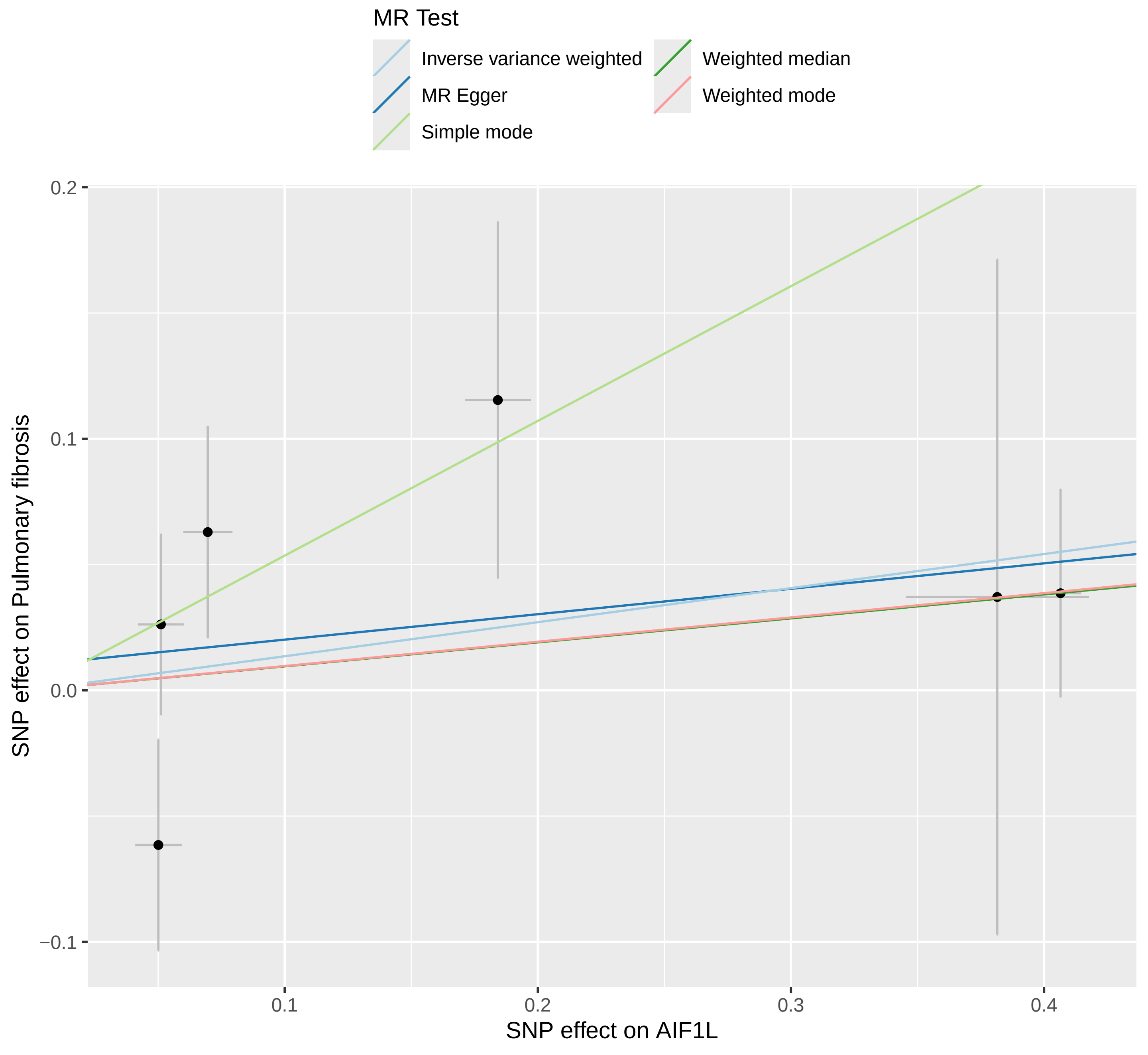

MR Test
Inverse variance weighted
MR Egger
Simple mode
Weighted median
Weighted mode
0.2
0.1
0.0
−0.1
SNP effect on Pulmonary fibrosis
0.1
0.2
0.3
0.4
SNP effect on AIF1L

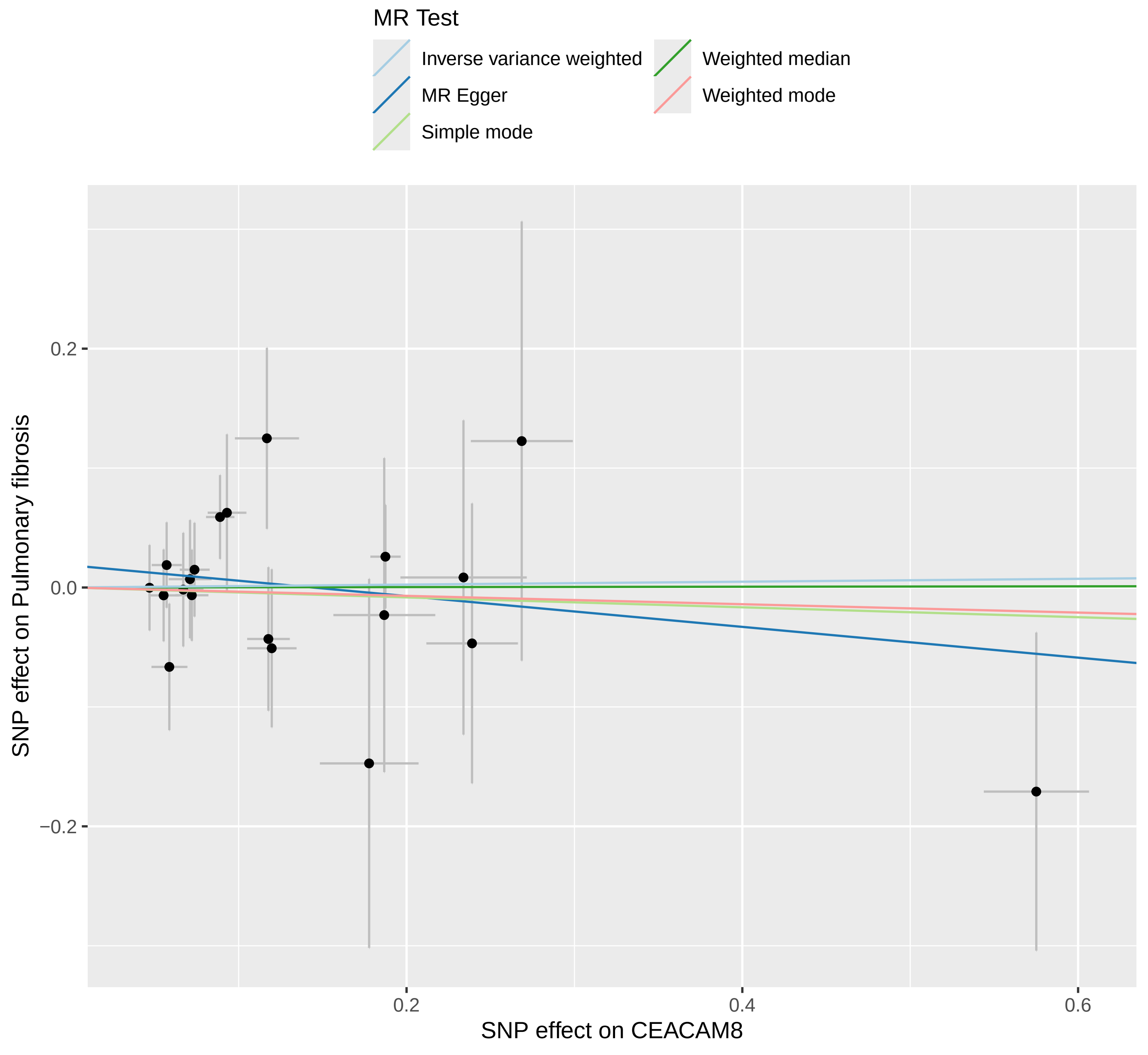
MR Test
Inverse variance weighted
MR Egger
Simple mode
Weighted median
Weighted mode
0.2
0.0
−0.2
0.2
0.4
0.6
SNP effect on Pulmonary fibrosis
SNP effect on CEACAM8

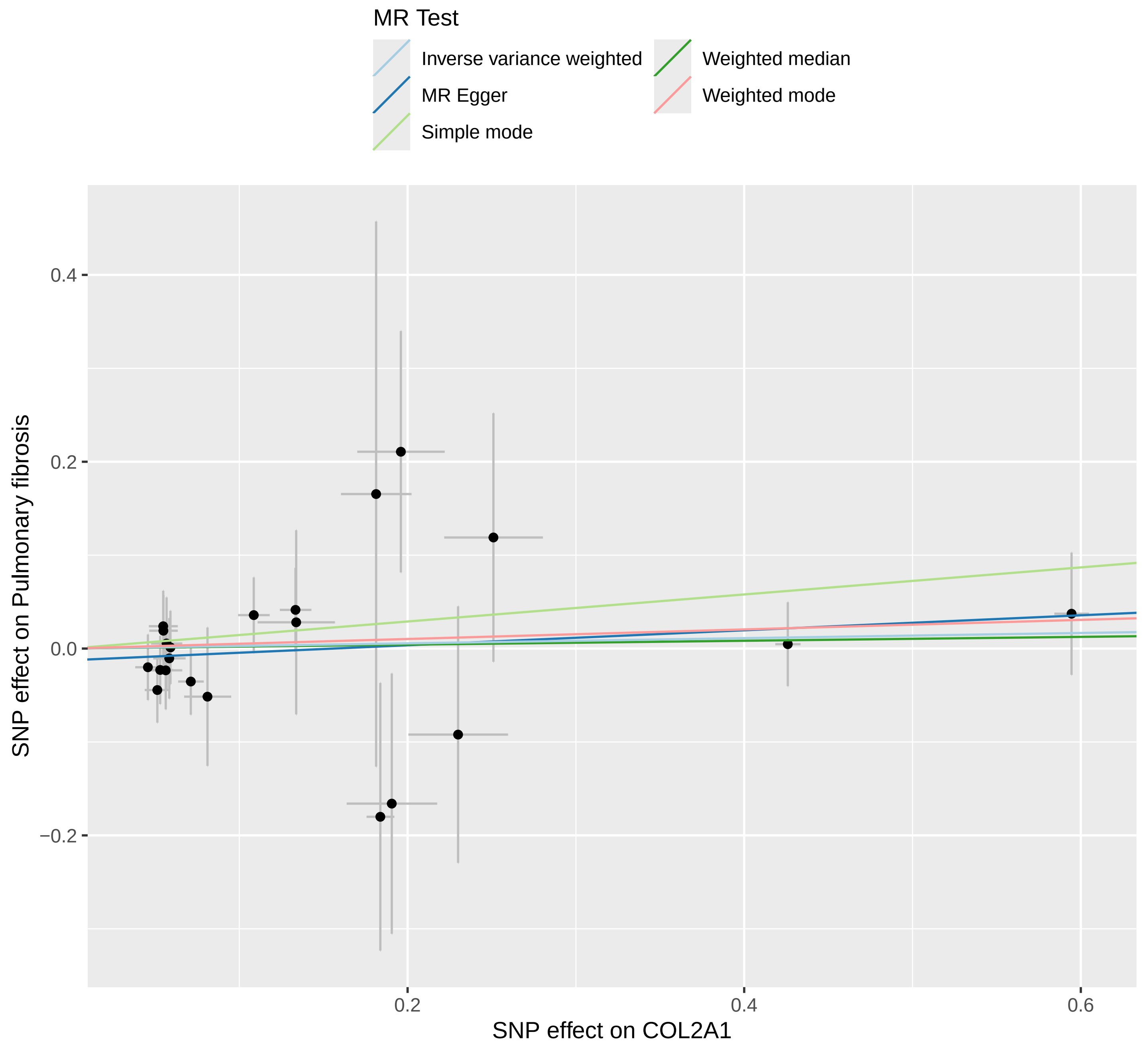

MR Test
Inverse variance weighted
MR Egger
Simple mode
Weighted median
Weighted mode
0.4
0.2
0.0
−0.2
0.2
0.4
0.6
SNP effect on Pulmonary fibrosis
SNP effect on COL2A1

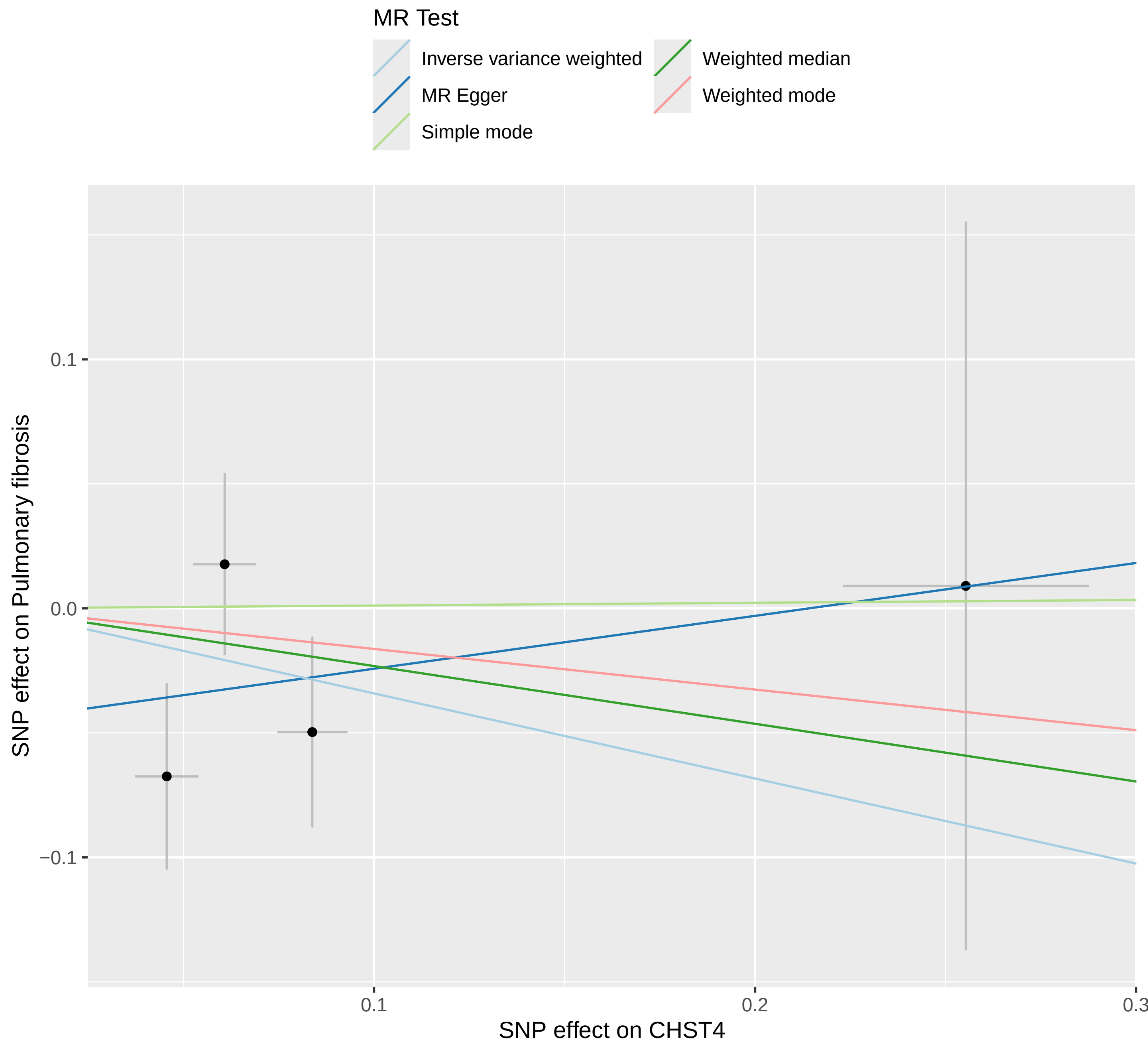

MR Test
Inverse variance weighted
MR Egger
Simple mode
Weighted median
Weighted mode
0.1
0.0
−0.1
SNP effect on Pulmonary fibrosis
0.1
0.2
0.3
SNP effect on CHST4

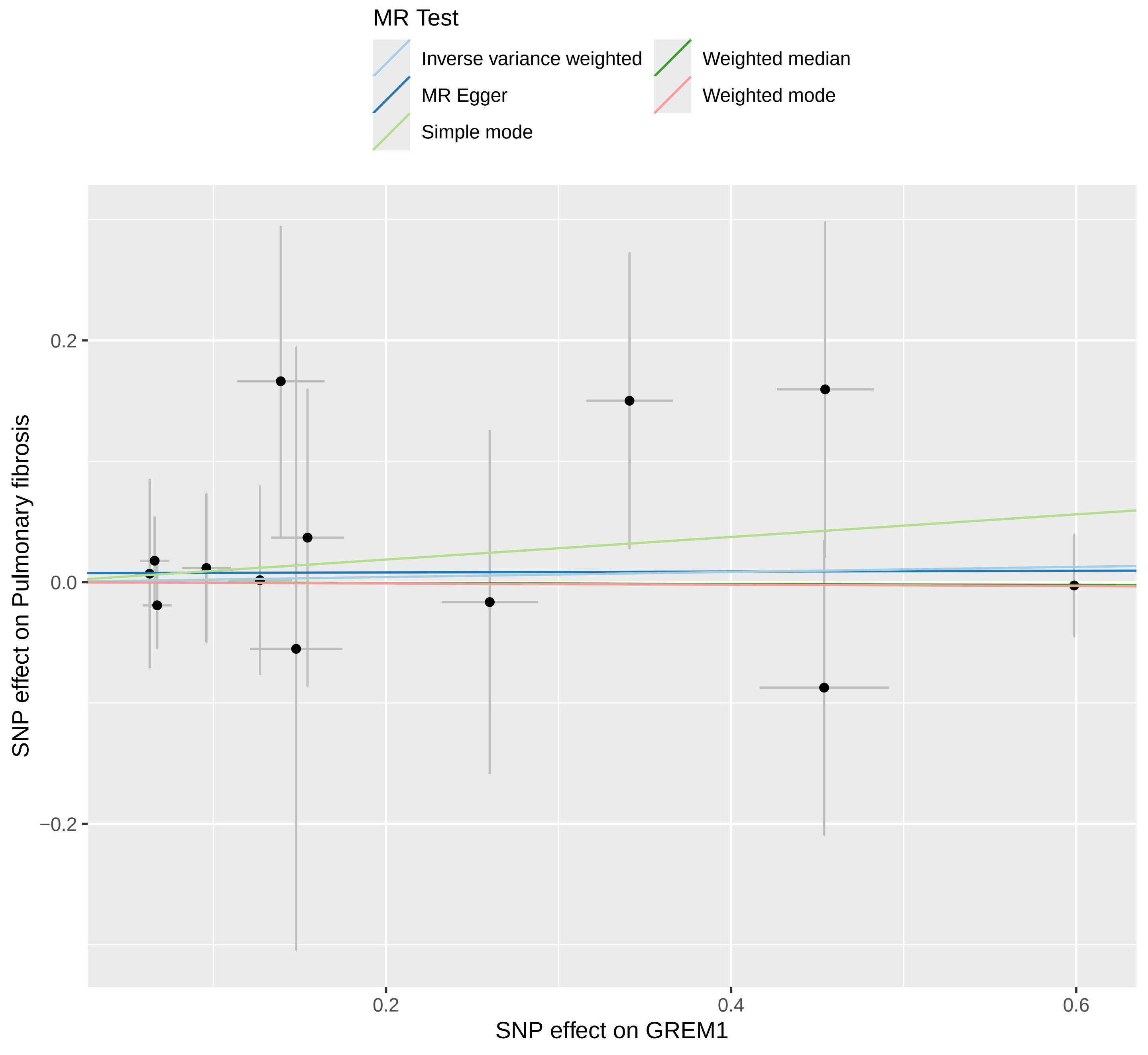

MR Test
Inverse variance weighted
MR Egger
Simple mode
Weighted median
Weighted mode
SNP effect on Pulmonary fibrosis
0.2
0.0
−0.2
0.2
0.4
0.6
SNP effect on GREM1

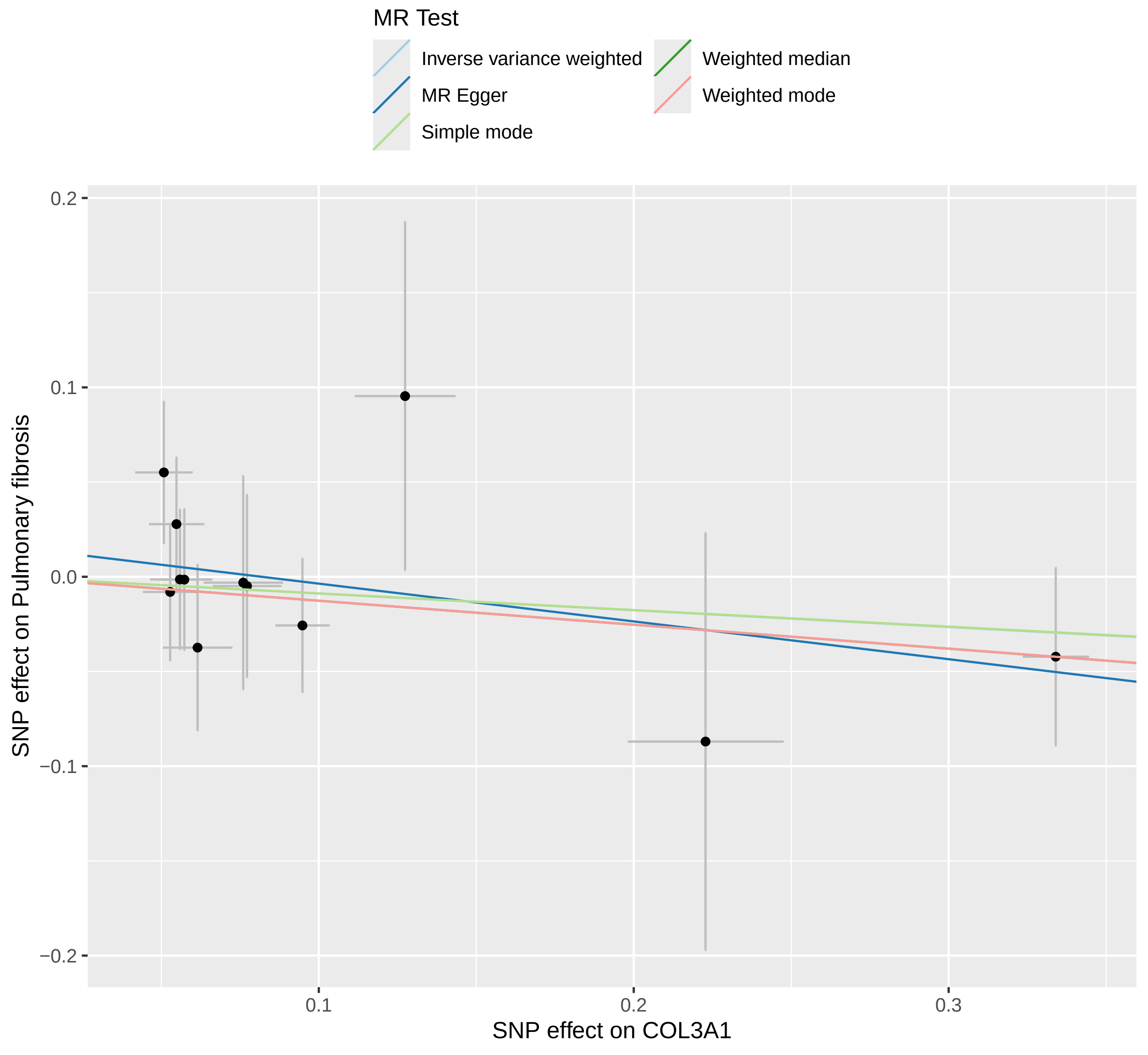
MR Test
Inverse variance weighted
MR Egger
Simple mode
Weighted median
Weighted mode
0.2
0.1
0.0
−0.1
−0.2
0.1
0.2
0.3
SNP effect on Pulmonary fibrosis
SNP effect on COL3A1

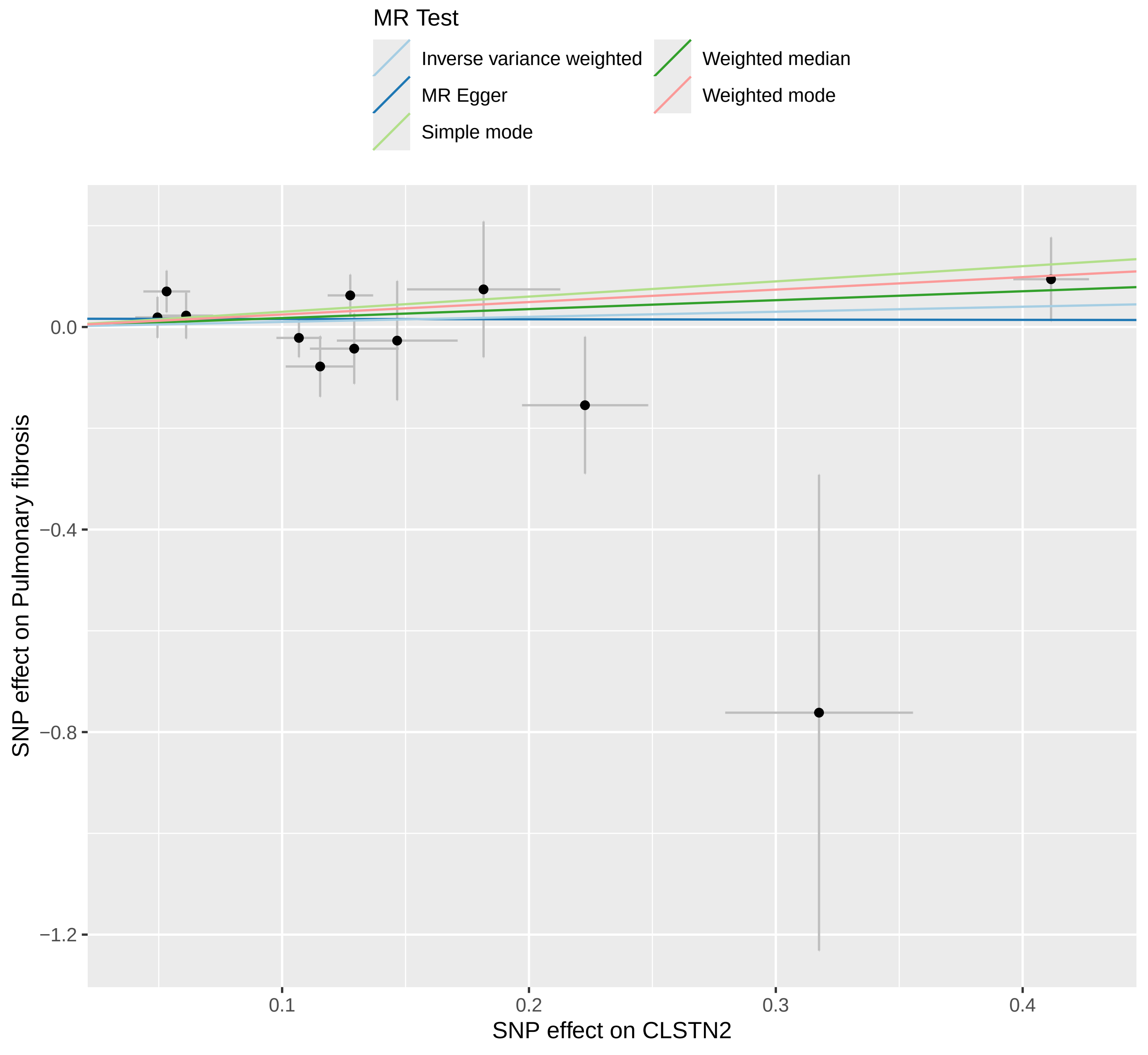
MR Test
Inverse variance weighted
MR Egger
Simple mode
Weighted median
Weighted mode
0.0
−0.4
−0.8
−1.2
0.1
0.2
0.3
0.4
SNP effect on Pulmonary fibrosis
SNP effect on CLSTN2

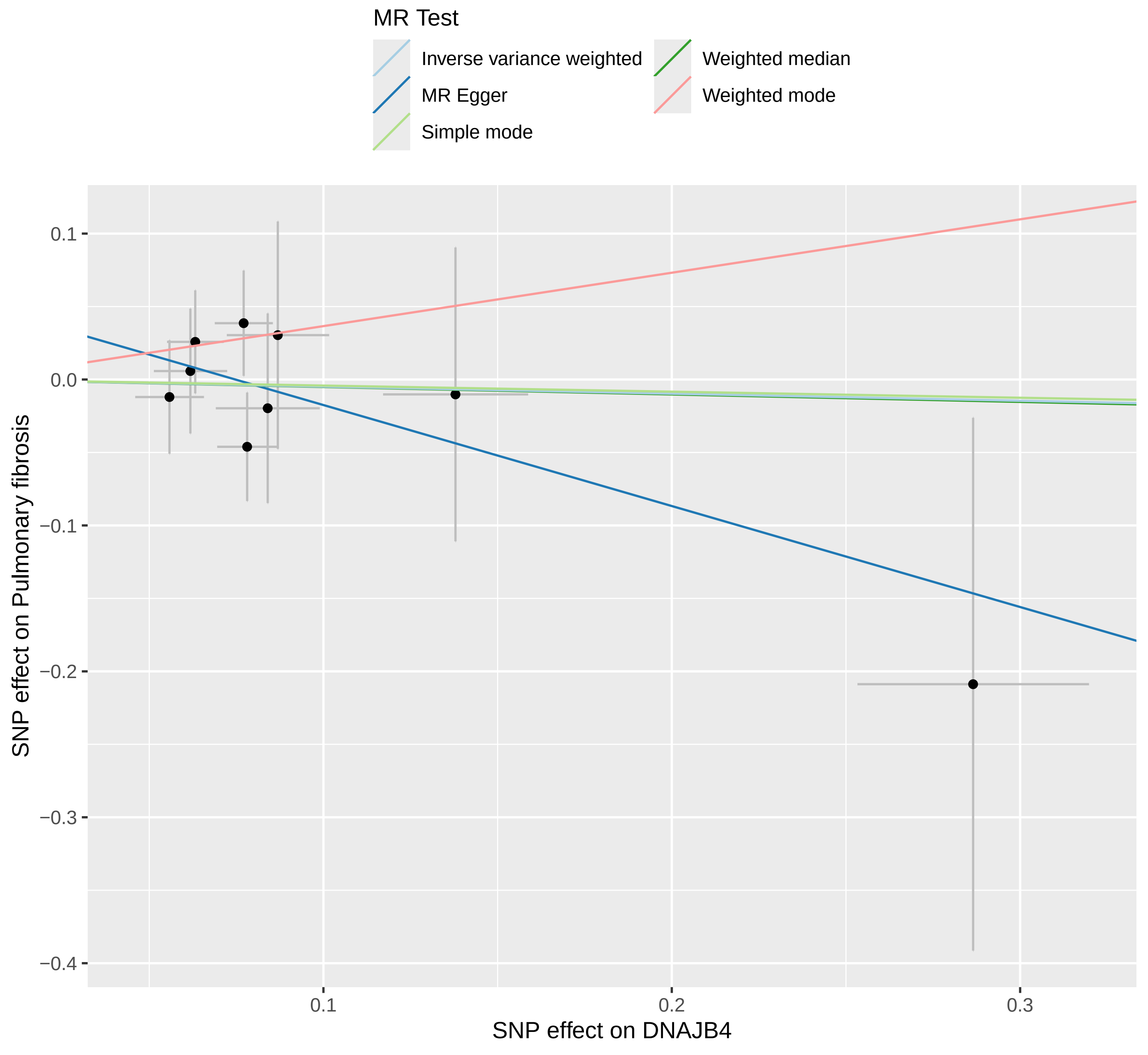

MR Test
Inverse variance weighted
MR Egger
Simple mode
Weighted median
Weighted mode
0.1
0.0
−0.1
−0.2
−0.3
−0.4
0.1
0.2
0.3
SNP effect on Pulmonary fibrosis
SNP effect on DNAJB4

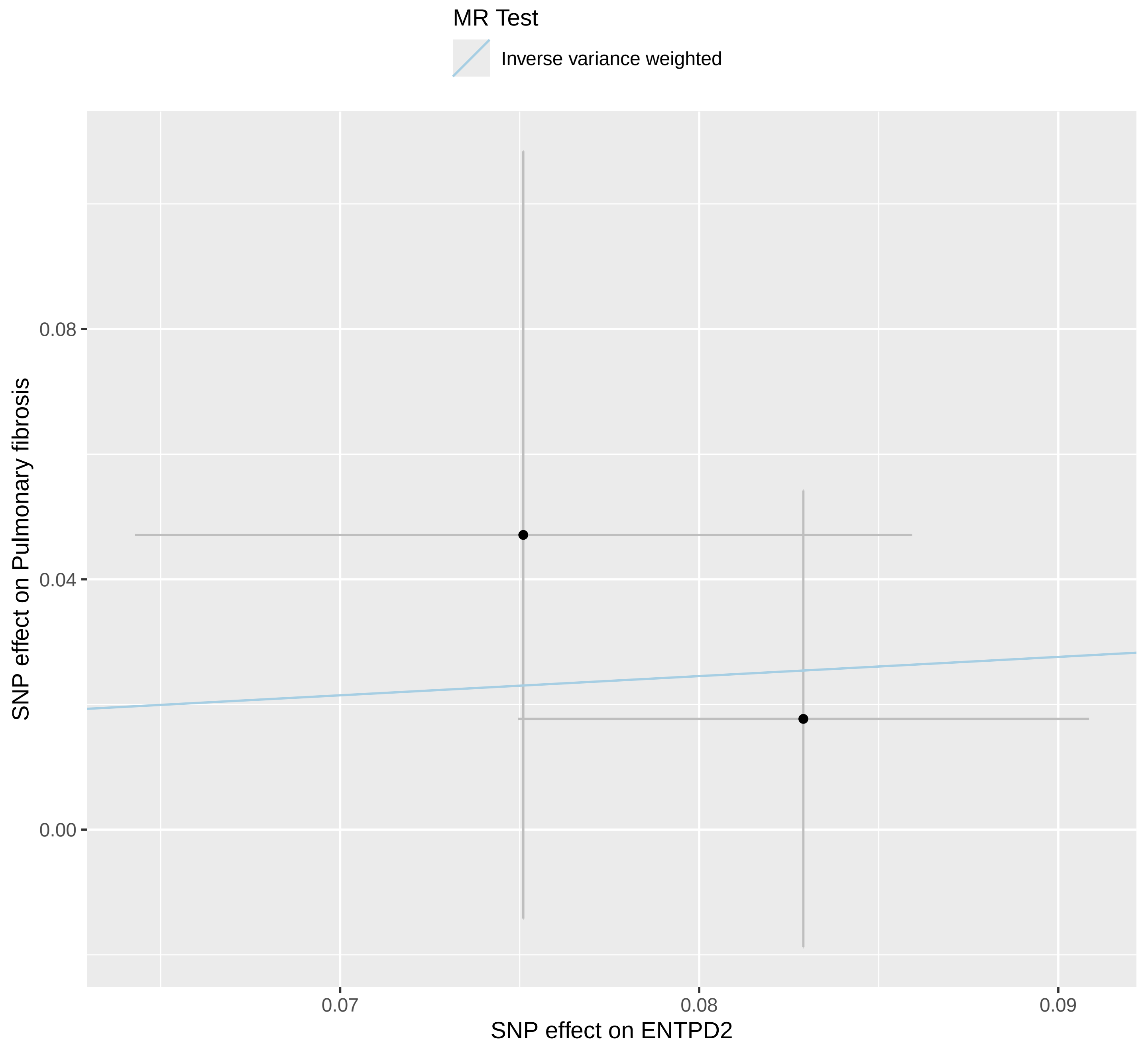
MR Test
Inverse variance weighted
0.08
0.04
0.00
0.07
0.08
0.09
SNP effect on ENTPD2
SNP effect on Pulmonary fibrosis

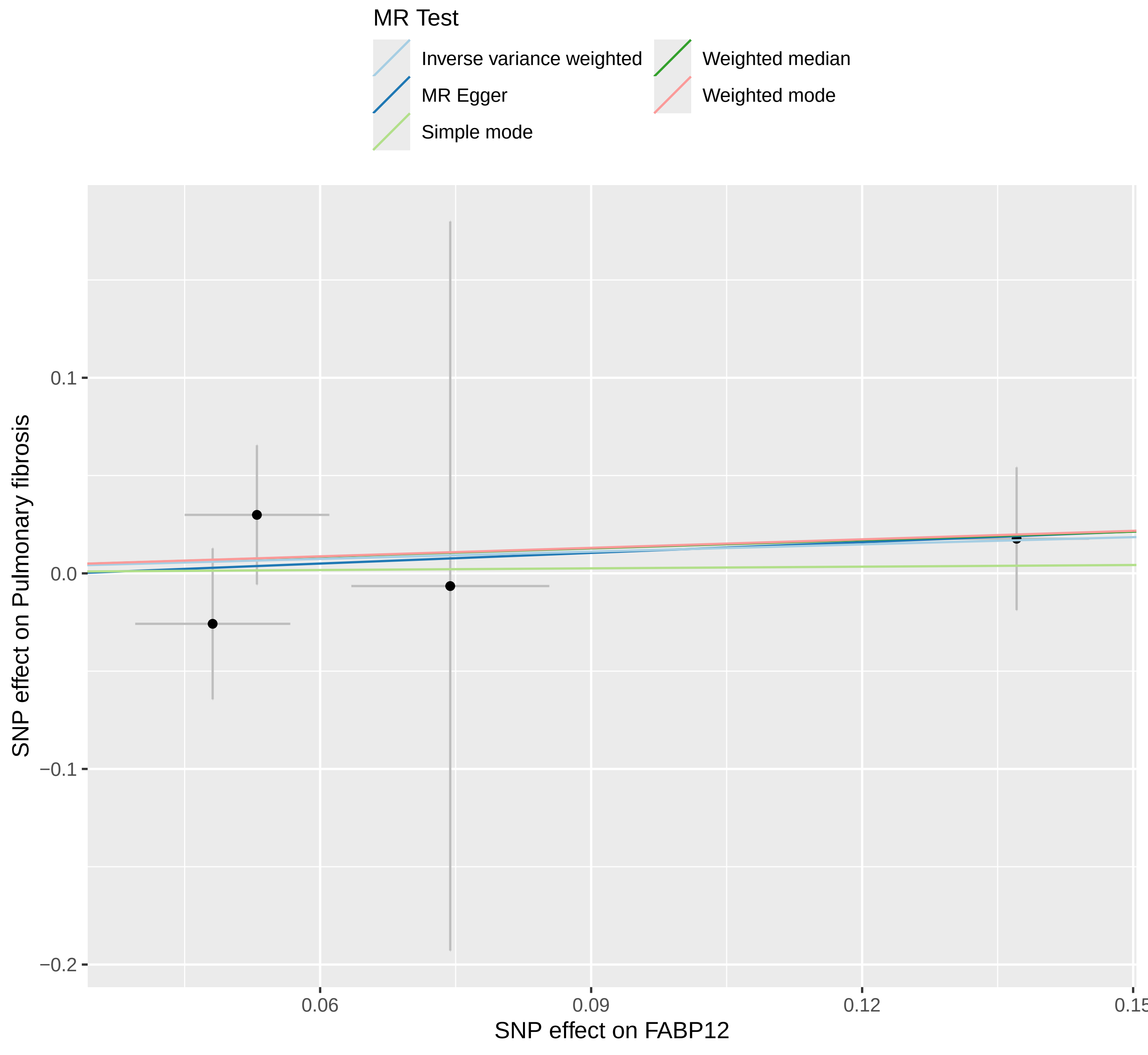
MR Test
Inverse variance weighted
MR Egger
Simple mode
Weighted median
Weighted mode
0.1
0.0
−0.1
−0.2
SNP effect on Pulmonary fibrosis
0.06
0.09
0.12
0.15
SNP effect on FABP12

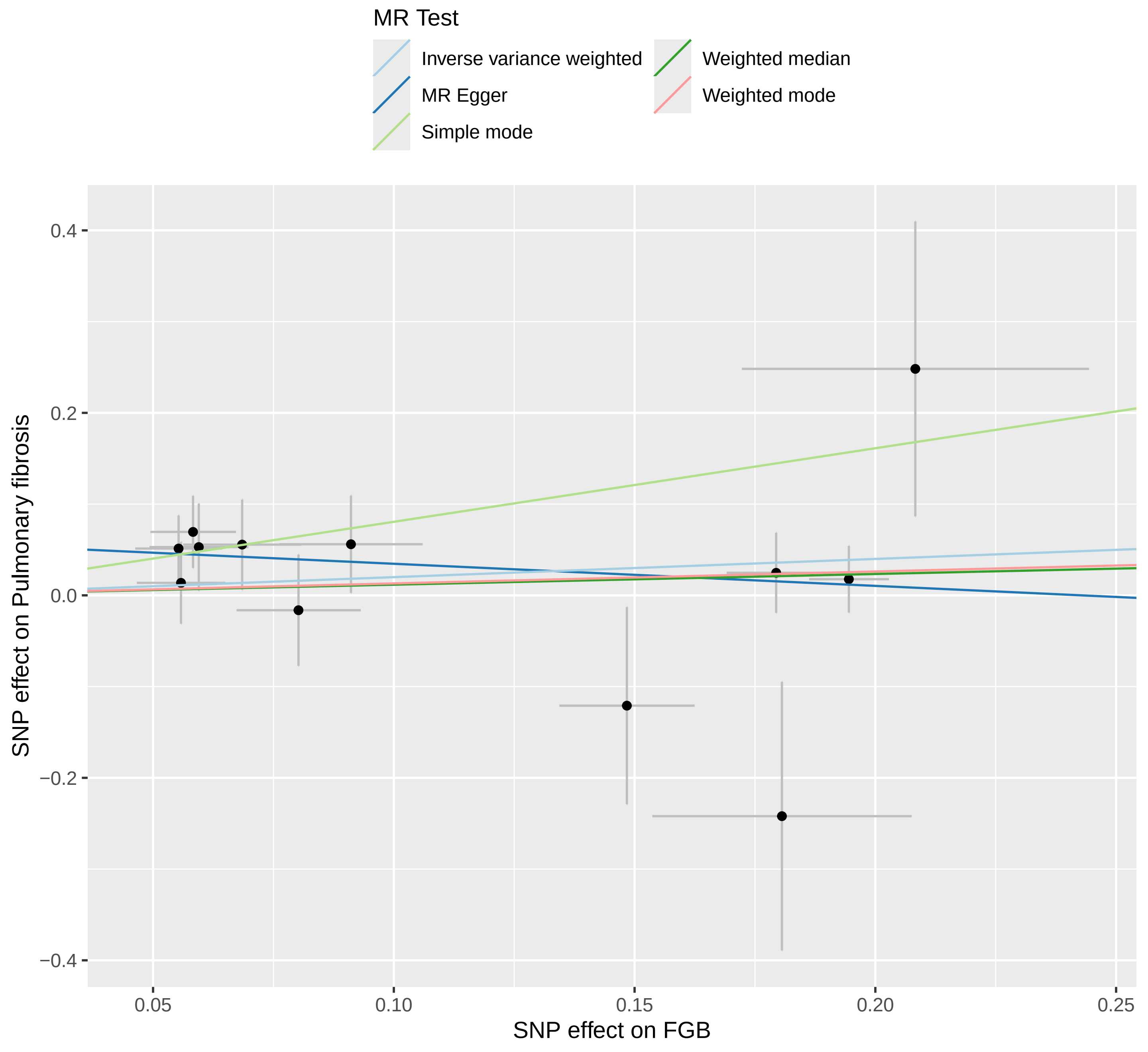

MR Test
Inverse variance weighted
MR Egger
Simple mode
Weighted median
Weighted mode
0.4
0.2
0.0
−0.2
−0.4
SNP effect on Pulmonary fibrosis
0.05
0.10
0.15
0.20
0.25
SNP effect on FGB

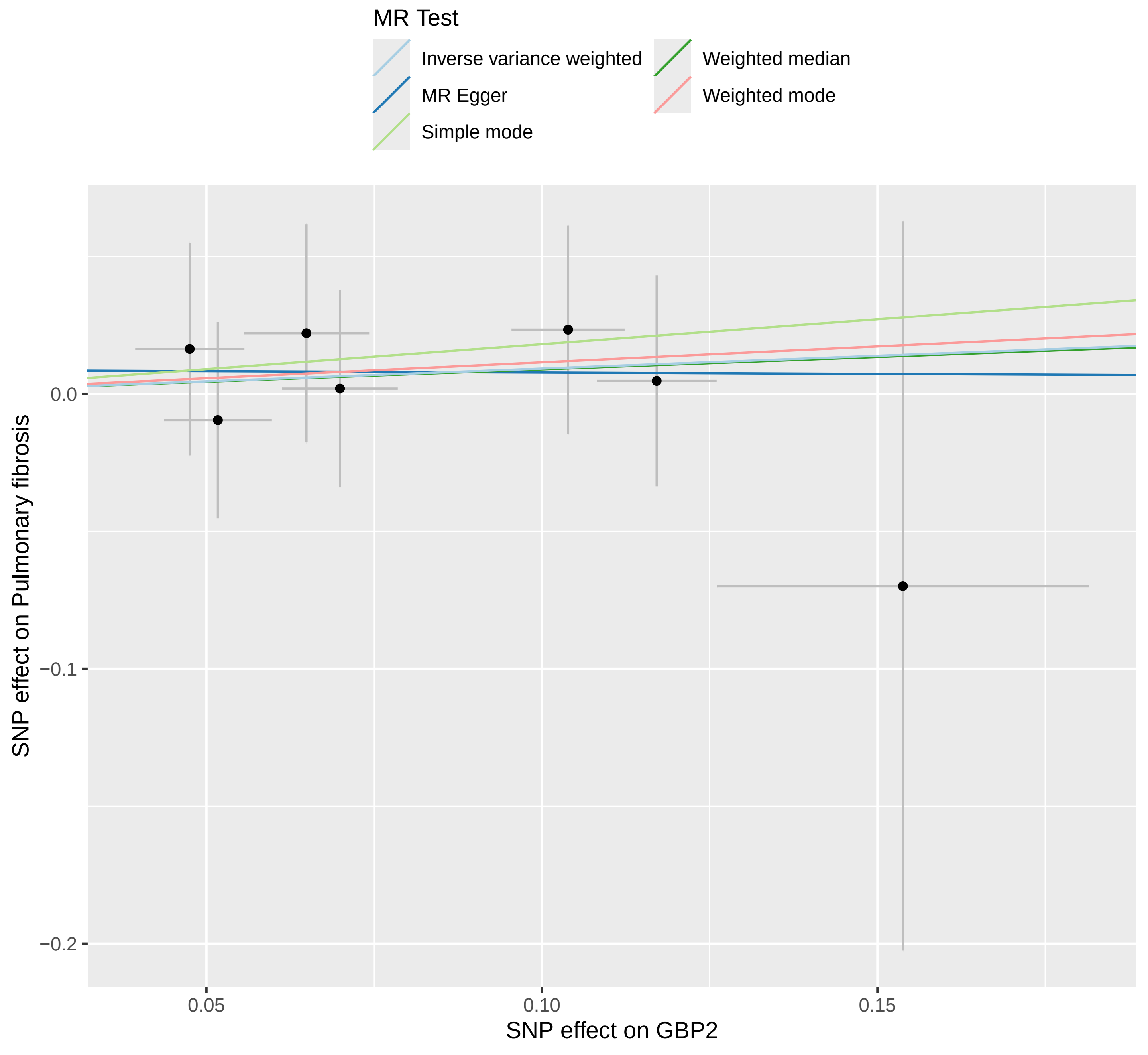

MR Test
Inverse variance weighted
MR Egger
Simple mode
Weighted median
Weighted mode
0.0
−0.1
−0.2
0.05
0.10
0.15
SNP effect on Pulmonary fibrosis
SNP effect on GBP2

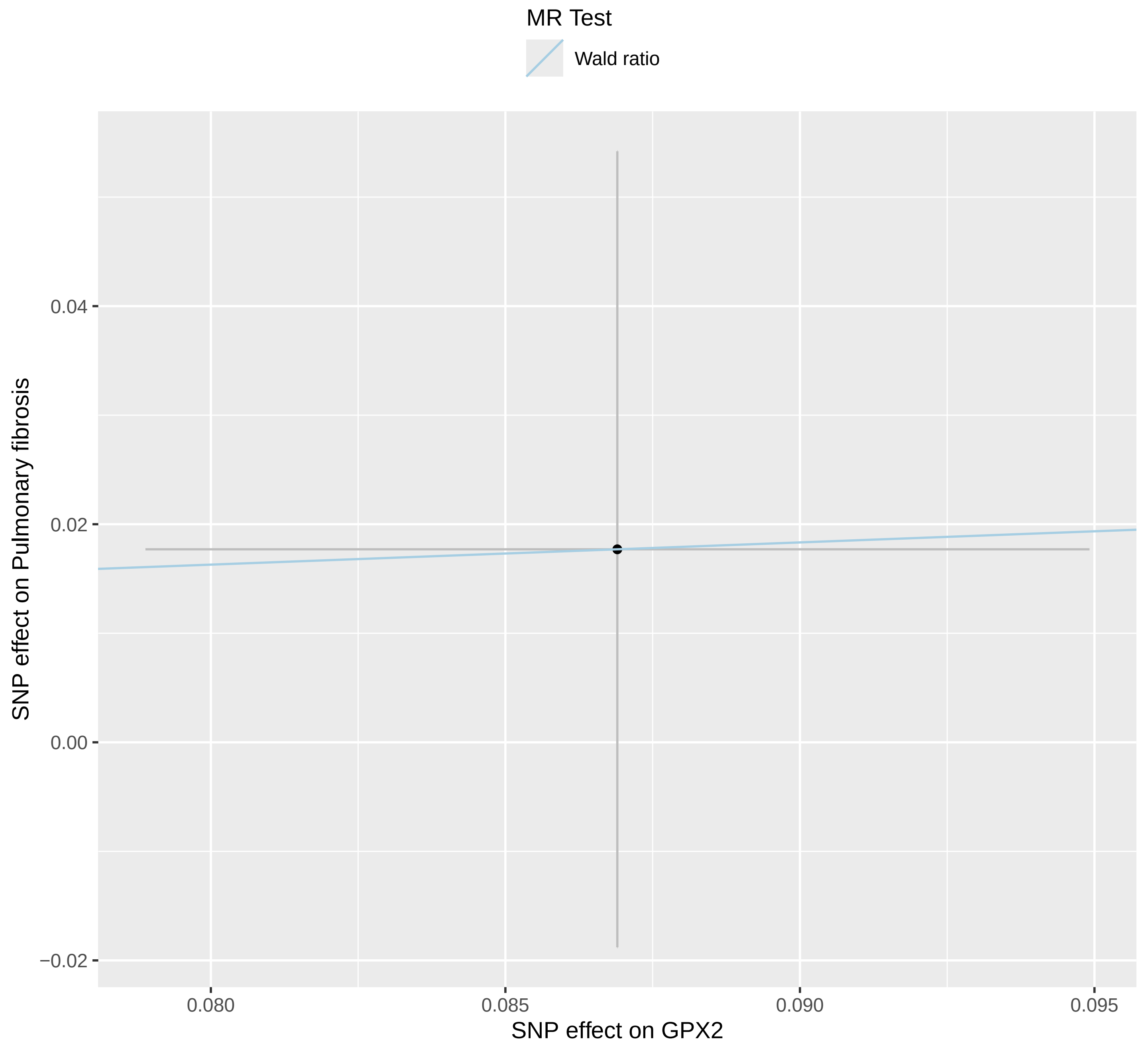

MR Test
Wald ratio
0.04
0.02
0.00
−0.02
SNP effect on Pulmonary fibrosis
0.080
0.085
0.090
0.095
SNP effect on GPX2

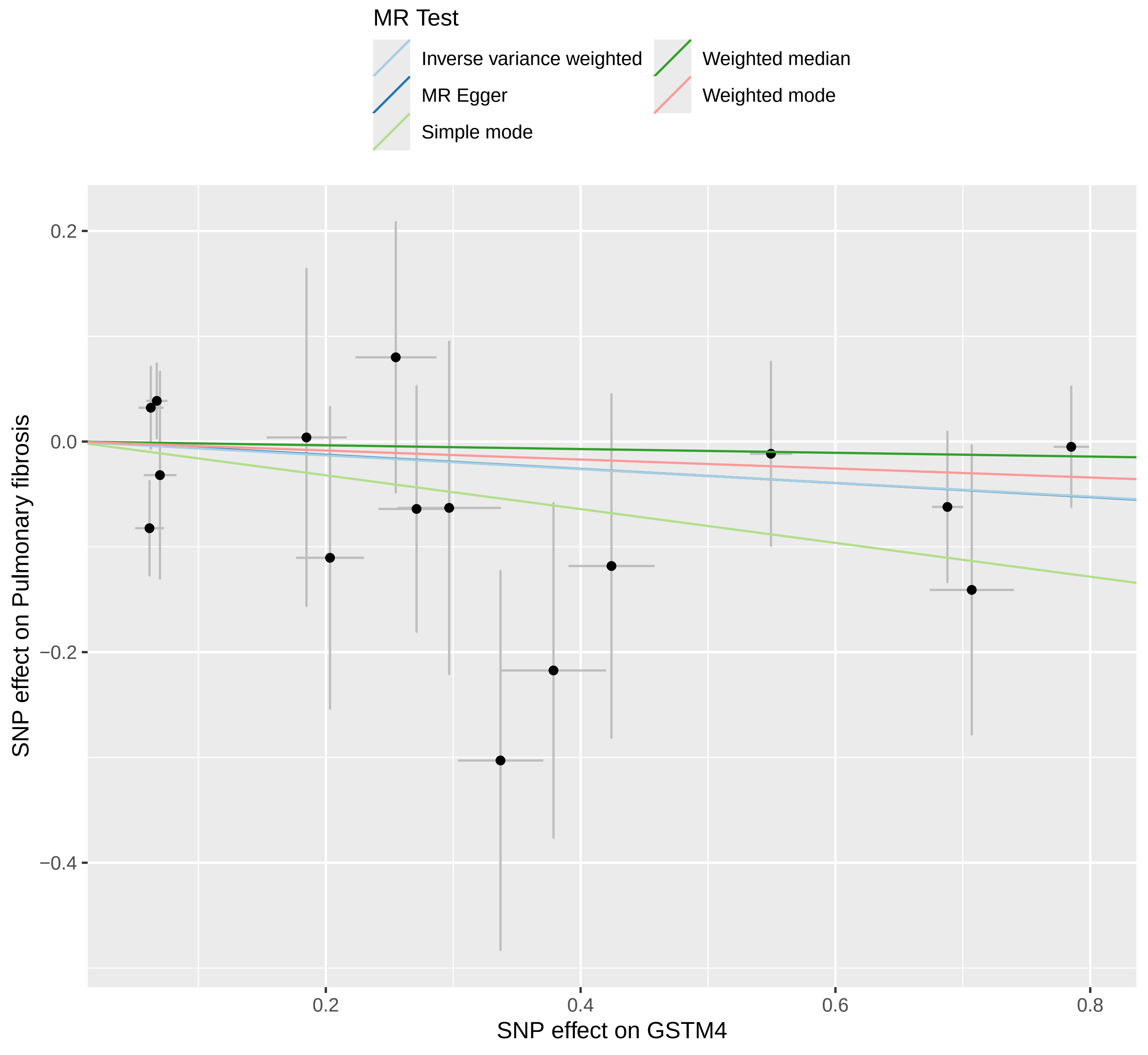

MR Test
Inverse variance weighted
MR Egger
Simple mode
Weighted median
Weighted mode
SNP effect on Pulmonary fibrosis
0.2
0.0
−0.2
−0.4
0.2
0.4
0.6
0.8
SNP effect on GSTM4

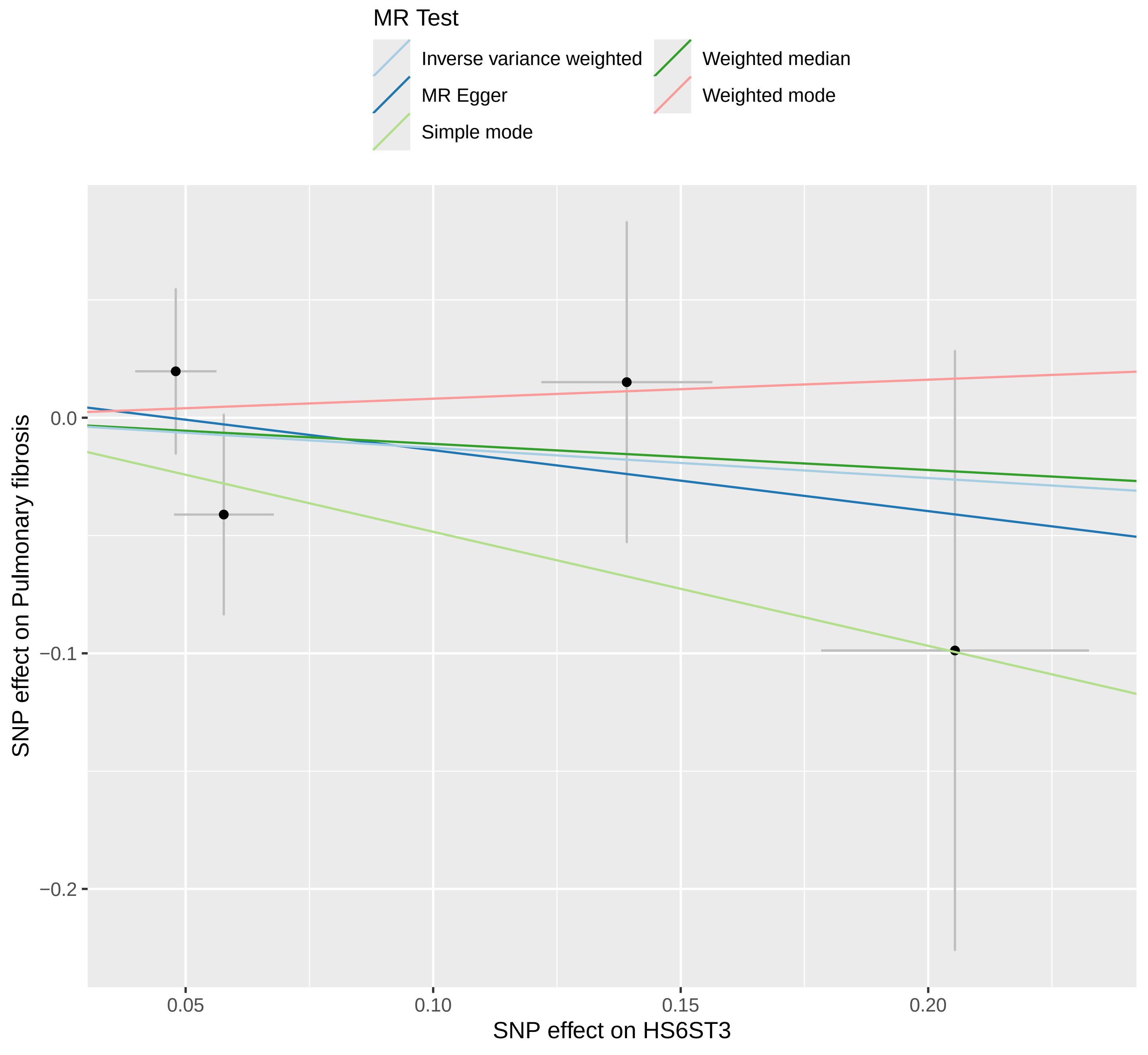
MR Test
Inverse variance weighted
MR Egger
Simple mode
Weighted median
Weighted mode
0.0
−0.1
−0.2
SNP effect on Pulmonary fibrosis
0.05
0.10
0.15
0.20
SNP effect on HS6ST3

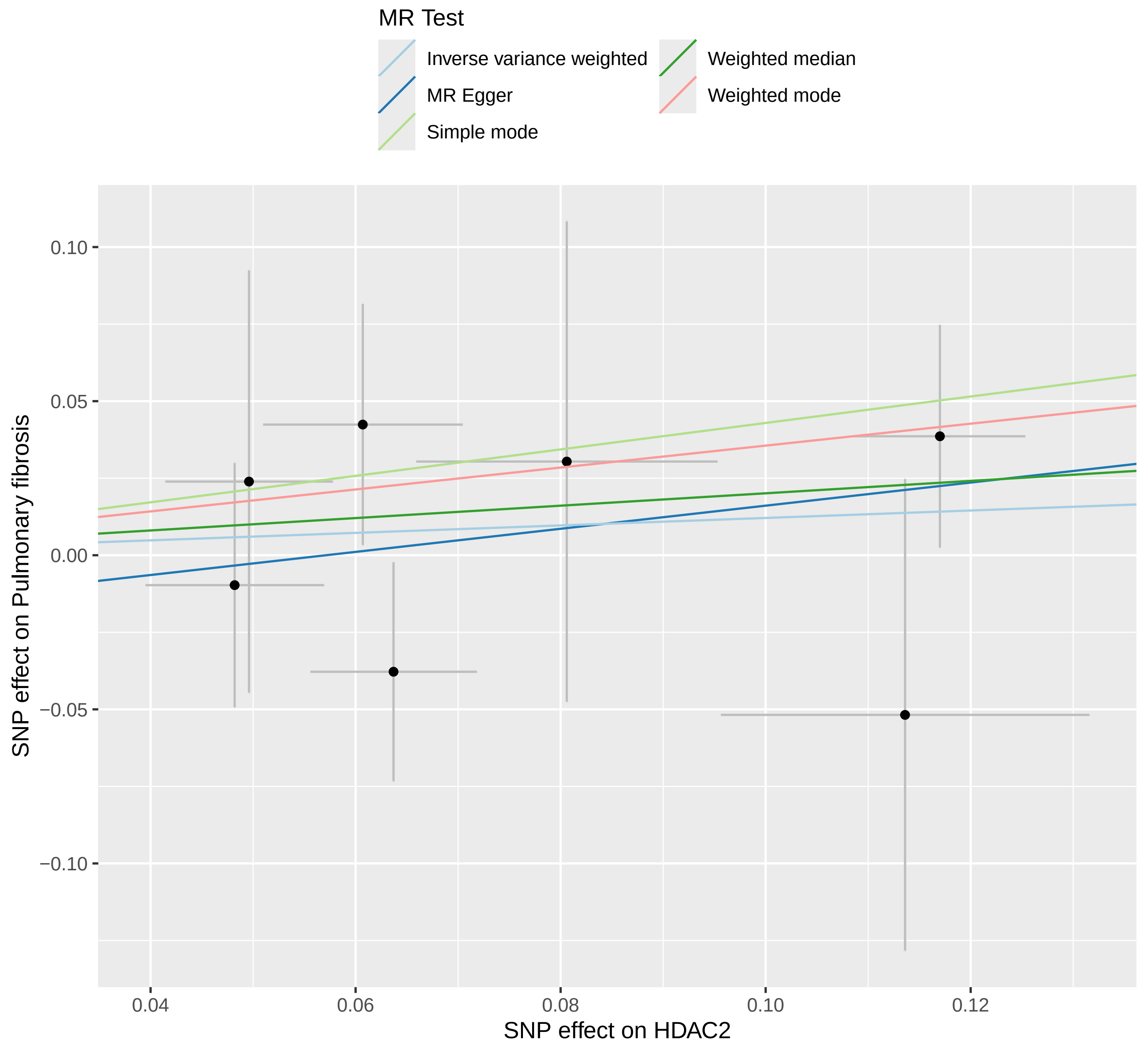
MR Test
Inverse variance weighted
MR Egger
Simple mode
Weighted median
Weighted mode
0.10
0.05
0.00
−0.05
−0.10
SNP effect on Pulmonary fibrosis
0.04
0.06
0.08
0.10
0.12
SNP effect on HDAC2

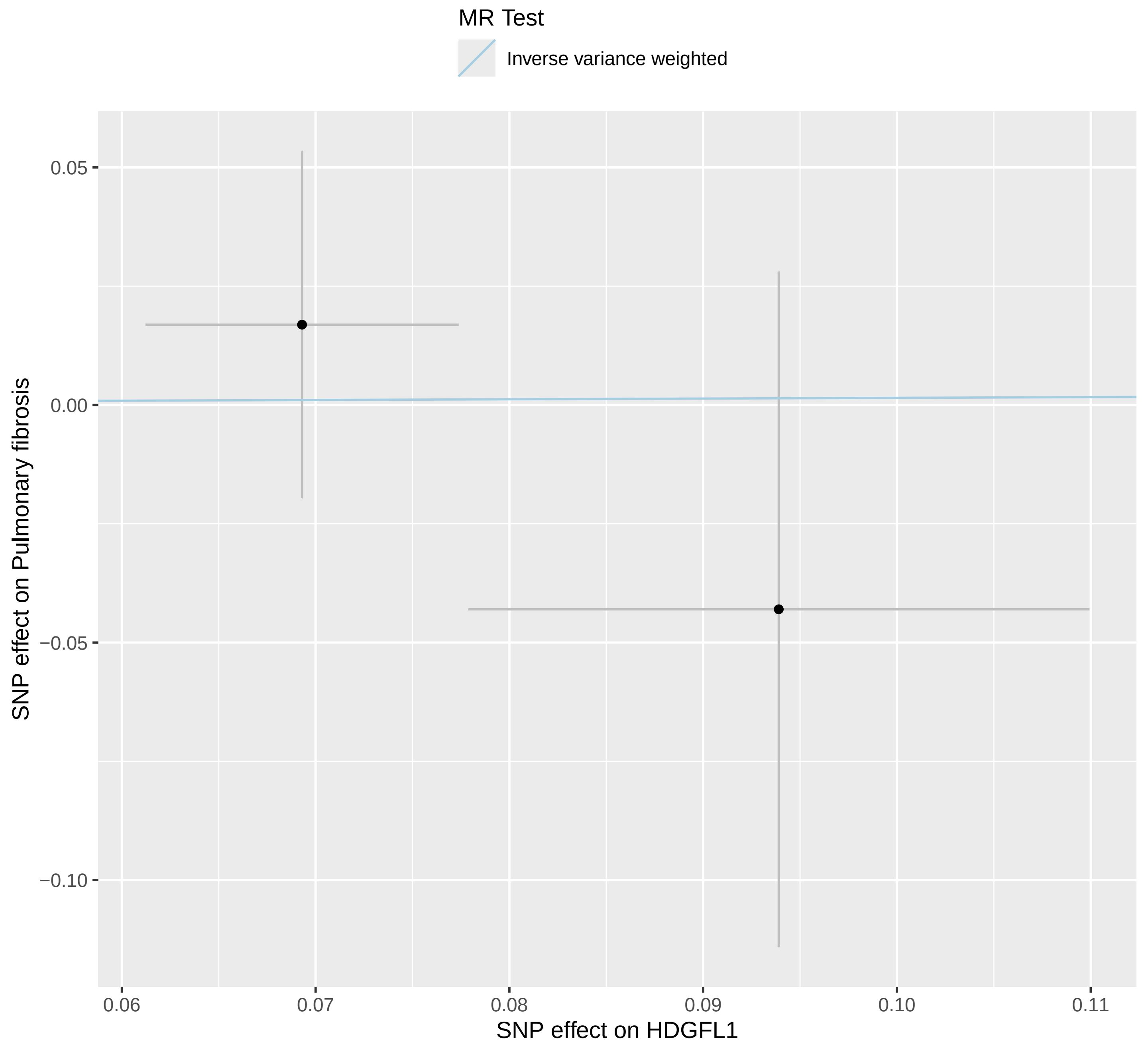
MR Test
Inverse variance weighted
0.05
0.00
−0.05
−0.10
SNP effect on Pulmonary fibrosis
0.06
0.07
0.08
0.09
0.10
0.11
SNP effect on HDGFL1

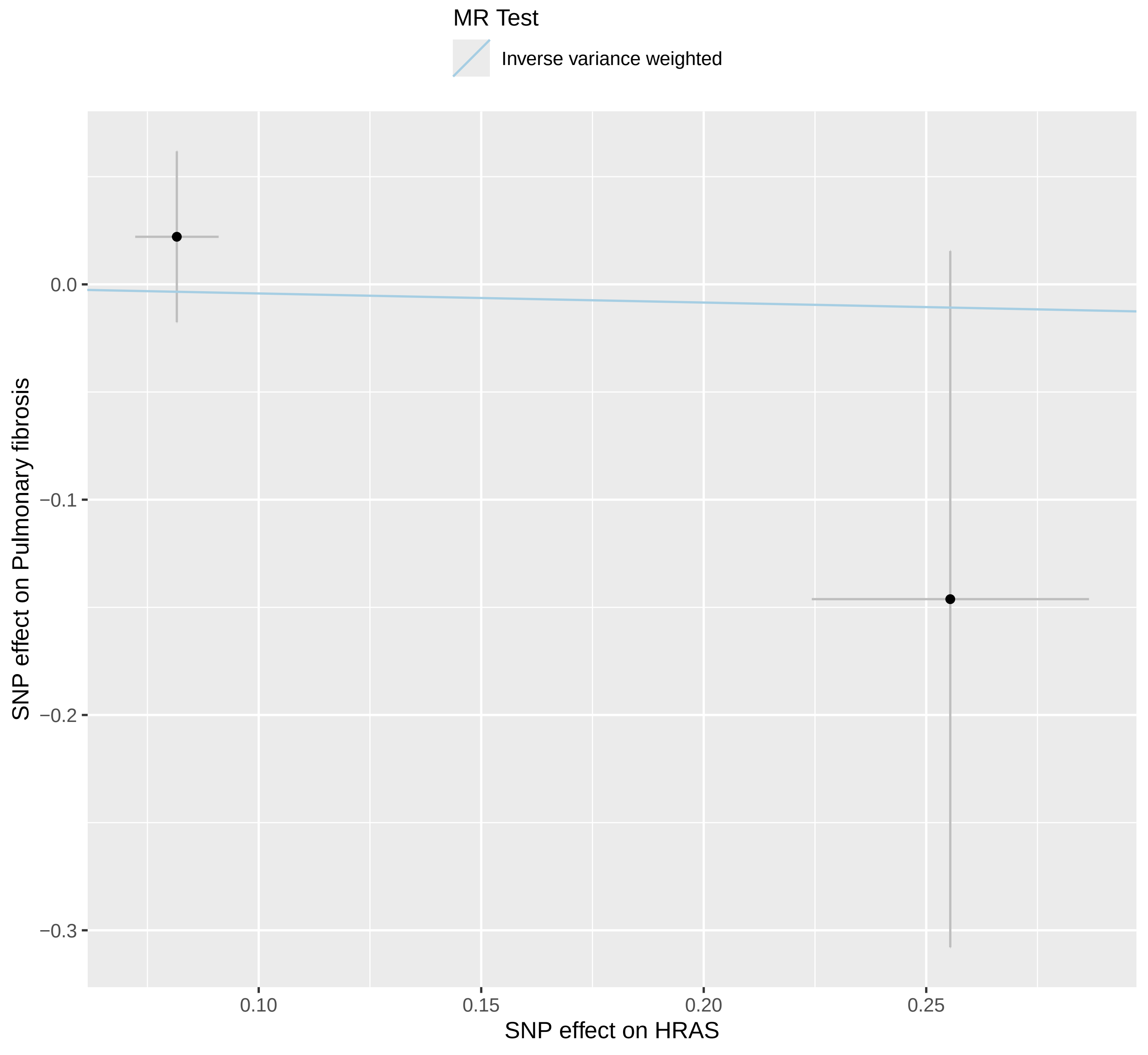
MR Test
Inverse variance weighted
0.0
−0.1
−0.2
−0.3
0.10
0.15
0.20
0.25
SNP effect on HRAS
SNP effect on Pulmonary fibrosis

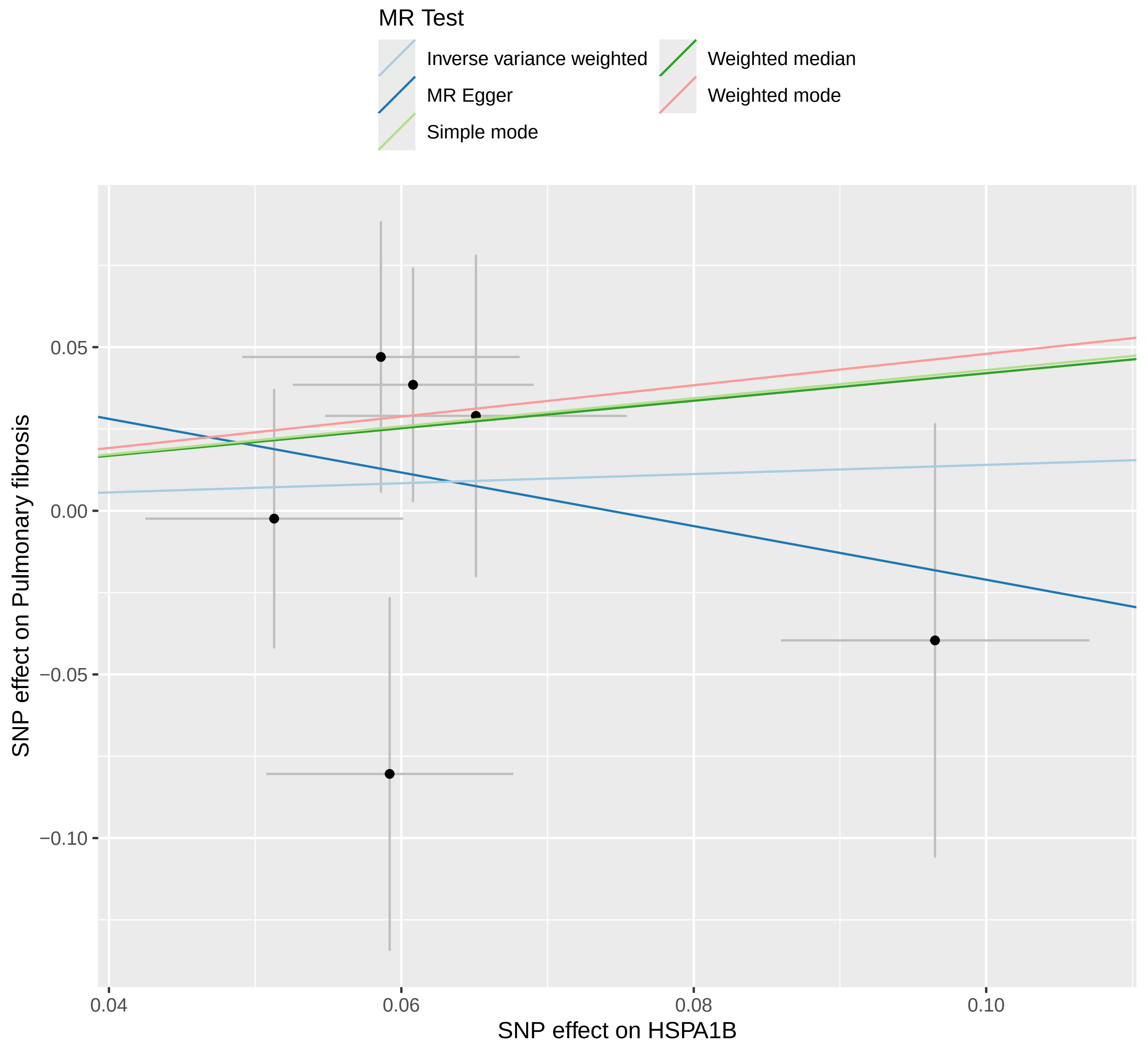
MR Test
Inverse variance weighted
MR Egger
Simple mode
Weighted median
Weighted mode
SNP effect on Pulmonary fibrosis
0.05
0.00
−0.05
−0.10
0.04
0.06
0.08
0.10
SNP effect on HSPA1B

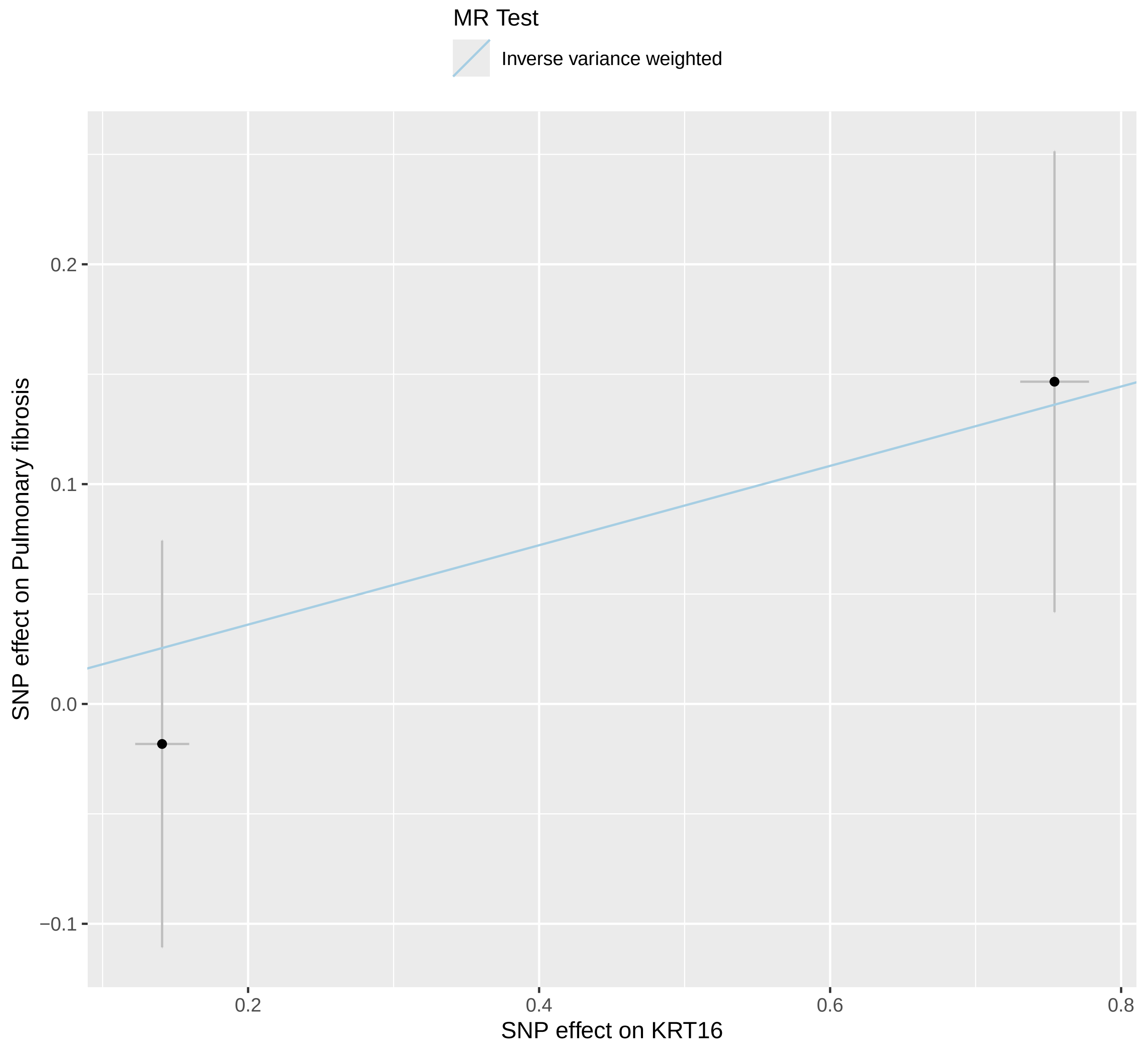
MR Test
Inverse variance weighted
SNP effect on Pulmonary fibrosis
0.2
0.1
0.0
−0.1
0.2
0.4
0.6
0.8
SNP effect on KRT16

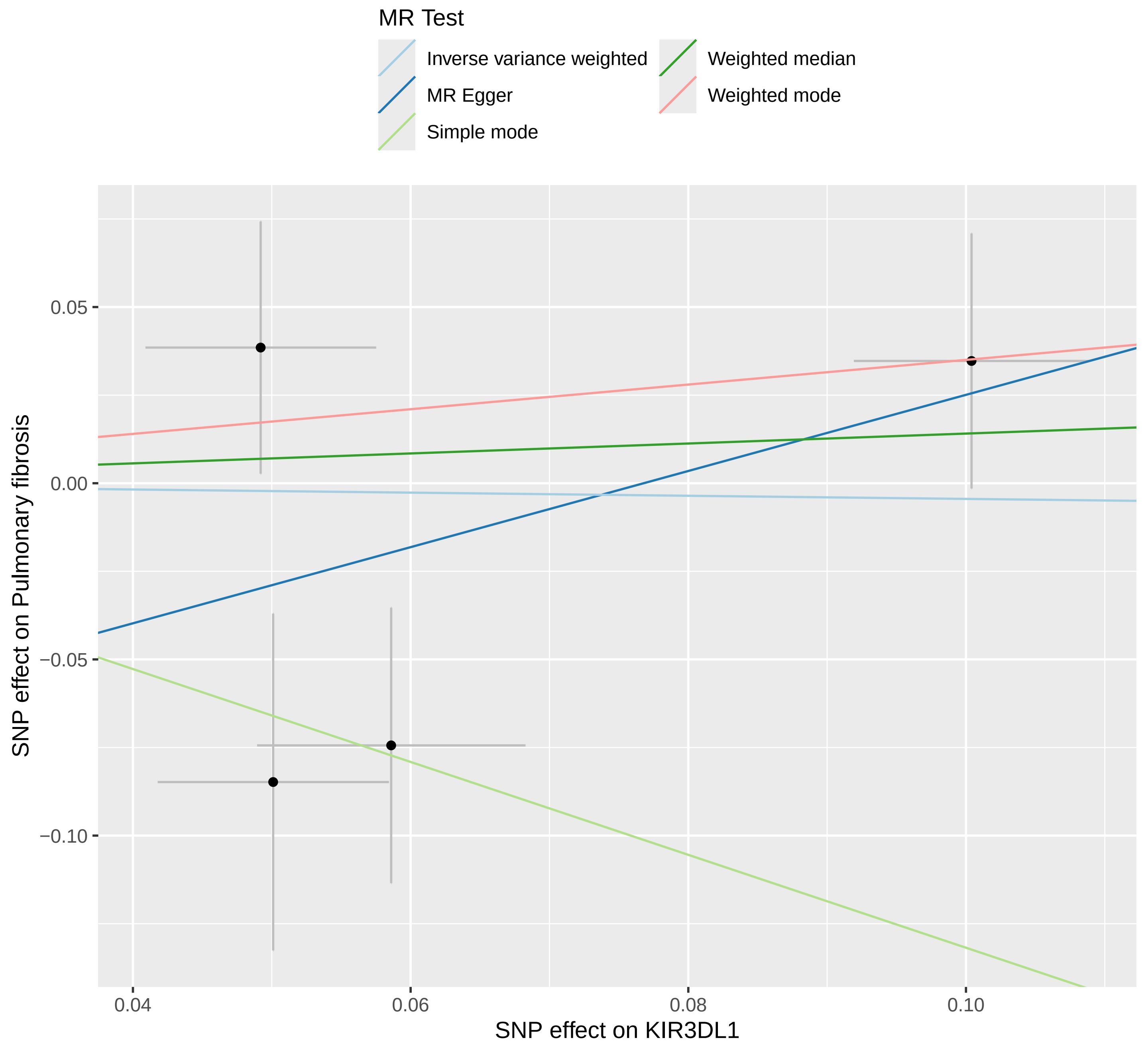
MR Test
Inverse variance weighted
MR Egger
Simple mode
Weighted median
Weighted mode
0.05
0.00
−0.05
−0.10
0.04
0.06
0.08
0.10
SNP effect on Pulmonary fibrosis
SNP effect on KIR3DL1

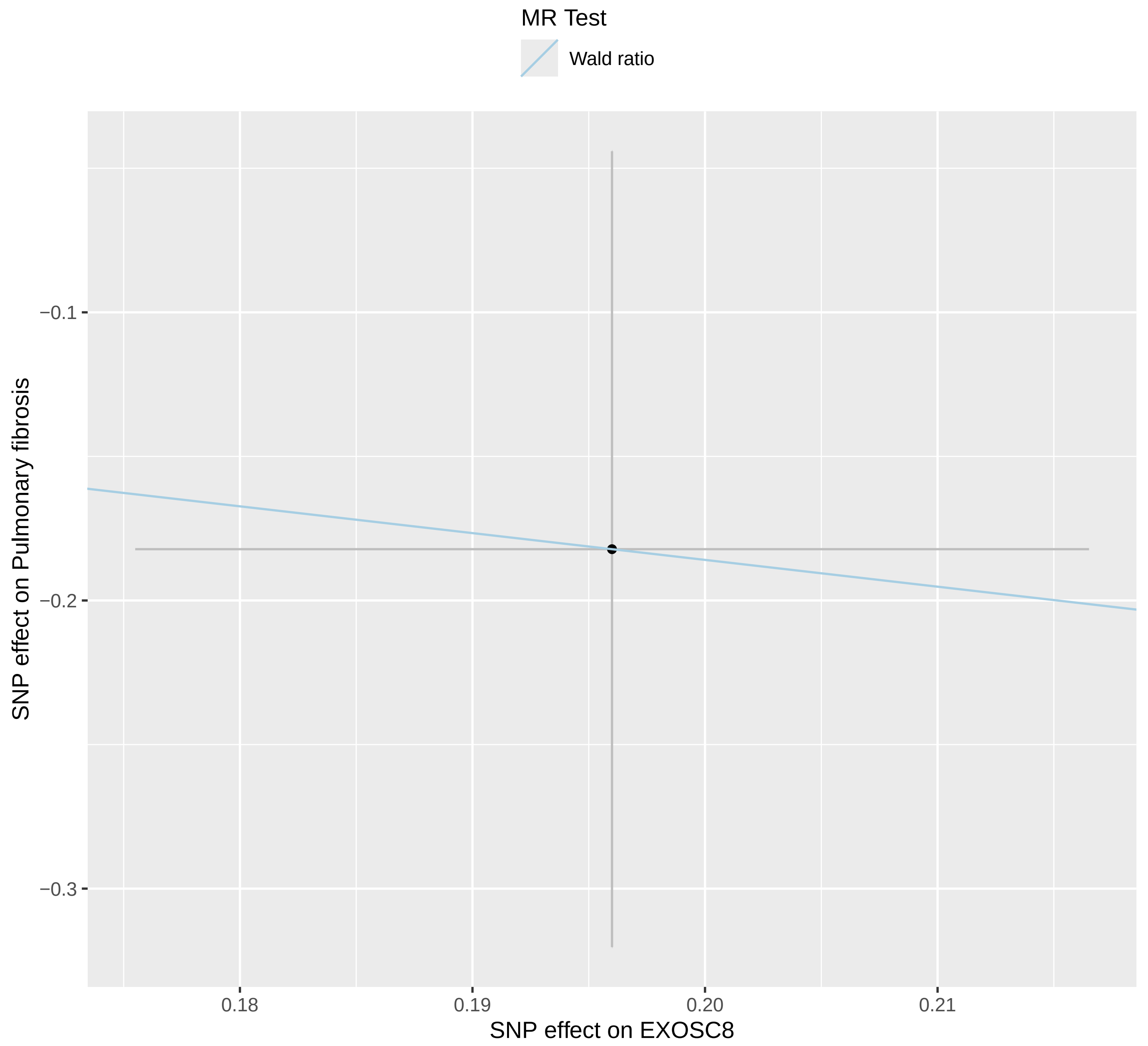

MR Test
Wald ratio
−0.1
−0.2
−0.3
SNP effect on Pulmonary fibrosis
0.18
0.19
0.20
0.21
SNP effect on EXOSC8

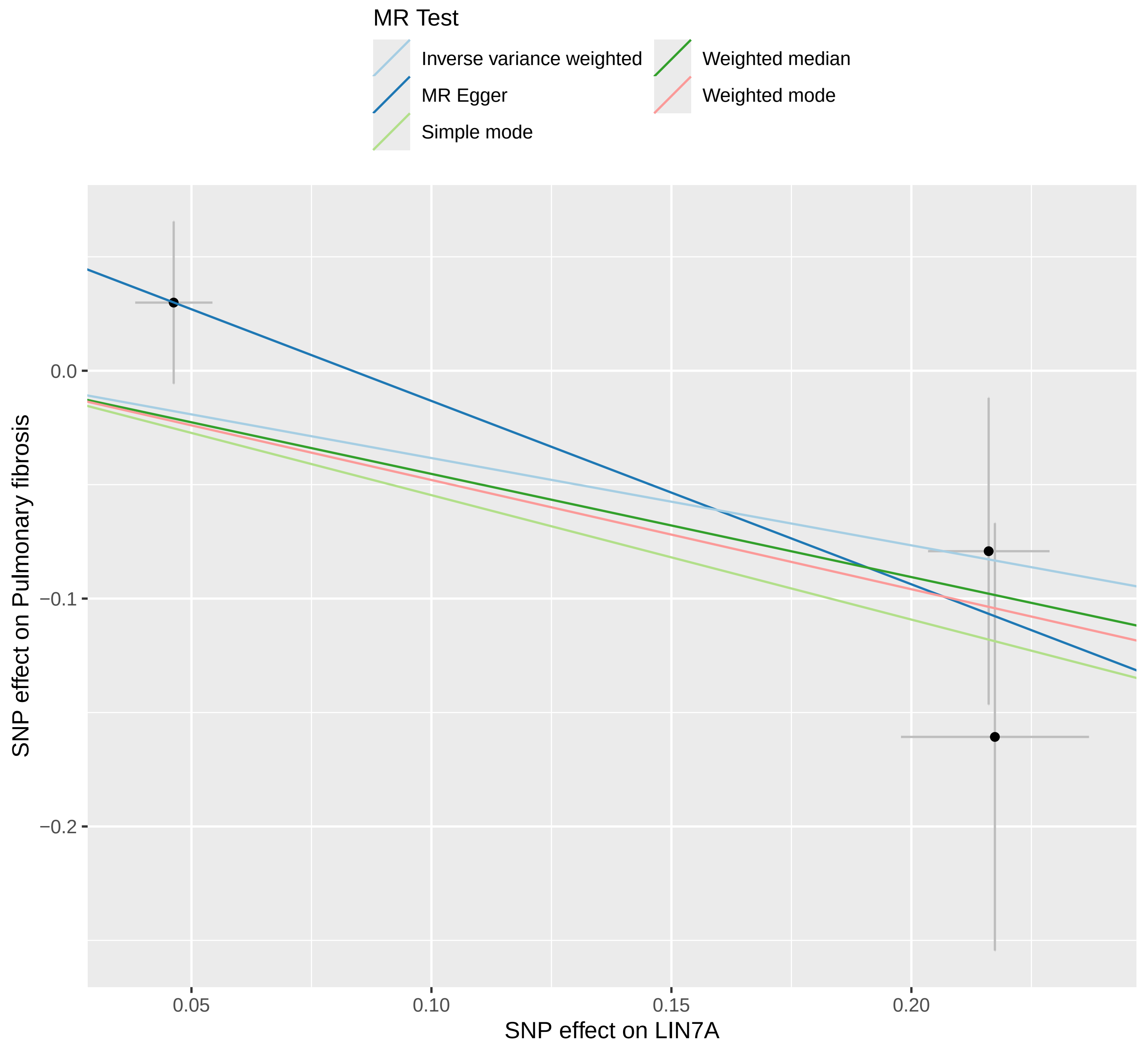

MR Test
Inverse variance weighted
MR Egger
Simple mode
Weighted median
Weighted mode
SNP effect on Pulmonary fibrosis
0.0
−0.1
−0.2
0.05
0.10
0.15
0.20
SNP effect on LIN7A

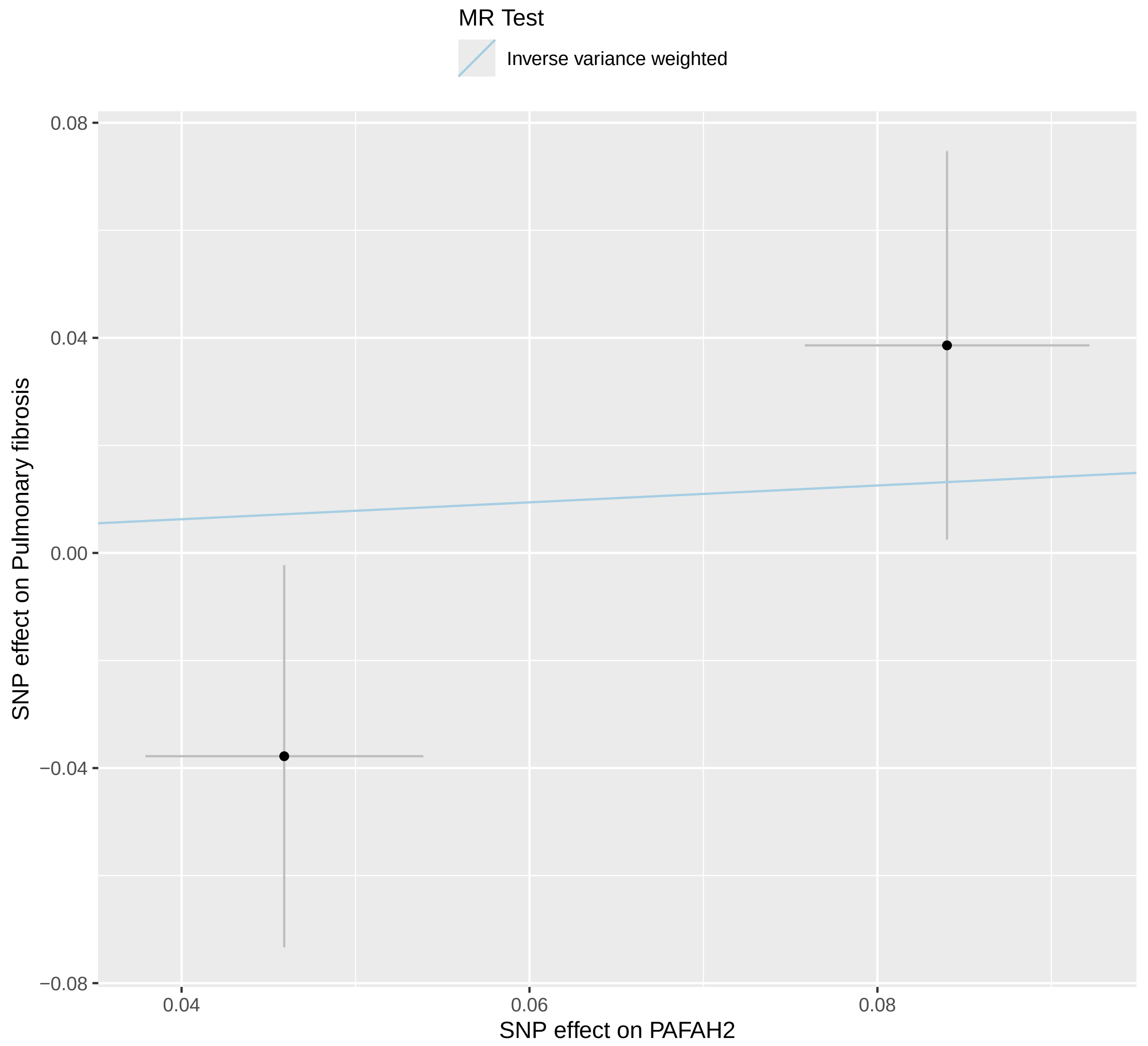

MR Test
Inverse variance weighted
0.08
0.04
0.00
−0.04
−0.08
SNP effect on Pulmonary fibrosis
0.04
0.06
0.08
SNP effect on PAFAH2

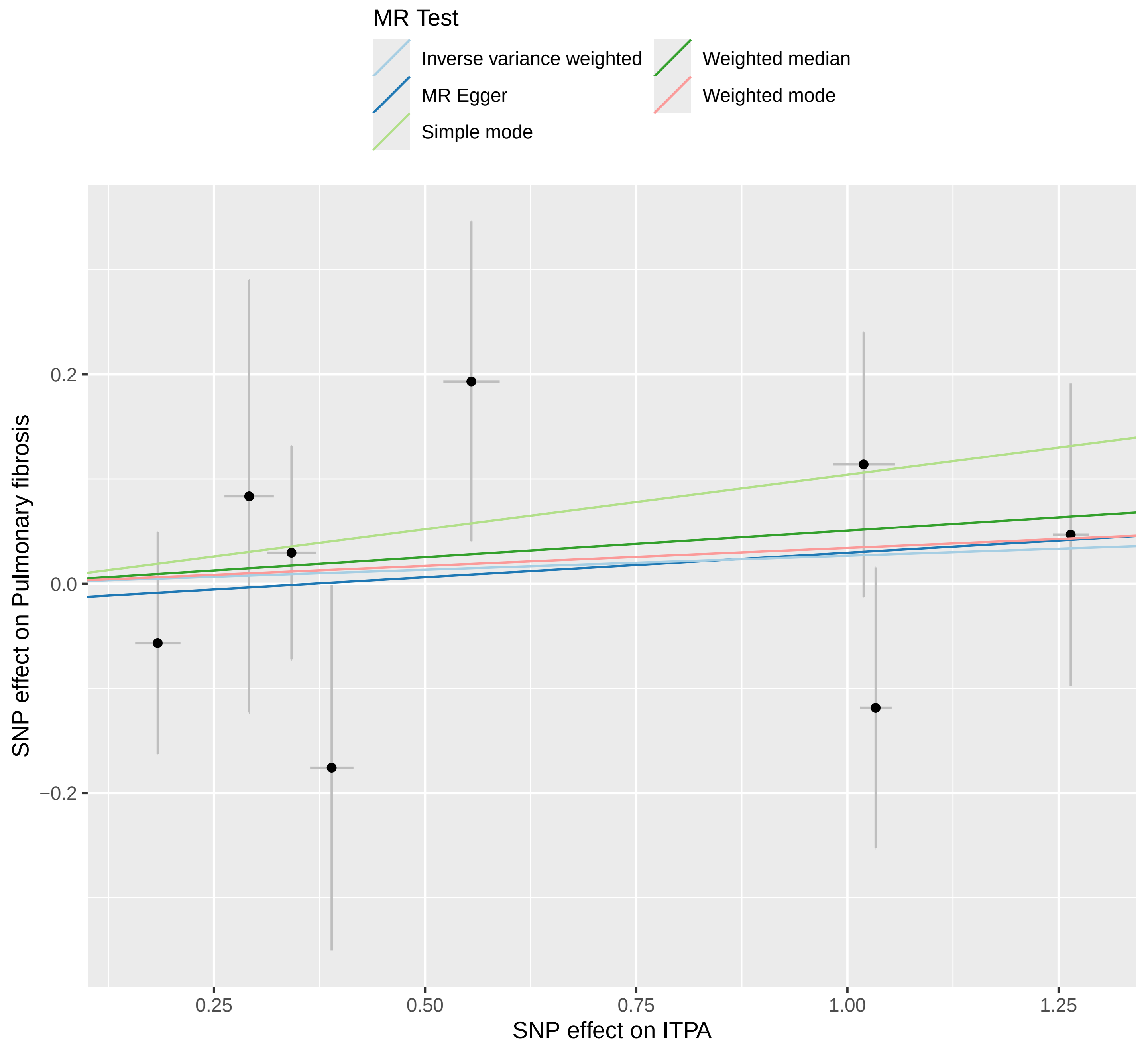
MR Test
Inverse variance weighted
MR Egger
Simple mode
Weighted median
Weighted mode
0.2
0.0
−0.2
0.25
0.50
0.75
1.00
1.25
SNP effect on Pulmonary fibrosis
SNP effect on ITPA

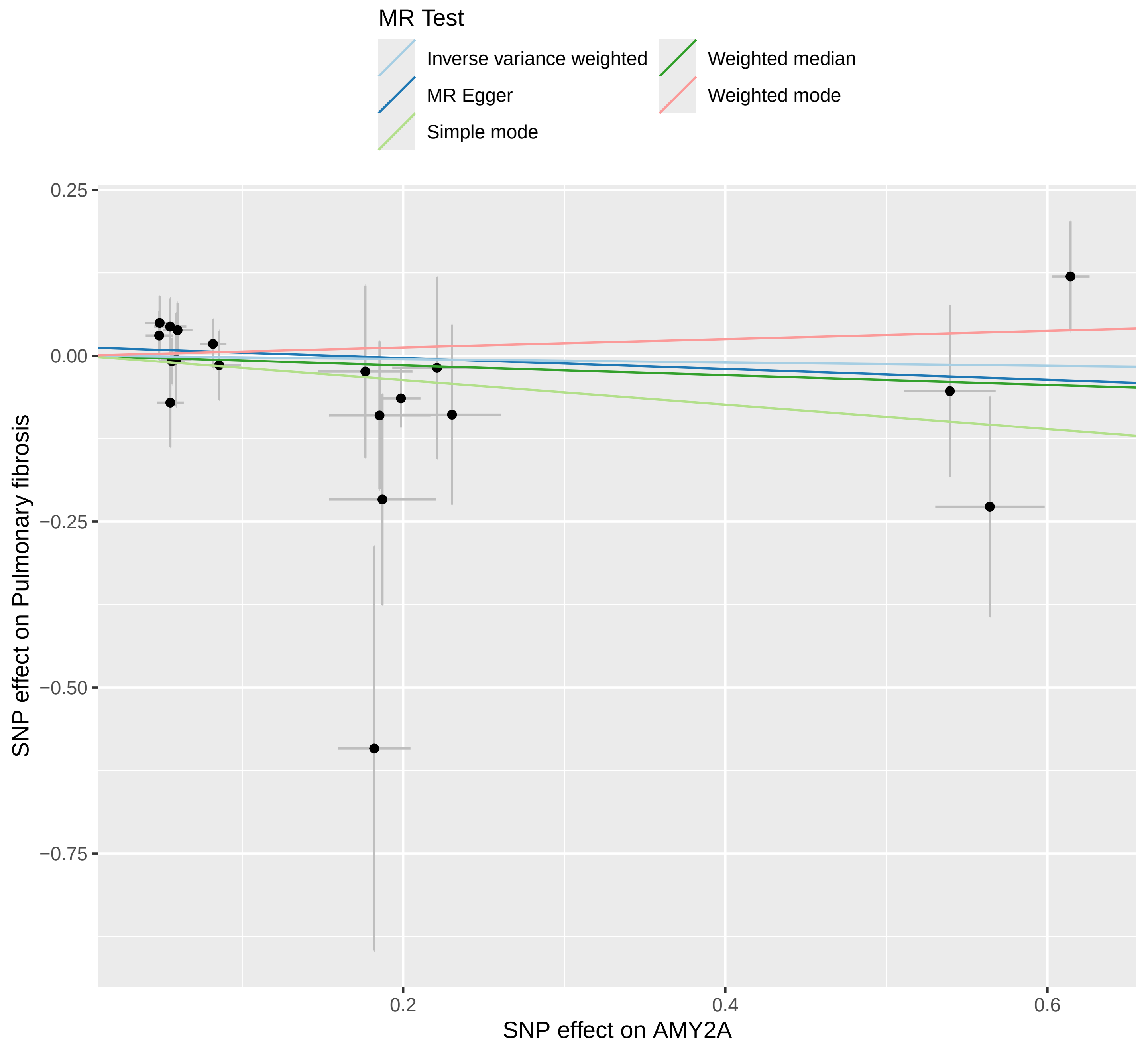

MR Test
Inverse variance weighted
MR Egger
Simple mode
Weighted median
Weighted mode
0.25
0.00
−0.25
−0.50
−0.75
0.2
0.4
0.6
SNP effect on Pulmonary fibrosis
SNP effect on AMY2A

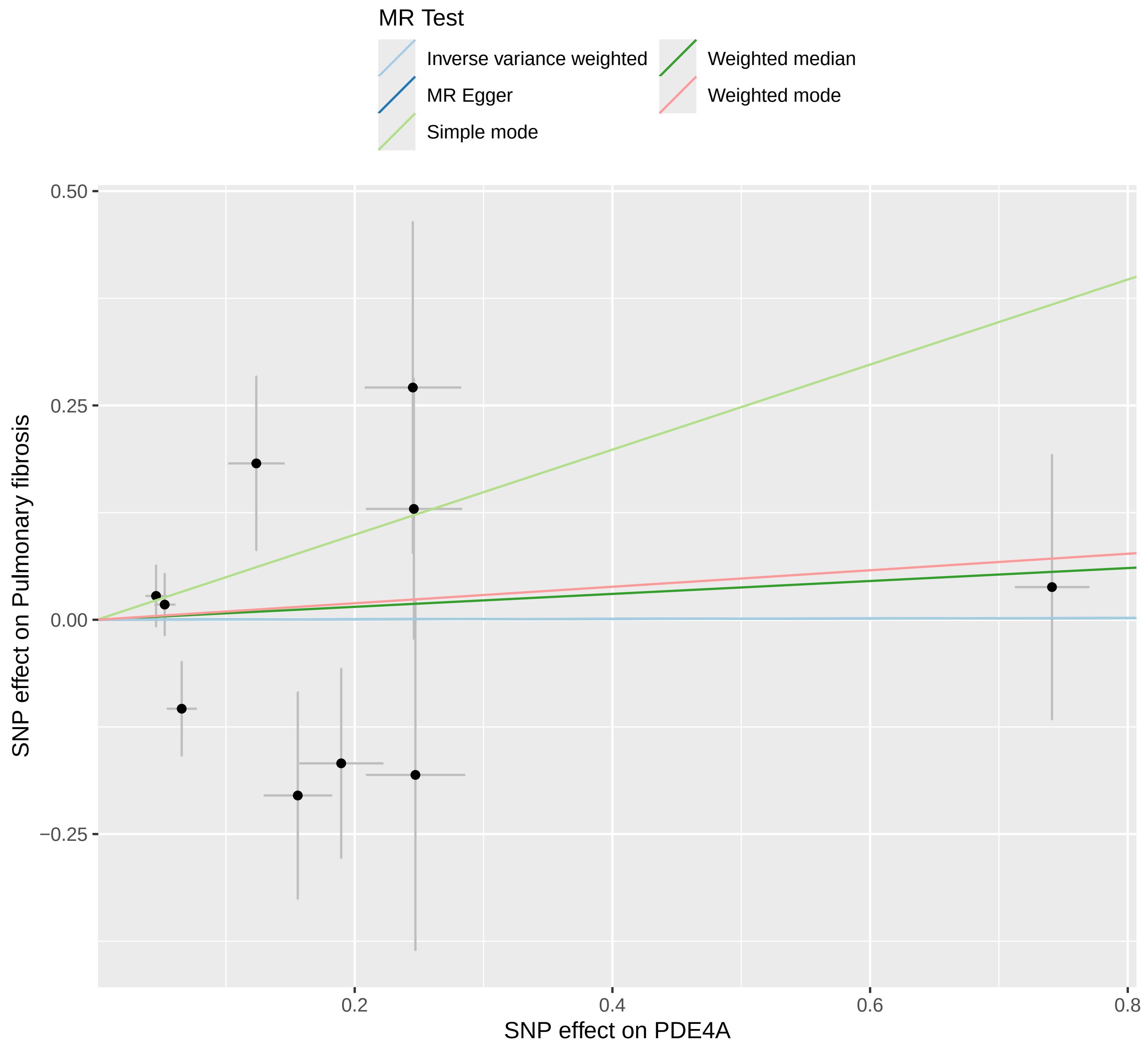
MR Test
Inverse variance weighted
MR Egger
Simple mode
Weighted median
Weighted mode
0.50
0.25
0.00
−0.25
SNP effect on Pulmonary fibrosis
0.2
0.4
0.6
0.8
SNP effect on PDE4A

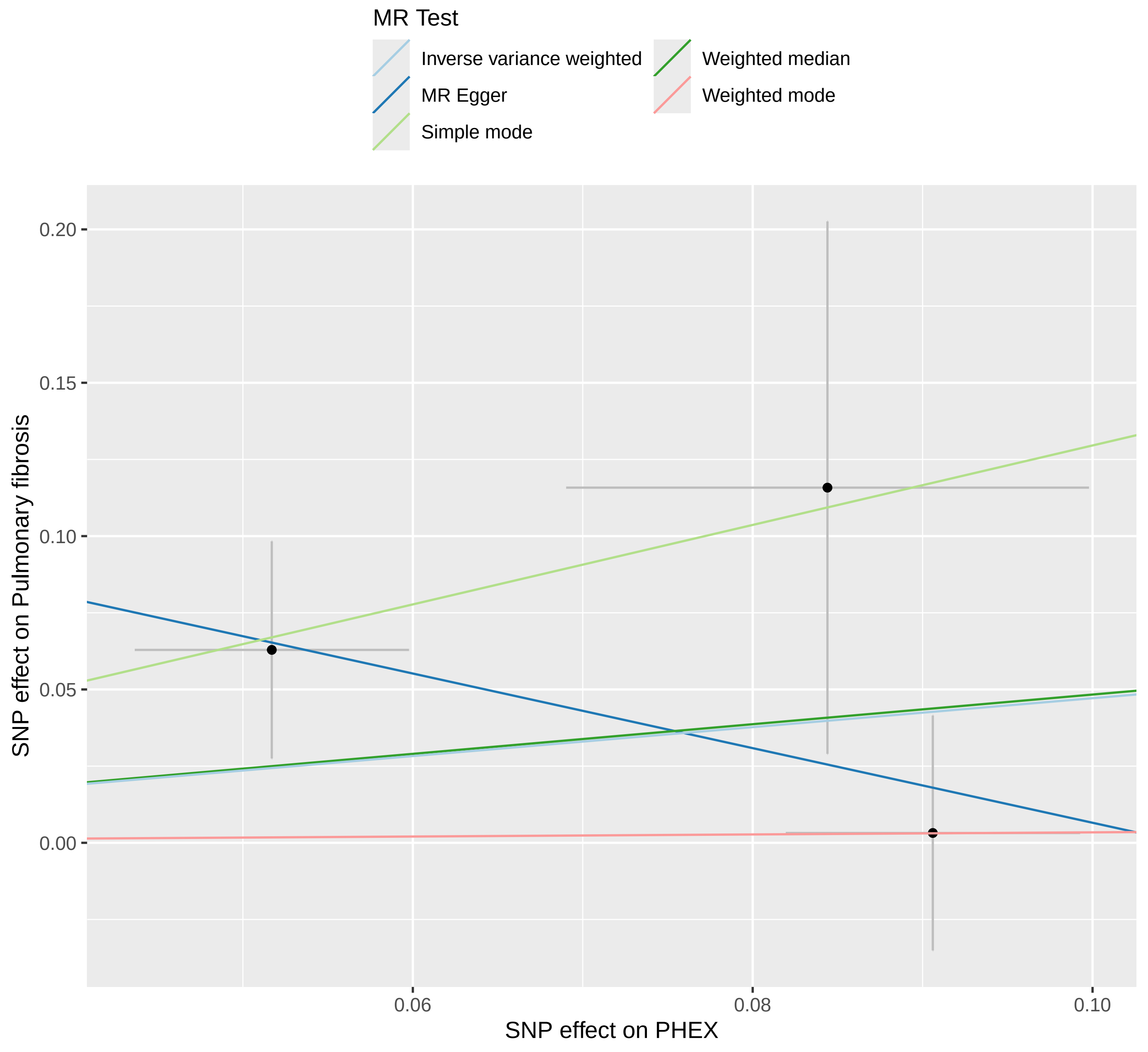
MR Test
Inverse variance weighted
MR Egger
Simple mode
Weighted median
Weighted mode
0.20
0.15
0.10
0.05
0.00
0.06
0.08
0.10
SNP effect on Pulmonary fibrosis
SNP effect on PHEX

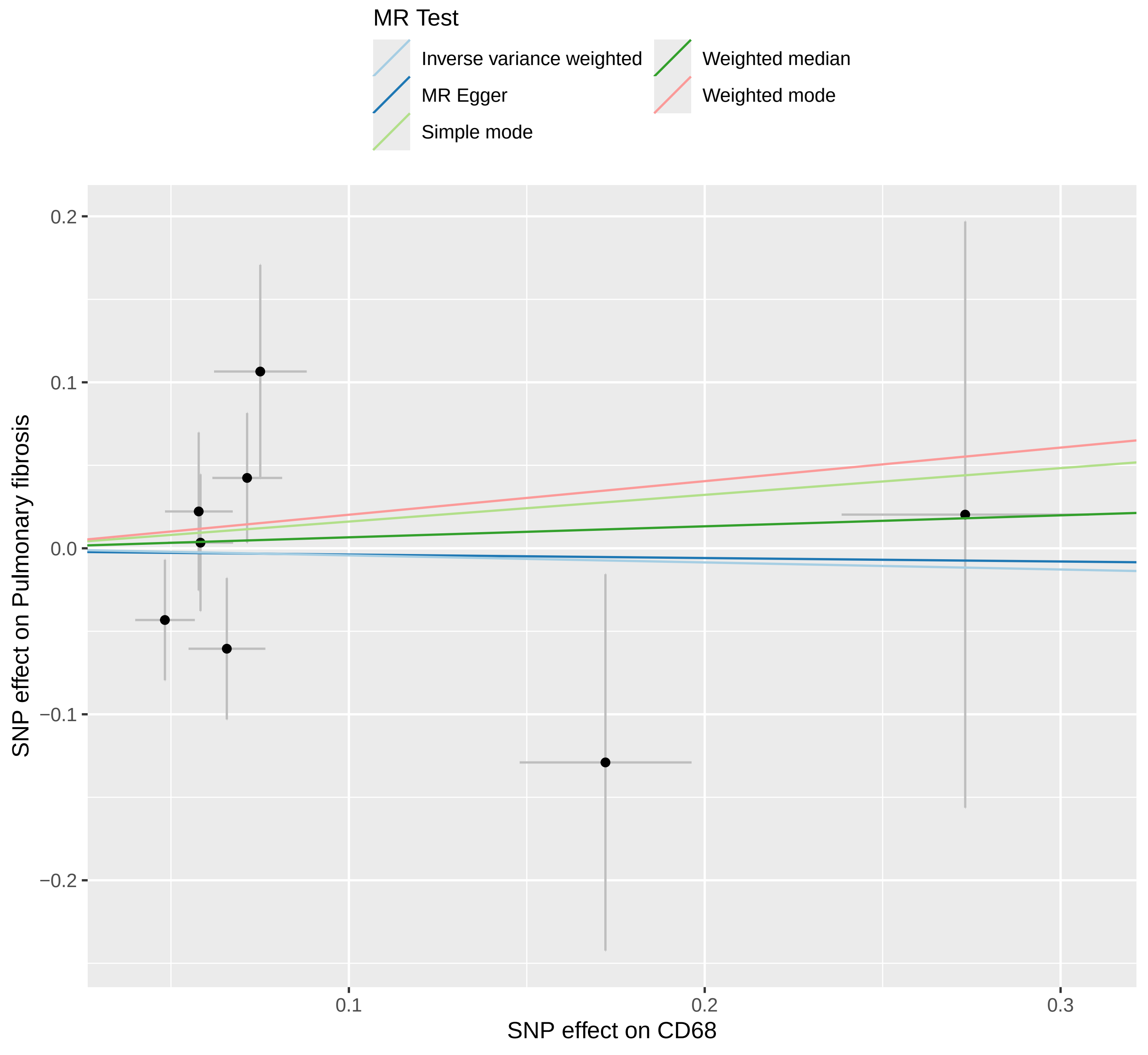

MR Test
Inverse variance weighted
MR Egger
Simple mode
Weighted median
Weighted mode
SNP effect on Pulmonary fibrosis
0.2
0.1
0.0
−0.1
−0.2
0.1
0.2
0.3
SNP effect on CD68

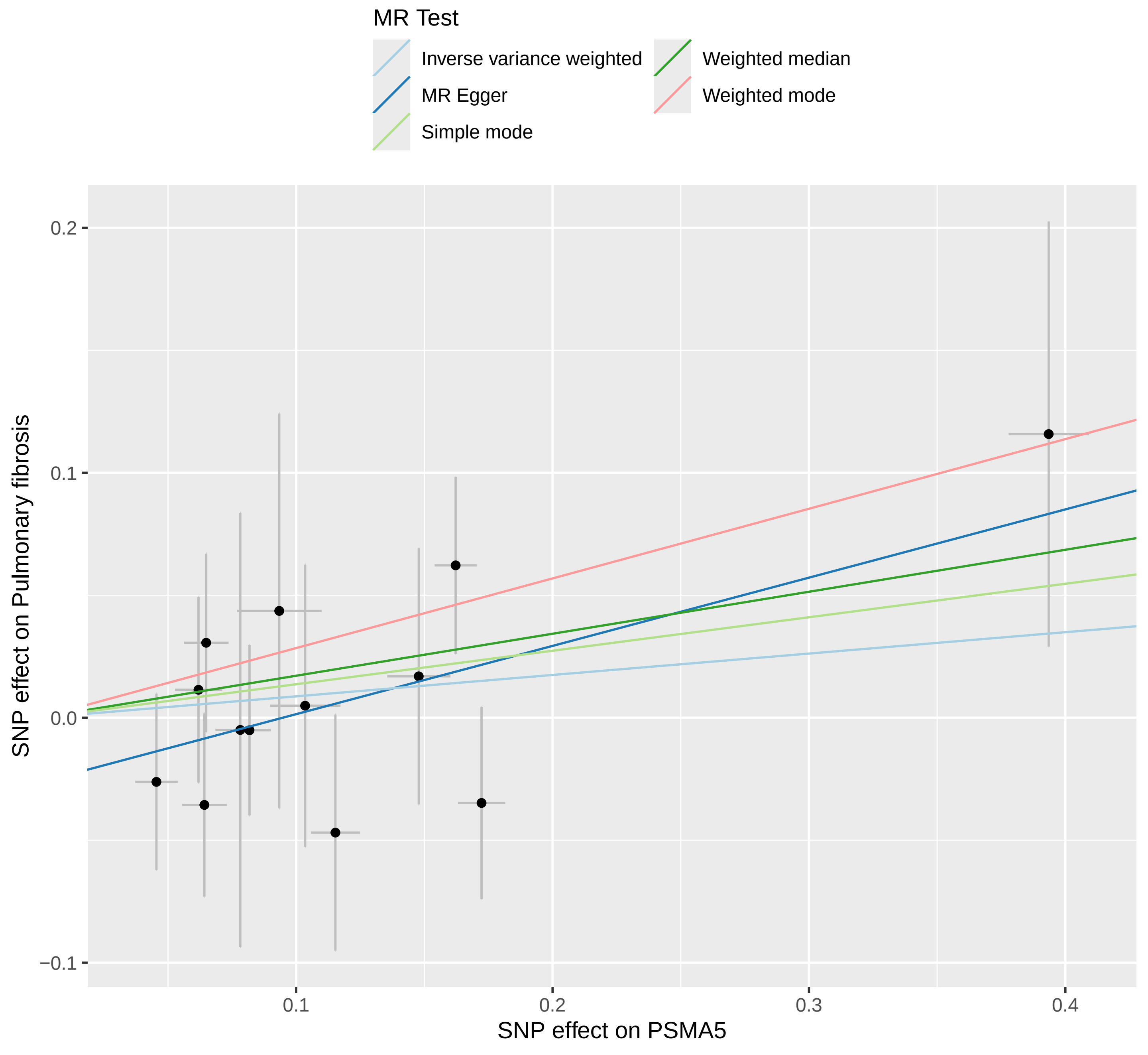

MR Test
Inverse variance weighted
MR Egger
Simple mode
Weighted median
Weighted mode
SNP effect on Pulmonary fibrosis
0.2
0.1
0.0
−0.1
0.1
0.2
0.3
0.4
SNP effect on PSMA5

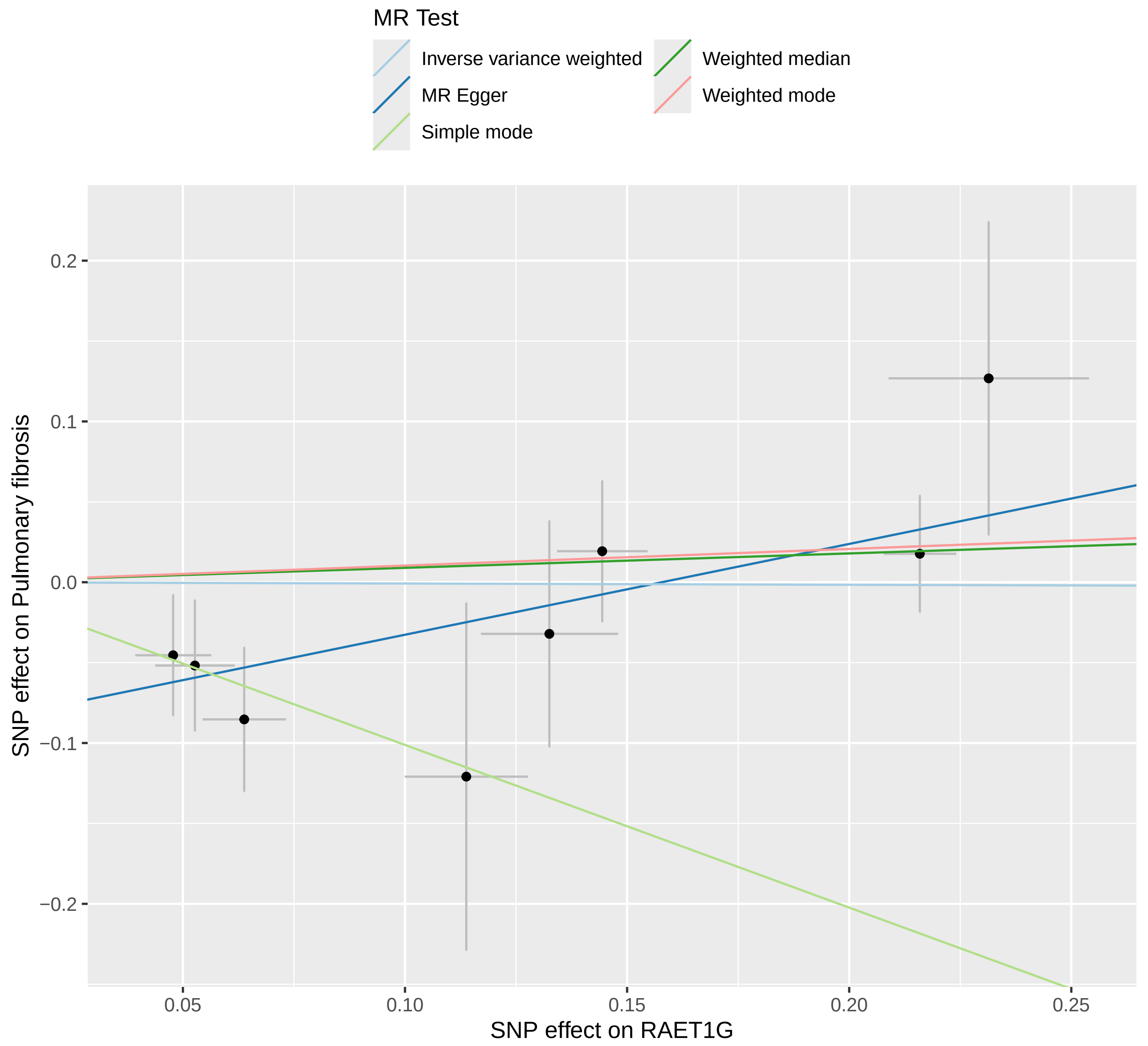

MR Test
Inverse variance weighted
MR Egger
Simple mode
Weighted median
Weighted mode
0.2
0.1
0.0
−0.1
−0.2
SNP effect on Pulmonary fibrosis
0.05
0.10
0.15
0.20
0.25
SNP effect on RAET1G

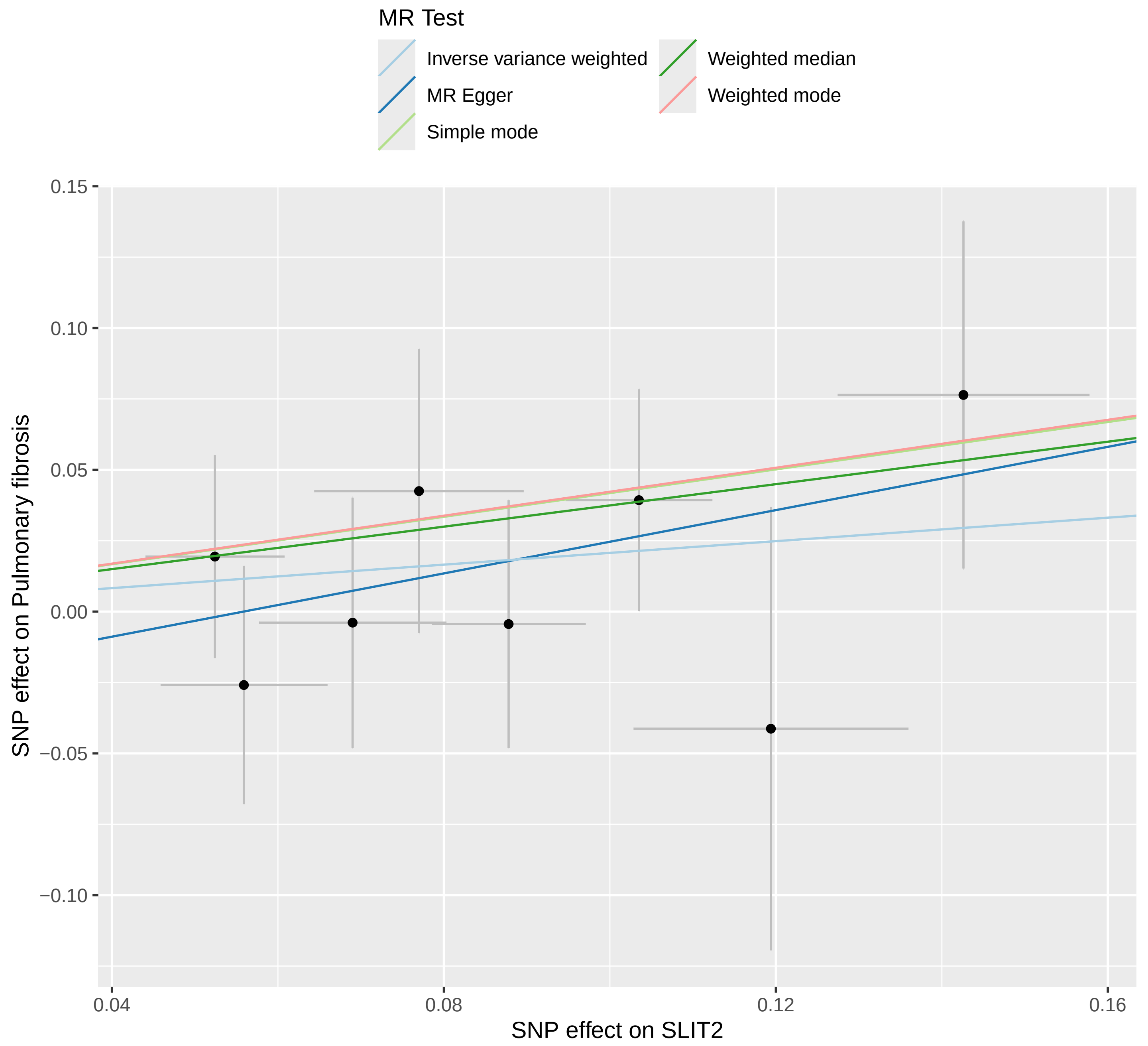
MR Test
Inverse variance weighted
MR Egger
Simple mode
Weighted median
Weighted mode
0.15
0.10
0.05
0.00
−0.05
−0.10
SNP effect on Pulmonary fibrosis
0.04
0.08
0.12
0.16
SNP effect on SLIT2

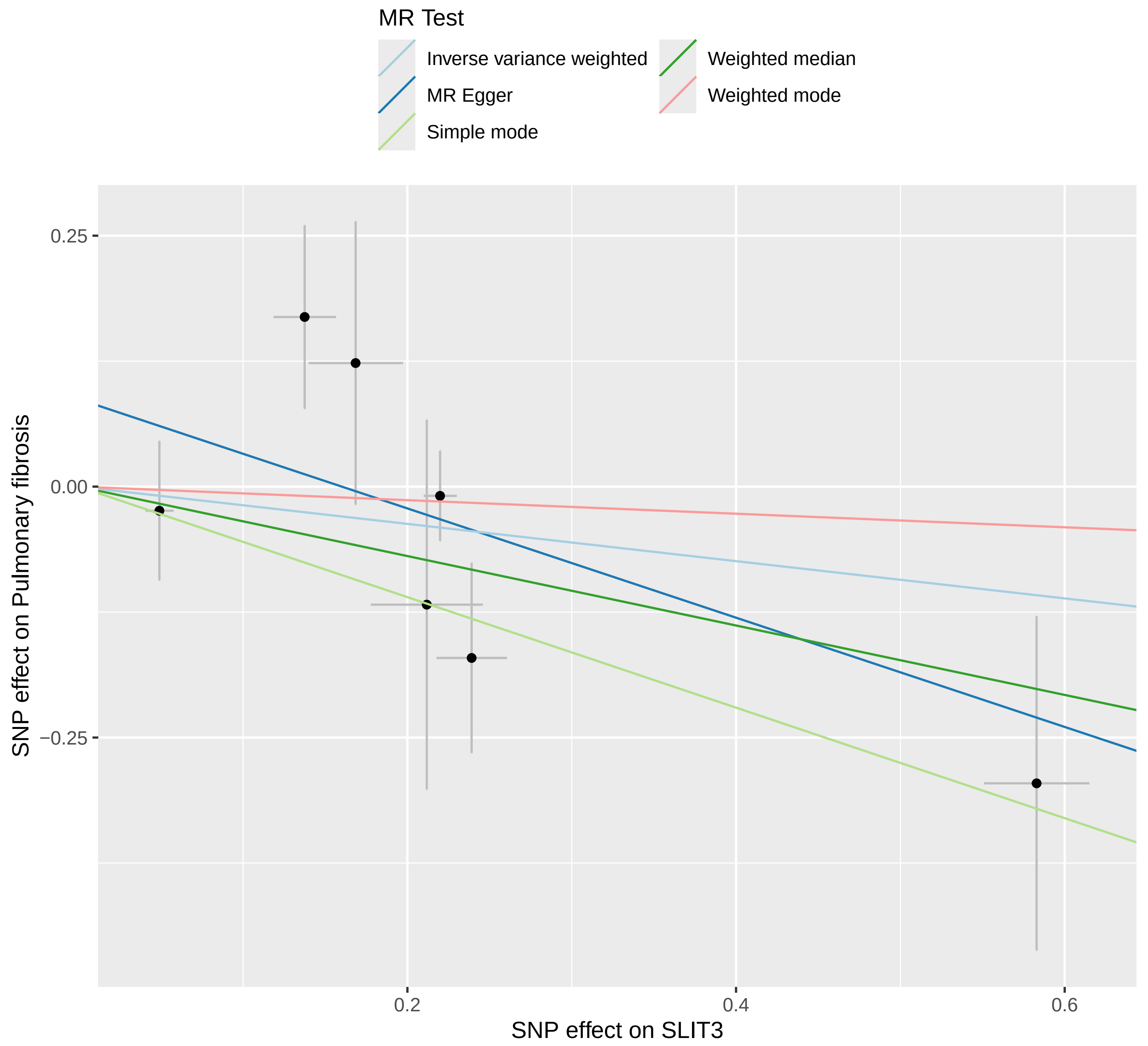
MR Test
Inverse variance weighted
MR Egger
Simple mode
Weighted median
Weighted mode
0.25
0.00
−0.25
0.2
0.4
0.6
SNP effect on Pulmonary fibrosis
SNP effect on SLIT3

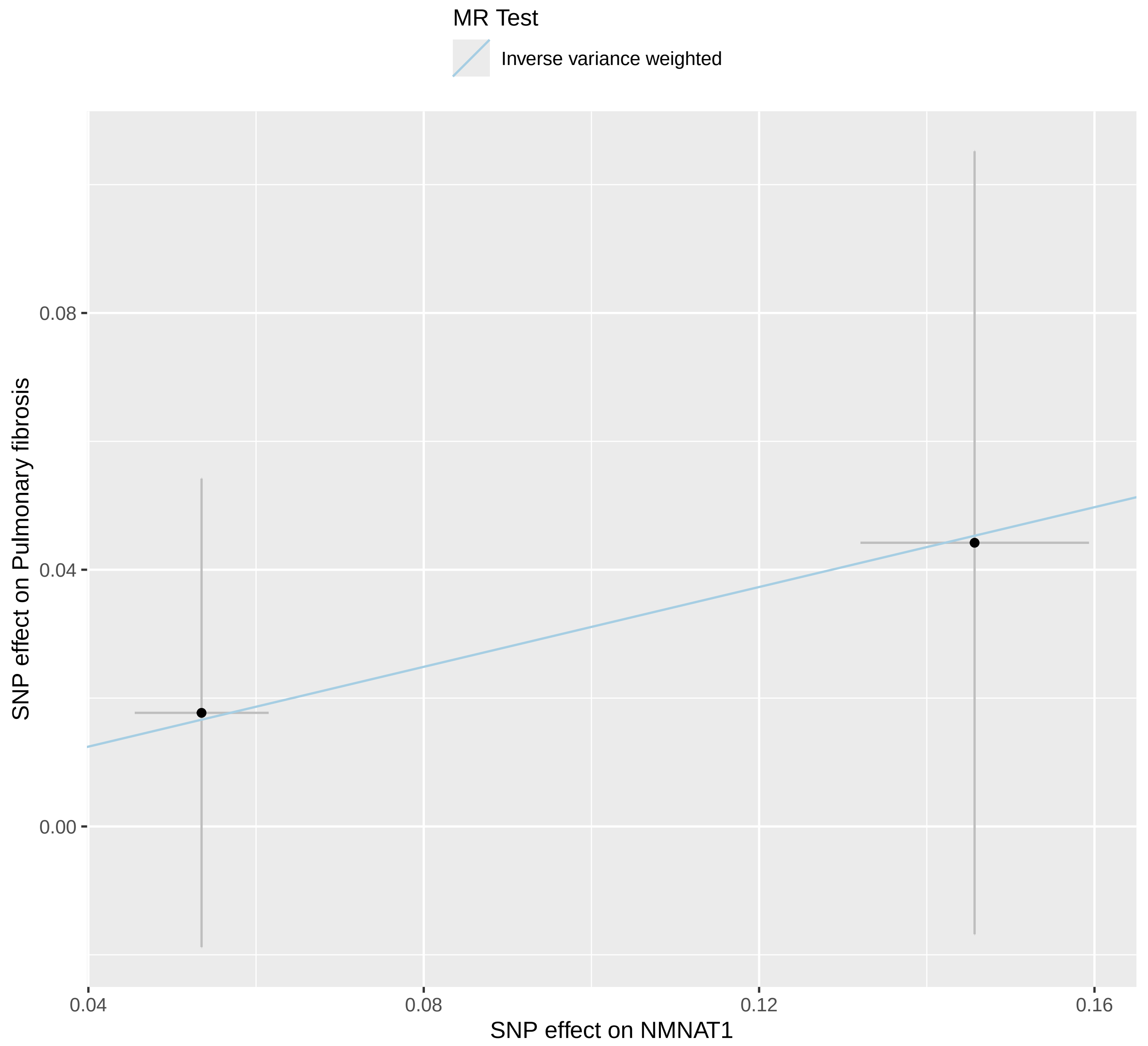

MR Test
Inverse variance weighted
0.08
0.04
0.00
0.04
0.08
0.12
0.16
SNP effect on Pulmonary fibrosis
SNP effect on NMNAT1

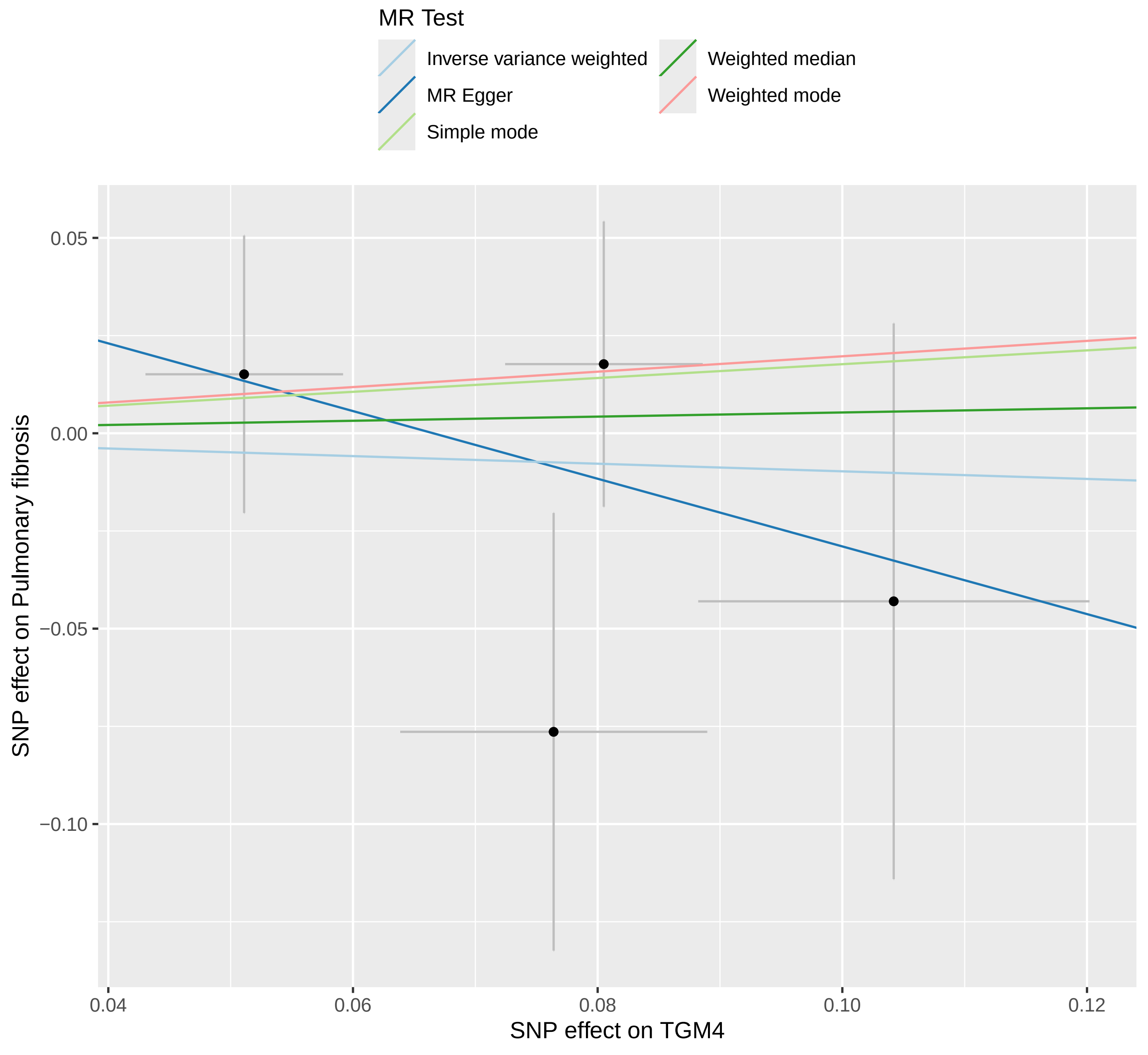

MR Test
Inverse variance weighted
MR Egger
Simple mode
Weighted median
Weighted mode
0.05
0.00
−0.05
−0.10
SNP effect on Pulmonary fibrosis
0.04
0.06
0.08
0.10
0.12
SNP effect on TGM4

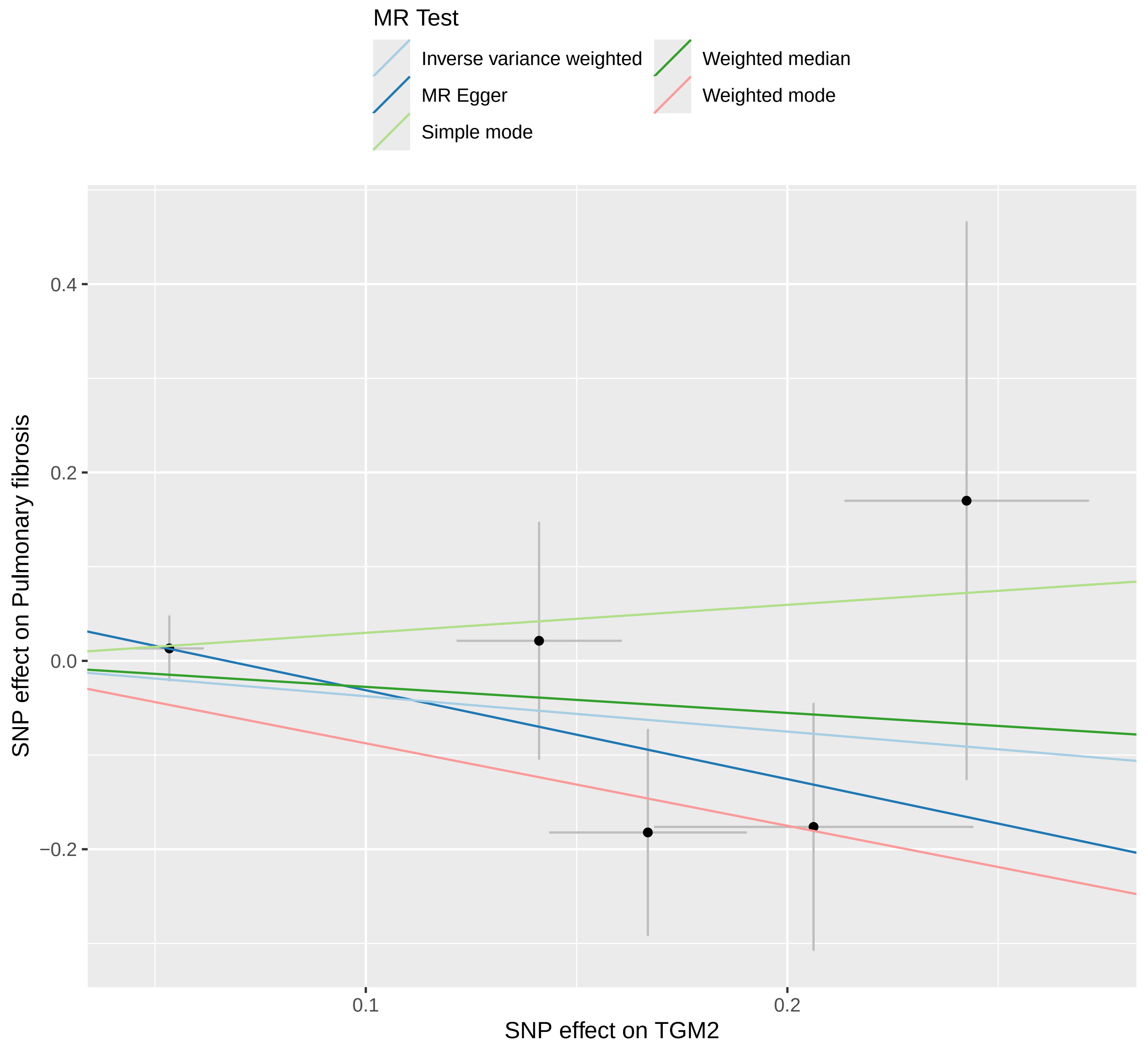
MR Test
Inverse variance weighted
MR Egger
Simple mode
Weighted median
Weighted mode
0.4
0.2
0.0
−0.2
0.1
0.2
SNP effect on Pulmonary fibrosis
SNP effect on TGM2

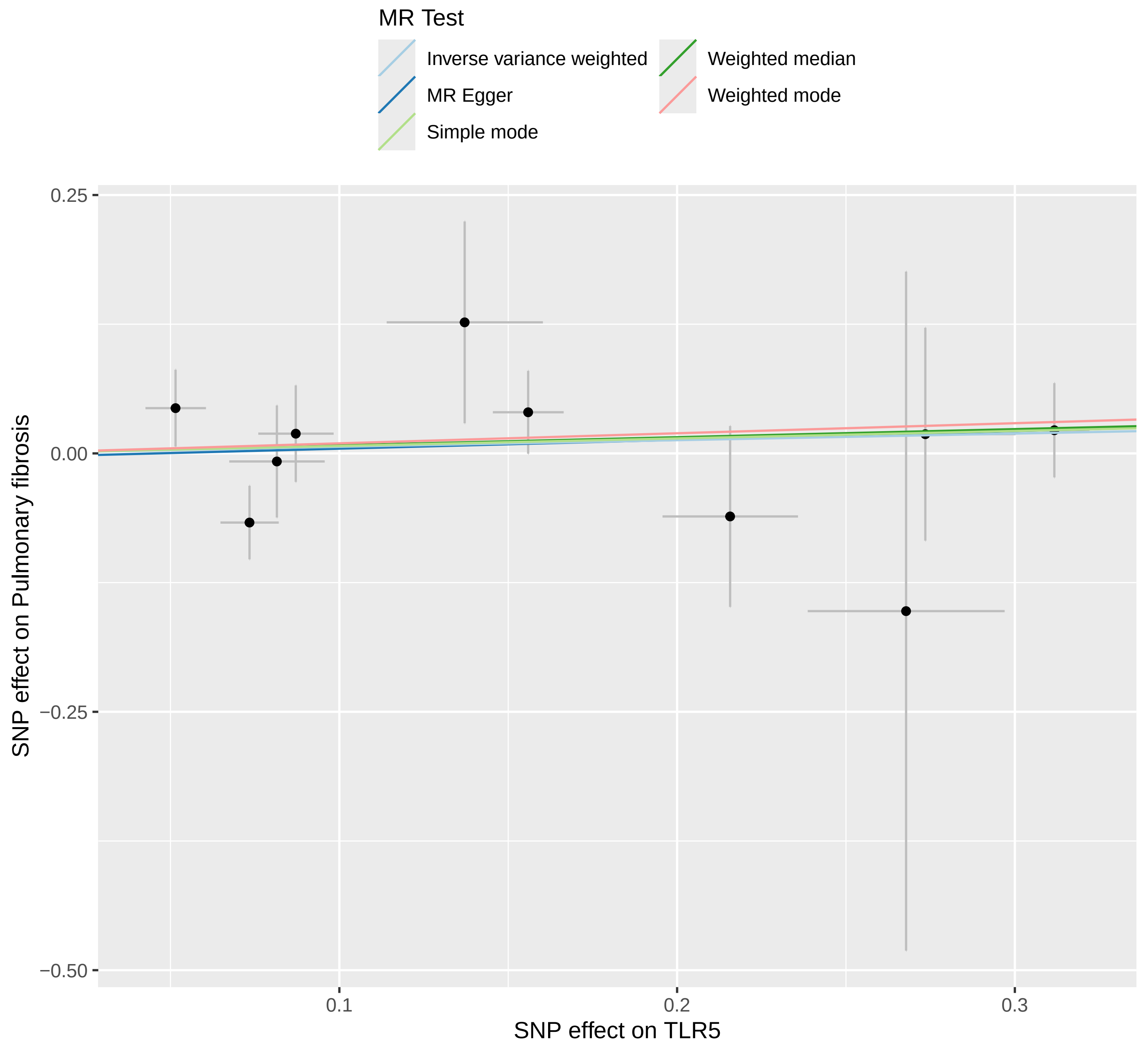

MR Test
Inverse variance weighted
MR Egger
Simple mode
Weighted median
Weighted mode
0.25
0.00
−0.25
−0.50
0.1
0.2
0.3
SNP effect on Pulmonary fibrosis
SNP effect on TLR5

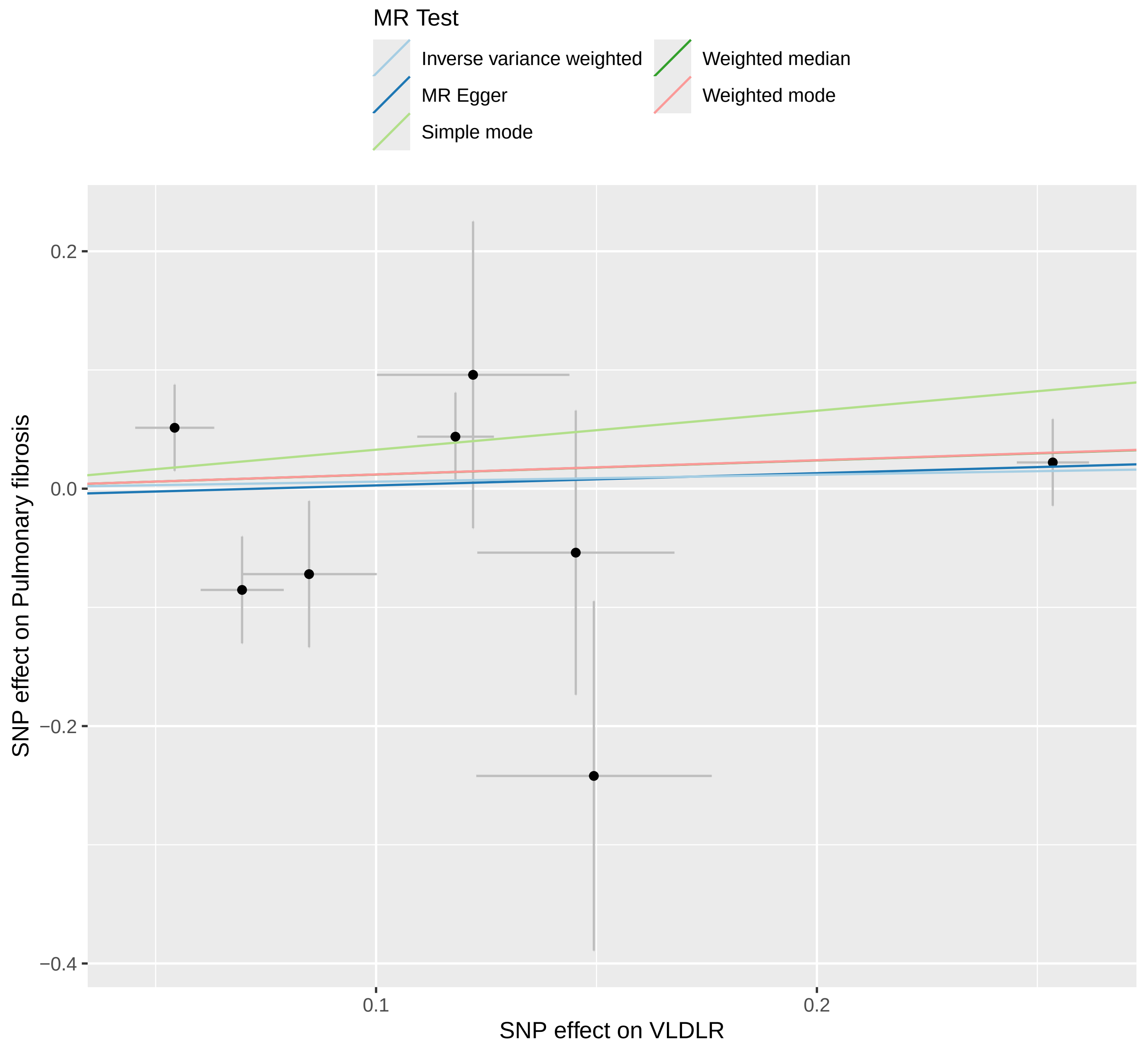

MR Test
Inverse variance weighted
MR Egger
Simple mode
Weighted median
Weighted mode
0.2
0.0
−0.2
−0.4
SNP effect on Pulmonary fibrosis
0.1
0.2
SNP effect on VLDLR

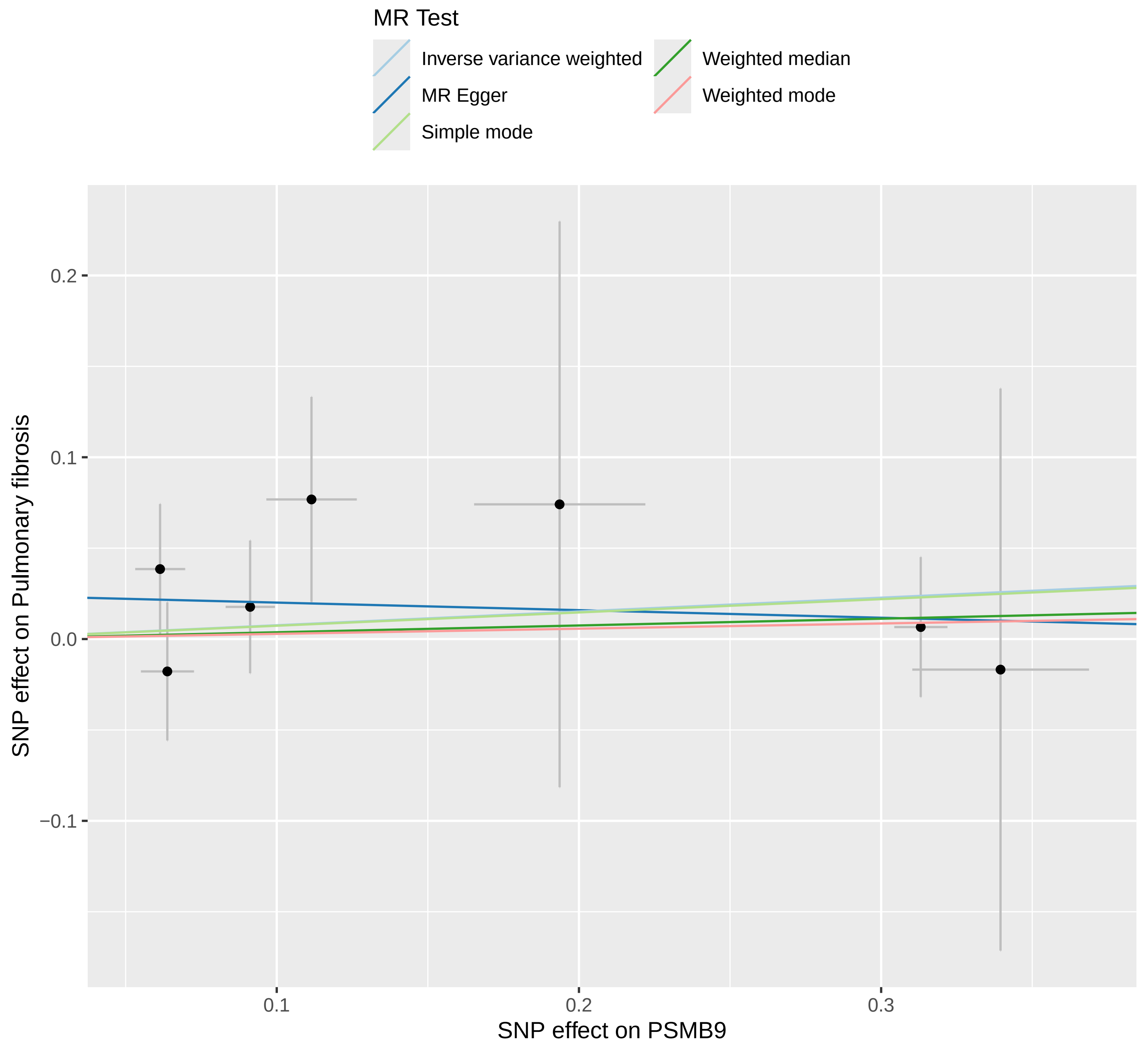

MR Test
Inverse variance weighted
MR Egger
Simple mode
Weighted median
Weighted mode
SNP effect on Pulmonary fibrosis
0.2
0.1
0.0
−0.1
0.1
0.2
0.3
SNP effect on PSMB9

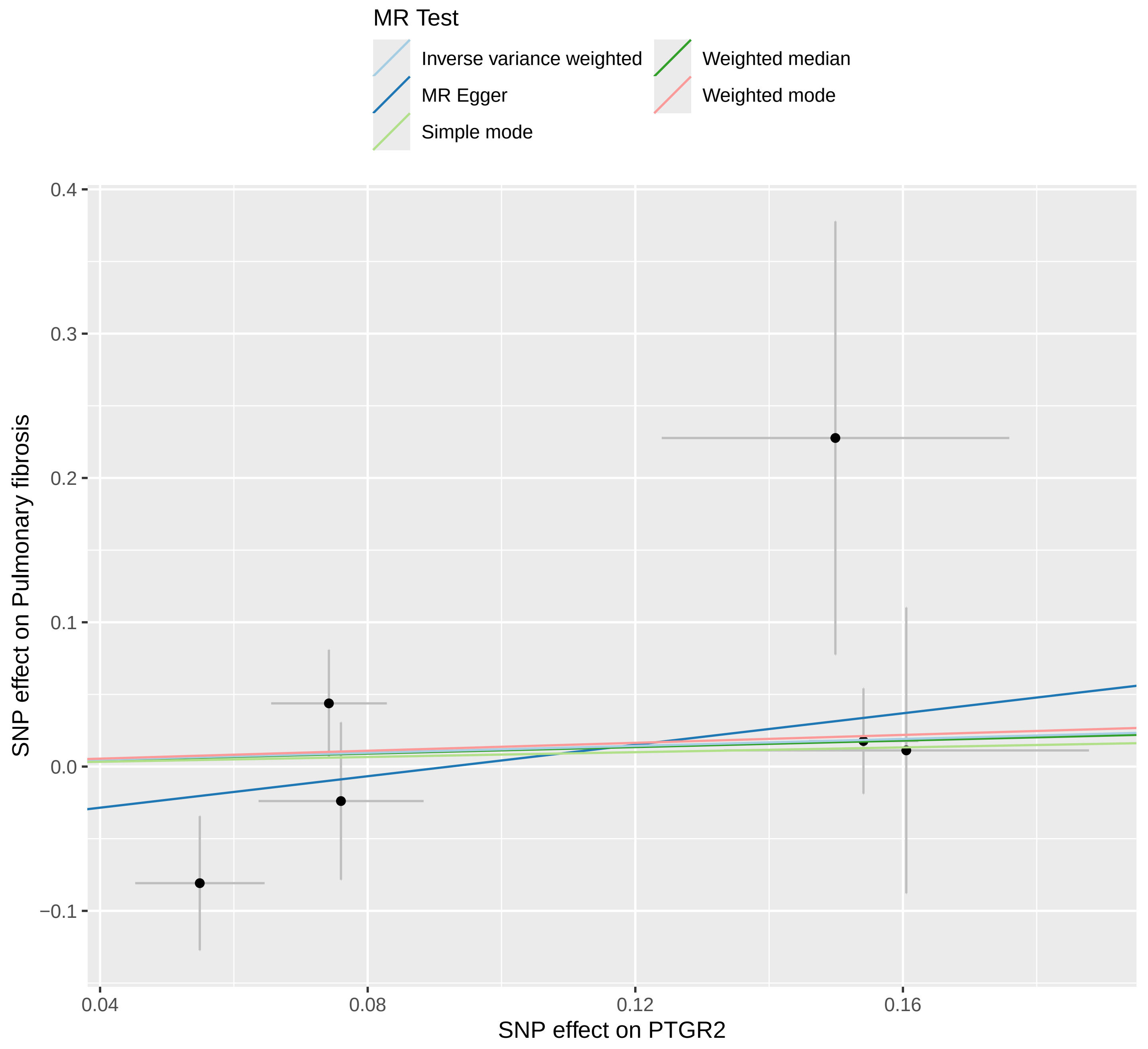

MR Test
Inverse variance weighted
MR Egger
Simple mode
Weighted median
Weighted mode
0.4
0.3
0.2
0.1
0.0
−0.1
SNP effect on Pulmonary fibrosis
0.04
0.08
0.12
0.16
SNP effect on PTGR2

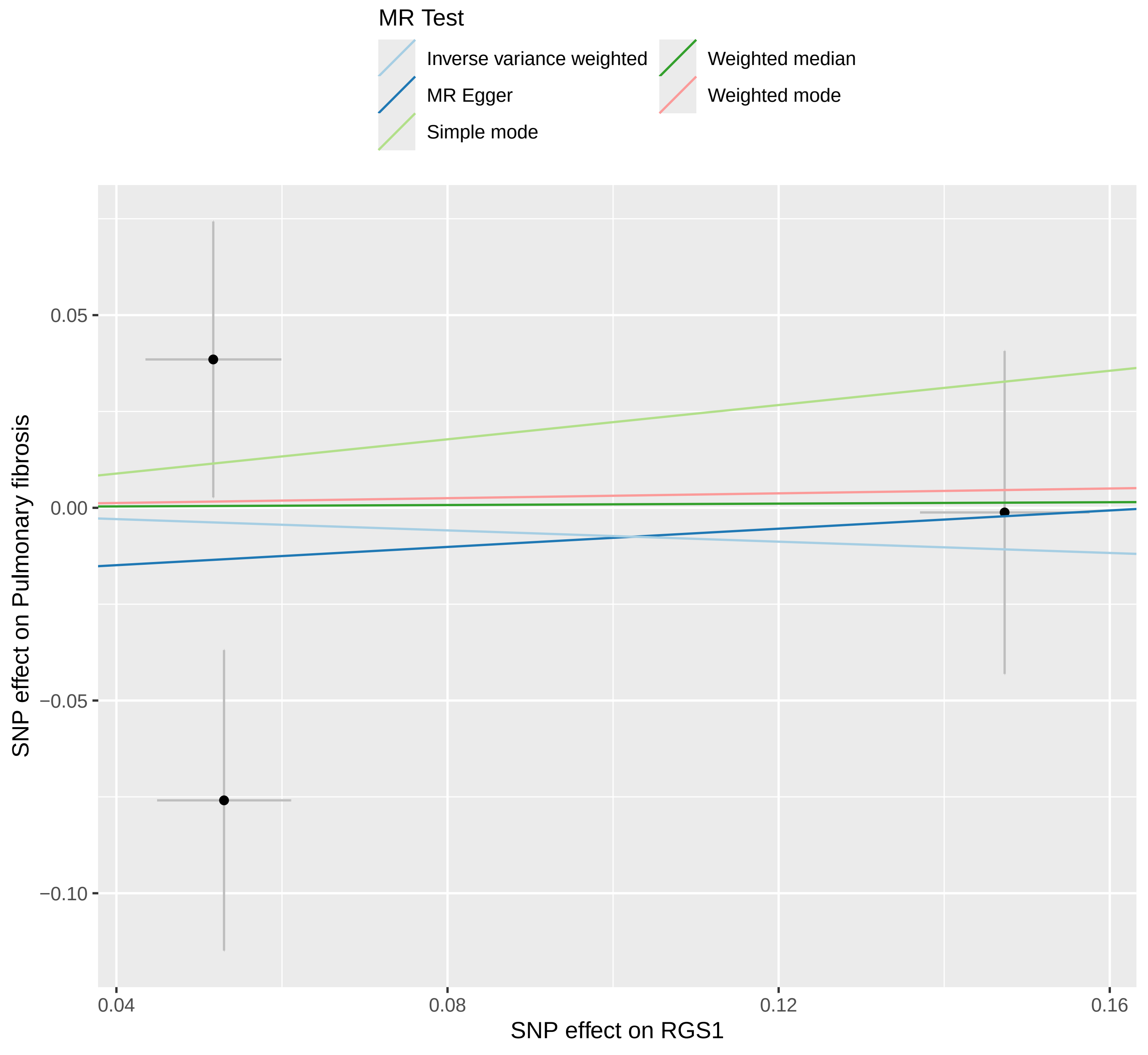

MR Test
Inverse variance weighted
MR Egger
Simple mode
Weighted median
Weighted mode
0.05
0.00
−0.05
−0.10
SNP effect on Pulmonary fibrosis
0.04
0.08
0.12
0.16
SNP effect on RGS1

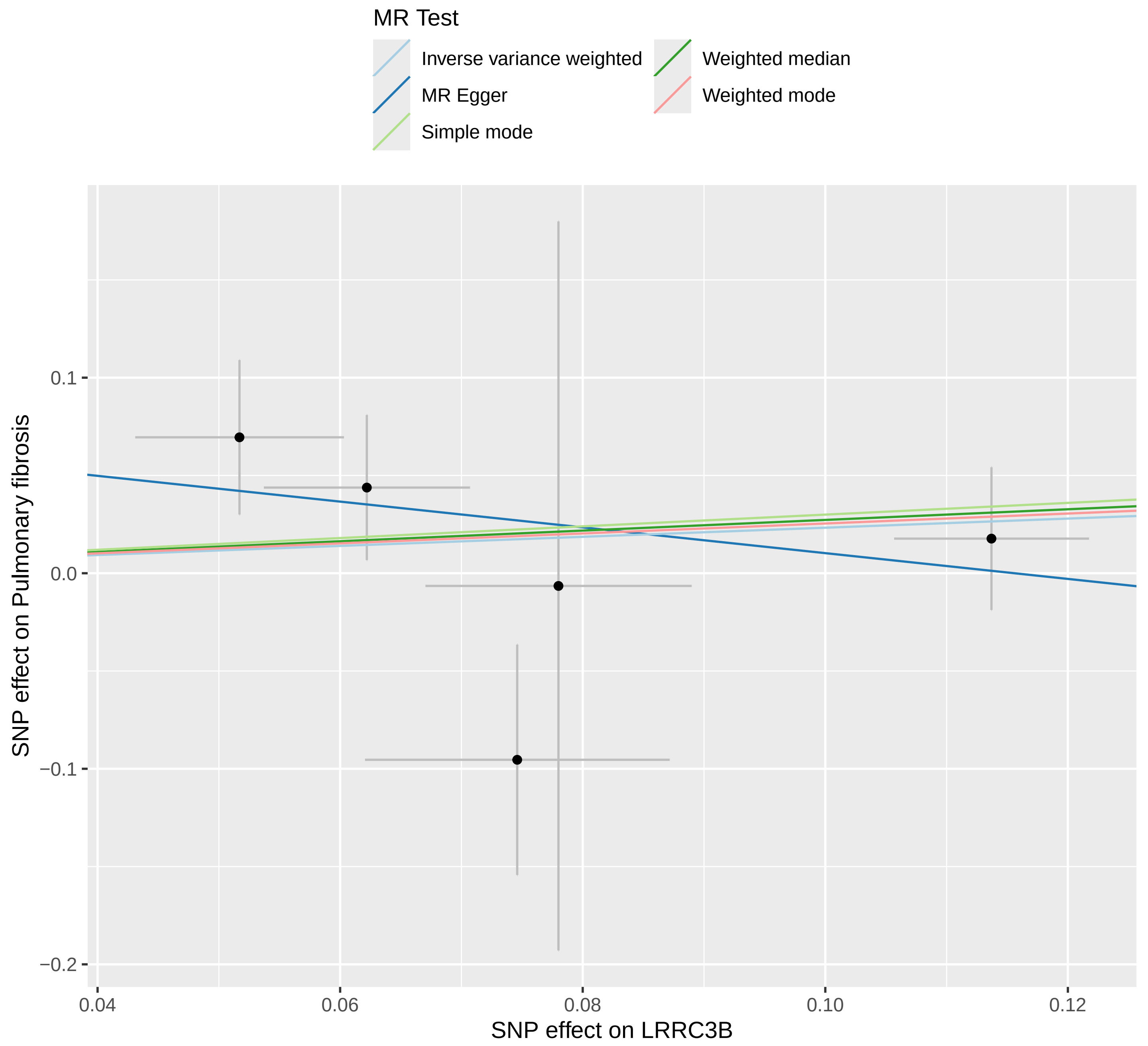

MR Test
Inverse variance weighted
MR Egger
Simple mode
Weighted median
Weighted mode
0.1
0.0
−0.1
−0.2
SNP effect on Pulmonary fibrosis
0.04
0.06
0.08
0.10
0.12
SNP effect on LRRC3B

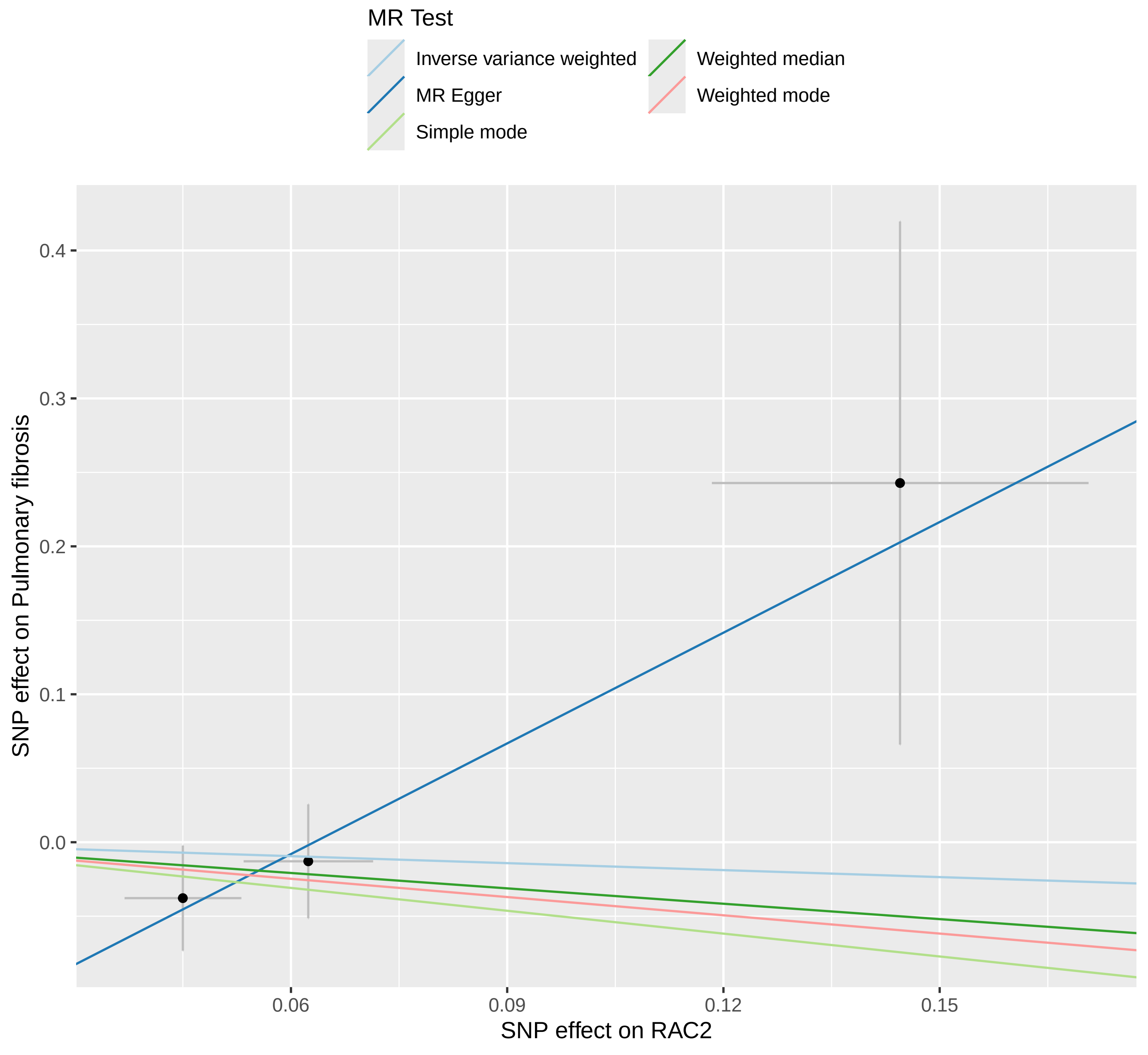
MR Test
Inverse variance weighted
MR Egger
Simple mode
Weighted median
Weighted mode
SNP effect on Pulmonary fibrosis
0.4
0.3
0.2
0.1
0.0
0.06
0.09
0.12
0.15
SNP effect on RAC2

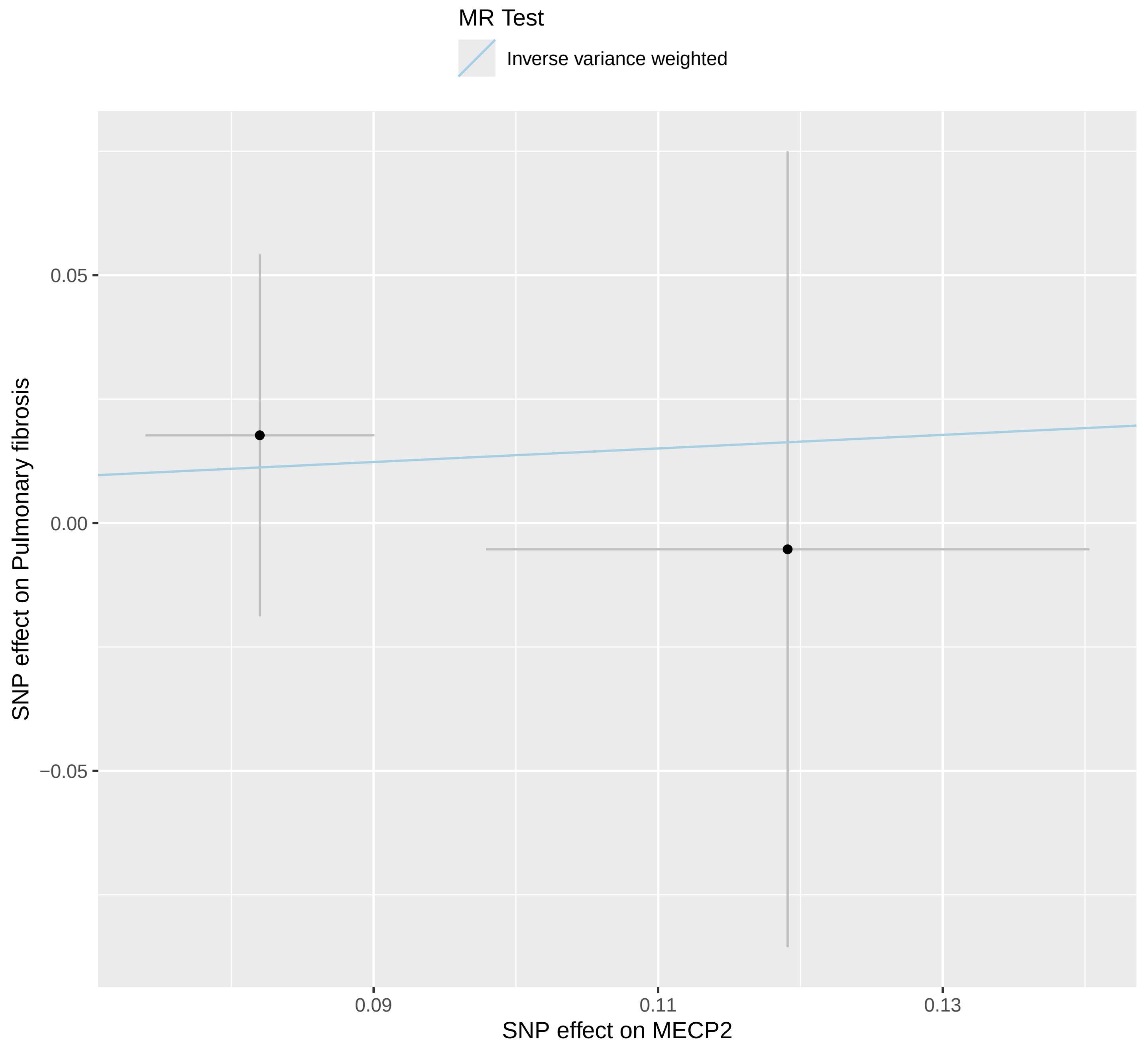
MR Test
Inverse variance weighted
0.05
0.00
−0.05
SNP effect on Pulmonary fibrosis
0.09
0.11
0.13
SNP effect on MECP2

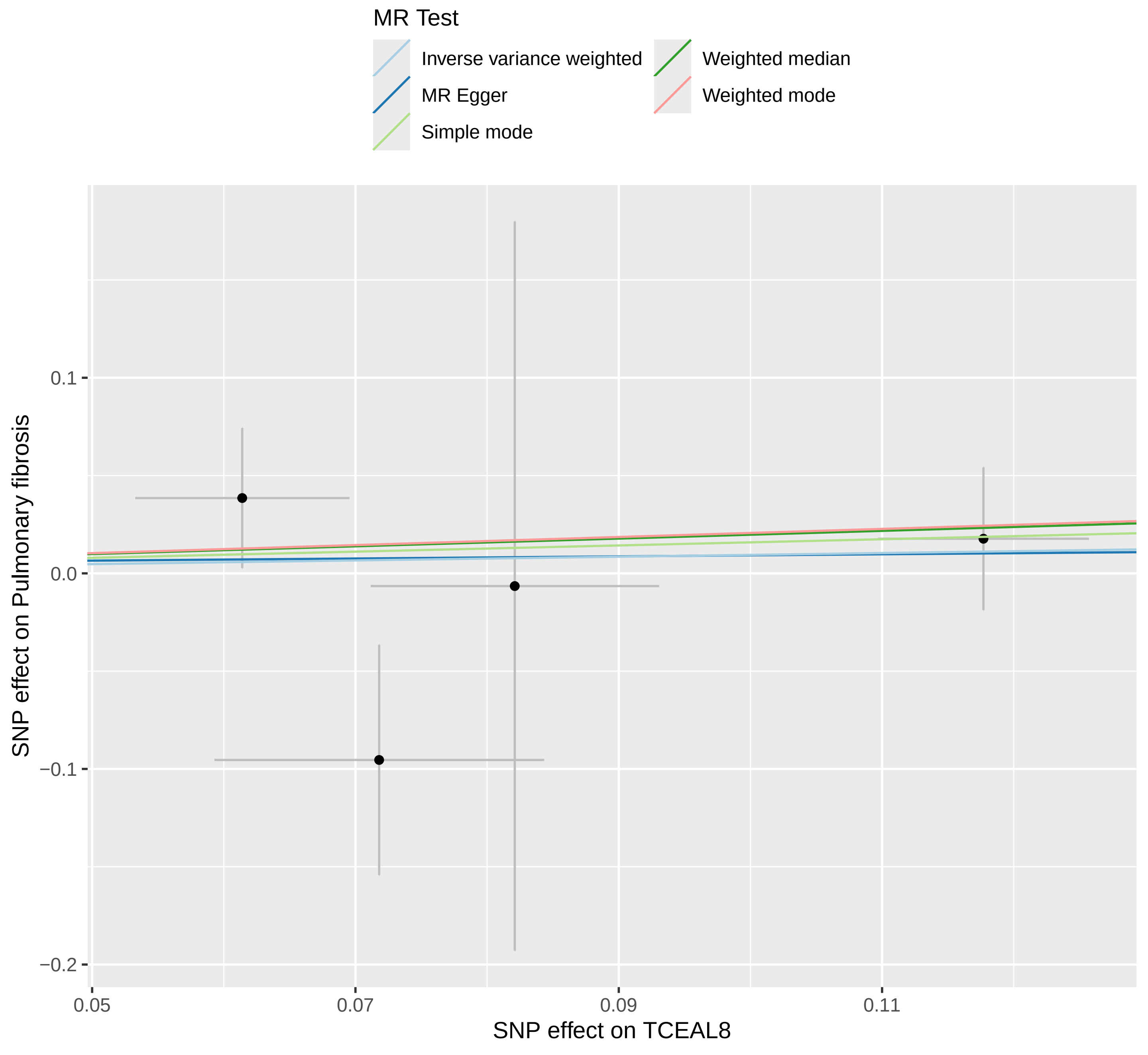
MR Test
Inverse variance weighted
MR Egger
Simple mode
Weighted median
Weighted mode
SNP effect on Pulmonary fibrosis
0.1
0.0
−0.1
−0.2
0.05
0.07
0.09
0.11
SNP effect on TCEAL8

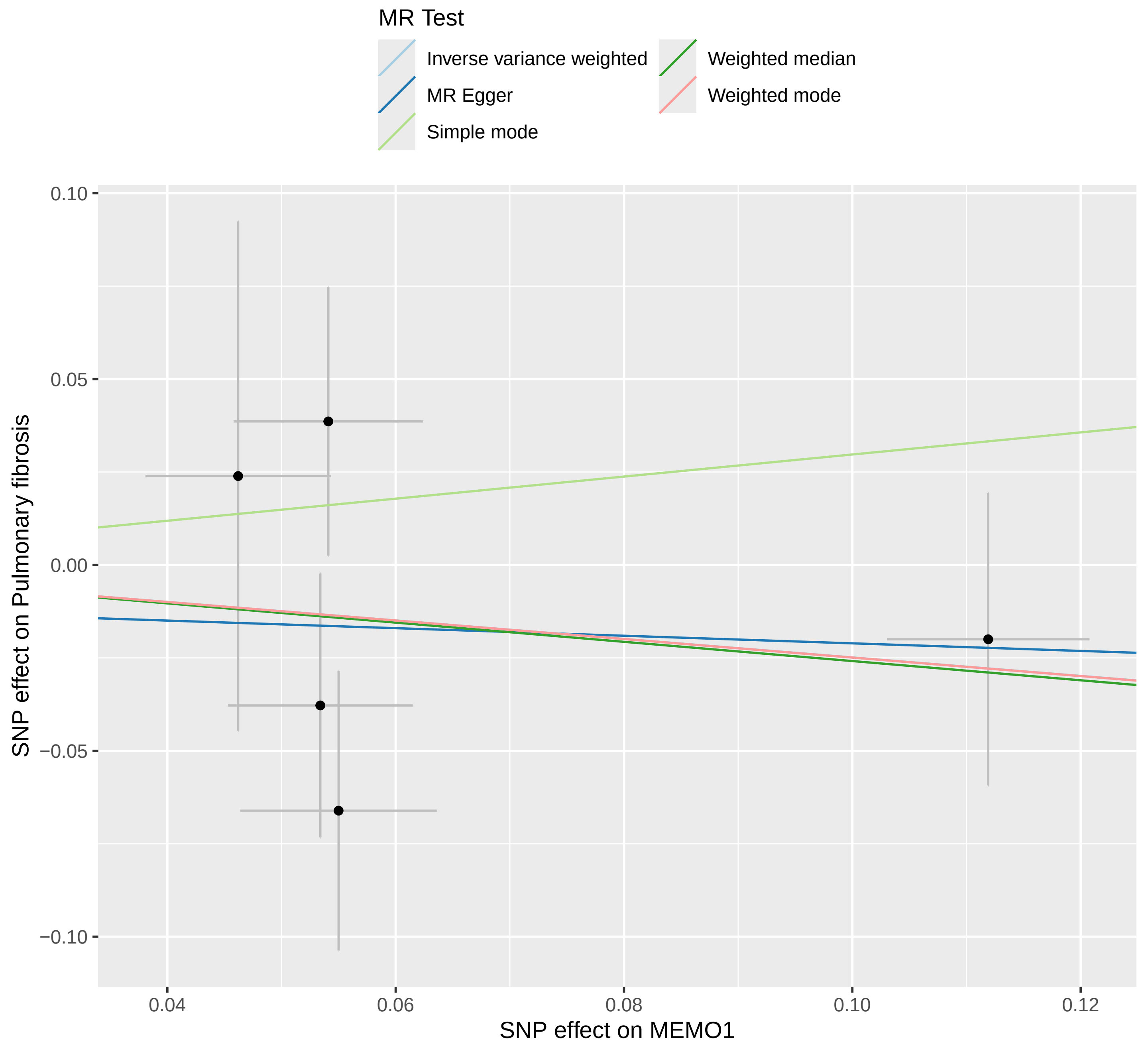

MR Test
Inverse variance weighted
MR Egger
Simple mode
Weighted median
Weighted mode
0.10
0.05
0.00
−0.05
−0.10
SNP effect on Pulmonary fibrosis
0.04
0.06
0.08
0.10
0.12
SNP effect on MEMO1

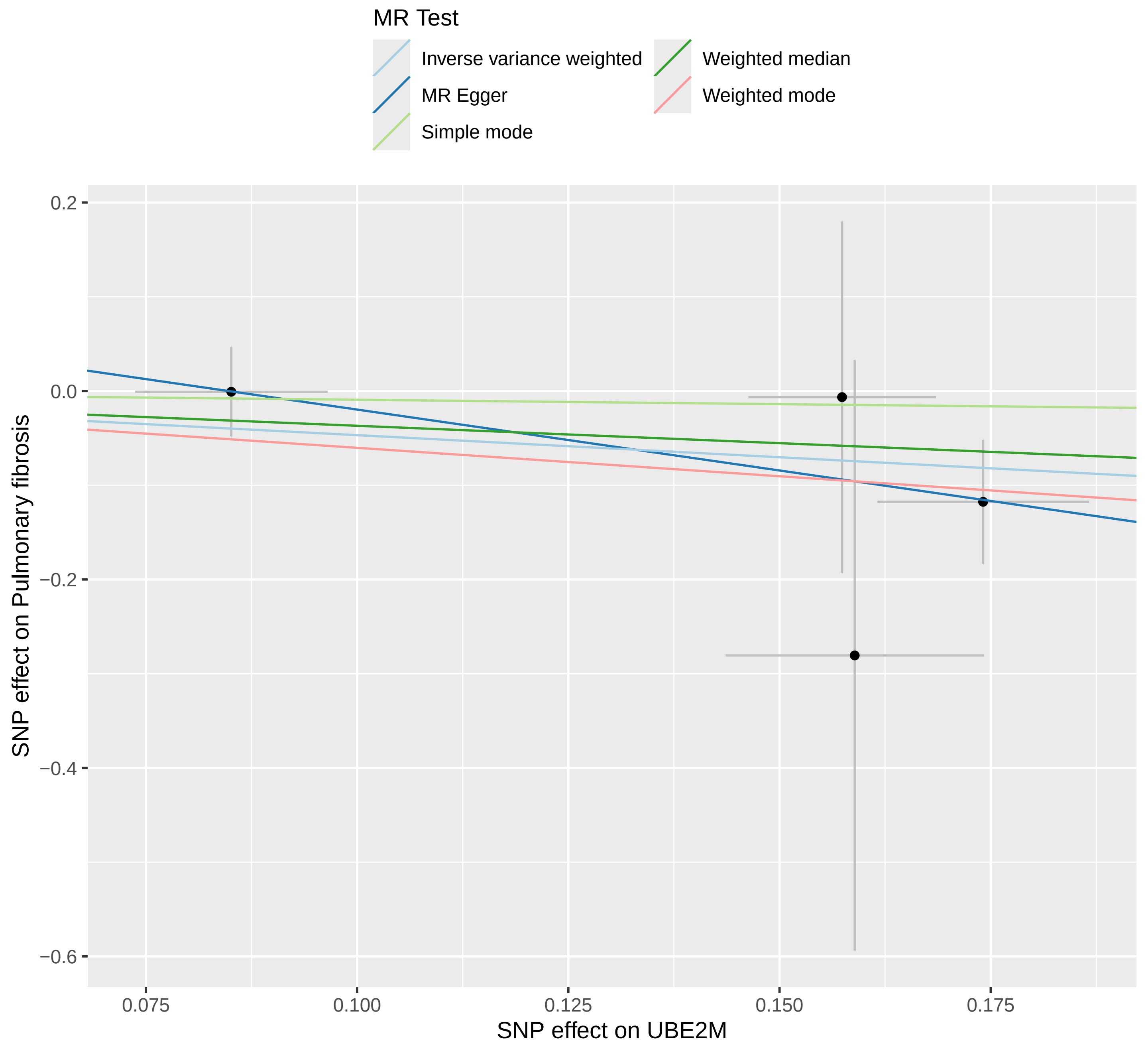

MR Test
Inverse variance weighted
MR Egger
Simple mode
Weighted median
Weighted mode
SNP effect on Pulmonary fibrosis
SNP effect on UBE2M
0.2
0.0
−0.2
−0.4
−0.6
0.075
0.100
0.125
0.150
0.175

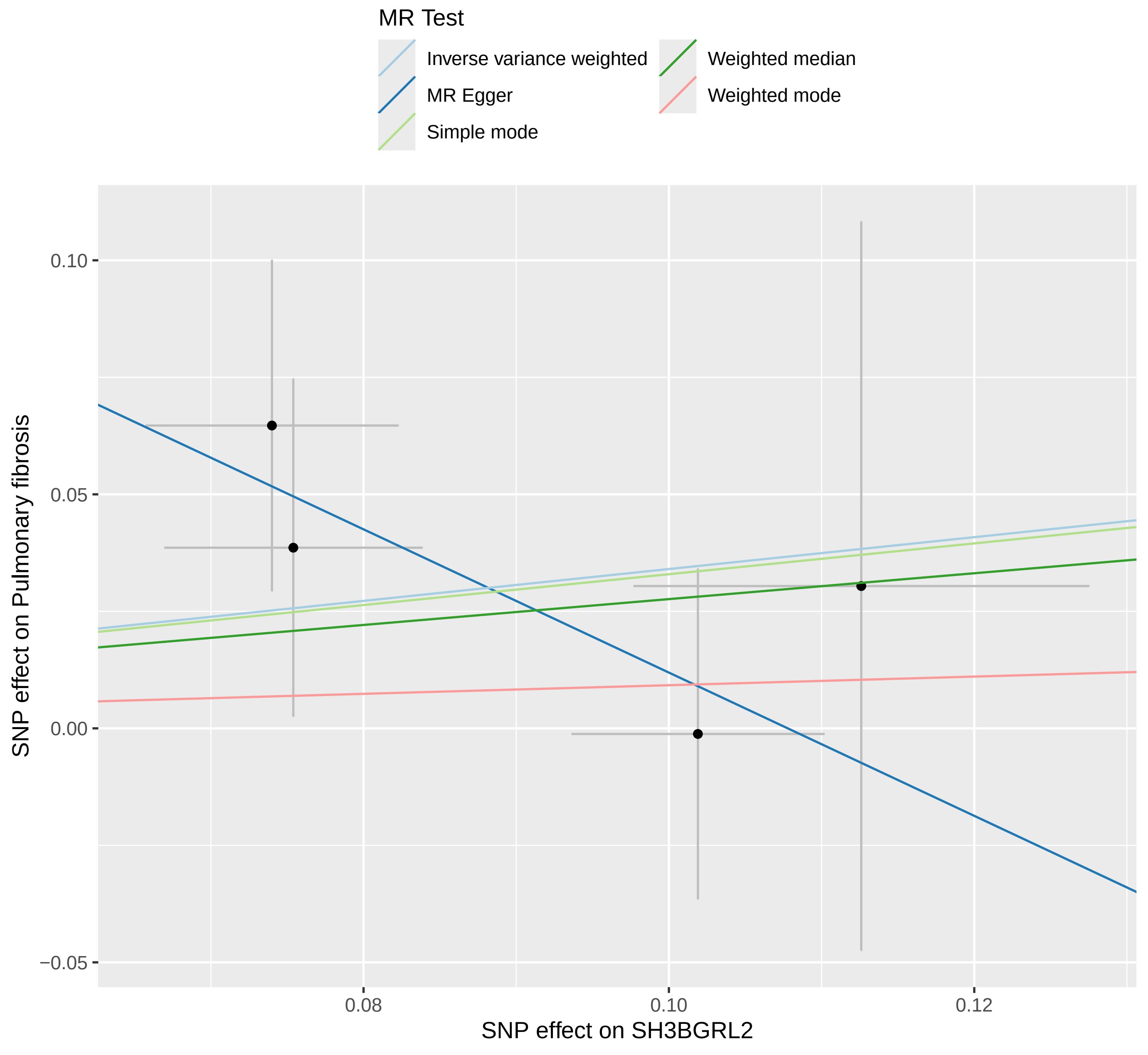

MR Test
Inverse variance weighted
MR Egger
Simple mode
Weighted median
Weighted mode
0.10
0.05
0.00
−0.05
0.08
0.10
0.12
SNP effect on Pulmonary fibrosis
SNP effect on SH3BGRL2

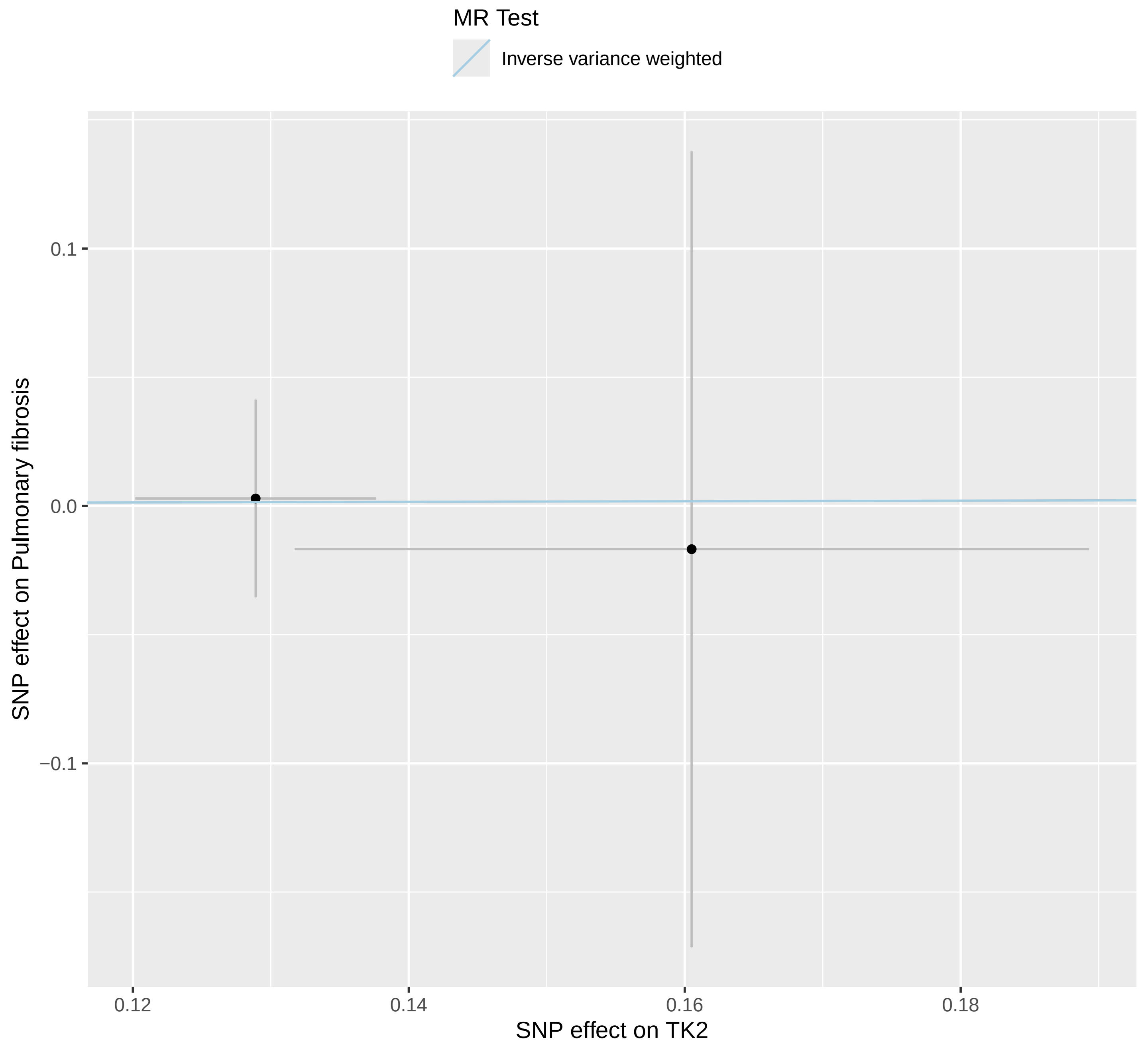

MR Test
Inverse variance weighted
0.1
0.0
−0.1
SNP effect on Pulmonary fibrosis
0.12
0.14
0.16
0.18
SNP effect on TK2

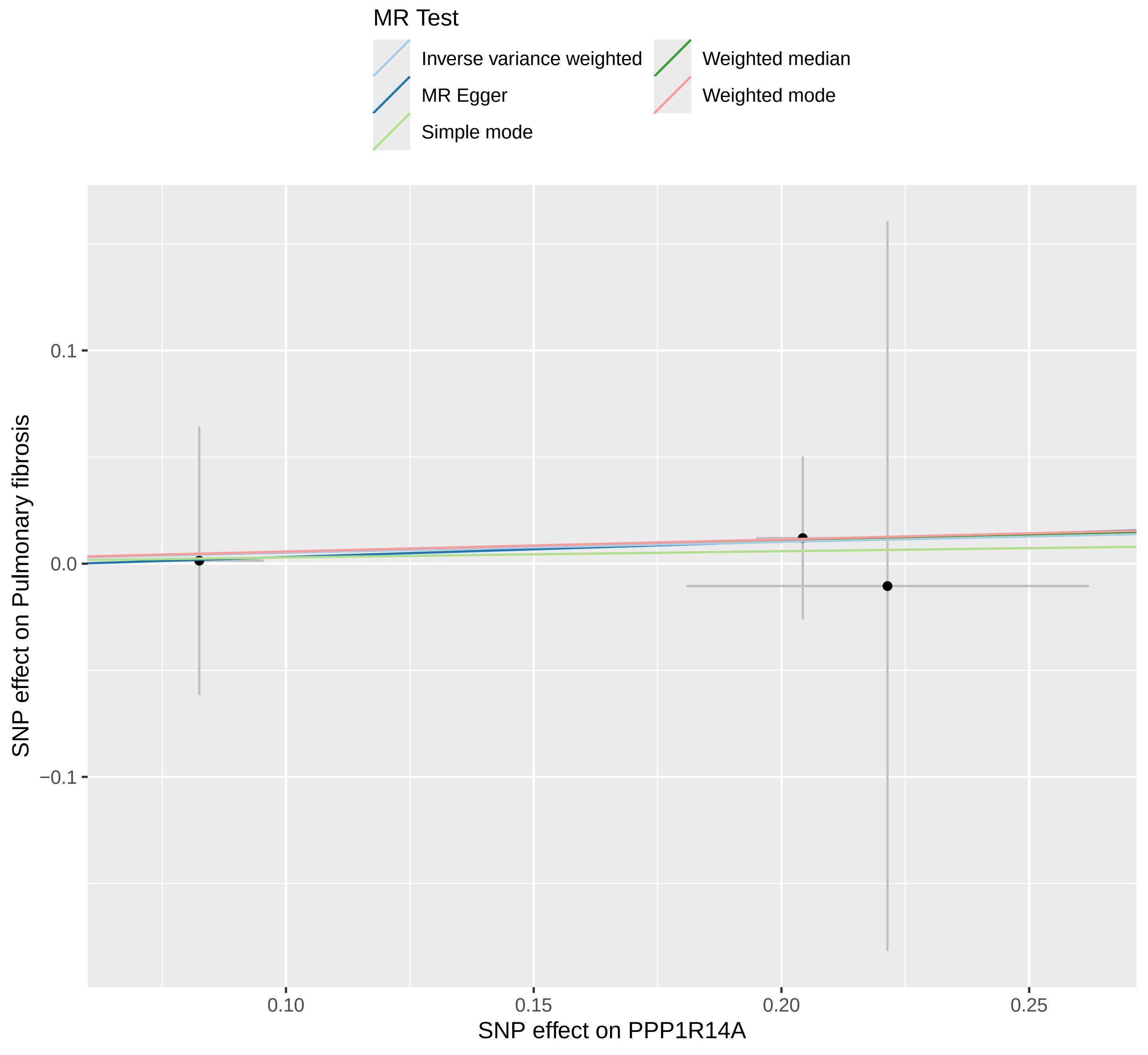
MR Test
Inverse variance weighted
MR Egger
Simple mode
Weighted median
Weighted mode
0.1
0.0
−0.1
0.10
0.15
0.20
0.25
SNP effect on Pulmonary fibrosis
SNP effect on PPP1R14A

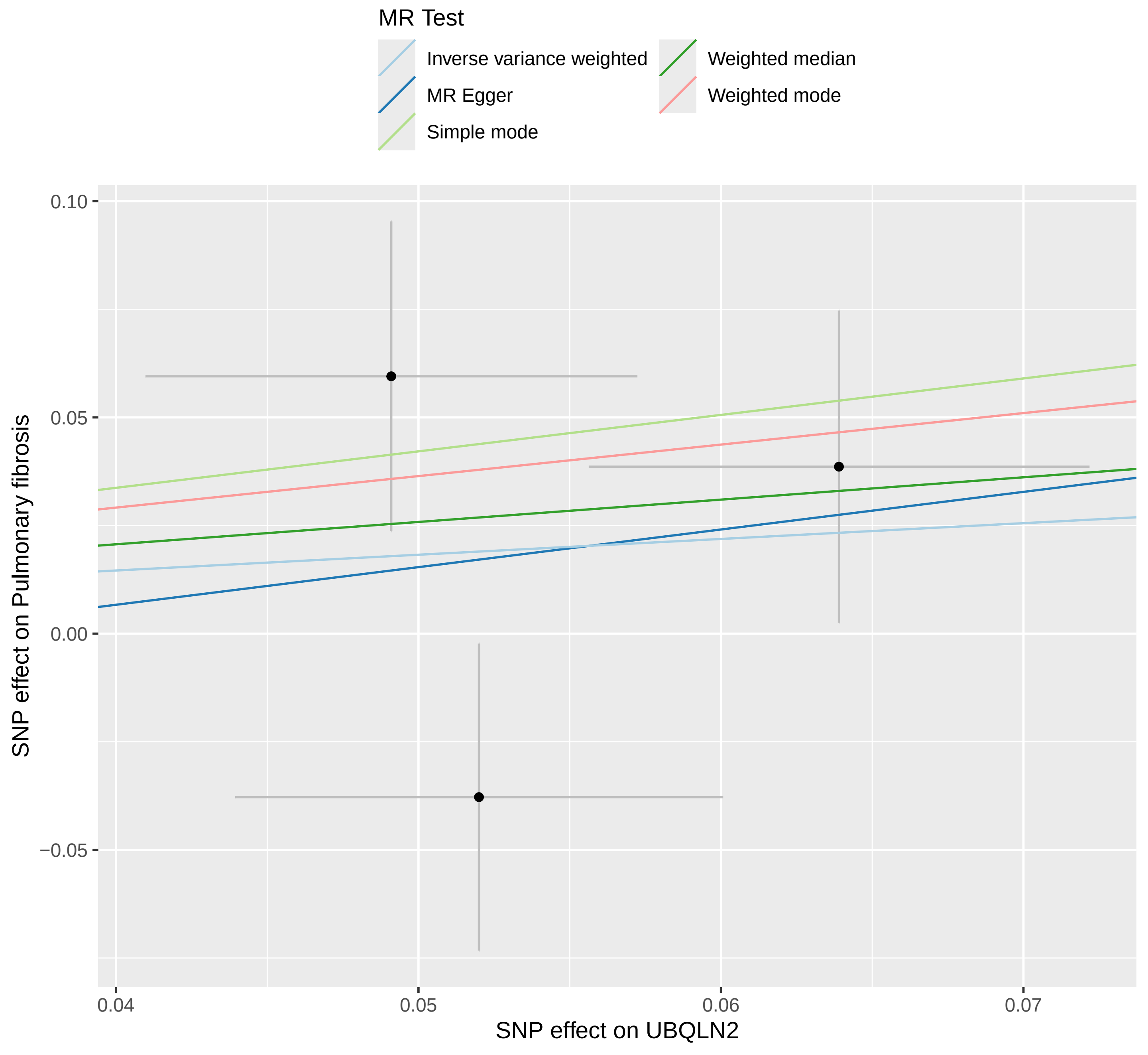
MR Test
Inverse variance weighted
MR Egger
Simple mode
Weighted median
Weighted mode
0.10
0.05
0.00
−0.05
SNP effect on Pulmonary fibrosis
0.04
0.05
0.06
0.07
SNP effect on UBQLN2

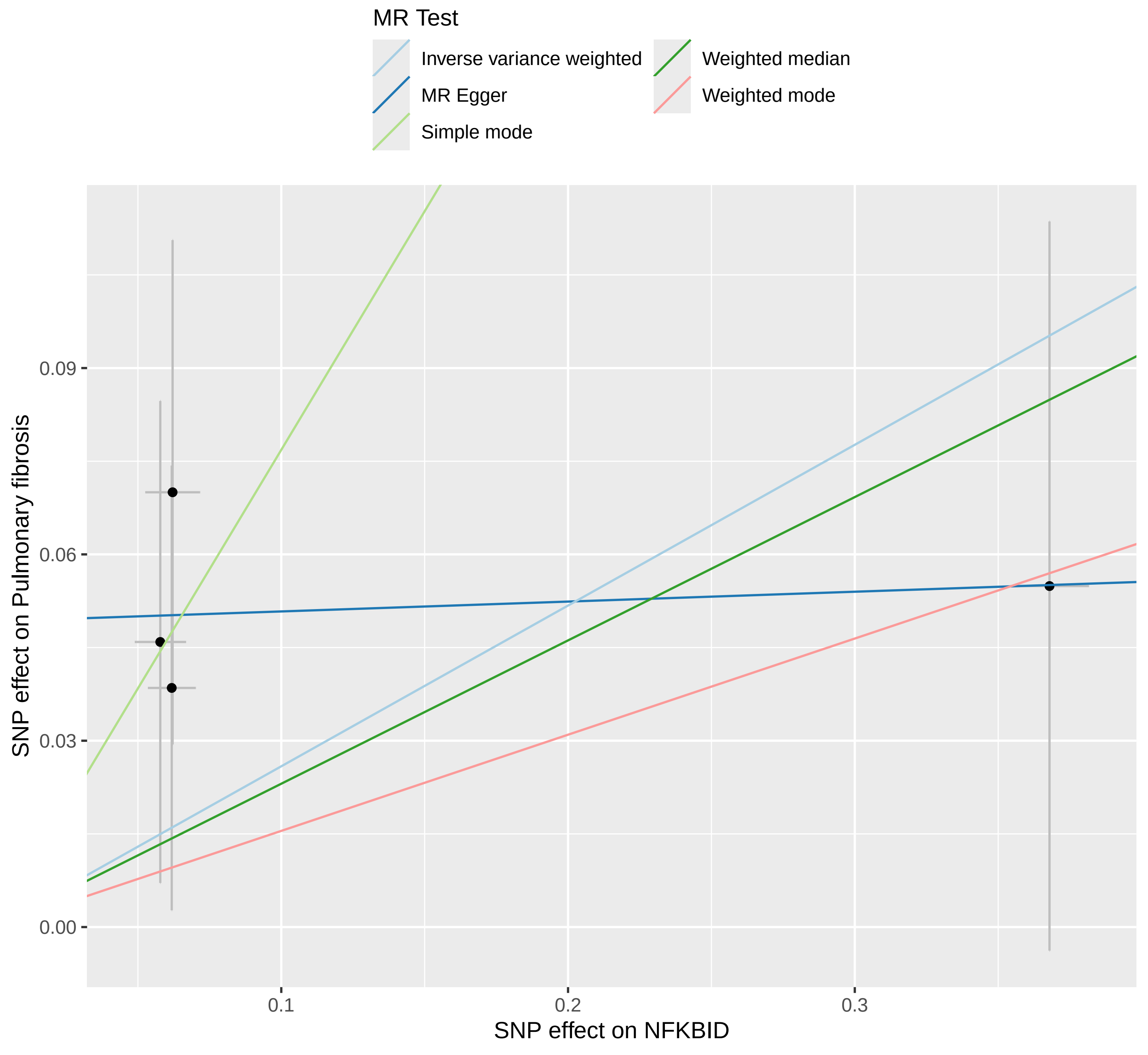
MR Test
Inverse variance weighted
MR Egger
Simple mode
Weighted median
Weighted mode
0.09
0.06
0.03
0.00
SNP effect on Pulmonary fibrosis
0.1
0.2
0.3
SNP effect on NFKBID

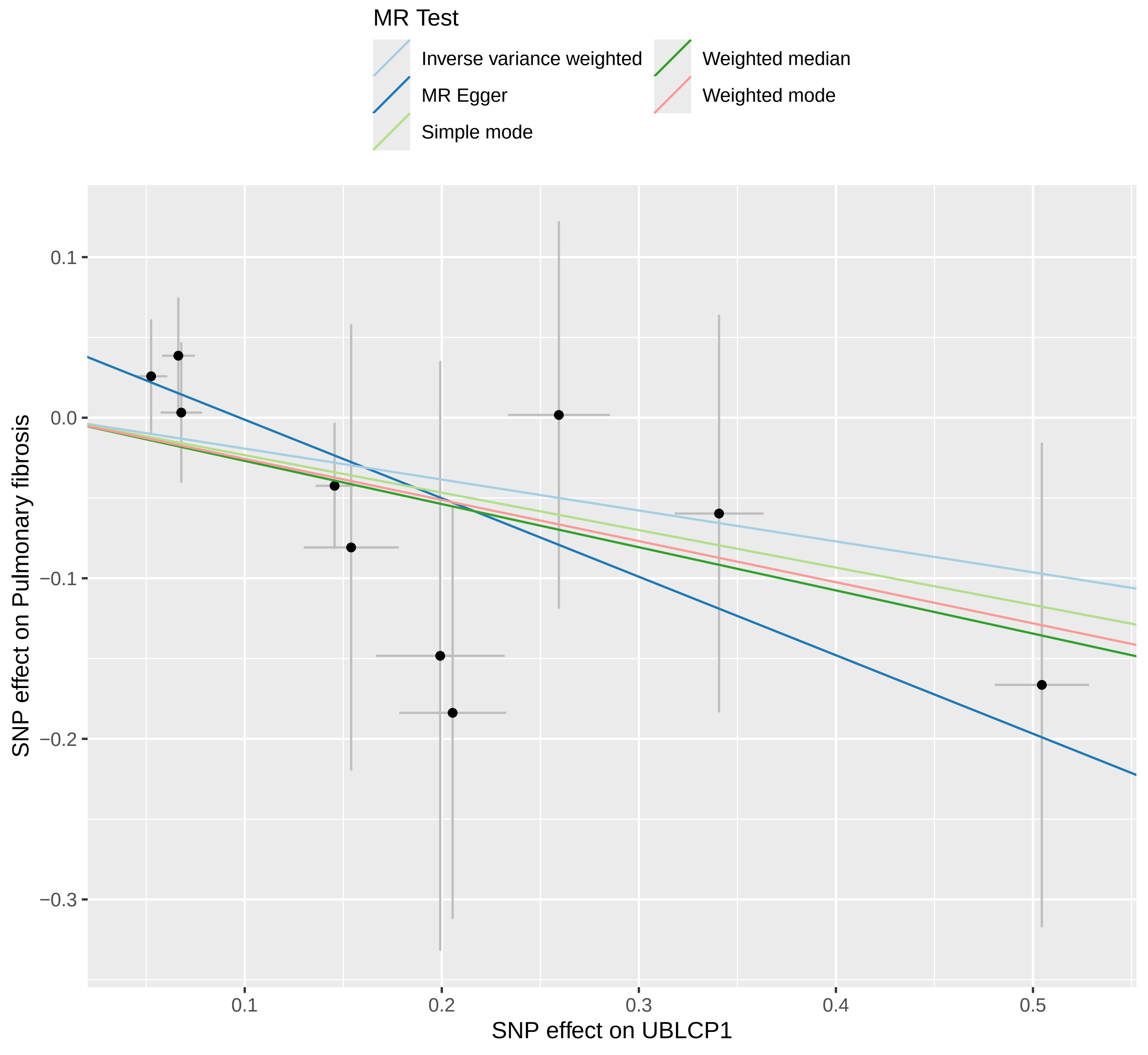
MR Test
Inverse variance weighted
MR Egger
Simple mode
Weighted median
Weighted mode
0.1
0.0
−0.1
−0.2
−0.3
0.1
0.2
0.3
0.4
0.5
SNP effect on Pulmonary fibrosis
SNP effect on UBLCP1

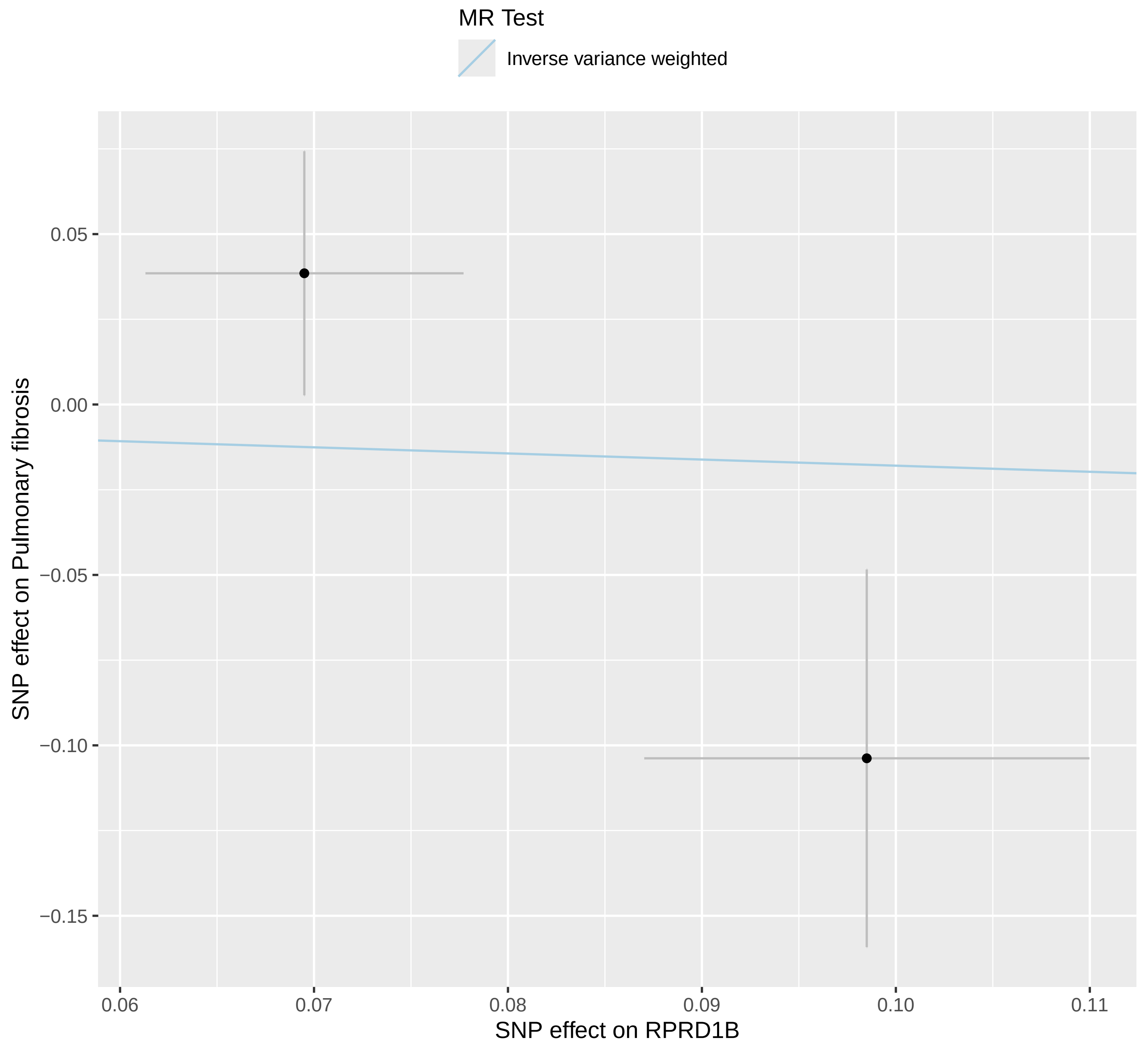

MR Test
Inverse variance weighted
SNP effect on Pulmonary fibrosis
0.05
0.00
−0.05
−0.10
−0.15
0.06
0.07
0.08
0.09
0.10
0.11
SNP effect on RPRD1B

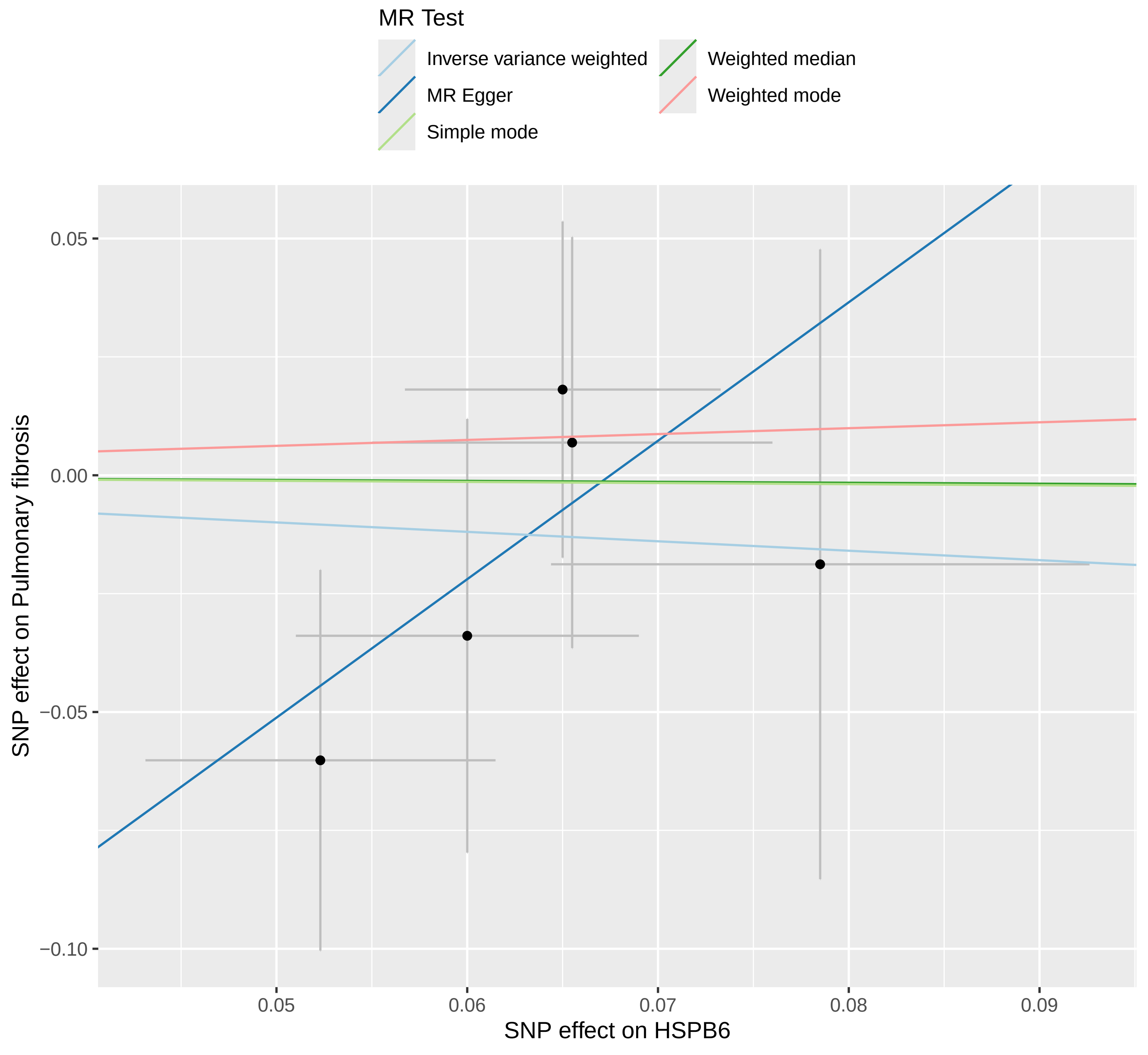

MR Test
Inverse variance weighted
MR Egger
Simple mode
Weighted median
Weighted mode
0.05
0.00
−0.05
−0.10
SNP effect on Pulmonary fibrosis
0.05
0.06
0.07
0.08
0.09
SNP effect on HSPB6

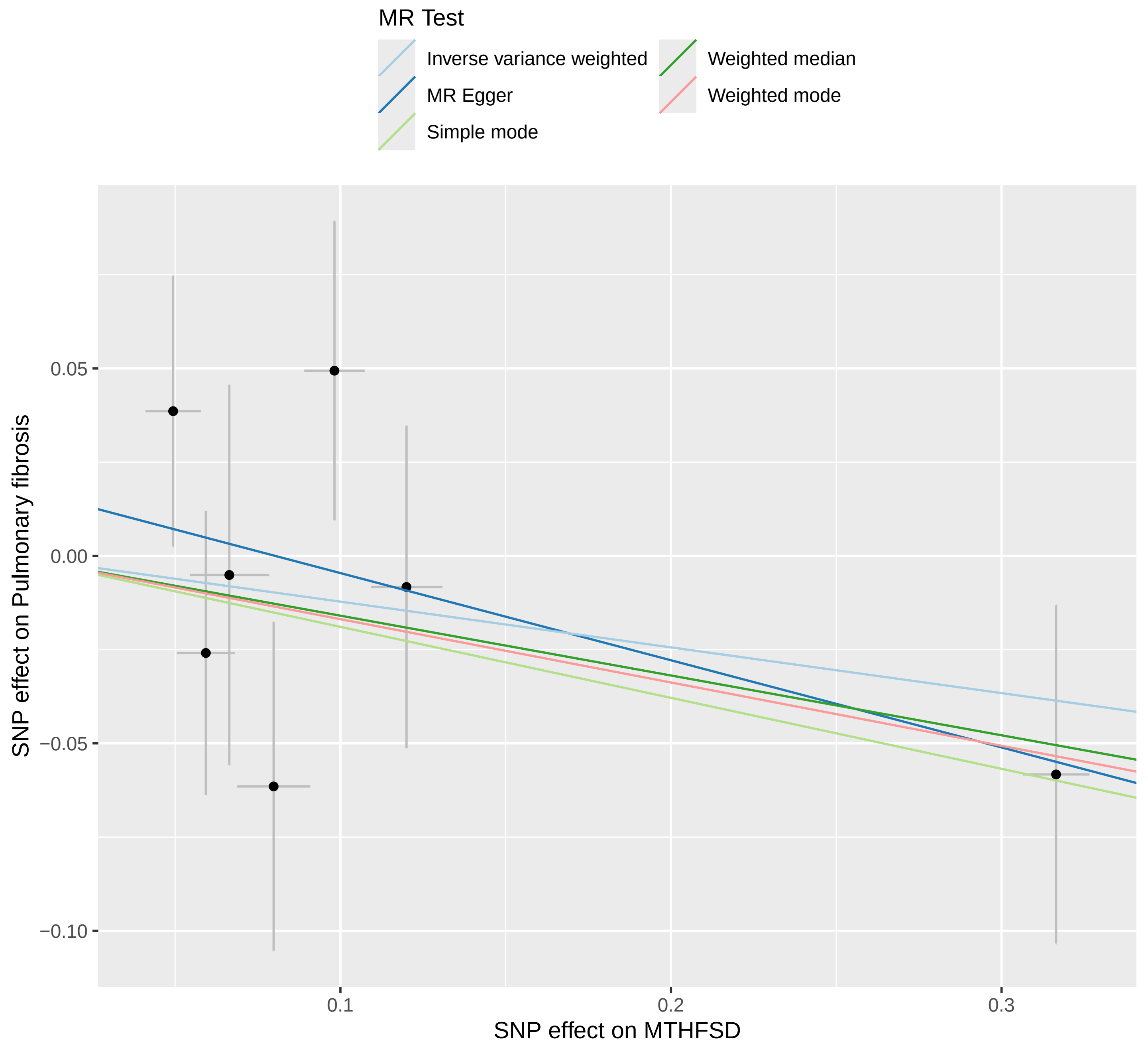
MR Test
Inverse variance weighted
MR Egger
Simple mode
Weighted median
Weighted mode
0.05
0.00
−0.05
−0.10
SNP effect on Pulmonary fibrosis
0.1
0.2
0.3
SNP effect on MTHFSD

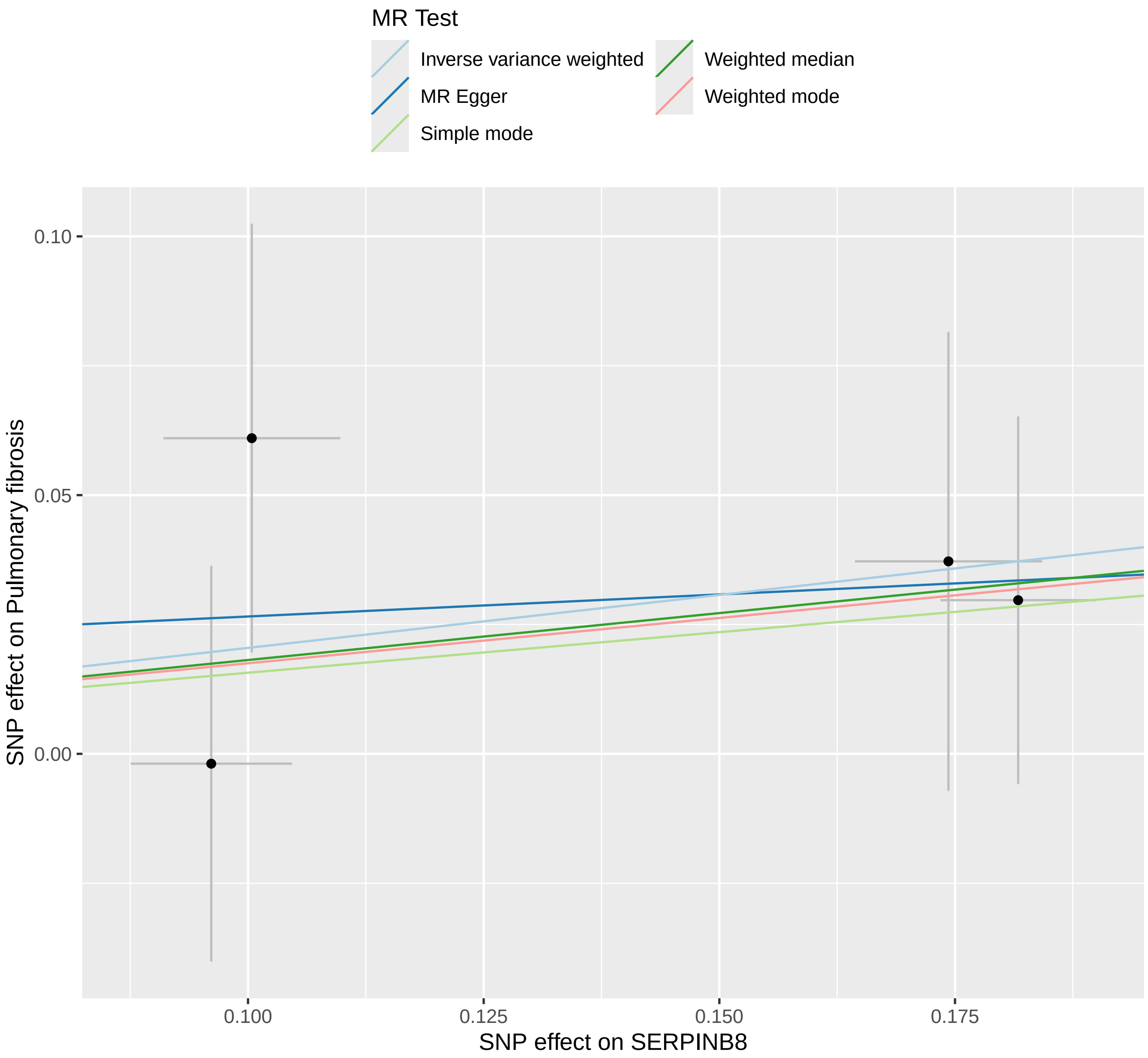

MR Test
Inverse variance weighted
MR Egger
Simple mode
Weighted median
Weighted mode
0.10
0.05
0.00
SNP effect on Pulmonary fibrosis
0.100
0.125
0.150
0.175
SNP effect on SERPINB8

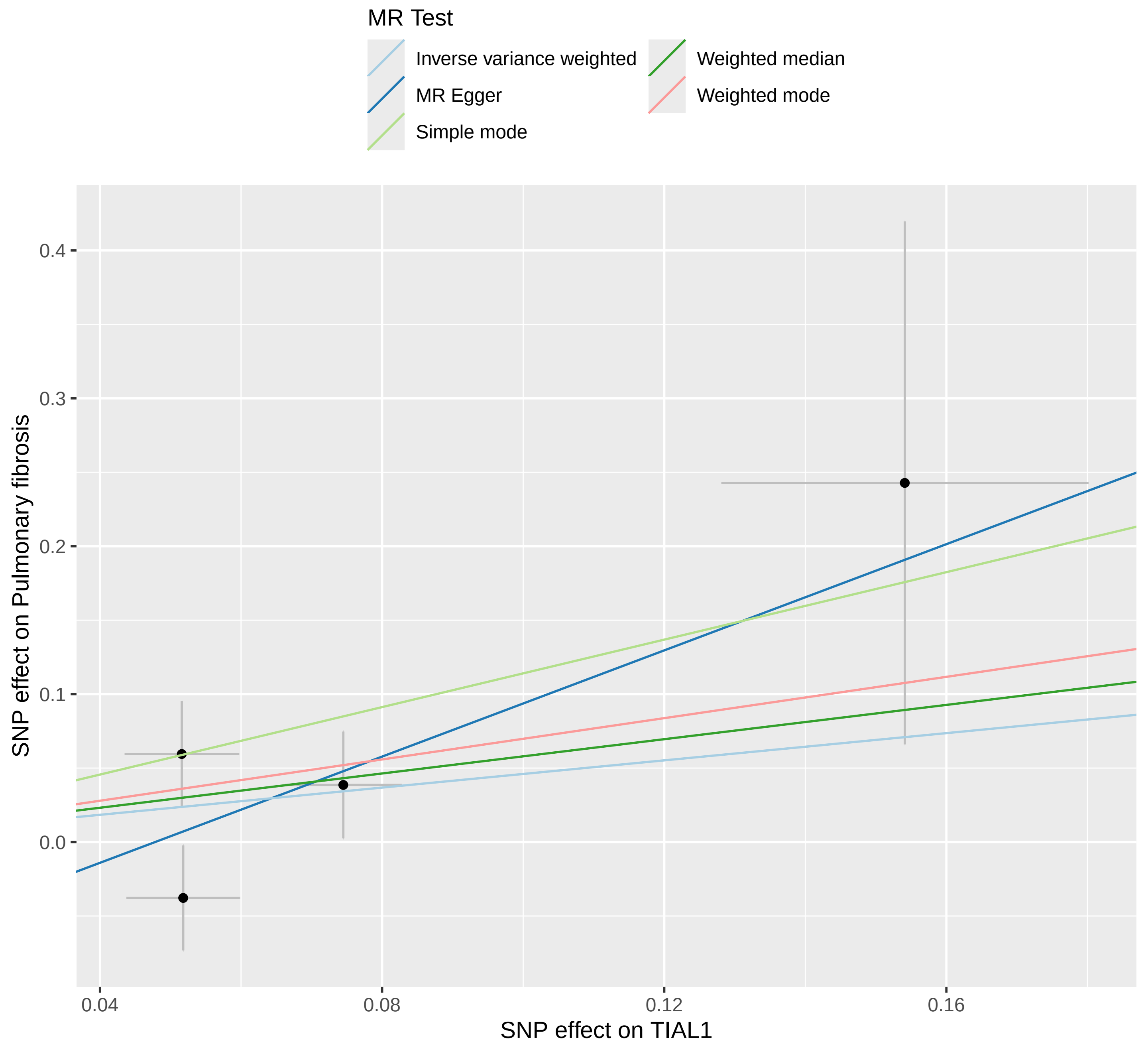

MR Test
Inverse variance weighted
MR Egger
Simple mode
Weighted median
Weighted mode
0.4
0.3
0.2
0.1
0.0
SNP effect on Pulmonary fibrosis
0.04
0.08
0.12
0.16
SNP effect on TIAL1

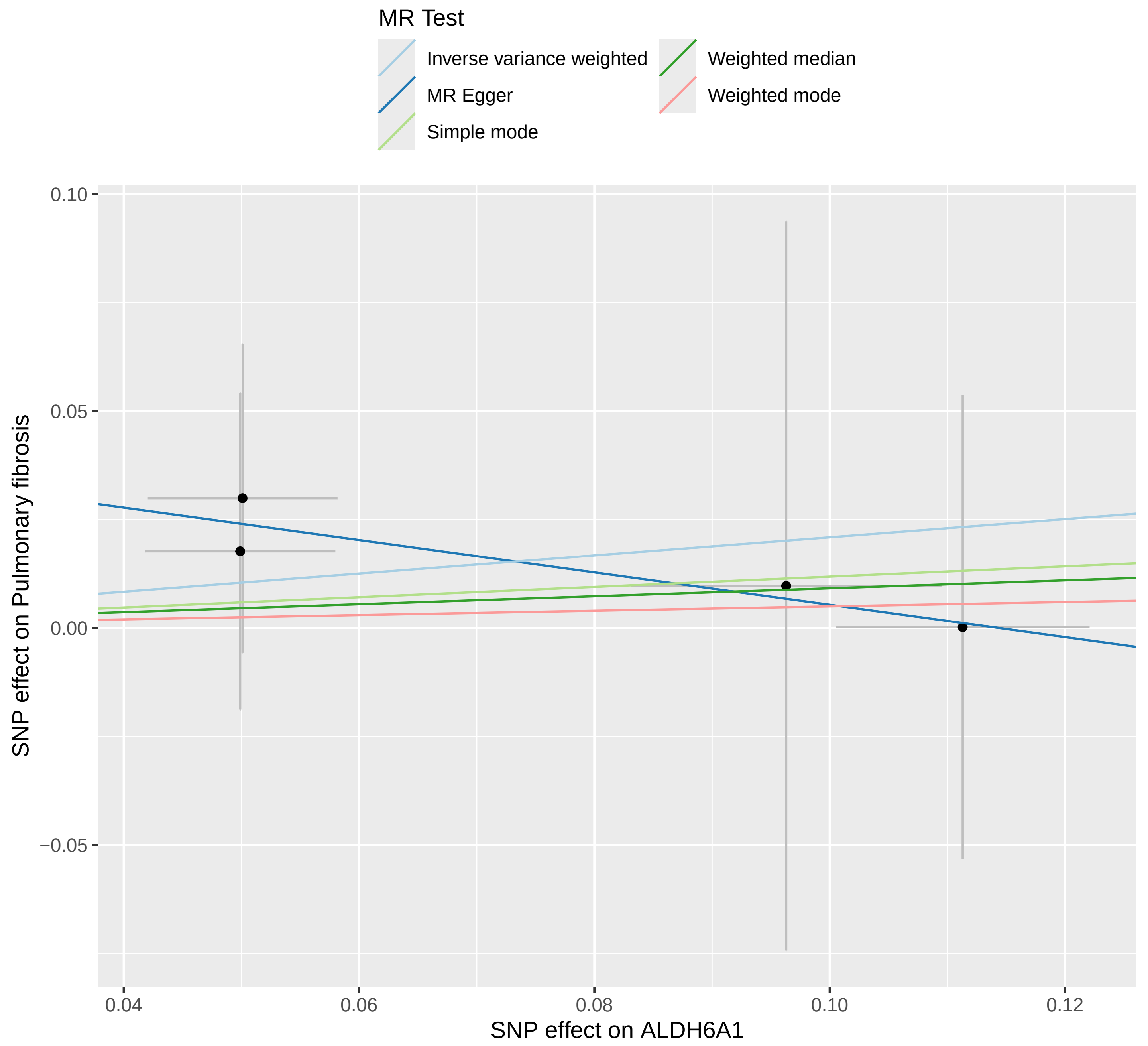
MR Test
Inverse variance weighted
MR Egger
Simple mode
Weighted median
Weighted mode
0.10
0.05
0.00
−0.05
0.04
0.06
0.08
0.10
0.12
SNP effect on Pulmonary fibrosis
SNP effect on ALDH6A1

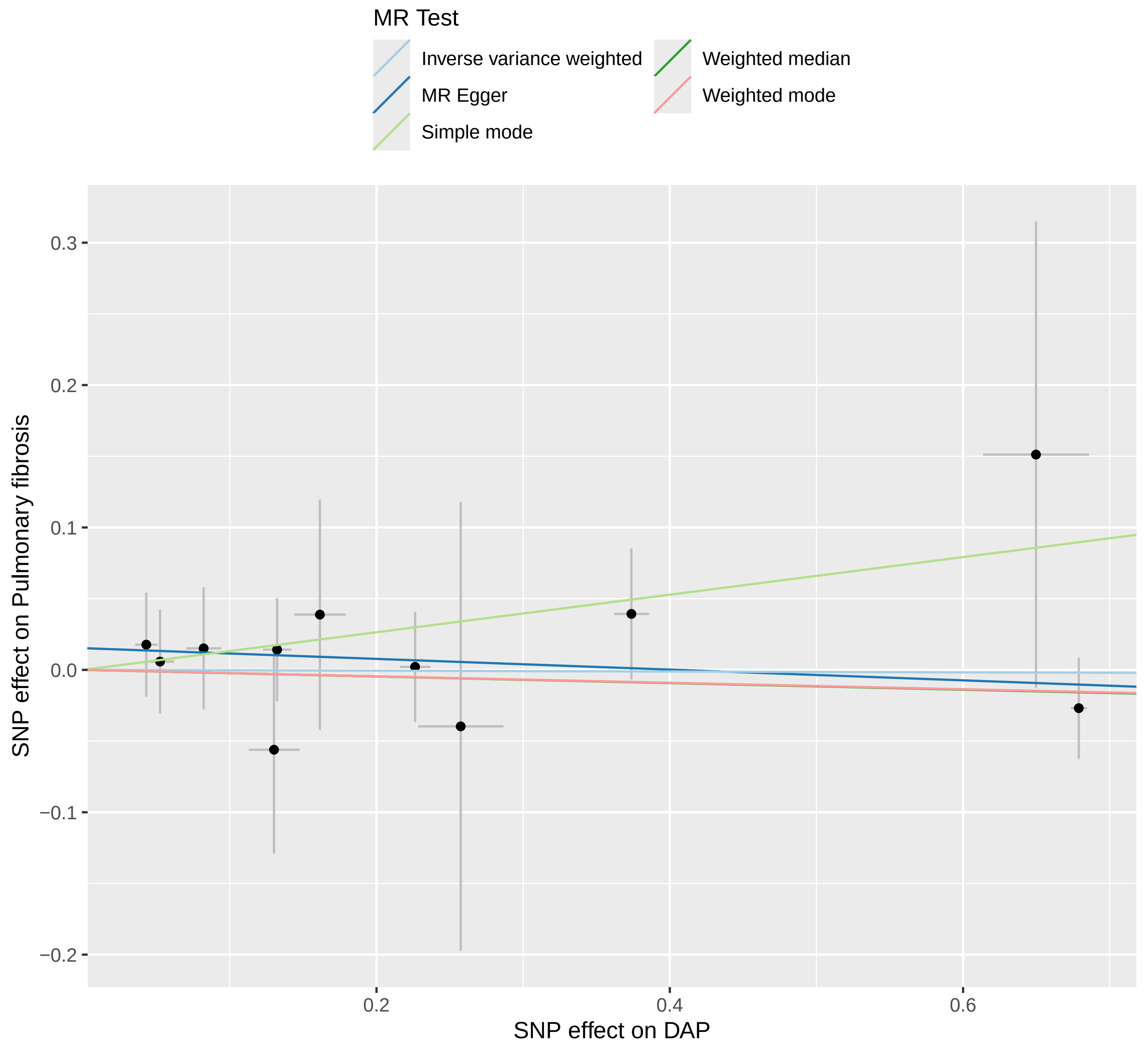

MR Test
Inverse variance weighted
MR Egger
Simple mode
Weighted median
Weighted mode
0.3
0.2
0.1
0.0
−0.1
−0.2
0.2
0.4
0.6
SNP effect on Pulmonary fibrosis
SNP effect on DAP

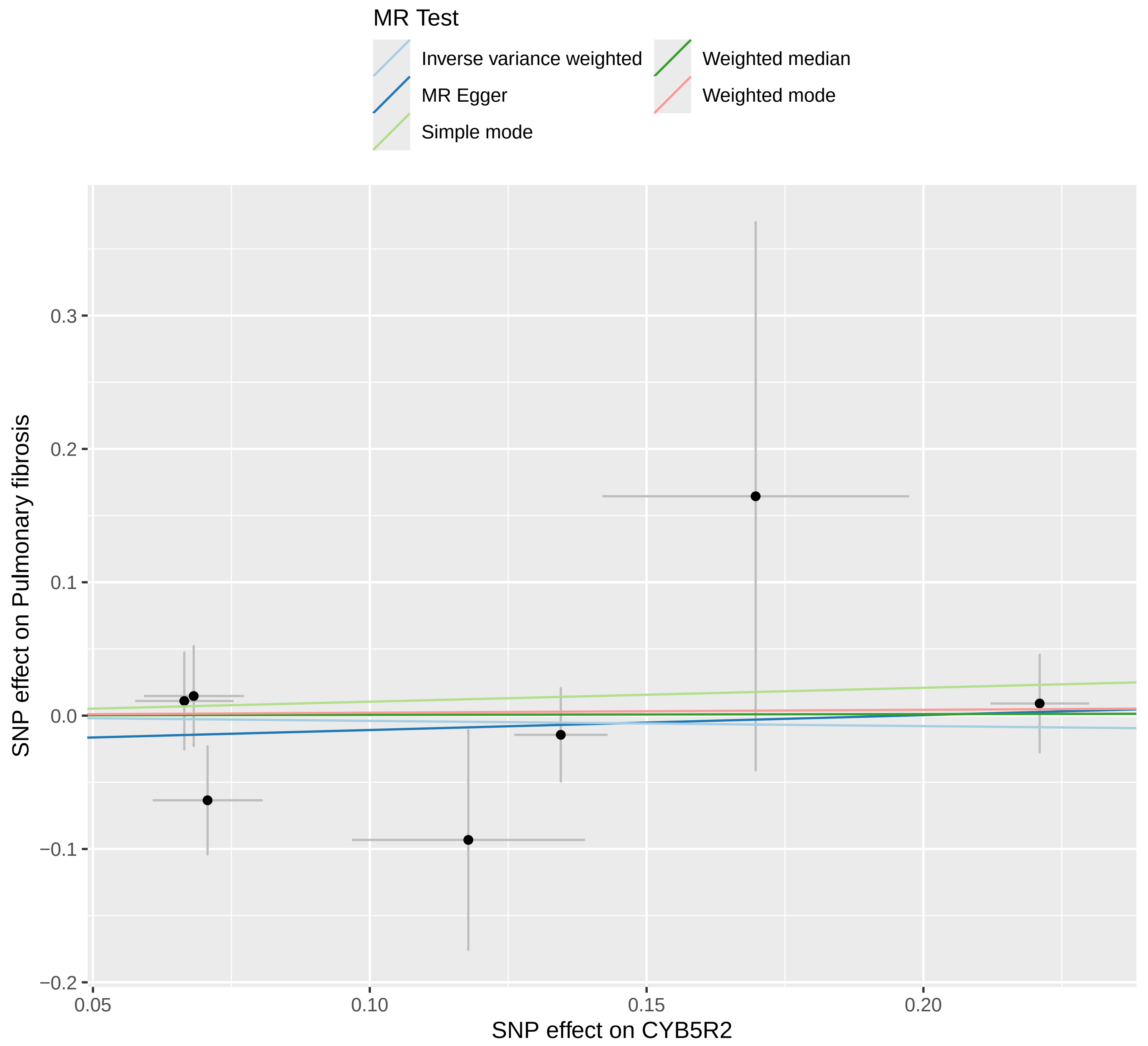

MR Test
Inverse variance weighted
MR Egger
Simple mode
Weighted median
Weighted mode
0.3
0.2
0.1
0.0
−0.1
−0.2
0.05
0.10
0.15
0.20
SNP effect on Pulmonary fibrosis
SNP effect on CYB5R2

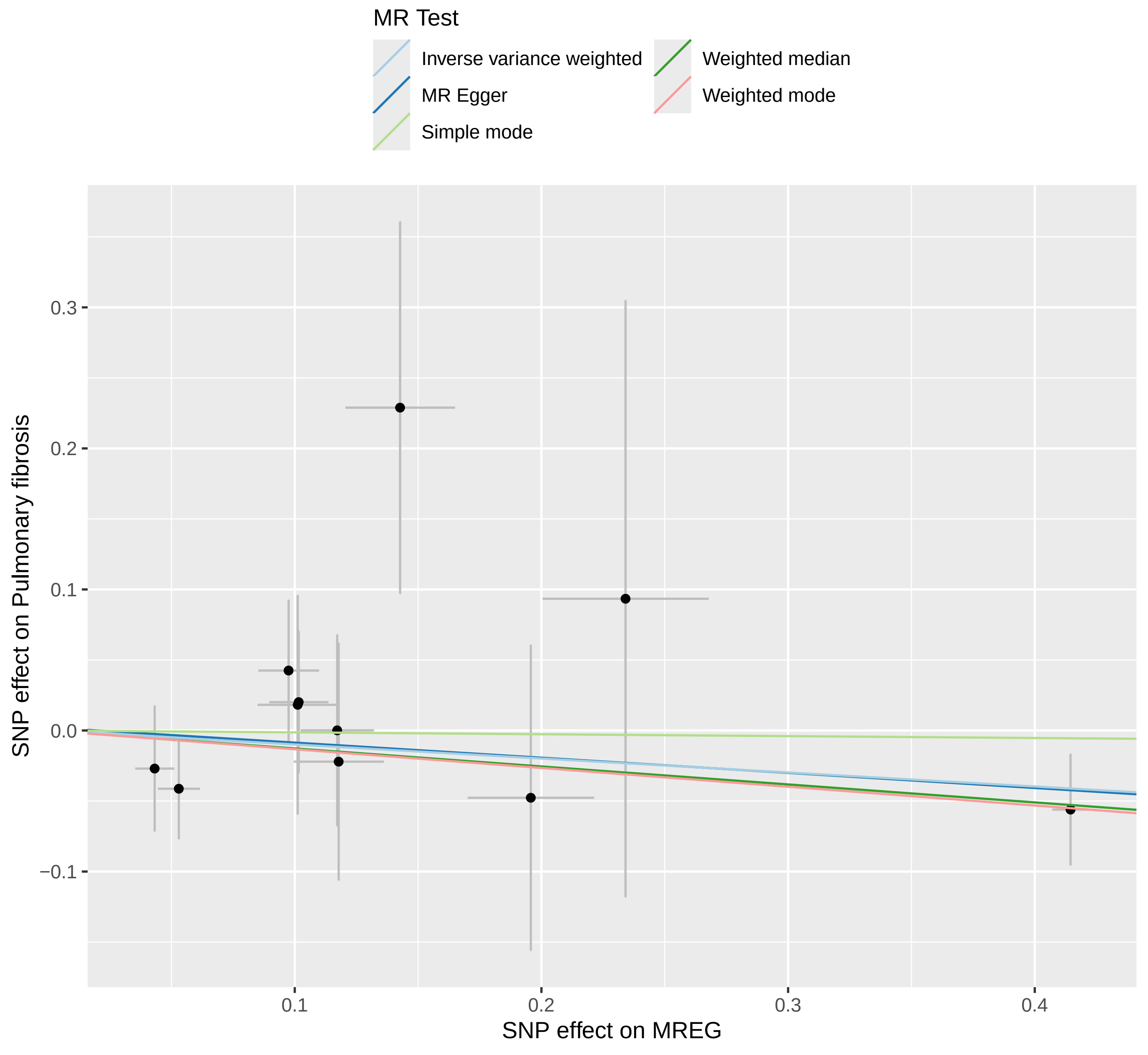
MR Test
Inverse variance weighted
MR Egger
Simple mode
Weighted median
Weighted mode
SNP effect on Pulmonary fibrosis
0.3
0.2
0.1
0.0
−0.1
0.1
0.2
0.3
0.4
SNP effect on MREG

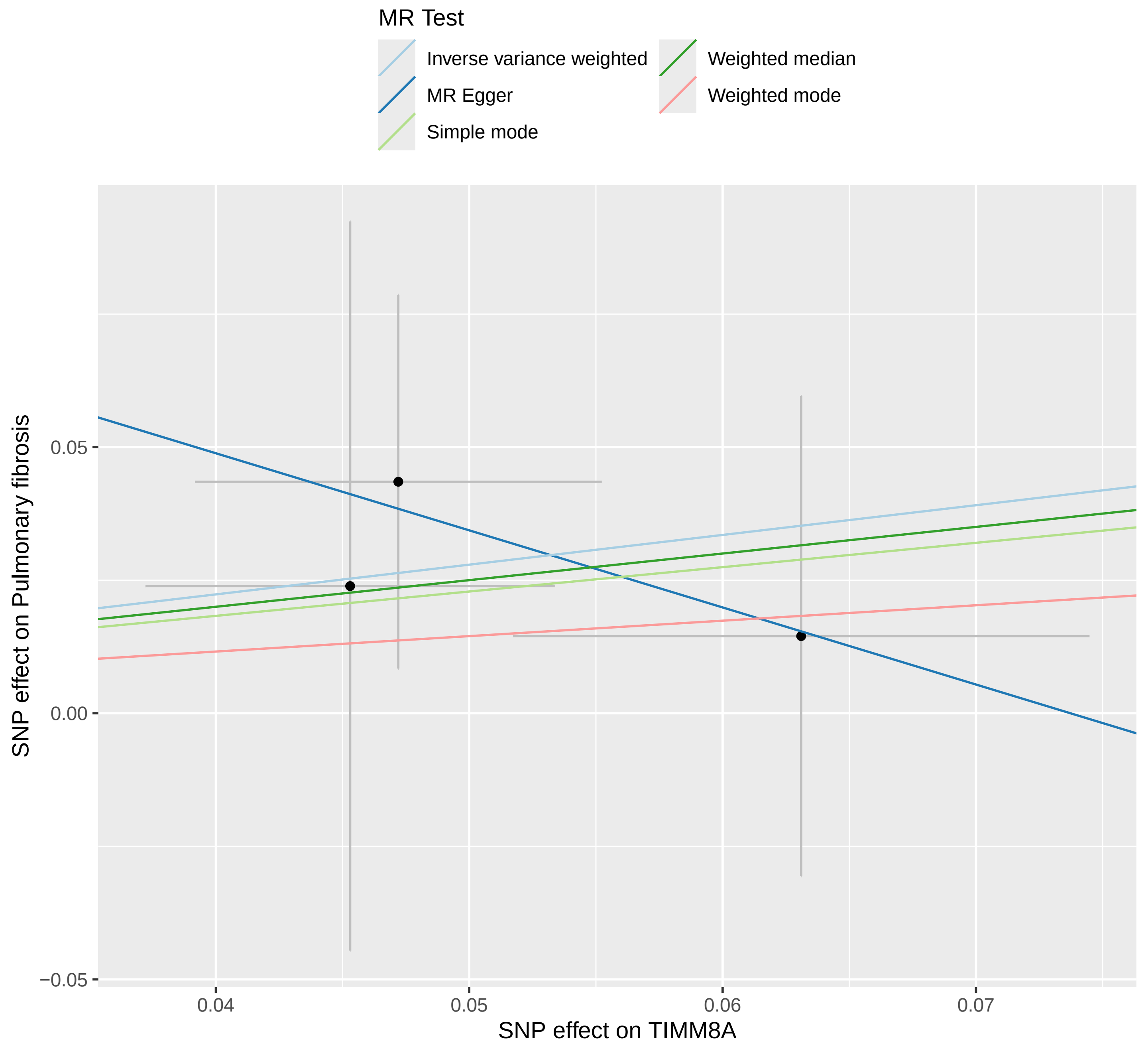

MR Test
Inverse variance weighted
MR Egger
Simple mode
Weighted median
Weighted mode
SNP effect on Pulmonary fibrosis
0.05
0.00
−0.05
0.04
0.05
0.06
0.07
SNP effect on TIMM8A

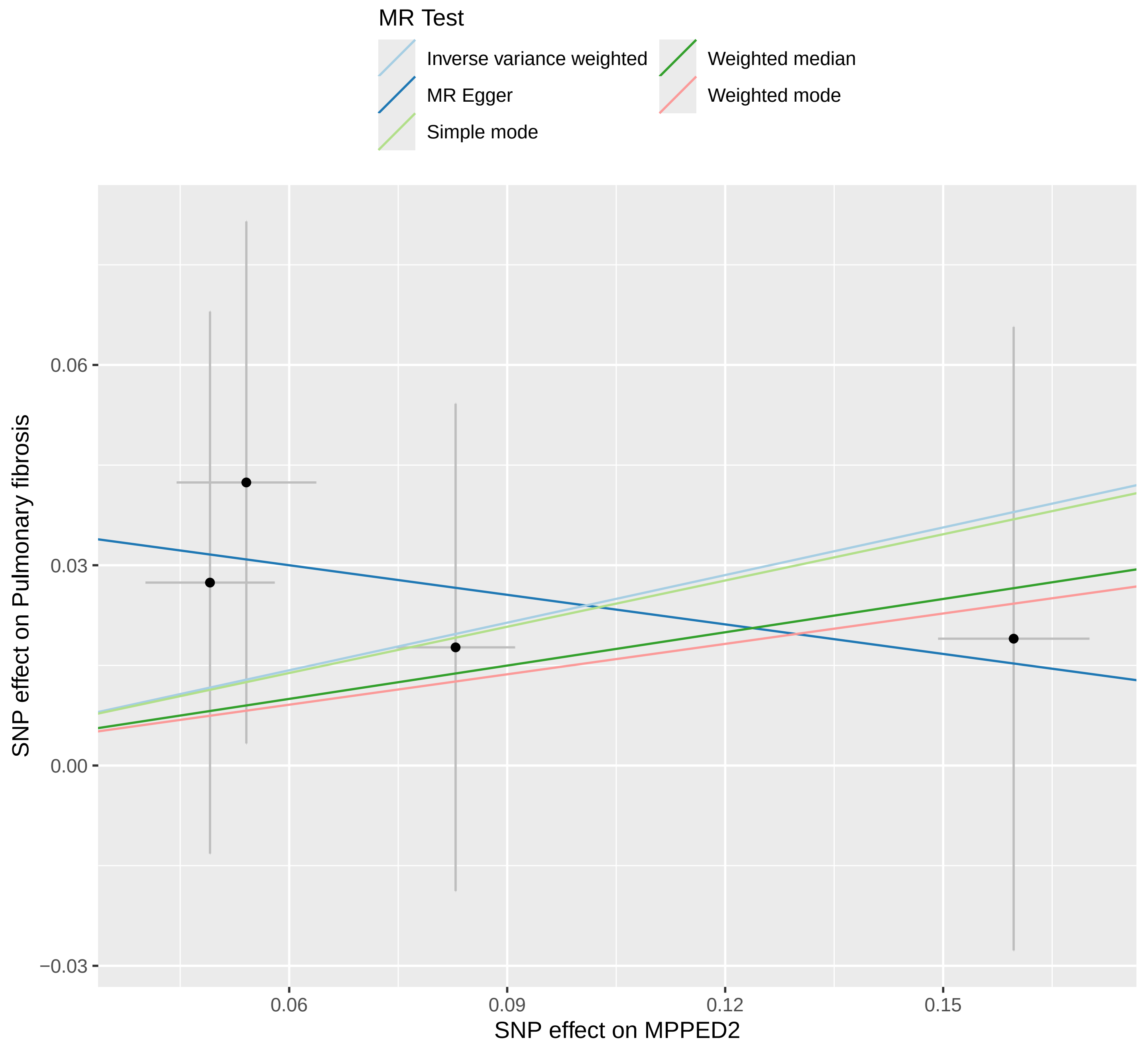
MR Test
Inverse variance weighted
MR Egger
Simple mode
Weighted median
Weighted mode
SNP effect on Pulmonary fibrosis
0.06
0.03
0.00
−0.03
0.06
0.09
0.12
0.15
SNP effect on MPPED2

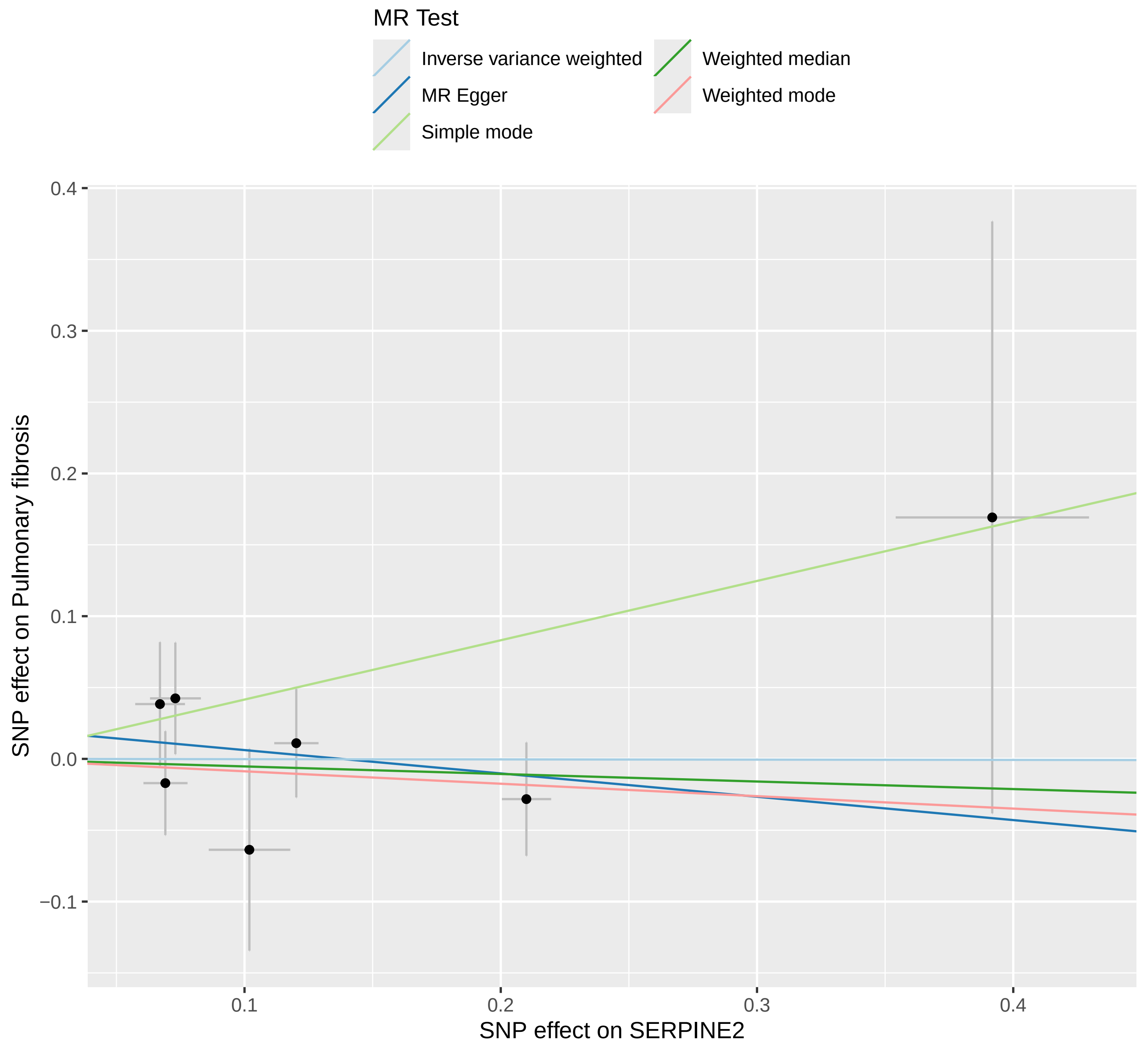

MR Test
Inverse variance weighted
MR Egger
Simple mode
Weighted median
Weighted mode
SNP effect on Pulmonary fibrosis
0.4
0.3
0.2
0.1
0.0
−0.1
0.1
0.2
0.3
0.4
SNP effect on SERPINE2

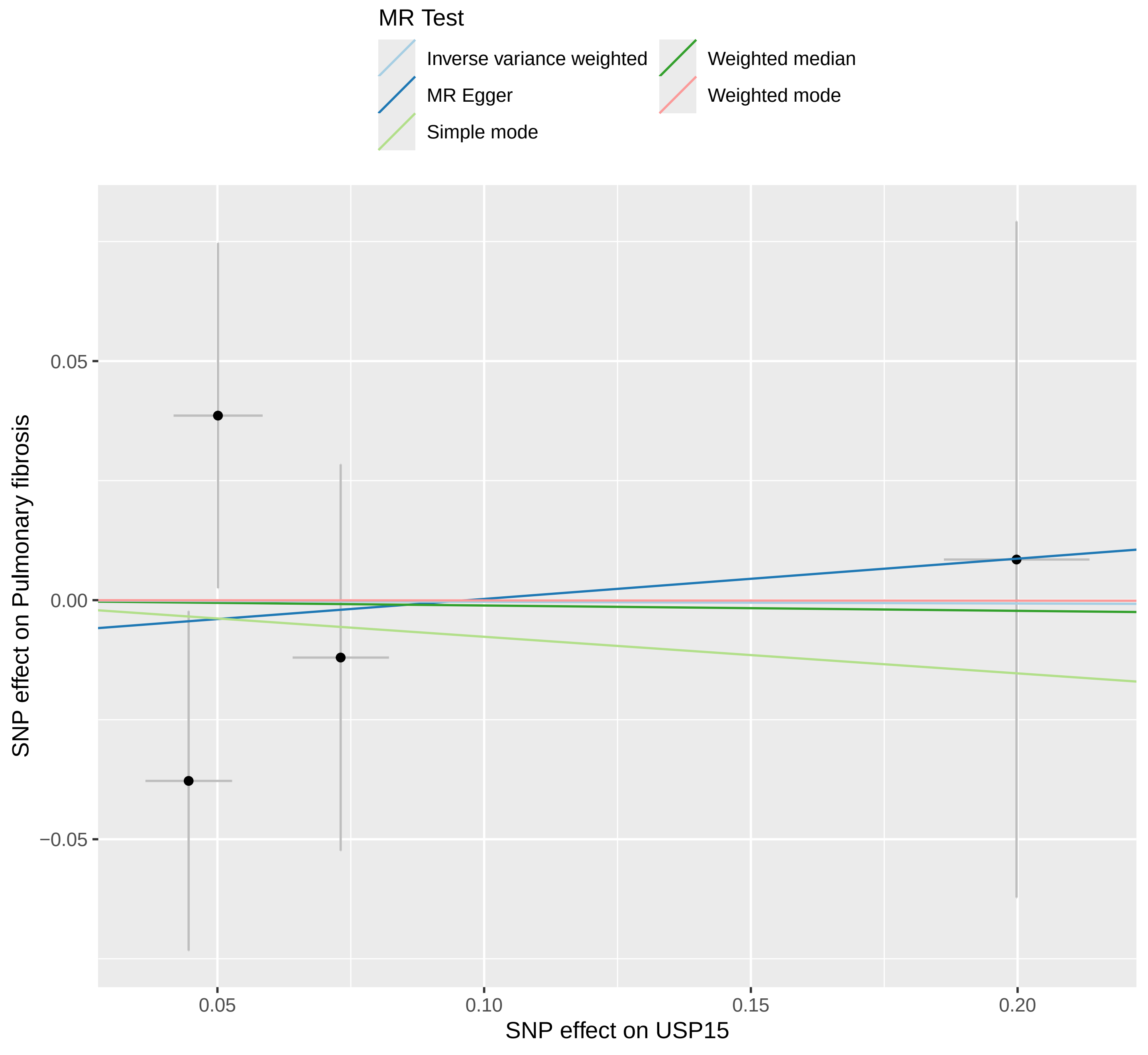

MR Test
Inverse variance weighted
MR Egger
Simple mode
Weighted median
Weighted mode
0.05
0.00
−0.05
SNP effect on Pulmonary fibrosis
0.05
0.10
0.15
0.20
SNP effect on USP15

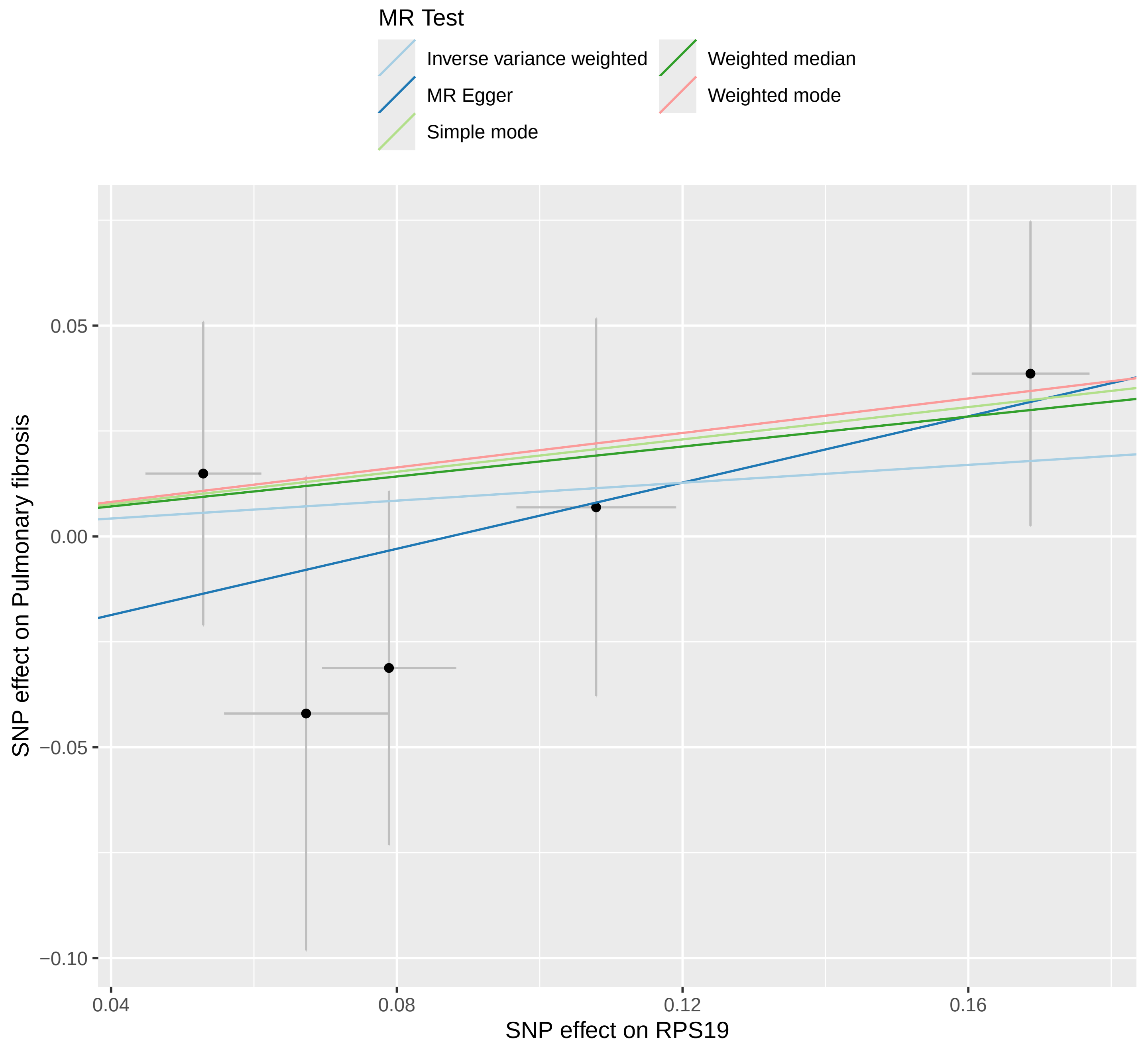

MR Test
Inverse variance weighted
MR Egger
Simple mode
Weighted median
Weighted mode
0.05
0.00
−0.05
−0.10
0.04
0.08
0.12
0.16
SNP effect on Pulmonary fibrosis
SNP effect on RPS19

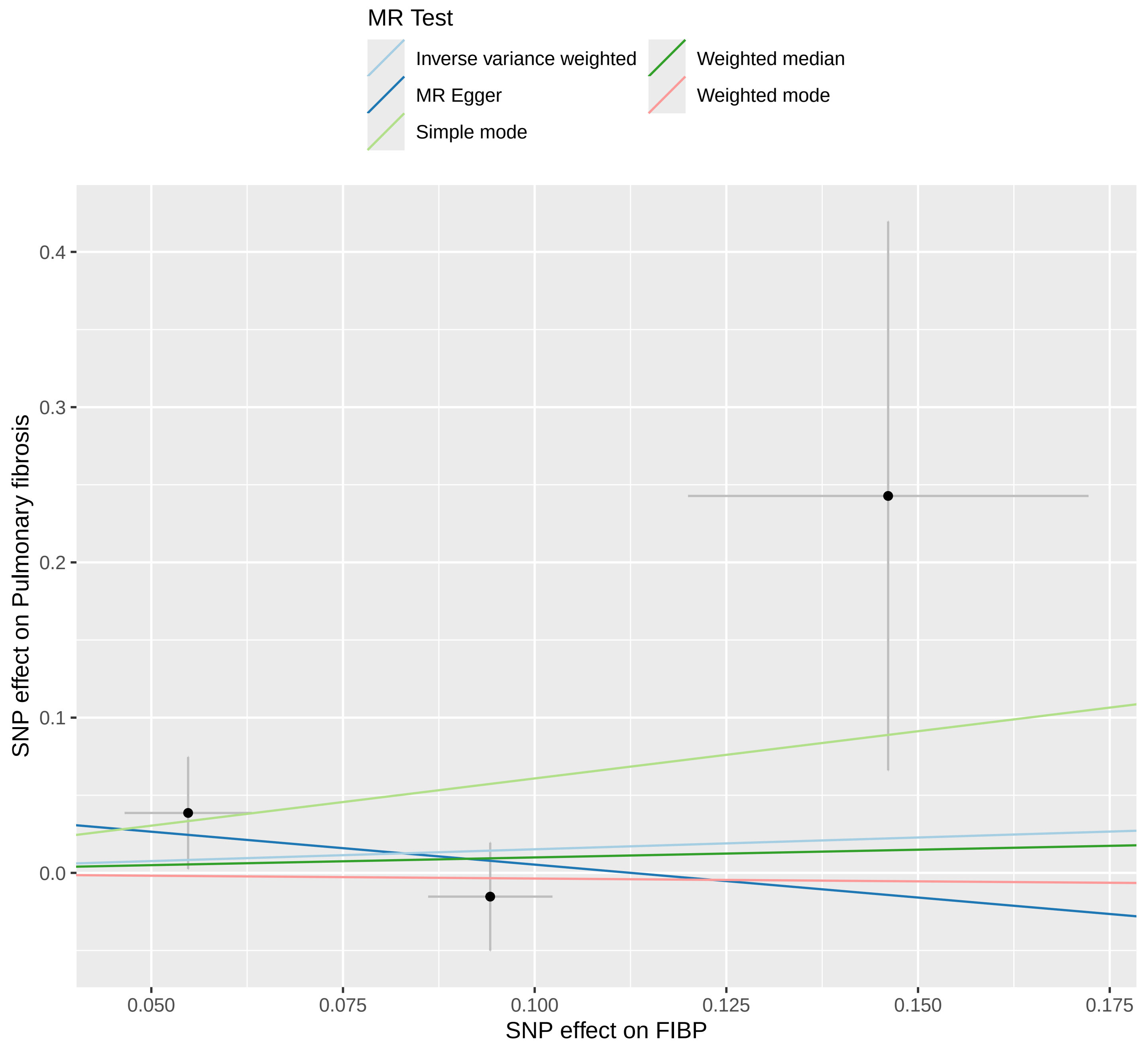
MR Test
Inverse variance weighted
MR Egger
Simple mode
Weighted median
Weighted mode
0.4
0.3
0.2
0.1
0.0
0.050
0.075
0.100
0.125
0.150
0.175
SNP effect on Pulmonary fibrosis
SNP effect on FIBP

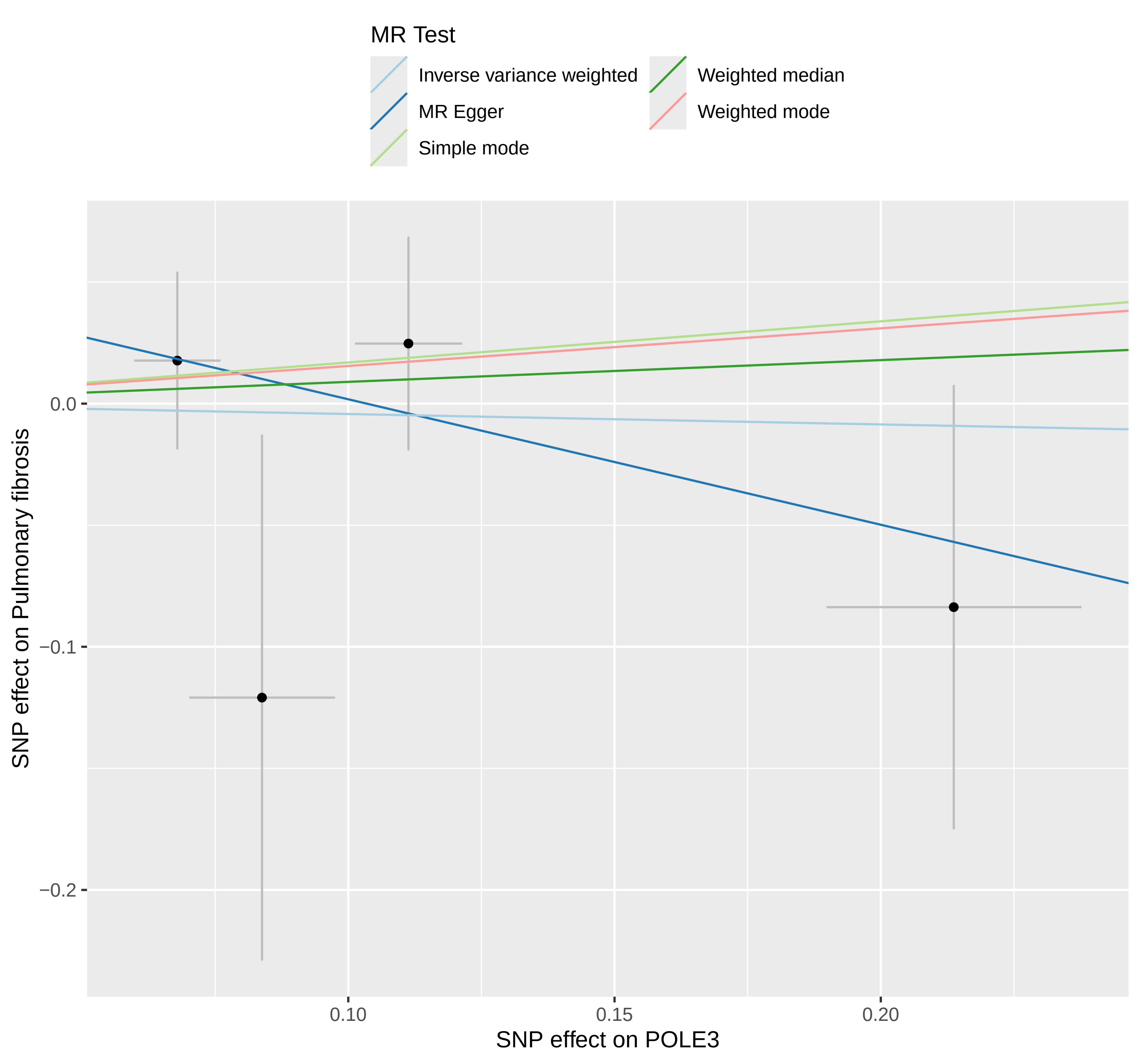
MR Test
Inverse variance weighted
MR Egger
Simple mode
Weighted median
Weighted mode
0.0
−0.1
−0.2
0.10
0.15
0.20
SNP effect on Pulmonary fibrosis
SNP effect on POLE3

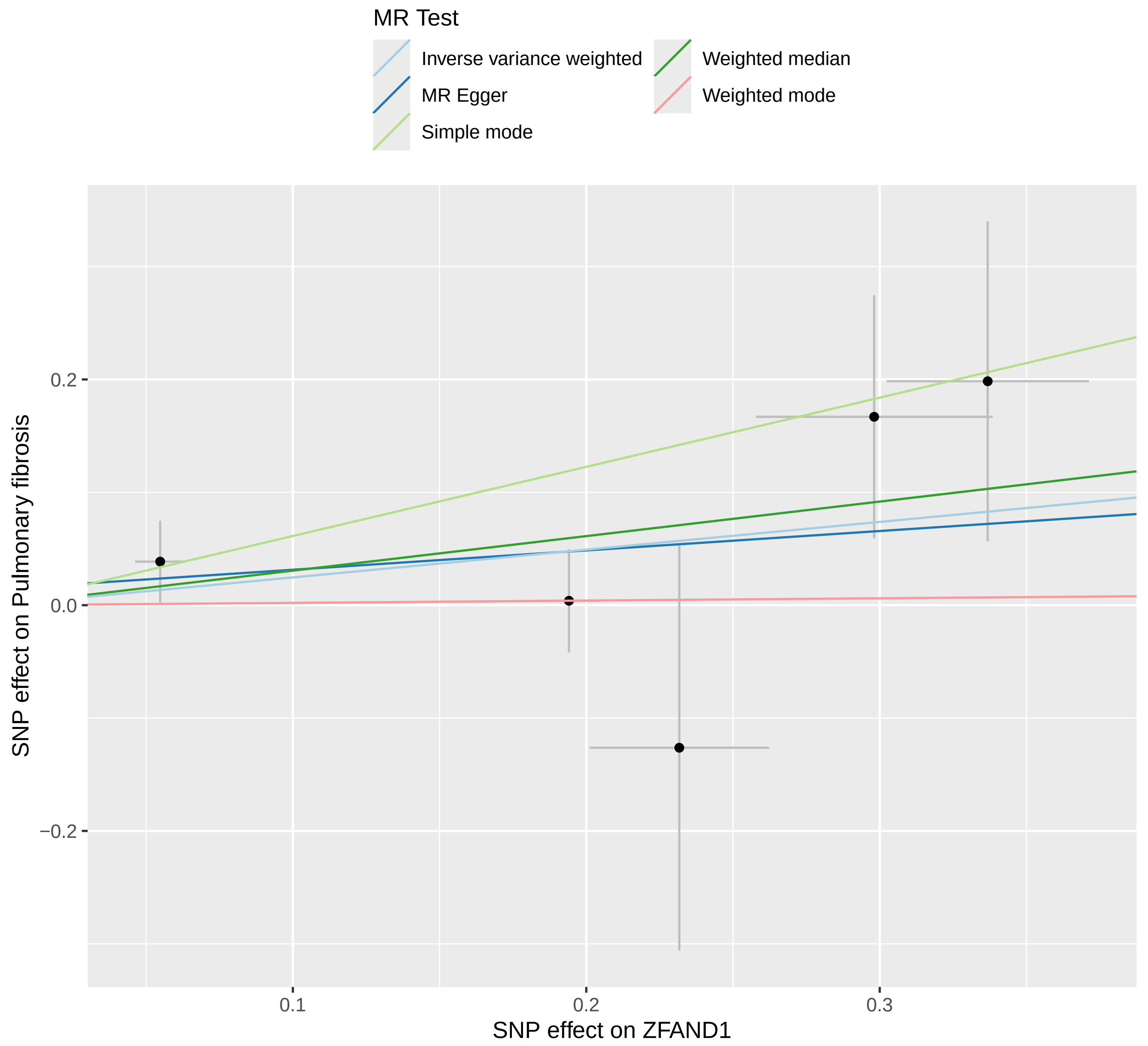

MR Test
Inverse variance weighted
MR Egger
Simple mode
Weighted median
Weighted mode
0.2
0.0
−0.2
SNP effect on Pulmonary fibrosis
0.1
0.2
0.3
SNP effect on ZFAND1

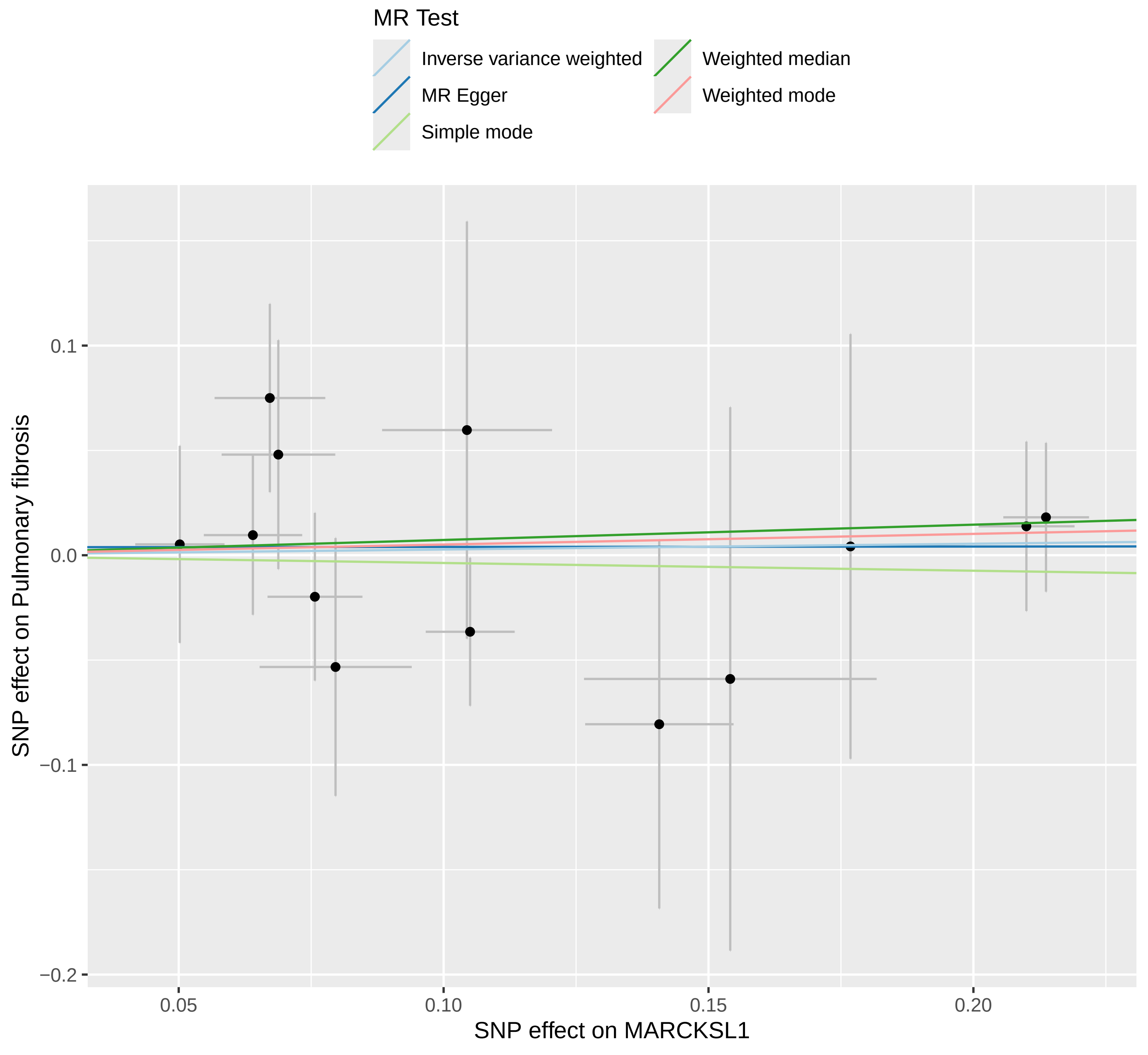

MR Test
Inverse variance weighted
MR Egger
Simple mode
Weighted median
Weighted mode
SNP effect on Pulmonary fibrosis
0.1
0.0
−0.1
−0.2
0.05
0.10
0.15
0.20
SNP effect on MARCKSL1

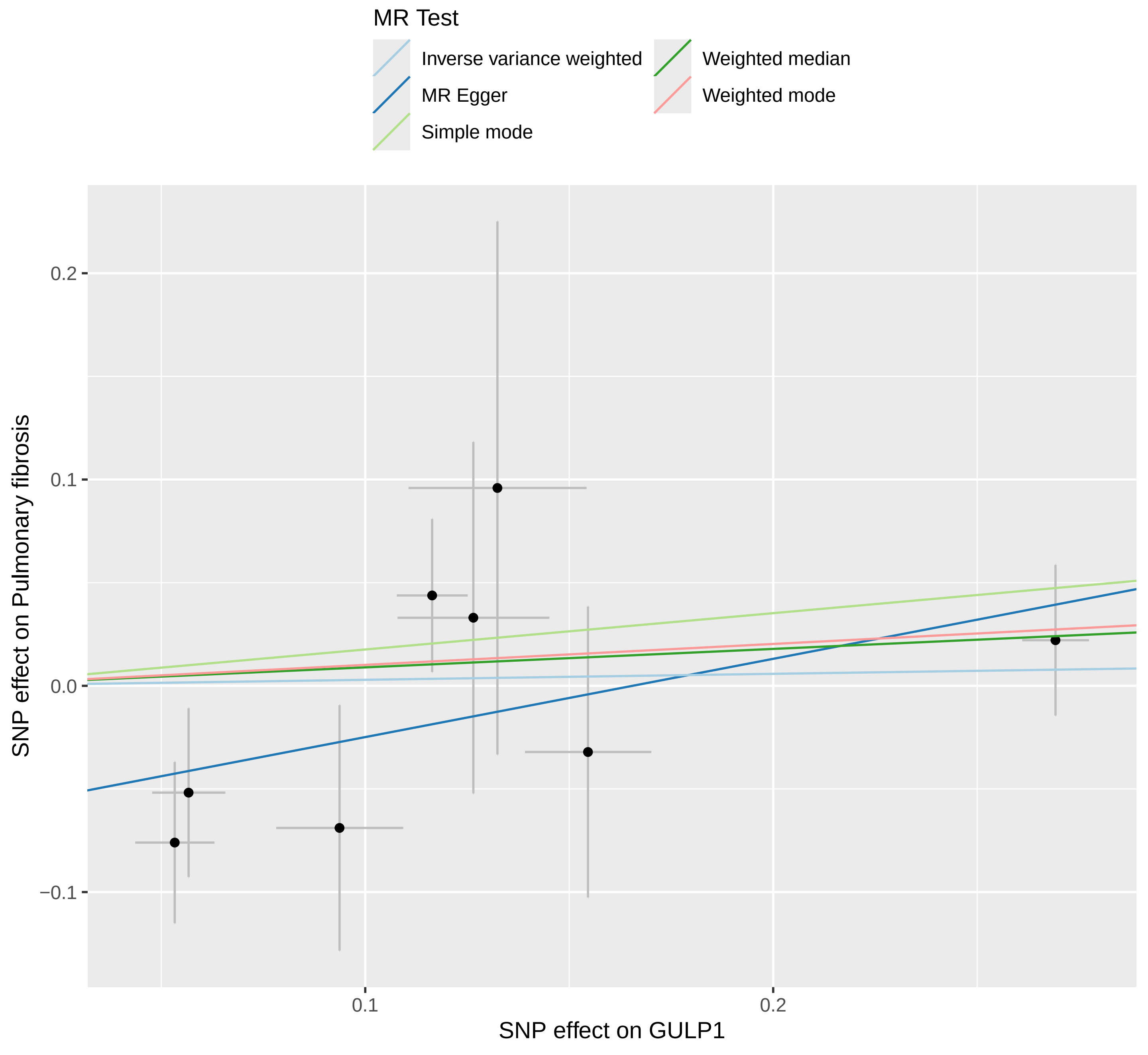

MR Test
Inverse variance weighted
MR Egger
Simple mode
Weighted median
Weighted mode
0.2
0.1
0.0
−0.1
0.1
0.2
SNP effect on Pulmonary fibrosis
SNP effect on GULP1

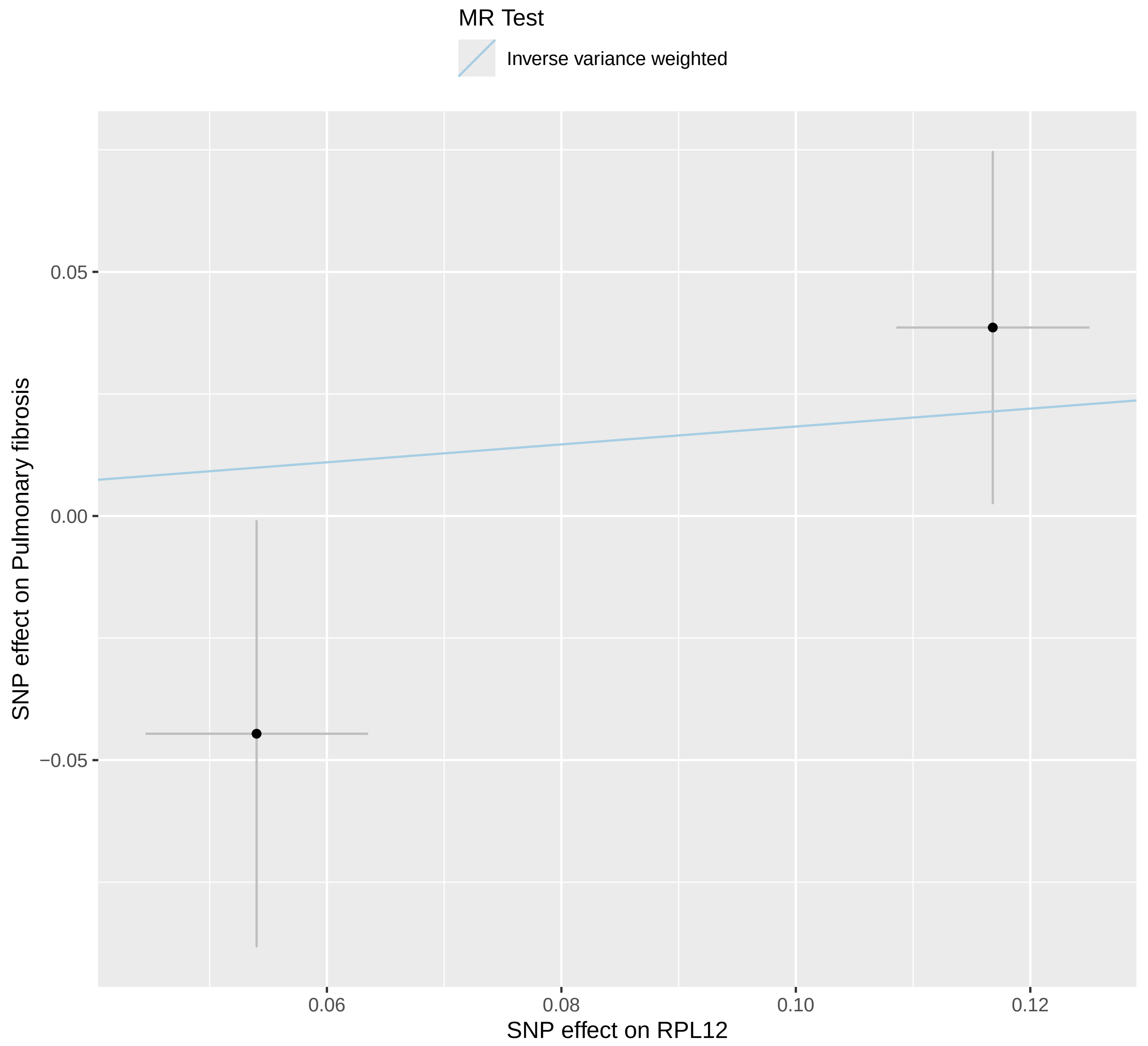
MR Test
Inverse variance weighted
0.05
0.00
−0.05
0.06
0.08
0.10
0.12
SNP effect on RPL12
SNP effect on Pulmonary fibrosis

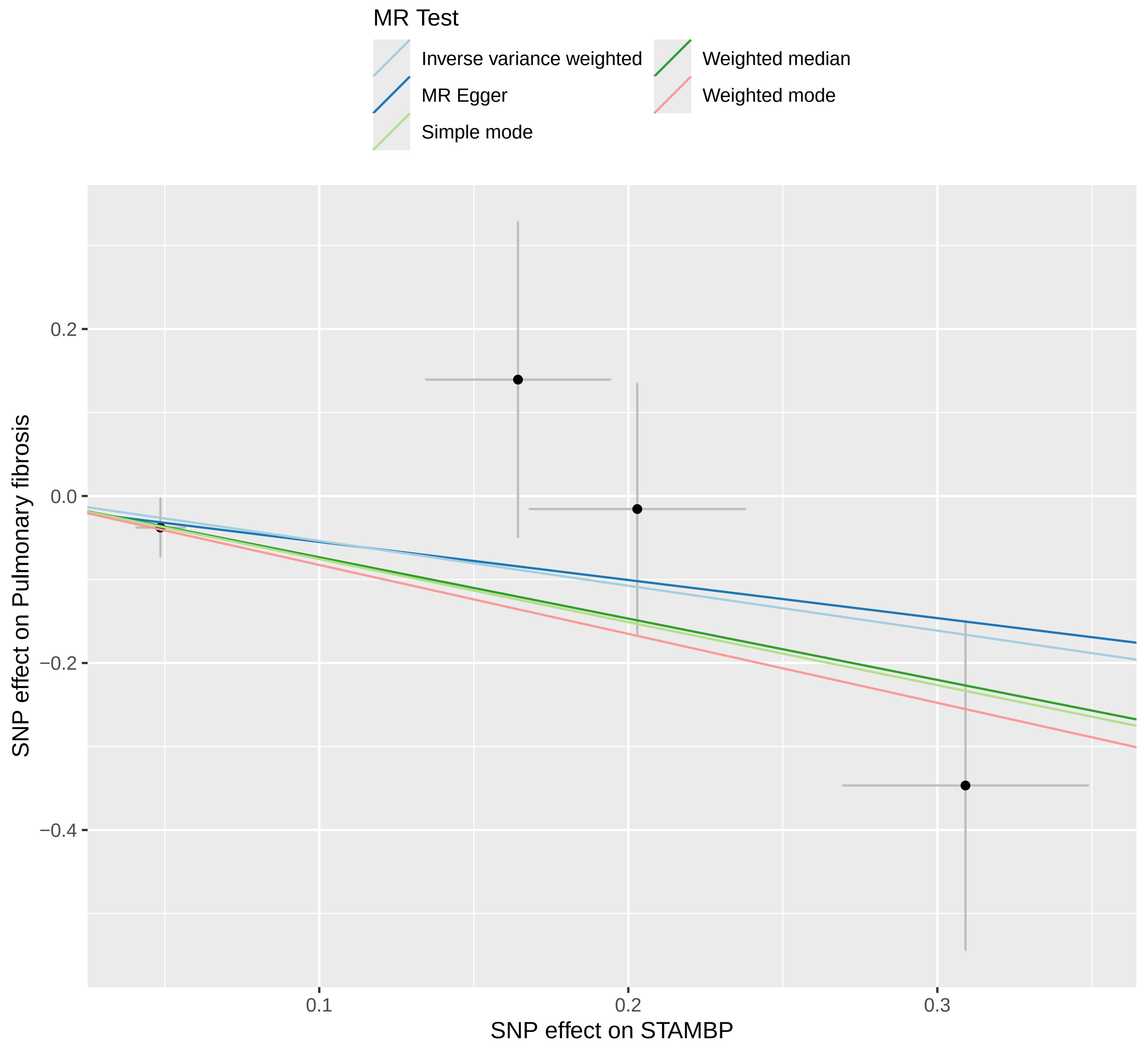

MR Test
Inverse variance weighted
MR Egger
Simple mode
Weighted median
Weighted mode
0.2
0.0
−0.2
−0.4
0.1
0.2
0.3
SNP effect on Pulmonary fibrosis
SNP effect on STAMBP

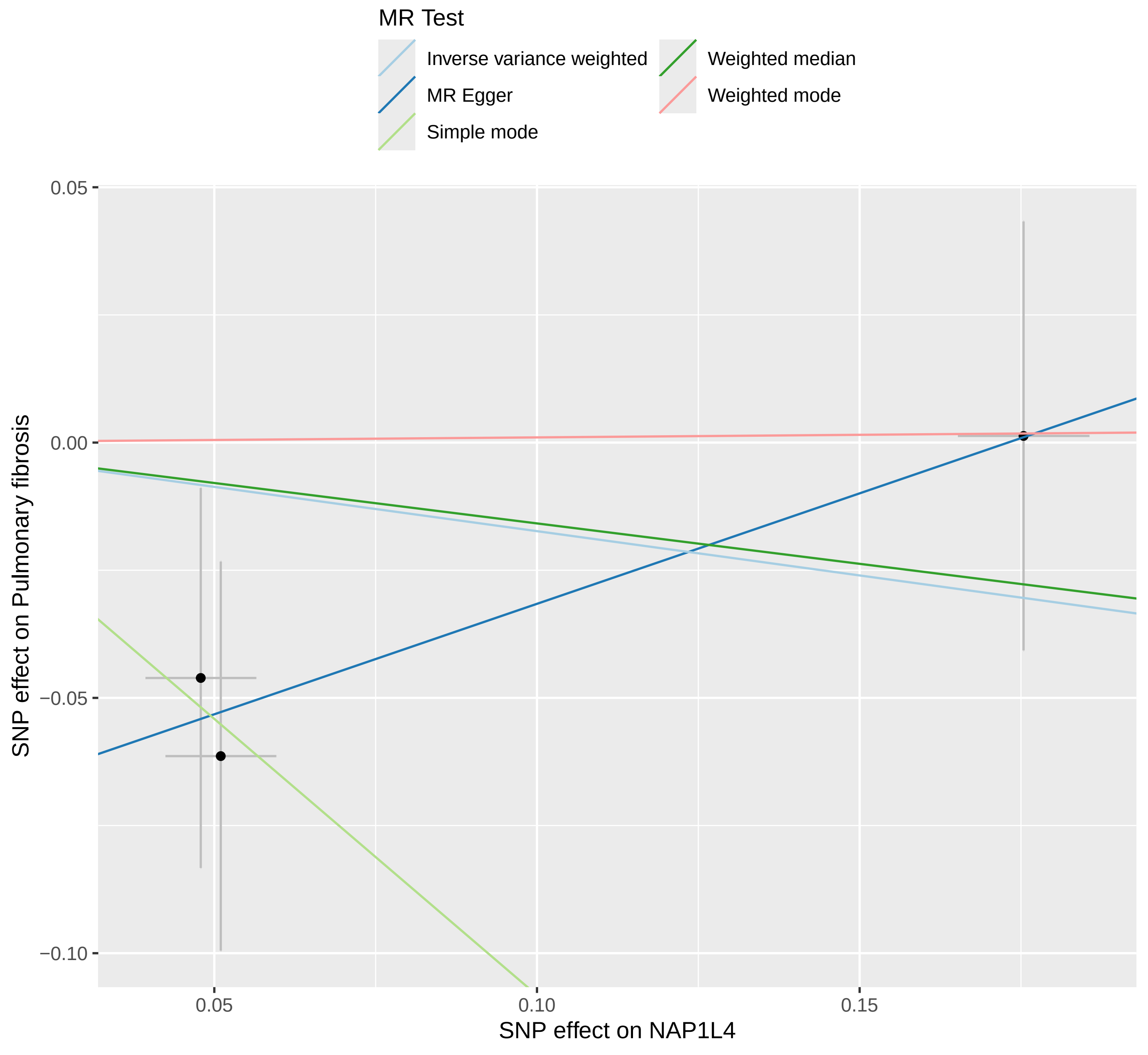
MR Test
Inverse variance weighted
MR Egger
Simple mode
Weighted median
Weighted mode
0.05
0.00
−0.05
−0.10
0.05
0.10
0.15
SNP effect on Pulmonary fibrosis
SNP effect on NAP1L4

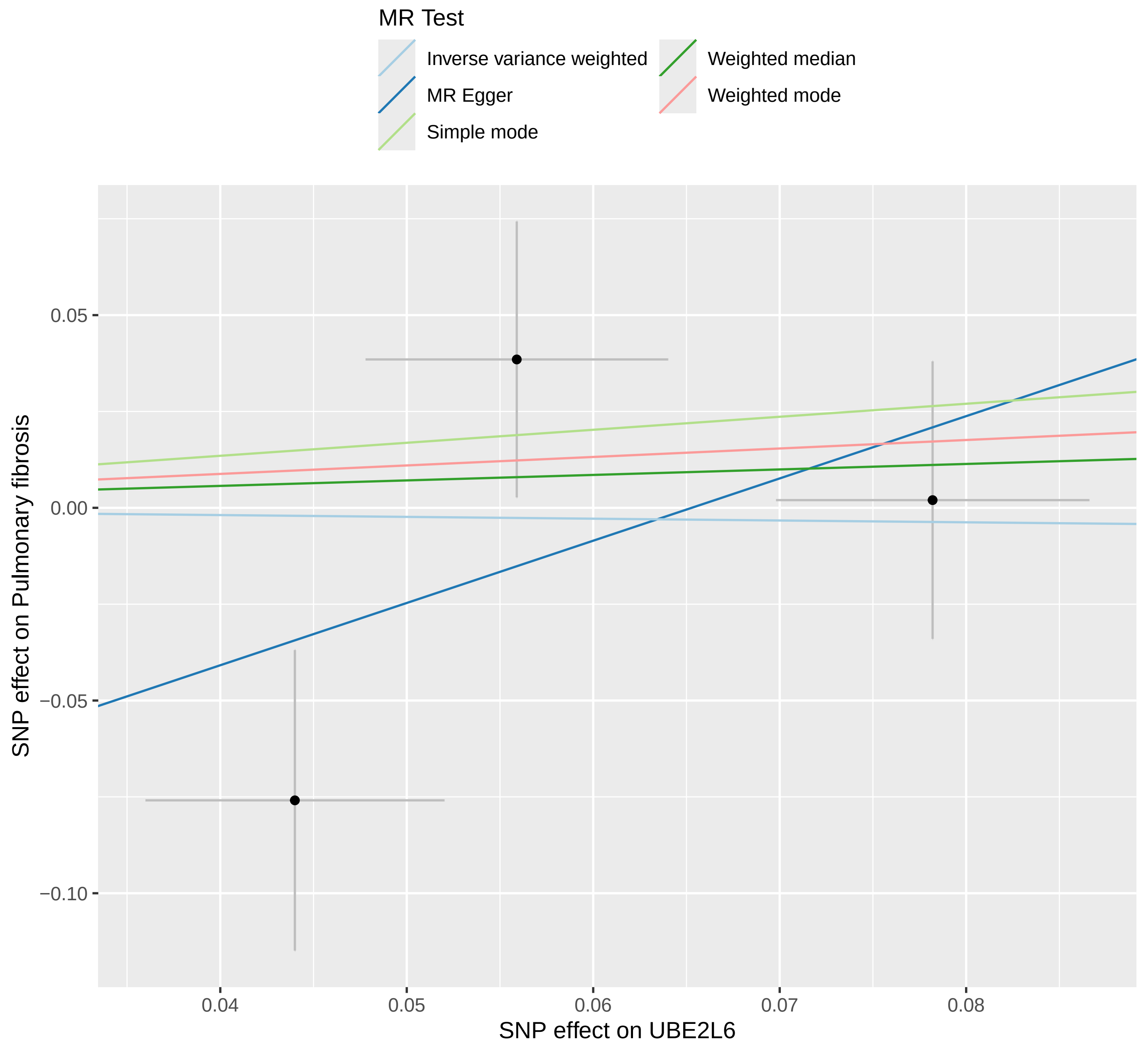

MR Test
Inverse variance weighted
MR Egger
Simple mode
Weighted median
Weighted mode
0.05
0.00
−0.05
−0.10
0.04
0.05
0.06
0.07
0.08
SNP effect on Pulmonary fibrosis
SNP effect on UBE2L6

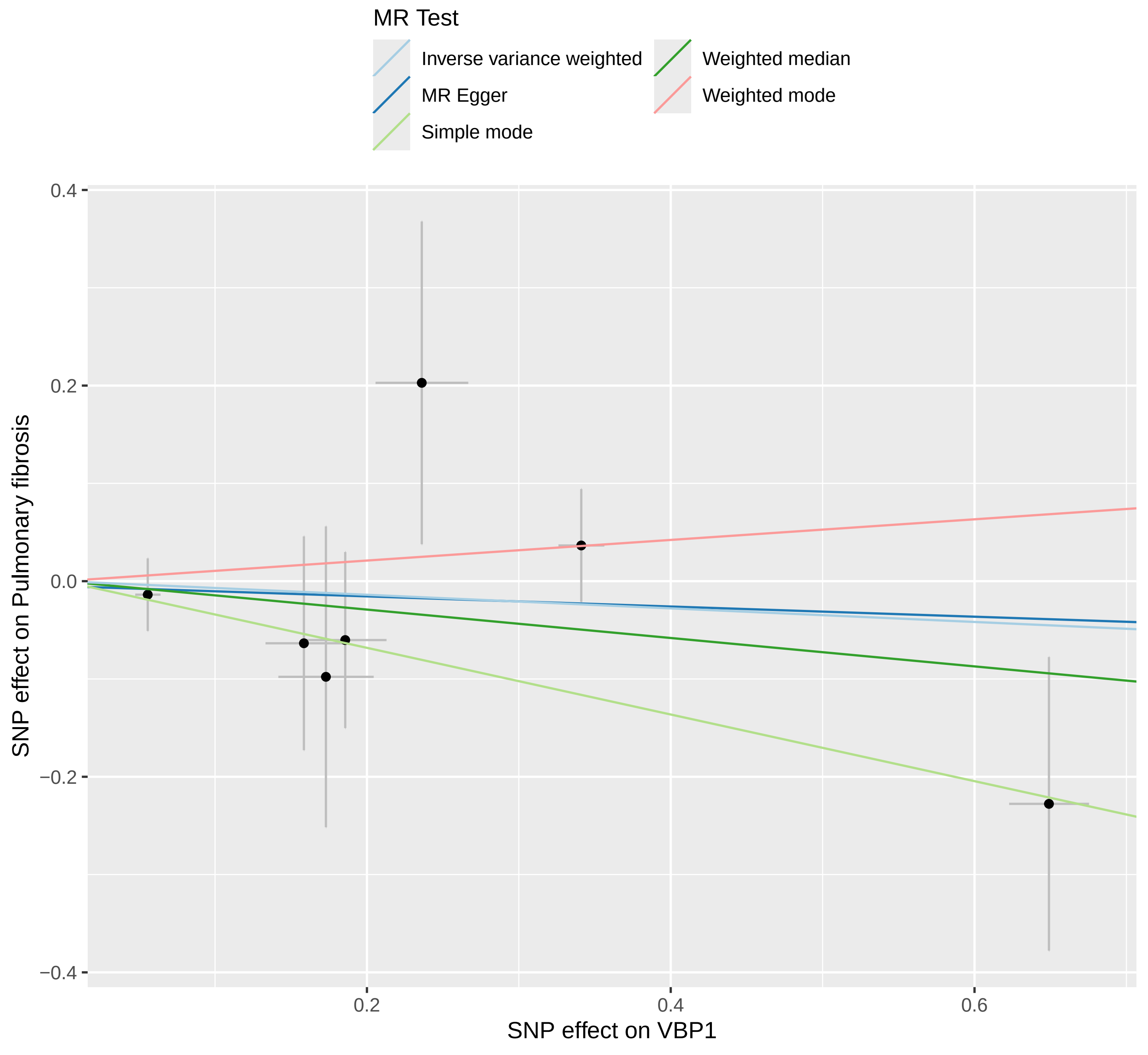

MR Test
Inverse variance weighted
MR Egger
Simple mode
Weighted median
Weighted mode
0.4
0.2
0.0
−0.2
−0.4
SNP effect on Pulmonary fibrosis
0.2
0.4
0.6
SNP effect on VBP1

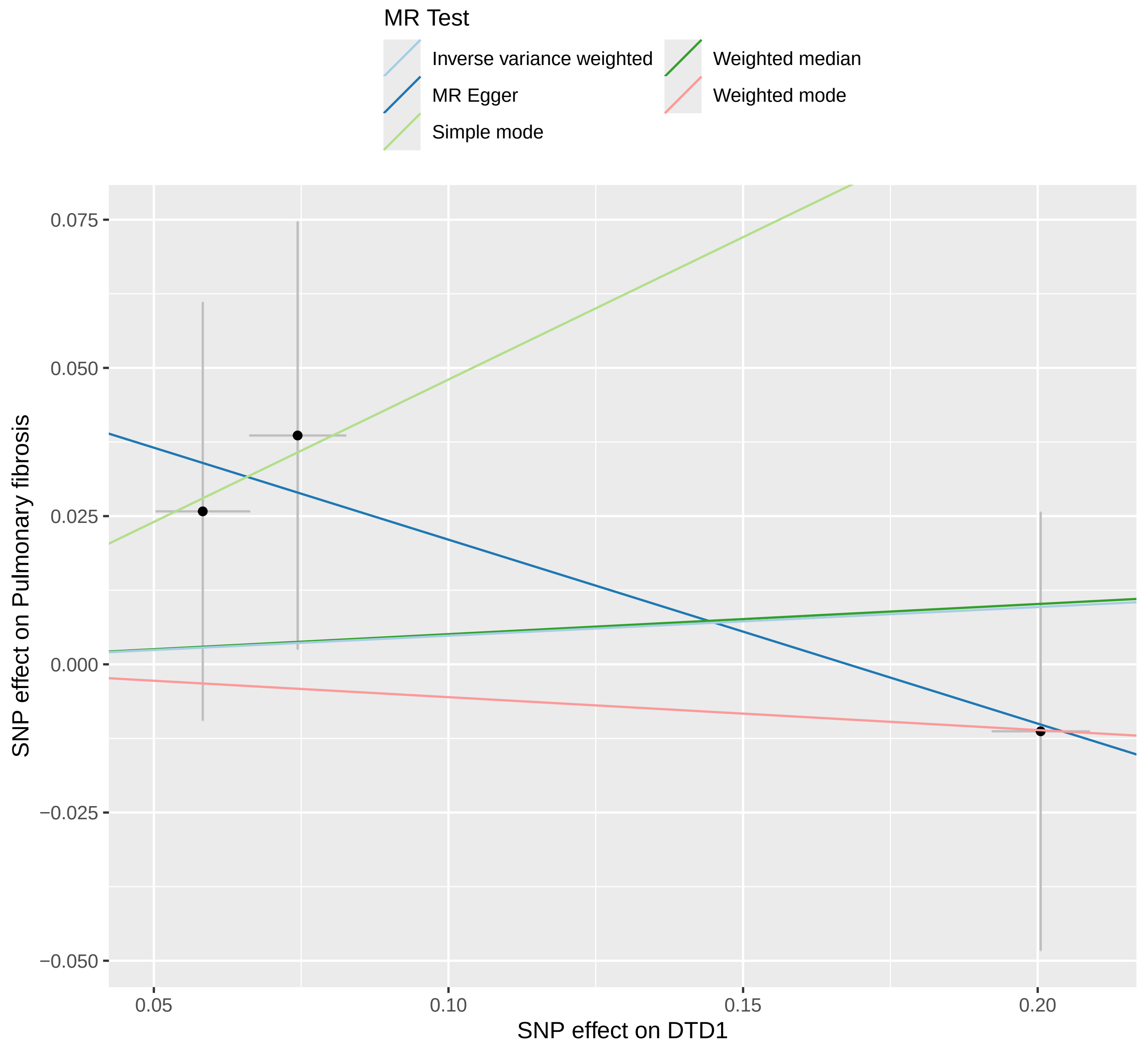
MR Test
Inverse variance weighted
MR Egger
Simple mode
Weighted median
Weighted mode
0.075
0.050
0.025
0.000
−0.025
−0.050
SNP effect on Pulmonary fibrosis
0.05
0.10
0.15
0.20
SNP effect on DTD1

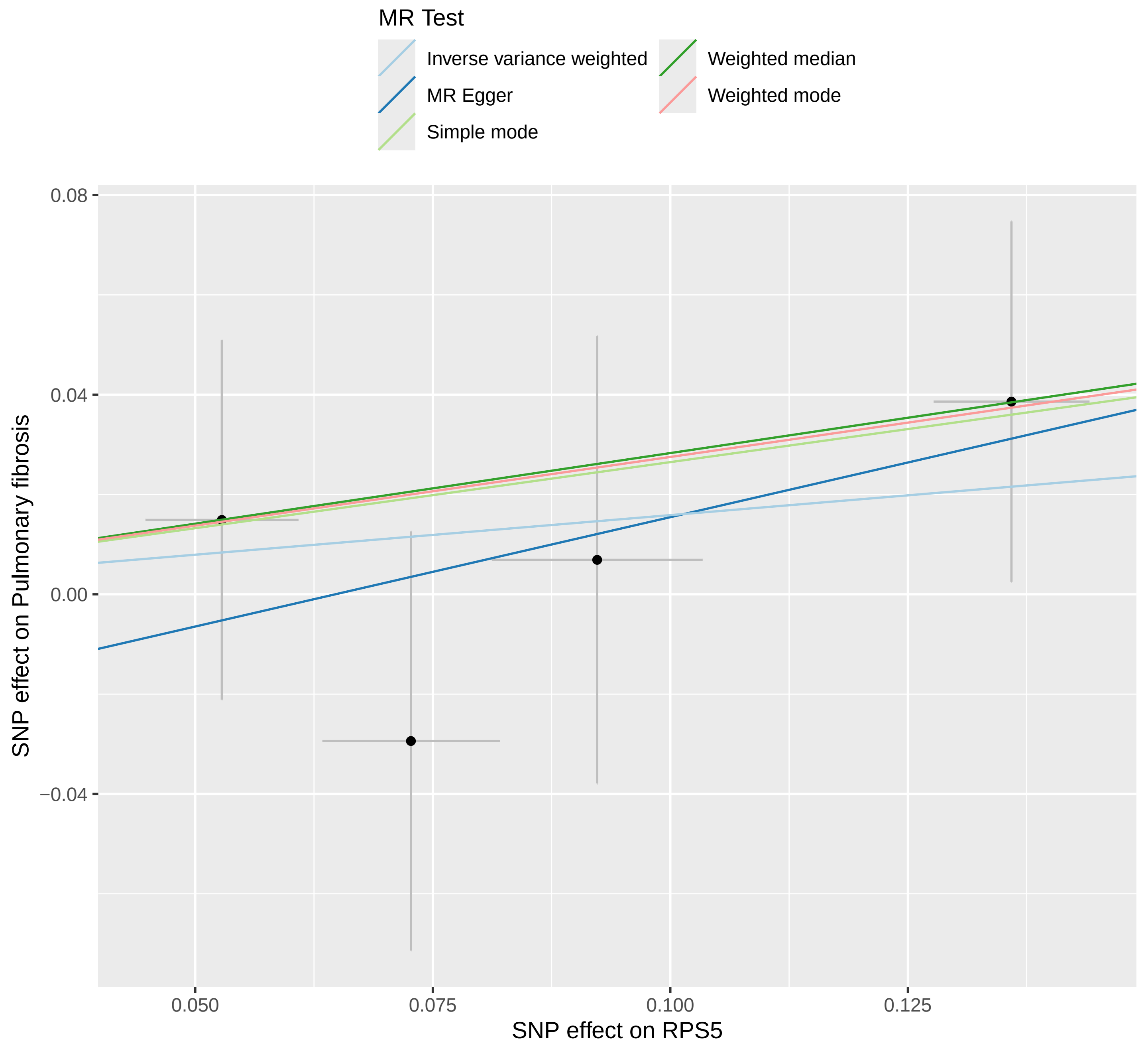

MR Test
Inverse variance weighted
MR Egger
Simple mode
Weighted median
Weighted mode
0.08
0.04
0.00
−0.04
SNP effect on Pulmonary fibrosis
0.050
0.075
0.100
0.125
SNP effect on RPS5

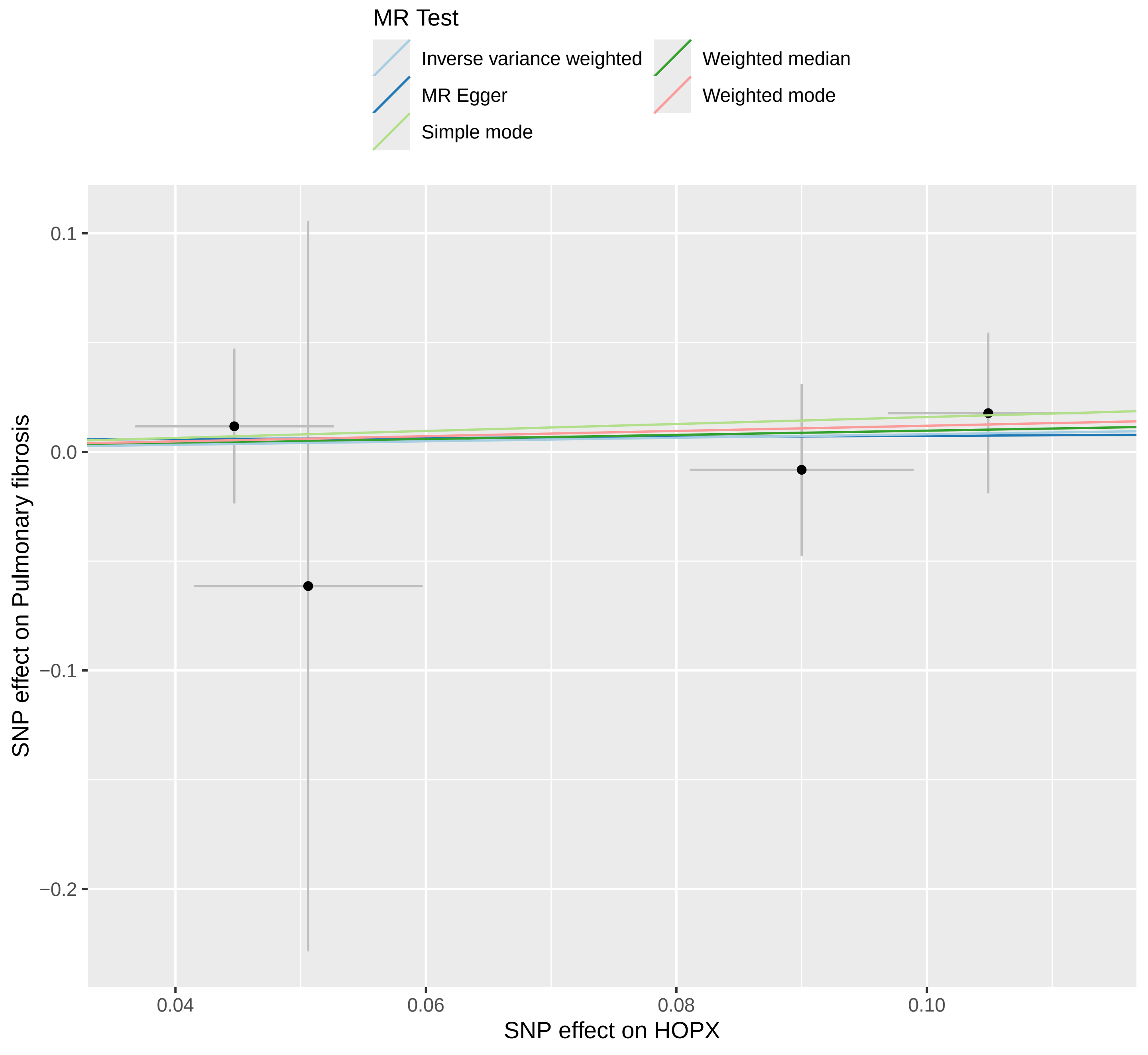
MR Test
Inverse variance weighted
MR Egger
Simple mode
Weighted median
Weighted mode
0.1
0.0
−0.1
−0.2
0.04
0.06
0.08
0.10
SNP effect on Pulmonary fibrosis
SNP effect on HOPX

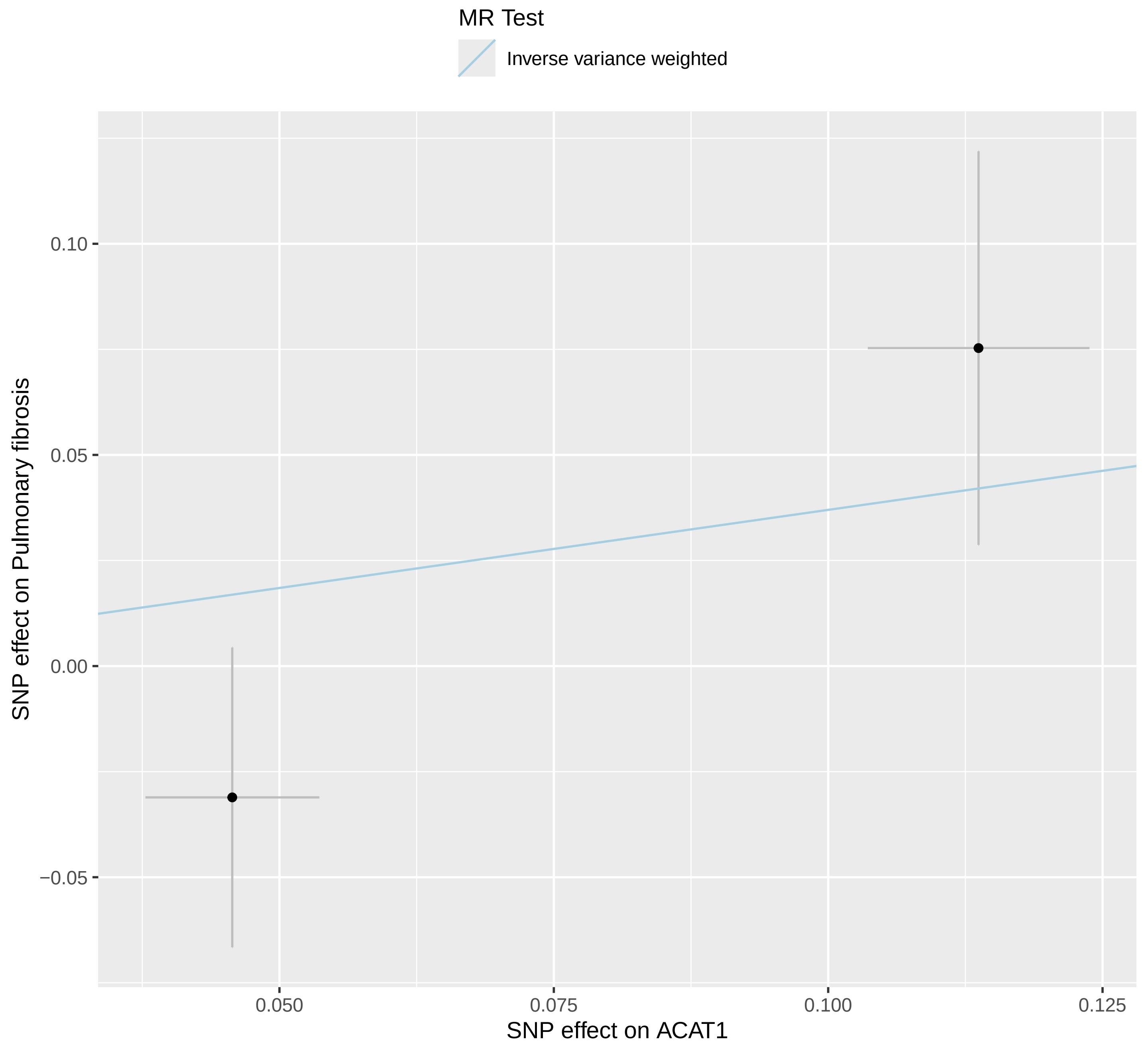
MR Test
Inverse variance weighted
0.10
0.05
0.00
−0.05
SNP effect on Pulmonary fibrosis
0.050
0.075
0.100
0.125
SNP effect on ACAT1

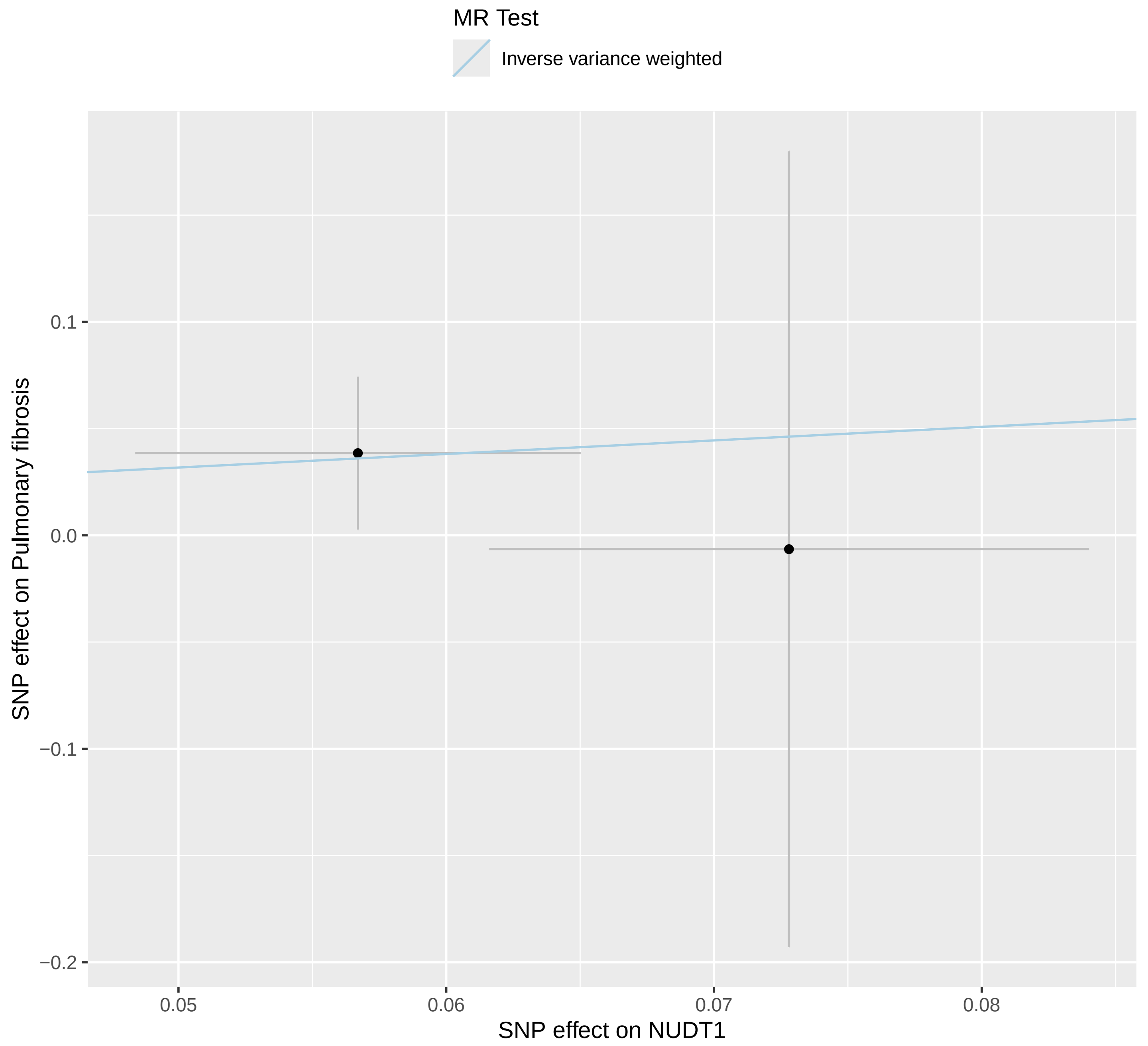
MR Test
Inverse variance weighted
0.1
0.0
−0.1
−0.2
SNP effect on Pulmonary fibrosis
0.05
0.06
0.07
0.08
SNP effect on NUDT1

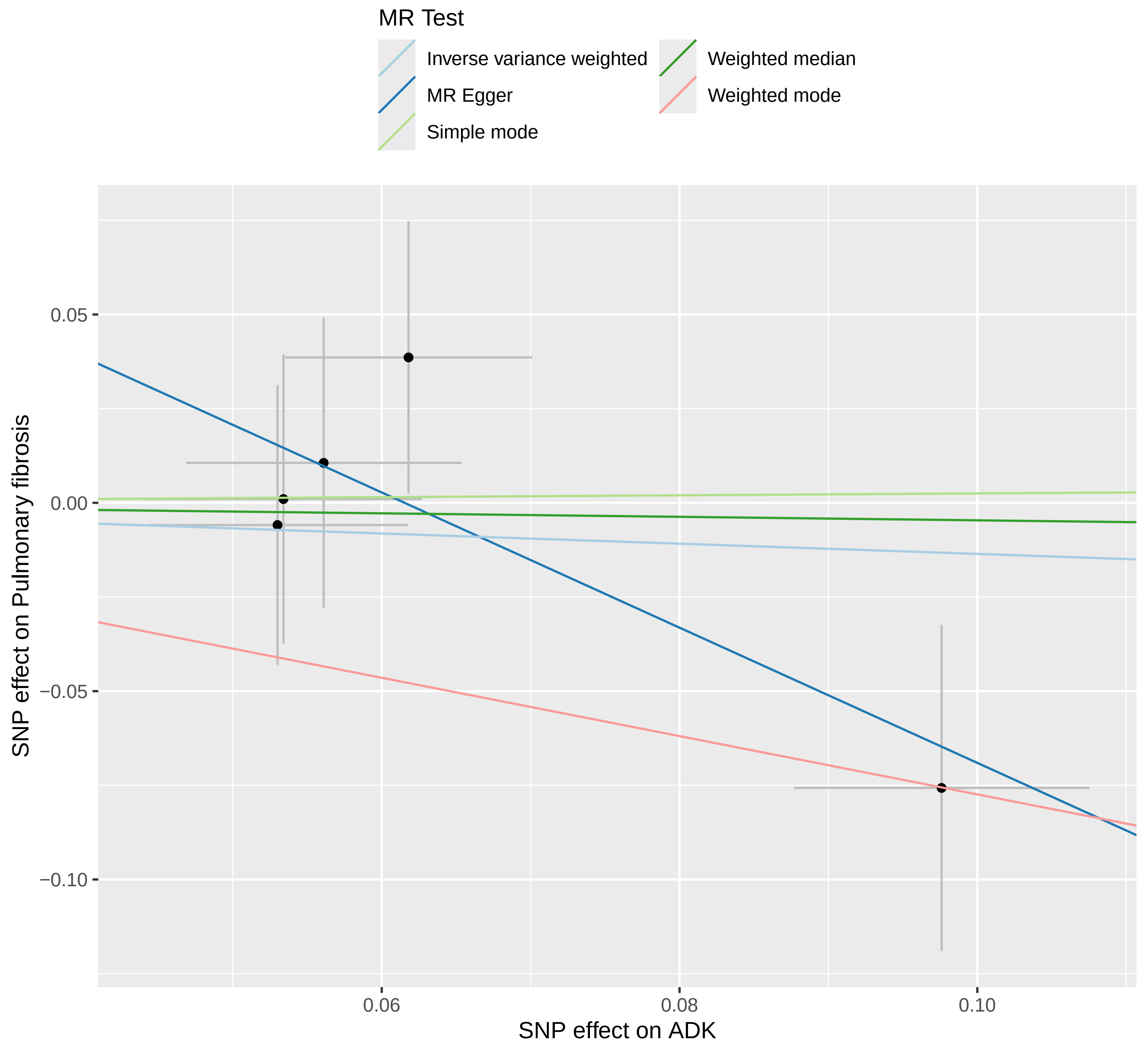

MR Test
Inverse variance weighted
MR Egger
Simple mode
Weighted median
Weighted mode
0.05
0.00
−0.05
−0.10
0.06
0.08
0.10
SNP effect on Pulmonary fibrosis
SNP effect on ADK

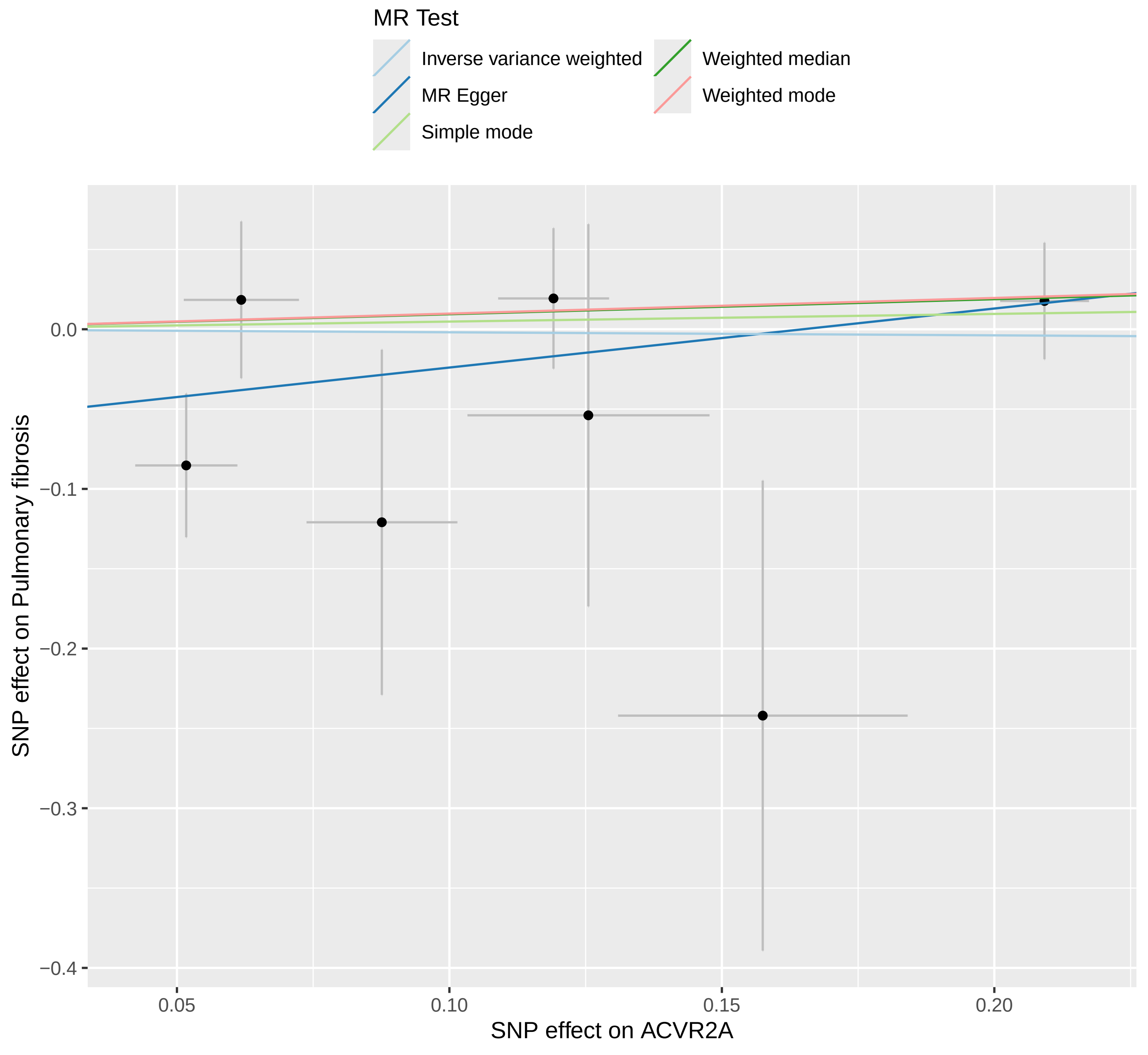

MR Test
Inverse variance weighted
MR Egger
Simple mode
Weighted median
Weighted mode
SNP effect on Pulmonary fibrosis
0.0
−0.1
−0.2
−0.3
−0.4
0.05
0.10
0.15
0.20
SNP effect on ACVR2A

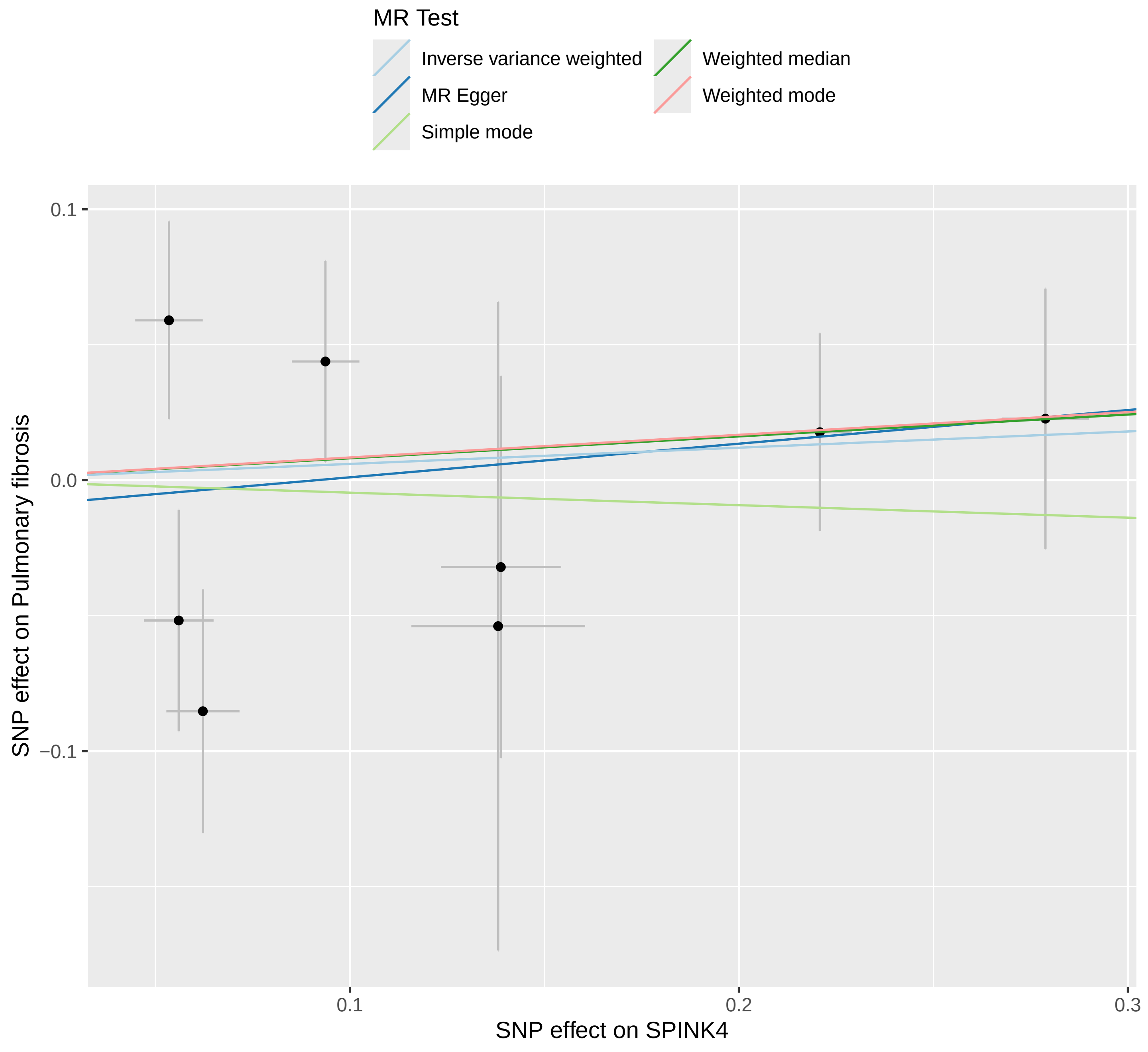

MR Test
Inverse variance weighted
MR Egger
Simple mode
Weighted median
Weighted mode
0.1
0.0
−0.1
0.1
0.2
0.3
SNP effect on Pulmonary fibrosis
SNP effect on SPINK4

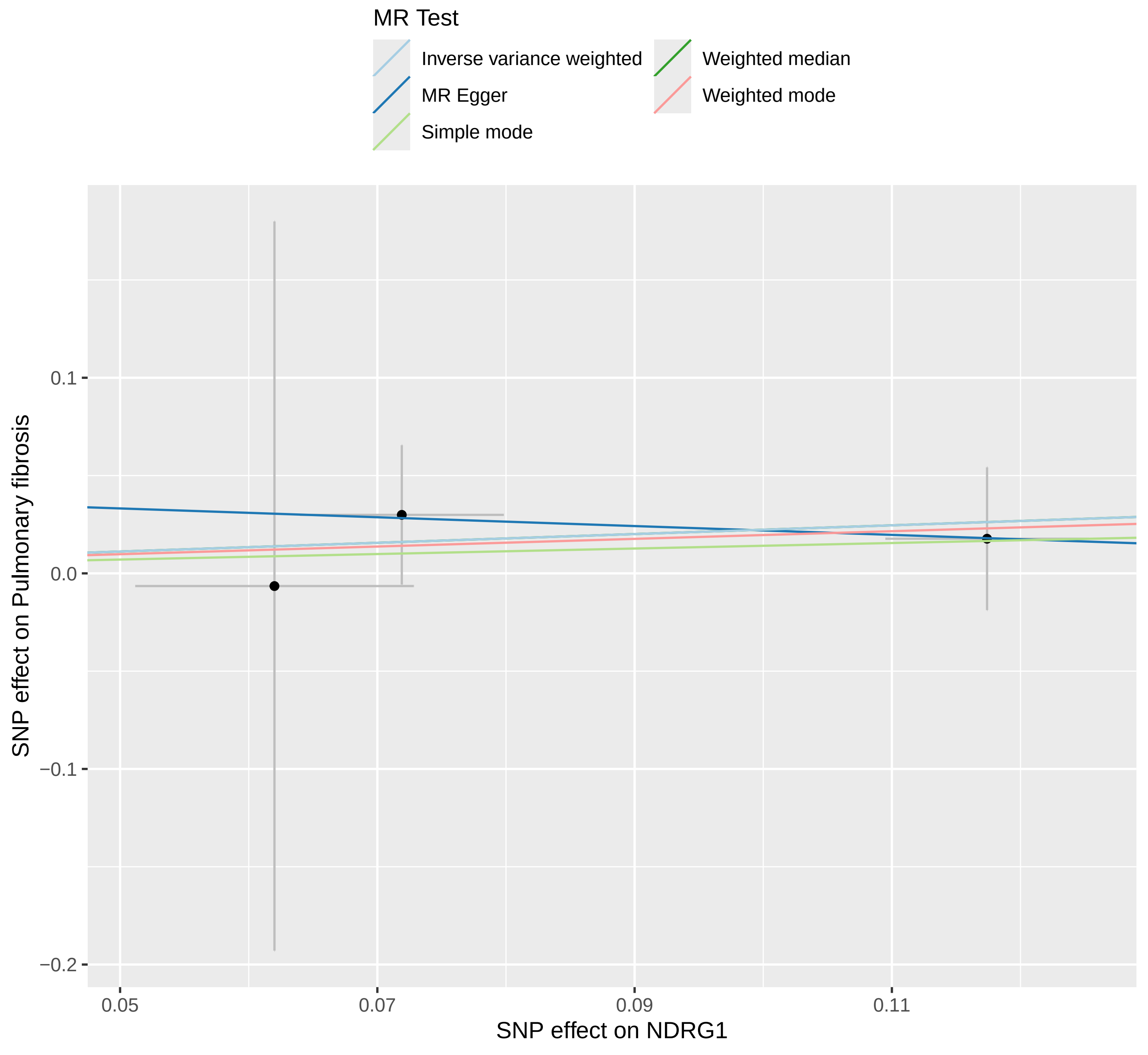

MR Test
Inverse variance weighted
MR Egger
Simple mode
Weighted median
Weighted mode
0.1
0.0
−0.1
−0.2
SNP effect on Pulmonary fibrosis
0.05
0.07
0.09
0.11
SNP effect on NDRG1

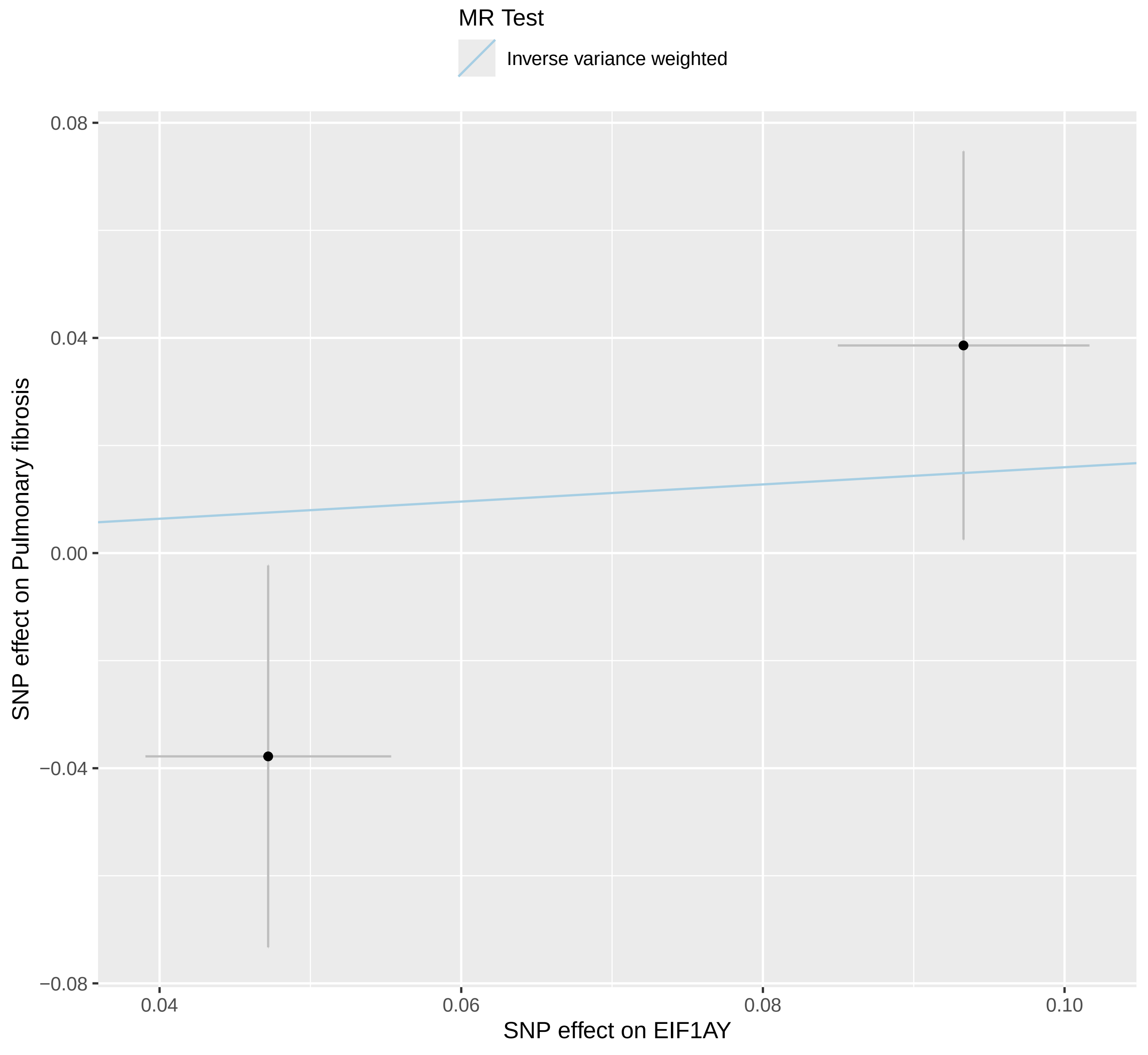
MR Test
Inverse variance weighted
0.08
0.04
0.00
−0.04
−0.08
SNP effect on Pulmonary fibrosis
0.04
0.06
0.08
0.10
SNP effect on EIF1AY

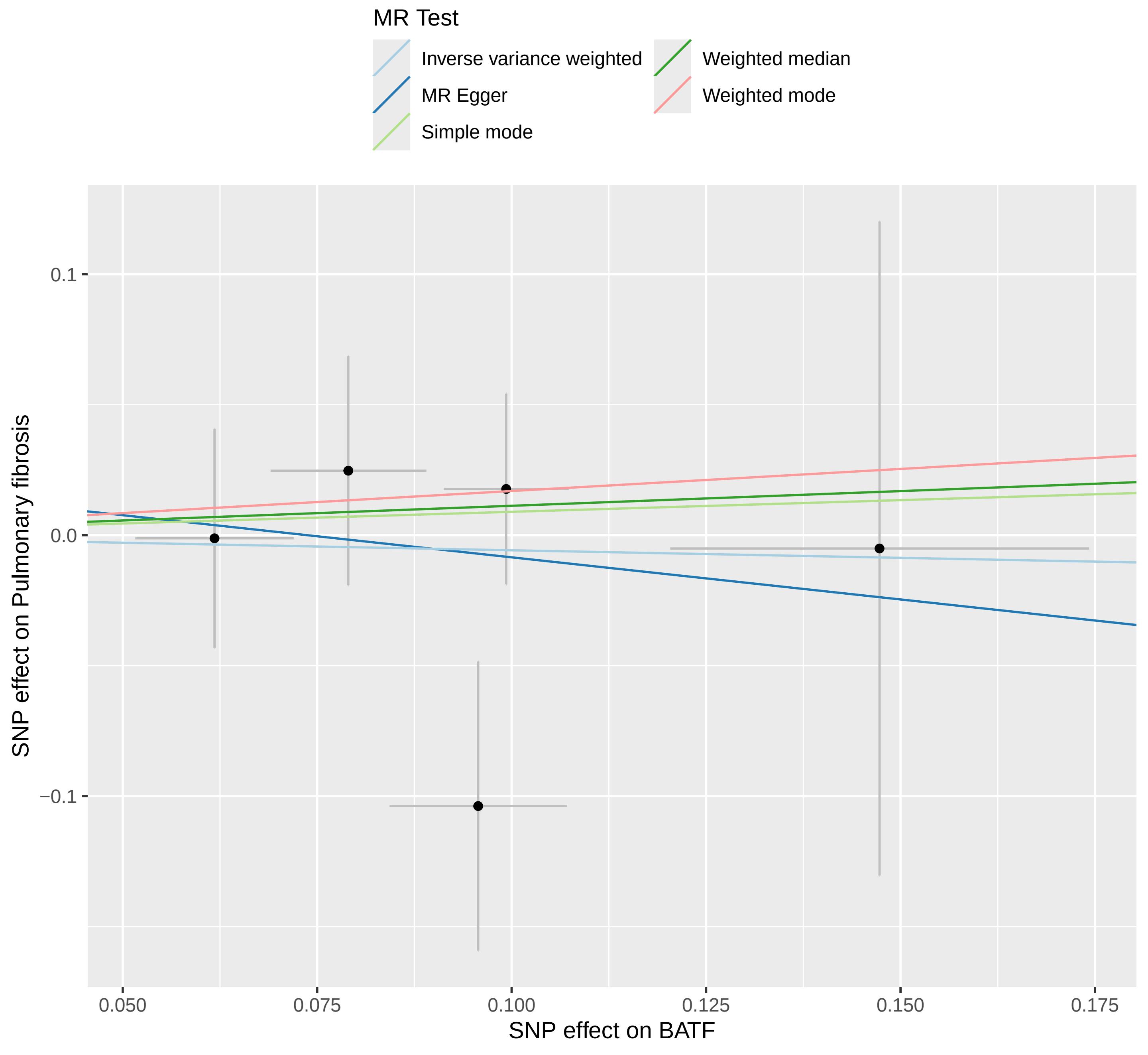
MR Test
Inverse variance weighted
MR Egger
Simple mode
Weighted median
Weighted mode
0.1
0.0
−0.1
0.050
0.075
0.100
0.125
0.150
0.175
SNP effect on BATF
SNP effect on Pulmonary fibrosis

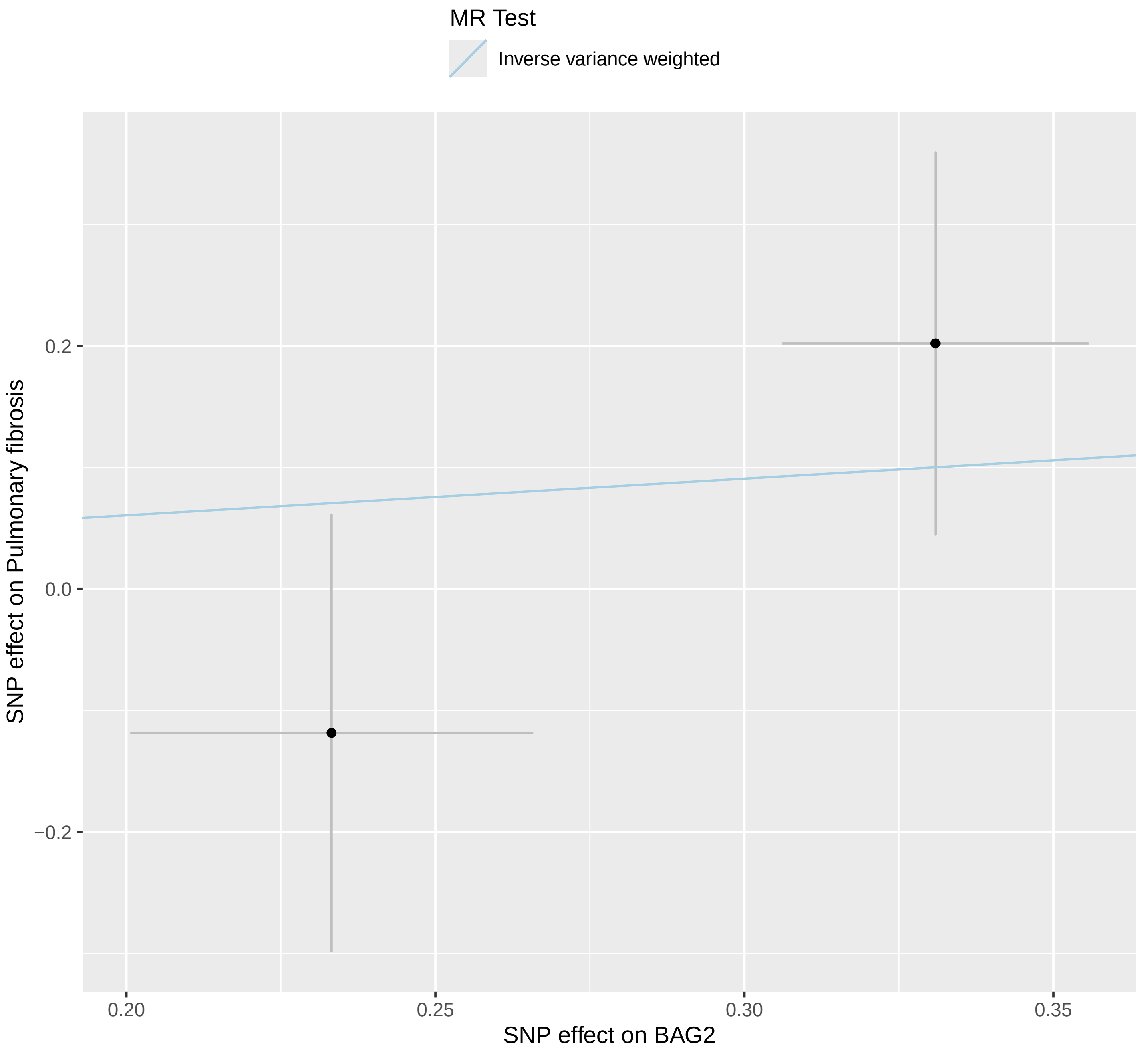

MR Test
Inverse variance weighted
0.2
0.0
−0.2
SNP effect on Pulmonary fibrosis
0.20
0.25
0.30
0.35
SNP effect on BAG2

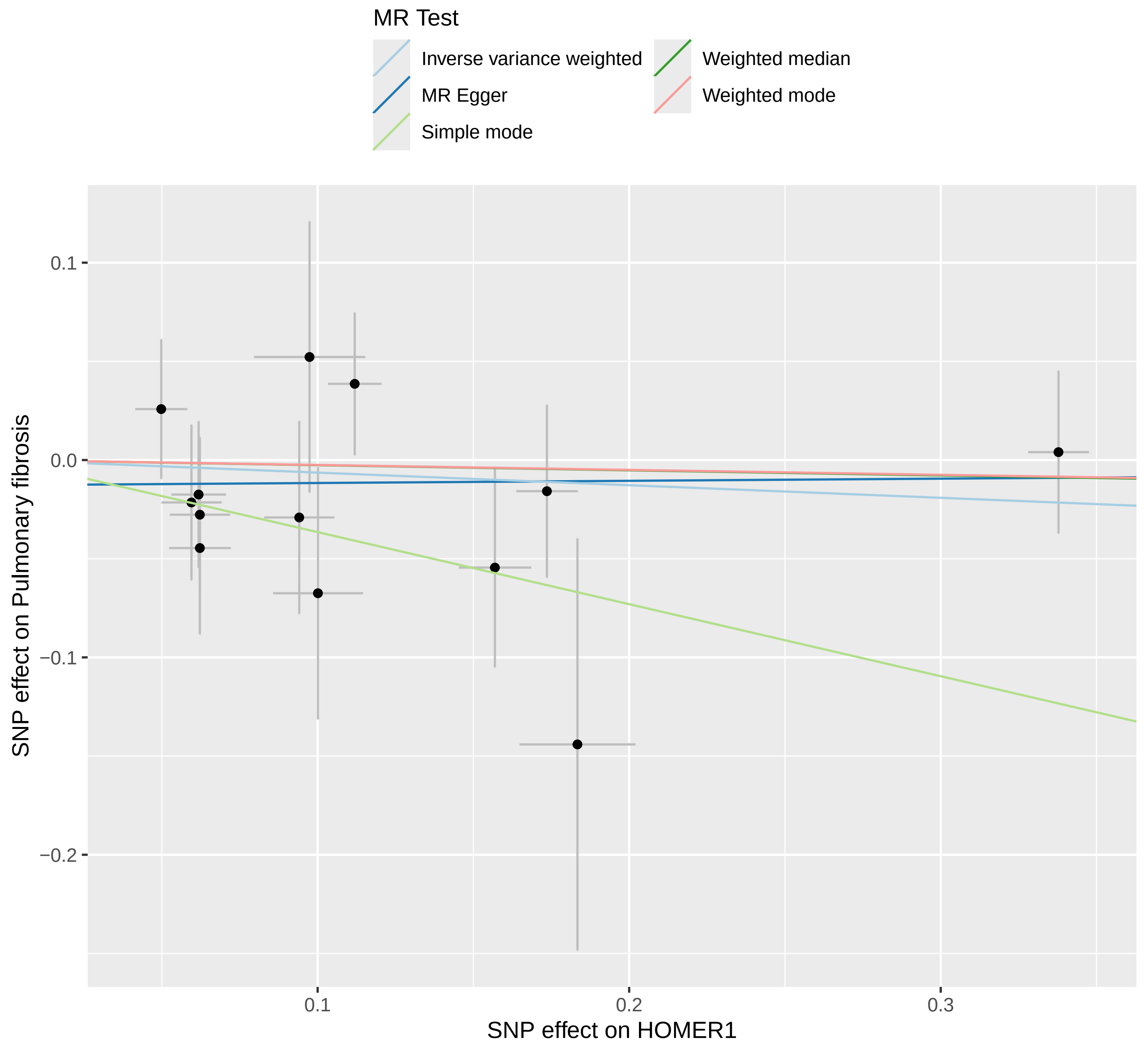
MR Test
Inverse variance weighted
MR Egger
Simple mode
Weighted median
Weighted mode
0.1
0.0
−0.1
−0.2
SNP effect on Pulmonary fibrosis
0.1
0.2
0.3
SNP effect on HOMER1

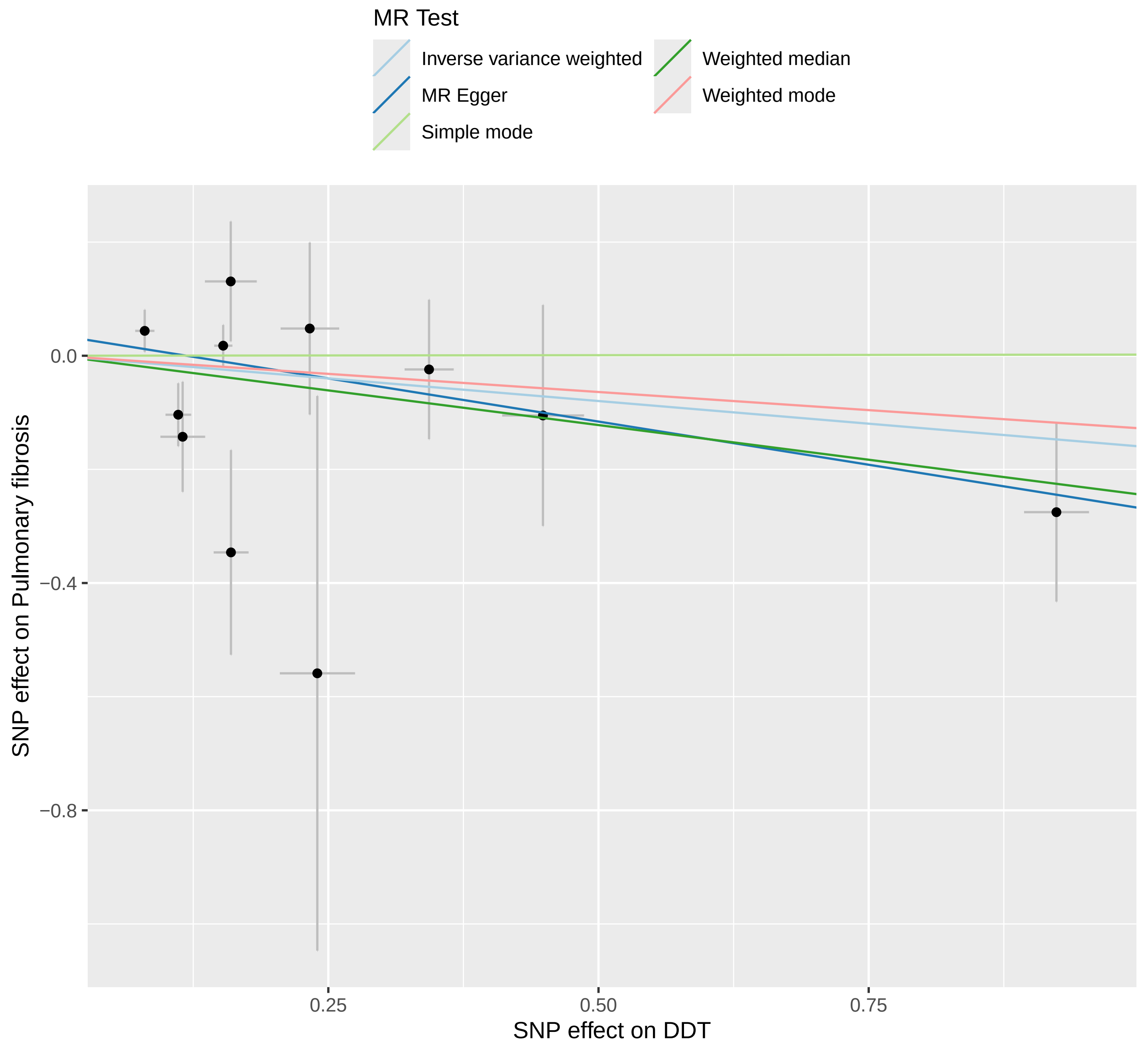
MR Test
Inverse variance weighted
MR Egger
Simple mode
Weighted median
Weighted mode
0.0
−0.4
−0.8
0.25
0.50
0.75
SNP effect on Pulmonary fibrosis
SNP effect on DDT

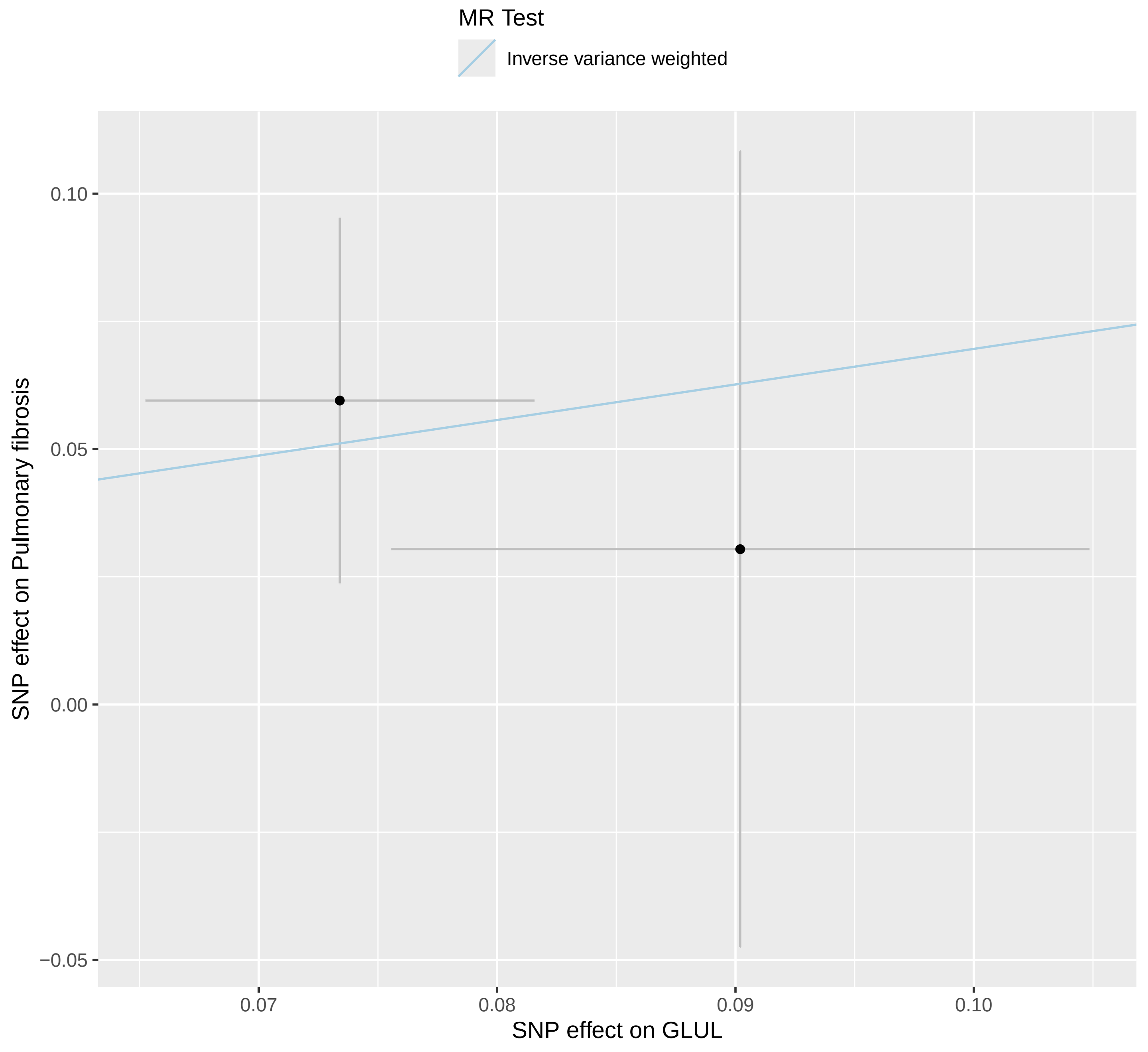

MR Test
Inverse variance weighted
0.10
0.05
0.00
−0.05
0.07
0.08
0.09
0.10
SNP effect on GLUL
SNP effect on Pulmonary fibrosis

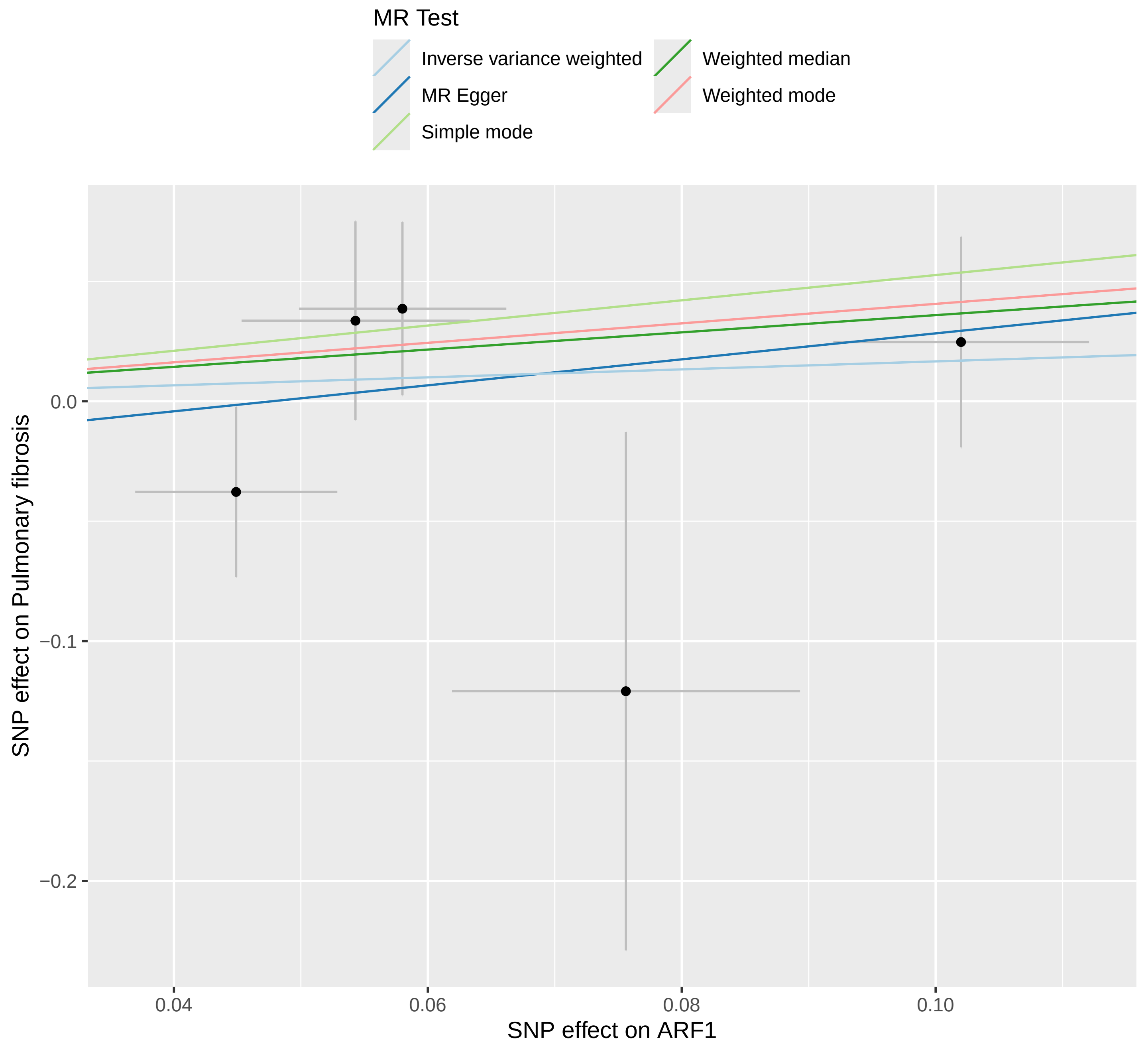
MR Test
Inverse variance weighted
MR Egger
Simple mode
Weighted median
Weighted mode
0.0
−0.1
−0.2
0.04
0.06
0.08
0.10
SNP effect on Pulmonary fibrosis
SNP effect on ARF1

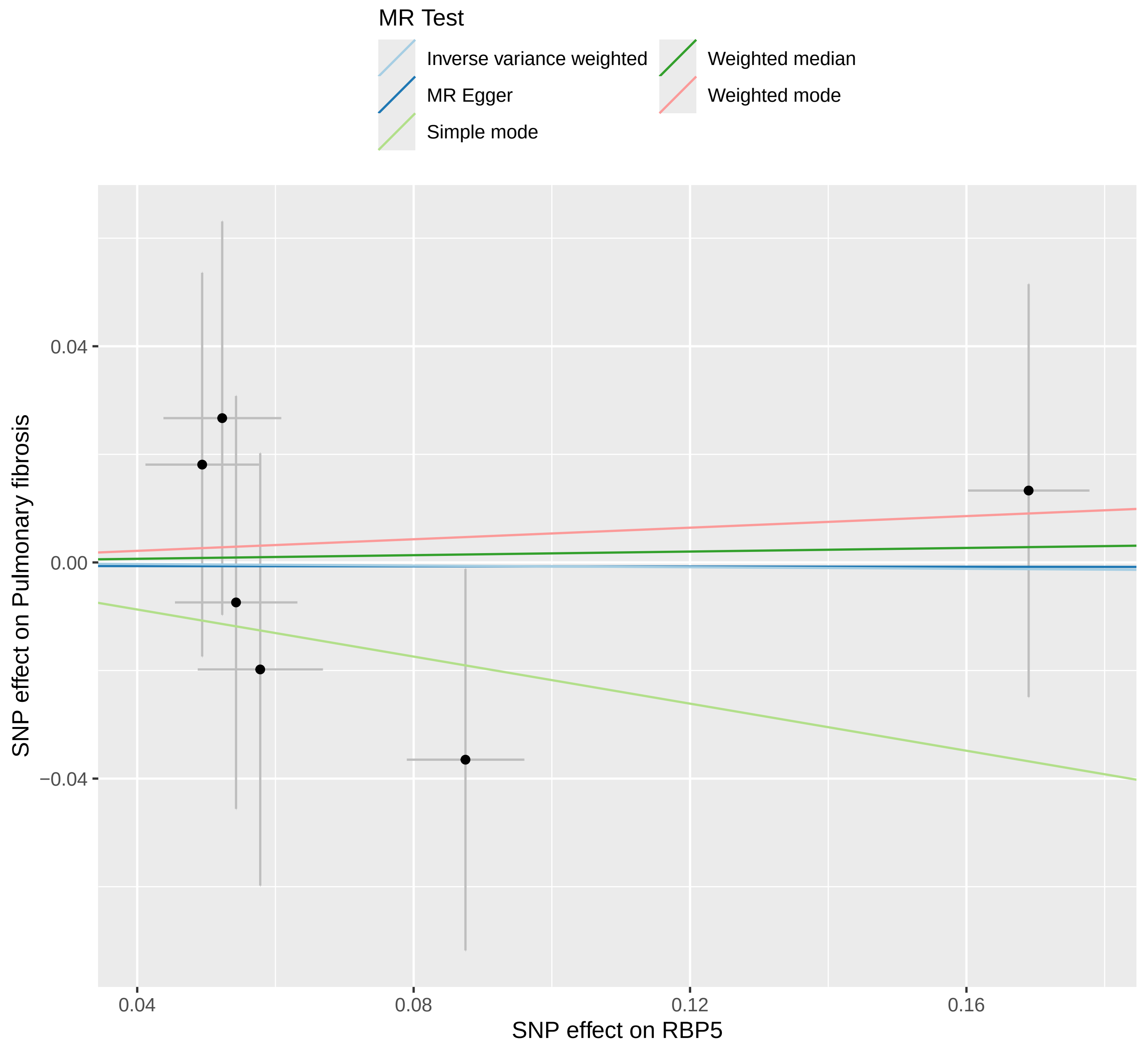
MR Test
Inverse variance weighted
MR Egger
Simple mode
Weighted median
Weighted mode
0.04
0.00
−0.04
SNP effect on Pulmonary fibrosis
0.04
0.08
0.12
0.16
SNP effect on RBP5

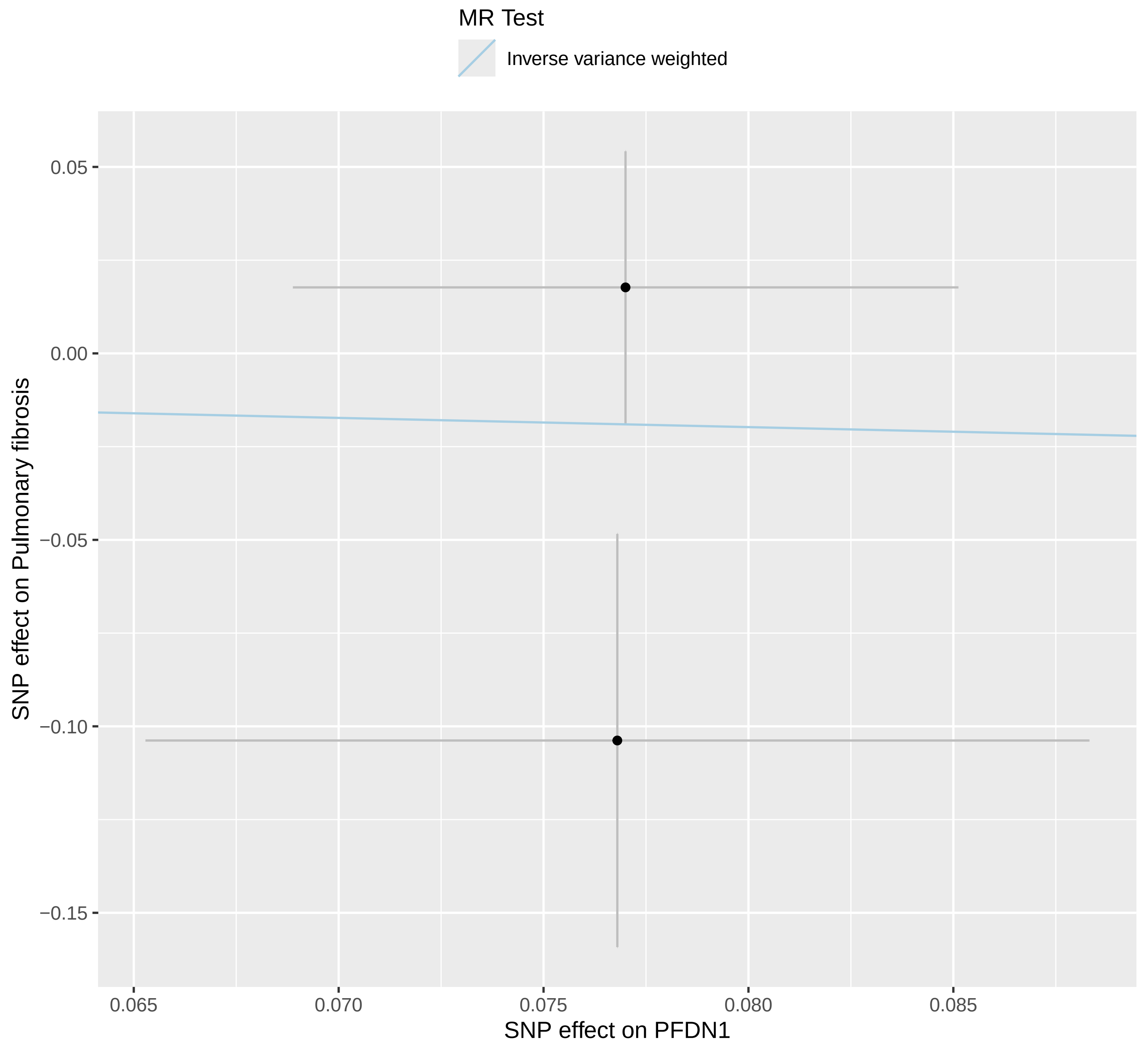
MR Test
Inverse variance weighted
0.05
0.00
−0.05
−0.10
−0.15
0.065
0.070
0.075
0.080
0.085
SNP effect on PFDN1
SNP effect on Pulmonary fibrosis

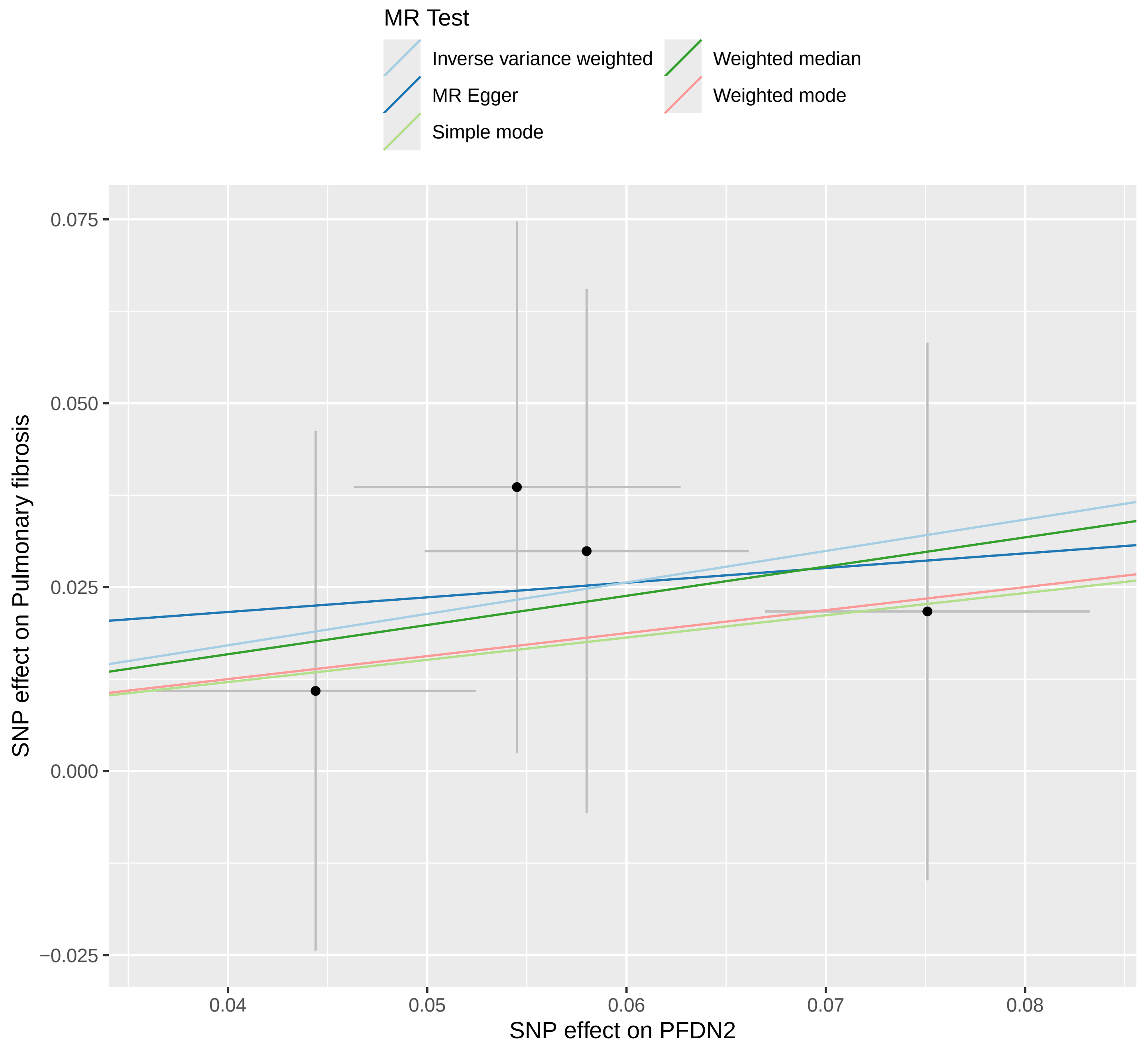
MR Test
Inverse variance weighted
MR Egger
Simple mode
Weighted median
Weighted mode
0.075
0.050
0.025
0.000
−0.025
0.04
0.05
0.06
0.07
0.08
SNP effect on Pulmonary fibrosis
SNP effect on PFDN2

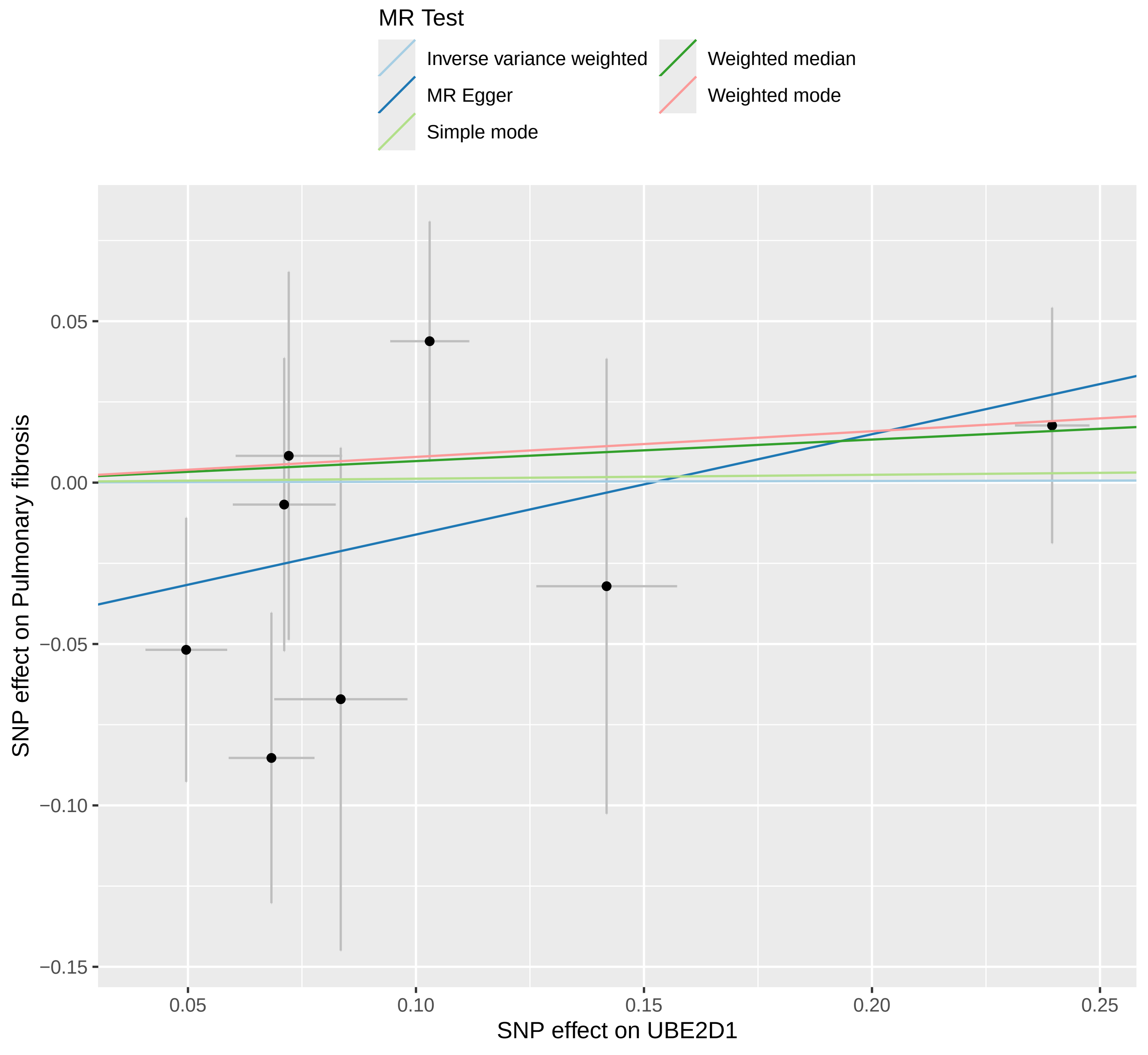

MR Test
Inverse variance weighted
MR Egger
Simple mode
Weighted median
Weighted mode
0.05
0.00
−0.05
−0.10
−0.15
SNP effect on Pulmonary fibrosis
0.05
0.10
0.15
0.20
0.25
SNP effect on UBE2D1

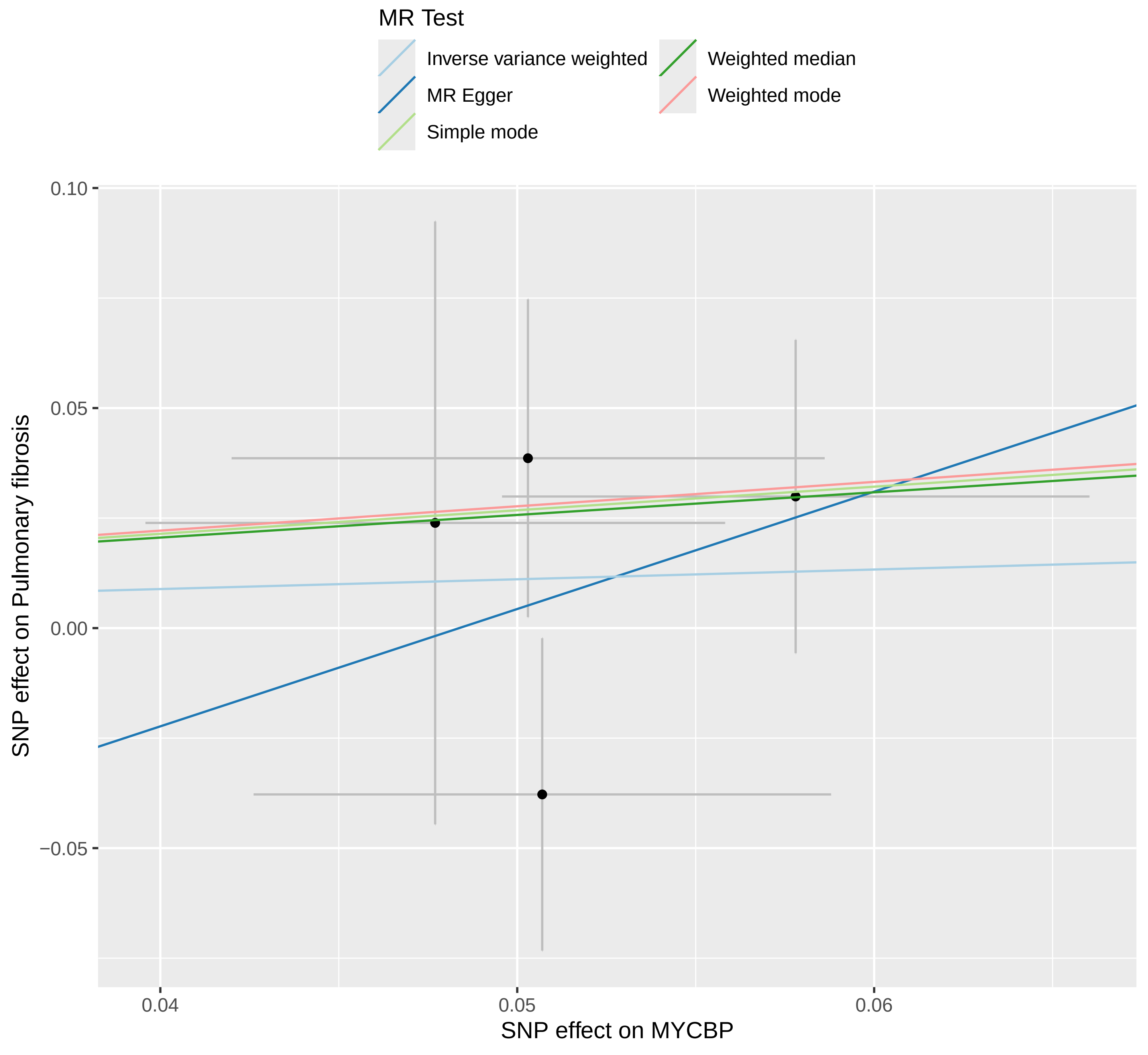

MR Test
Inverse variance weighted
MR Egger
Simple mode
Weighted median
Weighted mode
0.10
0.05
0.00
−0.05
0.04
0.05
0.06
SNP effect on MYCBP
SNP effect on Pulmonary fibrosis

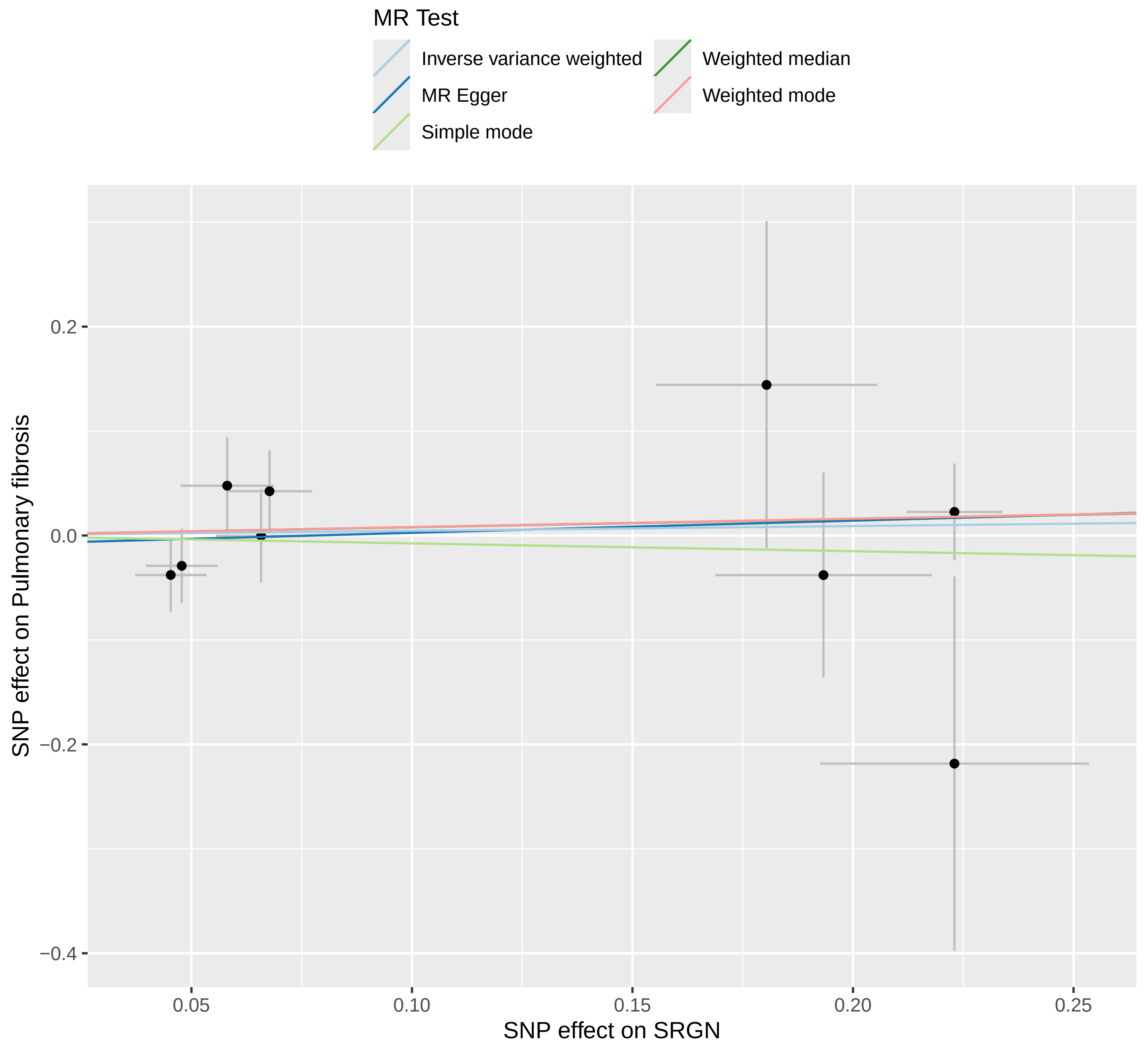

MR Test
Inverse variance weighted
MR Egger
Simple mode
Weighted median
Weighted mode
SNP effect on Pulmonary fibrosis
0.2
0.0
−0.2
−0.4
0.05
0.10
0.15
0.20
0.25
SNP effect on SRGN

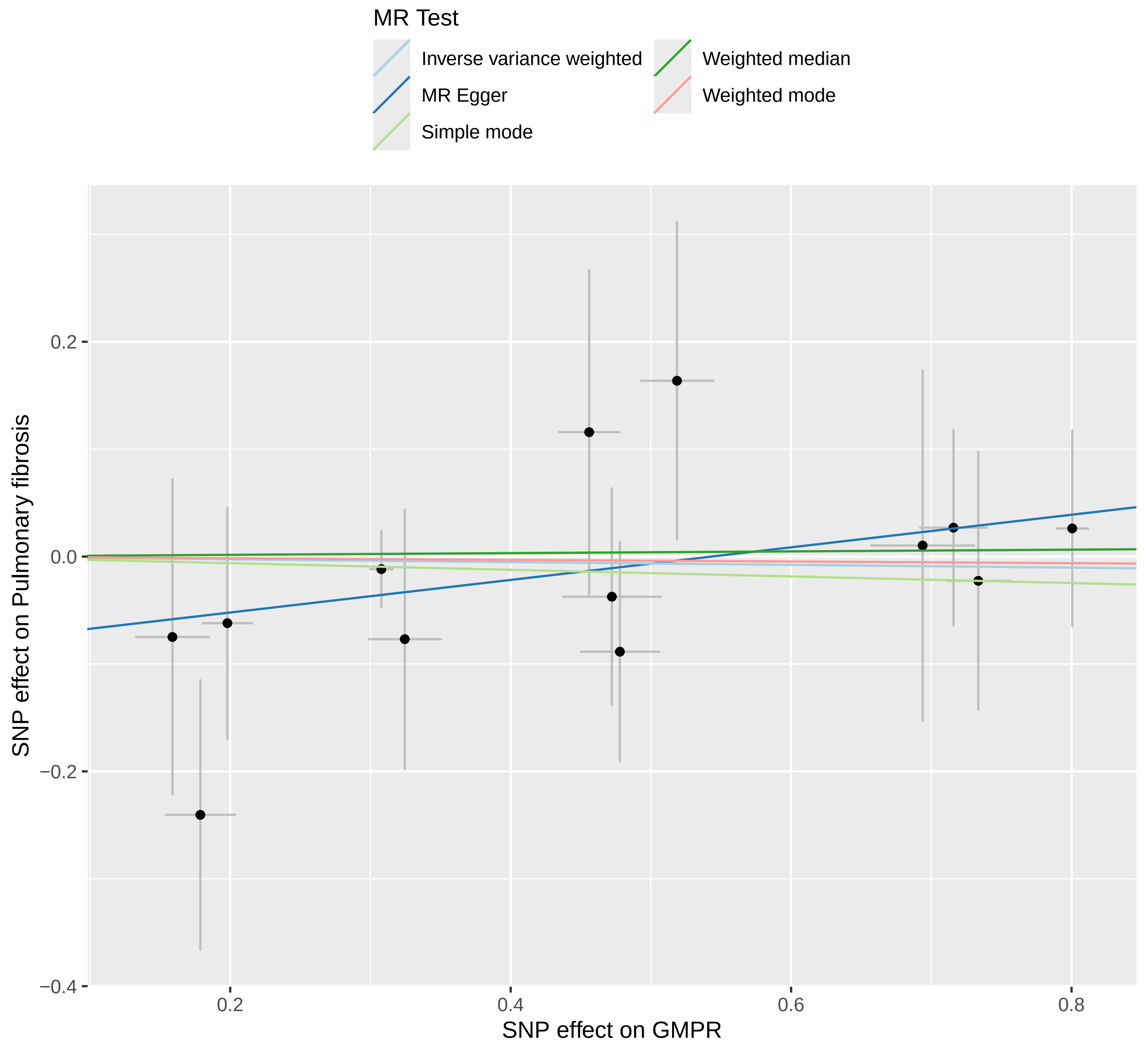

MR Test
Inverse variance weighted
MR Egger
Simple mode
Weighted median
Weighted mode
SNP effect on Pulmonary fibrosis
0.2
0.0
−0.2
−0.4
0.2
0.4
0.6
0.8
SNP effect on GMPR

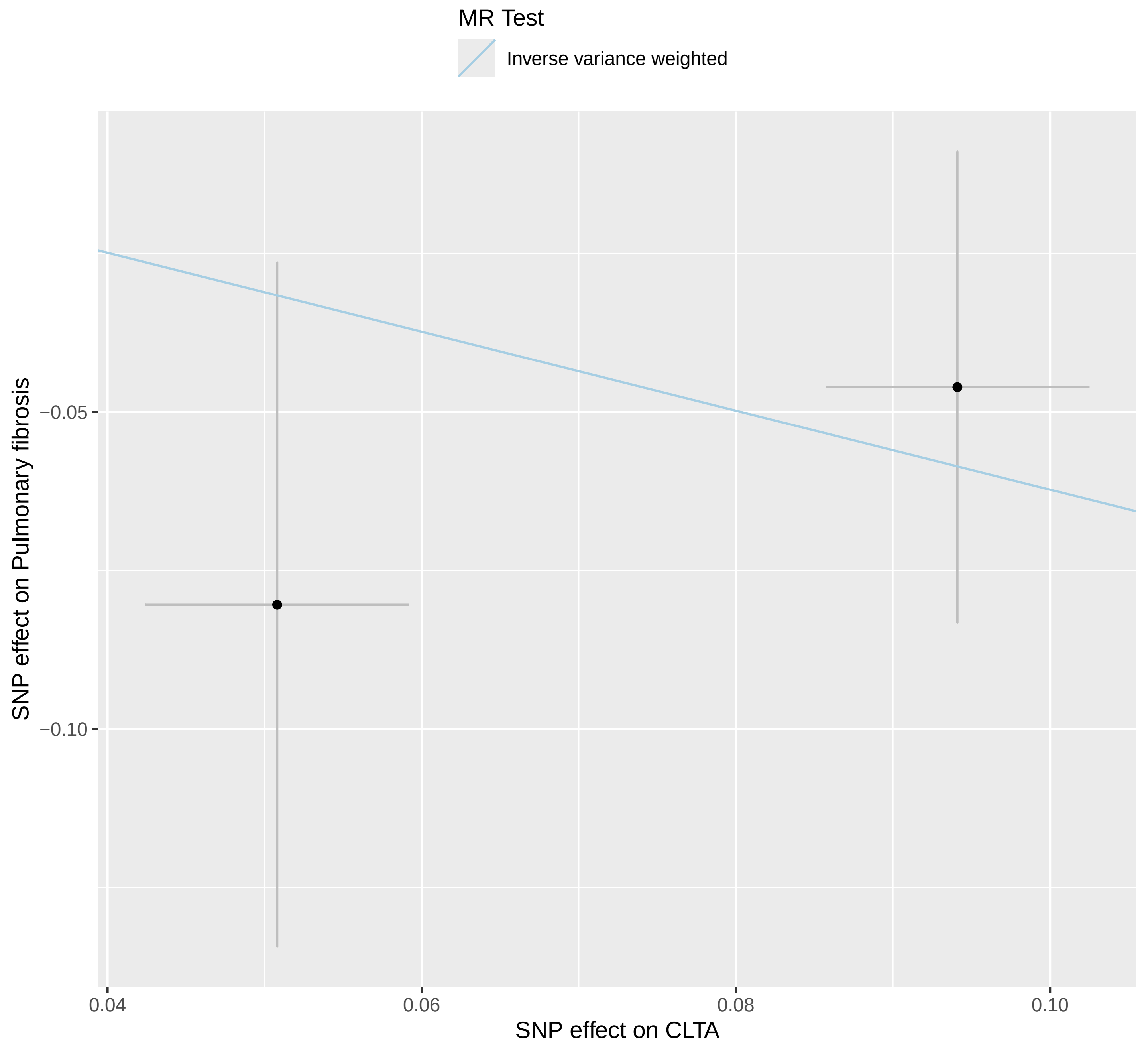
MR Test
Inverse variance weighted
SNP effect on Pulmonary fibrosis
−0.05
−0.10
0.04
0.06
0.08
0.10
SNP effect on CLTA

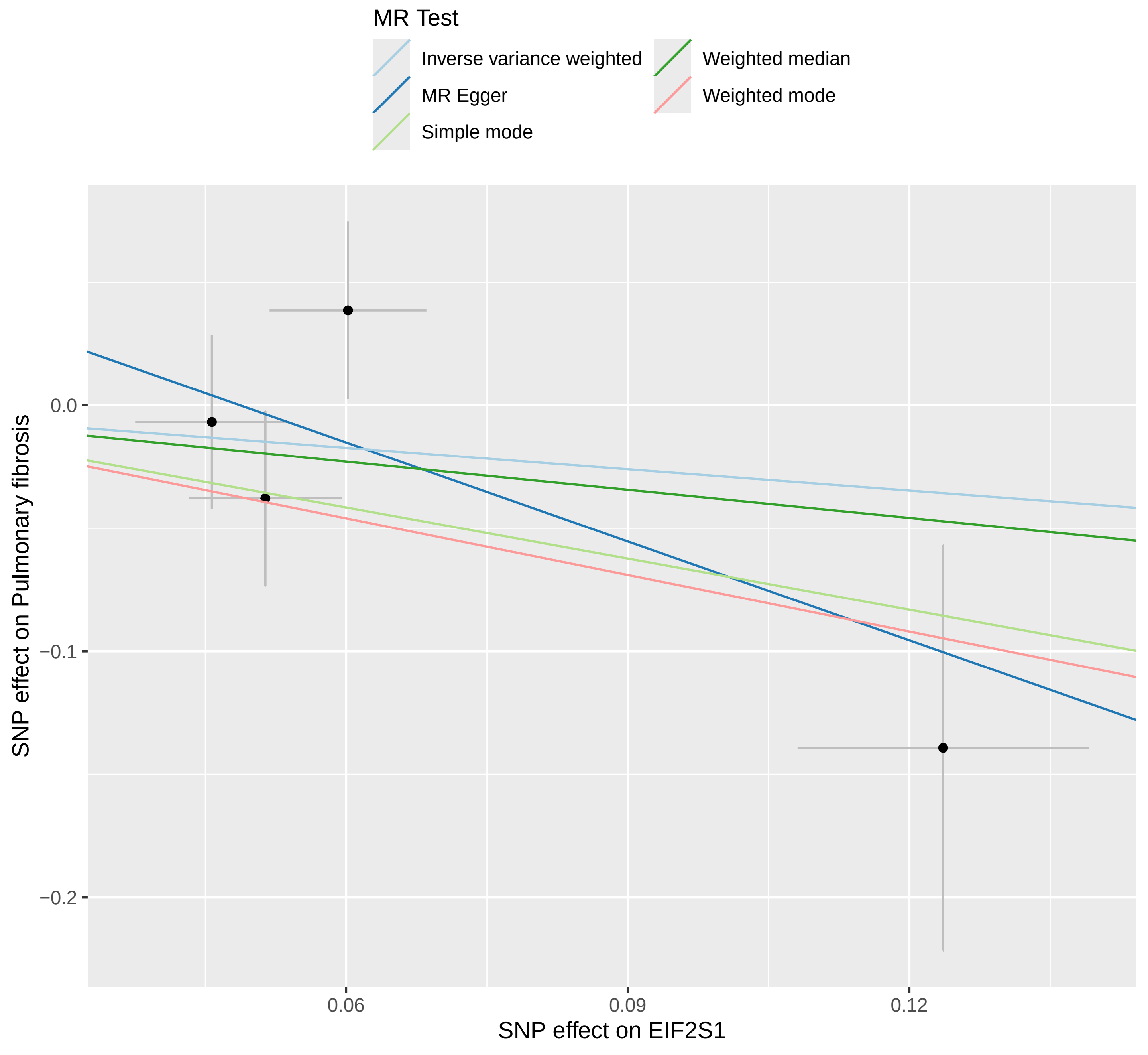

MR Test
Inverse variance weighted
MR Egger
Simple mode
Weighted median
Weighted mode
0.0
−0.1
−0.2
SNP effect on Pulmonary fibrosis
0.06
0.09
0.12
SNP effect on EIF2S1

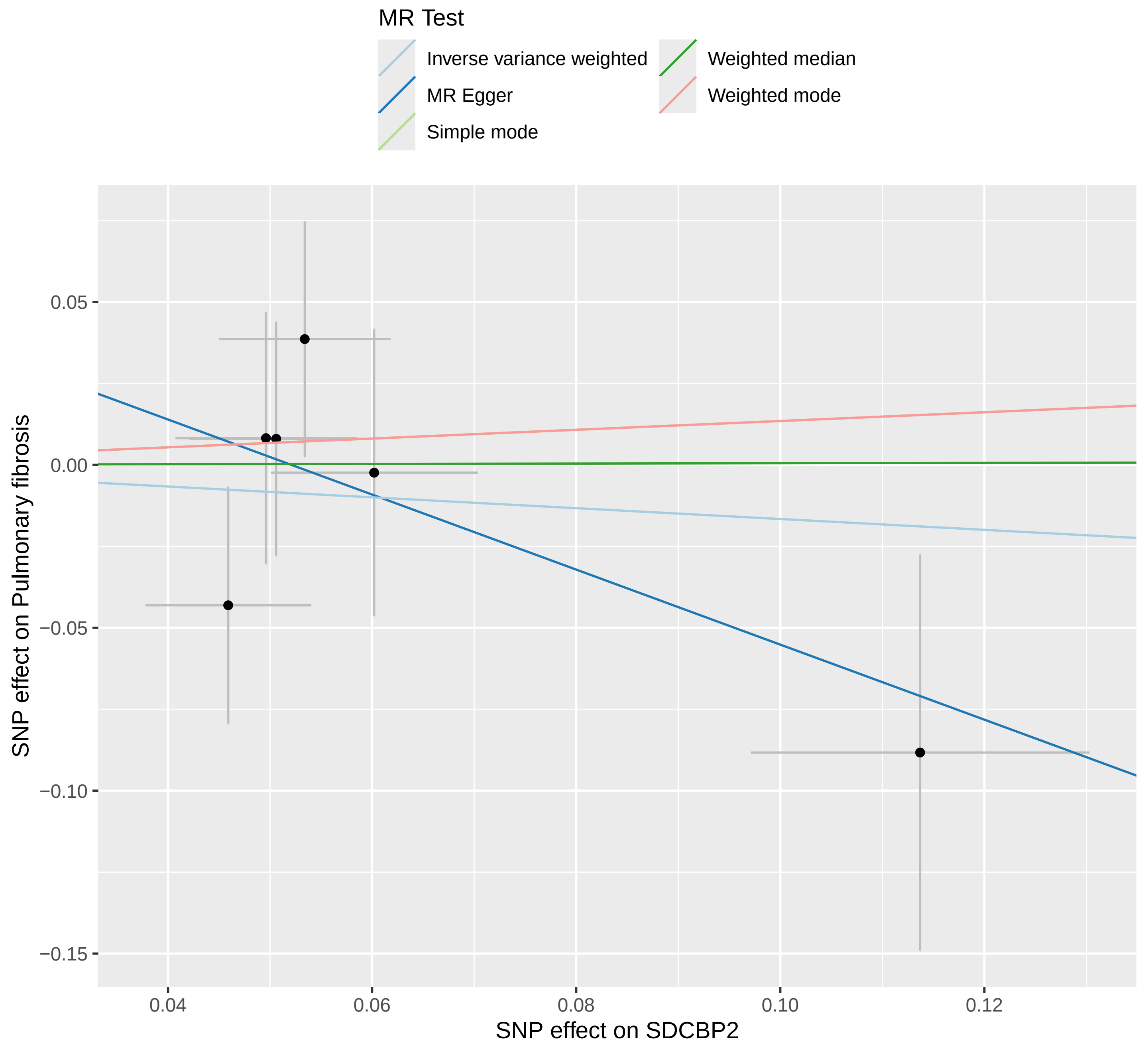

MR Test
Inverse variance weighted
MR Egger
Simple mode
Weighted median
Weighted mode
0.05
0.00
−0.05
−0.10
−0.15
0.04
0.06
0.08
0.10
0.12
SNP effect on SDCBP2
SNP effect on Pulmonary fibrosis

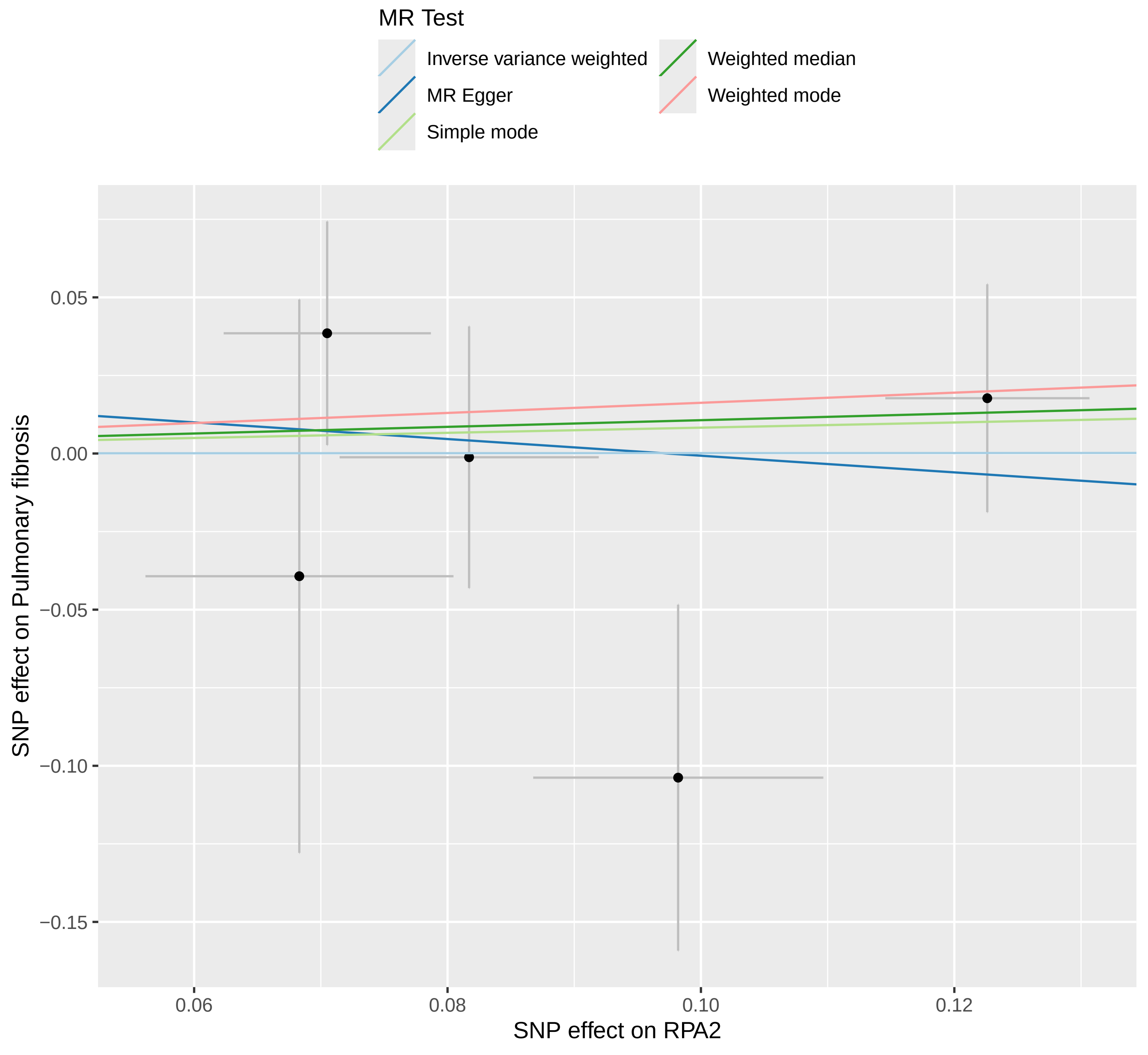

MR Test
Inverse variance weighted
MR Egger
Simple mode
Weighted median
Weighted mode
0.05
0.00
−0.05
−0.10
−0.15
0.06
0.08
0.10
0.12
SNP effect on Pulmonary fibrosis
SNP effect on RPA2

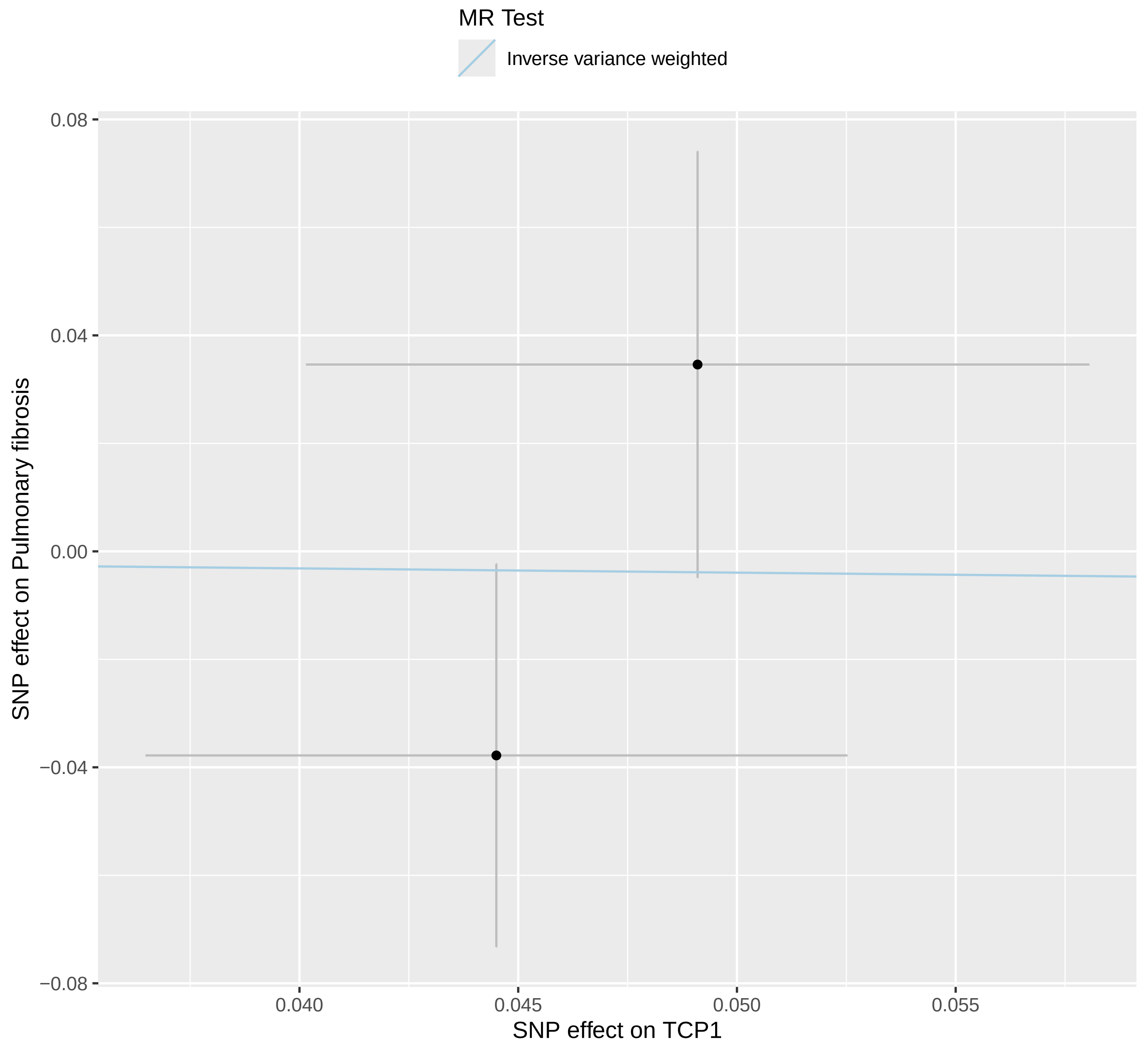

MR Test
Inverse variance weighted
SNP effect on Pulmonary fibrosis
0.08
0.04
0.00
−0.04
−0.08
0.040
0.045
0.050
0.055
SNP effect on TCP1

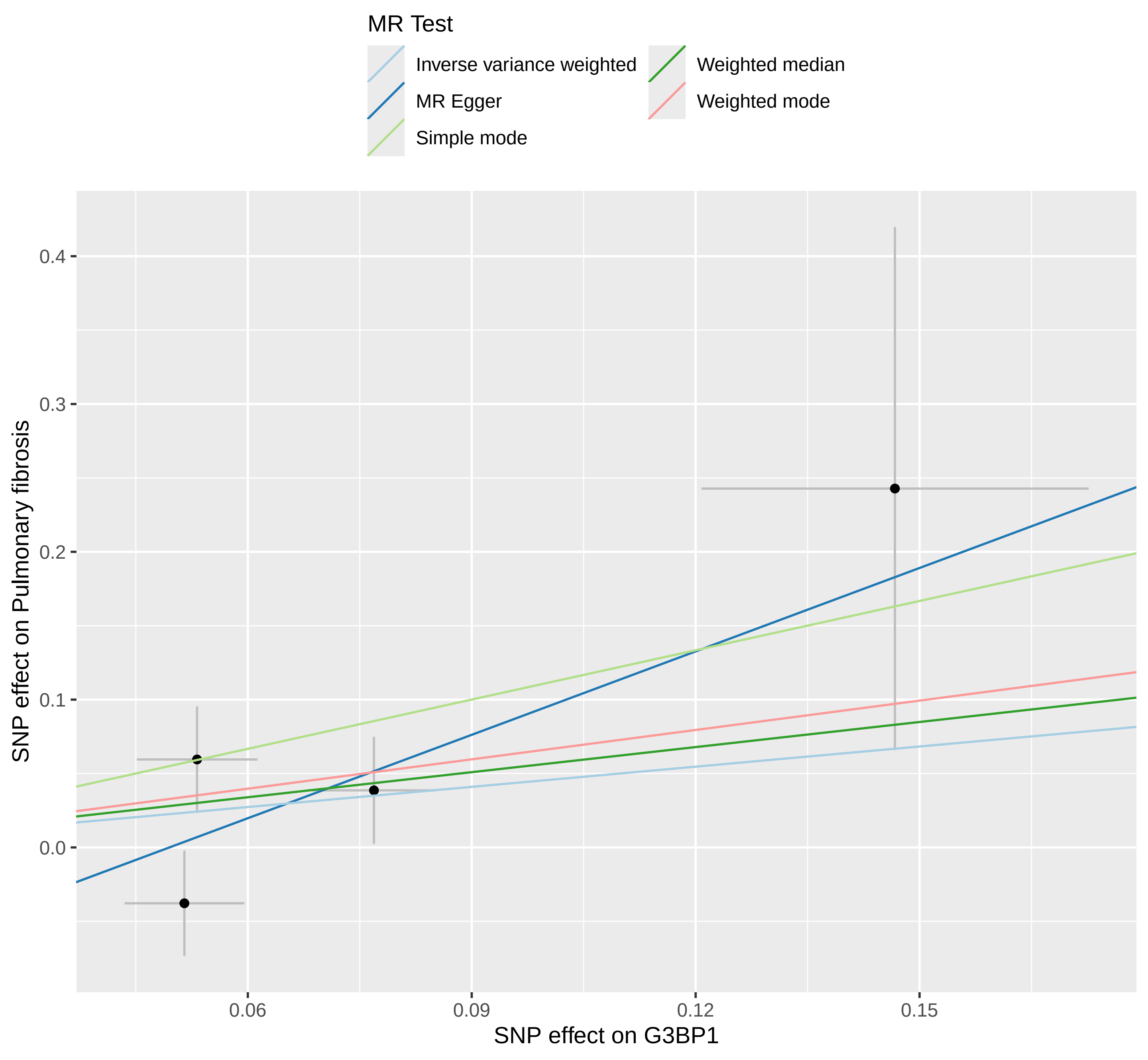

MR Test
Inverse variance weighted
MR Egger
Simple mode
Weighted median
Weighted mode
0.4
0.3
0.2
0.1
0.0
SNP effect on Pulmonary fibrosis
0.06
0.09
0.12
0.15
SNP effect on G3BP1

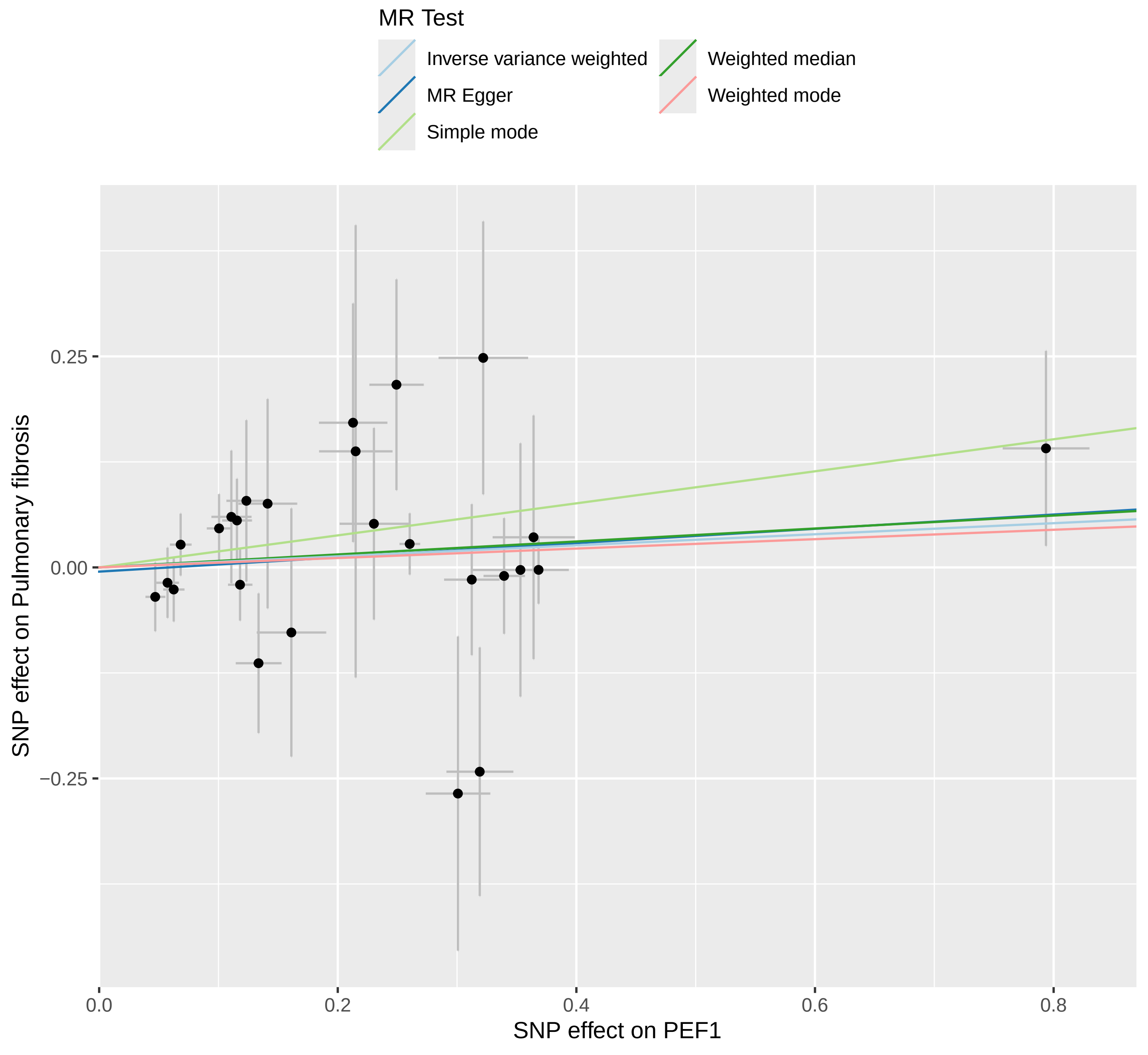

MR Test
Inverse variance weighted
MR Egger
Simple mode
Weighted median
Weighted mode
0.25
0.00
−0.25
SNP effect on Pulmonary fibrosis
0.0
0.2
0.4
0.6
0.8
SNP effect on PEF1

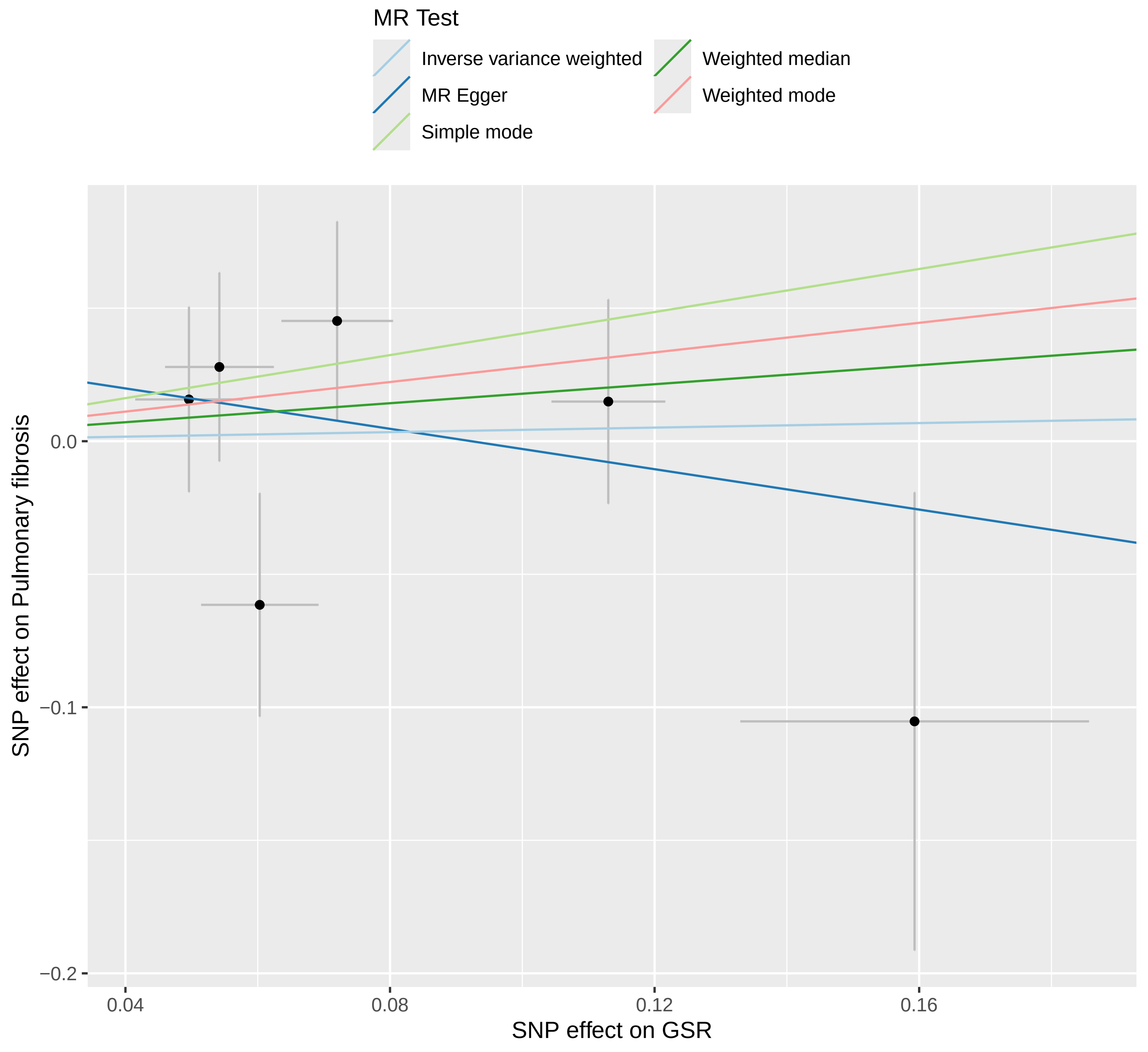
MR Test
Inverse variance weighted
MR Egger
Simple mode
Weighted median
Weighted mode
SNP effect on Pulmonary fibrosis
0.0
−0.1
−0.2
0.04
0.08
0.12
0.16
SNP effect on GSR

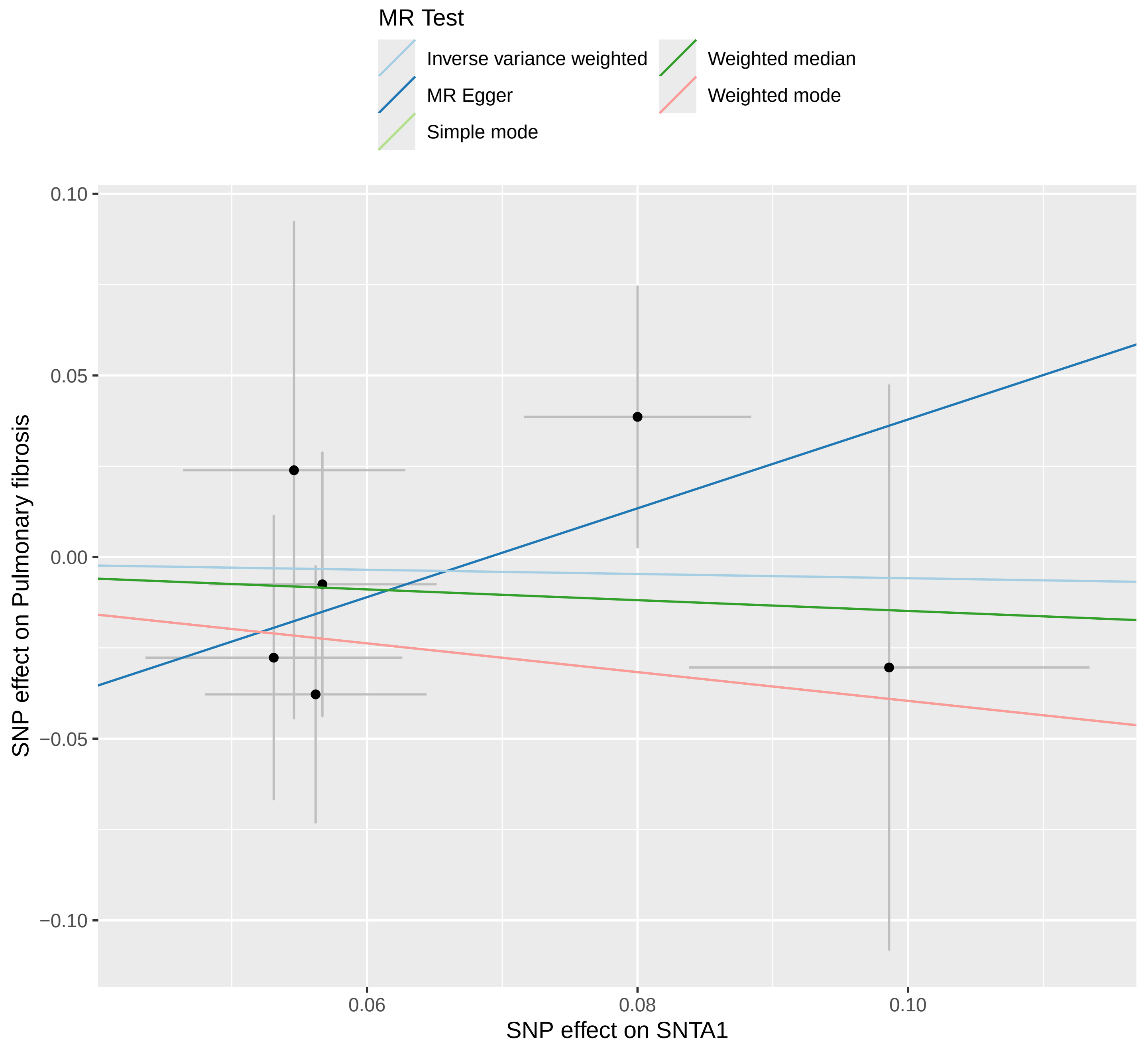
MR Test
Inverse variance weighted
MR Egger
Simple mode
Weighted median
Weighted mode
0.10
0.05
0.00
−0.05
−0.10
SNP effect on Pulmonary fibrosis
0.06
0.08
0.10
SNP effect on SNTA1

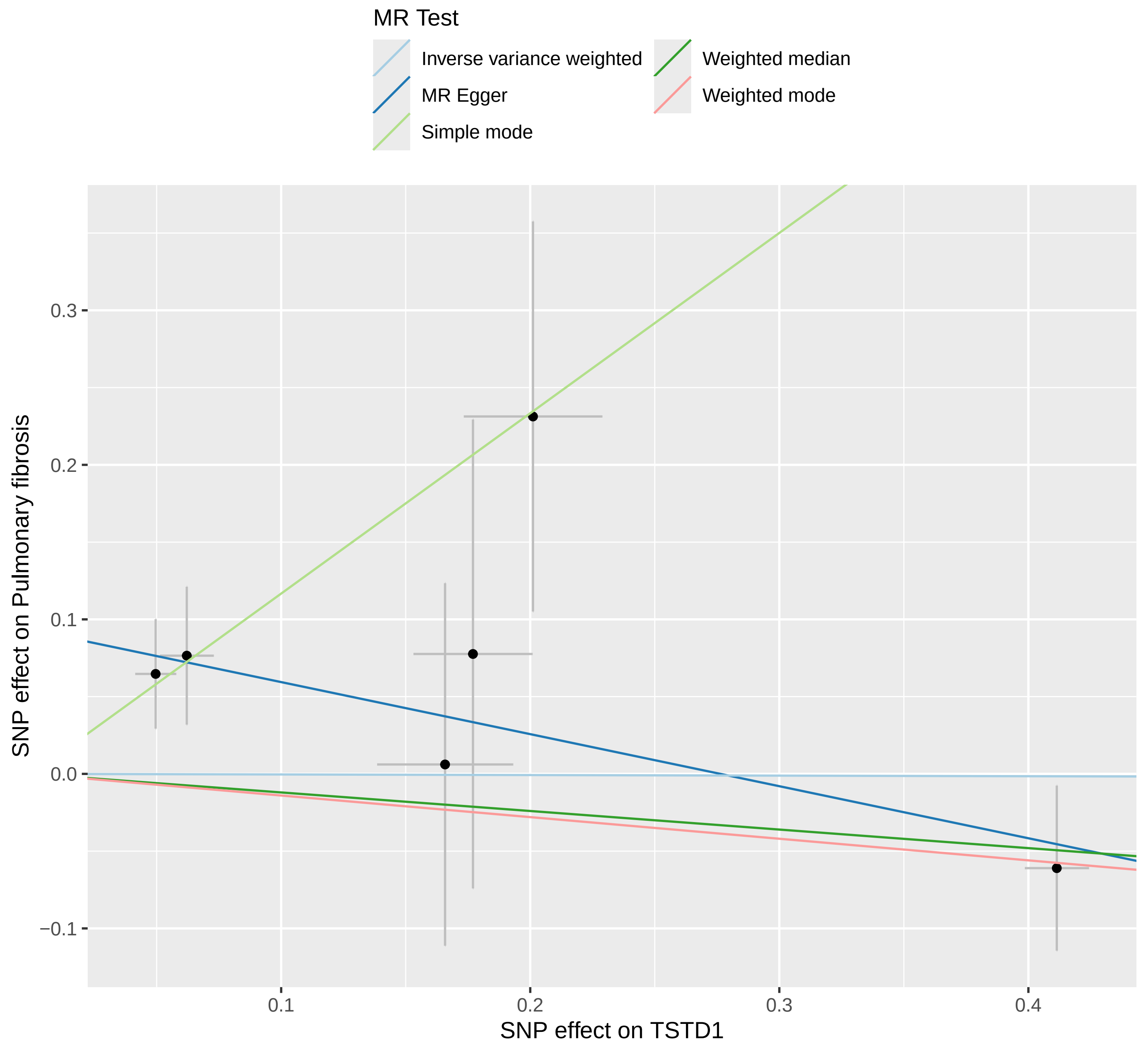
MR Test
Inverse variance weighted
MR Egger
Simple mode
Weighted median
Weighted mode
SNP effect on Pulmonary fibrosis
0.3
0.2
0.1
0.0
−0.1
0.1
0.2
0.3
0.4
SNP effect on TSTD1

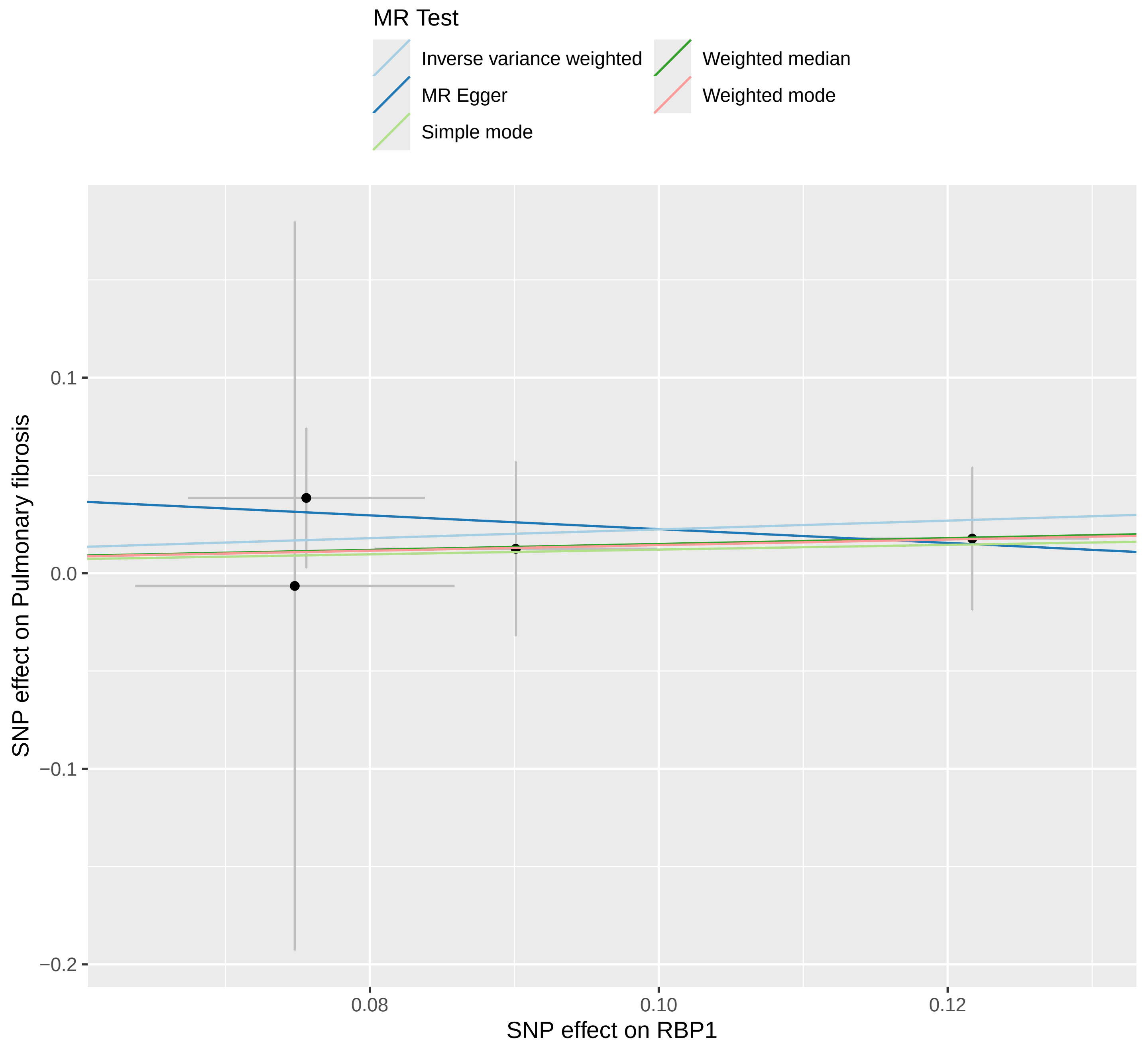
MR Test
Inverse variance weighted
MR Egger
Simple mode
Weighted median
Weighted mode
0.1
0.0
−0.1
−0.2
0.08
0.10
0.12
SNP effect on Pulmonary fibrosis
SNP effect on RBP1

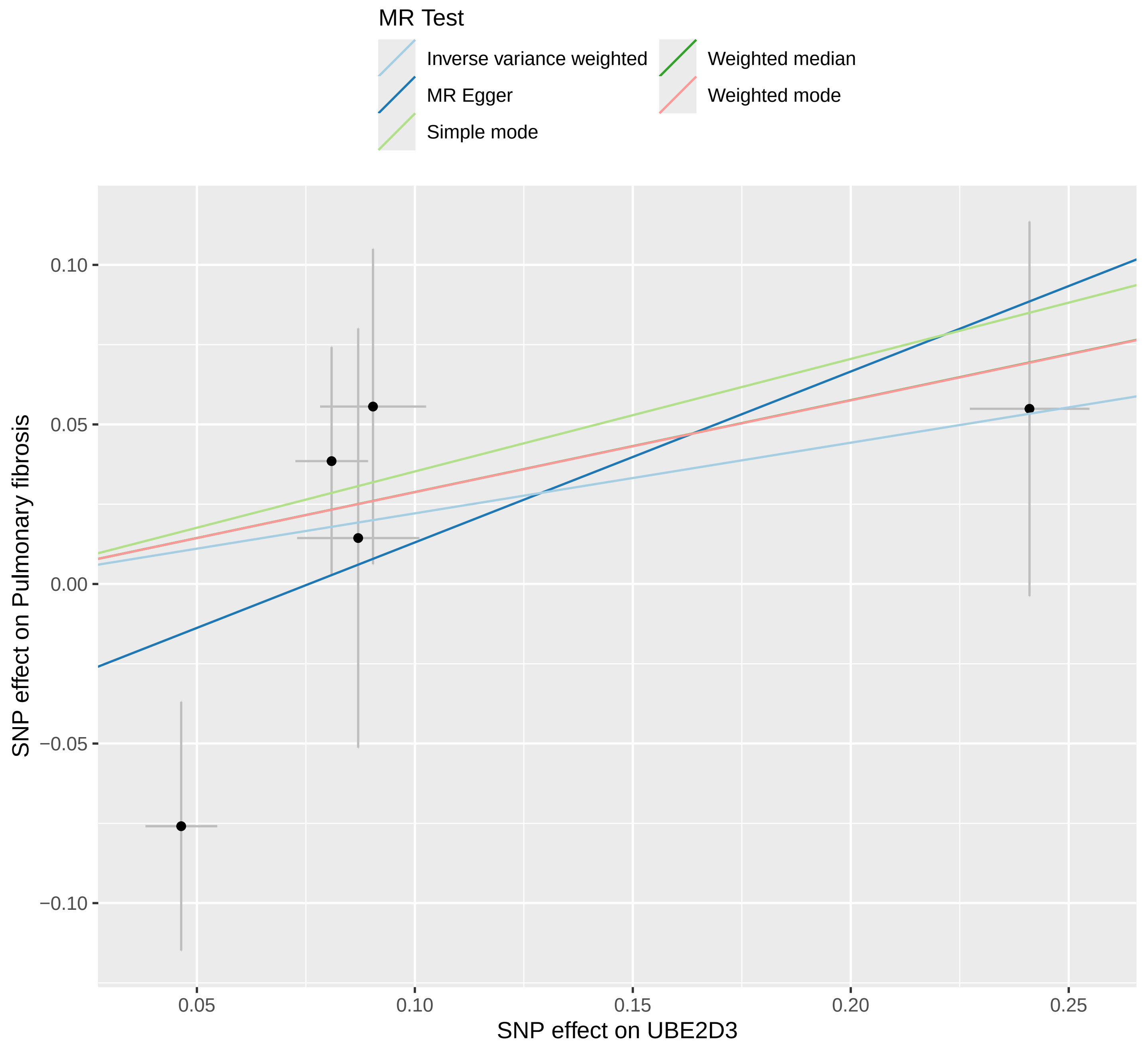
MR Test
Inverse variance weighted
MR Egger
Simple mode
Weighted median
Weighted mode
SNP effect on Pulmonary fibrosis
0.10
0.05
0.00
−0.05
−0.10
0.05
0.10
0.15
0.20
0.25
SNP effect on UBE2D3

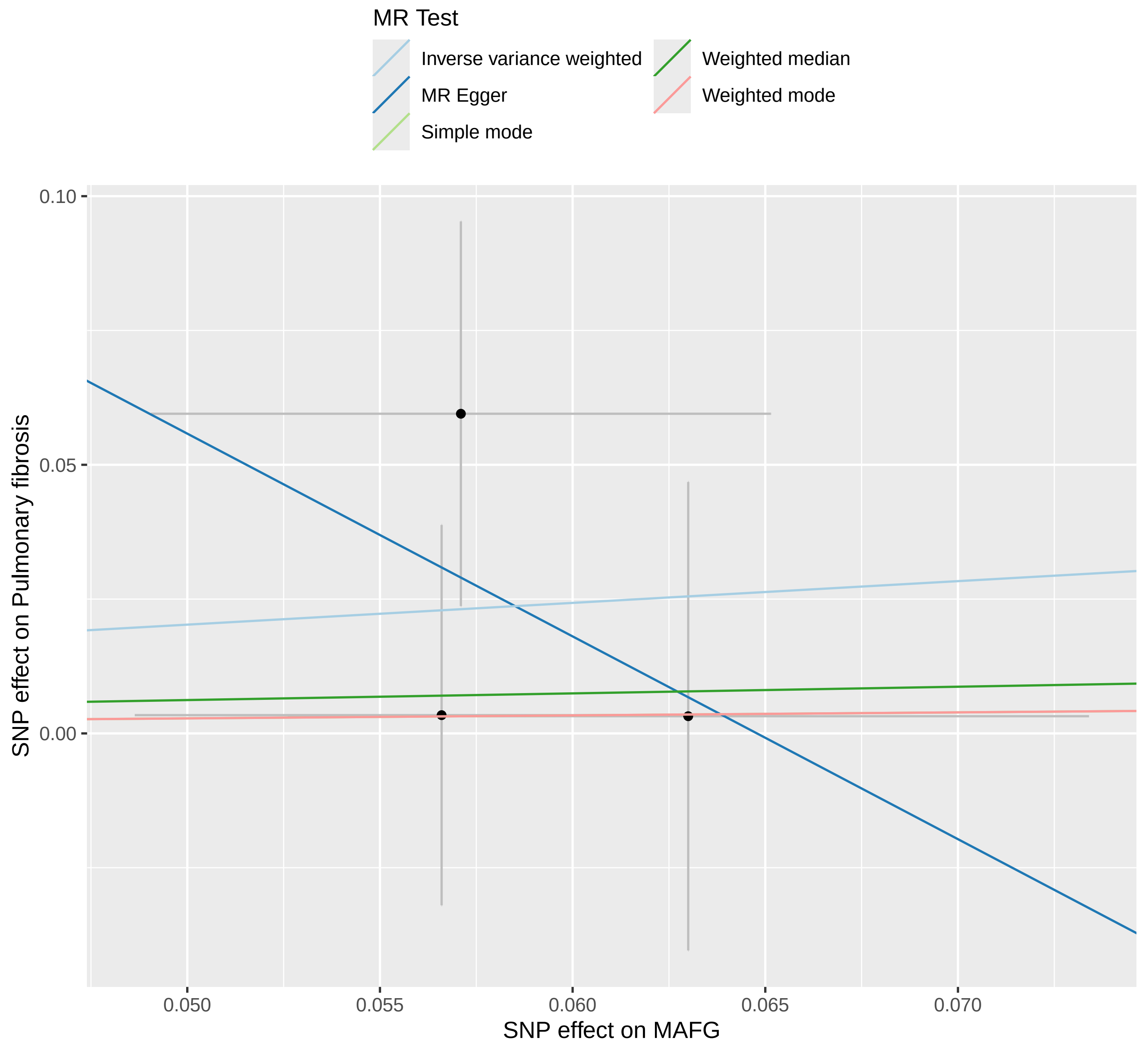
MR Test
Inverse variance weighted
MR Egger
Simple mode
Weighted median
Weighted mode
0.10
0.05
0.00
SNP effect on Pulmonary fibrosis
0.050
0.055
0.060
0.065
0.070
SNP effect on MAFG

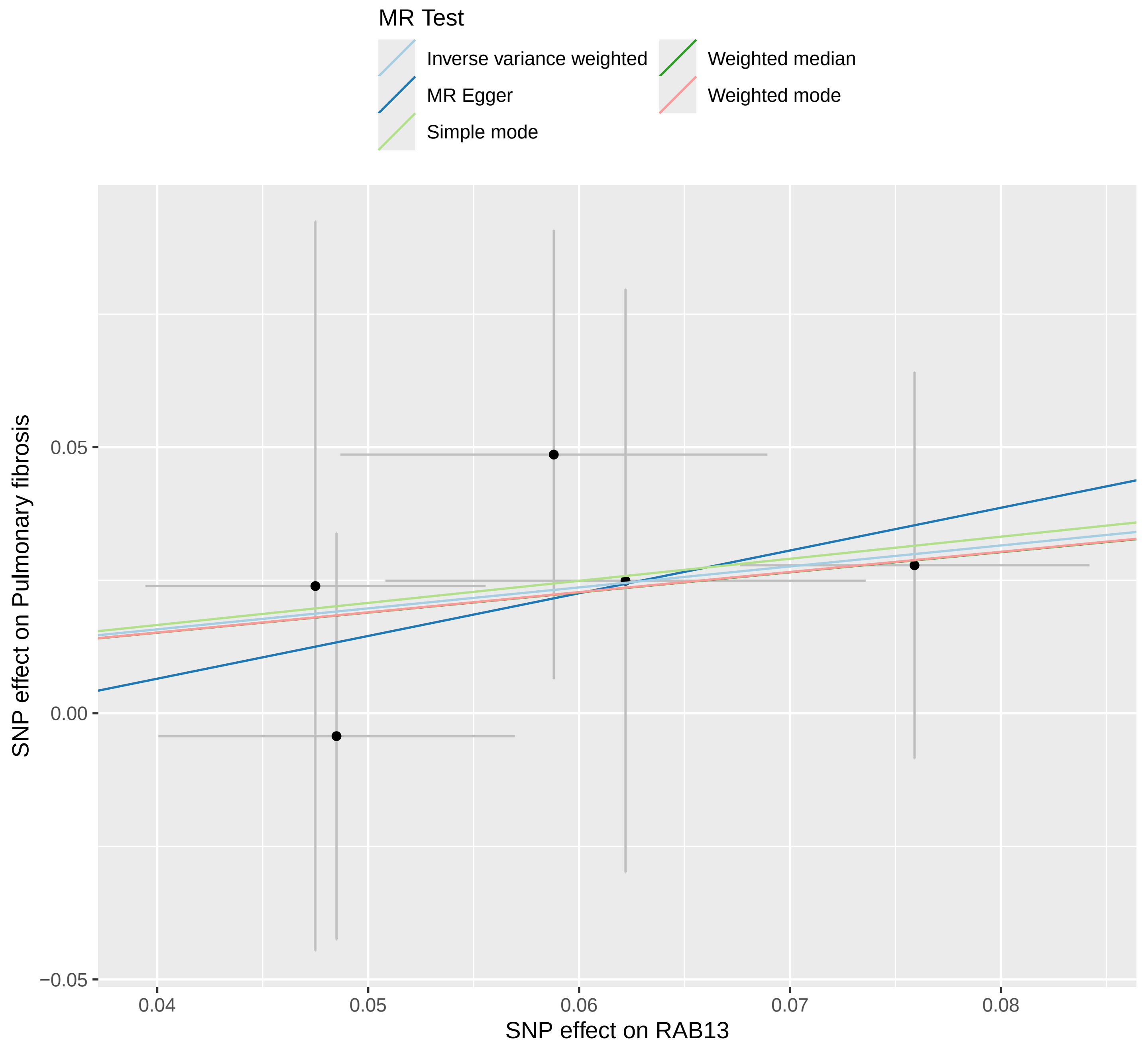

MR Test
Inverse variance weighted
MR Egger
Simple mode
Weighted median
Weighted mode
SNP effect on Pulmonary fibrosis
0.05
0.00
−0.05
0.04
0.05
0.06
0.07
0.08
SNP effect on RAB13

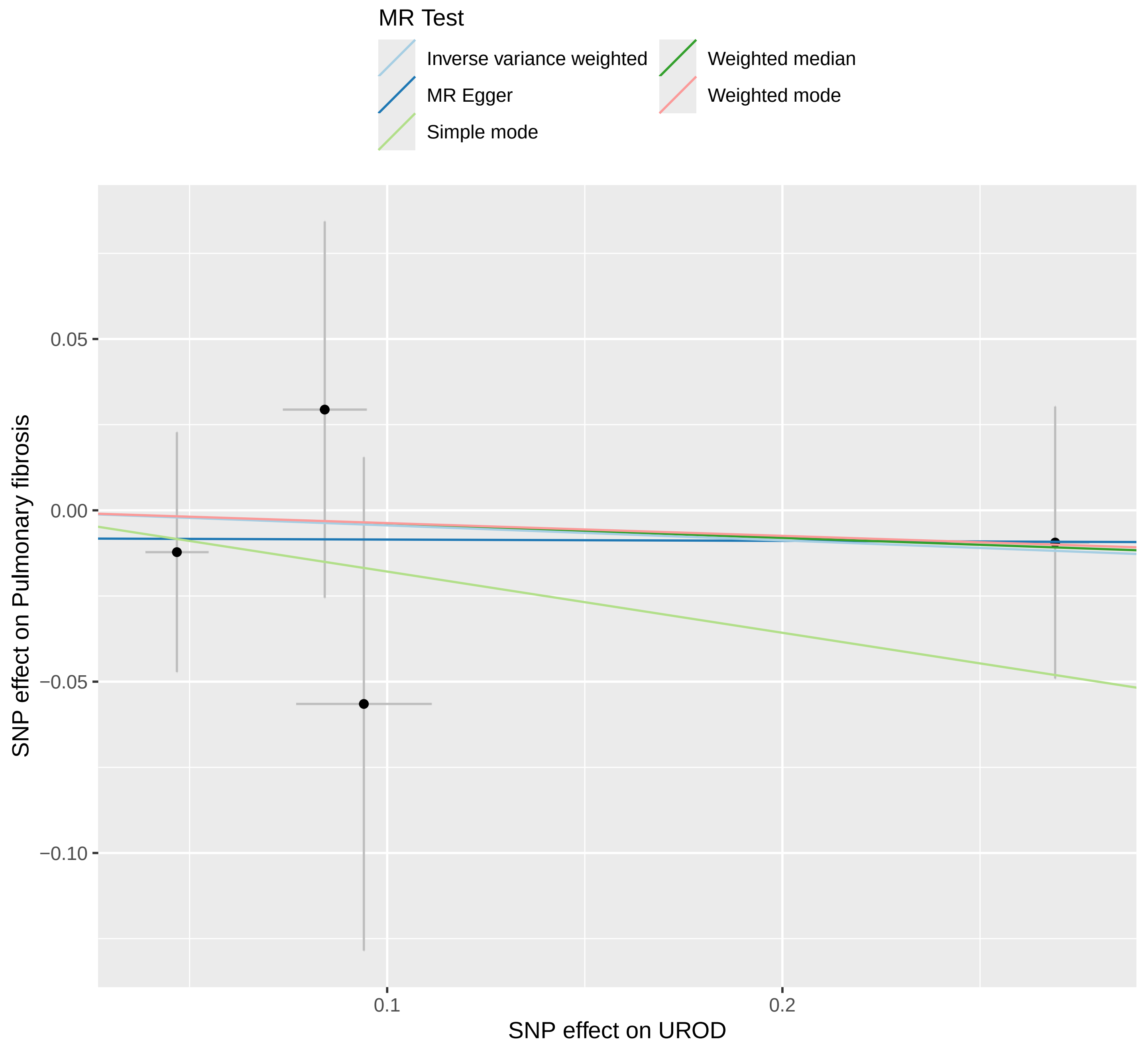

MR Test
Inverse variance weighted
MR Egger
Simple mode
Weighted median
Weighted mode
0.05
0.00
−0.05
−0.10
0.1
0.2
SNP effect on Pulmonary fibrosis
SNP effect on UROD

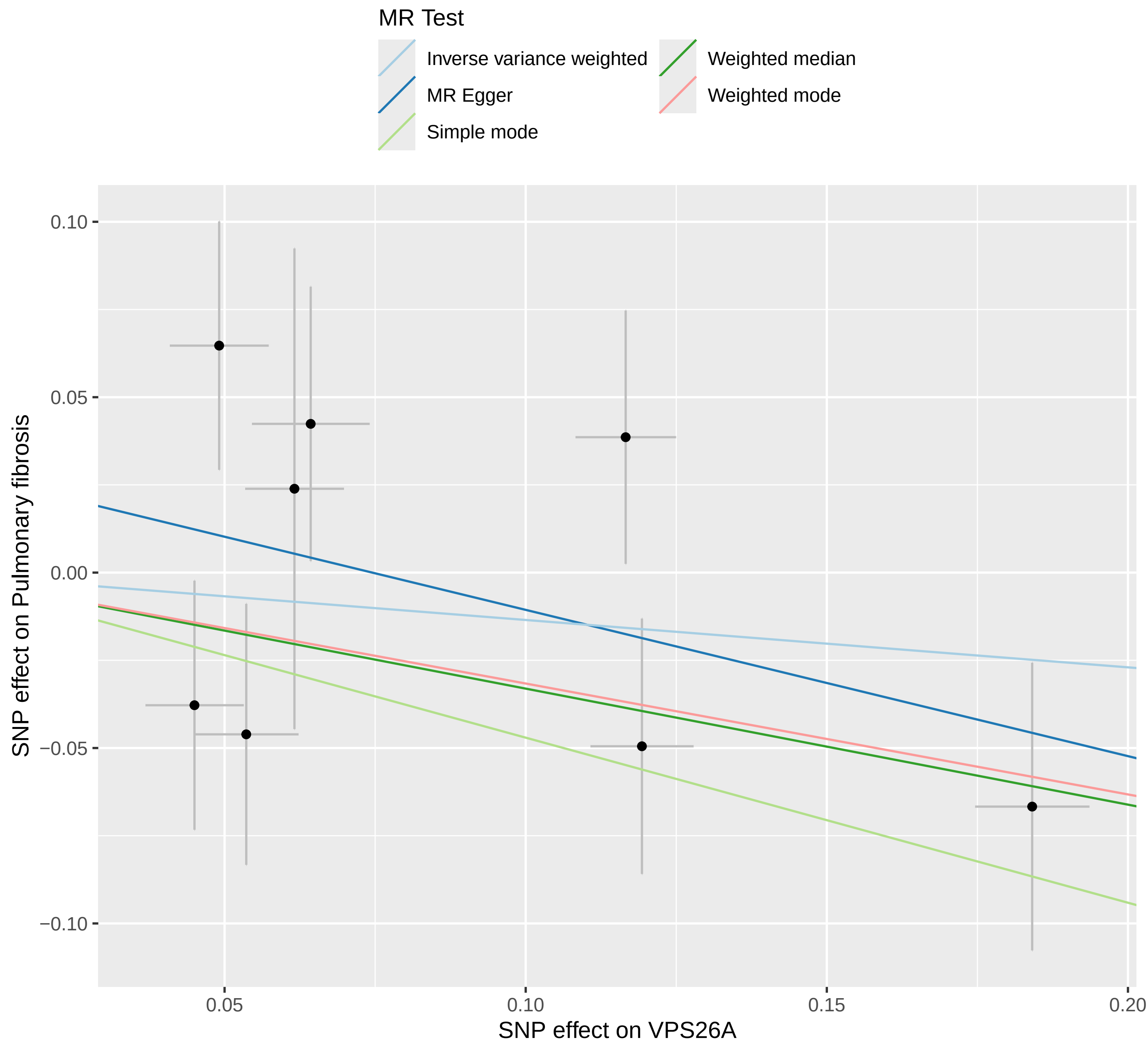

MR Test
Inverse variance weighted
MR Egger
Simple mode
Weighted median
Weighted mode
0.10
0.05
0.00
−0.05
−0.10
SNP effect on Pulmonary fibrosis
0.05
0.10
0.15
0.20
SNP effect on VPS26A

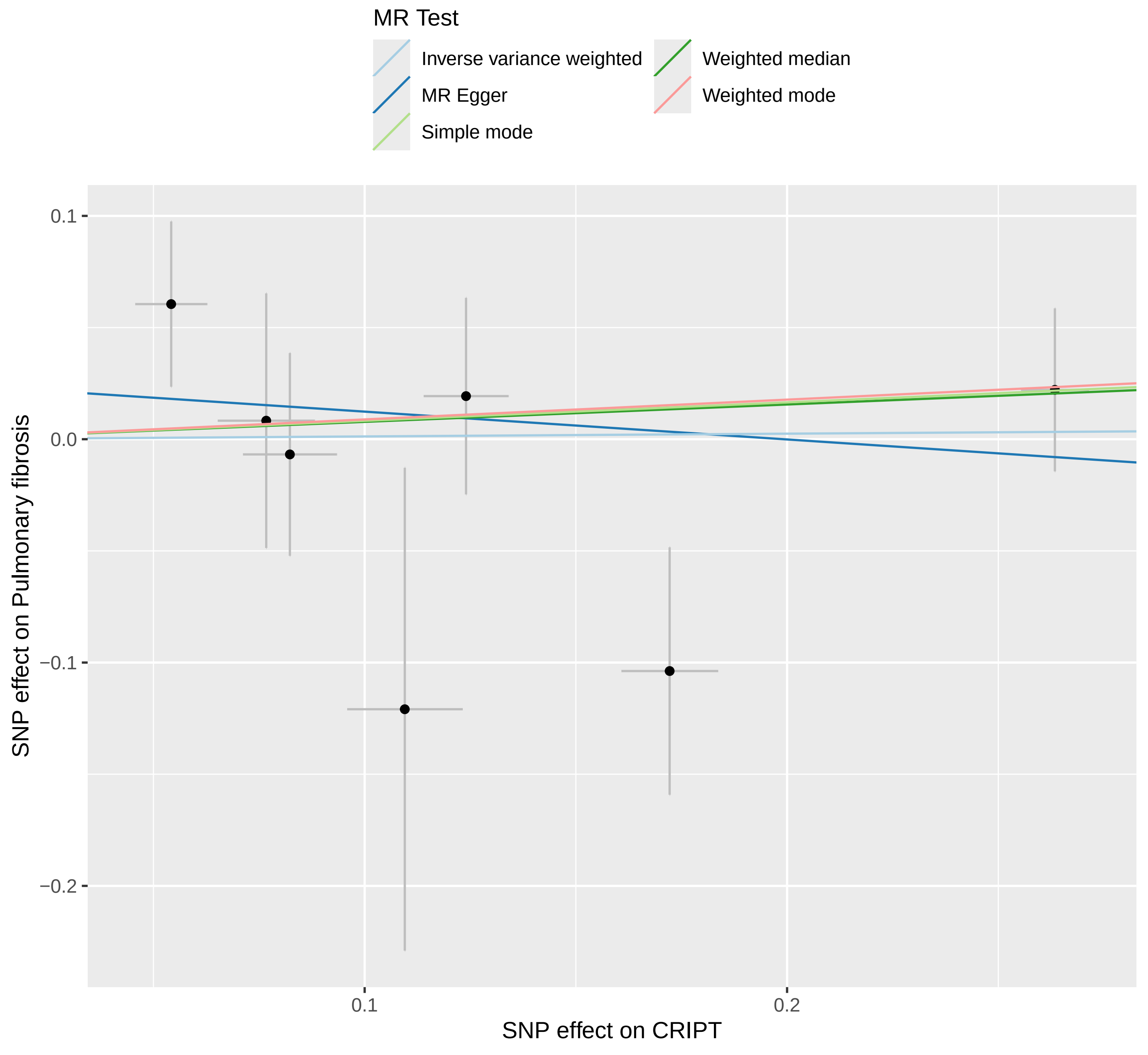
MR Test
Inverse variance weighted
MR Egger
Simple mode
Weighted median
Weighted mode
0.1
0.0
−0.1
−0.2
SNP effect on Pulmonary fibrosis
0.1
0.2
SNP effect on CRIPT

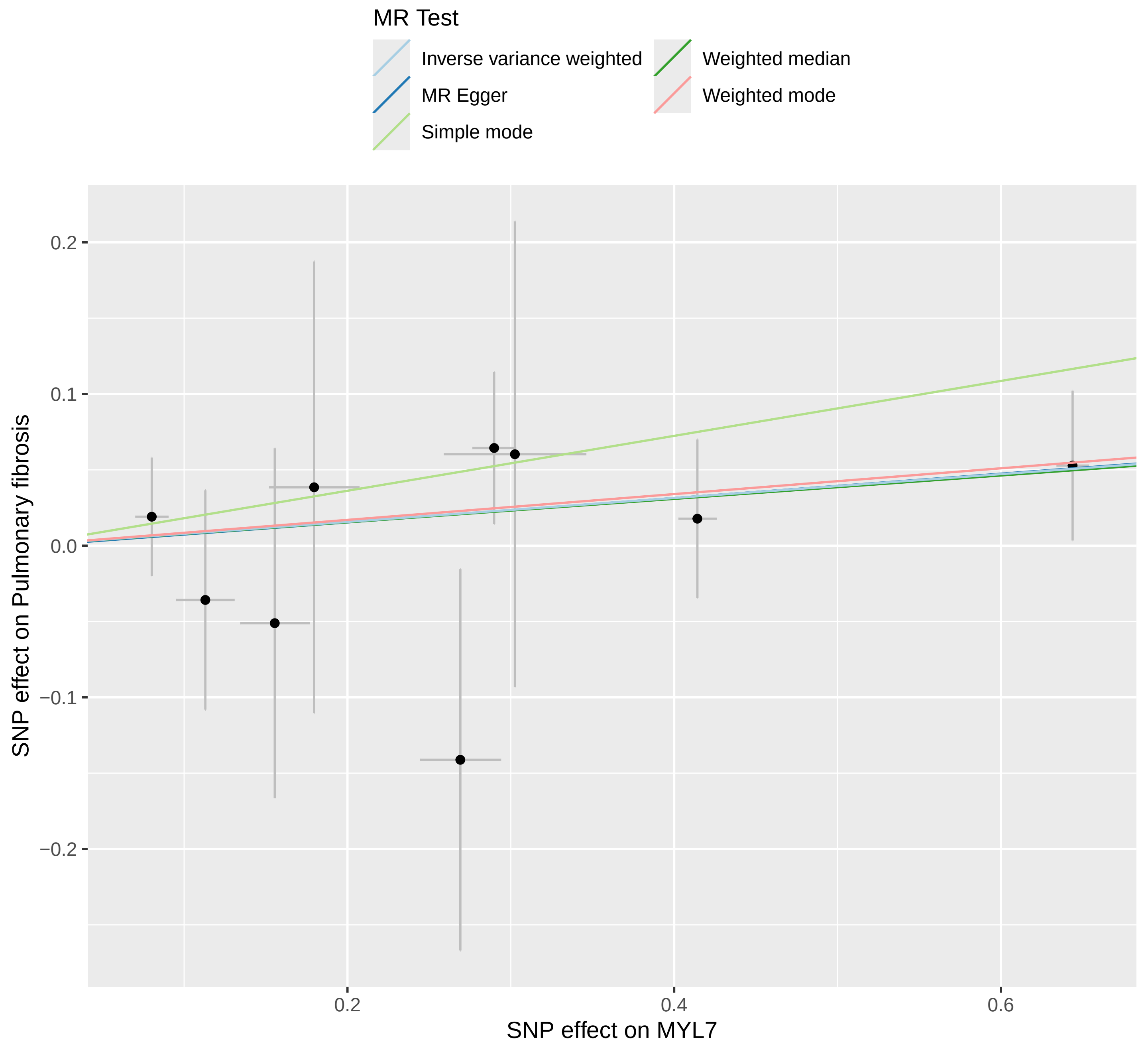
MR Test
Inverse variance weighted
MR Egger
Simple mode
Weighted median
Weighted mode
SNP effect on Pulmonary fibrosis
0.2
0.1
0.0
−0.1
−0.2
0.2
0.4
0.6
SNP effect on MYL7

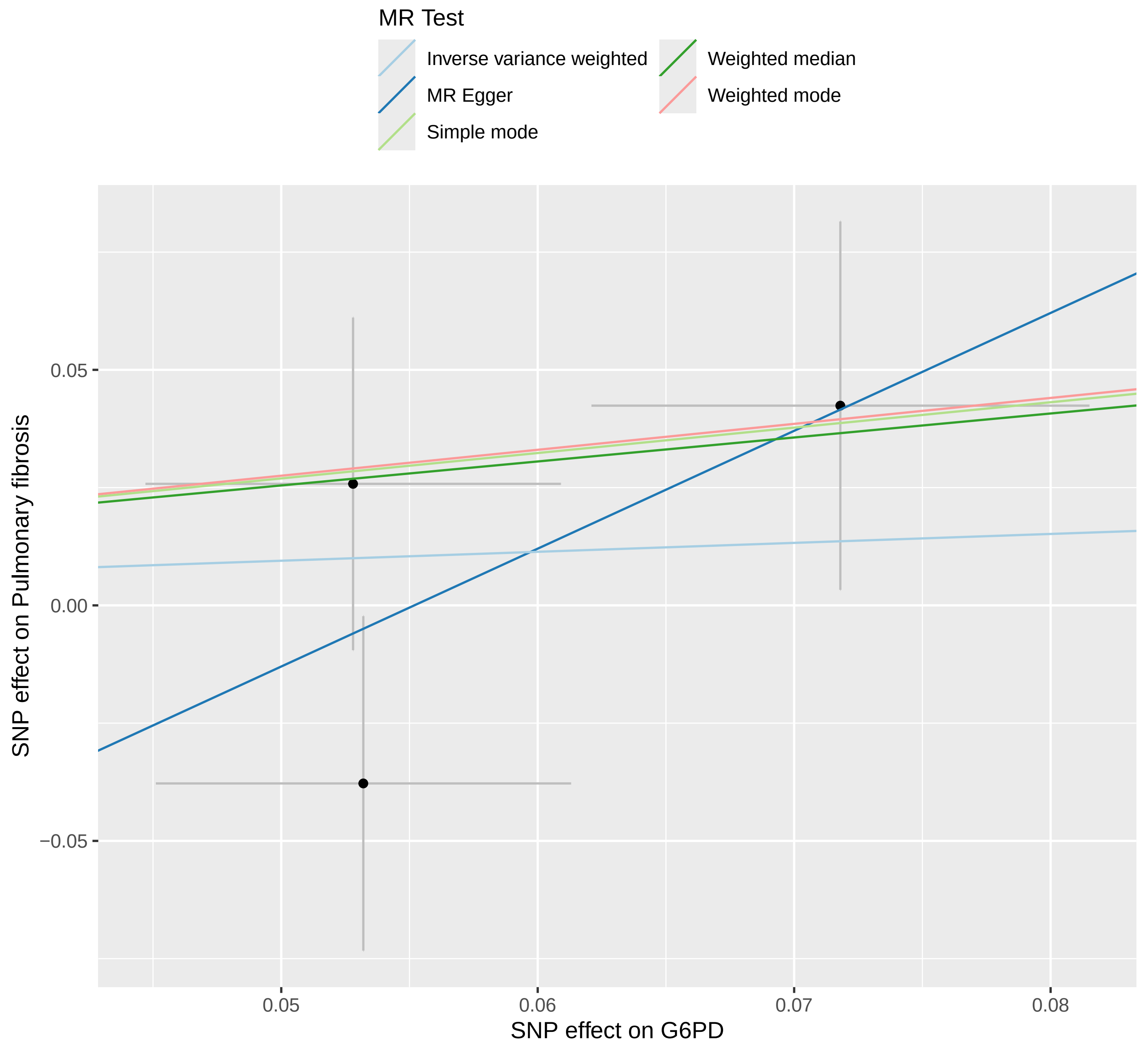

MR Test
Inverse variance weighted
MR Egger
Simple mode
Weighted median
Weighted mode
0.05
0.00
−0.05
SNP effect on Pulmonary fibrosis
0.05
0.06
0.07
0.08
SNP effect on G6PD

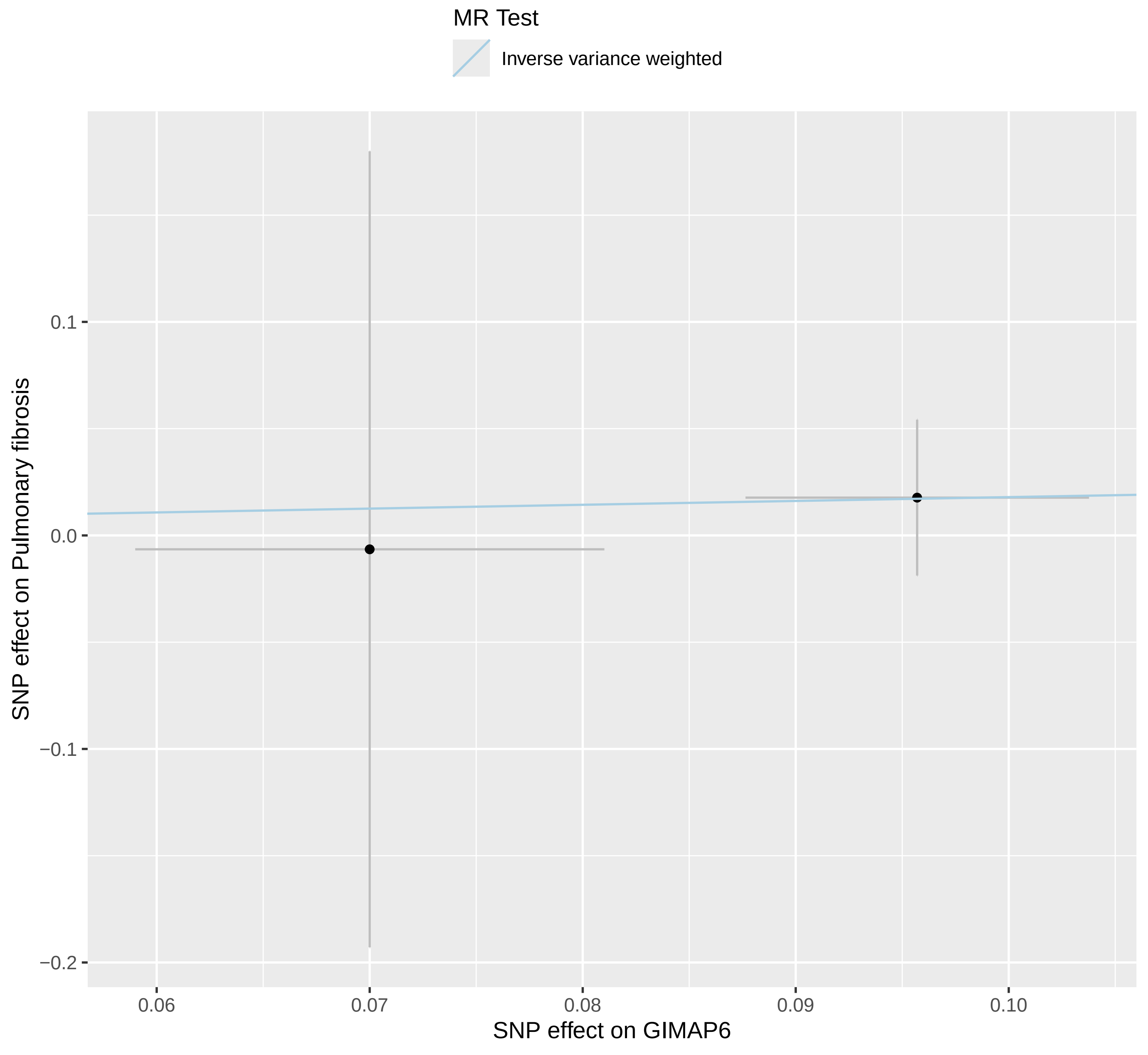

MR Test
Inverse variance weighted
0.1
0.0
−0.1
−0.2
SNP effect on Pulmonary fibrosis
0.06
0.07
0.08
0.09
0.10
SNP effect on GIMAP6

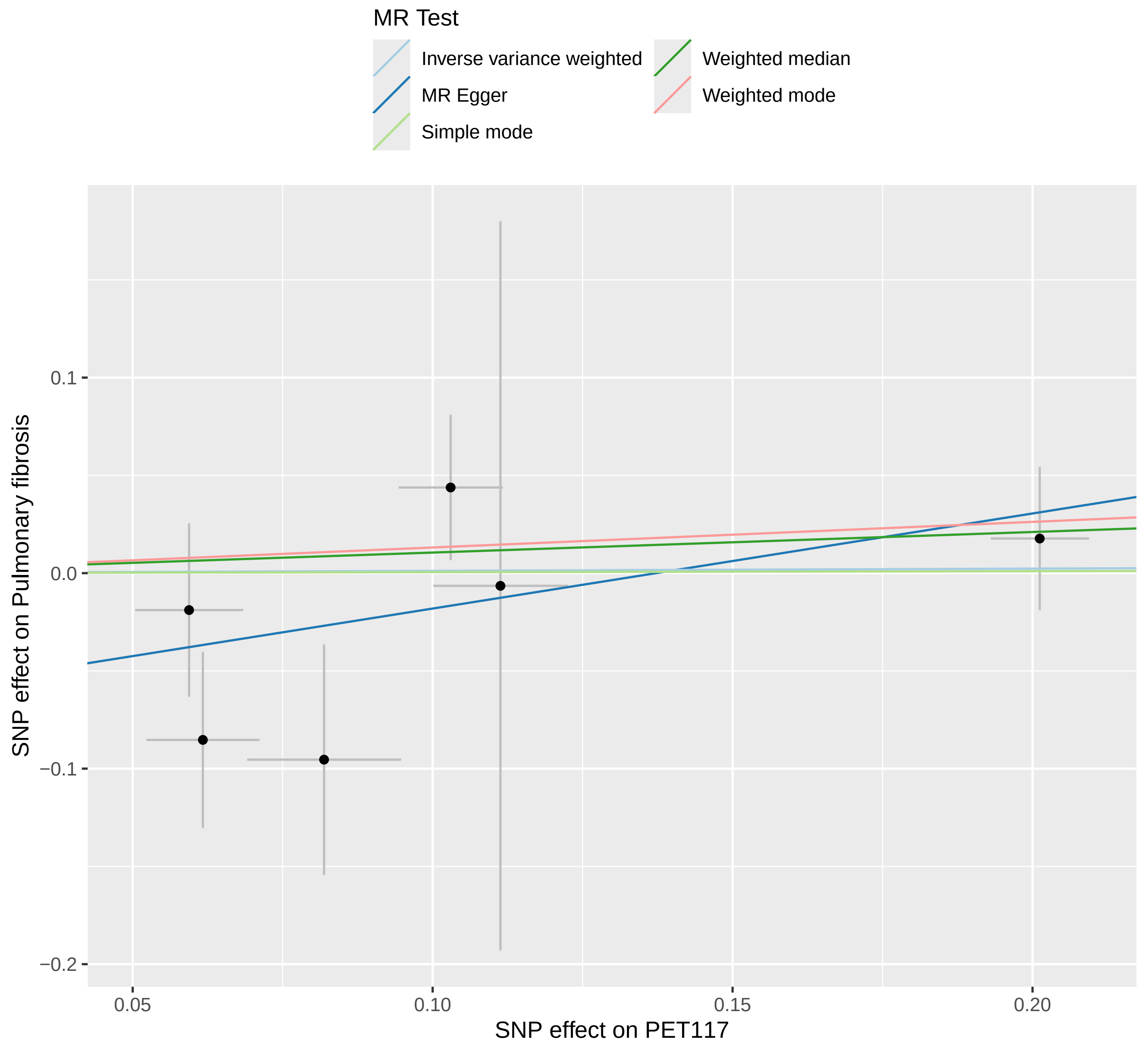
MR Test
Inverse variance weighted
MR Egger
Simple mode
Weighted median
Weighted mode
0.1
0.0
−0.1
−0.2
SNP effect on Pulmonary fibrosis
0.05
0.10
0.15
0.20
SNP effect on PET117

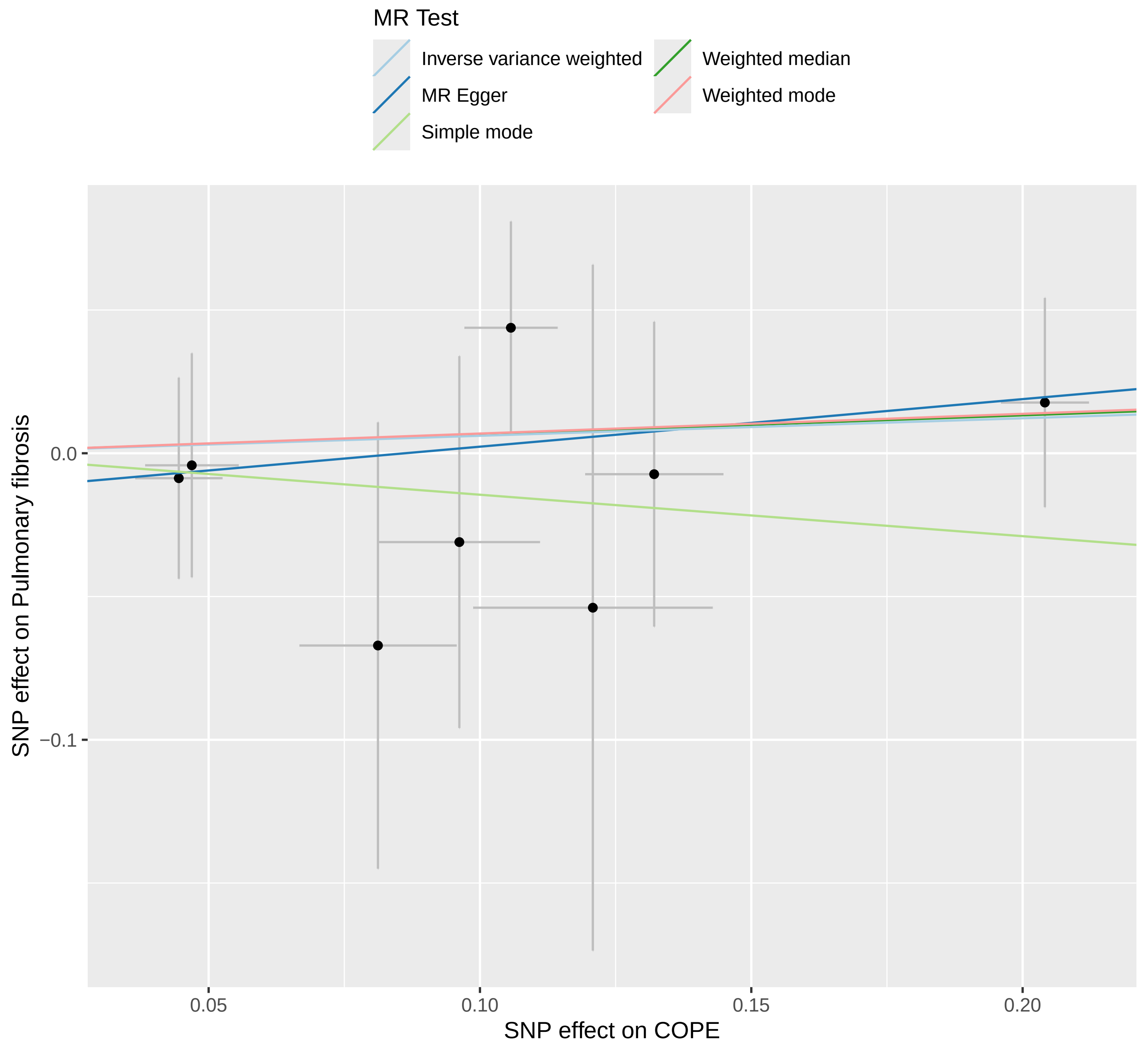

MR Test
Inverse variance weighted
MR Egger
Simple mode
Weighted median
Weighted mode
SNP effect on Pulmonary fibrosis
0.0
−0.1
0.05
0.10
0.15
0.20
SNP effect on COPE

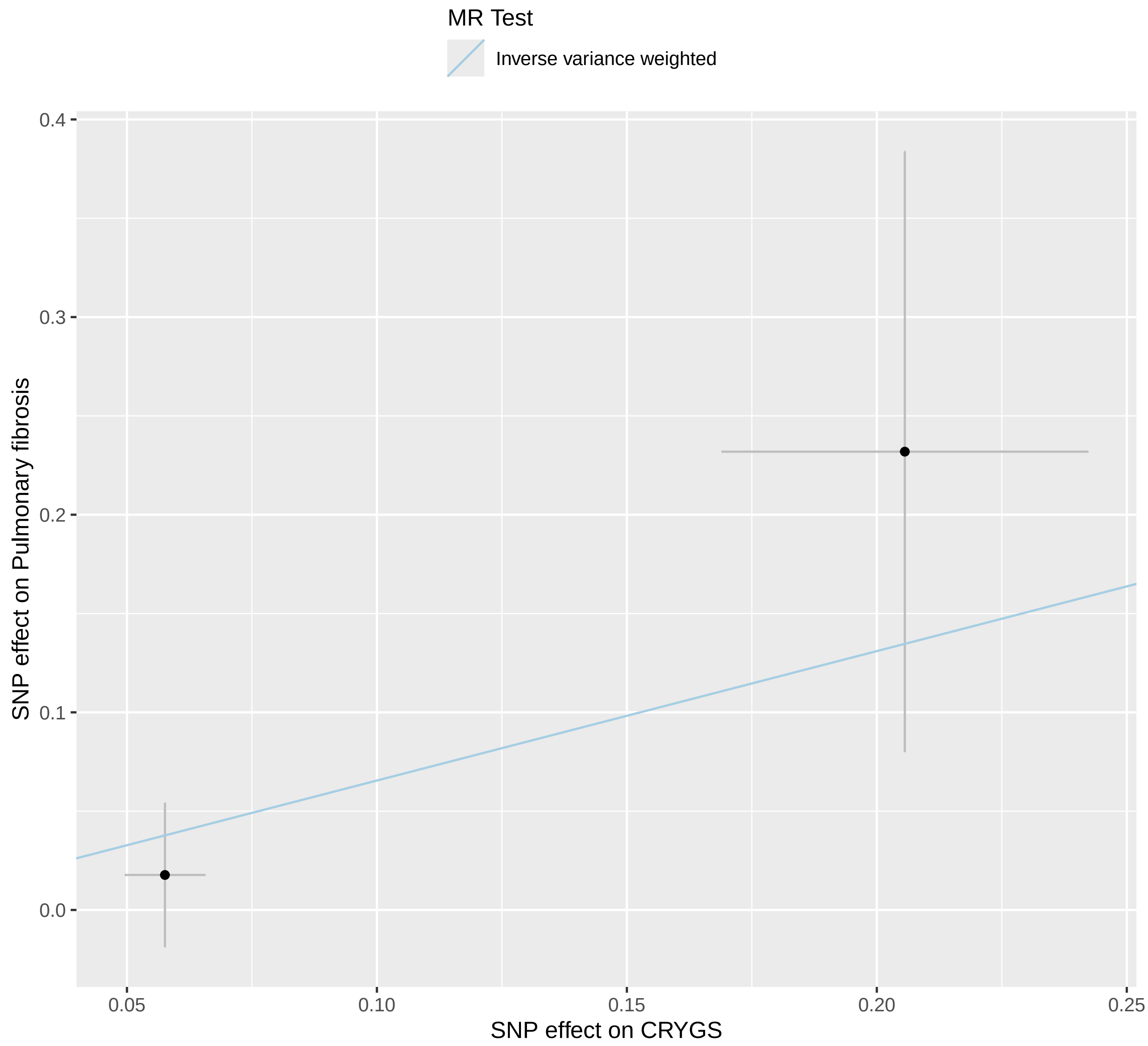

MR Test
Inverse variance weighted
0.4
0.3
0.2
0.1
0.0
SNP effect on Pulmonary fibrosis
0.05
0.10
0.15
0.20
0.25
SNP effect on CRYGS

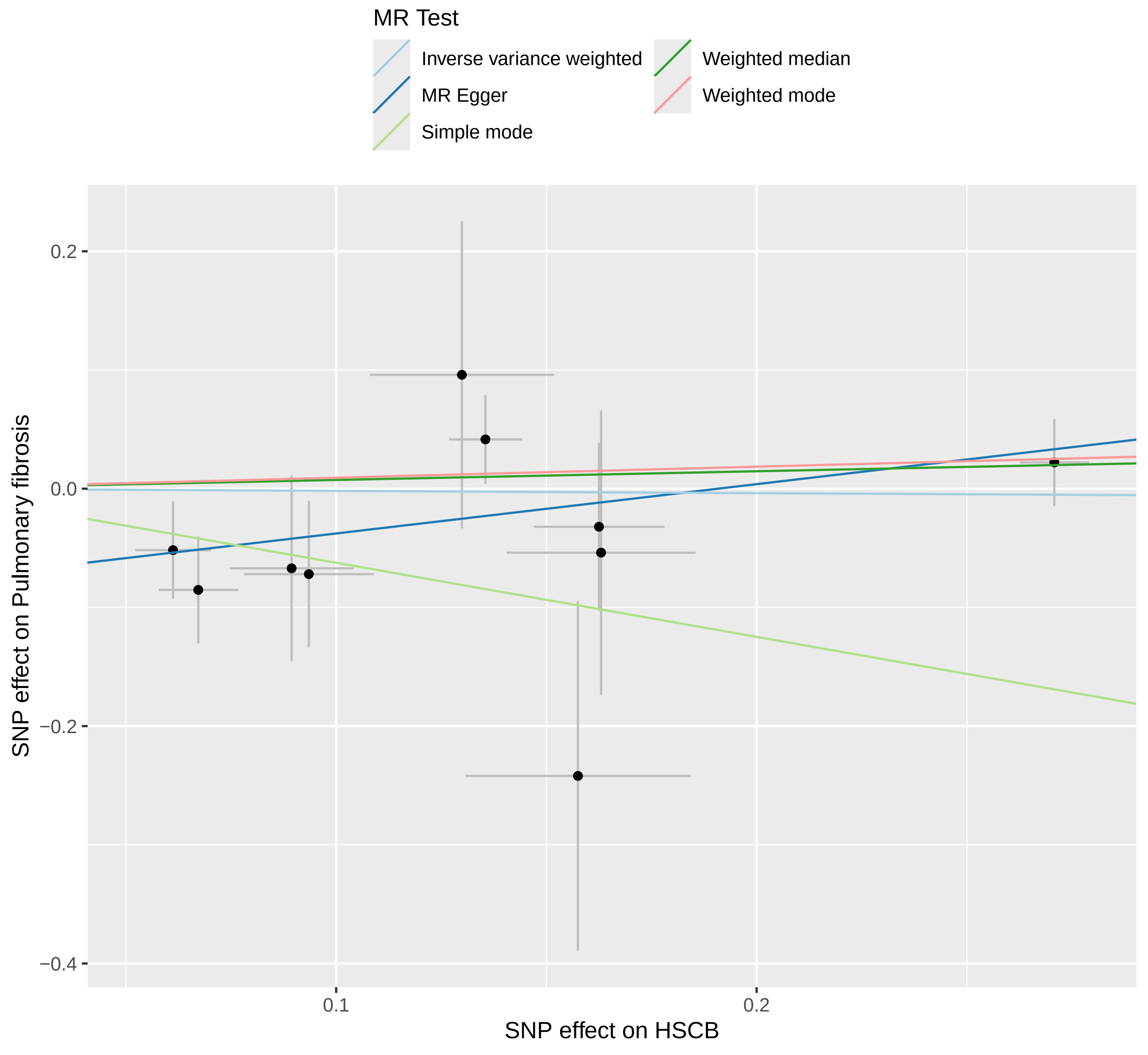

MR Test
Inverse variance weighted
MR Egger
Simple mode
Weighted median
Weighted mode
0.2
0.0
−0.2
−0.4
SNP effect on Pulmonary fibrosis
0.1
0.2
SNP effect on HSCB

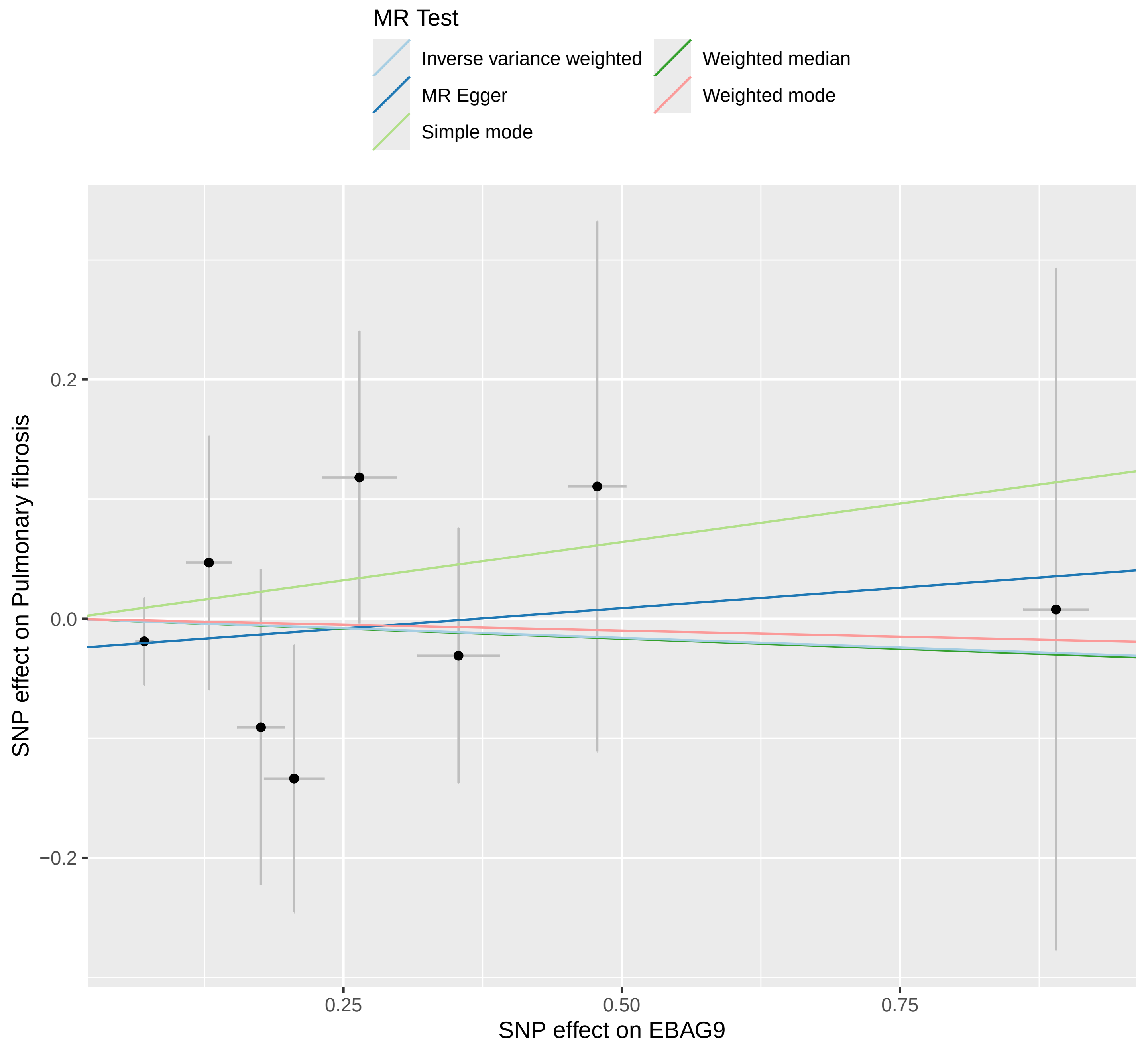
MR Test
Inverse variance weighted
MR Egger
Simple mode
Weighted median
Weighted mode
0.2
0.0
−0.2
0.25
0.50
0.75
SNP effect on Pulmonary fibrosis
SNP effect on EBAG9

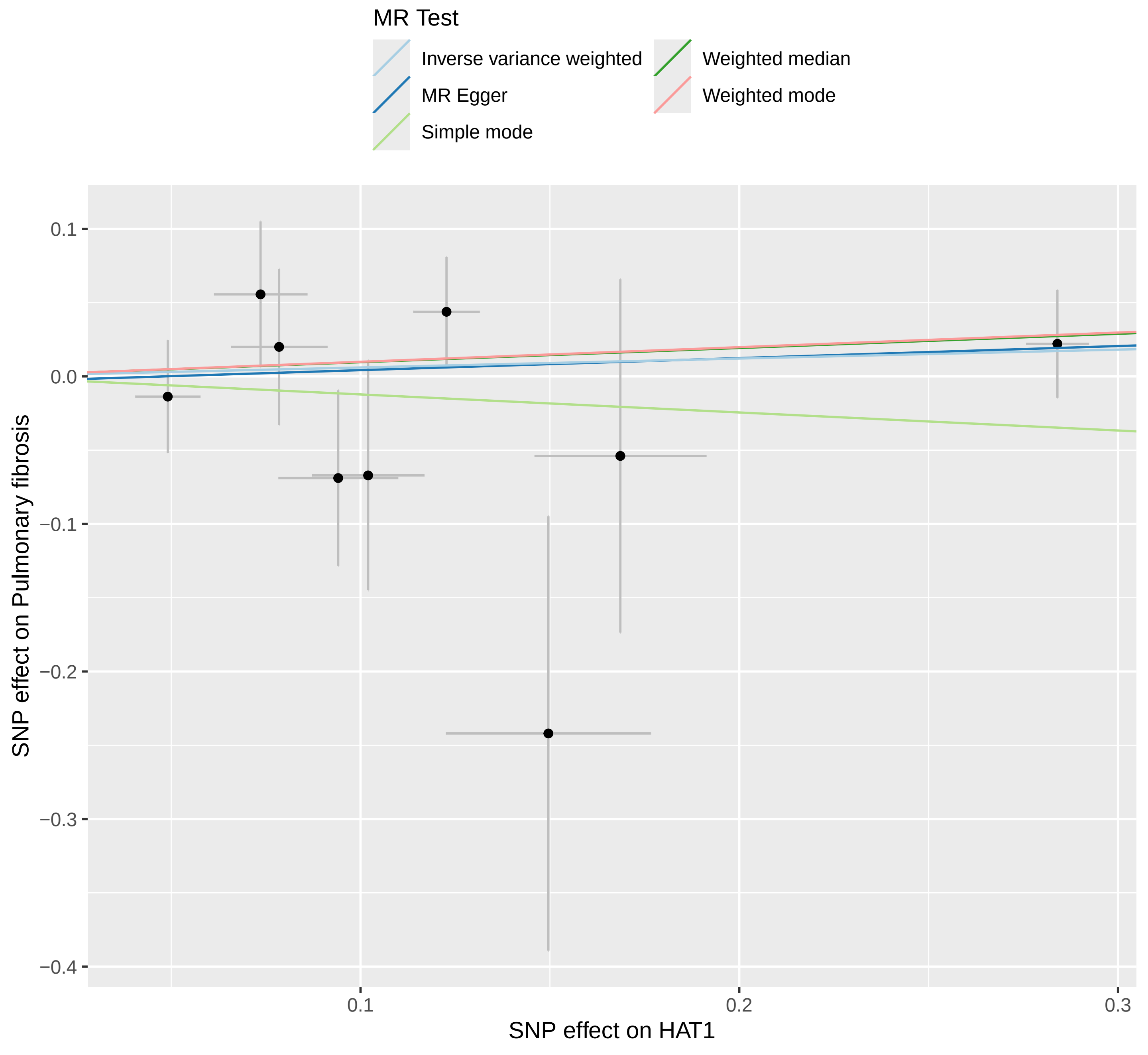
MR Test
Inverse variance weighted
MR Egger
Simple mode
Weighted median
Weighted mode
0.1
0.0
−0.1
−0.2
−0.3
−0.4
SNP effect on Pulmonary fibrosis
0.1
0.2
0.3
SNP effect on HAT1

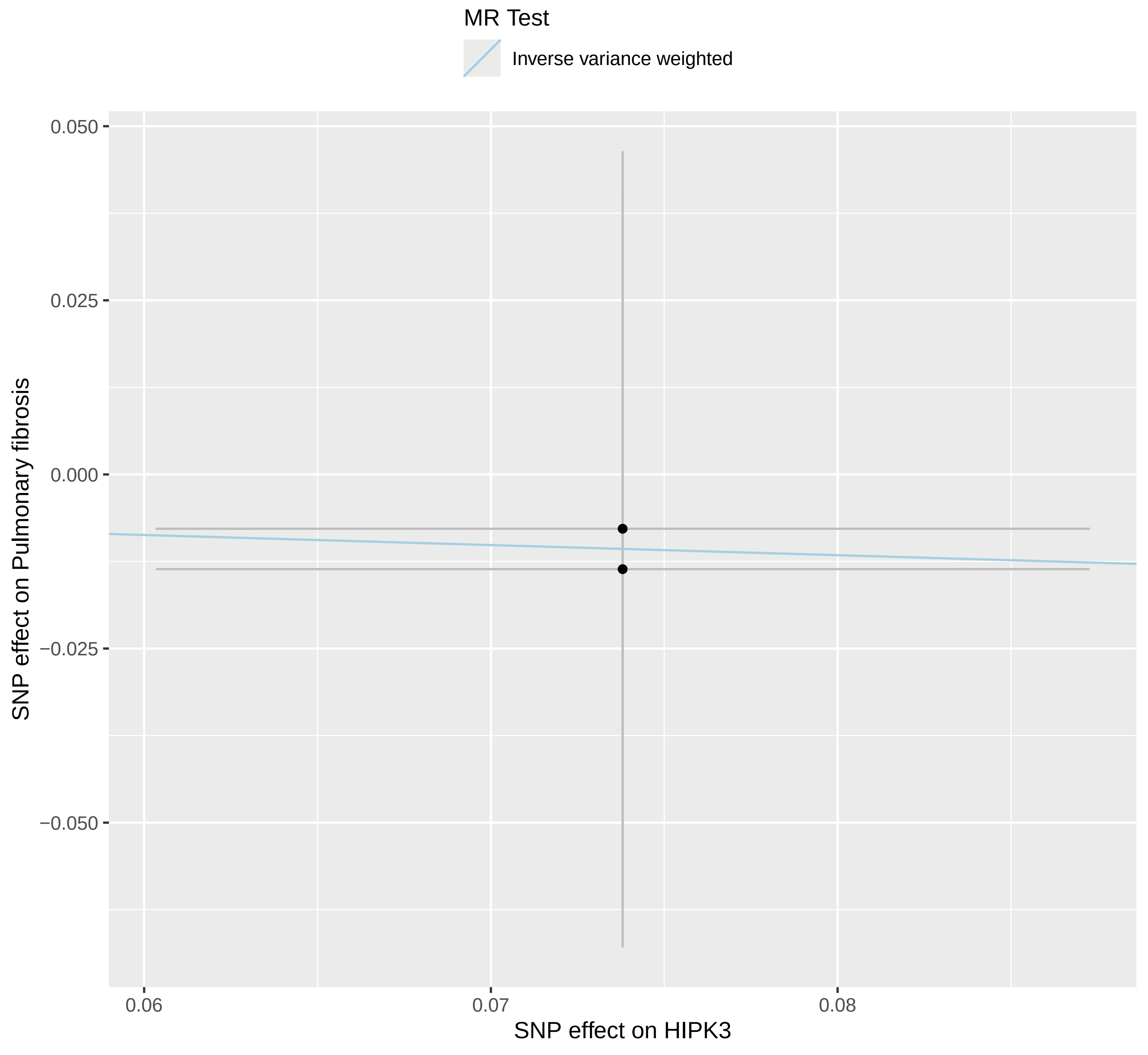

MR Test
Inverse variance weighted
0.050
0.025
0.000
−0.025
−0.050
SNP effect on Pulmonary fibrosis
0.06
0.07
0.08
SNP effect on HIPK3

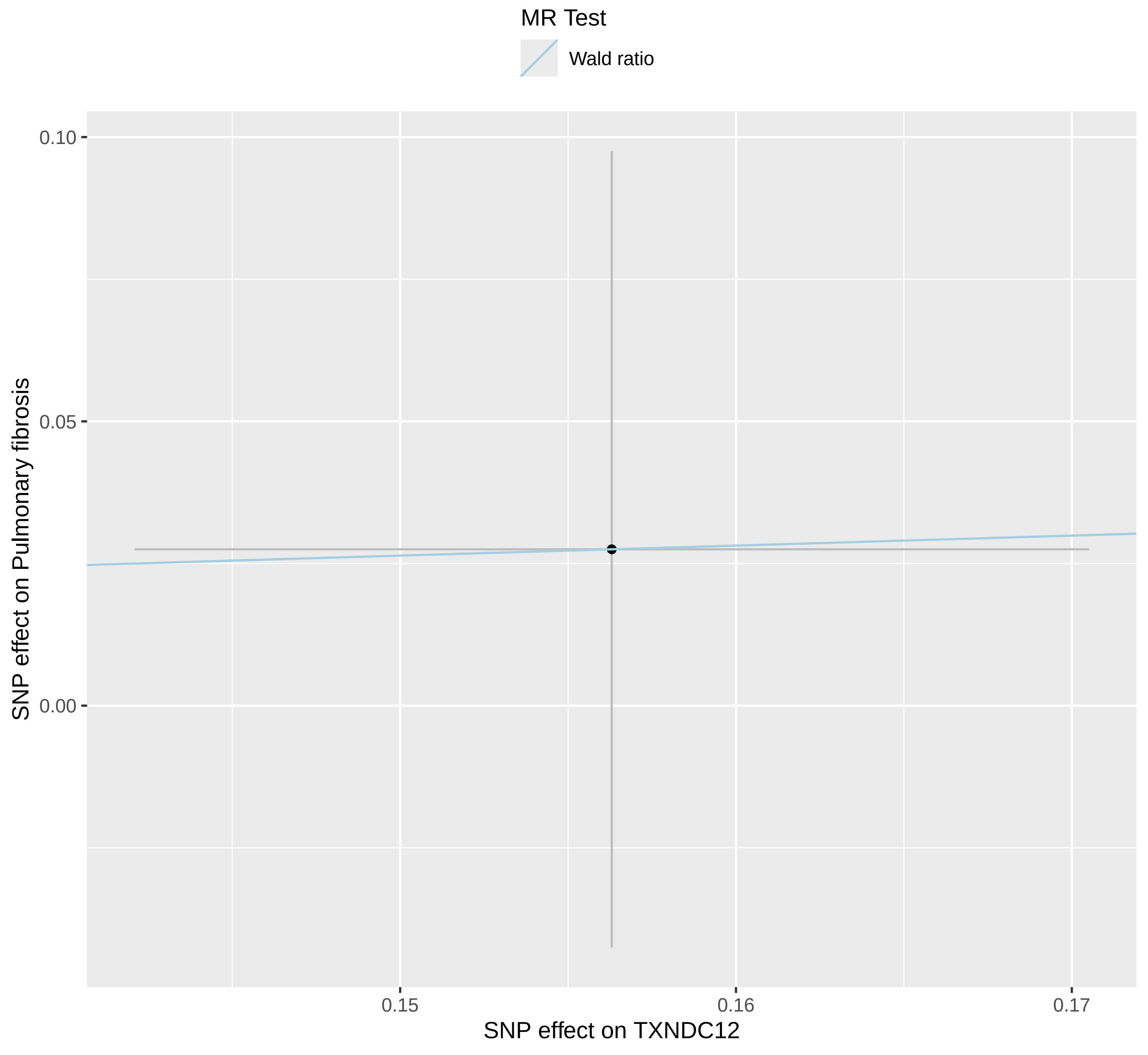
MR Test
Wald ratio
0.10
0.05
0.00
SNP effect on Pulmonary fibrosis
0.15
0.16
0.17
SNP effect on TXNDC12

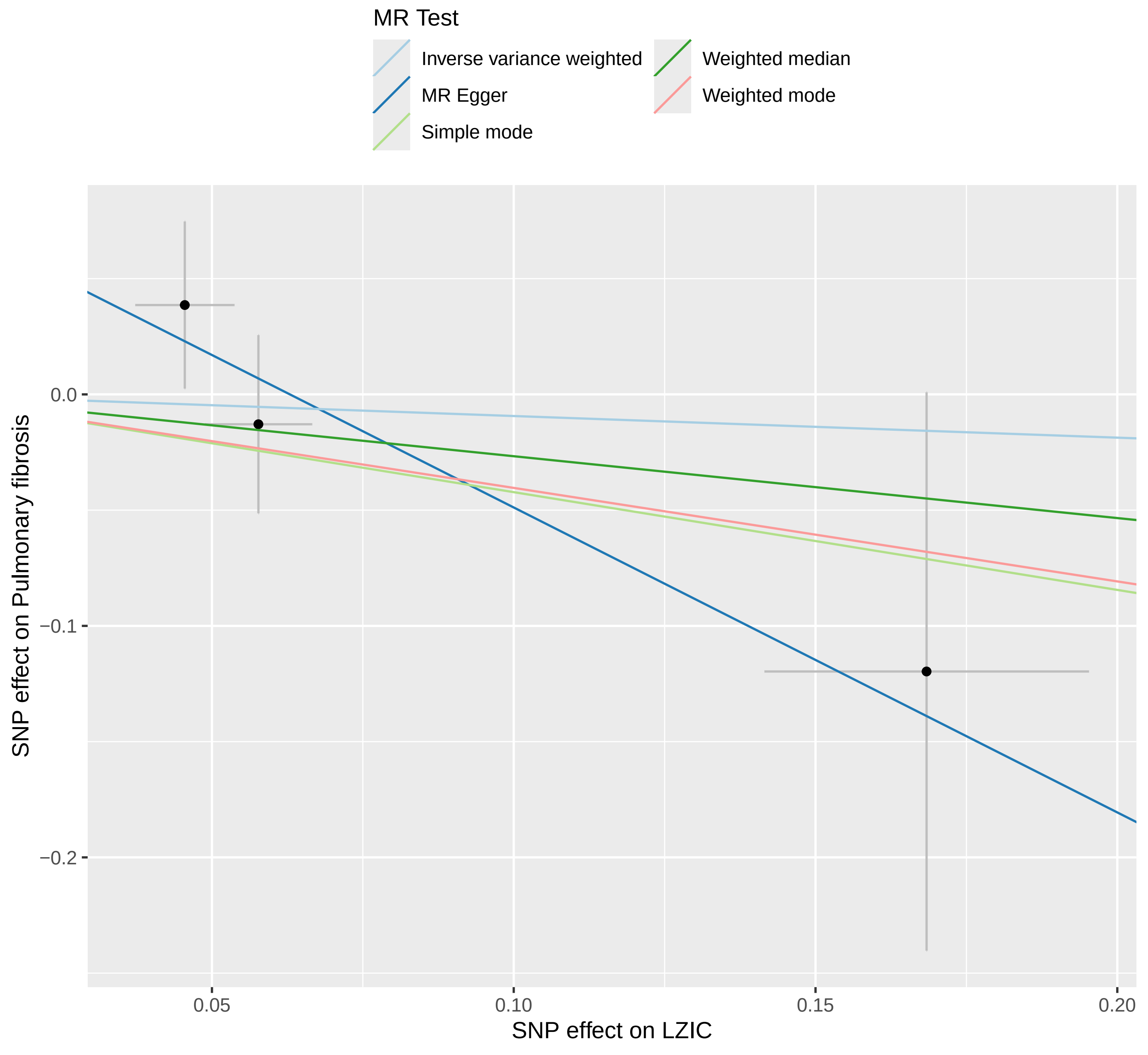

MR Test
Inverse variance weighted
MR Egger
Simple mode
Weighted median
Weighted mode
0.0
−0.1
−0.2
0.05
0.10
0.15
0.20
SNP effect on LZIC
SNP effect on Pulmonary fibrosis

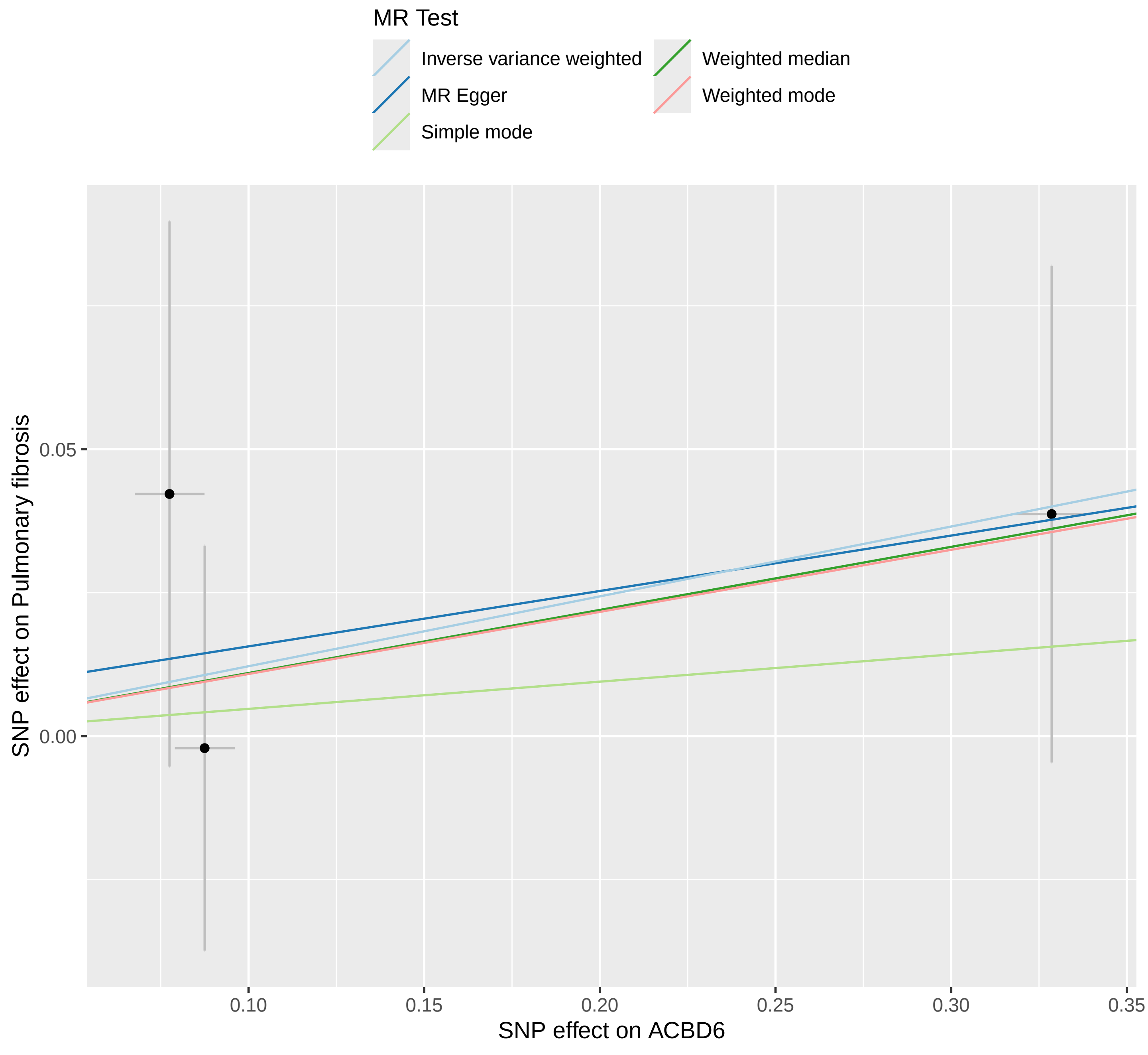
MR Test
Inverse variance weighted
MR Egger
Simple mode
Weighted median
Weighted mode
SNP effect on Pulmonary fibrosis
0.05
0.00
0.10
0.15
0.20
0.25
0.30
0.35
SNP effect on ACBD6

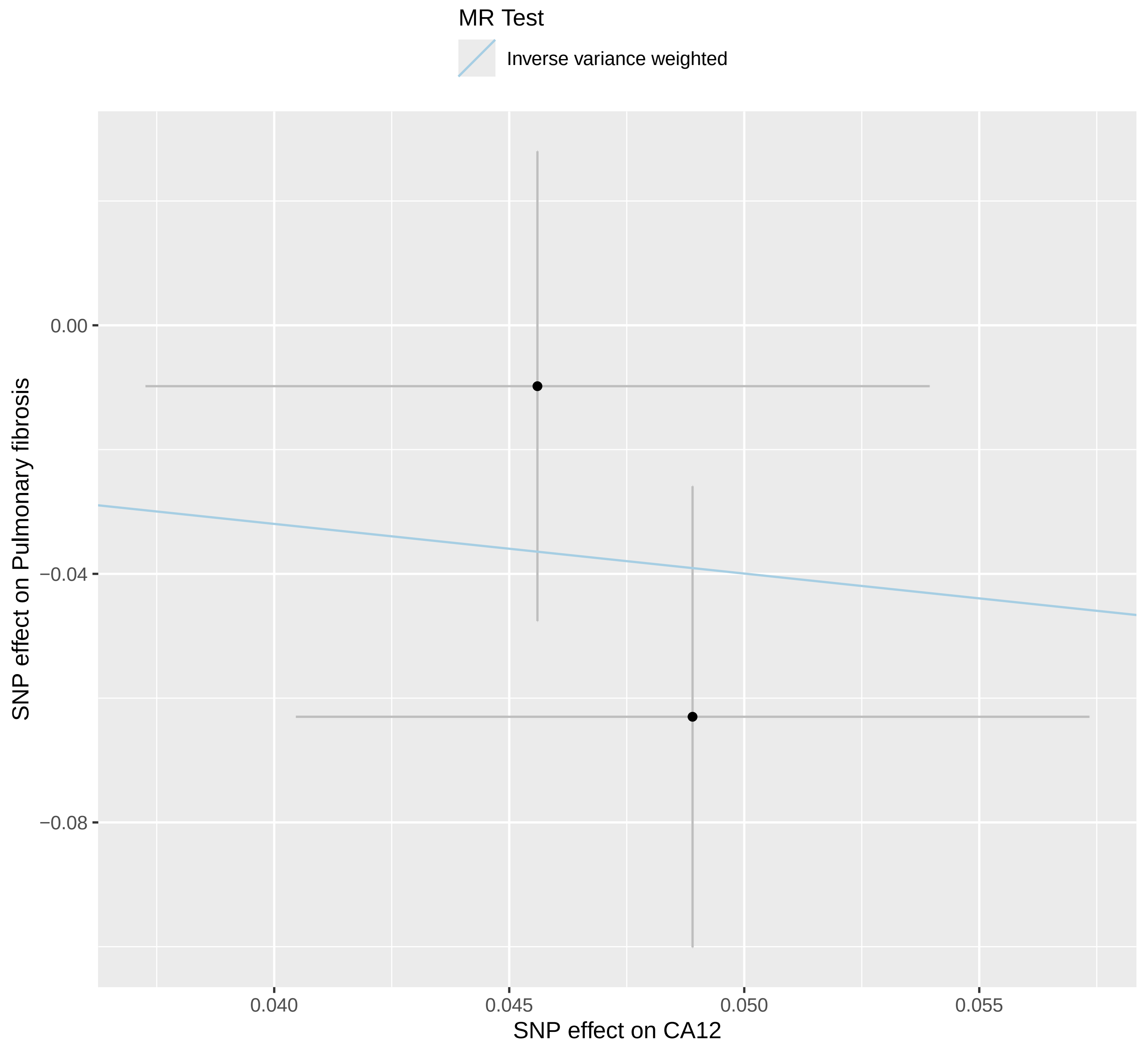
MR Test
Inverse variance weighted
SNP effect on Pulmonary fibrosis
0.00
−0.04
−0.08
0.040
0.045
0.050
0.055
SNP effect on CA12

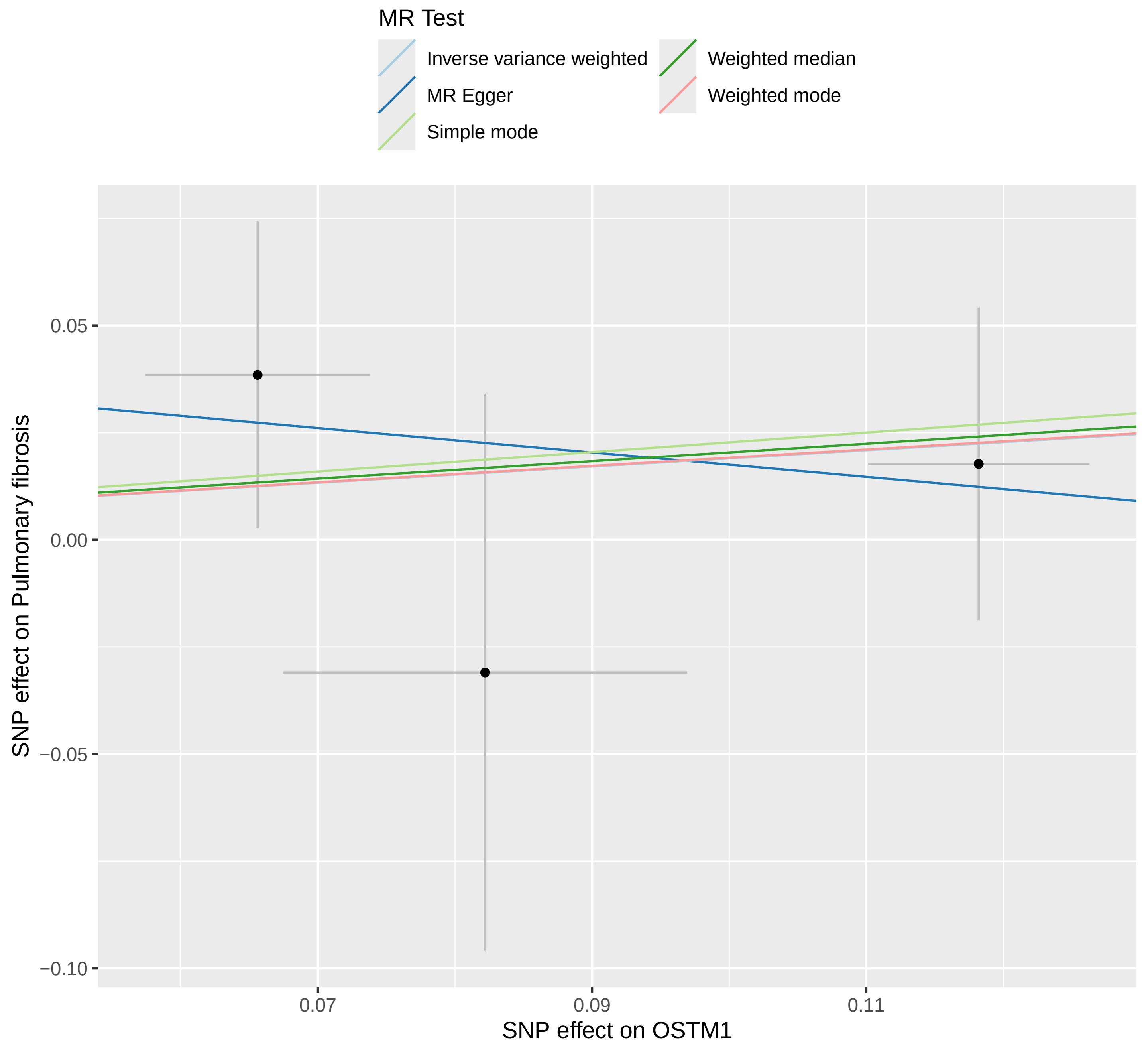

MR Test
Inverse variance weighted
MR Egger
Simple mode
Weighted median
Weighted mode
0.05
0.00
−0.05
−0.10
0.07
0.09
0.11
SNP effect on Pulmonary fibrosis
SNP effect on OSTM1

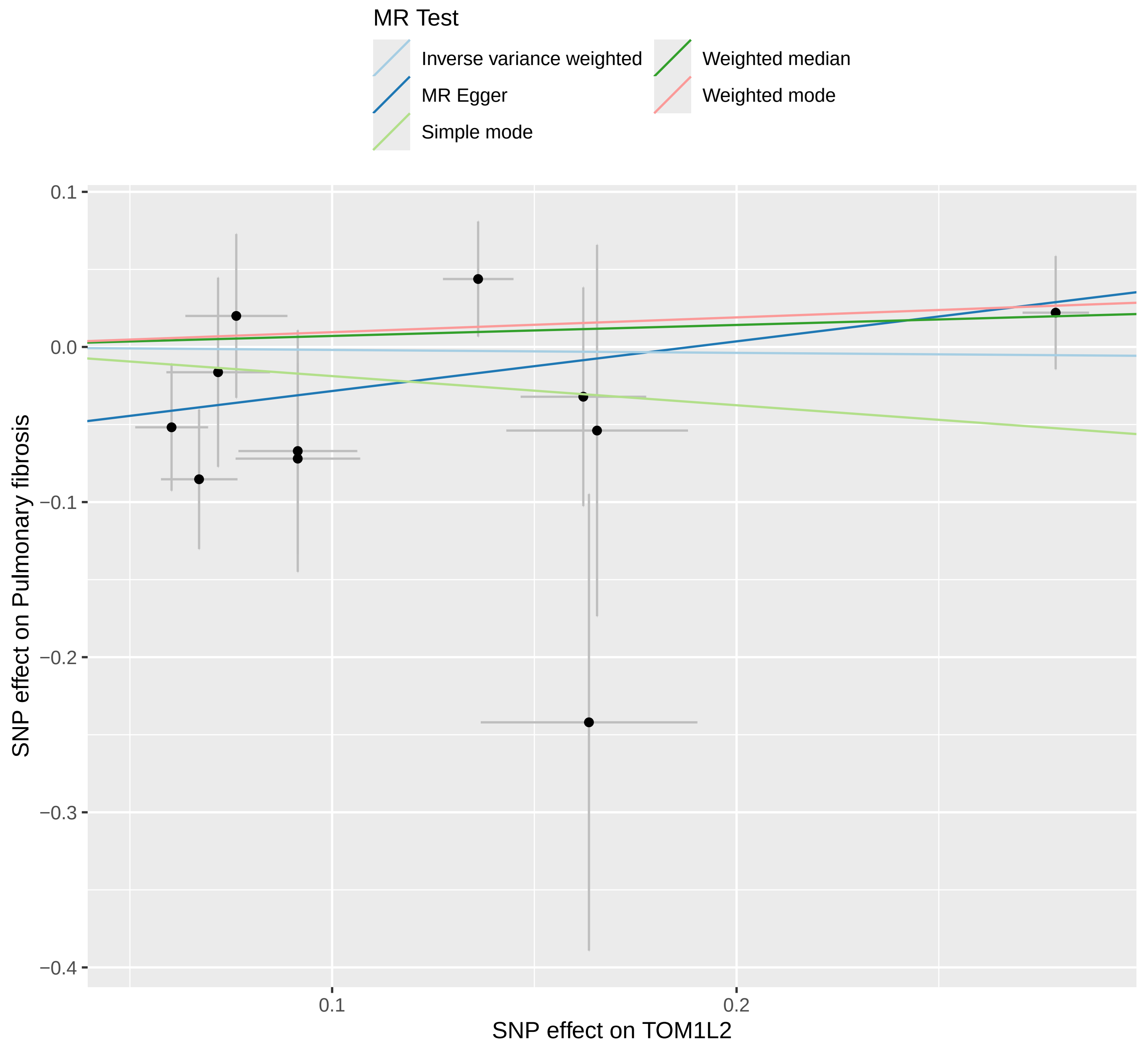
MR Test
Inverse variance weighted
MR Egger
Simple mode
Weighted median
Weighted mode
0.1
0.0
−0.1
−0.2
−0.3
−0.4
0.1
0.2
SNP effect on Pulmonary fibrosis
SNP effect on TOM1L2

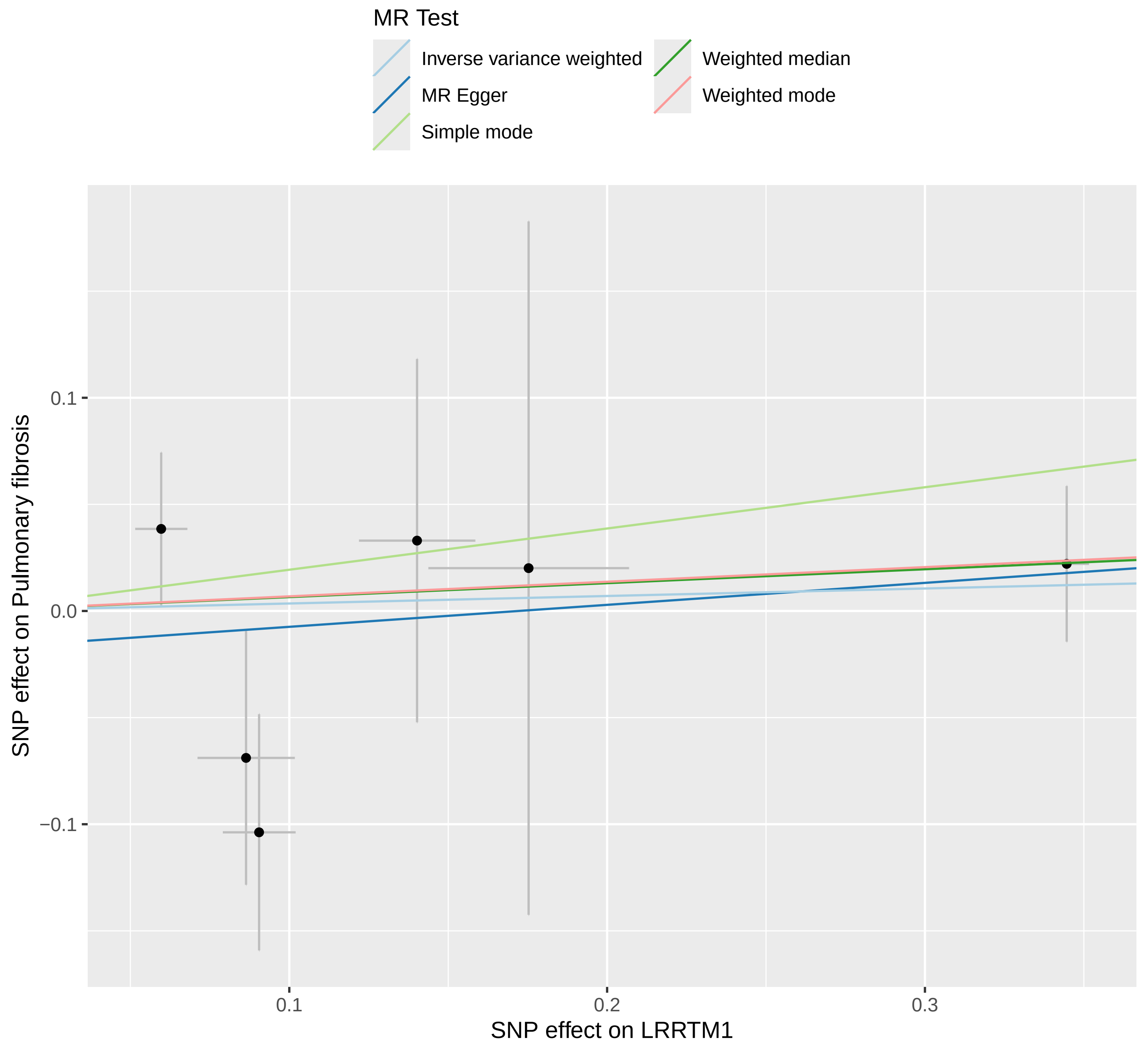

MR Test
Inverse variance weighted
MR Egger
Simple mode
Weighted median
Weighted mode
SNP effect on Pulmonary fibrosis
0.1
0.0
−0.1
0.1
0.2
0.3
SNP effect on LRRTM1

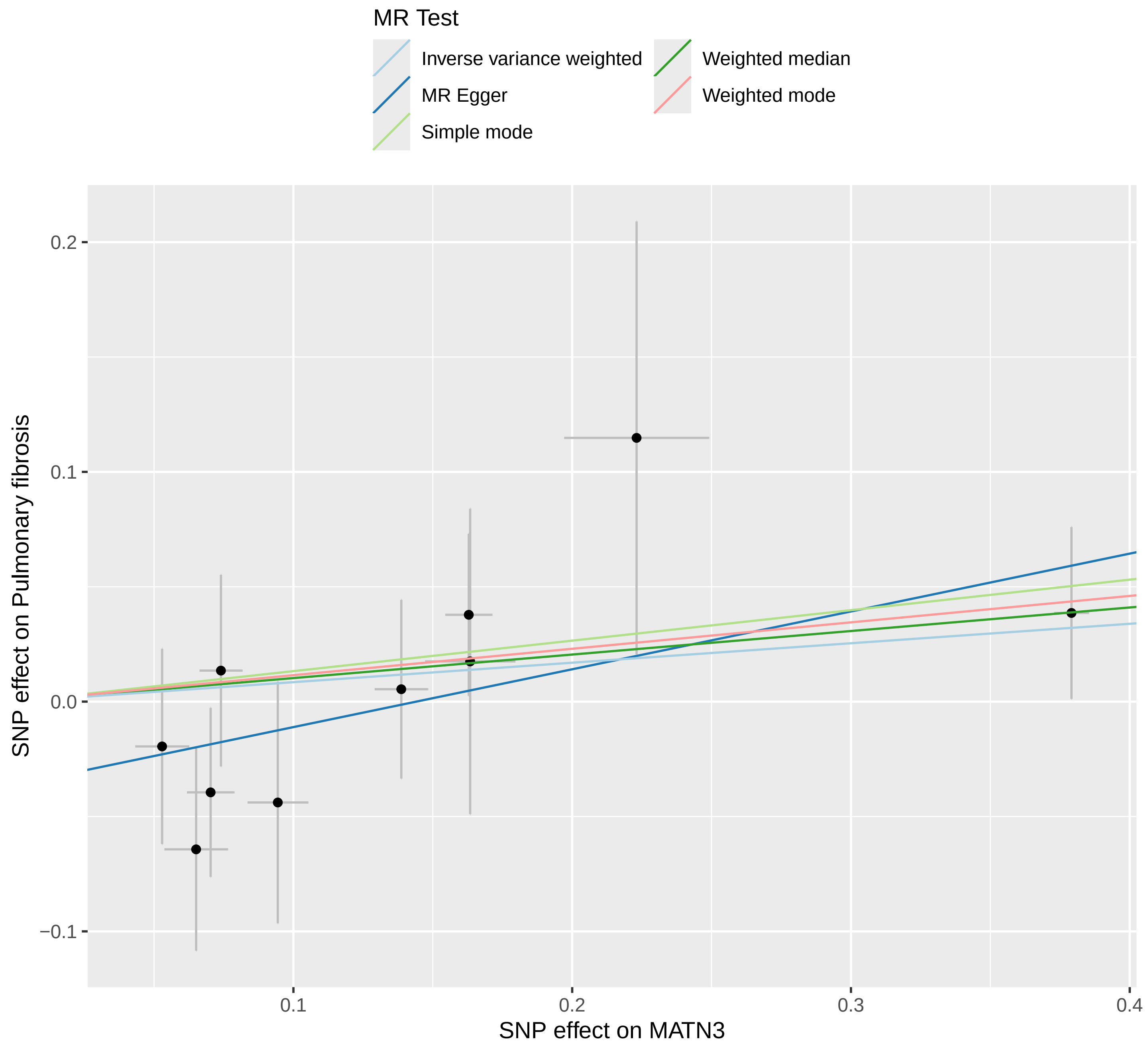
MR Test
Inverse variance weighted
MR Egger
Simple mode
Weighted median
Weighted mode
SNP effect on Pulmonary fibrosis
0.2
0.1
0.0
−0.1
0.1
0.2
0.3
0.4
SNP effect on MATN3

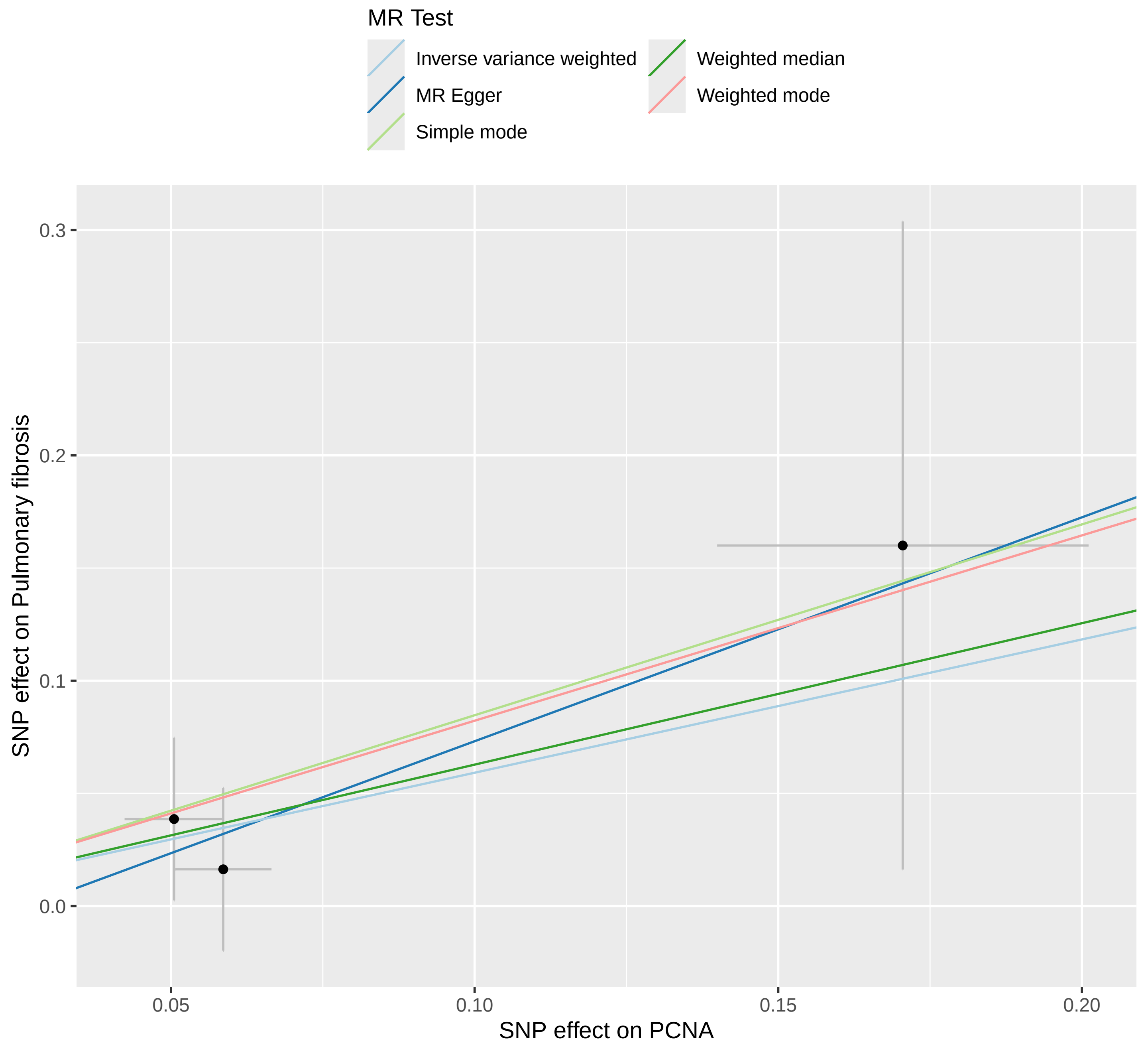

MR Test
Inverse variance weighted
MR Egger
Simple mode
Weighted median
Weighted mode
SNP effect on Pulmonary fibrosis
0.0
0.1
0.2
0.3
0.05
0.10
0.15
0.20
SNP effect on PCNA

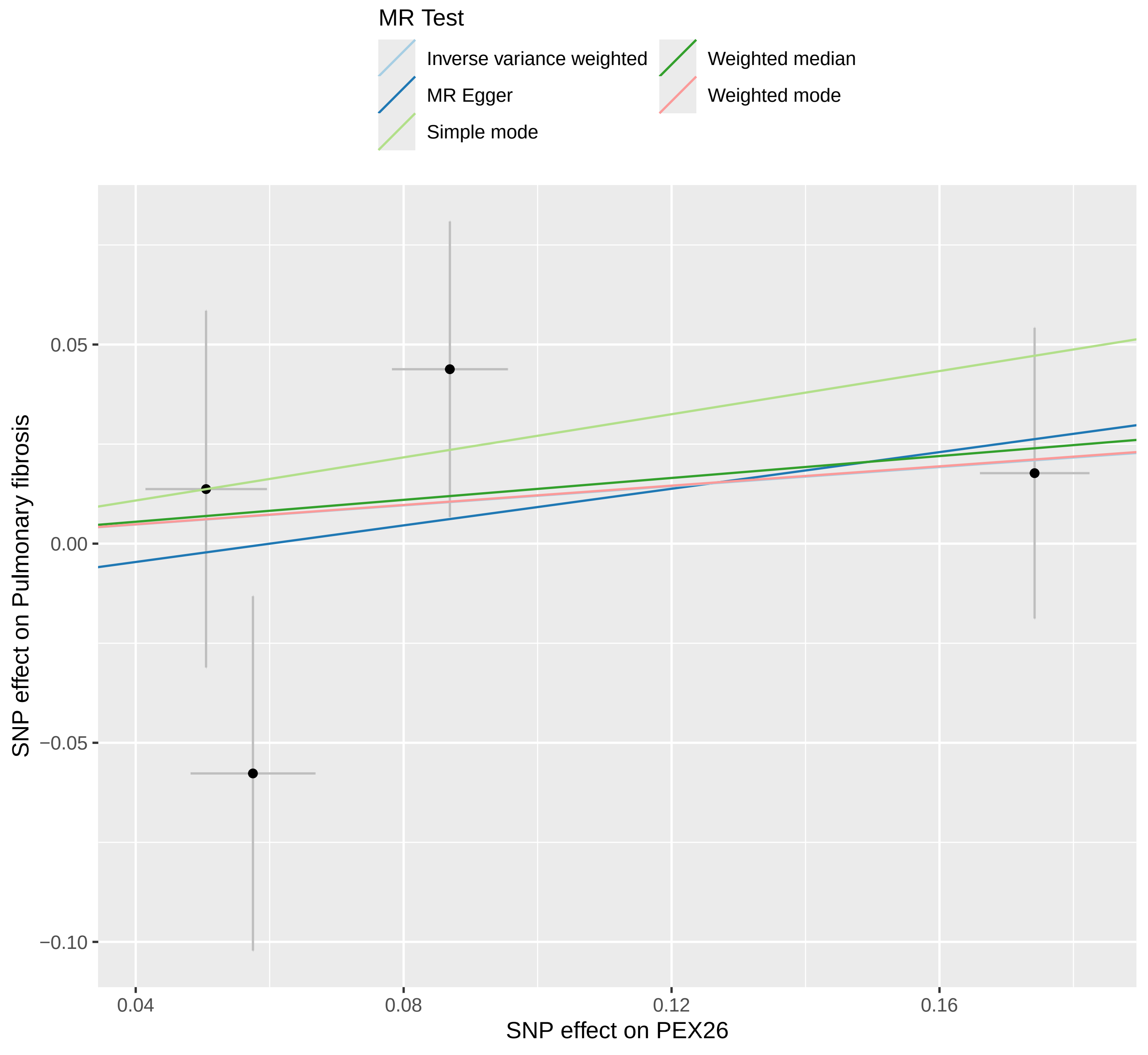
MR Test
Inverse variance weighted
MR Egger
Simple mode
Weighted median
Weighted mode
SNP effect on Pulmonary fibrosis
0.05
0.00
−0.05
−0.10
0.04
0.08
0.12
0.16
SNP effect on PEX26

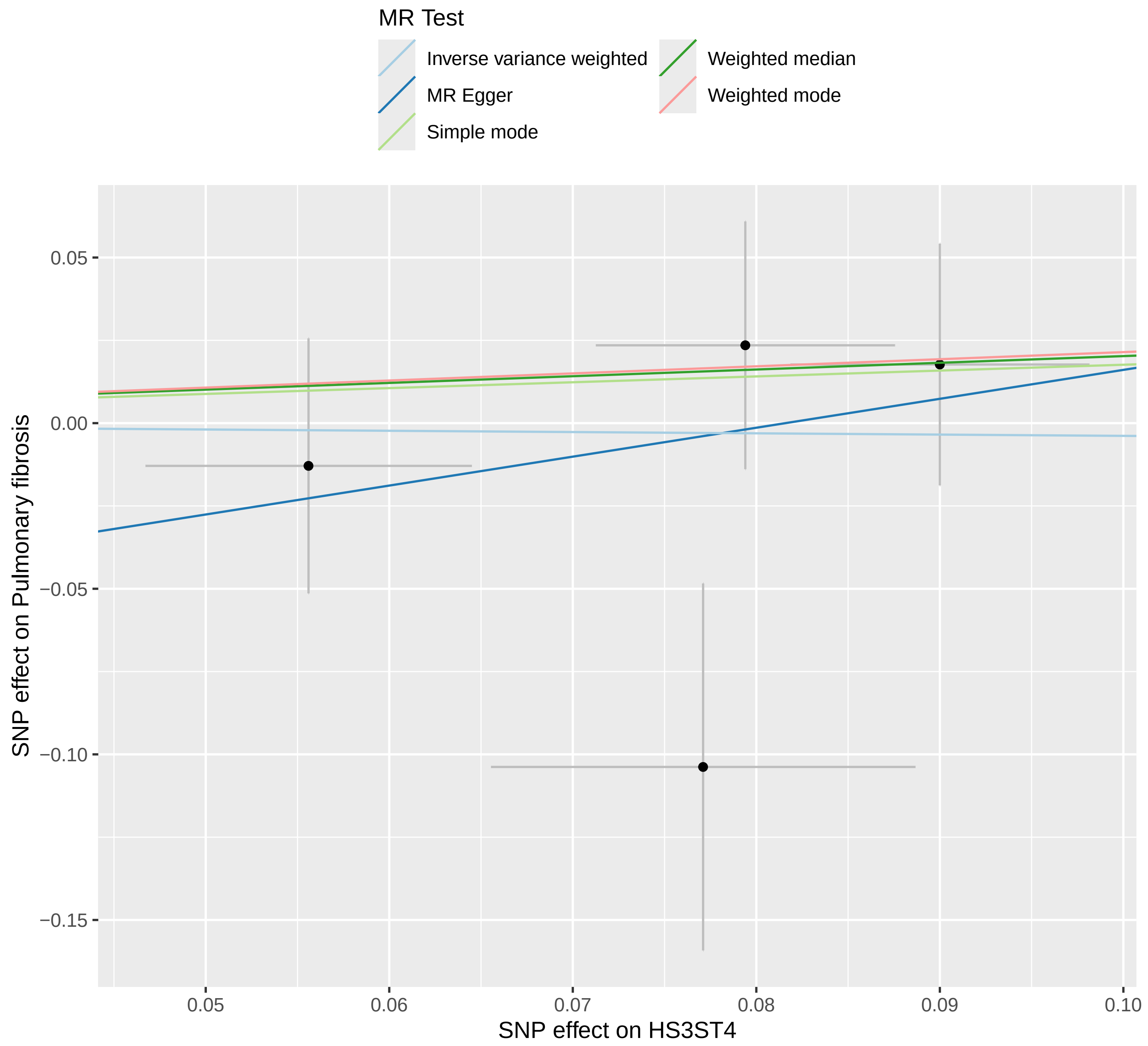
MR Test
Inverse variance weighted
MR Egger
Simple mode
Weighted median
Weighted mode
0.05
0.00
−0.05
−0.10
−0.15
SNP effect on Pulmonary fibrosis
0.05
0.06
0.07
0.08
0.09
0.10
SNP effect on HS3ST4

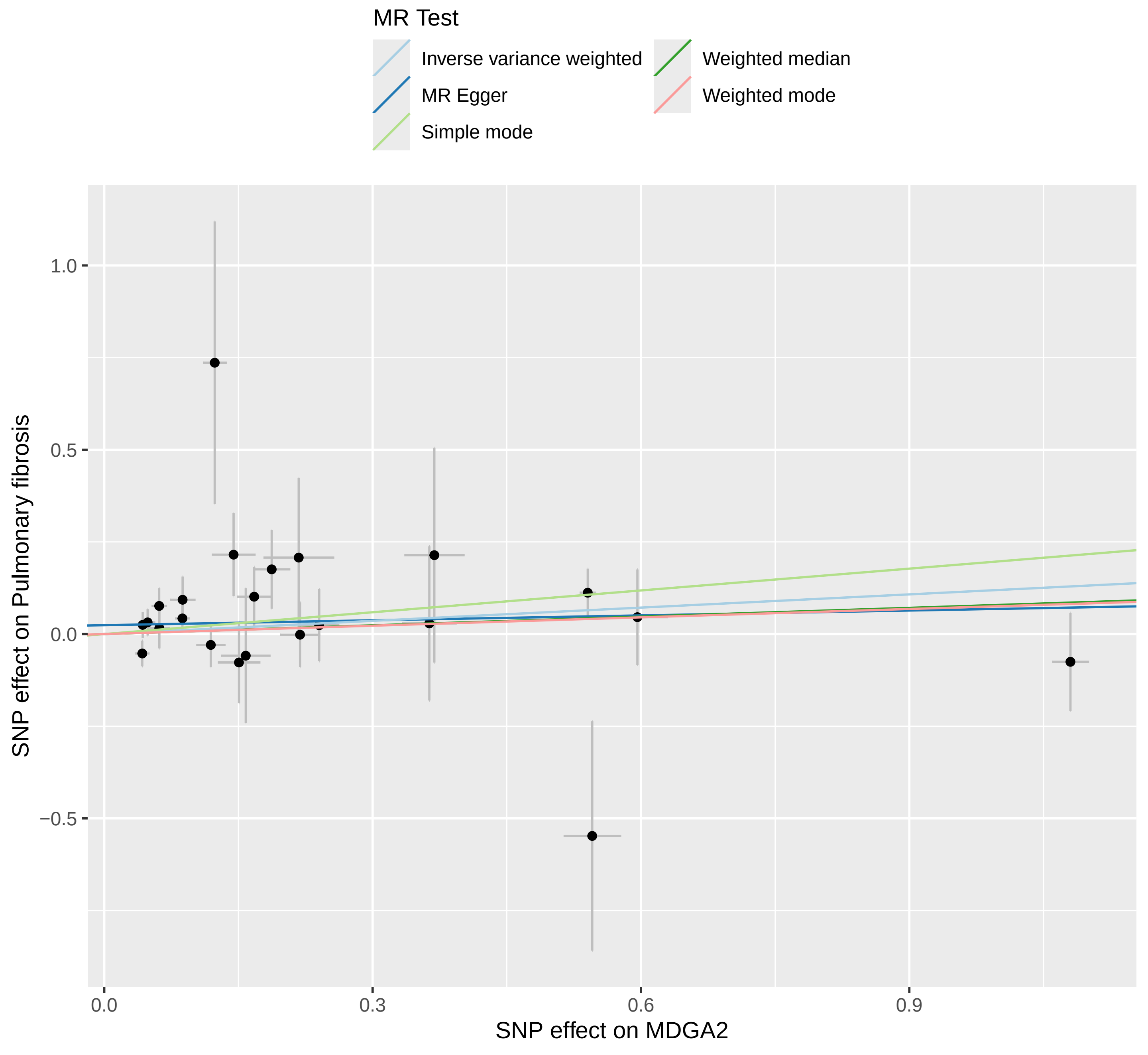
MR Test
Inverse variance weighted
MR Egger
Simple mode
Weighted median
Weighted mode
1.0
0.5
0.0
−0.5
0.0
0.3
0.6
0.9
SNP effect on Pulmonary fibrosis
SNP effect on MDGA2

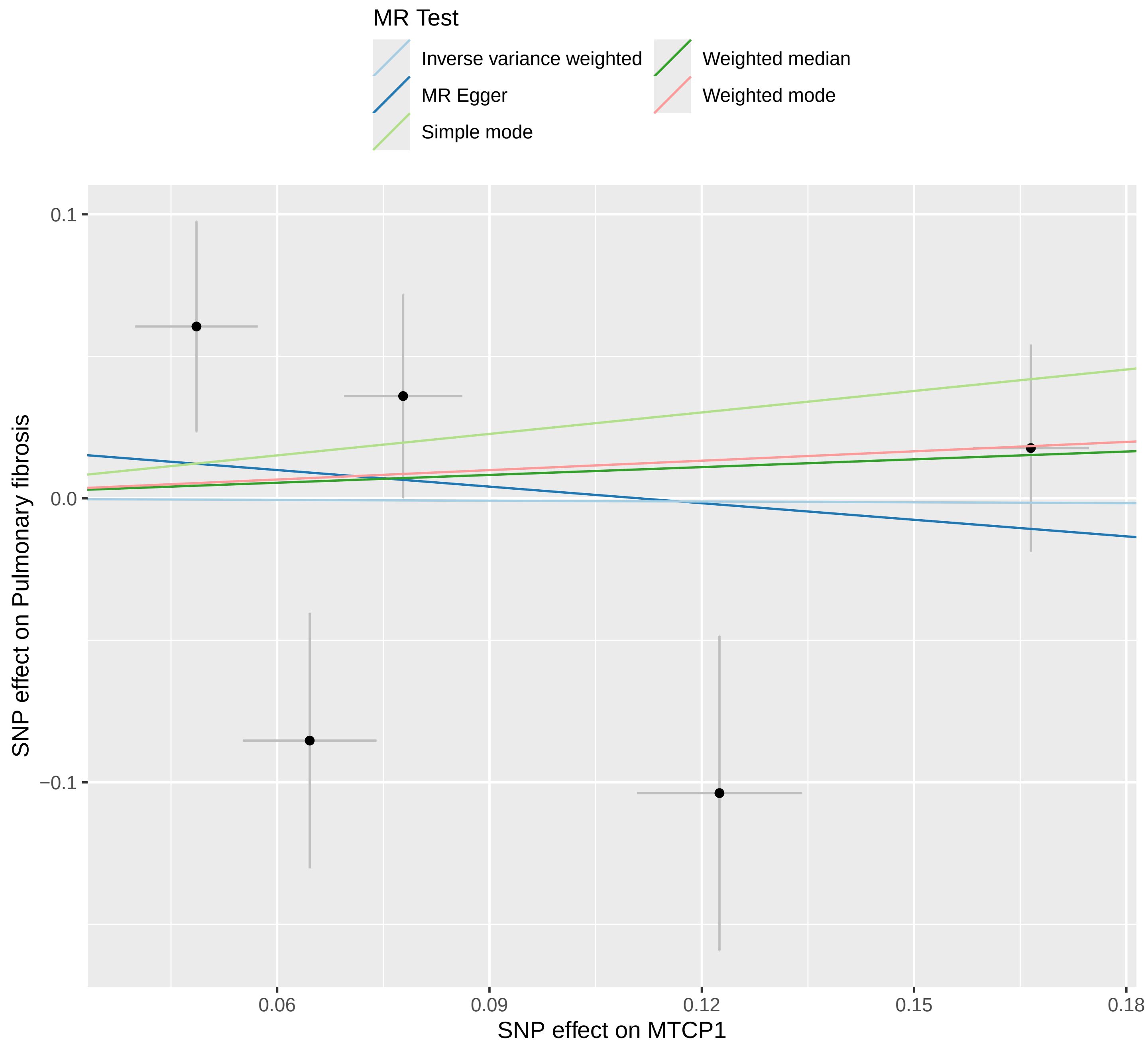

MR Test
Inverse variance weighted
MR Egger
Simple mode
Weighted median
Weighted mode
0.1
0.0
−0.1
0.06
0.09
0.12
0.15
0.18
SNP effect on Pulmonary fibrosis
SNP effect on MTCP1

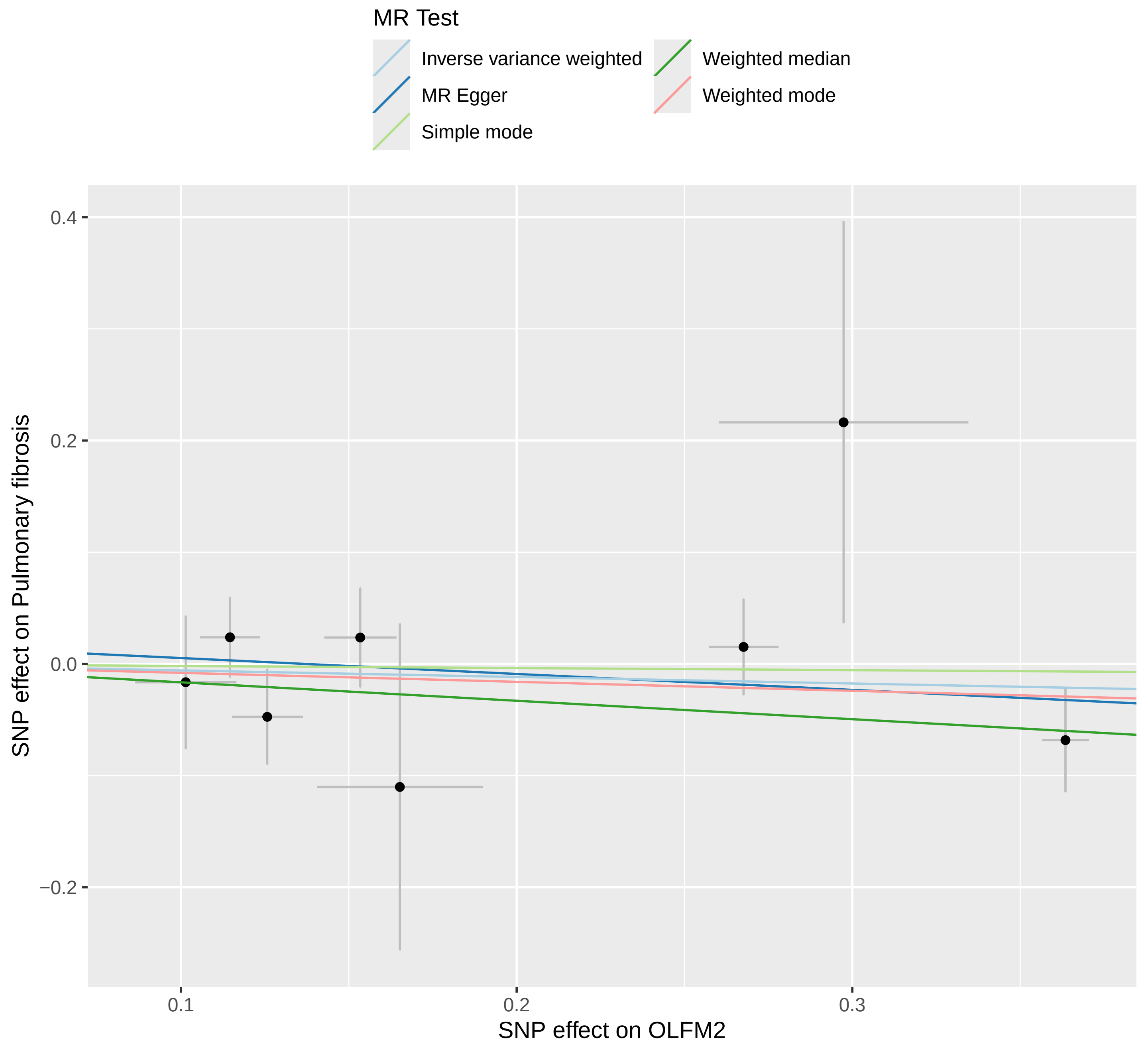

MR Test
Inverse variance weighted
MR Egger
Simple mode
Weighted median
Weighted mode
0.4
0.2
0.0
−0.2
0.1
0.2
0.3
SNP effect on Pulmonary fibrosis
SNP effect on OLFM2

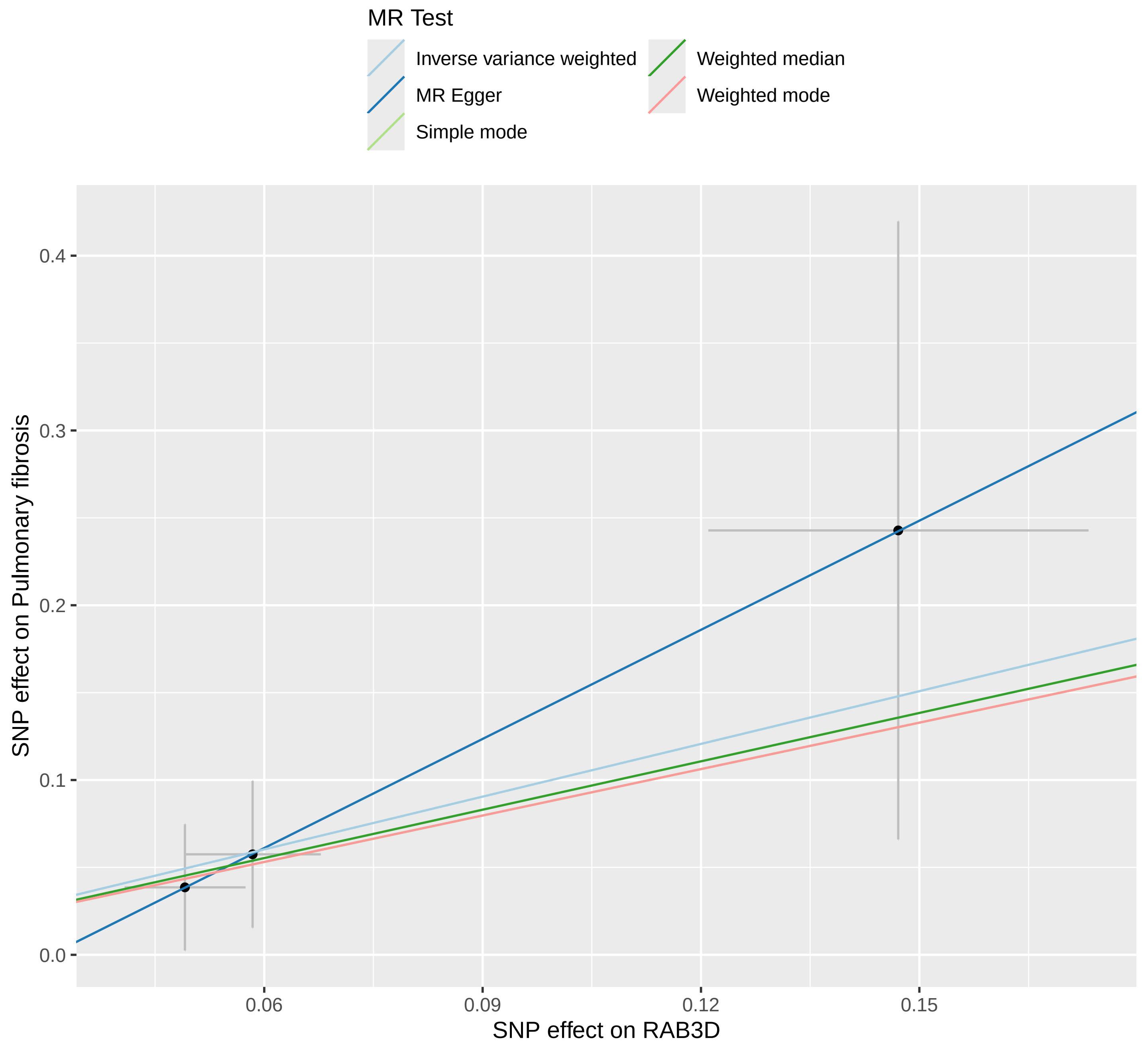

MR Test
Inverse variance weighted
MR Egger
Simple mode
Weighted median
Weighted mode
0.4
0.3
0.2
0.1
0.0
0.06
0.09
0.12
0.15
SNP effect on Pulmonary fibrosis
SNP effect on RAB3D

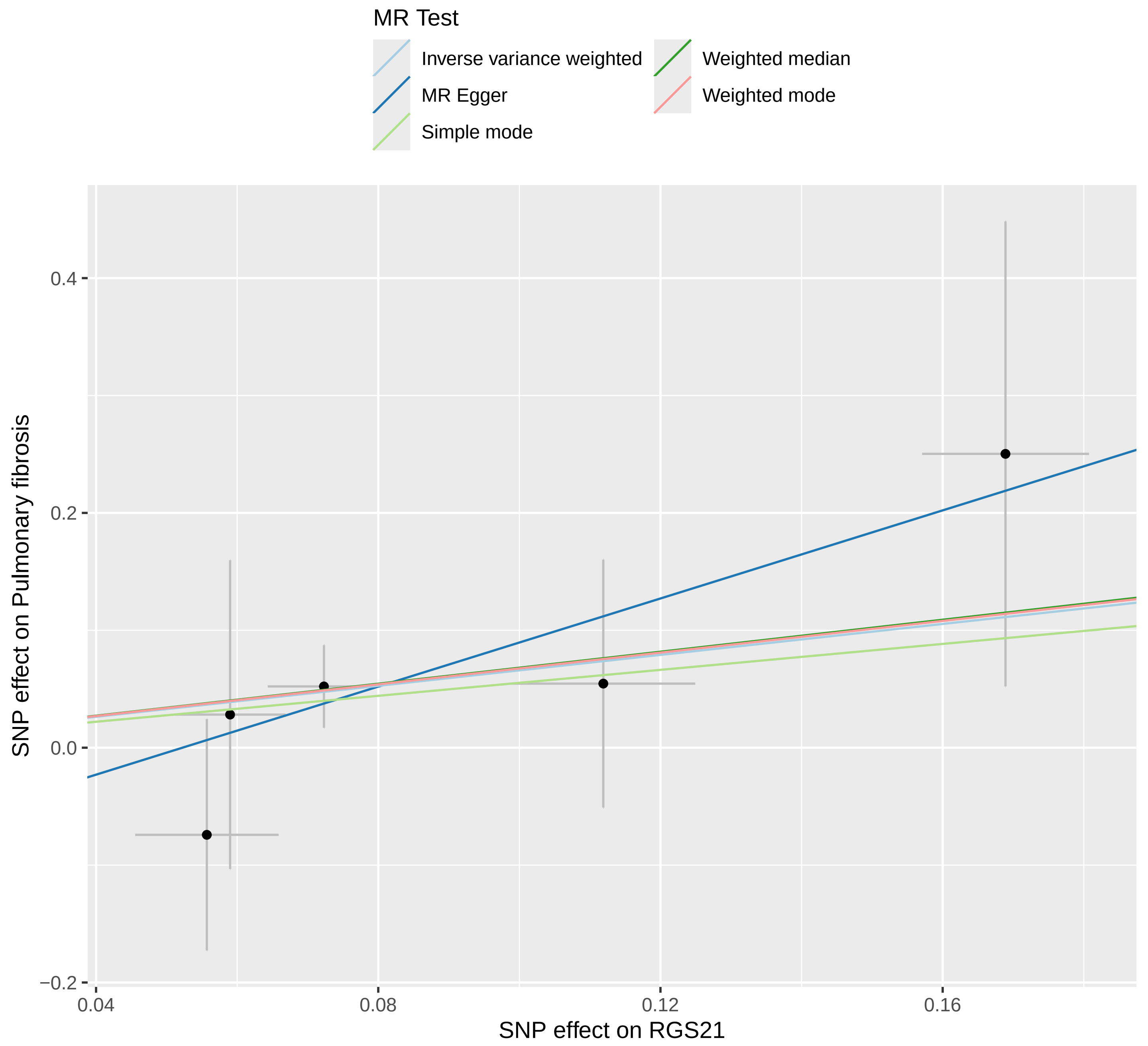

MR Test
Inverse variance weighted
MR Egger
Simple mode
Weighted median
Weighted mode
0.4
0.2
0.0
−0.2
0.04
0.08
0.12
0.16
SNP effect on Pulmonary fibrosis
SNP effect on RGS21

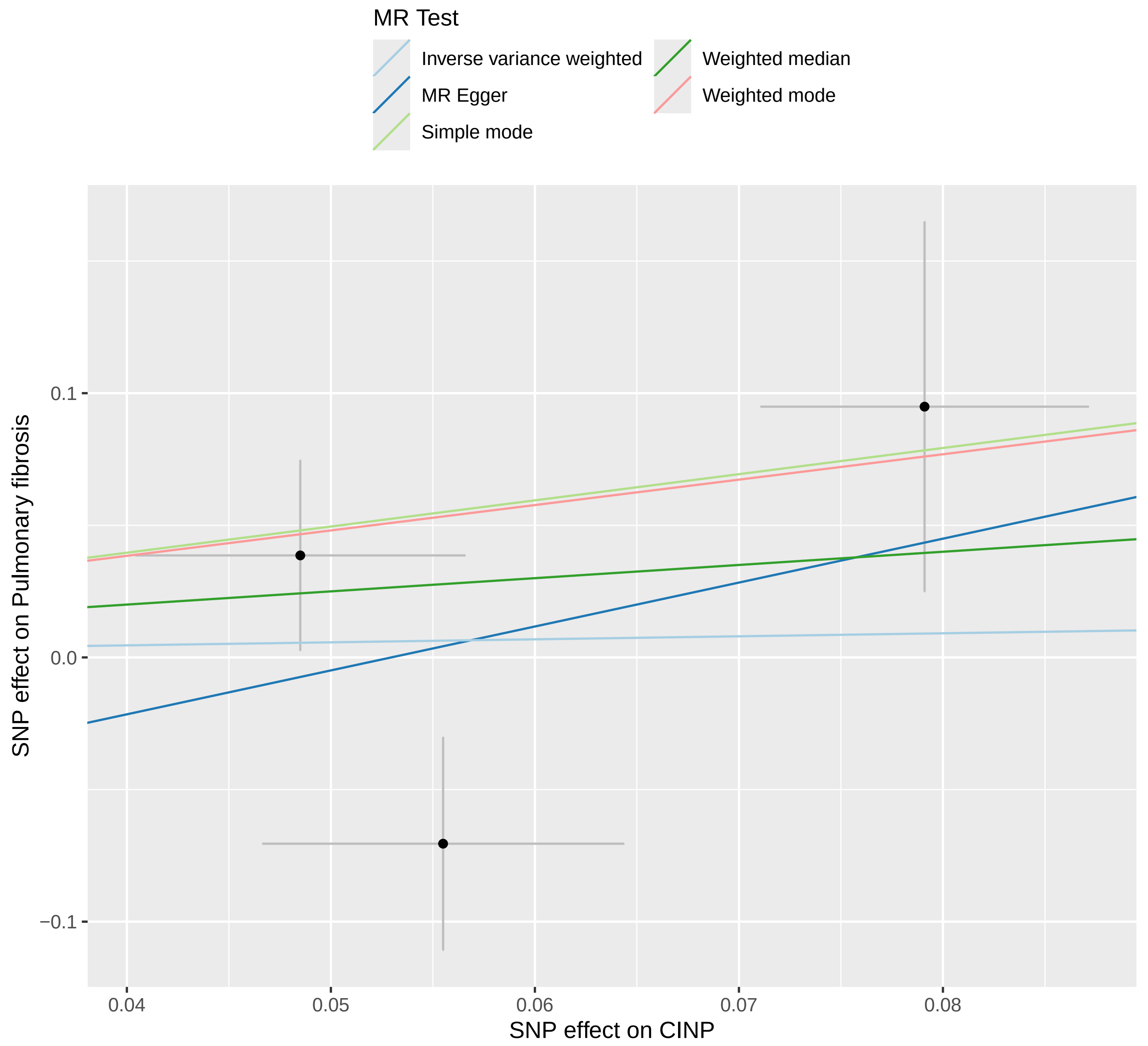

MR Test
Inverse variance weighted
MR Egger
Simple mode
Weighted median
Weighted mode
SNP effect on Pulmonary fibrosis
0.1
0.0
−0.1
0.04
0.05
0.06
0.07
0.08
SNP effect on CINP

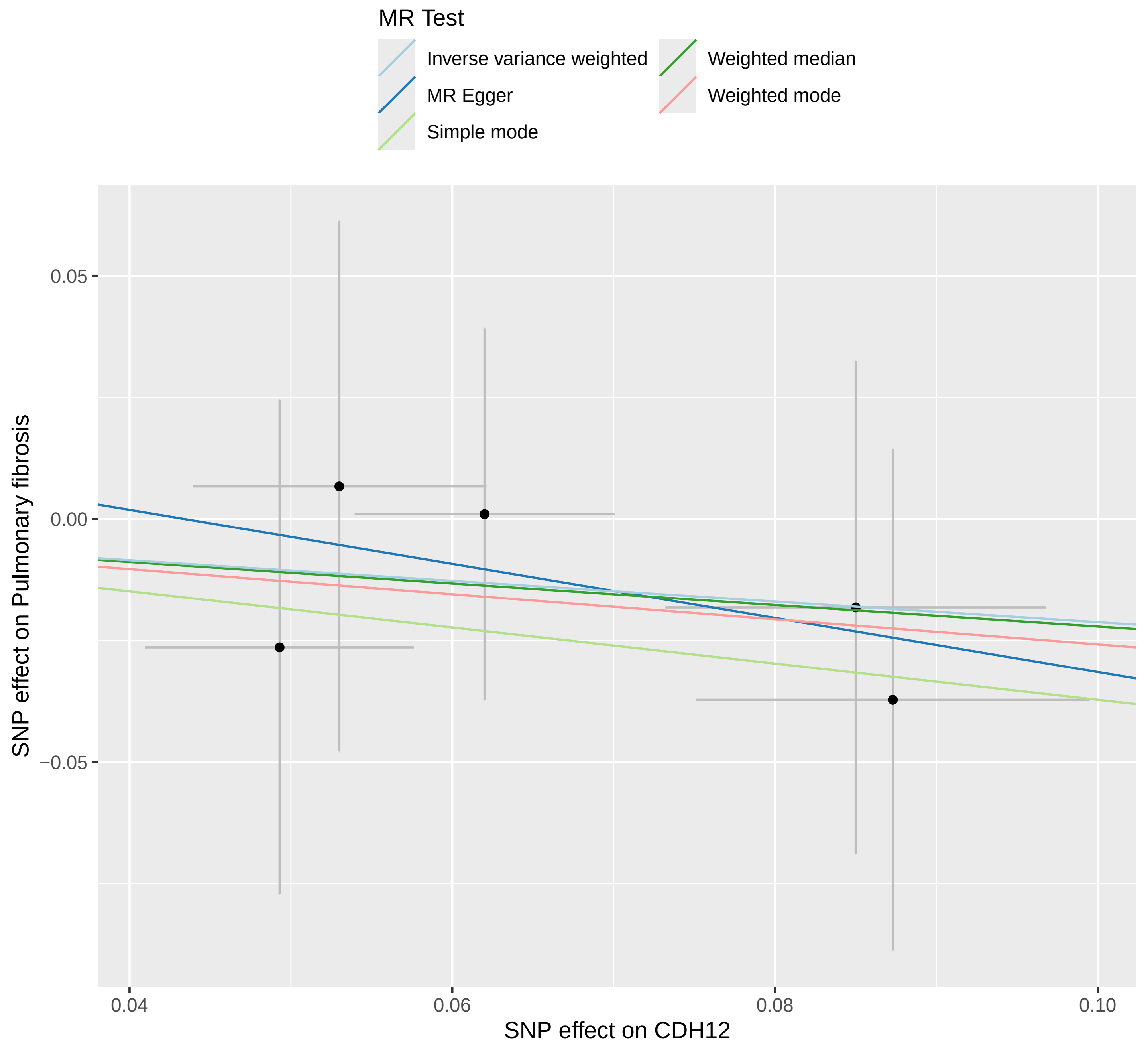

MR Test
Inverse variance weighted
MR Egger
Simple mode
Weighted median
Weighted mode
SNP effect on Pulmonary fibrosis
0.05
0.00
−0.05
0.04
0.06
0.08
0.10
SNP effect on CDH12

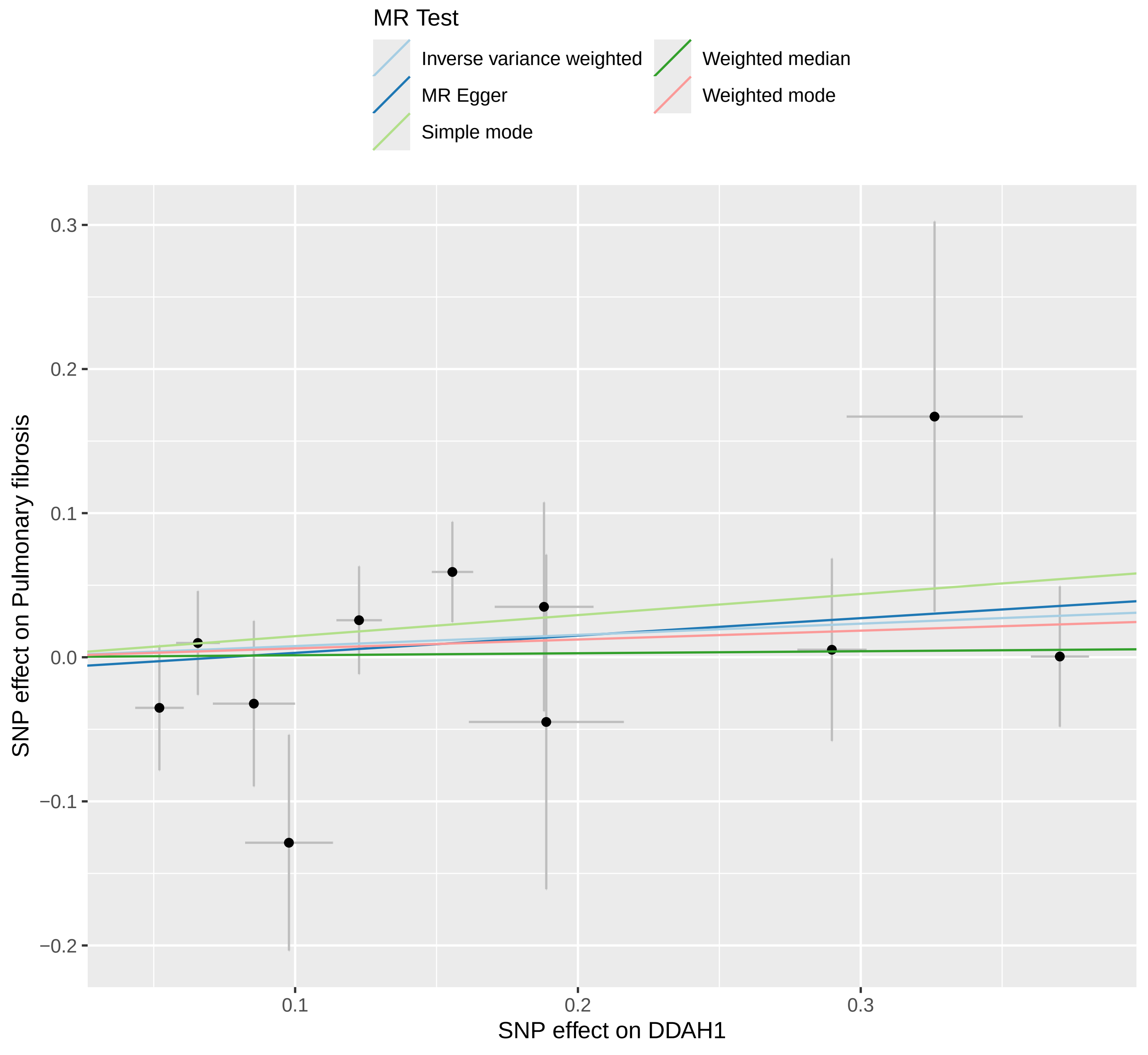
MR Test
Inverse variance weighted
MR Egger
Simple mode
Weighted median
Weighted mode
0.3
0.2
0.1
0.0
−0.1
−0.2
SNP effect on Pulmonary fibrosis
0.1
0.2
0.3
SNP effect on DDAH1

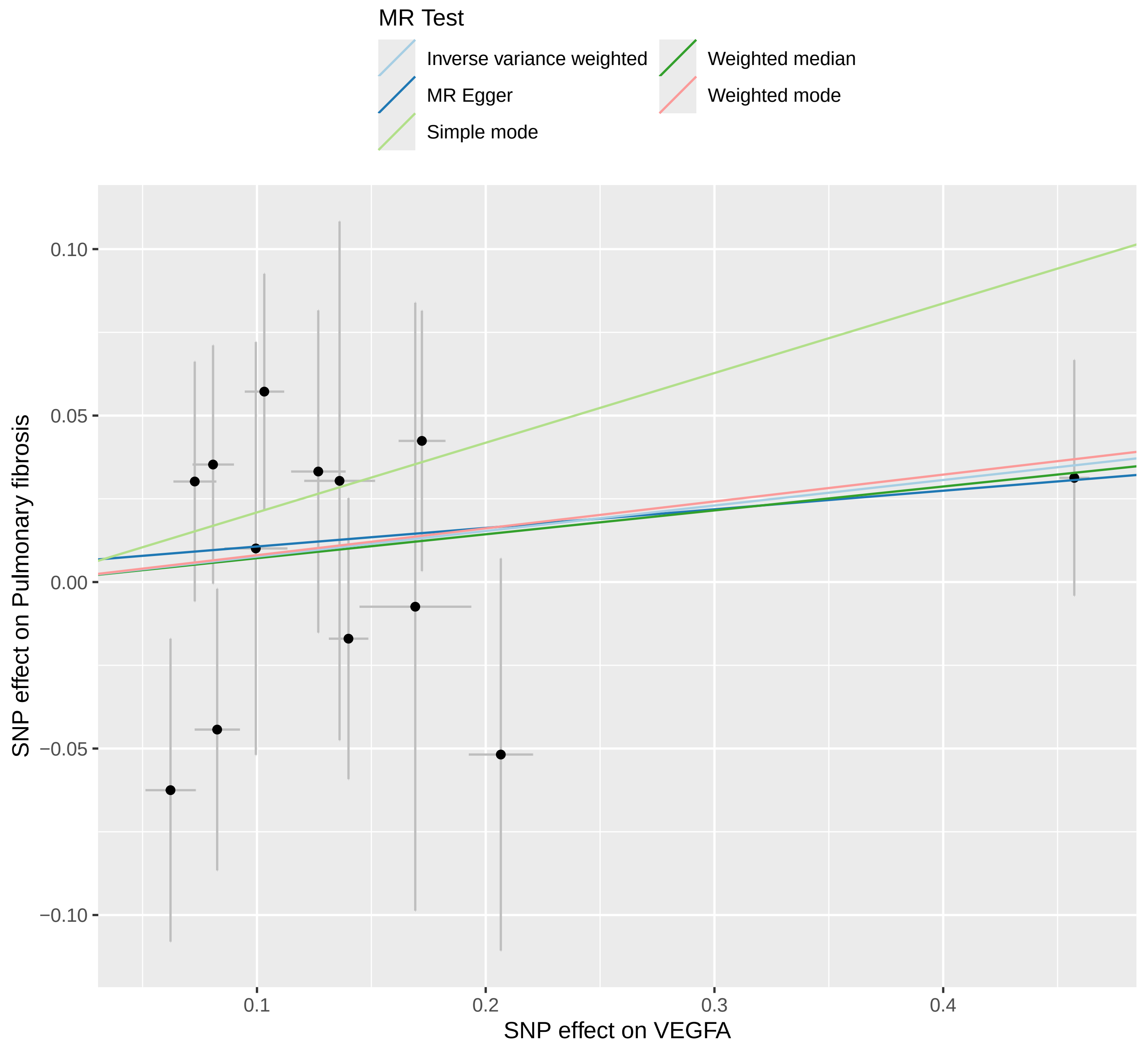
MR Test
Inverse variance weighted
MR Egger
Simple mode
Weighted median
Weighted mode
0.10
0.05
0.00
−0.05
−0.10
0.1
0.2
0.3
0.4
SNP effect on Pulmonary fibrosis
SNP effect on VEGFA

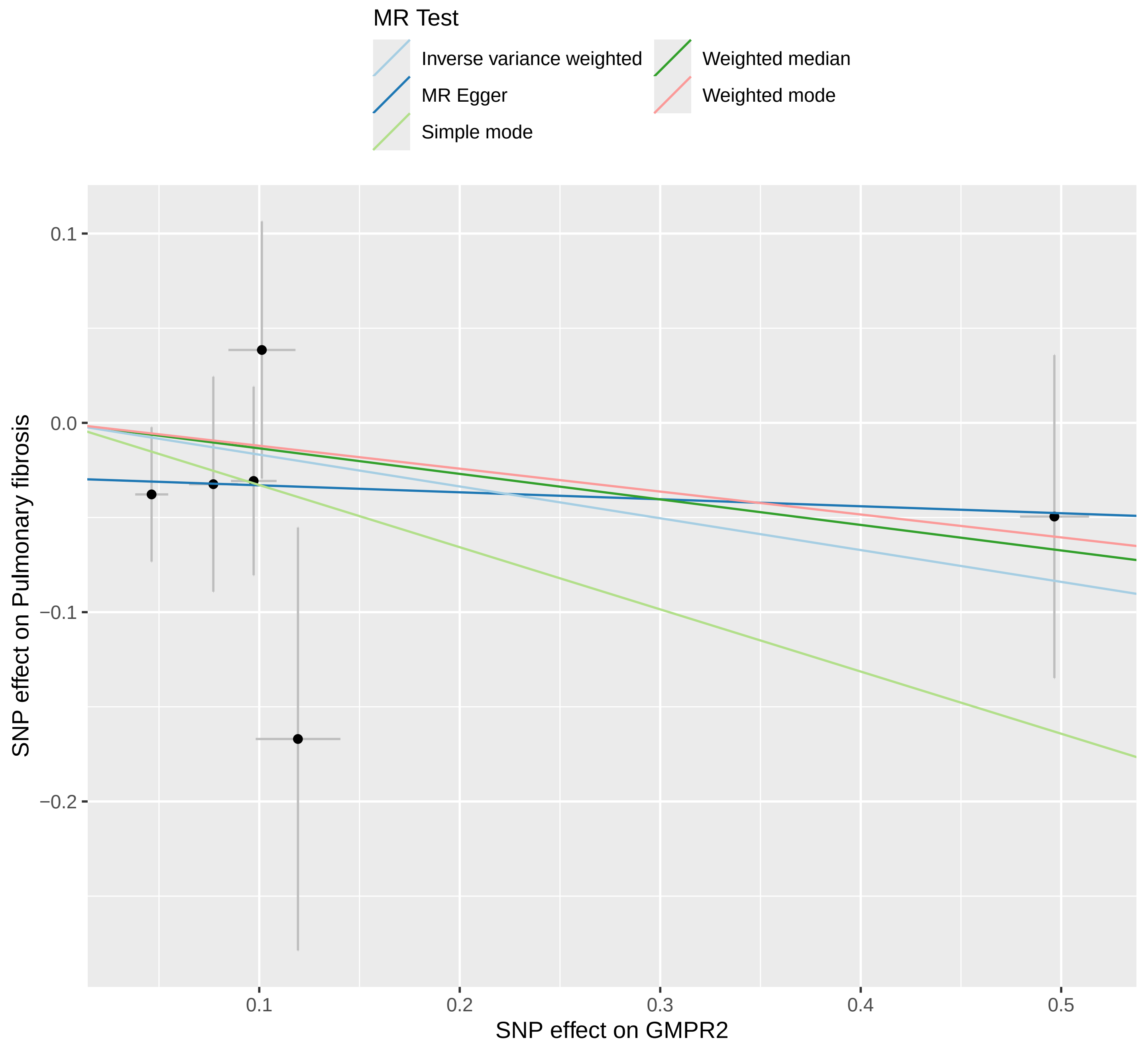

MR Test
Inverse variance weighted
MR Egger
Simple mode
Weighted median
Weighted mode
0.1
0.0
−0.1
−0.2
SNP effect on Pulmonary fibrosis
0.1
0.2
0.3
0.4
0.5
SNP effect on GMPR2

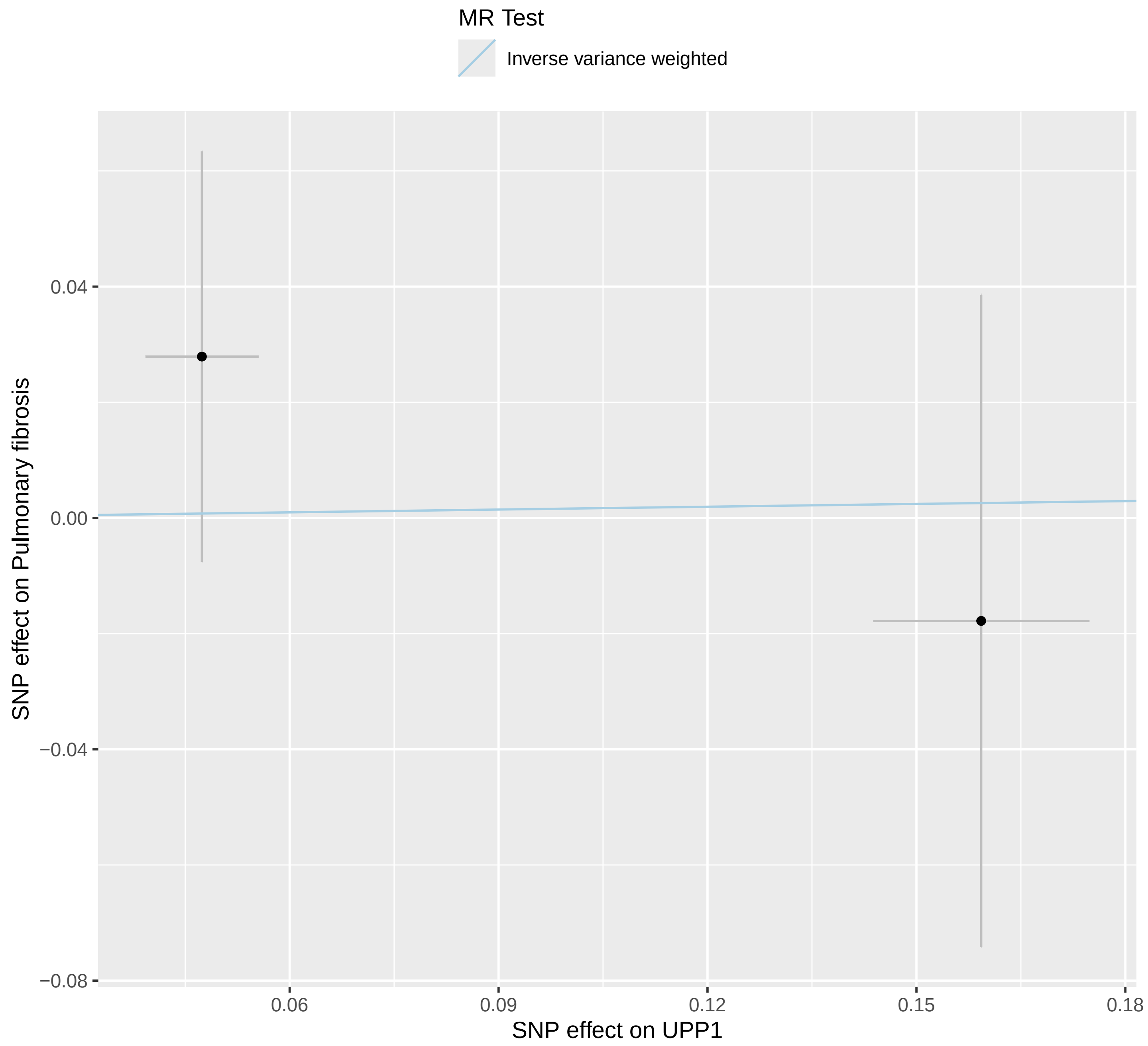
MR Test
Inverse variance weighted
0.04
0.00
−0.04
−0.08
SNP effect on Pulmonary fibrosis
0.06
0.09
0.12
0.15
0.18
SNP effect on UPP1

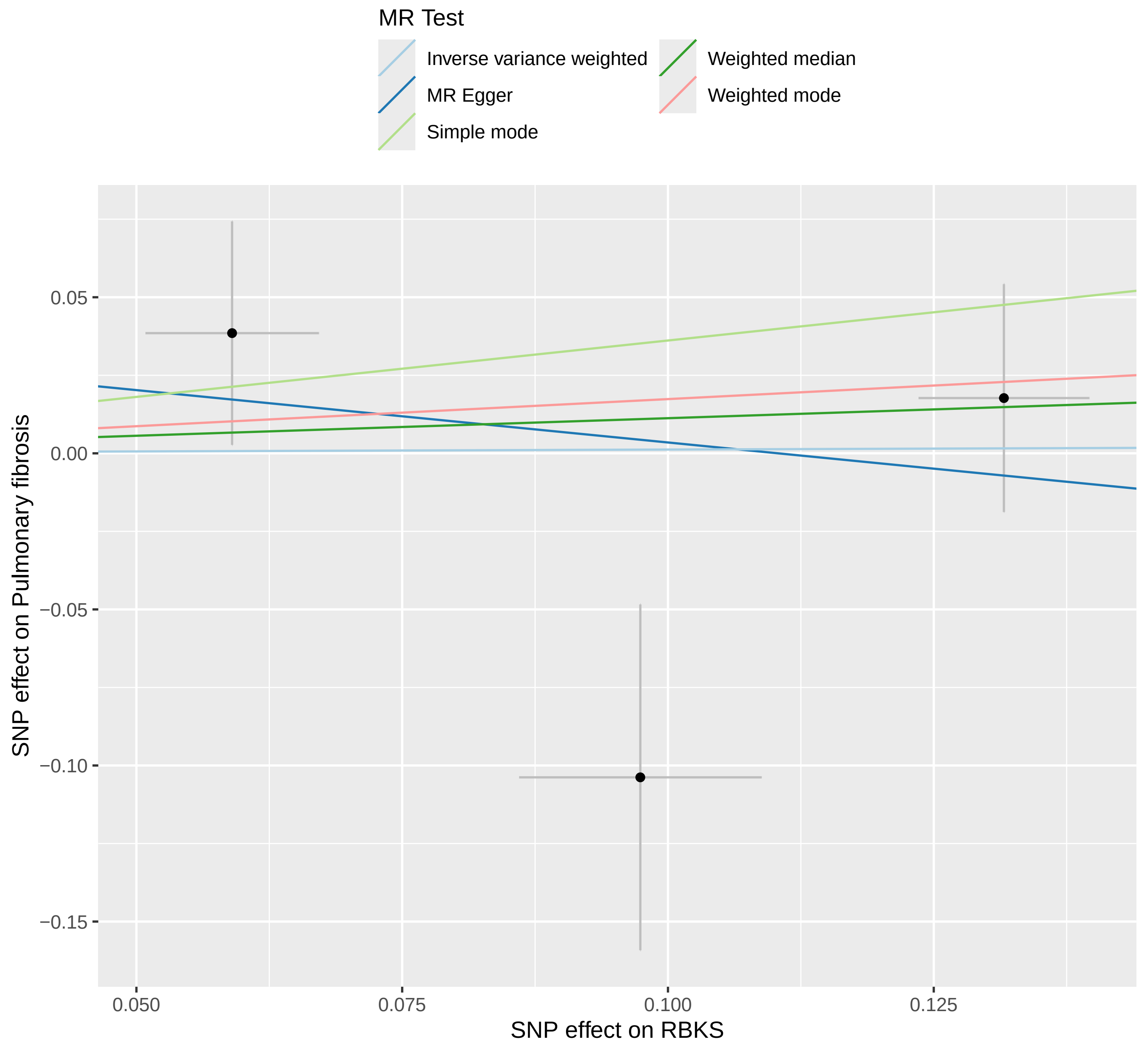

MR Test
Inverse variance weighted
MR Egger
Simple mode
Weighted median
Weighted mode
0.05
0.00
−0.05
−0.10
−0.15
SNP effect on Pulmonary fibrosis
0.050
0.075
0.100
0.125
SNP effect on RBKS

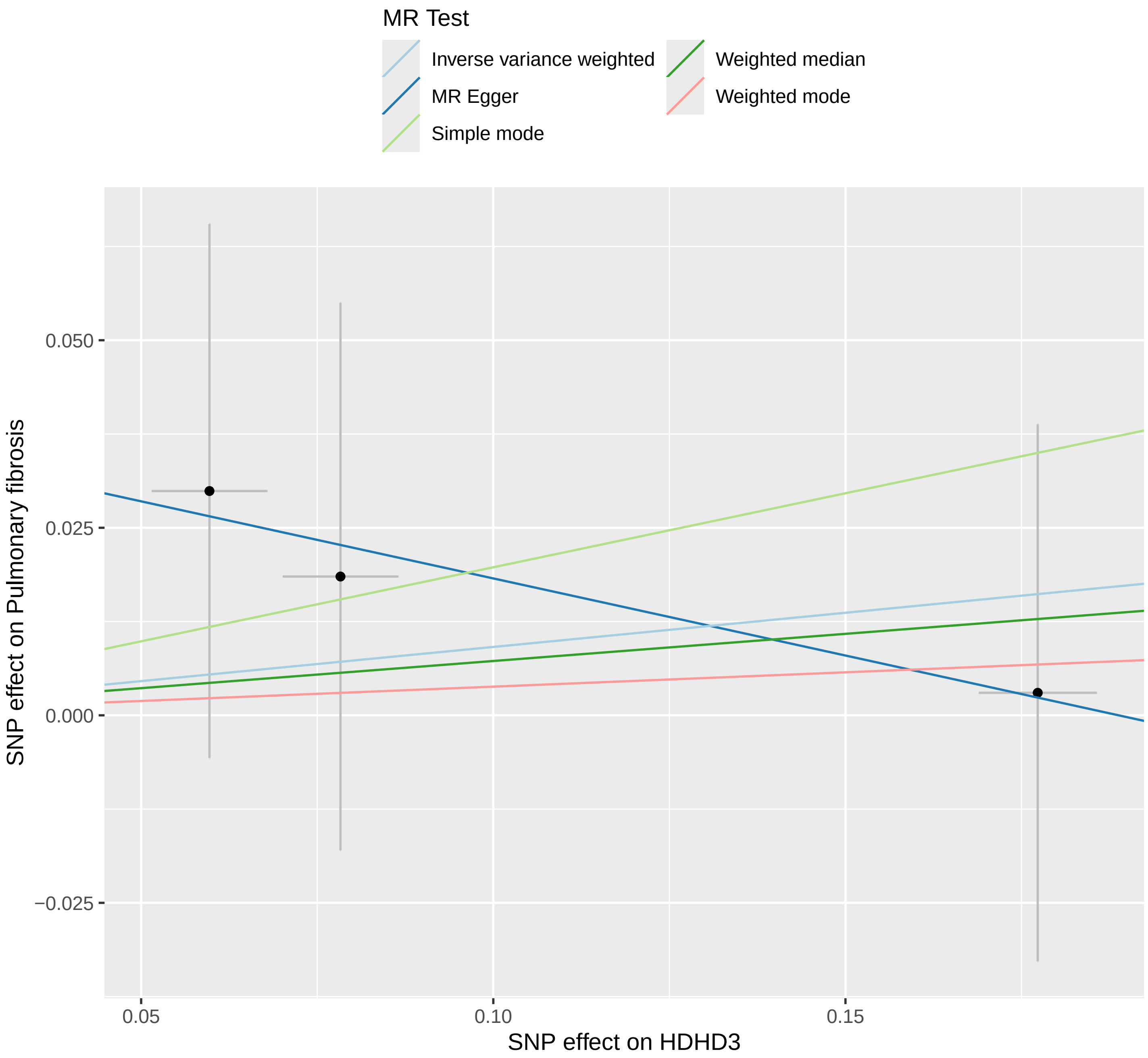

MR Test
Inverse variance weighted
MR Egger
Simple mode
Weighted median
Weighted mode
0.050
0.025
0.000
−0.025
0.05
0.10
0.15
SNP effect on Pulmonary fibrosis
SNP effect on HDHD3

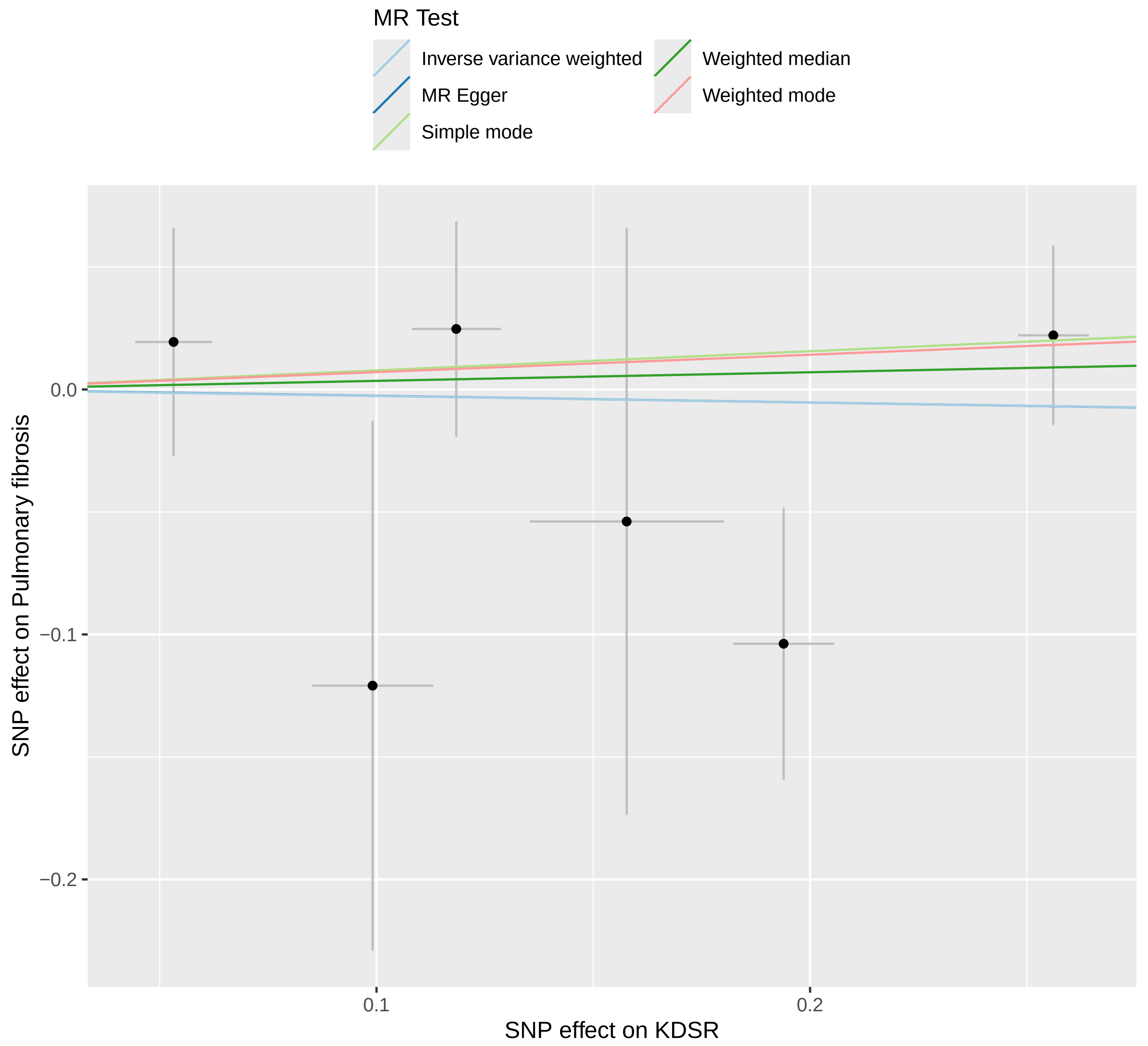
MR Test
Inverse variance weighted
MR Egger
Simple mode
Weighted median
Weighted mode
SNP effect on Pulmonary fibrosis
0.0
−0.1
−0.2
0.1
0.2
SNP effect on KDSR

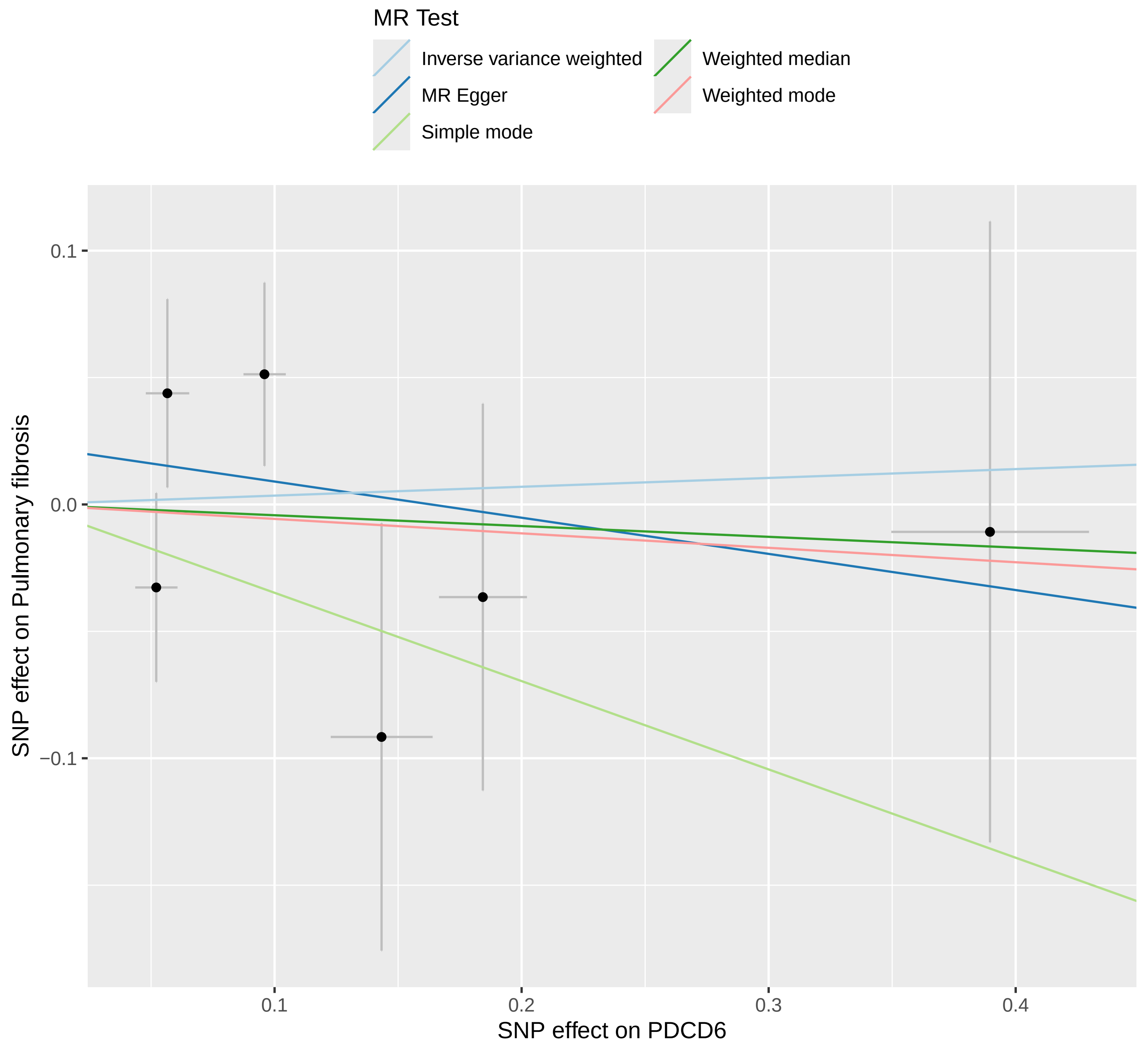

MR Test
Inverse variance weighted
MR Egger
Simple mode
Weighted median
Weighted mode
0.1
0.0
−0.1
SNP effect on Pulmonary fibrosis
0.1
0.2
0.3
0.4
SNP effect on PDCD6

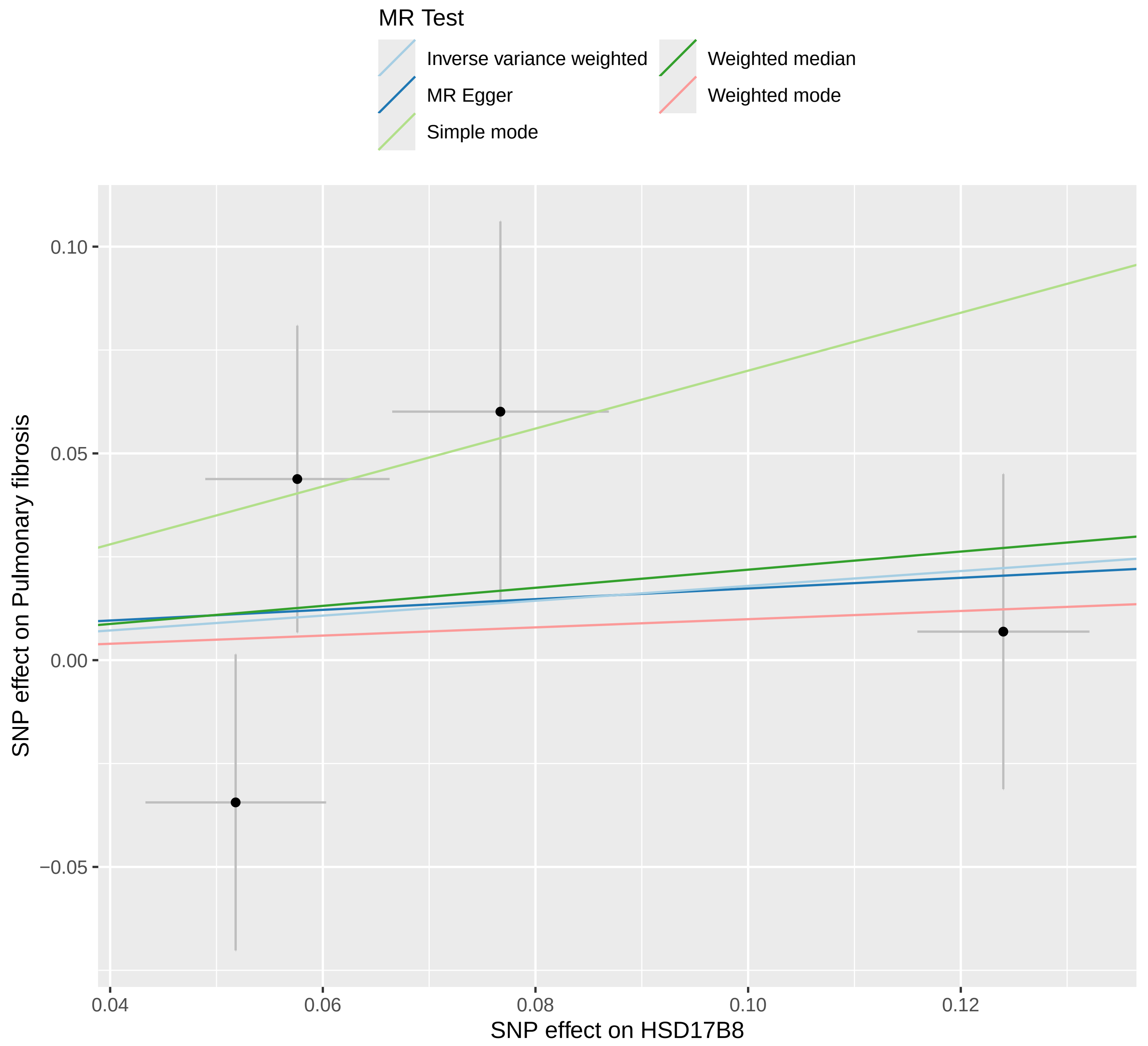

MR Test
Inverse variance weighted
MR Egger
Simple mode
Weighted median
Weighted mode
0.10
0.05
0.00
−0.05
SNP effect on Pulmonary fibrosis
0.04
0.06
0.08
0.10
0.12
SNP effect on HSD17B8

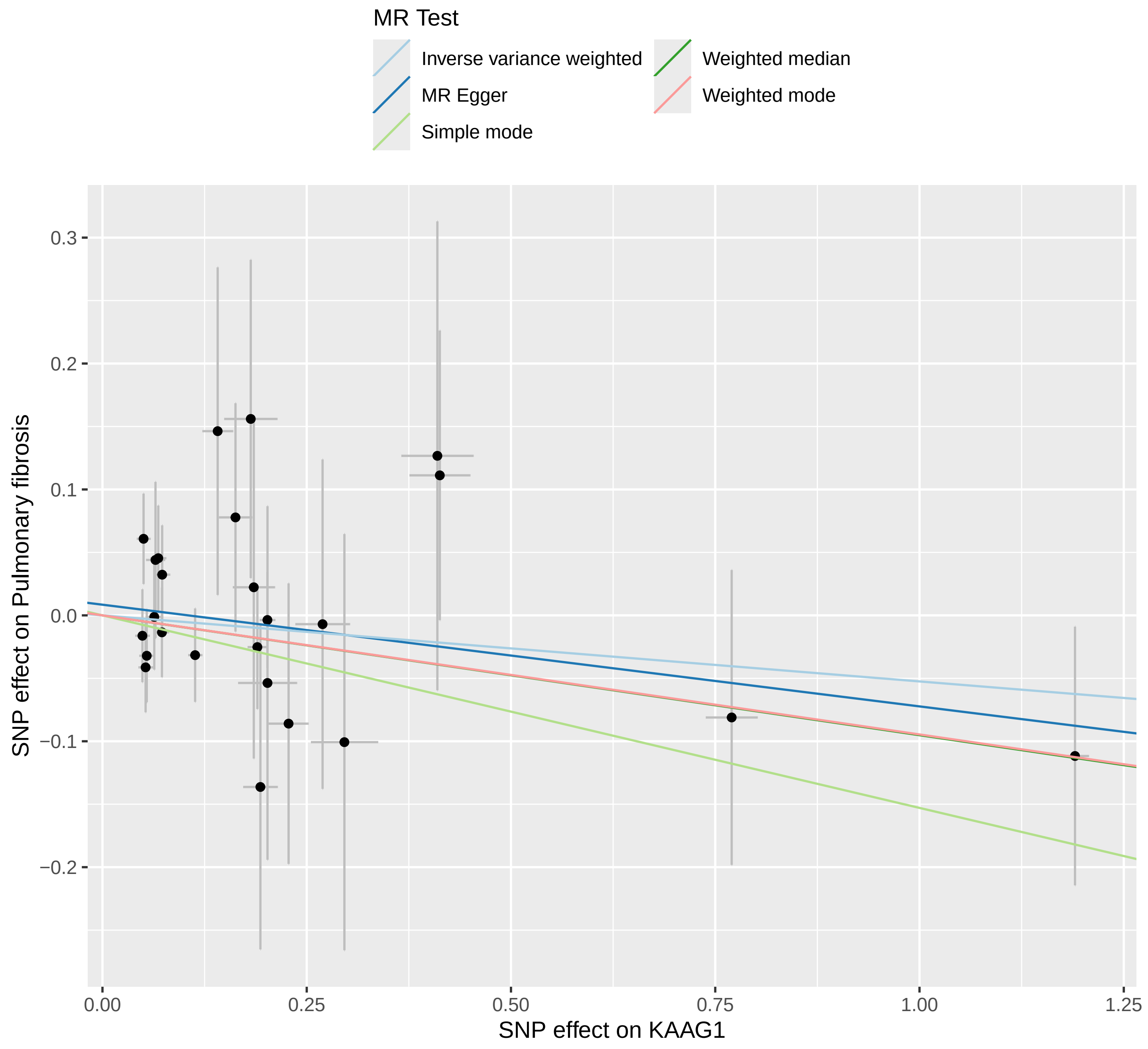

MR Test
Inverse variance weighted
MR Egger
Simple mode
Weighted median
Weighted mode
SNP effect on Pulmonary fibrosis
0.3
0.2
0.1
0.0
−0.1
−0.2
0.00
0.25
0.50
0.75
1.00
1.25
SNP effect on KAAG1

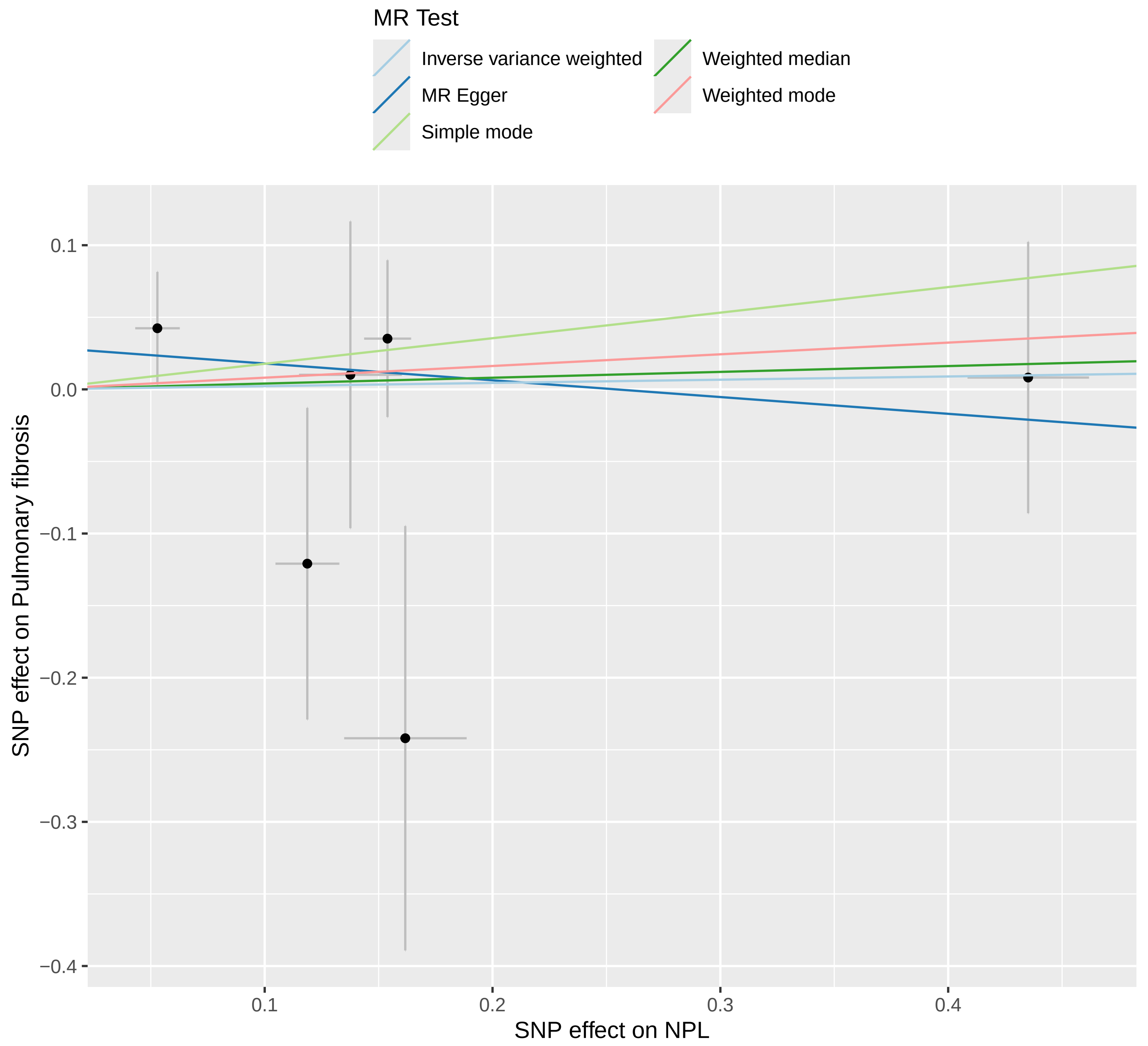
MR Test
Inverse variance weighted
MR Egger
Simple mode
Weighted median
Weighted mode
0.1
0.0
−0.1
−0.2
−0.3
−0.4
SNP effect on Pulmonary fibrosis
0.1
0.2
0.3
0.4
SNP effect on NPL

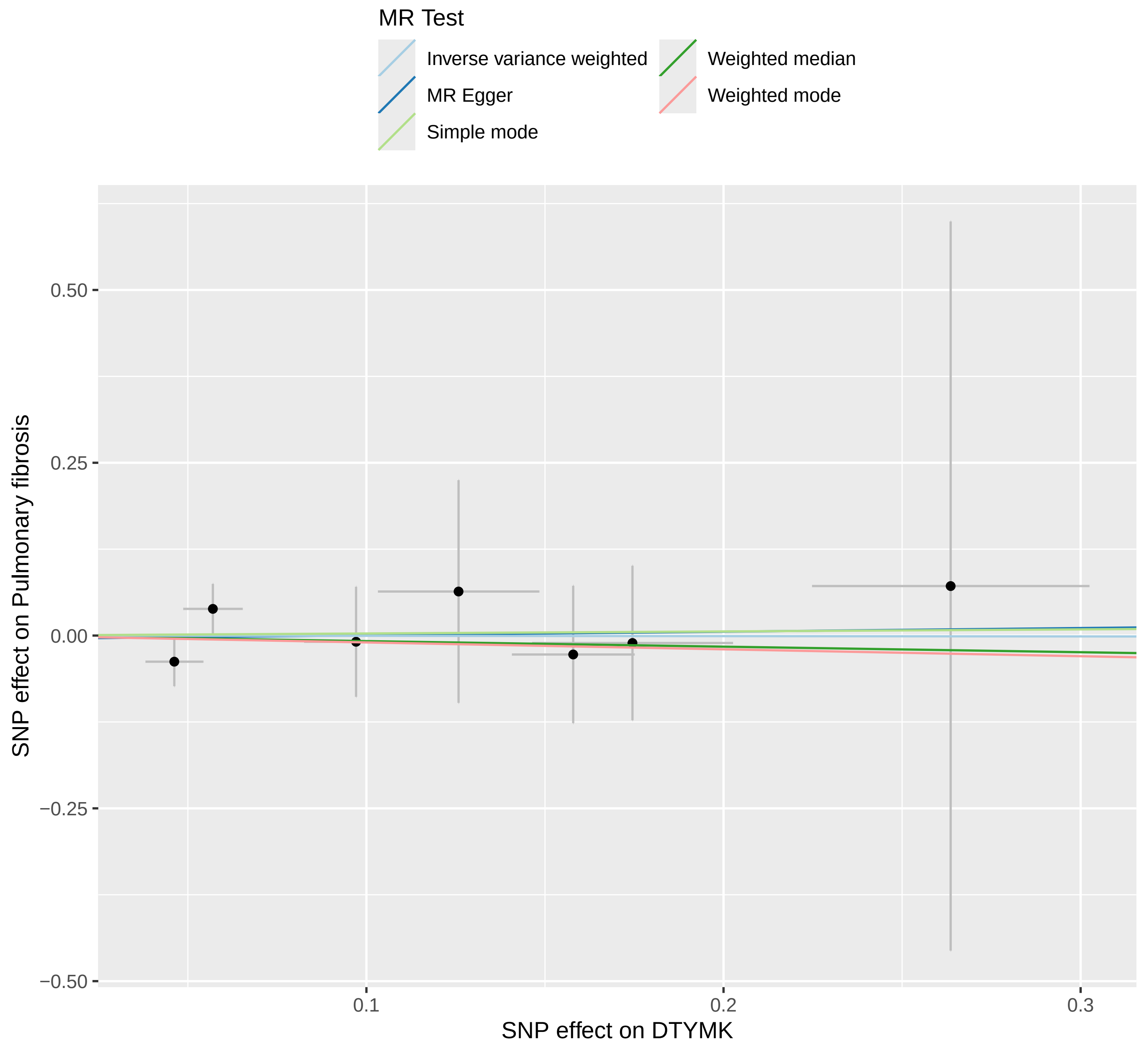
MR Test
Inverse variance weighted
MR Egger
Simple mode
Weighted median
Weighted mode
SNP effect on Pulmonary fibrosis
0.50
0.25
0.00
−0.25
−0.50
0.1
0.2
0.3
SNP effect on DTYMK

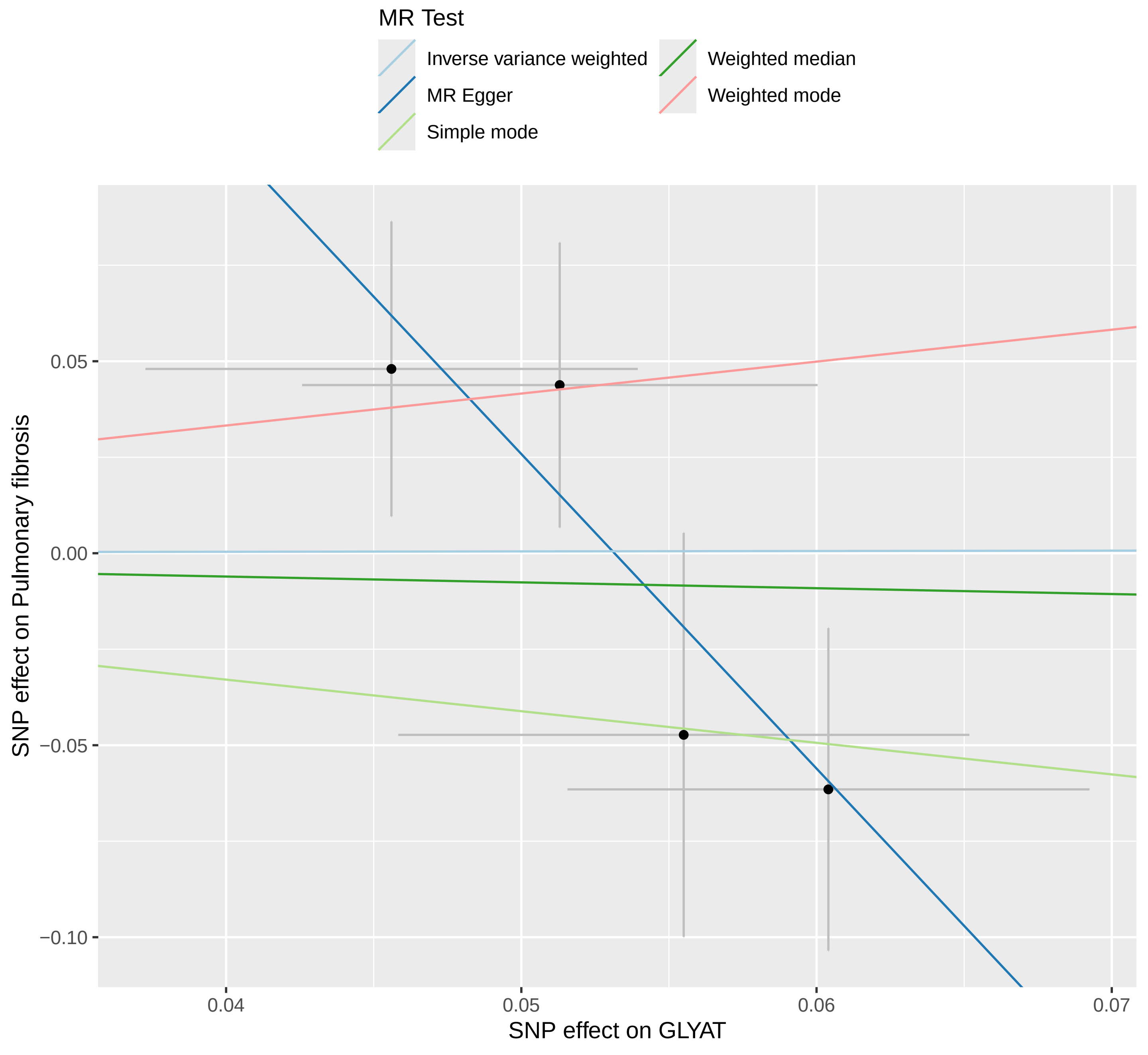
MR Test
Inverse variance weighted
MR Egger
Simple mode
Weighted median
Weighted mode
0.05
0.00
−0.05
−0.10
SNP effect on Pulmonary fibrosis
0.04
0.05
0.06
0.07
SNP effect on GLYAT

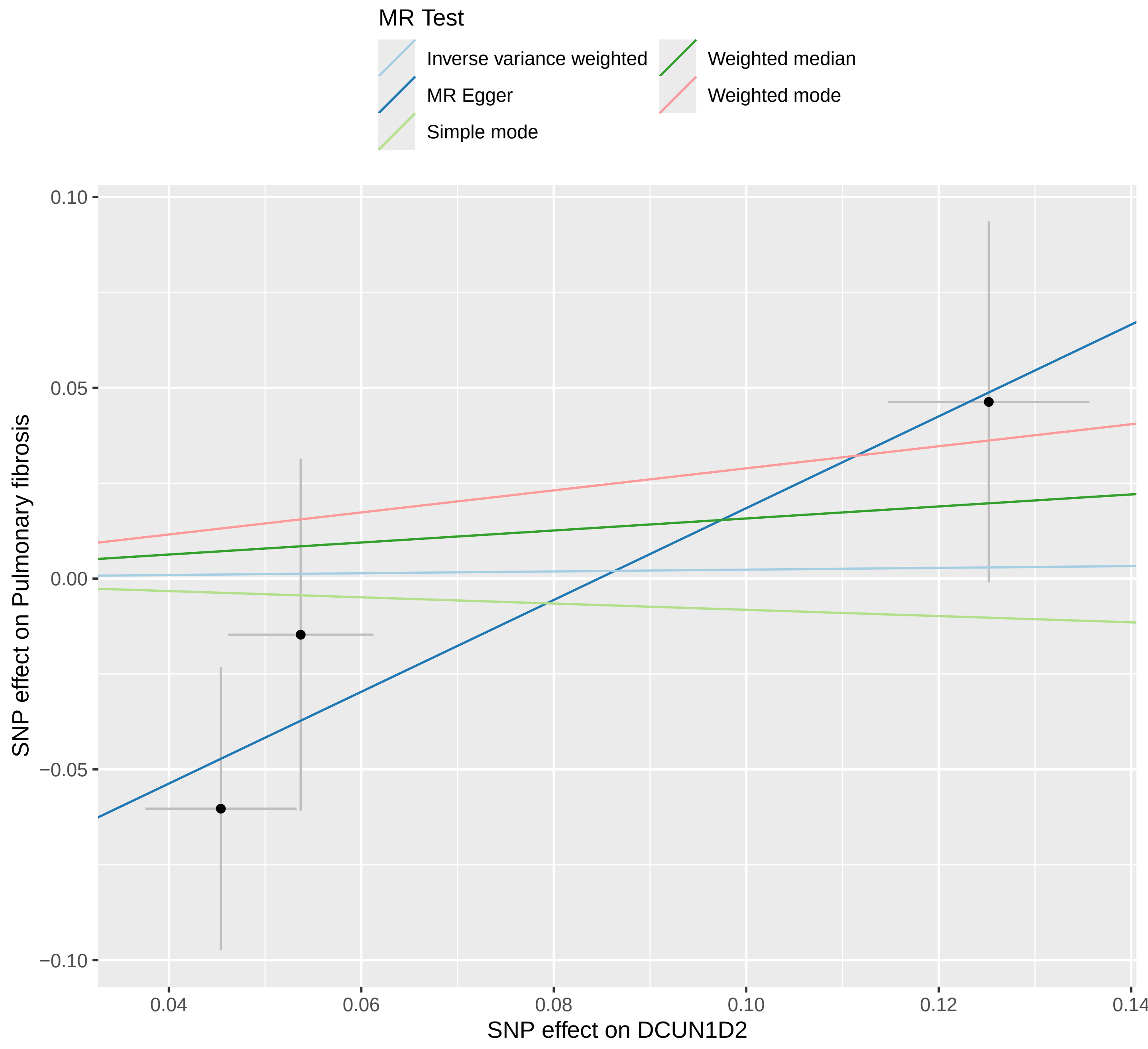

MR Test
Inverse variance weighted
MR Egger
Simple mode
Weighted median
Weighted mode
0.10
0.05
0.00
−0.05
−0.10
SNP effect on Pulmonary fibrosis
0.04
0.06
0.08
0.10
0.12
0.14
SNP effect on DCUN1D2

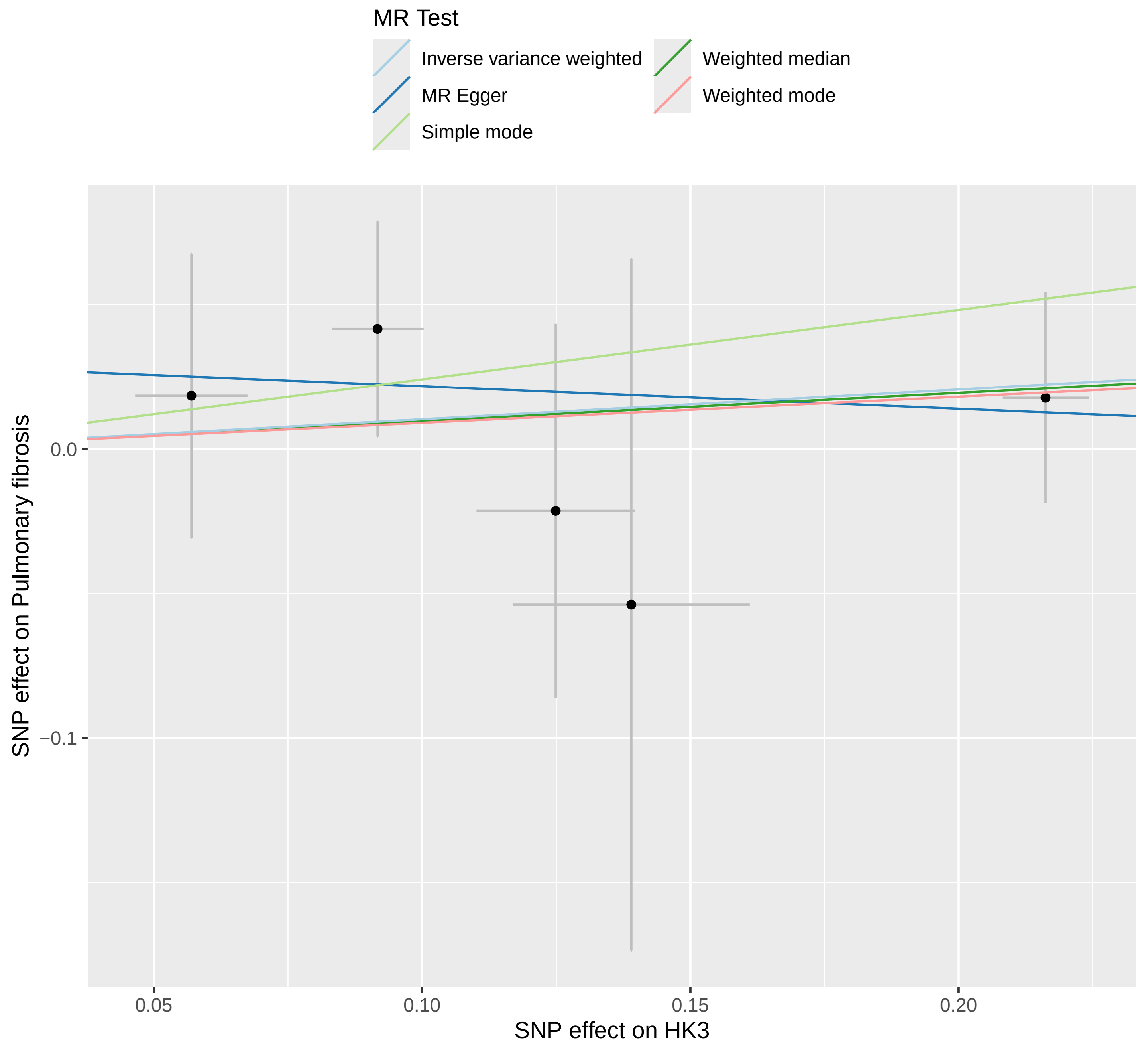

MR Test
Inverse variance weighted
MR Egger
Simple mode
Weighted median
Weighted mode
0.0
−0.1
0.05
0.10
0.15
0.20
SNP effect on HK3
SNP effect on Pulmonary fibrosis

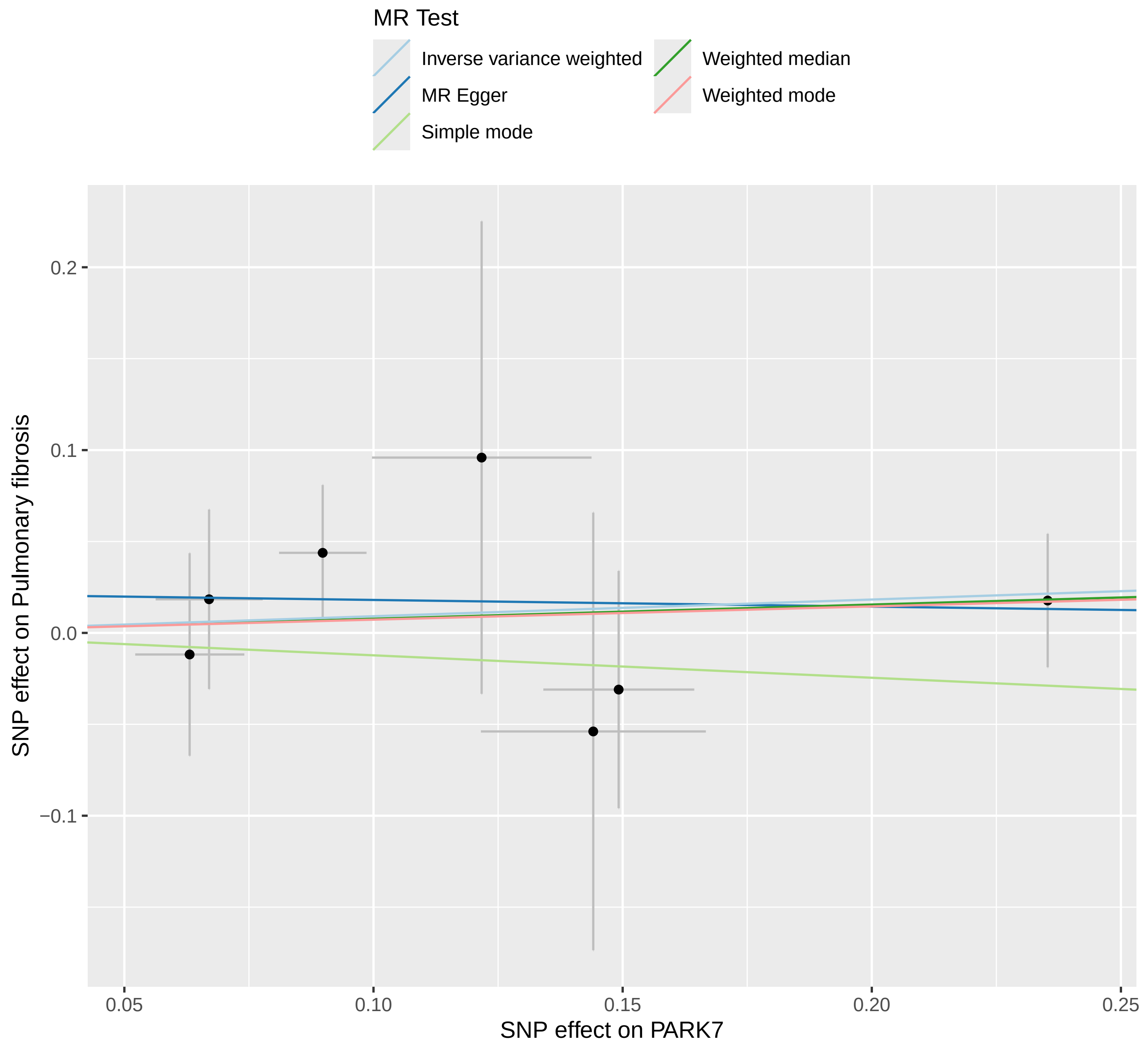

MR Test
Inverse variance weighted
MR Egger
Simple mode
Weighted median
Weighted mode
0.2
0.1
0.0
−0.1
0.05
0.10
0.15
0.20
0.25
SNP effect on PARK7
SNP effect on Pulmonary fibrosis

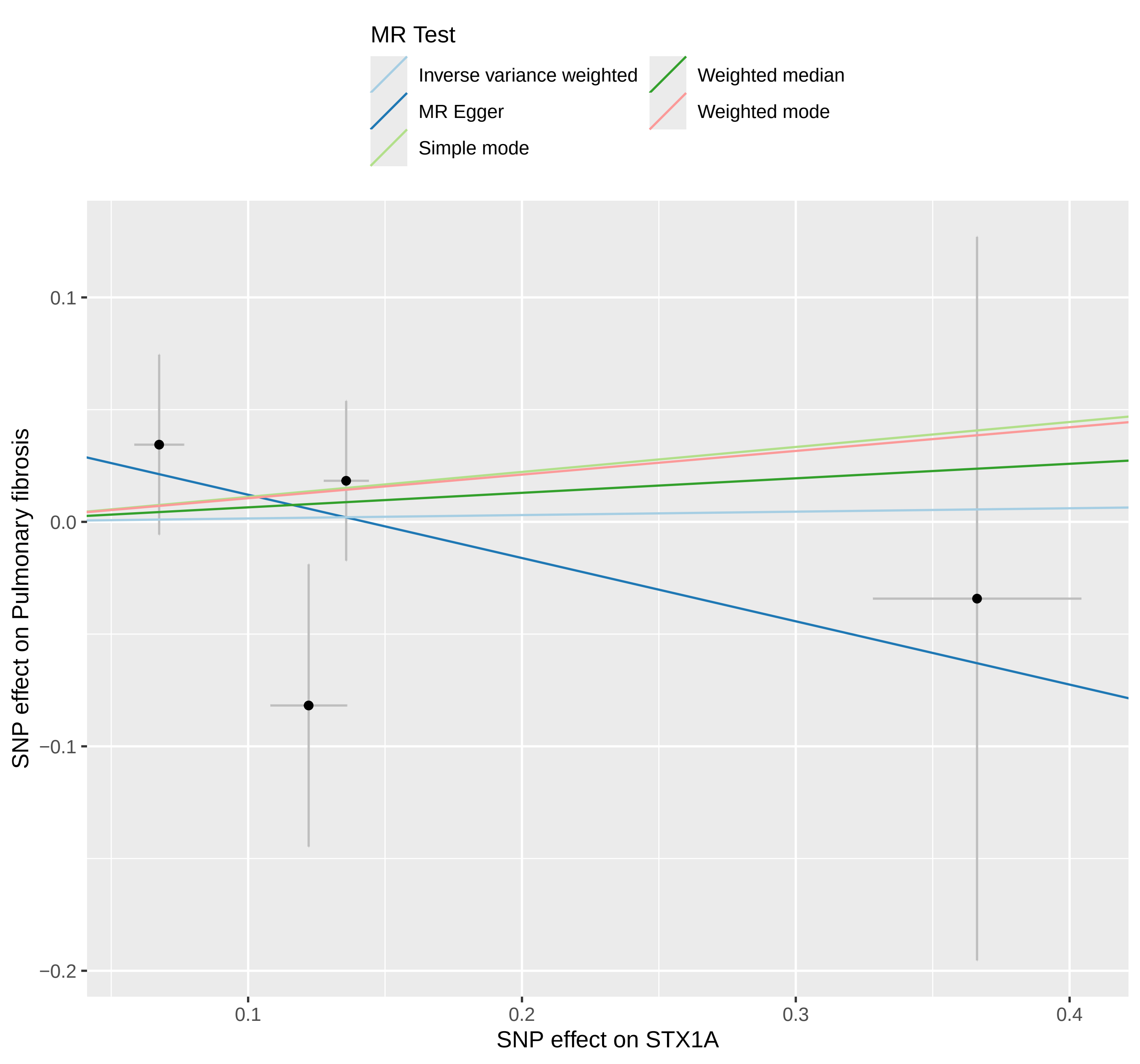

MR Test
Inverse variance weighted
MR Egger
Simple mode
Weighted median
Weighted mode
0.1
0.0
−0.1
−0.2
SNP effect on Pulmonary fibrosis
0.1
0.2
0.3
0.4
SNP effect on STX1A

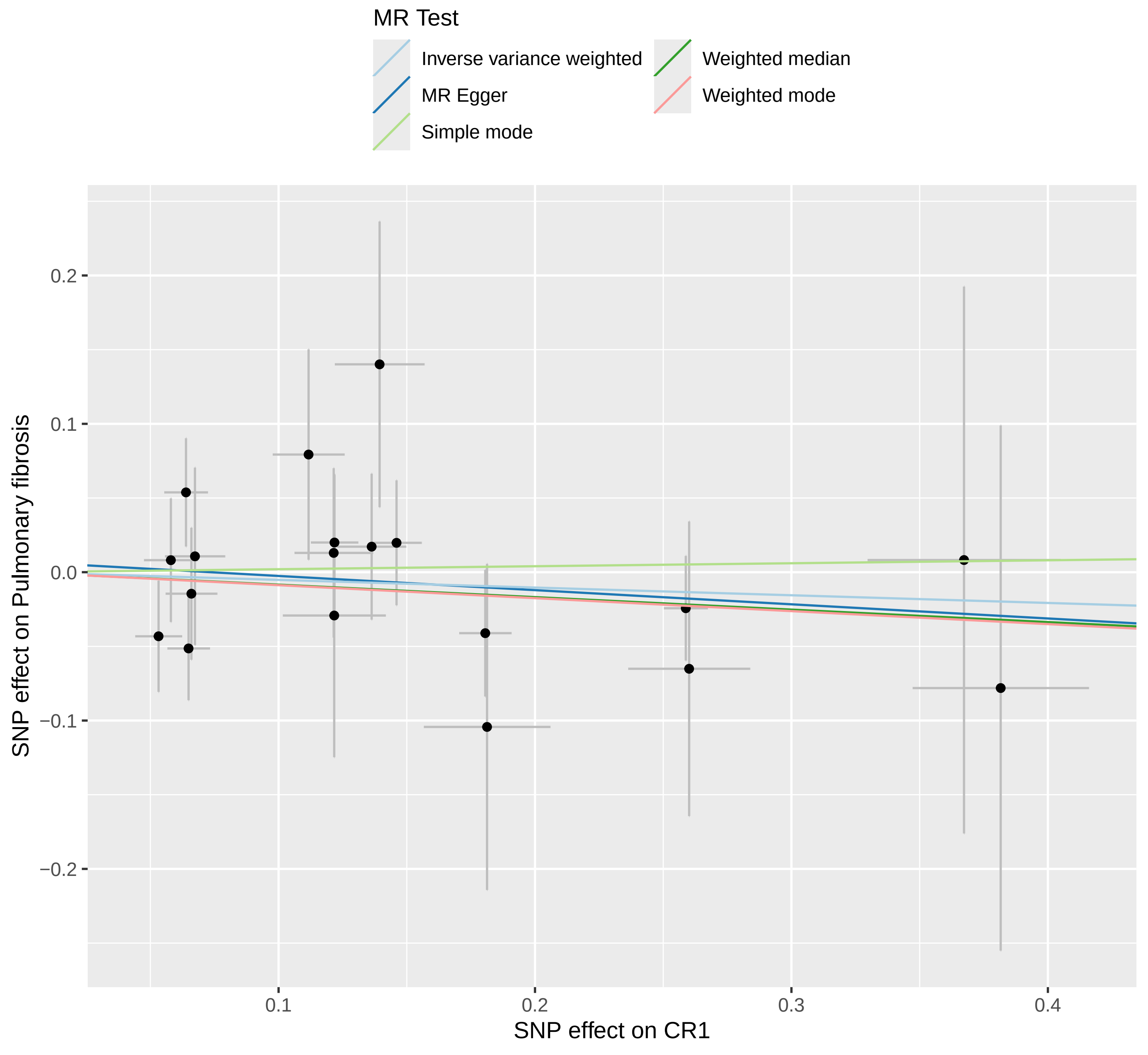

MR Test
Inverse variance weighted
MR Egger
Simple mode
Weighted median
Weighted mode
SNP effect on Pulmonary fibrosis
0.2
0.1
0.0
−0.1
−0.2
0.1
0.2
0.3
0.4
SNP effect on CR1

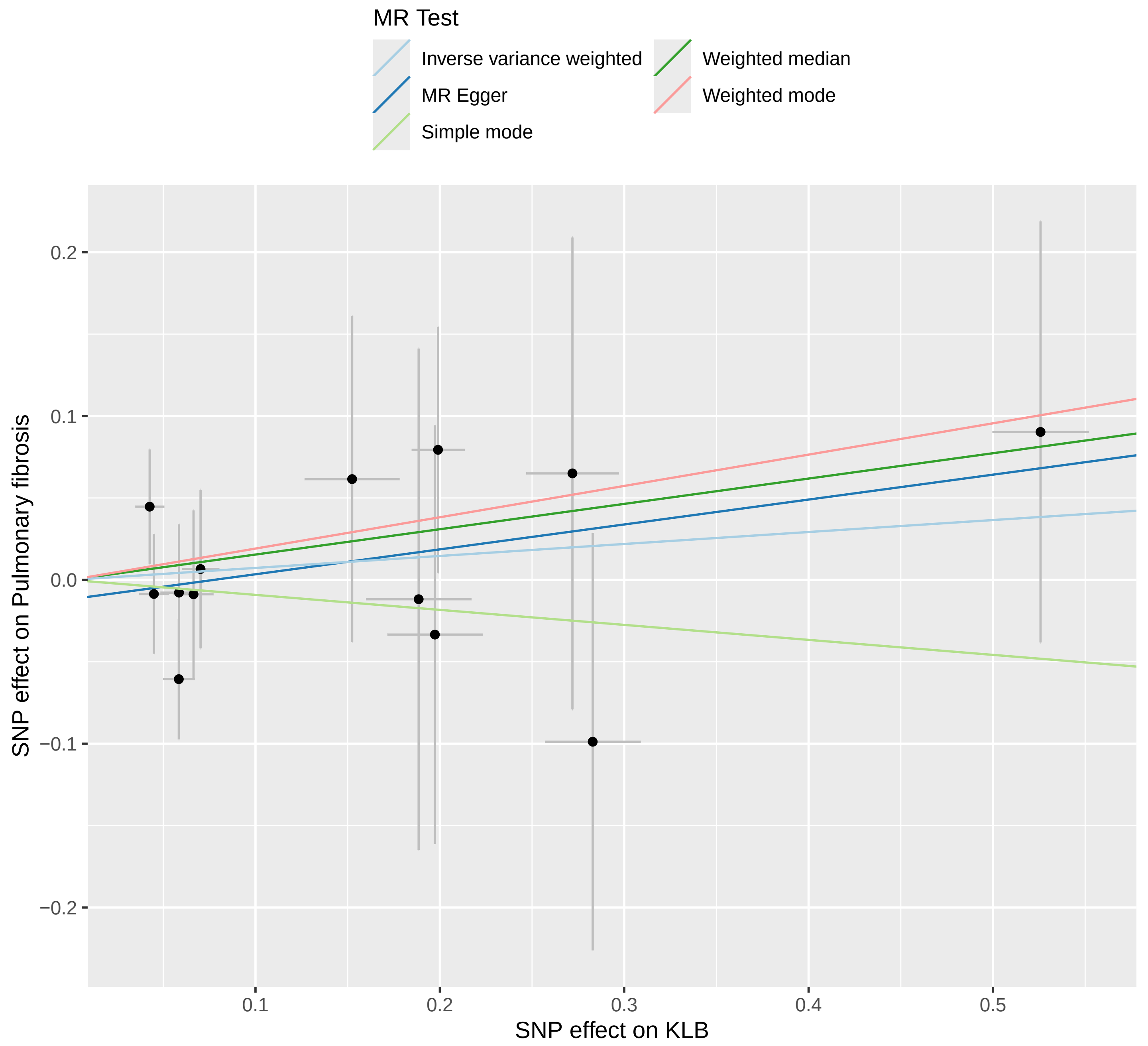
MR Test
Inverse variance weighted
MR Egger
Simple mode
Weighted median
Weighted mode
0.2
0.1
0.0
−0.1
−0.2
SNP effect on Pulmonary fibrosis
0.1
0.2
0.3
0.4
0.5
SNP effect on KLB

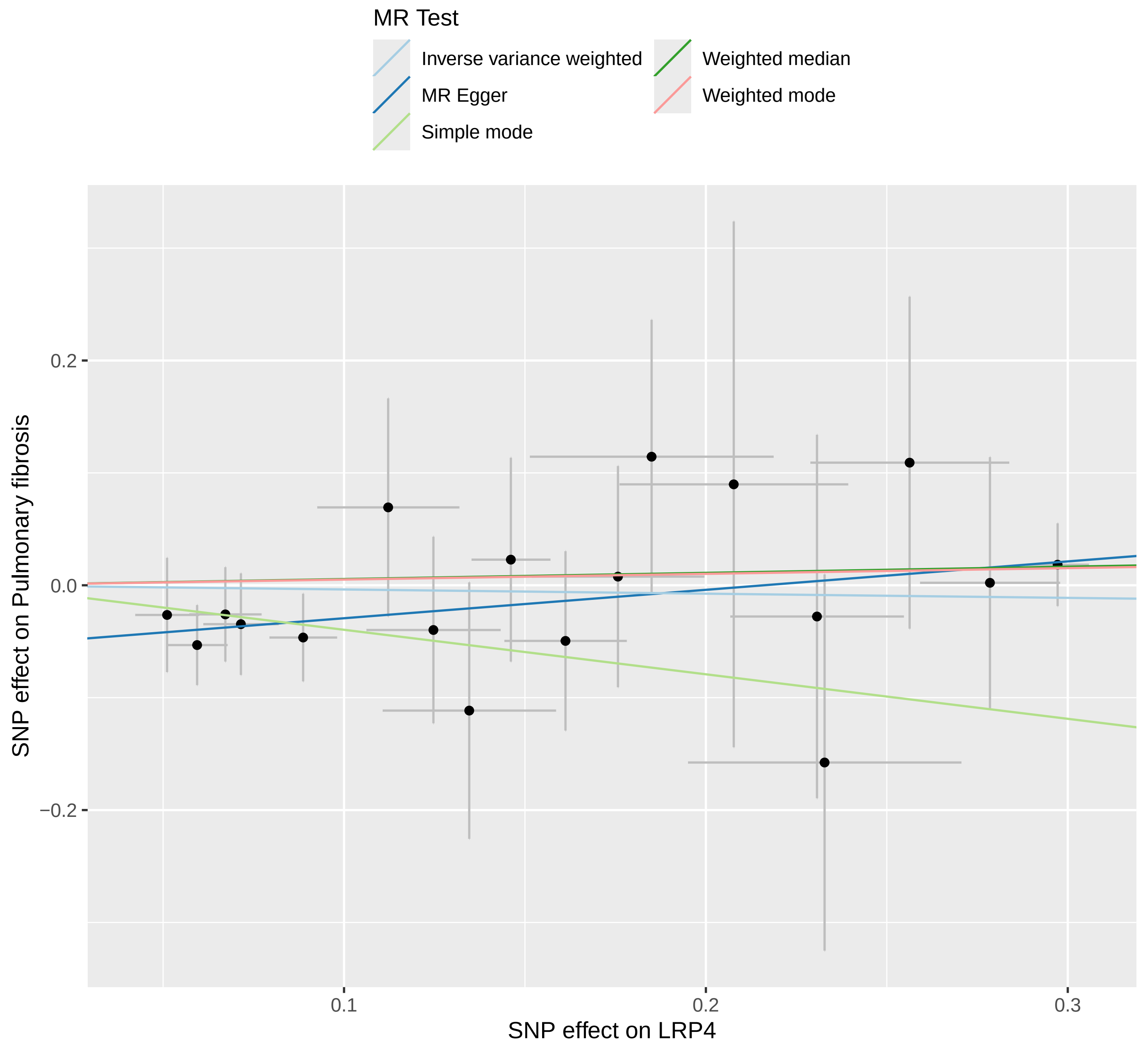
MR Test
Inverse variance weighted
MR Egger
Simple mode
Weighted median
Weighted mode
SNP effect on Pulmonary fibrosis
0.2
0.0
−0.2
0.1
0.2
0.3
SNP effect on LRP4

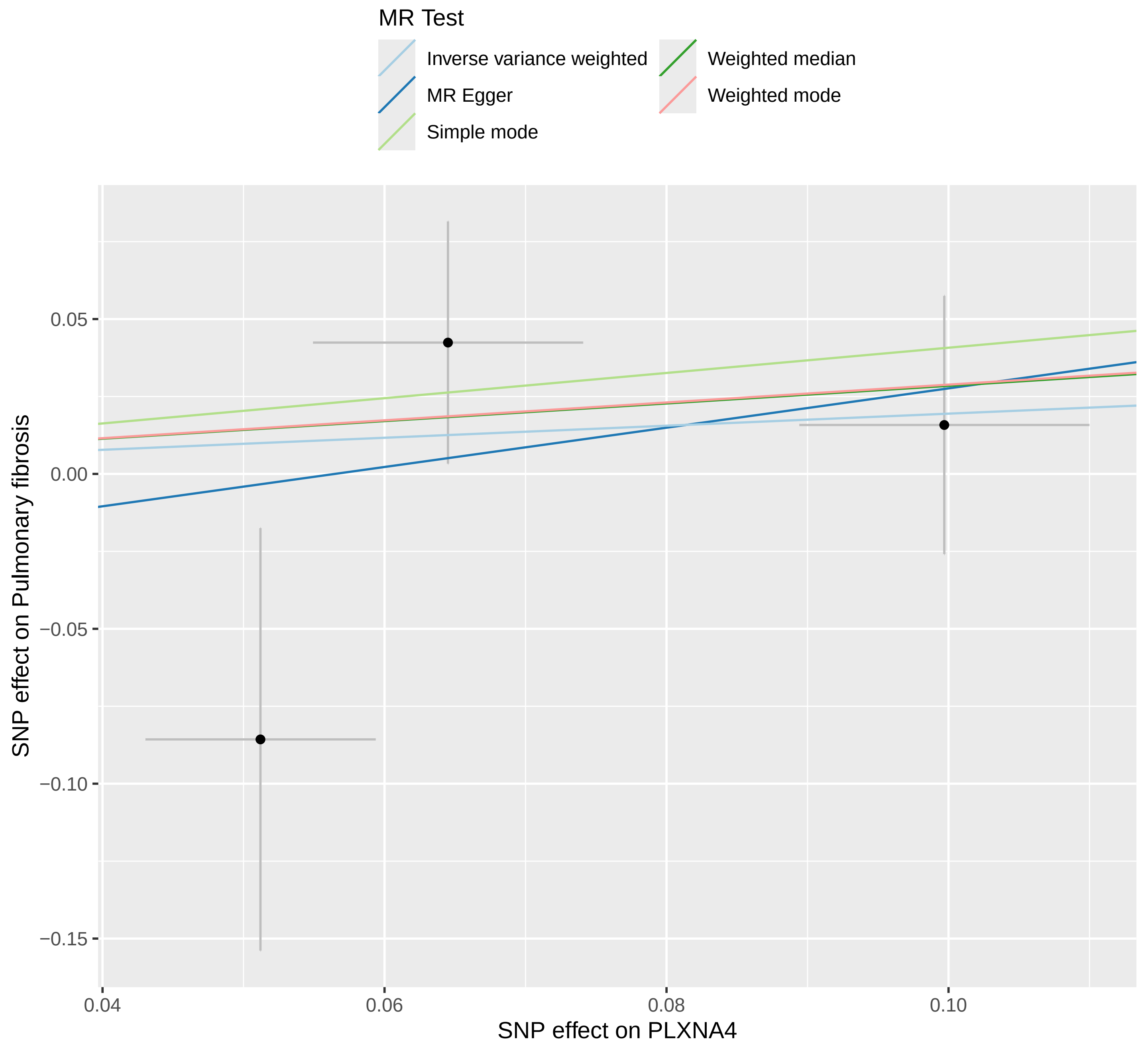

MR Test
Inverse variance weighted
MR Egger
Simple mode
Weighted median
Weighted mode
0.05
0.00
−0.05
−0.10
−0.15
0.04
0.06
0.08
0.10
SNP effect on Pulmonary fibrosis
SNP effect on PLXNA4

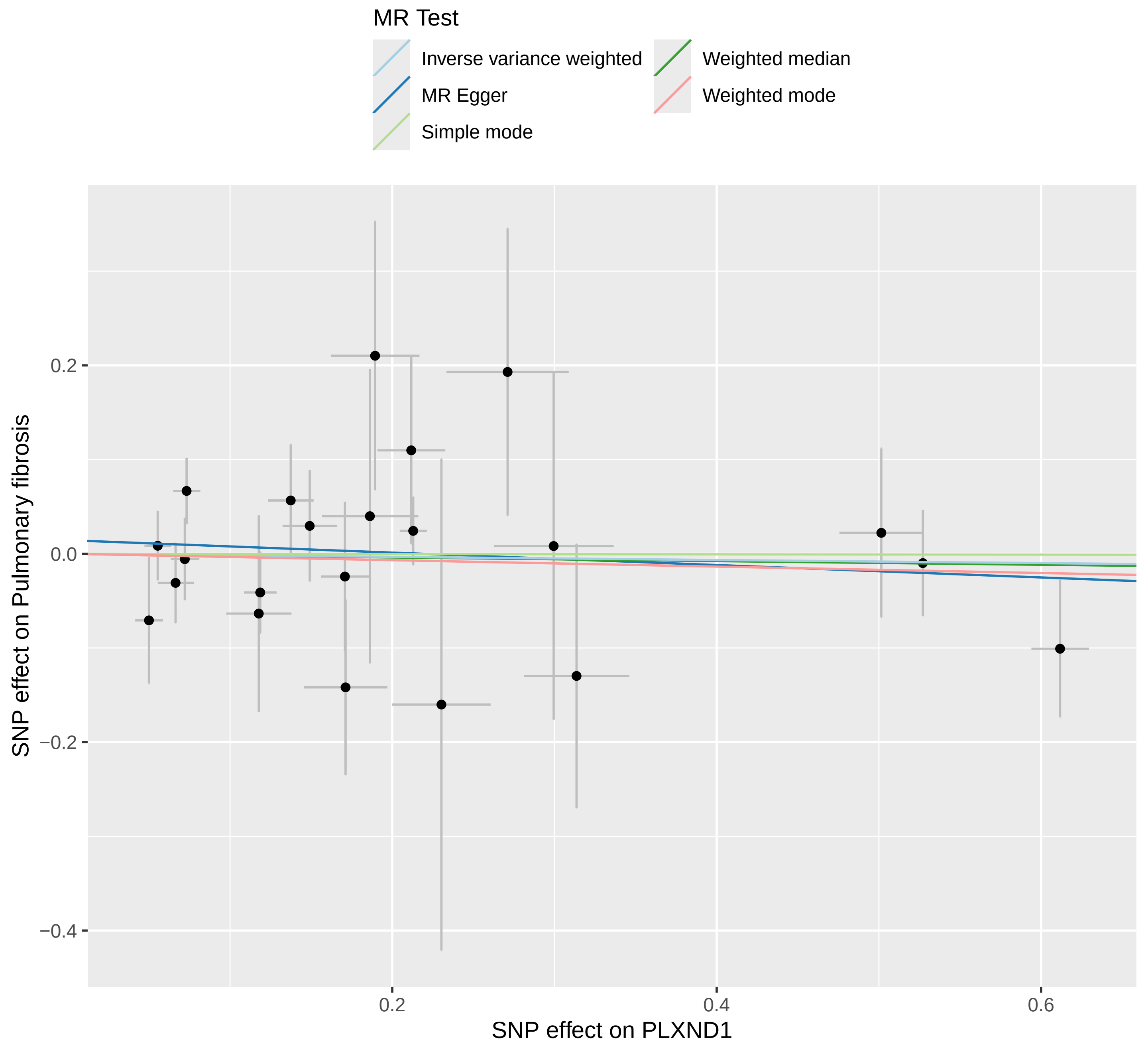

MR Test
Inverse variance weighted
MR Egger
Simple mode
Weighted median
Weighted mode
0.2
0.0
−0.2
−0.4
SNP effect on Pulmonary fibrosis
0.2
0.4
0.6
SNP effect on PLXND1

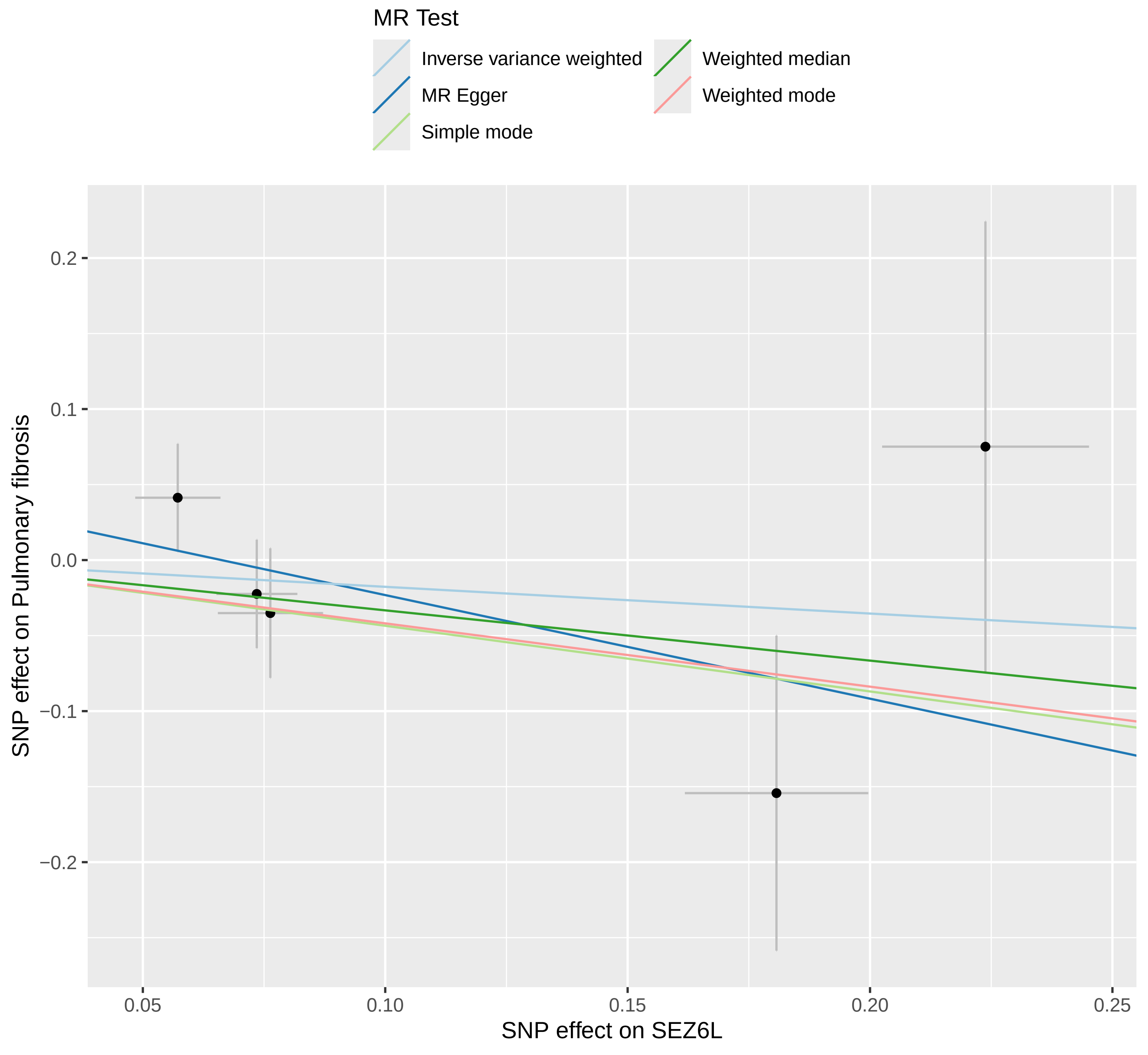

MR Test
Inverse variance weighted
MR Egger
Simple mode
Weighted median
Weighted mode
0.2
0.1
0.0
−0.1
−0.2
SNP effect on Pulmonary fibrosis
0.05
0.10
0.15
0.20
0.25
SNP effect on SEZ6L

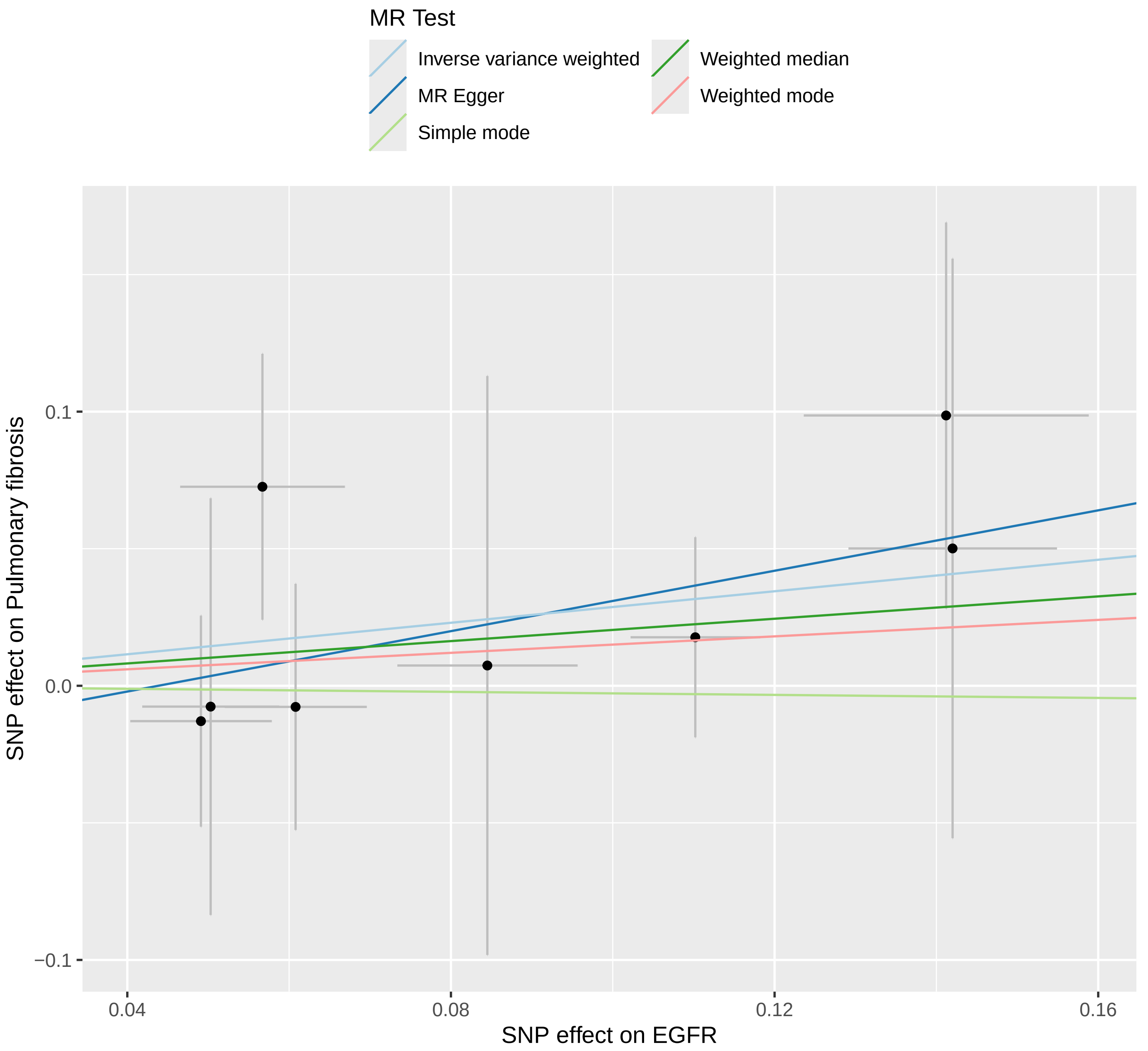

MR Test
Inverse variance weighted
MR Egger
Simple mode
Weighted median
Weighted mode
SNP effect on Pulmonary fibrosis
0.1
0.0
−0.1
0.04
0.08
0.12
0.16
SNP effect on EGFR

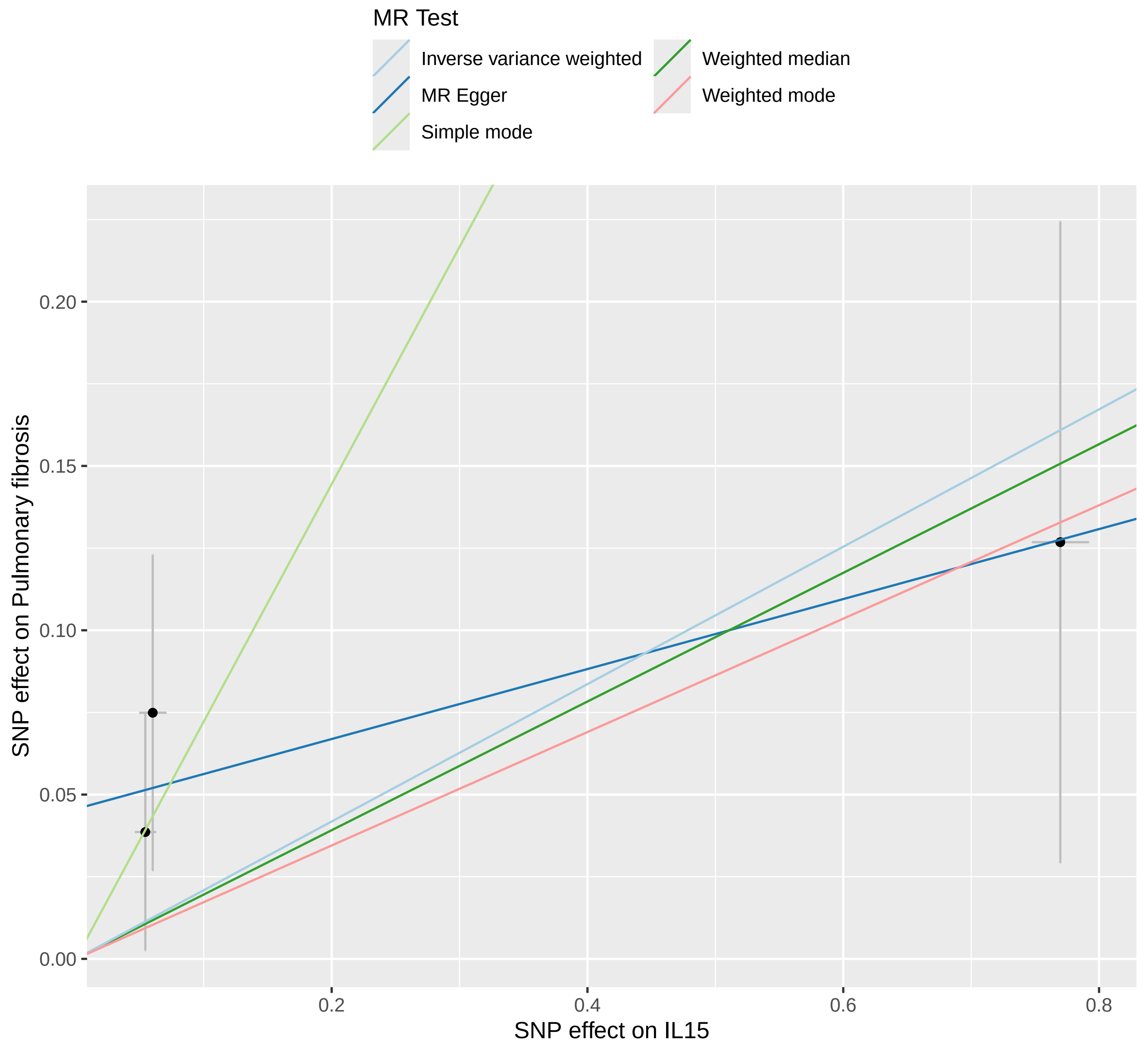

MR Test
Inverse variance weighted
MR Egger
Simple mode
Weighted median
Weighted mode
0.20
0.15
0.10
0.05
0.00
SNP effect on Pulmonary fibrosis
0.2
0.4
0.6
0.8
SNP effect on IL15

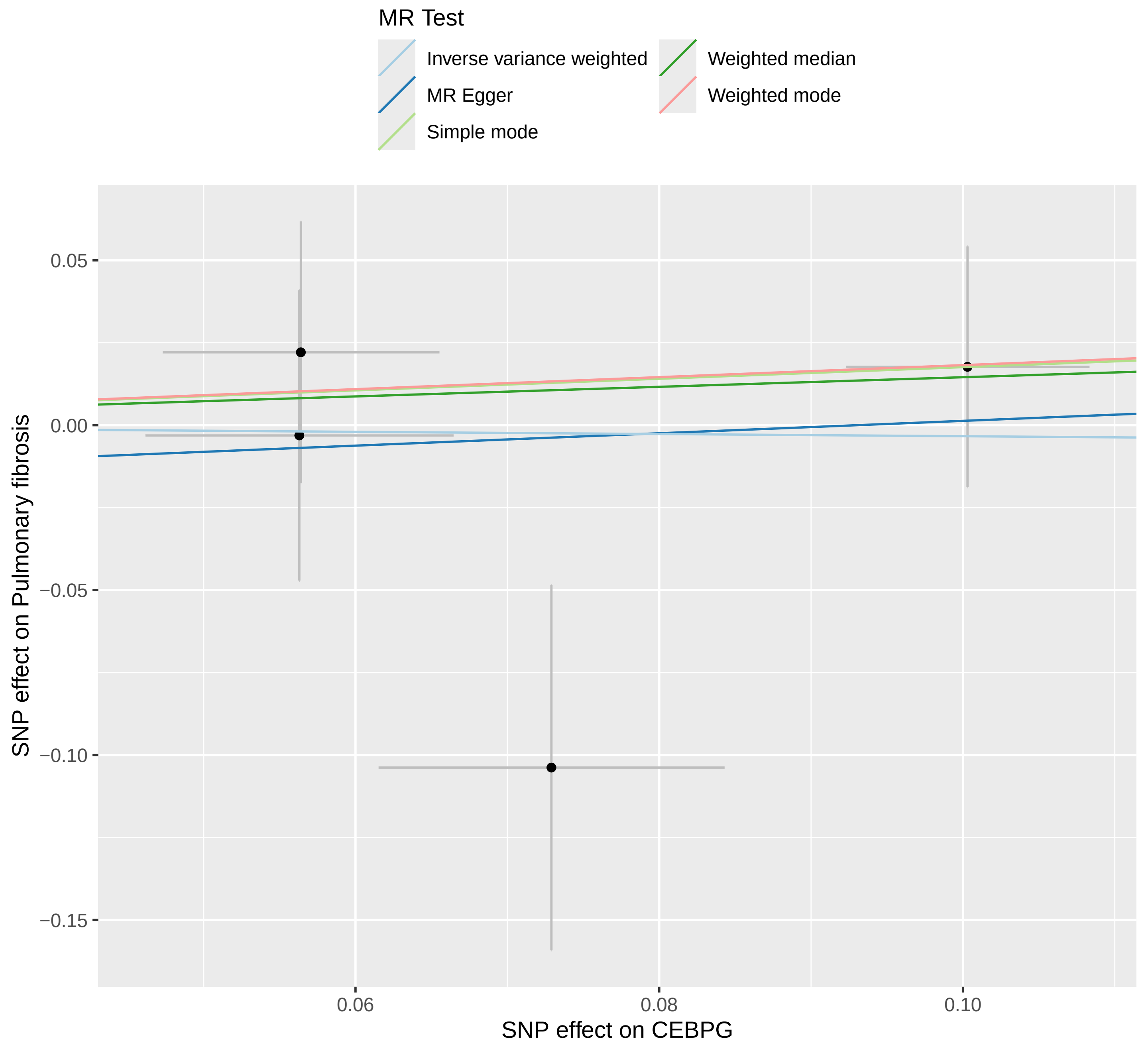
MR Test
Inverse variance weighted
MR Egger
Simple mode
Weighted median
Weighted mode
0.05
0.00
−0.05
−0.10
−0.15
SNP effect on Pulmonary fibrosis
0.06
0.08
0.10
SNP effect on CEBPG

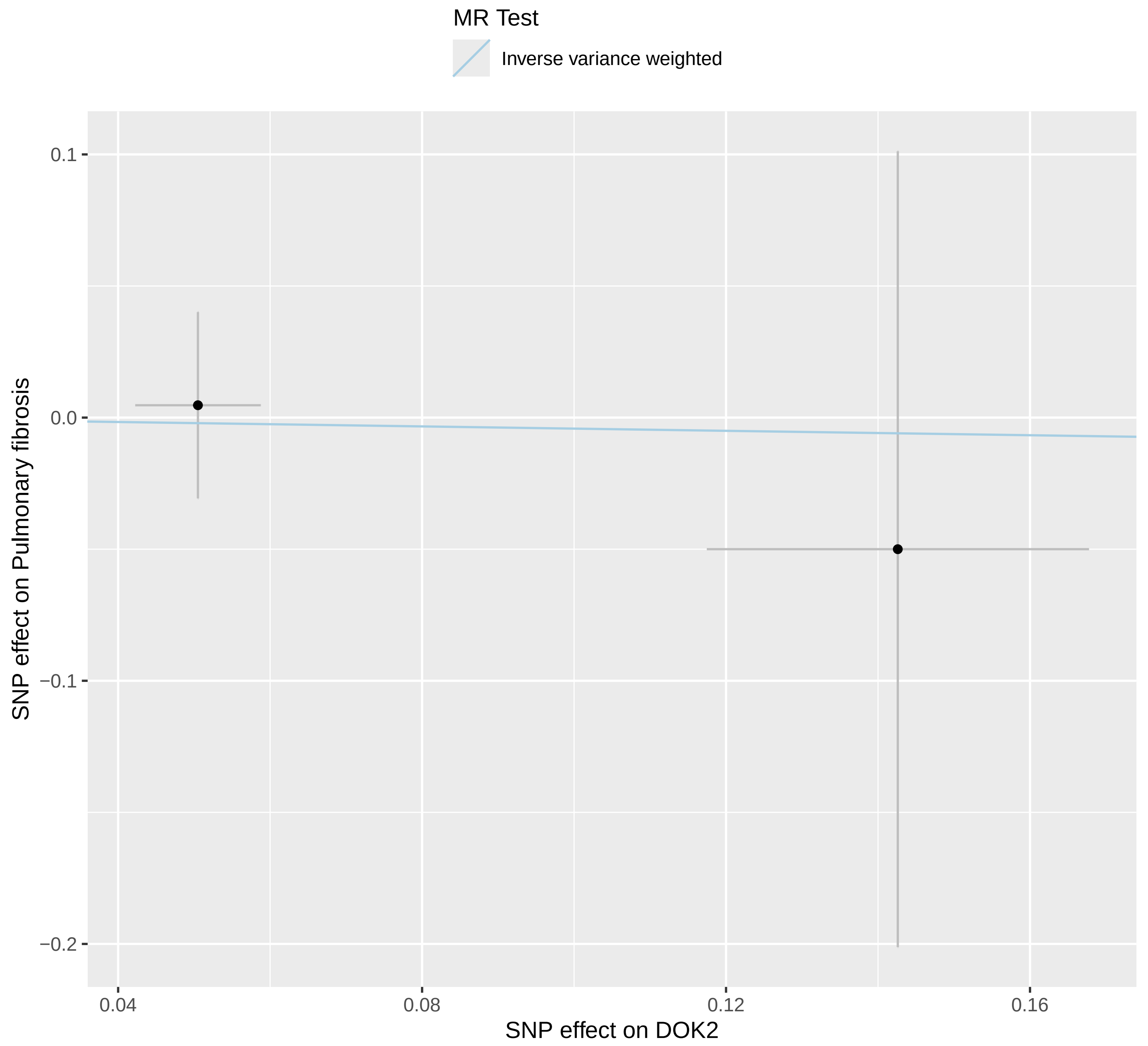

MR Test
Inverse variance weighted
0.1
0.0
−0.1
−0.2
0.04
0.08
0.12
0.16
SNP effect on Pulmonary fibrosis
SNP effect on DOK2

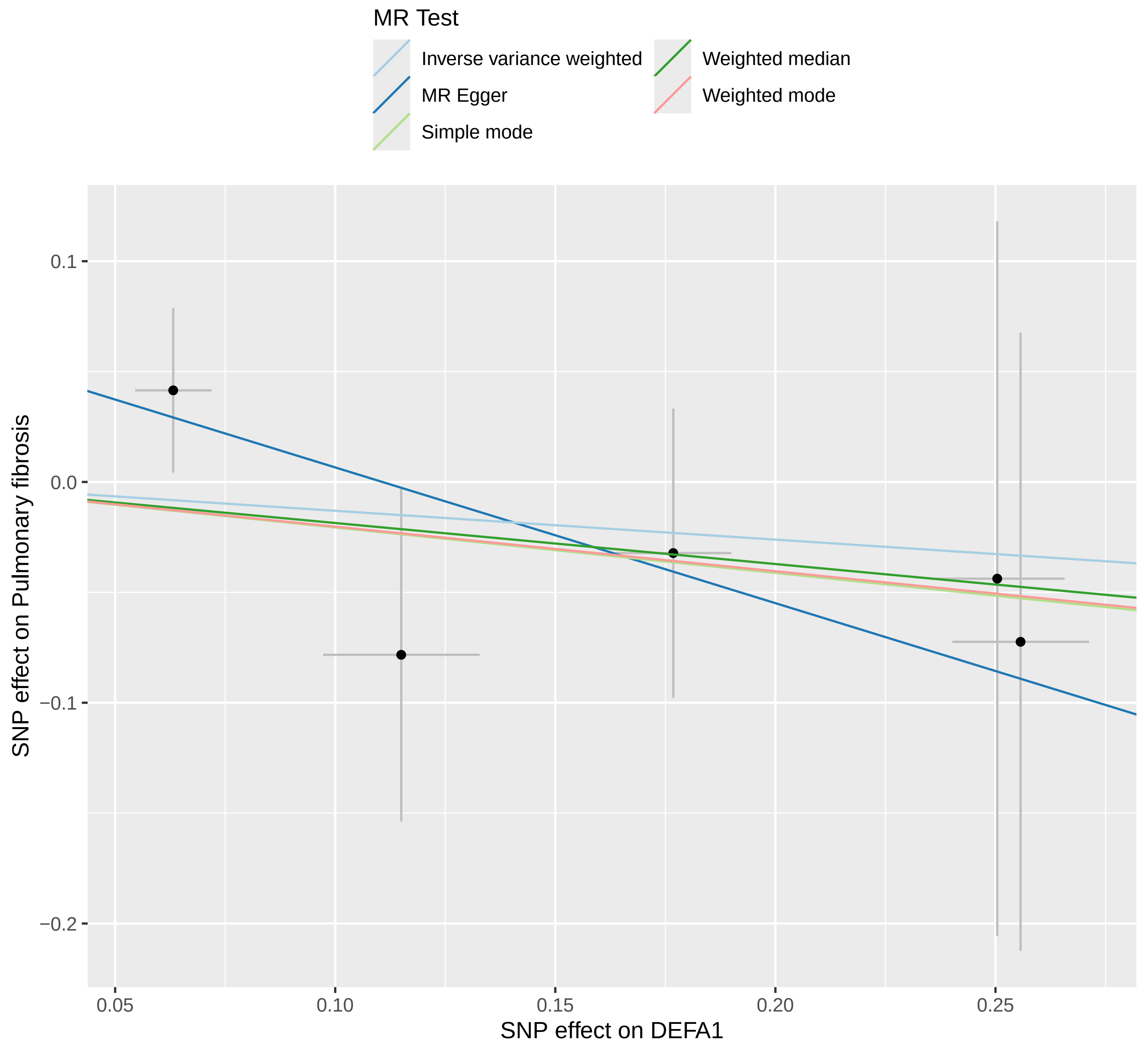
MR Test
Inverse variance weighted
MR Egger
Simple mode
Weighted median
Weighted mode
0.1
0.0
−0.1
−0.2
SNP effect on Pulmonary fibrosis
0.05
0.10
0.15
0.20
0.25
SNP effect on DEFA1

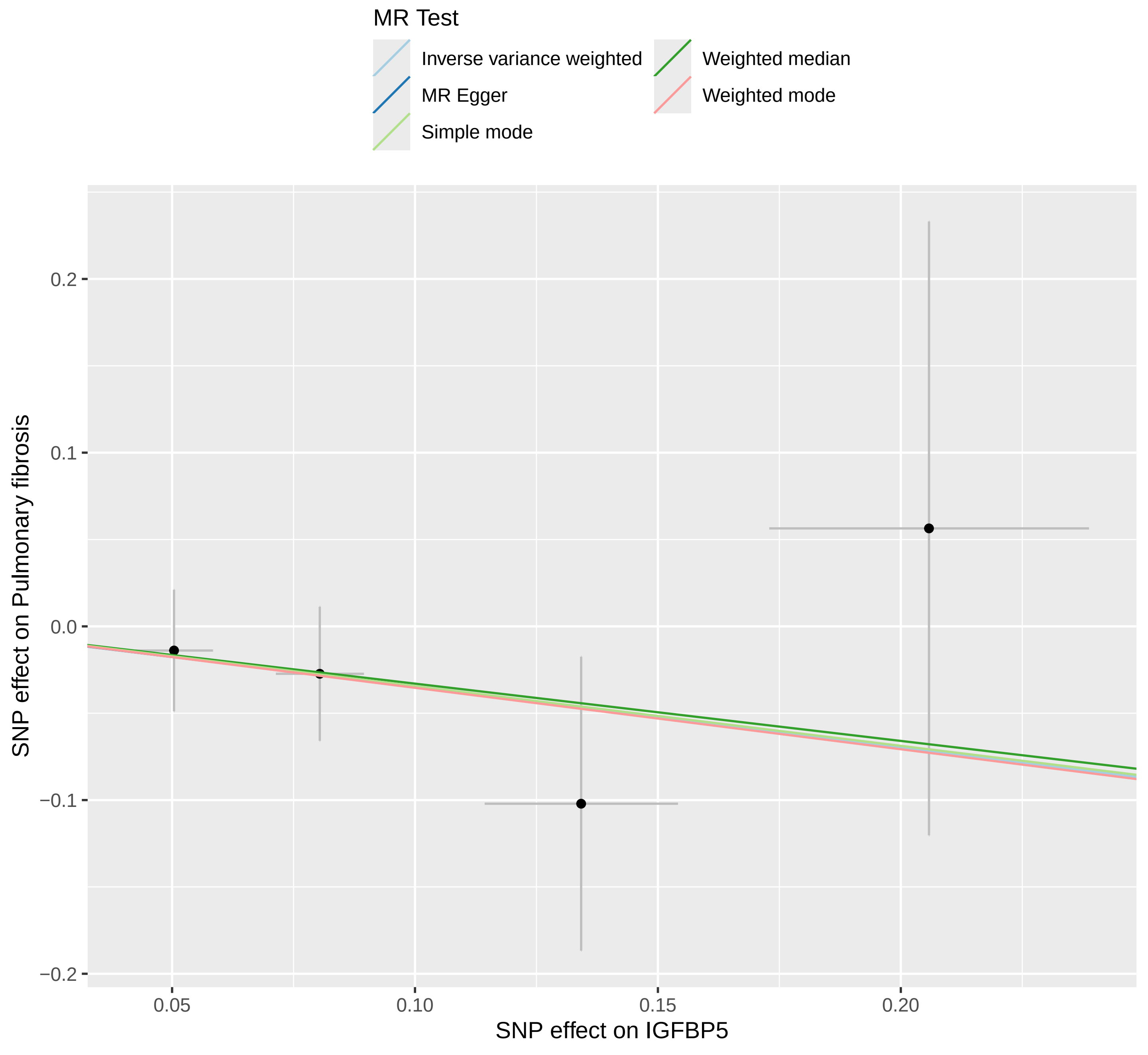
MR Test
Inverse variance weighted
MR Egger
Simple mode
Weighted median
Weighted mode
0.2
0.1
0.0
−0.1
−0.2
0.05
0.10
0.15
0.20
SNP effect on Pulmonary fibrosis
SNP effect on IGFBP5

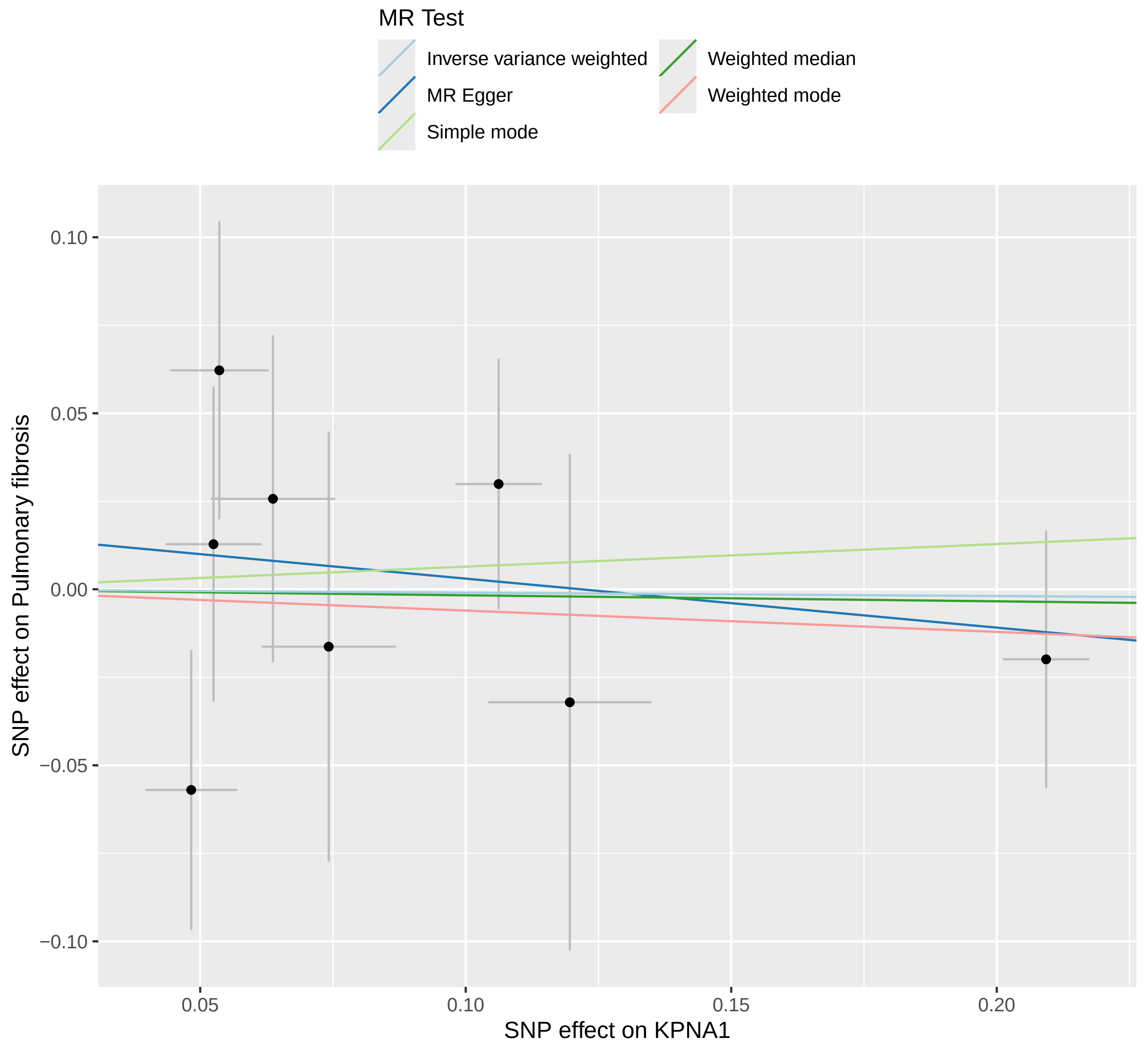
MR Test
Inverse variance weighted
MR Egger
Simple mode
Weighted median
Weighted mode
0.10
0.05
0.00
−0.05
−0.10
SNP effect on Pulmonary fibrosis
0.05
0.10
0.15
0.20
SNP effect on KPNA1

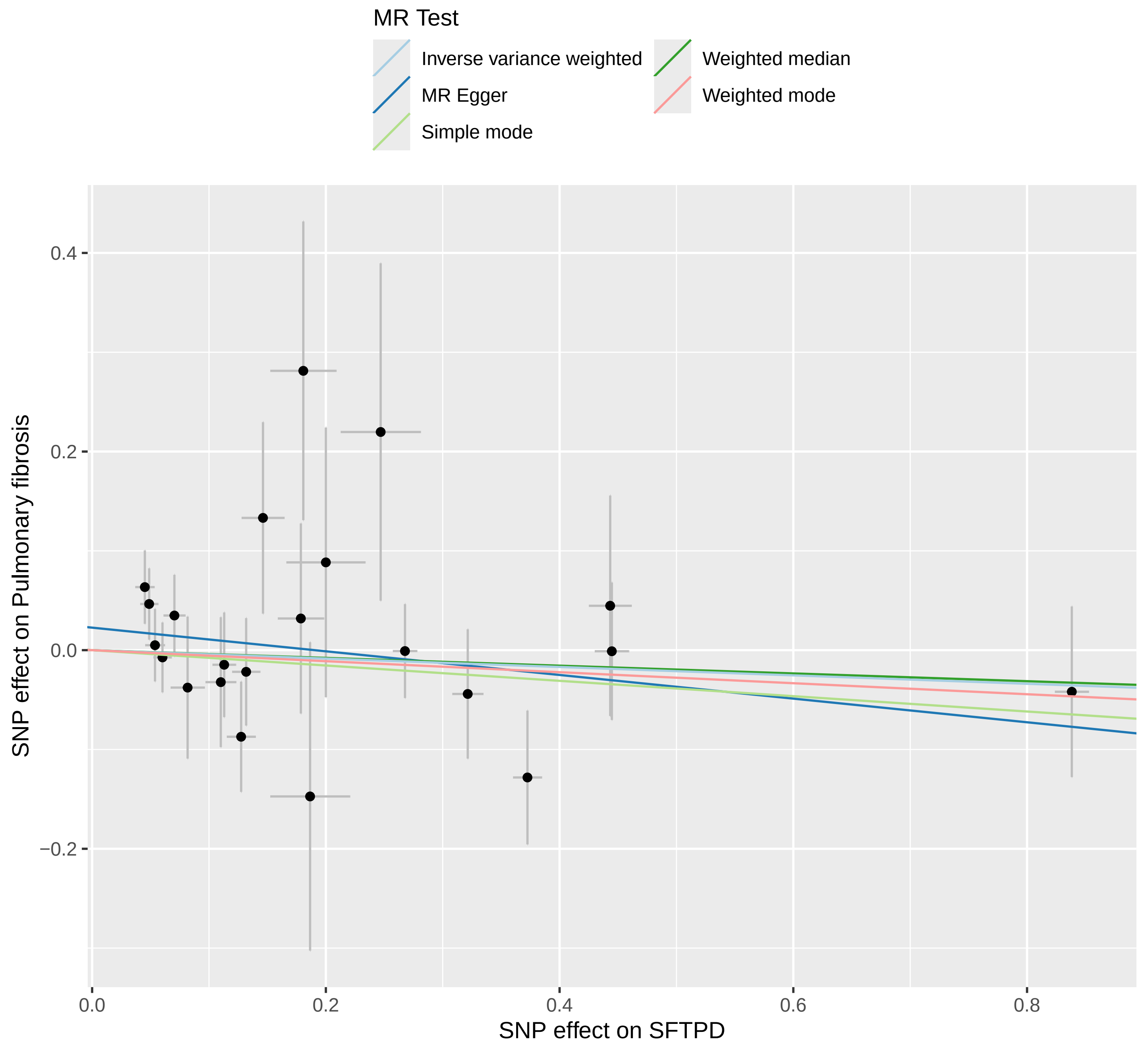

MR Test
Inverse variance weighted
MR Egger
Simple mode
Weighted median
Weighted mode
SNP effect on Pulmonary fibrosis
0.4
0.2
0.0
−0.2
0.0
0.2
0.4
0.6
0.8
SNP effect on SFTPD

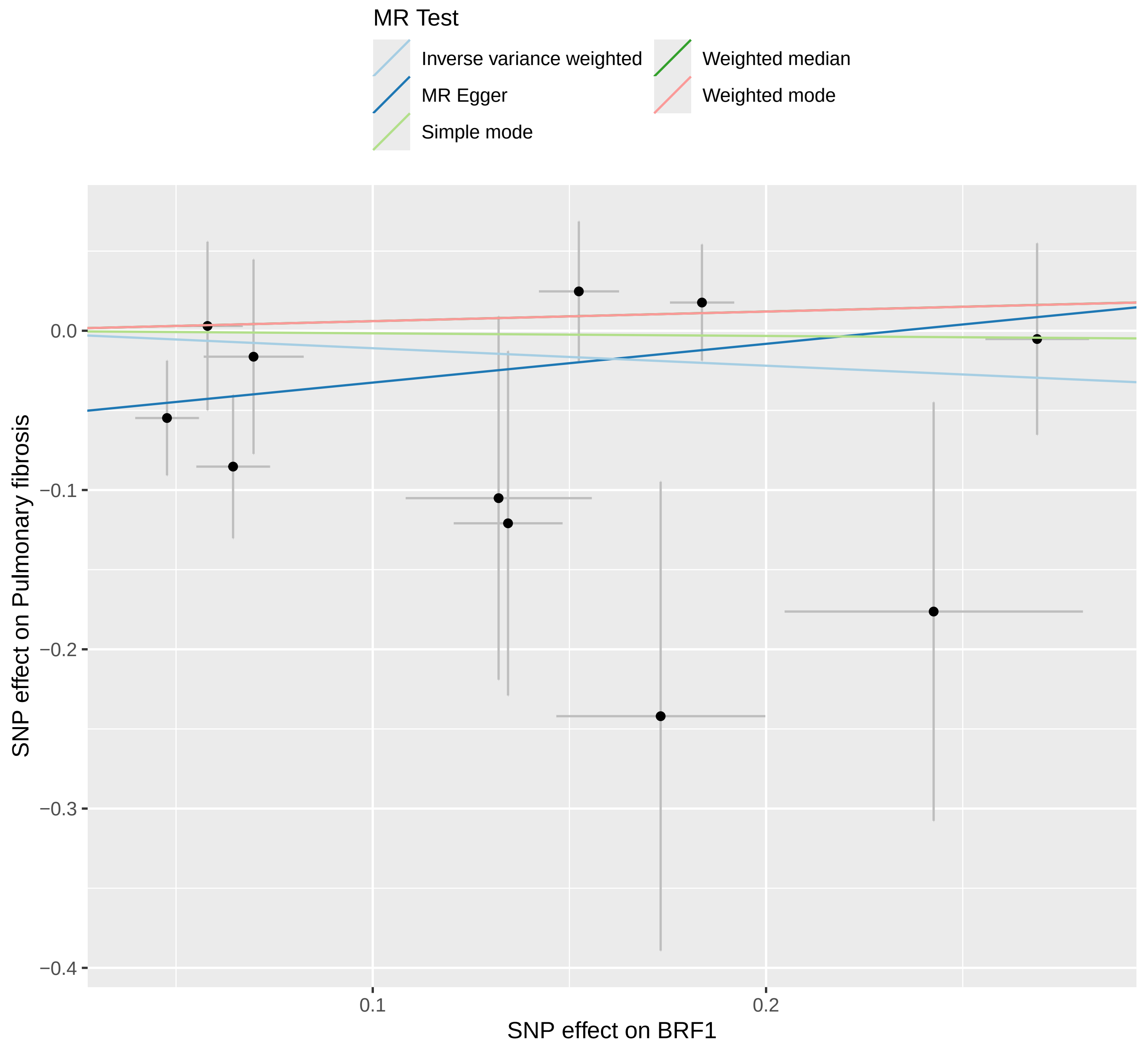

MR Test
Inverse variance weighted
MR Egger
Simple mode
Weighted median
Weighted mode
0.0
−0.1
−0.2
−0.3
−0.4
0.1
0.2
SNP effect on Pulmonary fibrosis
SNP effect on BRF1

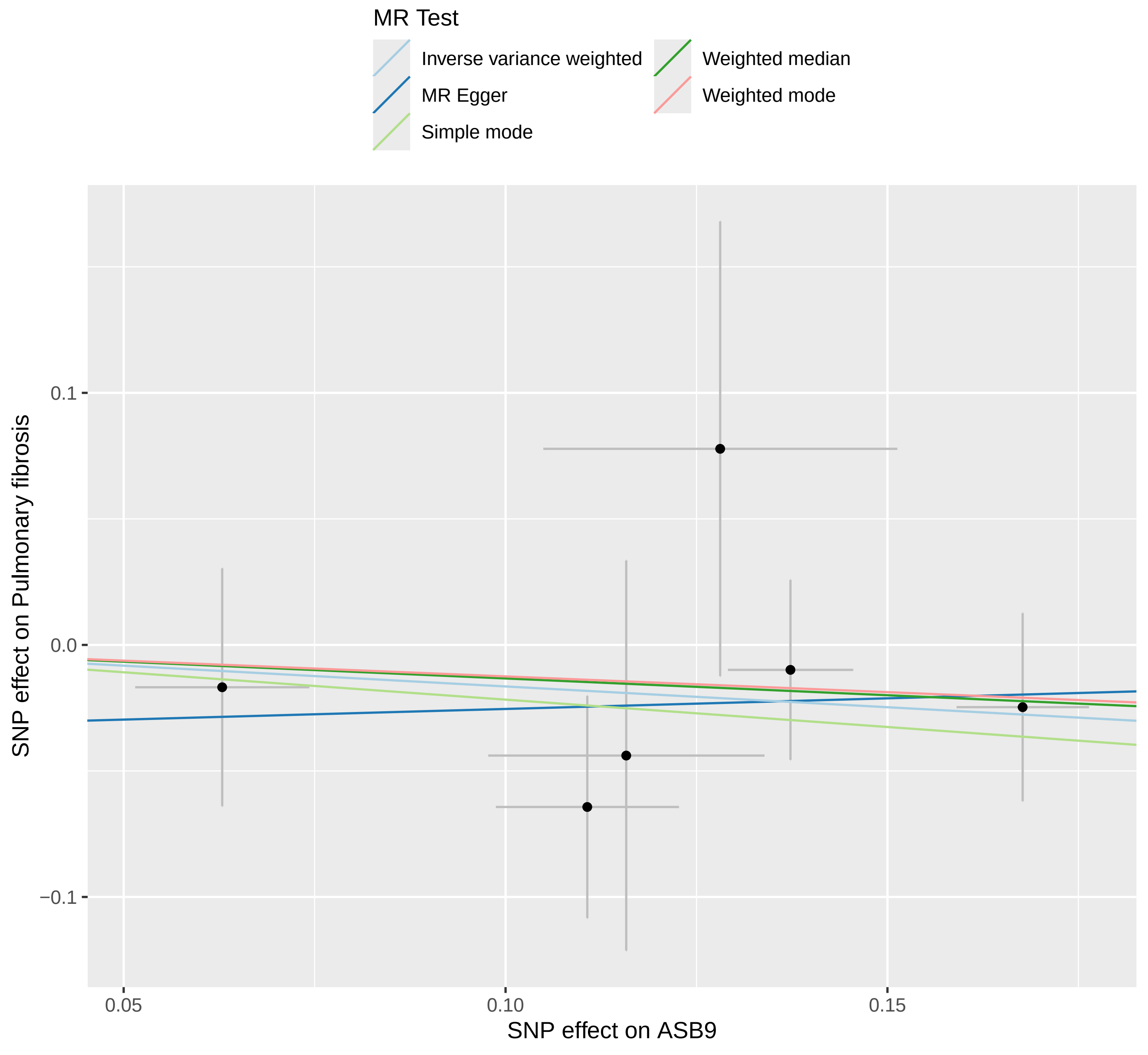
MR Test
Inverse variance weighted
MR Egger
Simple mode
Weighted median
Weighted mode
SNP effect on Pulmonary fibrosis
0.1
0.0
−0.1
0.05
0.10
0.15
SNP effect on ASB9

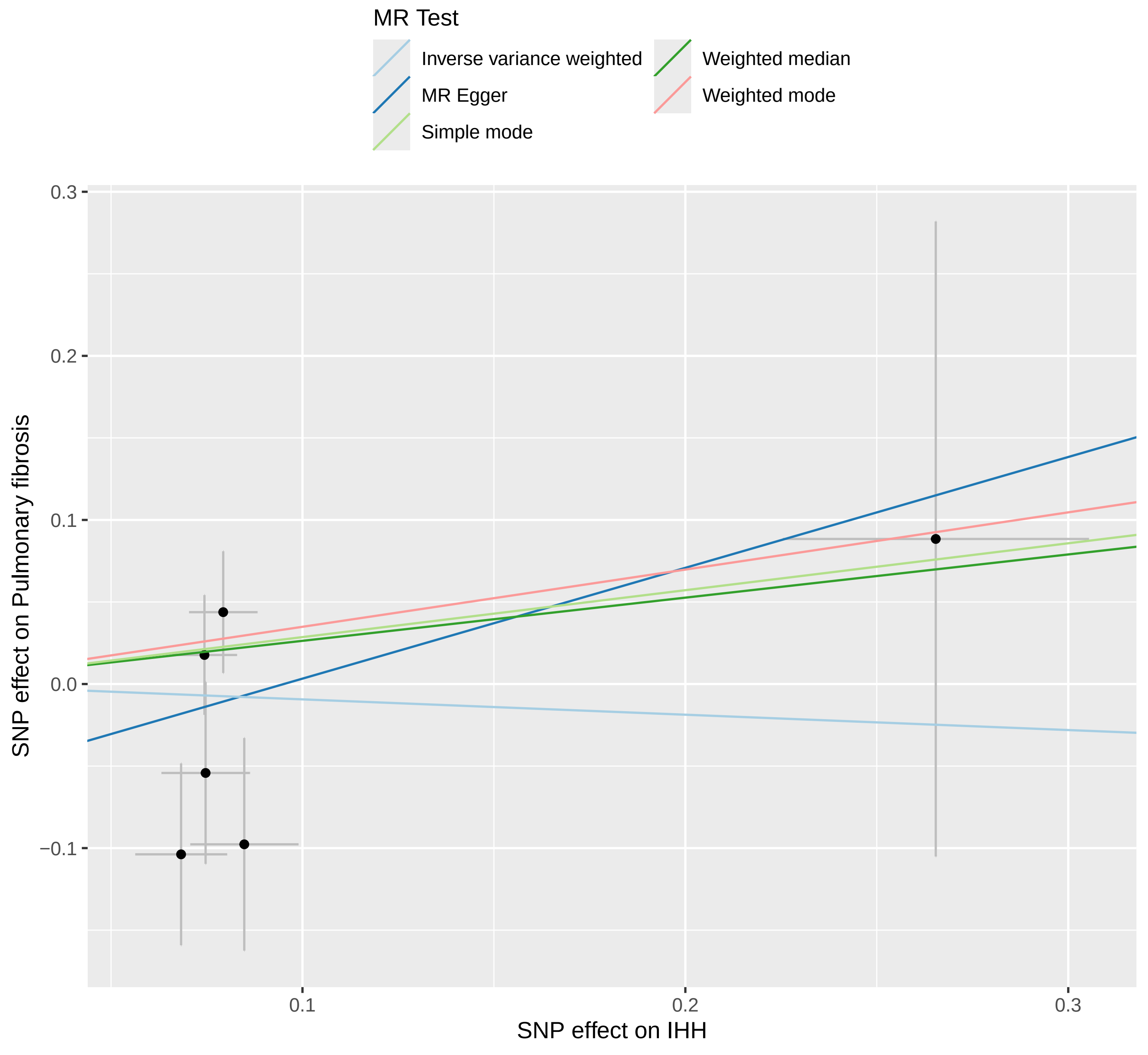

MR Test
Inverse variance weighted
MR Egger
Simple mode
Weighted median
Weighted mode
0.3
0.2
0.1
0.0
−0.1
SNP effect on Pulmonary fibrosis
0.1
0.2
0.3
SNP effect on IHH

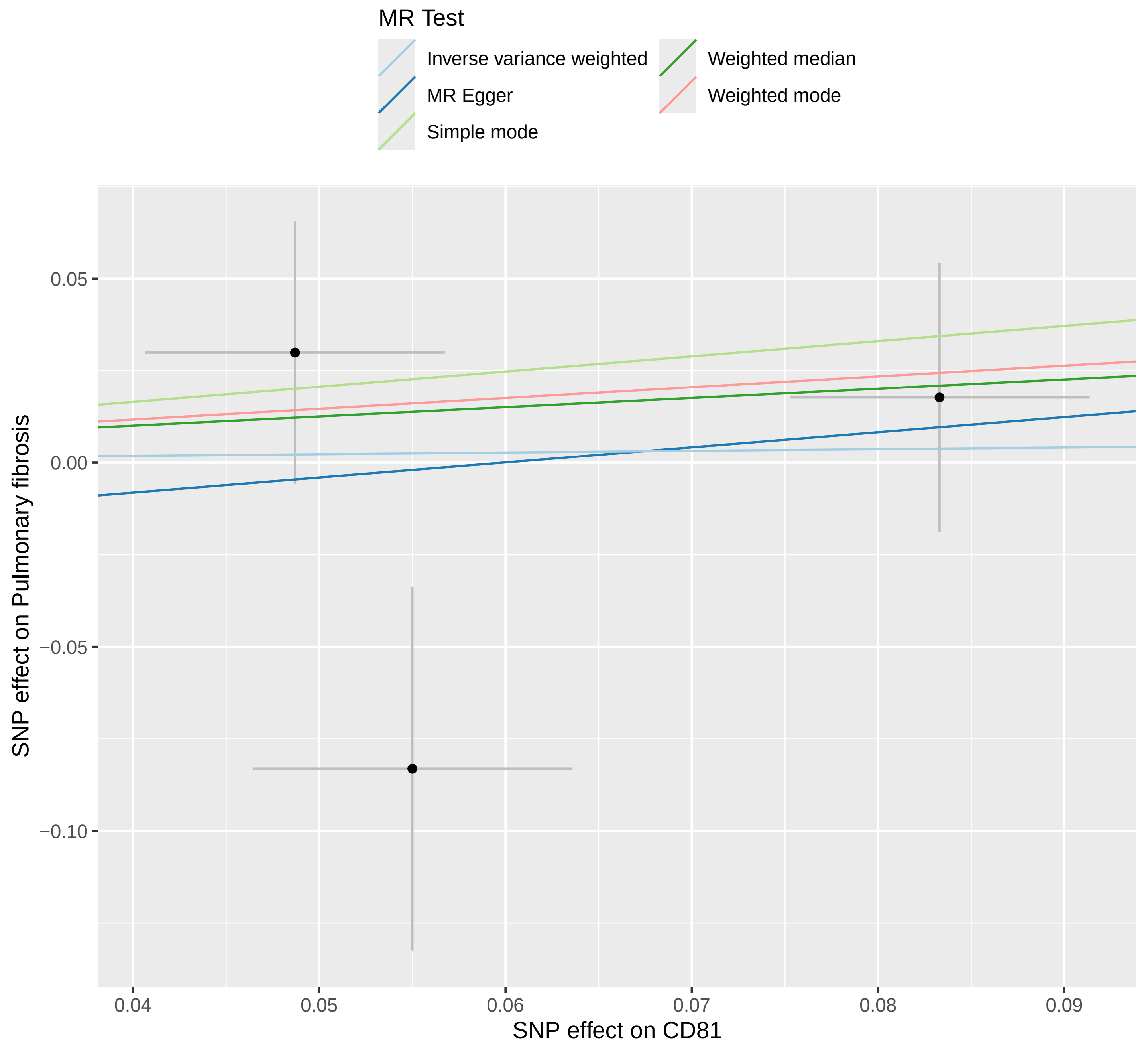
MR Test
Inverse variance weighted
MR Egger
Simple mode
Weighted median
Weighted mode
SNP effect on Pulmonary fibrosis
0.05
0.00
−0.05
−0.10
0.04
0.05
0.06
0.07
0.08
0.09
SNP effect on CD81

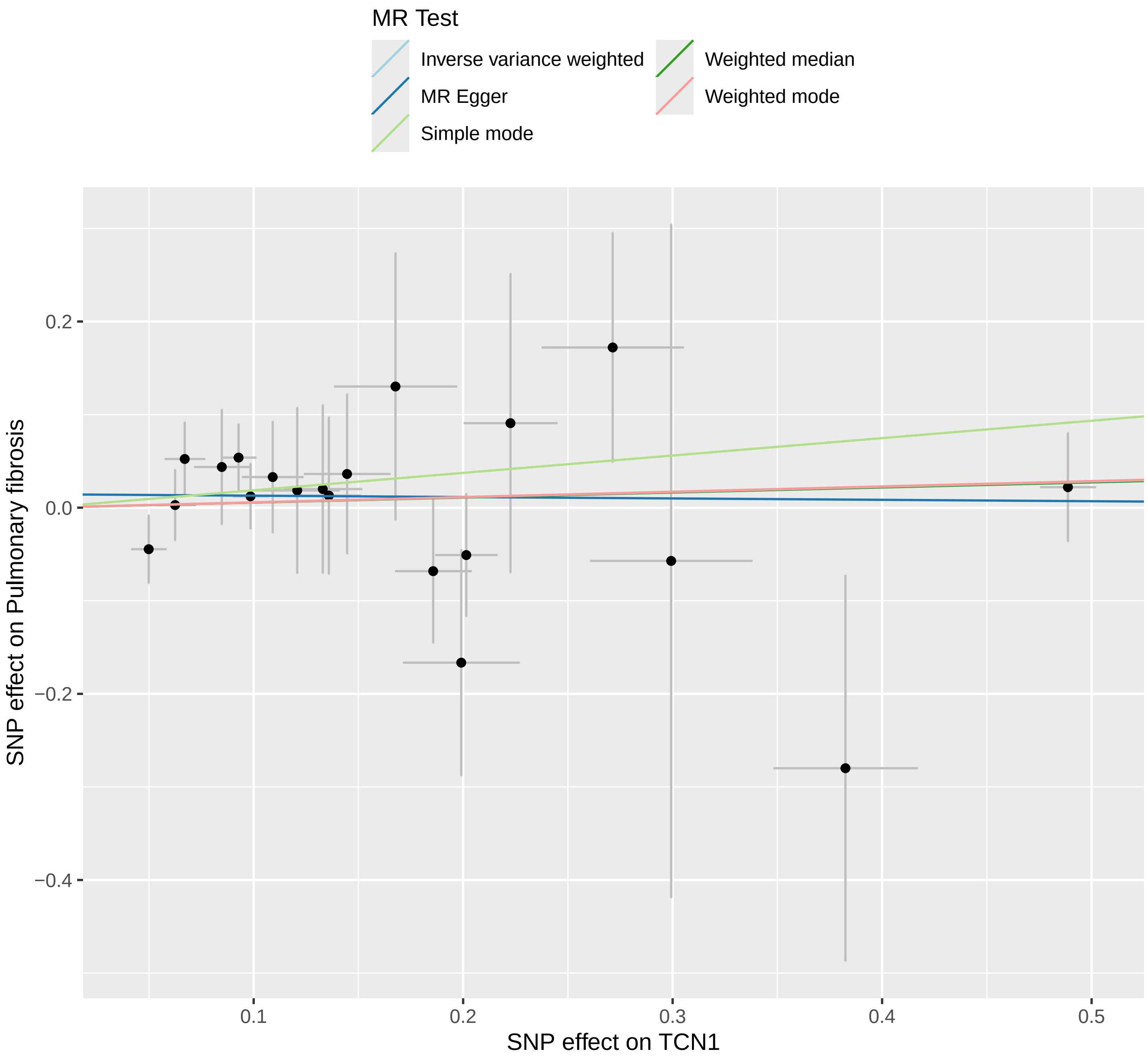

MR Test
Inverse variance weighted
MR Egger
Simple mode
Weighted median
Weighted mode
0.2
0.0
−0.2
−0.4
SNP effect on Pulmonary fibrosis
0.1
0.2
0.3
0.4
0.5
SNP effect on TCN1

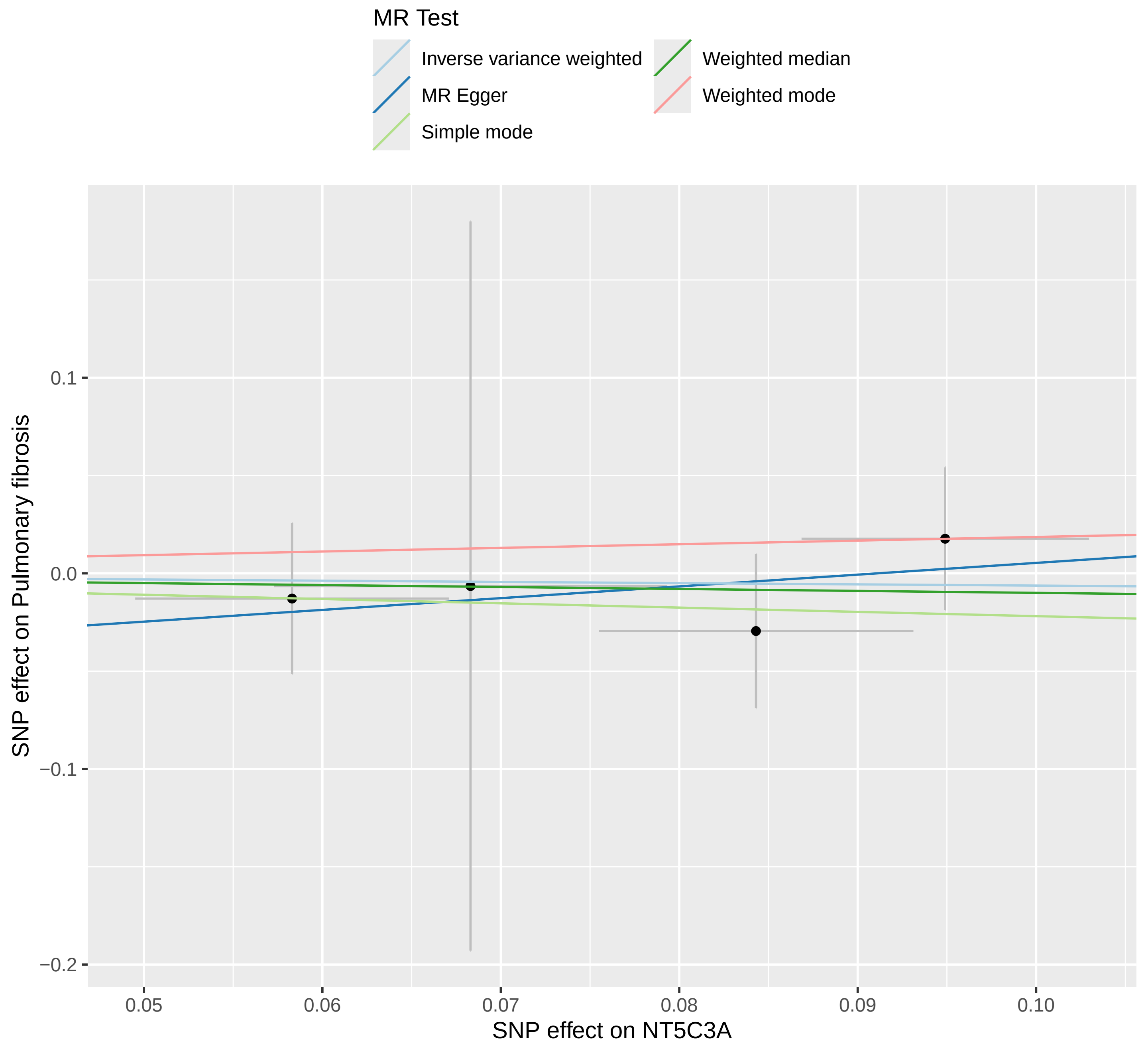

MR Test
Inverse variance weighted
MR Egger
Simple mode
Weighted median
Weighted mode
SNP effect on Pulmonary fibrosis
0.1
0.0
−0.1
−0.2
0.05
0.06
0.07
0.08
0.09
0.10
SNP effect on NT5C3A

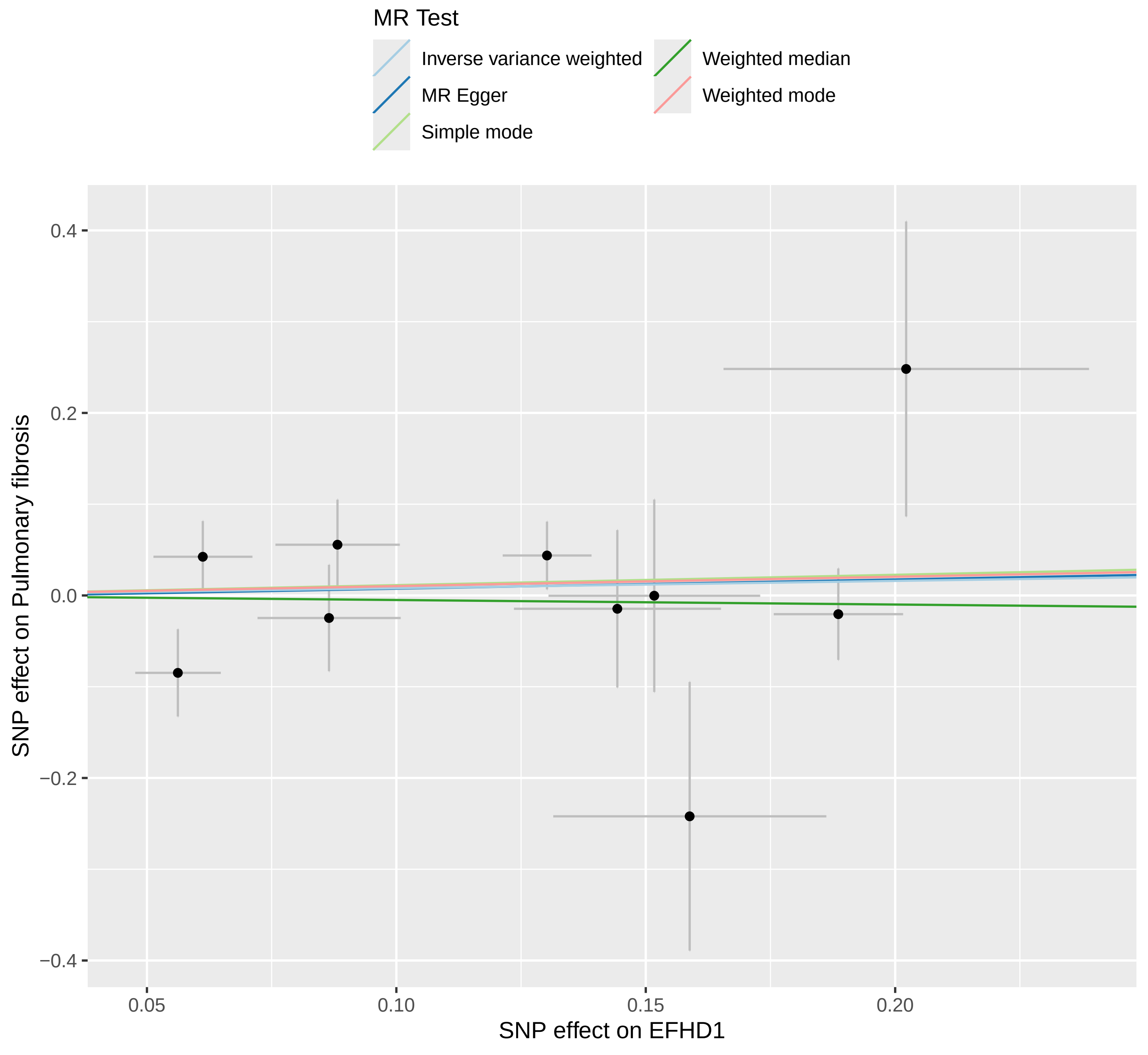
MR Test
Inverse variance weighted
MR Egger
Simple mode
Weighted median
Weighted mode
0.4
0.2
0.0
−0.2
−0.4
SNP effect on Pulmonary fibrosis
0.05
0.10
0.15
0.20
SNP effect on EFHD1

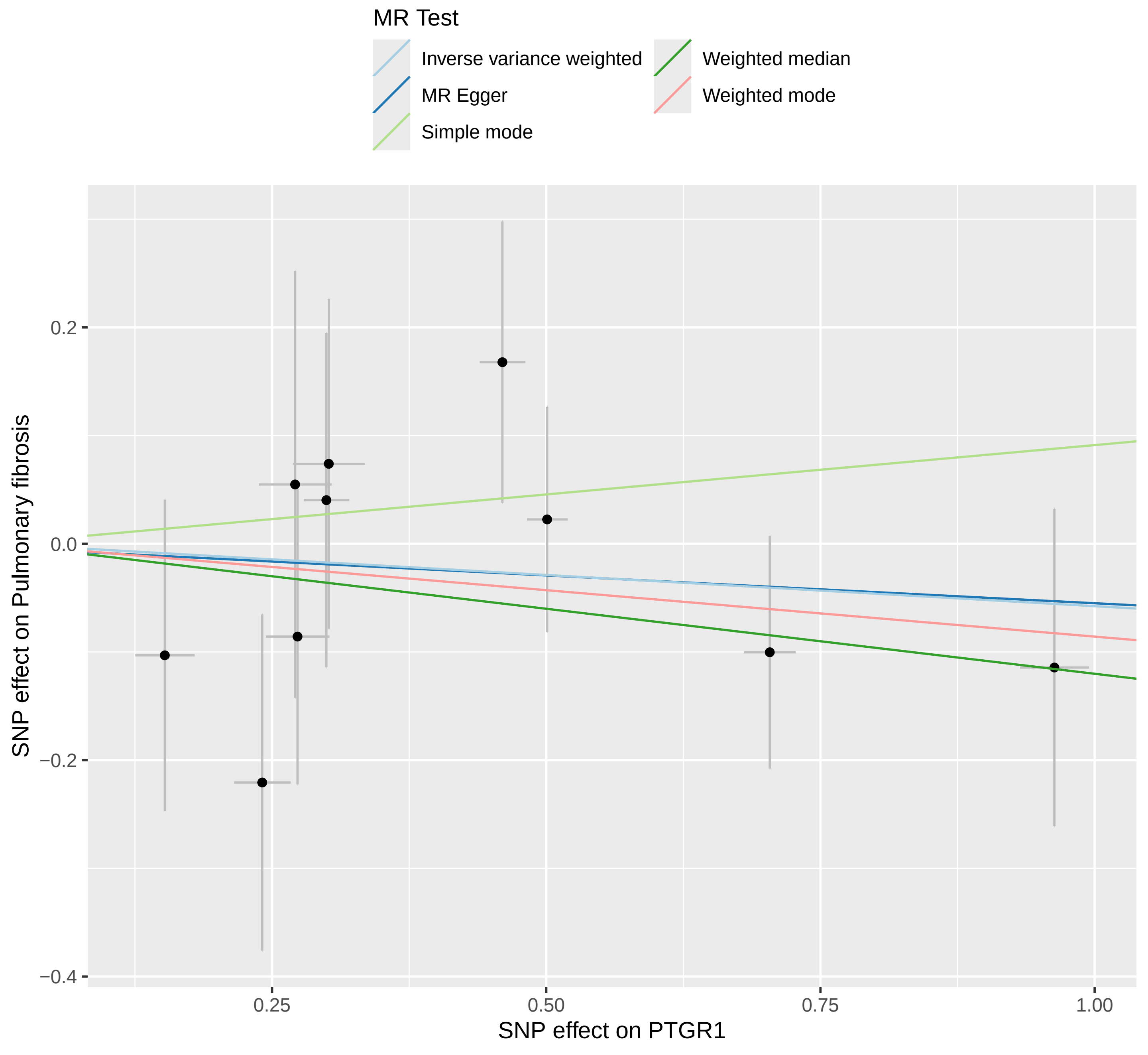
MR Test
Inverse variance weighted
MR Egger
Simple mode
Weighted median
Weighted mode
0.2
0.0
−0.2
−0.4
0.25
0.50
0.75
1.00
SNP effect on Pulmonary fibrosis
SNP effect on PTGR1

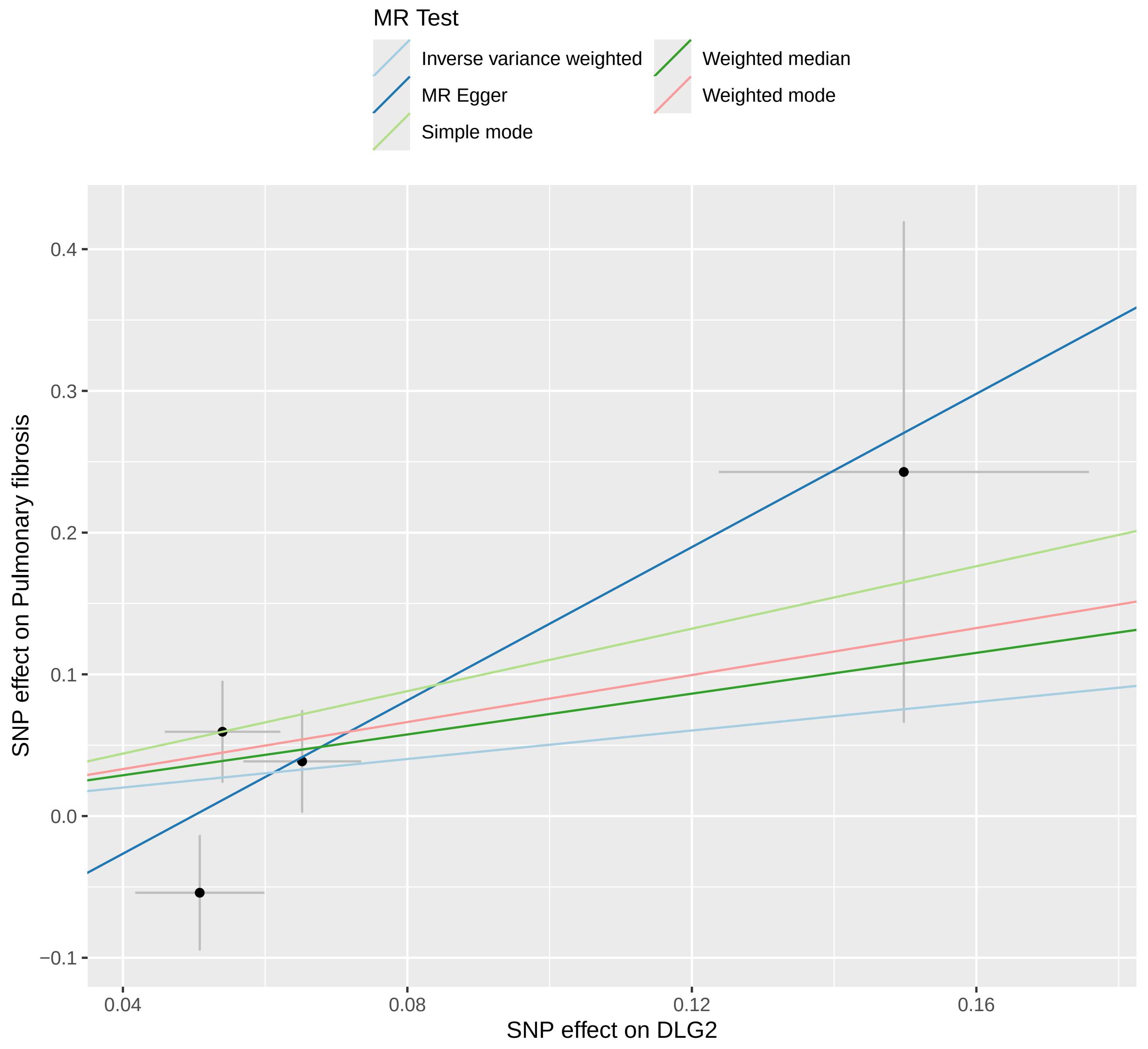

MR Test
Inverse variance weighted
MR Egger
Simple mode
Weighted median
Weighted mode
SNP effect on Pulmonary fibrosis
0.4
0.3
0.2
0.1
0.0
−0.1
0.04
0.08
0.12
0.16
SNP effect on DLG2

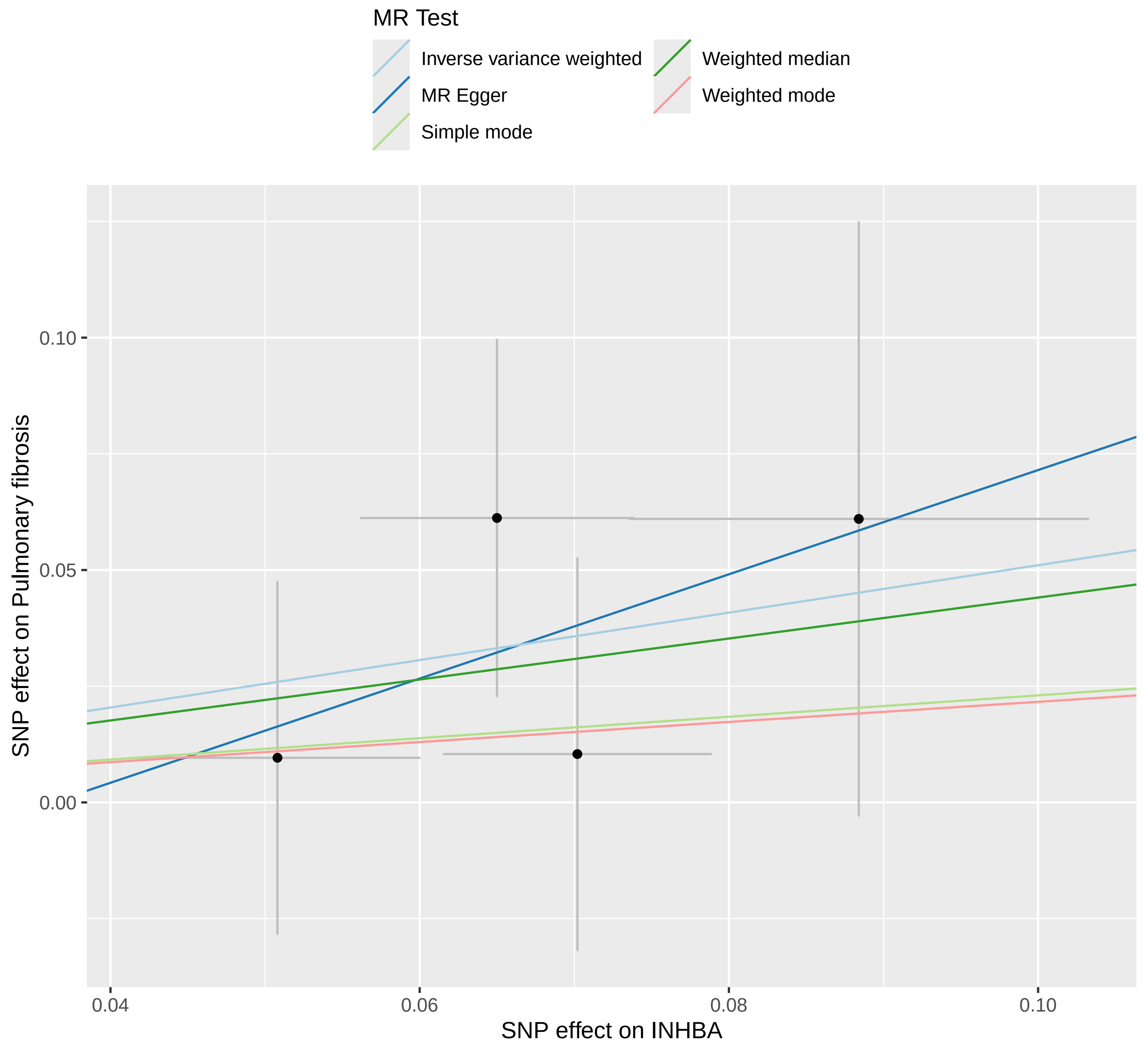
MR Test
Inverse variance weighted
MR Egger
Simple mode
Weighted median
Weighted mode
0.10
0.05
0.00
SNP effect on Pulmonary fibrosis
0.04
0.06
0.08
0.10
SNP effect on INHBA

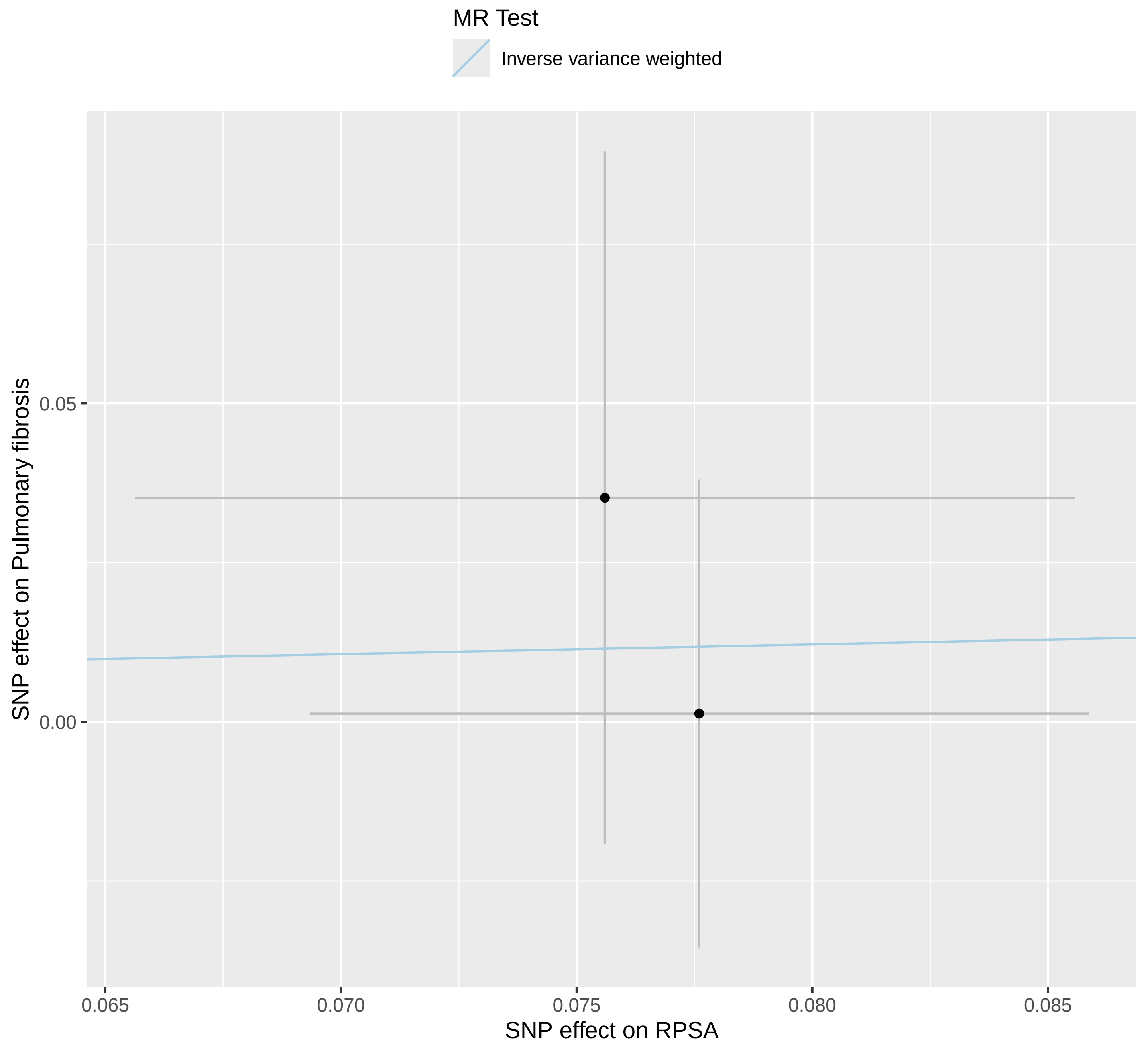

MR Test
Inverse variance weighted
0.05
0.00
0.065
0.070
0.075
0.080
0.085
SNP effect on RPSA
SNP effect on Pulmonary fibrosis

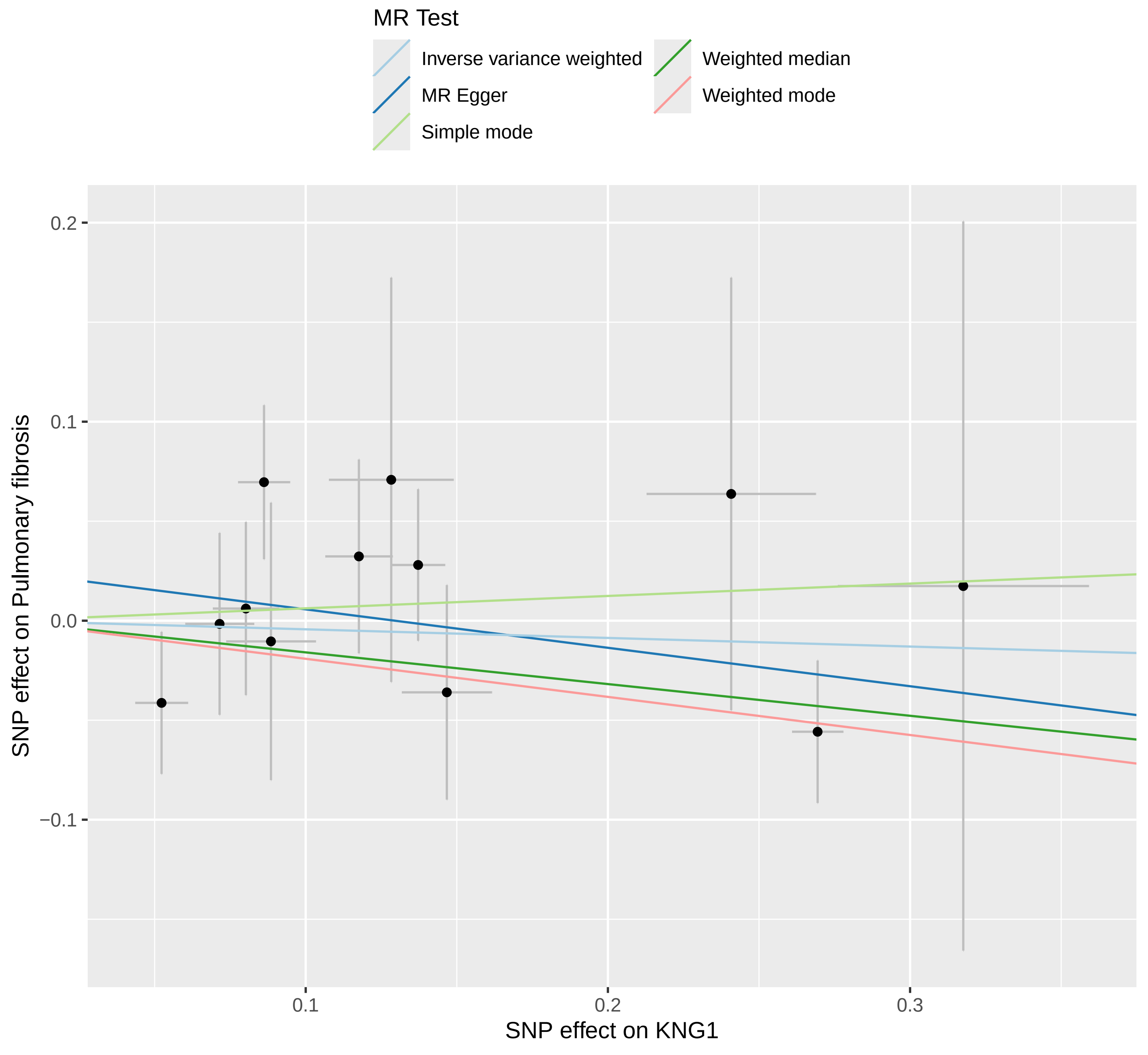

MR Test
Inverse variance weighted
MR Egger
Simple mode
Weighted median
Weighted mode
SNP effect on Pulmonary fibrosis
0.2
0.1
0.0
−0.1
0.1
0.2
0.3
SNP effect on KNG1

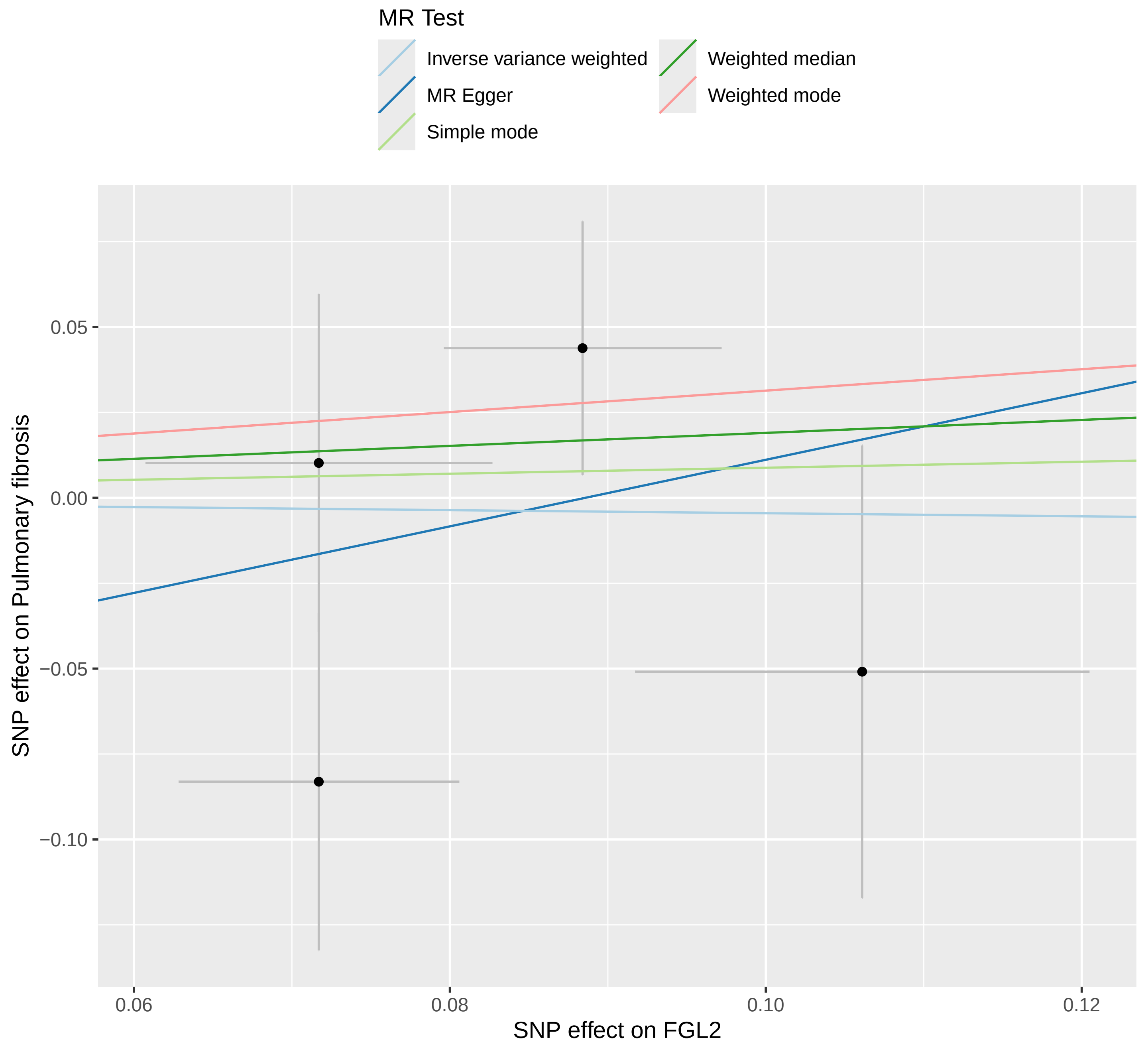
MR Test
Inverse variance weighted
MR Egger
Simple mode
Weighted median
Weighted mode
SNP effect on Pulmonary fibrosis
0.05
0.00
−0.05
−0.10
0.06
0.08
0.10
0.12
SNP effect on FGL2

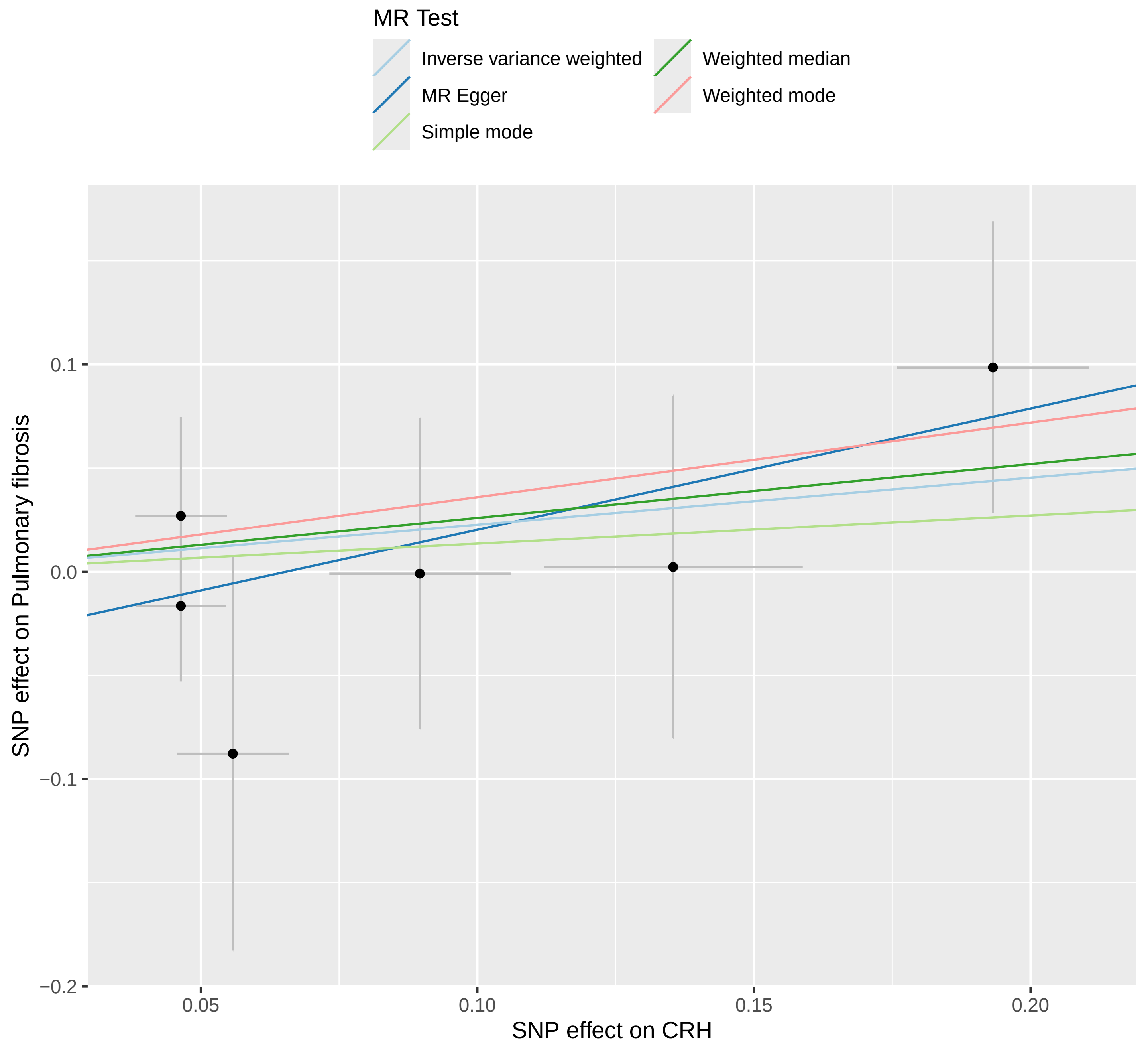
MR Test
Inverse variance weighted
MR Egger
Simple mode
Weighted median
Weighted mode
0.1
0.0
−0.1
−0.2
0.05
0.10
0.15
0.20
SNP effect on CRH
SNP effect on Pulmonary fibrosis

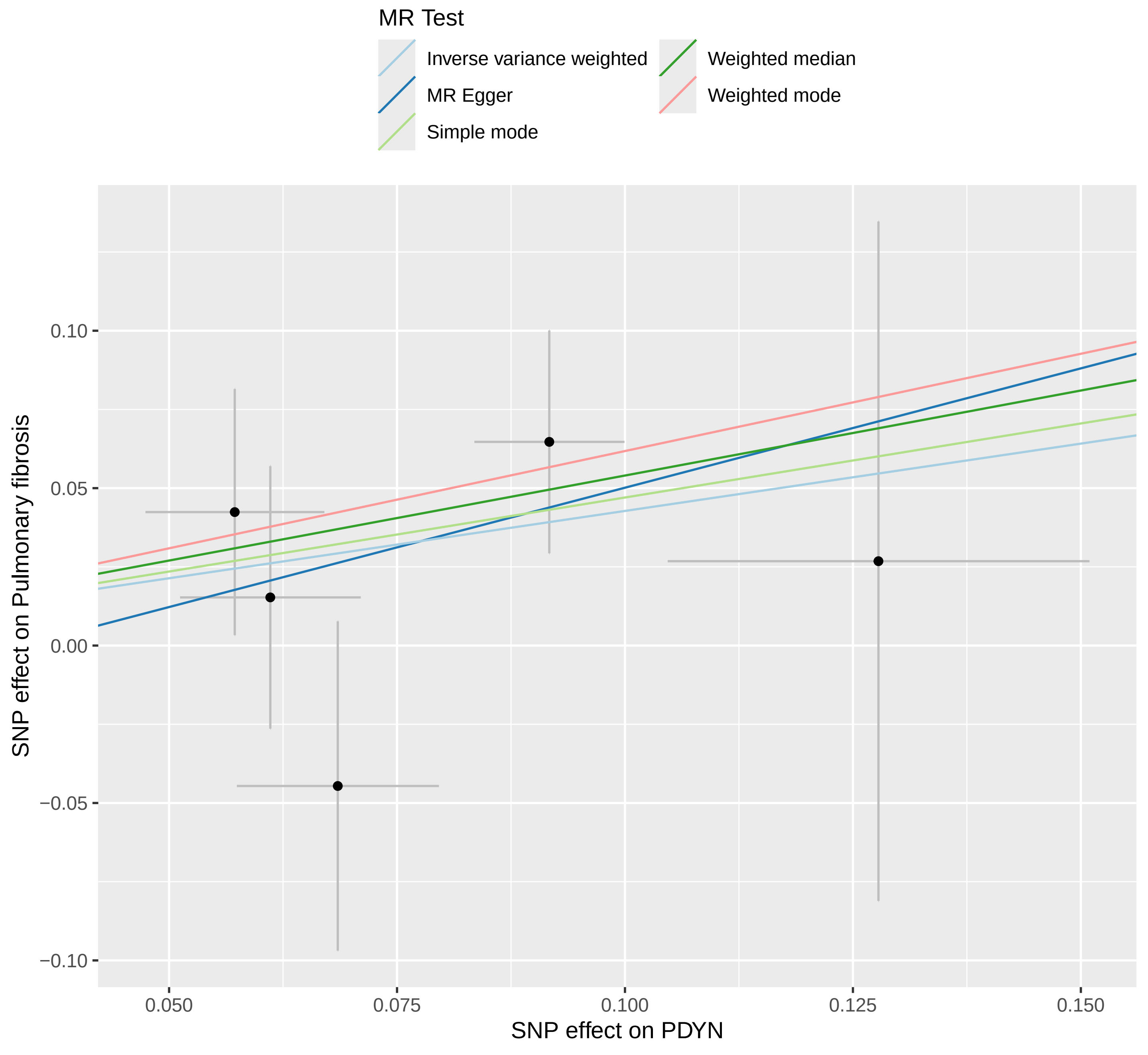
MR Test
Inverse variance weighted
MR Egger
Simple mode
Weighted median
Weighted mode
0.10
0.05
0.00
−0.05
−0.10
SNP effect on Pulmonary fibrosis
0.050
0.075
0.100
0.125
0.150
SNP effect on PDYN

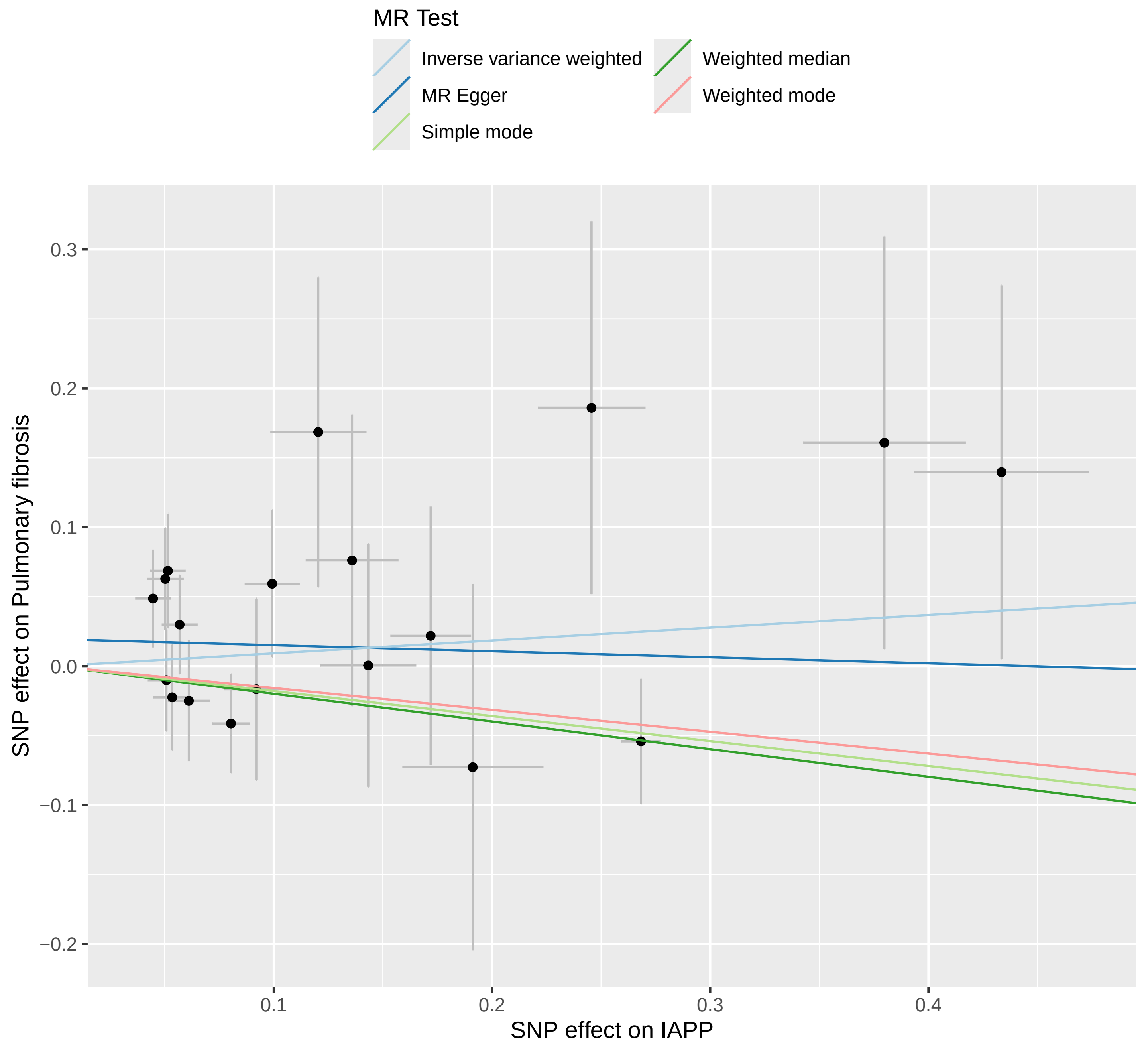

MR Test
Inverse variance weighted
MR Egger
Simple mode
Weighted median
Weighted mode
SNP effect on Pulmonary fibrosis
0.3
0.2
0.1
0.0
−0.1
−0.2
0.1
0.2
0.3
0.4
SNP effect on IAPP

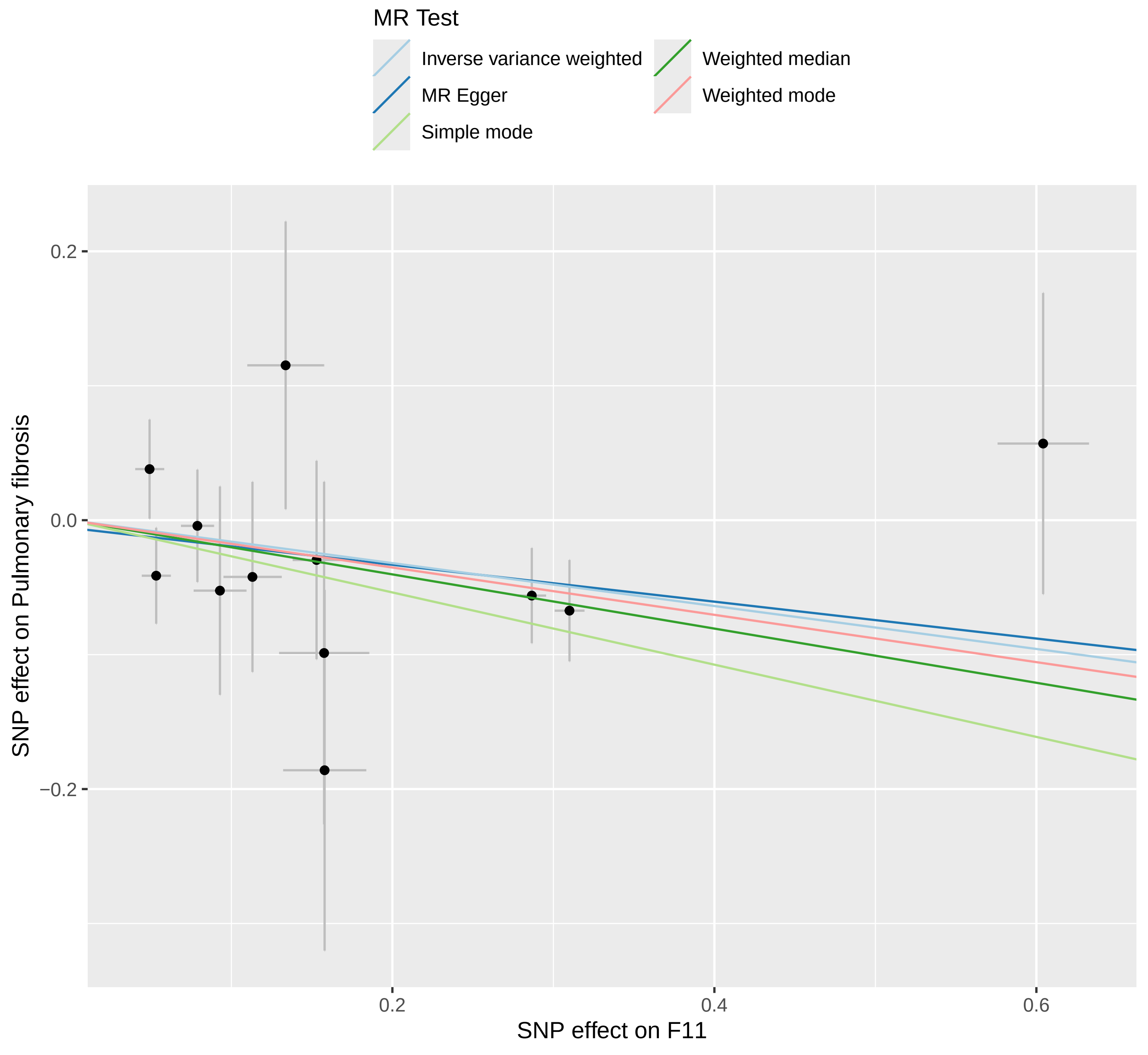

MR Test
Inverse variance weighted
MR Egger
Simple mode
Weighted median
Weighted mode
0.2
0.0
−0.2
SNP effect on Pulmonary fibrosis
0.2
0.4
0.6
SNP effect on F11

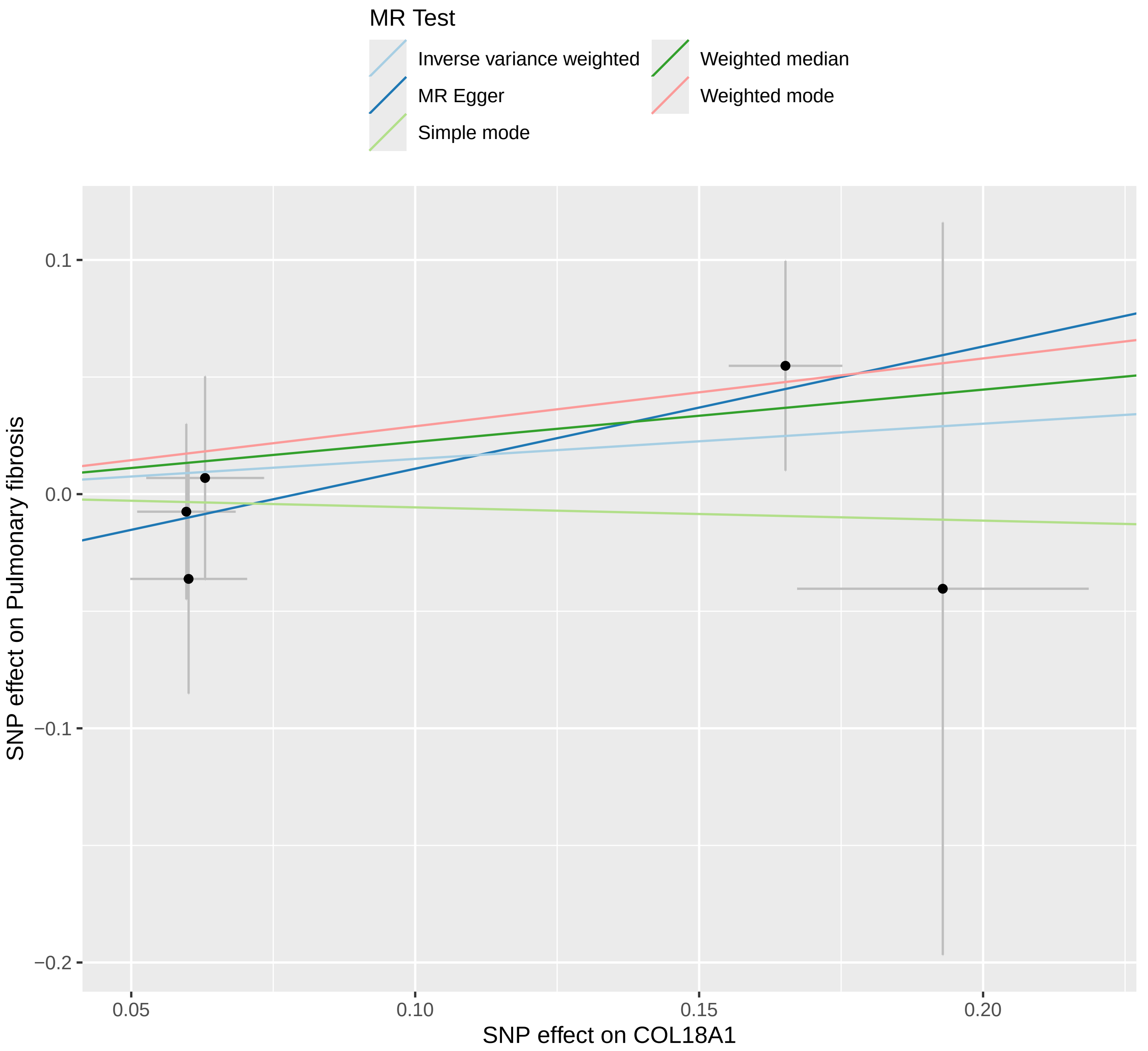

MR Test
Inverse variance weighted
MR Egger
Simple mode
Weighted median
Weighted mode
0.1
0.0
−0.1
−0.2
SNP effect on Pulmonary fibrosis
0.05
0.10
0.15
0.20
SNP effect on COL18A1

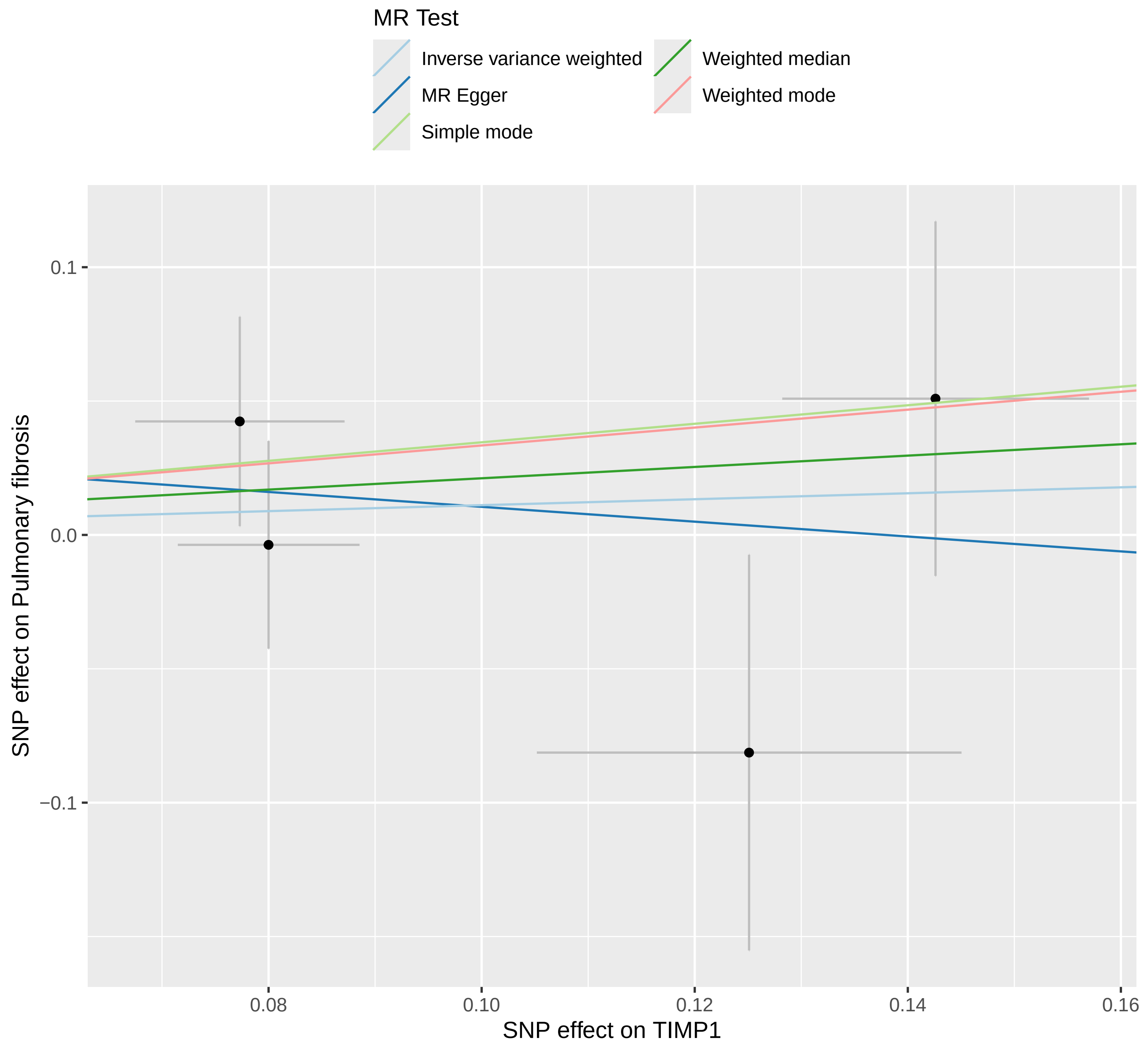

MR Test
Inverse variance weighted
MR Egger
Simple mode
Weighted median
Weighted mode
0.1
0.0
−0.1
0.08
0.10
0.12
0.14
0.16
SNP effect on TIMP1
SNP effect on Pulmonary fibrosis

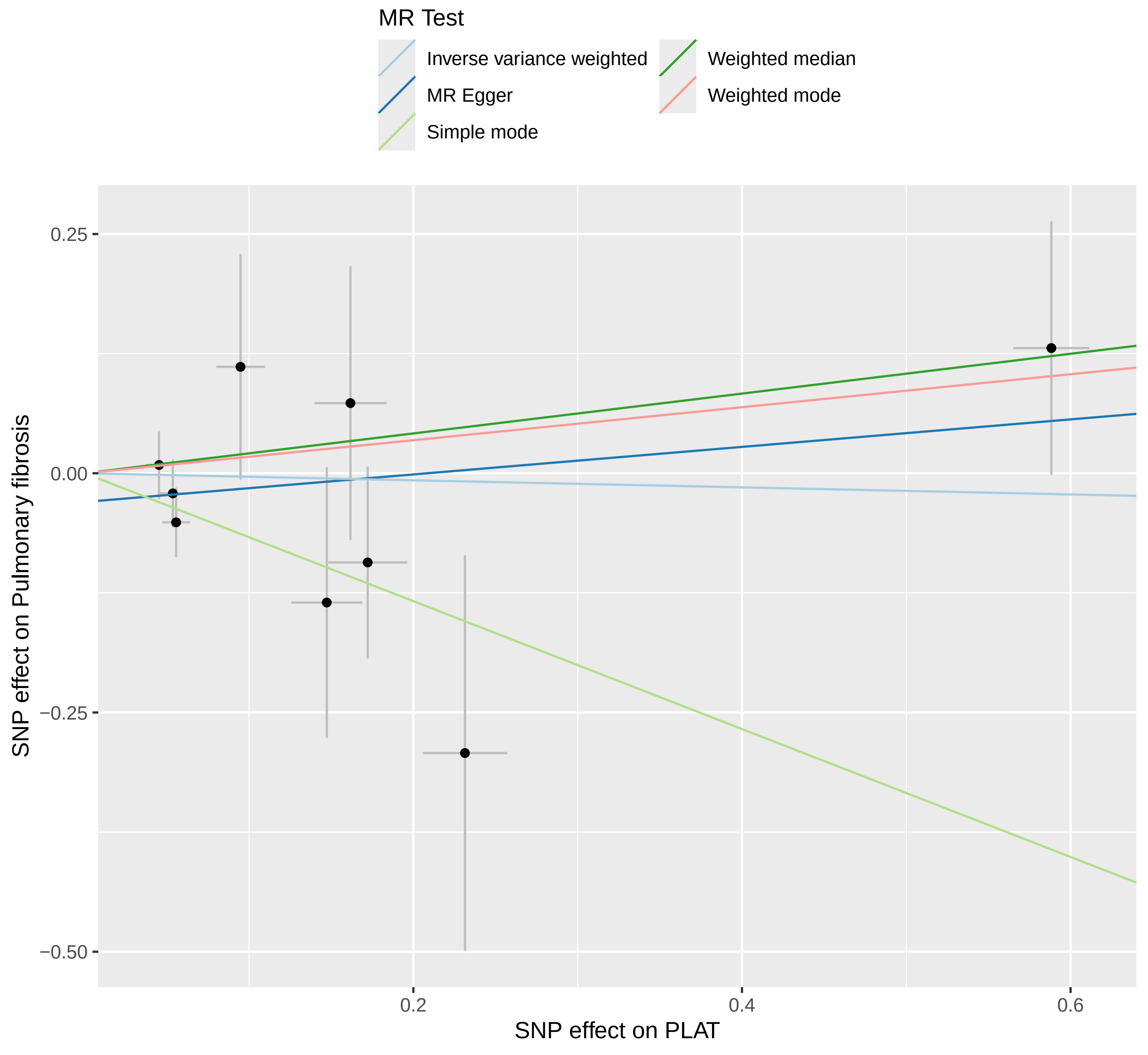
MR Test
Inverse variance weighted
MR Egger
Simple mode
Weighted median
Weighted mode
0.25
0.00
−0.25
−0.50
0.2
0.4
0.6
SNP effect on Pulmonary fibrosis
SNP effect on PLAT

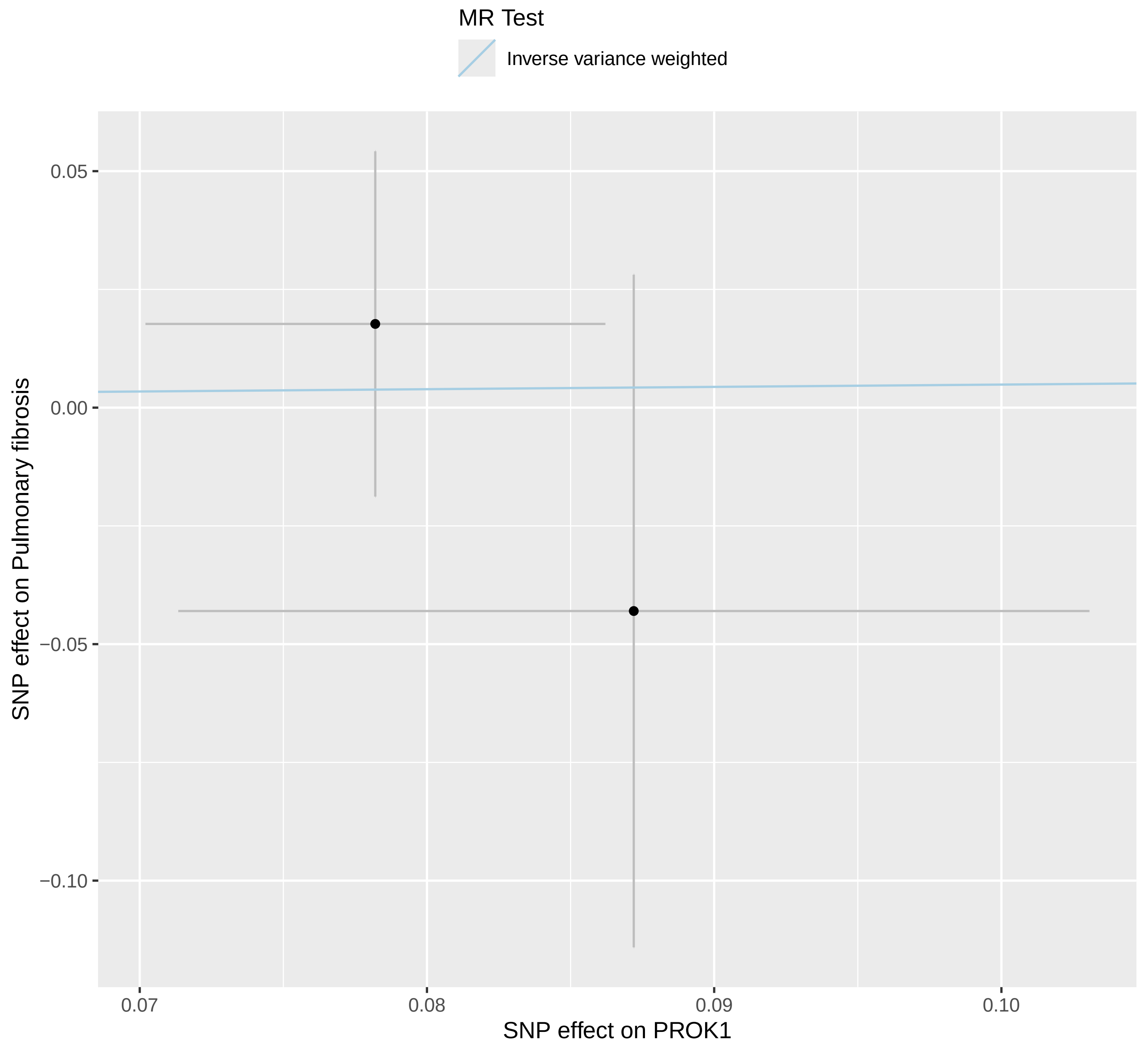
MR Test
Inverse variance weighted
0.05
0.00
−0.05
−0.10
0.07
0.08
0.09
0.10
SNP effect on Pulmonary fibrosis
SNP effect on PROK1

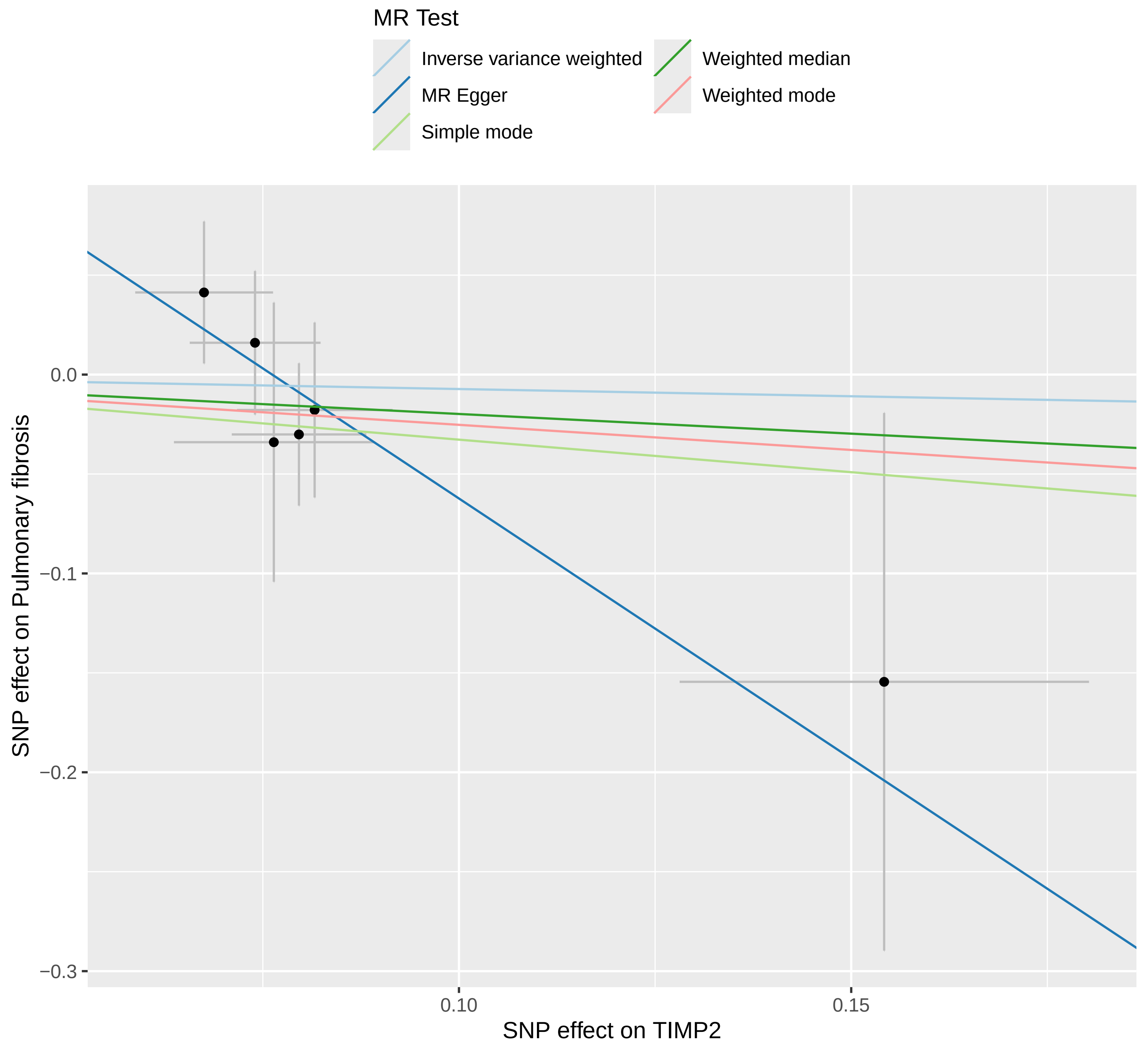

MR Test
Inverse variance weighted
MR Egger
Simple mode
Weighted median
Weighted mode
0.0
−0.1
−0.2
−0.3
0.10
0.15
SNP effect on TIMP2
SNP effect on Pulmonary fibrosis

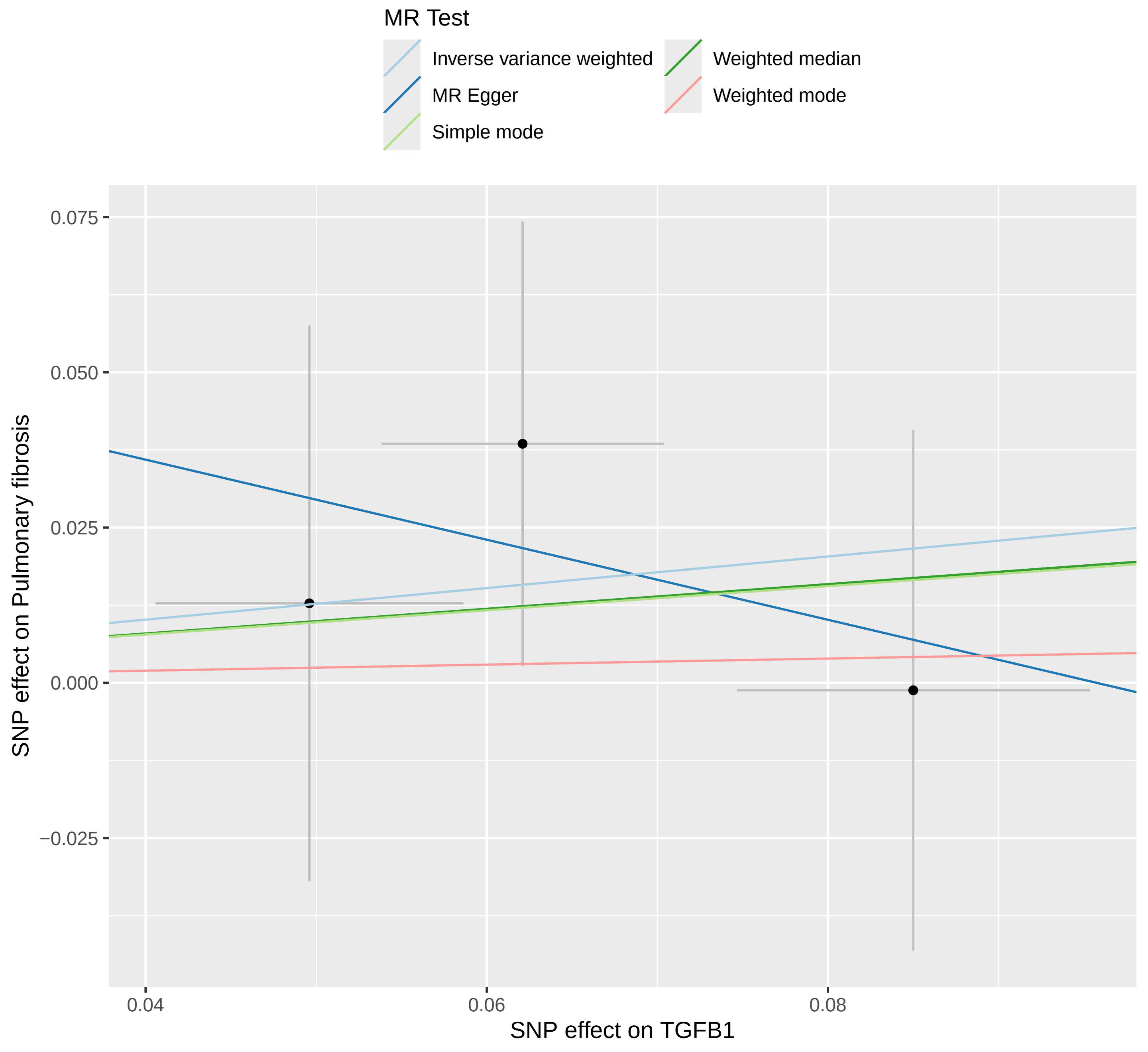

MR Test
Inverse variance weighted
MR Egger
Simple mode
Weighted median
Weighted mode
0.075
0.050
0.025
0.000
−0.025
SNP effect on Pulmonary fibrosis
0.04
0.06
0.08
SNP effect on TGFB1

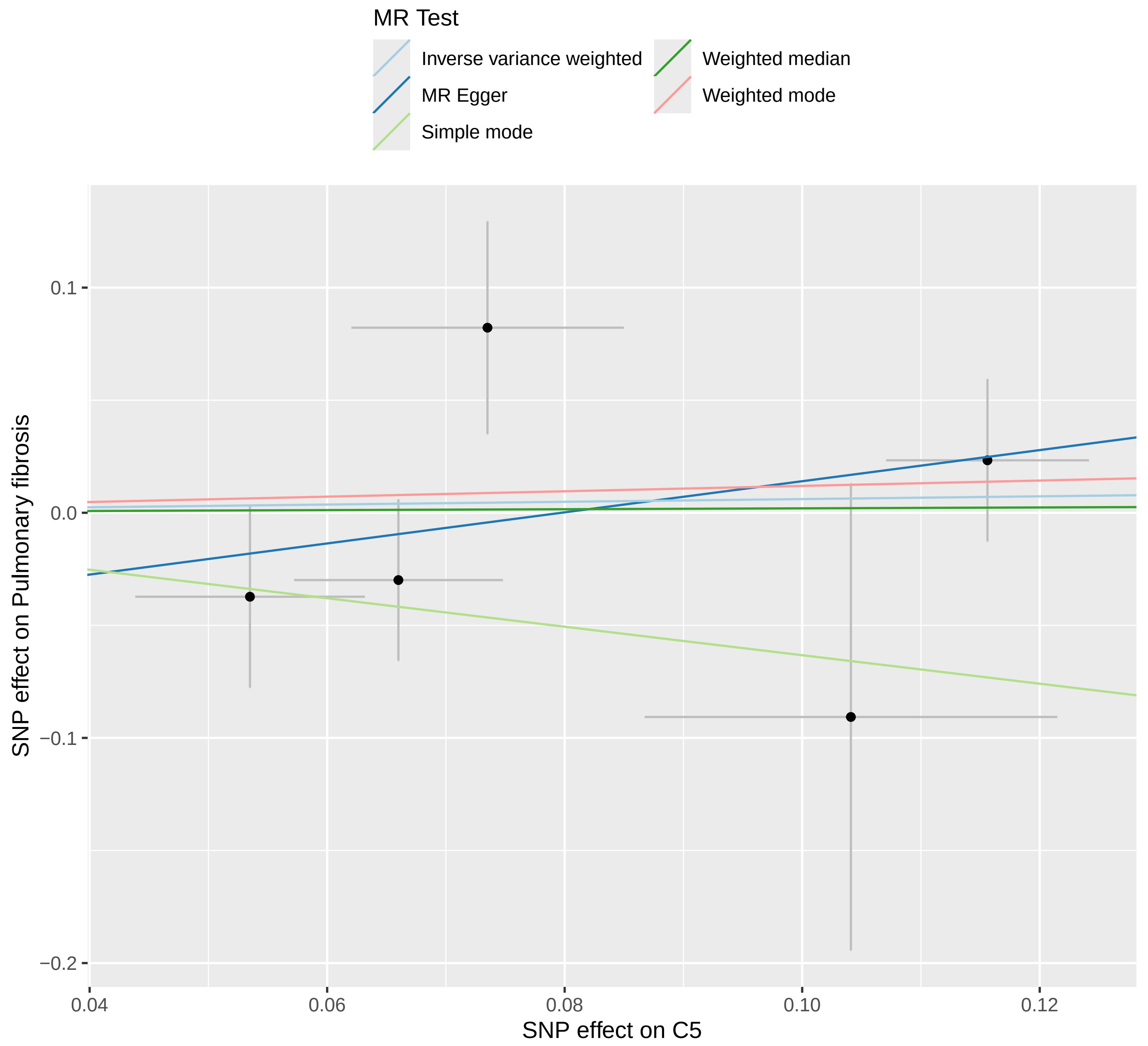

MR Test
Inverse variance weighted
MR Egger
Simple mode
Weighted median
Weighted mode
0.1
0.0
−0.1
−0.2
SNP effect on Pulmonary fibrosis
0.04
0.06
0.08
0.10
0.12
SNP effect on C5

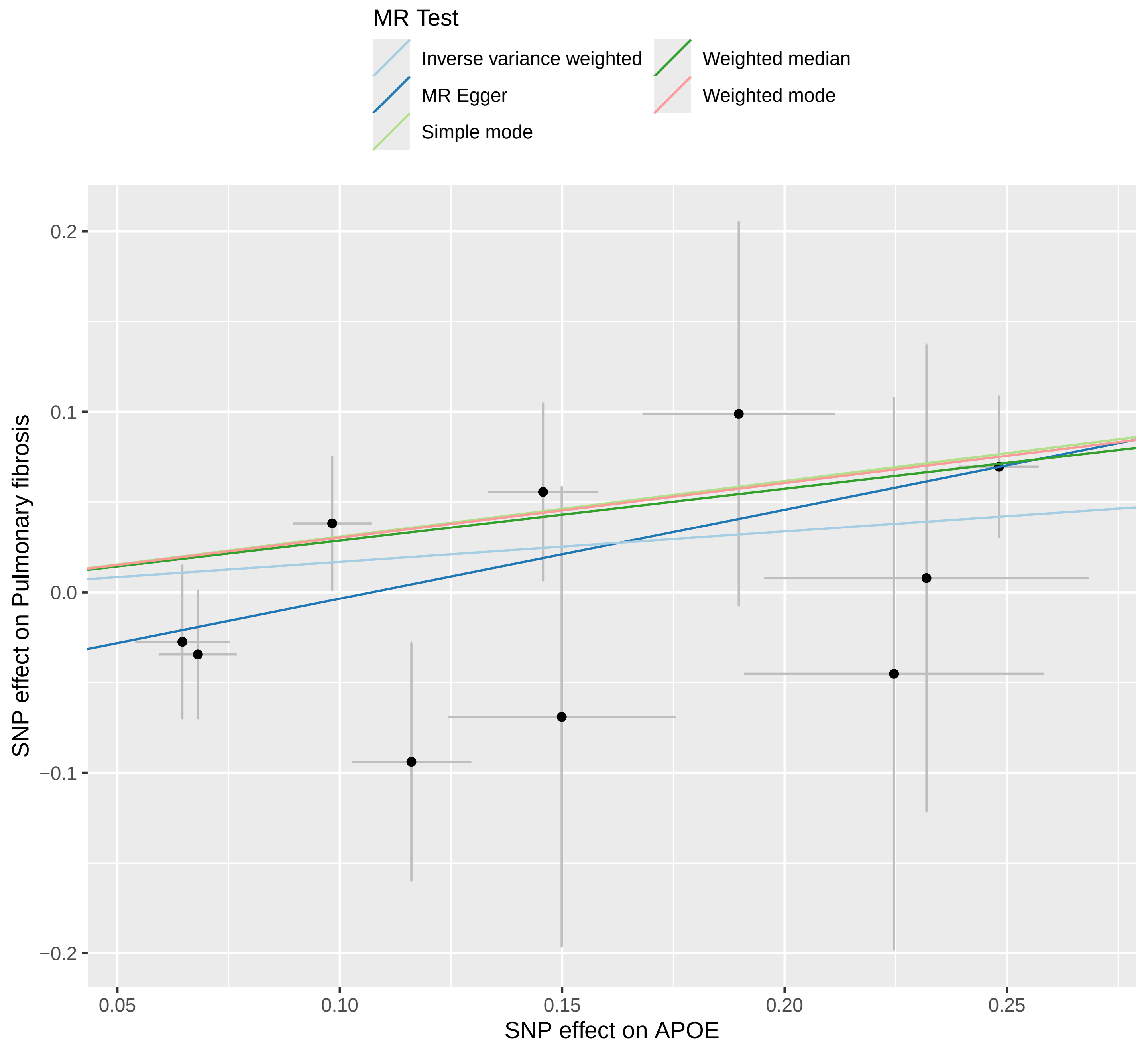

MR Test
Inverse variance weighted
MR Egger
Simple mode
Weighted median
Weighted mode
0.2
0.1
0.0
−0.1
−0.2
SNP effect on Pulmonary fibrosis
0.05
0.10
0.15
0.20
0.25
SNP effect on APOE

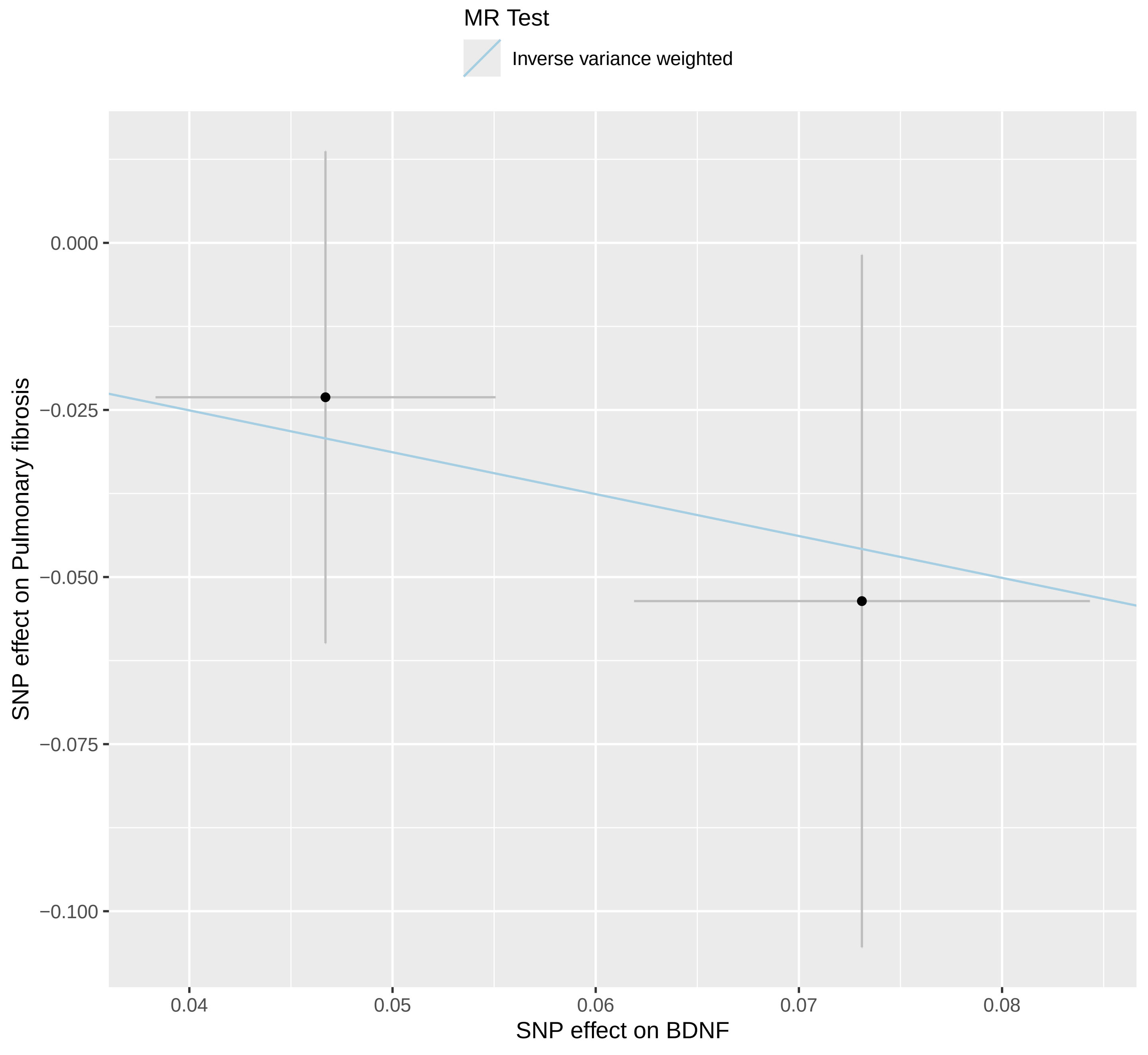

MR Test
Inverse variance weighted
0.000
−0.025
−0.050
−0.075
−0.100
SNP effect on Pulmonary fibrosis
0.04
0.05
0.06
0.07
0.08
SNP effect on BDNF

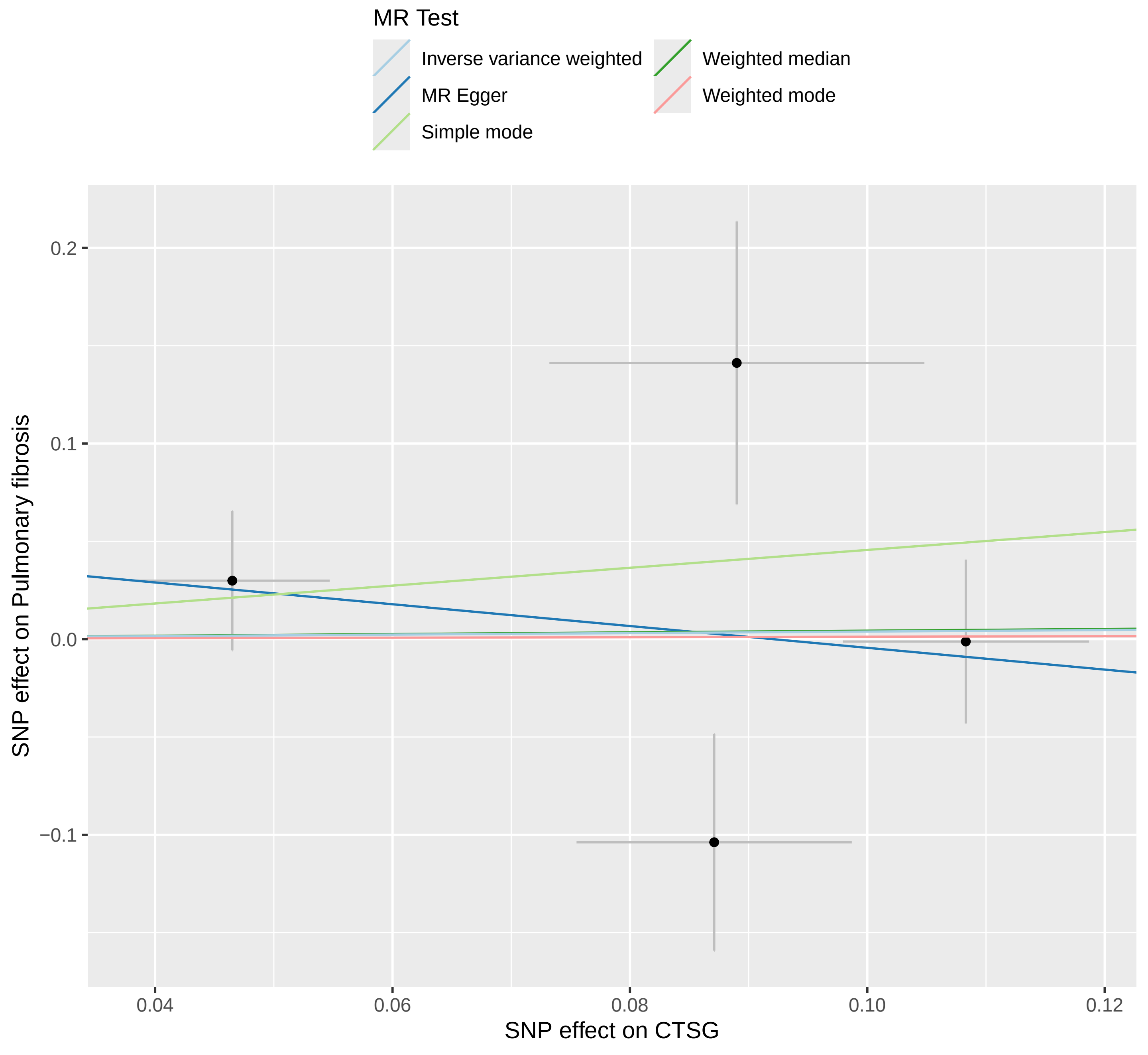
MR Test
Inverse variance weighted
MR Egger
Simple mode
Weighted median
Weighted mode
SNP effect on Pulmonary fibrosis
0.2
0.1
0.0
−0.1
0.04
0.06
0.08
0.10
0.12
SNP effect on CTSG

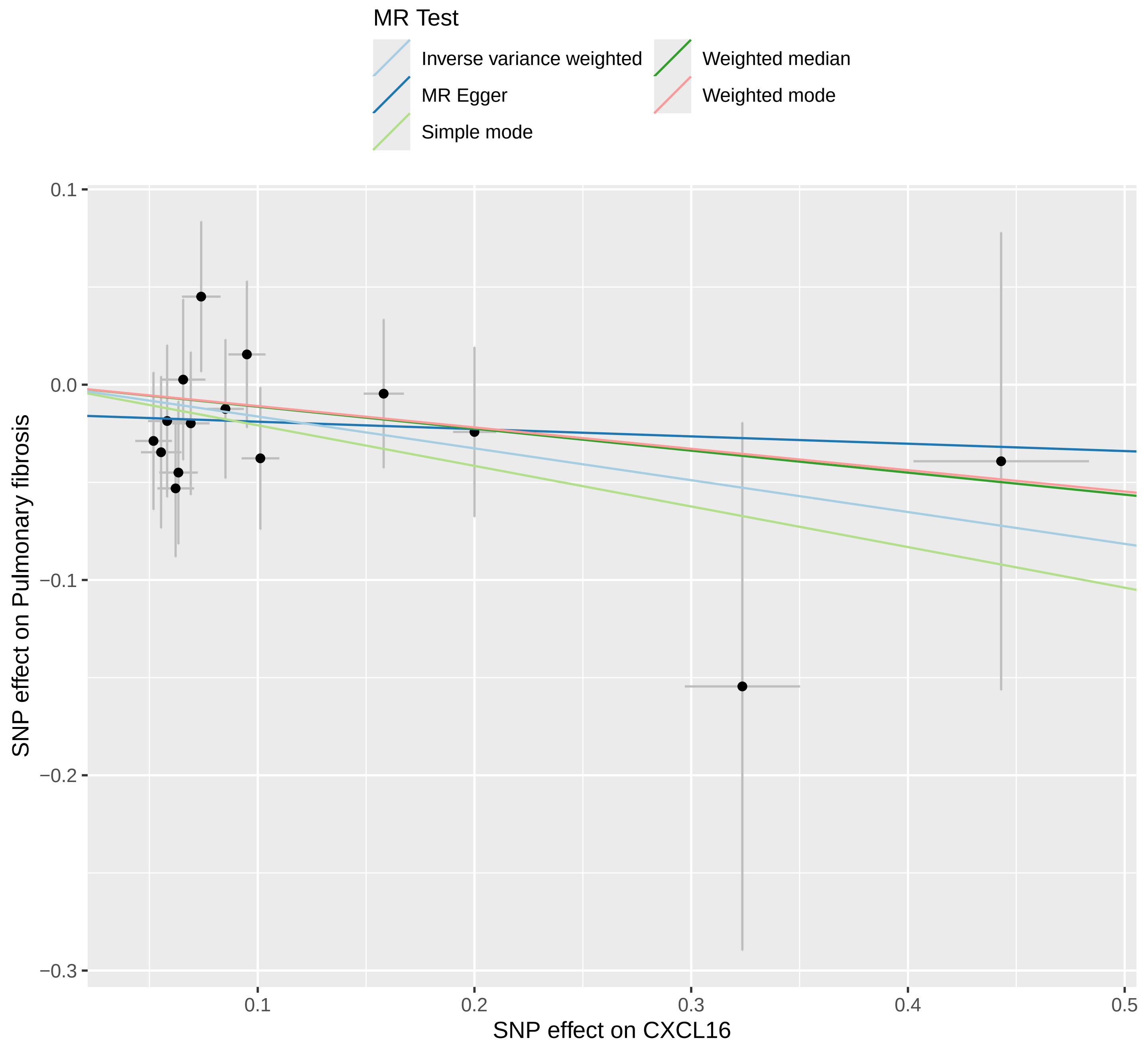

MR Test
Inverse variance weighted
MR Egger
Simple mode
Weighted median
Weighted mode
0.1
0.0
−0.1
−0.2
−0.3
0.1
0.2
0.3
0.4
0.5
SNP effect on CXCL16
SNP effect on Pulmonary fibrosis

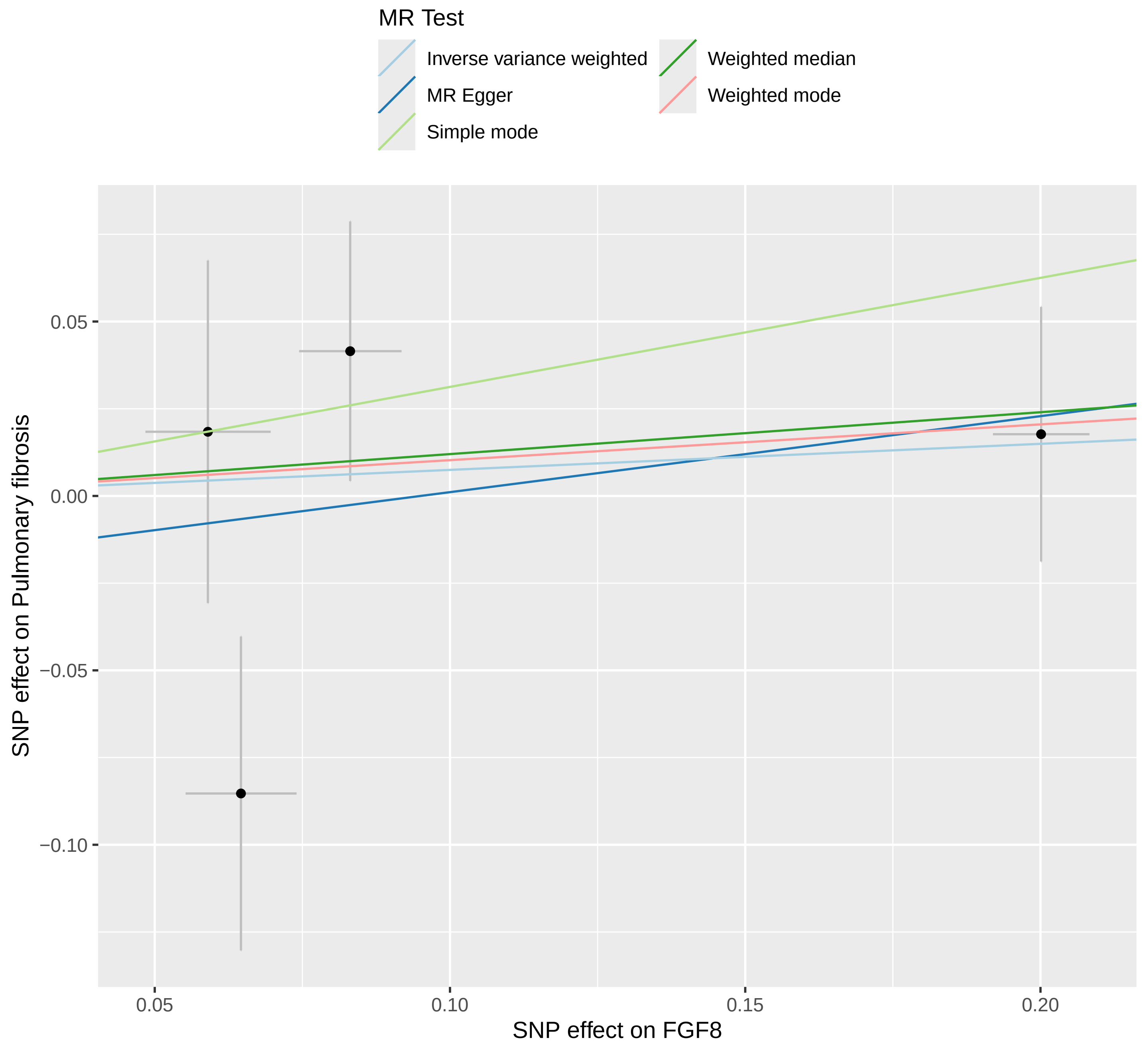

MR Test
Inverse variance weighted
MR Egger
Simple mode
Weighted median
Weighted mode
0.05
0.00
−0.05
−0.10
SNP effect on Pulmonary fibrosis
0.05
0.10
0.15
0.20
SNP effect on FGF8

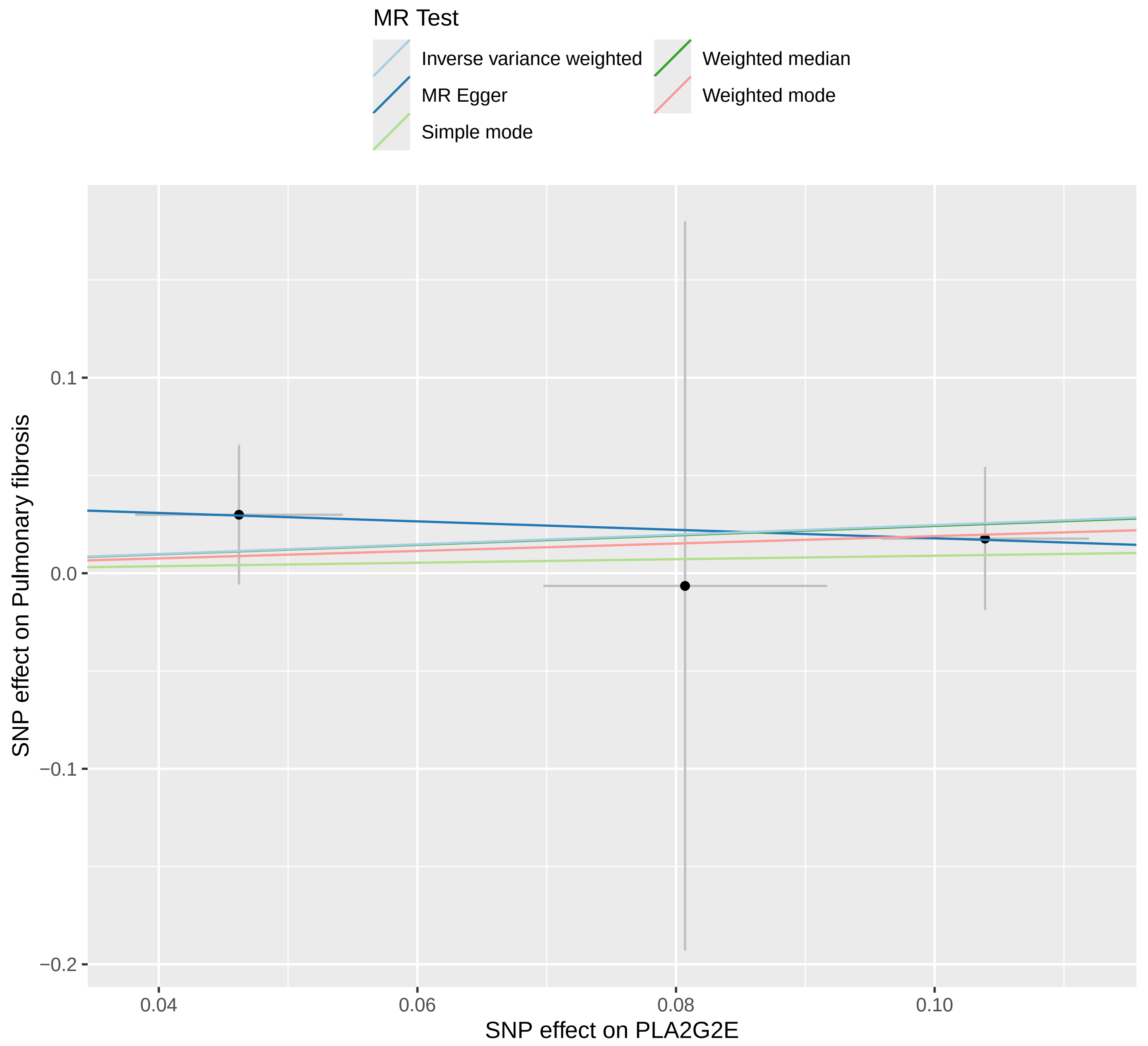

MR Test
Inverse variance weighted
MR Egger
Simple mode
Weighted median
Weighted mode
0.1
0.0
−0.1
−0.2
SNP effect on Pulmonary fibrosis
0.04
0.06
0.08
0.10
SNP effect on PLA2G2E

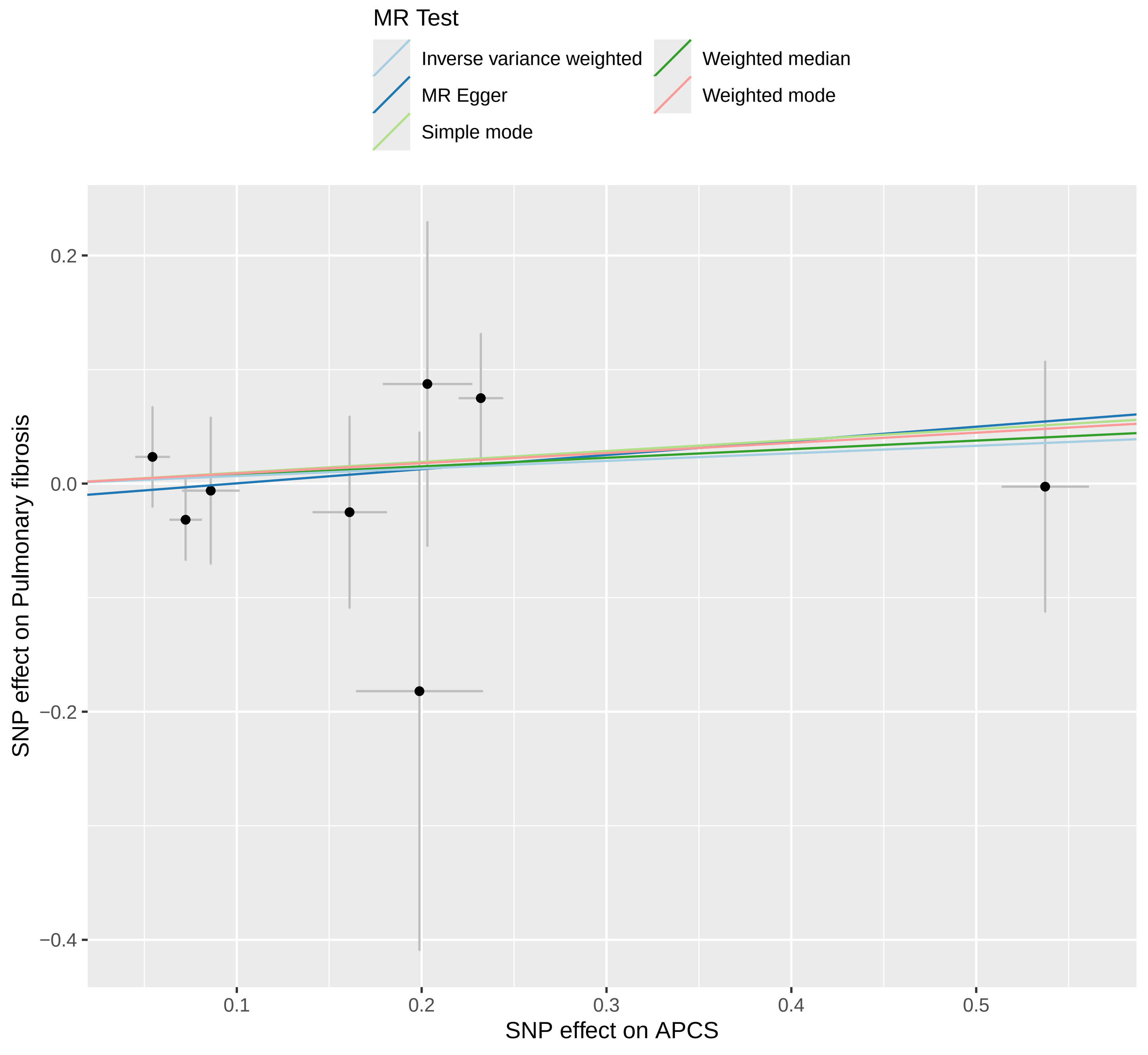

MR Test
Inverse variance weighted
MR Egger
Simple mode
Weighted median
Weighted mode
0.2
0.0
−0.2
−0.4
0.1
0.2
0.3
0.4
0.5
SNP effect on APCS
SNP effect on Pulmonary fibrosis

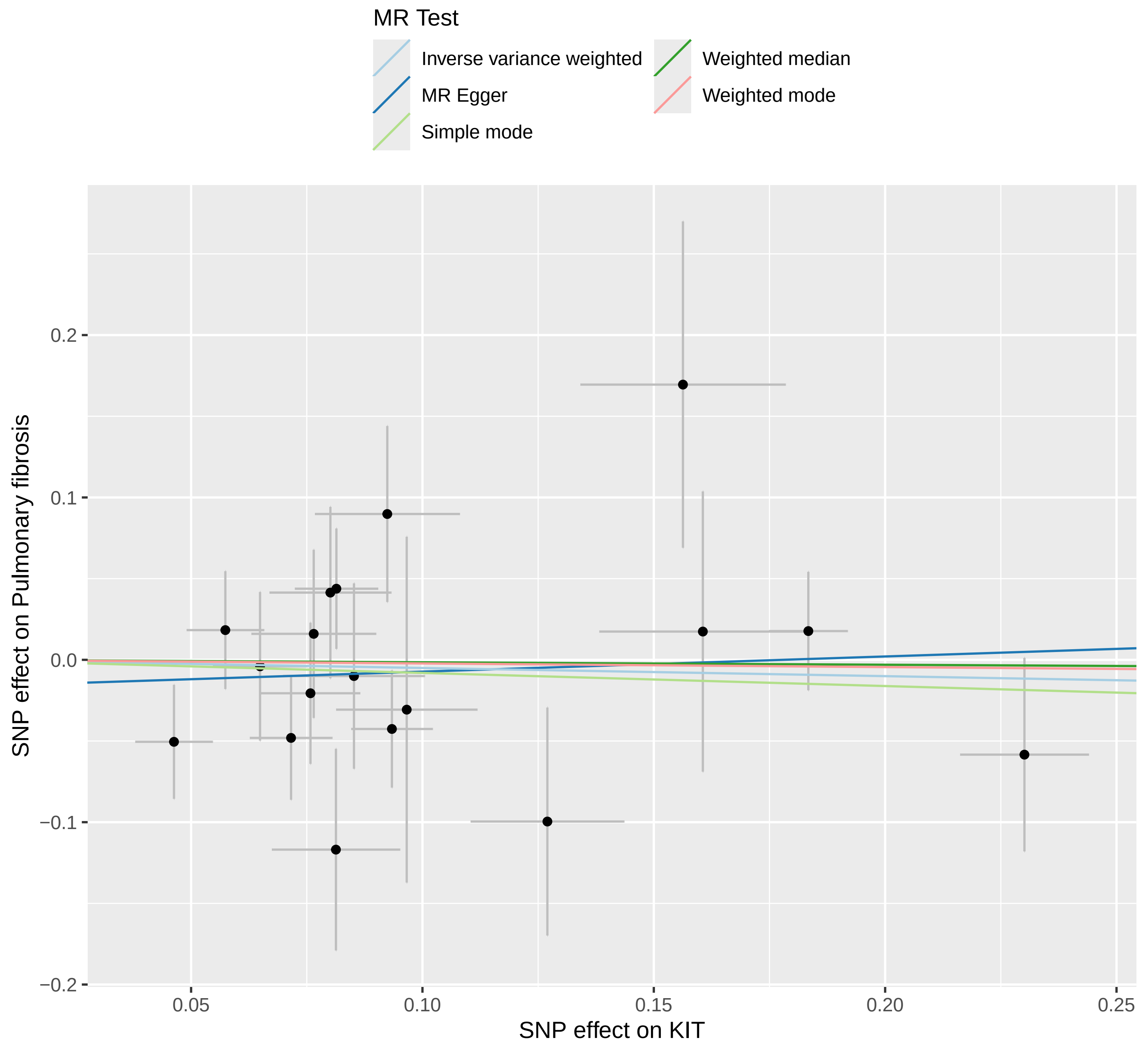
MR Test
Inverse variance weighted
MR Egger
Simple mode
Weighted median
Weighted mode
0.2
0.1
0.0
−0.1
−0.2
0.05
0.10
0.15
0.20
0.25
SNP effect on KIT
SNP effect on Pulmonary fibrosis

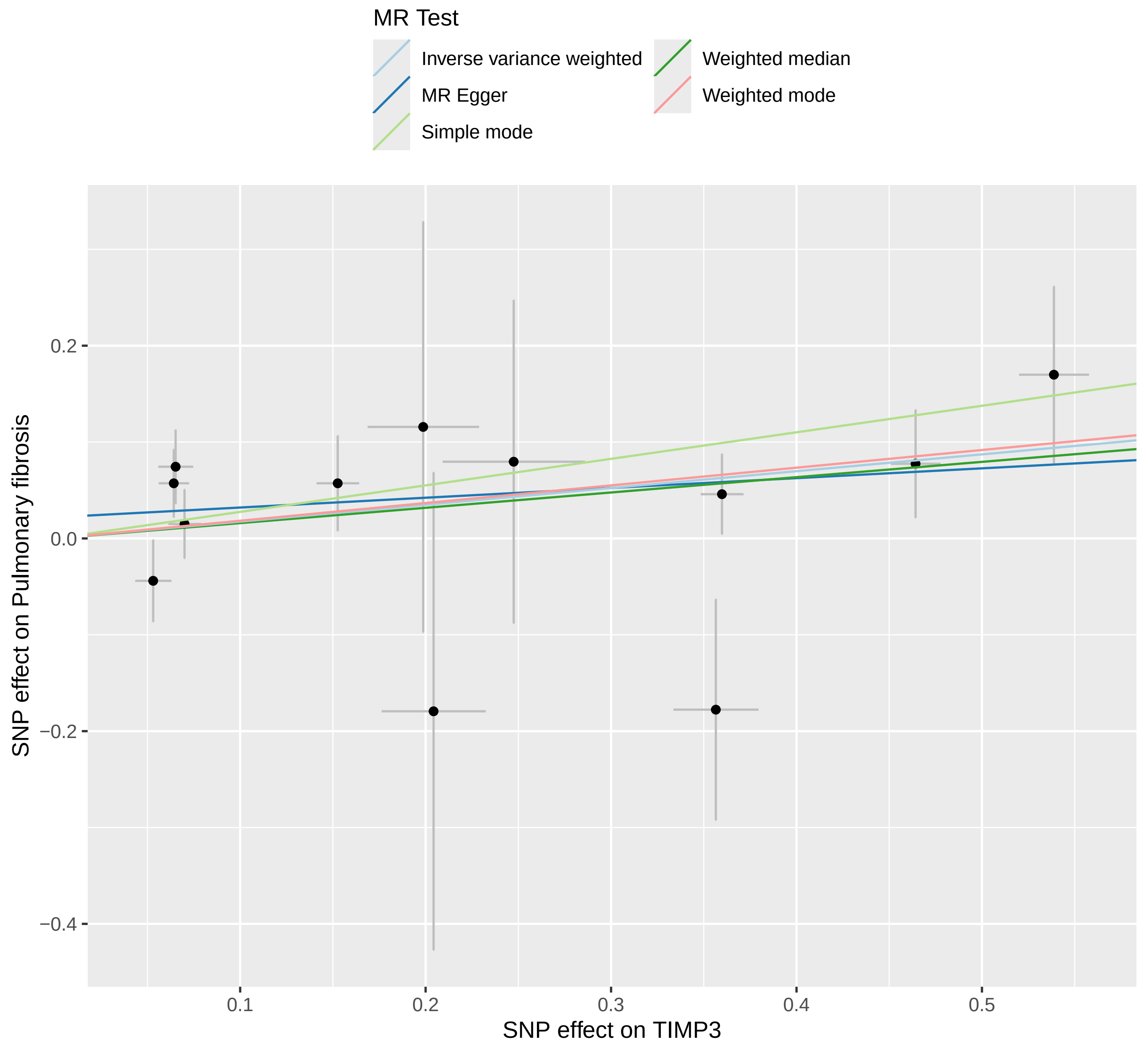

MR Test
Inverse variance weighted
MR Egger
Simple mode
Weighted median
Weighted mode
0.2
0.0
−0.2
−0.4
SNP effect on Pulmonary fibrosis
0.1
0.2
0.3
0.4
0.5
SNP effect on TIMP3

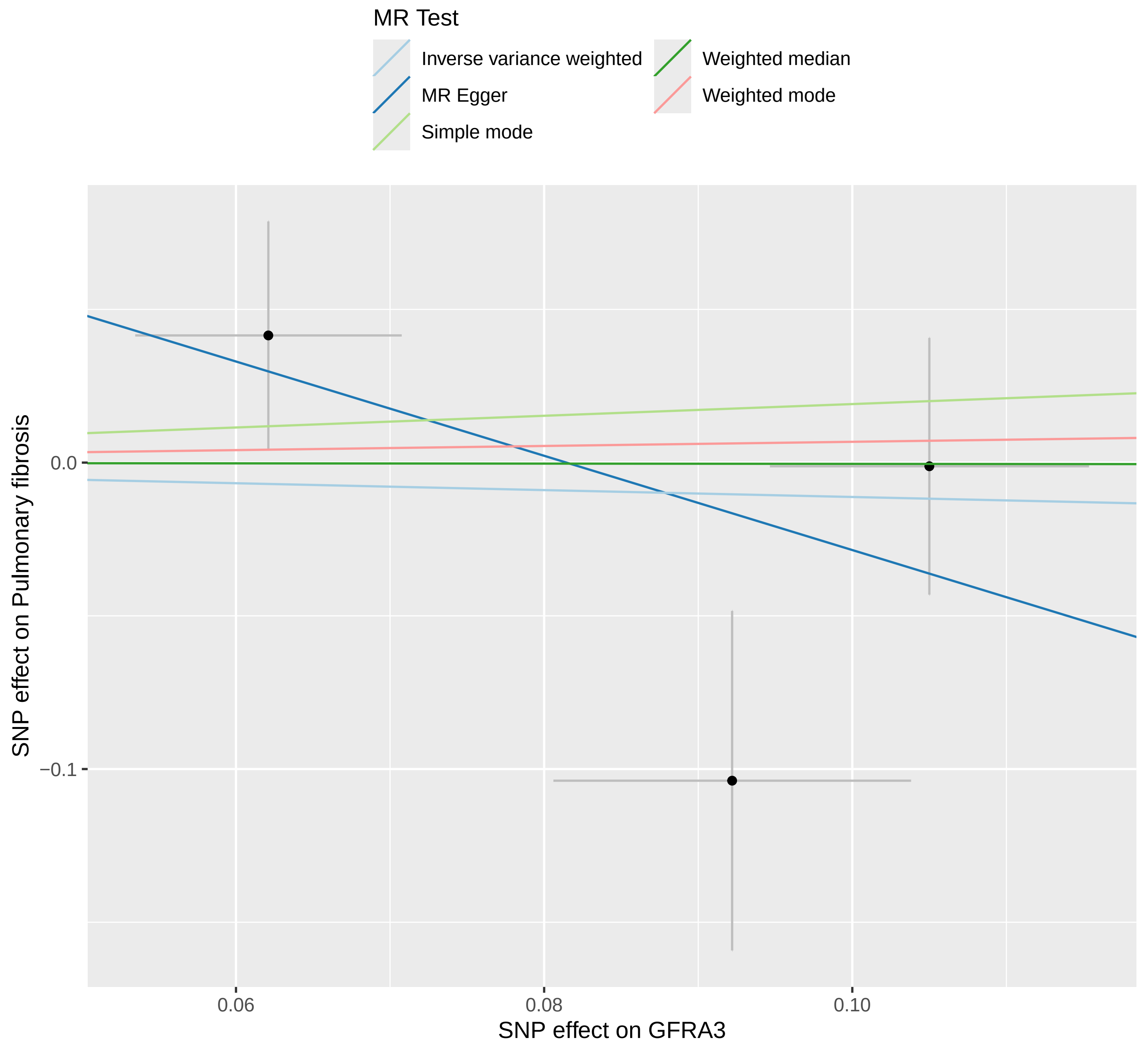

MR Test
Inverse variance weighted
MR Egger
Simple mode
Weighted median
Weighted mode
0.0
−0.1
SNP effect on Pulmonary fibrosis
0.06
0.08
0.10
SNP effect on GFRA3

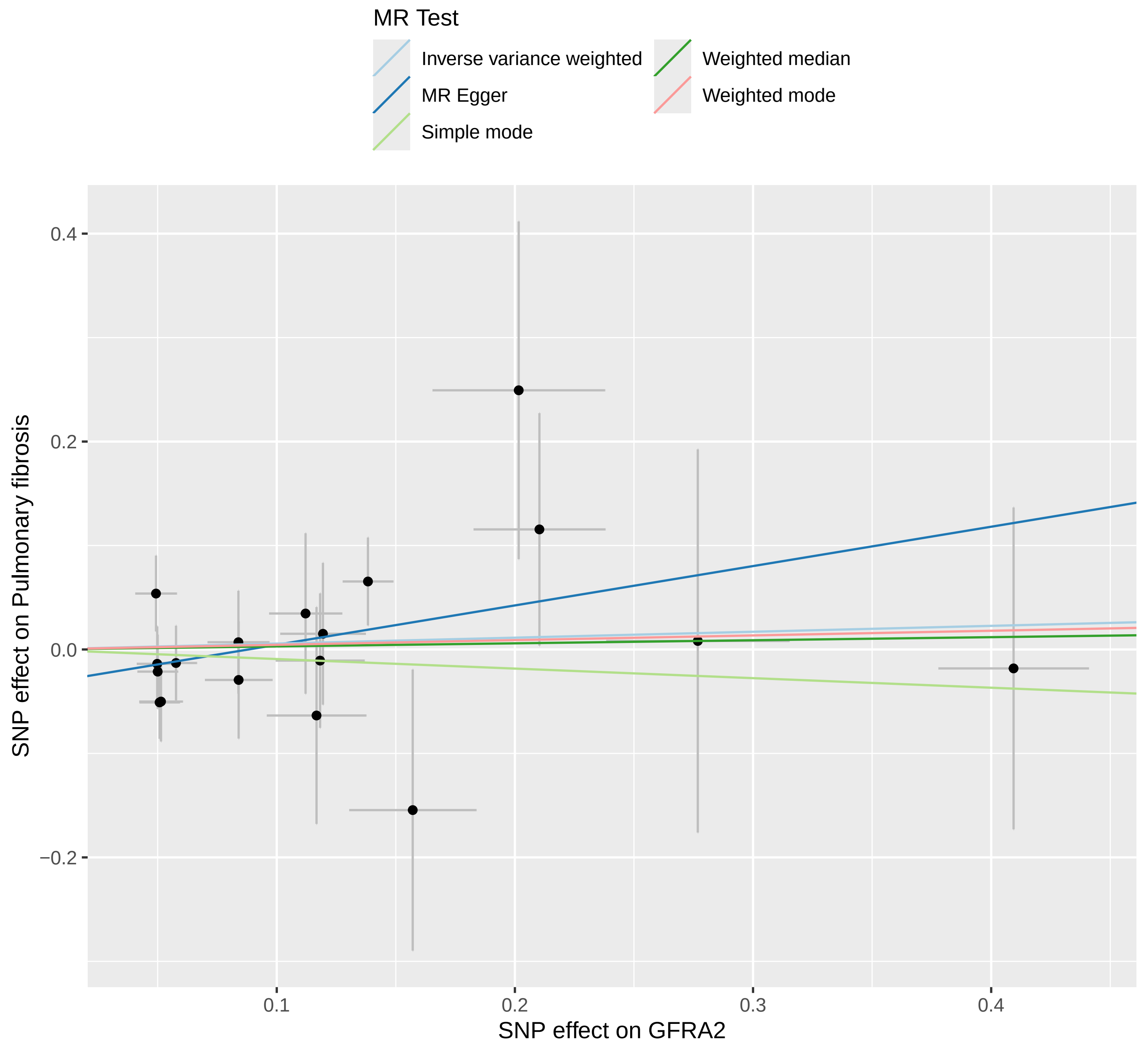

MR Test
Inverse variance weighted
MR Egger
Simple mode
Weighted median
Weighted mode
0.4
0.2
0.0
−0.2
SNP effect on Pulmonary fibrosis
0.1
0.2
0.3
0.4
SNP effect on GFRA2

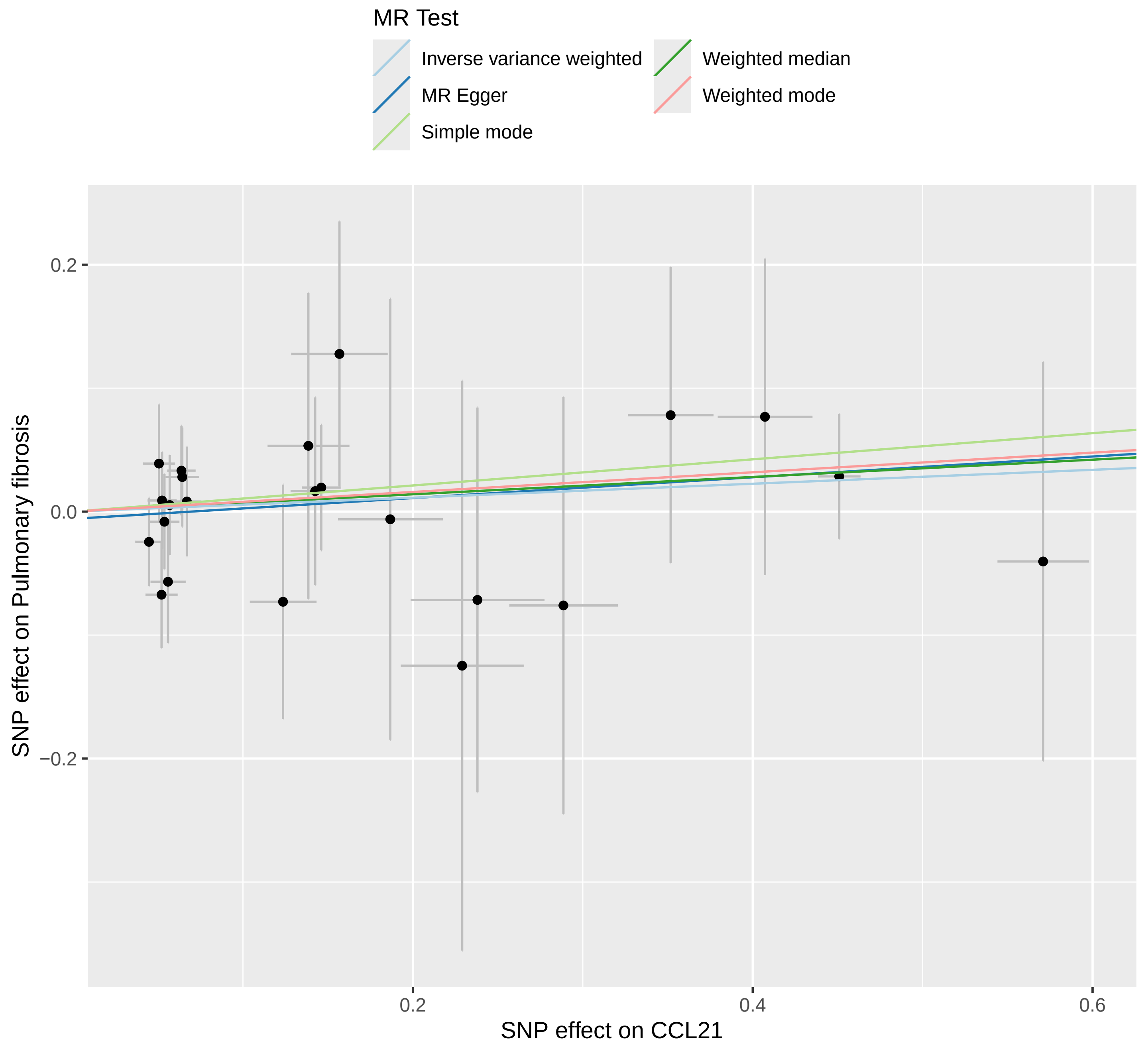

MR Test
Inverse variance weighted
MR Egger
Simple mode
Weighted median
Weighted mode
0.2
0.0
−0.2
0.2
0.4
0.6
SNP effect on Pulmonary fibrosis
SNP effect on CCL21

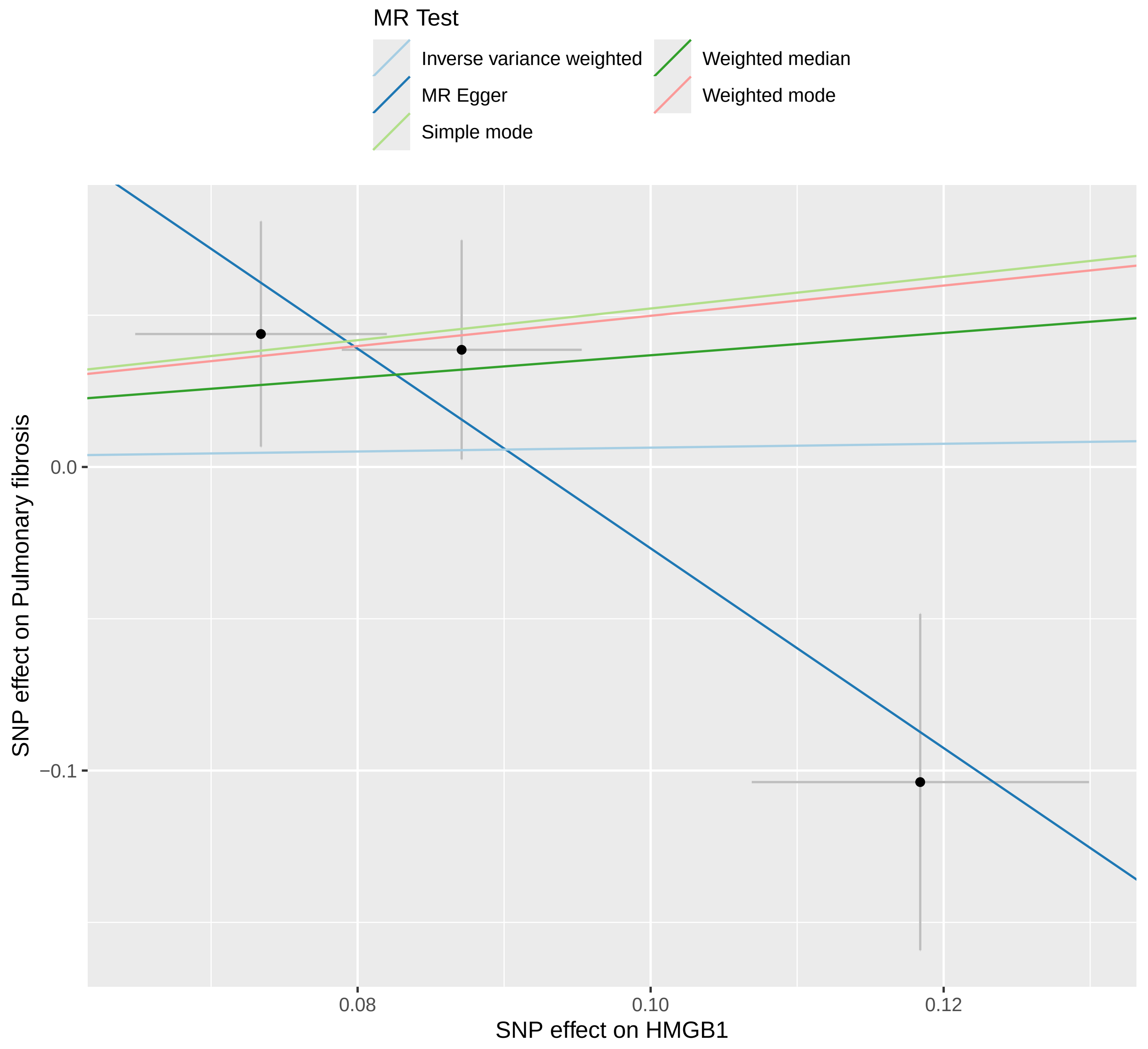

MR Test
Inverse variance weighted
MR Egger
Simple mode
Weighted median
Weighted mode
0.0
−0.1
0.08
0.10
0.12
SNP effect on HMGB1
SNP effect on Pulmonary fibrosis

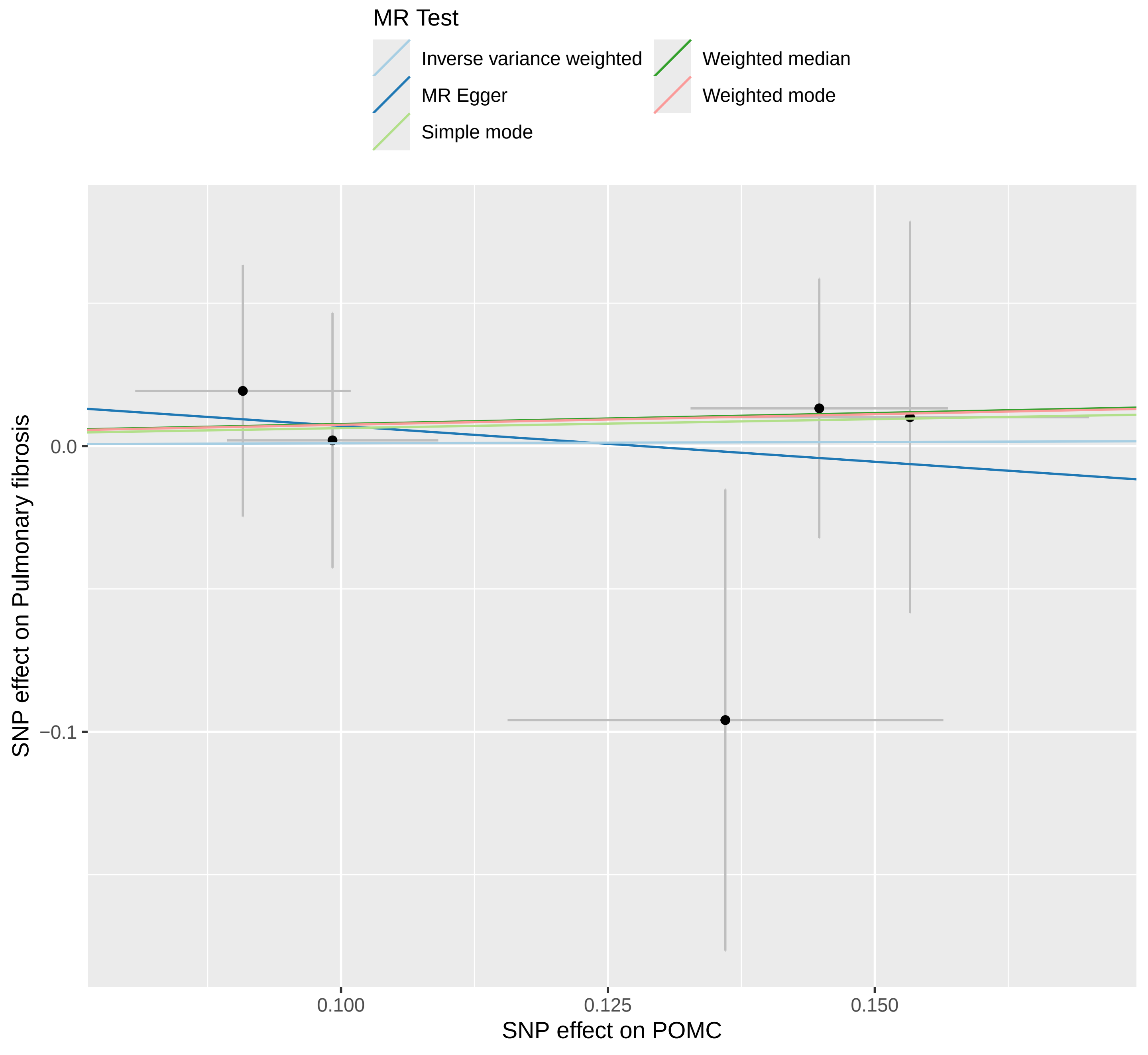
MR Test
Inverse variance weighted
MR Egger
Simple mode
Weighted median
Weighted mode
0.0
−0.1
0.100
0.125
0.150
SNP effect on POMC
SNP effect on Pulmonary fibrosis

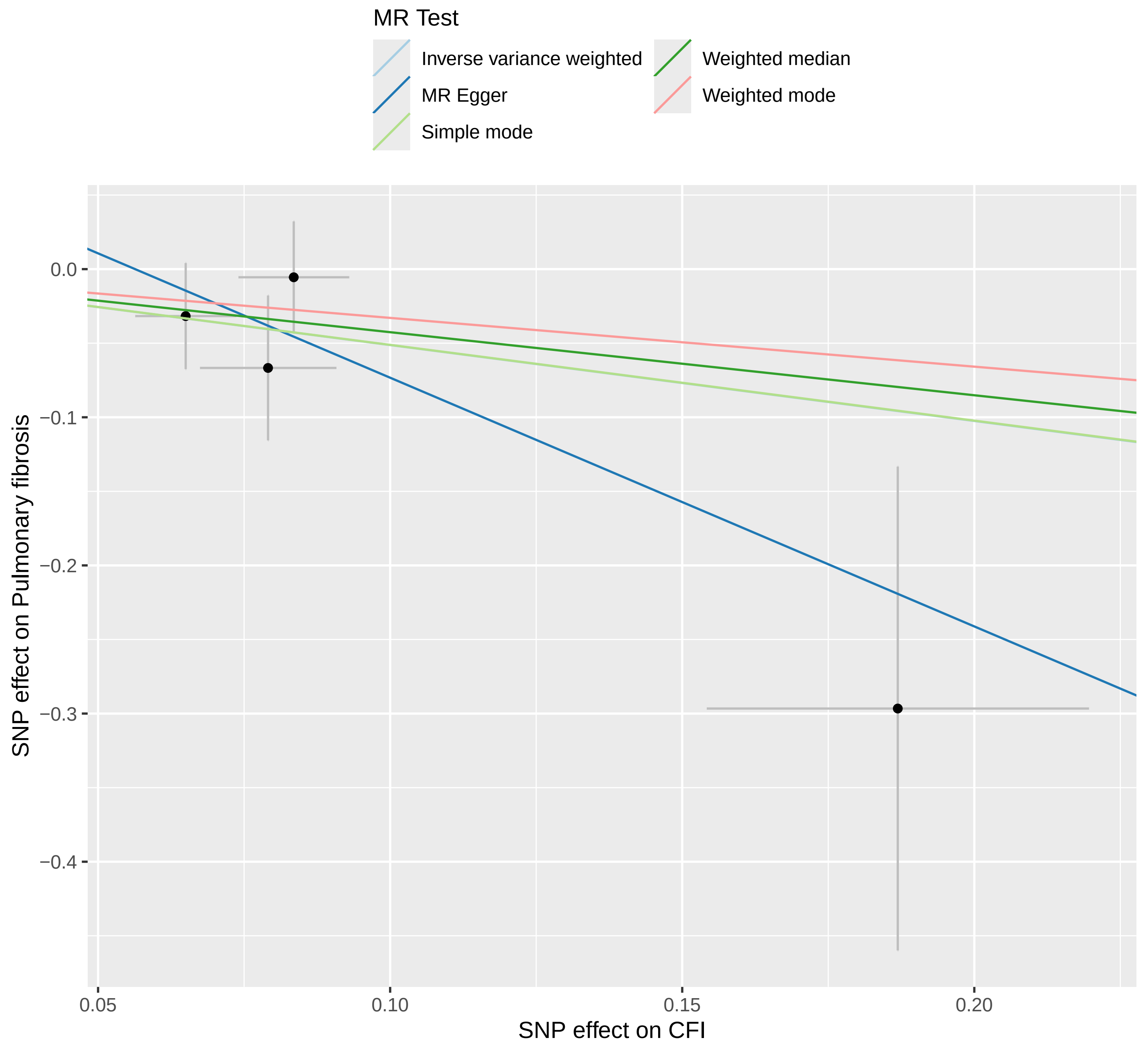

MR Test
Inverse variance weighted
MR Egger
Simple mode
Weighted median
Weighted mode
0.0
−0.1
−0.2
−0.3
−0.4
SNP effect on Pulmonary fibrosis
0.05
0.10
0.15
0.20
SNP effect on CFI

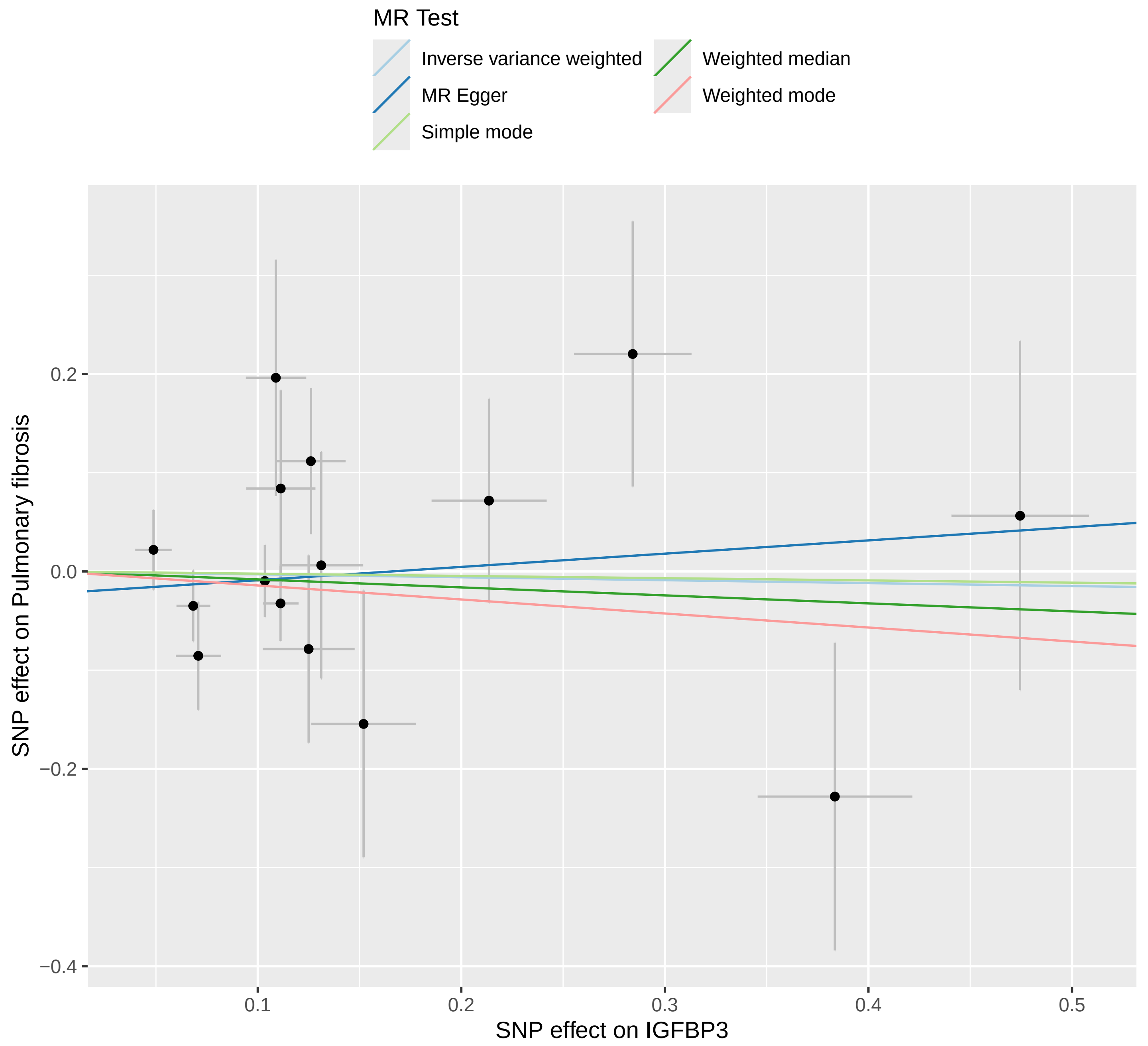

MR Test
Inverse variance weighted
MR Egger
Simple mode
Weighted median
Weighted mode
SNP effect on Pulmonary fibrosis
0.2
0.0
−0.2
−0.4
0.1
0.2
0.3
0.4
0.5
SNP effect on IGFBP3

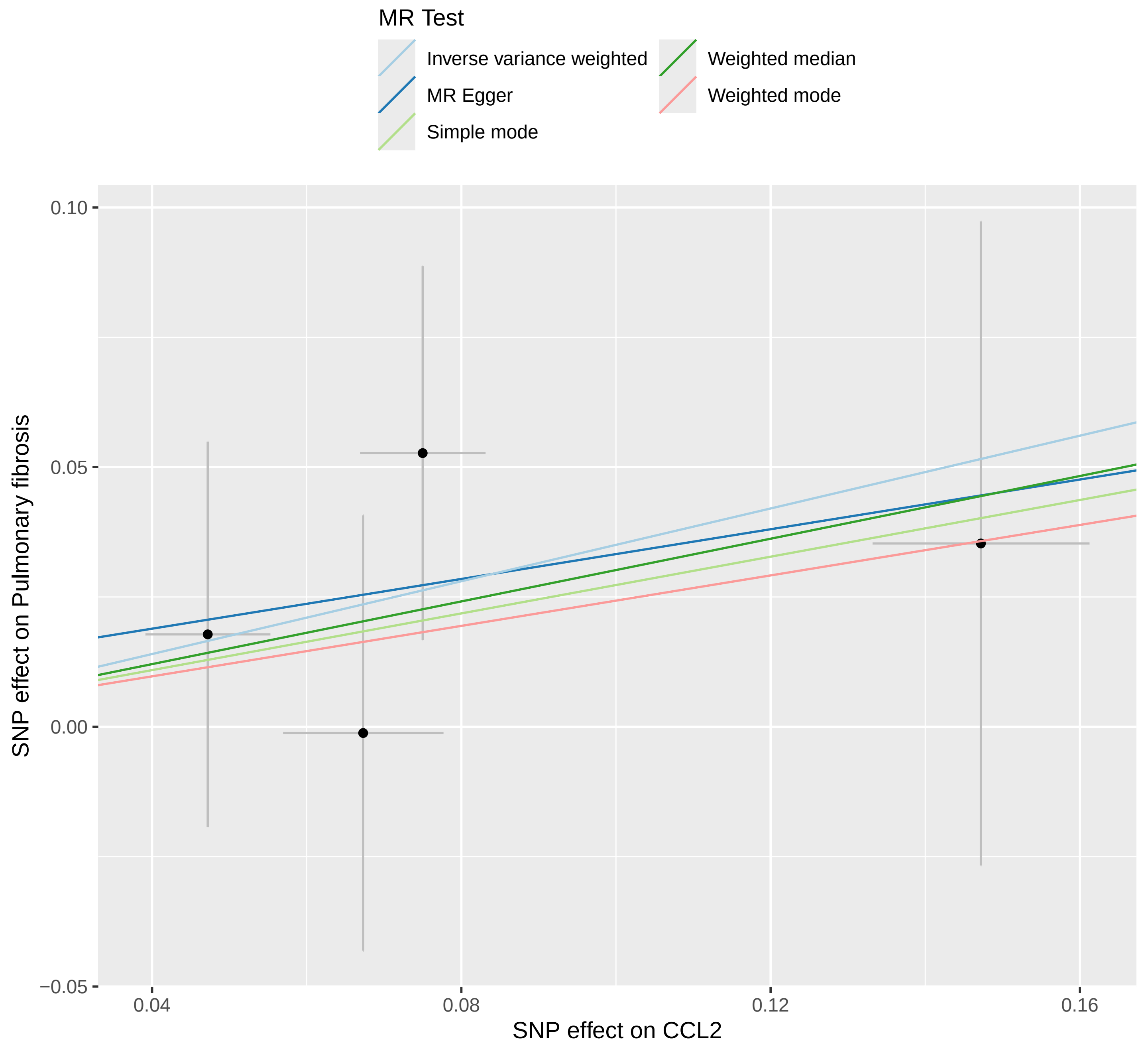
MR Test
Inverse variance weighted
MR Egger
Simple mode
Weighted median
Weighted mode
0.10
0.05
0.00
−0.05
0.04
0.08
0.12
0.16
SNP effect on Pulmonary fibrosis
SNP effect on CCL2

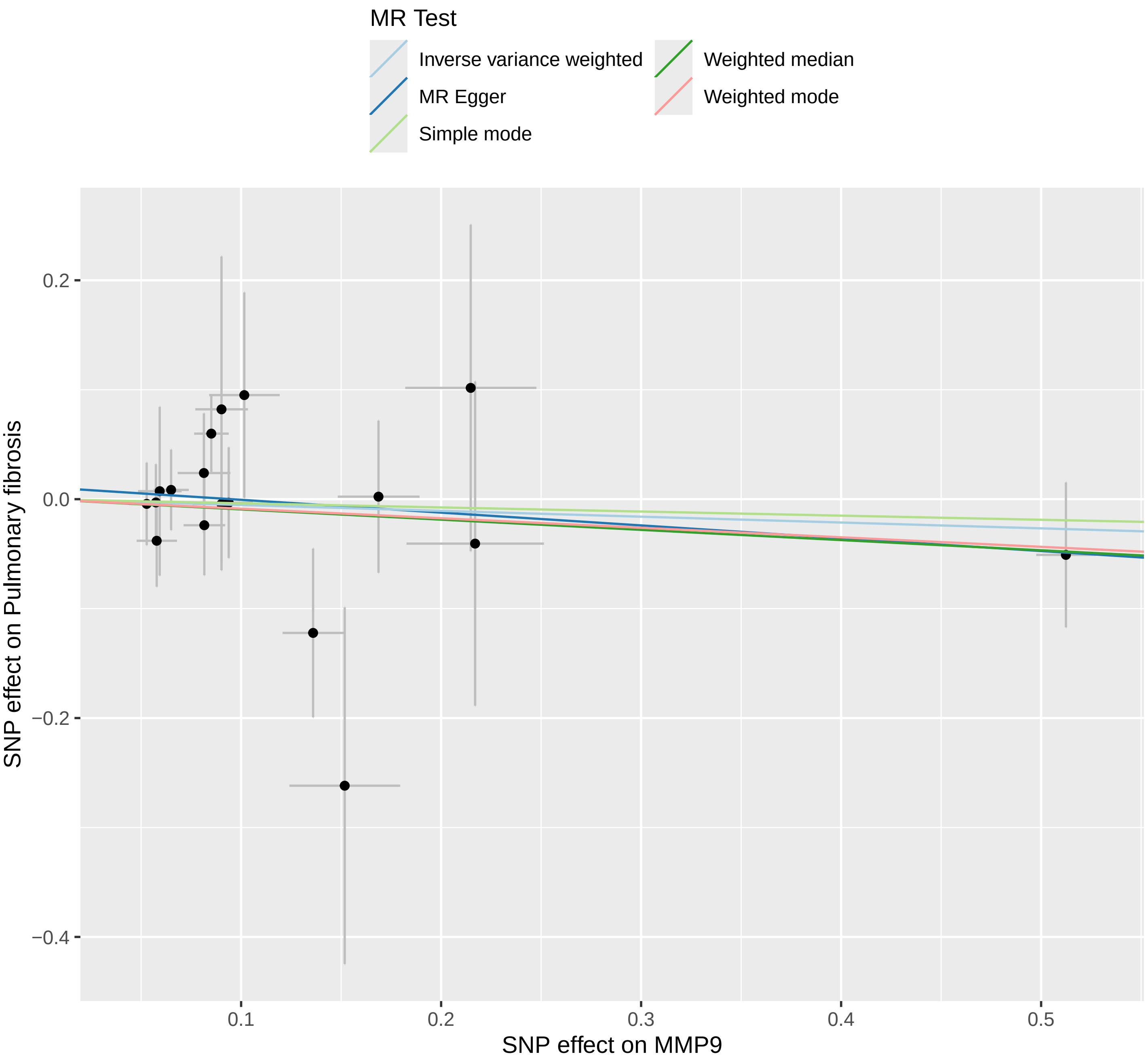

MR Test
Inverse variance weighted
MR Egger
Simple mode
Weighted median
Weighted mode
SNP effect on Pulmonary fibrosis
0.2
0.0
−0.2
−0.4
0.1
0.2
0.3
0.4
0.5
SNP effect on MMP9

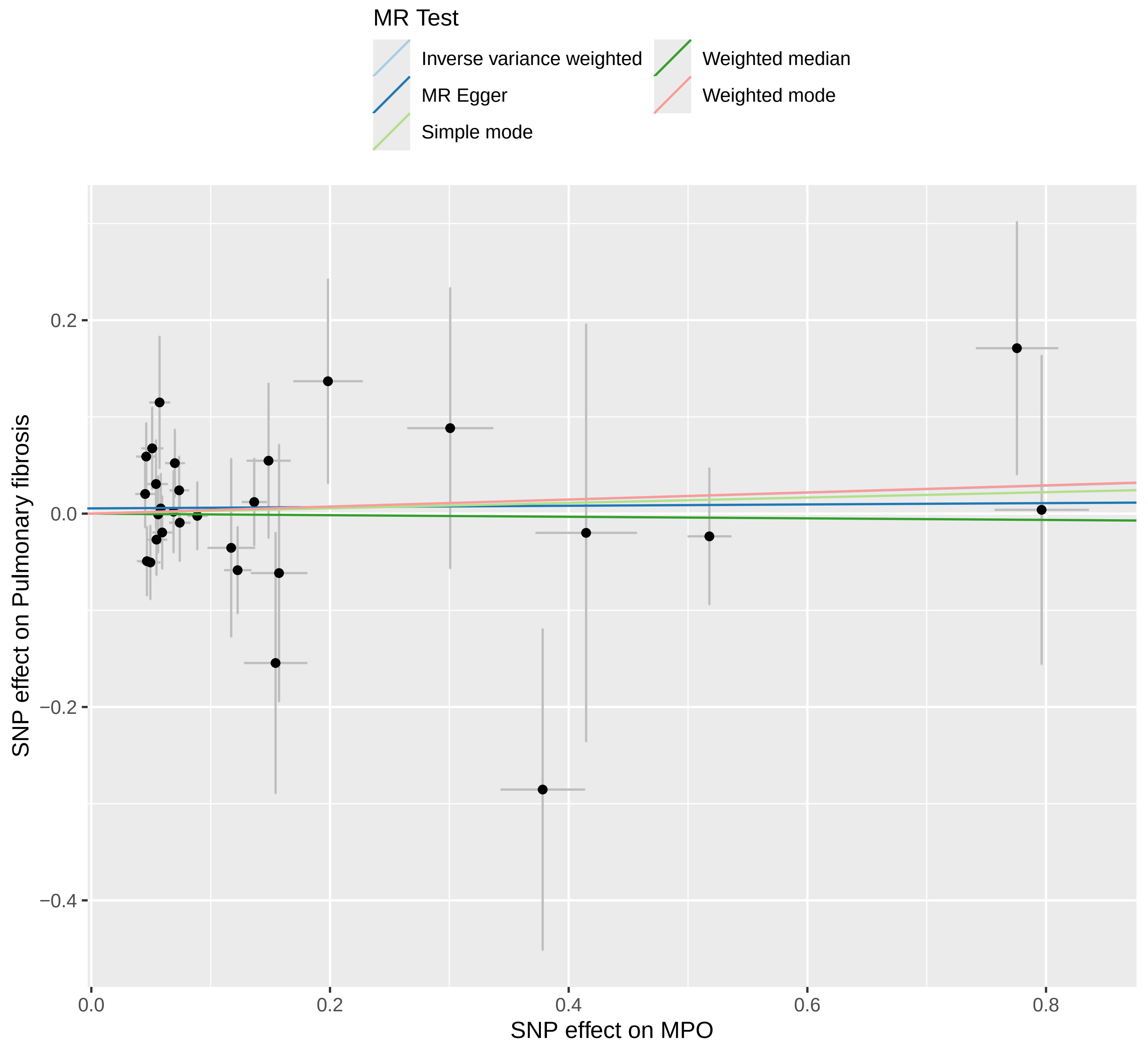

MR Test
Inverse variance weighted
MR Egger
Simple mode
Weighted median
Weighted mode
0.2
0.0
−0.2
−0.4
SNP effect on Pulmonary fibrosis
0.0
0.2
0.4
0.6
0.8
SNP effect on MPO

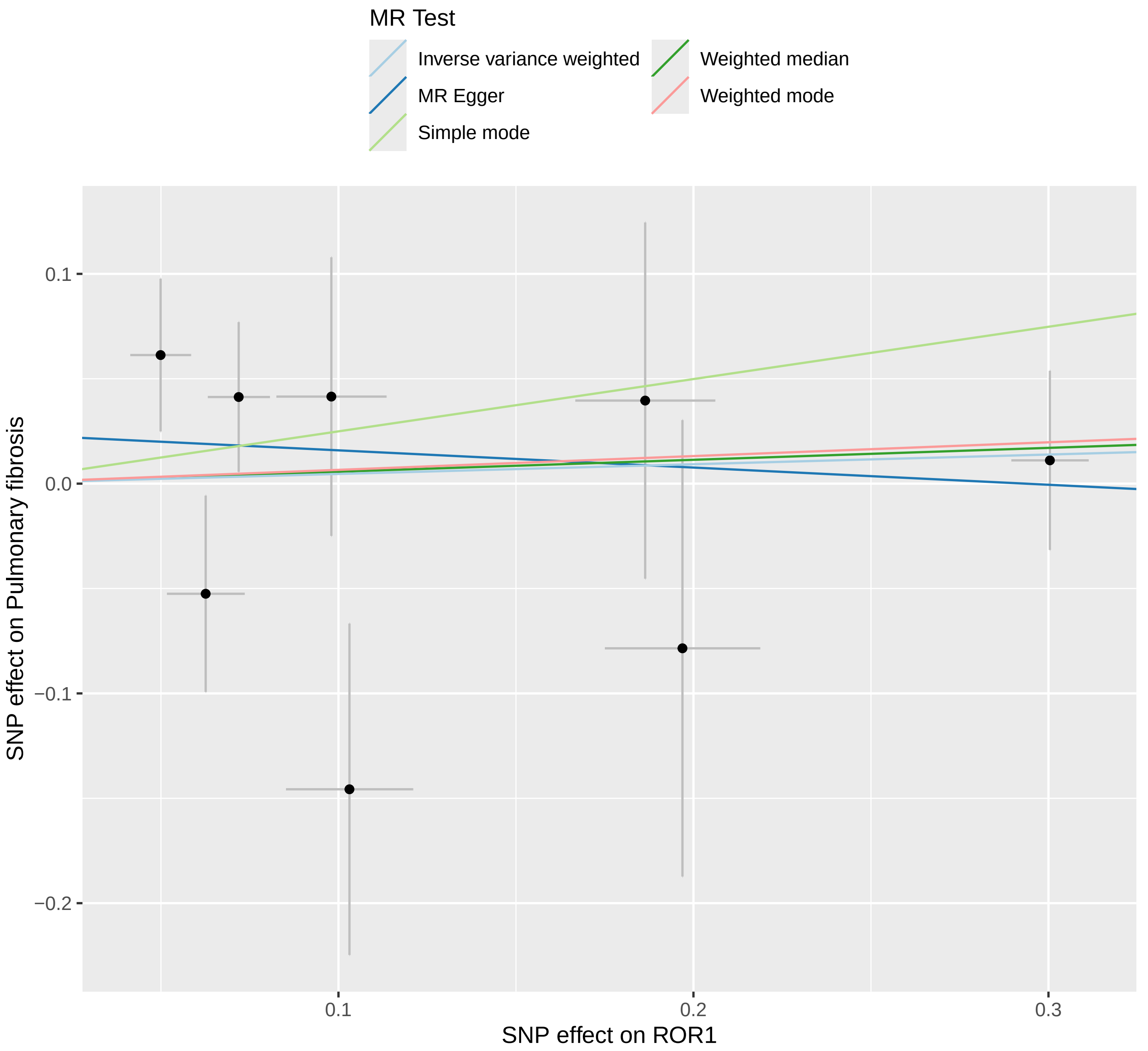

MR Test
Inverse variance weighted
MR Egger
Simple mode
Weighted median
Weighted mode
0.1
0.0
−0.1
−0.2
SNP effect on Pulmonary fibrosis
0.1
0.2
0.3
SNP effect on ROR1

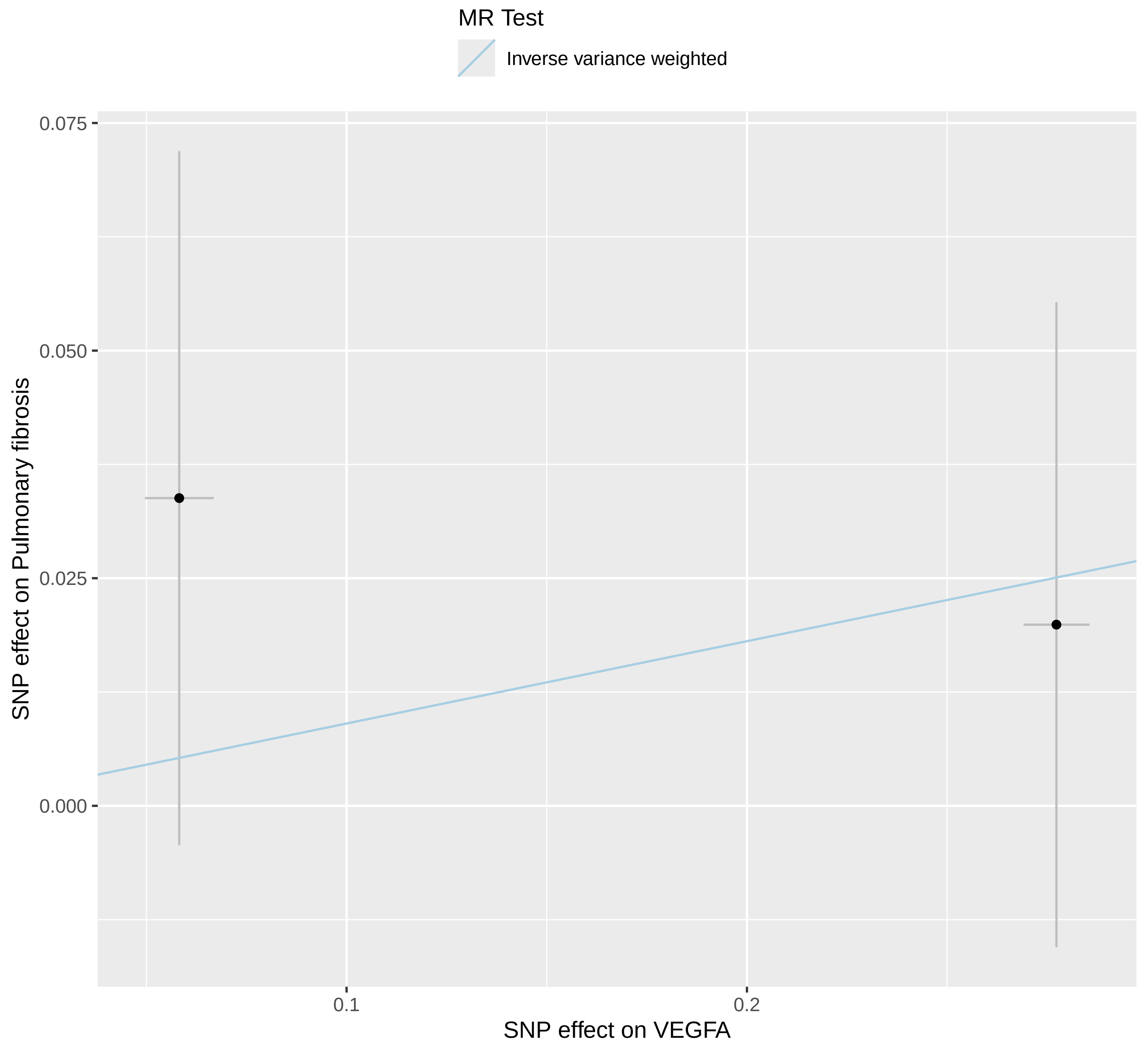

MR Test
Inverse variance weighted
0.075
0.050
0.025
0.000
0.1
0.2
SNP effect on Pulmonary fibrosis
SNP effect on VEGFA

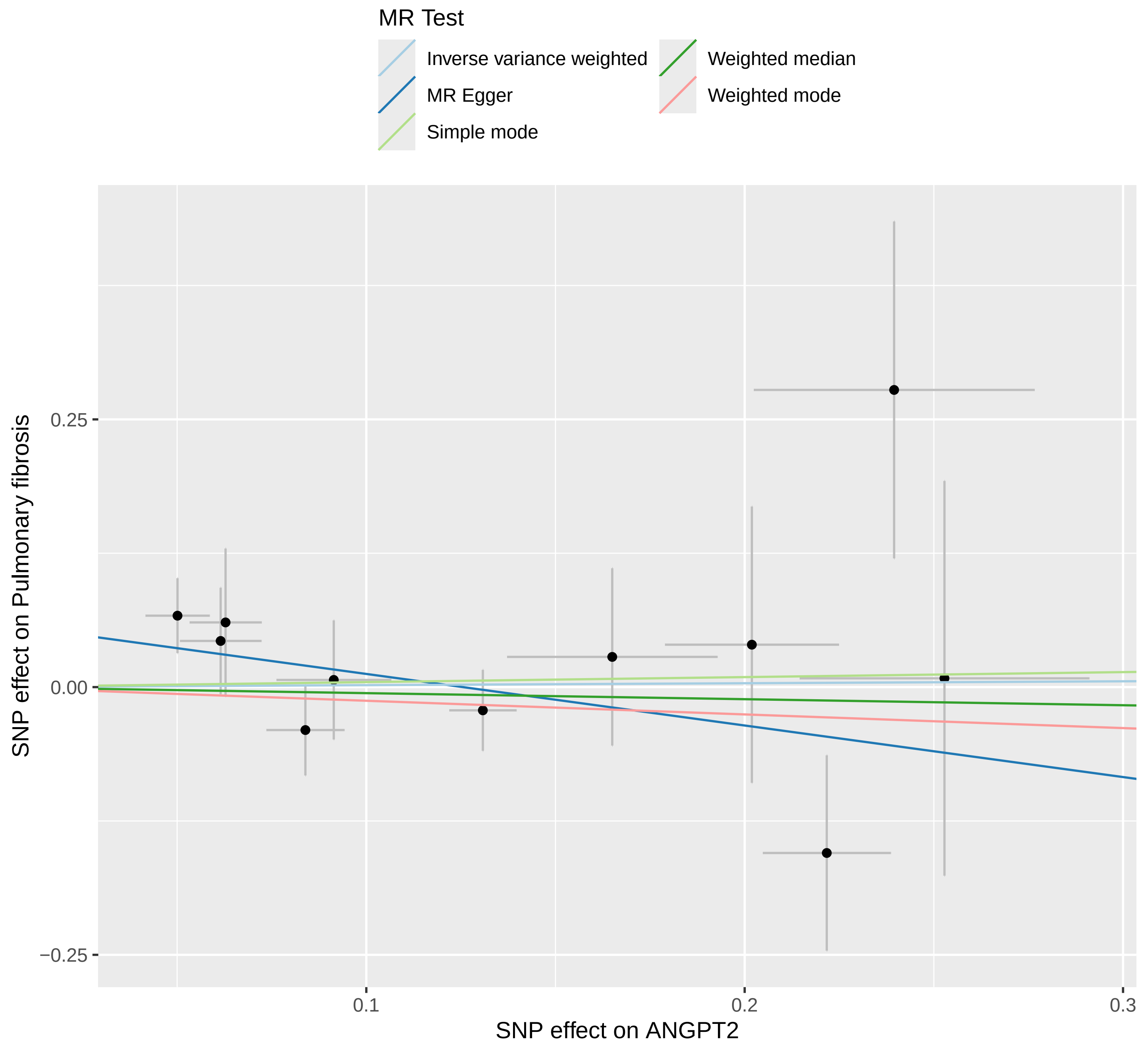
MR Test
Inverse variance weighted
MR Egger
Simple mode
Weighted median
Weighted mode
0.25
0.00
−0.25
0.1
0.2
0.3
SNP effect on Pulmonary fibrosis
SNP effect on ANGPT2

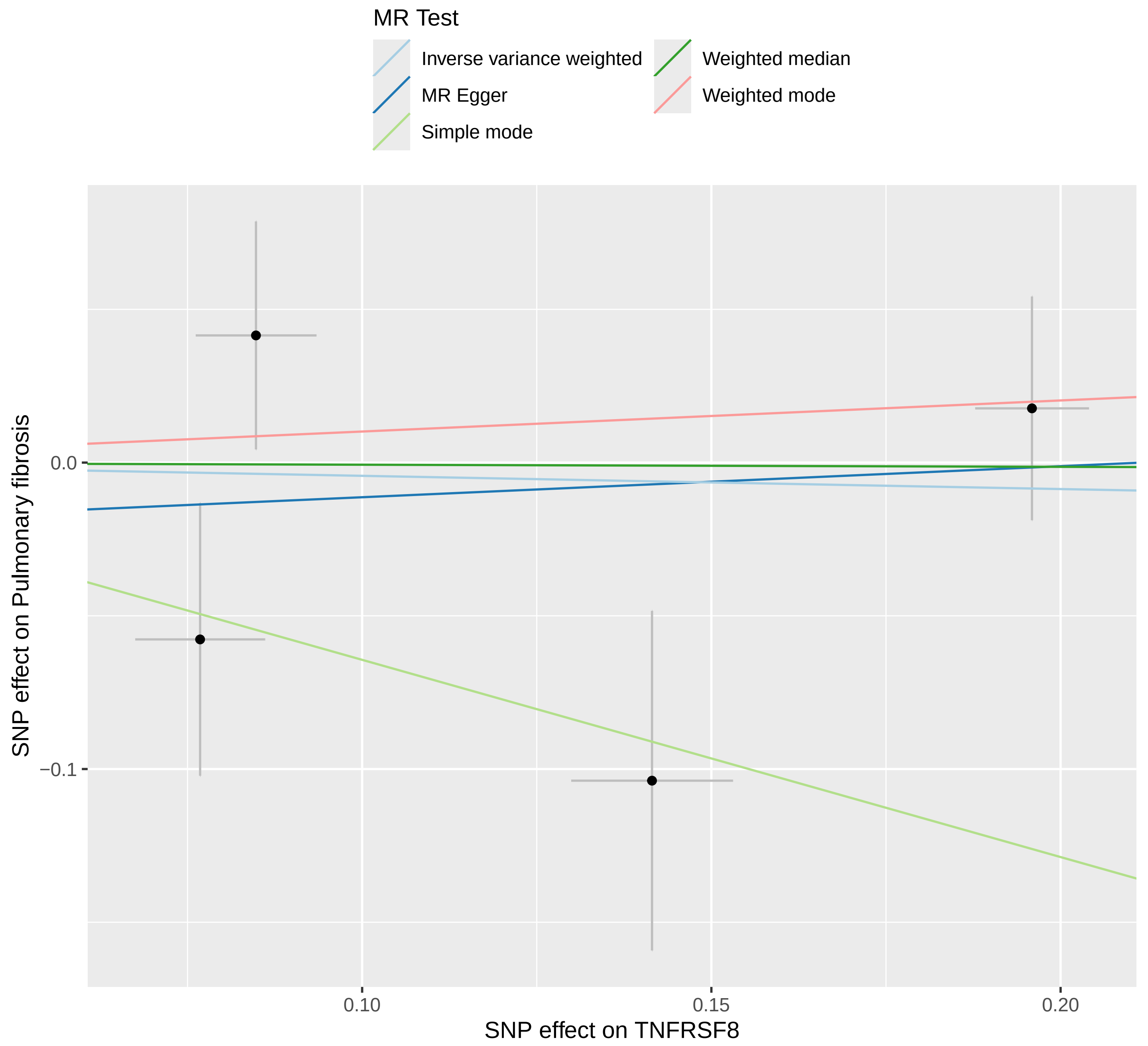
MR Test
Inverse variance weighted
MR Egger
Simple mode
Weighted median
Weighted mode
SNP effect on Pulmonary fibrosis
0.0
−0.1
0.10
0.15
0.20
SNP effect on TNFRSF8

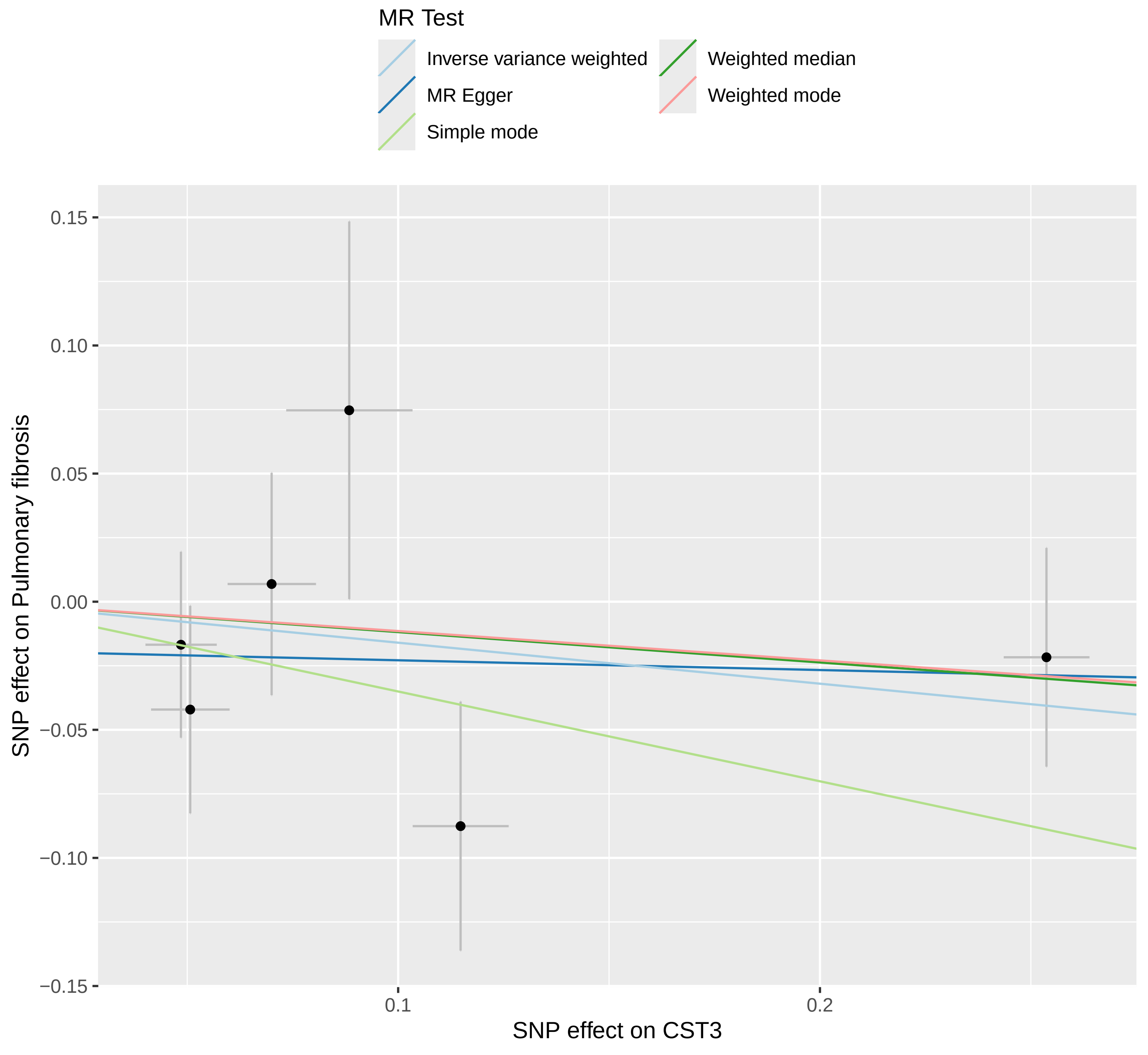
MR Test
Inverse variance weighted
MR Egger
Simple mode
Weighted median
Weighted mode
0.15
0.10
0.05
0.00
−0.05
−0.10
−0.15
SNP effect on Pulmonary fibrosis
0.1
0.2
SNP effect on CST3

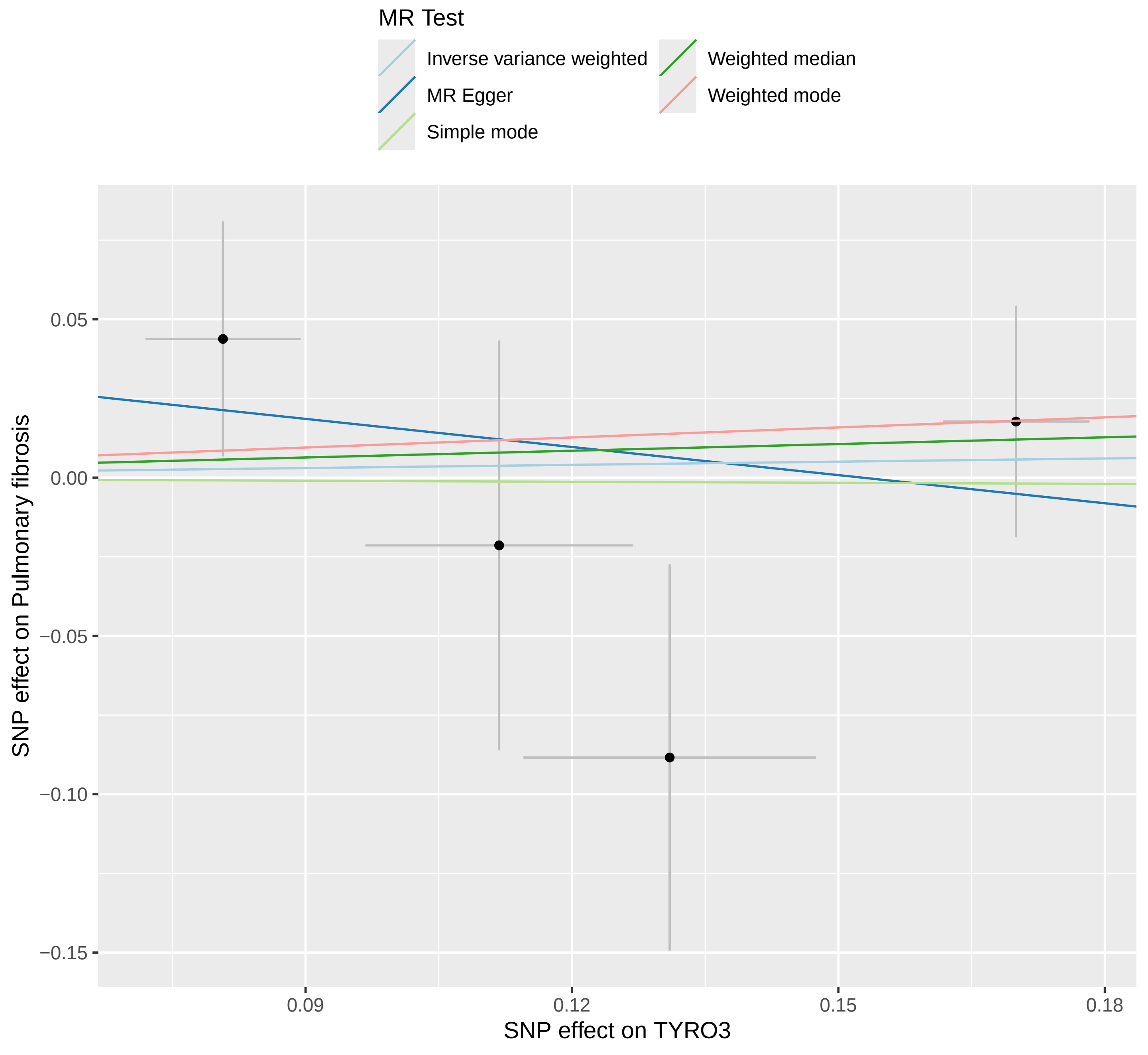

MR Test
Inverse variance weighted
MR Egger
Simple mode
Weighted median
Weighted mode
0.05
0.00
−0.05
−0.10
−0.15
0.09
0.12
0.15
0.18
SNP effect on Pulmonary fibrosis
SNP effect on TYRO3

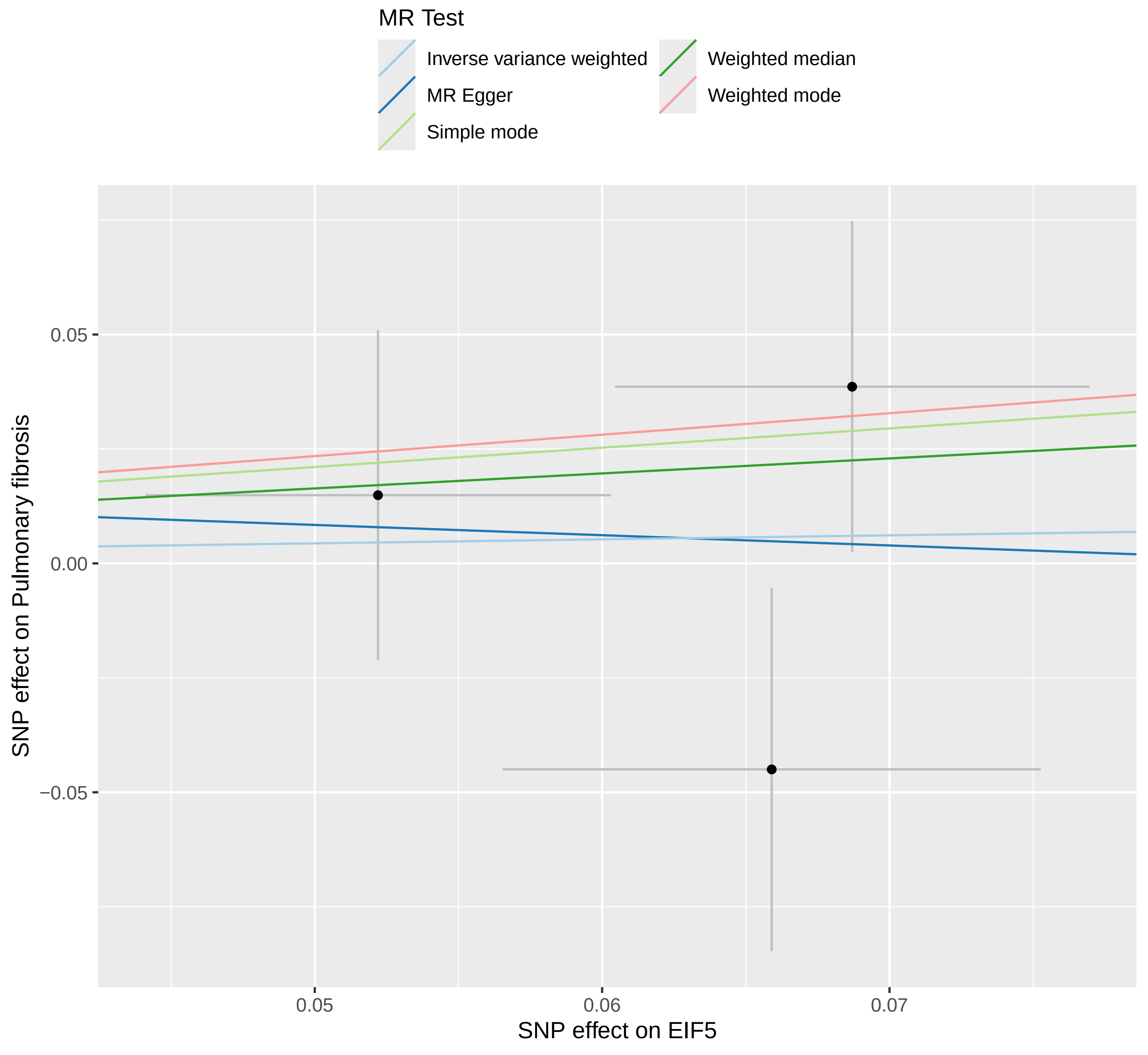
MR Test
Inverse variance weighted
MR Egger
Simple mode
Weighted median
Weighted mode
0.05
0.00
−0.05
SNP effect on Pulmonary fibrosis
0.05
0.06
0.07
SNP effect on EIF5

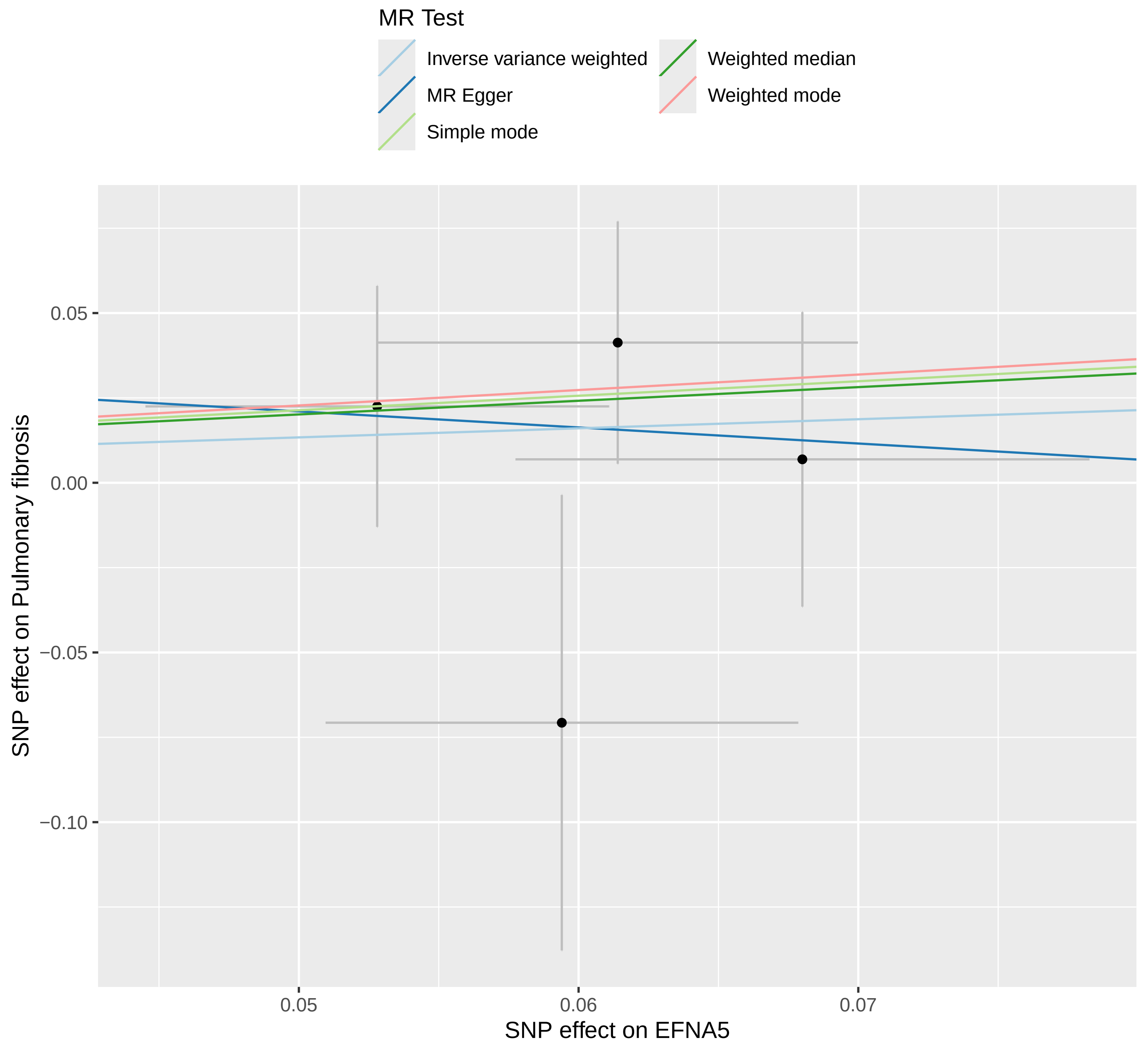

MR Test
Inverse variance weighted
MR Egger
Simple mode
Weighted median
Weighted mode
0.05
0.00
−0.05
−0.10
0.05
0.06
0.07
SNP effect on Pulmonary fibrosis
SNP effect on EFNA5

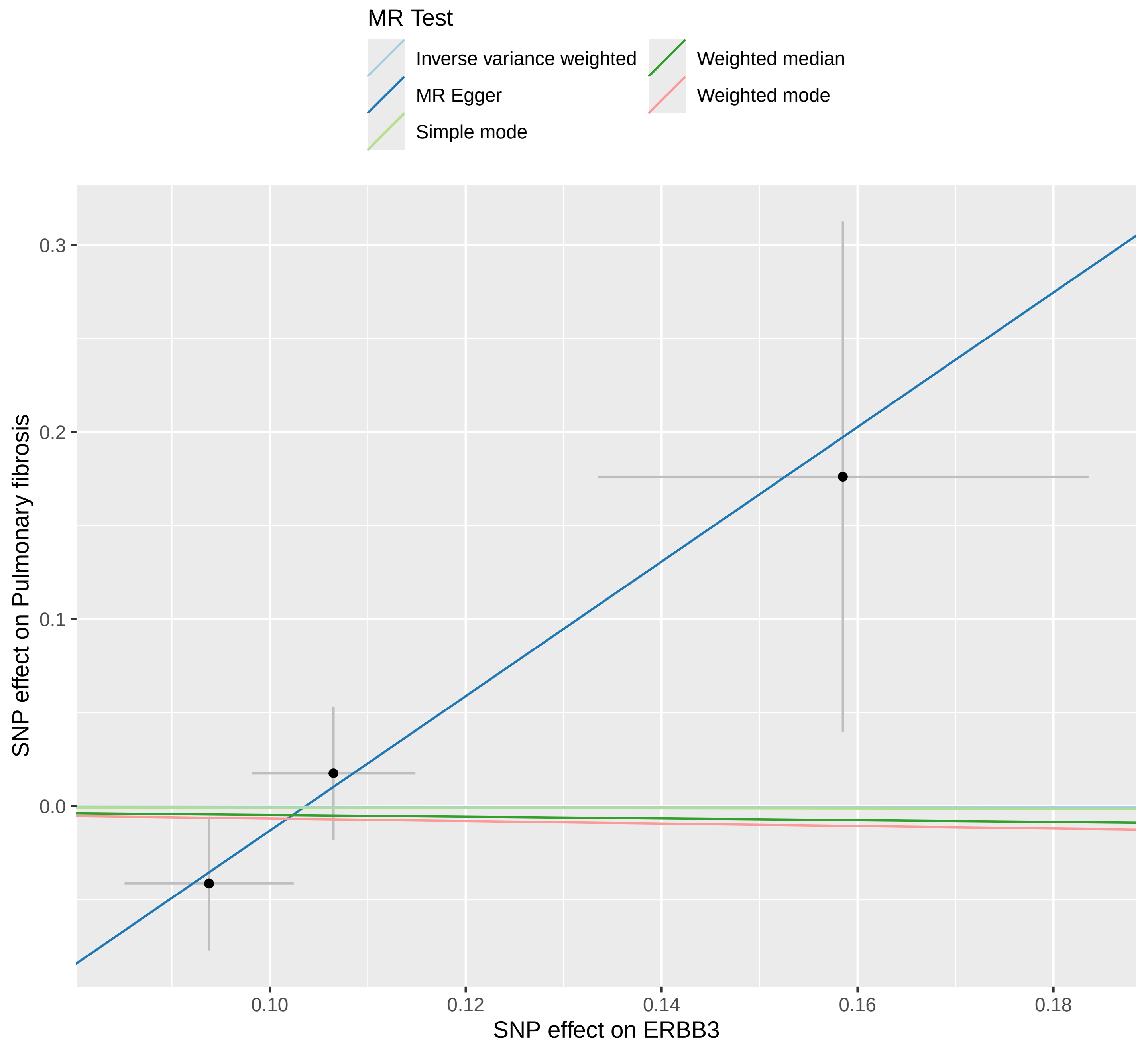

MR Test
Inverse variance weighted
MR Egger
Simple mode
Weighted median
Weighted mode
SNP effect on Pulmonary fibrosis
0.3
0.2
0.1
0.0
0.10
0.12
0.14
0.16
0.18
SNP effect on ERBB3

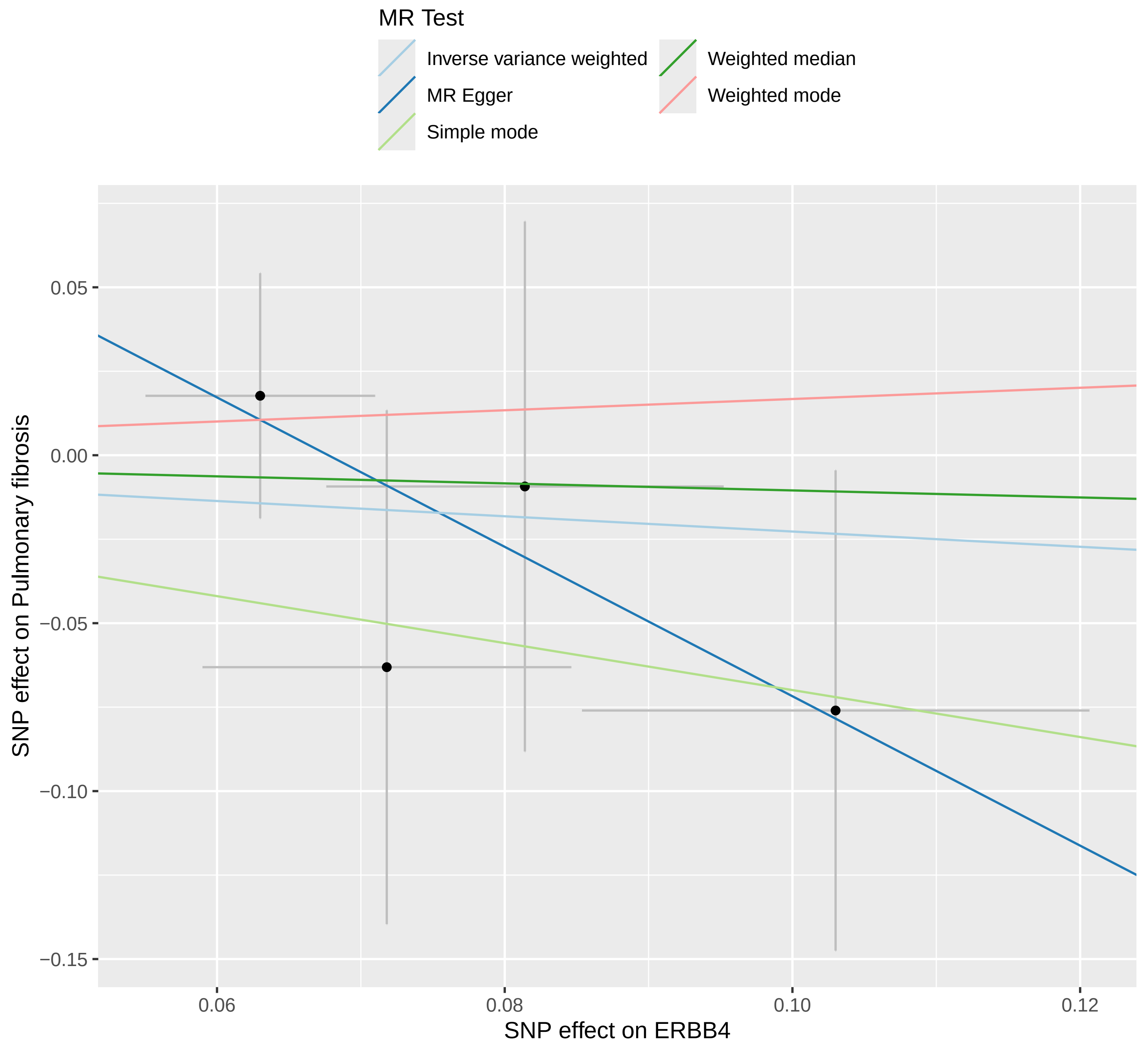
MR Test
Inverse variance weighted
MR Egger
Simple mode
Weighted median
Weighted mode
SNP effect on Pulmonary fibrosis
0.05
0.00
−0.05
−0.10
−0.15
0.06
0.08
0.10
0.12
SNP effect on ERBB4

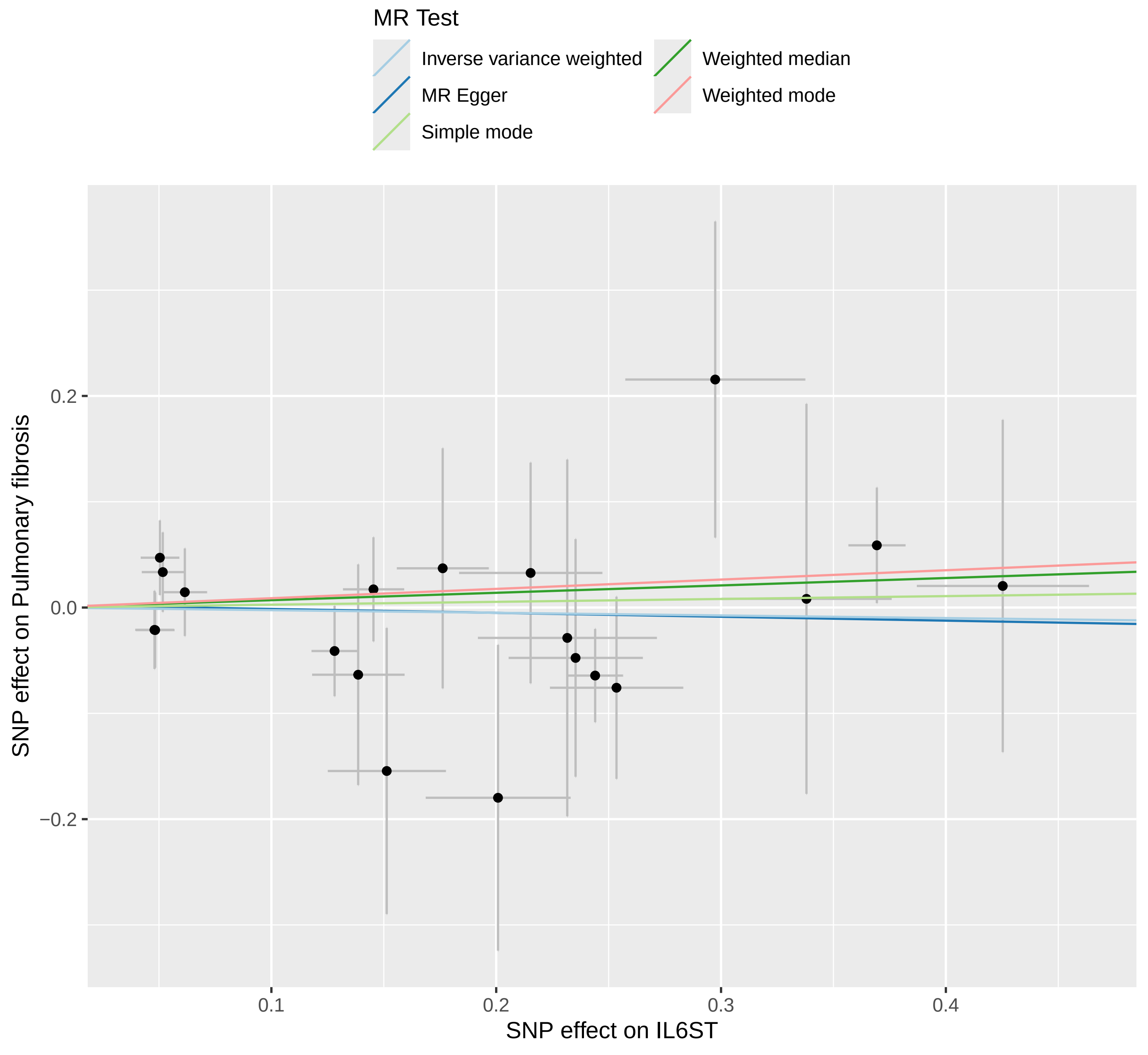
MR Test
Inverse variance weighted
MR Egger
Simple mode
Weighted median
Weighted mode
SNP effect on Pulmonary fibrosis
0.2
0.0
−0.2
0.1
0.2
0.3
0.4
SNP effect on IL6ST

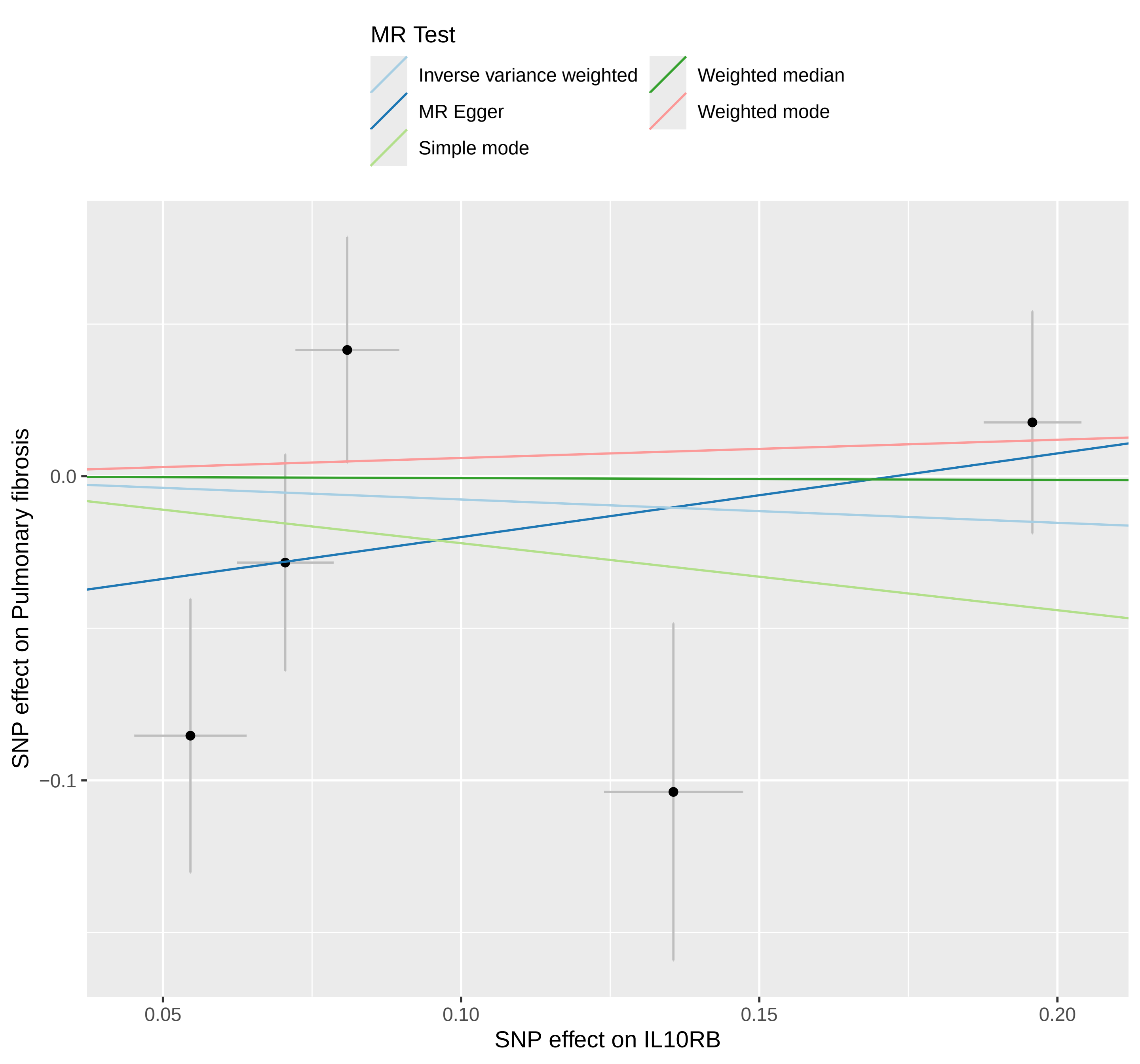
MR Test
Inverse variance weighted
MR Egger
Simple mode
Weighted median
Weighted mode
SNP effect on Pulmonary fibrosis
0.0
−0.1
0.05
0.10
0.15
0.20
SNP effect on IL10RB

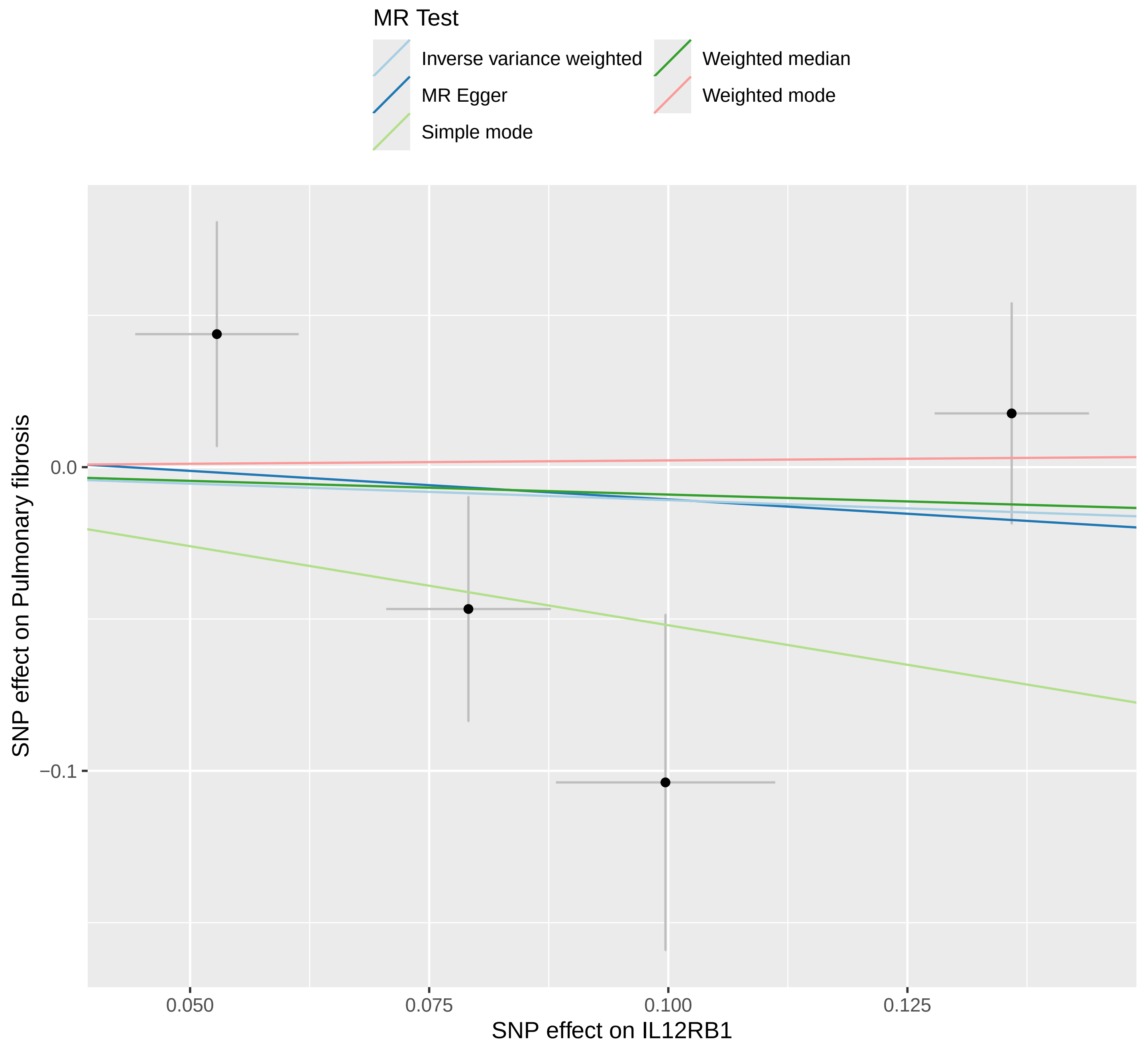

MR Test
Inverse variance weighted
MR Egger
Simple mode
Weighted median
Weighted mode
SNP effect on Pulmonary fibrosis
0.0
−0.1
0.050
0.075
0.100
0.125
SNP effect on IL12RB1

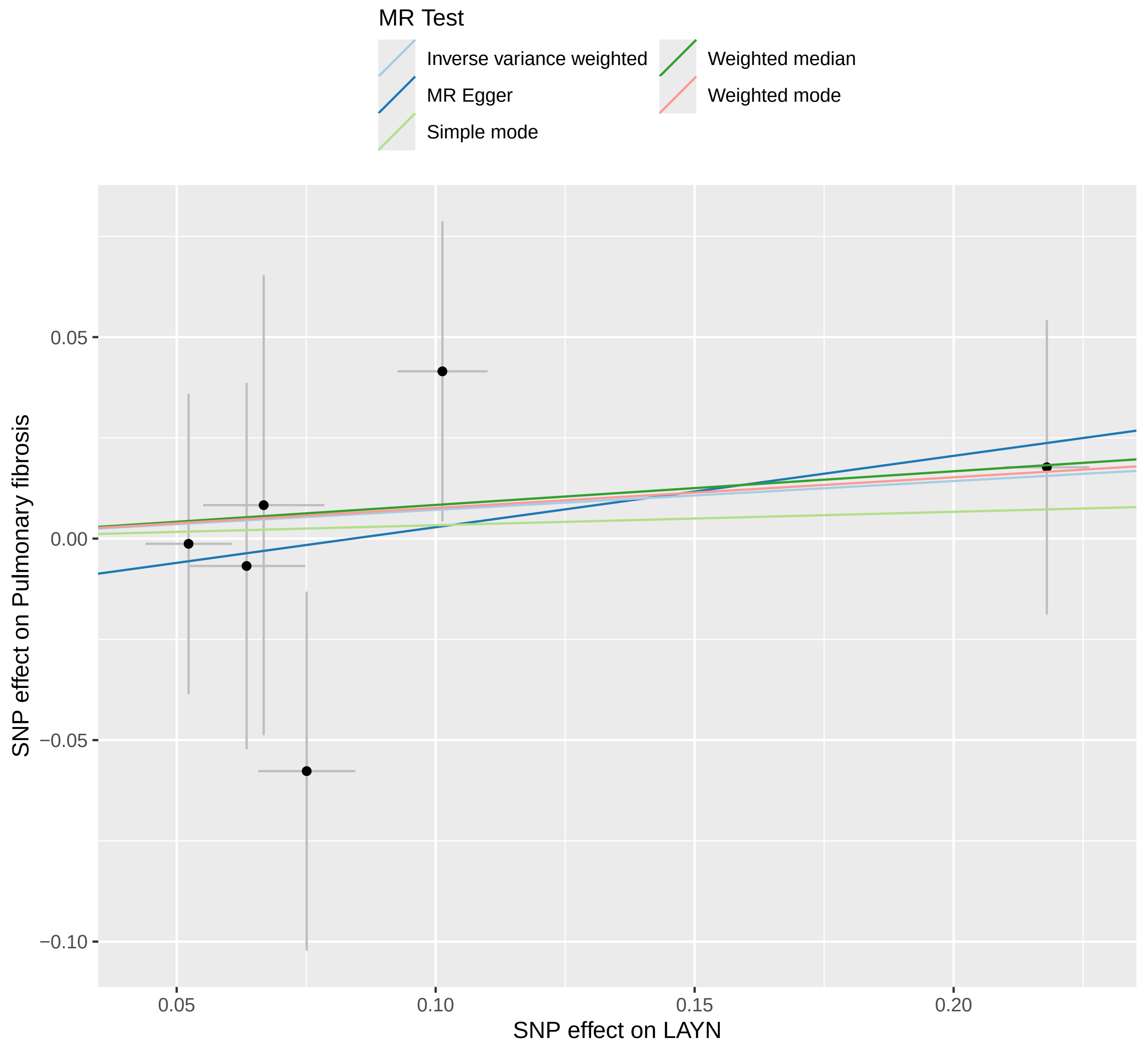

MR Test
Inverse variance weighted
MR Egger
Simple mode
Weighted median
Weighted mode
0.05
0.00
−0.05
−0.10
0.05
0.10
0.15
0.20
SNP effect on Pulmonary fibrosis
SNP effect on LAYN

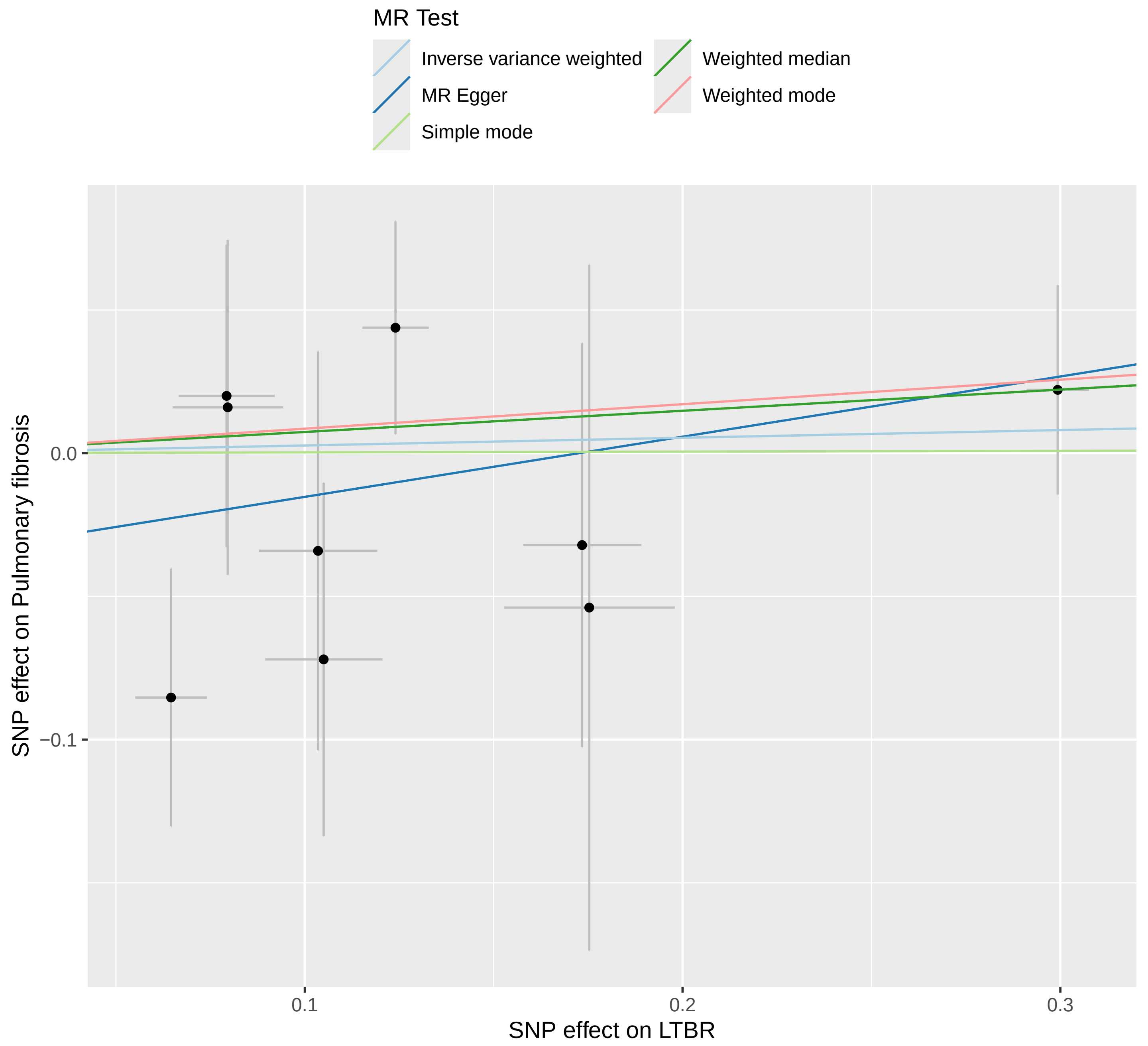

MR Test
Inverse variance weighted
MR Egger
Simple mode
Weighted median
Weighted mode
SNP effect on Pulmonary fibrosis
0.0
−0.1
0.1
0.2
0.3
SNP effect on LTBR

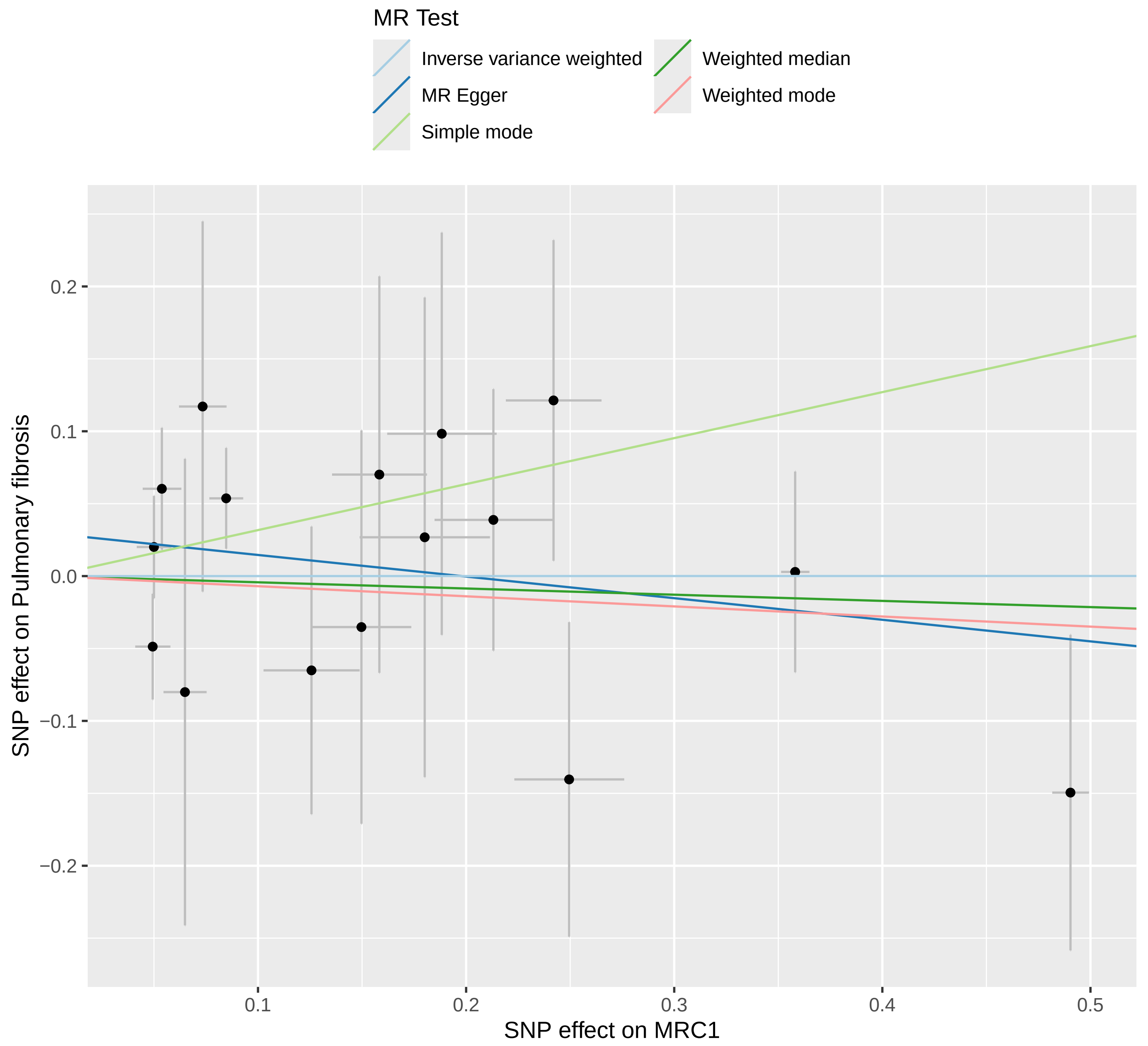
MR Test
Inverse variance weighted
MR Egger
Simple mode
Weighted median
Weighted mode
0.2
0.1
0.0
−0.1
−0.2
SNP effect on Pulmonary fibrosis
0.1
0.2
0.3
0.4
0.5
SNP effect on MRC1

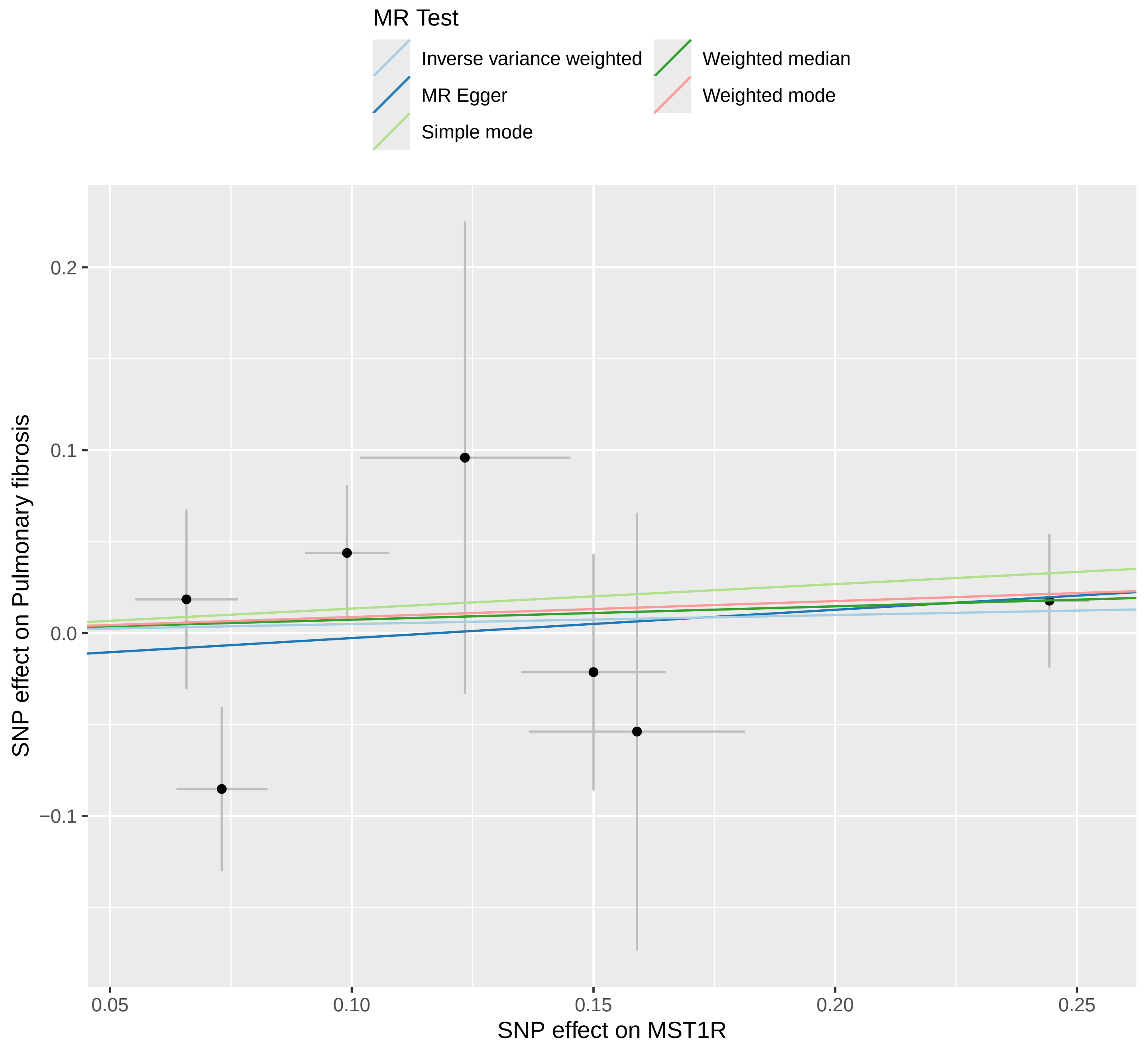
MR Test
Inverse variance weighted
MR Egger
Simple mode
Weighted median
Weighted mode
SNP effect on Pulmonary fibrosis
0.2
0.1
0.0
−0.1
0.05
0.10
0.15
0.20
0.25
SNP effect on MST1R

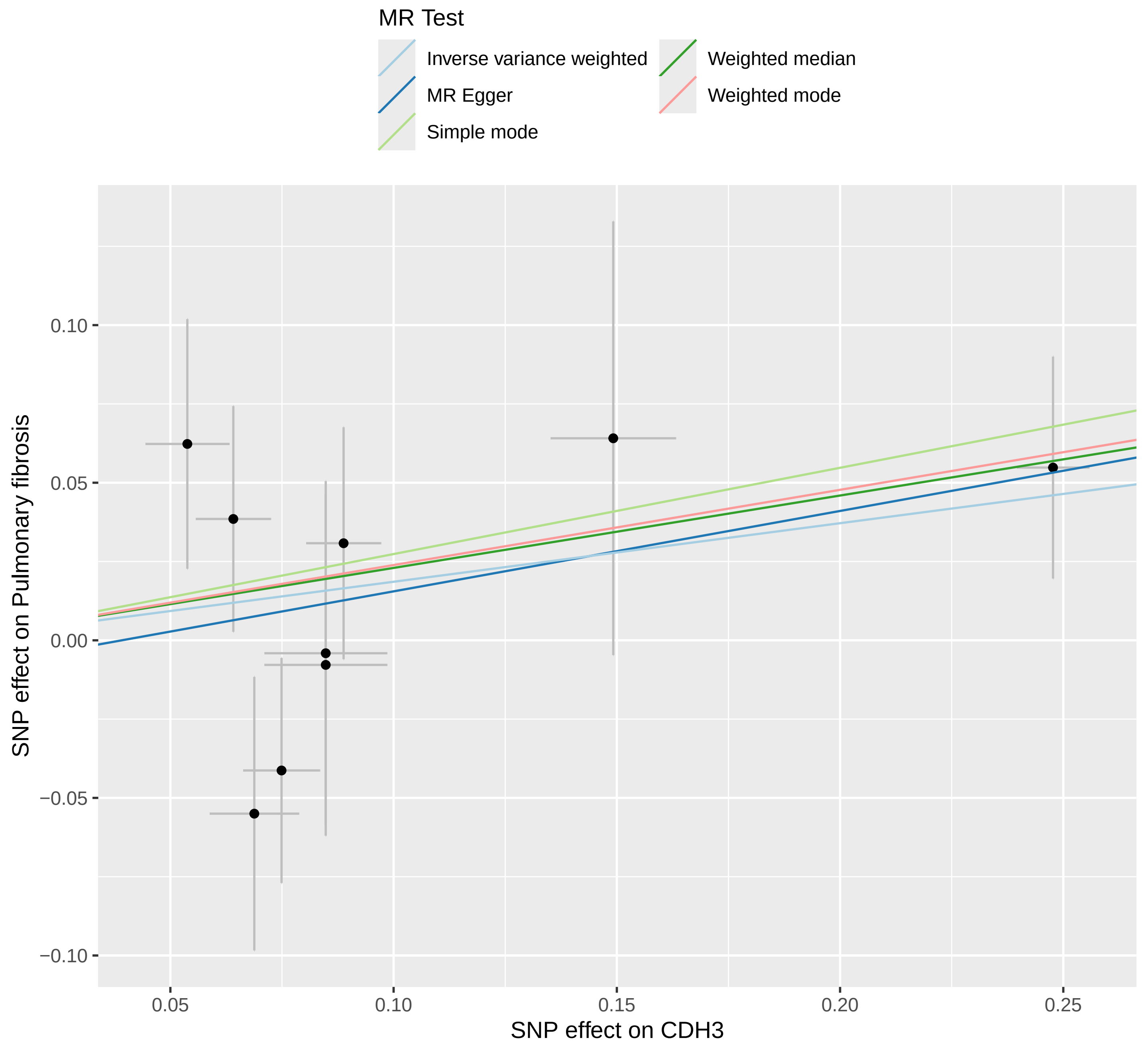

MR Test
Inverse variance weighted
MR Egger
Simple mode
Weighted median
Weighted mode
SNP effect on Pulmonary fibrosis
0.10
0.05
0.00
−0.05
−0.10
0.05
0.10
0.15
0.20
0.25
SNP effect on CDH3

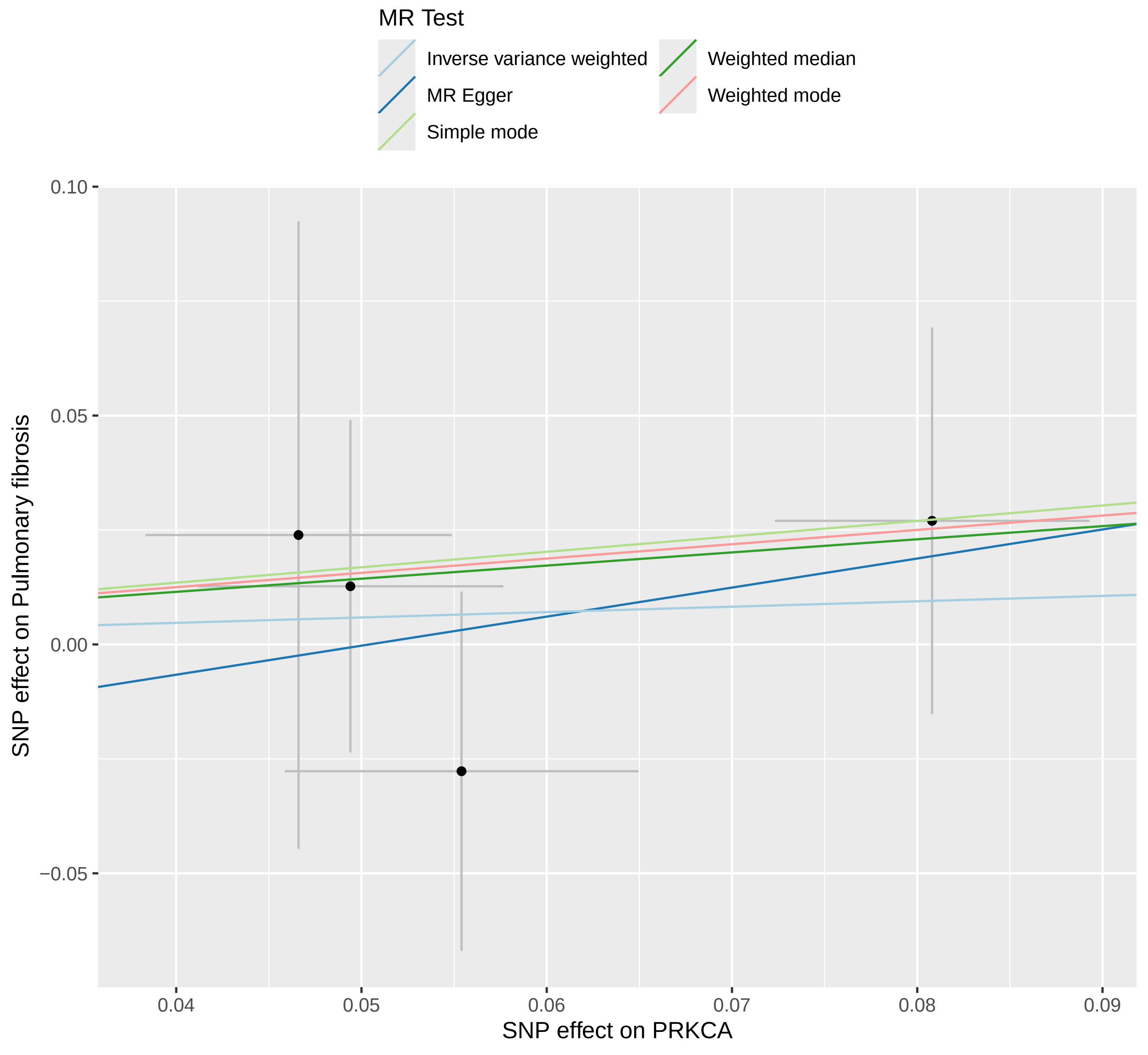
MR Test
Inverse variance weighted
MR Egger
Simple mode
Weighted median
Weighted mode
0.10
0.05
0.00
−0.05
SNP effect on Pulmonary fibrosis
0.04
0.05
0.06
0.07
0.08
0.09
SNP effect on PRKCA

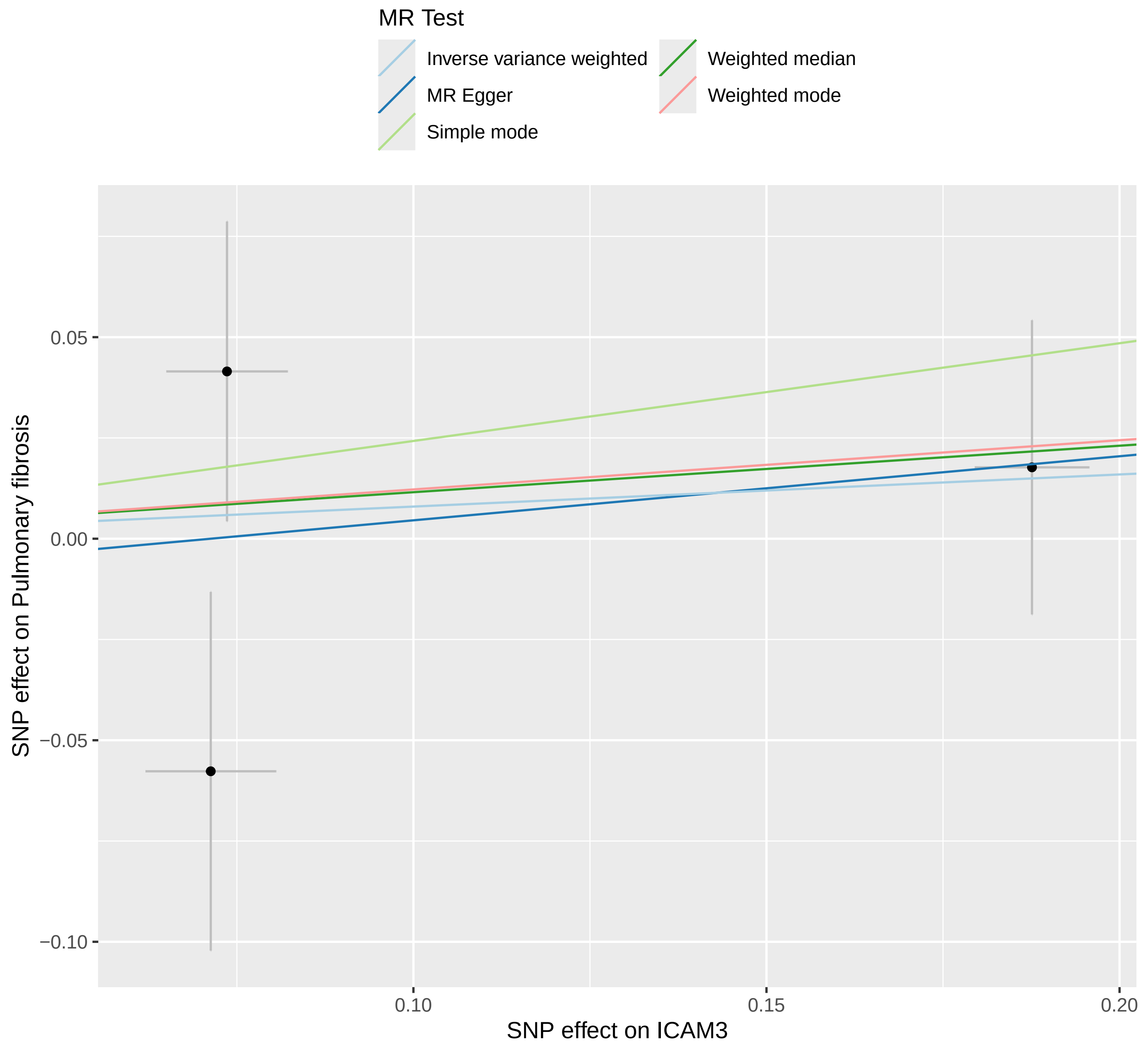
MR Test
Inverse variance weighted
MR Egger
Simple mode
Weighted median
Weighted mode
0.05
0.00
−0.05
−0.10
0.10
0.15
0.20
SNP effect on Pulmonary fibrosis
SNP effect on ICAM3

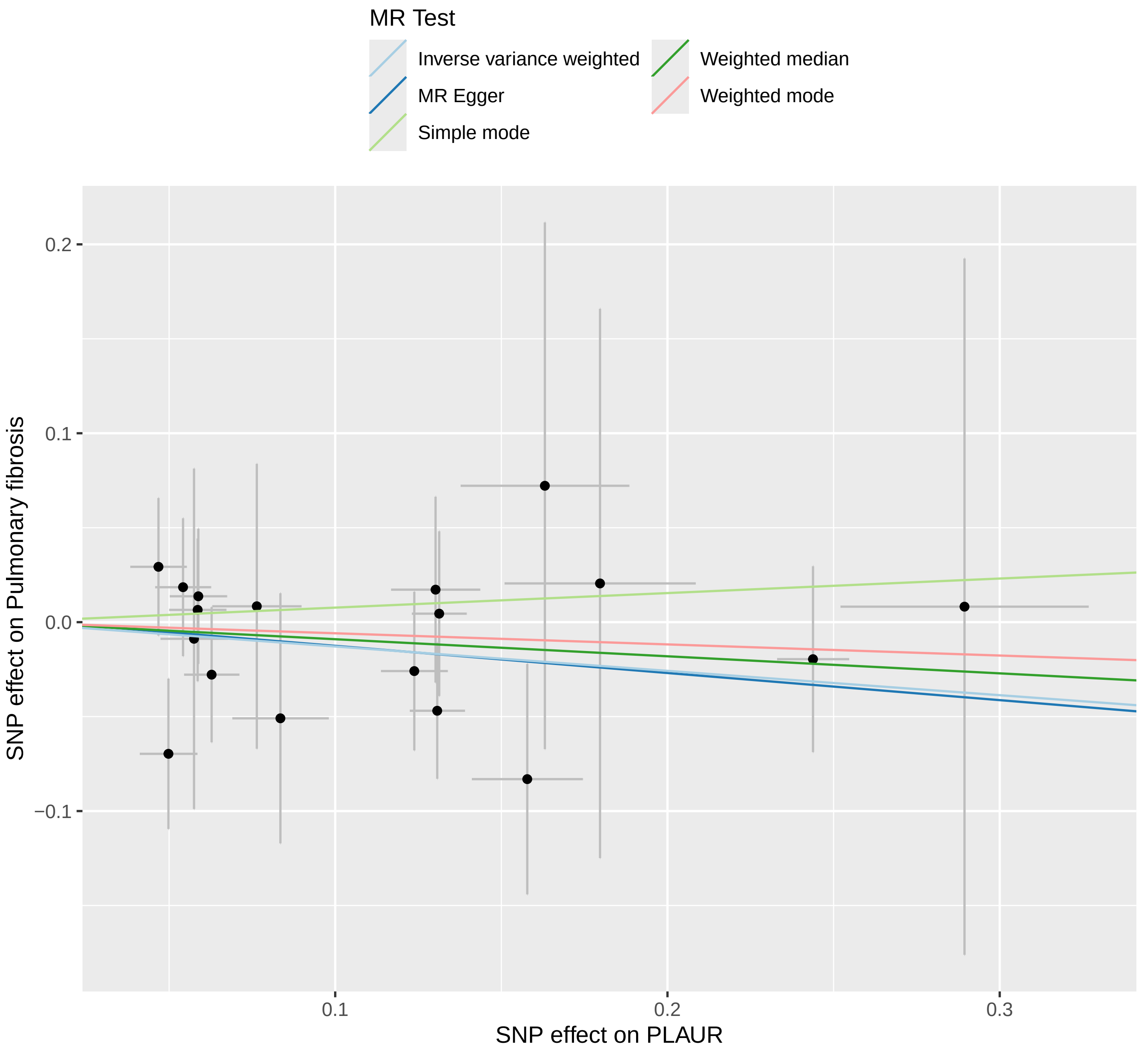
MR Test
Inverse variance weighted
MR Egger
Simple mode
Weighted median
Weighted mode
0.2
0.1
0.0
−0.1
SNP effect on Pulmonary fibrosis
0.1
0.2
0.3
SNP effect on PLAUR

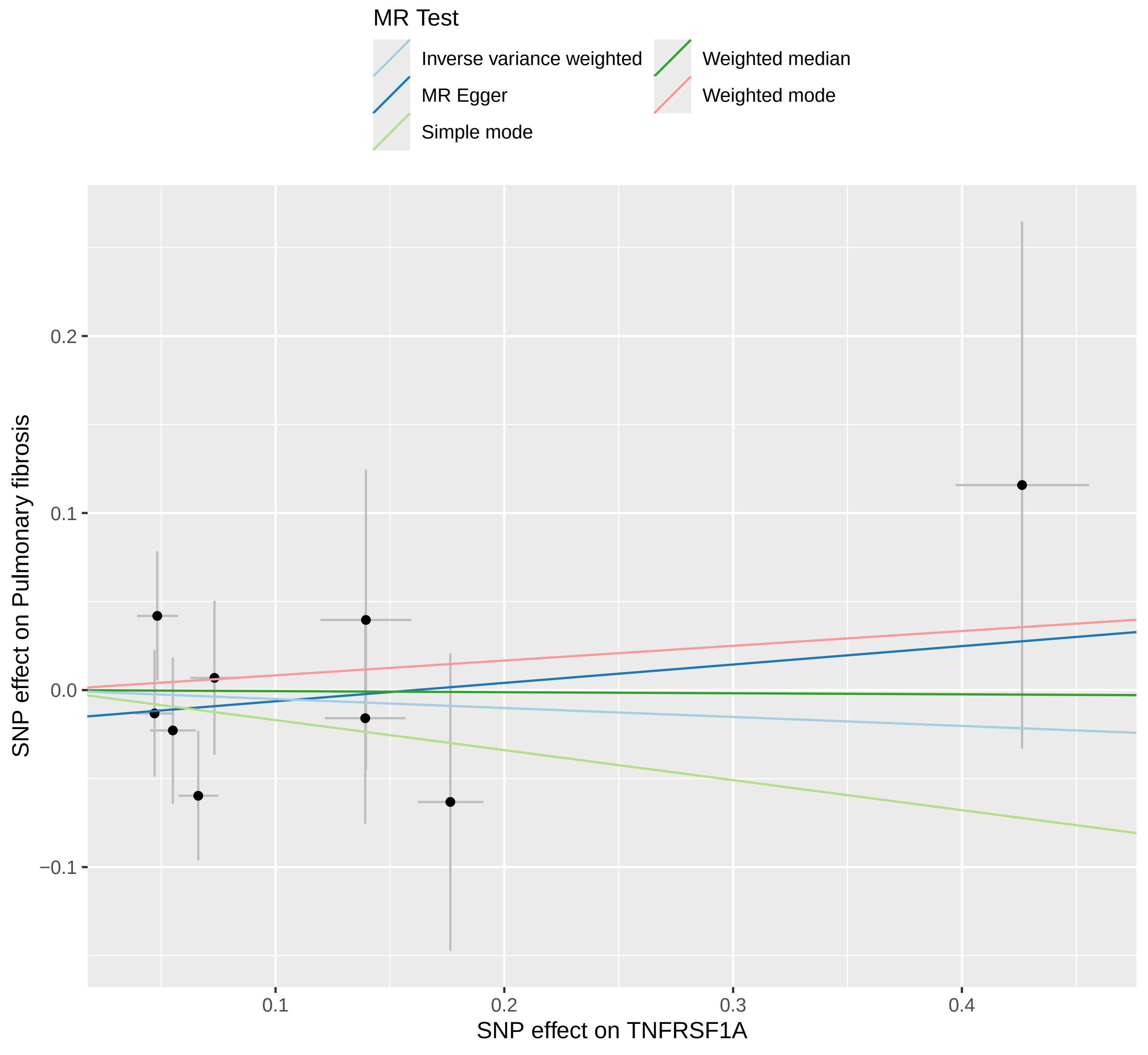
MR Test
Inverse variance weighted
MR Egger
Simple mode
Weighted median
Weighted mode
SNP effect on Pulmonary fibrosis
0.2
0.1
0.0
−0.1
0.1
0.2
0.3
0.4
SNP effect on TNFRSF1A

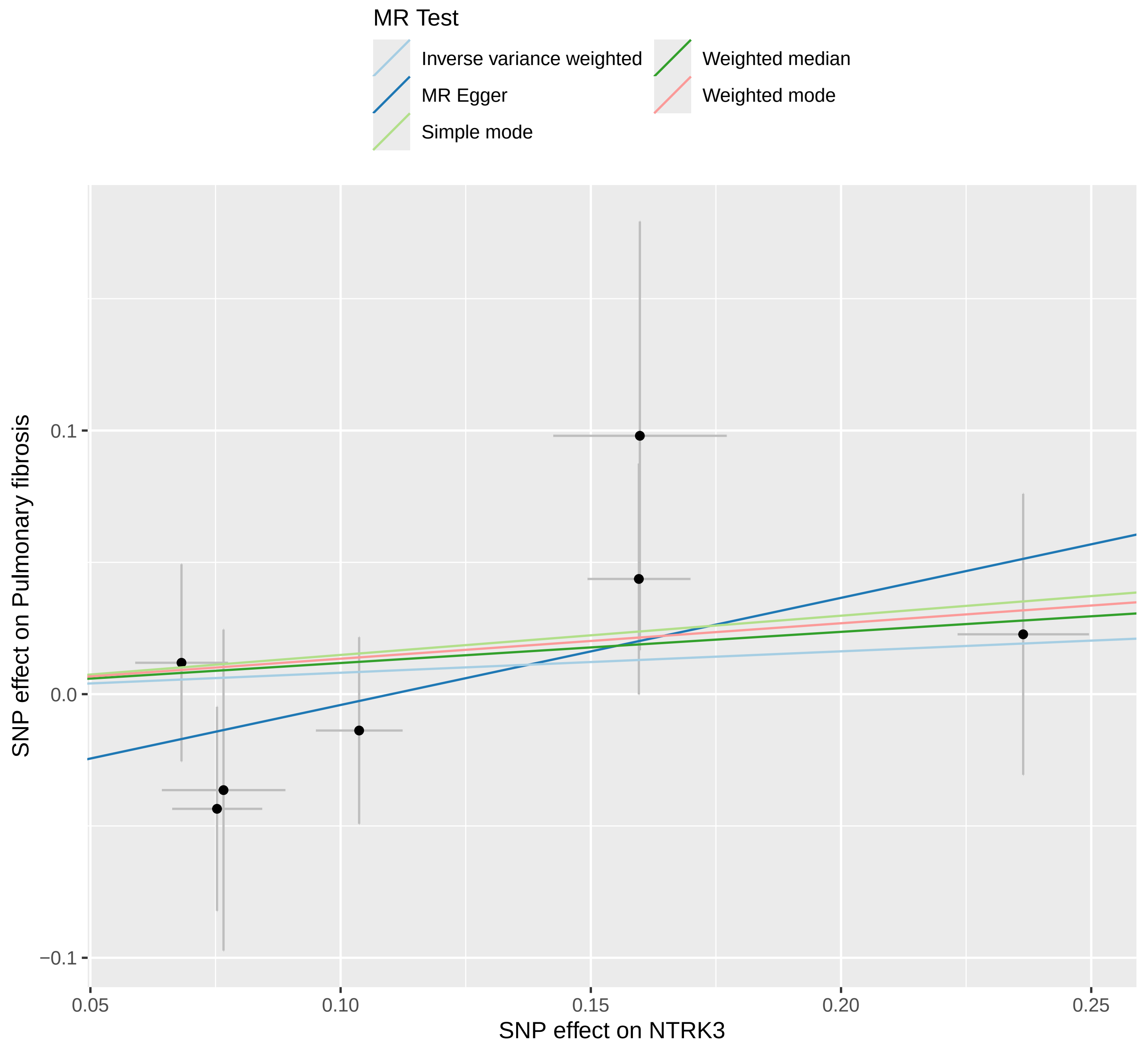

MR Test
Inverse variance weighted
MR Egger
Simple mode
Weighted median
Weighted mode
SNP effect on Pulmonary fibrosis
0.1
0.0
−0.1
0.05
0.10
0.15
0.20
0.25
SNP effect on NTRK3

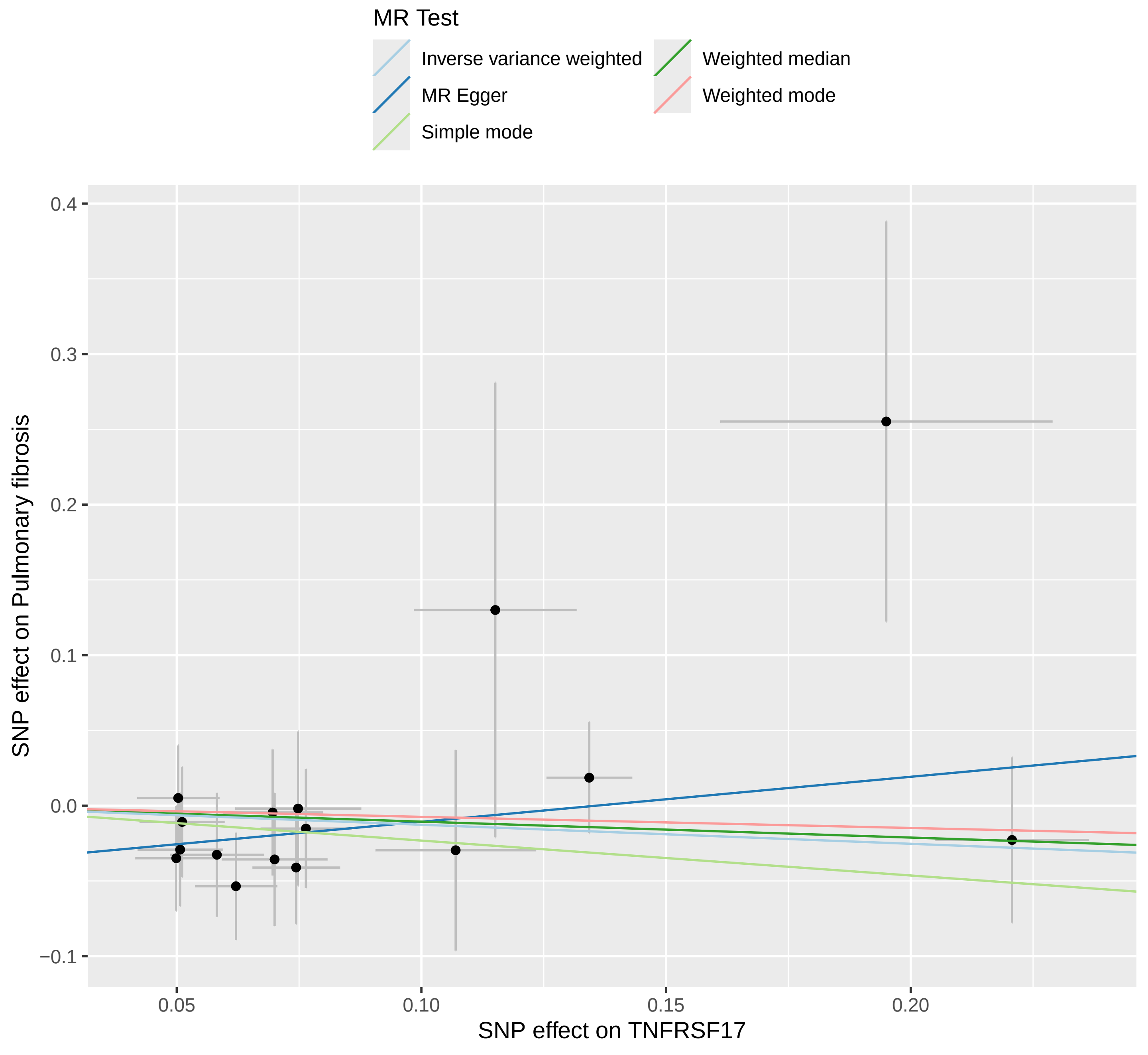

MR Test
Inverse variance weighted
MR Egger
Simple mode
Weighted median
Weighted mode
0.4
0.3
0.2
0.1
0.0
−0.1
0.05
0.10
0.15
0.20
SNP effect on Pulmonary fibrosis
SNP effect on TNFRSF17

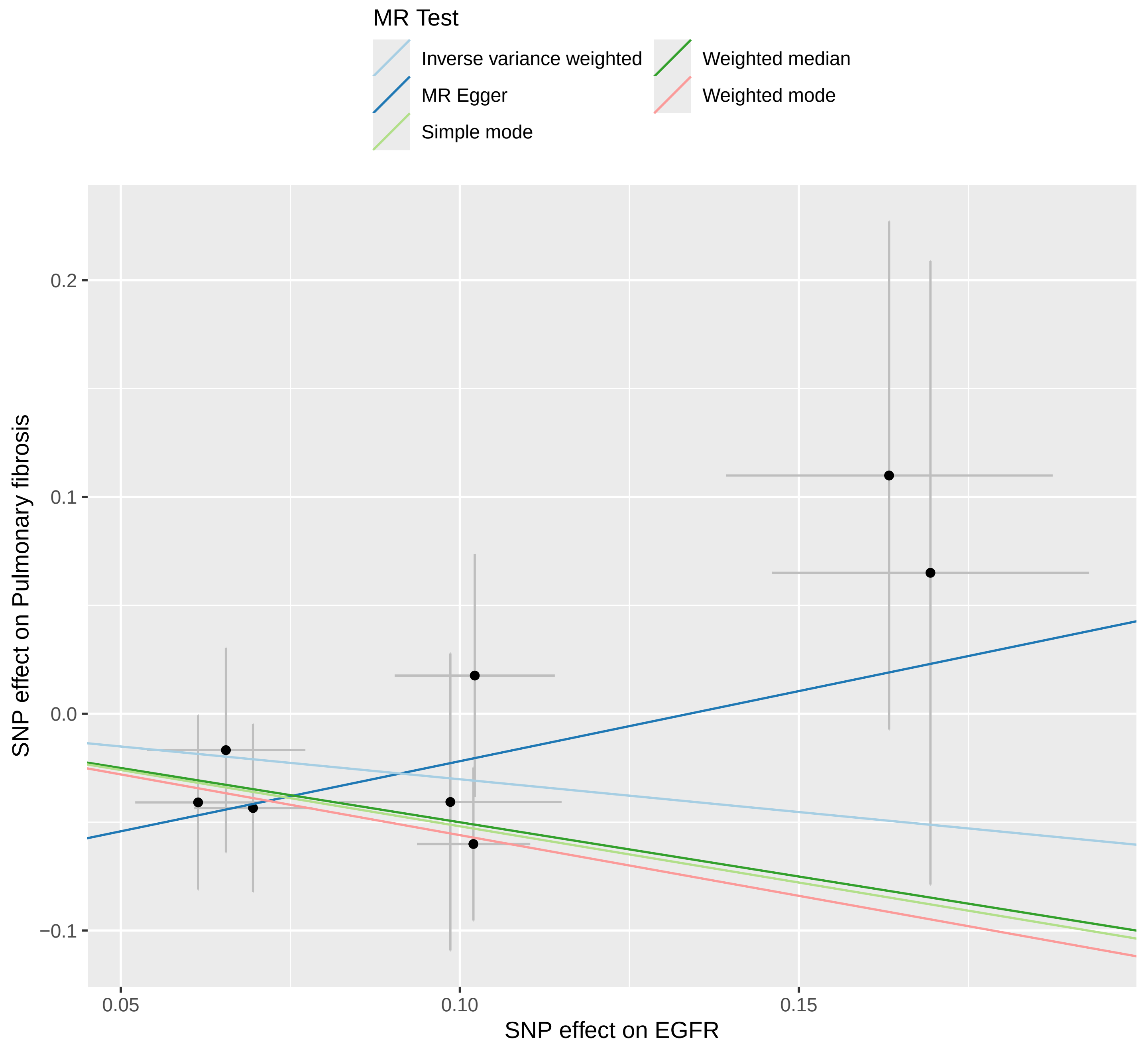

MR Test
Inverse variance weighted
MR Egger
Simple mode
Weighted median
Weighted mode
SNP effect on Pulmonary fibrosis
0.2
0.1
0.0
−0.1
0.05
0.10
0.15
SNP effect on EGFR

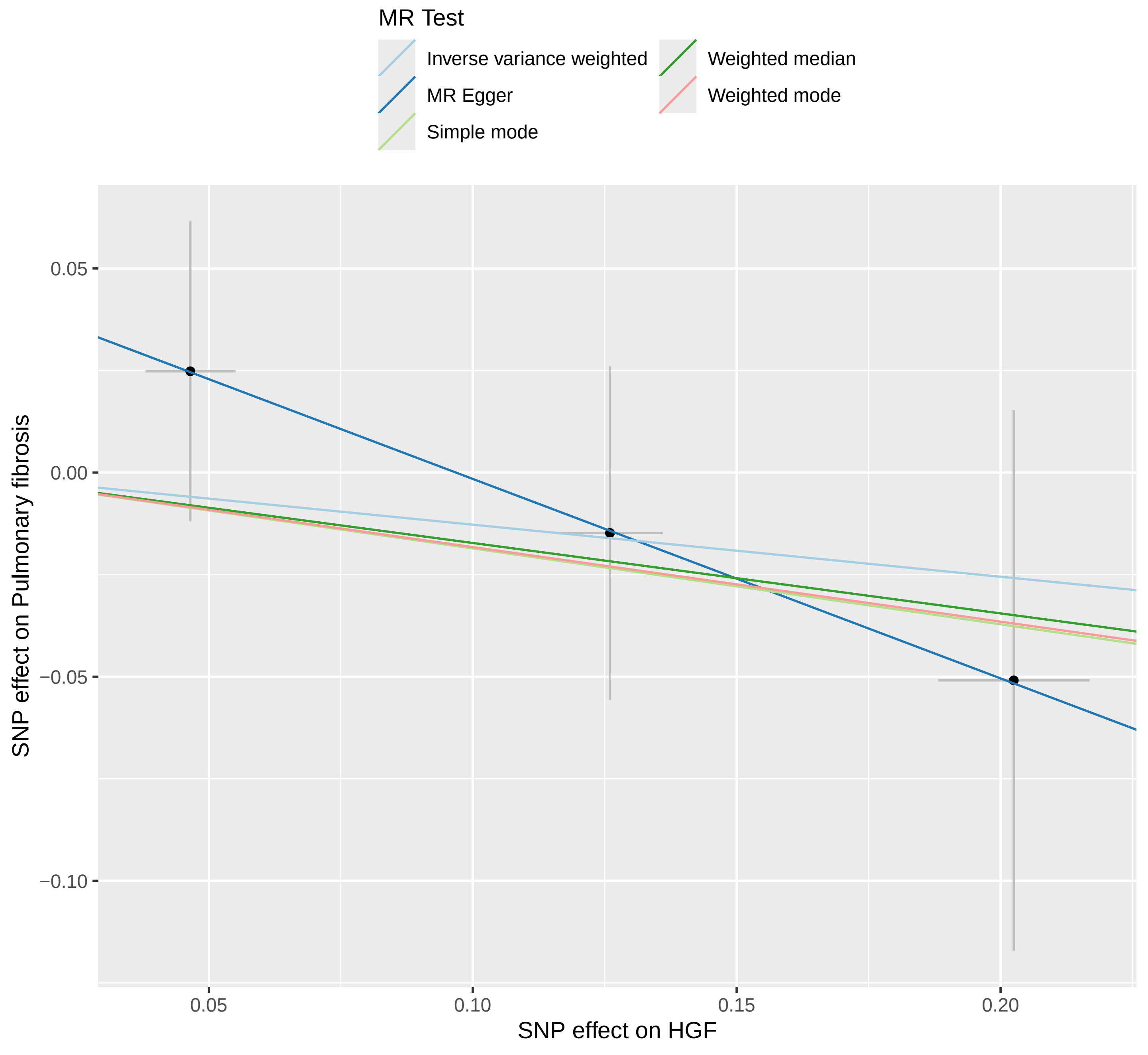
MR Test
Inverse variance weighted
MR Egger
Simple mode
Weighted median
Weighted mode
0.05
0.00
−0.05
−0.10
SNP effect on Pulmonary fibrosis
0.05
0.10
0.15
0.20
SNP effect on HGF

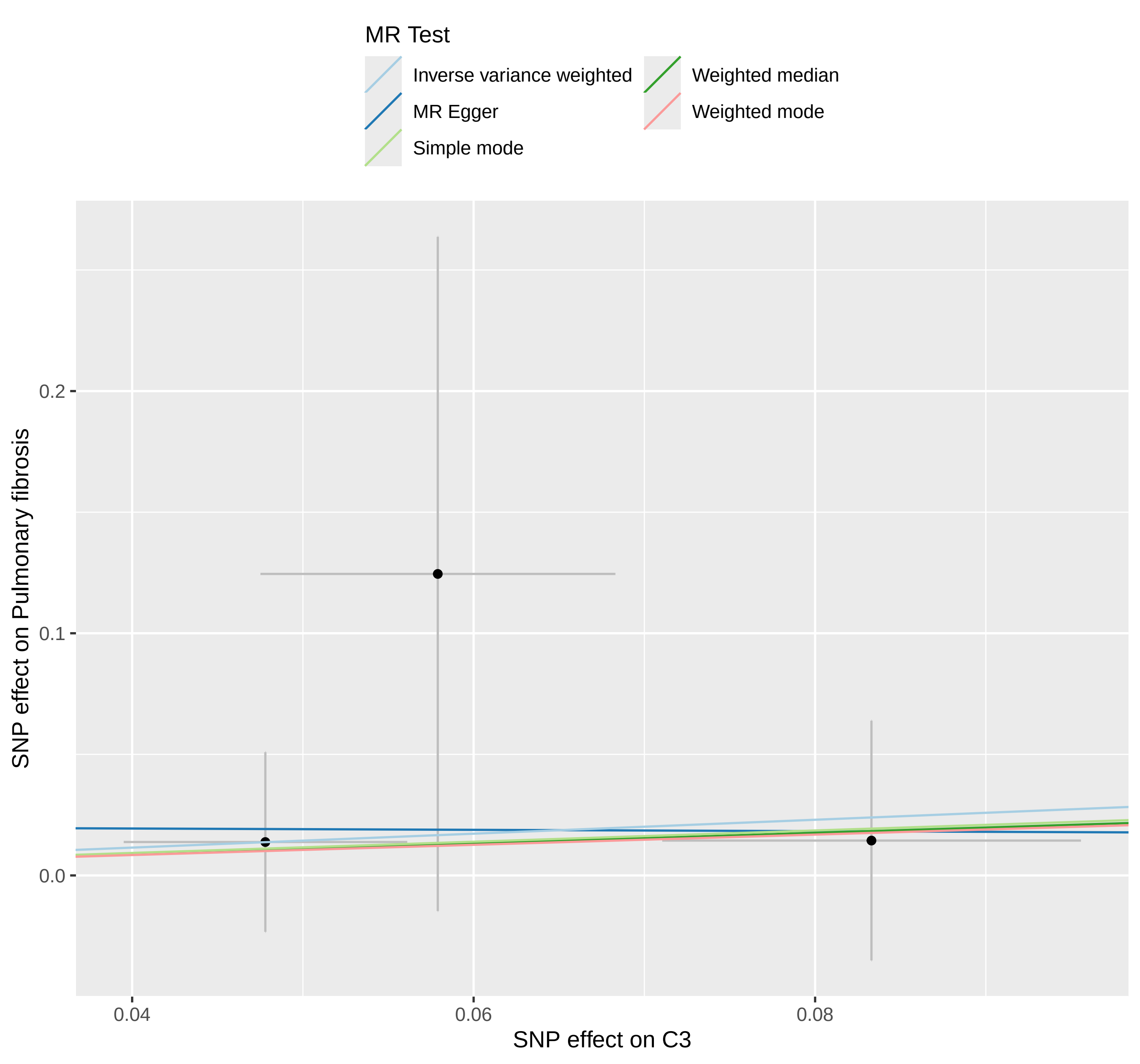

MR Test
Inverse variance weighted
MR Egger
Simple mode
Weighted median
Weighted mode
0.2
0.1
0.0
SNP effect on Pulmonary fibrosis
0.04
0.06
0.08
SNP effect on C3

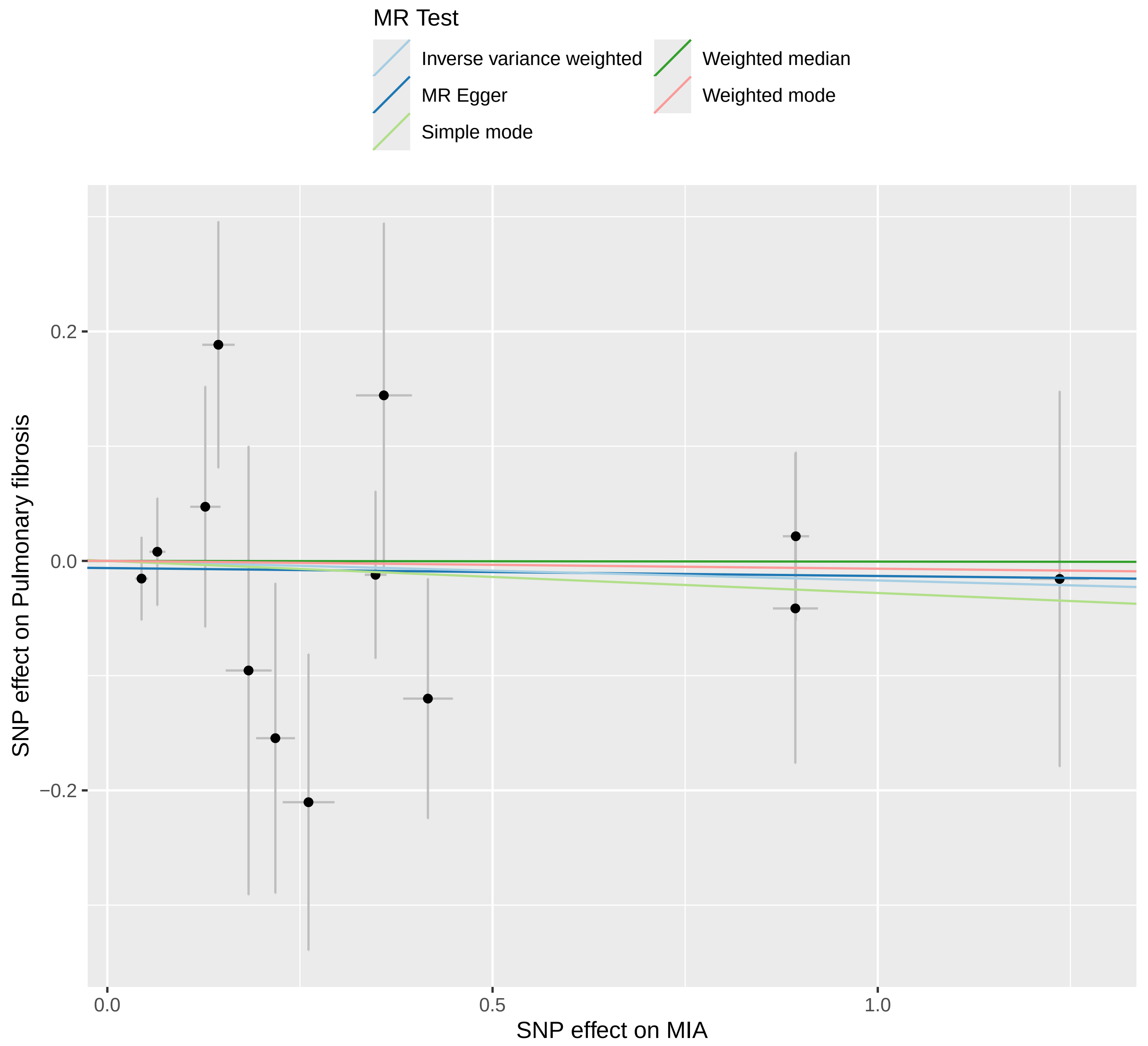

MR Test
Inverse variance weighted
MR Egger
Simple mode
Weighted median
Weighted mode
SNP effect on Pulmonary fibrosis
0.2
0.0
−0.2
0.0
0.5
1.0
SNP effect on MIA

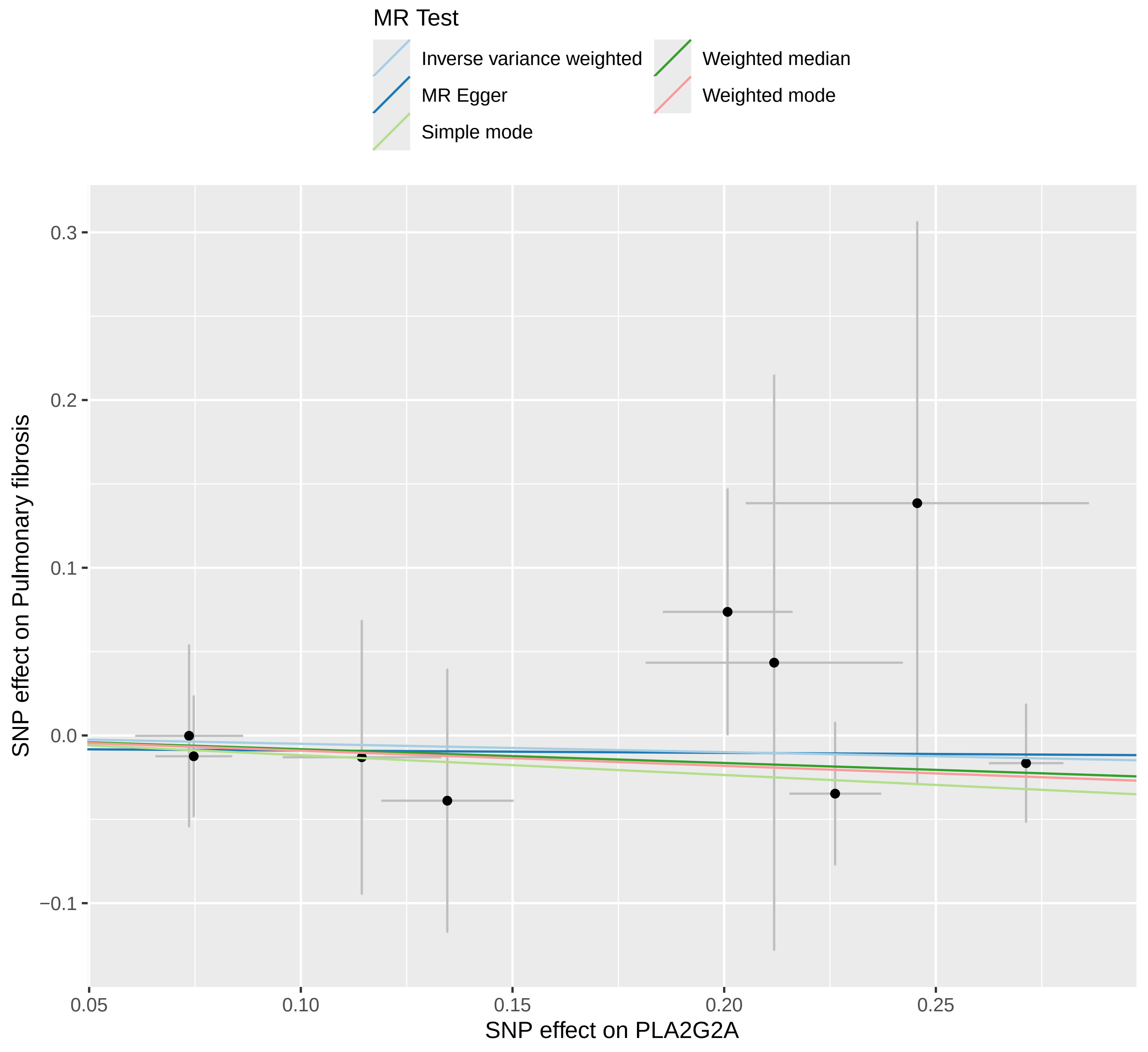
MR Test
Inverse variance weighted
MR Egger
Simple mode
Weighted median
Weighted mode
0.3
0.2
0.1
0.0
−0.1
SNP effect on Pulmonary fibrosis
0.05
0.10
0.15
0.20
0.25
SNP effect on PLA2G2A

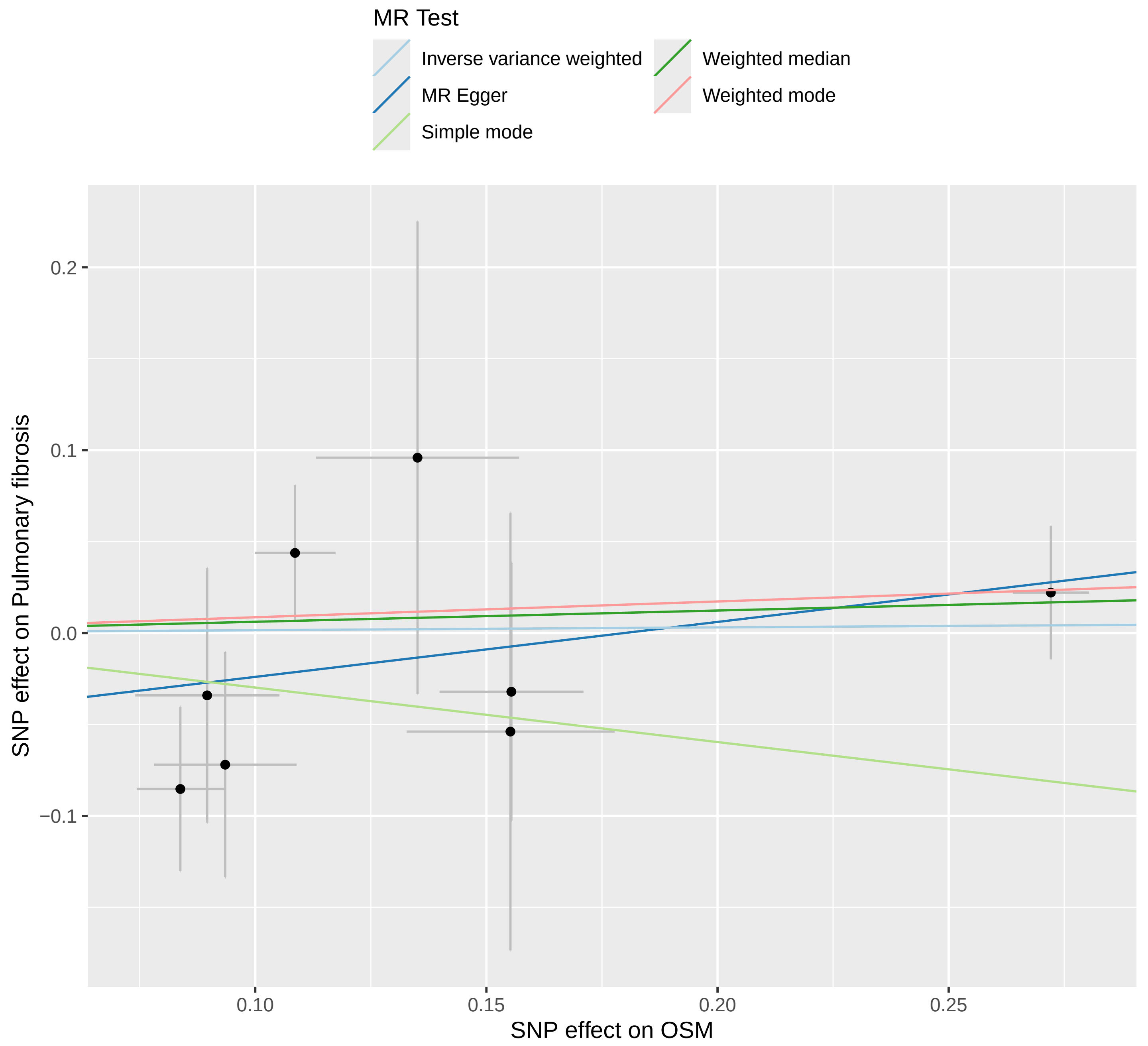
MR Test
Inverse variance weighted
MR Egger
Simple mode
Weighted median
Weighted mode
0.2
0.1
0.0
−0.1
0.10
0.15
0.20
0.25
SNP effect on Pulmonary fibrosis
SNP effect on OSM

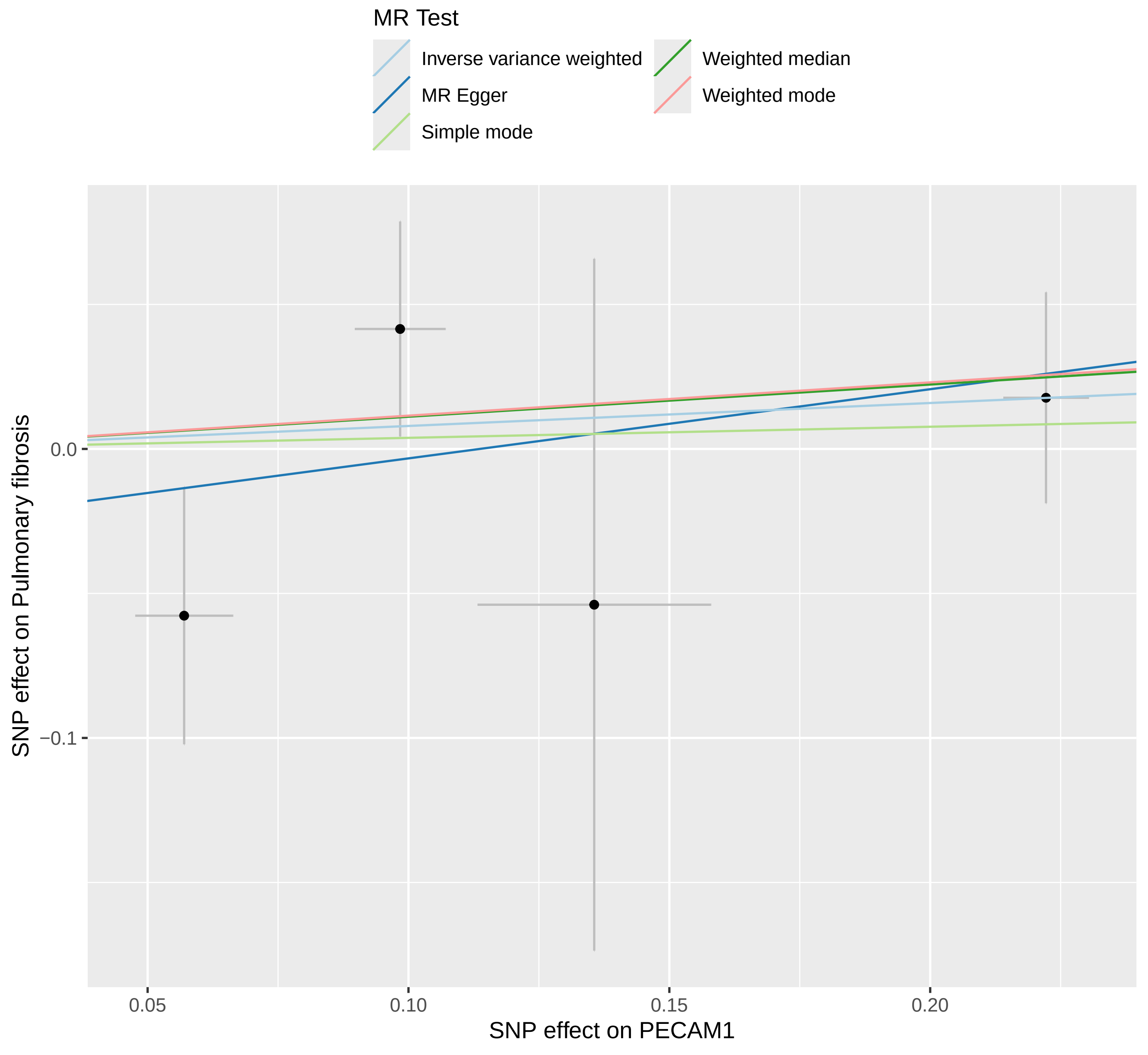

MR Test
Inverse variance weighted
MR Egger
Simple mode
Weighted median
Weighted mode
SNP effect on Pulmonary fibrosis
0.0
−0.1
0.05
0.10
0.15
0.20
SNP effect on PECAM1

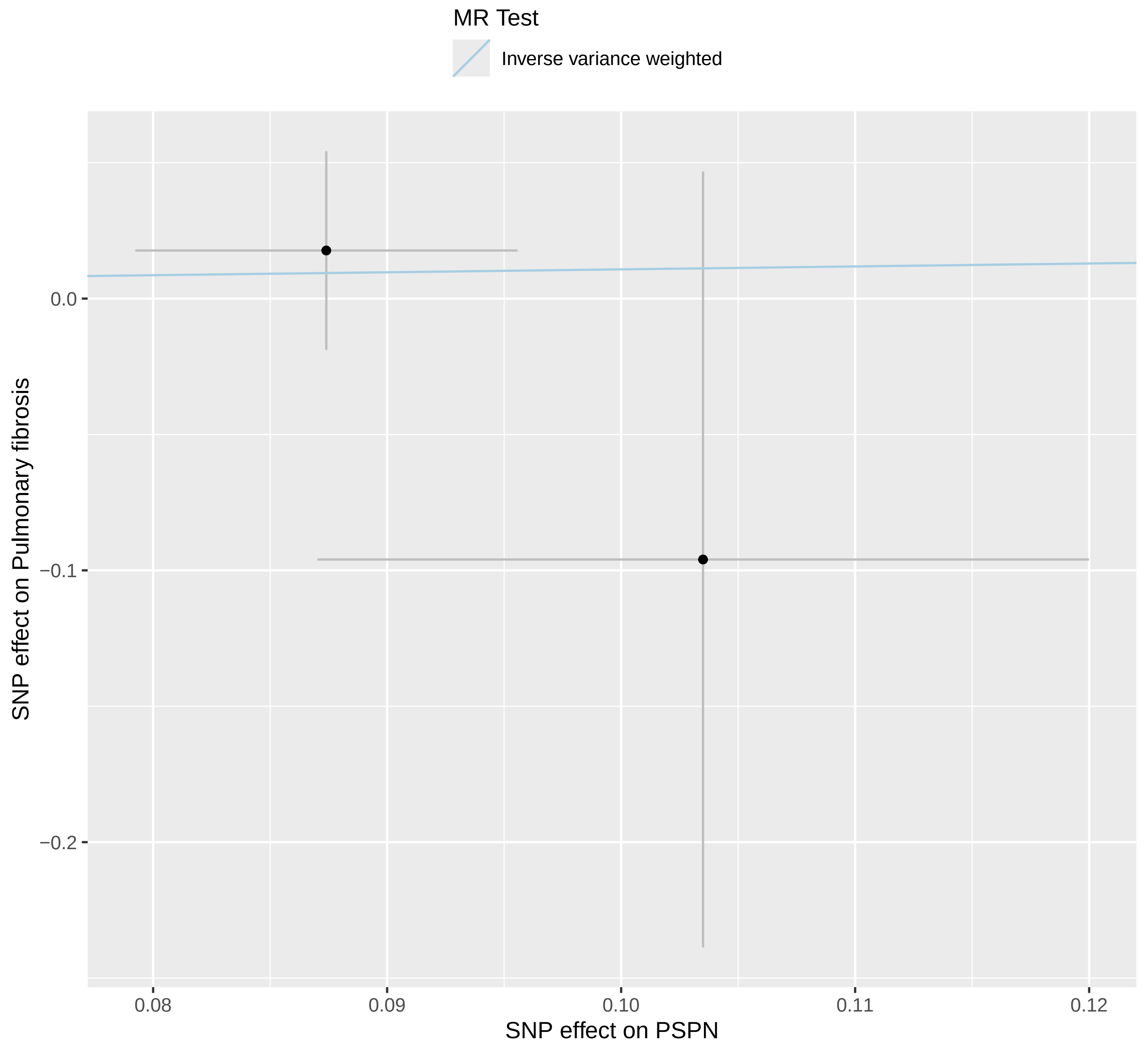
MR Test
Inverse variance weighted
0.0
−0.1
−0.2
SNP effect on Pulmonary fibrosis
0.08
0.09
0.10
0.11
0.12
SNP effect on PSPN

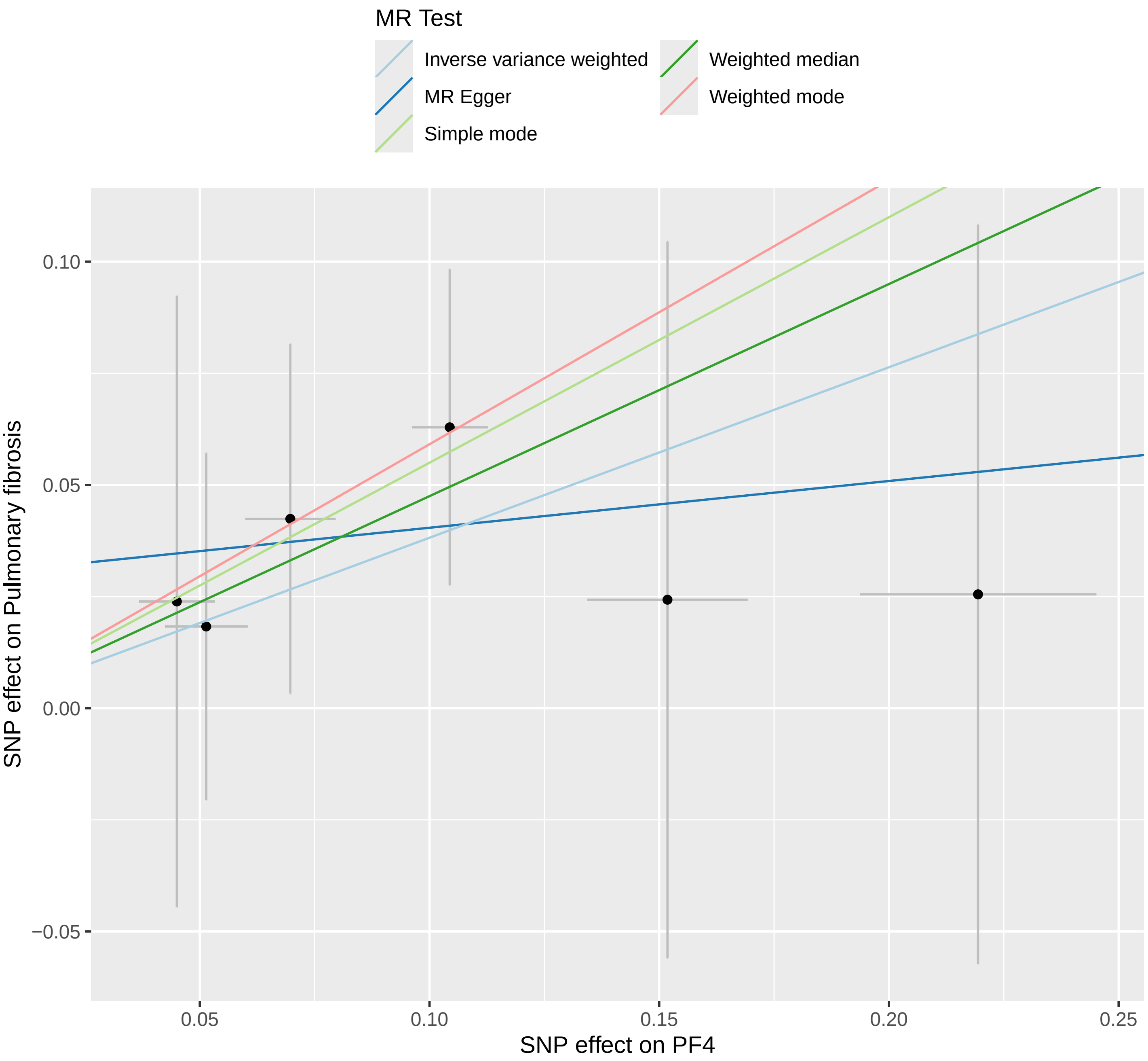
MR Test
Inverse variance weighted
MR Egger
Simple mode
Weighted median
Weighted mode
0.10
0.05
0.00
−0.05
SNP effect on Pulmonary fibrosis
0.05
0.10
0.15
0.20
0.25
SNP effect on PF4

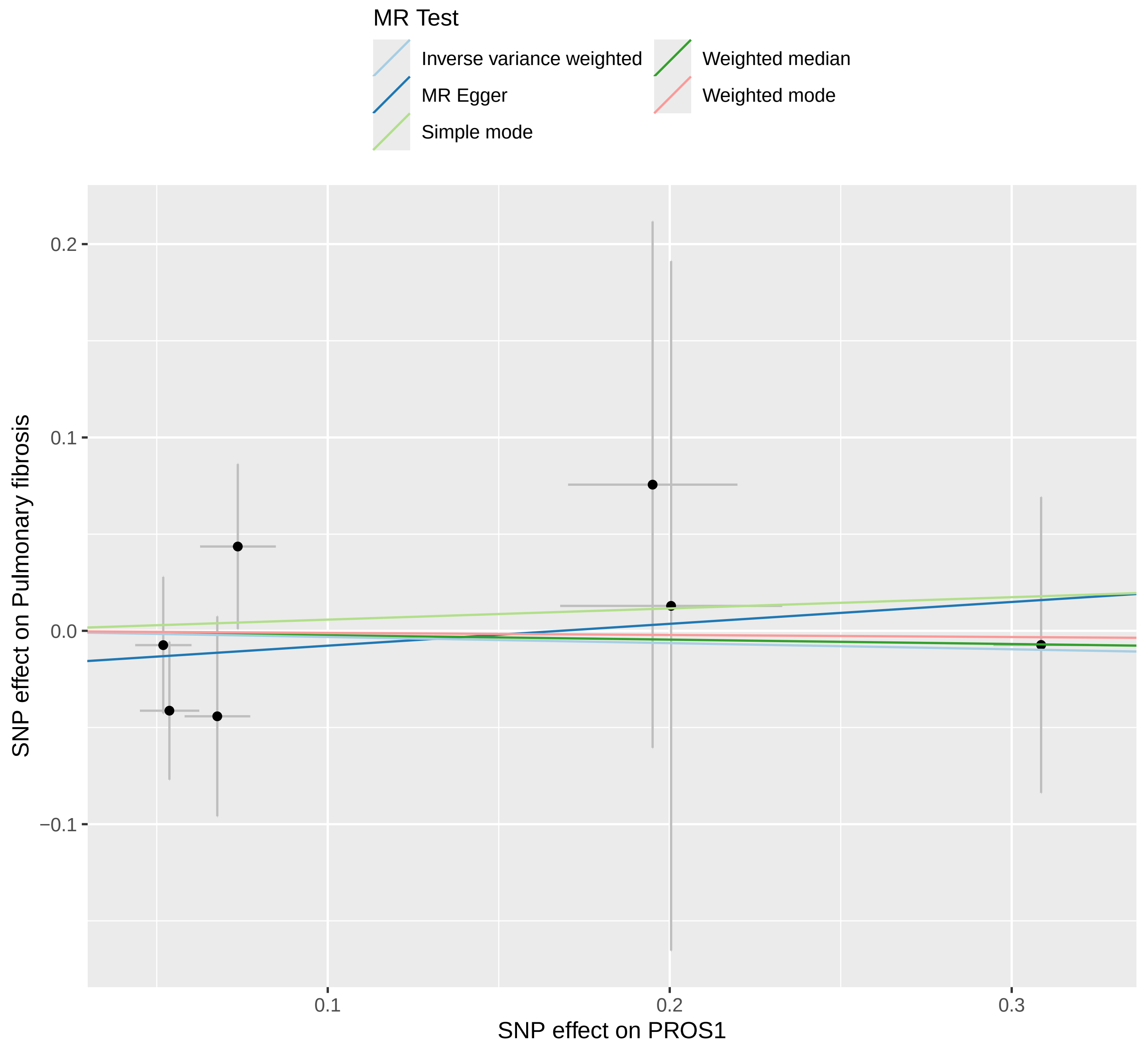
MR Test
Inverse variance weighted
MR Egger
Simple mode
Weighted median
Weighted mode
0.2
0.1
0.0
−0.1
0.1
0.2
0.3
SNP effect on Pulmonary fibrosis
SNP effect on PROS1

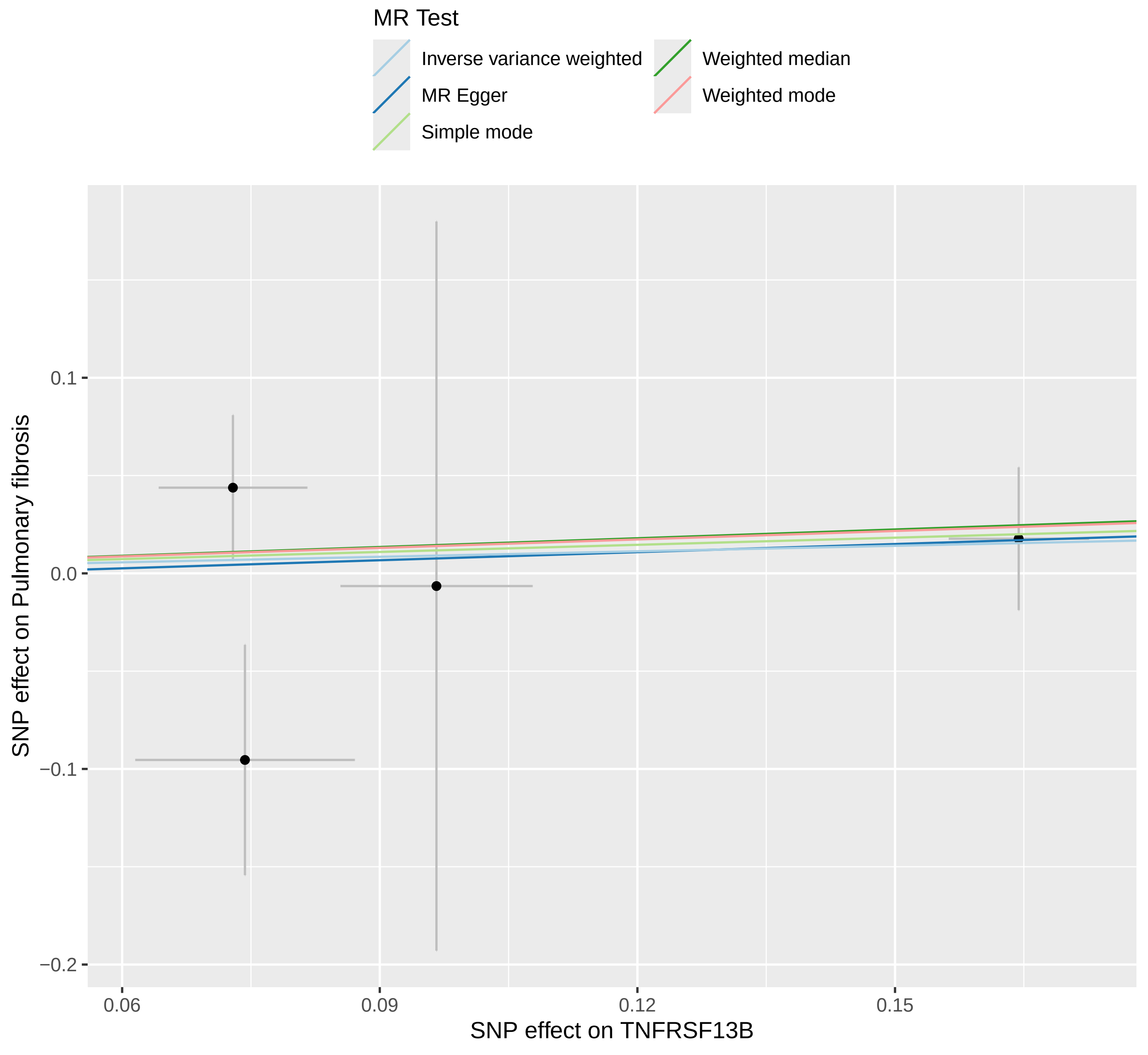

MR Test
Inverse variance weighted
MR Egger
Simple mode
Weighted median
Weighted mode
0.1
0.0
−0.1
−0.2
0.06
0.09
0.12
0.15
SNP effect on TNFRSF13B
SNP effect on Pulmonary fibrosis

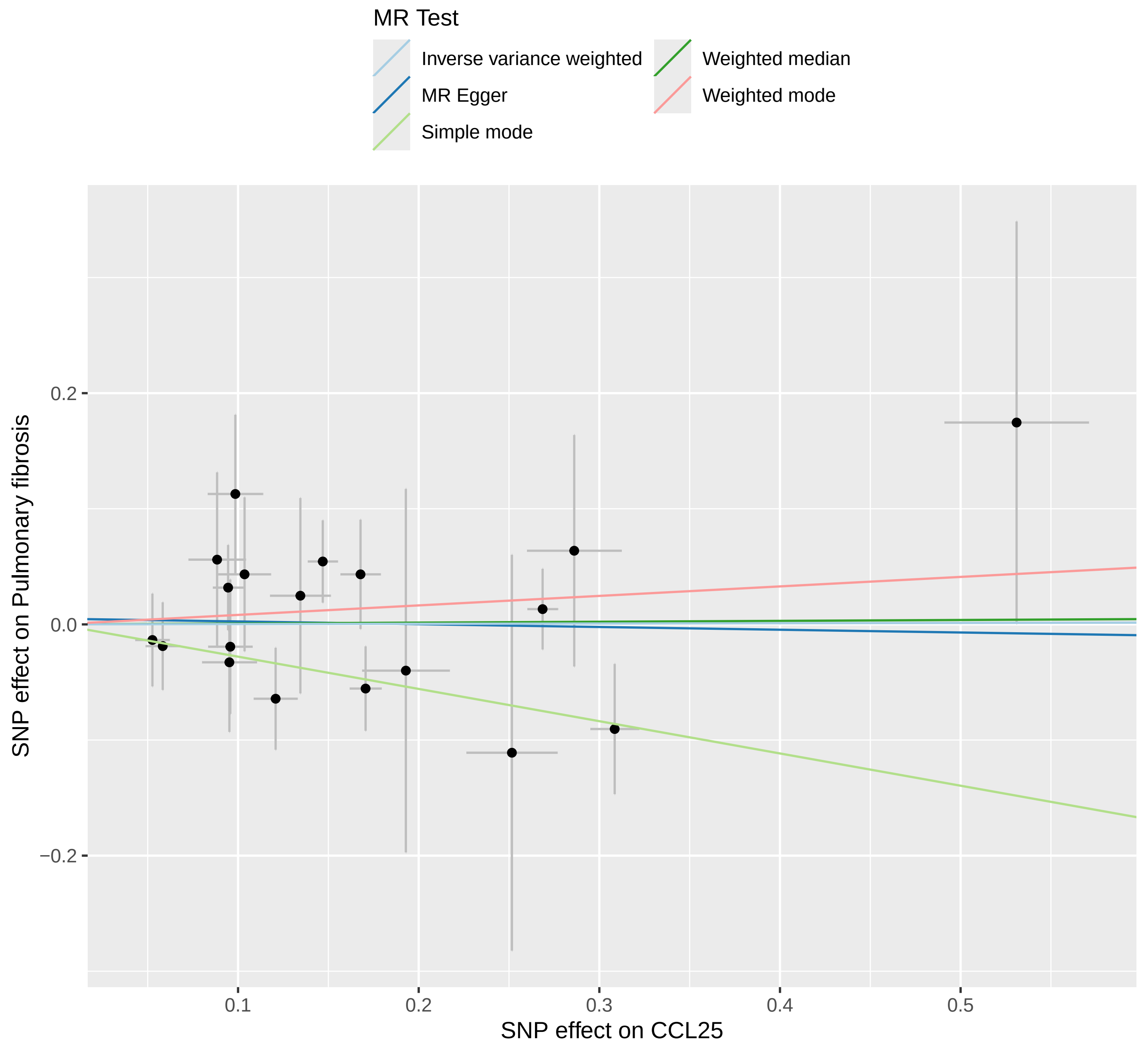
MR Test
Inverse variance weighted
MR Egger
Simple mode
Weighted median
Weighted mode
0.2
0.0
−0.2
SNP effect on Pulmonary fibrosis
0.1
0.2
0.3
0.4
0.5
SNP effect on CCL25

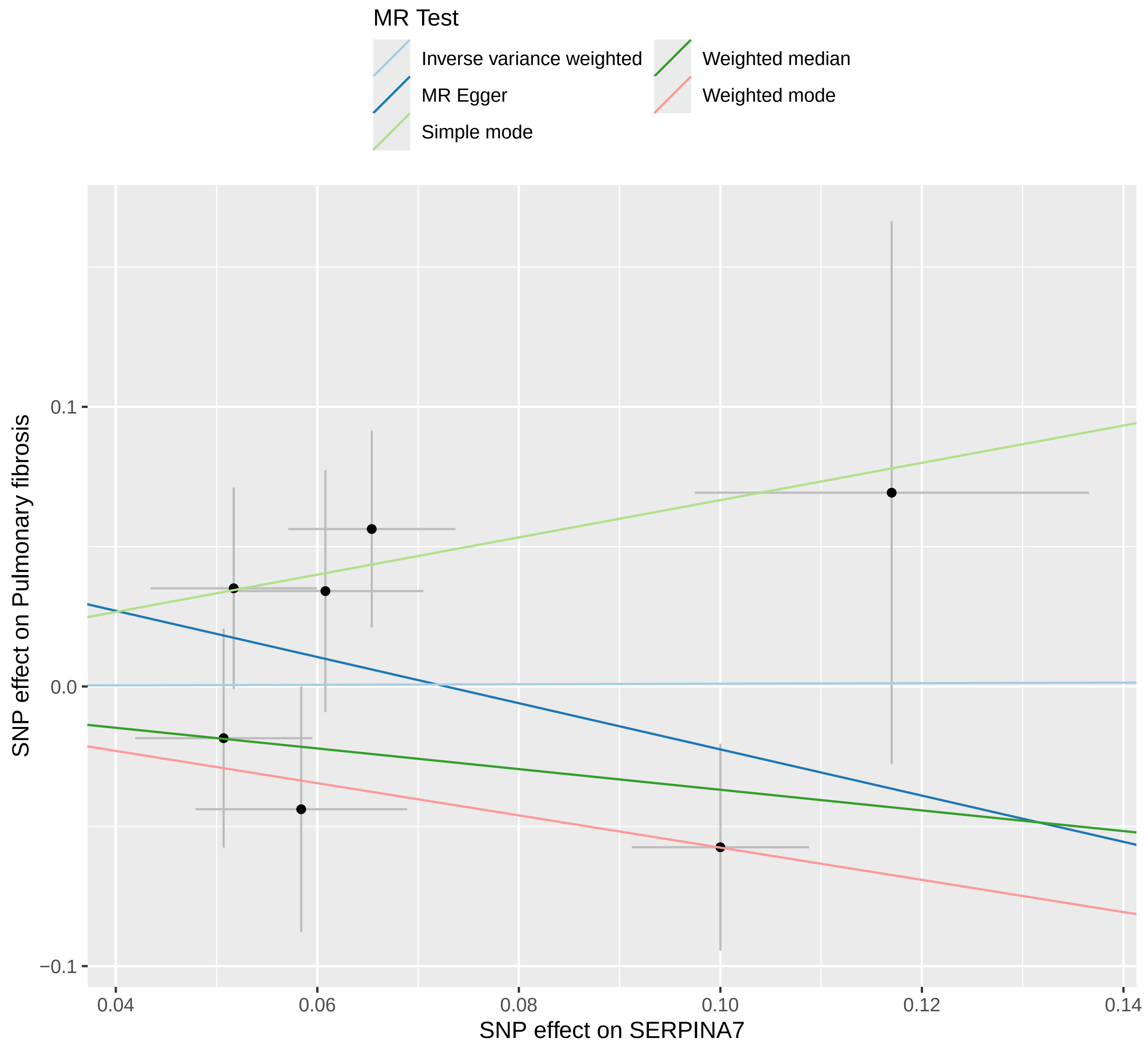

MR Test
Inverse variance weighted
MR Egger
Simple mode
Weighted median
Weighted mode
0.1
0.0
−0.1
0.04
0.06
0.08
0.10
0.12
0.14
SNP effect on SERPINA7
SNP effect on Pulmonary fibrosis

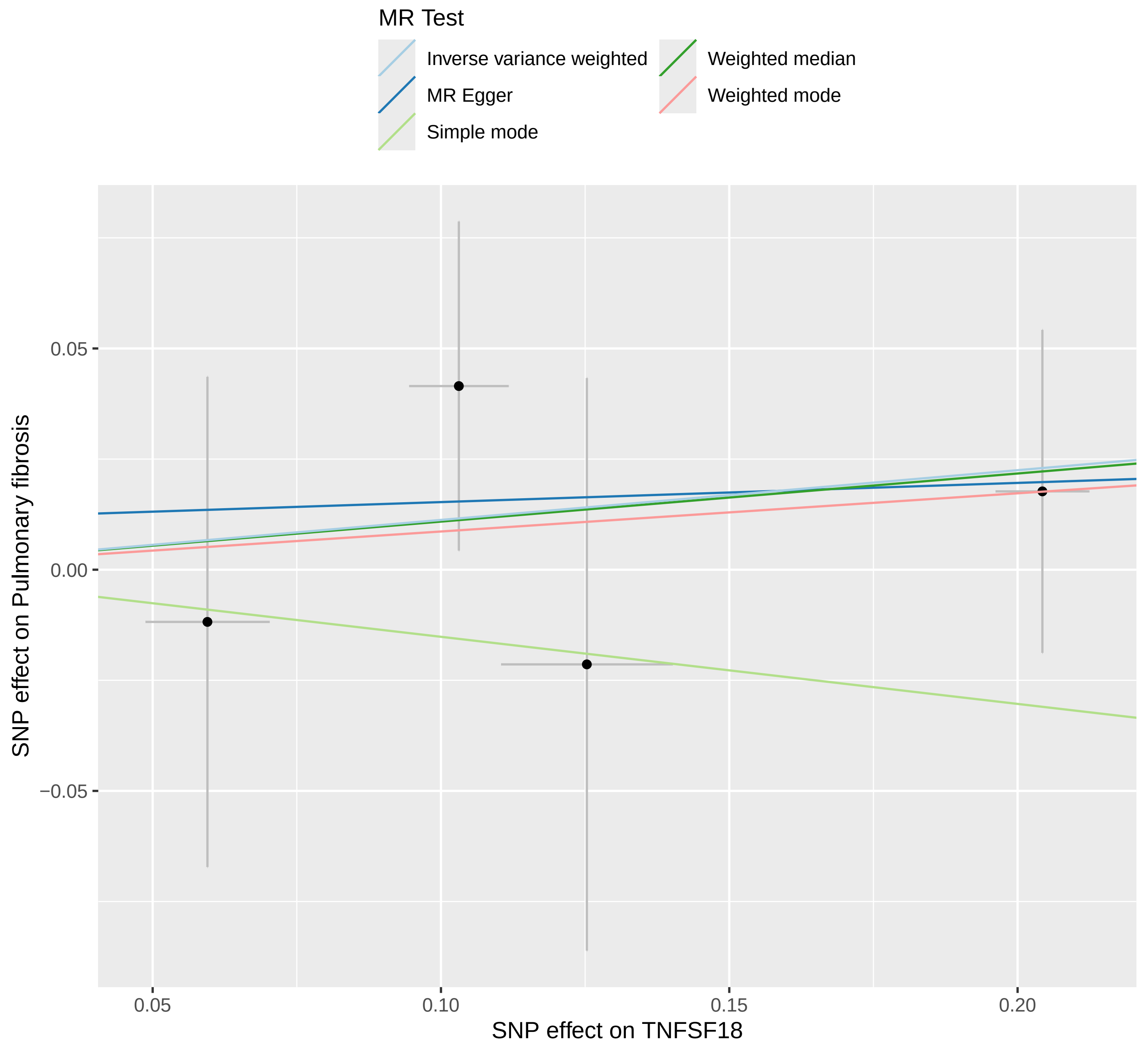

MR Test
Inverse variance weighted
MR Egger
Simple mode
Weighted median
Weighted mode
0.05
0.00
−0.05
0.05
0.10
0.15
0.20
SNP effect on Pulmonary fibrosis
SNP effect on TNFSF18

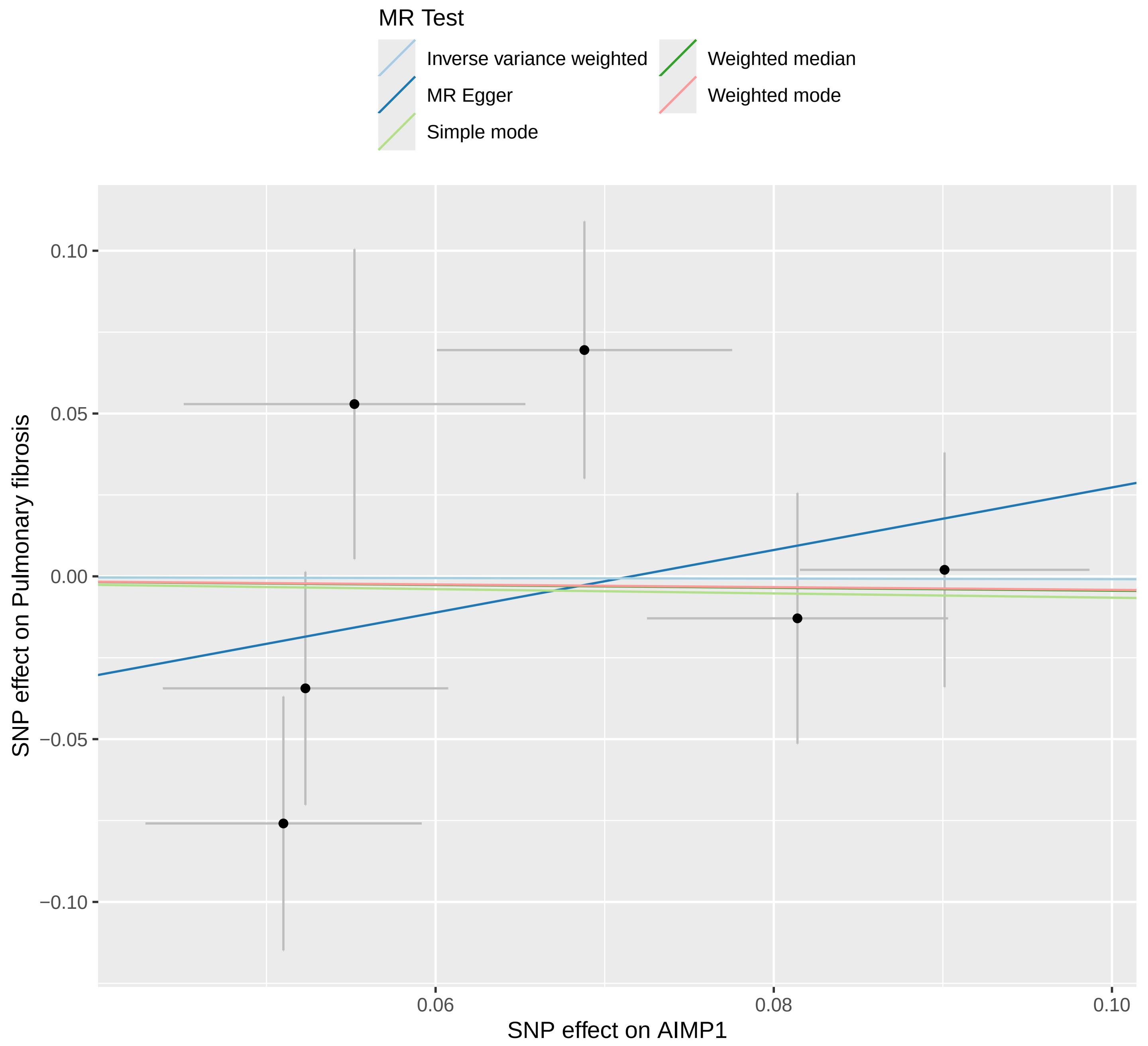
MR Test
Inverse variance weighted
MR Egger
Simple mode
Weighted median
Weighted mode
0.10
0.05
0.00
−0.05
−0.10
SNP effect on Pulmonary fibrosis
0.06
0.08
0.10
SNP effect on AIMP1

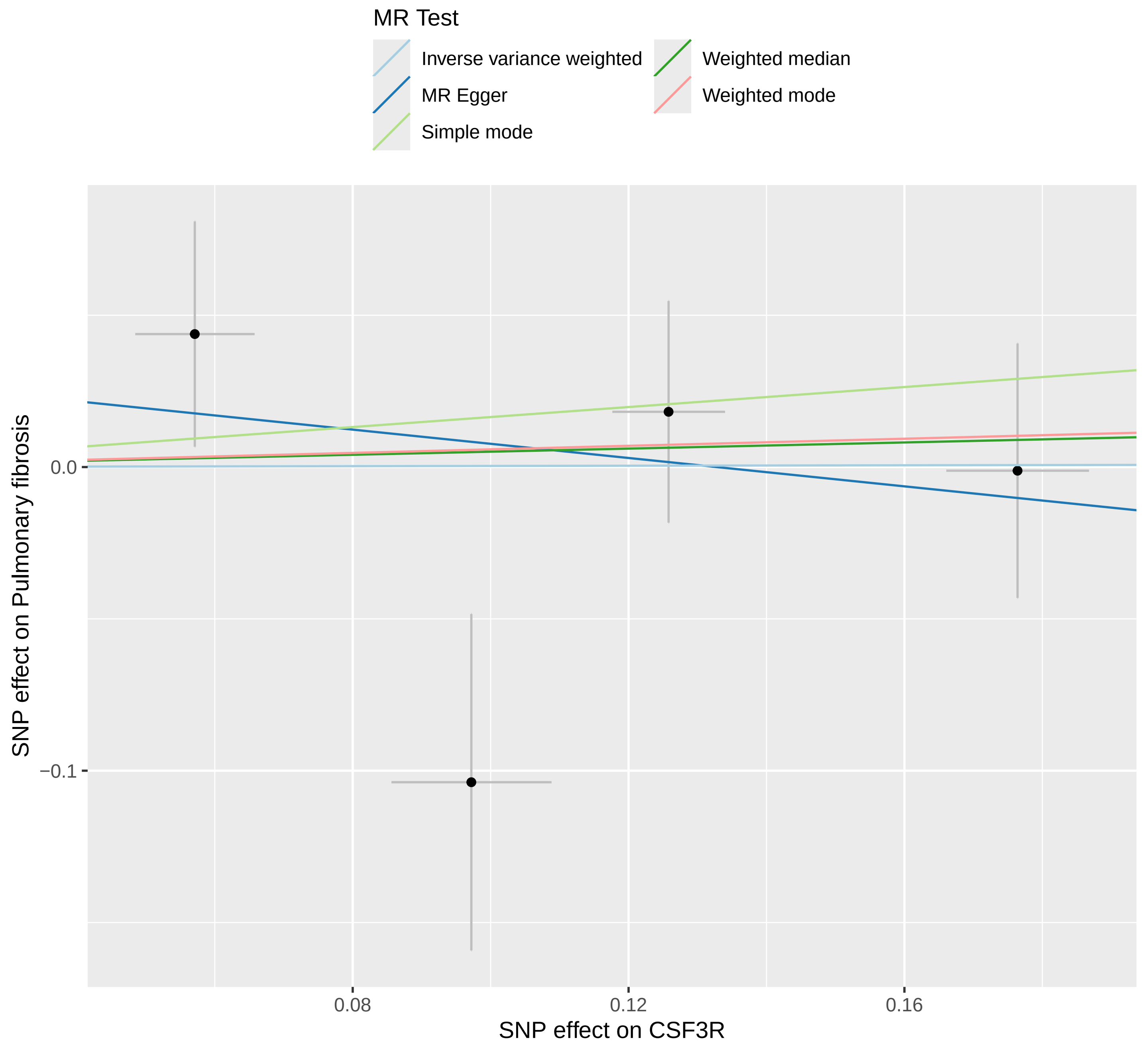
MR Test
Inverse variance weighted
MR Egger
Simple mode
Weighted median
Weighted mode
0.0
−0.1
0.08
0.12
0.16
SNP effect on CSF3R
SNP effect on Pulmonary fibrosis

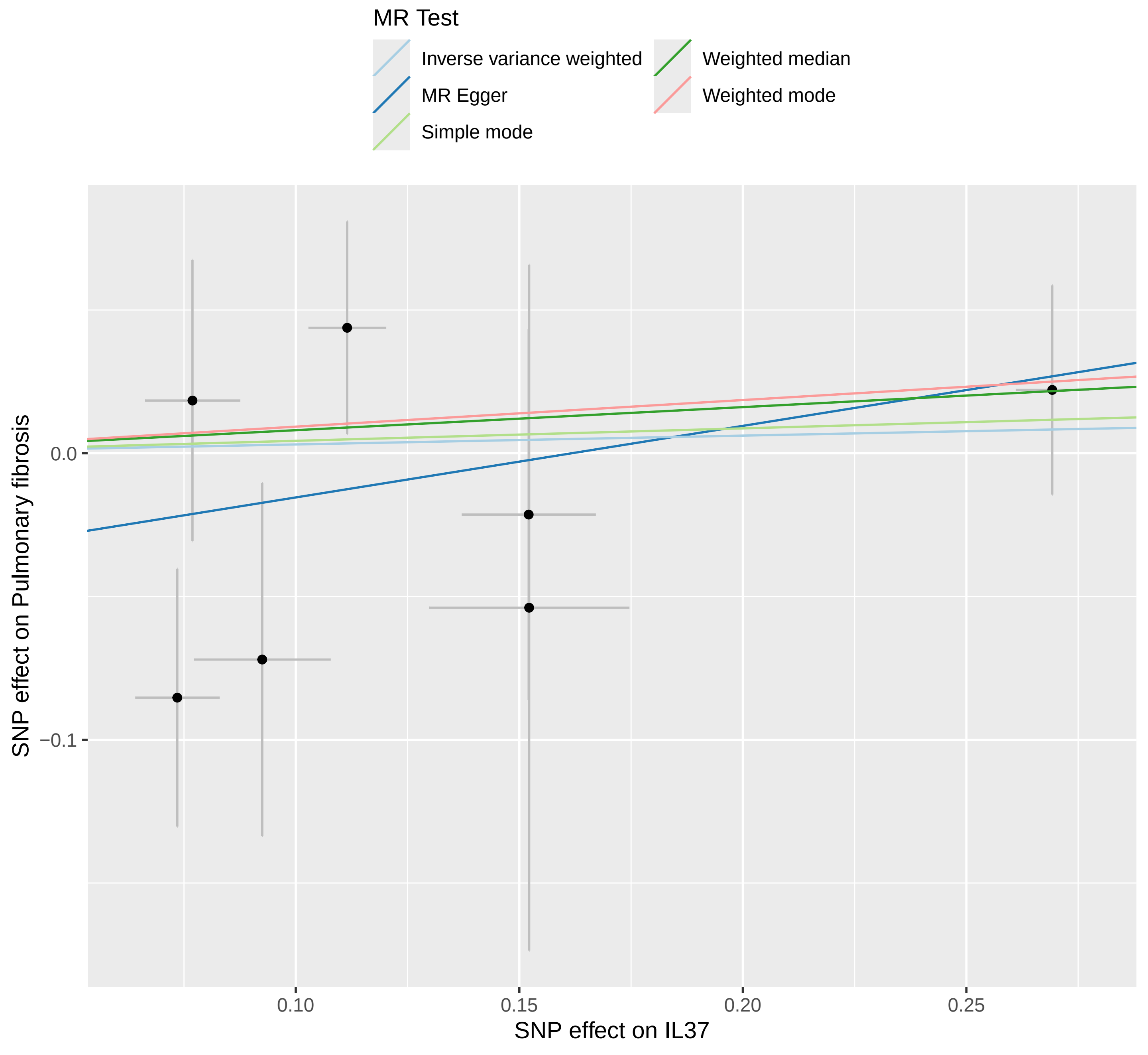
MR Test
Inverse variance weighted
MR Egger
Simple mode
Weighted median
Weighted mode
0.0
−0.1
0.10
0.15
0.20
0.25
SNP effect on IL37
SNP effect on Pulmonary fibrosis

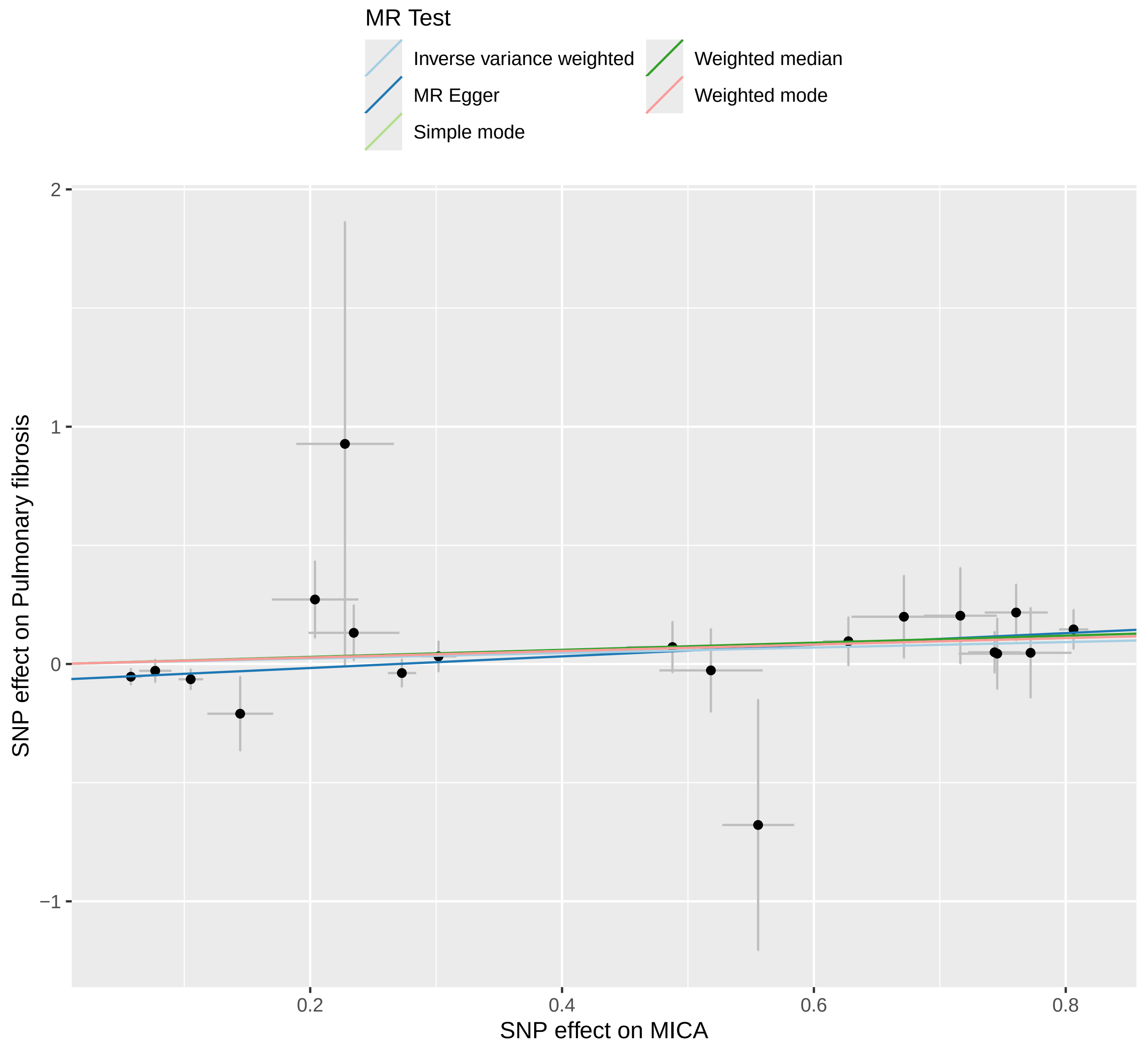
MR Test
Inverse variance weighted
MR Egger
Simple mode
Weighted median
Weighted mode
2
1
0
−1
SNP effect on Pulmonary fibrosis
0.2
0.4
0.6
0.8
SNP effect on MICA

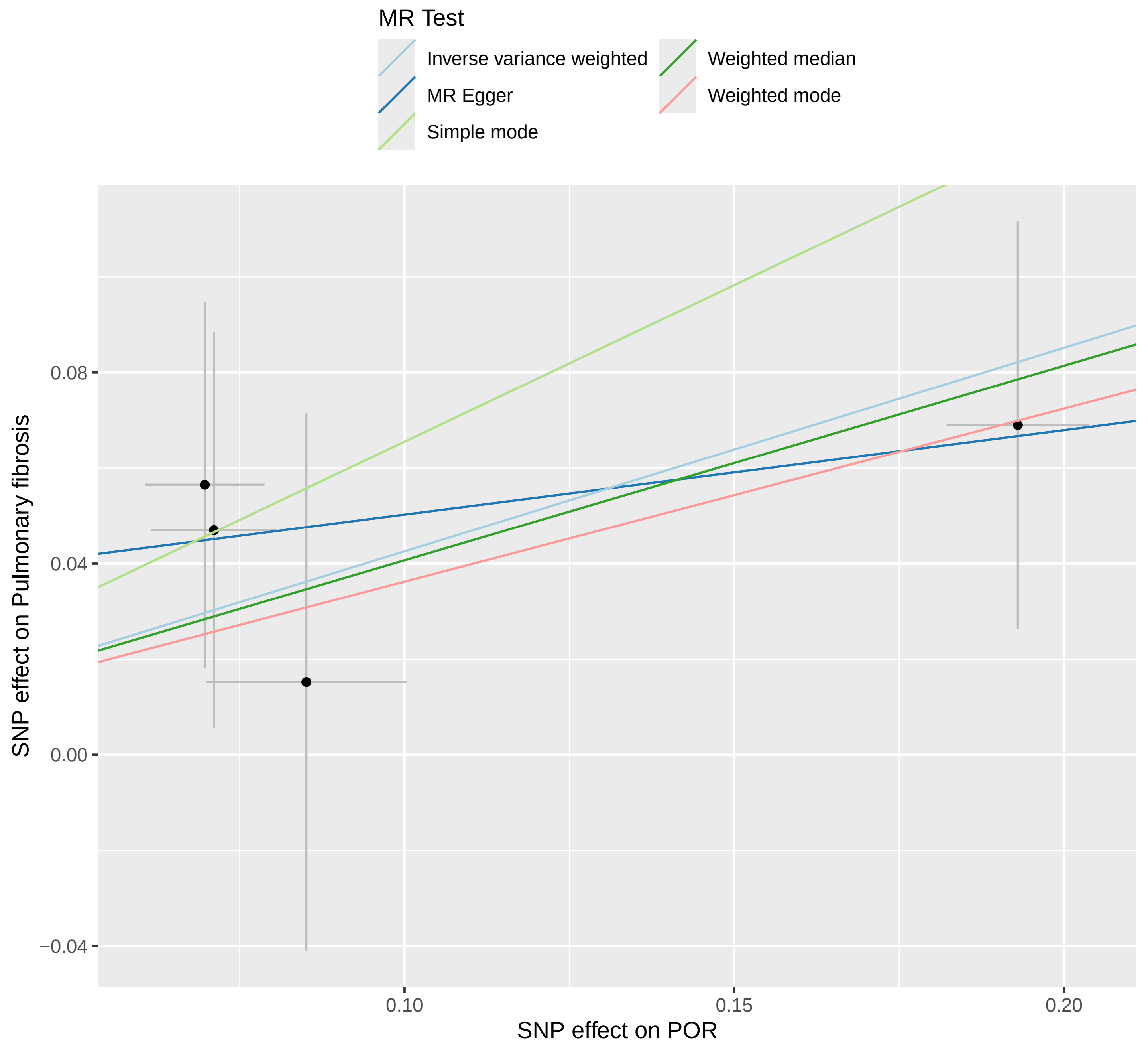

MR Test
Inverse variance weighted
MR Egger
Simple mode
Weighted median
Weighted mode
SNP effect on Pulmonary fibrosis
0.08
0.04
0.00
−0.04
0.10
0.15
0.20
SNP effect on POR

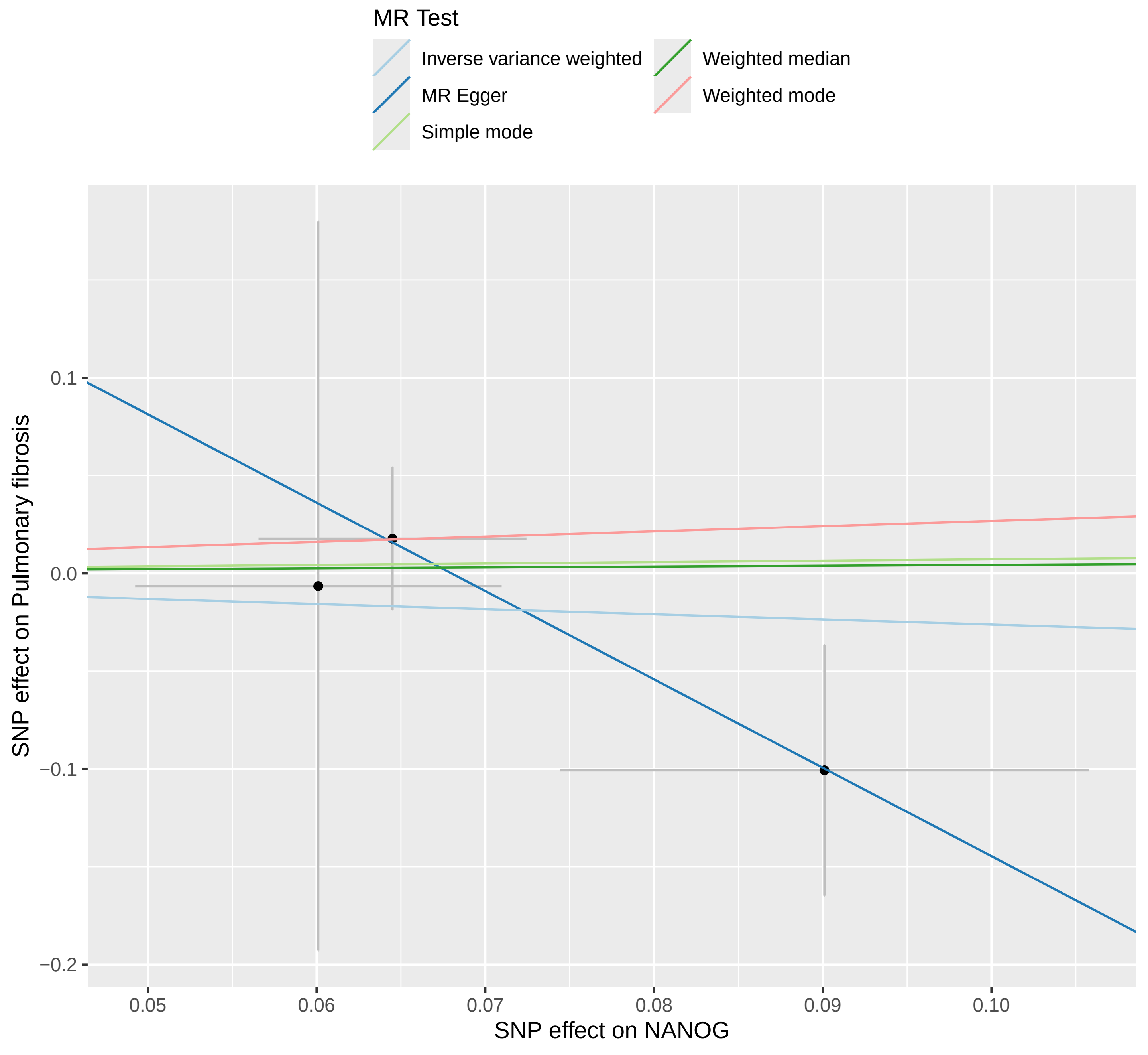
MR Test
Inverse variance weighted
MR Egger
Simple mode
Weighted median
Weighted mode
0.1
0.0
−0.1
−0.2
SNP effect on Pulmonary fibrosis
0.05
0.06
0.07
0.08
0.09
0.10
SNP effect on NANOG

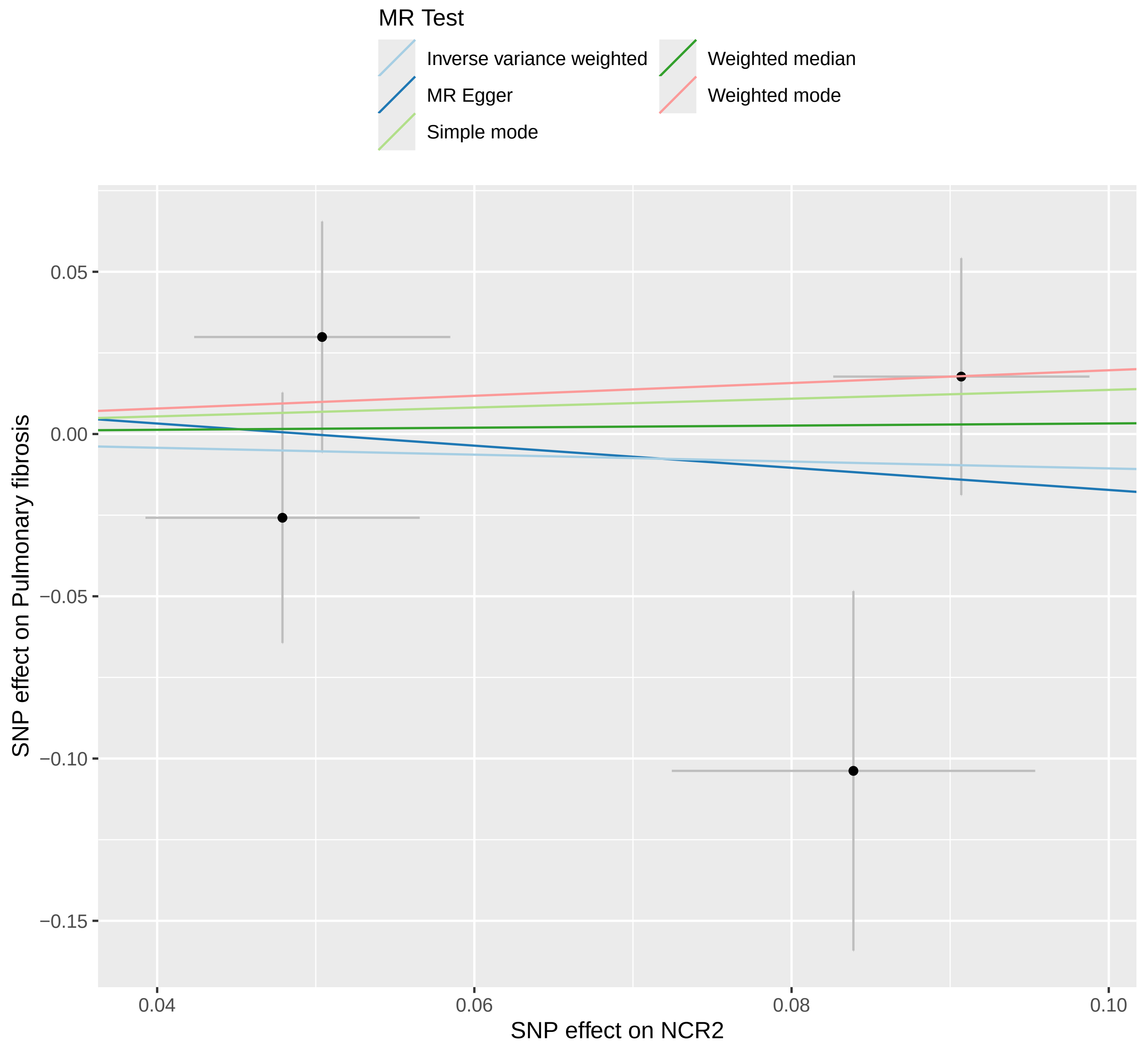

MR Test
Inverse variance weighted
MR Egger
Simple mode
Weighted median
Weighted mode
0.05
0.00
−0.05
−0.10
−0.15
SNP effect on Pulmonary fibrosis
0.04
0.06
0.08
0.10
SNP effect on NCR2

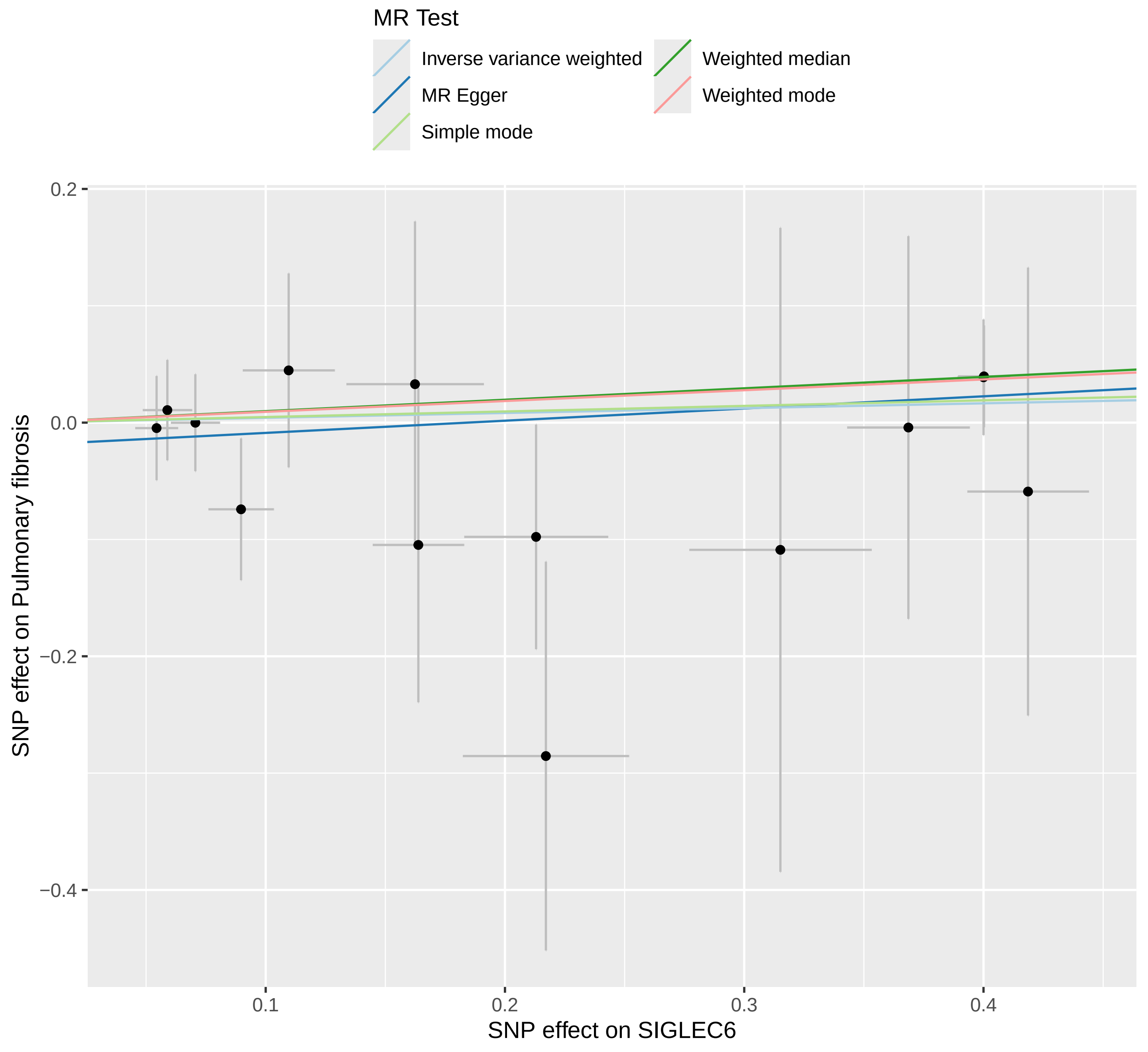
MR Test
Inverse variance weighted
MR Egger
Simple mode
Weighted median
Weighted mode
0.2
0.0
−0.2
−0.4
0.1
0.2
0.3
0.4
SNP effect on Pulmonary fibrosis
SNP effect on SIGLEC6

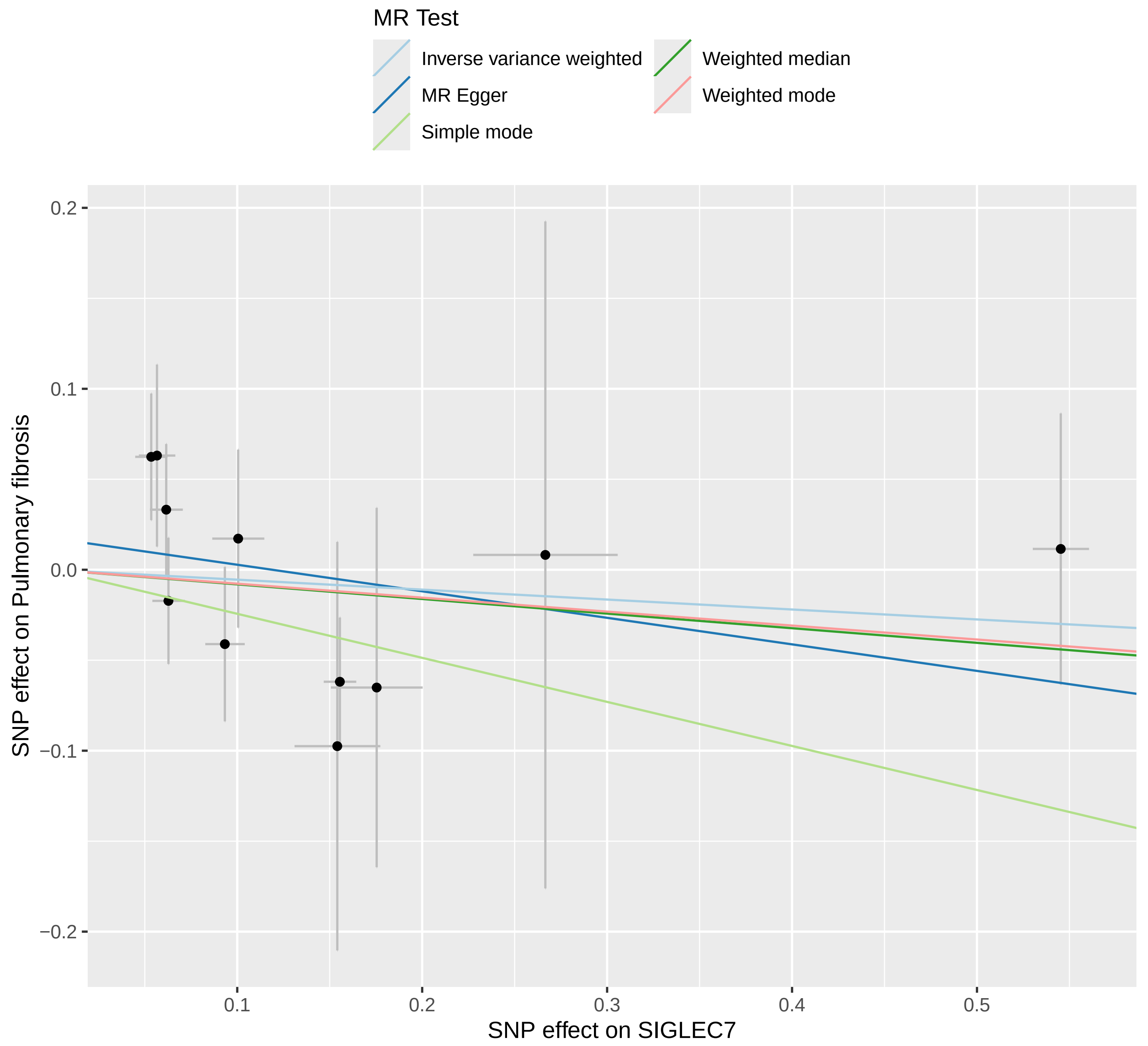

MR Test
Inverse variance weighted
MR Egger
Simple mode
Weighted median
Weighted mode
0.2
0.1
0.0
−0.1
−0.2
0.1
0.2
0.3
0.4
0.5
SNP effect on SIGLEC7
SNP effect on Pulmonary fibrosis

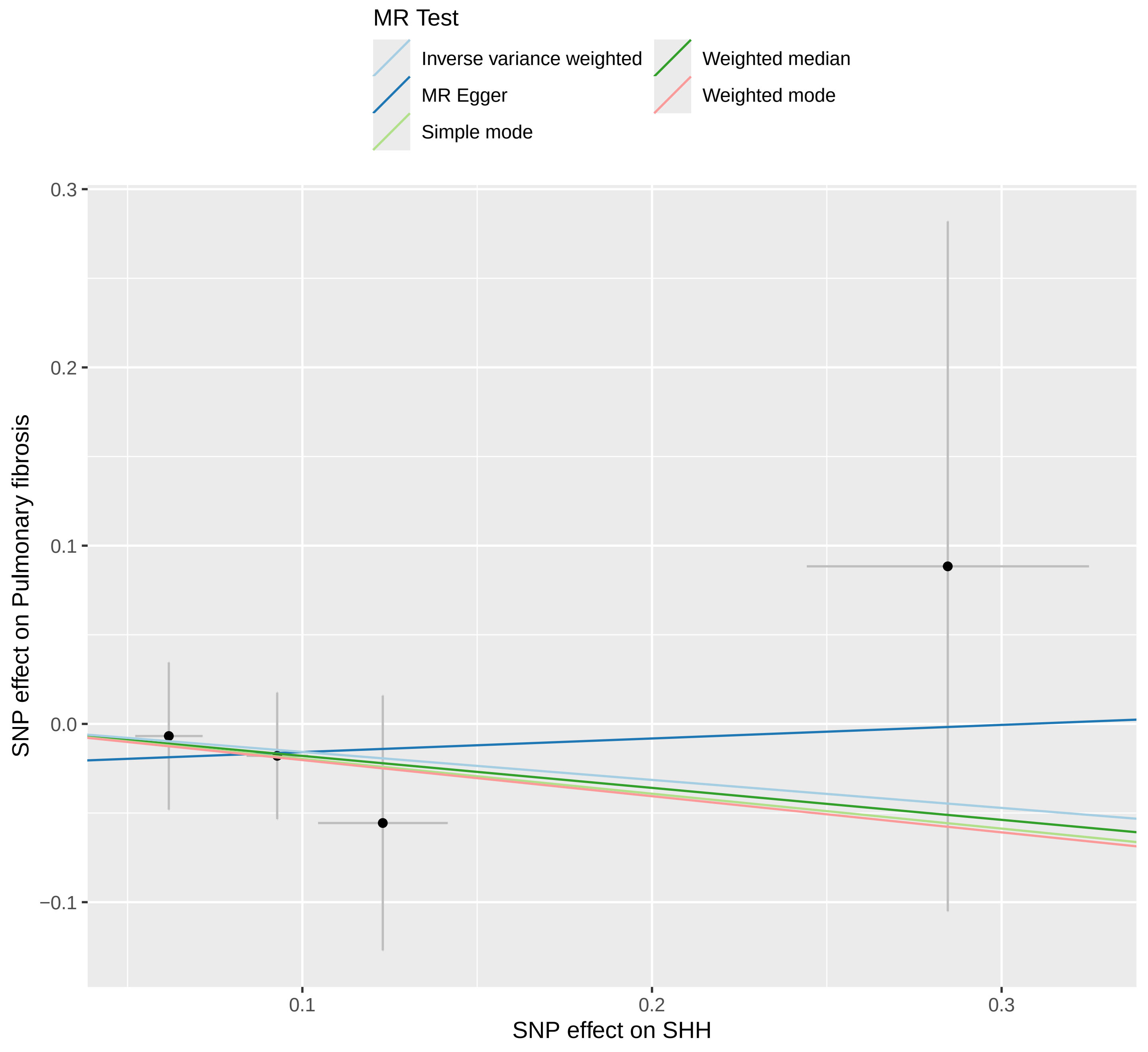
MR Test
Inverse variance weighted
MR Egger
Simple mode
Weighted median
Weighted mode
0.3
0.2
0.1
0.0
−0.1
SNP effect on Pulmonary fibrosis
0.1
0.2
0.3
SNP effect on SHH

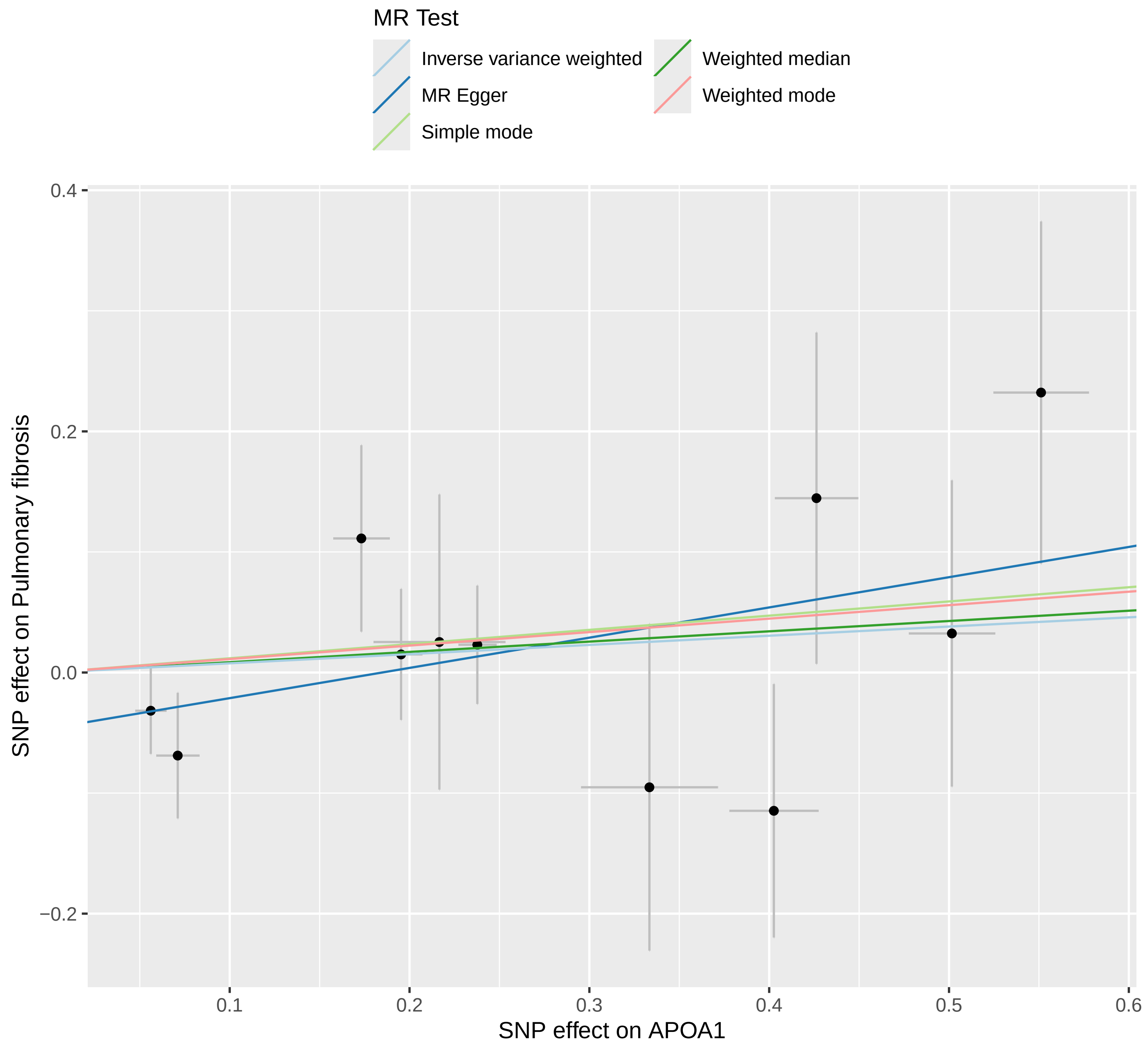
MR Test
Inverse variance weighted
MR Egger
Simple mode
Weighted median
Weighted mode
0.4
0.2
0.0
−0.2
SNP effect on Pulmonary fibrosis
0.1
0.2
0.3
0.4
0.5
0.6
SNP effect on APOA1

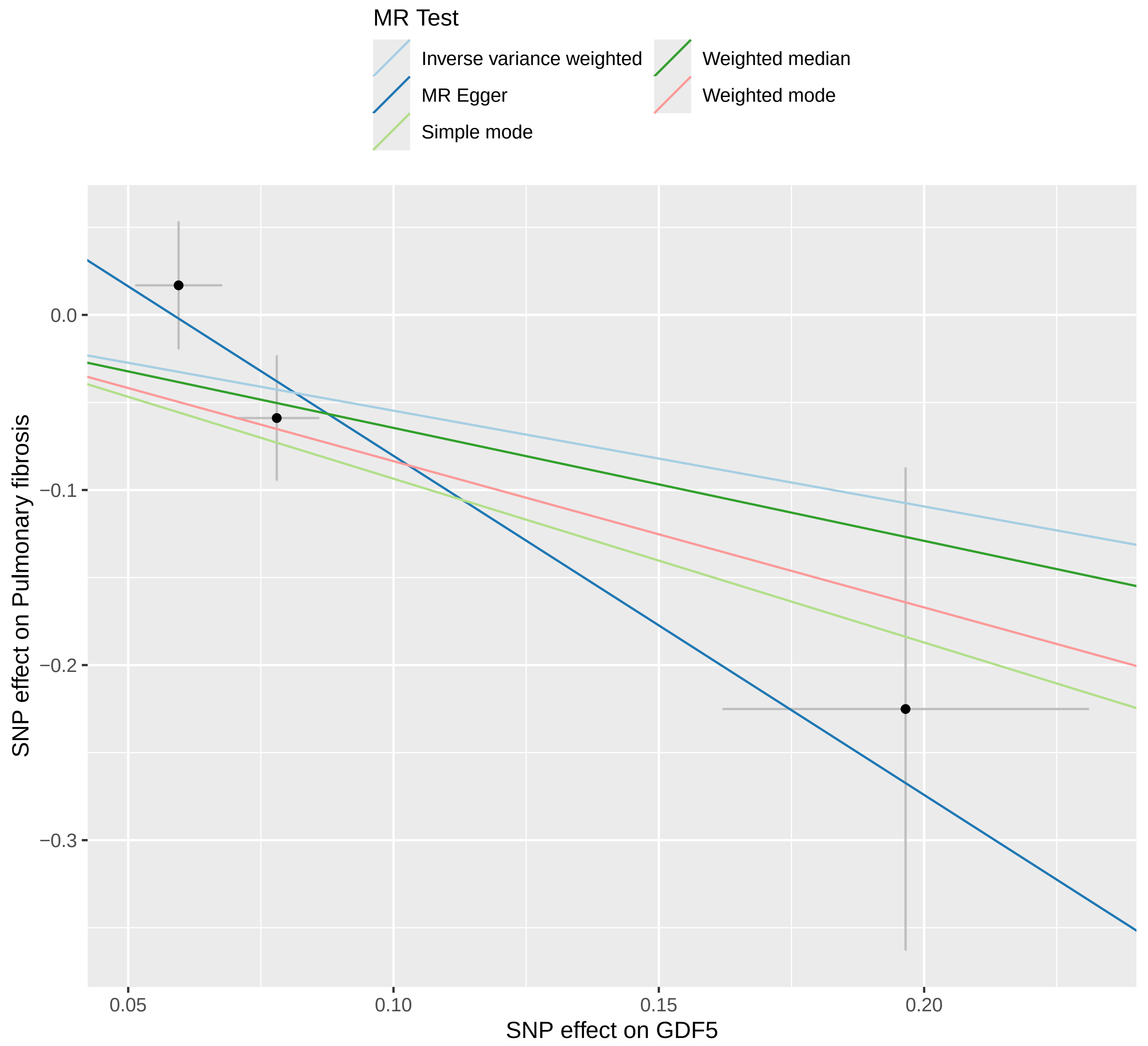

MR Test
Inverse variance weighted
MR Egger
Simple mode
Weighted median
Weighted mode
SNP effect on Pulmonary fibrosis
0.0
−0.1
−0.2
−0.3
0.05
0.10
0.15
0.20
SNP effect on GDF5

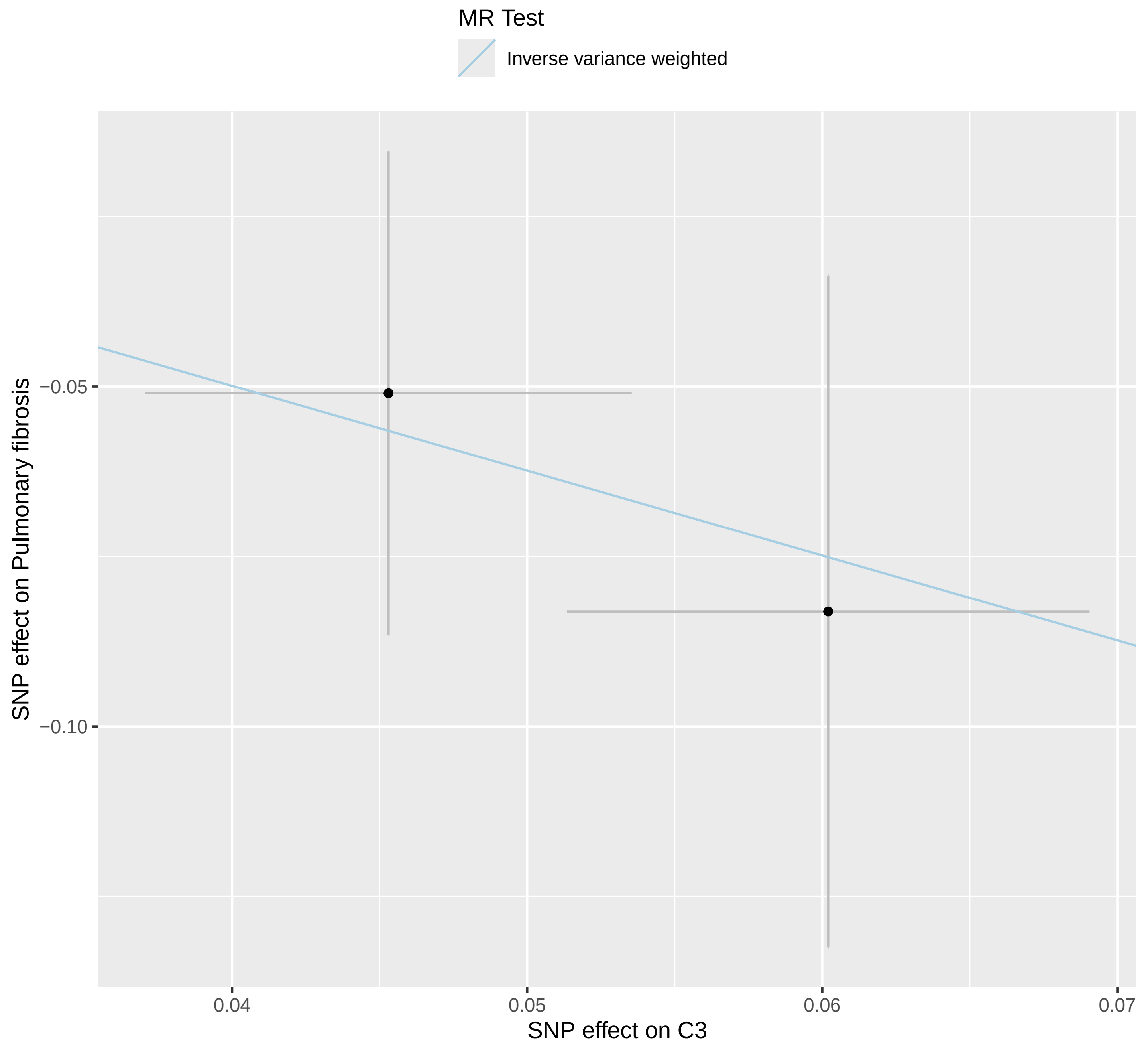

MR Test
Inverse variance weighted
−0.05
−0.10
0.04
0.05
0.06
0.07
SNP effect on C3
SNP effect on Pulmonary fibrosis

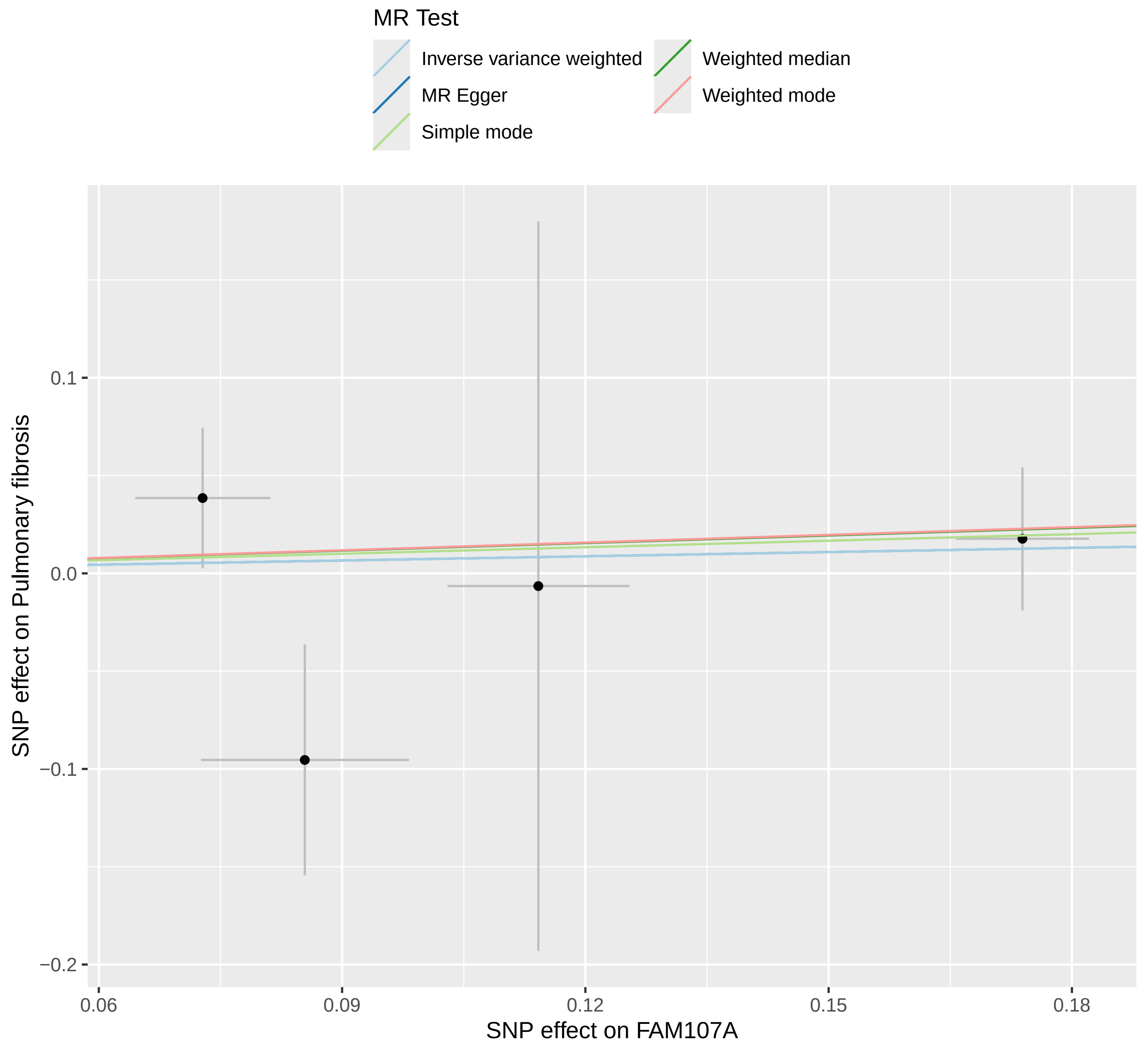

MR Test
Inverse variance weighted
MR Egger
Simple mode
Weighted median
Weighted mode
SNP effect on Pulmonary fibrosis
0.1
0.0
−0.1
−0.2
0.06
0.09
0.12
0.15
0.18
SNP effect on FAM107A

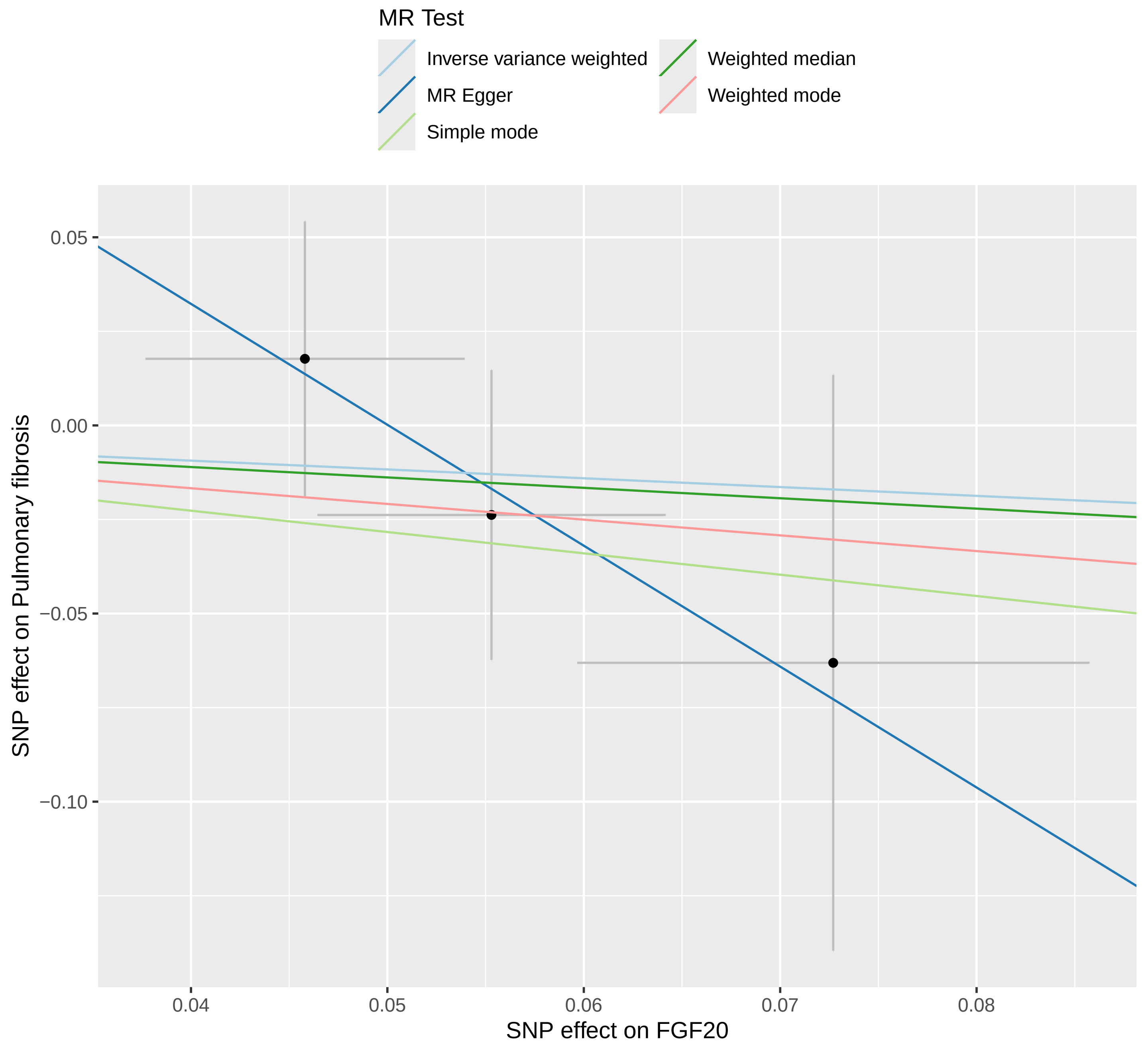

MR Test
Inverse variance weighted
MR Egger
Simple mode
Weighted median
Weighted mode
0.05
0.00
−0.05
−0.10
SNP effect on Pulmonary fibrosis
0.04
0.05
0.06
0.07
0.08
SNP effect on FGF20

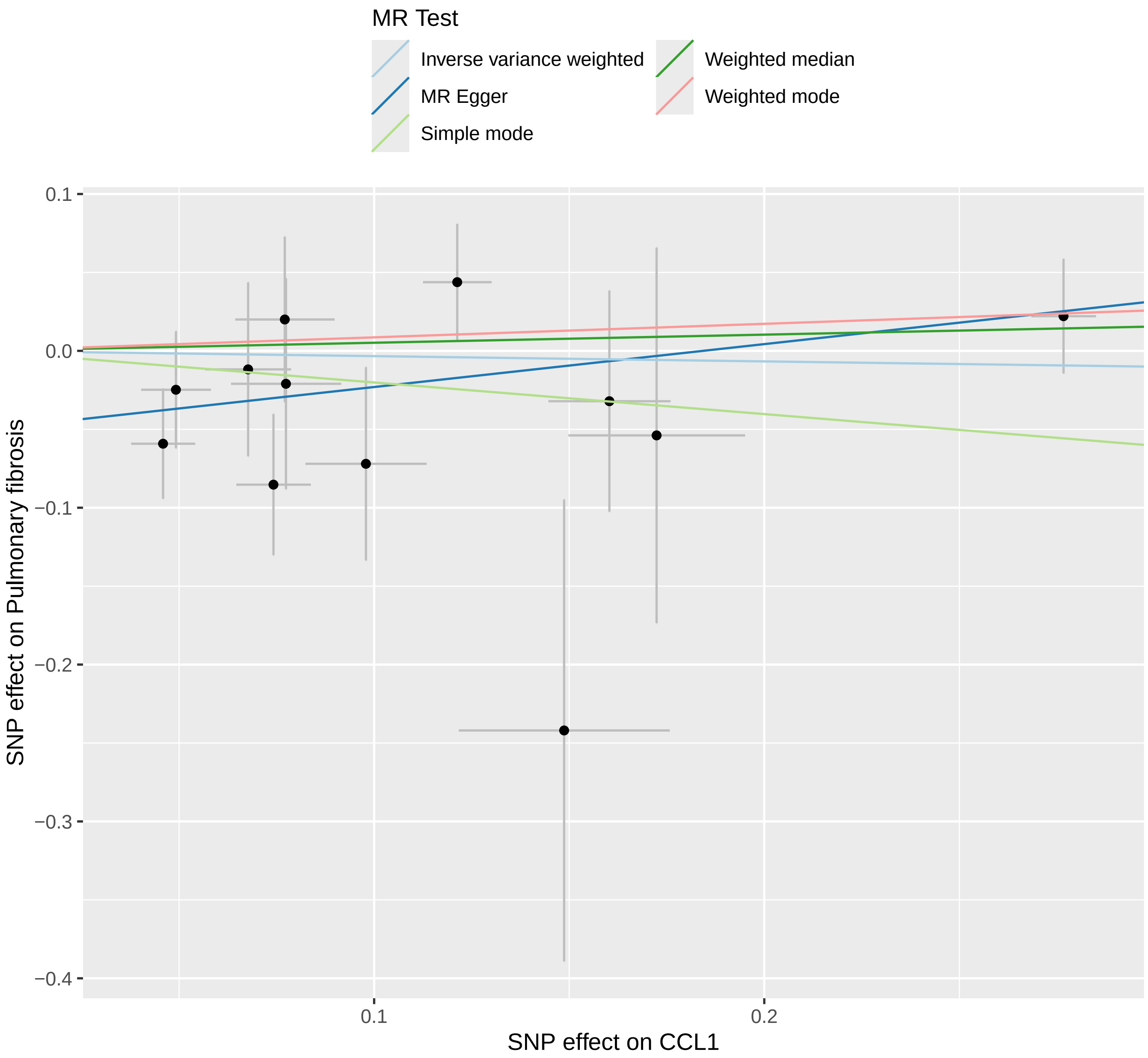

MR Test
Inverse variance weighted
MR Egger
Simple mode
Weighted median
Weighted mode
0.1
0.0
−0.1
−0.2
−0.3
−0.4
SNP effect on Pulmonary fibrosis
0.1
0.2
SNP effect on CCL1

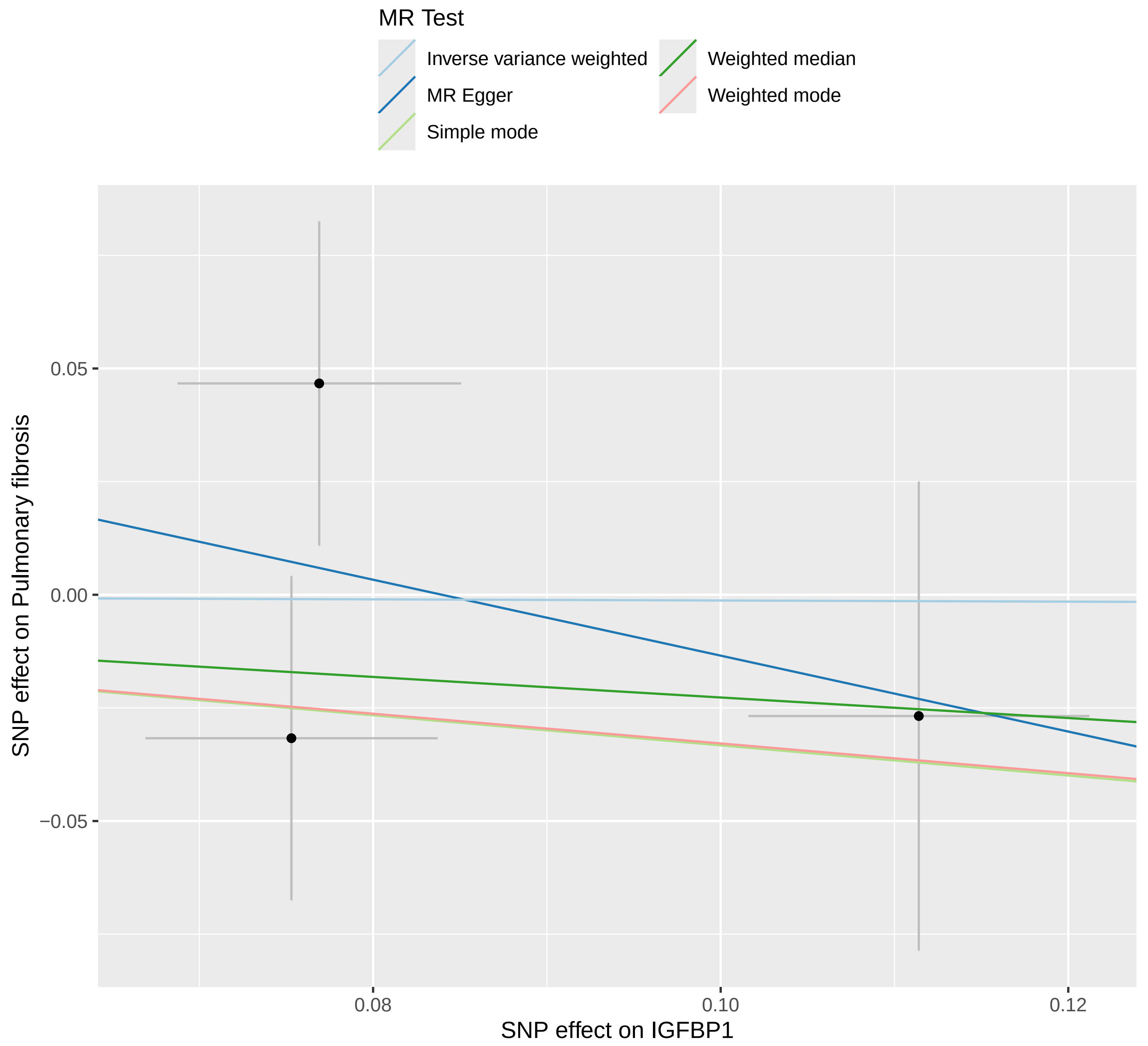

MR Test
Inverse variance weighted
MR Egger
Simple mode
Weighted median
Weighted mode
0.05
0.00
−0.05
SNP effect on Pulmonary fibrosis
0.08
0.10
0.12
SNP effect on IGFBP1

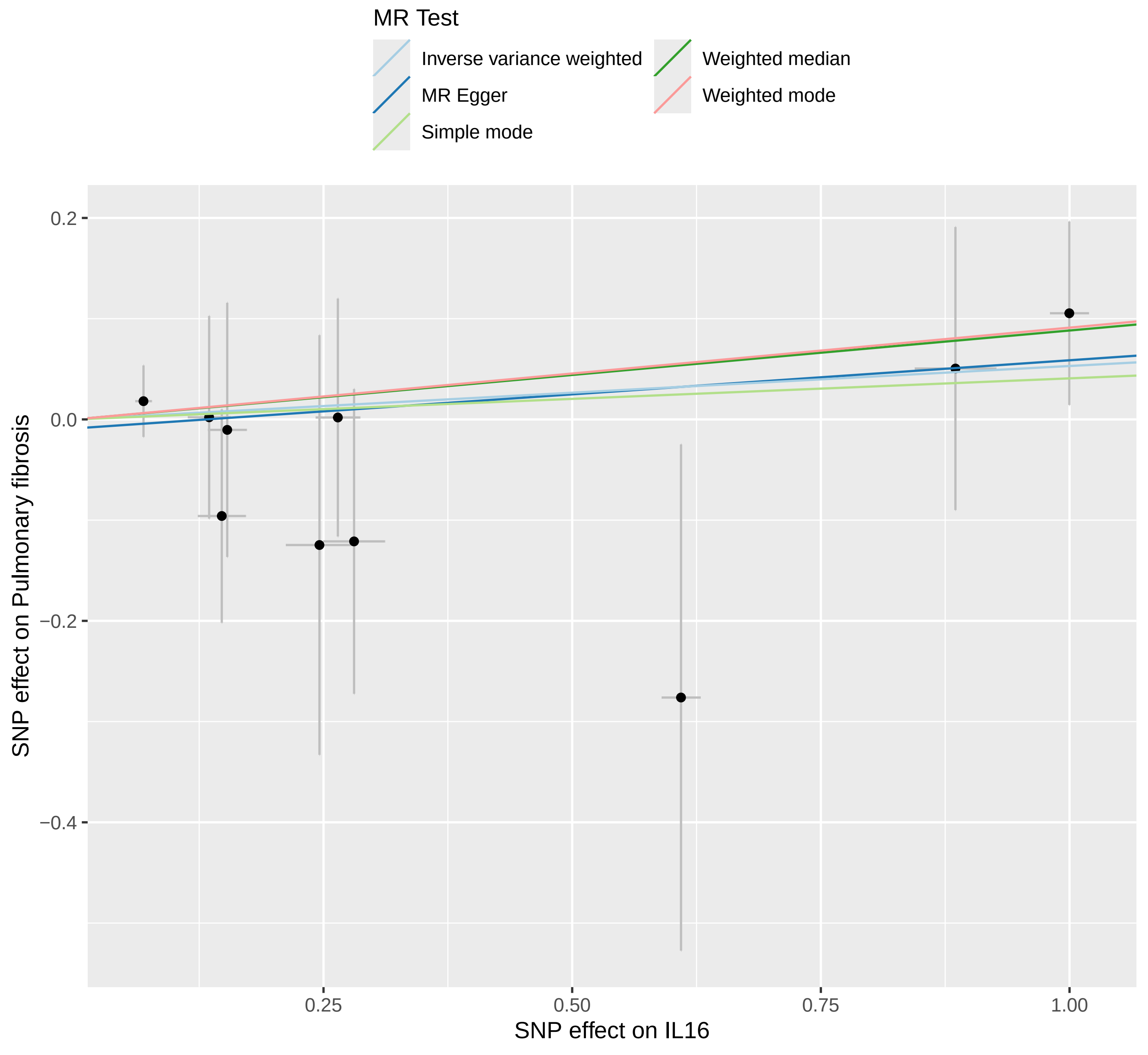
MR Test
Inverse variance weighted
MR Egger
Simple mode
Weighted median
Weighted mode
0.2
0.0
−0.2
−0.4
SNP effect on Pulmonary fibrosis
0.25
0.50
0.75
1.00
SNP effect on IL16

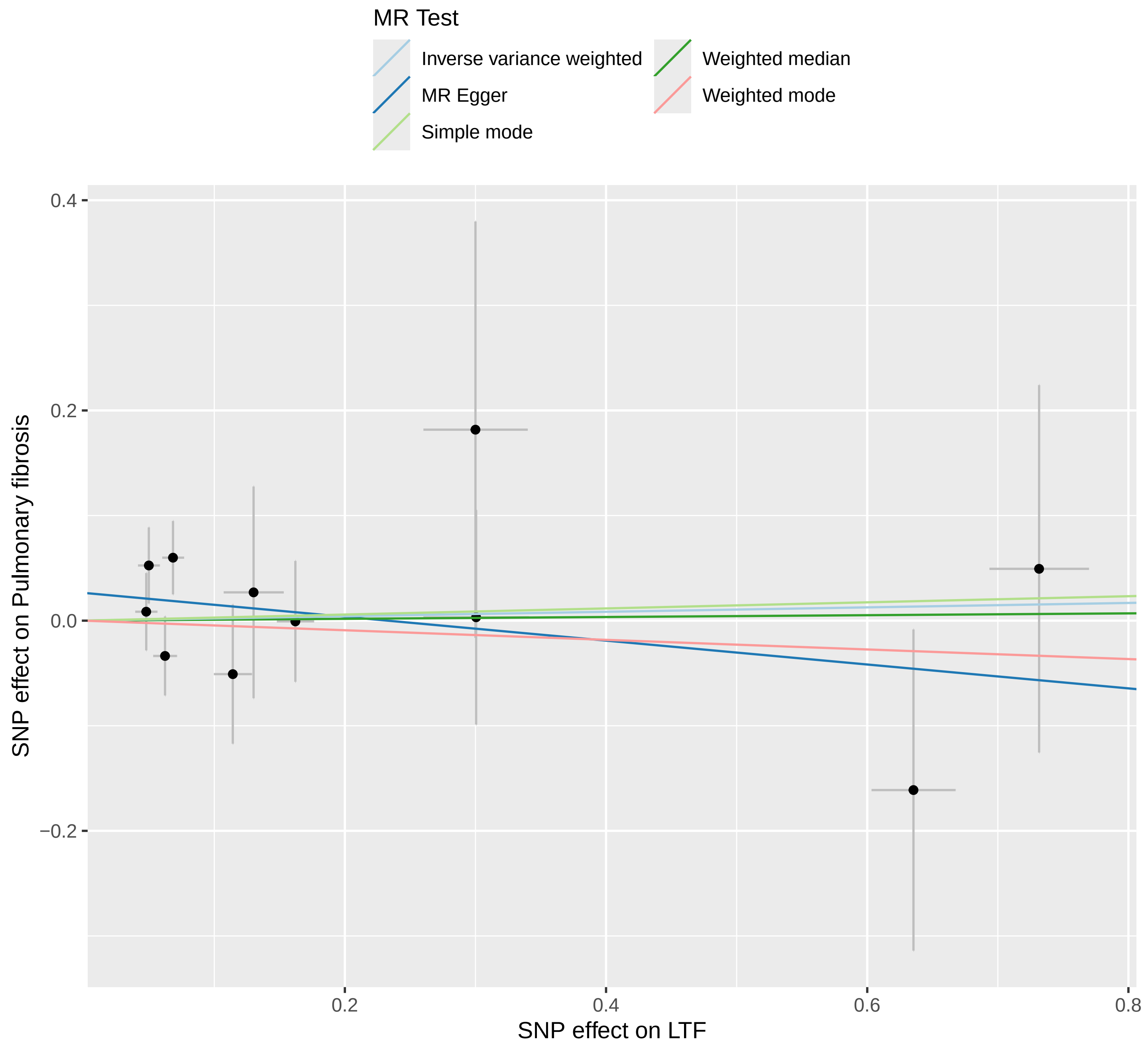

MR Test
Inverse variance weighted
MR Egger
Simple mode
Weighted median
Weighted mode
SNP effect on Pulmonary fibrosis
0.4
0.2
0.0
−0.2
SNP effect on LTF
0.2
0.4
0.6
0.8

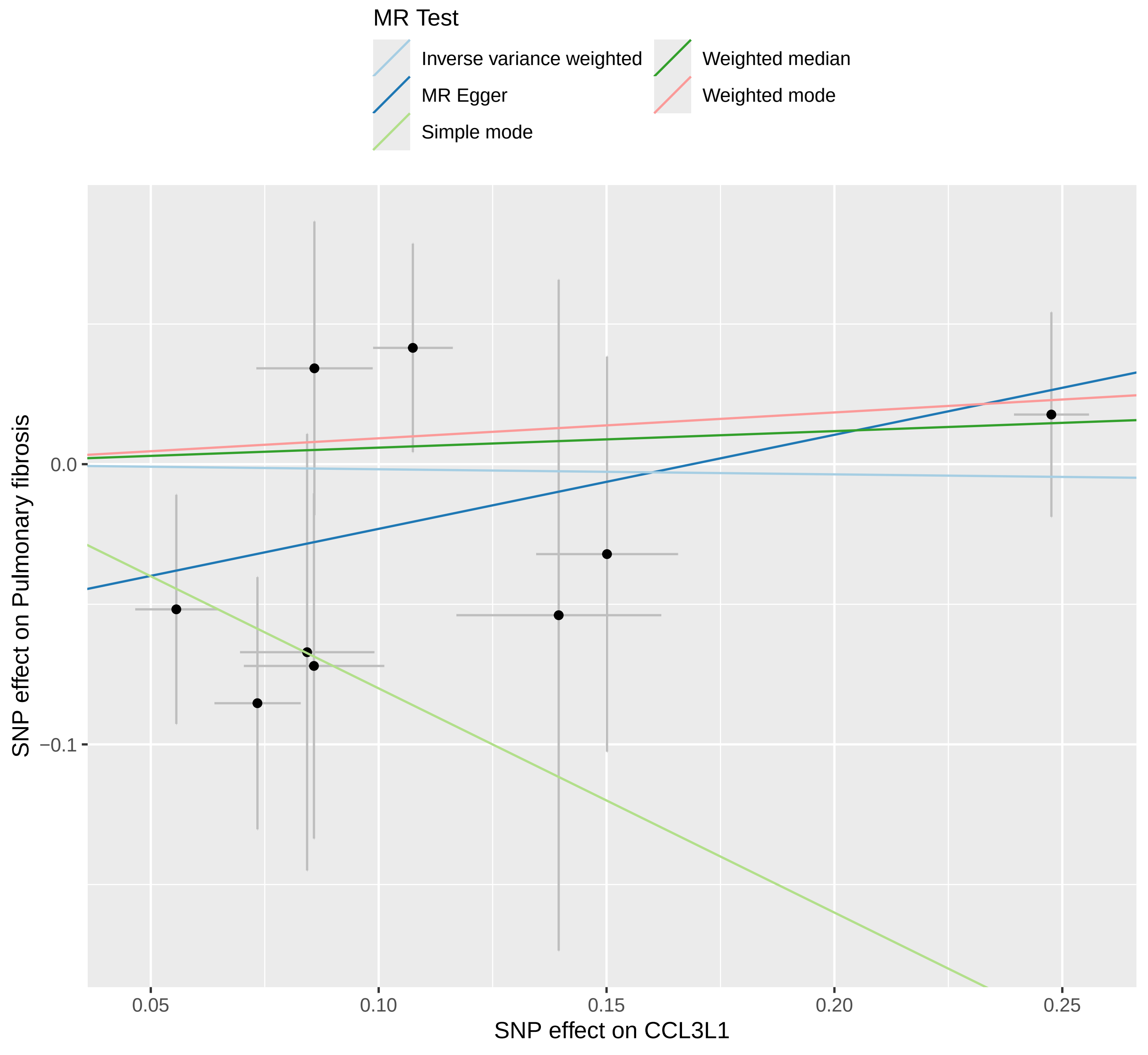

MR Test
Inverse variance weighted
MR Egger
Simple mode
Weighted median
Weighted mode
0.0
−0.1
SNP effect on Pulmonary fibrosis
0.05
0.10
0.15
0.20
0.25
SNP effect on CCL3L1

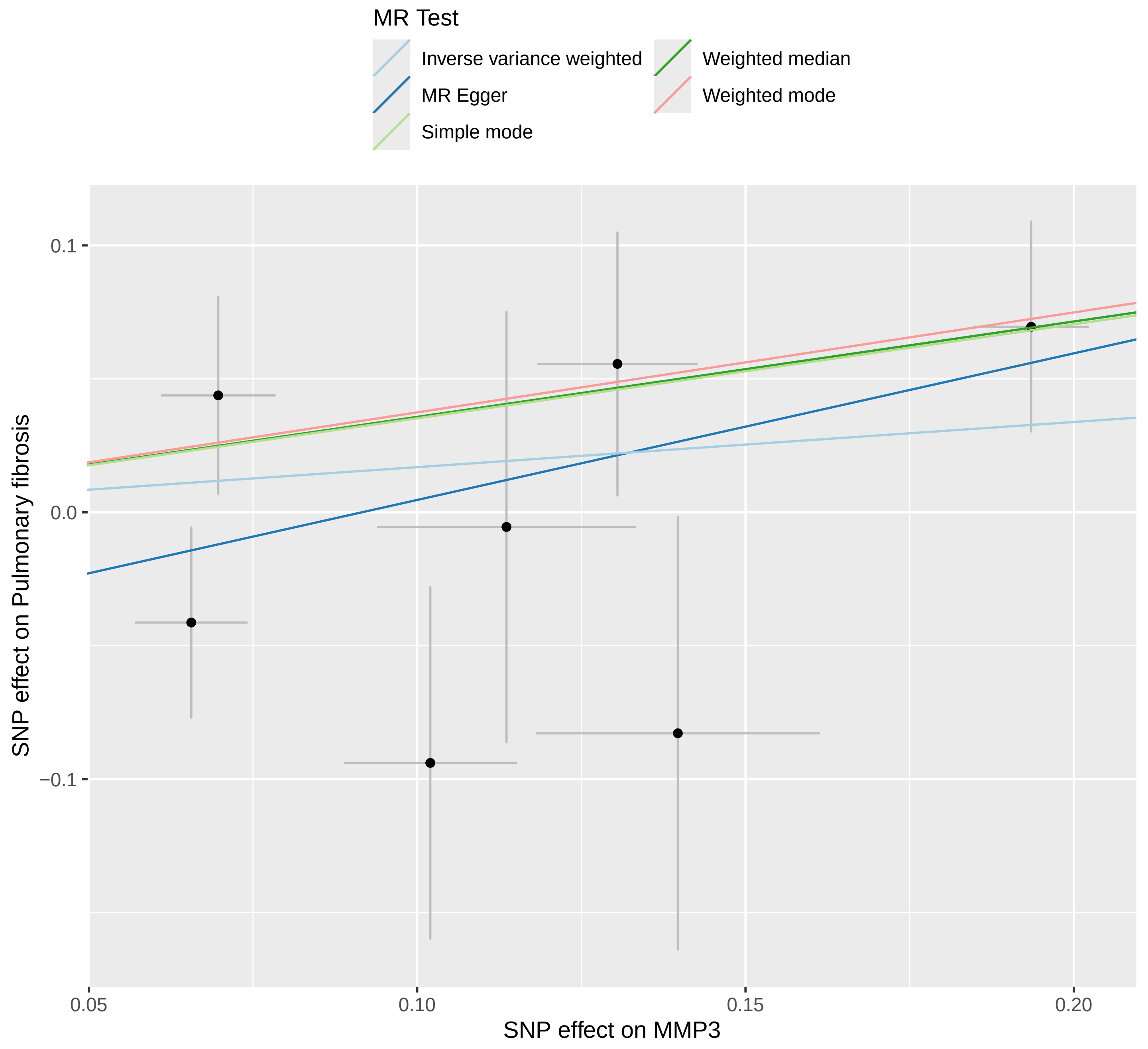
MR Test
Inverse variance weighted
MR Egger
Simple mode
Weighted median
Weighted mode
0.1
0.0
−0.1
0.05
0.10
0.15
0.20
SNP effect on Pulmonary fibrosis
SNP effect on MMP3

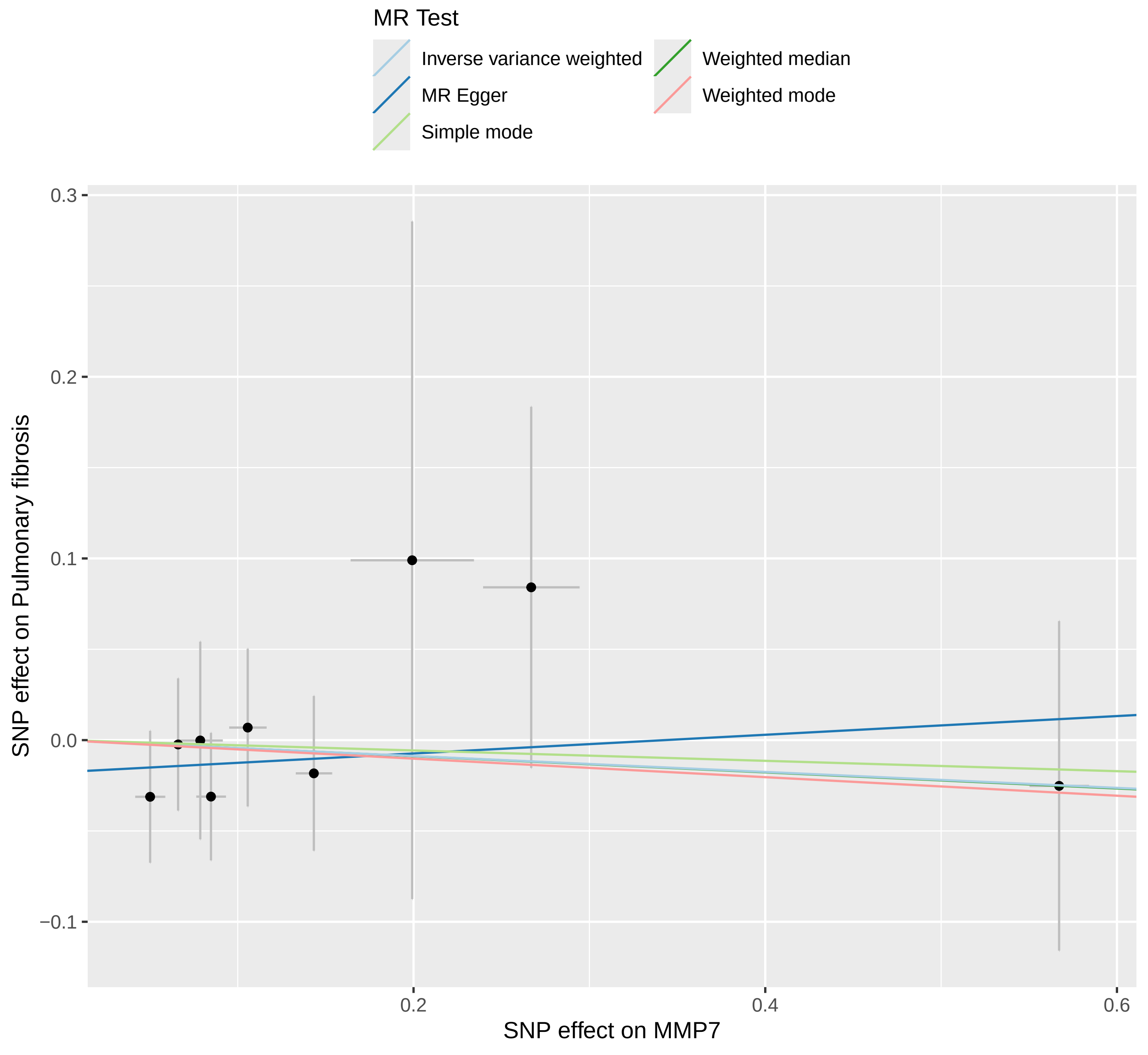
MR Test
Inverse variance weighted
MR Egger
Simple mode
Weighted median
Weighted mode
0.3
0.2
0.1
0.0
−0.1
SNP effect on Pulmonary fibrosis
0.2
0.4
0.6
SNP effect on MMP7

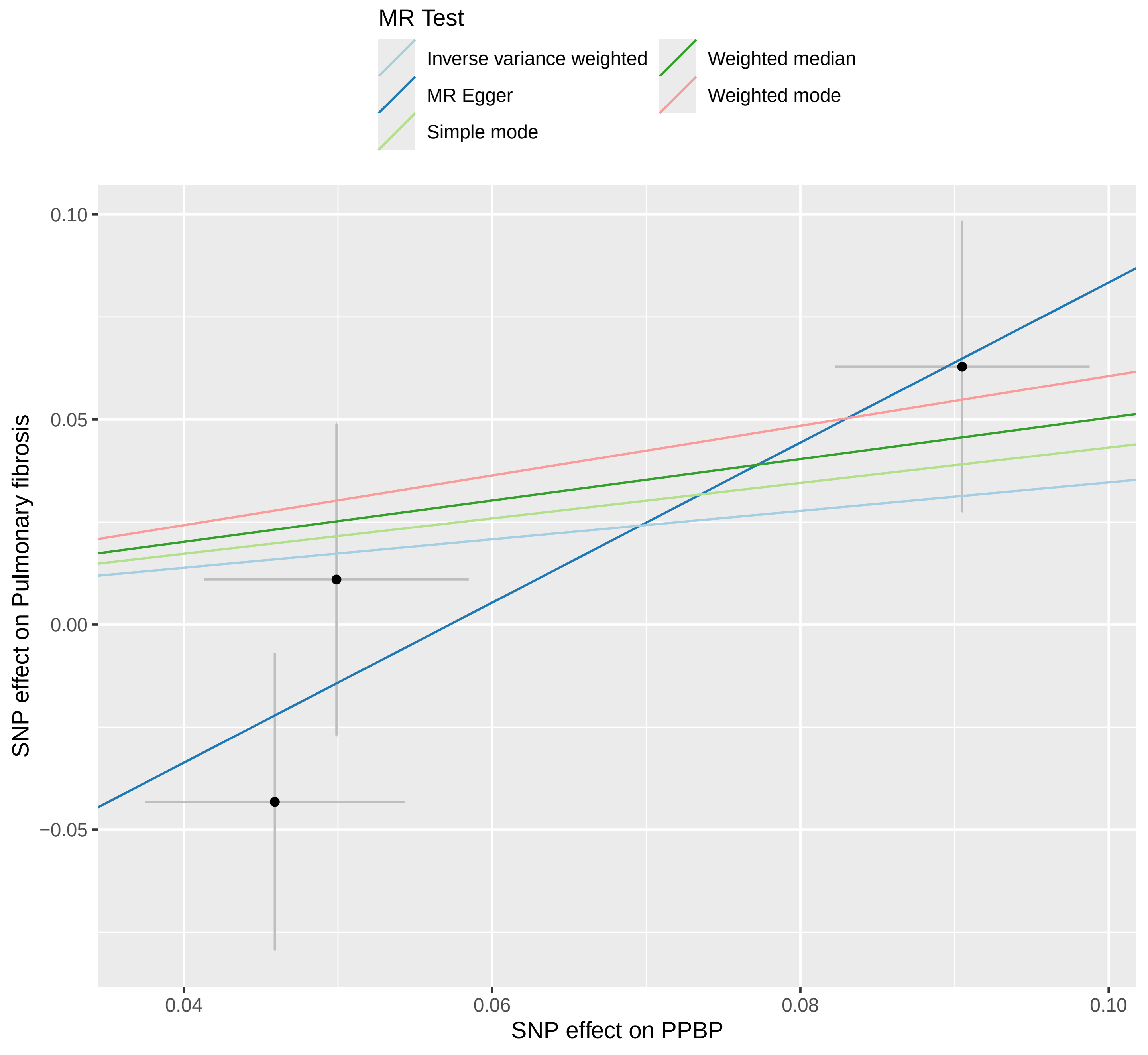

MR Test
Inverse variance weighted
MR Egger
Simple mode
Weighted median
Weighted mode
0.10
0.05
0.00
−0.05
SNP effect on Pulmonary fibrosis
0.04
0.06
0.08
0.10
SNP effect on PPBP

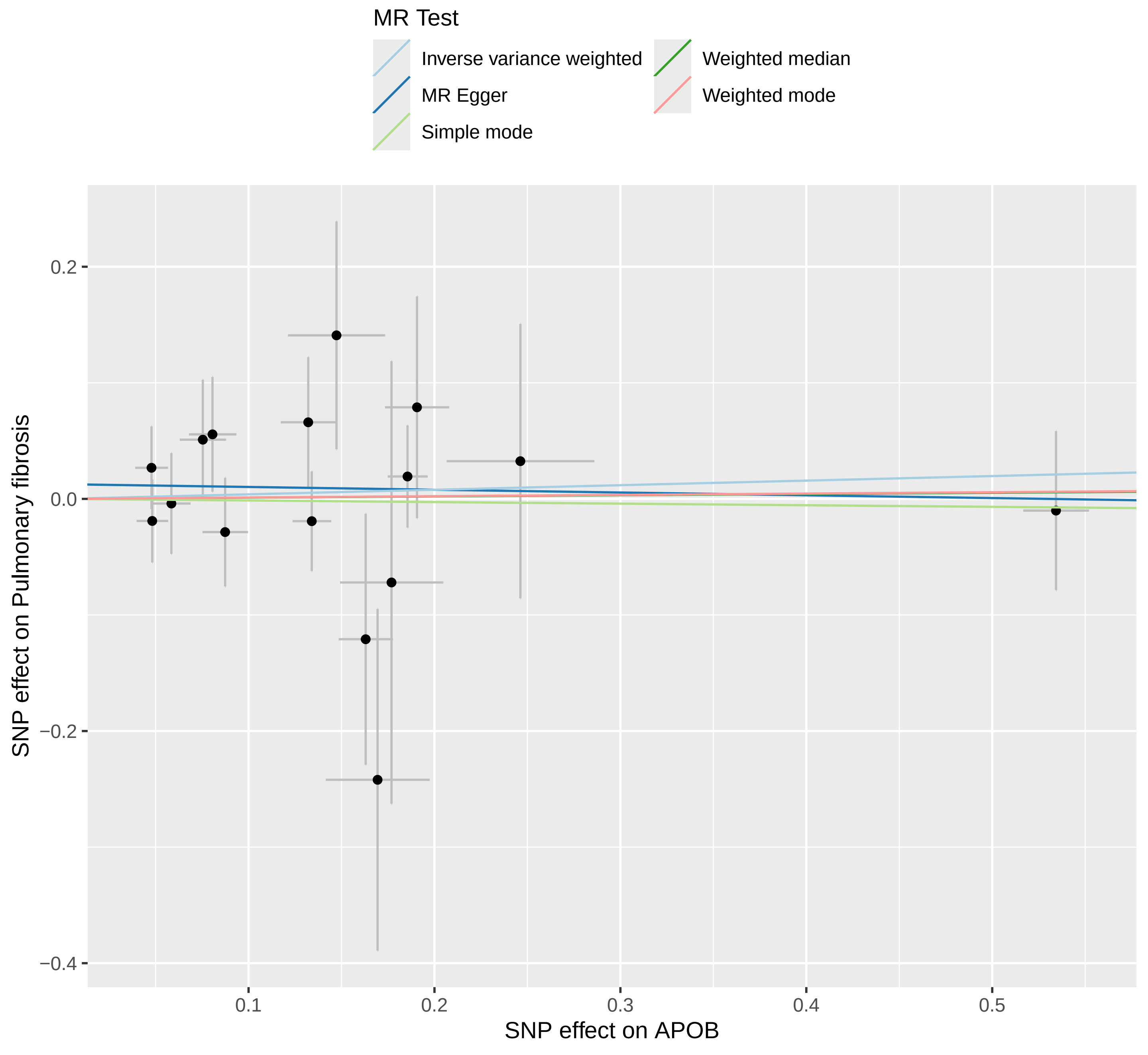

MR Test
Inverse variance weighted
MR Egger
Simple mode
Weighted median
Weighted mode
0.2
0.0
−0.2
−0.4
SNP effect on Pulmonary fibrosis
0.1
0.2
0.3
0.4
0.5
SNP effect on APOB

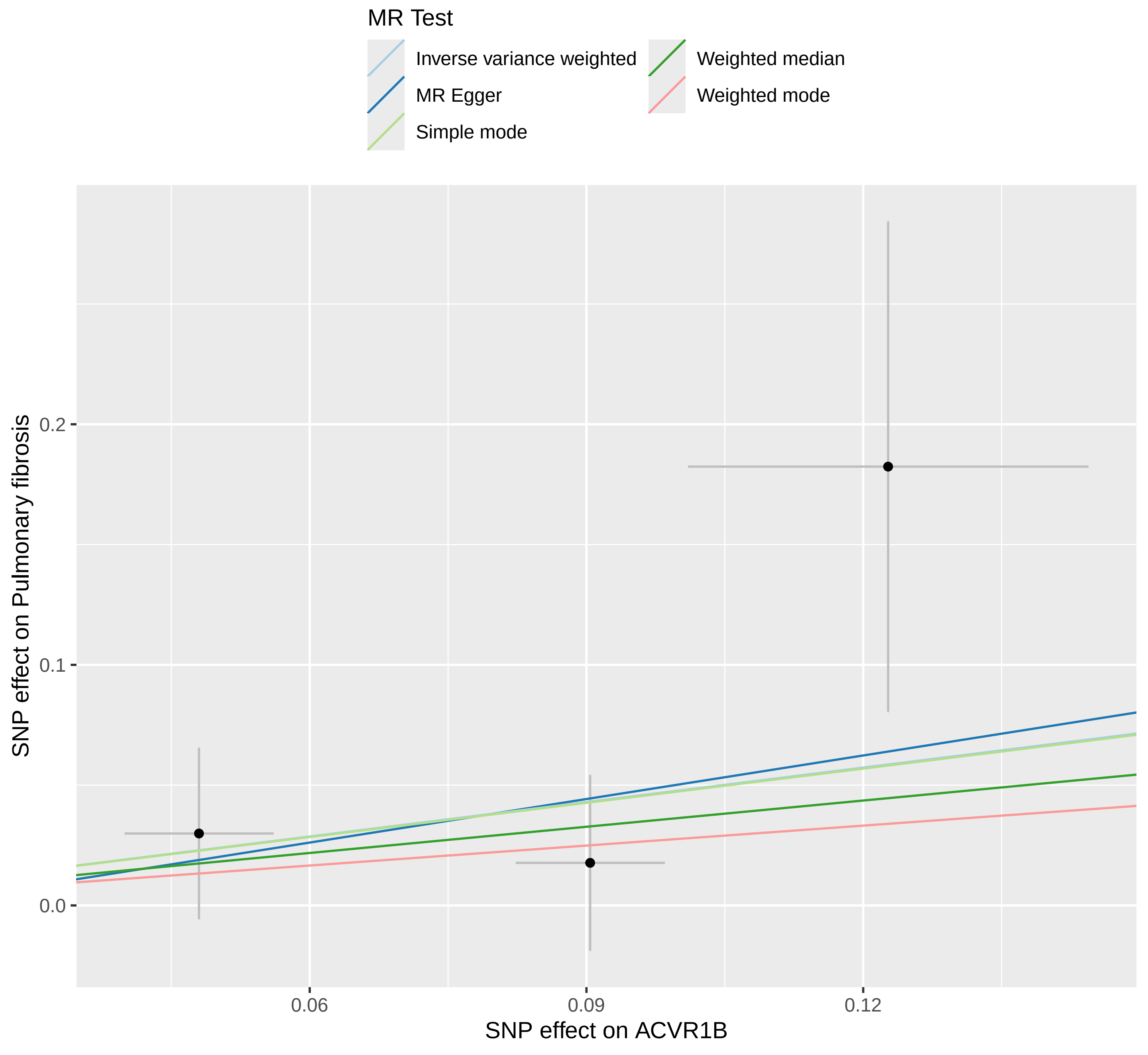
MR Test
Inverse variance weighted
MR Egger
Simple mode
Weighted median
Weighted mode
0.2
0.1
0.0
SNP effect on Pulmonary fibrosis
0.06
0.09
0.12
SNP effect on ACVR1B

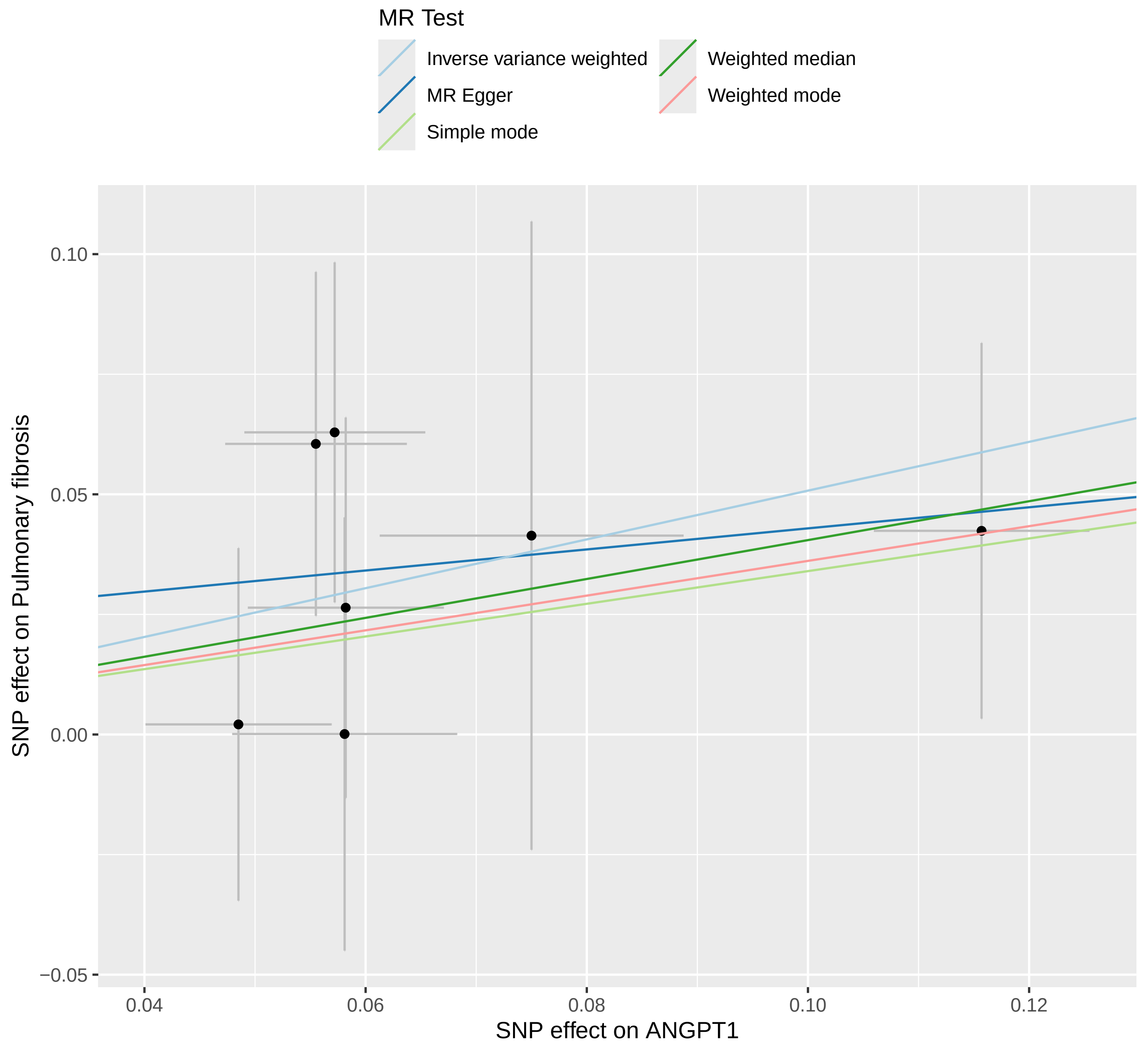
MR Test
Inverse variance weighted
MR Egger
Simple mode
Weighted median
Weighted mode
SNP effect on Pulmonary fibrosis
0.10
0.05
0.00
−0.05
0.04
0.06
0.08
0.10
0.12
SNP effect on ANGPT1

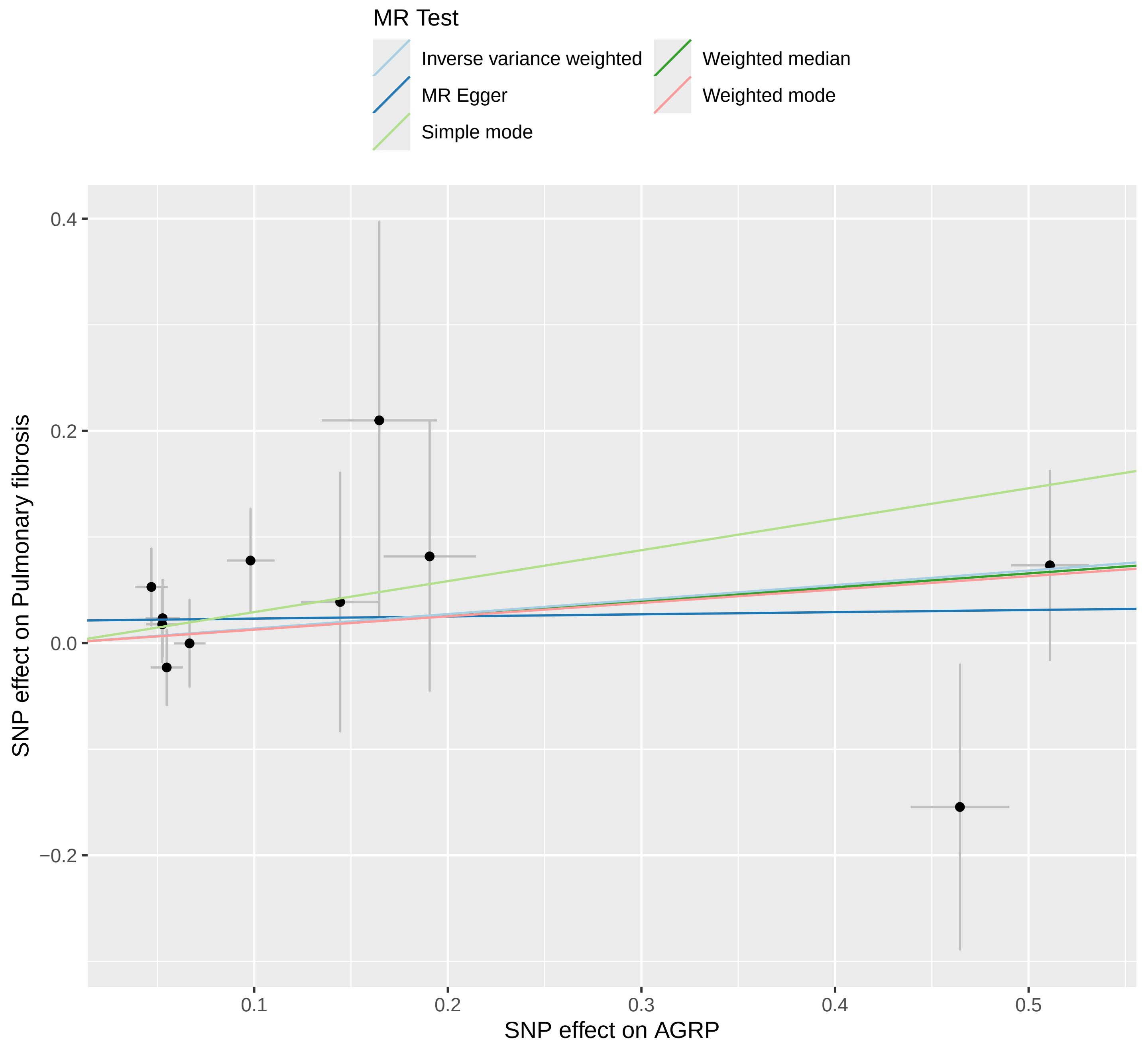
MR Test
Inverse variance weighted
MR Egger
Simple mode
Weighted median
Weighted mode
SNP effect on Pulmonary fibrosis
0.4
0.2
0.0
−0.2
0.1
0.2
0.3
0.4
0.5
SNP effect on AGRP

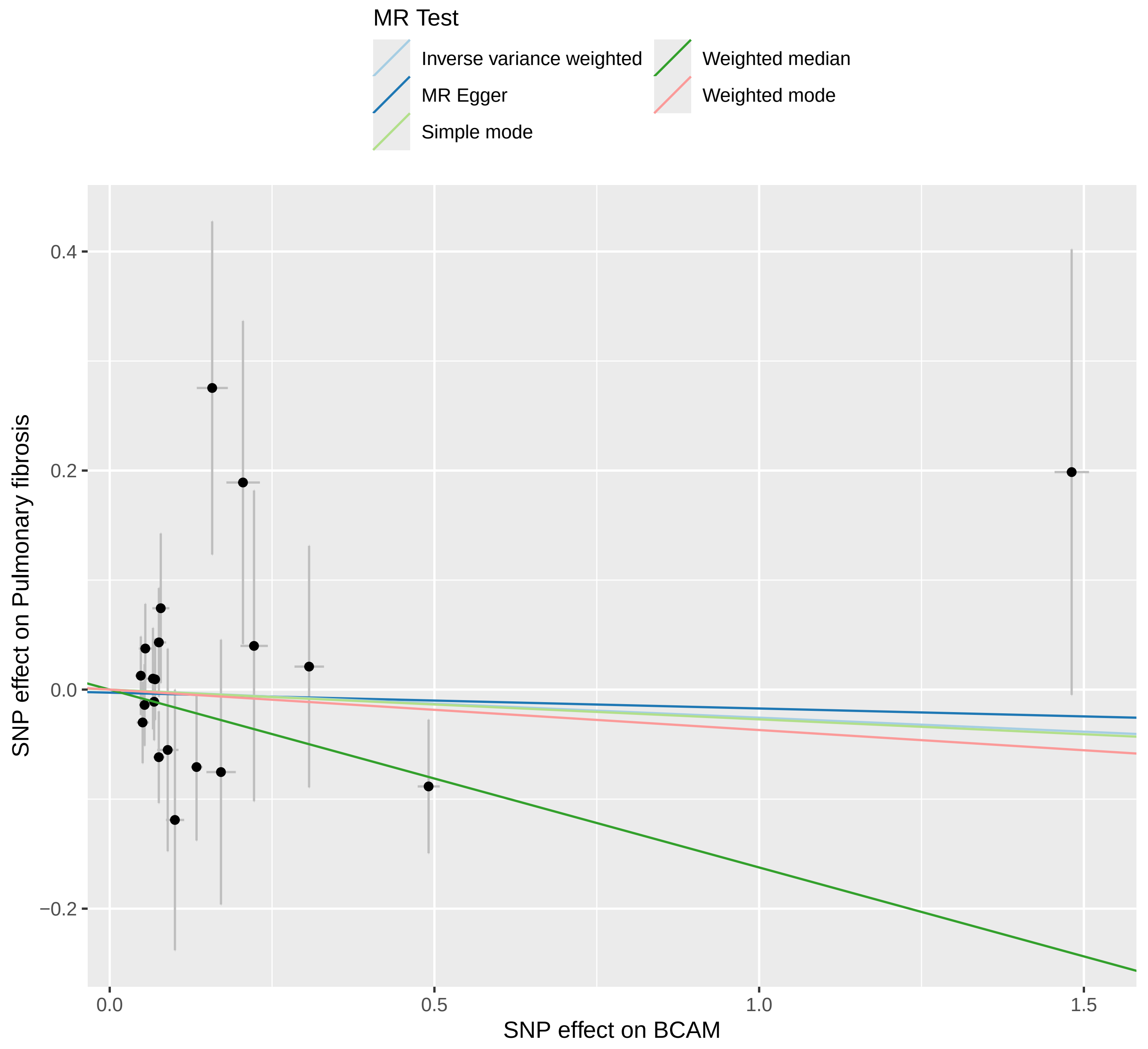
MR Test
Inverse variance weighted
MR Egger
Simple mode
Weighted median
Weighted mode
SNP effect on Pulmonary fibrosis
0.4
0.2
0.0
−0.2
0.0
0.5
1.0
1.5
SNP effect on BCAM

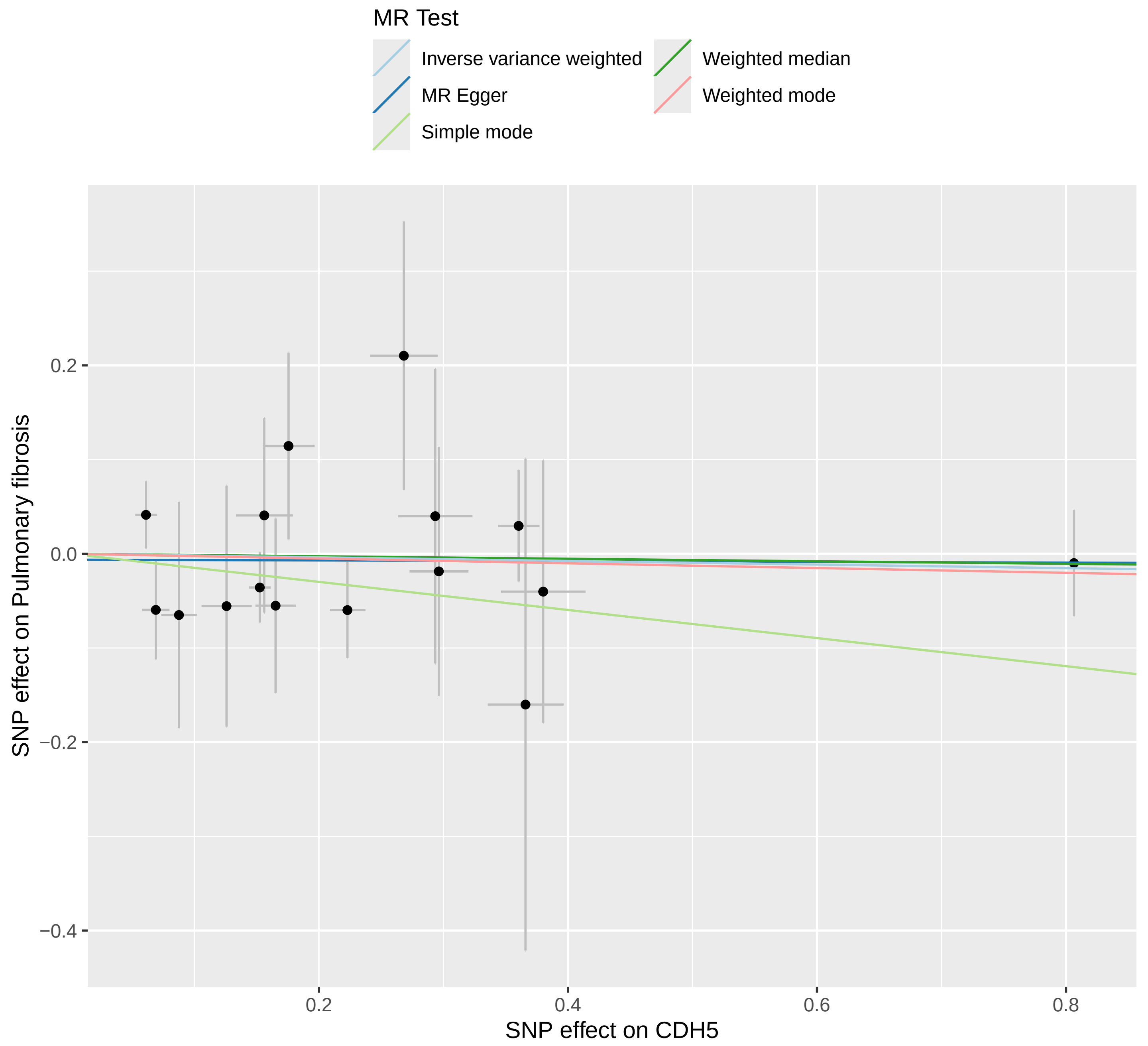

MR Test
Inverse variance weighted
MR Egger
Simple mode
Weighted median
Weighted mode
0.2
0.0
−0.2
−0.4
0.2
0.4
0.6
0.8
SNP effect on Pulmonary fibrosis
SNP effect on CDH5

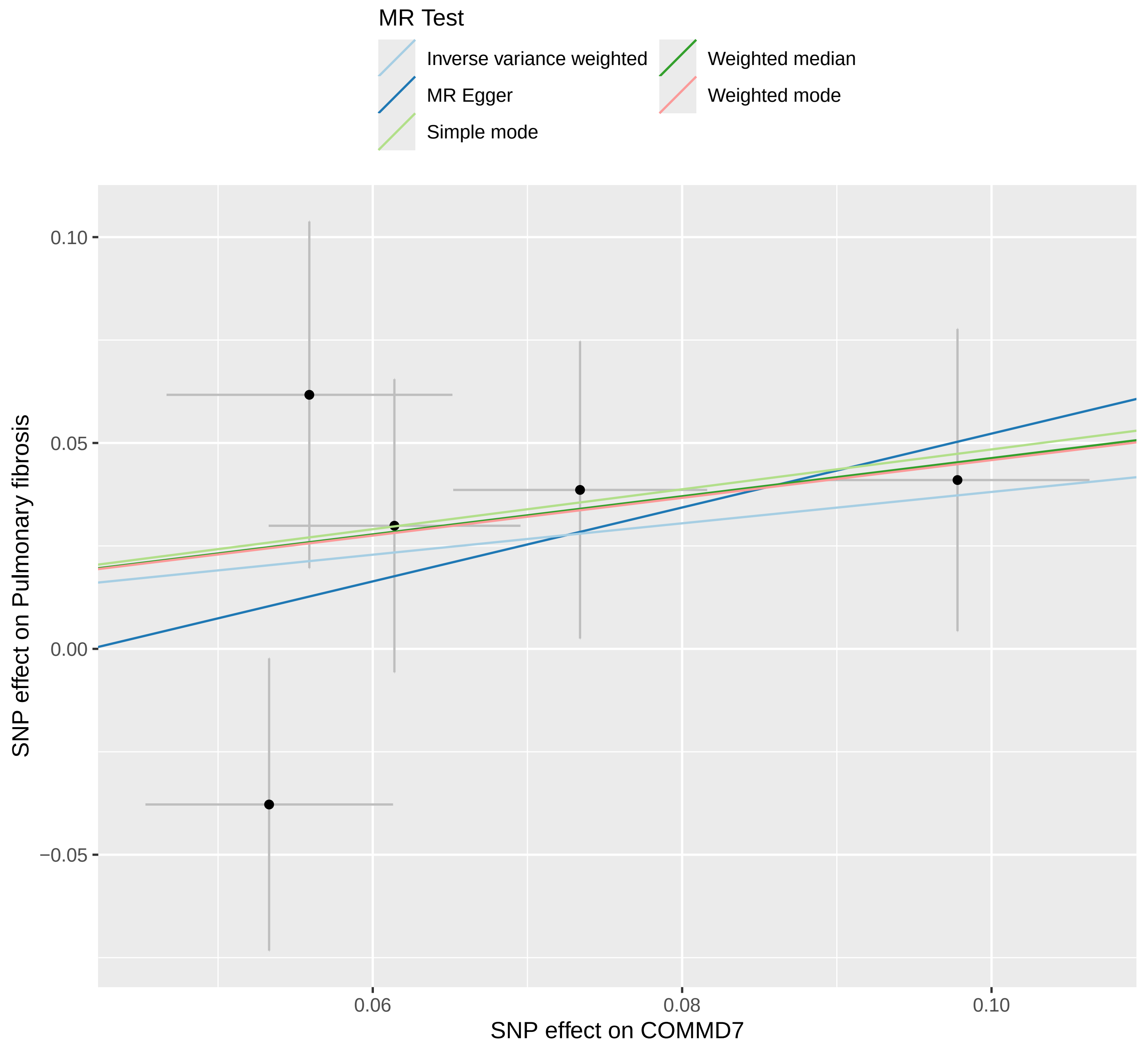

MR Test
Inverse variance weighted
MR Egger
Simple mode
Weighted median
Weighted mode
0.10
0.05
0.00
−0.05
SNP effect on Pulmonary fibrosis
0.06
0.08
0.10
SNP effect on COMMD7

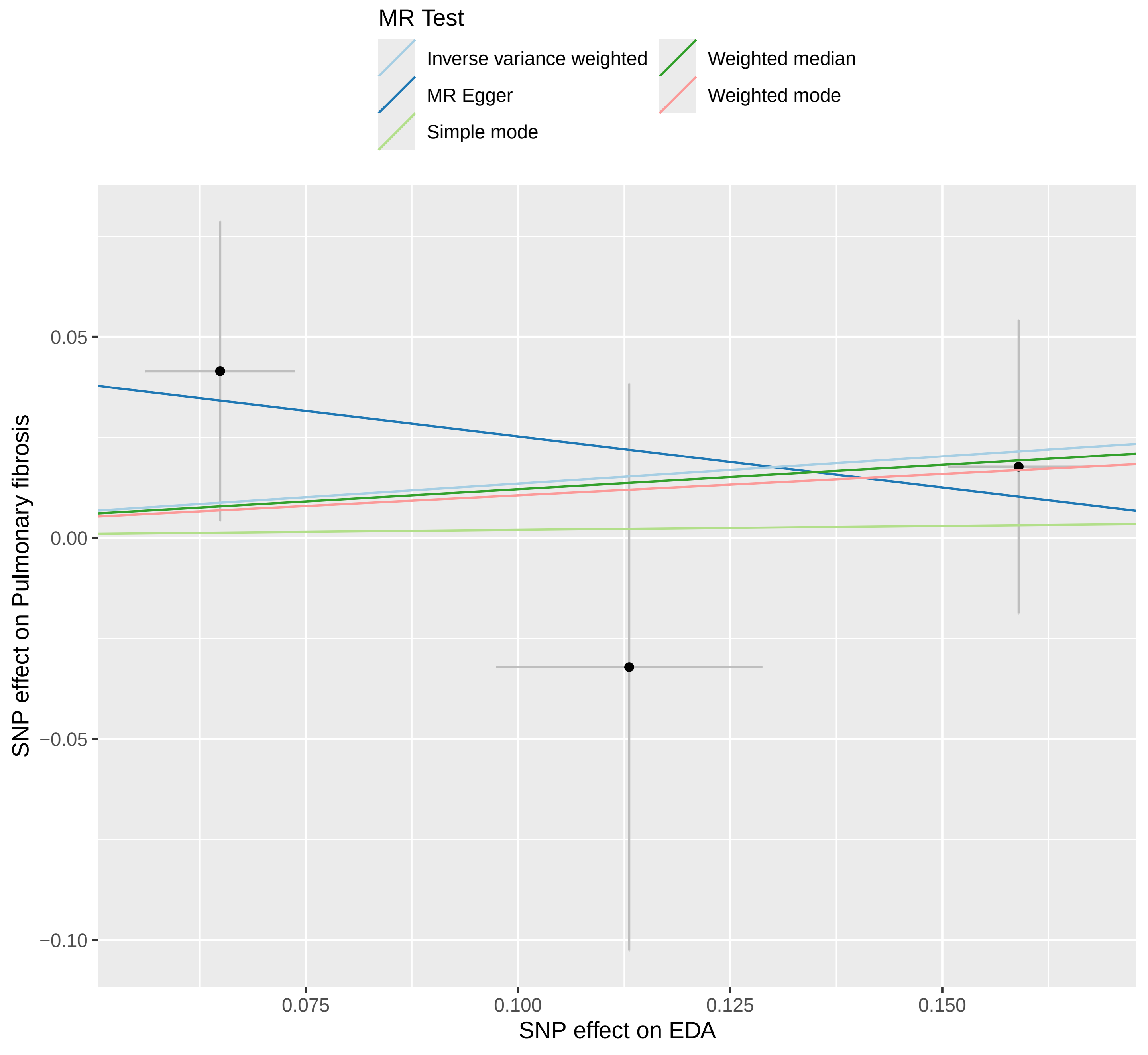
MR Test
Inverse variance weighted
MR Egger
Simple mode
Weighted median
Weighted mode
0.05
0.00
−0.05
−0.10
SNP effect on Pulmonary fibrosis
0.075
0.100
0.125
0.150
SNP effect on EDA

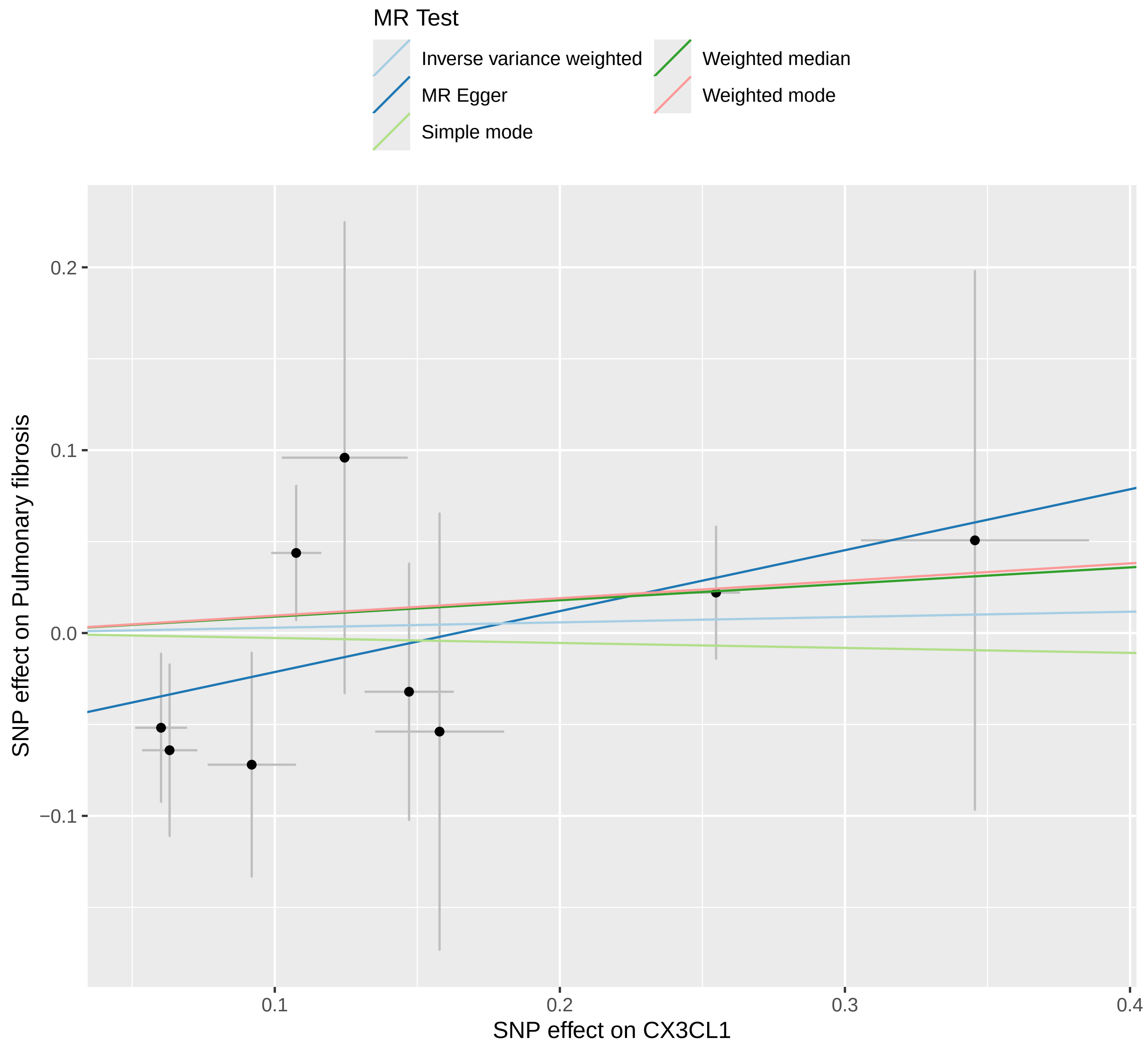

MR Test
Inverse variance weighted
MR Egger
Simple mode
Weighted median
Weighted mode
SNP effect on Pulmonary fibrosis
0.2
0.1
0.0
−0.1
0.1
0.2
0.3
0.4
SNP effect on CX3CL1

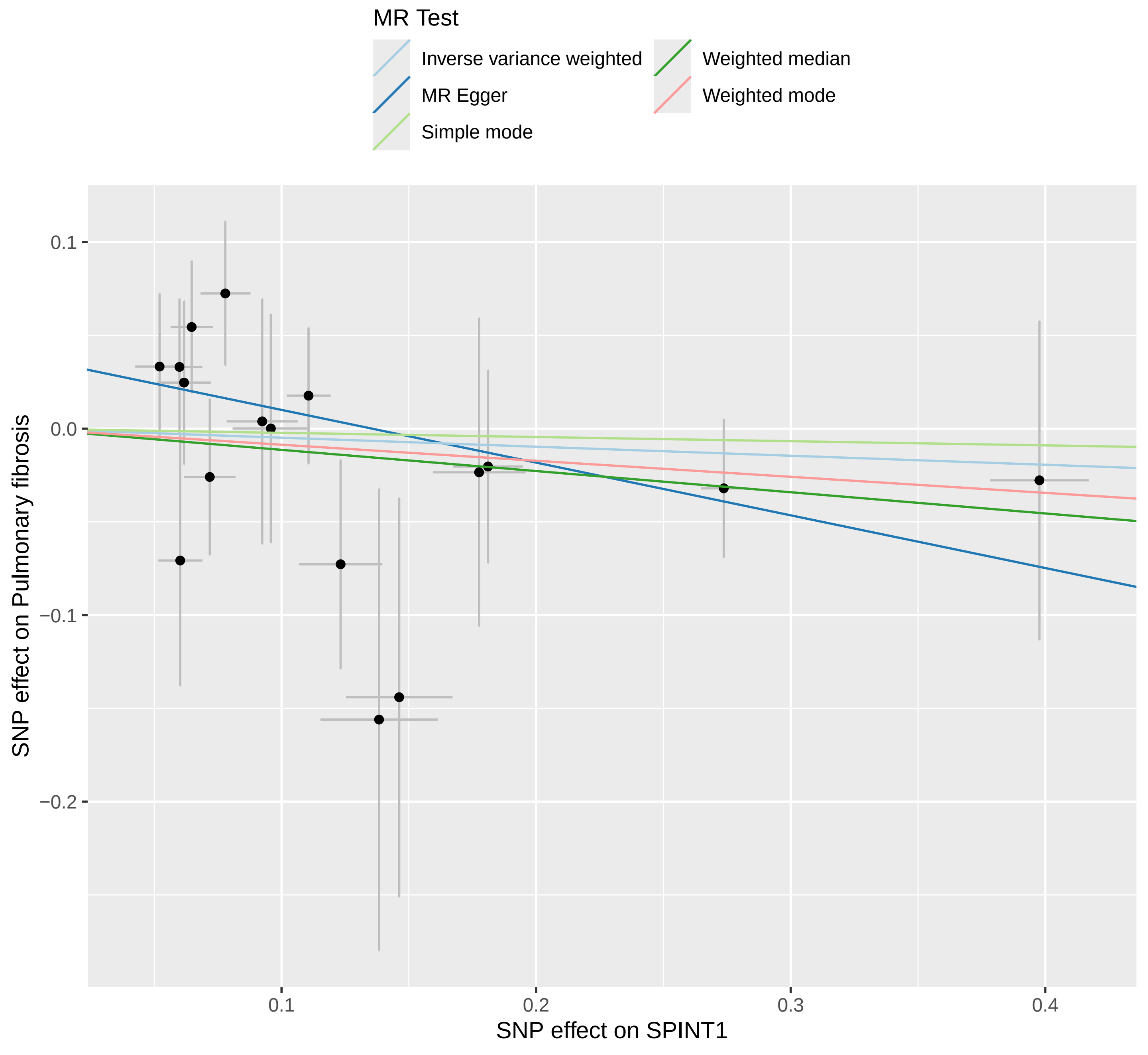

MR Test
Inverse variance weighted
MR Egger
Simple mode
Weighted median
Weighted mode
0.1
0.0
−0.1
−0.2
SNP effect on Pulmonary fibrosis
0.1
0.2
0.3
0.4
SNP effect on SPINT1

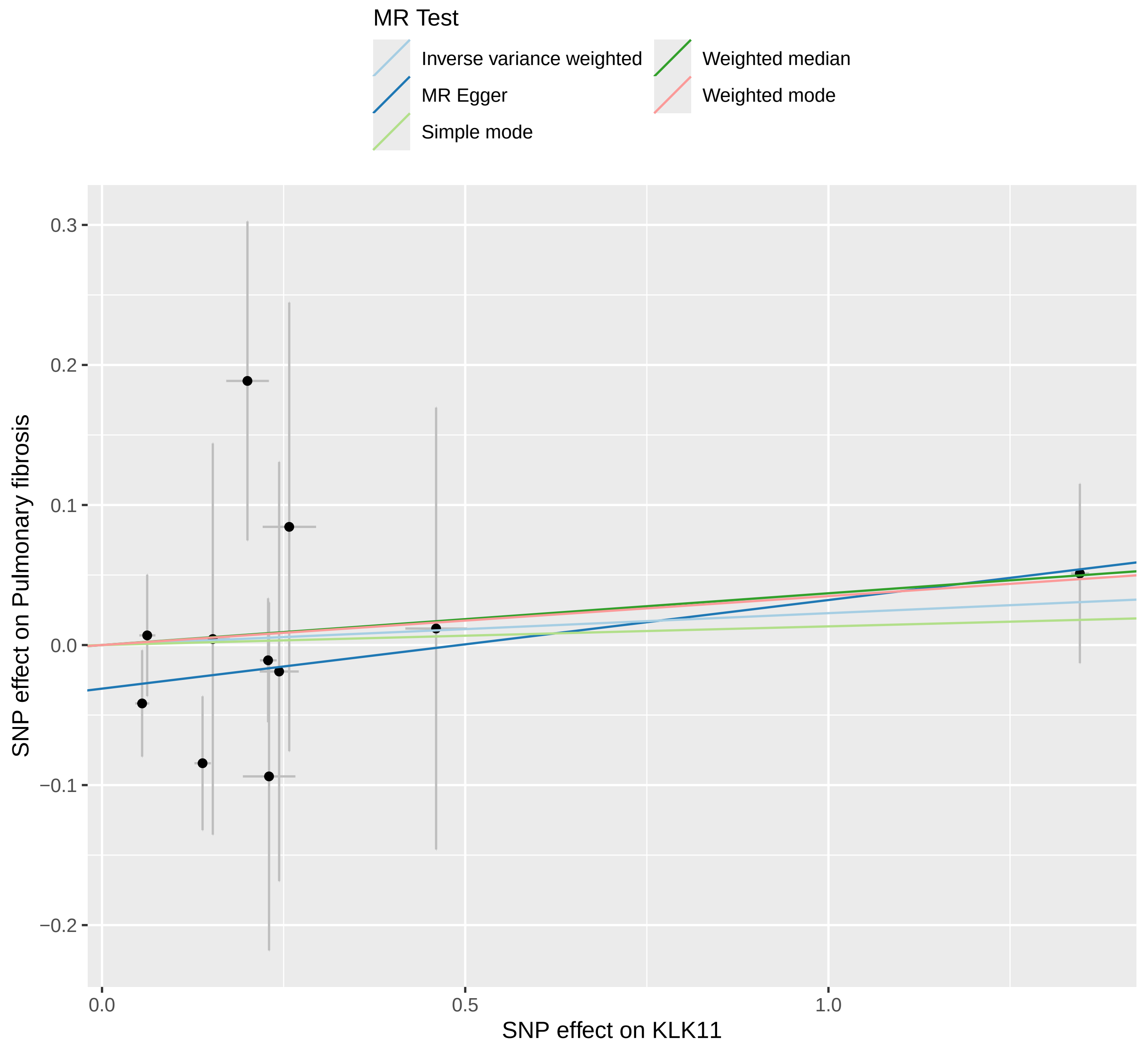

MR Test
Inverse variance weighted
MR Egger
Simple mode
Weighted median
Weighted mode
0.3
0.2
0.1
0.0
−0.1
−0.2
0.0
0.5
1.0
SNP effect on Pulmonary fibrosis
SNP effect on KLK11

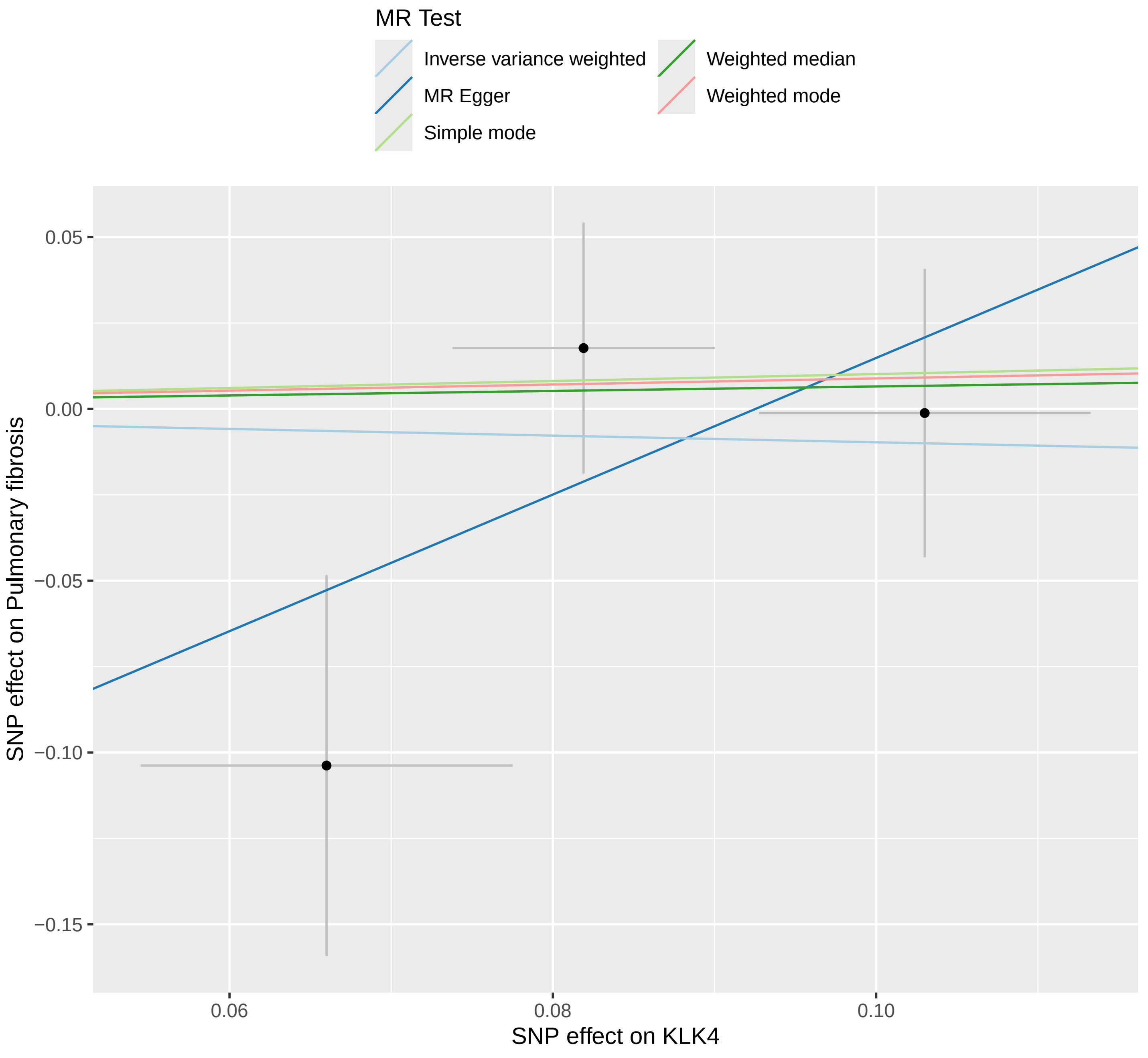
MR Test
Inverse variance weighted
MR Egger
Simple mode
Weighted median
Weighted mode
0.05
0.00
−0.05
−0.10
−0.15
0.06
0.08
0.10
SNP effect on KLK4
SNP effect on Pulmonary fibrosis

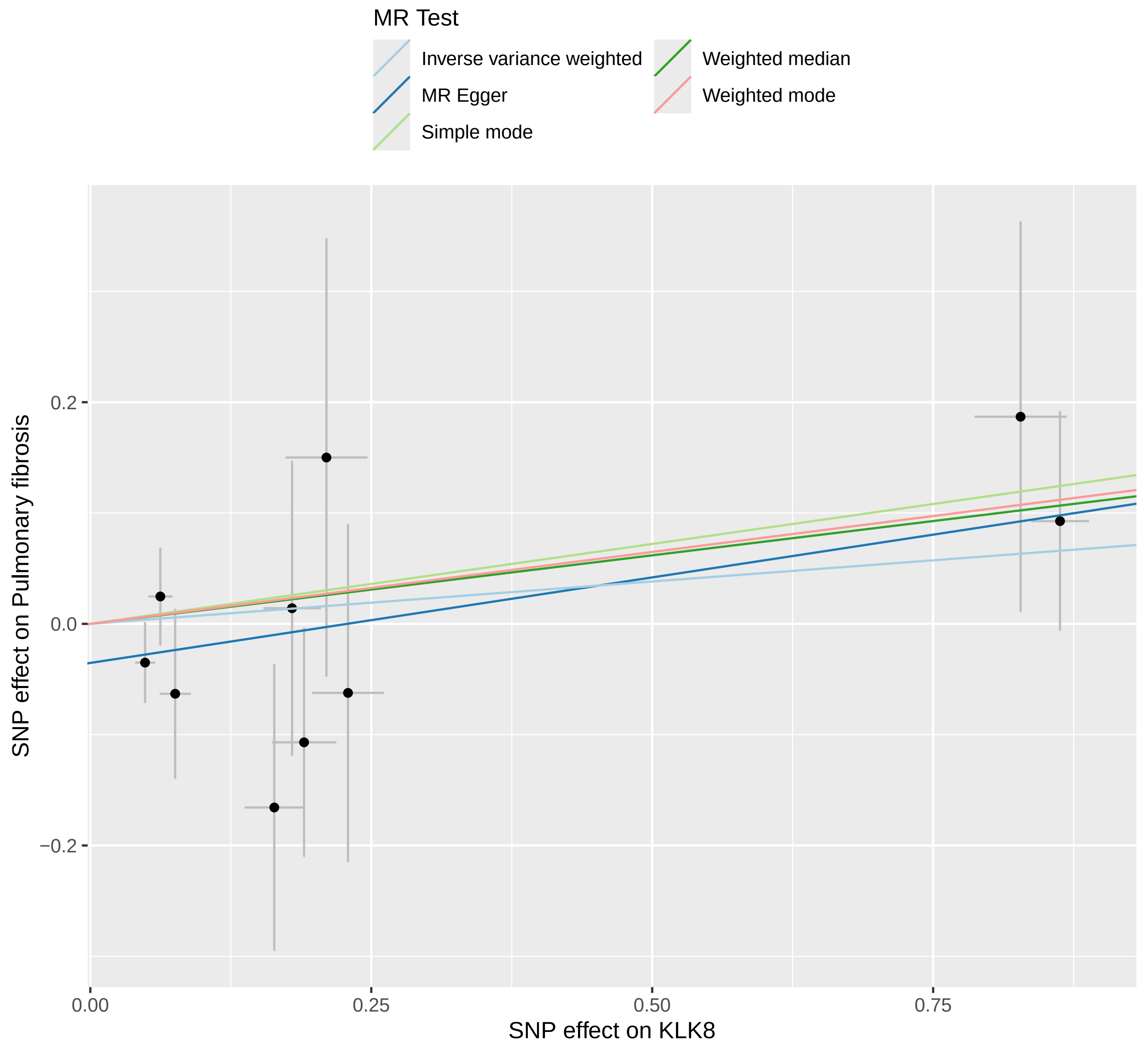

MR Test
Inverse variance weighted
MR Egger
Simple mode
Weighted median
Weighted mode
SNP effect on Pulmonary fibrosis
0.2
0.0
−0.2
0.00
0.25
0.50
0.75
SNP effect on KLK8

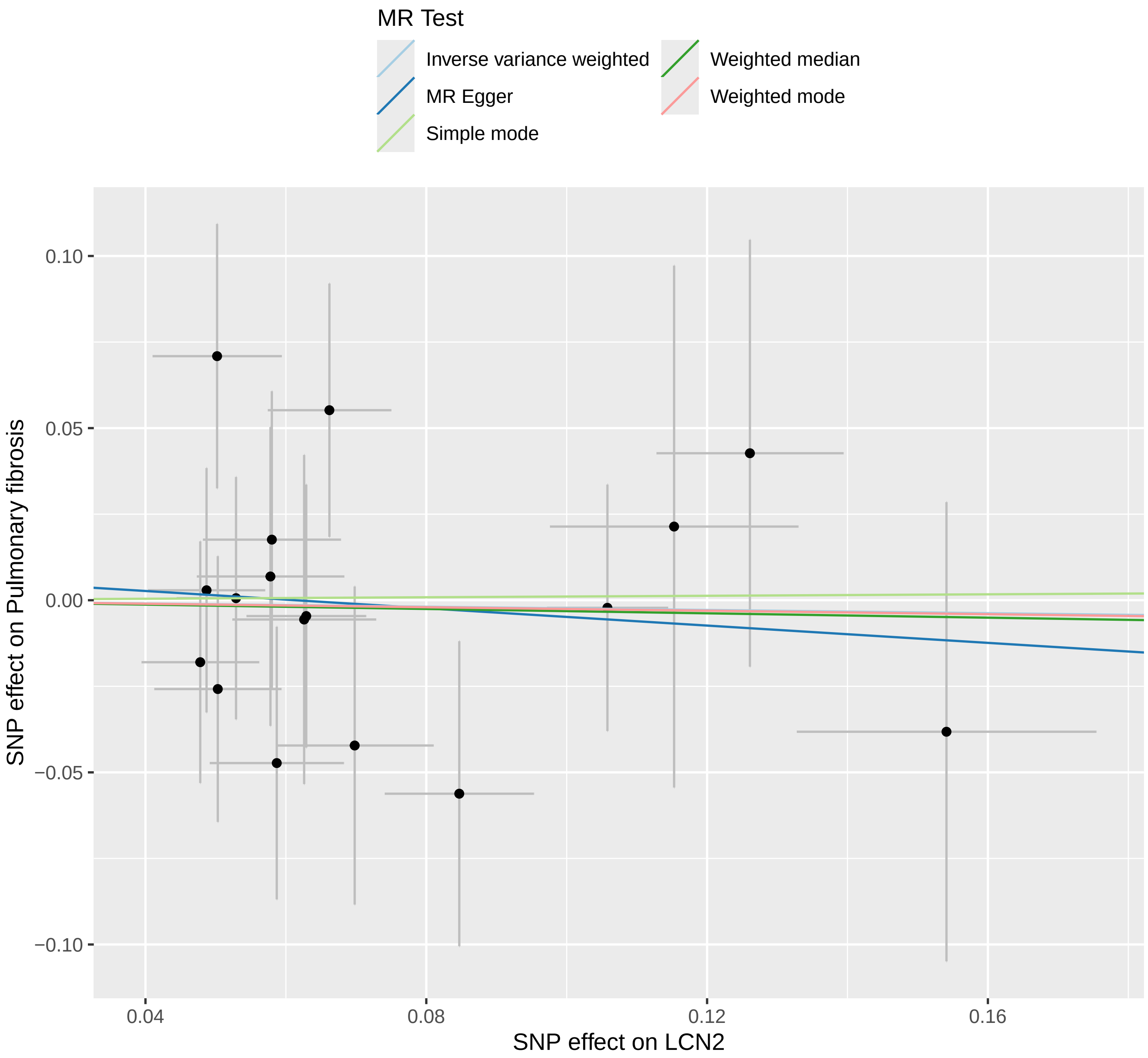

MR Test
Inverse variance weighted
MR Egger
Simple mode
Weighted median
Weighted mode
0.10
0.05
0.00
−0.05
−0.10
SNP effect on Pulmonary fibrosis
0.04
0.08
0.12
0.16
SNP effect on LCN2

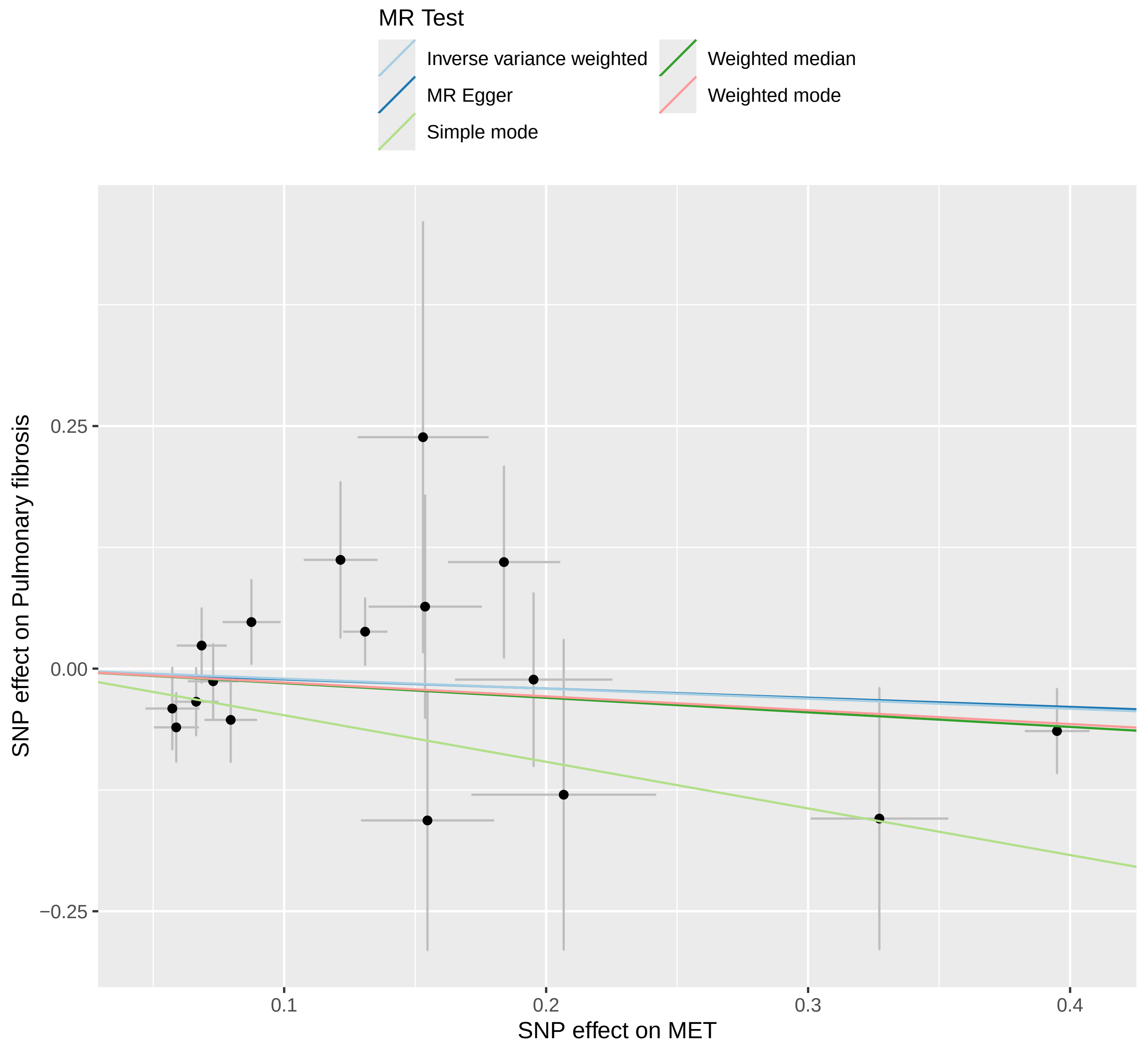

MR Test
Inverse variance weighted
MR Egger
Simple mode
Weighted median
Weighted mode
SNP effect on Pulmonary fibrosis
0.25
0.00
−0.25
0.1
0.2
0.3
0.4
SNP effect on MET

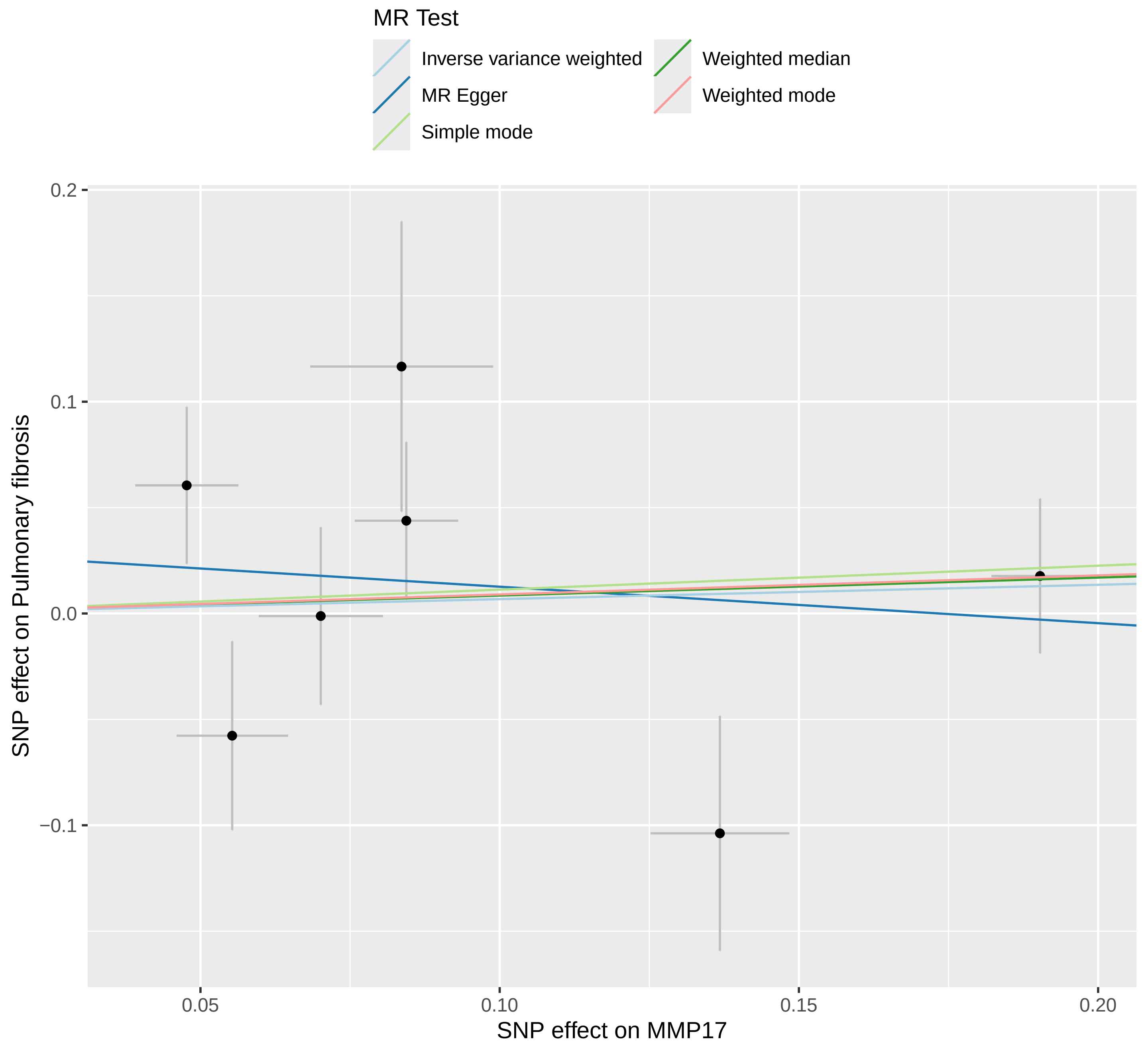
MR Test
Inverse variance weighted
MR Egger
Simple mode
Weighted median
Weighted mode
SNP effect on Pulmonary fibrosis
0.2
0.1
0.0
−0.1
0.05
0.10
0.15
0.20
SNP effect on MMP17

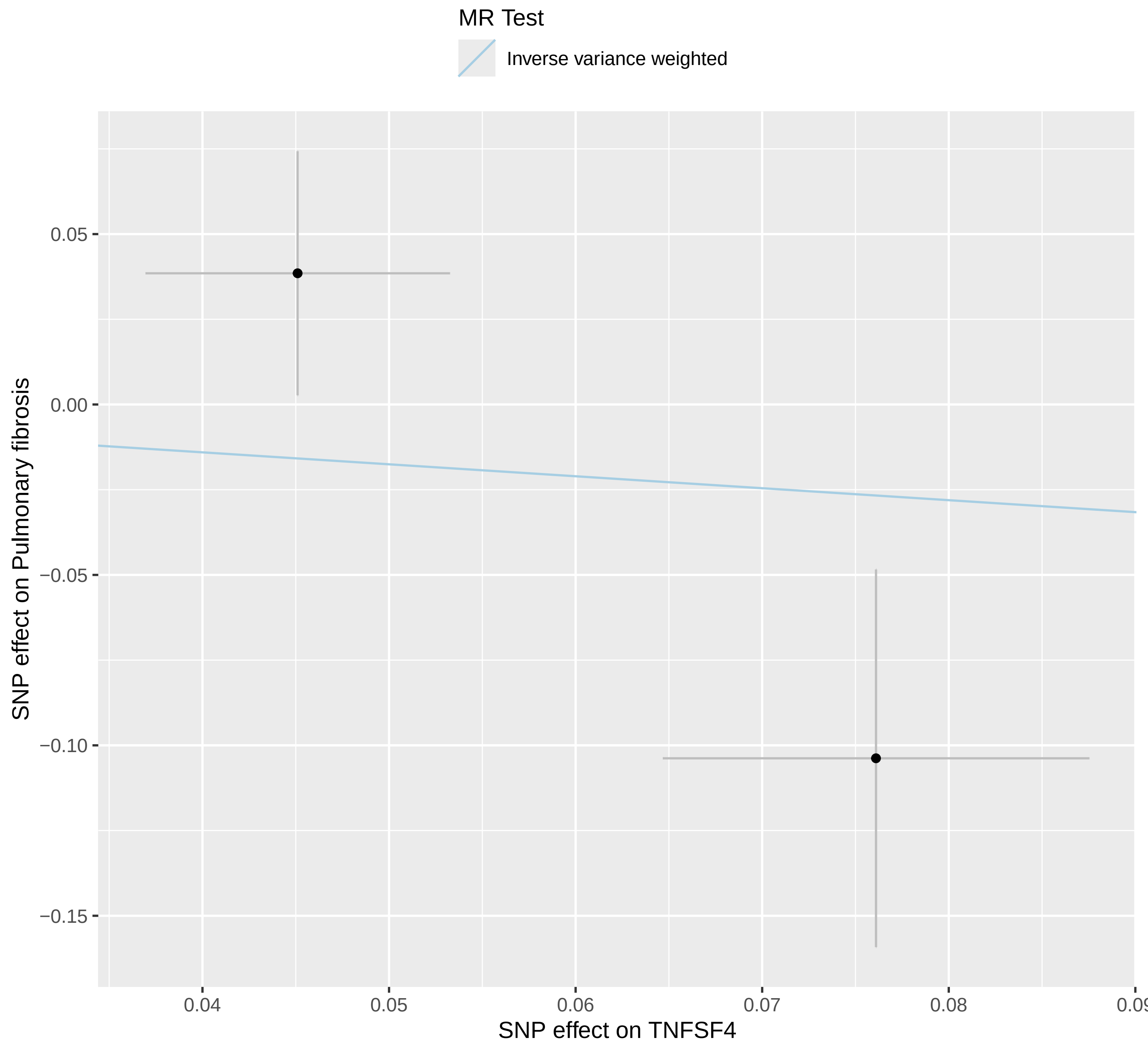

MR Test
Inverse variance weighted
0.05
0.00
−0.05
−0.10
−0.15
SNP effect on Pulmonary fibrosis
0.04
0.05
0.06
0.07
0.08
SNP effect on TNFSF4

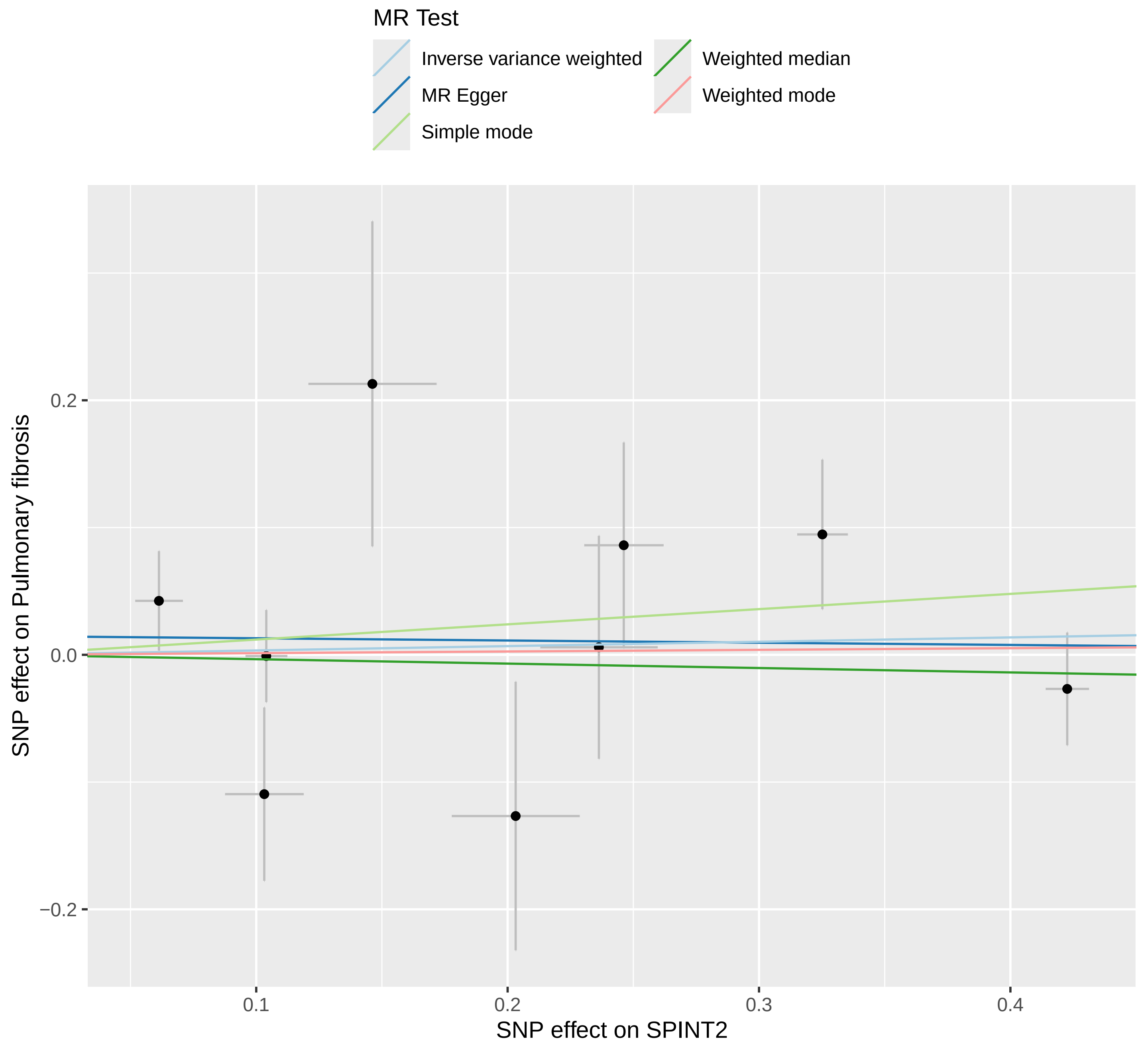
MR Test
Inverse variance weighted
MR Egger
Simple mode
Weighted median
Weighted mode
0.2
0.0
−0.2
0.1
0.2
0.3
0.4
SNP effect on Pulmonary fibrosis
SNP effect on SPINT2

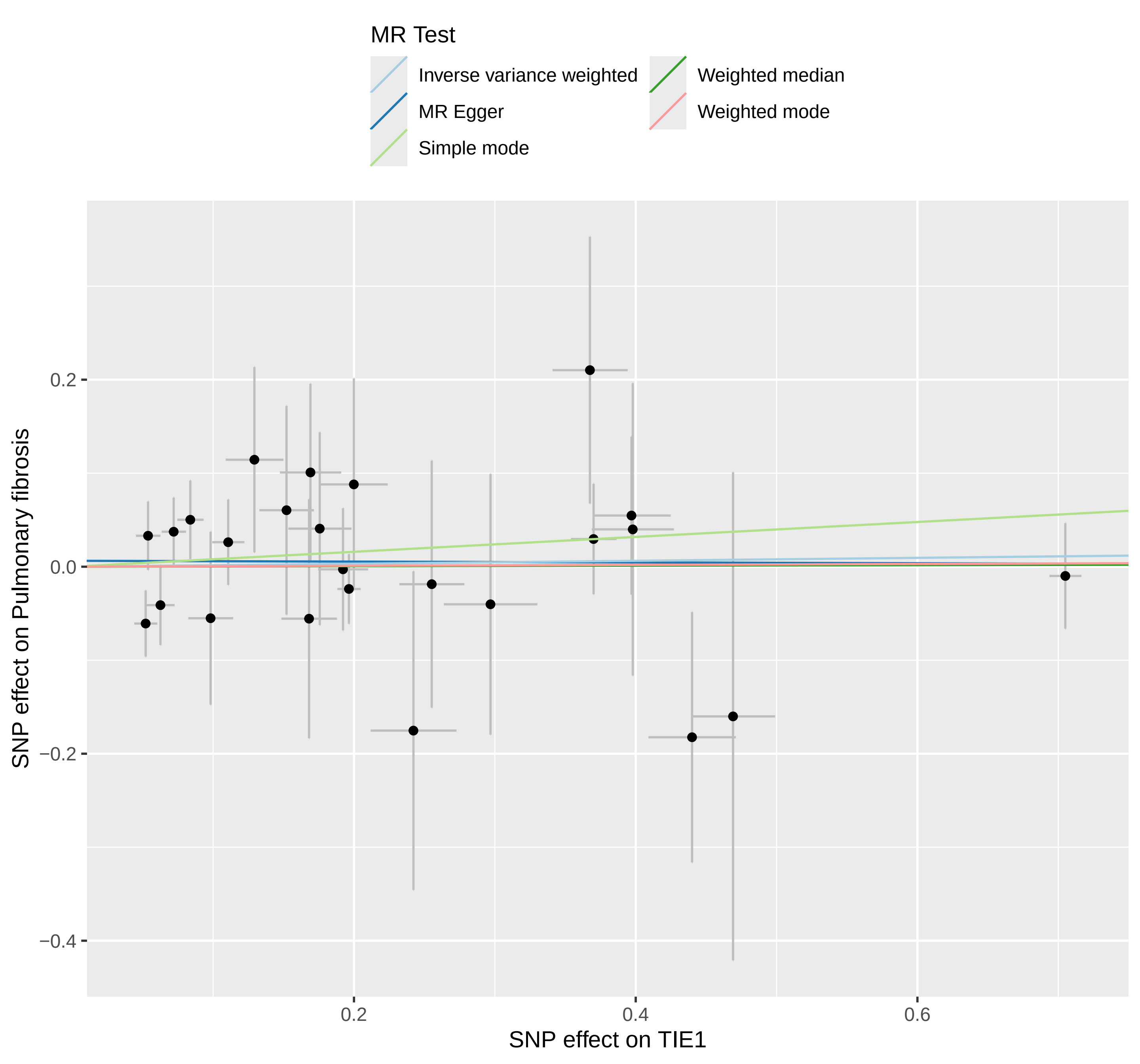

MR Test
Inverse variance weighted
MR Egger
Simple mode
Weighted median
Weighted mode
0.2
0.0
−0.2
−0.4
SNP effect on Pulmonary fibrosis
0.2
0.4
0.6
SNP effect on TIE1

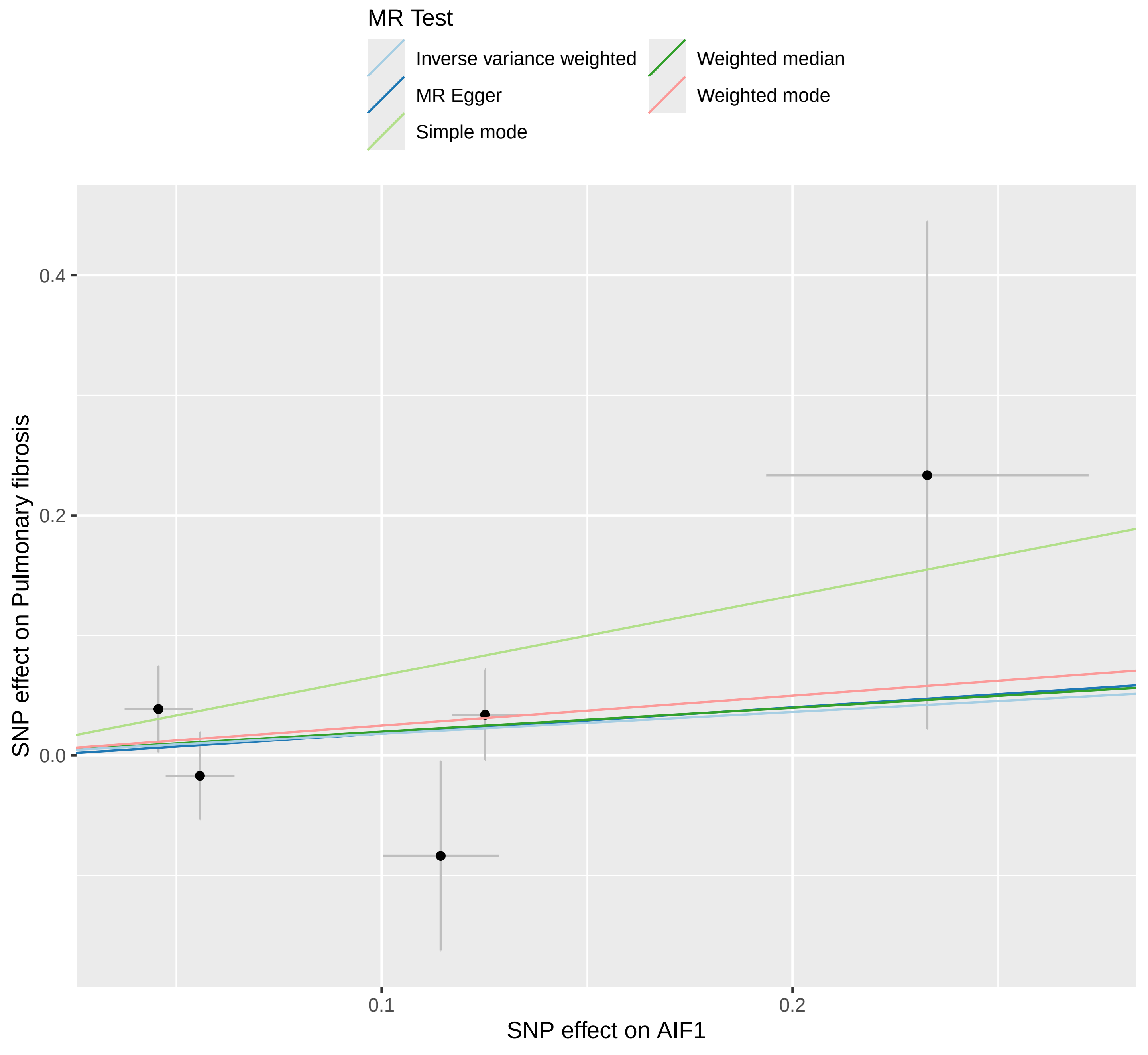

MR Test
Inverse variance weighted
MR Egger
Simple mode
Weighted median
Weighted mode
0.4
0.2
0.0
0.1
0.2
SNP effect on Pulmonary fibrosis
SNP effect on AIF1

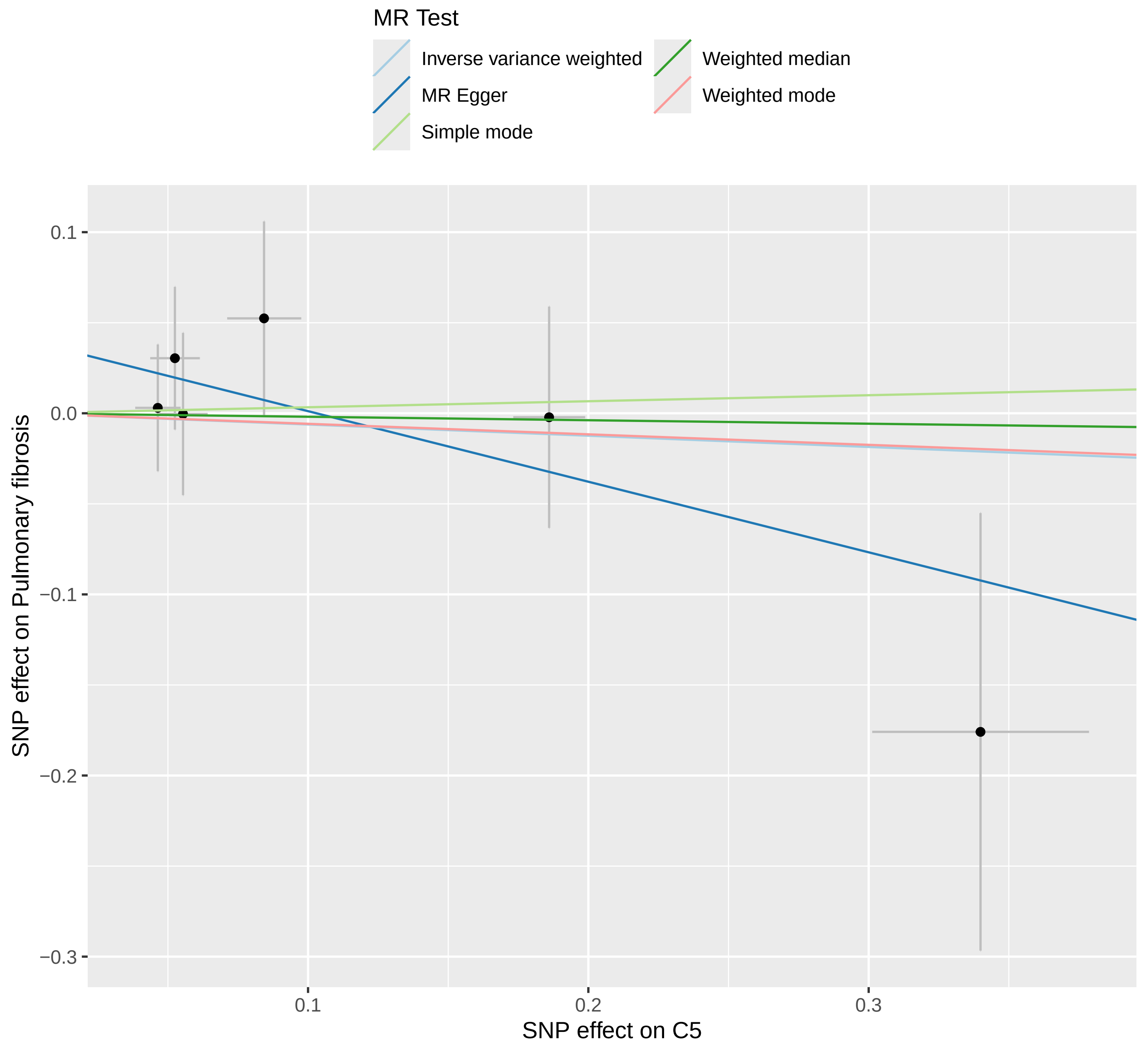

MR Test
Inverse variance weighted
MR Egger
Simple mode
Weighted median
Weighted mode
0.1
0.0
−0.1
−0.2
−0.3
SNP effect on Pulmonary fibrosis
0.1
0.2
0.3
SNP effect on C5

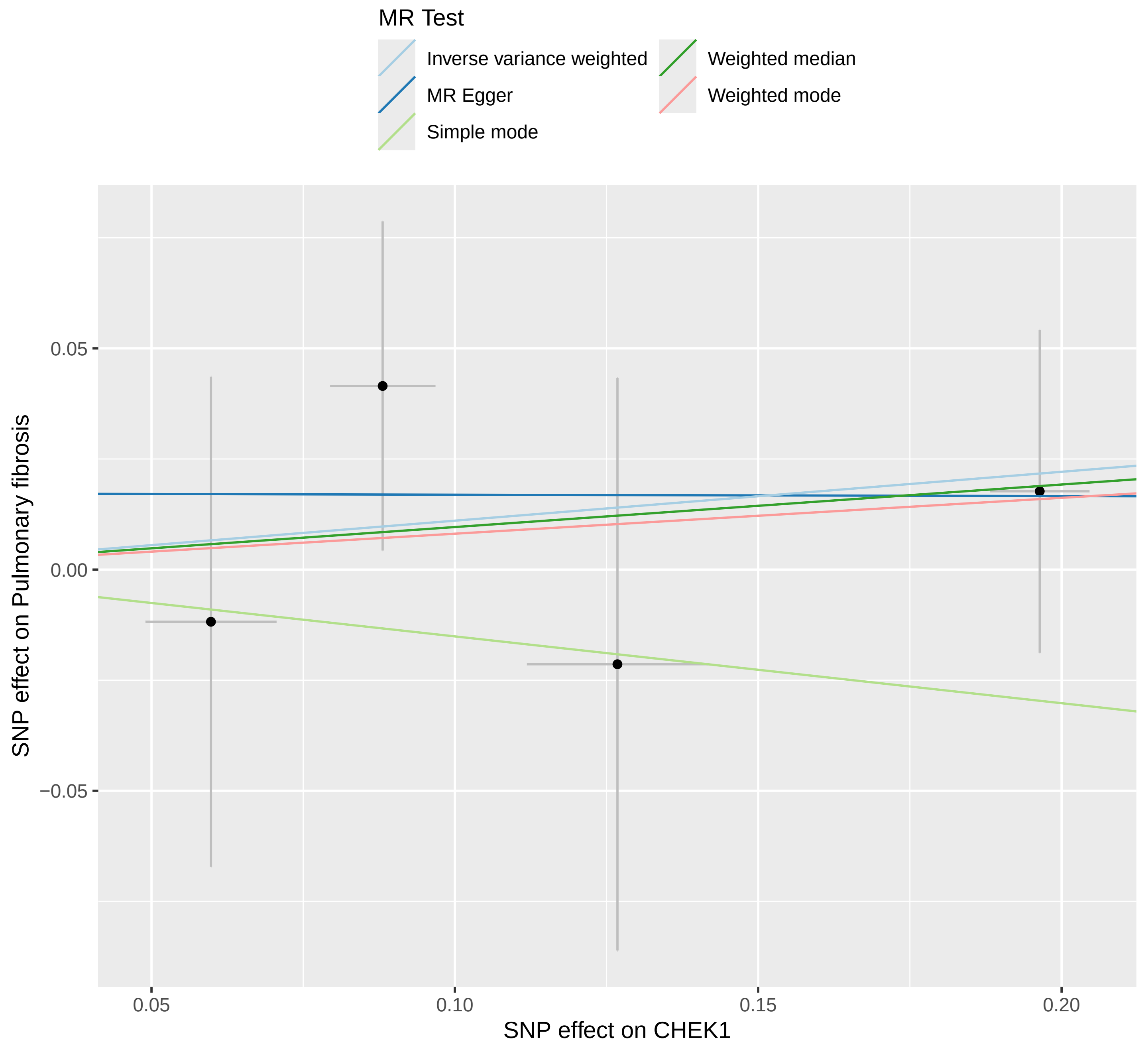
MR Test
Inverse variance weighted
MR Egger
Simple mode
Weighted median
Weighted mode
SNP effect on Pulmonary fibrosis
0.05
0.00
−0.05
0.05
0.10
0.15
0.20
SNP effect on CHEK1

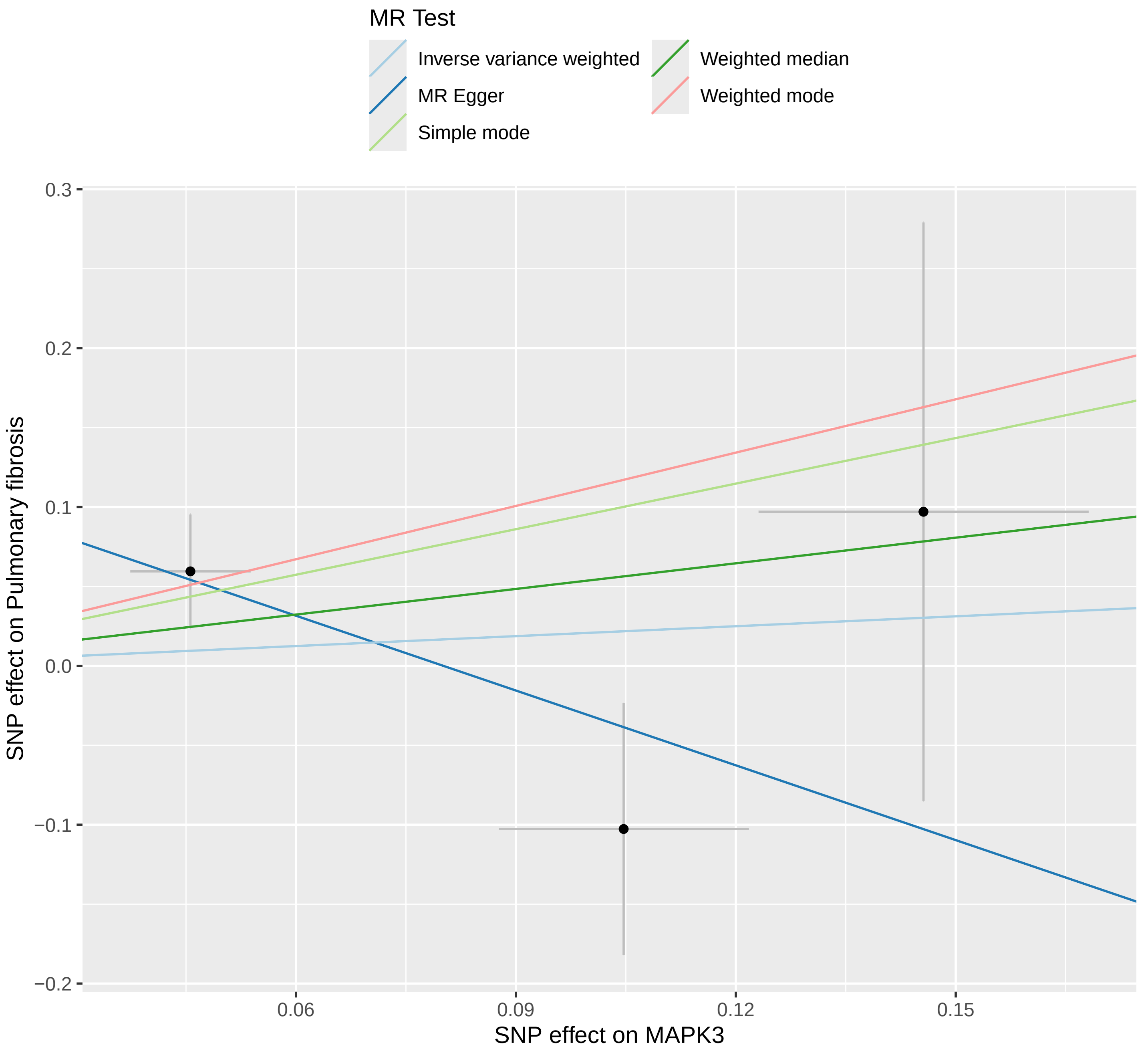
MR Test
Inverse variance weighted
MR Egger
Simple mode
Weighted median
Weighted mode
0.3
0.2
0.1
0.0
−0.1
−0.2
SNP effect on Pulmonary fibrosis
0.06
0.09
0.12
0.15
SNP effect on MAPK3

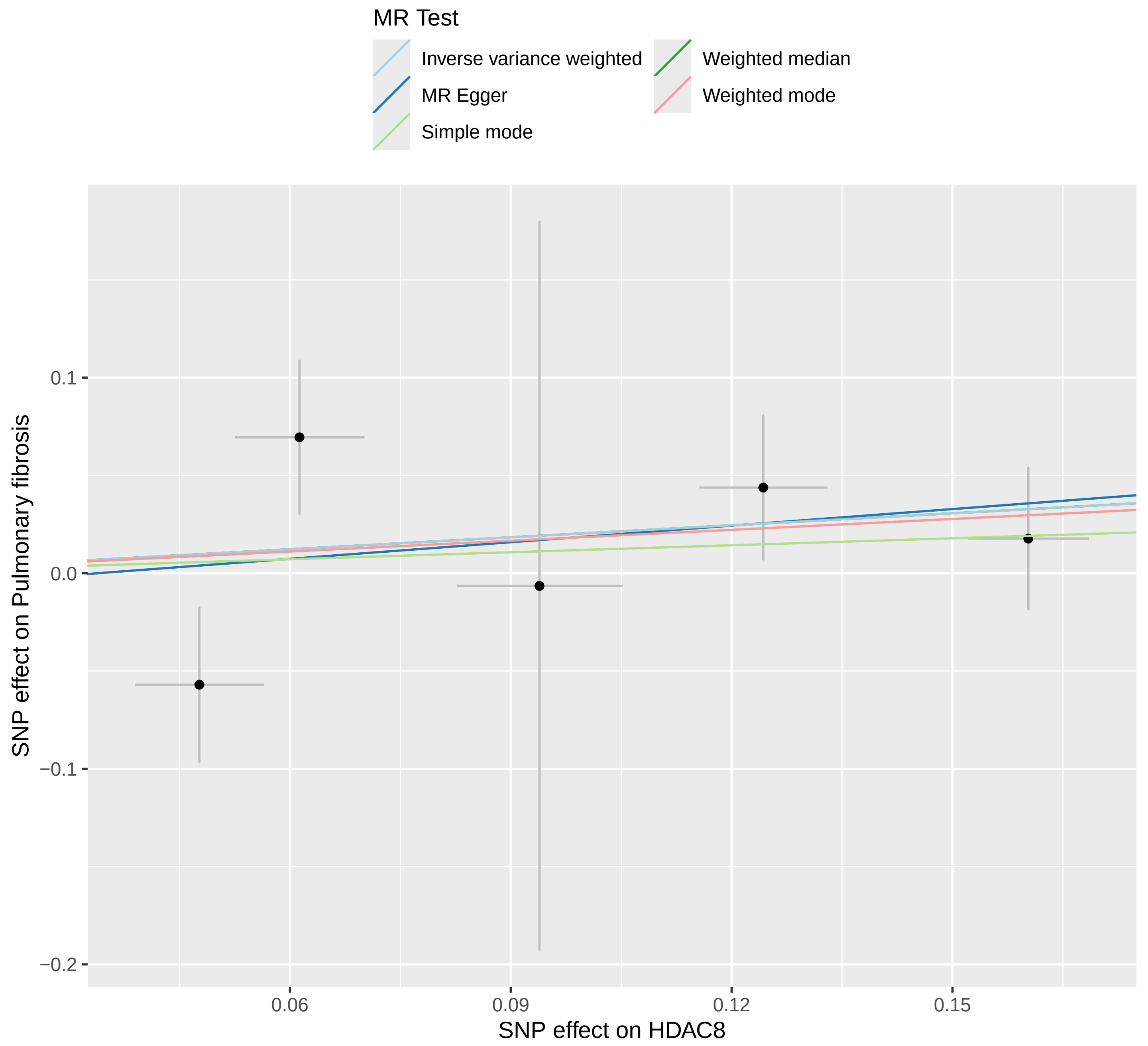
MR Test
Inverse variance weighted
MR Egger
Simple mode
Weighted median
Weighted mode
0.1
0.0
−0.1
−0.2
SNP effect on Pulmonary fibrosis
0.06
0.09
0.12
0.15
SNP effect on HDAC8

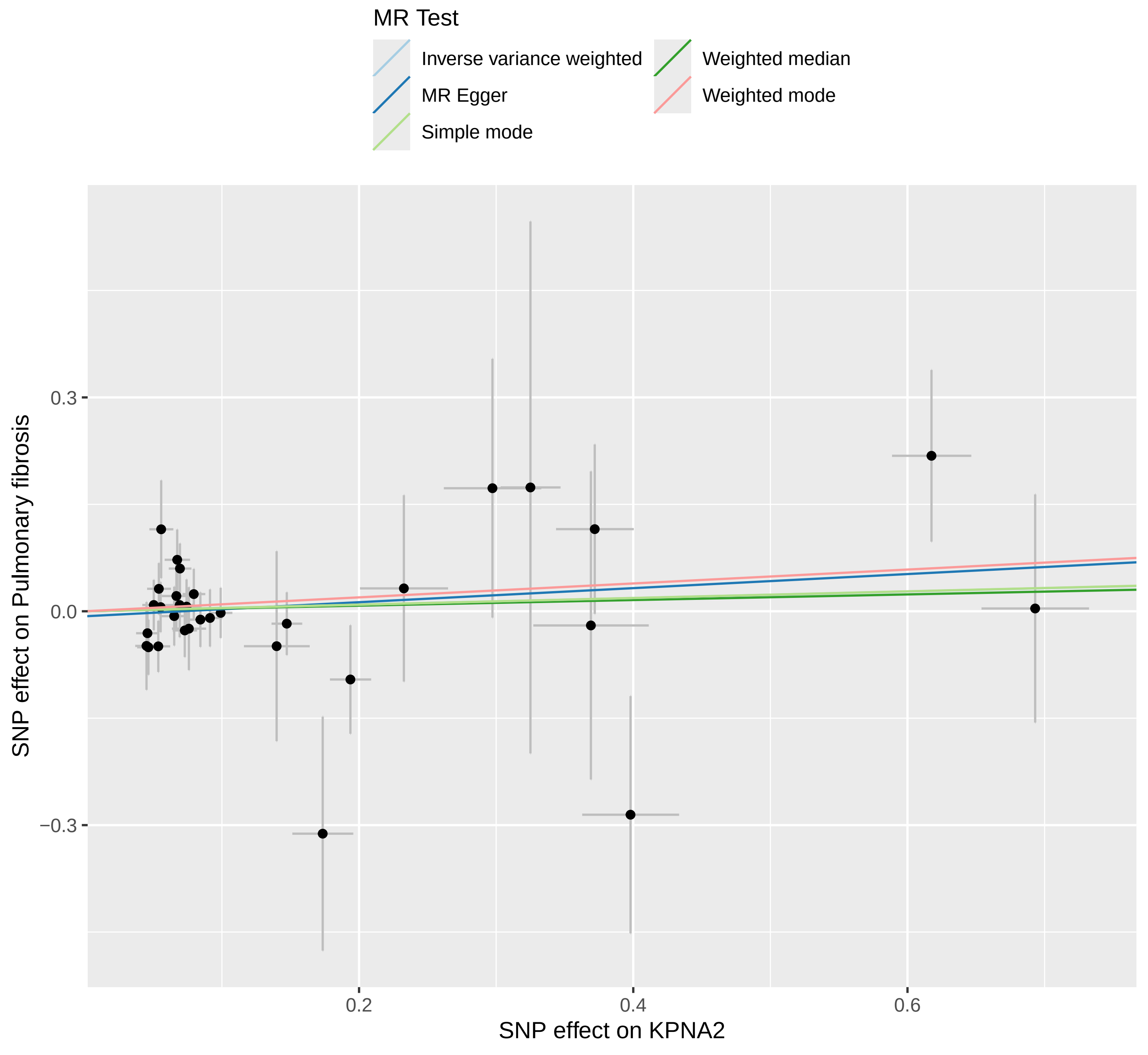

MR Test
Inverse variance weighted
MR Egger
Simple mode
Weighted median
Weighted mode
0.3
0.0
−0.3
SNP effect on Pulmonary fibrosis
0.2
0.4
0.6
SNP effect on KPNA2

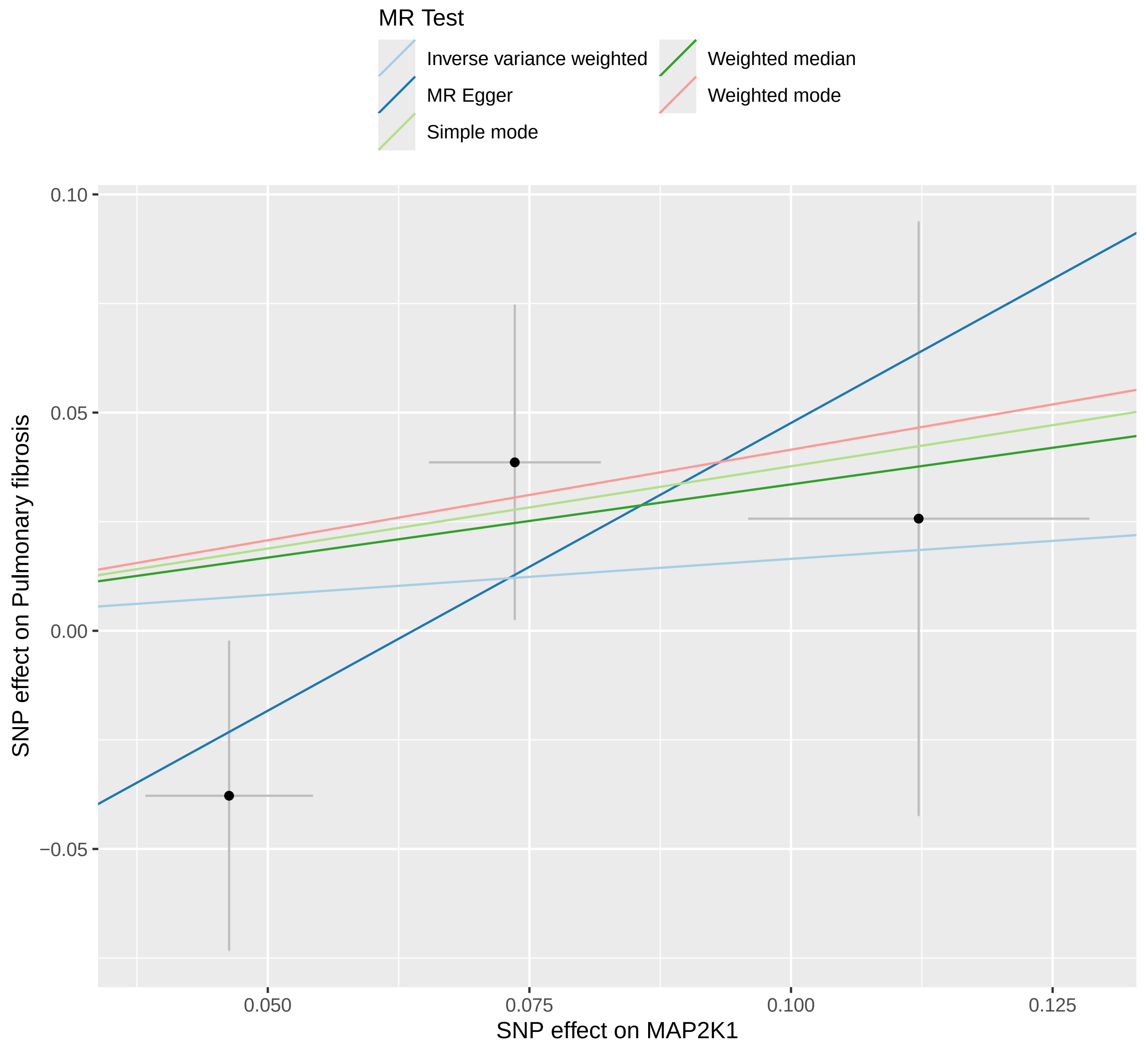

MR Test
Inverse variance weighted
MR Egger
Simple mode
Weighted median
Weighted mode
0.10
0.05
0.00
−0.05
SNP effect on Pulmonary fibrosis
0.050
0.075
0.100
0.125
SNP effect on MAP2K1

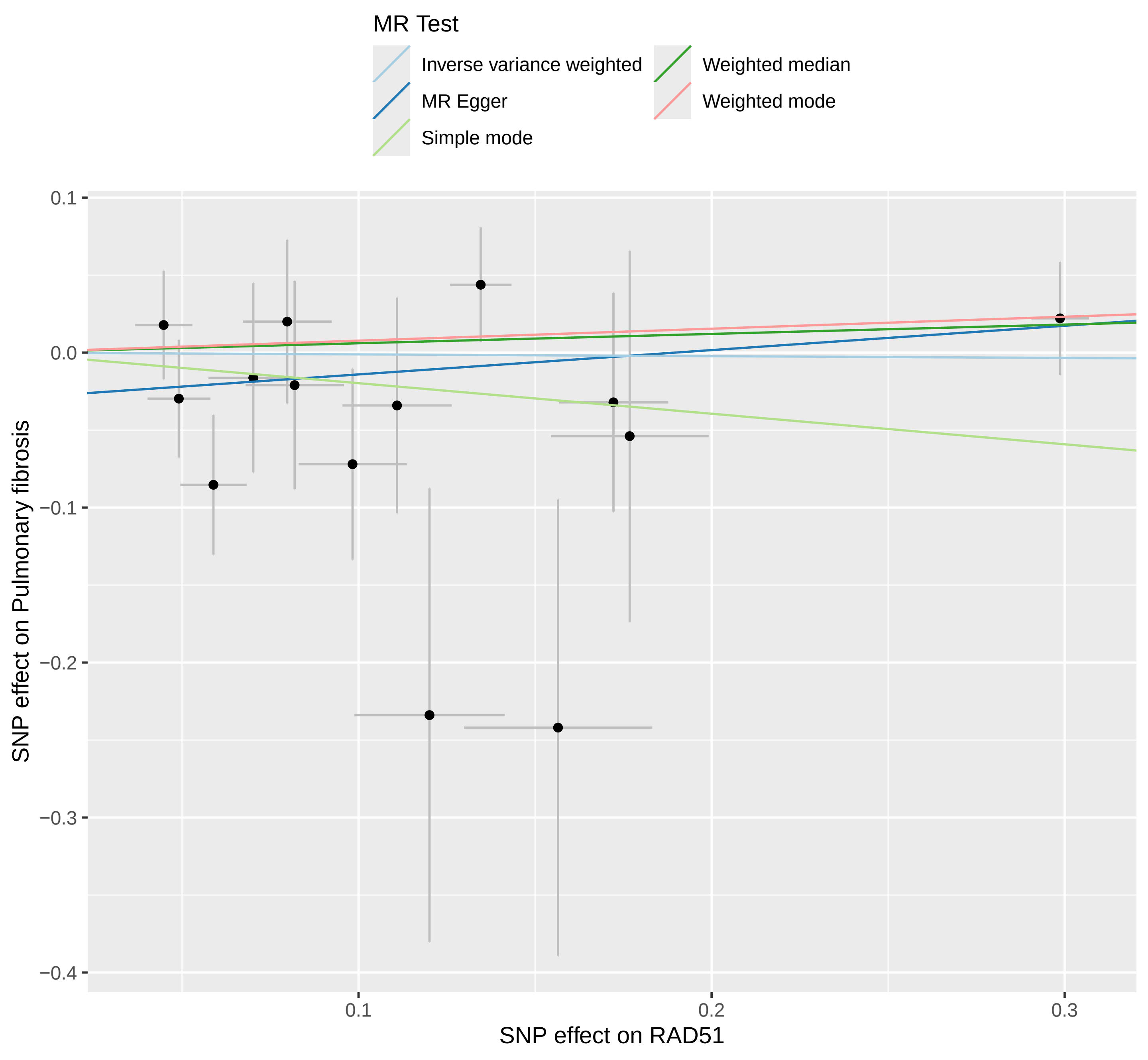
MR Test
Inverse variance weighted
MR Egger
Simple mode
Weighted median
Weighted mode
0.1
0.0
−0.1
−0.2
−0.3
−0.4
0.1
0.2
0.3
SNP effect on Pulmonary fibrosis
SNP effect on RAD51

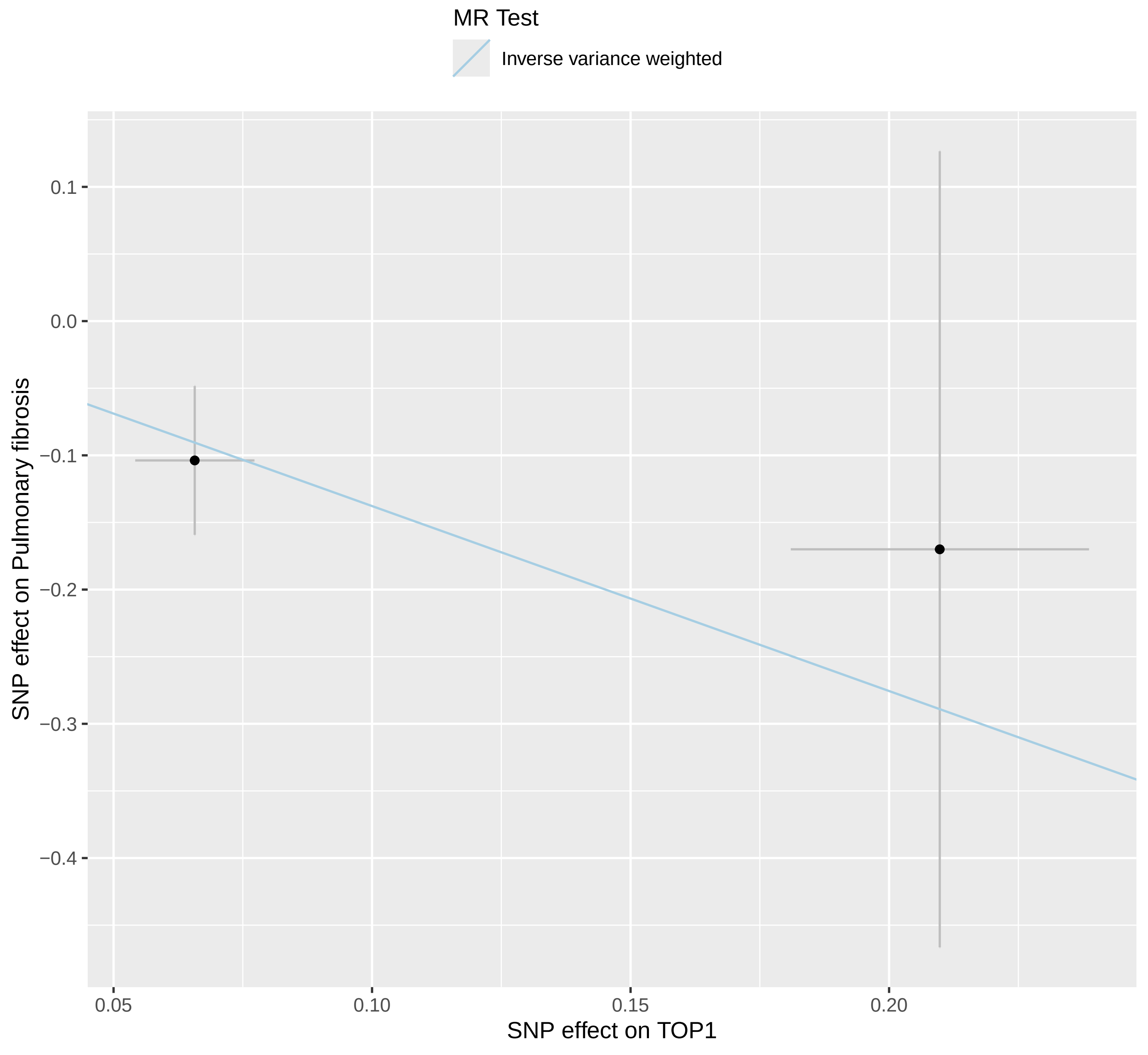

MR Test
Inverse variance weighted
0.1
0.0
−0.1
−0.2
−0.3
−0.4
SNP effect on Pulmonary fibrosis
0.05
0.10
0.15
0.20
SNP effect on TOP1

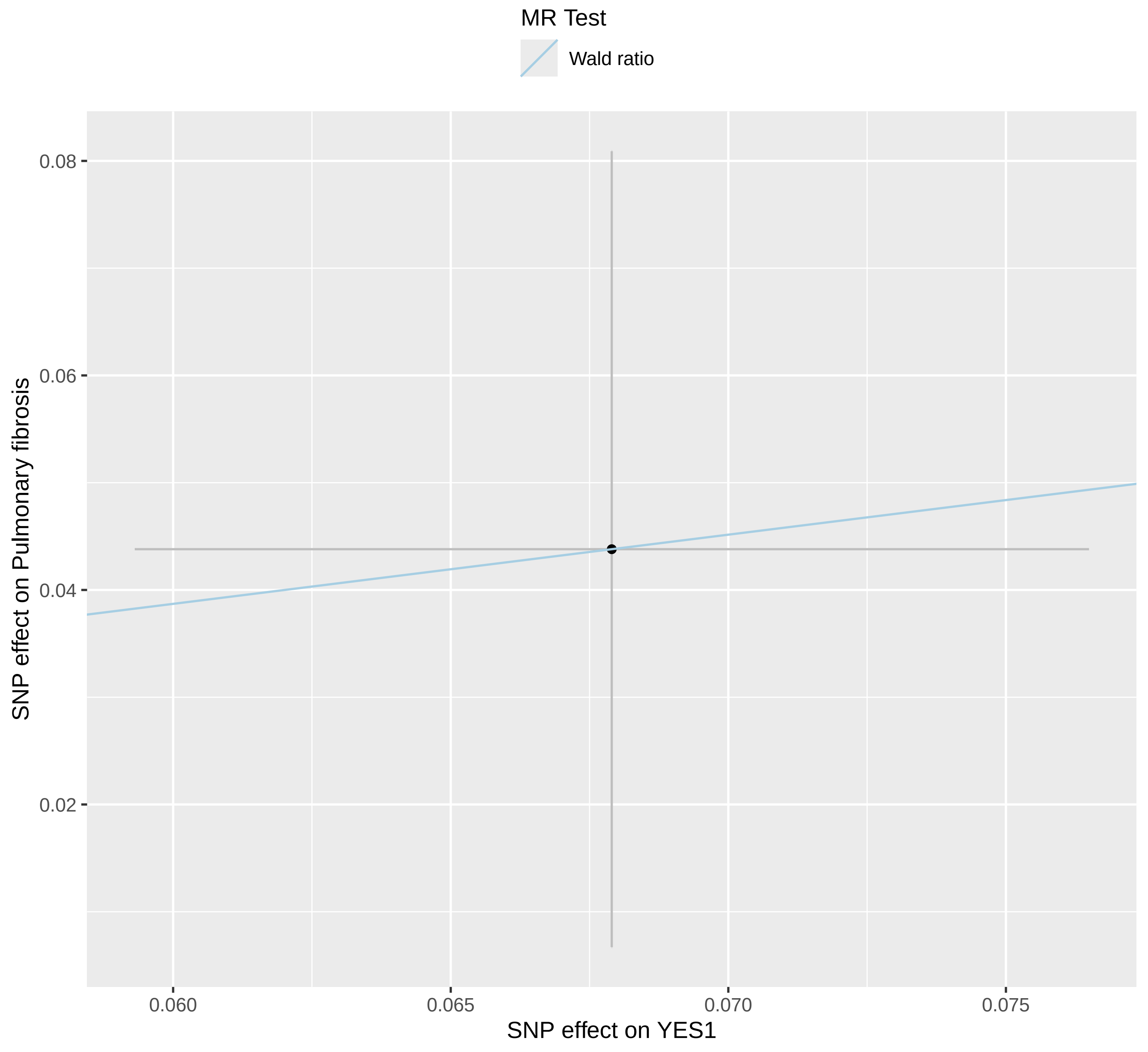

MR Test
Wald ratio
0.08
0.06
0.04
0.02
SNP effect on Pulmonary fibrosis
0.060
0.065
0.070
0.075
SNP effect on YES1

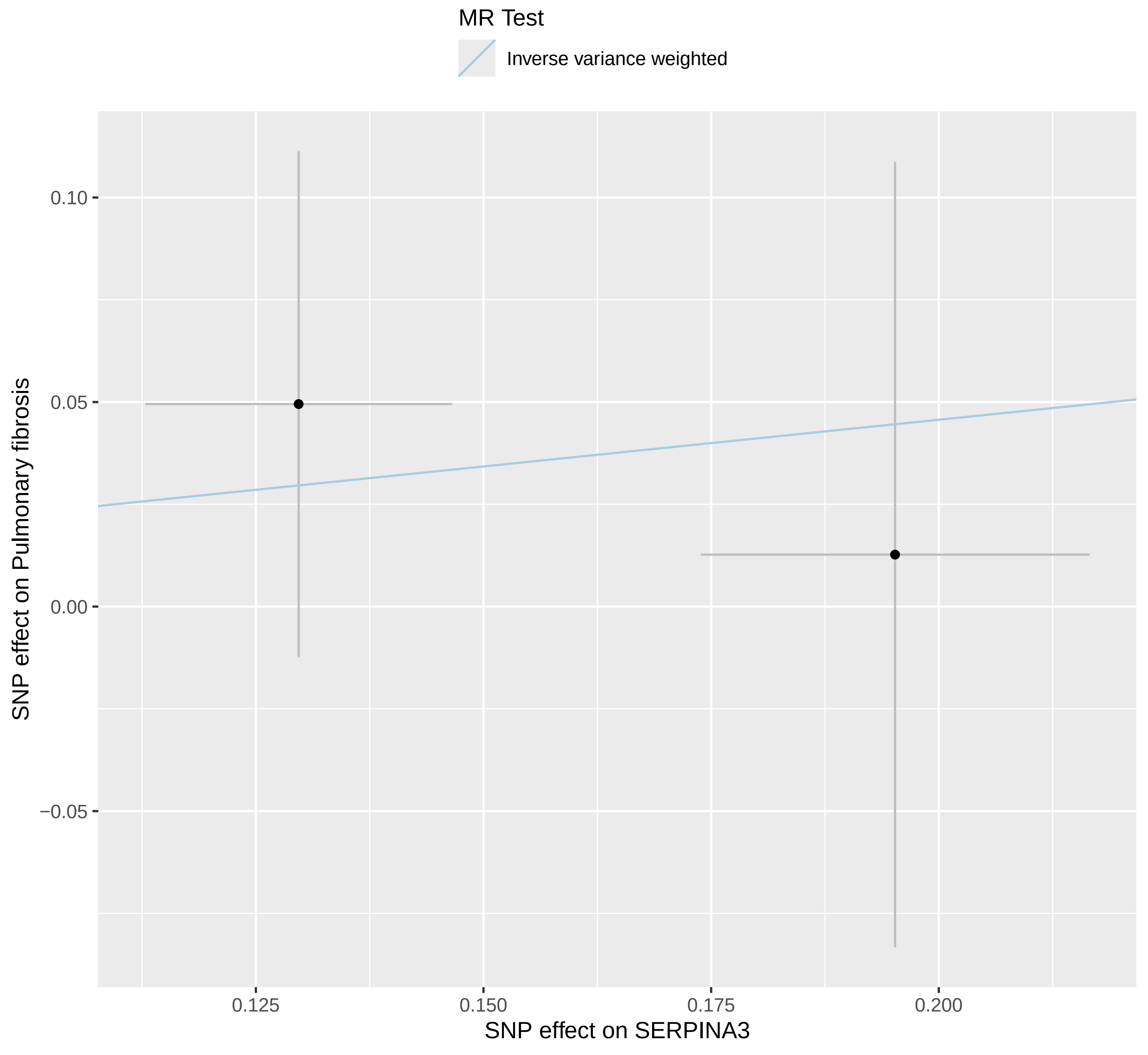
MR Test
Inverse variance weighted
0.10
0.05
0.00
−0.05
SNP effect on Pulmonary fibrosis
0.125
0.150
0.175
0.200
SNP effect on SERPINA3

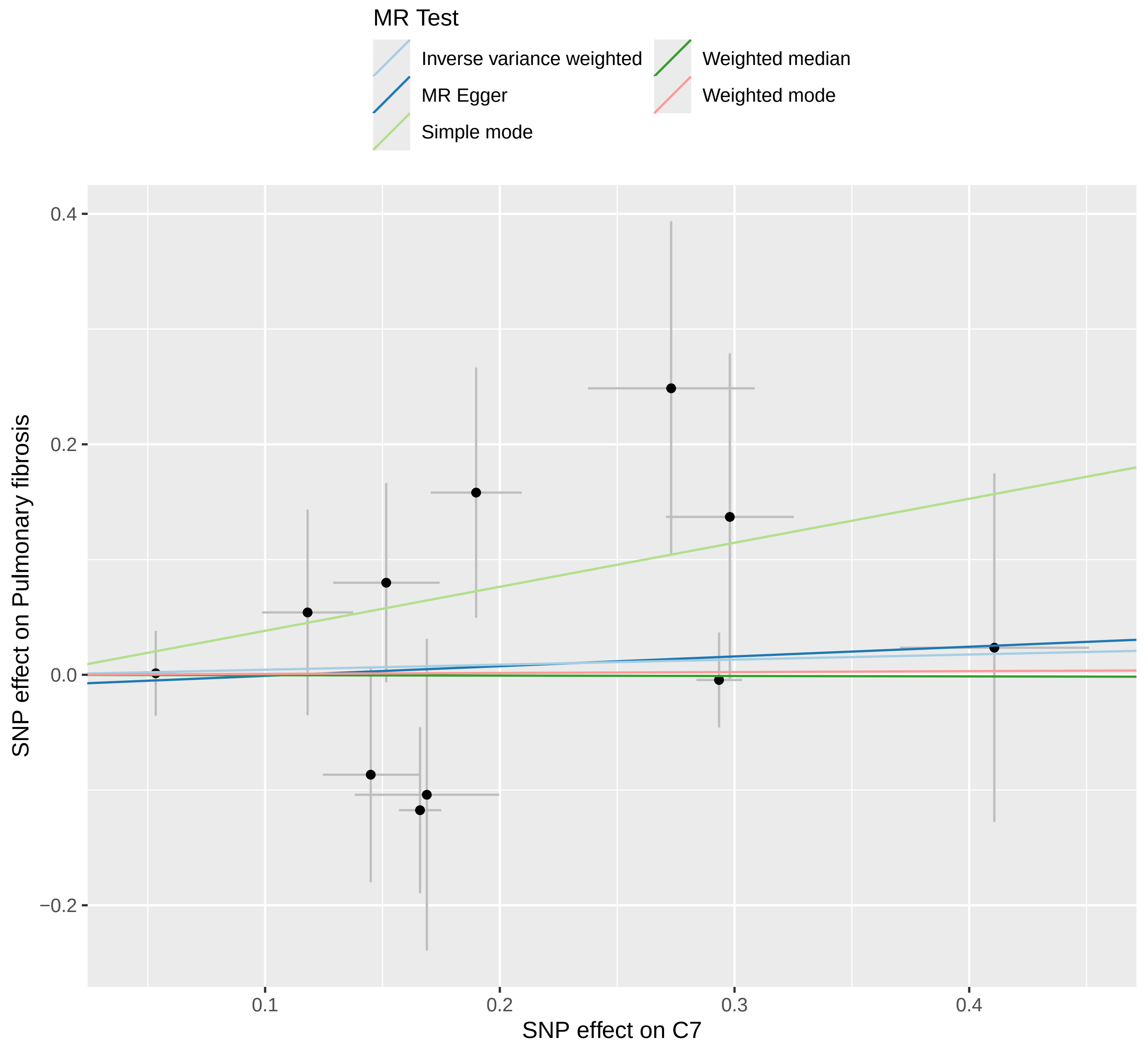
MR Test
Inverse variance weighted
MR Egger
Simple mode
Weighted median
Weighted mode
0.4
0.2
0.0
−0.2
SNP effect on Pulmonary fibrosis
0.1
0.2
0.3
0.4
SNP effect on C7

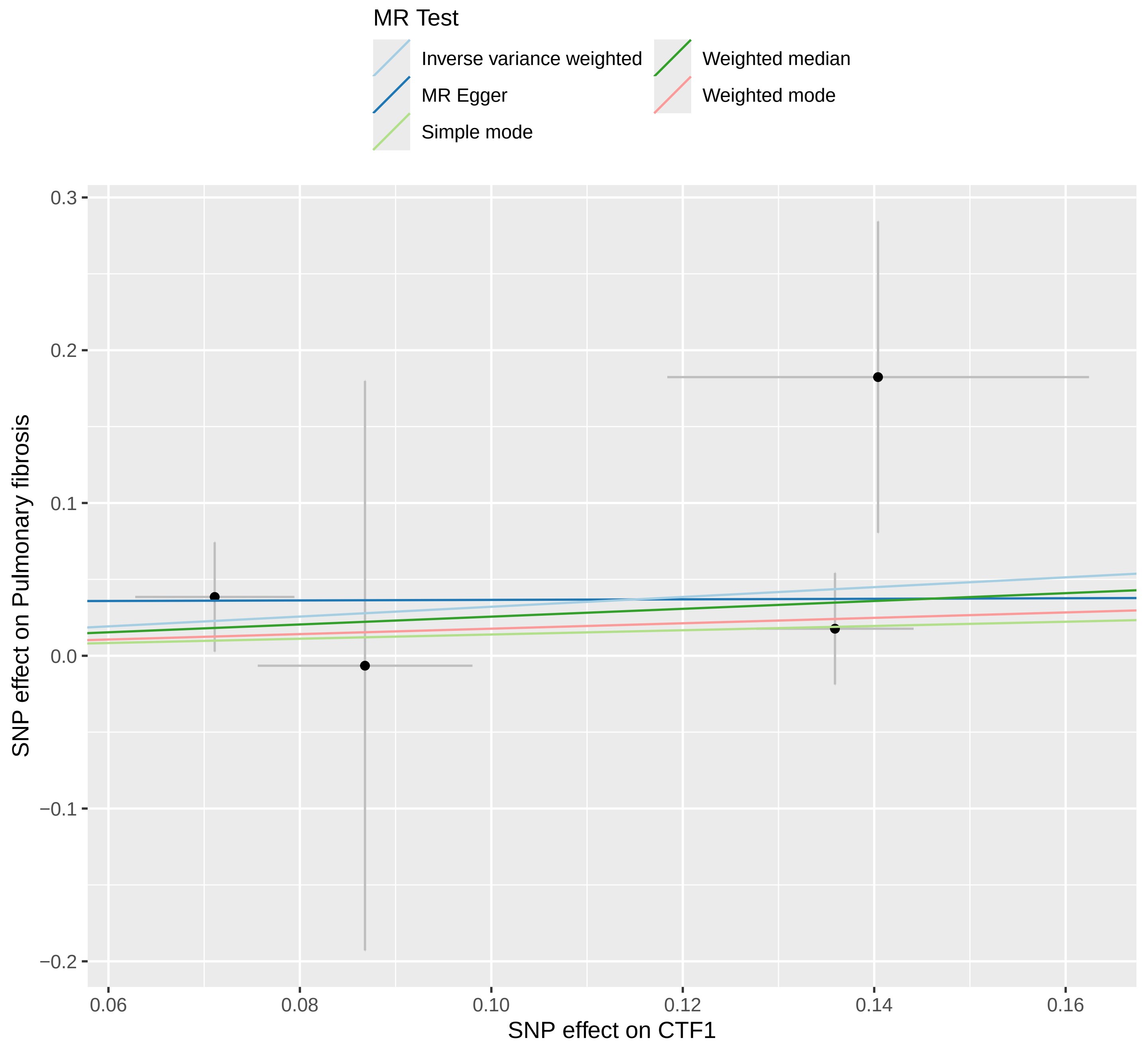

MR Test
Inverse variance weighted
MR Egger
Simple mode
Weighted median
Weighted mode
SNP effect on Pulmonary fibrosis
0.3
0.2
0.1
0.0
−0.1
−0.2
0.06
0.08
0.10
0.12
0.14
0.16
SNP effect on CTF1

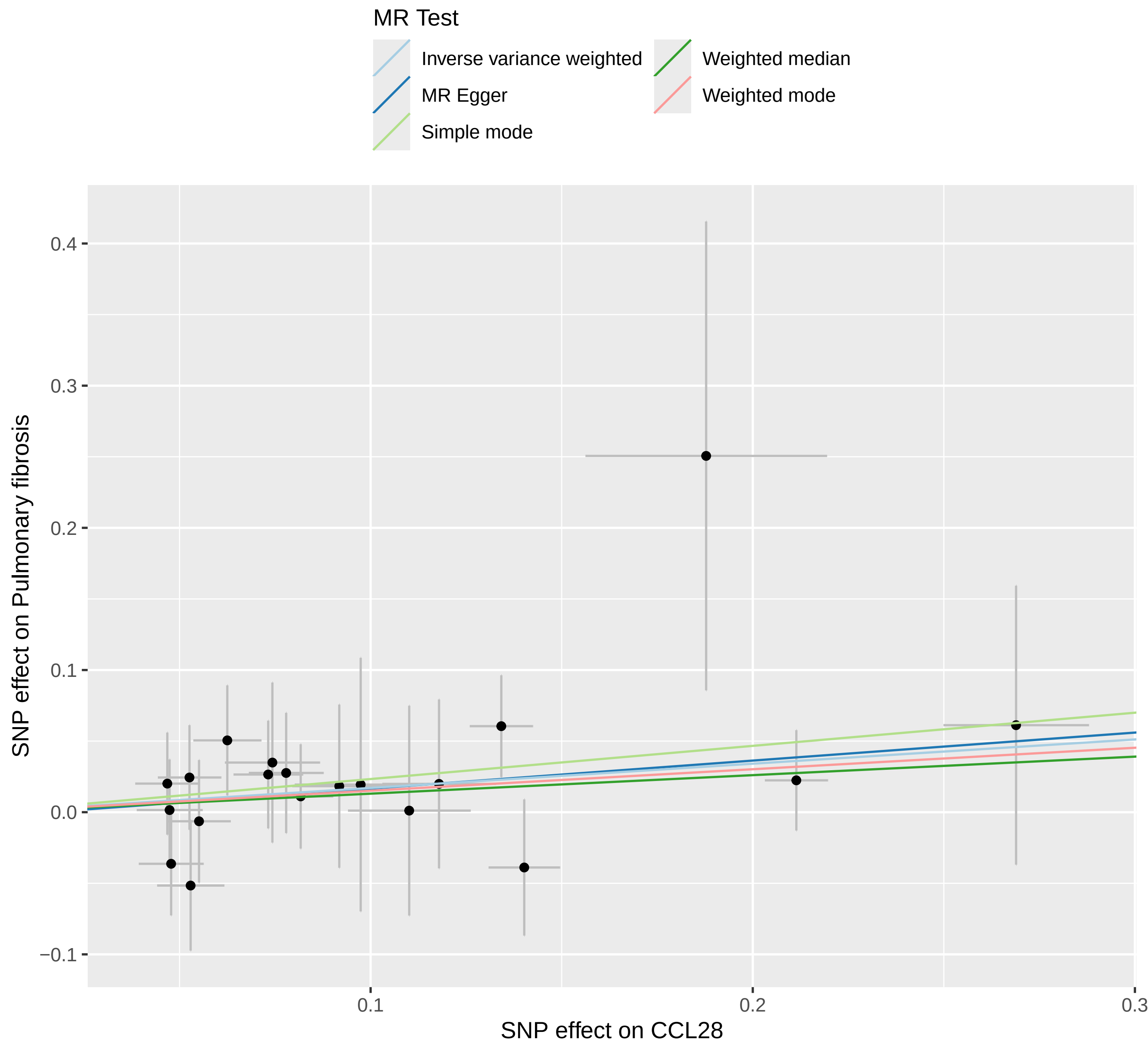

MR Test
Inverse variance weighted
MR Egger
Simple mode
Weighted median
Weighted mode
SNP effect on Pulmonary fibrosis
0.4
0.3
0.2
0.1
0.0
−0.1
0.1
0.2
0.3
SNP effect on CCL28

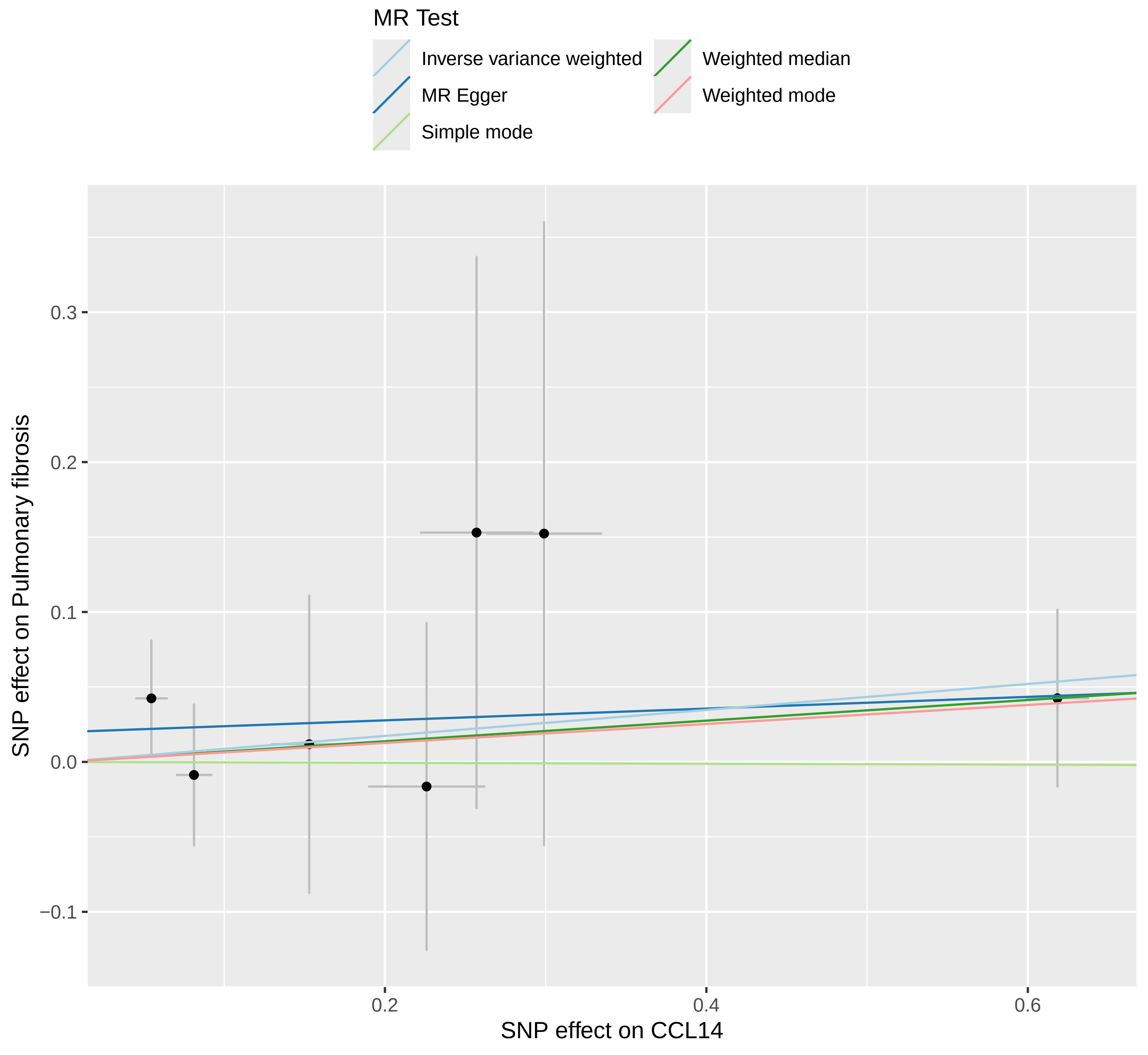
MR Test
Inverse variance weighted
MR Egger
Simple mode
Weighted median
Weighted mode
0.3
0.2
0.1
0.0
−0.1
SNP effect on Pulmonary fibrosis
0.2
0.4
0.6
SNP effect on CCL14

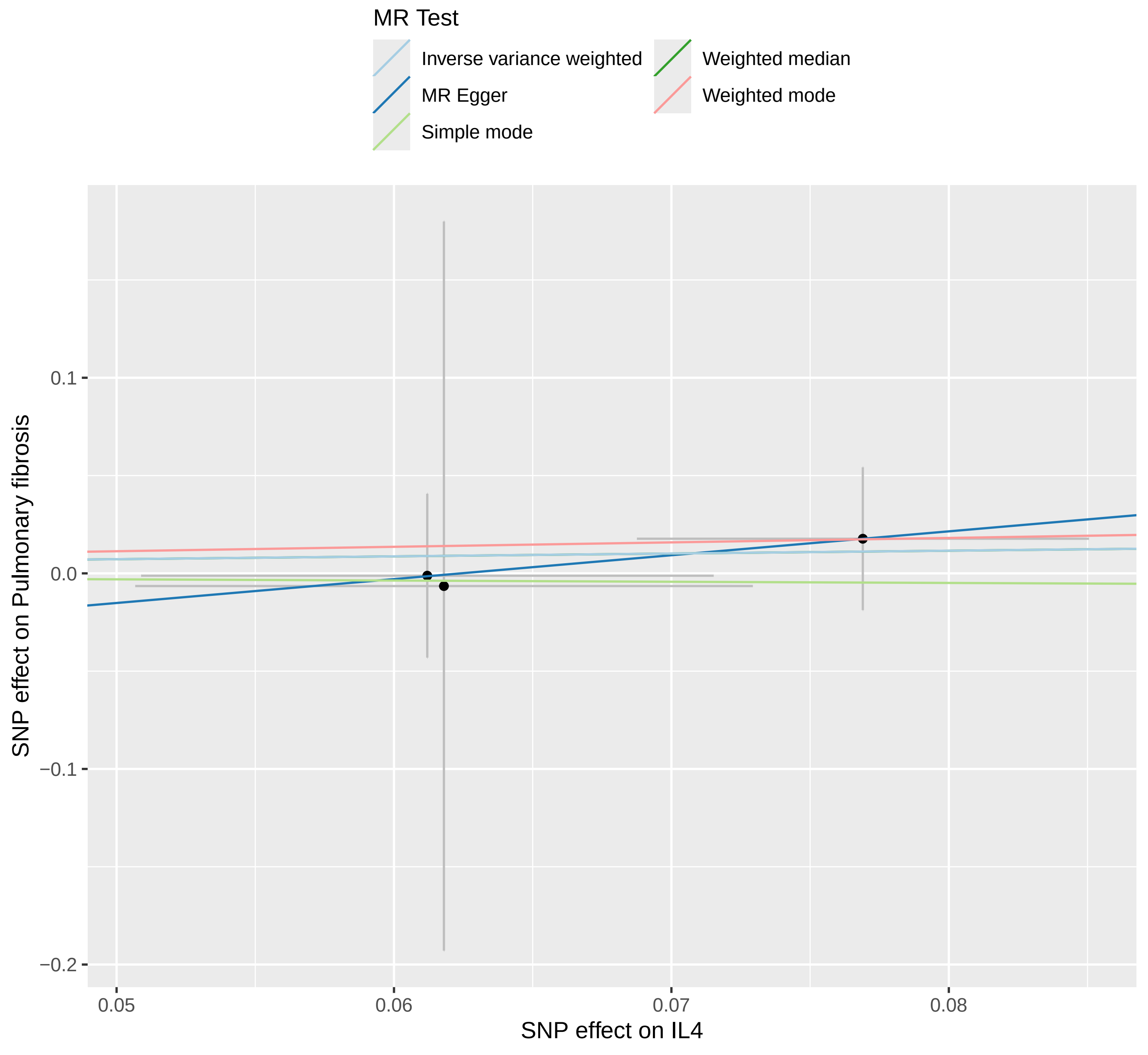

MR Test
Inverse variance weighted
MR Egger
Simple mode
Weighted median
Weighted mode
0.1
0.0
−0.1
−0.2
SNP effect on Pulmonary fibrosis
0.05
0.06
0.07
0.08
SNP effect on IL4

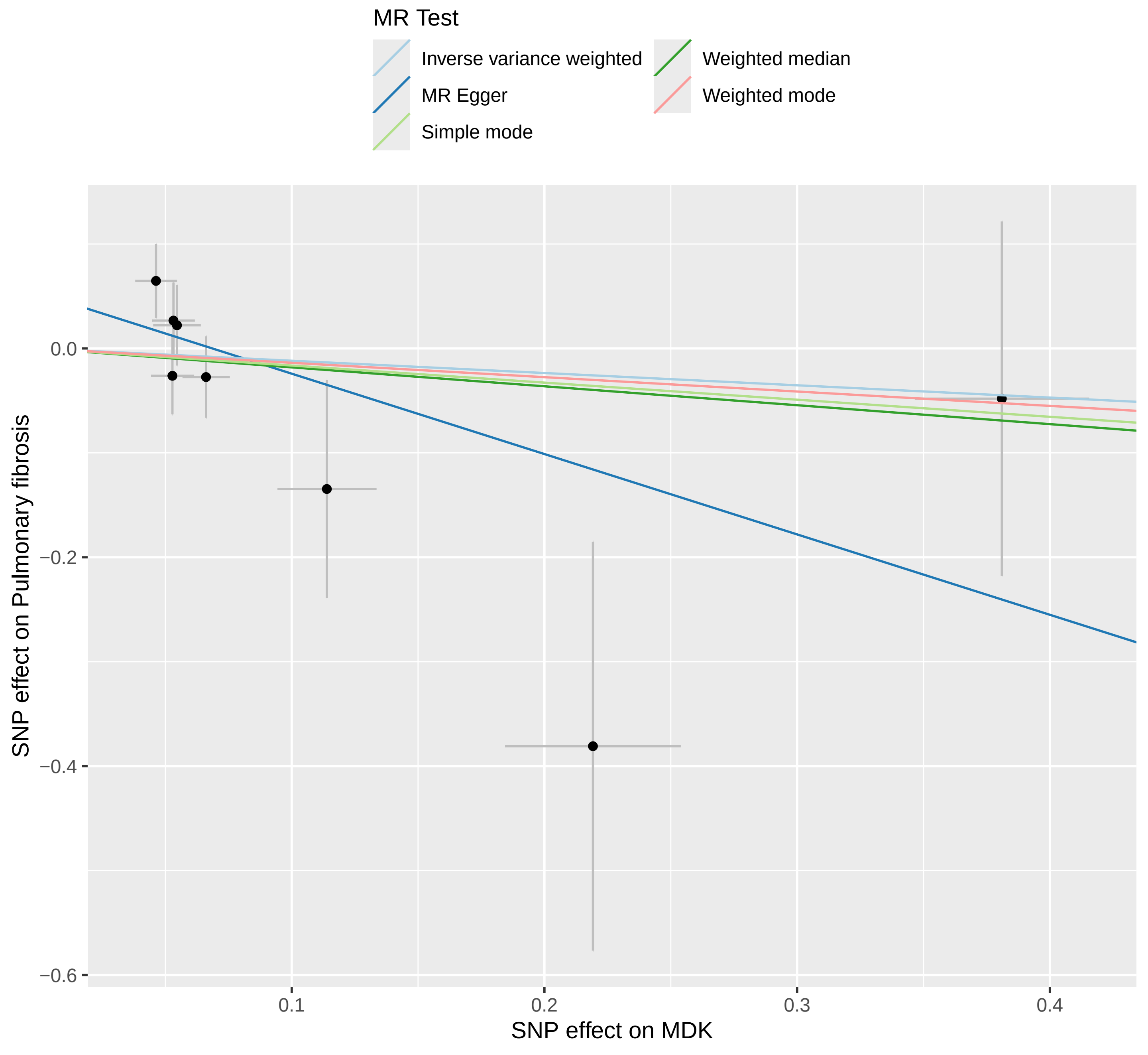
MR Test
Inverse variance weighted
MR Egger
Simple mode
Weighted median
Weighted mode
0.0
−0.2
−0.4
−0.6
0.1
0.2
0.3
0.4
SNP effect on Pulmonary fibrosis
SNP effect on MDK

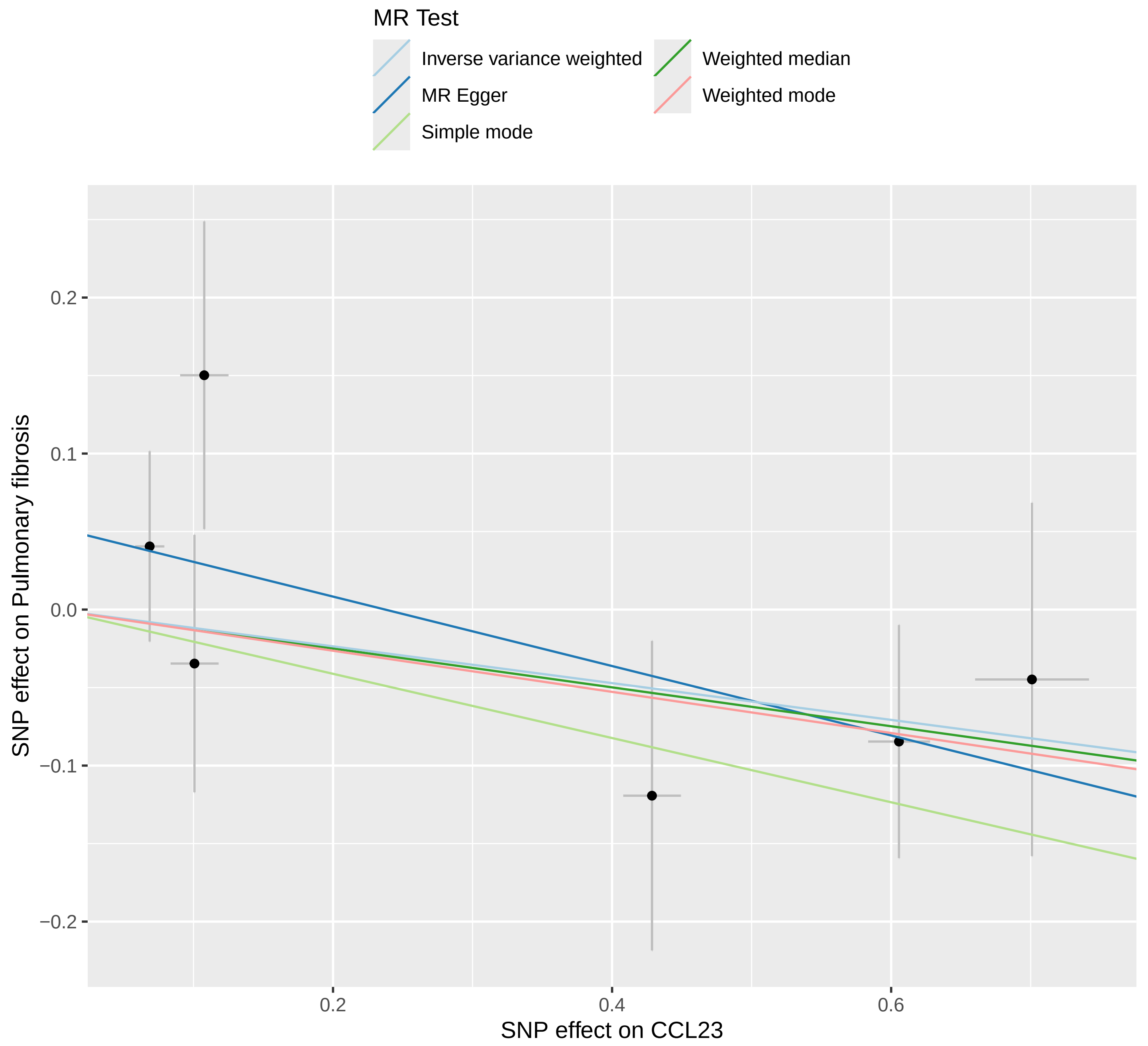
MR Test
Inverse variance weighted
MR Egger
Simple mode
Weighted median
Weighted mode
SNP effect on Pulmonary fibrosis
0.2
0.1
0.0
−0.1
−0.2
0.2
0.4
0.6
SNP effect on CCL23

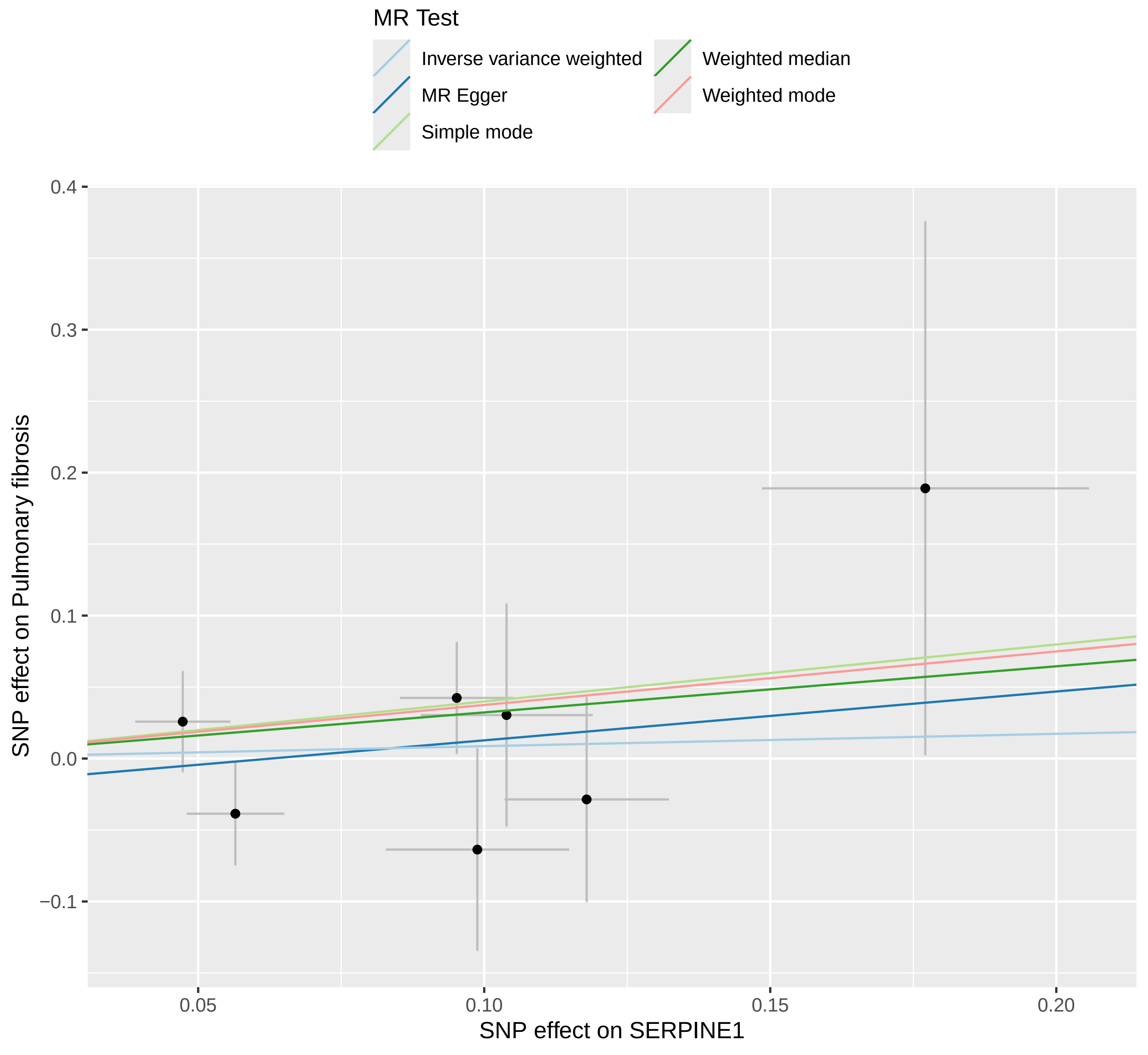
MR Test
Inverse variance weighted
MR Egger
Simple mode
Weighted median
Weighted mode
SNP effect on Pulmonary fibrosis
0.4
0.3
0.2
0.1
0.0
−0.1
0.05
0.10
0.15
0.20
SNP effect on SERPINE1

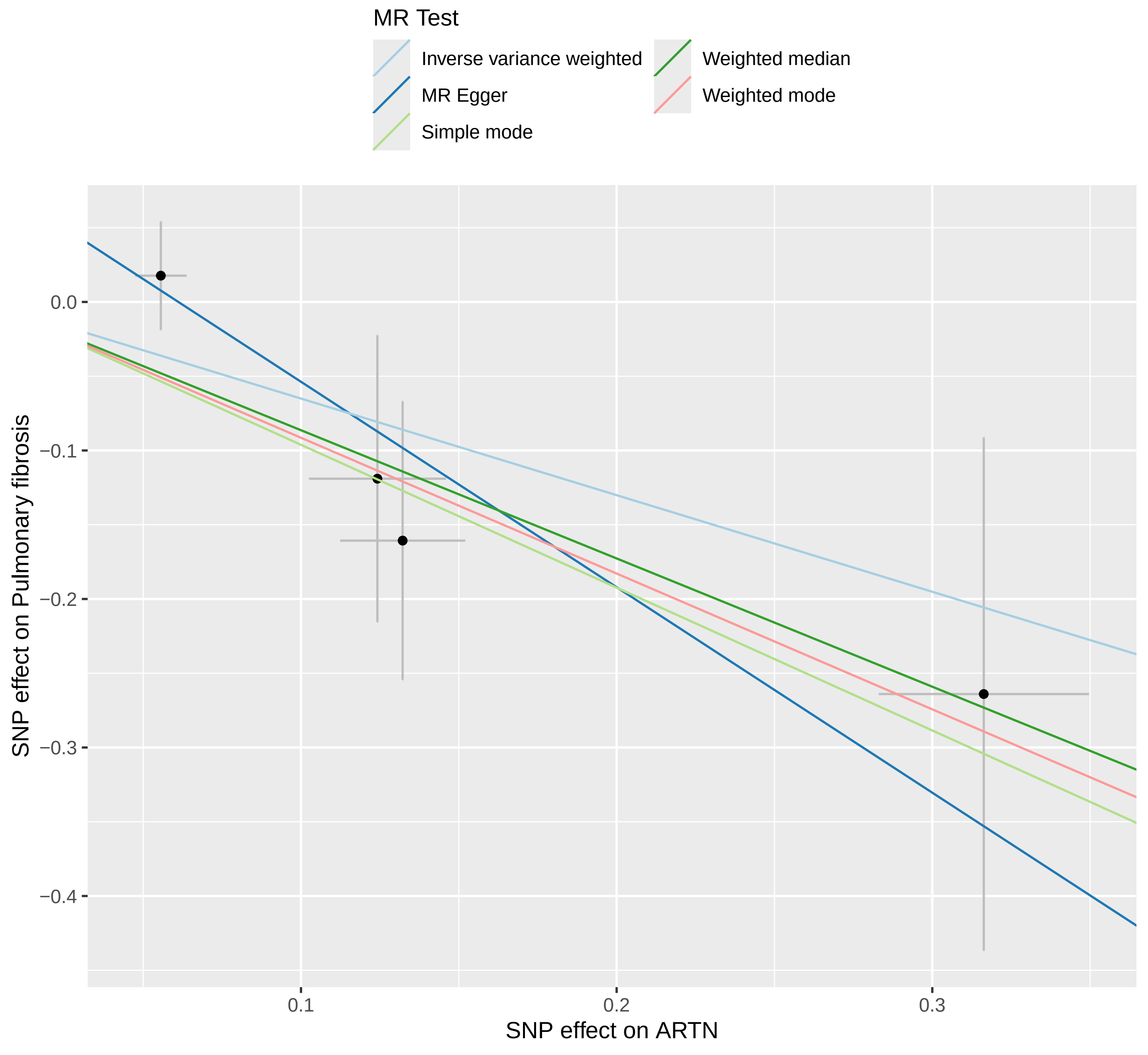
MR Test
Inverse variance weighted
MR Egger
Simple mode
Weighted median
Weighted mode
SNP effect on Pulmonary fibrosis
0.0
−0.1
−0.2
−0.3
−0.4
0.1
0.2
0.3
SNP effect on ARTN

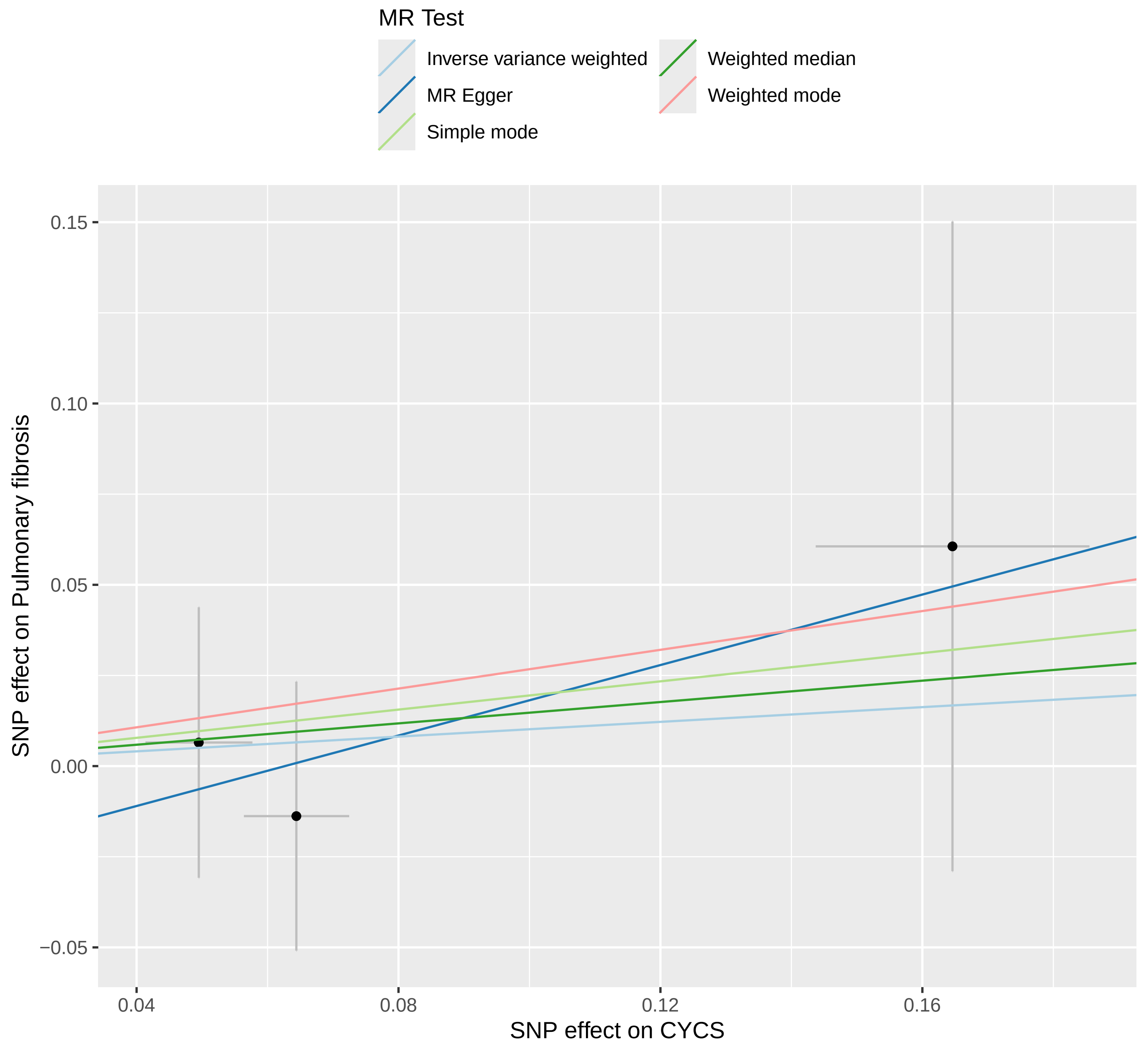

MR Test
Inverse variance weighted
MR Egger
Simple mode
Weighted median
Weighted mode
0.15
0.10
0.05
0.00
−0.05
0.04
0.08
0.12
0.16
SNP effect on Pulmonary fibrosis
SNP effect on CYCS

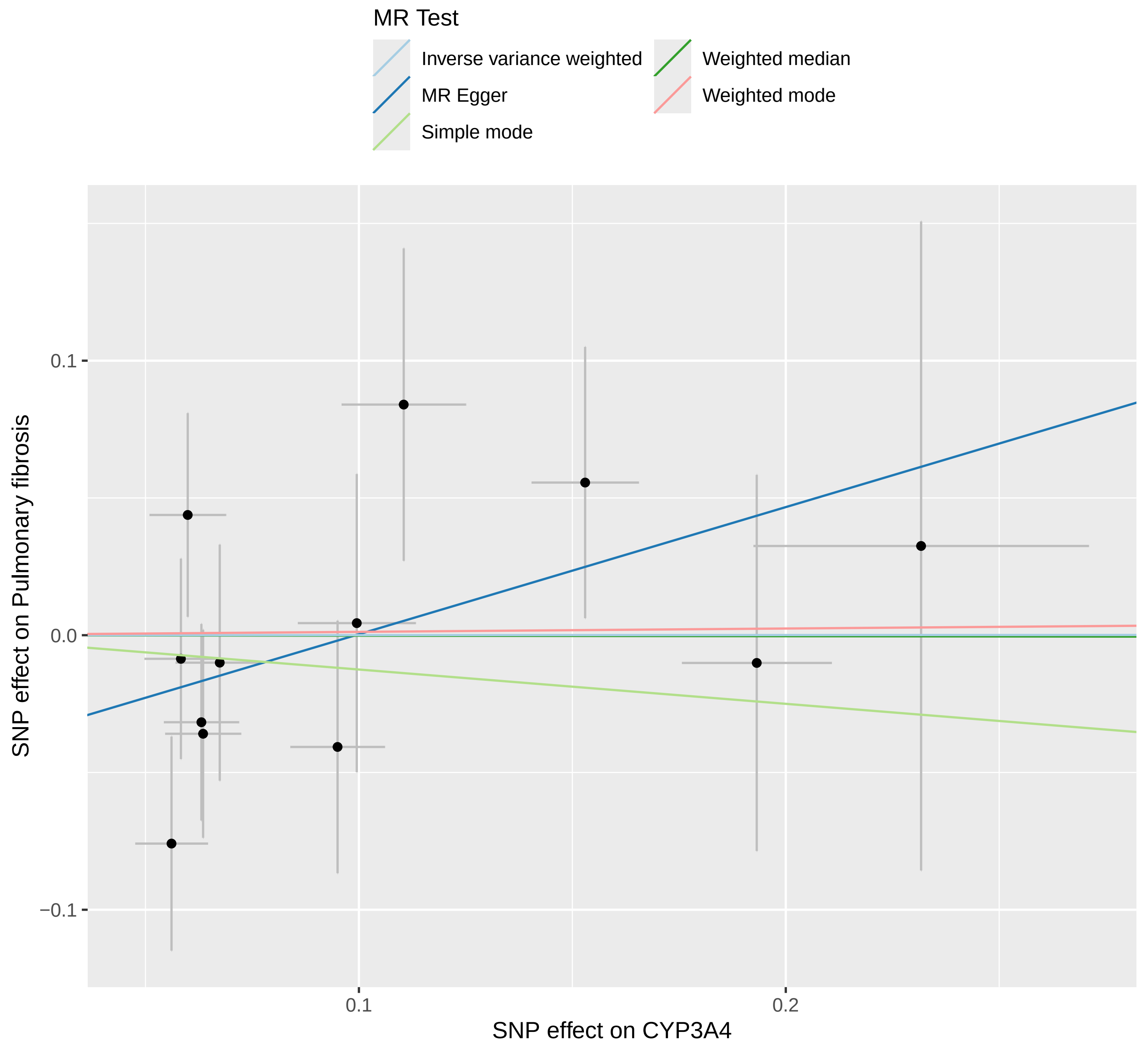

MR Test
Inverse variance weighted
MR Egger
Simple mode
Weighted median
Weighted mode
0.1
0.0
−0.1
SNP effect on Pulmonary fibrosis
0.1
0.2
SNP effect on CYP3A4

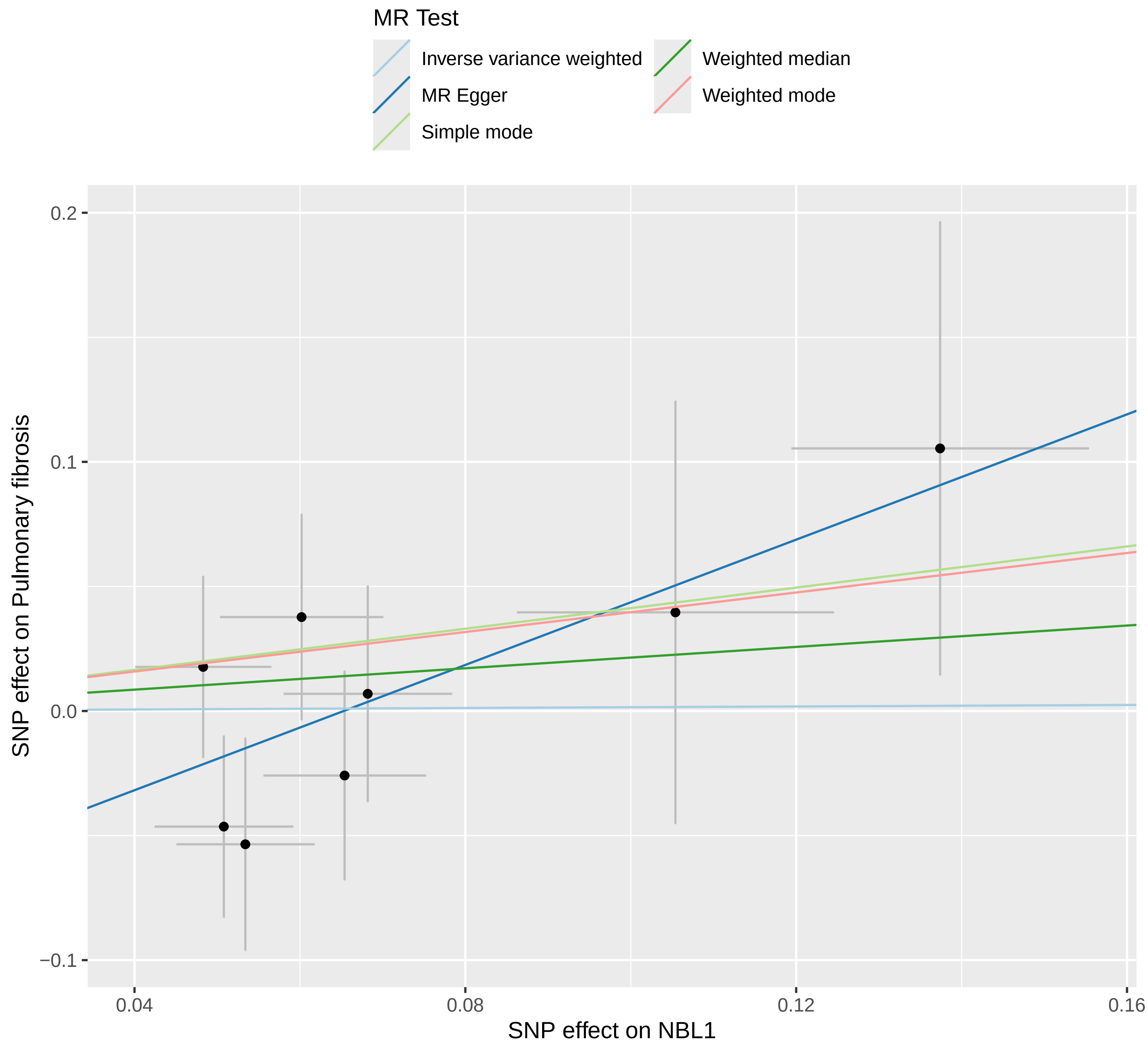

MR Test
Inverse variance weighted
MR Egger
Simple mode
Weighted median
Weighted mode
SNP effect on Pulmonary fibrosis
0.2
0.1
0.0
−0.1
0.04
0.08
0.12
0.16
SNP effect on NBL1

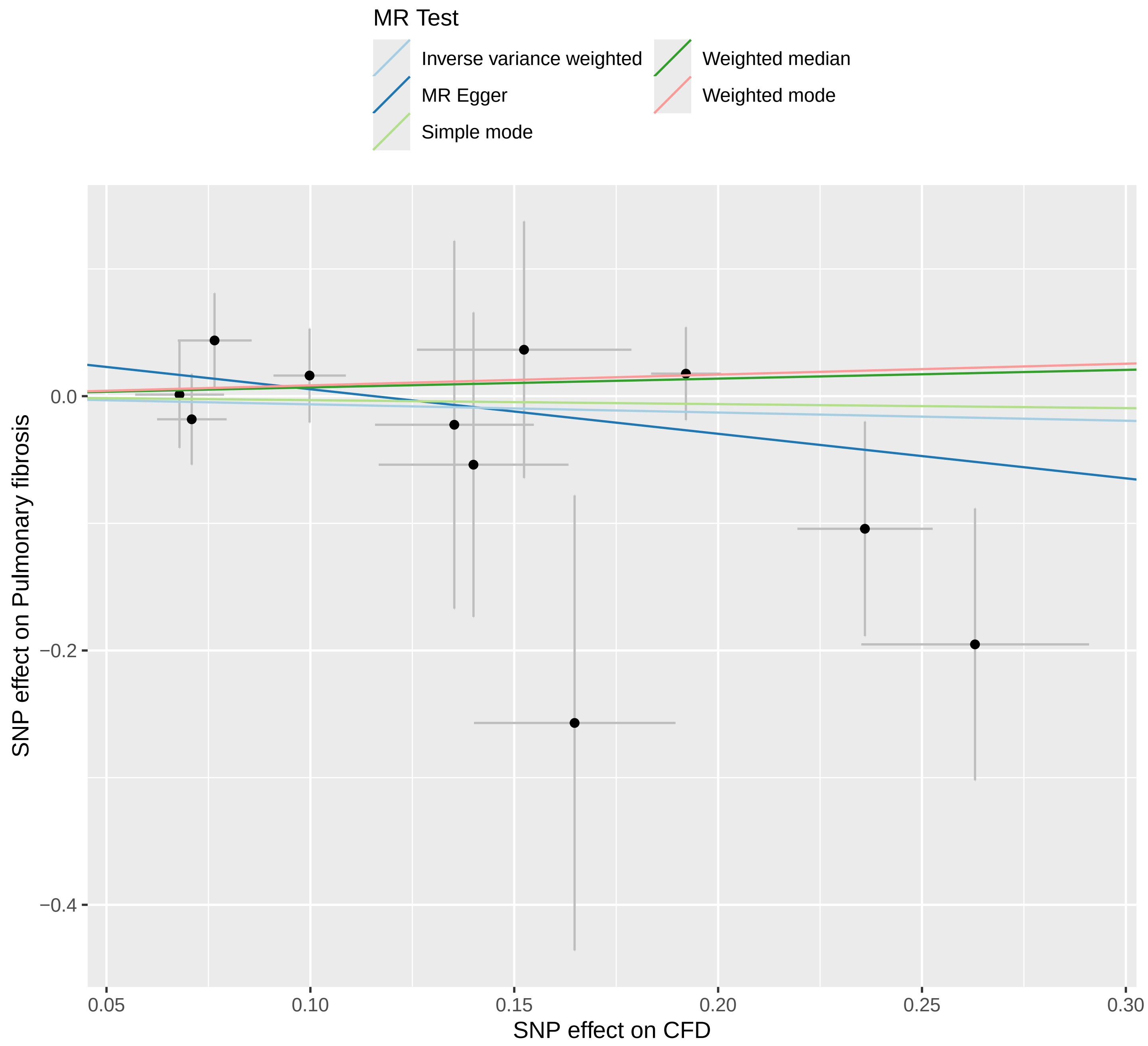
MR Test
Inverse variance weighted
MR Egger
Simple mode
Weighted median
Weighted mode
SNP effect on Pulmonary fibrosis
0.0
−0.2
−0.4
0.05
0.10
0.15
0.20
0.25
0.30
SNP effect on CFD

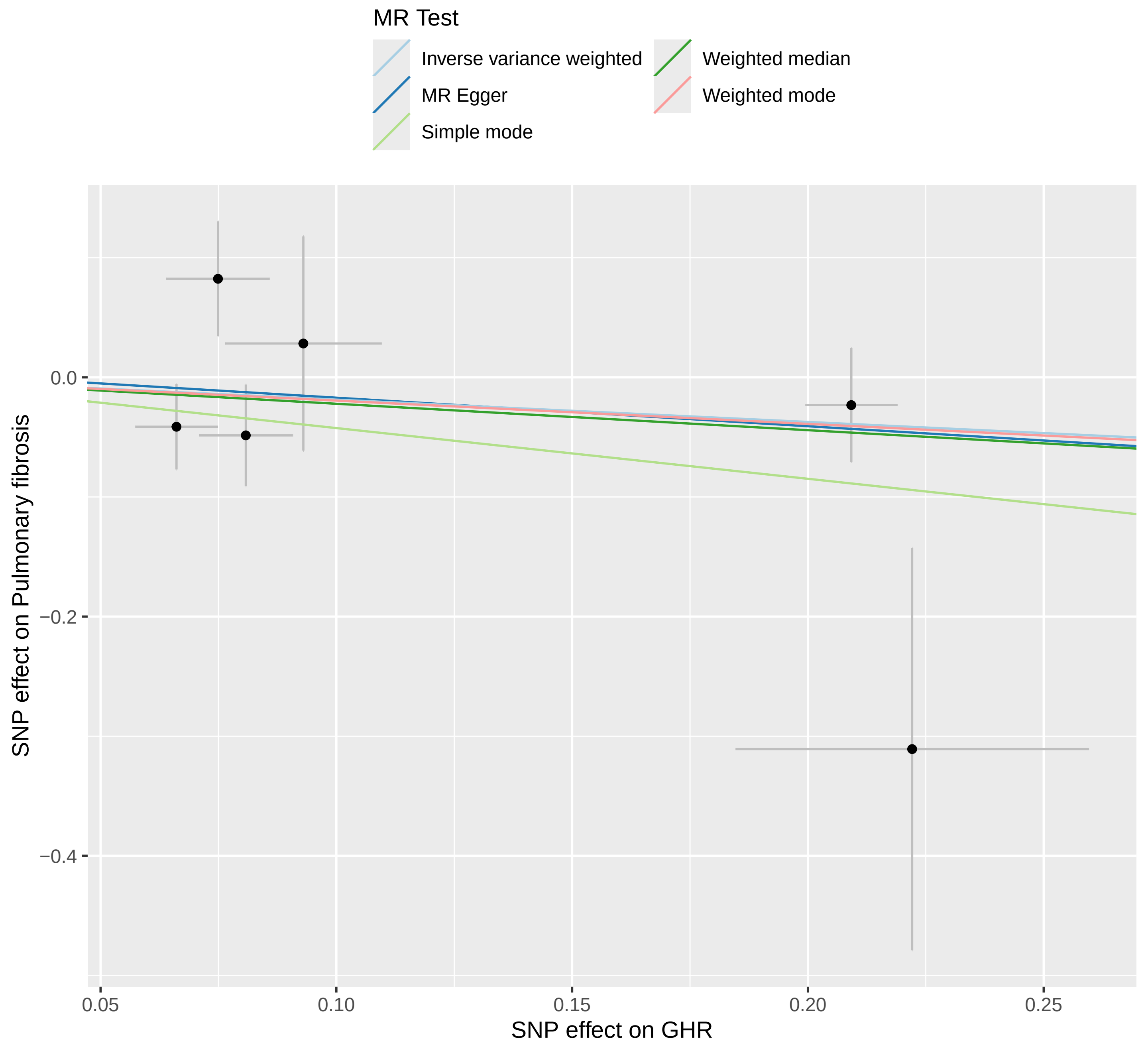

MR Test
Inverse variance weighted
MR Egger
Simple mode
Weighted median
Weighted mode
0.0
−0.2
−0.4
0.05
0.10
0.15
0.20
0.25
SNP effect on Pulmonary fibrosis
SNP effect on GHR

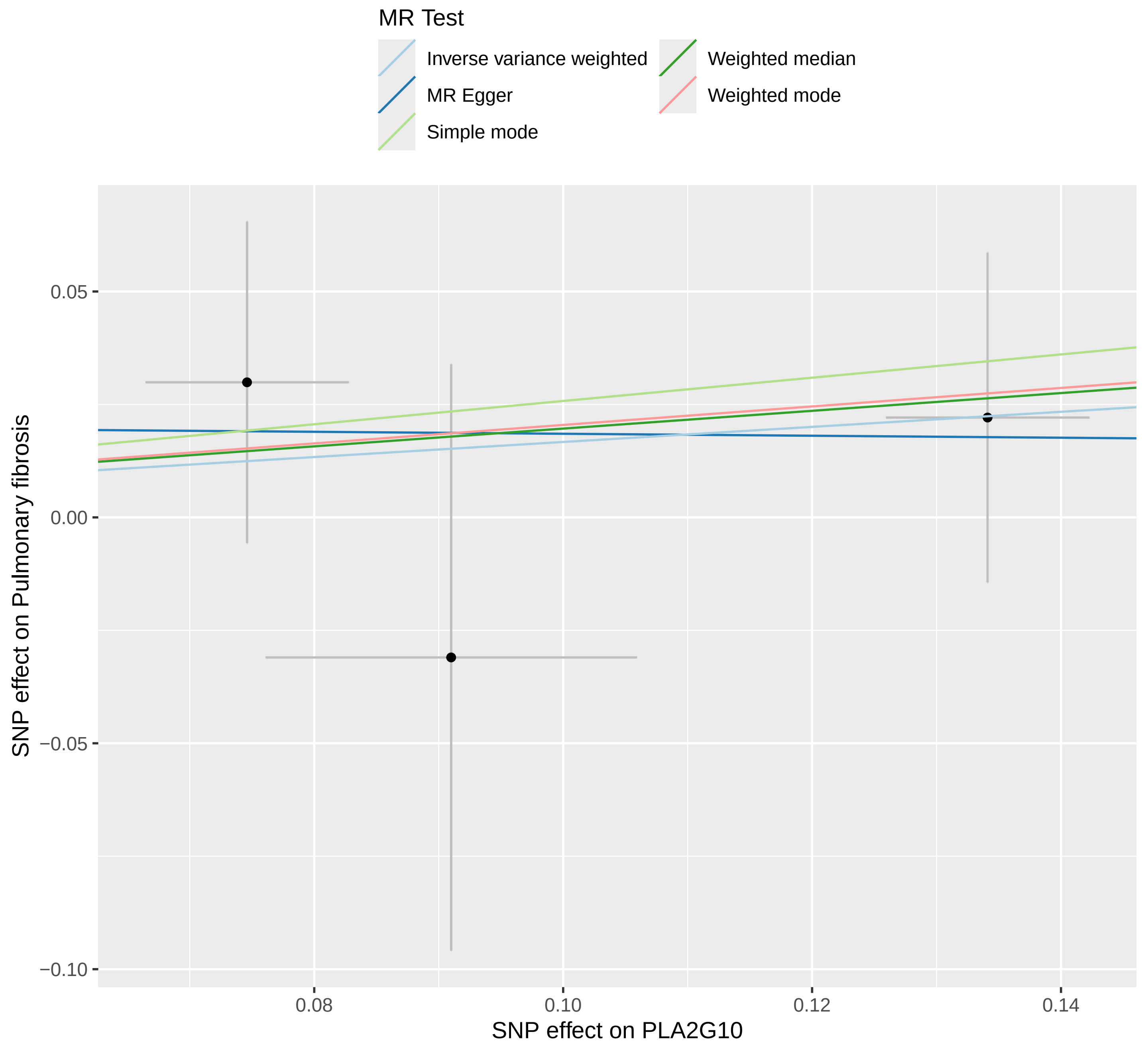
MR Test
Inverse variance weighted
MR Egger
Simple mode
Weighted median
Weighted mode
0.05
0.00
−0.05
−0.10
0.08
0.10
0.12
0.14
SNP effect on Pulmonary fibrosis
SNP effect on PLA2G10

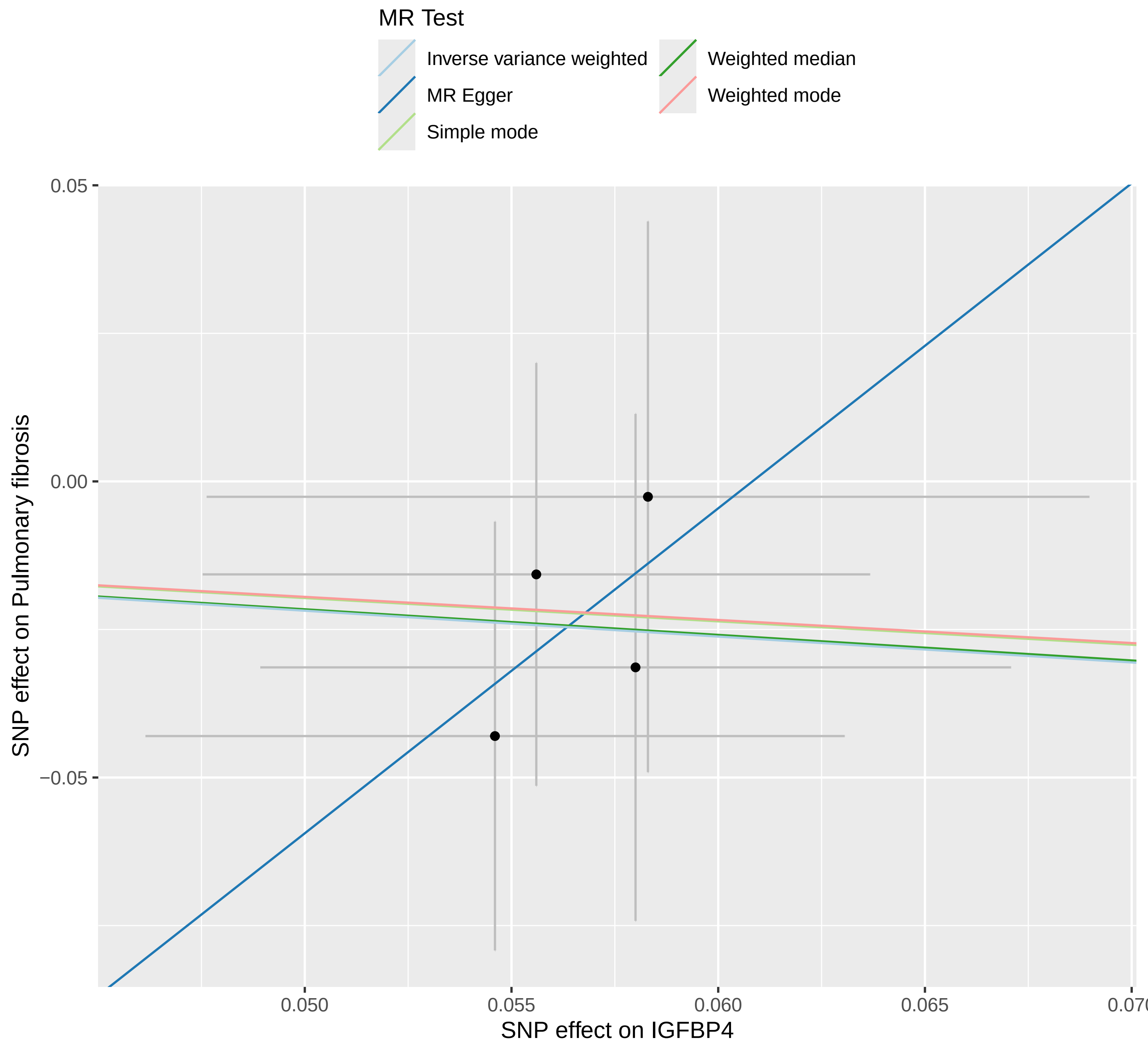

MR Test
Inverse variance weighted
MR Egger
Simple mode
Weighted median
Weighted mode
0.05
0.00
−0.05
SNP effect on Pulmonary fibrosis
0.050
0.055
0.060
0.065
0.070
SNP effect on IGFBP4

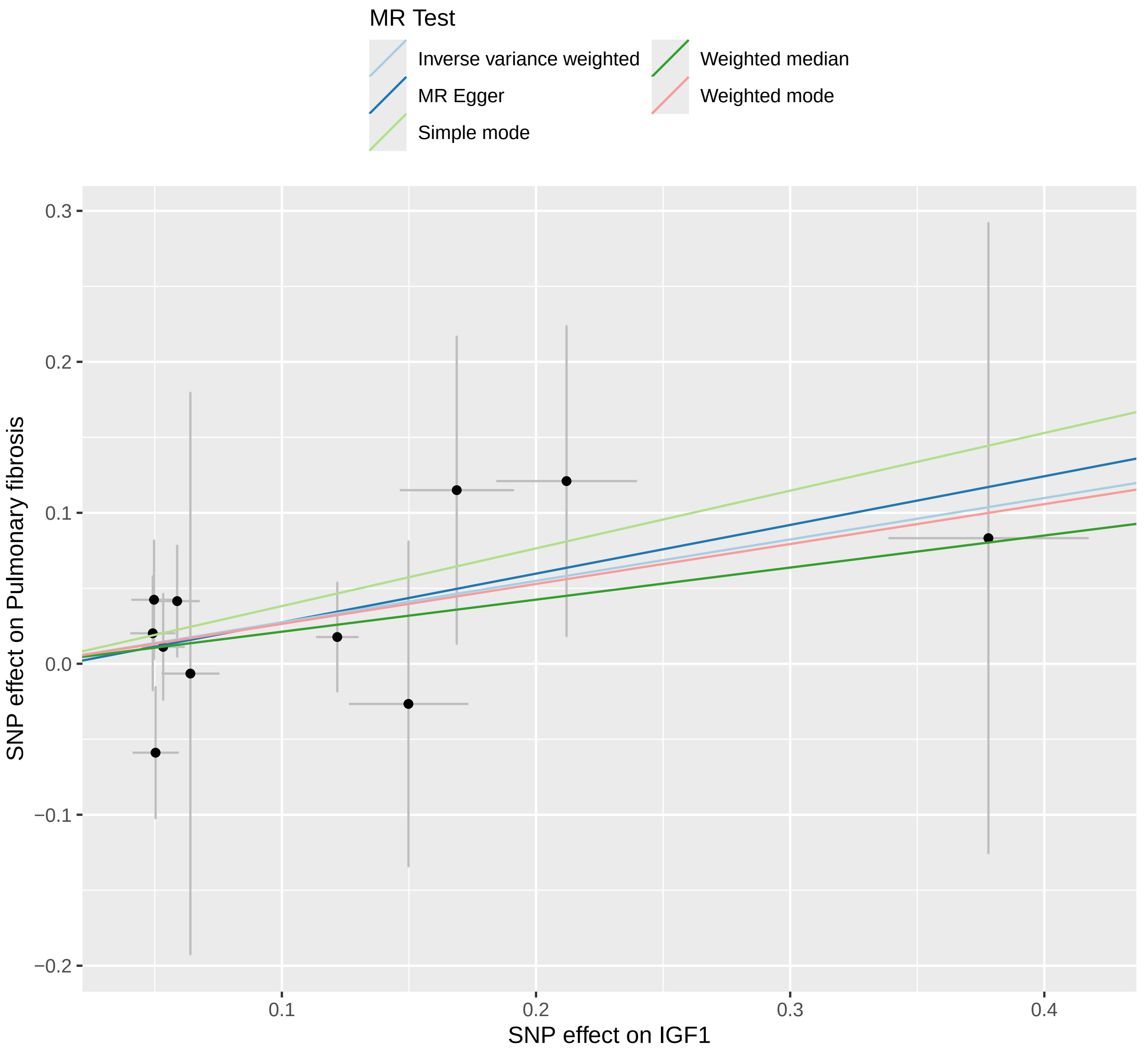

MR Test
Inverse variance weighted
MR Egger
Simple mode
Weighted median
Weighted mode
0.3
0.2
0.1
0.0
−0.1
−0.2
0.1
0.2
0.3
0.4
SNP effect on IGF1
SNP effect on Pulmonary fibrosis

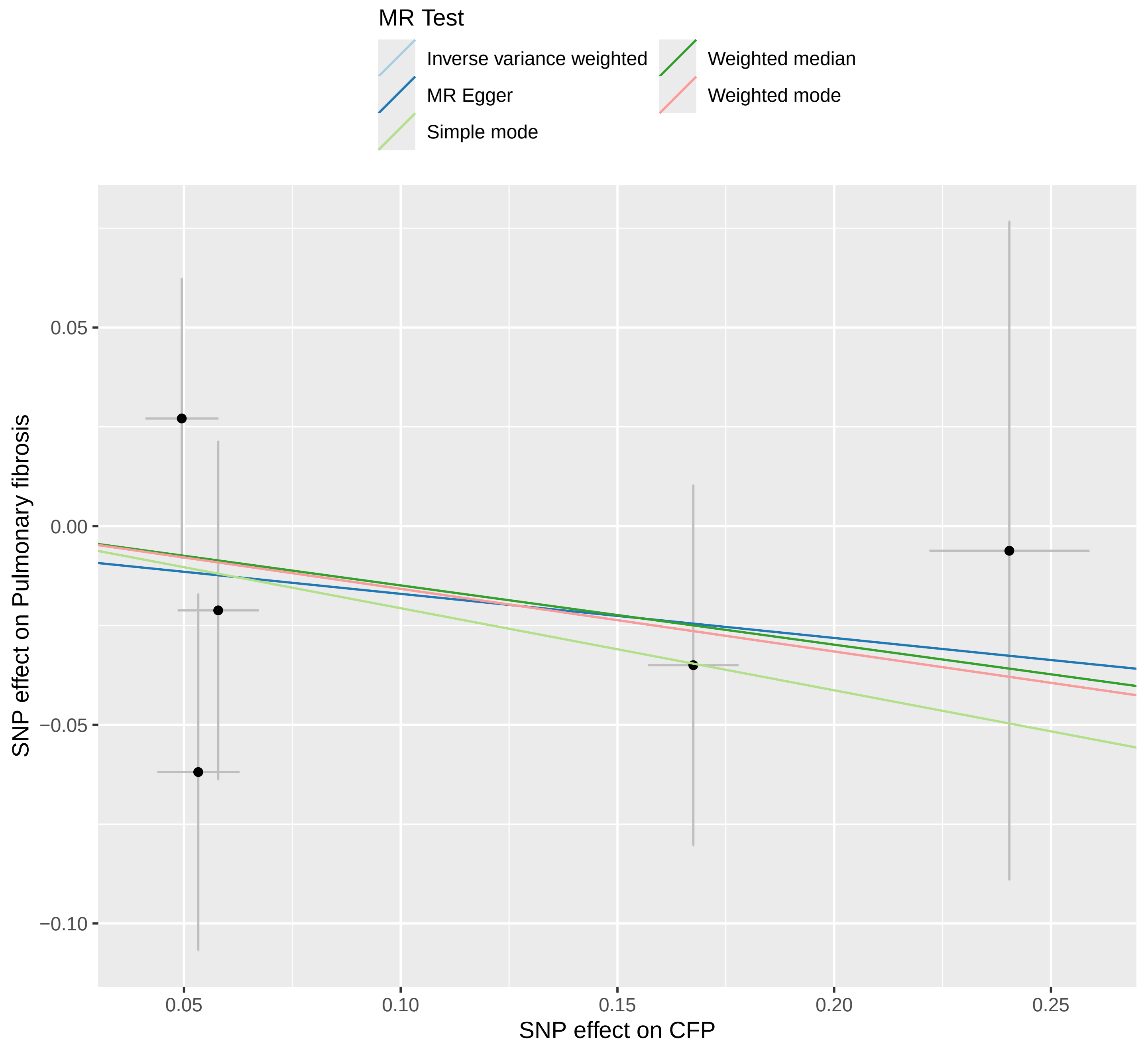

MR Test
Inverse variance weighted
MR Egger
Simple mode
Weighted median
Weighted mode
0.05
0.00
−0.05
−0.10
0.05
0.10
0.15
0.20
0.25
SNP effect on Pulmonary fibrosis
SNP effect on CFP

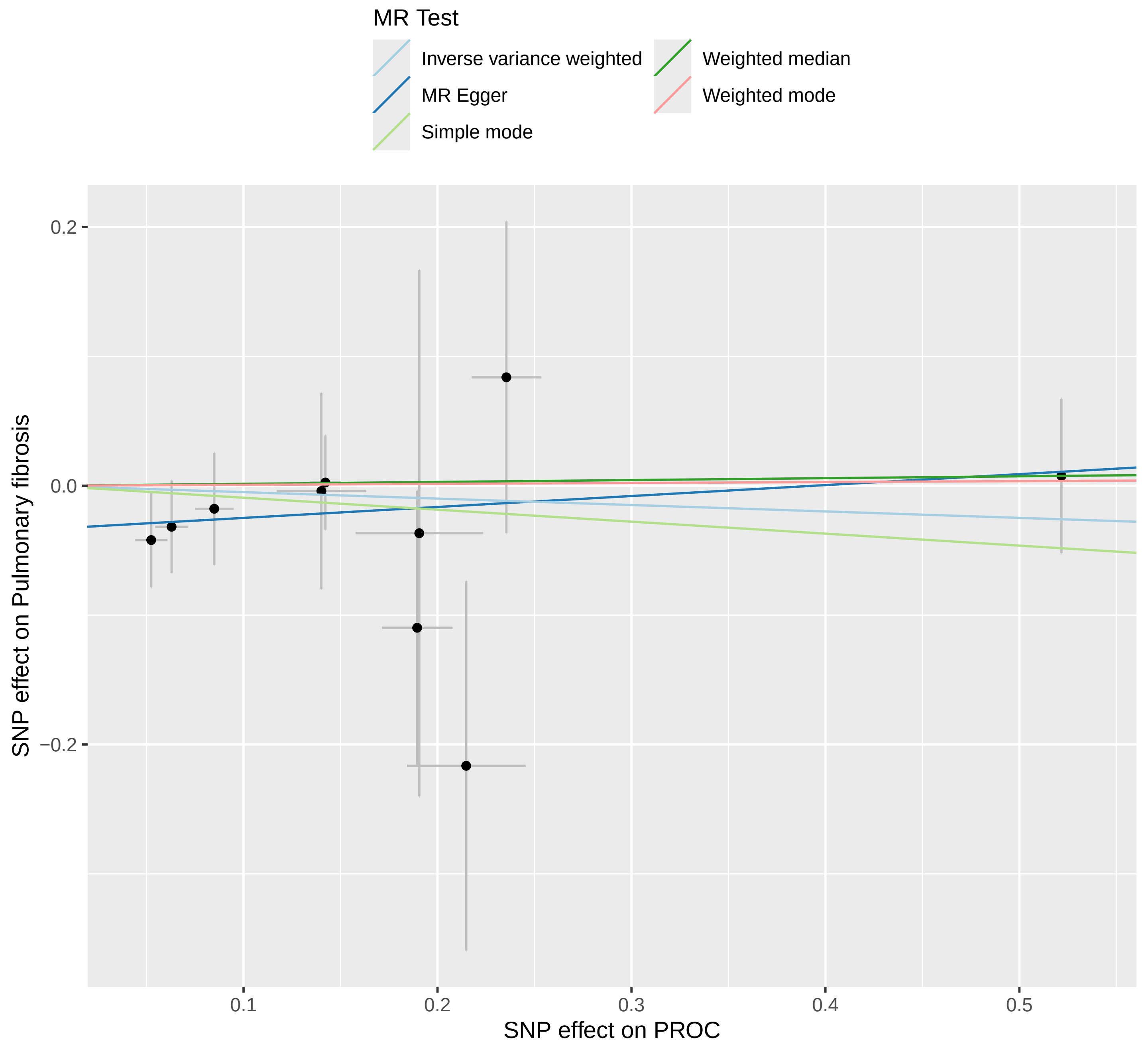
MR Test
Inverse variance weighted
MR Egger
Simple mode
Weighted median
Weighted mode
0.2
0.0
−0.2
0.1
0.2
0.3
0.4
0.5
SNP effect on PROC
SNP effect on Pulmonary fibrosis

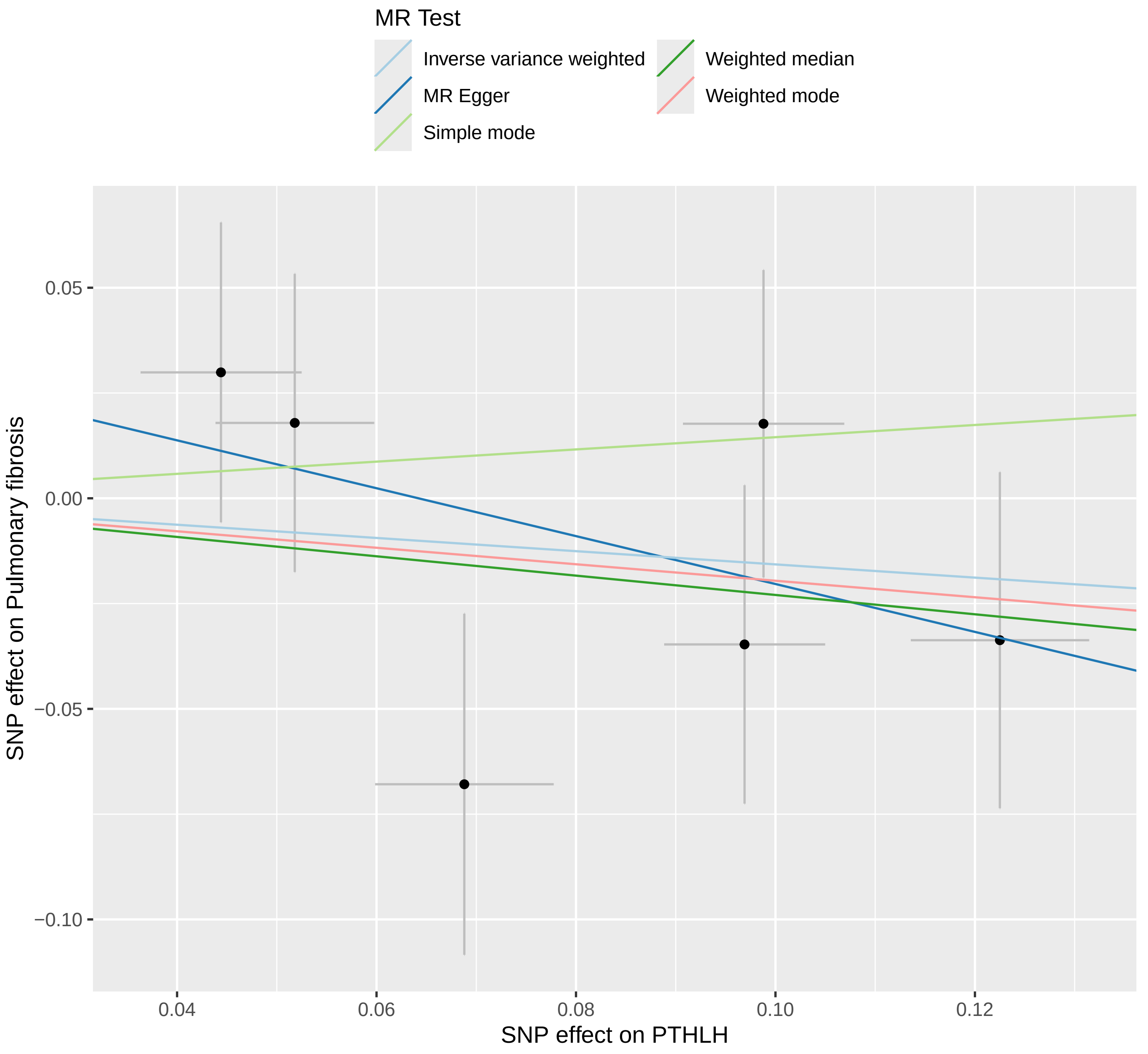
MR Test
Inverse variance weighted
MR Egger
Simple mode
Weighted median
Weighted mode
0.05
0.00
−0.05
−0.10
SNP effect on Pulmonary fibrosis
0.04
0.06
0.08
0.10
0.12
SNP effect on PTHLH

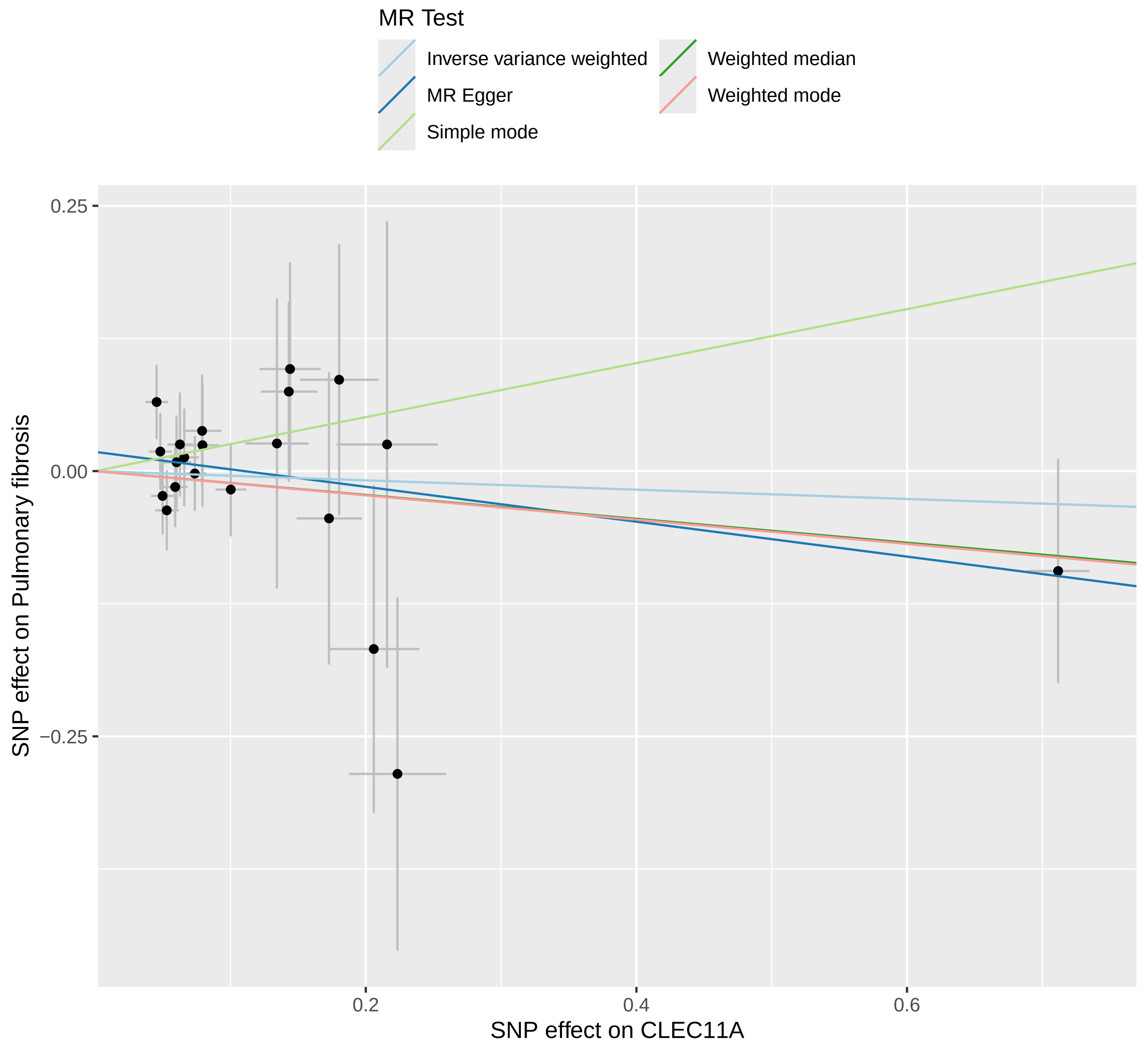

MR Test
Inverse variance weighted
MR Egger
Simple mode
Weighted median
Weighted mode
0.25
0.00
−0.25
0.2
0.4
0.6
SNP effect on Pulmonary fibrosis
SNP effect on CLEC11A

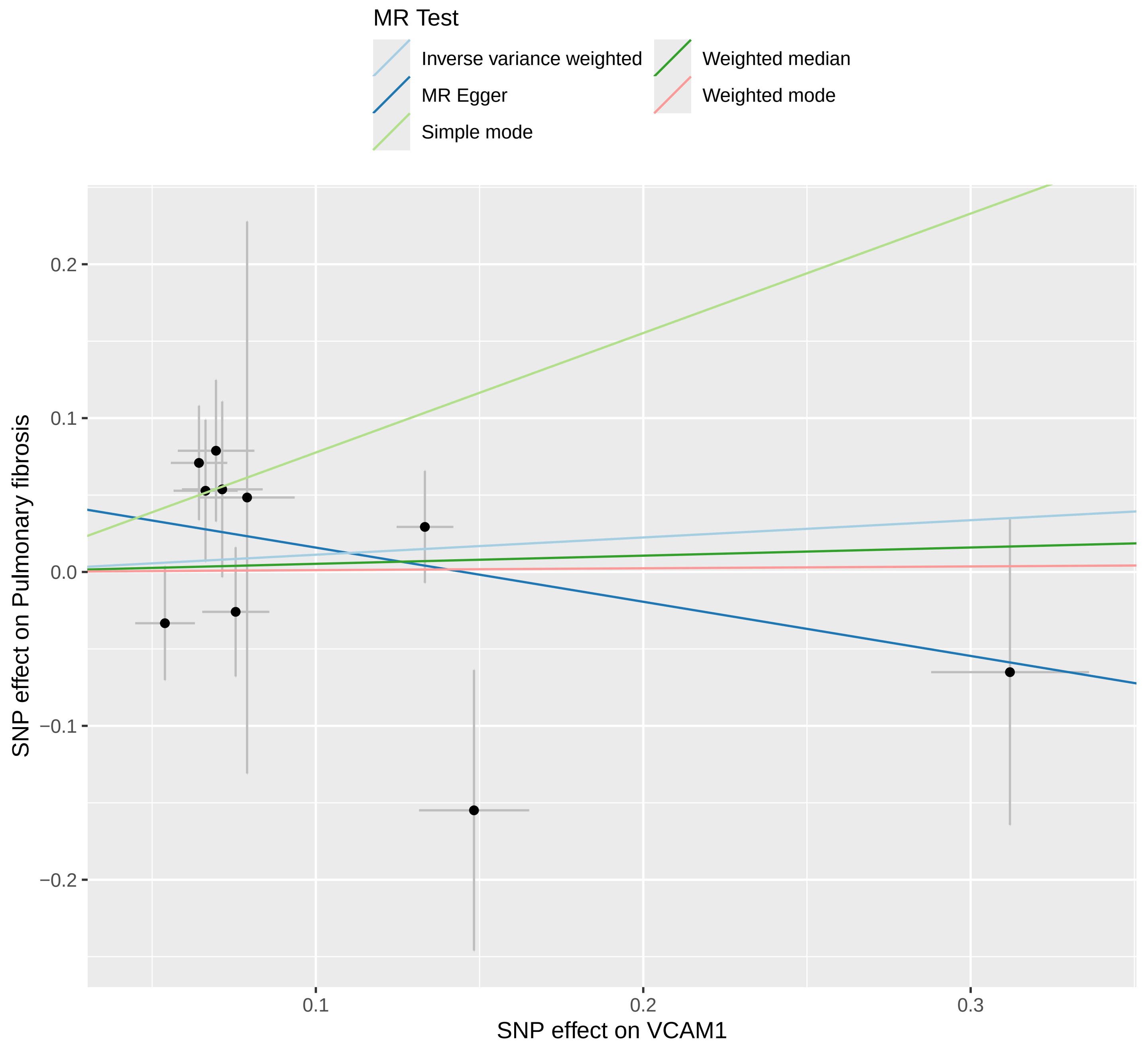
MR Test
Inverse variance weighted
MR Egger
Simple mode
Weighted median
Weighted mode
0.2
0.1
0.0
−0.1
−0.2
SNP effect on Pulmonary fibrosis
0.1
0.2
0.3
SNP effect on VCAM1

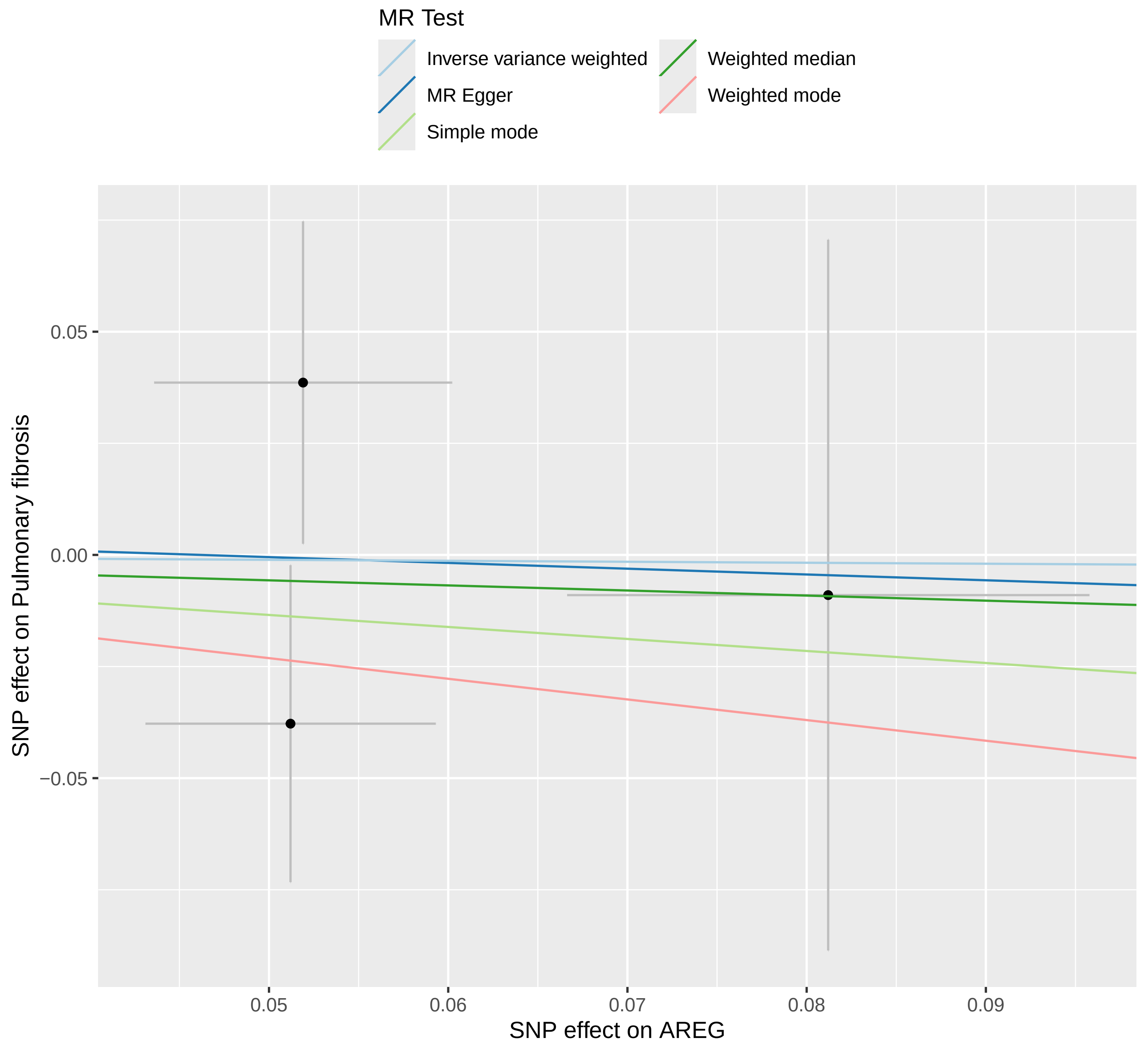
MR Test
Inverse variance weighted
MR Egger
Simple mode
Weighted median
Weighted mode
0.05
0.00
−0.05
SNP effect on Pulmonary fibrosis
0.05
0.06
0.07
0.08
0.09
SNP effect on AREG

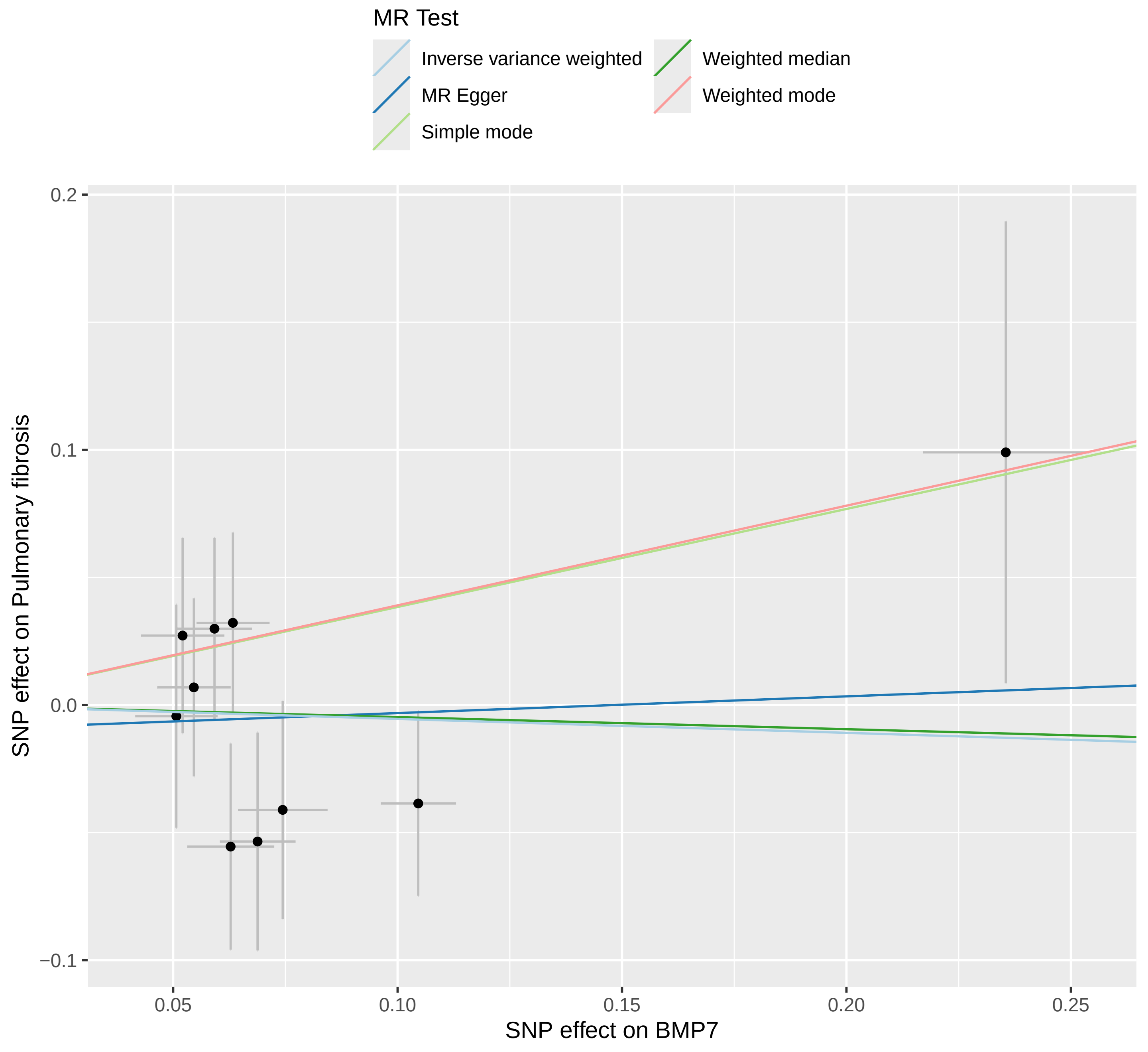
MR Test
Inverse variance weighted
MR Egger
Simple mode
Weighted median
Weighted mode
0.2
0.1
0.0
−0.1
SNP effect on Pulmonary fibrosis
0.05
0.10
0.15
0.20
0.25
SNP effect on BMP7

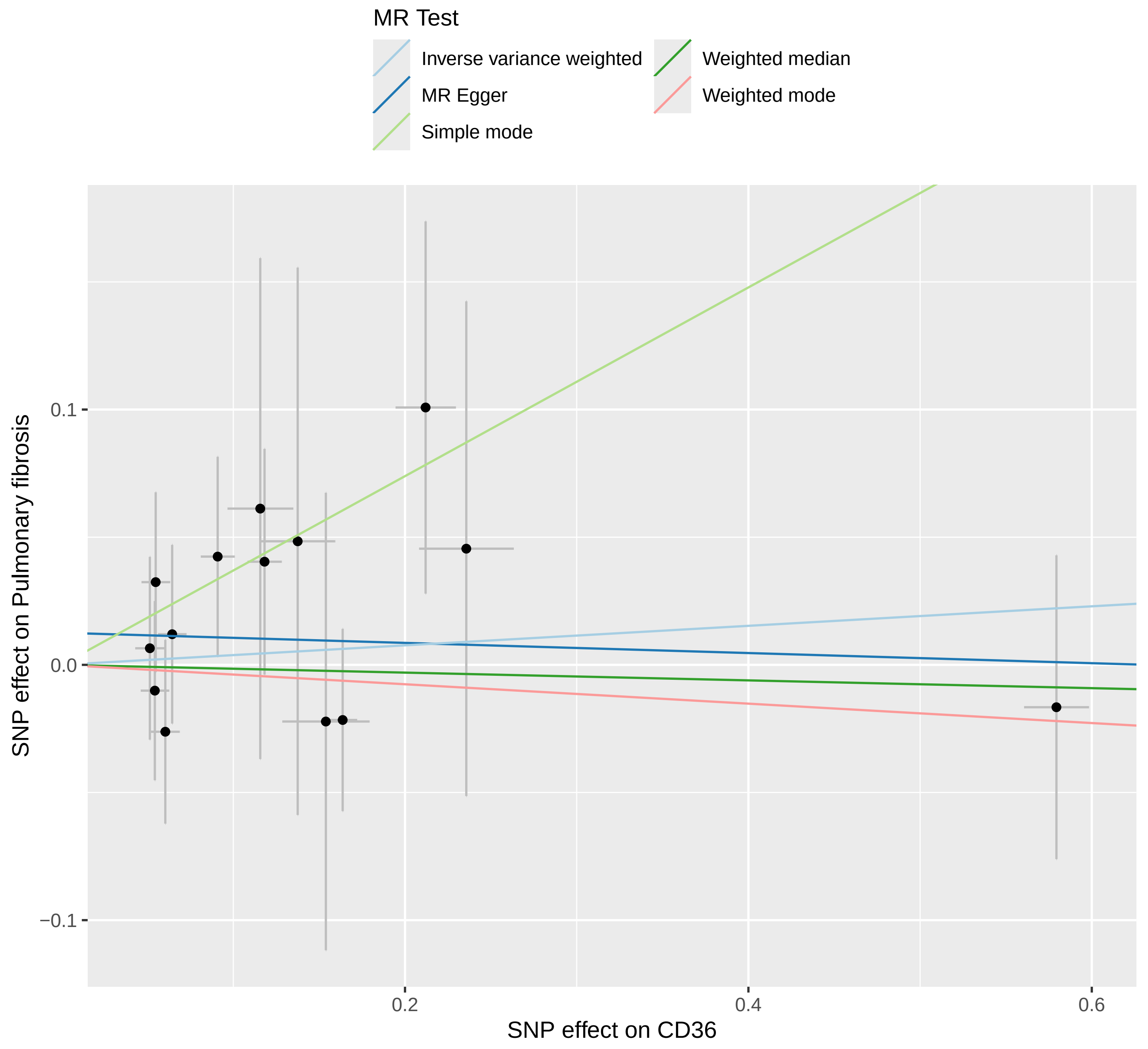

MR Test
Inverse variance weighted
MR Egger
Simple mode
Weighted median
Weighted mode
SNP effect on Pulmonary fibrosis
0.1
0.0
−0.1
0.2
0.4
0.6
SNP effect on CD36

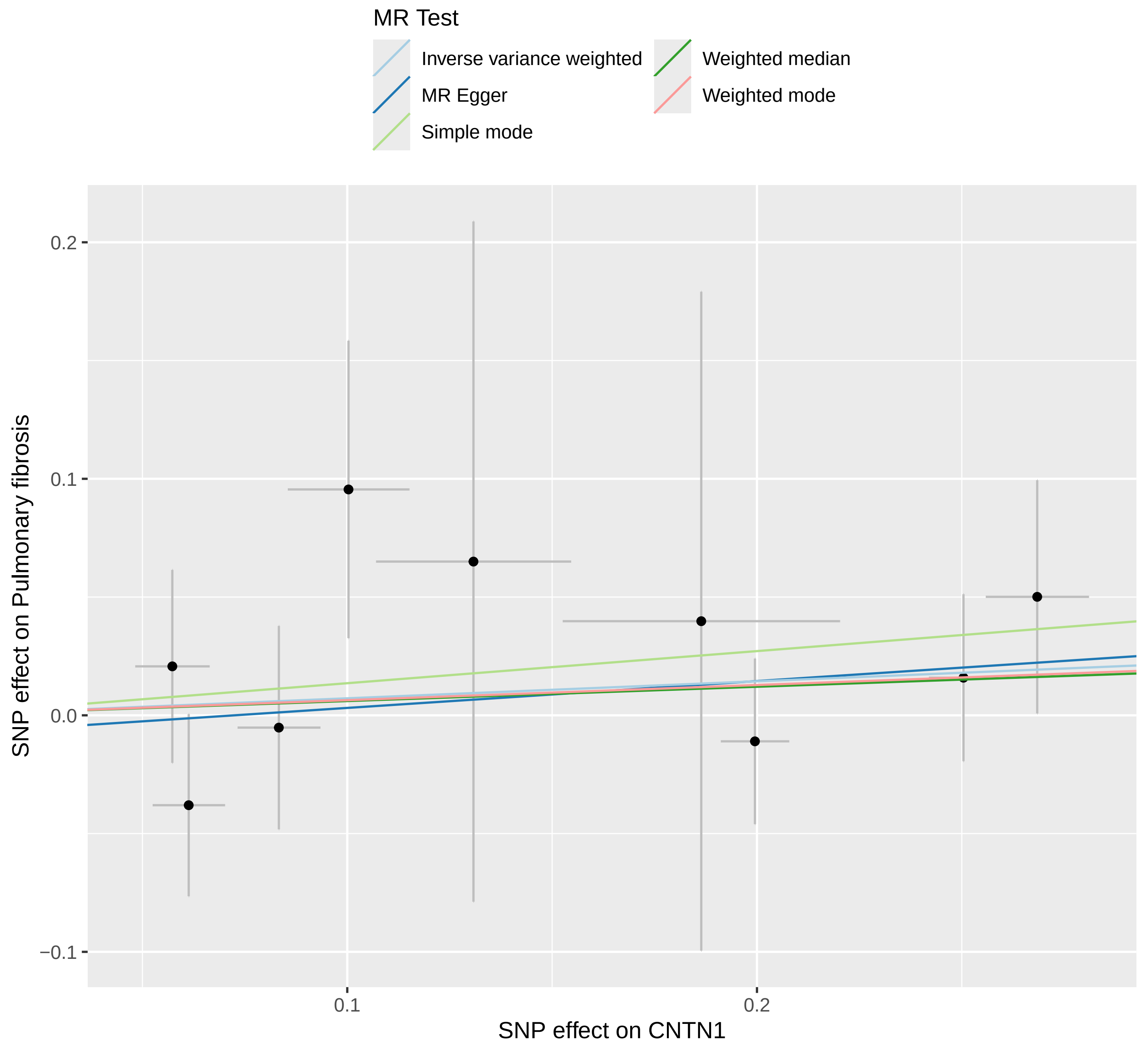
MR Test
Inverse variance weighted
MR Egger
Simple mode
Weighted median
Weighted mode
SNP effect on Pulmonary fibrosis
0.2
0.1
0.0
−0.1
0.1
0.2
SNP effect on CNTN1

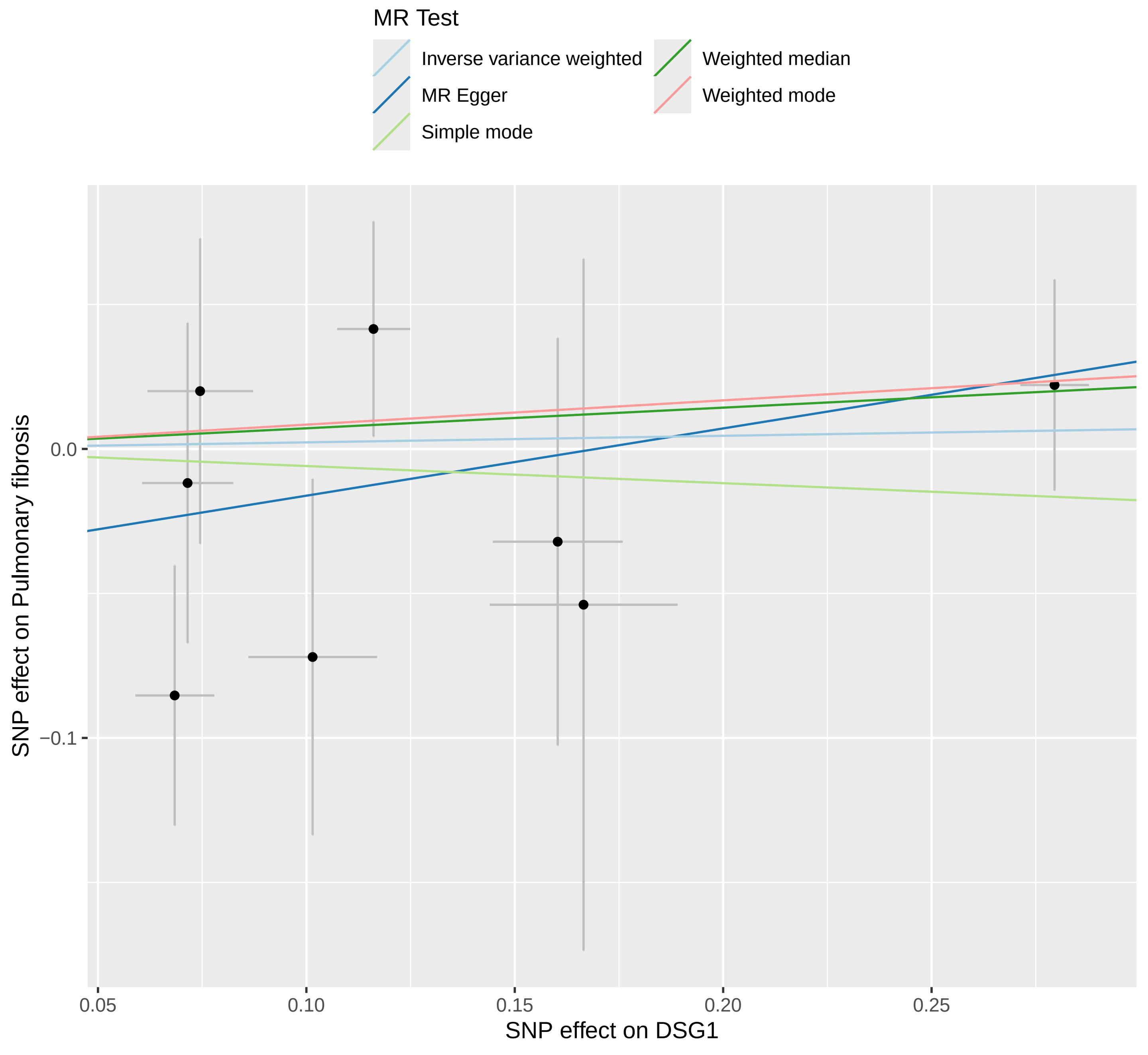

MR Test
Inverse variance weighted
MR Egger
Simple mode
Weighted median
Weighted mode
SNP effect on Pulmonary fibrosis
0.0
−0.1
0.05
0.10
0.15
0.20
0.25
SNP effect on DSG1

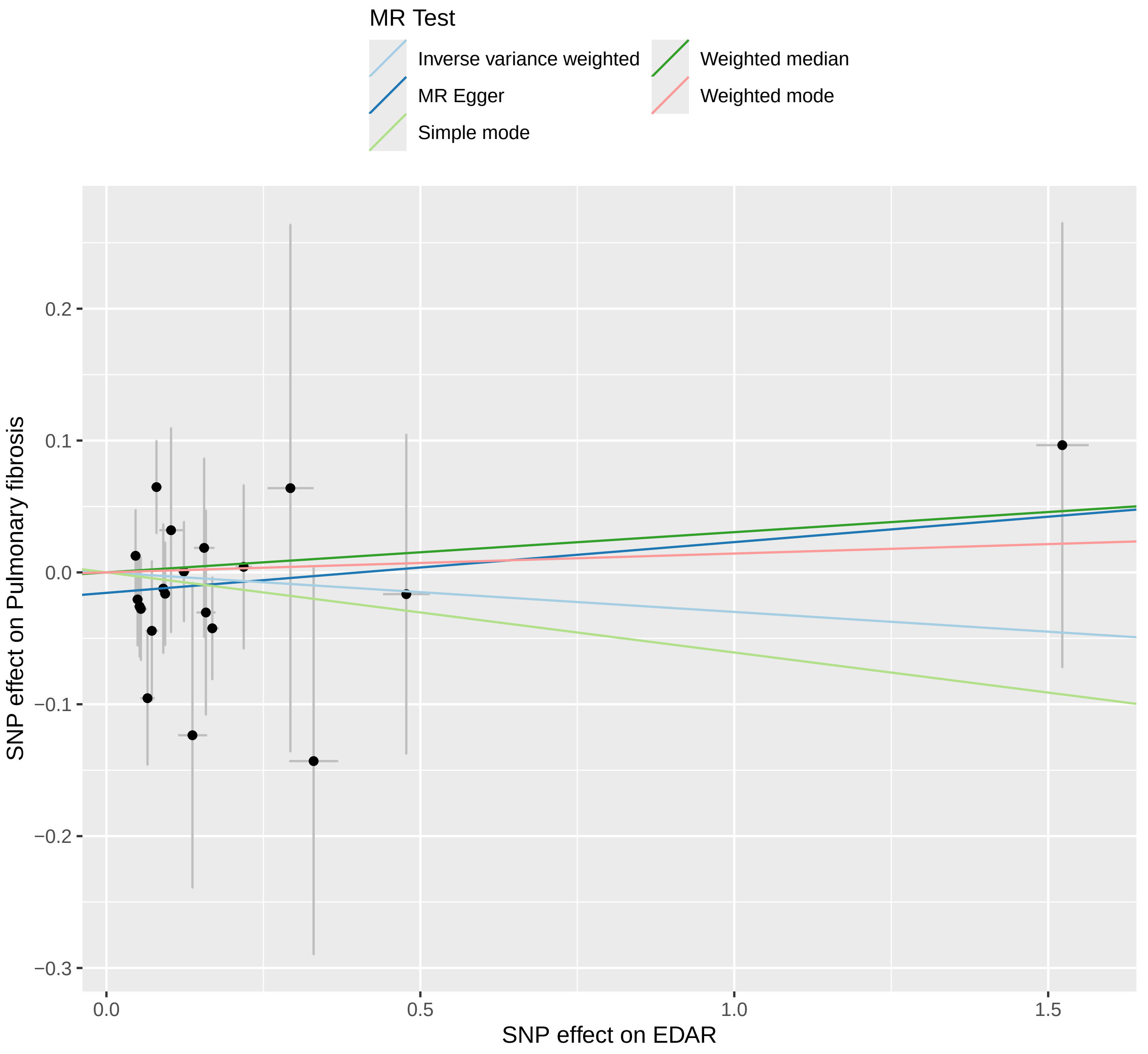

MR Test
Inverse variance weighted
MR Egger
Simple mode
Weighted median
Weighted mode
0.2
0.1
0.0
−0.1
−0.2
−0.3
0.0
0.5
1.0
1.5
SNP effect on Pulmonary fibrosis
SNP effect on EDAR

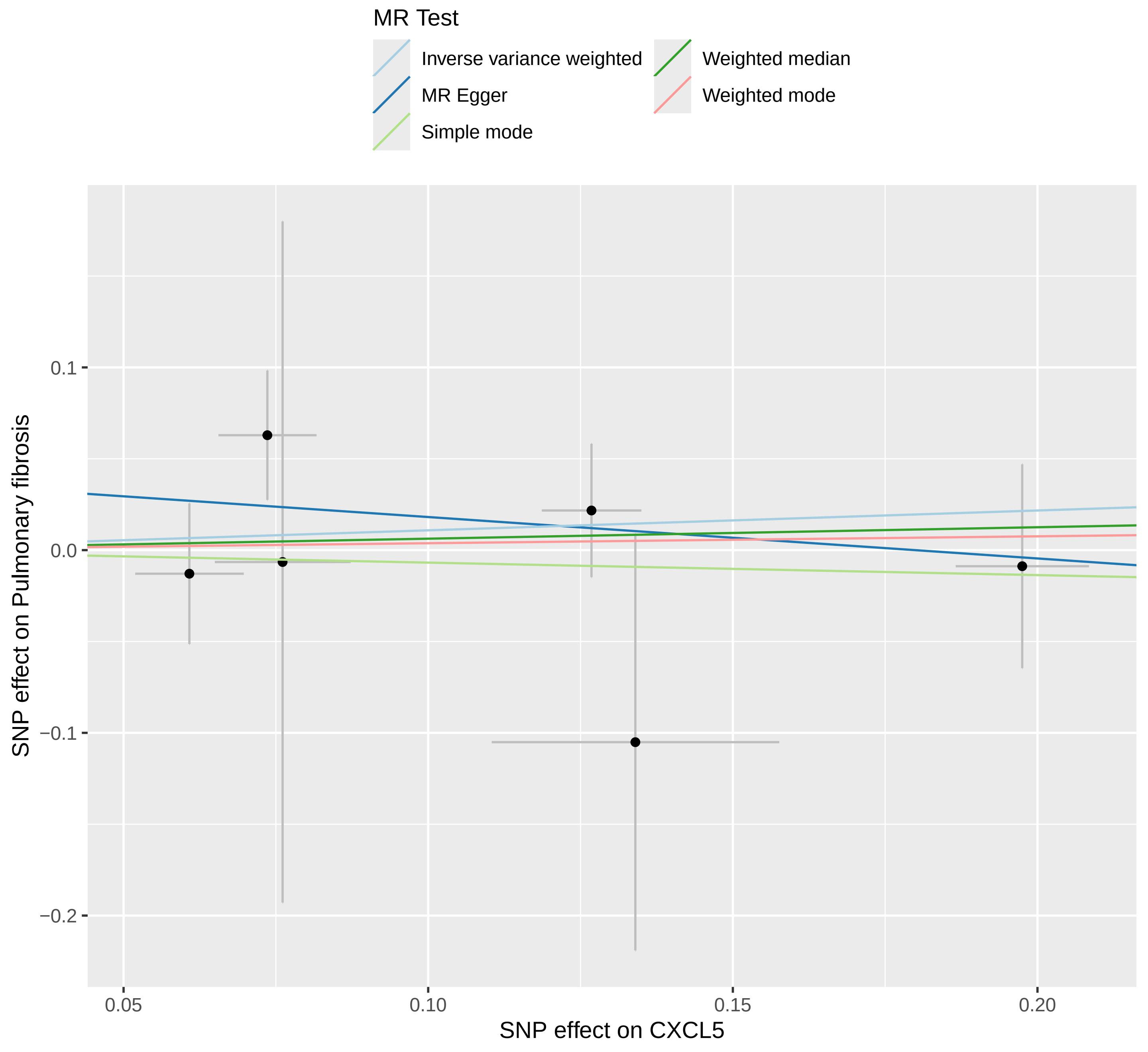

MR Test
Inverse variance weighted
MR Egger
Simple mode
Weighted median
Weighted mode
0.1
0.0
−0.1
−0.2
SNP effect on Pulmonary fibrosis
0.05
0.10
0.15
0.20
SNP effect on CXCL5

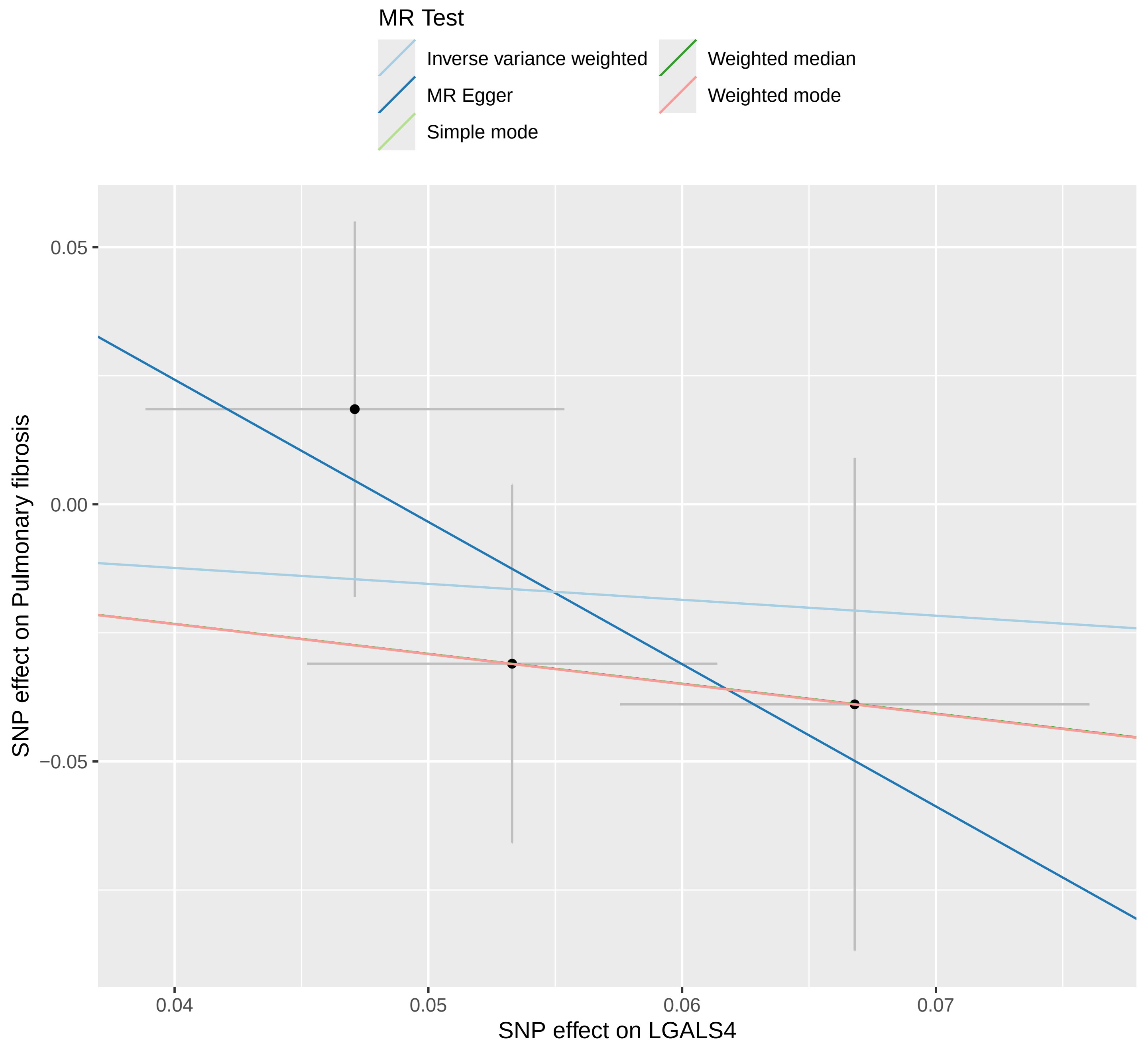
MR Test
Inverse variance weighted
MR Egger
Simple mode
Weighted median
Weighted mode
0.05
0.00
−0.05
SNP effect on Pulmonary fibrosis
0.04
0.05
0.06
0.07
SNP effect on LGALS4

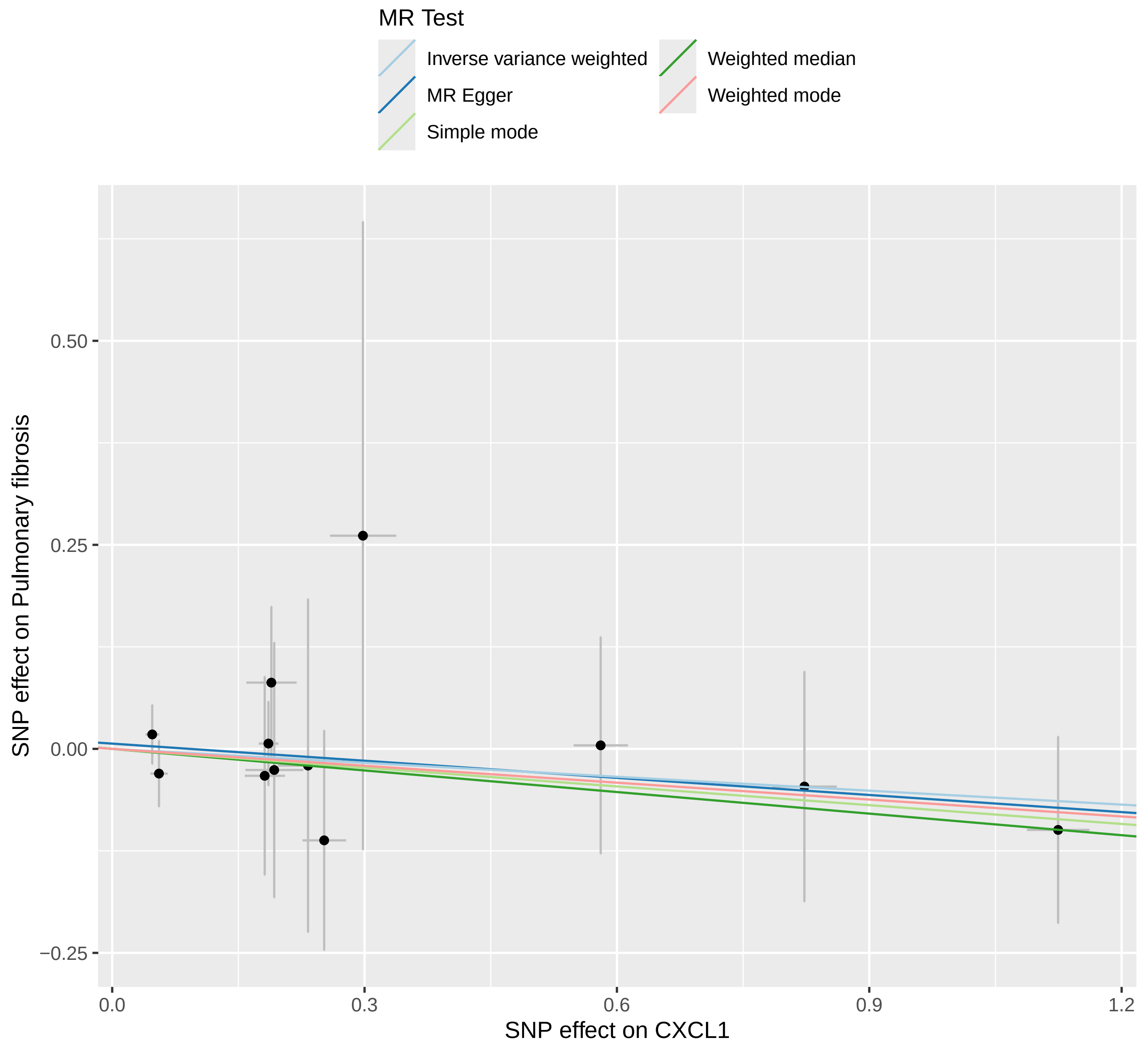

MR Test
Inverse variance weighted
MR Egger
Simple mode
Weighted median
Weighted mode
SNP effect on Pulmonary fibrosis
0.50
0.25
0.00
−0.25
0.0
0.3
0.6
0.9
1.2
SNP effect on CXCL1

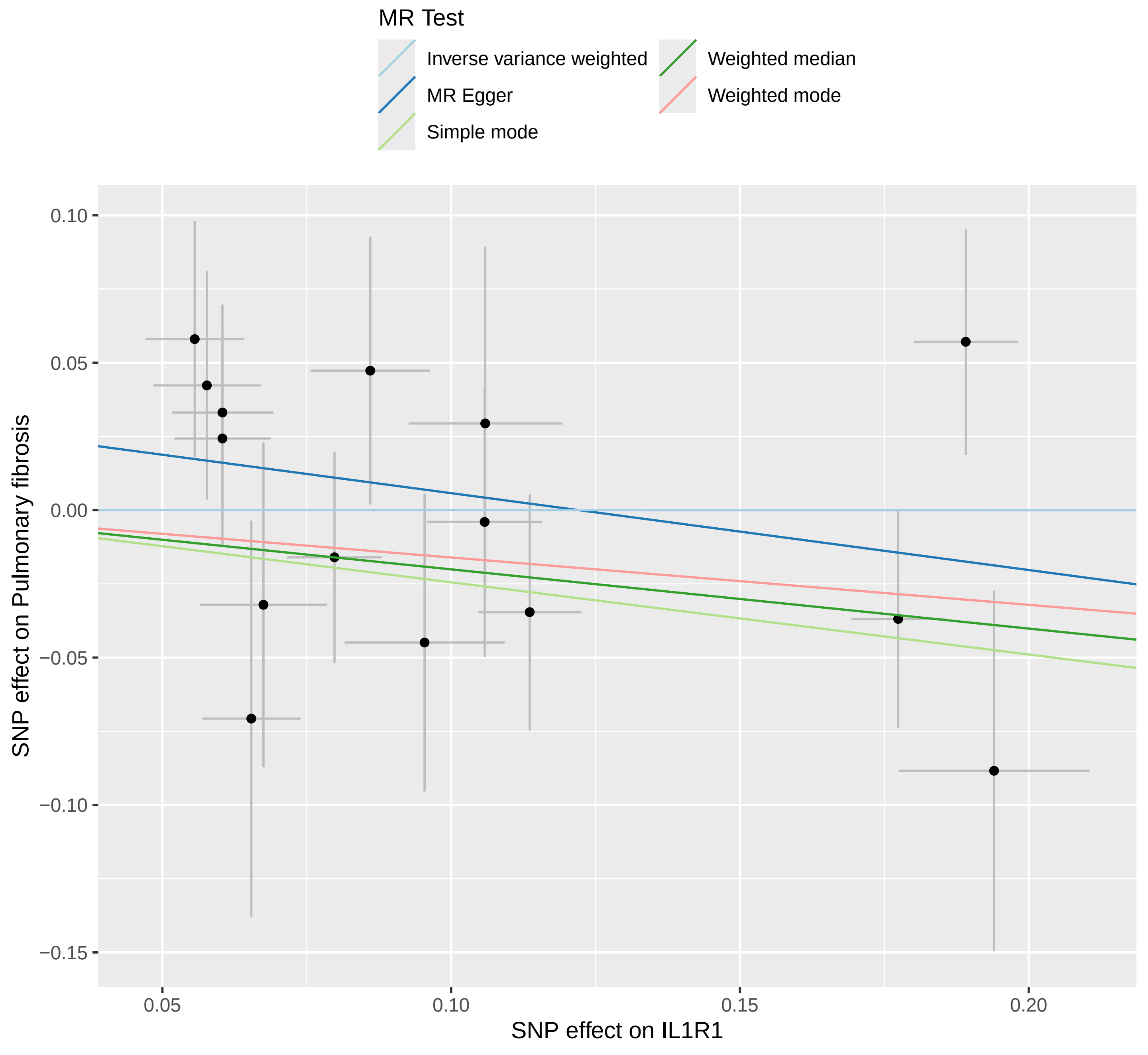

MR Test
Inverse variance weighted
MR Egger
Simple mode
Weighted median
Weighted mode
0.10
0.05
0.00
−0.05
−0.10
−0.15
0.05
0.10
0.15
0.20
SNP effect on Pulmonary fibrosis
SNP effect on IL1R1

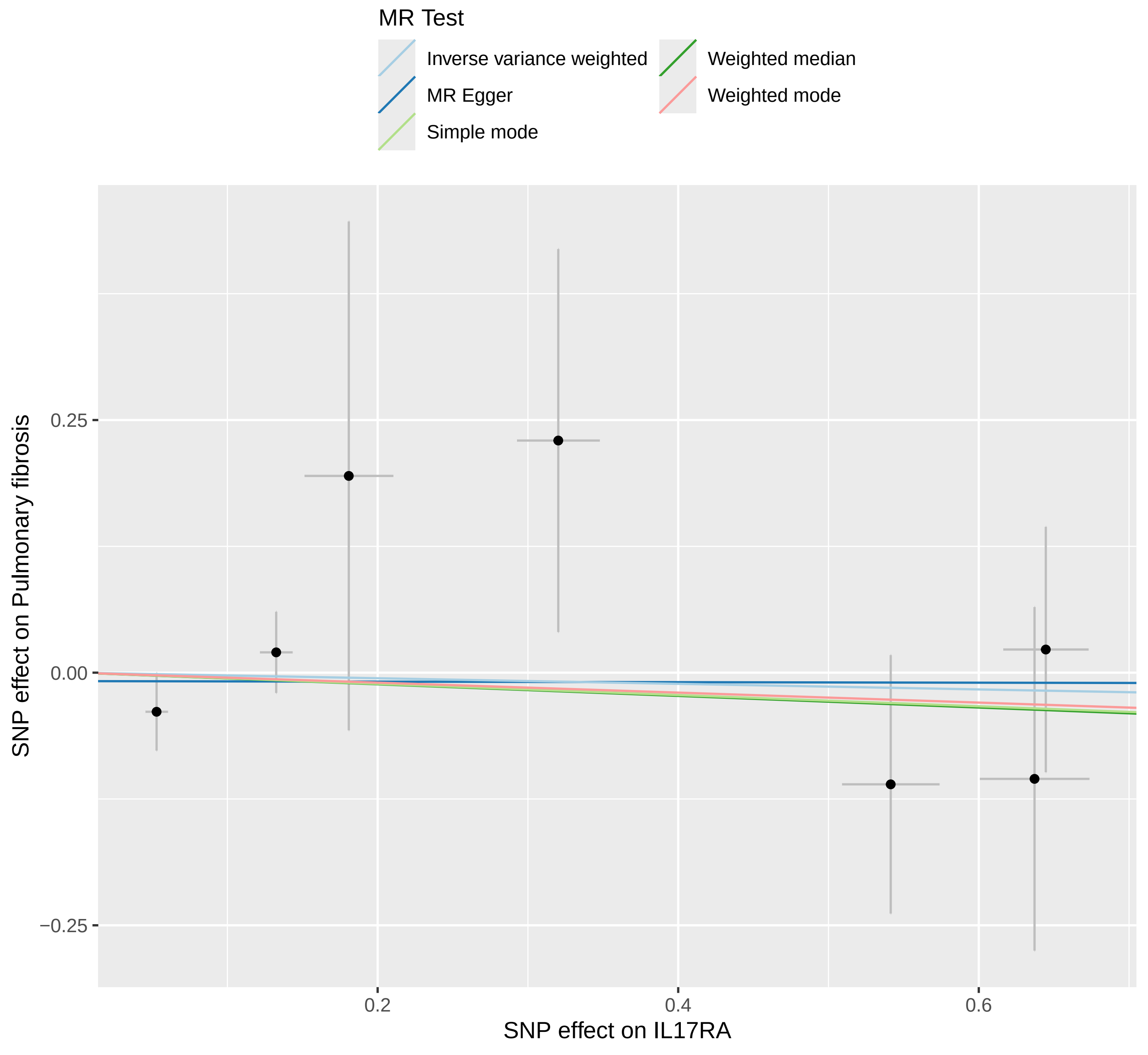
MR Test
Inverse variance weighted
MR Egger
Simple mode
Weighted median
Weighted mode
SNP effect on Pulmonary fibrosis
0.25
0.00
−0.25
0.2
0.4
0.6
SNP effect on IL17RA

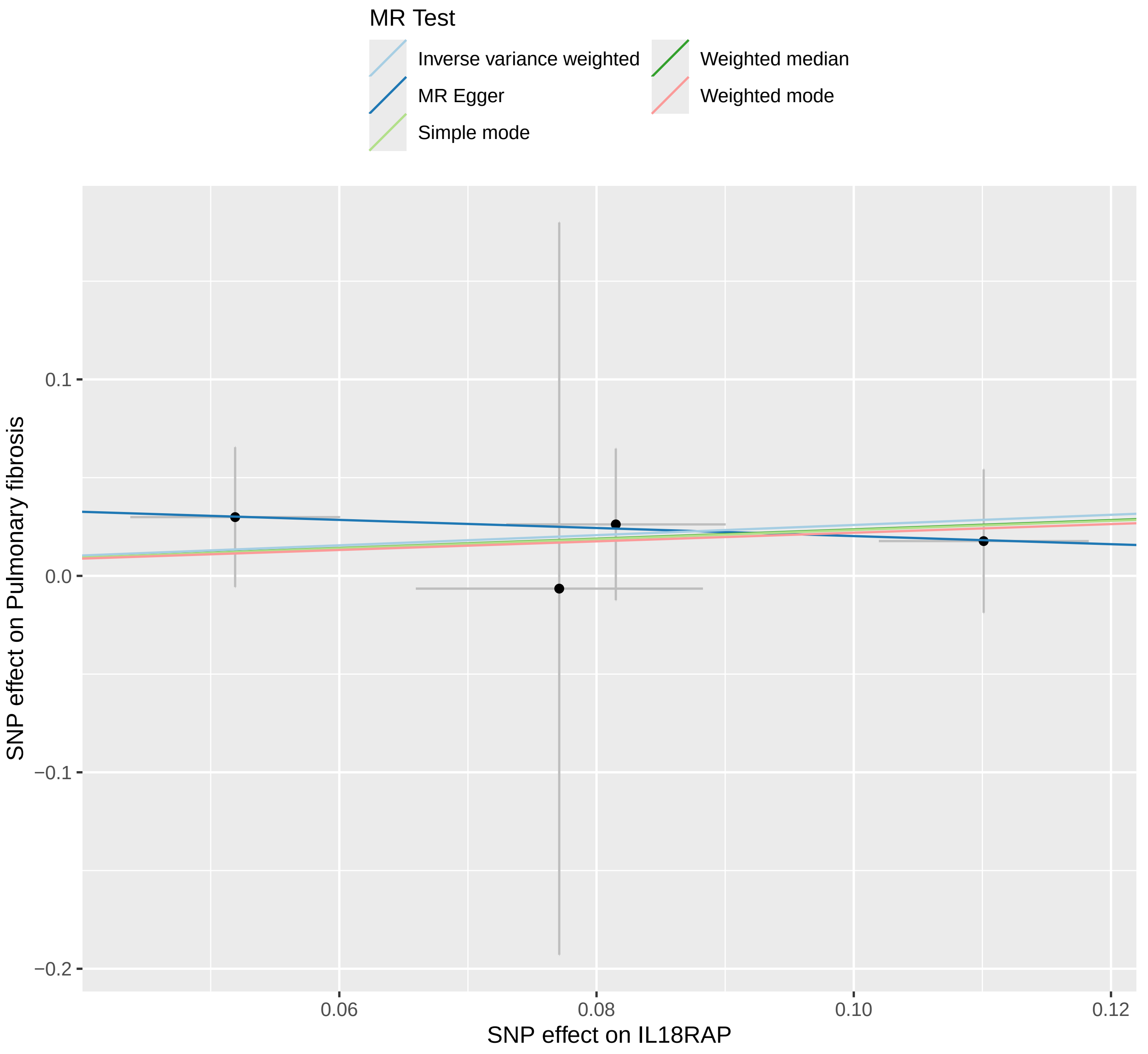

MR Test
Inverse variance weighted
MR Egger
Simple mode
Weighted median
Weighted mode
0.1
0.0
−0.1
−0.2
0.06
0.08
0.10
0.12
SNP effect on IL18RAP
SNP effect on Pulmonary fibrosis

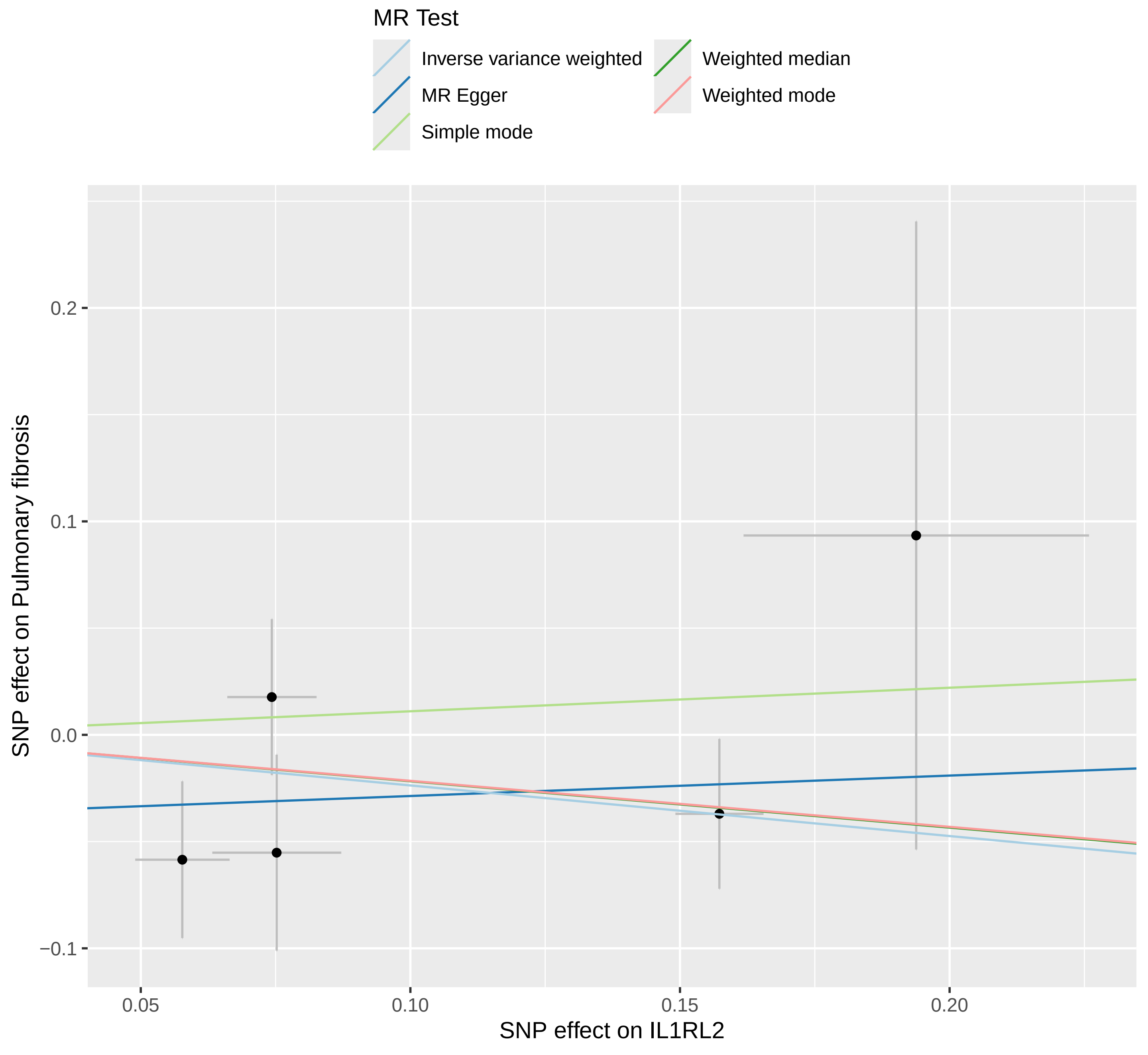
MR Test
Inverse variance weighted
MR Egger
Simple mode
Weighted median
Weighted mode
0.2
0.1
0.0
−0.1
0.05
0.10
0.15
0.20
SNP effect on Pulmonary fibrosis
SNP effect on IL1RL2

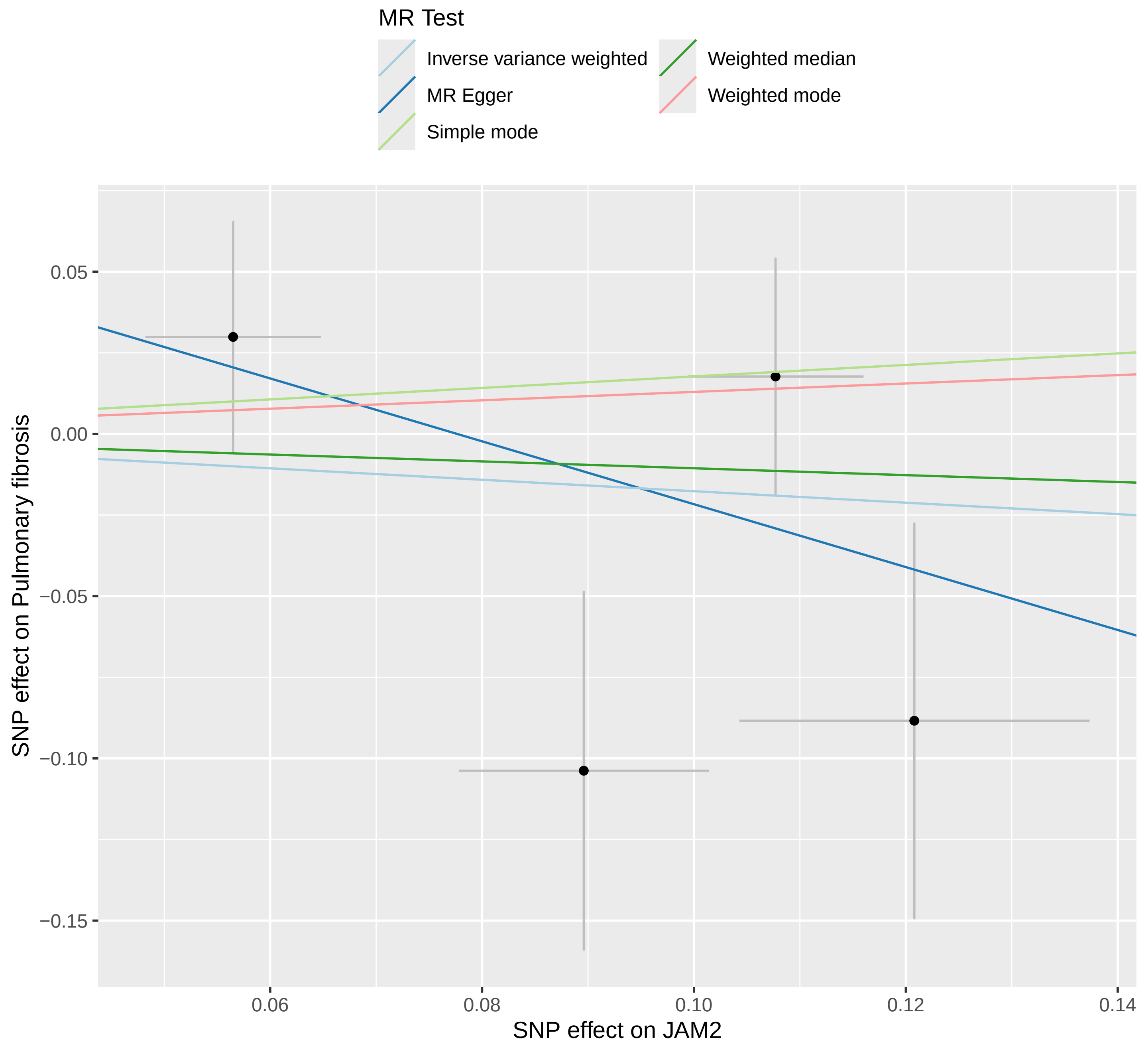
MR Test
Inverse variance weighted
MR Egger
Simple mode
Weighted median
Weighted mode
0.05
0.00
−0.05
−0.10
−0.15
0.06
0.08
0.10
0.12
0.14
SNP effect on JAM2
SNP effect on Pulmonary fibrosis

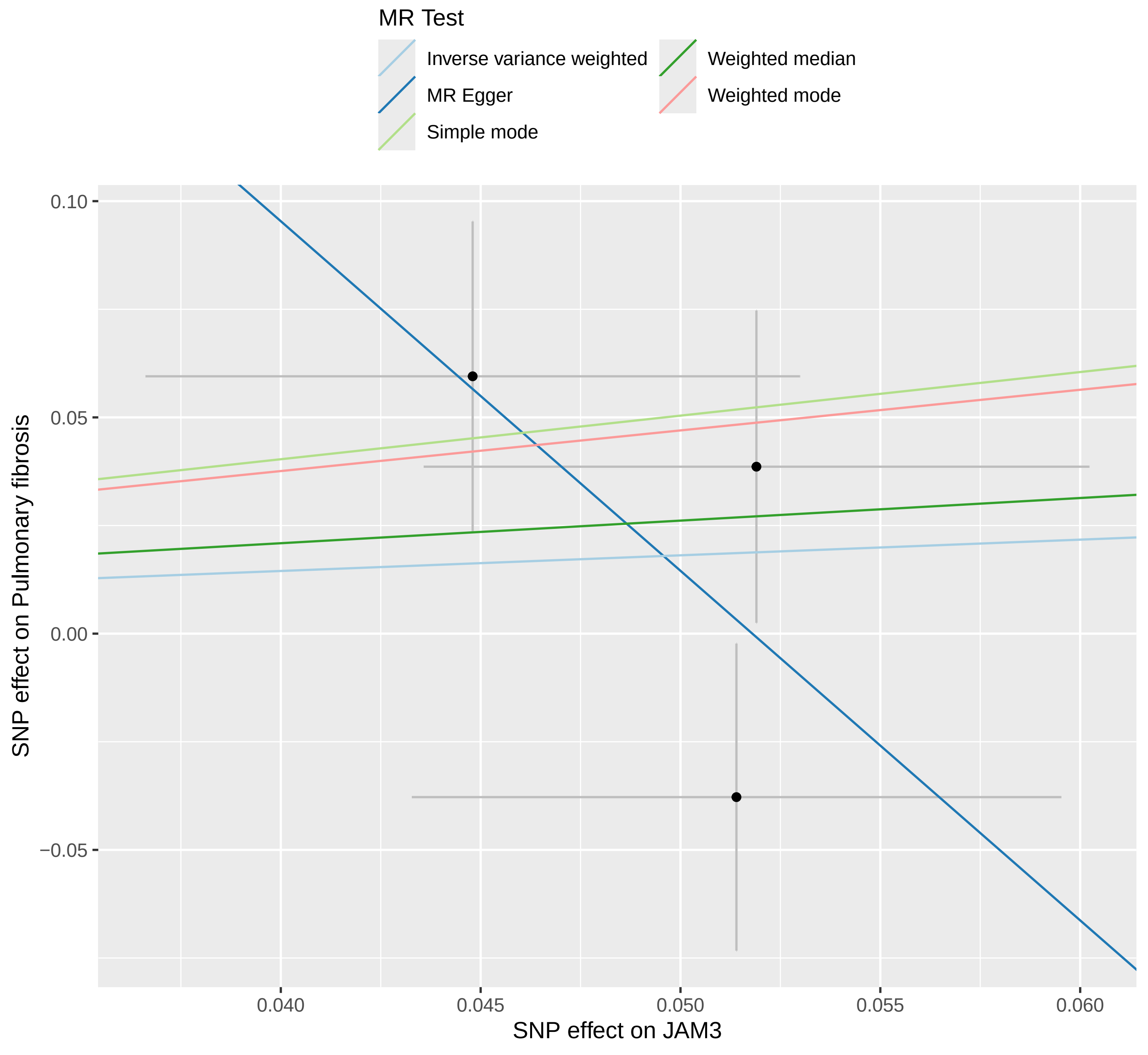
MR Test
Inverse variance weighted
MR Egger
Simple mode
Weighted median
Weighted mode
0.10
0.05
0.00
−0.05
SNP effect on Pulmonary fibrosis
0.040
0.045
0.050
0.055
0.060
SNP effect on JAM3

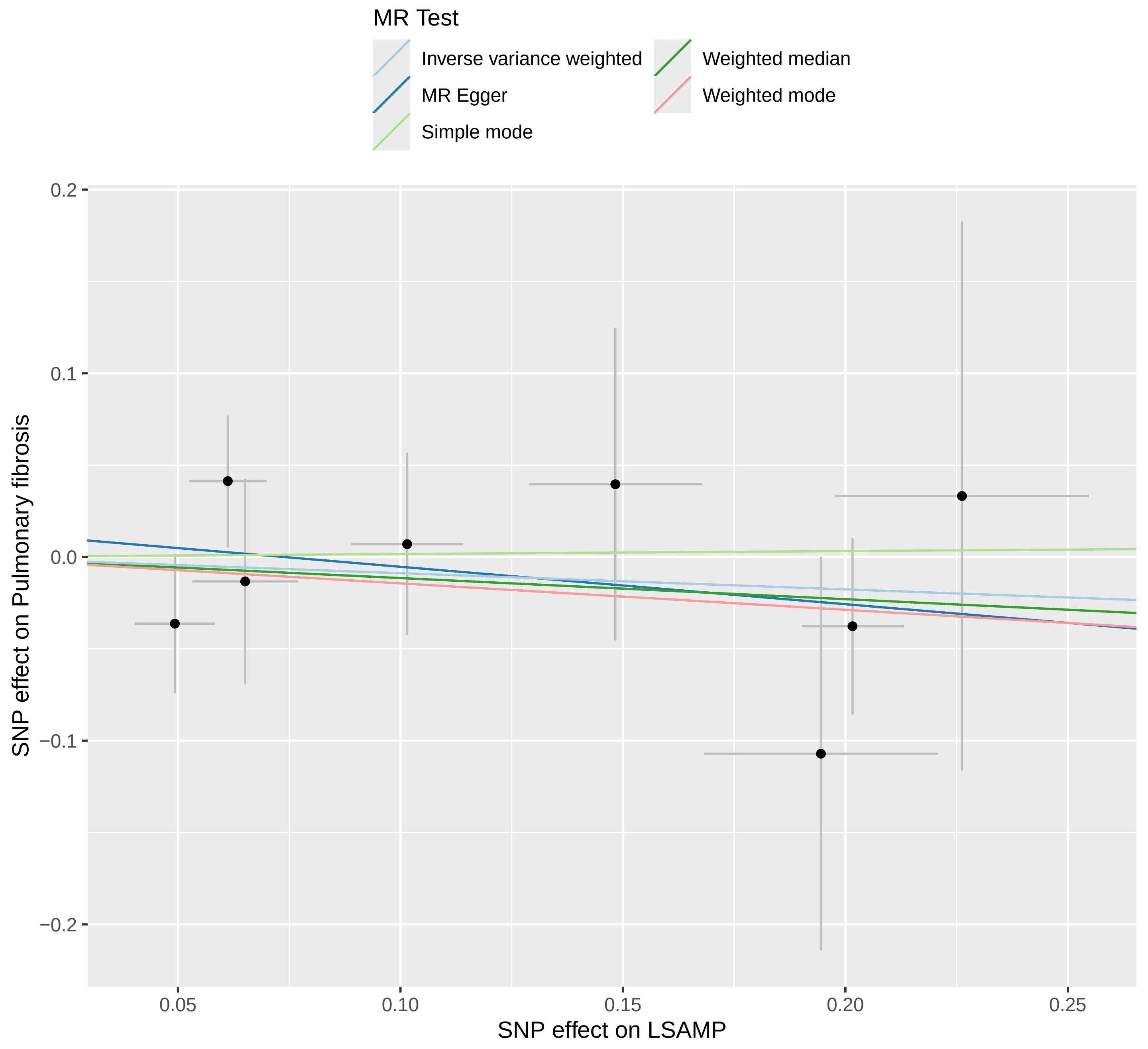
MR Test
Inverse variance weighted
MR Egger
Simple mode
Weighted median
Weighted mode
0.2
0.1
0.0
−0.1
−0.2
SNP effect on Pulmonary fibrosis
0.05
0.10
0.15
0.20
0.25
SNP effect on LSAMP

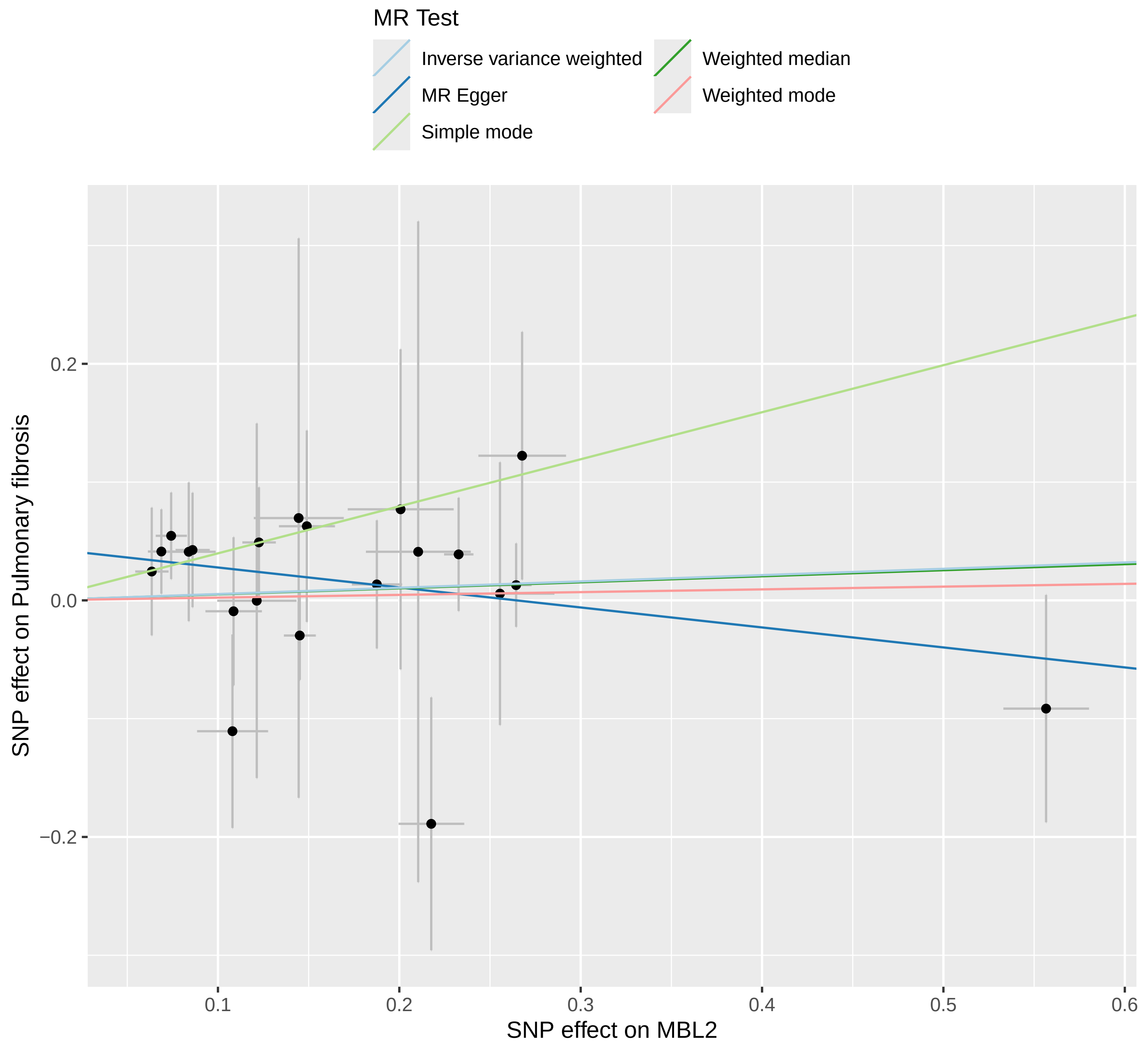

MR Test
Inverse variance weighted
MR Egger
Simple mode
Weighted median
Weighted mode
0.2
0.0
−0.2
0.1
0.2
0.3
0.4
0.5
0.6
SNP effect on Pulmonary fibrosis
SNP effect on MBL2

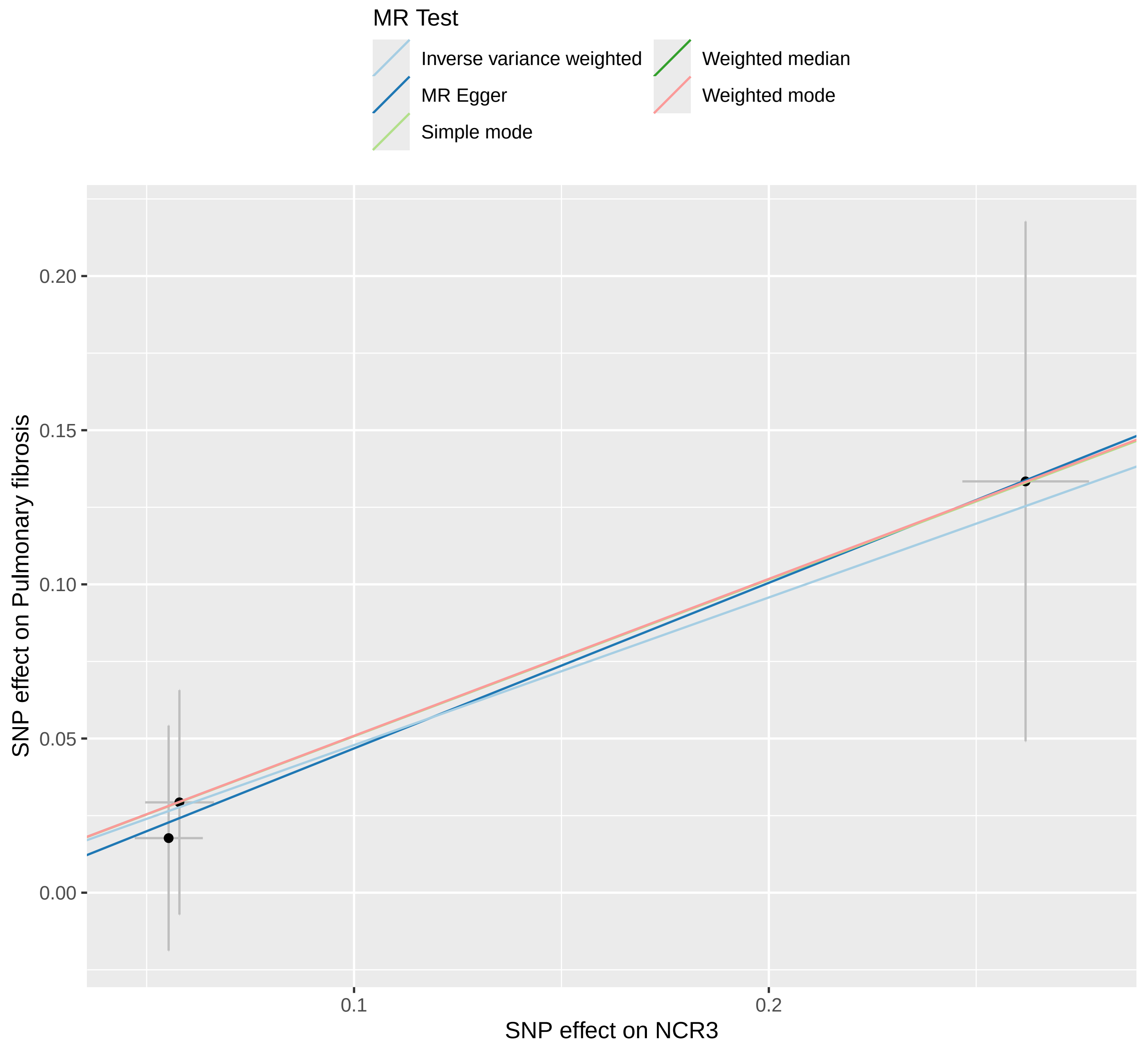

MR Test
Inverse variance weighted
MR Egger
Simple mode
Weighted median
Weighted mode
SNP effect on Pulmonary fibrosis
0.20
0.15
0.10
0.05
0.00
0.1
0.2
SNP effect on NCR3

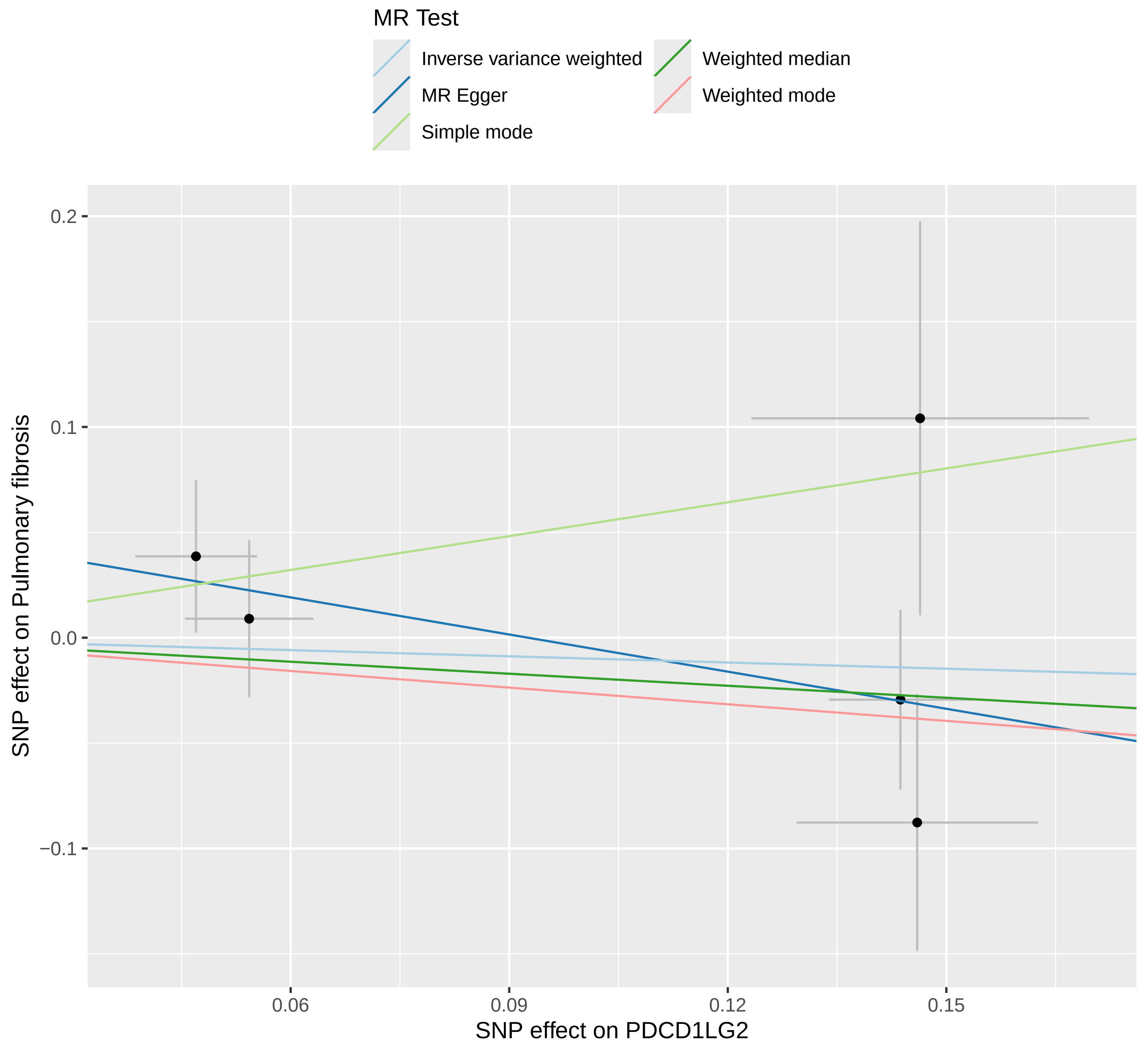

MR Test
Inverse variance weighted
MR Egger
Simple mode
Weighted median
Weighted mode
0.2
0.1
0.0
−0.1
0.06
0.09
0.12
0.15
SNP effect on PDCD1LG2
SNP effect on Pulmonary fibrosis

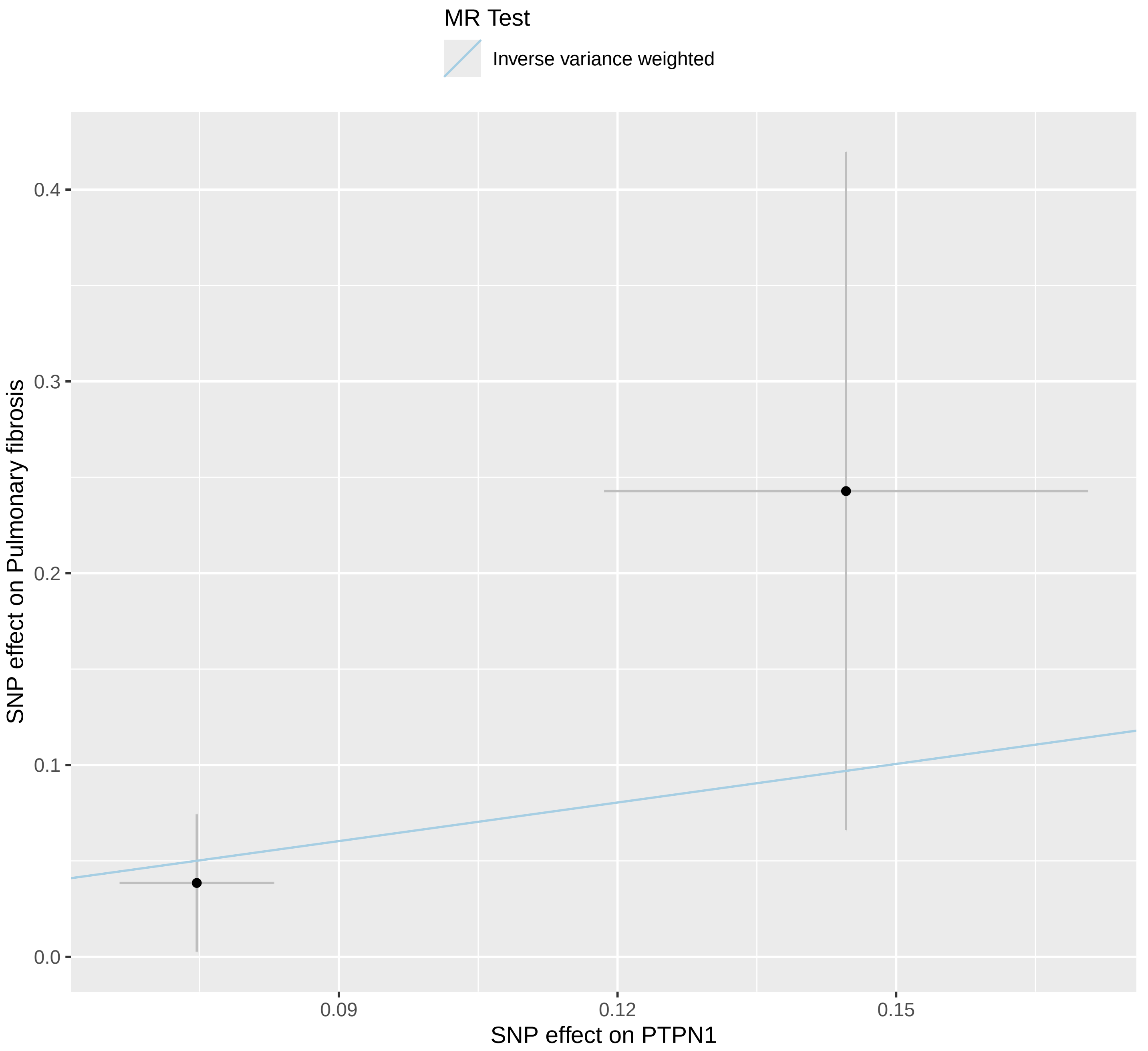

MR Test
Inverse variance weighted
SNP effect on Pulmonary fibrosis
SNP effect on PTPN1
0.0
0.1
0.2
0.3
0.4
0.09
0.12
0.15

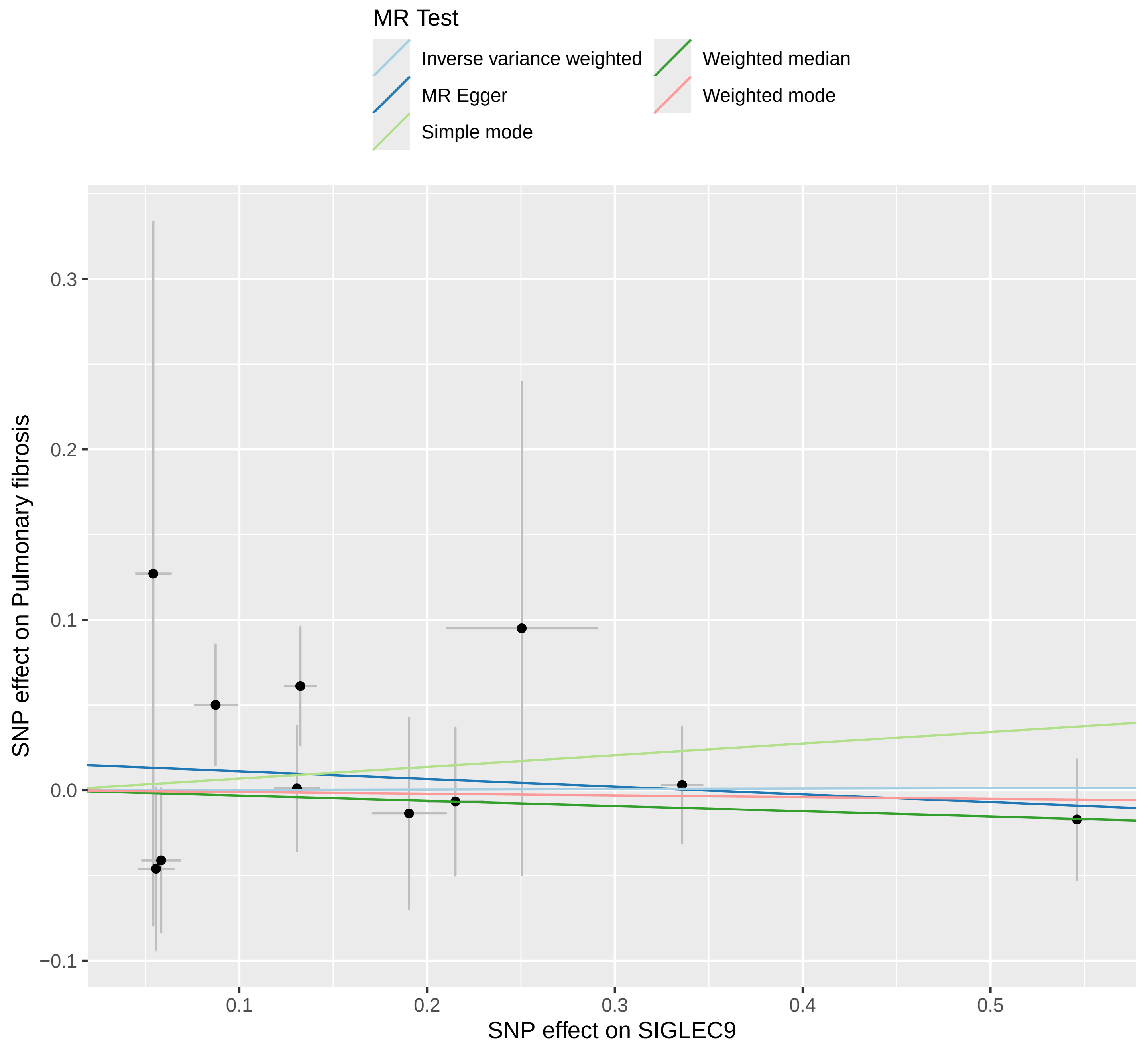
MR Test
Inverse variance weighted
MR Egger
Simple mode
Weighted median
Weighted mode
SNP effect on Pulmonary fibrosis
0.3
0.2
0.1
0.0
−0.1
0.1
0.2
0.3
0.4
0.5
SNP effect on SIGLEC9

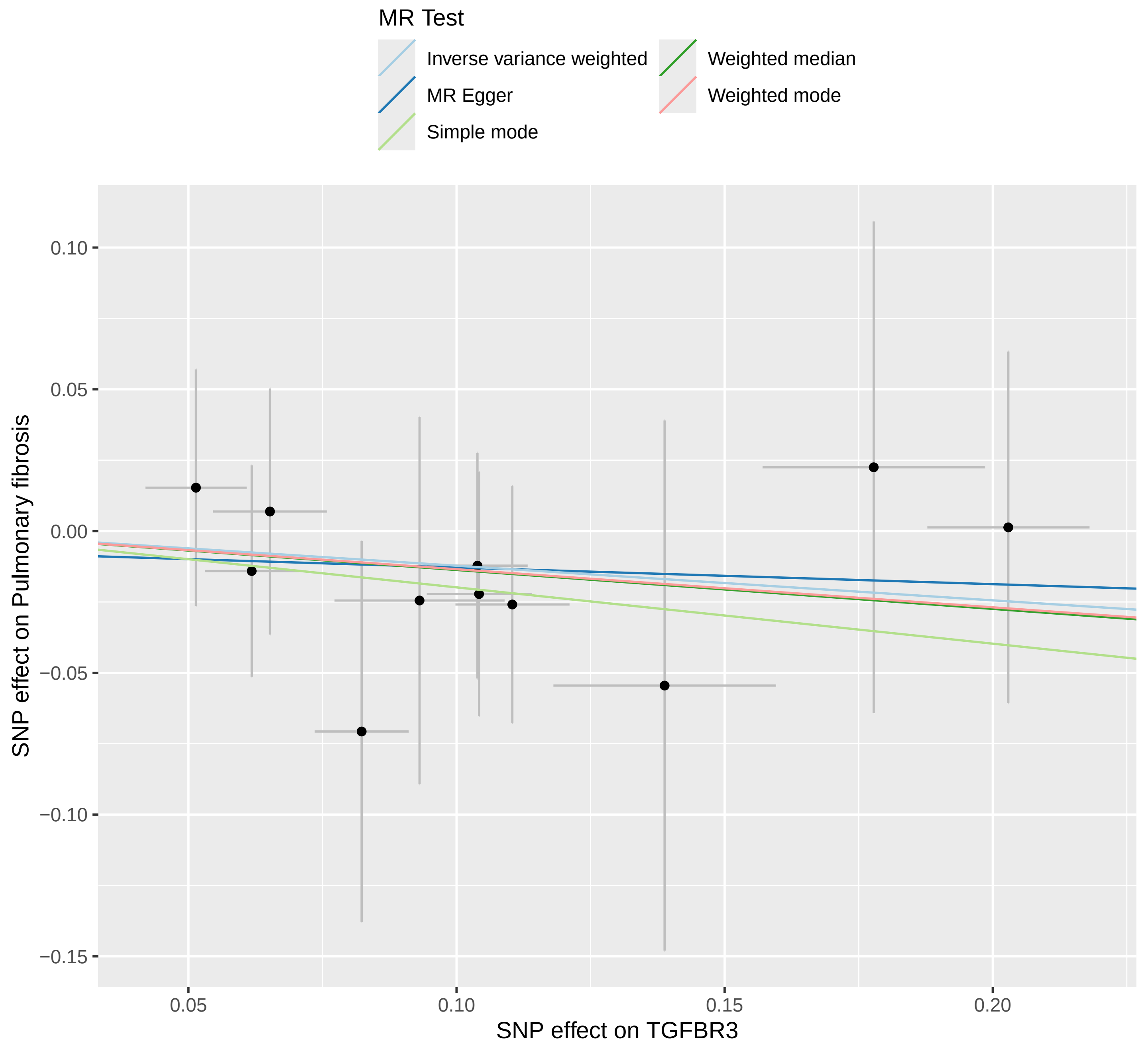
MR Test
Inverse variance weighted
MR Egger
Simple mode
Weighted median
Weighted mode
0.10
0.05
0.00
−0.05
−0.10
−0.15
SNP effect on Pulmonary fibrosis
0.05
0.10
0.15
0.20
SNP effect on TGFBR3

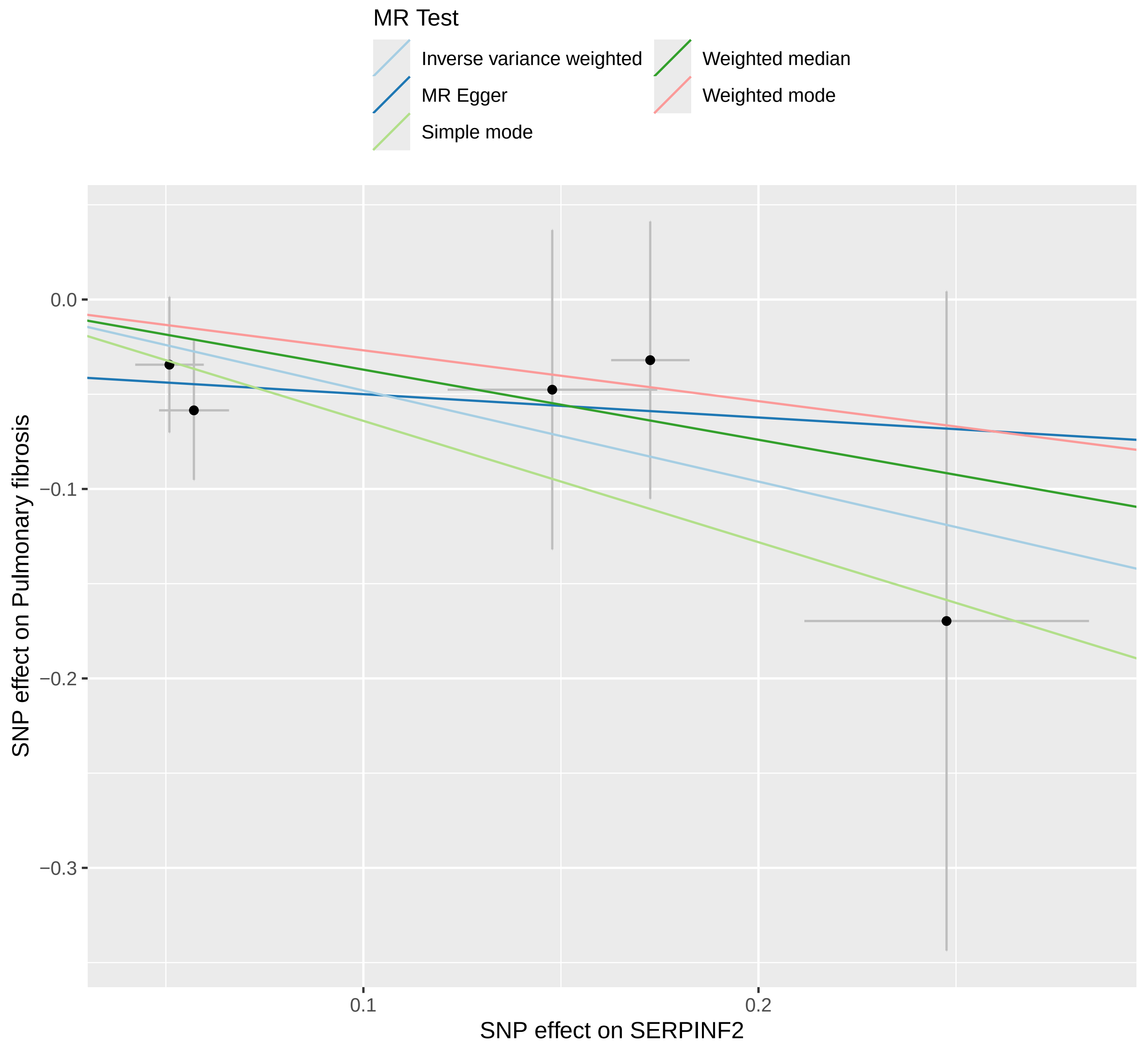

MR Test
Inverse variance weighted
MR Egger
Simple mode
Weighted median
Weighted mode
0.0
−0.1
−0.2
−0.3
0.1
0.2
SNP effect on Pulmonary fibrosis
SNP effect on SERPINF2

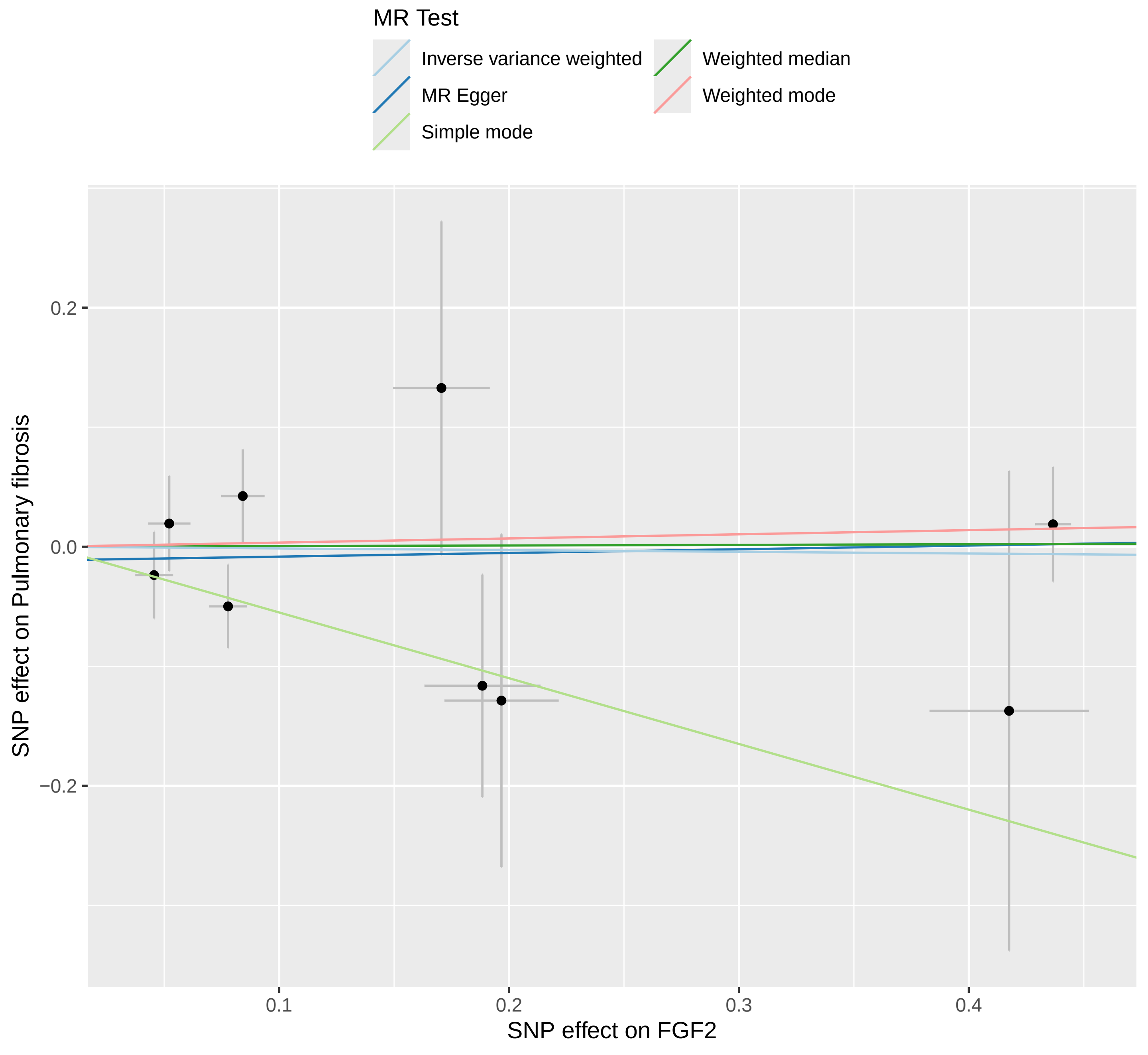
MR Test
Inverse variance weighted
Weighted median
MR Egger
Weighted mode
Simple mode
0.2
0.0
−0.2
SNP effect on Pulmonary fibrosis
0.1
0.2
0.3
0.4
SNP effect on FGF2

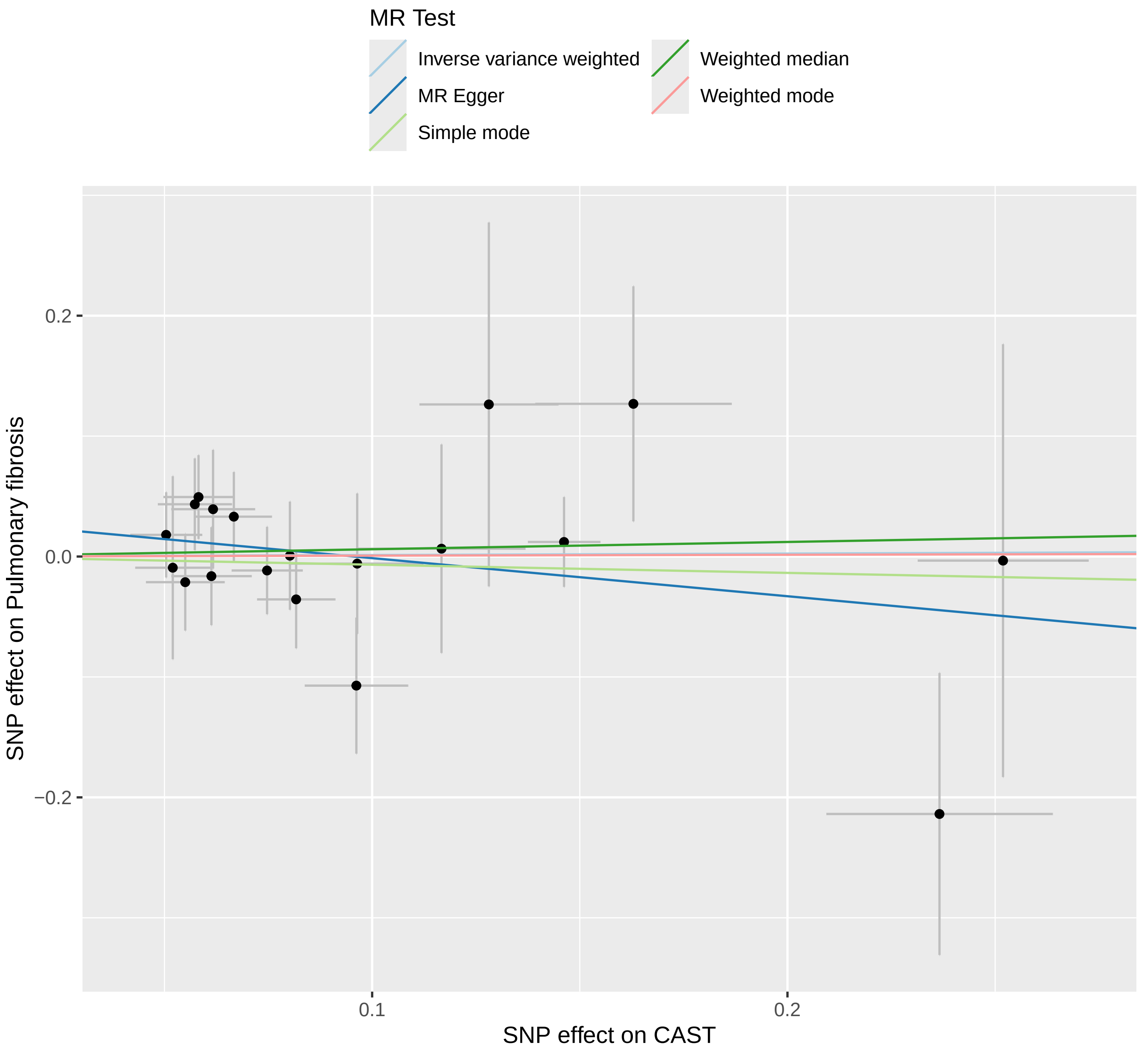

MR Test
Inverse variance weighted
MR Egger
Simple mode
Weighted median
Weighted mode
0.2
0.0
−0.2
0.1
0.2
SNP effect on Pulmonary fibrosis
SNP effect on CAST

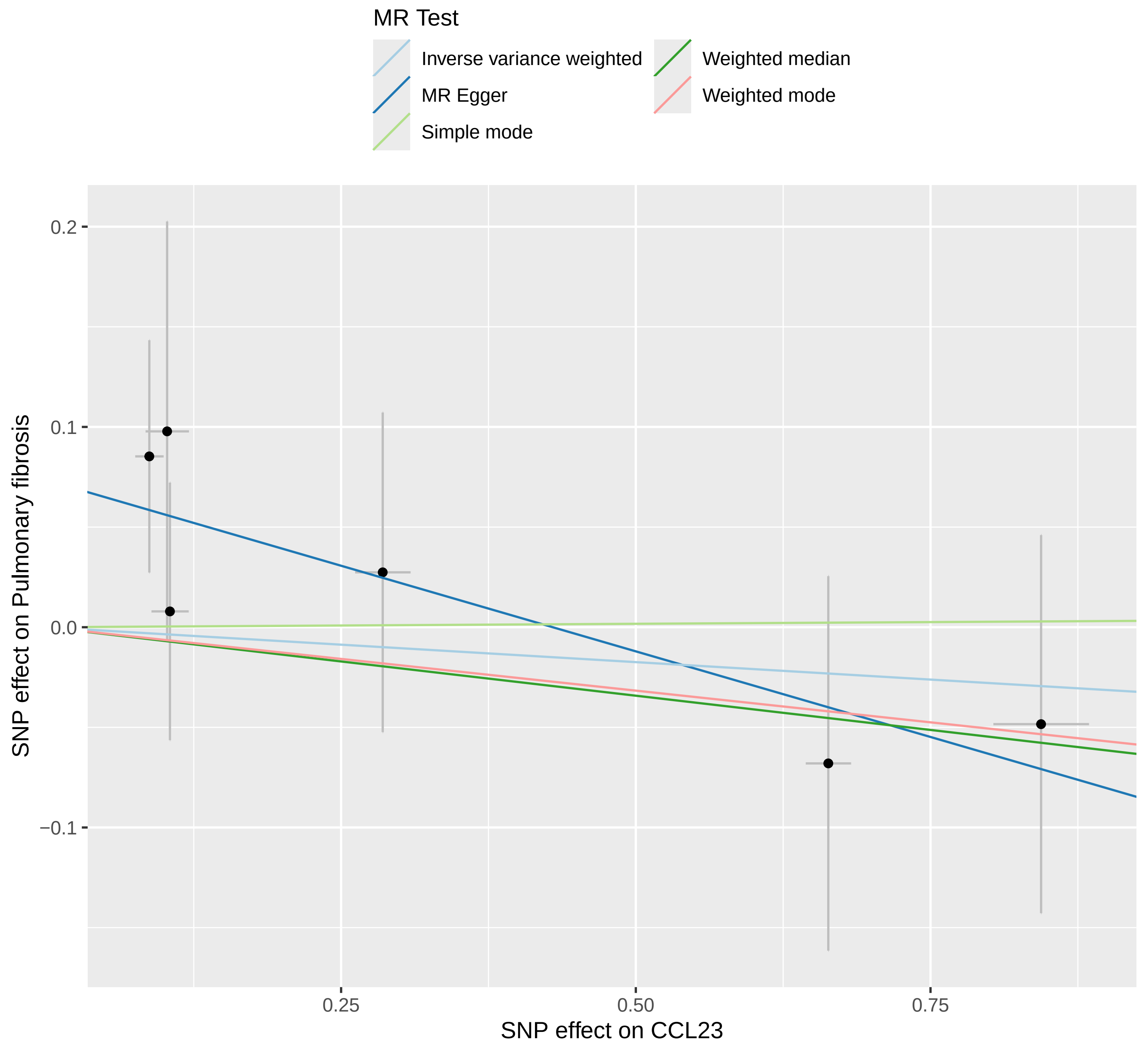

MR Test
Inverse variance weighted
MR Egger
Simple mode
Weighted median
Weighted mode
0.2
0.1
0.0
−0.1
0.25
0.50
0.75
SNP effect on Pulmonary fibrosis
SNP effect on CCL23

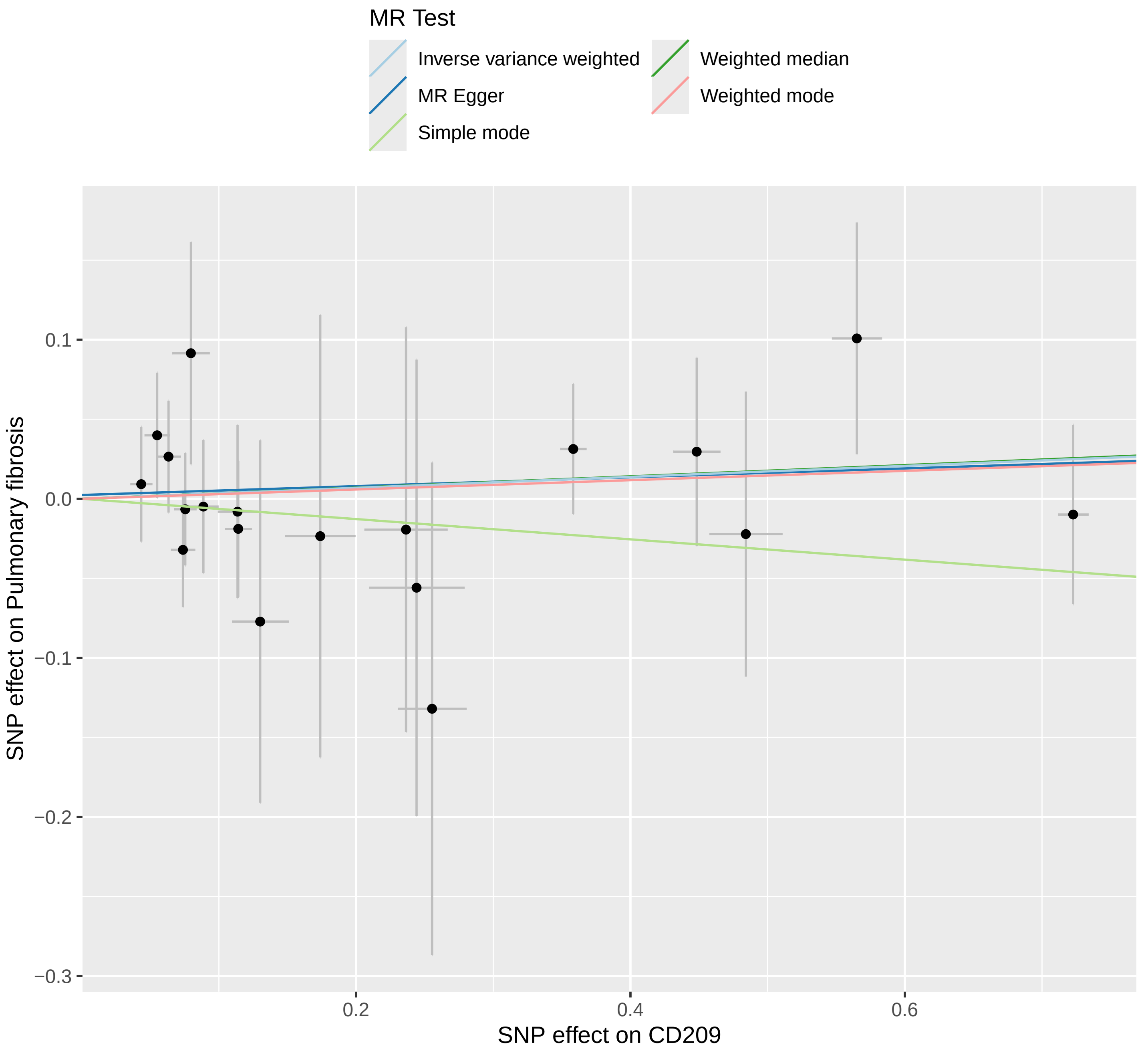
MR Test
Inverse variance weighted
MR Egger
Simple mode
Weighted median
Weighted mode
0.1
0.0
−0.1
−0.2
−0.3
0.2
0.4
0.6
SNP effect on Pulmonary fibrosis
SNP effect on CD209

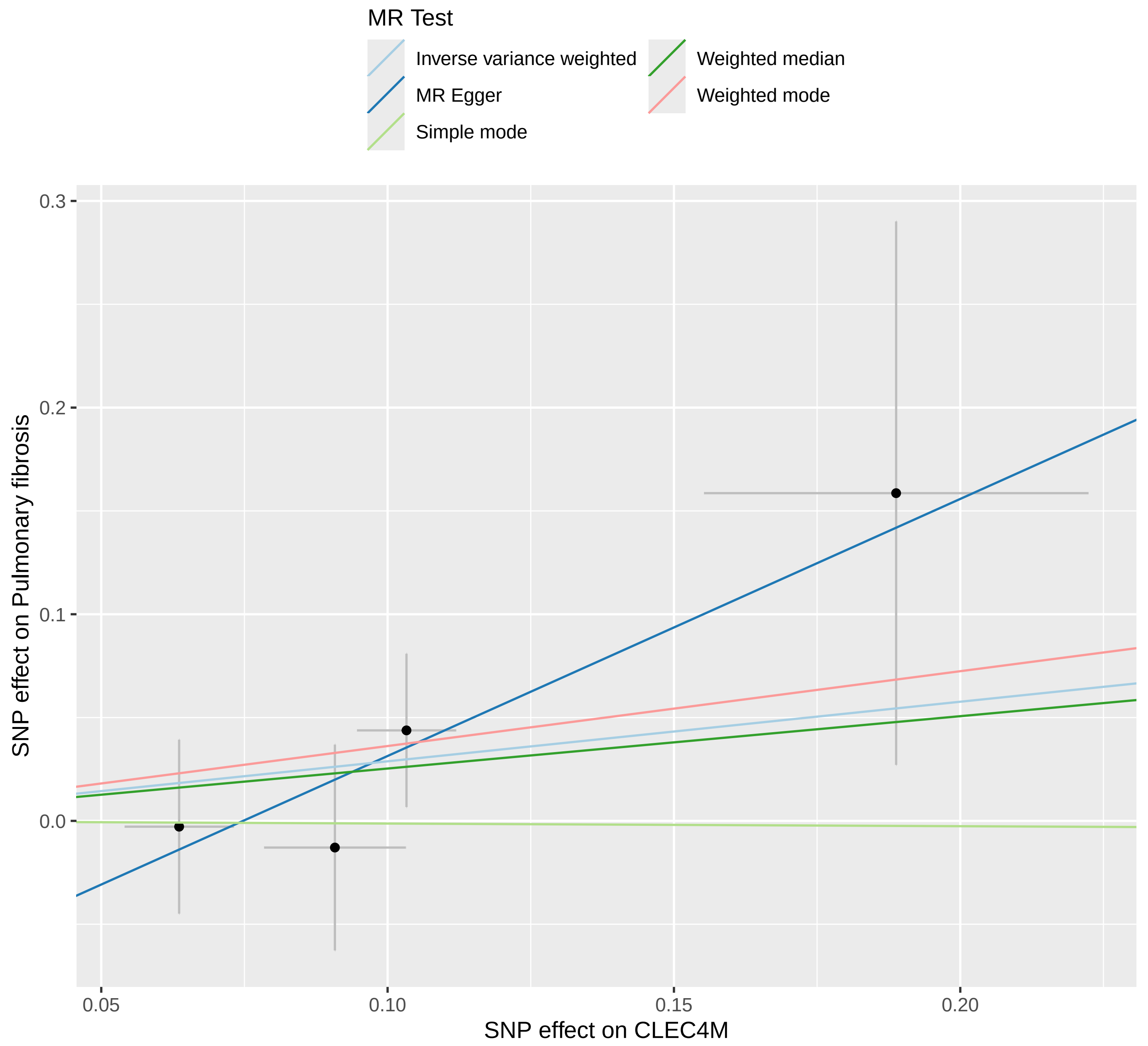

MR Test
Inverse variance weighted
MR Egger
Simple mode
Weighted median
Weighted mode
0.3
0.2
0.1
0.0
0.05
0.10
0.15
0.20
SNP effect on Pulmonary fibrosis
SNP effect on CLEC4M

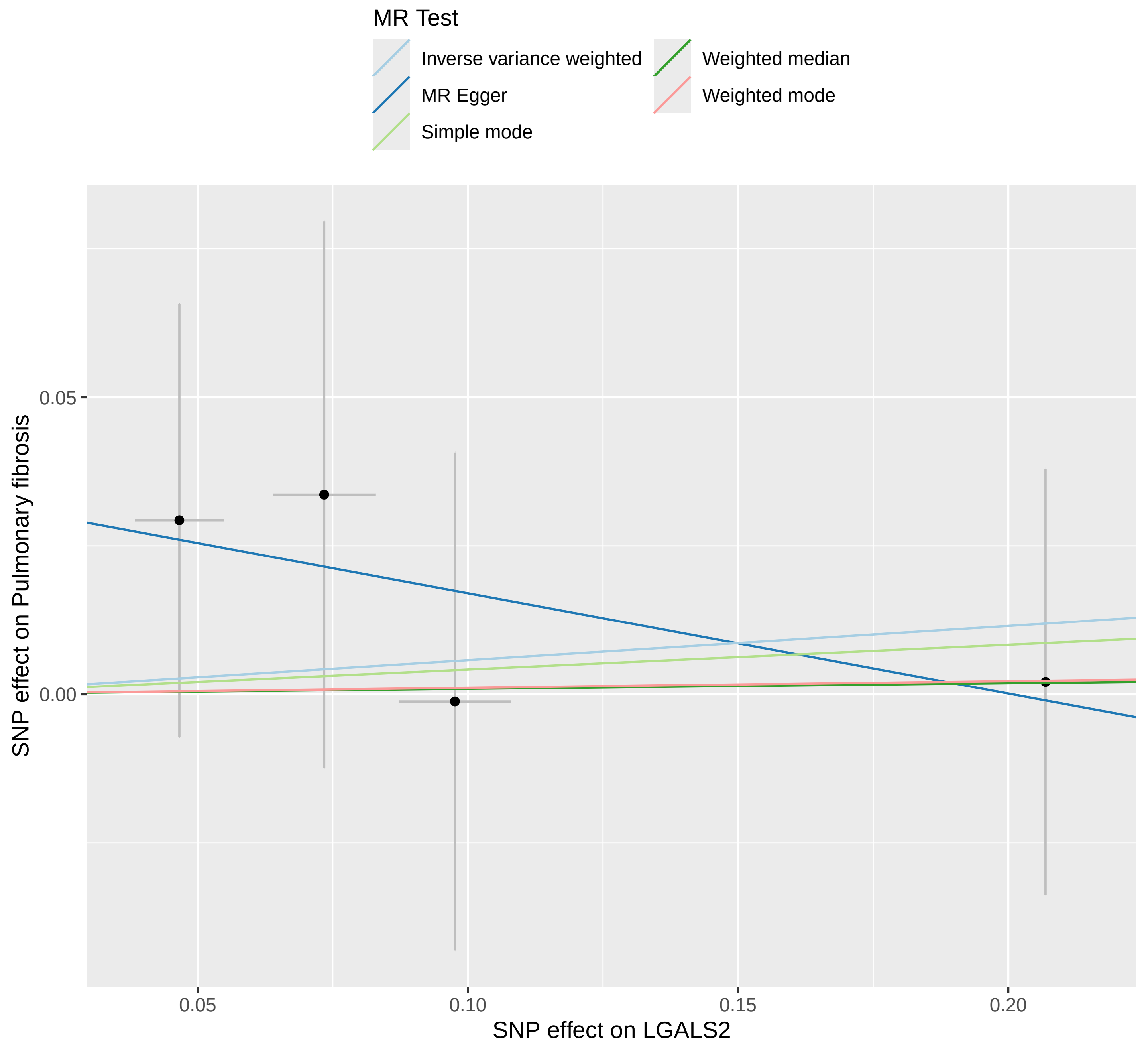
MR Test
Inverse variance weighted
MR Egger
Simple mode
Weighted median
Weighted mode
0.05
0.00
SNP effect on Pulmonary fibrosis
0.05
0.10
0.15
0.20
SNP effect on LGALS2

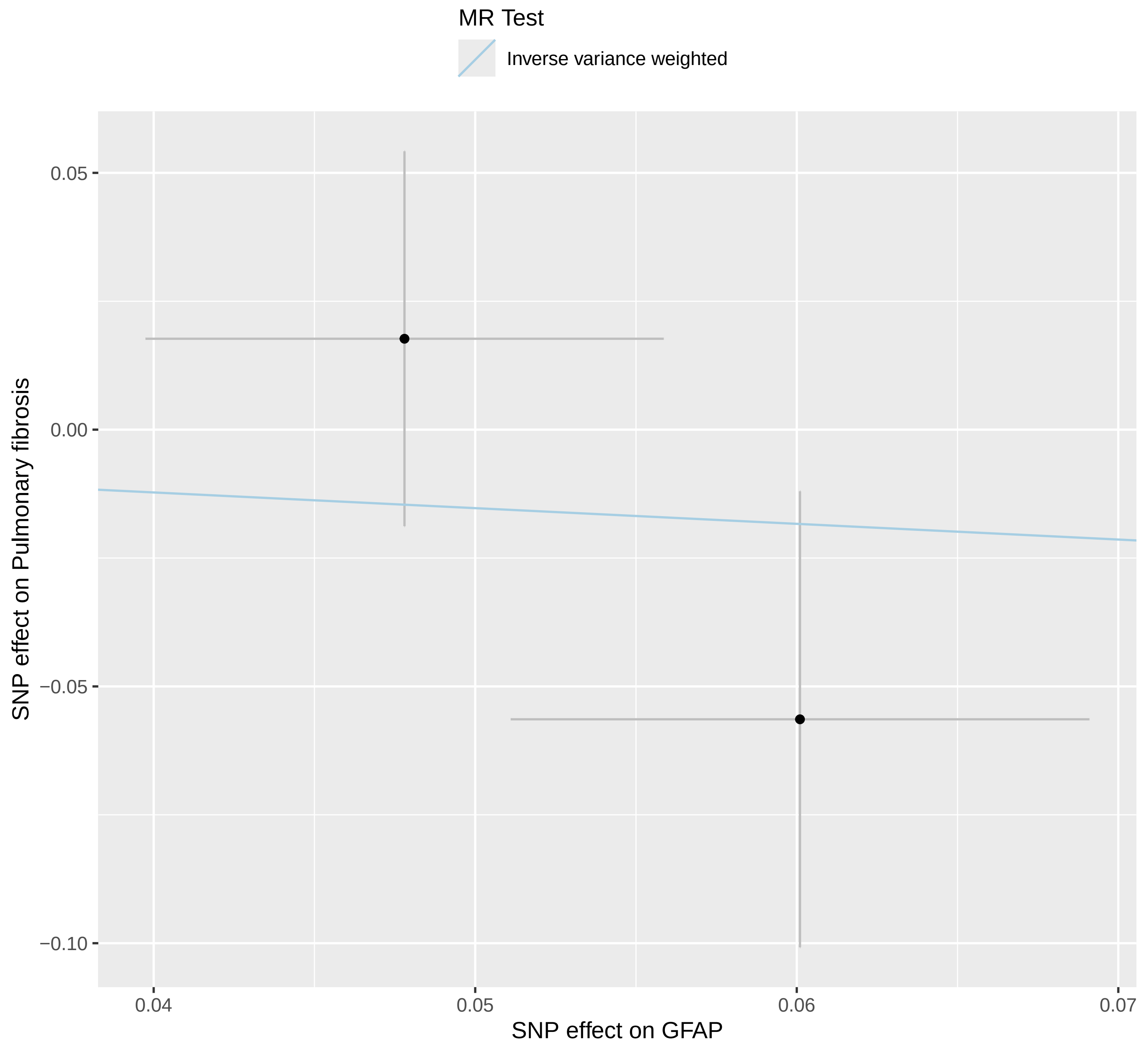
MR Test
Inverse variance weighted
0.05
0.00
−0.05
−0.10
SNP effect on Pulmonary fibrosis
0.04
0.05
0.06
0.07
SNP effect on GFAP

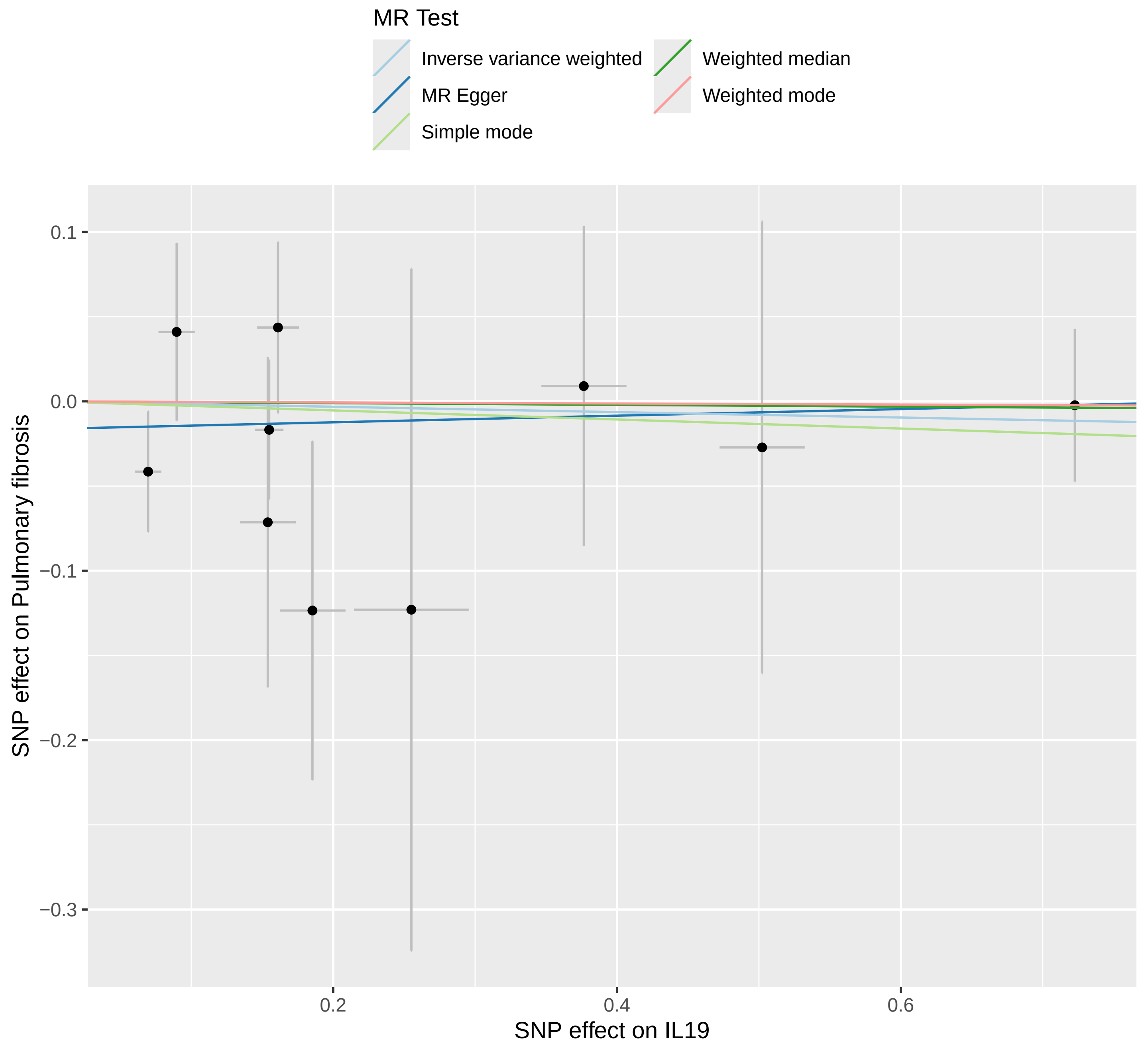
MR Test
Inverse variance weighted
MR Egger
Simple mode
Weighted median
Weighted mode
0.1
0.0
−0.1
−0.2
−0.3
SNP effect on Pulmonary fibrosis
0.2
0.4
0.6
SNP effect on IL19

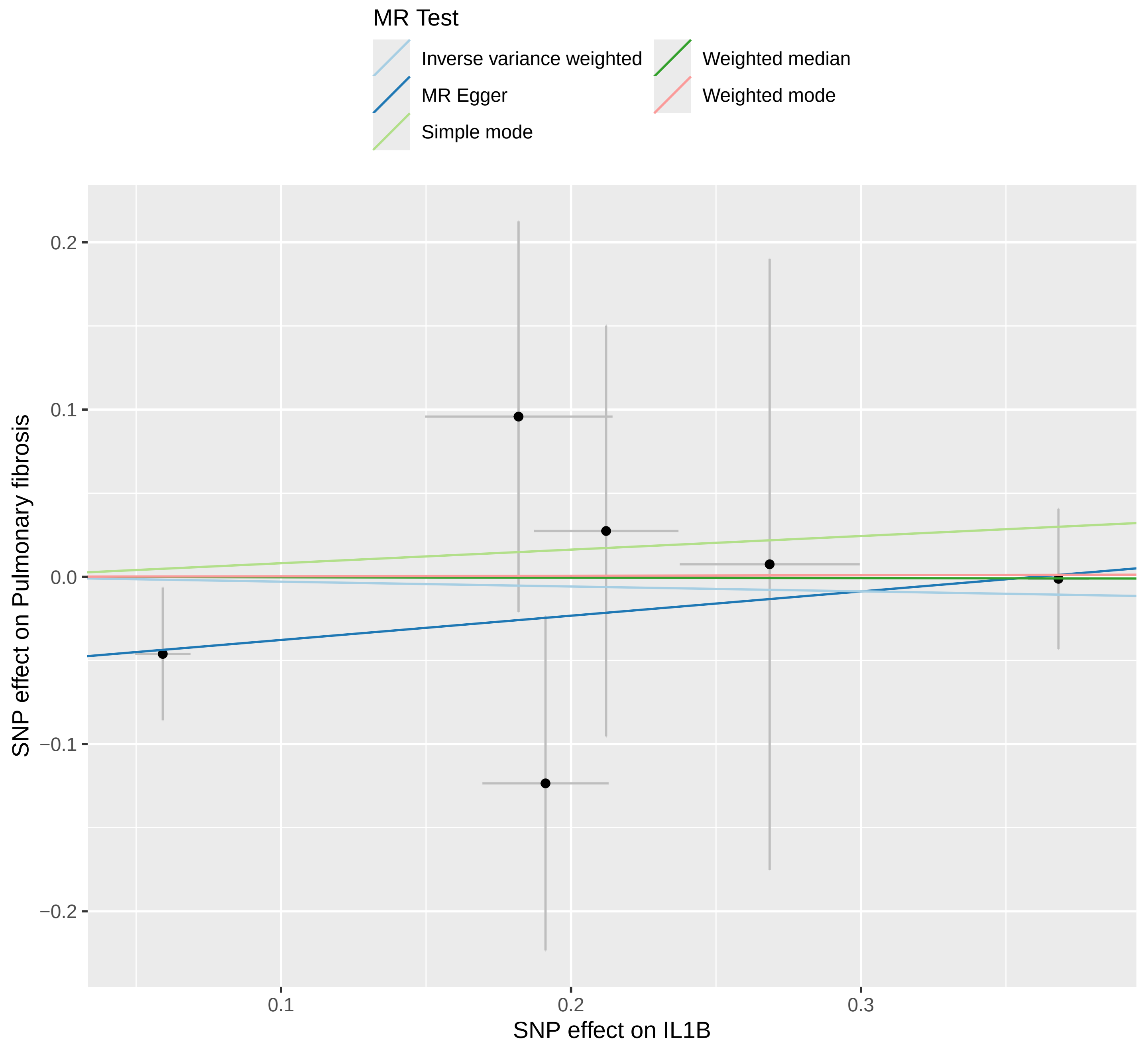

MR Test
Inverse variance weighted
MR Egger
Simple mode
Weighted median
Weighted mode
0.2
0.1
0.0
−0.1
−0.2
0.1
0.2
0.3
SNP effect on Pulmonary fibrosis
SNP effect on IL1B

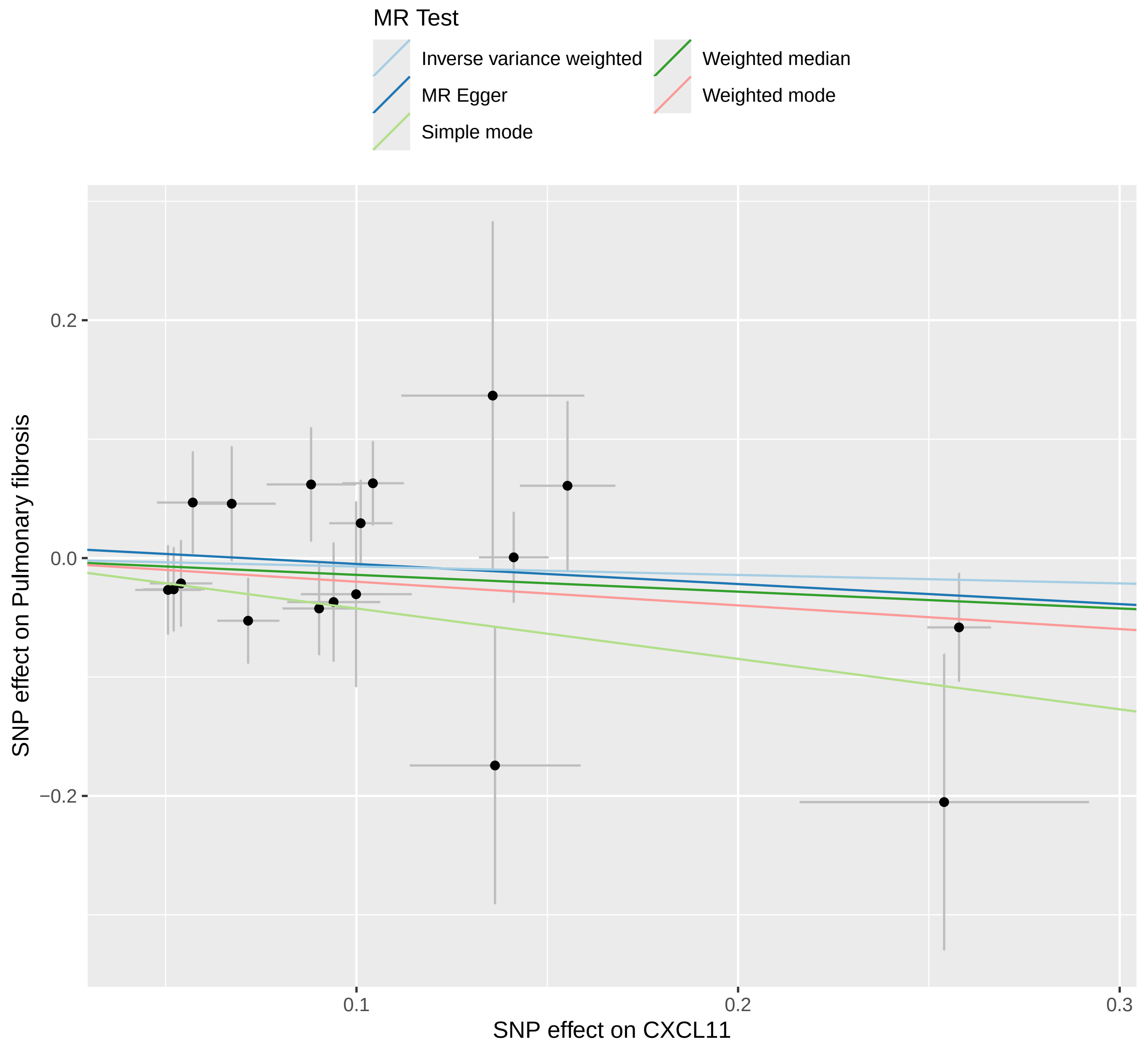
MR Test
Inverse variance weighted
MR Egger
Simple mode
Weighted median
Weighted mode
0.2
0.0
−0.2
0.1
0.2
0.3
SNP effect on Pulmonary fibrosis
SNP effect on CXCL11

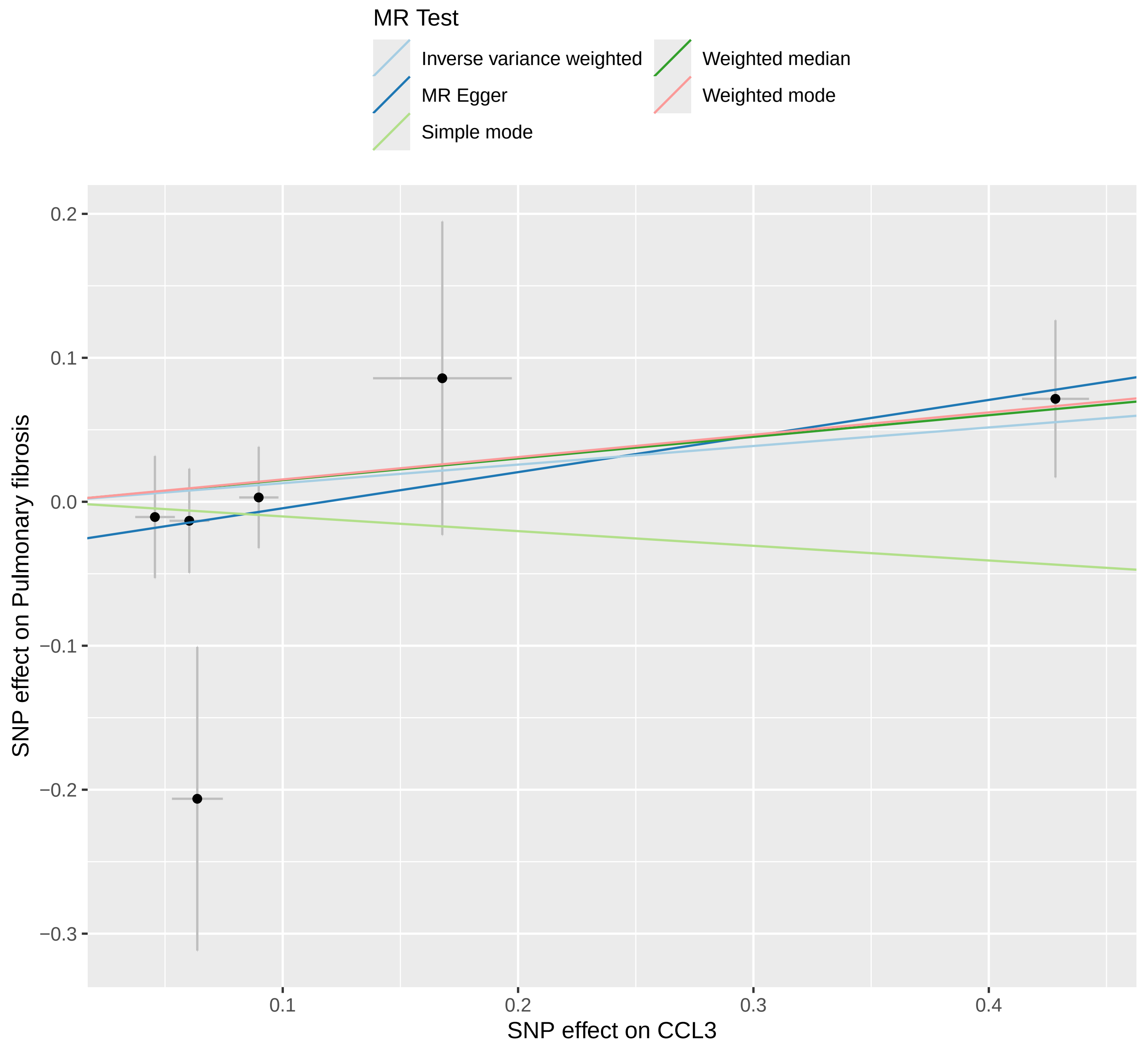

MR Test
Inverse variance weighted
MR Egger
Simple mode
Weighted median
Weighted mode
0.2
0.1
0.0
−0.1
−0.2
−0.3
SNP effect on Pulmonary fibrosis
0.1
0.2
0.3
0.4
SNP effect on CCL3

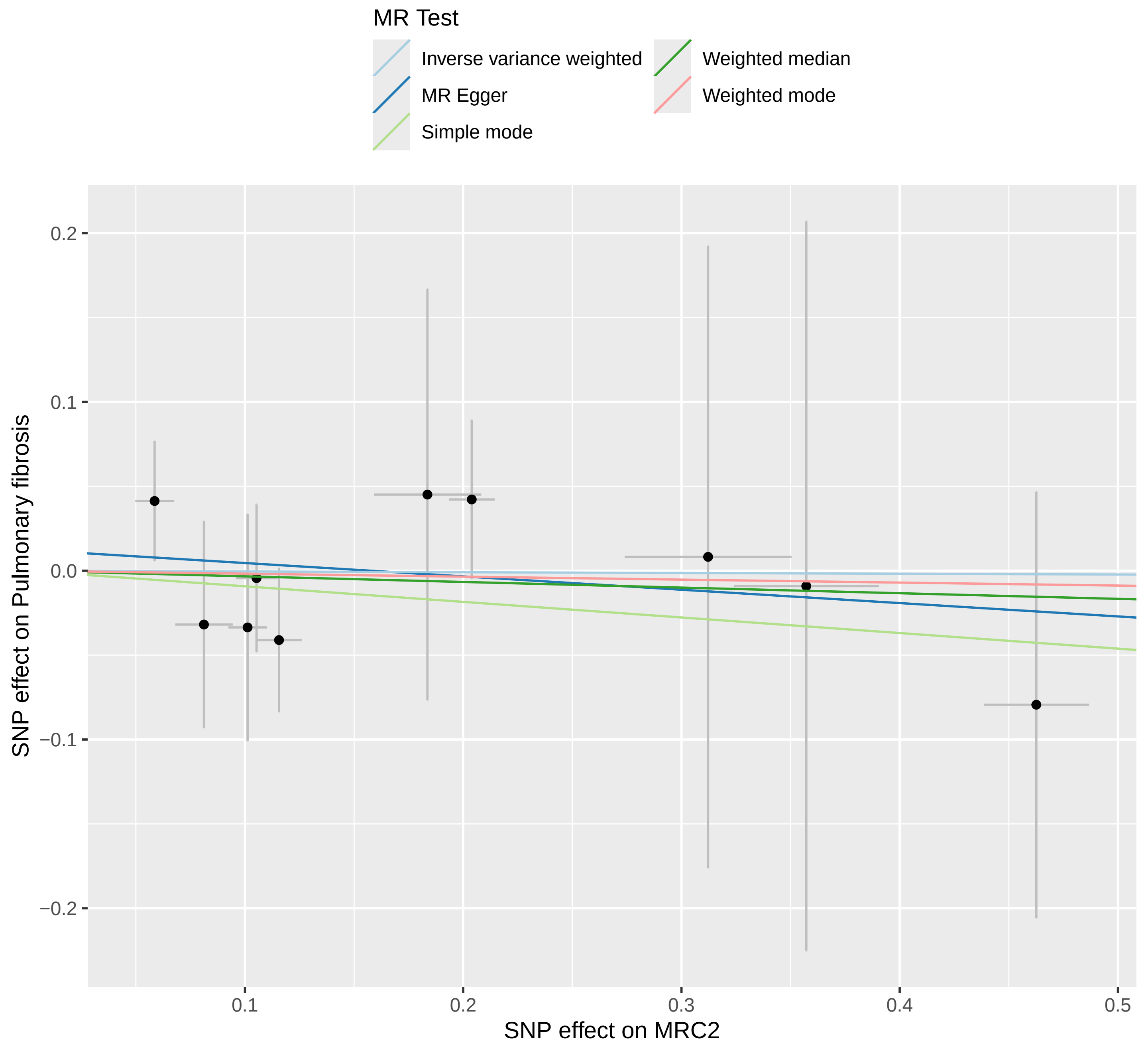

MR Test
Inverse variance weighted
MR Egger
Simple mode
Weighted median
Weighted mode
SNP effect on Pulmonary fibrosis
0.2
0.1
0.0
−0.1
−0.2
0.1
0.2
0.3
0.4
0.5
SNP effect on MRC2

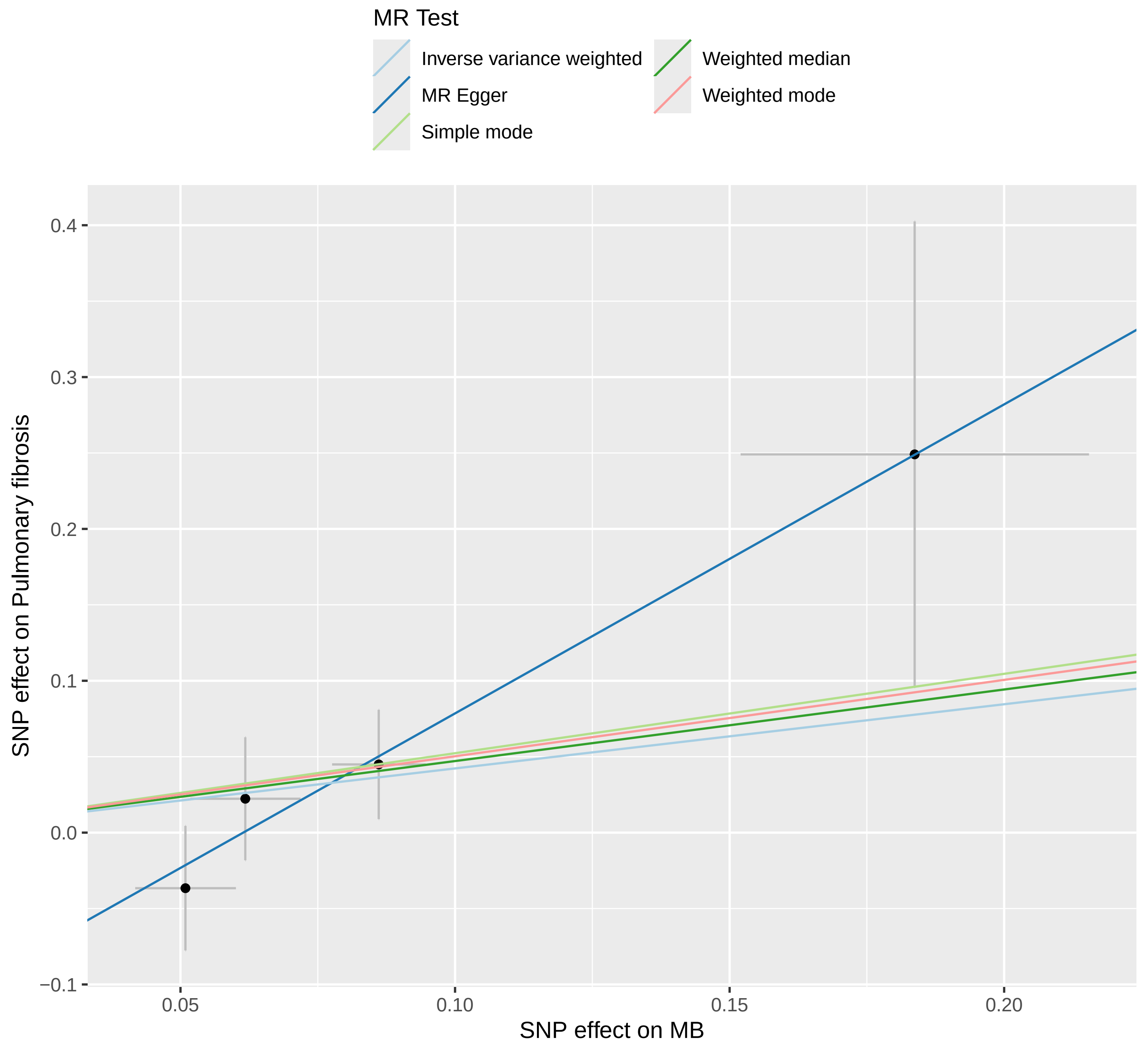

MR Test
Inverse variance weighted
MR Egger
Simple mode
Weighted median
Weighted mode
SNP effect on Pulmonary fibrosis
0.4
0.3
0.2
0.1
0.0
−0.1
0.05
0.10
0.15
0.20
SNP effect on MB

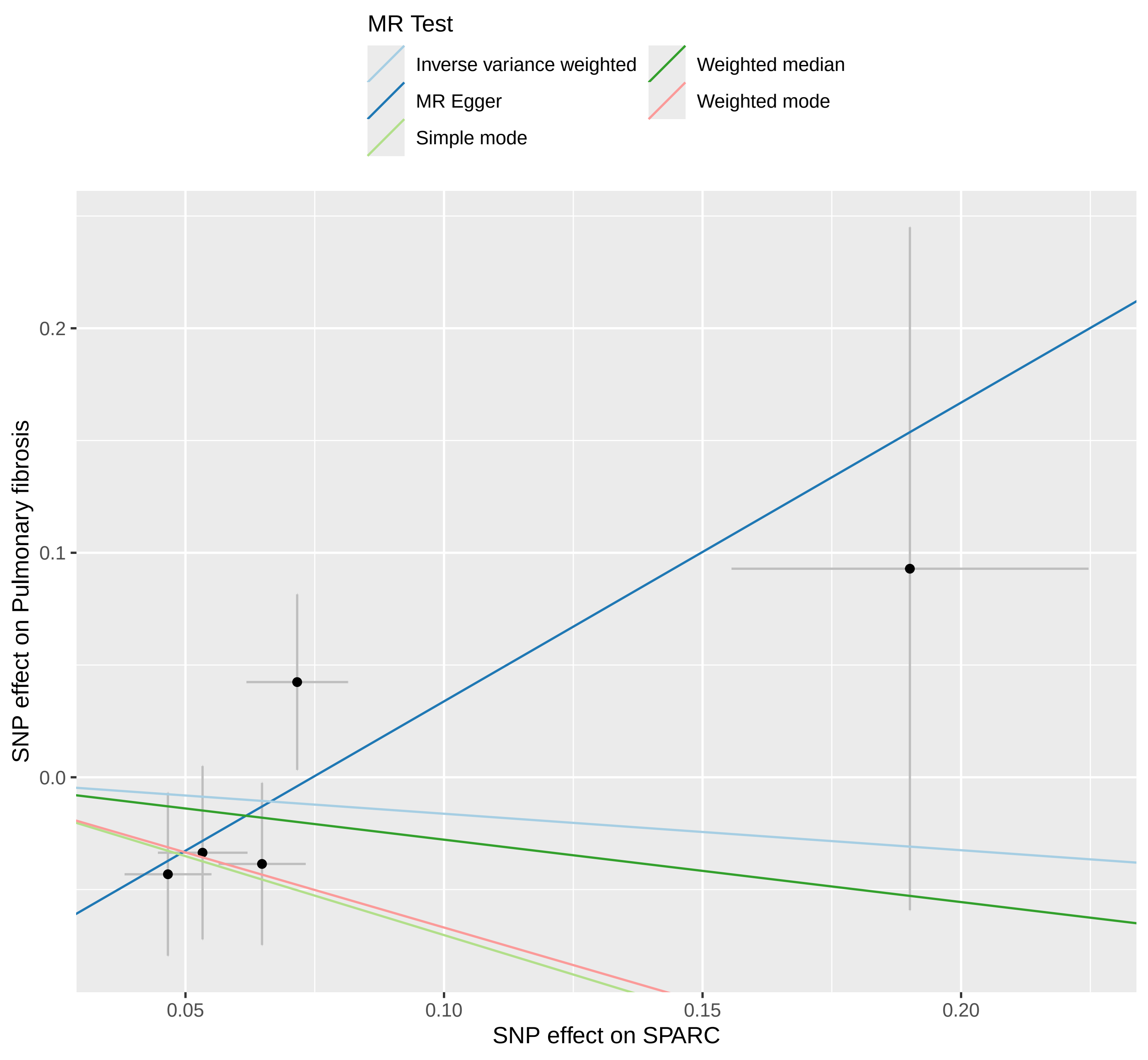

MR Test
Inverse variance weighted
MR Egger
Simple mode
Weighted median
Weighted mode
SNP effect on Pulmonary fibrosis
0.2
0.1
0.0
0.05
0.10
0.15
0.20
SNP effect on SPARC

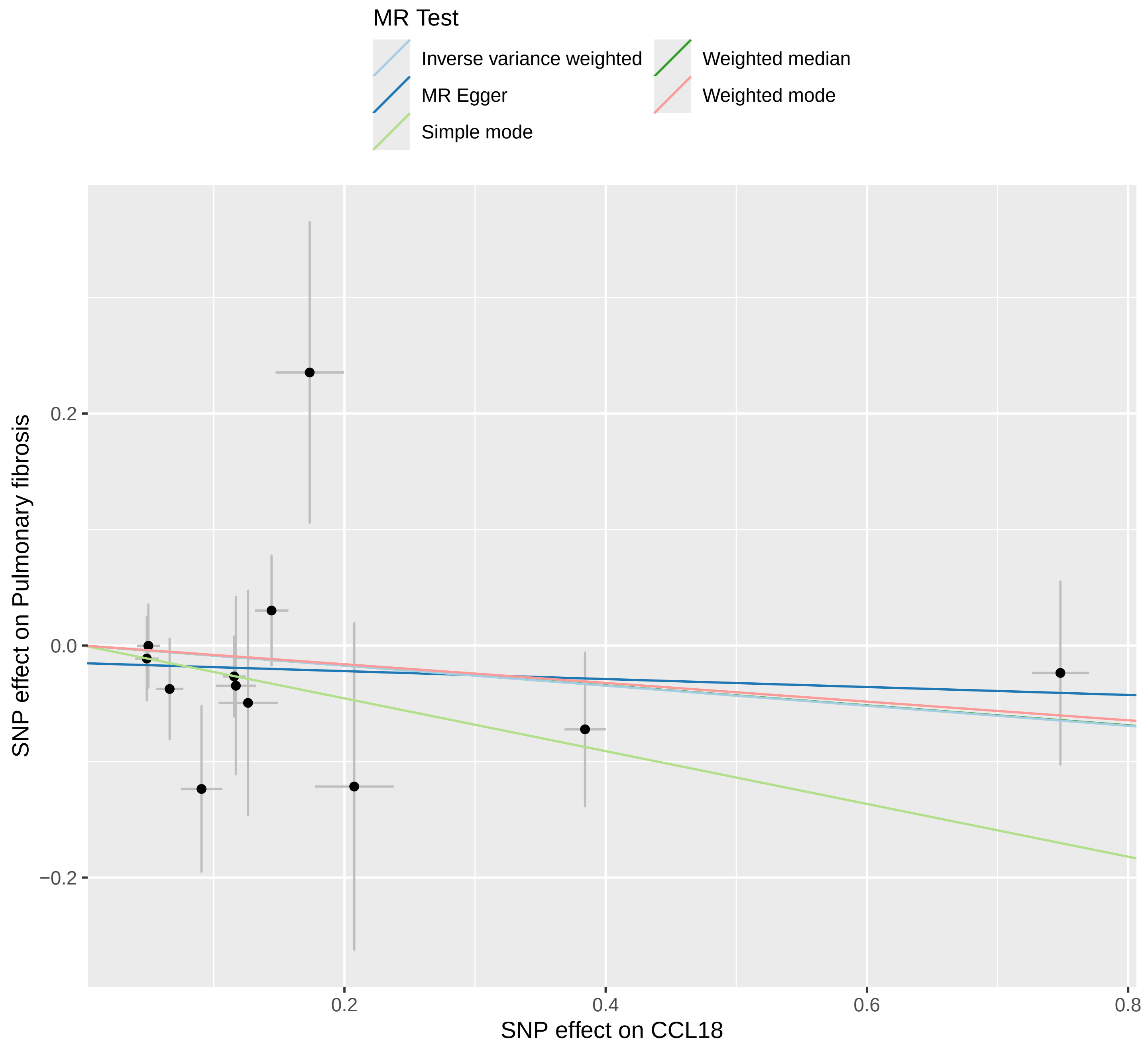

MR Test
Inverse variance weighted
MR Egger
Simple mode
Weighted median
Weighted mode
0.2
0.0
−0.2
SNP effect on Pulmonary fibrosis
0.2
0.4
0.6
0.8
SNP effect on CCL18

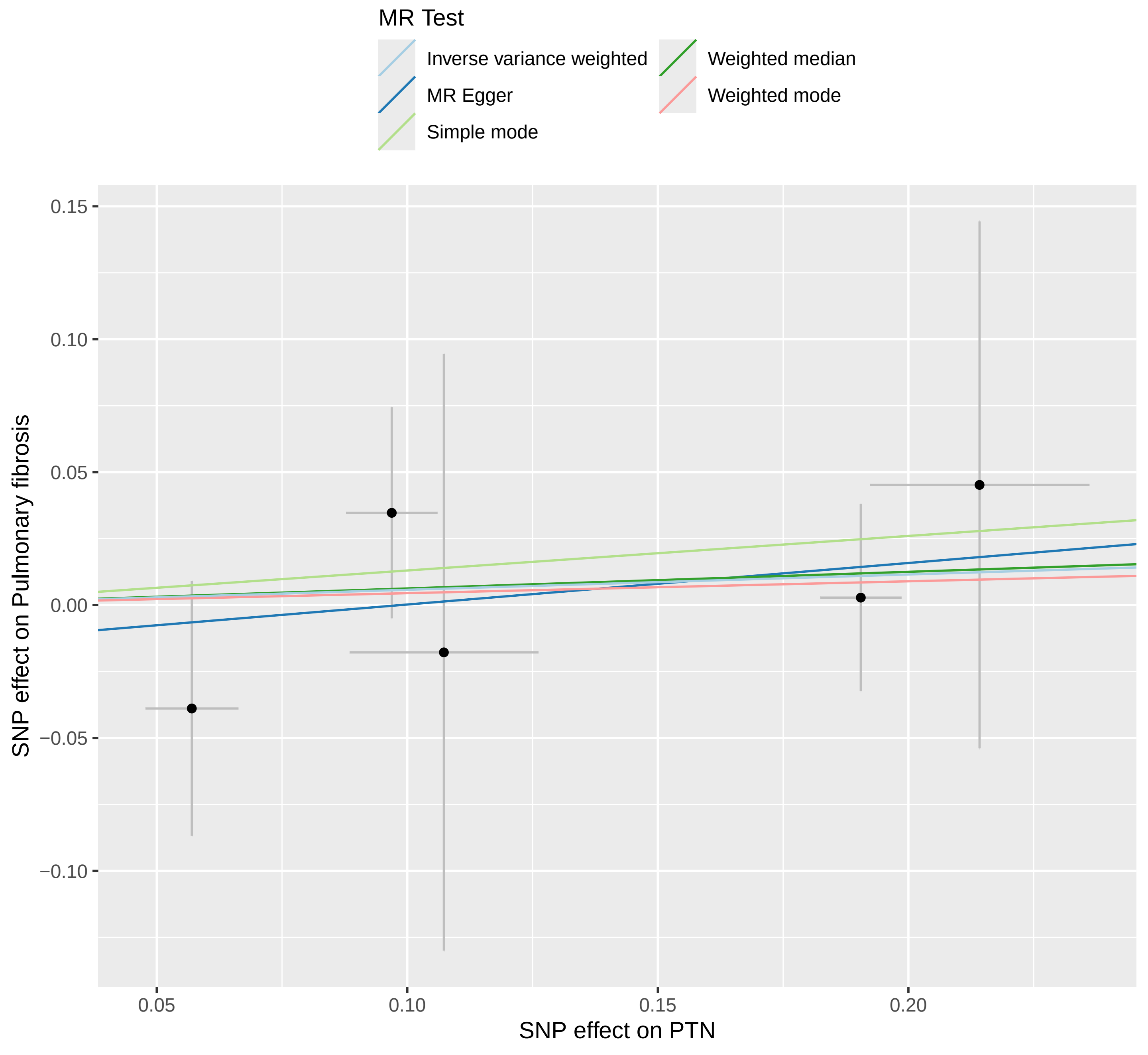
MR Test
Inverse variance weighted
MR Egger
Simple mode
Weighted median
Weighted mode
0.15
0.10
0.05
0.00
−0.05
−0.10
SNP effect on Pulmonary fibrosis
0.05
0.10
0.15
0.20
SNP effect on PTN

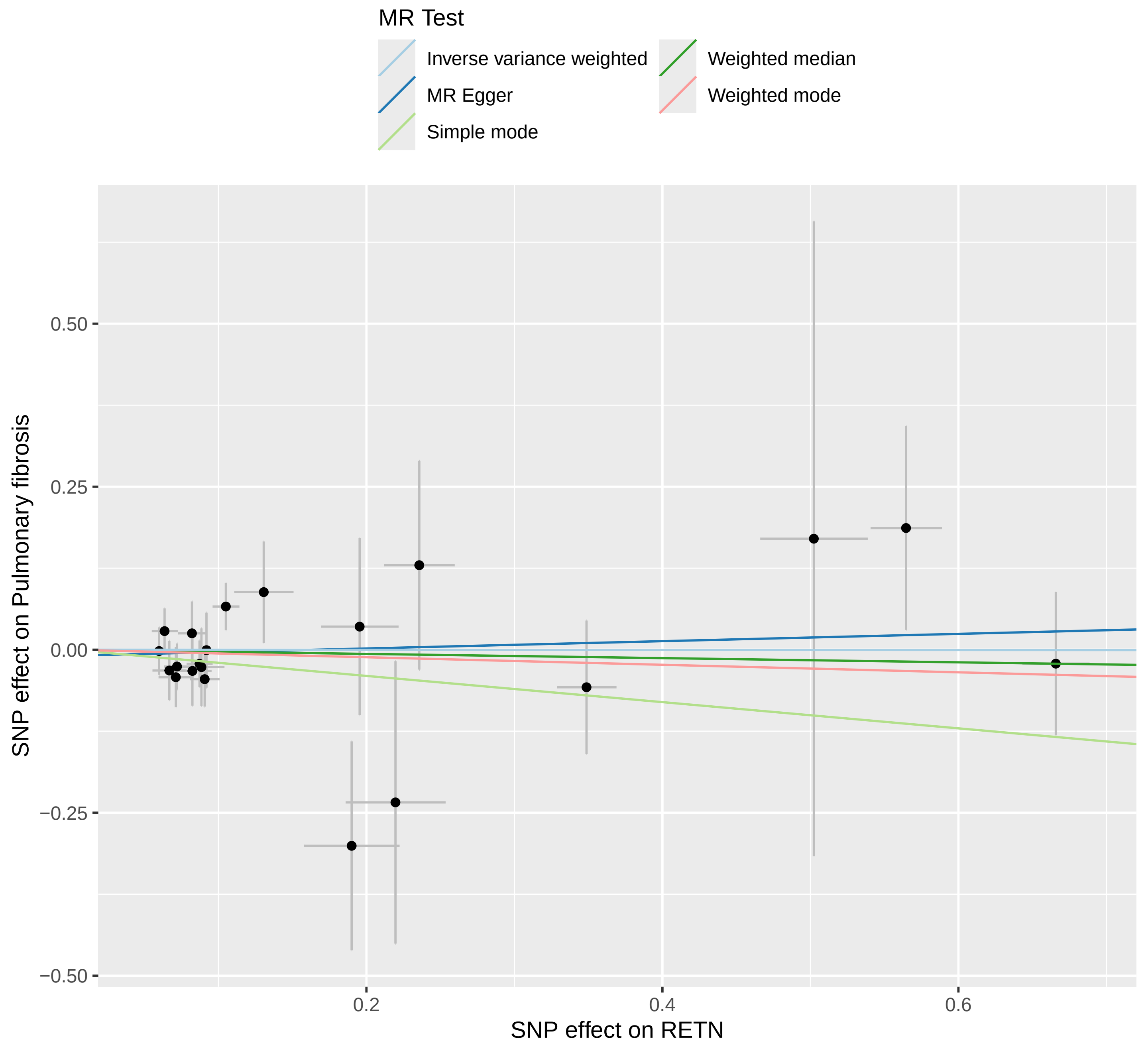

MR Test
Inverse variance weighted
MR Egger
Simple mode
Weighted median
Weighted mode
0.50
0.25
0.00
−0.25
−0.50
SNP effect on Pulmonary fibrosis
0.2
0.4
0.6
SNP effect on RETN

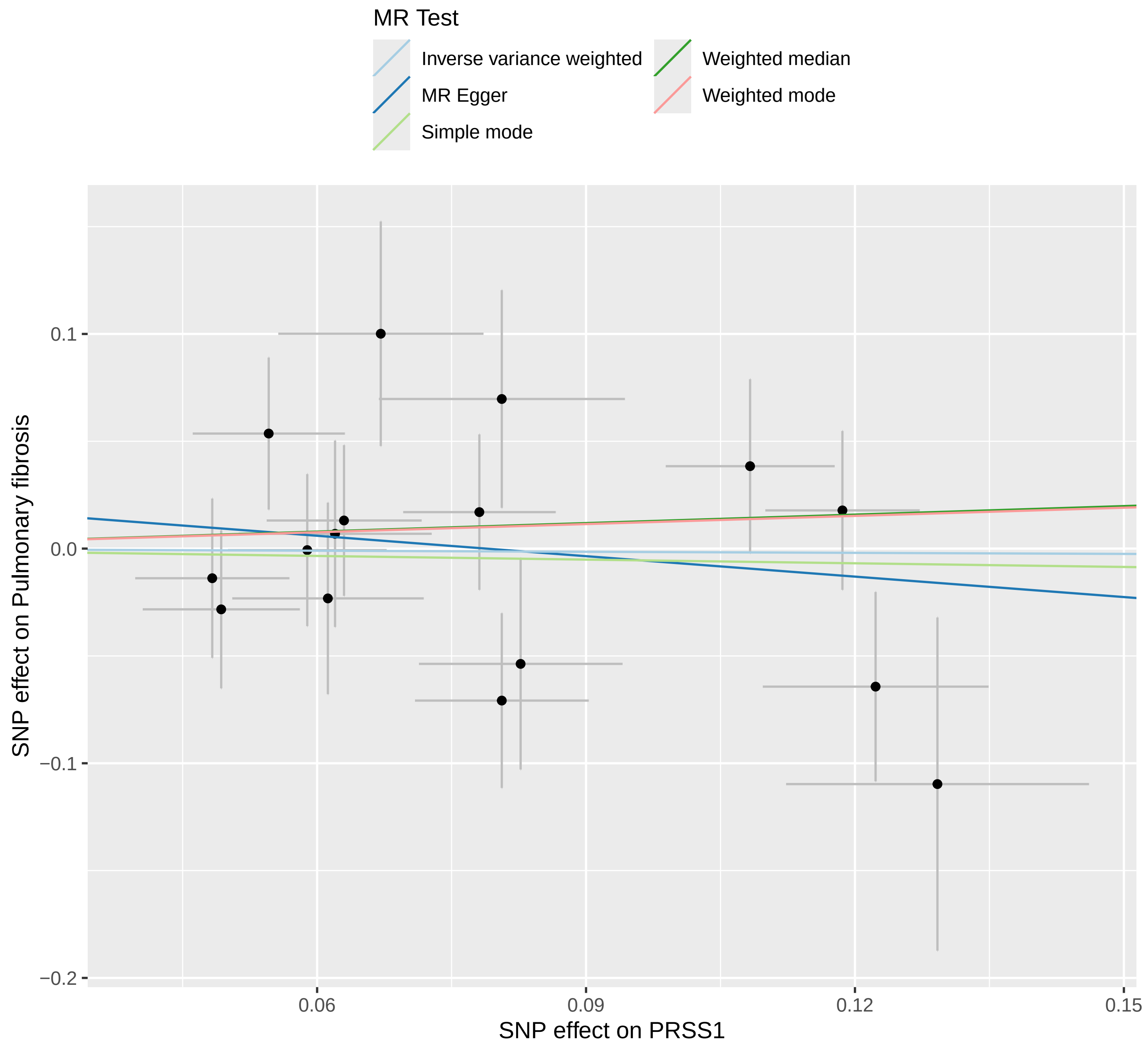

MR Test
Inverse variance weighted
MR Egger
Simple mode
Weighted median
Weighted mode
0.1
0.0
−0.1
−0.2
0.06
0.09
0.12
0.15
SNP effect on Pulmonary fibrosis
SNP effect on PRSS1

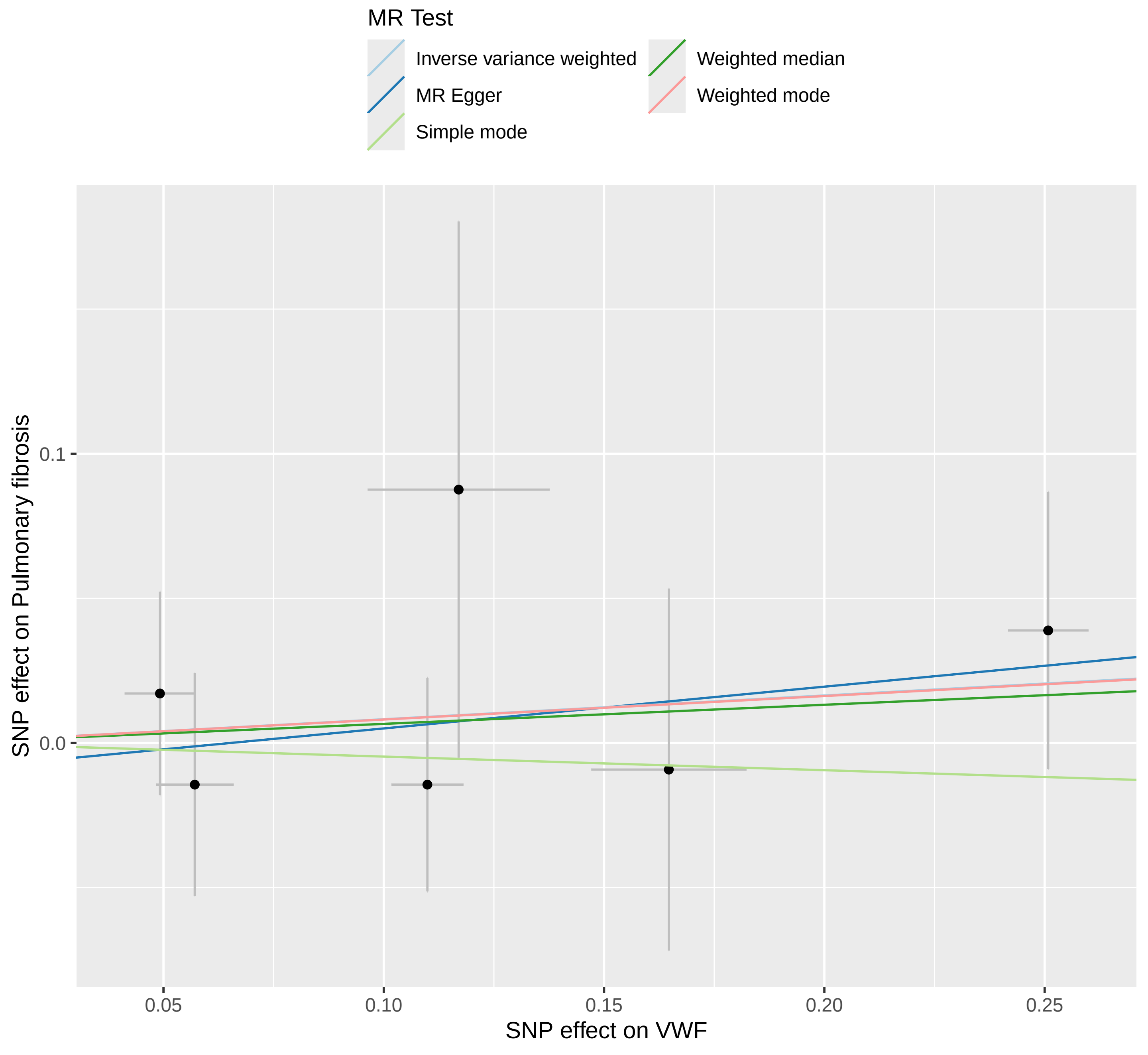
MR Test
Inverse variance weighted
MR Egger
Simple mode
Weighted median
Weighted mode
0.1
0.0
0.05
0.10
0.15
0.20
0.25
SNP effect on VWF
SNP effect on Pulmonary fibrosis

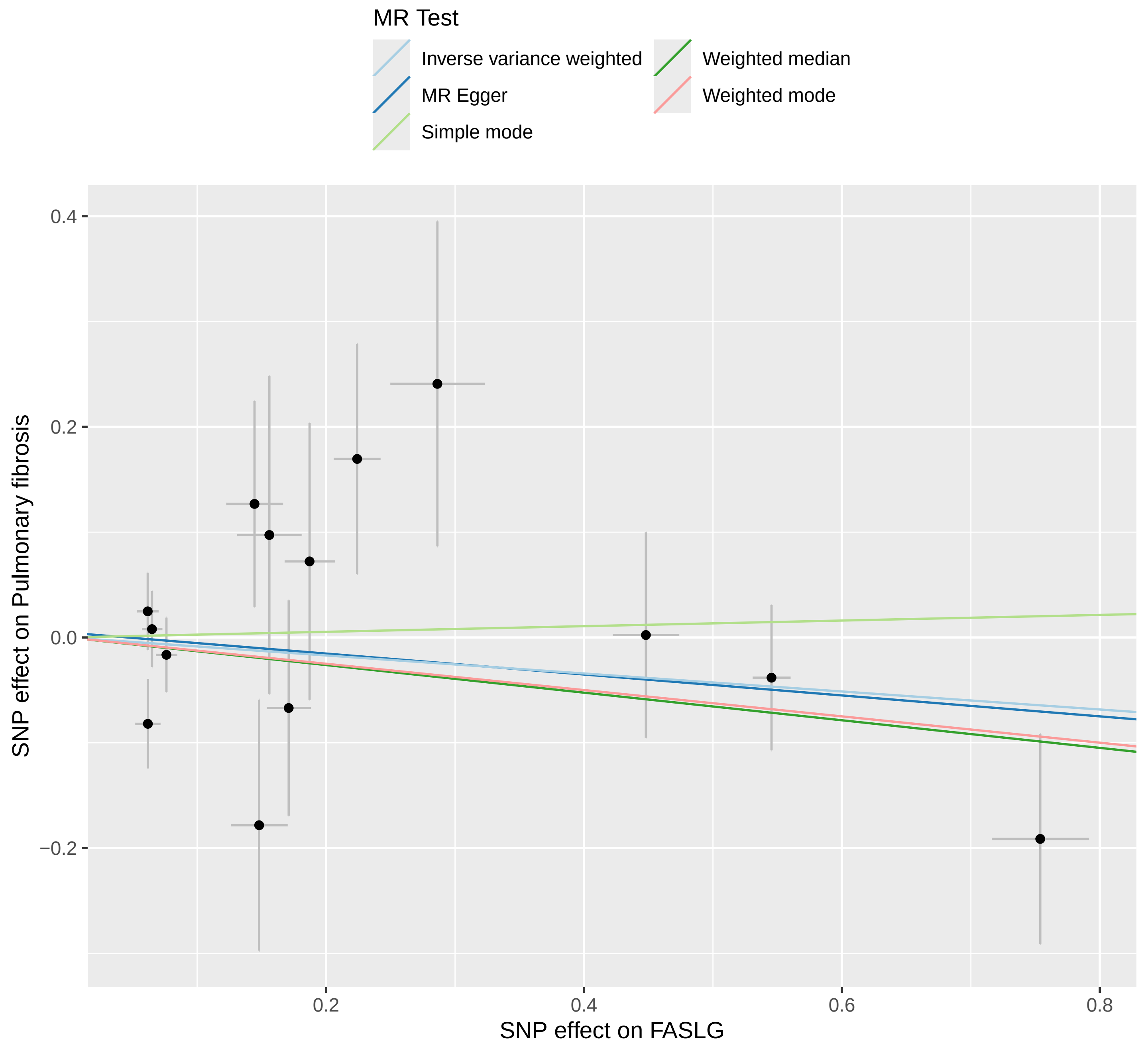
MR Test
Inverse variance weighted
MR Egger
Simple mode
Weighted median
Weighted mode
0.4
0.2
0.0
−0.2
0.2
0.4
0.6
0.8
SNP effect on Pulmonary fibrosis
SNP effect on FASLG

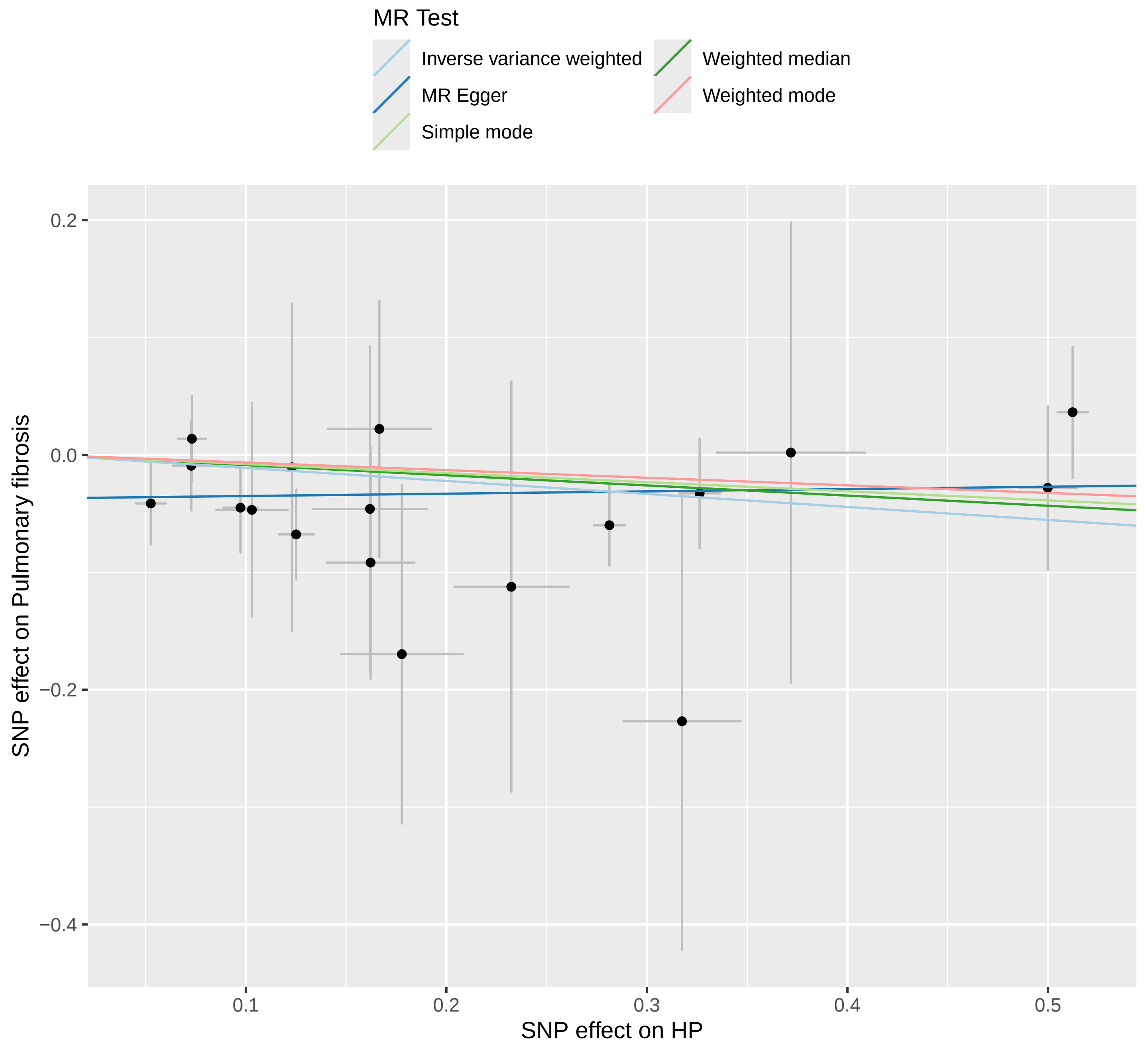
MR Test
Inverse variance weighted
MR Egger
Simple mode
Weighted median
Weighted mode
0.2
0.0
−0.2
−0.4
0.1
0.2
0.3
0.4
0.5
SNP effect on Pulmonary fibrosis
SNP effect on HP

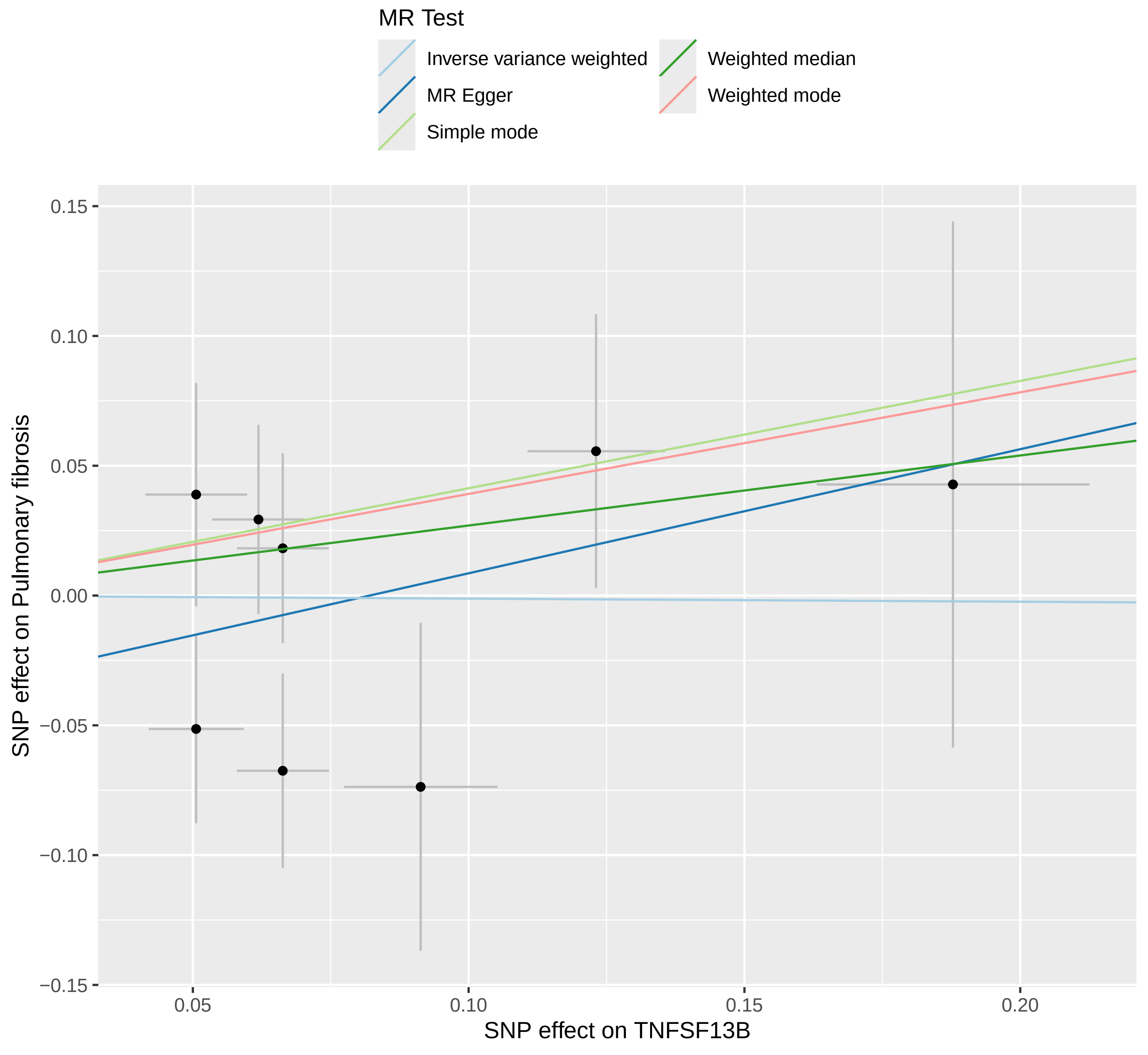
MR Test
Inverse variance weighted
MR Egger
Simple mode
Weighted median
Weighted mode
0.15
0.10
0.05
0.00
−0.05
−0.10
−0.15
SNP effect on Pulmonary fibrosis
0.05
0.10
0.15
0.20
SNP effect on TNFSF13B

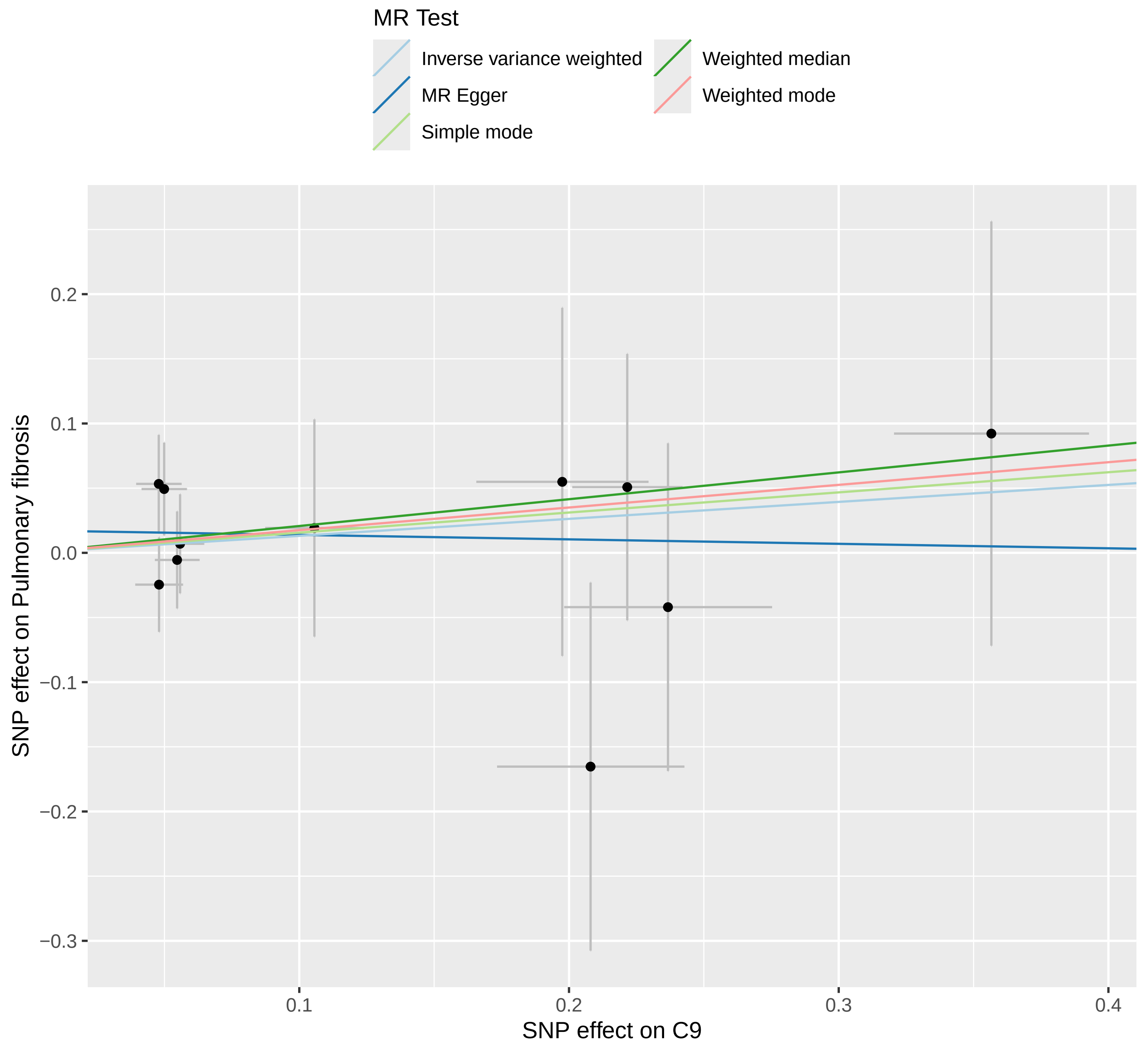

MR Test
Inverse variance weighted
MR Egger
Simple mode
Weighted median
Weighted mode
SNP effect on Pulmonary fibrosis
0.2
0.1
0.0
−0.1
−0.2
−0.3
0.1
0.2
0.3
0.4
SNP effect on C9

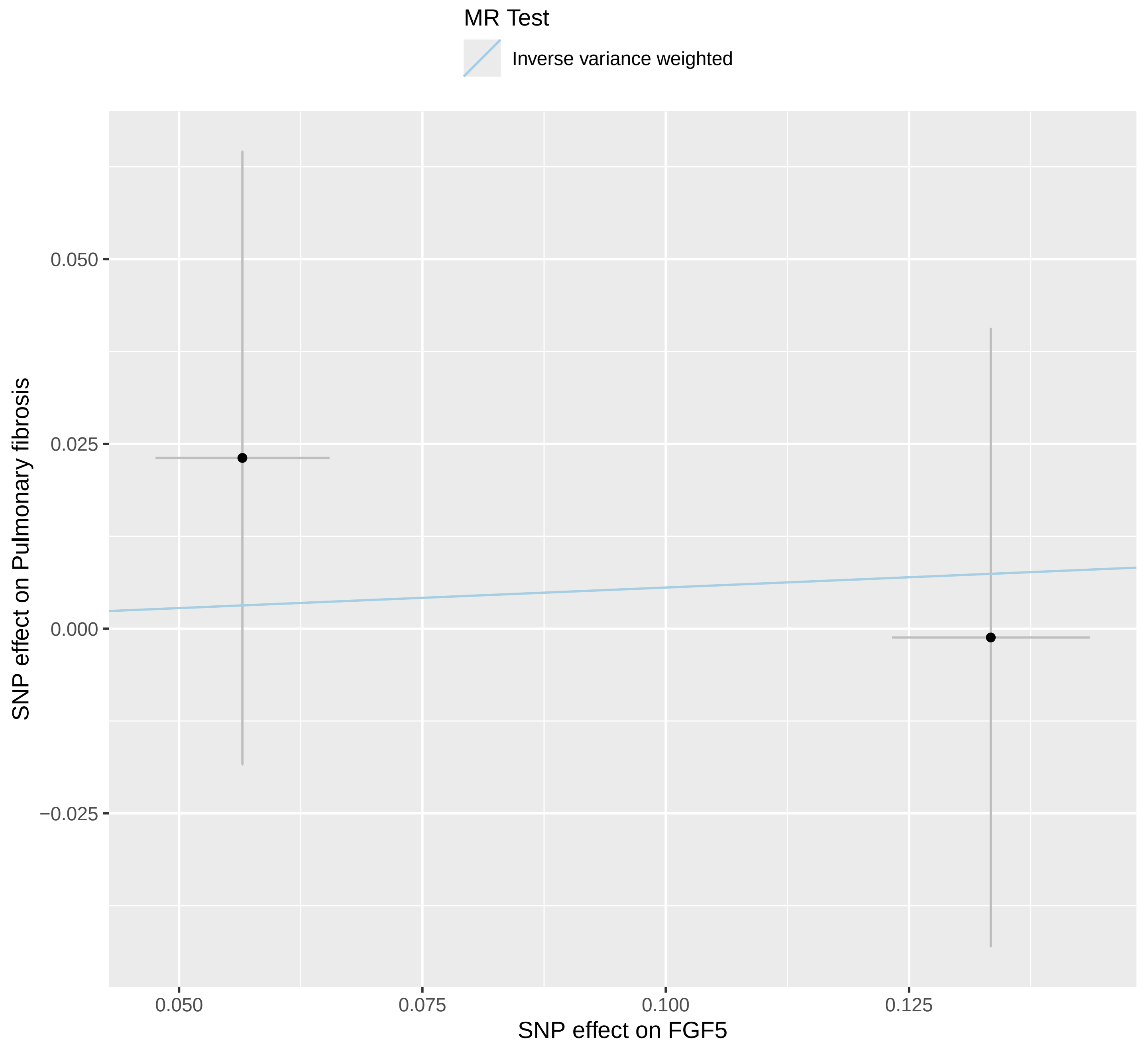
MR Test
Inverse variance weighted
0.050
0.025
0.000
−0.025
SNP effect on Pulmonary fibrosis
0.050
0.075
0.100
0.125
SNP effect on FGF5

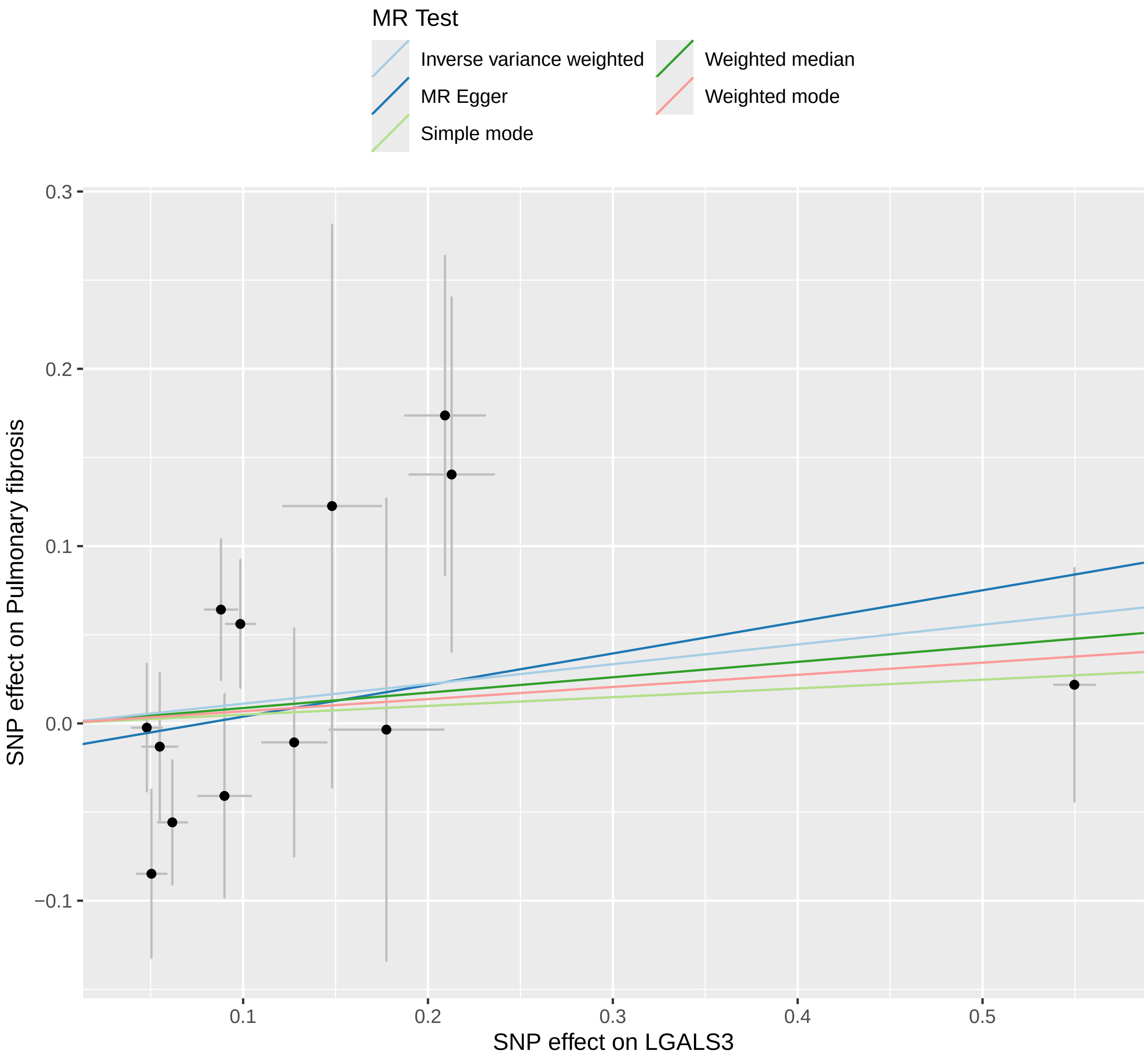

MR Test
Inverse variance weighted
MR Egger
Simple mode
Weighted median
Weighted mode
0.3
0.2
0.1
0.0
−0.1
0.1
0.2
0.3
0.4
0.5
SNP effect on Pulmonary fibrosis
SNP effect on LGALS3

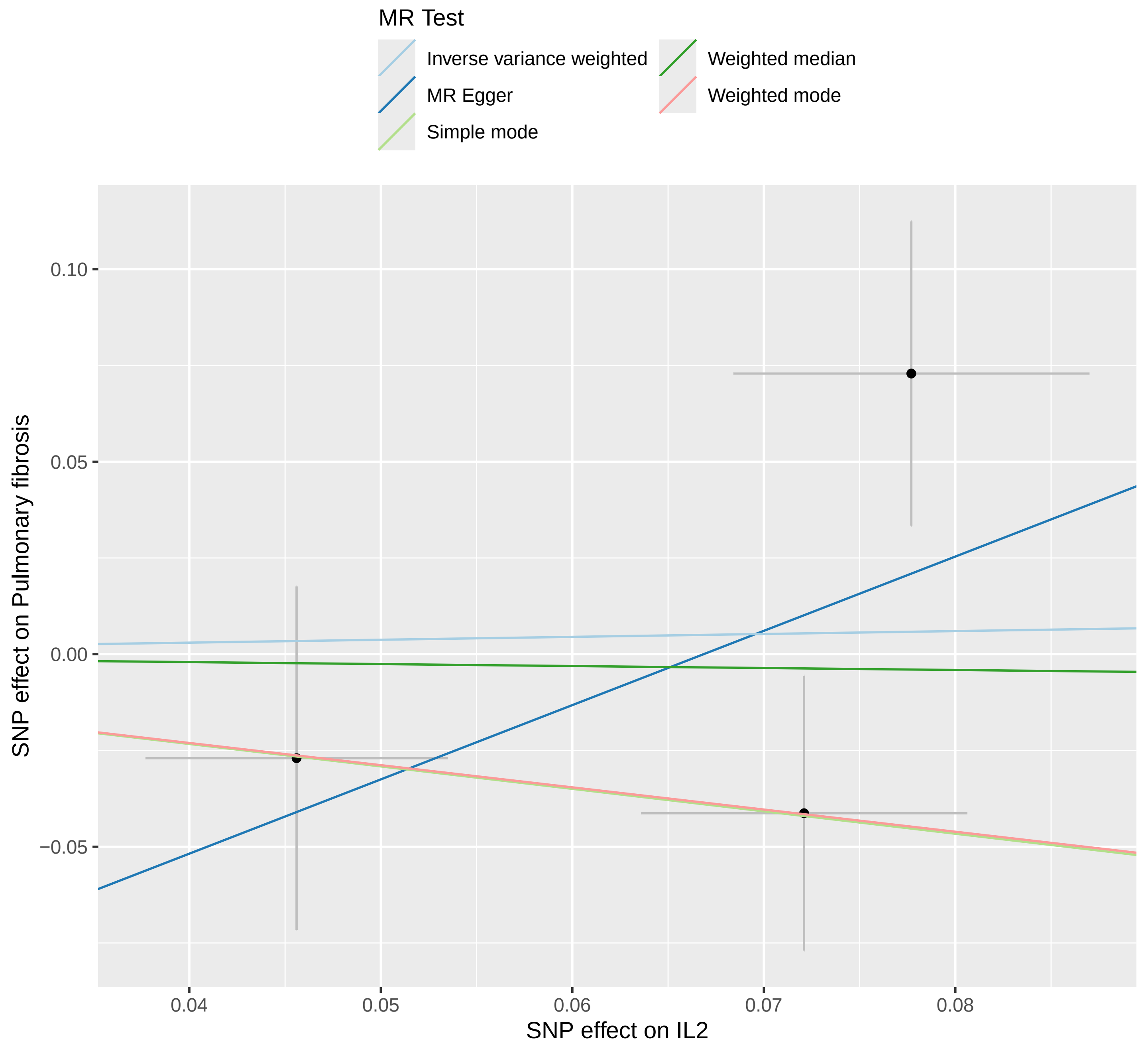
MR Test
Inverse variance weighted
MR Egger
Simple mode
Weighted median
Weighted mode
0.10
0.05
0.00
−0.05
0.04
0.05
0.06
0.07
0.08
SNP effect on Pulmonary fibrosis
SNP effect on IL2

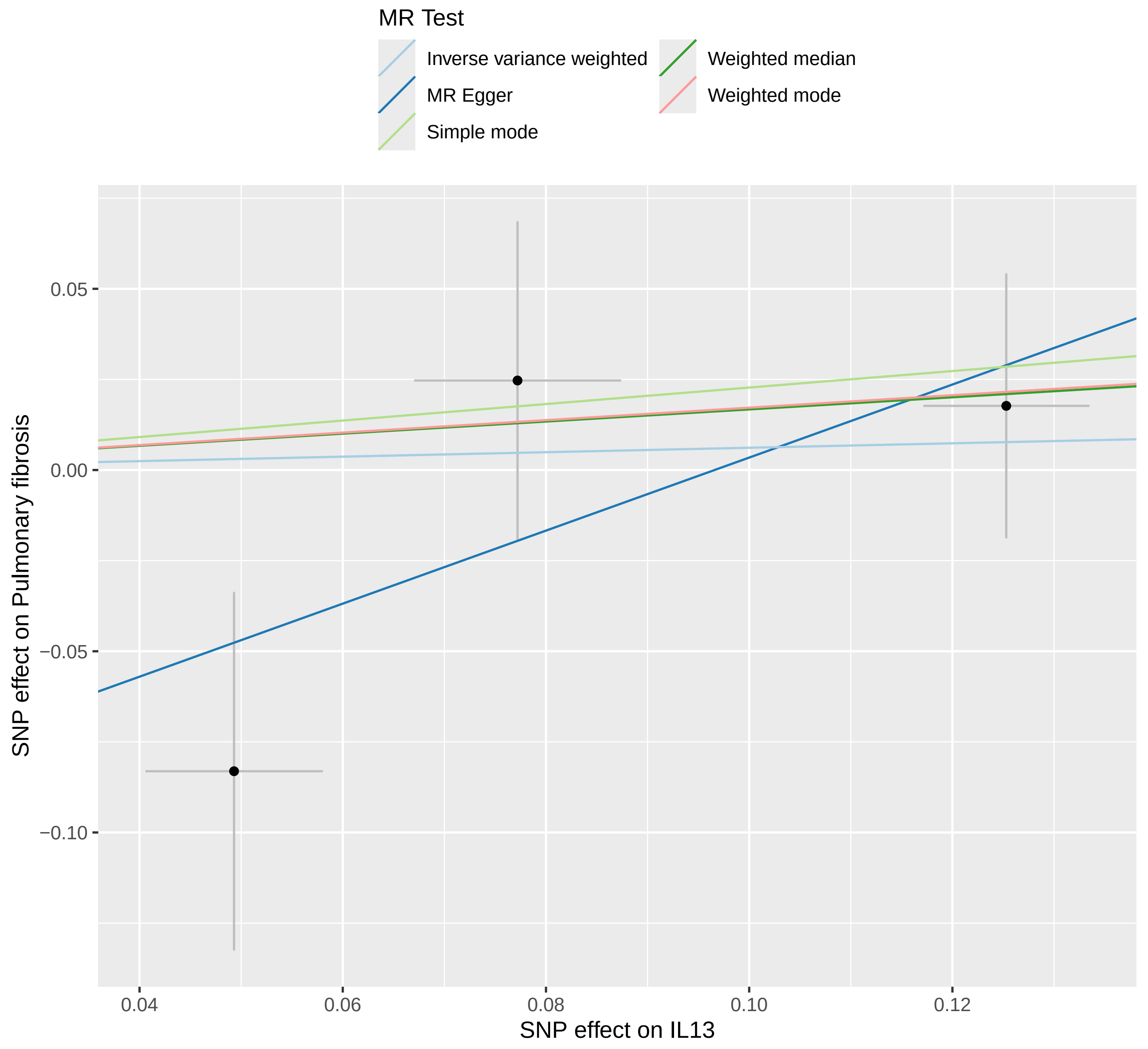
MR Test
Inverse variance weighted
MR Egger
Simple mode
Weighted median
Weighted mode
0.05
0.00
−0.05
−0.10
SNP effect on Pulmonary fibrosis
0.04
0.06
0.08
0.10
0.12
SNP effect on IL13

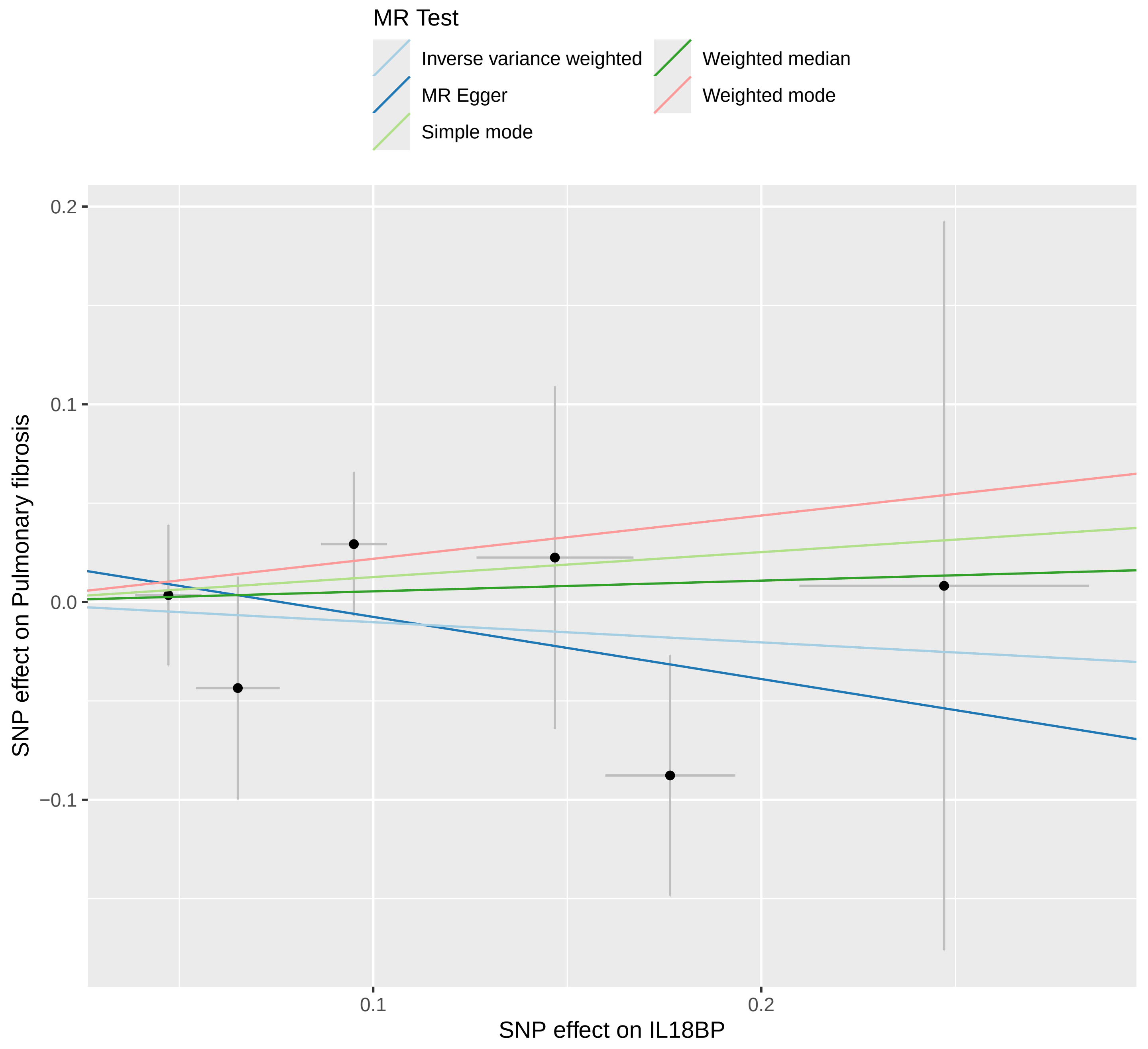
MR Test
Inverse variance weighted
MR Egger
Simple mode
Weighted median
Weighted mode
0.2
0.1
0.0
−0.1
0.1
0.2
SNP effect on Pulmonary fibrosis
SNP effect on IL18BP

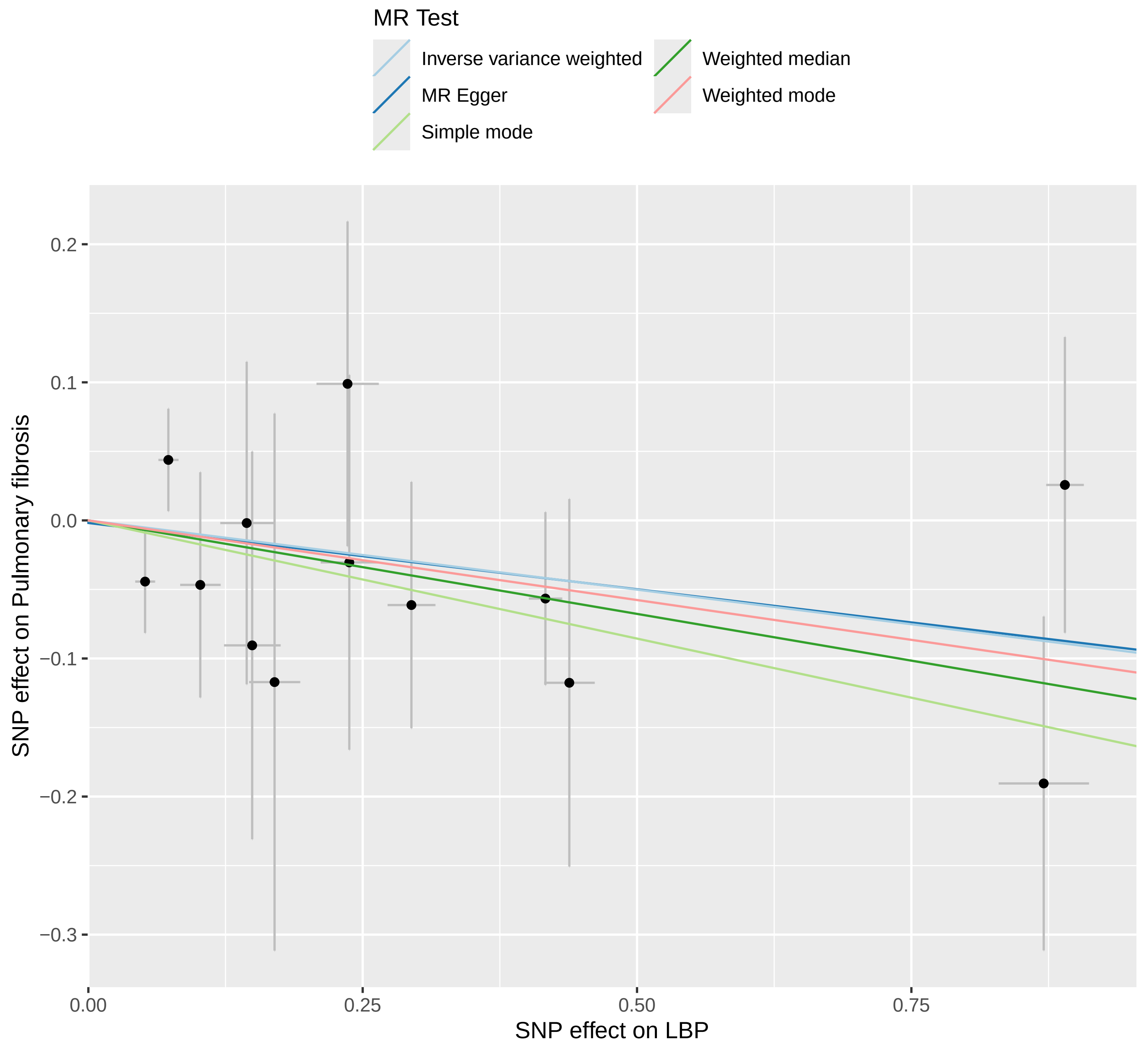

MR Test
Inverse variance weighted
MR Egger
Simple mode
Weighted median
Weighted mode
SNP effect on Pulmonary fibrosis
0.2
0.1
0.0
−0.1
−0.2
−0.3
0.00
0.25
0.50
0.75
SNP effect on LBP

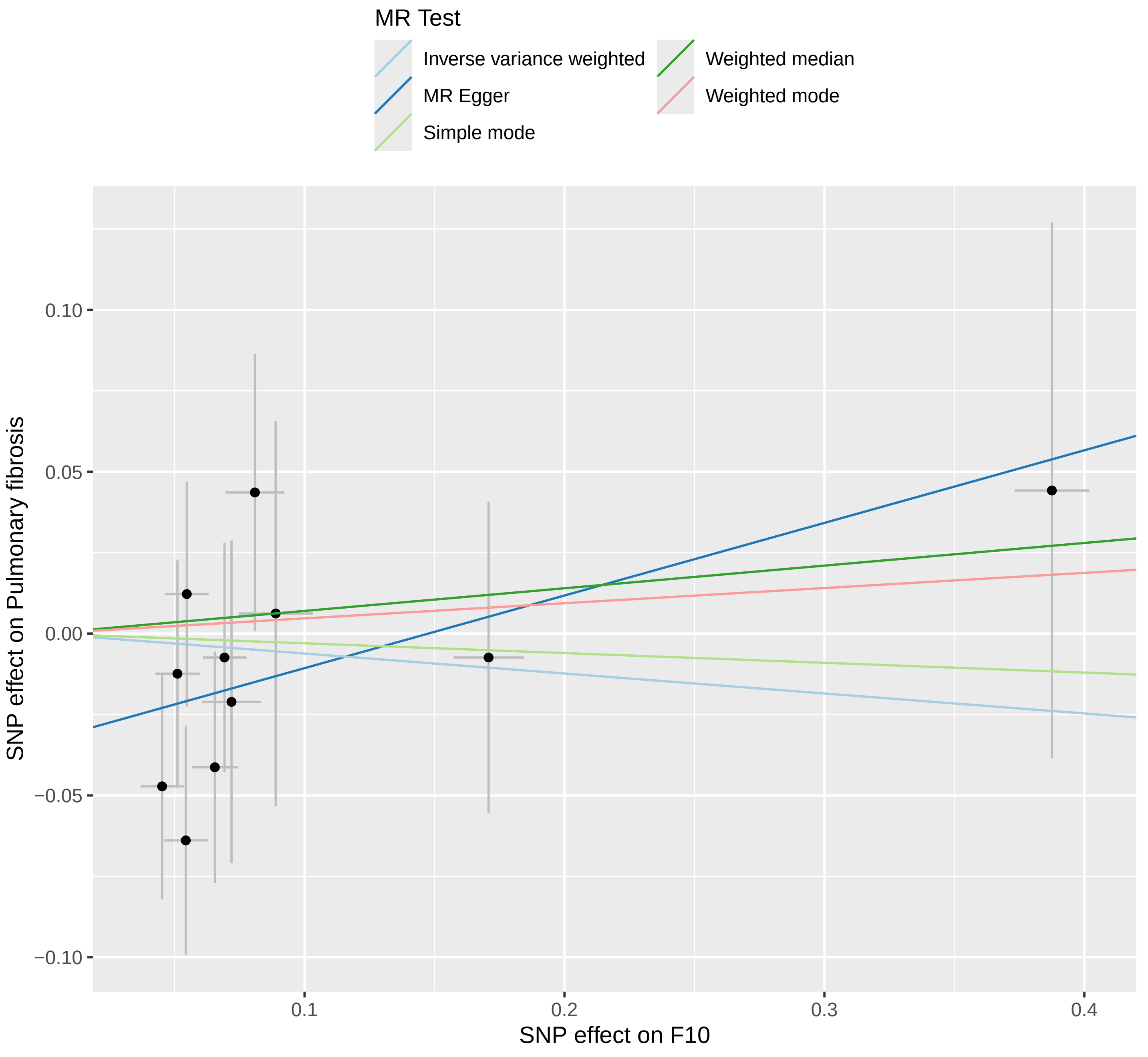
MR Test
Inverse variance weighted
MR Egger
Simple mode
Weighted median
Weighted mode
0.10
0.05
0.00
−0.05
−0.10
SNP effect on Pulmonary fibrosis
0.1
0.2
0.3
0.4
SNP effect on F10

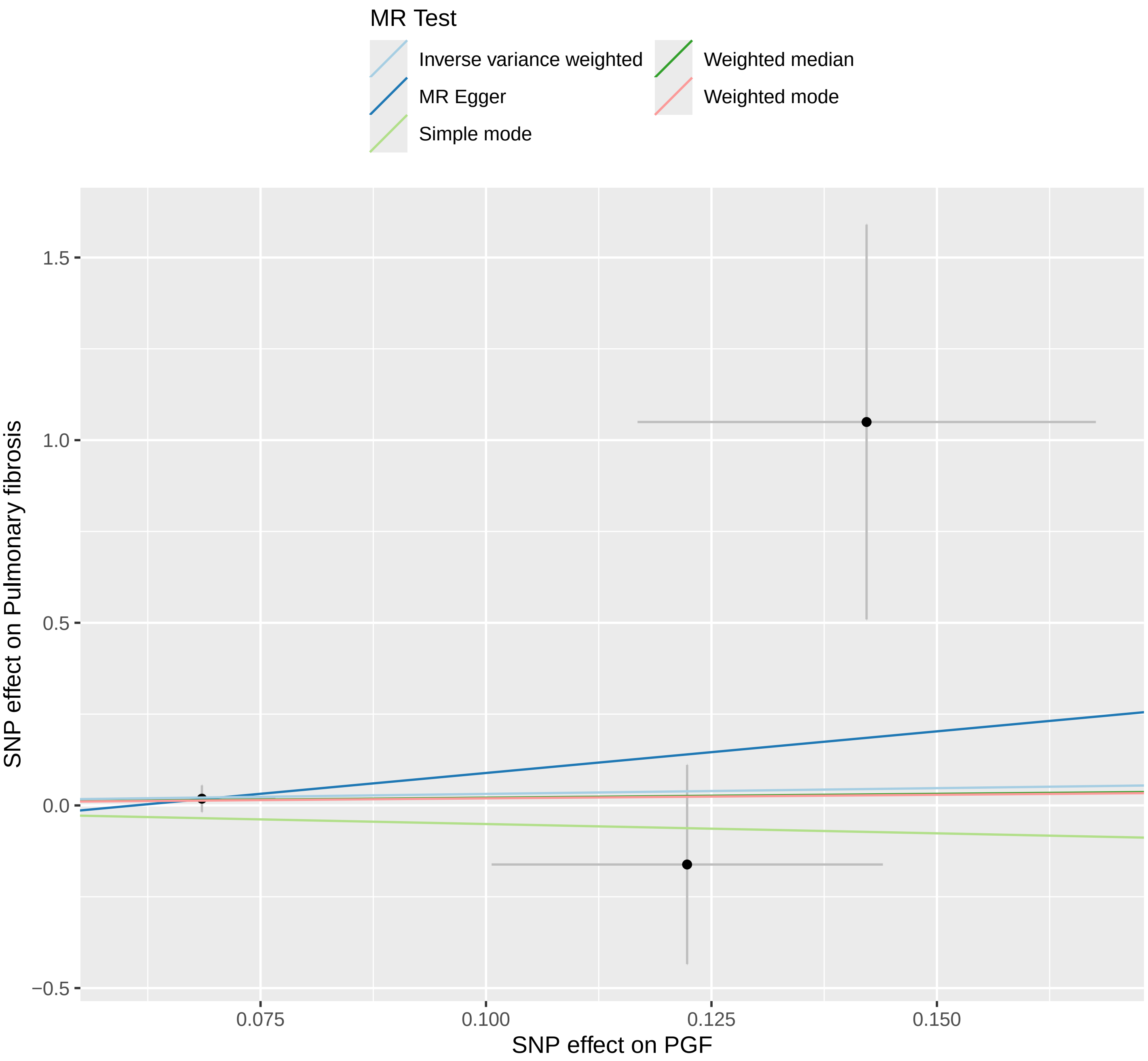

MR Test
Inverse variance weighted
MR Egger
Simple mode
Weighted median
Weighted mode
1.5
1.0
0.5
0.0
−0.5
0.075
0.100
0.125
0.150
SNP effect on Pulmonary fibrosis
SNP effect on PGF

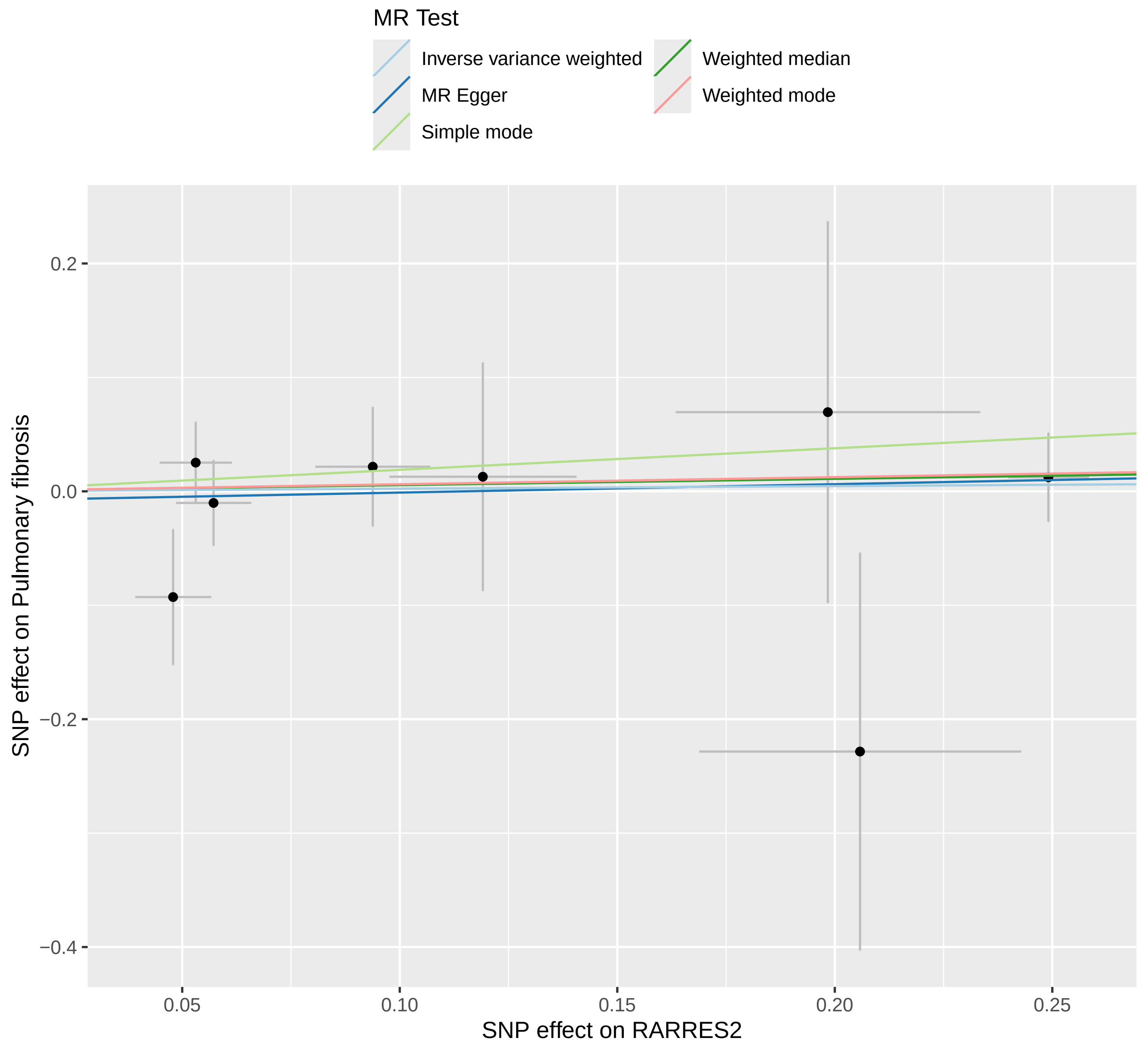
MR Test
Inverse variance weighted
MR Egger
Simple mode
Weighted median
Weighted mode
0.2
0.0
−0.2
−0.4
SNP effect on Pulmonary fibrosis
0.05
0.10
0.15
0.20
0.25
SNP effect on RARRES2

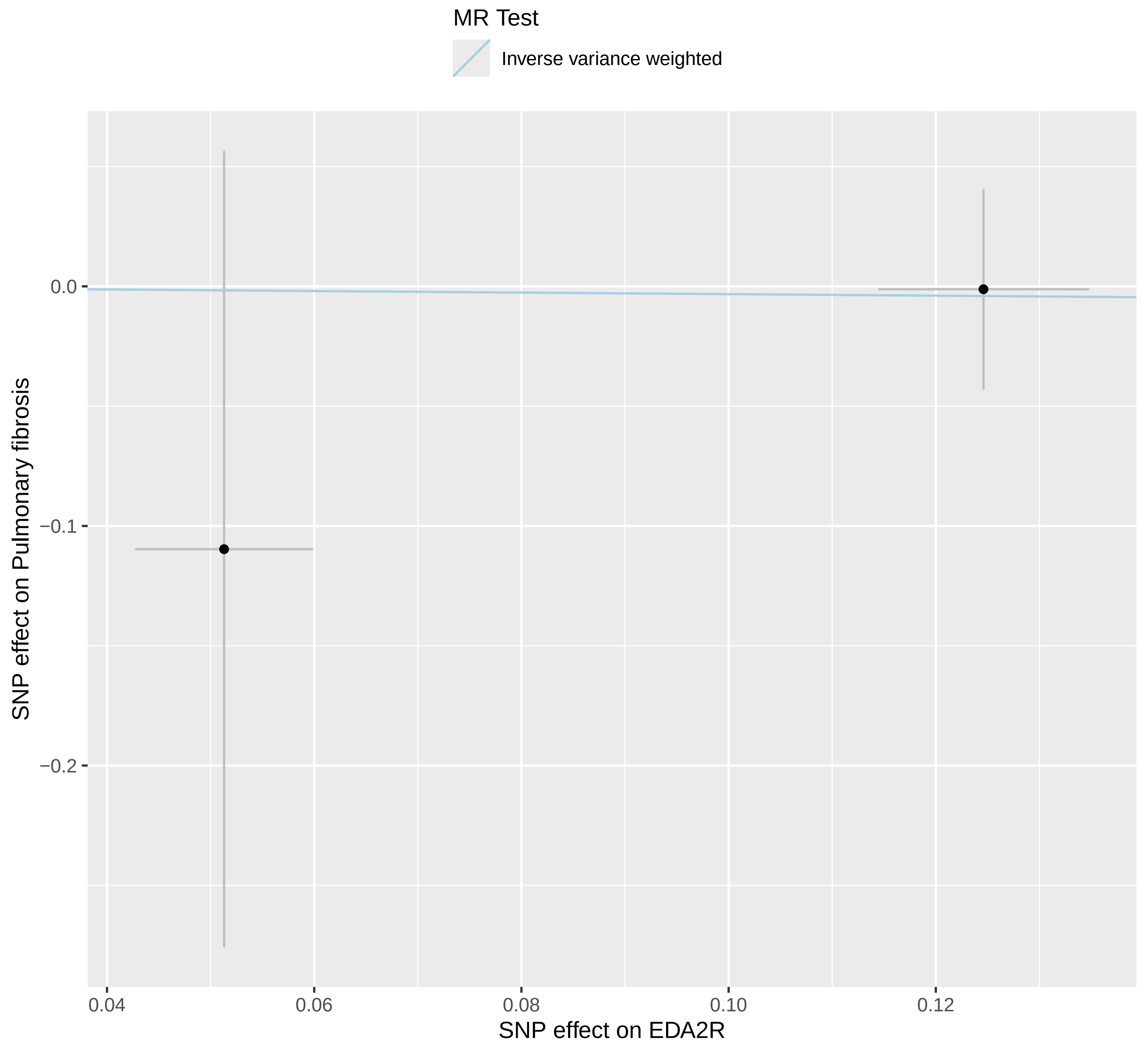
MR Test
Inverse variance weighted
0.0
−0.1
−0.2
SNP effect on Pulmonary fibrosis
0.04
0.06
0.08
0.10
0.12
SNP effect on EDA2R

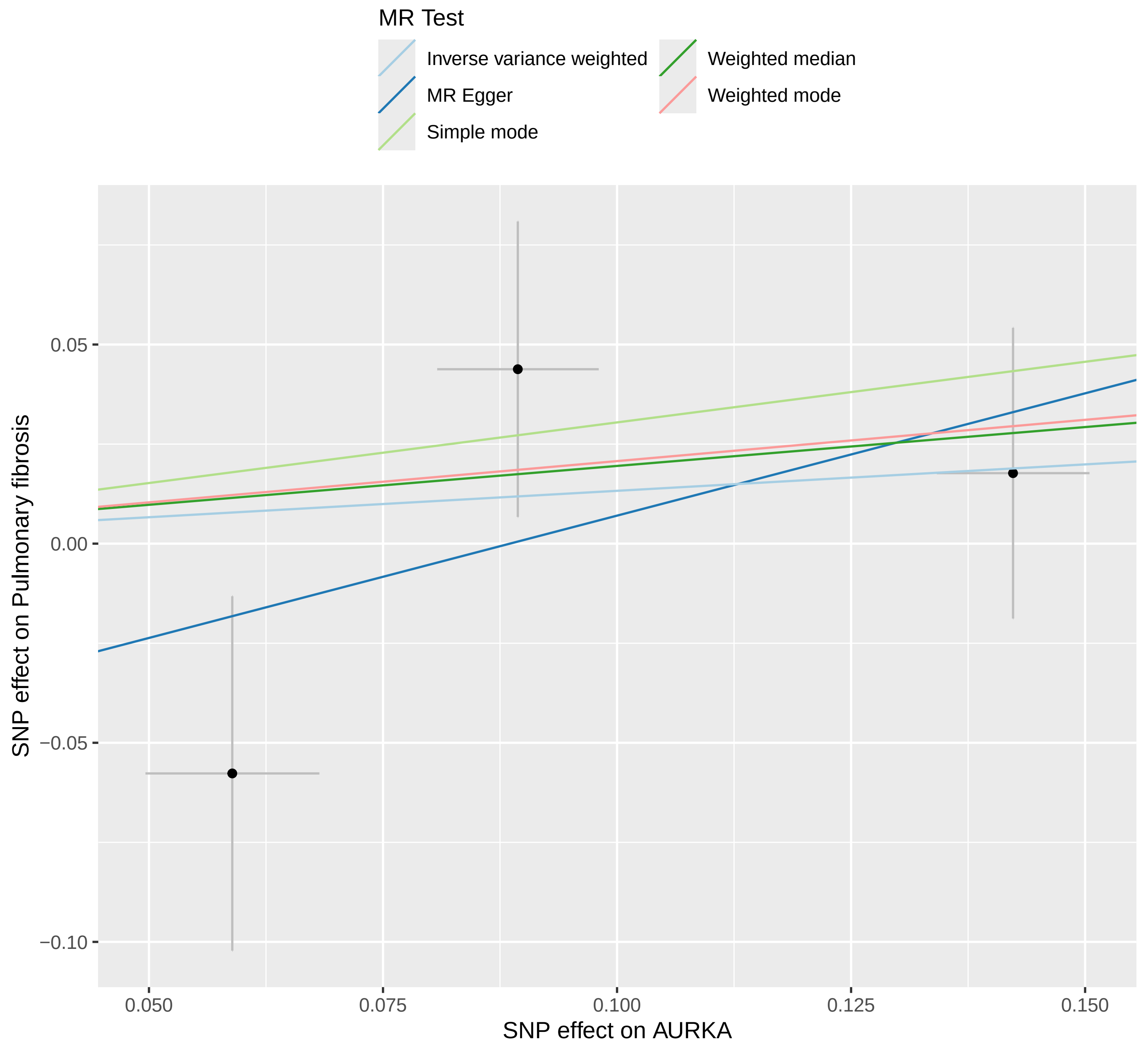
MR Test
Inverse variance weighted
MR Egger
Simple mode
Weighted median
Weighted mode
0.05
0.00
−0.05
−0.10
0.050
0.075
0.100
0.125
0.150
SNP effect on AURKA
SNP effect on Pulmonary fibrosis

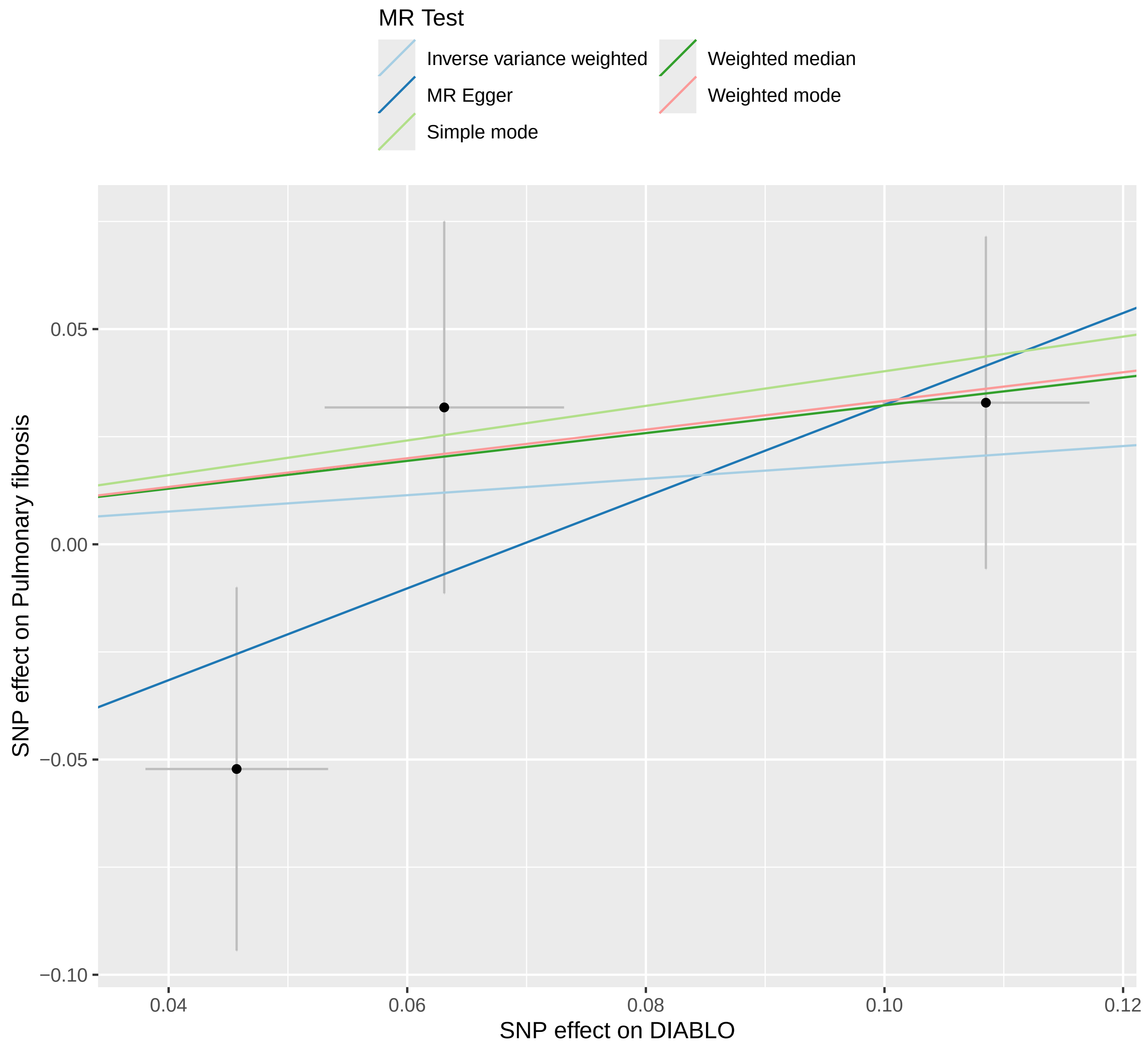
MR Test
Inverse variance weighted
MR Egger
Simple mode
Weighted median
Weighted mode
0.05
0.00
−0.05
−0.10
0.04
0.06
0.08
0.10
0.12
SNP effect on DIABLO
SNP effect on Pulmonary fibrosis

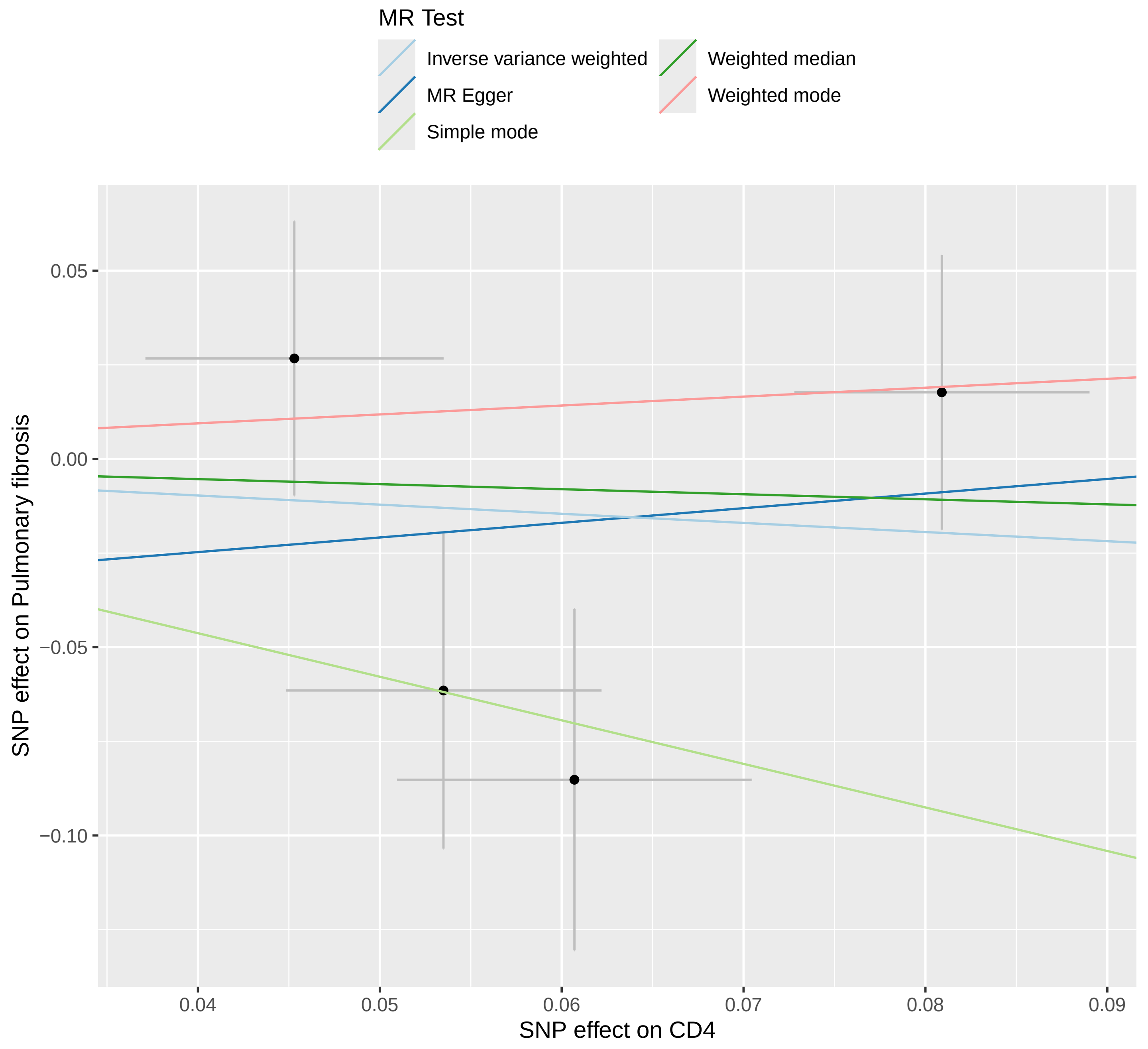
MR Test
Inverse variance weighted
MR Egger
Simple mode
Weighted median
Weighted mode
0.05
0.00
−0.05
−0.10
0.04
0.05
0.06
0.07
0.08
0.09
SNP effect on CD4
SNP effect on Pulmonary fibrosis

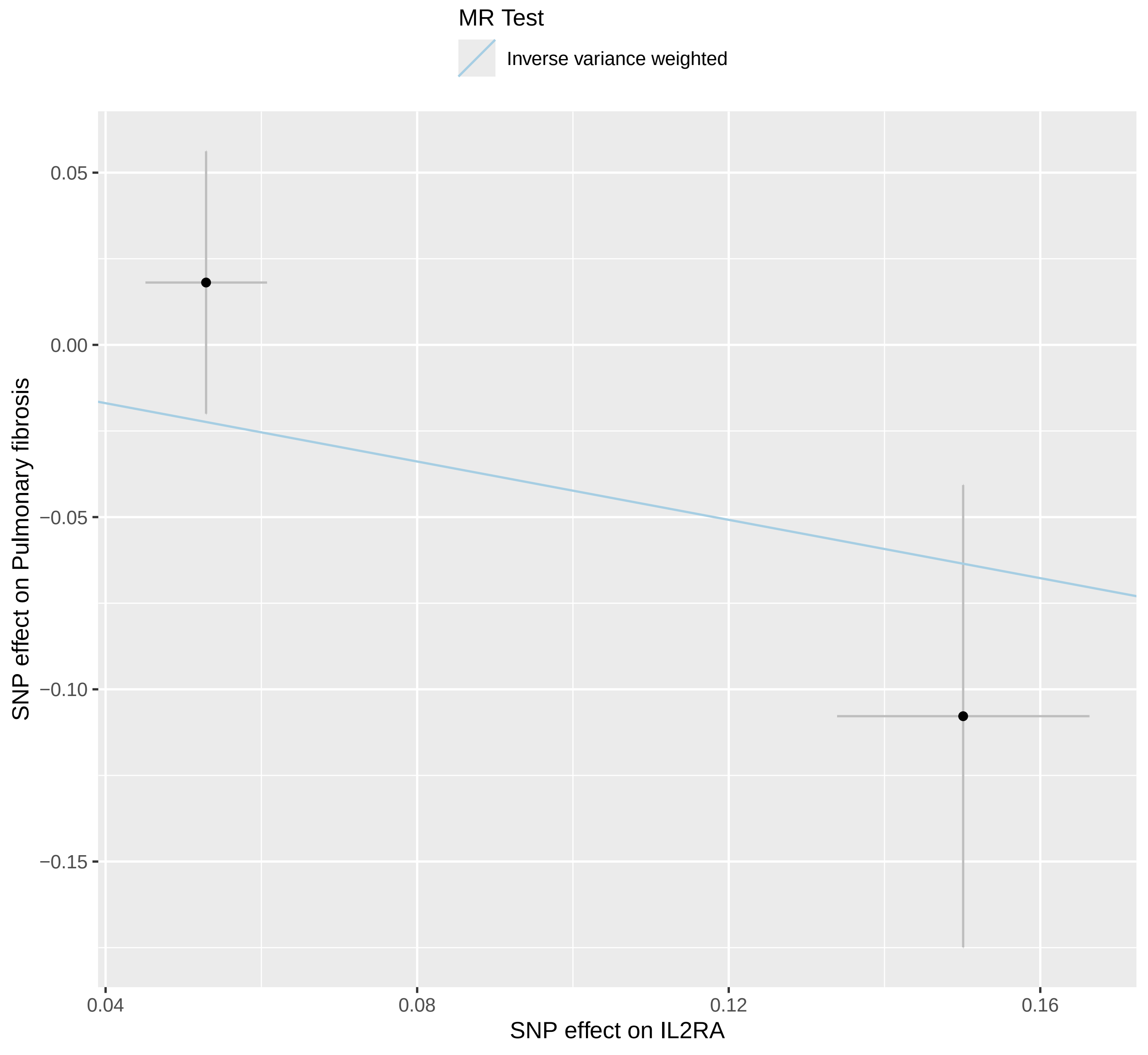

MR Test
Inverse variance weighted
0.05
0.00
−0.05
−0.10
−0.15
SNP effect on Pulmonary fibrosis
0.04
0.08
0.12
0.16
SNP effect on IL2RA

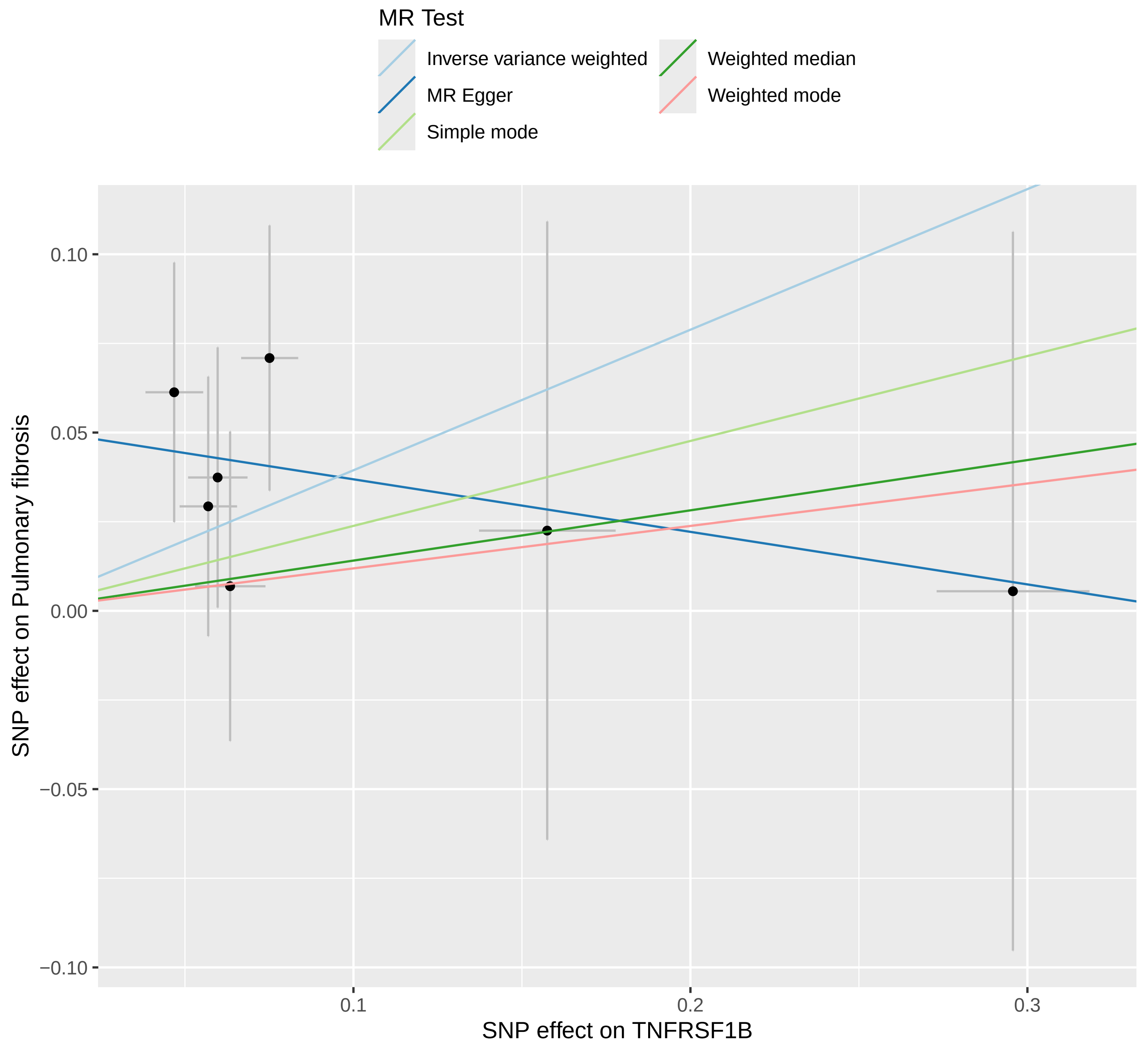

MR Test
Inverse variance weighted
MR Egger
Simple mode
Weighted median
Weighted mode
SNP effect on Pulmonary fibrosis
0.10
0.05
0.00
−0.05
−0.10
0.1
0.2
0.3
SNP effect on TNFRSF1B

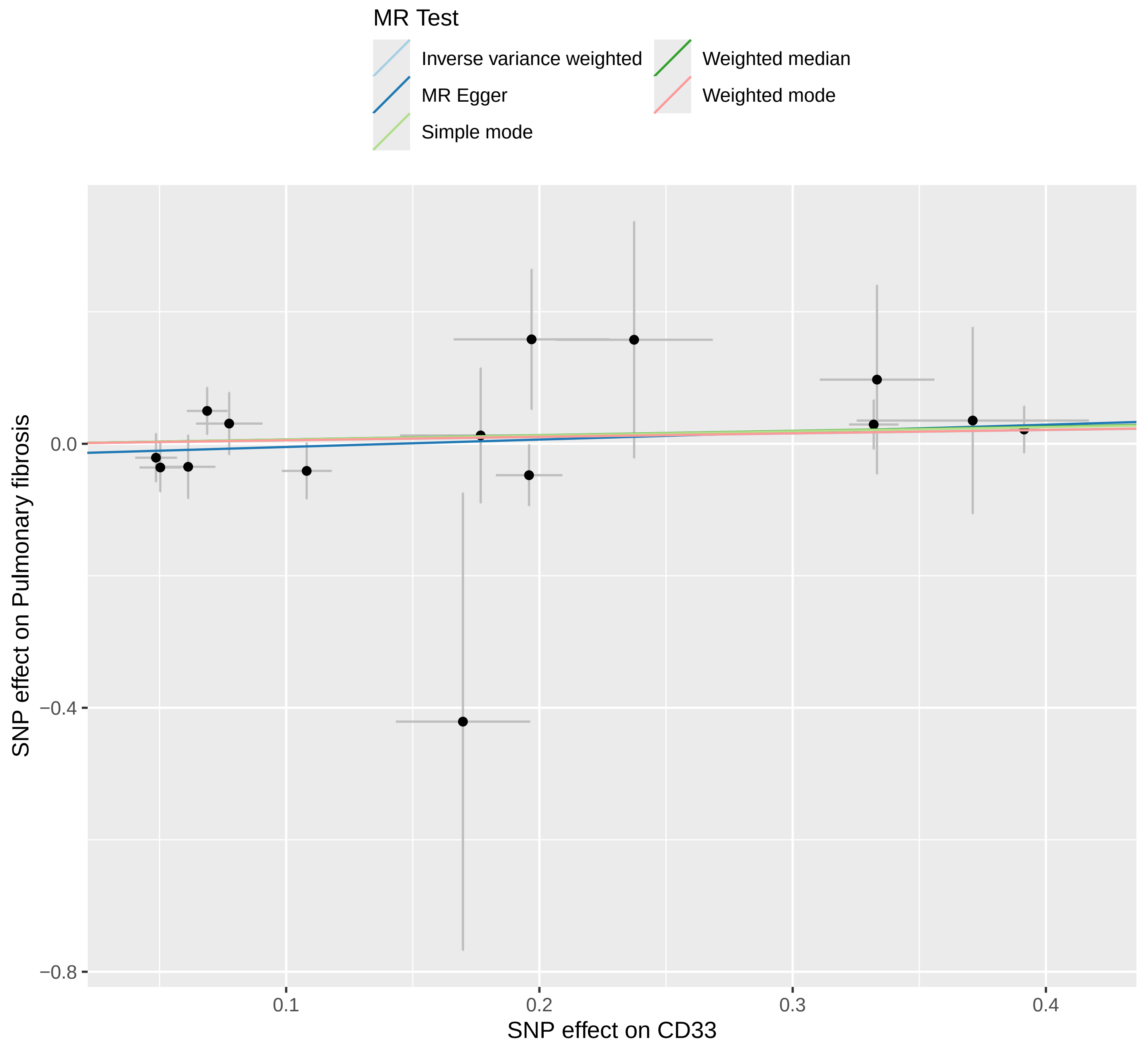

MR Test
Inverse variance weighted
MR Egger
Simple mode
Weighted median
Weighted mode
SNP effect on Pulmonary fibrosis
0.0
−0.4
−0.8
0.1
0.2
0.3
0.4
SNP effect on CD33

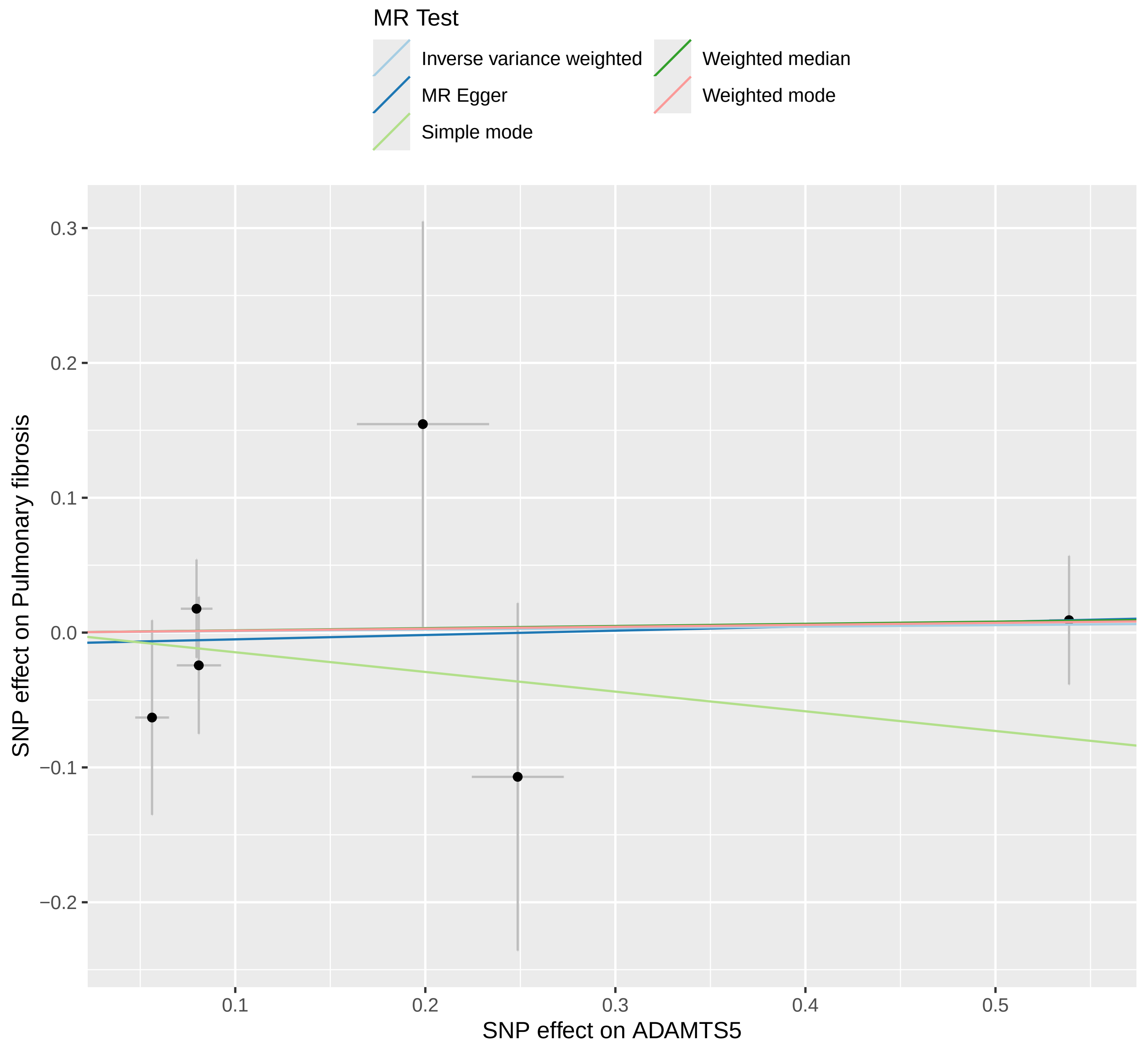
MR Test
Inverse variance weighted
MR Egger
Simple mode
Weighted median
Weighted mode
0.3
0.2
0.1
0.0
−0.1
−0.2
SNP effect on Pulmonary fibrosis
0.1
0.2
0.3
0.4
0.5
SNP effect on ADAMTS5

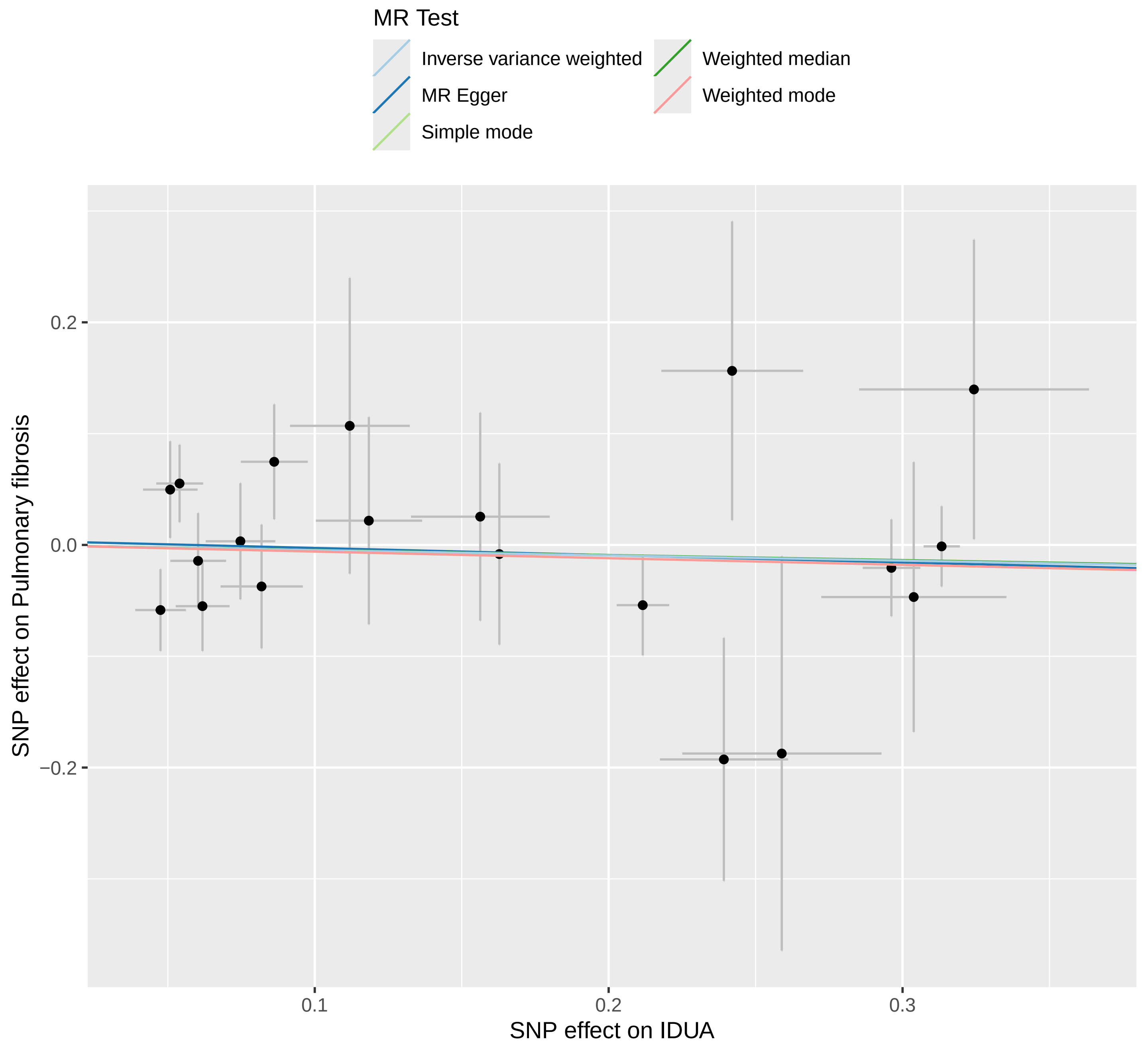

MR Test
Inverse variance weighted
MR Egger
Simple mode
Weighted median
Weighted mode
0.2
0.0
−0.2
0.1
0.2
0.3
SNP effect on Pulmonary fibrosis
SNP effect on IDUA

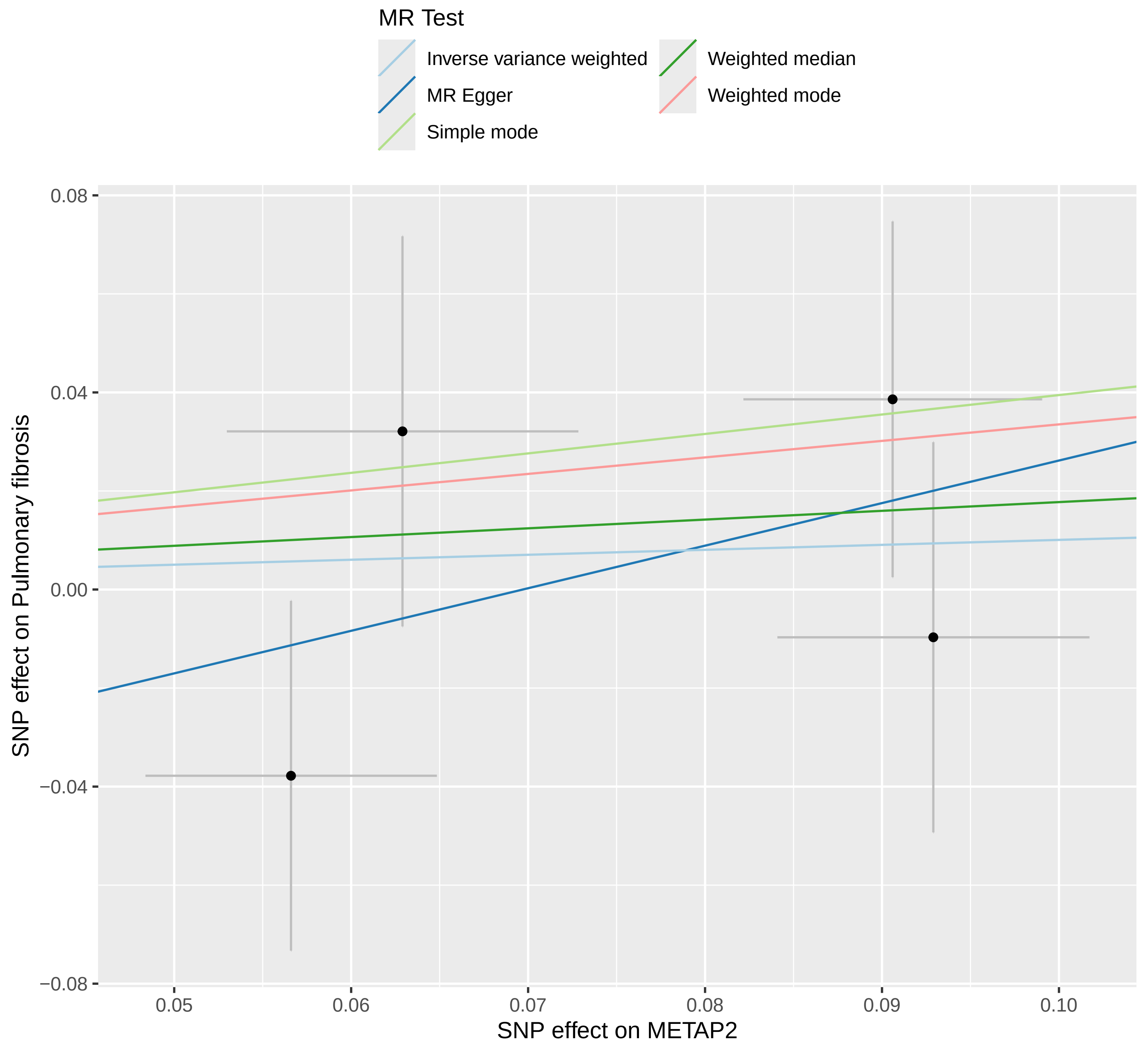

MR Test
Inverse variance weighted
MR Egger
Simple mode
Weighted median
Weighted mode
0.08
0.04
0.00
−0.04
−0.08
SNP effect on Pulmonary fibrosis
0.05
0.06
0.07
0.08
0.09
0.10
SNP effect on METAP2

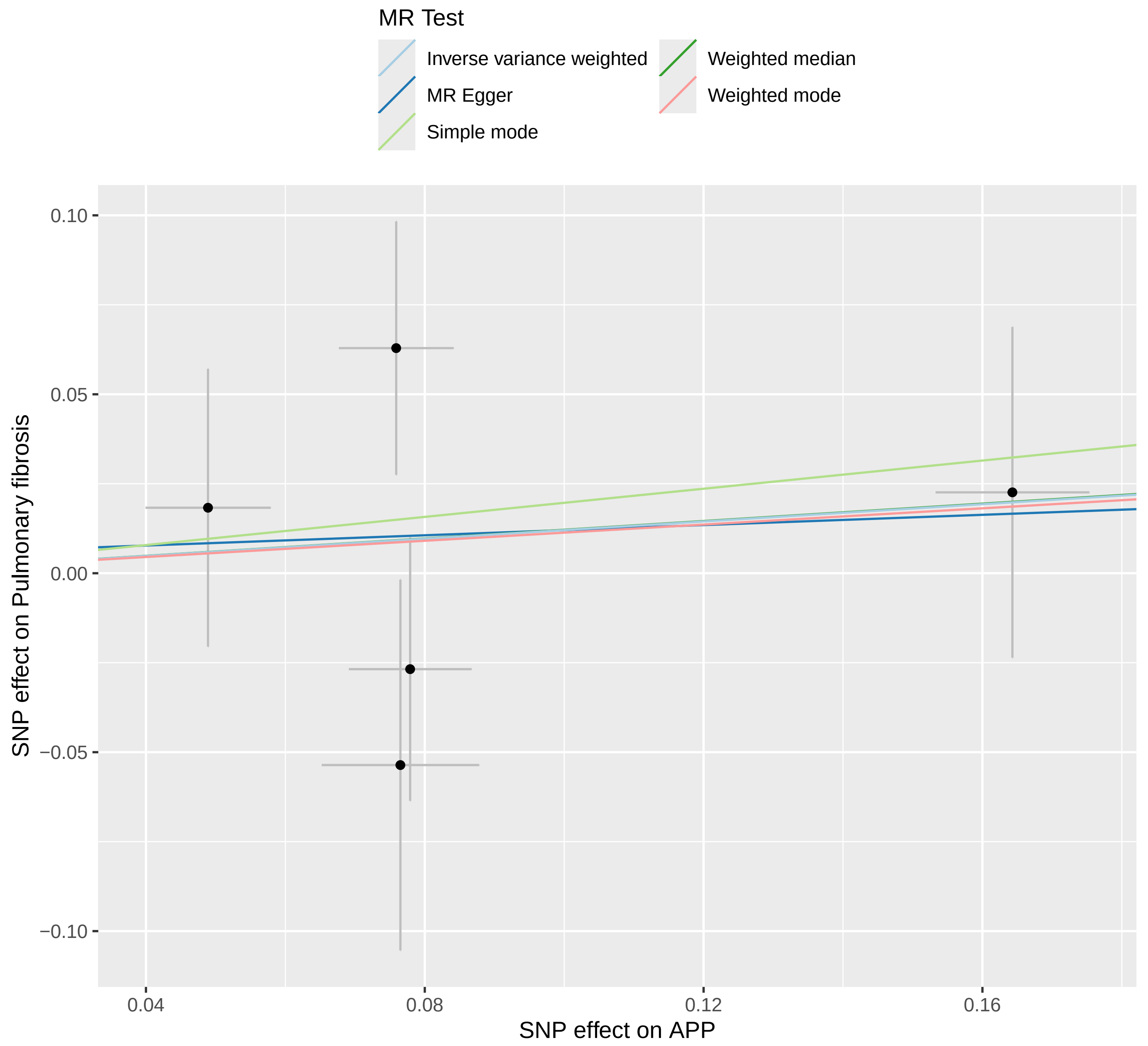

MR Test
Inverse variance weighted
MR Egger
Simple mode
Weighted median
Weighted mode
0.10
0.05
0.00
−0.05
−0.10
SNP effect on Pulmonary fibrosis
0.04
0.08
0.12
0.16
SNP effect on APP

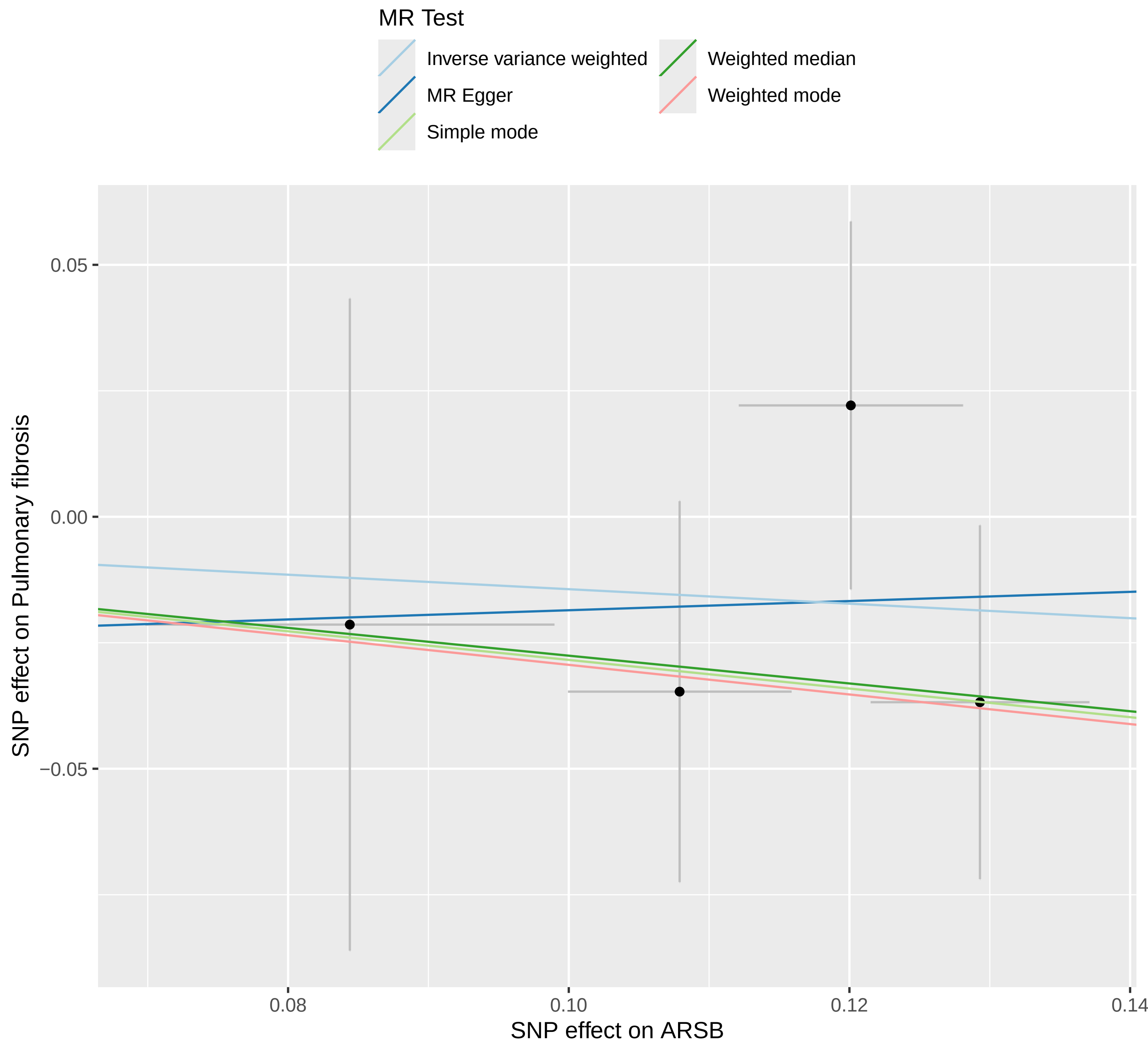
MR Test
Inverse variance weighted
MR Egger
Simple mode
Weighted median
Weighted mode
0.05
0.00
−0.05
SNP effect on Pulmonary fibrosis
0.08
0.10
0.12
0.14
SNP effect on ARSB

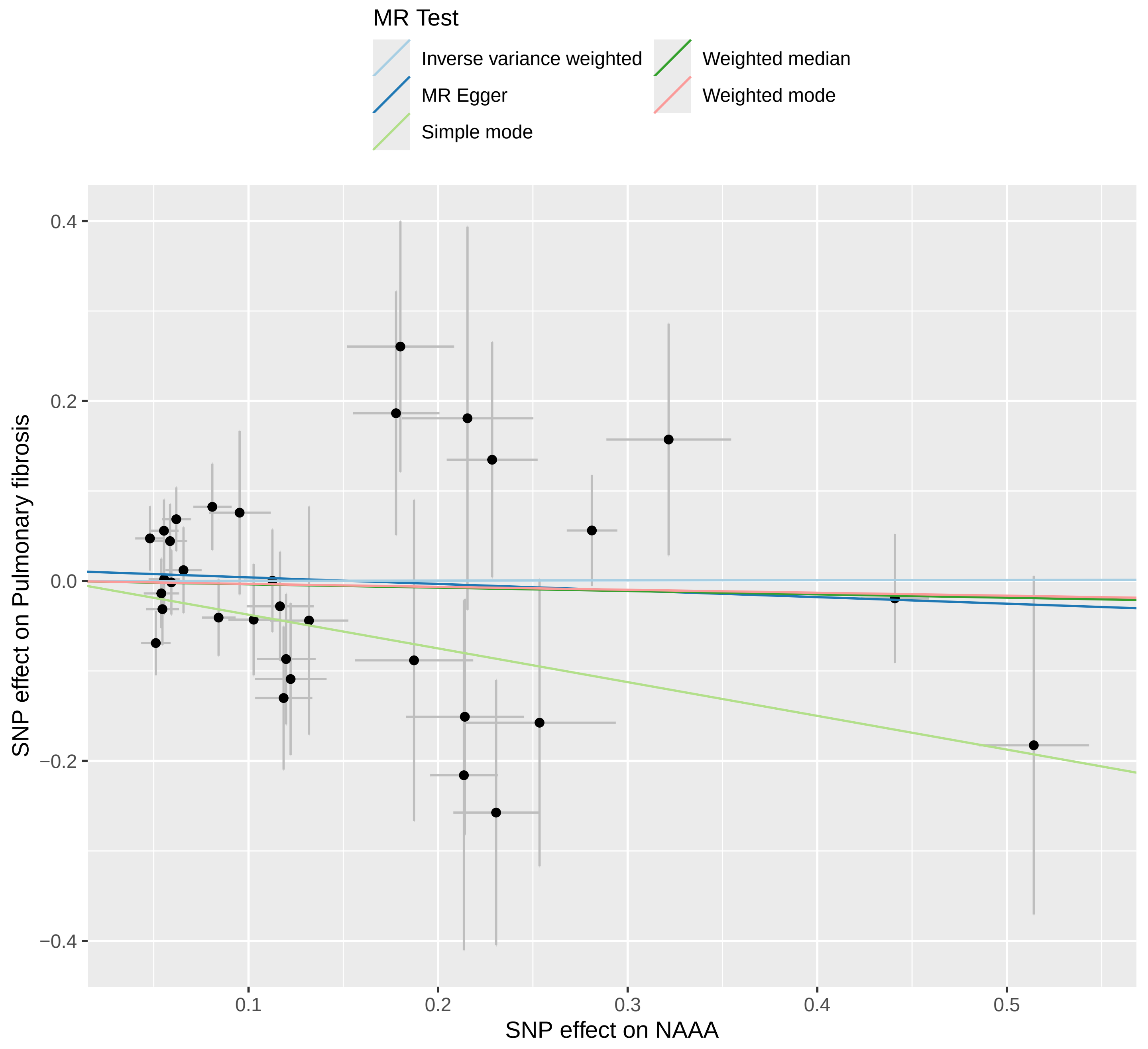
MR Test
Inverse variance weighted
MR Egger
Simple mode
Weighted median
Weighted mode
0.4
0.2
0.0
−0.2
−0.4
SNP effect on Pulmonary fibrosis
0.1
0.2
0.3
0.4
0.5
SNP effect on NAAA

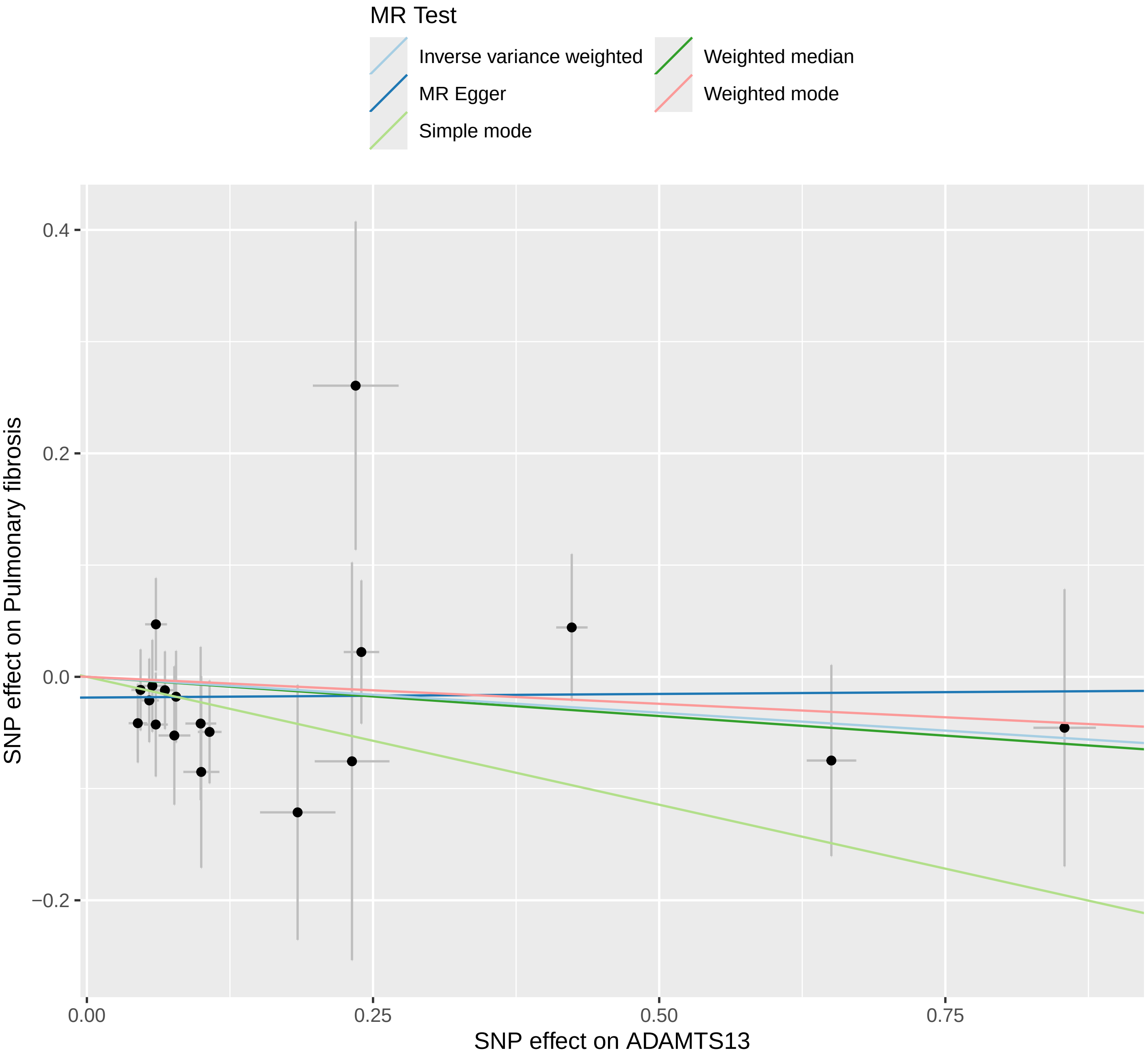

MR Test
Inverse variance weighted
MR Egger
Simple mode
Weighted median
Weighted mode
0.4
0.2
0.0
−0.2
0.00
0.25
0.50
0.75
SNP effect on Pulmonary fibrosis
SNP effect on ADAMTS13

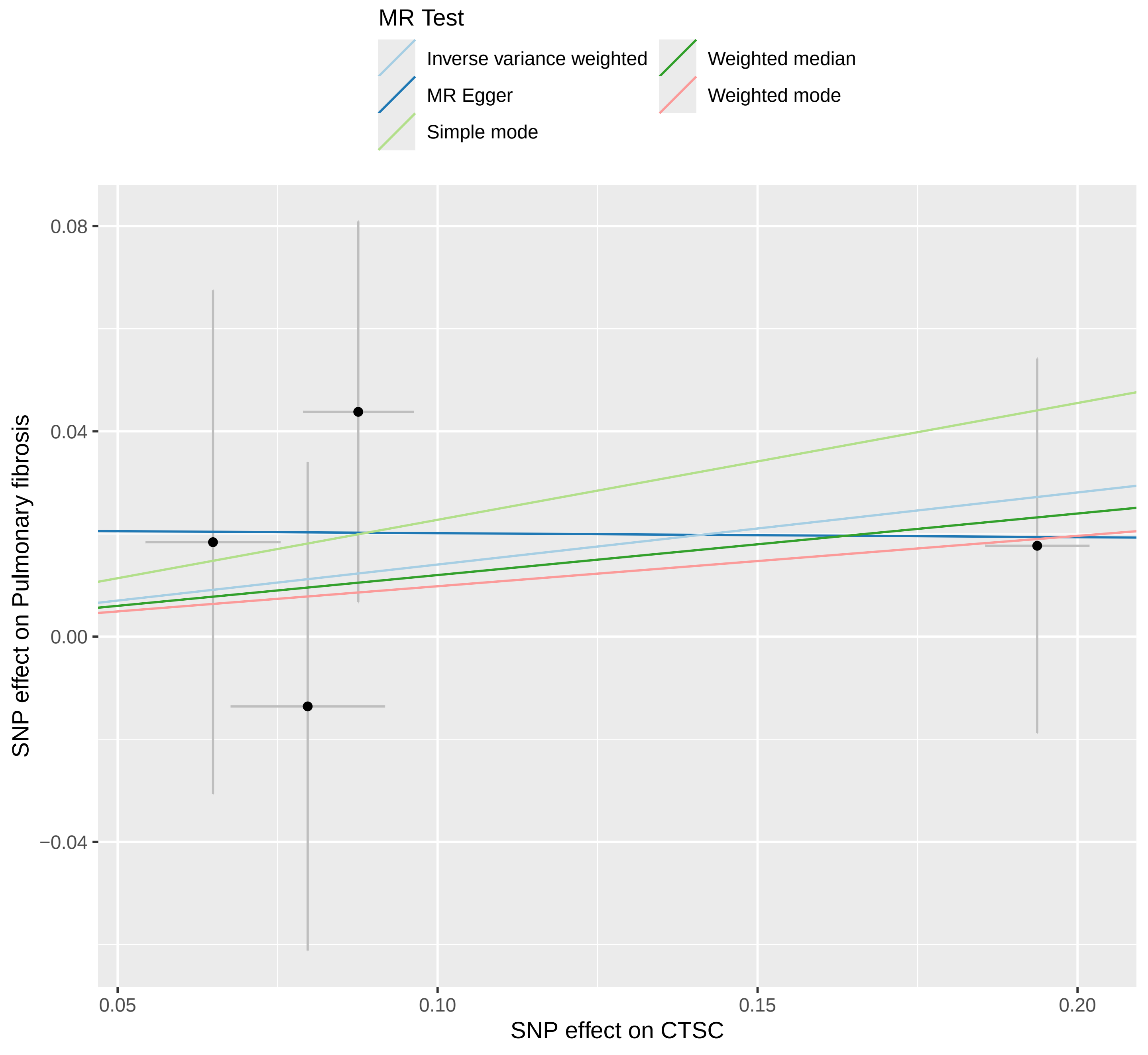

MR Test
Inverse variance weighted
MR Egger
Simple mode
Weighted median
Weighted mode
0.08
0.04
0.00
−0.04
0.05
0.10
0.15
0.20
SNP effect on CTSC
SNP effect on Pulmonary fibrosis

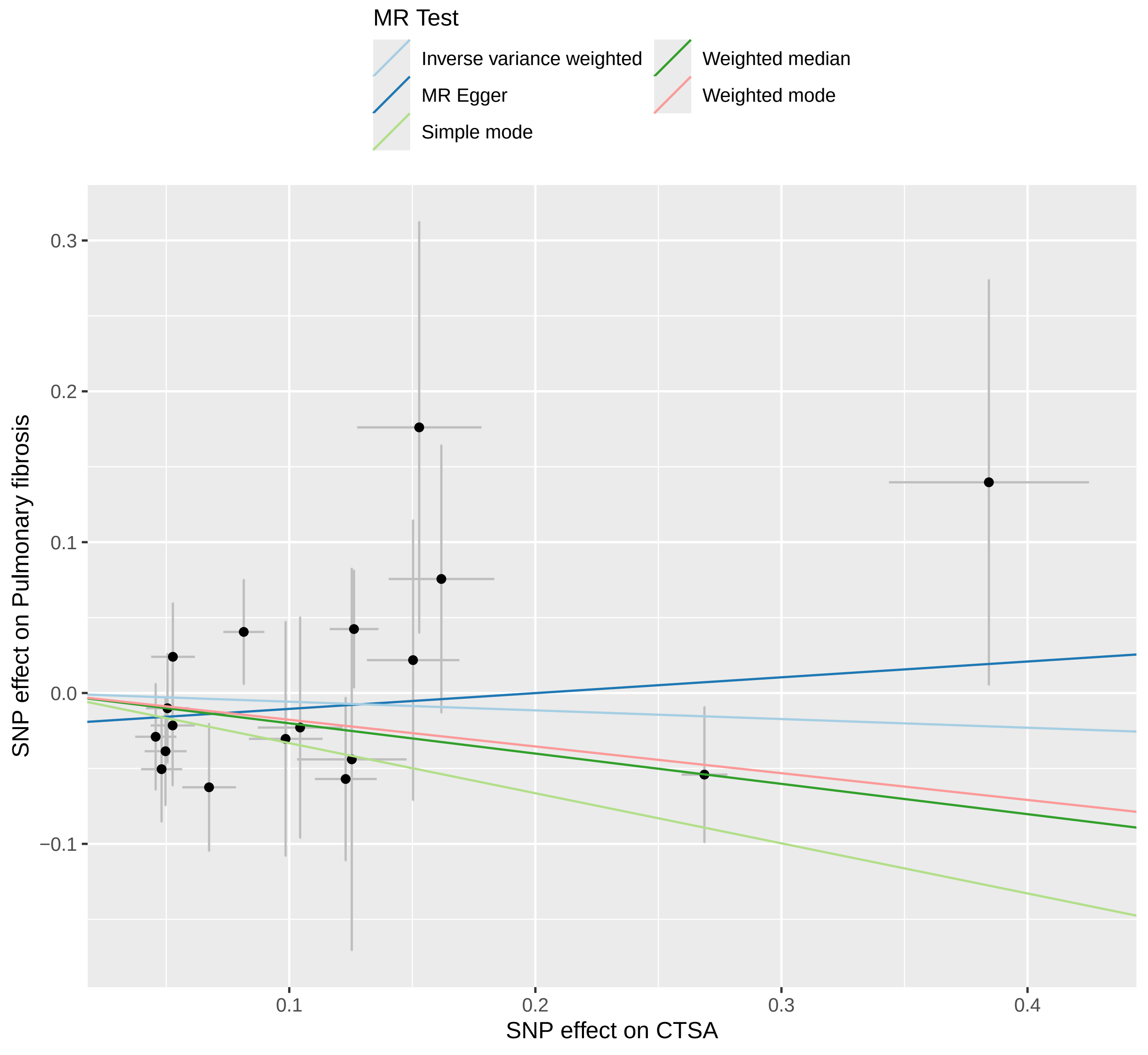

MR Test
Inverse variance weighted
MR Egger
Simple mode
Weighted median
Weighted mode
SNP effect on Pulmonary fibrosis
0.3
0.2
0.1
0.0
−0.1
0.1
0.2
0.3
0.4
SNP effect on CTSA

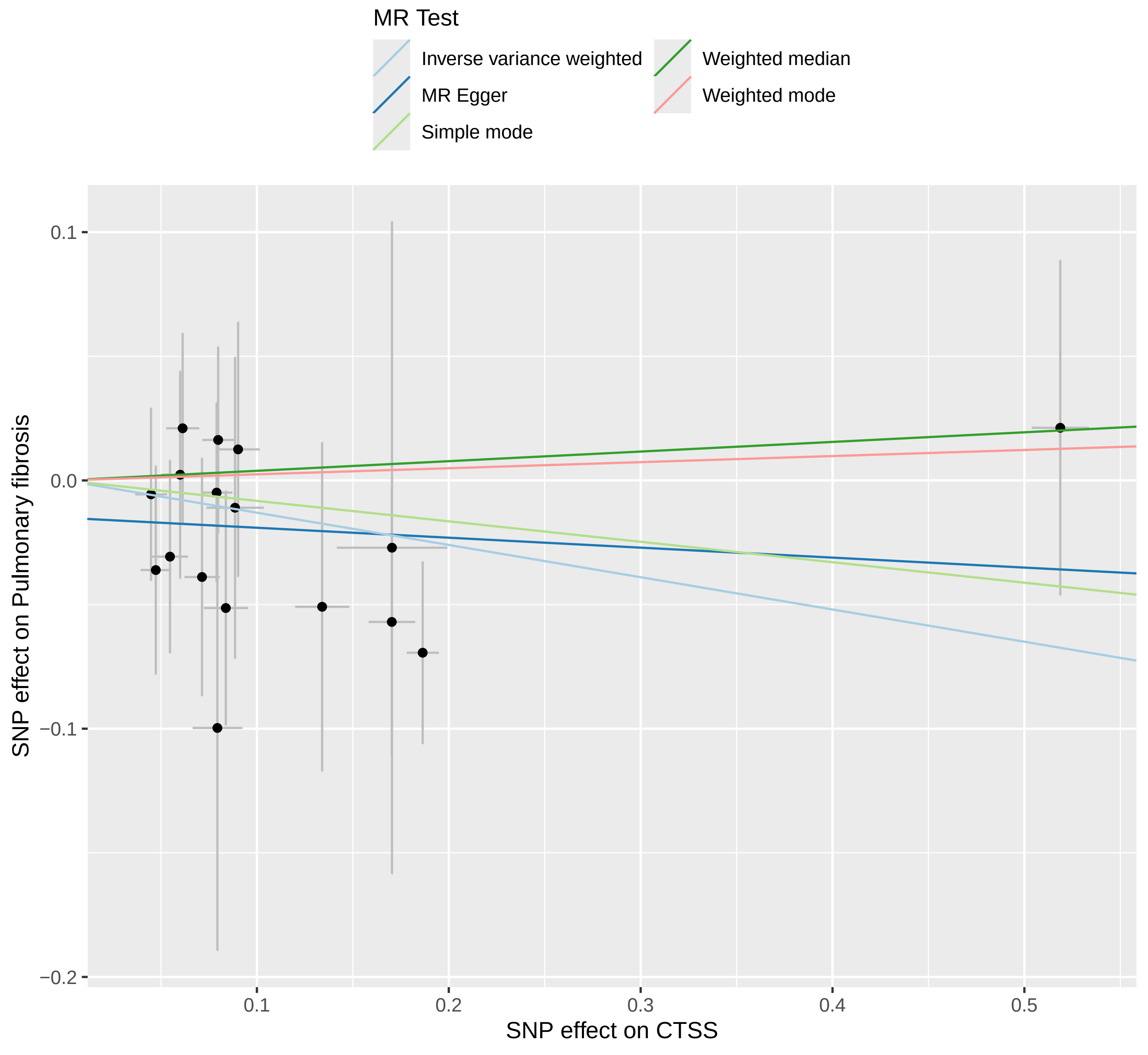

MR Test
Inverse variance weighted
MR Egger
Simple mode
Weighted median
Weighted mode
0.1
0.0
−0.1
−0.2
SNP effect on Pulmonary fibrosis
0.1
0.2
0.3
0.4
0.5
SNP effect on CTSS

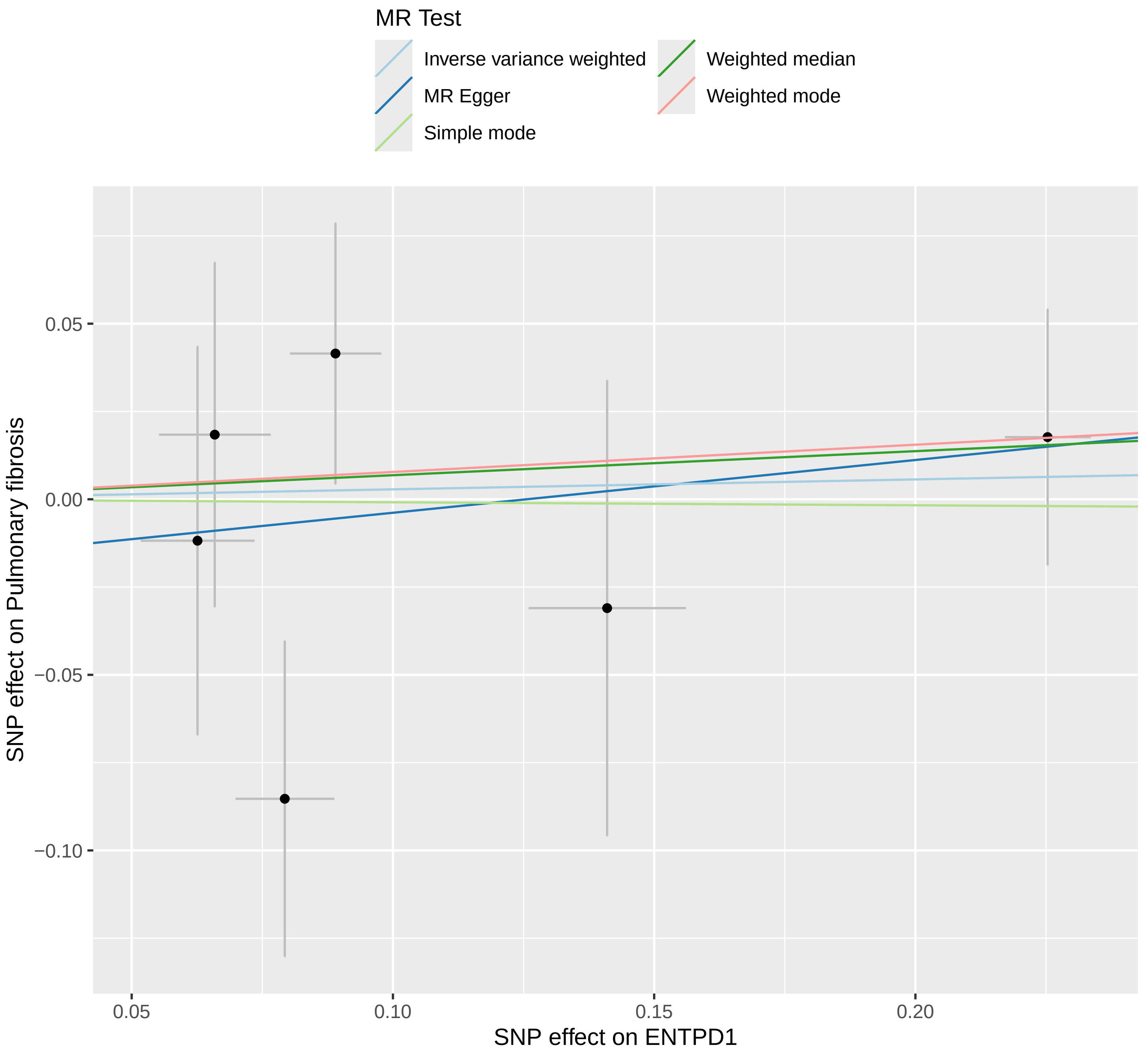
MR Test
Inverse variance weighted
MR Egger
Simple mode
Weighted median
Weighted mode
0.05
0.00
−0.05
−0.10
0.05
0.10
0.15
0.20
SNP effect on Pulmonary fibrosis
SNP effect on ENTPD1

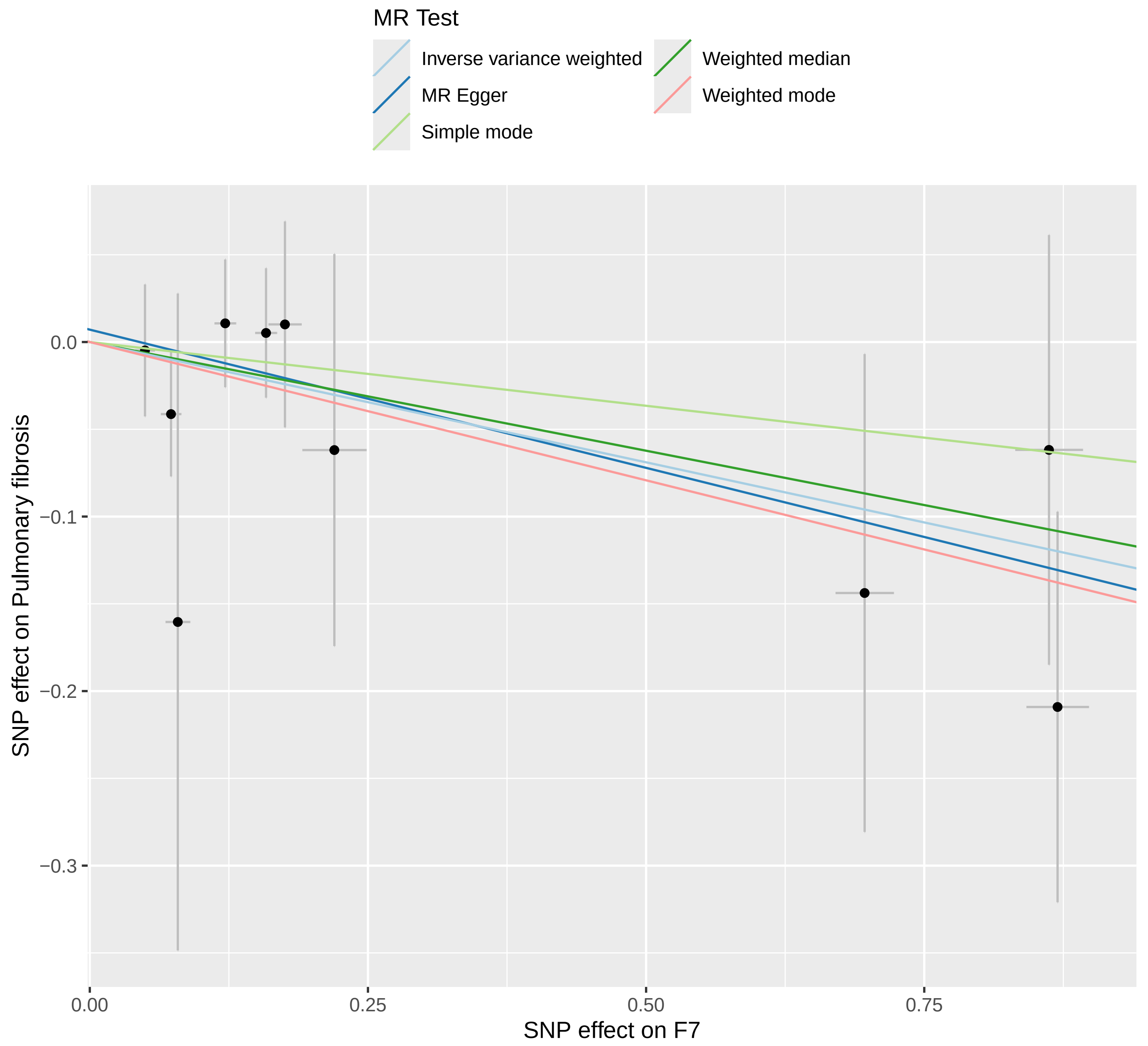
MR Test
Inverse variance weighted
MR Egger
Simple mode
Weighted median
Weighted mode
0.0
−0.1
−0.2
−0.3
0.00
0.25
0.50
0.75
SNP effect on F7
SNP effect on Pulmonary fibrosis

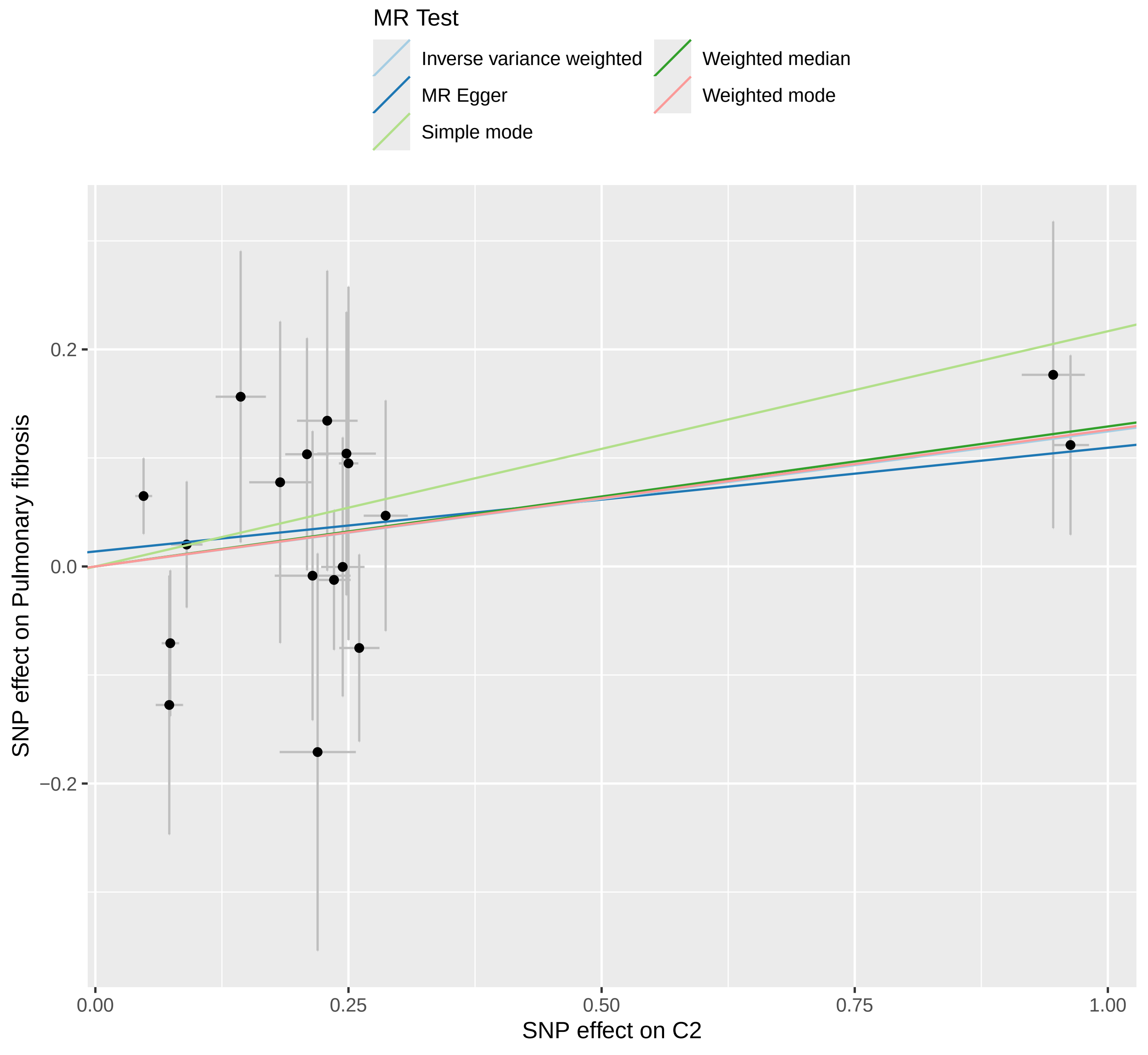

MR Test
Inverse variance weighted
MR Egger
Simple mode
Weighted median
Weighted mode
0.2
0.0
−0.2
SNP effect on Pulmonary fibrosis
0.00
0.25
0.50
0.75
1.00
SNP effect on C2

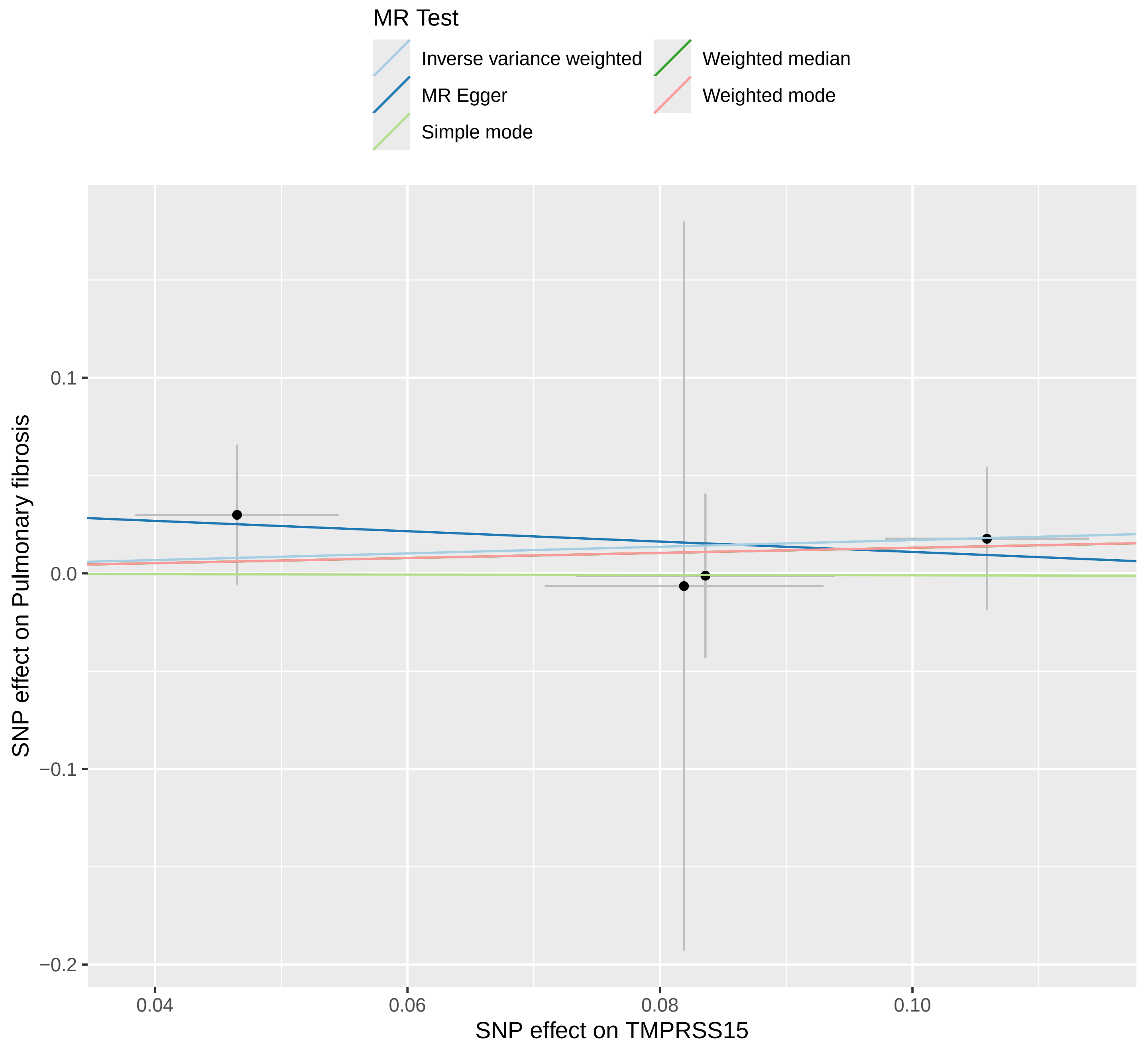

MR Test
Inverse variance weighted
MR Egger
Simple mode
Weighted median
Weighted mode
0.1
0.0
−0.1
−0.2
SNP effect on Pulmonary fibrosis
0.04
0.06
0.08
0.10
SNP effect on TMPRSS15

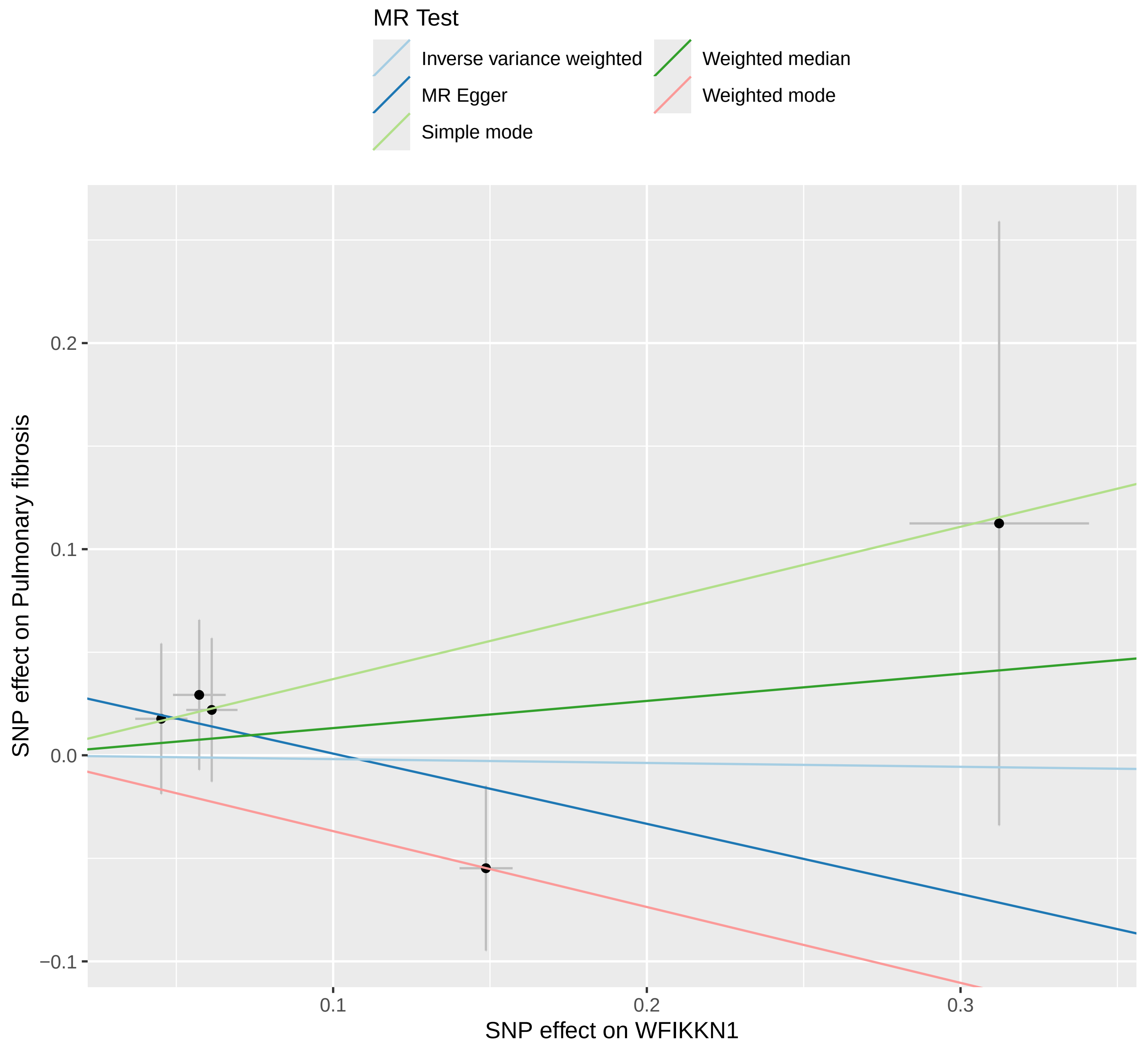
MR Test
Inverse variance weighted
MR Egger
Simple mode
Weighted median
Weighted mode
SNP effect on Pulmonary fibrosis
0.2
0.1
0.0
−0.1
0.1
0.2
0.3
SNP effect on WFIKKN1

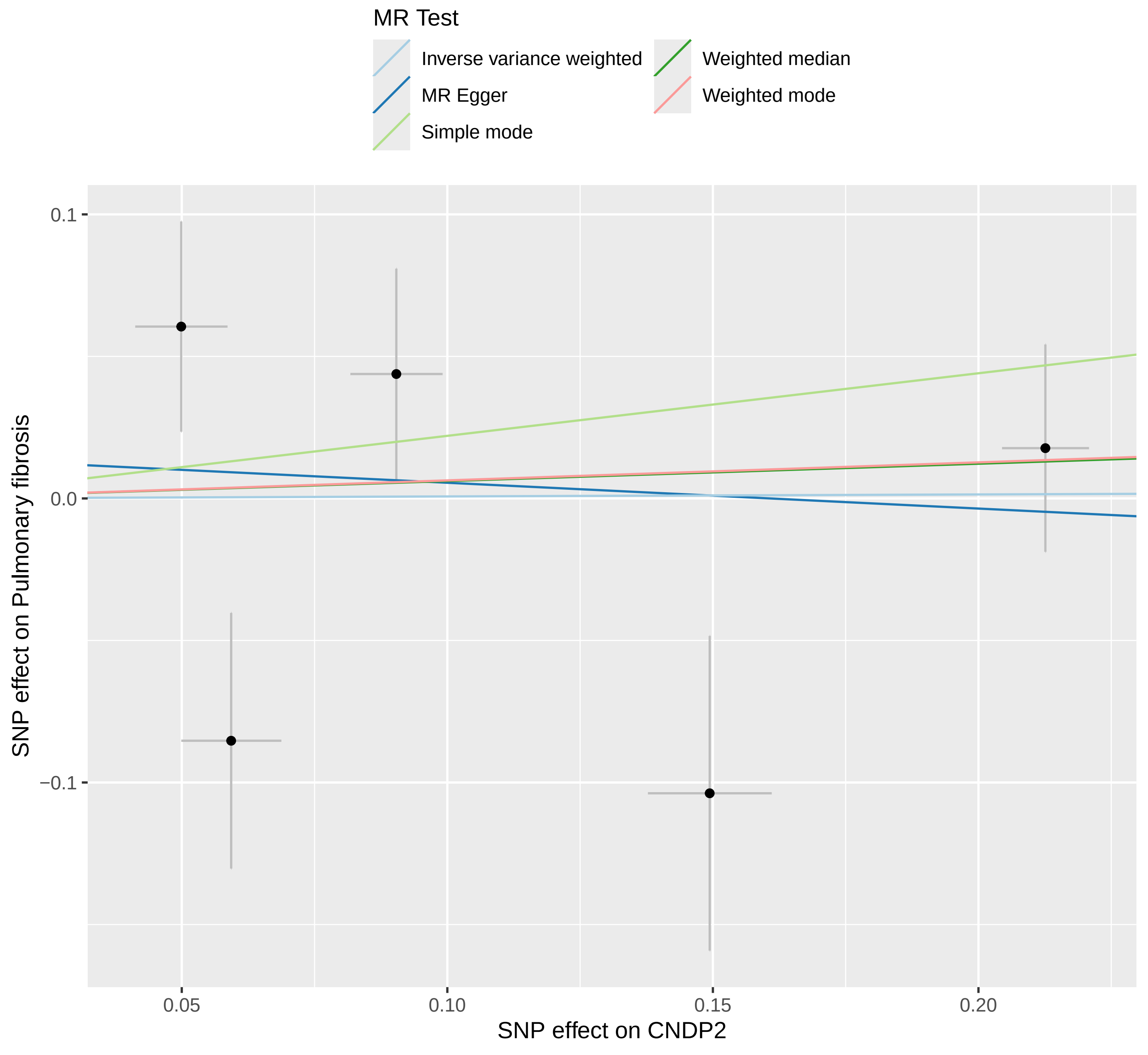
MR Test
Inverse variance weighted
MR Egger
Simple mode
Weighted median
Weighted mode
0.1
0.0
−0.1
SNP effect on Pulmonary fibrosis
0.05
0.10
0.15
0.20
SNP effect on CNDP2

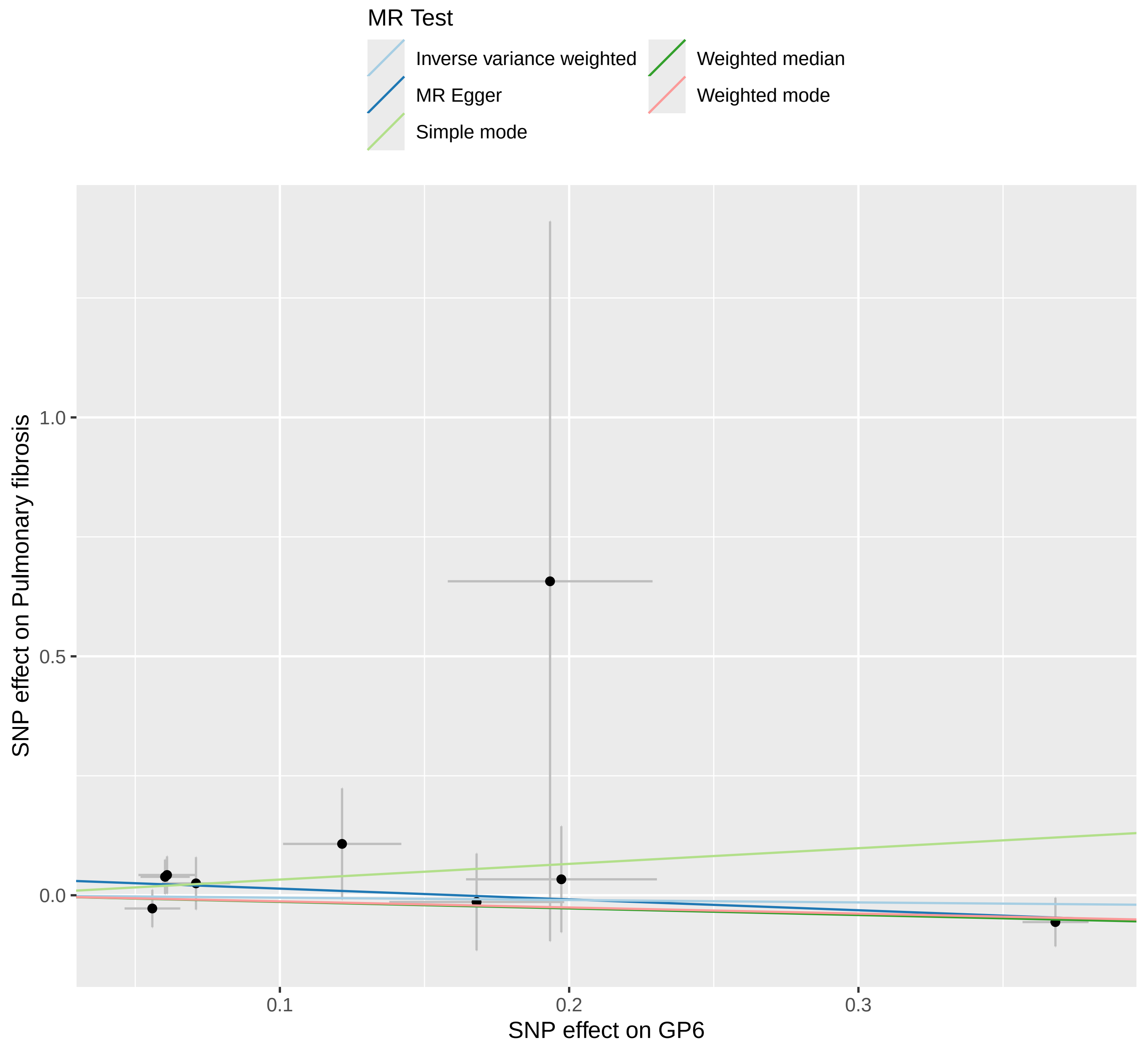
MR Test
Inverse variance weighted
MR Egger
Simple mode
Weighted median
Weighted mode
SNP effect on Pulmonary fibrosis
1.0
0.5
0.0
0.1
0.2
0.3
SNP effect on GP6

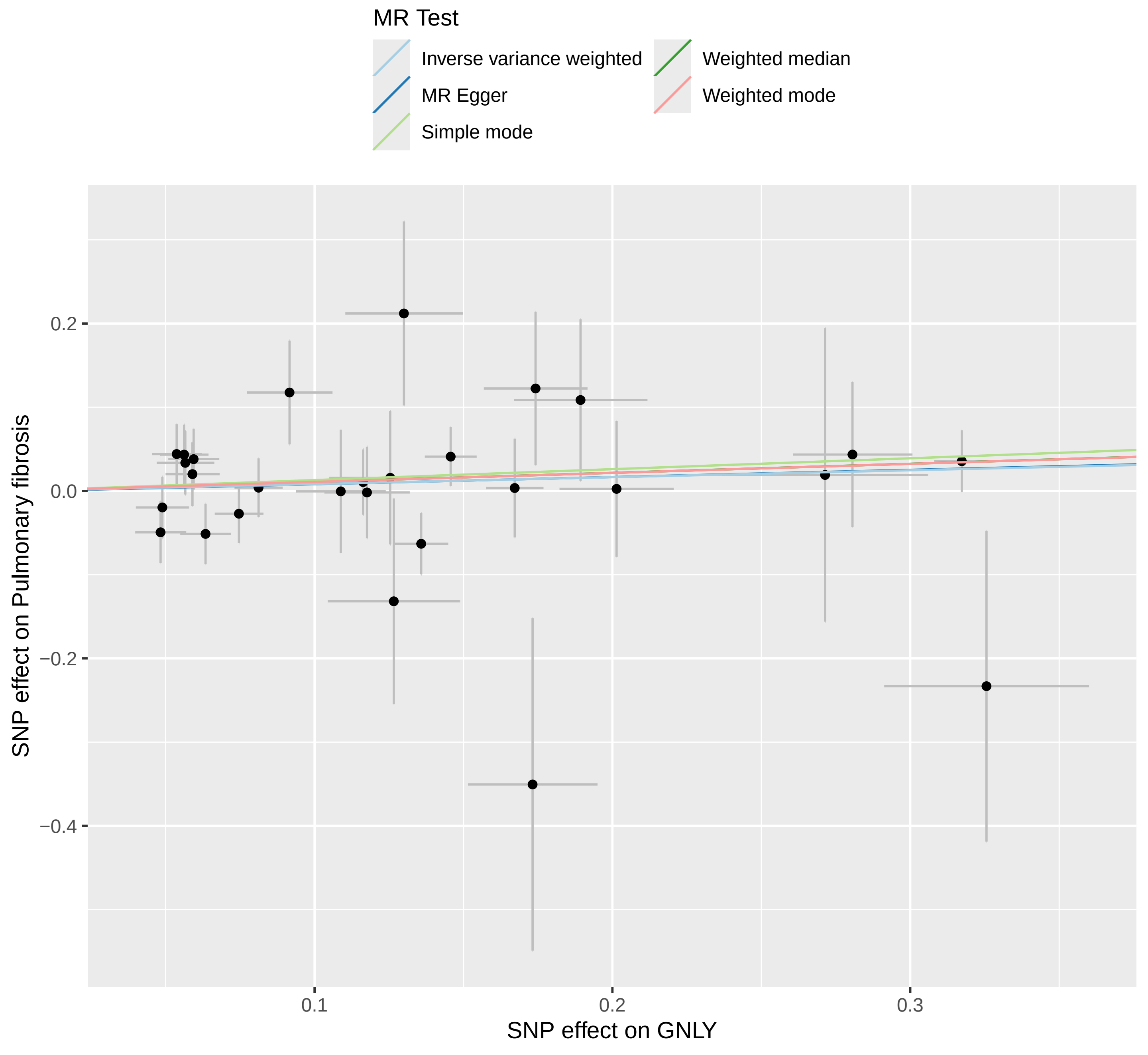
MR Test
Inverse variance weighted
MR Egger
Simple mode
Weighted median
Weighted mode
0.2
0.0
−0.2
−0.4
SNP effect on Pulmonary fibrosis
0.1
0.2
0.3
SNP effect on GNLY

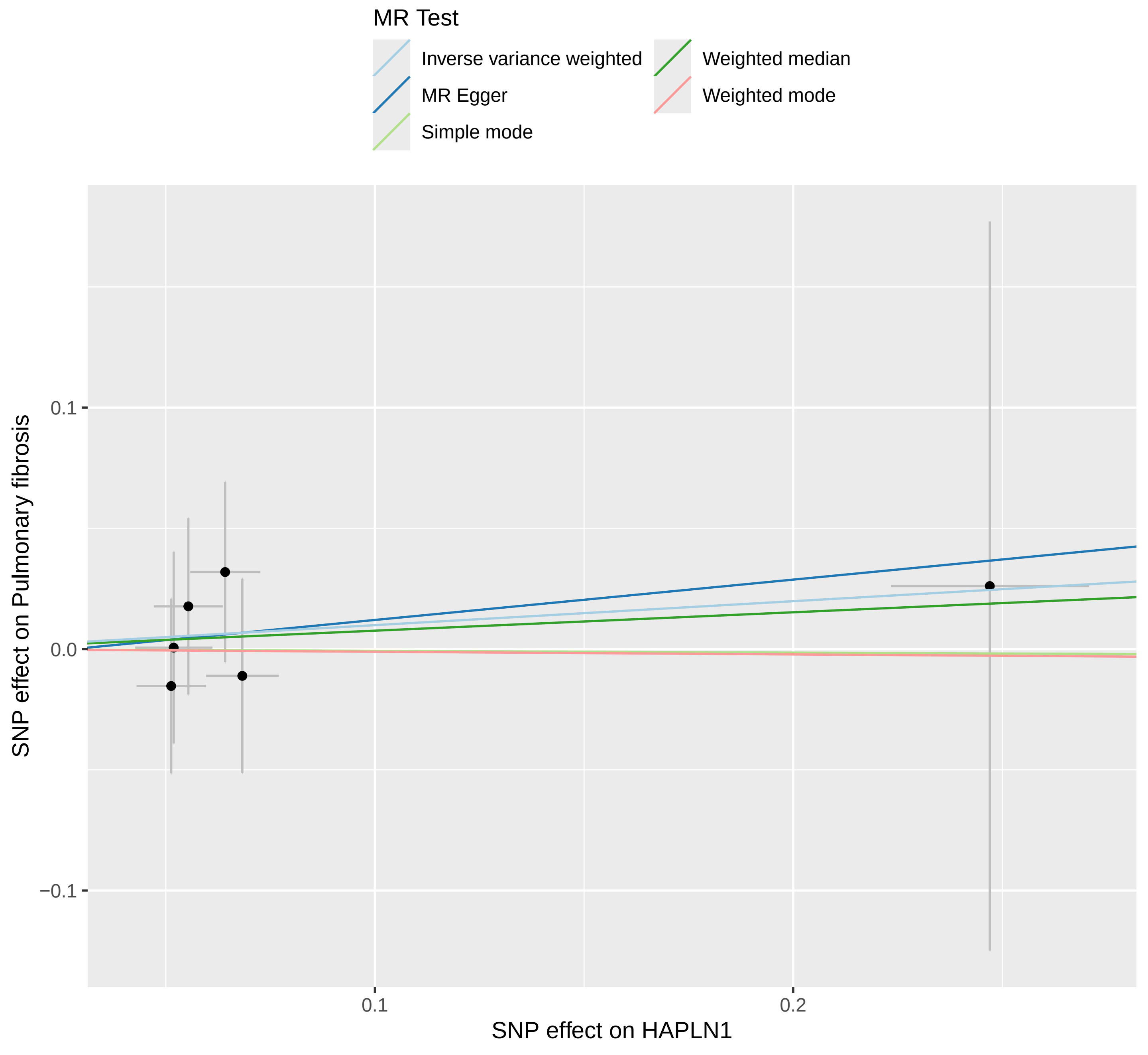

MR Test
Inverse variance weighted
MR Egger
Simple mode
Weighted median
Weighted mode
0.1
0.0
−0.1
0.1
0.2
SNP effect on Pulmonary fibrosis
SNP effect on HAPLN1

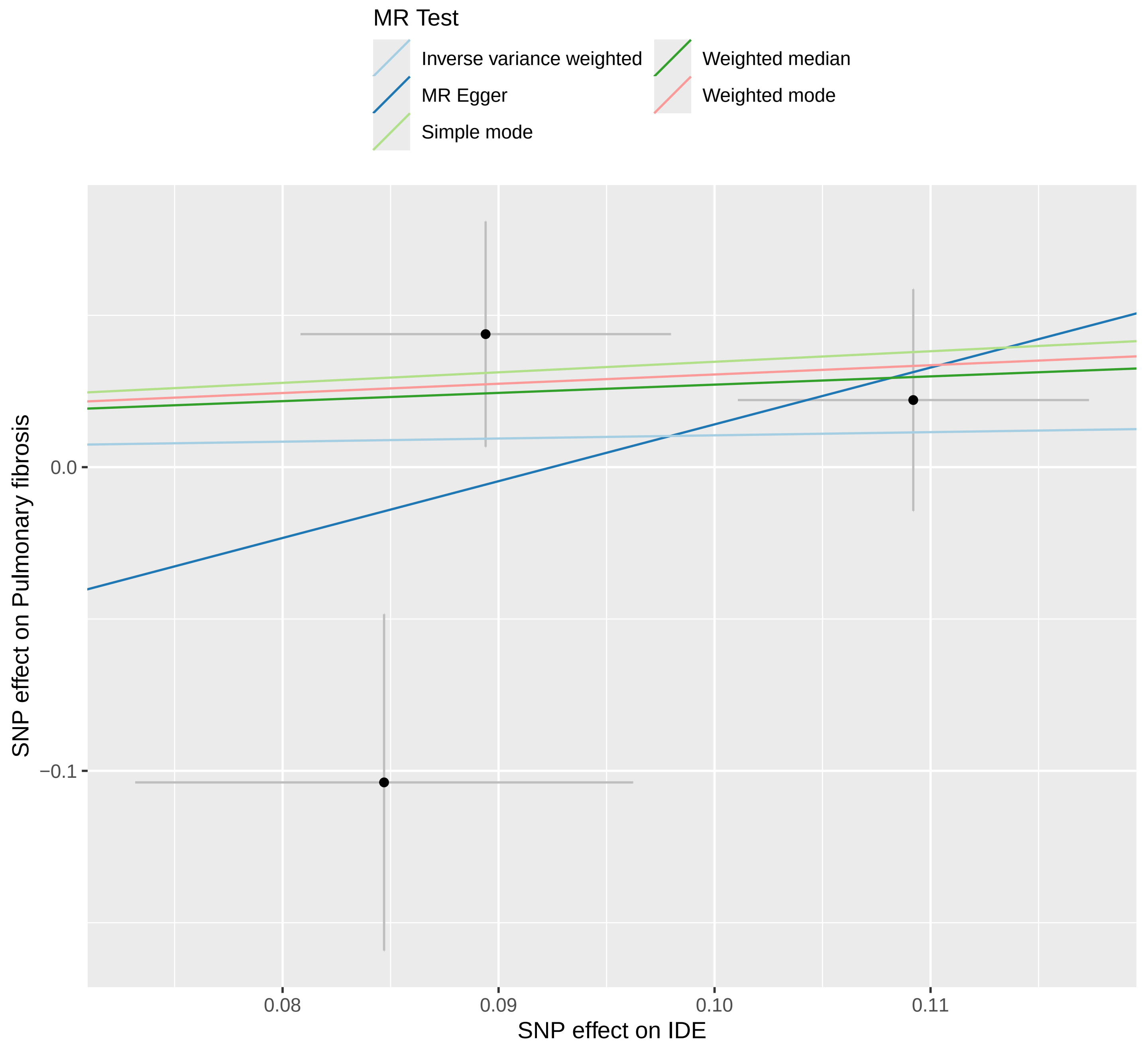

MR Test
Inverse variance weighted
MR Egger
Simple mode
Weighted median
Weighted mode
SNP effect on Pulmonary fibrosis
0.0
−0.1
0.08
0.09
0.10
0.11
SNP effect on IDE

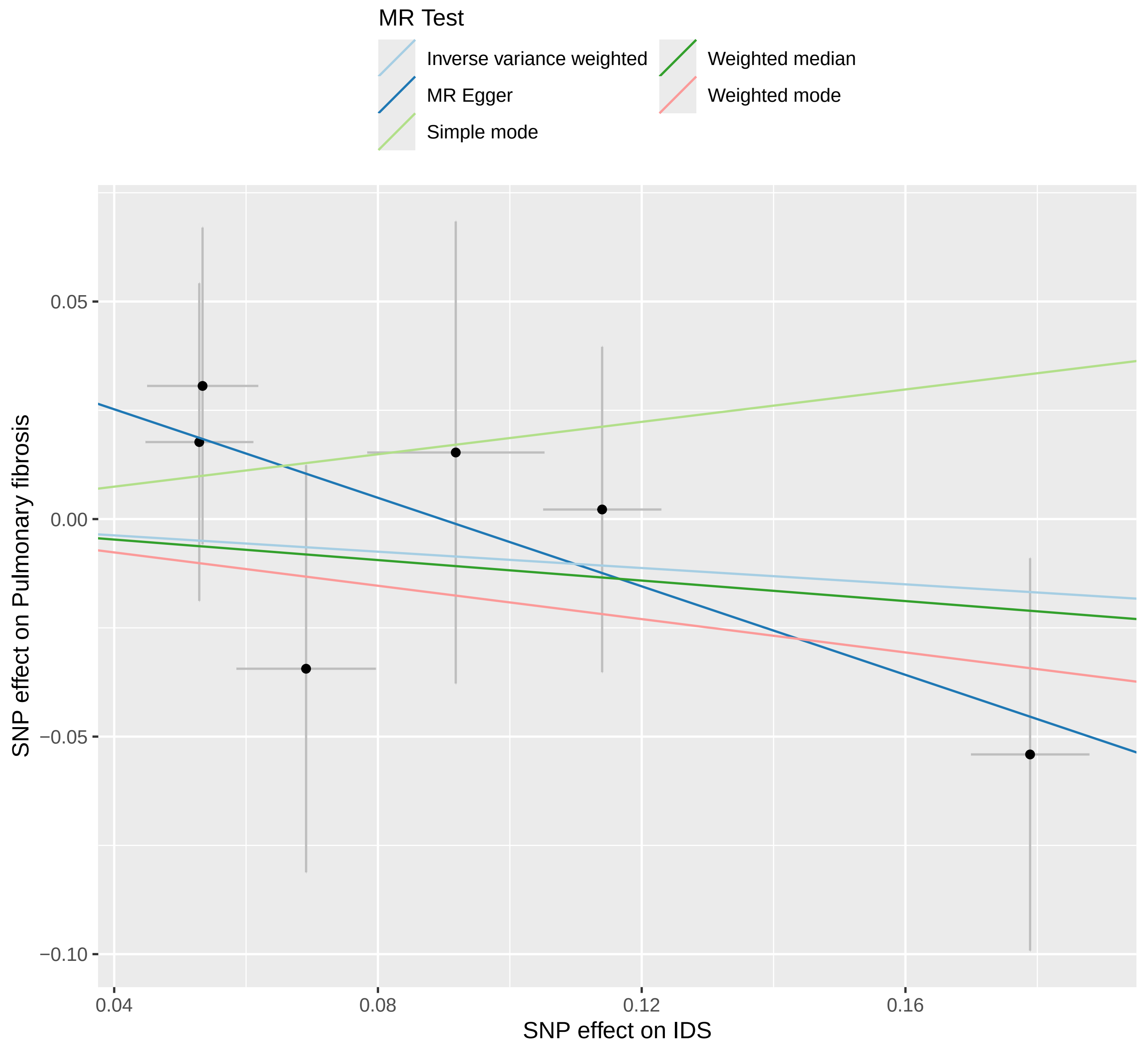

MR Test
Inverse variance weighted
MR Egger
Simple mode
Weighted median
Weighted mode
0.05
0.00
−0.05
−0.10
0.04
0.08
0.12
0.16
SNP effect on Pulmonary fibrosis
SNP effect on IDS

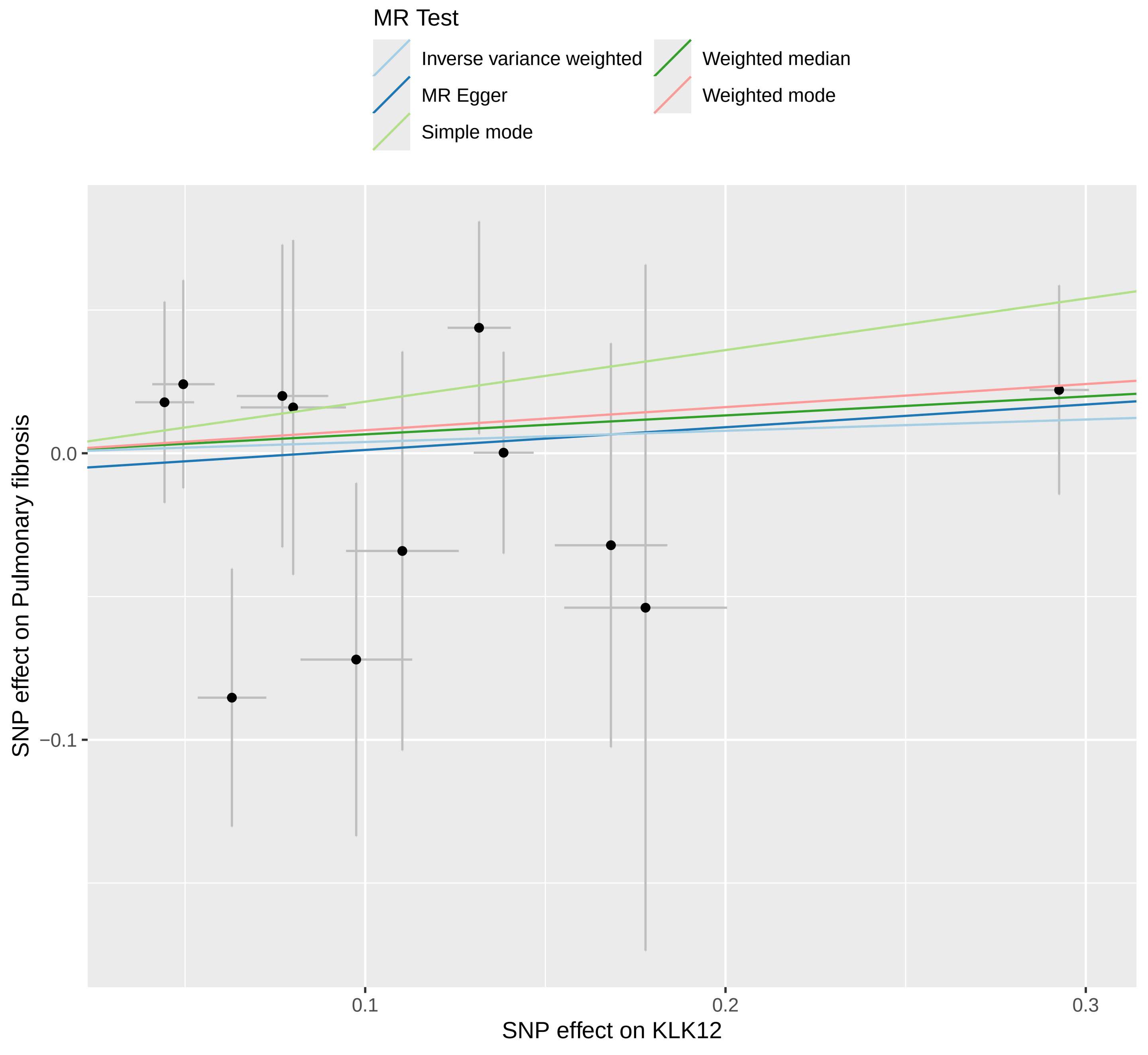

MR Test
Inverse variance weighted
MR Egger
Simple mode
Weighted median
Weighted mode
0.0
−0.1
0.1
0.2
0.3
SNP effect on Pulmonary fibrosis
SNP effect on KLK12

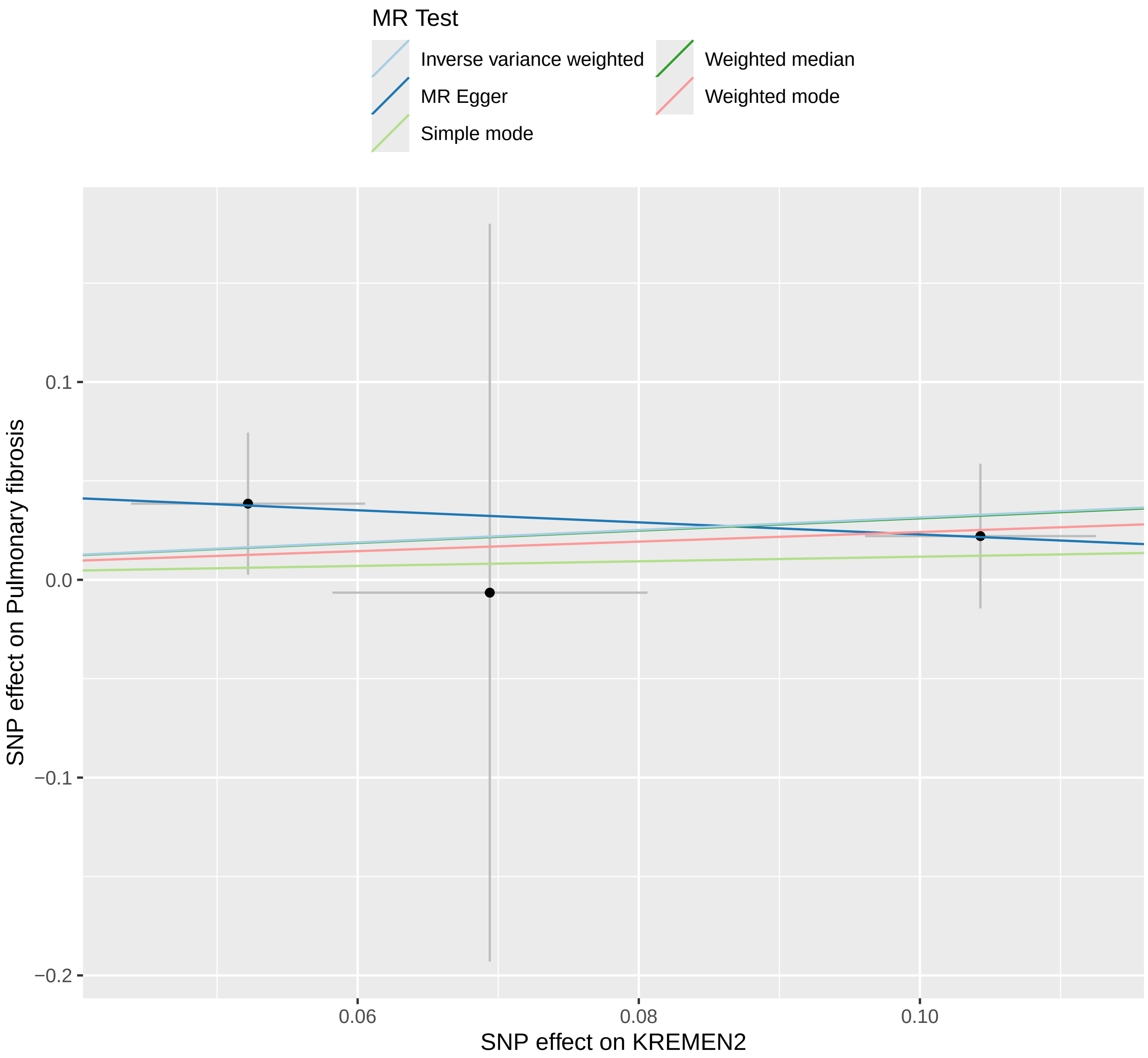
MR Test
Inverse variance weighted
MR Egger
Simple mode
Weighted median
Weighted mode
0.1
0.0
−0.1
−0.2
0.06
0.08
0.10
SNP effect on KREMEN2
SNP effect on Pulmonary fibrosis

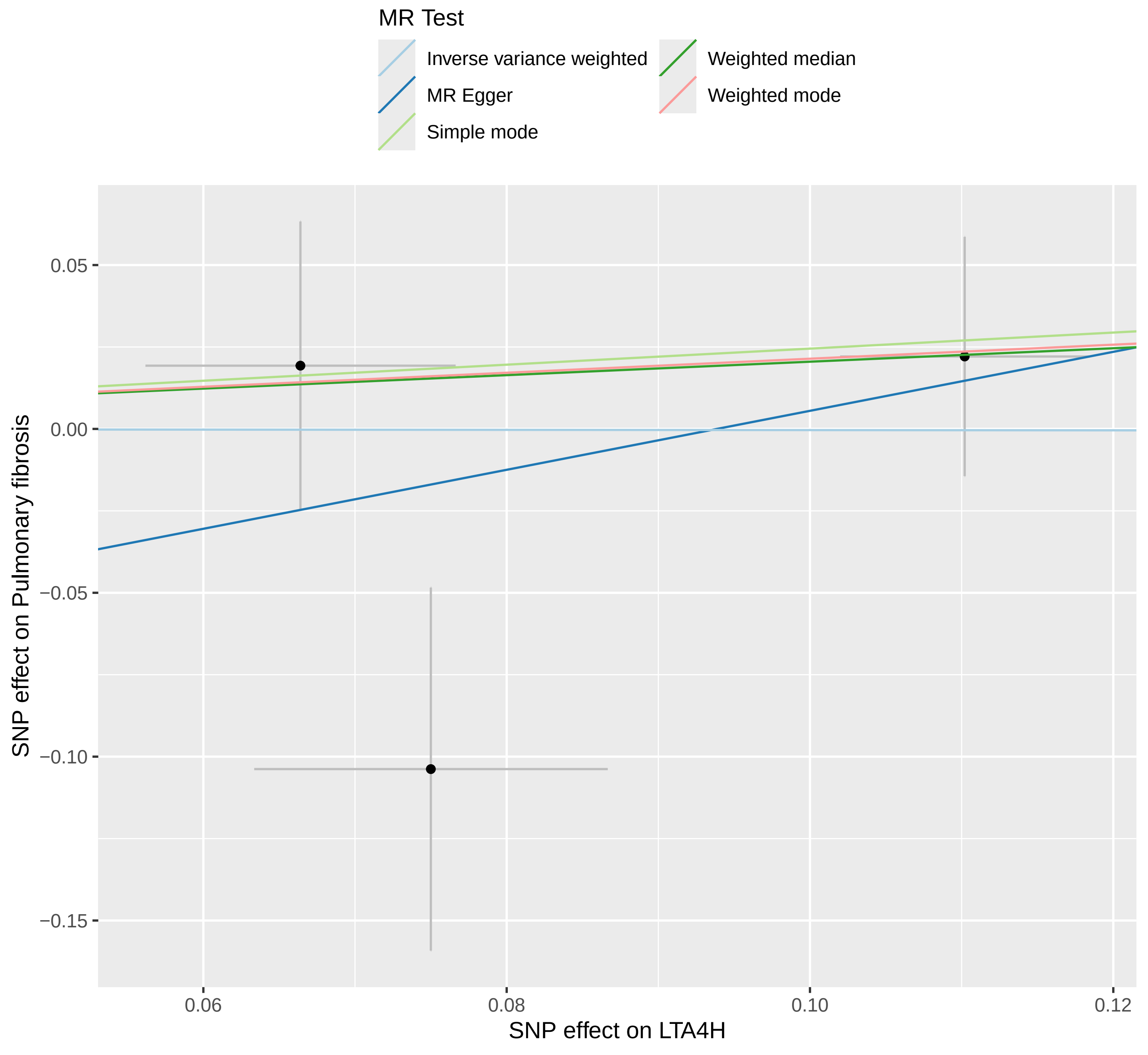

MR Test
Inverse variance weighted
MR Egger
Simple mode
Weighted median
Weighted mode
SNP effect on Pulmonary fibrosis
0.05
0.00
−0.05
−0.10
−0.15
0.06
0.08
0.10
0.12
SNP effect on LTA4H

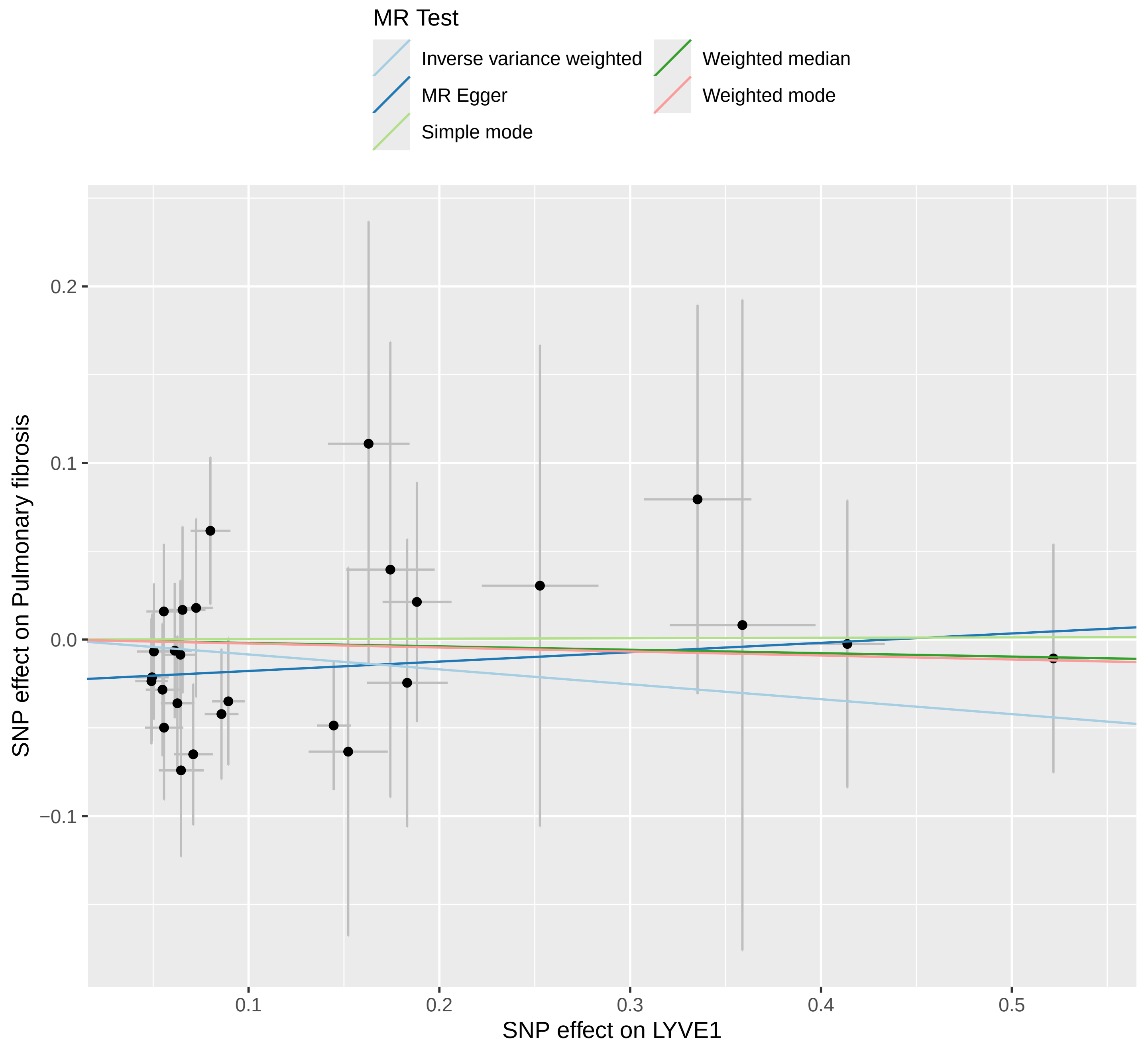
MR Test
Inverse variance weighted
MR Egger
Simple mode
Weighted median
Weighted mode
0.2
0.1
0.0
−0.1
0.1
0.2
0.3
0.4
0.5
SNP effect on LYVE1
SNP effect on Pulmonary fibrosis

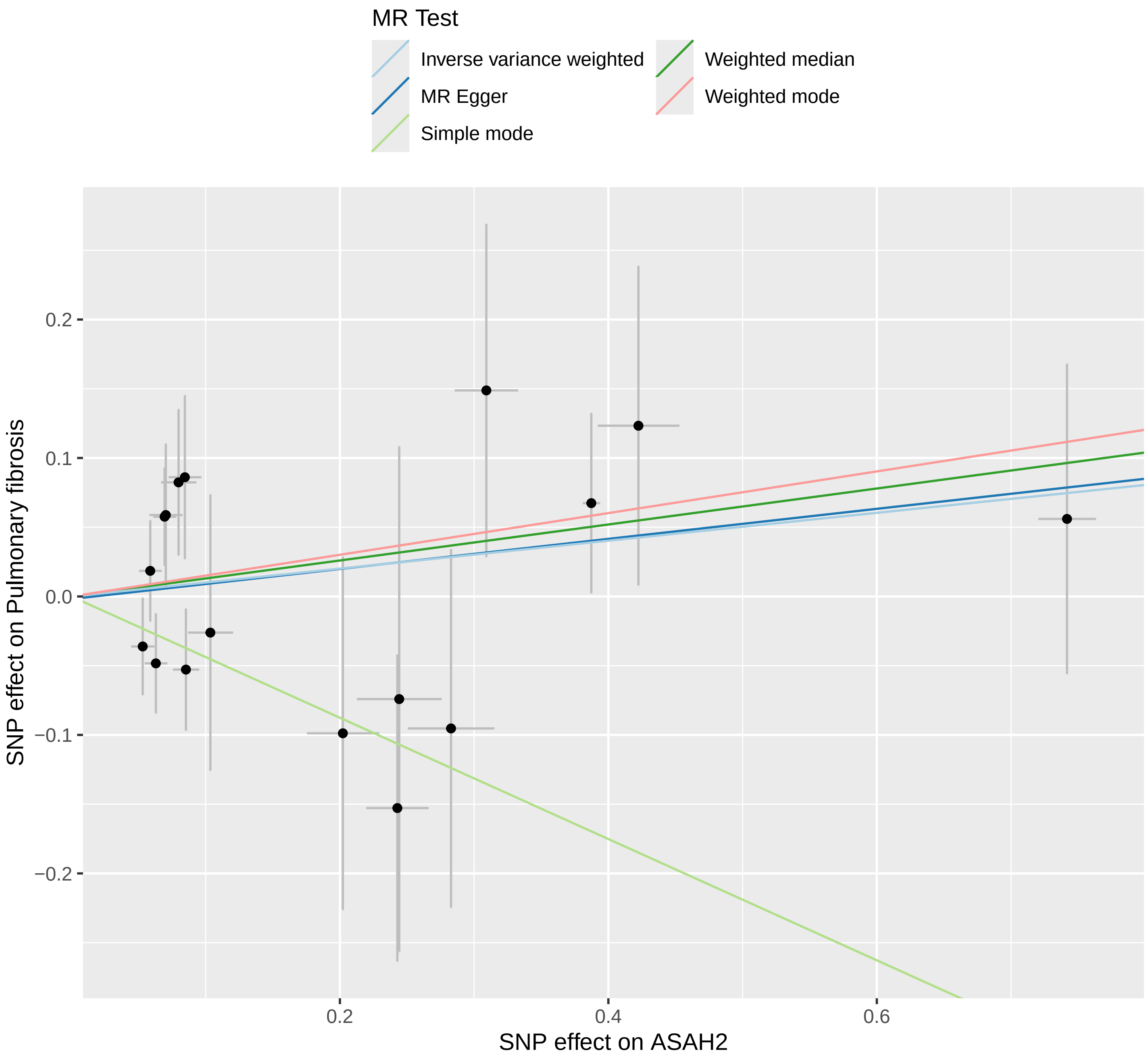
MR Test
Inverse variance weighted
MR Egger
Simple mode
Weighted median
Weighted mode
0.2
0.1
0.0
−0.1
−0.2
SNP effect on Pulmonary fibrosis
0.2
0.4
0.6
SNP effect on ASAH2

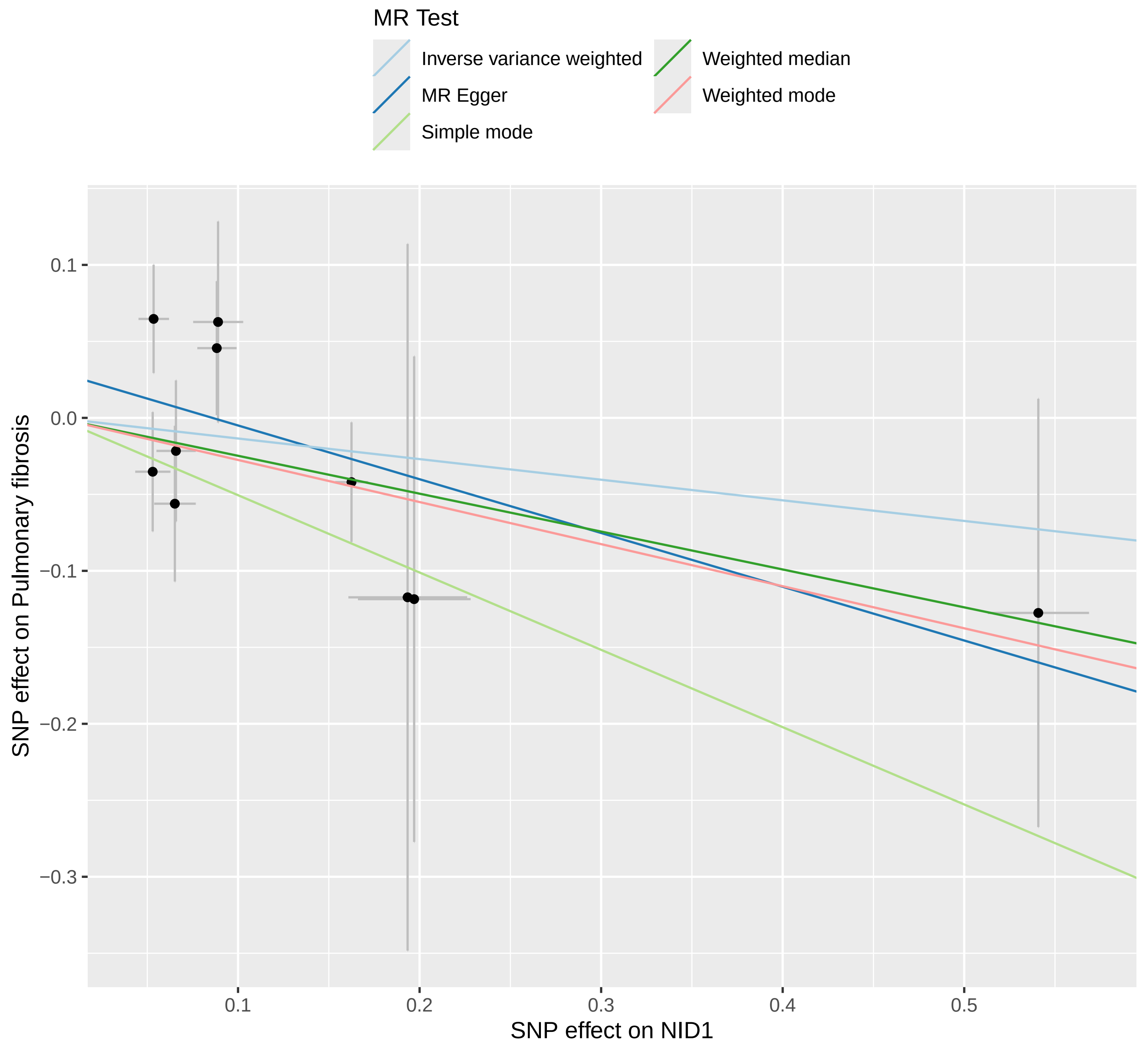
MR Test
Inverse variance weighted
MR Egger
Simple mode
Weighted median
Weighted mode
SNP effect on Pulmonary fibrosis
0.1
0.0
−0.1
−0.2
−0.3
0.1
0.2
0.3
0.4
0.5
SNP effect on NID1

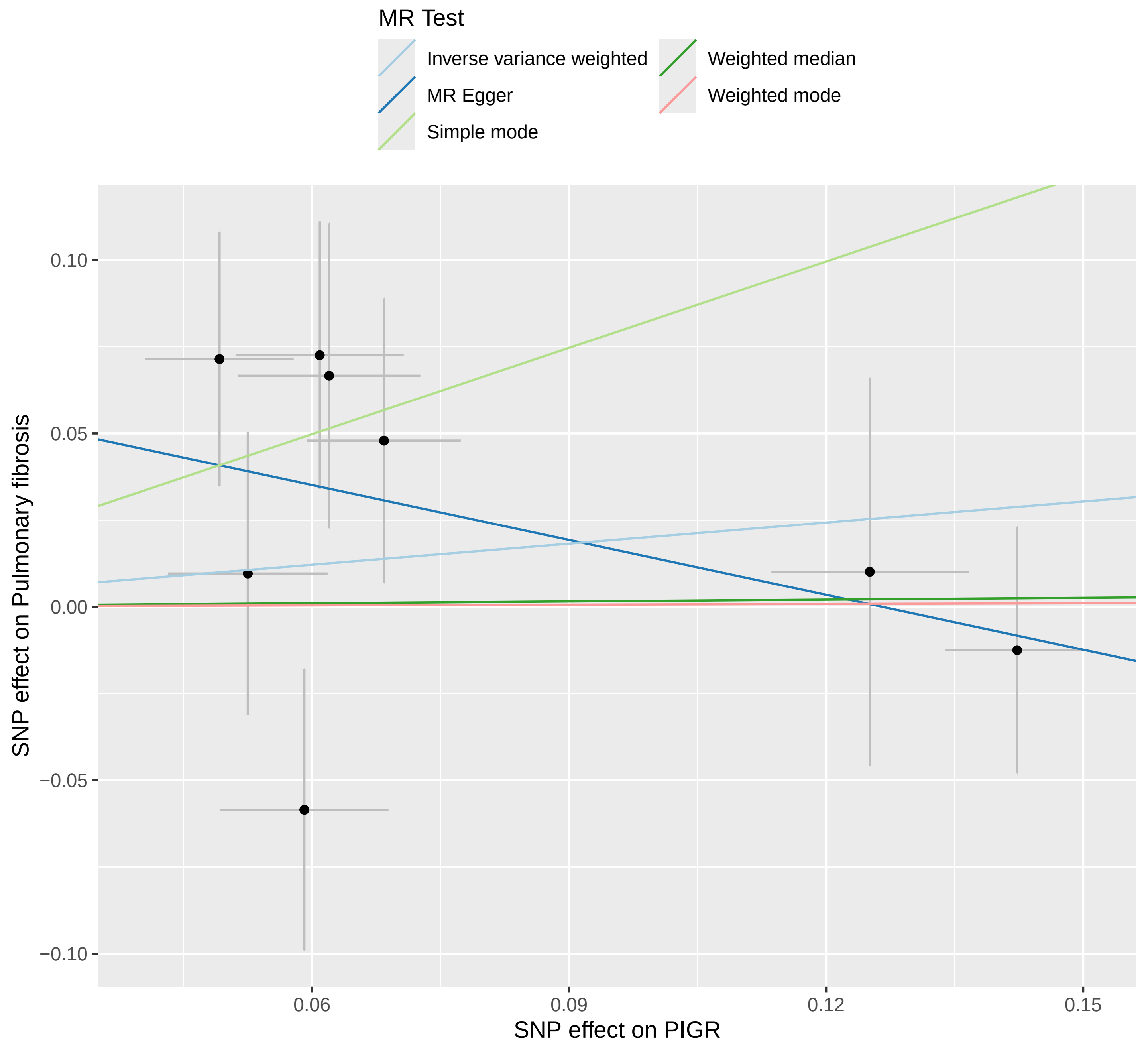
MR Test
Inverse variance weighted
MR Egger
Simple mode
Weighted median
Weighted mode
0.10
0.05
0.00
−0.05
−0.10
SNP effect on Pulmonary fibrosis
0.06
0.09
0.12
0.15
SNP effect on PIGR

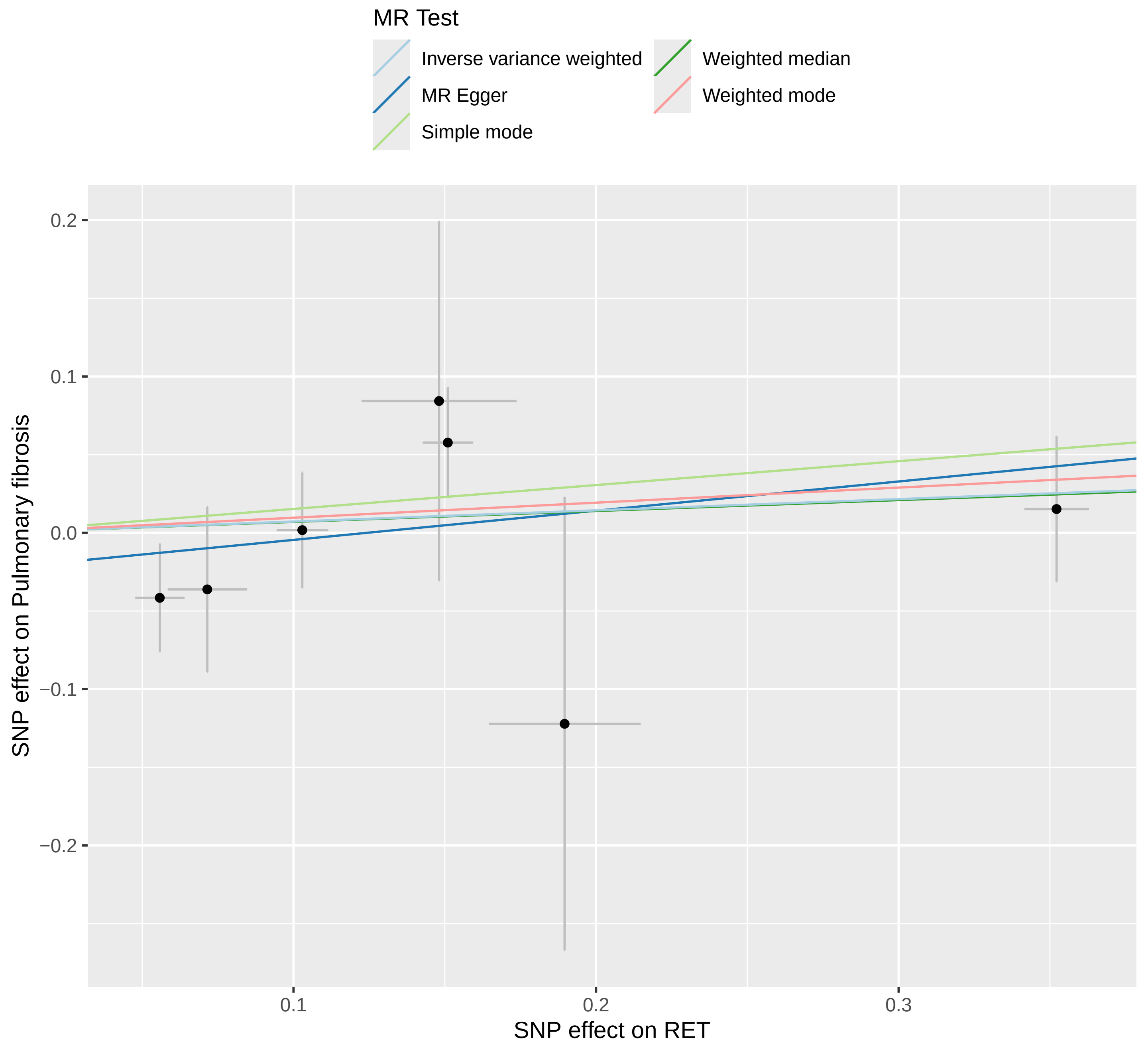
MR Test
Inverse variance weighted
MR Egger
Simple mode
Weighted median
Weighted mode
0.2
0.1
0.0
−0.1
−0.2
0.1
0.2
0.3
SNP effect on Pulmonary fibrosis
SNP effect on RET

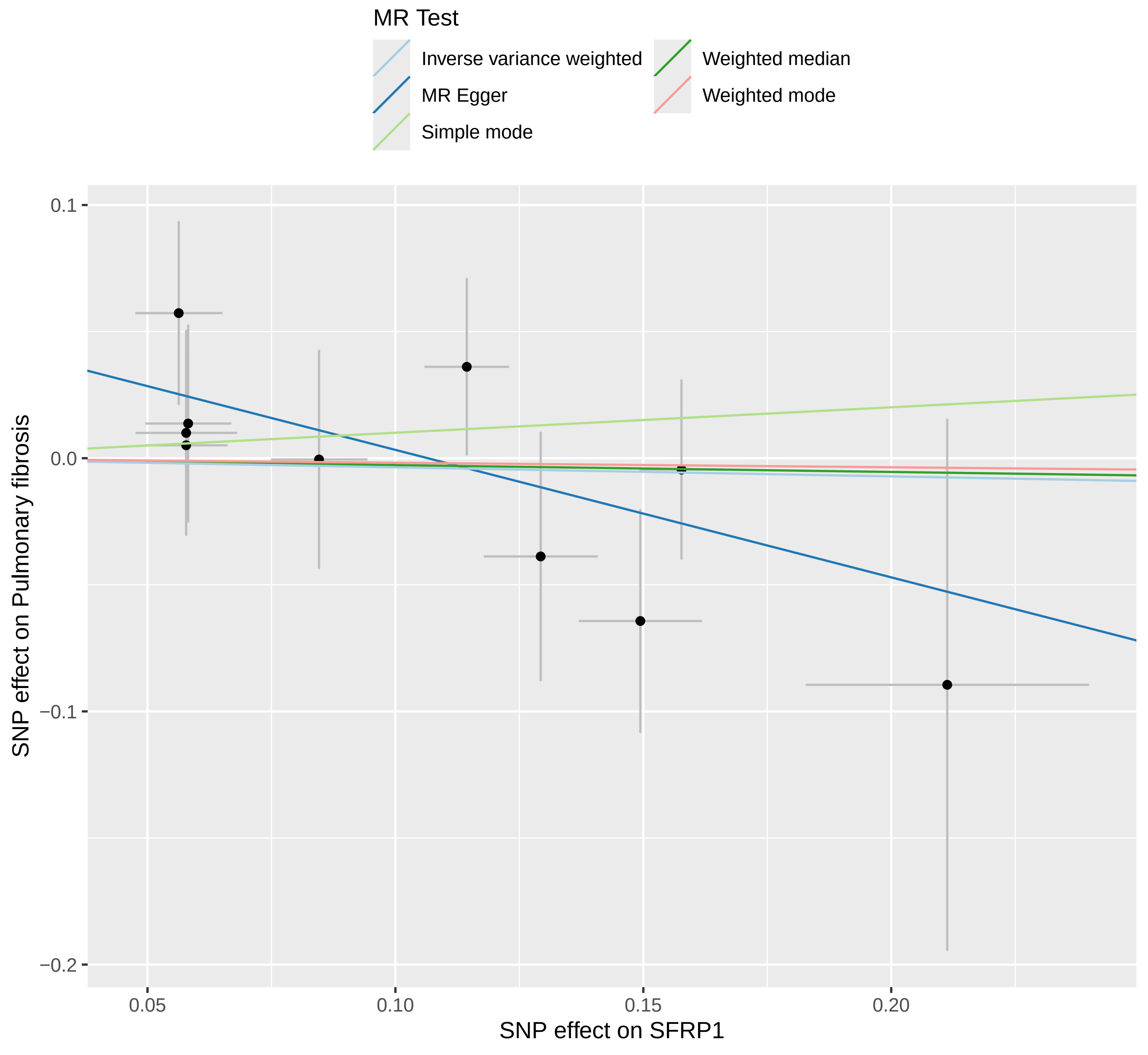
MR Test
Inverse variance weighted
MR Egger
Simple mode
Weighted median
Weighted mode
0.1
0.0
−0.1
−0.2
0.05
0.10
0.15
0.20
SNP effect on SFRP1
SNP effect on Pulmonary fibrosis

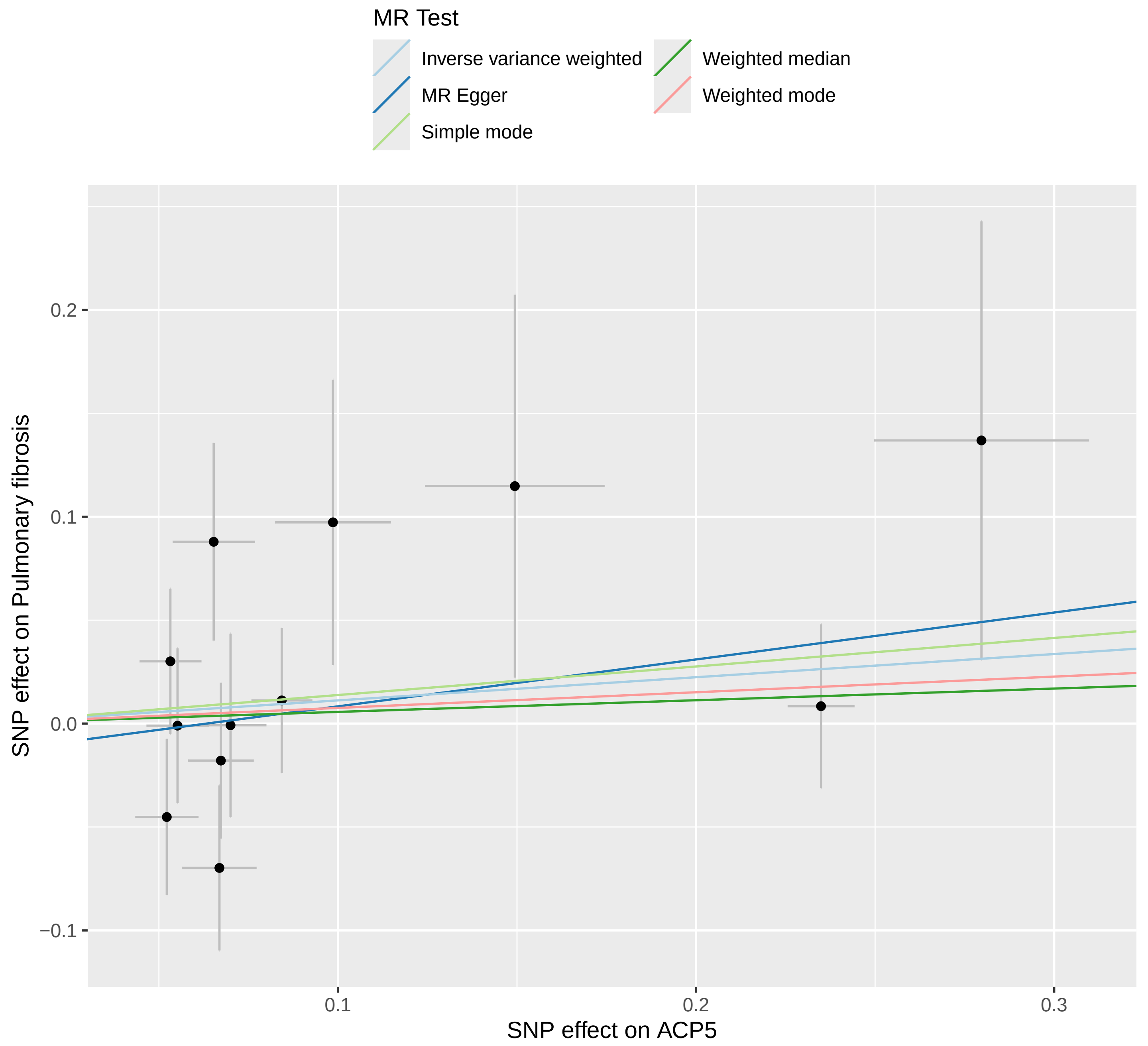

MR Test
Inverse variance weighted
MR Egger
Simple mode
Weighted median
Weighted mode
0.2
0.1
0.0
−0.1
0.1
0.2
0.3
SNP effect on Pulmonary fibrosis
SNP effect on ACP5

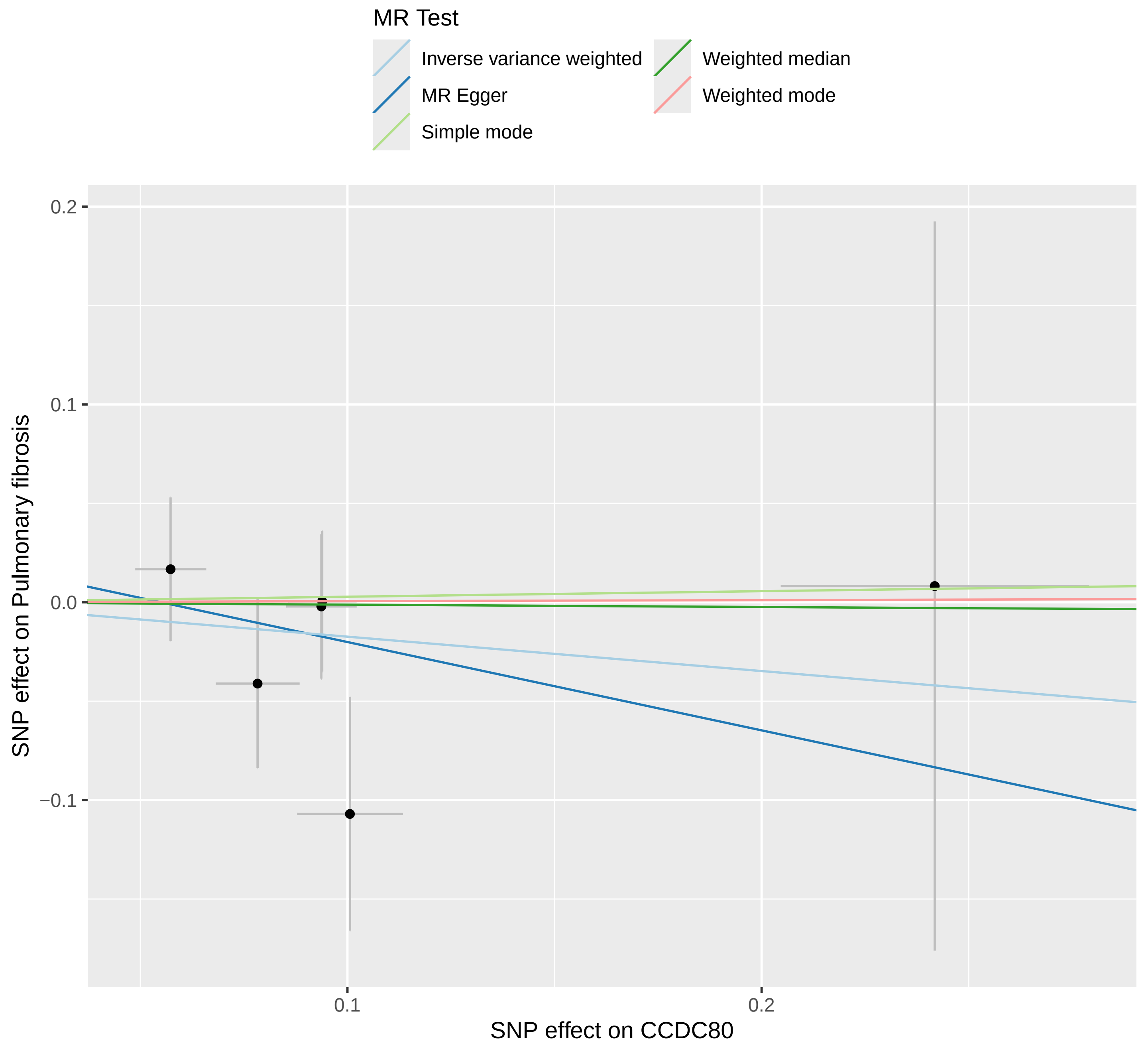
MR Test
Inverse variance weighted
MR Egger
Simple mode
Weighted median
Weighted mode
0.2
0.1
0.0
−0.1
SNP effect on Pulmonary fibrosis
0.1
0.2
SNP effect on CCDC80

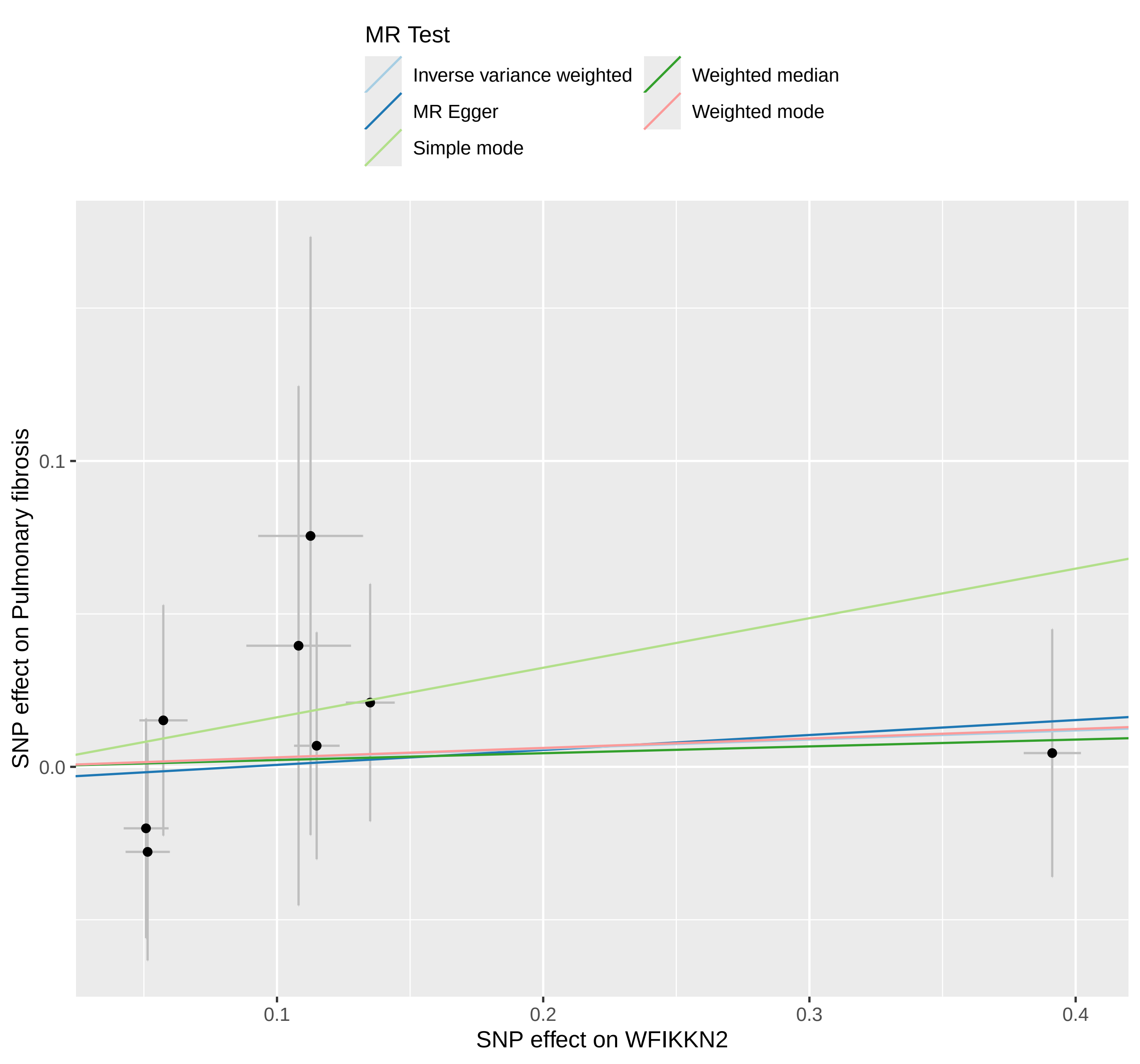
MR Test
Inverse variance weighted
MR Egger
Simple mode
Weighted median
Weighted mode
SNP effect on Pulmonary fibrosis
0.1
0.0
0.1
0.2
0.3
0.4
SNP effect on WFIKKN2

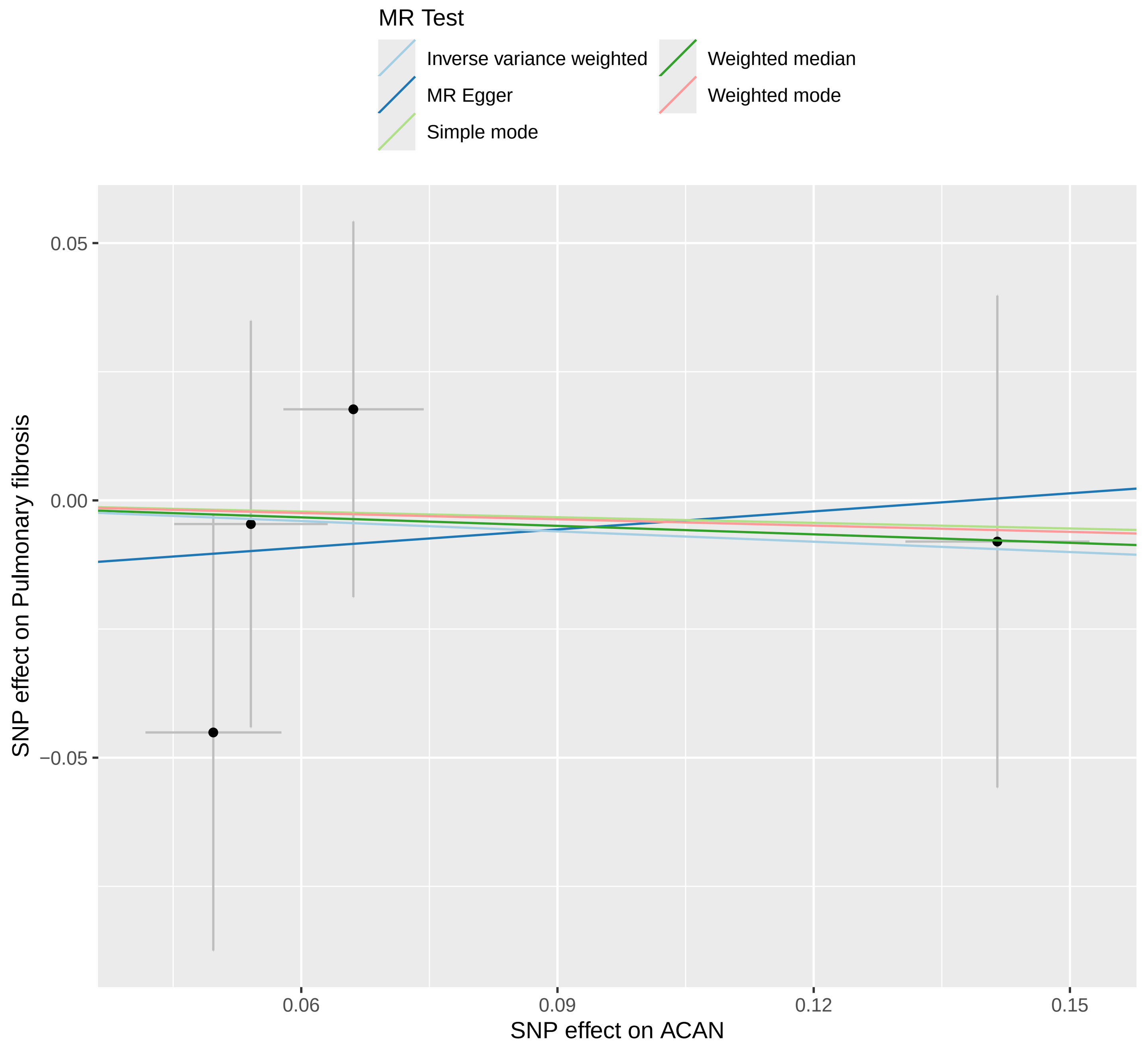

MR Test
Inverse variance weighted
MR Egger
Simple mode
Weighted median
Weighted mode
0.05
0.00
−0.05
0.06
0.09
0.12
0.15
SNP effect on Pulmonary fibrosis
SNP effect on ACAN

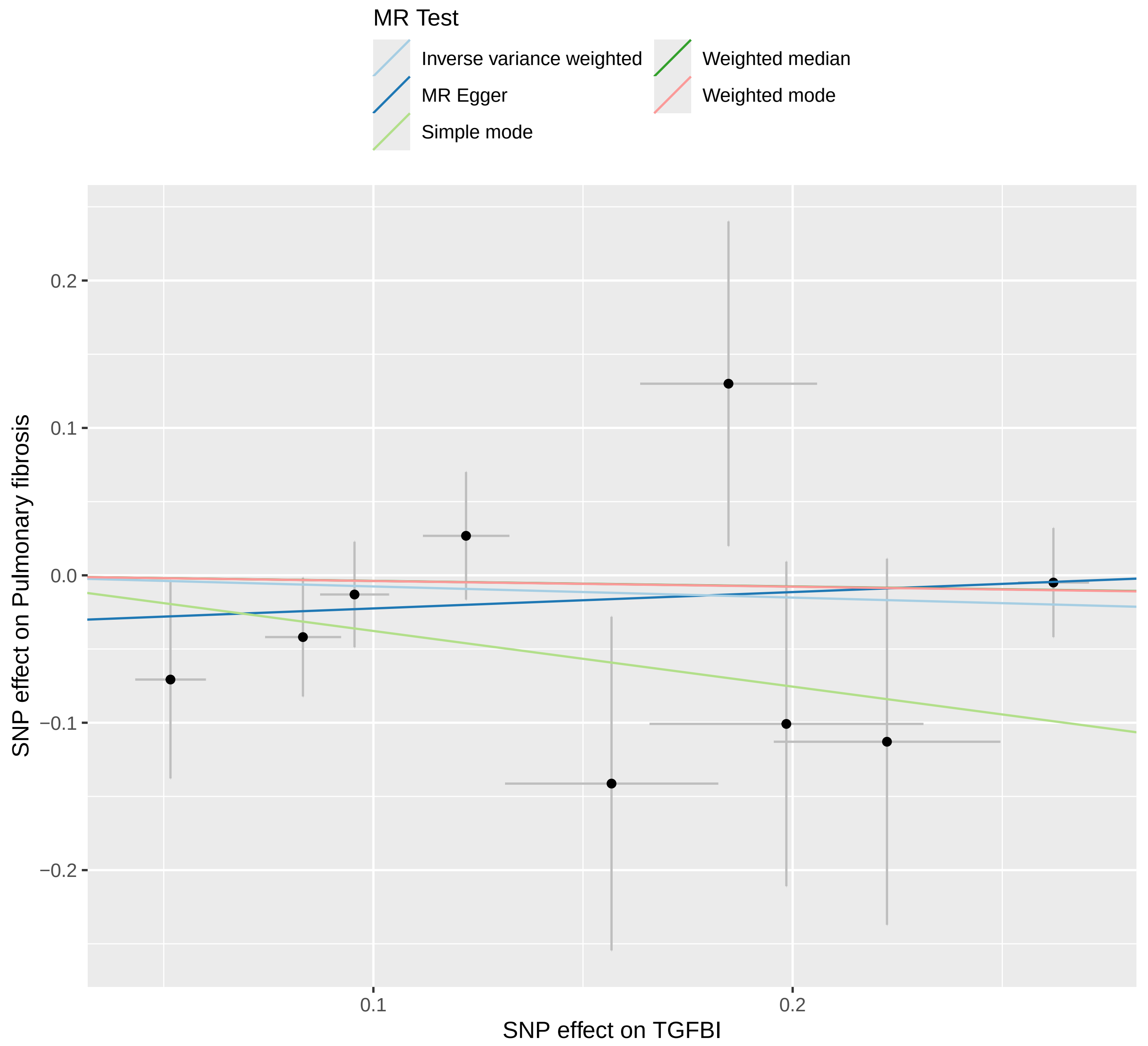
MR Test
Inverse variance weighted
MR Egger
Simple mode
Weighted median
Weighted mode
0.2
0.1
0.0
−0.1
−0.2
SNP effect on Pulmonary fibrosis
0.1
0.2
SNP effect on TGFBI

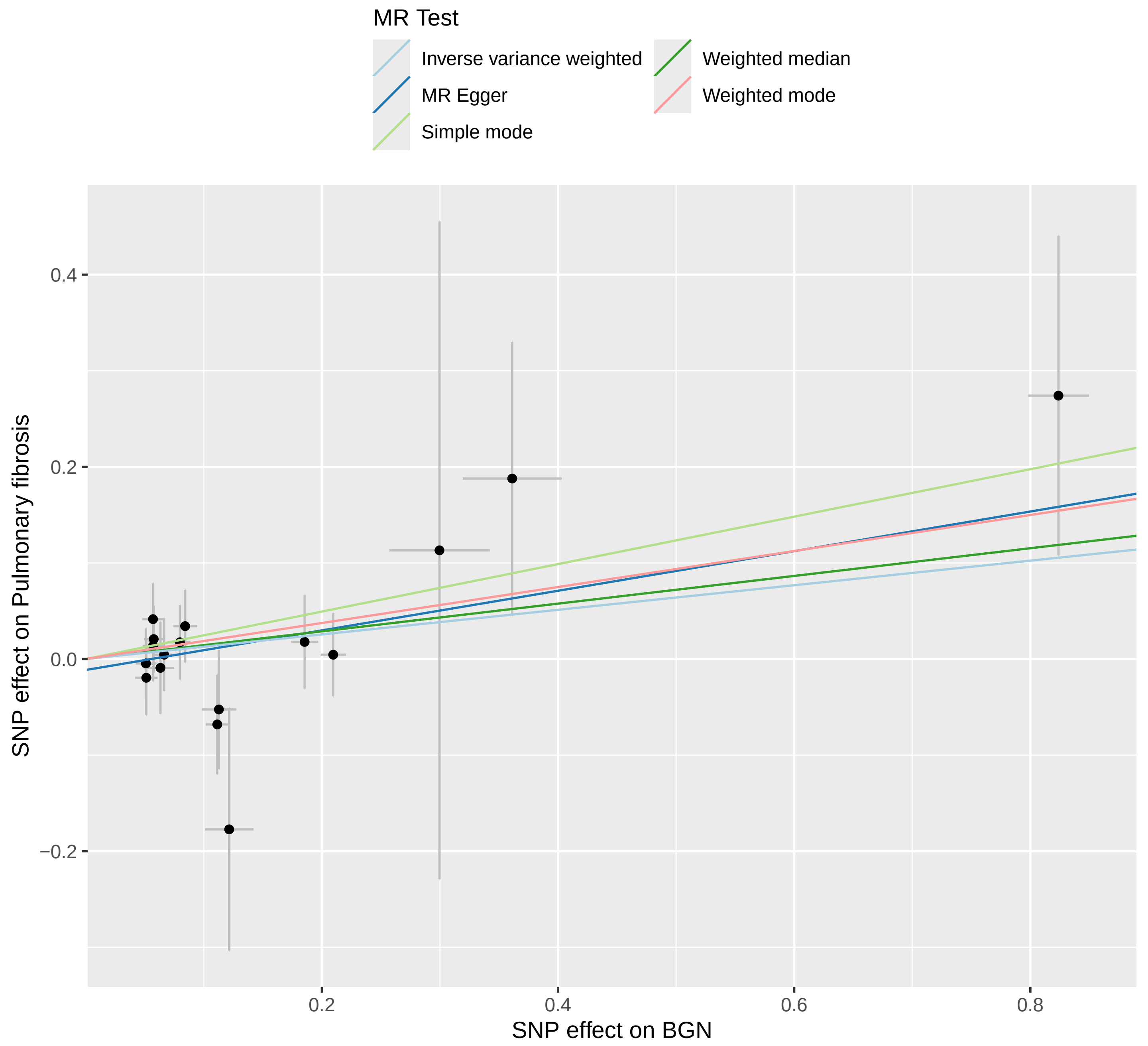
MR Test
Inverse variance weighted
MR Egger
Simple mode
Weighted median
Weighted mode
0.4
0.2
0.0
−0.2
0.2
0.4
0.6
0.8
SNP effect on Pulmonary fibrosis
SNP effect on BGN

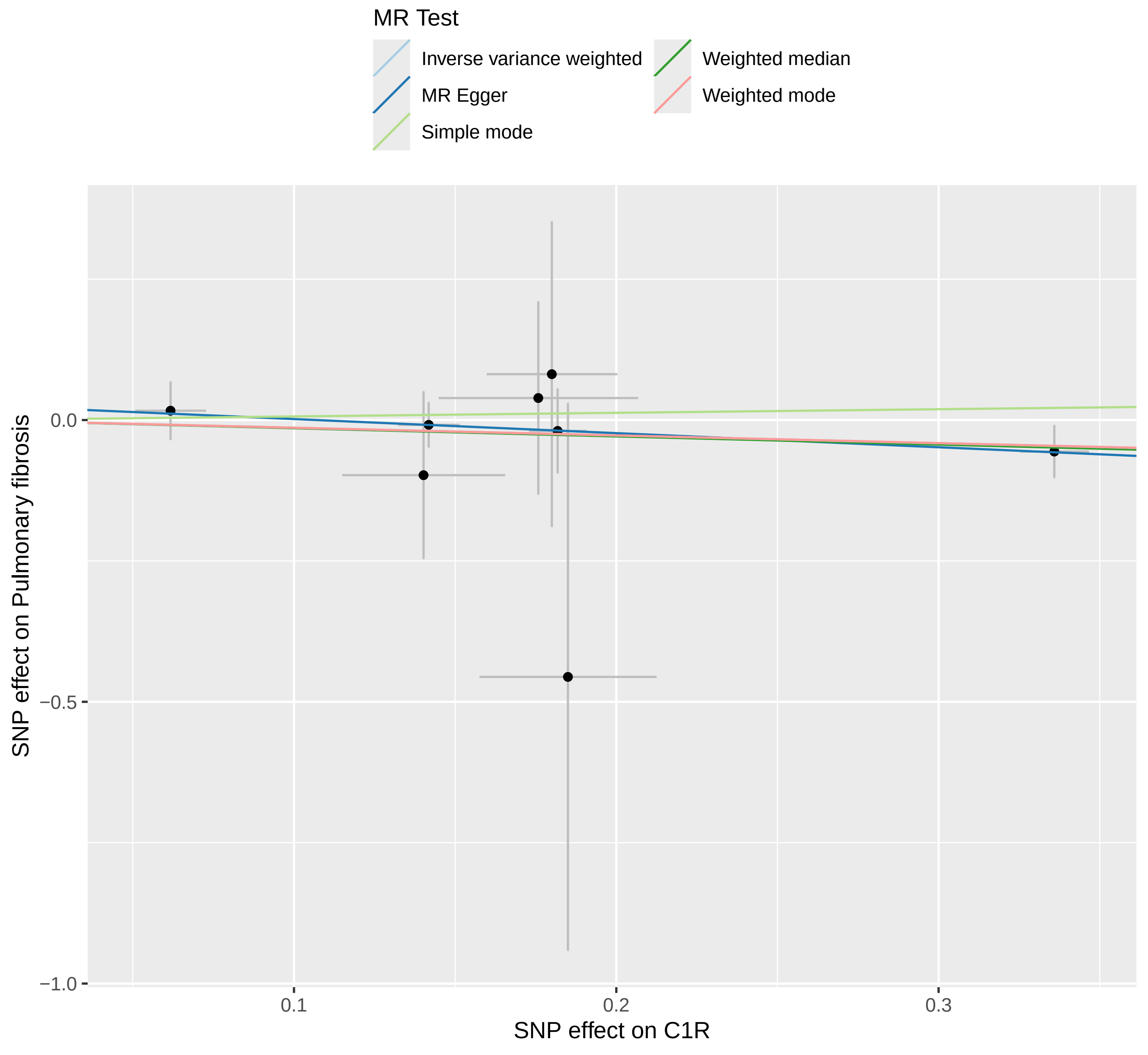
MR Test
Inverse variance weighted
MR Egger
Simple mode
Weighted median
Weighted mode
0.0
−0.5
−1.0
0.1
0.2
0.3
SNP effect on Pulmonary fibrosis
SNP effect on C1R

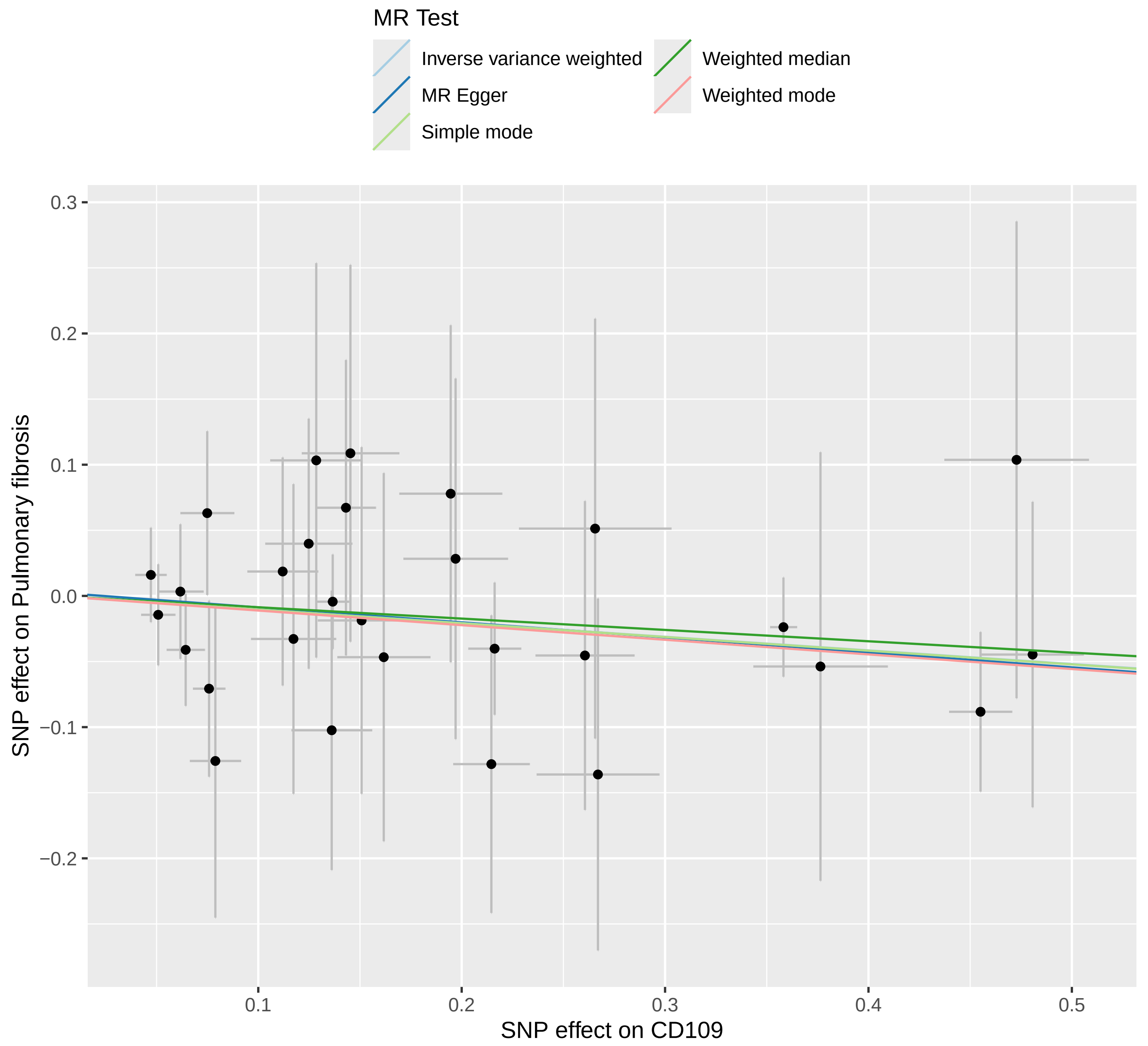
MR Test
Inverse variance weighted
MR Egger
Simple mode
Weighted median
Weighted mode
SNP effect on Pulmonary fibrosis
0.3
0.2
0.1
0.0
−0.1
−0.2
0.1
0.2
0.3
0.4
0.5
SNP effect on CD109

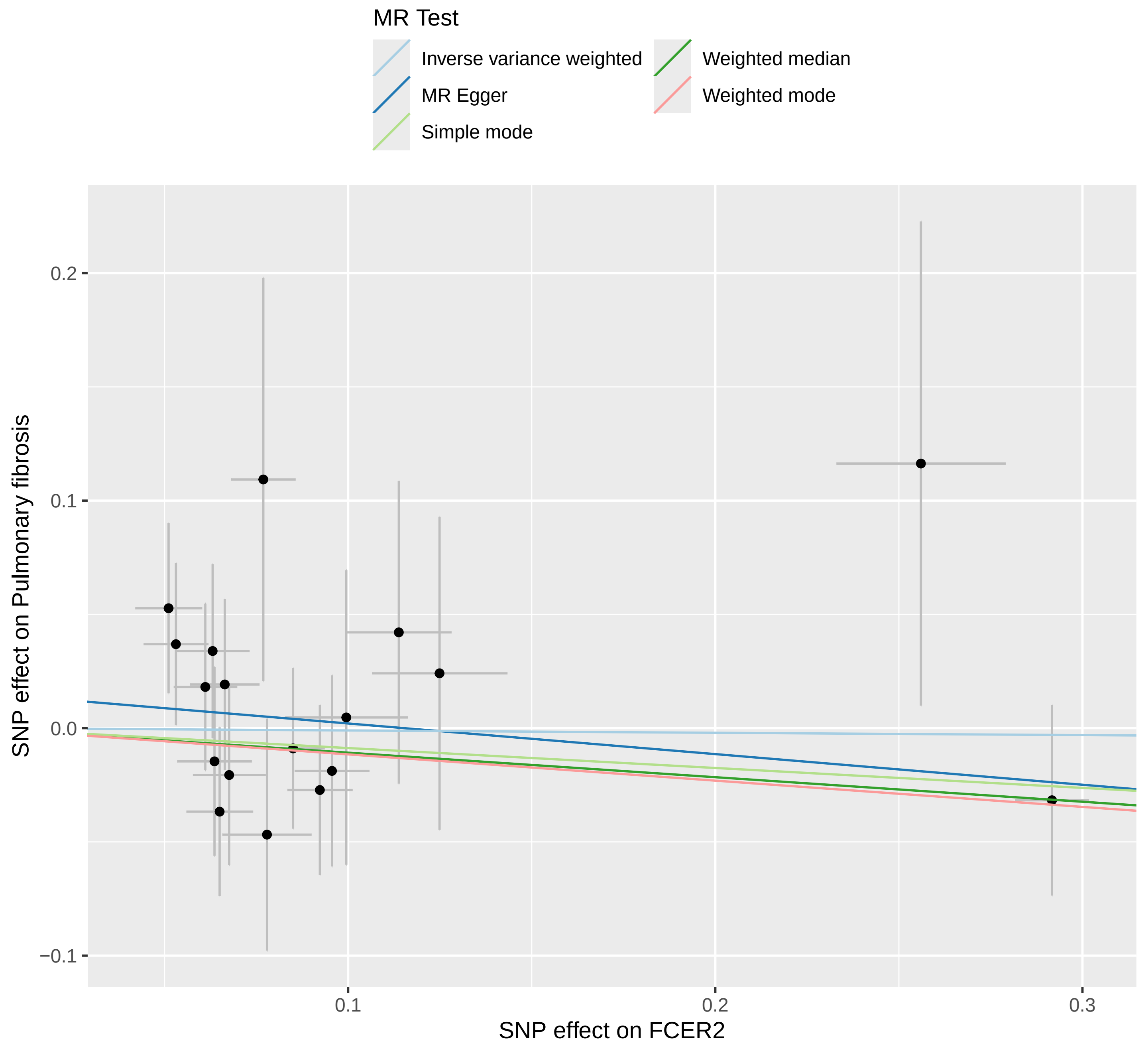
MR Test
Inverse variance weighted
MR Egger
Simple mode
Weighted median
Weighted mode
0.2
0.1
0.0
−0.1
0.1
0.2
0.3
SNP effect on Pulmonary fibrosis
SNP effect on FCER2

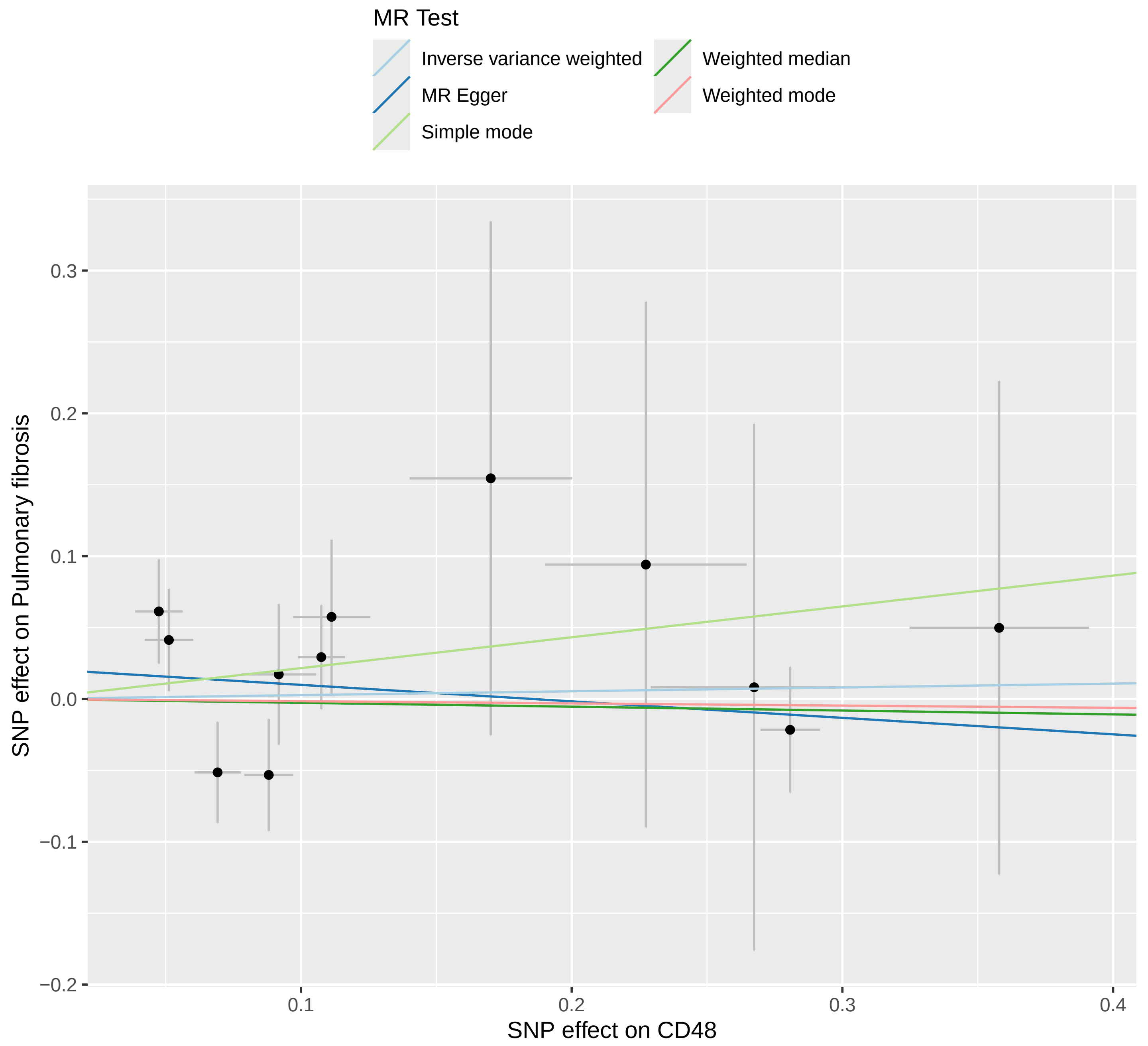

MR Test
Inverse variance weighted
MR Egger
Simple mode
Weighted median
Weighted mode
SNP effect on Pulmonary fibrosis
SNP effect on CD48
0.3
0.2
0.1
0.0
−0.1
−0.2
0.1
0.2
0.3
0.4

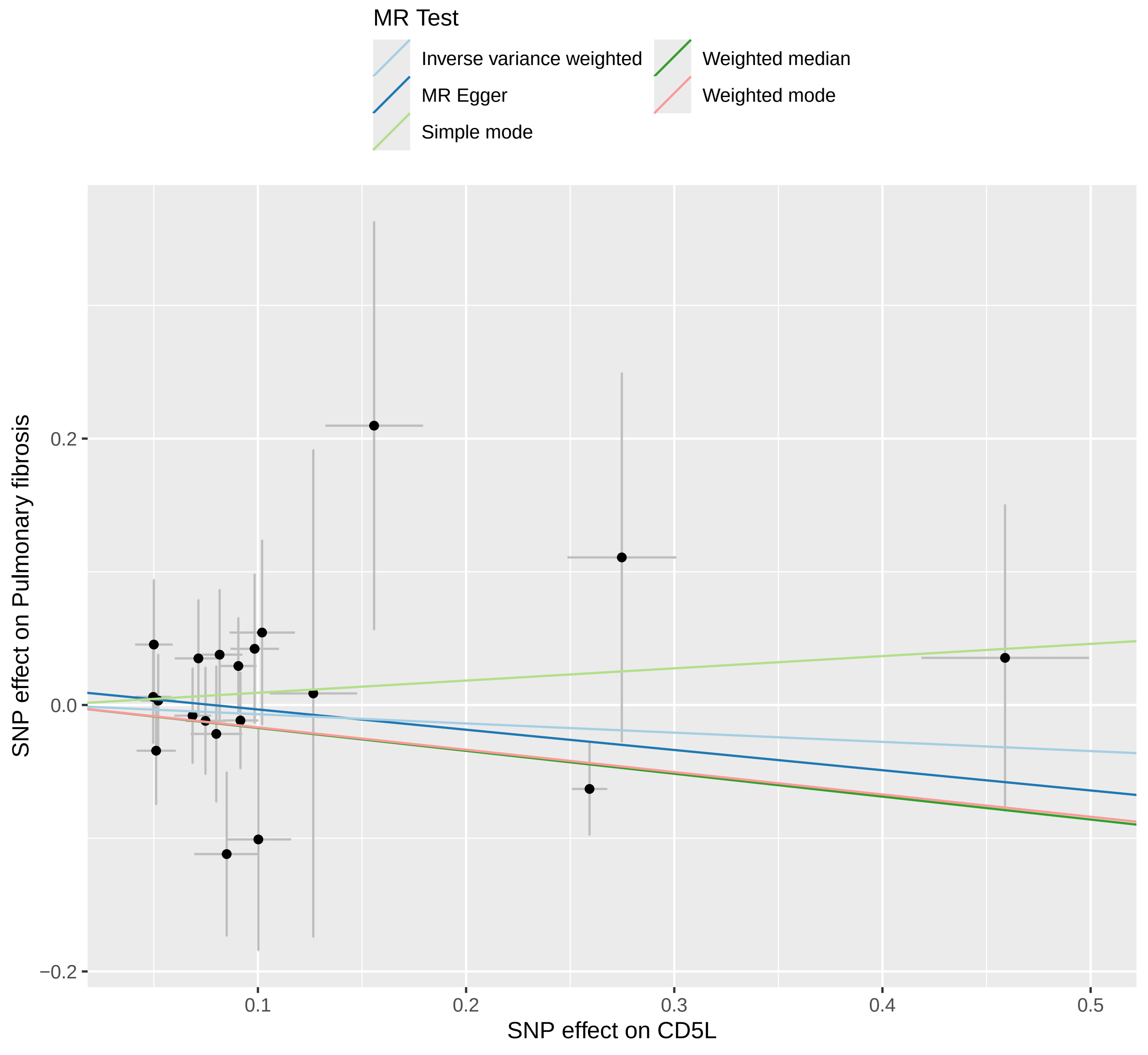

MR Test
Inverse variance weighted
MR Egger
Simple mode
Weighted median
Weighted mode
SNP effect on Pulmonary fibrosis
0.2
0.0
−0.2
0.1
0.2
0.3
0.4
0.5
SNP effect on CD5L

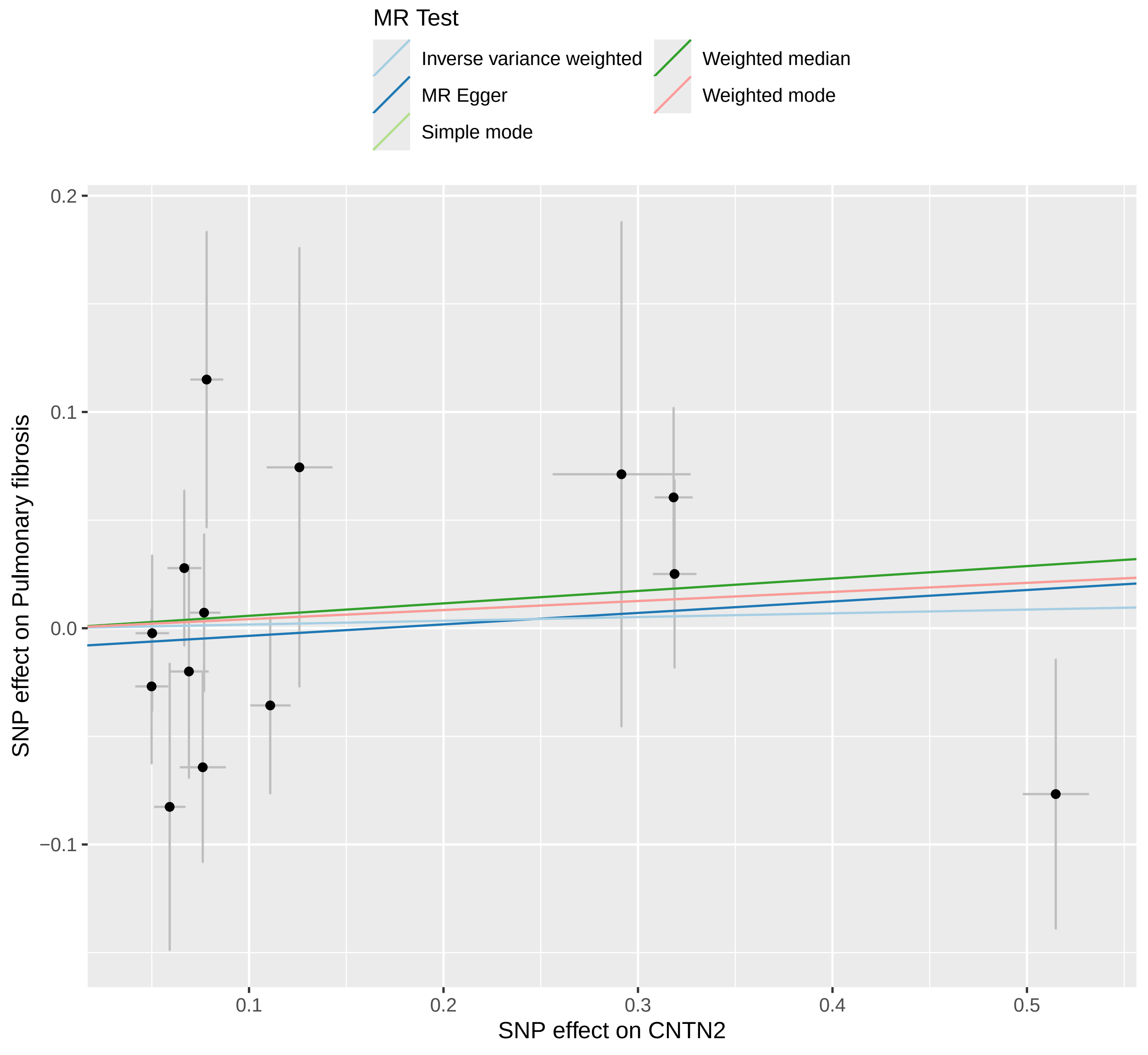

MR Test
Inverse variance weighted
MR Egger
Simple mode
Weighted median
Weighted mode
0.2
0.1
0.0
−0.1
SNP effect on Pulmonary fibrosis
0.1
0.2
0.3
0.4
0.5
SNP effect on CNTN2

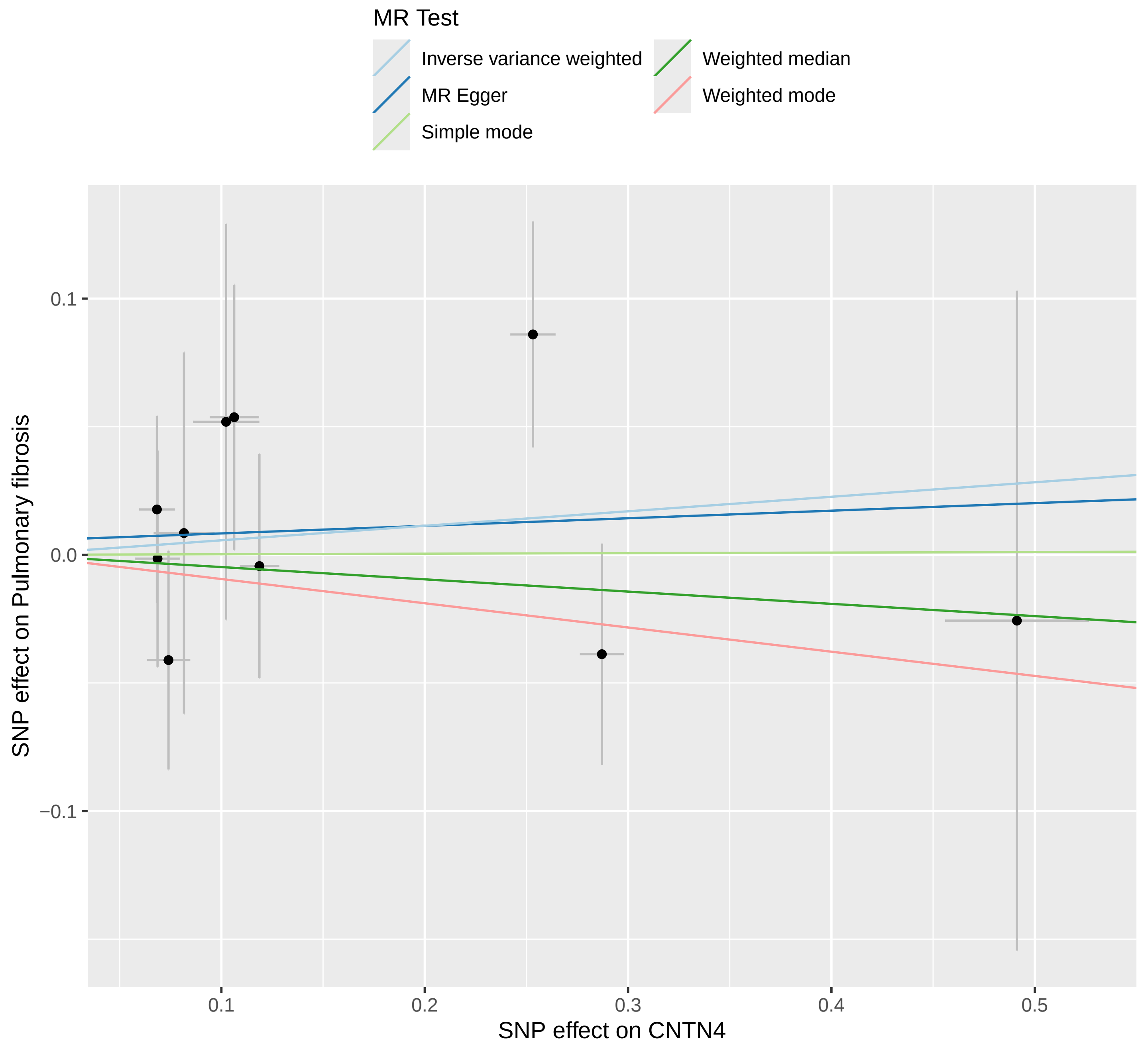

MR Test
Inverse variance weighted
MR Egger
Simple mode
Weighted median
Weighted mode
0.1
0.0
−0.1
SNP effect on Pulmonary fibrosis
0.1
0.2
0.3
0.4
0.5
SNP effect on CNTN4

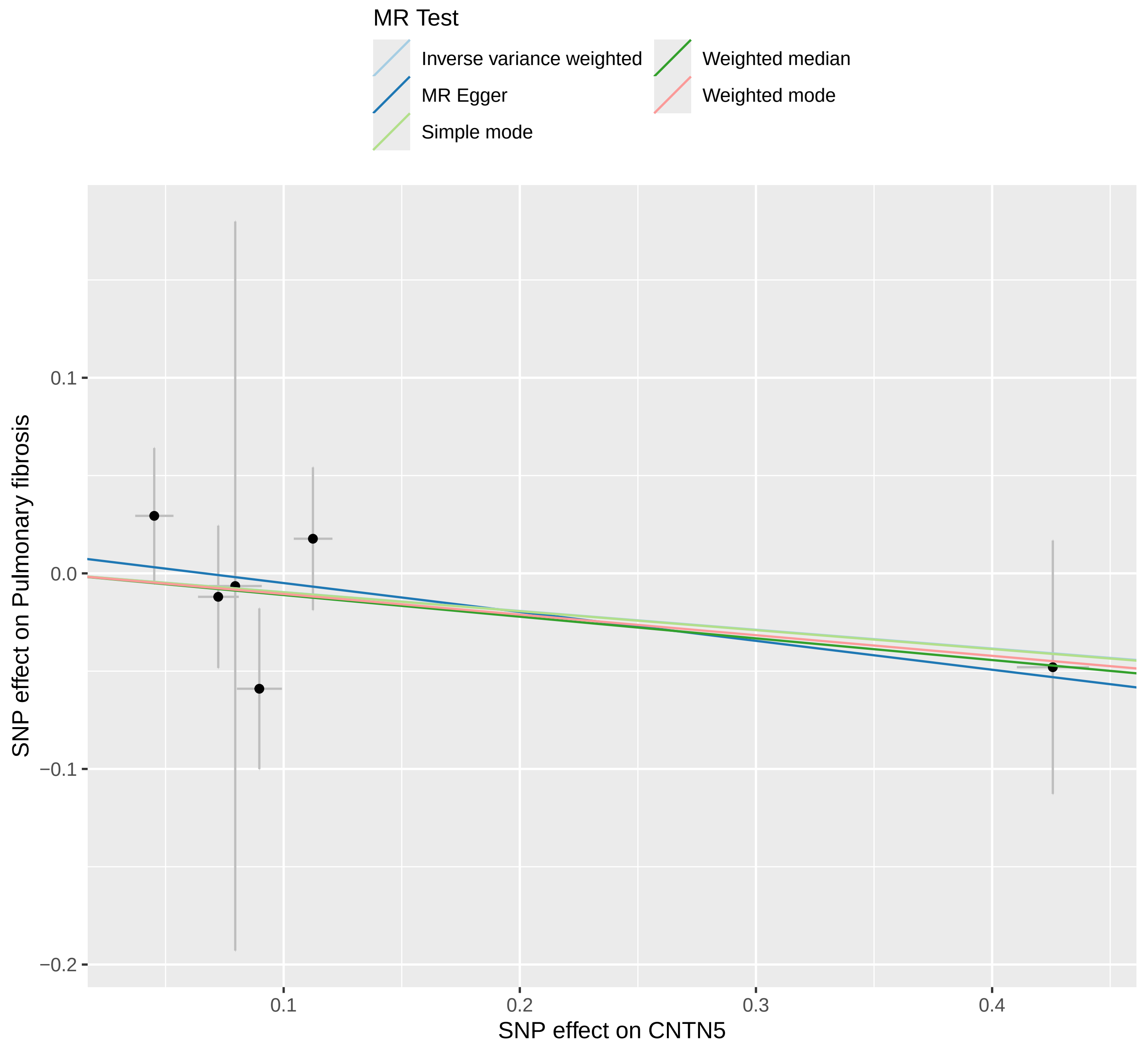

MR Test
Inverse variance weighted
MR Egger
Simple mode
Weighted median
Weighted mode
SNP effect on Pulmonary fibrosis
0.1
0.0
−0.1
−0.2
0.1
0.2
0.3
0.4
SNP effect on CNTN5

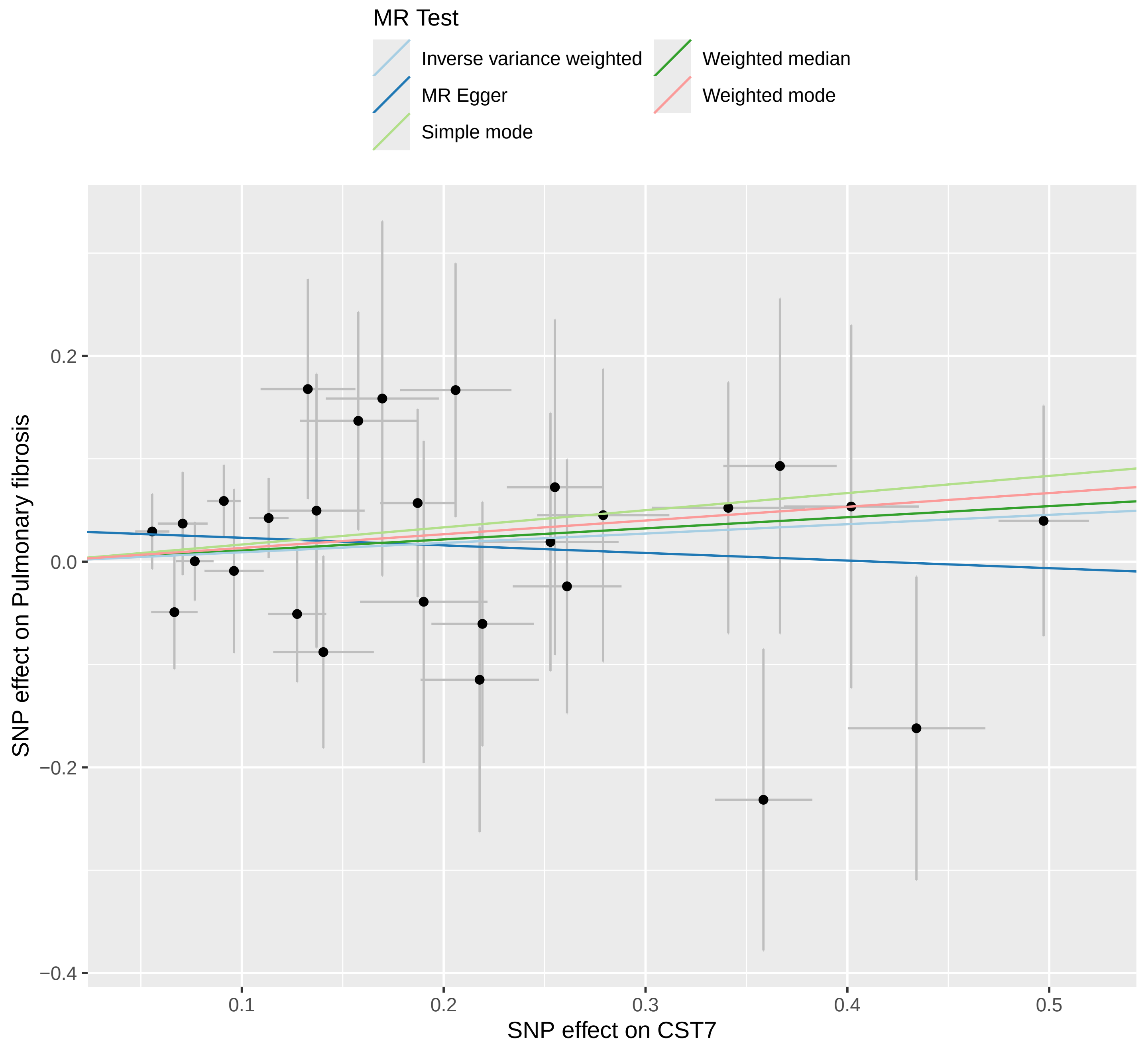
MR Test
Inverse variance weighted
MR Egger
Simple mode
Weighted median
Weighted mode
SNP effect on Pulmonary fibrosis
0.2
0.0
−0.2
−0.4
0.1
0.2
0.3
0.4
0.5
SNP effect on CST7

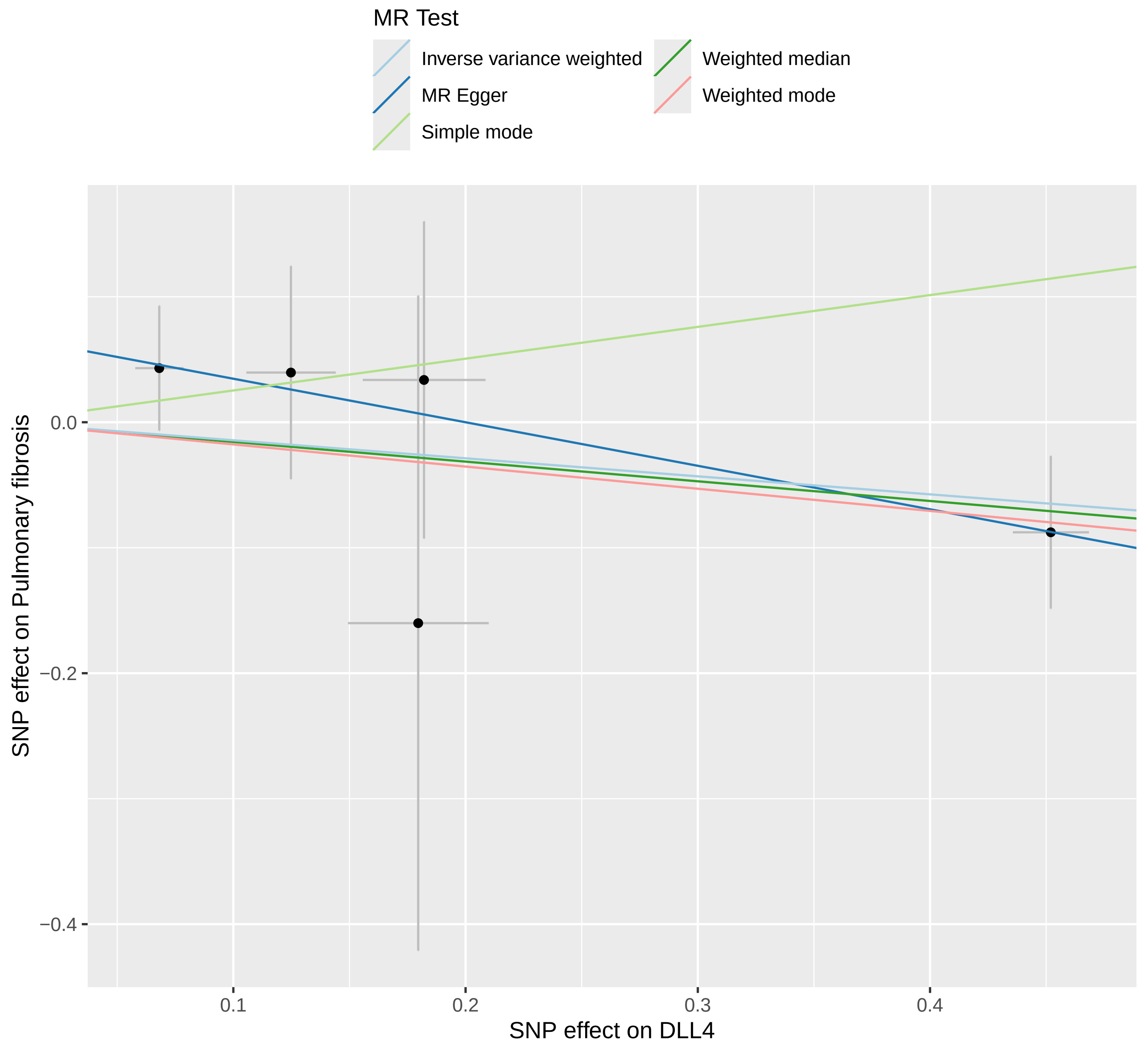

MR Test
Inverse variance weighted
MR Egger
Simple mode
Weighted median
Weighted mode
SNP effect on Pulmonary fibrosis
0.0
−0.2
−0.4
0.1
0.2
0.3
0.4
SNP effect on DLL4

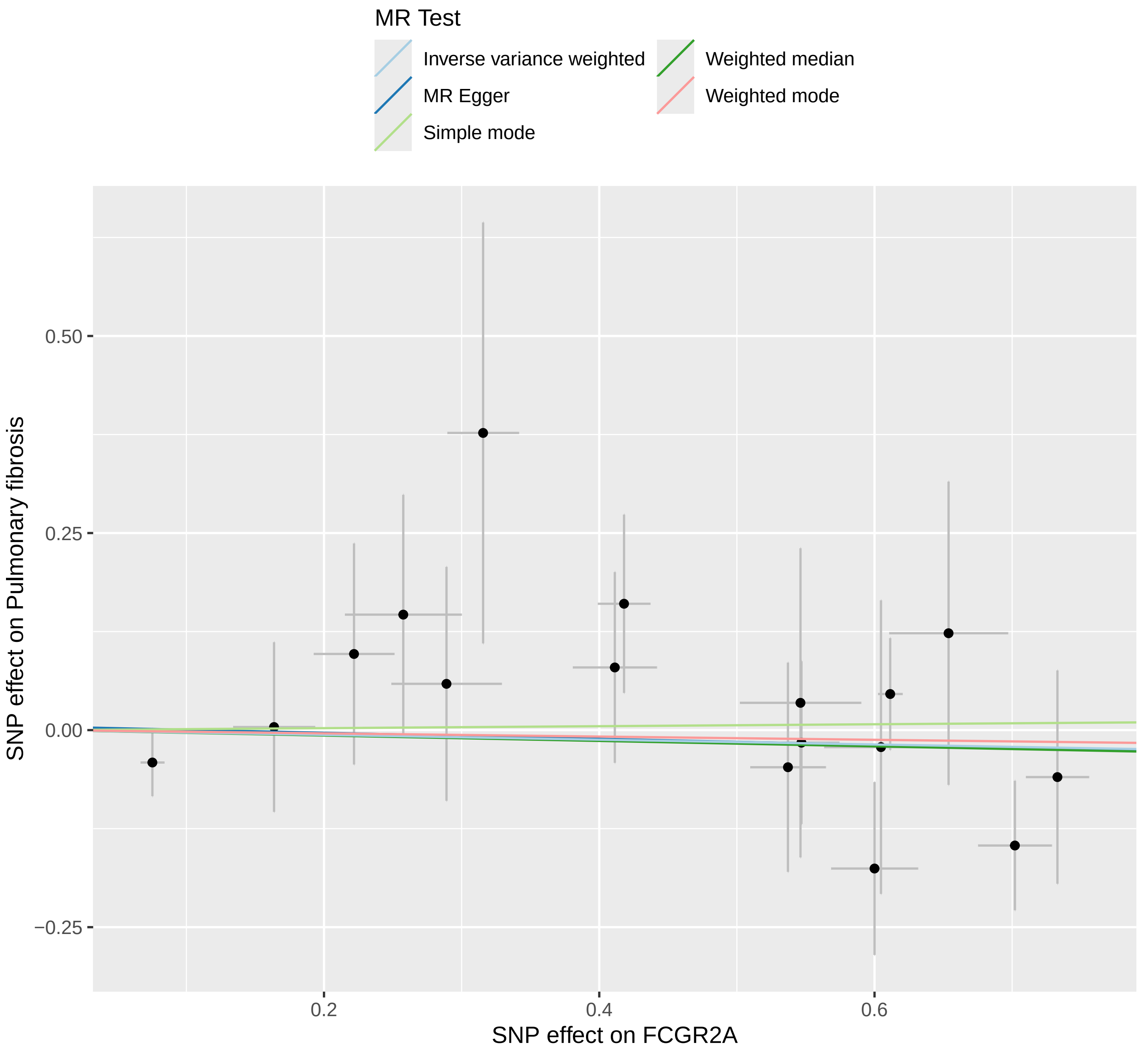

MR Test
Inverse variance weighted
MR Egger
Simple mode
Weighted median
Weighted mode
SNP effect on Pulmonary fibrosis
0.50
0.25
0.00
−0.25
0.2
0.4
0.6
SNP effect on FCGR2A

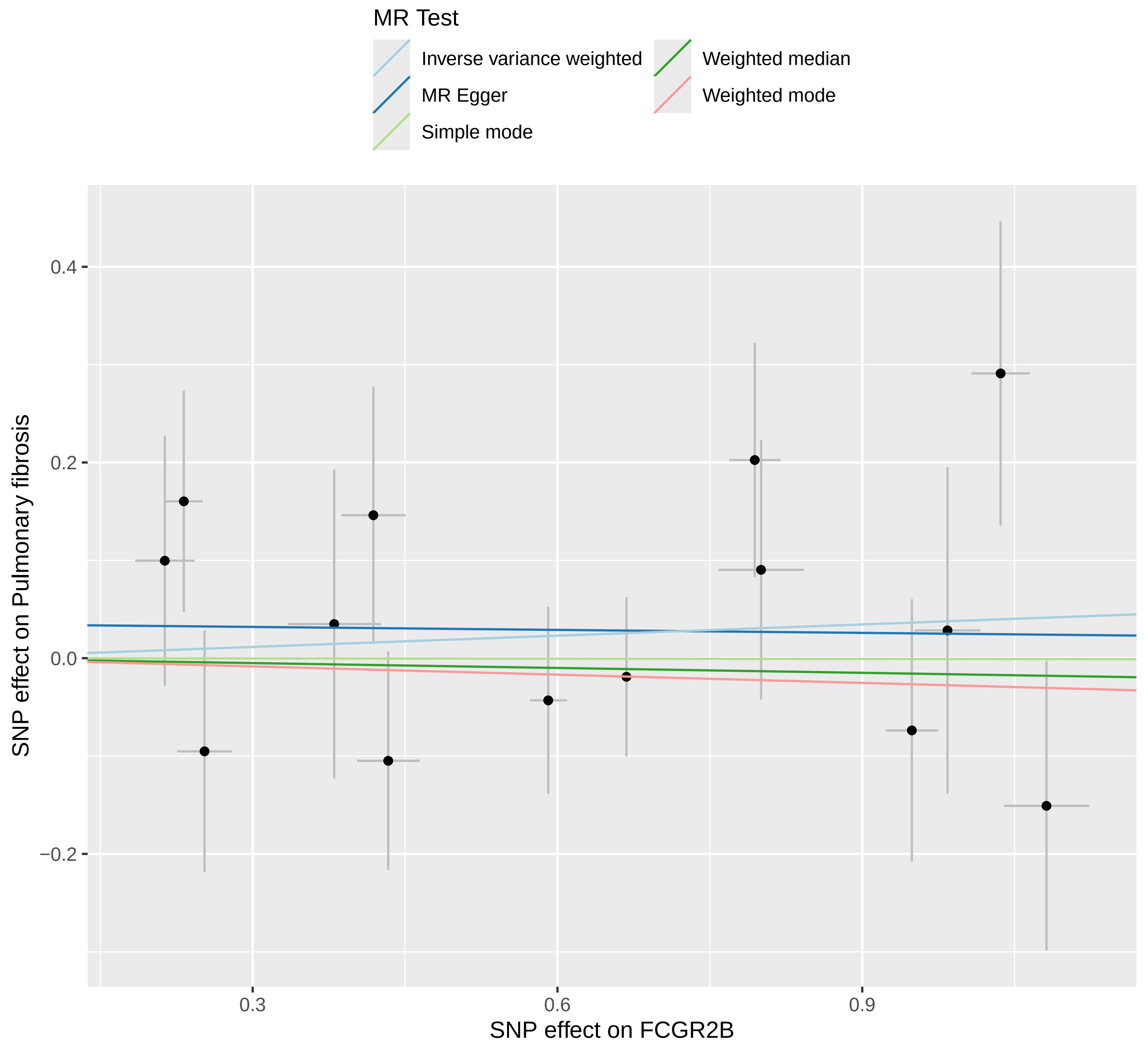

MR Test
Inverse variance weighted
MR Egger
Simple mode
Weighted median
Weighted mode
SNP effect on Pulmonary fibrosis
0.4
0.2
0.0
−0.2
0.3
0.6
0.9
SNP effect on FCGR2B

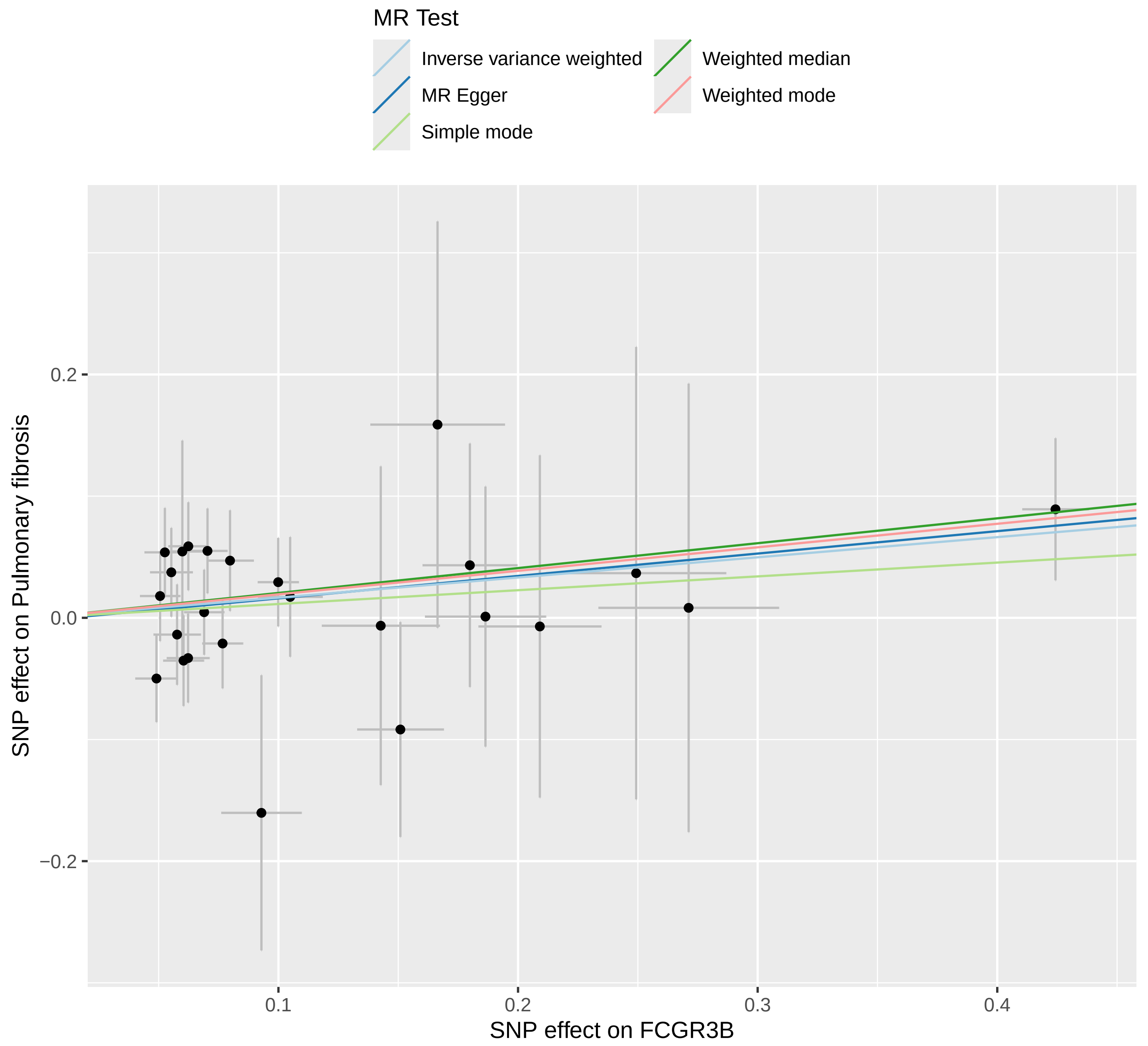
MR Test
Inverse variance weighted
MR Egger
Simple mode
Weighted median
Weighted mode
SNP effect on Pulmonary fibrosis
0.2
0.0
−0.2
0.1
0.2
0.3
0.4
SNP effect on FCGR3B

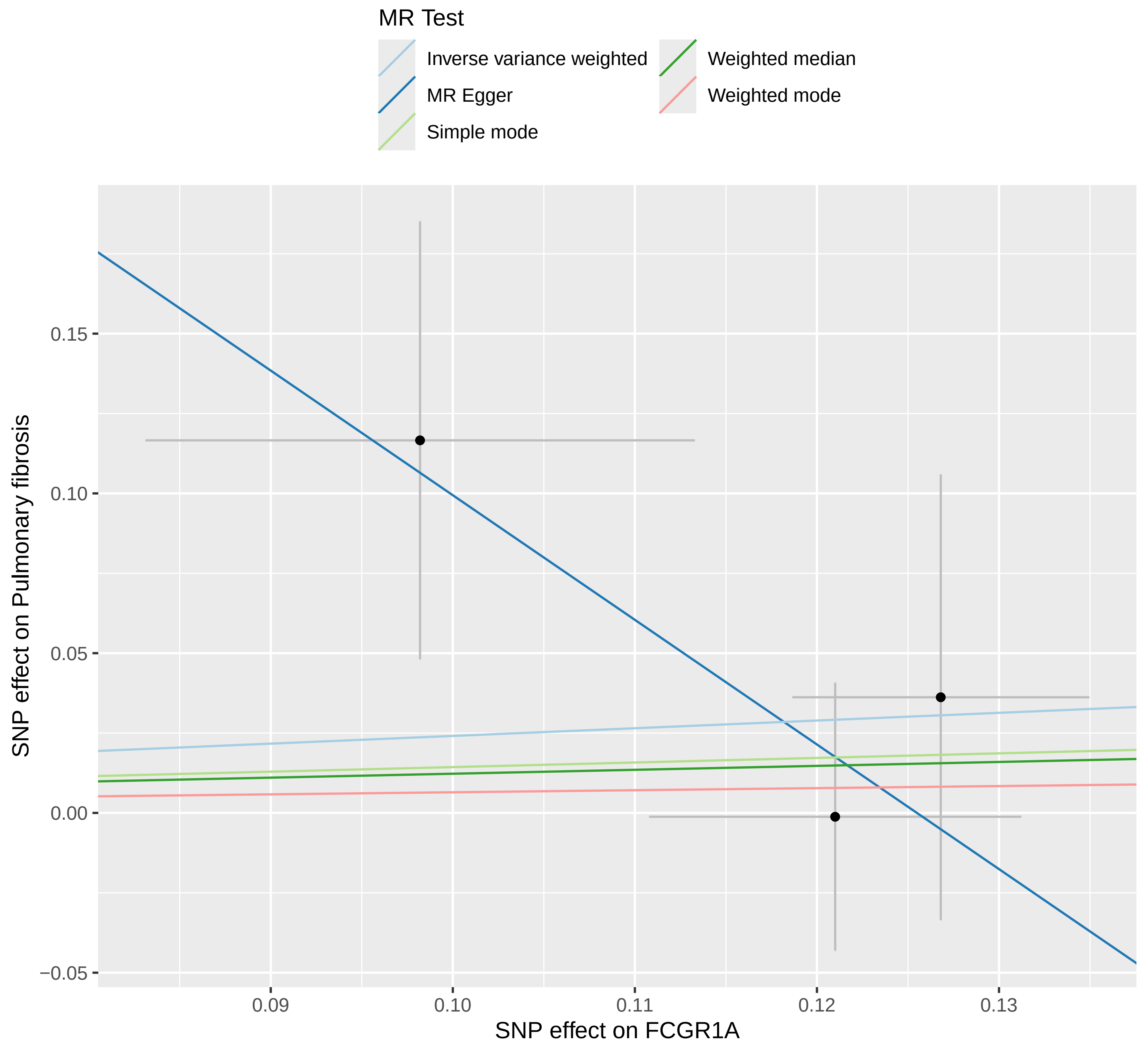
MR Test
Inverse variance weighted
MR Egger
Simple mode
Weighted median
Weighted mode
SNP effect on Pulmonary fibrosis
0.15
0.10
0.05
0.00
−0.05
0.09
0.10
0.11
0.12
0.13
SNP effect on FCGR1A

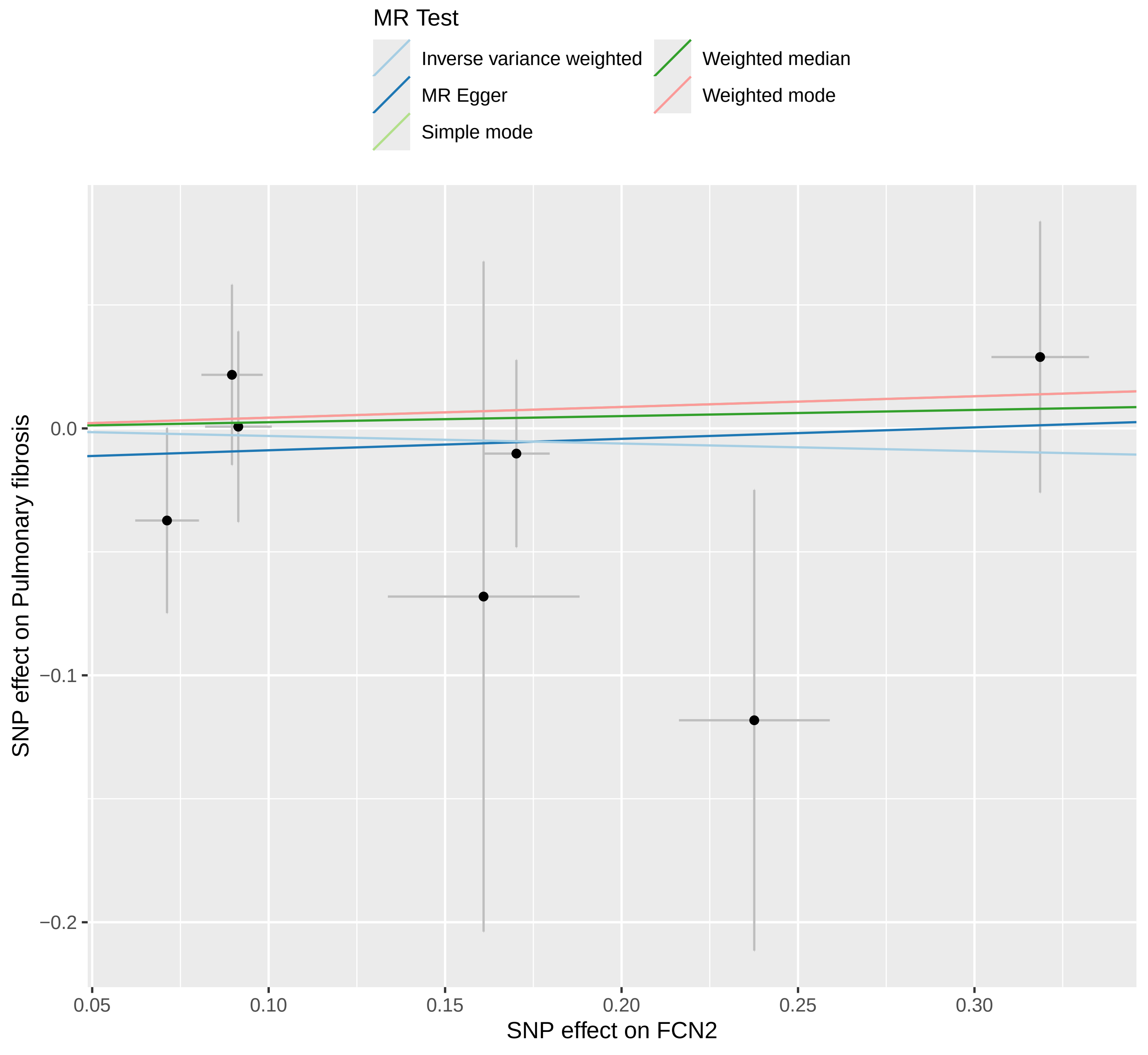
MR Test
Inverse variance weighted
MR Egger
Simple mode
Weighted median
Weighted mode
0.0
−0.1
−0.2
SNP effect on Pulmonary fibrosis
0.05
0.10
0.15
0.20
0.25
0.30
SNP effect on FCN2

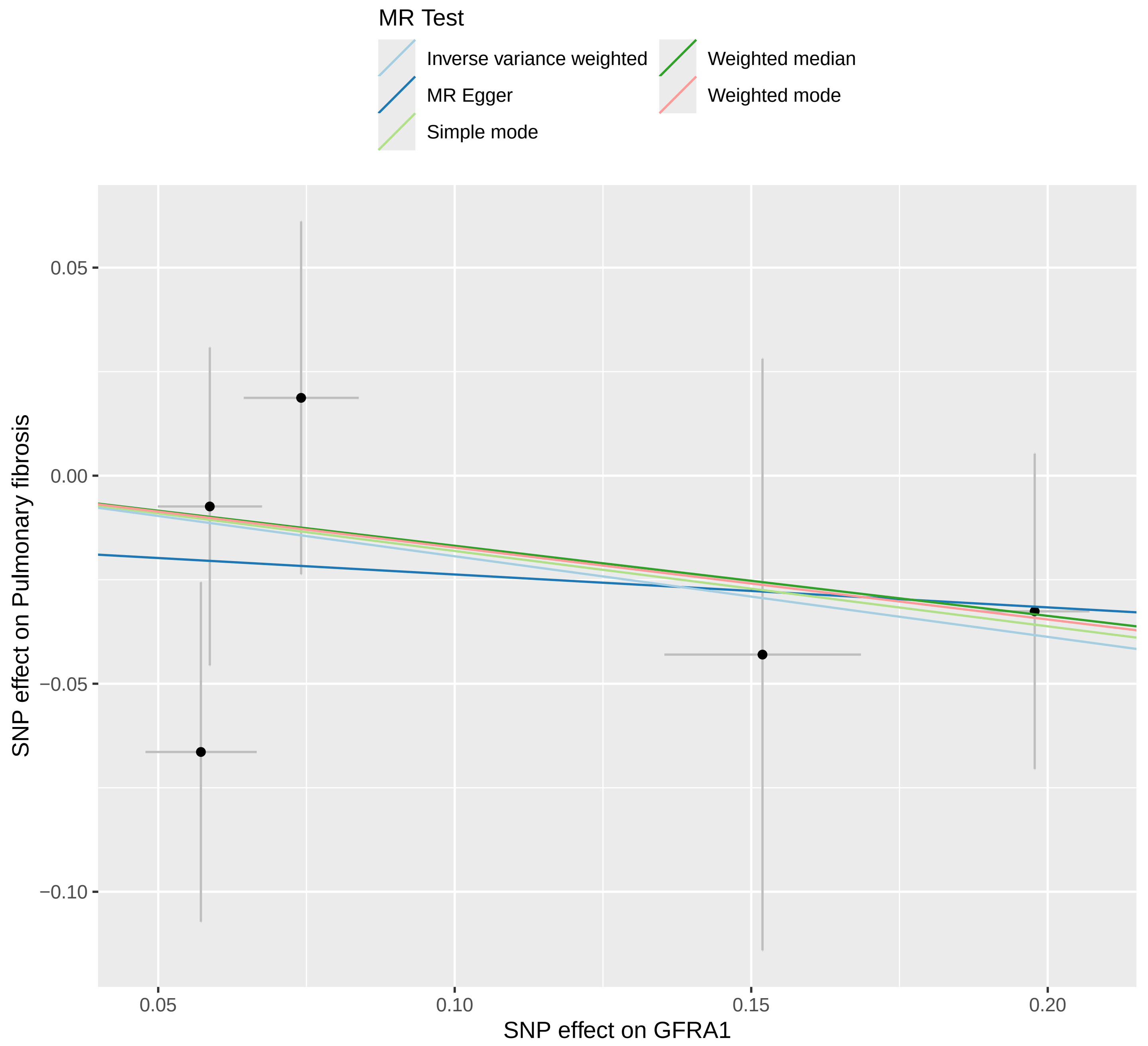
MR Test
Inverse variance weighted
MR Egger
Simple mode
Weighted median
Weighted mode
0.05
0.00
−0.05
−0.10
0.05
0.10
0.15
0.20
SNP effect on Pulmonary fibrosis
SNP effect on GFRA1

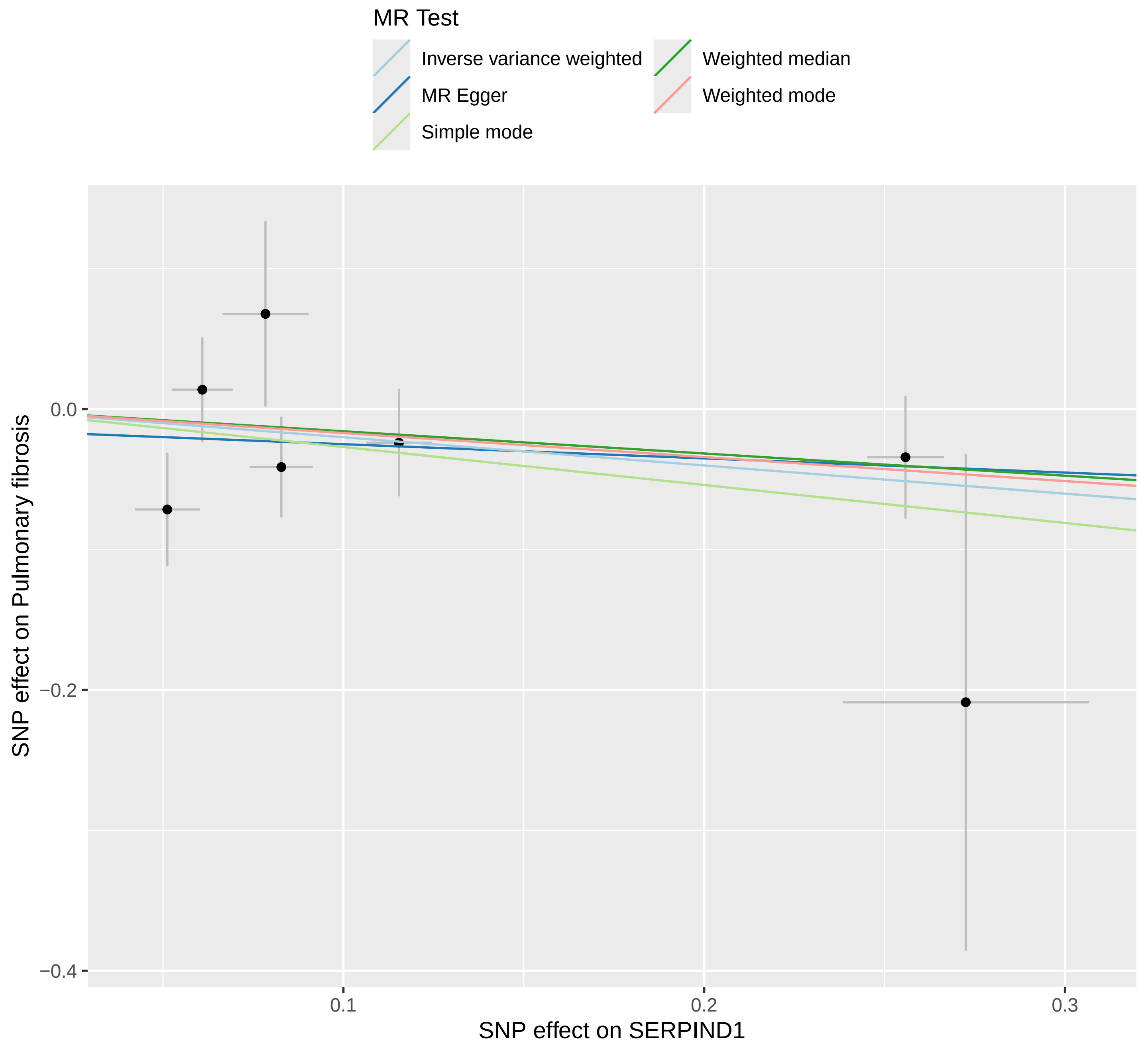

MR Test
Inverse variance weighted
MR Egger
Simple mode
Weighted median
Weighted mode
SNP effect on Pulmonary fibrosis
0.0
−0.2
−0.4
0.1
0.2
0.3
SNP effect on SERPIND1

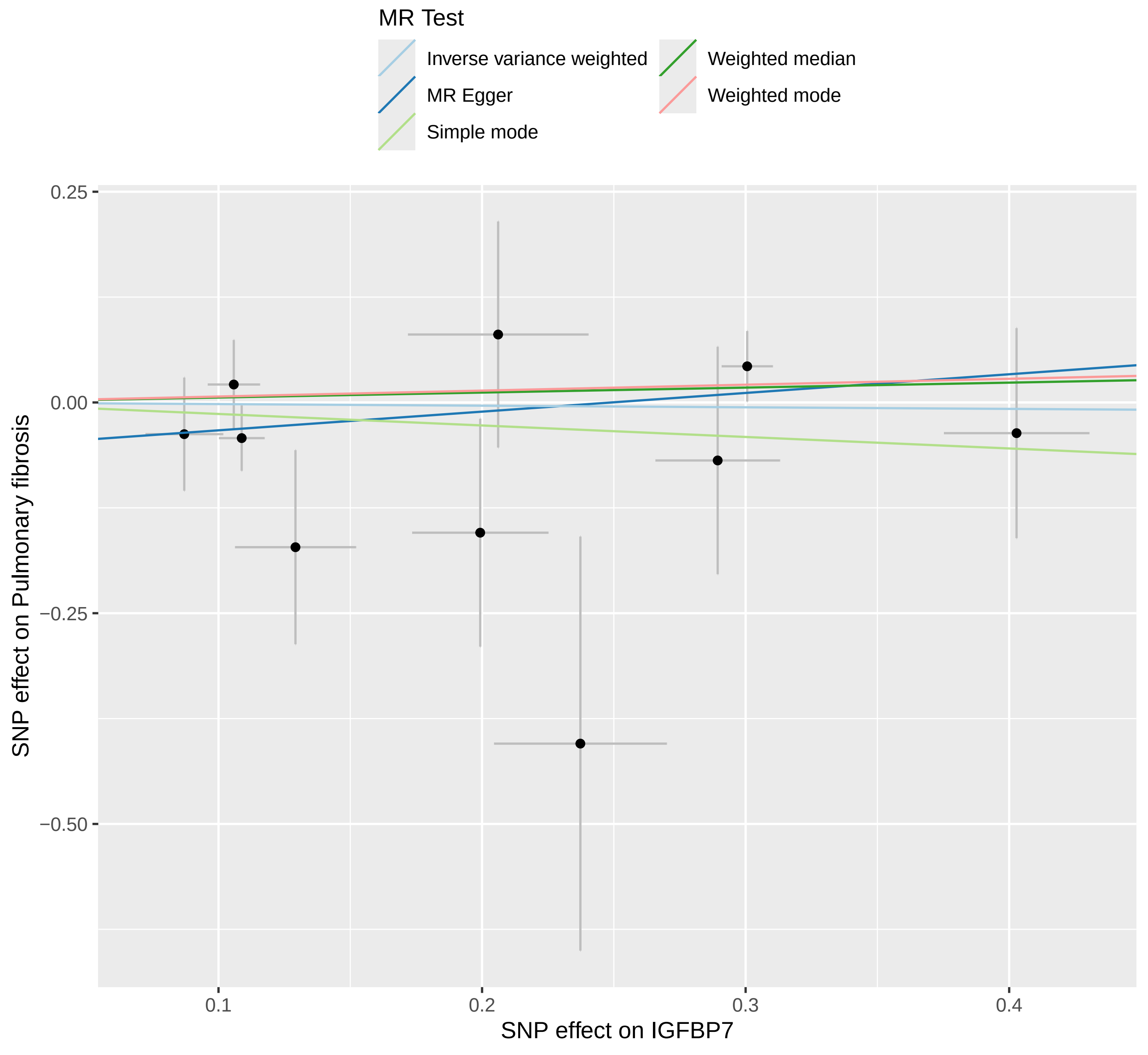

MR Test
Inverse variance weighted
MR Egger
Simple mode
Weighted median
Weighted mode
0.25
0.00
−0.25
−0.50
SNP effect on Pulmonary fibrosis
0.1
0.2
0.3
0.4
SNP effect on IGFBP7

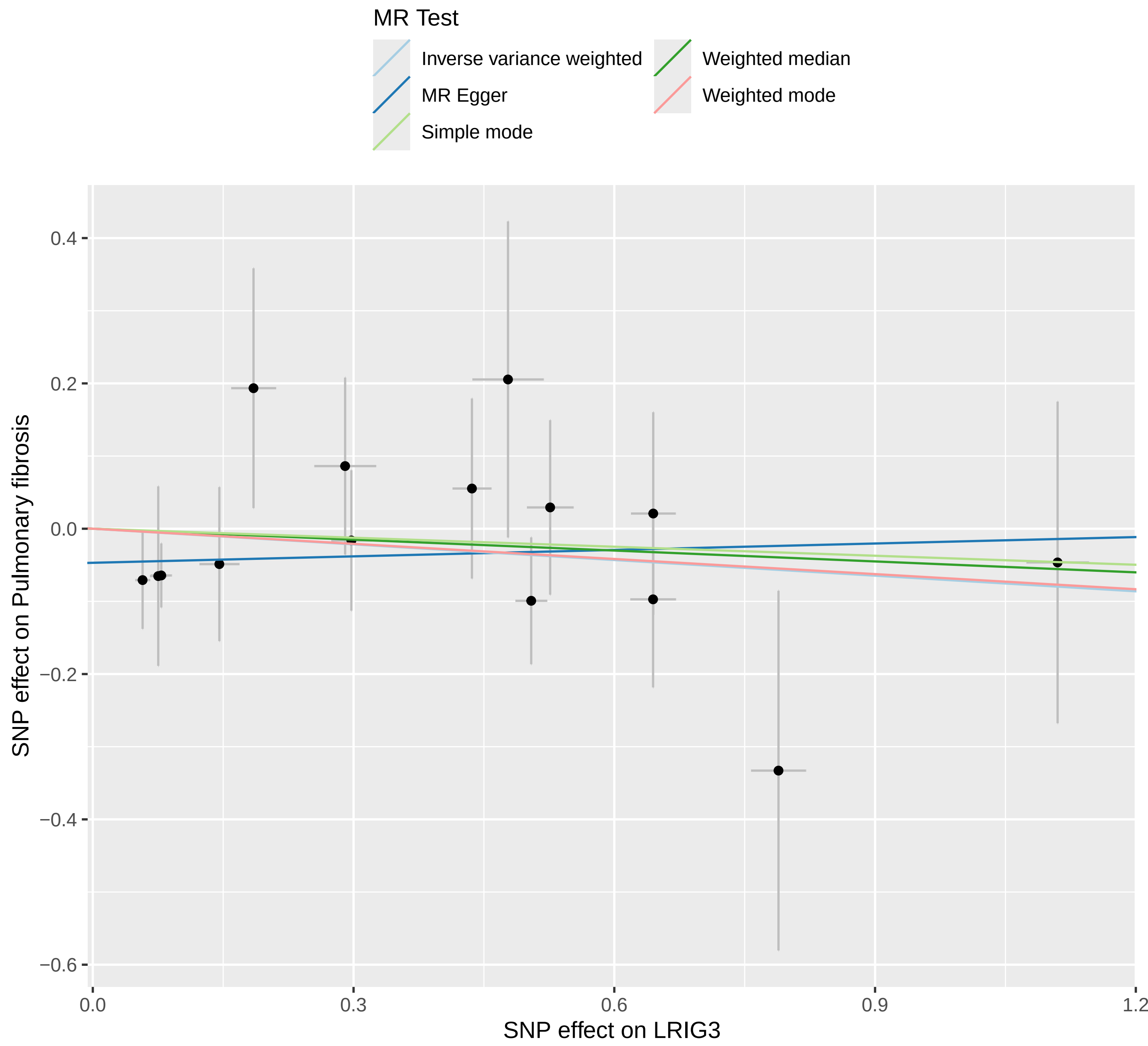
MR Test
Inverse variance weighted
MR Egger
Simple mode
Weighted median
Weighted mode
0.4
0.2
0.0
−0.2
−0.4
−0.6
SNP effect on Pulmonary fibrosis
0.0
0.3
0.6
0.9
1.2
SNP effect on LRIG3

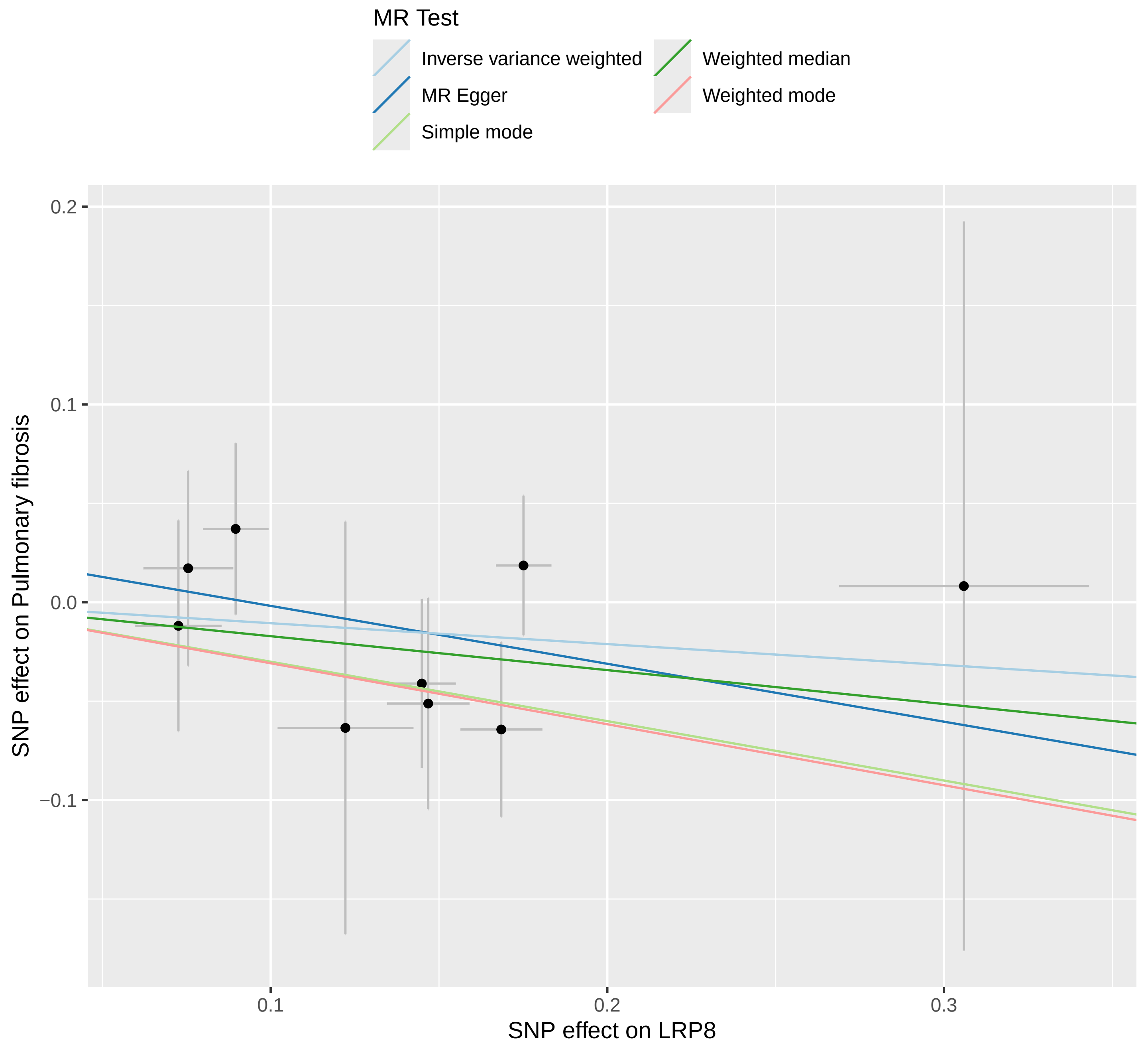
MR Test
Inverse variance weighted
MR Egger
Simple mode
Weighted median
Weighted mode
0.2
0.1
0.0
−0.1
SNP effect on Pulmonary fibrosis
0.1
0.2
0.3
SNP effect on LRP8

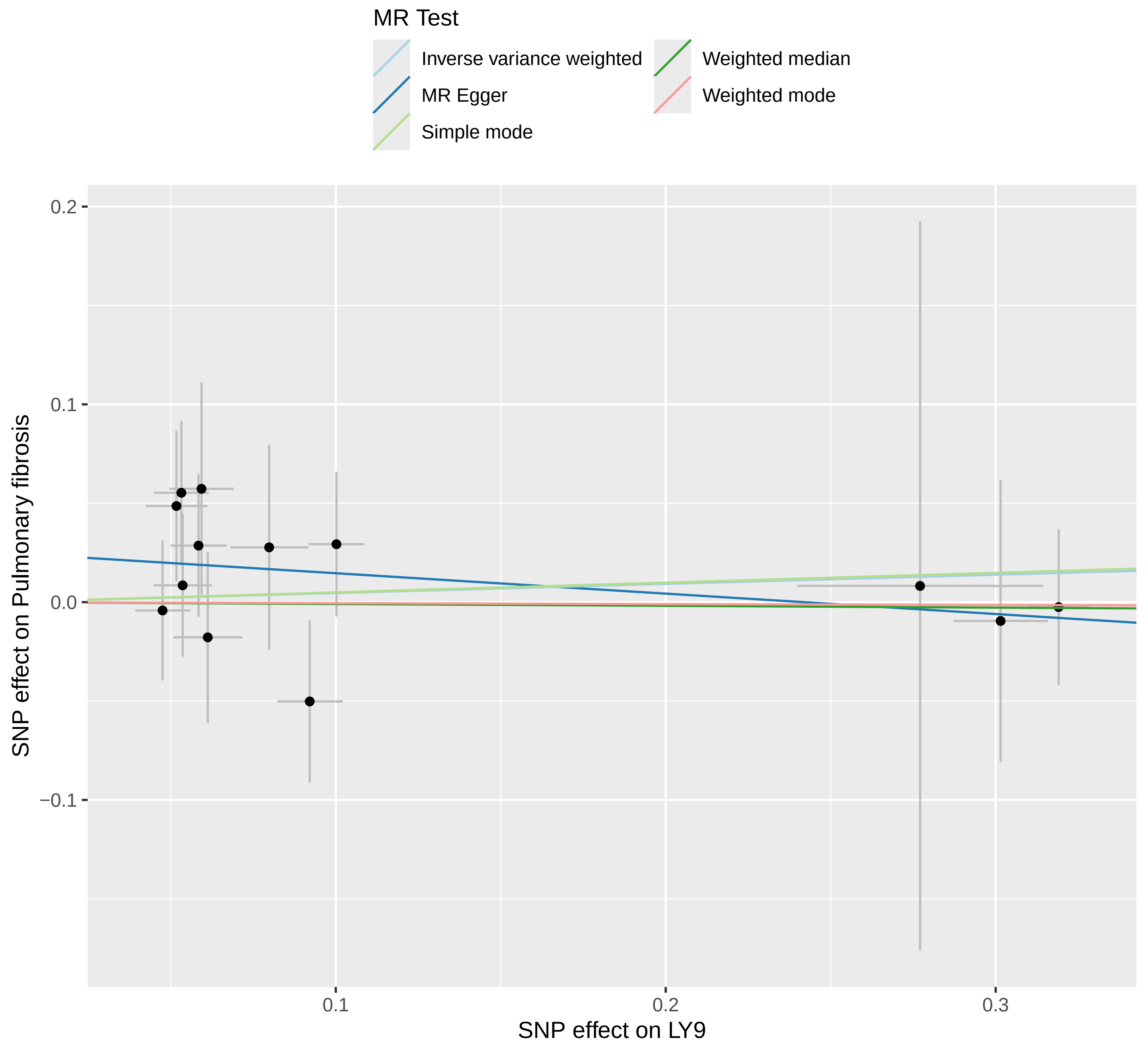

MR Test
Inverse variance weighted
MR Egger
Simple mode
Weighted median
Weighted mode
0.2
0.1
0.0
−0.1
SNP effect on Pulmonary fibrosis
0.1
0.2
0.3
SNP effect on LY9

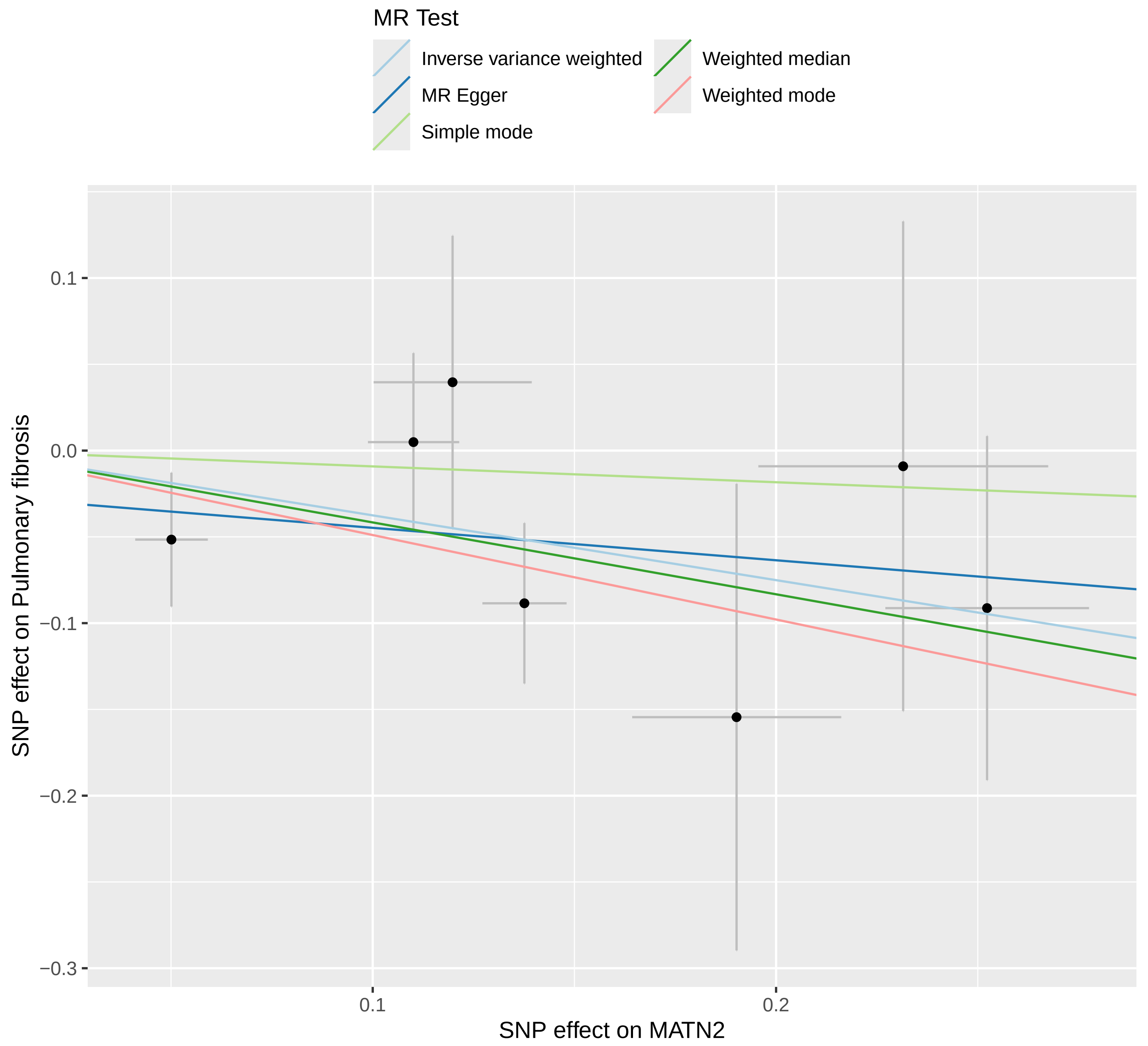
MR Test
Inverse variance weighted
MR Egger
Simple mode
Weighted median
Weighted mode
0.1
0.0
−0.1
−0.2
−0.3
SNP effect on Pulmonary fibrosis
0.1
0.2
SNP effect on MATN2

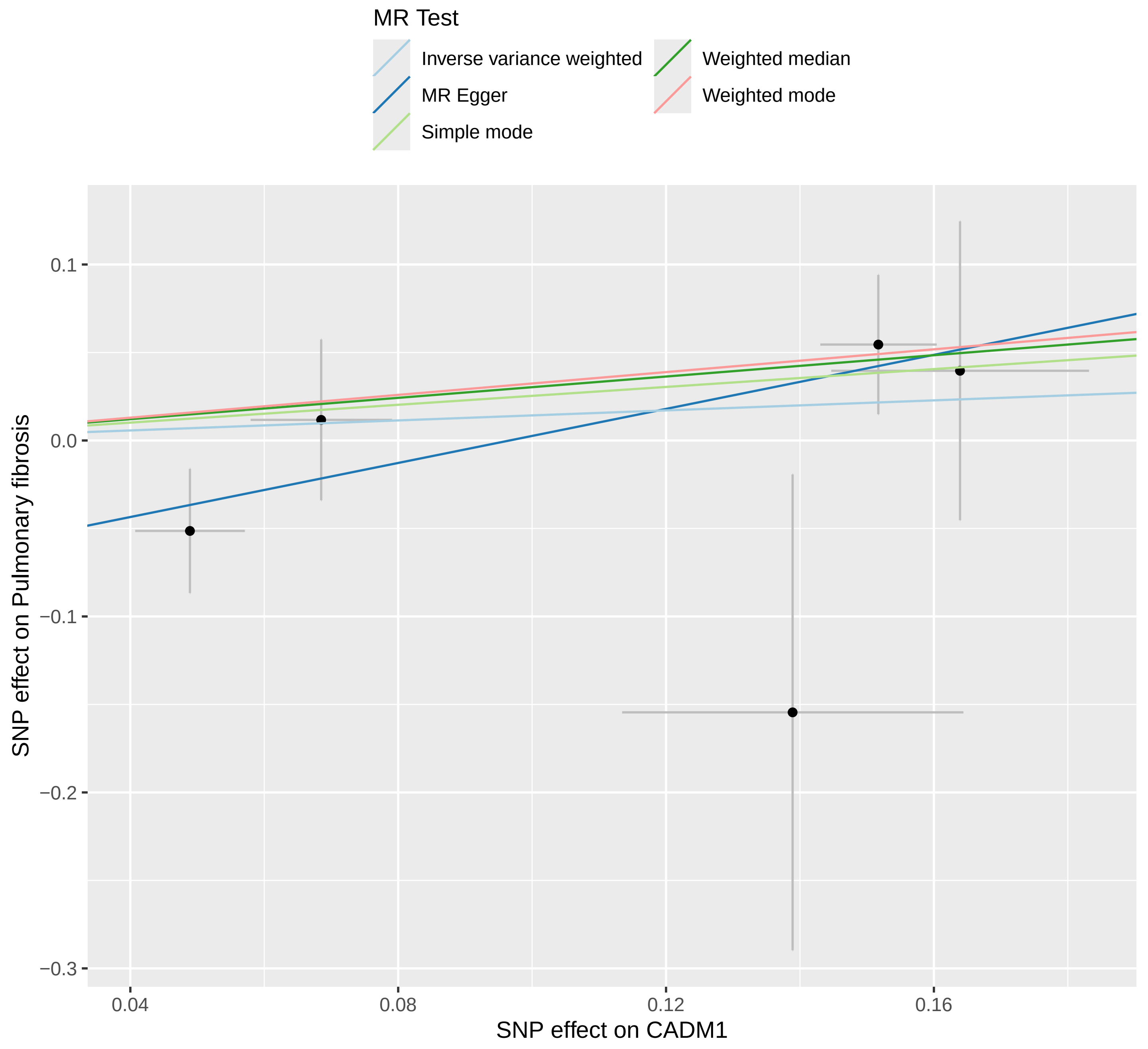
MR Test
Inverse variance weighted
MR Egger
Simple mode
Weighted median
Weighted mode
0.1
0.0
−0.1
−0.2
−0.3
SNP effect on Pulmonary fibrosis
0.04
0.08
0.12
0.16
SNP effect on CADM1

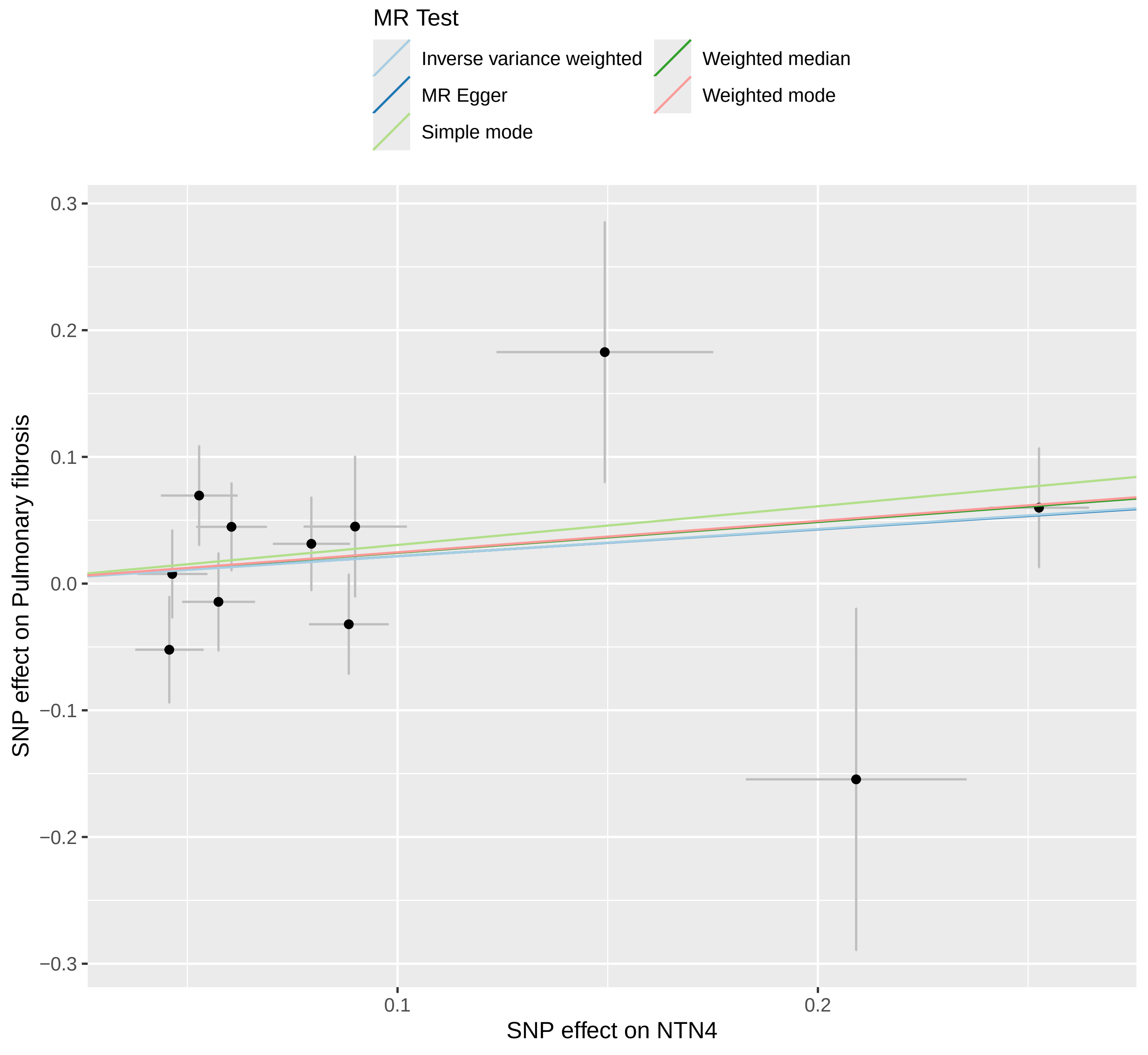

MR Test
Inverse variance weighted
MR Egger
Simple mode
Weighted median
Weighted mode
0.3
0.2
0.1
0.0
−0.1
−0.2
−0.3
SNP effect on Pulmonary fibrosis
0.1
0.2
SNP effect on NTN4

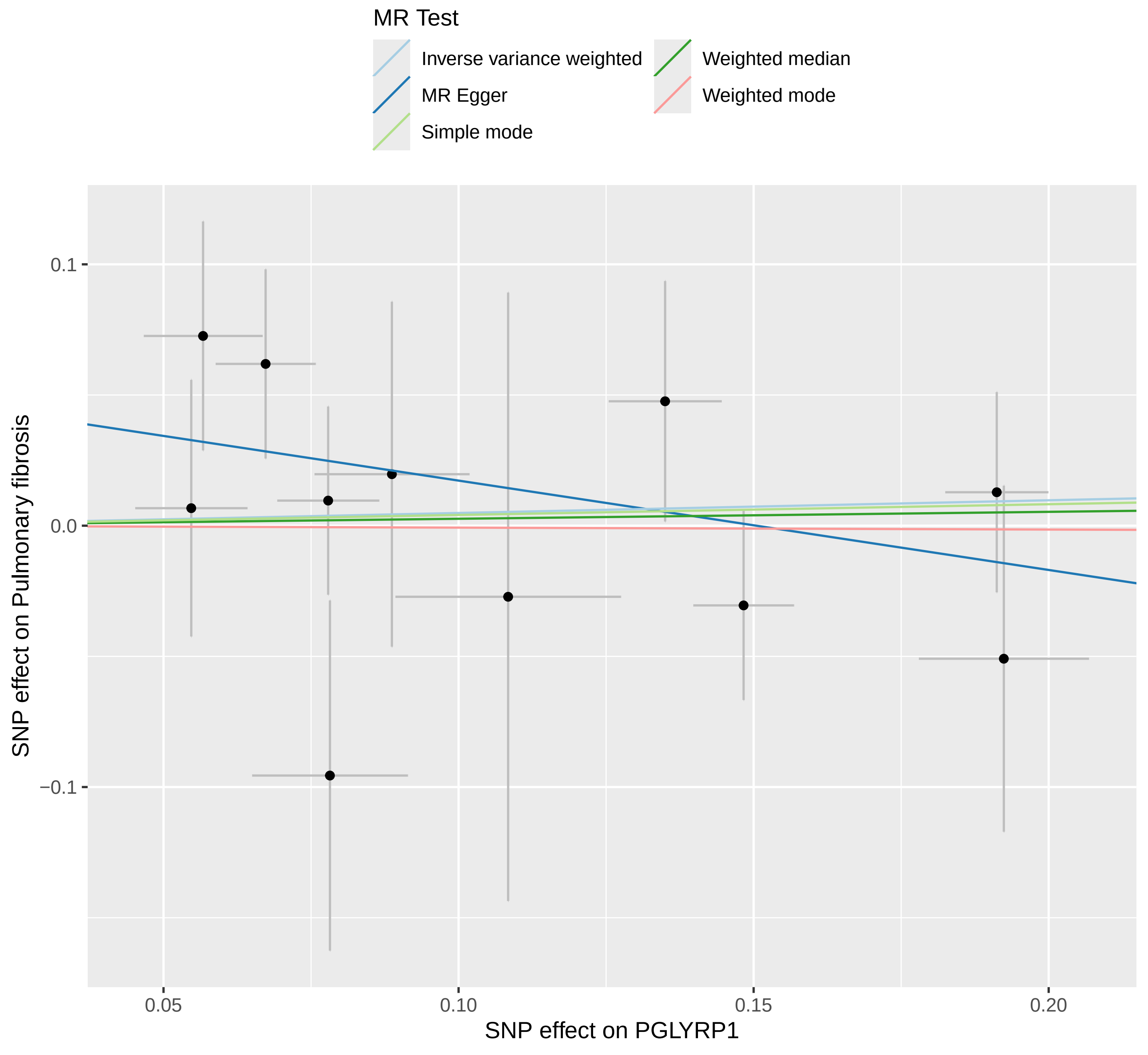

MR Test
Inverse variance weighted
MR Egger
Simple mode
Weighted median
Weighted mode
0.1
0.0
−0.1
0.05
0.10
0.15
0.20
SNP effect on PGLYRP1
SNP effect on Pulmonary fibrosis

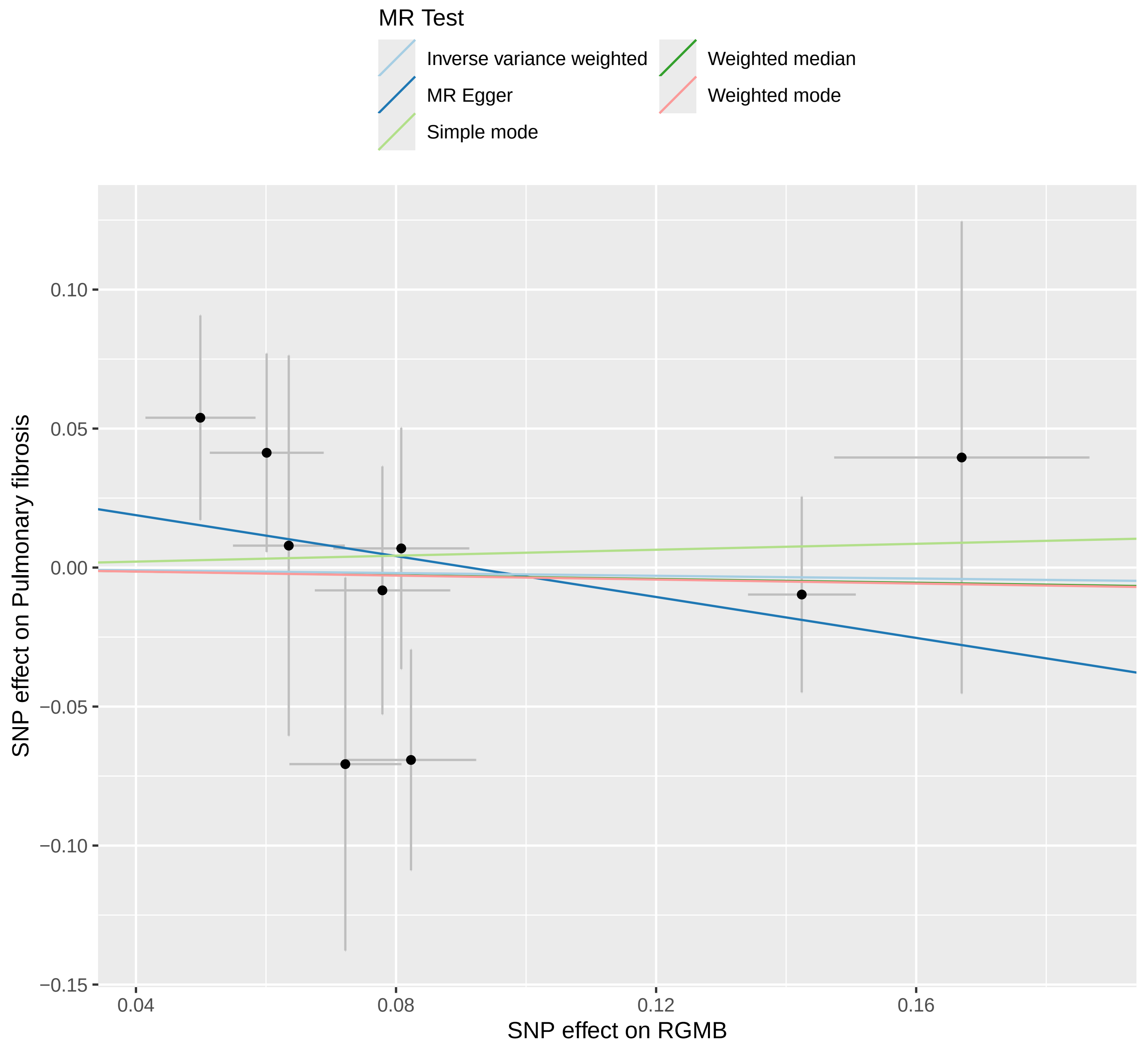

MR Test
Inverse variance weighted
MR Egger
Simple mode
Weighted median
Weighted mode
SNP effect on Pulmonary fibrosis
0.10
0.05
0.00
−0.05
−0.10
−0.15
0.04
0.08
0.12
0.16
SNP effect on RGMB

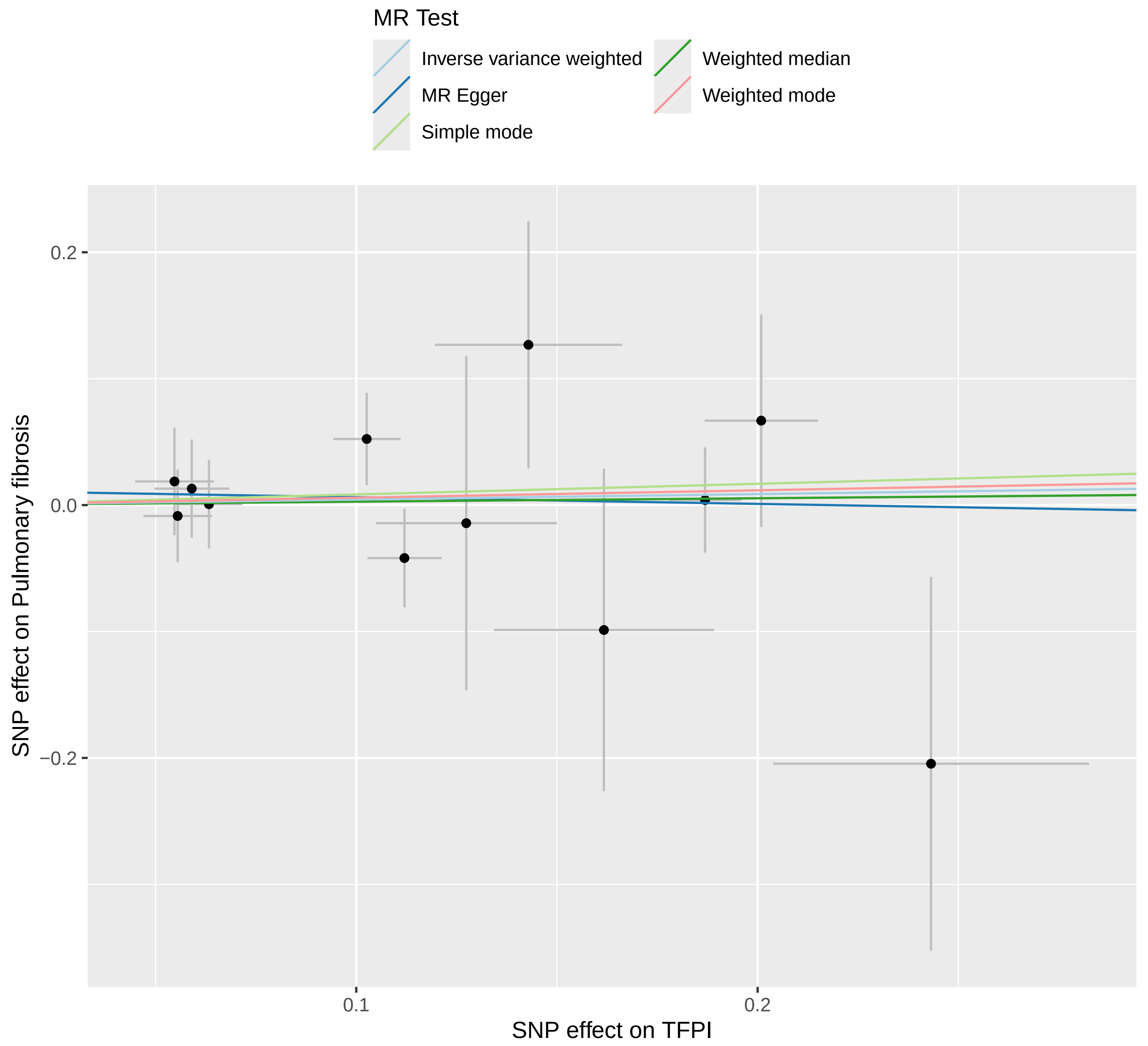

MR Test
Inverse variance weighted
MR Egger
Simple mode
Weighted median
Weighted mode
0.2
0.0
−0.2
0.1
0.2
SNP effect on Pulmonary fibrosis
SNP effect on TFPI

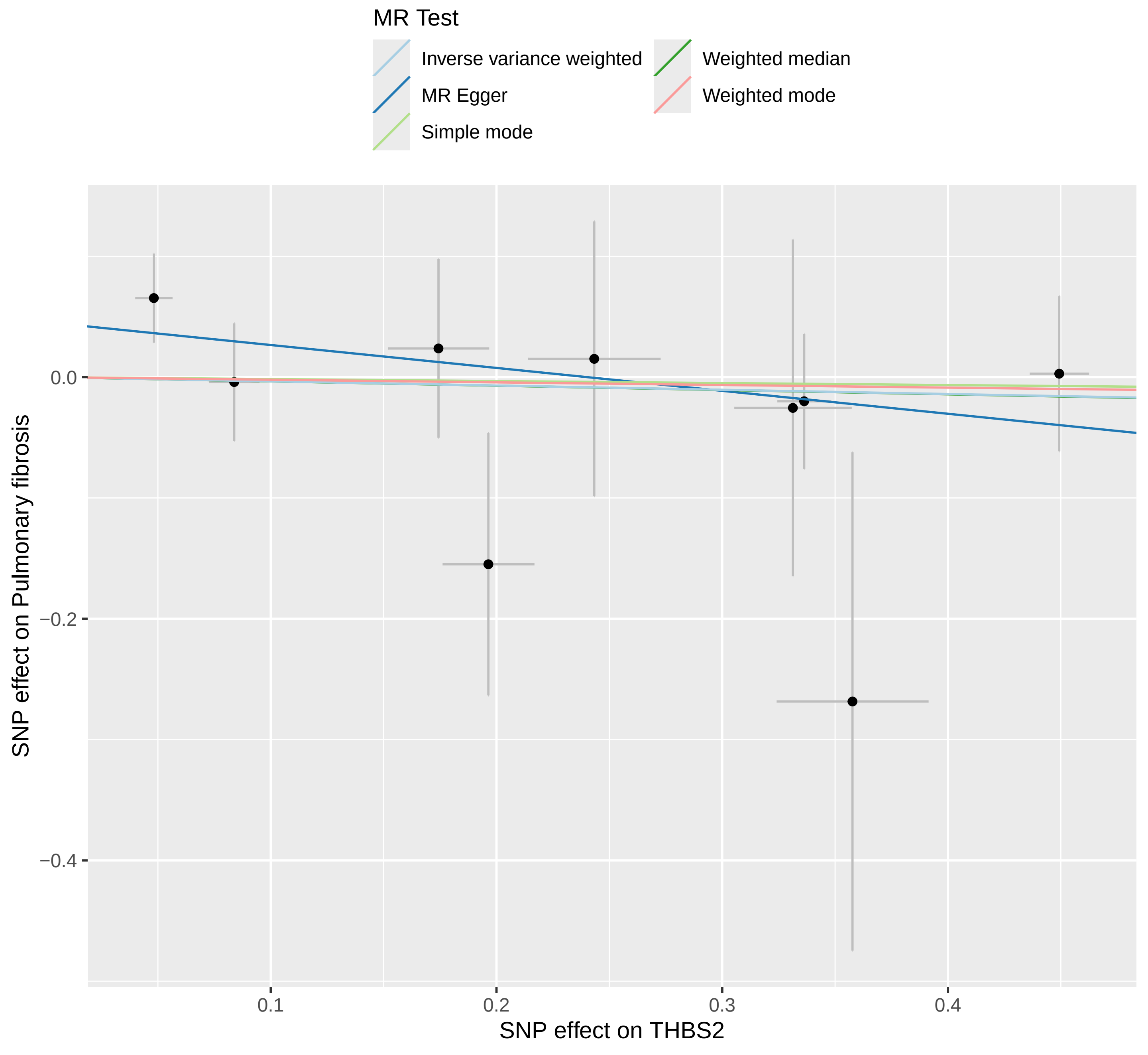
MR Test
Inverse variance weighted
MR Egger
Simple mode
Weighted median
Weighted mode
0.0
−0.2
−0.4
0.1
0.2
0.3
0.4
SNP effect on THBS2
SNP effect on Pulmonary fibrosis

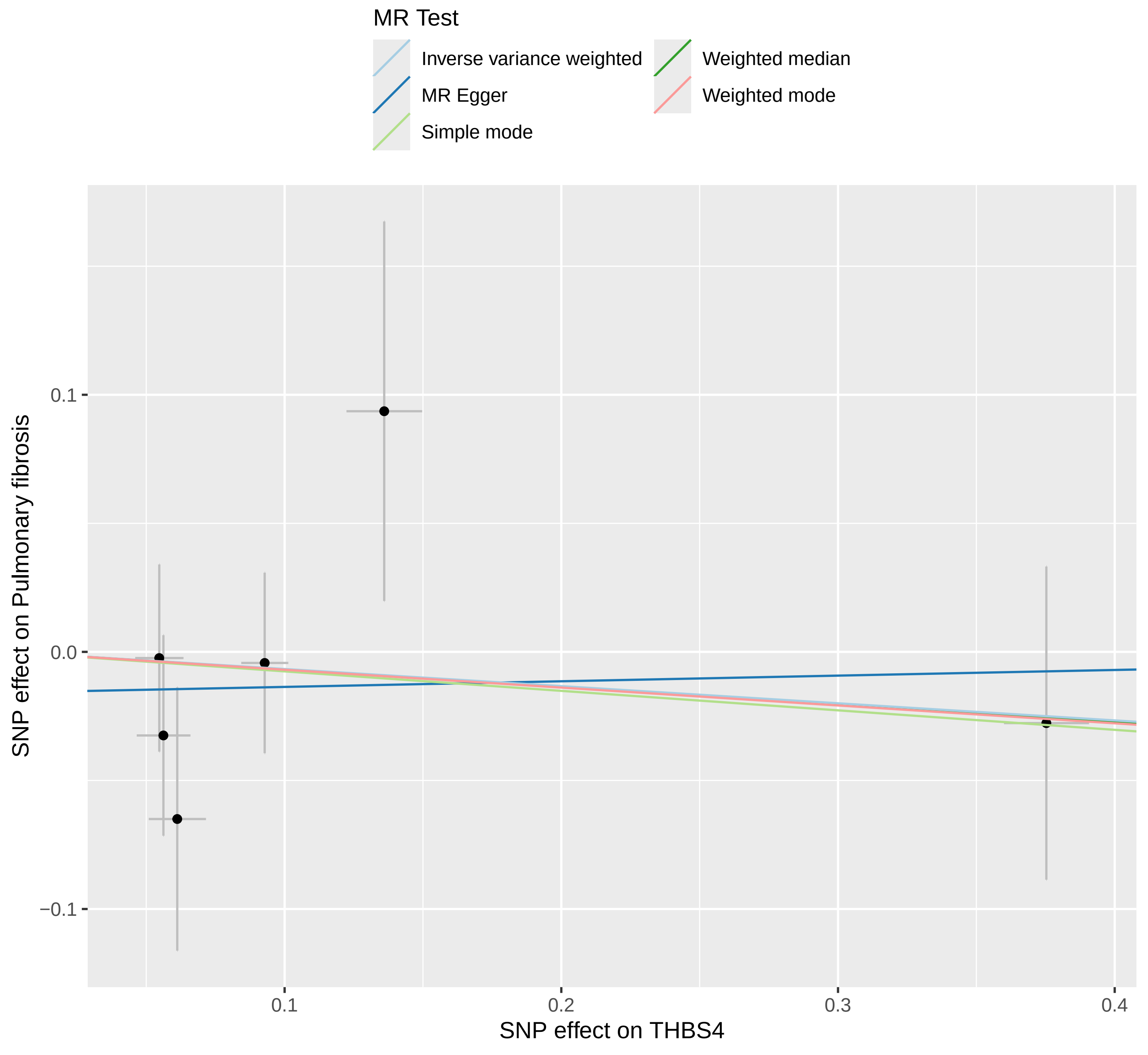
MR Test
Inverse variance weighted
MR Egger
Simple mode
Weighted median
Weighted mode
0.1
0.0
−0.1
0.1
0.2
0.3
0.4
SNP effect on Pulmonary fibrosis
SNP effect on THBS4

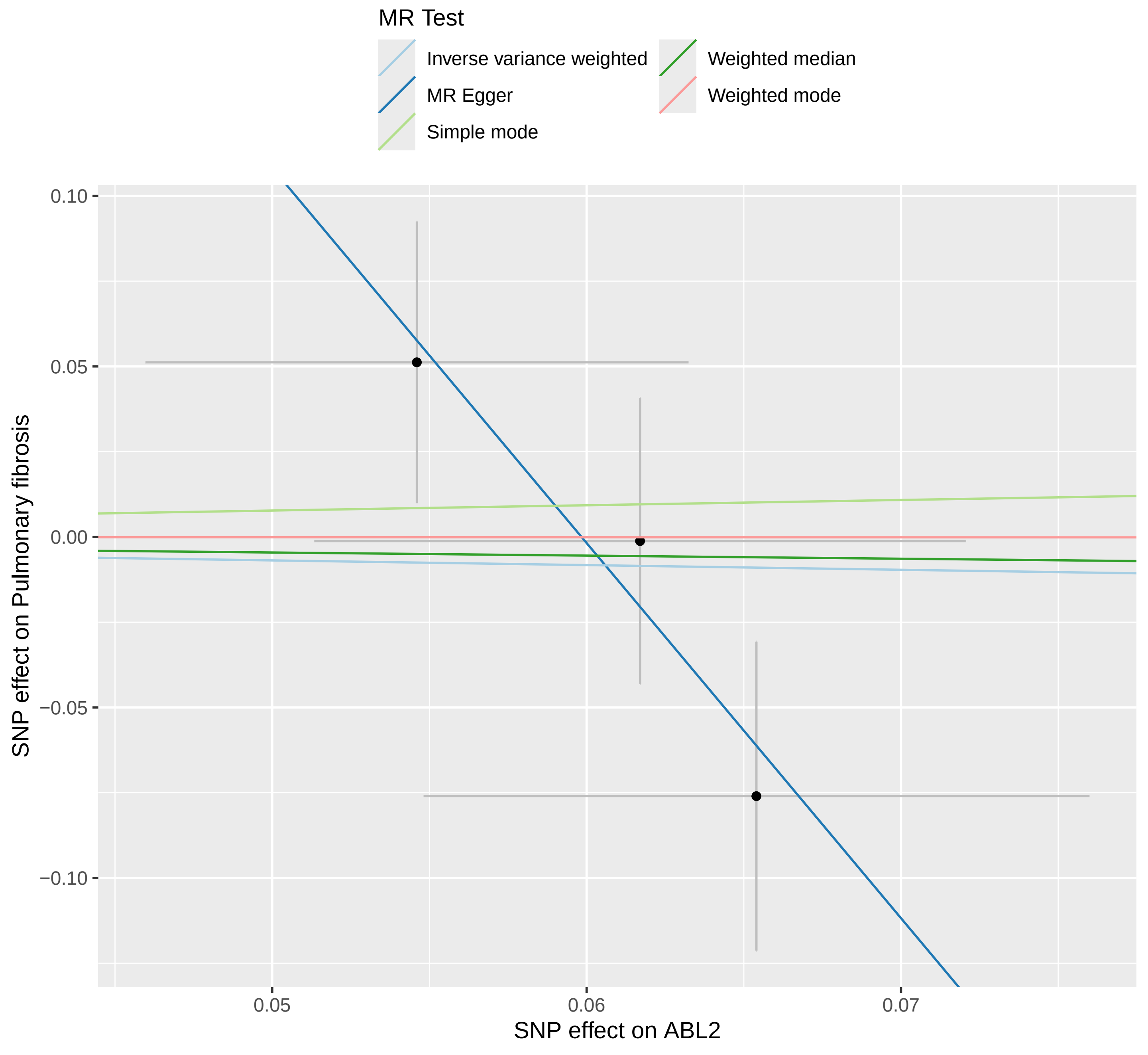
MR Test
Inverse variance weighted
MR Egger
Simple mode
Weighted median
Weighted mode
0.10
0.05
0.00
−0.05
−0.10
0.05
0.06
0.07
SNP effect on ABL2
SNP effect on Pulmonary fibrosis

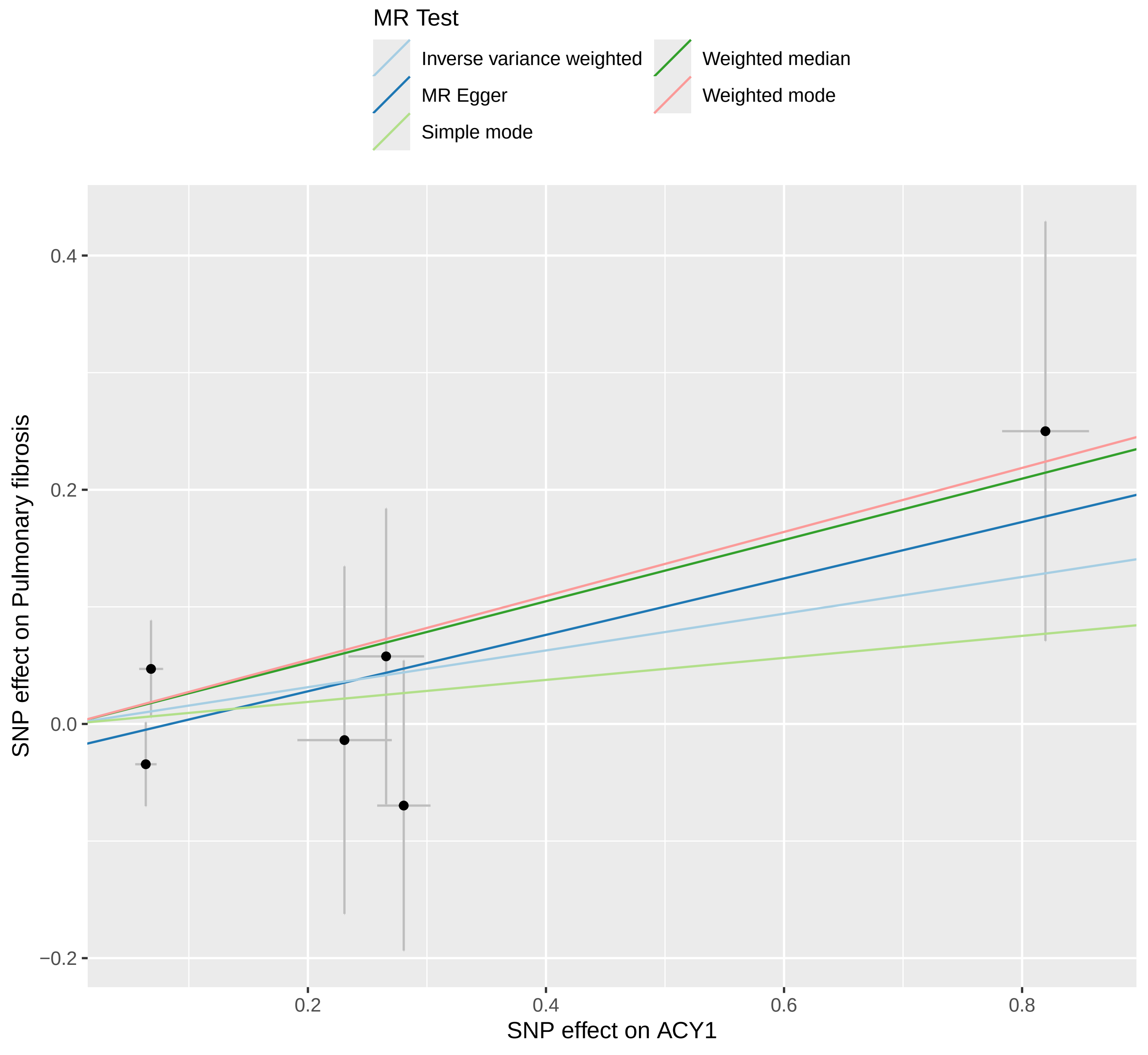

MR Test
Inverse variance weighted
MR Egger
Simple mode
Weighted median
Weighted mode
SNP effect on Pulmonary fibrosis
0.4
0.2
0.0
−0.2
0.2
0.4
0.6
0.8
SNP effect on ACY1

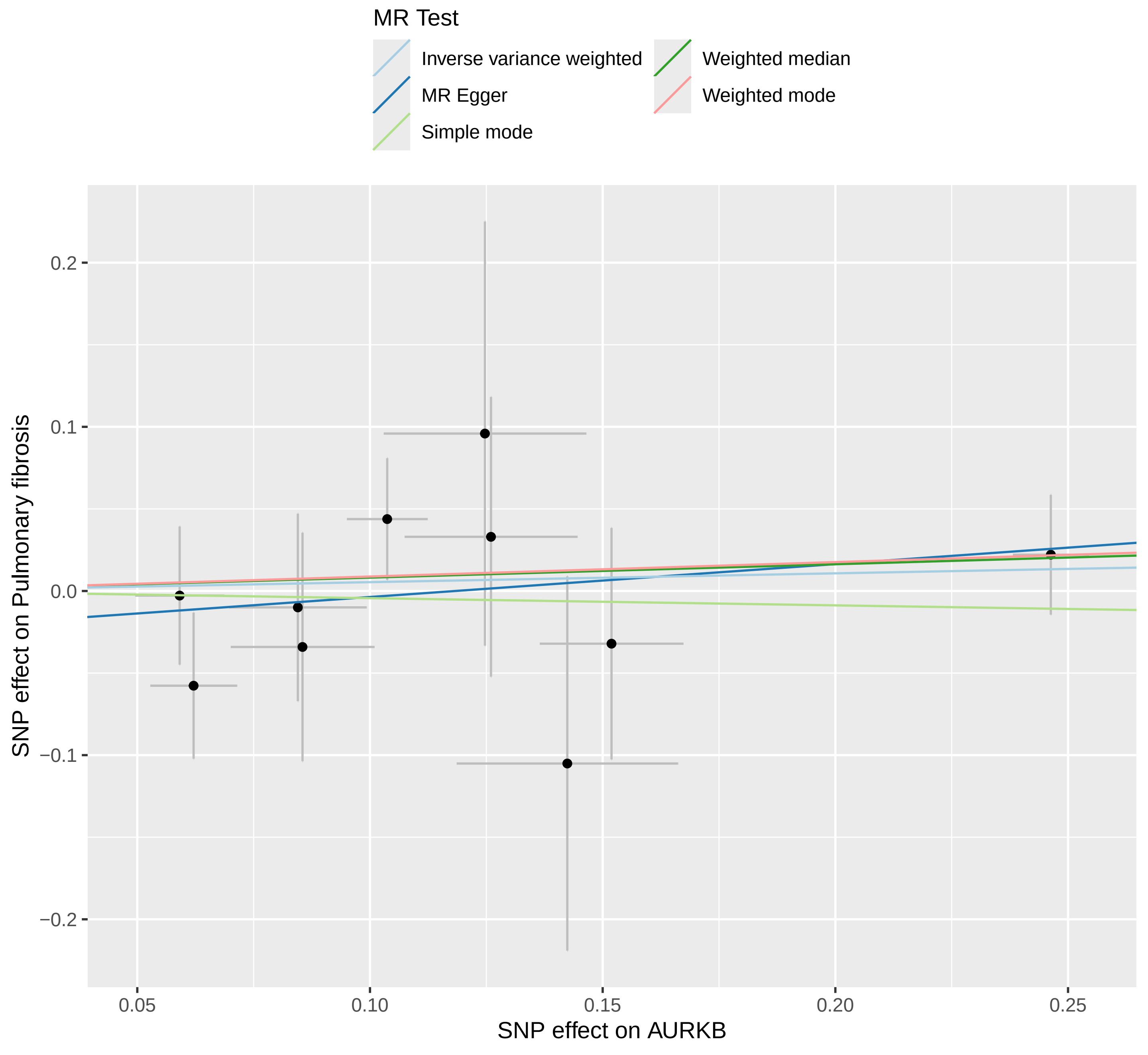
MR Test
Inverse variance weighted
MR Egger
Simple mode
Weighted median
Weighted mode
0.2
0.1
0.0
−0.1
−0.2
SNP effect on Pulmonary fibrosis
0.05
0.10
0.15
0.20
0.25
SNP effect on AURKB

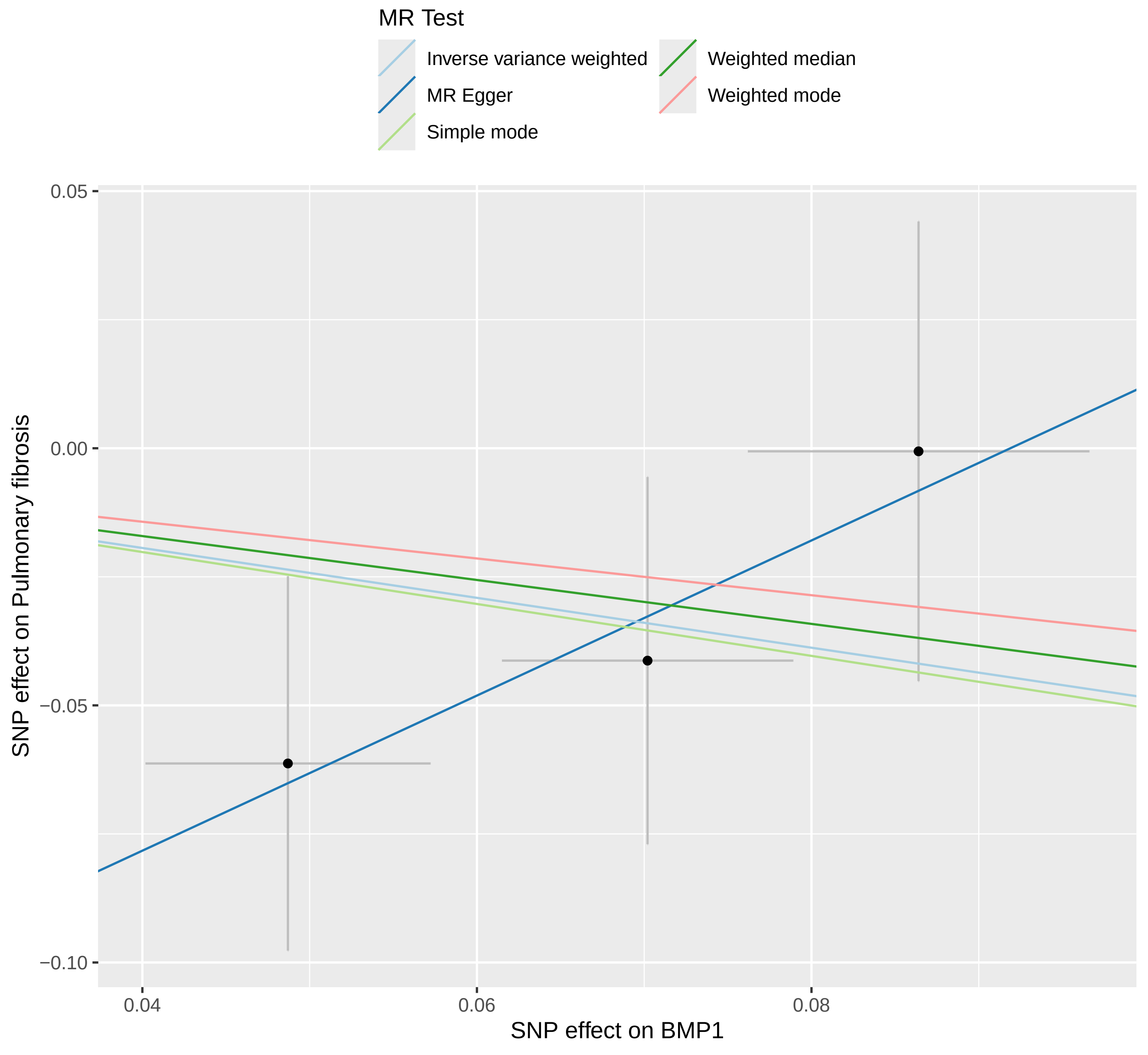

MR Test
Inverse variance weighted
MR Egger
Simple mode
Weighted median
Weighted mode
0.05
0.00
−0.05
−0.10
0.04
0.06
0.08
SNP effect on Pulmonary fibrosis
SNP effect on BMP1

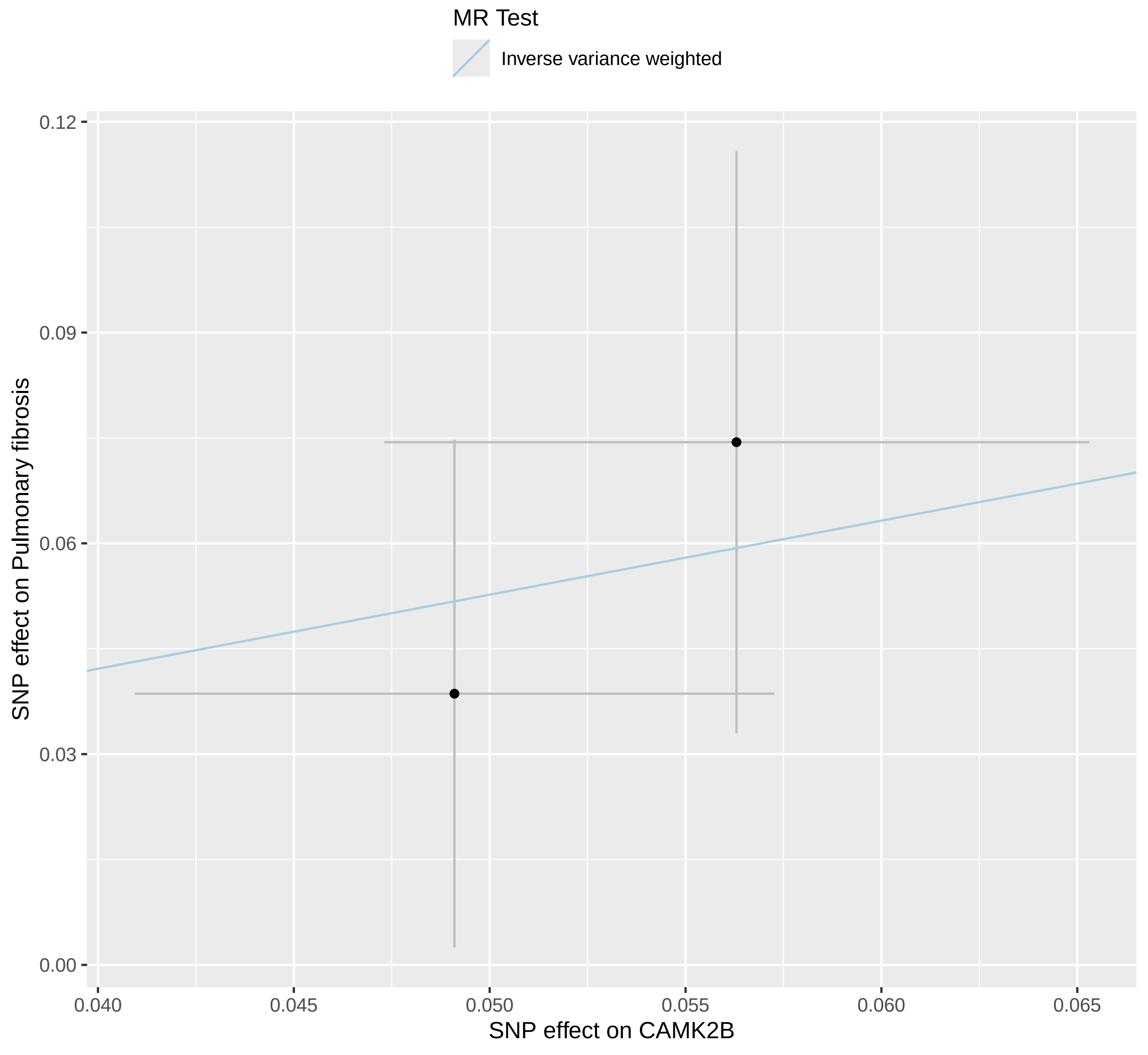

MR Test
Inverse variance weighted
0.12
0.09
0.06
0.03
0.00
SNP effect on Pulmonary fibrosis
0.040
0.045
0.050
0.055
0.060
0.065
SNP effect on CAMK2B

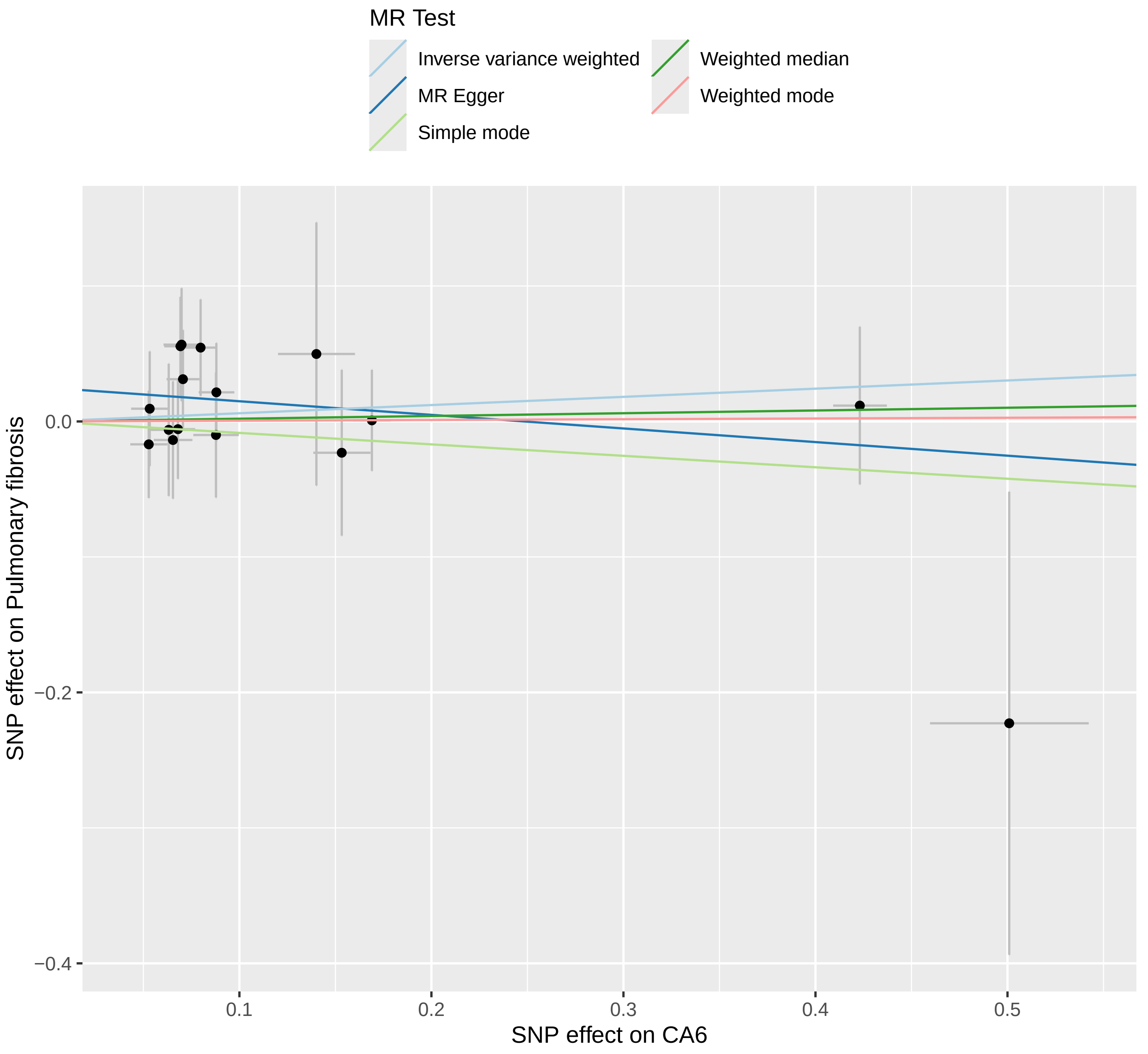
MR Test
Inverse variance weighted
MR Egger
Simple mode
Weighted median
Weighted mode
SNP effect on Pulmonary fibrosis
0.0
−0.2
−0.4
0.1
0.2
0.3
0.4
0.5
SNP effect on CA6

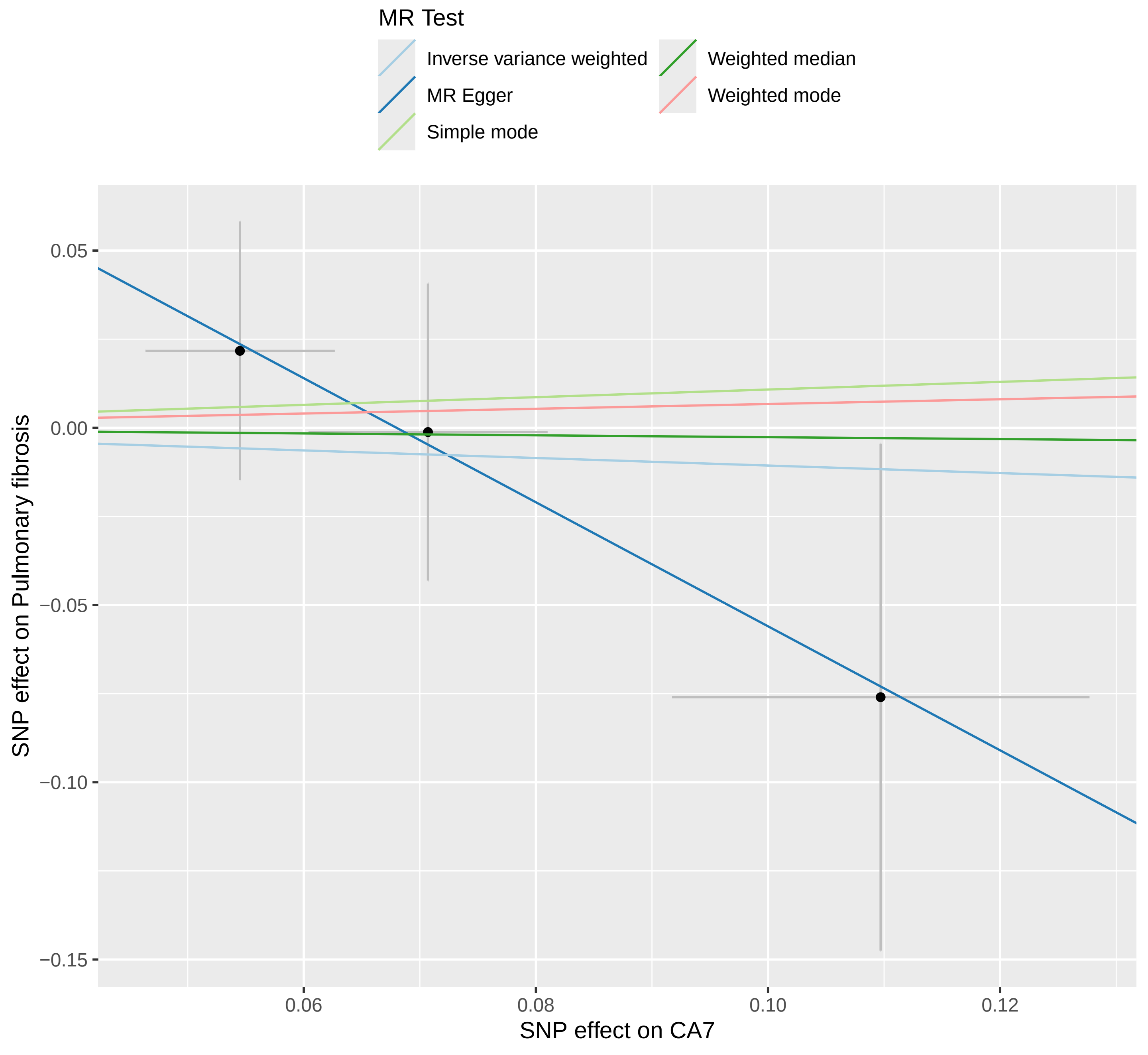
MR Test
Inverse variance weighted
MR Egger
Simple mode
Weighted median
Weighted mode
0.05
0.00
−0.05
−0.10
−0.15
0.06
0.08
0.10
0.12
SNP effect on Pulmonary fibrosis
SNP effect on CA7

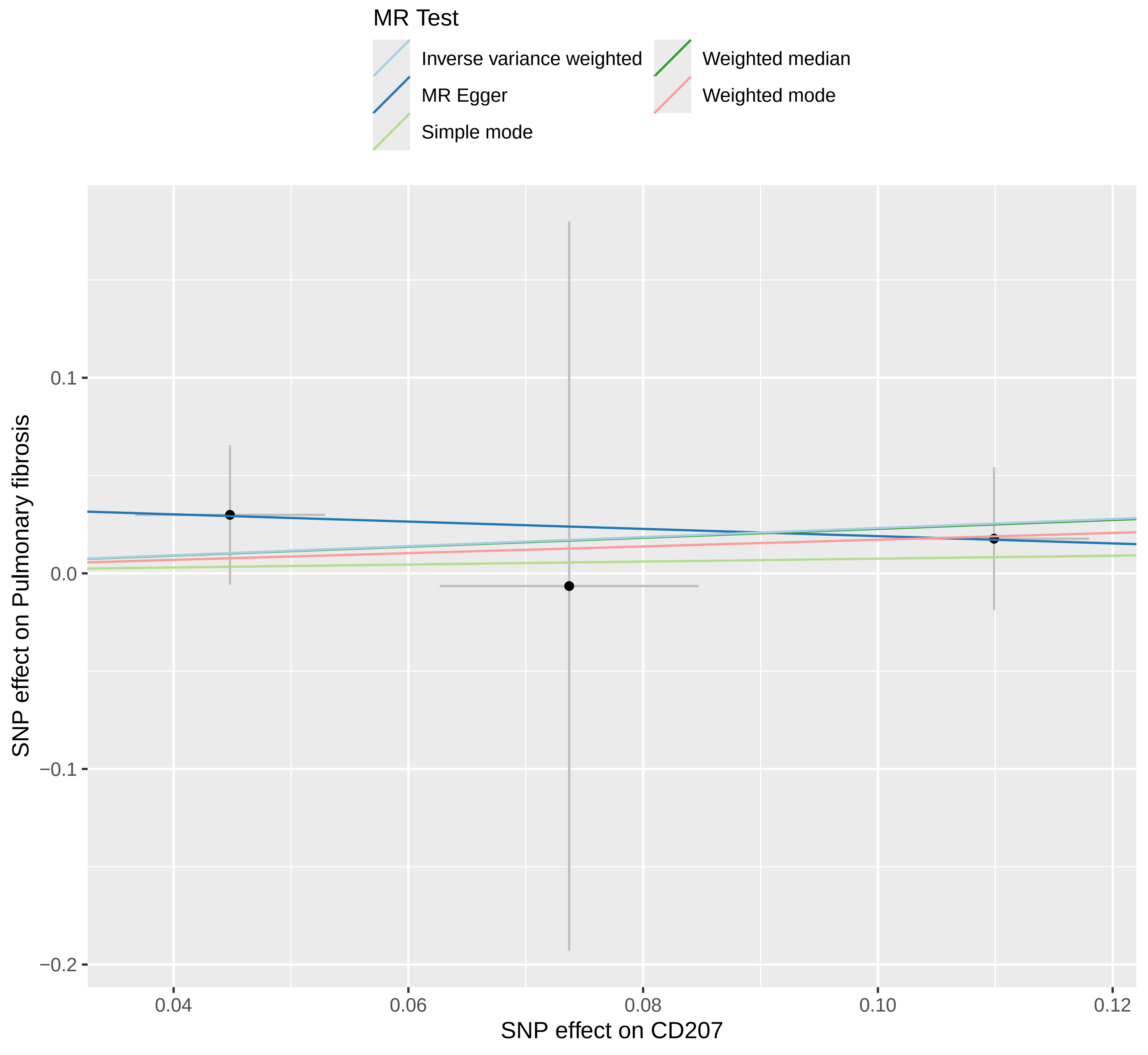

MR Test
Inverse variance weighted
MR Egger
Simple mode
Weighted median
Weighted mode
SNP effect on Pulmonary fibrosis
0.1
0.0
−0.1
−0.2
0.04
0.06
0.08
0.10
0.12
SNP effect on CD207

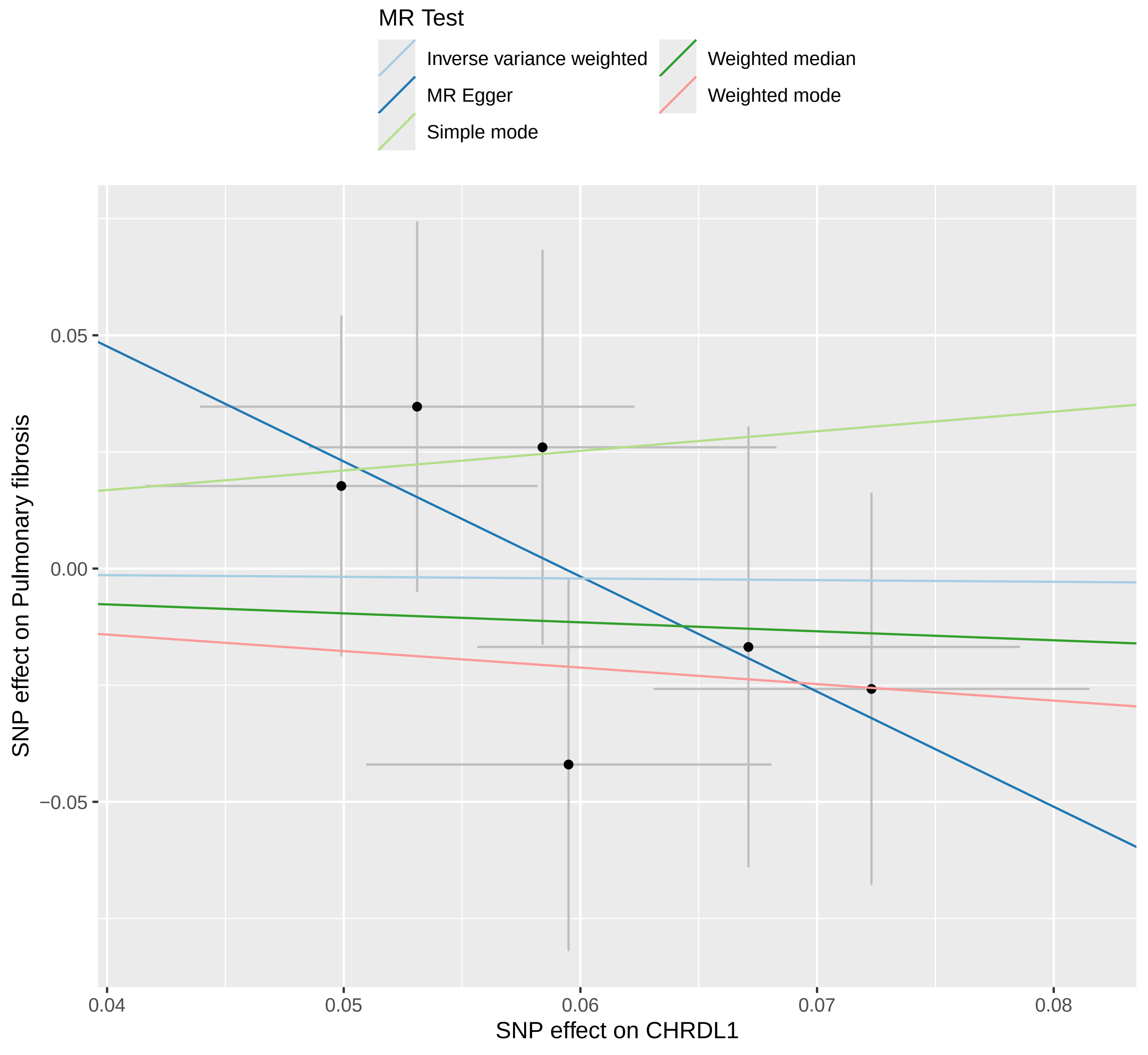
MR Test
Inverse variance weighted
MR Egger
Simple mode
Weighted median
Weighted mode
0.05
0.00
−0.05
SNP effect on Pulmonary fibrosis
0.04
0.05
0.06
0.07
0.08
SNP effect on CHRDL1

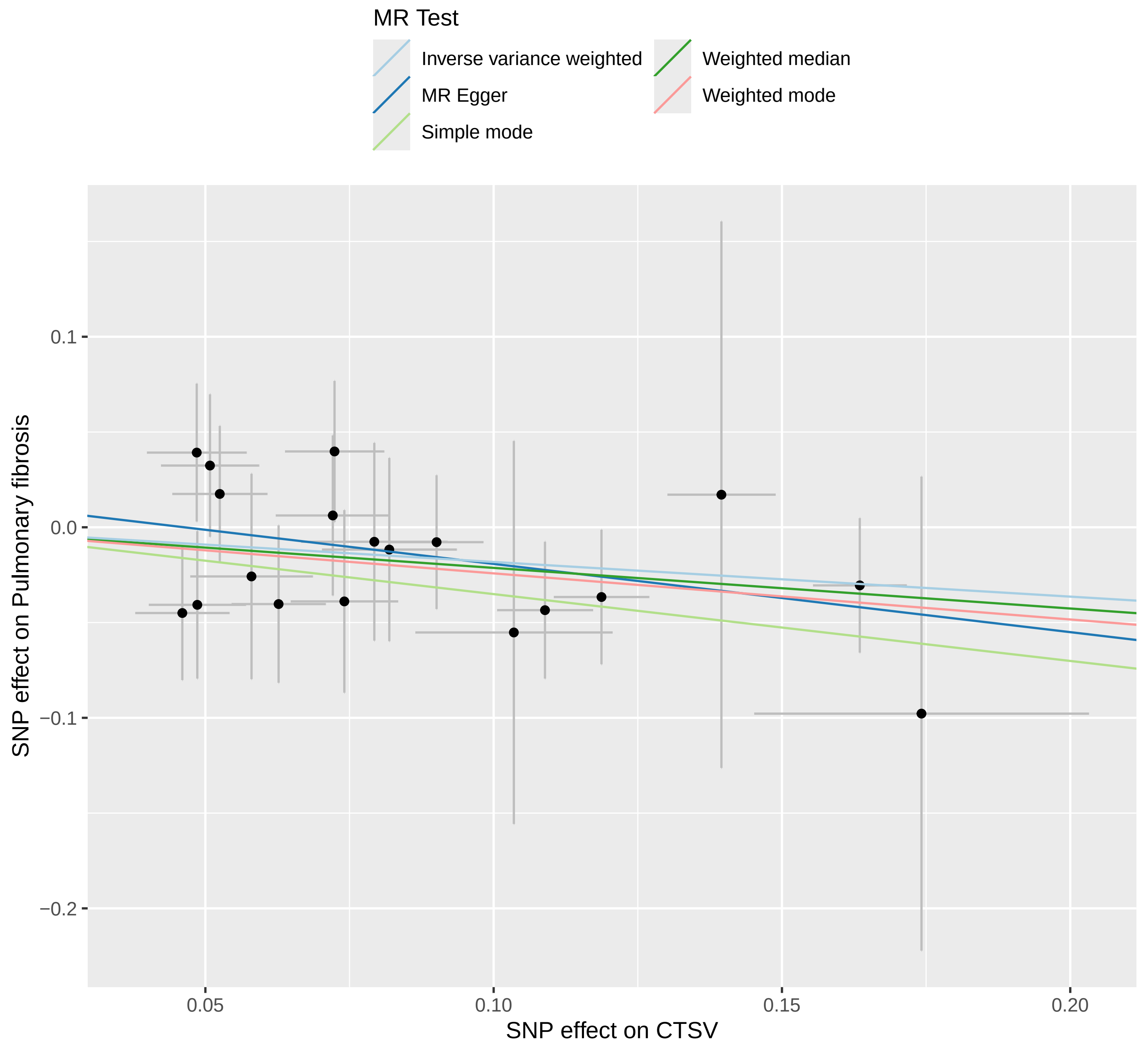

MR Test
Inverse variance weighted
MR Egger
Simple mode
Weighted median
Weighted mode
0.1
0.0
−0.1
−0.2
SNP effect on Pulmonary fibrosis
0.05
0.10
0.15
0.20
SNP effect on CTSV

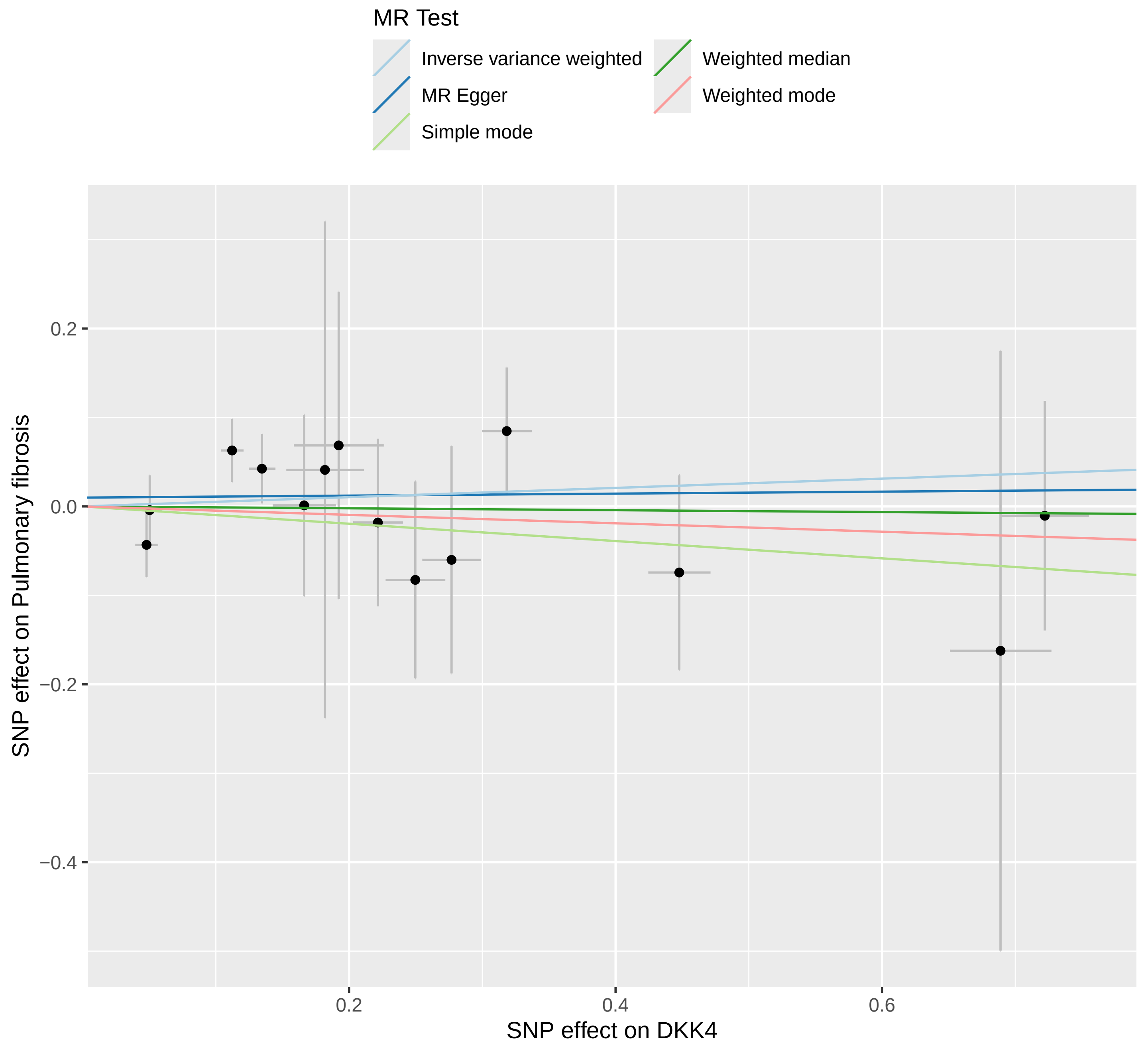

MR Test
Inverse variance weighted
MR Egger
Simple mode
Weighted median
Weighted mode
0.2
0.0
−0.2
−0.4
SNP effect on Pulmonary fibrosis
0.2
0.4
0.6
SNP effect on DKK4

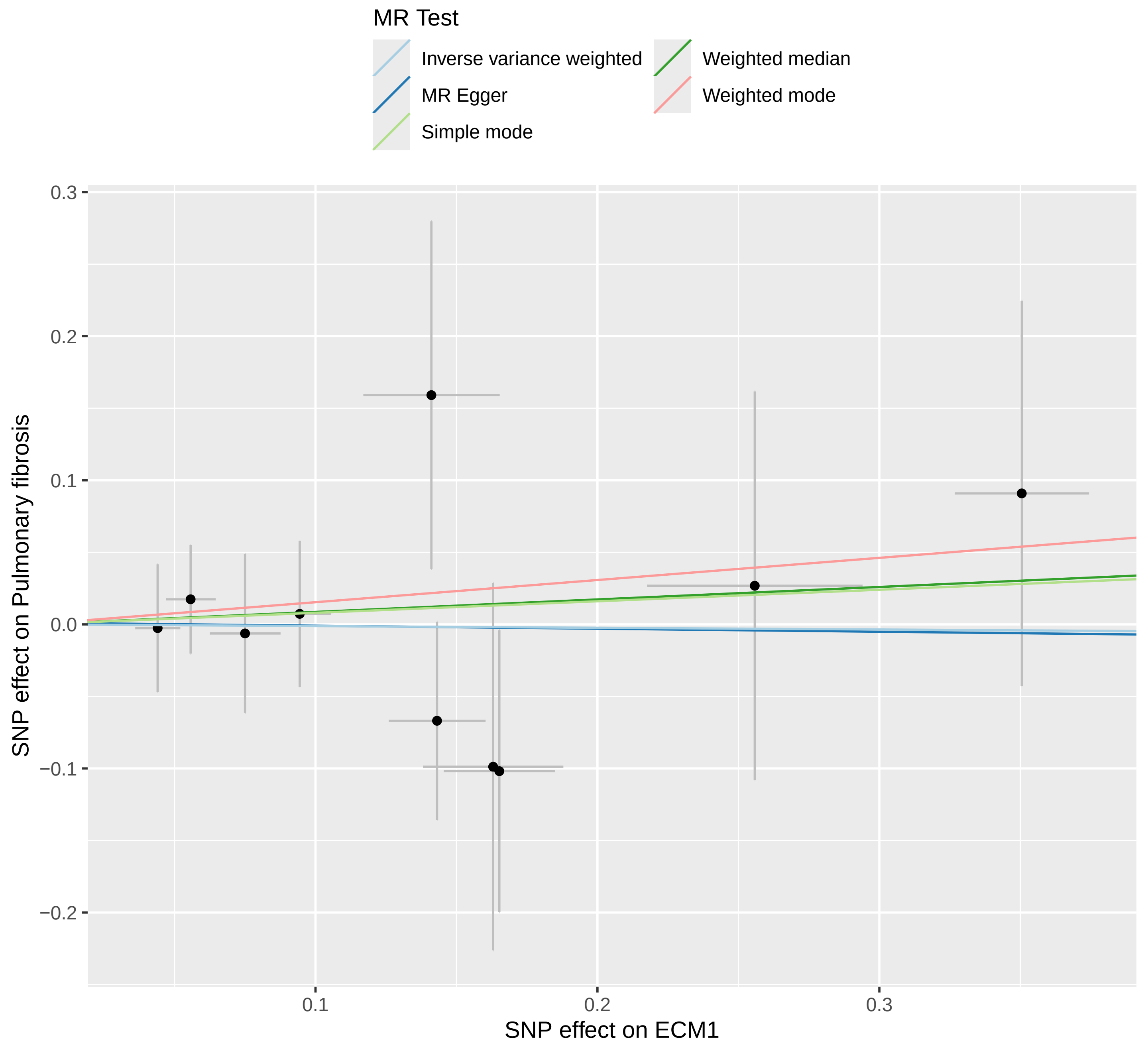
MR Test
Inverse variance weighted
MR Egger
Simple mode
Weighted median
Weighted mode
0.3
0.2
0.1
0.0
−0.1
−0.2
SNP effect on Pulmonary fibrosis
0.1
0.2
0.3
SNP effect on ECM1

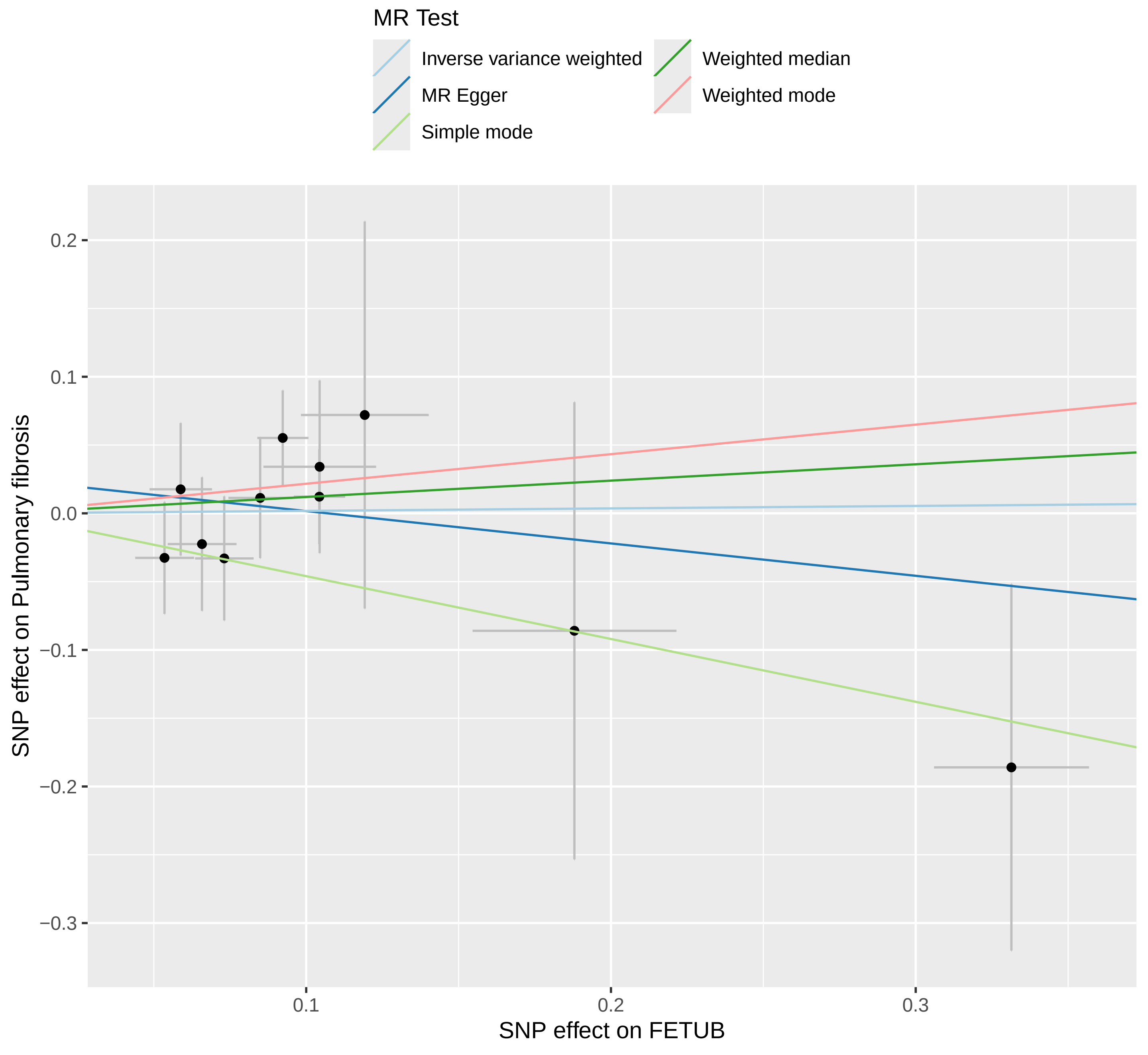

MR Test
Inverse variance weighted
MR Egger
Simple mode
Weighted median
Weighted mode
SNP effect on Pulmonary fibrosis
SNP effect on FETUB
0.2
0.1
0.0
−0.1
−0.2
−0.3
0.1
0.2
0.3

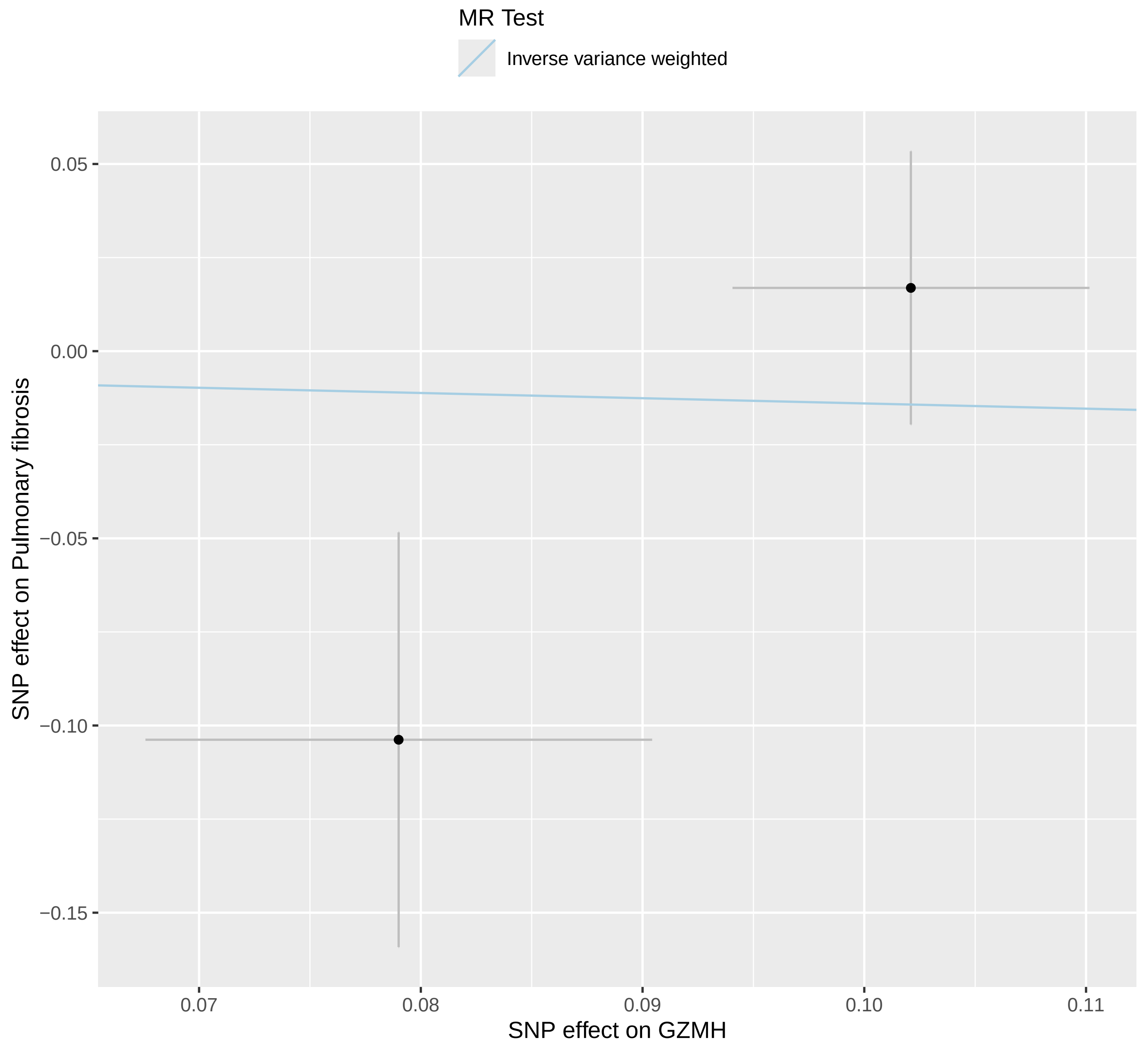
MR Test
Inverse variance weighted
0.05
0.00
−0.05
−0.10
−0.15
SNP effect on Pulmonary fibrosis
0.07
0.08
0.09
0.10
0.11
SNP effect on GZMH

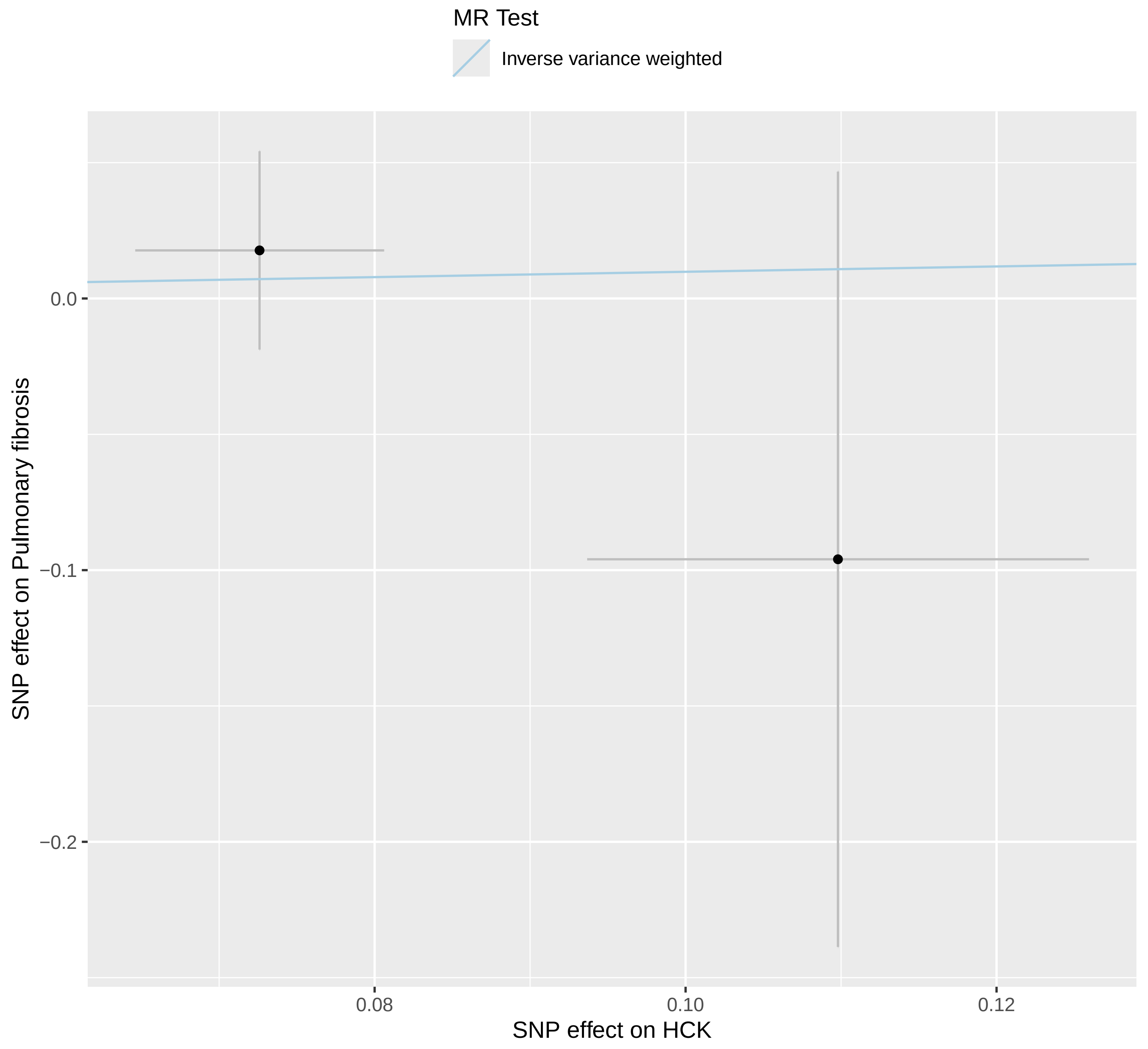

MR Test
Inverse variance weighted
SNP effect on Pulmonary fibrosis
0.0
−0.1
−0.2
0.08
0.10
0.12
SNP effect on HCK

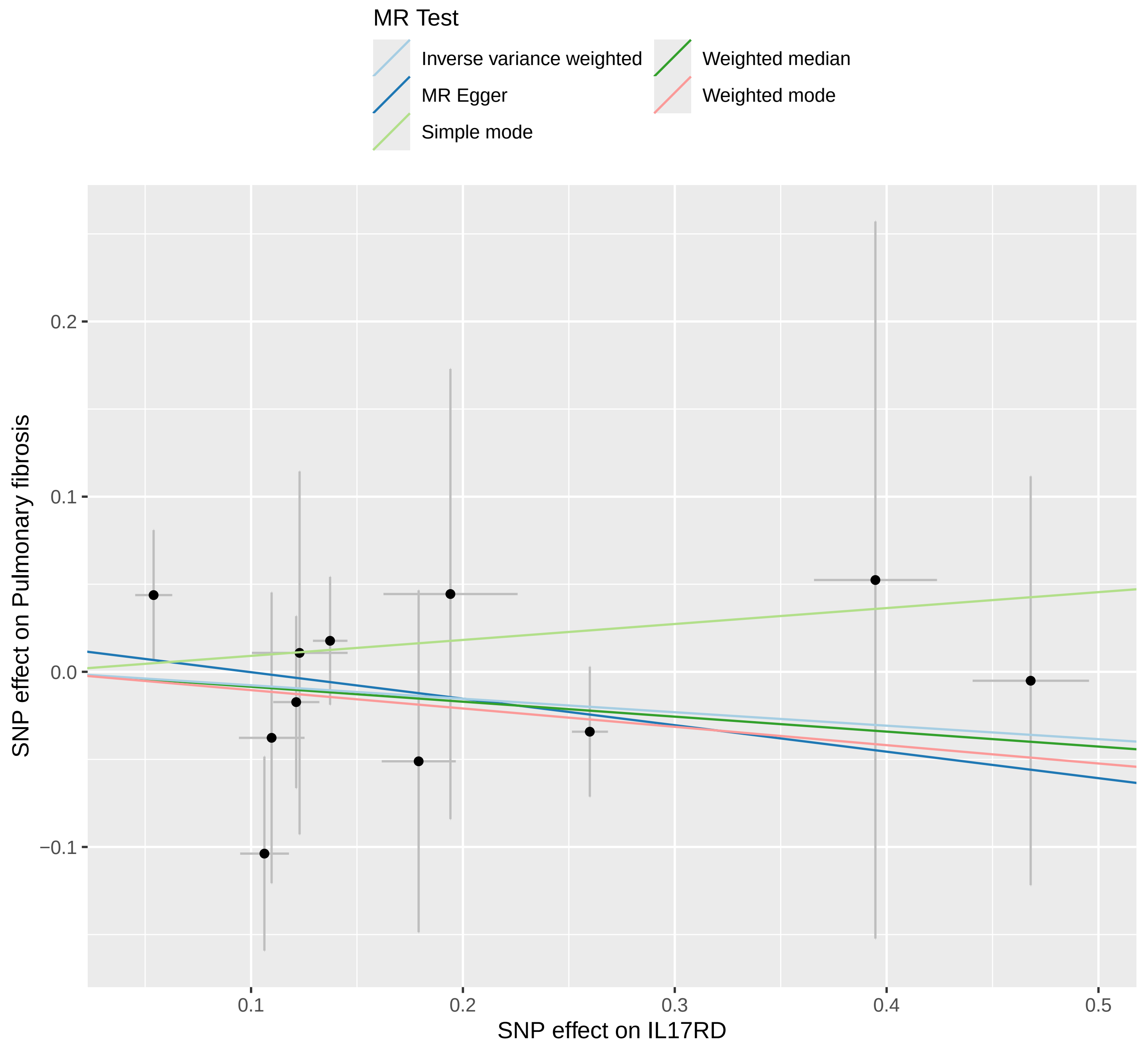

MR Test
Inverse variance weighted
MR Egger
Simple mode
Weighted median
Weighted mode
SNP effect on Pulmonary fibrosis
0.2
0.1
0.0
−0.1
0.1
0.2
0.3
0.4
0.5
SNP effect on IL17RD

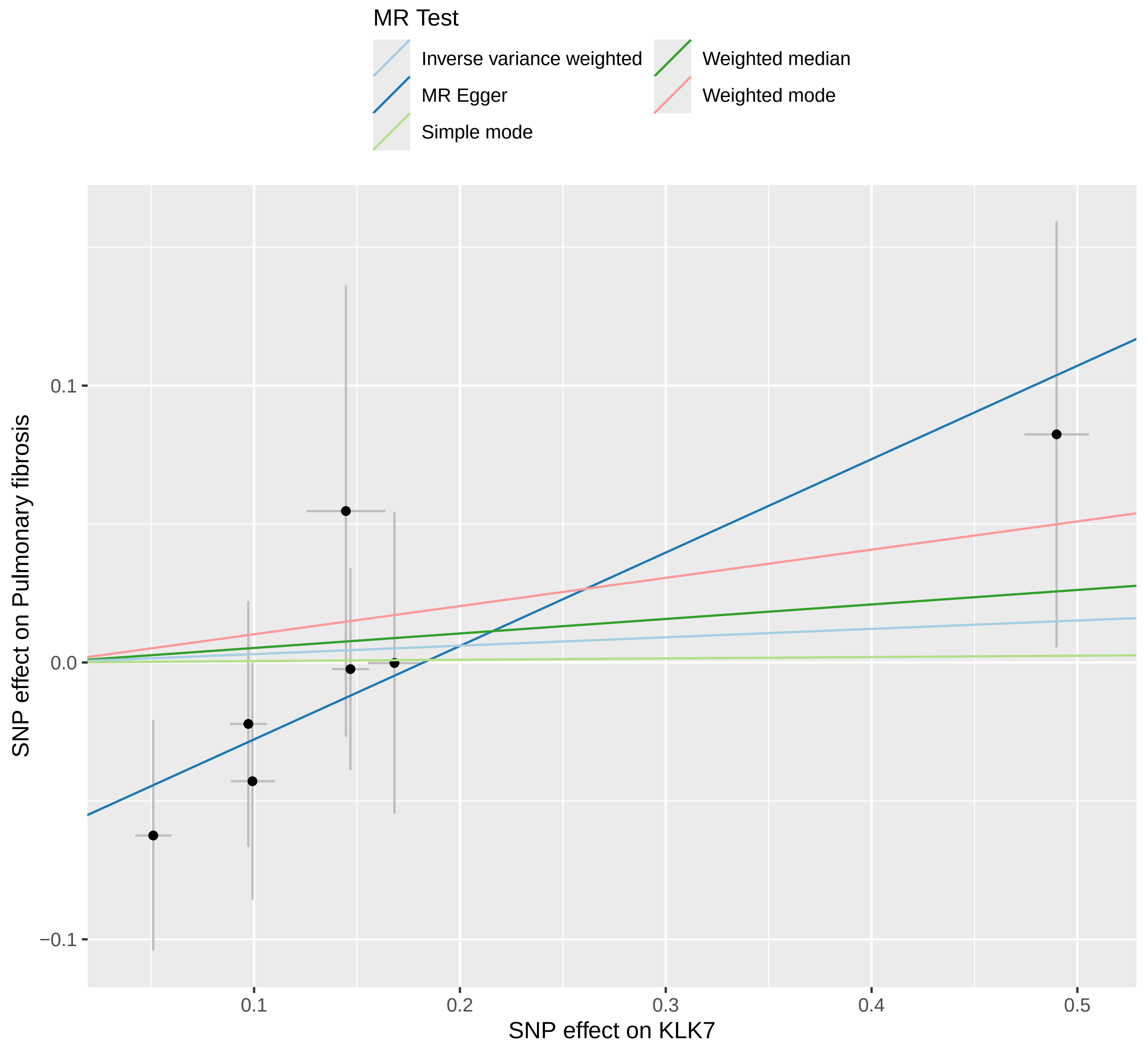
MR Test
Inverse variance weighted
MR Egger
Simple mode
Weighted median
Weighted mode
SNP effect on Pulmonary fibrosis
0.1
0.0
−0.1
0.1
0.2
0.3
0.4
0.5
SNP effect on KLK7

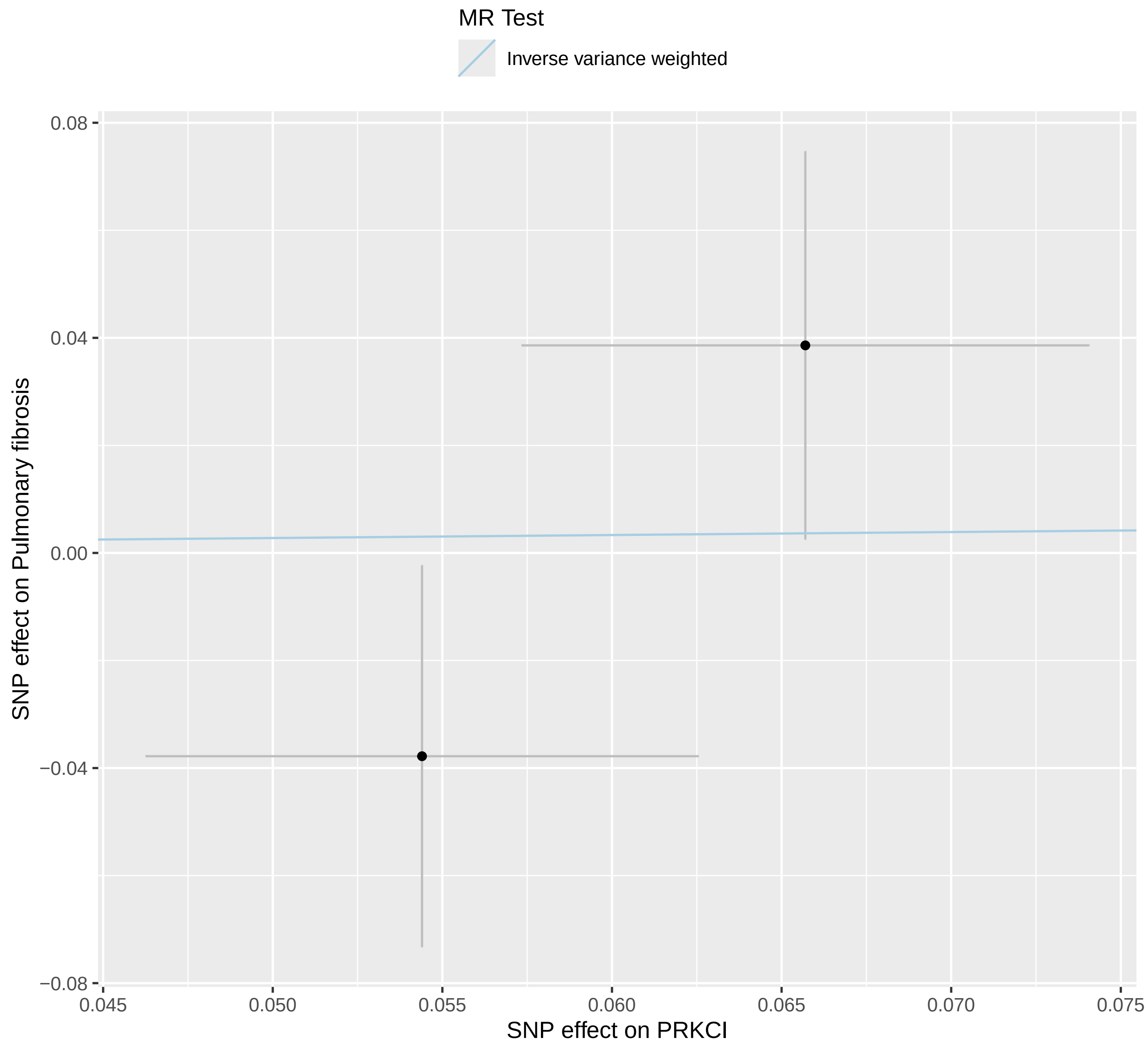

MR Test
Inverse variance weighted
0.08
0.04
0.00
−0.04
−0.08
SNP effect on Pulmonary fibrosis
0.045
0.050
0.055
0.060
0.065
0.070
0.075
SNP effect on PRKCI

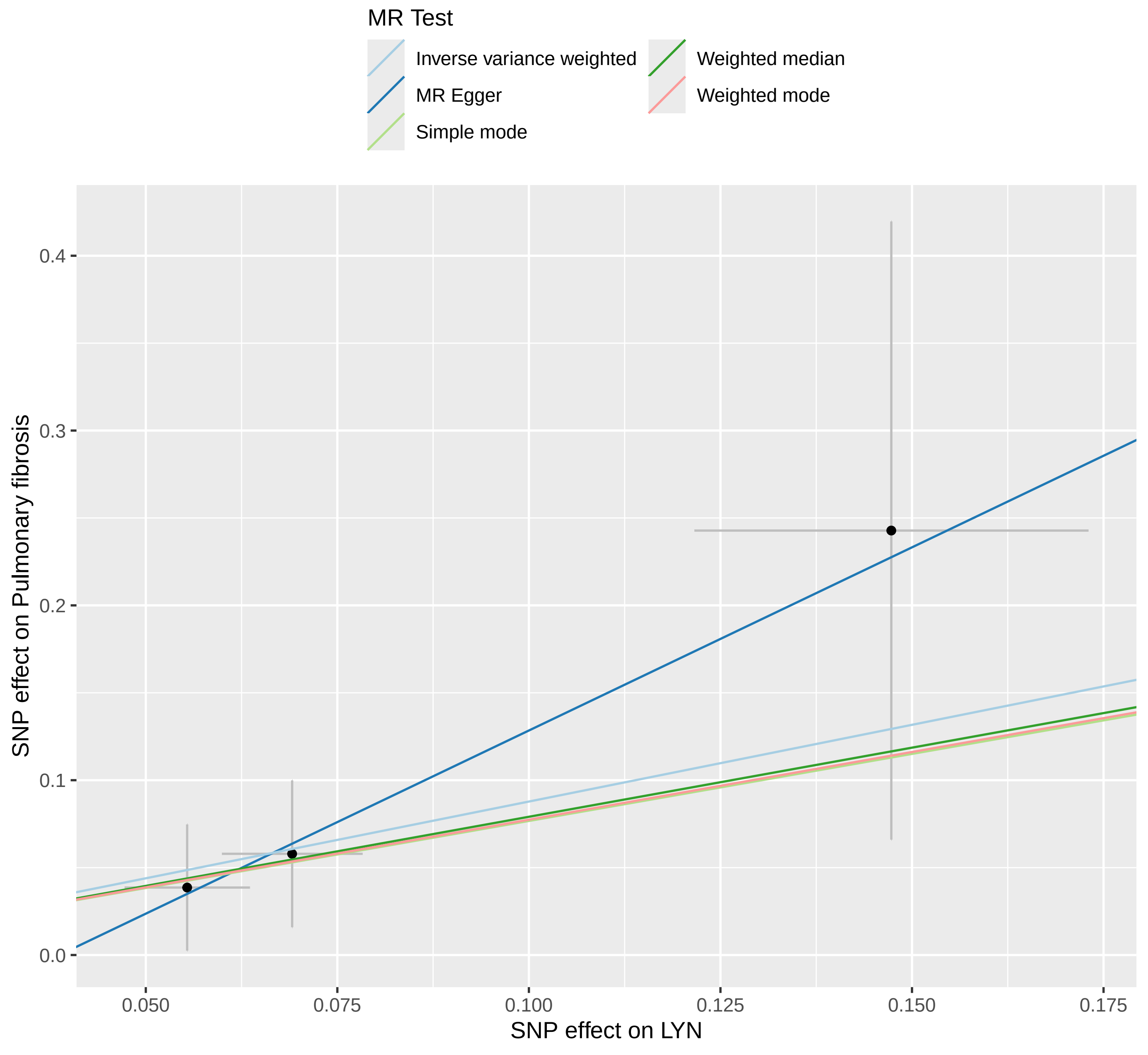

MR Test
Inverse variance weighted
MR Egger
Simple mode
Weighted median
Weighted mode
SNP effect on Pulmonary fibrosis
0.0
0.1
0.2
0.3
0.4
0.050
0.075
0.100
0.125
0.150
0.175
SNP effect on LYN

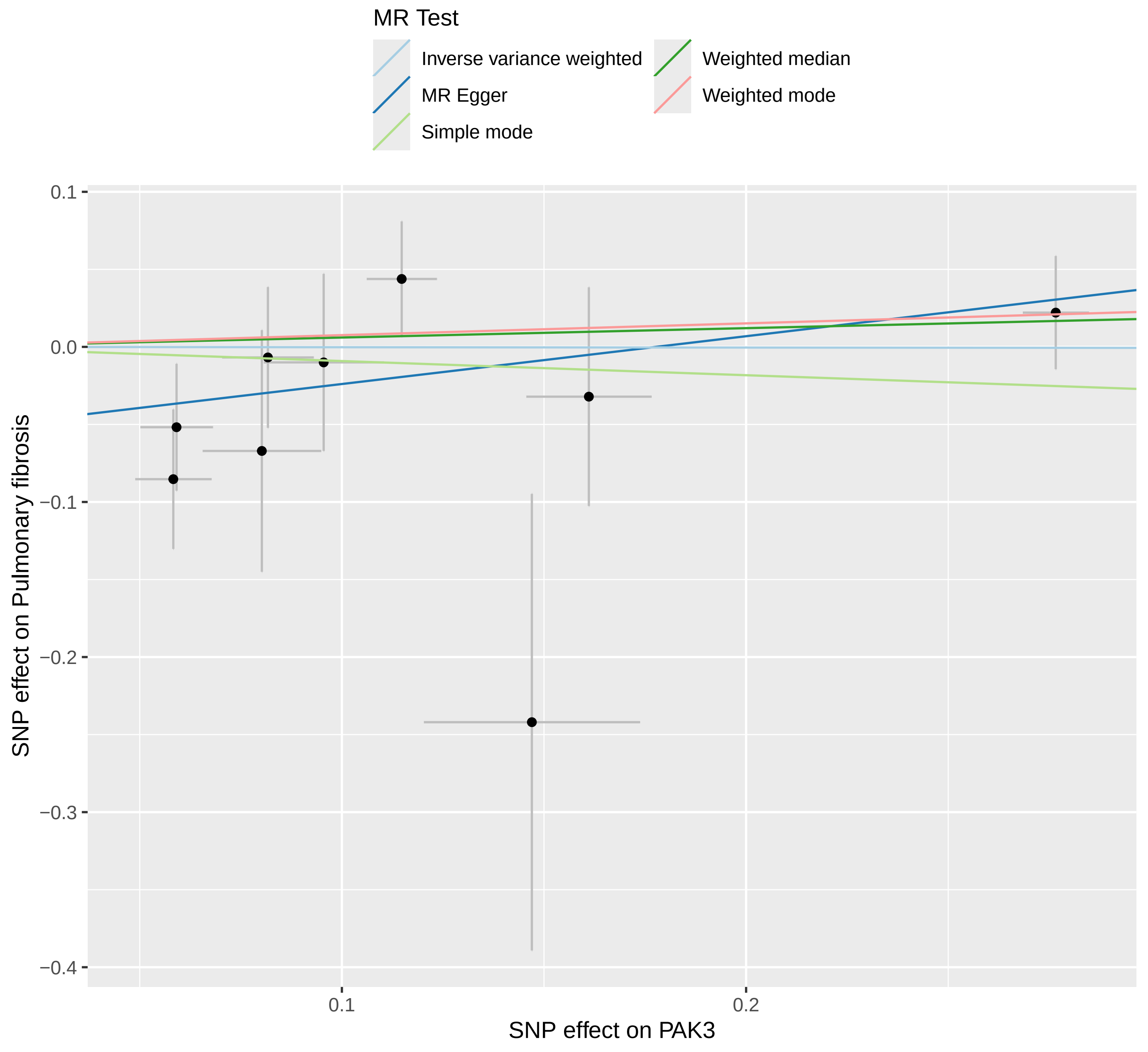
MR Test
Inverse variance weighted
MR Egger
Simple mode
Weighted median
Weighted mode
0.1
0.0
−0.1
−0.2
−0.3
−0.4
SNP effect on Pulmonary fibrosis
0.1
0.2
SNP effect on PAK3

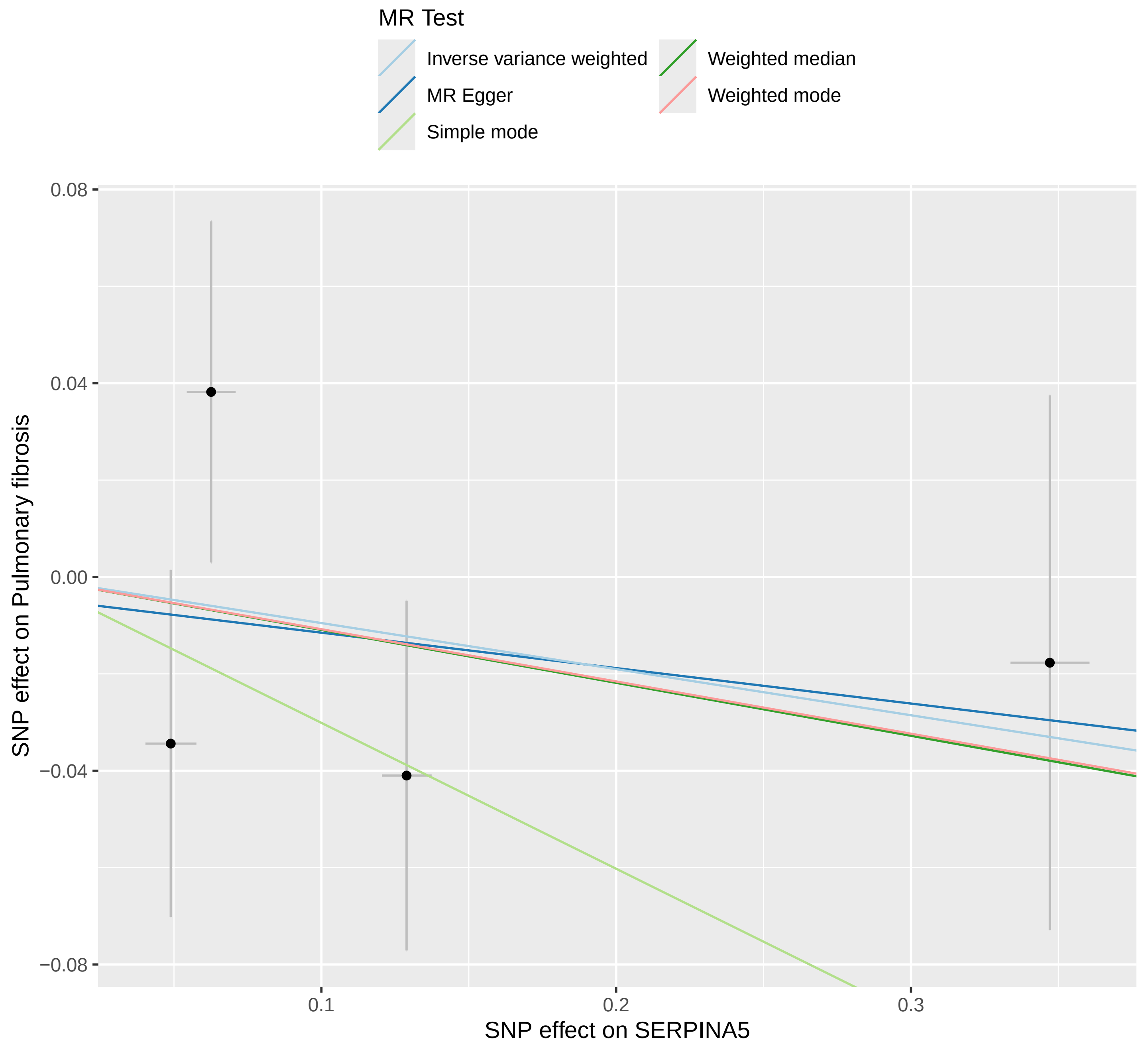

MR Test
Inverse variance weighted
MR Egger
Simple mode
Weighted median
Weighted mode
0.08
0.04
0.00
−0.04
−0.08
SNP effect on Pulmonary fibrosis
0.1
0.2
0.3
SNP effect on SERPINA5

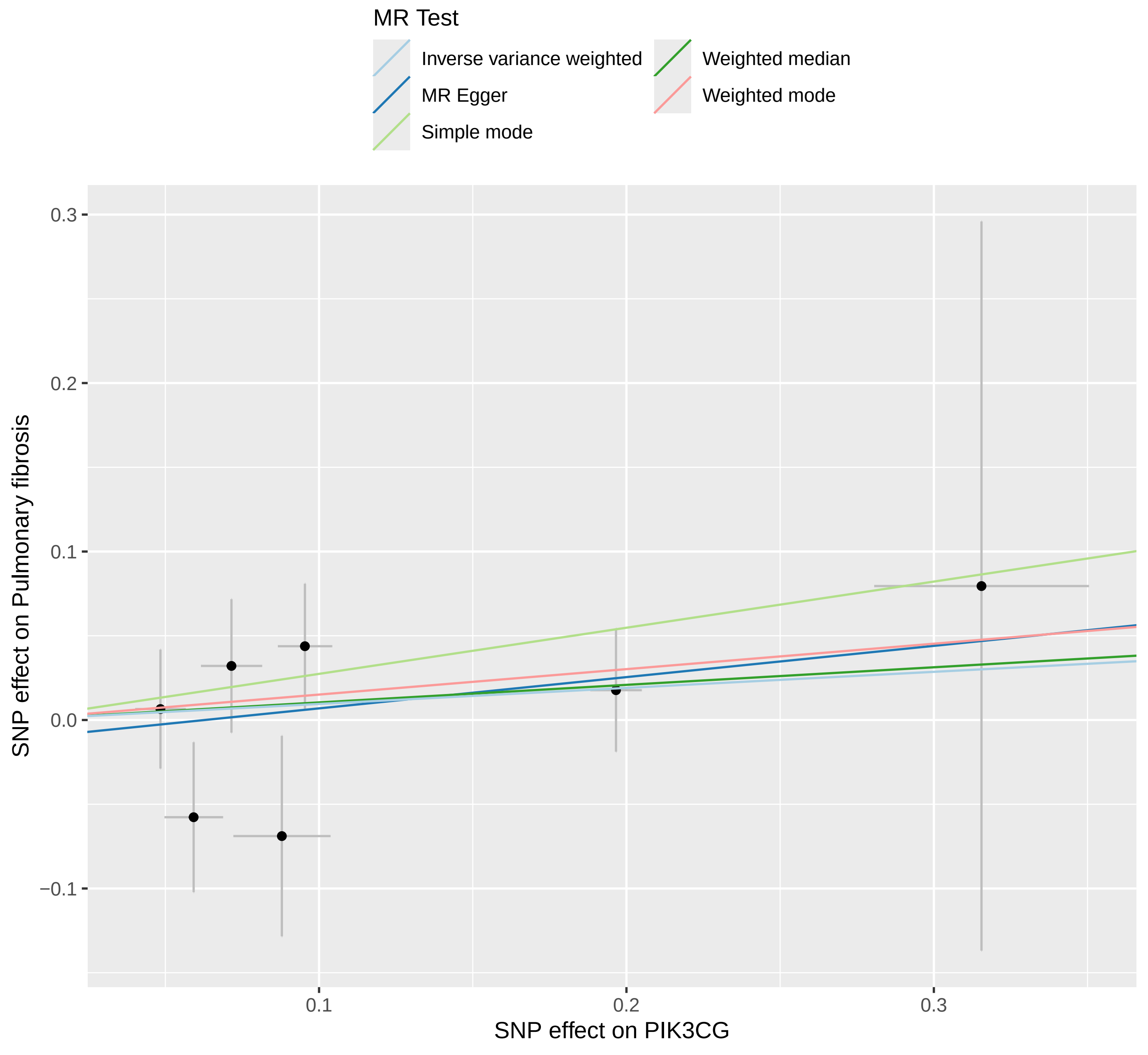

MR Test
Inverse variance weighted
MR Egger
Simple mode
Weighted median
Weighted mode
0.3
0.2
0.1
0.0
−0.1
0.1
0.2
0.3
SNP effect on Pulmonary fibrosis
SNP effect on PIK3CG

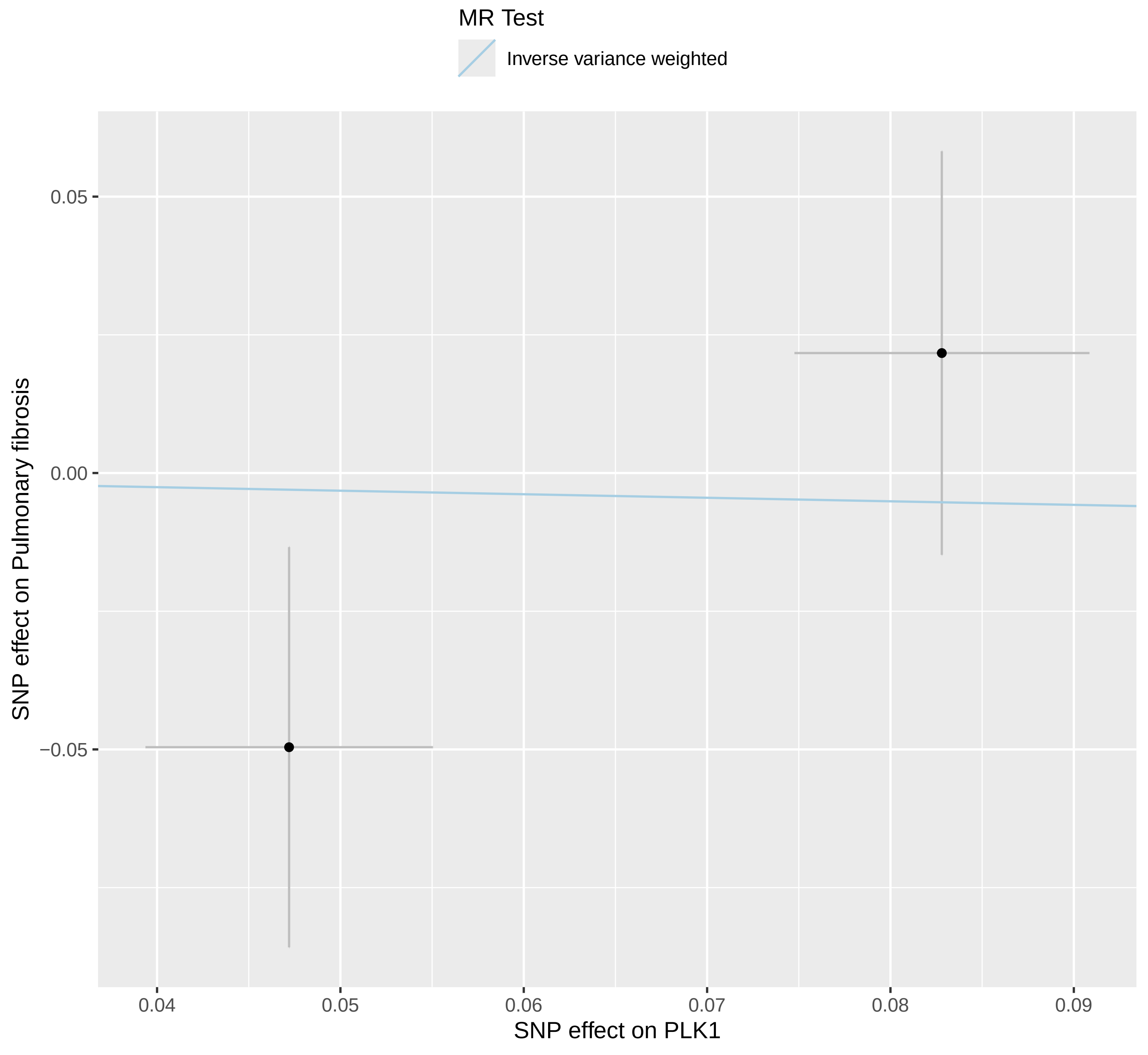

MR Test
Inverse variance weighted
0.05
0.00
−0.05
0.04
0.05
0.06
0.07
0.08
0.09
SNP effect on PLK1
SNP effect on Pulmonary fibrosis

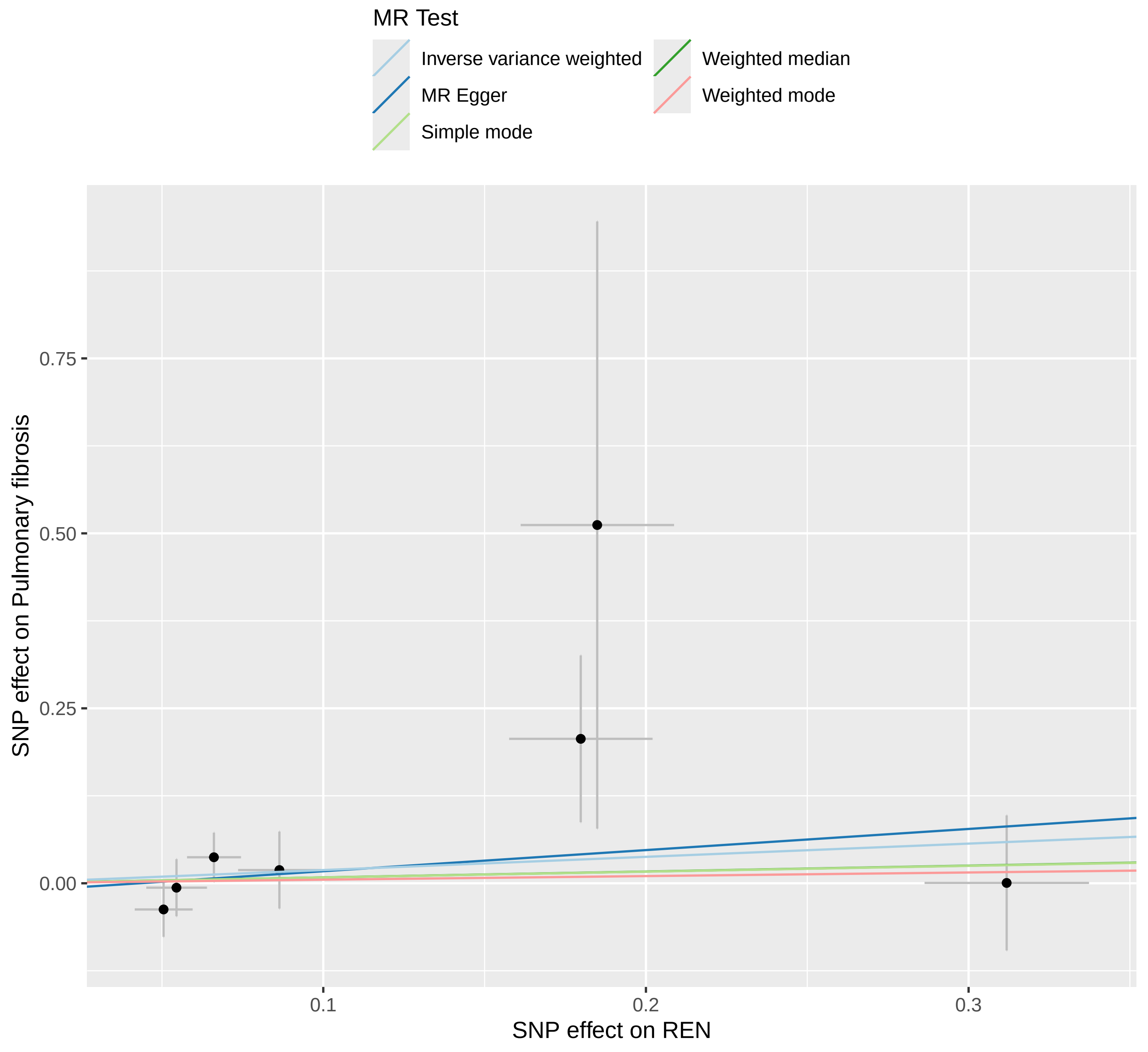
MR Test
Inverse variance weighted
MR Egger
Simple mode
Weighted median
Weighted mode
SNP effect on Pulmonary fibrosis
0.75
0.50
0.25
0.00
0.1
0.2
0.3
SNP effect on REN

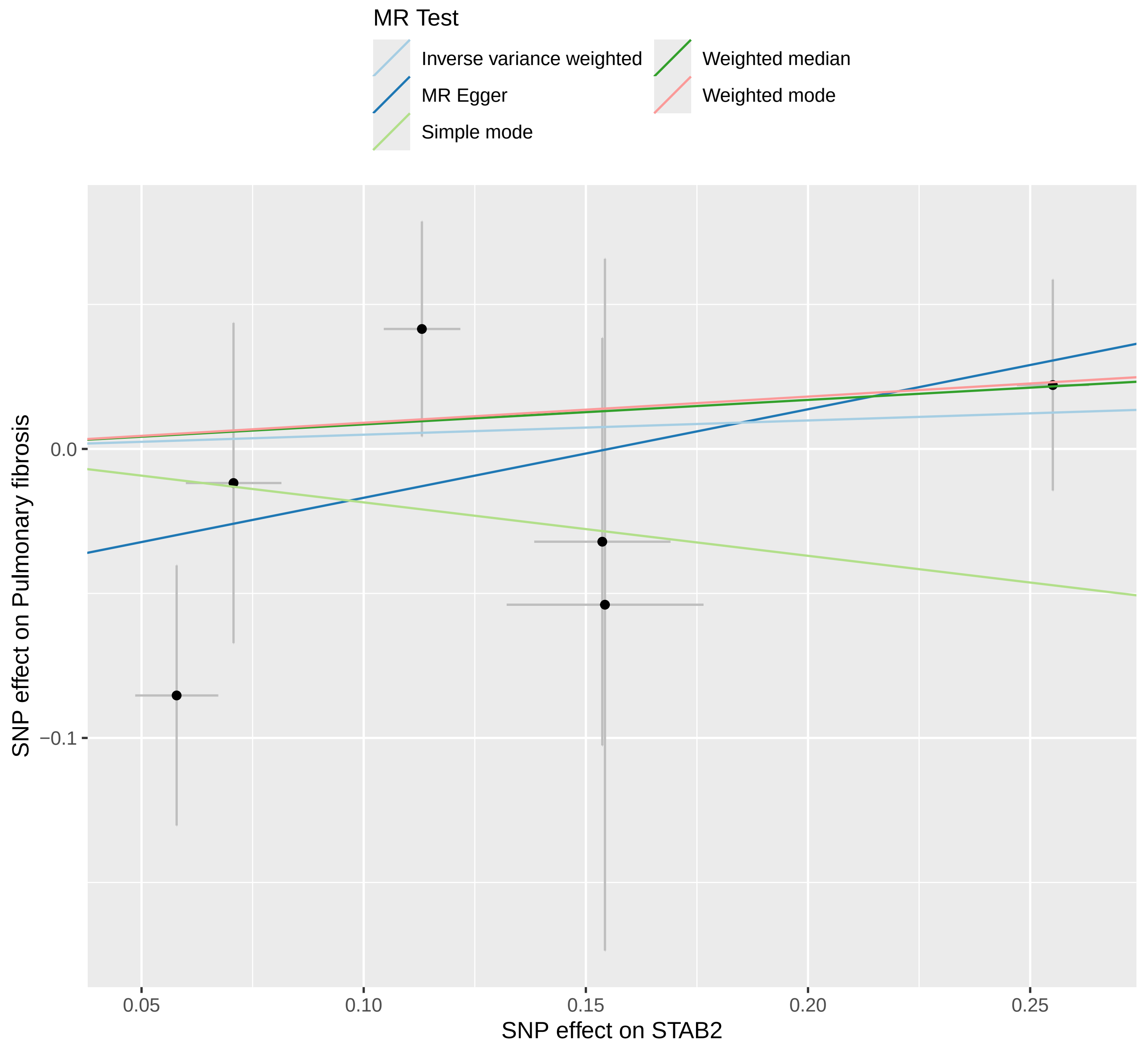

MR Test
Inverse variance weighted
MR Egger
Simple mode
Weighted median
Weighted mode
SNP effect on Pulmonary fibrosis
0.0
−0.1
0.05
0.10
0.15
0.20
0.25
SNP effect on STAB2

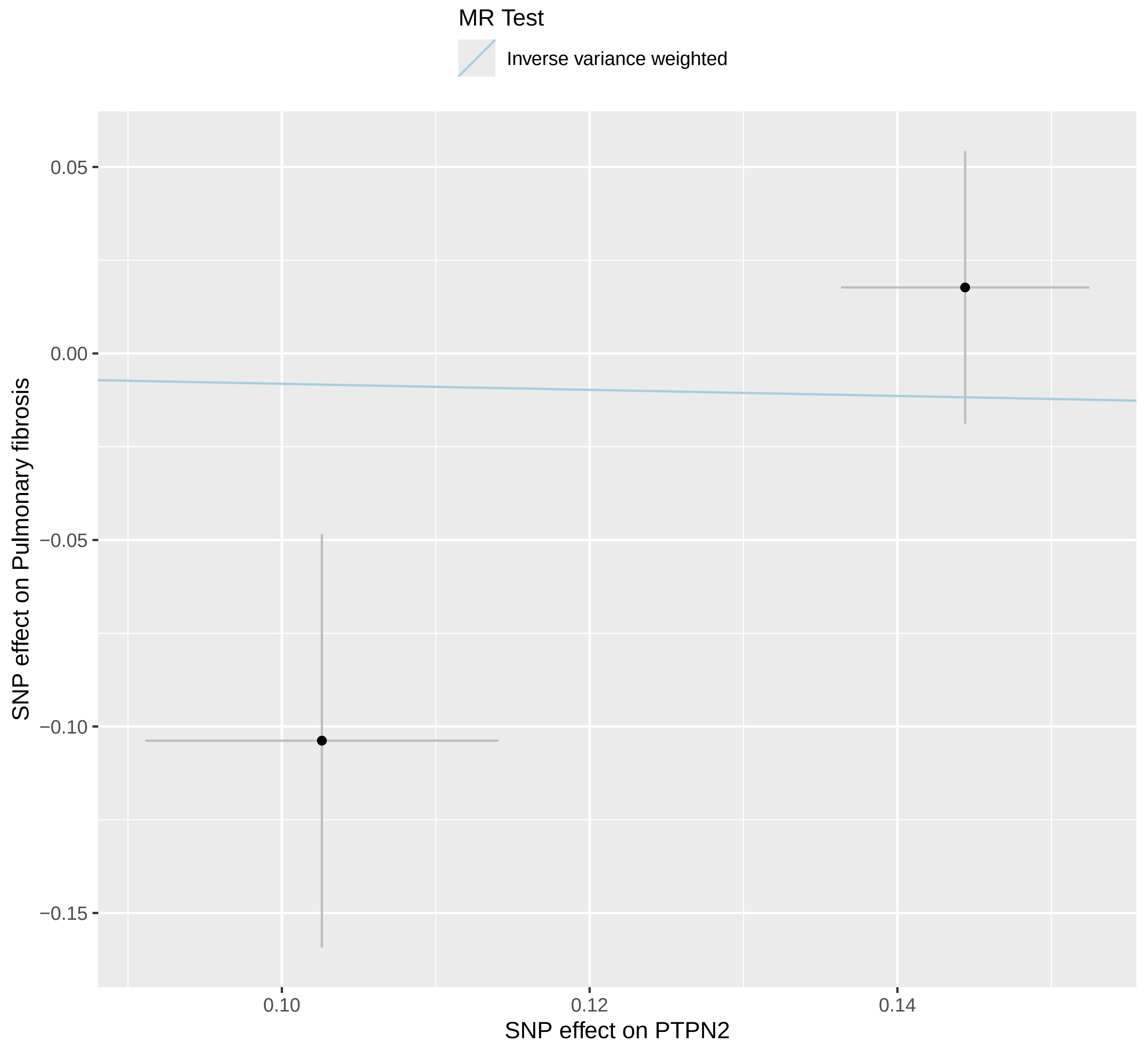
MR Test
Inverse variance weighted
0.05
0.00
−0.05
−0.10
−0.15
0.10
0.12
0.14
SNP effect on PTPN2
SNP effect on Pulmonary fibrosis

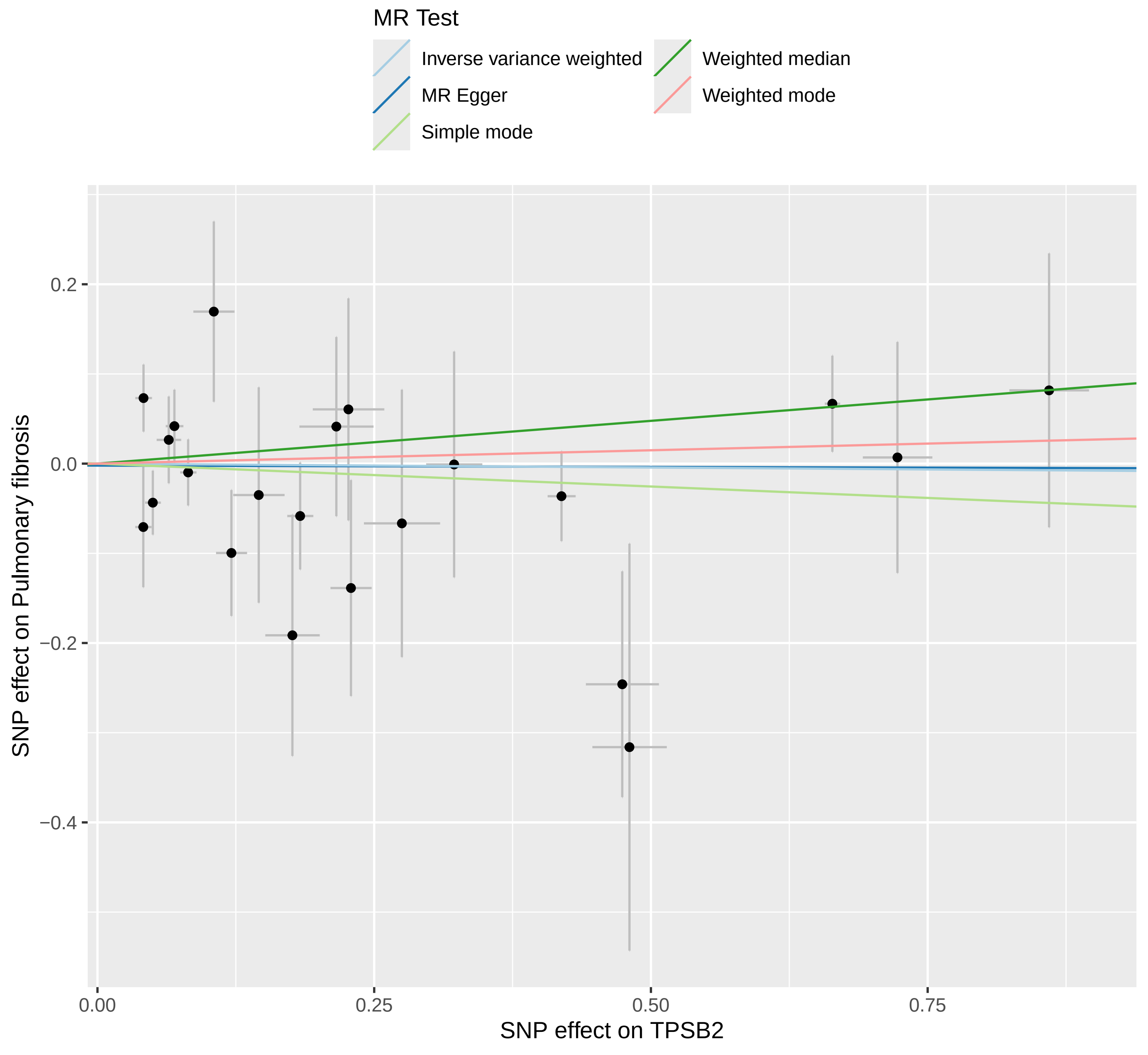

MR Test
Inverse variance weighted
MR Egger
Simple mode
Weighted median
Weighted mode
SNP effect on Pulmonary fibrosis
0.2
0.0
−0.2
−0.4
0.00
0.25
0.50
0.75
SNP effect on TPSB2

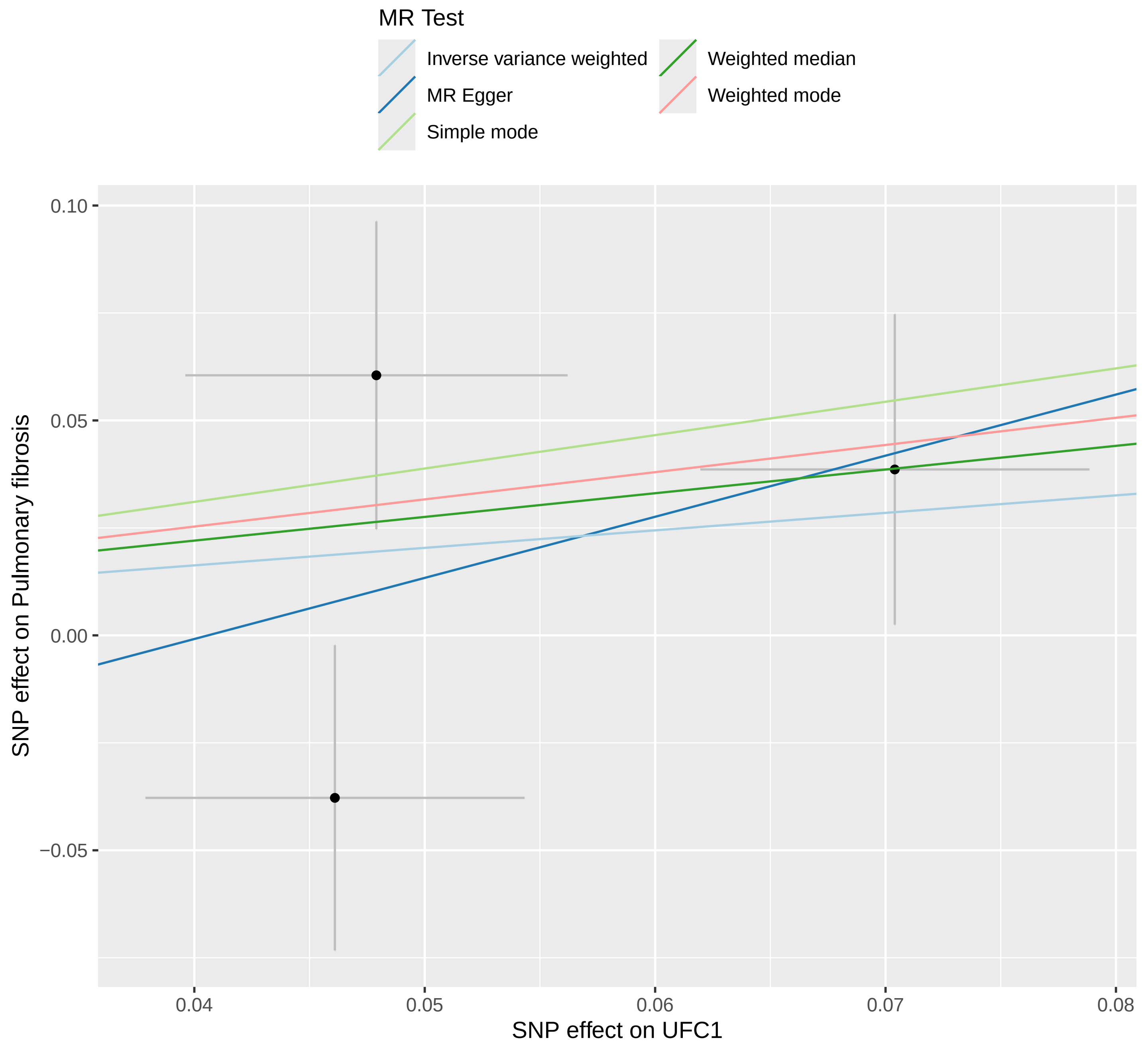

MR Test
Inverse variance weighted
MR Egger
Simple mode
Weighted median
Weighted mode
0.10
0.05
0.00
−0.05
SNP effect on Pulmonary fibrosis
0.04
0.05
0.06
0.07
0.08
SNP effect on UFC1

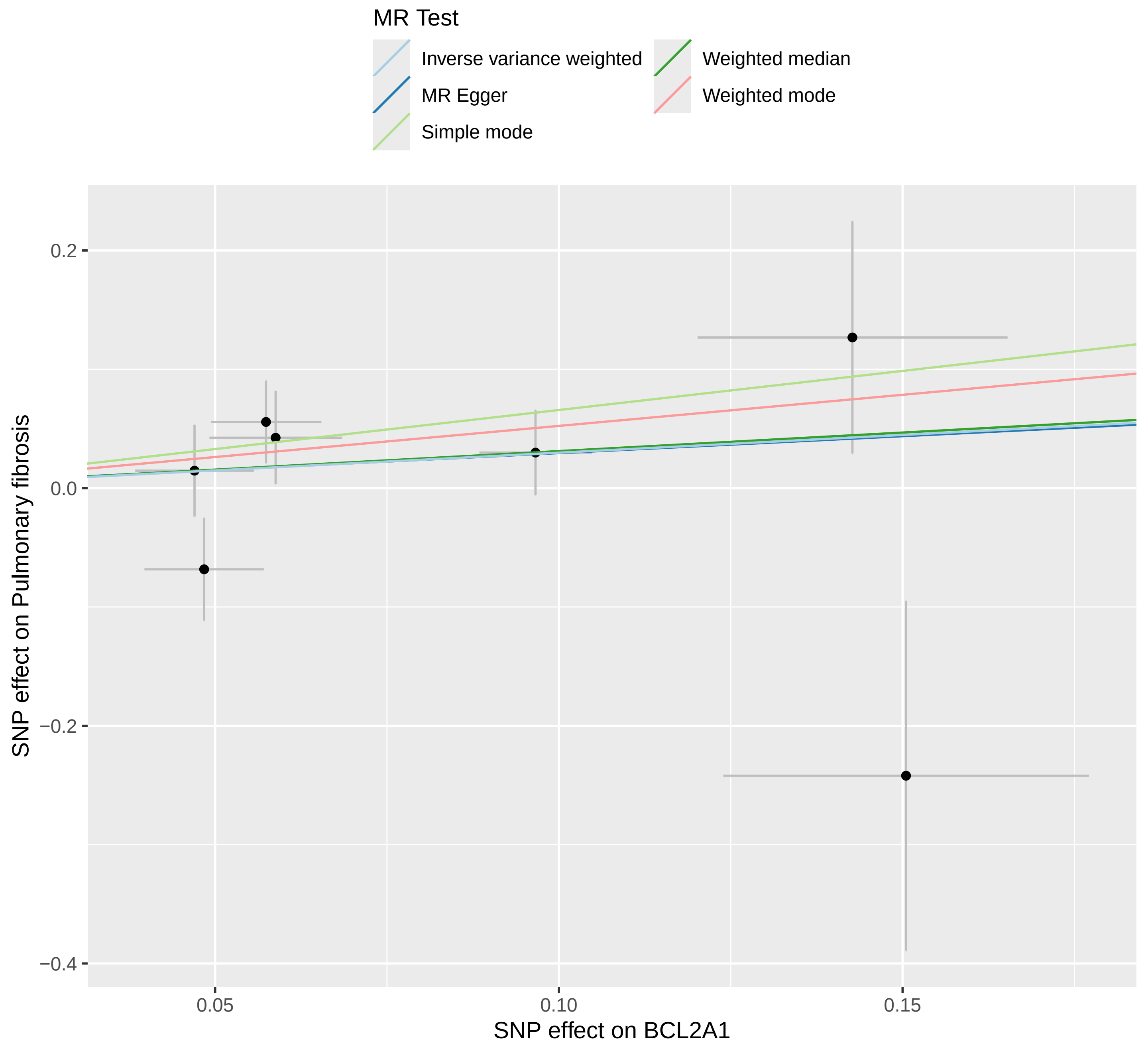
MR Test
Inverse variance weighted
MR Egger
Simple mode
Weighted median
Weighted mode
0.2
0.0
−0.2
−0.4
0.05
0.10
0.15
SNP effect on Pulmonary fibrosis
SNP effect on BCL2A1

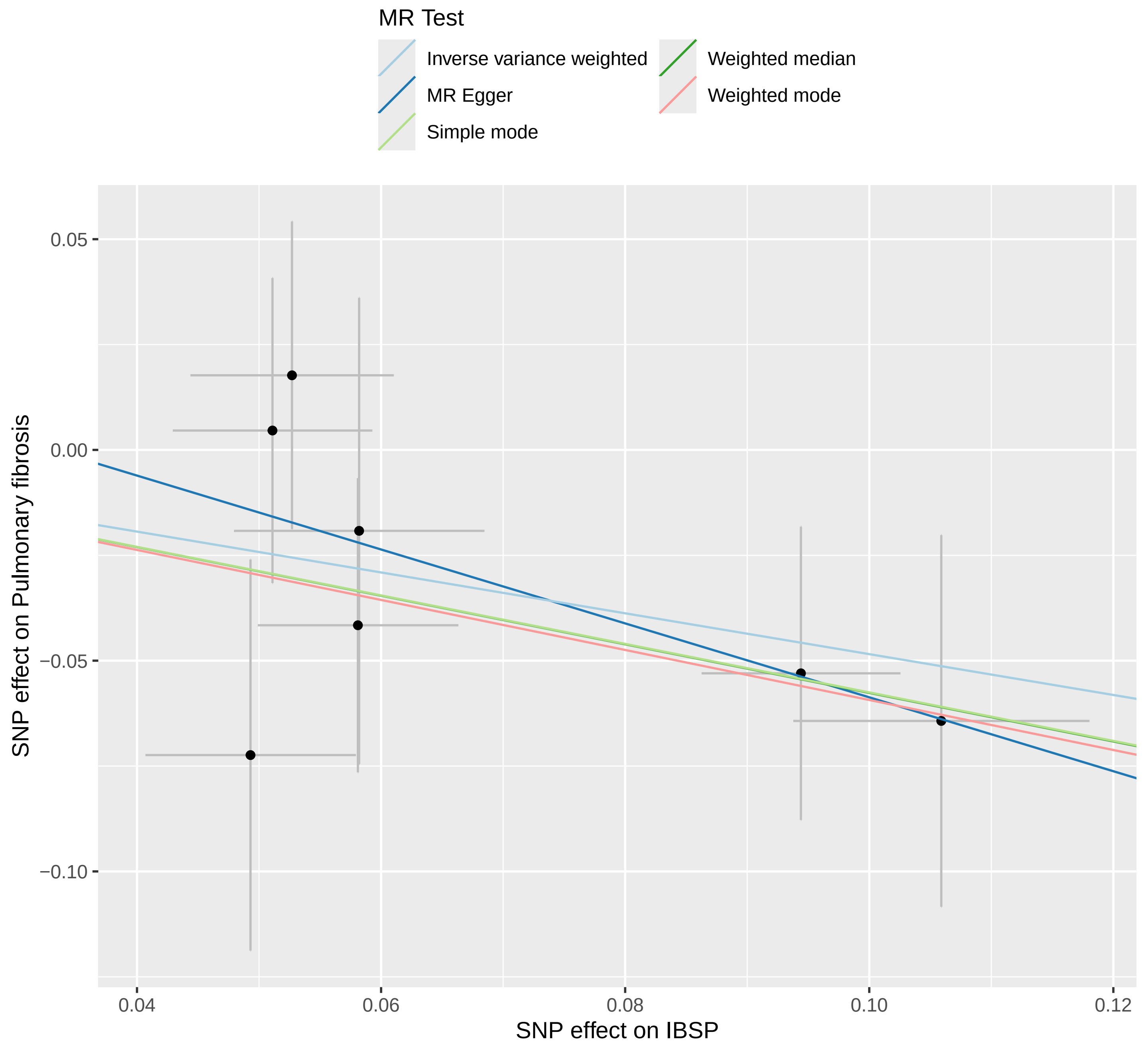

MR Test
Inverse variance weighted
MR Egger
Simple mode
Weighted median
Weighted mode
0.05
0.00
−0.05
−0.10
SNP effect on Pulmonary fibrosis
0.04
0.06
0.08
0.10
0.12
SNP effect on IBSP

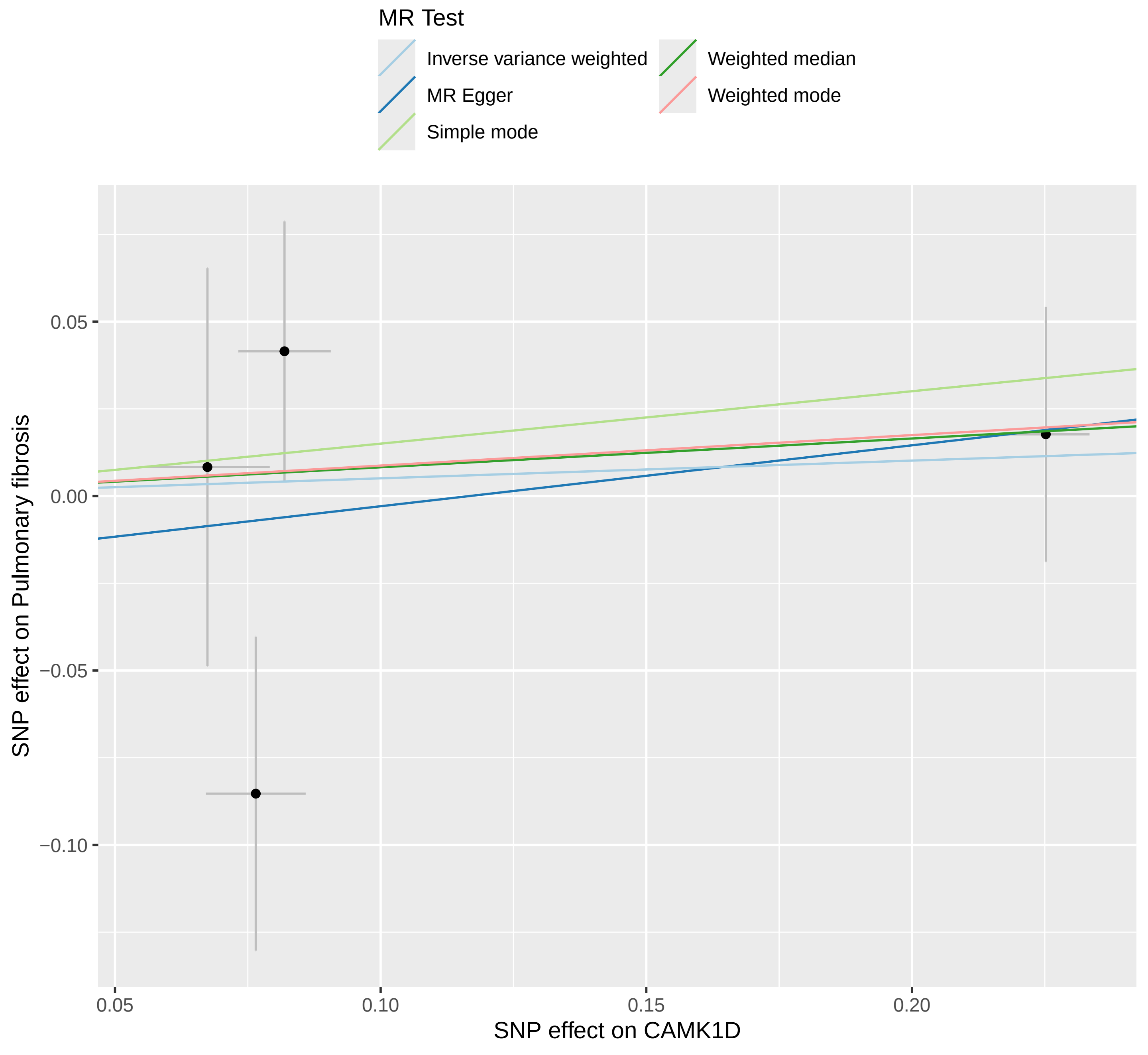

MR Test
Inverse variance weighted
MR Egger
Simple mode
Weighted median
Weighted mode
SNP effect on Pulmonary fibrosis
0.05
0.00
−0.05
−0.10
0.05
0.10
0.15
0.20
SNP effect on CAMK1D

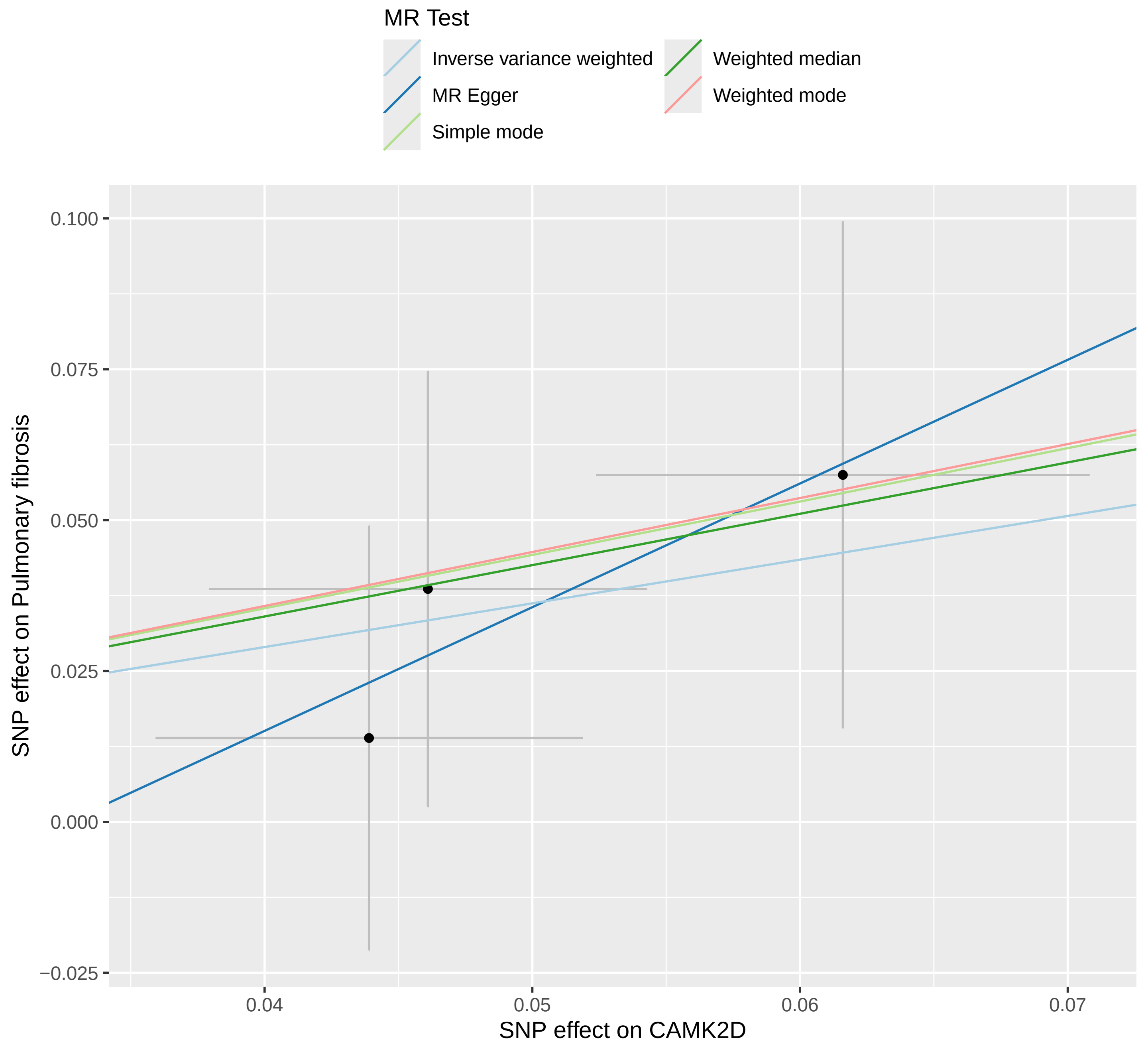
MR Test
Inverse variance weighted
MR Egger
Simple mode
Weighted median
Weighted mode
0.100
0.075
0.050
0.025
0.000
−0.025
0.04
0.05
0.06
0.07
SNP effect on Pulmonary fibrosis
SNP effect on CAMK2D

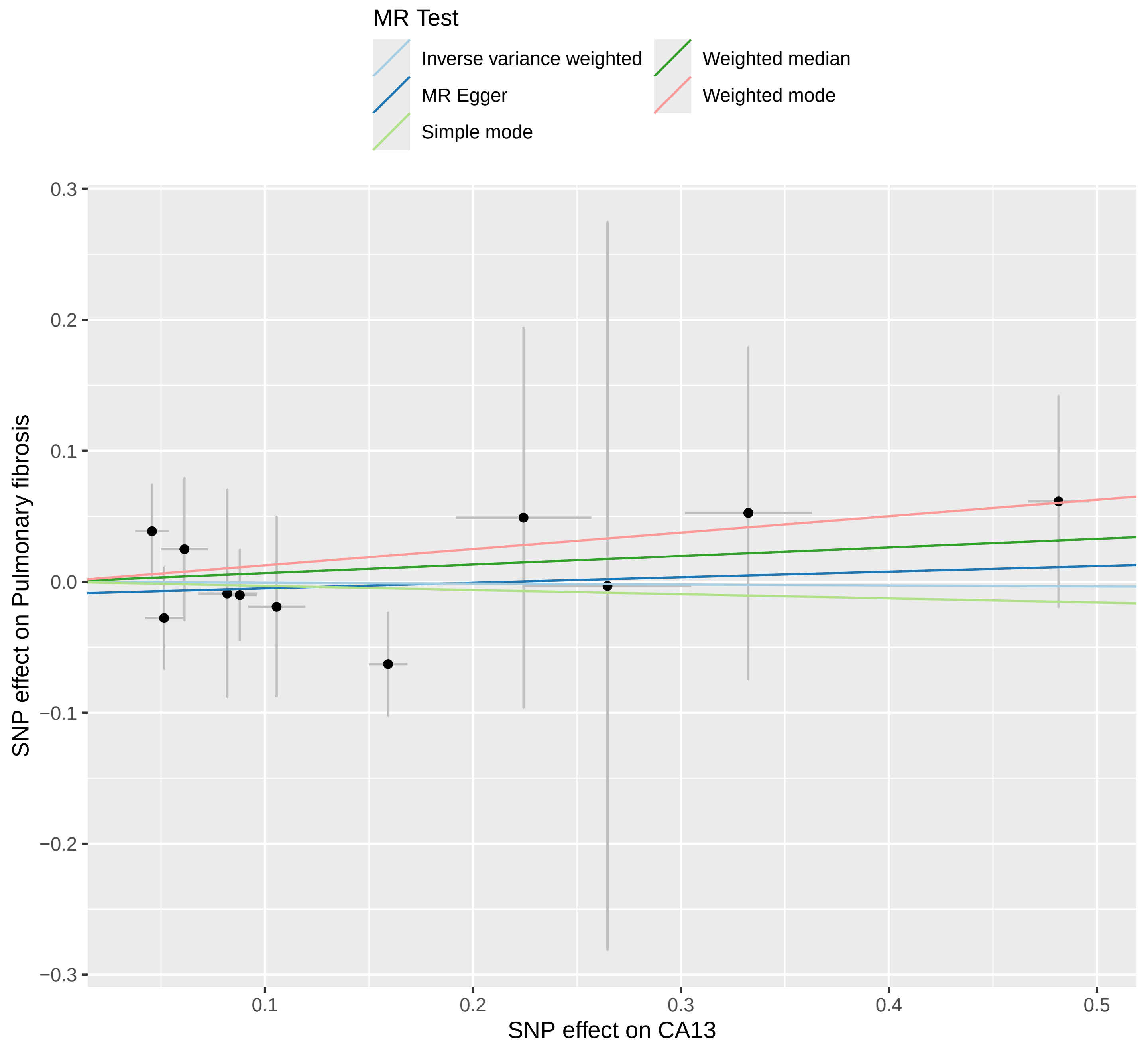

MR Test
Inverse variance weighted
MR Egger
Simple mode
Weighted median
Weighted mode
0.3
0.2
0.1
0.0
−0.1
−0.2
−0.3
0.1
0.2
0.3
0.4
0.5
SNP effect on Pulmonary fibrosis
SNP effect on CA13

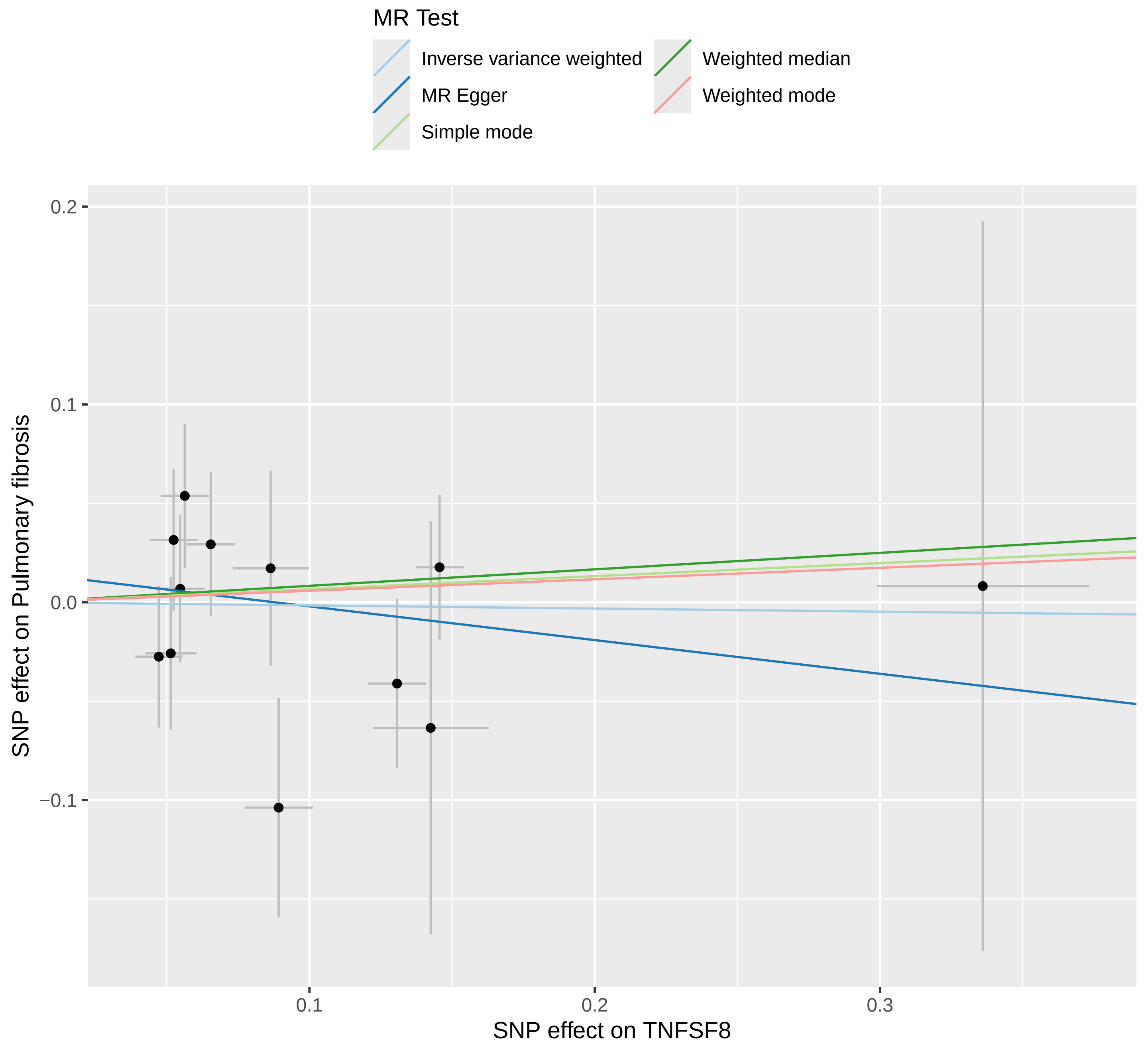
MR Test
Inverse variance weighted
MR Egger
Simple mode
Weighted median
Weighted mode
0.2
0.1
0.0
−0.1
0.1
0.2
0.3
SNP effect on Pulmonary fibrosis
SNP effect on TNFSF8

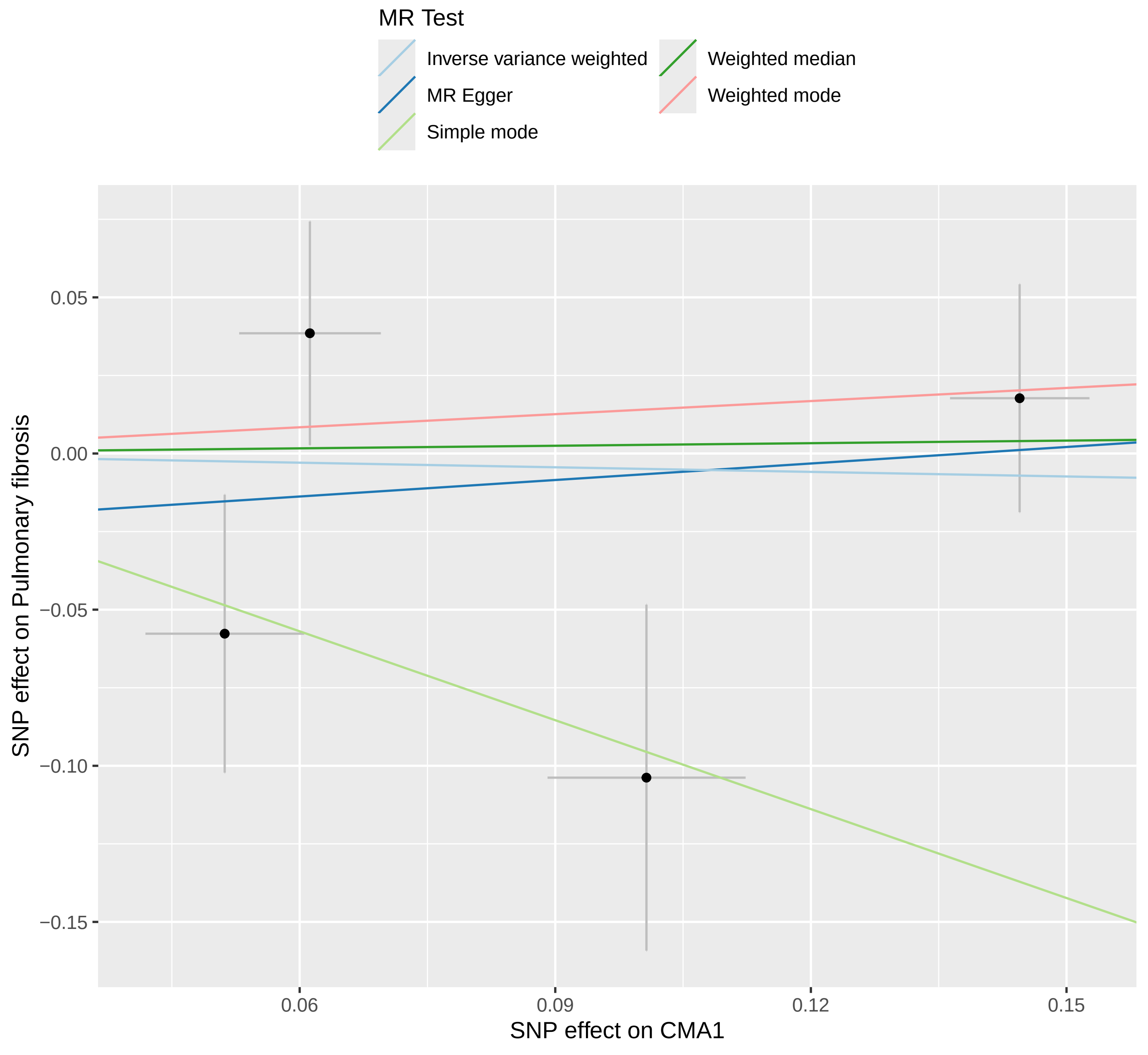

MR Test
Inverse variance weighted
MR Egger
Simple mode
Weighted median
Weighted mode
0.05
0.00
−0.05
−0.10
−0.15
0.06
0.09
0.12
0.15
SNP effect on CMA1
SNP effect on Pulmonary fibrosis

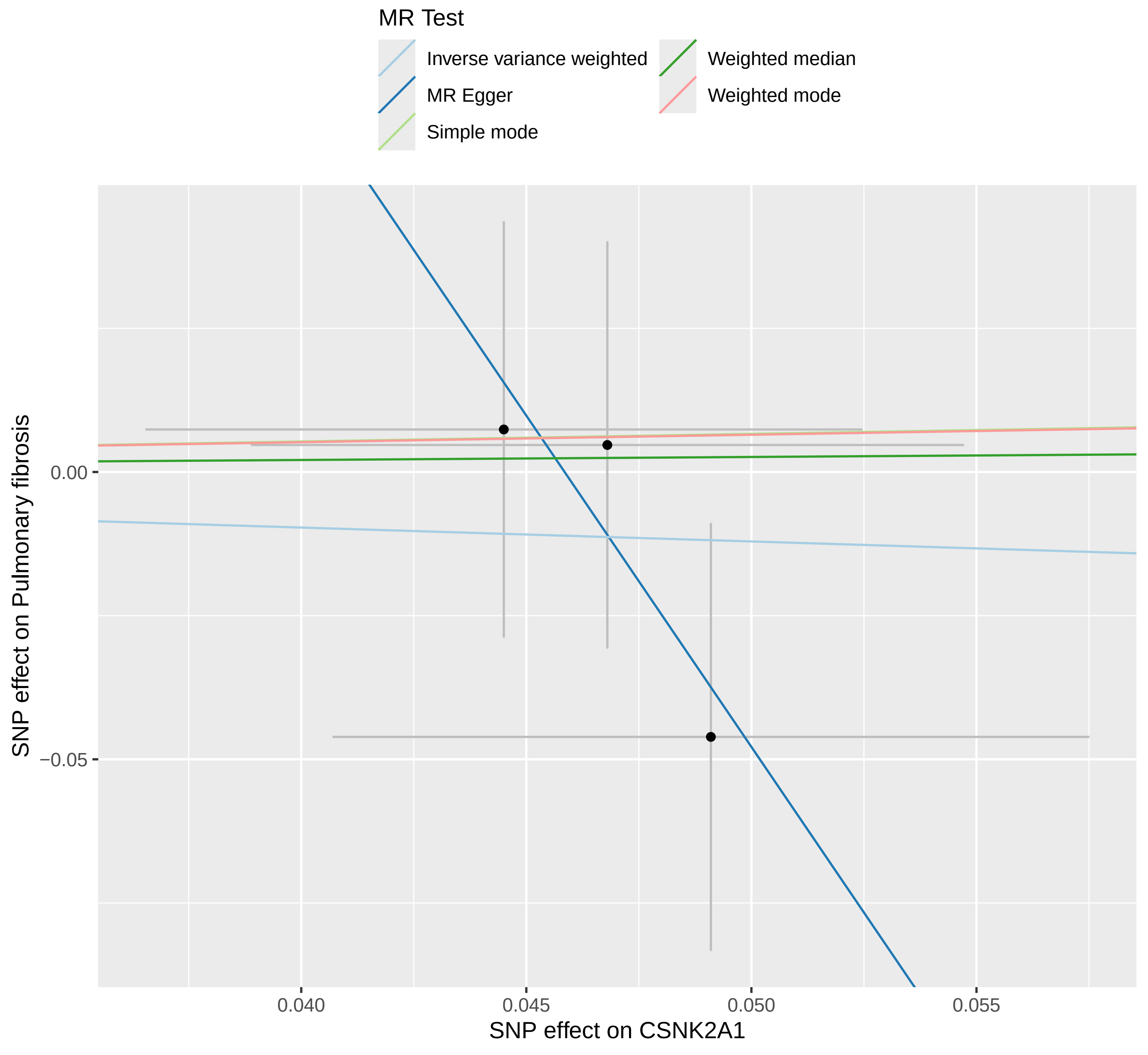

MR Test
Inverse variance weighted
MR Egger
Simple mode
Weighted median
Weighted mode
0.00
−0.05
0.040
0.045
0.050
0.055
SNP effect on CSNK2A1
SNP effect on Pulmonary fibrosis

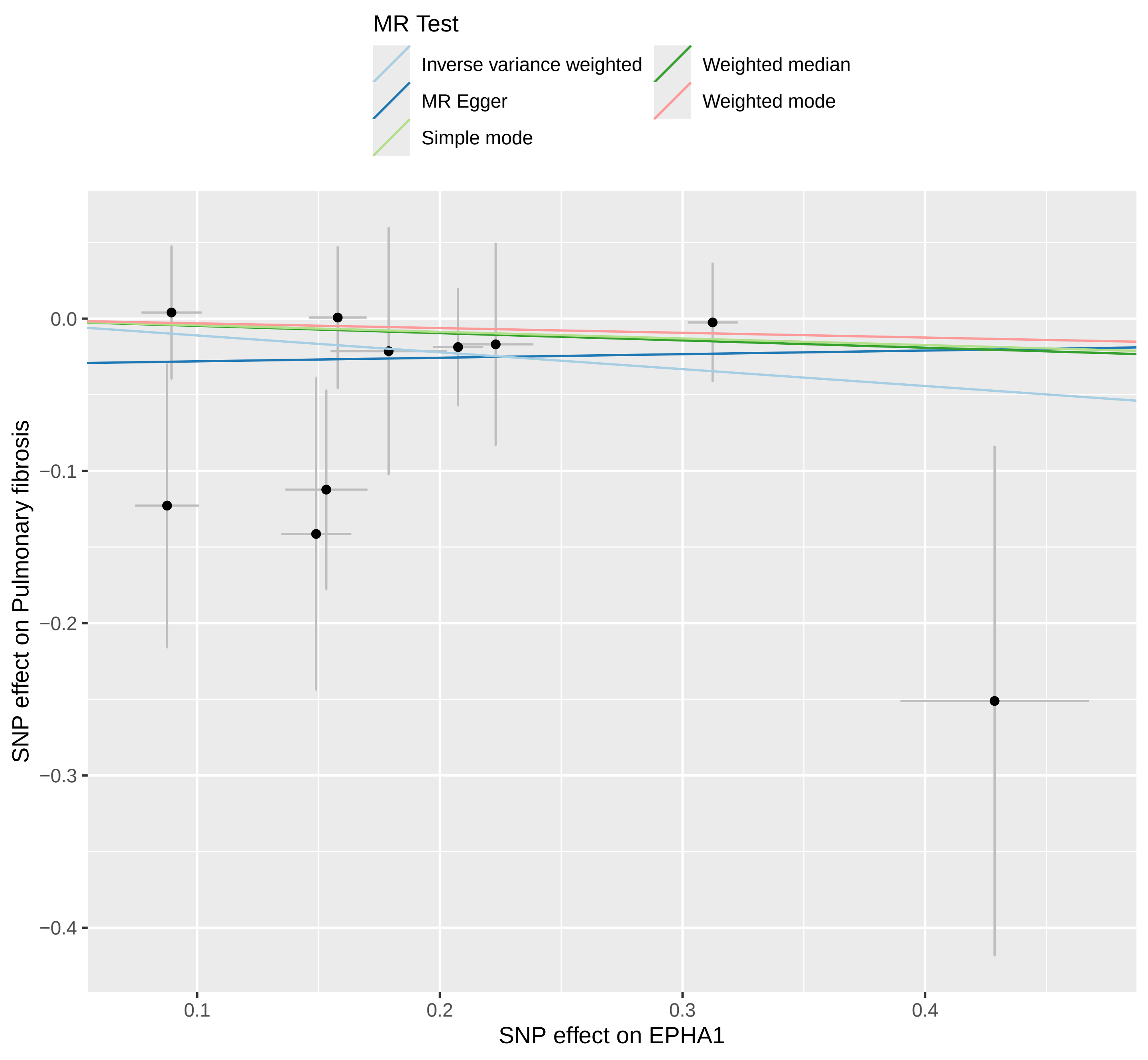
MR Test
Inverse variance weighted
MR Egger
Simple mode
Weighted median
Weighted mode
0.0
−0.1
−0.2
−0.3
−0.4
SNP effect on Pulmonary fibrosis
0.1
0.2
0.3
0.4
SNP effect on EPHA1

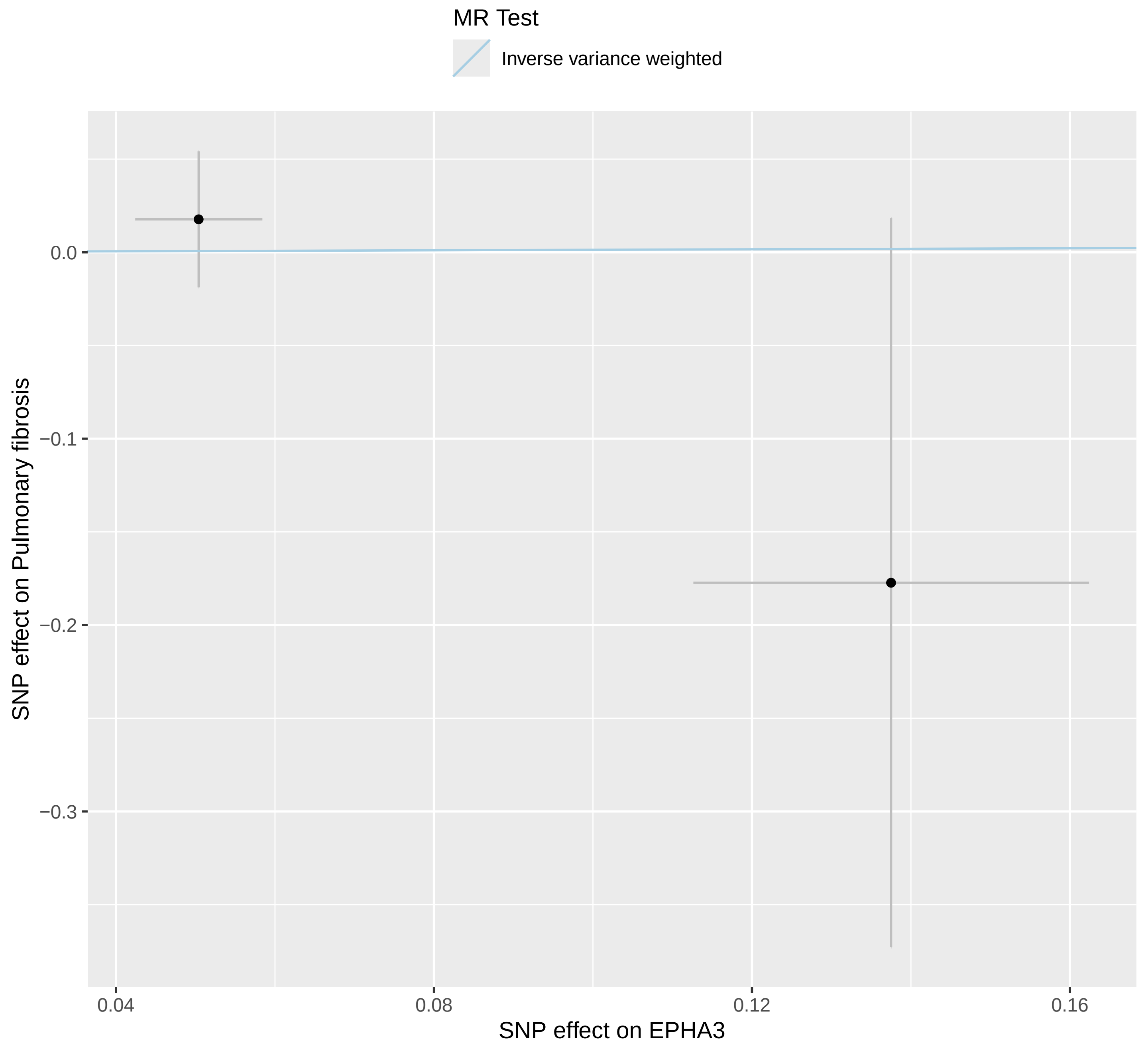

MR Test
Inverse variance weighted
0.0
−0.1
−0.2
−0.3
0.04
0.08
0.12
0.16
SNP effect on EPHA3
SNP effect on Pulmonary fibrosis

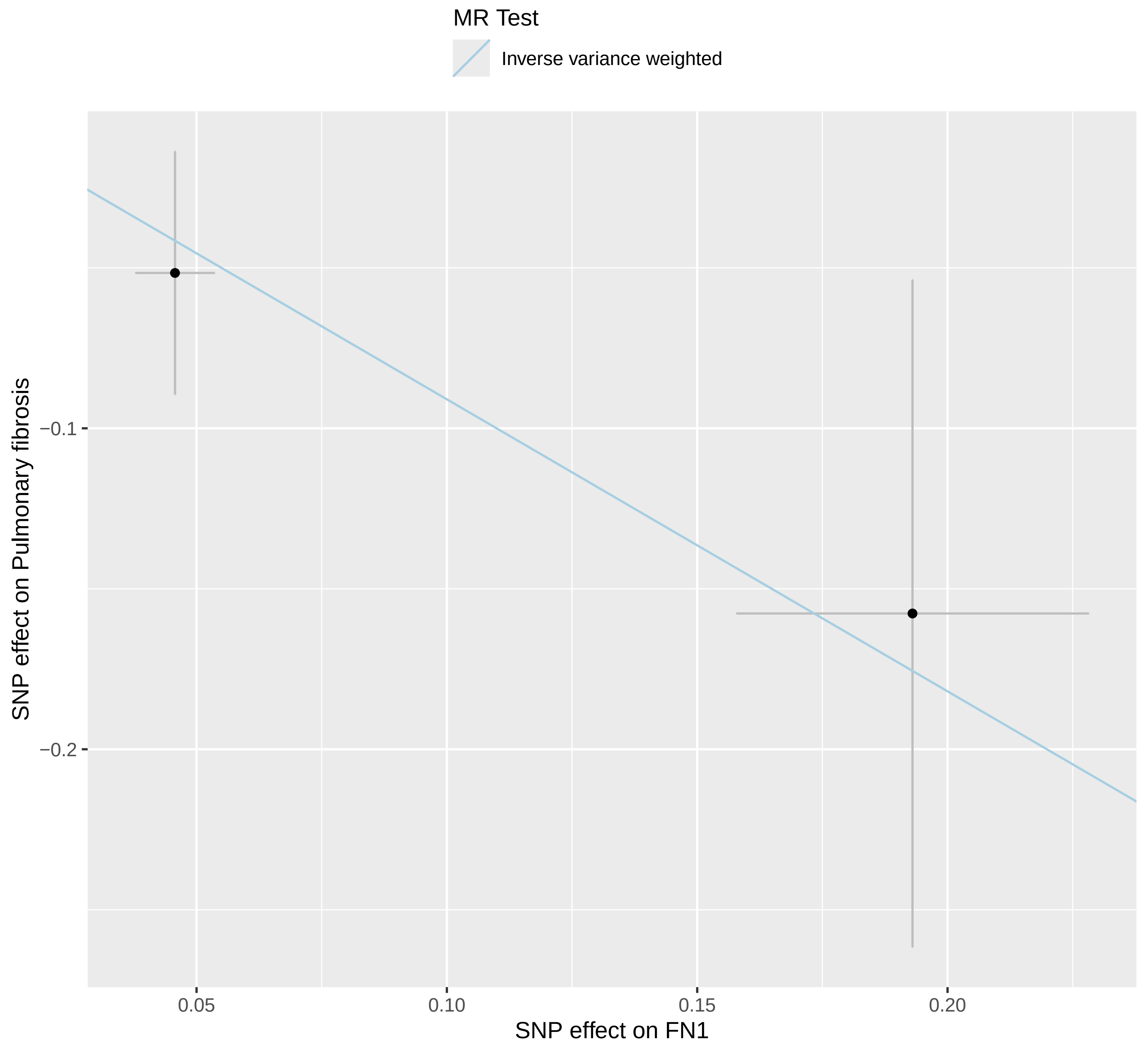
MR Test
Inverse variance weighted
−0.1
−0.2
0.05
0.10
0.15
0.20
SNP effect on Pulmonary fibrosis
SNP effect on FN1

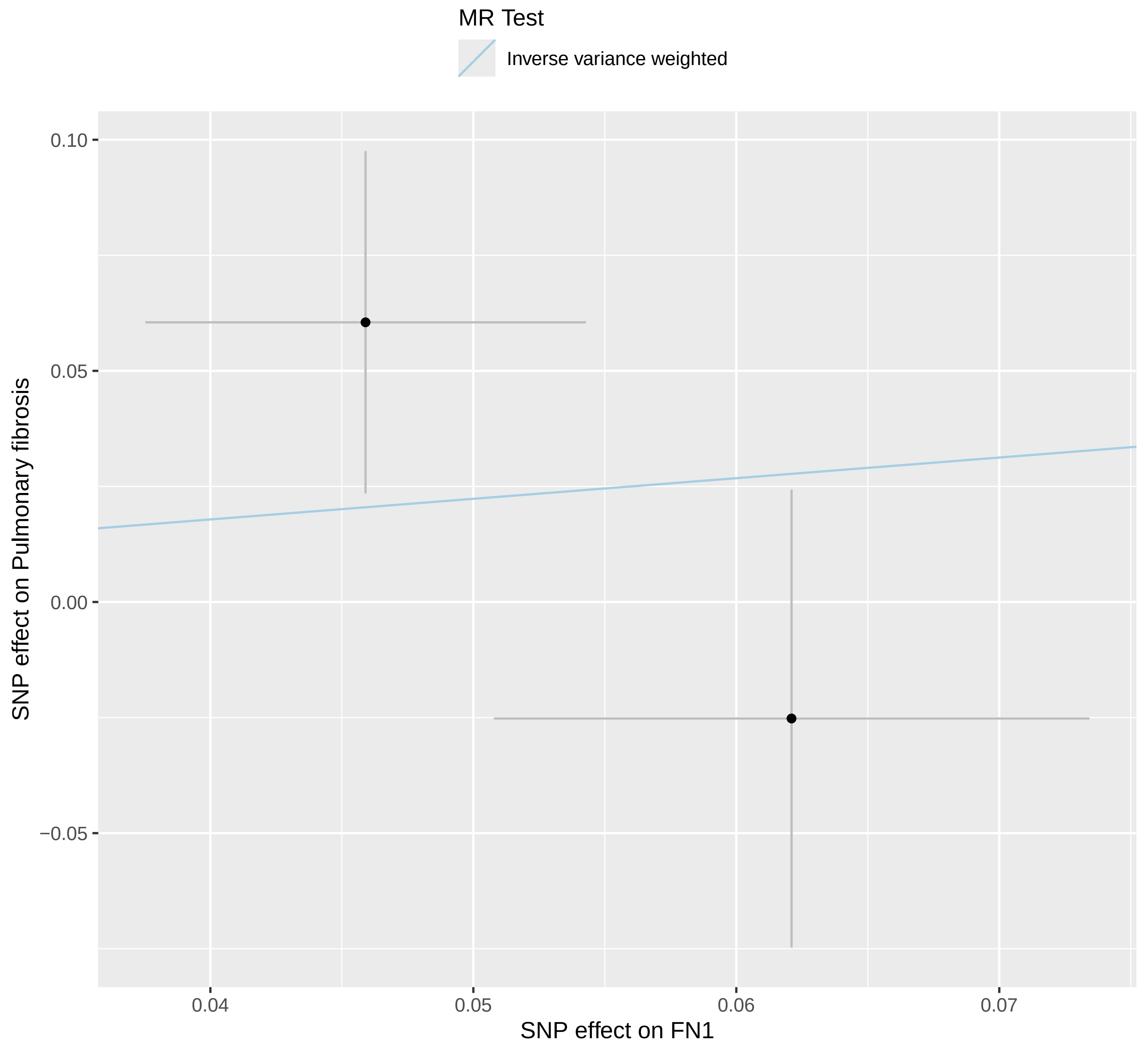

MR Test
Inverse variance weighted
0.10
0.05
0.00
−0.05
0.04
0.05
0.06
0.07
SNP effect on FN1
SNP effect on Pulmonary fibrosis

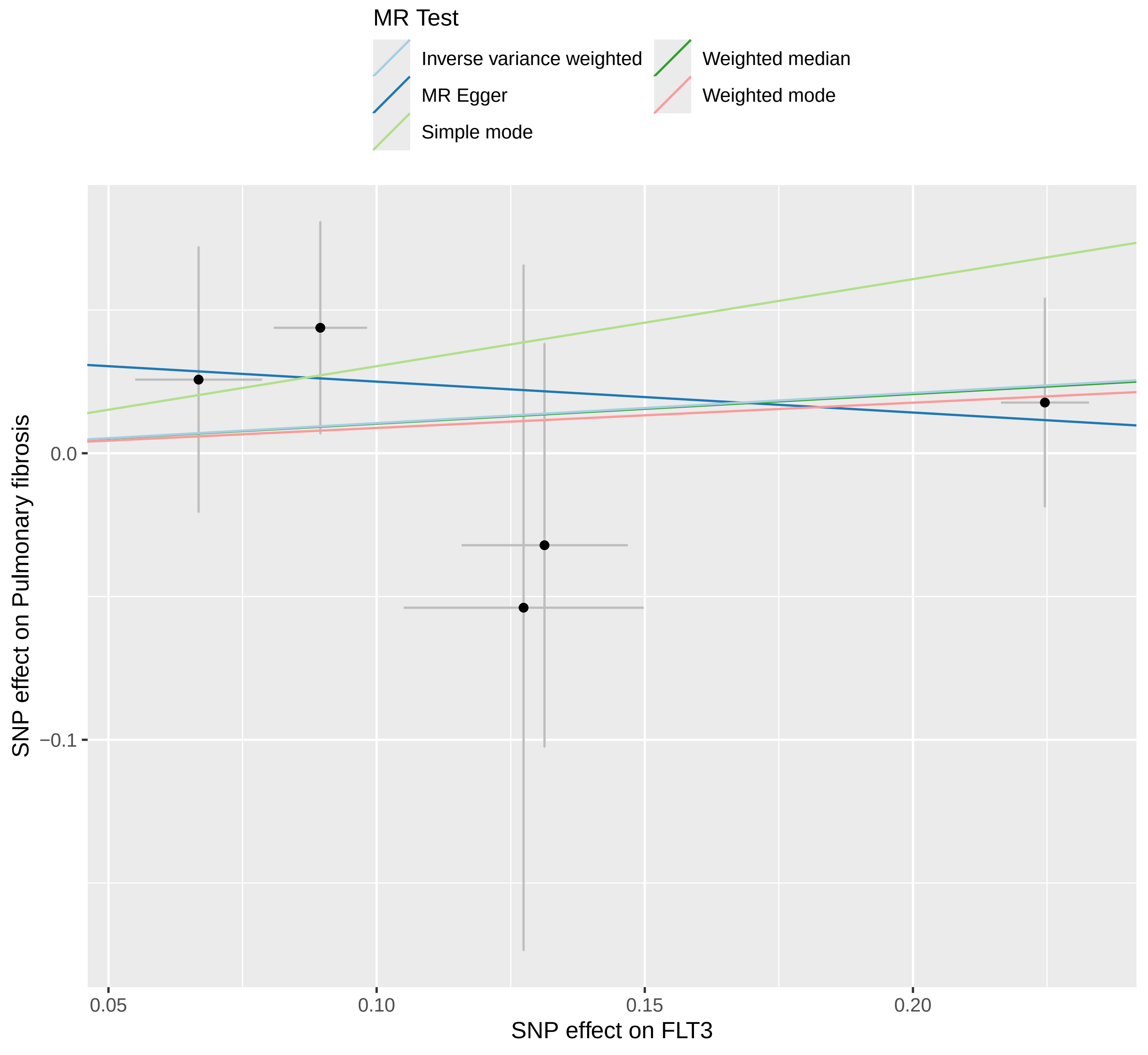

MR Test
Inverse variance weighted
MR Egger
Simple mode
Weighted median
Weighted mode
SNP effect on Pulmonary fibrosis
0.0
−0.1
0.05
0.10
0.15
0.20
SNP effect on FLT3

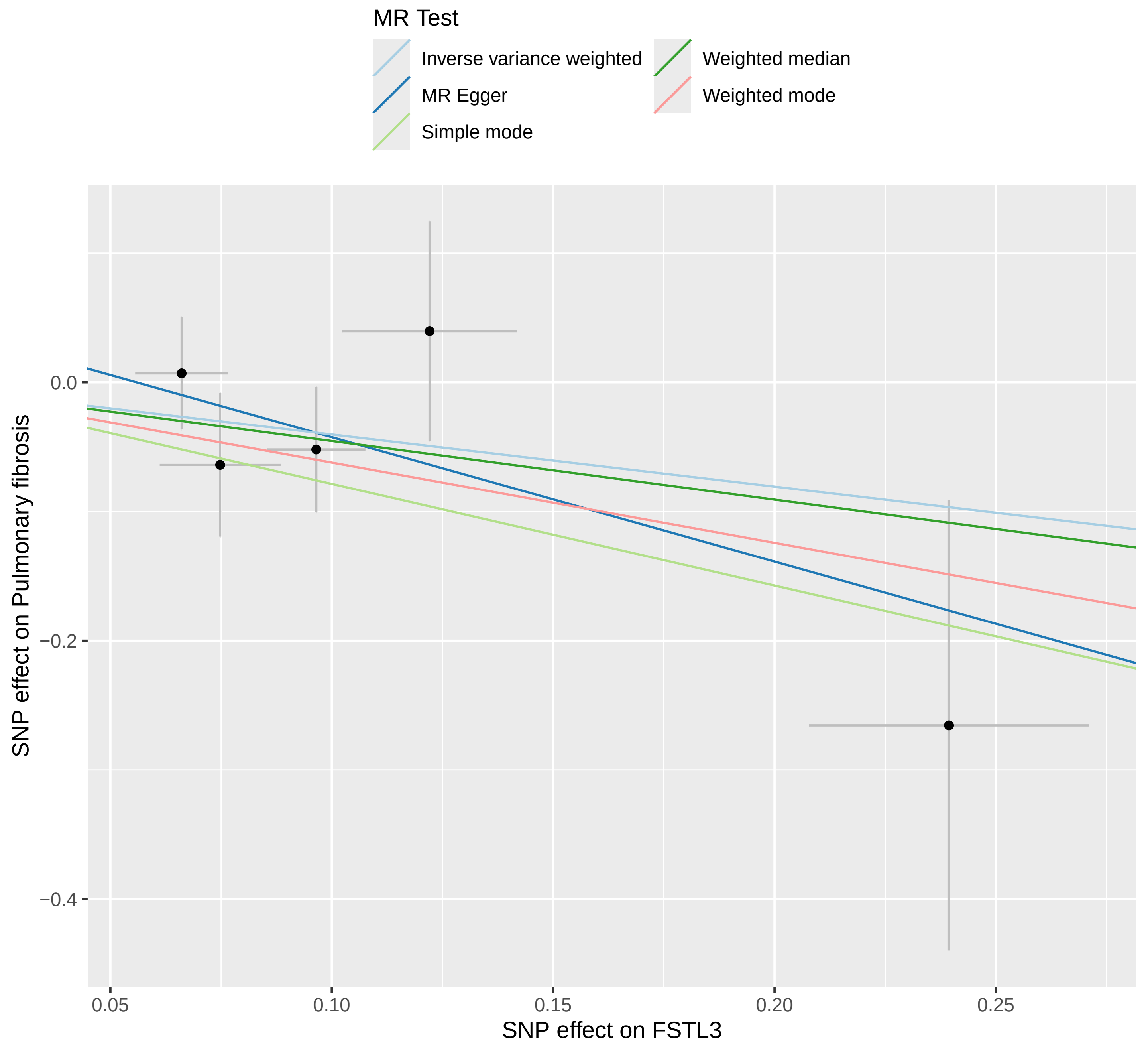
MR Test
Inverse variance weighted
MR Egger
Simple mode
Weighted median
Weighted mode
0.0
−0.2
−0.4
0.05
0.10
0.15
0.20
0.25
SNP effect on Pulmonary fibrosis
SNP effect on FSTL3

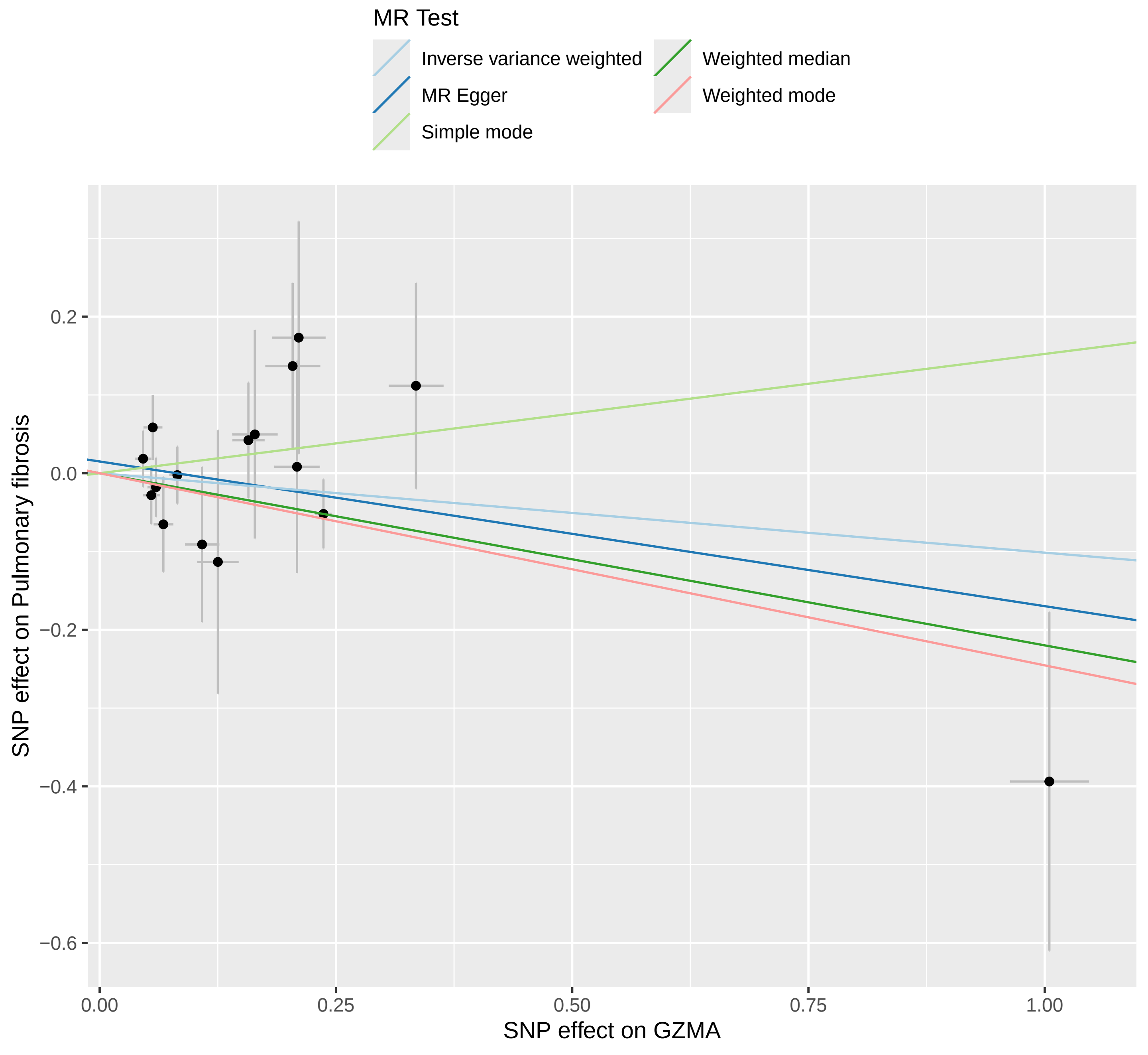
MR Test
Inverse variance weighted
MR Egger
Simple mode
Weighted median
Weighted mode
0.2
0.0
−0.2
−0.4
−0.6
SNP effect on Pulmonary fibrosis
0.00
0.25
0.50
0.75
1.00
SNP effect on GZMA

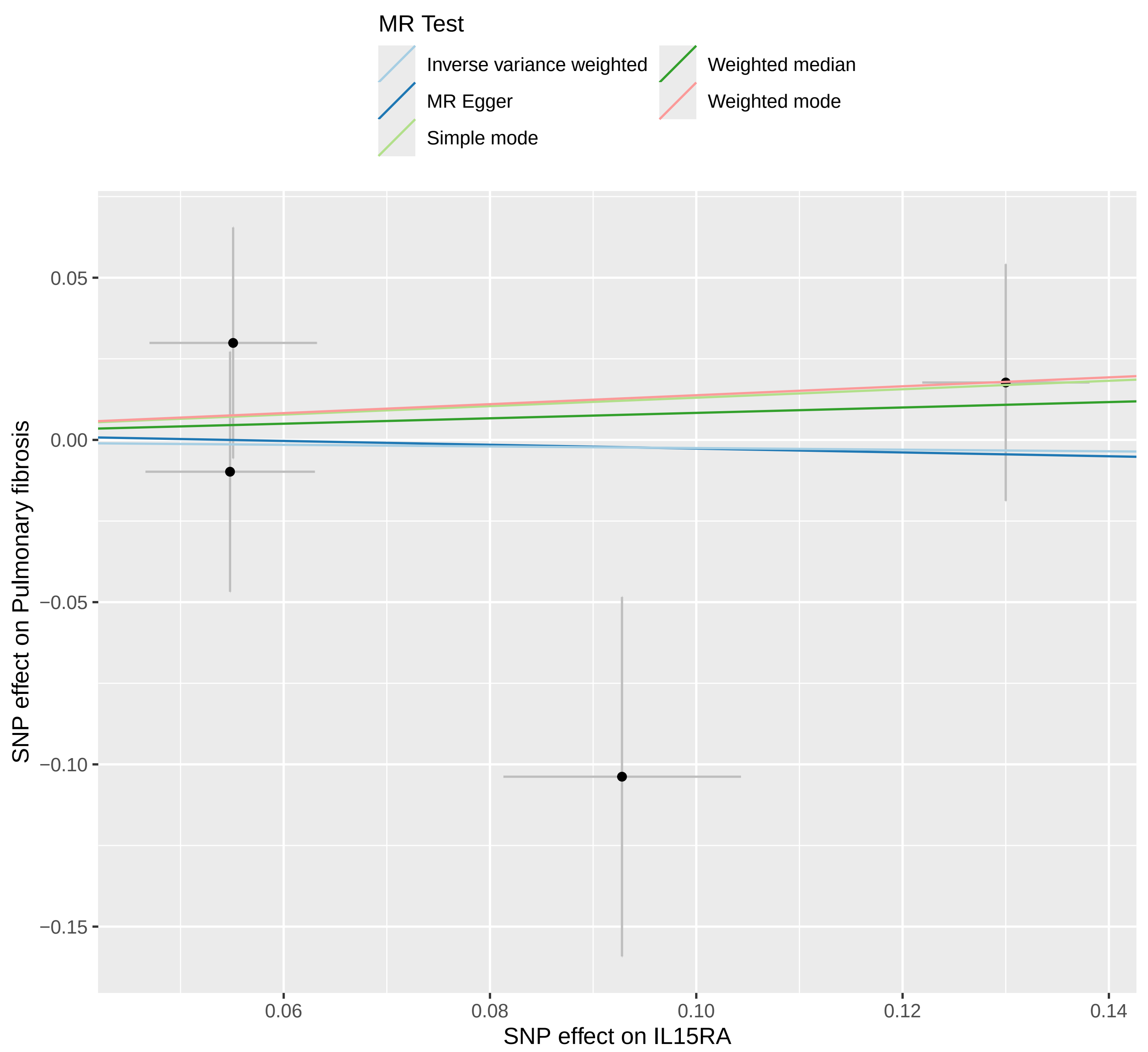

MR Test
Inverse variance weighted
MR Egger
Simple mode
Weighted median
Weighted mode
0.05
0.00
−0.05
−0.10
−0.15
SNP effect on Pulmonary fibrosis
0.06
0.08
0.10
0.12
0.14
SNP effect on IL15RA

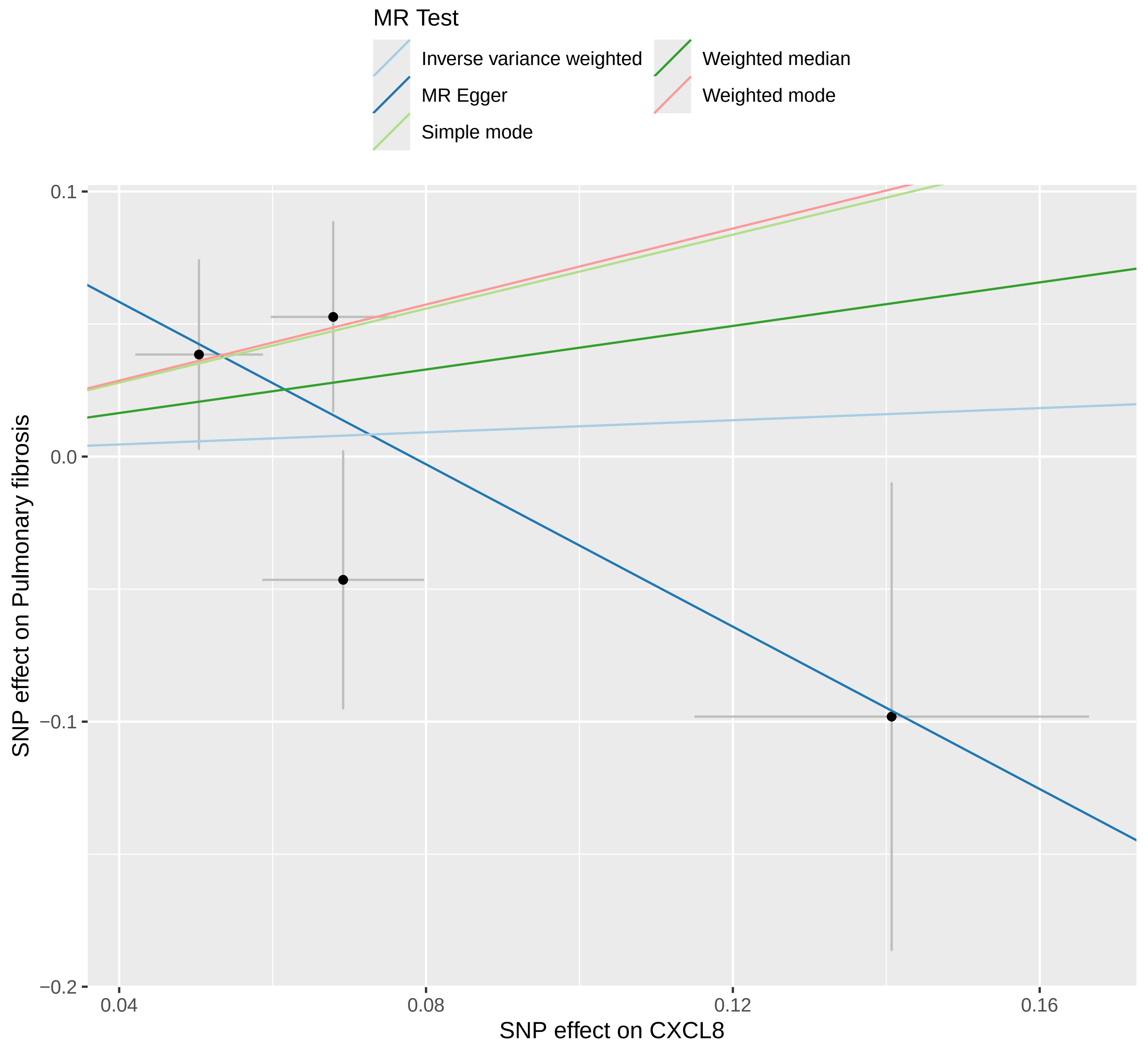

MR Test
Inverse variance weighted
MR Egger
Simple mode
Weighted median
Weighted mode
0.1
0.0
−0.1
−0.2
0.04
0.08
0.12
0.16
SNP effect on CXCL8
SNP effect on Pulmonary fibrosis

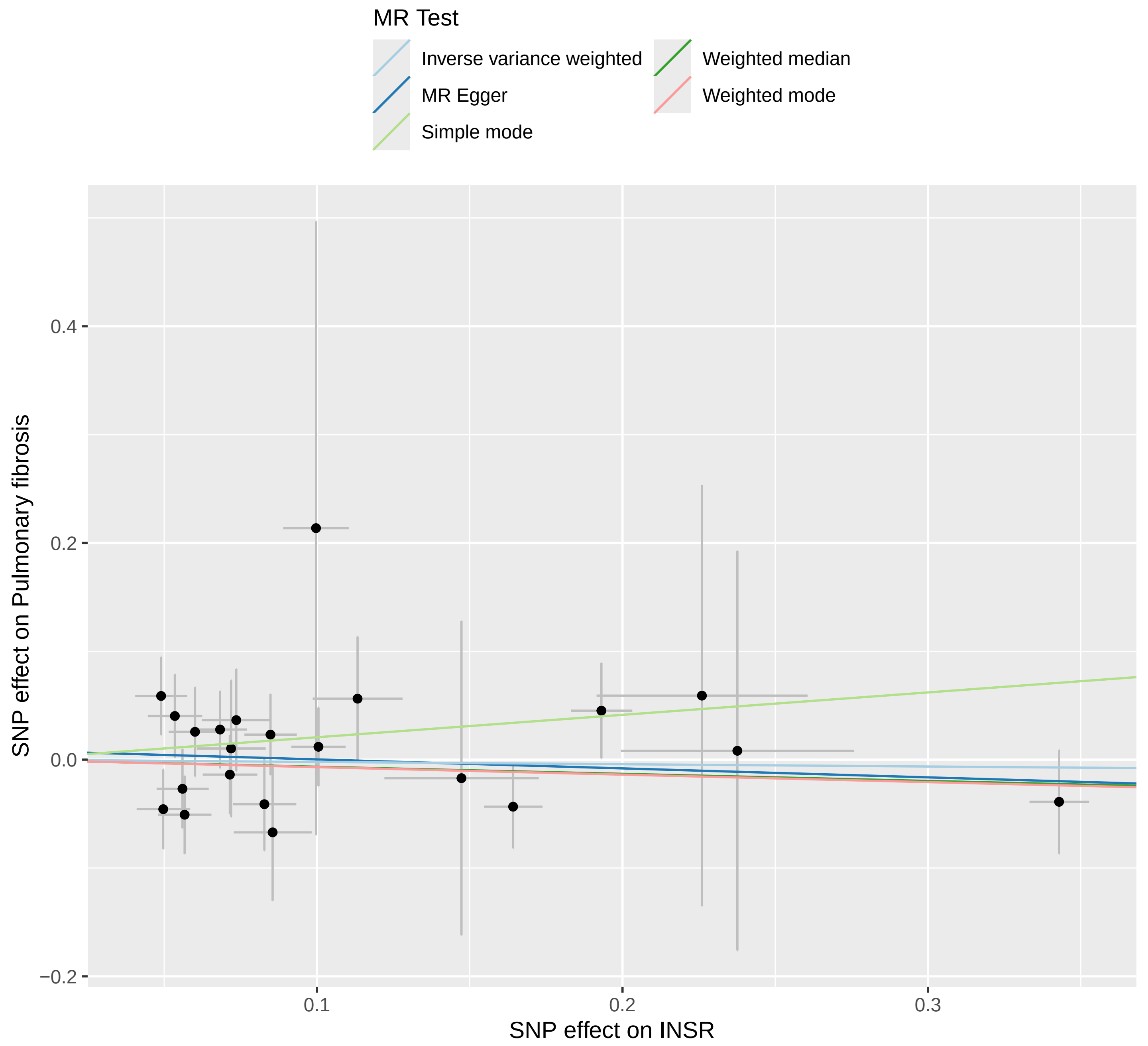

MR Test
Inverse variance weighted
MR Egger
Simple mode
Weighted median
Weighted mode
SNP effect on Pulmonary fibrosis
0.4
0.2
0.0
−0.2
0.1
0.2
0.3
SNP effect on INSR

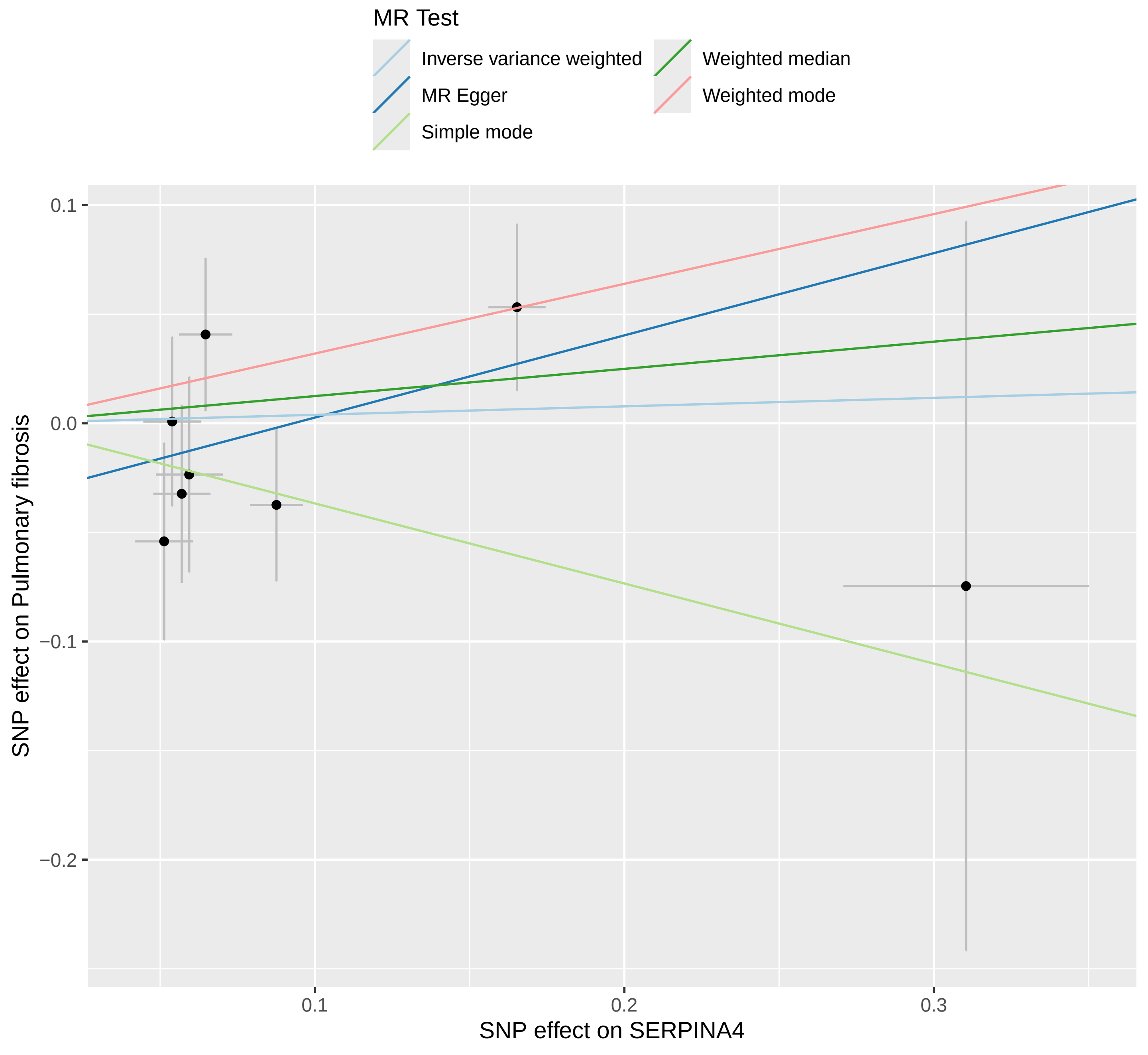

MR Test
Inverse variance weighted
MR Egger
Simple mode
Weighted median
Weighted mode
0.1
0.0
−0.1
−0.2
SNP effect on Pulmonary fibrosis
0.1
0.2
0.3
SNP effect on SERPINA4

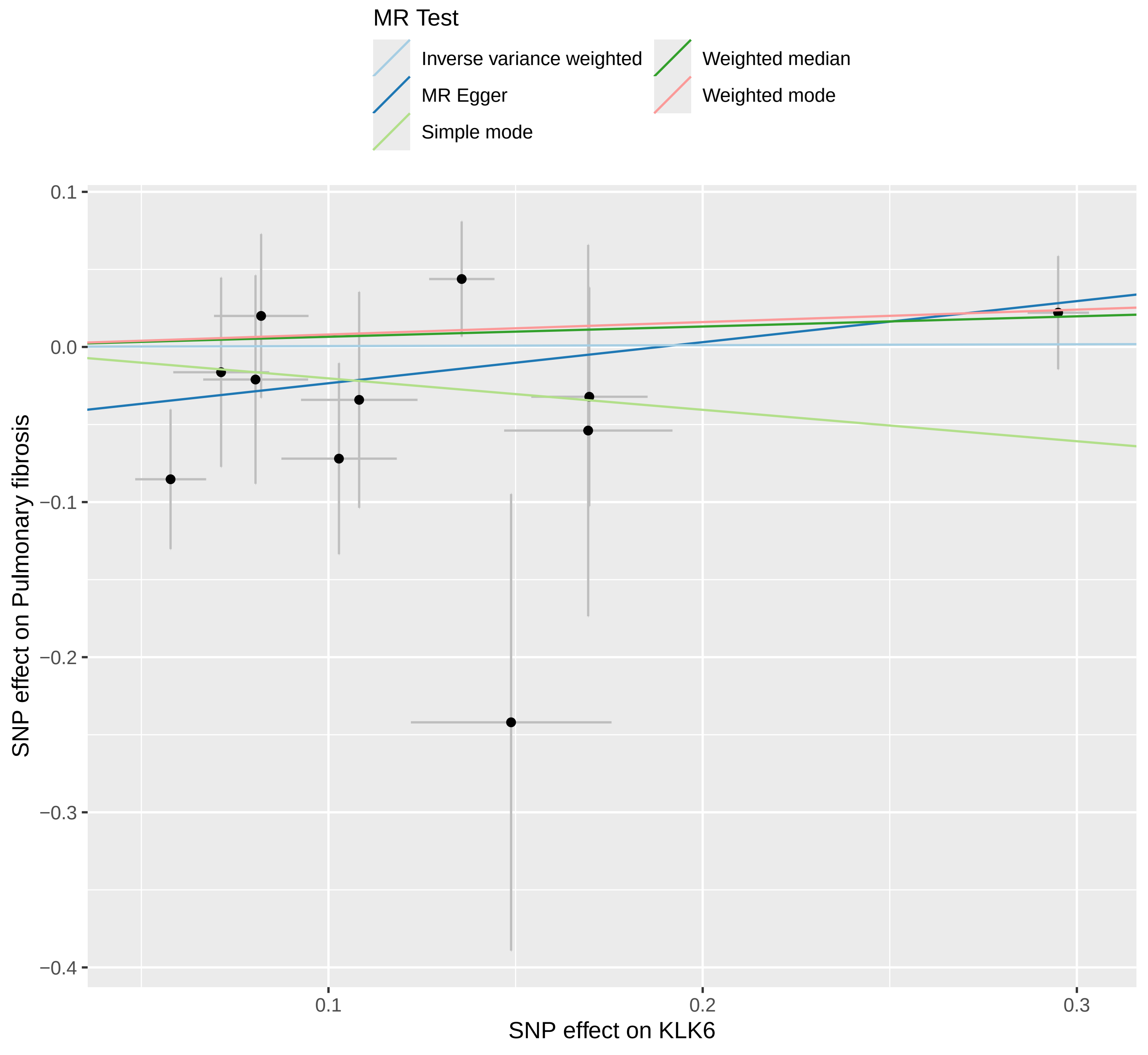

MR Test
Inverse variance weighted
MR Egger
Simple mode
Weighted median
Weighted mode
0.1
0.0
−0.1
−0.2
−0.3
−0.4
SNP effect on Pulmonary fibrosis
0.1
0.2
0.3
SNP effect on KLK6

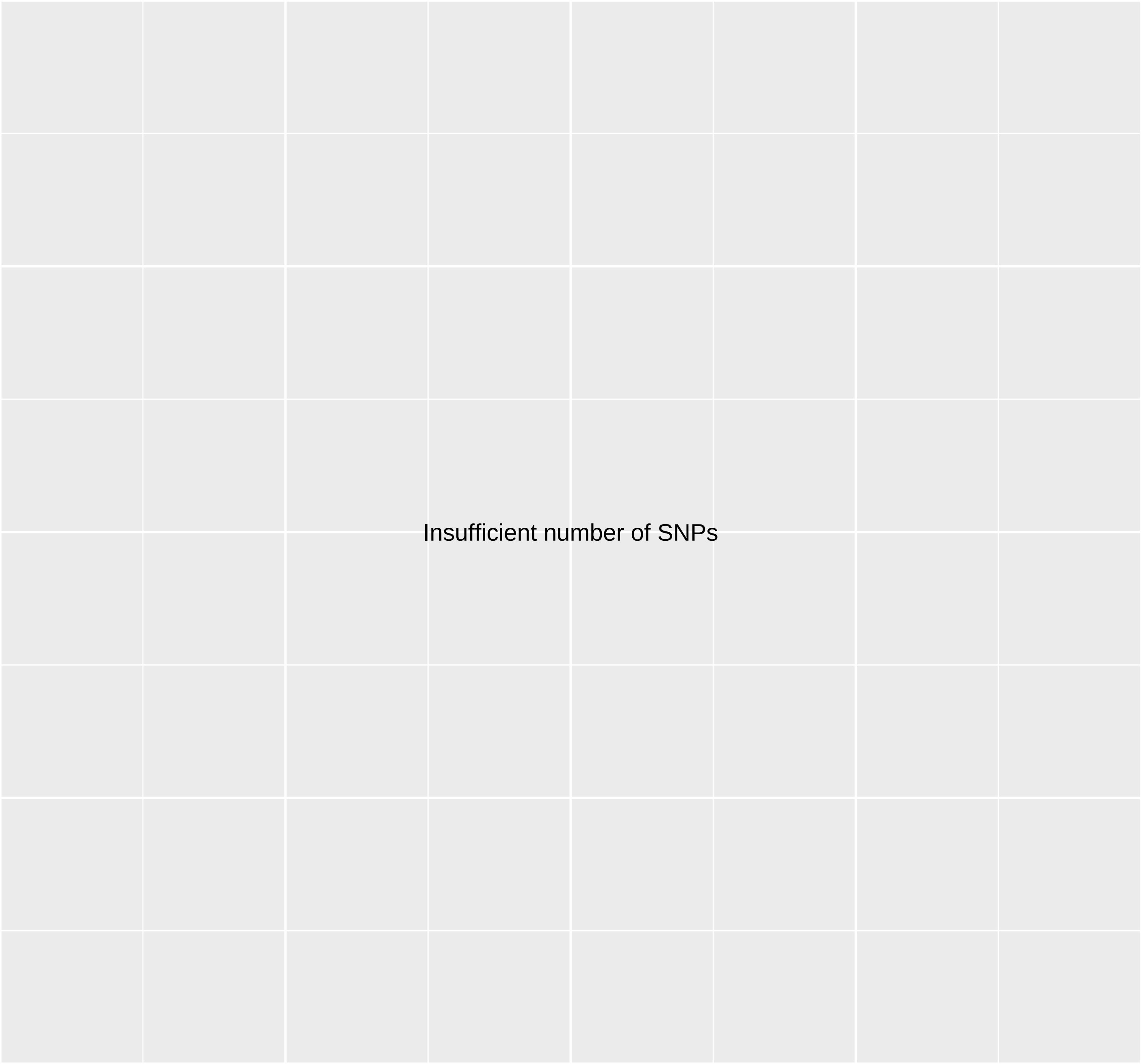
Insufficient number of SNPs

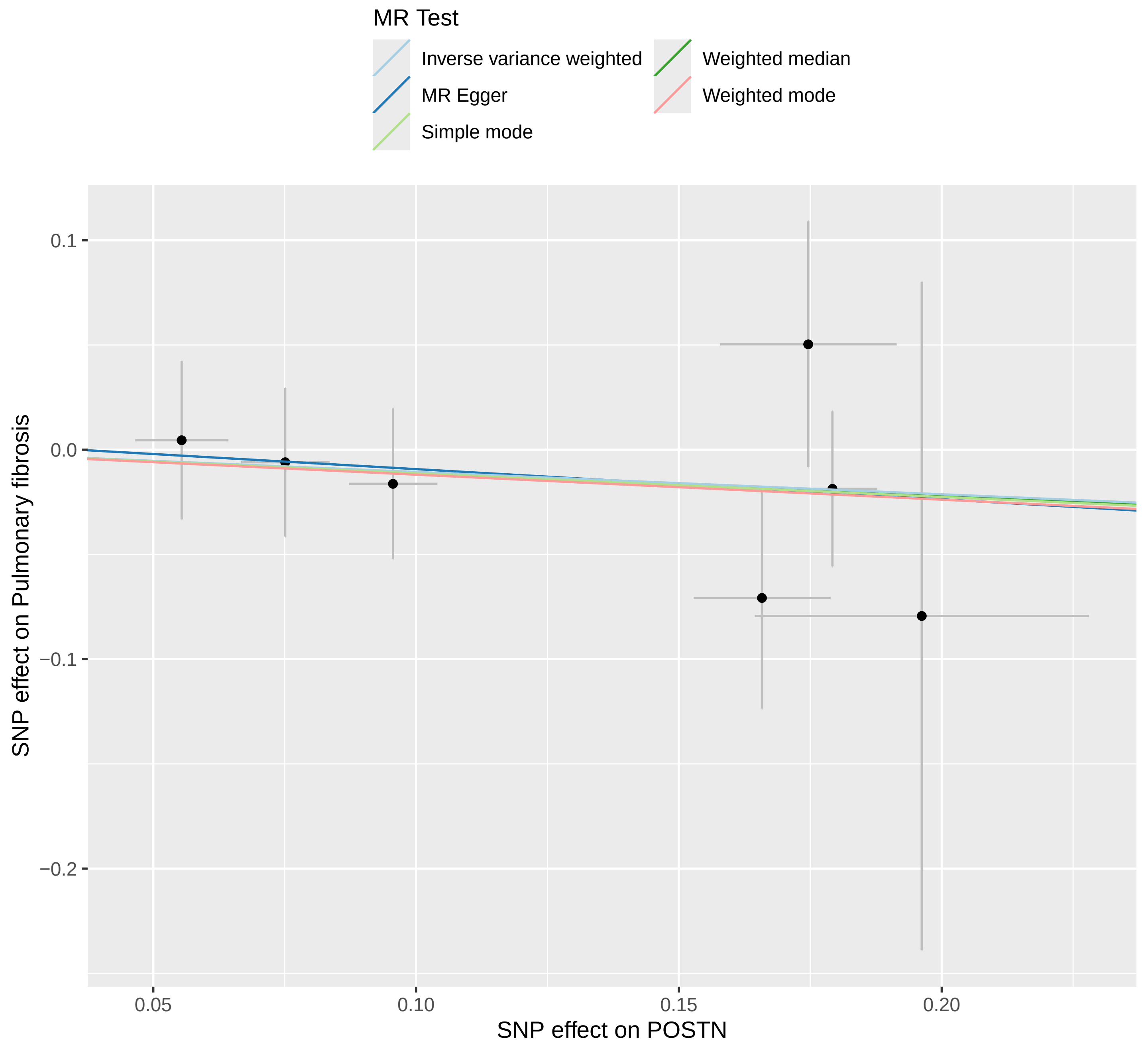
MR Test
Inverse variance weighted
MR Egger
Simple mode
Weighted median
Weighted mode
0.1
0.0
−0.1
−0.2
SNP effect on Pulmonary fibrosis
0.05
0.10
0.15
0.20
SNP effect on POSTN

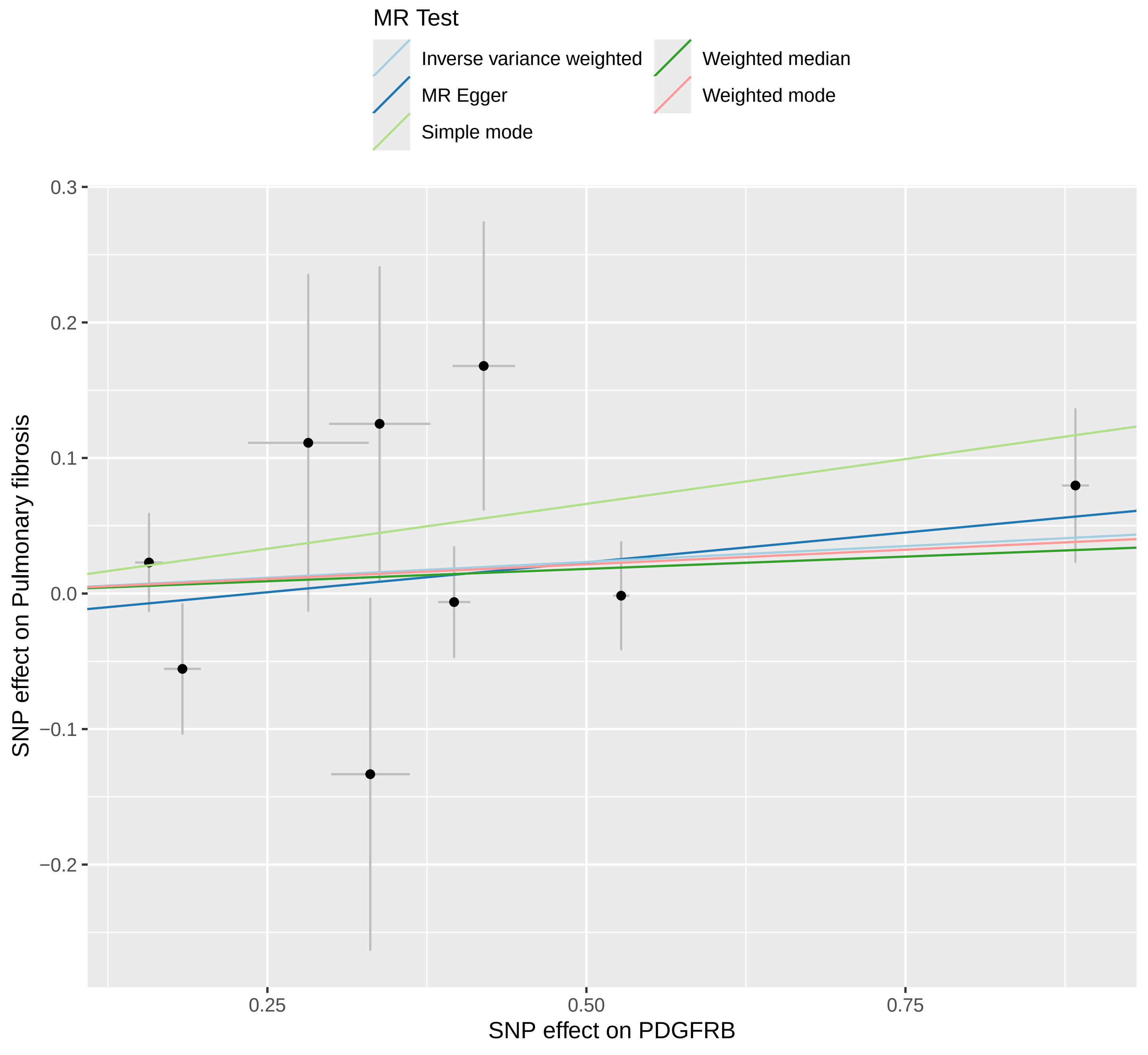
MR Test
Inverse variance weighted
MR Egger
Simple mode
Weighted median
Weighted mode
0.3
0.2
0.1
0.0
−0.1
−0.2
0.25
0.50
0.75
SNP effect on PDGFRB
SNP effect on Pulmonary fibrosis

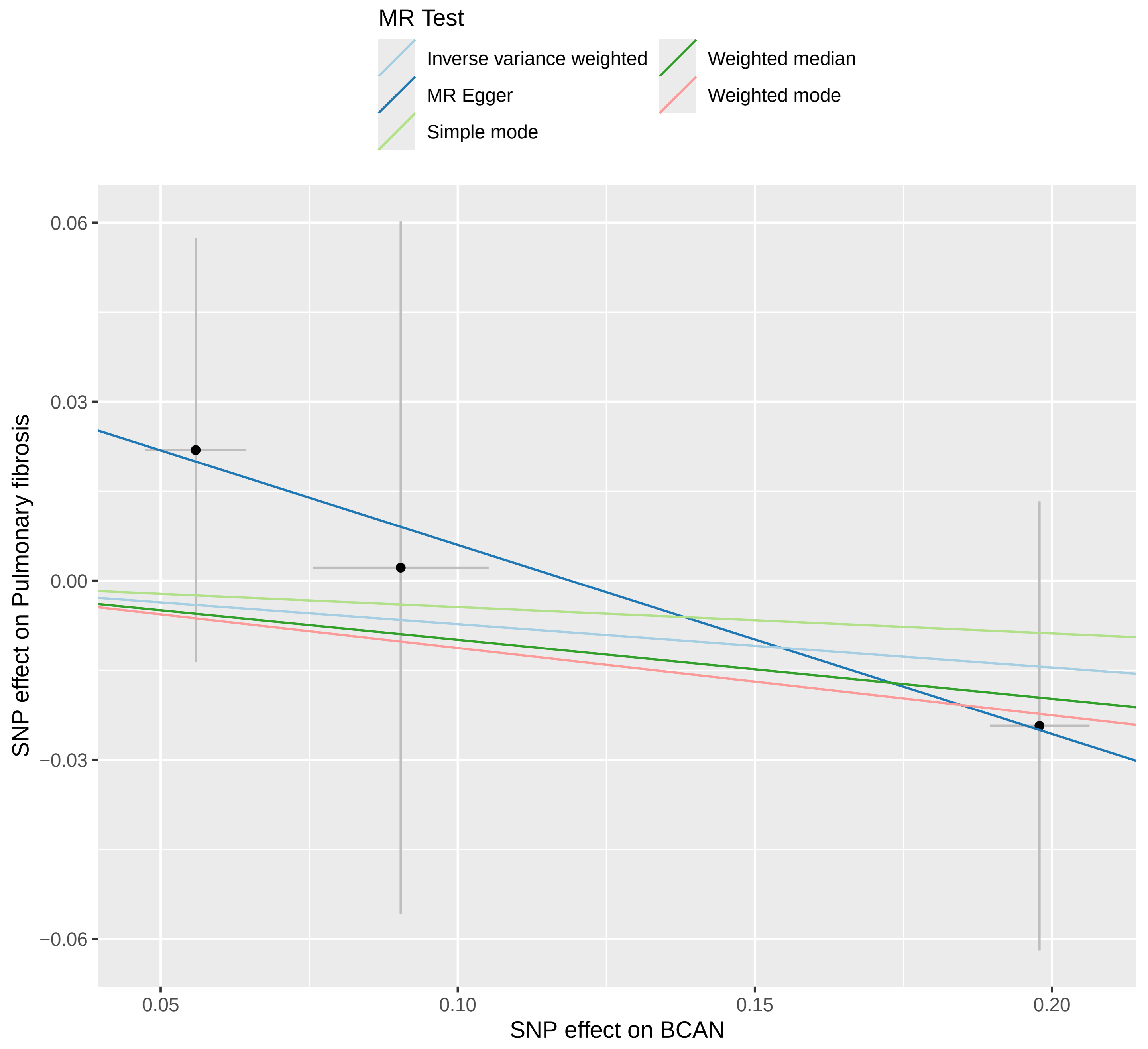

MR Test
Inverse variance weighted
MR Egger
Simple mode
Weighted median
Weighted mode
0.06
0.03
0.00
−0.03
−0.06
SNP effect on Pulmonary fibrosis
0.05
0.10
0.15
0.20
SNP effect on BCAN

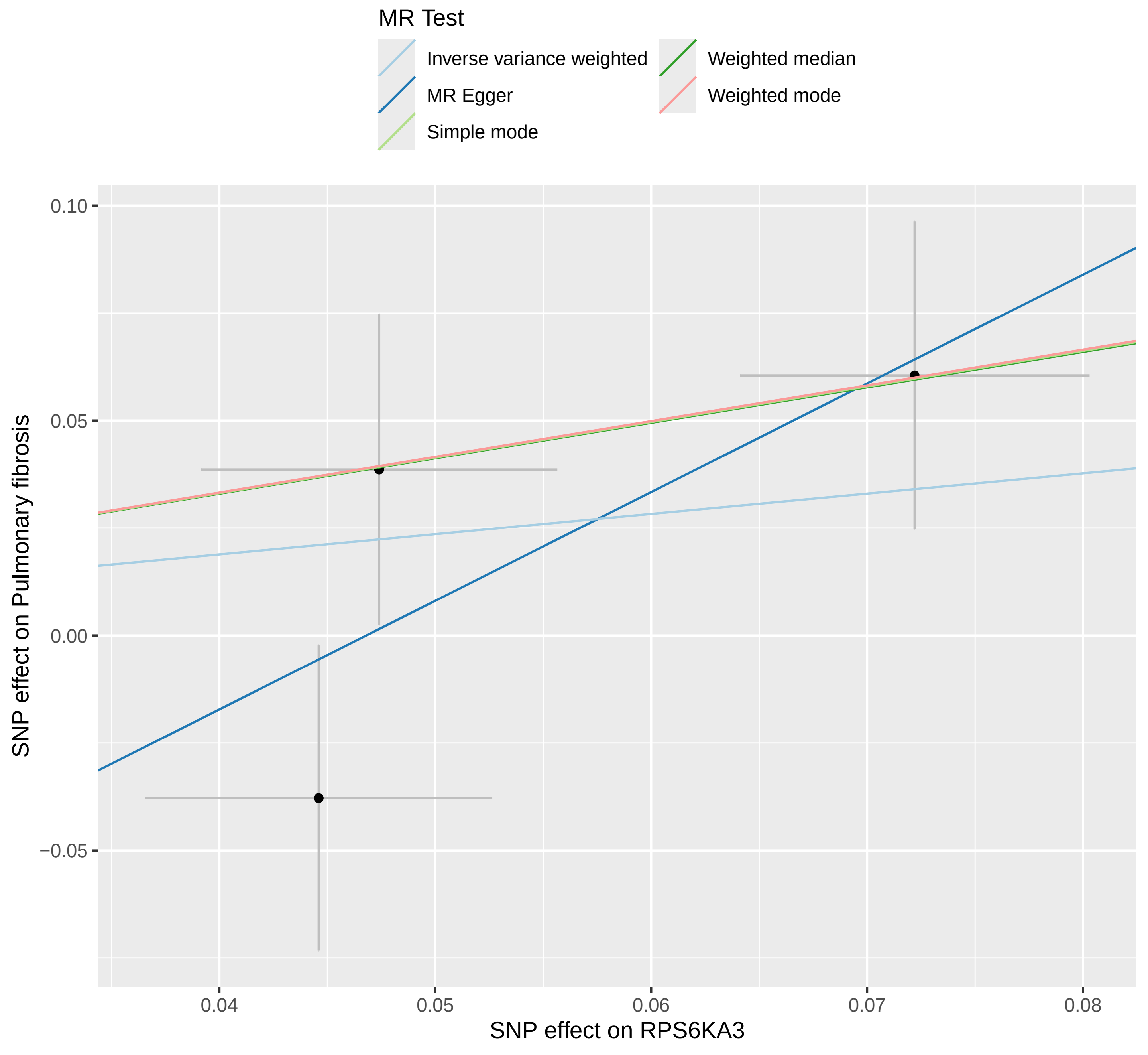

MR Test
Inverse variance weighted
MR Egger
Simple mode
Weighted median
Weighted mode
0.10
0.05
0.00
−0.05
0.04
0.05
0.06
0.07
0.08
SNP effect on Pulmonary fibrosis
SNP effect on RPS6KA3

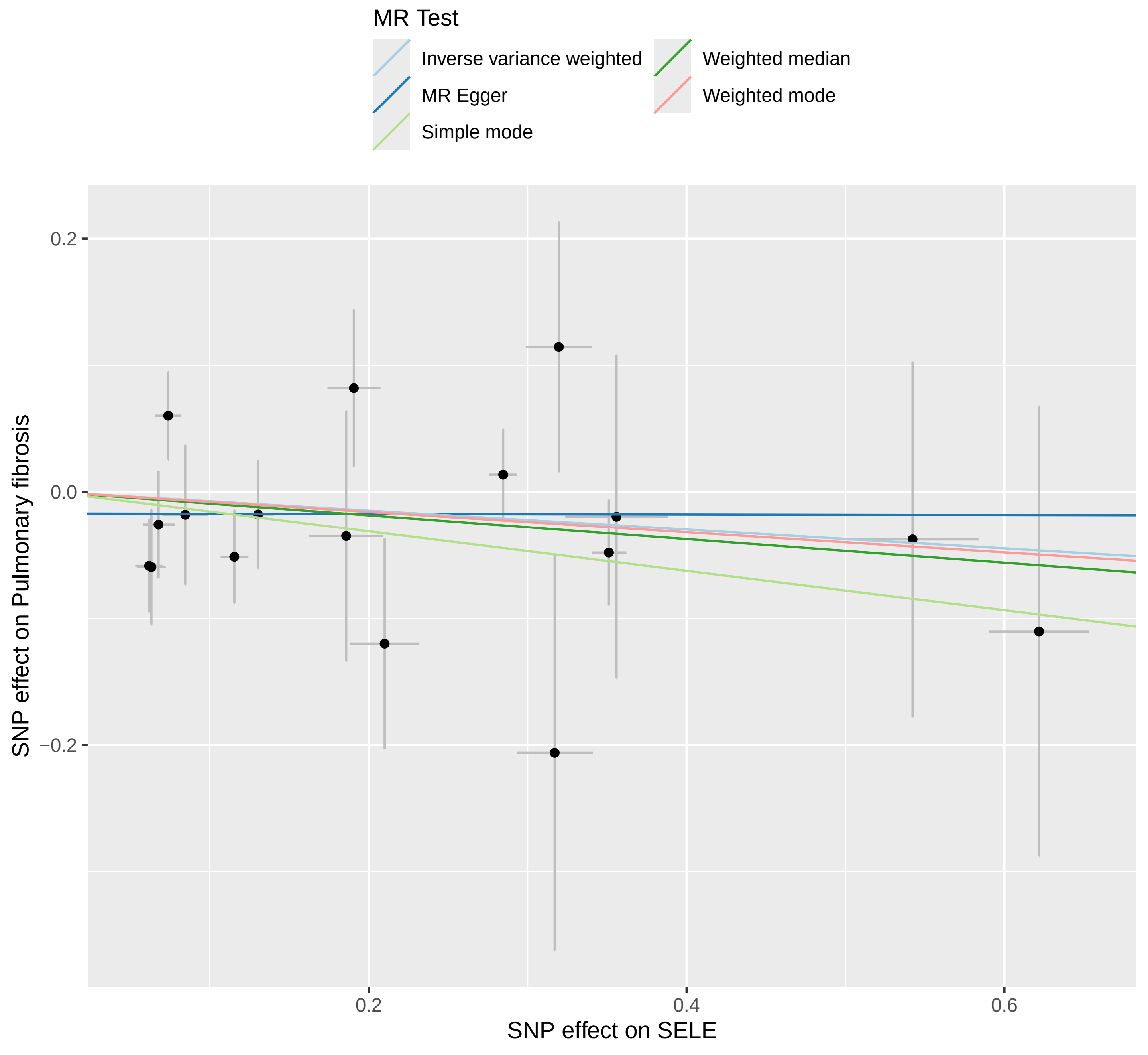

MR Test
Inverse variance weighted
MR Egger
Simple mode
Weighted median
Weighted mode
0.2
0.0
−0.2
0.2
0.4
0.6
SNP effect on Pulmonary fibrosis
SNP effect on SELE

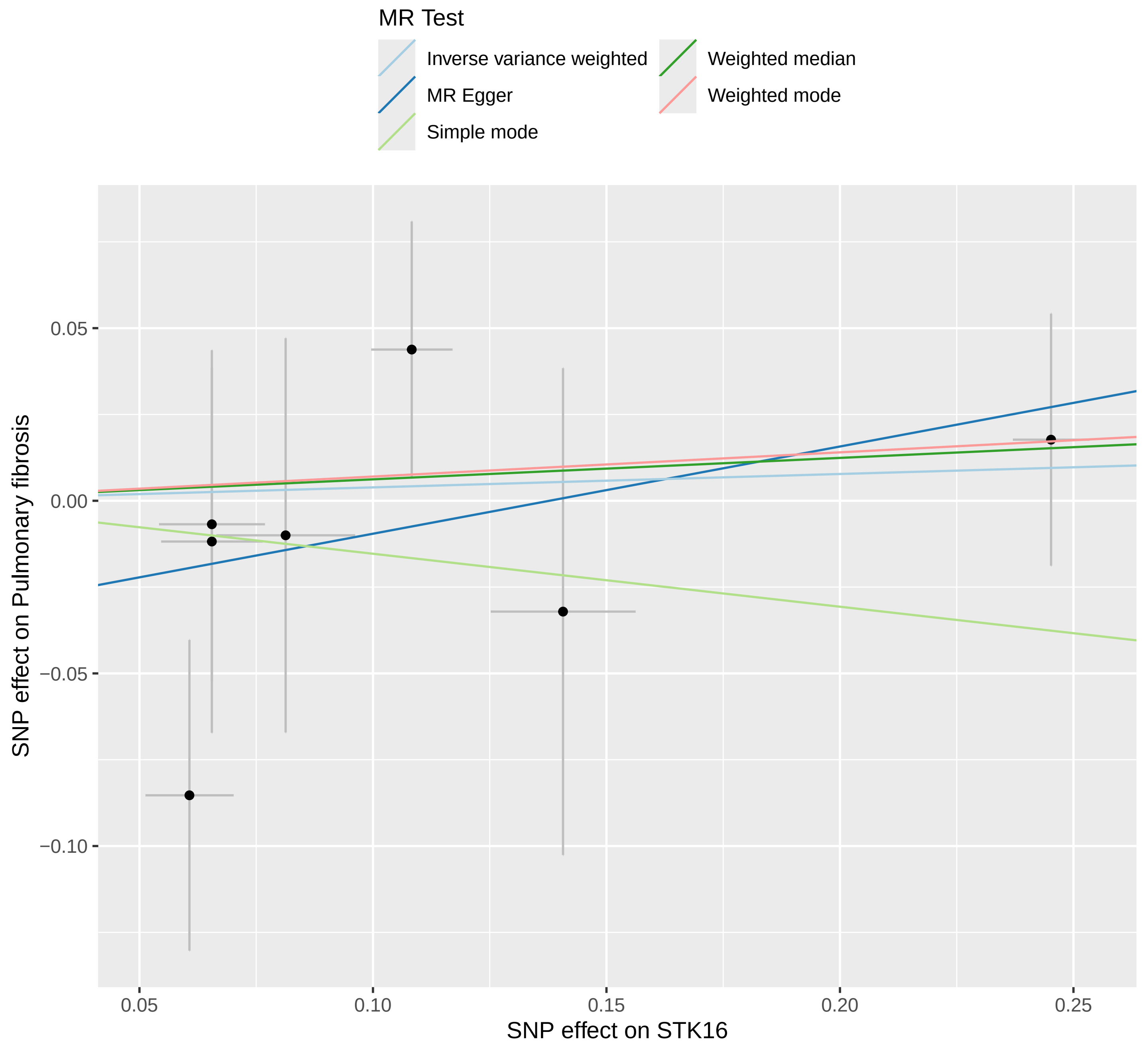
MR Test
Inverse variance weighted
MR Egger
Simple mode
Weighted median
Weighted mode
SNP effect on Pulmonary fibrosis
0.05
0.00
−0.05
−0.10
0.05
0.10
0.15
0.20
0.25
SNP effect on STK16

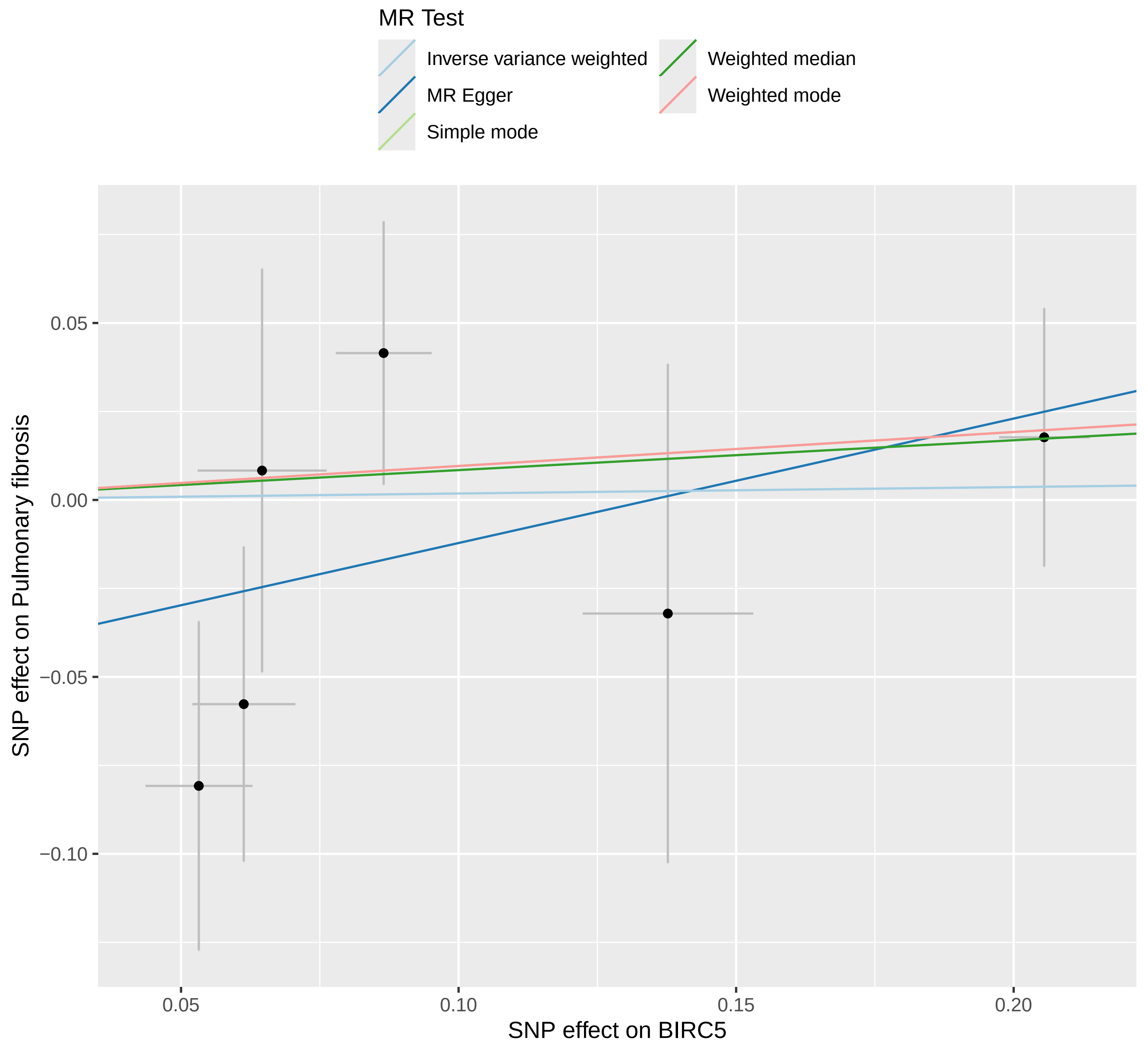
MR Test
Inverse variance weighted
MR Egger
Simple mode
Weighted median
Weighted mode
0.05
0.00
−0.05
−0.10
SNP effect on Pulmonary fibrosis
0.05
0.10
0.15
0.20
SNP effect on BIRC5

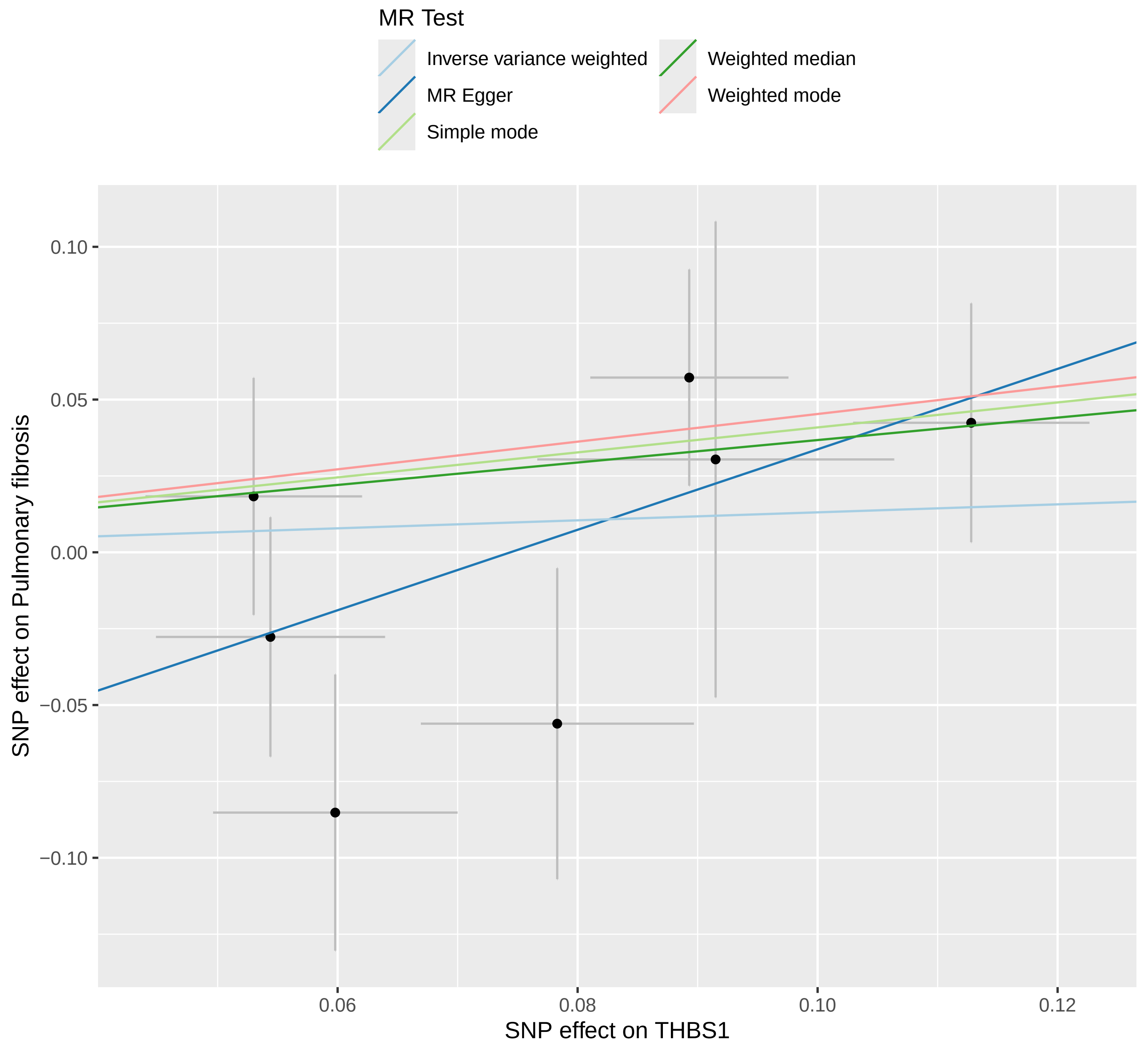
MR Test
Inverse variance weighted
MR Egger
Simple mode
Weighted median
Weighted mode
0.10
0.05
0.00
−0.05
−0.10
SNP effect on Pulmonary fibrosis
0.06
0.08
0.10
0.12
SNP effect on THBS1

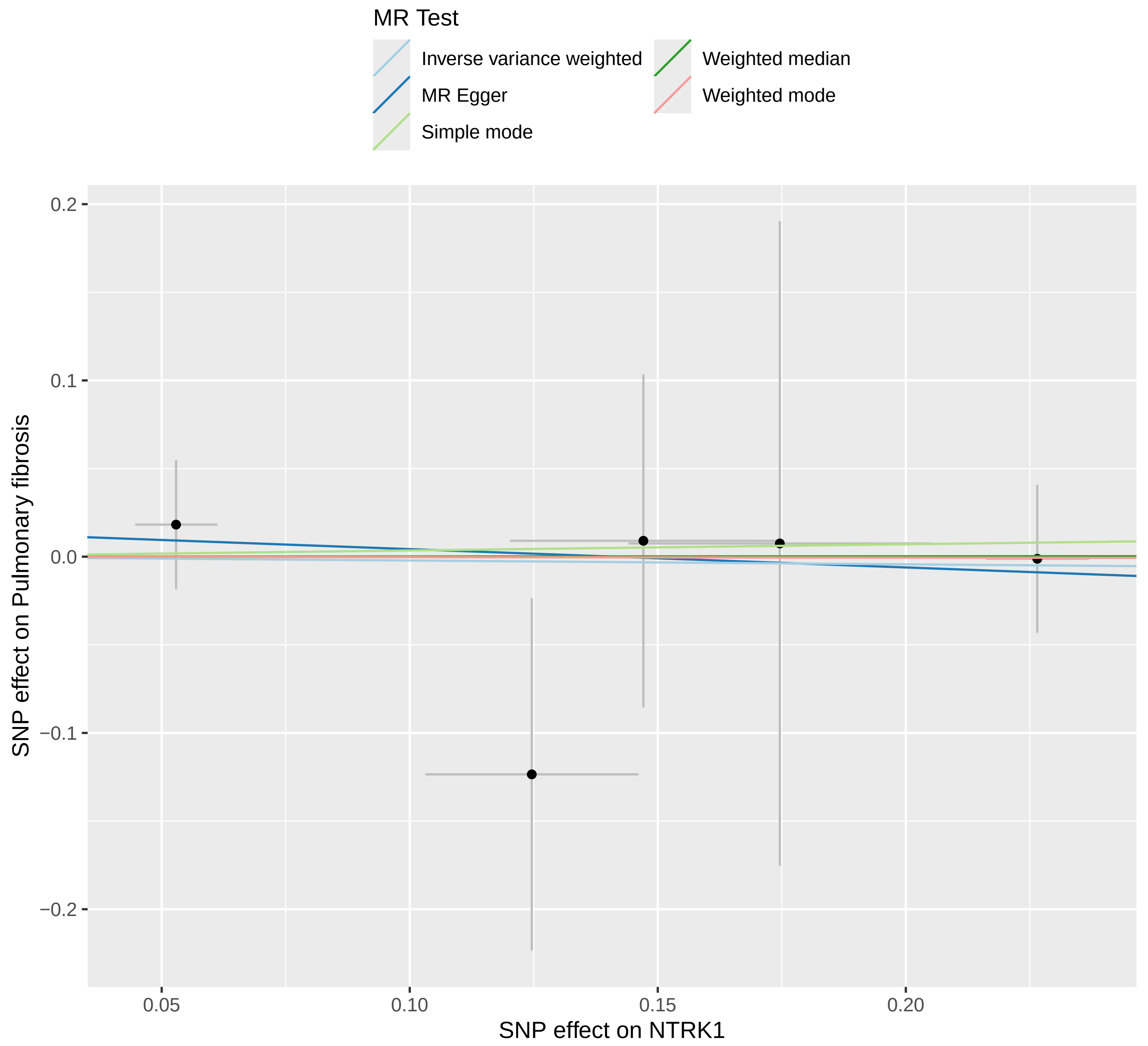
MR Test
Inverse variance weighted
MR Egger
Simple mode
Weighted median
Weighted mode
0.2
0.1
0.0
−0.1
−0.2
0.05
0.10
0.15
0.20
SNP effect on Pulmonary fibrosis
SNP effect on NTRK1

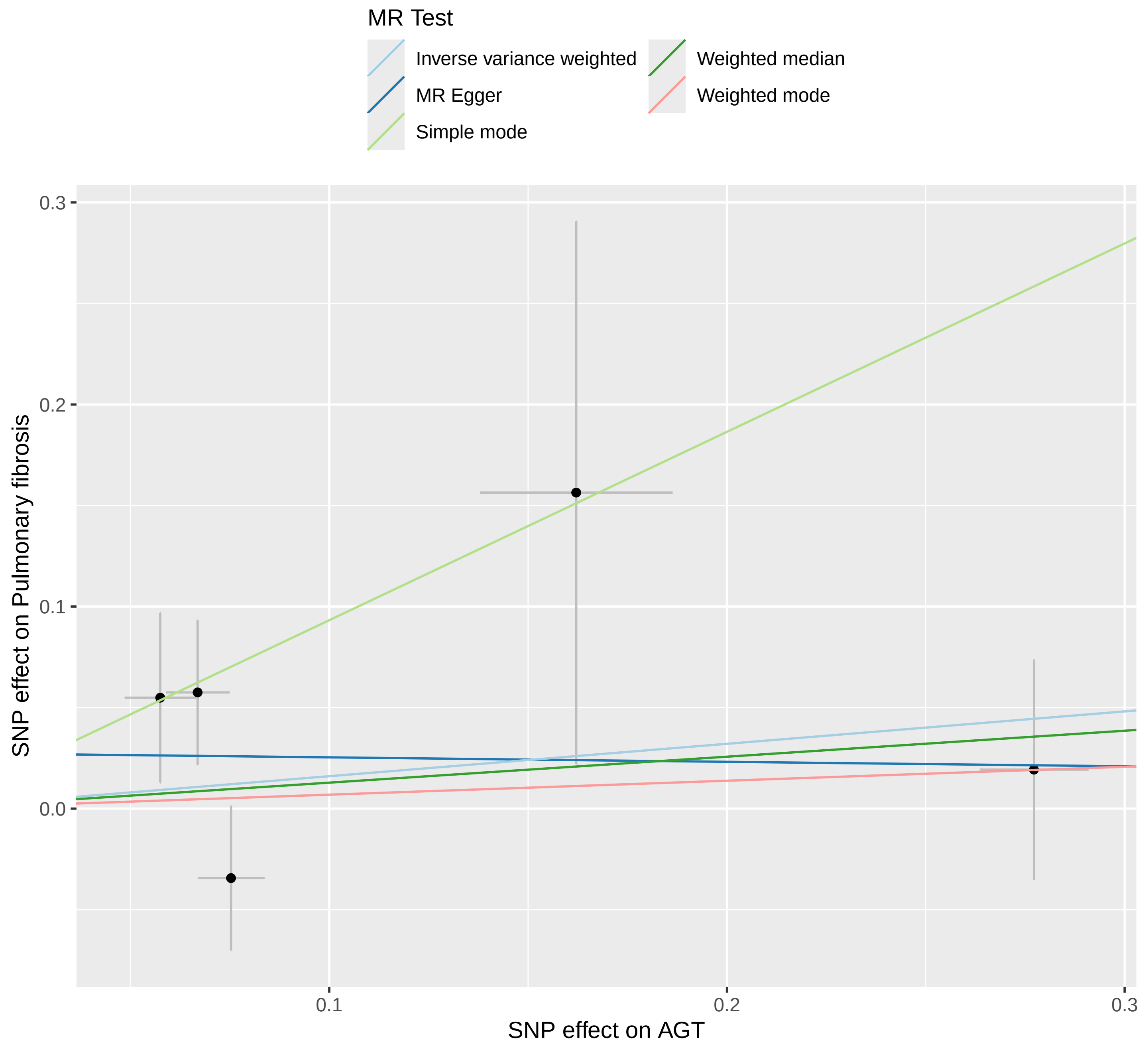
MR Test
Inverse variance weighted
MR Egger
Simple mode
Weighted median
Weighted mode
0.3
0.2
0.1
0.0
0.1
0.2
0.3
SNP effect on Pulmonary fibrosis
SNP effect on AGT

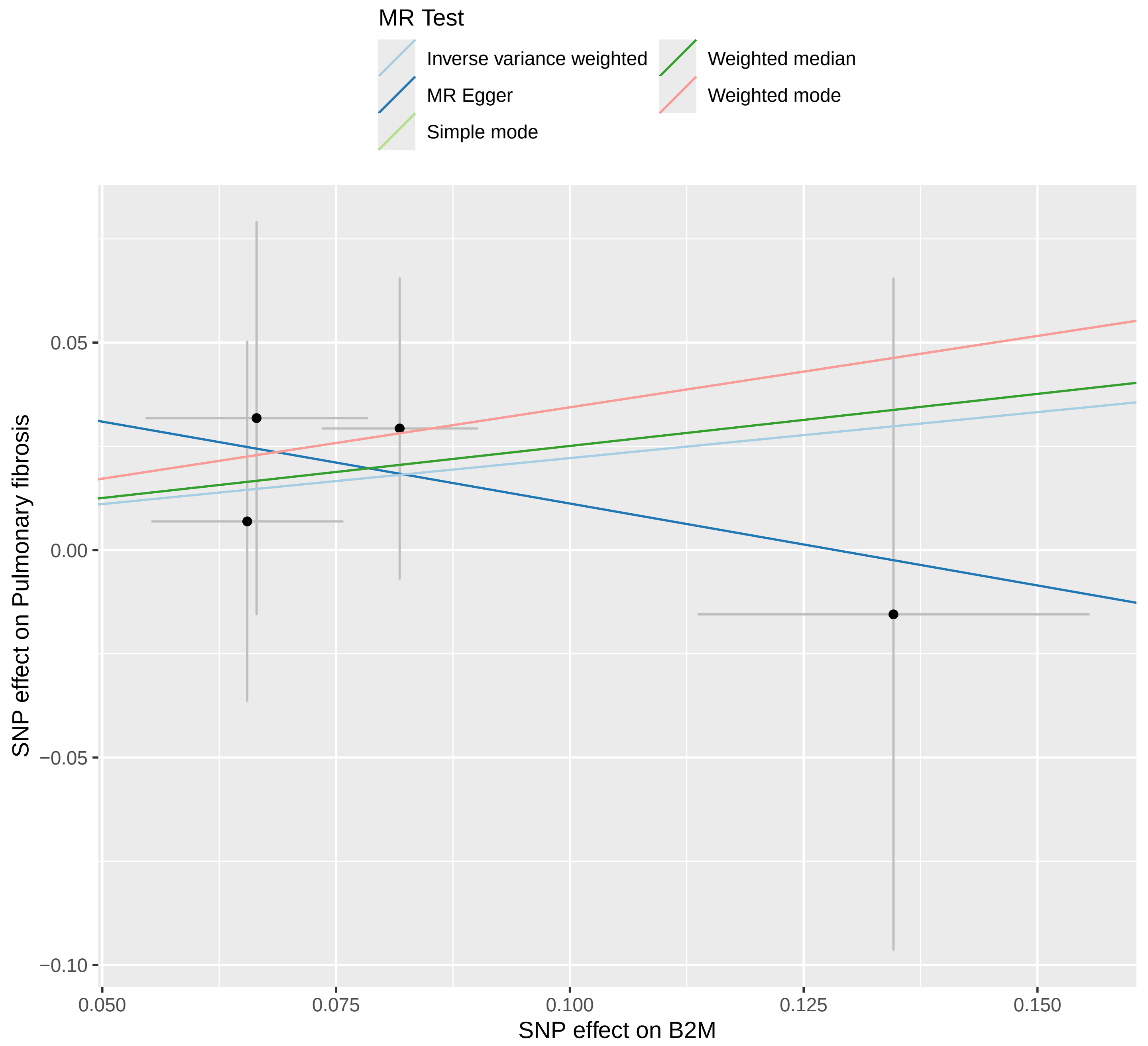
MR Test
Inverse variance weighted
MR Egger
Simple mode
Weighted median
Weighted mode
SNP effect on Pulmonary fibrosis
0.05
0.00
−0.05
−0.10
0.050
0.075
0.100
0.125
0.150
SNP effect on B2M

Insufficient number of SNPs

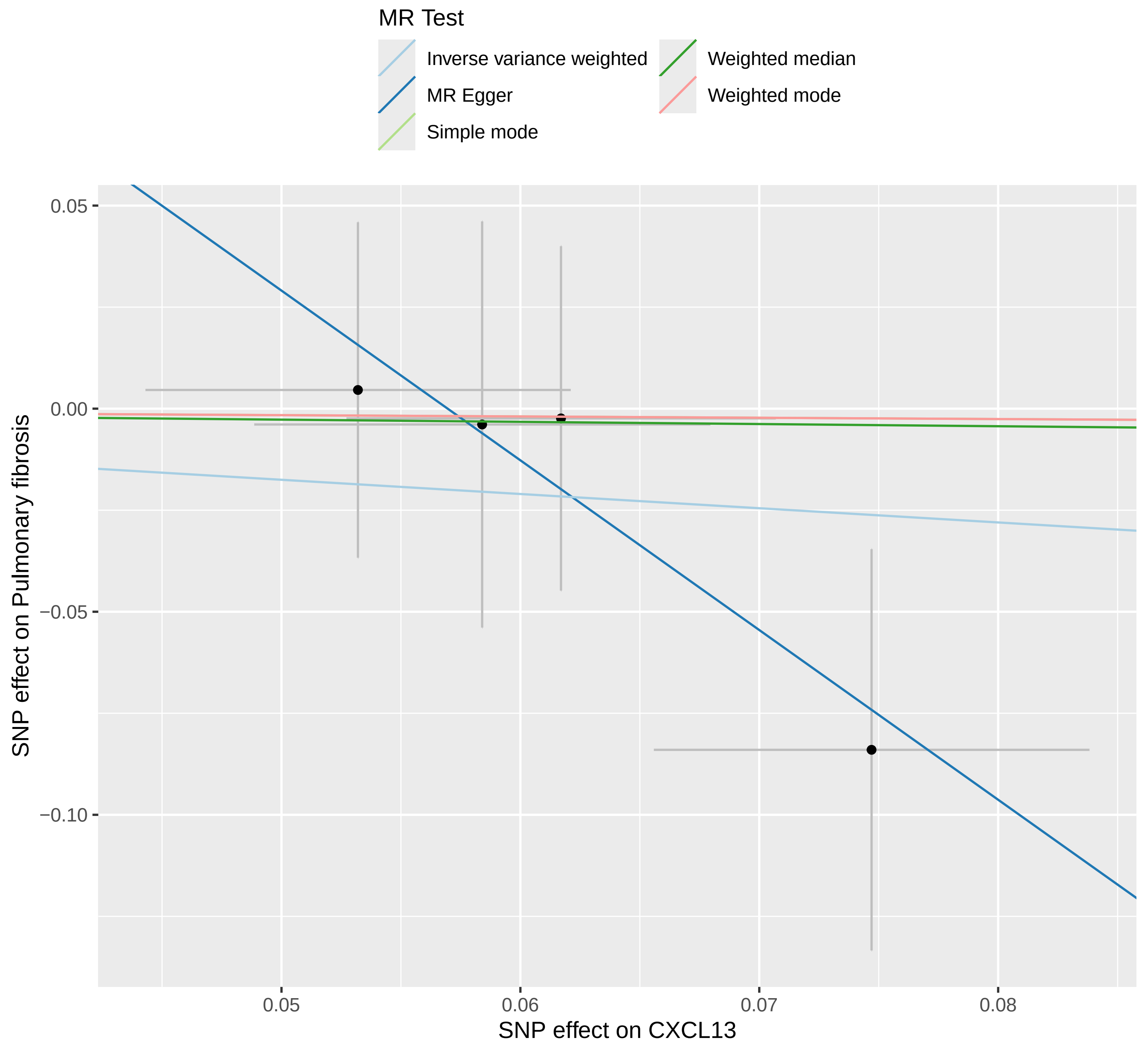
MR Test
Inverse variance weighted
MR Egger
Simple mode
Weighted median
Weighted mode
SNP effect on Pulmonary fibrosis
0.05
0.00
−0.05
−0.10
0.05
0.06
0.07
0.08
SNP effect on CXCL13

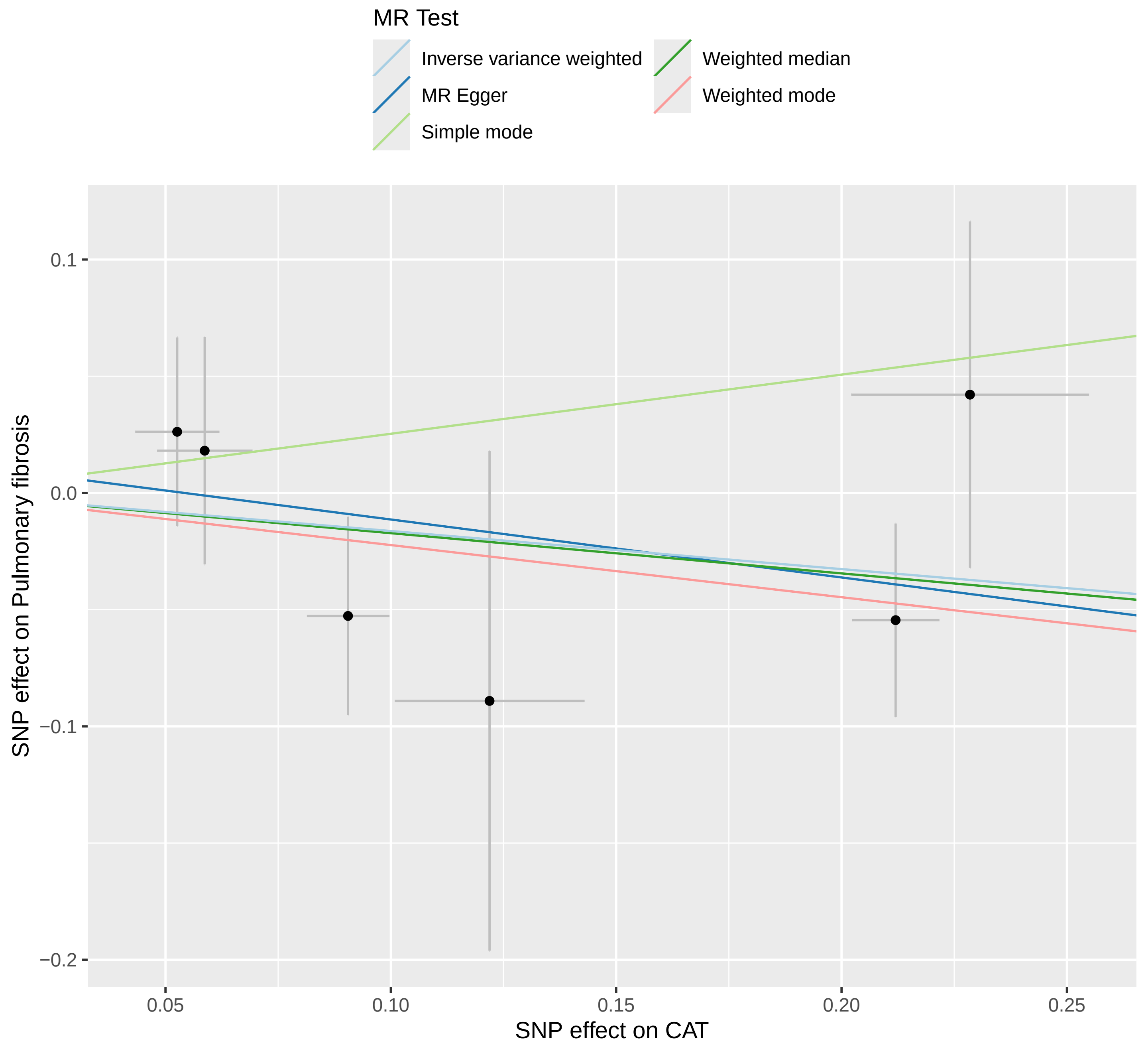
MR Test
Inverse variance weighted
MR Egger
Simple mode
Weighted median
Weighted mode
SNP effect on Pulmonary fibrosis
0.1
0.0
−0.1
−0.2
0.05
0.10
0.15
0.20
0.25
SNP effect on CAT

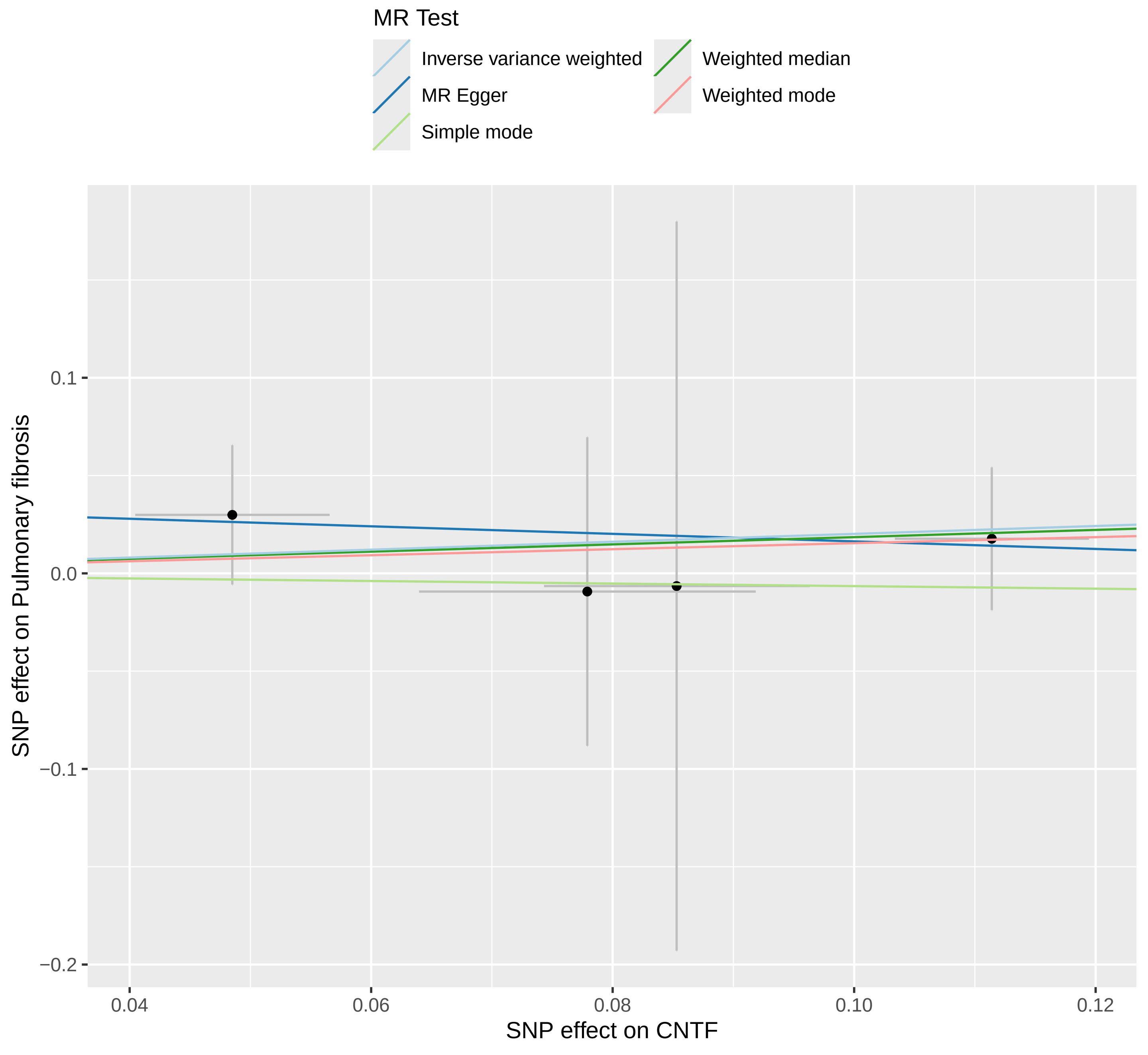

MR Test
Inverse variance weighted
MR Egger
Simple mode
Weighted median
Weighted mode
0.1
0.0
−0.1
−0.2
SNP effect on Pulmonary fibrosis
0.04
0.06
0.08
0.10
0.12
SNP effect on CNTF

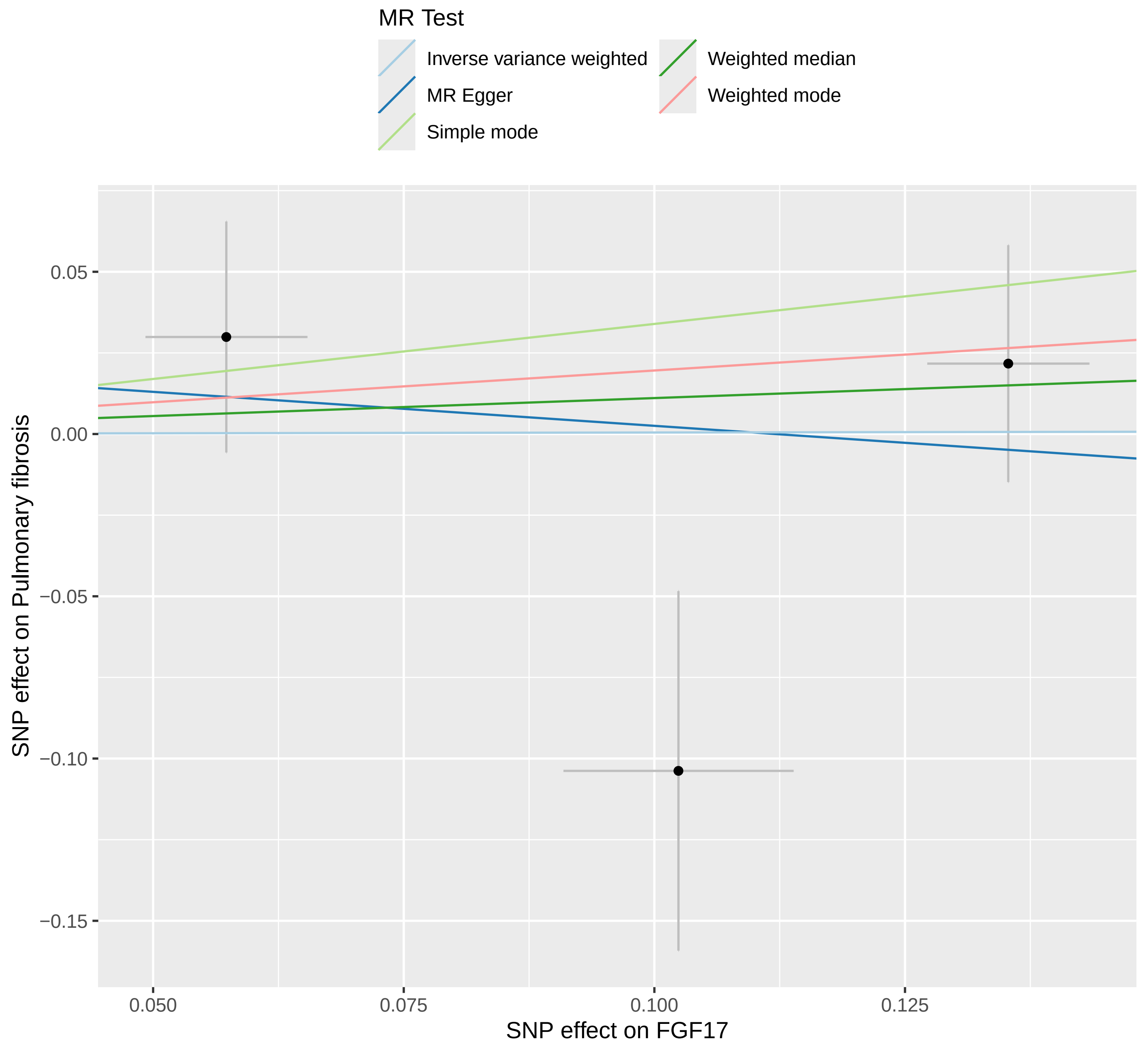

MR Test
Inverse variance weighted
MR Egger
Simple mode
Weighted median
Weighted mode
0.05
0.00
−0.05
−0.10
−0.15
SNP effect on Pulmonary fibrosis
0.050
0.075
0.100
0.125
SNP effect on FGF17

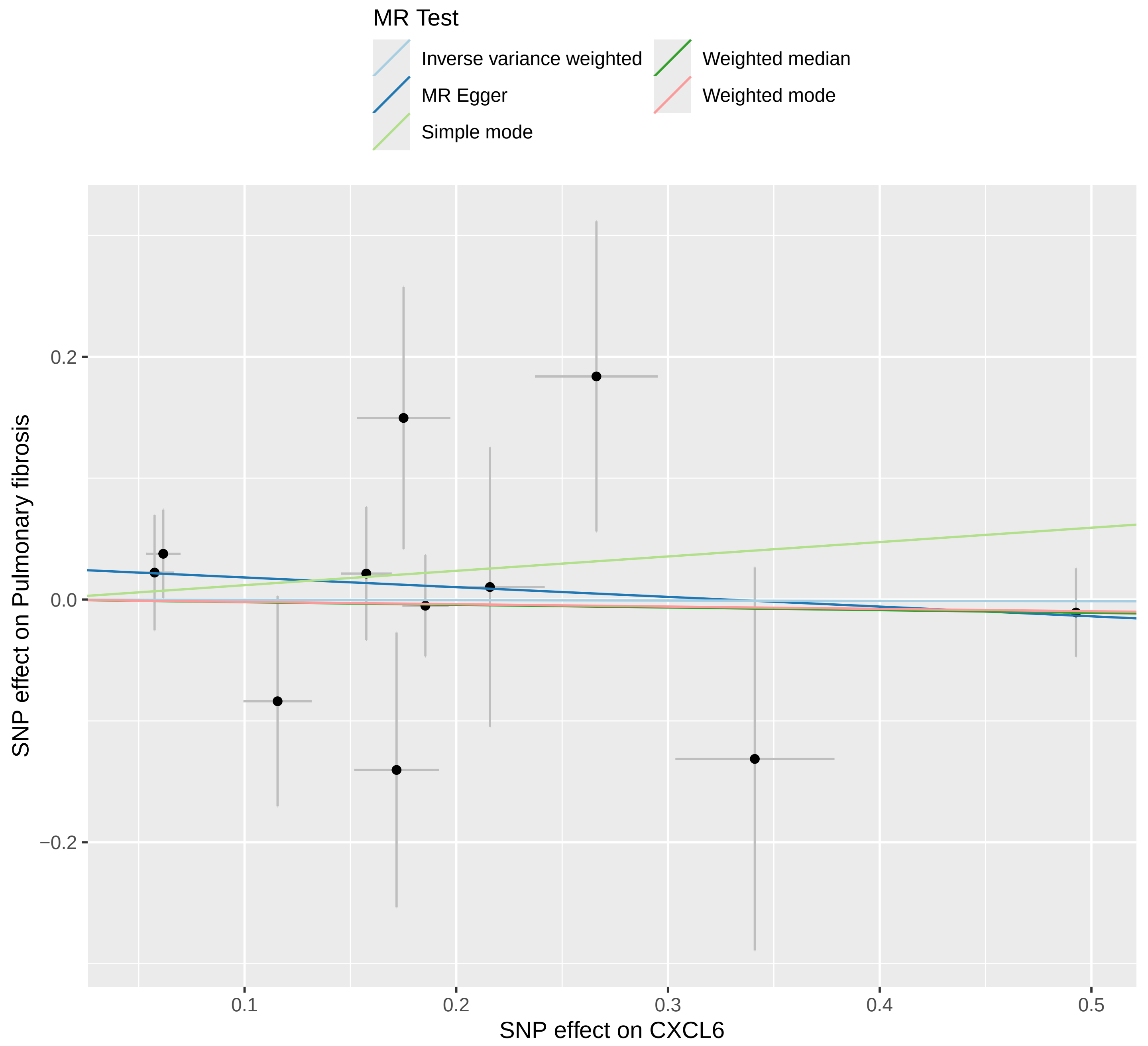
MR Test
Inverse variance weighted
MR Egger
Simple mode
Weighted median
Weighted mode
SNP effect on Pulmonary fibrosis
0.2
0.0
−0.2
0.1
0.2
0.3
0.4
0.5
SNP effect on CXCL6

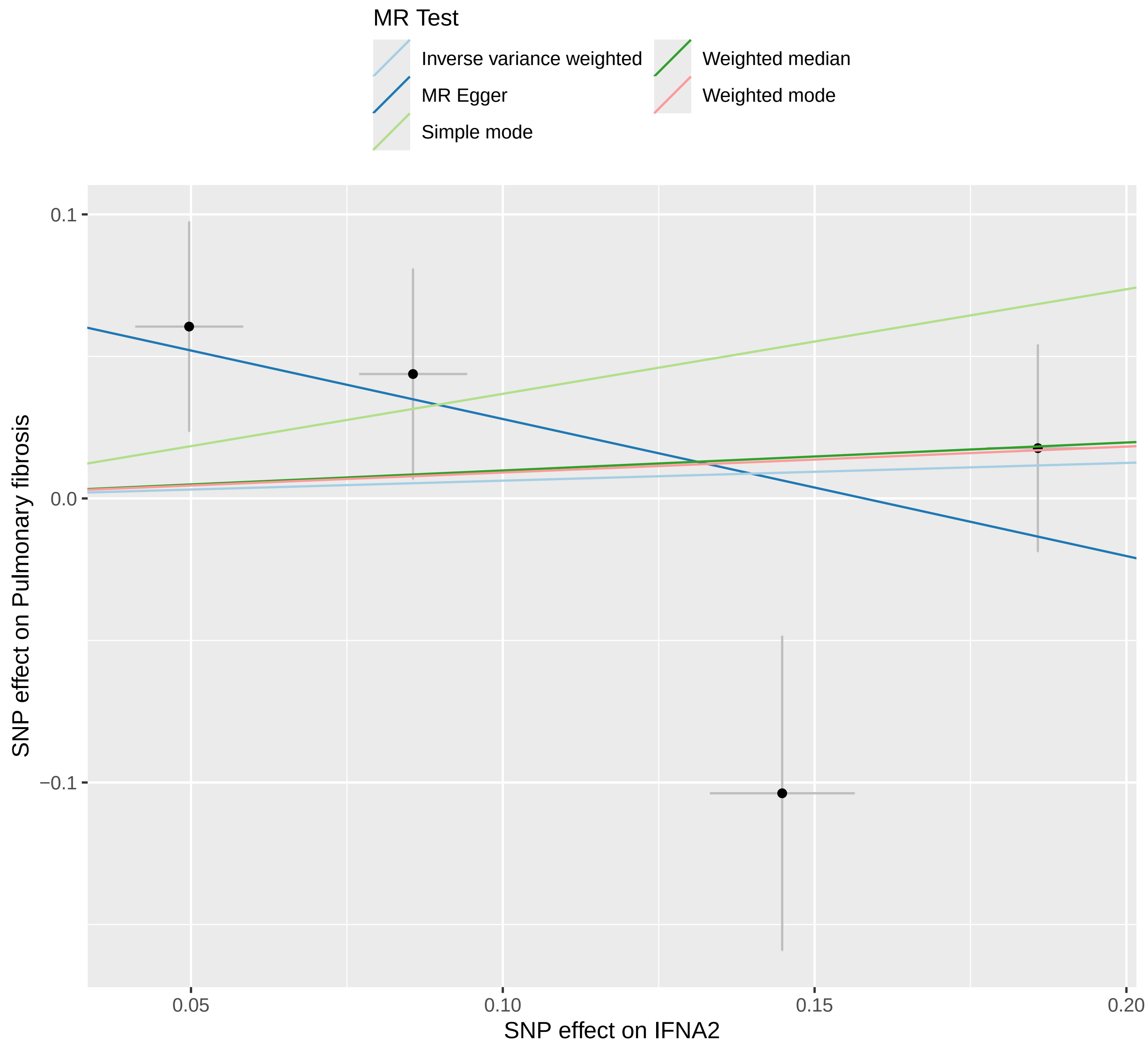
MR Test
Inverse variance weighted
MR Egger
Simple mode
Weighted median
Weighted mode
0.1
0.0
−0.1
SNP effect on Pulmonary fibrosis
0.05
0.10
0.15
0.20
SNP effect on IFNA2

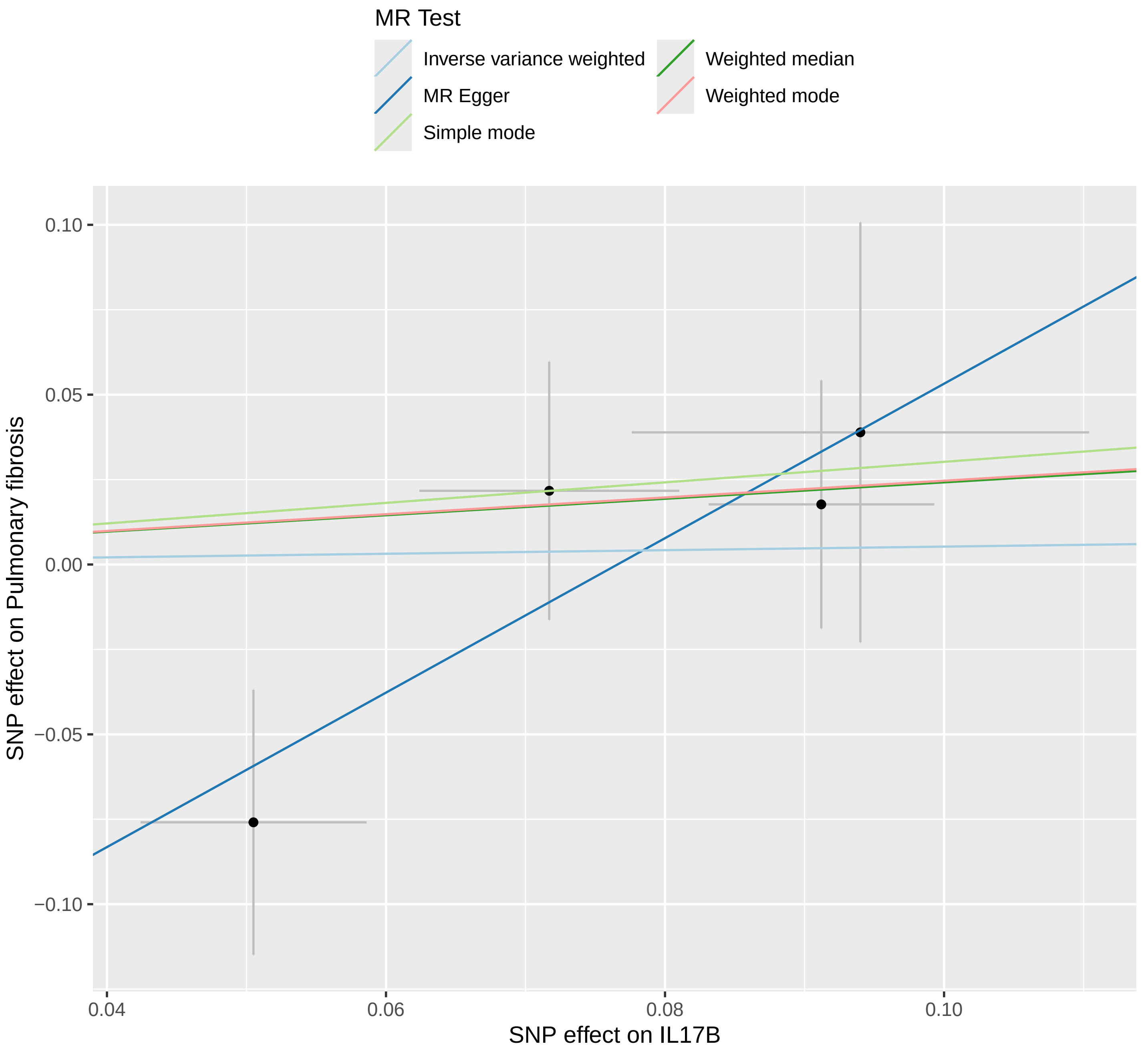
MR Test
Inverse variance weighted
MR Egger
Simple mode
Weighted median
Weighted mode
0.10
0.05
0.00
−0.05
−0.10
SNP effect on Pulmonary fibrosis
0.04
0.06
0.08
0.10
SNP effect on IL17B

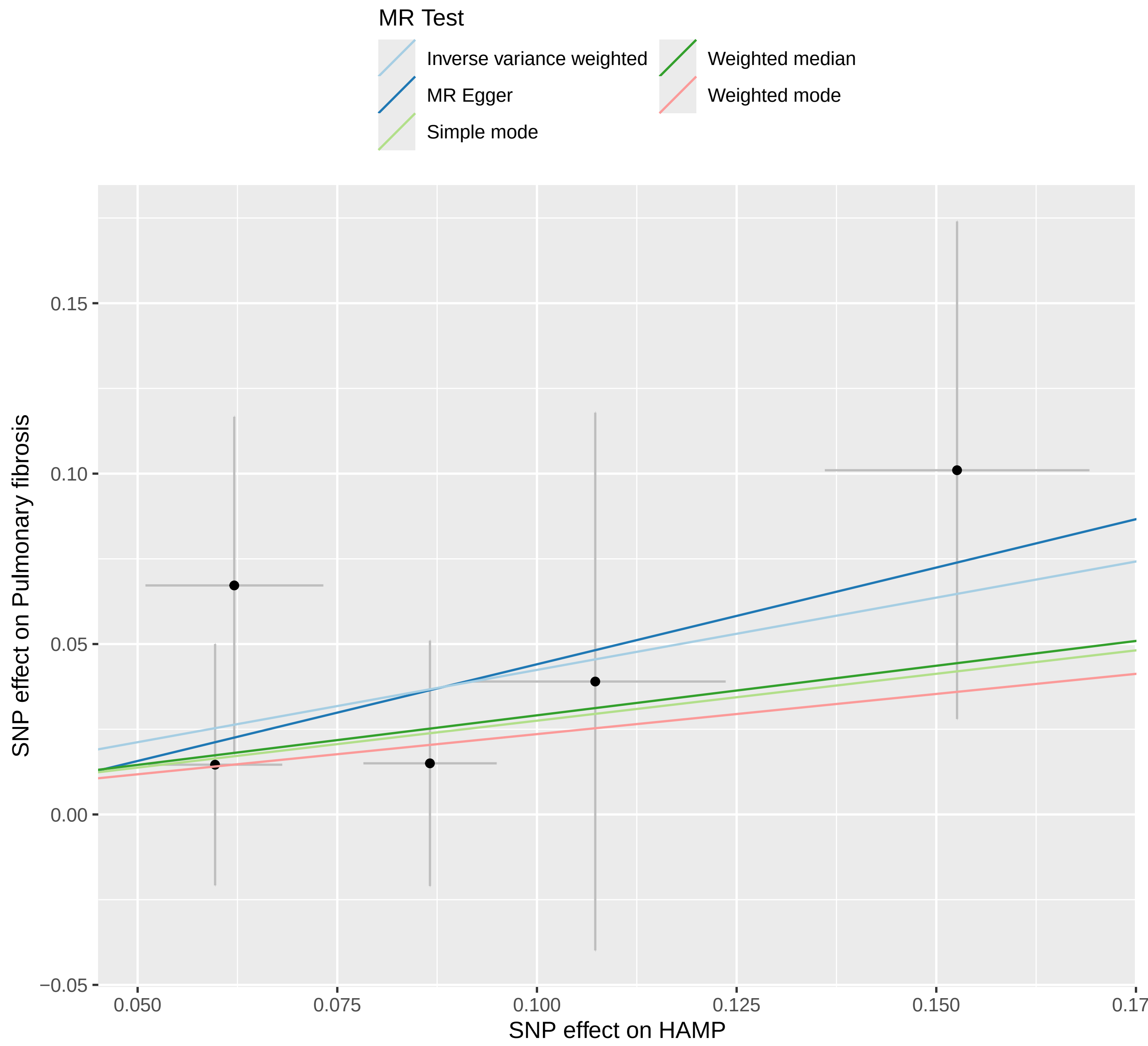

MR Test
Inverse variance weighted
MR Egger
Simple mode
Weighted median
Weighted mode
SNP effect on Pulmonary fibrosis
0.15
0.10
0.05
0.00
−0.05
0.050
0.075
0.100
0.125
0.150
SNP effect on HAMP

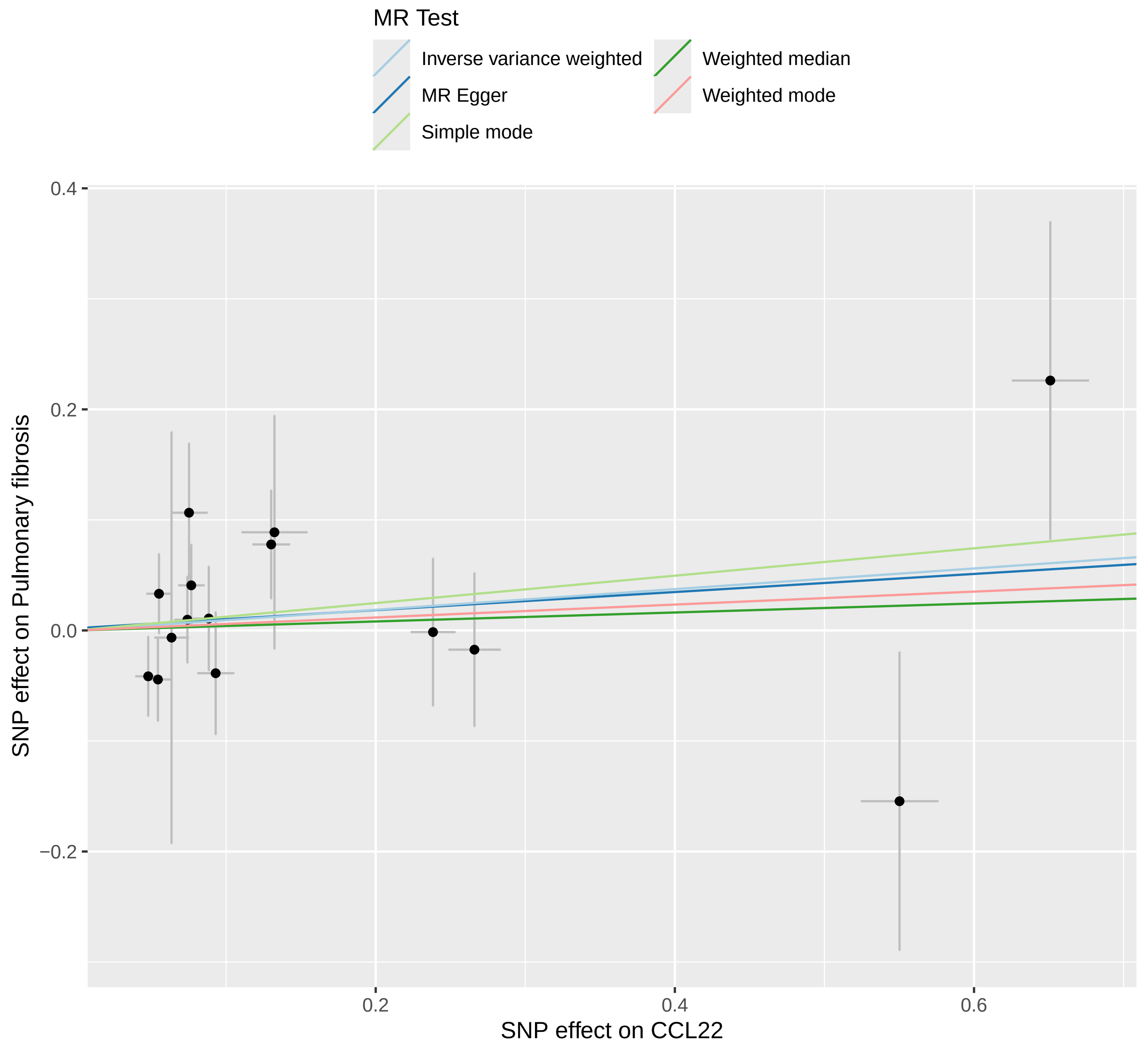
MR Test
Inverse variance weighted
MR Egger
Simple mode
Weighted median
Weighted mode
0.4
0.2
0.0
−0.2
0.2
0.4
0.6
SNP effect on Pulmonary fibrosis
SNP effect on CCL22

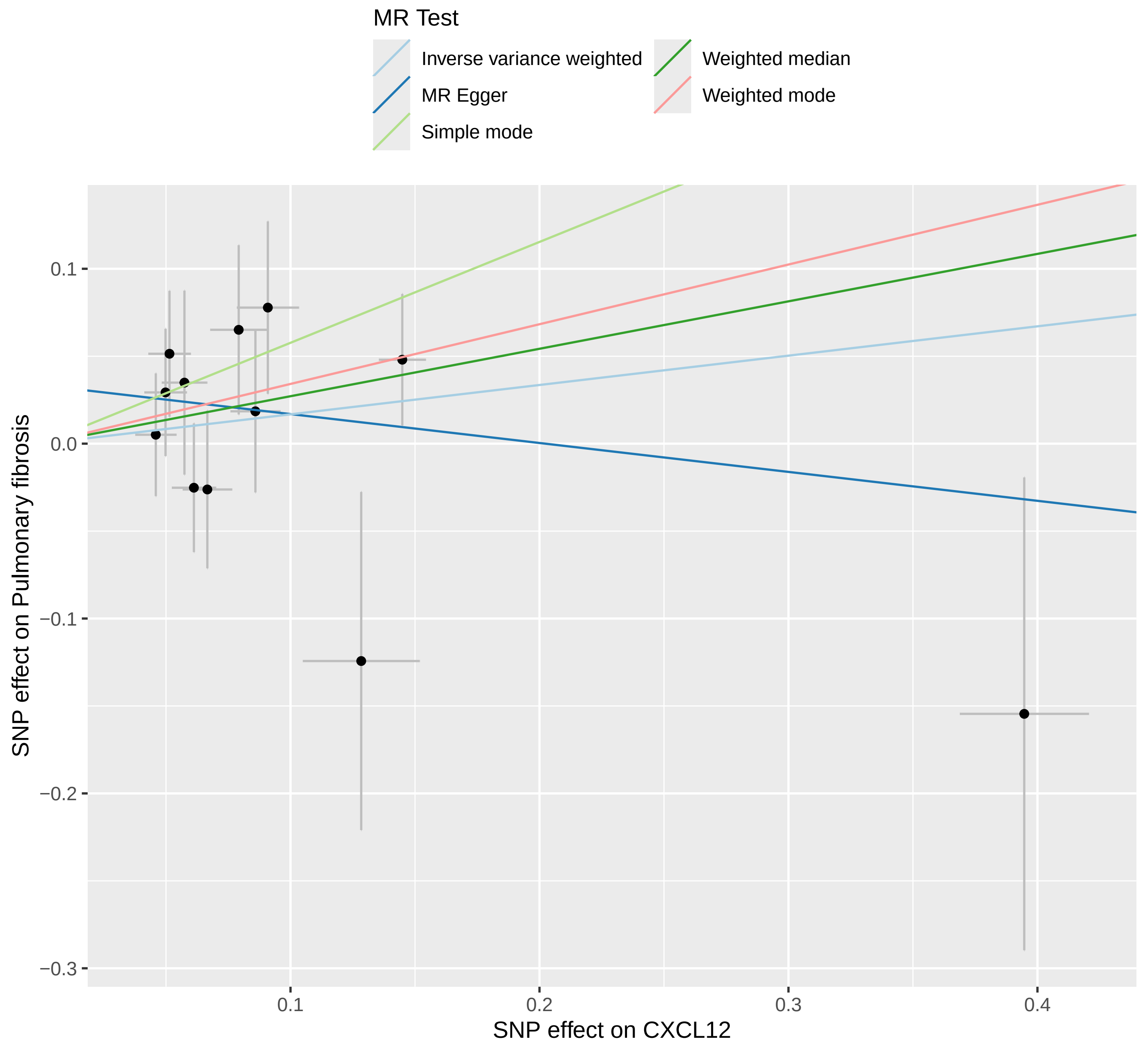

MR Test
Inverse variance weighted
MR Egger
Simple mode
Weighted median
Weighted mode
SNP effect on Pulmonary fibrosis
0.1
0.0
−0.1
−0.2
−0.3
0.1
0.2
0.3
0.4
SNP effect on CXCL12

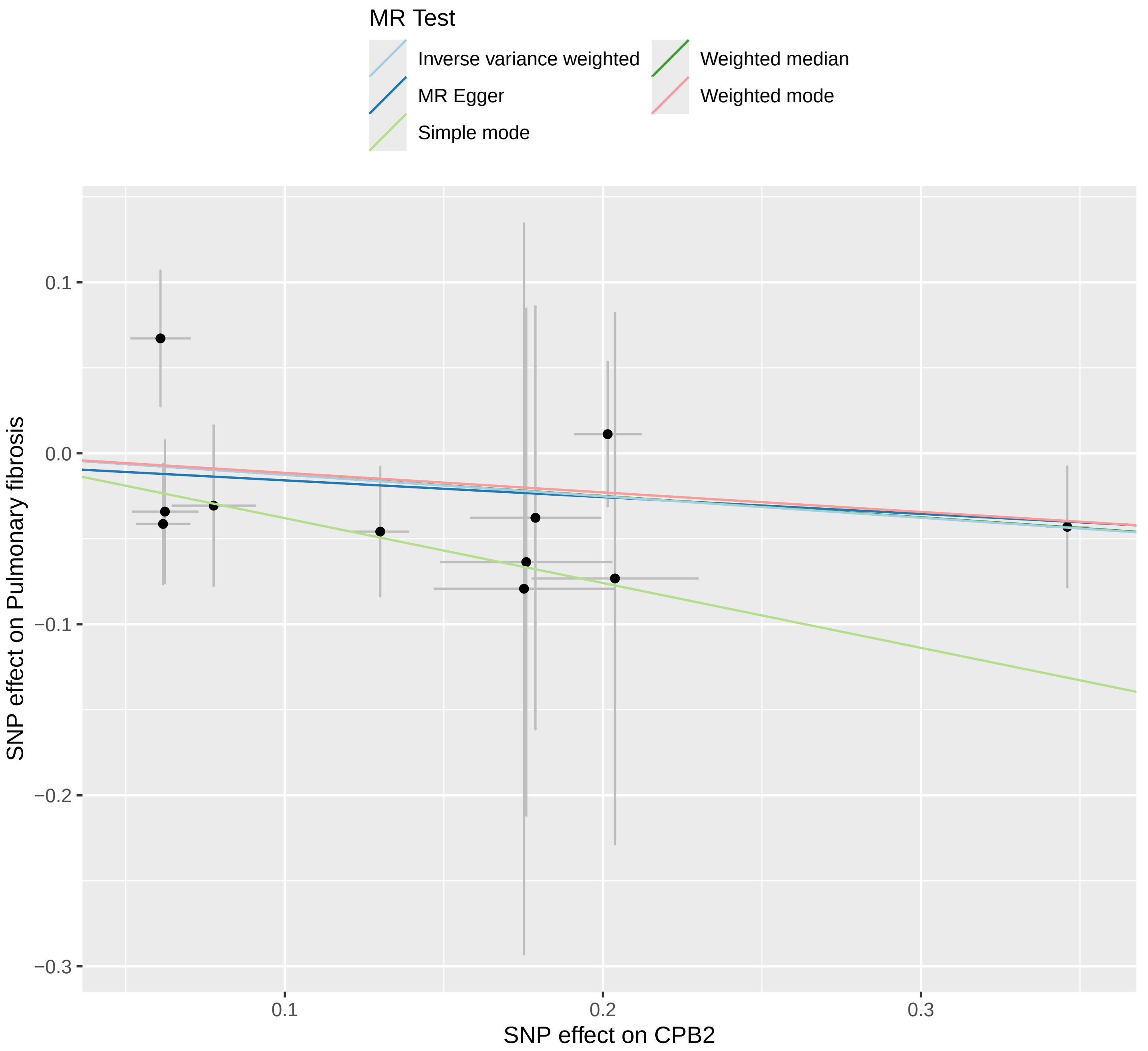

MR Test
Inverse variance weighted
MR Egger
Simple mode
Weighted median
Weighted mode
0.1
0.0
−0.1
−0.2
−0.3
SNP effect on Pulmonary fibrosis
0.1
0.2
0.3
SNP effect on CPB2

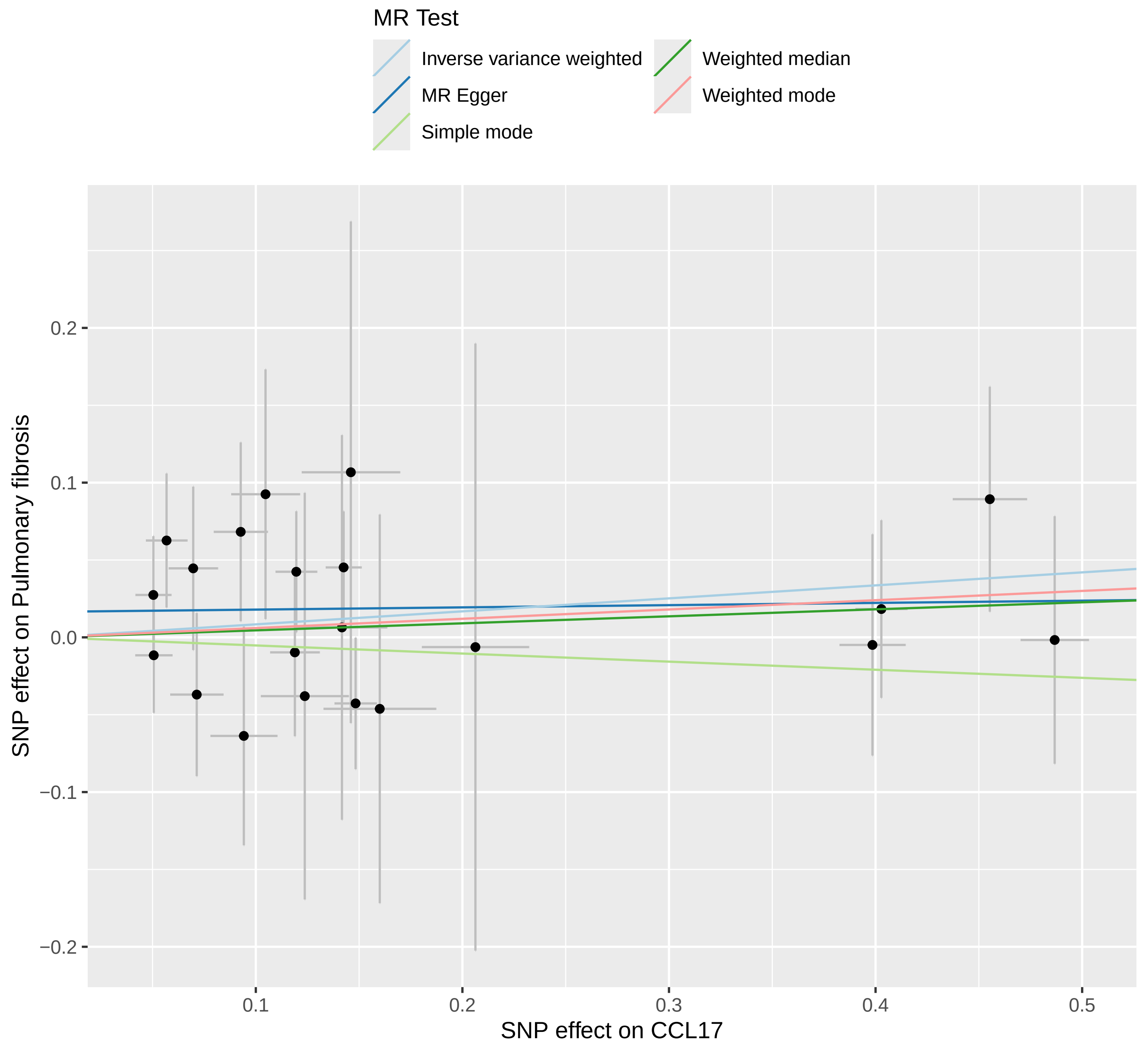
MR Test
Inverse variance weighted
MR Egger
Simple mode
Weighted median
Weighted mode
SNP effect on Pulmonary fibrosis
0.2
0.1
0.0
−0.1
−0.2
0.1
0.2
0.3
0.4
0.5
SNP effect on CCL17

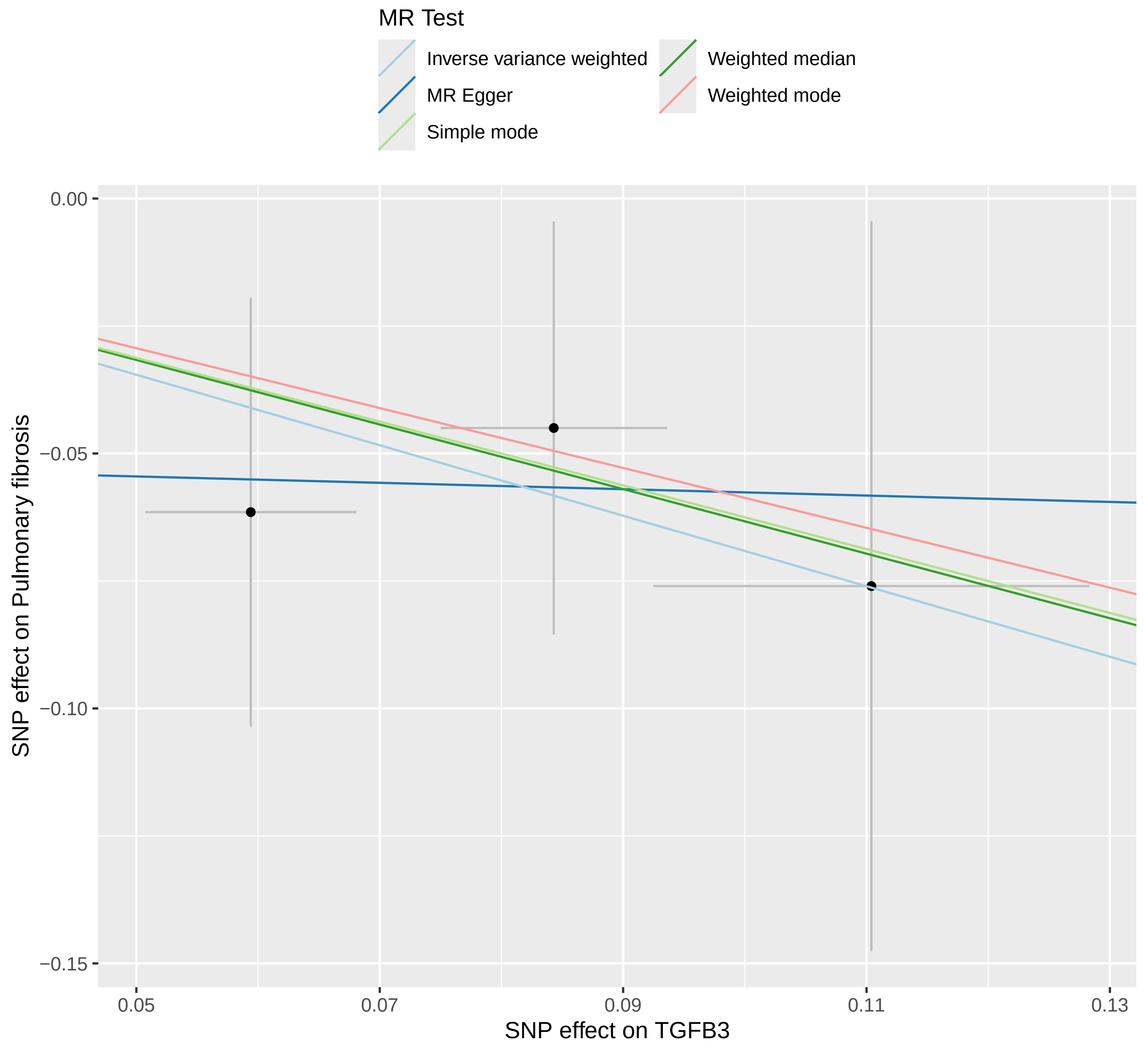
MR Test
Inverse variance weighted
MR Egger
Simple mode
Weighted median
Weighted mode
0.00
−0.05
−0.10
−0.15
0.05
0.07
0.09
0.11
0.13
SNP effect on Pulmonary fibrosis
SNP effect on TGFB3

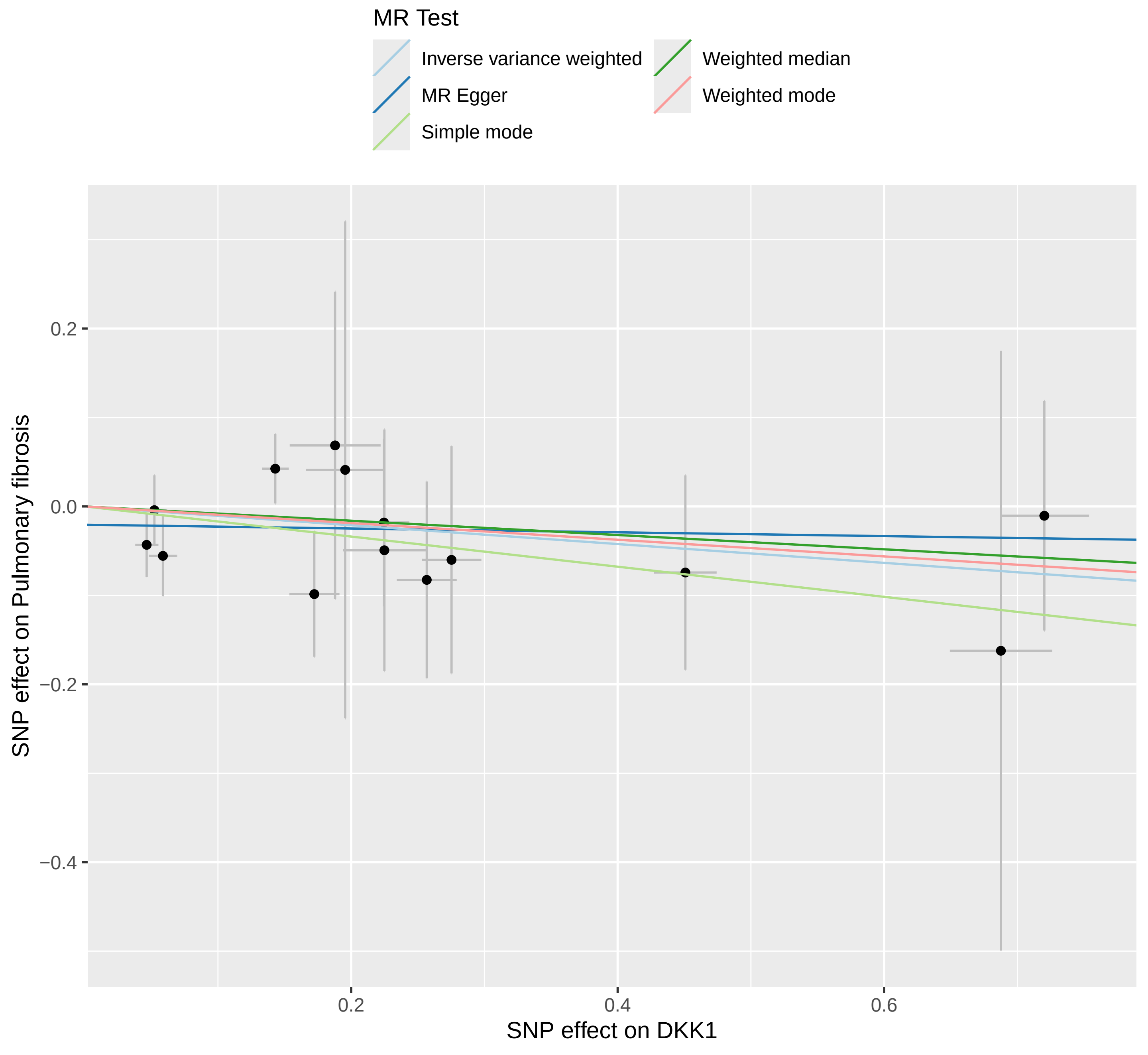

MR Test
Inverse variance weighted
MR Egger
Simple mode
Weighted median
Weighted mode
0.2
0.0
−0.2
−0.4
0.2
0.4
0.6
SNP effect on Pulmonary fibrosis
SNP effect on DKK1

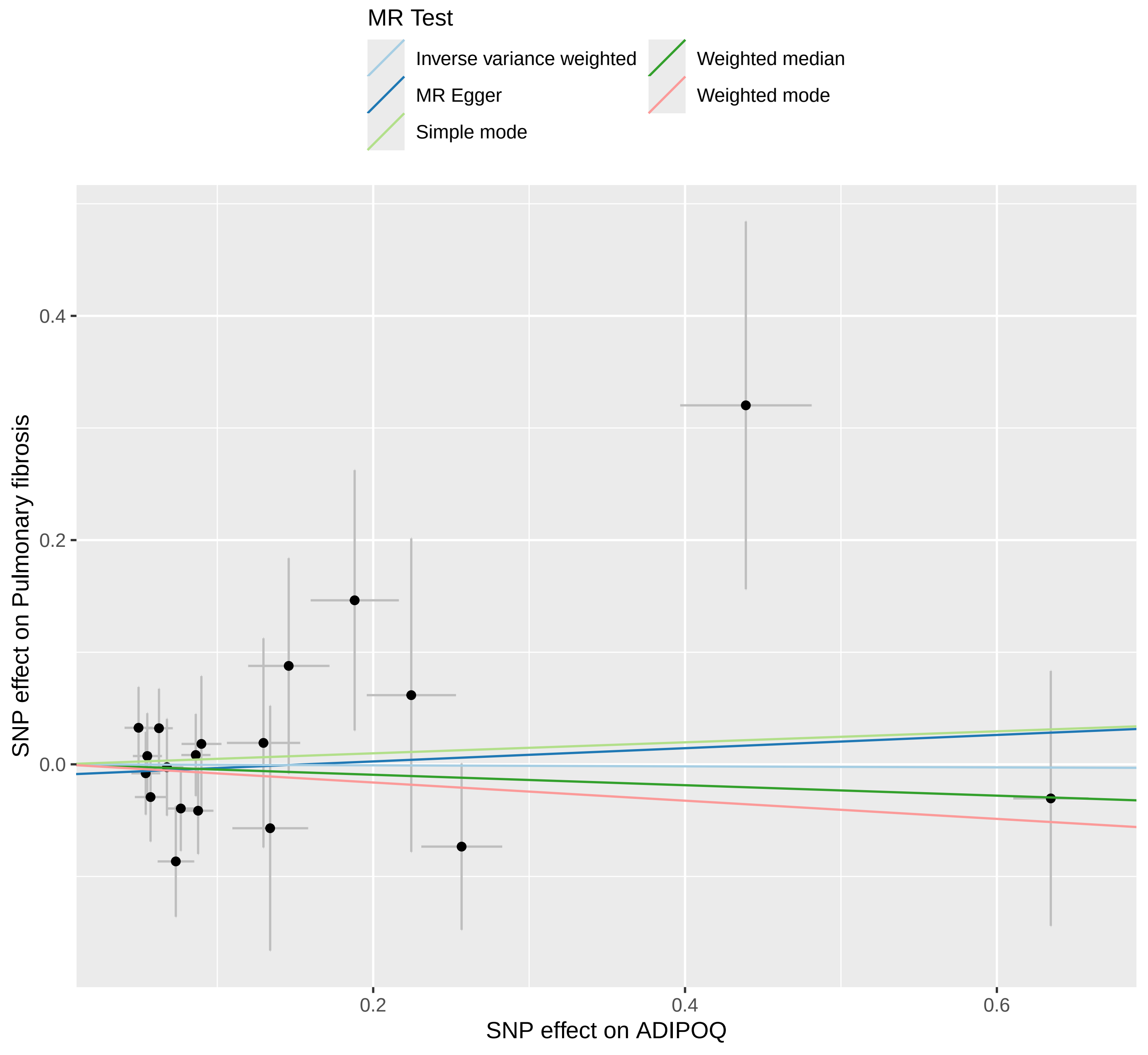

MR Test
Inverse variance weighted
MR Egger
Simple mode
Weighted median
Weighted mode
SNP effect on Pulmonary fibrosis
0.4
0.2
0.0
0.2
0.4
0.6
SNP effect on ADIPOQ

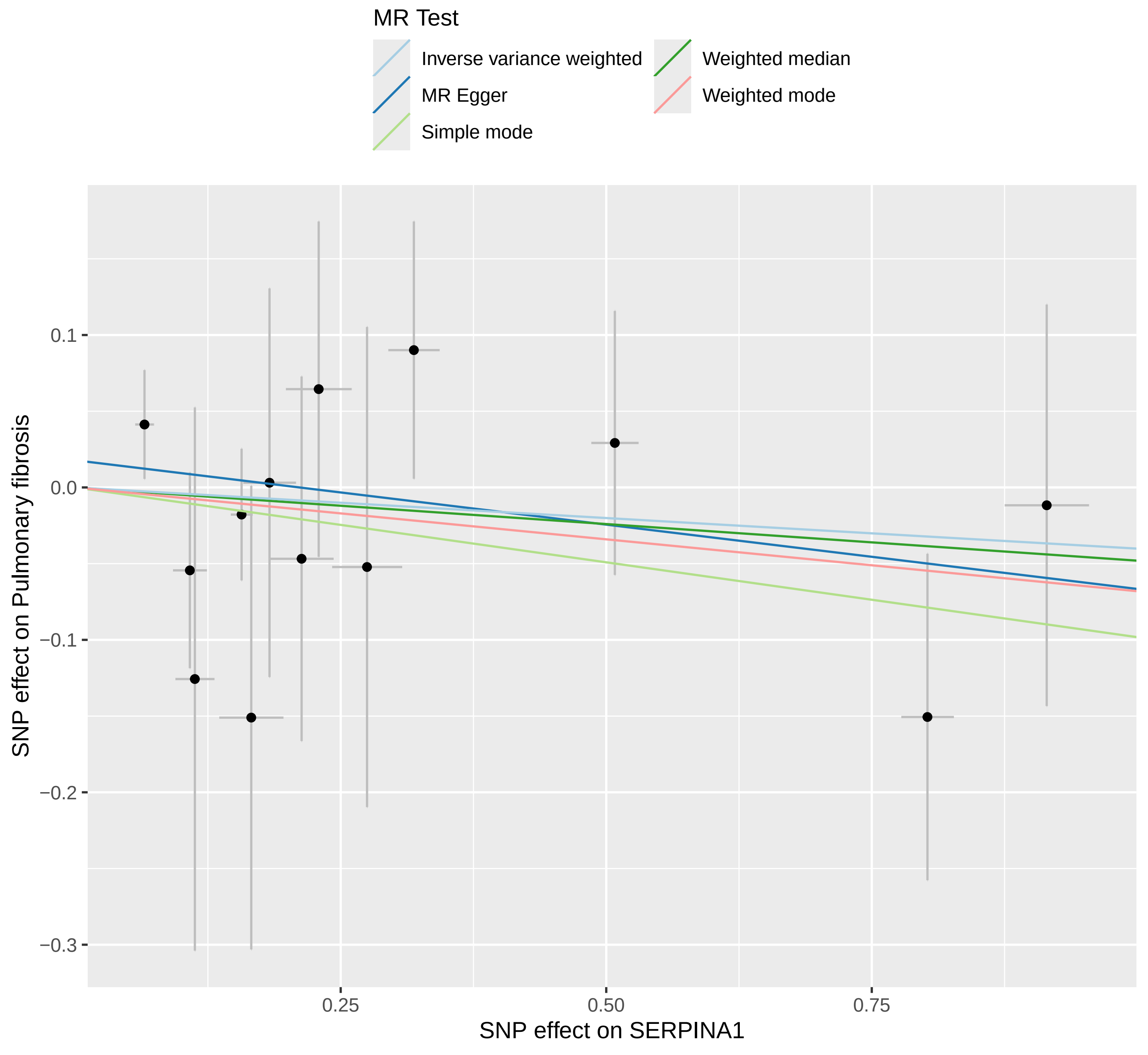

MR Test
Inverse variance weighted
MR Egger
Simple mode
Weighted median
Weighted mode
0.1
0.0
−0.1
−0.2
−0.3
SNP effect on Pulmonary fibrosis
0.25
0.50
0.75
SNP effect on SERPINA1

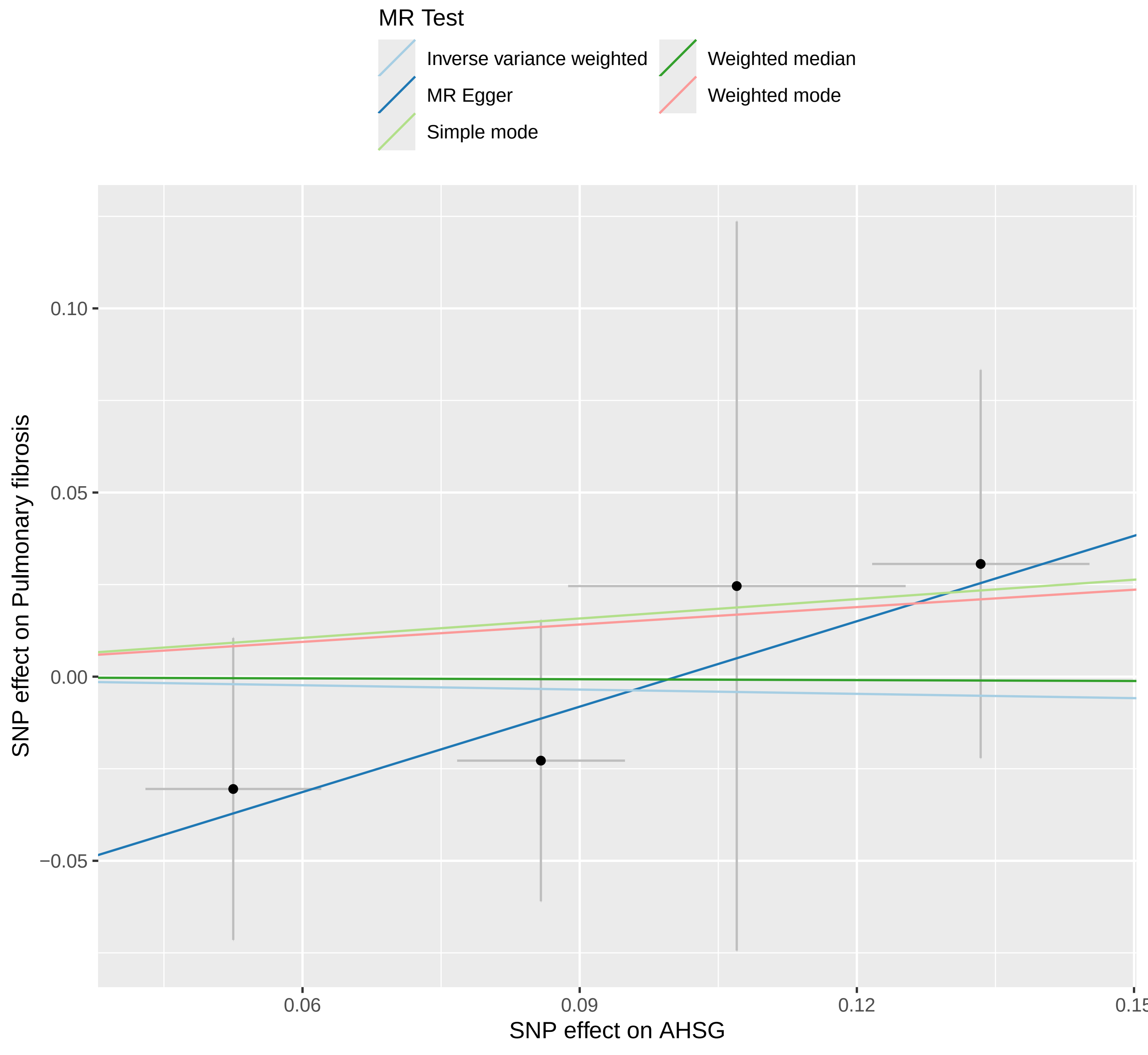
MR Test
Inverse variance weighted
MR Egger
Simple mode
Weighted median
Weighted mode
0.10
0.05
0.00
−0.05
0.06
0.09
0.12
0.15
SNP effect on AHSG
SNP effect on Pulmonary fibrosis

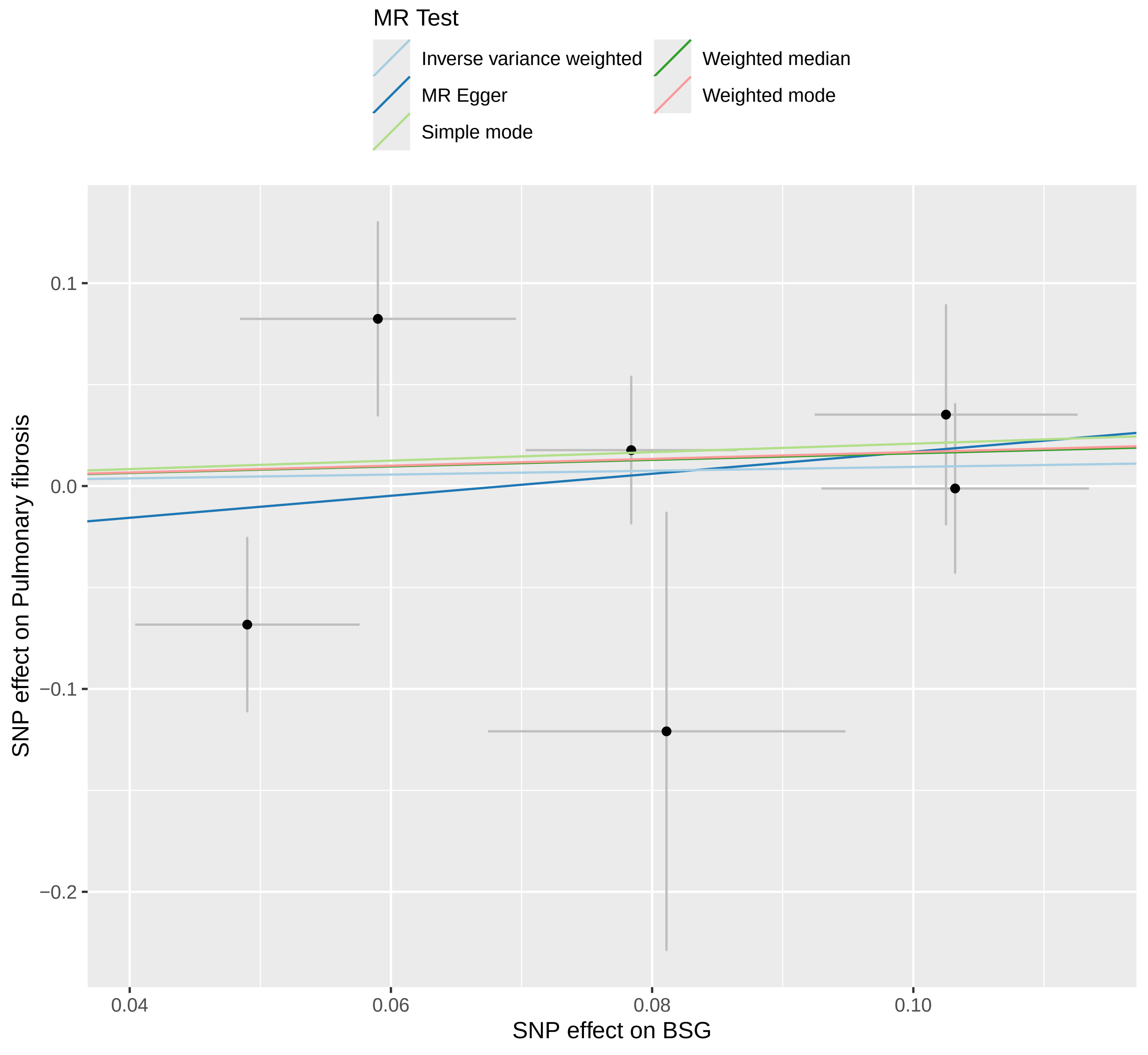
MR Test
Inverse variance weighted
MR Egger
Simple mode
Weighted median
Weighted mode
0.1
0.0
−0.1
−0.2
0.04
0.06
0.08
0.10
SNP effect on BSG
SNP effect on Pulmonary fibrosis

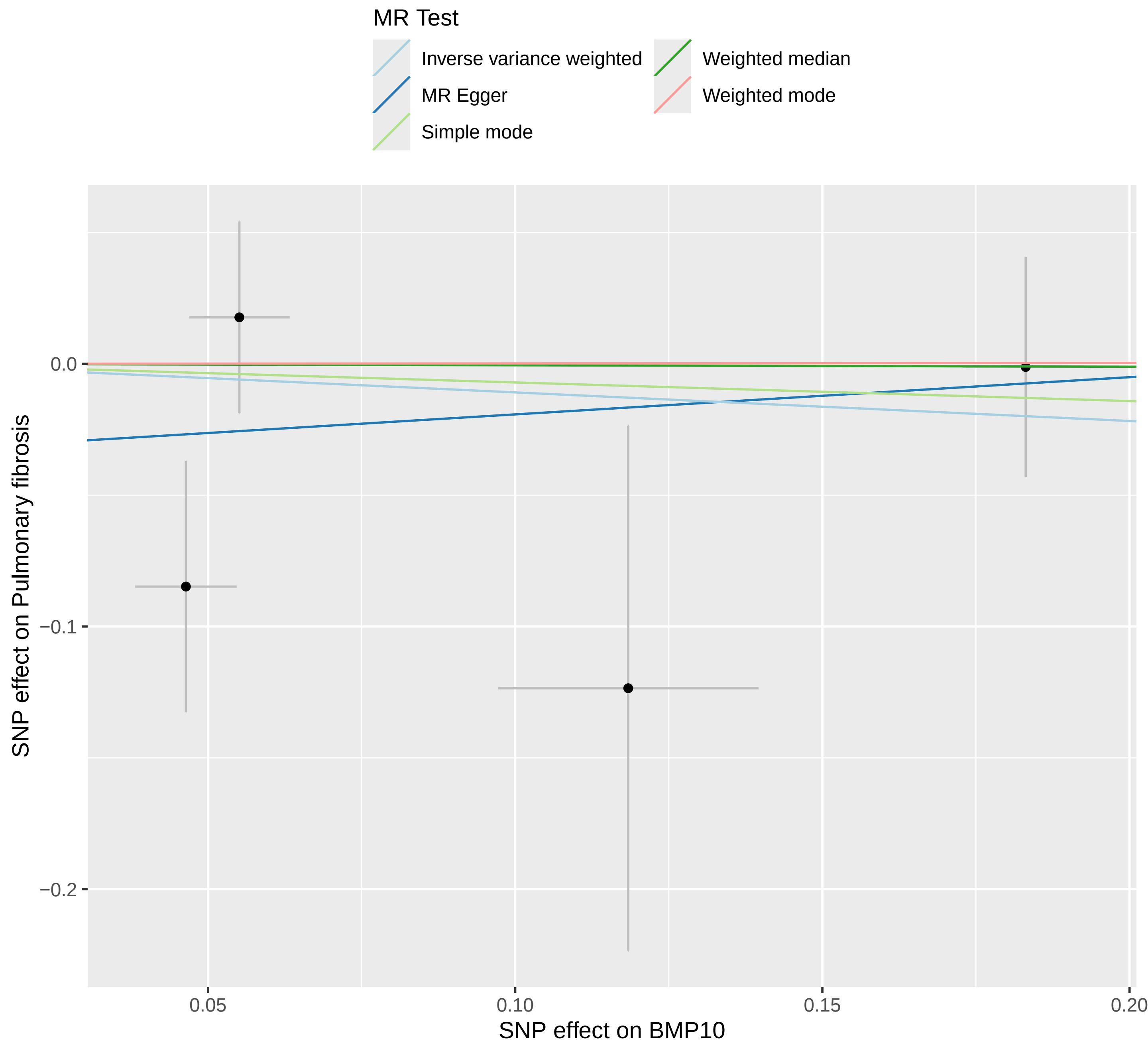

MR Test
Inverse variance weighted
MR Egger
Simple mode
Weighted median
Weighted mode
0.0
−0.1
−0.2
SNP effect on Pulmonary fibrosis
0.05
0.10
0.15
0.20
SNP effect on BMP10

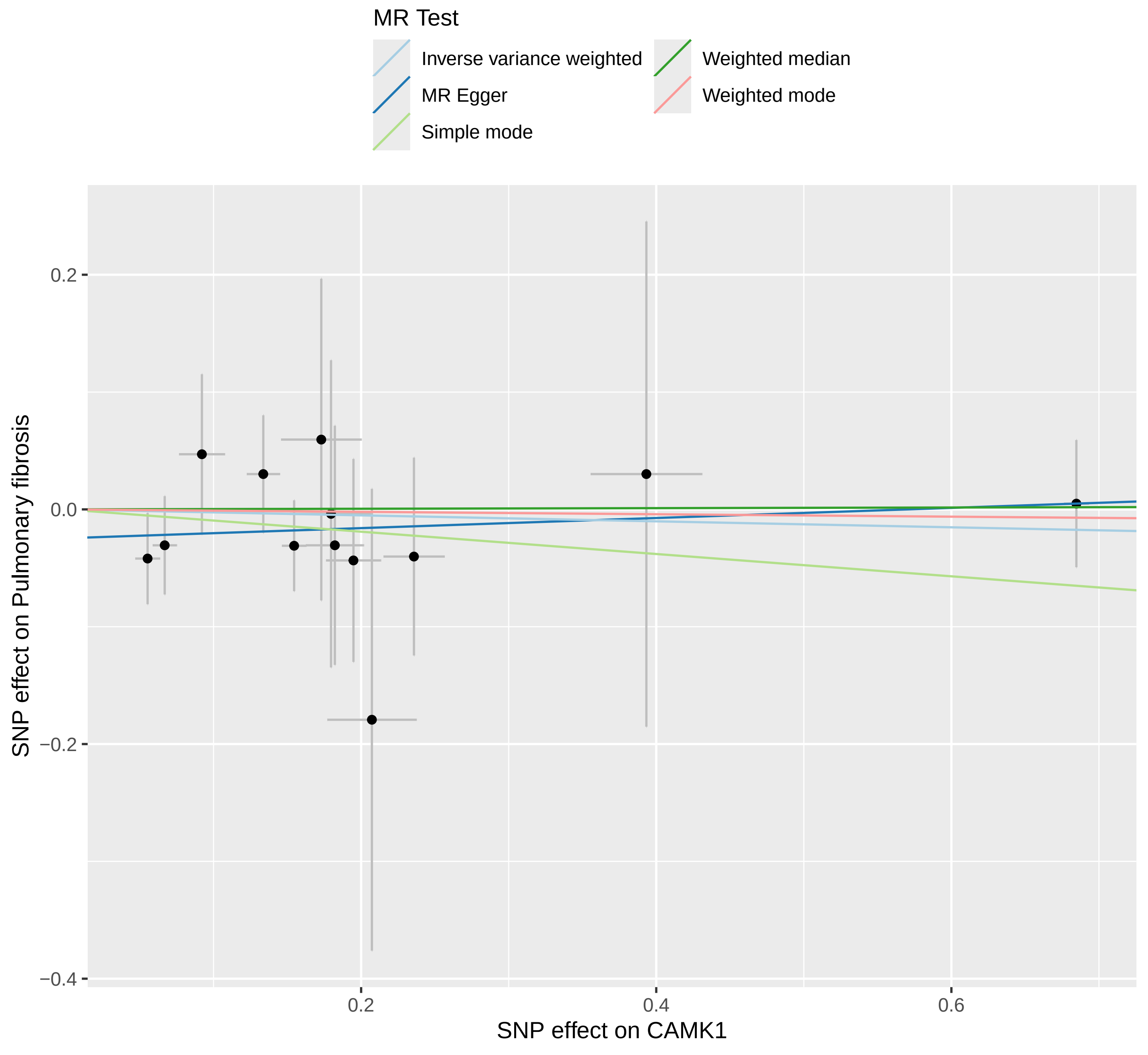
MR Test
Inverse variance weighted
MR Egger
Simple mode
Weighted median
Weighted mode
0.2
0.0
−0.2
−0.4
0.2
0.4
0.6
SNP effect on Pulmonary fibrosis
SNP effect on CAMK1

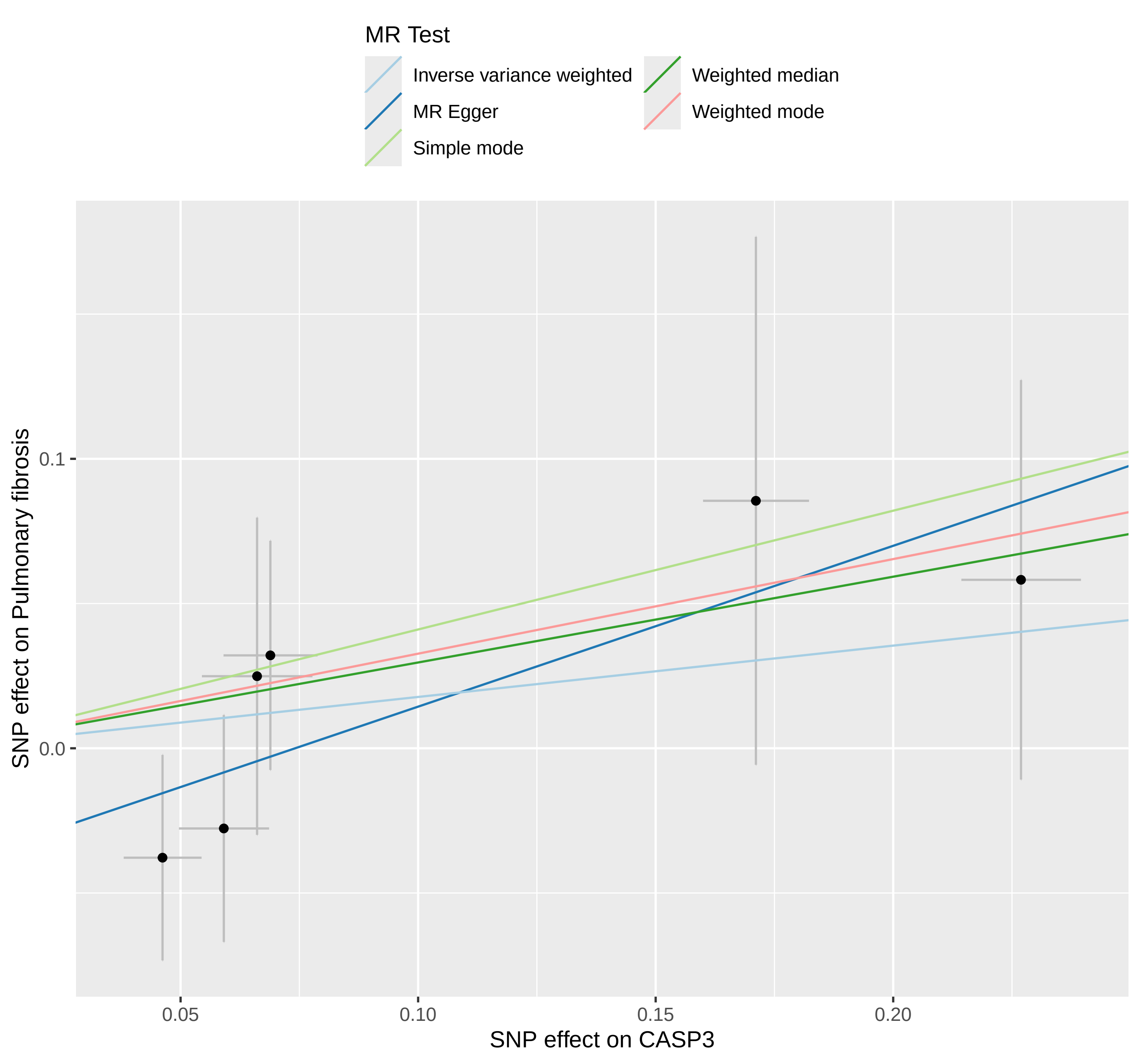

MR Test
Inverse variance weighted
MR Egger
Simple mode
Weighted median
Weighted mode
0.1
0.0
0.05
0.10
0.15
0.20
SNP effect on CASP3
SNP effect on Pulmonary fibrosis

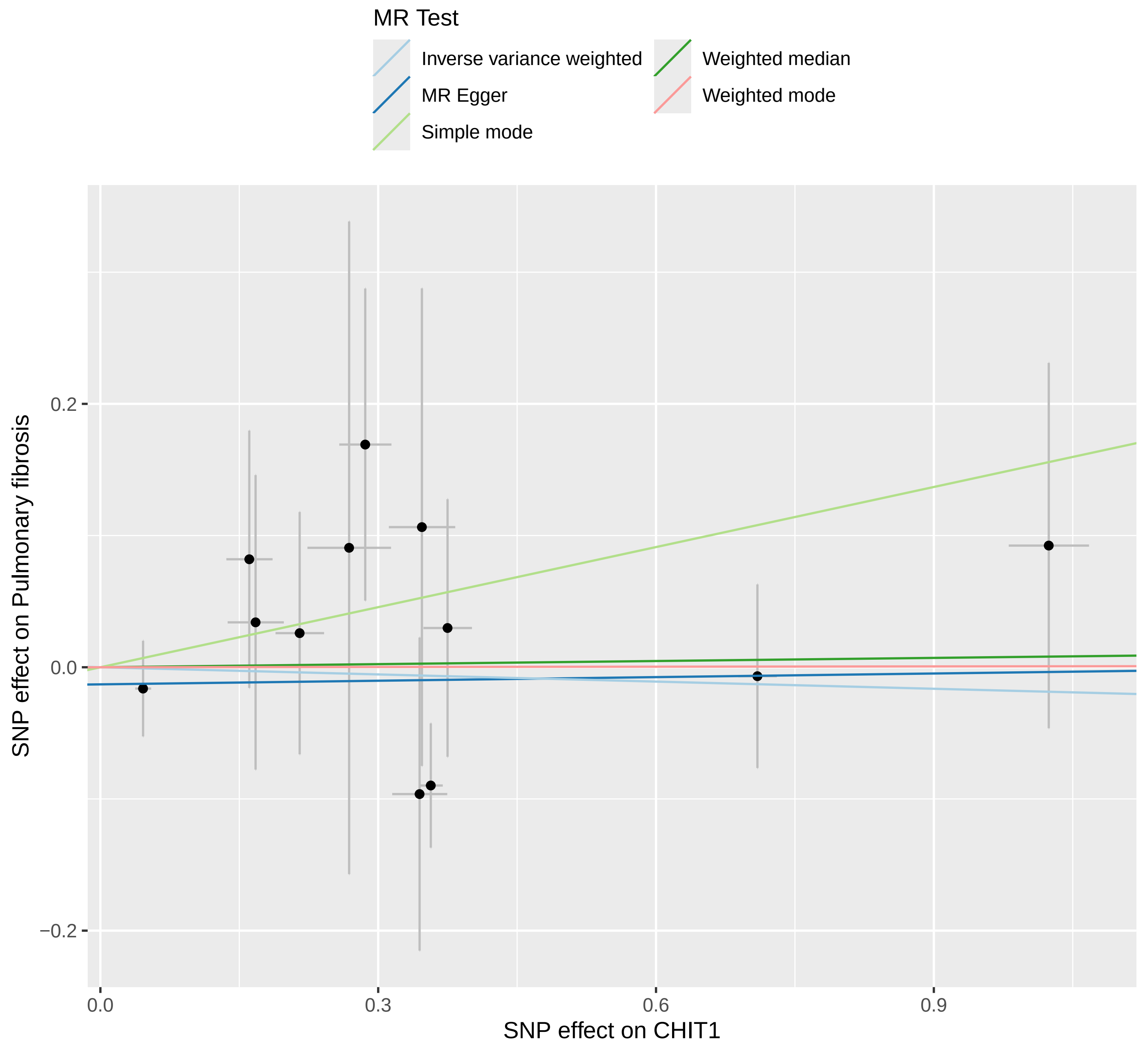

MR Test
Inverse variance weighted
MR Egger
Simple mode
Weighted median
Weighted mode
0.2
0.0
−0.2
0.0
0.3
0.6
0.9
SNP effect on Pulmonary fibrosis
SNP effect on CHIT1

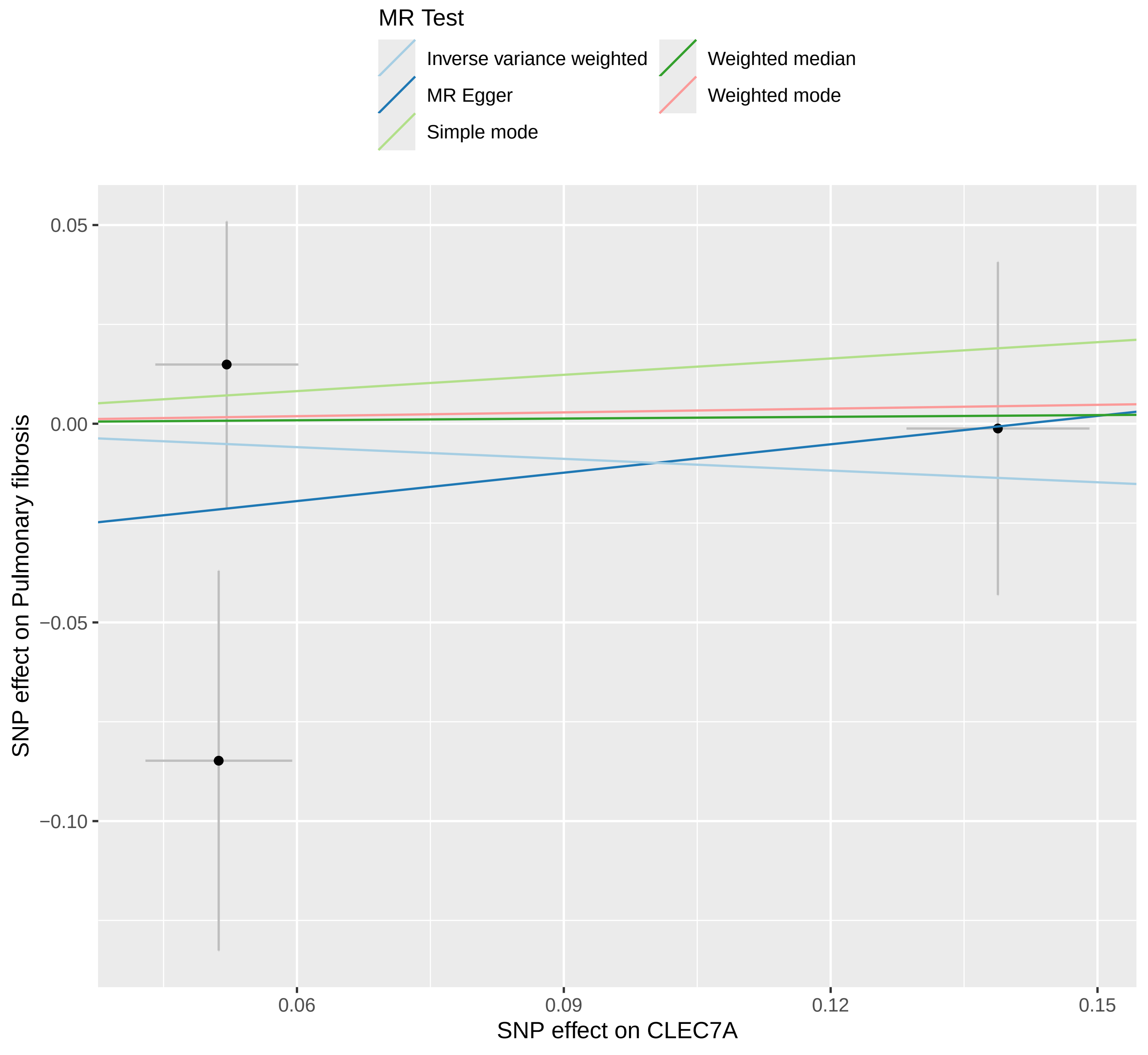

MR Test
Inverse variance weighted
MR Egger
Simple mode
Weighted median
Weighted mode
0.05
0.00
−0.05
−0.10
0.06
0.09
0.12
0.15
SNP effect on Pulmonary fibrosis
SNP effect on CLEC7A

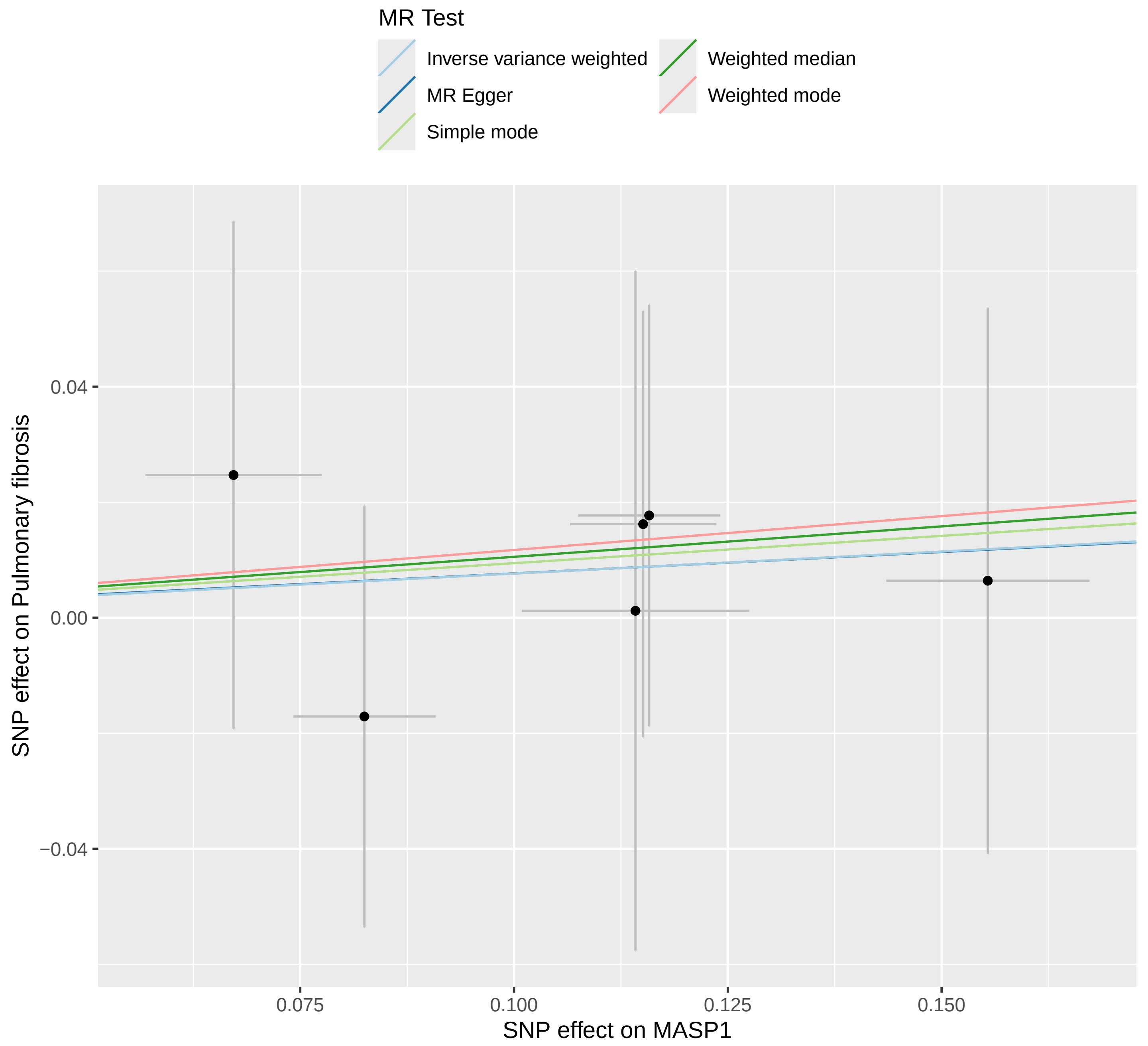

MR Test
Inverse variance weighted
MR Egger
Simple mode
Weighted median
Weighted mode
0.04
0.00
−0.04
SNP effect on Pulmonary fibrosis
0.075
0.100
0.125
0.150
SNP effect on MASP1

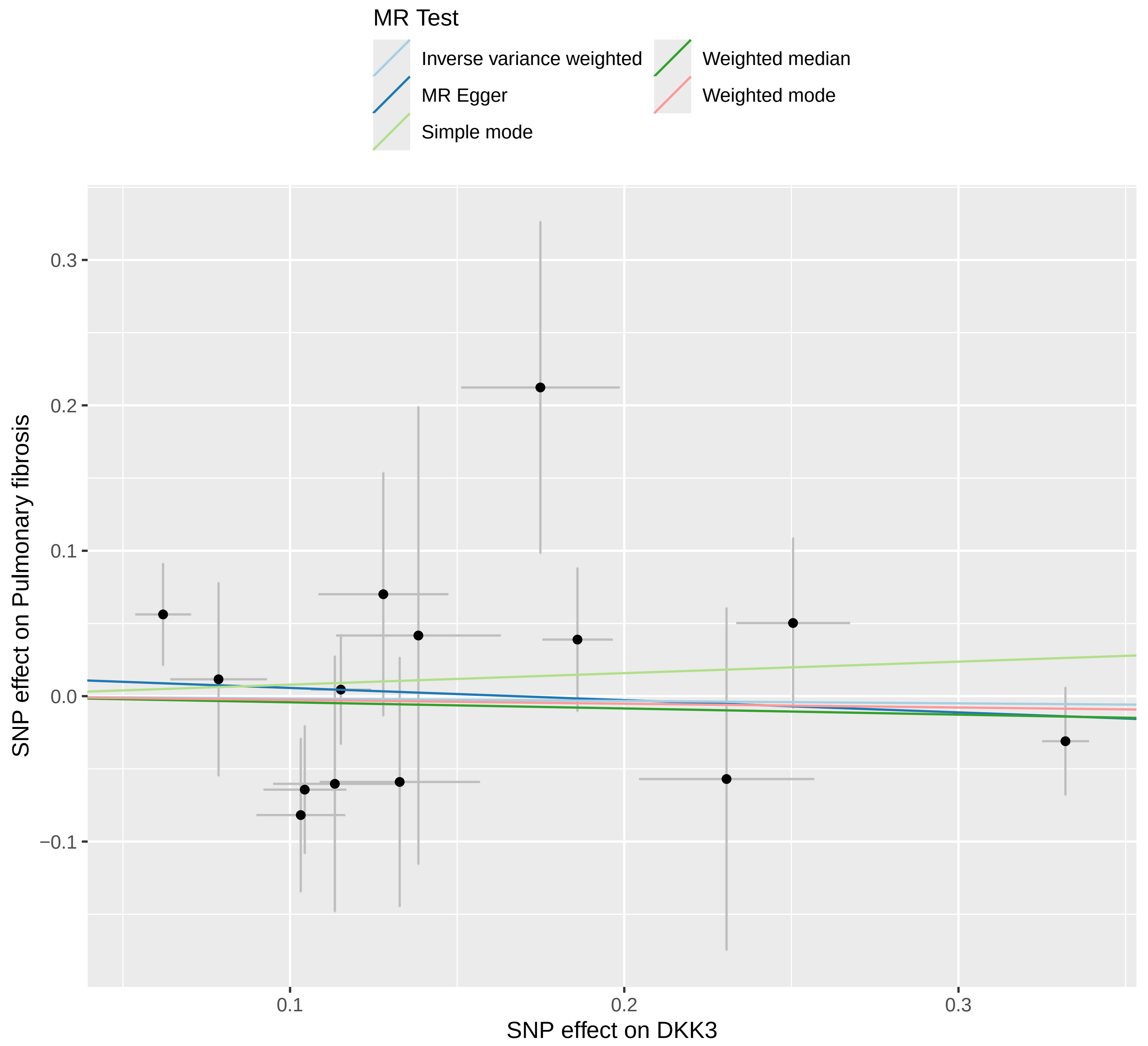
MR Test
Inverse variance weighted
MR Egger
Simple mode
Weighted median
Weighted mode
0.3
0.2
0.1
0.0
−0.1
SNP effect on Pulmonary fibrosis
0.1
0.2
0.3
SNP effect on DKK3

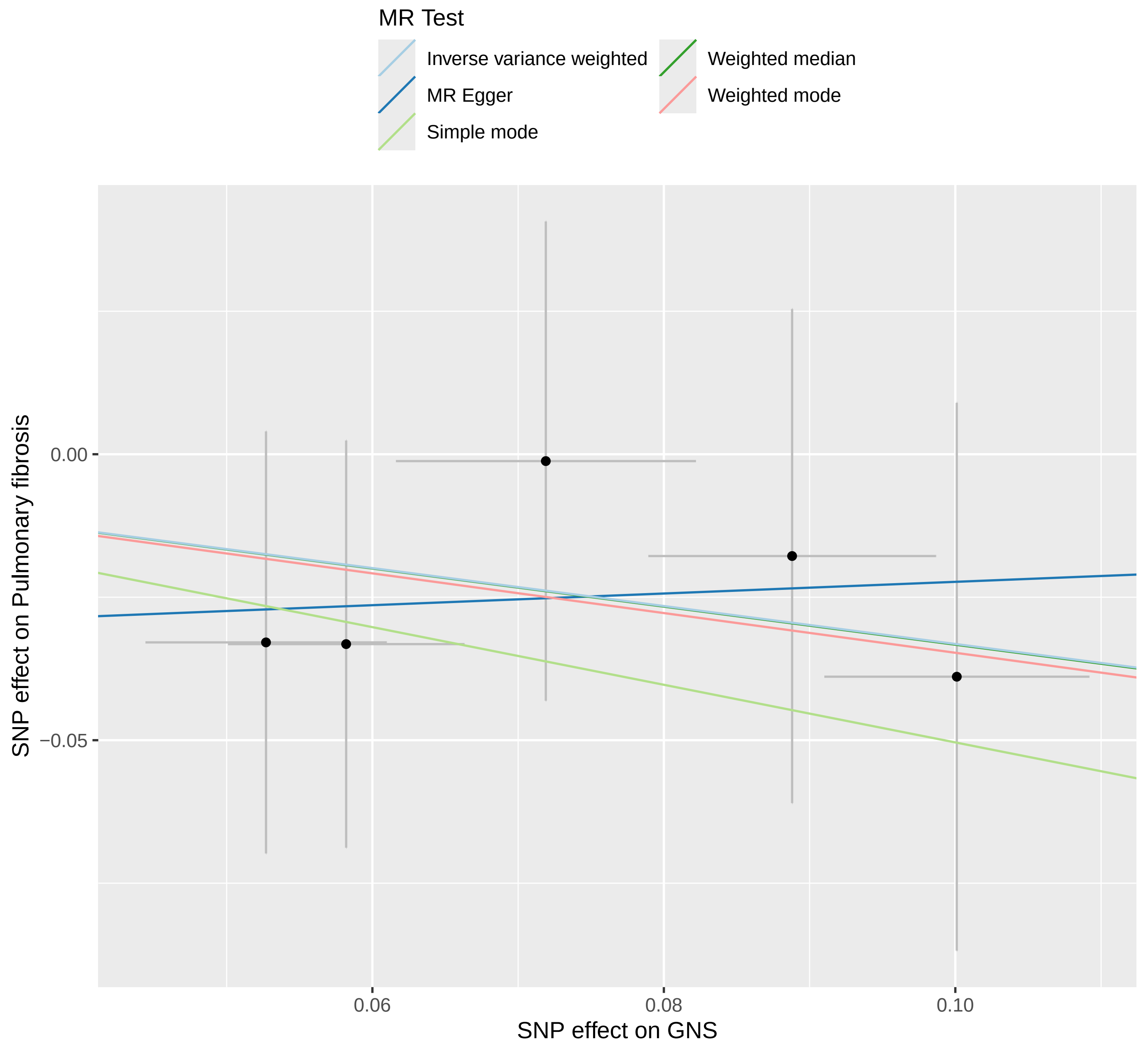
MR Test
Inverse variance weighted
MR Egger
Simple mode
Weighted median
Weighted mode
0.00
−0.05
0.06
0.08
0.10
SNP effect on Pulmonary fibrosis
SNP effect on GNS

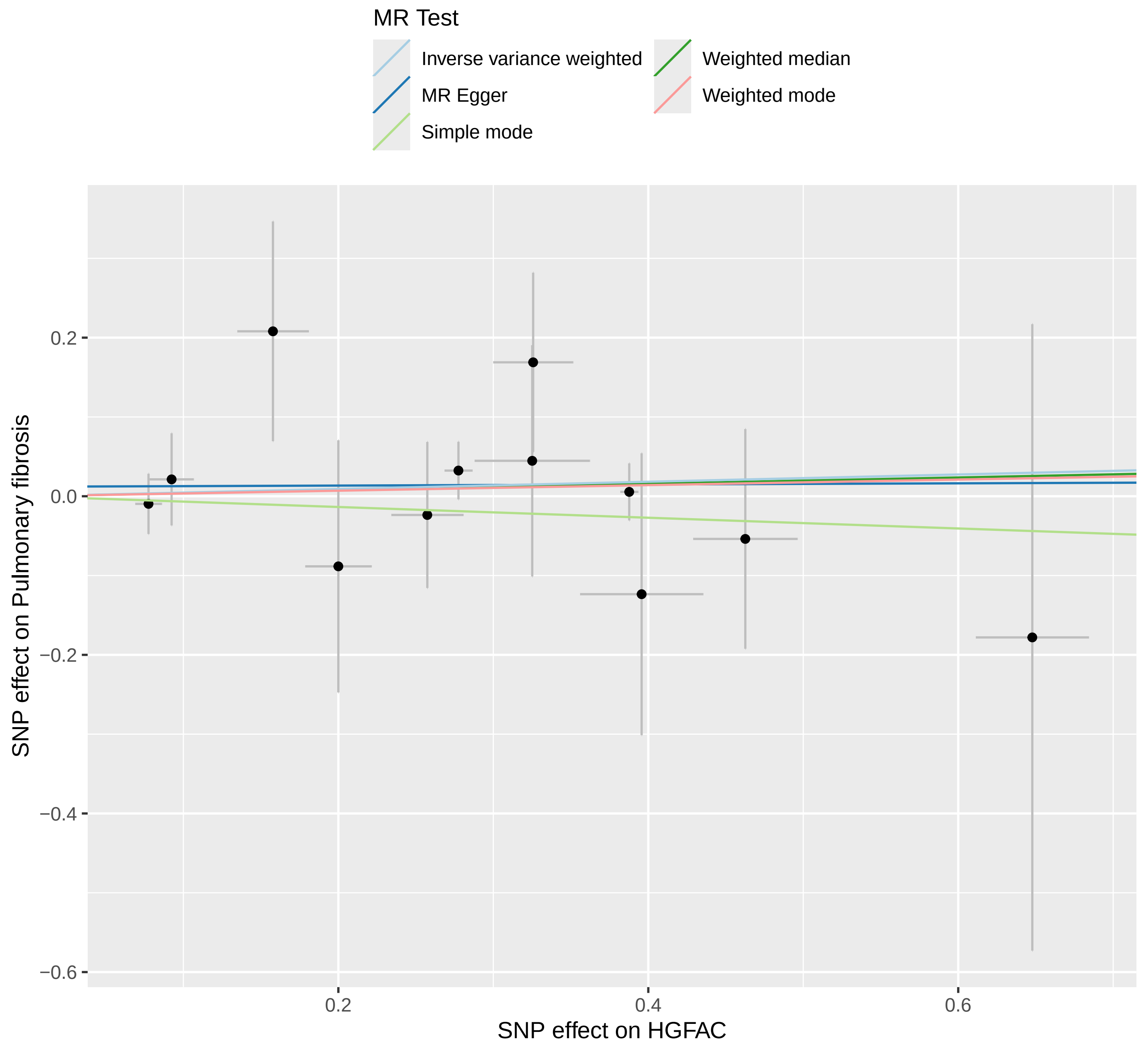

MR Test
Inverse variance weighted
MR Egger
Simple mode
Weighted median
Weighted mode
0.2
0.0
−0.2
−0.4
−0.6
SNP effect on Pulmonary fibrosis
0.2
0.4
0.6
SNP effect on HGFAC

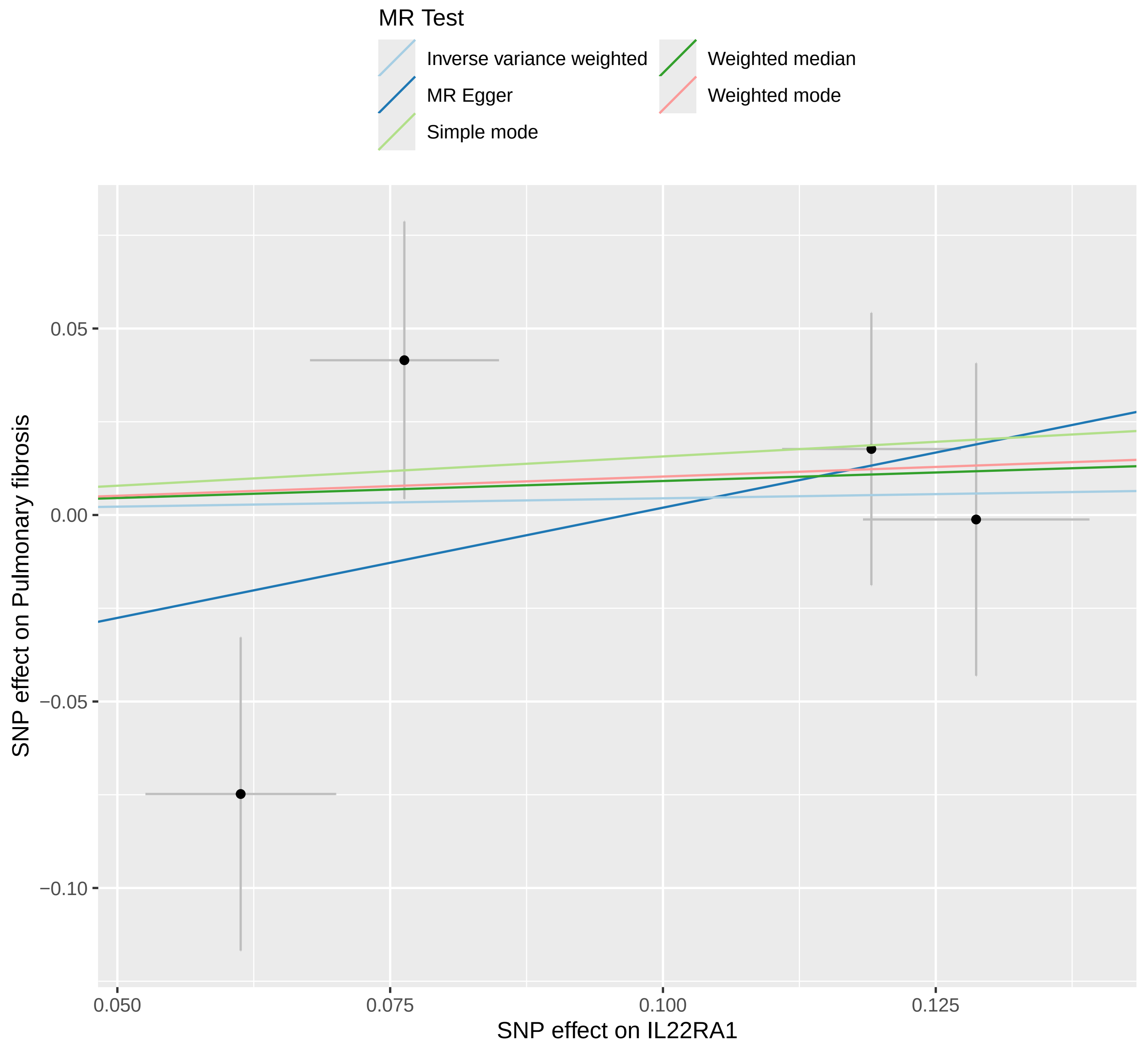
MR Test
Inverse variance weighted
MR Egger
Simple mode
Weighted median
Weighted mode
0.05
0.00
−0.05
−0.10
0.050
0.075
0.100
0.125
SNP effect on IL22RA1
SNP effect on Pulmonary fibrosis

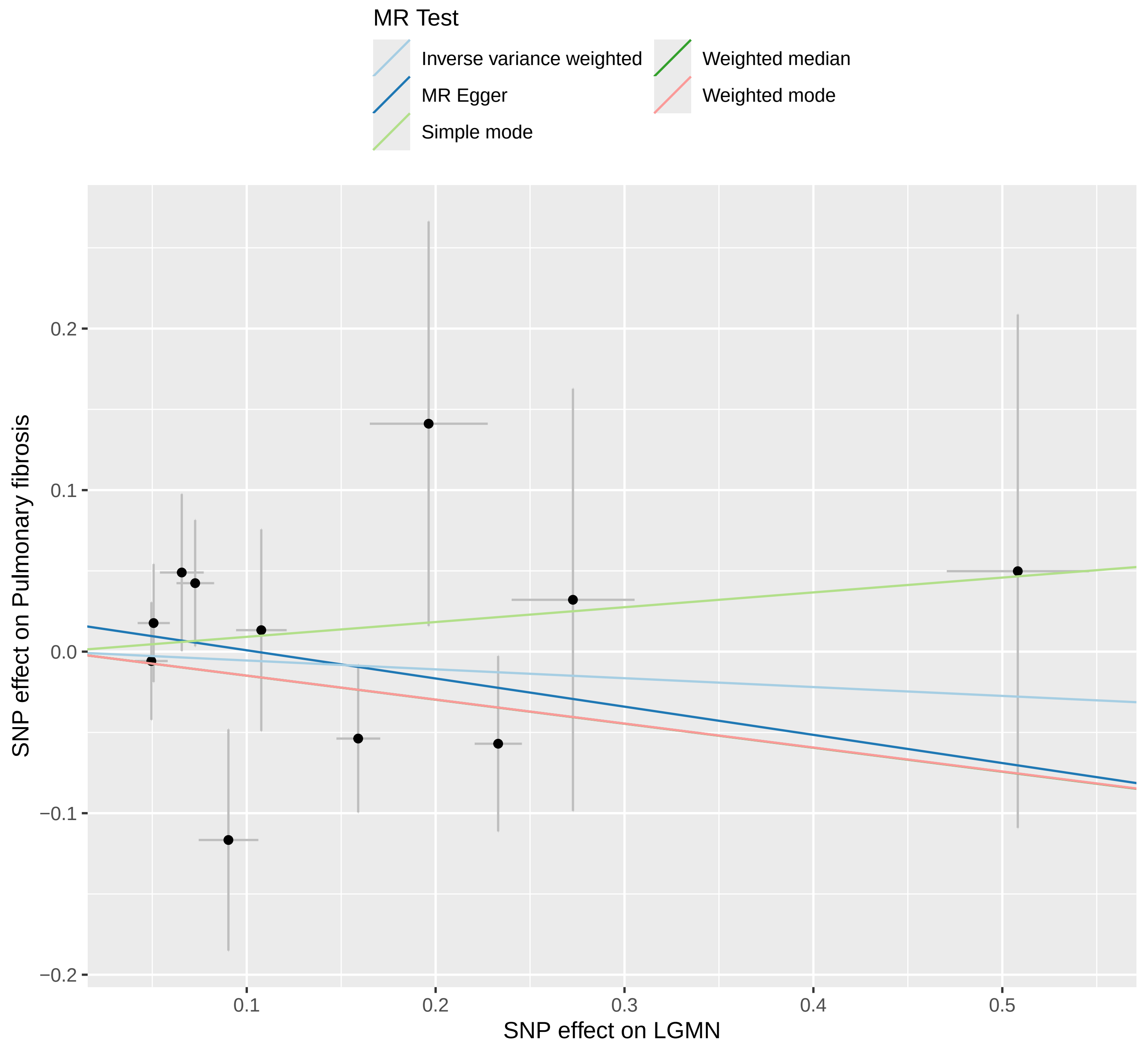
MR Test
Inverse variance weighted
MR Egger
Simple mode
Weighted median
Weighted mode
0.2
0.1
0.0
−0.1
−0.2
0.1
0.2
0.3
0.4
0.5
SNP effect on LGMN
SNP effect on Pulmonary fibrosis

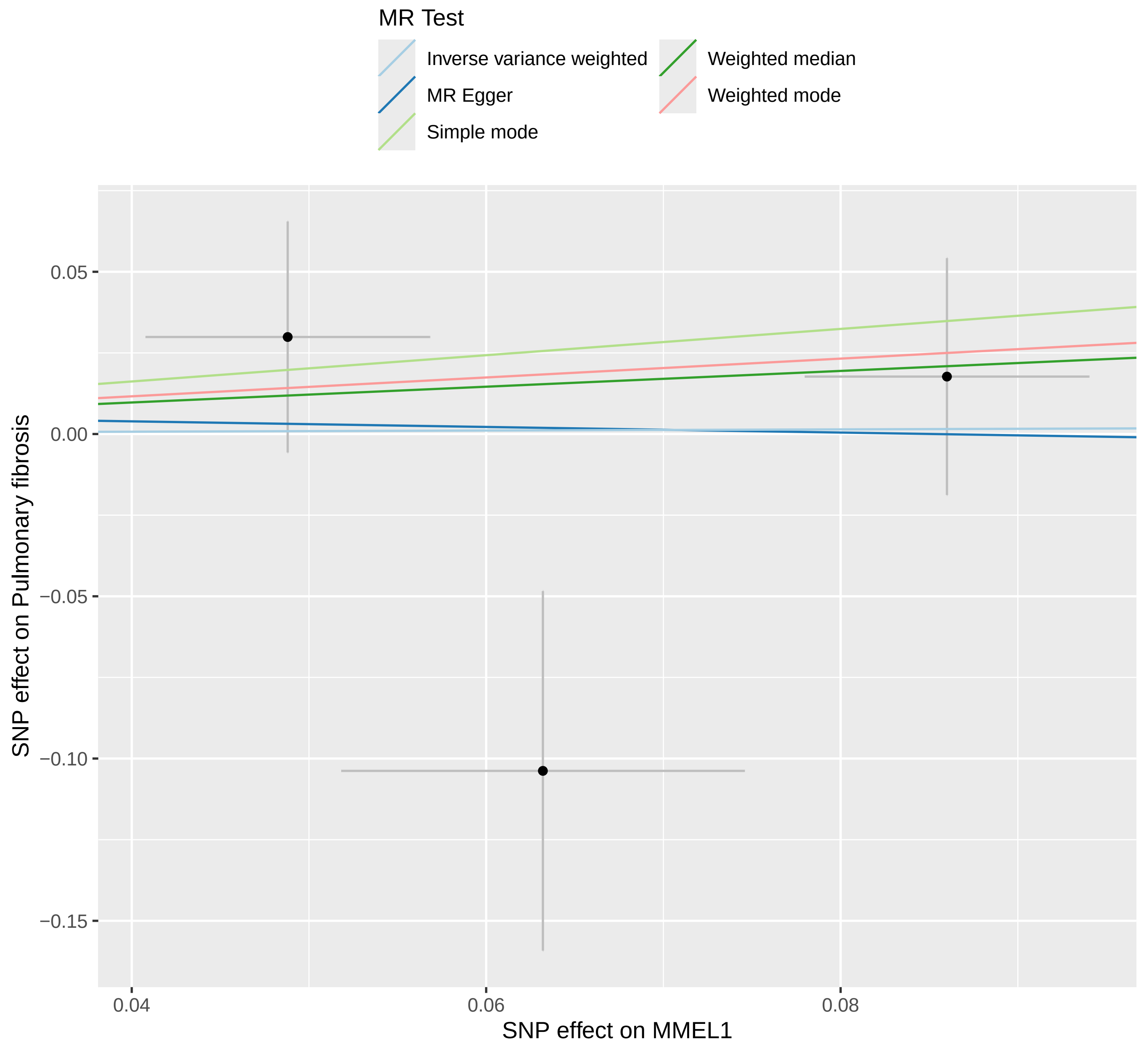

MR Test
Inverse variance weighted
MR Egger
Simple mode
Weighted median
Weighted mode
0.05
0.00
−0.05
−0.10
−0.15
0.04
0.06
0.08
SNP effect on Pulmonary fibrosis
SNP effect on MMEL1

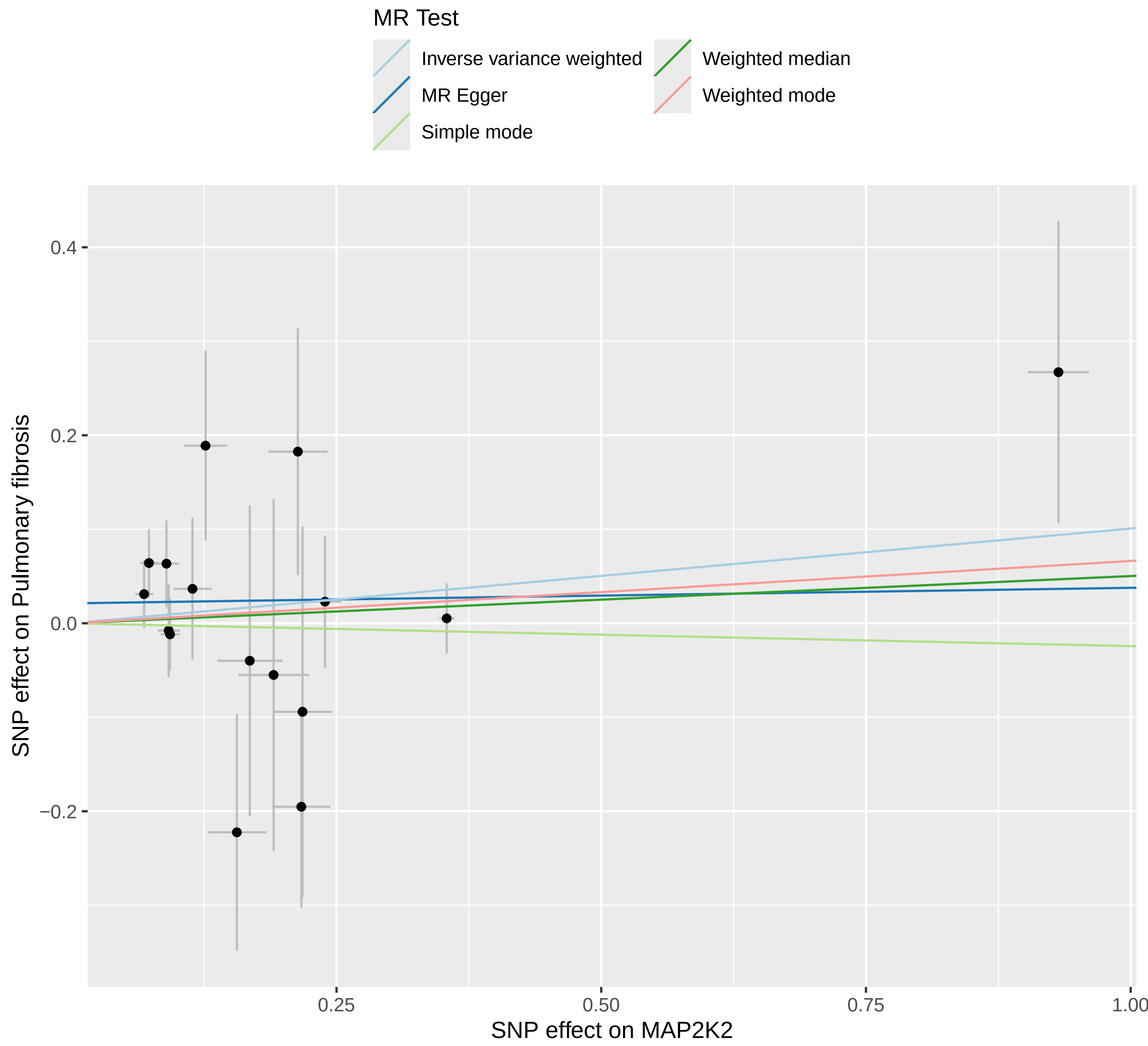
MR Test
Inverse variance weighted
MR Egger
Simple mode
Weighted median
Weighted mode
0.4
0.2
0.0
−0.2
0.25
0.50
0.75
1.00
SNP effect on Pulmonary fibrosis
SNP effect on MAP2K2

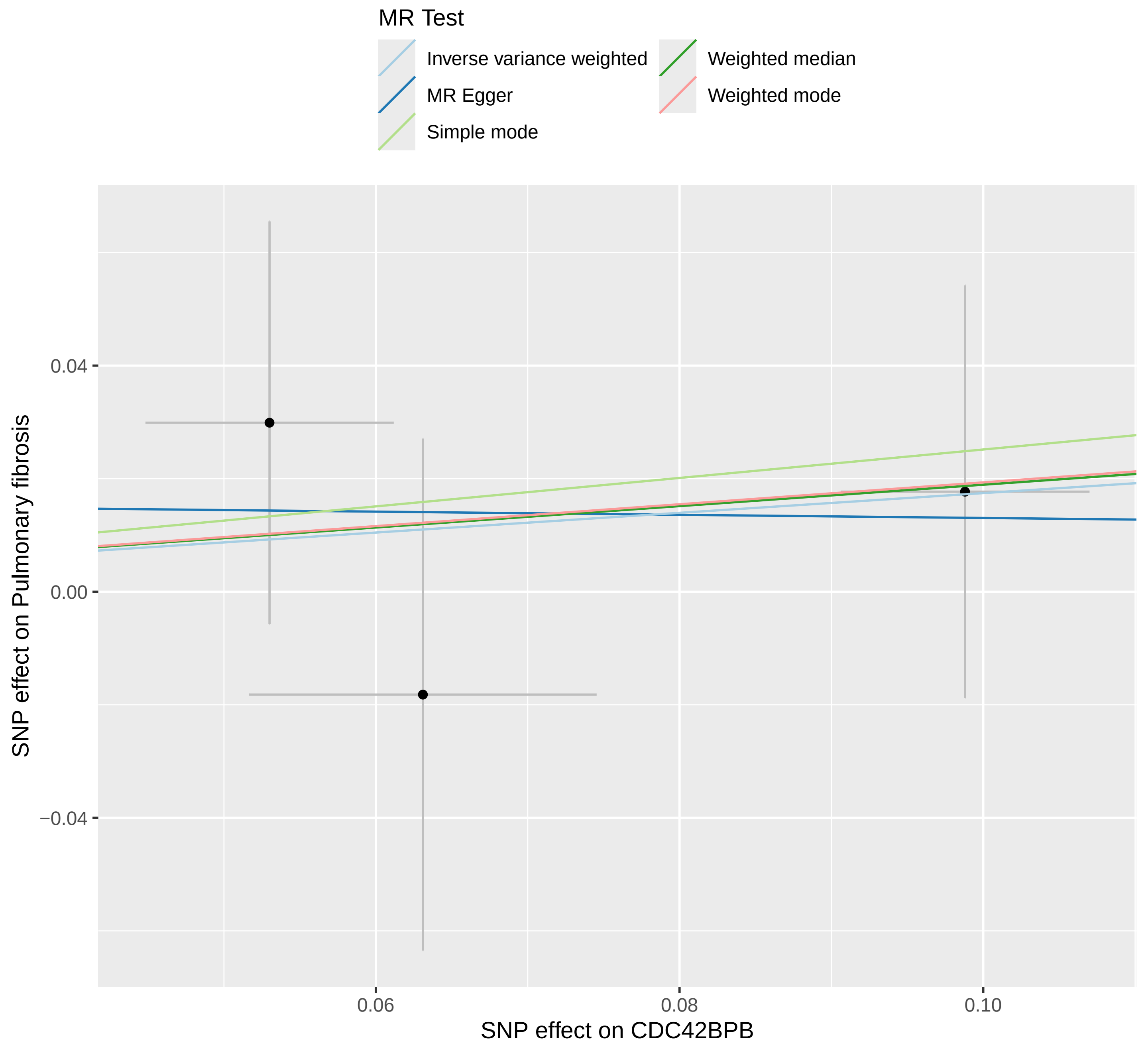
MR Test
Inverse variance weighted
MR Egger
Simple mode
Weighted median
Weighted mode
SNP effect on Pulmonary fibrosis
0.04
0.00
−0.04
0.06
0.08
0.10
SNP effect on CDC42BPB

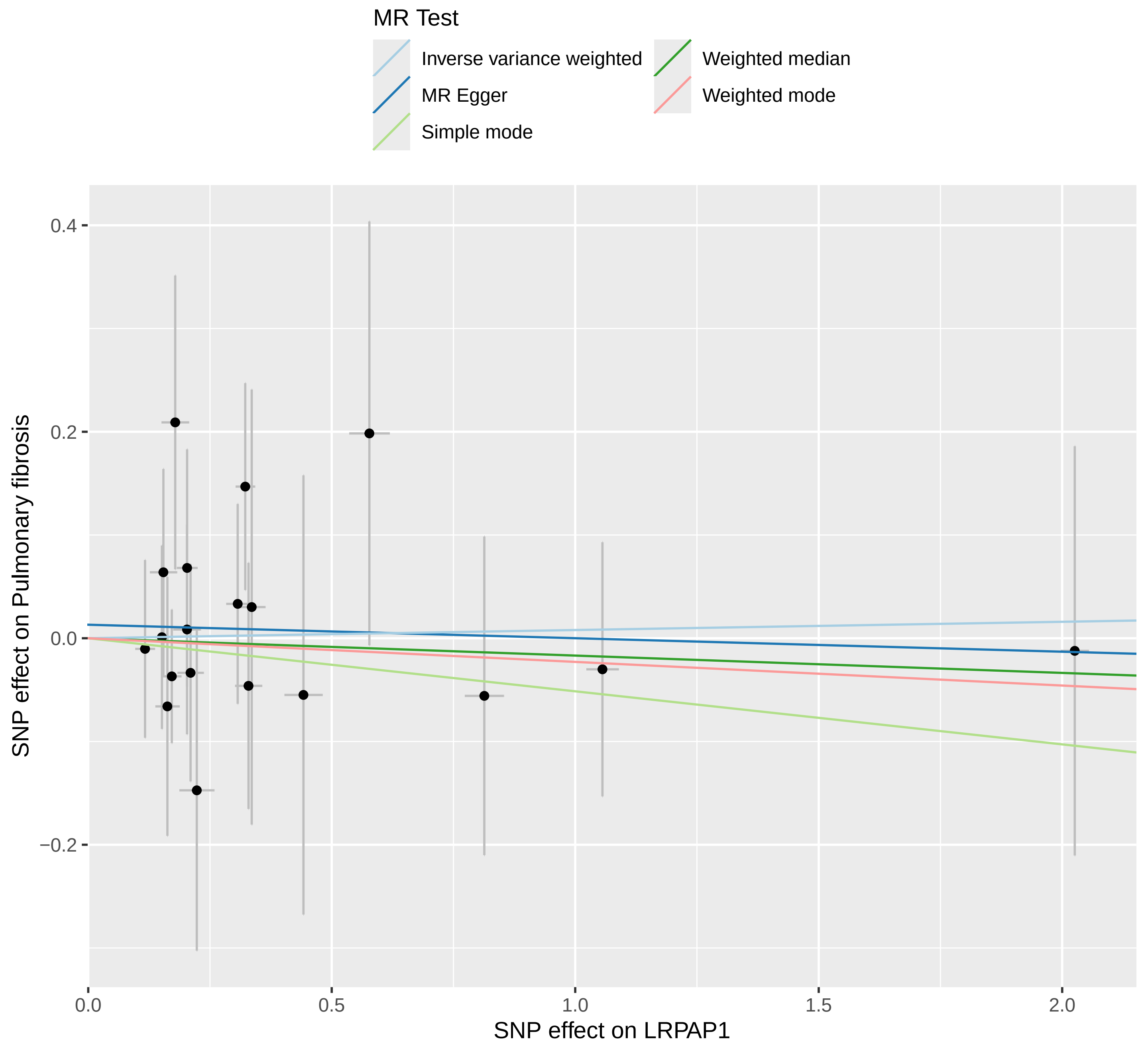
MR Test
Inverse variance weighted
MR Egger
Simple mode
Weighted median
Weighted mode
0.4
0.2
0.0
−0.2
SNP effect on Pulmonary fibrosis
0.0
0.5
1.0
1.5
2.0
SNP effect on LRPAP1

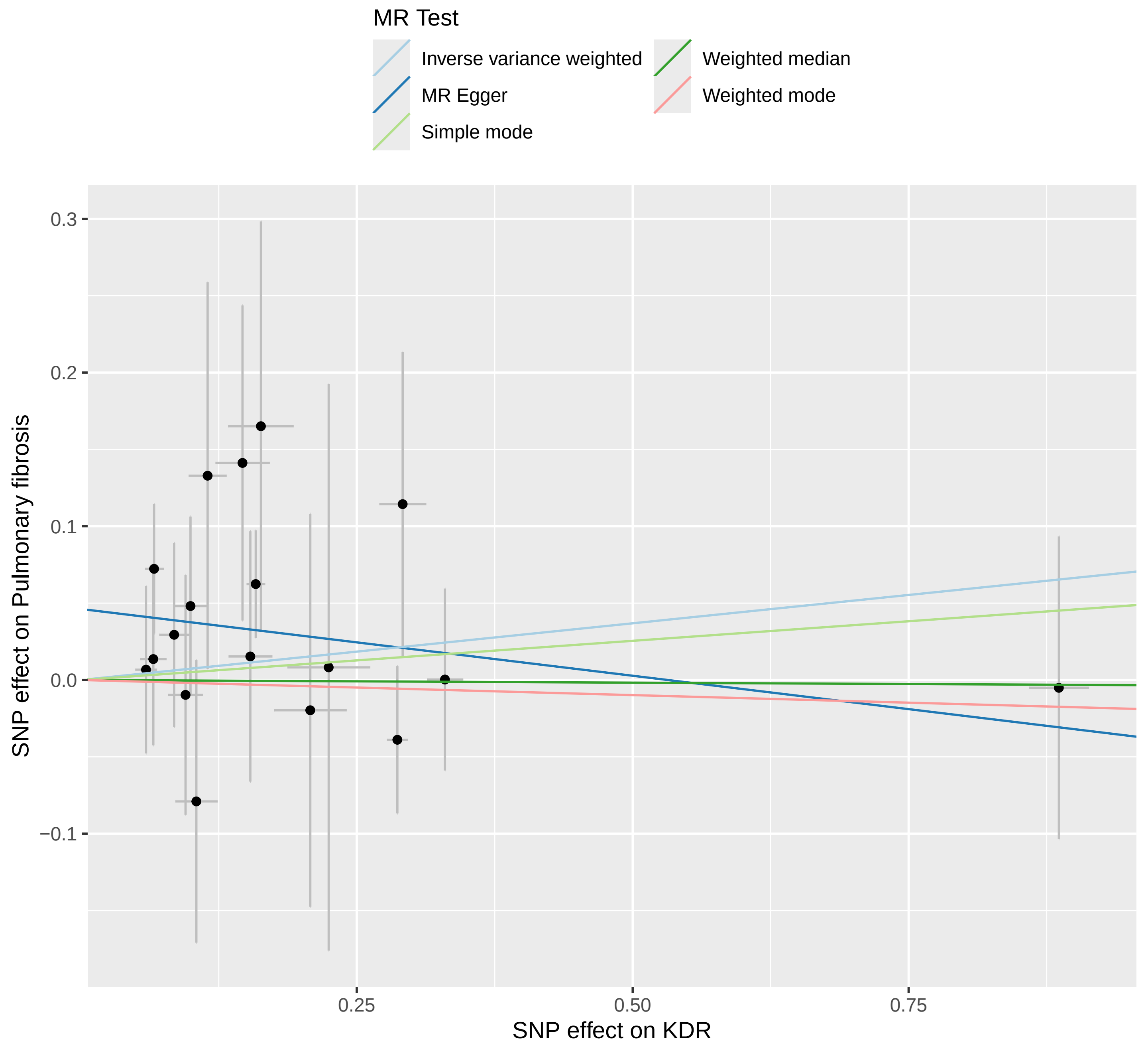

MR Test
Inverse variance weighted
MR Egger
Simple mode
Weighted median
Weighted mode
0.3
0.2
0.1
0.0
−0.1
SNP effect on Pulmonary fibrosis
0.25
0.50
0.75
SNP effect on KDR

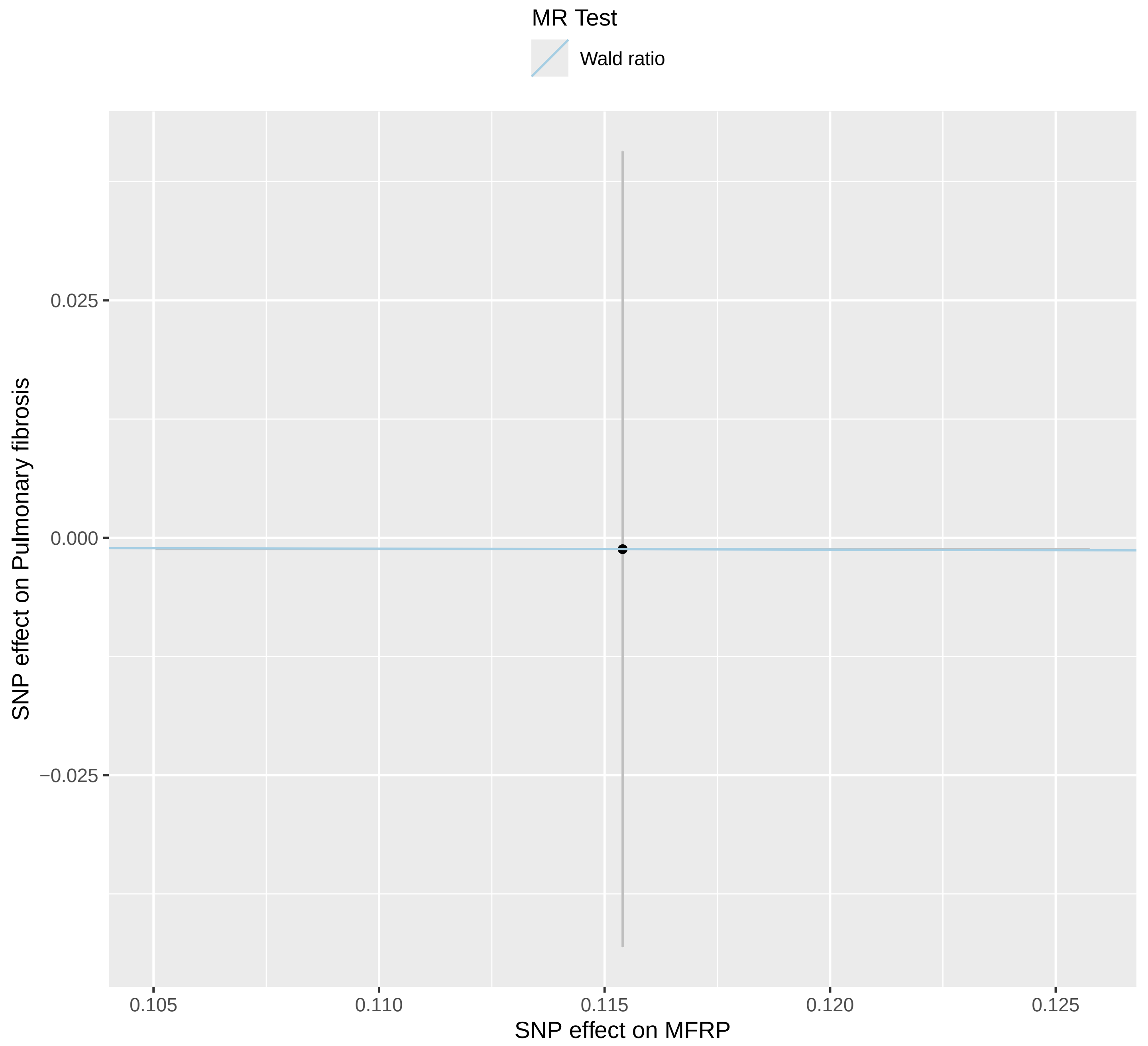
MR Test
Wald ratio
0.025
0.000
−0.025
0.105
0.110
0.115
0.120
0.125
SNP effect on Pulmonary fibrosis
SNP effect on MFRP

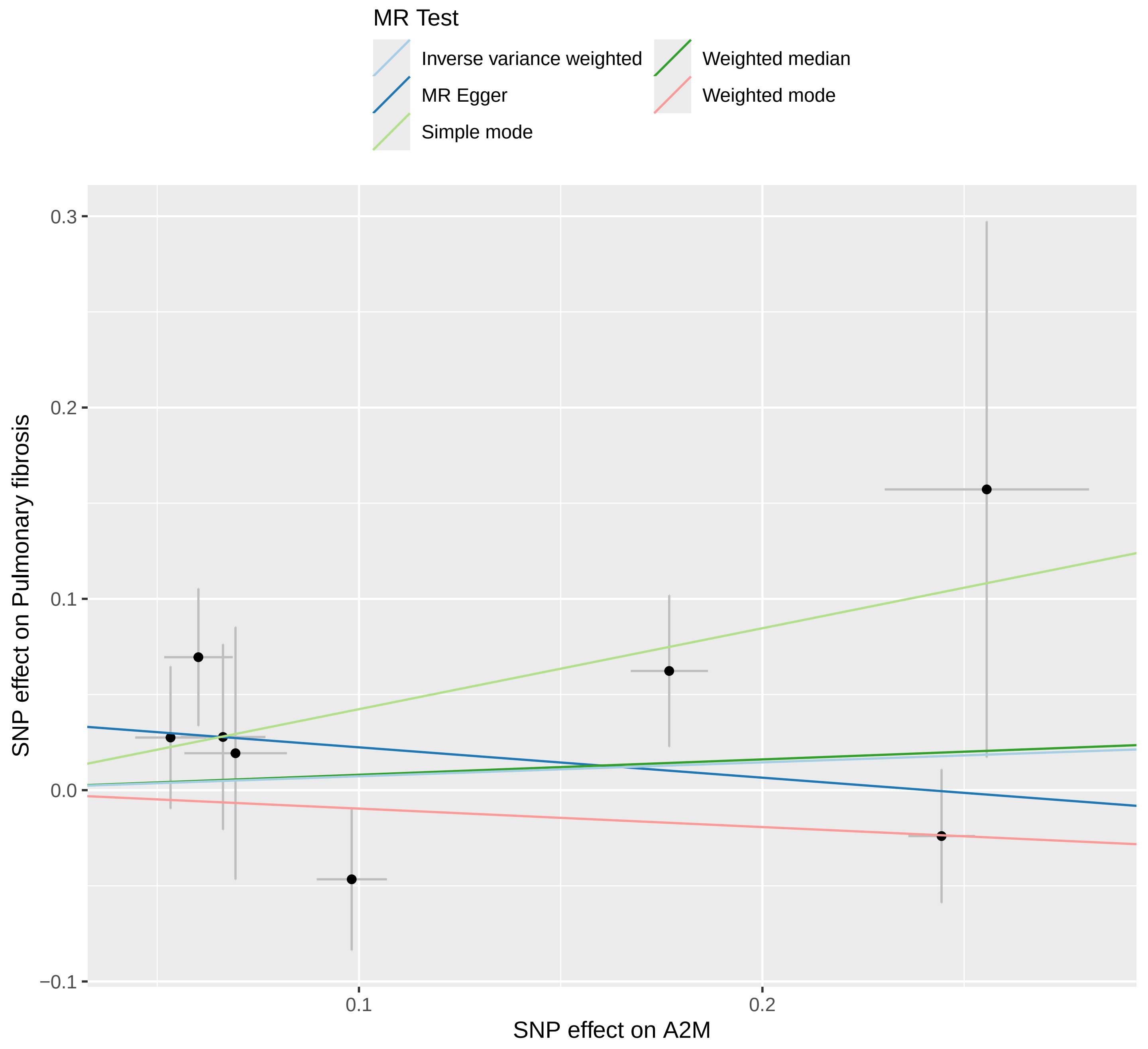

MR Test
Inverse variance weighted
MR Egger
Simple mode
Weighted median
Weighted mode
0.3
0.2
0.1
0.0
−0.1
0.1
0.2
SNP effect on Pulmonary fibrosis
SNP effect on A2M

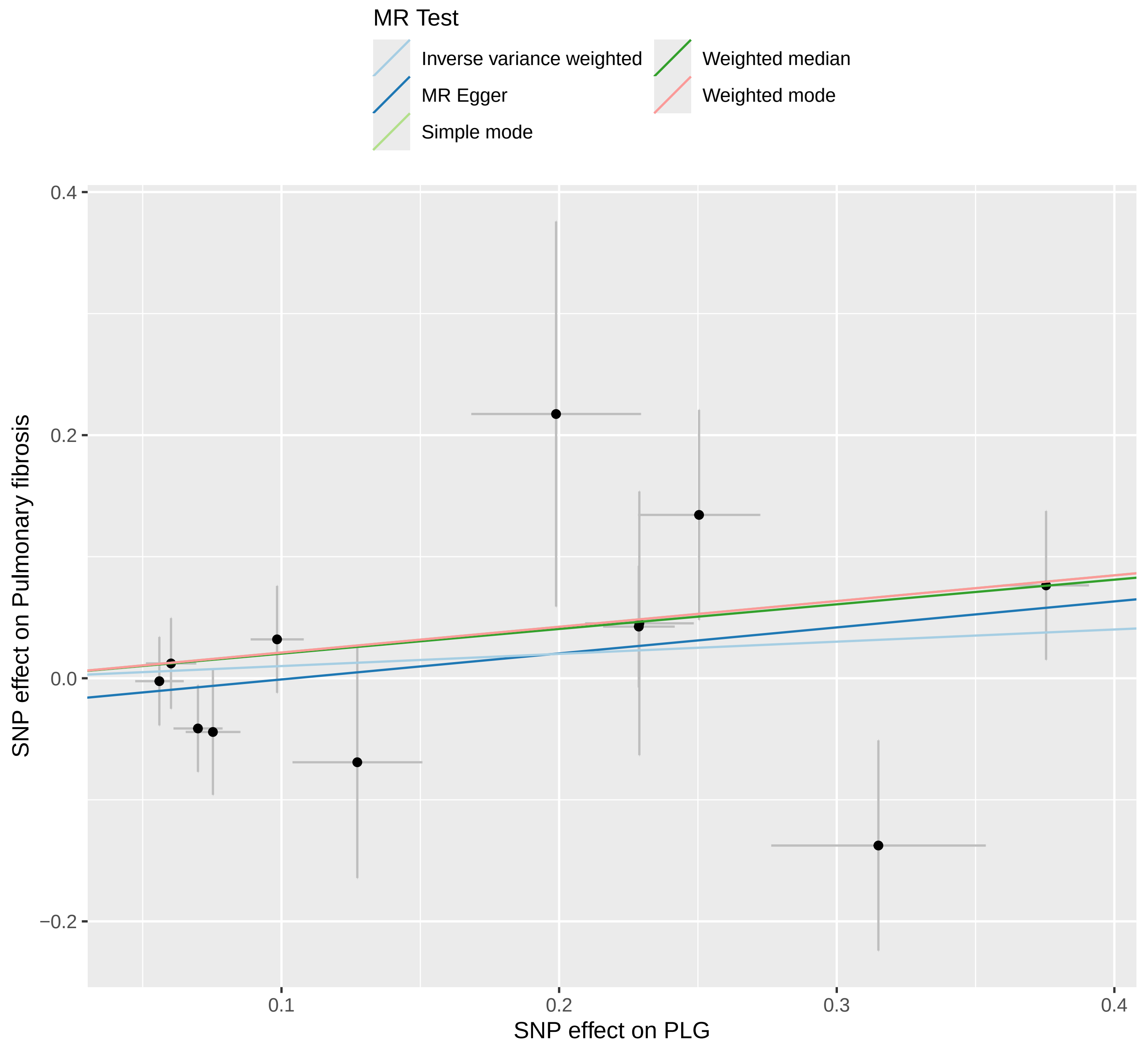

MR Test
Inverse variance weighted
MR Egger
Simple mode
Weighted median
Weighted mode
0.4
0.2
0.0
−0.2
SNP effect on Pulmonary fibrosis
0.1
0.2
0.3
0.4
SNP effect on PLG

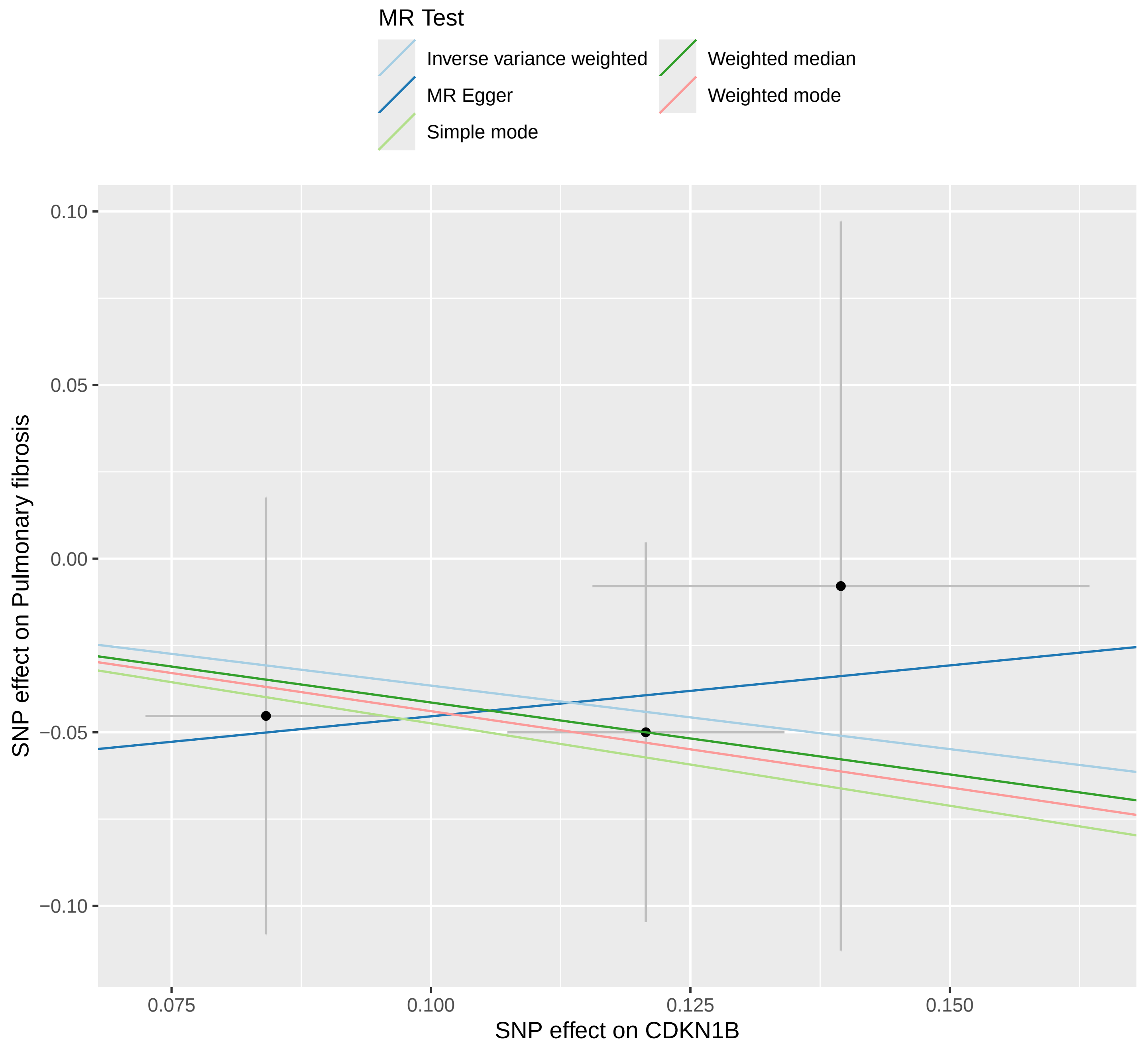
MR Test
Inverse variance weighted
MR Egger
Simple mode
Weighted median
Weighted mode
0.10
0.05
0.00
−0.05
−0.10
0.075
0.100
0.125
0.150
SNP effect on Pulmonary fibrosis
SNP effect on CDKN1B

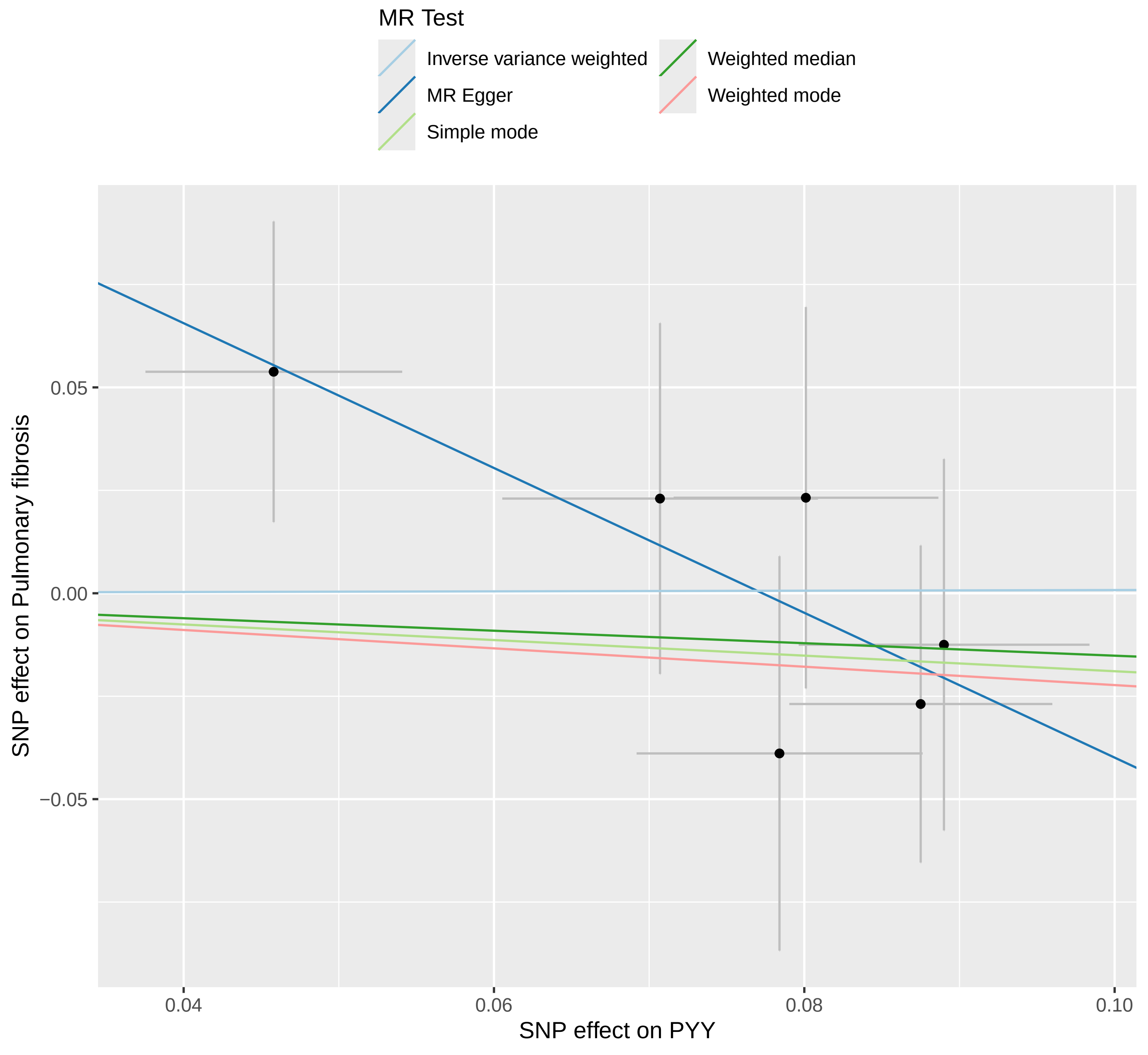
MR Test
Inverse variance weighted
MR Egger
Simple mode
Weighted median
Weighted mode
SNP effect on Pulmonary fibrosis
0.05
0.00
−0.05
0.04
0.06
0.08
0.10
SNP effect on PYY

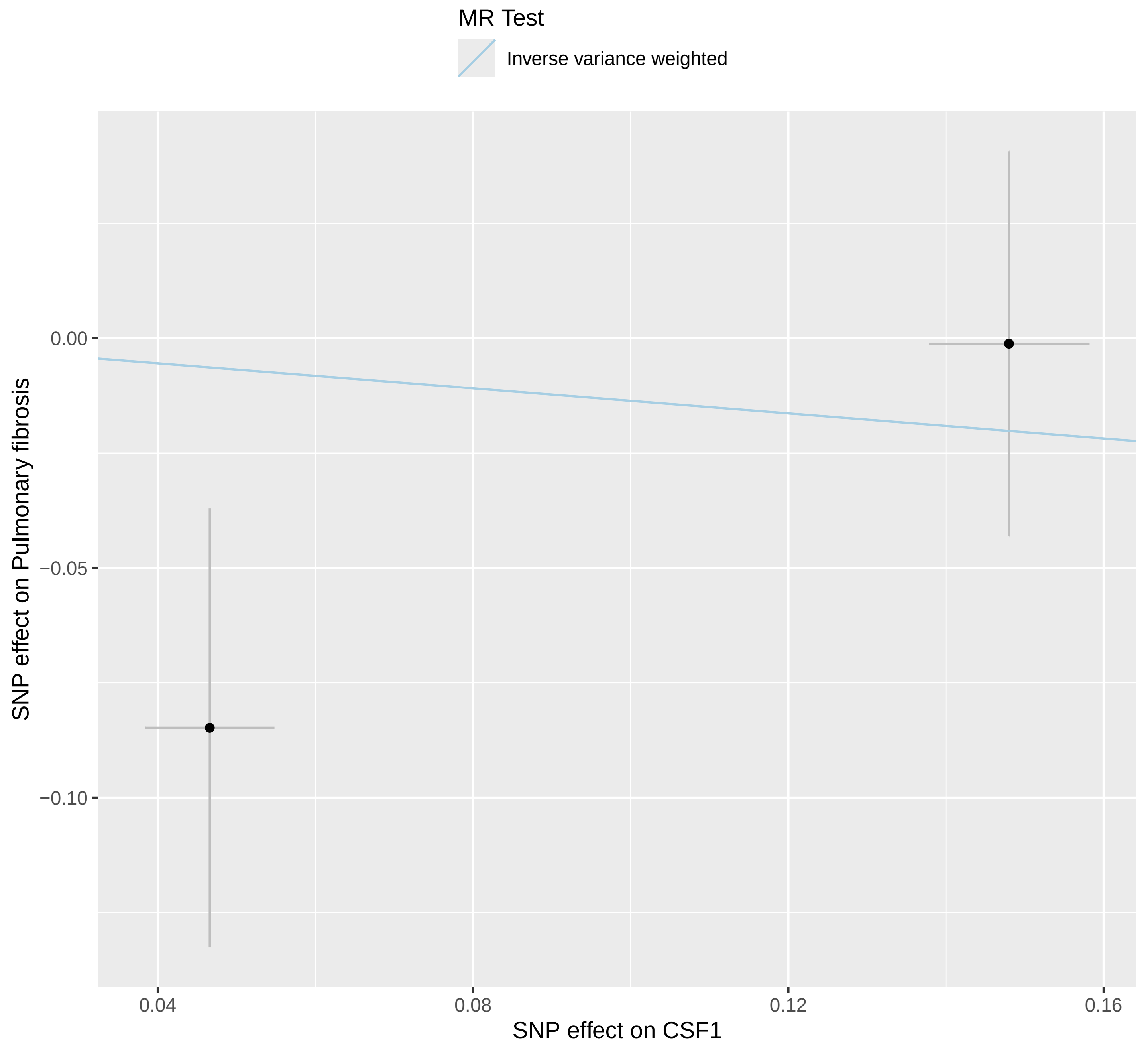

MR Test
Inverse variance weighted
0.00
−0.05
−0.10
SNP effect on Pulmonary fibrosis
0.04
0.08
0.12
0.16
SNP effect on CSF1

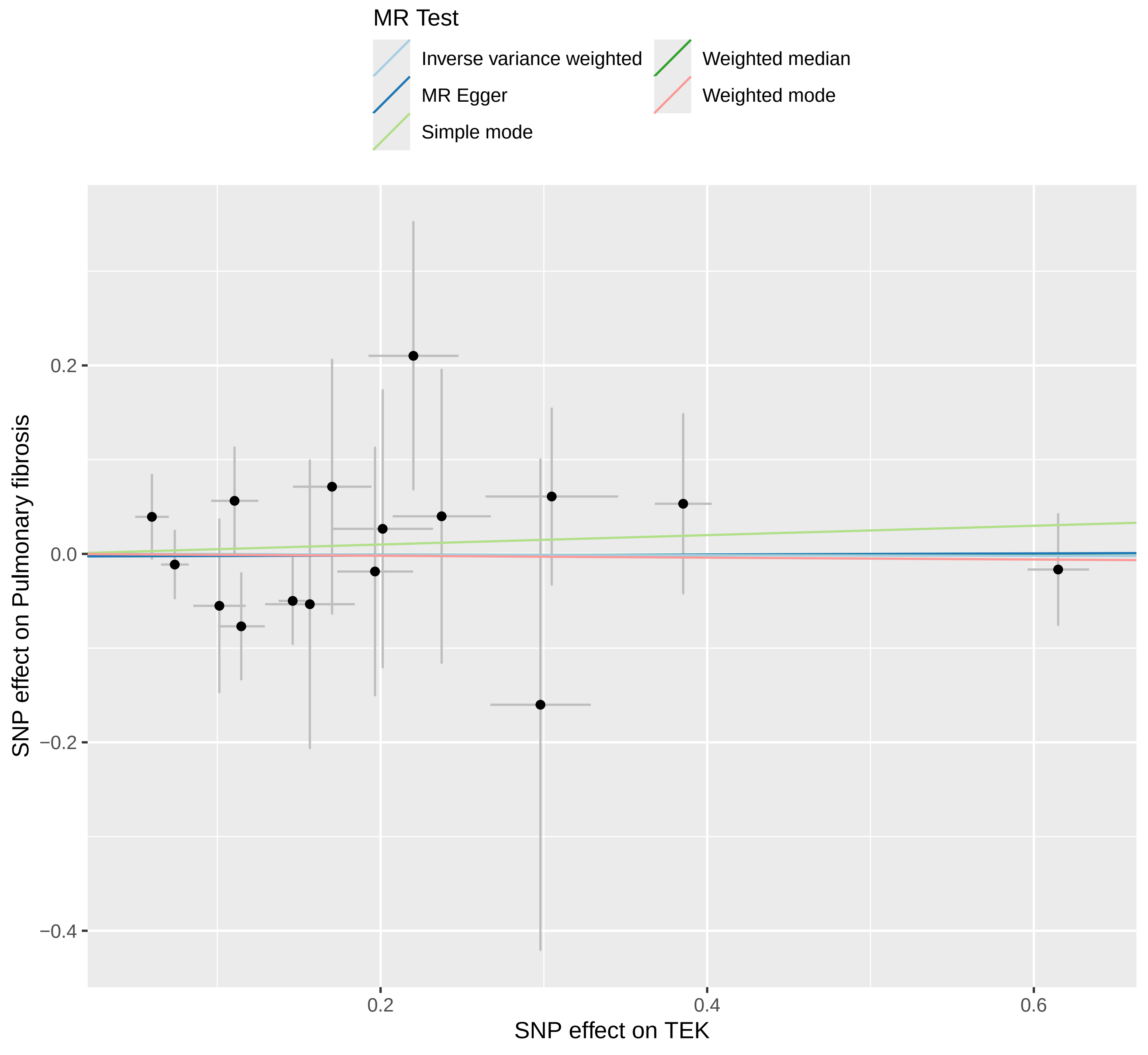
MR Test
Inverse variance weighted
MR Egger
Simple mode
Weighted median
Weighted mode
SNP effect on Pulmonary fibrosis
0.2
0.0
−0.2
−0.4
0.2
0.4
0.6
SNP effect on TEK

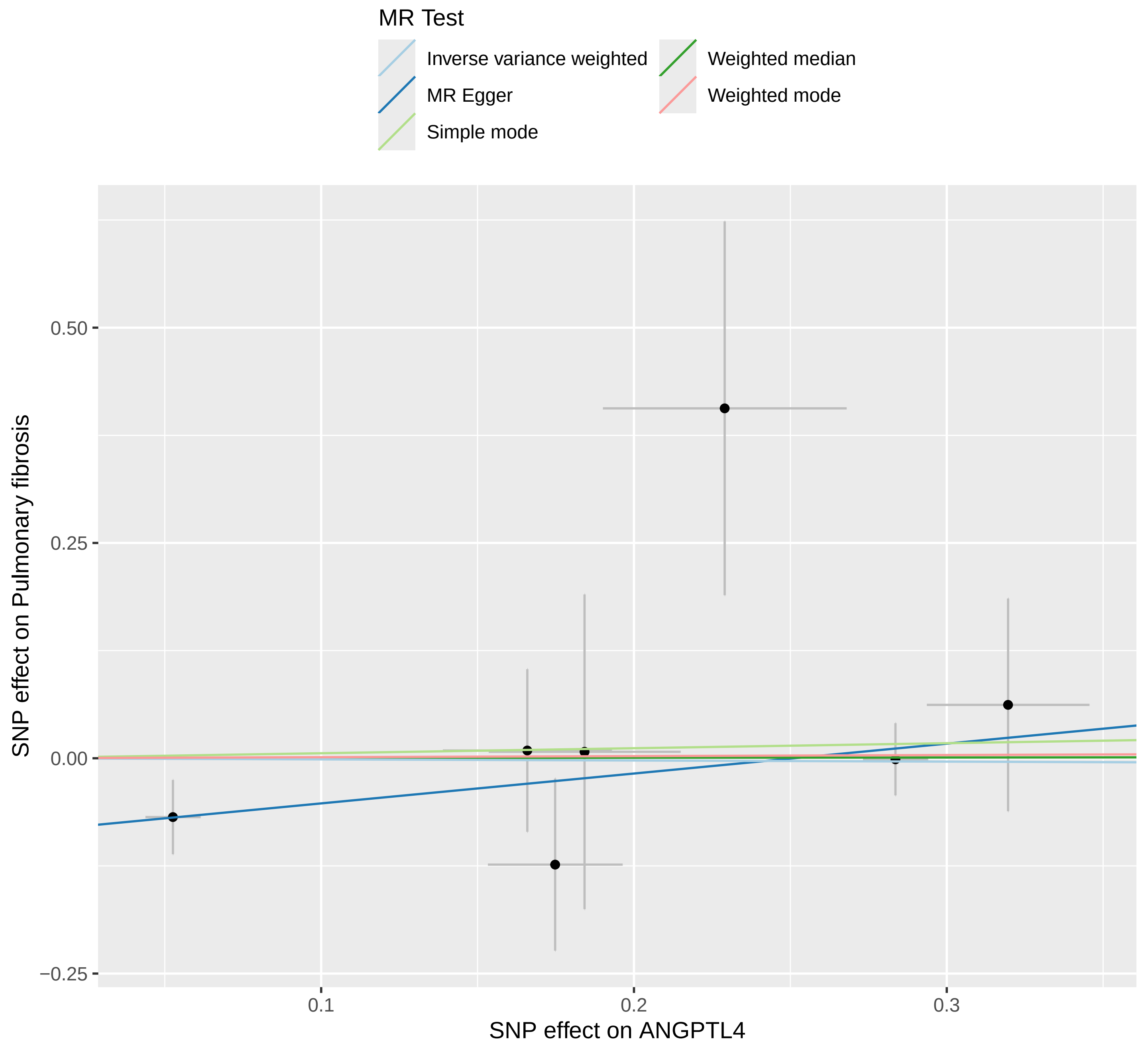
MR Test
Inverse variance weighted
MR Egger
Simple mode
Weighted median
Weighted mode
SNP effect on Pulmonary fibrosis
0.50
0.25
0.00
−0.25
0.1
0.2
0.3
SNP effect on ANGPTL4

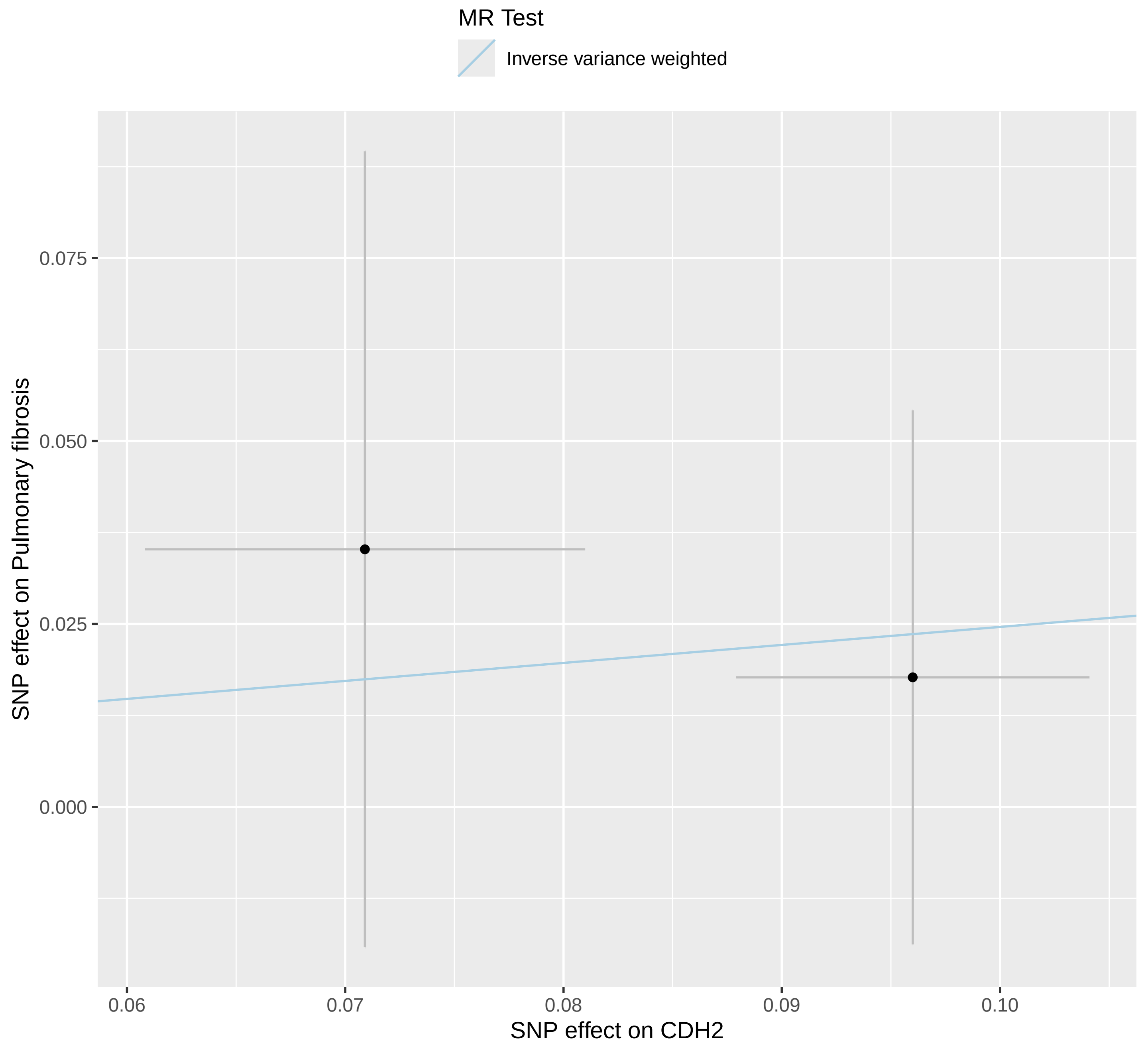

MR Test
Inverse variance weighted
0.075
0.050
0.025
0.000
SNP effect on Pulmonary fibrosis
0.06
0.07
0.08
0.09
0.10
SNP effect on CDH2

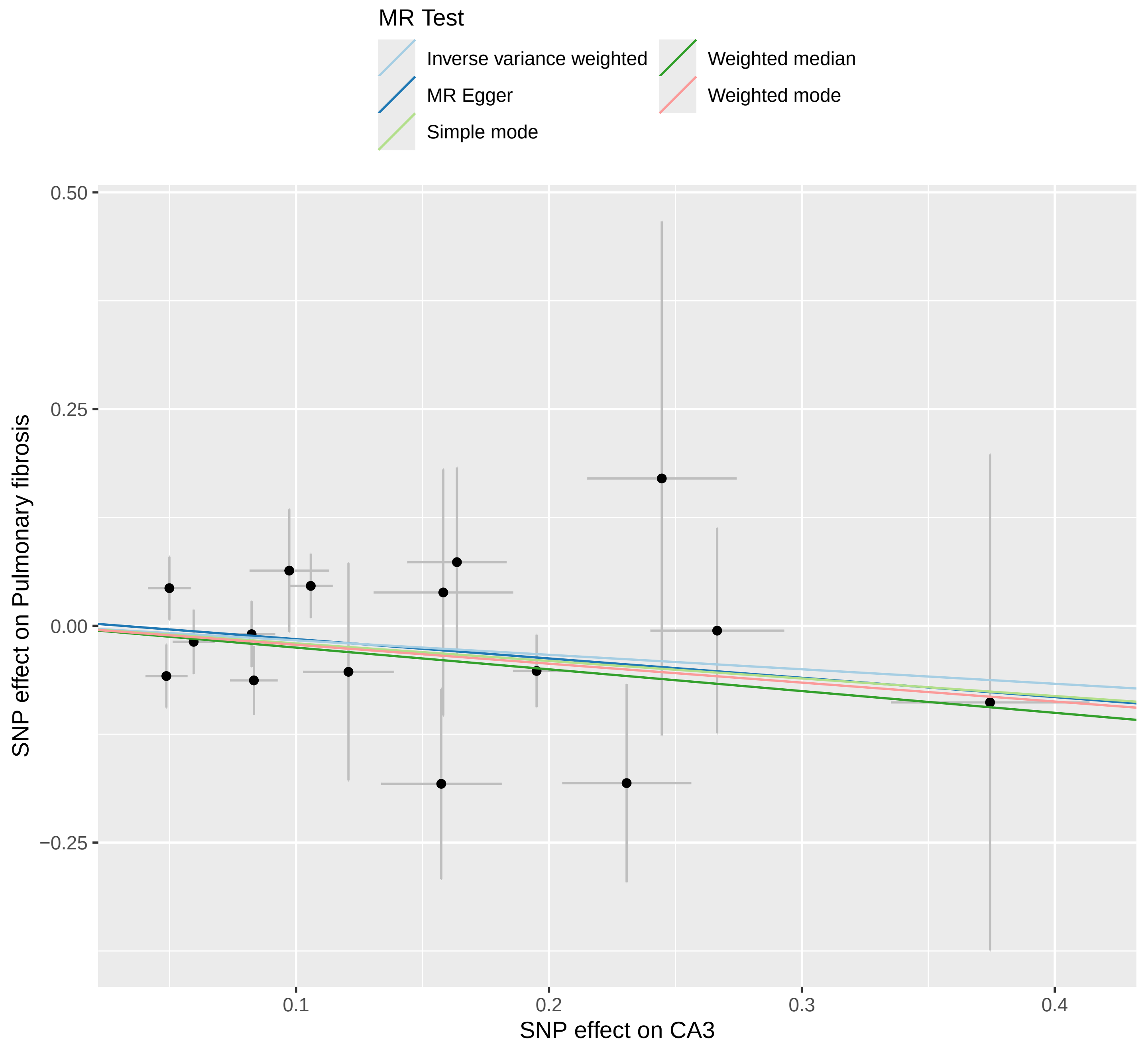

MR Test
Inverse variance weighted
MR Egger
Simple mode
Weighted median
Weighted mode
0.50
0.25
0.00
−0.25
0.1
0.2
0.3
0.4
SNP effect on Pulmonary fibrosis
SNP effect on CA3

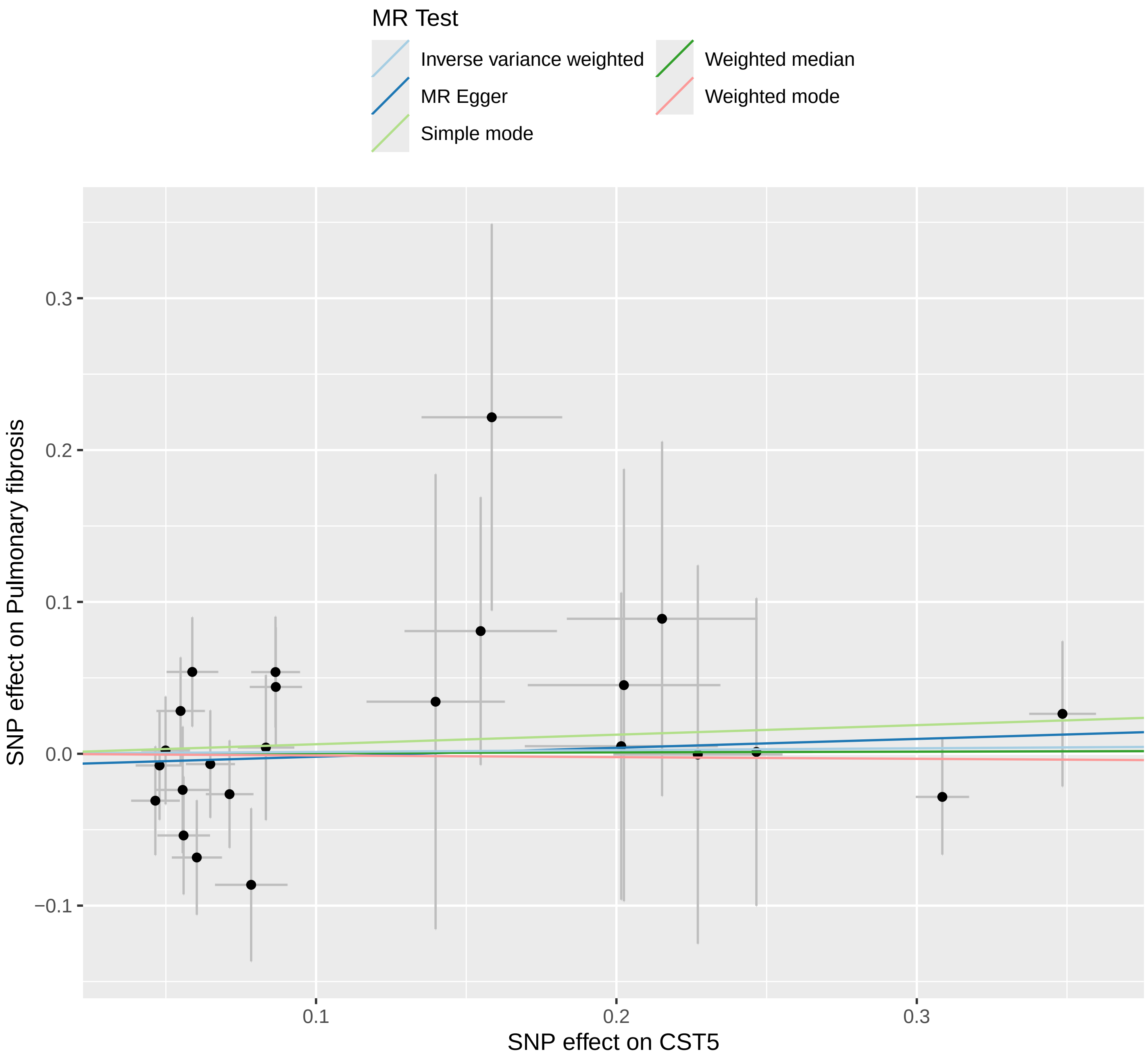

MR Test
Inverse variance weighted
MR Egger
Simple mode
Weighted median
Weighted mode
0.3
0.2
0.1
0.0
−0.1
SNP effect on Pulmonary fibrosis
0.1
0.2
0.3
SNP effect on CST5

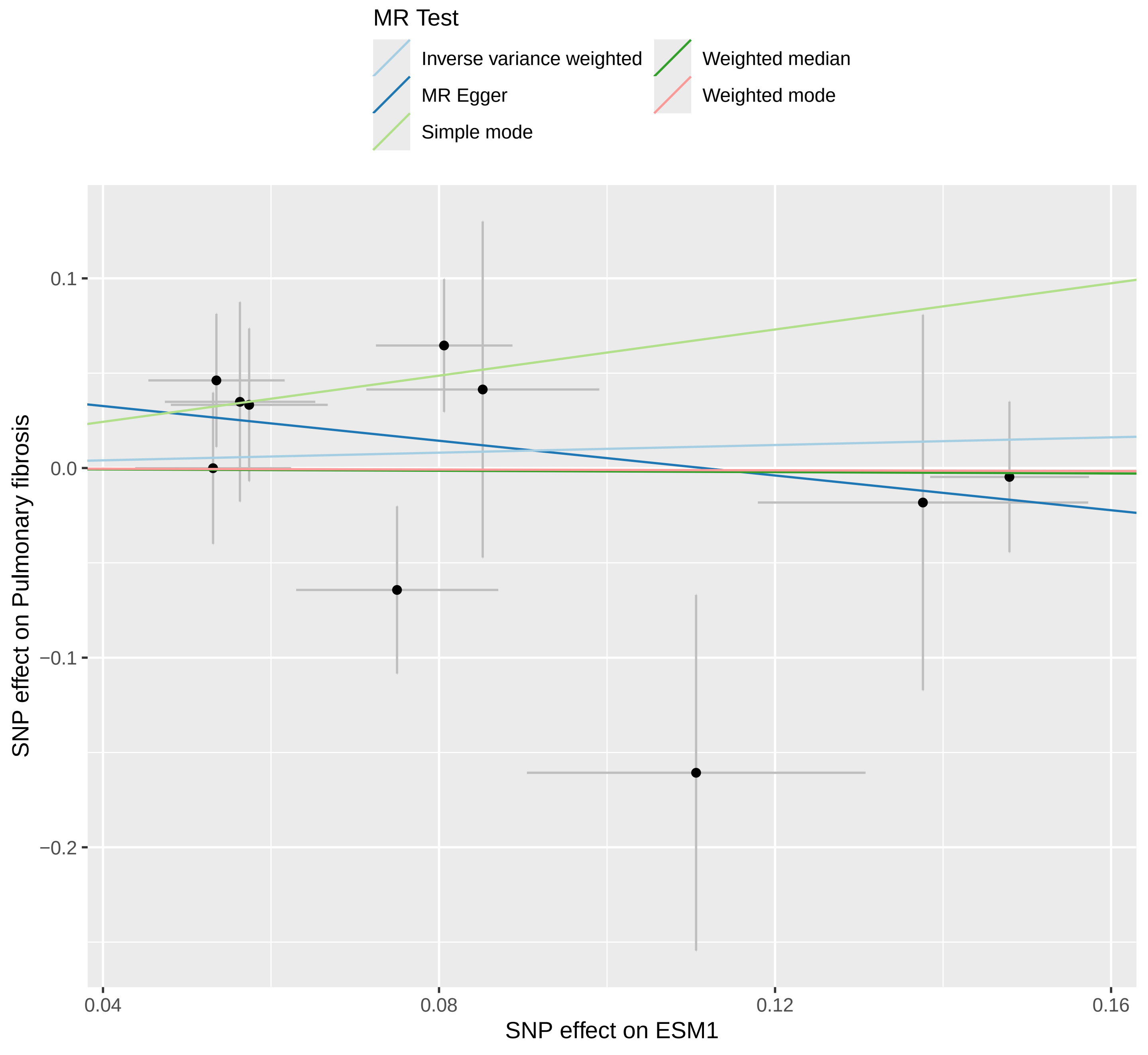
MR Test
Inverse variance weighted
MR Egger
Simple mode
Weighted median
Weighted mode
0.1
0.0
−0.1
−0.2
SNP effect on Pulmonary fibrosis
0.04
0.08
0.12
0.16
SNP effect on ESM1

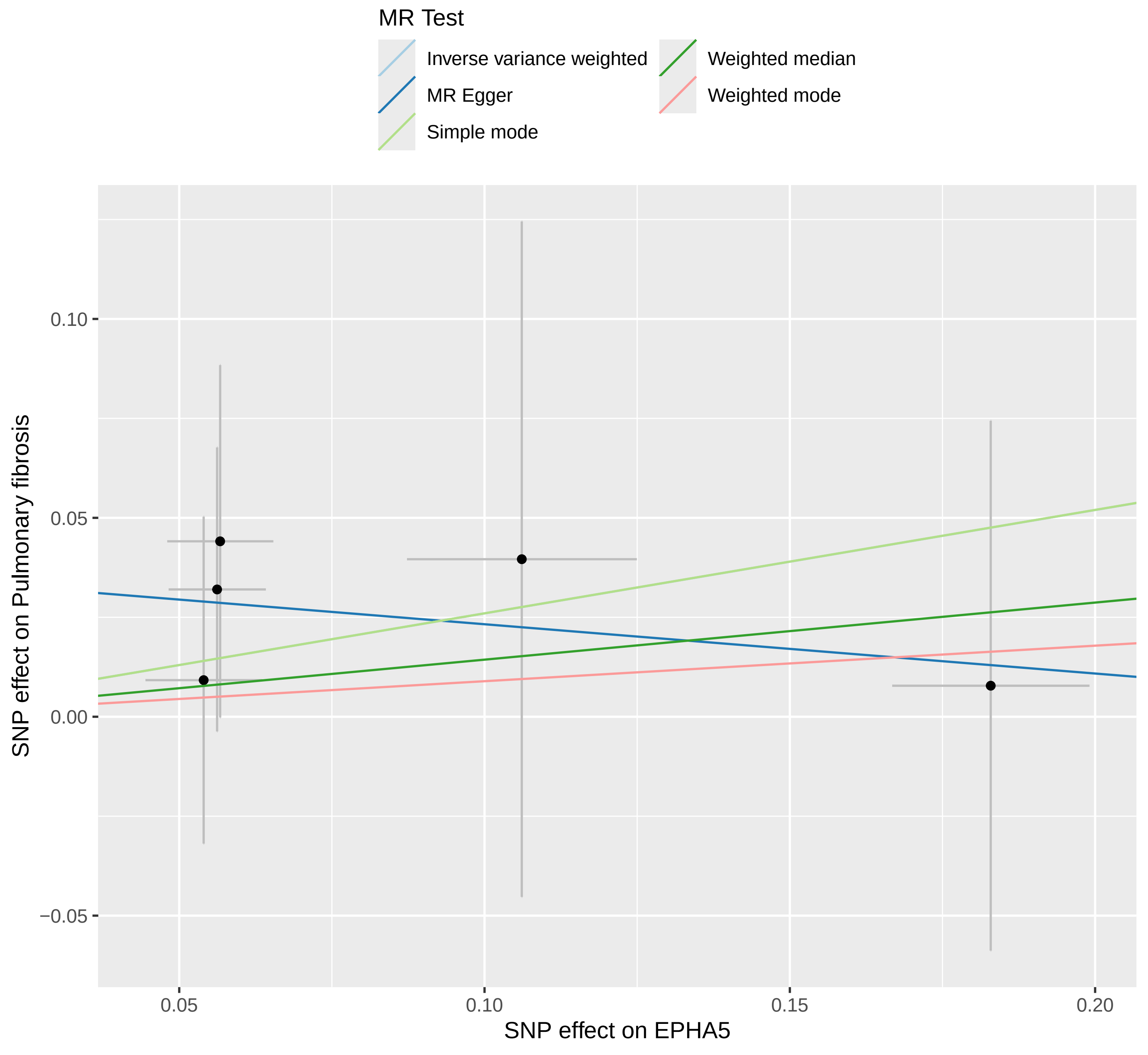

MR Test
Inverse variance weighted
MR Egger
Simple mode
Weighted median
Weighted mode
SNP effect on Pulmonary fibrosis
0.10
0.05
0.00
−0.05
0.05
0.10
0.15
0.20
SNP effect on EPHA5

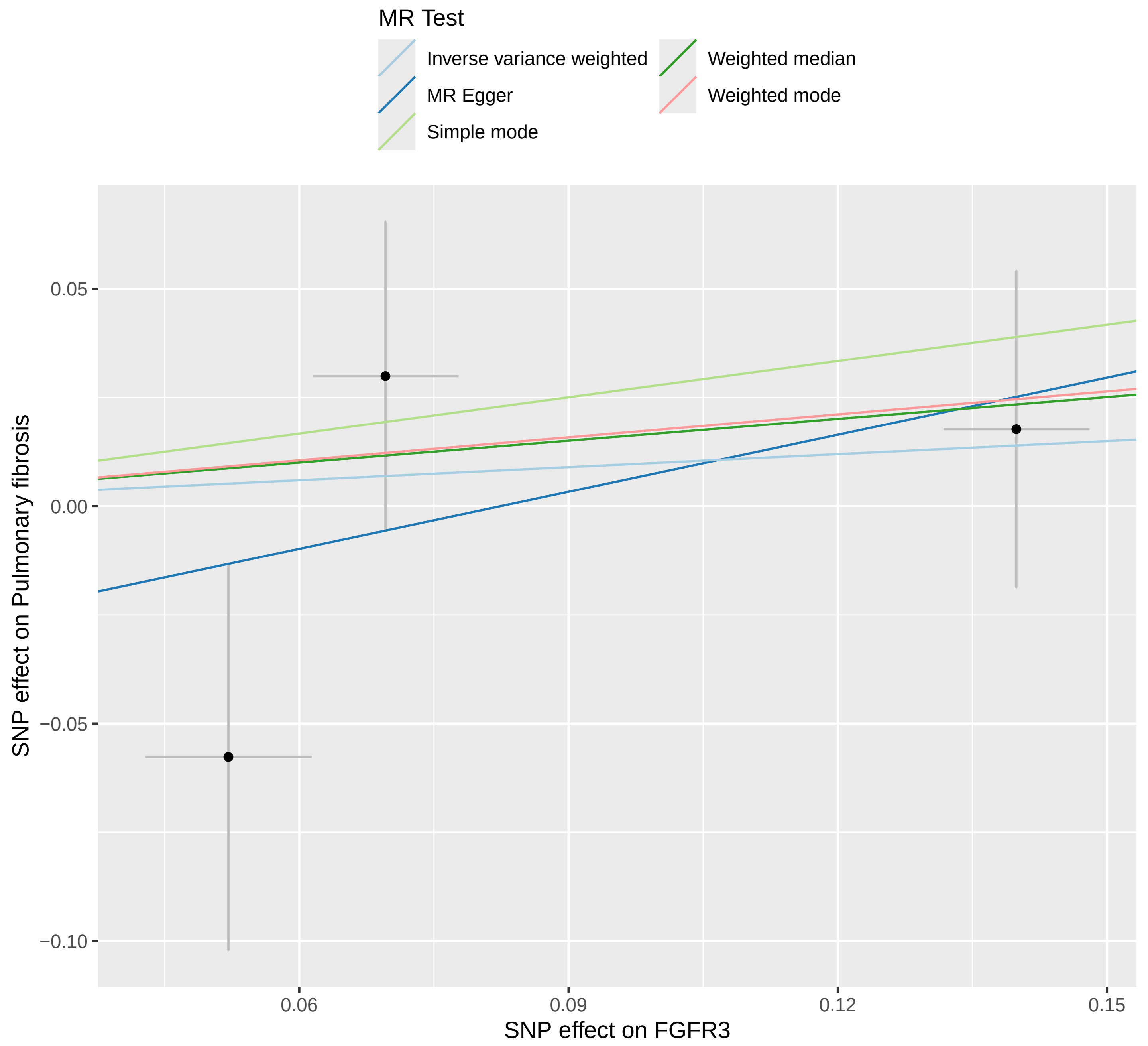

MR Test
Inverse variance weighted
MR Egger
Simple mode
Weighted median
Weighted mode
0.05
0.00
−0.05
−0.10
0.06
0.09
0.12
0.15
SNP effect on FGFR3
SNP effect on Pulmonary fibrosis

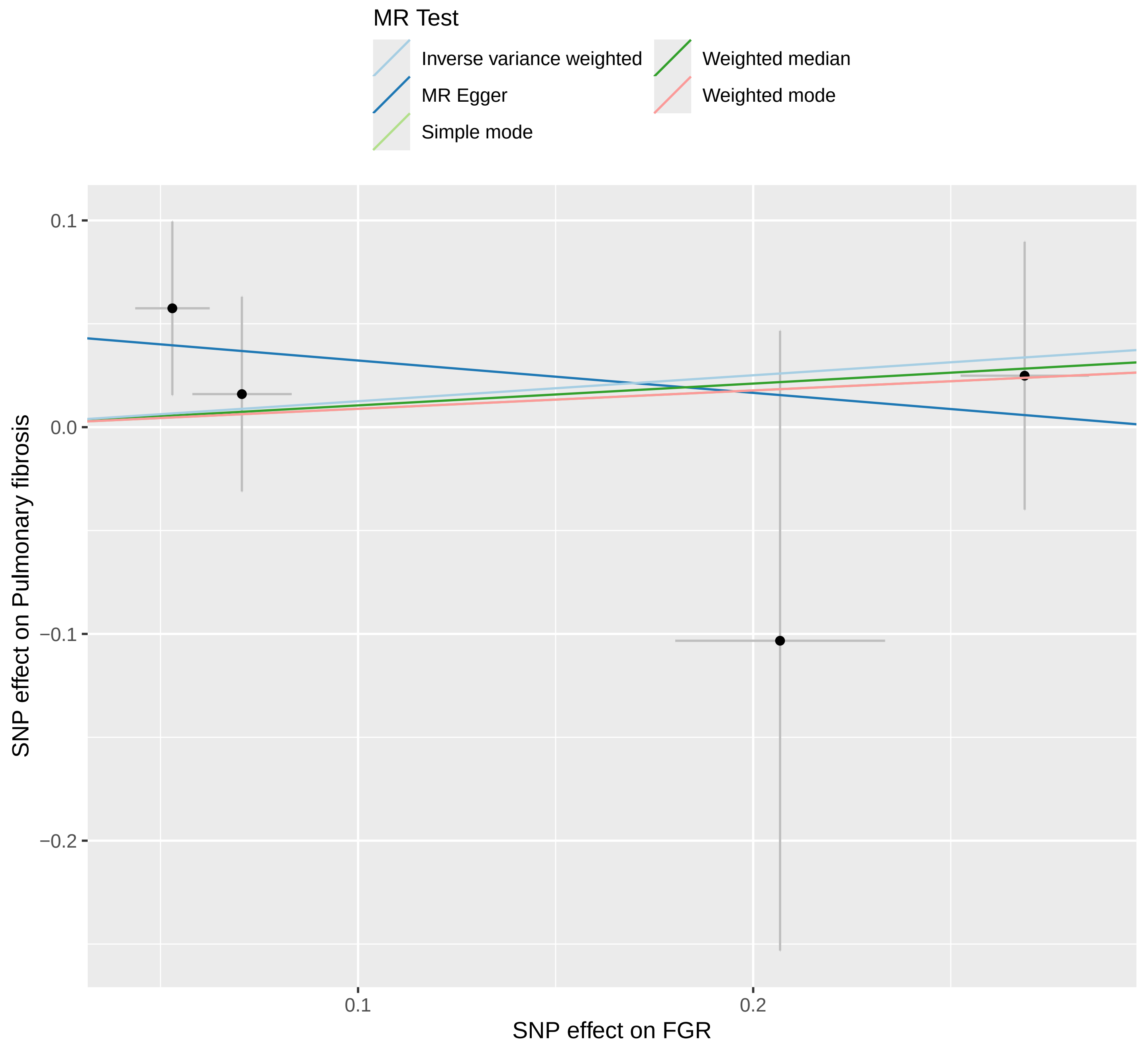

MR Test
Inverse variance weighted
MR Egger
Simple mode
Weighted median
Weighted mode
0.1
0.0
−0.1
−0.2
SNP effect on Pulmonary fibrosis
0.1
0.2
SNP effect on FGR

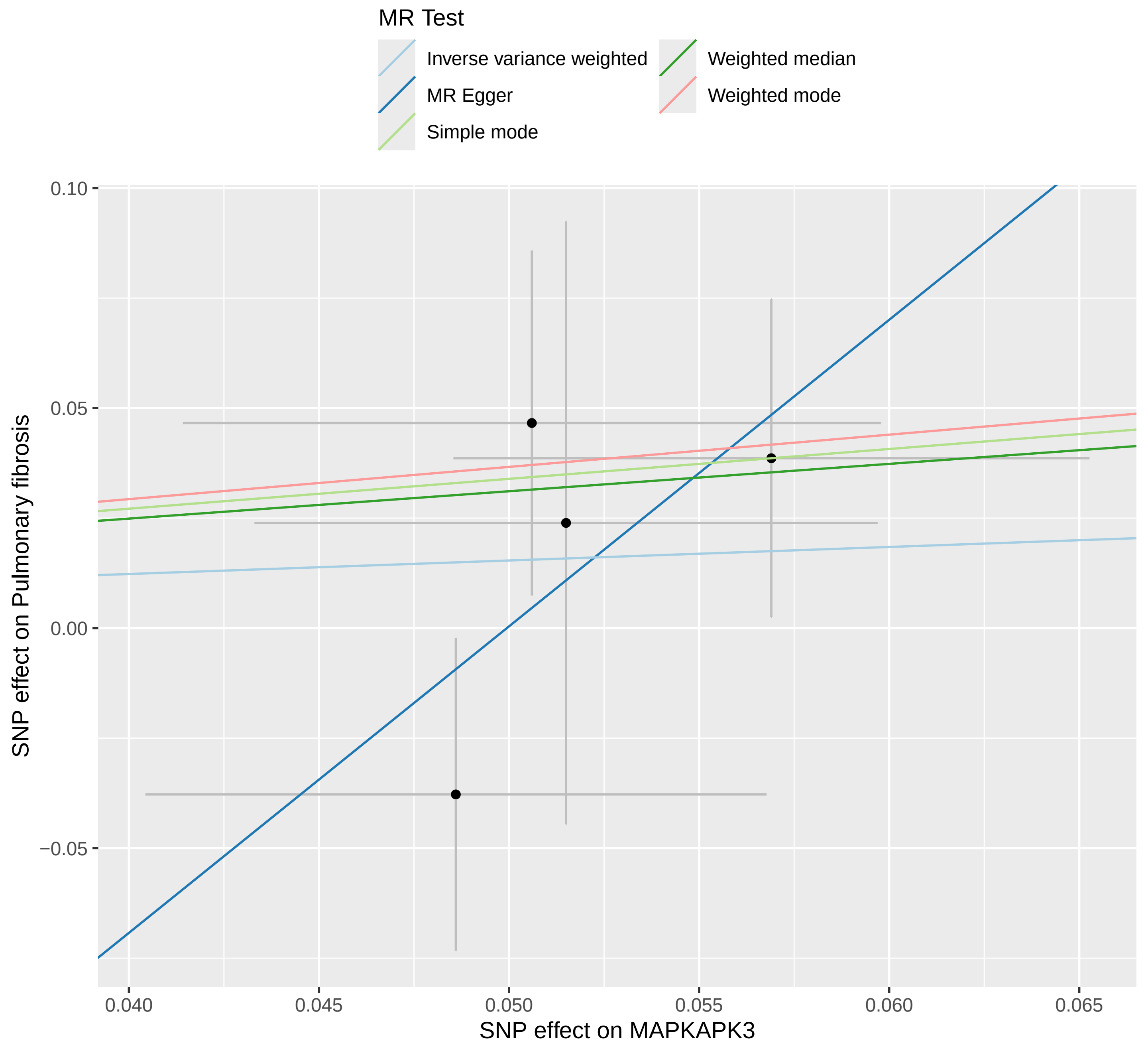
MR Test
Inverse variance weighted
MR Egger
Simple mode
Weighted median
Weighted mode
0.10
0.05
0.00
−0.05
0.040
0.045
0.050
0.055
0.060
0.065
SNP effect on MAPKAPK3
SNP effect on Pulmonary fibrosis

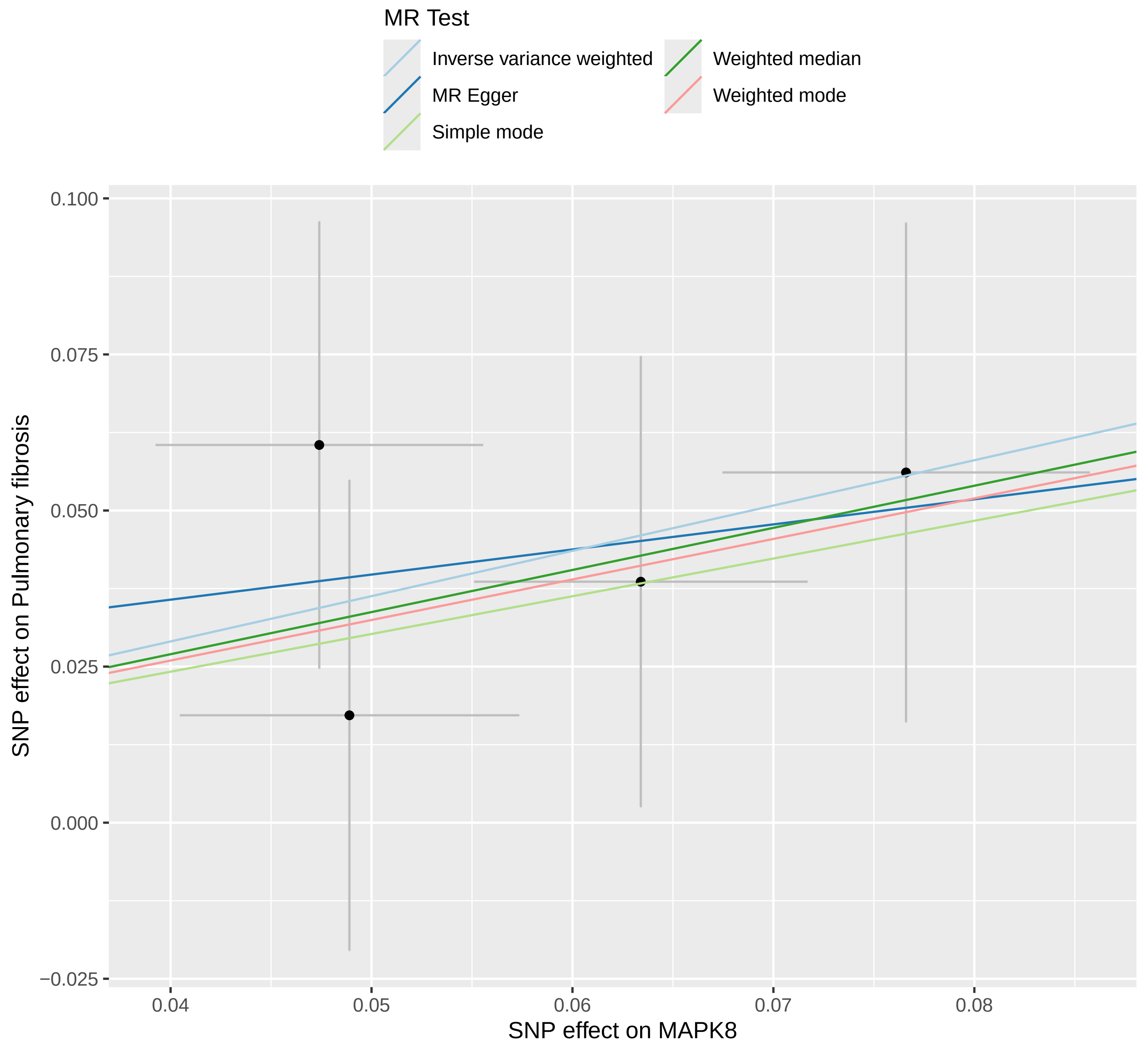

MR Test
Inverse variance weighted
MR Egger
Simple mode
Weighted median
Weighted mode
0.100
0.075
0.050
0.025
0.000
−0.025
SNP effect on Pulmonary fibrosis
0.04
0.05
0.06
0.07
0.08
SNP effect on MAPK8

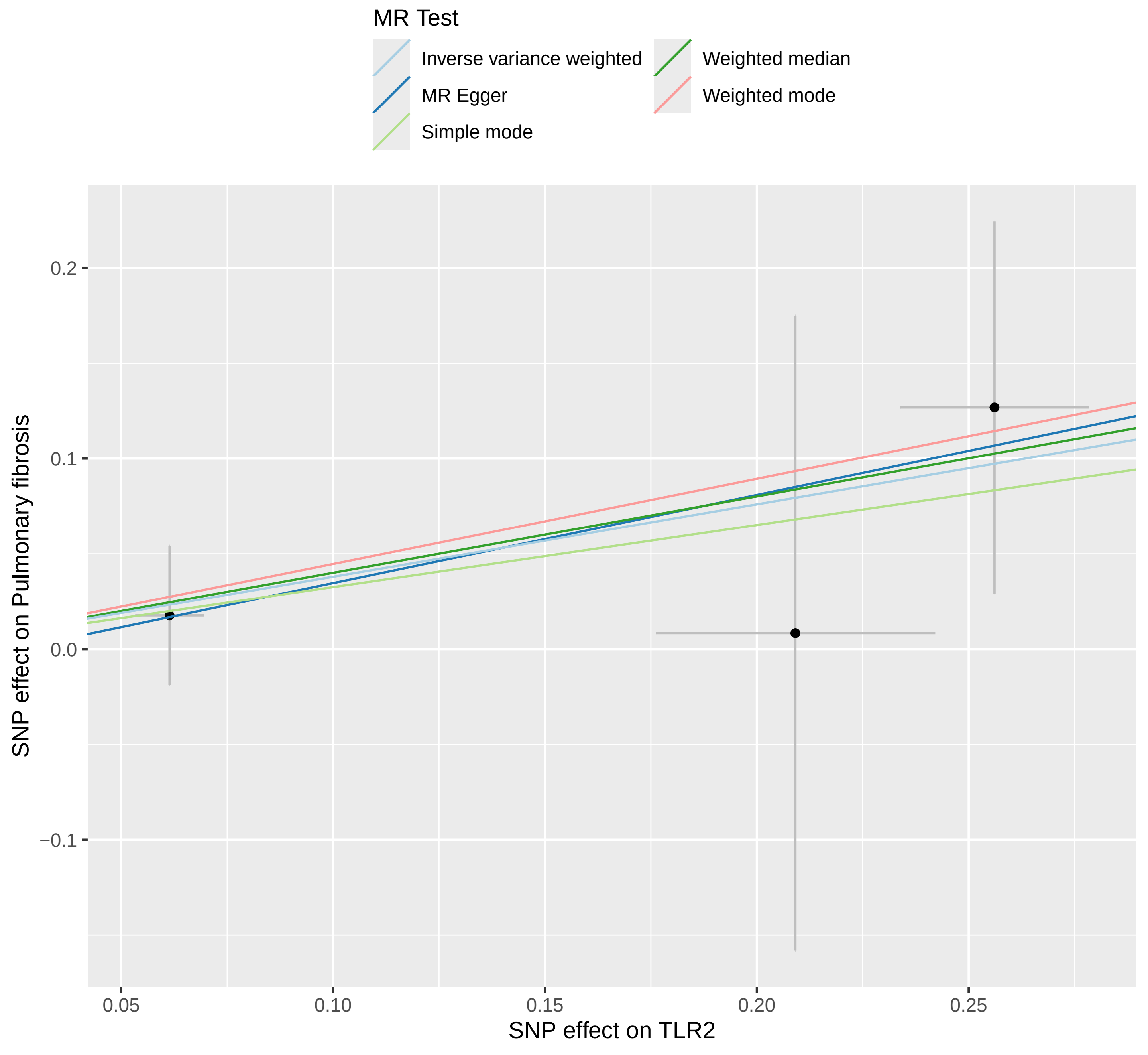
MR Test
Inverse variance weighted
MR Egger
Simple mode
Weighted median
Weighted mode
0.2
0.1
0.0
−0.1
0.05
0.10
0.15
0.20
0.25
SNP effect on Pulmonary fibrosis
SNP effect on TLR2

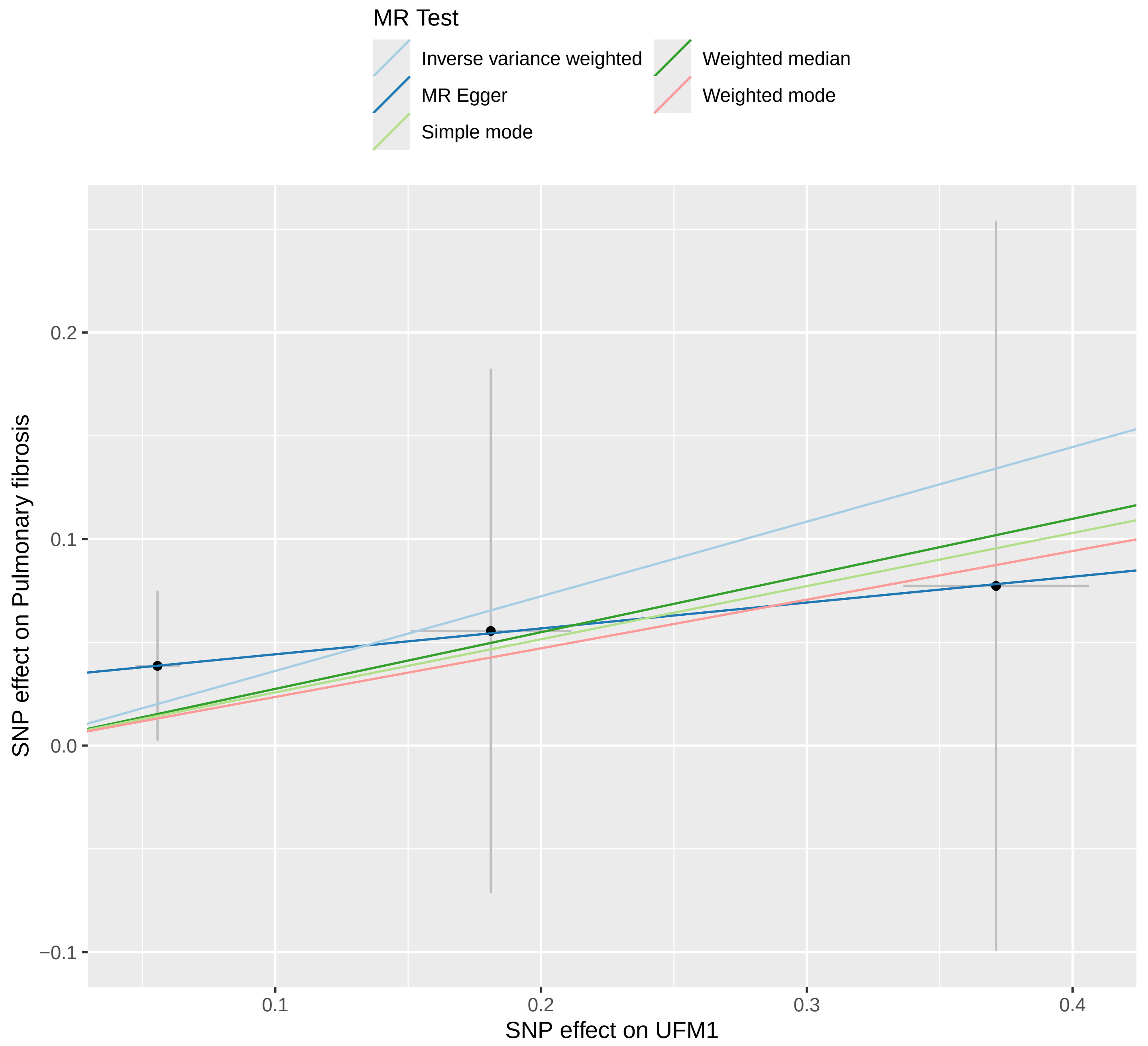
MR Test
Inverse variance weighted
MR Egger
Simple mode
Weighted median
Weighted mode
SNP effect on Pulmonary fibrosis
0.2
0.1
0.0
−0.1
0.1
0.2
0.3
0.4
SNP effect on UFM1

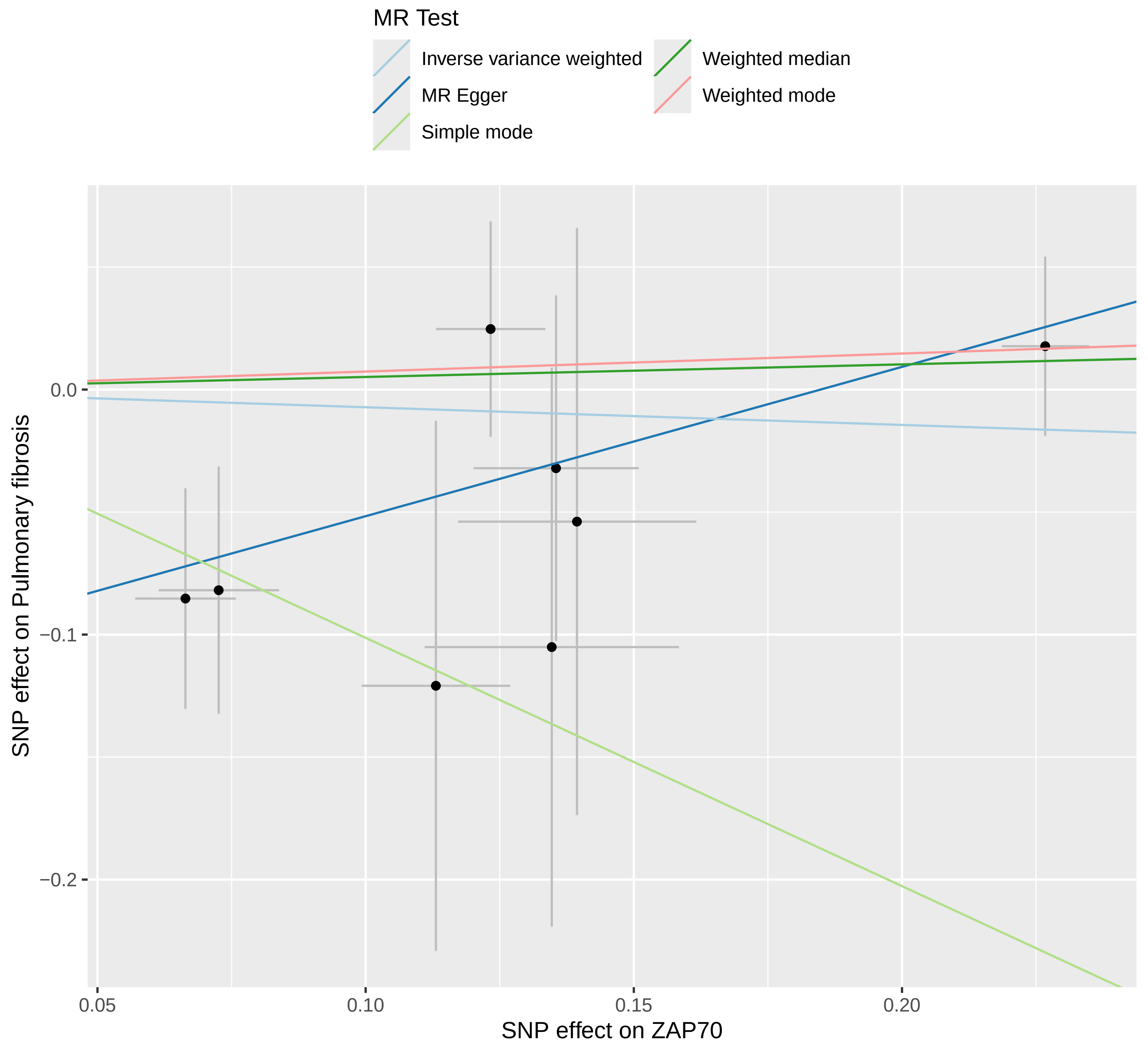
MR Test
Inverse variance weighted
MR Egger
Simple mode
Weighted median
Weighted mode
0.0
−0.1
−0.2
SNP effect on Pulmonary fibrosis
0.05
0.10
0.15
0.20
SNP effect on ZAP70

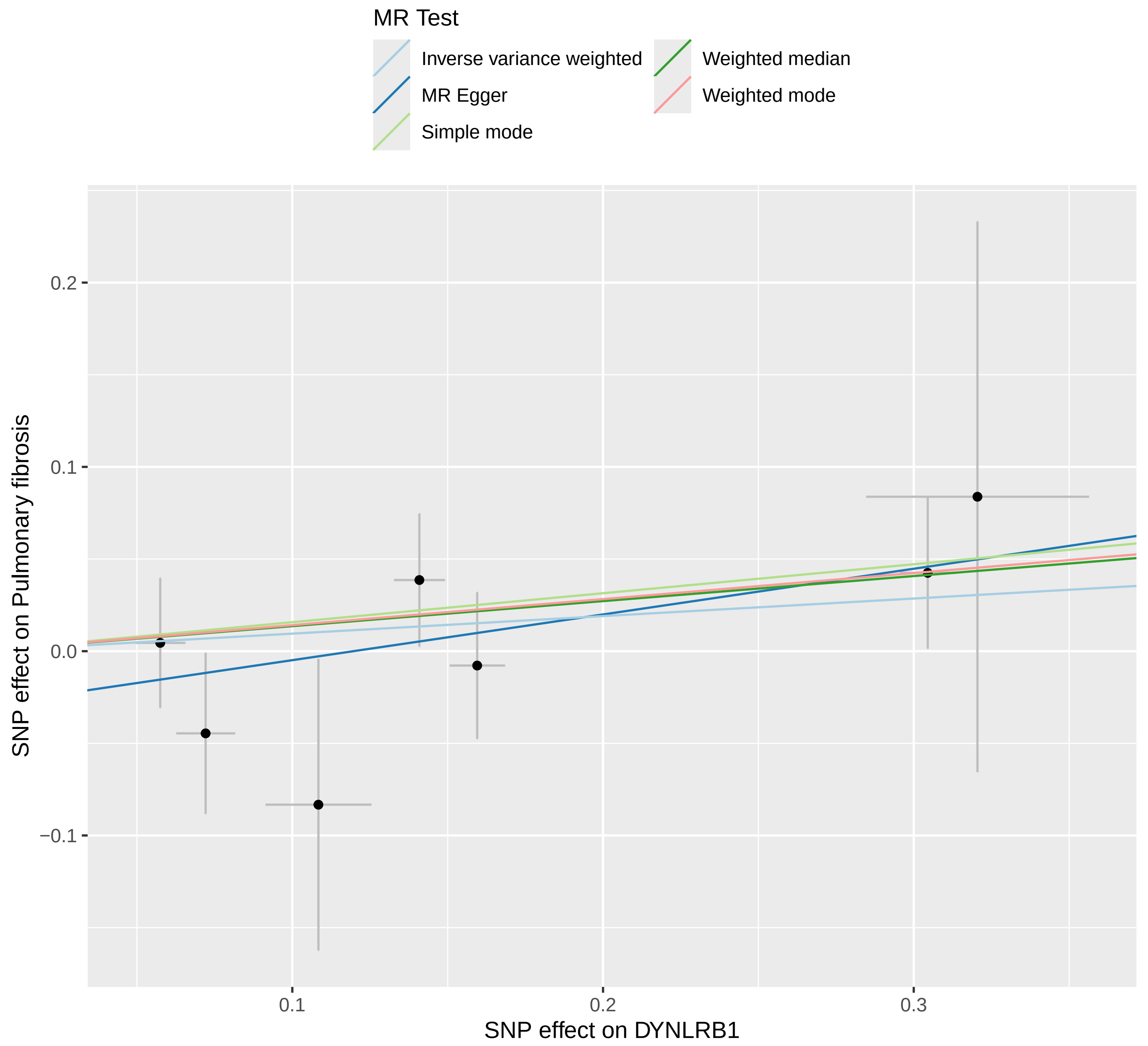
MR Test
Inverse variance weighted
MR Egger
Simple mode
Weighted median
Weighted mode
0.2
0.1
0.0
−0.1
0.1
0.2
0.3
SNP effect on Pulmonary fibrosis
SNP effect on DYNLRB1

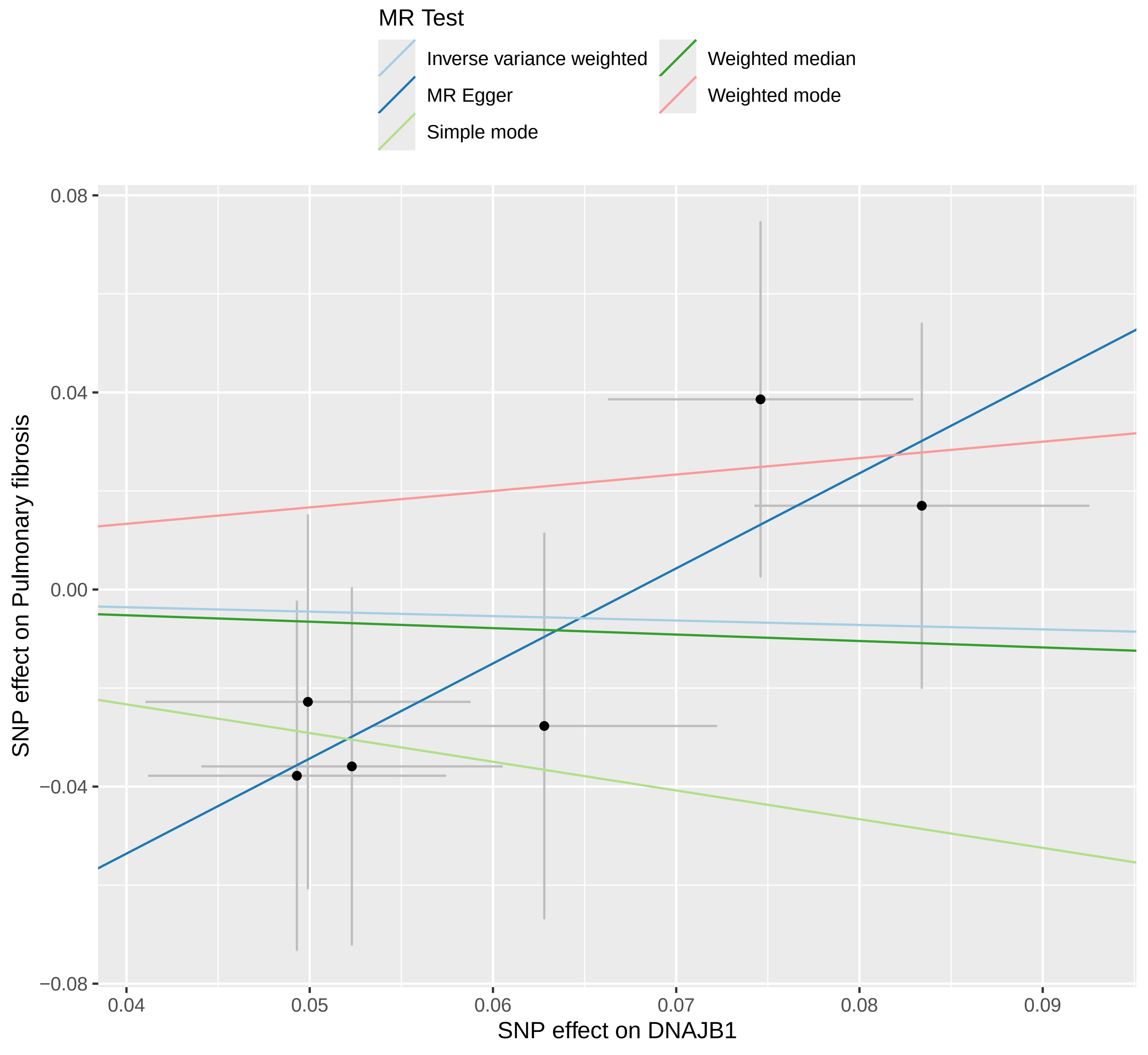
MR Test
Inverse variance weighted
MR Egger
Simple mode
Weighted median
Weighted mode
0.08
0.04
0.00
−0.04
−0.08
0.04
0.05
0.06
0.07
0.08
0.09
SNP effect on Pulmonary fibrosis
SNP effect on DNAJB1

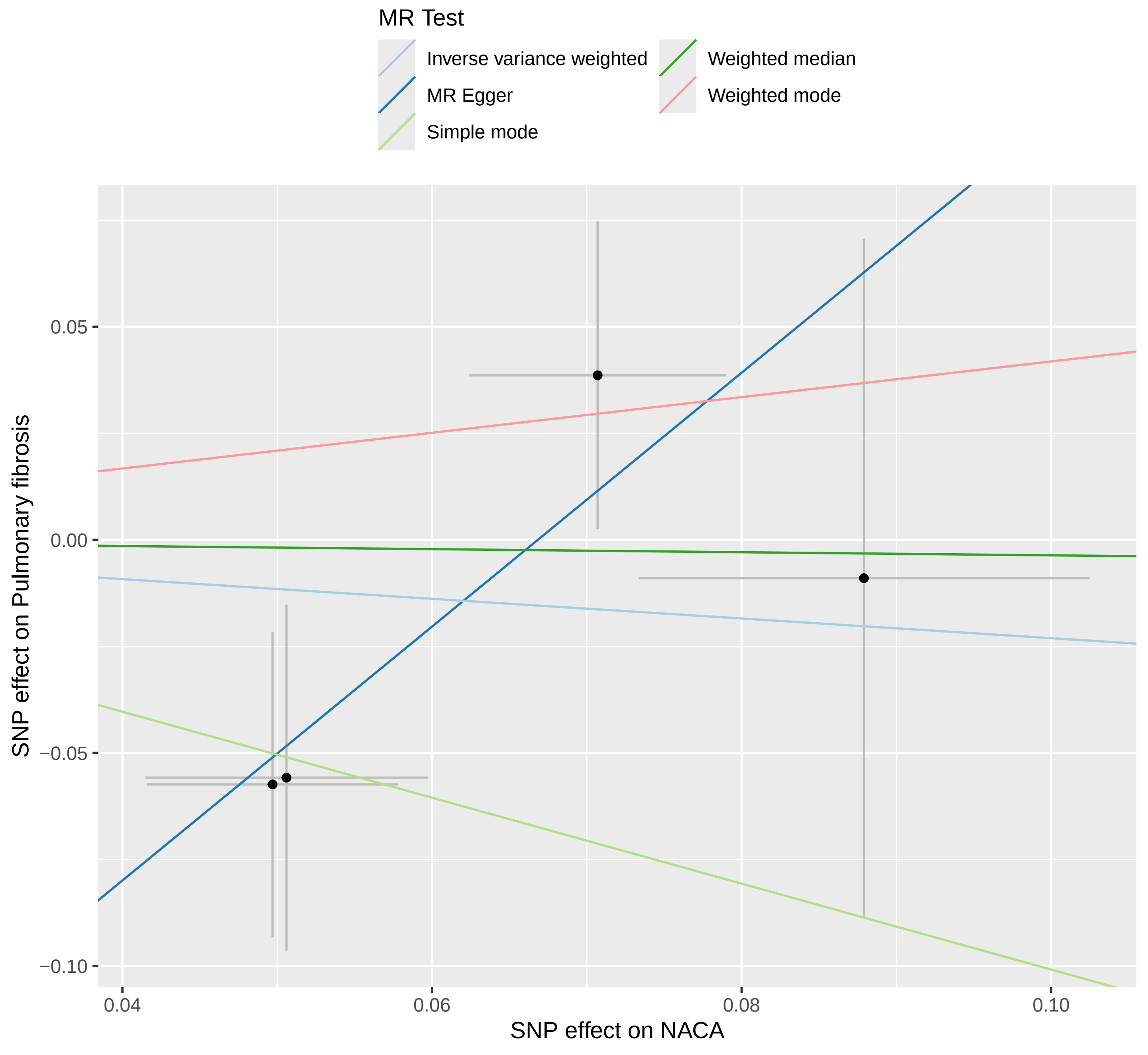
MR Test
Inverse variance weighted
MR Egger
Simple mode
Weighted median
Weighted mode
SNP effect on Pulmonary fibrosis
0.05
0.00
−0.05
−0.10
0.04
0.06
0.08
0.10
SNP effect on NACA

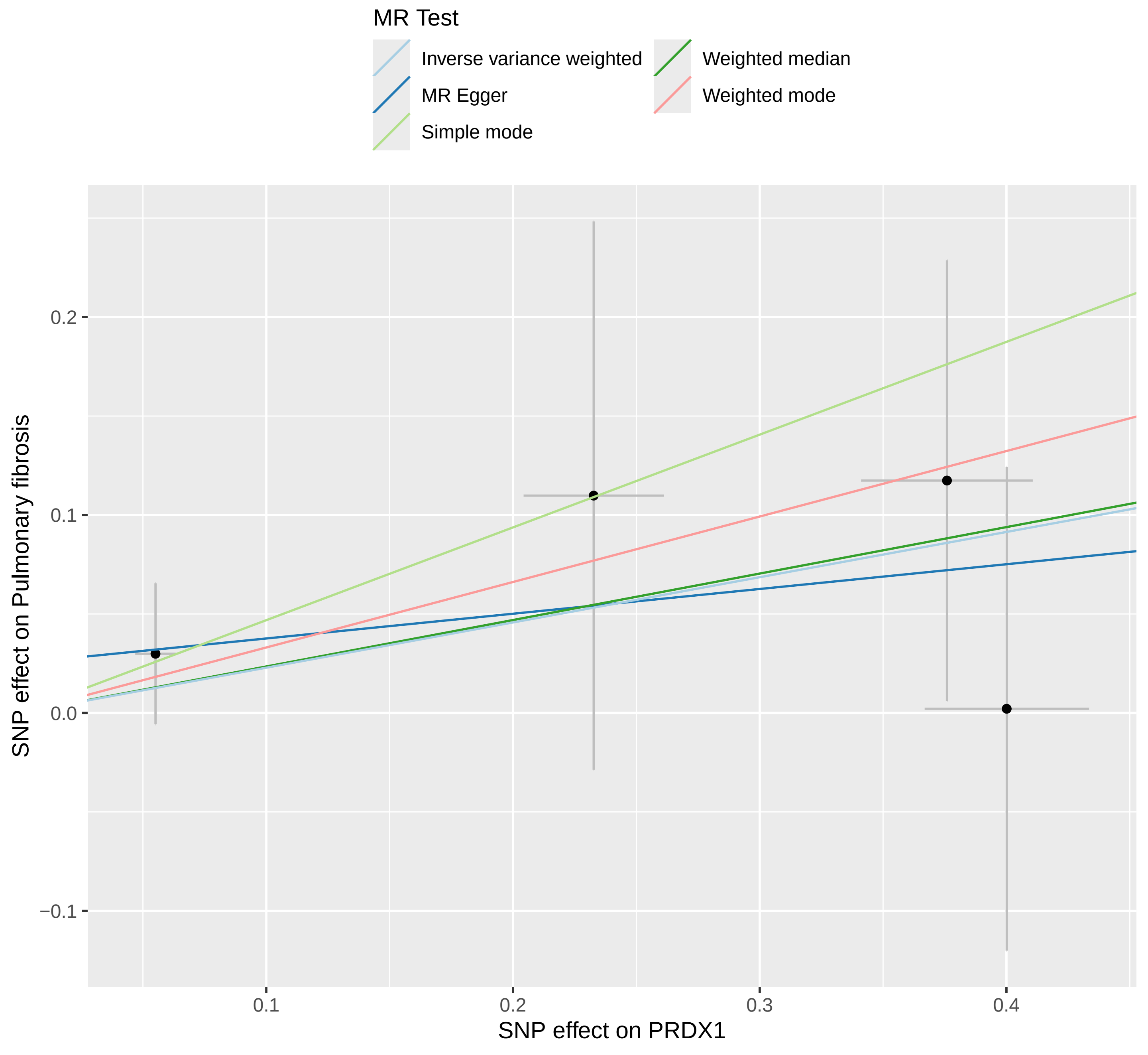

MR Test
Inverse variance weighted
MR Egger
Simple mode
Weighted median
Weighted mode
SNP effect on Pulmonary fibrosis
0.2
0.1
0.0
−0.1
0.1
0.2
0.3
0.4
SNP effect on PRDX1

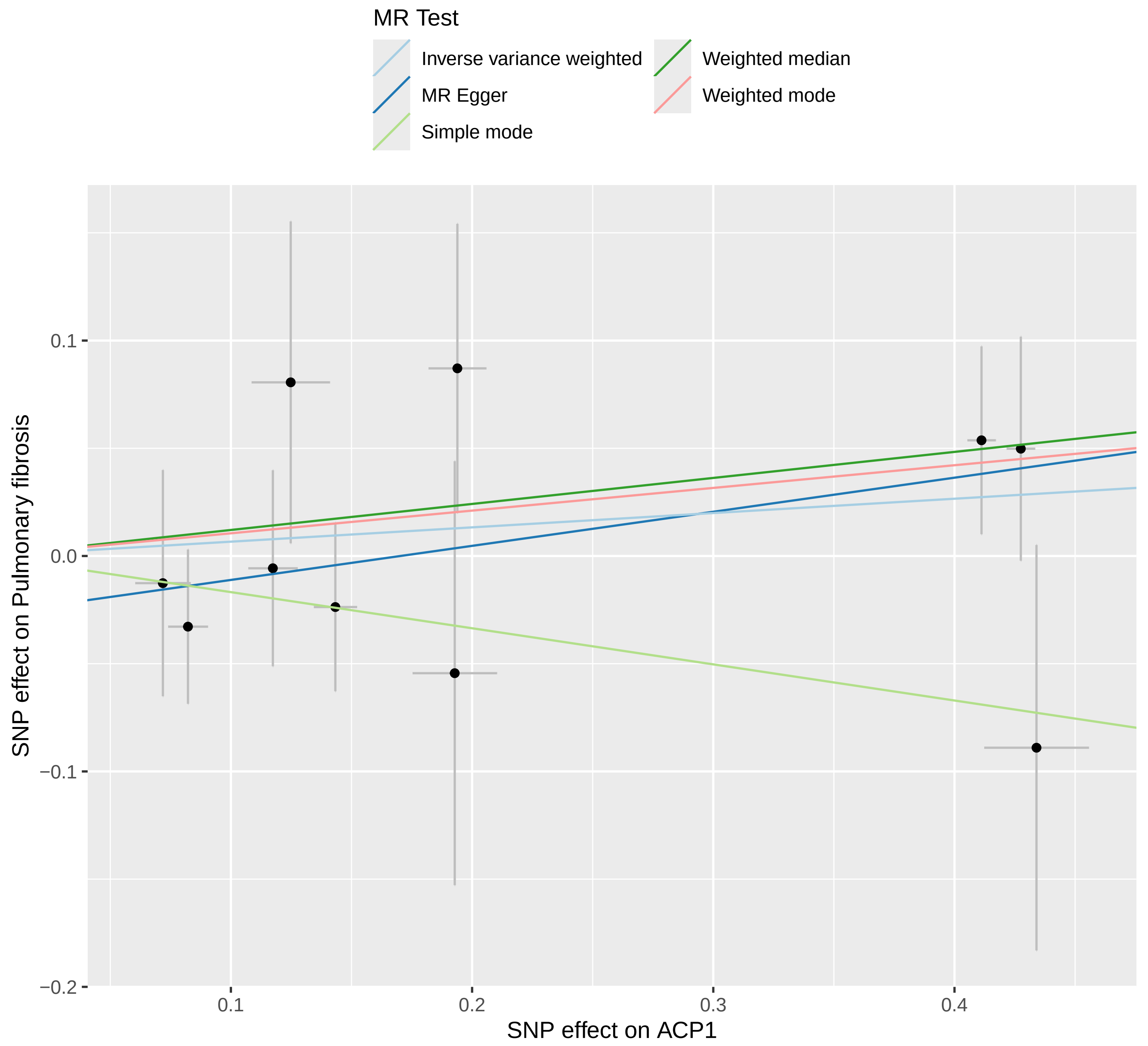

MR Test
Inverse variance weighted
MR Egger
Simple mode
Weighted median
Weighted mode
0.1
0.0
−0.1
−0.2
SNP effect on Pulmonary fibrosis
0.1
0.2
0.3
0.4
SNP effect on ACP1

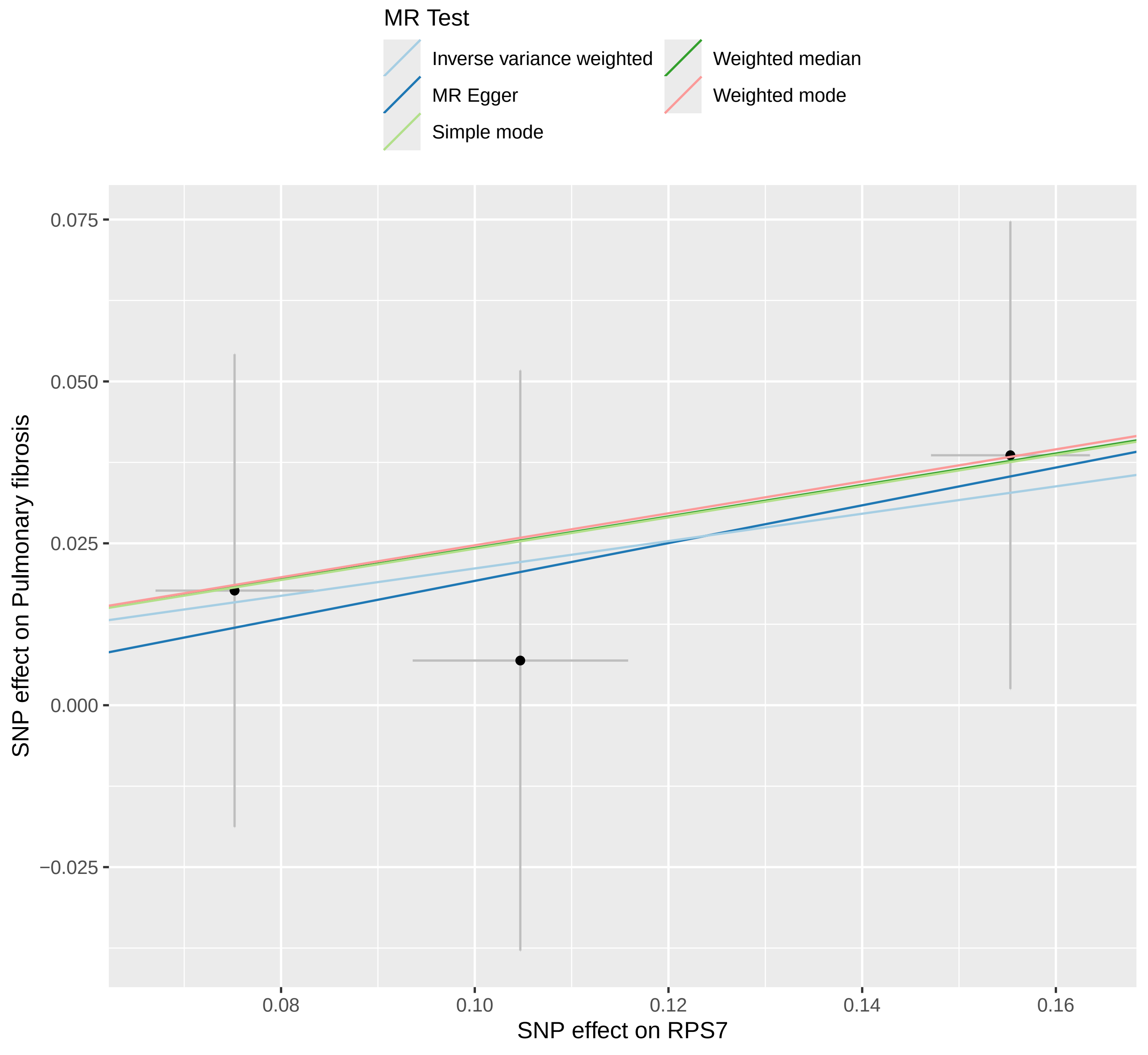

MR Test
Inverse variance weighted
MR Egger
Simple mode
Weighted median
Weighted mode
0.075
0.050
0.025
0.000
−0.025
0.08
0.10
0.12
0.14
0.16
SNP effect on RPS7
SNP effect on Pulmonary fibrosis

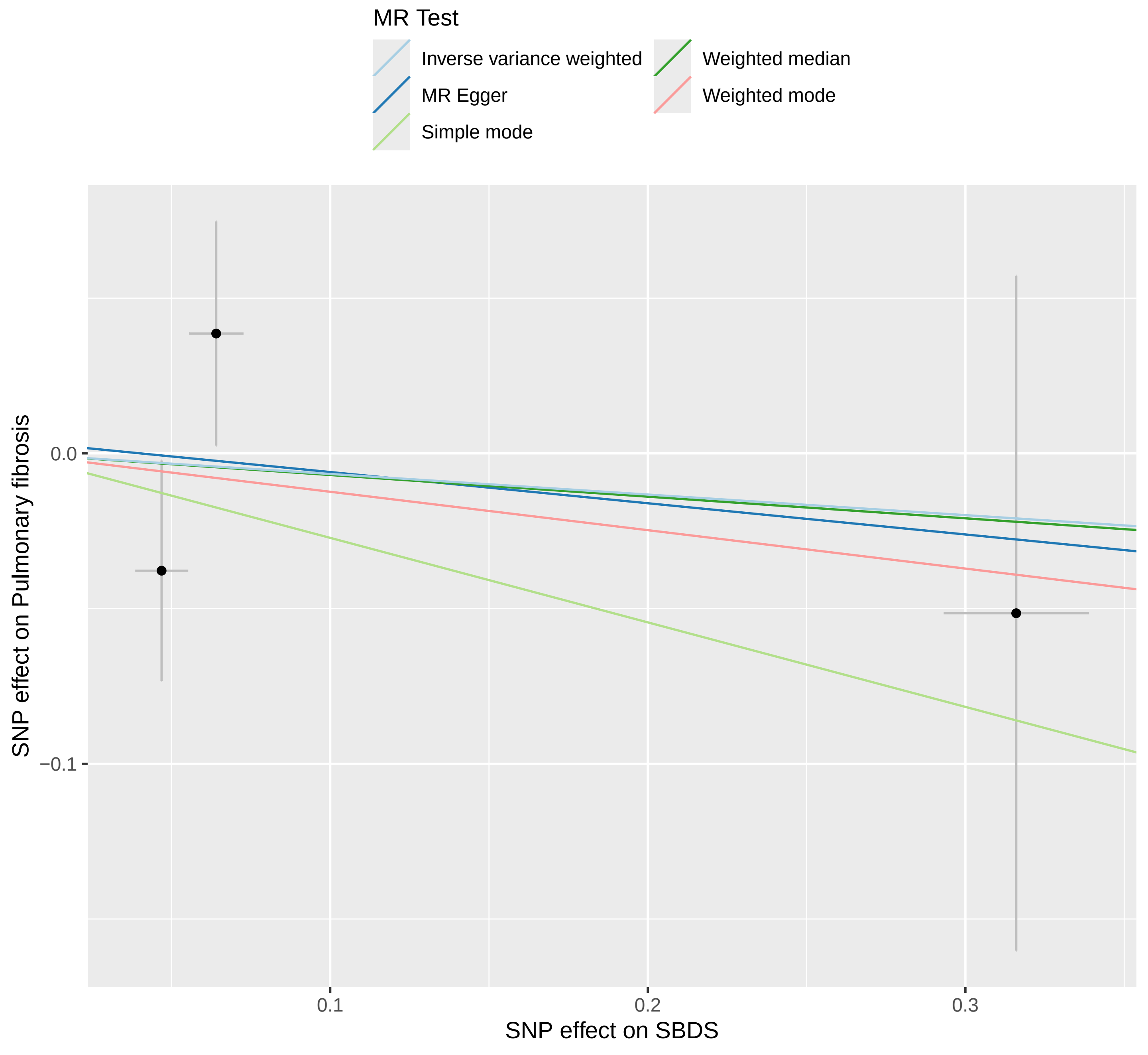

MR Test
Inverse variance weighted
MR Egger
Simple mode
Weighted median
Weighted mode
SNP effect on Pulmonary fibrosis
0.0
−0.1
0.1
0.2
0.3
SNP effect on SBDS

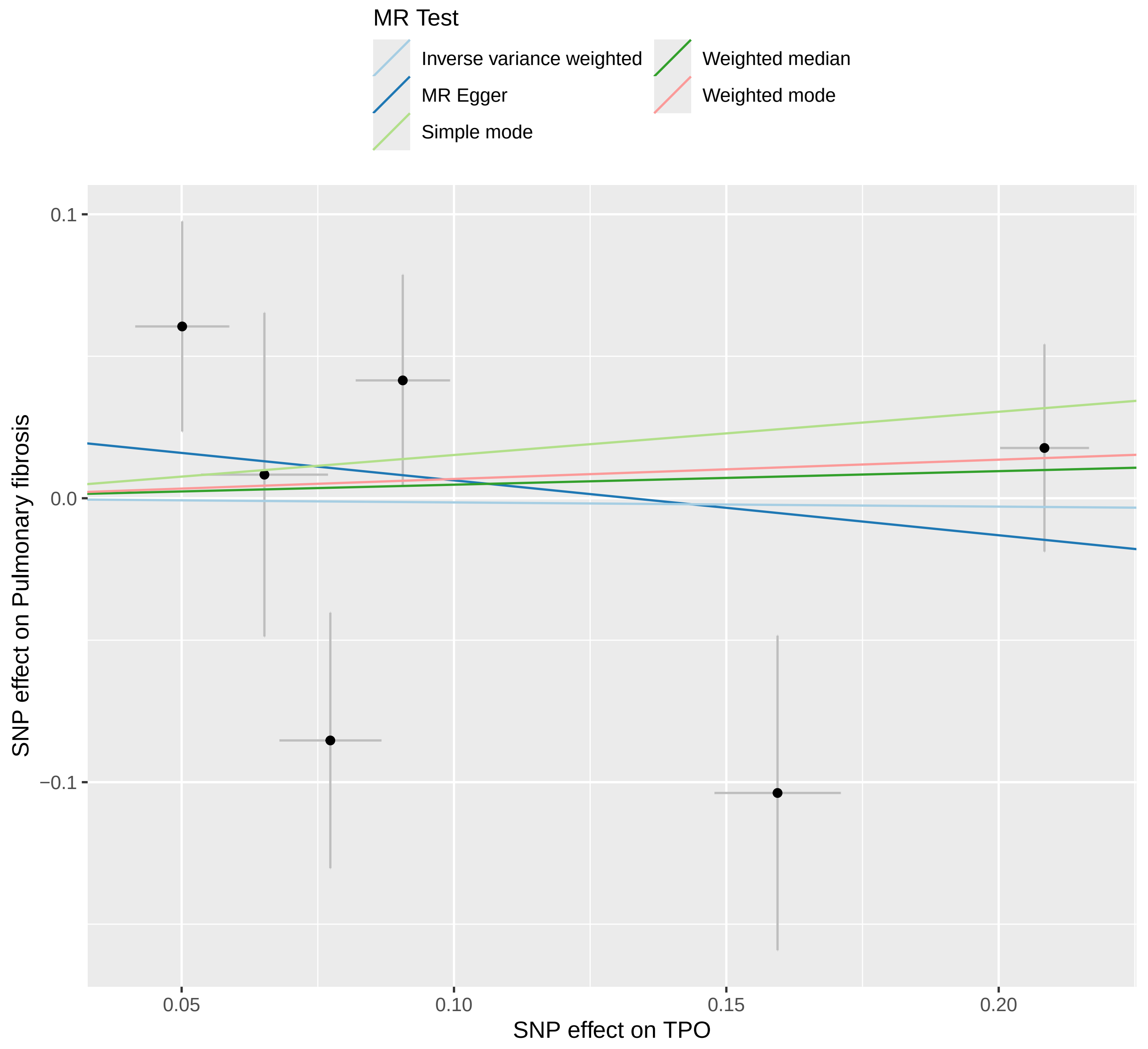
MR Test
Inverse variance weighted
MR Egger
Simple mode
Weighted median
Weighted mode
0.1
0.0
−0.1
0.05
0.10
0.15
0.20
SNP effect on TPO
SNP effect on Pulmonary fibrosis

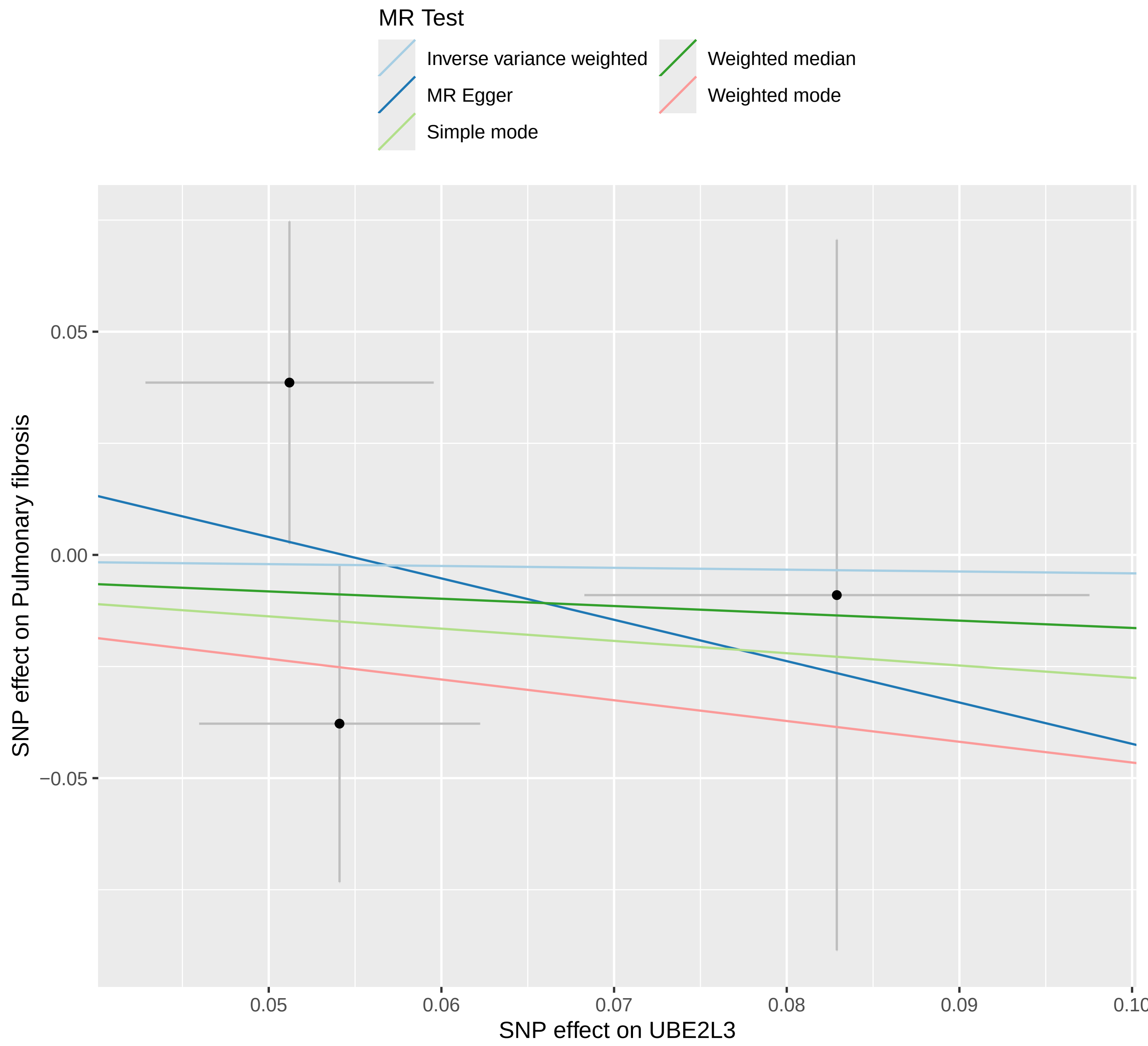
MR Test
Inverse variance weighted
MR Egger
Simple mode
Weighted median
Weighted mode
0.05
0.00
−0.05
SNP effect on Pulmonary fibrosis
0.05
0.06
0.07
0.08
0.09
0.10
SNP effect on UBE2L3

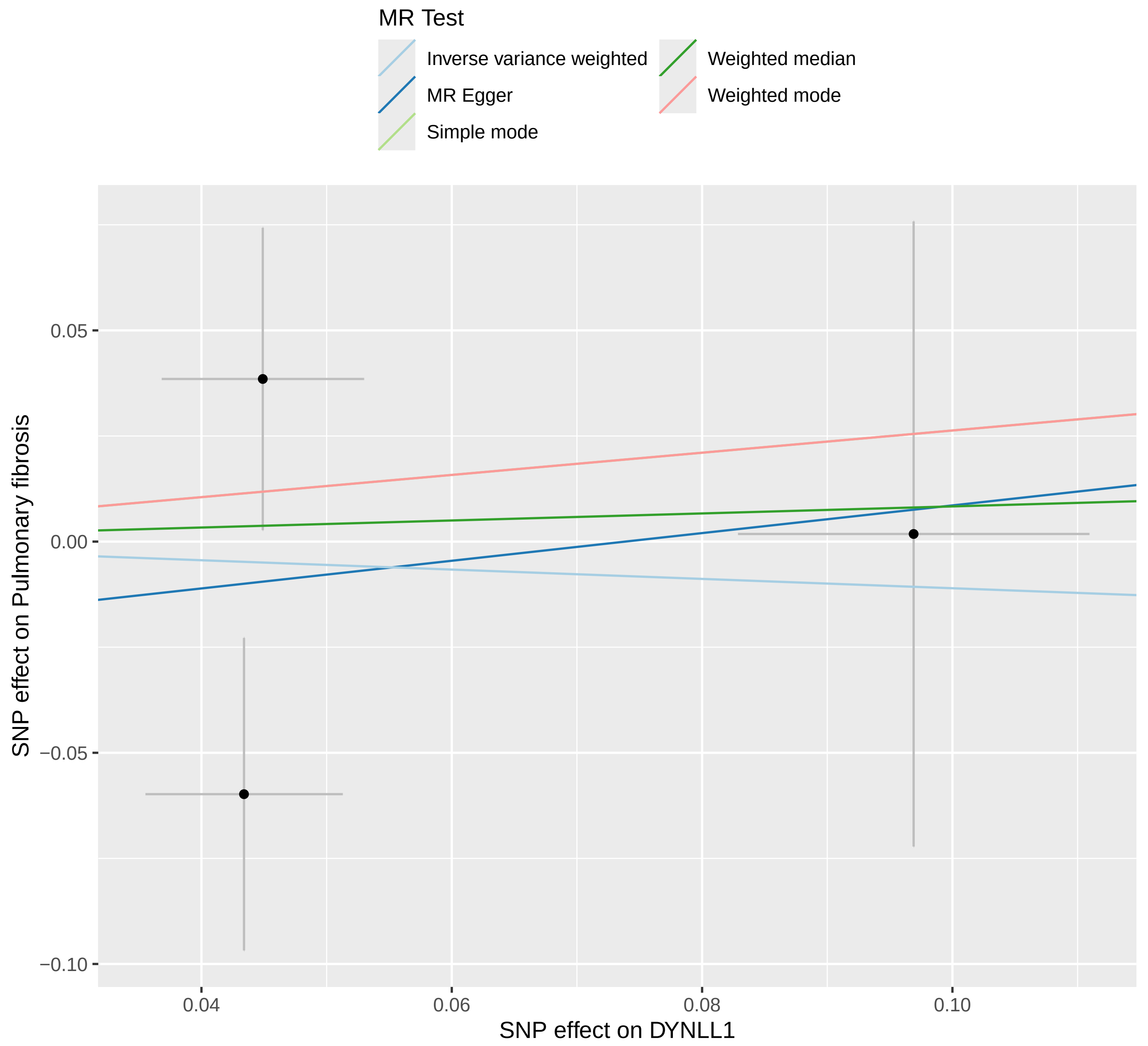
MR Test
Inverse variance weighted
MR Egger
Simple mode
Weighted median
Weighted mode
SNP effect on Pulmonary fibrosis
0.05
0.00
−0.05
−0.10
0.04
0.06
0.08
0.10
SNP effect on DYNLL1

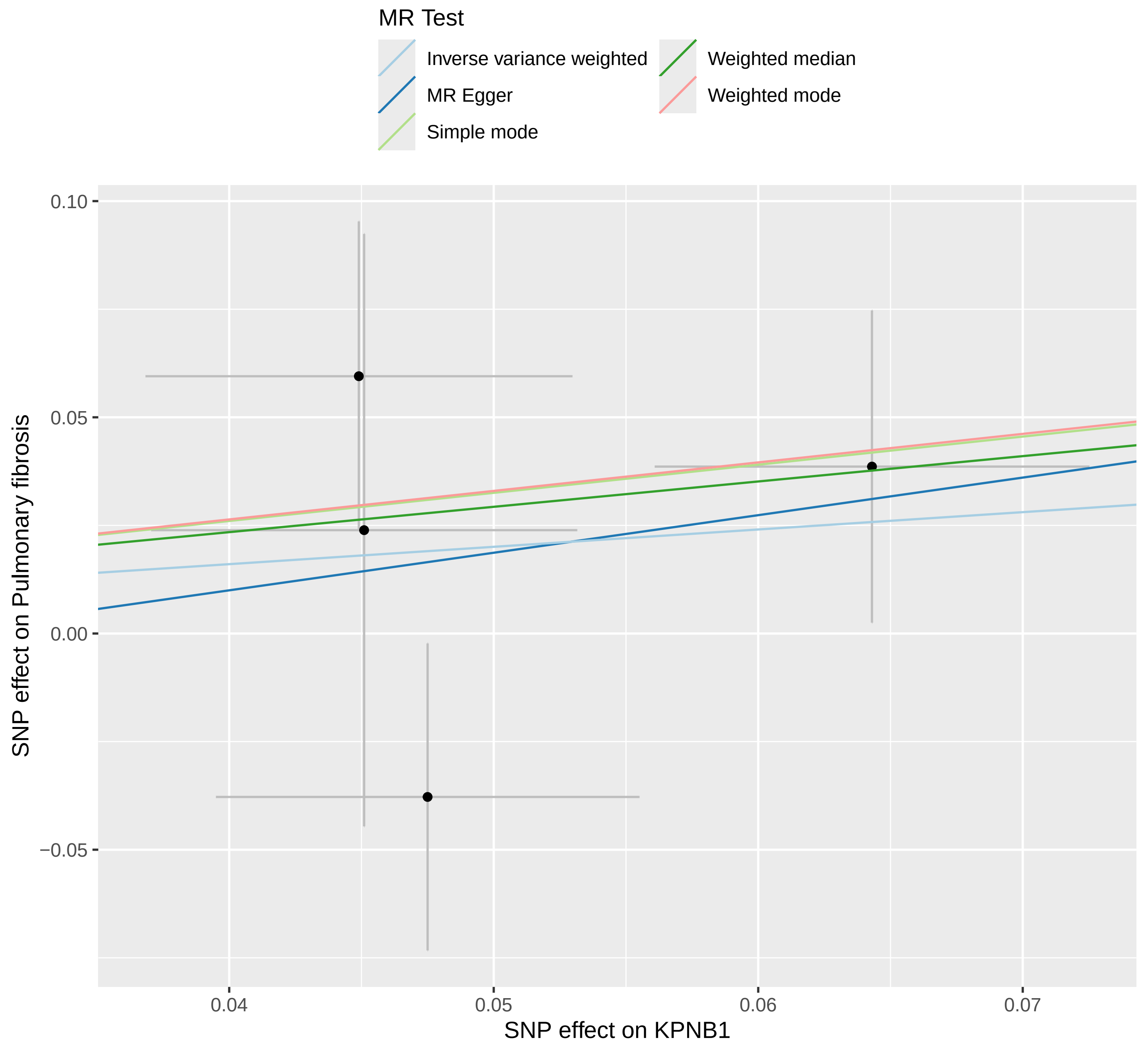

MR Test
Inverse variance weighted
MR Egger
Simple mode
Weighted median
Weighted mode
0.10
0.05
0.00
−0.05
0.04
0.05
0.06
0.07
SNP effect on Pulmonary fibrosis
SNP effect on KPNB1

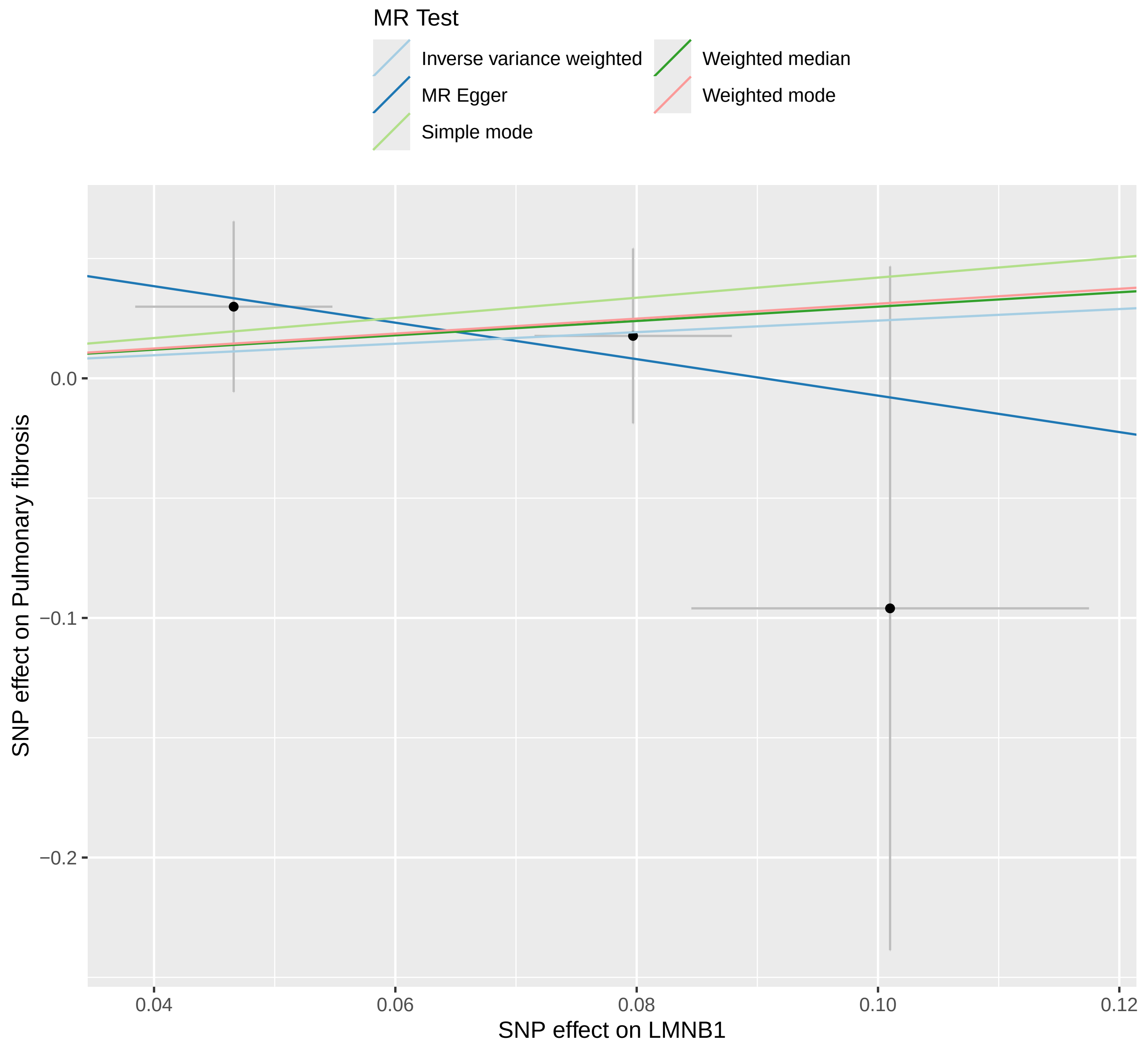
MR Test
Inverse variance weighted
MR Egger
Simple mode
Weighted median
Weighted mode
0.0
−0.1
−0.2
SNP effect on Pulmonary fibrosis
0.04
0.06
0.08
0.10
0.12
SNP effect on LMNB1

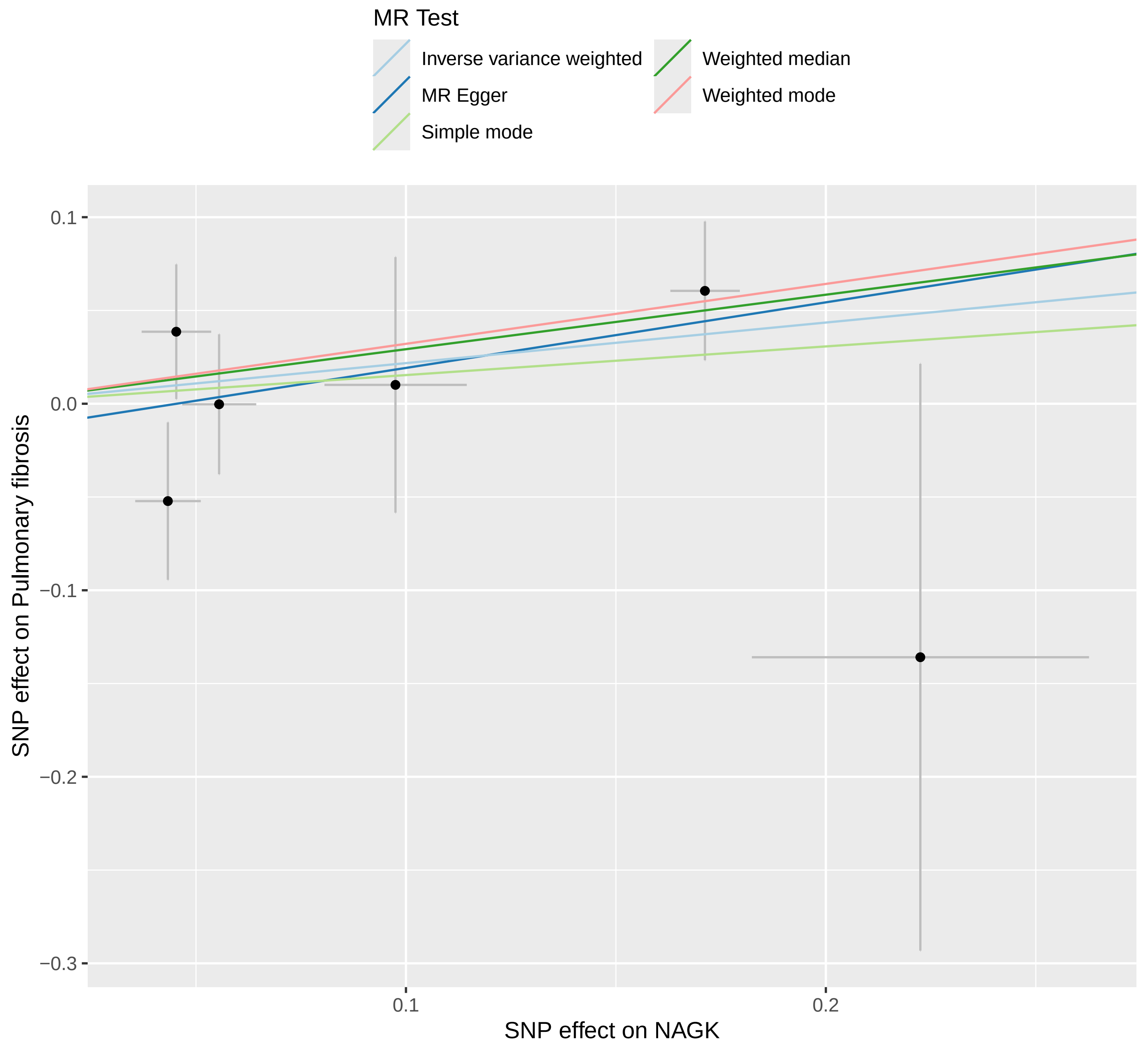

MR Test
Inverse variance weighted
MR Egger
Simple mode
Weighted median
Weighted mode
0.1
0.0
−0.1
−0.2
−0.3
SNP effect on Pulmonary fibrosis
0.1
0.2
SNP effect on NAGK

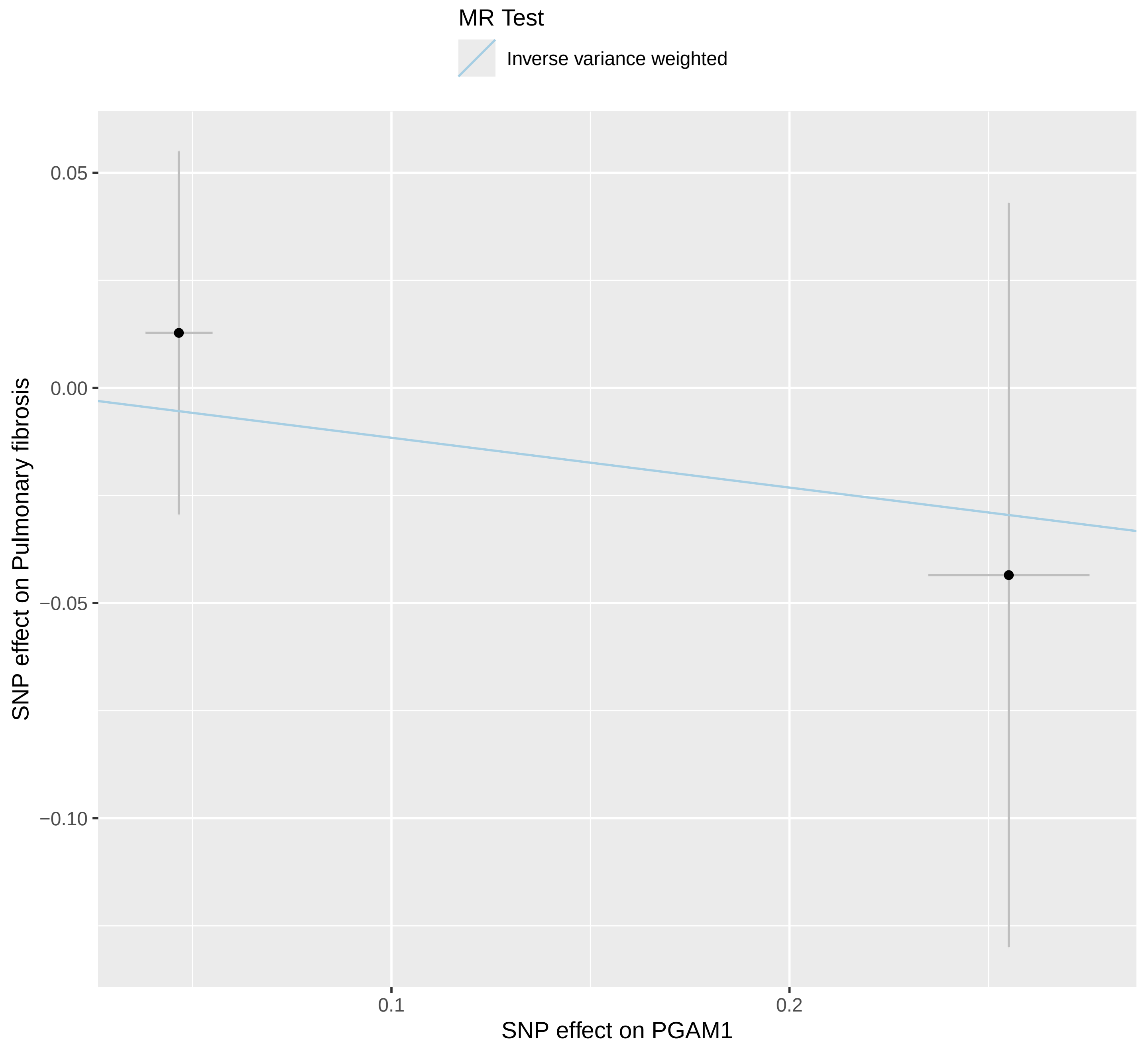

MR Test
Inverse variance weighted
0.05
0.00
−0.05
−0.10
0.1
0.2
SNP effect on Pulmonary fibrosis
SNP effect on PGAM1

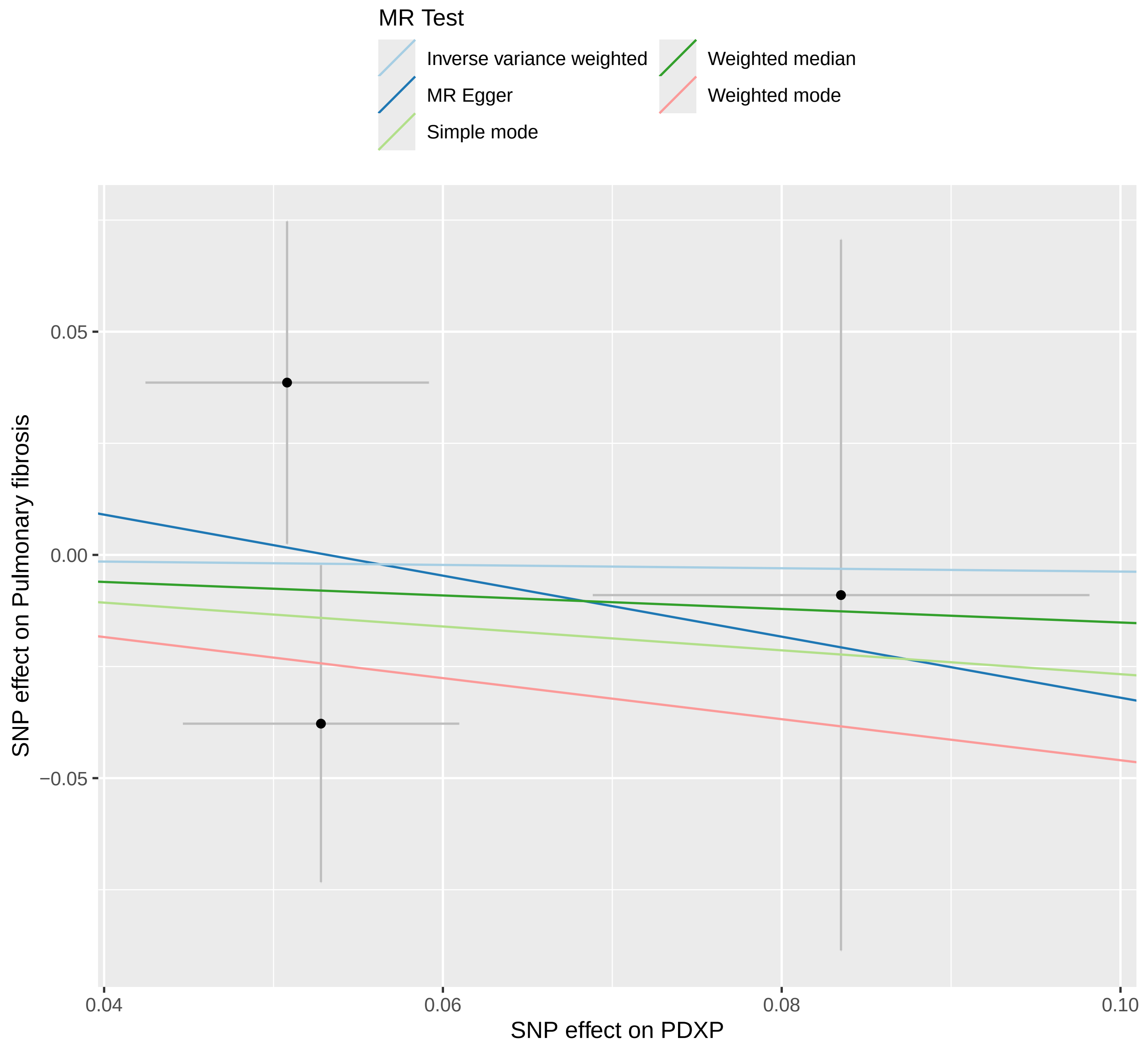
MR Test
Inverse variance weighted
MR Egger
Simple mode
Weighted median
Weighted mode
0.05
0.00
−0.05
SNP effect on Pulmonary fibrosis
0.04
0.06
0.08
0.10
SNP effect on PDXP

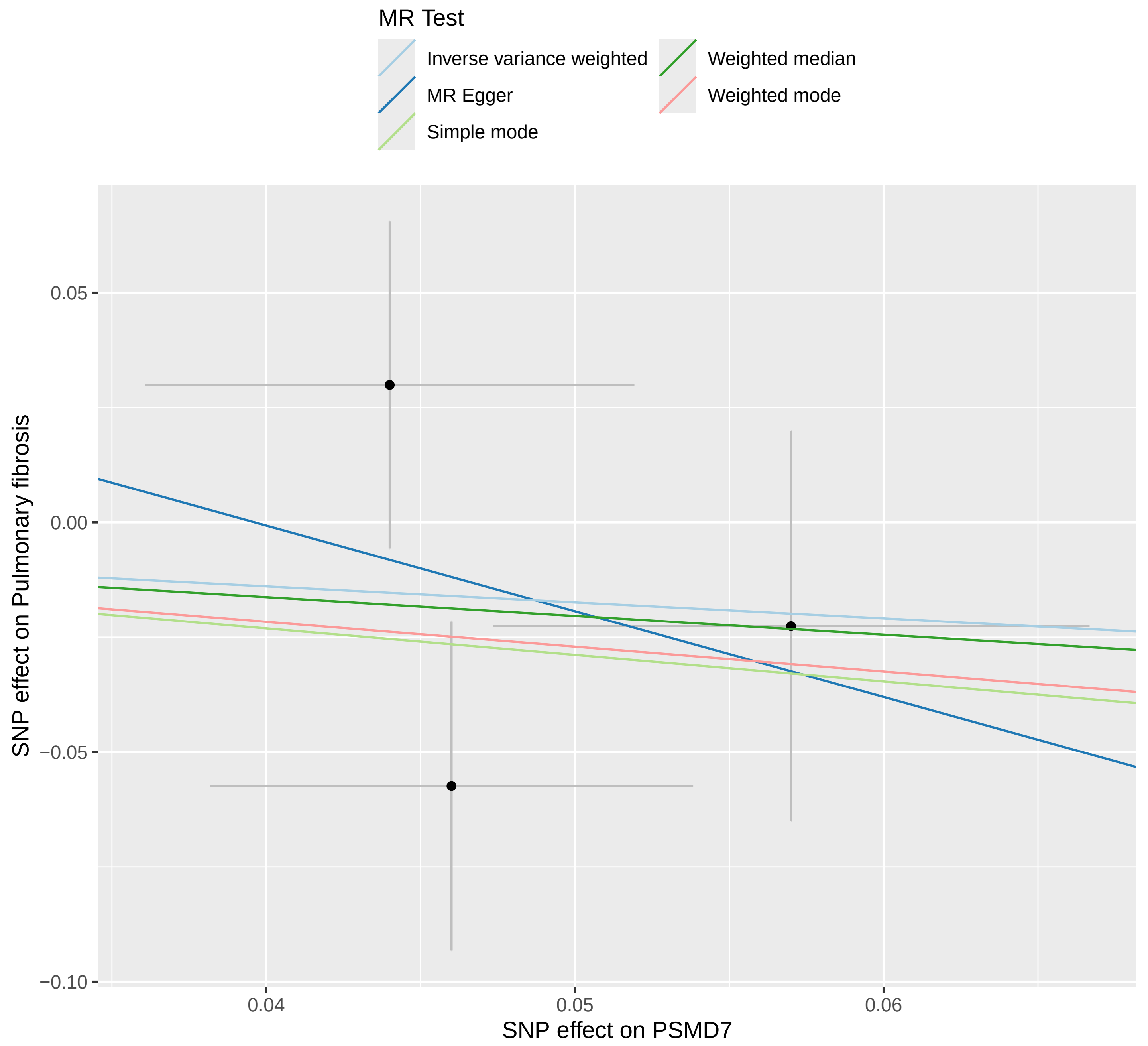

MR Test
Inverse variance weighted
MR Egger
Simple mode
Weighted median
Weighted mode
0.05
0.00
−0.05
−0.10
0.04
0.05
0.06
SNP effect on PSMD7
SNP effect on Pulmonary fibrosis

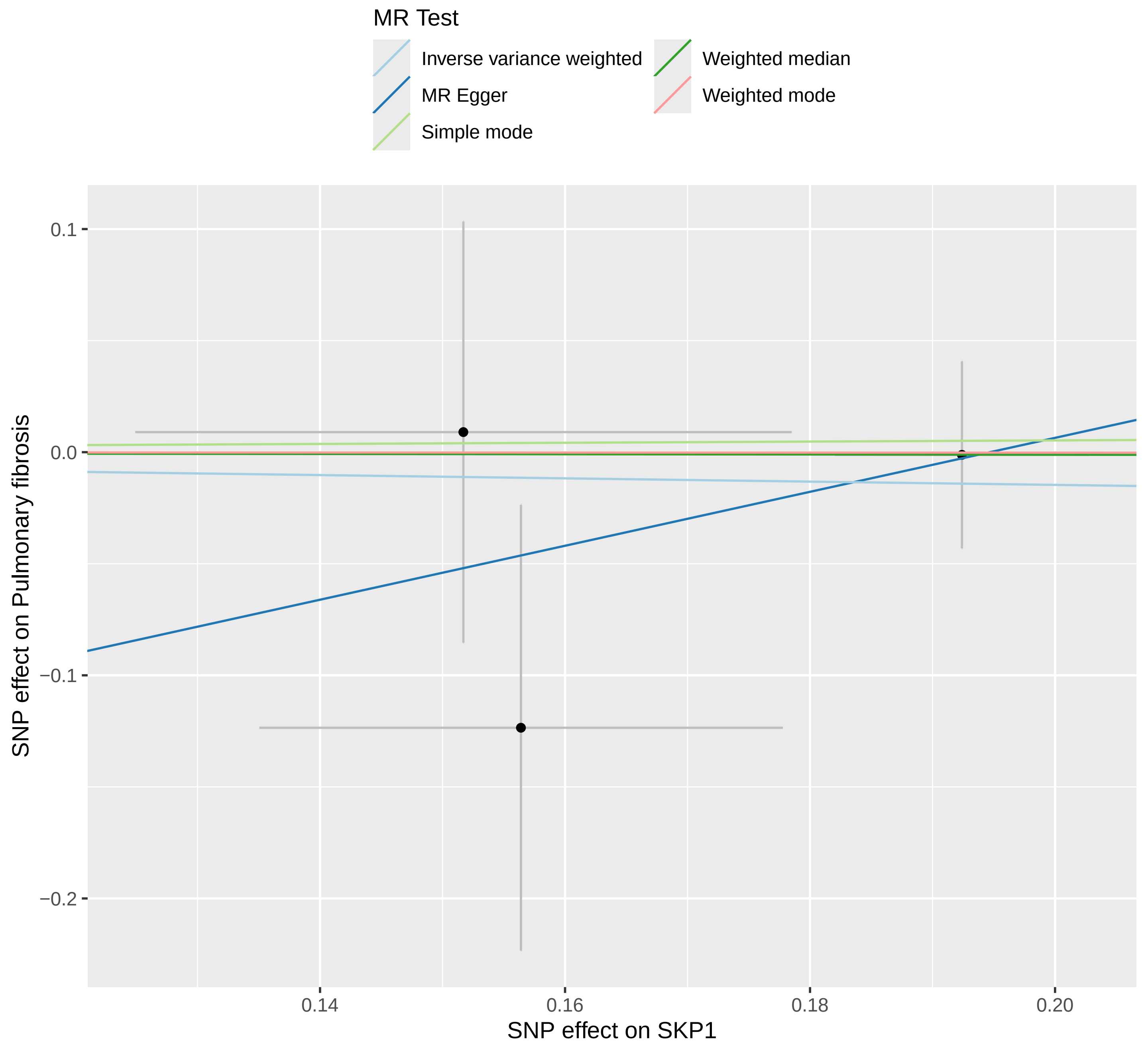

MR Test
Inverse variance weighted
MR Egger
Simple mode
Weighted median
Weighted mode
0.1
0.0
−0.1
−0.2
0.14
0.16
0.18
0.20
SNP effect on Pulmonary fibrosis
SNP effect on SKP1

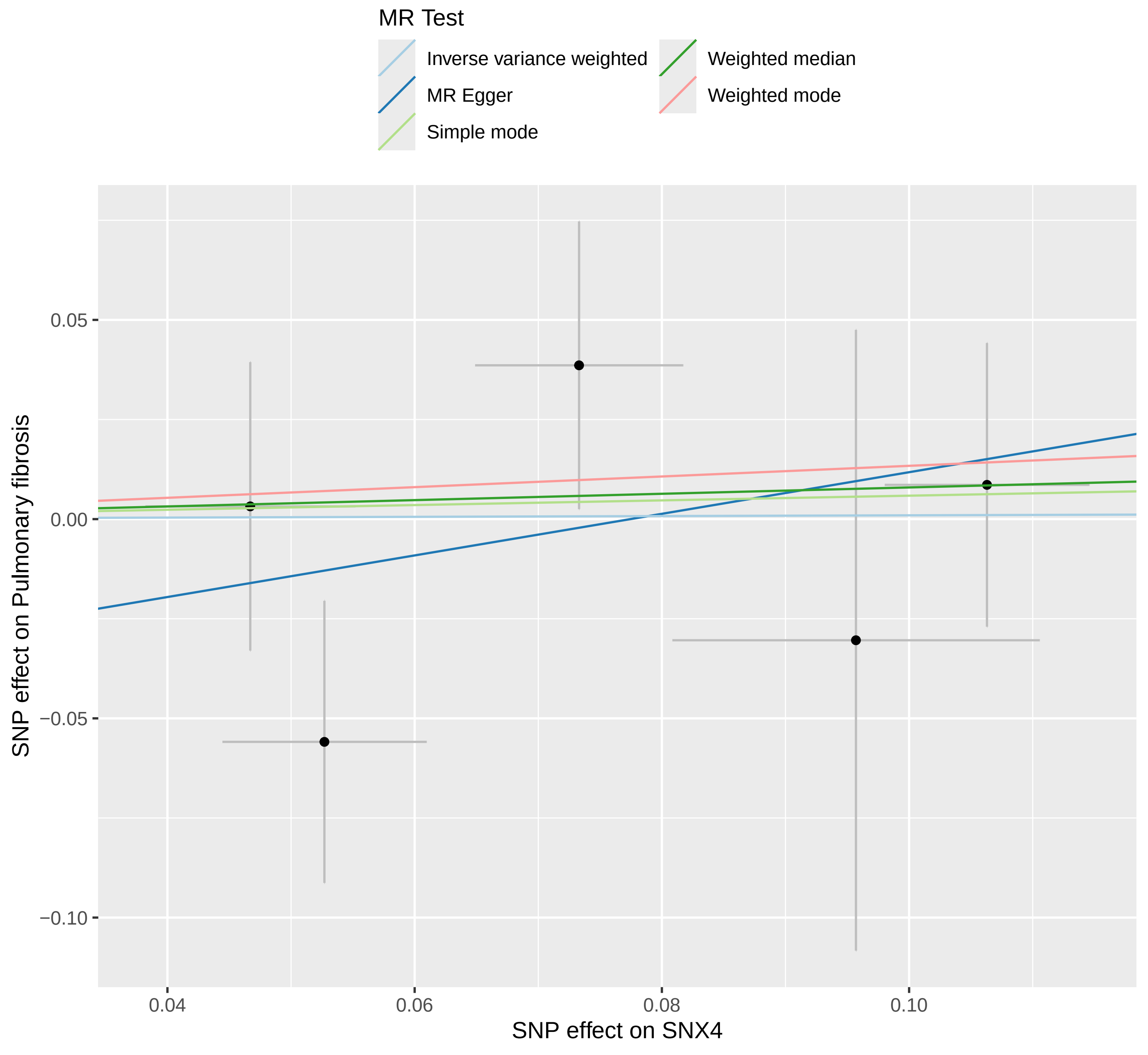

MR Test
Inverse variance weighted
MR Egger
Simple mode
Weighted median
Weighted mode
0.05
0.00
−0.05
−0.10
0.04
0.06
0.08
0.10
SNP effect on SNX4
SNP effect on Pulmonary fibrosis

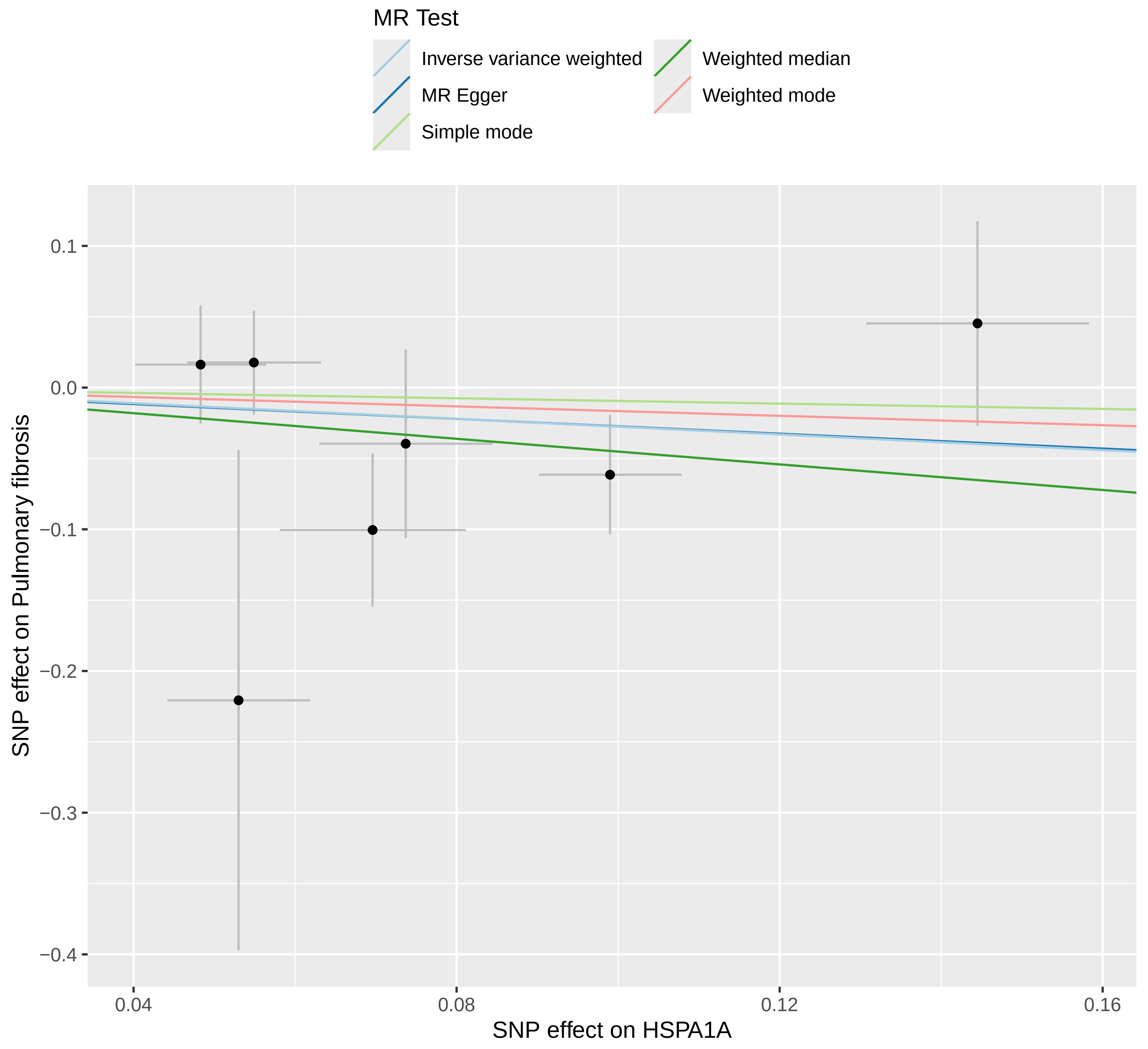

MR Test
Inverse variance weighted
MR Egger
Simple mode
Weighted median
Weighted mode
0.1
0.0
−0.1
−0.2
−0.3
−0.4
0.04
0.08
0.12
0.16
SNP effect on Pulmonary fibrosis
SNP effect on HSPA1A

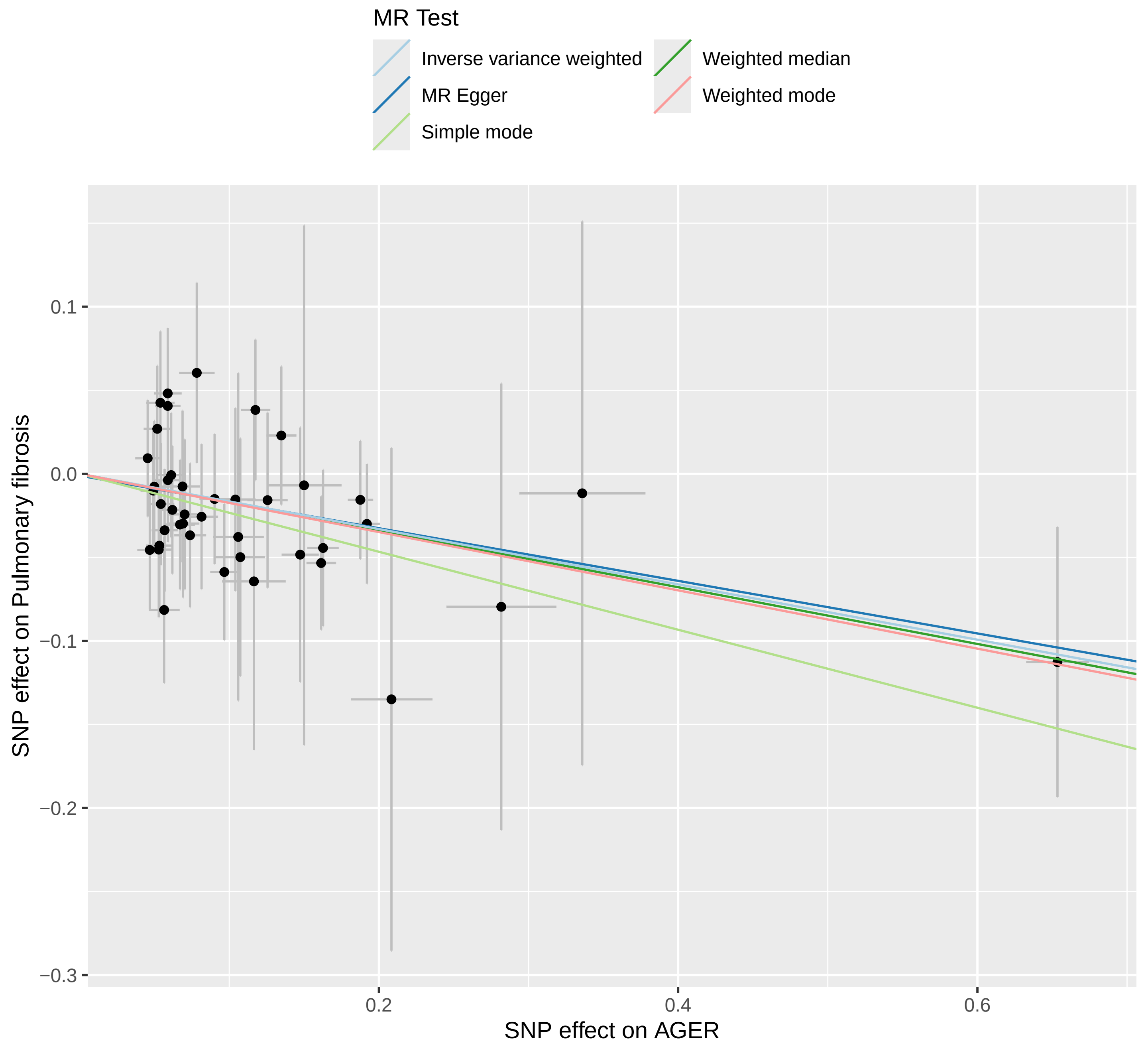

MR Test
Inverse variance weighted
MR Egger
Simple mode
Weighted median
Weighted mode
SNP effect on Pulmonary fibrosis
0.1
0.0
−0.1
−0.2
−0.3
0.2
0.4
0.6
SNP effect on AGER

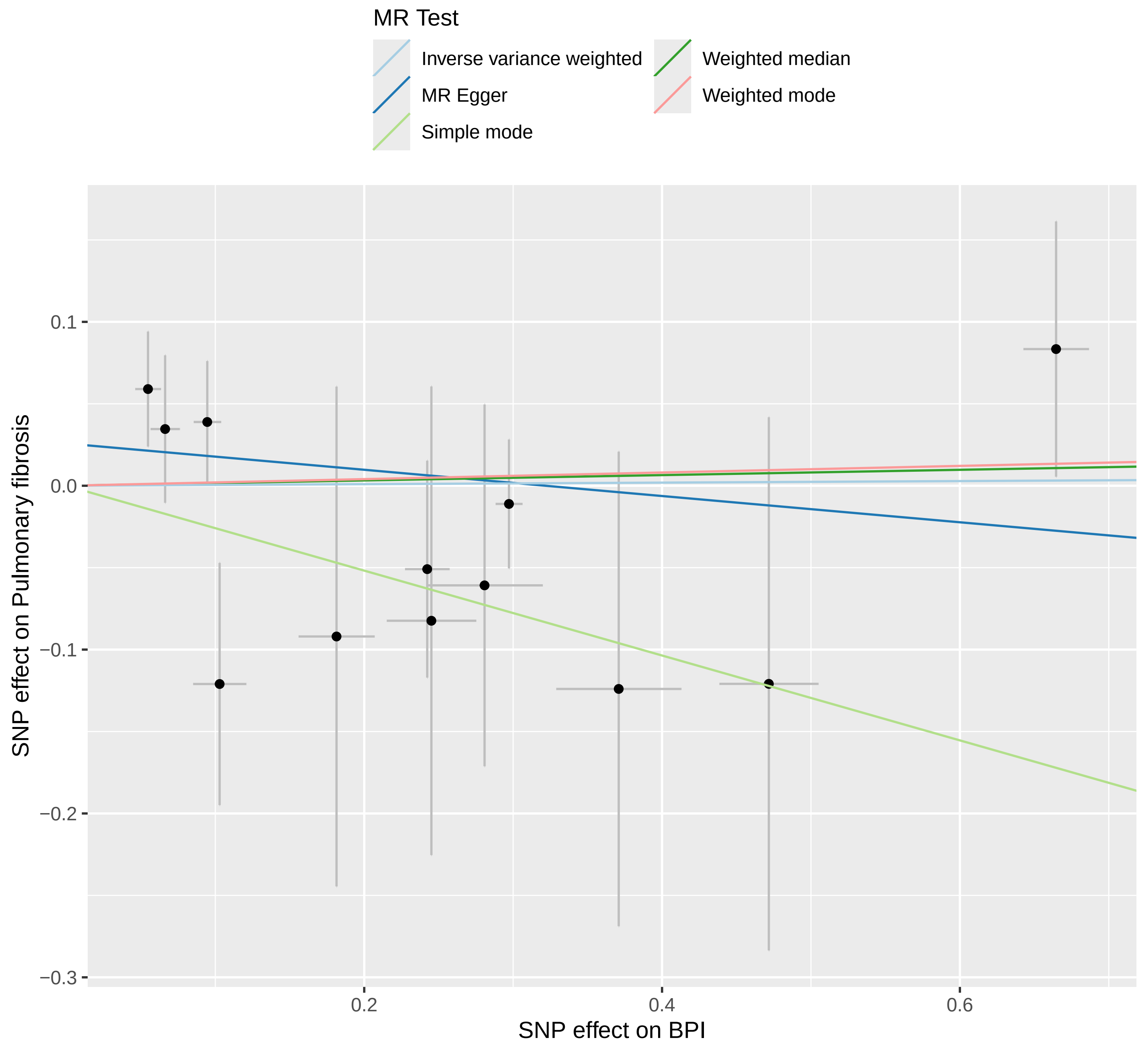

MR Test
Inverse variance weighted
MR Egger
Simple mode
Weighted median
Weighted mode
0.1
0.0
−0.1
−0.2
−0.3
SNP effect on Pulmonary fibrosis
0.2
0.4
0.6
SNP effect on BPI

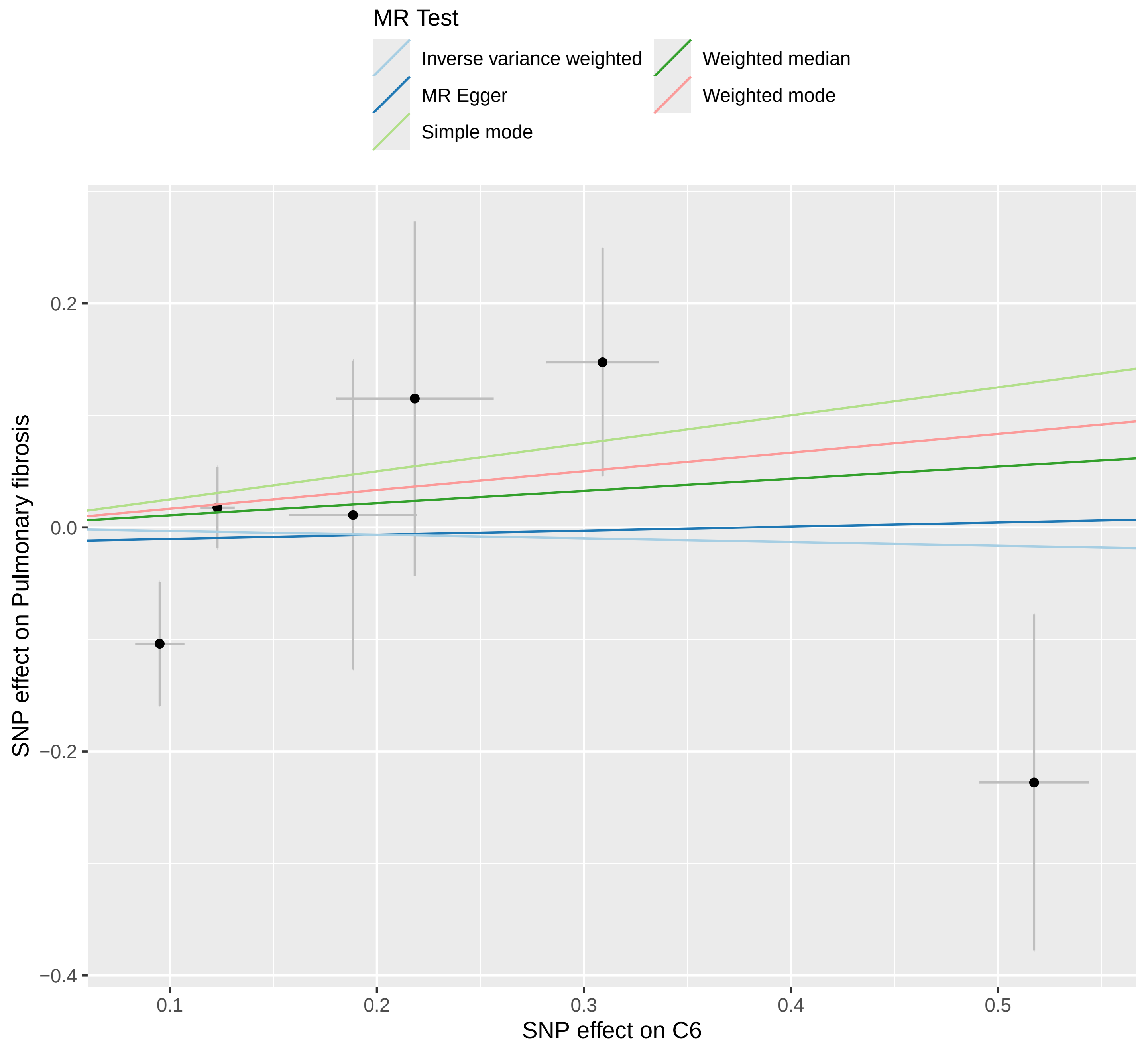
MR Test
Inverse variance weighted
MR Egger
Simple mode
Weighted median
Weighted mode
SNP effect on Pulmonary fibrosis
0.2
0.0
−0.2
−0.4
0.1
0.2
0.3
0.4
0.5
SNP effect on C6

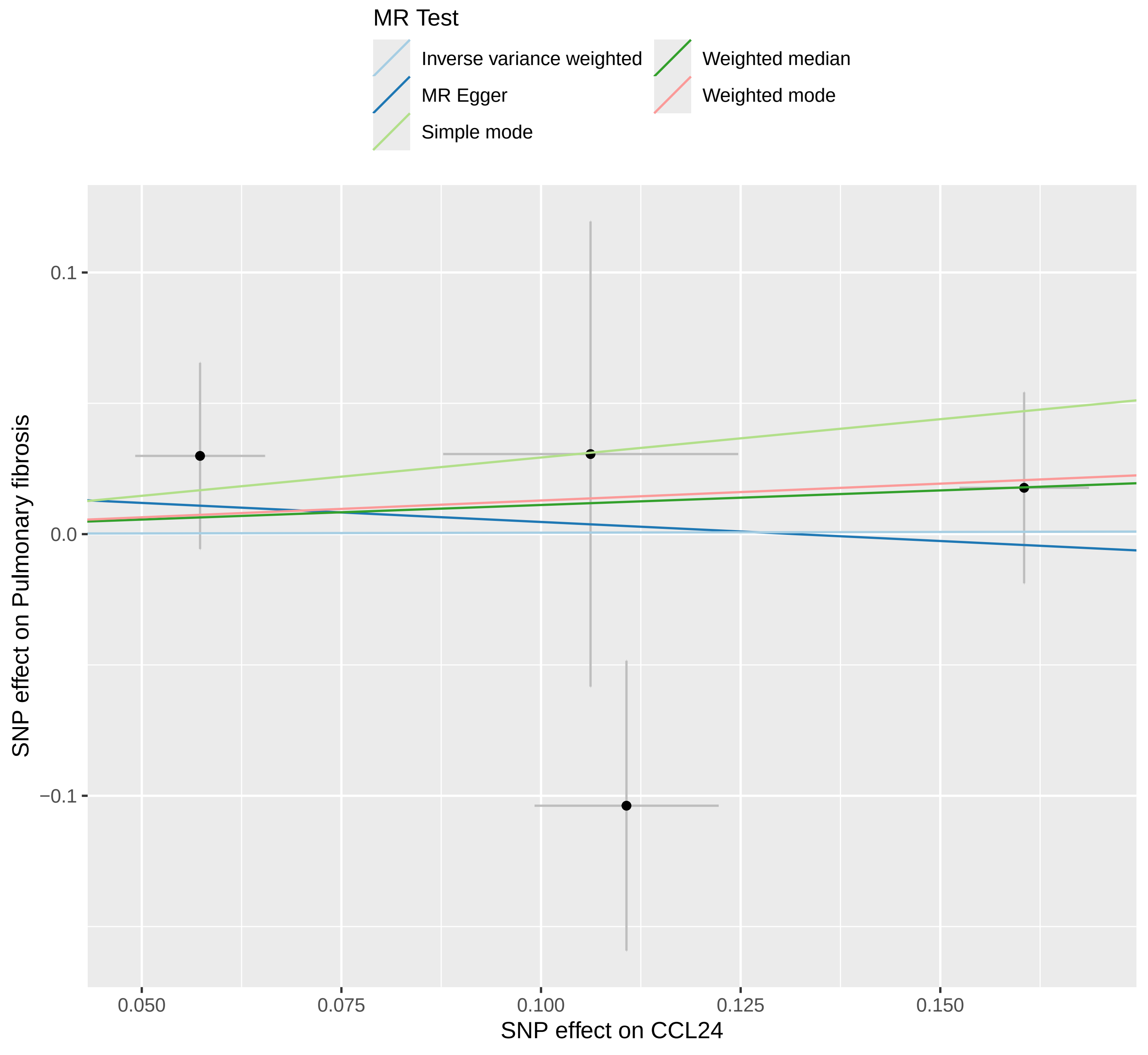

MR Test
Inverse variance weighted
MR Egger
Simple mode
Weighted median
Weighted mode
SNP effect on Pulmonary fibrosis
0.1
0.0
−0.1
0.050
0.075
0.100
0.125
0.150
SNP effect on CCL24

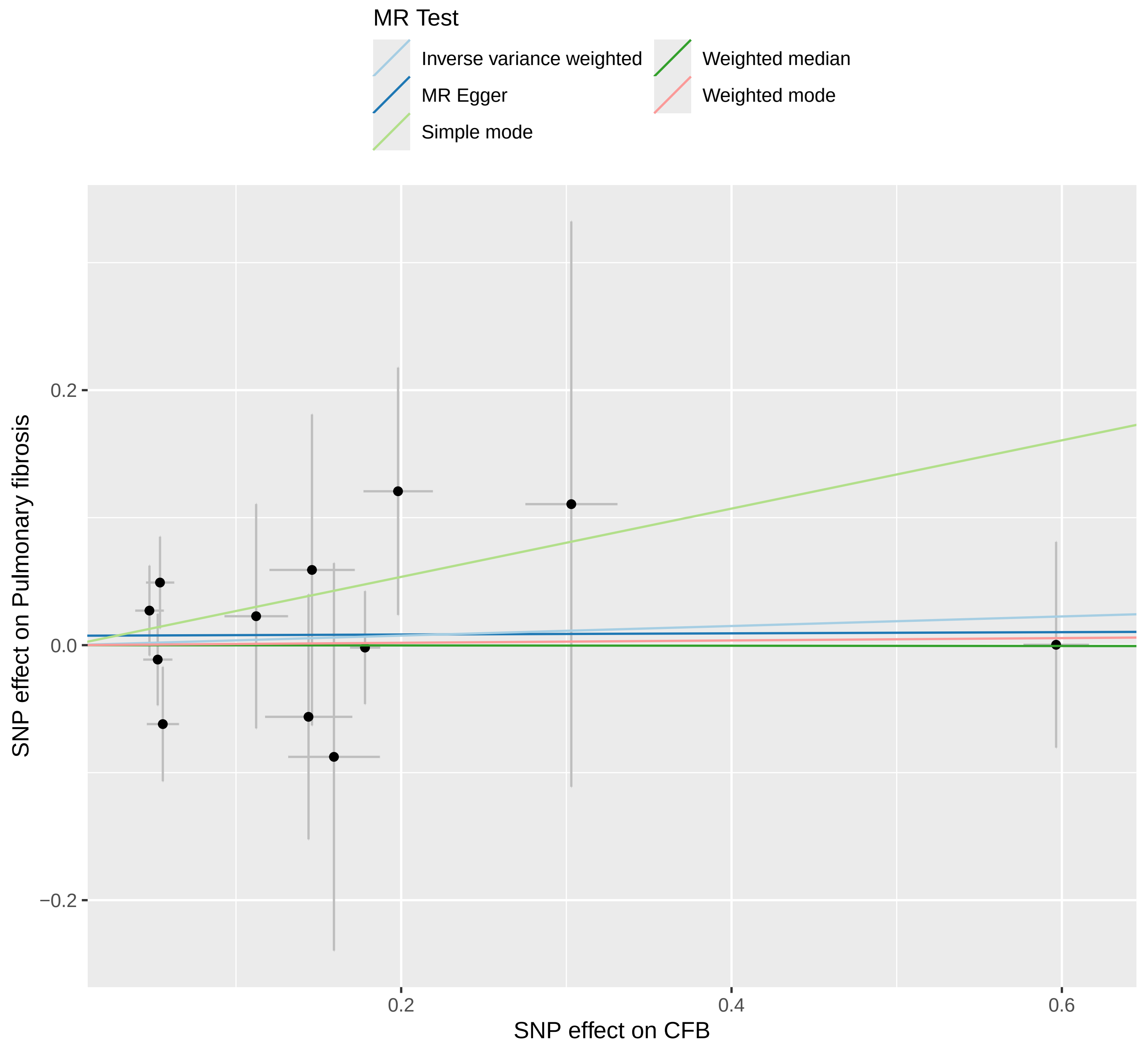
MR Test
Inverse variance weighted
MR Egger
Simple mode
Weighted median
Weighted mode
0.2
0.0
−0.2
0.2
0.4
0.6
SNP effect on Pulmonary fibrosis
SNP effect on CFB

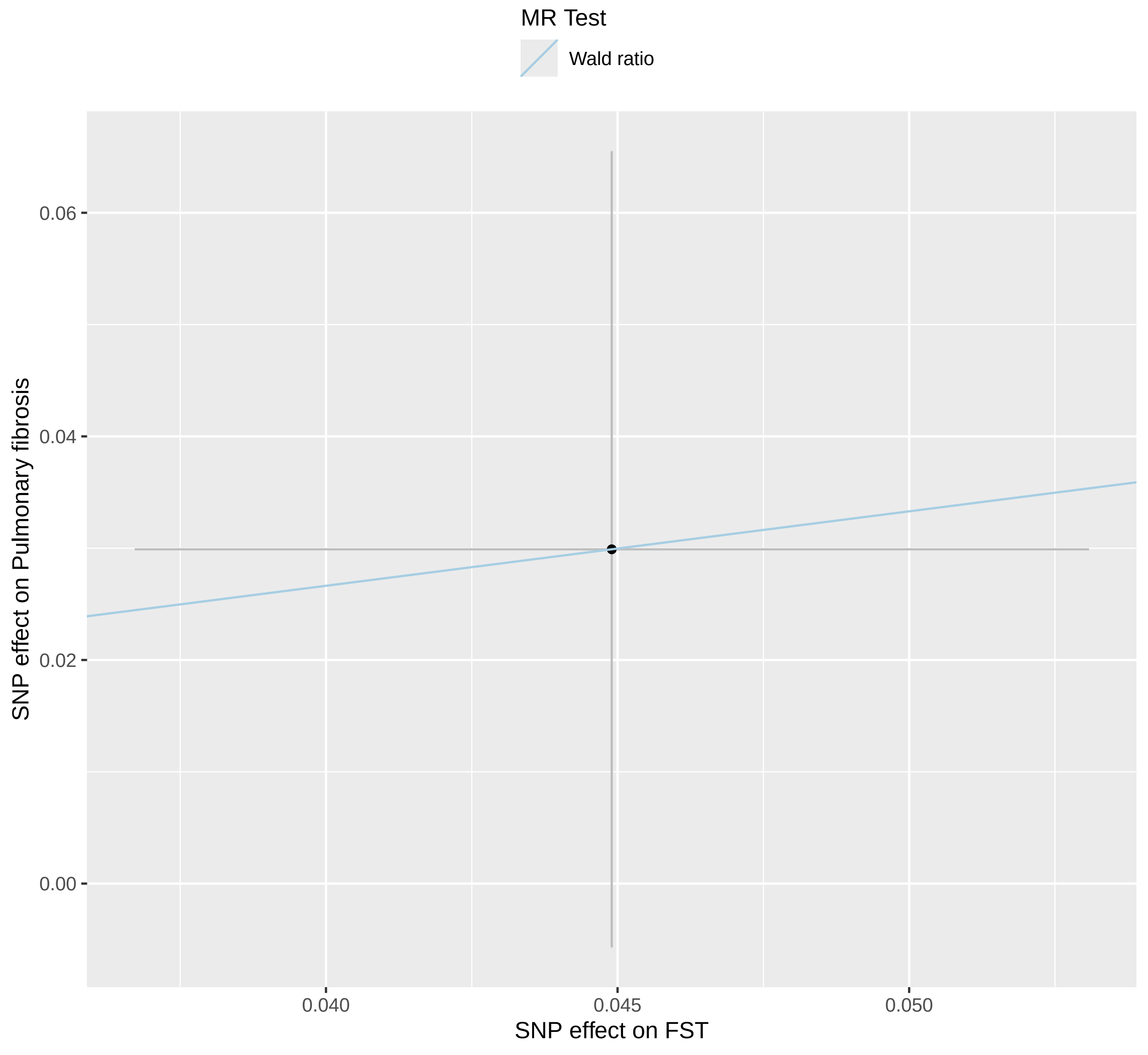

MR Test
Wald ratio
0.06
0.04
0.02
0.00
SNP effect on Pulmonary fibrosis
0.040
0.045
0.050
SNP effect on FST

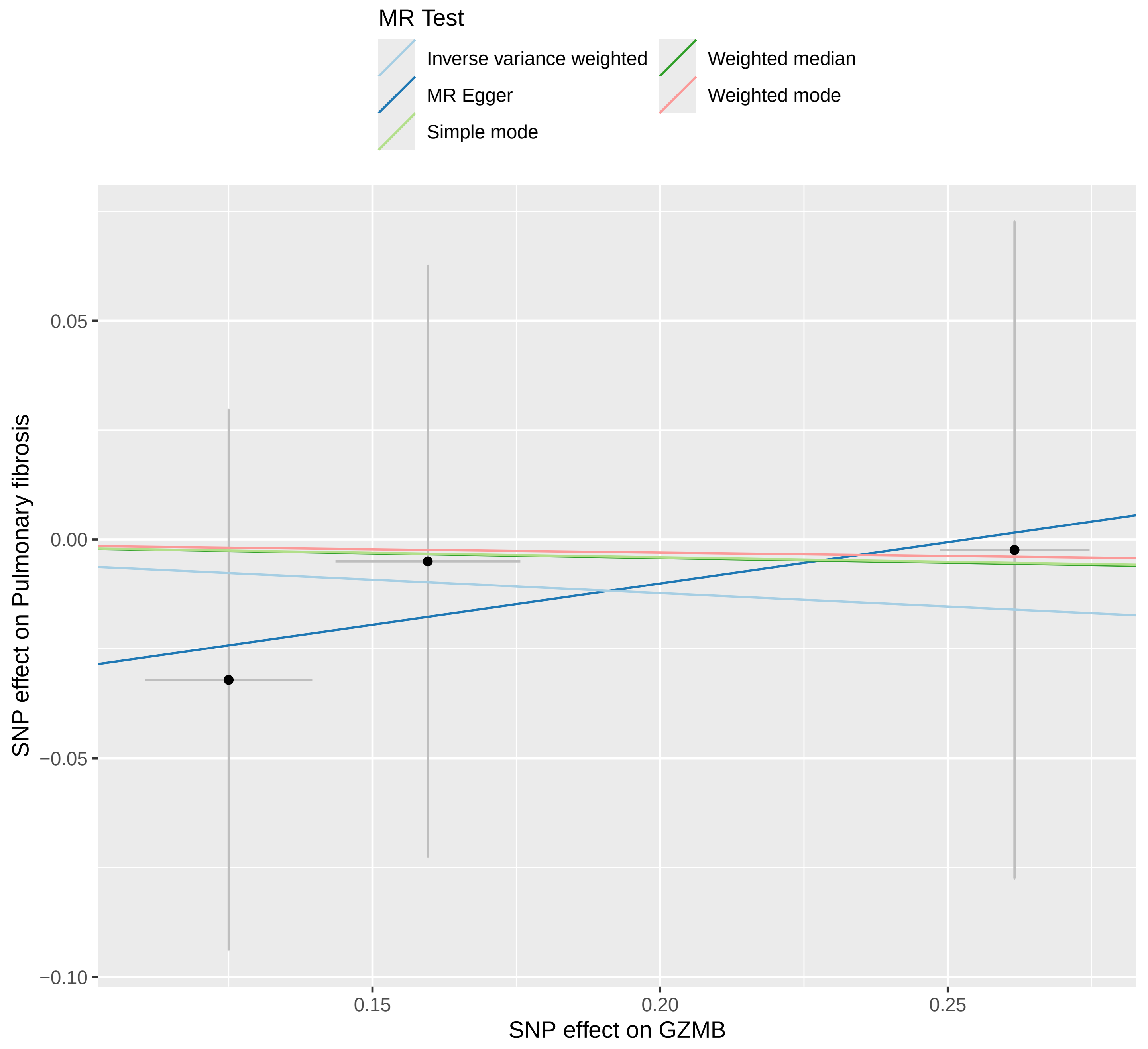
MR Test
Inverse variance weighted
MR Egger
Simple mode
Weighted median
Weighted mode
0.05
0.00
−0.05
−0.10
0.15
0.20
0.25
SNP effect on Pulmonary fibrosis
SNP effect on GZMB

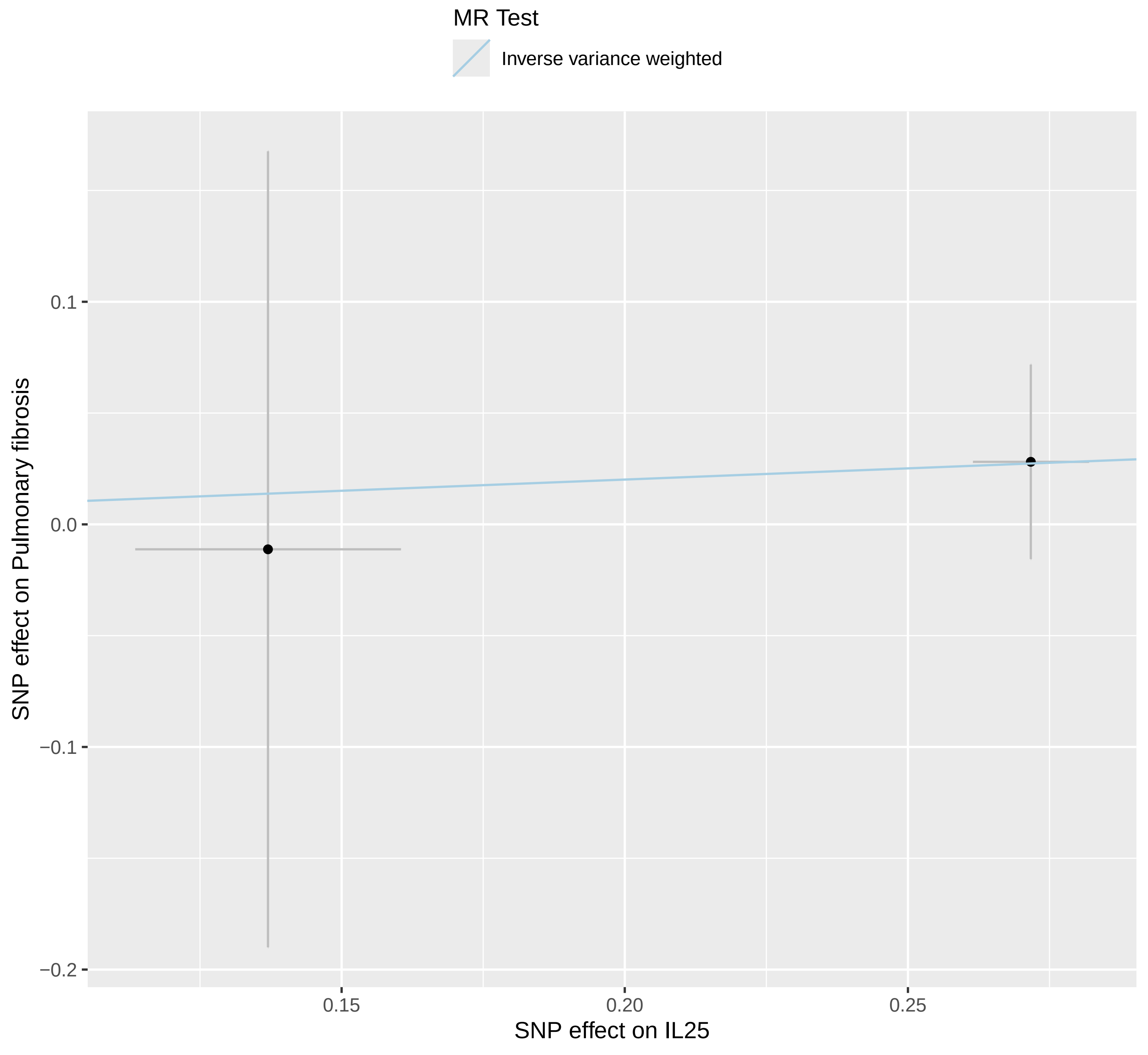

MR Test
Inverse variance weighted
0.1
0.0
−0.1
−0.2
SNP effect on Pulmonary fibrosis
0.15
0.20
0.25
SNP effect on IL25

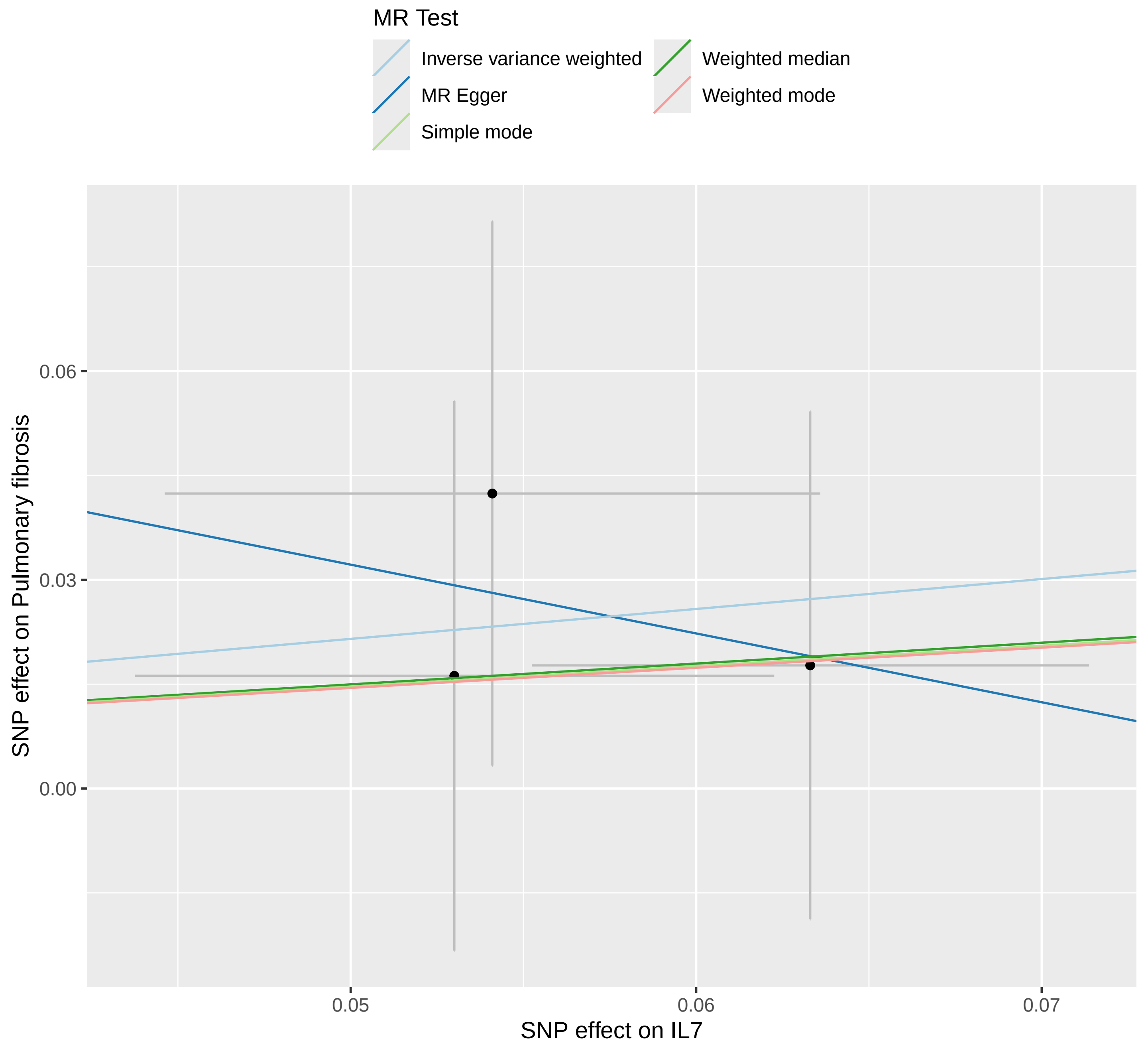

MR Test
Inverse variance weighted
MR Egger
Simple mode
Weighted median
Weighted mode
0.06
0.03
0.00
0.05
0.06
0.07
SNP effect on Pulmonary fibrosis
SNP effect on IL7

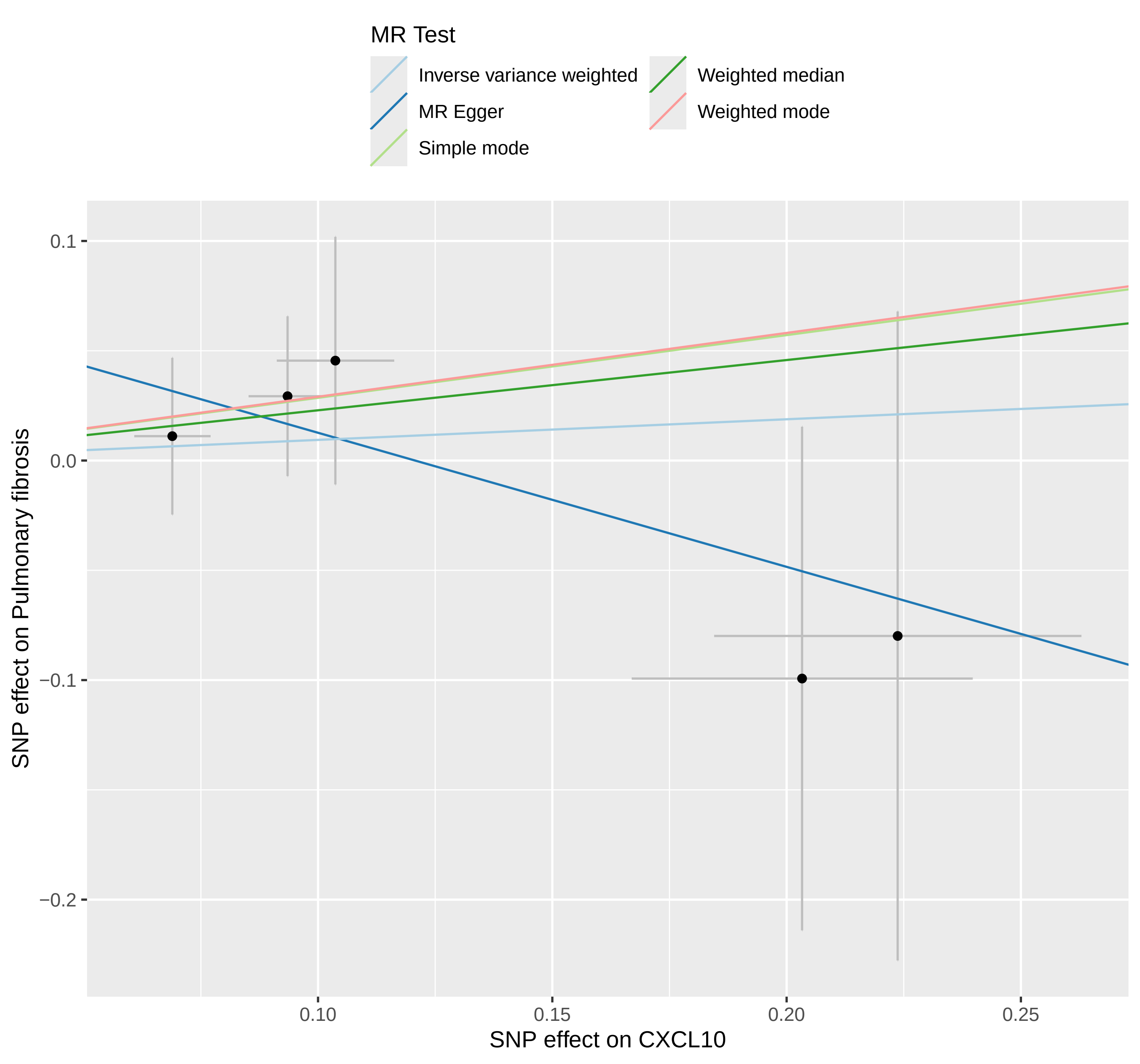
MR Test
Inverse variance weighted
MR Egger
Simple mode
Weighted median
Weighted mode
0.1
0.0
−0.1
−0.2
0.10
0.15
0.20
0.25
SNP effect on Pulmonary fibrosis
SNP effect on CXCL10

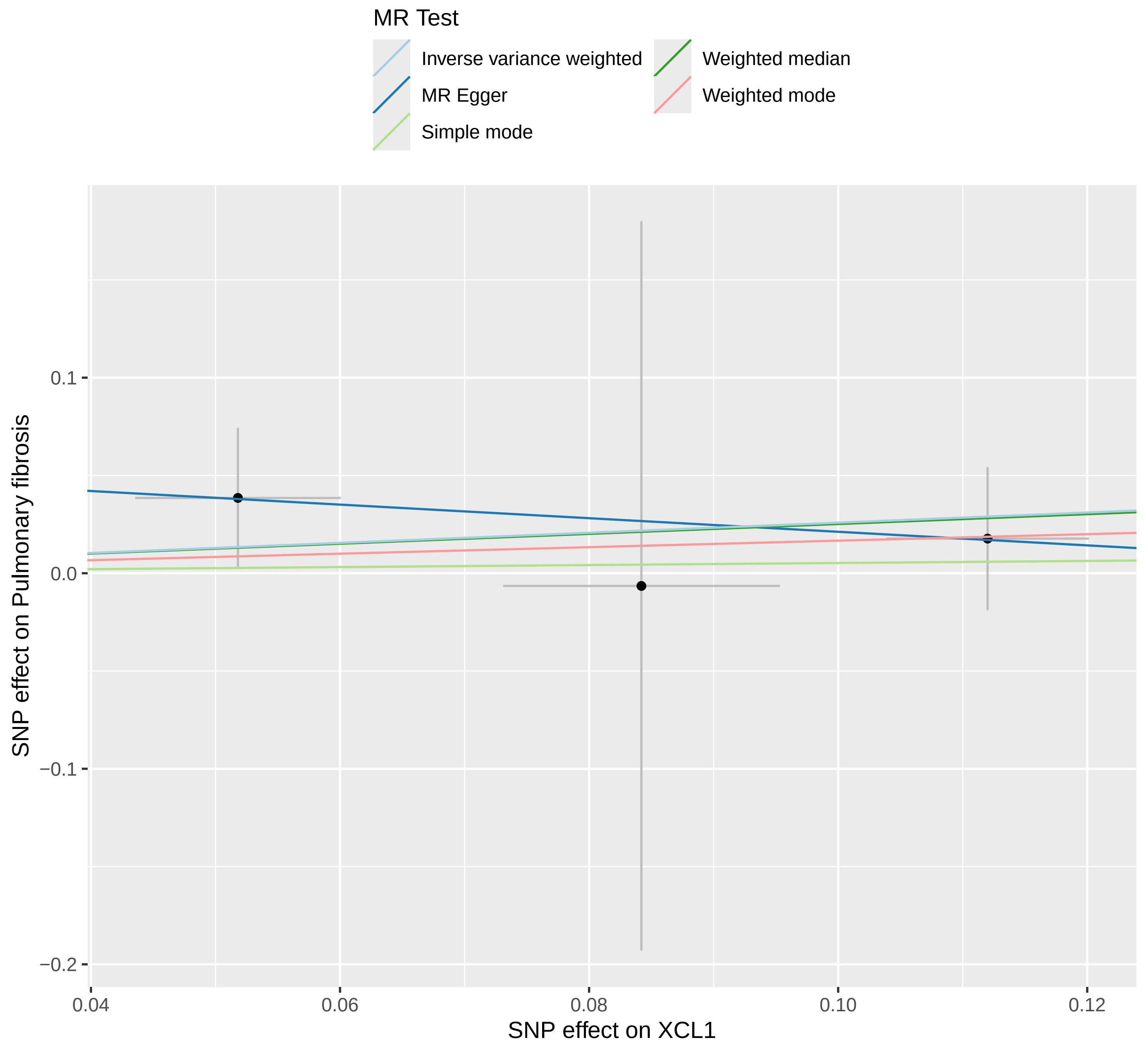

MR Test
Inverse variance weighted
MR Egger
Simple mode
Weighted median
Weighted mode
SNP effect on Pulmonary fibrosis
0.1
0.0
−0.1
−0.2
0.04
0.06
0.08
0.10
0.12
SNP effect on XCL1

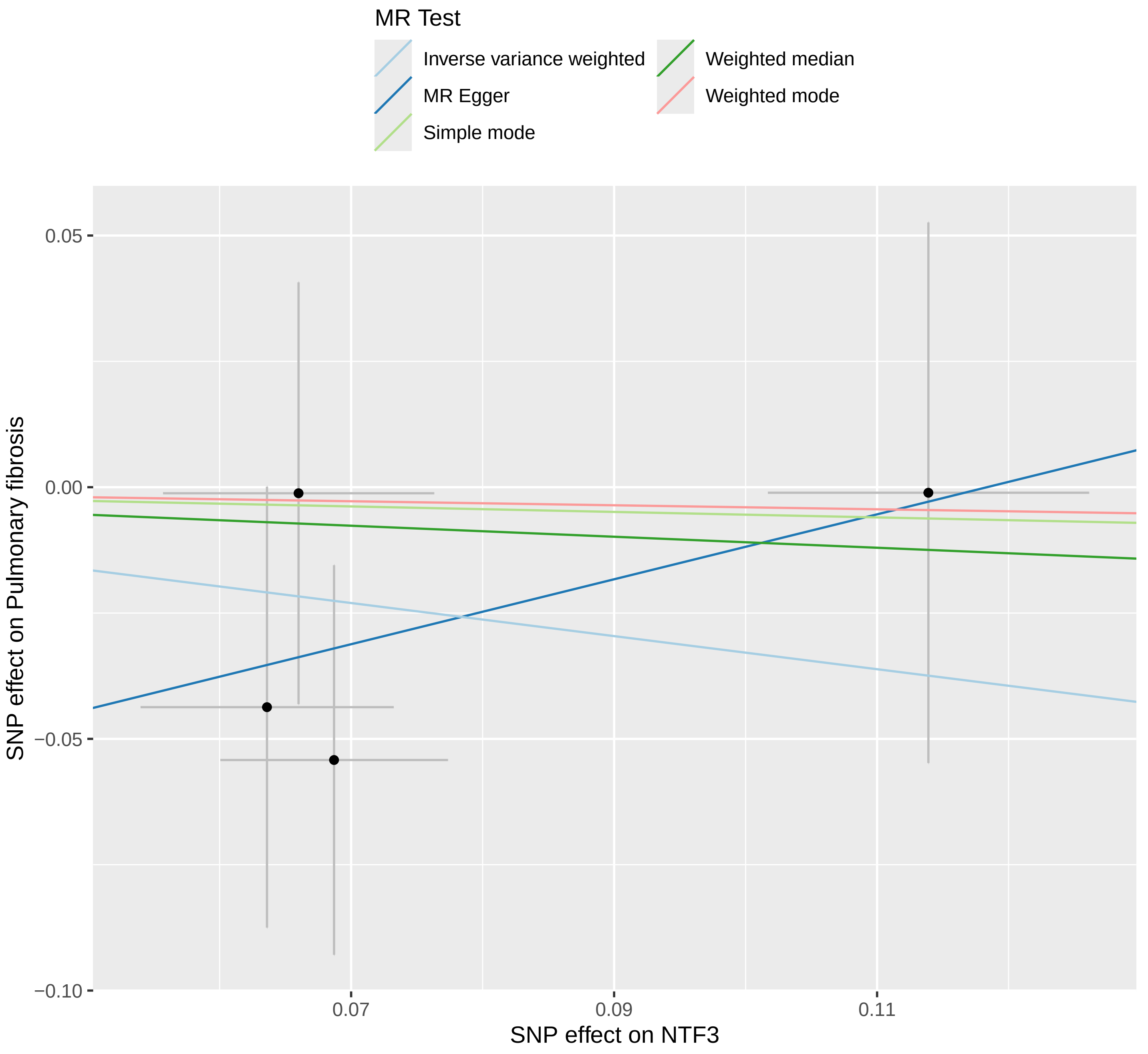
MR Test
Inverse variance weighted
MR Egger
Simple mode
Weighted median
Weighted mode
0.05
0.00
−0.05
−0.10
0.07
0.09
0.11
SNP effect on Pulmonary fibrosis
SNP effect on NTF3

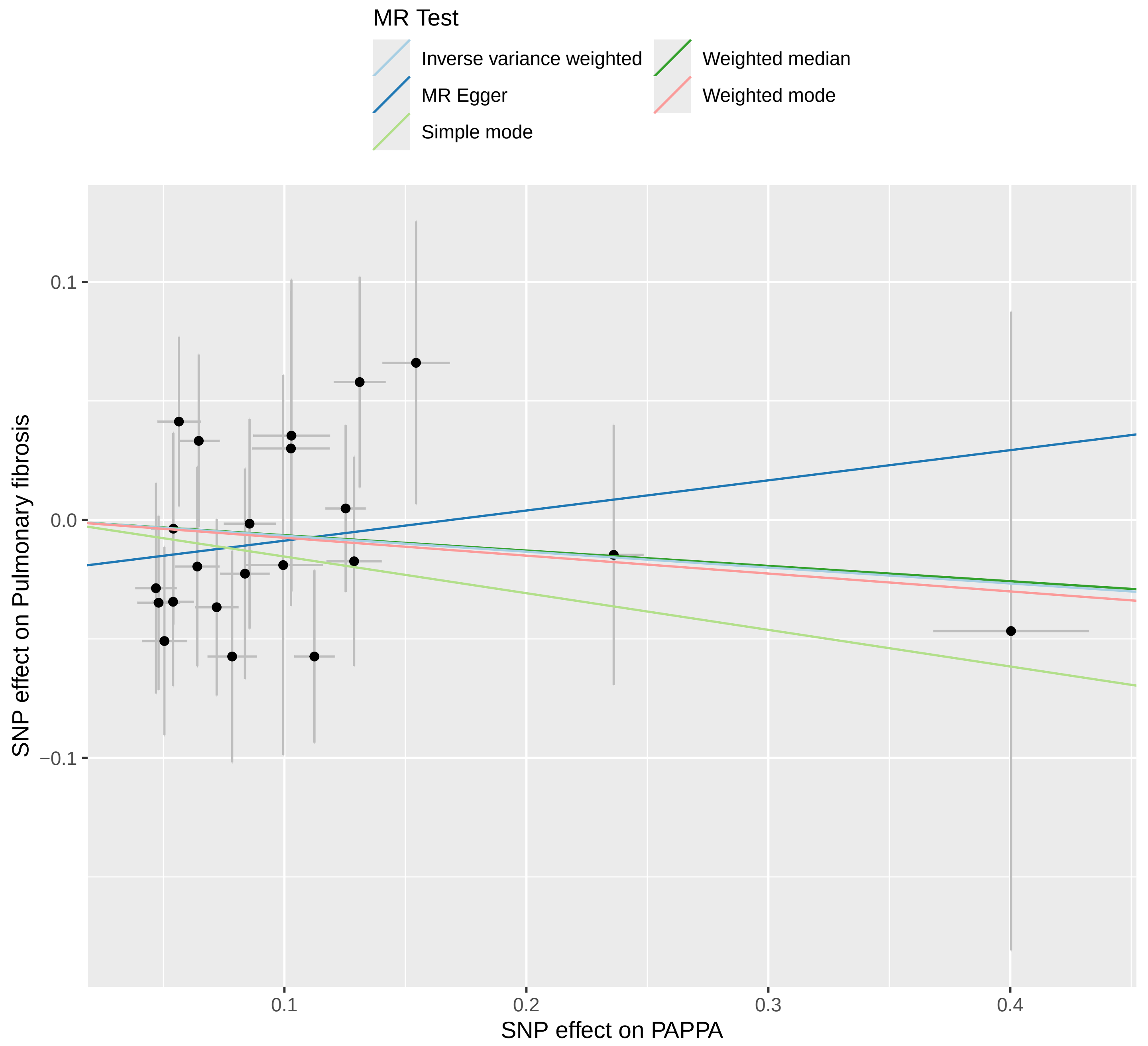

MR Test
Inverse variance weighted
MR Egger
Simple mode
Weighted median
Weighted mode
0.1
0.0
−0.1
SNP effect on Pulmonary fibrosis
0.1
0.2
0.3
0.4
SNP effect on PAPPA

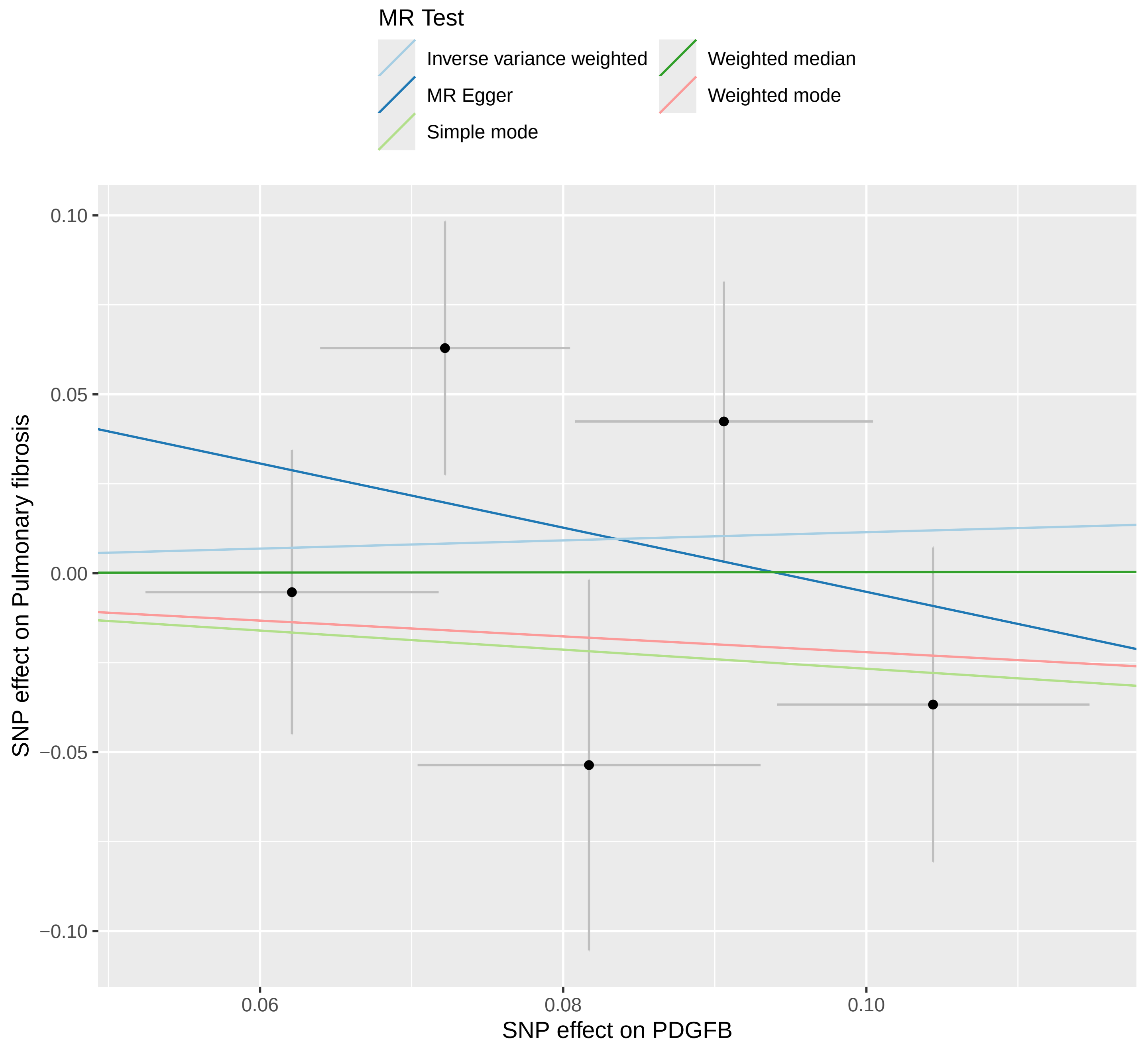

MR Test
Inverse variance weighted
MR Egger
Simple mode
Weighted median
Weighted mode
0.10
0.05
0.00
−0.05
−0.10
SNP effect on Pulmonary fibrosis
0.06
0.08
0.10
SNP effect on PDGFB

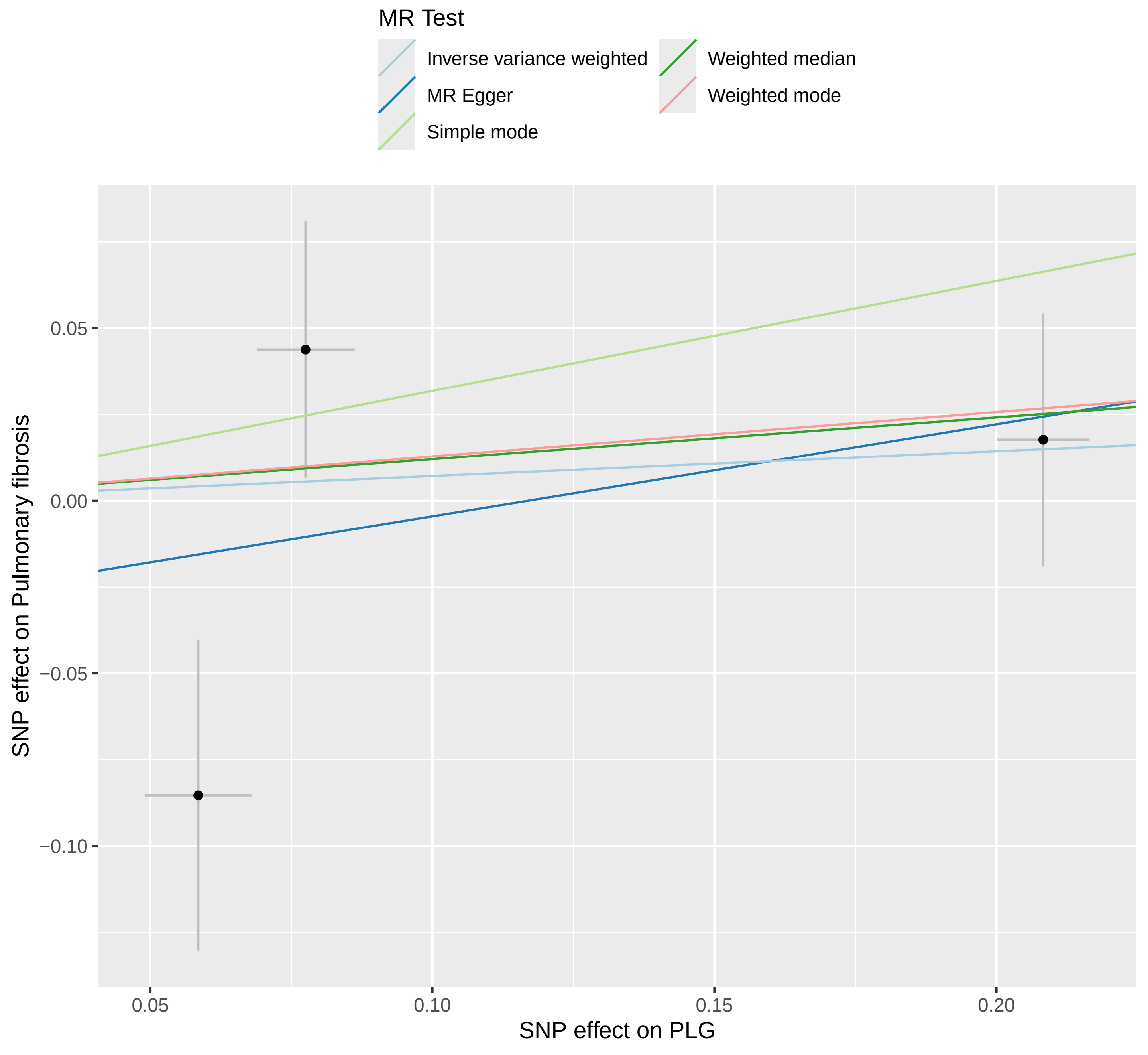
MR Test
Inverse variance weighted
MR Egger
Simple mode
Weighted median
Weighted mode
0.05
0.00
−0.05
−0.10
SNP effect on Pulmonary fibrosis
0.05
0.10
0.15
0.20
SNP effect on PLG

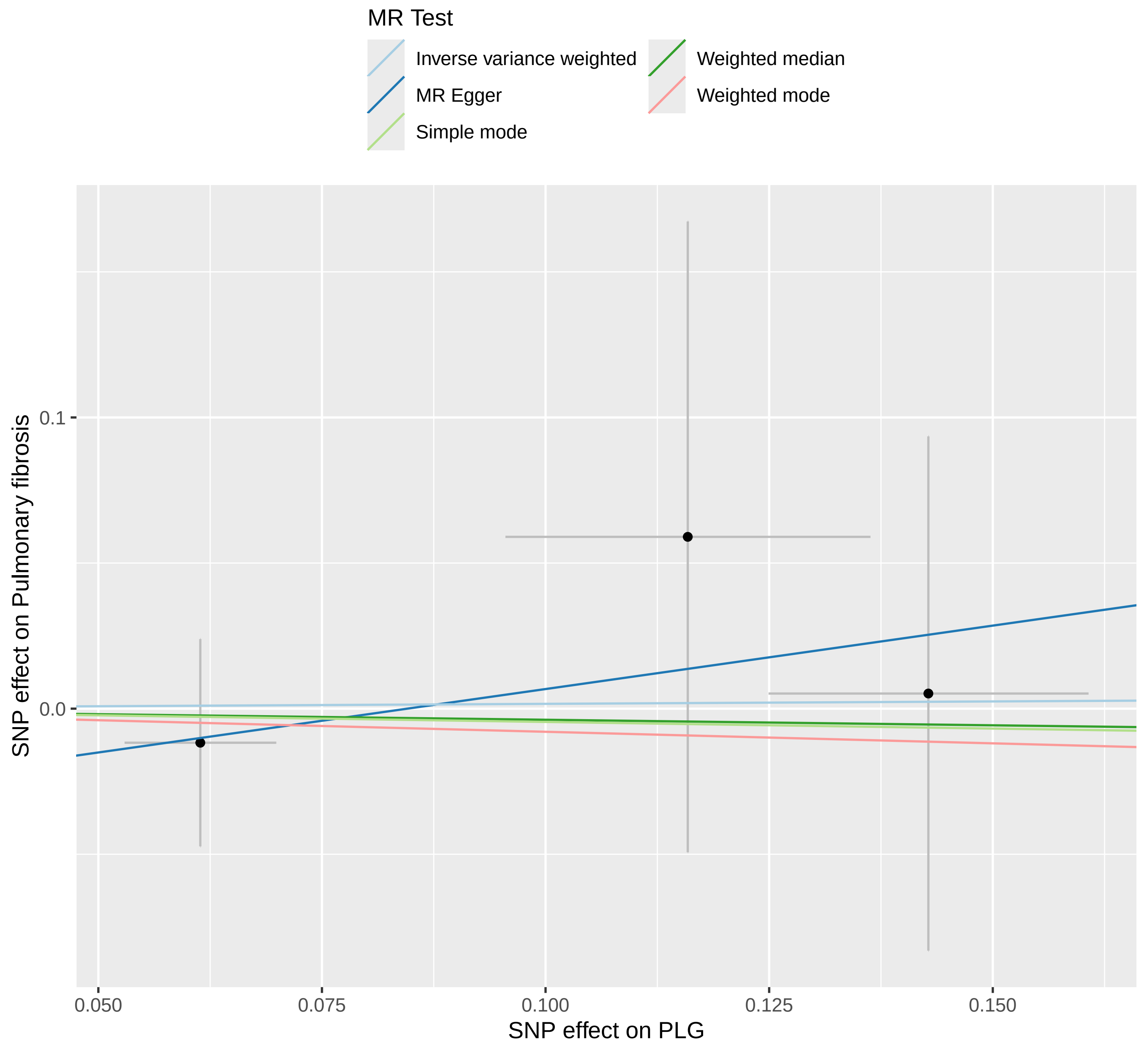

MR Test
Inverse variance weighted
MR Egger
Simple mode
Weighted median
Weighted mode
SNP effect on Pulmonary fibrosis
0.1
0.0
0.050
0.075
0.100
0.125
0.150
SNP effect on PLG

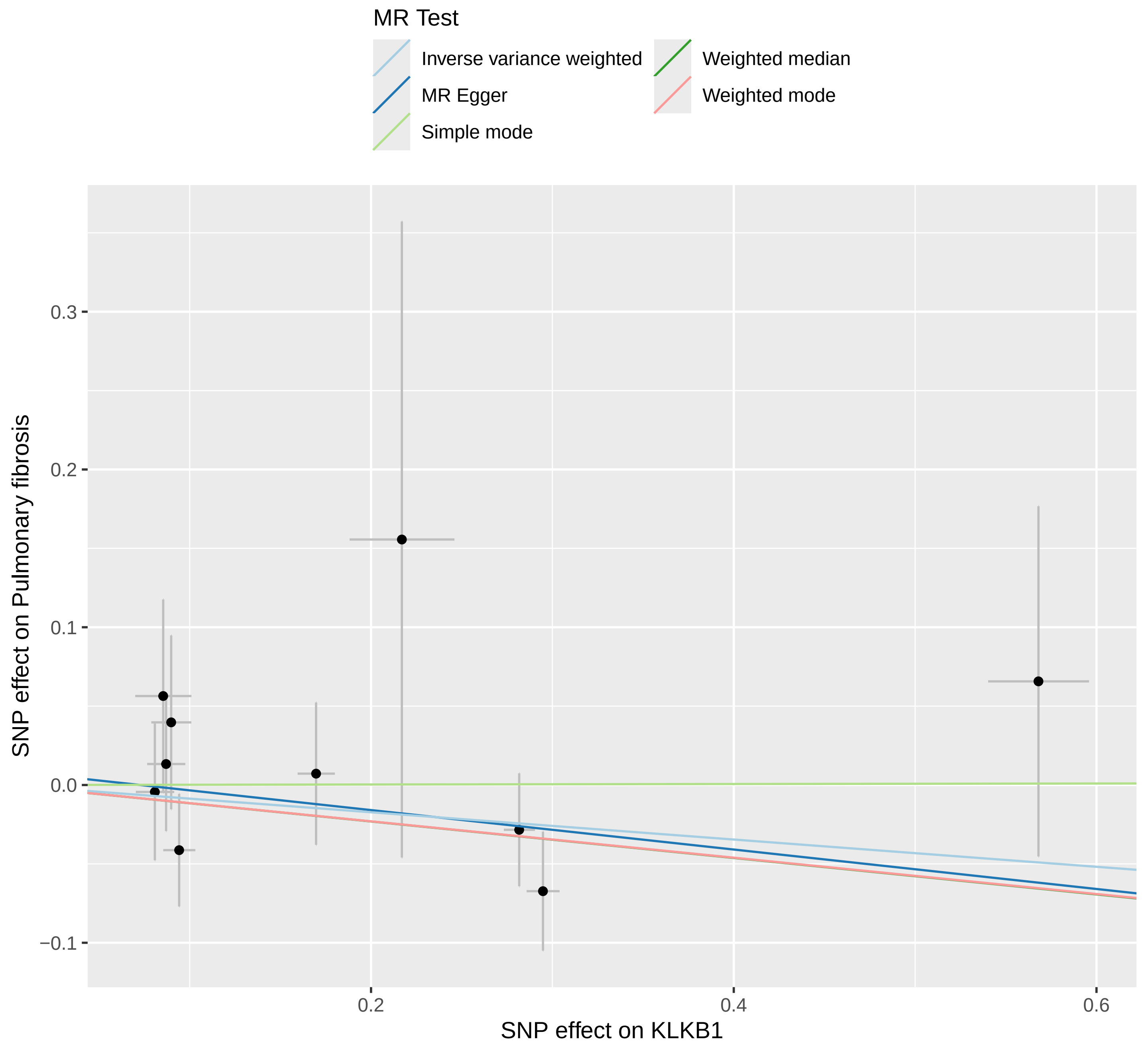
MR Test
Inverse variance weighted
MR Egger
Simple mode
Weighted median
Weighted mode
SNP effect on Pulmonary fibrosis
SNP effect on KLKB1
0.3
0.2
0.1
0.0
−0.1
0.2
0.4
0.6

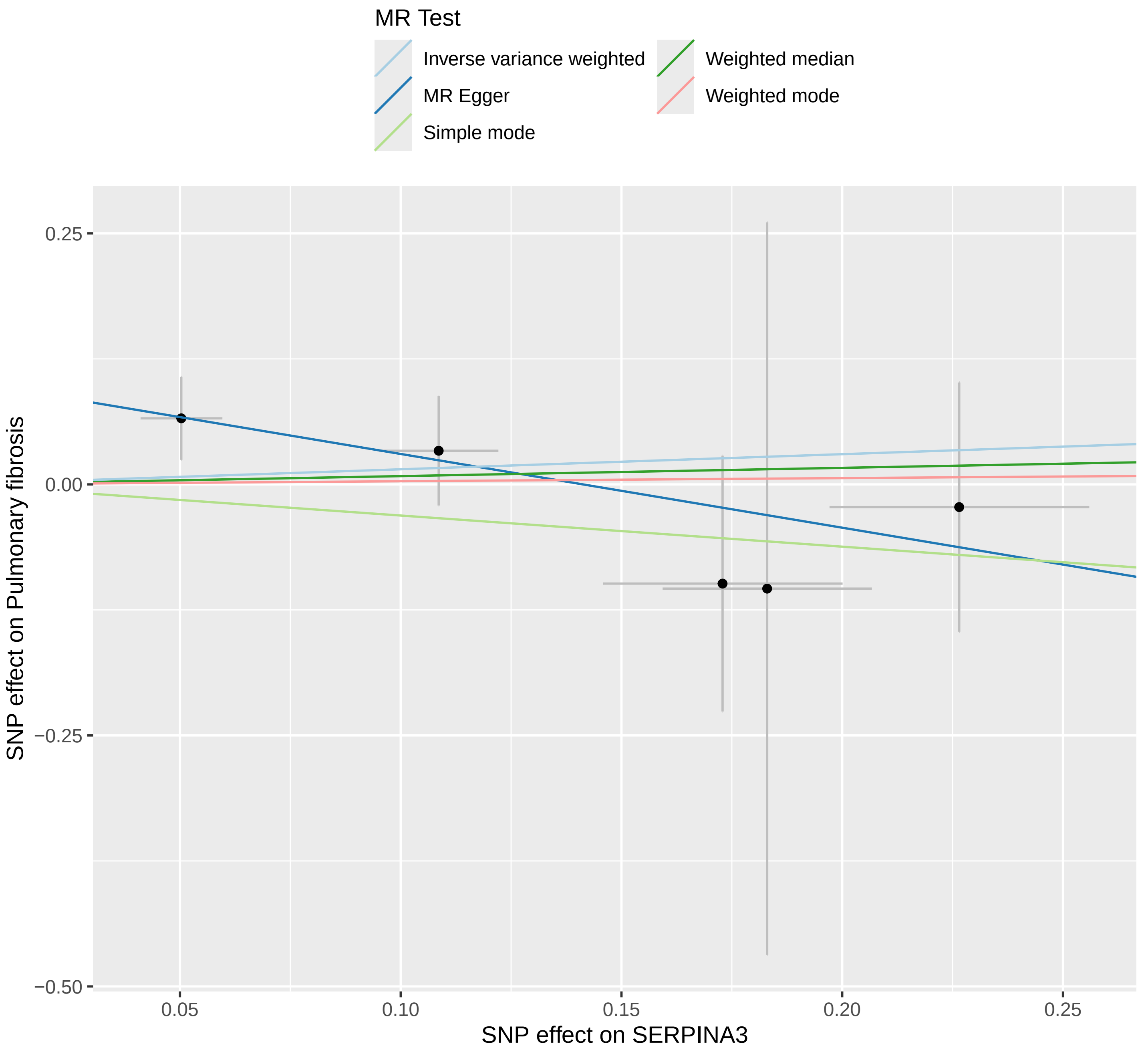

MR Test
Inverse variance weighted
MR Egger
Simple mode
Weighted median
Weighted mode
SNP effect on Pulmonary fibrosis
0.25
0.00
−0.25
−0.50
0.05
0.10
0.15
0.20
0.25
SNP effect on SERPINA3

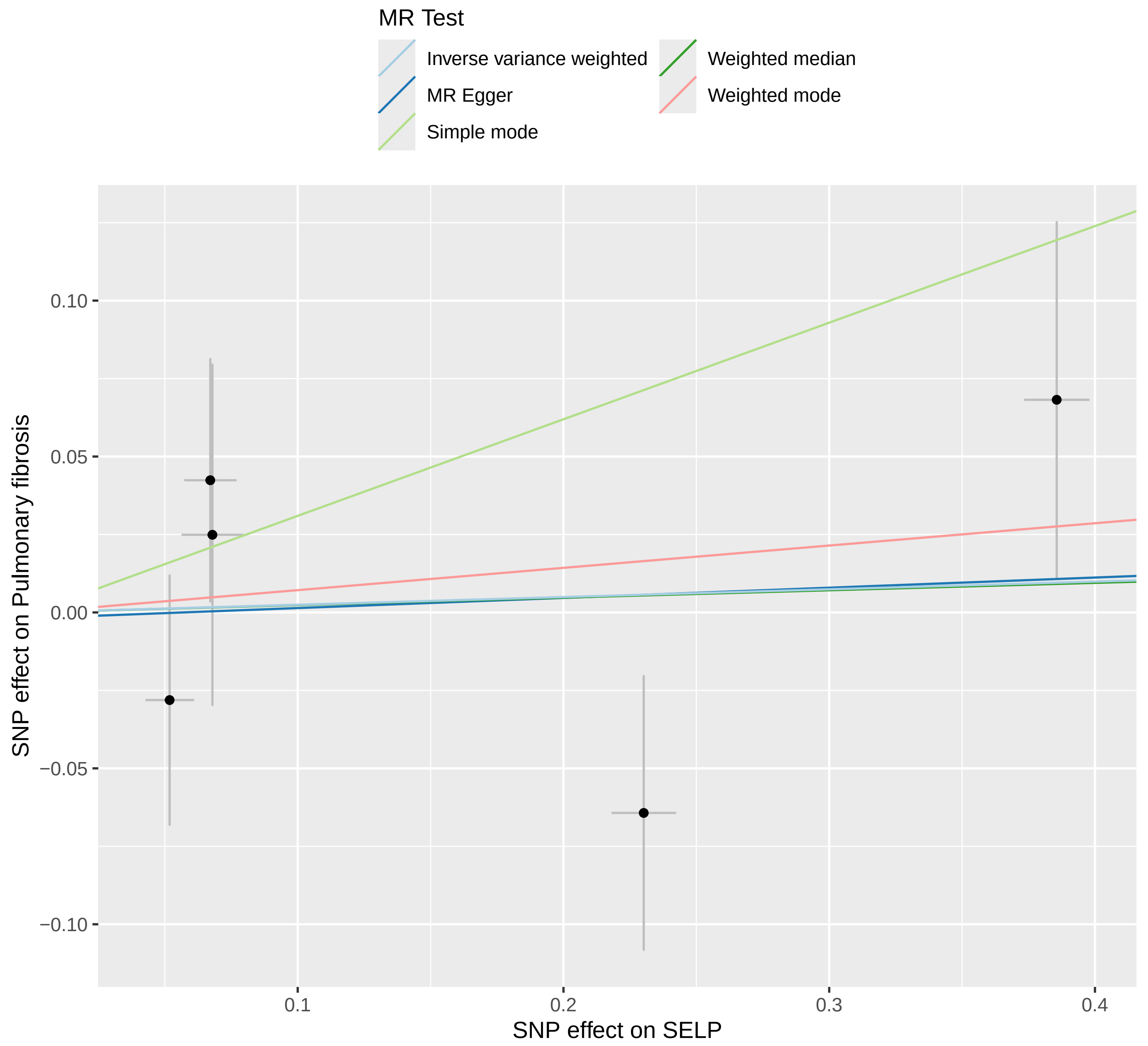

MR Test
Inverse variance weighted
MR Egger
Simple mode
Weighted median
Weighted mode
SNP effect on Pulmonary fibrosis
0.10
0.05
0.00
−0.05
−0.10
0.1
0.2
0.3
0.4
SNP effect on SELP

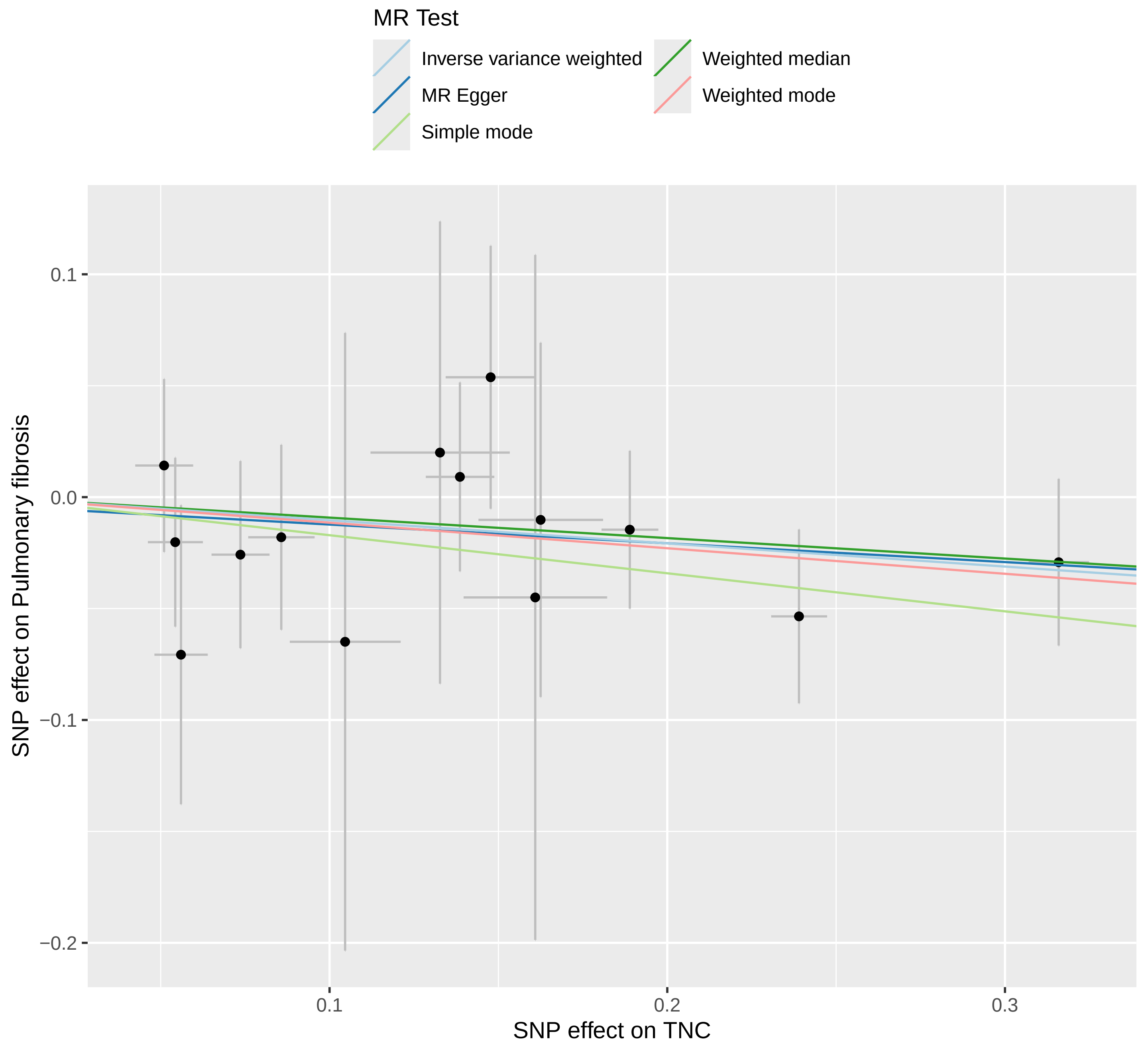
MR Test
Inverse variance weighted
MR Egger
Simple mode
Weighted median
Weighted mode
SNP effect on Pulmonary fibrosis
0.1
0.0
−0.1
−0.2
0.1
0.2
0.3
SNP effect on TNC

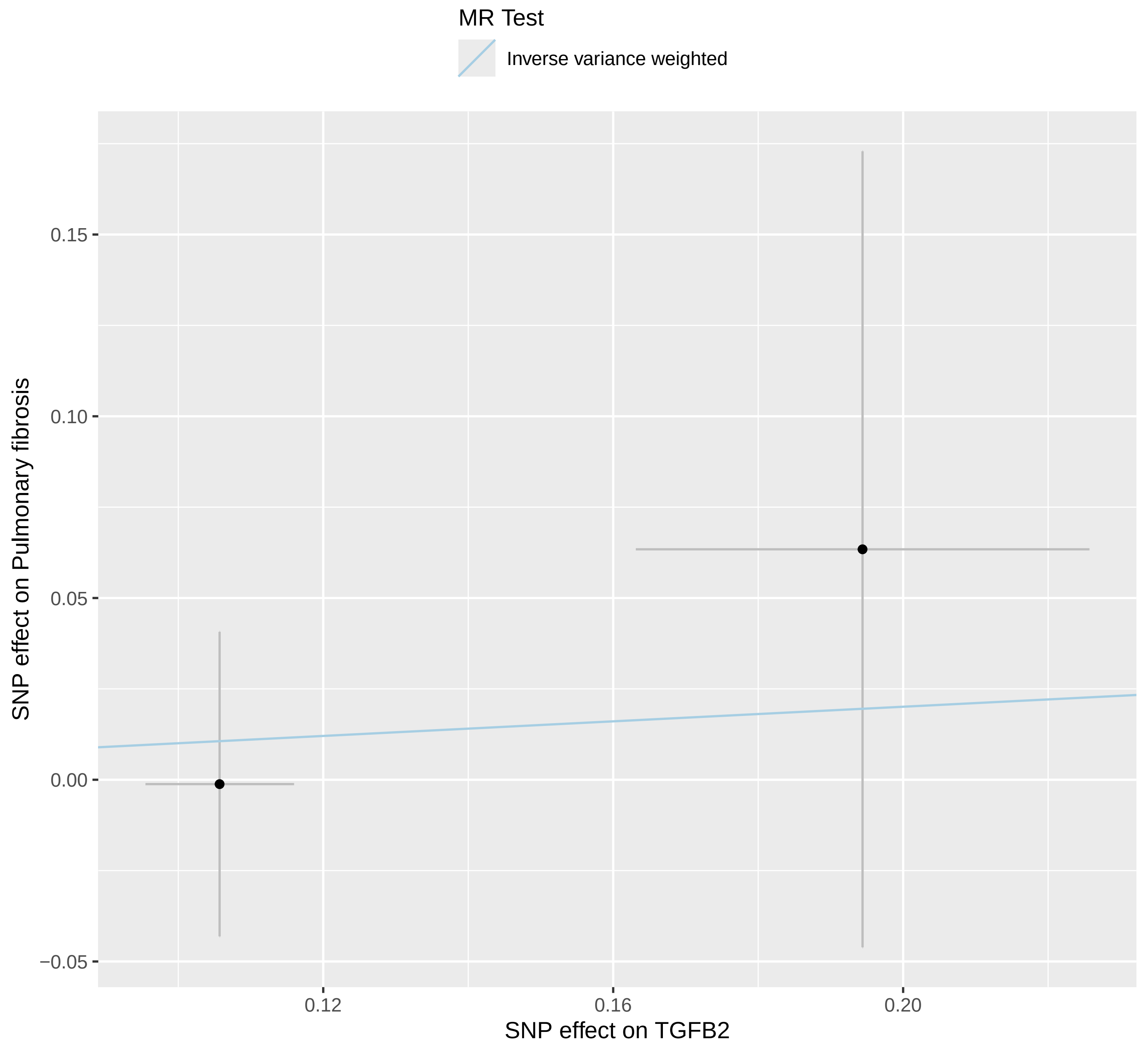
MR Test
Inverse variance weighted
SNP effect on Pulmonary fibrosis
0.15
0.10
0.05
0.00
−0.05
0.12
0.16
0.20
SNP effect on TGFB2

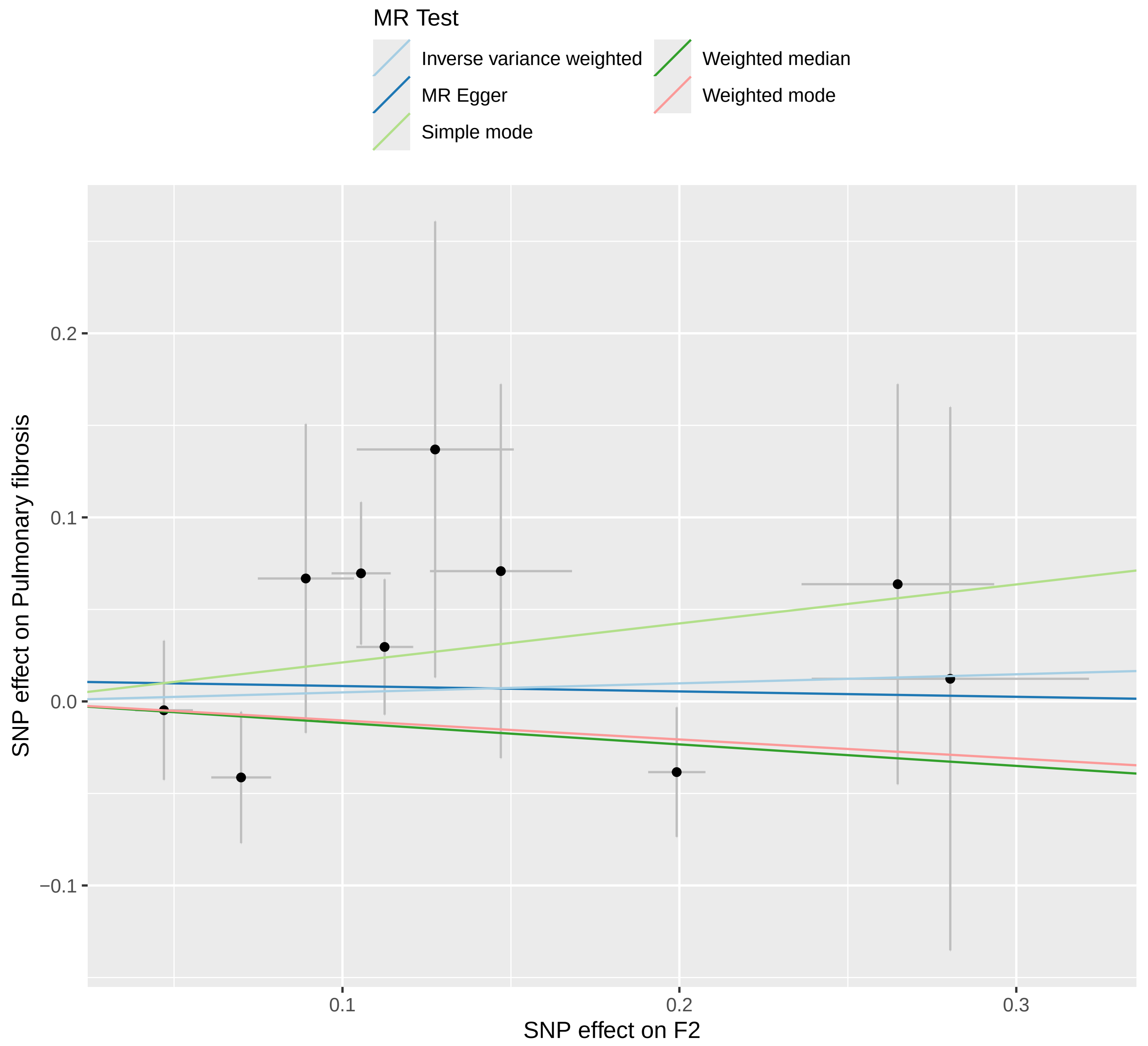
MR Test
Inverse variance weighted
MR Egger
Simple mode
Weighted median
Weighted mode
0.2
0.1
0.0
−0.1
0.1
0.2
0.3
SNP effect on Pulmonary fibrosis
SNP effect on F2

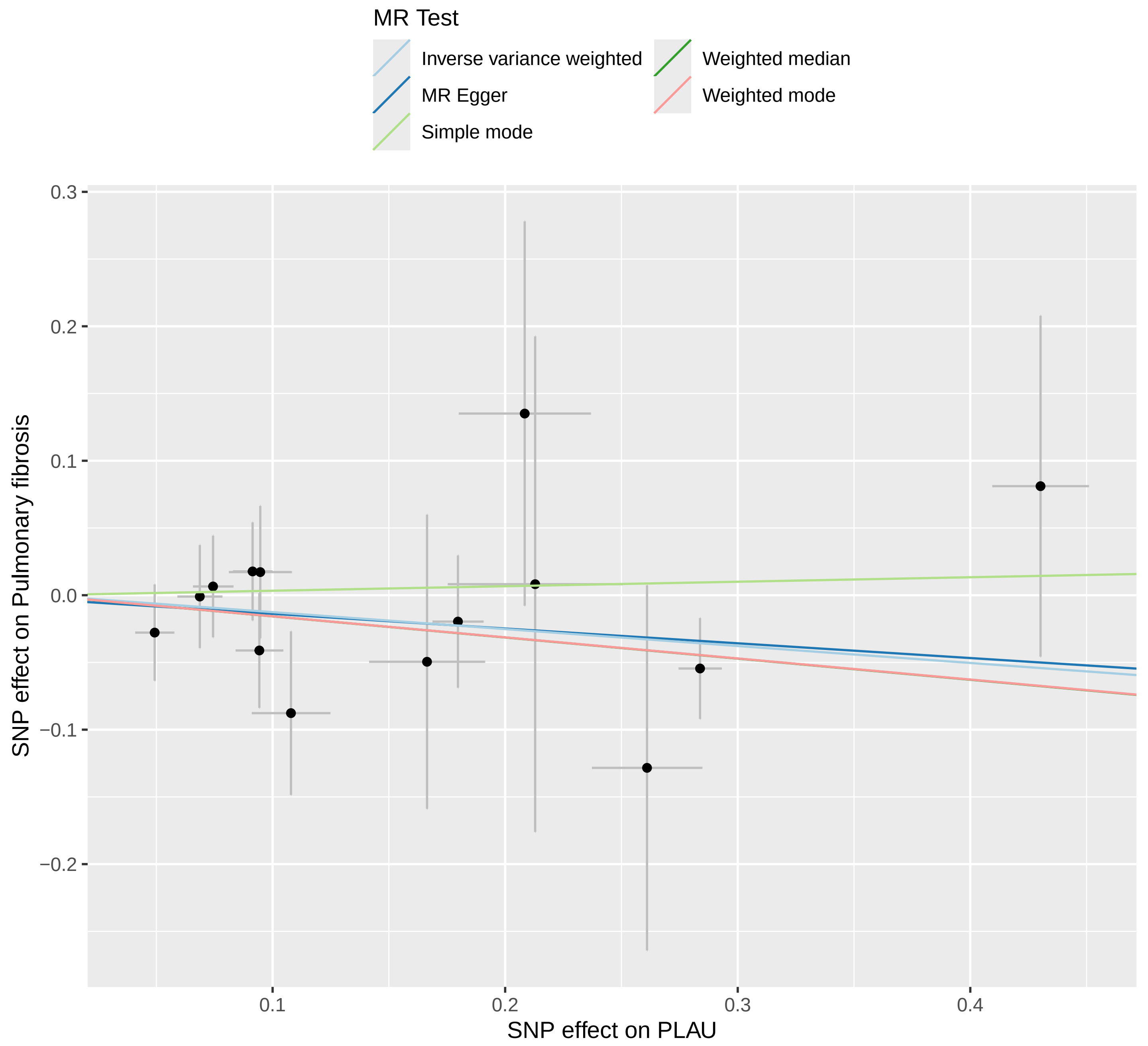
MR Test
Inverse variance weighted
MR Egger
Simple mode
Weighted median
Weighted mode
0.3
0.2
0.1
0.0
−0.1
−0.2
0.1
0.2
0.3
0.4
SNP effect on Pulmonary fibrosis
SNP effect on PLAU

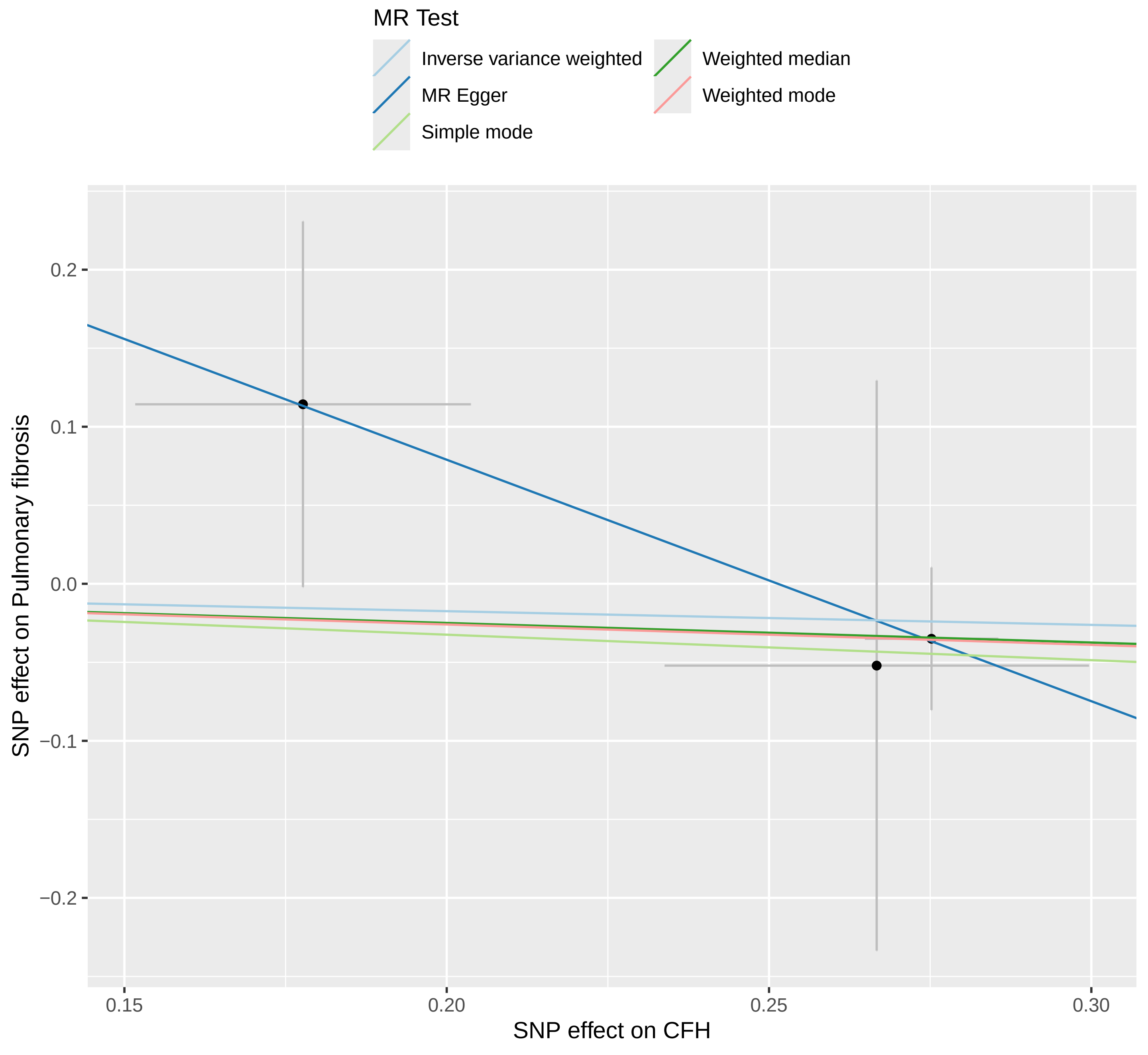

MR Test
Inverse variance weighted
MR Egger
Simple mode
Weighted median
Weighted mode
0.2
0.1
0.0
−0.1
−0.2
0.15
0.20
0.25
0.30
SNP effect on Pulmonary fibrosis
SNP effect on CFH

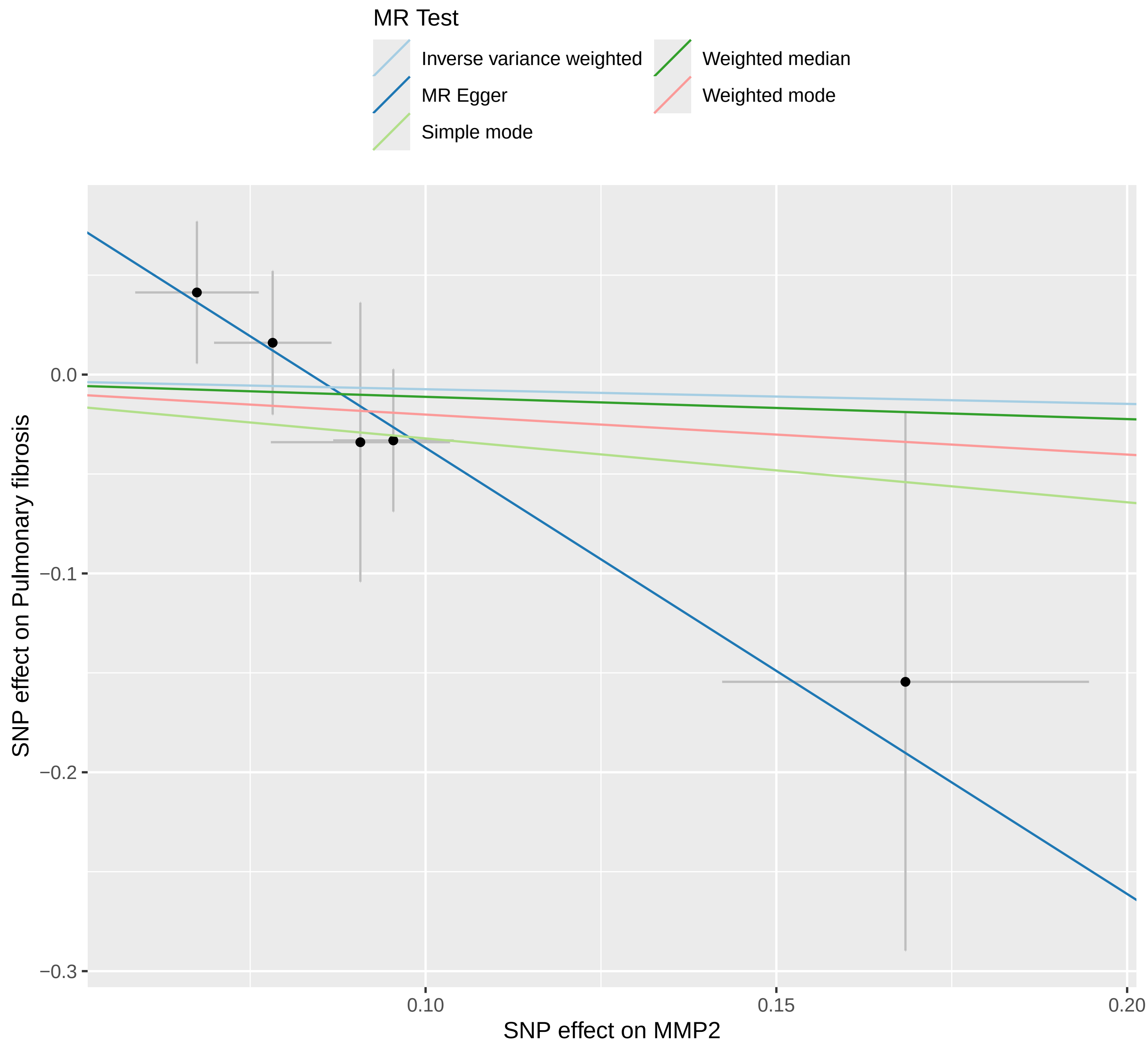

MR Test
Inverse variance weighted
MR Egger
Simple mode
Weighted median
Weighted mode
0.0
−0.1
−0.2
−0.3
0.10
0.15
0.20
SNP effect on MMP2
SNP effect on Pulmonary fibrosis

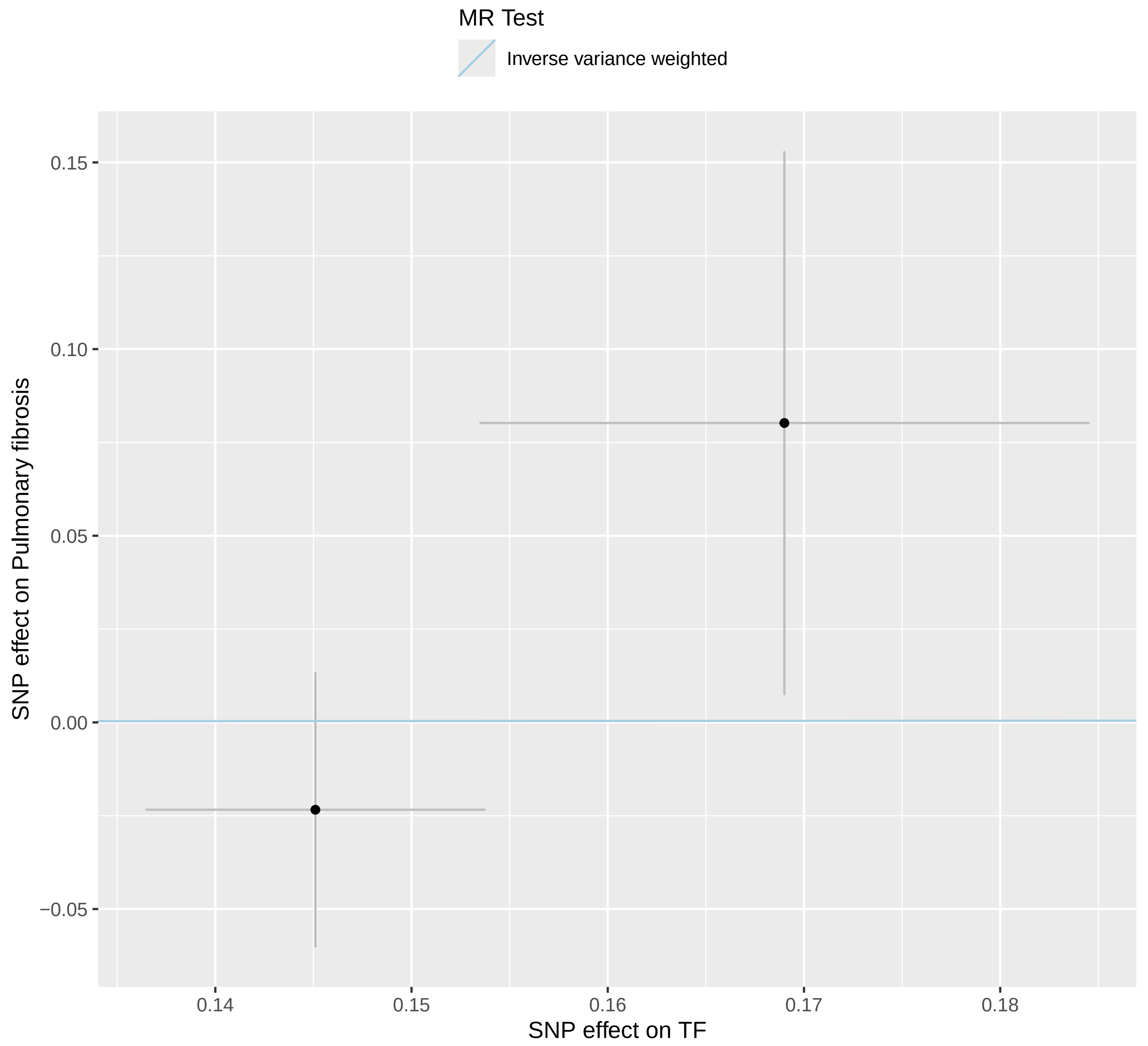

MR Test
Inverse variance weighted
0.15
0.10
0.05
0.00
−0.05
SNP effect on Pulmonary fibrosis
0.14
0.15
0.16
0.17
0.18
SNP effect on TF

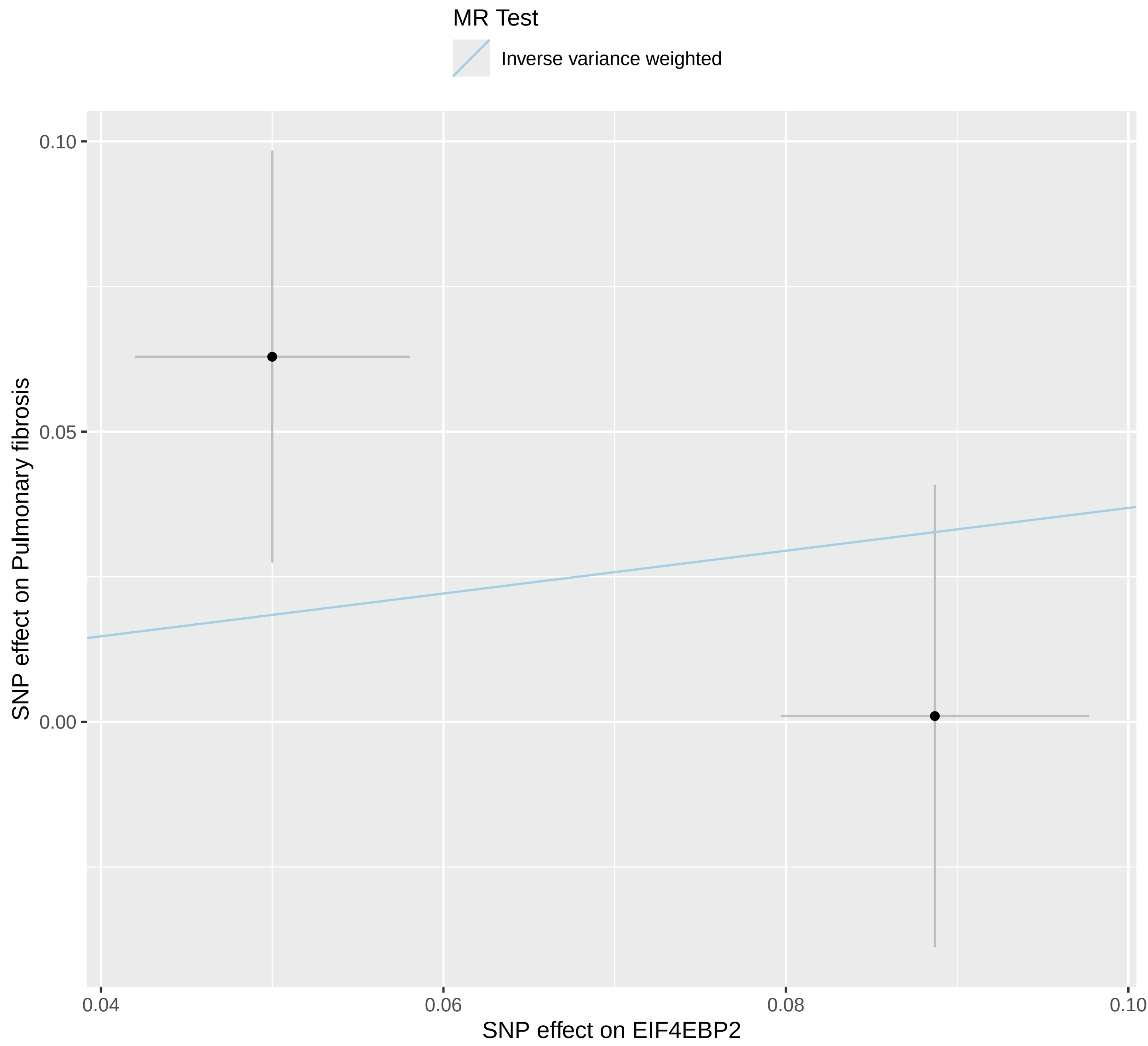
MR Test
Inverse variance weighted
0.10
0.05
0.00
0.04
0.06
0.08
0.10
SNP effect on EIF4EBP2
SNP effect on Pulmonary fibrosis

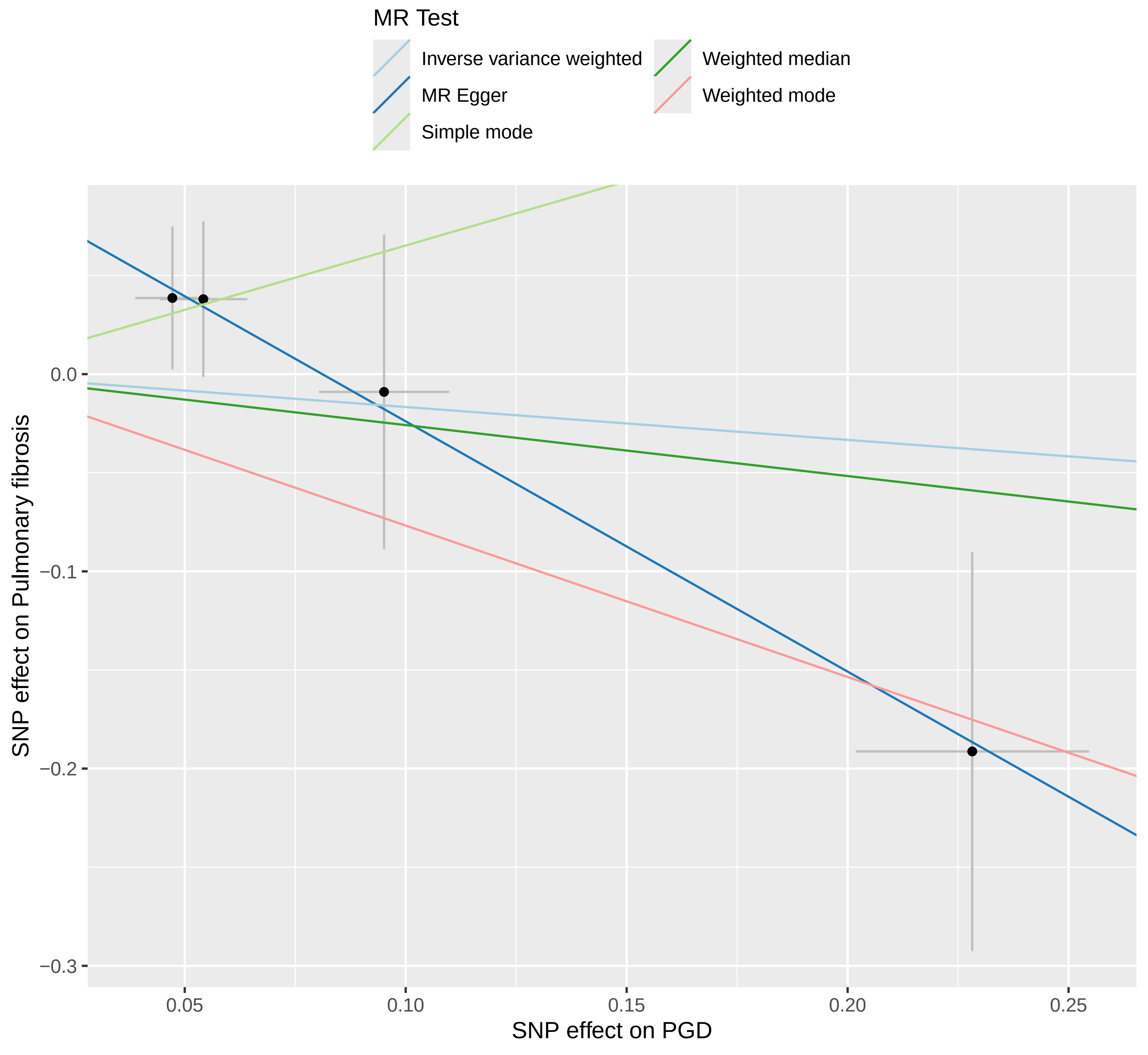
MR Test
Inverse variance weighted
MR Egger
Simple mode
Weighted median
Weighted mode
0.0
−0.1
−0.2
−0.3
SNP effect on Pulmonary fibrosis
0.05
0.10
0.15
0.20
0.25
SNP effect on PGD

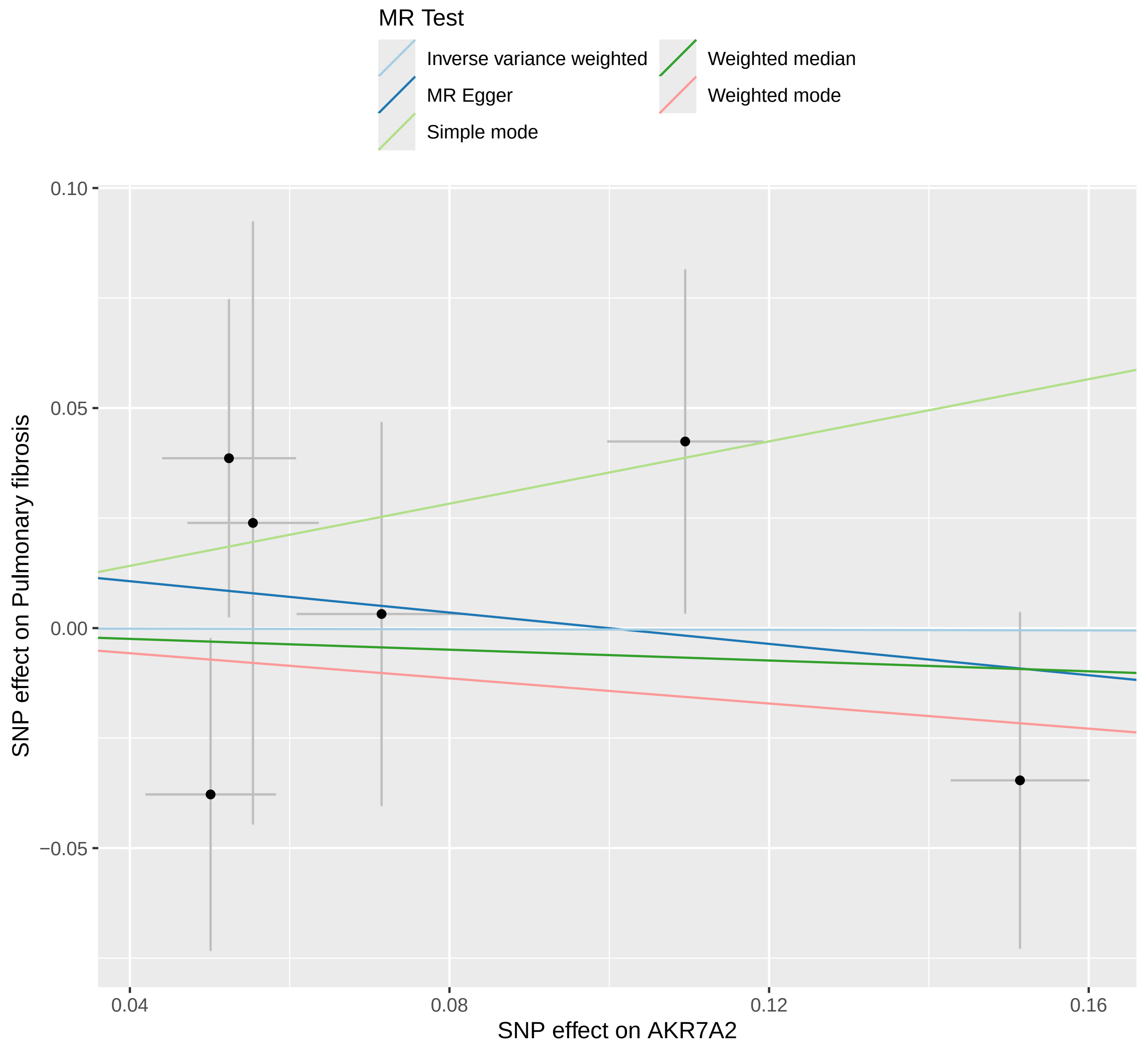
MR Test
Inverse variance weighted
MR Egger
Simple mode
Weighted median
Weighted mode
0.10
0.05
0.00
−0.05
SNP effect on Pulmonary fibrosis
0.04
0.08
0.12
0.16
SNP effect on AKR7A2

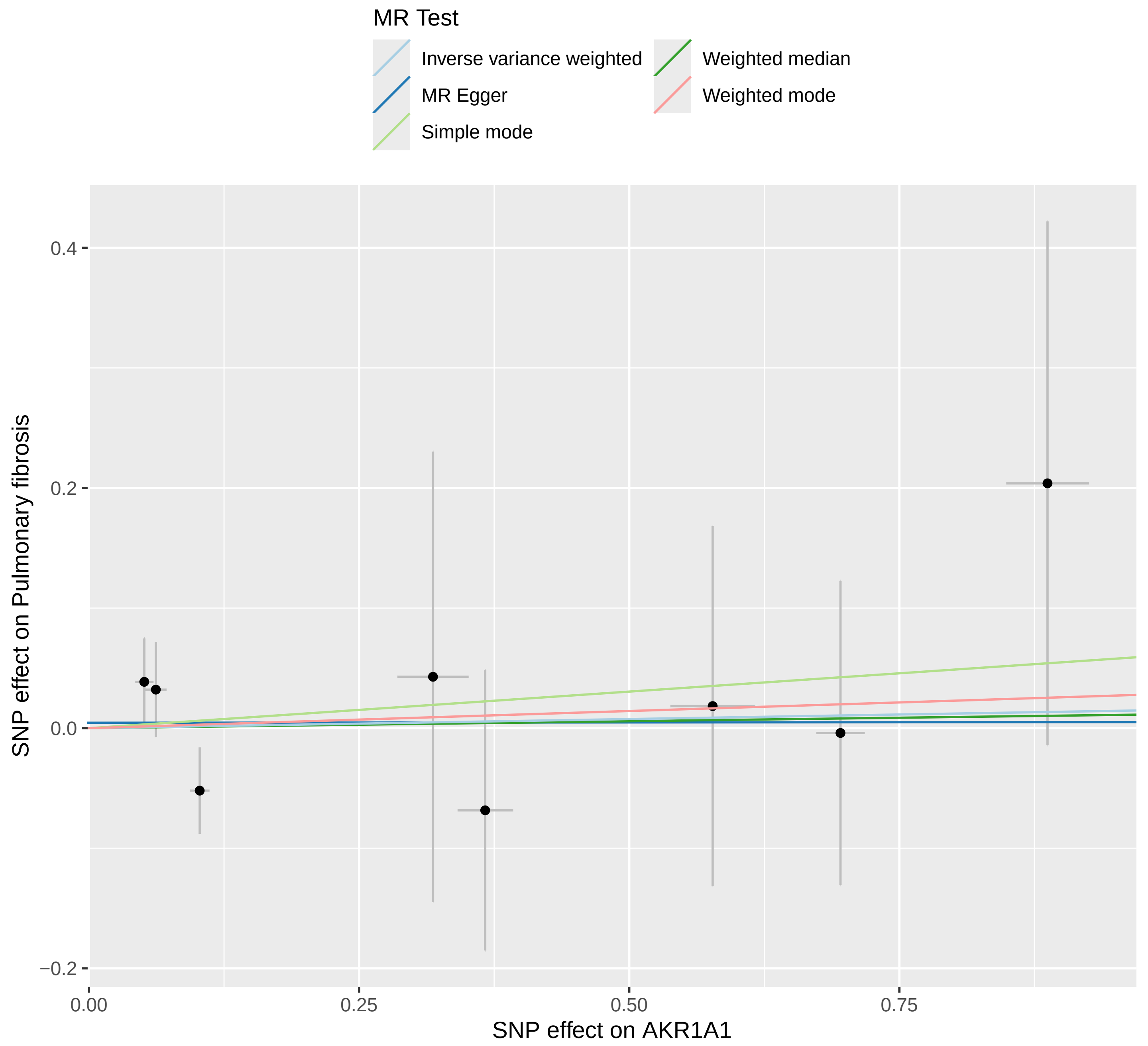

MR Test
Inverse variance weighted
MR Egger
Simple mode
Weighted median
Weighted mode
0.4
0.2
0.0
−0.2
0.00
0.25
0.50
0.75
SNP effect on Pulmonary fibrosis
SNP effect on AKR1A1

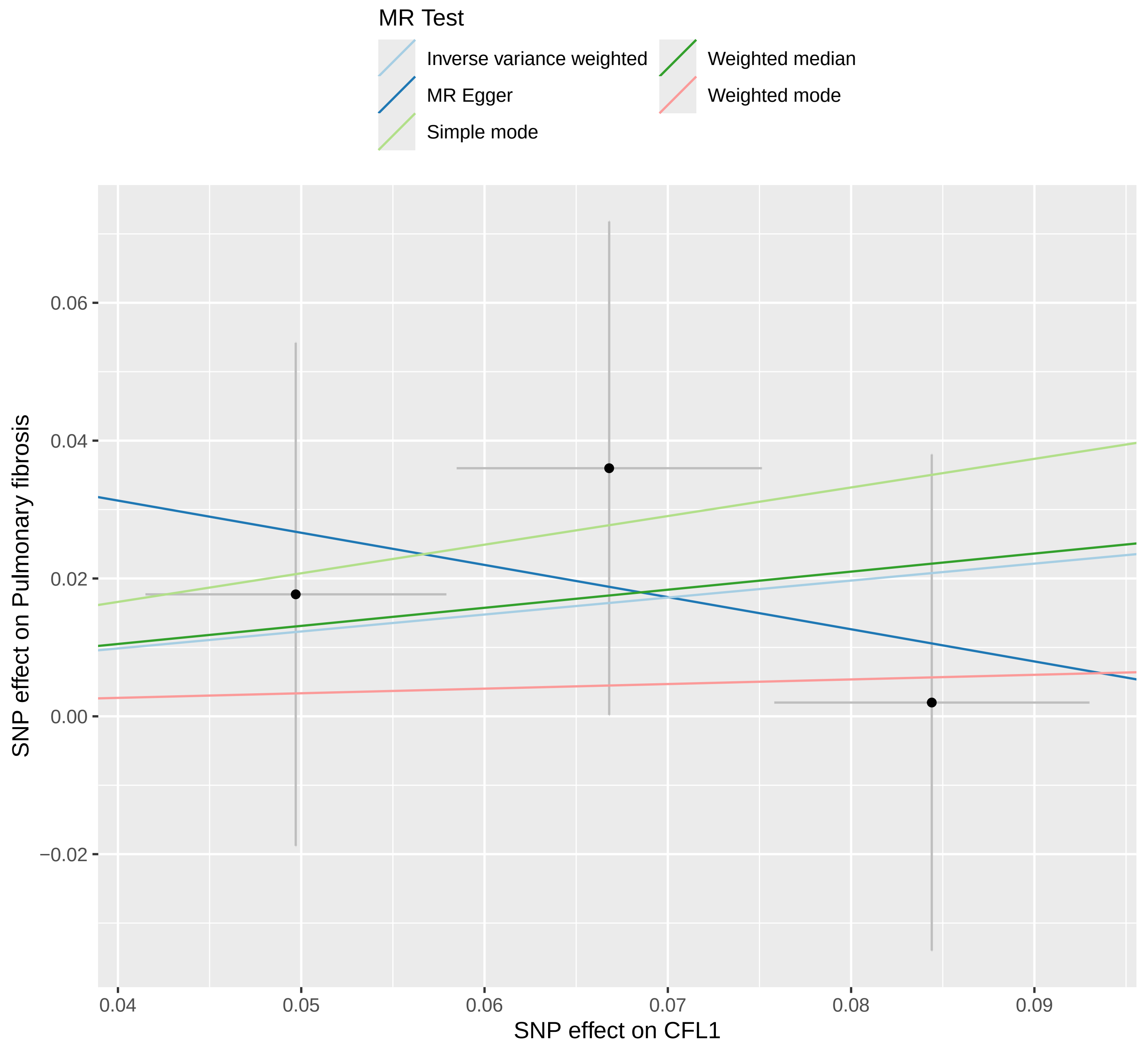
MR Test
Inverse variance weighted
MR Egger
Simple mode
Weighted median
Weighted mode
SNP effect on Pulmonary fibrosis
0.06
0.04
0.02
0.00
−0.02
0.04
0.05
0.06
0.07
0.08
0.09
SNP effect on CFL1

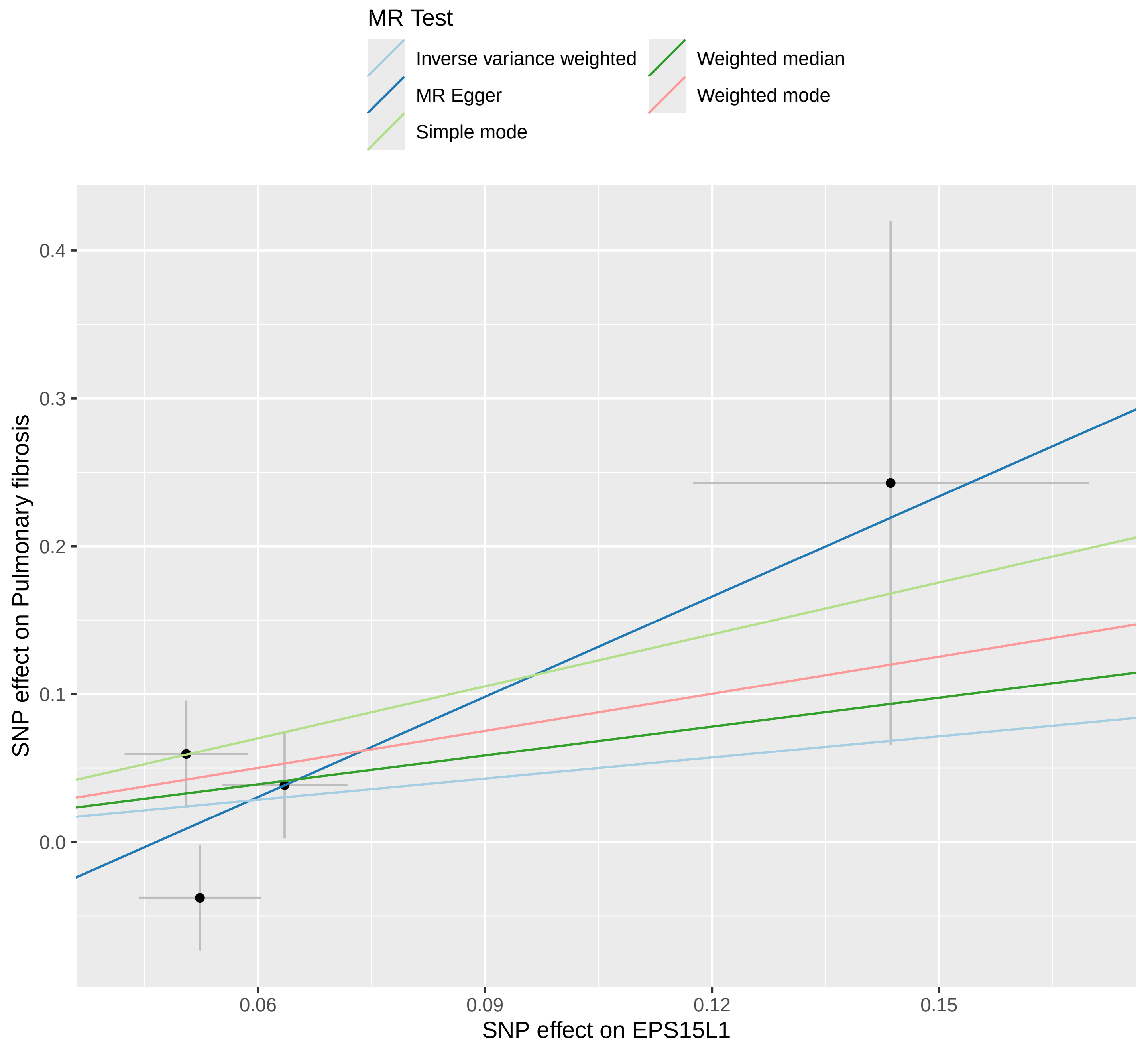

MR Test
Inverse variance weighted
MR Egger
Simple mode
Weighted median
Weighted mode
0.4
0.3
0.2
0.1
0.0
SNP effect on Pulmonary fibrosis
0.06
0.09
0.12
0.15
SNP effect on EPS15L1

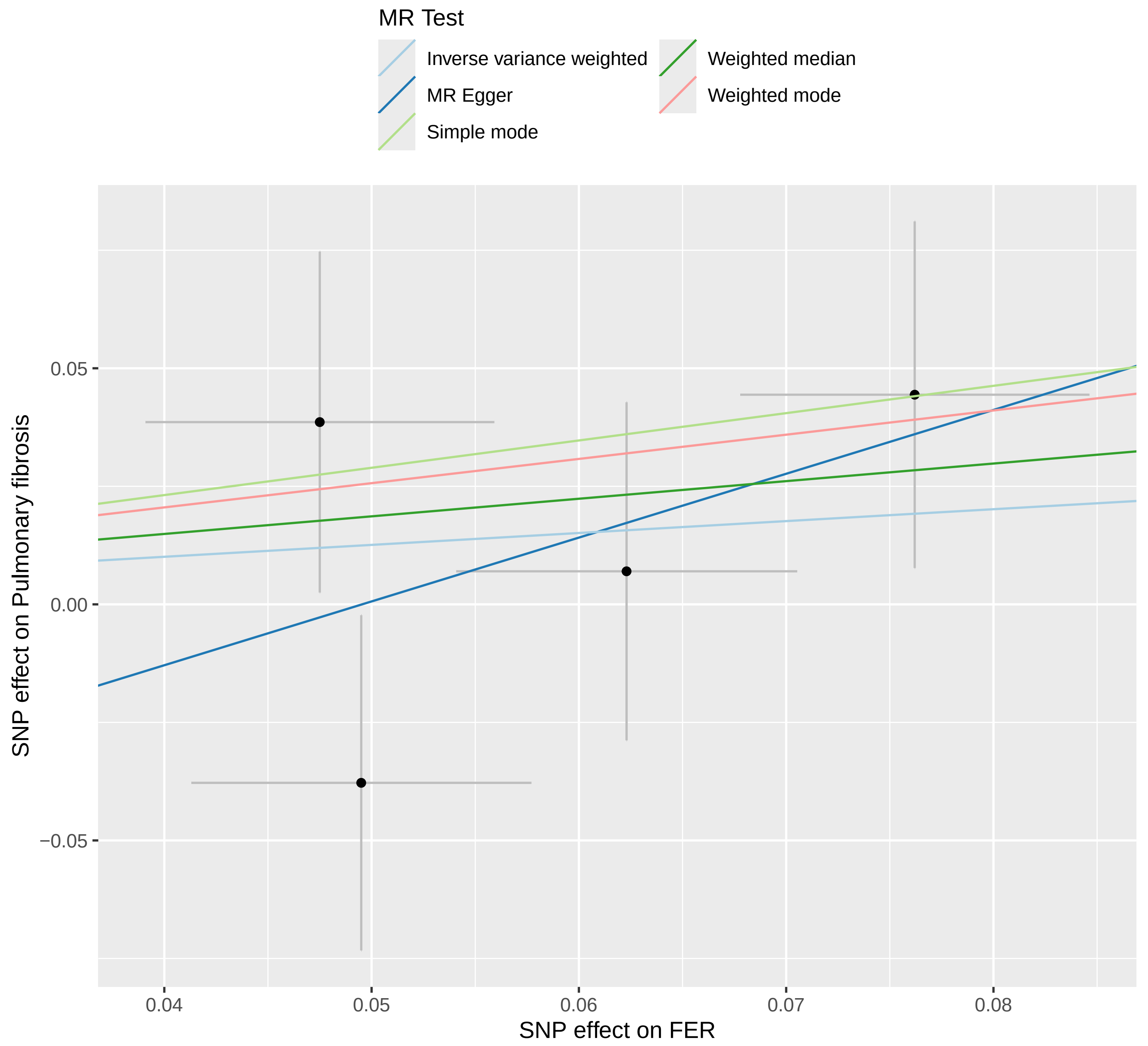

MR Test
Inverse variance weighted
MR Egger
Simple mode
Weighted median
Weighted mode
SNP effect on Pulmonary fibrosis
0.05
0.00
−0.05
0.04
0.05
0.06
0.07
0.08
SNP effect on FER

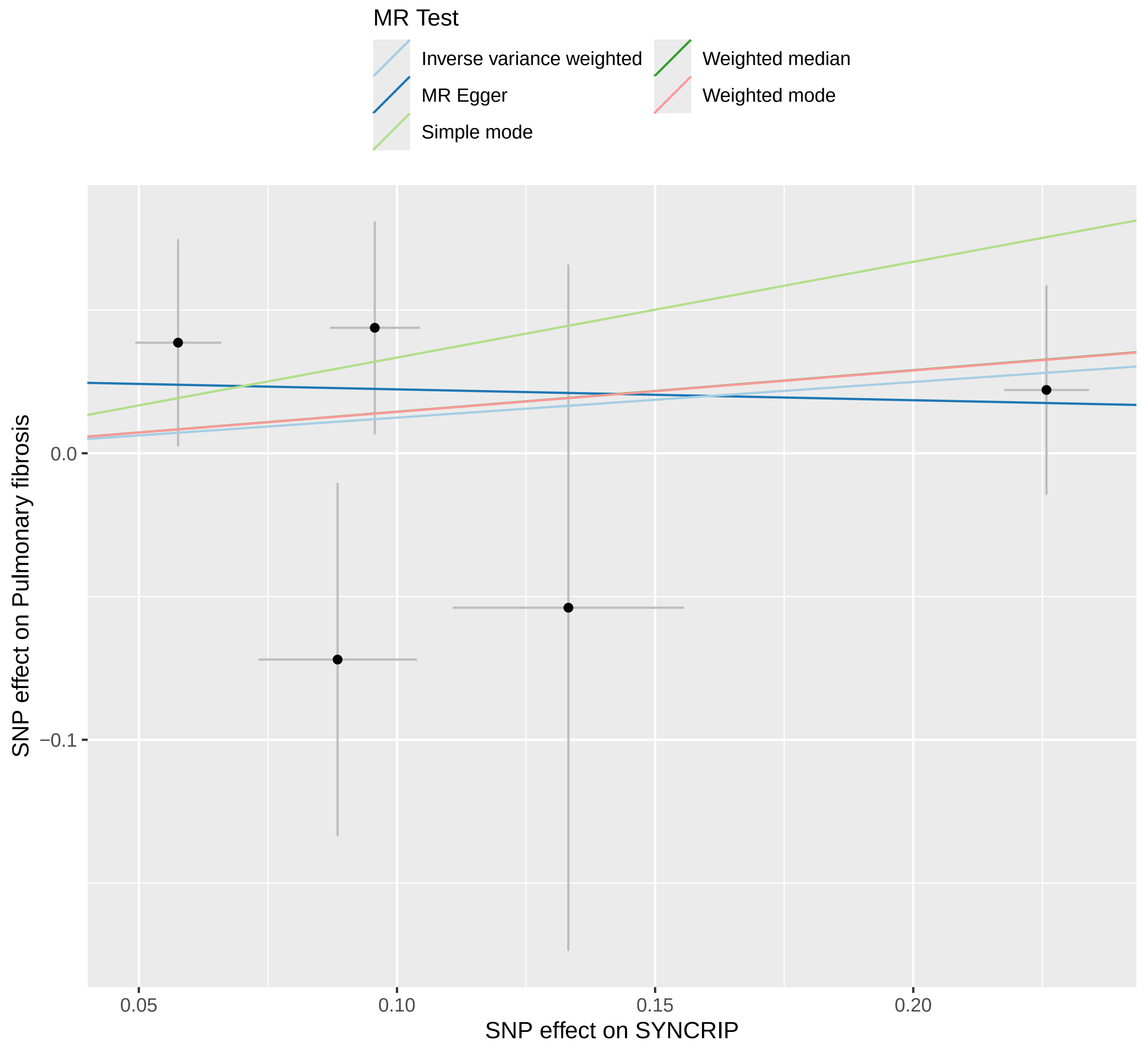

MR Test
Inverse variance weighted
MR Egger
Simple mode
Weighted median
Weighted mode
0.0
−0.1
0.05
0.10
0.15
0.20
SNP effect on SYNCRIP
SNP effect on Pulmonary fibrosis

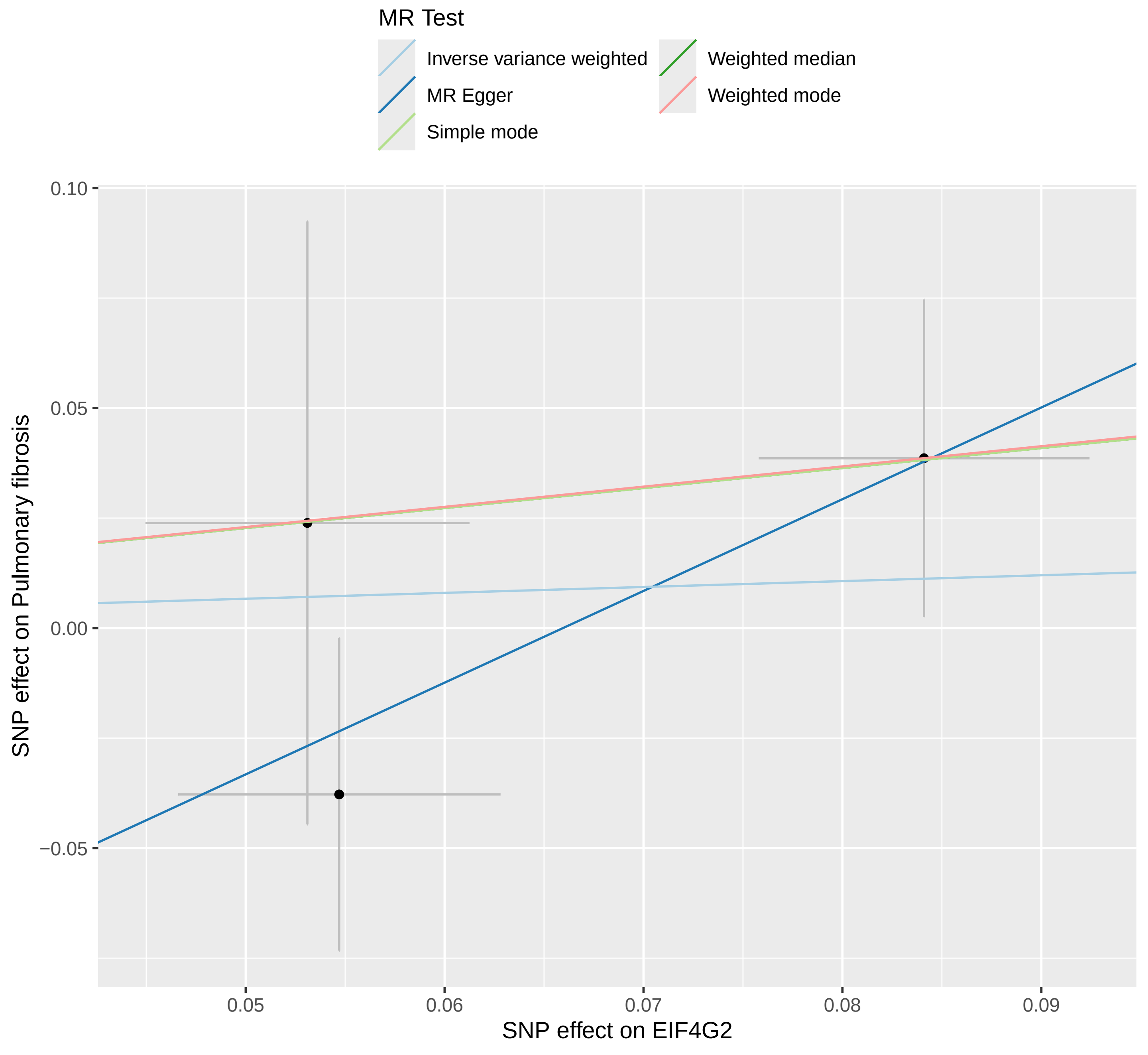
MR Test
Inverse variance weighted
MR Egger
Simple mode
Weighted median
Weighted mode
0.10
0.05
0.00
−0.05
0.05
0.06
0.07
0.08
0.09
SNP effect on Pulmonary fibrosis
SNP effect on EIF4G2

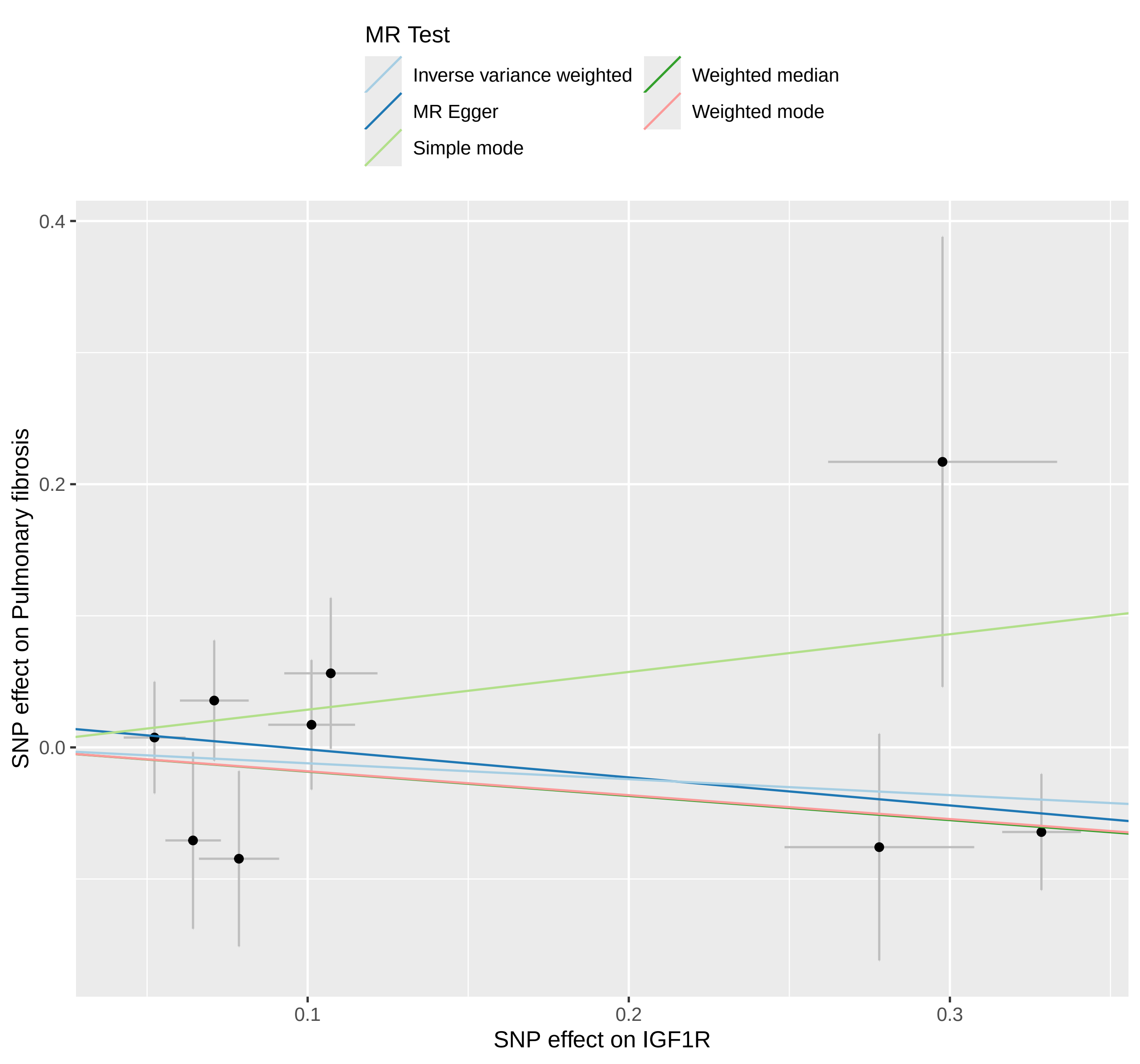

MR Test
Inverse variance weighted
MR Egger
Simple mode
Weighted median
Weighted mode
0.4
0.2
0.0
0.1
0.2
0.3
SNP effect on Pulmonary fibrosis
SNP effect on IGF1R

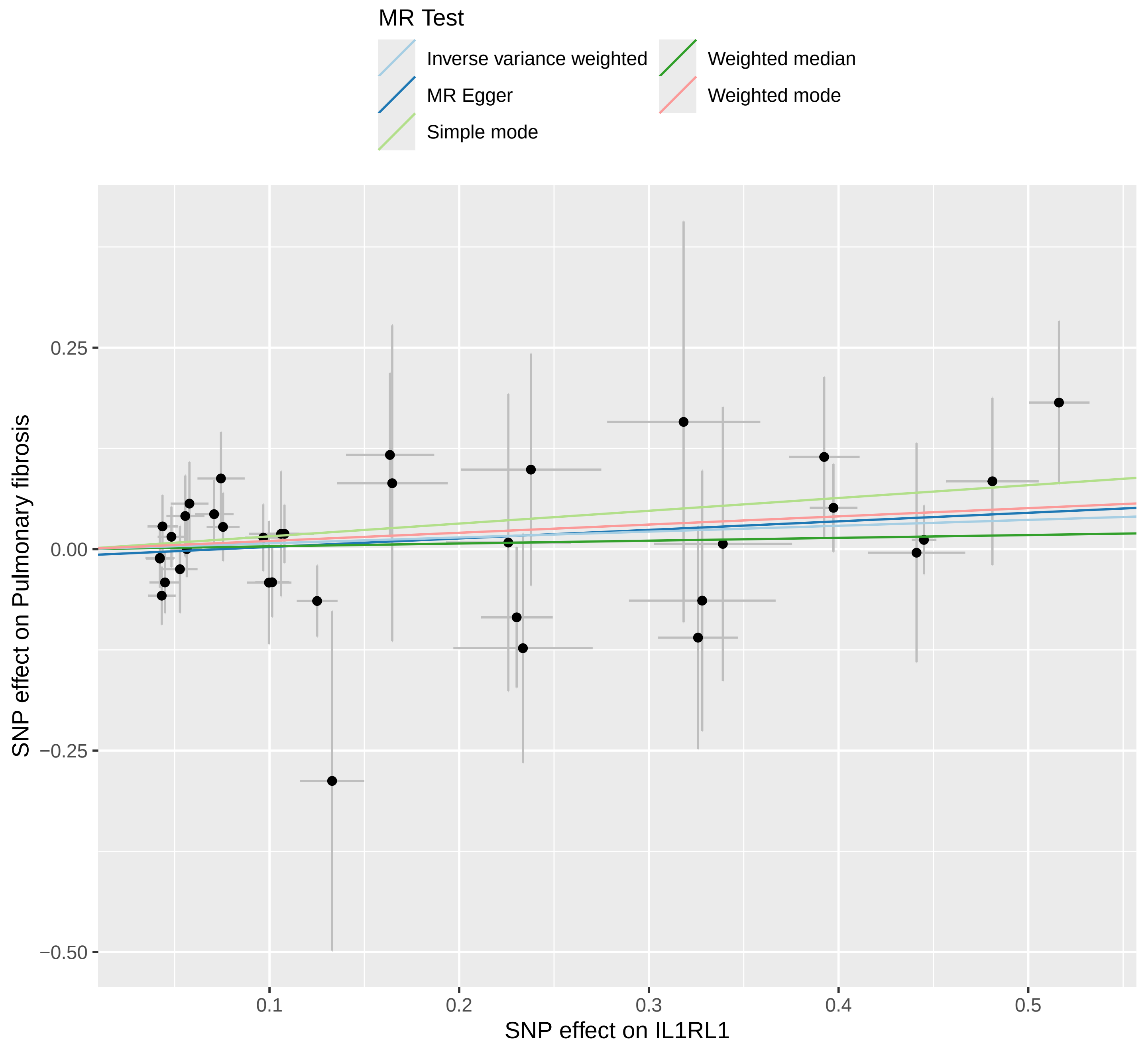

MR Test
Inverse variance weighted
MR Egger
Simple mode
Weighted median
Weighted mode
SNP effect on Pulmonary fibrosis
0.25
0.00
−0.25
−0.50
0.1
0.2
0.3
0.4
0.5
SNP effect on IL1RL1

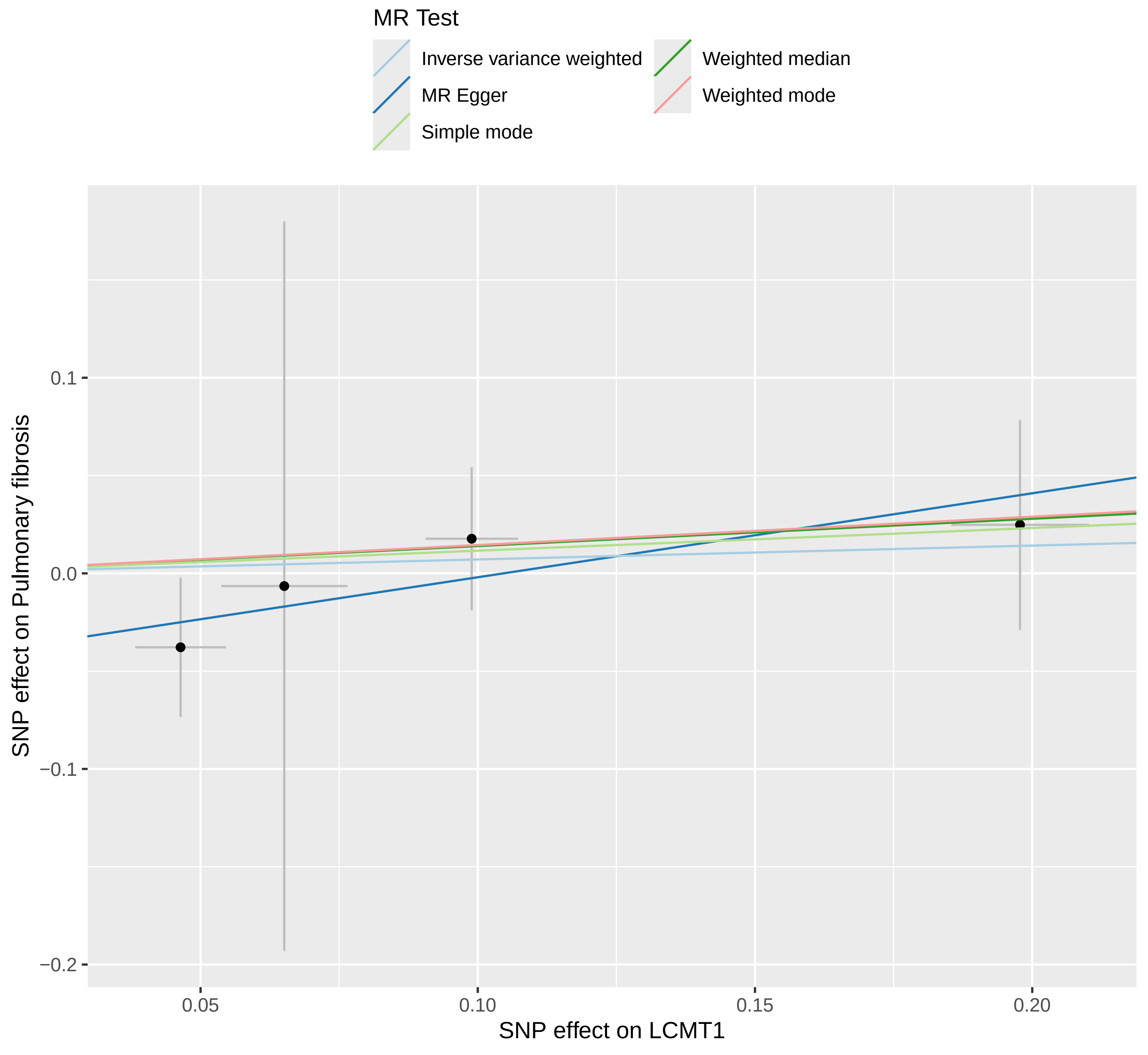
MR Test
Inverse variance weighted
MR Egger
Simple mode
Weighted median
Weighted mode
SNP effect on Pulmonary fibrosis
0.1
0.0
−0.1
−0.2
0.05
0.10
0.15
0.20
SNP effect on LCMT1

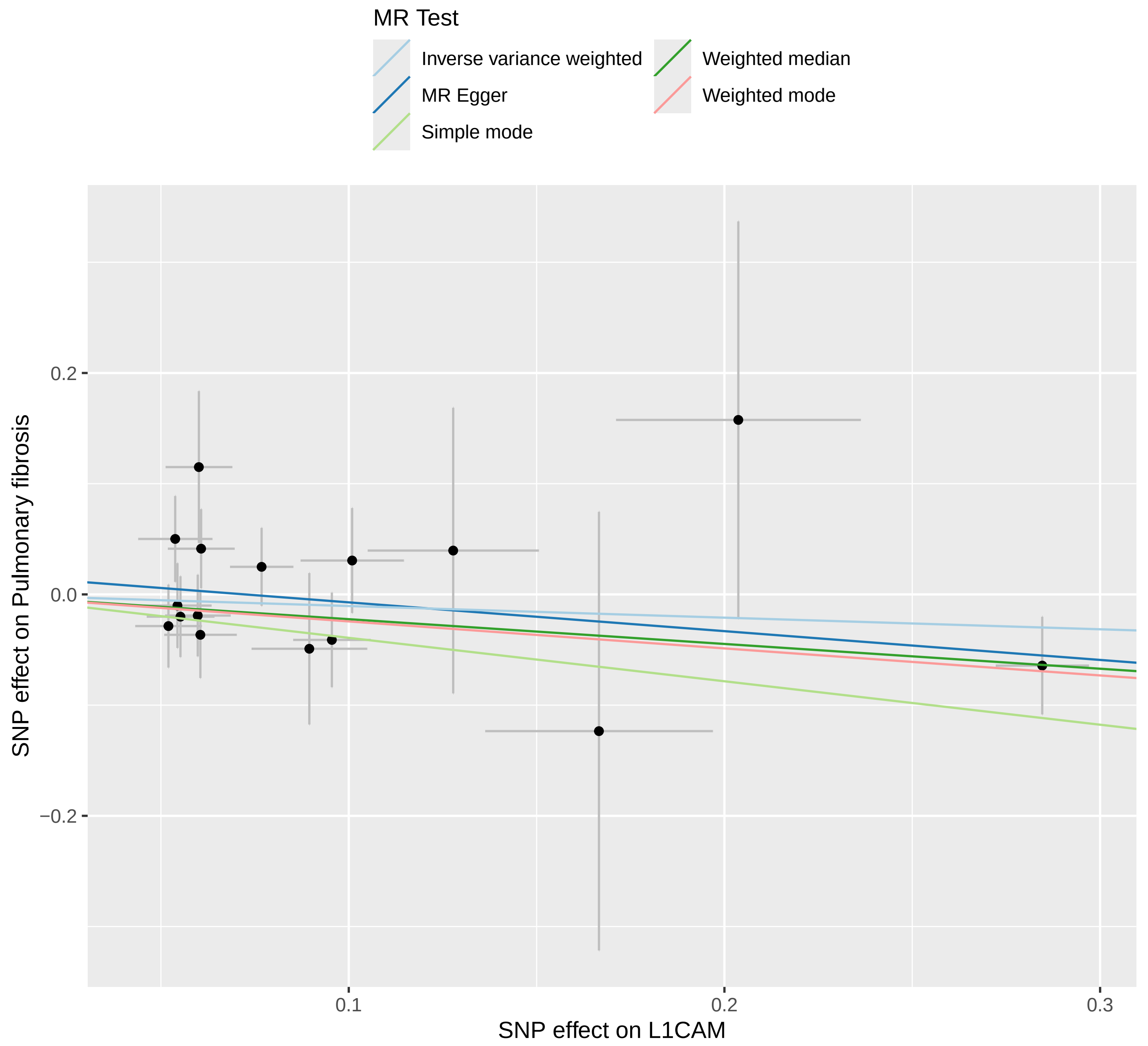

MR Test
Inverse variance weighted
Weighted median
MR Egger
Weighted mode
Simple mode
0.2
0.0
−0.2
0.1
0.2
0.3
SNP effect on Pulmonary fibrosis
SNP effect on L1CAM

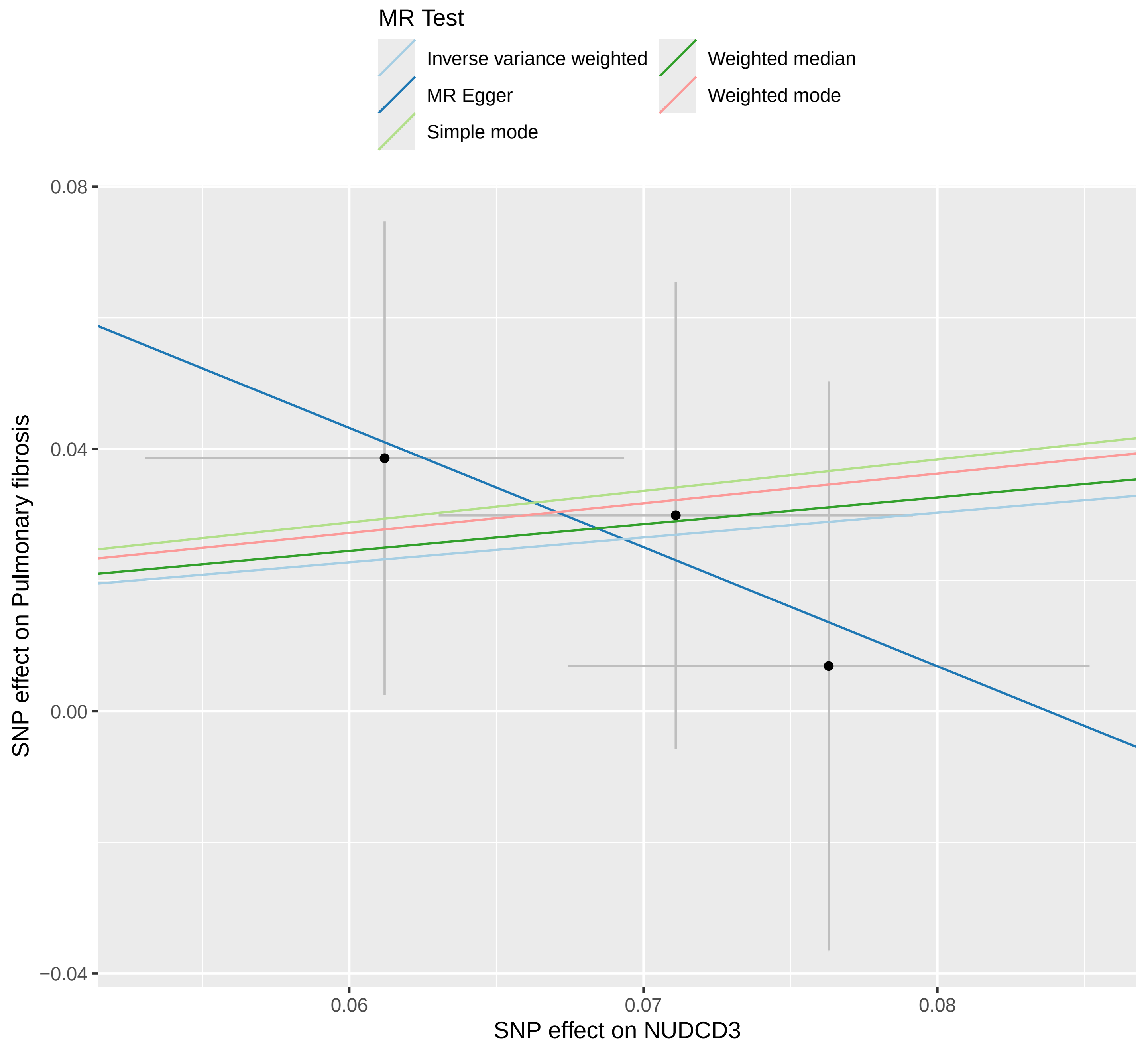
MR Test
Inverse variance weighted
MR Egger
Simple mode
Weighted median
Weighted mode
0.08
0.04
0.00
−0.04
0.06
0.07
0.08
SNP effect on Pulmonary fibrosis
SNP effect on NUDCD3

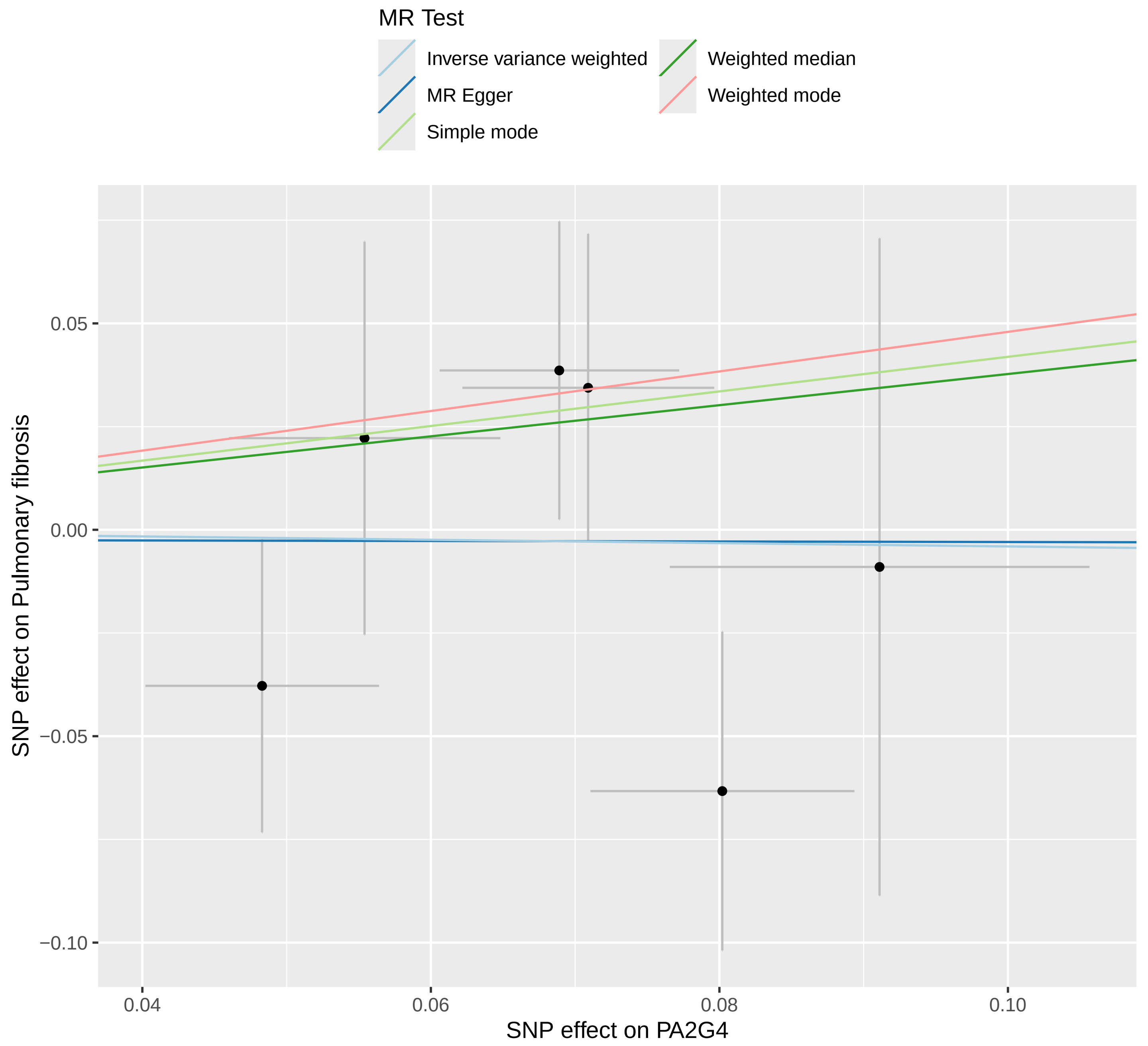
MR Test
Inverse variance weighted
MR Egger
Simple mode
Weighted median
Weighted mode
SNP effect on Pulmonary fibrosis
0.05
0.00
−0.05
−0.10
0.04
0.06
0.08
0.10
SNP effect on PA2G4

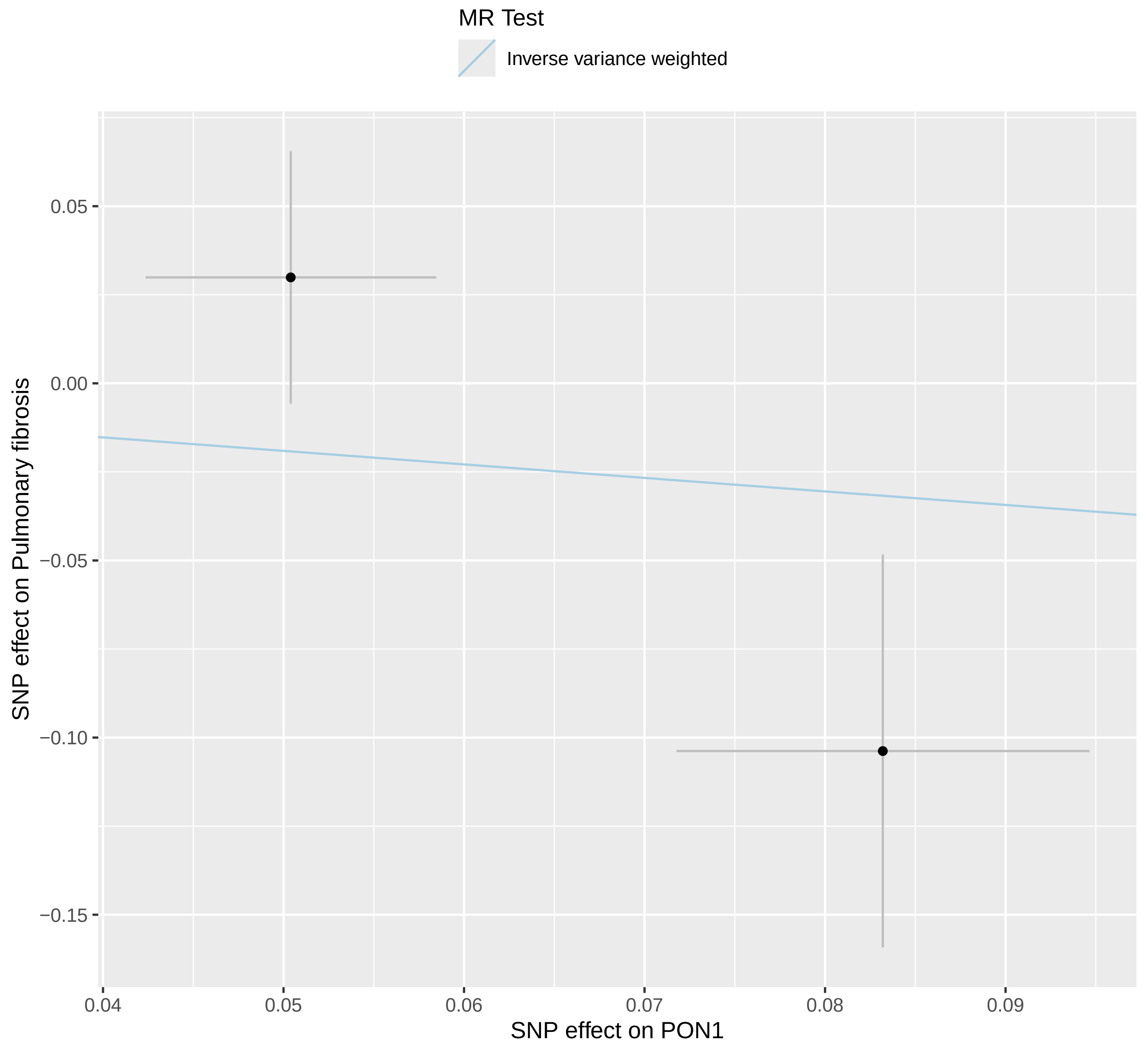
MR Test
Inverse variance weighted
0.05
0.00
−0.05
−0.10
−0.15
SNP effect on Pulmonary fibrosis
0.04
0.05
0.06
0.07
0.08
0.09
SNP effect on PON1

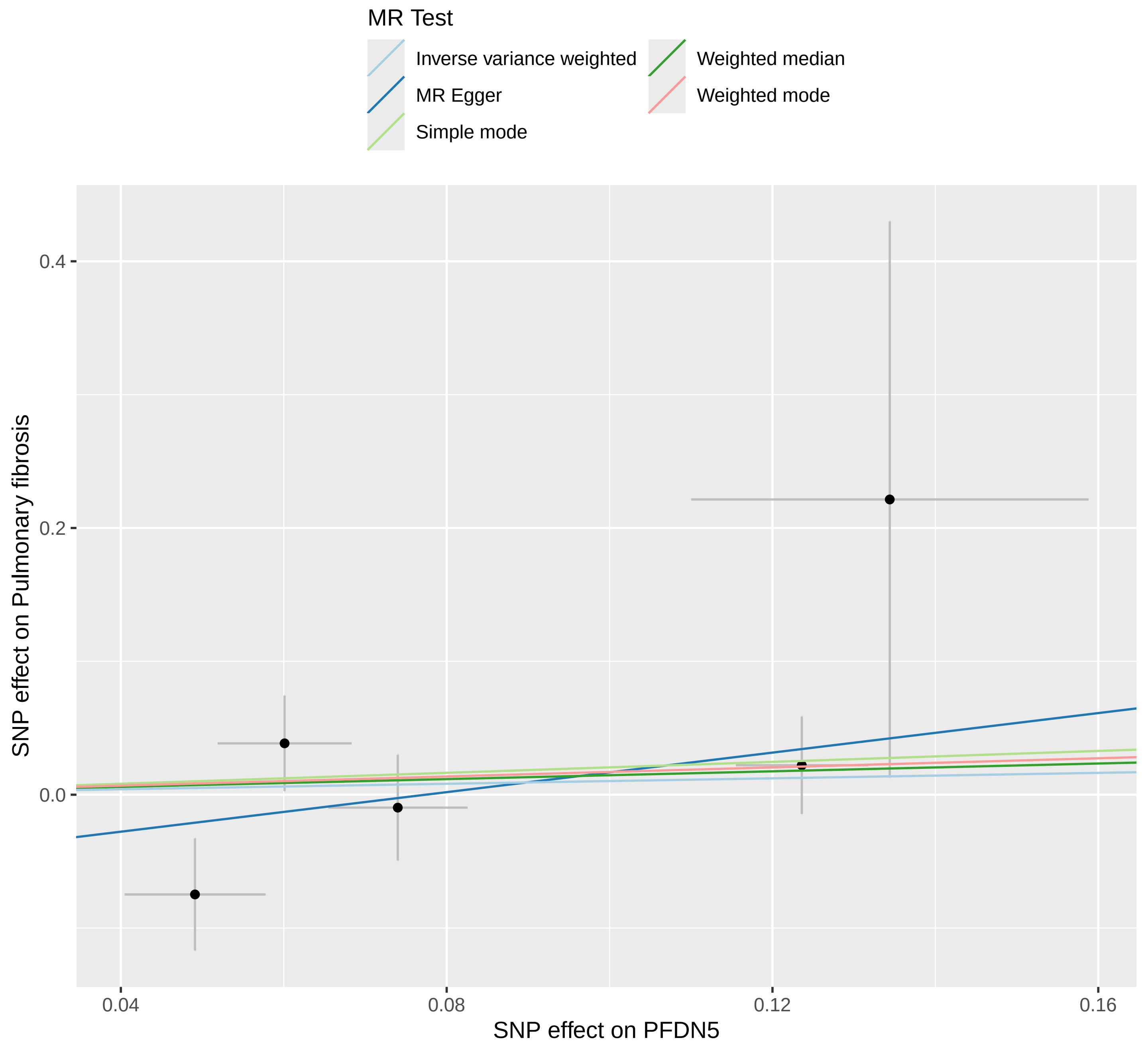
MR Test
Inverse variance weighted
MR Egger
Simple mode
Weighted median
Weighted mode
0.4
0.2
0.0
0.04
0.08
0.12
0.16
SNP effect on Pulmonary fibrosis
SNP effect on PFDN5

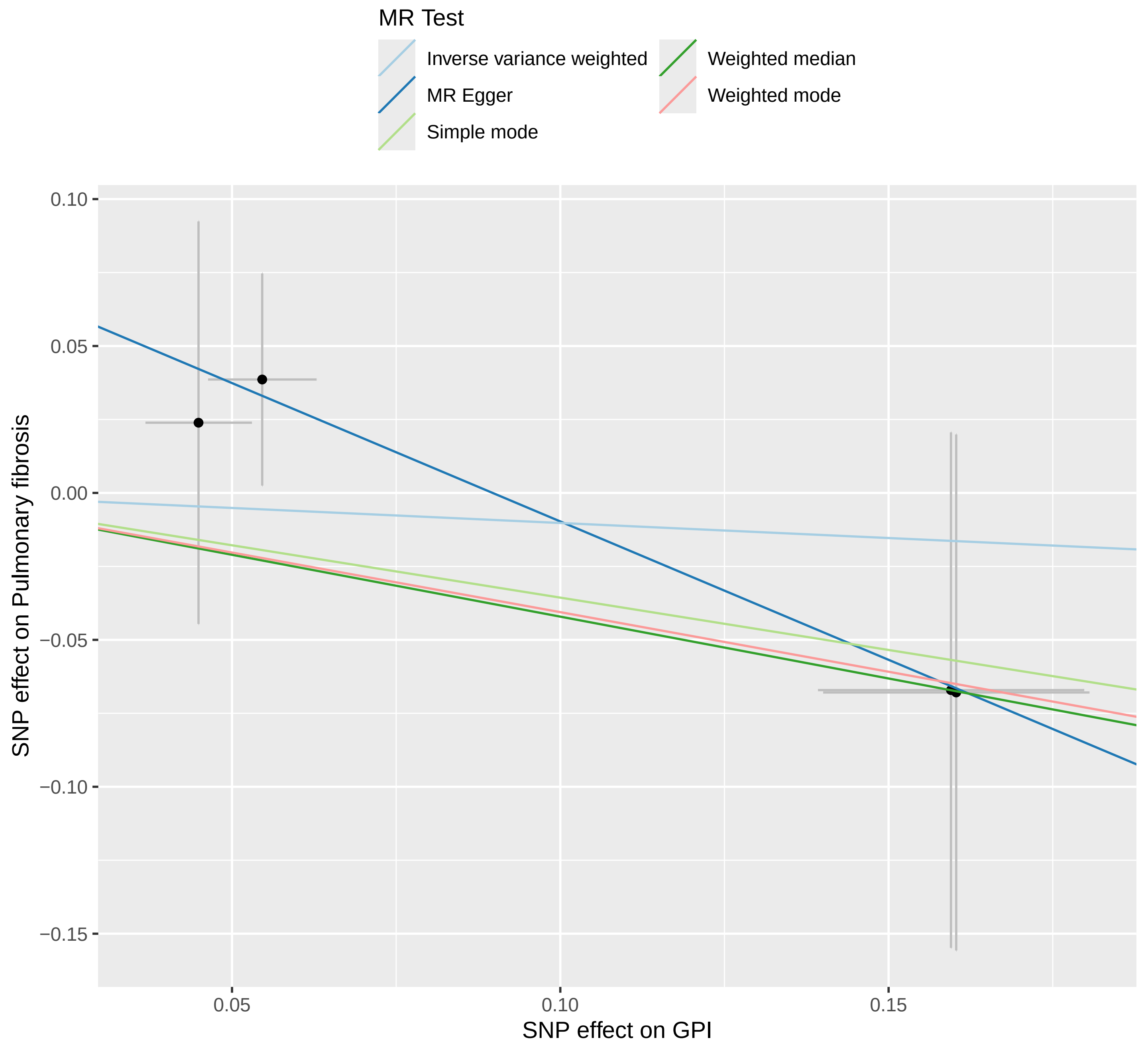
MR Test
Inverse variance weighted
MR Egger
Simple mode
Weighted median
Weighted mode
0.10
0.05
0.00
−0.05
−0.10
−0.15
SNP effect on Pulmonary fibrosis
0.05
0.10
0.15
SNP effect on GPI

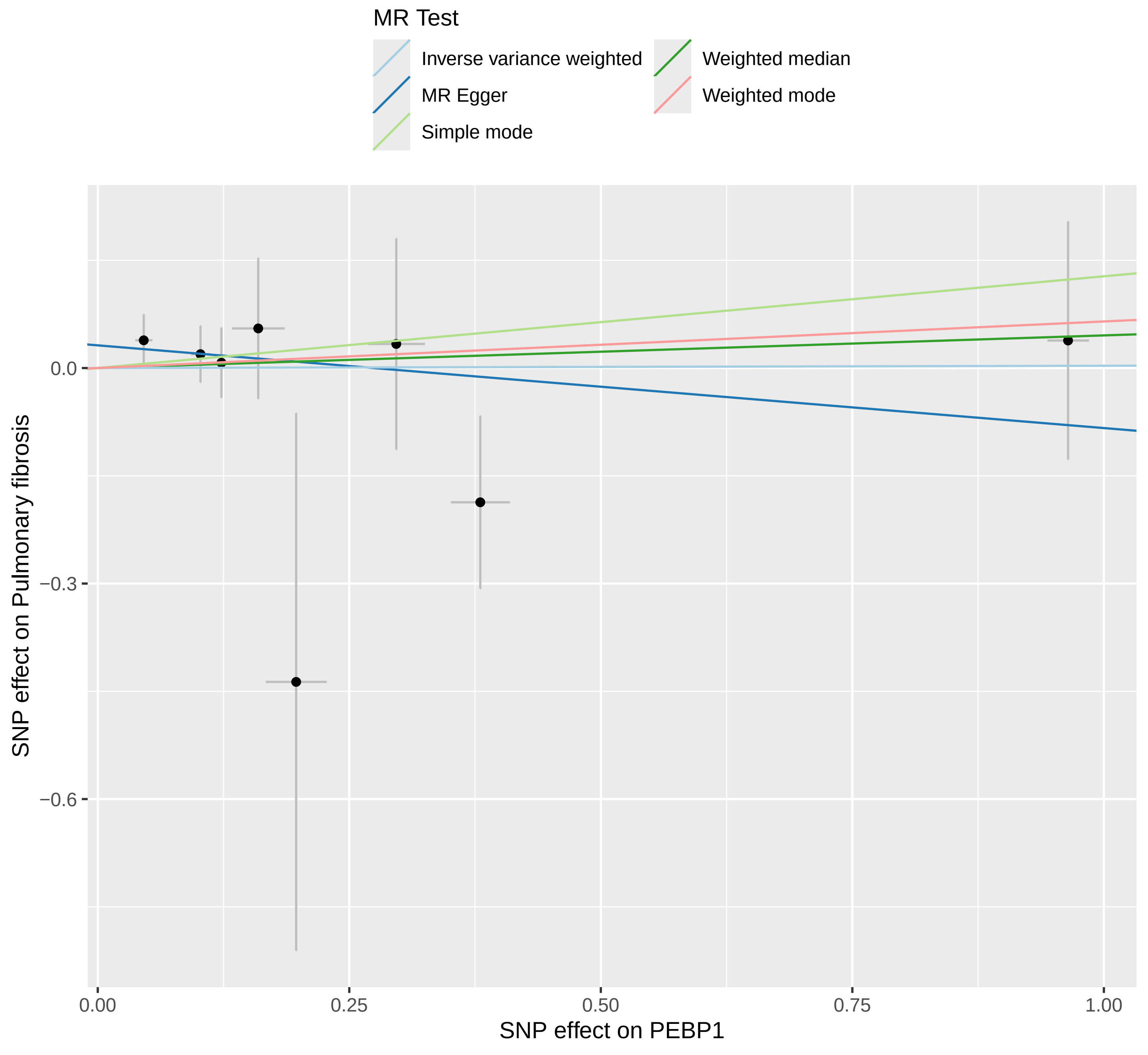

MR Test
Inverse variance weighted
MR Egger
Simple mode
Weighted median
Weighted mode
0.0
−0.3
−0.6
0.00
0.25
0.50
0.75
1.00
SNP effect on PEBP1
SNP effect on Pulmonary fibrosis

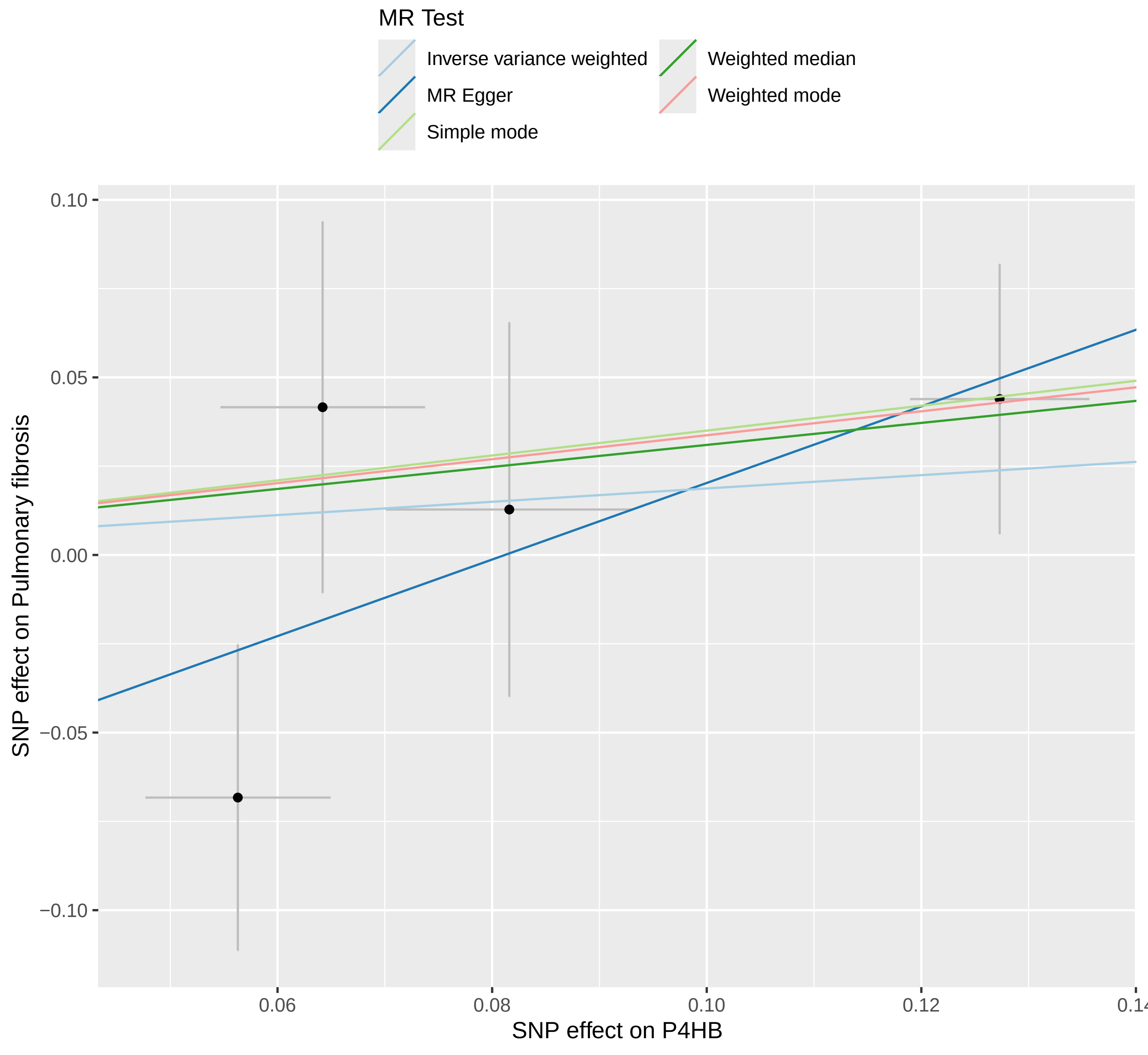

MR Test
Inverse variance weighted
MR Egger
Simple mode
Weighted median
Weighted mode
0.10
0.05
0.00
−0.05
−0.10
SNP effect on Pulmonary fibrosis
0.06
0.08
0.10
0.12
0.14
SNP effect on P4HB

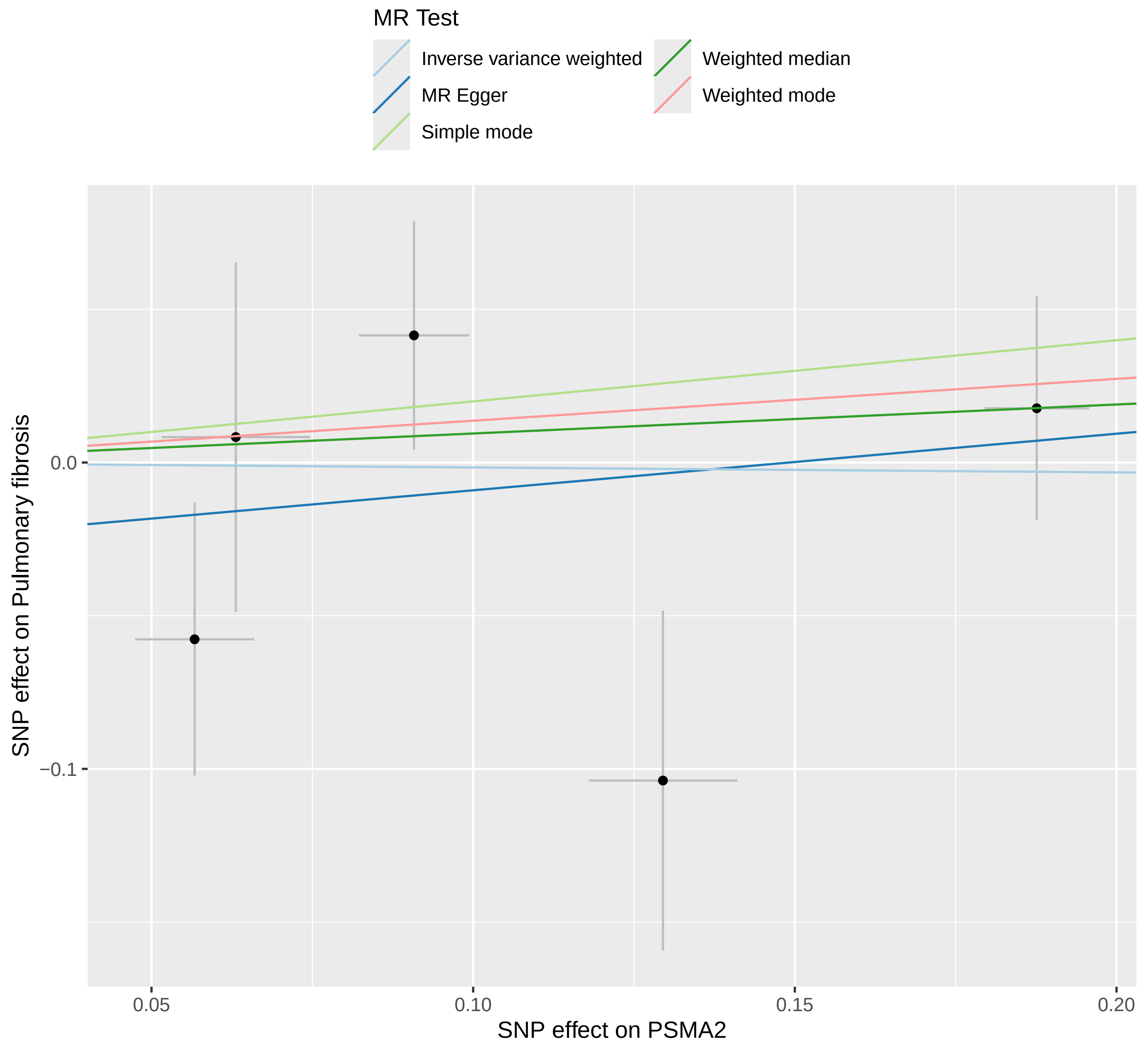

MR Test
Inverse variance weighted
MR Egger
Simple mode
Weighted median
Weighted mode
SNP effect on Pulmonary fibrosis
0.0
−0.1
0.05
0.10
0.15
0.20
SNP effect on PSMA2

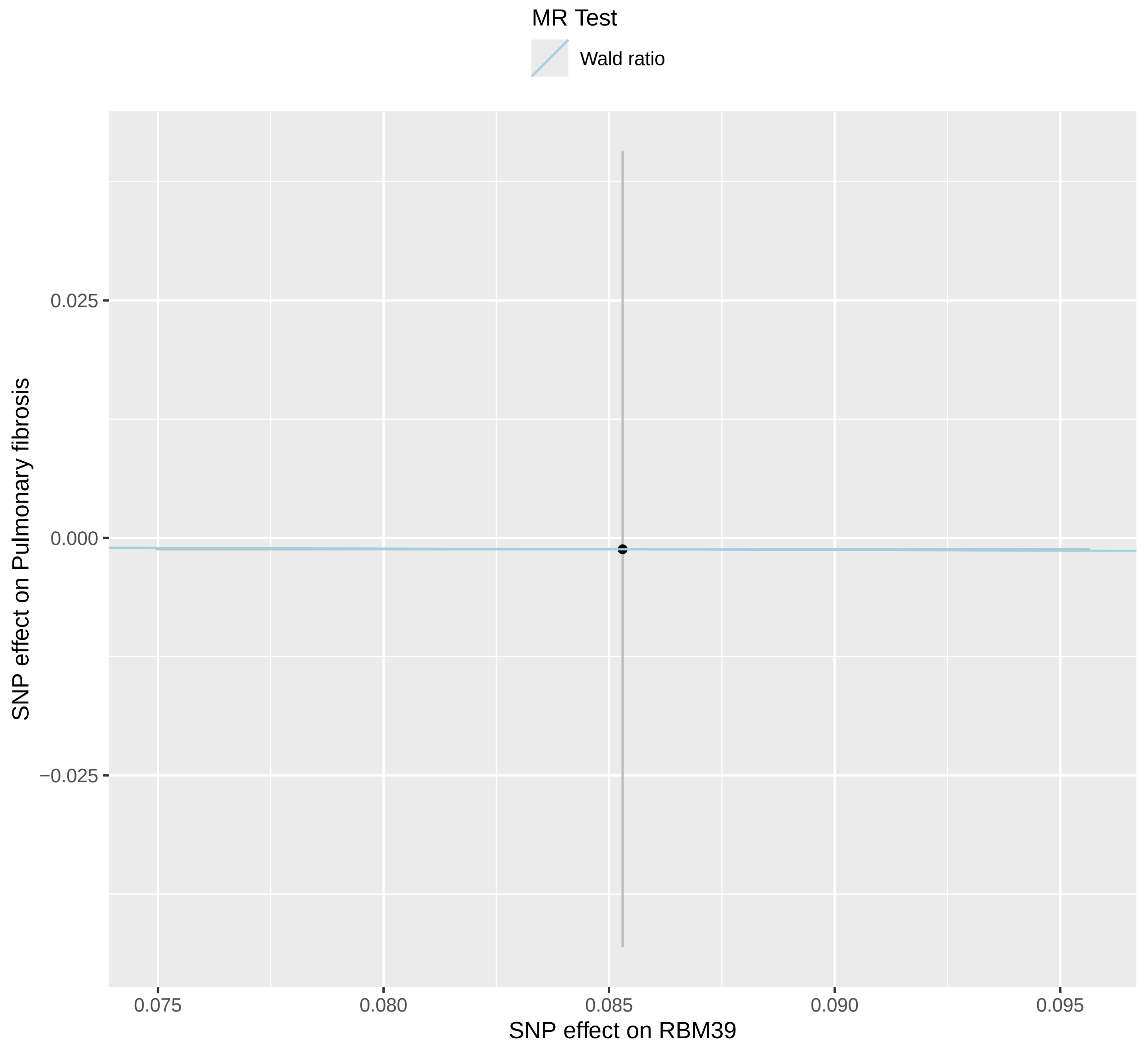
MR Test
Wald ratio
SNP effect on Pulmonary fibrosis
0.025
0.000
−0.025
0.075
0.080
0.085
0.090
0.095
SNP effect on RBM39

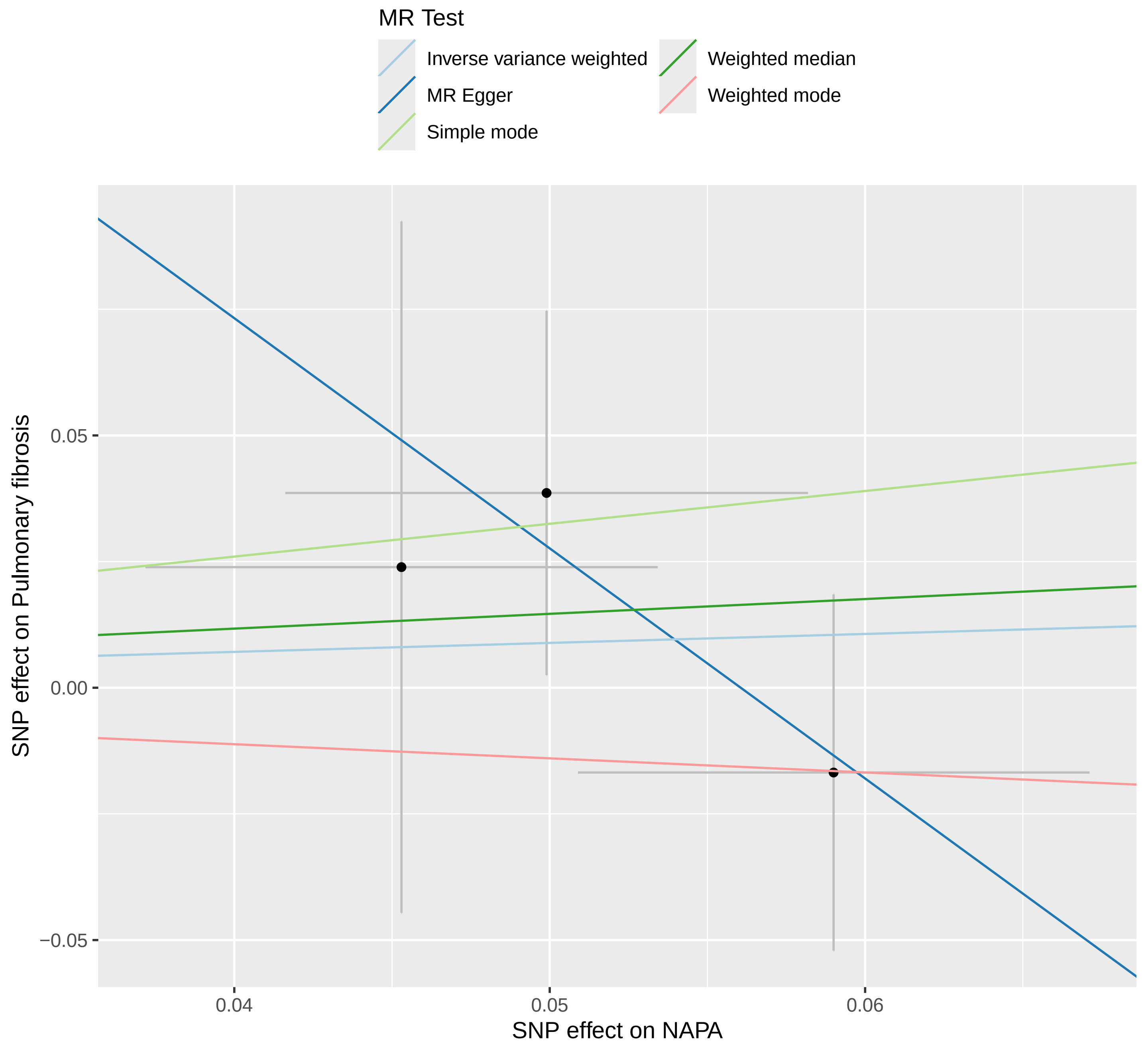
MR Test
Inverse variance weighted
MR Egger
Simple mode
Weighted median
Weighted mode
0.05
0.00
−0.05
SNP effect on Pulmonary fibrosis
0.04
0.05
0.06
SNP effect on NAPA

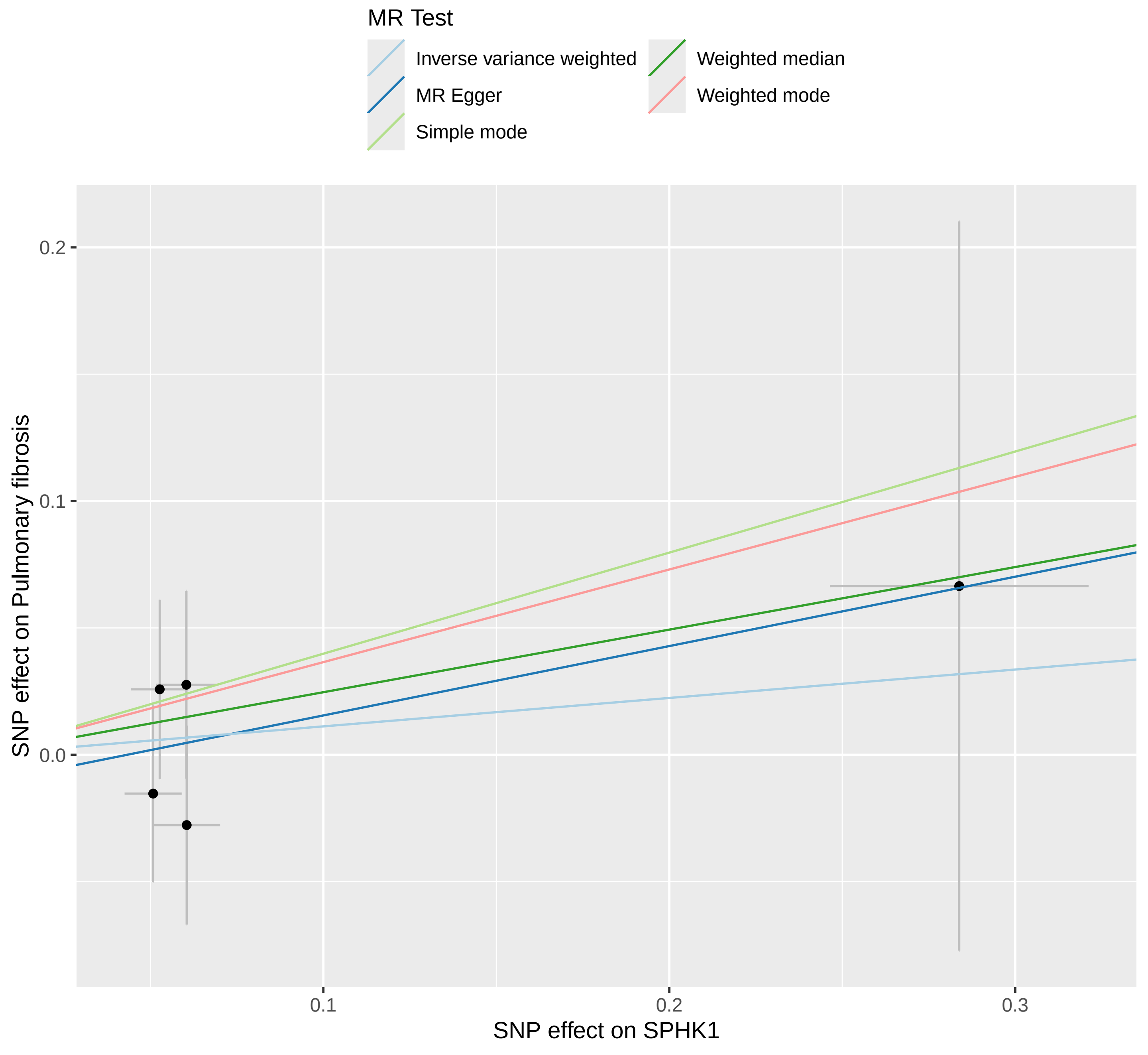

MR Test
Inverse variance weighted
MR Egger
Simple mode
Weighted median
Weighted mode
SNP effect on Pulmonary fibrosis
0.2
0.1
0.0
0.1
0.2
0.3
SNP effect on SPHK1

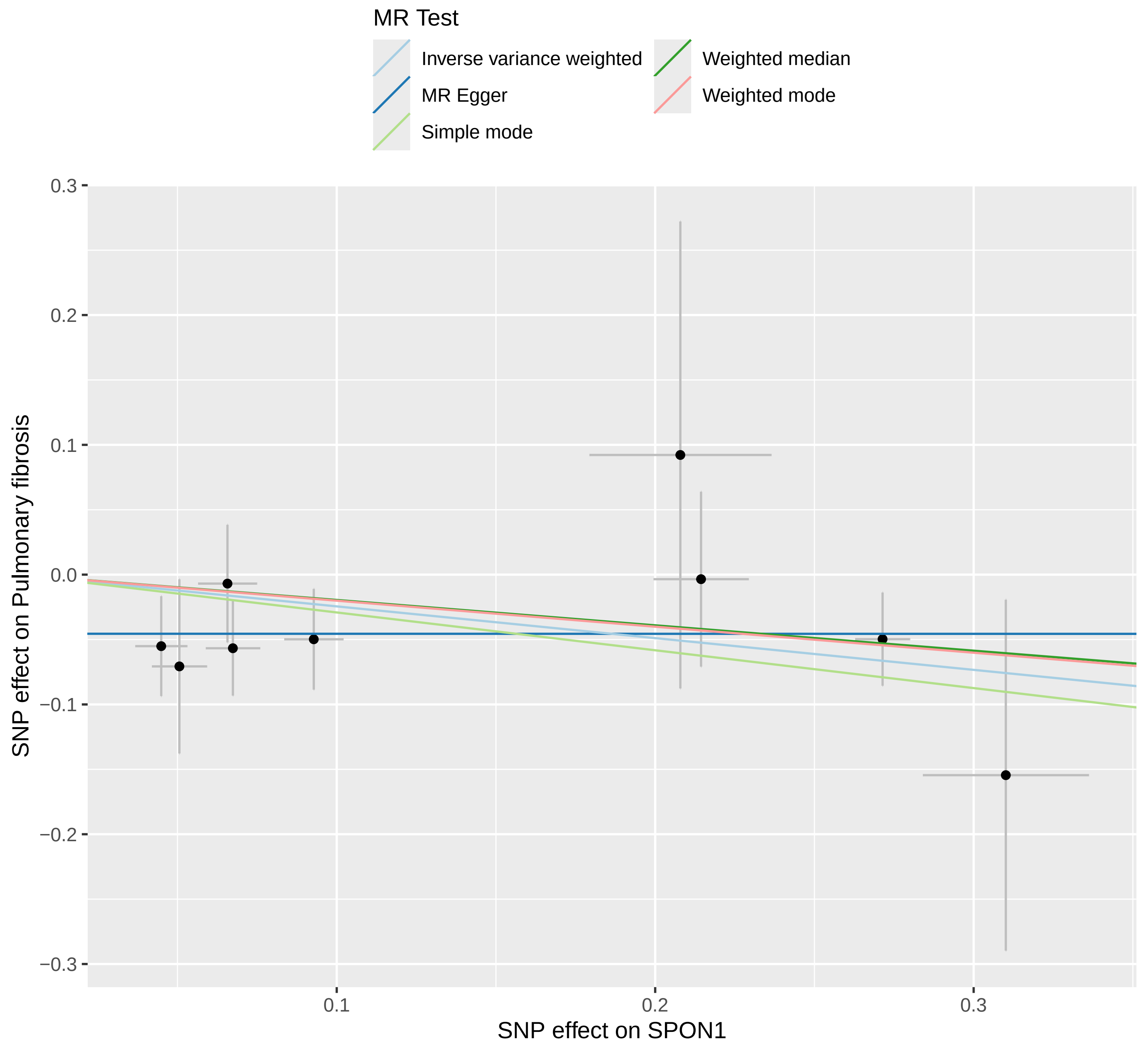

MR Test
Inverse variance weighted
MR Egger
Simple mode
Weighted median
Weighted mode
0.3
0.2
0.1
0.0
−0.1
−0.2
−0.3
SNP effect on Pulmonary fibrosis
0.1
0.2
0.3
SNP effect on SPON1

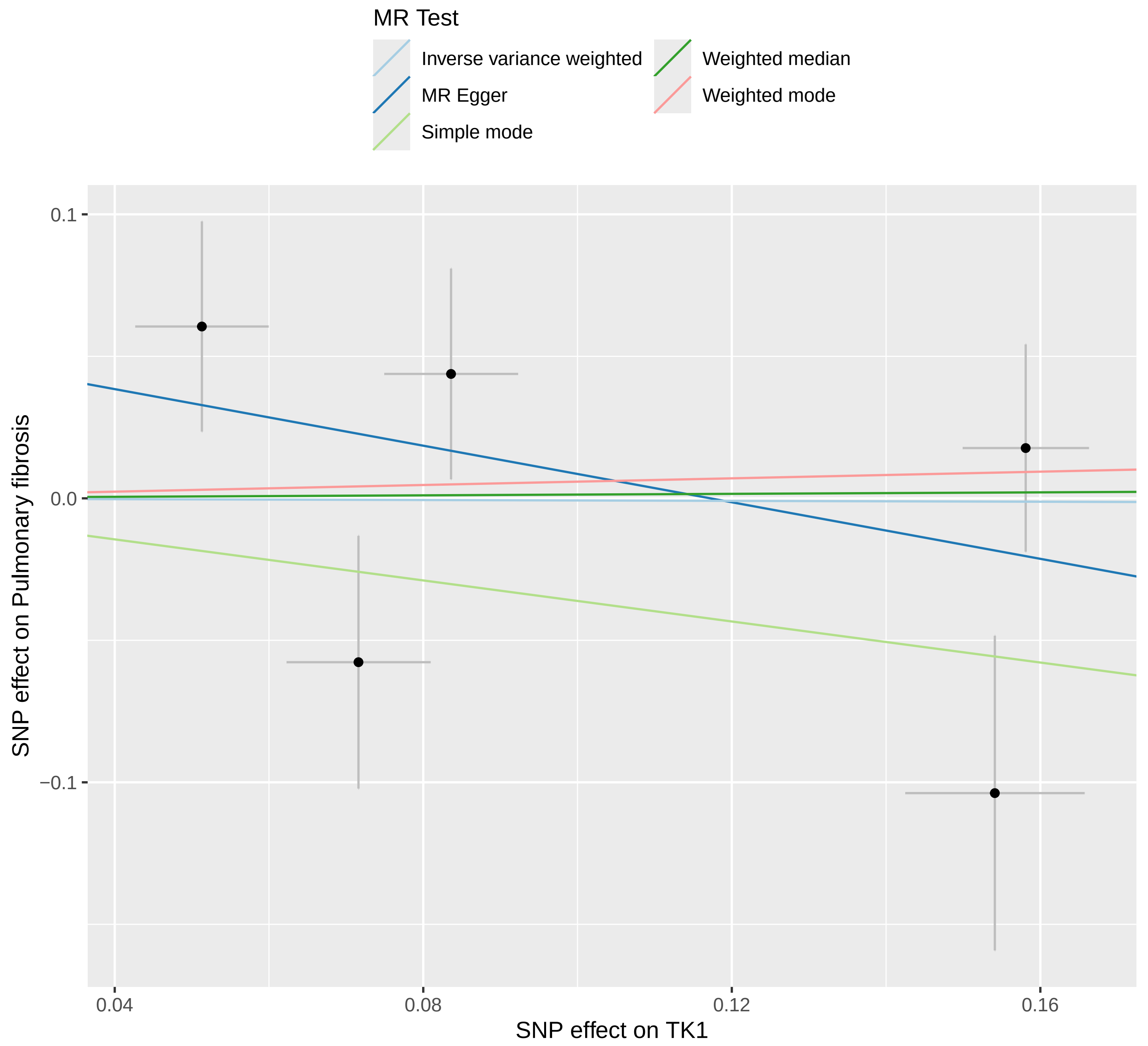
MR Test
Inverse variance weighted
MR Egger
Simple mode
Weighted median
Weighted mode
0.1
0.0
−0.1
0.04
0.08
0.12
0.16
SNP effect on Pulmonary fibrosis
SNP effect on TK1

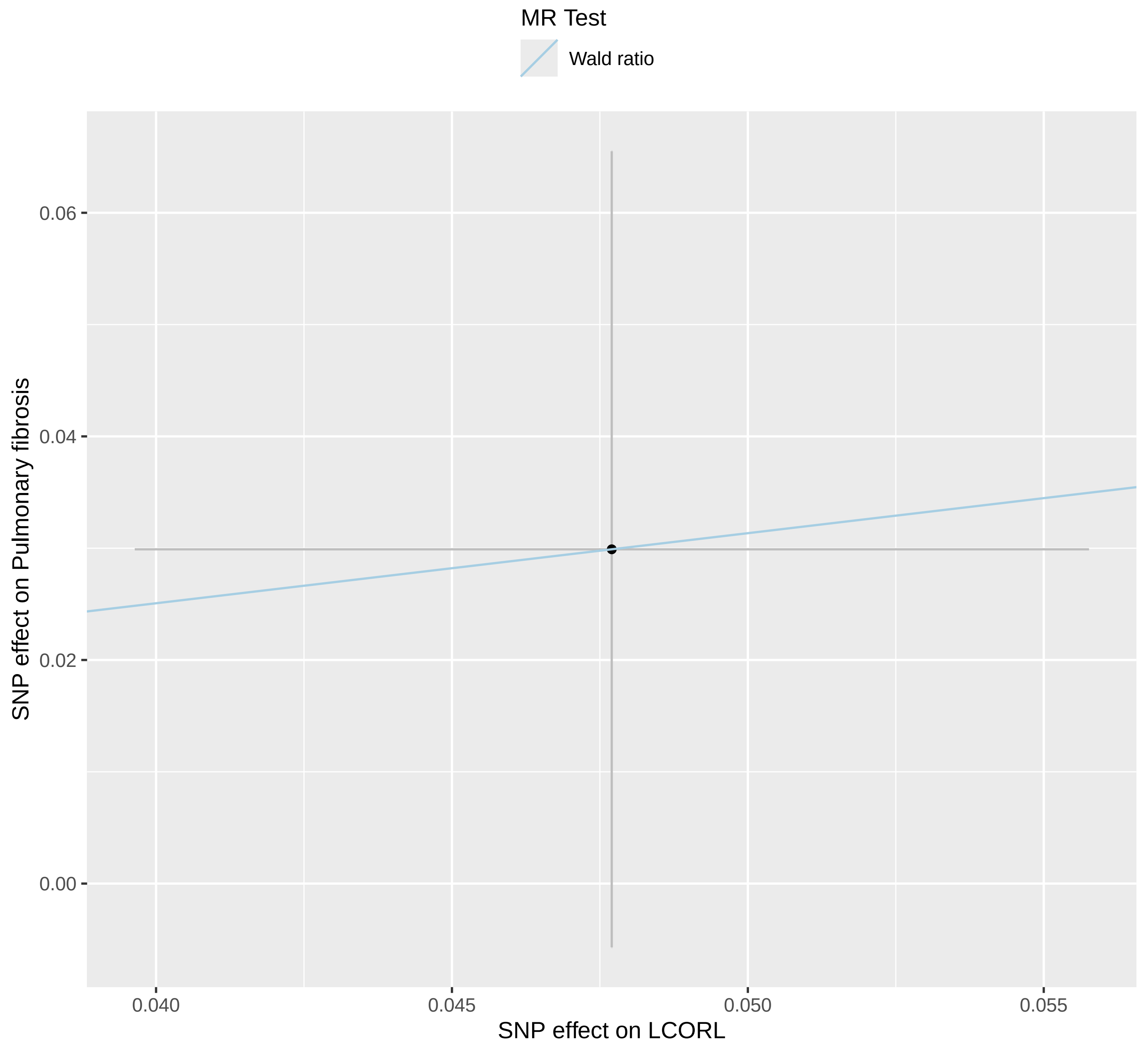

MR Test
Wald ratio
0.06
0.04
0.02
0.00
SNP effect on Pulmonary fibrosis
0.040
0.045
0.050
0.055
SNP effect on LCORL

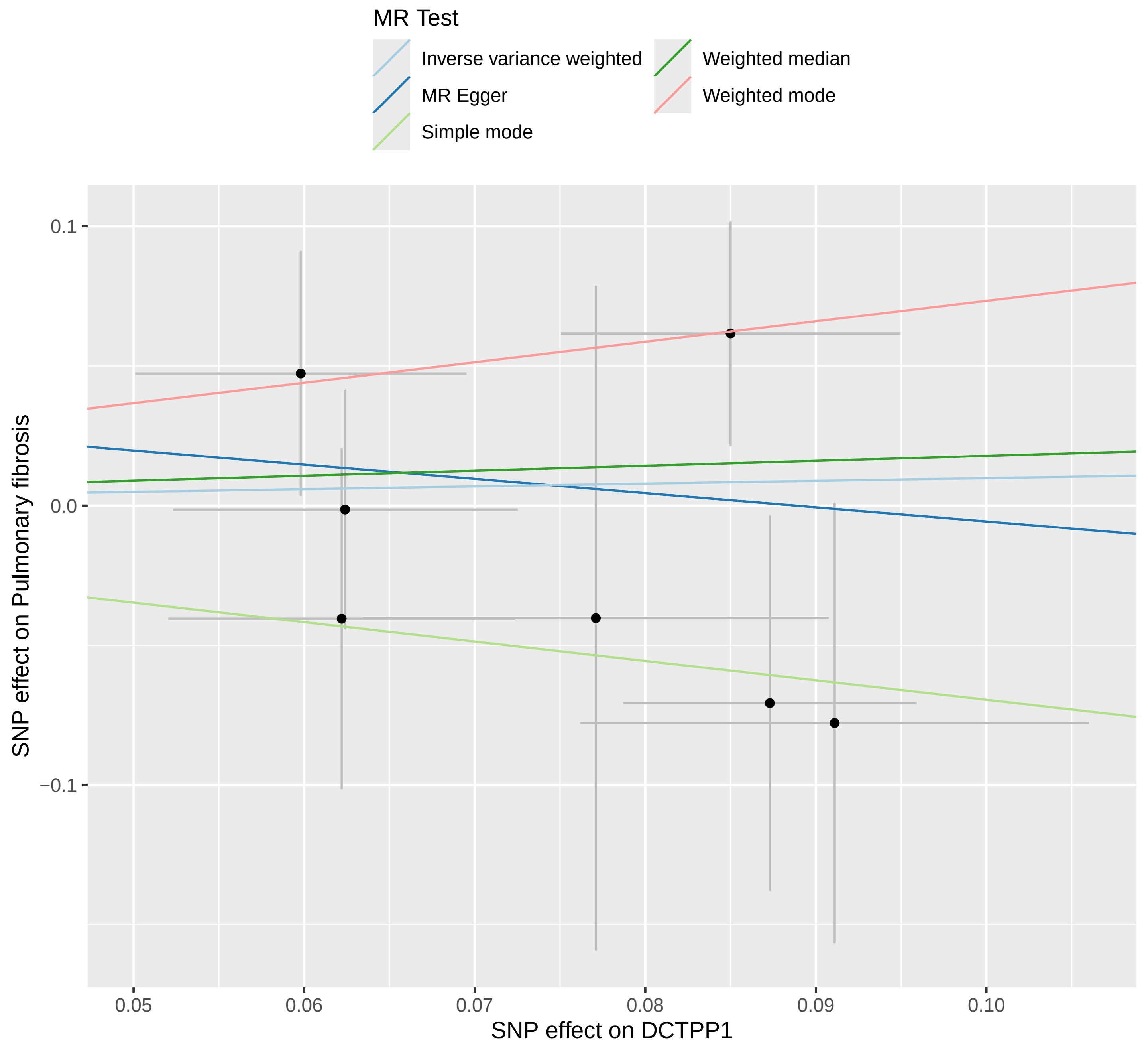

MR Test
Inverse variance weighted
MR Egger
Simple mode
Weighted median
Weighted mode
SNP effect on Pulmonary fibrosis
0.1
0.0
−0.1
0.05
0.06
0.07
0.08
0.09
0.10
SNP effect on DCTPP1

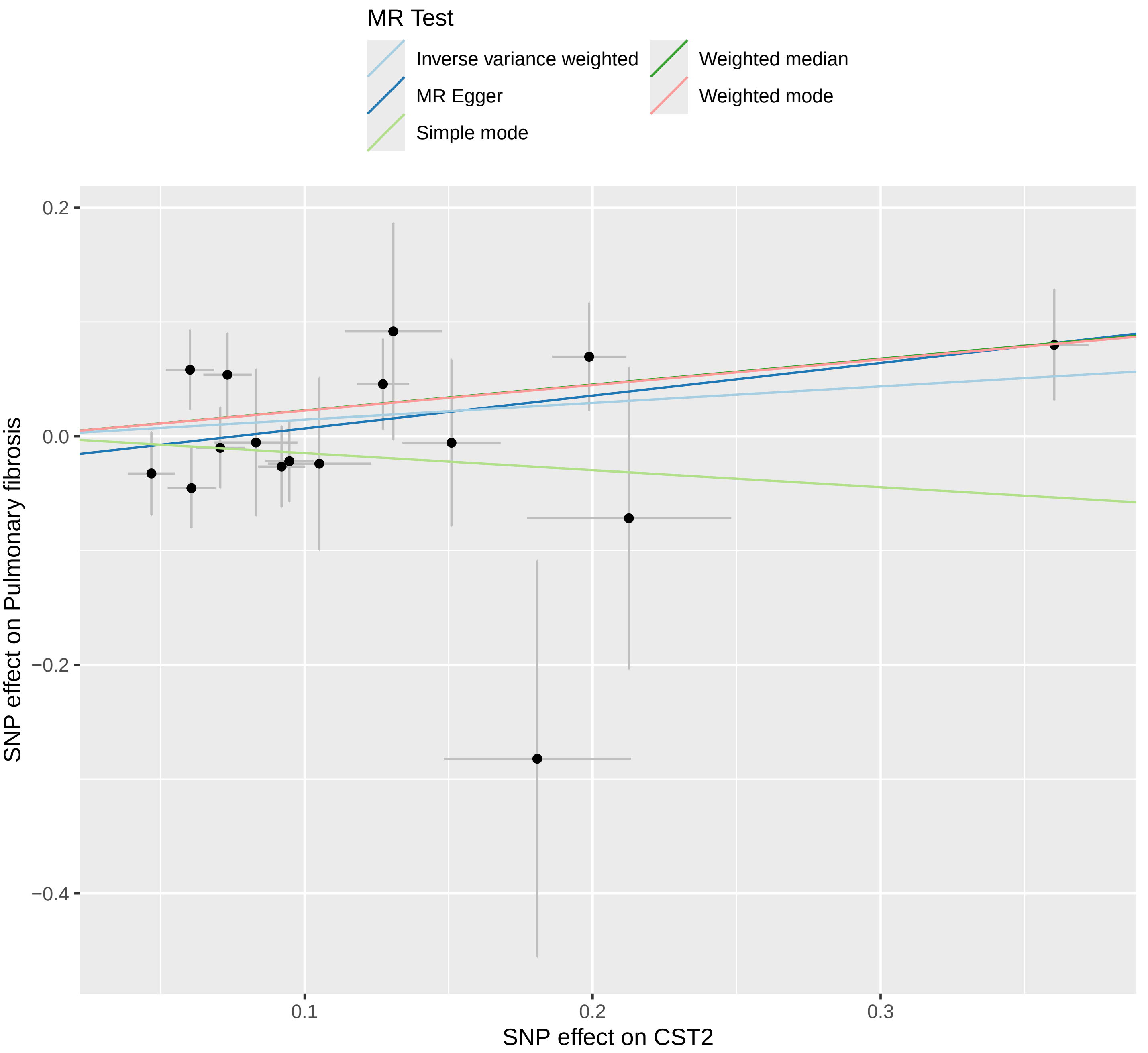
MR Test
Inverse variance weighted
MR Egger
Simple mode
Weighted median
Weighted mode
0.2
0.0
−0.2
−0.4
SNP effect on Pulmonary fibrosis
0.1
0.2
0.3
SNP effect on CST2

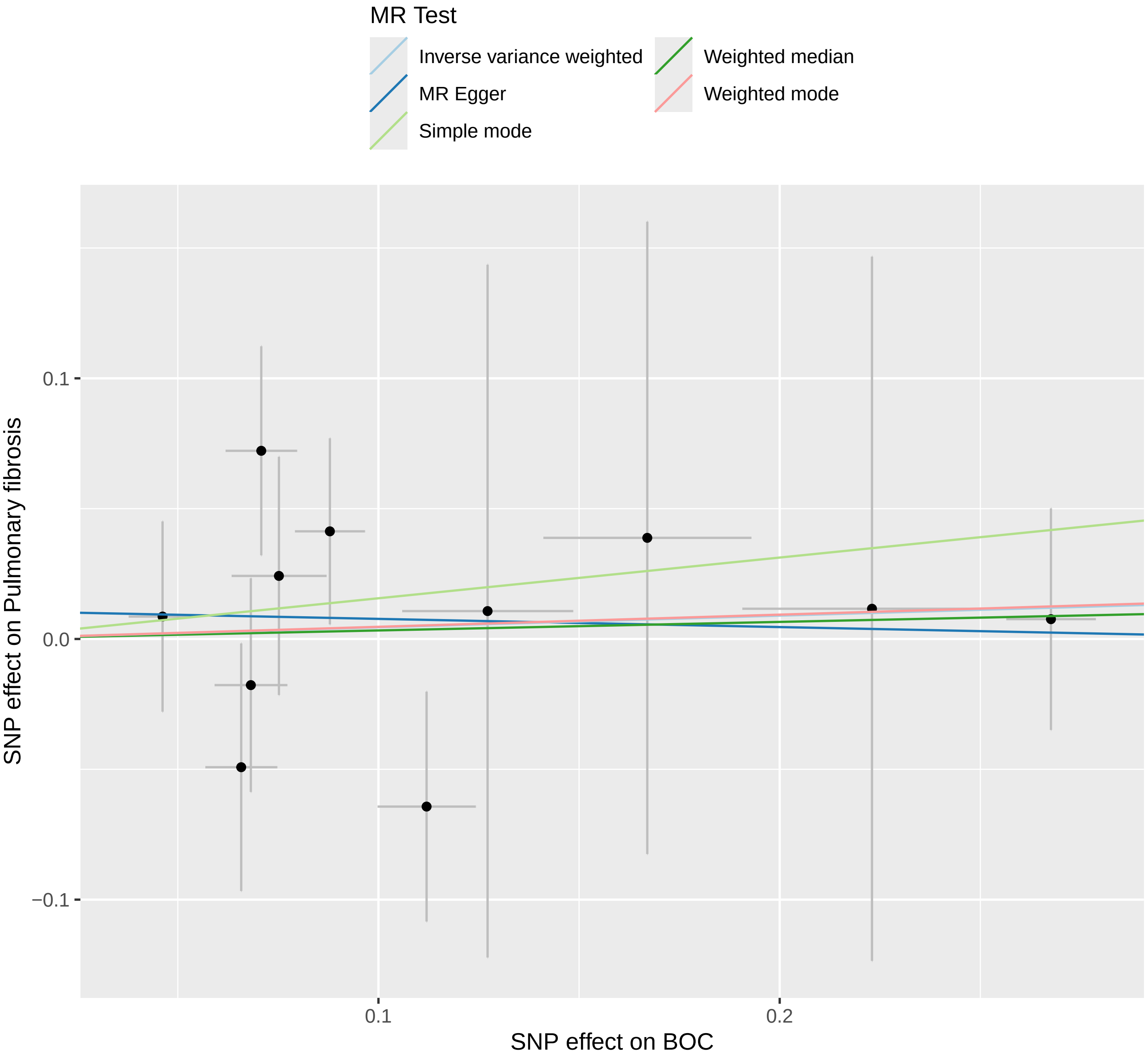

MR Test
Inverse variance weighted
MR Egger
Simple mode
Weighted median
Weighted mode
0.1
0.0
−0.1
0.1
0.2
SNP effect on Pulmonary fibrosis
SNP effect on BOC

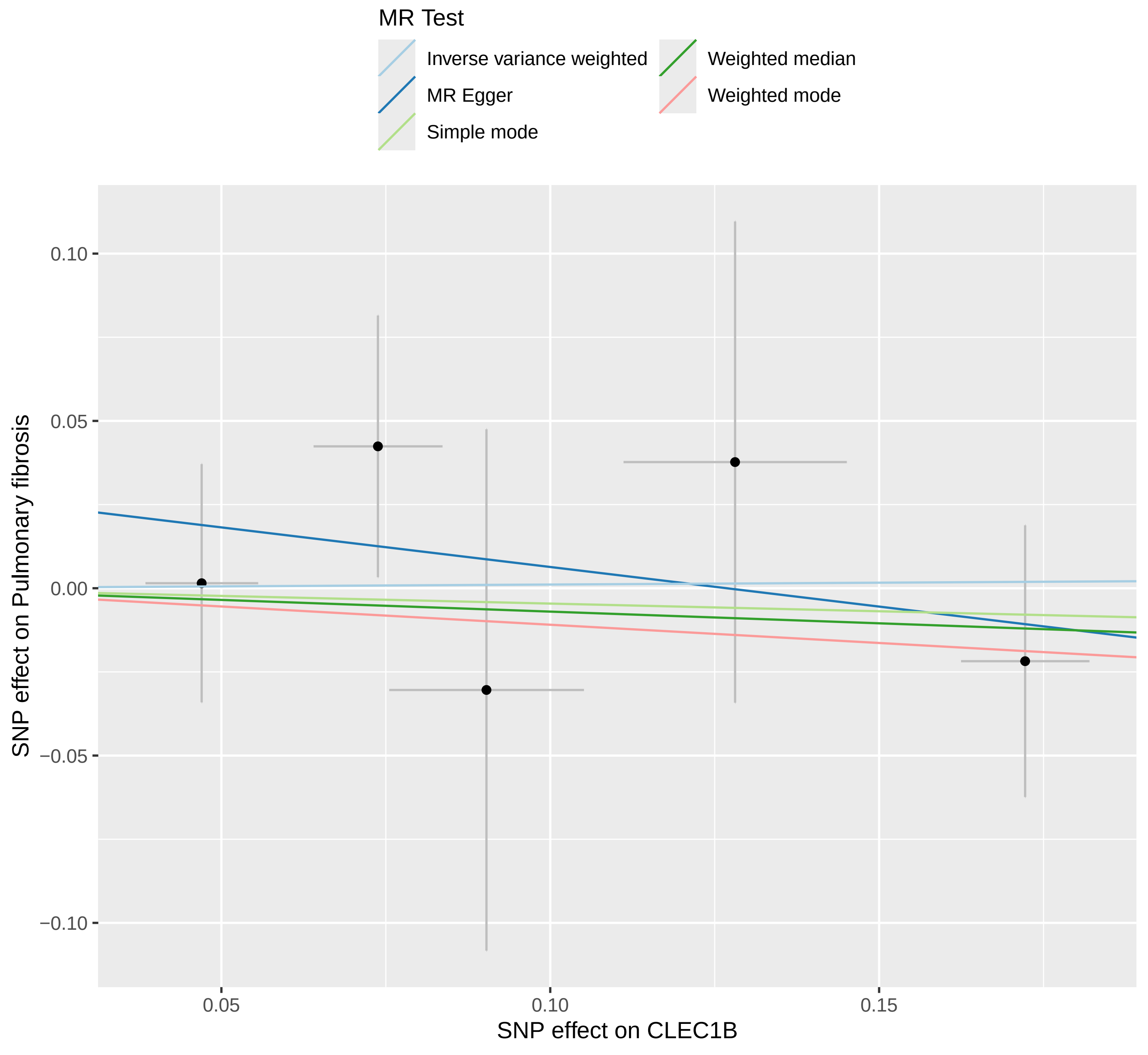

MR Test
Inverse variance weighted
MR Egger
Simple mode
Weighted median
Weighted mode
0.10
0.05
0.00
−0.05
−0.10
0.05
0.10
0.15
SNP effect on Pulmonary fibrosis
SNP effect on CLEC1B

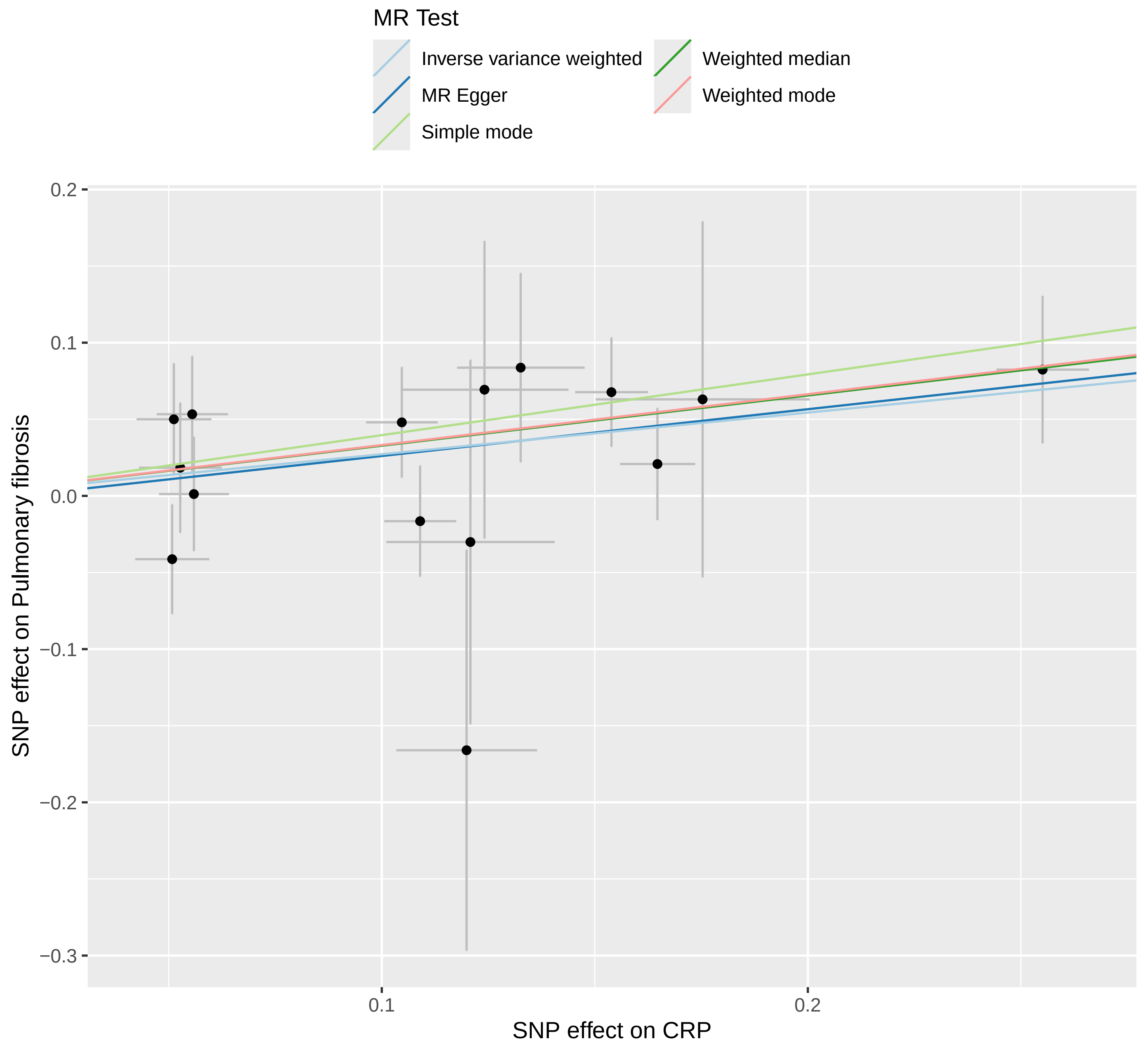

MR Test
Inverse variance weighted
MR Egger
Simple mode
Weighted median
Weighted mode
0.2
0.1
0.0
−0.1
−0.2
−0.3
0.1
0.2
SNP effect on CRP
SNP effect on Pulmonary fibrosis

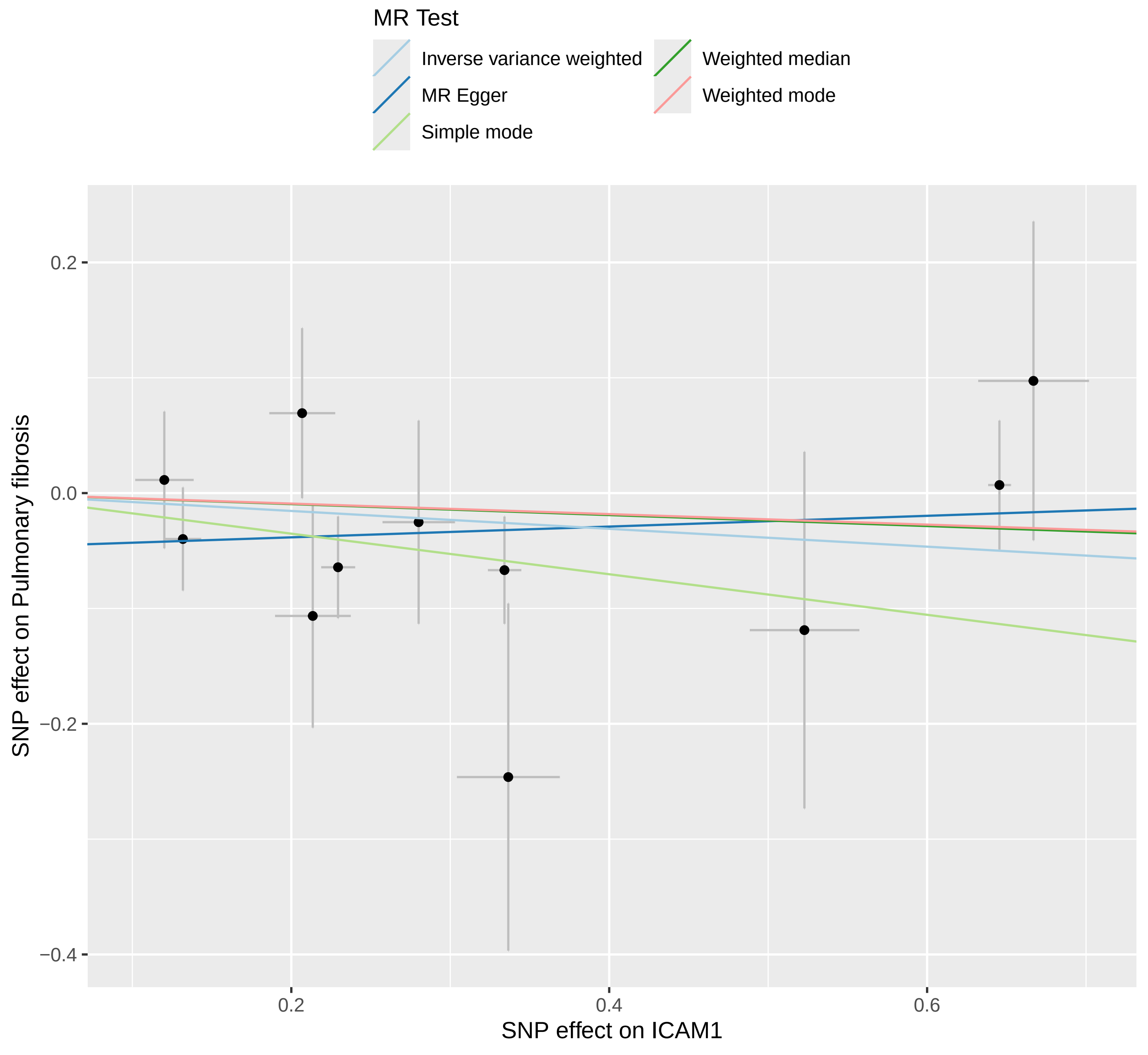
MR Test
Inverse variance weighted
MR Egger
Simple mode
Weighted median
Weighted mode
0.2
0.0
−0.2
−0.4
SNP effect on Pulmonary fibrosis
0.2
0.4
0.6
SNP effect on ICAM1

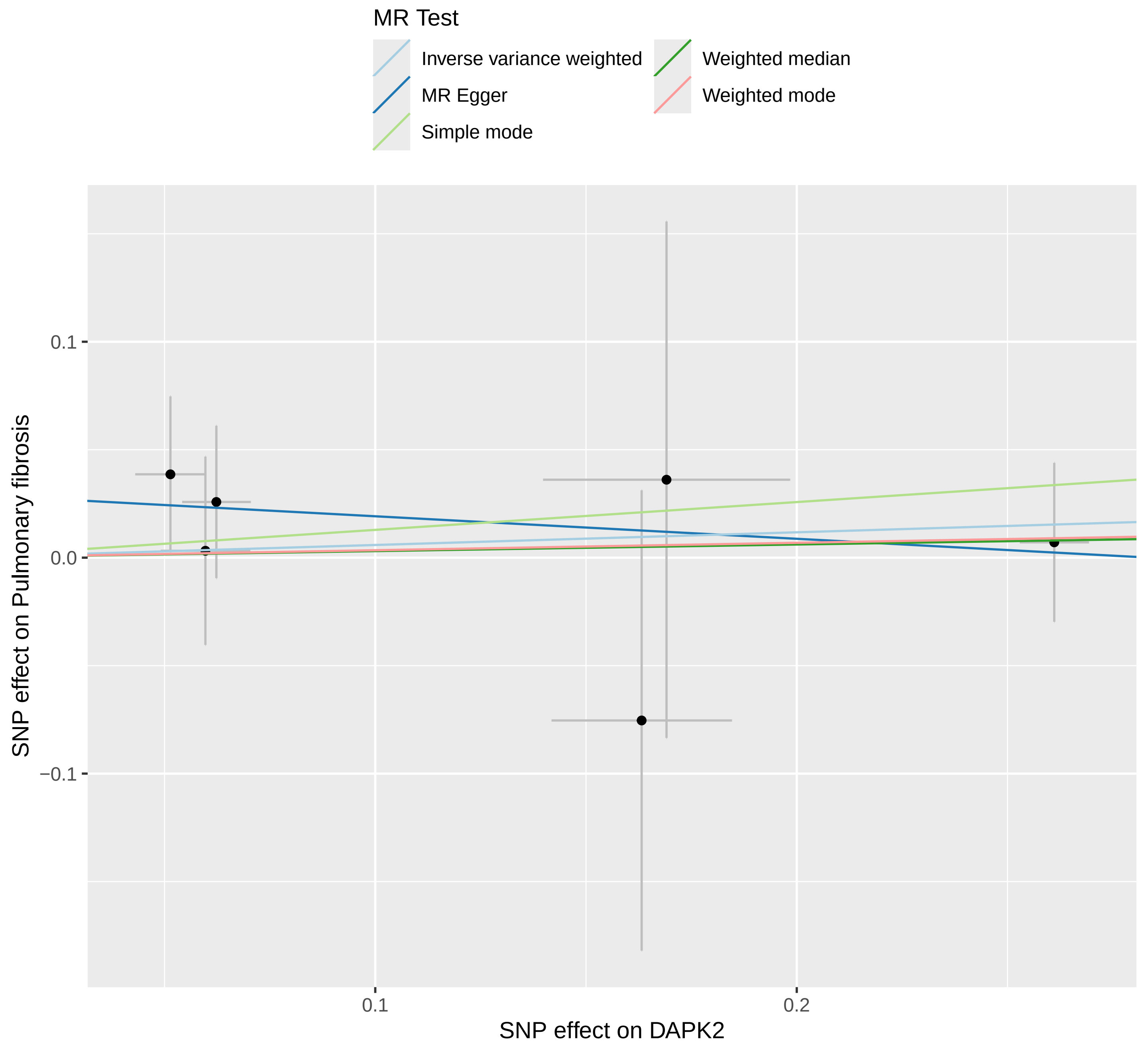
MR Test
Inverse variance weighted
MR Egger
Simple mode
Weighted median
Weighted mode
0.1
0.0
−0.1
0.1
0.2
SNP effect on Pulmonary fibrosis
SNP effect on DAPK2

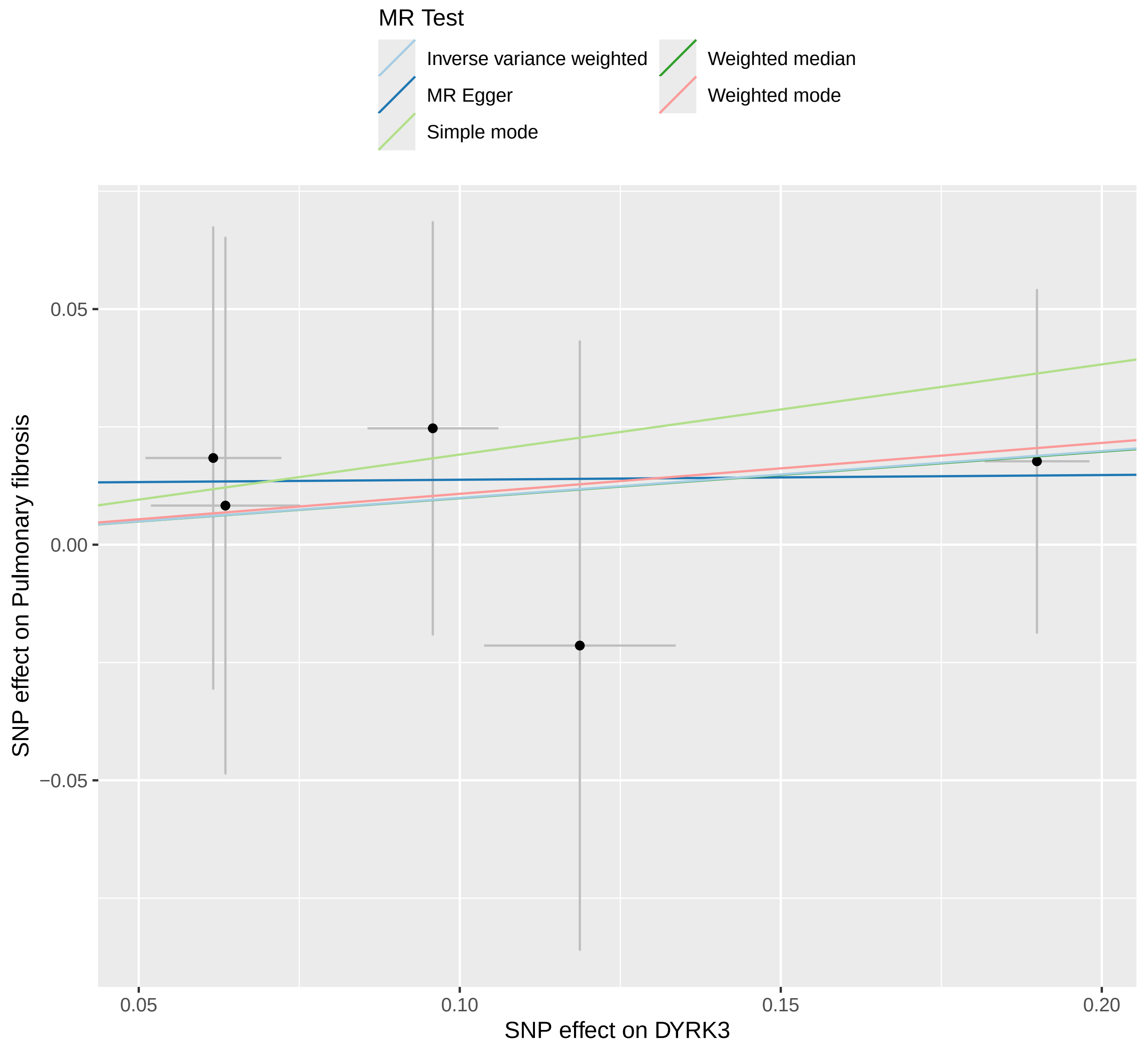

MR Test
Inverse variance weighted
MR Egger
Simple mode
Weighted median
Weighted mode
SNP effect on Pulmonary fibrosis
0.05
0.00
−0.05
0.05
0.10
0.15
0.20
SNP effect on DYRK3

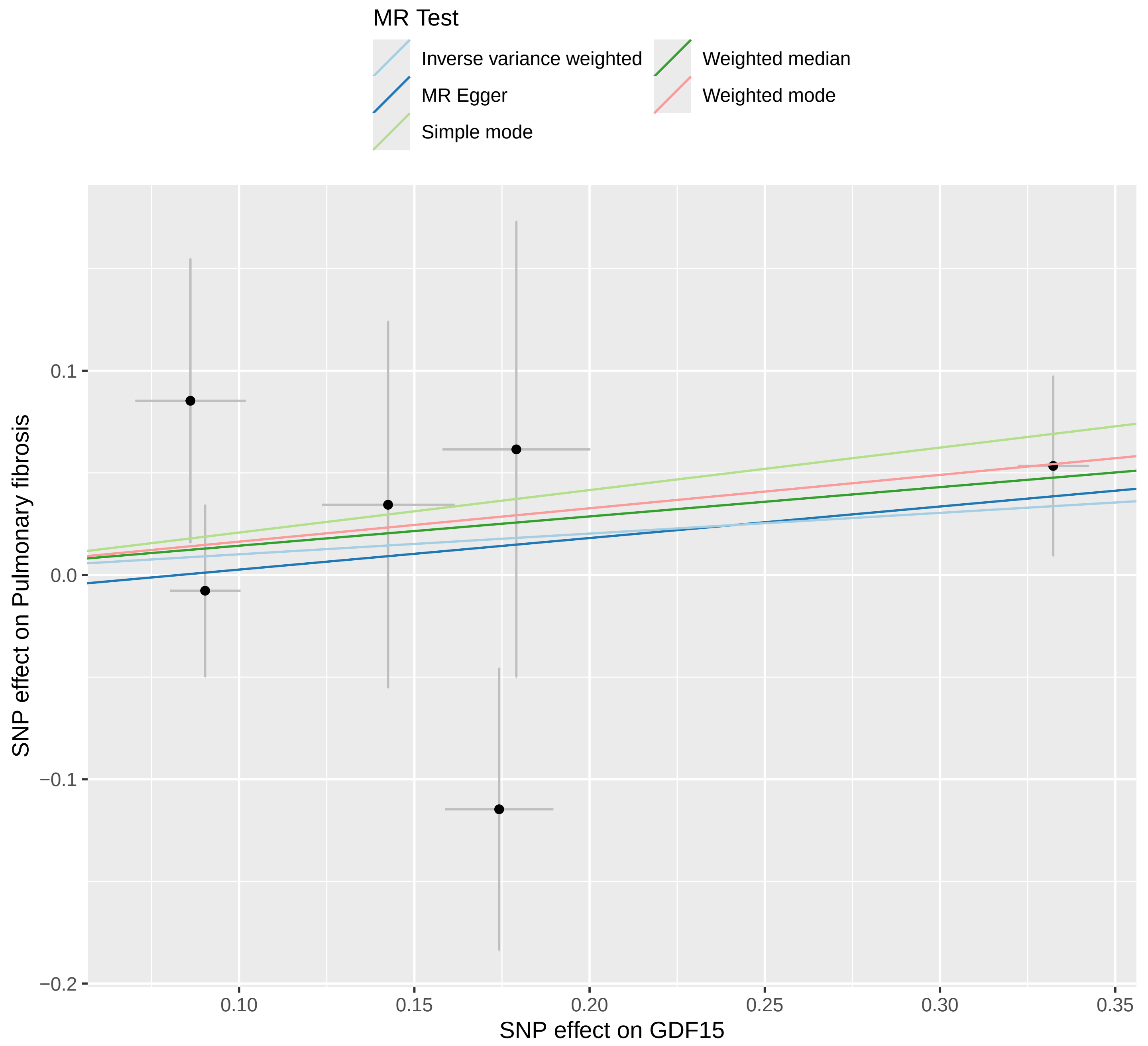

MR Test
Inverse variance weighted
MR Egger
Simple mode
Weighted median
Weighted mode
SNP effect on Pulmonary fibrosis
0.1
0.0
−0.1
−0.2
0.10
0.15
0.20
0.25
0.30
0.35
SNP effect on GDF15

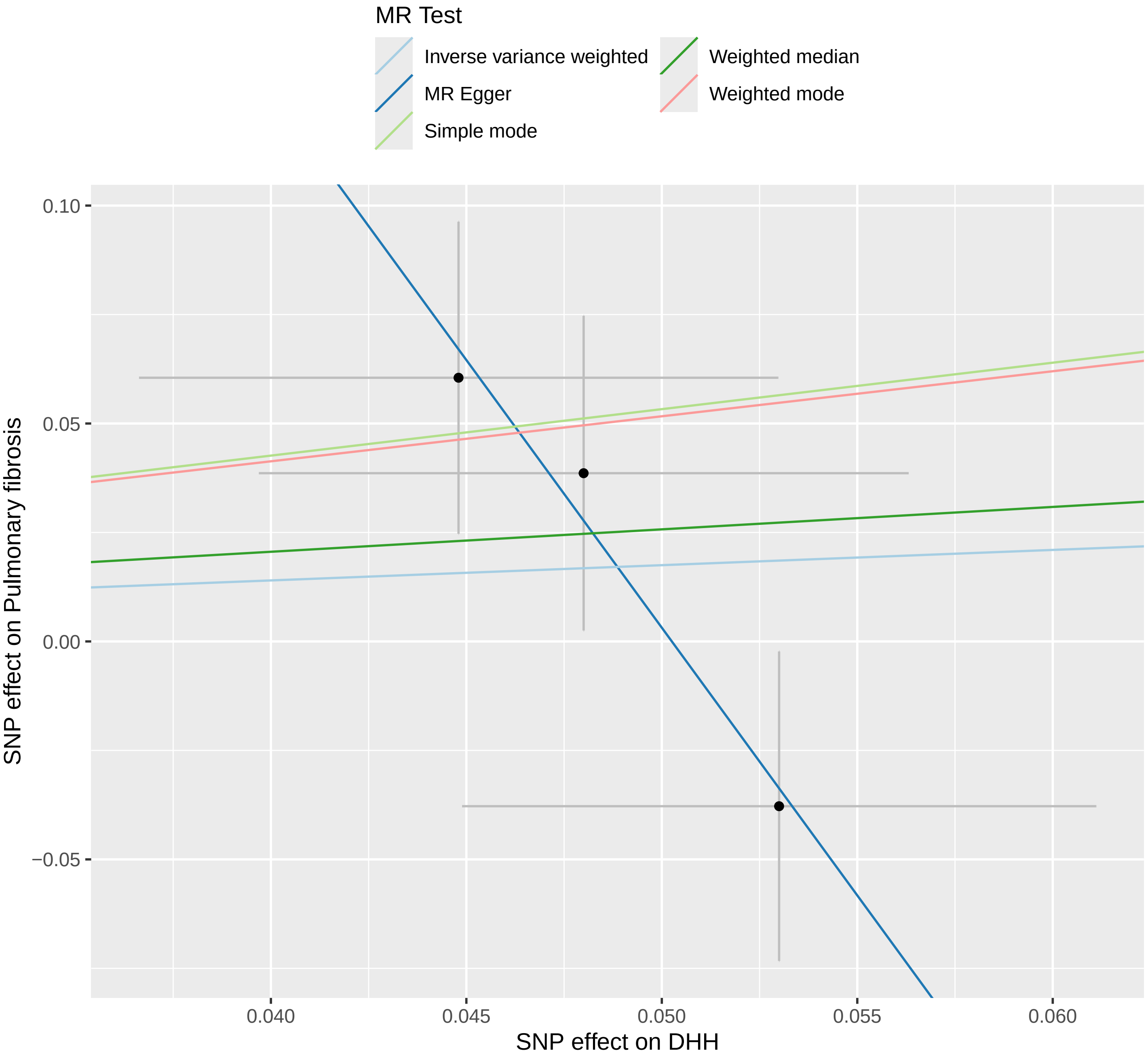

MR Test
Inverse variance weighted
MR Egger
Simple mode
Weighted median
Weighted mode
0.10
0.05
0.00
−0.05
SNP effect on Pulmonary fibrosis
0.040
0.045
0.050
0.055
0.060
SNP effect on DHH

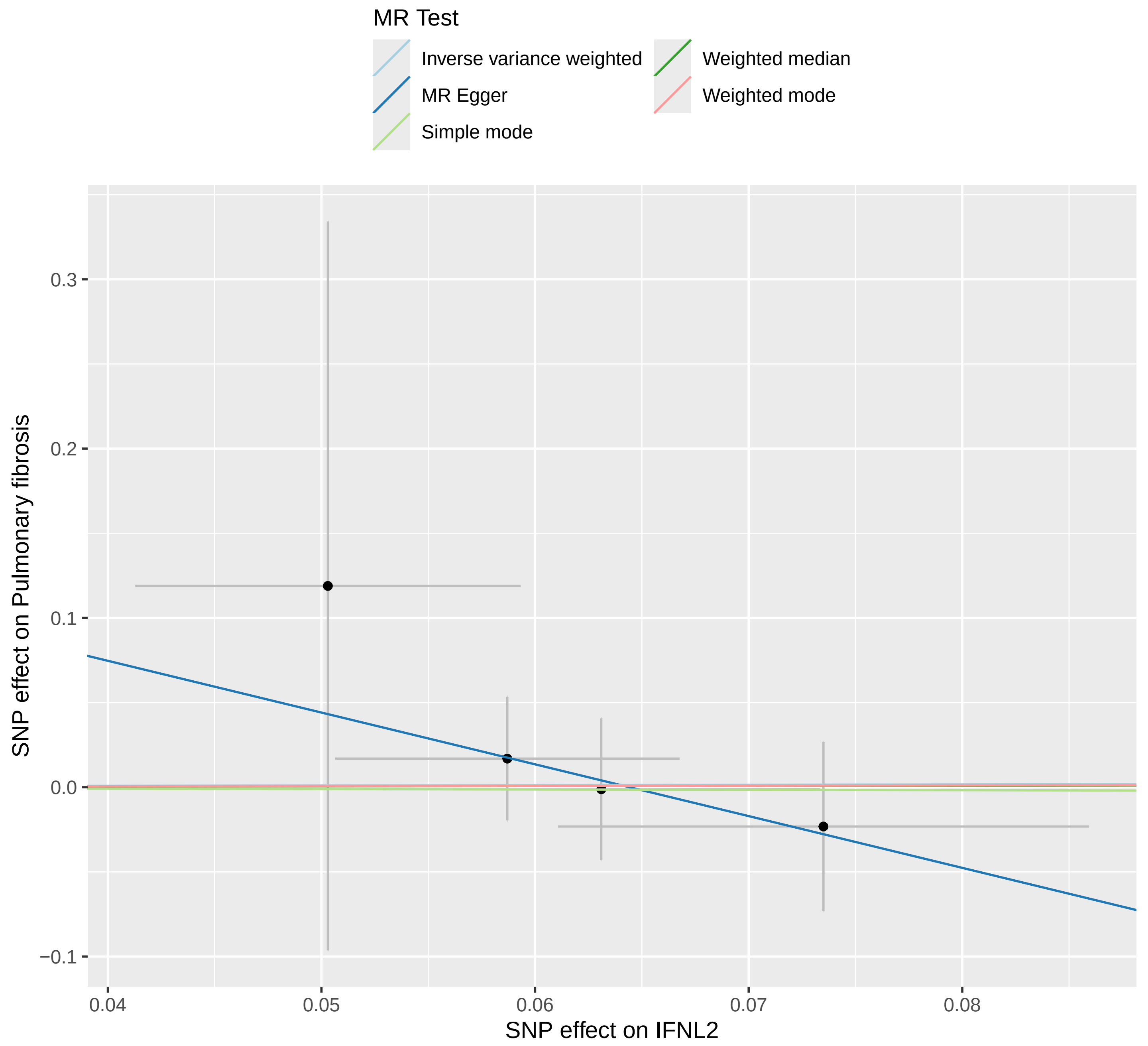

MR Test
Inverse variance weighted
MR Egger
Simple mode
Weighted median
Weighted mode
SNP effect on Pulmonary fibrosis
0.3
0.2
0.1
0.0
−0.1
0.04
0.05
0.06
0.07
0.08
SNP effect on IFNL2

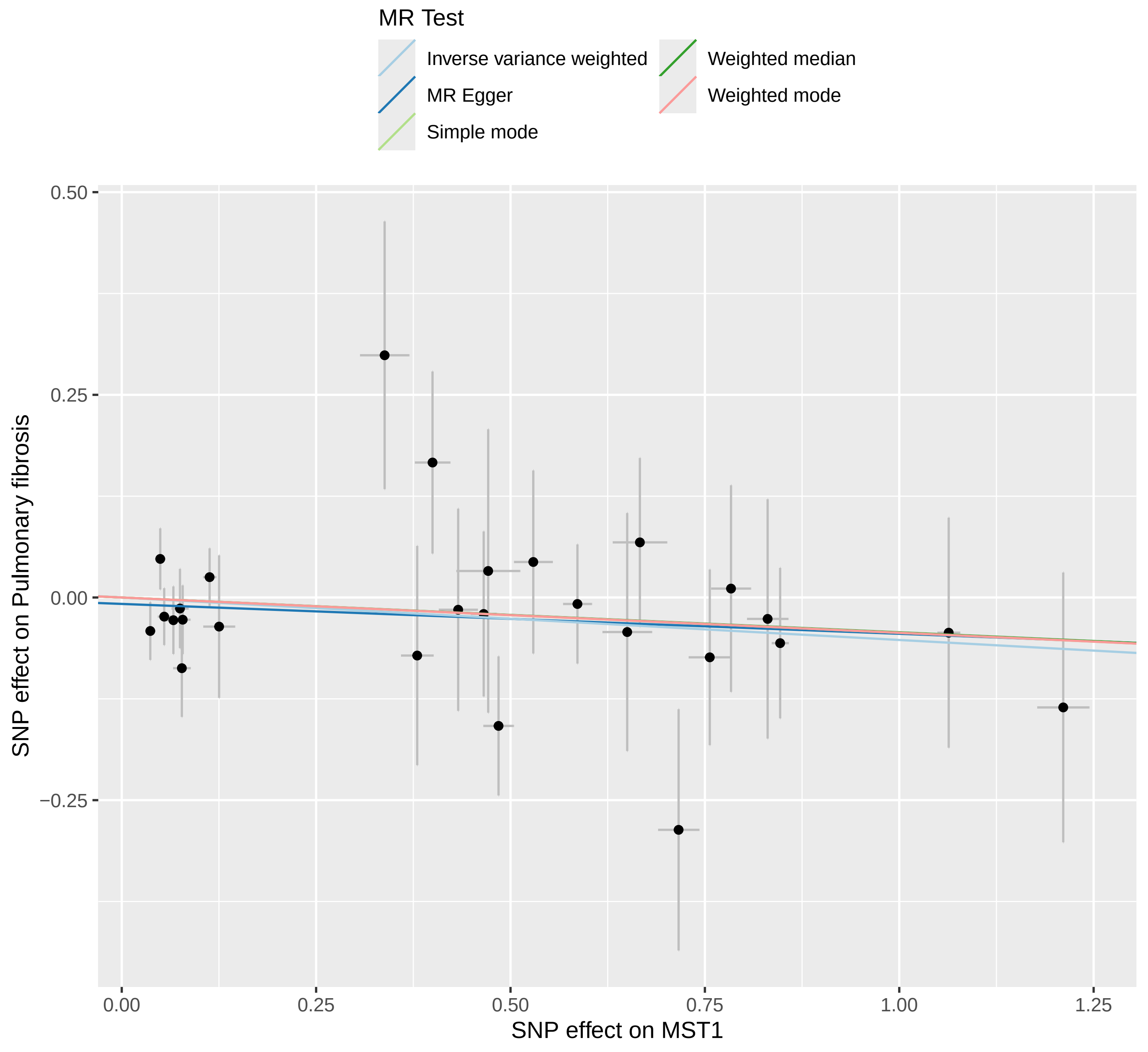

MR Test
Inverse variance weighted
MR Egger
Simple mode
Weighted median
Weighted mode
0.50
0.25
0.00
−0.25
SNP effect on Pulmonary fibrosis
0.00
0.25
0.50
0.75
1.00
1.25
SNP effect on MST1

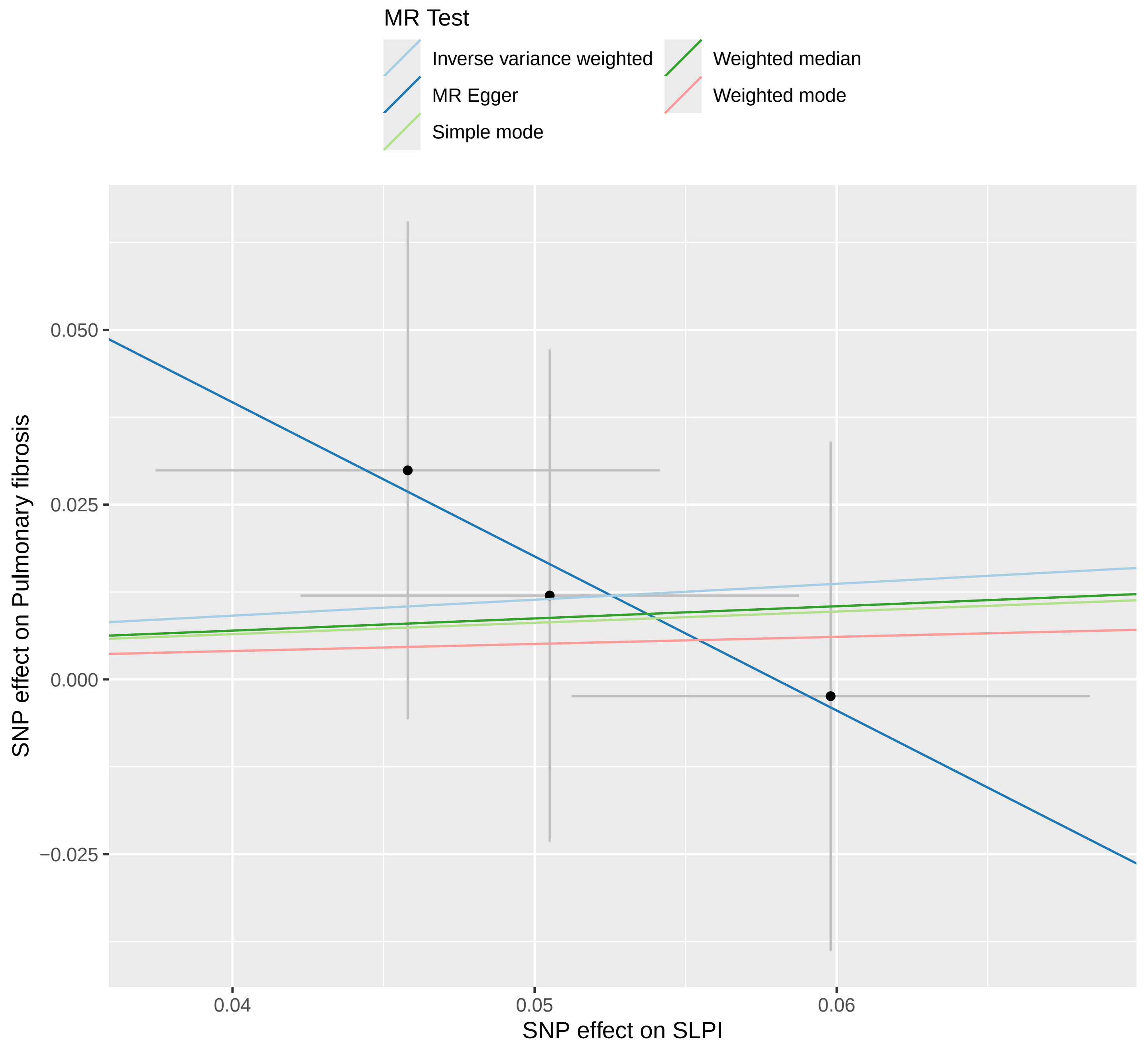

MR Test
Inverse variance weighted
MR Egger
Simple mode
Weighted median
Weighted mode
0.050
0.025
0.000
−0.025
SNP effect on Pulmonary fibrosis
0.04
0.05
0.06
SNP effect on SLPI

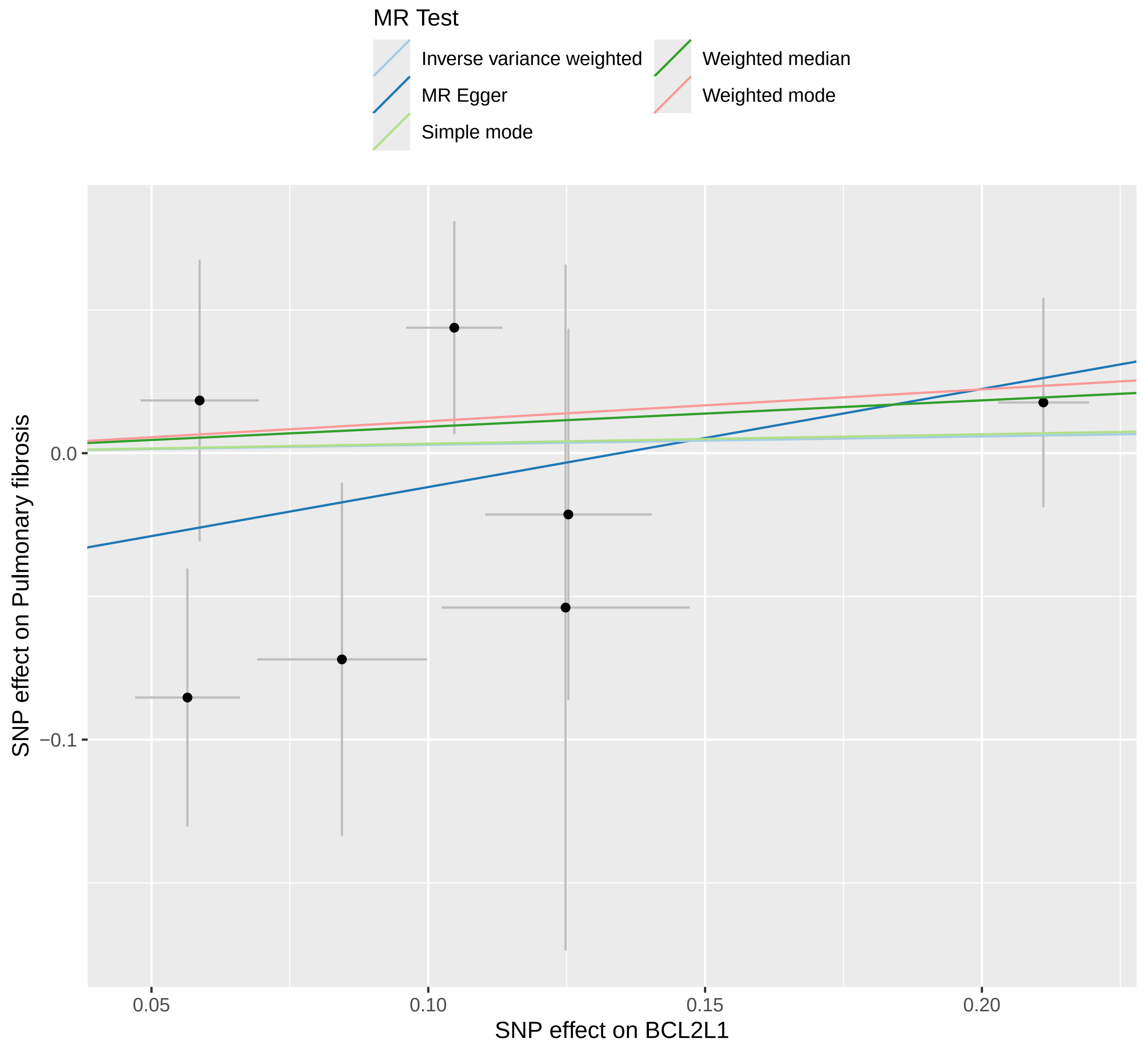

MR Test
Inverse variance weighted
MR Egger
Simple mode
Weighted median
Weighted mode
SNP effect on Pulmonary fibrosis
0.0
−0.1
0.05
0.10
0.15
0.20
SNP effect on BCL2L1

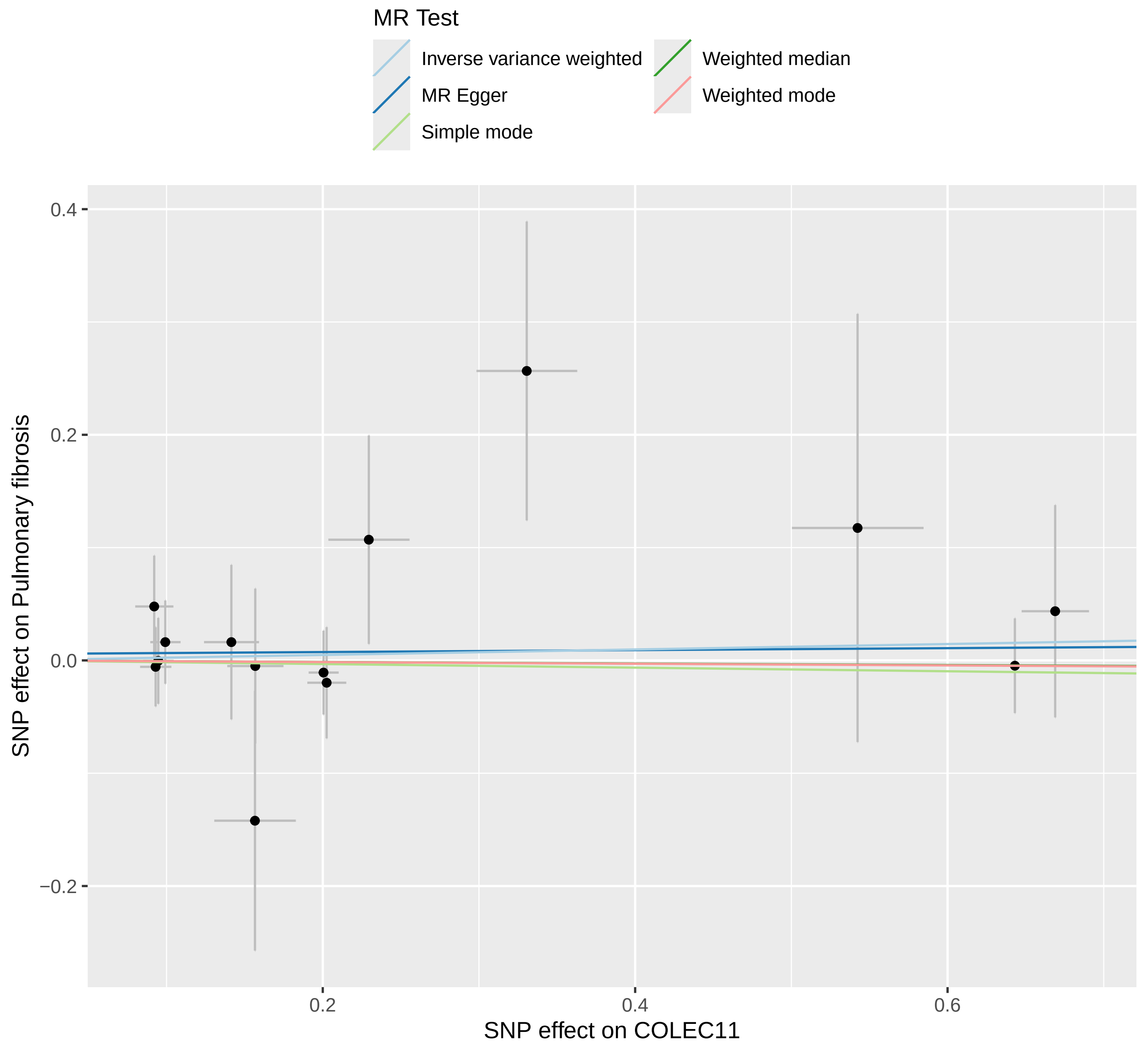

MR Test
Inverse variance weighted
MR Egger
Simple mode
Weighted median
Weighted mode
0.4
0.2
0.0
−0.2
0.2
0.4
0.6
SNP effect on Pulmonary fibrosis
SNP effect on COLEC11

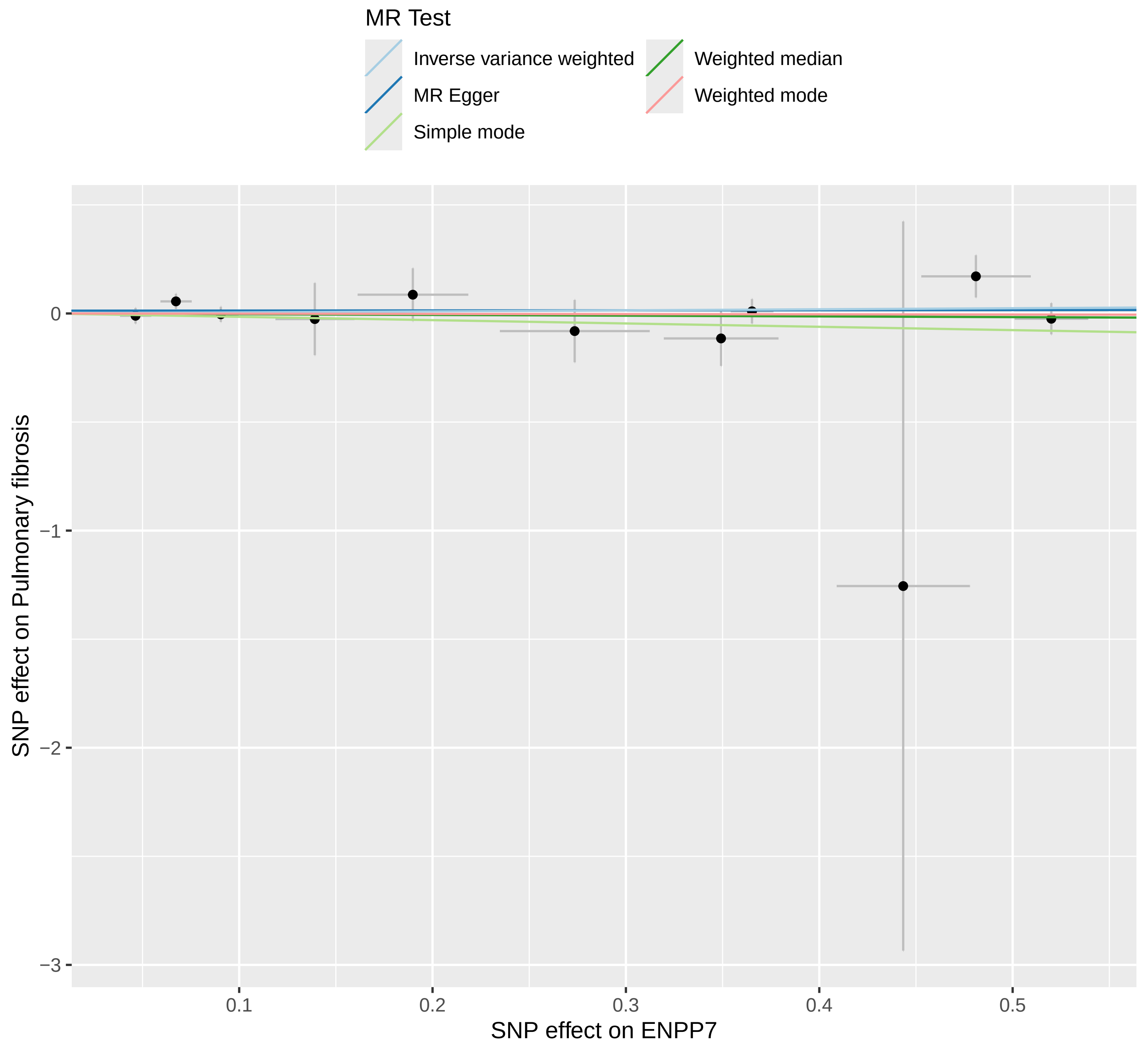
MR Test
Inverse variance weighted
MR Egger
Simple mode
Weighted median
Weighted mode
0
−1
−2
−3
0.1
0.2
0.3
0.4
0.5
SNP effect on Pulmonary fibrosis
SNP effect on ENPP7

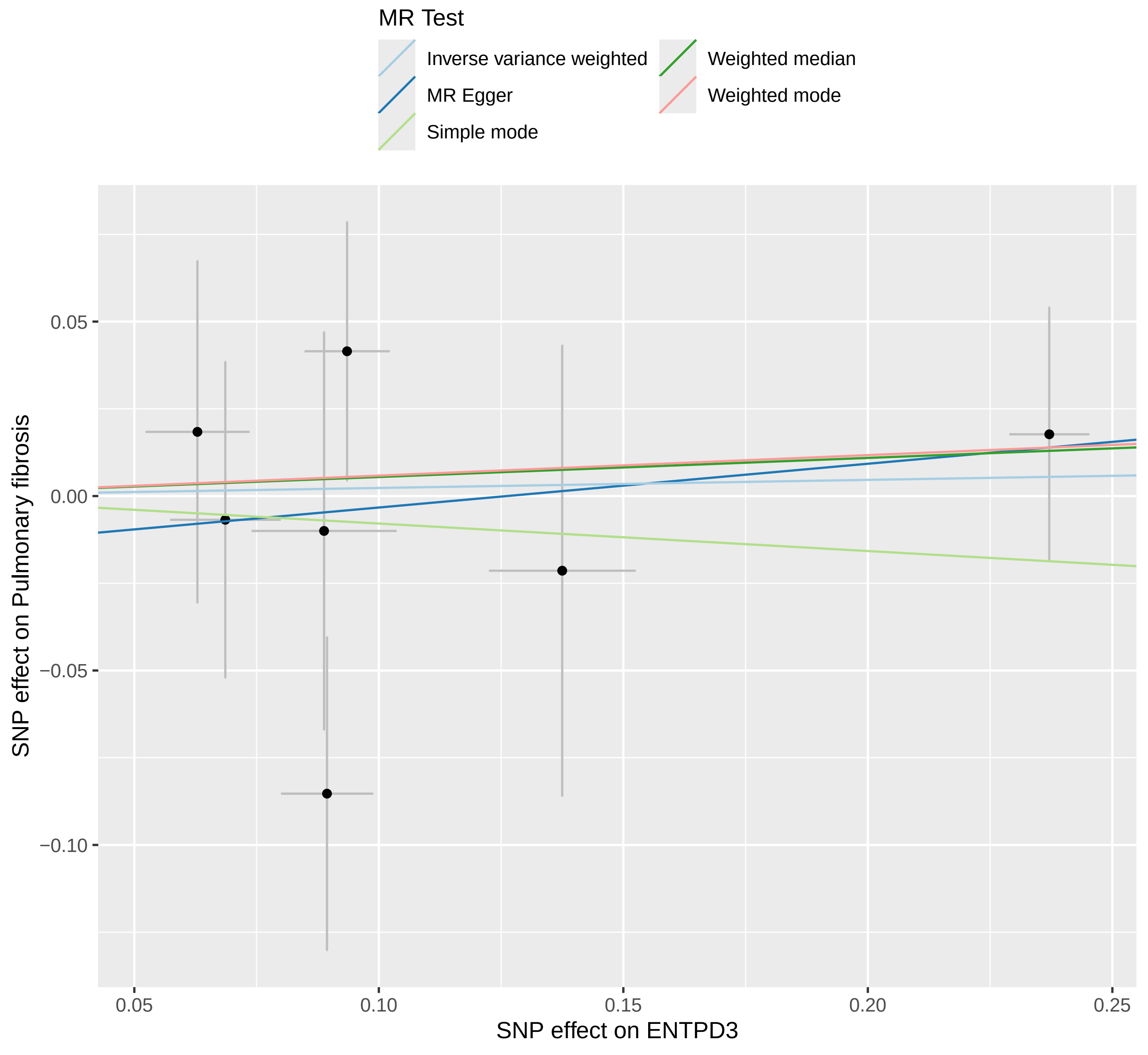

MR Test
Inverse variance weighted
MR Egger
Simple mode
Weighted median
Weighted mode
0.05
0.00
−0.05
−0.10
0.05
0.10
0.15
0.20
0.25
SNP effect on ENTPD3
SNP effect on Pulmonary fibrosis

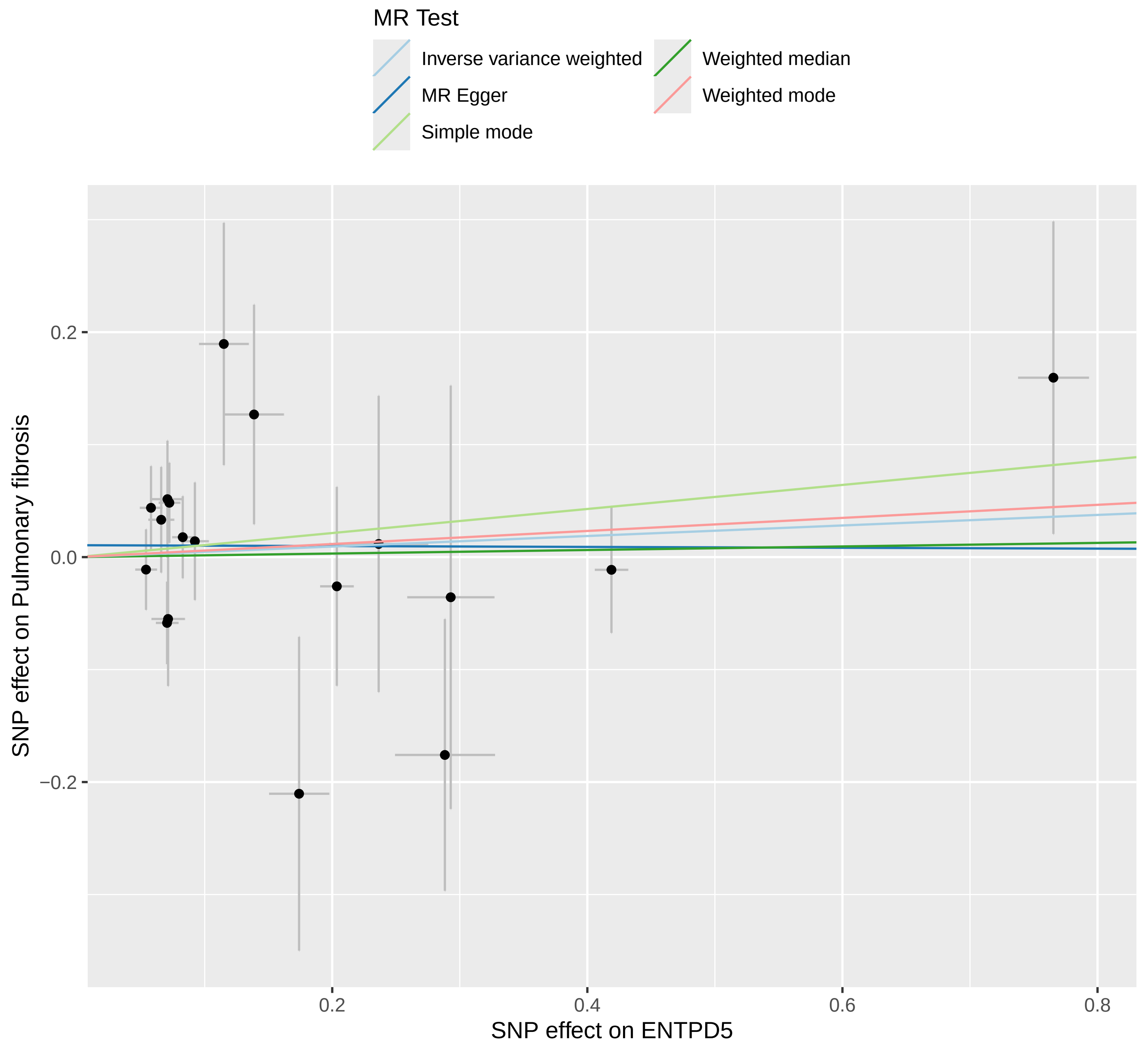

MR Test
Inverse variance weighted
MR Egger
Simple mode
Weighted median
Weighted mode
0.2
0.0
−0.2
0.2
0.4
0.6
0.8
SNP effect on Pulmonary fibrosis
SNP effect on ENTPD5

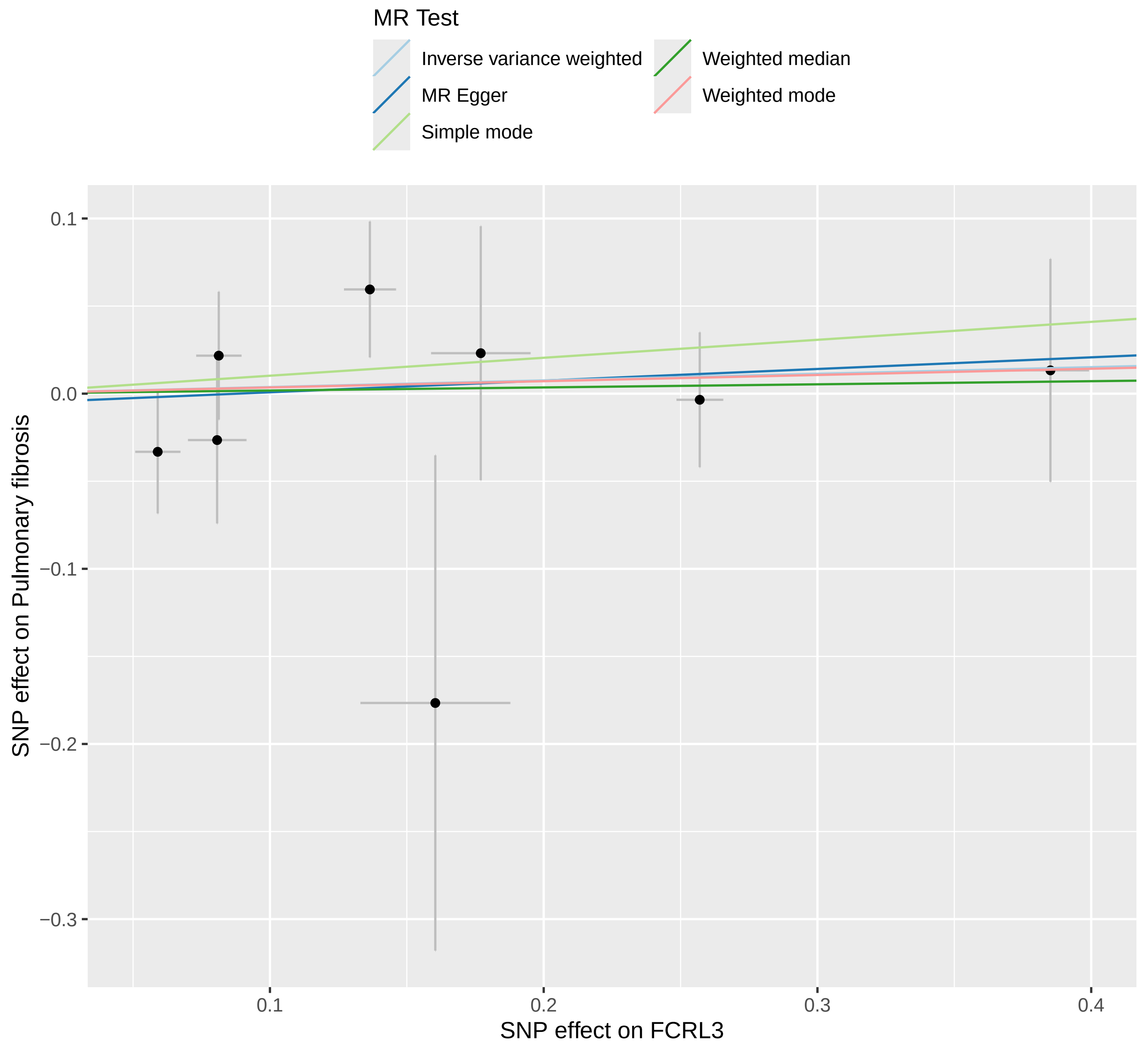

MR Test
Inverse variance weighted
MR Egger
Simple mode
Weighted median
Weighted mode
0.1
0.0
−0.1
−0.2
−0.3
SNP effect on Pulmonary fibrosis
0.1
0.2
0.3
0.4
SNP effect on FCRL3

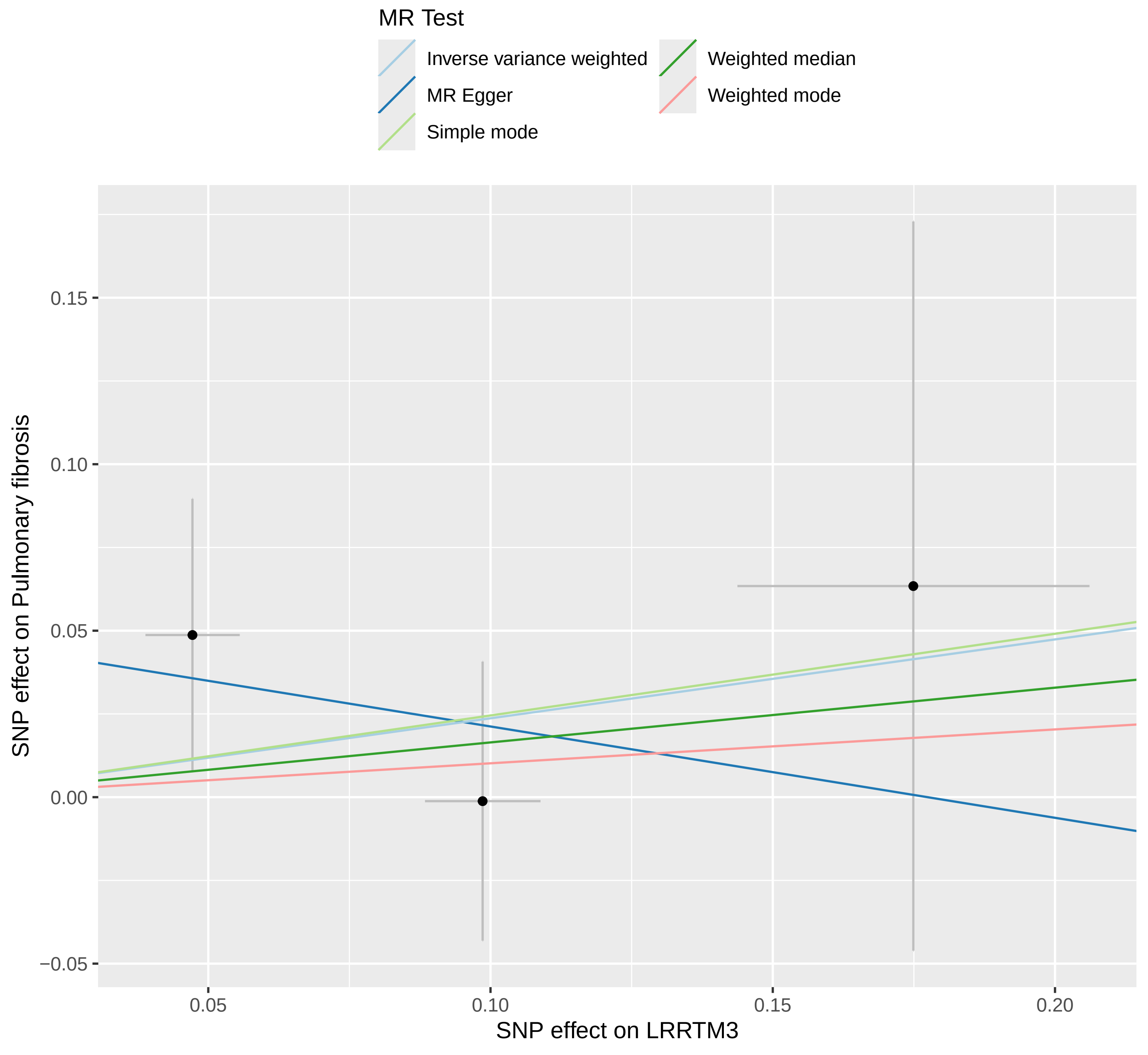

MR Test
Inverse variance weighted
MR Egger
Simple mode
Weighted median
Weighted mode
SNP effect on Pulmonary fibrosis
0.15
0.10
0.05
0.00
−0.05
0.05
0.10
0.15
0.20
SNP effect on LRRTM3

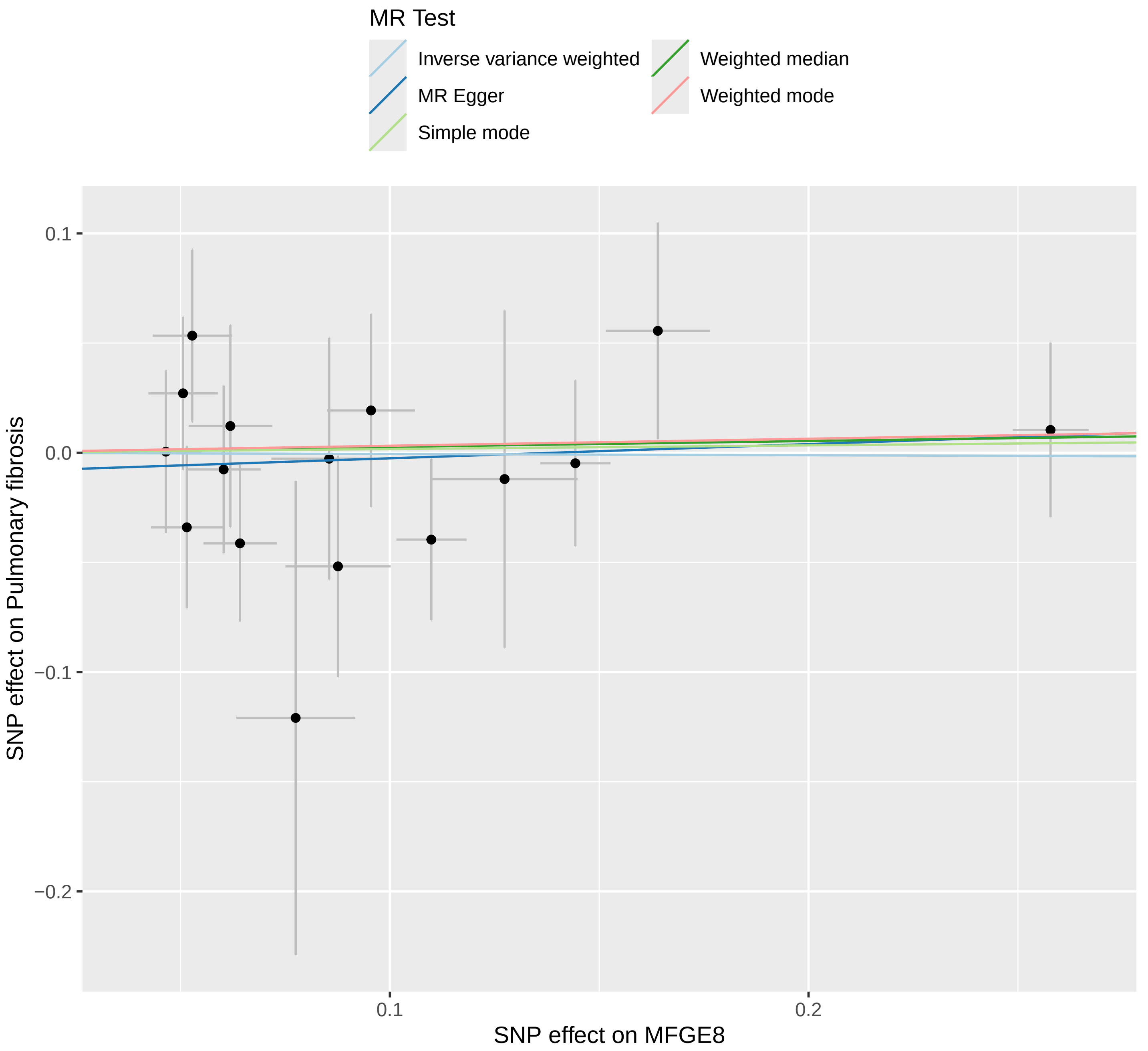

MR Test
Inverse variance weighted
MR Egger
Simple mode
Weighted median
Weighted mode
0.1
0.0
−0.1
−0.2
SNP effect on Pulmonary fibrosis
0.1
0.2
SNP effect on MFGE8

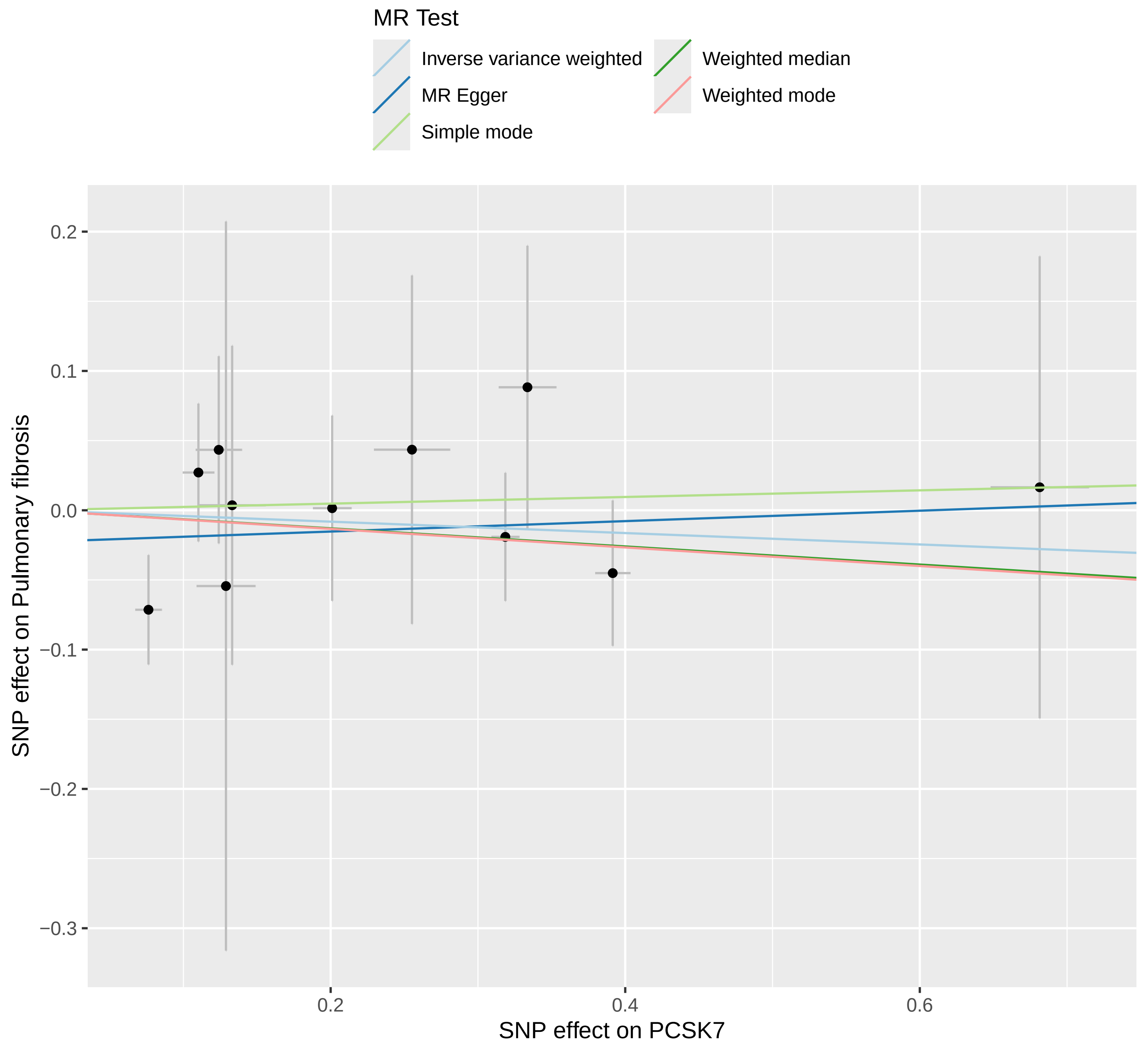

MR Test
Inverse variance weighted
MR Egger
Simple mode
Weighted median
Weighted mode
SNP effect on Pulmonary fibrosis
0.2
0.1
0.0
−0.1
−0.2
−0.3
0.2
0.4
0.6
SNP effect on PCSK7

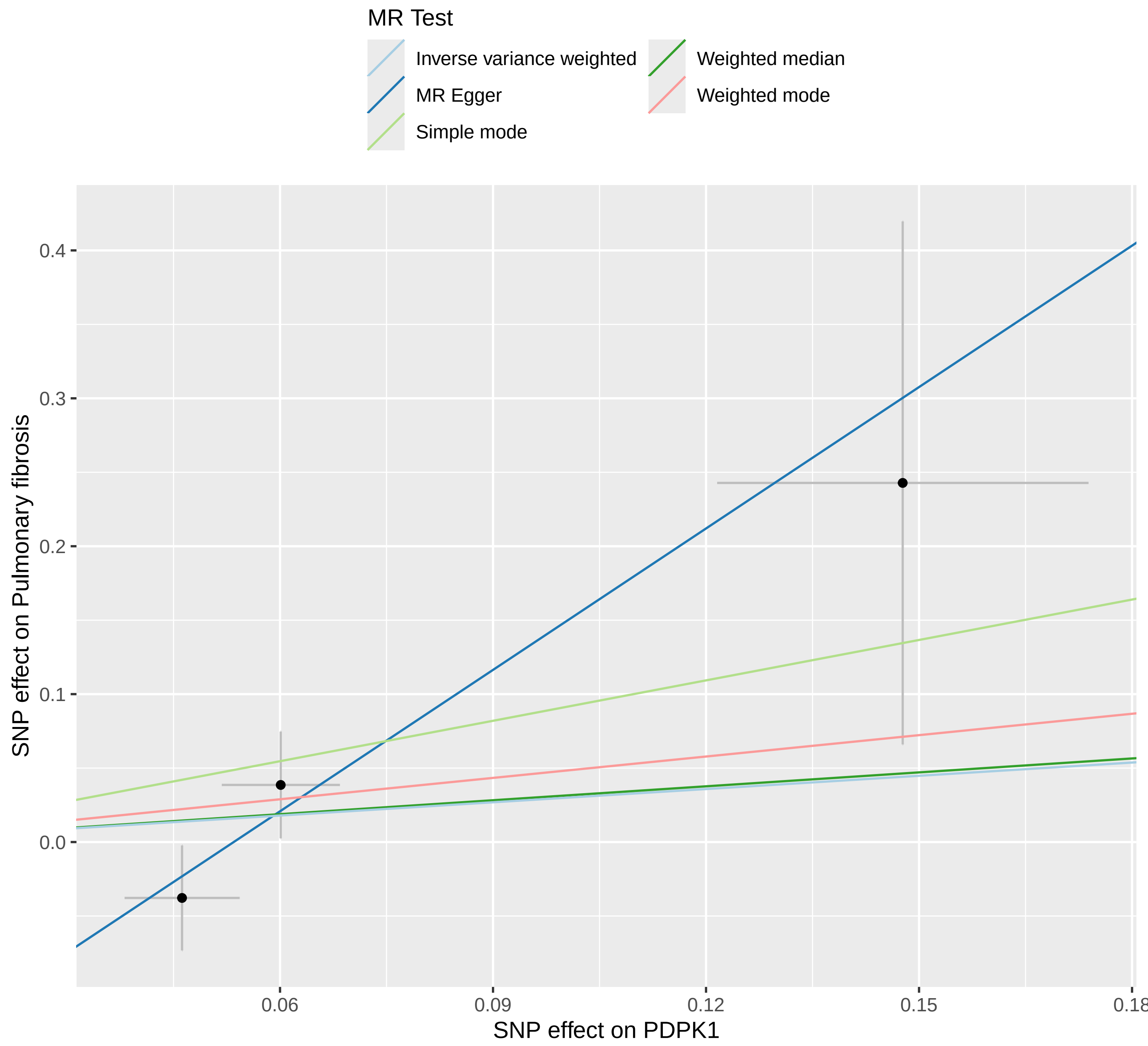
MR Test
Inverse variance weighted
MR Egger
Simple mode
Weighted median
Weighted mode
SNP effect on Pulmonary fibrosis
0.4
0.3
0.2
0.1
0.0
0.06
0.09
0.12
0.15
0.18
SNP effect on PDPK1

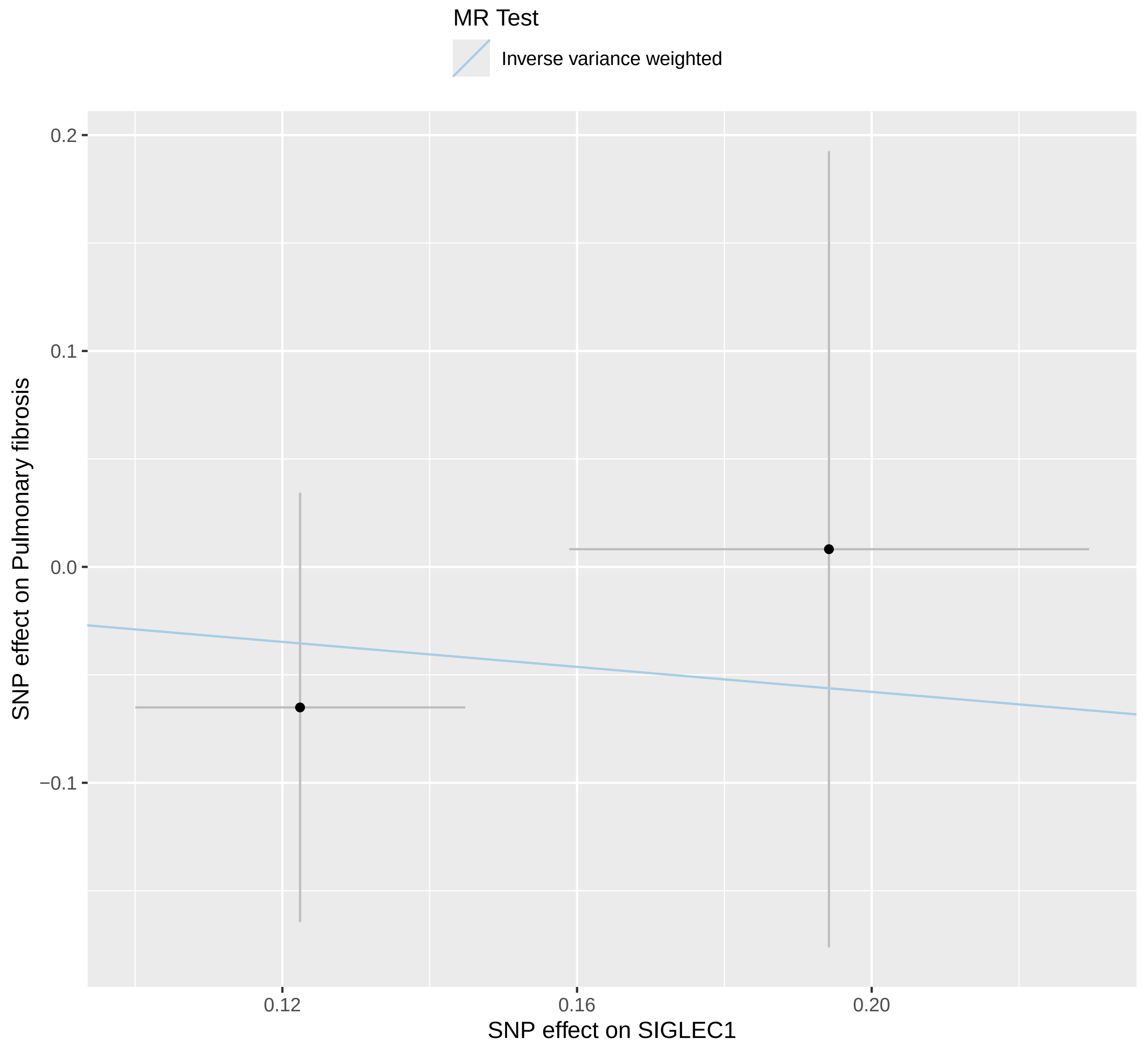

MR Test
Inverse variance weighted
0.2
0.1
0.0
−0.1
SNP effect on Pulmonary fibrosis
0.12
0.16
0.20
SNP effect on SIGLEC1

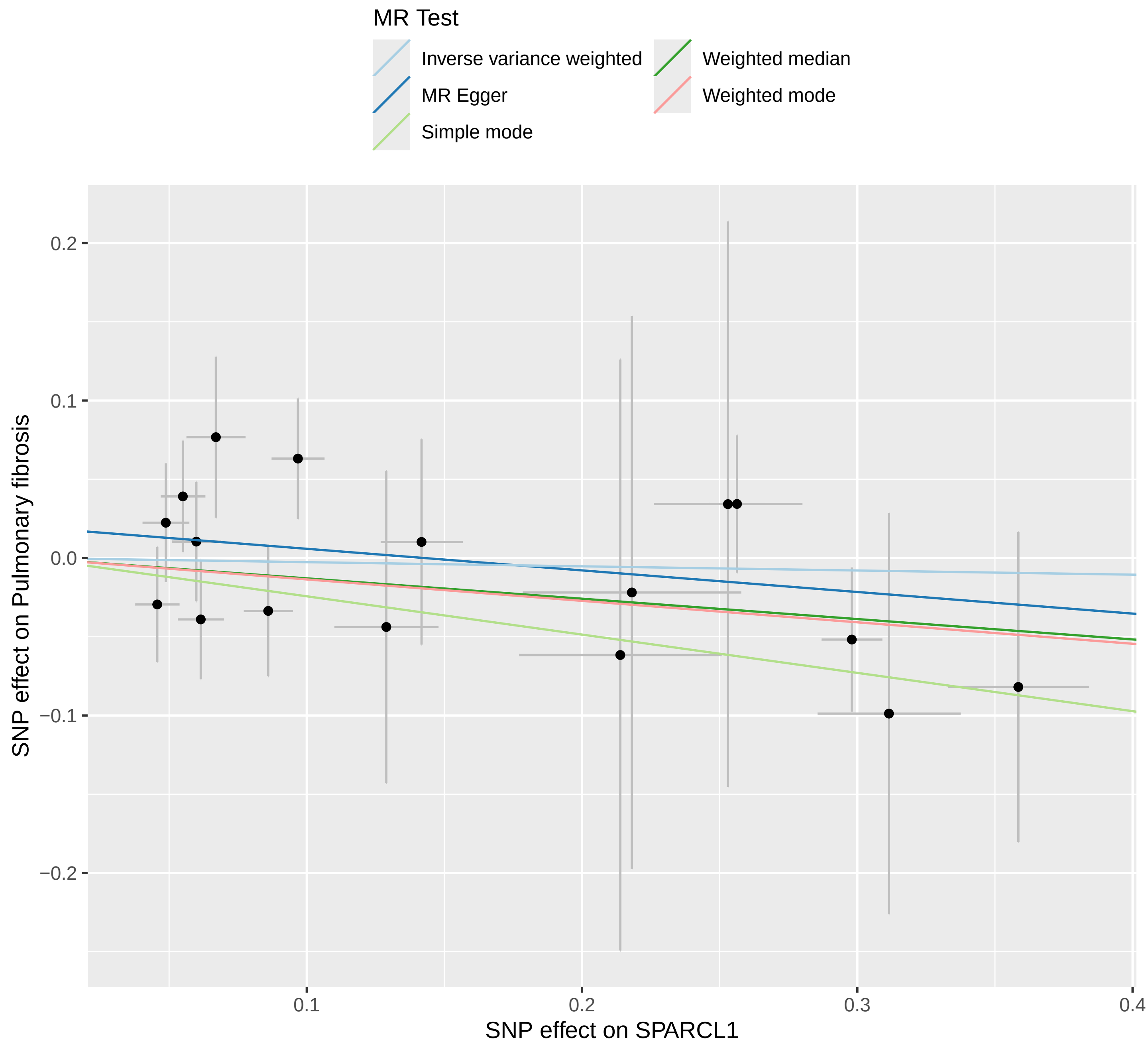

MR Test
Inverse variance weighted
MR Egger
Simple mode
Weighted median
Weighted mode
0.2
0.1
0.0
−0.1
−0.2
SNP effect on Pulmonary fibrosis
0.1
0.2
0.3
0.4
SNP effect on SPARCL1

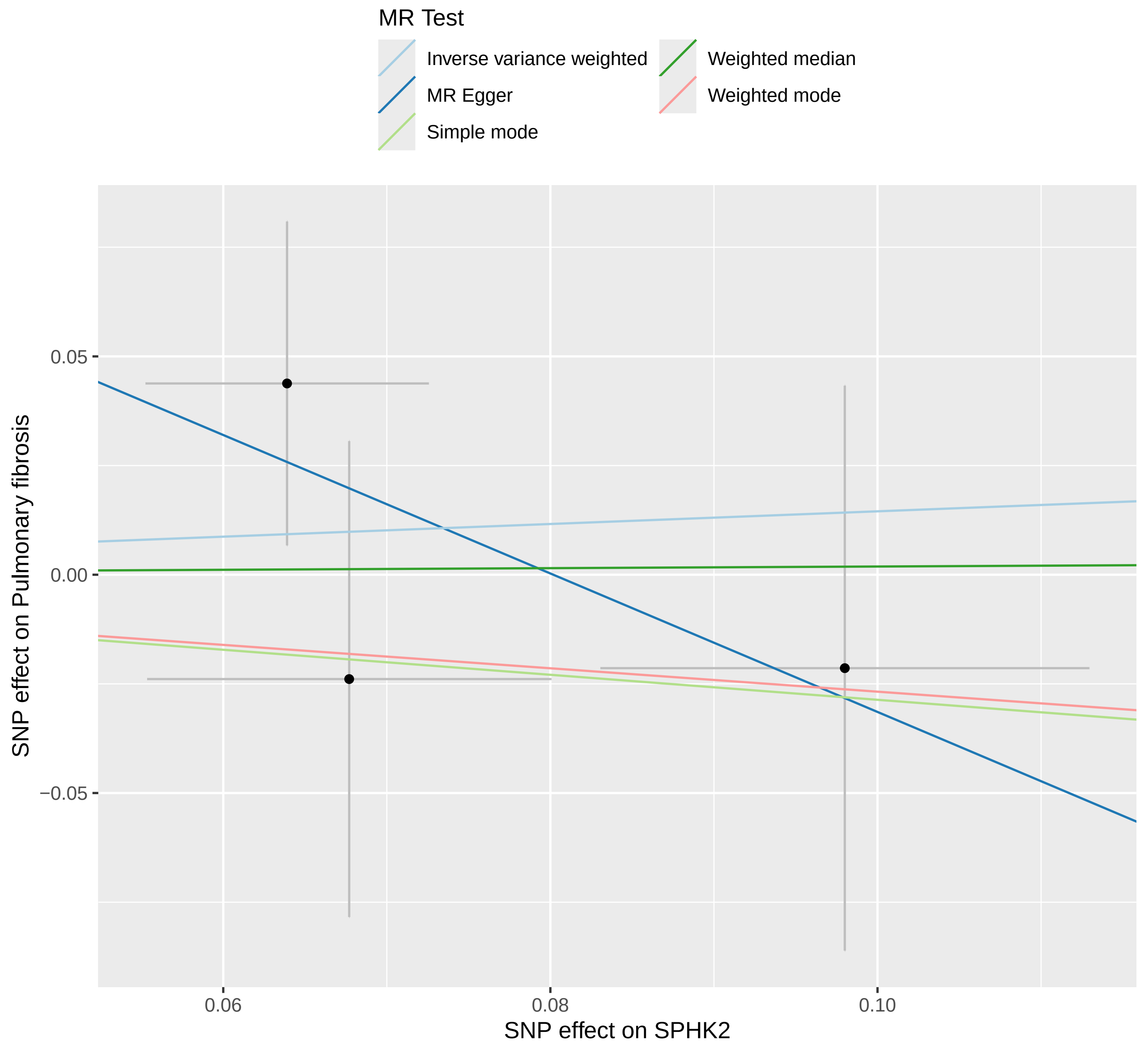
MR Test
Inverse variance weighted
MR Egger
Simple mode
Weighted median
Weighted mode
0.05
0.00
−0.05
SNP effect on Pulmonary fibrosis
0.06
0.08
0.10
SNP effect on SPHK2

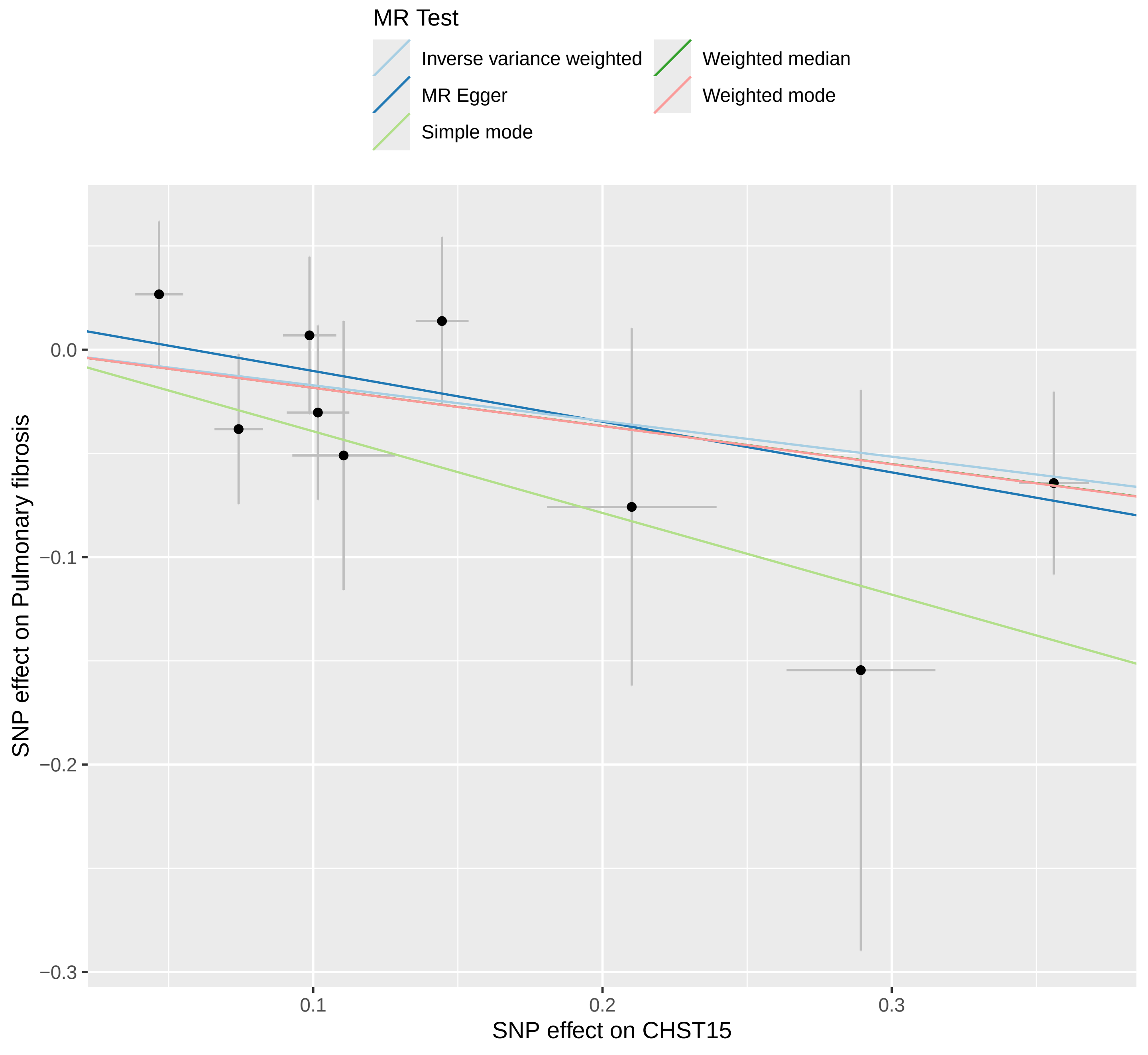

MR Test
Inverse variance weighted
MR Egger
Simple mode
Weighted median
Weighted mode
0.0
−0.1
−0.2
−0.3
0.1
0.2
0.3
SNP effect on Pulmonary fibrosis
SNP effect on CHST15

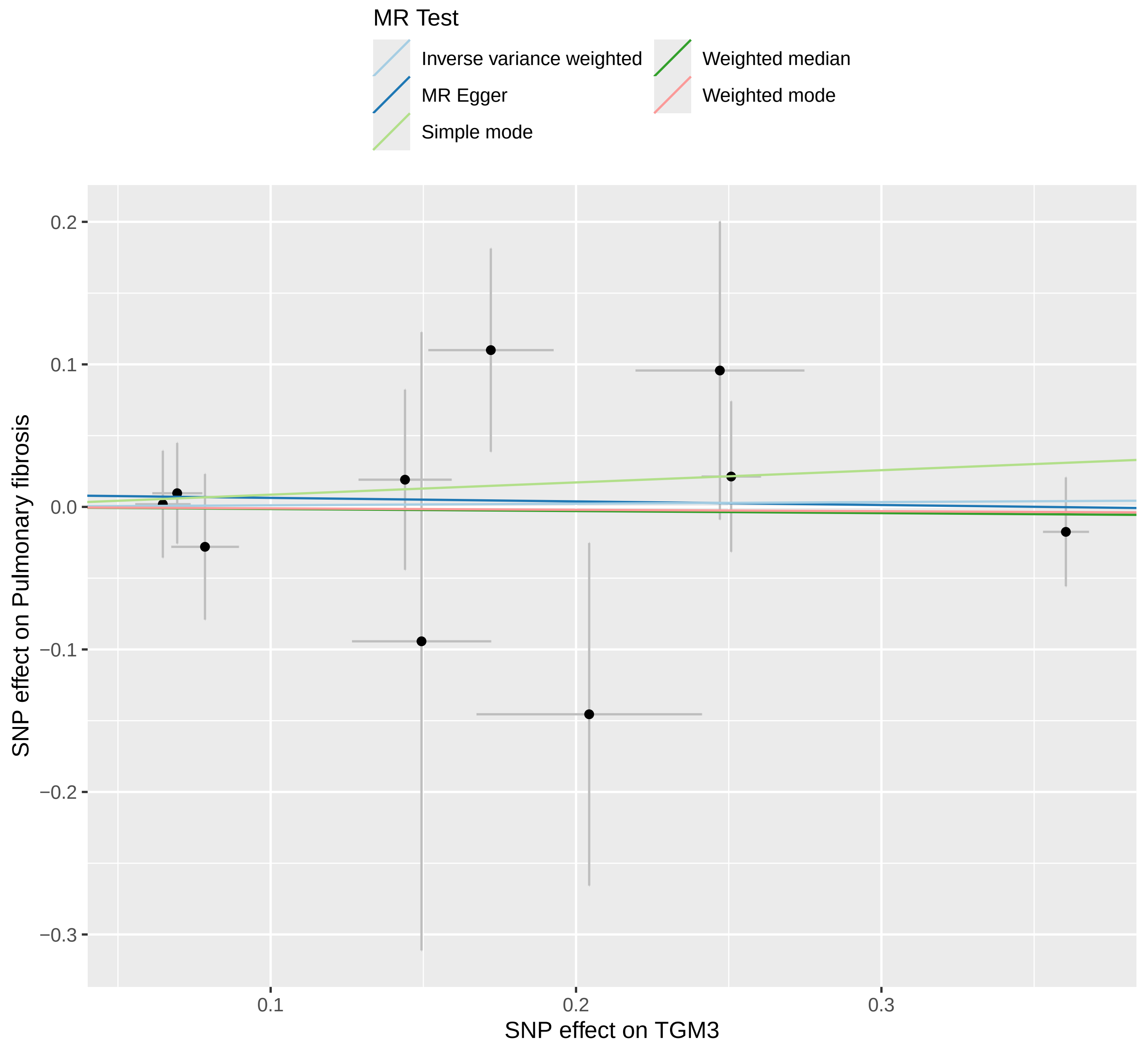
MR Test
Inverse variance weighted
MR Egger
Simple mode
Weighted median
Weighted mode
0.2
0.1
0.0
−0.1
−0.2
−0.3
0.1
0.2
0.3
SNP effect on Pulmonary fibrosis
SNP effect on TGM3

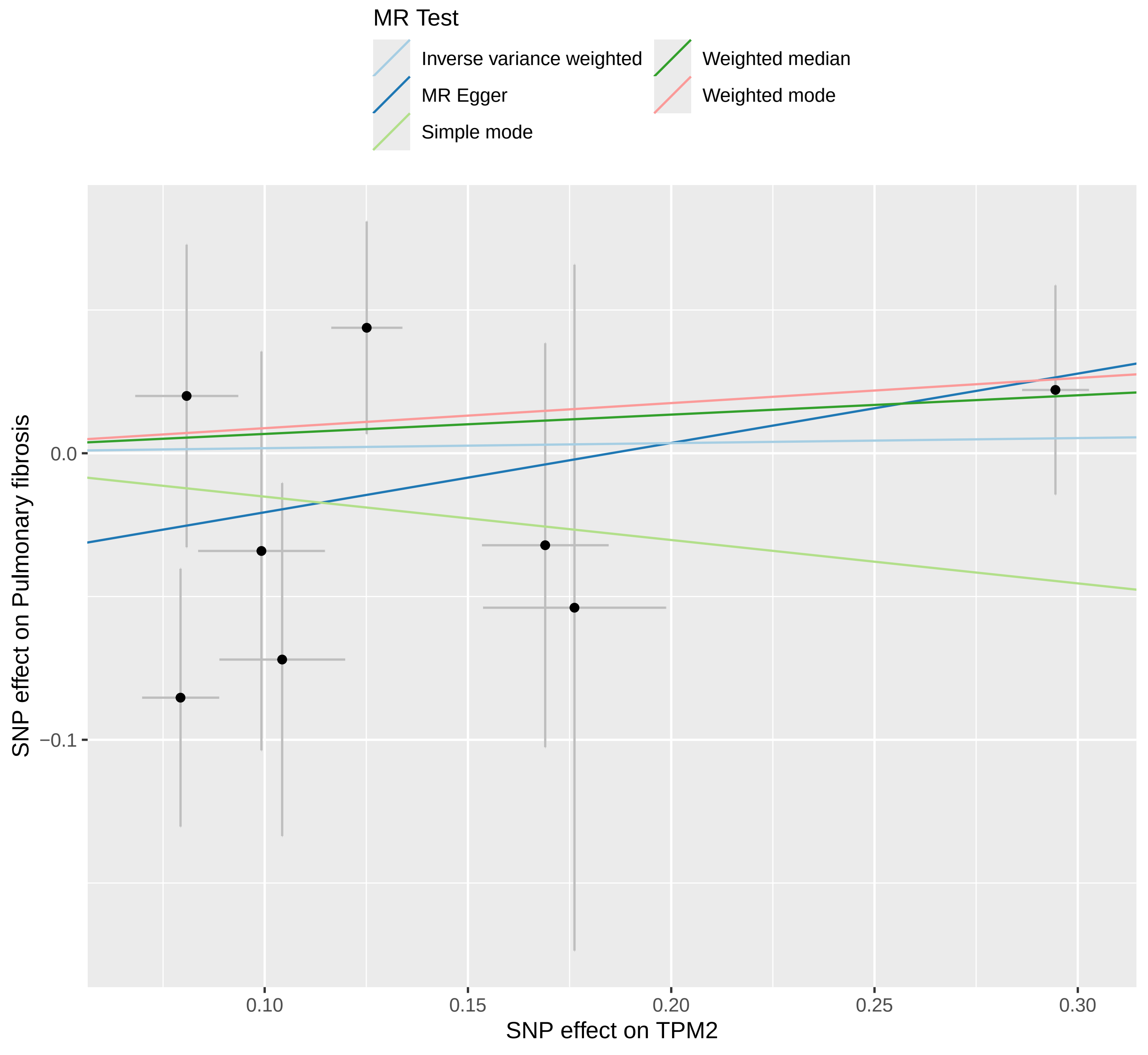
MR Test
Inverse variance weighted
MR Egger
Simple mode
Weighted median
Weighted mode
0.0
−0.1
0.10
0.15
0.20
0.25
0.30
SNP effect on TPM2
SNP effect on Pulmonary fibrosis

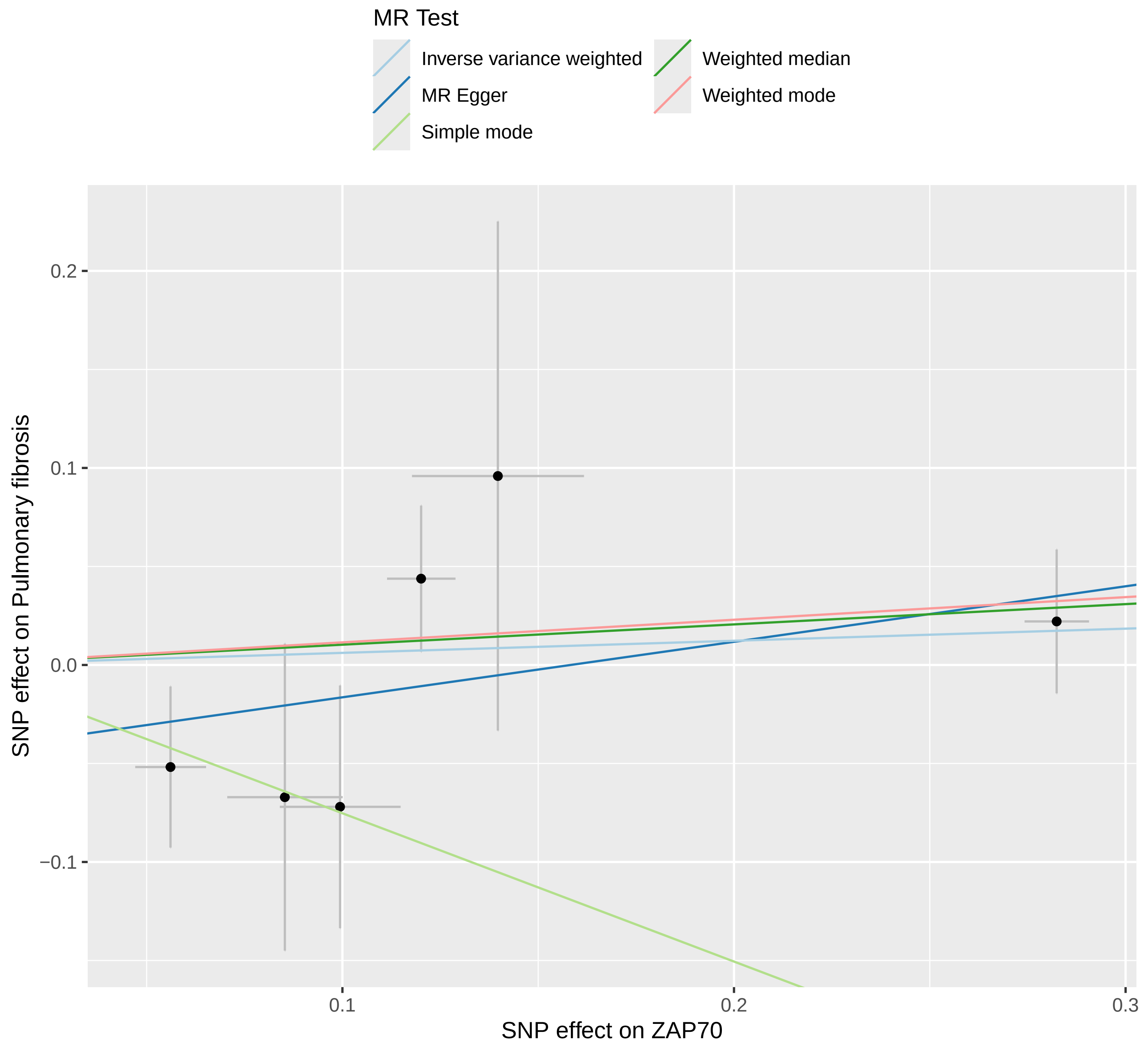
MR Test
Inverse variance weighted
MR Egger
Simple mode
Weighted median
Weighted mode
0.2
0.1
0.0
−0.1
0.1
0.2
0.3
SNP effect on Pulmonary fibrosis
SNP effect on ZAP70

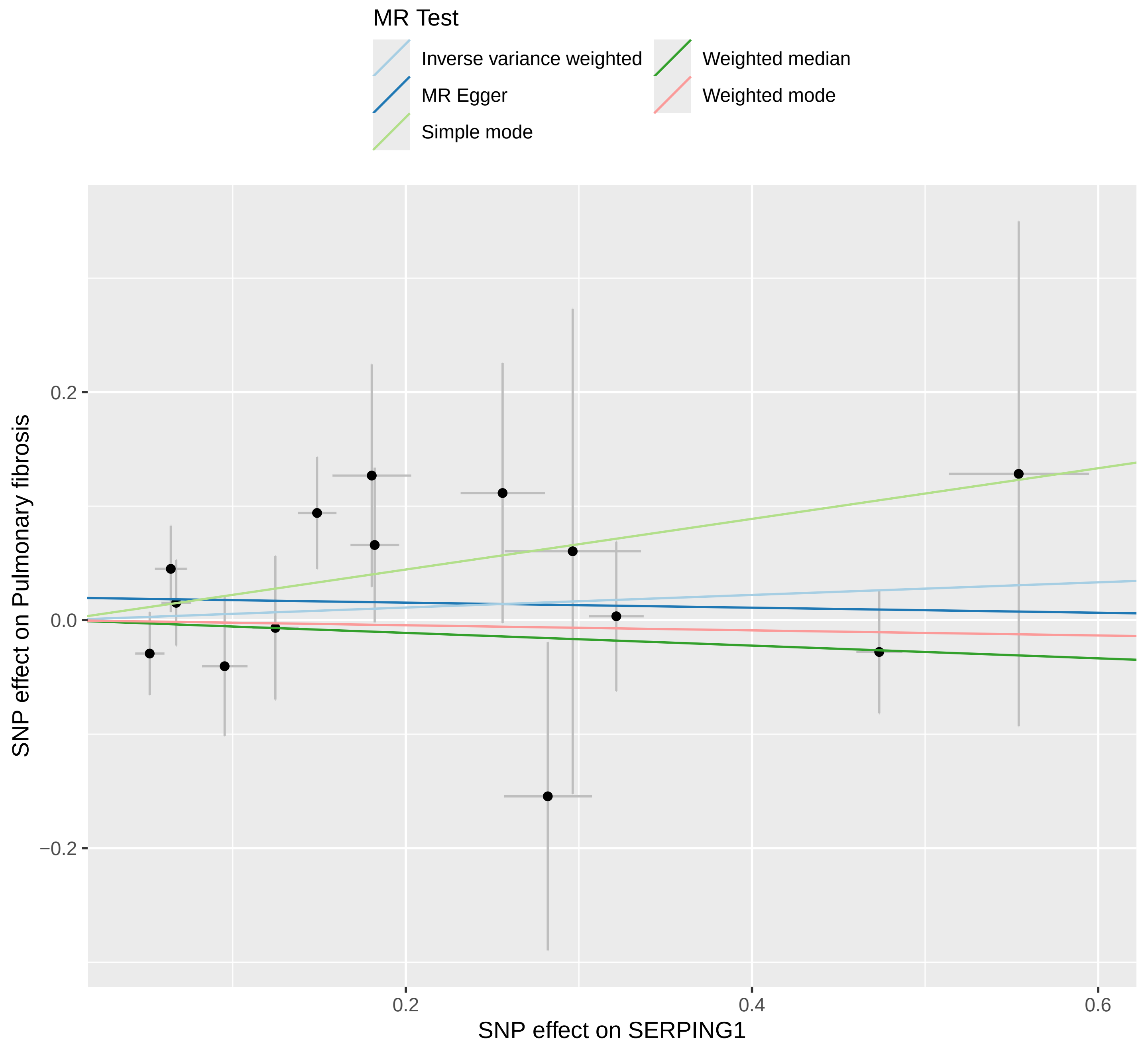

MR Test
Inverse variance weighted
MR Egger
Simple mode
Weighted median
Weighted mode
0.2
0.0
−0.2
SNP effect on Pulmonary fibrosis
0.2
0.4
0.6
SNP effect on SERPING1

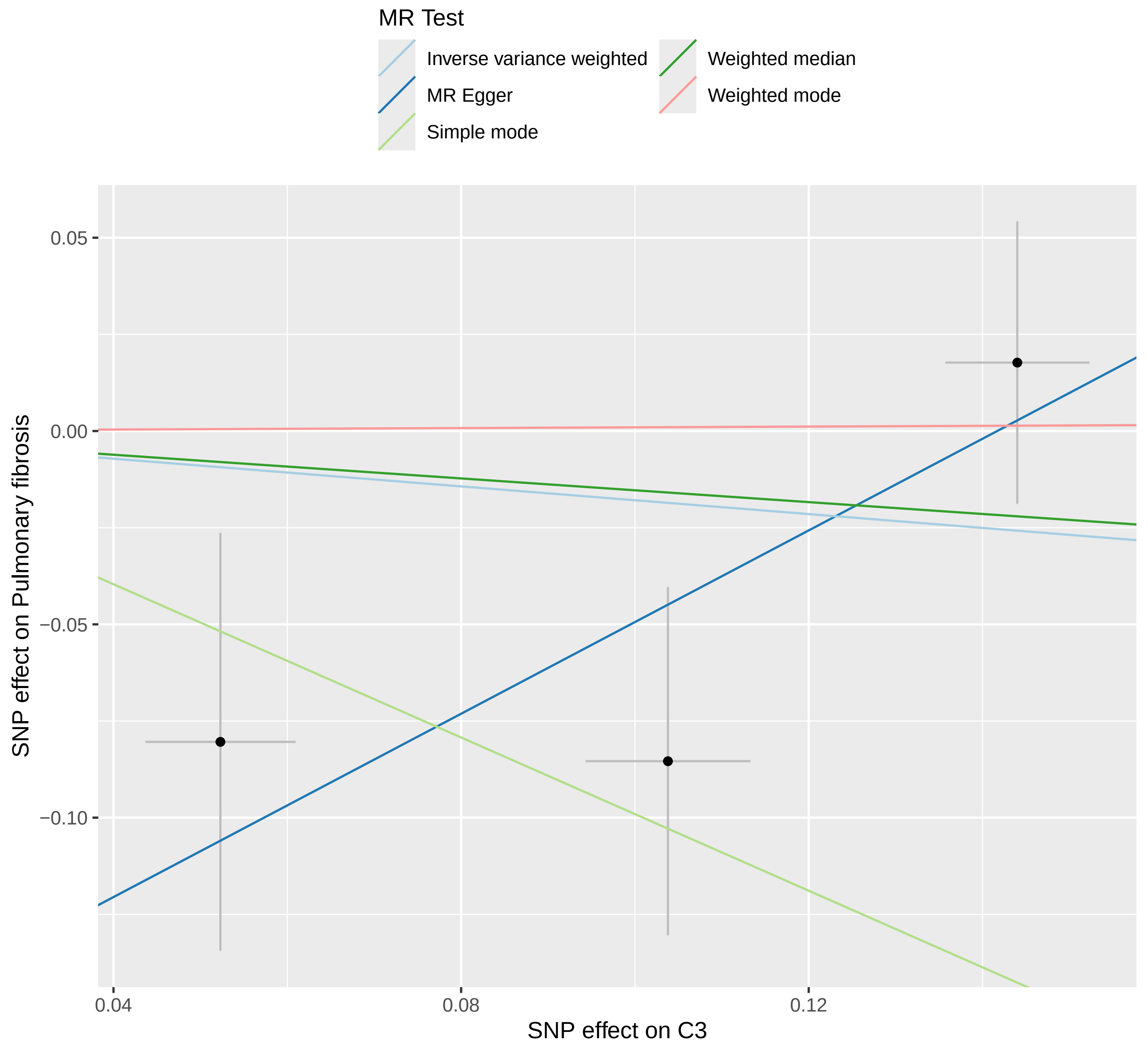

MR Test
Inverse variance weighted
MR Egger
Simple mode
Weighted median
Weighted mode
0.05
0.00
−0.05
−0.10
SNP effect on Pulmonary fibrosis
0.04
0.08
0.12
SNP effect on C3

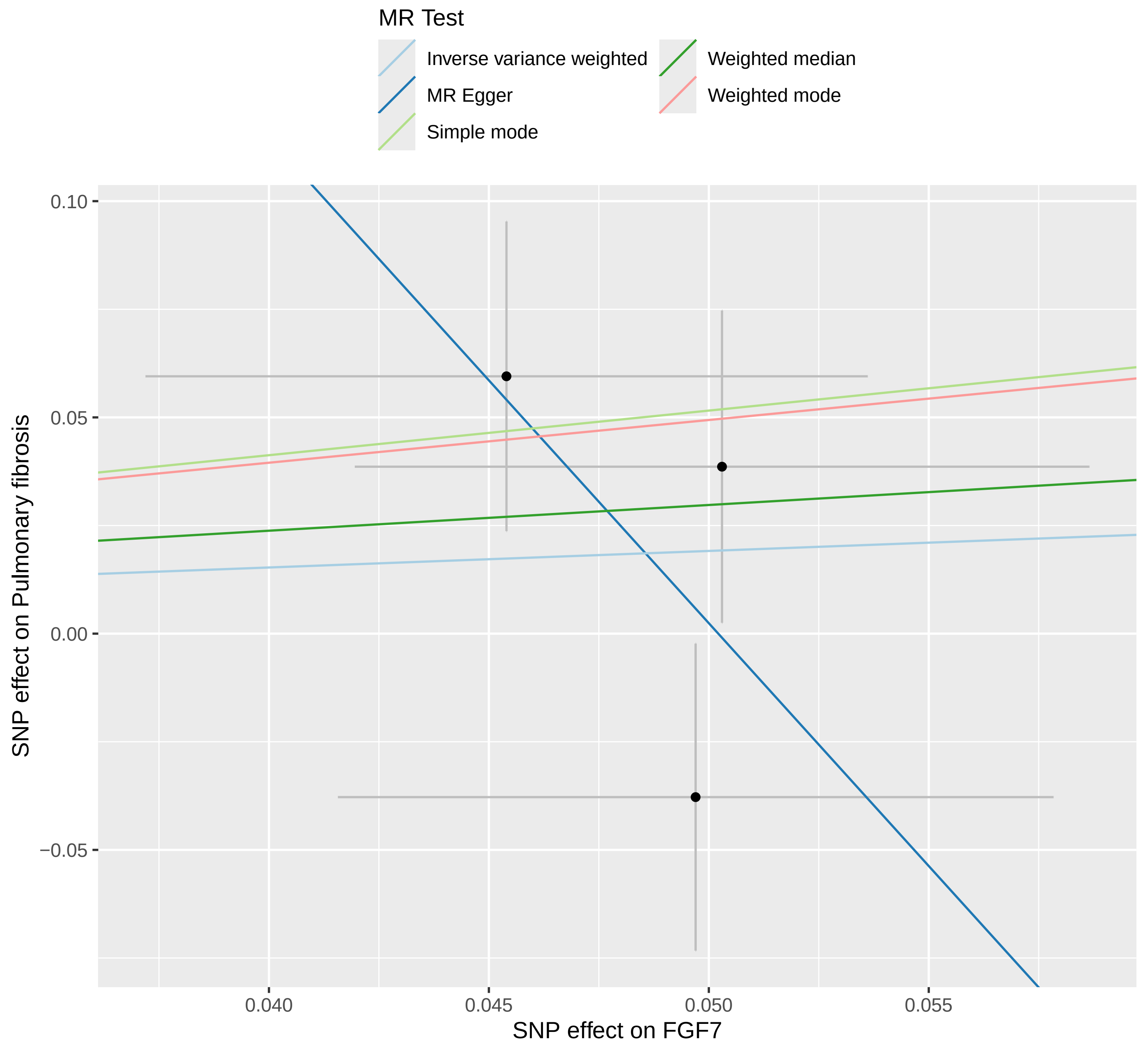
MR Test
Inverse variance weighted
MR Egger
Simple mode
Weighted median
Weighted mode
0.10
0.05
0.00
−0.05
SNP effect on Pulmonary fibrosis
0.040
0.045
0.050
0.055
SNP effect on FGF7

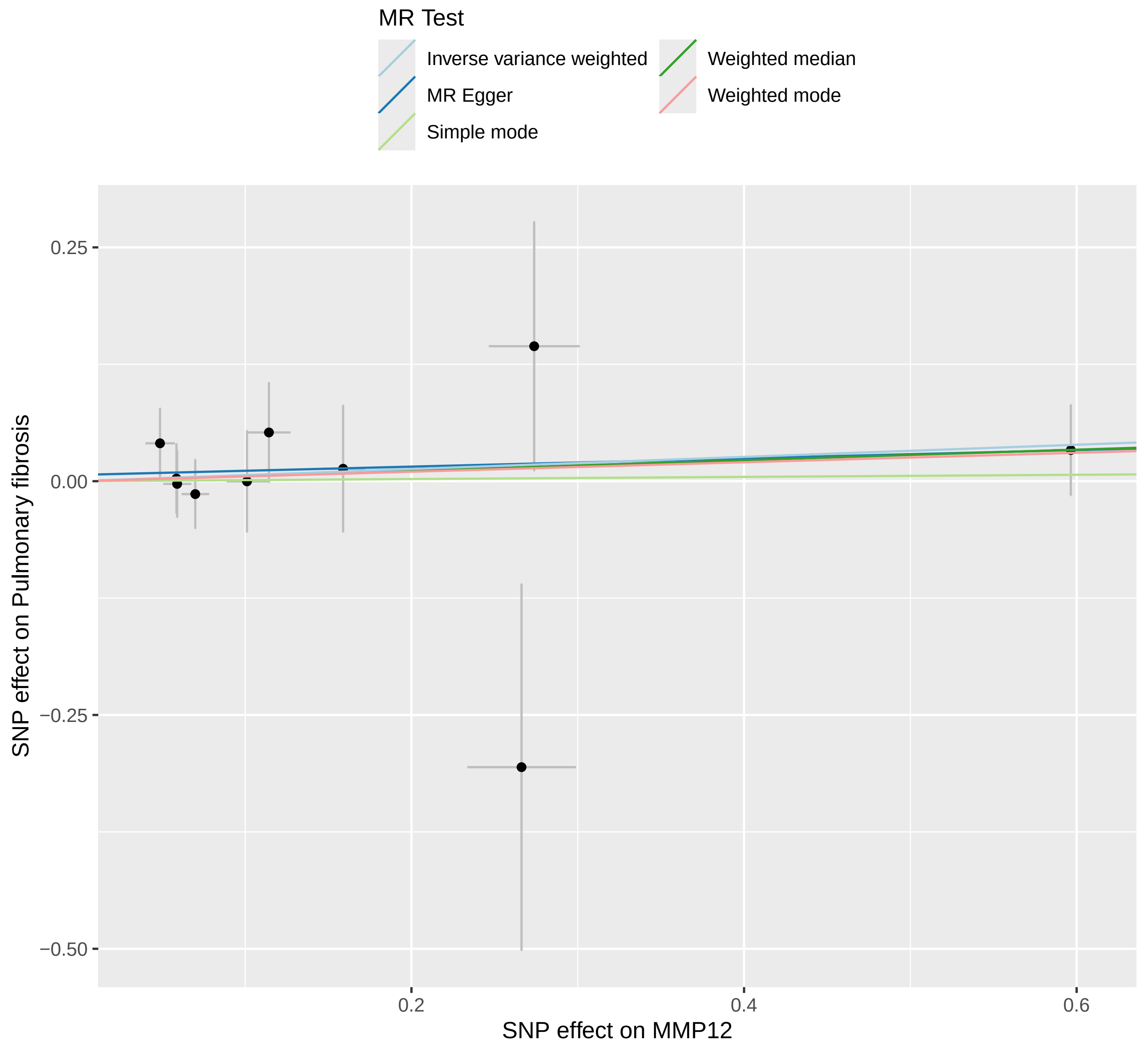
MR Test
Inverse variance weighted
MR Egger
Simple mode
Weighted median
Weighted mode
0.25
0.00
−0.25
−0.50
0.2
0.4
0.6
SNP effect on Pulmonary fibrosis
SNP effect on MMP12

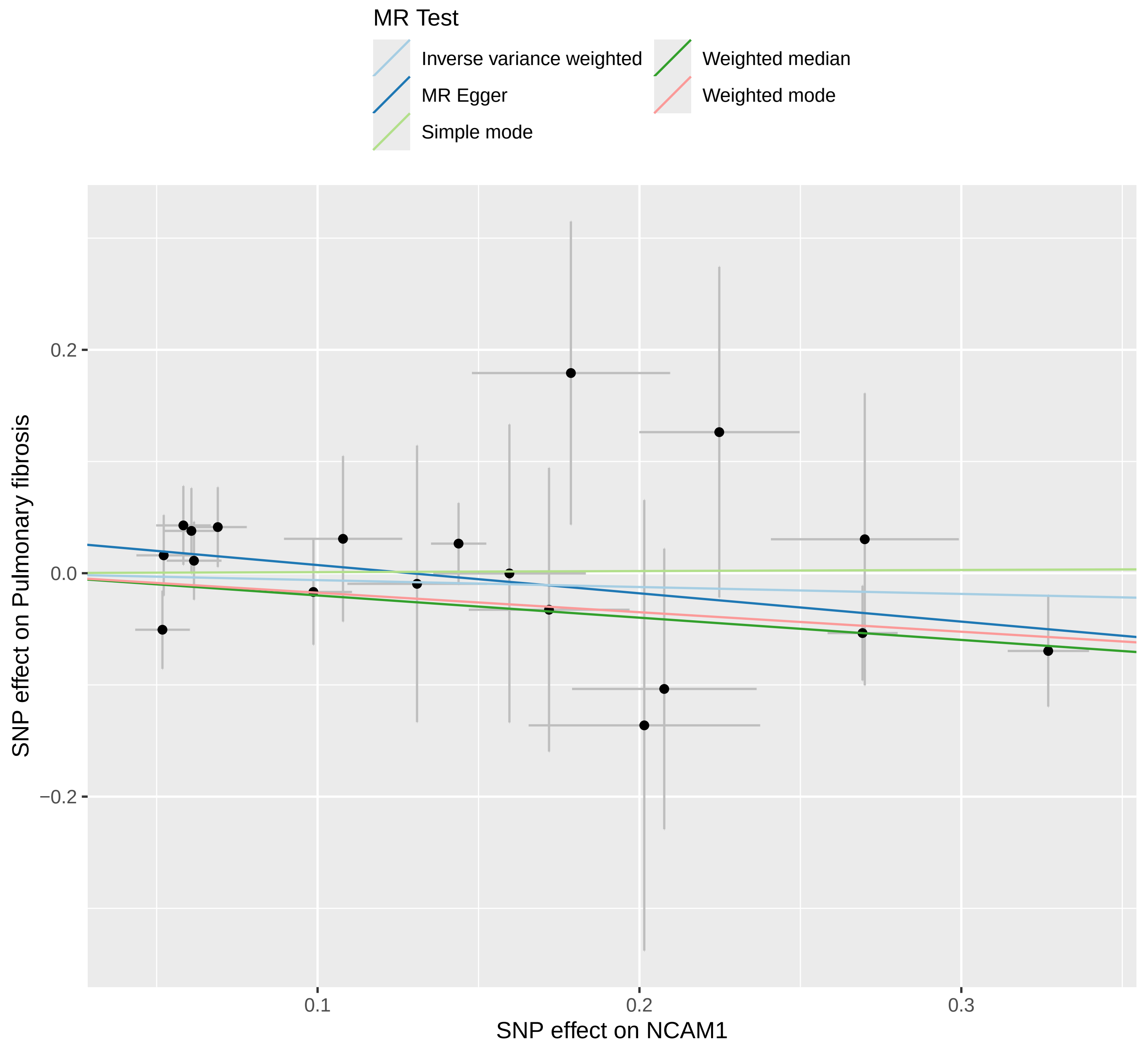

MR Test
Inverse variance weighted
MR Egger
Simple mode
Weighted median
Weighted mode
SNP effect on Pulmonary fibrosis
0.2
0.0
−0.2
0.1
0.2
0.3
SNP effect on NCAM1

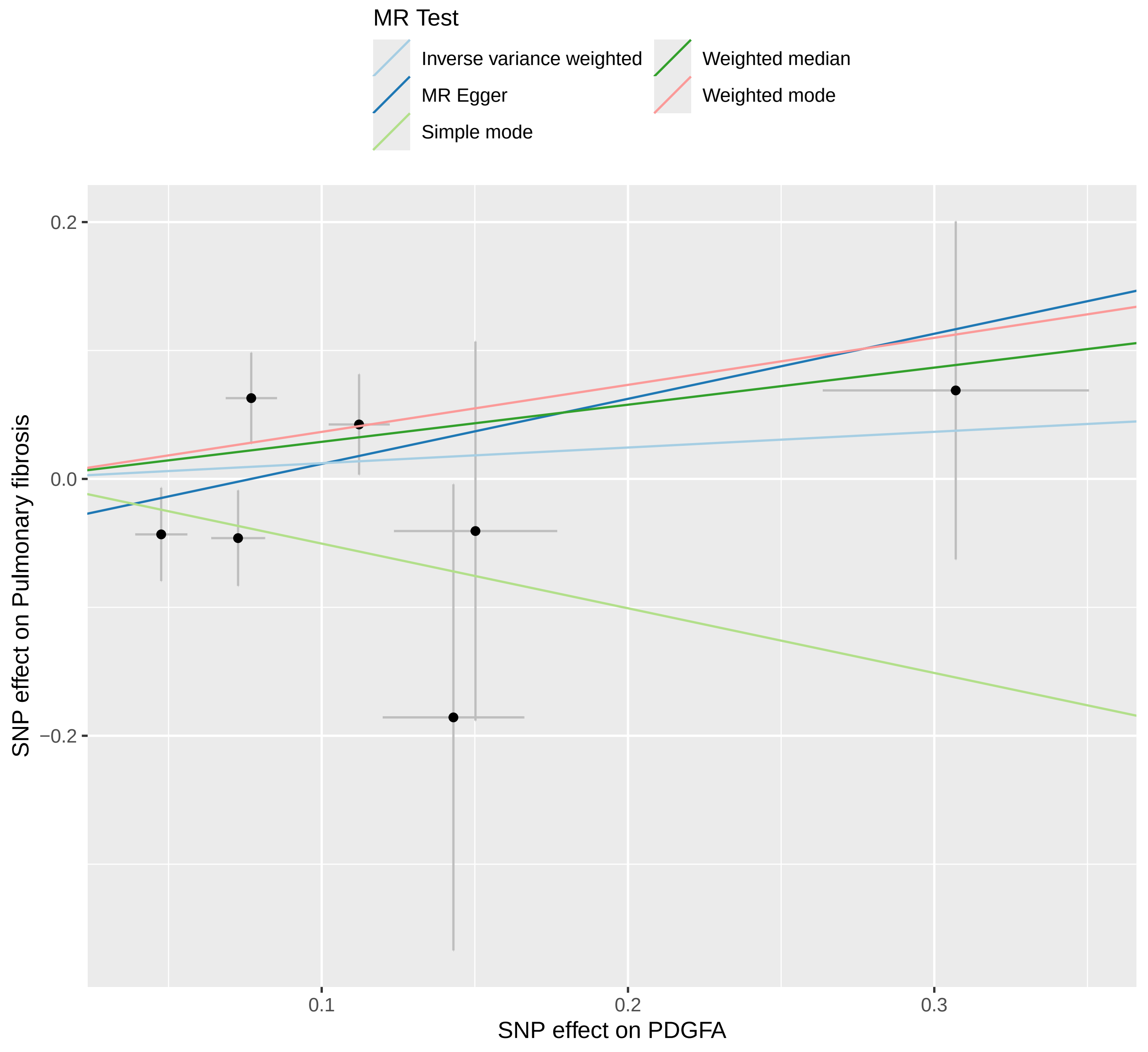

MR Test
Inverse variance weighted
MR Egger
Simple mode
Weighted median
Weighted mode
0.2
0.0
−0.2
0.1
0.2
0.3
SNP effect on Pulmonary fibrosis
SNP effect on PDGFA

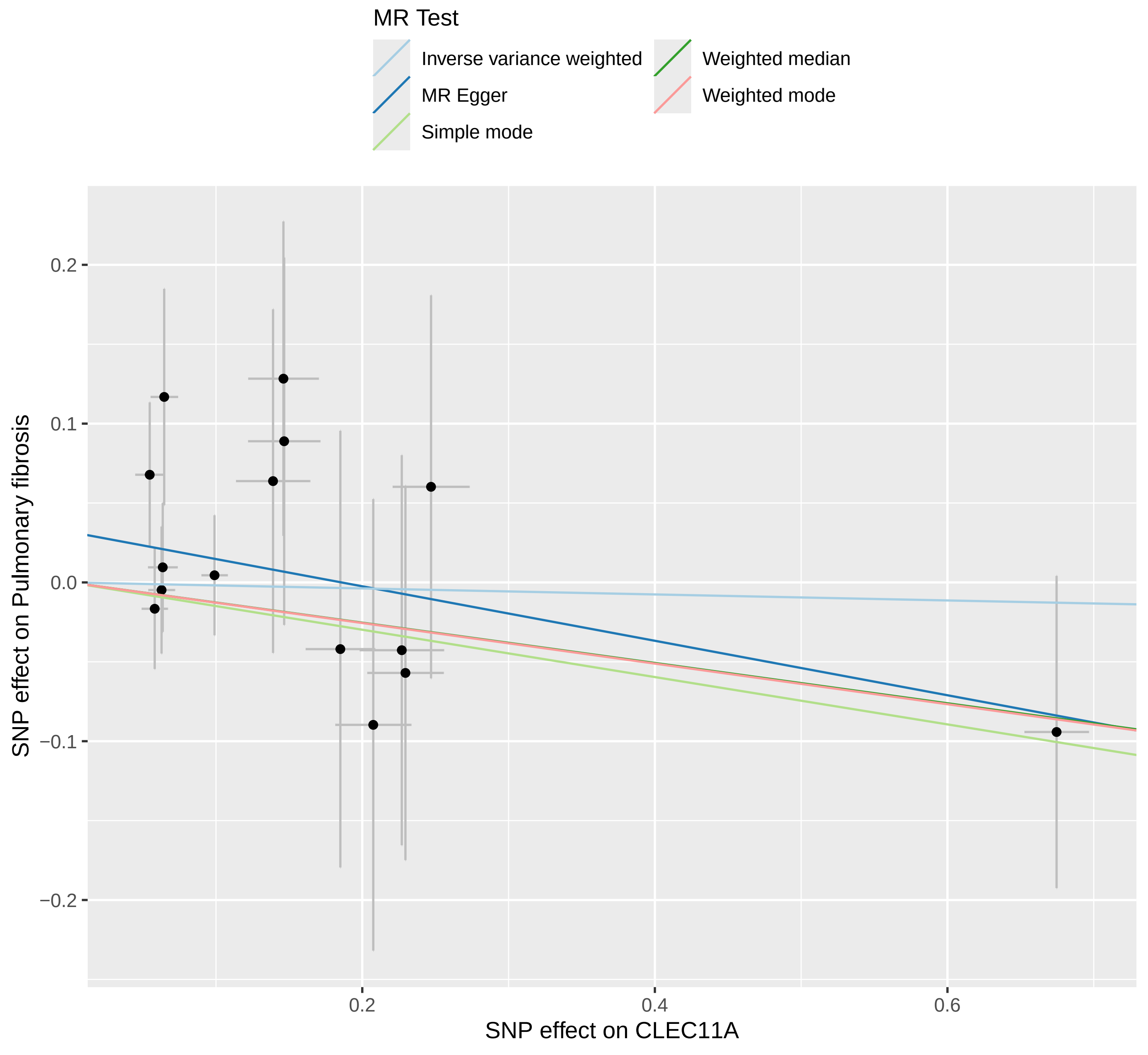

MR Test
Inverse variance weighted
MR Egger
Simple mode
Weighted median
Weighted mode
0.2
0.1
0.0
−0.1
−0.2
0.2
0.4
0.6
SNP effect on Pulmonary fibrosis
SNP effect on CLEC11A

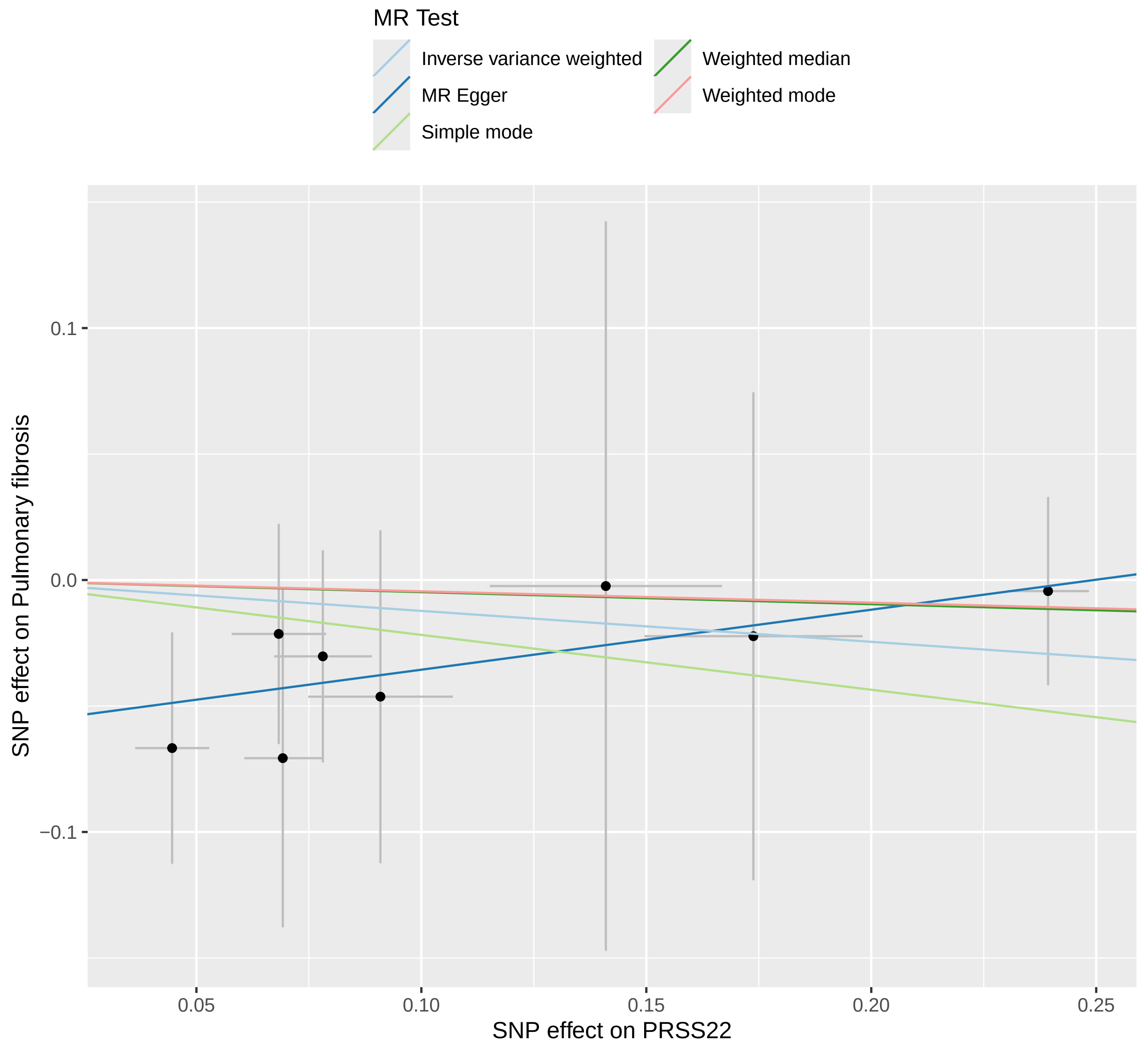

MR Test
Inverse variance weighted
MR Egger
Simple mode
Weighted median
Weighted mode
0.1
0.0
−0.1
SNP effect on Pulmonary fibrosis
0.05
0.10
0.15
0.20
0.25
SNP effect on PRSS22

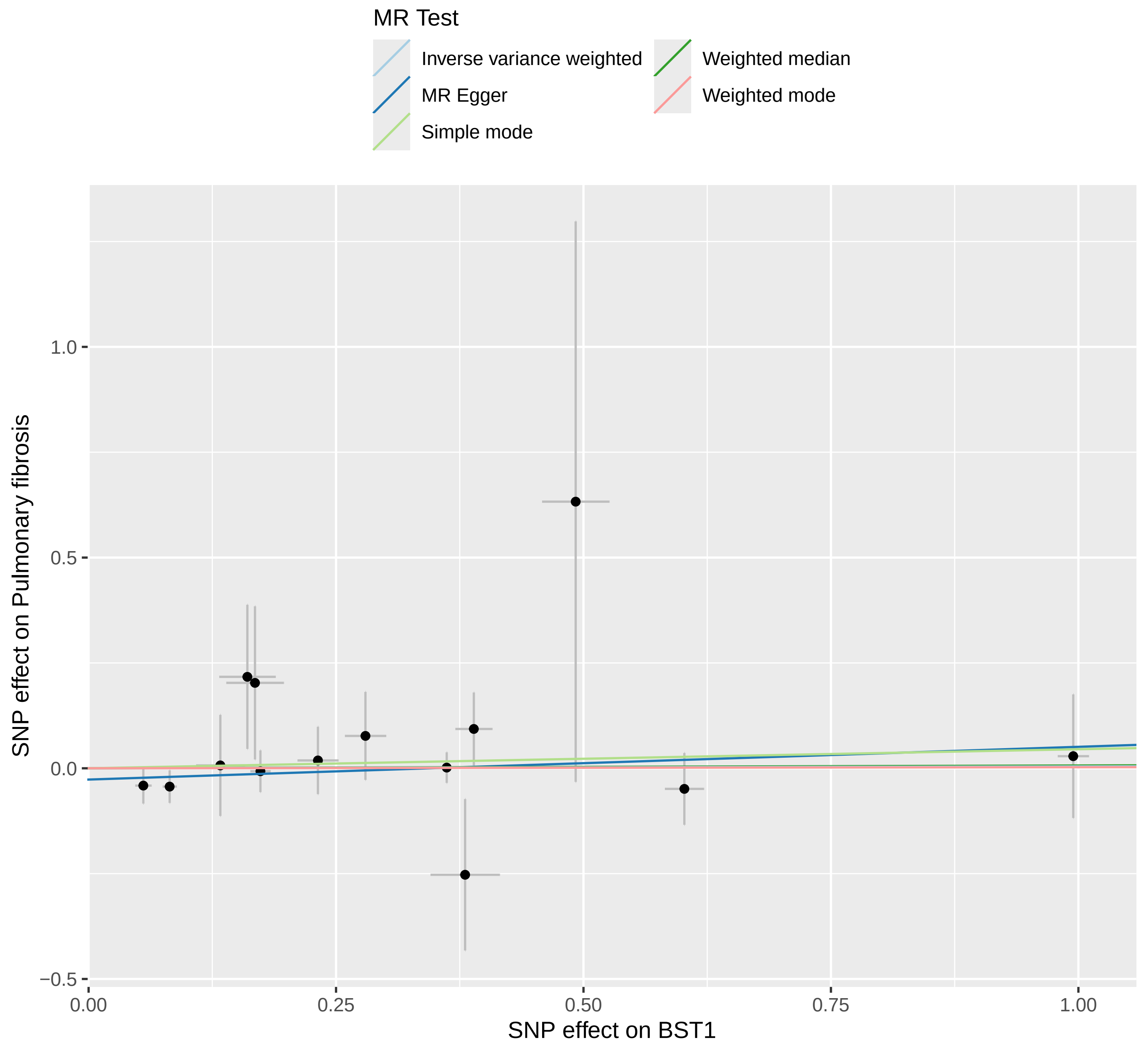

MR Test
Inverse variance weighted
MR Egger
Simple mode
Weighted median
Weighted mode
1.0
0.5
0.0
−0.5
0.00
0.25
0.50
0.75
1.00
SNP effect on BST1
SNP effect on Pulmonary fibrosis

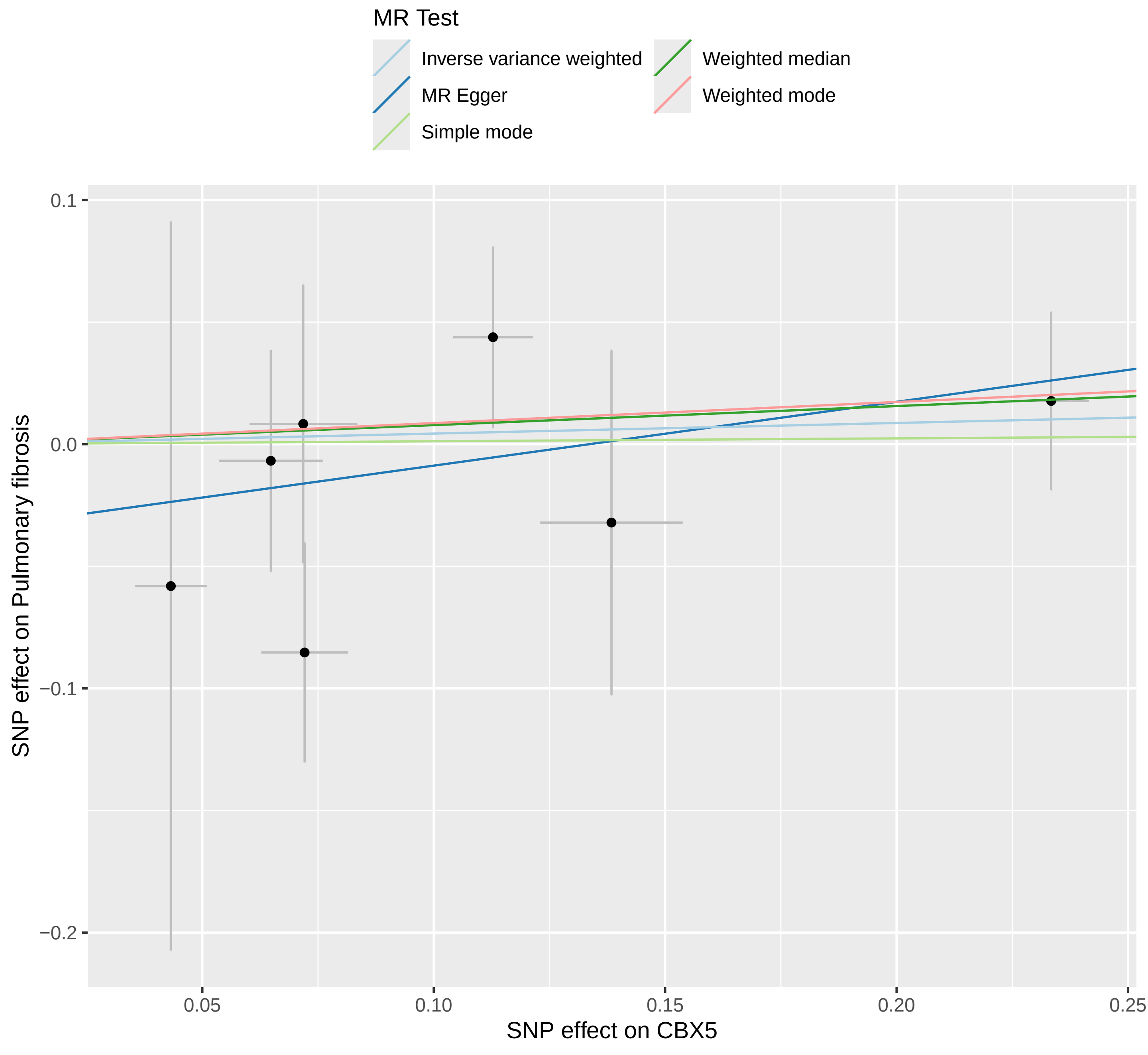
MR Test
Inverse variance weighted
MR Egger
Simple mode
Weighted median
Weighted mode
0.1
0.0
−0.1
−0.2
SNP effect on Pulmonary fibrosis
0.05
0.10
0.15
0.20
0.25
SNP effect on CBX5

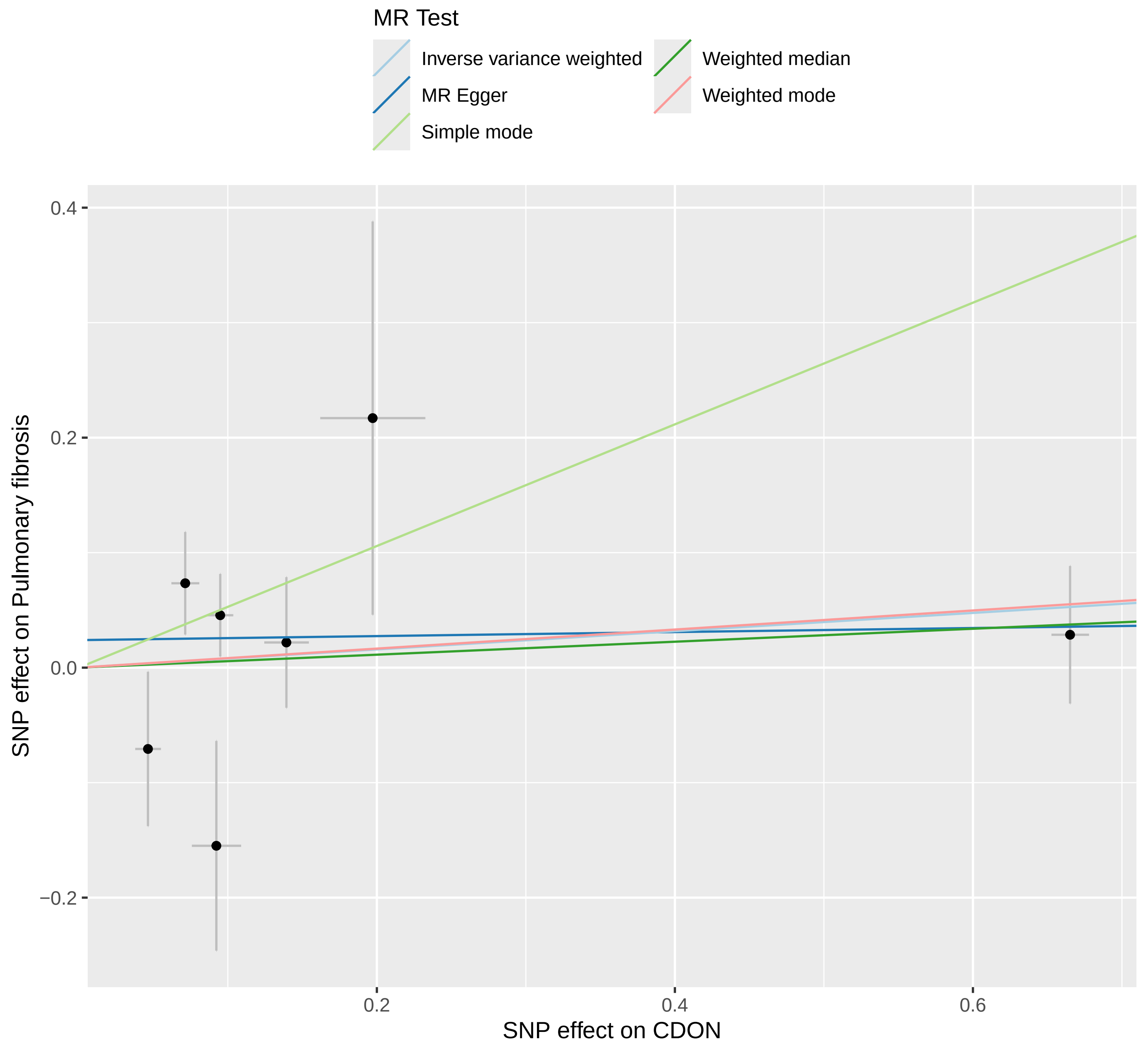
MR Test
Inverse variance weighted
MR Egger
Simple mode
Weighted median
Weighted mode
0.4
0.2
0.0
−0.2
0.2
0.4
0.6
SNP effect on Pulmonary fibrosis
SNP effect on CDON

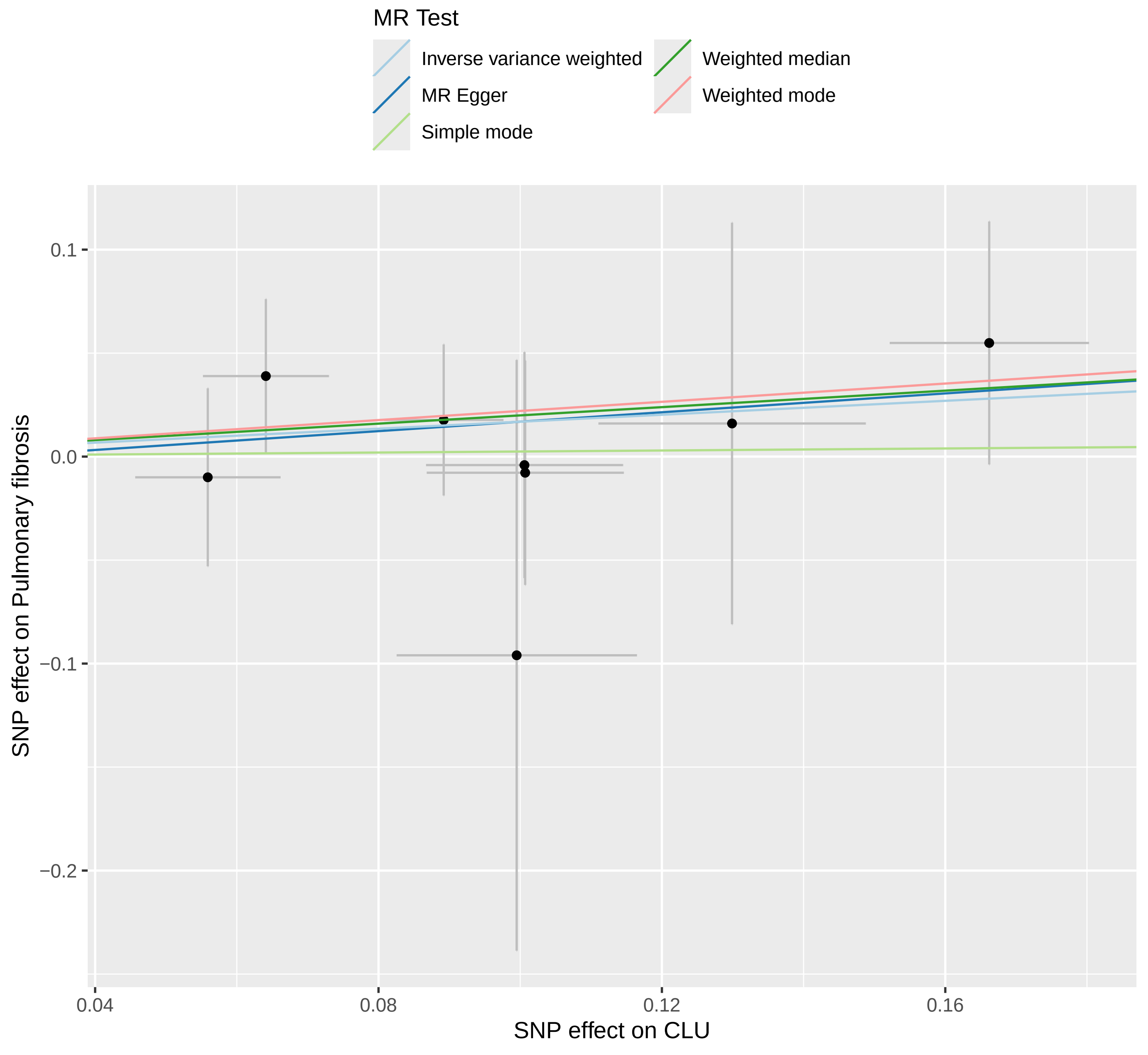

MR Test
Inverse variance weighted
MR Egger
Simple mode
Weighted median
Weighted mode
0.1
0.0
−0.1
−0.2
0.04
0.08
0.12
0.16
SNP effect on CLU
SNP effect on Pulmonary fibrosis

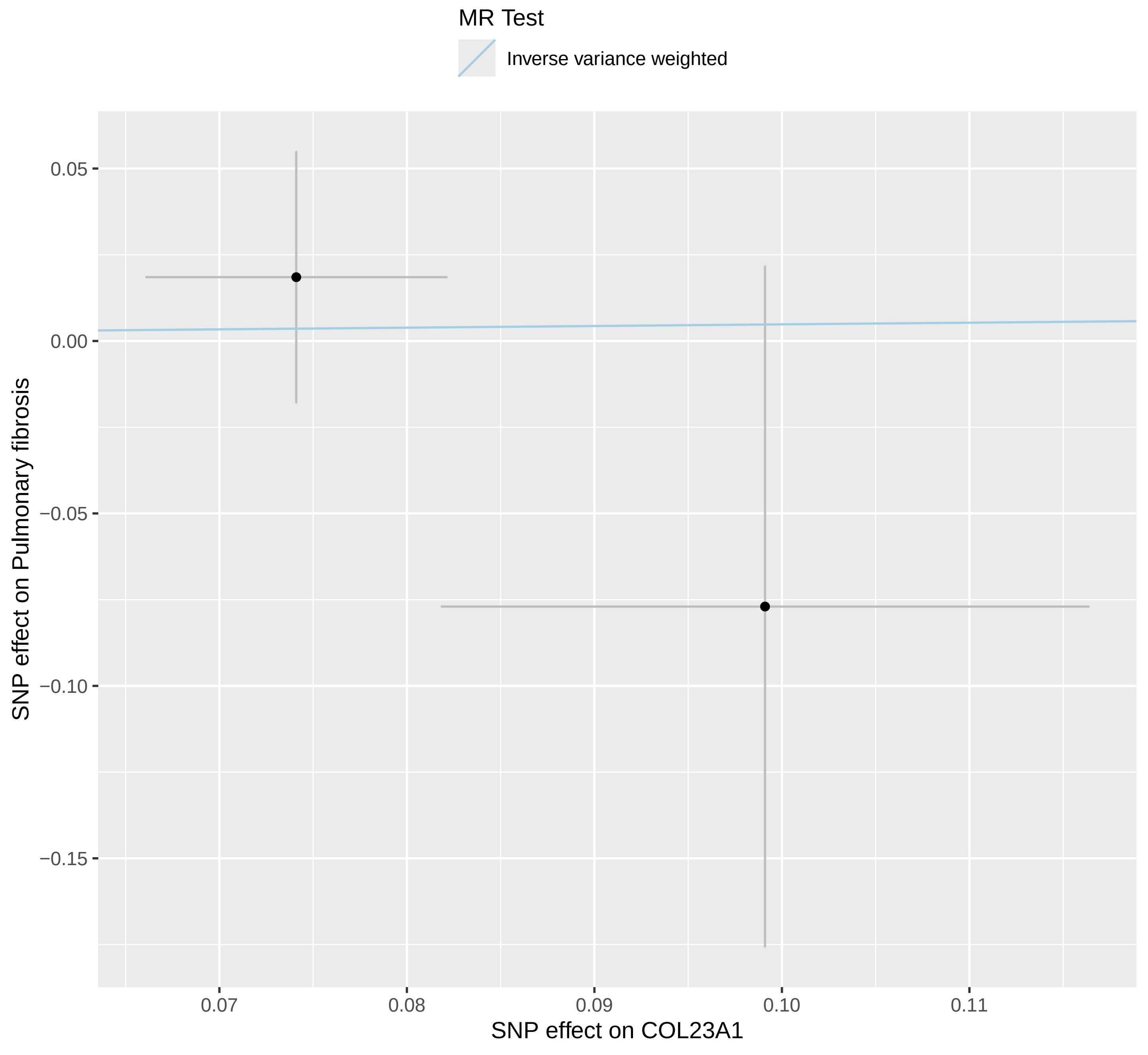

MR Test
Inverse variance weighted
SNP effect on Pulmonary fibrosis
0.05
0.00
−0.05
−0.10
−0.15
0.07
0.08
0.09
0.10
0.11
SNP effect on COL23A1

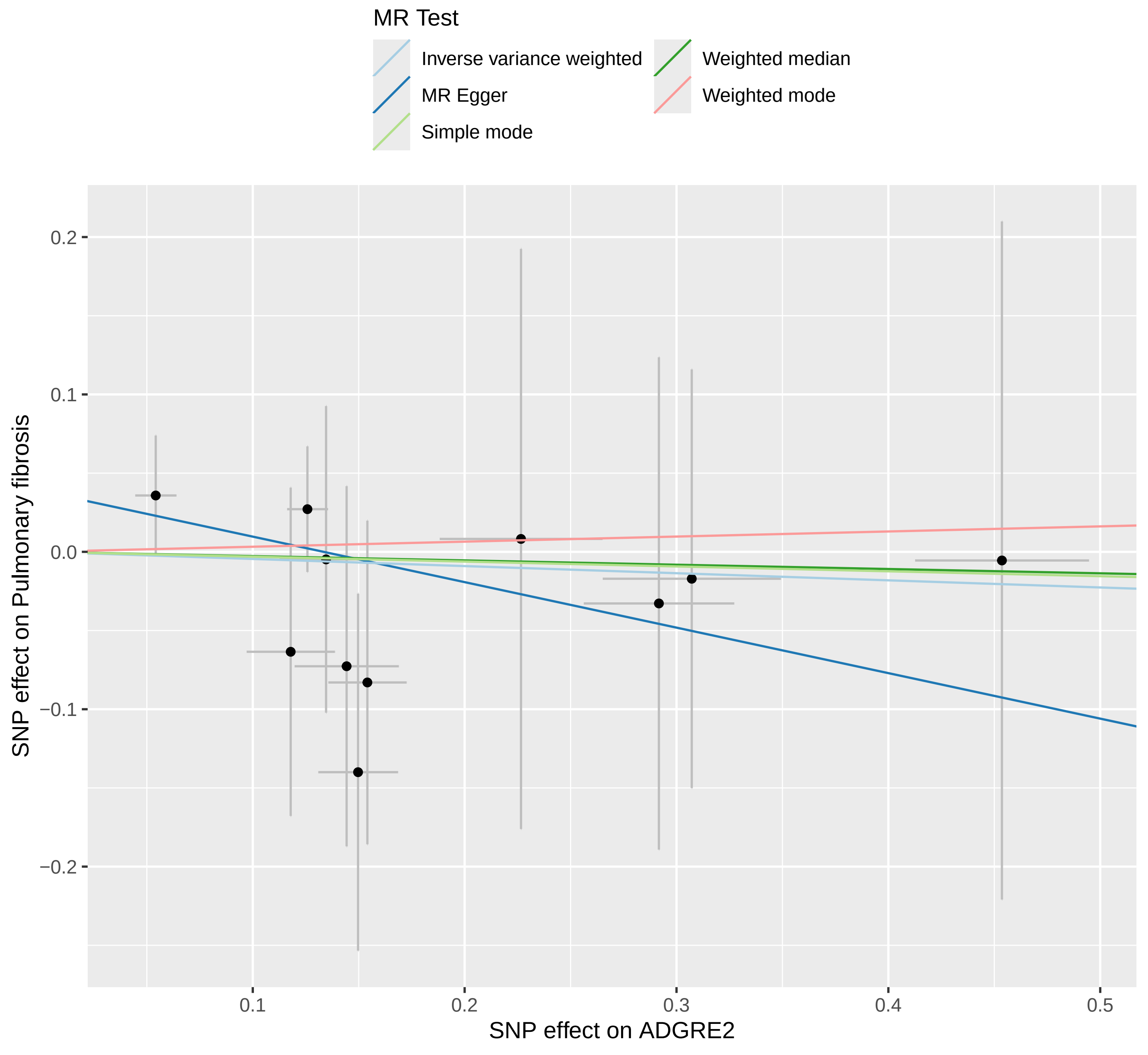

MR Test
Inverse variance weighted
MR Egger
Simple mode
Weighted median
Weighted mode
0.2
0.1
0.0
−0.1
−0.2
SNP effect on Pulmonary fibrosis
0.1
0.2
0.3
0.4
0.5
SNP effect on ADGRE2

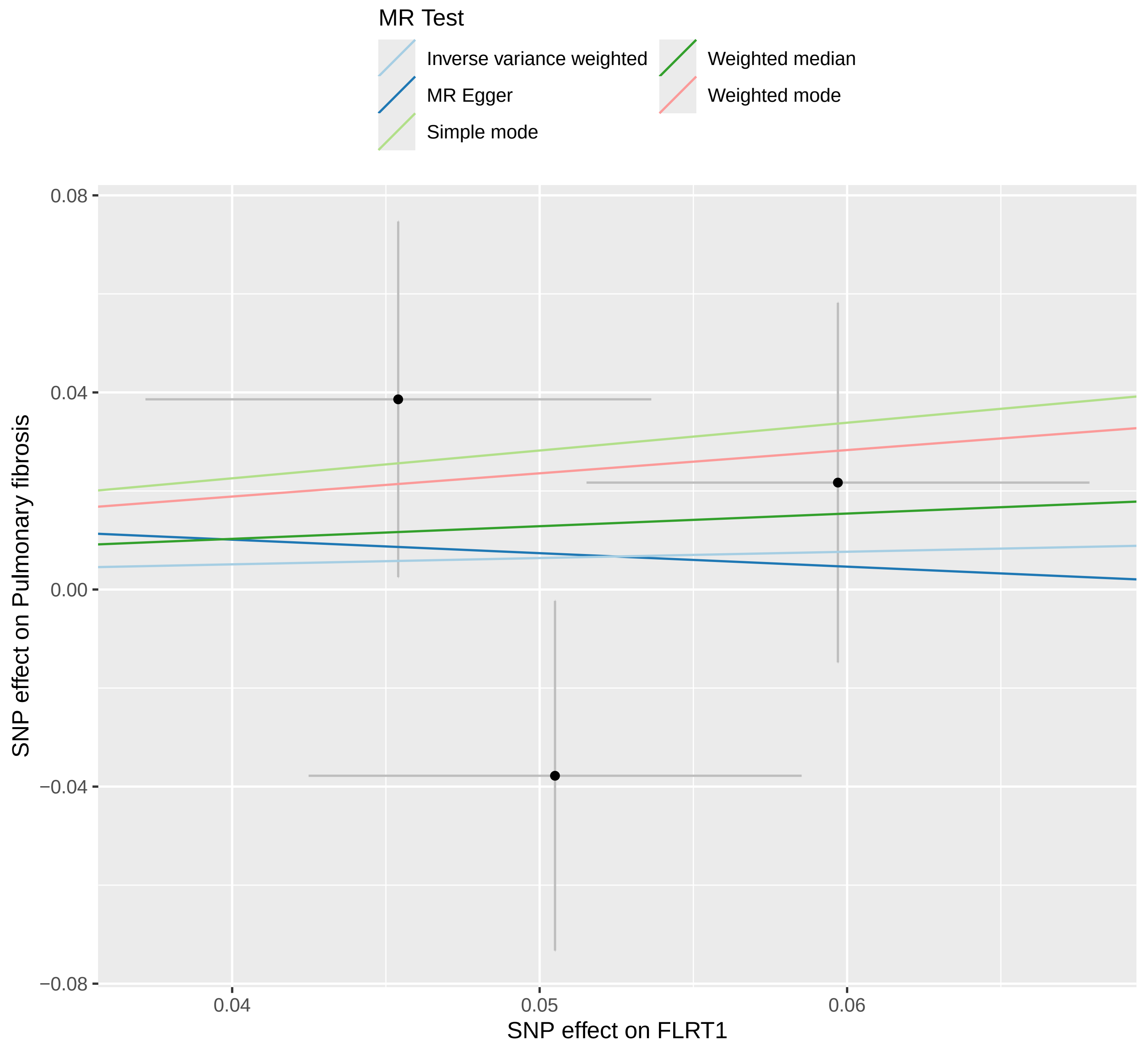

MR Test
Inverse variance weighted
MR Egger
Simple mode
Weighted median
Weighted mode
0.08
0.04
0.00
−0.04
−0.08
0.04
0.05
0.06
SNP effect on Pulmonary fibrosis
SNP effect on FLRT1

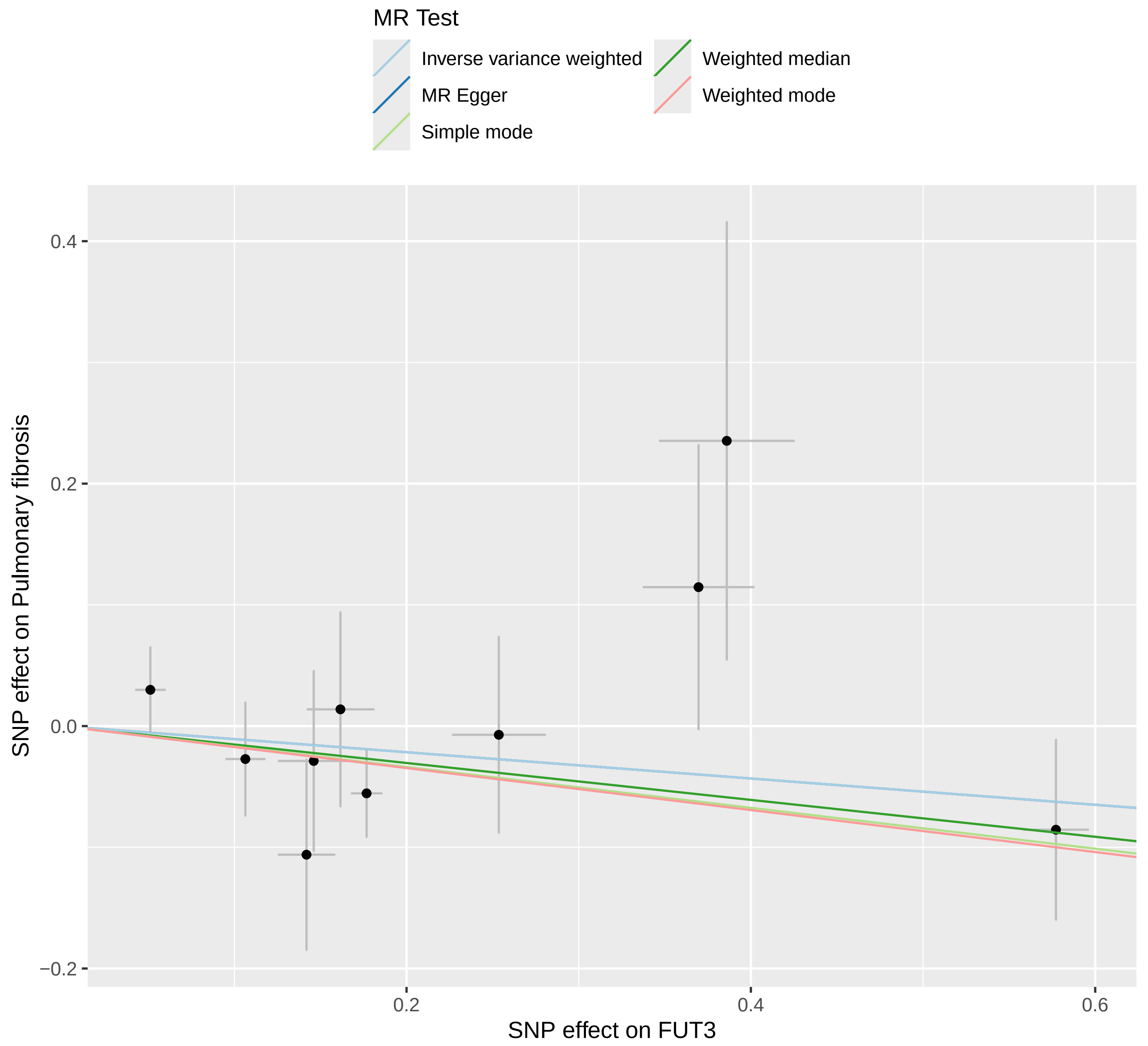
MR Test
Inverse variance weighted
MR Egger
Simple mode
Weighted median
Weighted mode
0.4
0.2
0.0
−0.2
0.2
0.4
0.6
SNP effect on Pulmonary fibrosis
SNP effect on FUT3

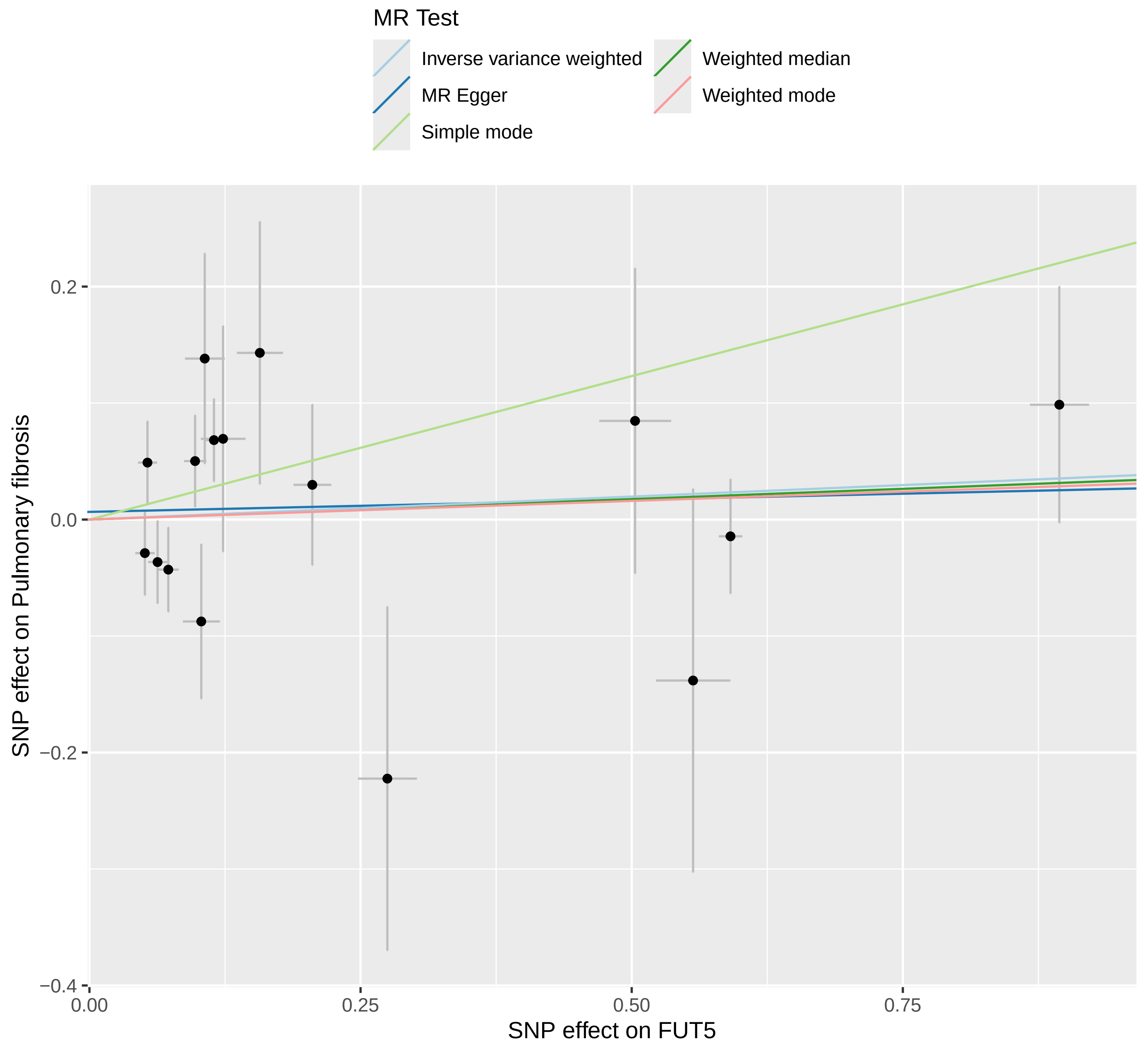
MR Test
Inverse variance weighted
MR Egger
Simple mode
Weighted median
Weighted mode
0.2
0.0
−0.2
−0.4
SNP effect on Pulmonary fibrosis
0.00
0.25
0.50
0.75
SNP effect on FUT5

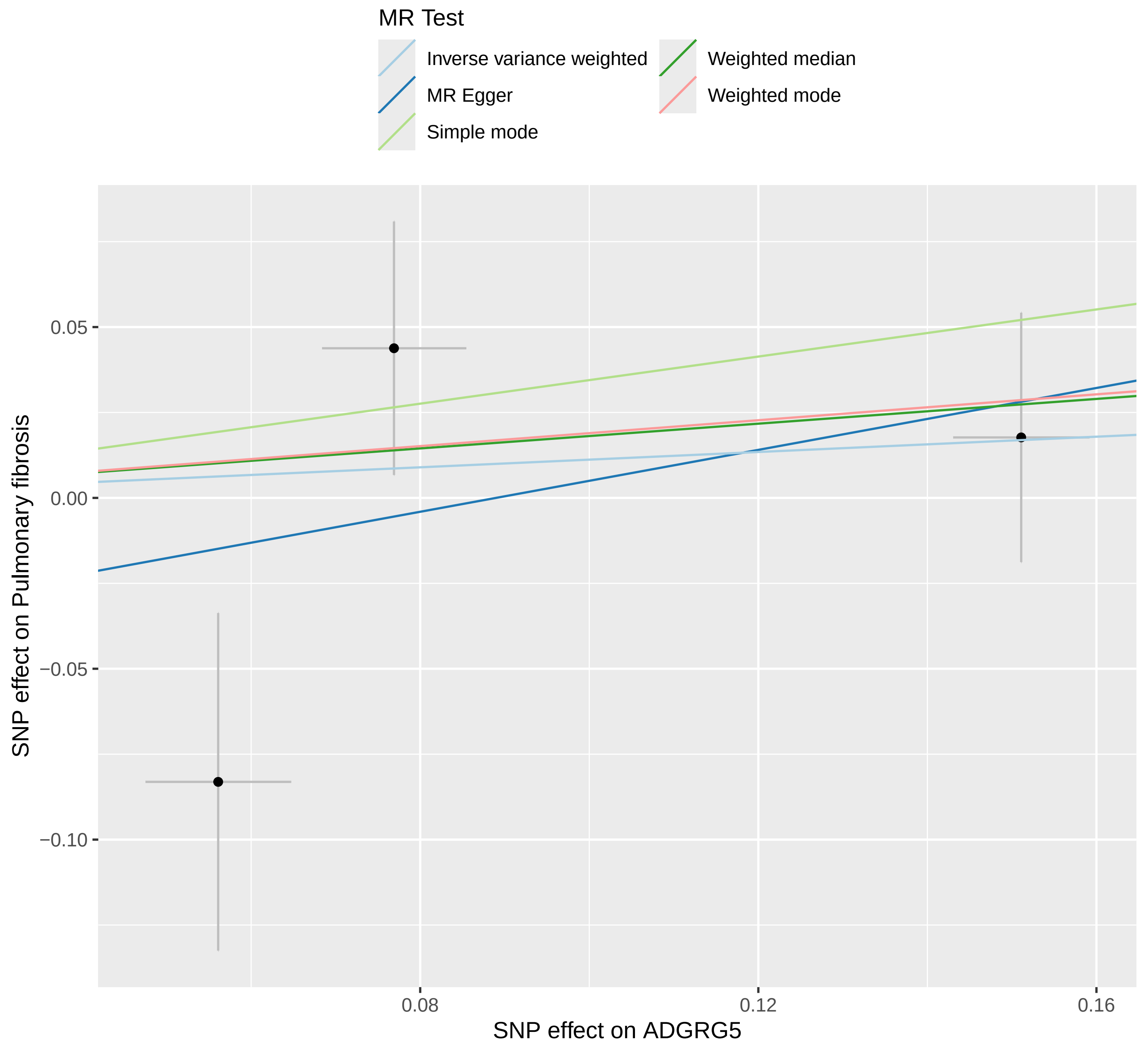

MR Test
Inverse variance weighted
MR Egger
Simple mode
Weighted median
Weighted mode
0.05
0.00
−0.05
−0.10
SNP effect on Pulmonary fibrosis
0.08
0.12
0.16
SNP effect on ADGRG5

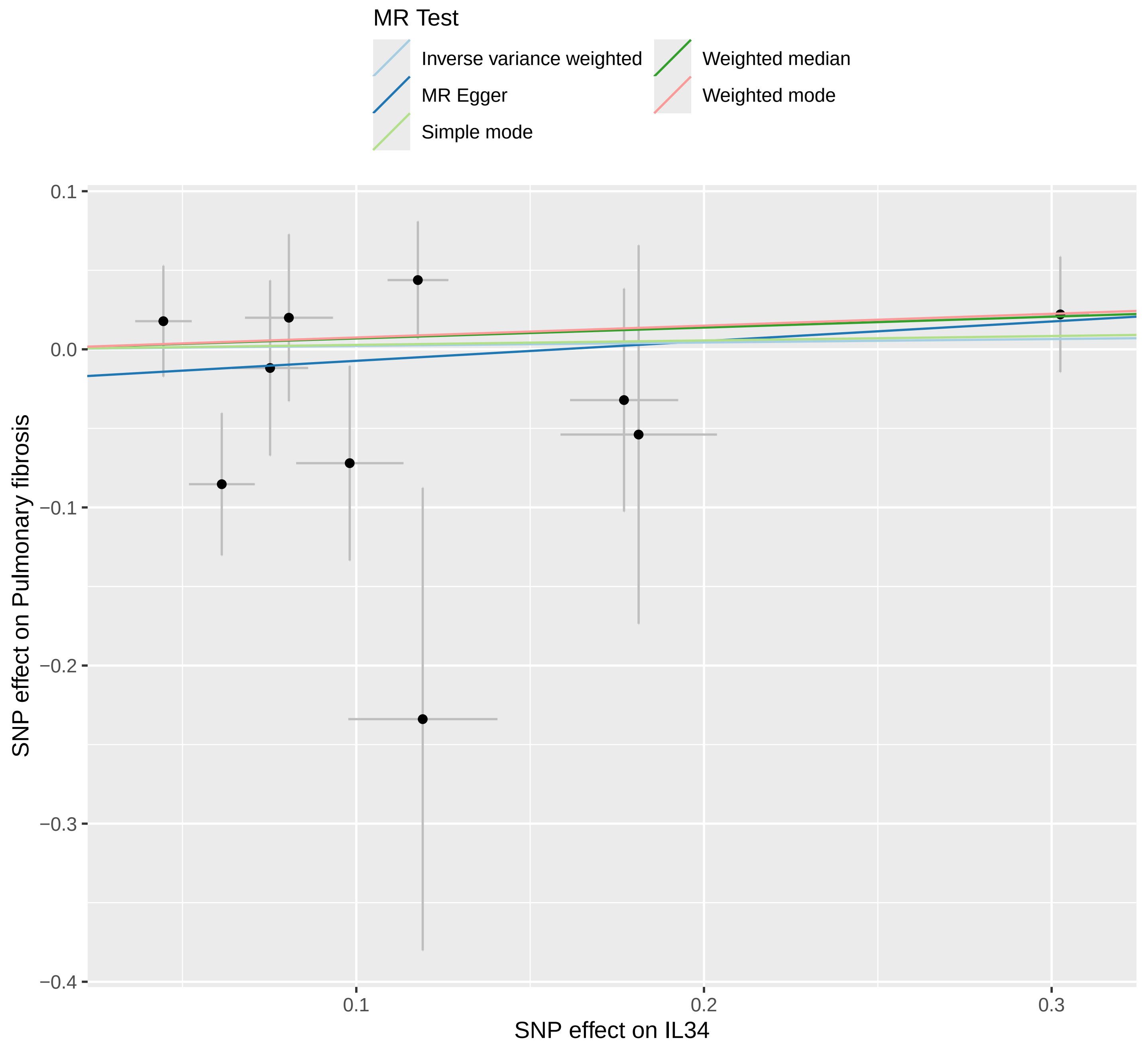
MR Test
Inverse variance weighted
MR Egger
Simple mode
Weighted median
Weighted mode
0.1
0.0
−0.1
−0.2
−0.3
−0.4
SNP effect on Pulmonary fibrosis
0.1
0.2
0.3
SNP effect on IL34

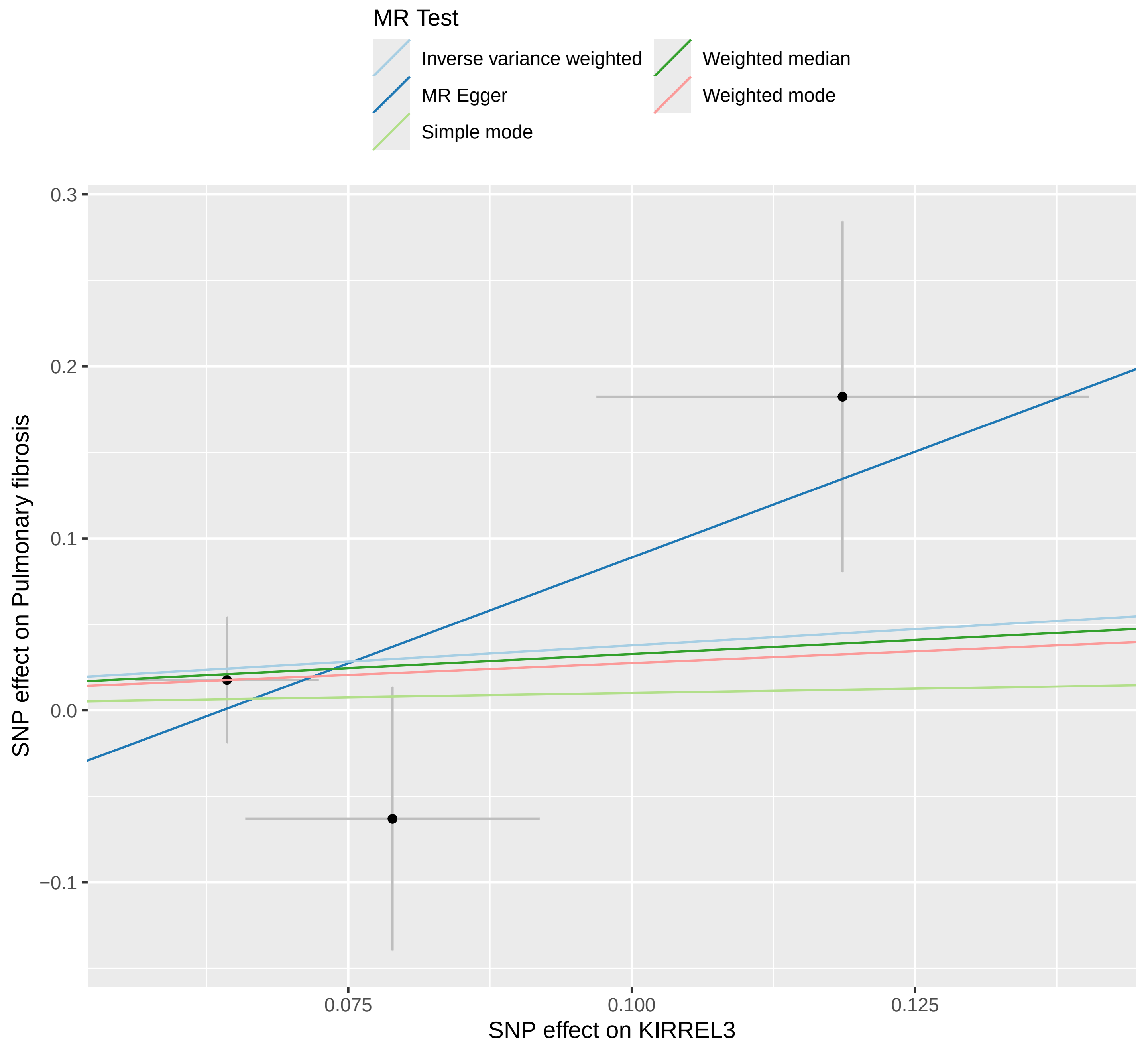
MR Test
Inverse variance weighted
MR Egger
Simple mode
Weighted median
Weighted mode
0.3
0.2
0.1
0.0
−0.1
0.075
0.100
0.125
SNP effect on Pulmonary fibrosis
SNP effect on KIRREL3

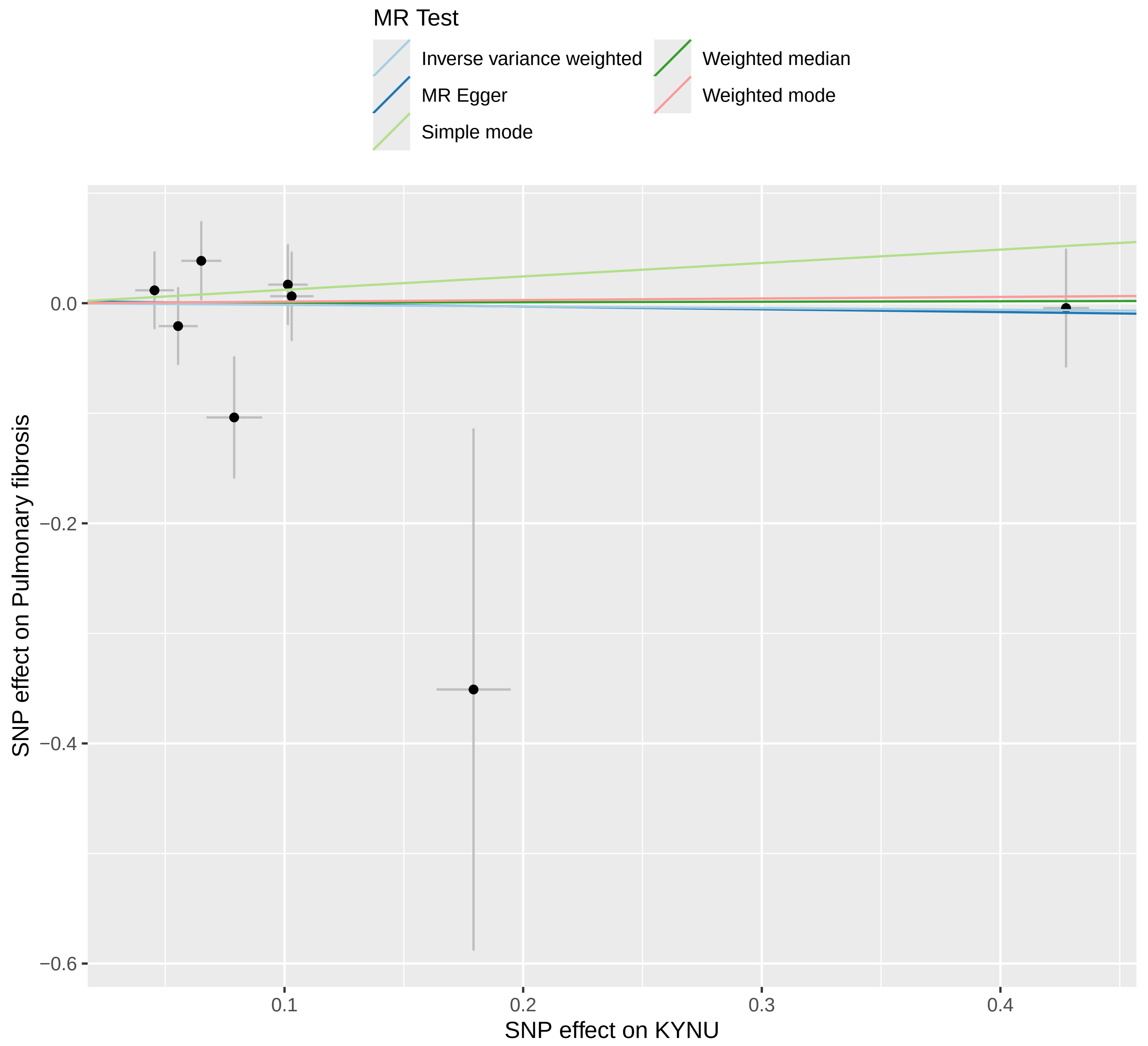

MR Test
Inverse variance weighted
MR Egger
Simple mode
Weighted median
Weighted mode
0.0
−0.2
−0.4
−0.6
0.1
0.2
0.3
0.4
SNP effect on Pulmonary fibrosis
SNP effect on KYNU

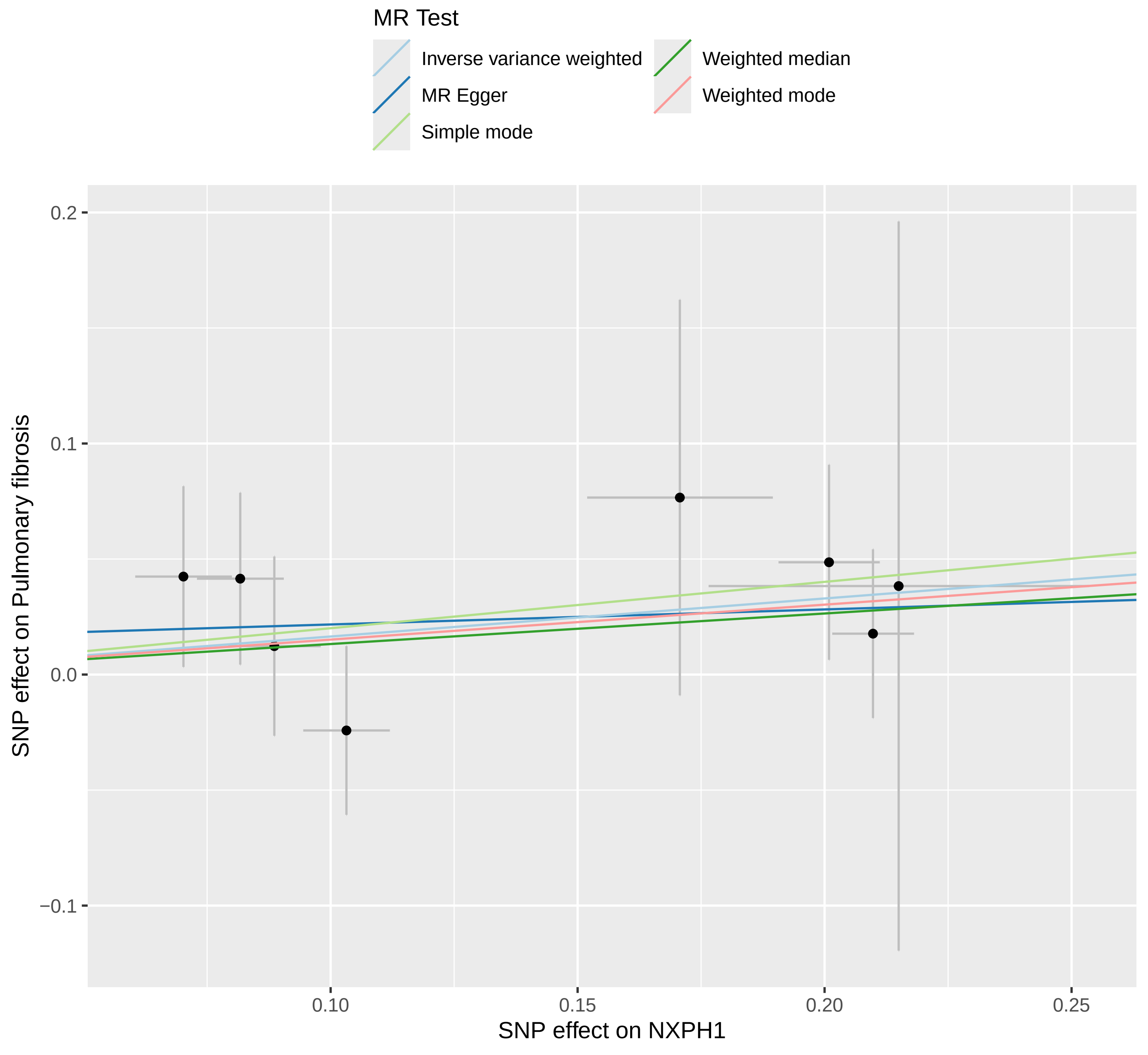

MR Test
Inverse variance weighted
MR Egger
Simple mode
Weighted median
Weighted mode
0.2
0.1
0.0
−0.1
0.10
0.15
0.20
0.25
SNP effect on Pulmonary fibrosis
SNP effect on NXPH1

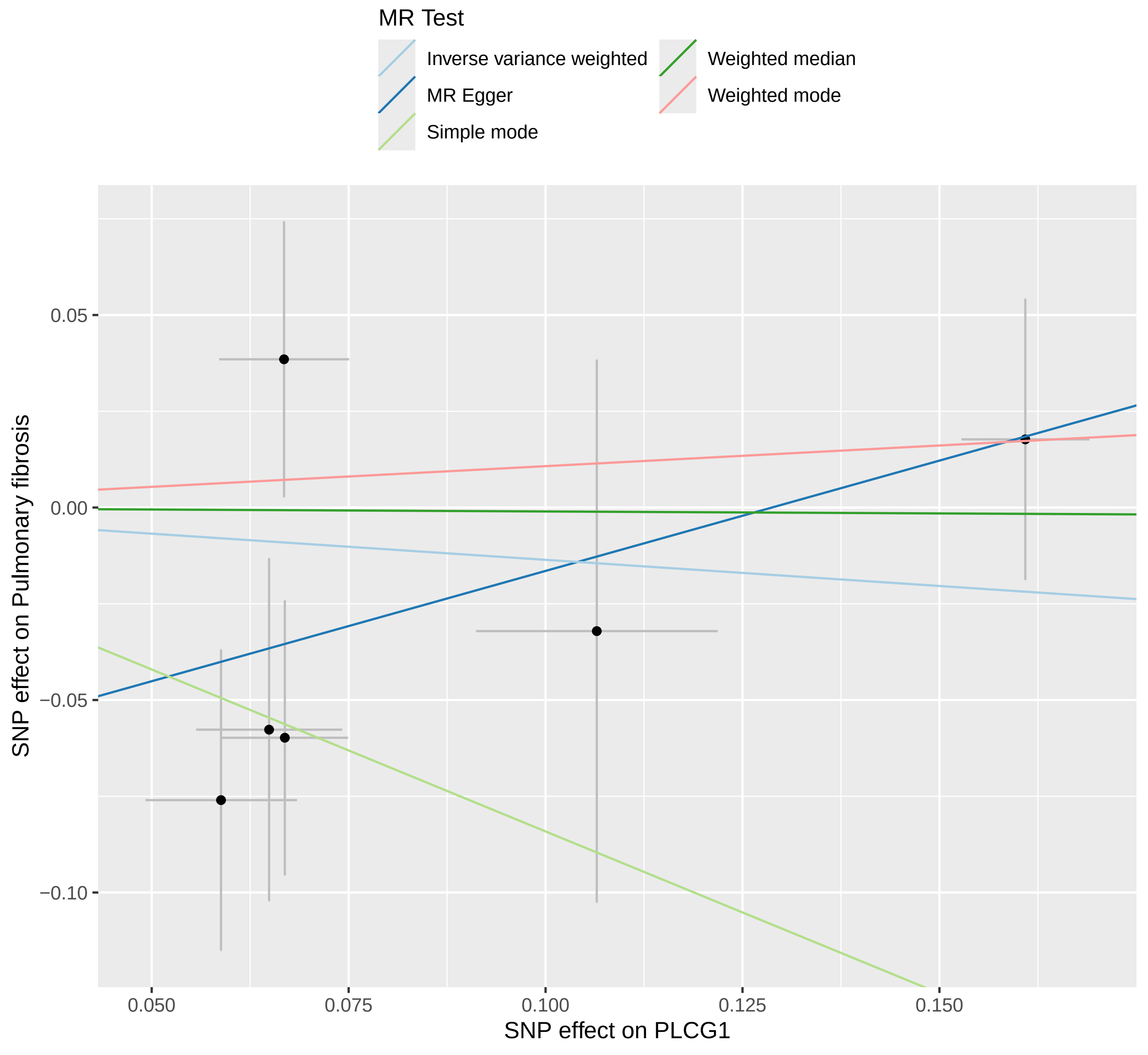

MR Test
Inverse variance weighted
MR Egger
Simple mode
Weighted median
Weighted mode
0.05
0.00
−0.05
−0.10
SNP effect on Pulmonary fibrosis
0.050
0.075
0.100
0.125
0.150
SNP effect on PLCG1

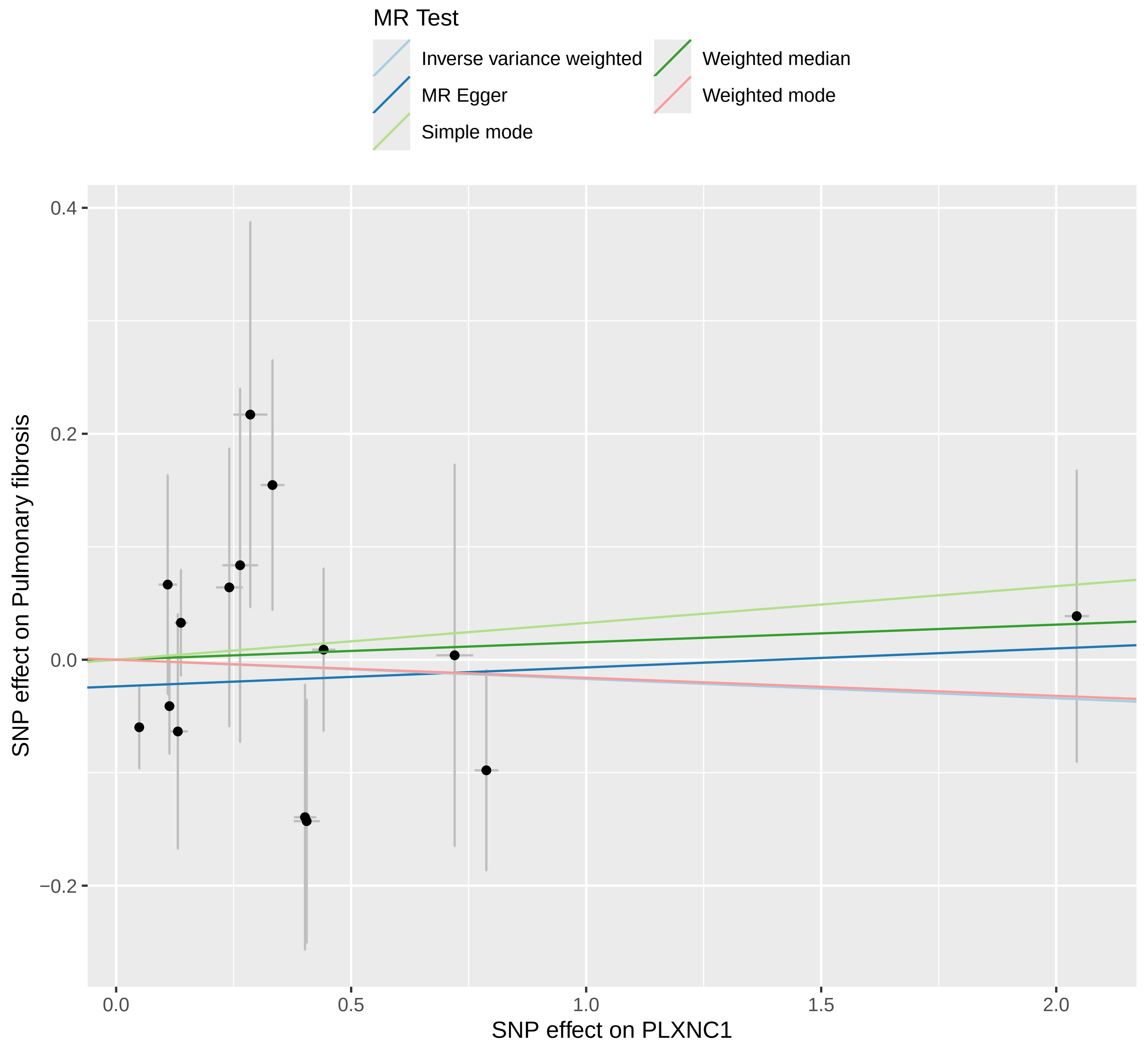

MR Test
Inverse variance weighted
MR Egger
Simple mode
Weighted median
Weighted mode
0.4
0.2
0.0
−0.2
0.0
0.5
1.0
1.5
2.0
SNP effect on Pulmonary fibrosis
SNP effect on PLXNC1

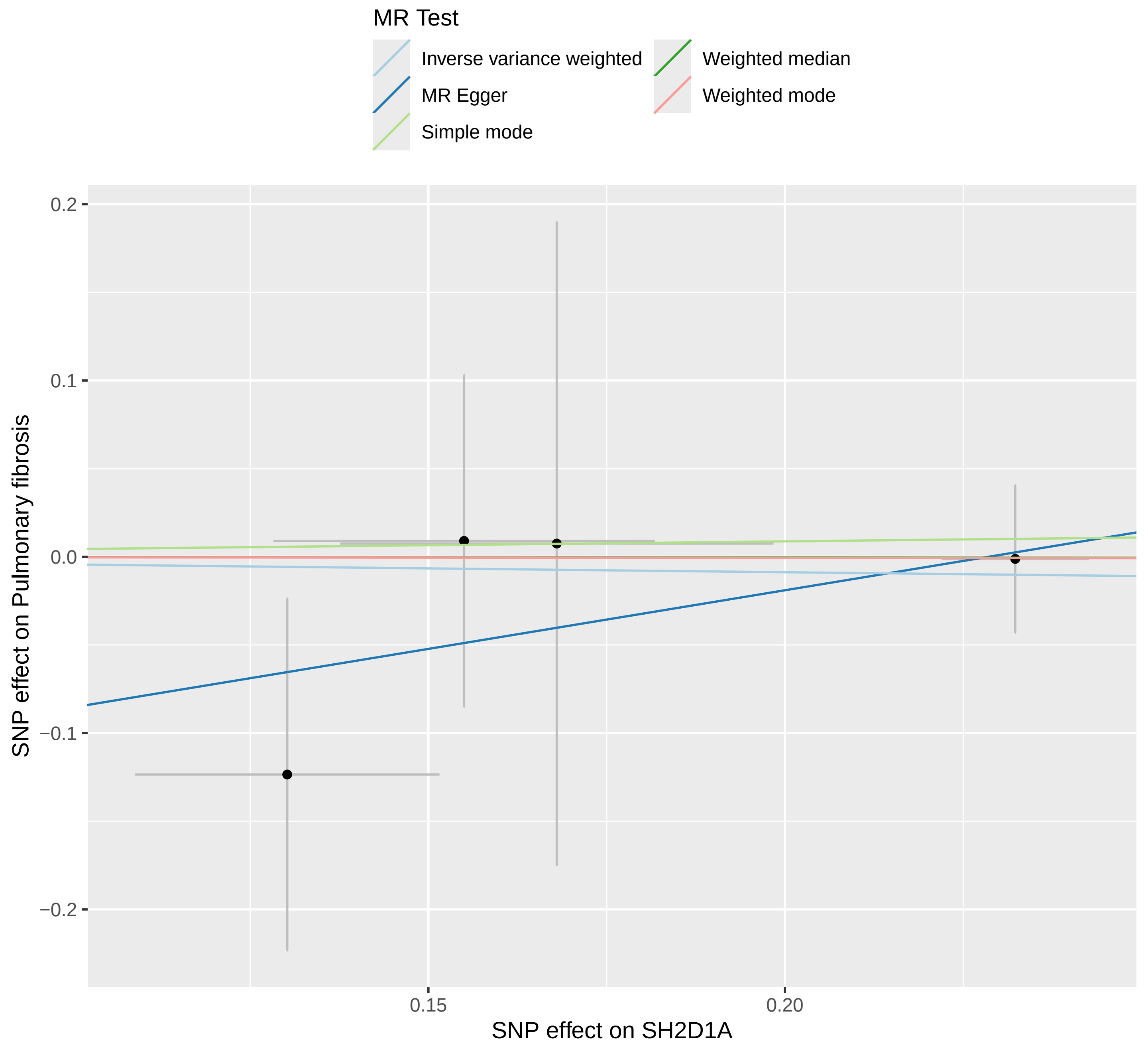
MR Test
Inverse variance weighted
MR Egger
Simple mode
Weighted median
Weighted mode
0.2
0.1
0.0
−0.1
−0.2
0.15
0.20
SNP effect on SH2D1A
SNP effect on Pulmonary fibrosis

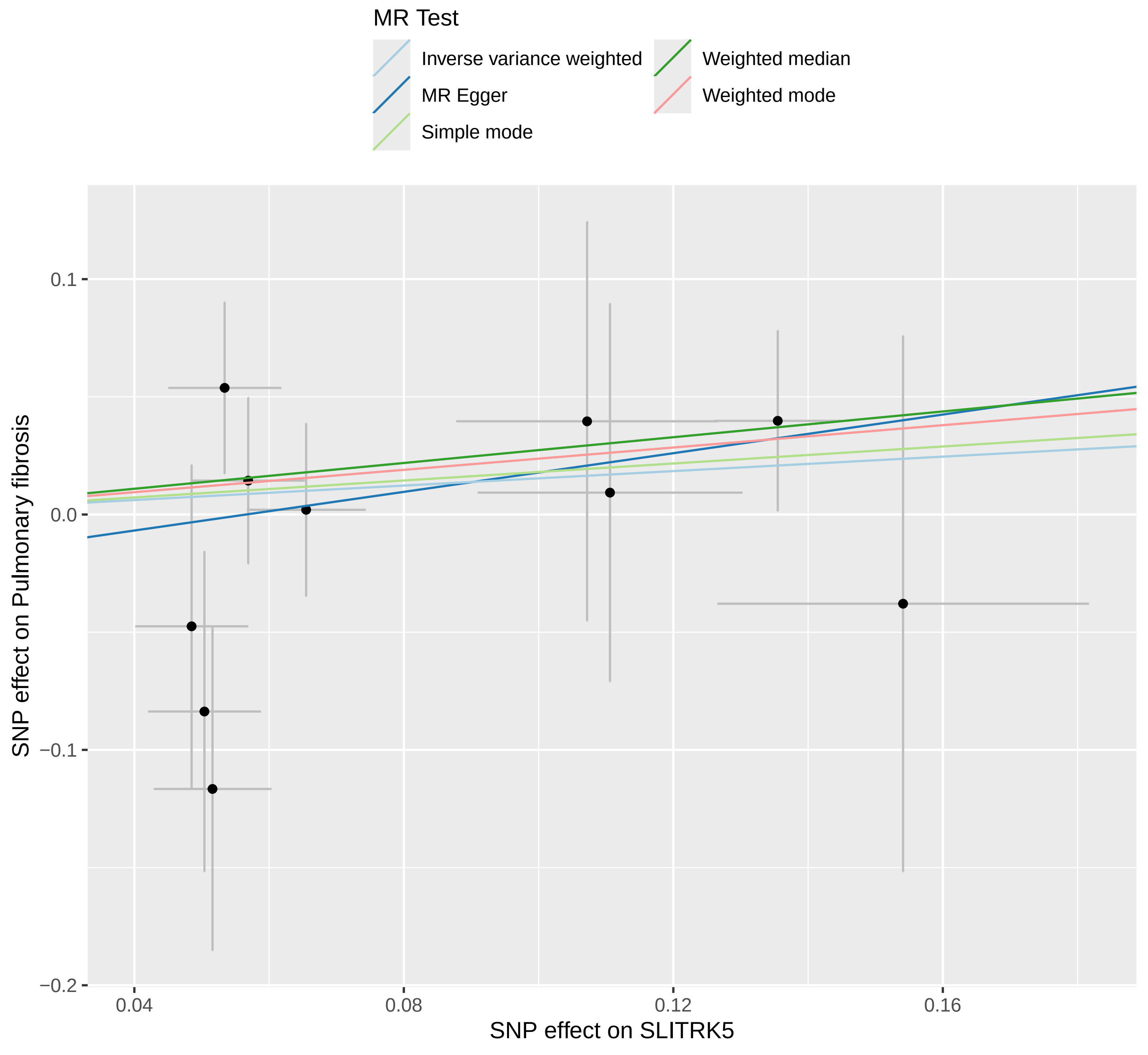
MR Test
Inverse variance weighted
MR Egger
Simple mode
Weighted median
Weighted mode
0.1
0.0
−0.1
−0.2
0.04
0.08
0.12
0.16
SNP effect on Pulmonary fibrosis
SNP effect on SLITRK5

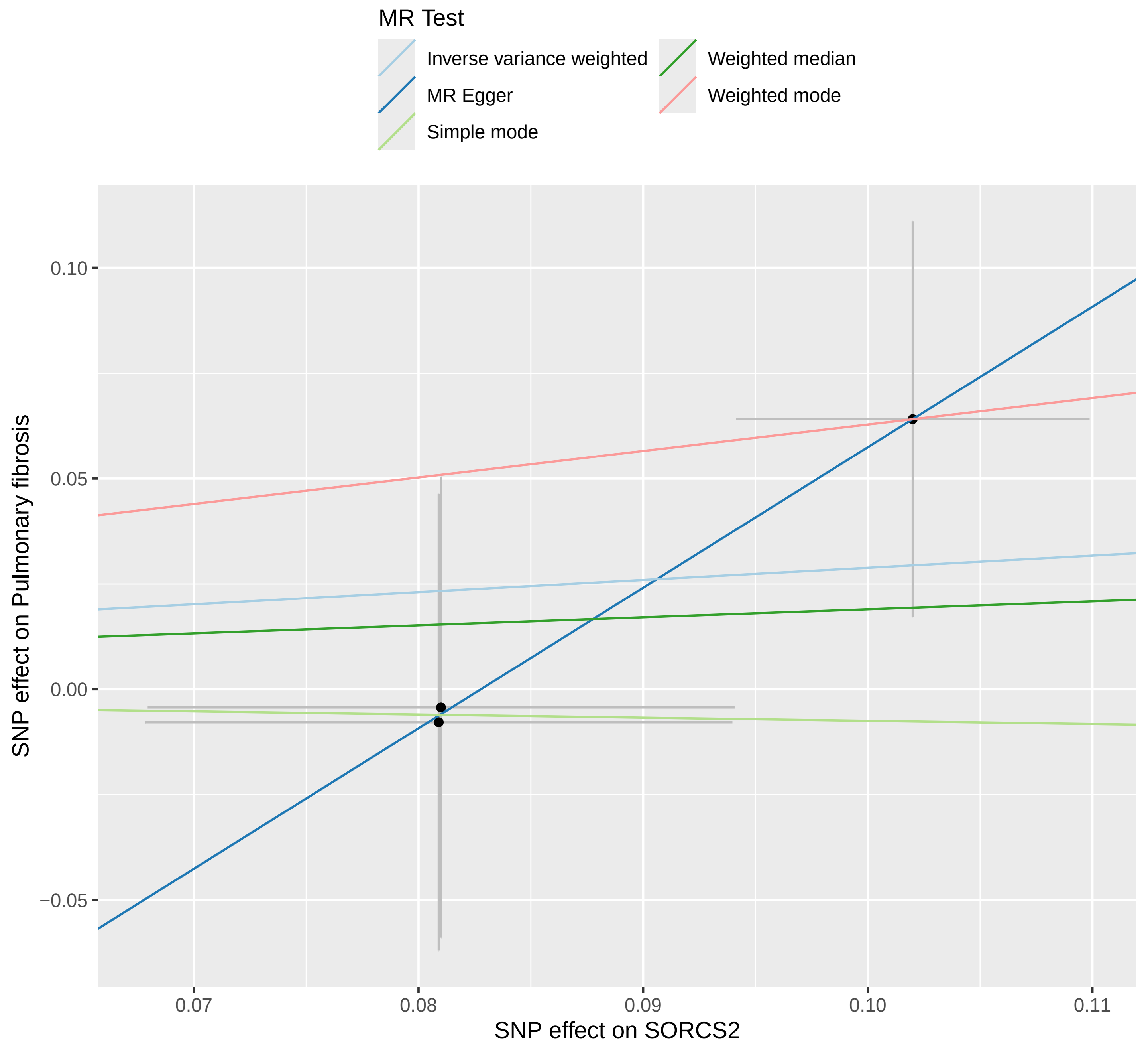
MR Test
Inverse variance weighted
MR Egger
Simple mode
Weighted median
Weighted mode
0.10
0.05
0.00
−0.05
0.07
0.08
0.09
0.10
0.11
SNP effect on Pulmonary fibrosis
SNP effect on SORCS2

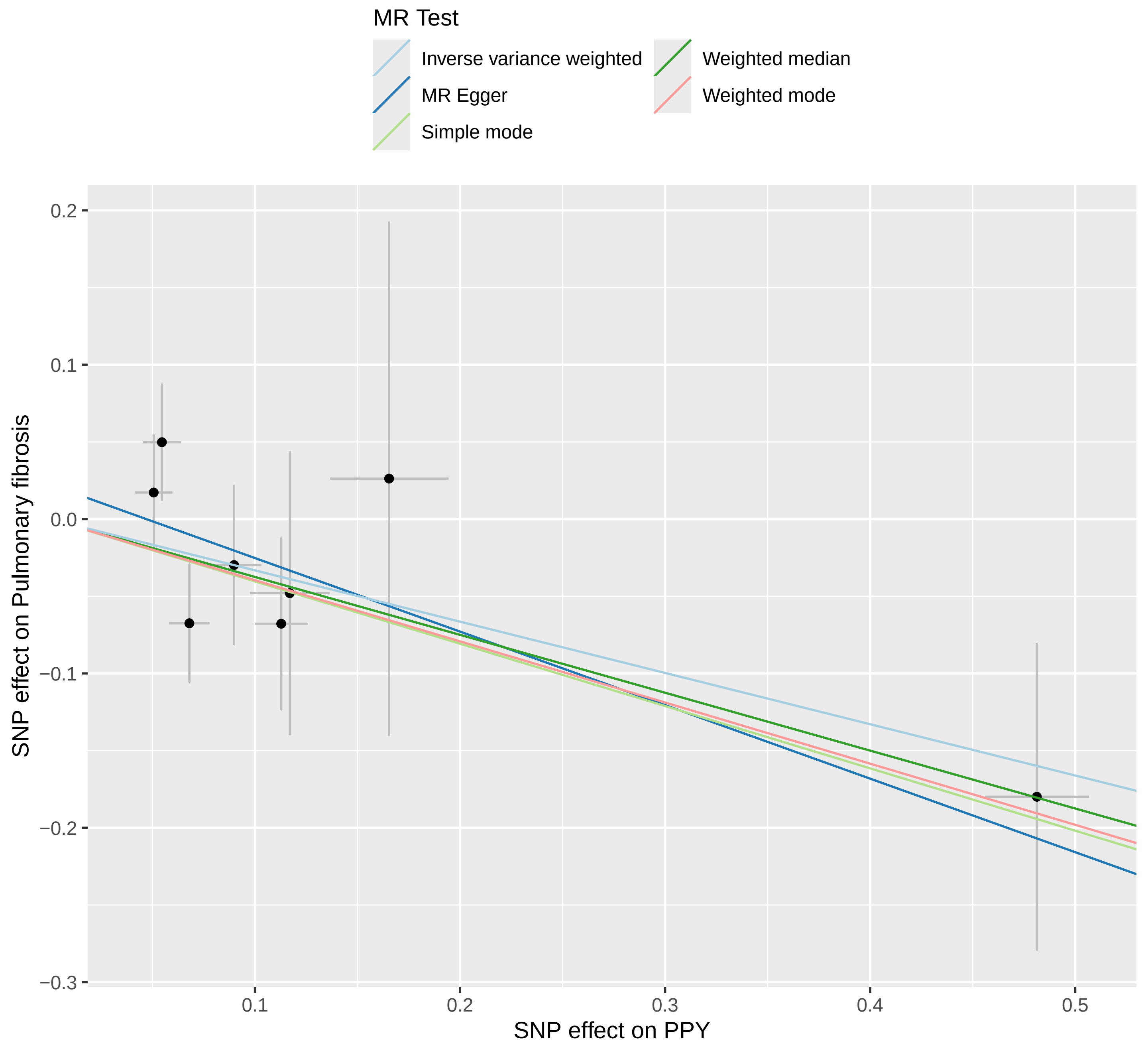
MR Test
Inverse variance weighted
MR Egger
Simple mode
Weighted median
Weighted mode
0.2
0.1
0.0
−0.1
−0.2
−0.3
0.1
0.2
0.3
0.4
0.5
SNP effect on Pulmonary fibrosis
SNP effect on PPY

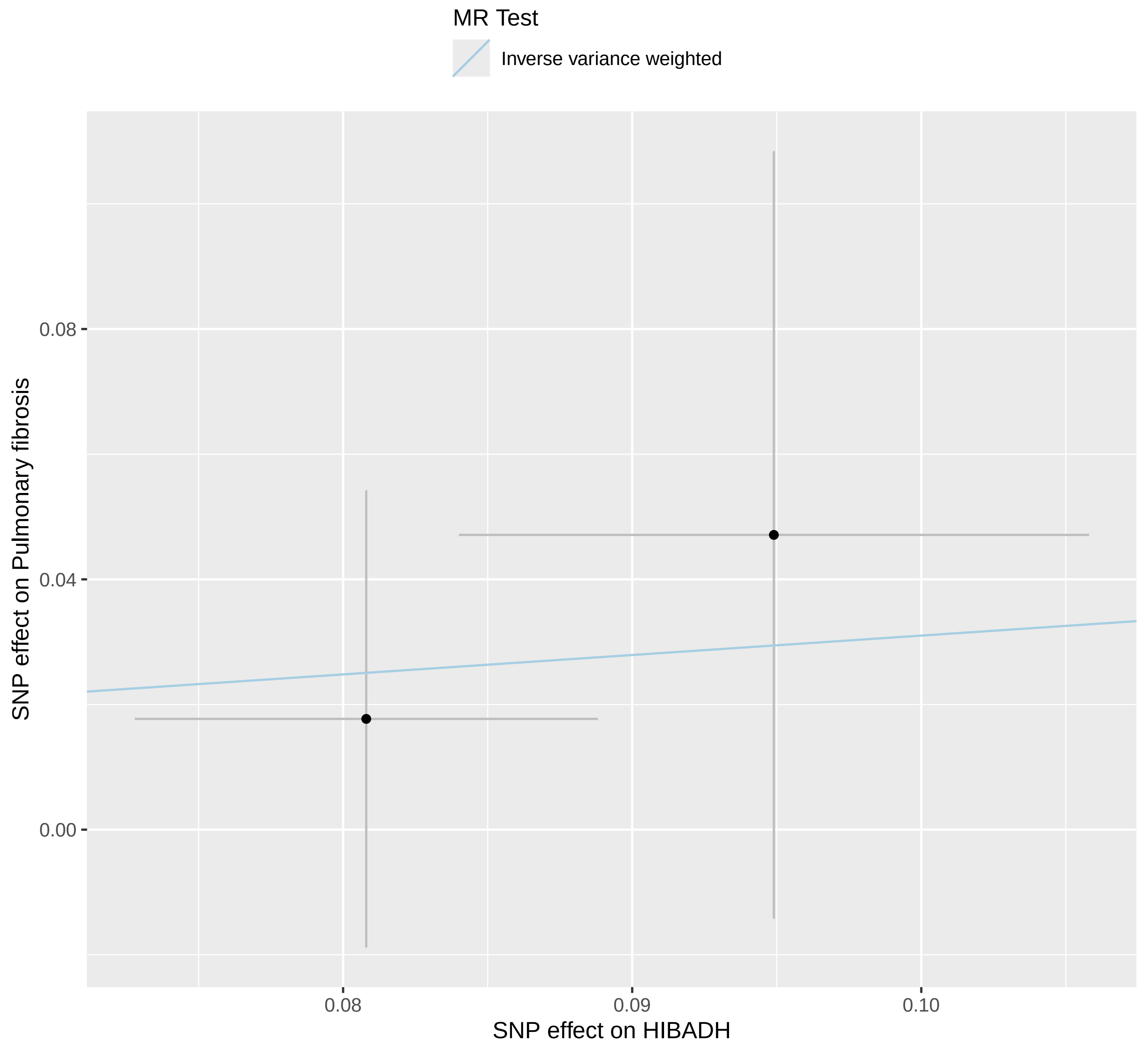
MR Test
Inverse variance weighted
0.08
0.04
0.00
SNP effect on Pulmonary fibrosis
0.08
0.09
0.10
SNP effect on HIBADH

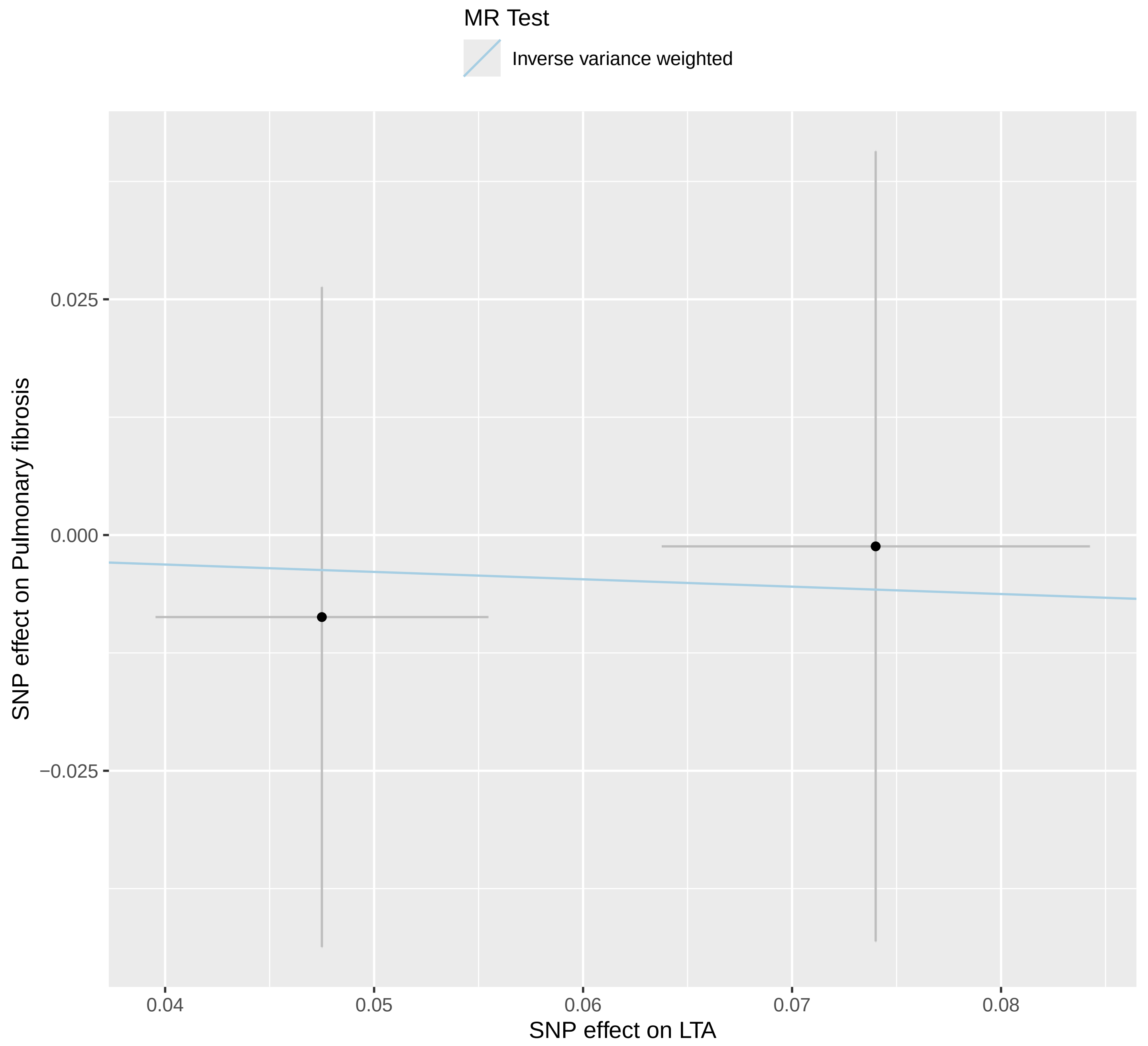
MR Test
Inverse variance weighted
0.025
0.000
−0.025
0.04
0.05
0.06
0.07
0.08
SNP effect on Pulmonary fibrosis
SNP effect on LTA

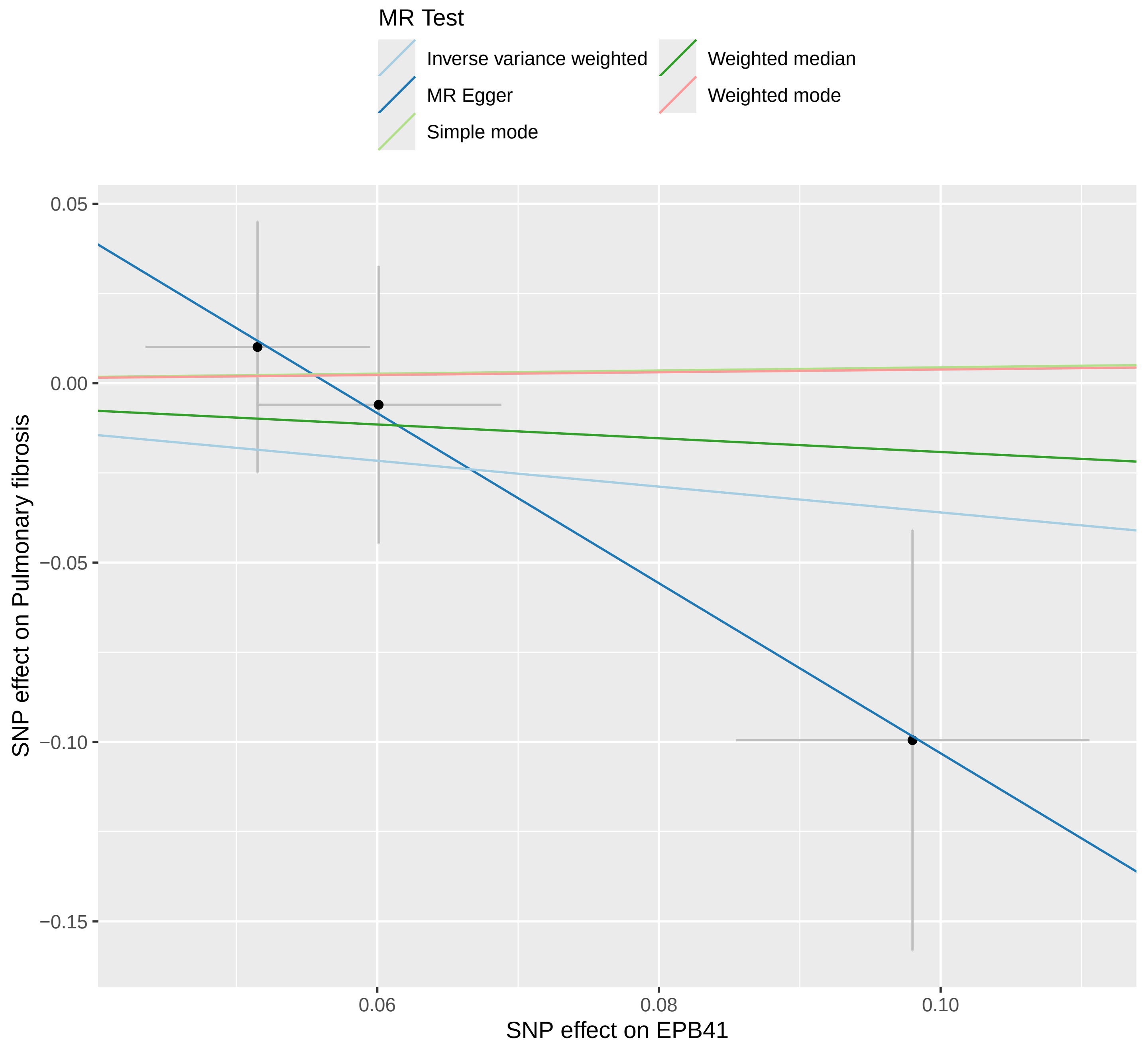
MR Test
Inverse variance weighted
MR Egger
Simple mode
Weighted median
Weighted mode
0.05
0.00
−0.05
−0.10
−0.15
0.06
0.08
0.10
SNP effect on Pulmonary fibrosis
SNP effect on EPB41

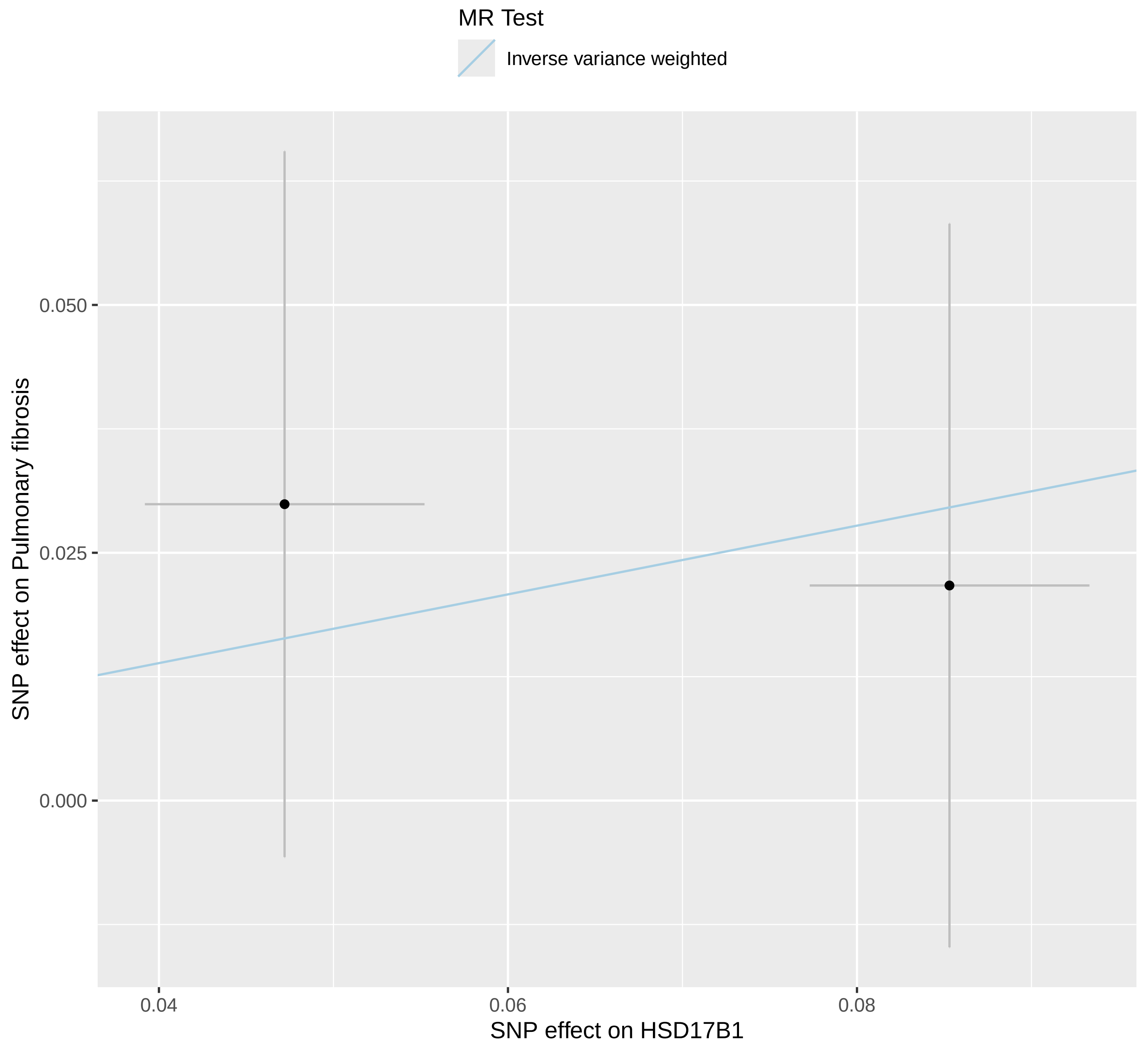
MR Test
Inverse variance weighted
0.050
0.025
0.000
0.04
0.06
0.08
SNP effect on HSD17B1
SNP effect on Pulmonary fibrosis

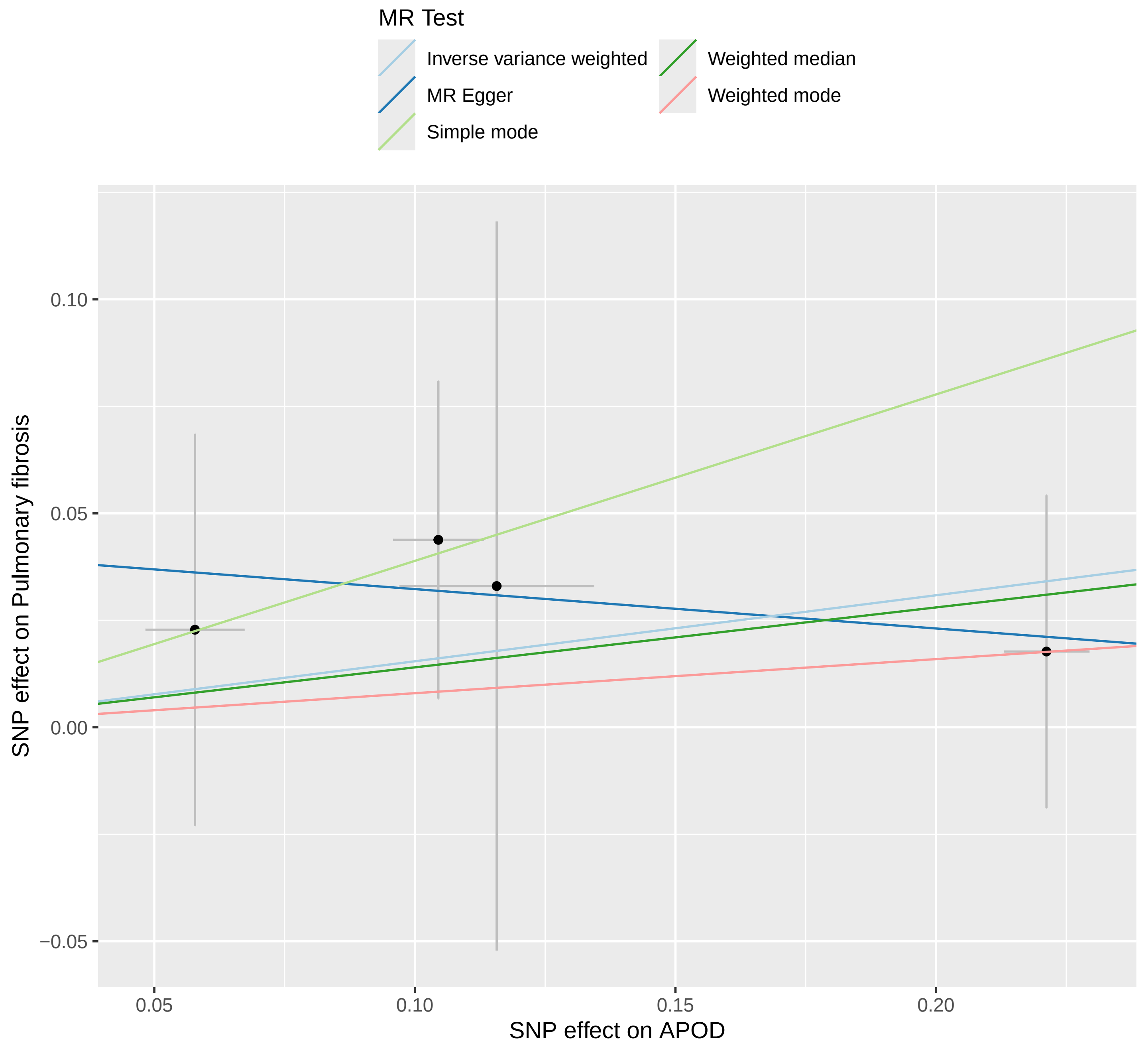

MR Test
Inverse variance weighted
MR Egger
Simple mode
Weighted median
Weighted mode
SNP effect on Pulmonary fibrosis
0.10
0.05
0.00
−0.05
0.05
0.10
0.15
0.20
SNP effect on APOD

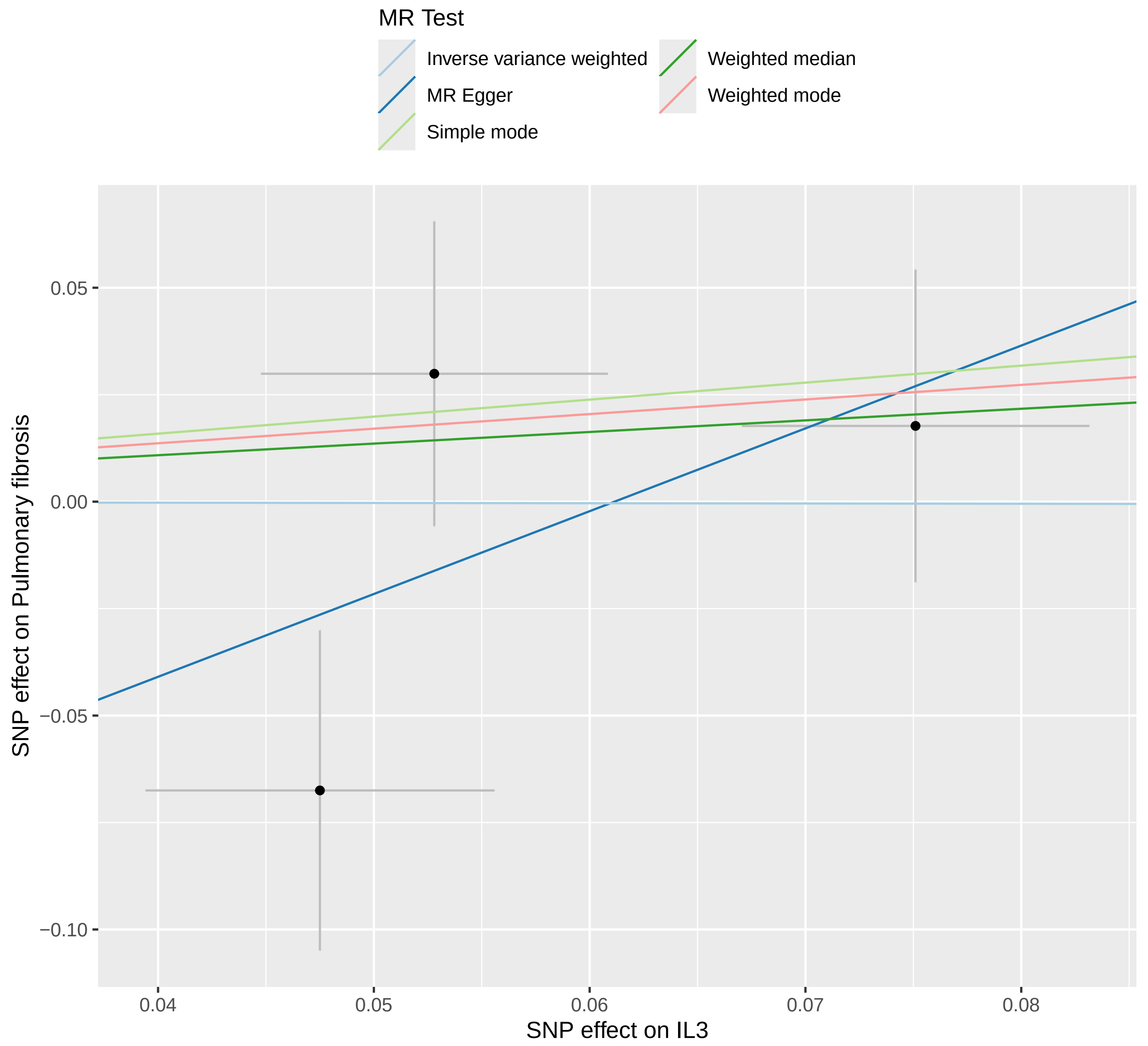

MR Test
Inverse variance weighted
MR Egger
Simple mode
Weighted median
Weighted mode
0.05
0.00
−0.05
−0.10
SNP effect on Pulmonary fibrosis
0.04
0.05
0.06
0.07
0.08
SNP effect on IL3

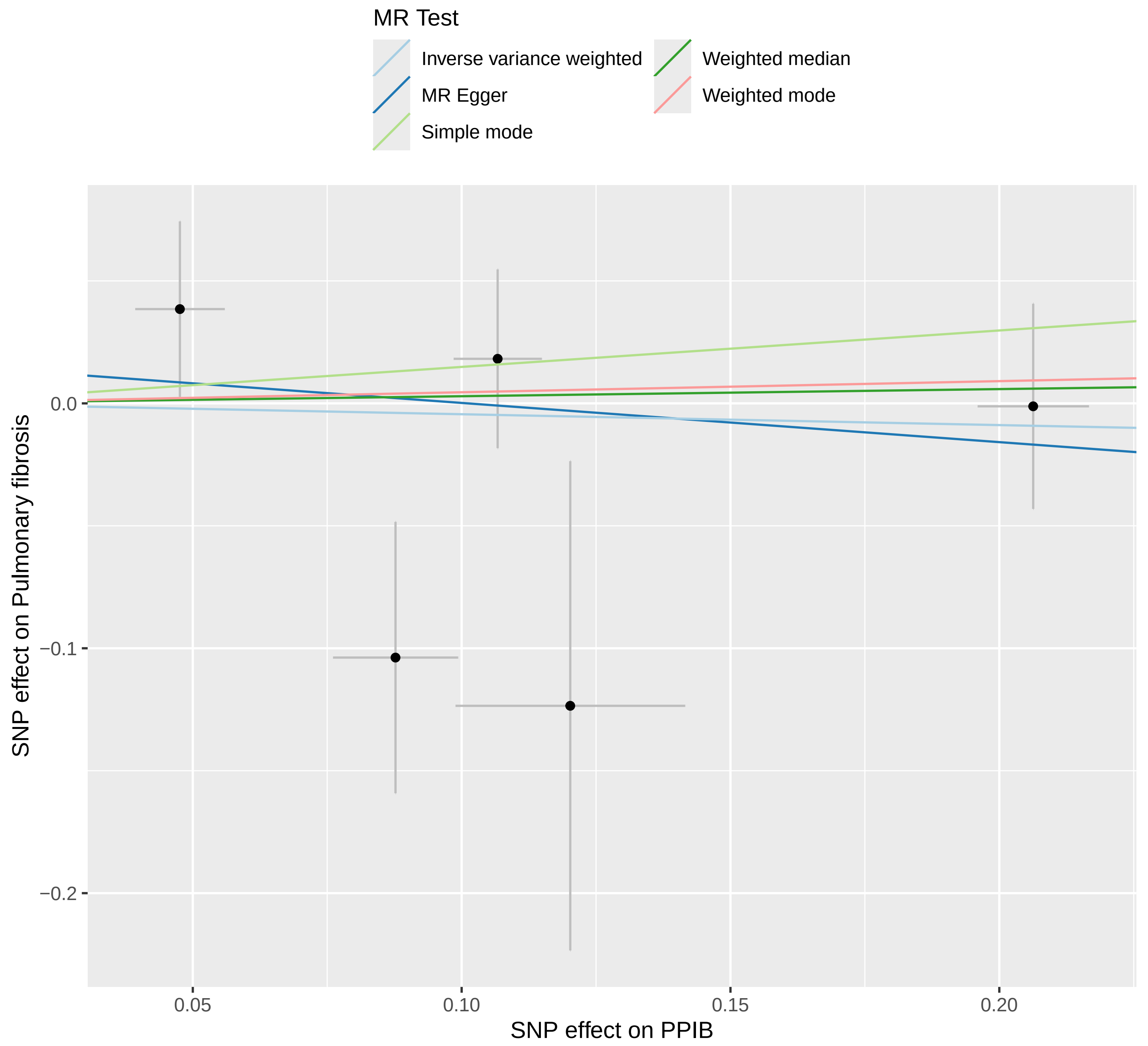

MR Test
Inverse variance weighted
MR Egger
Simple mode
Weighted median
Weighted mode
0.0
−0.1
−0.2
SNP effect on Pulmonary fibrosis
0.05
0.10
0.15
0.20
SNP effect on PPIB

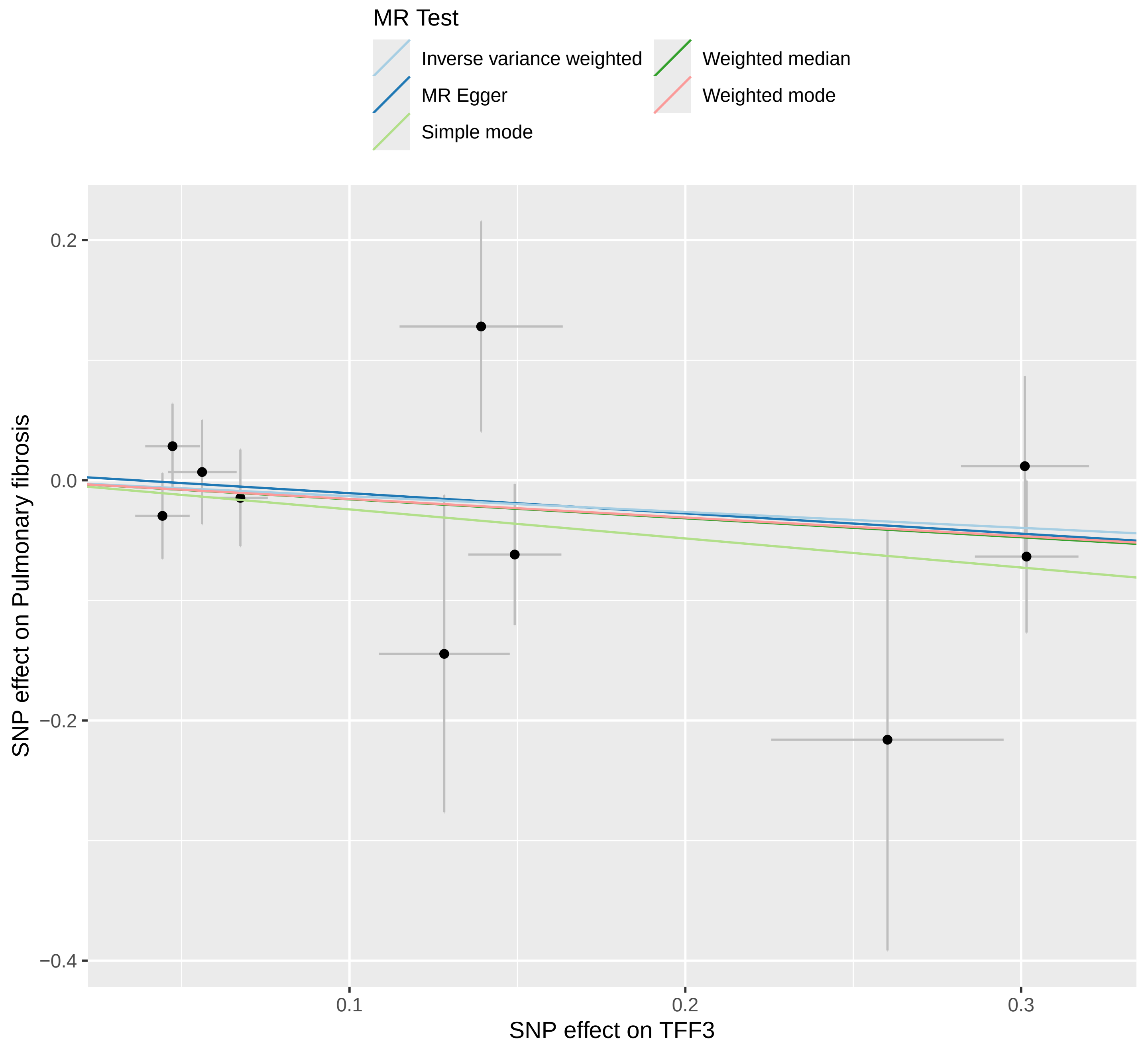

MR Test
Inverse variance weighted
MR Egger
Simple mode
Weighted median
Weighted mode
0.2
0.0
−0.2
−0.4
SNP effect on Pulmonary fibrosis
0.1
0.2
0.3
SNP effect on TFF3

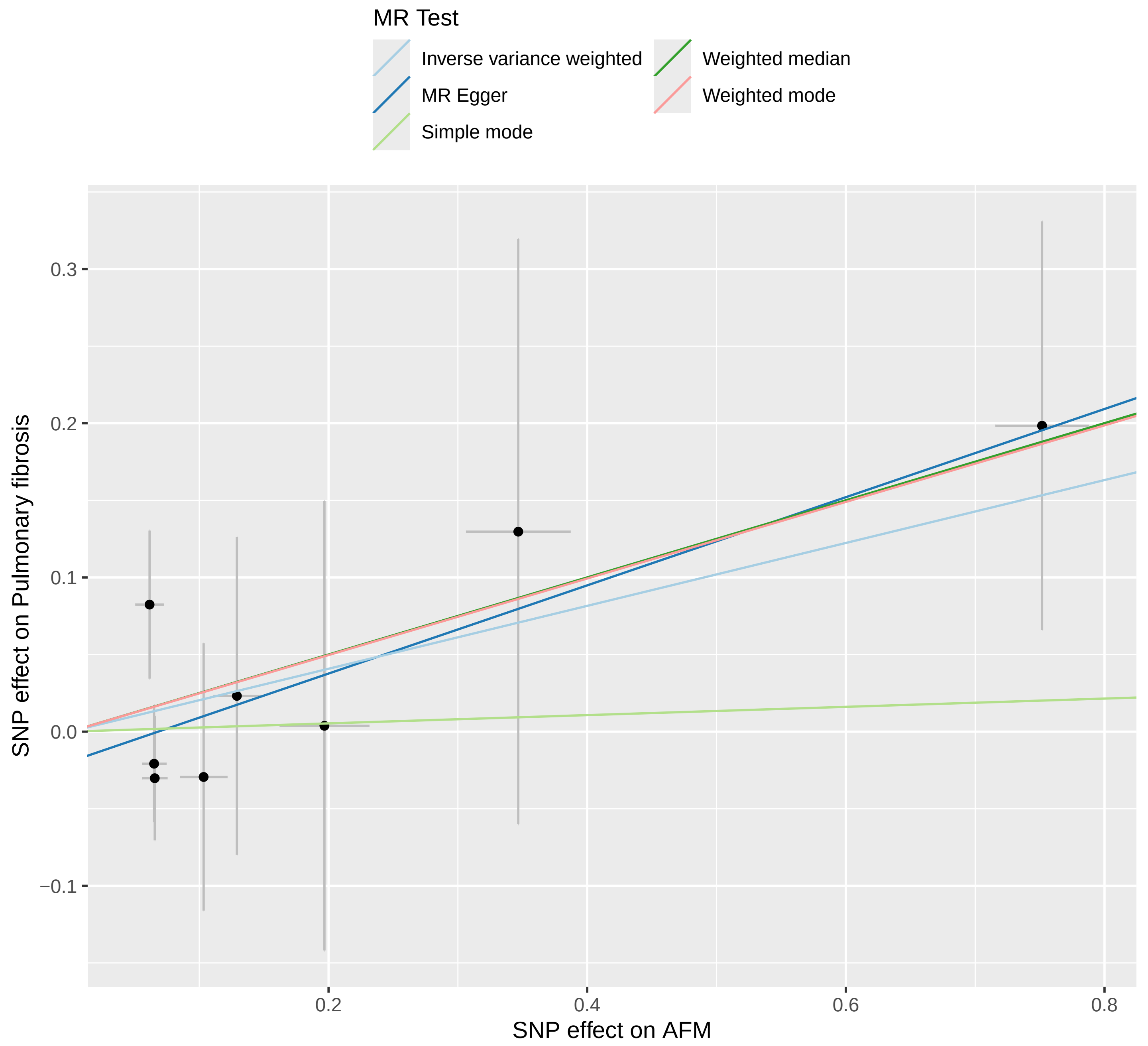

MR Test
Inverse variance weighted
MR Egger
Simple mode
Weighted median
Weighted mode
0.3
0.2
0.1
0.0
−0.1
0.2
0.4
0.6
0.8
SNP effect on Pulmonary fibrosis
SNP effect on AFM

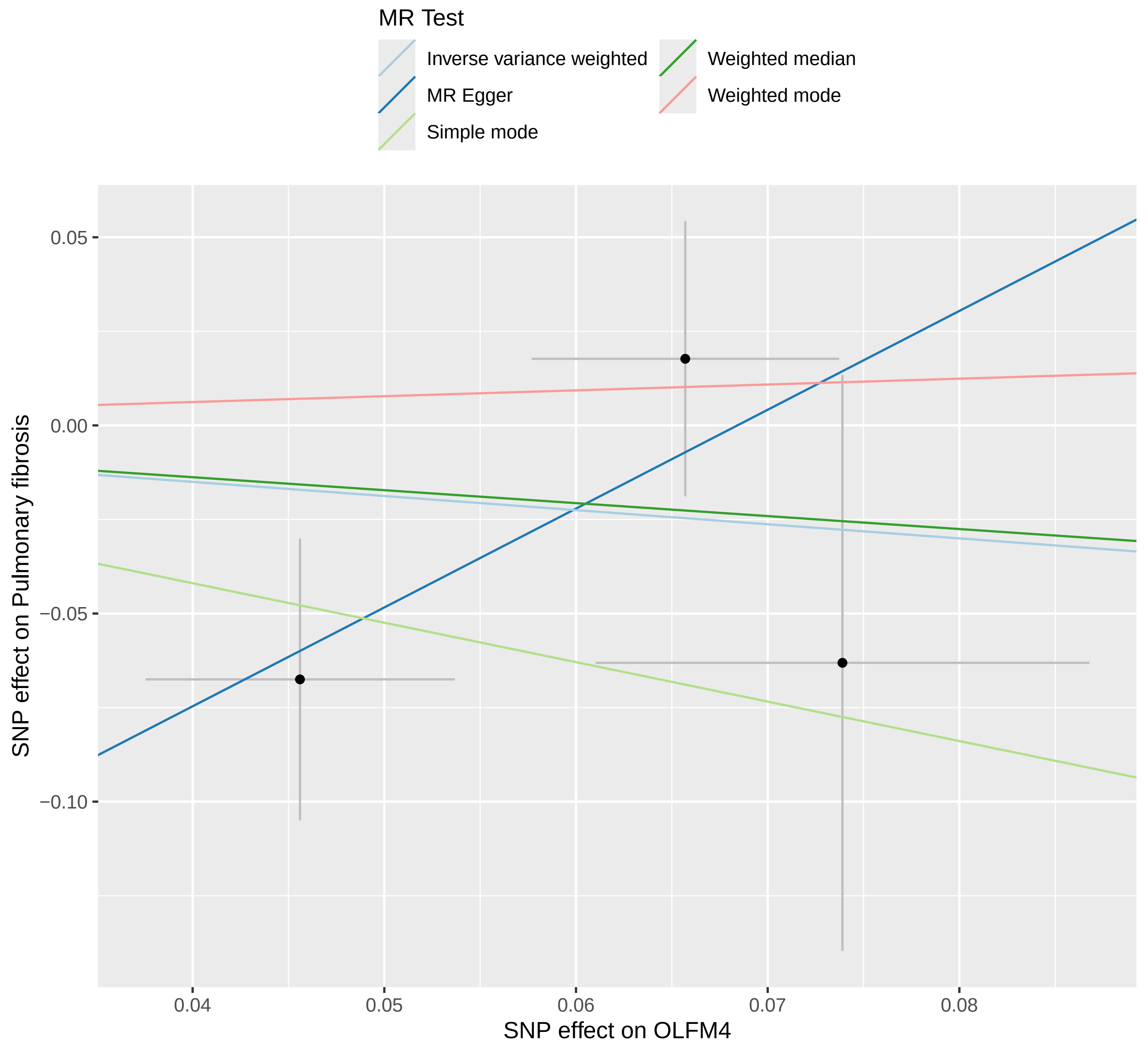

MR Test
Inverse variance weighted
MR Egger
Simple mode
Weighted median
Weighted mode
0.05
0.00
−0.05
−0.10
0.04
0.05
0.06
0.07
0.08
SNP effect on OLFM4
SNP effect on Pulmonary fibrosis

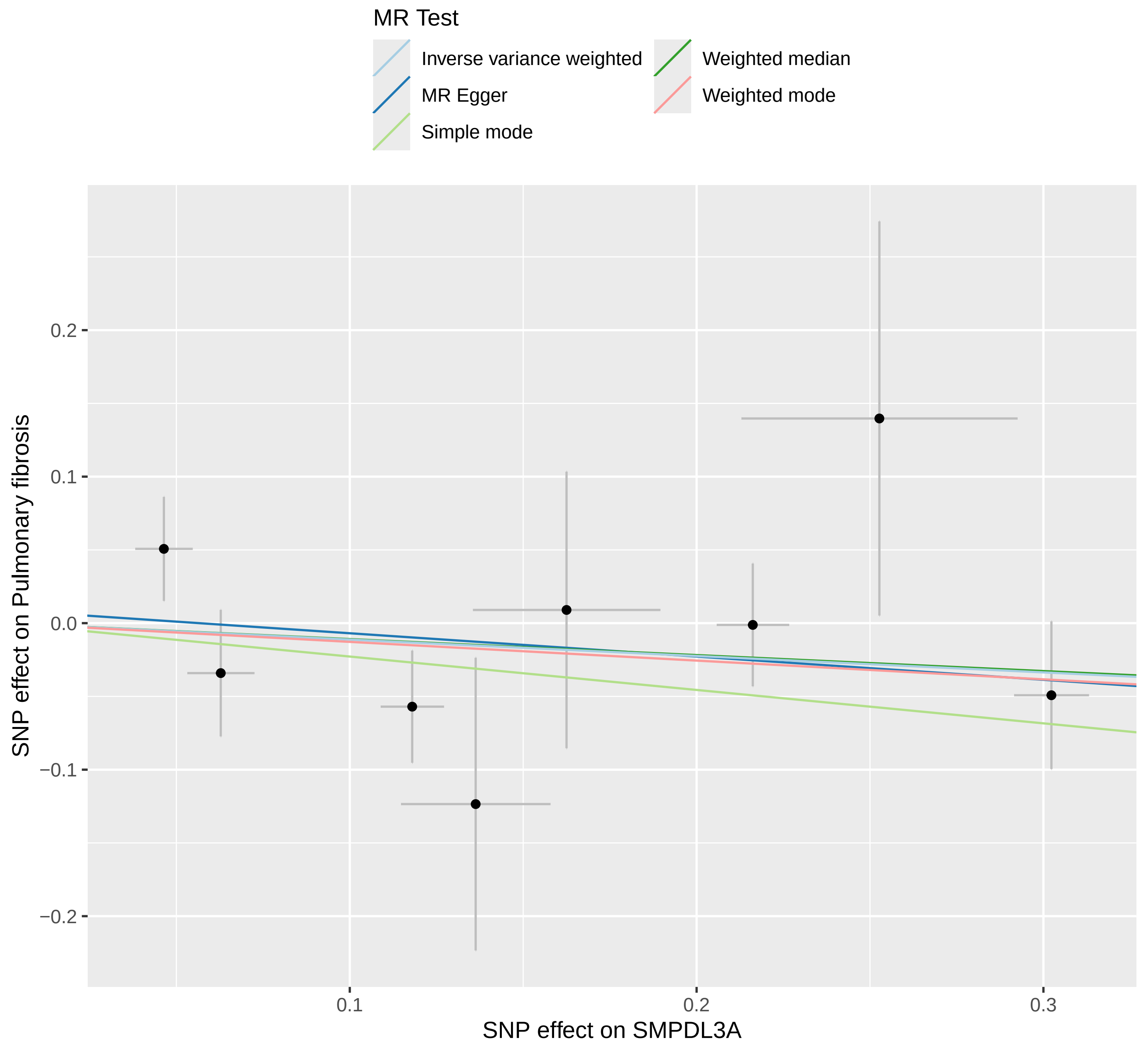
MR Test
Inverse variance weighted
MR Egger
Simple mode
Weighted median
Weighted mode
0.2
0.1
0.0
−0.1
−0.2
SNP effect on Pulmonary fibrosis
0.1
0.2
0.3
SNP effect on SMPDL3A

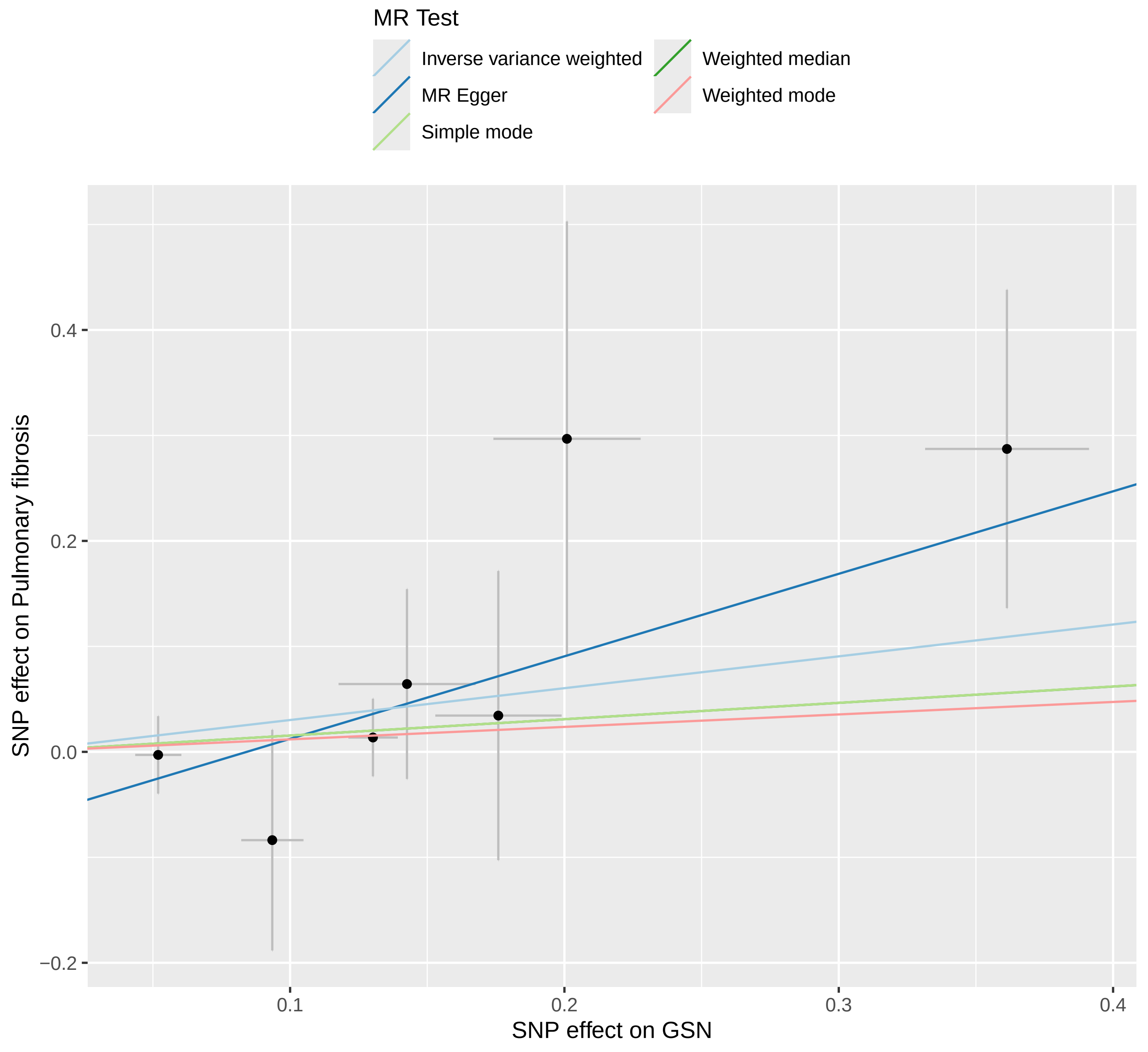
MR Test
Inverse variance weighted
MR Egger
Simple mode
Weighted median
Weighted mode
SNP effect on Pulmonary fibrosis
0.4
0.2
0.0
−0.2
0.1
0.2
0.3
0.4
SNP effect on GSN

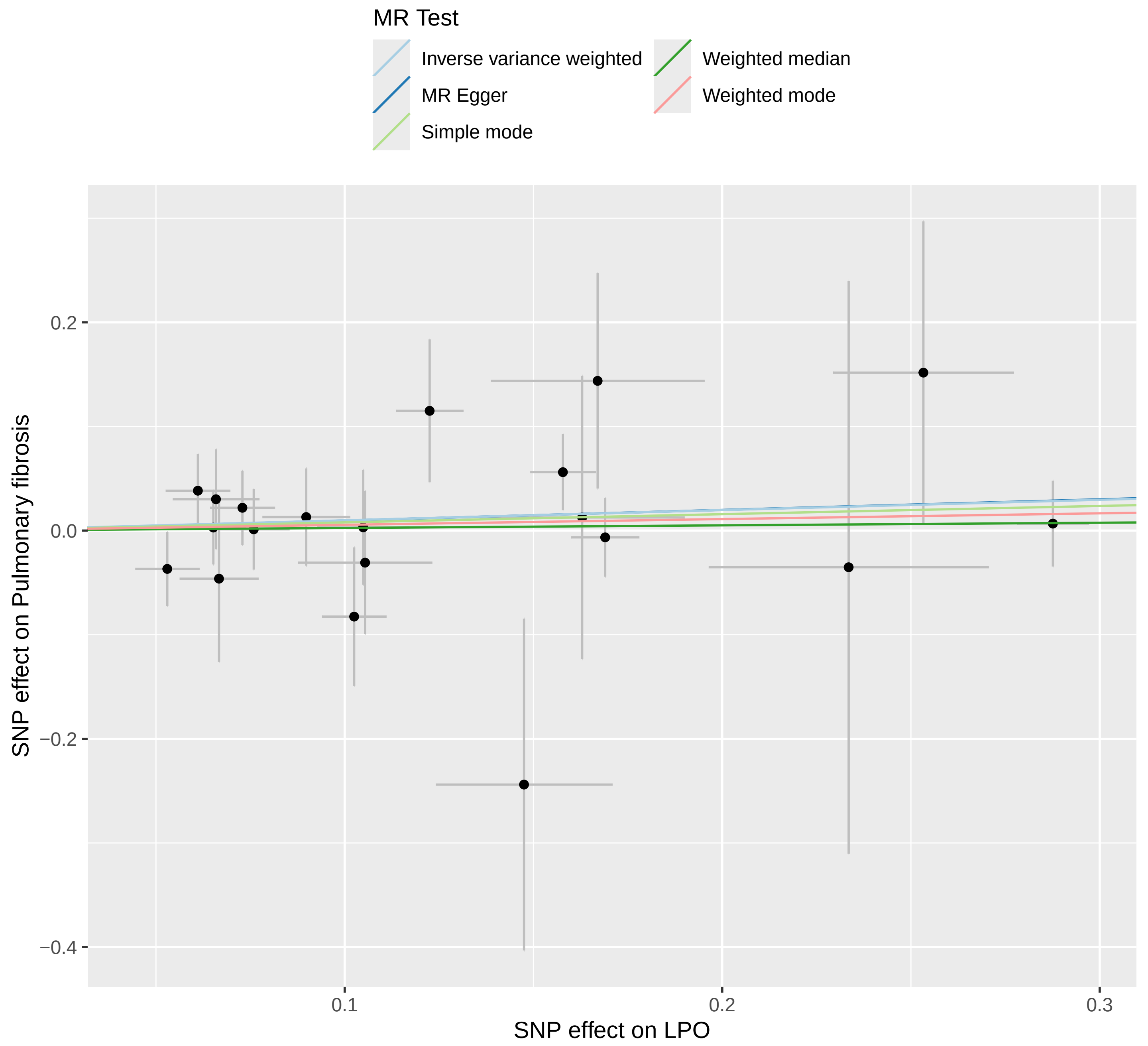

MR Test
Inverse variance weighted
MR Egger
Simple mode
Weighted median
Weighted mode
0.2
0.0
−0.2
−0.4
0.1
0.2
0.3
SNP effect on Pulmonary fibrosis
SNP effect on LPO

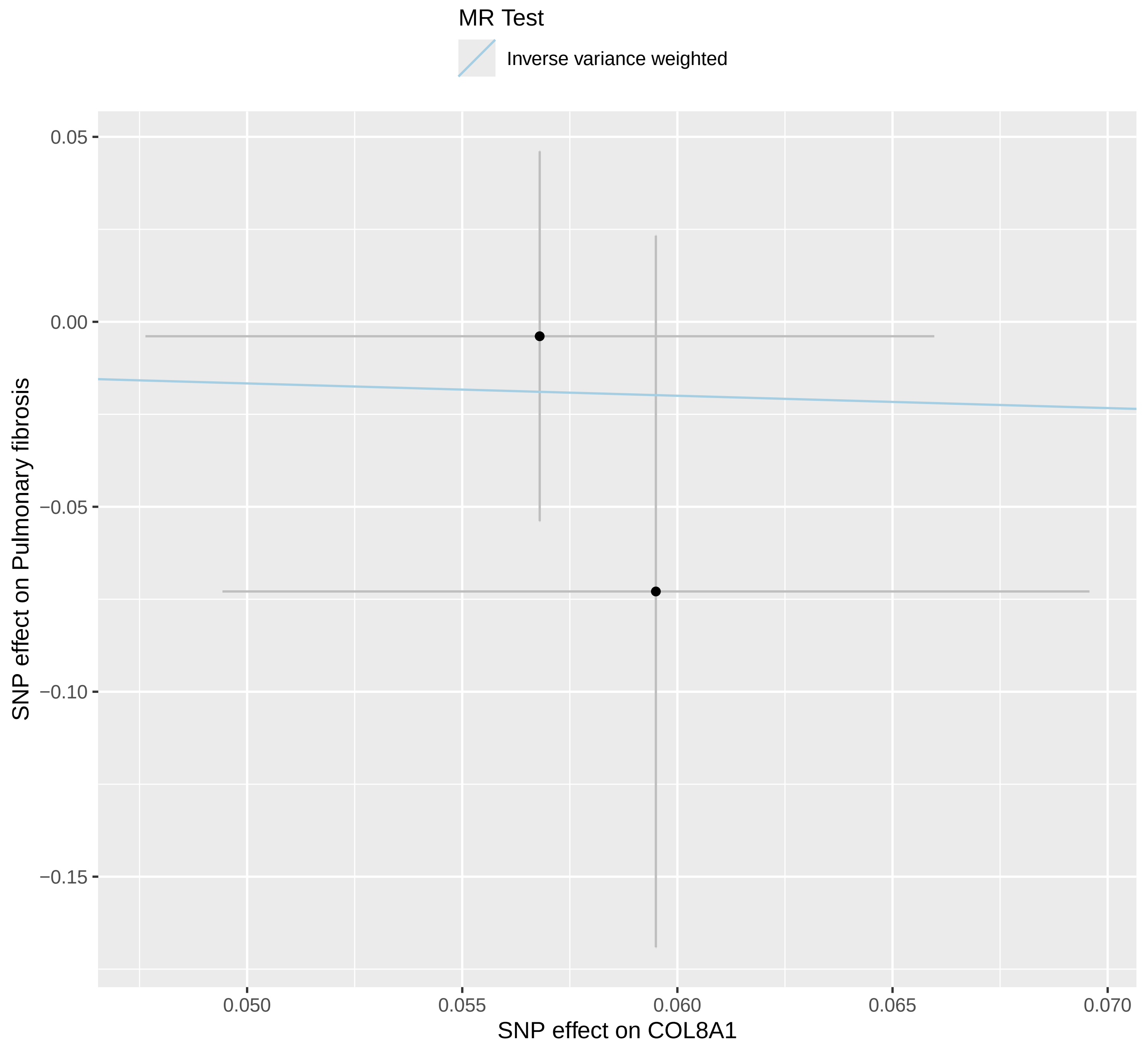
MR Test
Inverse variance weighted
0.05
0.00
−0.05
−0.10
−0.15
SNP effect on Pulmonary fibrosis
0.050
0.055
0.060
0.065
0.070
SNP effect on COL8A1

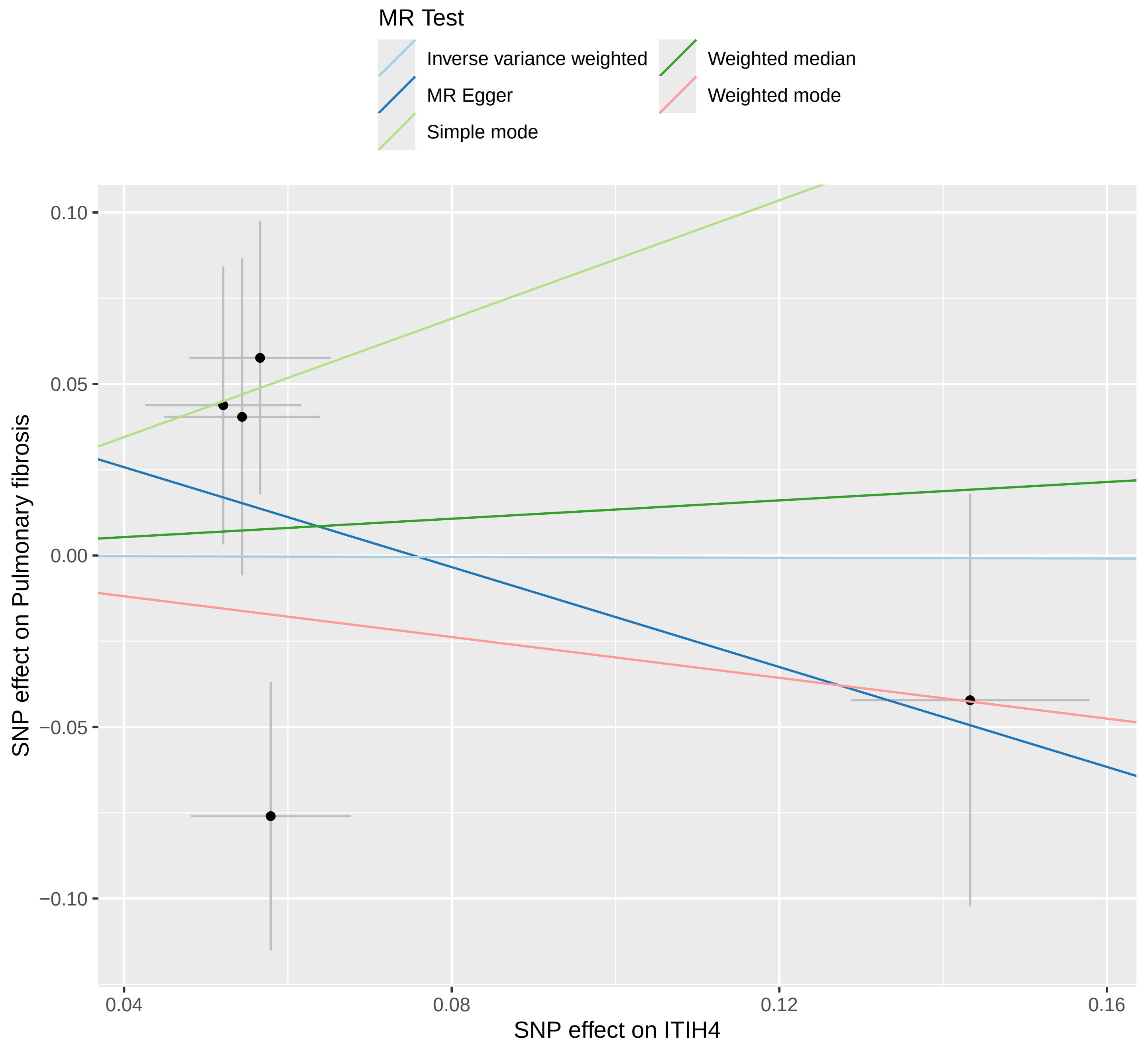
MR Test
Inverse variance weighted
MR Egger
Simple mode
Weighted median
Weighted mode
0.10
0.05
0.00
−0.05
−0.10
SNP effect on Pulmonary fibrosis
0.04
0.08
0.12
0.16
SNP effect on ITIH4

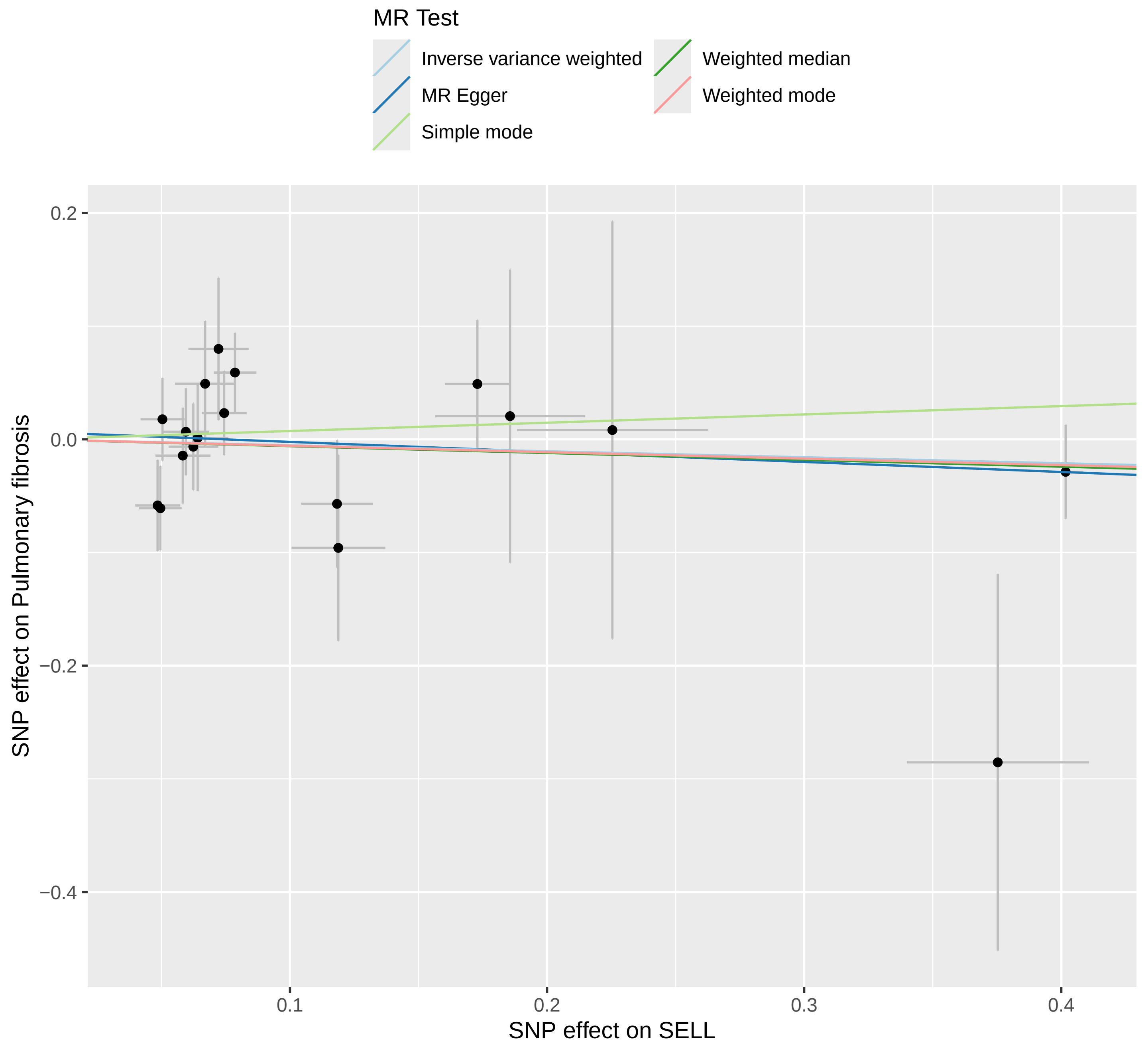

MR Test
Inverse variance weighted
MR Egger
Simple mode
Weighted median
Weighted mode
0.2
0.0
−0.2
−0.4
0.1
0.2
0.3
0.4
SNP effect on Pulmonary fibrosis
SNP effect on SELL

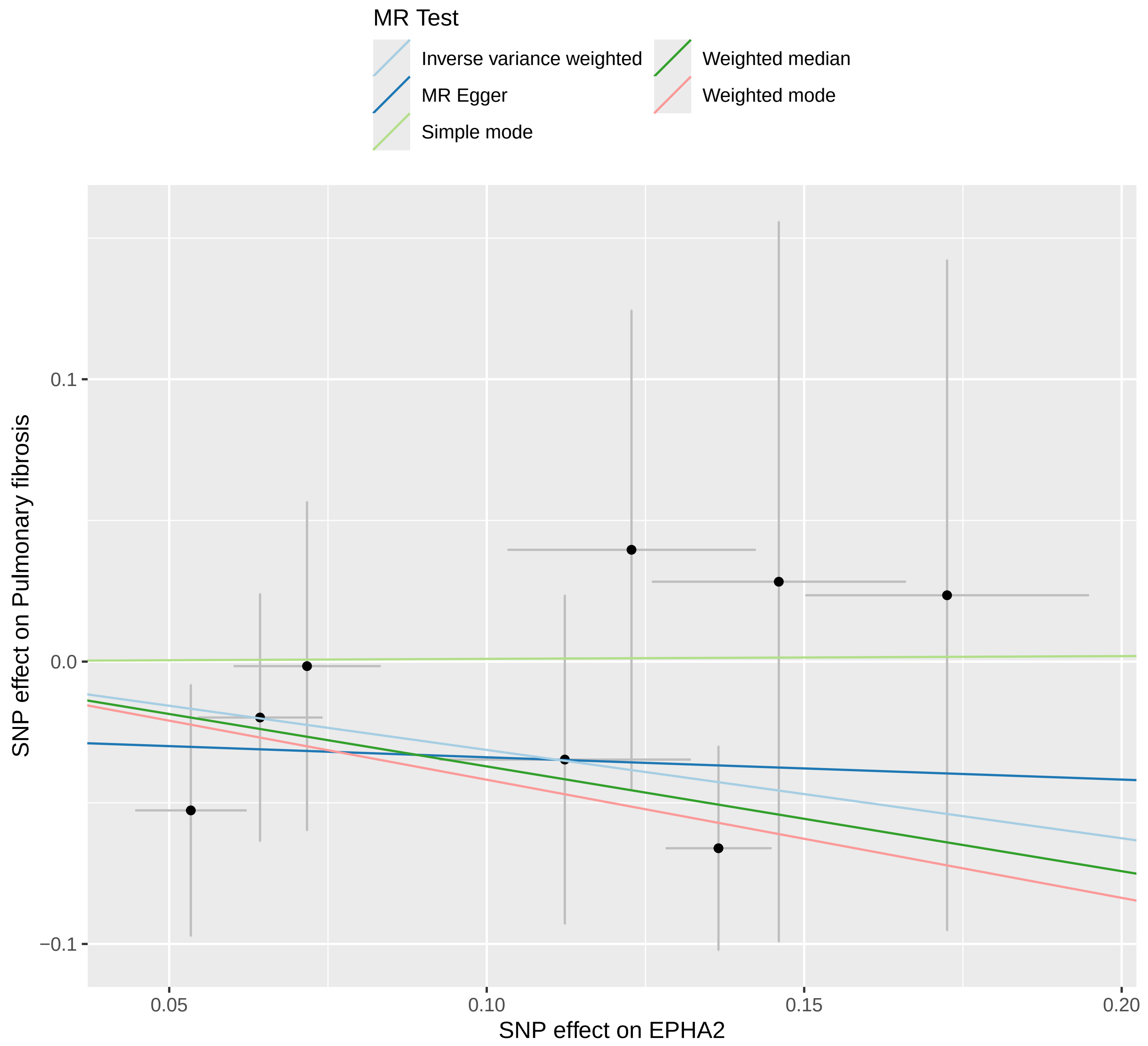

MR Test
Inverse variance weighted
MR Egger
Simple mode
Weighted median
Weighted mode
SNP effect on Pulmonary fibrosis
0.1
0.0
−0.1
0.05
0.10
0.15
0.20
SNP effect on EPHA2

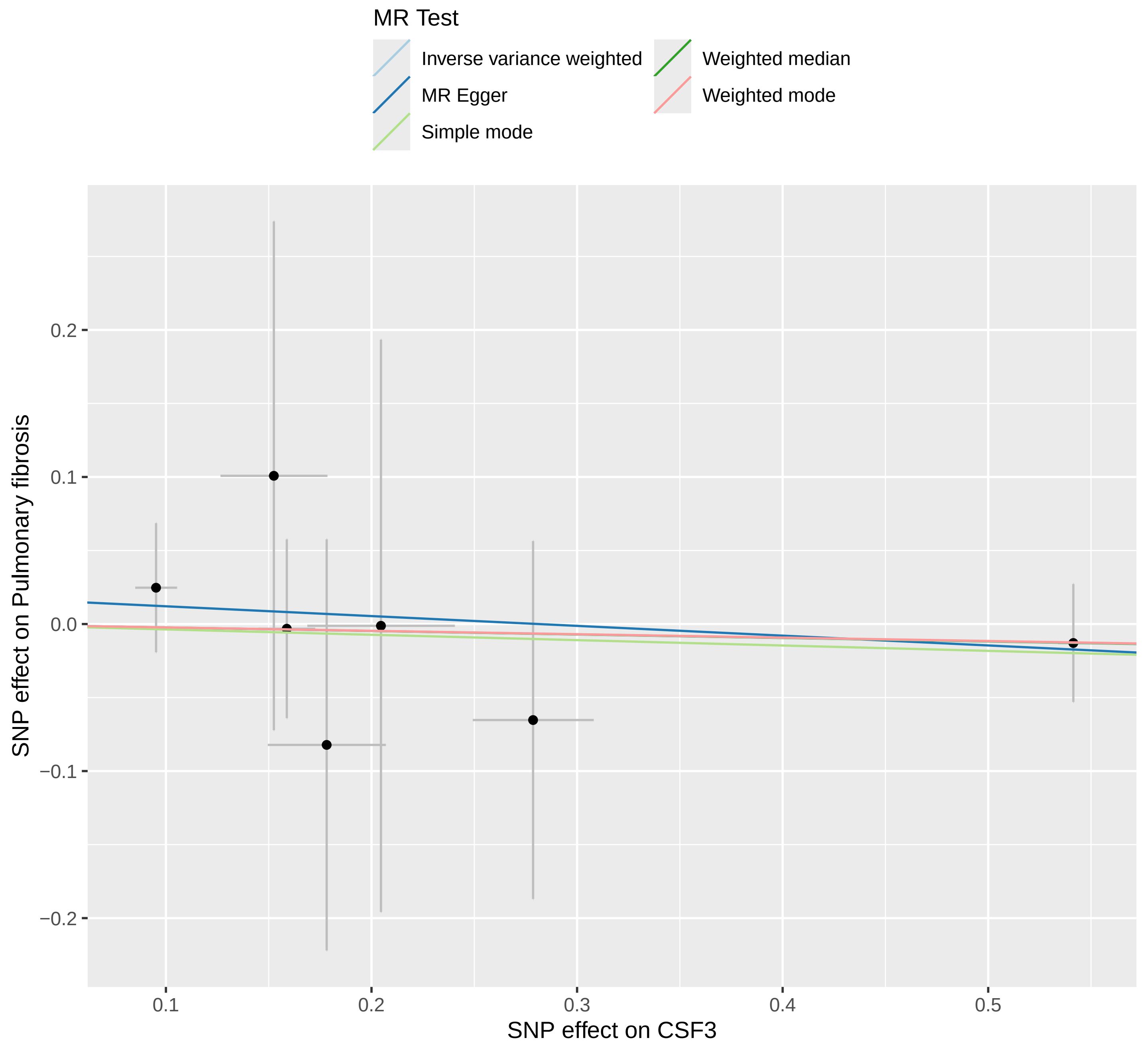

MR Test
Inverse variance weighted
MR Egger
Simple mode
Weighted median
Weighted mode
SNP effect on Pulmonary fibrosis
0.2
0.1
0.0
−0.1
−0.2
0.1
0.2
0.3
0.4
0.5
SNP effect on CSF3

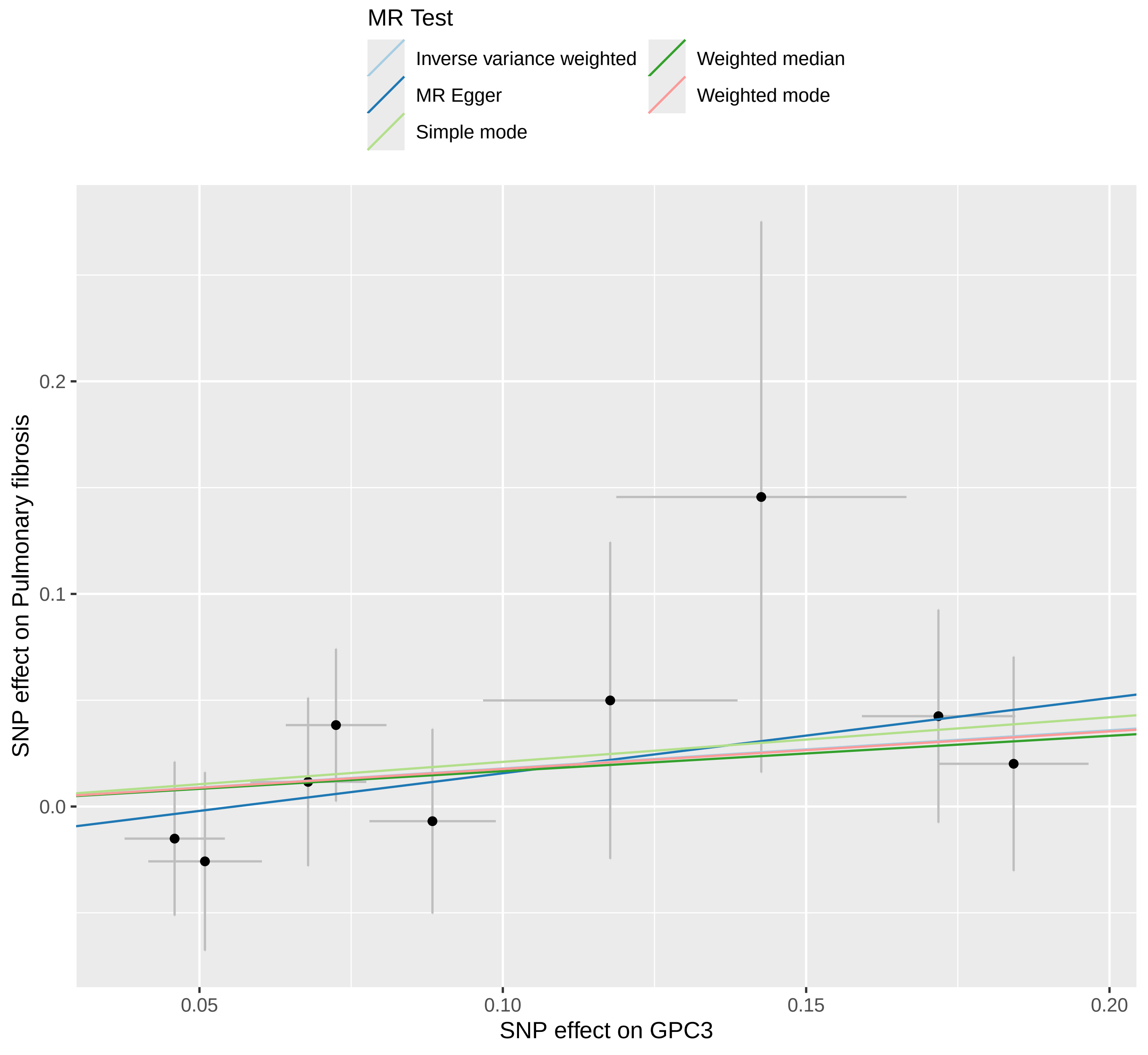

MR Test
Inverse variance weighted
MR Egger
Simple mode
Weighted median
Weighted mode
0.2
0.1
0.0
0.05
0.10
0.15
0.20
SNP effect on Pulmonary fibrosis
SNP effect on GPC3

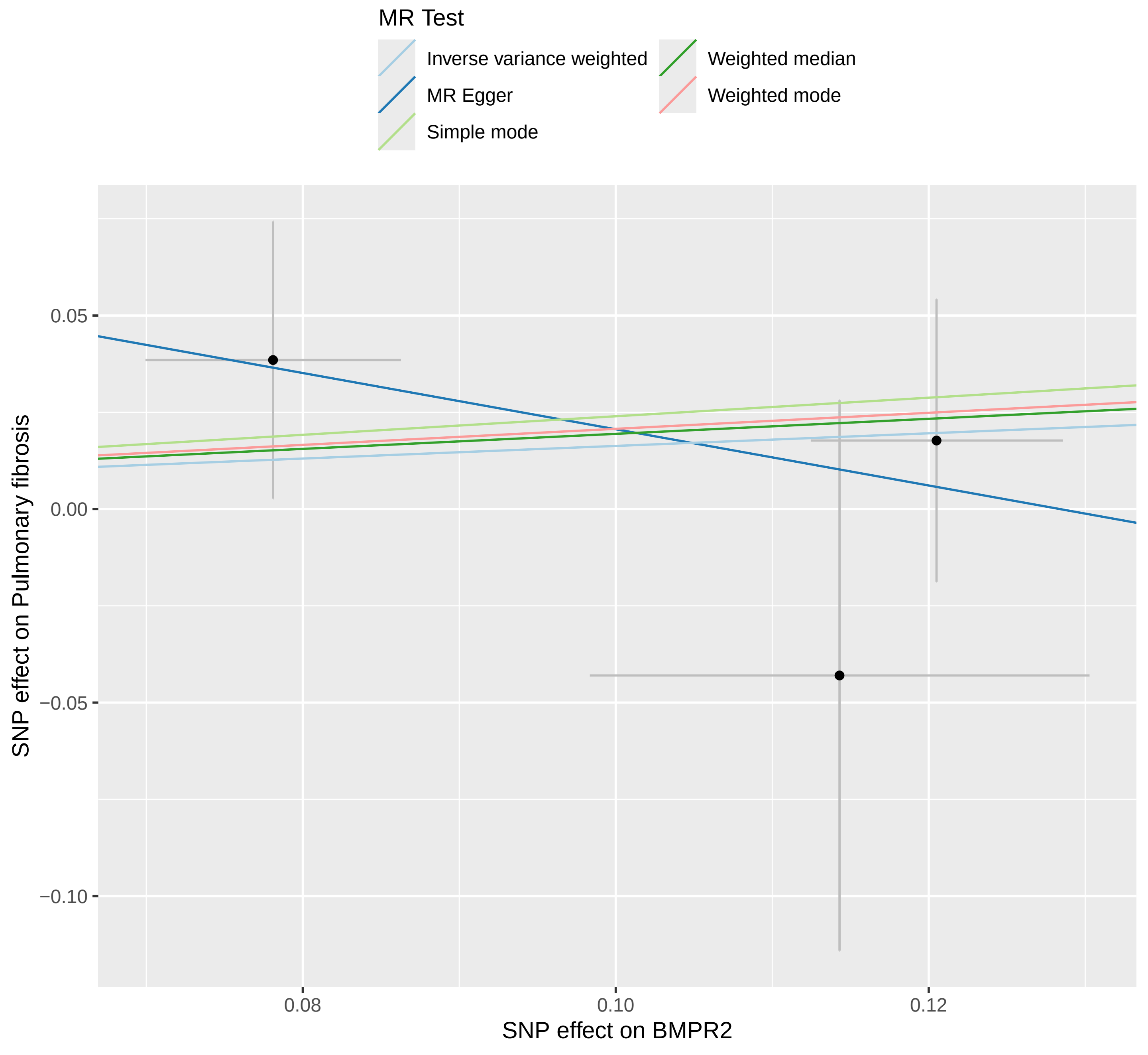

MR Test
Inverse variance weighted
MR Egger
Simple mode
Weighted median
Weighted mode
SNP effect on Pulmonary fibrosis
0.05
0.00
−0.05
−0.10
0.08
0.10
0.12
SNP effect on BMPR2

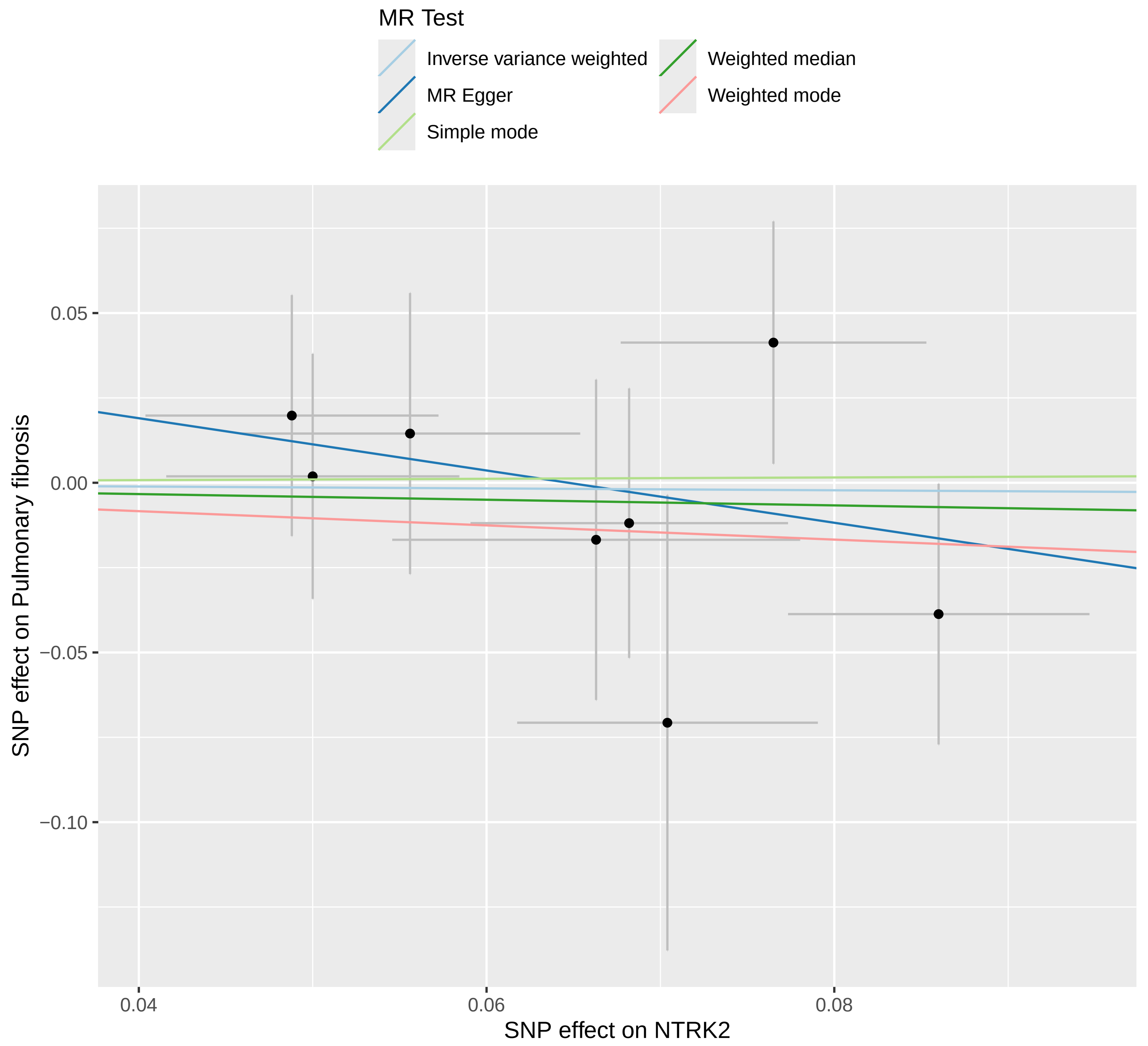
MR Test
Inverse variance weighted
MR Egger
Simple mode
Weighted median
Weighted mode
0.05
0.00
−0.05
−0.10
SNP effect on Pulmonary fibrosis
0.04
0.06
0.08
SNP effect on NTRK2

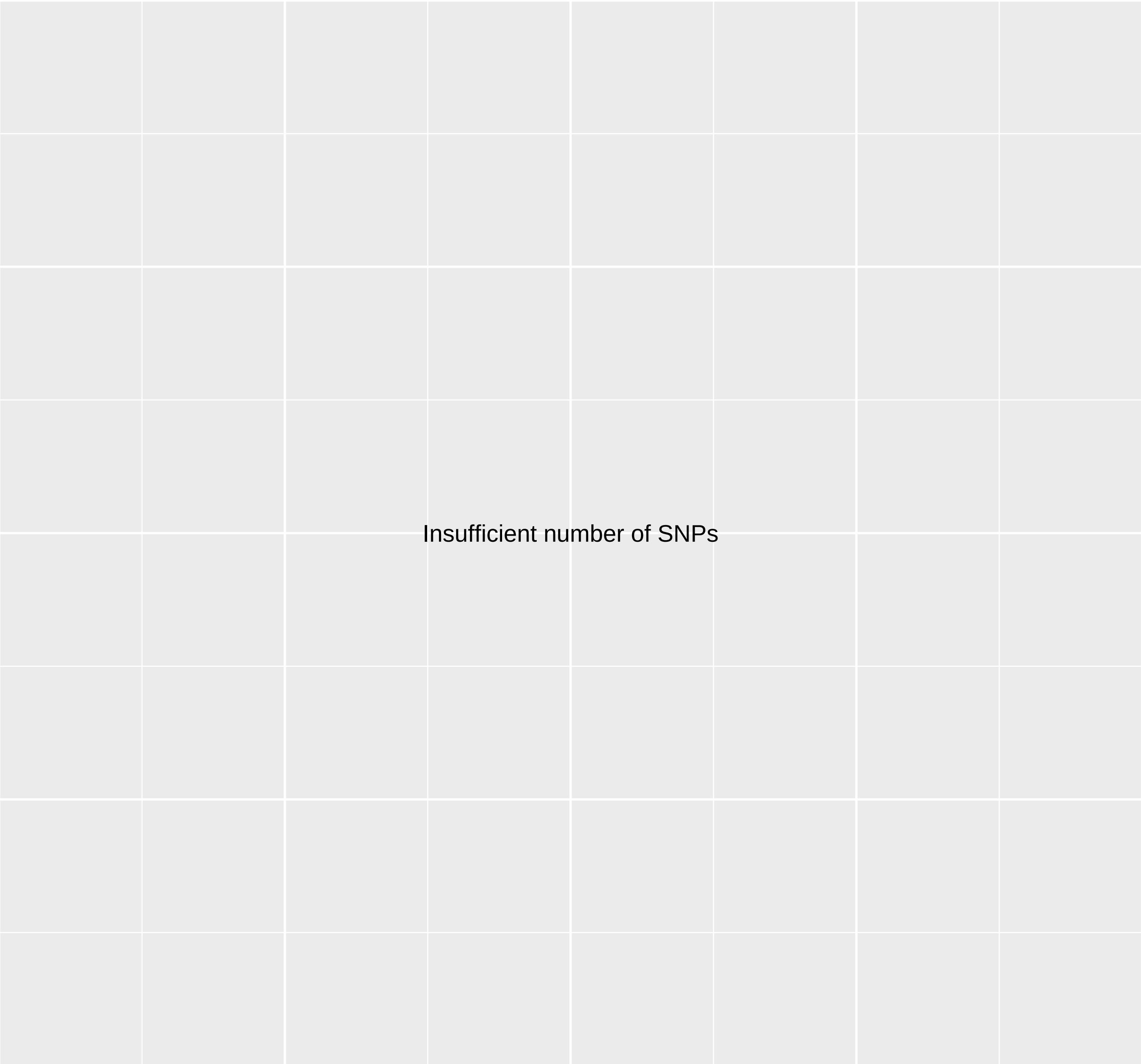
Insufficient number of SNPs

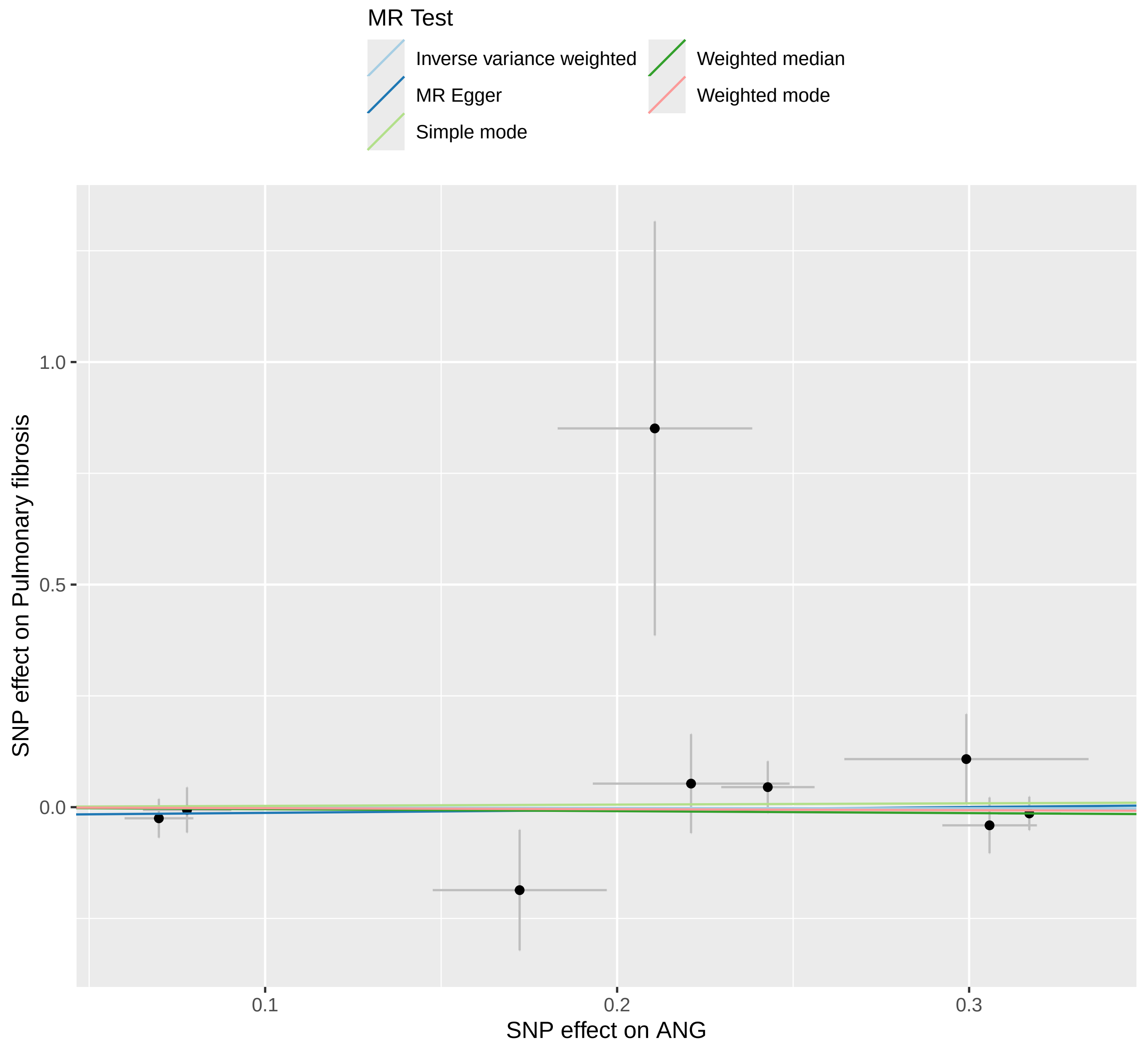

MR Test
Inverse variance weighted
MR Egger
Simple mode
Weighted median
Weighted mode
SNP effect on Pulmonary fibrosis
1.0
0.5
0.0
0.1
0.2
0.3
SNP effect on ANG

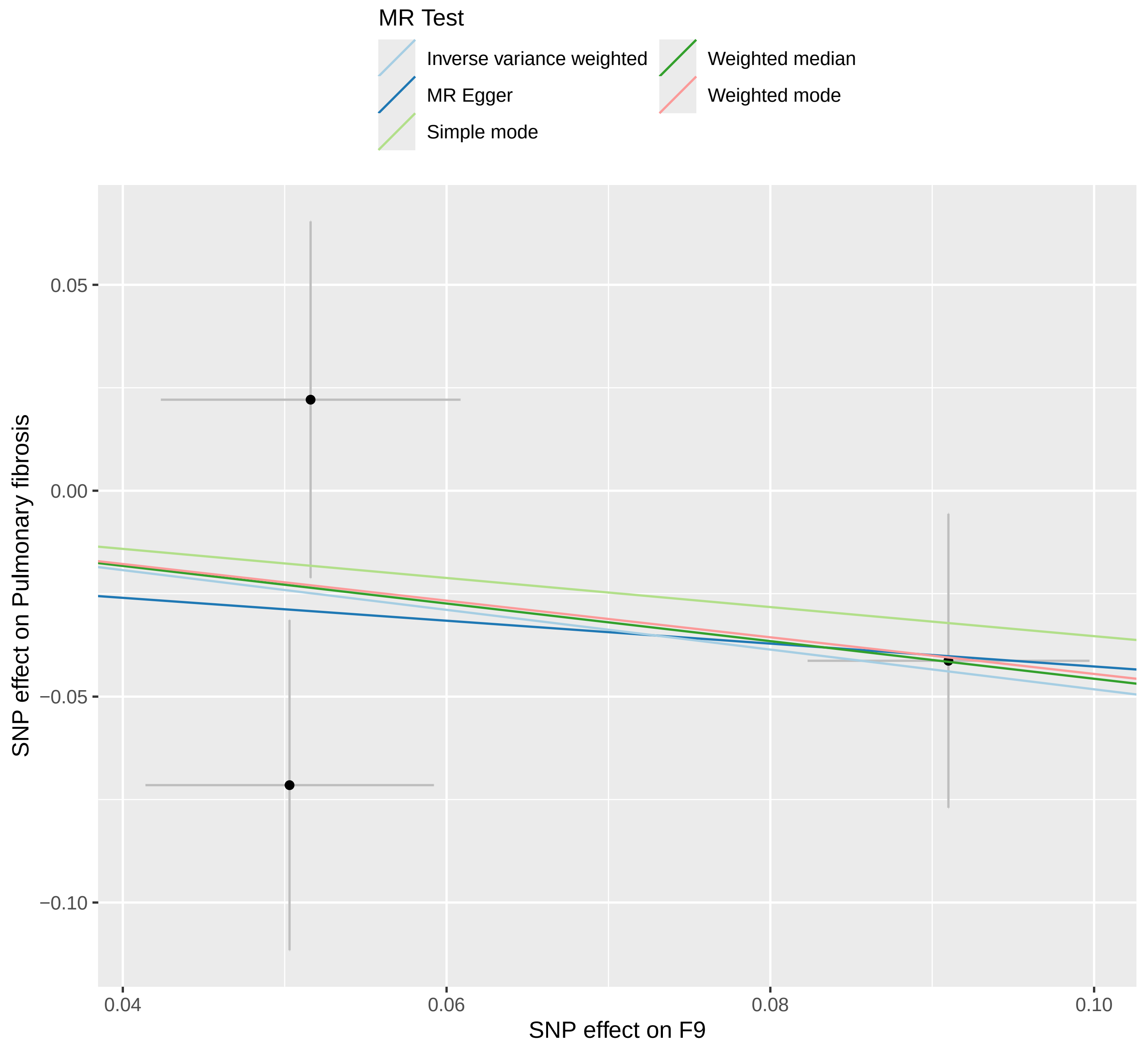

MR Test
Inverse variance weighted
MR Egger
Simple mode
Weighted median
Weighted mode
0.05
0.00
−0.05
−0.10
SNP effect on Pulmonary fibrosis
0.04
0.06
0.08
0.10
SNP effect on F9

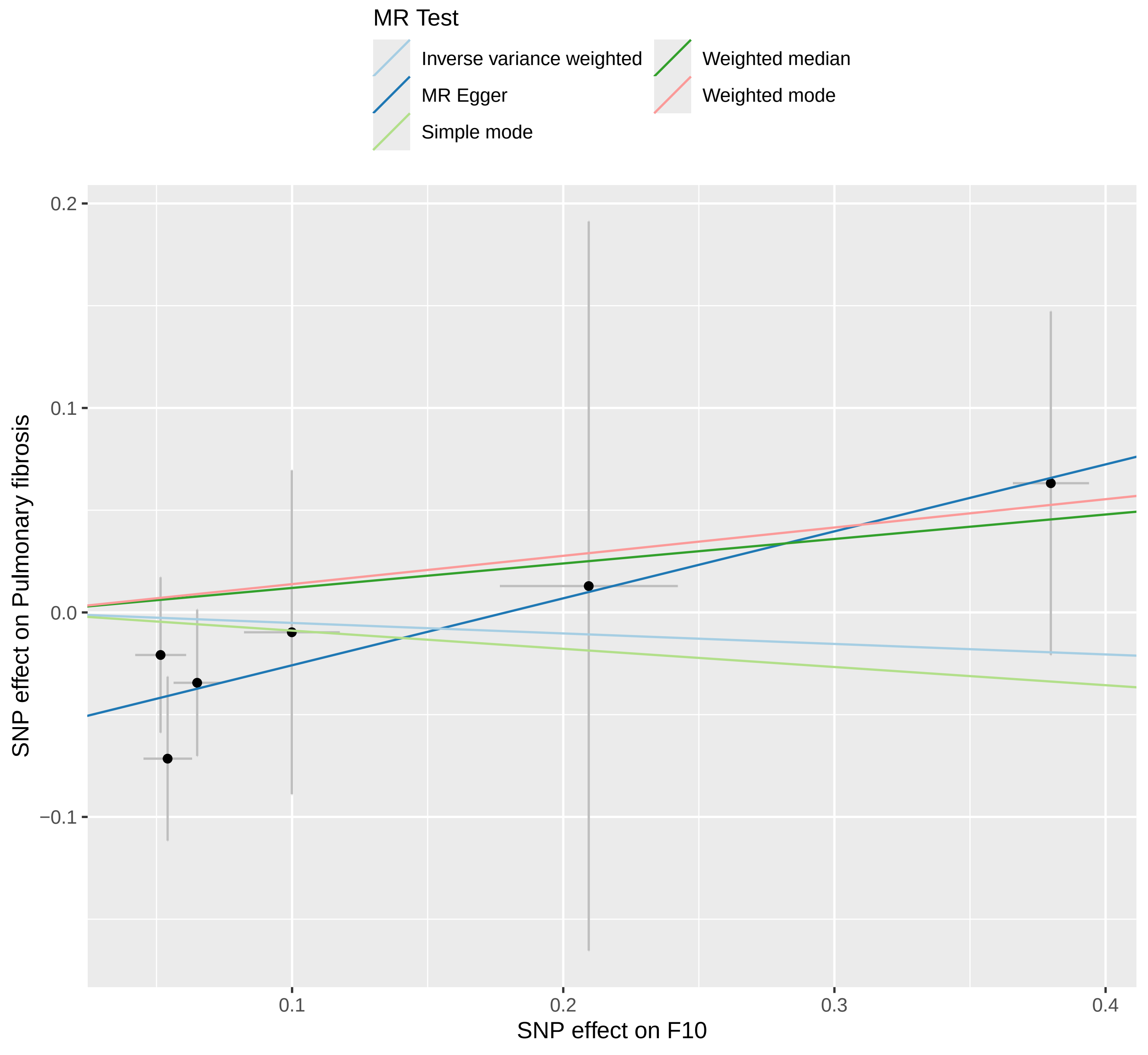

MR Test
Inverse variance weighted
MR Egger
Simple mode
Weighted median
Weighted mode
0.2
0.1
0.0
−0.1
SNP effect on Pulmonary fibrosis
0.1
0.2
0.3
0.4
SNP effect on F10

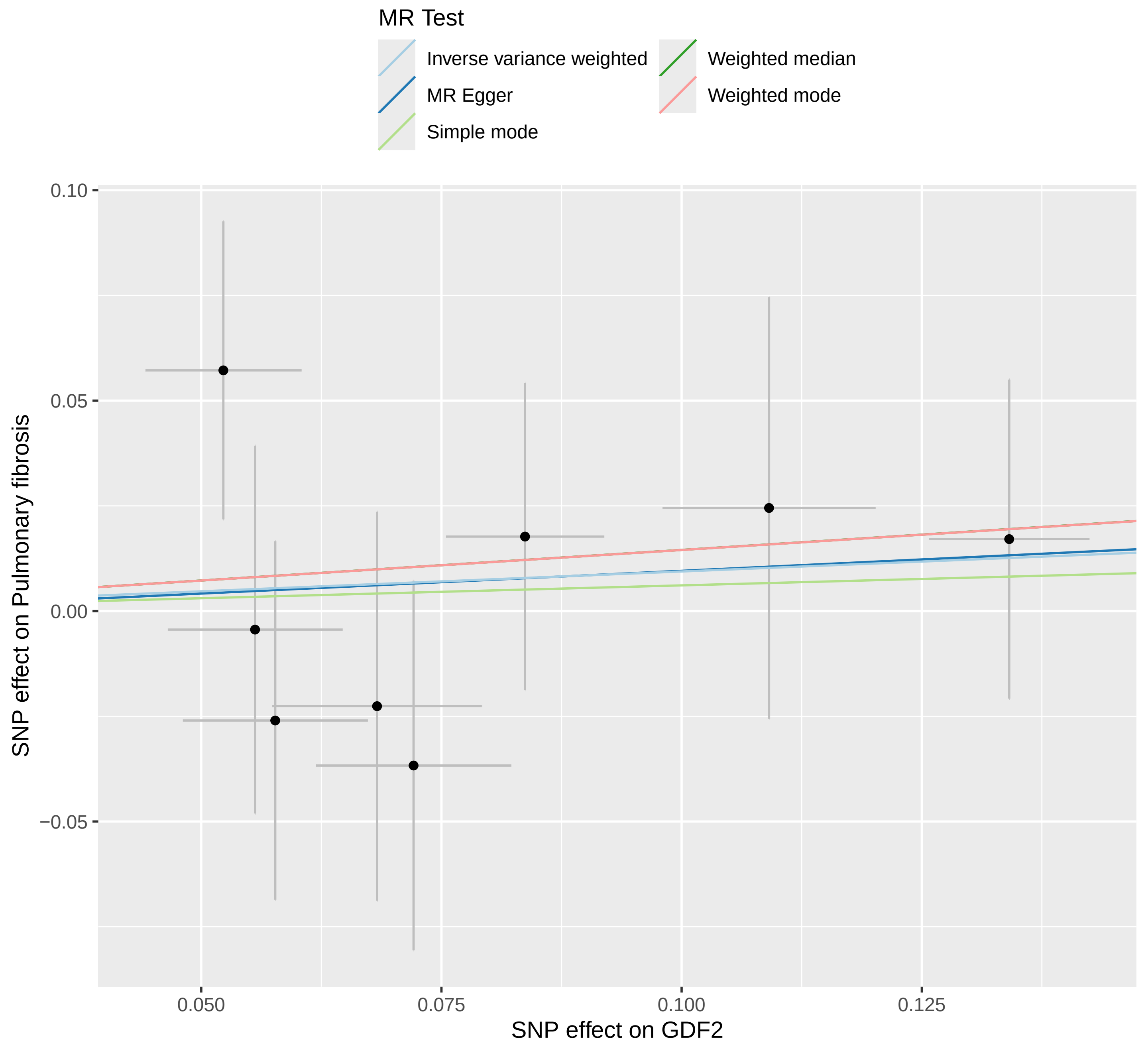

MR Test
Inverse variance weighted
MR Egger
Simple mode
Weighted median
Weighted mode
0.10
0.05
0.00
−0.05
SNP effect on Pulmonary fibrosis
0.050
0.075
0.100
0.125
SNP effect on GDF2

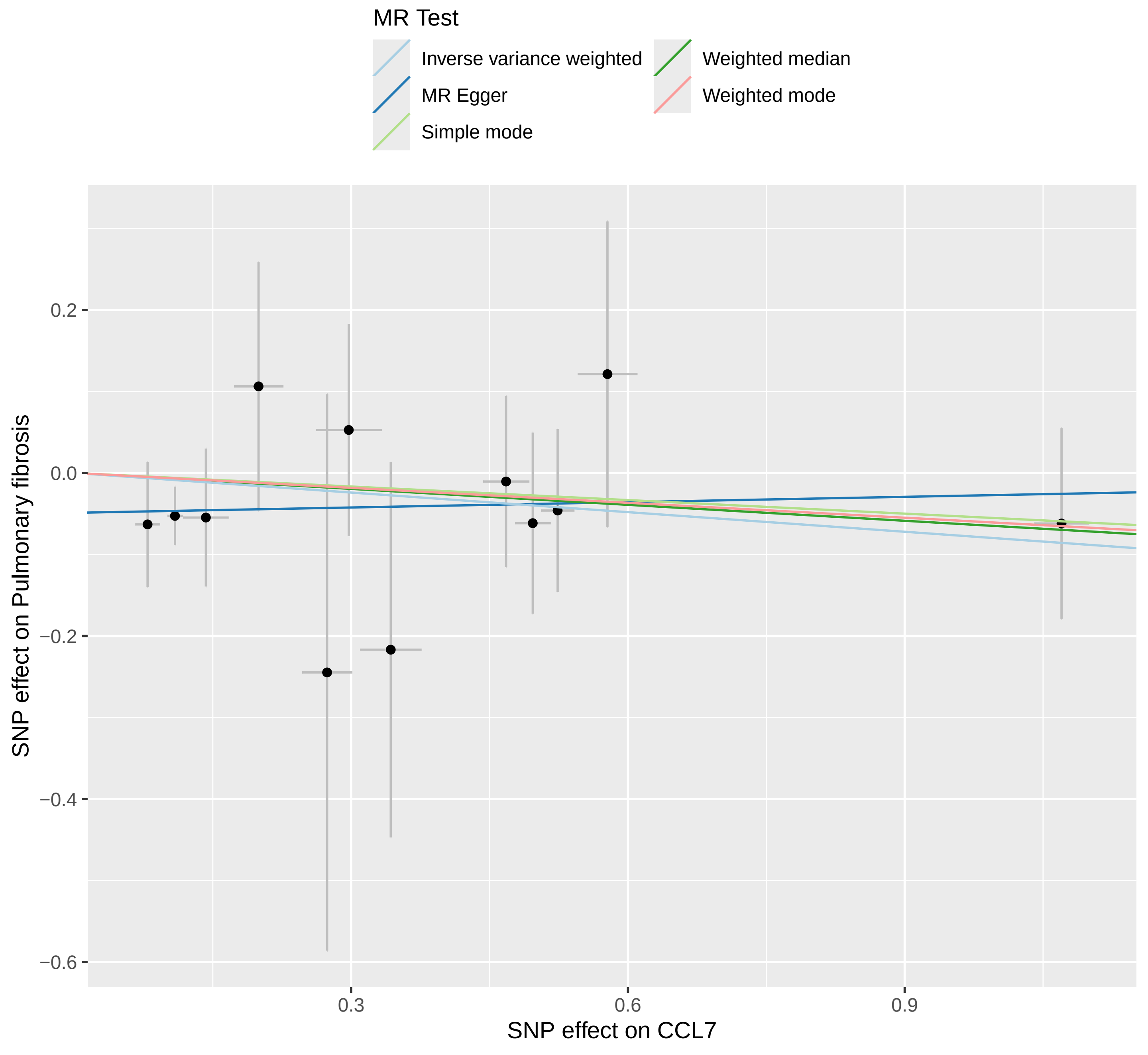

MR Test
Inverse variance weighted
MR Egger
Simple mode
Weighted median
Weighted mode
0.2
0.0
−0.2
−0.4
−0.6
SNP effect on Pulmonary fibrosis
0.3
0.6
0.9
SNP effect on CCL7

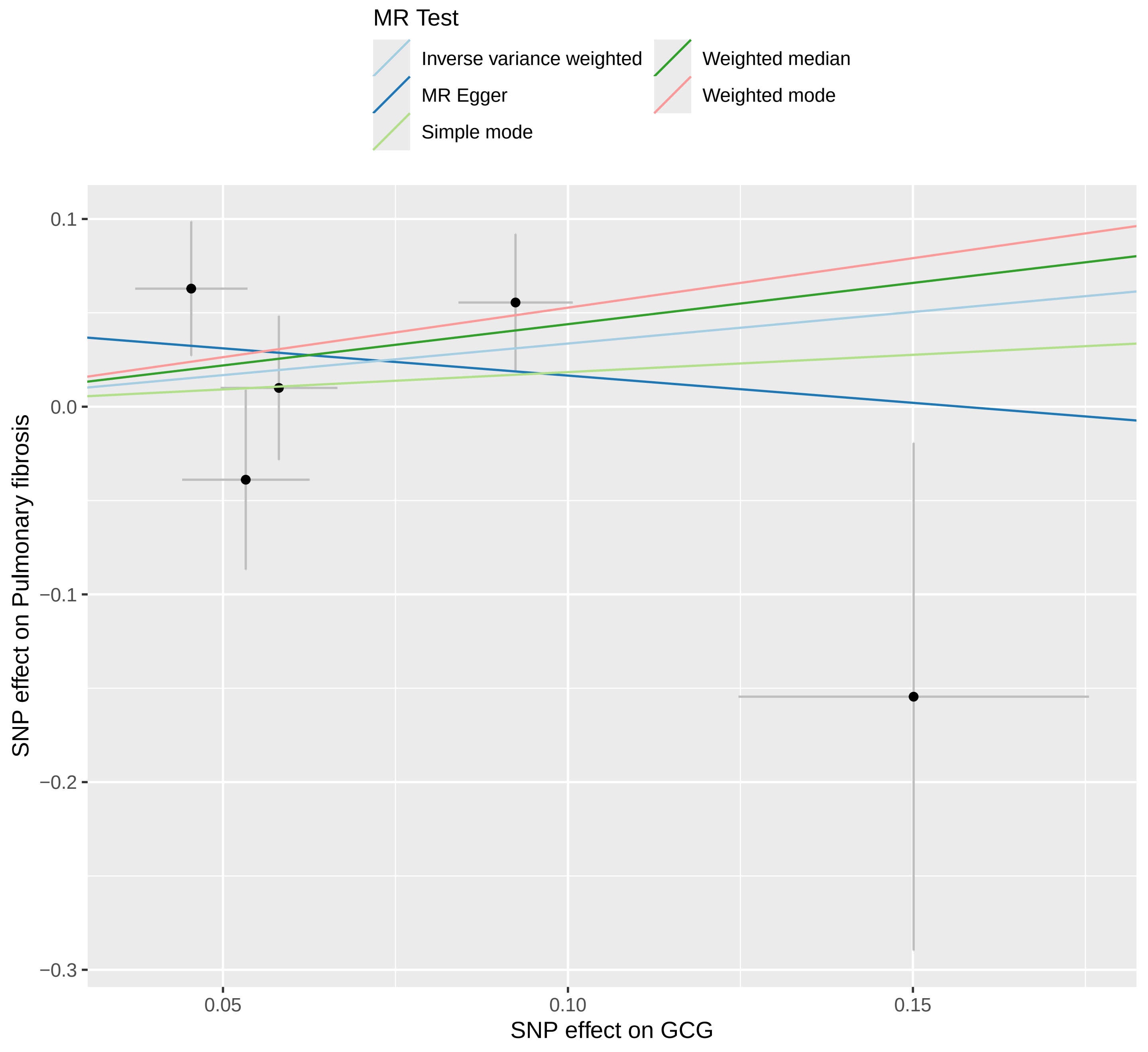
MR Test
Inverse variance weighted
MR Egger
Simple mode
Weighted median
Weighted mode
0.1
0.0
−0.1
−0.2
−0.3
SNP effect on Pulmonary fibrosis
0.05
0.10
0.15
SNP effect on GCG

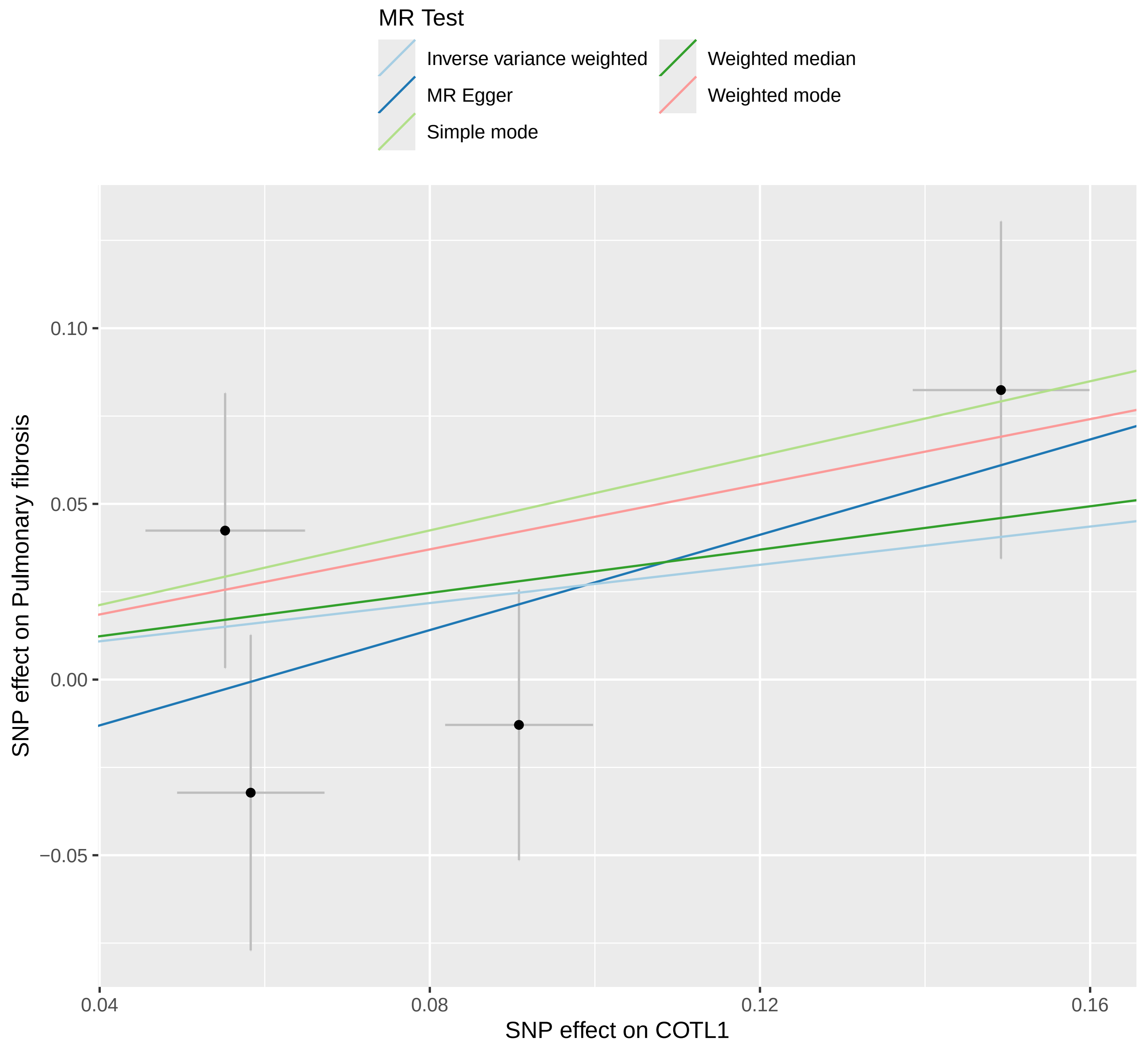

MR Test
Inverse variance weighted
MR Egger
Simple mode
Weighted median
Weighted mode
0.10
0.05
0.00
−0.05
SNP effect on Pulmonary fibrosis
0.04
0.08
0.12
0.16
SNP effect on COTL1

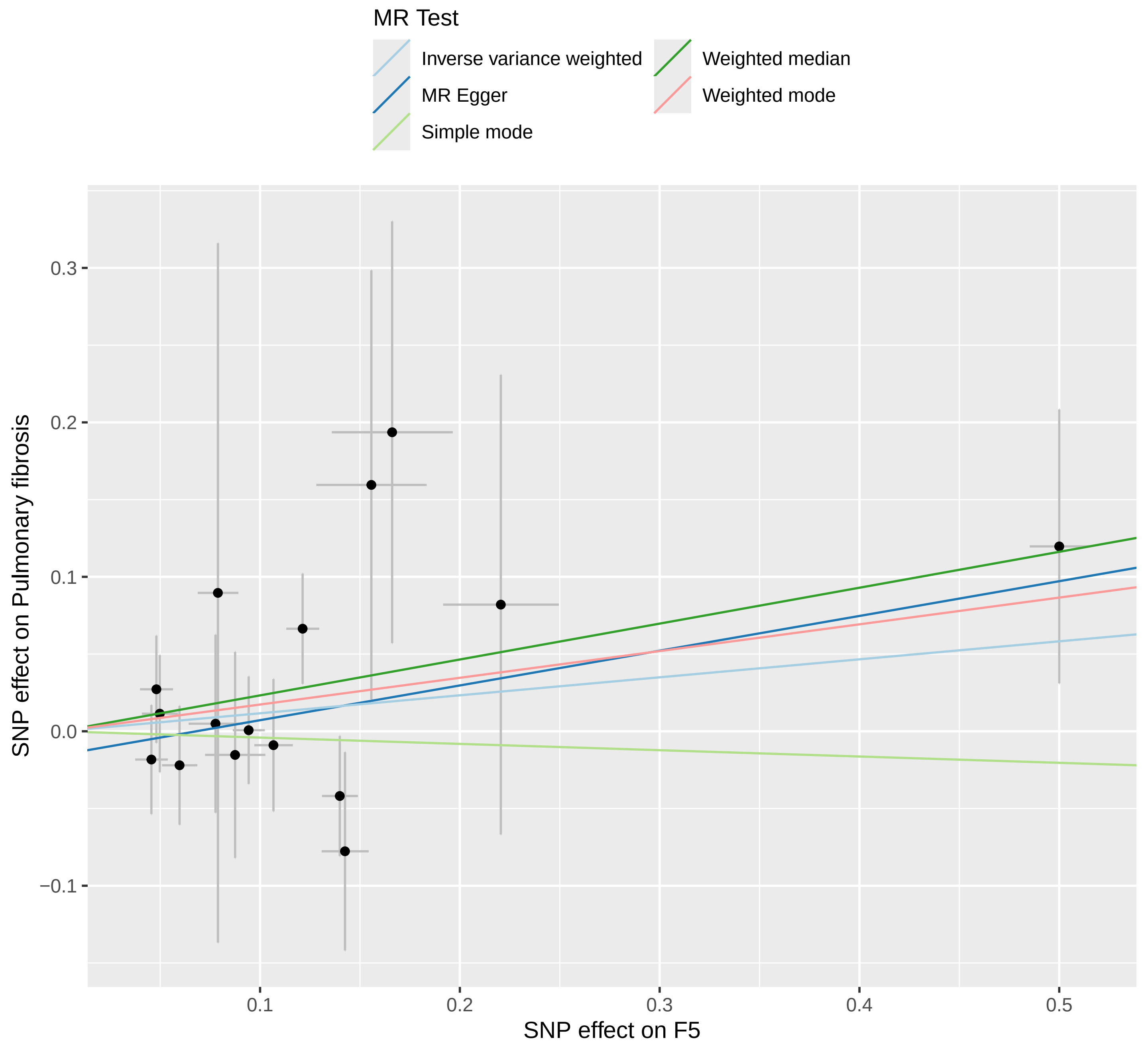
MR Test
Inverse variance weighted
MR Egger
Simple mode
Weighted median
Weighted mode
SNP effect on Pulmonary fibrosis
0.3
0.2
0.1
0.0
−0.1
0.1
0.2
0.3
0.4
0.5
SNP effect on F5

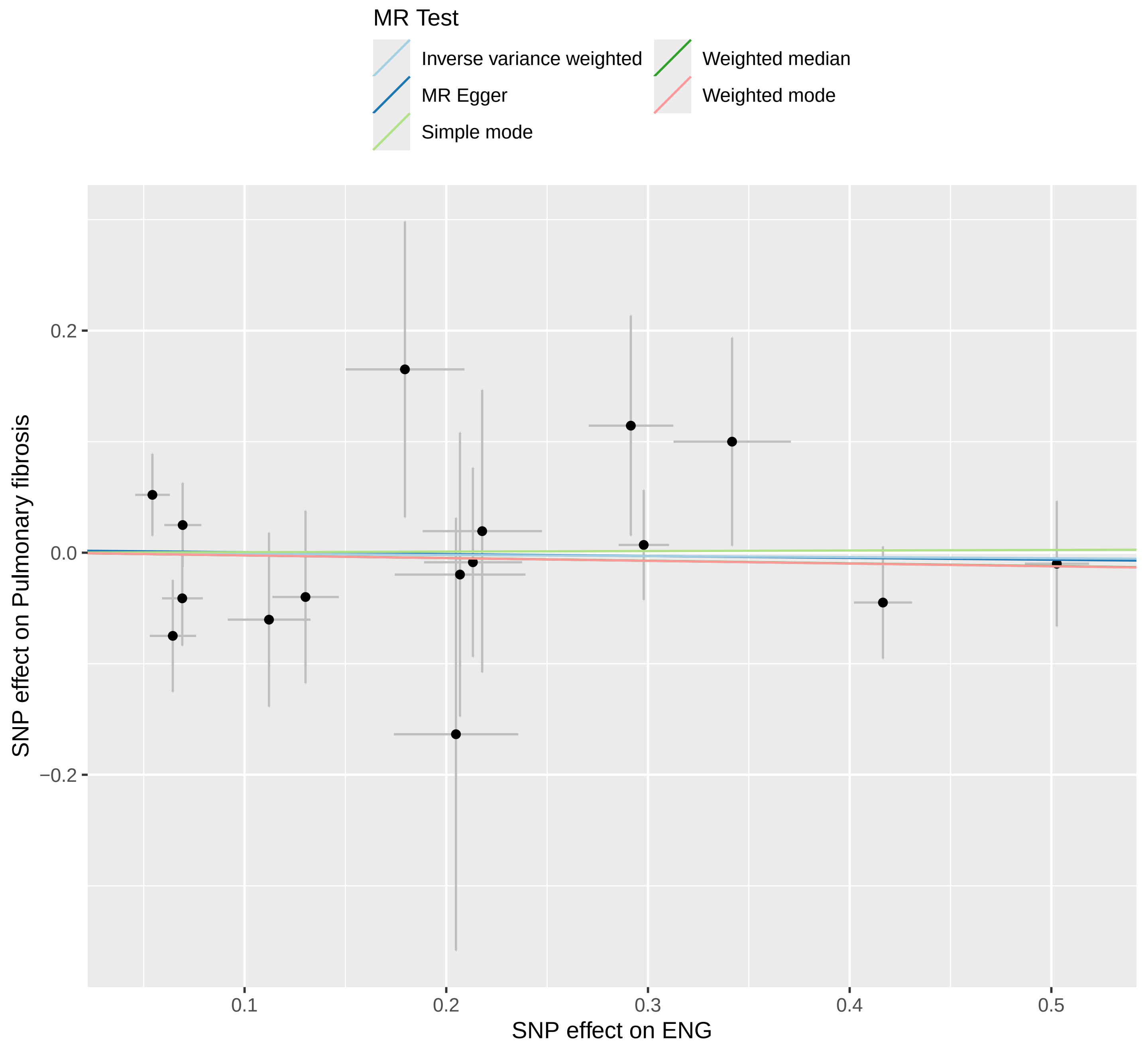

MR Test
Inverse variance weighted
MR Egger
Simple mode
Weighted median
Weighted mode
0.2
0.0
−0.2
0.1
0.2
0.3
0.4
0.5
SNP effect on Pulmonary fibrosis
SNP effect on ENG

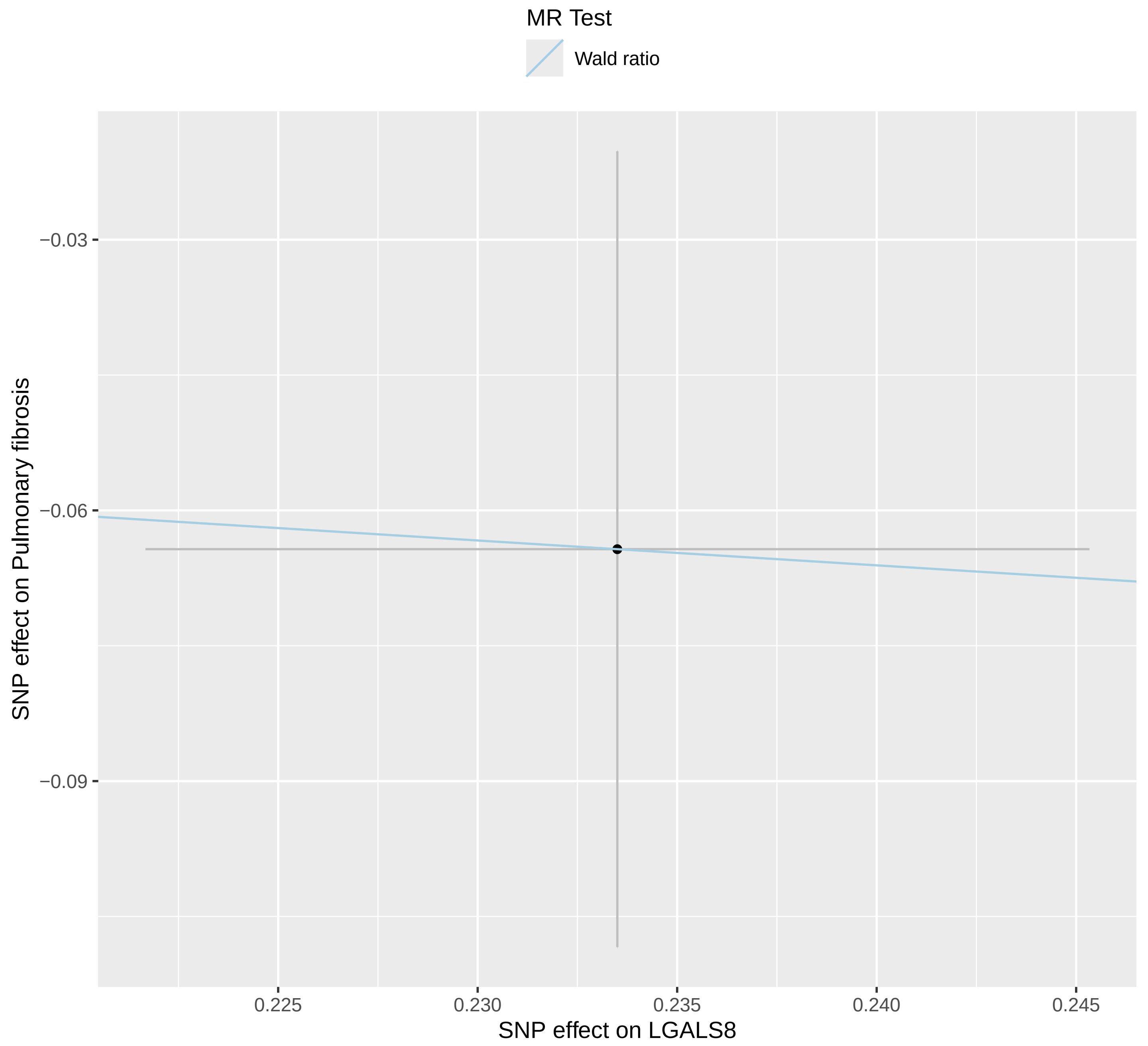
MR Test
Wald ratio
−0.03
−0.06
−0.09
SNP effect on Pulmonary fibrosis
0.225
0.230
0.235
0.240
0.245
SNP effect on LGALS8

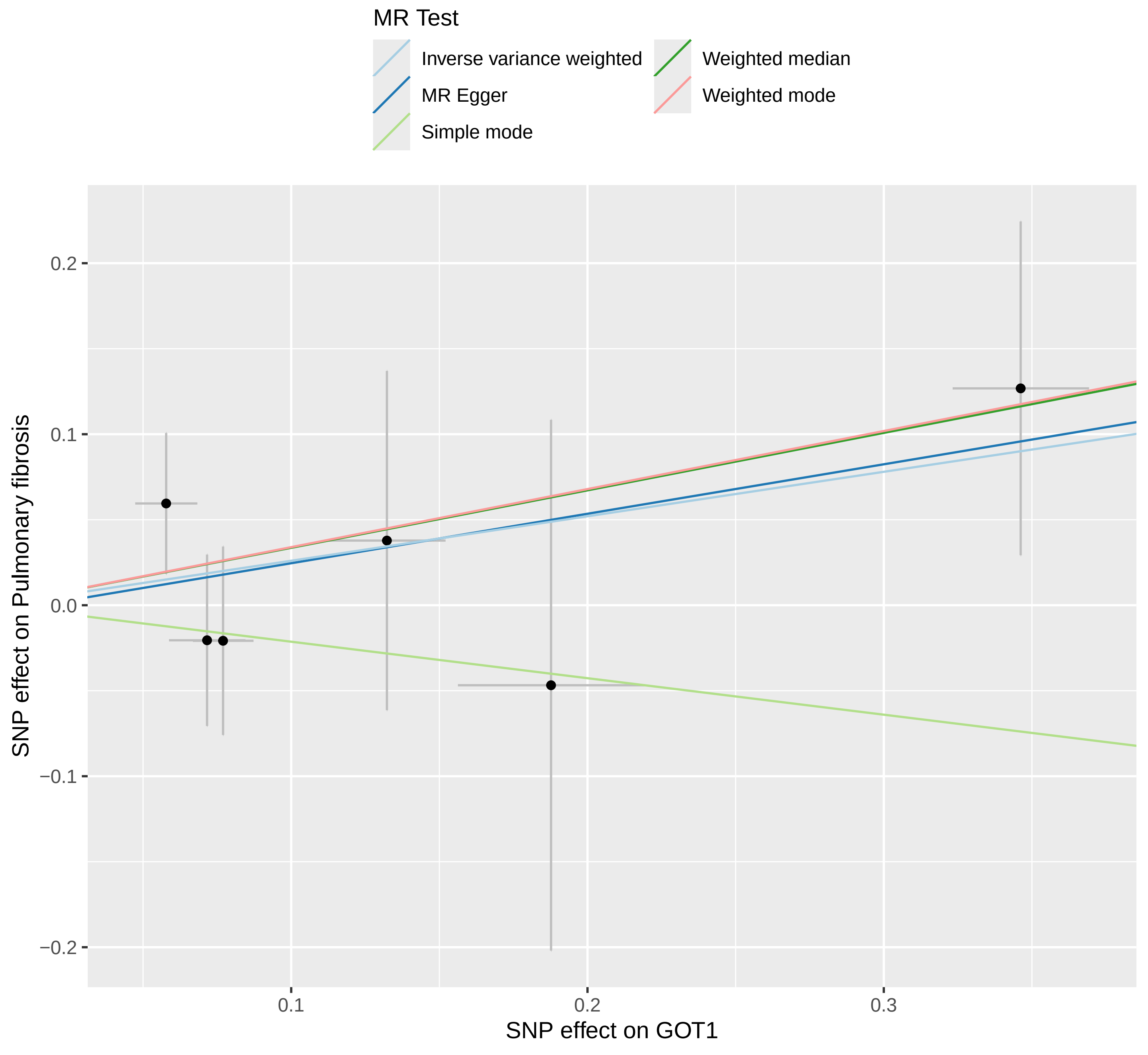

MR Test
Inverse variance weighted
MR Egger
Simple mode
Weighted median
Weighted mode
0.2
0.1
0.0
−0.1
−0.2
SNP effect on Pulmonary fibrosis
0.1
0.2
0.3
SNP effect on GOT1

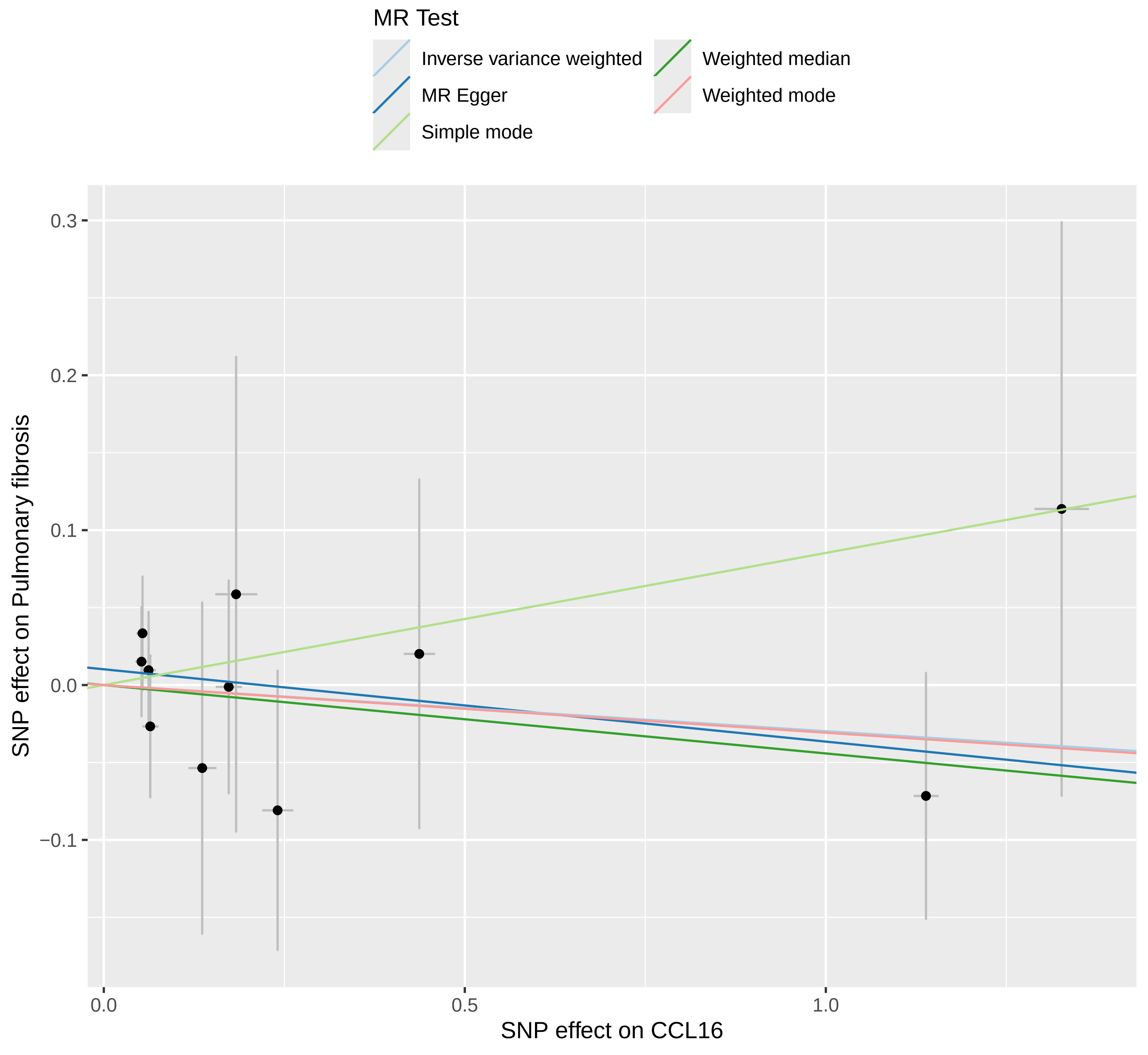
MR Test
Inverse variance weighted
MR Egger
Simple mode
Weighted median
Weighted mode
SNP effect on Pulmonary fibrosis
0.3
0.2
0.1
0.0
−0.1
0.0
0.5
1.0
SNP effect on CCL16

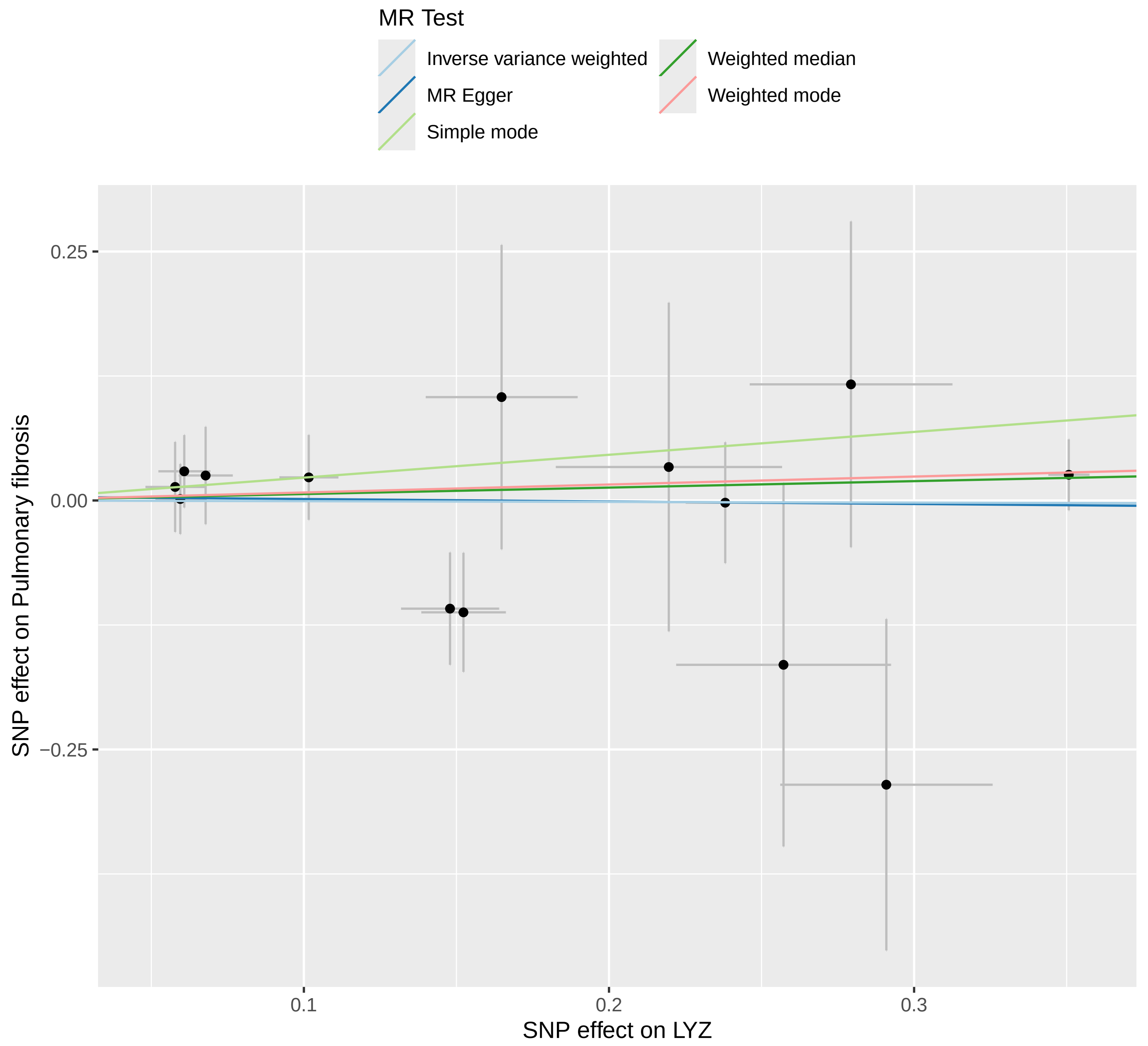

MR Test
Inverse variance weighted
MR Egger
Simple mode
Weighted median
Weighted mode
0.25
0.00
−0.25
SNP effect on Pulmonary fibrosis
0.1
0.2
0.3
SNP effect on LYZ

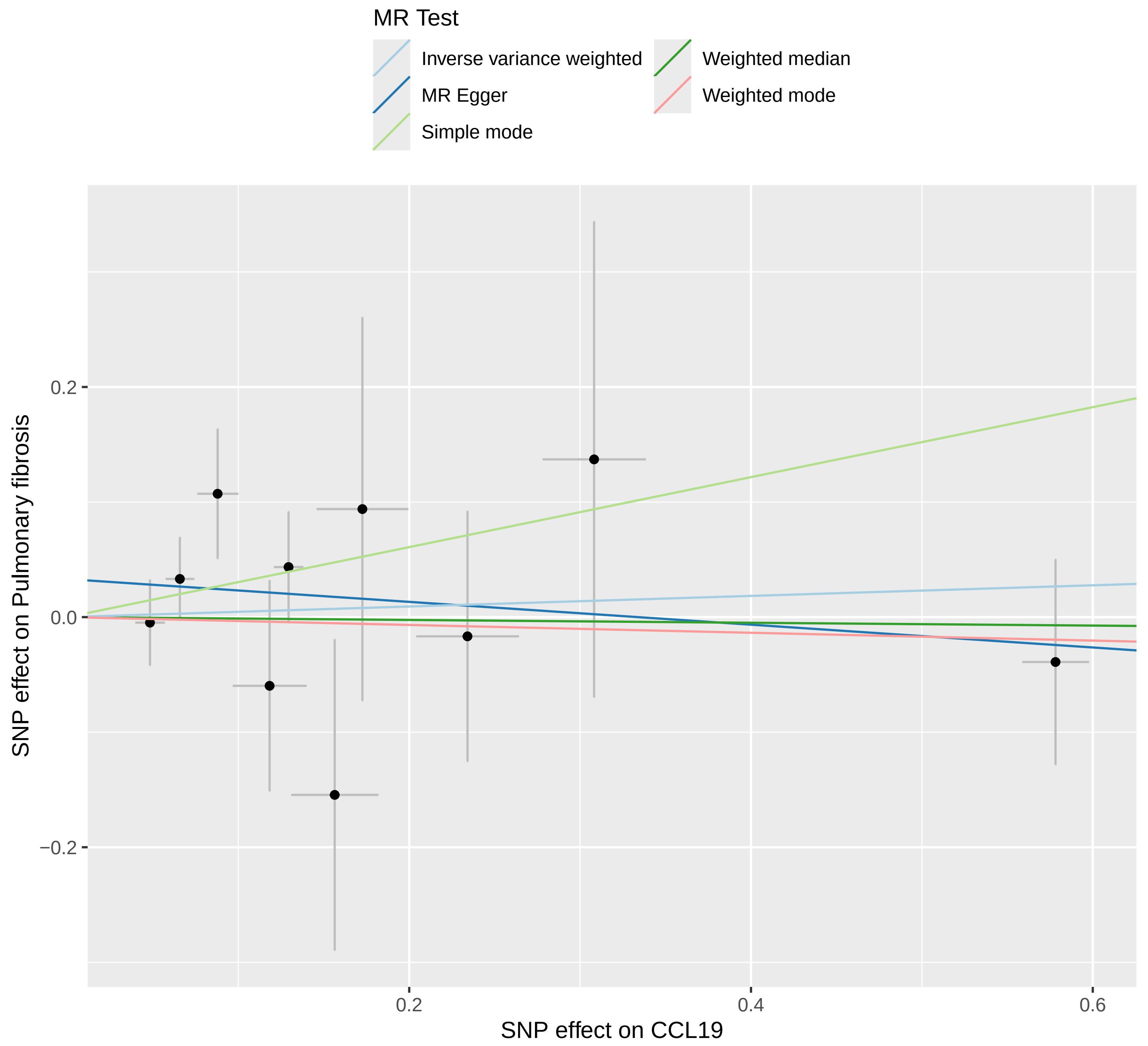

MR Test
Inverse variance weighted
MR Egger
Simple mode
Weighted median
Weighted mode
0.2
0.0
−0.2
0.2
0.4
0.6
SNP effect on Pulmonary fibrosis
SNP effect on CCL19

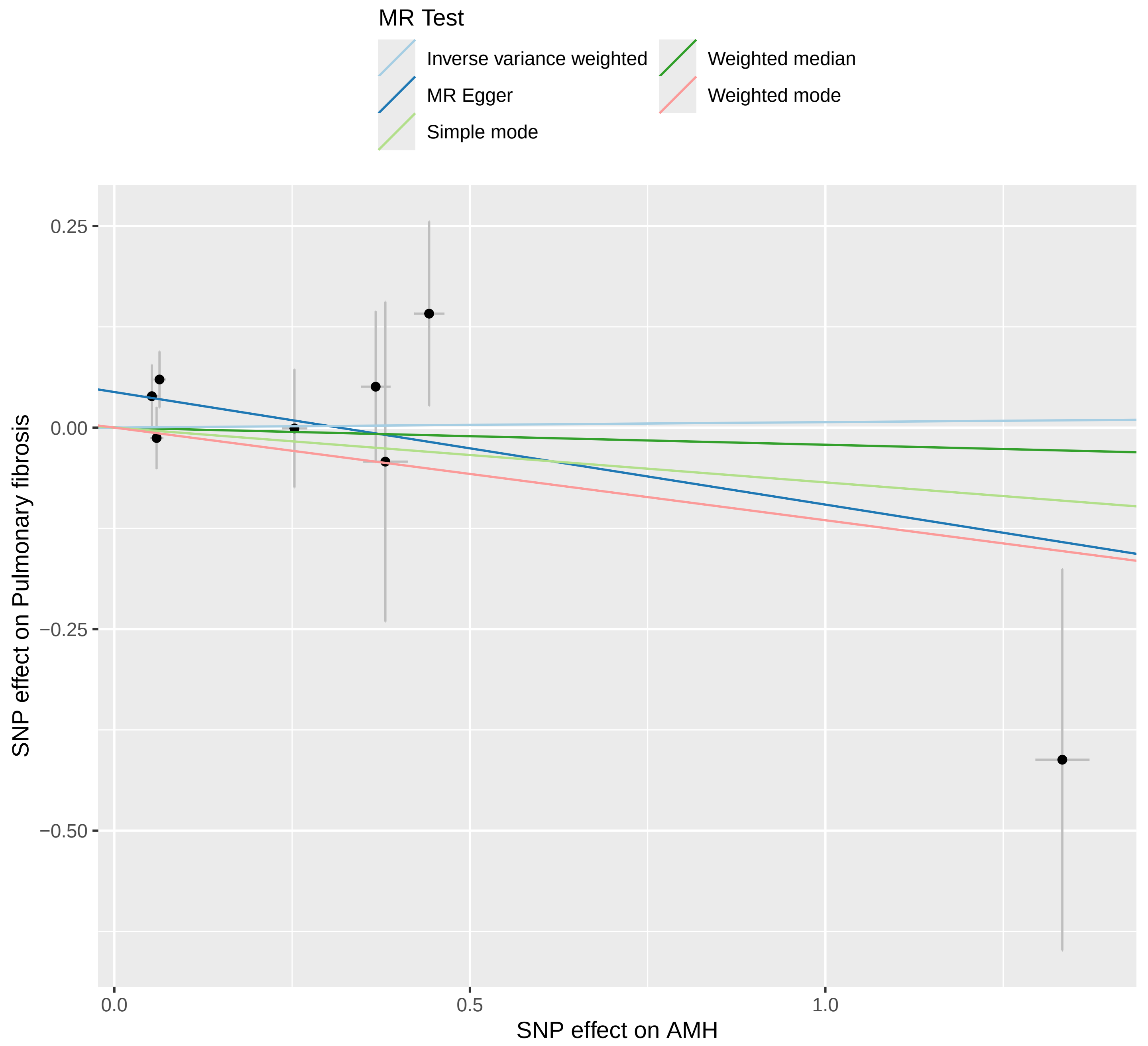
MR Test
Inverse variance weighted
MR Egger
Simple mode
Weighted median
Weighted mode
0.25
0.00
−0.25
−0.50
SNP effect on Pulmonary fibrosis
0.0
0.5
1.0
SNP effect on AMH

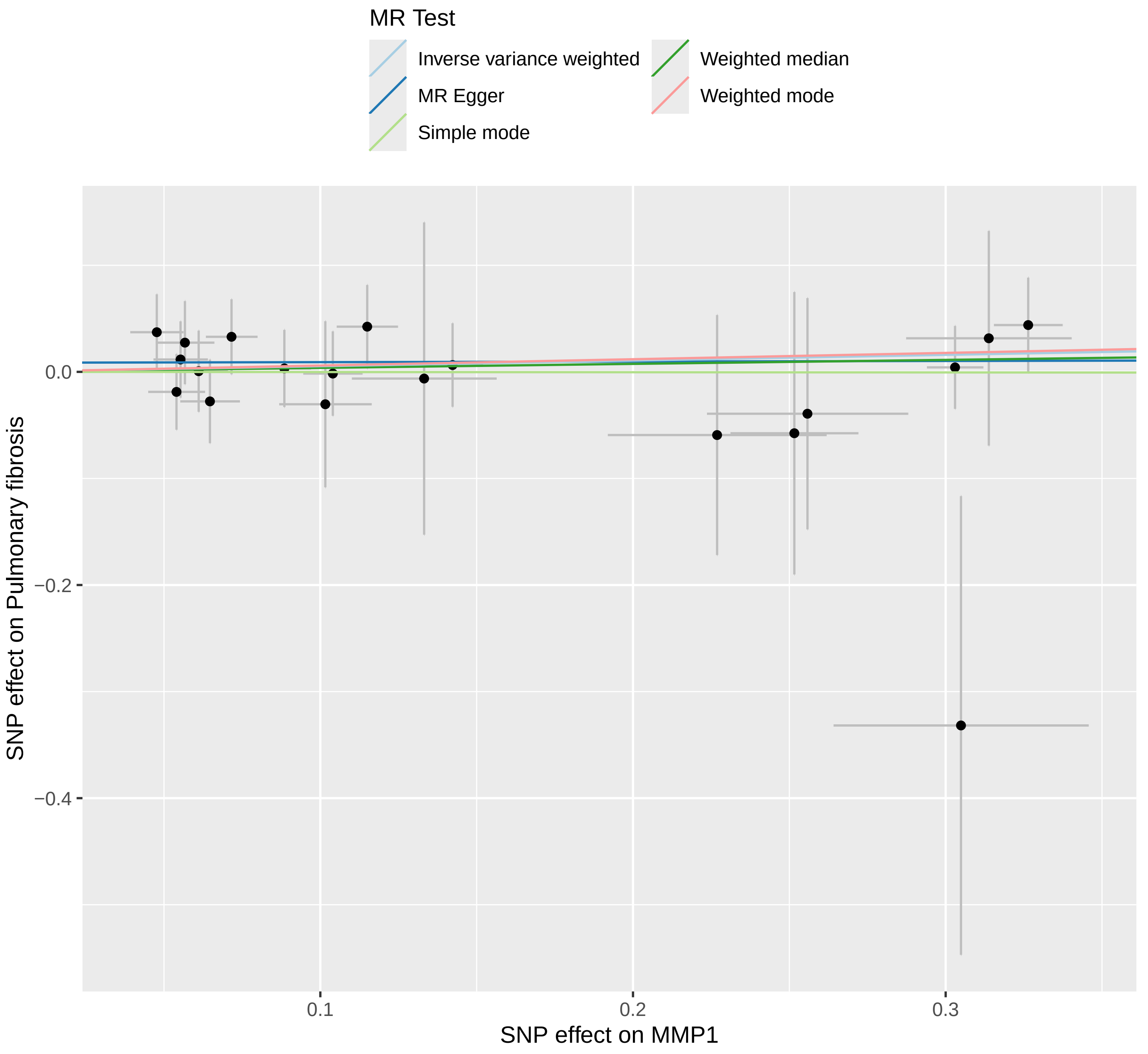
MR Test
Inverse variance weighted
MR Egger
Simple mode
Weighted median
Weighted mode
0.0
−0.2
−0.4
0.1
0.2
0.3
SNP effect on Pulmonary fibrosis
SNP effect on MMP1

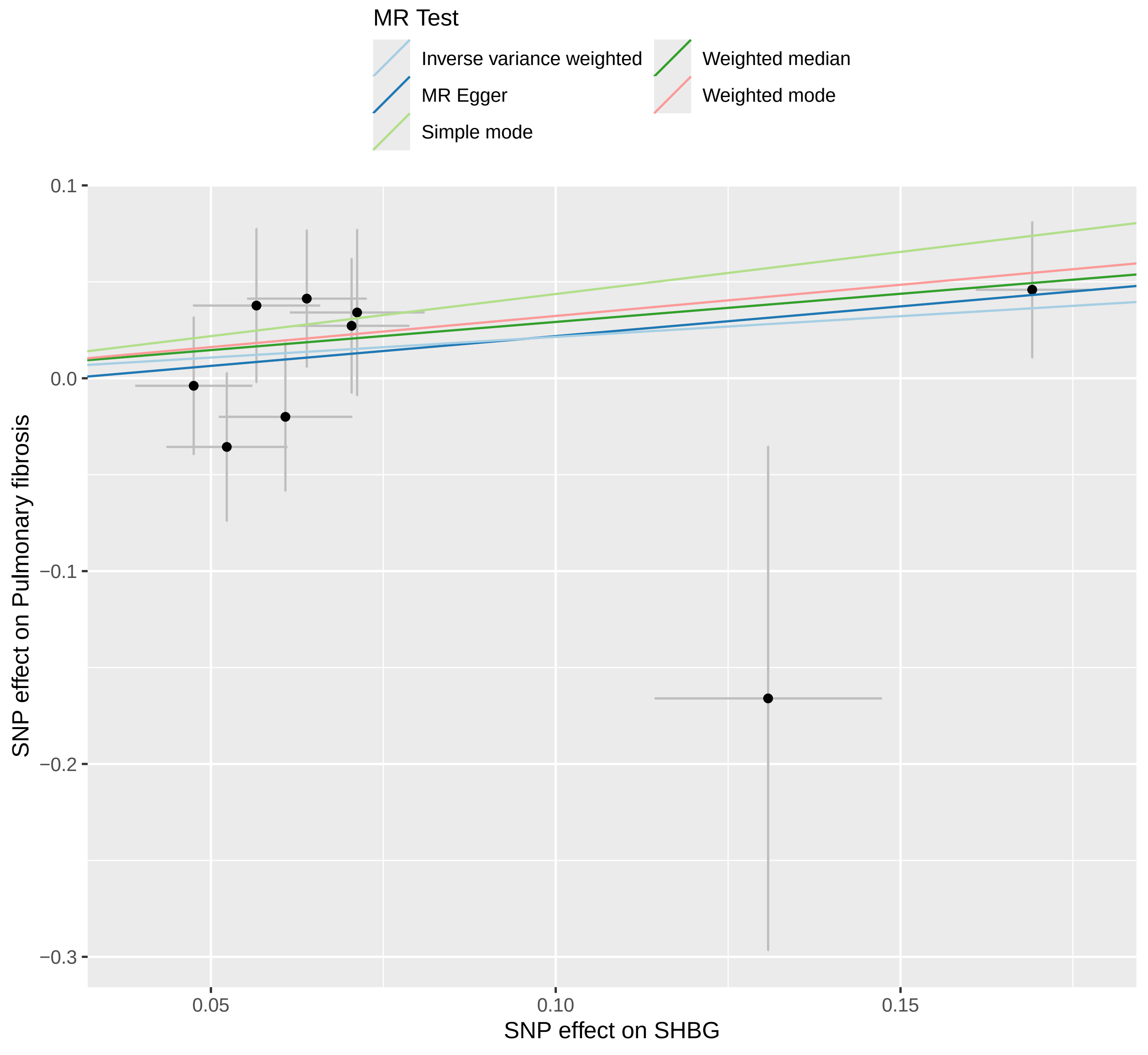
MR Test
Inverse variance weighted
MR Egger
Simple mode
Weighted median
Weighted mode
0.1
0.0
−0.1
−0.2
−0.3
SNP effect on Pulmonary fibrosis
0.05
0.10
0.15
SNP effect on SHBG

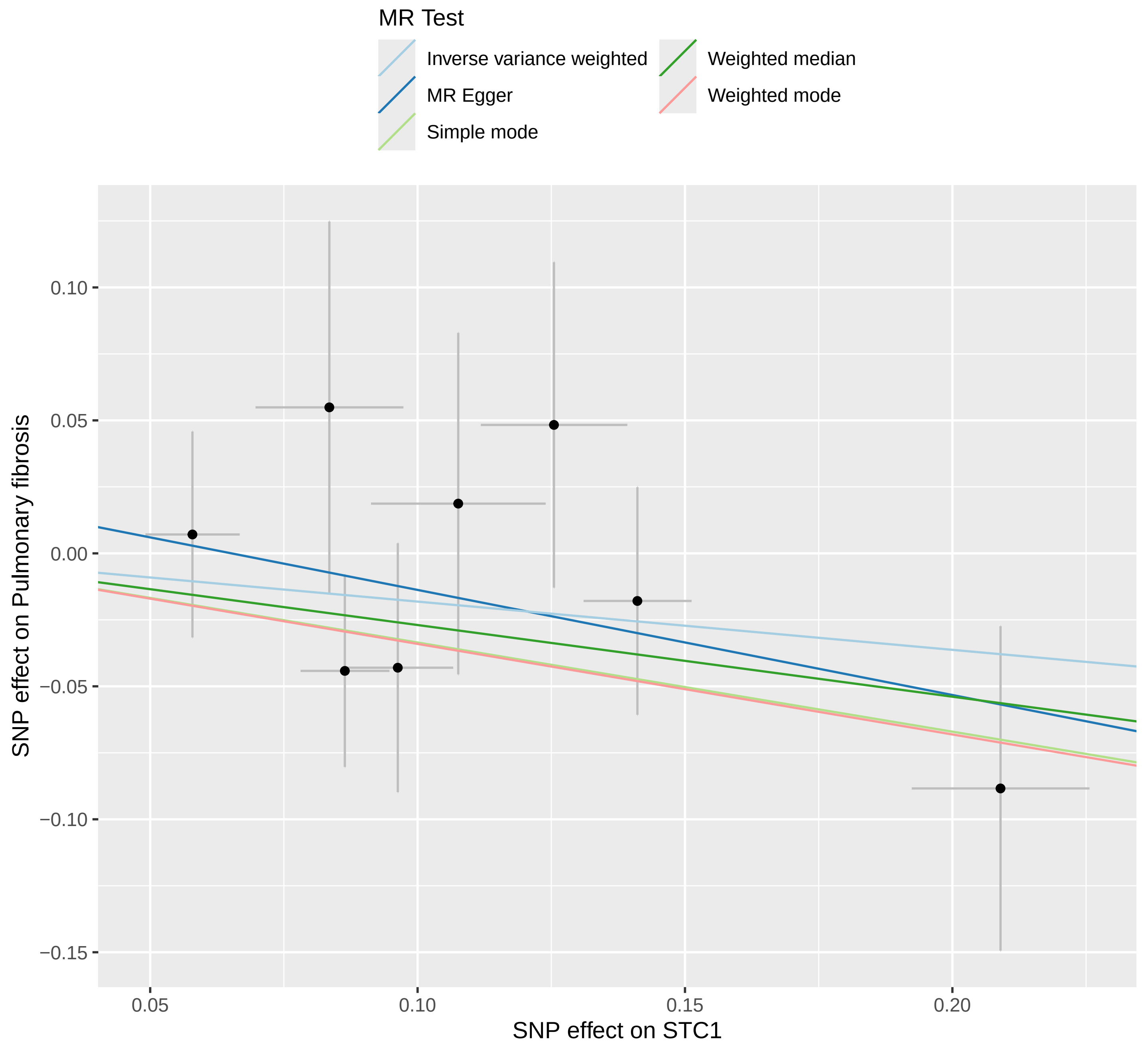

MR Test
Inverse variance weighted
MR Egger
Simple mode
Weighted median
Weighted mode
SNP effect on Pulmonary fibrosis
0.10
0.05
0.00
−0.05
−0.10
−0.15
0.05
0.10
0.15
0.20
SNP effect on STC1

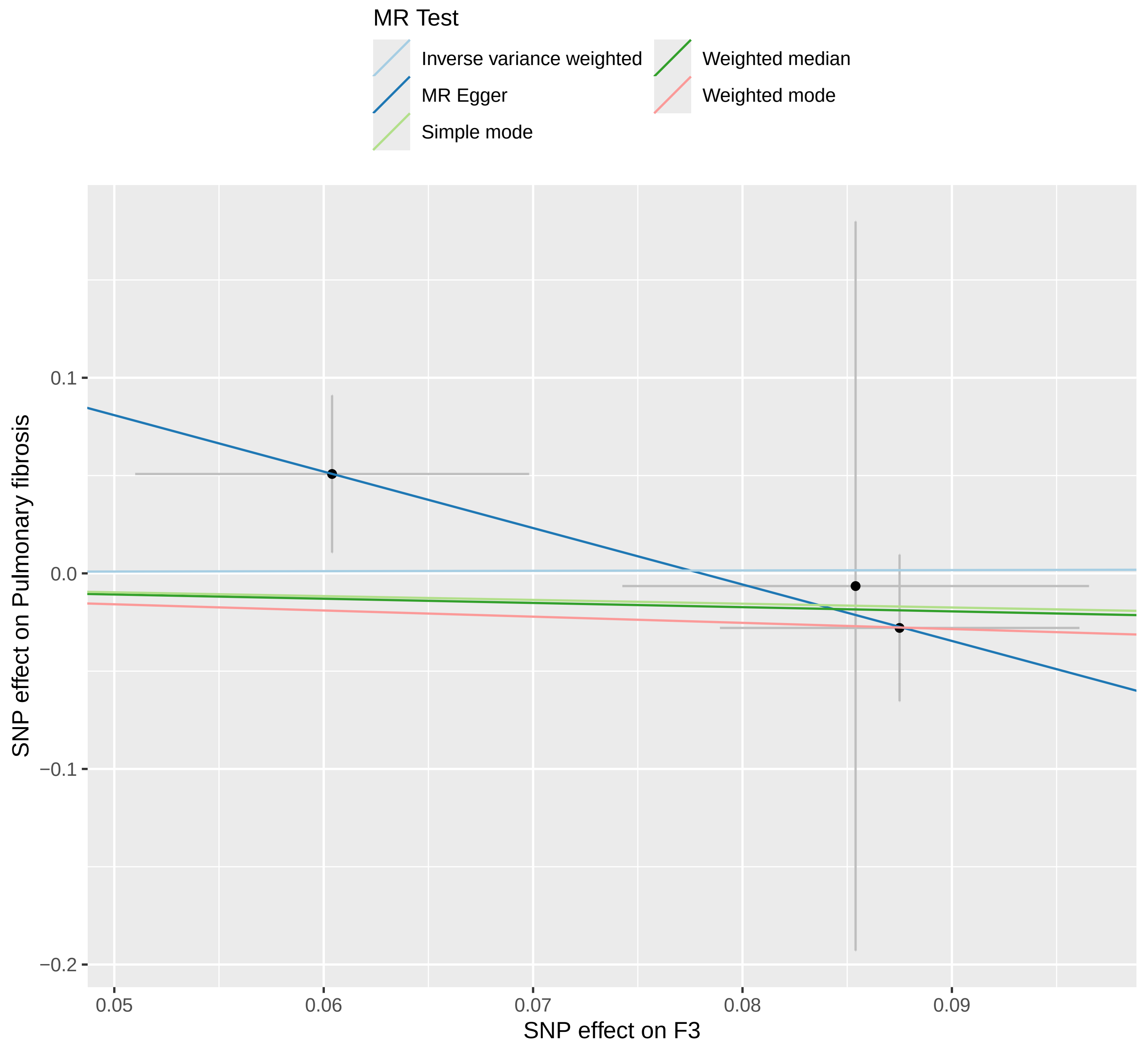
MR Test
Inverse variance weighted
MR Egger
Simple mode
Weighted median
Weighted mode
0.1
0.0
−0.1
−0.2
SNP effect on Pulmonary fibrosis
0.05
0.06
0.07
0.08
0.09
SNP effect on F3

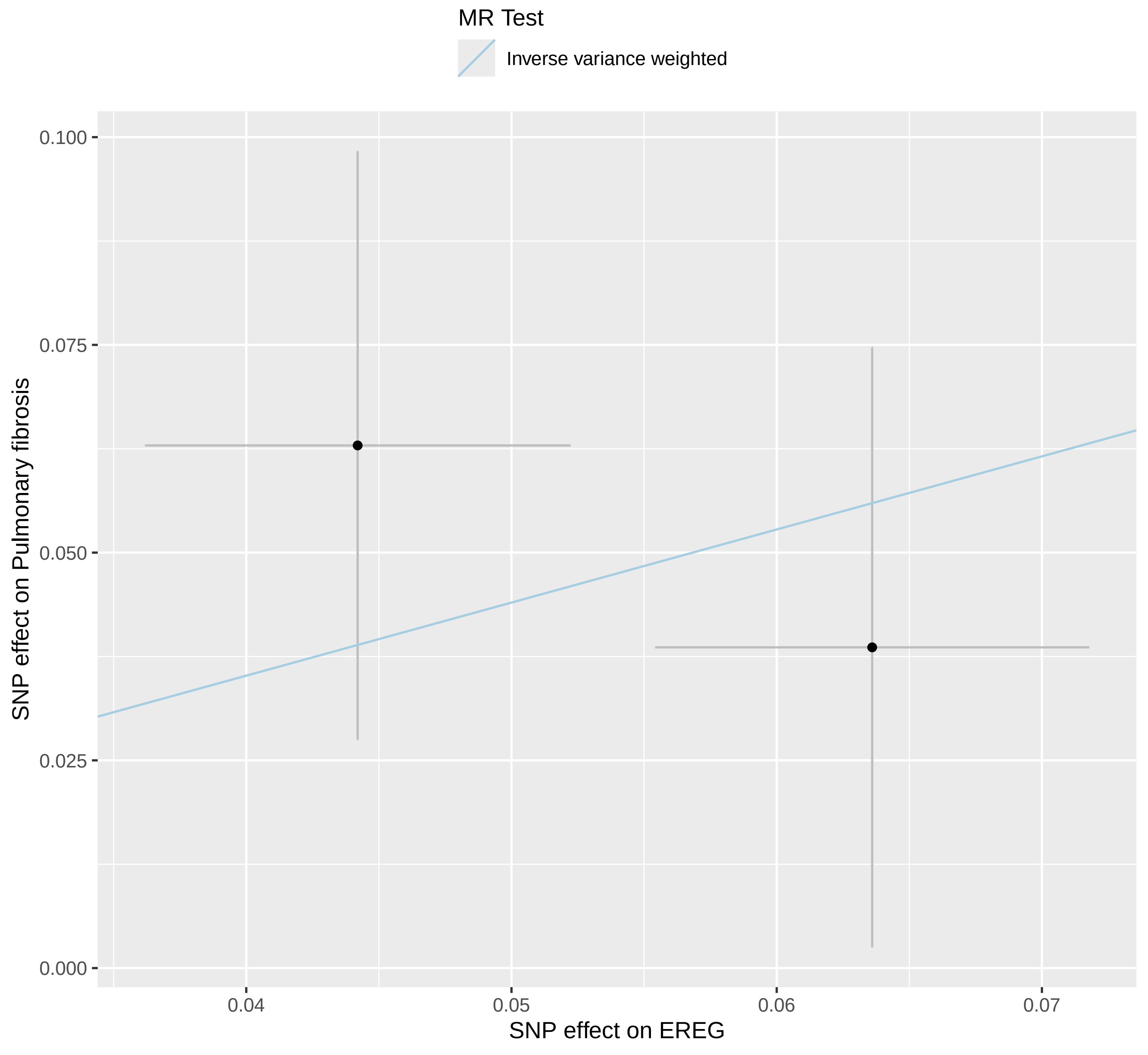
MR Test
Inverse variance weighted
SNP effect on Pulmonary fibrosis
0.100
0.075
0.050
0.025
0.000
0.04
0.05
0.06
0.07
SNP effect on EREG

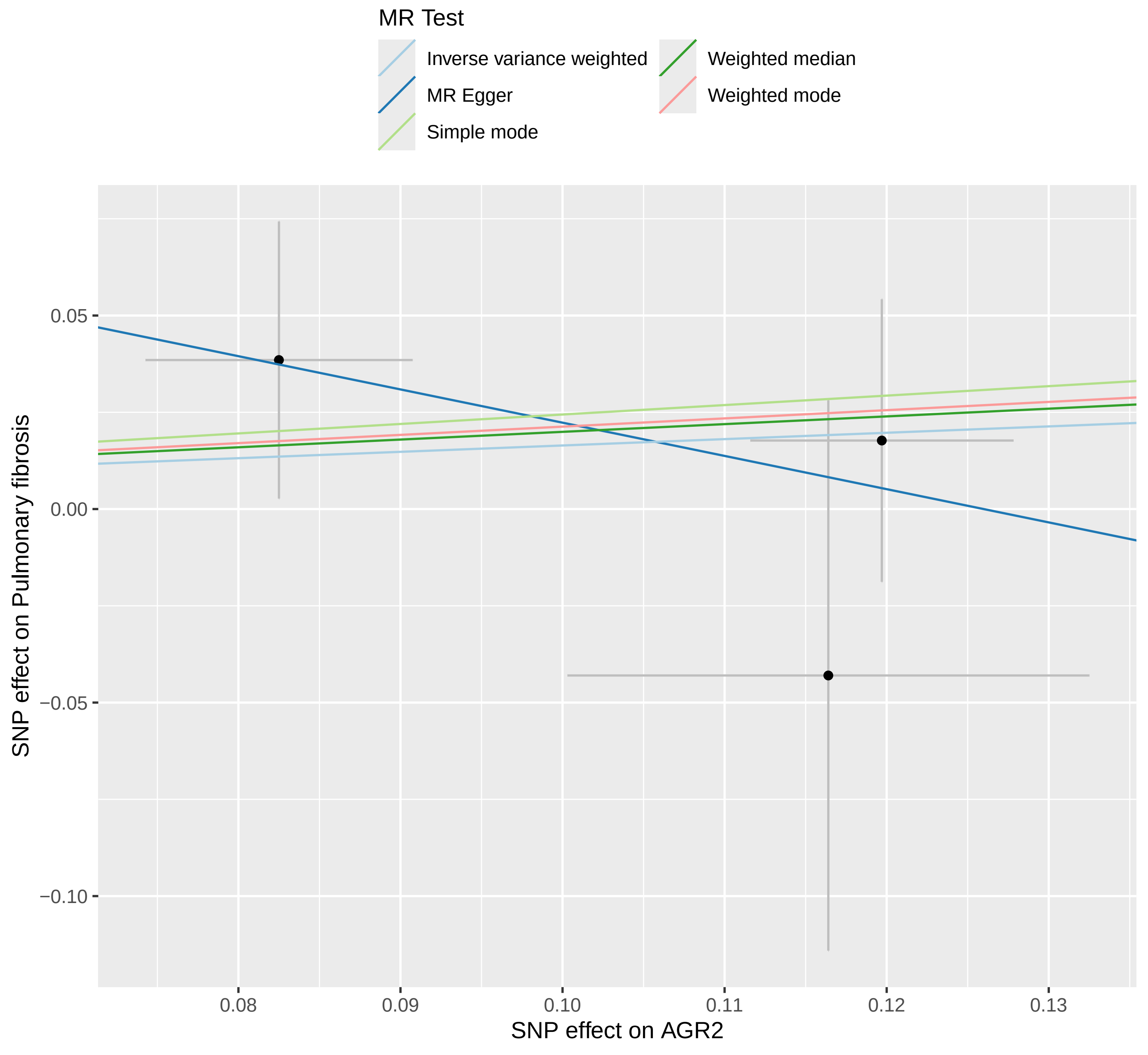

MR Test
Inverse variance weighted
MR Egger
Simple mode
Weighted median
Weighted mode
0.05
0.00
−0.05
−0.10
SNP effect on Pulmonary fibrosis
0.08
0.09
0.10
0.11
0.12
0.13
SNP effect on AGR2

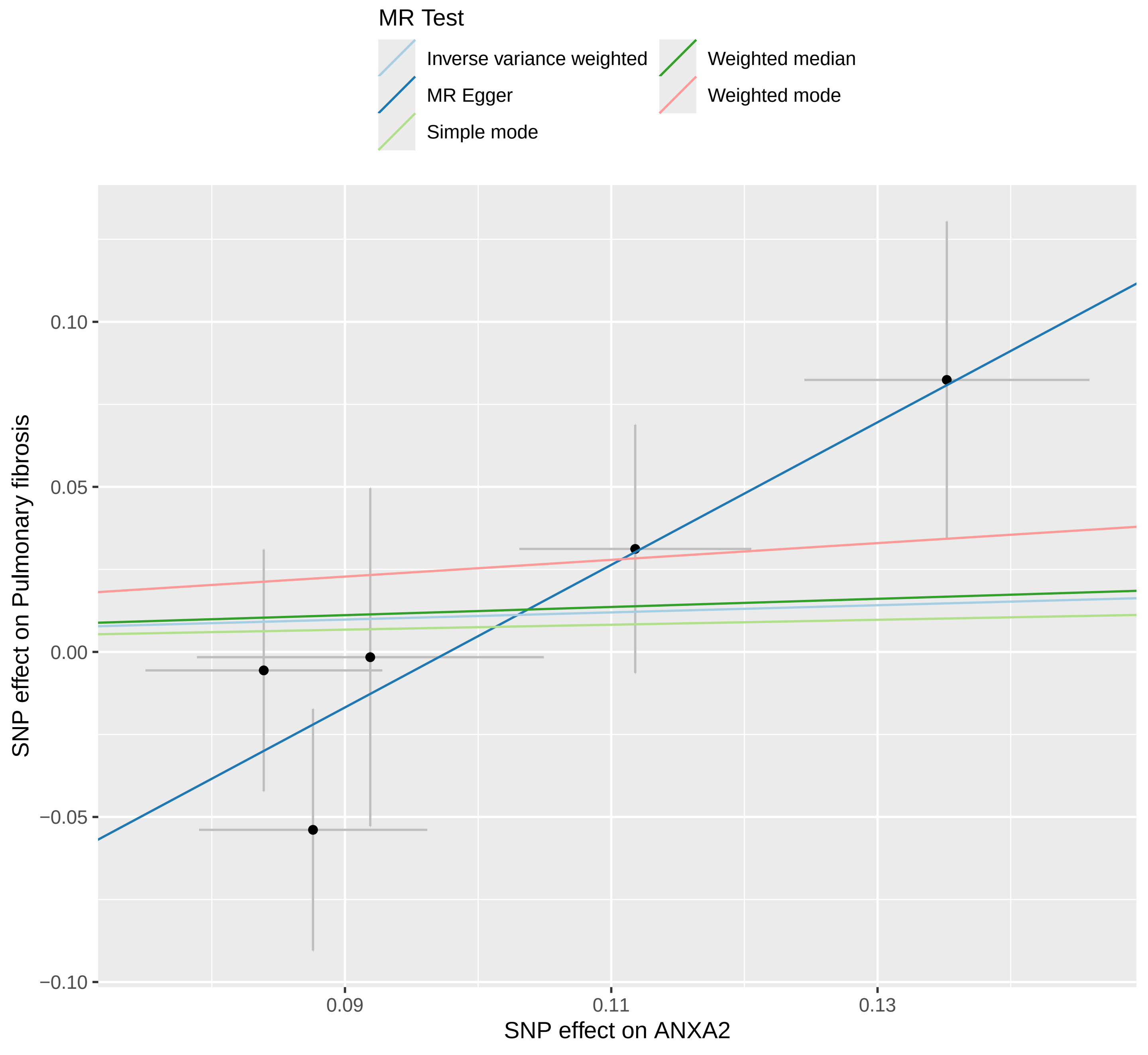

MR Test
Inverse variance weighted
MR Egger
Simple mode
Weighted median
Weighted mode
0.10
0.05
0.00
−0.05
−0.10
SNP effect on Pulmonary fibrosis
0.09
0.11
0.13
SNP effect on ANXA2

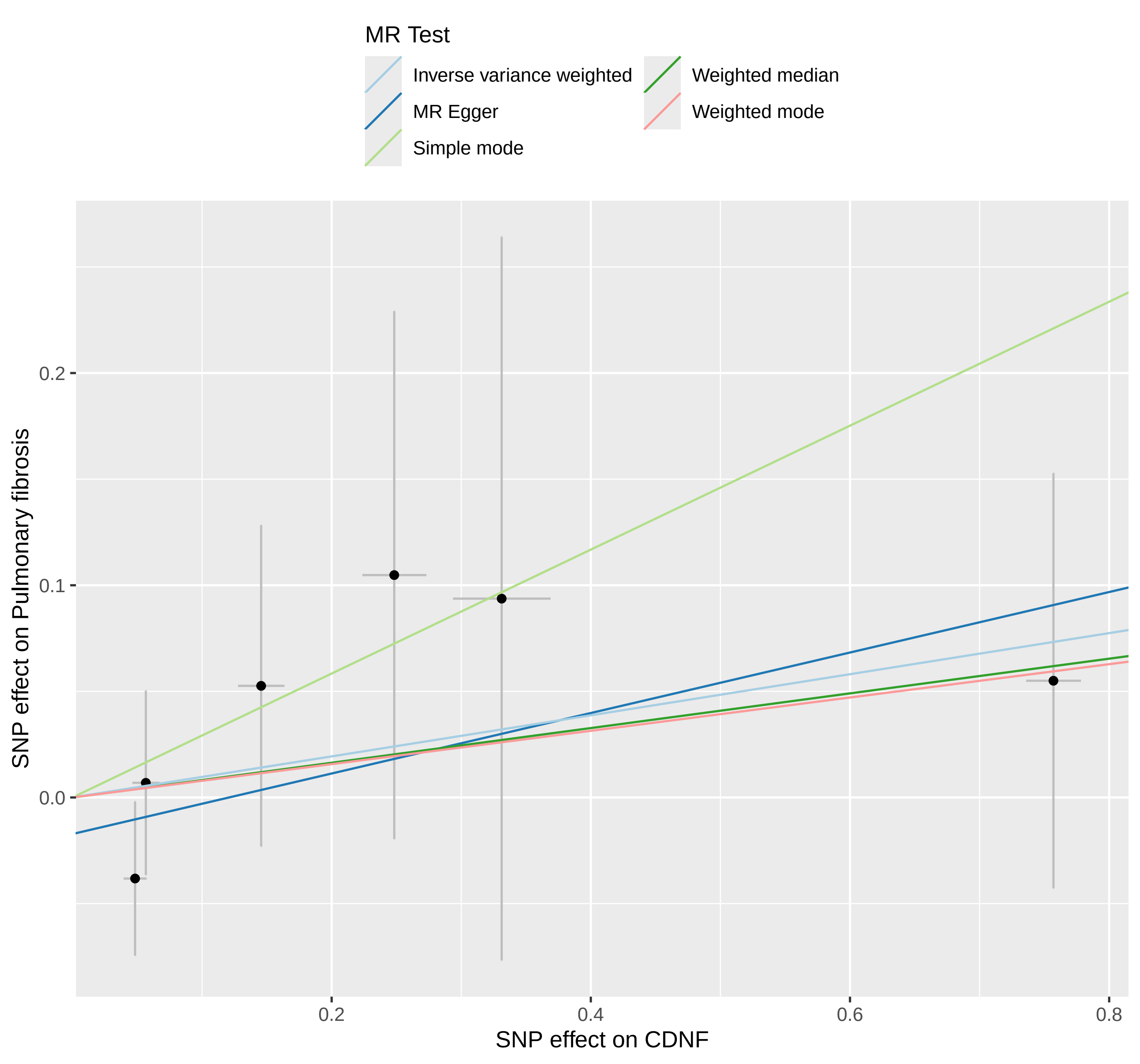
MR Test
Inverse variance weighted
MR Egger
Simple mode
Weighted median
Weighted mode
0.2
0.1
0.0
SNP effect on Pulmonary fibrosis
0.2
0.4
0.6
0.8
SNP effect on CDNF

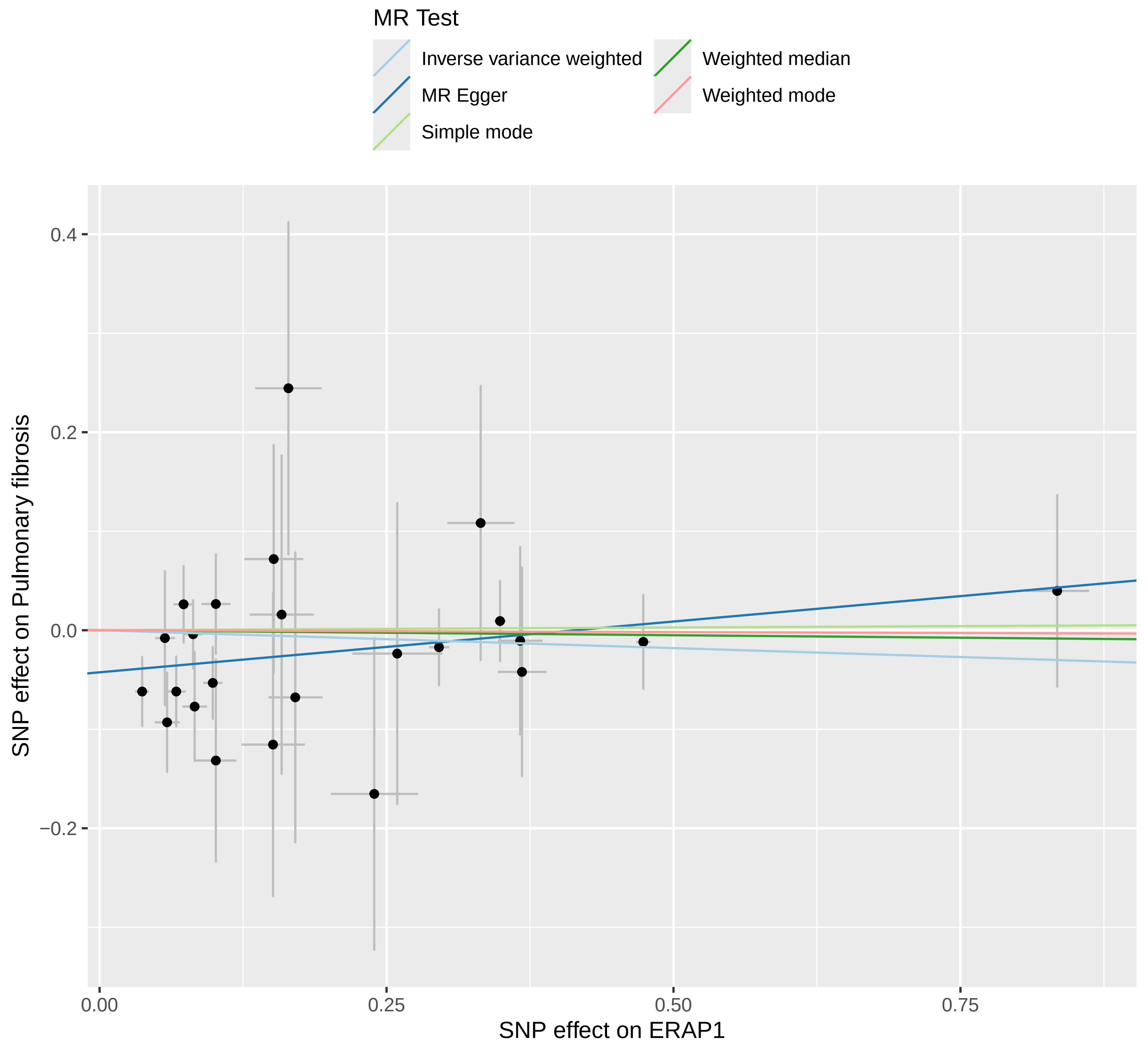

MR Test
Inverse variance weighted
MR Egger
Simple mode
Weighted median
Weighted mode
SNP effect on Pulmonary fibrosis
0.4
0.2
0.0
−0.2
0.00
0.25
0.50
0.75
SNP effect on ERAP1

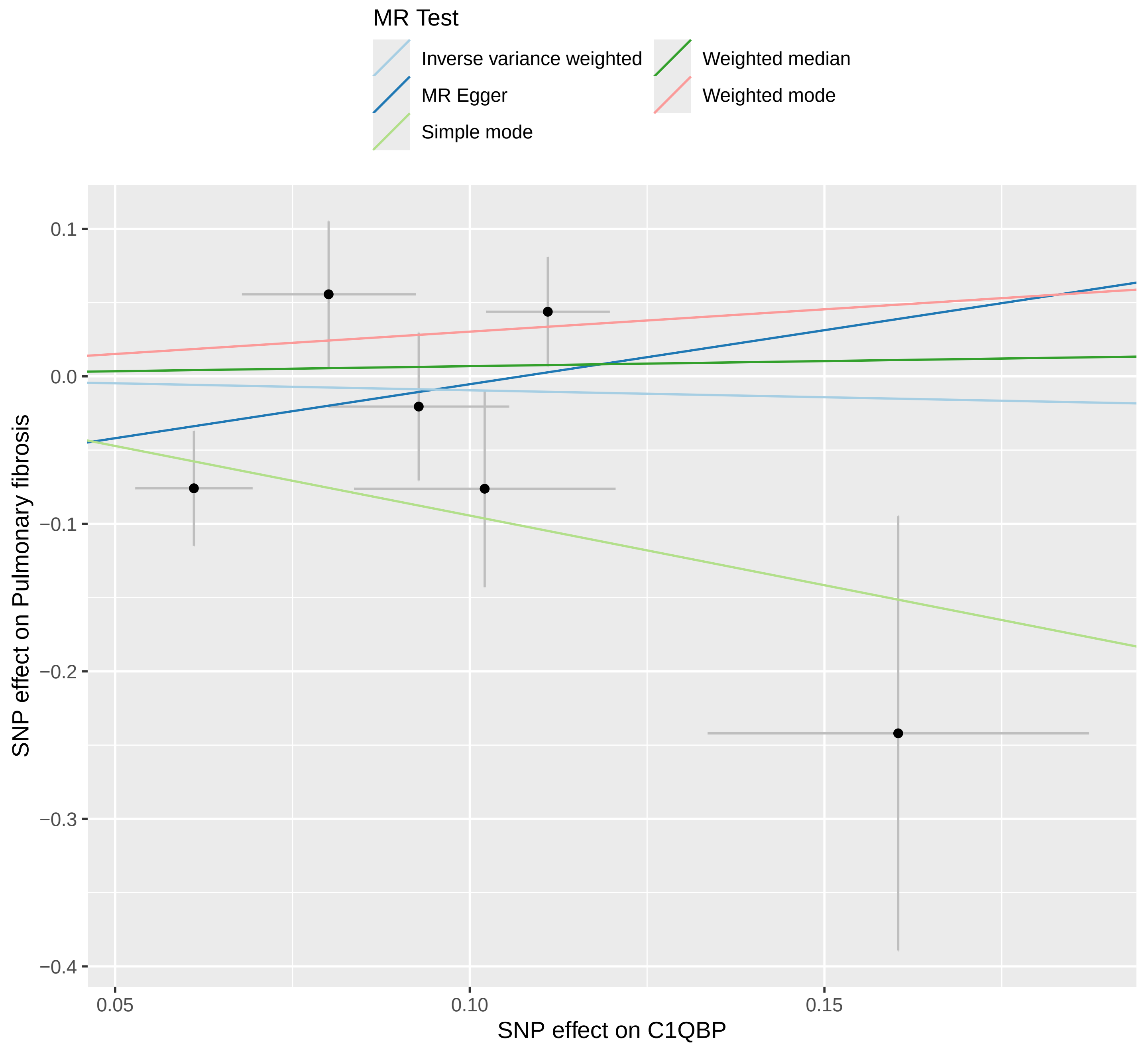
MR Test
Inverse variance weighted
MR Egger
Simple mode
Weighted median
Weighted mode
0.1
0.0
−0.1
−0.2
−0.3
−0.4
0.05
0.10
0.15
SNP effect on C1QBP
SNP effect on Pulmonary fibrosis

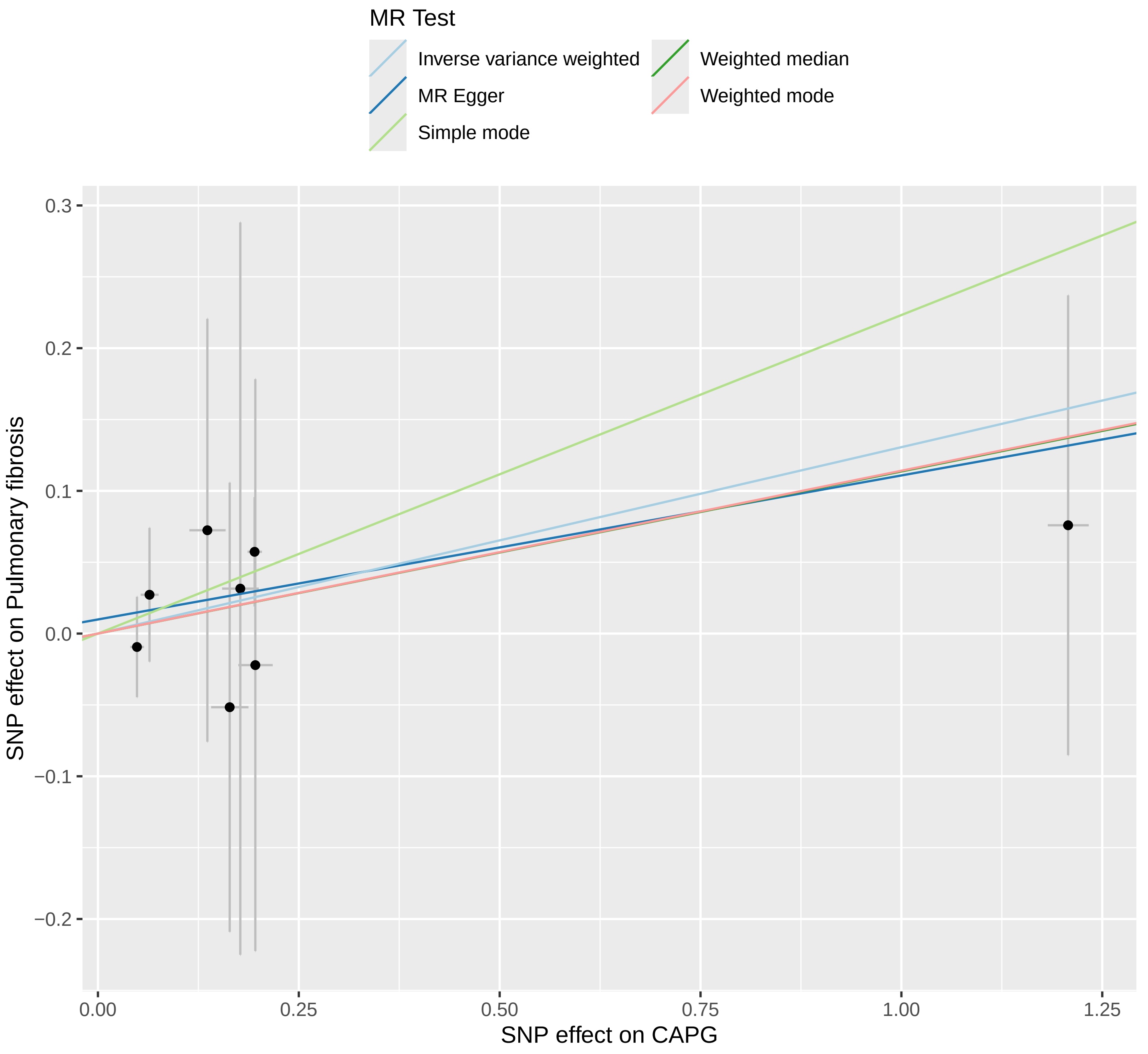

MR Test
Inverse variance weighted
MR Egger
Simple mode
Weighted median
Weighted mode
0.3
0.2
0.1
0.0
−0.1
−0.2
SNP effect on Pulmonary fibrosis
0.00
0.25
0.50
0.75
1.00
1.25
SNP effect on CAPG

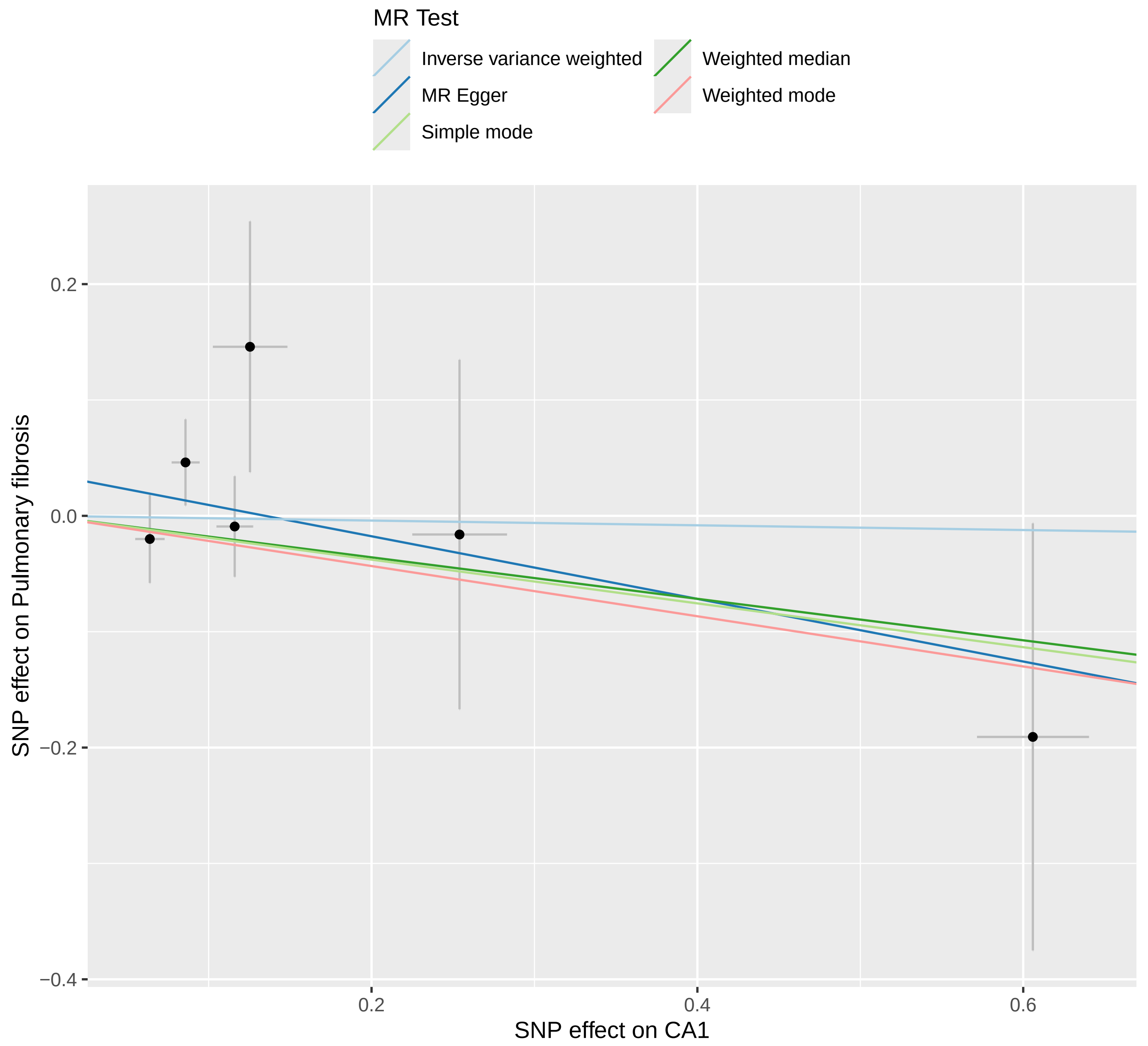
MR Test
Inverse variance weighted
MR Egger
Simple mode
Weighted median
Weighted mode
0.2
0.0
−0.2
−0.4
0.2
0.4
0.6
SNP effect on Pulmonary fibrosis
SNP effect on CA1

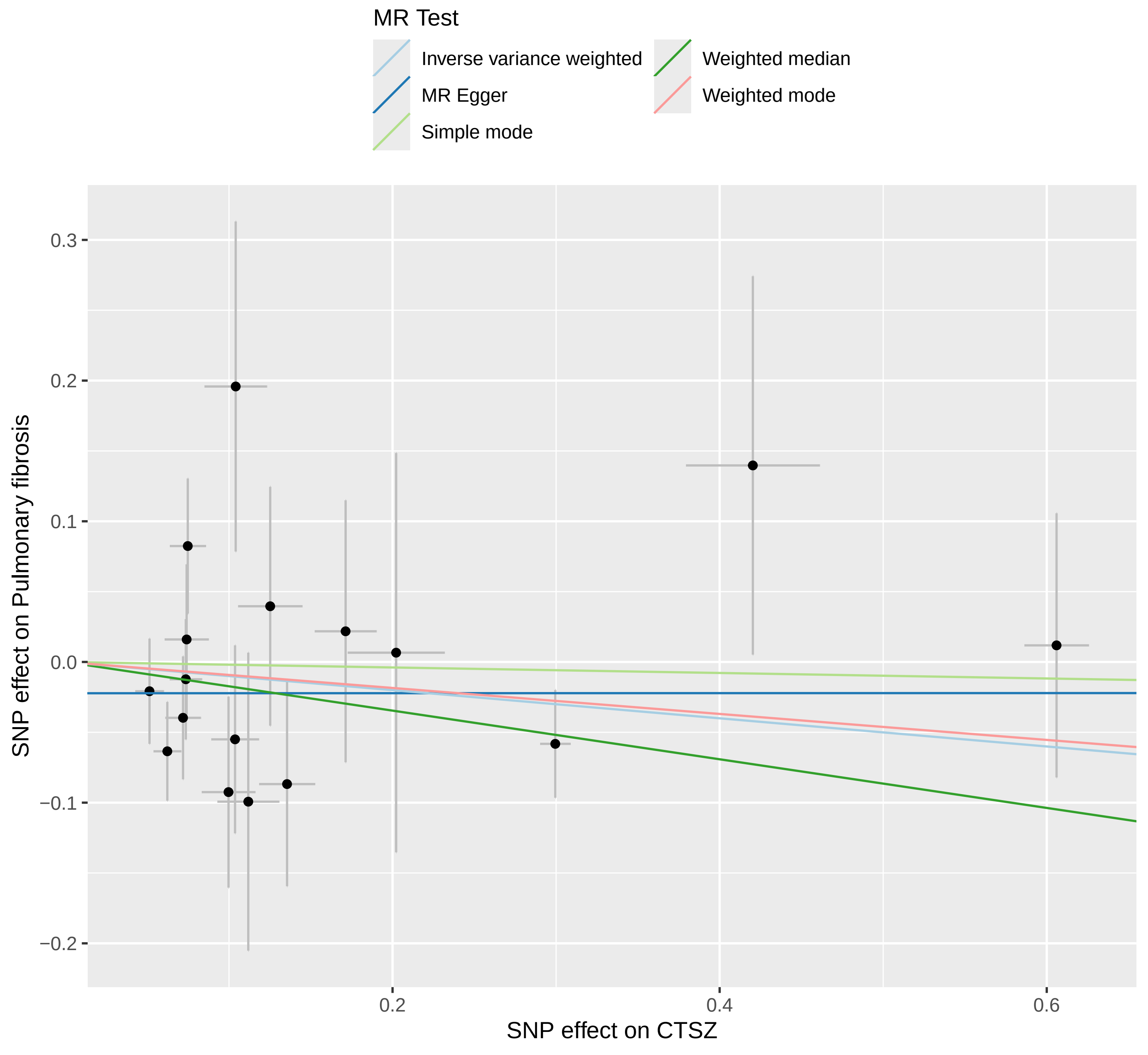

MR Test
Inverse variance weighted
MR Egger
Simple mode
Weighted median
Weighted mode
SNP effect on Pulmonary fibrosis
0.3
0.2
0.1
0.0
−0.1
−0.2
0.2
0.4
0.6
SNP effect on CTSZ

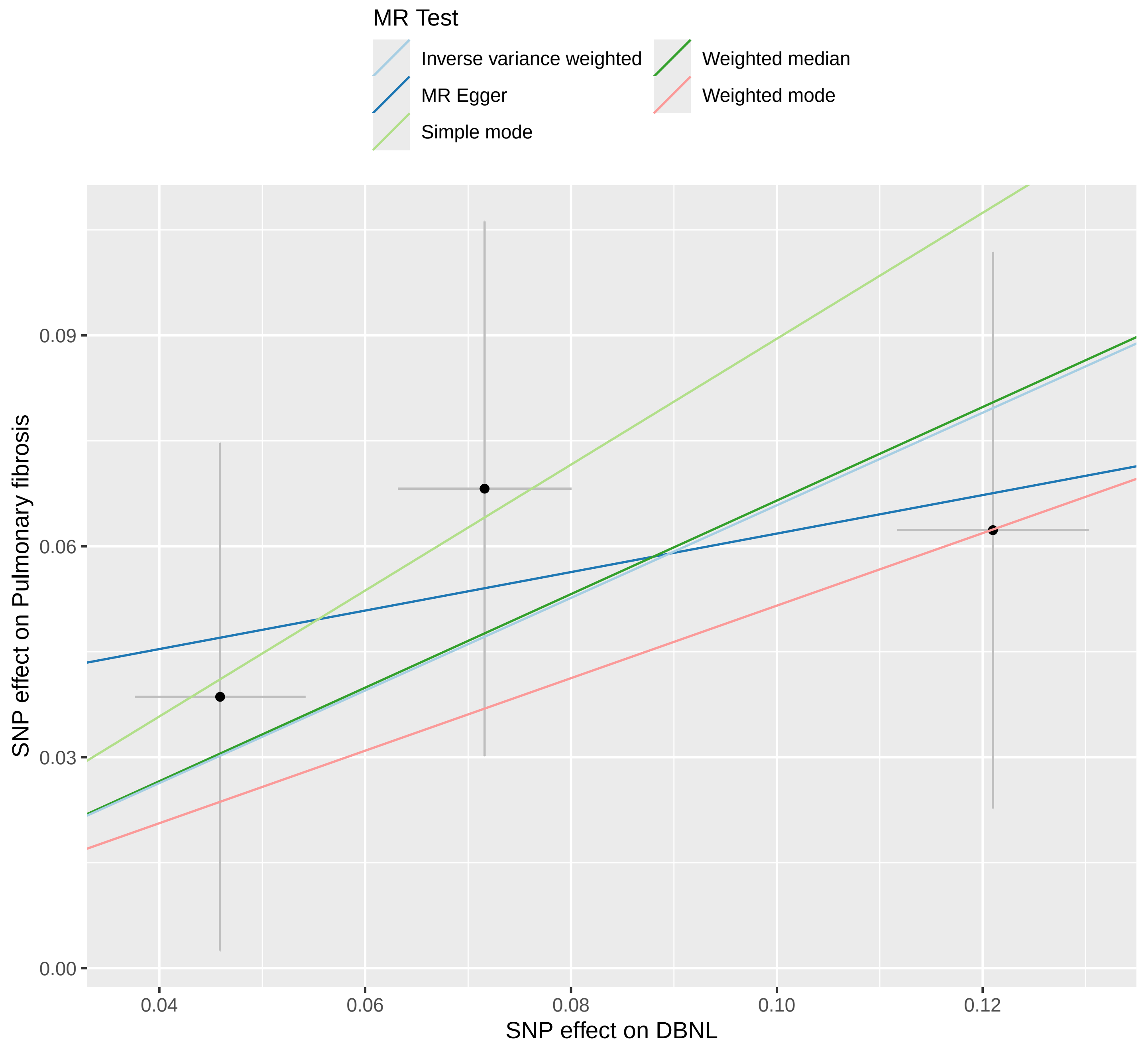
MR Test
Inverse variance weighted
MR Egger
Simple mode
Weighted median
Weighted mode
SNP effect on Pulmonary fibrosis
0.09
0.06
0.03
0.00
0.04
0.06
0.08
0.10
0.12
SNP effect on DBNL

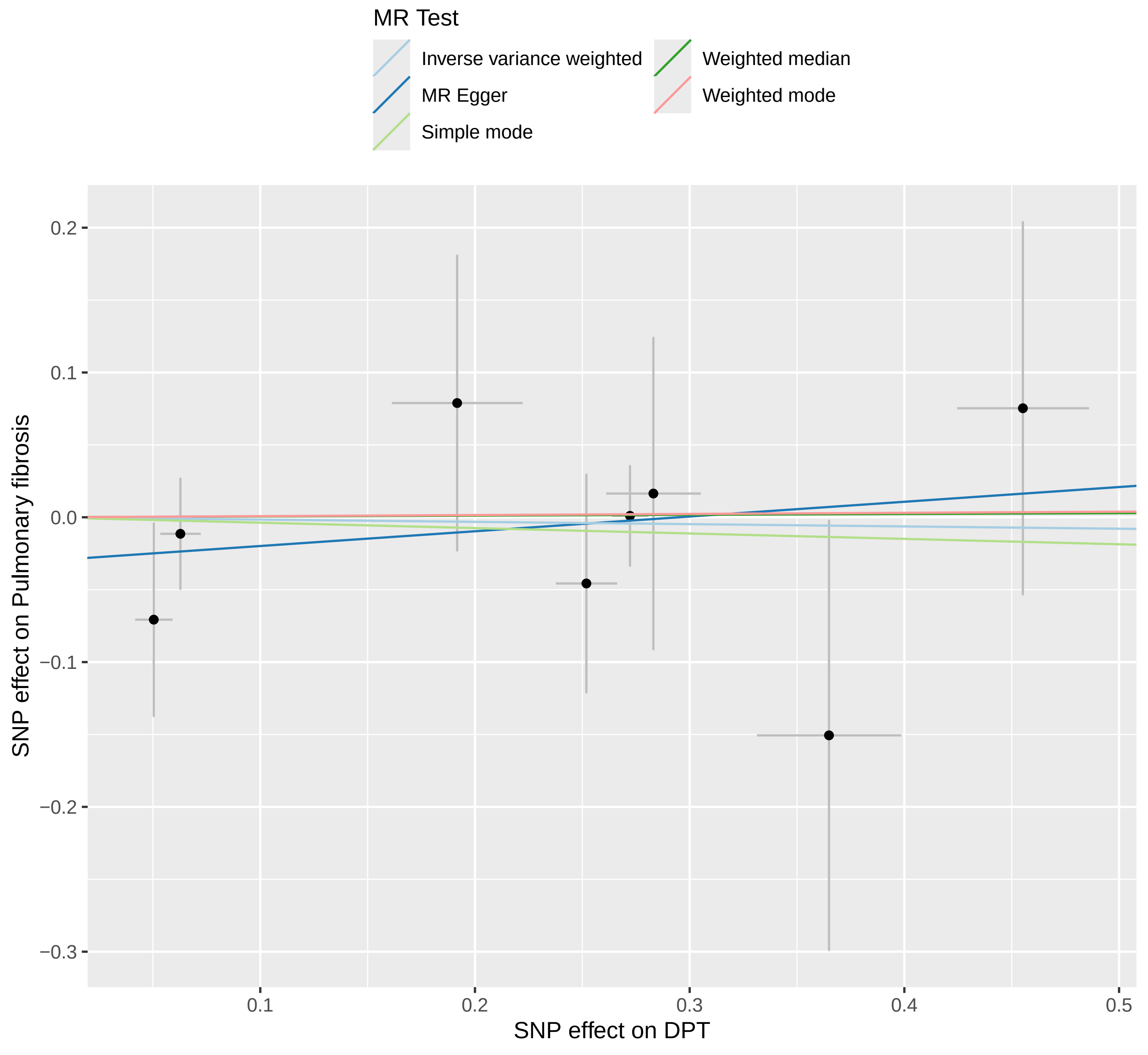

MR Test
Inverse variance weighted
MR Egger
Simple mode
Weighted median
Weighted mode
0.2
0.1
0.0
−0.1
−0.2
−0.3
SNP effect on Pulmonary fibrosis
0.1
0.2
0.3
0.4
0.5
SNP effect on DPT

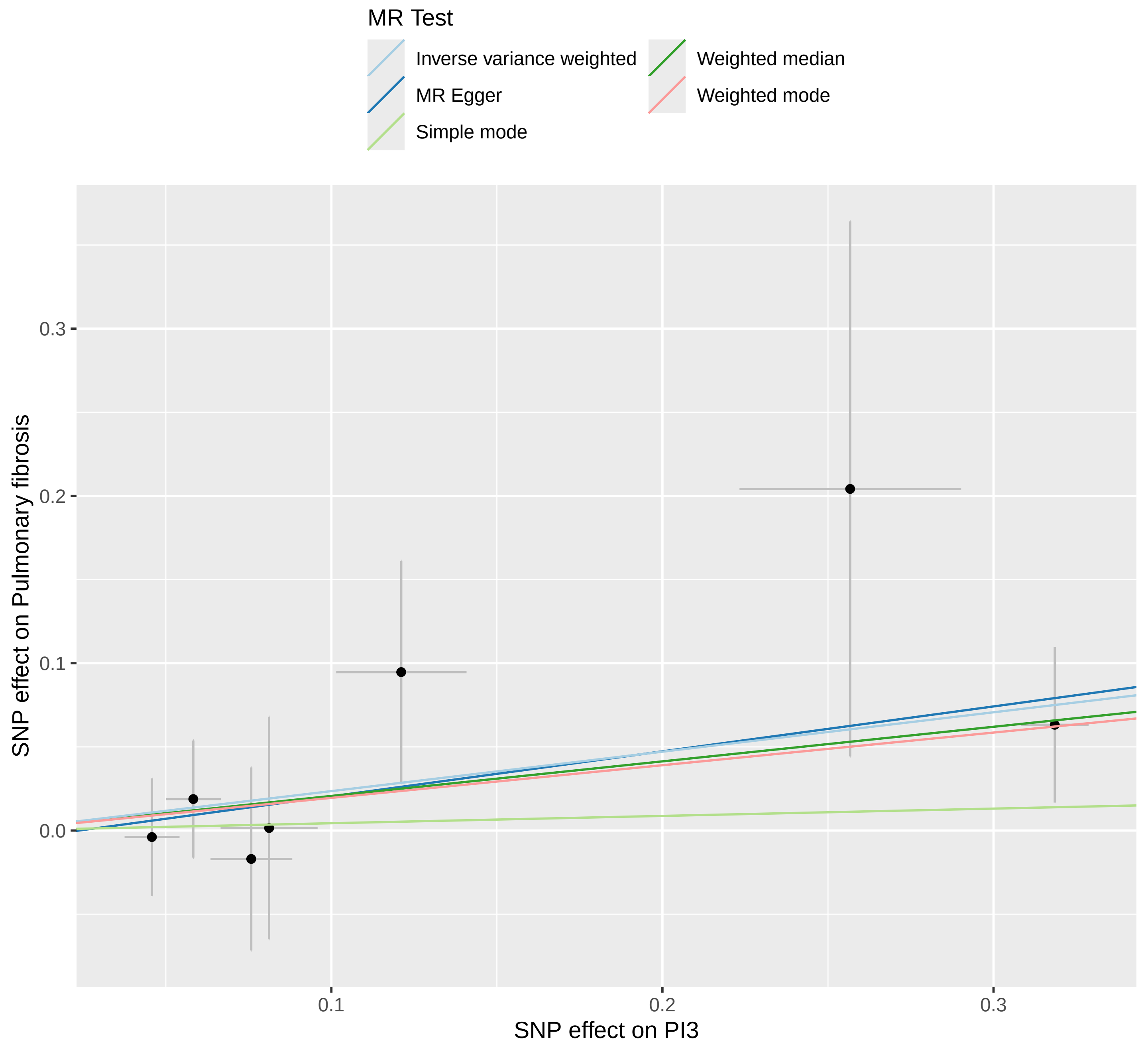

MR Test
Inverse variance weighted
MR Egger
Simple mode
Weighted median
Weighted mode
0.3
0.2
0.1
0.0
SNP effect on Pulmonary fibrosis
0.1
0.2
0.3
SNP effect on PI3

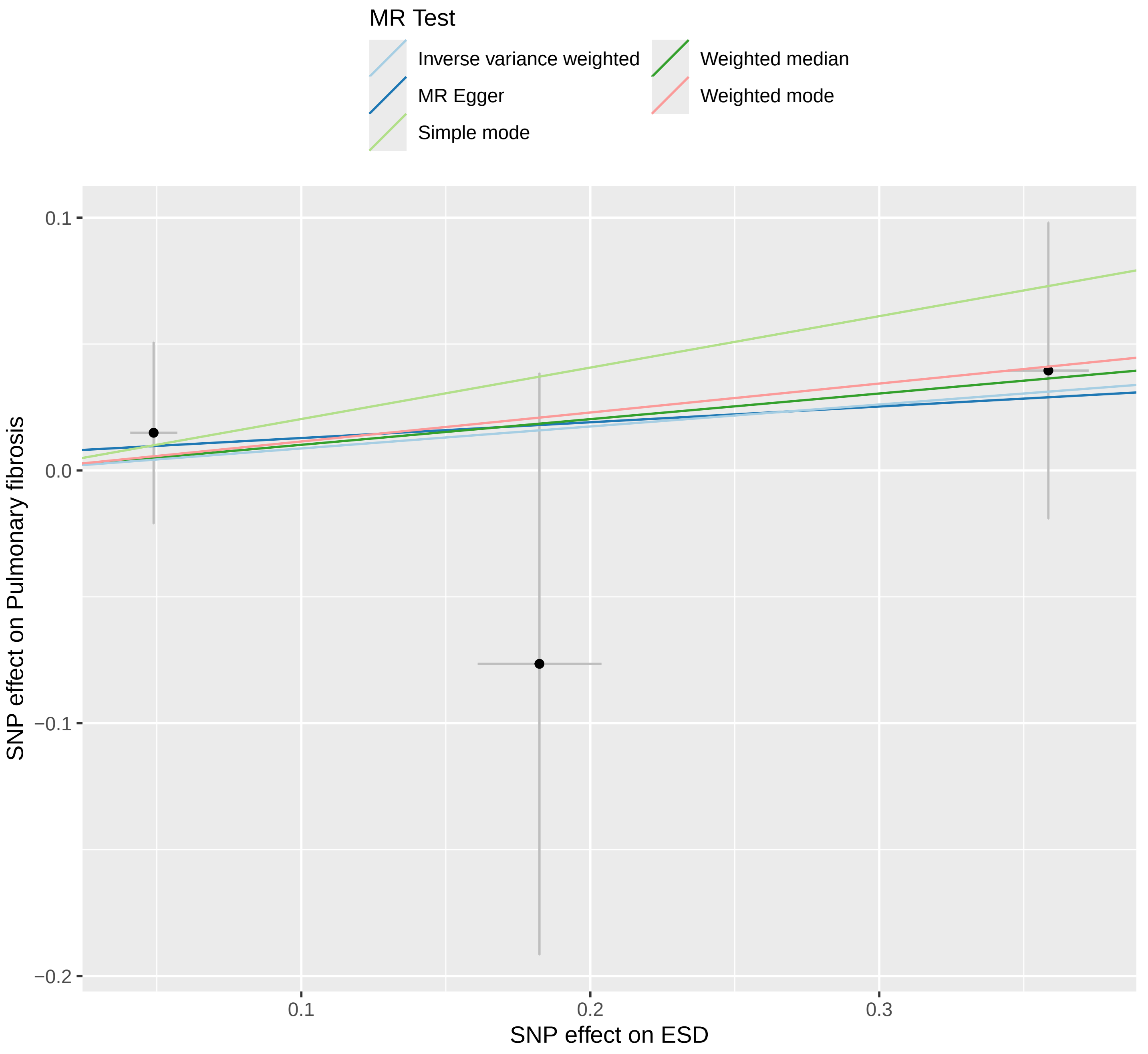
MR Test
Inverse variance weighted
MR Egger
Simple mode
Weighted median
Weighted mode
0.1
0.0
−0.1
−0.2
0.1
0.2
0.3
SNP effect on Pulmonary fibrosis
SNP effect on ESD

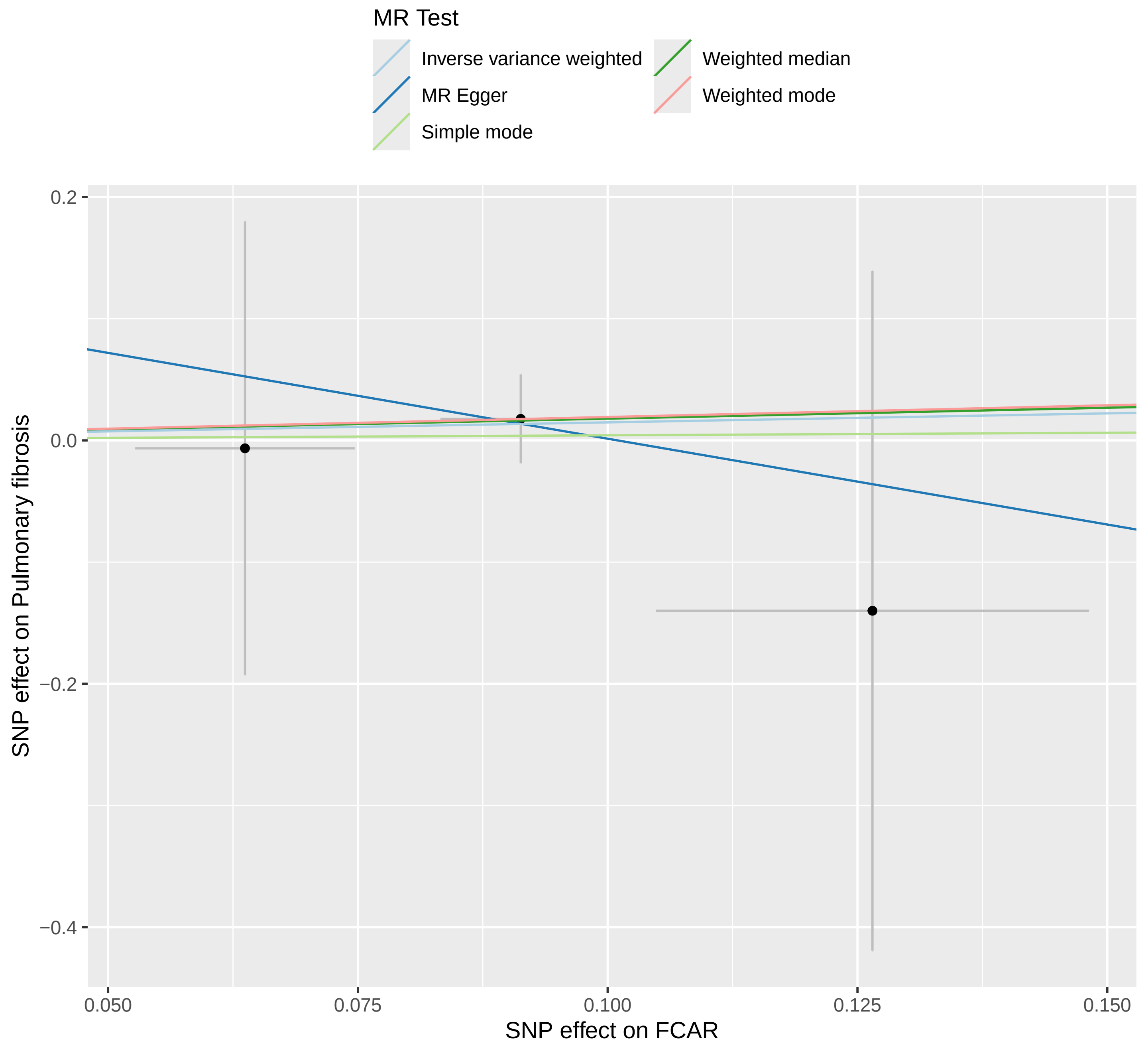
MR Test
Inverse variance weighted
MR Egger
Simple mode
Weighted median
Weighted mode
0.2
0.0
−0.2
−0.4
SNP effect on Pulmonary fibrosis
0.050
0.075
0.100
0.125
0.150
SNP effect on FCAR

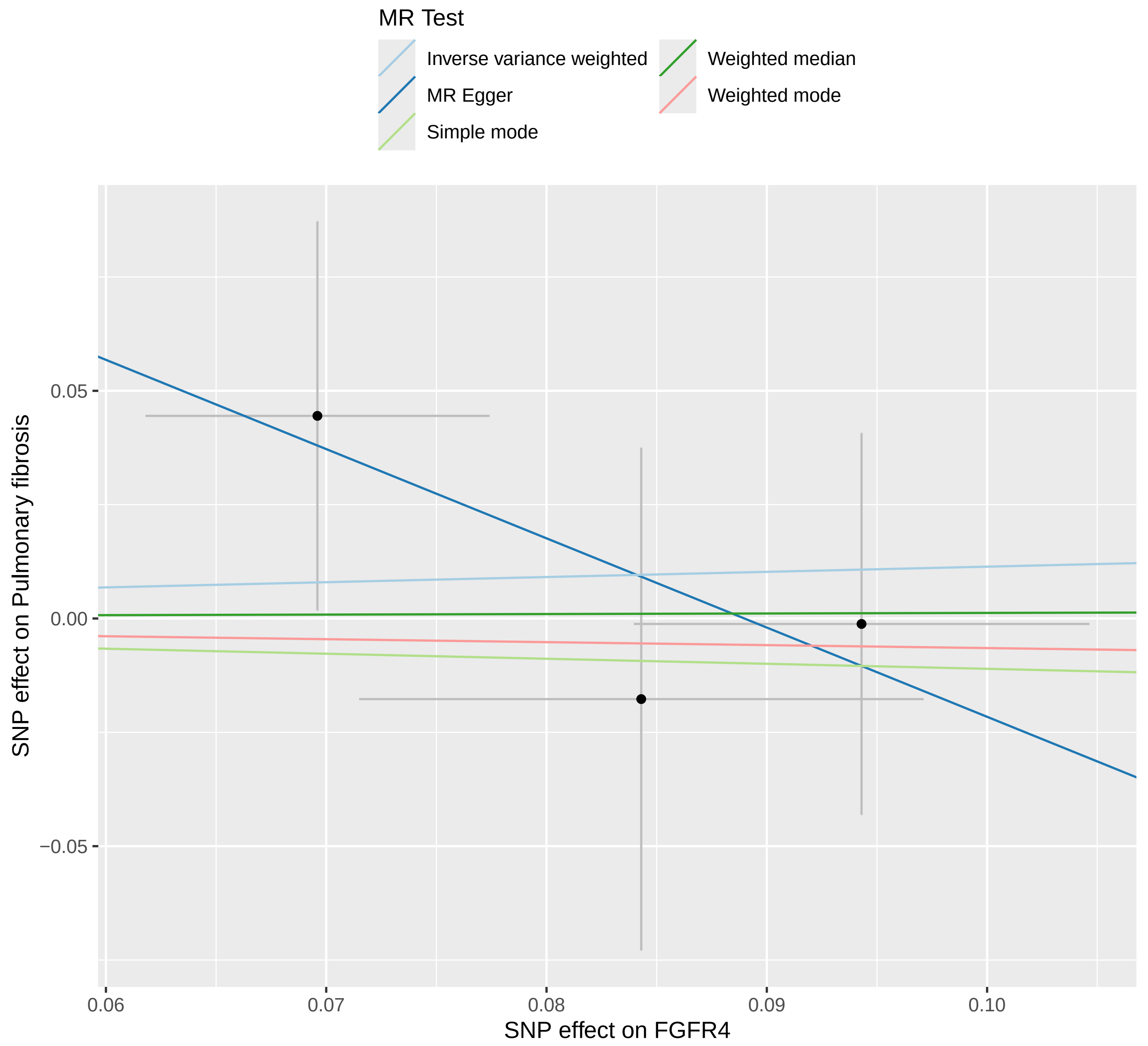

MR Test
Inverse variance weighted
MR Egger
Simple mode
Weighted median
Weighted mode
0.05
0.00
−0.05
SNP effect on Pulmonary fibrosis
0.06
0.07
0.08
0.09
0.10
SNP effect on FGFR4

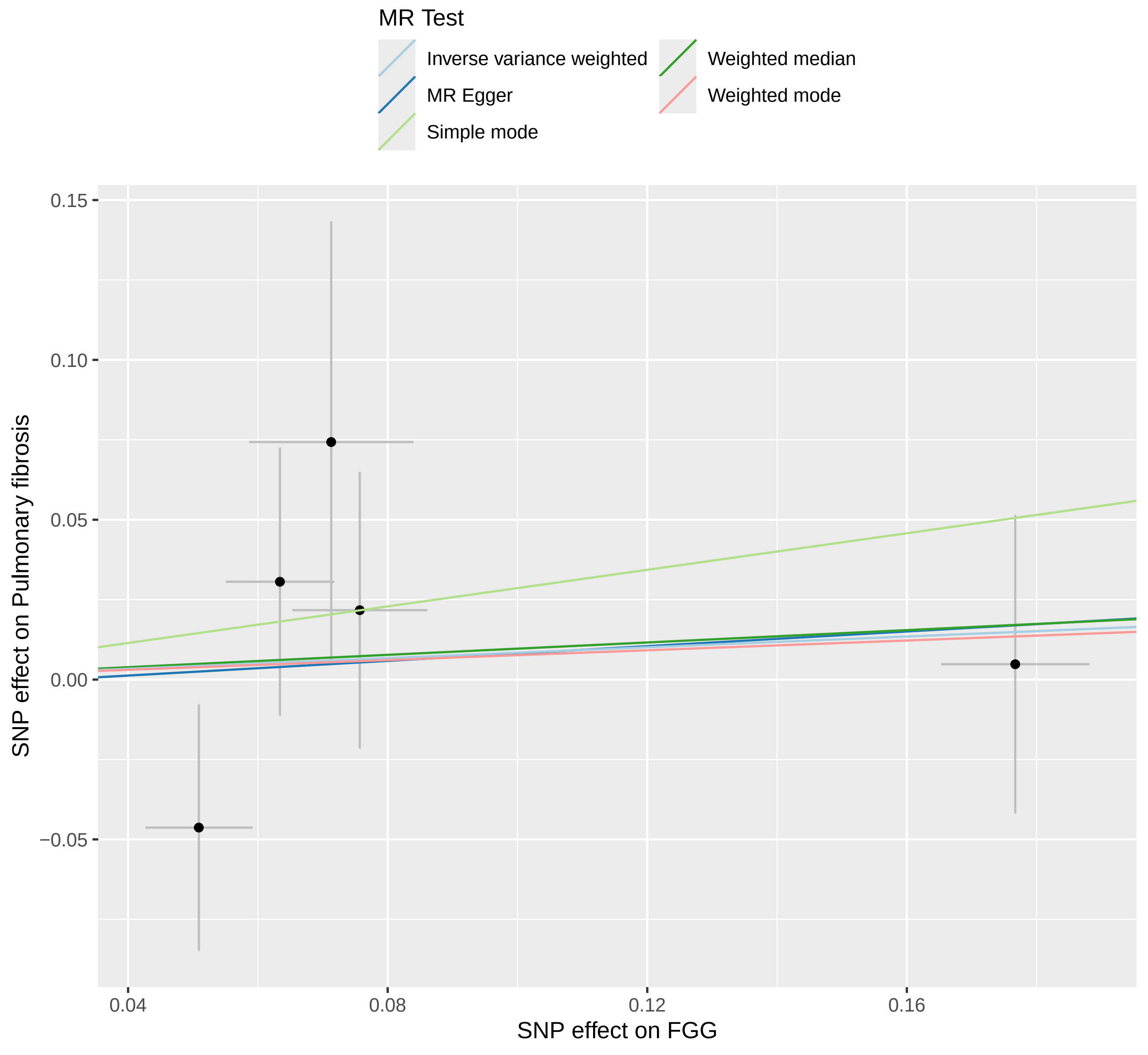
MR Test
Inverse variance weighted
MR Egger
Simple mode
Weighted median
Weighted mode
0.15
0.10
0.05
0.00
−0.05
0.04
0.08
0.12
0.16
SNP effect on FGG
SNP effect on Pulmonary fibrosis

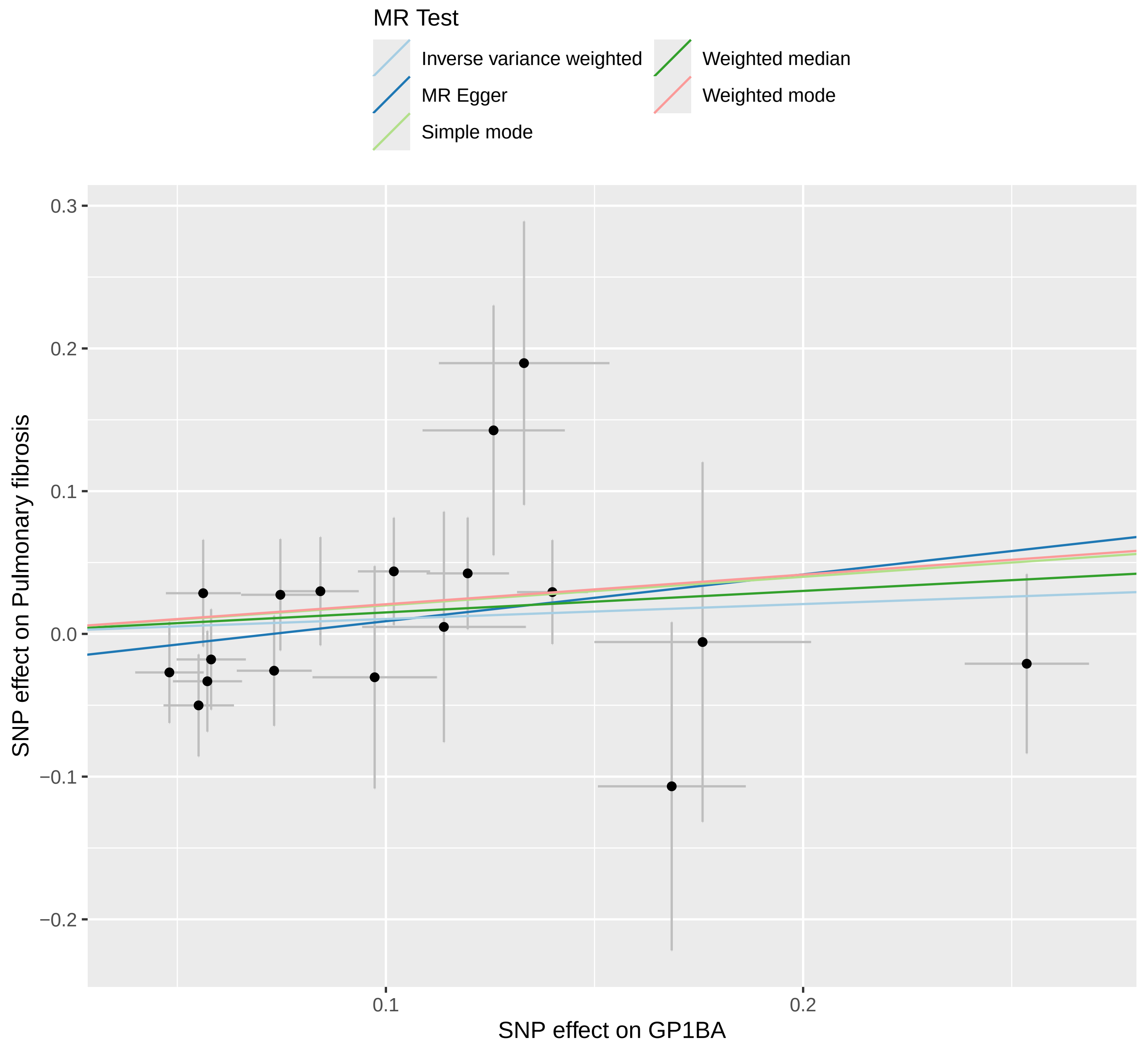

MR Test
Inverse variance weighted
MR Egger
Simple mode
Weighted median
Weighted mode
0.3
0.2
0.1
0.0
−0.1
−0.2
0.1
0.2
SNP effect on Pulmonary fibrosis
SNP effect on GP1BA

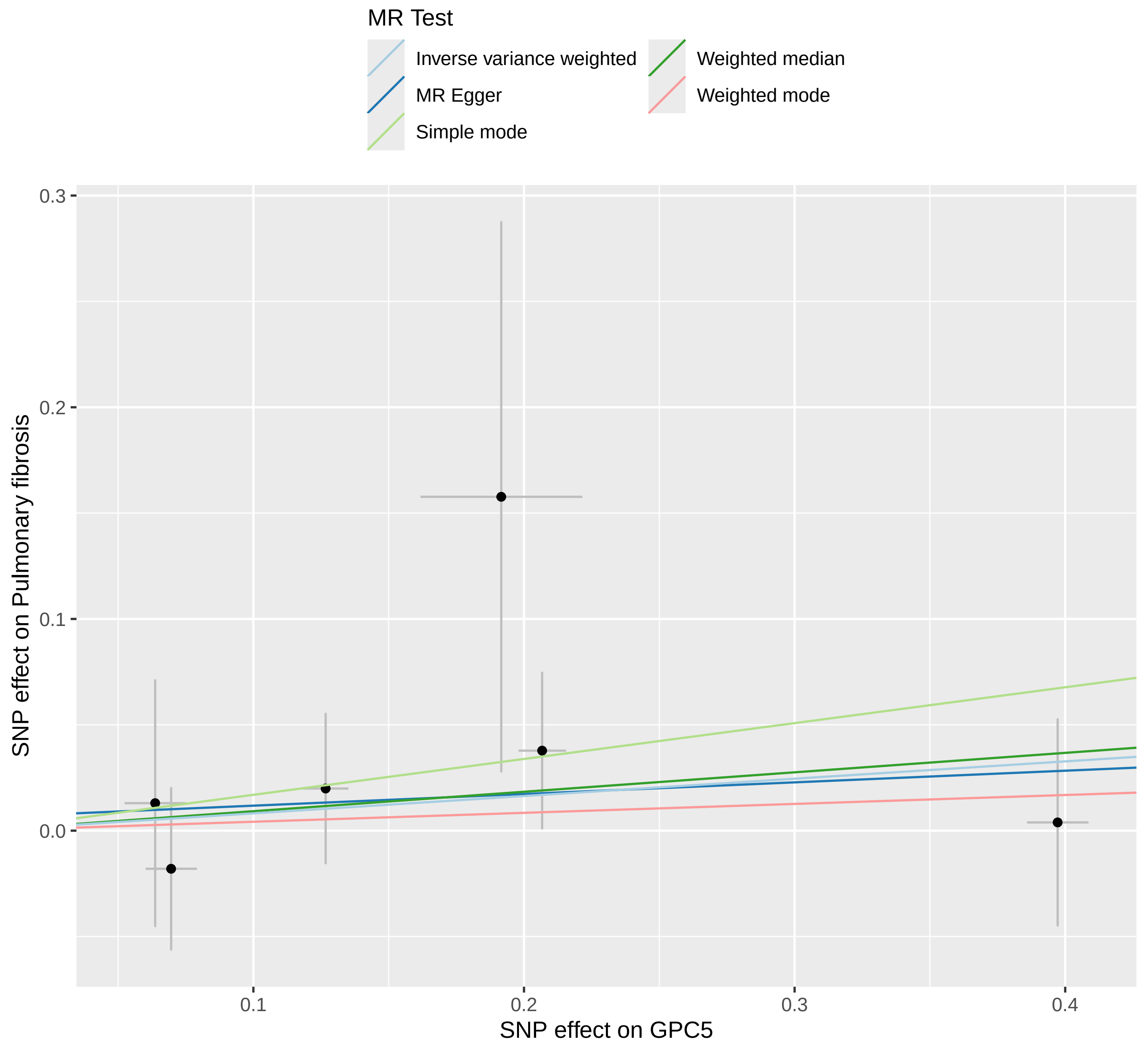

MR Test
Inverse variance weighted
MR Egger
Simple mode
Weighted median
Weighted mode
0.3
0.2
0.1
0.0
0.1
0.2
0.3
0.4
SNP effect on Pulmonary fibrosis
SNP effect on GPC5

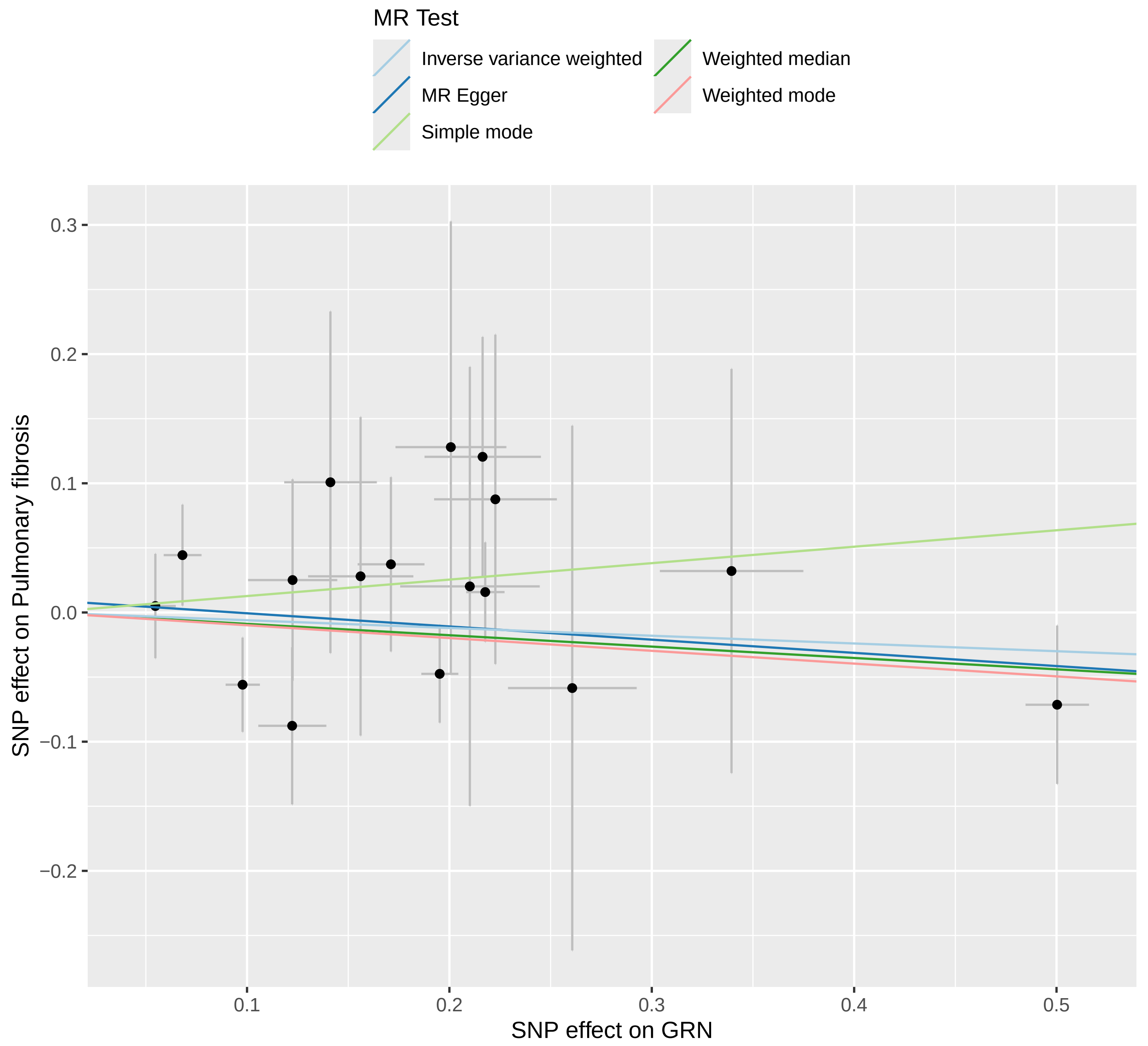

MR Test
Inverse variance weighted
MR Egger
Simple mode
Weighted median
Weighted mode
SNP effect on Pulmonary fibrosis
0.3
0.2
0.1
0.0
−0.1
−0.2
0.1
0.2
0.3
0.4
0.5
SNP effect on GRN

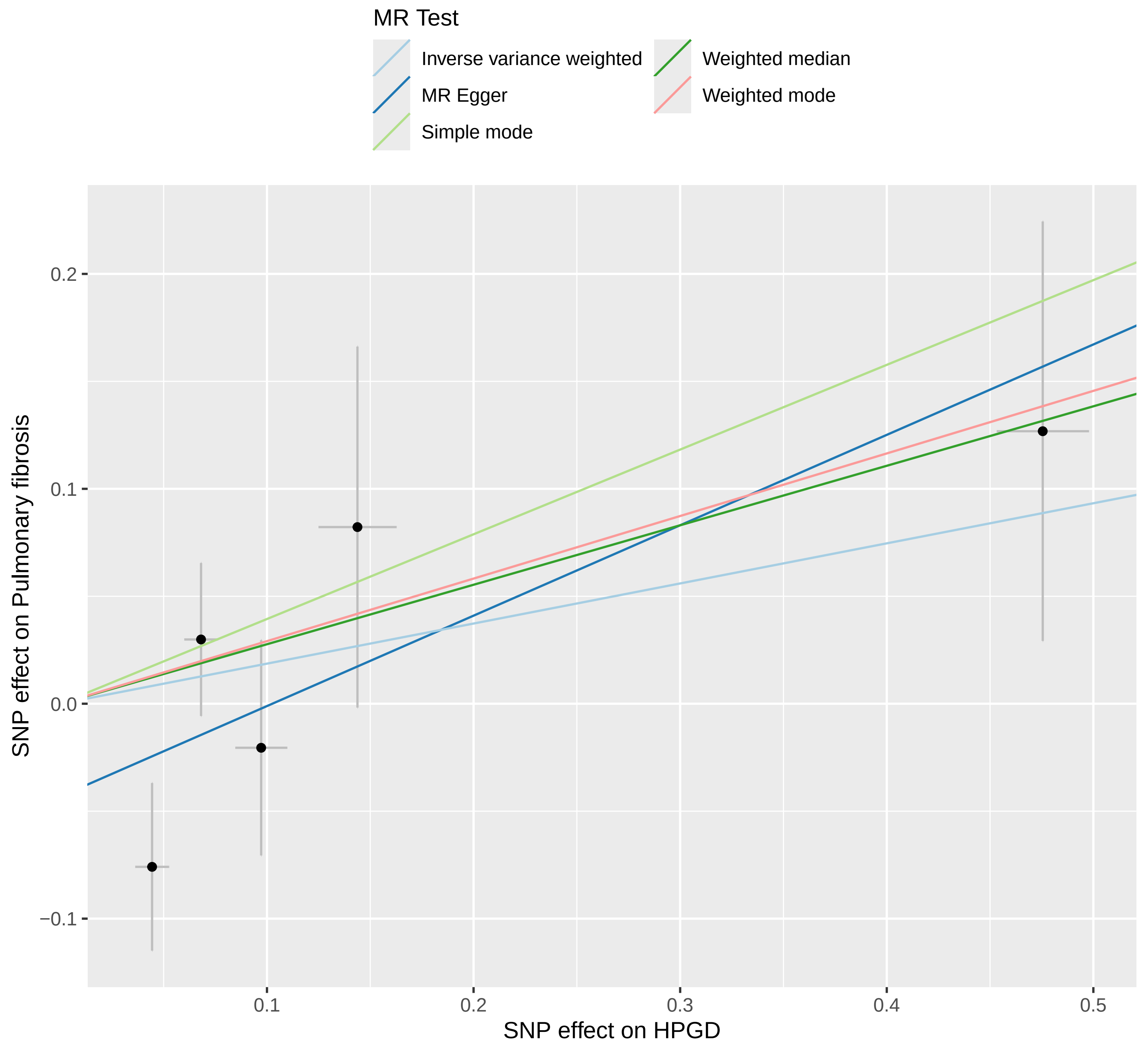
MR Test
Inverse variance weighted
MR Egger
Simple mode
Weighted median
Weighted mode
0.2
0.1
0.0
−0.1
0.1
0.2
0.3
0.4
0.5
SNP effect on HPGD
SNP effect on Pulmonary fibrosis

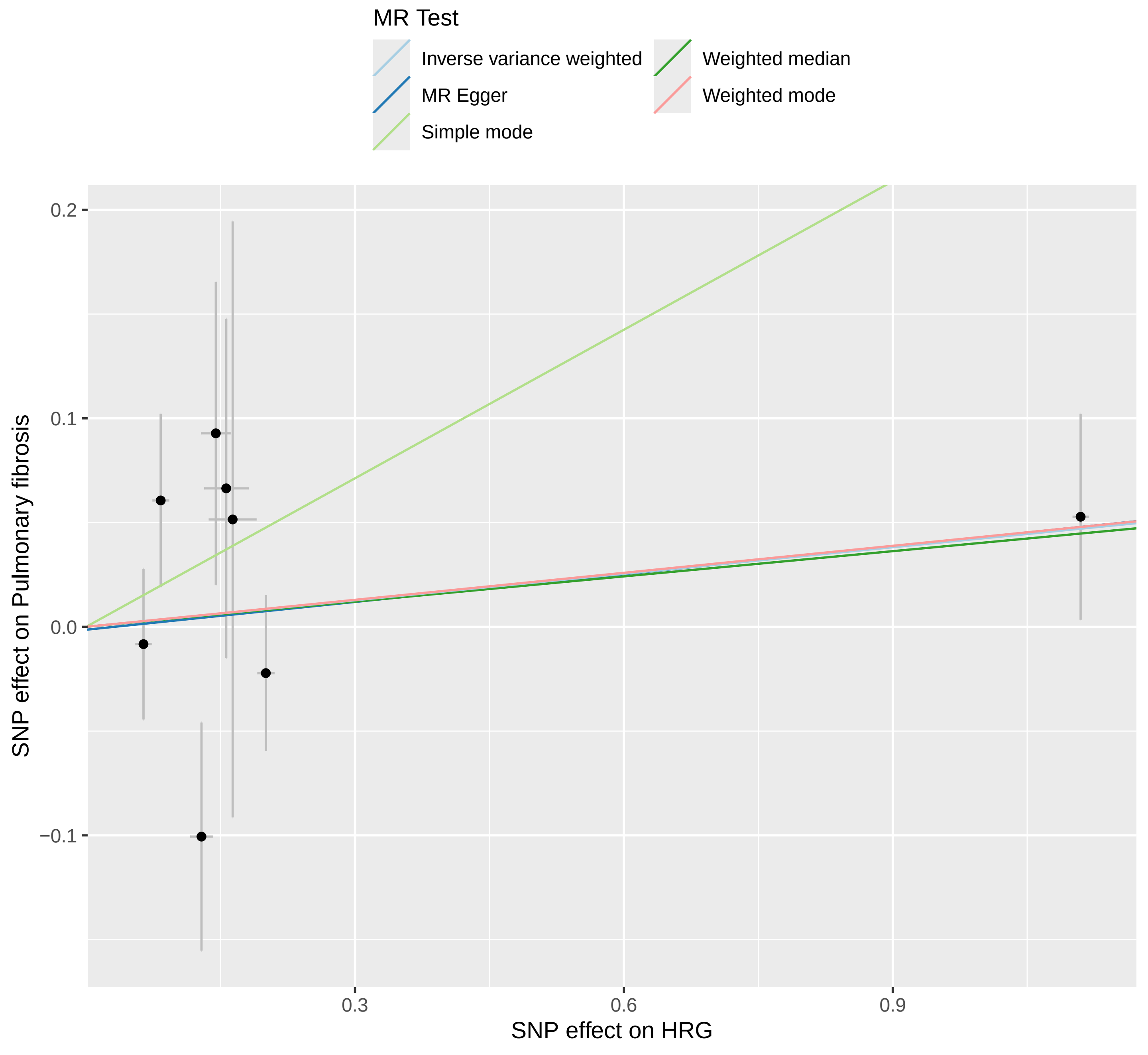

MR Test
Inverse variance weighted
MR Egger
Simple mode
Weighted median
Weighted mode
0.2
0.1
0.0
−0.1
SNP effect on Pulmonary fibrosis
0.3
0.6
0.9
SNP effect on HRG

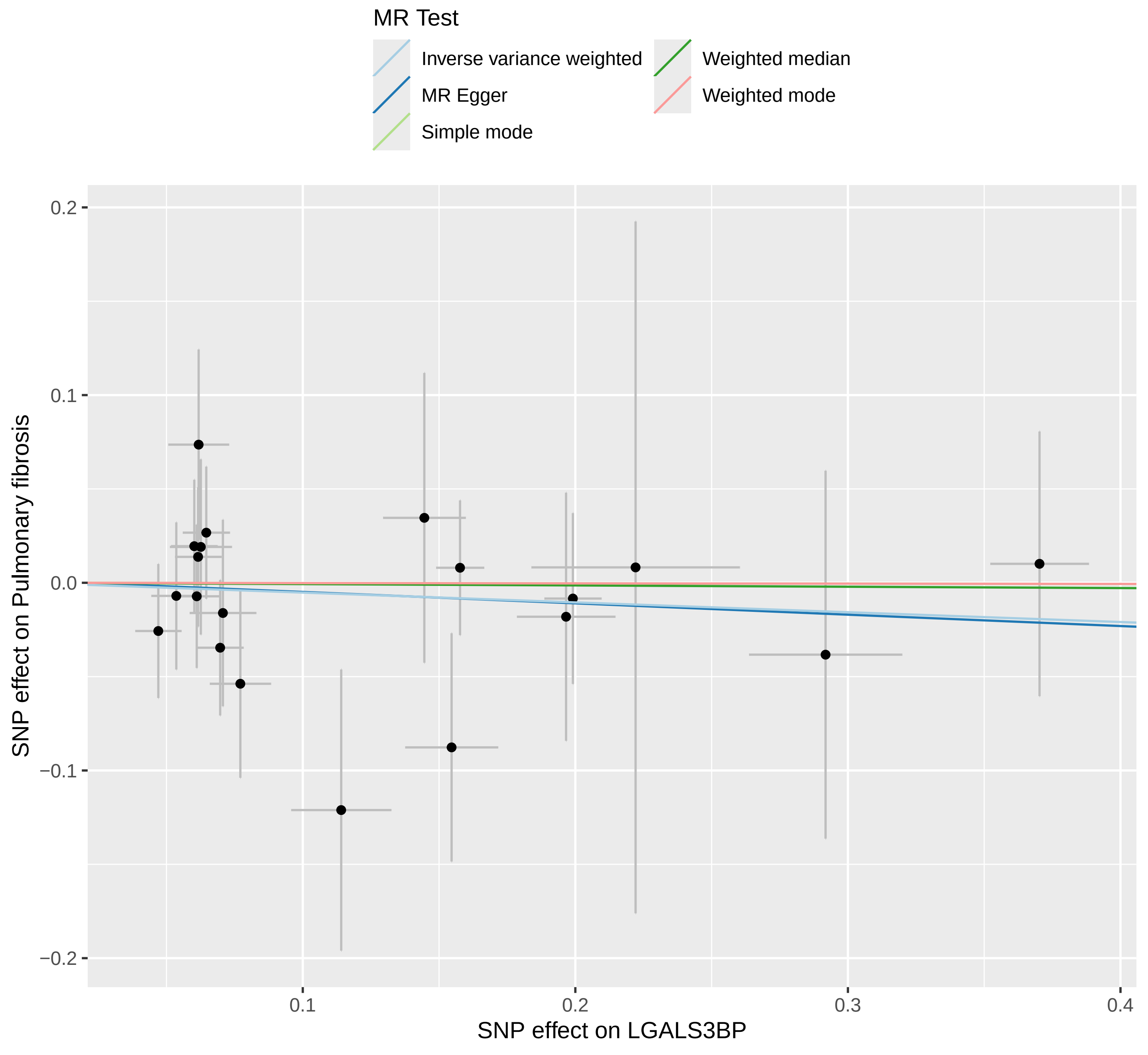

MR Test
Inverse variance weighted
MR Egger
Simple mode
Weighted median
Weighted mode
0.2
0.1
0.0
−0.1
−0.2
SNP effect on Pulmonary fibrosis
0.1
0.2
0.3
0.4
SNP effect on LGALS3BP

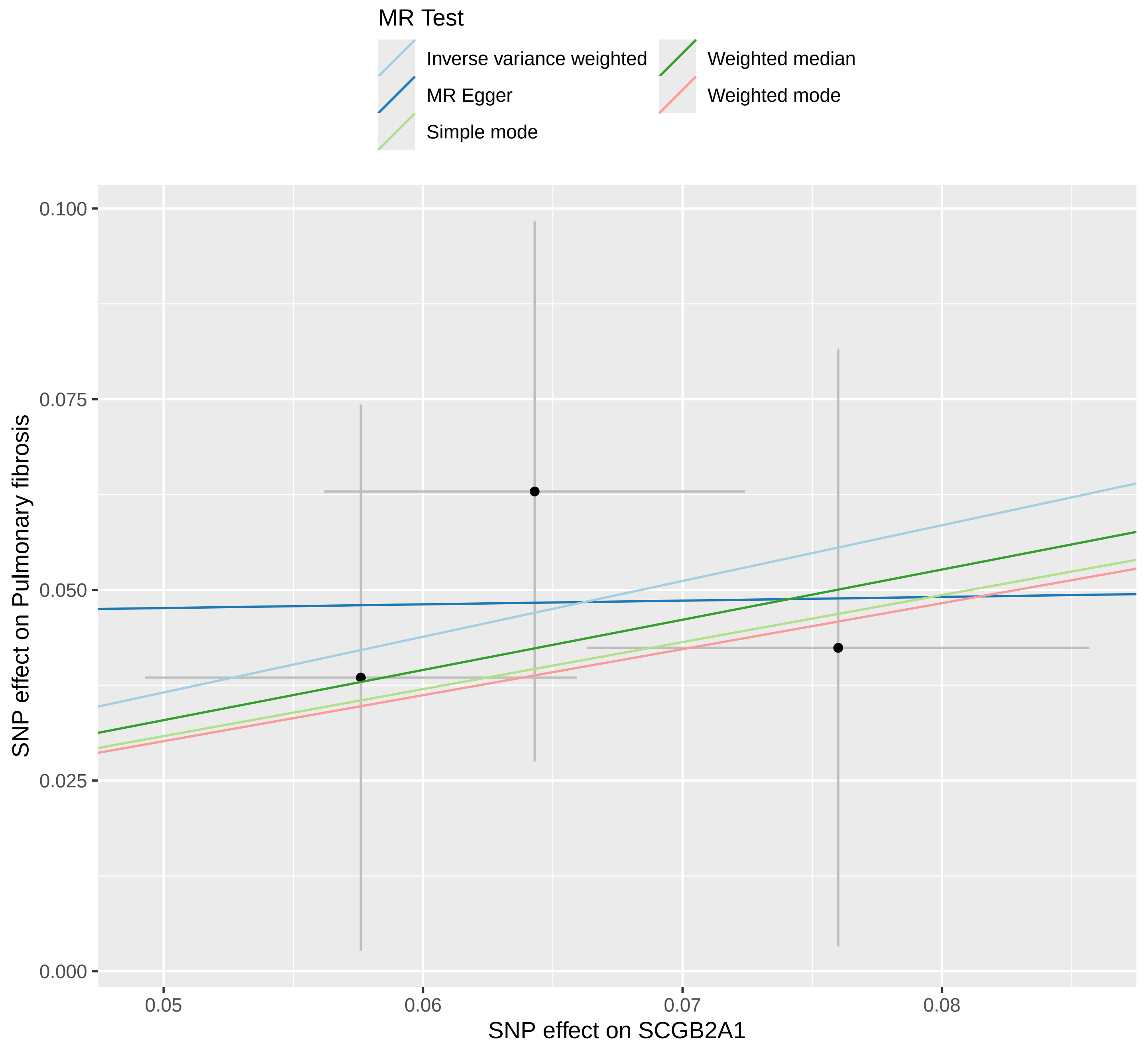
MR Test
Inverse variance weighted
MR Egger
Simple mode
Weighted median
Weighted mode
0.100
0.075
0.050
0.025
0.000
SNP effect on Pulmonary fibrosis
0.05
0.06
0.07
0.08
SNP effect on SCGB2A1

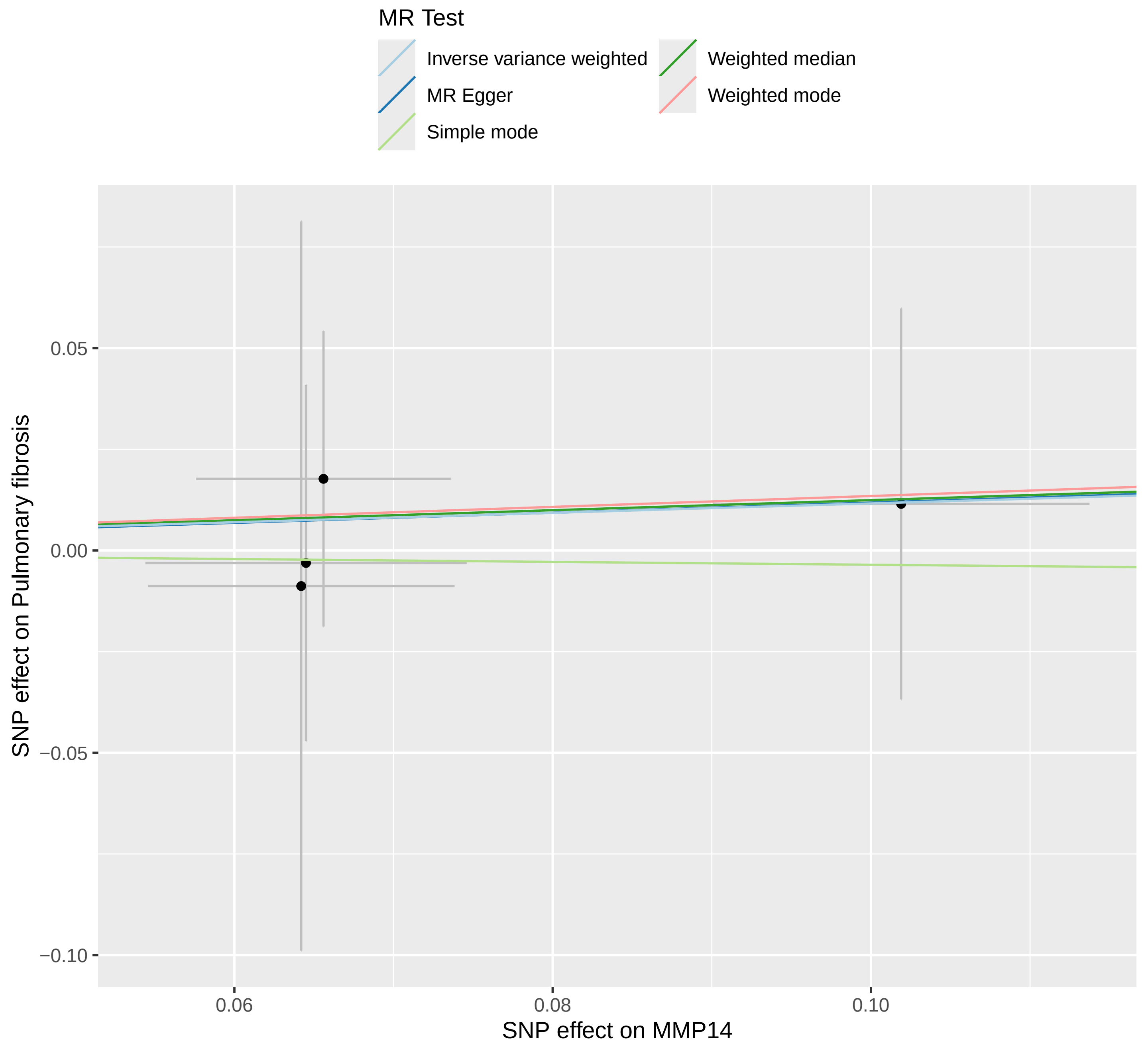

MR Test
Inverse variance weighted
MR Egger
Simple mode
Weighted median
Weighted mode
0.05
0.00
−0.05
−0.10
SNP effect on Pulmonary fibrosis
0.06
0.08
0.10
SNP effect on MMP14

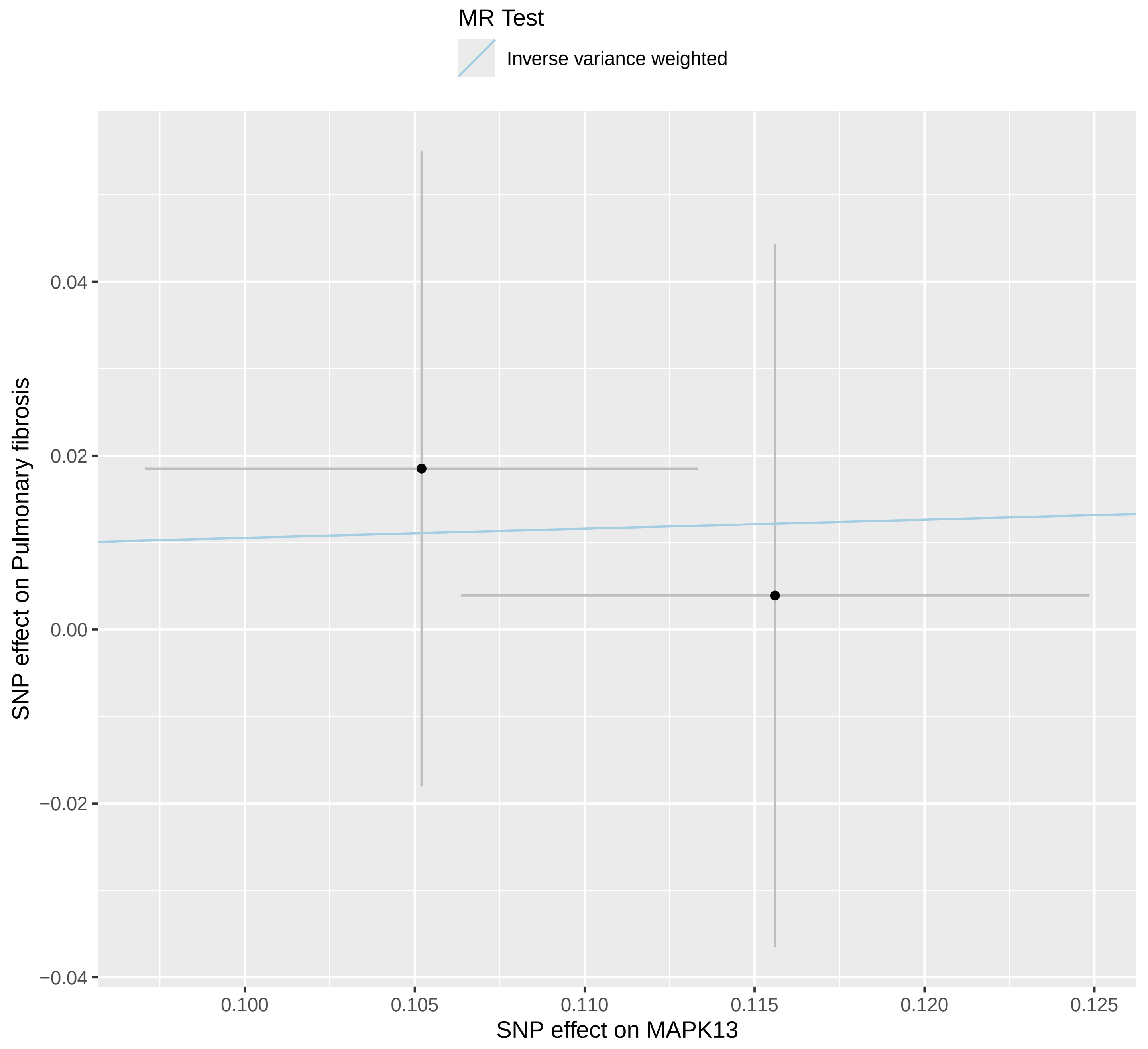
MR Test
Inverse variance weighted
0.04
0.02
0.00
−0.02
−0.04
0.100
0.105
0.110
0.115
0.120
0.125
SNP effect on MAPK13
SNP effect on Pulmonary fibrosis

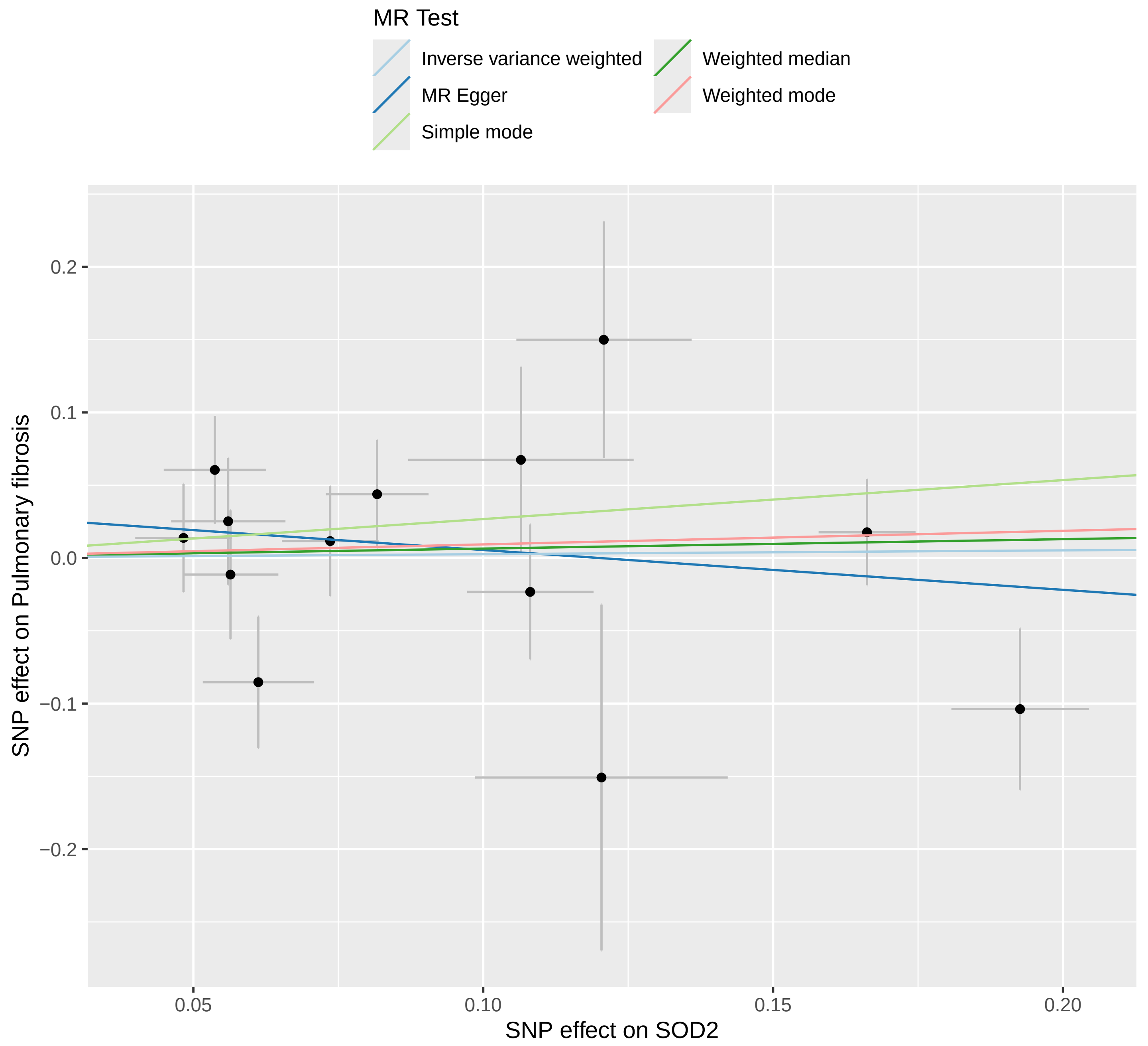

MR Test
Inverse variance weighted
MR Egger
Simple mode
Weighted median
Weighted mode
SNP effect on Pulmonary fibrosis
0.2
0.1
0.0
−0.1
−0.2
0.05
0.10
0.15
0.20
SNP effect on SOD2

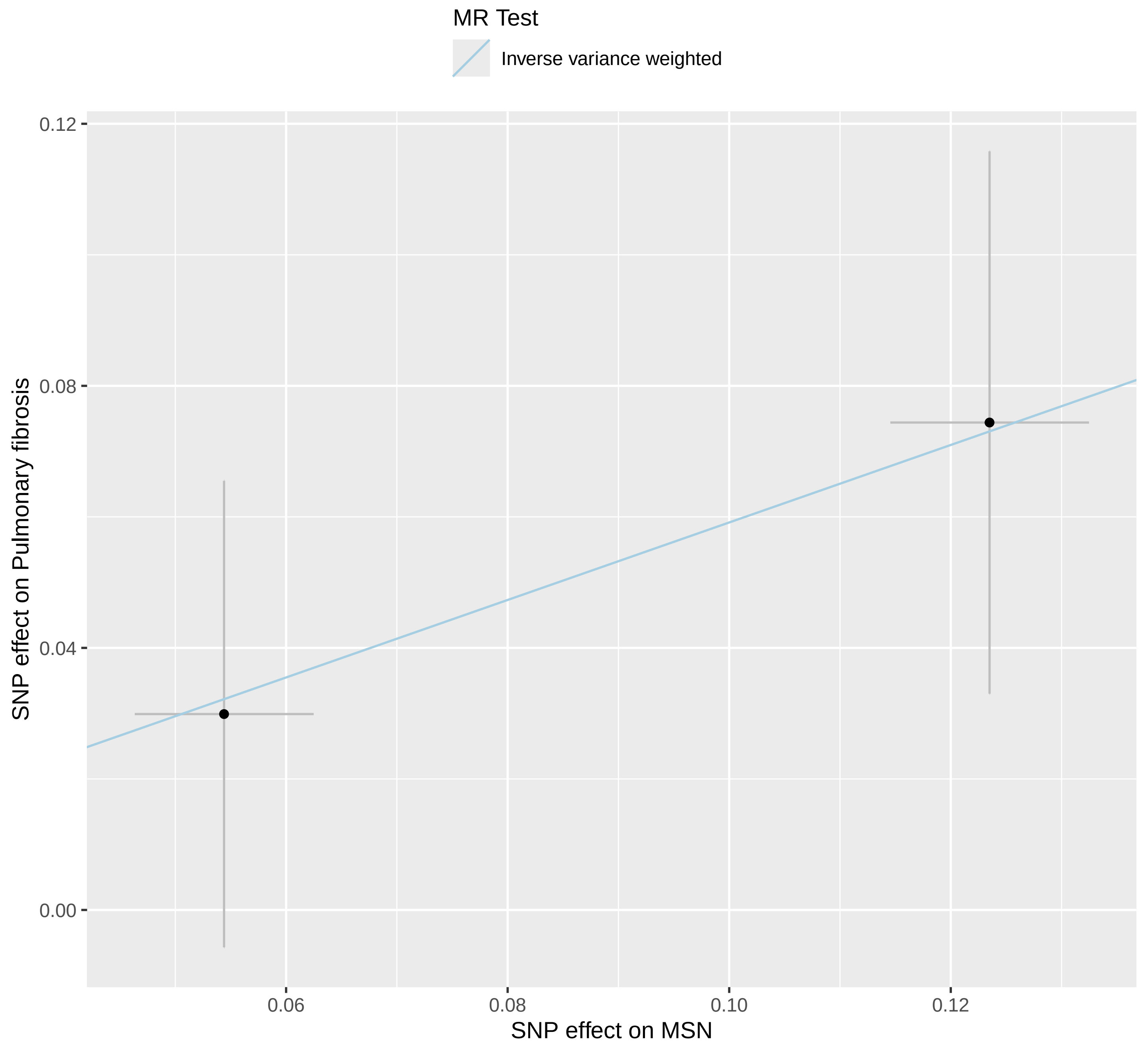

MR Test
Inverse variance weighted
0.12
0.08
0.04
0.00
SNP effect on Pulmonary fibrosis
0.06
0.08
0.10
0.12
SNP effect on MSN

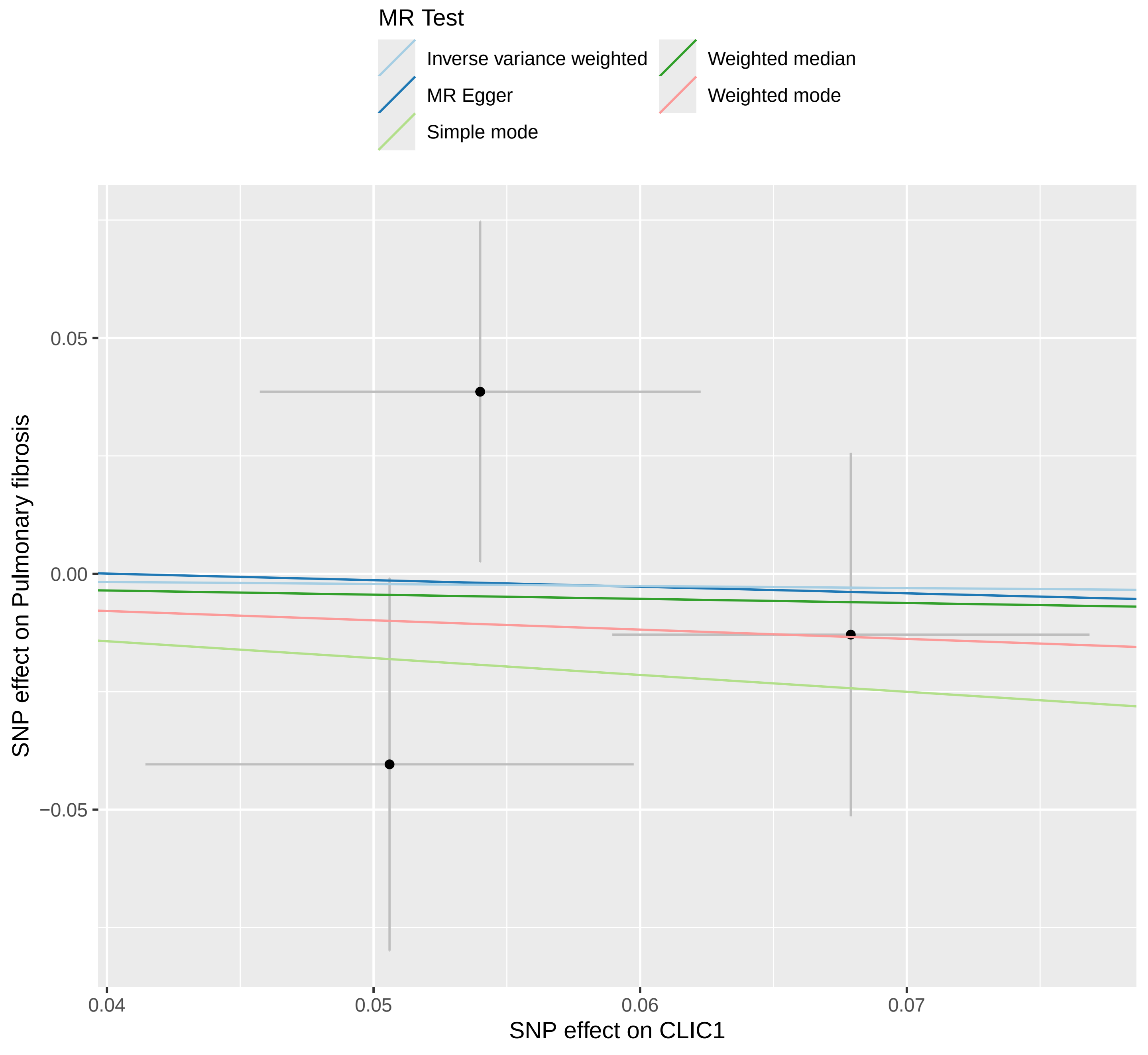
MR Test
Inverse variance weighted
MR Egger
Simple mode
Weighted median
Weighted mode
0.05
0.00
−0.05
0.04
0.05
0.06
0.07
SNP effect on Pulmonary fibrosis
SNP effect on CLIC1

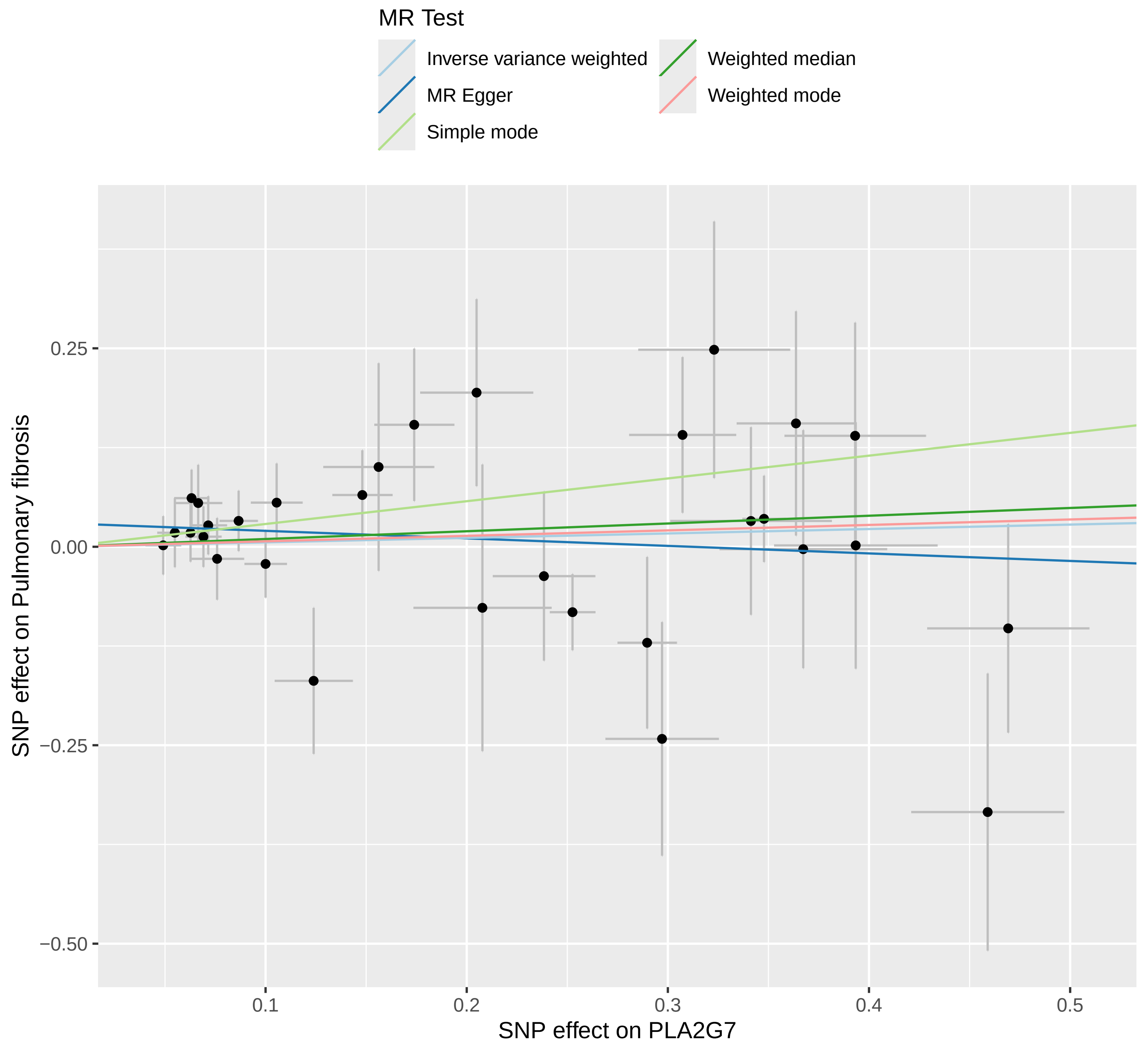

MR Test
Inverse variance weighted
MR Egger
Simple mode
Weighted median
Weighted mode
SNP effect on Pulmonary fibrosis
0.25
0.00
−0.25
−0.50
0.1
0.2
0.3
0.4
0.5
SNP effect on PLA2G7

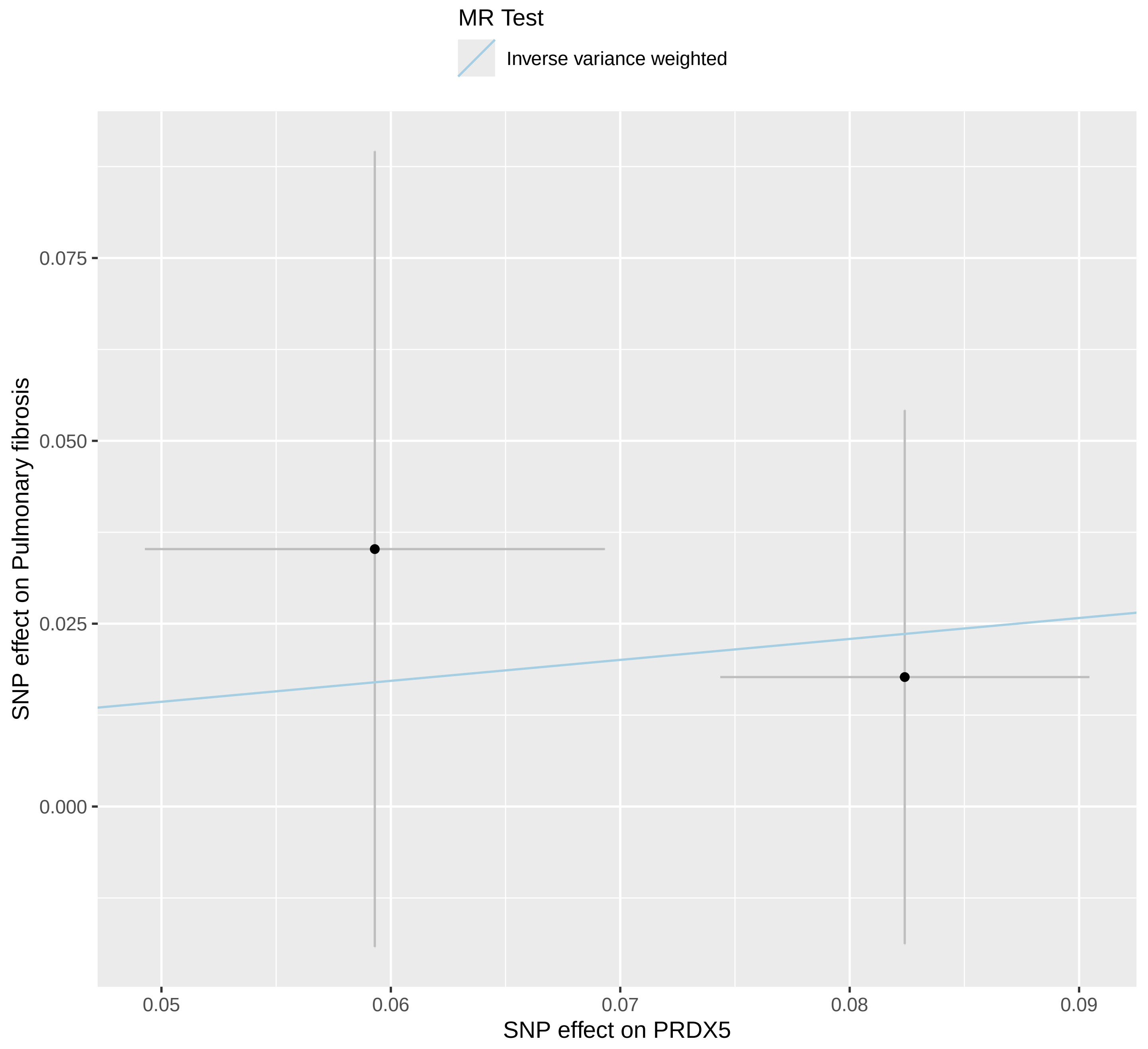

MR Test
Inverse variance weighted
SNP effect on Pulmonary fibrosis
0.075
0.050
0.025
0.000
0.05
0.06
0.07
0.08
0.09
SNP effect on PRDX5

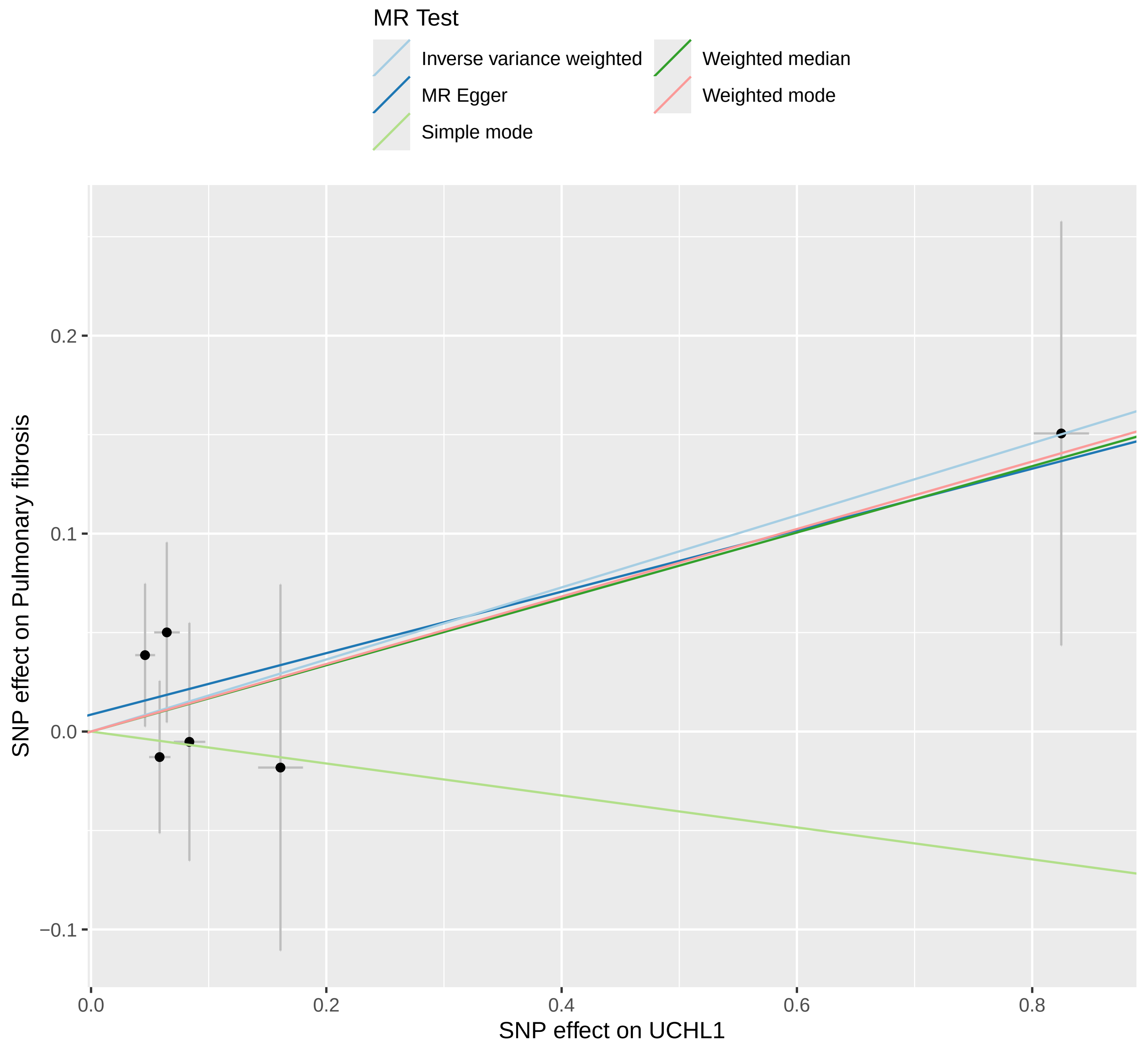

MR Test
Inverse variance weighted
MR Egger
Simple mode
Weighted median
Weighted mode
0.2
0.1
0.0
−0.1
0.0
0.2
0.4
0.6
0.8
SNP effect on Pulmonary fibrosis
SNP effect on UCHL1

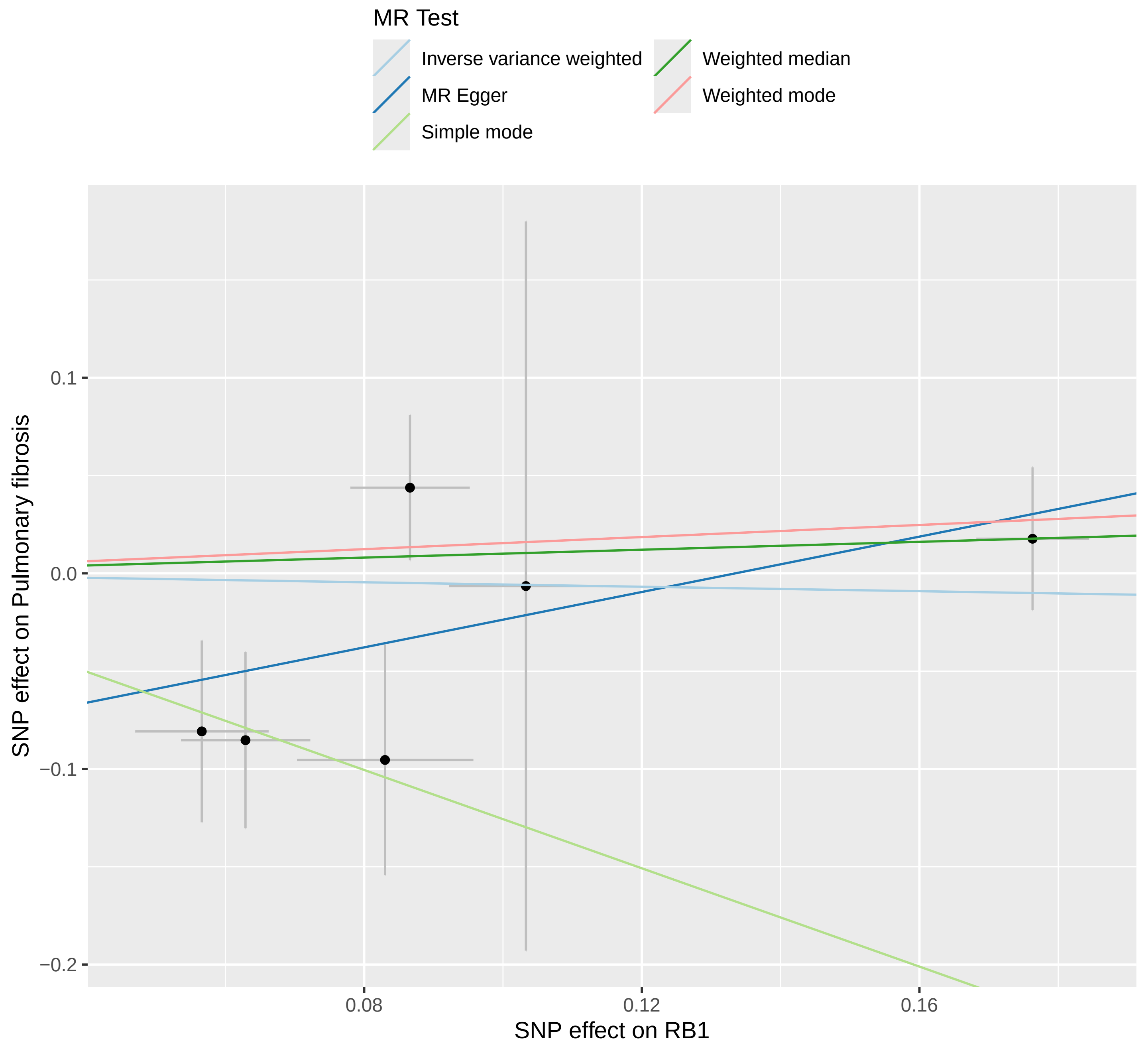

MR Test
Inverse variance weighted
MR Egger
Simple mode
Weighted median
Weighted mode
0.1
0.0
−0.1
−0.2
SNP effect on Pulmonary fibrosis
0.08
0.12
0.16
SNP effect on RB1

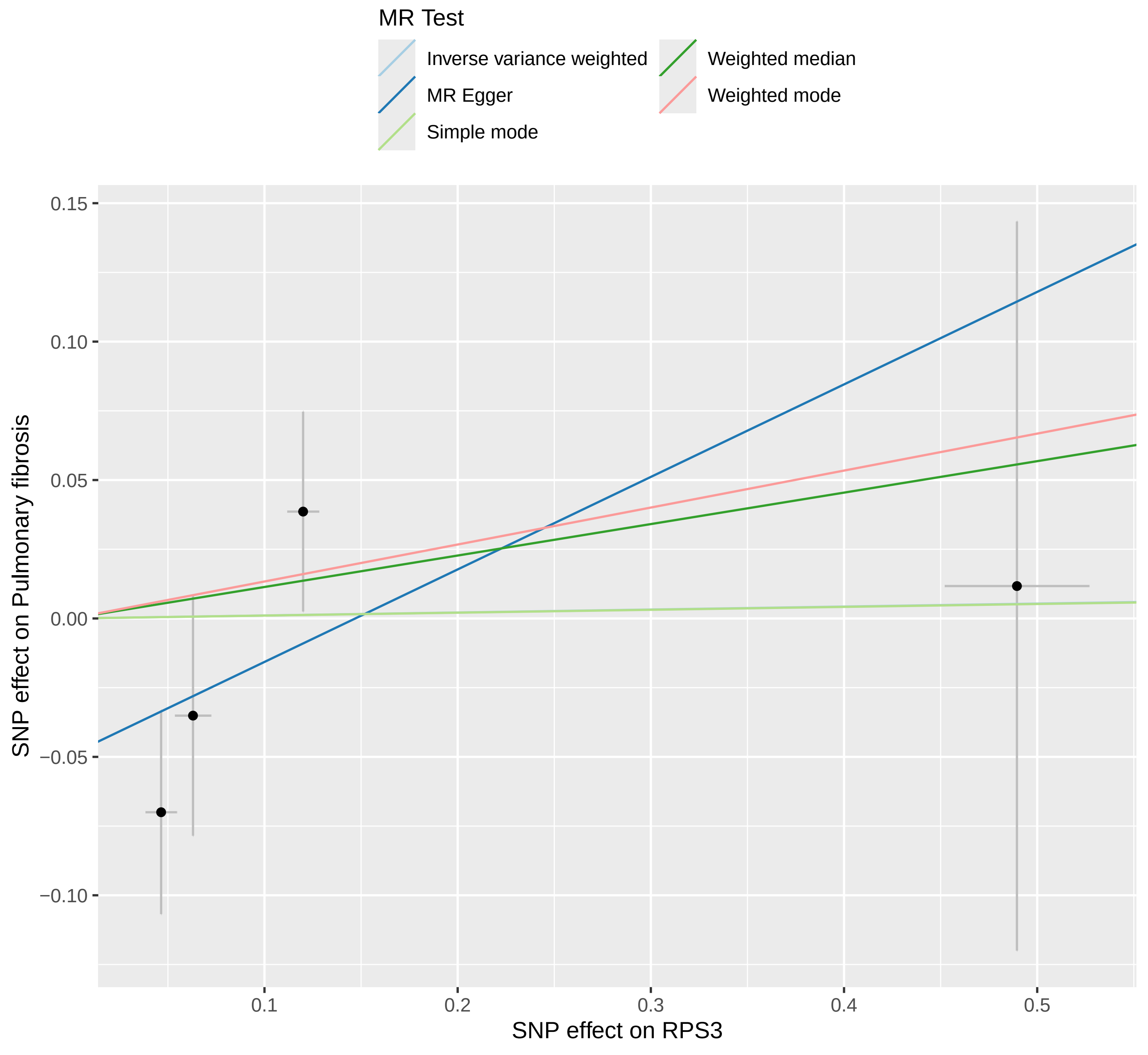

MR Test
Inverse variance weighted
MR Egger
Simple mode
Weighted median
Weighted mode
0.15
0.10
0.05
0.00
−0.05
−0.10
SNP effect on Pulmonary fibrosis
0.1
0.2
0.3
0.4
0.5
SNP effect on RPS3

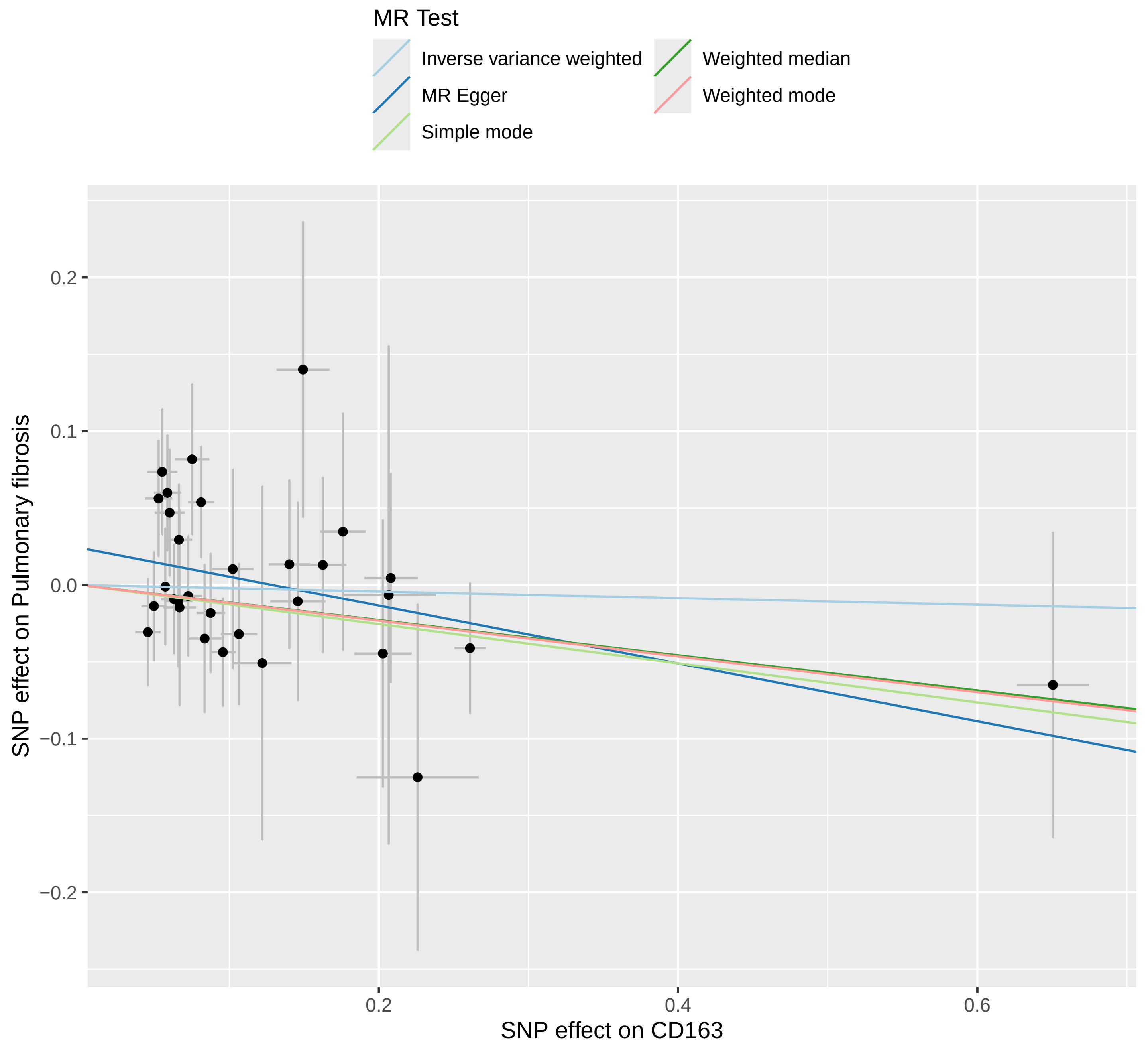
MR Test
Inverse variance weighted
MR Egger
Simple mode
Weighted median
Weighted mode
0.2
0.1
0.0
−0.1
−0.2
SNP effect on Pulmonary fibrosis
0.2
0.4
0.6
SNP effect on CD163

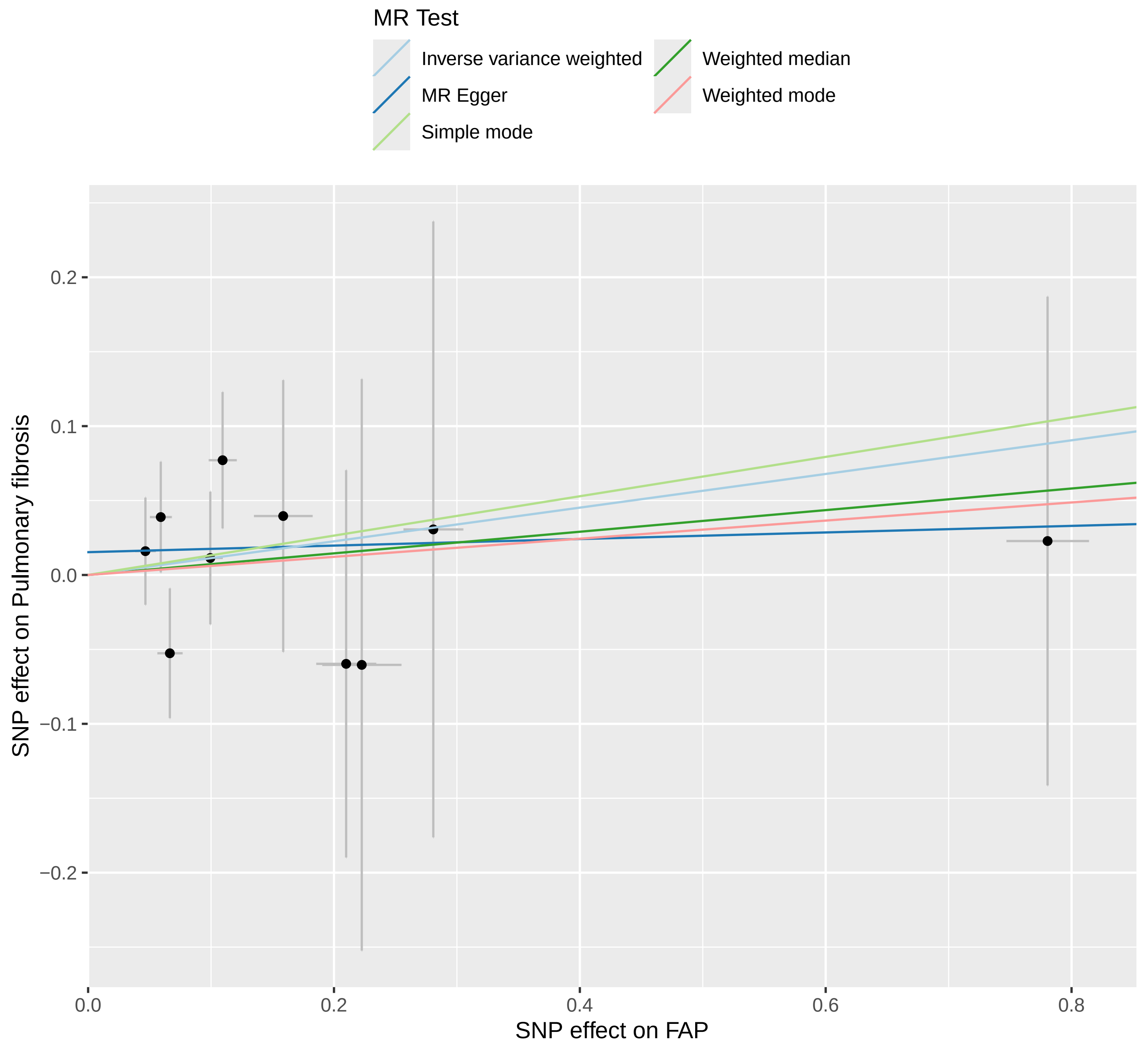

MR Test
Inverse variance weighted
MR Egger
Simple mode
Weighted median
Weighted mode
SNP effect on Pulmonary fibrosis
0.2
0.1
0.0
−0.1
−0.2
0.0
0.2
0.4
0.6
0.8
SNP effect on FAP

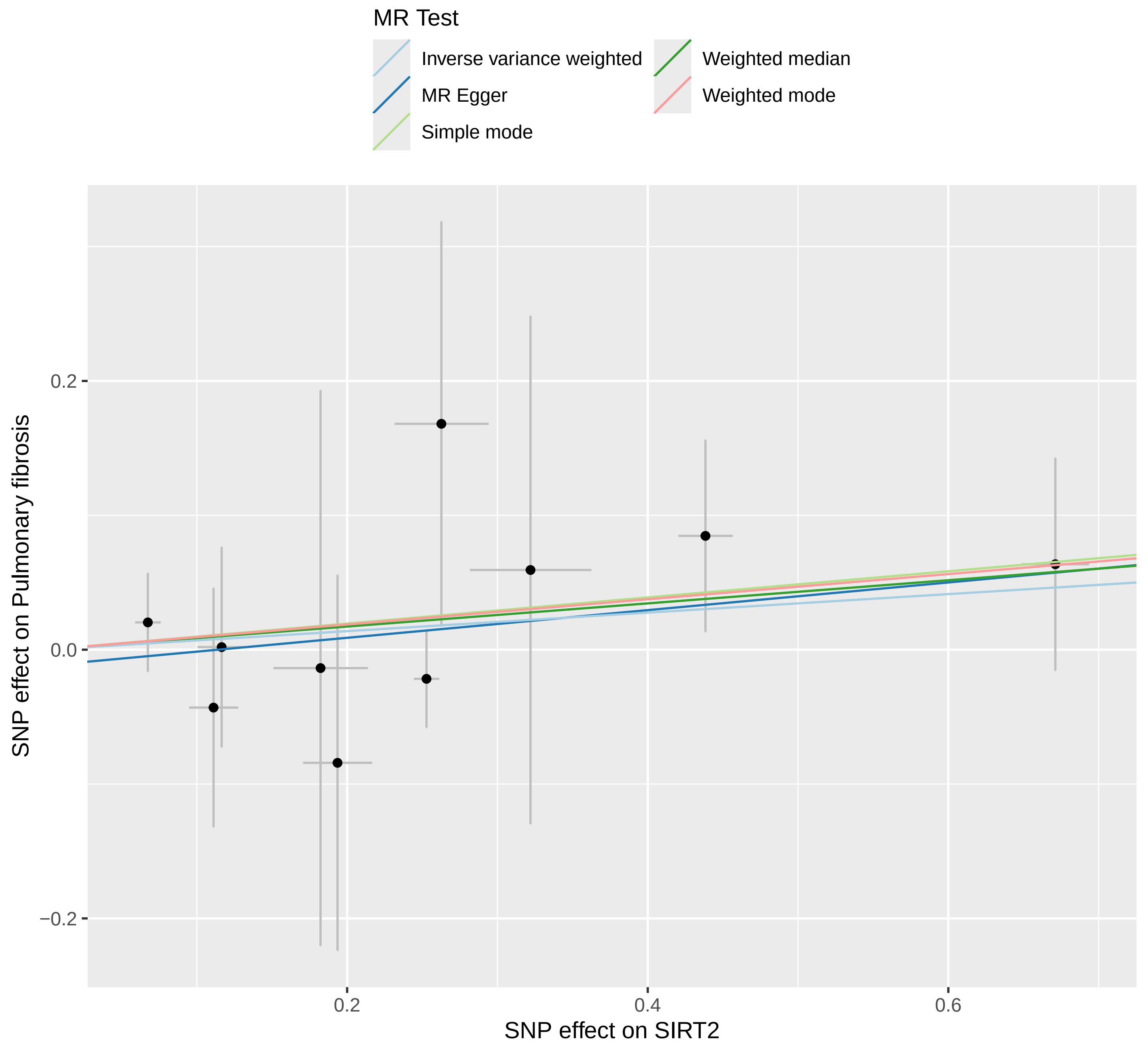
MR Test
Inverse variance weighted
MR Egger
Simple mode
Weighted median
Weighted mode
SNP effect on Pulmonary fibrosis
0.2
0.0
−0.2
0.2
0.4
0.6
SNP effect on SIRT2

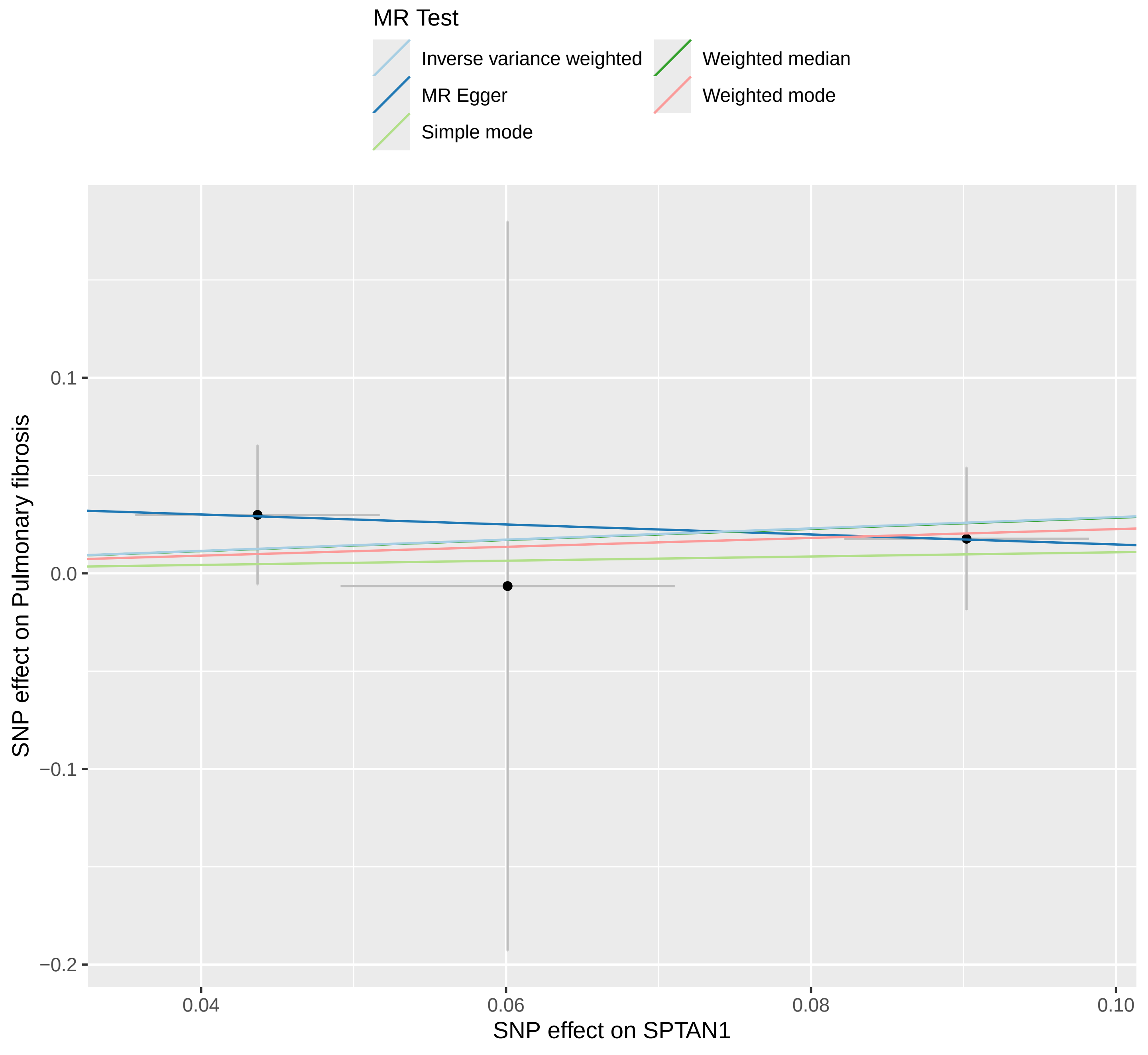

MR Test
Inverse variance weighted
MR Egger
Simple mode
Weighted median
Weighted mode
0.1
0.0
−0.1
−0.2
SNP effect on Pulmonary fibrosis
0.04
0.06
0.08
0.10
SNP effect on SPTAN1

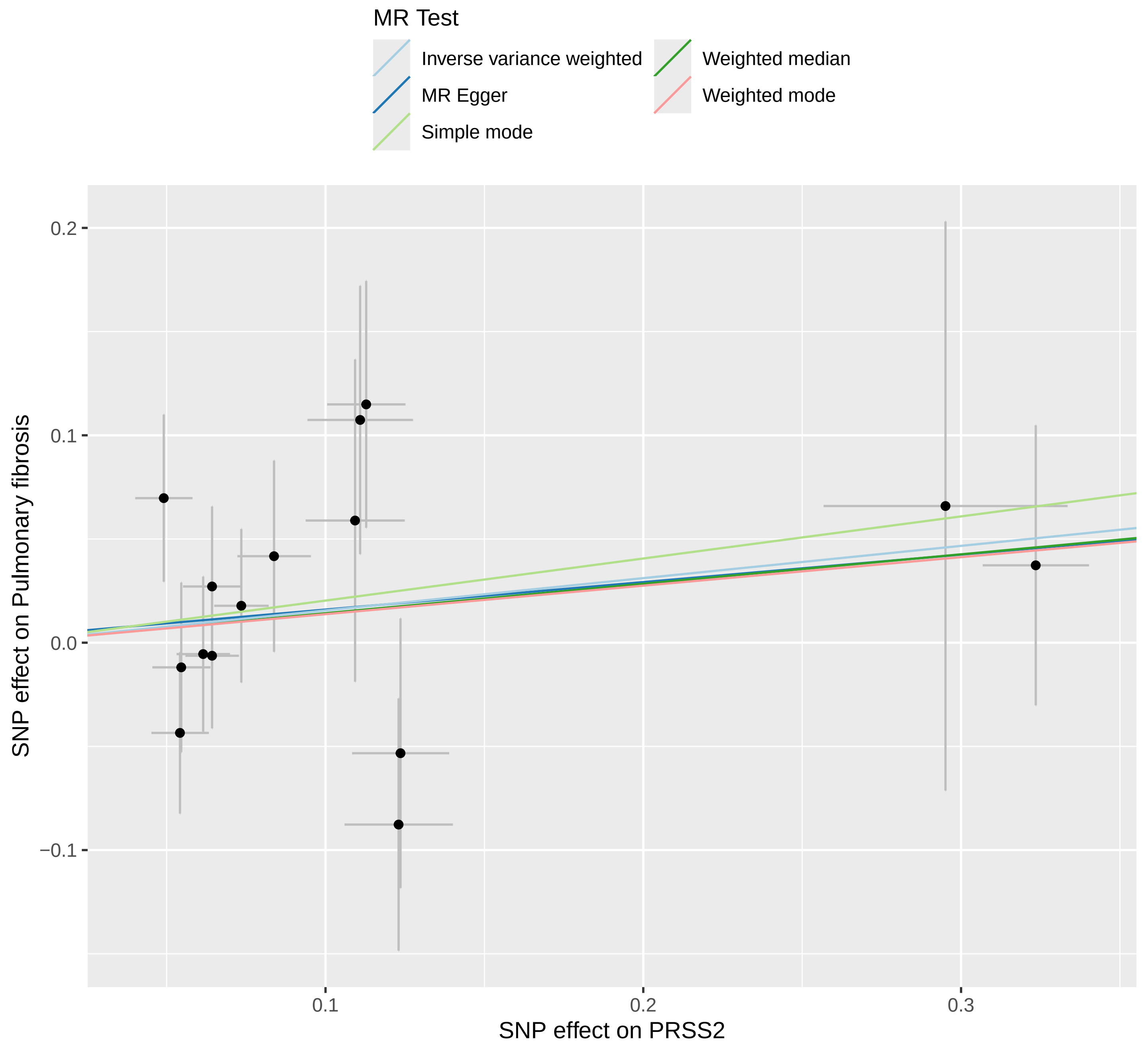

MR Test
Inverse variance weighted
MR Egger
Simple mode
Weighted median
Weighted mode
0.2
0.1
0.0
−0.1
SNP effect on Pulmonary fibrosis
0.1
0.2
0.3
SNP effect on PRSS2

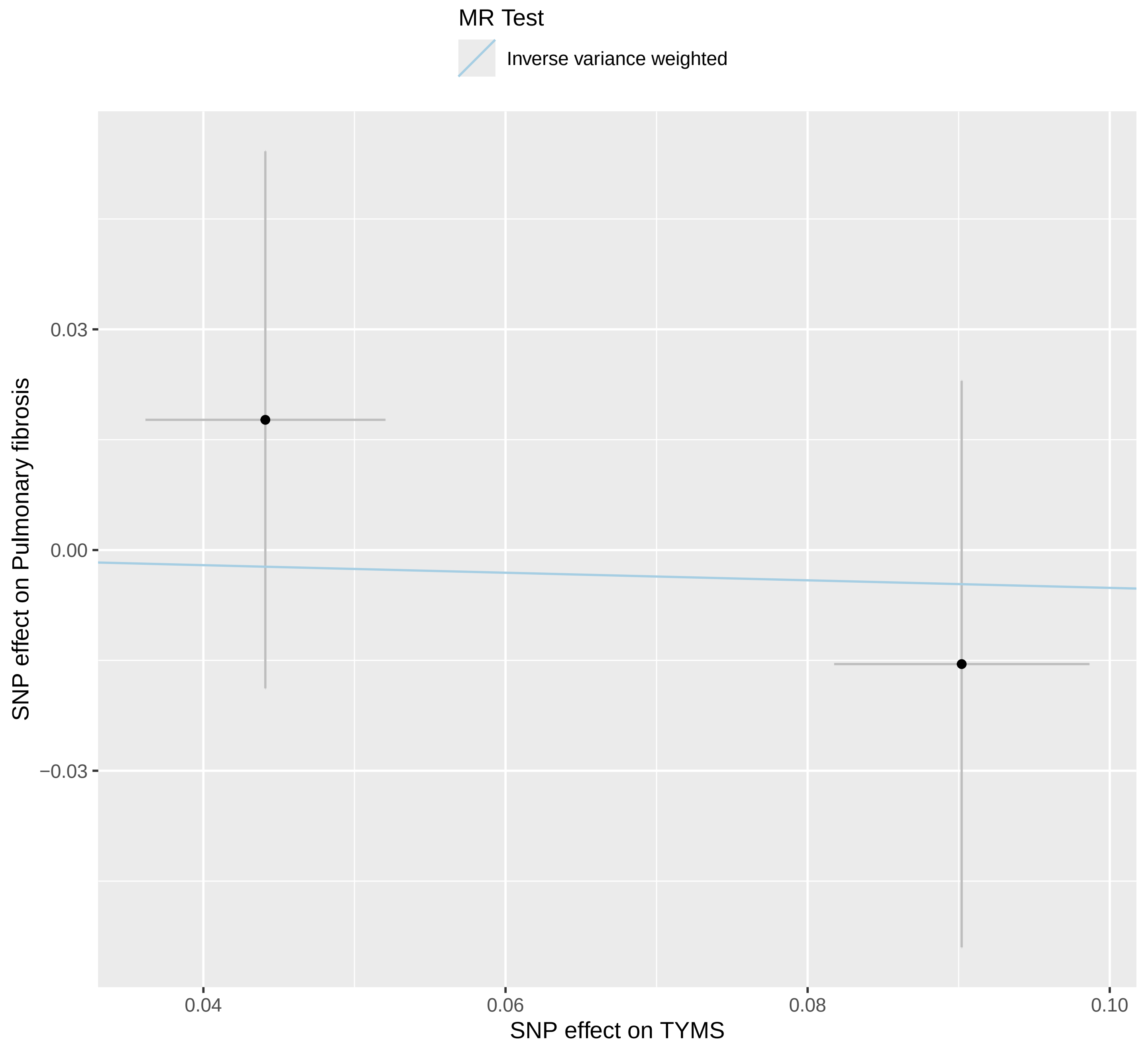
MR Test
Inverse variance weighted
0.03
0.00
−0.03
SNP effect on Pulmonary fibrosis
0.04
0.06
0.08
0.10
SNP effect on TYMS

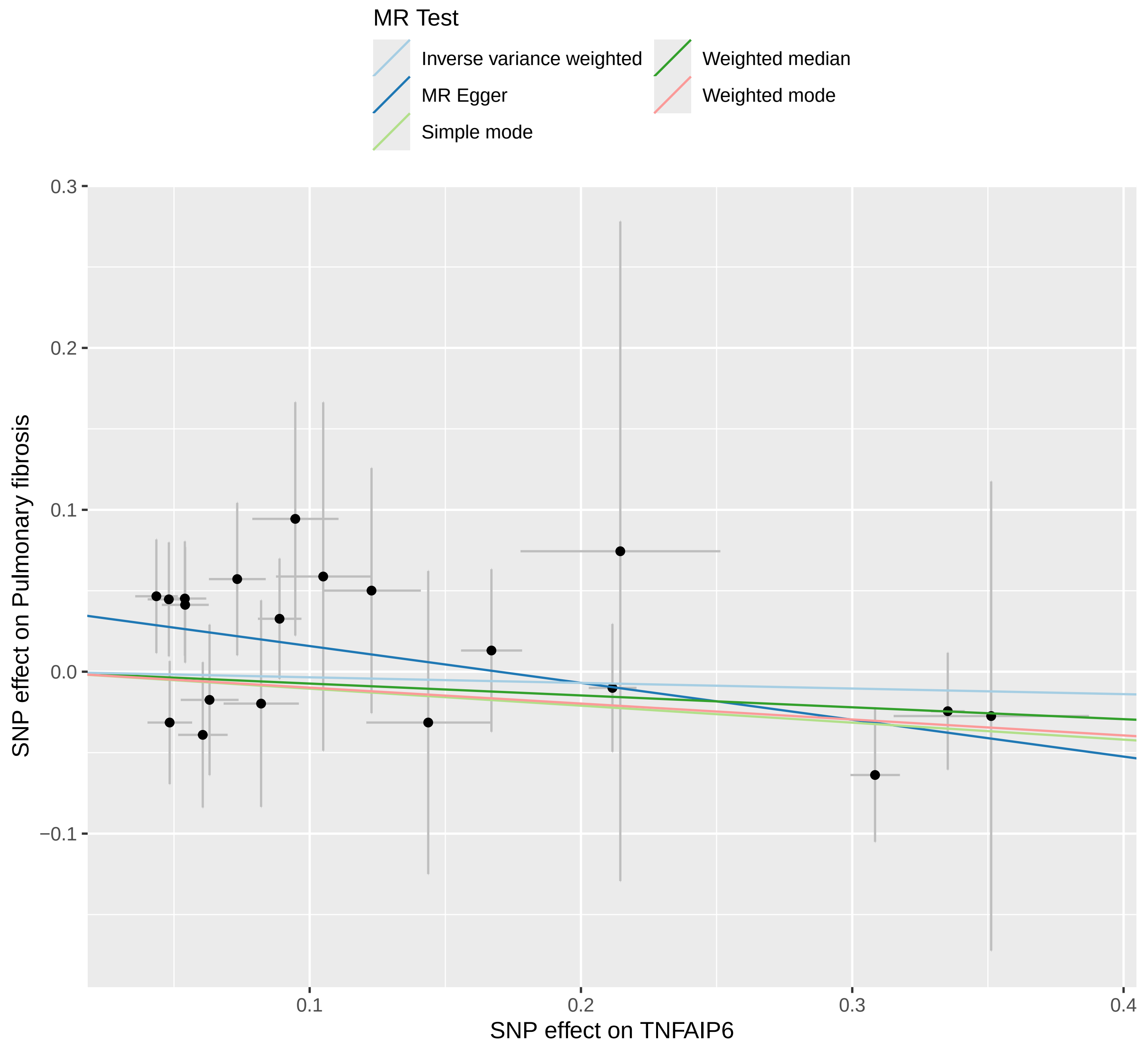

MR Test
Inverse variance weighted
MR Egger
Simple mode
Weighted median
Weighted mode
0.3
0.2
0.1
0.0
−0.1
0.1
0.2
0.3
0.4
SNP effect on TNFAIP6
SNP effect on Pulmonary fibrosis

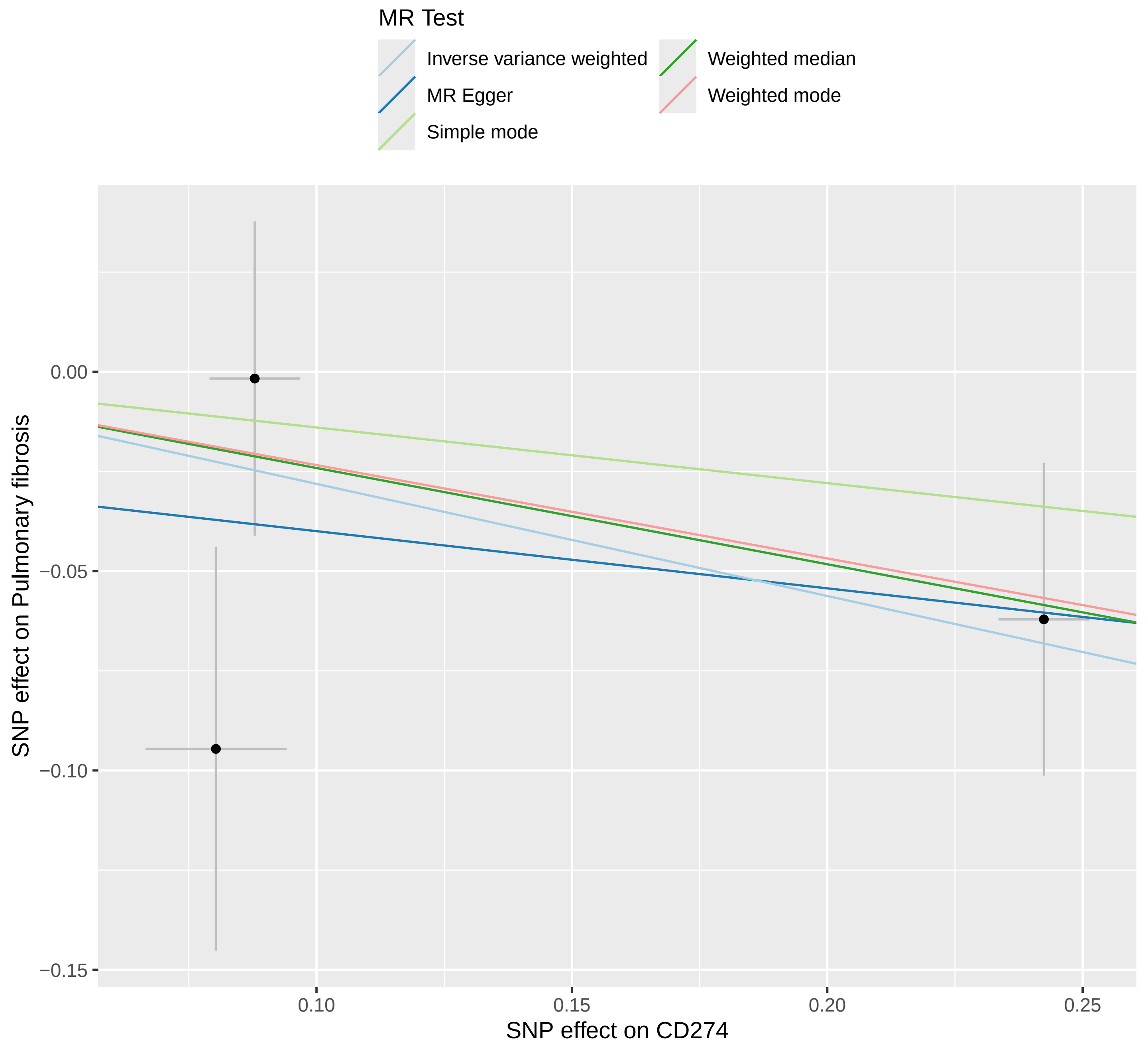

MR Test
Inverse variance weighted
MR Egger
Simple mode
Weighted median
Weighted mode
0.00
−0.05
−0.10
−0.15
0.10
0.15
0.20
0.25
SNP effect on Pulmonary fibrosis
SNP effect on CD274

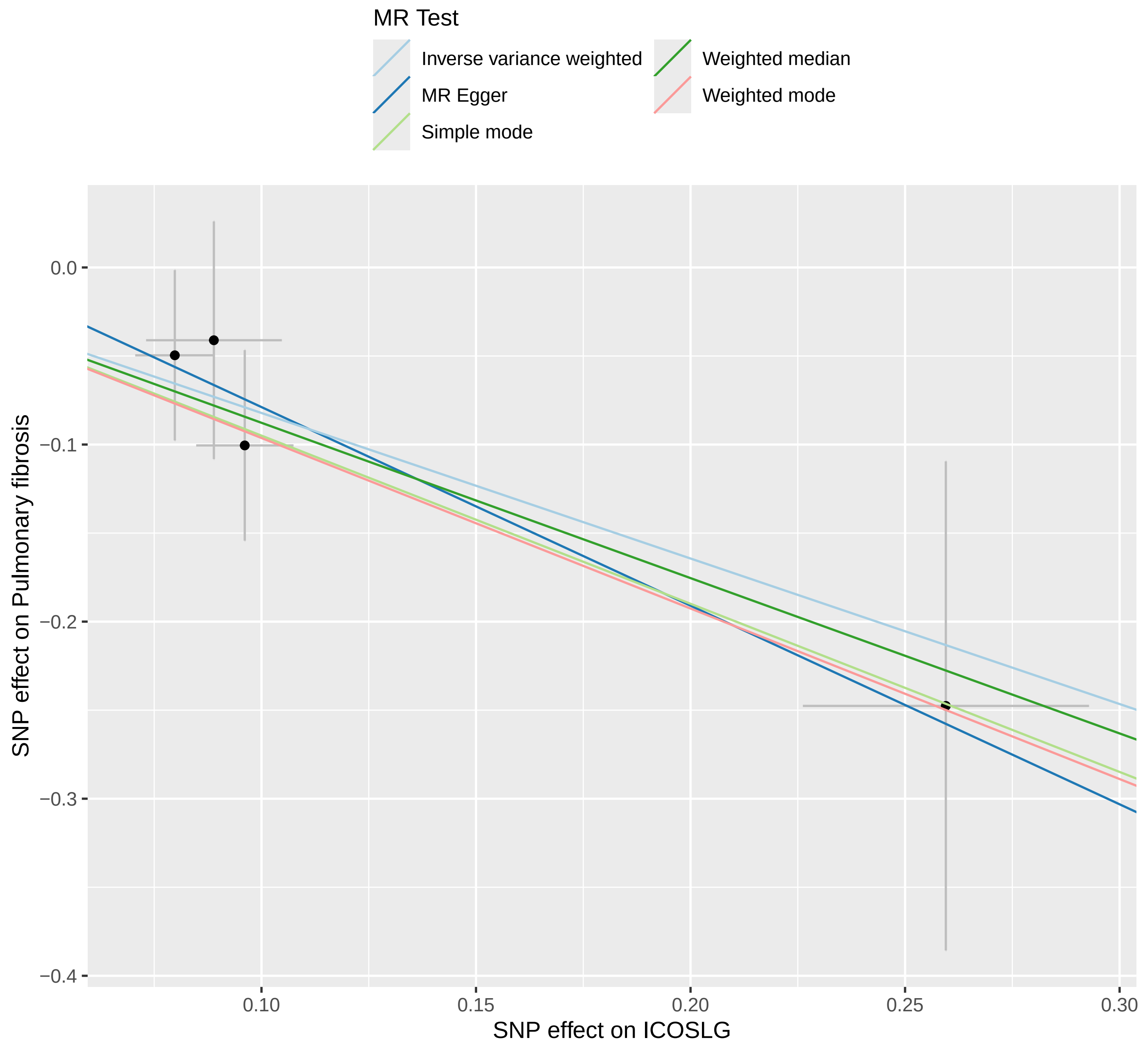

MR Test
Inverse variance weighted
MR Egger
Simple mode
Weighted median
Weighted mode
0.0
−0.1
−0.2
−0.3
−0.4
0.10
0.15
0.20
0.25
0.30
SNP effect on Pulmonary fibrosis
SNP effect on ICOSLG

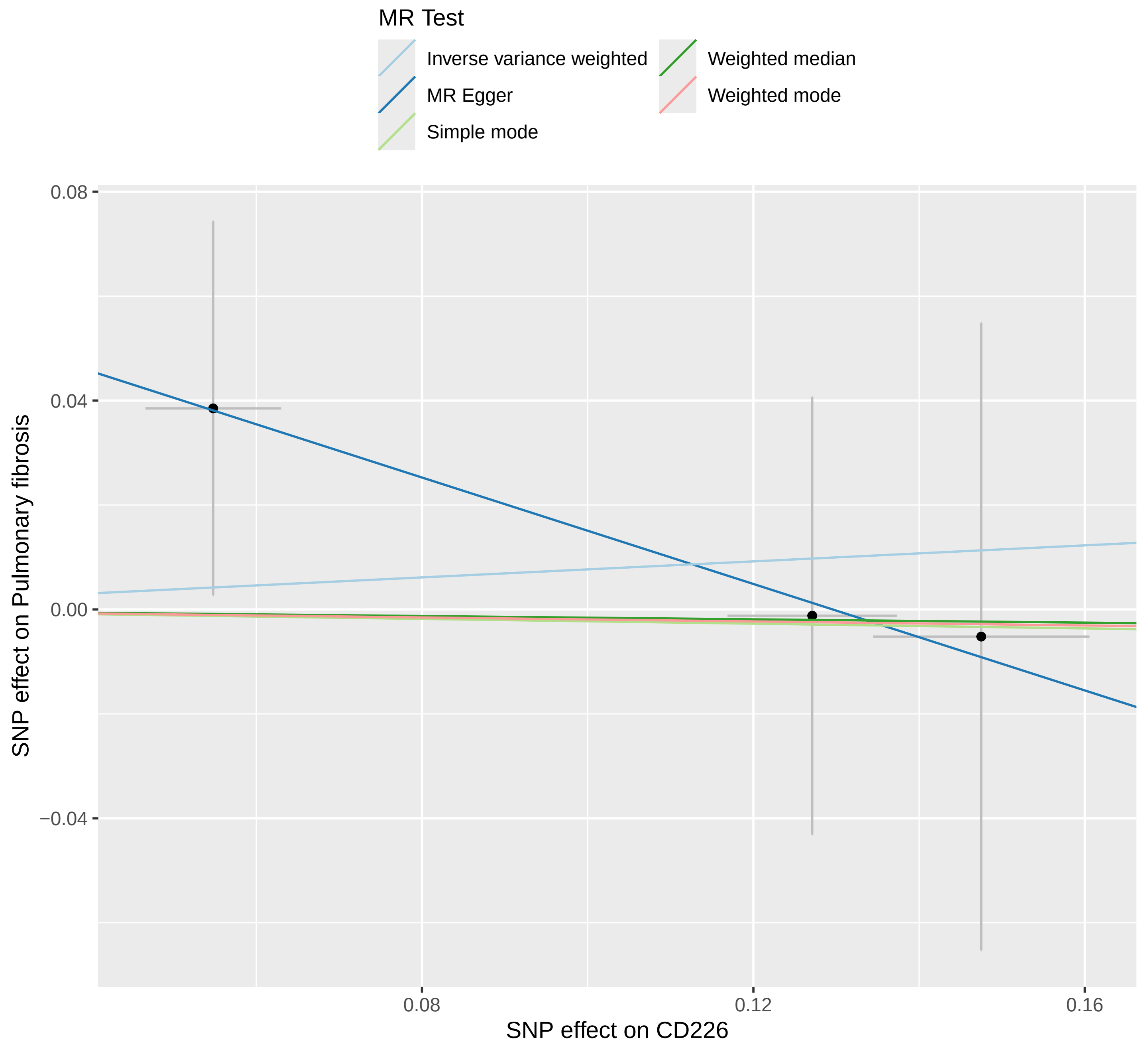
MR Test
Inverse variance weighted
MR Egger
Simple mode
Weighted median
Weighted mode
0.08
0.04
0.00
−0.04
0.08
0.12
0.16
SNP effect on Pulmonary fibrosis
SNP effect on CD226

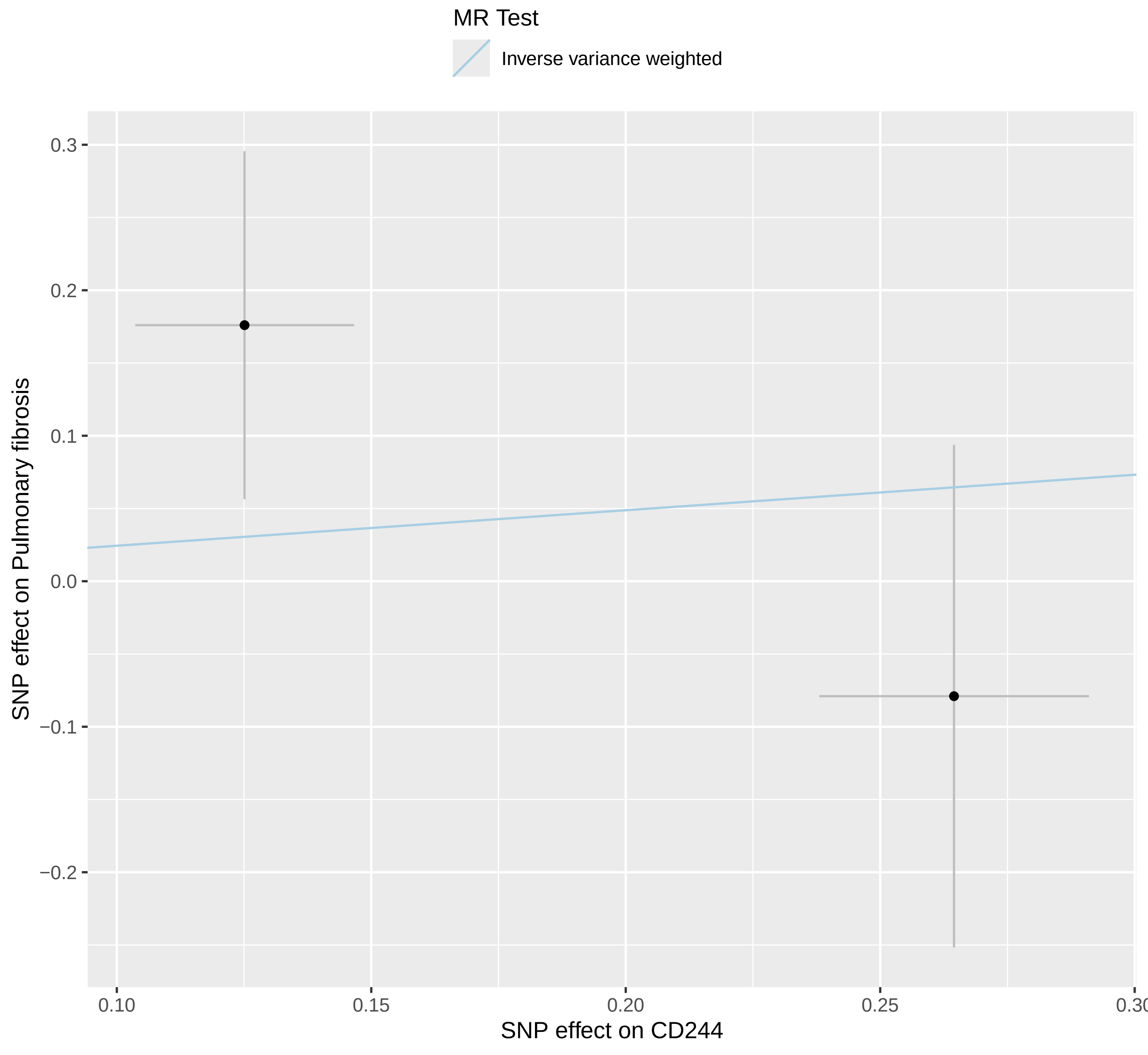
MR Test
Inverse variance weighted
0.3
0.2
0.1
0.0
−0.1
−0.2
SNP effect on Pulmonary fibrosis
0.10
0.15
0.20
0.25
0.30
SNP effect on CD244

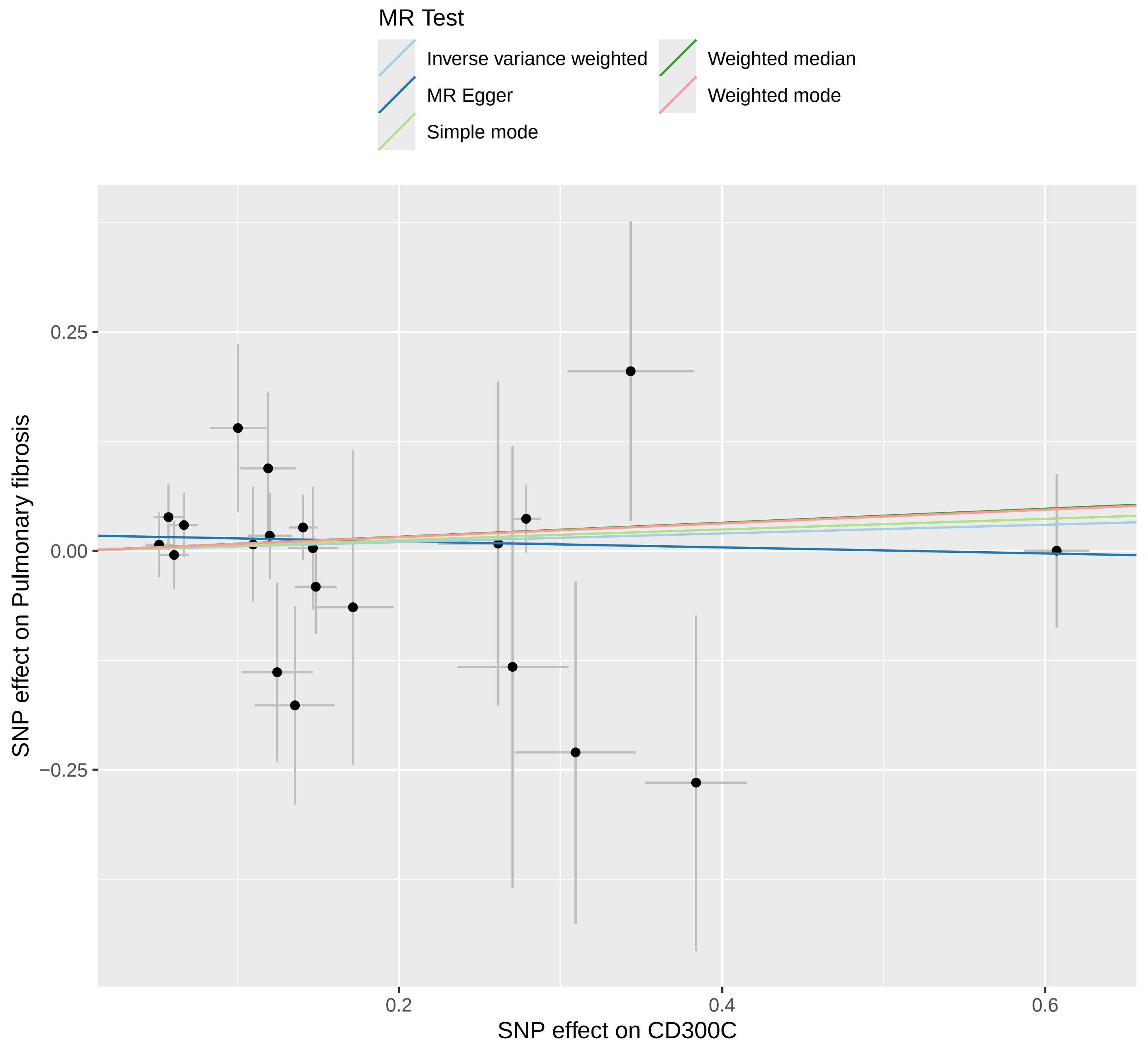

MR Test
Inverse variance weighted
MR Egger
Simple mode
Weighted median
Weighted mode
0.25
0.00
−0.25
0.2
0.4
0.6
SNP effect on Pulmonary fibrosis
SNP effect on CD300C

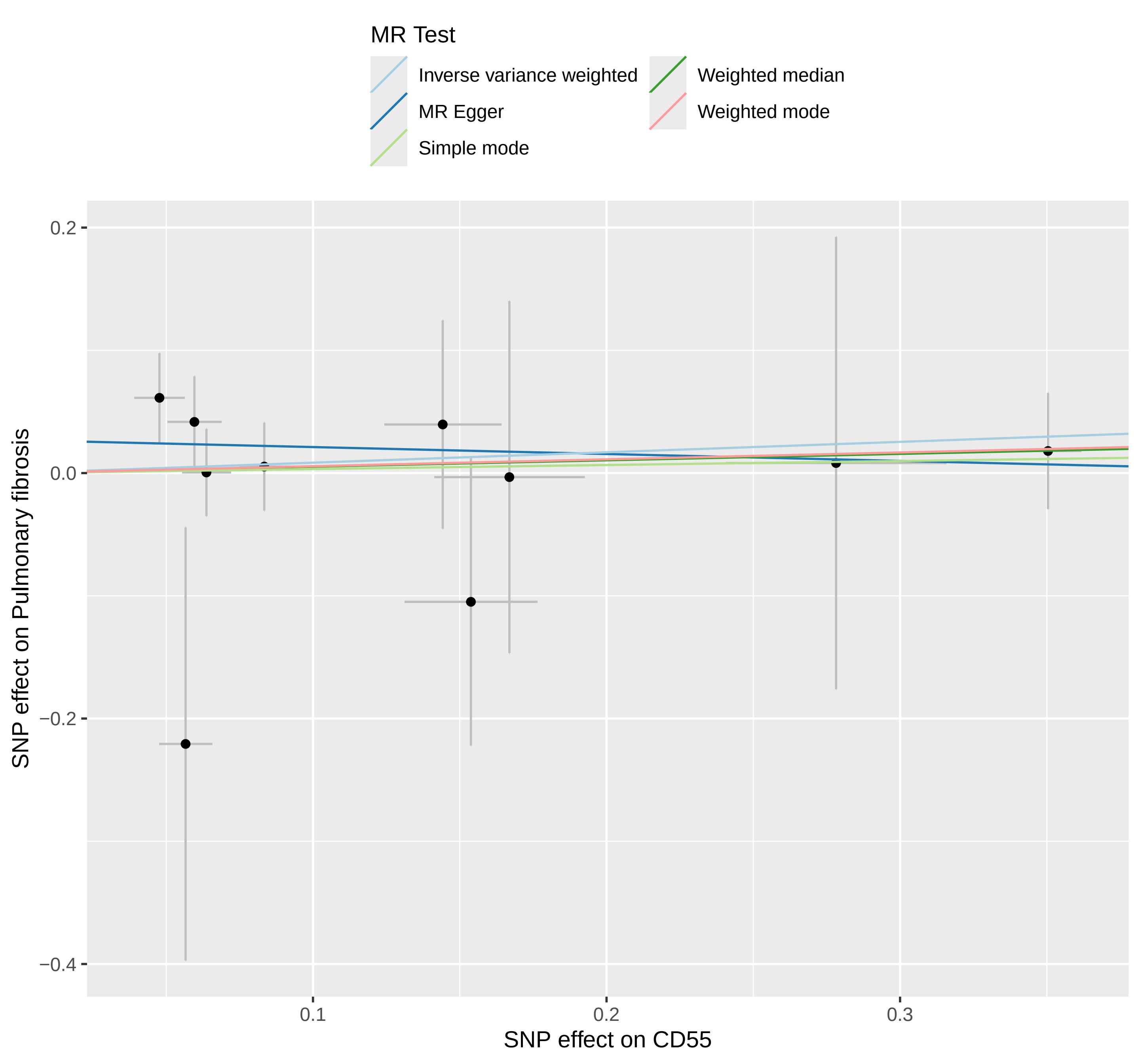
MR Test
Inverse variance weighted
MR Egger
Simple mode
Weighted median
Weighted mode
0.2
0.0
−0.2
−0.4
0.1
0.2
0.3
SNP effect on Pulmonary fibrosis
SNP effect on CD55

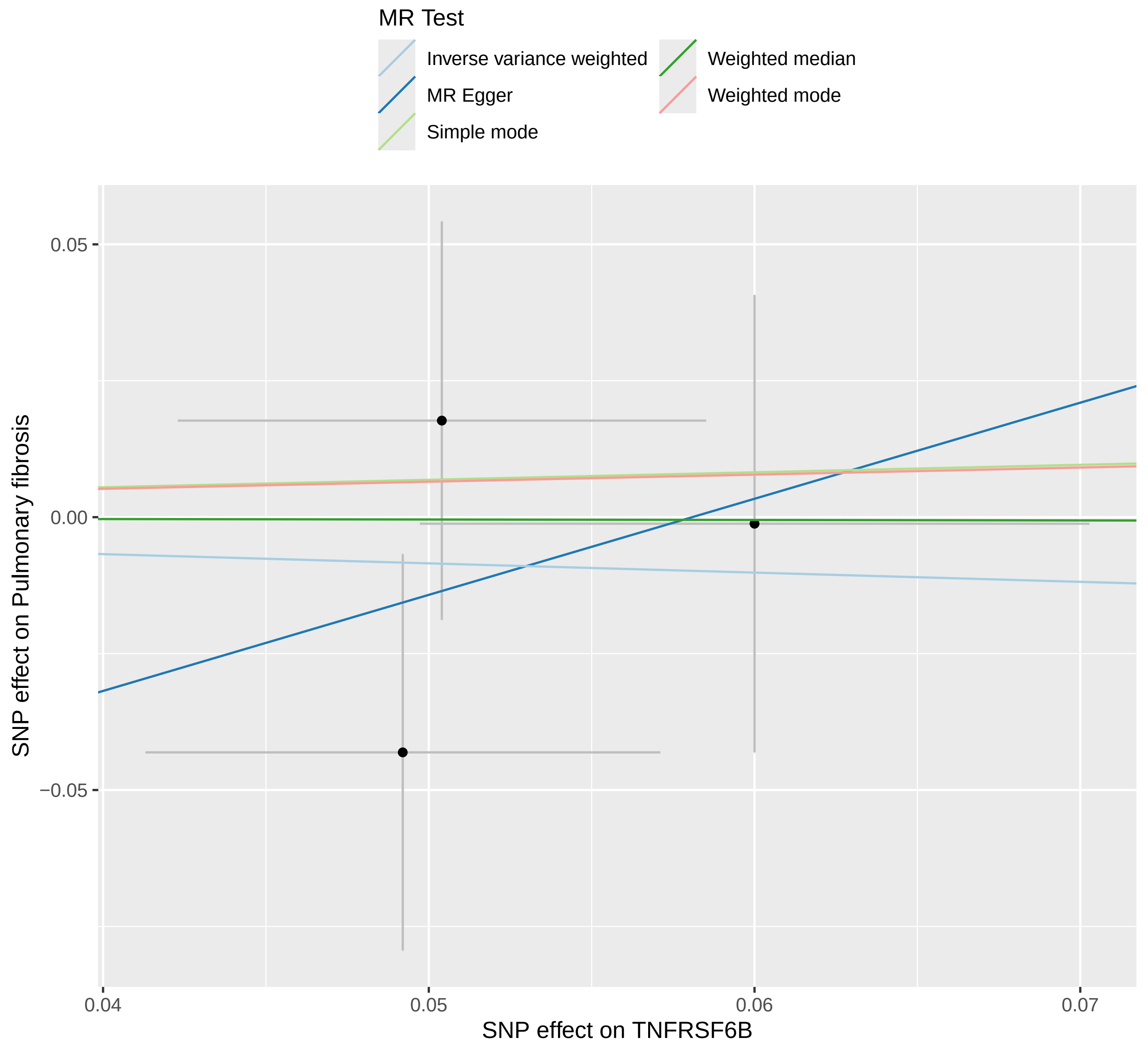

MR Test
Inverse variance weighted
MR Egger
Simple mode
Weighted median
Weighted mode
0.05
0.00
−0.05
SNP effect on Pulmonary fibrosis
0.04
0.05
0.06
0.07
SNP effect on TNFRSF6B

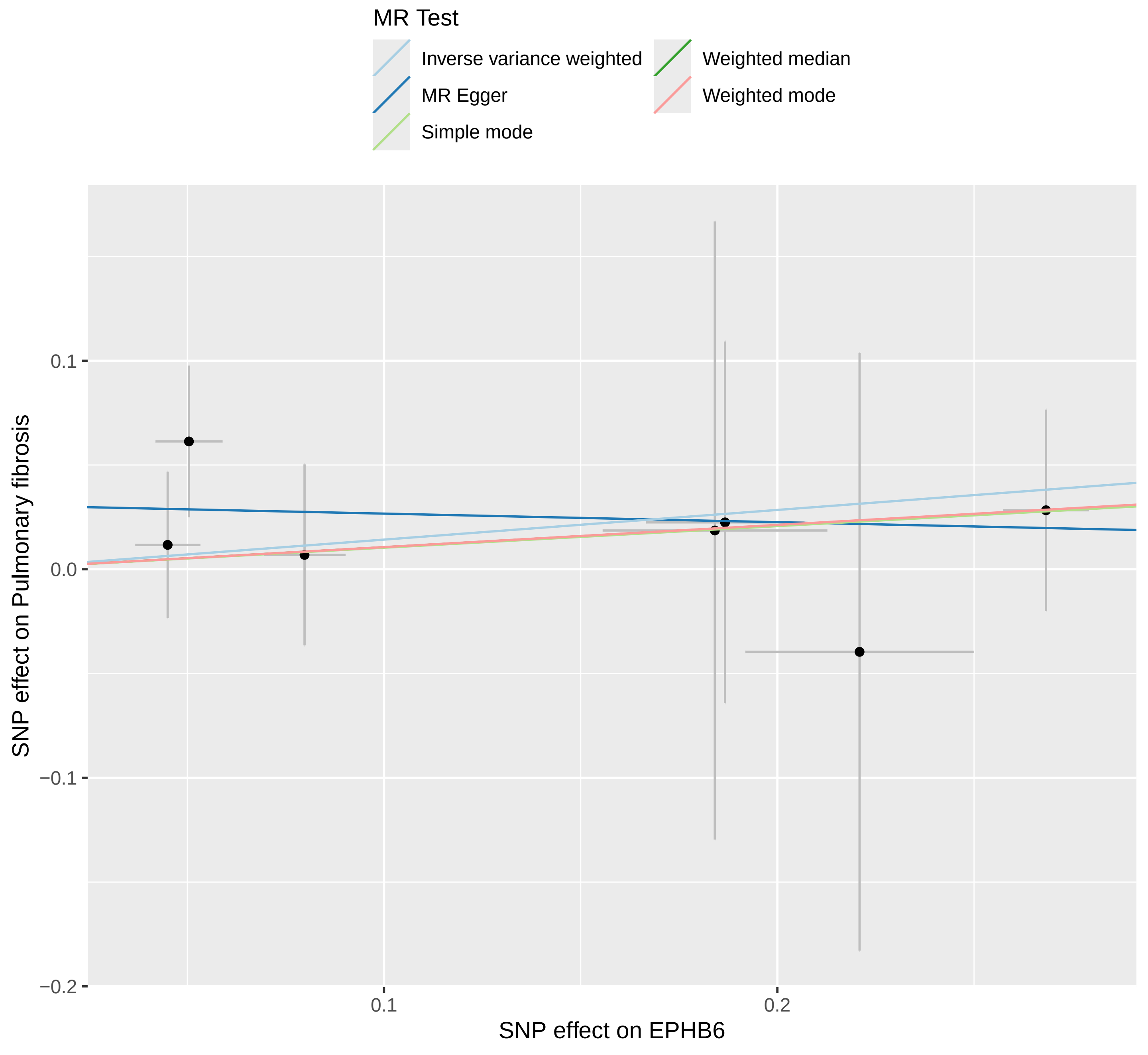
MR Test
Inverse variance weighted
MR Egger
Simple mode
Weighted median
Weighted mode
0.1
0.0
−0.1
−0.2
SNP effect on Pulmonary fibrosis
0.1
0.2
SNP effect on EPHB6

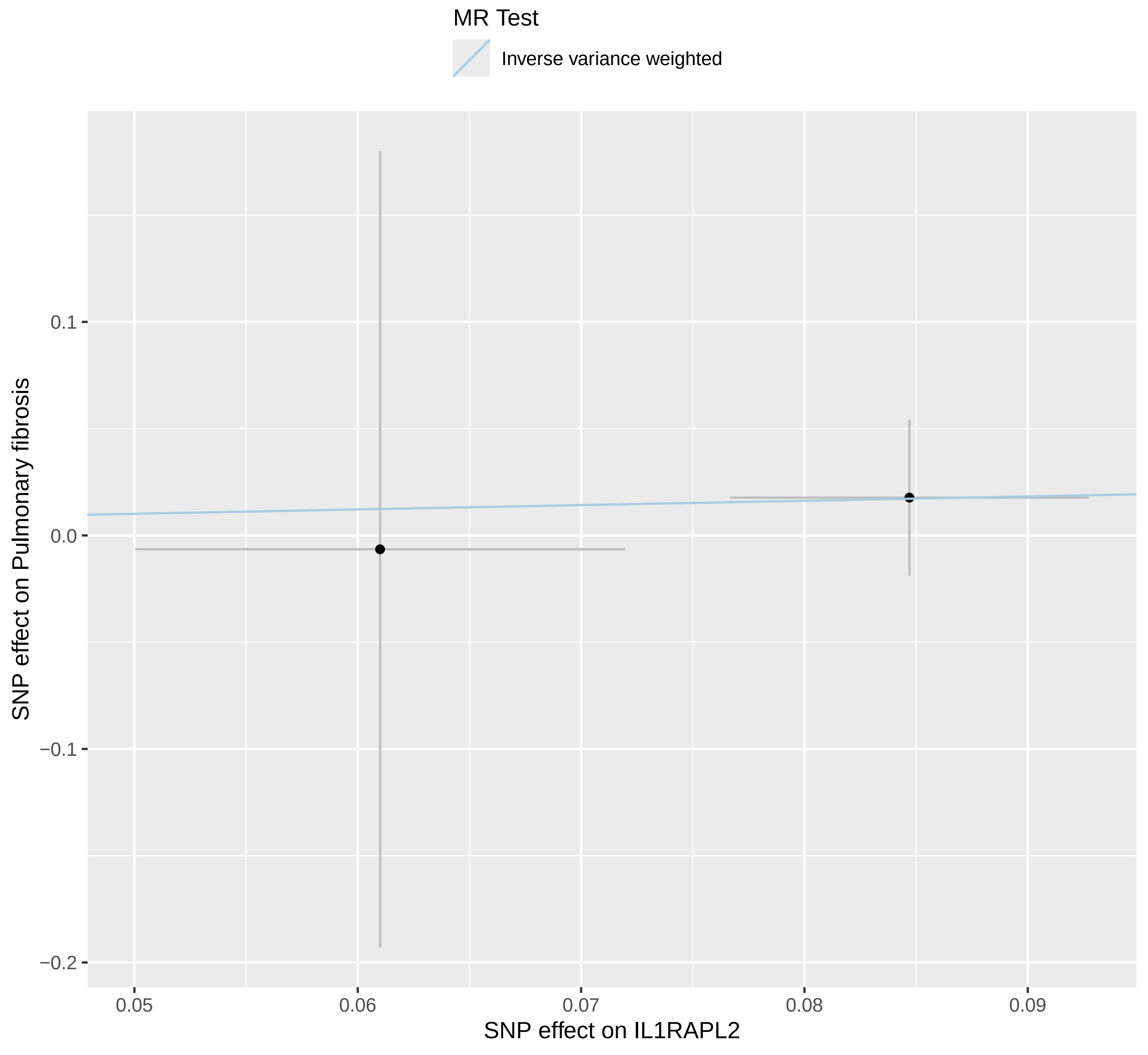

MR Test
Inverse variance weighted
0.1
0.0
−0.1
−0.2
SNP effect on Pulmonary fibrosis
0.05
0.06
0.07
0.08
0.09
SNP effect on IL1RAPL2

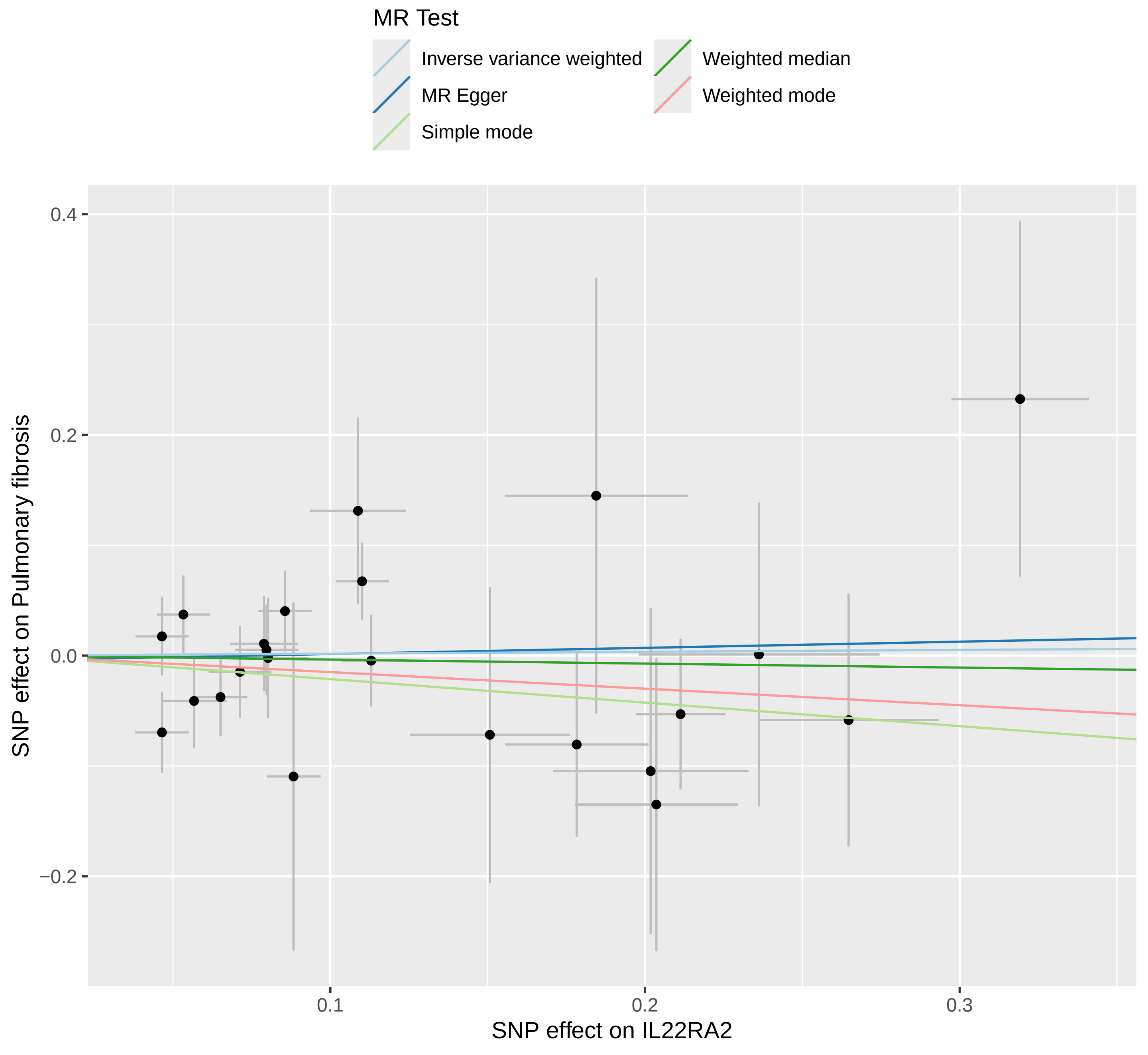

MR Test
Inverse variance weighted
MR Egger
Simple mode
Weighted median
Weighted mode
0.4
0.2
0.0
−0.2
0.1
0.2
0.3
SNP effect on Pulmonary fibrosis
SNP effect on IL22RA2

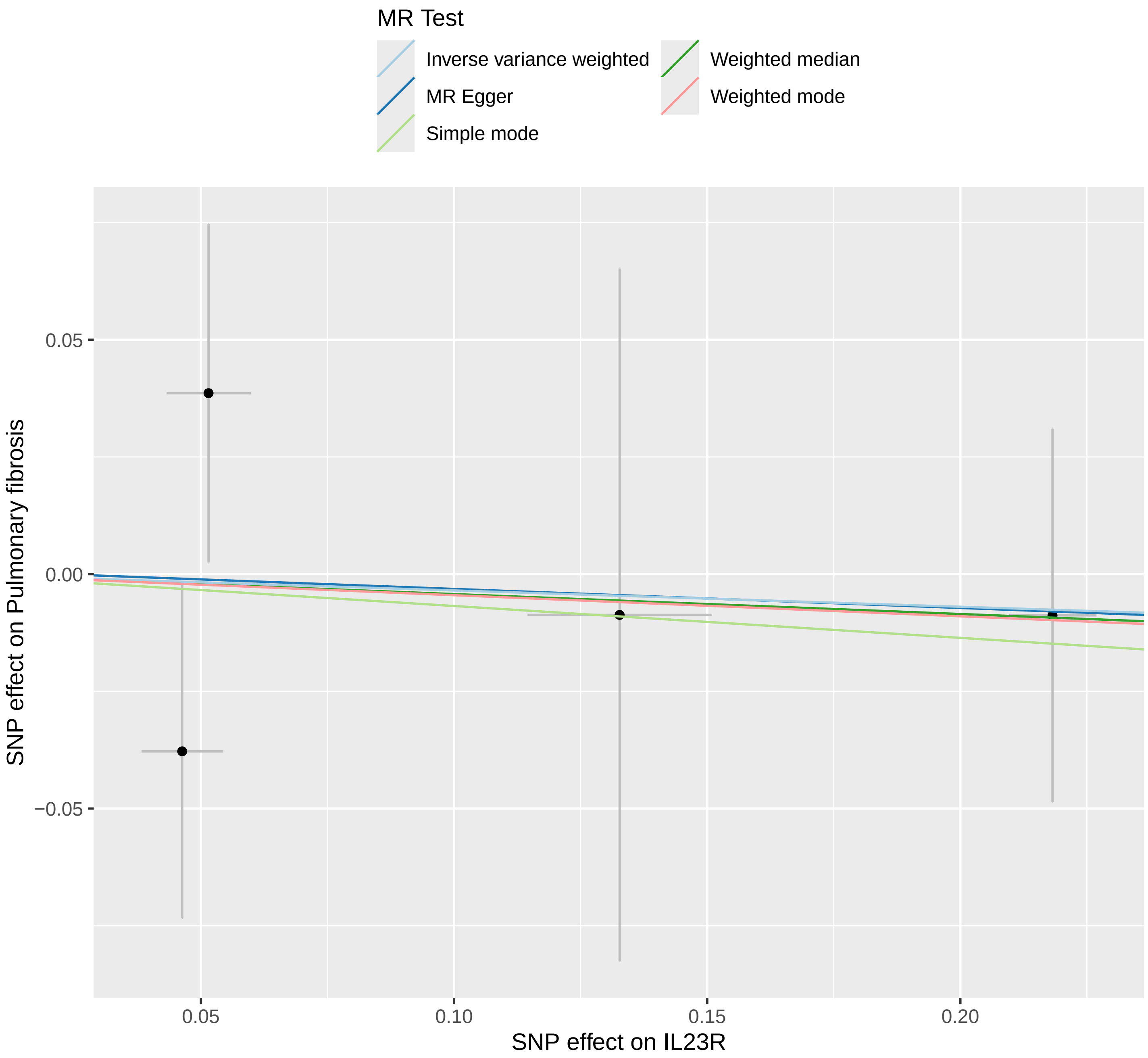

MR Test
Inverse variance weighted
MR Egger
Simple mode
Weighted median
Weighted mode
0.05
0.00
−0.05
0.05
0.10
0.15
0.20
SNP effect on Pulmonary fibrosis
SNP effect on IL23R

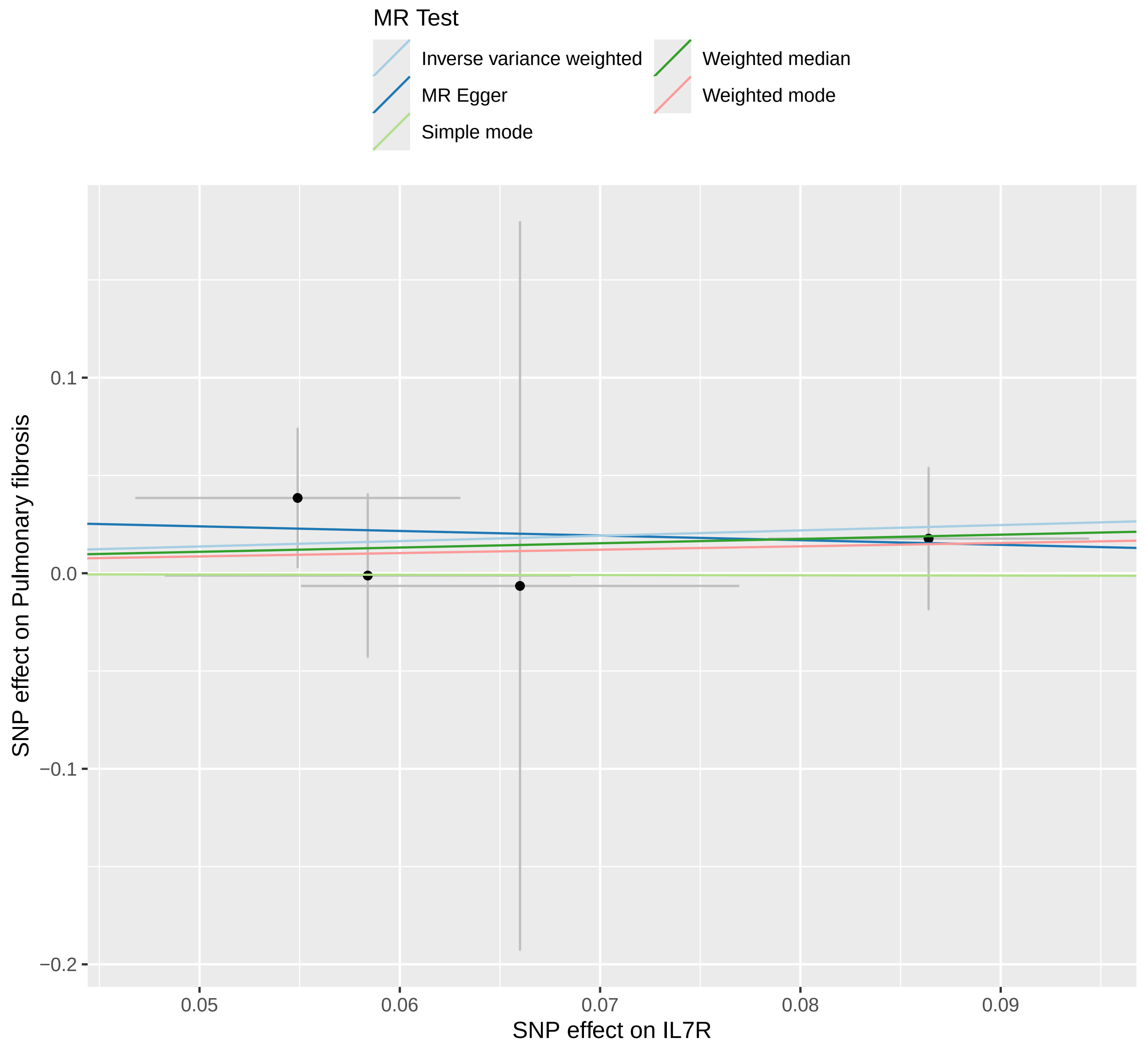
MR Test
Inverse variance weighted
MR Egger
Simple mode
Weighted median
Weighted mode
0.1
0.0
−0.1
−0.2
SNP effect on Pulmonary fibrosis
0.05
0.06
0.07
0.08
0.09
SNP effect on IL7R

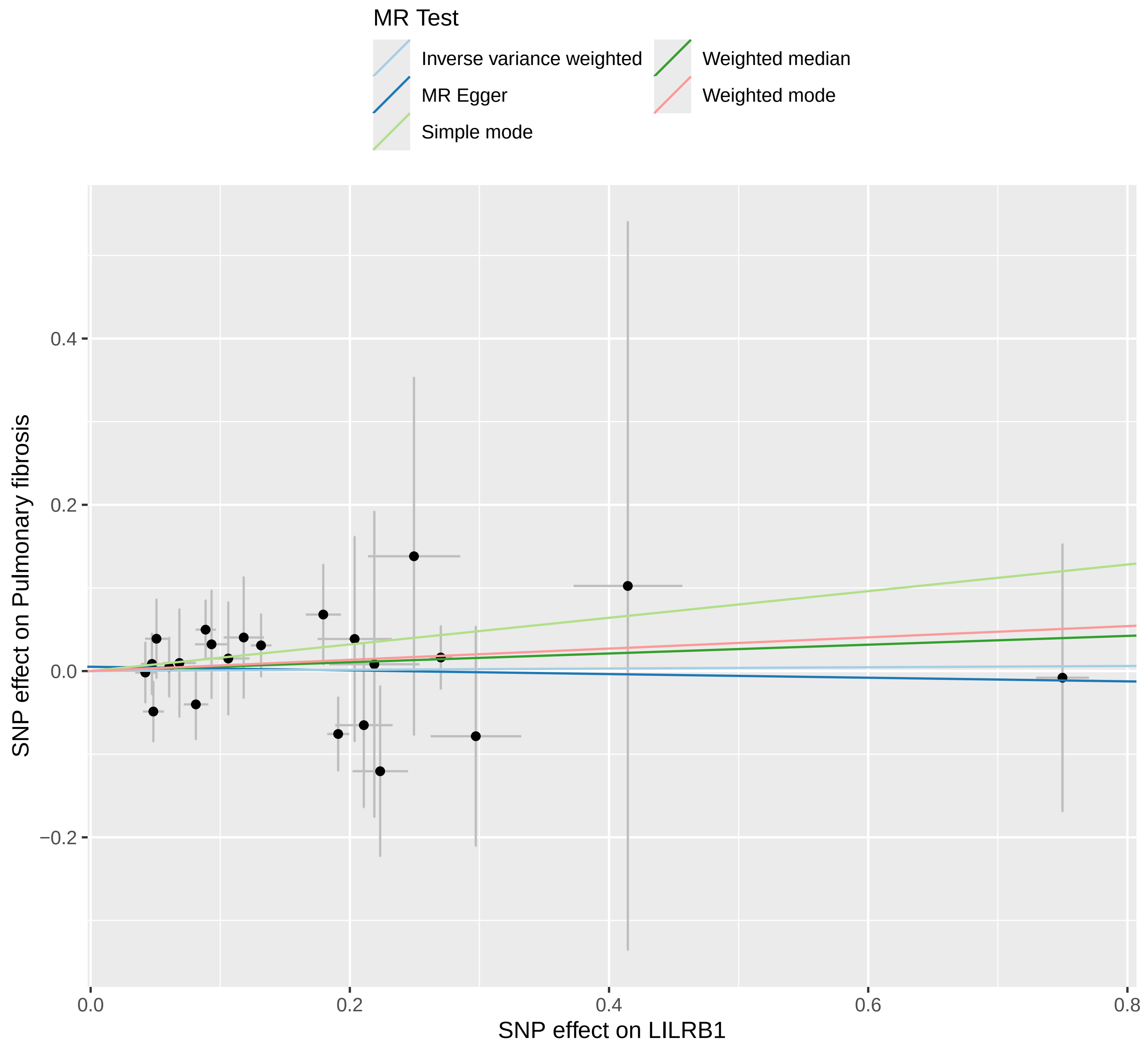

MR Test
Inverse variance weighted
MR Egger
Simple mode
Weighted median
Weighted mode
0.4
0.2
0.0
−0.2
0.0
0.2
0.4
0.6
0.8
SNP effect on Pulmonary fibrosis
SNP effect on LILRB1

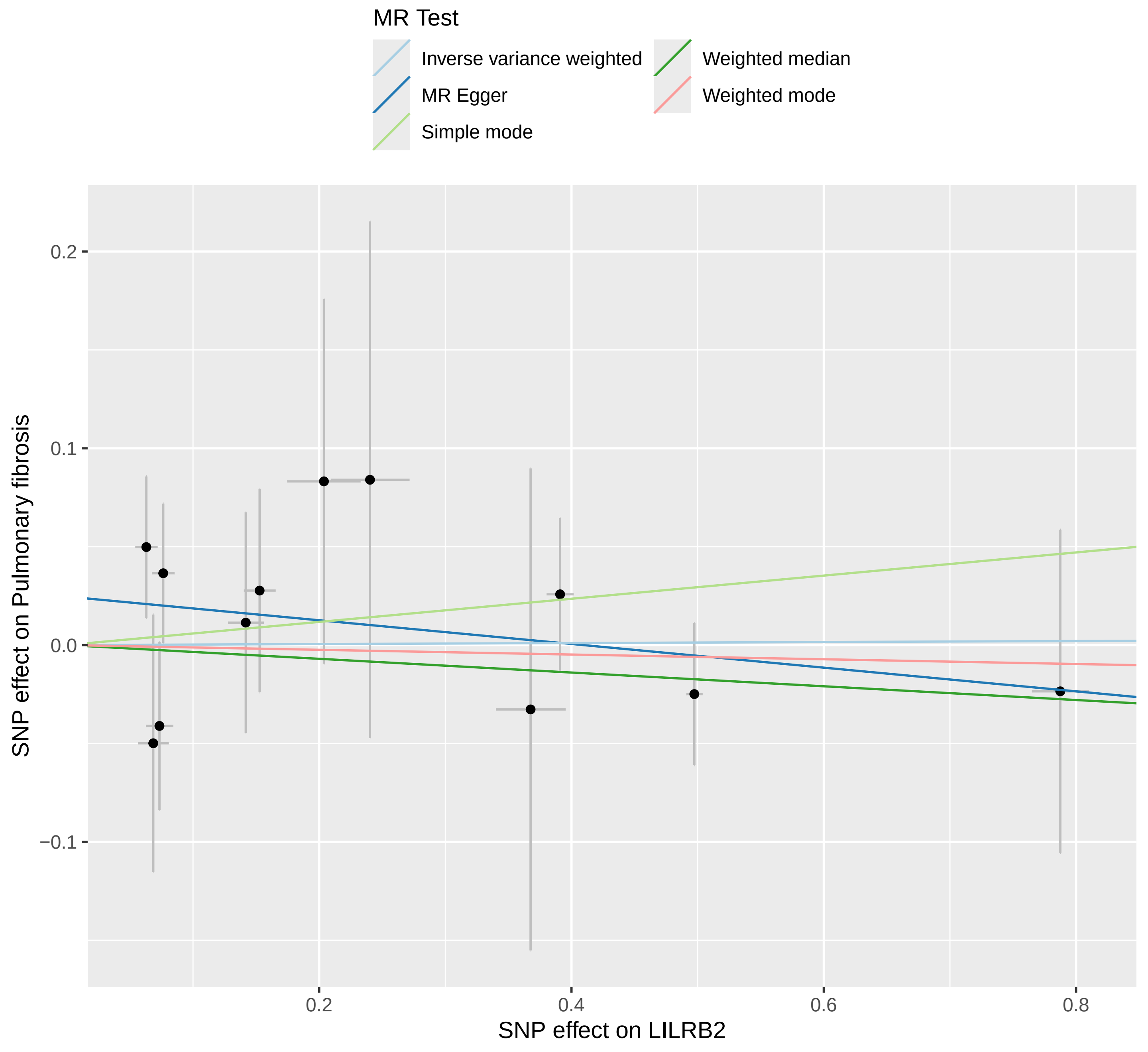

MR Test
Inverse variance weighted
MR Egger
Simple mode
Weighted median
Weighted mode
0.2
0.1
0.0
−0.1
SNP effect on Pulmonary fibrosis
0.2
0.4
0.6
0.8
SNP effect on LILRB2

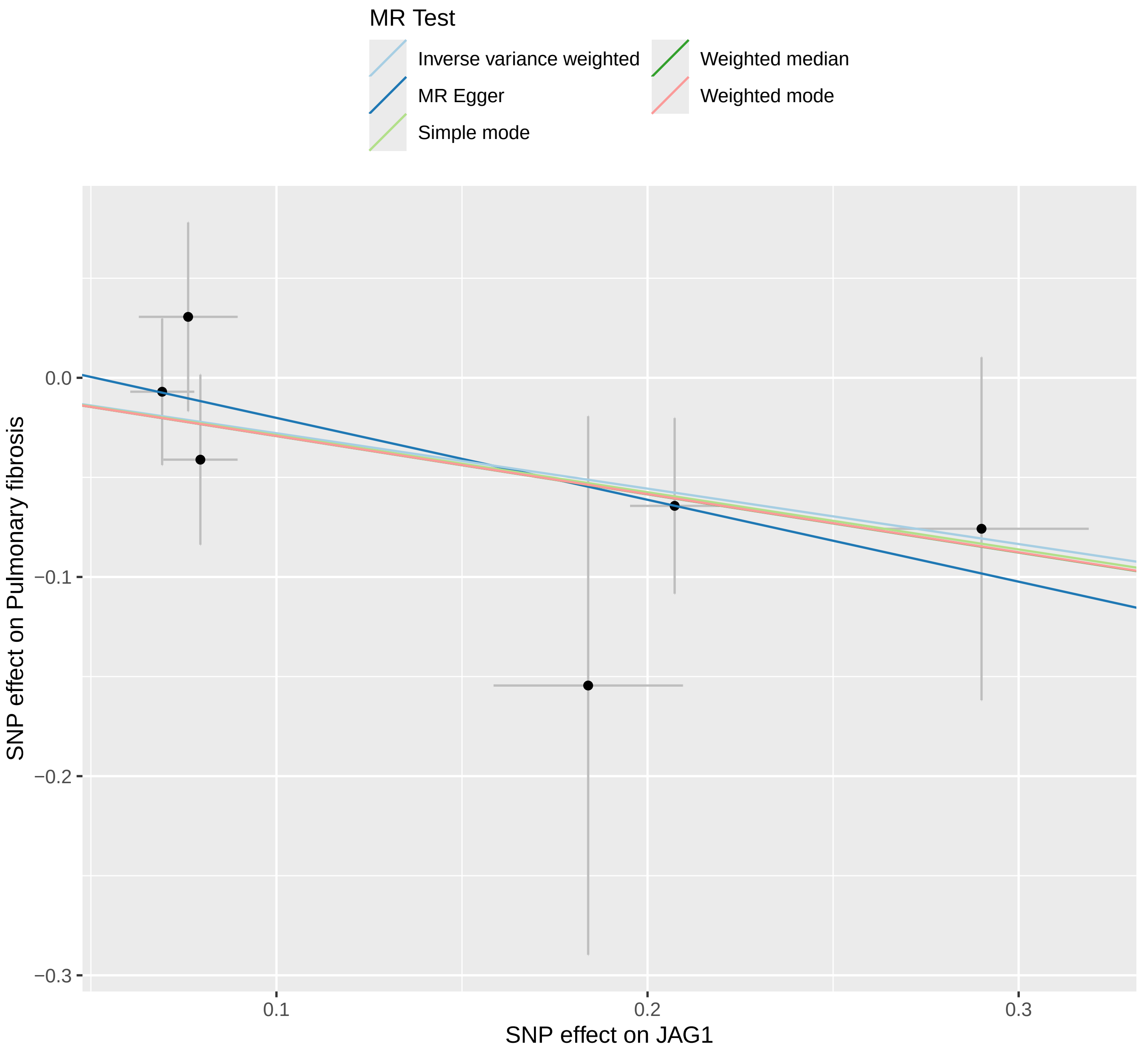

MR Test
Inverse variance weighted
MR Egger
Simple mode
Weighted median
Weighted mode
0.0
−0.1
−0.2
−0.3
0.1
0.2
0.3
SNP effect on Pulmonary fibrosis
SNP effect on JAG1

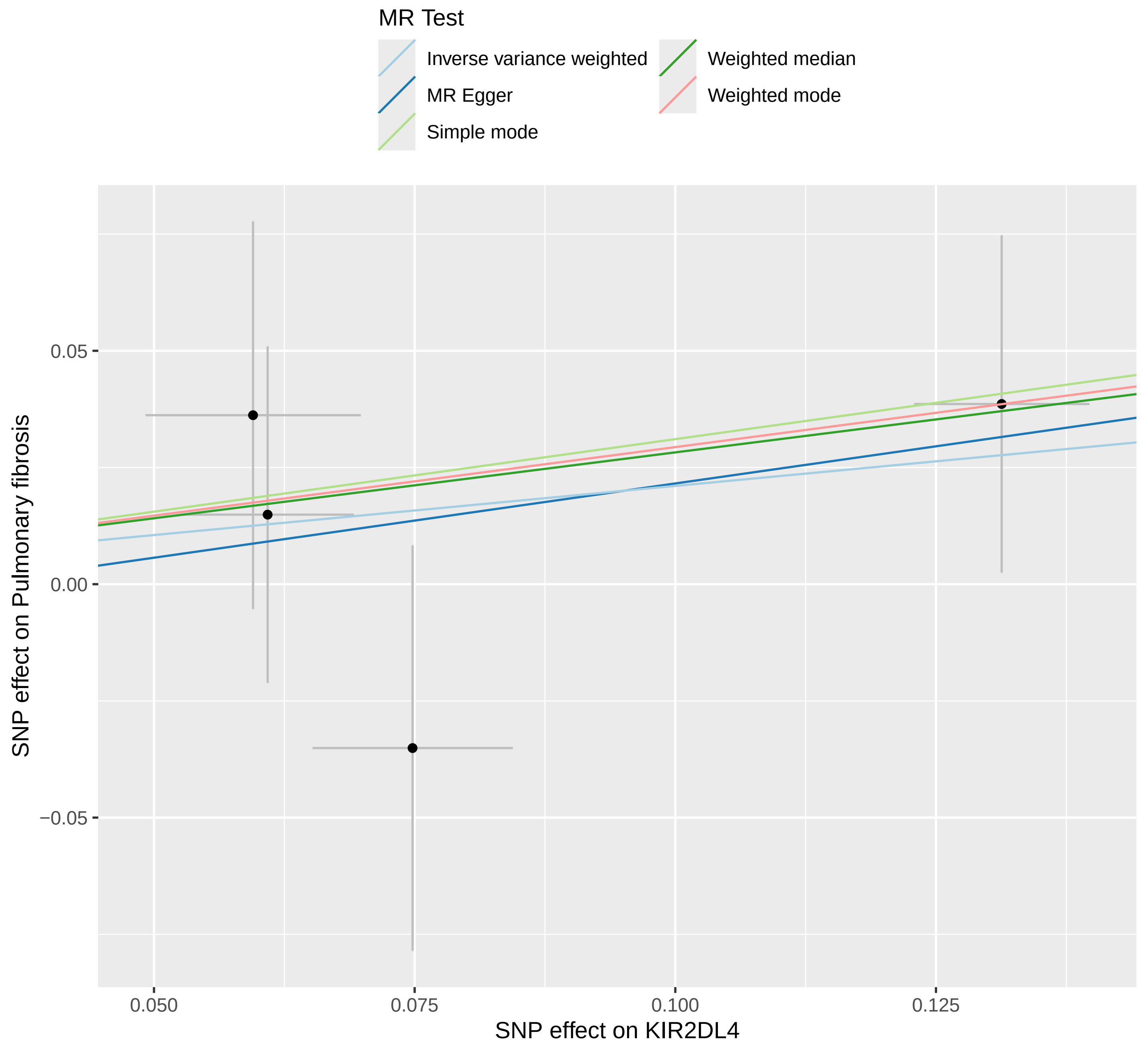
MR Test
Inverse variance weighted
MR Egger
Simple mode
Weighted median
Weighted mode
SNP effect on Pulmonary fibrosis
0.05
0.00
−0.05
0.050
0.075
0.100
0.125
SNP effect on KIR2DL4

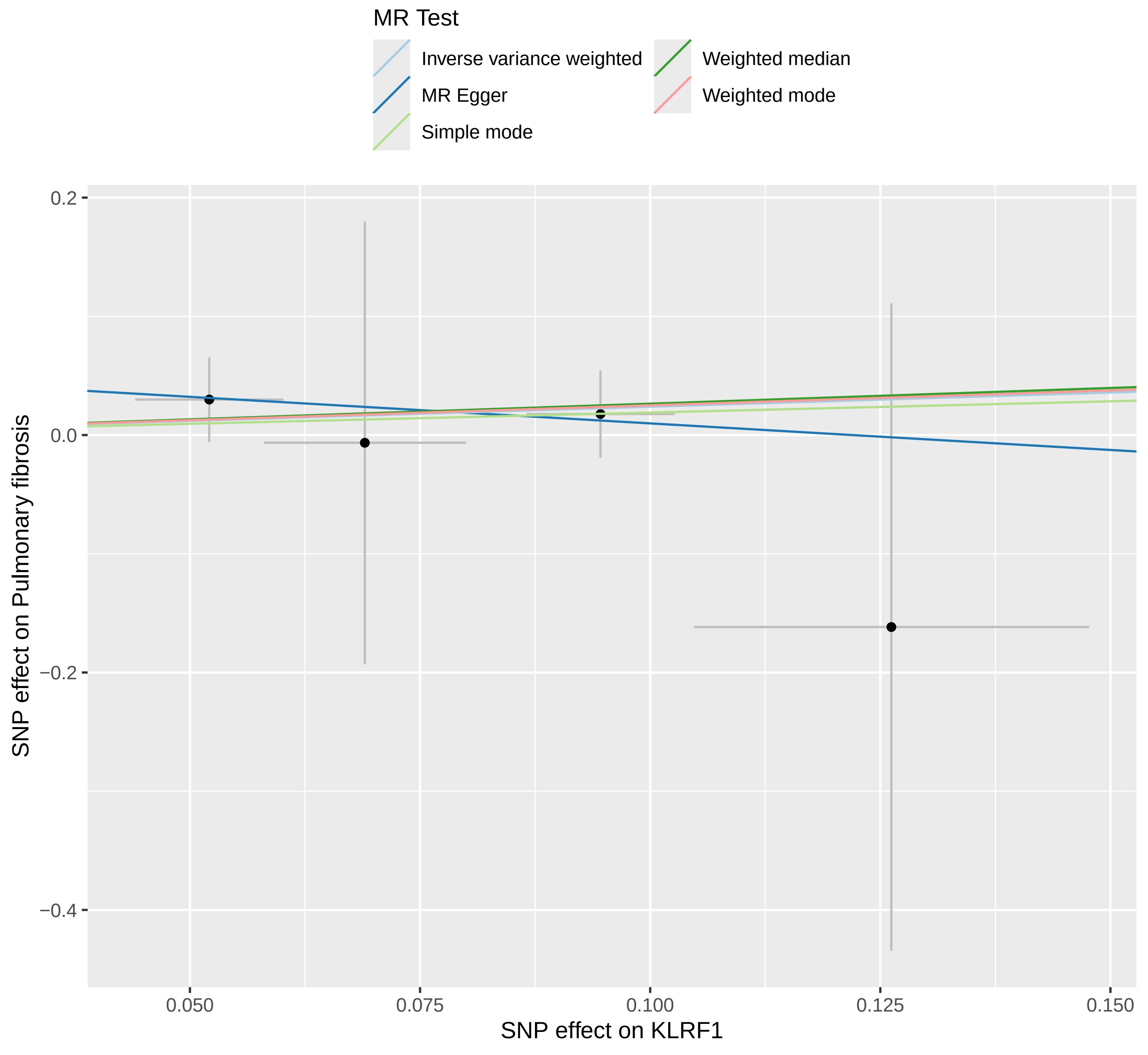

MR Test
Inverse variance weighted
MR Egger
Simple mode
Weighted median
Weighted mode
0.2
0.0
−0.2
−0.4
SNP effect on Pulmonary fibrosis
0.050
0.075
0.100
0.125
0.150
SNP effect on KLRF1

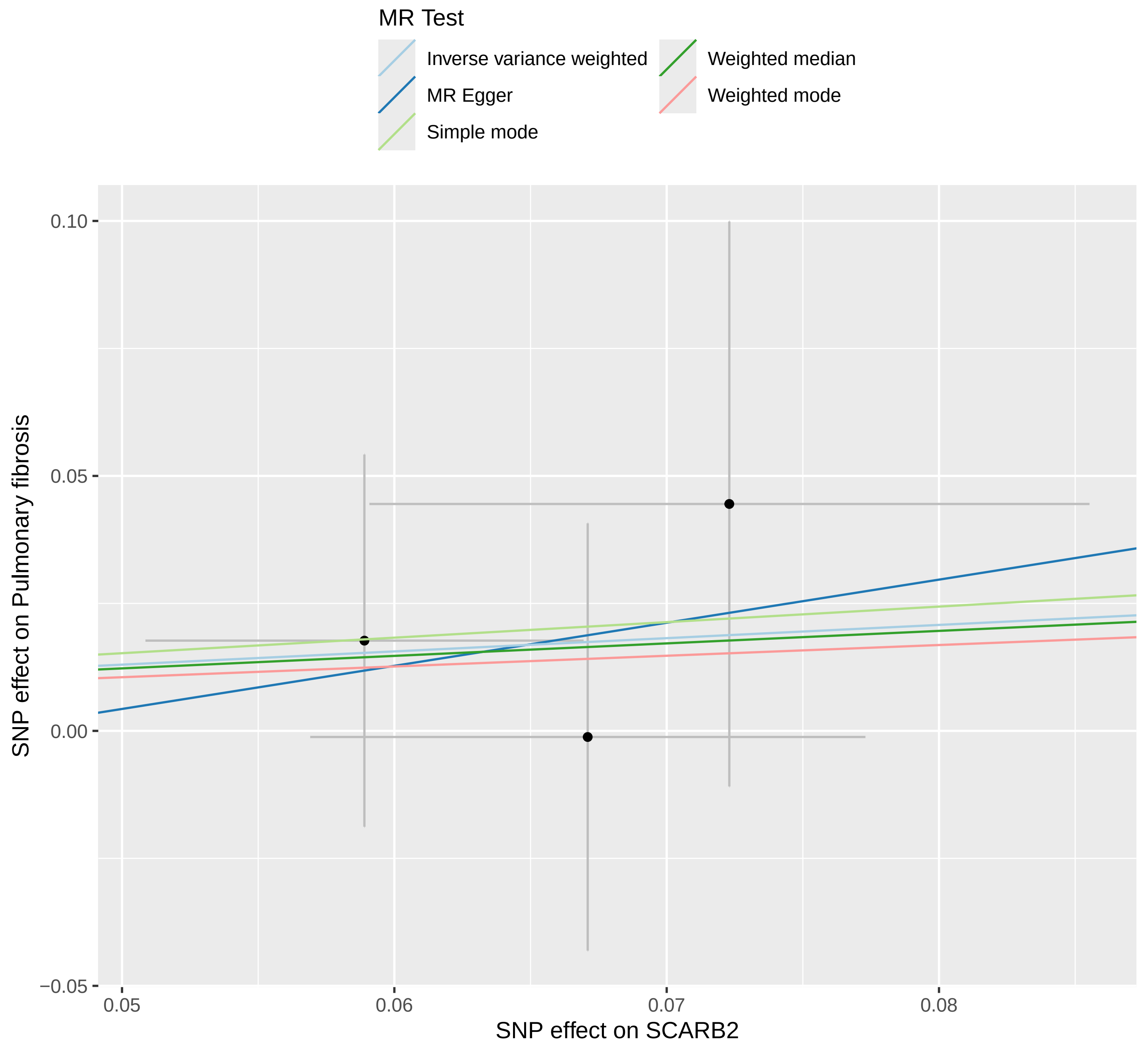

MR Test
Inverse variance weighted
MR Egger
Simple mode
Weighted median
Weighted mode
0.10
0.05
0.00
−0.05
SNP effect on Pulmonary fibrosis
0.05
0.06
0.07
0.08
SNP effect on SCARB2

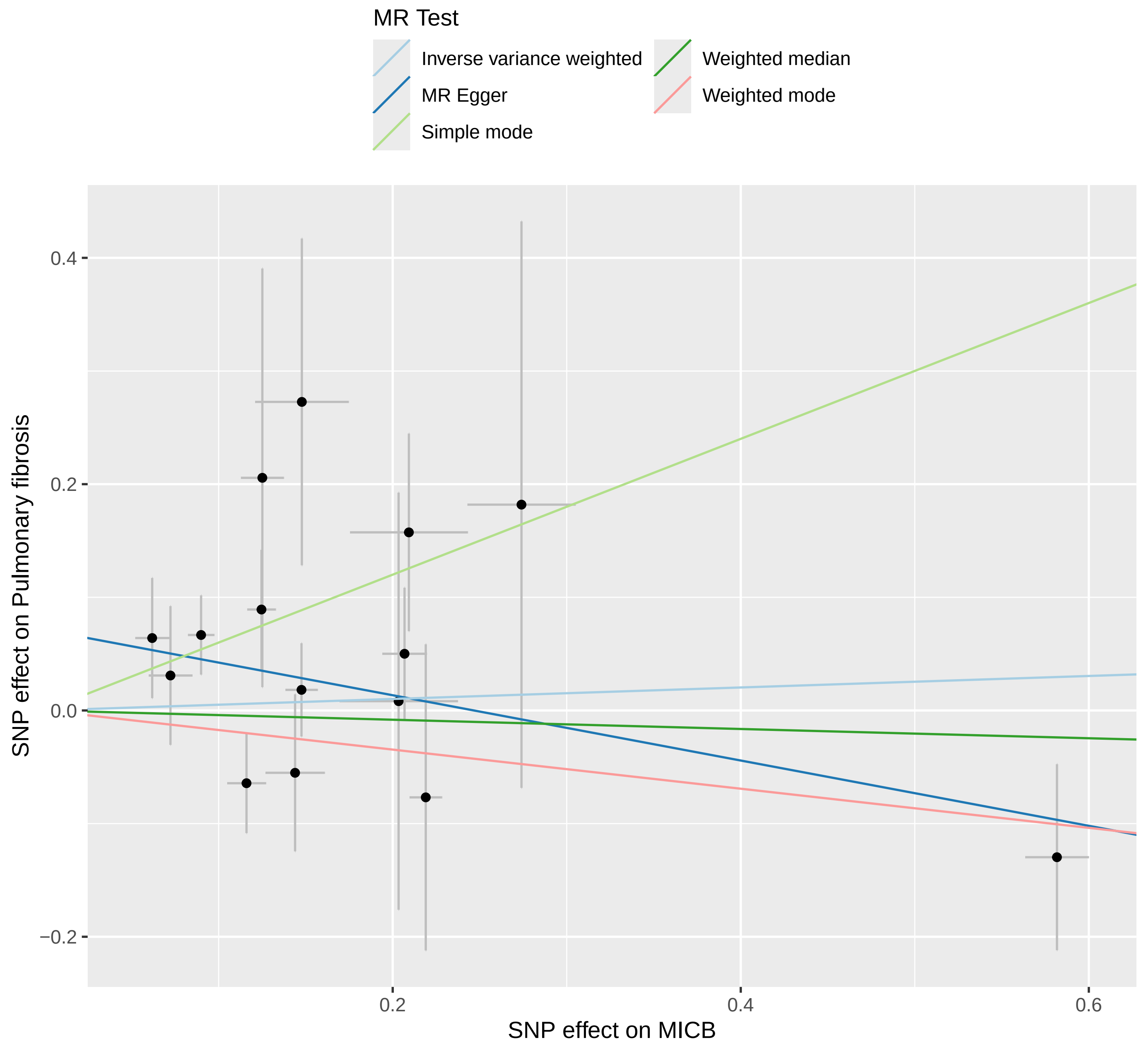

MR Test
Inverse variance weighted
MR Egger
Simple mode
Weighted median
Weighted mode
0.4
0.2
0.0
−0.2
SNP effect on Pulmonary fibrosis
0.2
0.4
0.6
SNP effect on MICB

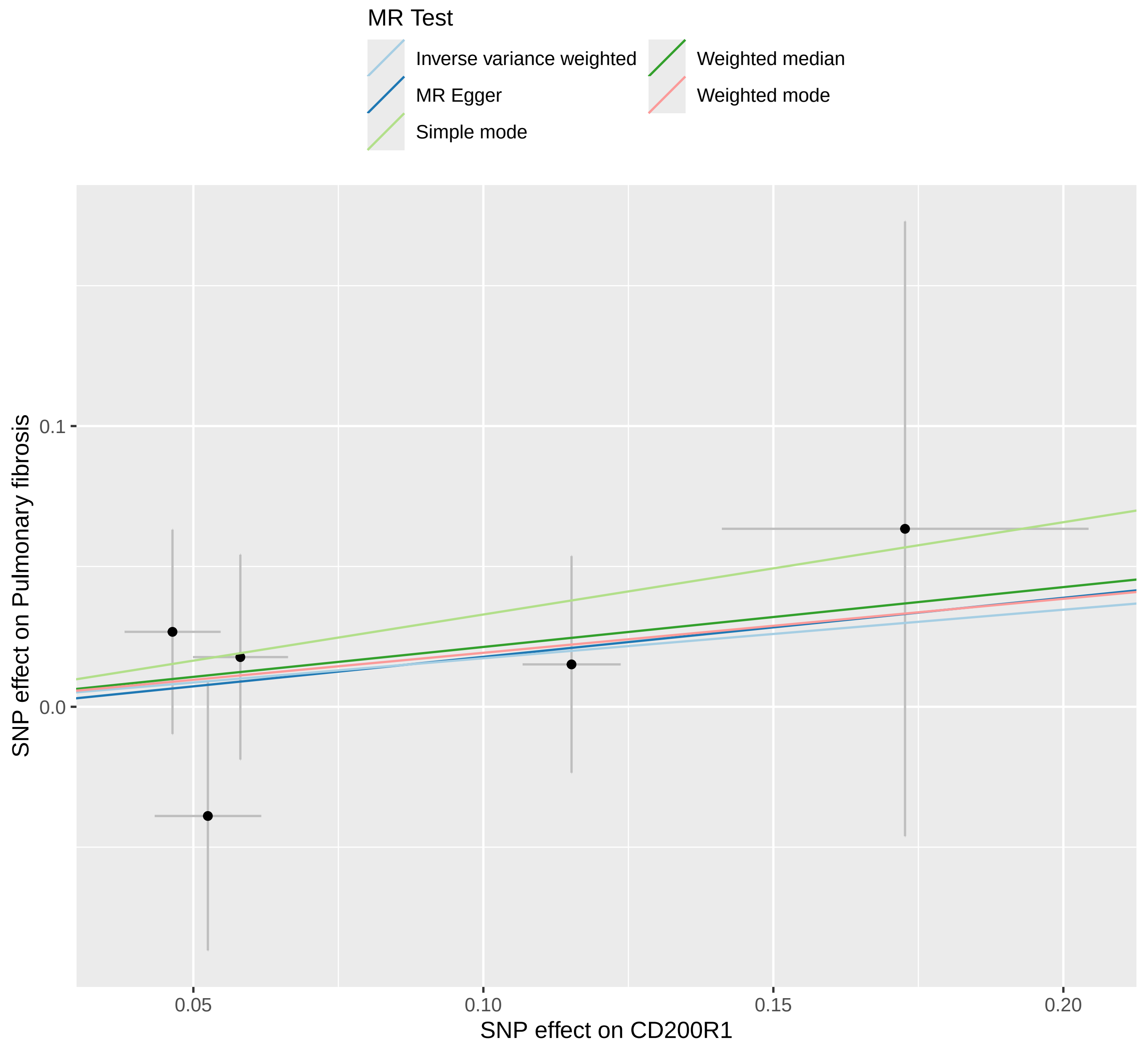
MR Test
Inverse variance weighted
MR Egger
Simple mode
Weighted median
Weighted mode
0.1
0.0
0.05
0.10
0.15
0.20
SNP effect on Pulmonary fibrosis
SNP effect on CD200R1

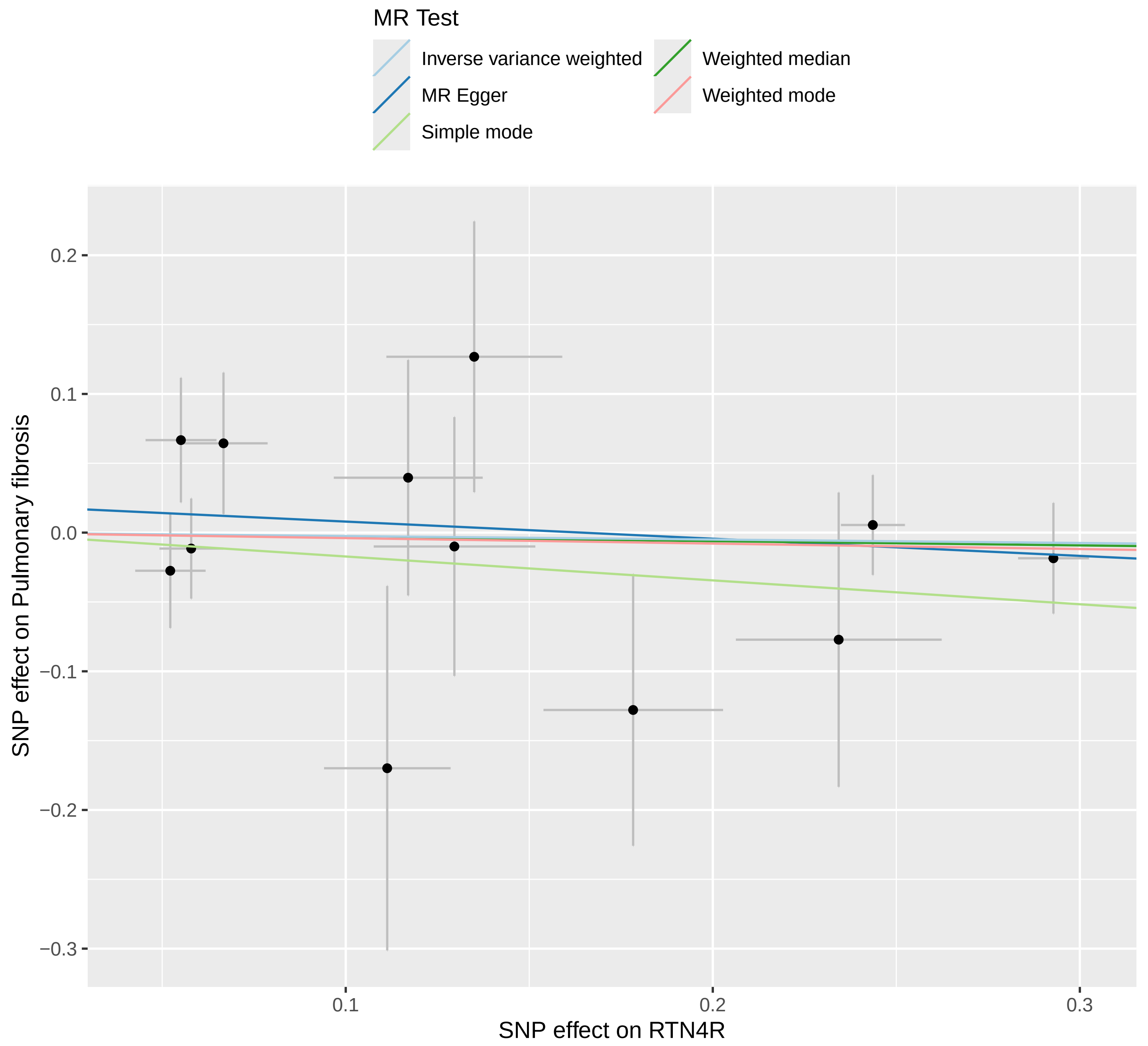
MR Test
Inverse variance weighted
MR Egger
Simple mode
Weighted median
Weighted mode
0.2
0.1
0.0
−0.1
−0.2
−0.3
SNP effect on Pulmonary fibrosis
0.1
0.2
0.3
SNP effect on RTN4R

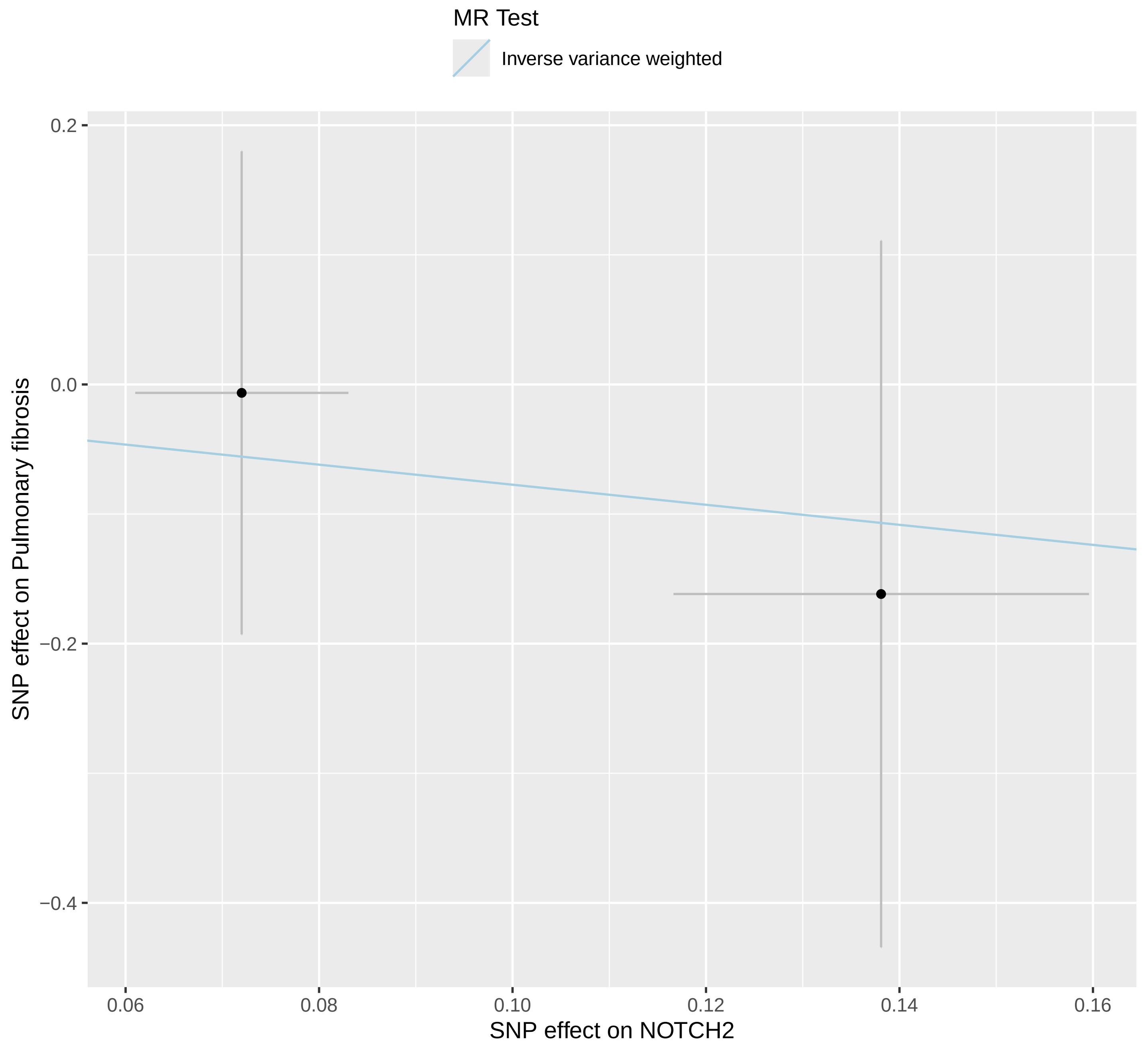
MR Test
Inverse variance weighted
0.2
0.0
−0.2
−0.4
SNP effect on Pulmonary fibrosis
0.06
0.08
0.10
0.12
0.14
0.16
SNP effect on NOTCH2

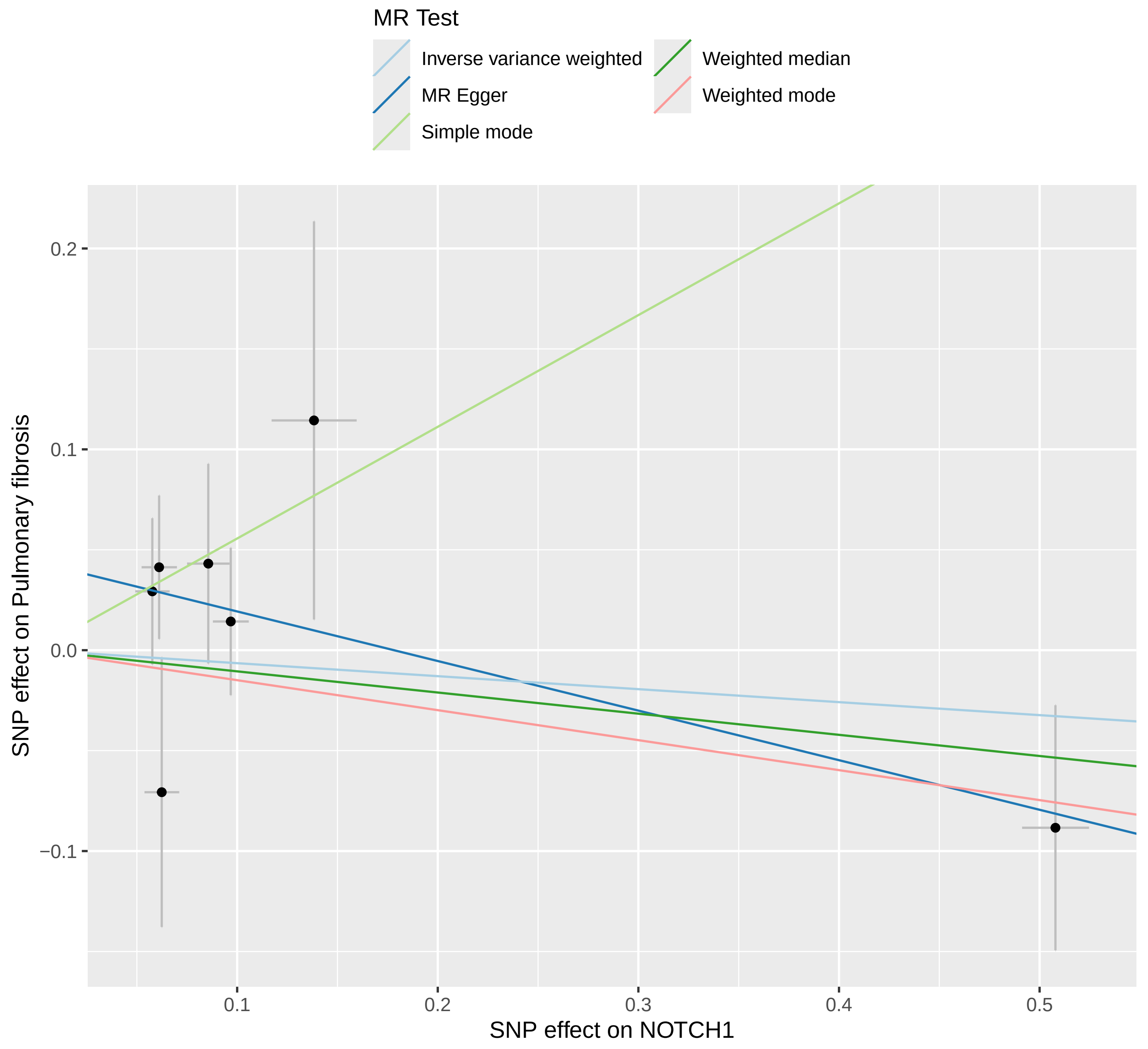
MR Test
Inverse variance weighted
MR Egger
Simple mode
Weighted median
Weighted mode
0.2
0.1
0.0
−0.1
SNP effect on Pulmonary fibrosis
0.1
0.2
0.3
0.4
0.5
SNP effect on NOTCH1

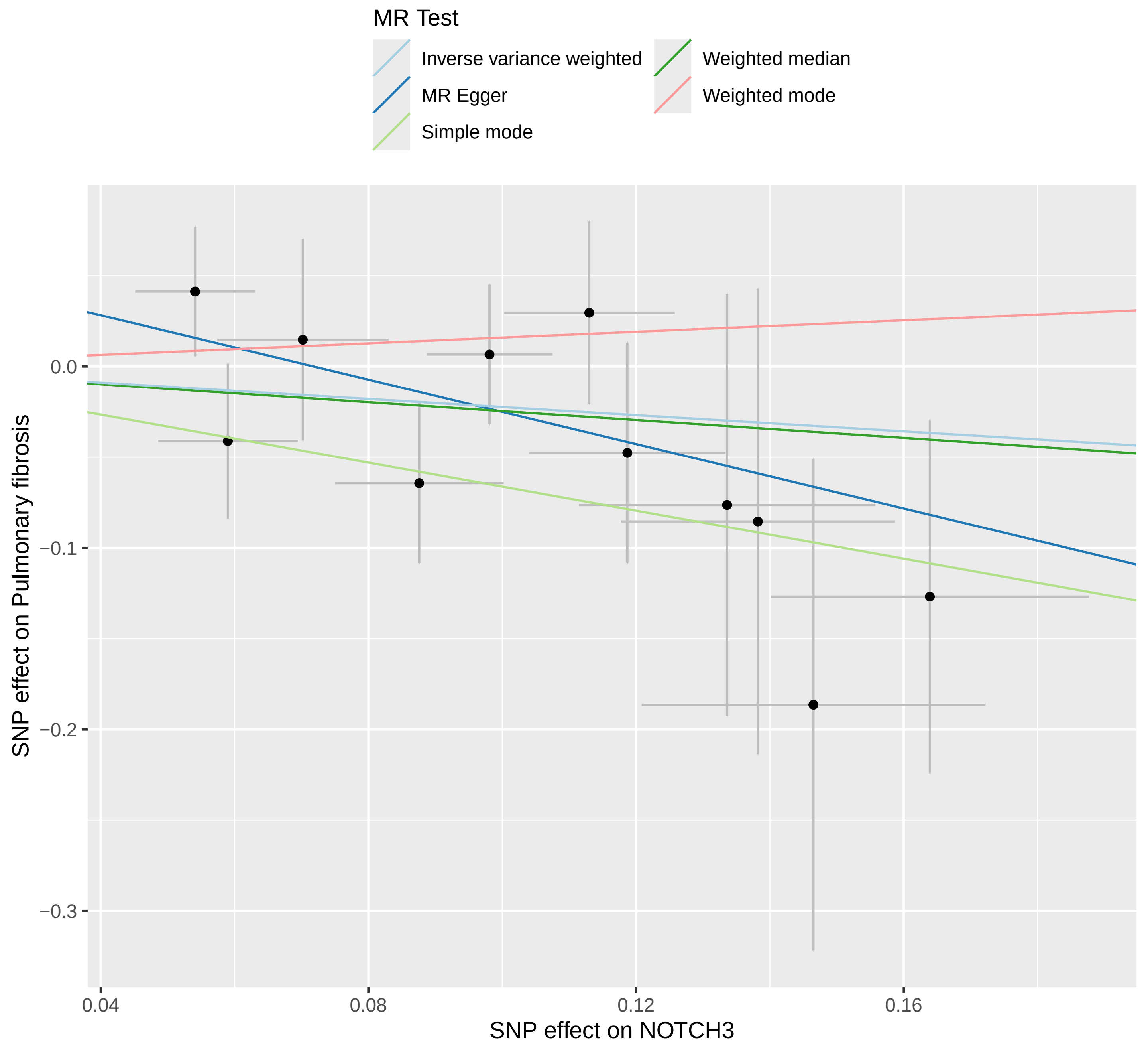
MR Test
Inverse variance weighted
MR Egger
Simple mode
Weighted median
Weighted mode
SNP effect on Pulmonary fibrosis
0.0
−0.1
−0.2
−0.3
0.04
0.08
0.12
0.16
SNP effect on NOTCH3

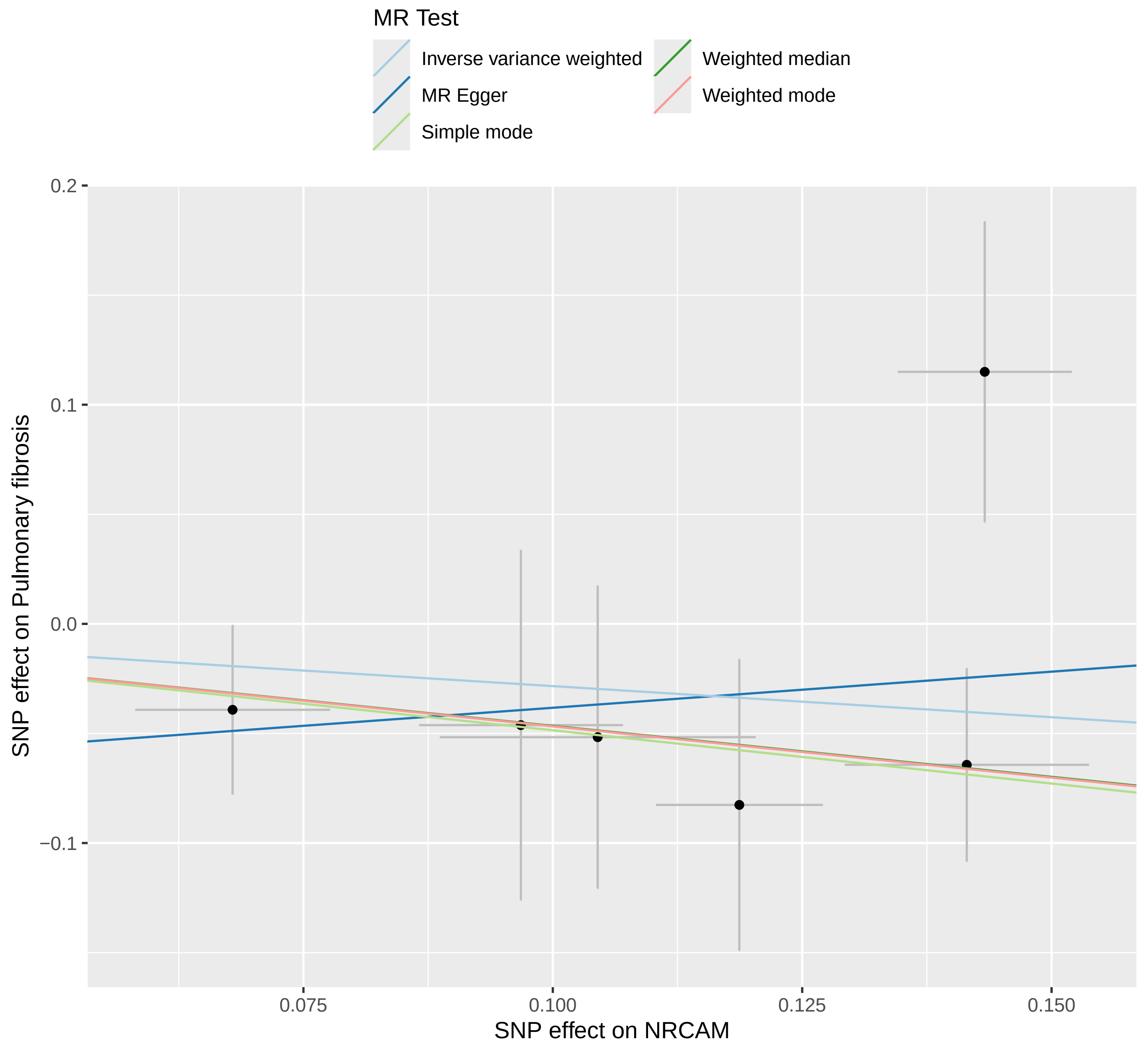
MR Test
Inverse variance weighted
MR Egger
Simple mode
Weighted median
Weighted mode
0.2
0.1
0.0
−0.1
SNP effect on Pulmonary fibrosis
0.075
0.100
0.125
0.150
SNP effect on NRCAM

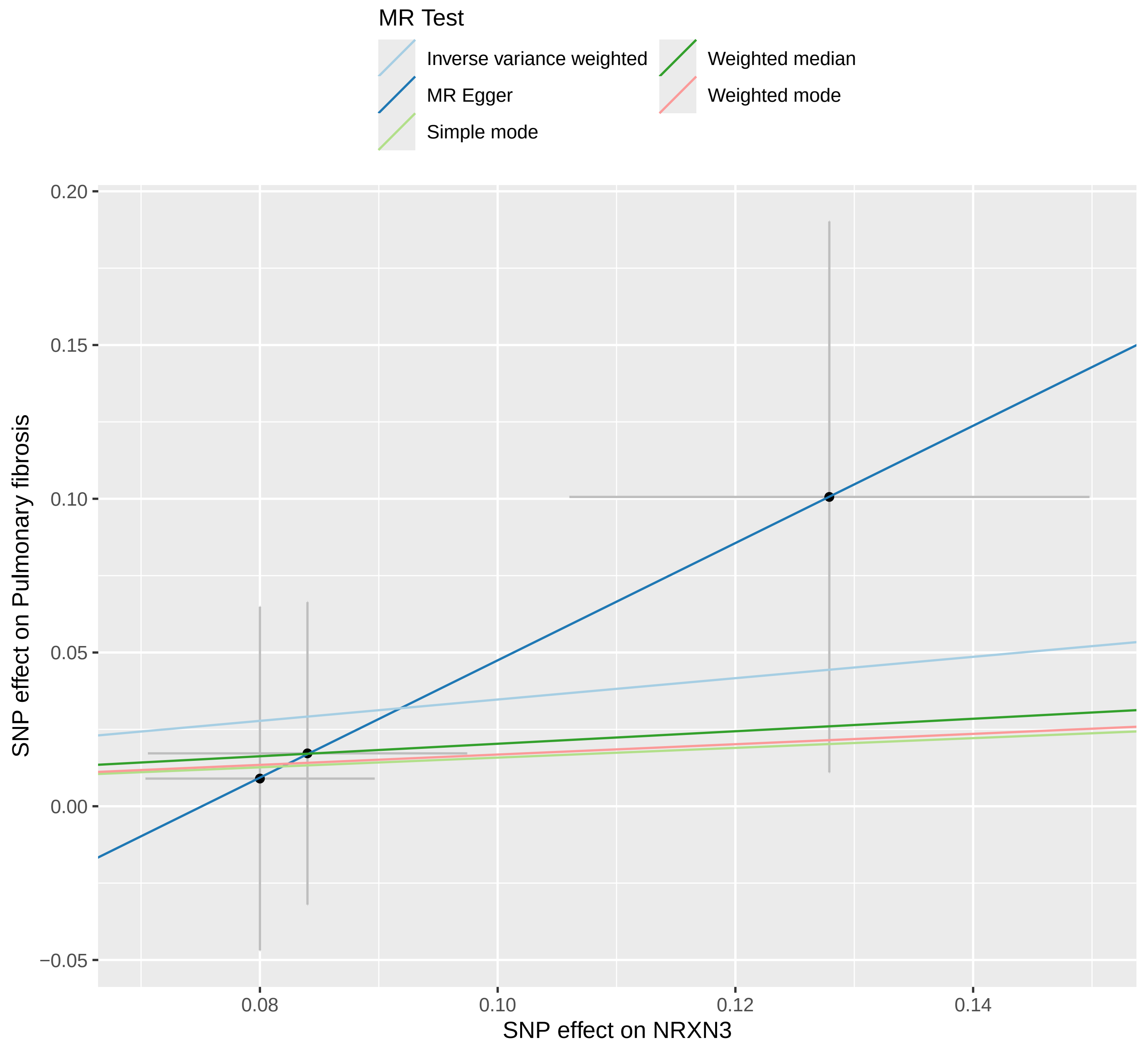

MR Test
Inverse variance weighted
MR Egger
Simple mode
Weighted median
Weighted mode
0.20
0.15
0.10
0.05
0.00
−0.05
SNP effect on Pulmonary fibrosis
0.08
0.10
0.12
0.14
SNP effect on NRXN3

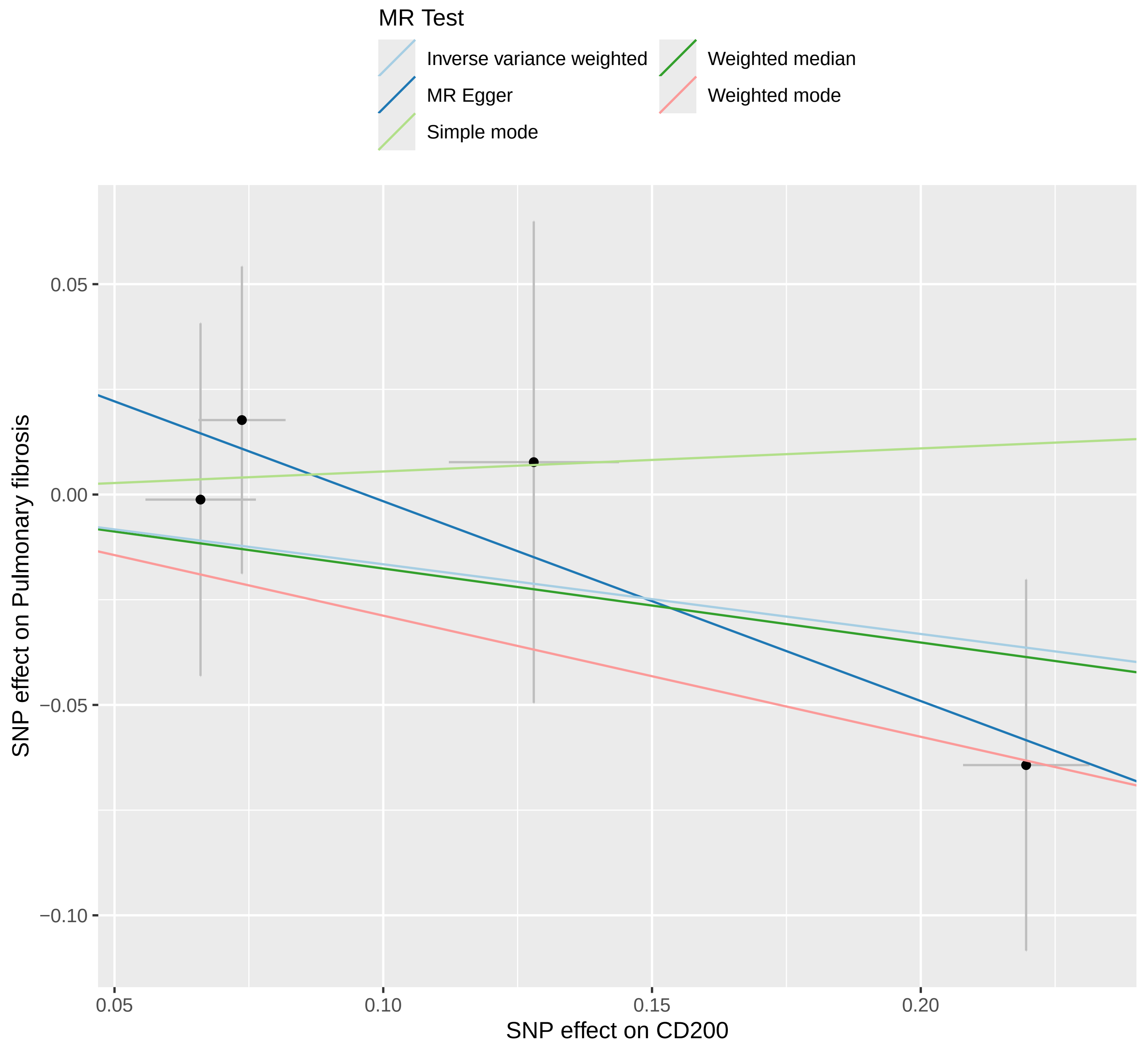

MR Test
Inverse variance weighted
MR Egger
Simple mode
Weighted median
Weighted mode
0.05
0.00
−0.05
−0.10
0.05
0.10
0.15
0.20
SNP effect on Pulmonary fibrosis
SNP effect on CD200

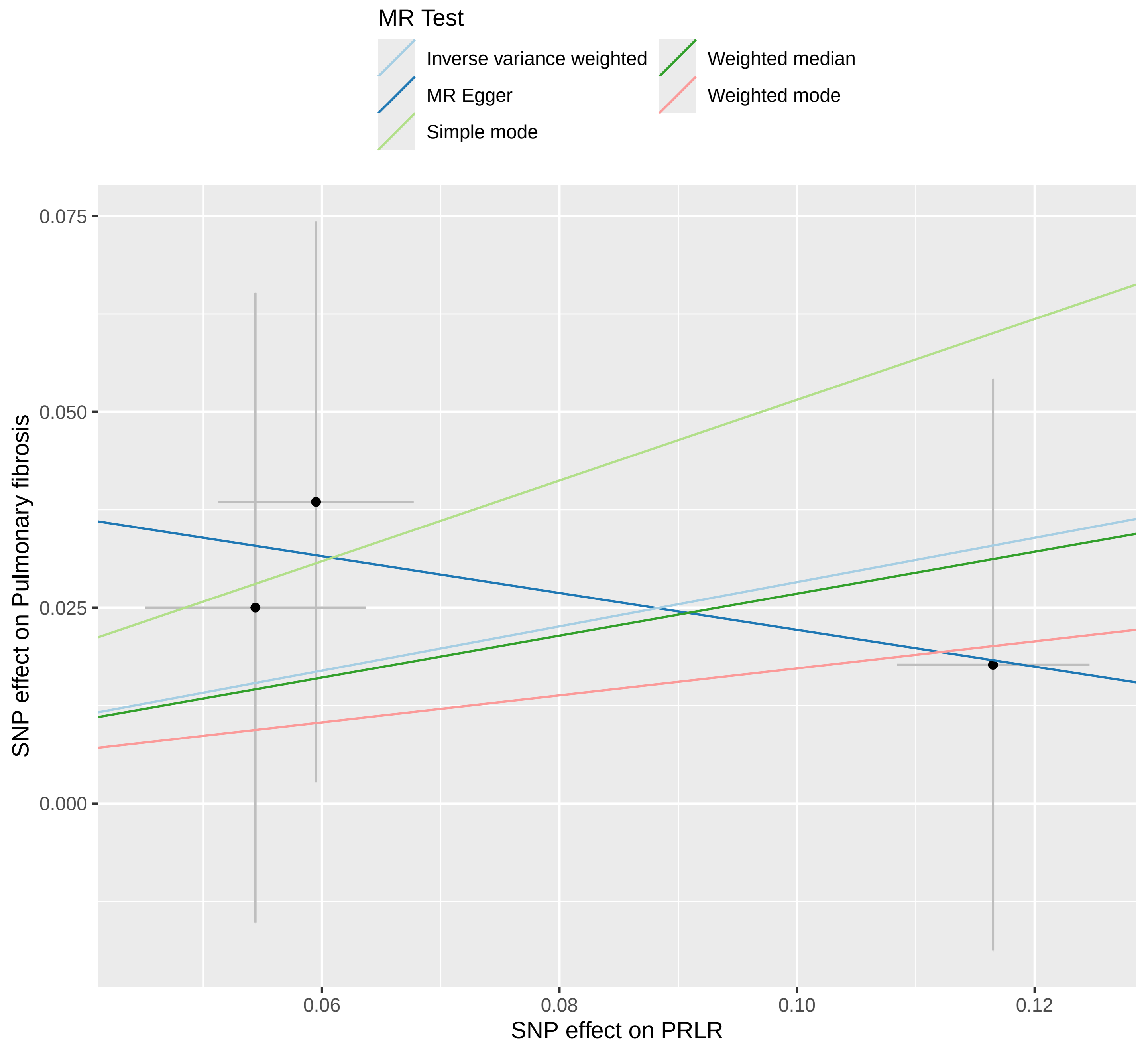
MR Test
Inverse variance weighted
MR Egger
Simple mode
Weighted median
Weighted mode
SNP effect on Pulmonary fibrosis
0.075
0.050
0.025
0.000
0.06
0.08
0.10
0.12
SNP effect on PRLR

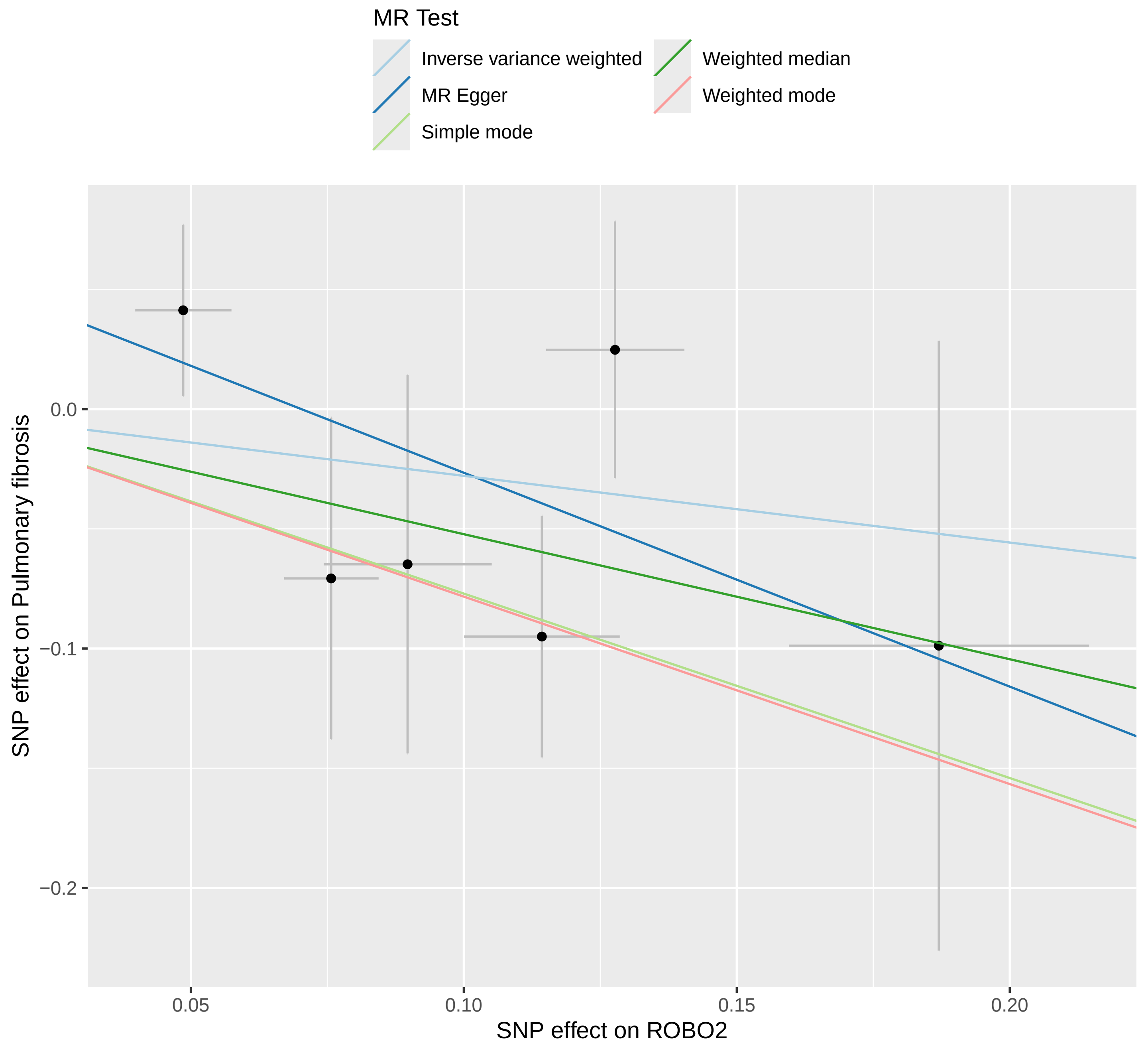
MR Test
Inverse variance weighted
MR Egger
Simple mode
Weighted median
Weighted mode
0.0
−0.1
−0.2
SNP effect on Pulmonary fibrosis
0.05
0.10
0.15
0.20
SNP effect on ROBO2

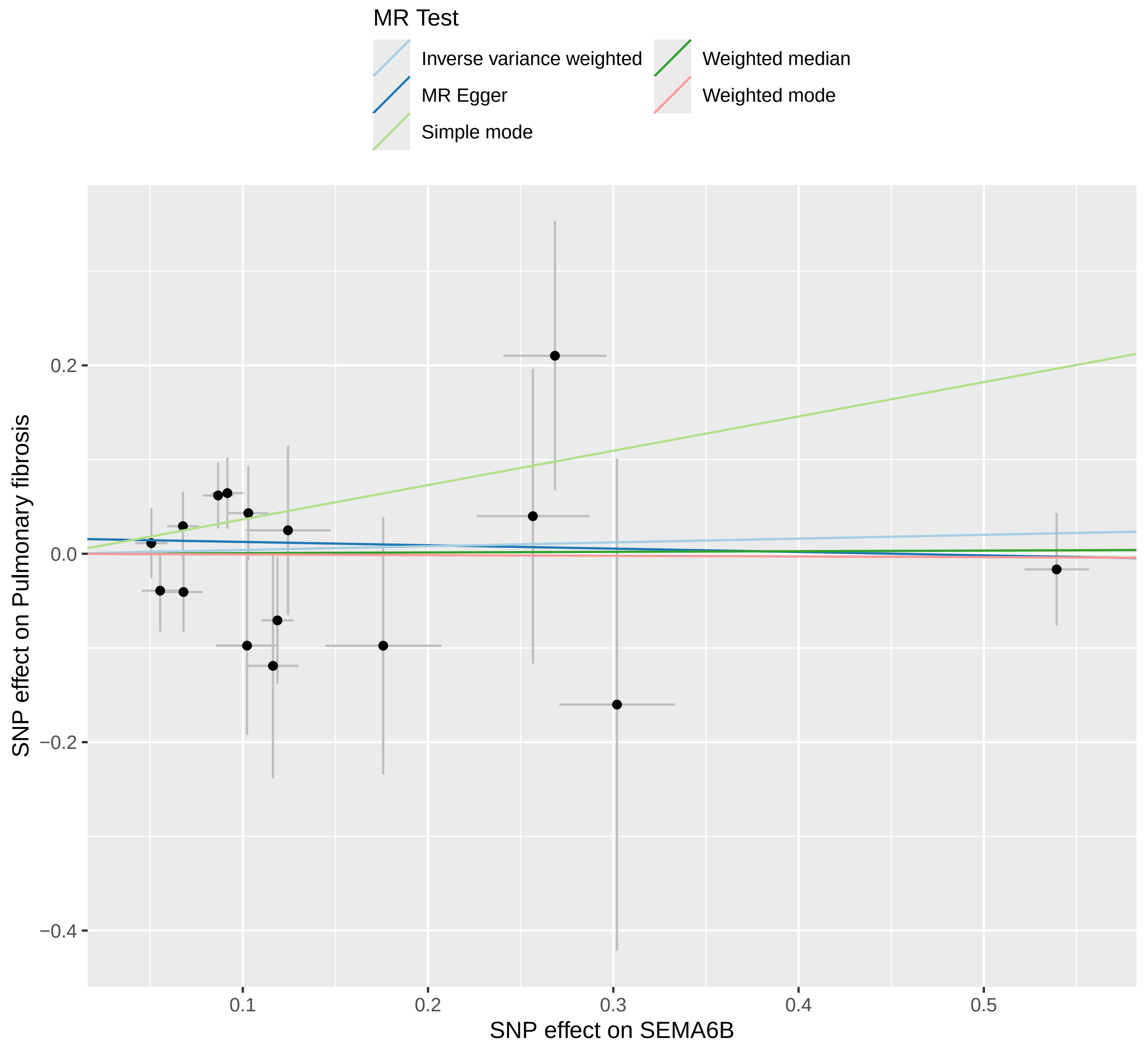

MR Test
Inverse variance weighted
MR Egger
Simple mode
Weighted median
Weighted mode
0.2
0.0
−0.2
−0.4
0.1
0.2
0.3
0.4
0.5
SNP effect on Pulmonary fibrosis
SNP effect on SEMA6B

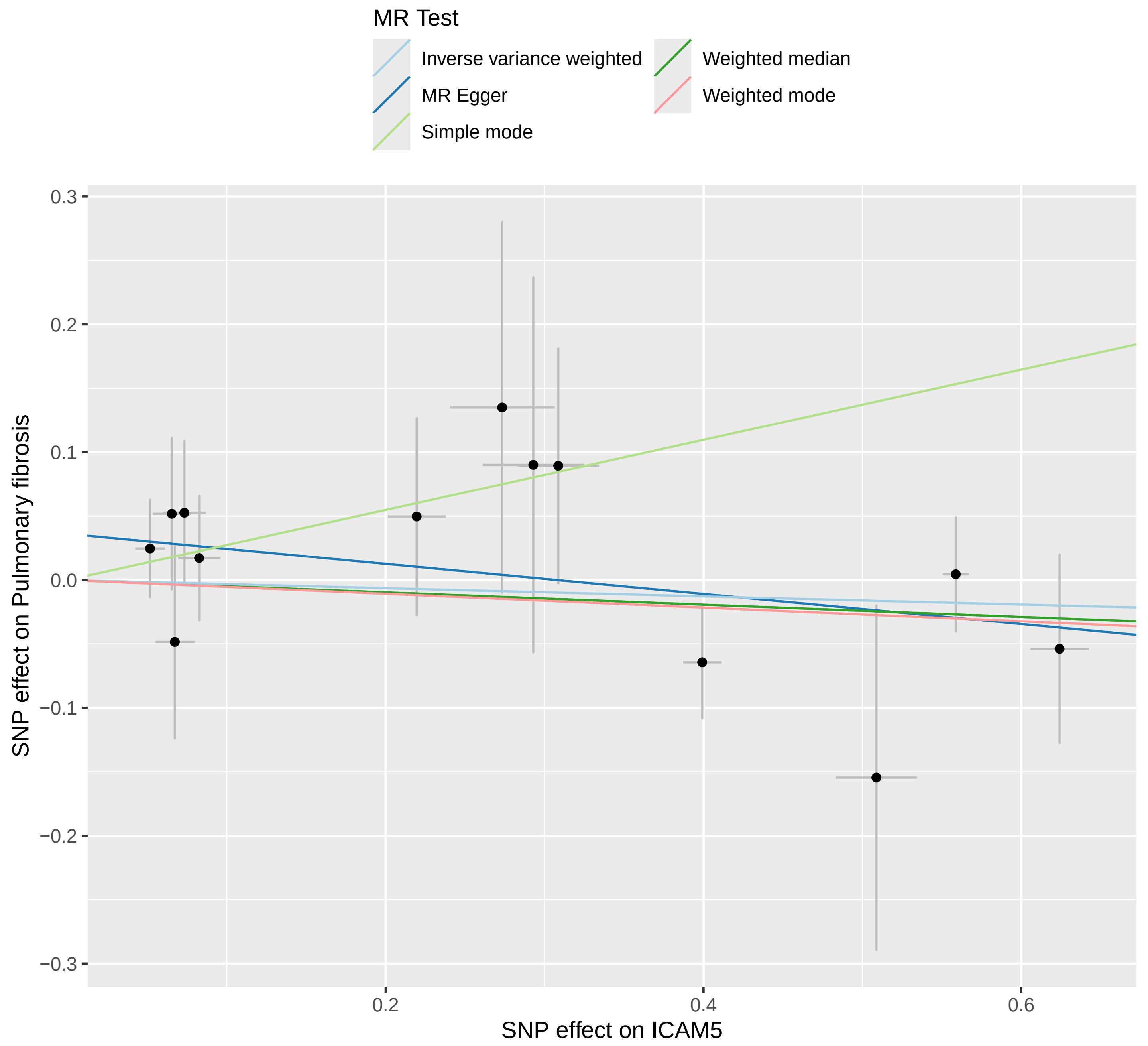

MR Test
Inverse variance weighted
MR Egger
Simple mode
Weighted median
Weighted mode
SNP effect on Pulmonary fibrosis
0.3
0.2
0.1
0.0
−0.1
−0.2
−0.3
0.2
0.4
0.6
SNP effect on ICAM5

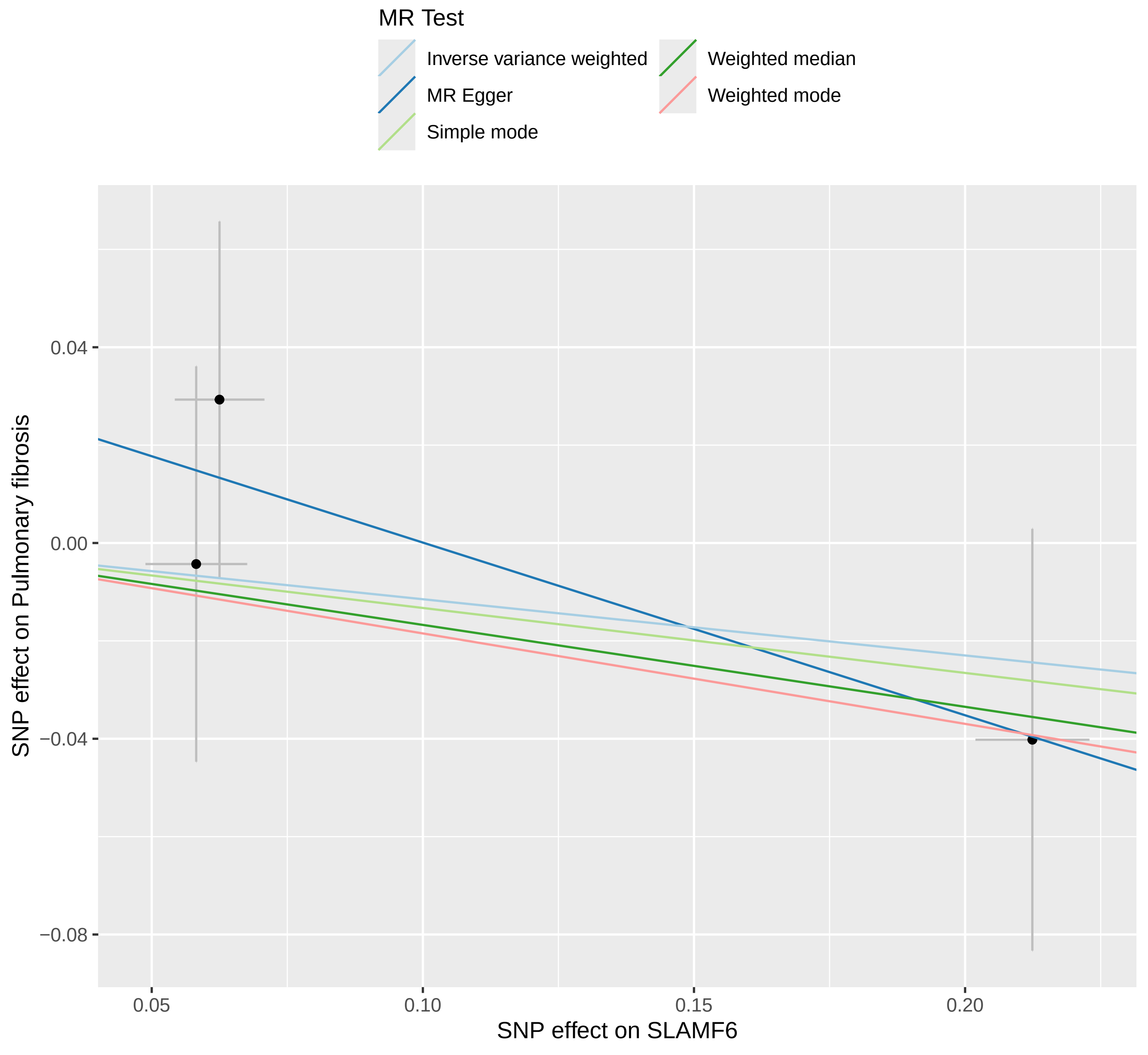

MR Test
Inverse variance weighted
MR Egger
Simple mode
Weighted median
Weighted mode
SNP effect on Pulmonary fibrosis
0.04
0.00
−0.04
−0.08
0.05
0.10
0.15
0.20
SNP effect on SLAMF6

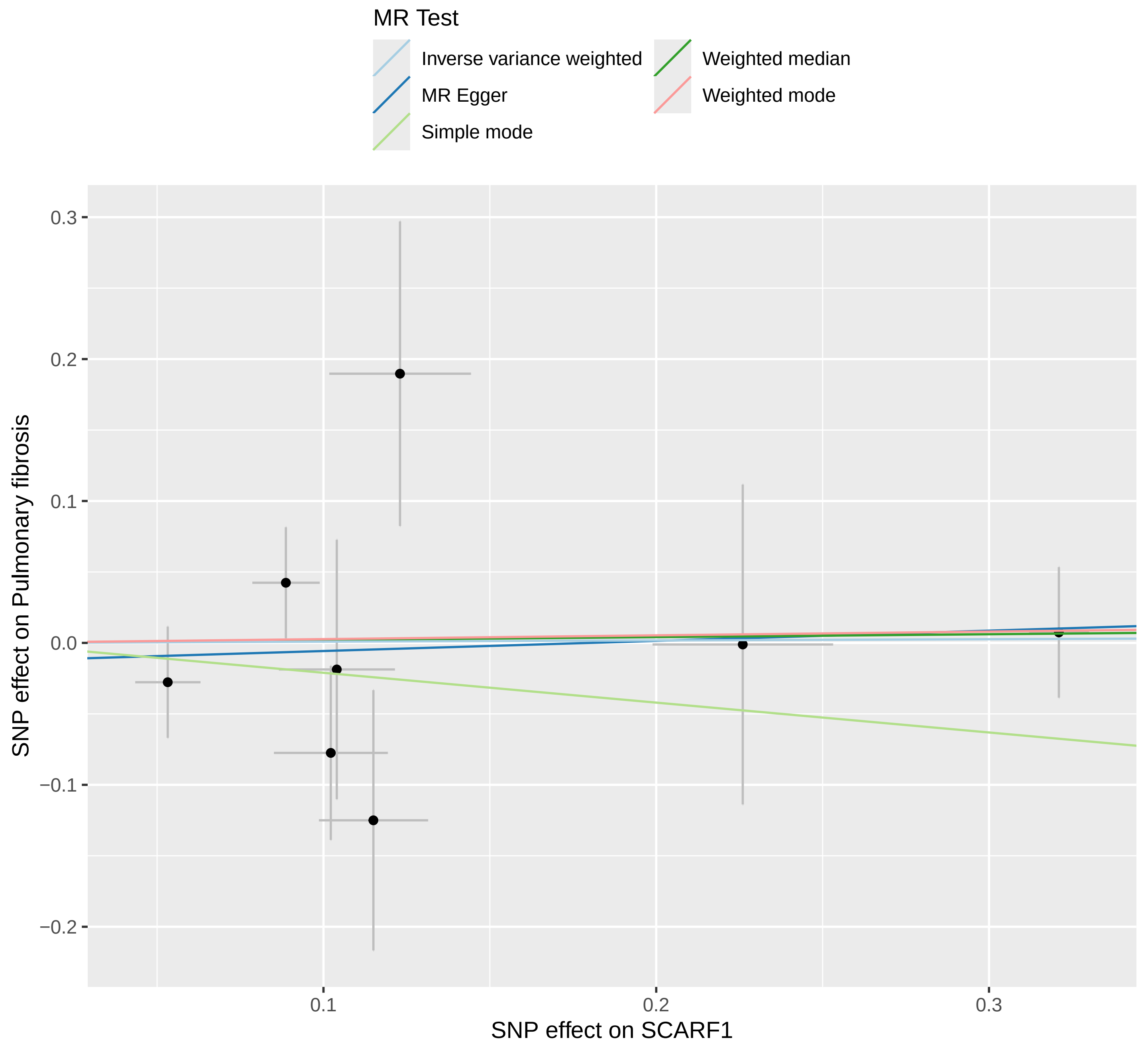

MR Test
Inverse variance weighted
MR Egger
Simple mode
Weighted median
Weighted mode
0.3
0.2
0.1
0.0
−0.1
−0.2
SNP effect on Pulmonary fibrosis
0.1
0.2
0.3
SNP effect on SCARF1

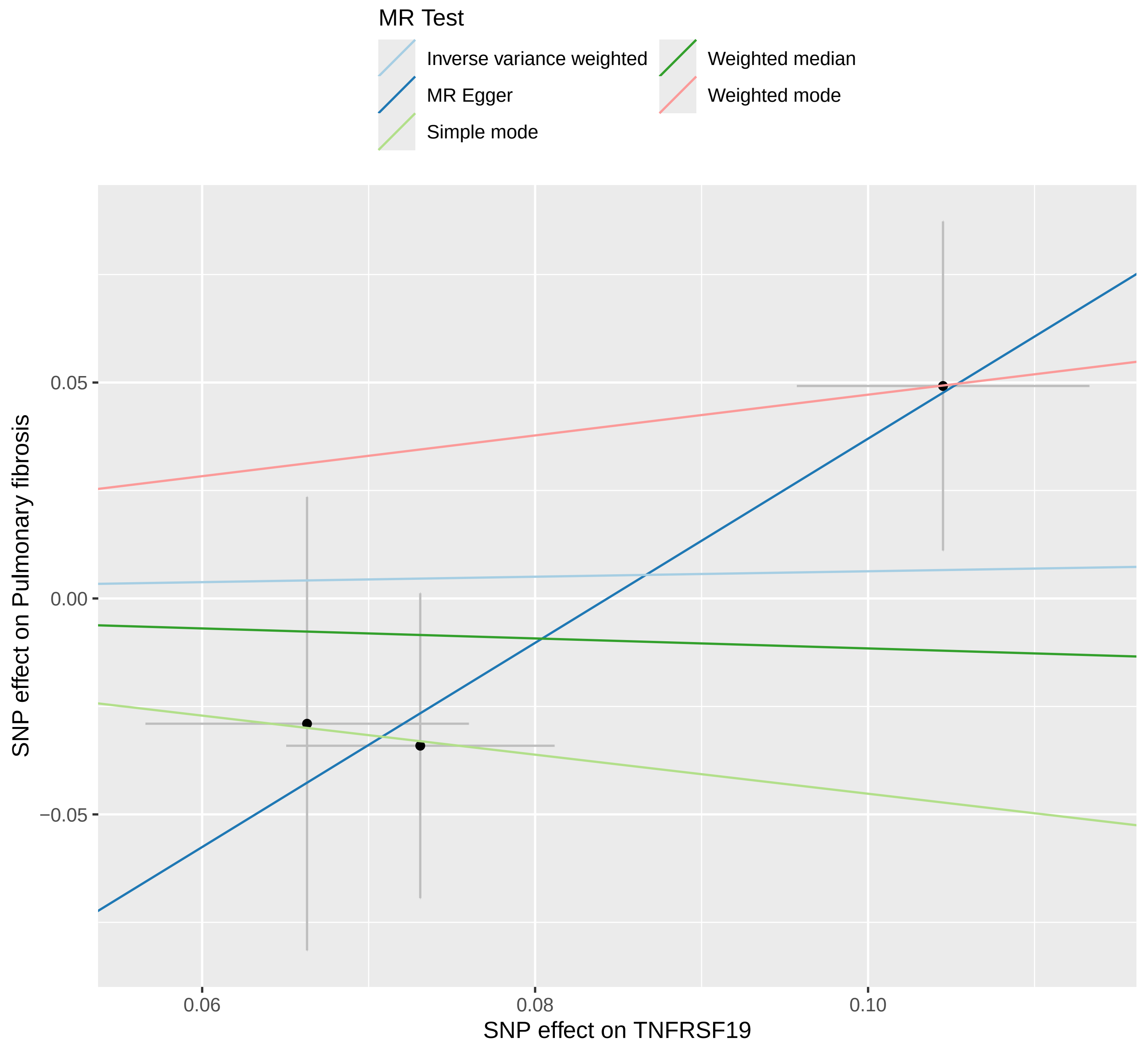

MR Test
Inverse variance weighted
MR Egger
Simple mode
Weighted median
Weighted mode
SNP effect on Pulmonary fibrosis
0.05
0.00
−0.05
0.06
0.08
0.10
SNP effect on TNFRSF19

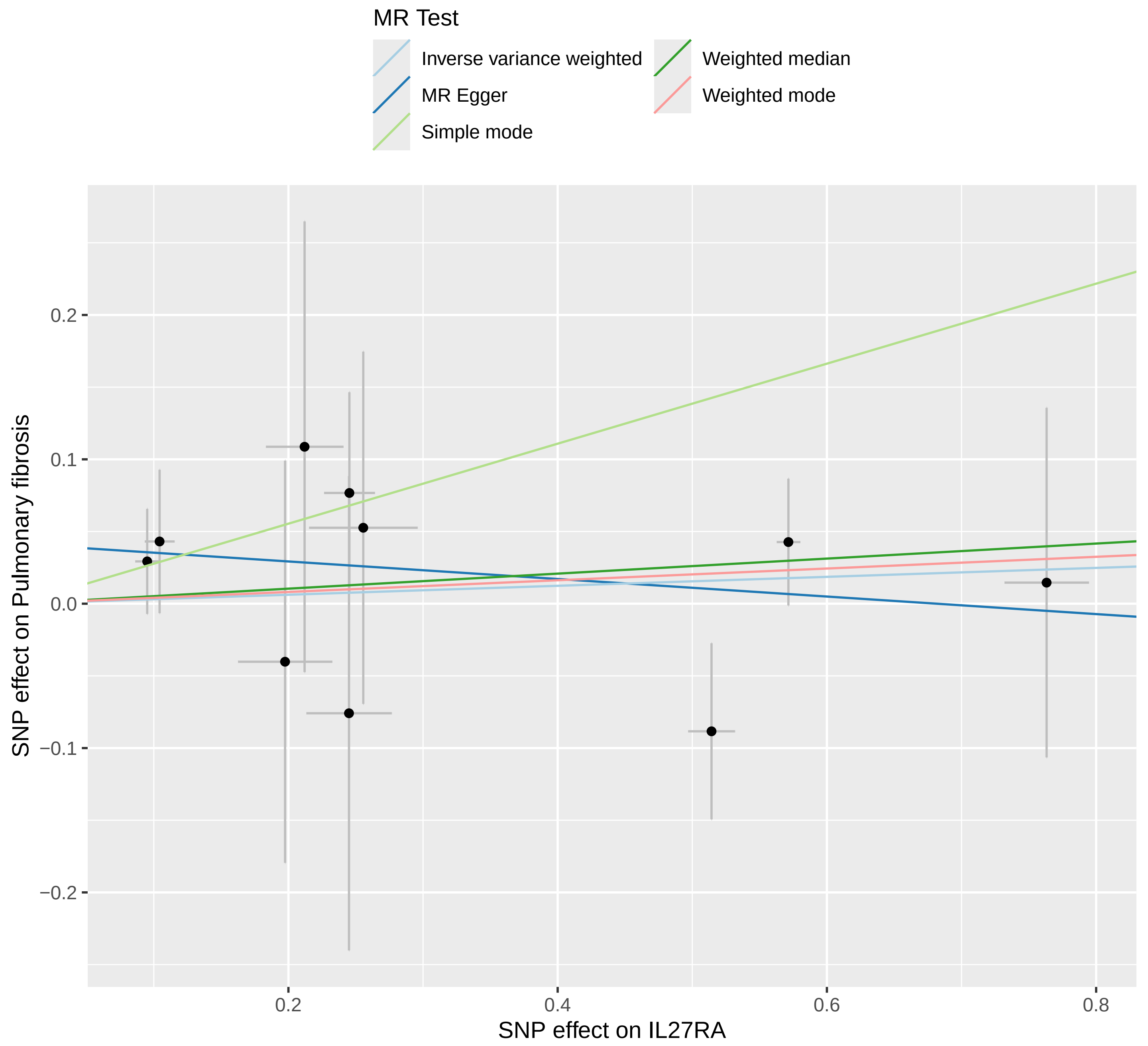

MR Test
Inverse variance weighted
MR Egger
Simple mode
Weighted median
Weighted mode
SNP effect on Pulmonary fibrosis
0.2
0.1
0.0
−0.1
−0.2
0.2
0.4
0.6
0.8
SNP effect on IL27RA

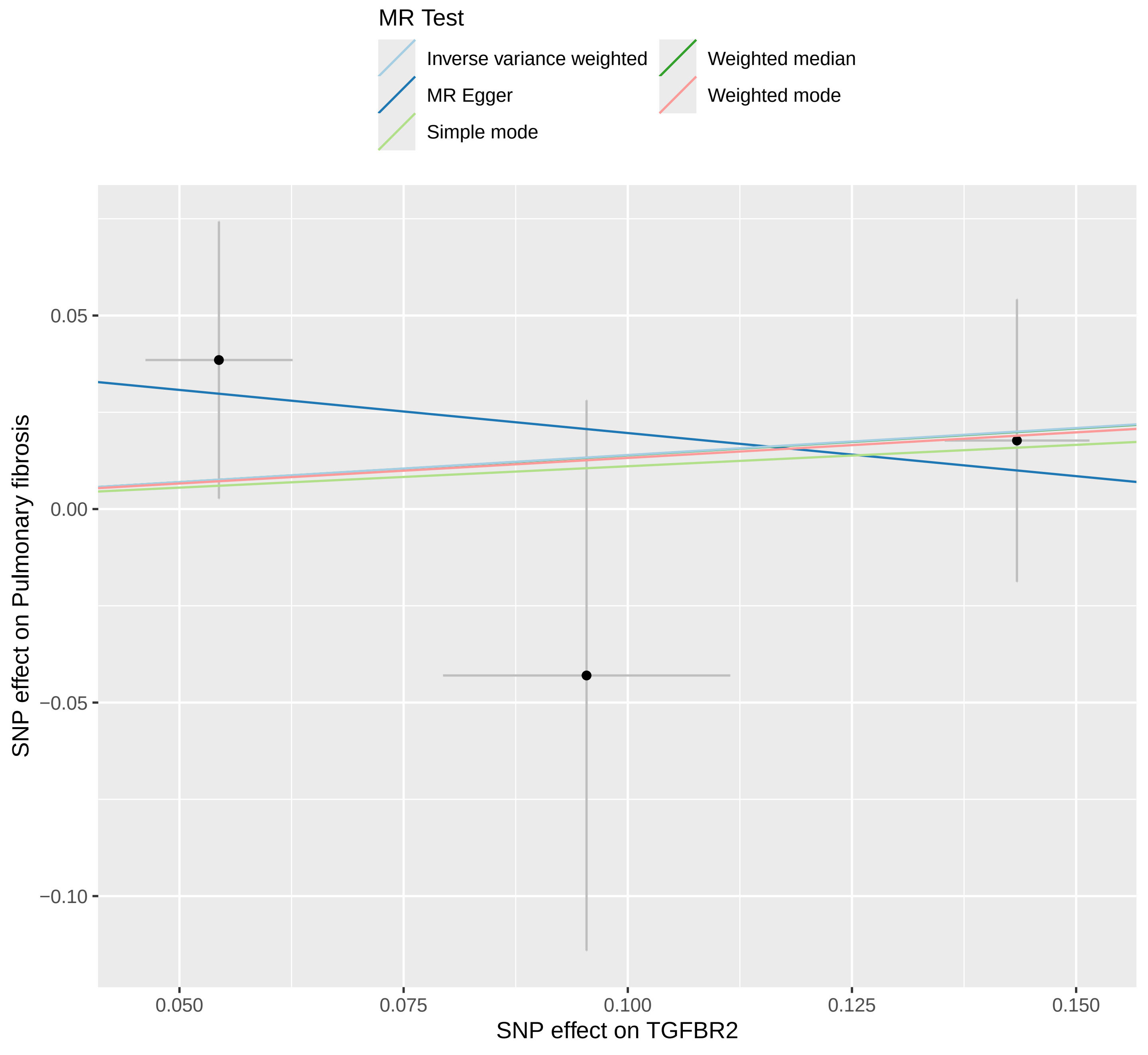

MR Test
Inverse variance weighted
MR Egger
Simple mode
Weighted median
Weighted mode
0.05
0.00
−0.05
−0.10
0.050
0.075
0.100
0.125
0.150
SNP effect on Pulmonary fibrosis
SNP effect on TGFBR2

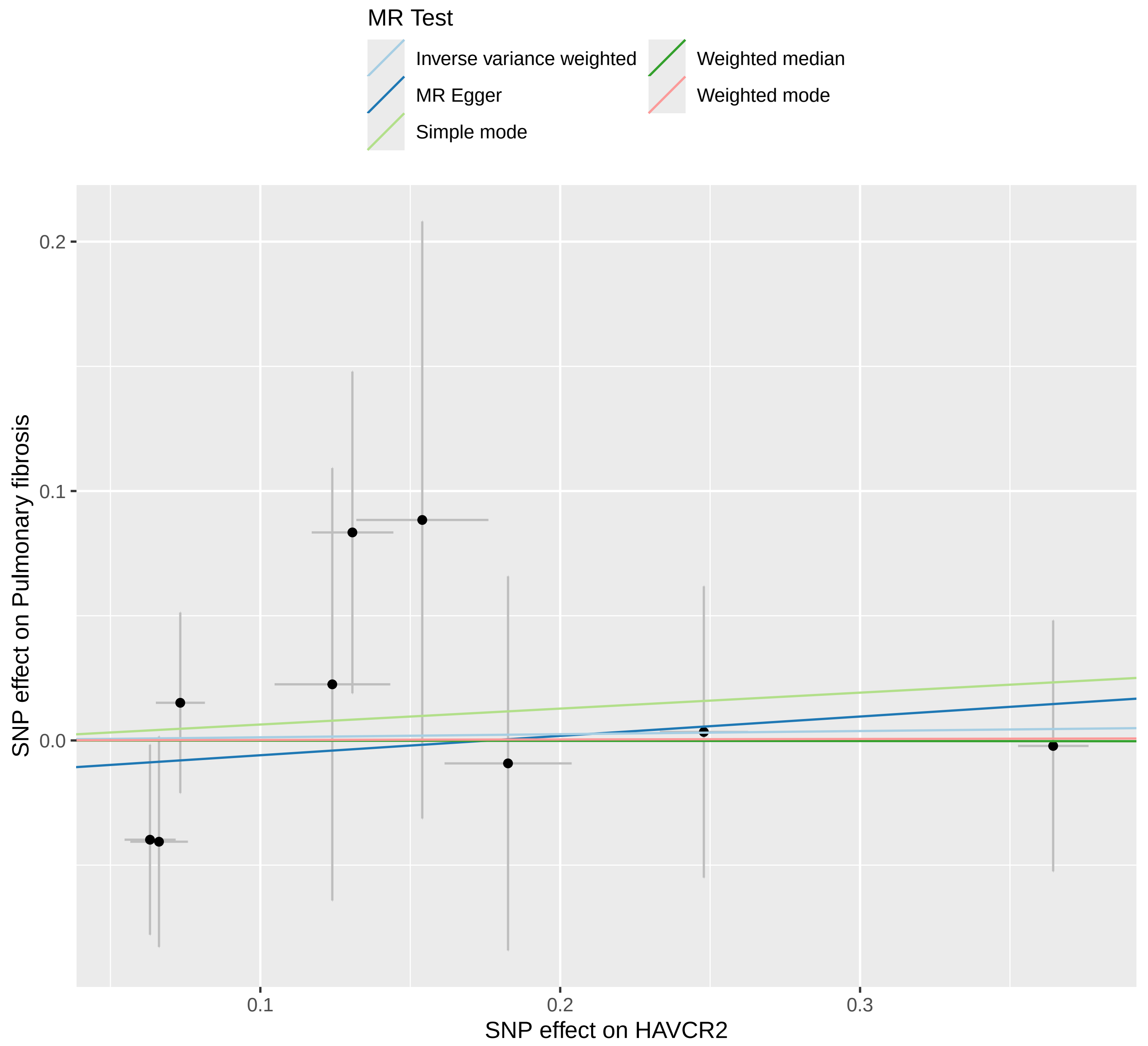

MR Test
Inverse variance weighted
MR Egger
Simple mode
Weighted median
Weighted mode
0.2
0.1
0.0
SNP effect on Pulmonary fibrosis
0.1
0.2
0.3
SNP effect on HAVCR2

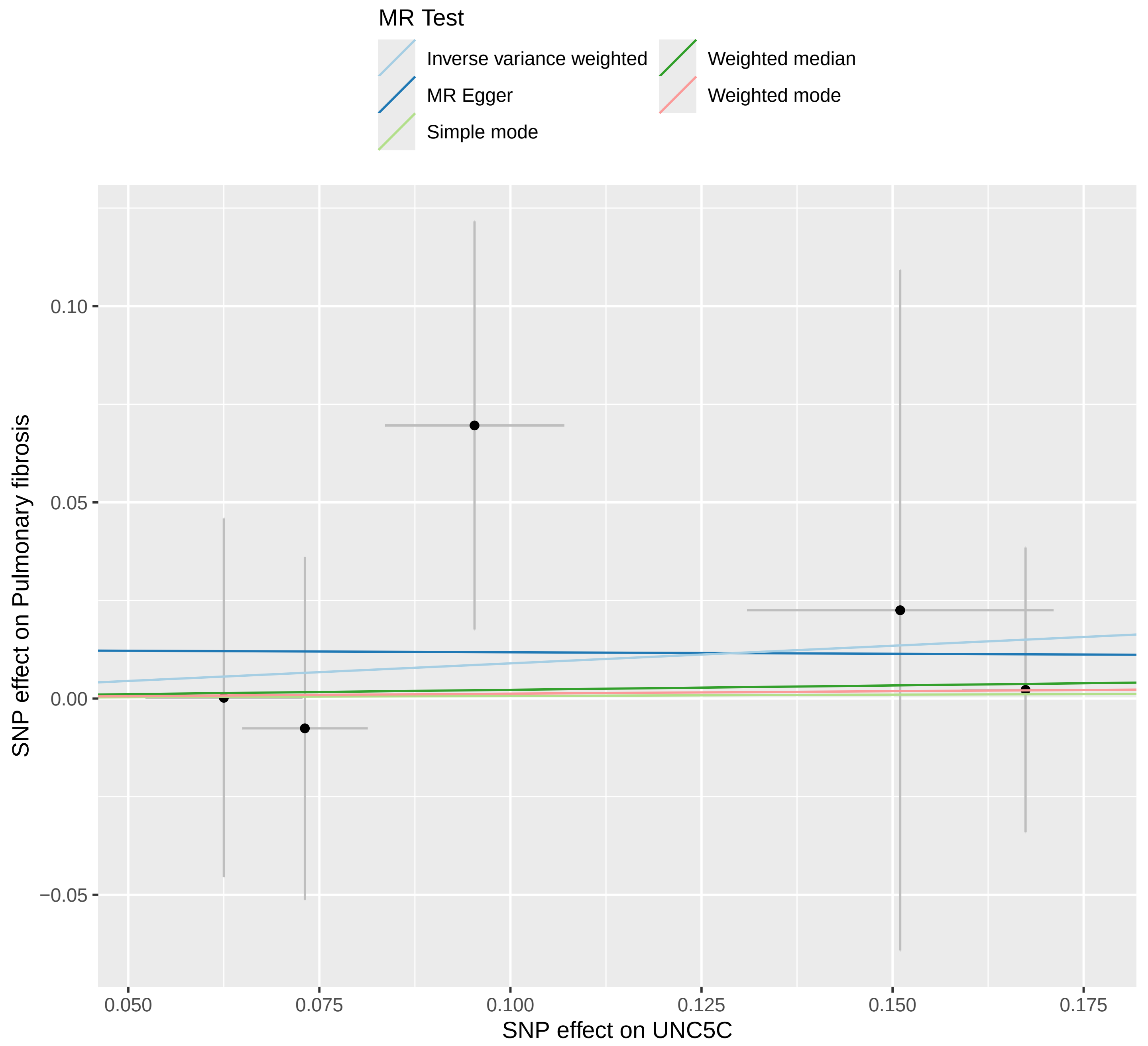

MR Test
Inverse variance weighted
MR Egger
Simple mode
Weighted median
Weighted mode
0.10
0.05
0.00
−0.05
SNP effect on Pulmonary fibrosis
0.050
0.075
0.100
0.125
0.150
0.175
SNP effect on UNC5C

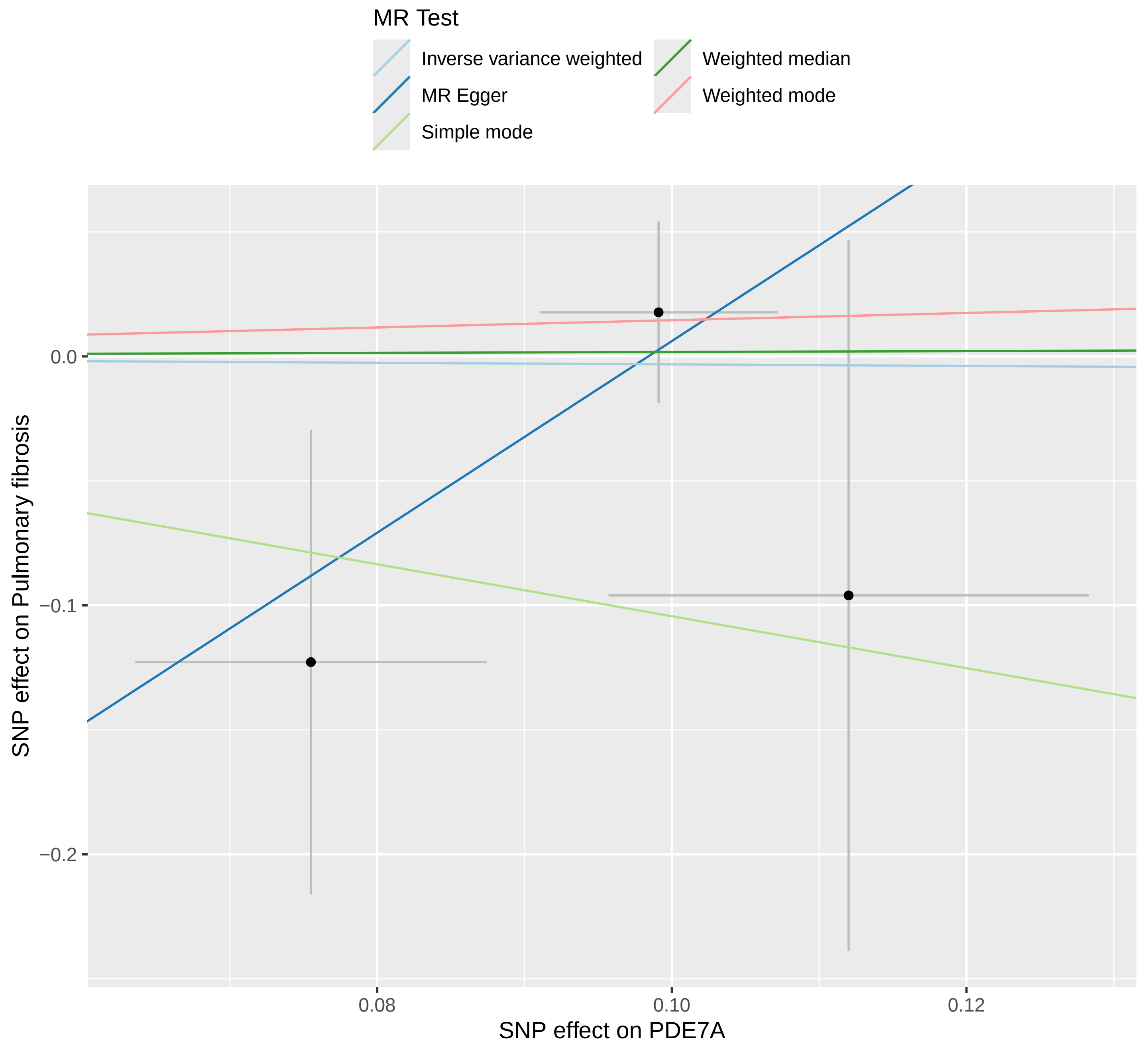

MR Test
Inverse variance weighted
MR Egger
Simple mode
Weighted median
Weighted mode
SNP effect on Pulmonary fibrosis
0.0
−0.1
−0.2
0.08
0.10
0.12
SNP effect on PDE7A

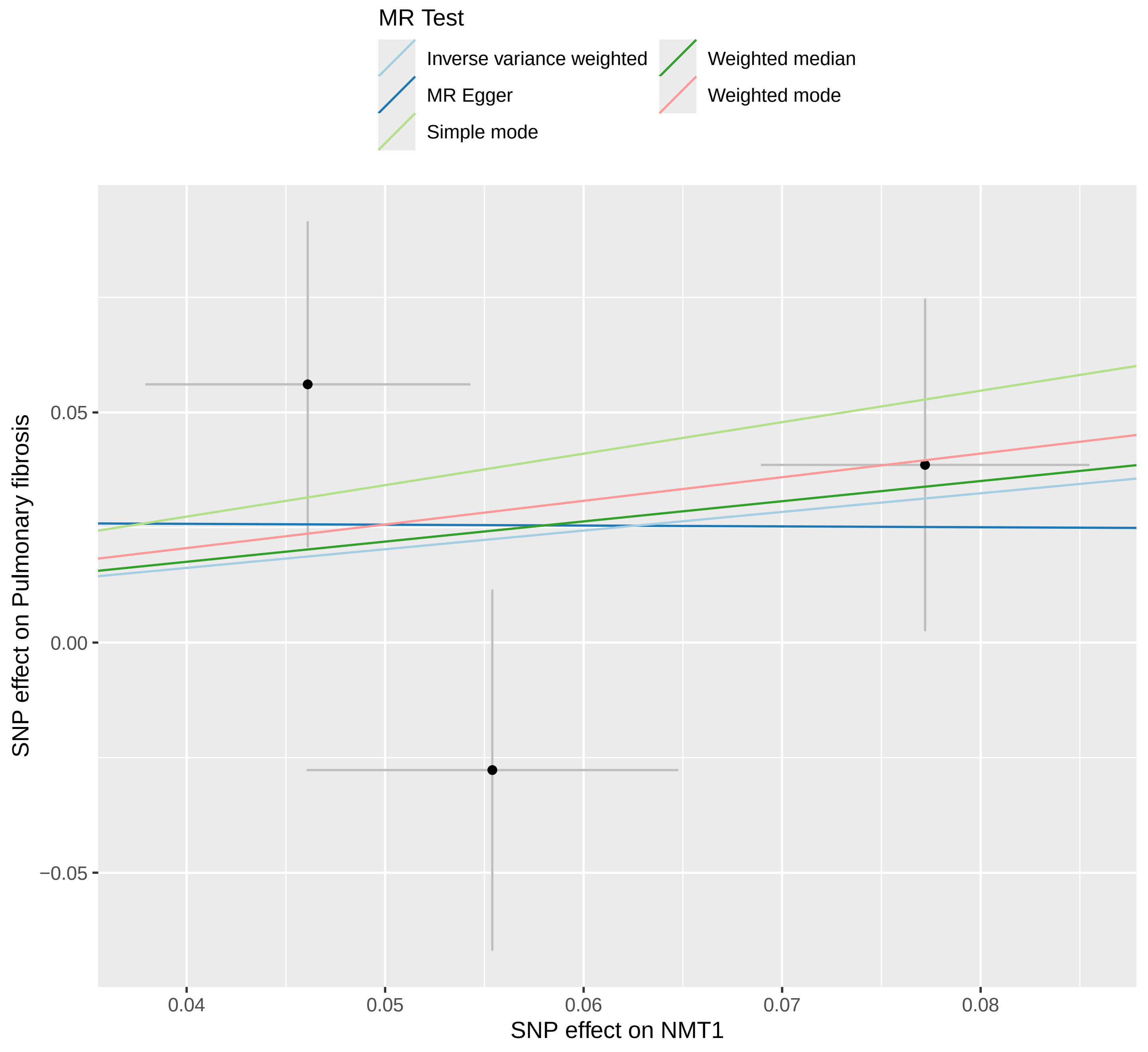

MR Test
Inverse variance weighted
MR Egger
Simple mode
Weighted median
Weighted mode
SNP effect on Pulmonary fibrosis
0.05
0.00
−0.05
0.04
0.05
0.06
0.07
0.08
SNP effect on NMT1

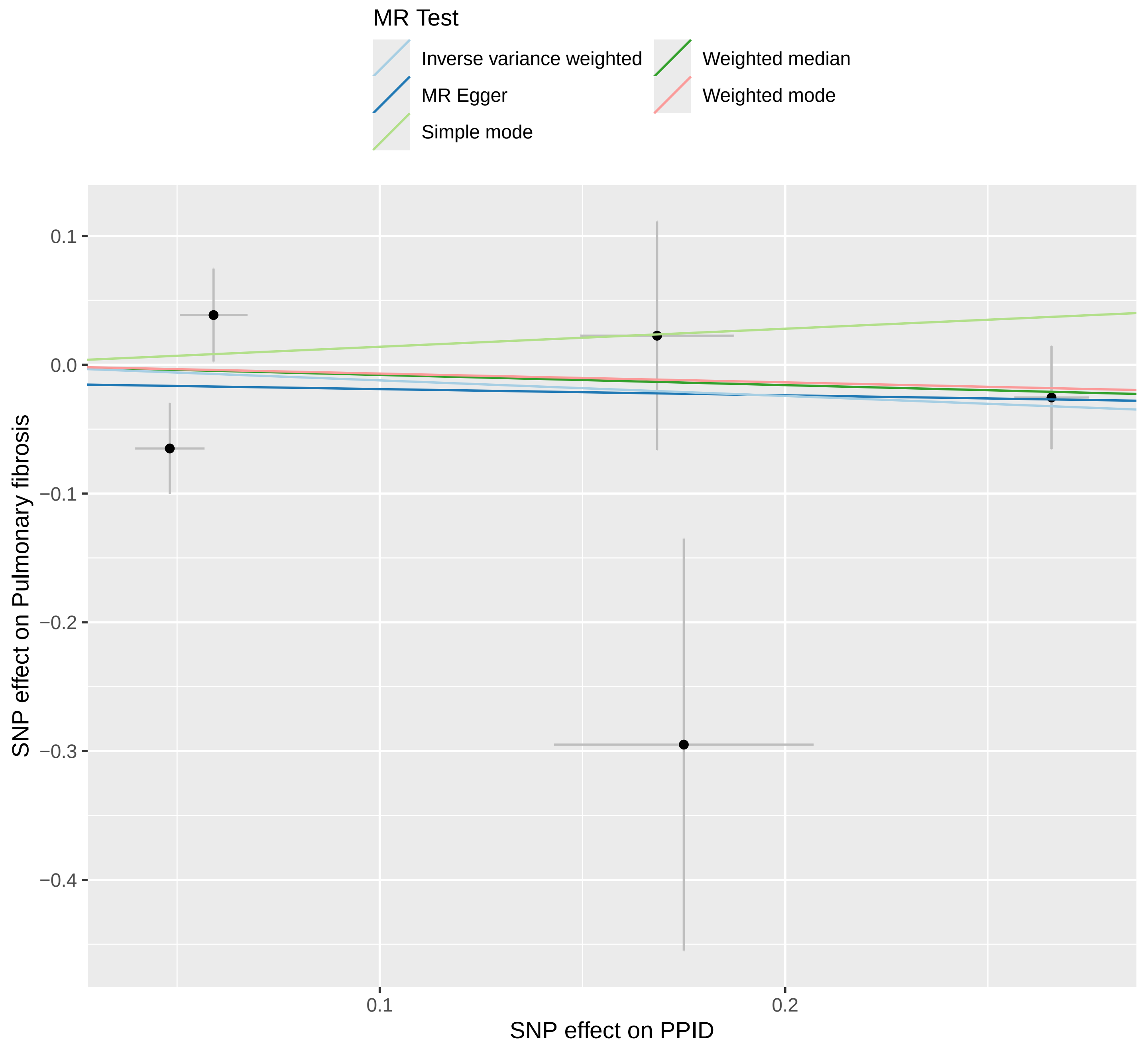
MR Test
Inverse variance weighted
MR Egger
Simple mode
Weighted median
Weighted mode
0.1
0.0
−0.1
−0.2
−0.3
−0.4
SNP effect on Pulmonary fibrosis
0.1
0.2
SNP effect on PPID

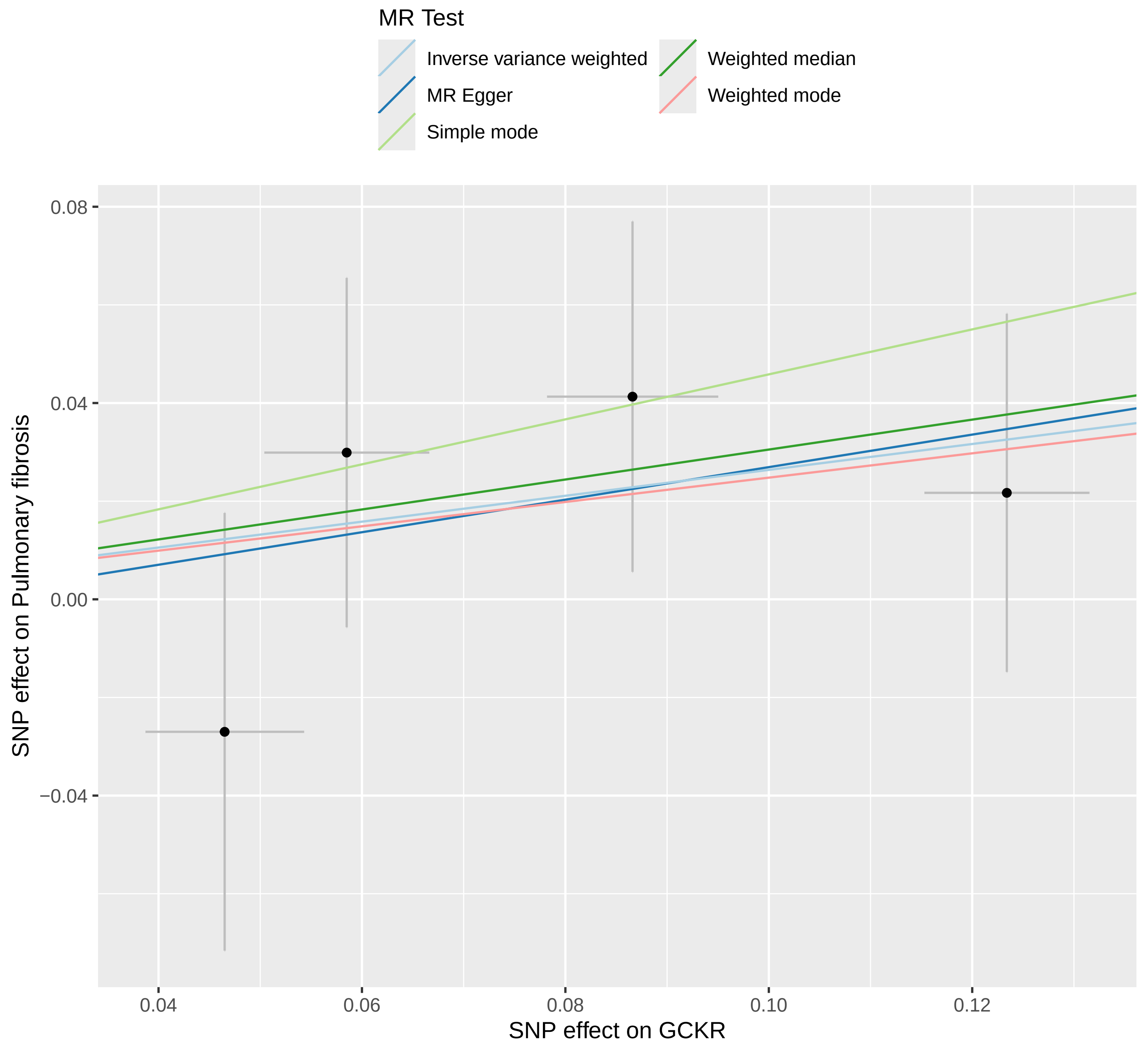

MR Test
Inverse variance weighted
MR Egger
Simple mode
Weighted median
Weighted mode
0.08
0.04
0.00
−0.04
0.04
0.06
0.08
0.10
0.12
SNP effect on GCKR
SNP effect on Pulmonary fibrosis

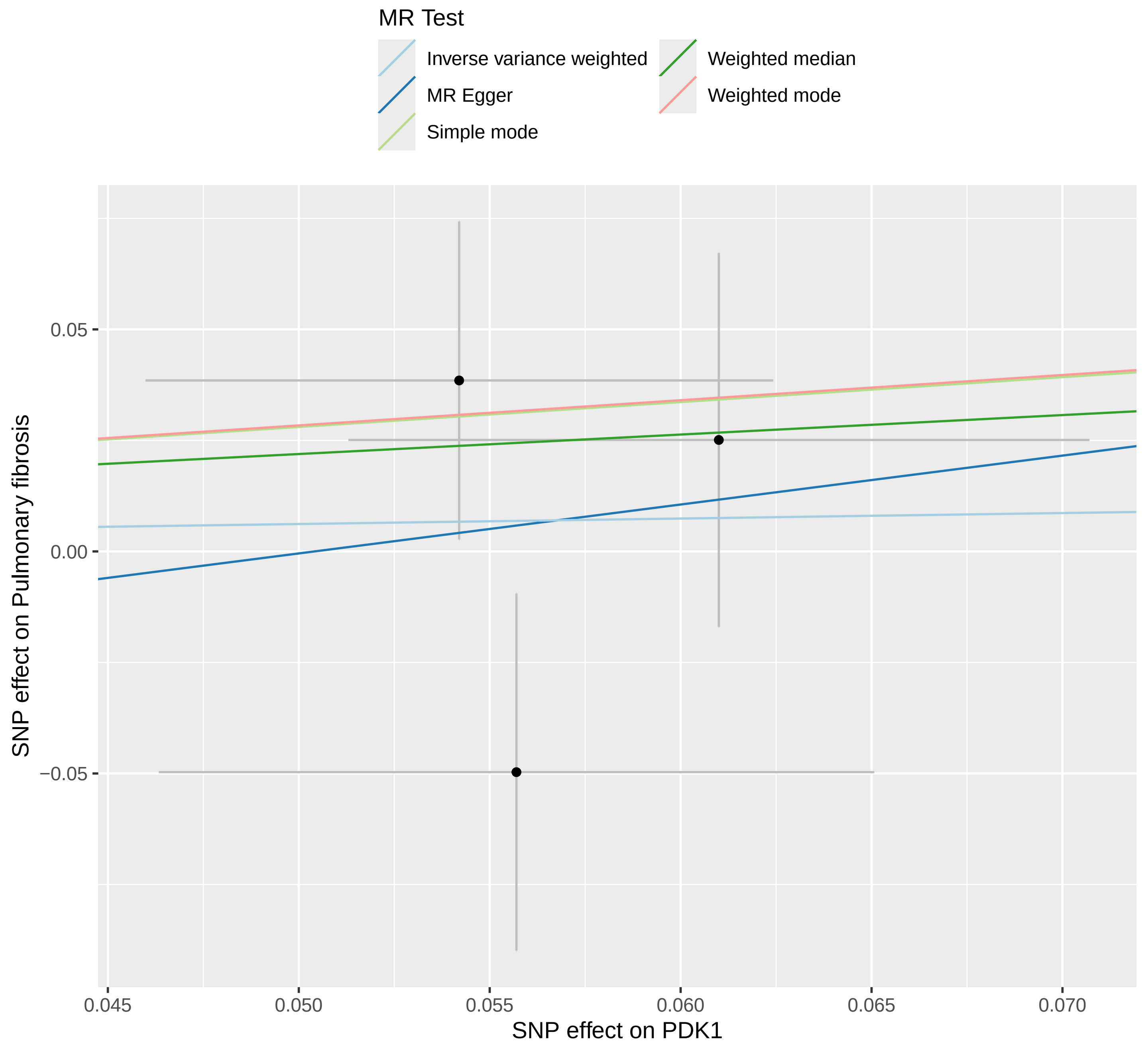

MR Test
Inverse variance weighted
MR Egger
Simple mode
Weighted median
Weighted mode
0.05
0.00
−0.05
SNP effect on Pulmonary fibrosis
0.045
0.050
0.055
0.060
0.065
0.070
SNP effect on PDK1

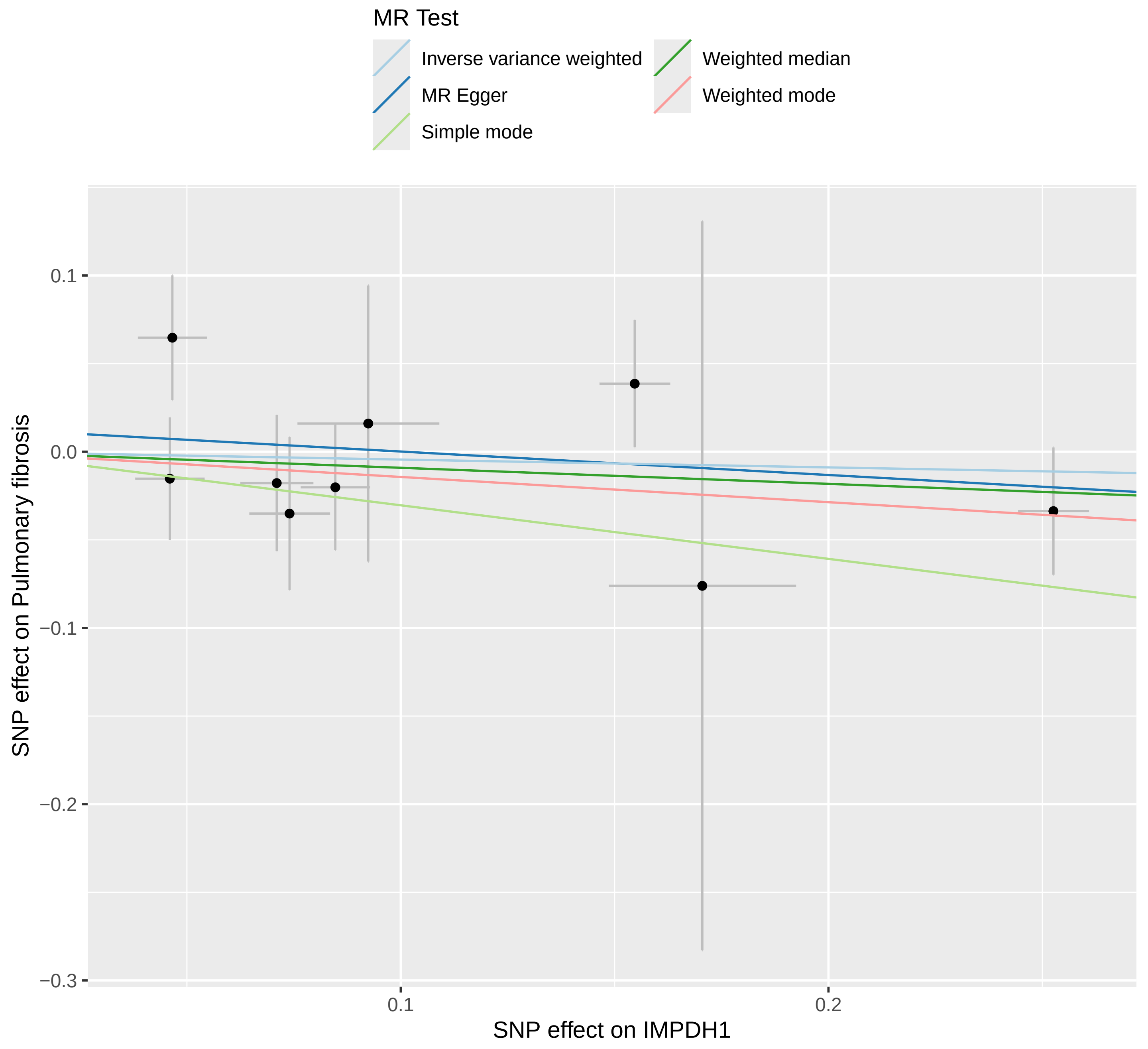

MR Test
Inverse variance weighted
MR Egger
Simple mode
Weighted median
Weighted mode
0.1
0.0
−0.1
−0.2
−0.3
SNP effect on Pulmonary fibrosis
0.1
0.2
SNP effect on IMPDH1

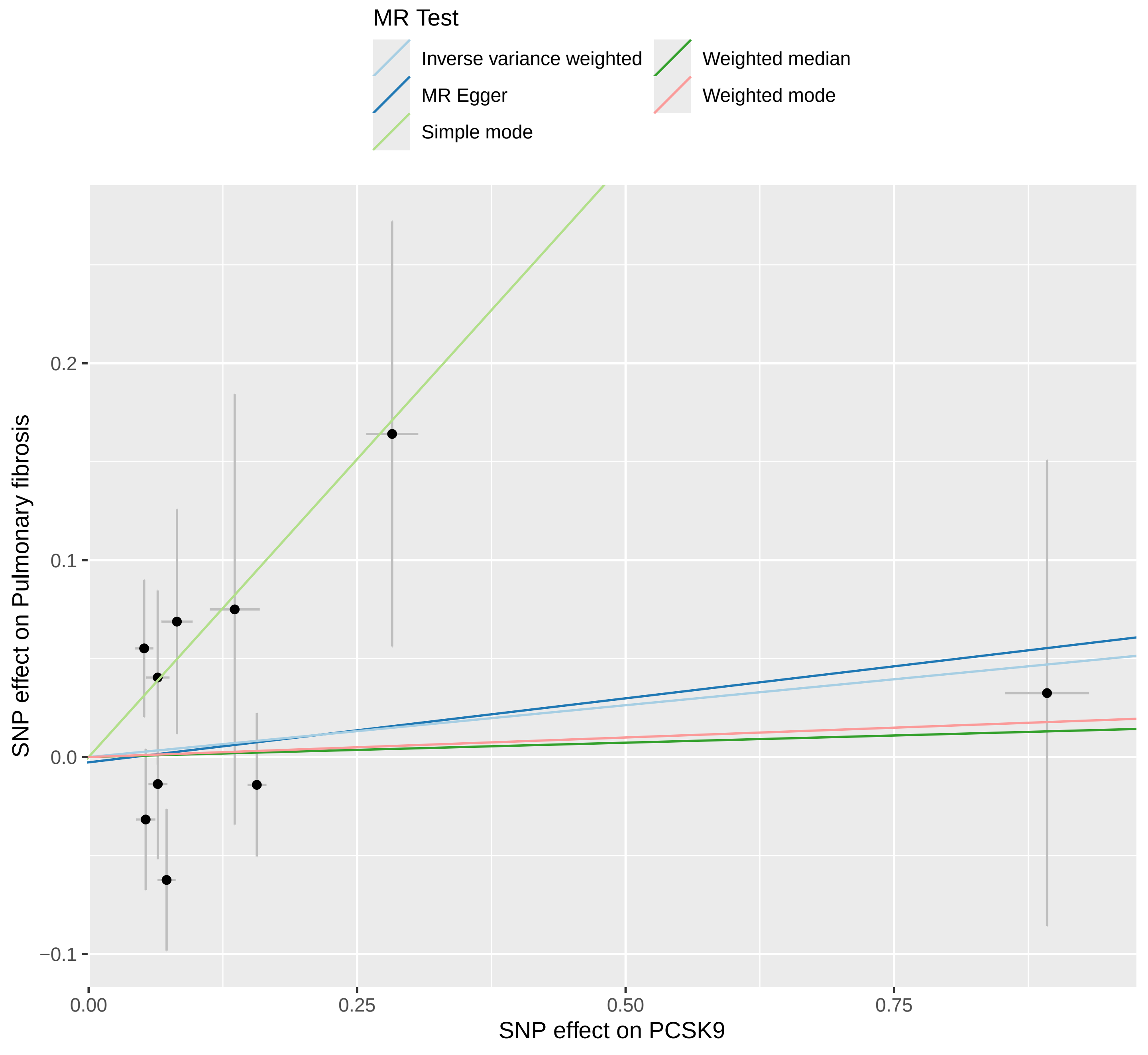

MR Test
Inverse variance weighted
MR Egger
Simple mode
Weighted median
Weighted mode
SNP effect on Pulmonary fibrosis
0.2
0.1
0.0
−0.1
0.00
0.25
0.50
0.75
SNP effect on PCSK9

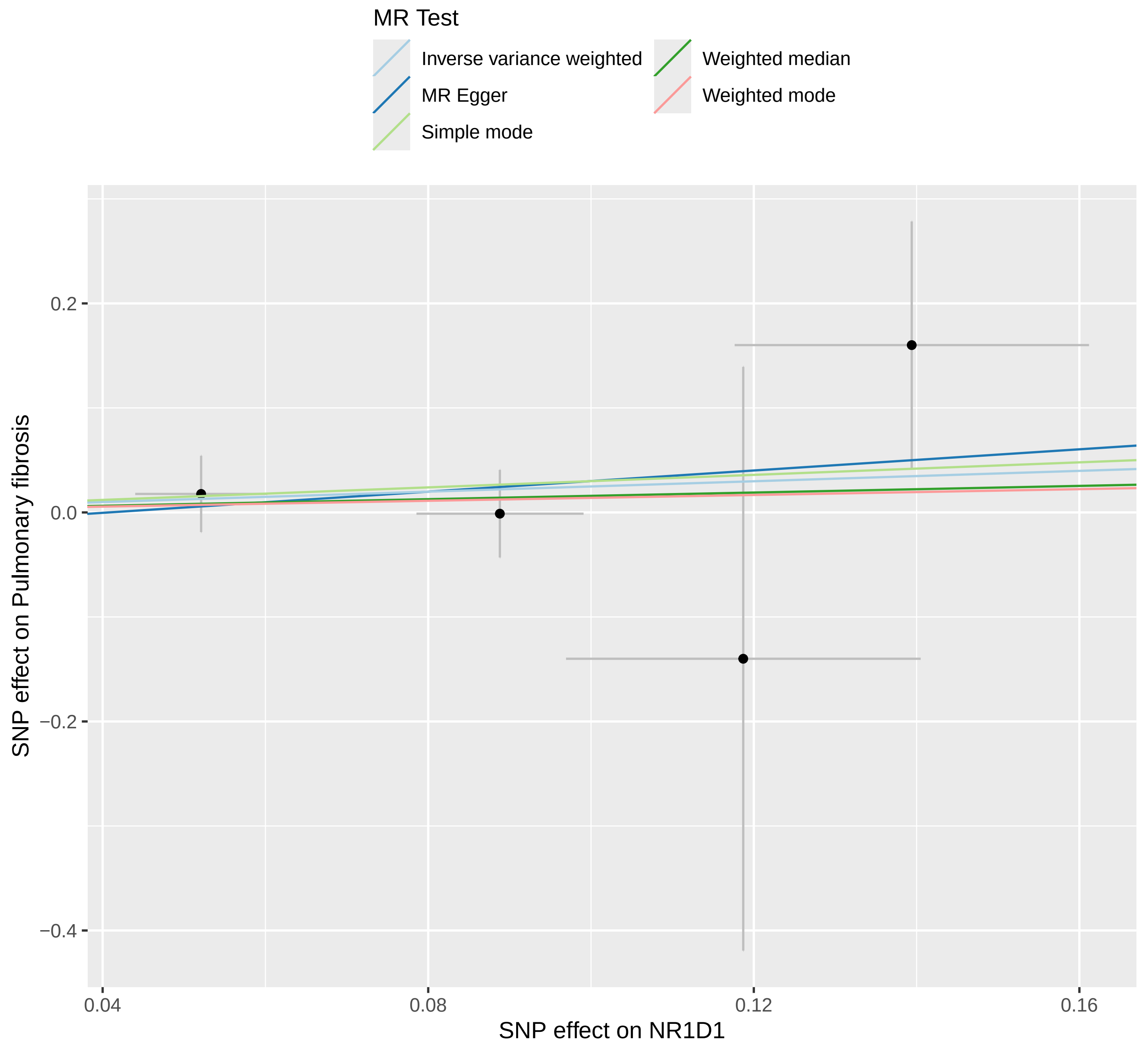

MR Test
Inverse variance weighted
MR Egger
Simple mode
Weighted median
Weighted mode
0.2
0.0
−0.2
−0.4
0.04
0.08
0.12
0.16
SNP effect on NR1D1
SNP effect on Pulmonary fibrosis

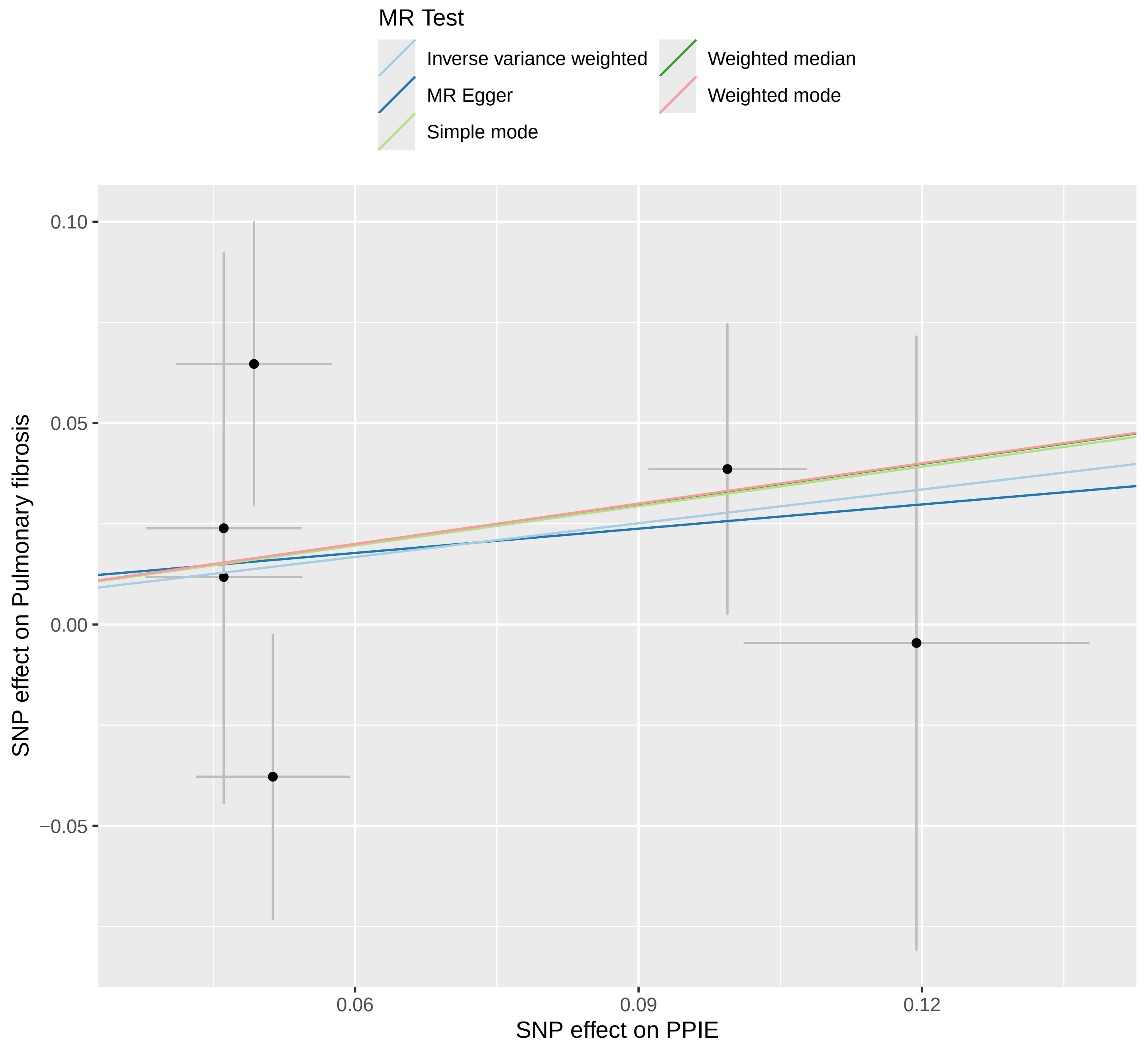
MR Test
Inverse variance weighted
MR Egger
Simple mode
Weighted median
Weighted mode
0.10
0.05
0.00
−0.05
SNP effect on Pulmonary fibrosis
0.06
0.09
0.12
SNP effect on PPIE

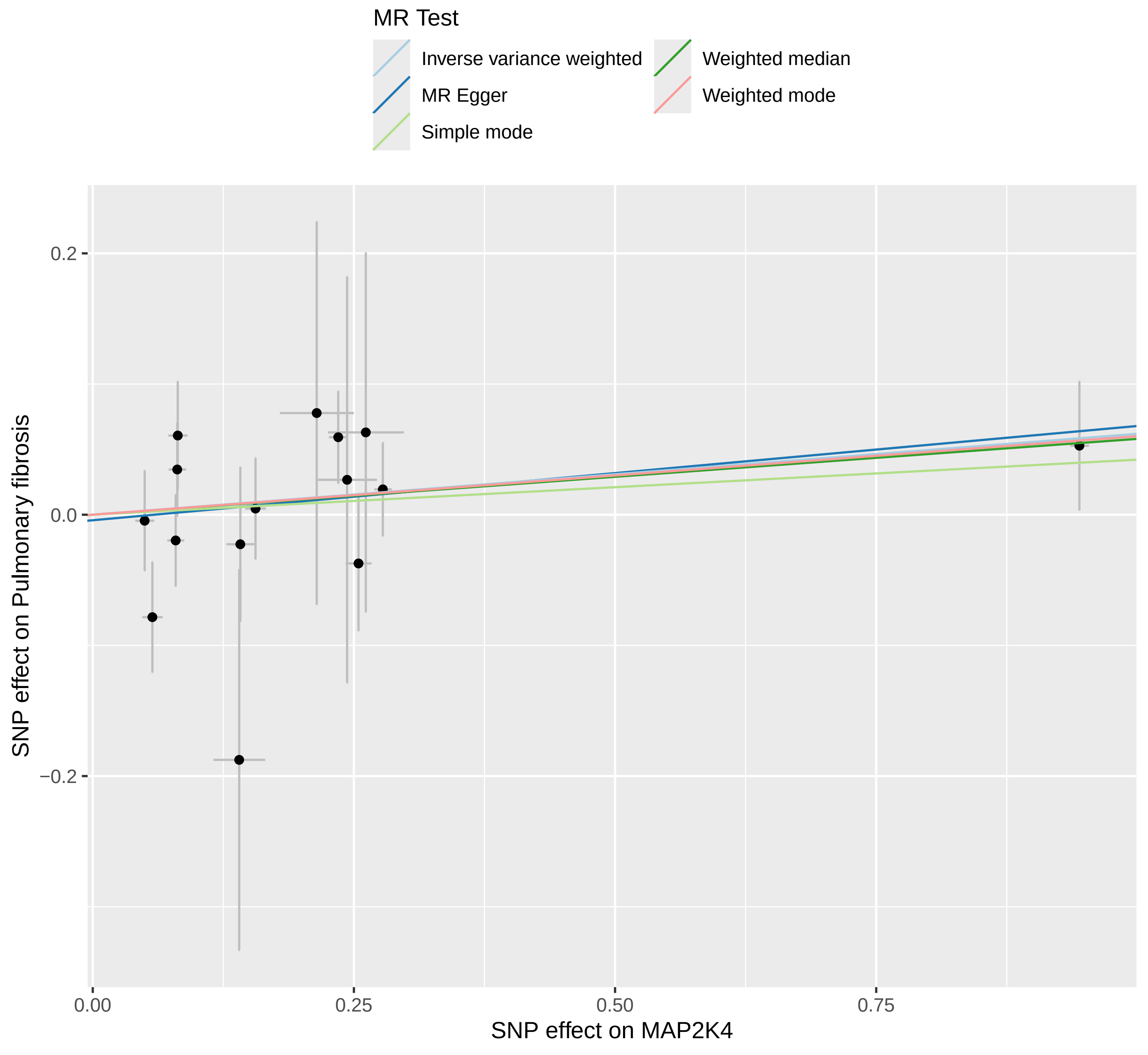

MR Test
Inverse variance weighted
MR Egger
Simple mode
Weighted median
Weighted mode
0.2
0.0
−0.2
0.00
0.25
0.50
0.75
SNP effect on MAP2K4
SNP effect on Pulmonary fibrosis

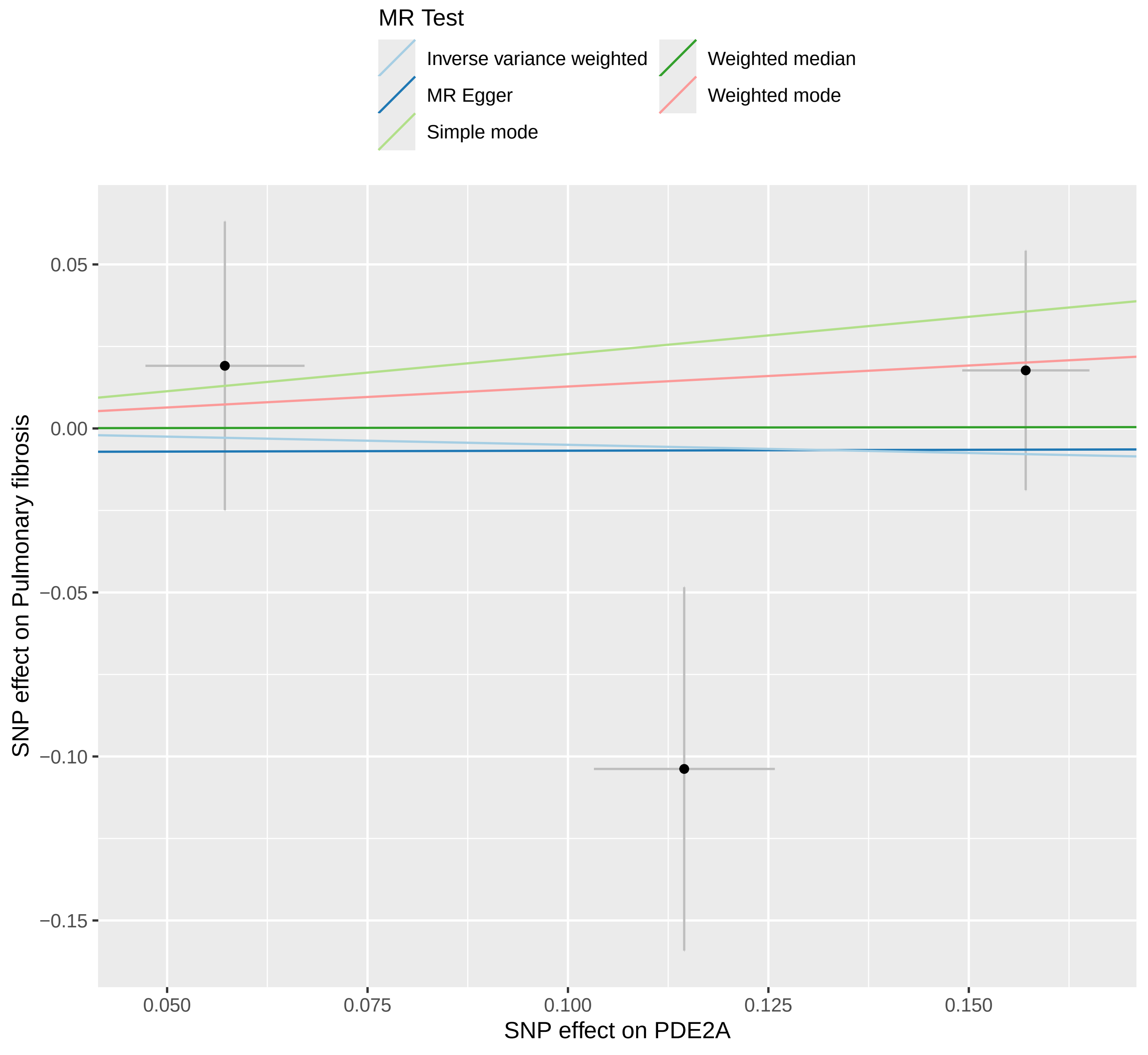
MR Test
Inverse variance weighted
MR Egger
Simple mode
Weighted median
Weighted mode
0.05
0.00
−0.05
−0.10
−0.15
SNP effect on Pulmonary fibrosis
0.050
0.075
0.100
0.125
0.150
SNP effect on PDE2A

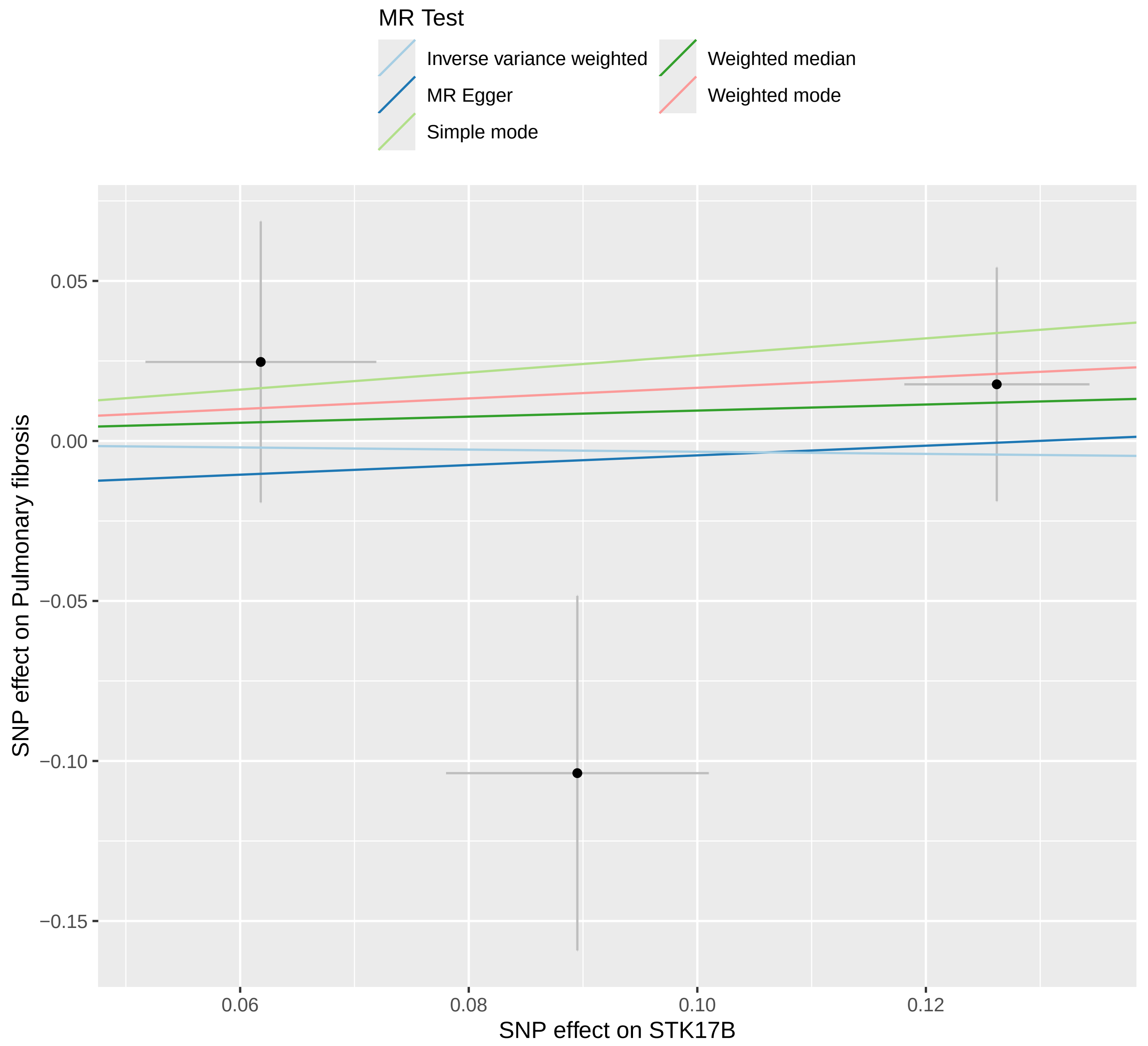

MR Test
Inverse variance weighted
MR Egger
Simple mode
Weighted median
Weighted mode
0.05
0.00
−0.05
−0.10
−0.15
SNP effect on Pulmonary fibrosis
0.06
0.08
0.10
0.12
SNP effect on STK17B

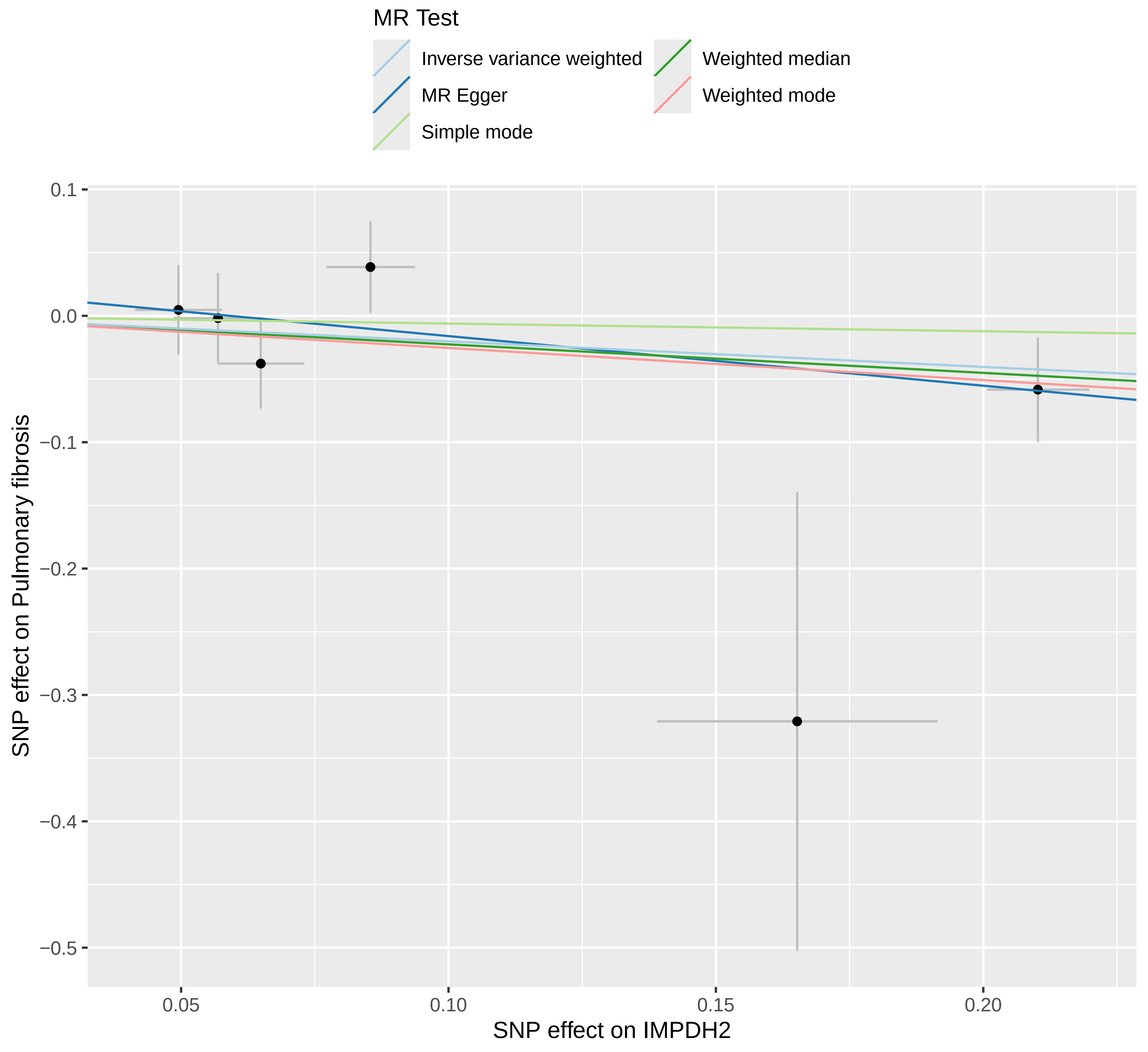

MR Test
Inverse variance weighted
MR Egger
Simple mode
Weighted median
Weighted mode
0.1
0.0
−0.1
−0.2
−0.3
−0.4
−0.5
SNP effect on Pulmonary fibrosis
0.05
0.10
0.15
0.20
SNP effect on IMPDH2

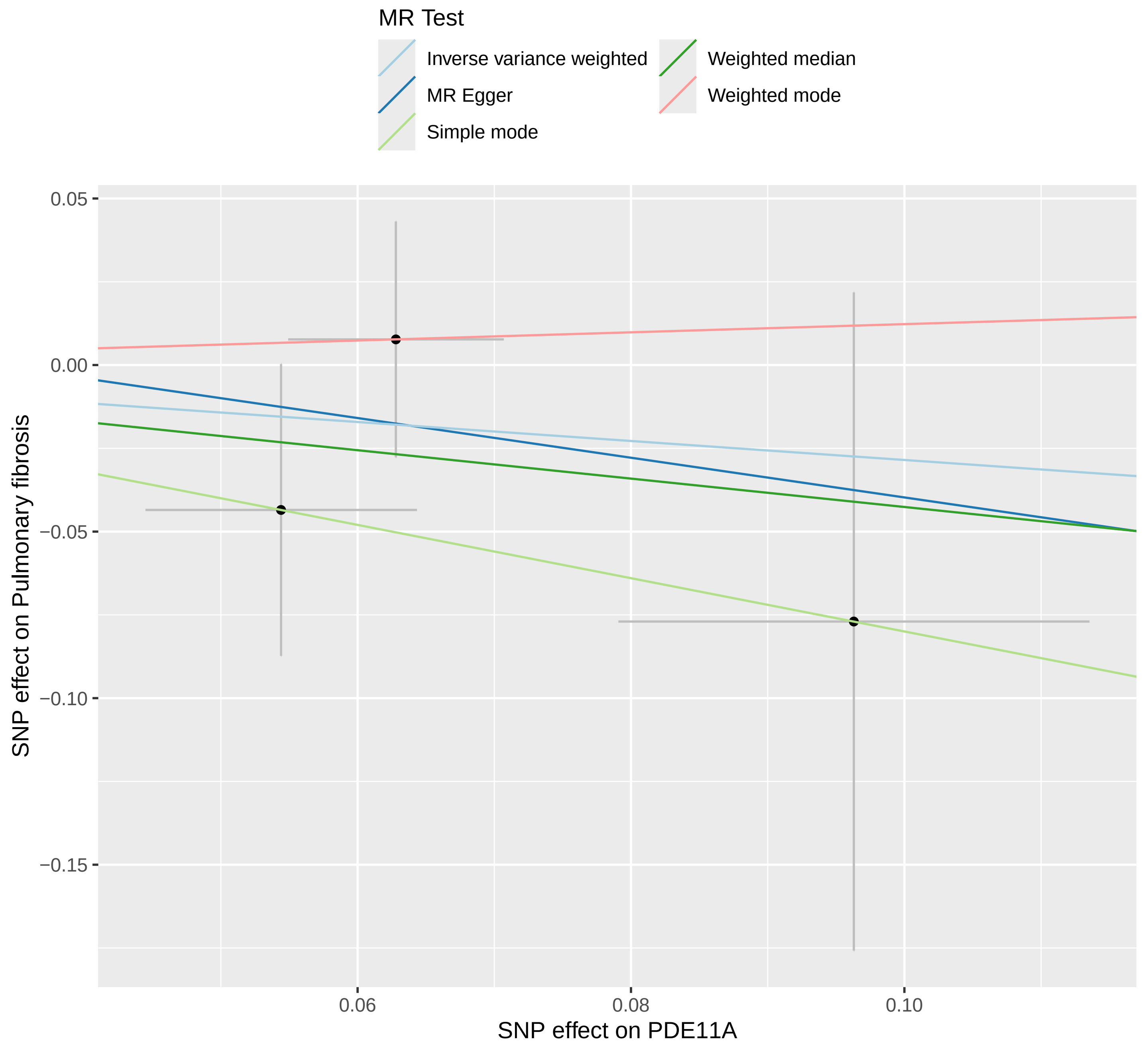
MR Test
Inverse variance weighted
MR Egger
Simple mode
Weighted median
Weighted mode
0.05
0.00
−0.05
−0.10
−0.15
SNP effect on Pulmonary fibrosis
0.06
0.08
0.10
SNP effect on PDE11A

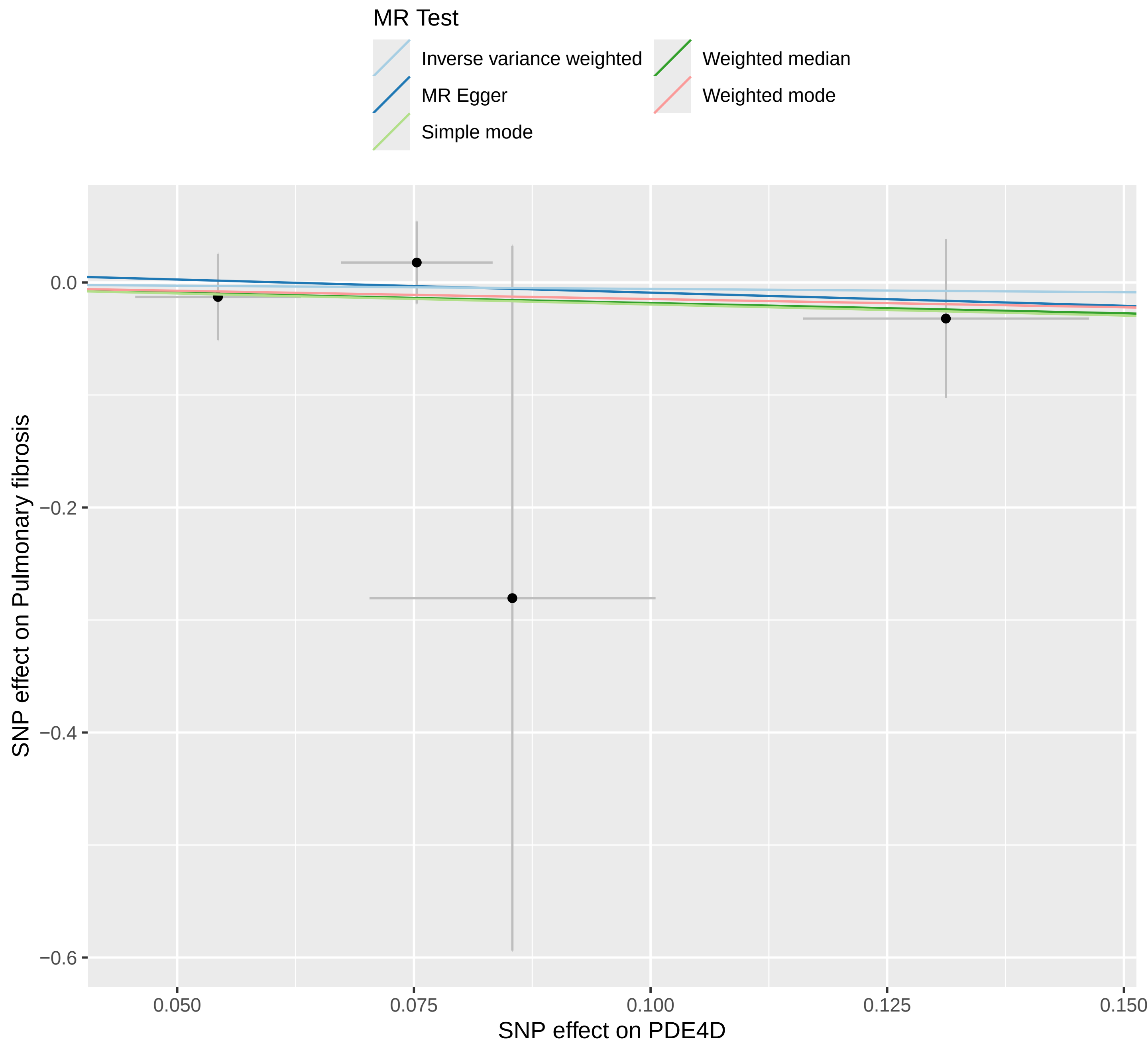

MR Test
Inverse variance weighted
MR Egger
Simple mode
Weighted median
Weighted mode
0.0
−0.2
−0.4
−0.6
SNP effect on Pulmonary fibrosis
0.050
0.075
0.100
0.125
0.150
SNP effect on PDE4D

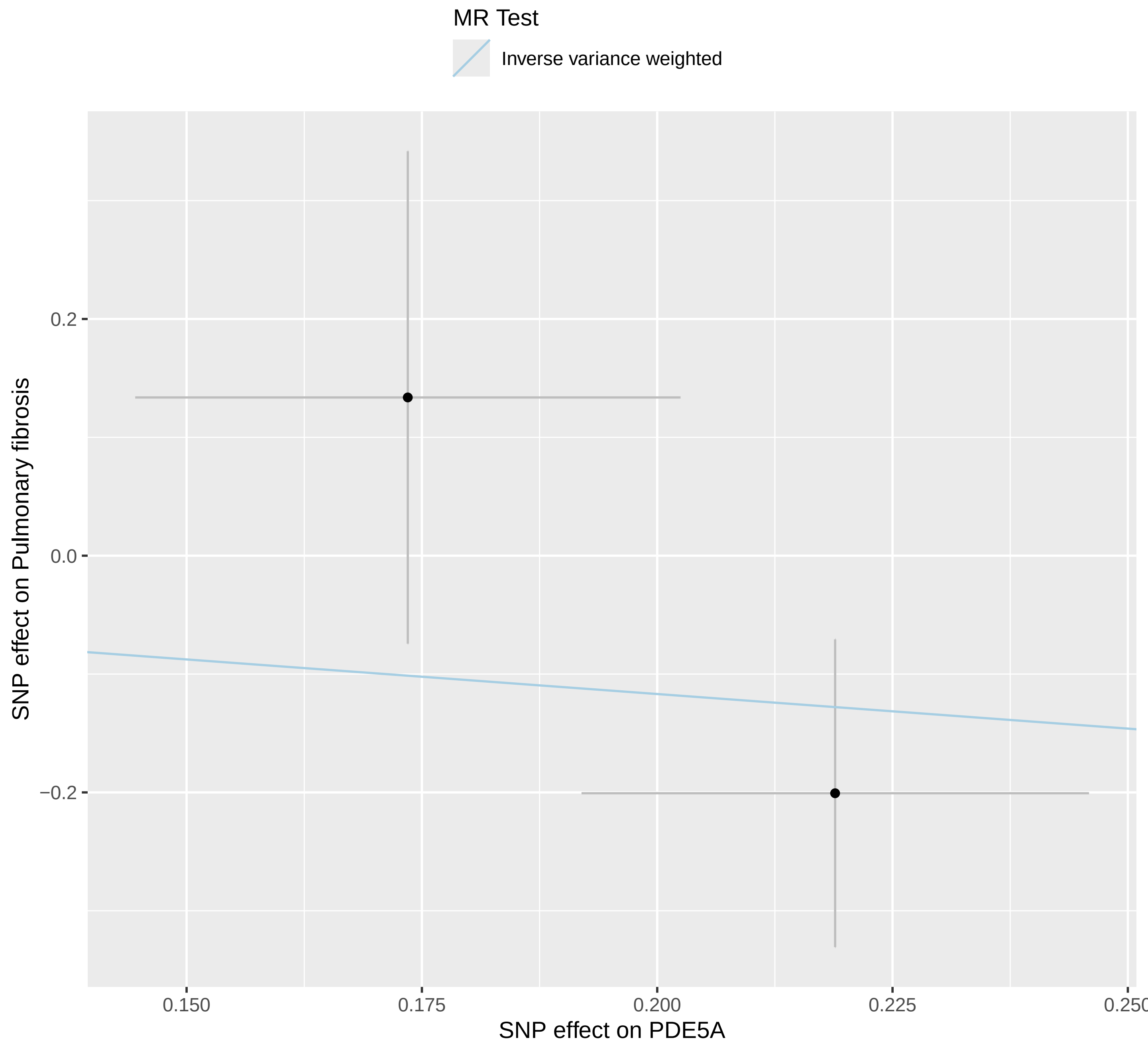
MR Test
Inverse variance weighted
0.2
0.0
−0.2
0.150
0.175
0.200
0.225
0.250
SNP effect on Pulmonary fibrosis
SNP effect on PDE5A

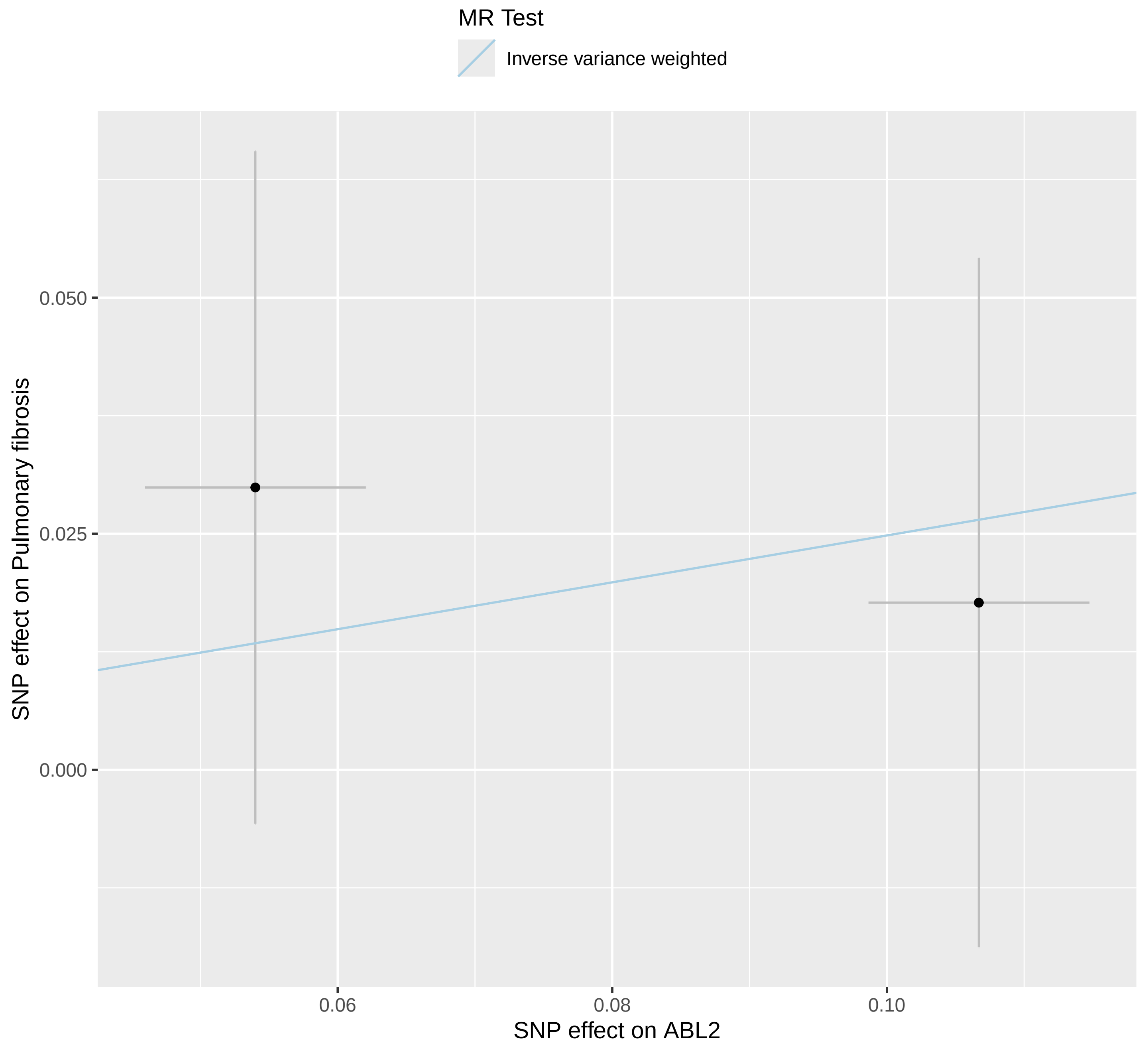

MR Test
Inverse variance weighted
0.050
0.025
0.000
SNP effect on Pulmonary fibrosis
0.06
0.08
0.10
SNP effect on ABL2

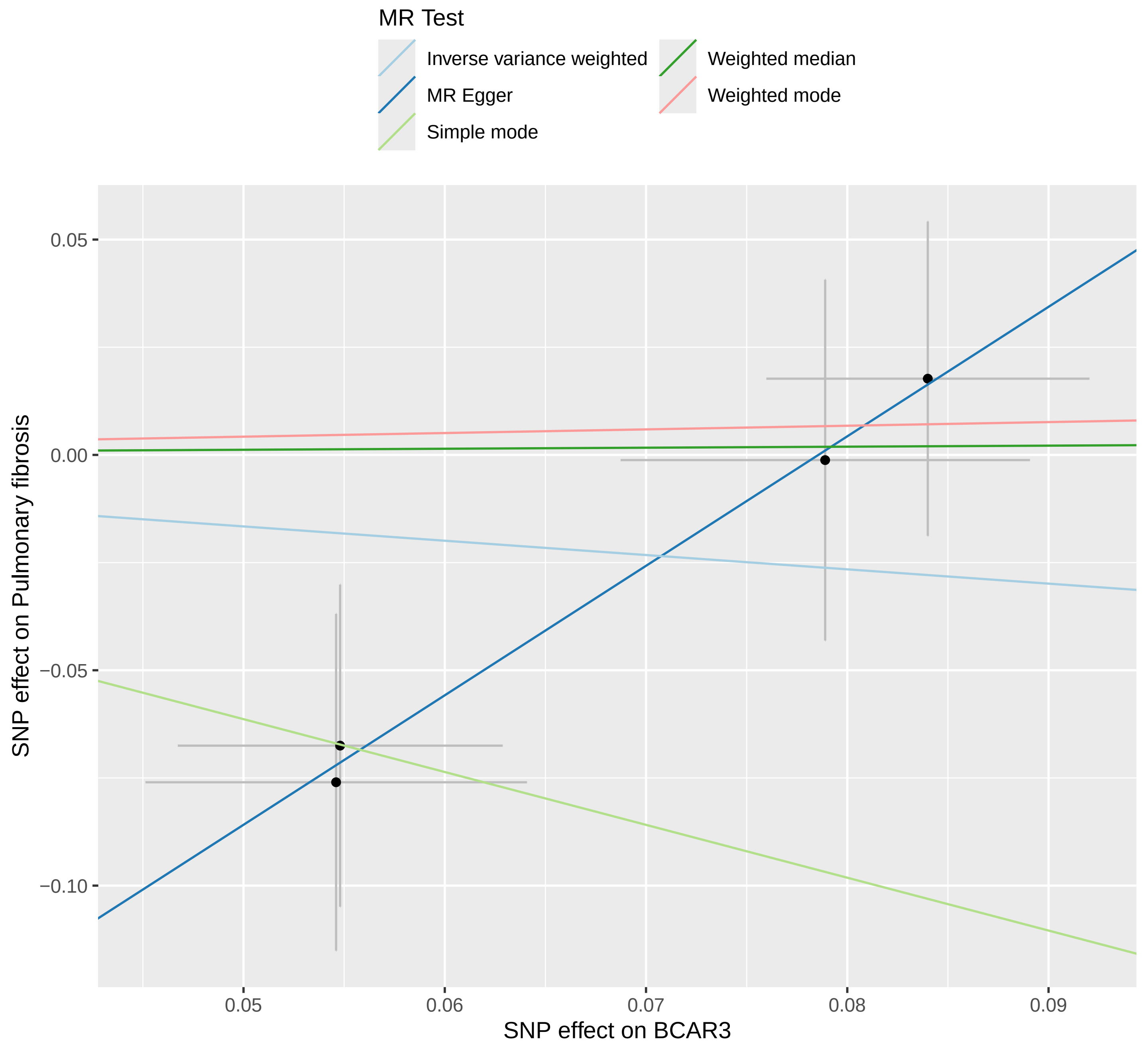

MR Test
Inverse variance weighted
MR Egger
Simple mode
Weighted median
Weighted mode
0.05
0.00
−0.05
−0.10
0.05
0.06
0.07
0.08
0.09
SNP effect on BCAR3
SNP effect on Pulmonary fibrosis

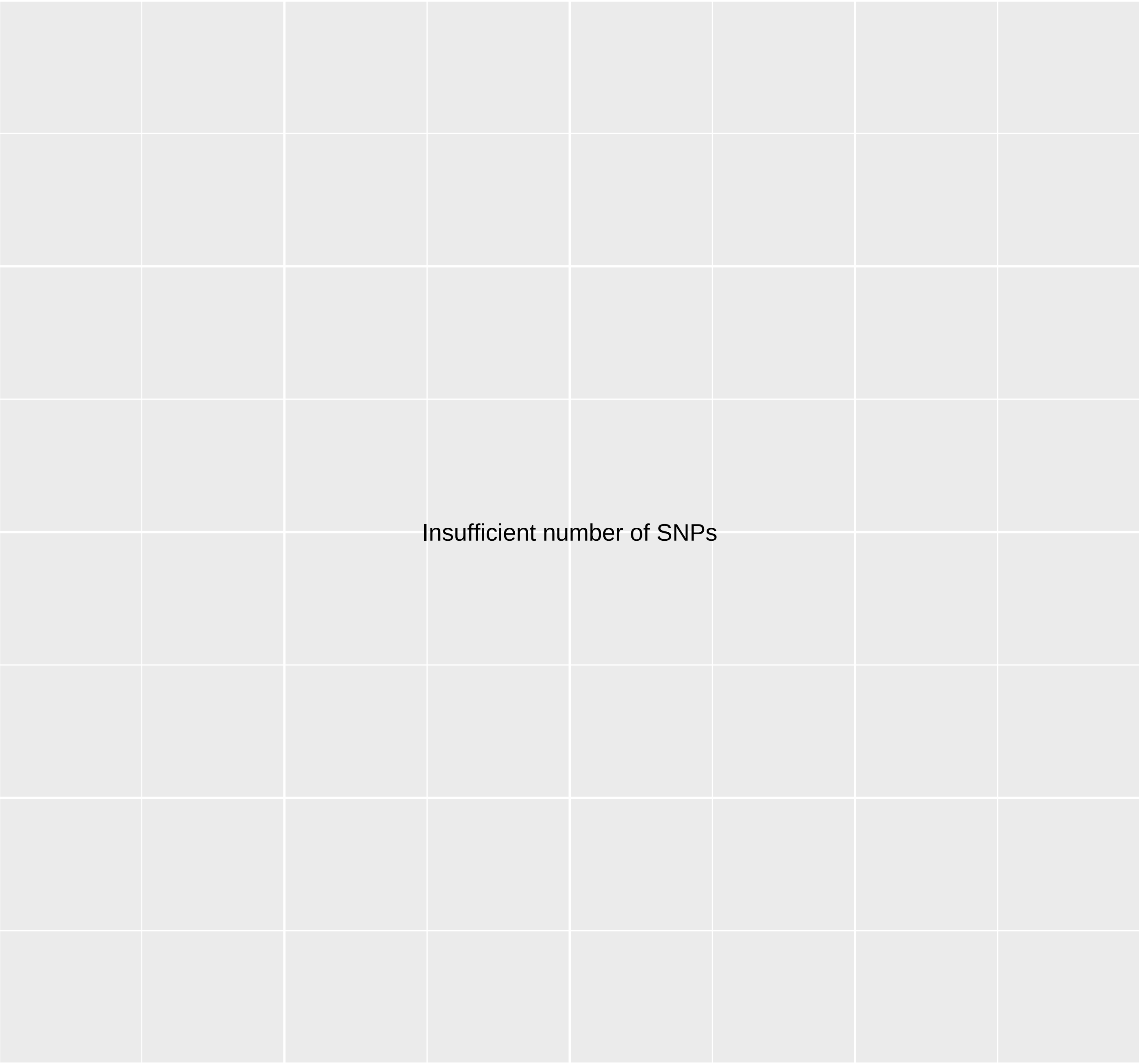
Insufficient number of SNPs

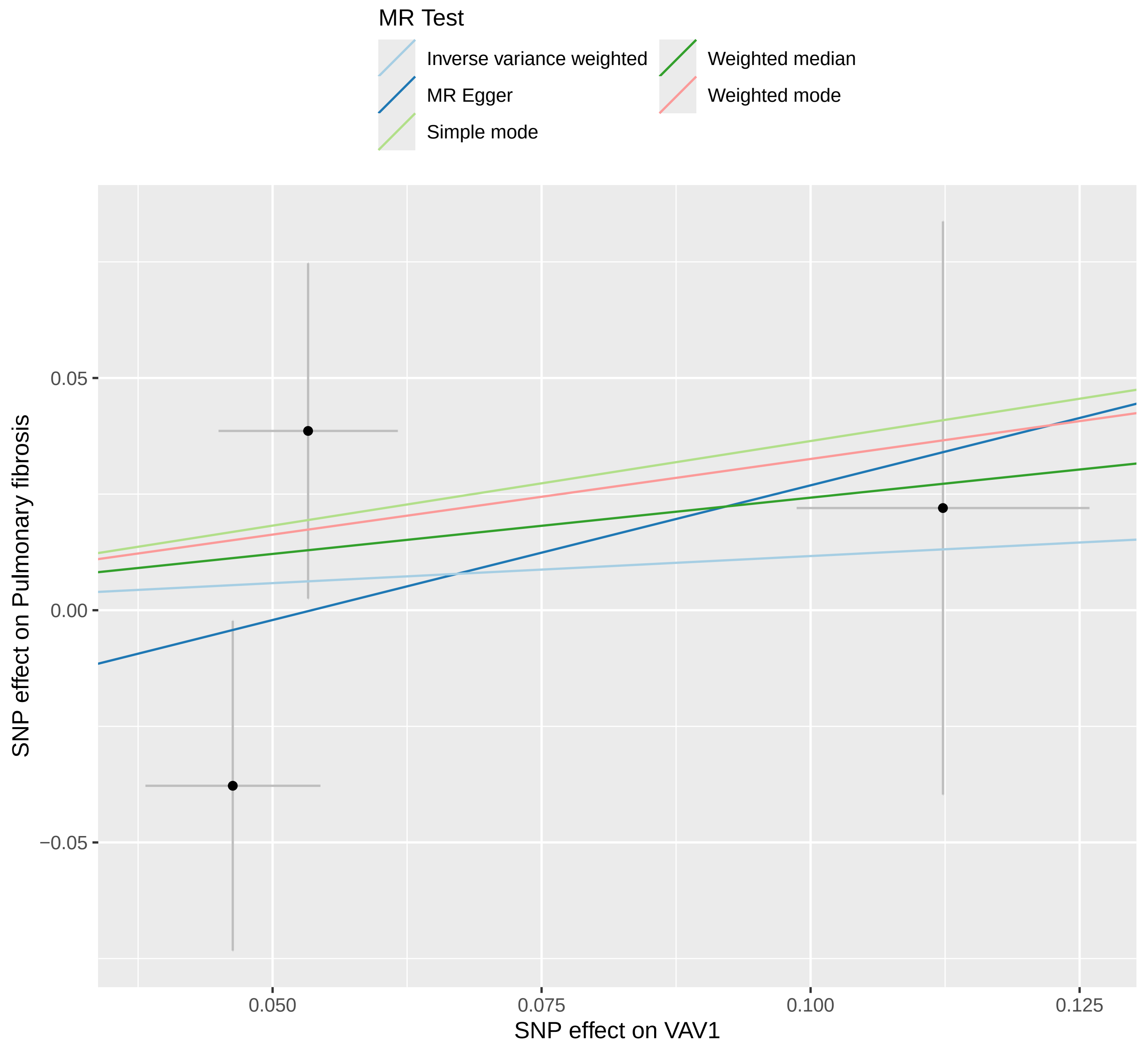
MR Test
Inverse variance weighted
MR Egger
Simple mode
Weighted median
Weighted mode
0.05
0.00
−0.05
SNP effect on Pulmonary fibrosis
0.050
0.075
0.100
0.125
SNP effect on VAV1

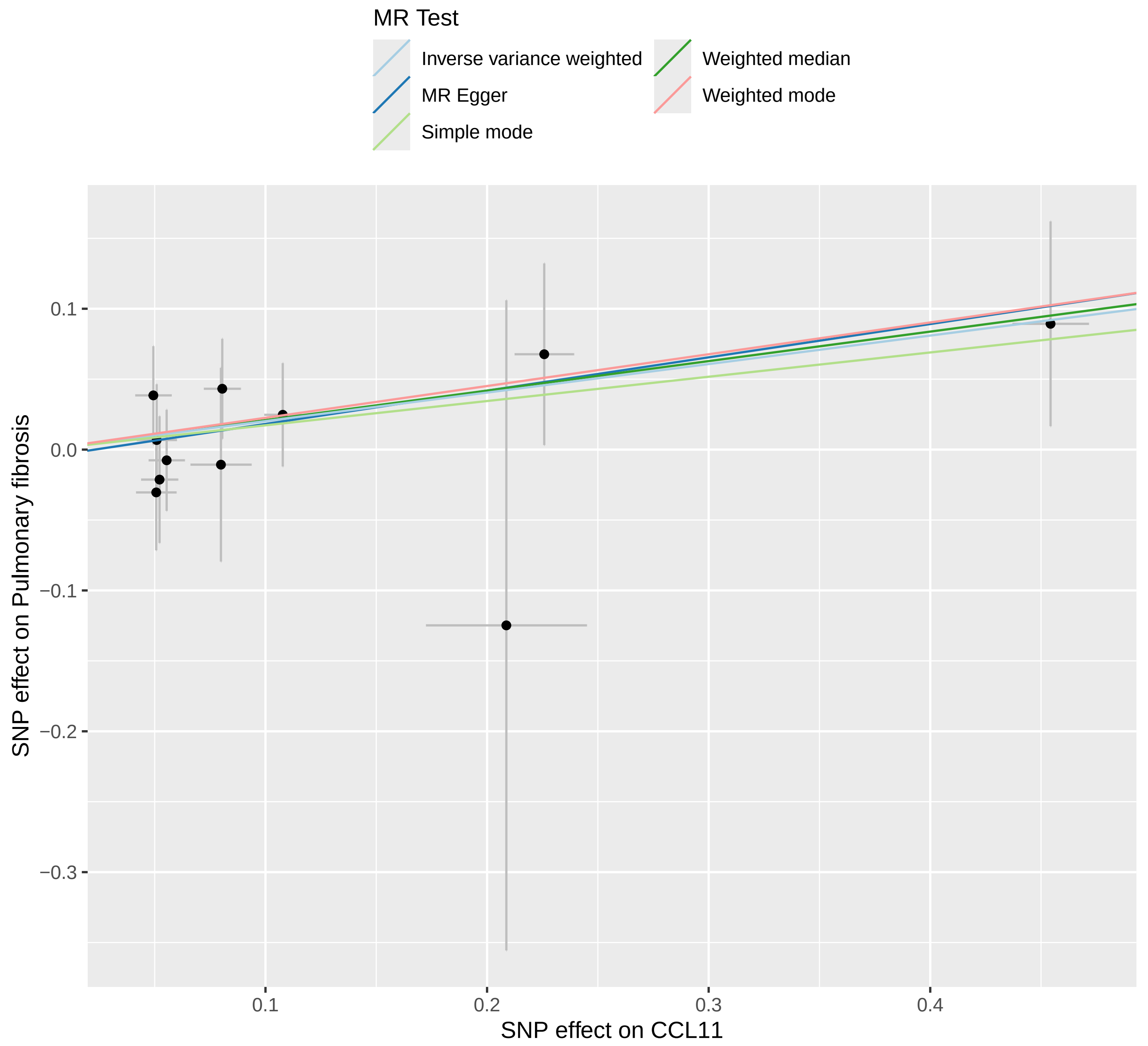
MR Test
Inverse variance weighted
MR Egger
Simple mode
Weighted median
Weighted mode
0.1
0.0
−0.1
−0.2
−0.3
SNP effect on Pulmonary fibrosis
0.1
0.2
0.3
0.4
SNP effect on CCL11

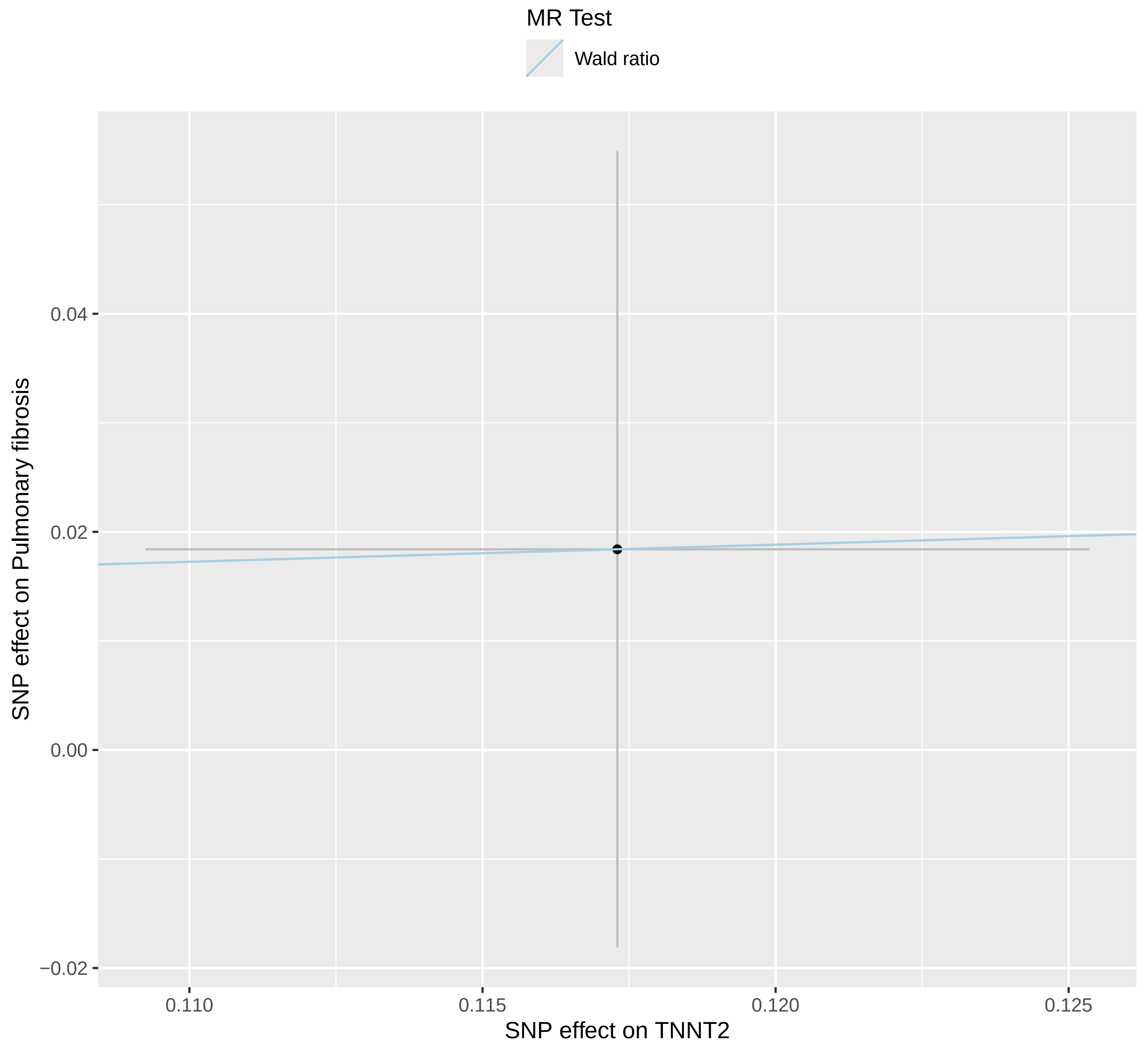
MR Test
Wald ratio
0.04
0.02
0.00
−0.02
SNP effect on Pulmonary fibrosis
0.110
0.115
0.120
0.125
SNP effect on TNNT2

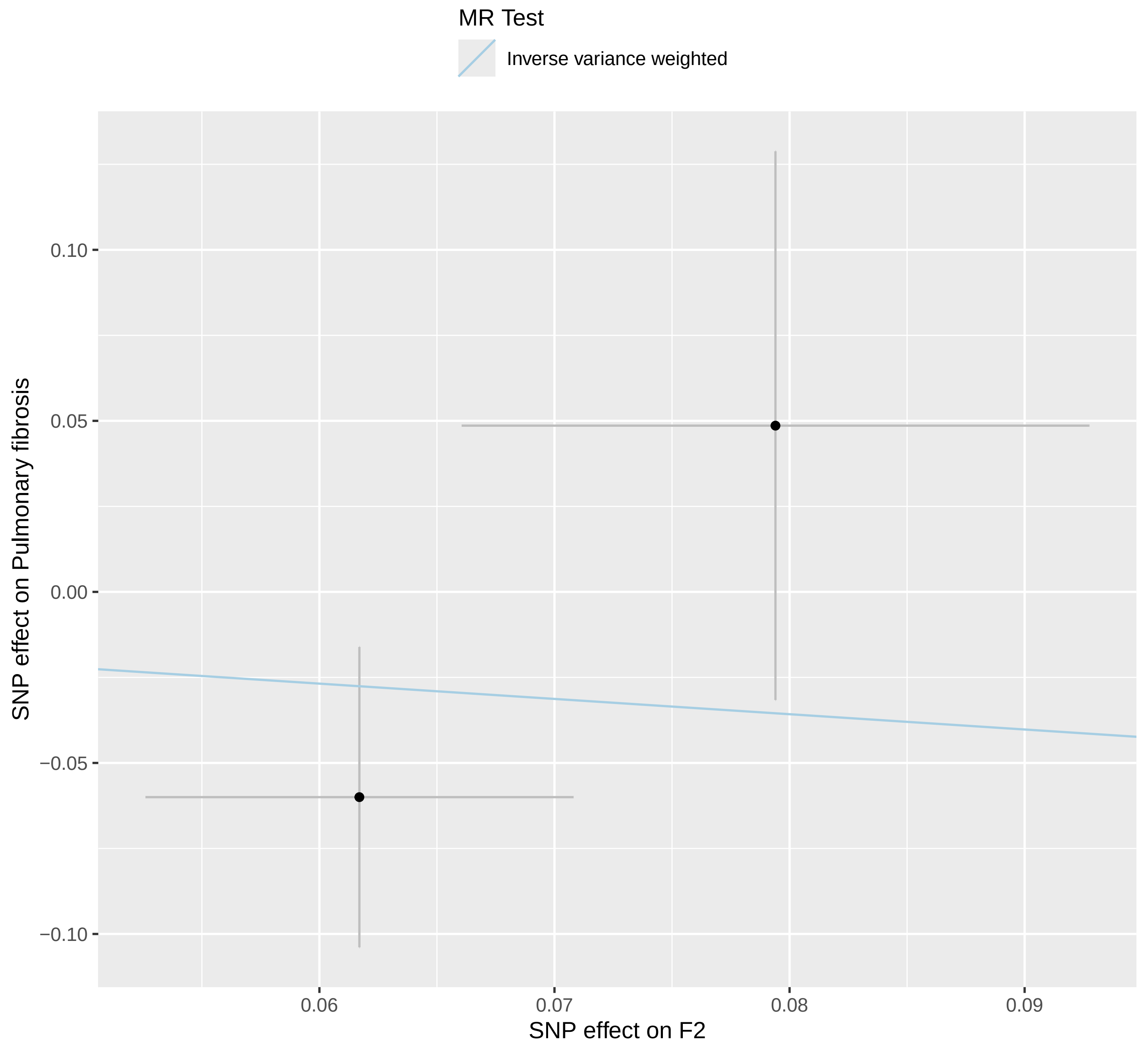
MR Test
Inverse variance weighted
0.10
0.05
0.00
−0.05
−0.10
SNP effect on Pulmonary fibrosis
0.06
0.07
0.08
0.09
SNP effect on F2

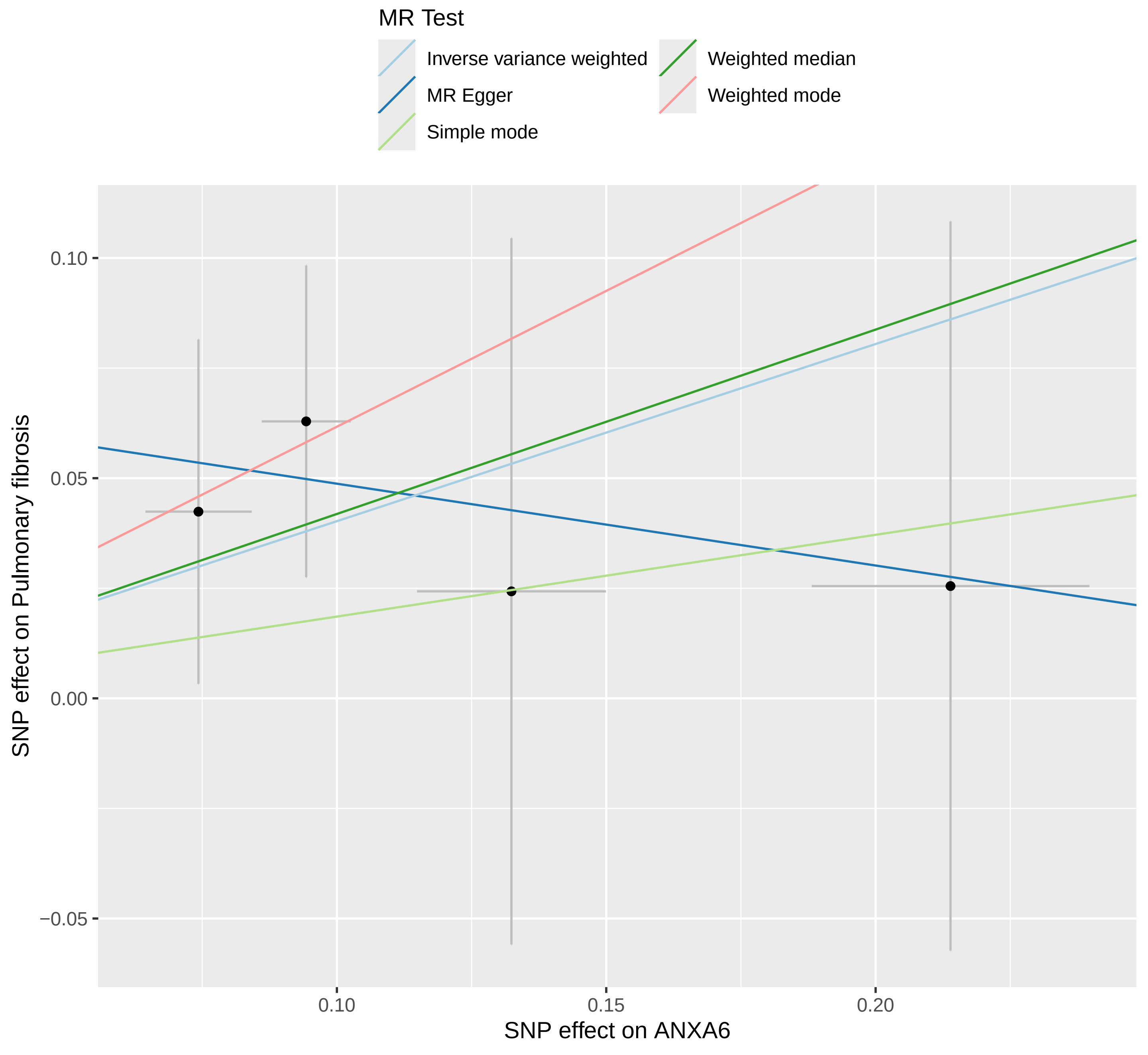

MR Test
Inverse variance weighted
MR Egger
Simple mode
Weighted median
Weighted mode
0.10
0.05
0.00
−0.05
0.10
0.15
0.20
SNP effect on Pulmonary fibrosis
SNP effect on ANXA6

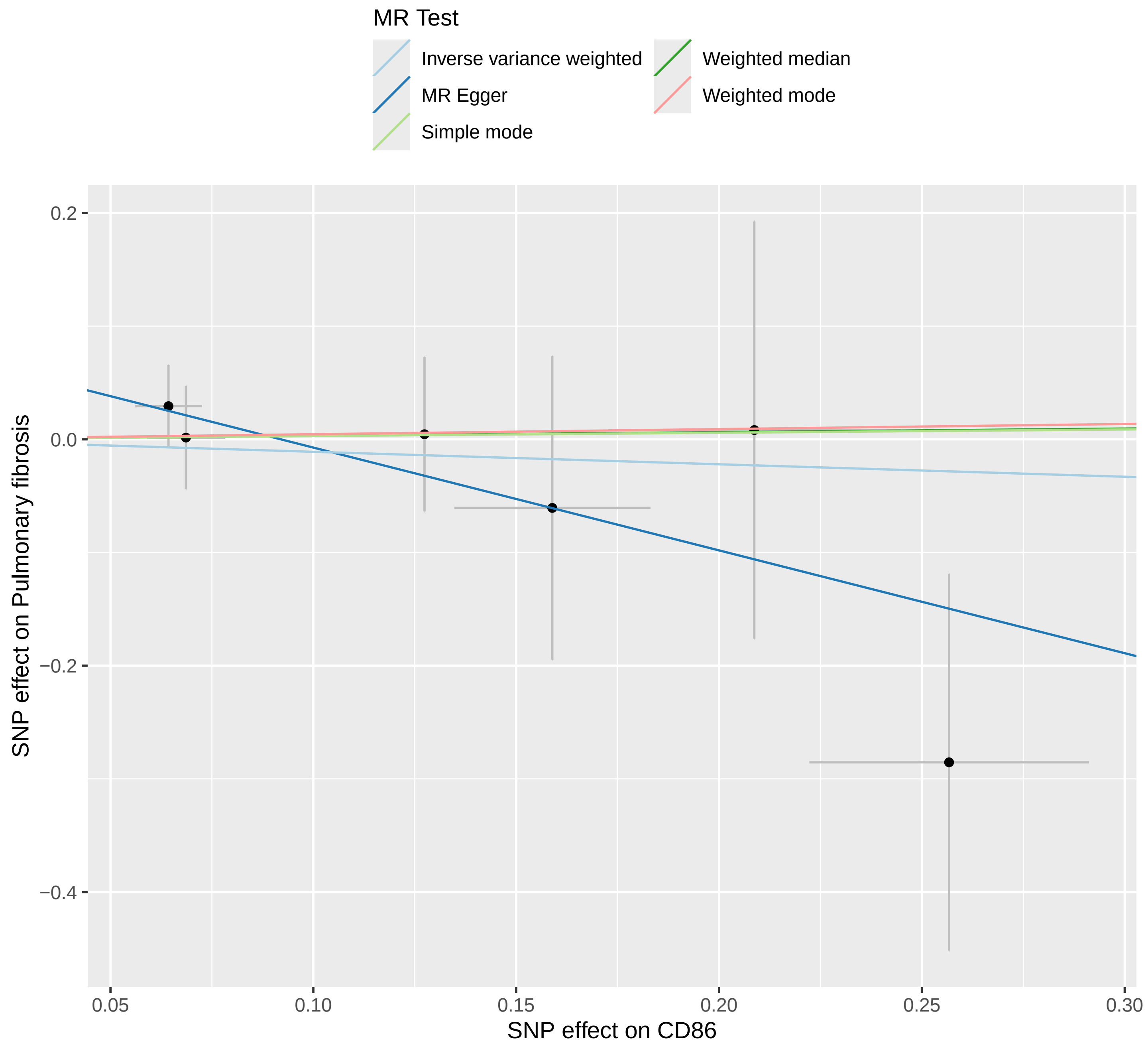

MR Test
Inverse variance weighted
MR Egger
Simple mode
Weighted median
Weighted mode
0.2
0.0
−0.2
−0.4
SNP effect on Pulmonary fibrosis
0.05
0.10
0.15
0.20
0.25
0.30
SNP effect on CD86

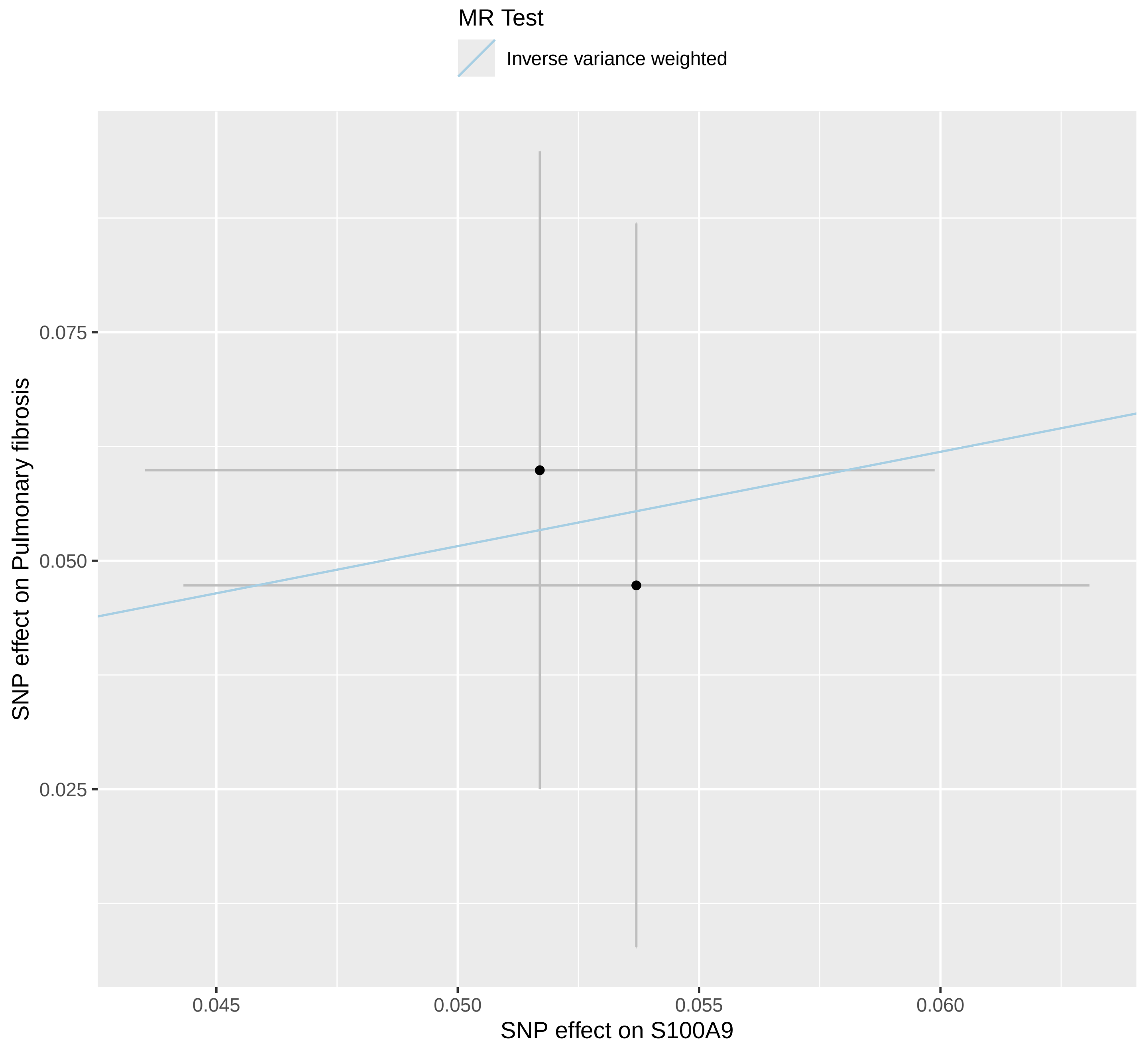
MR Test
Inverse variance weighted
0.075
0.050
0.025
SNP effect on Pulmonary fibrosis
0.045
0.050
0.055
0.060
SNP effect on S100A9

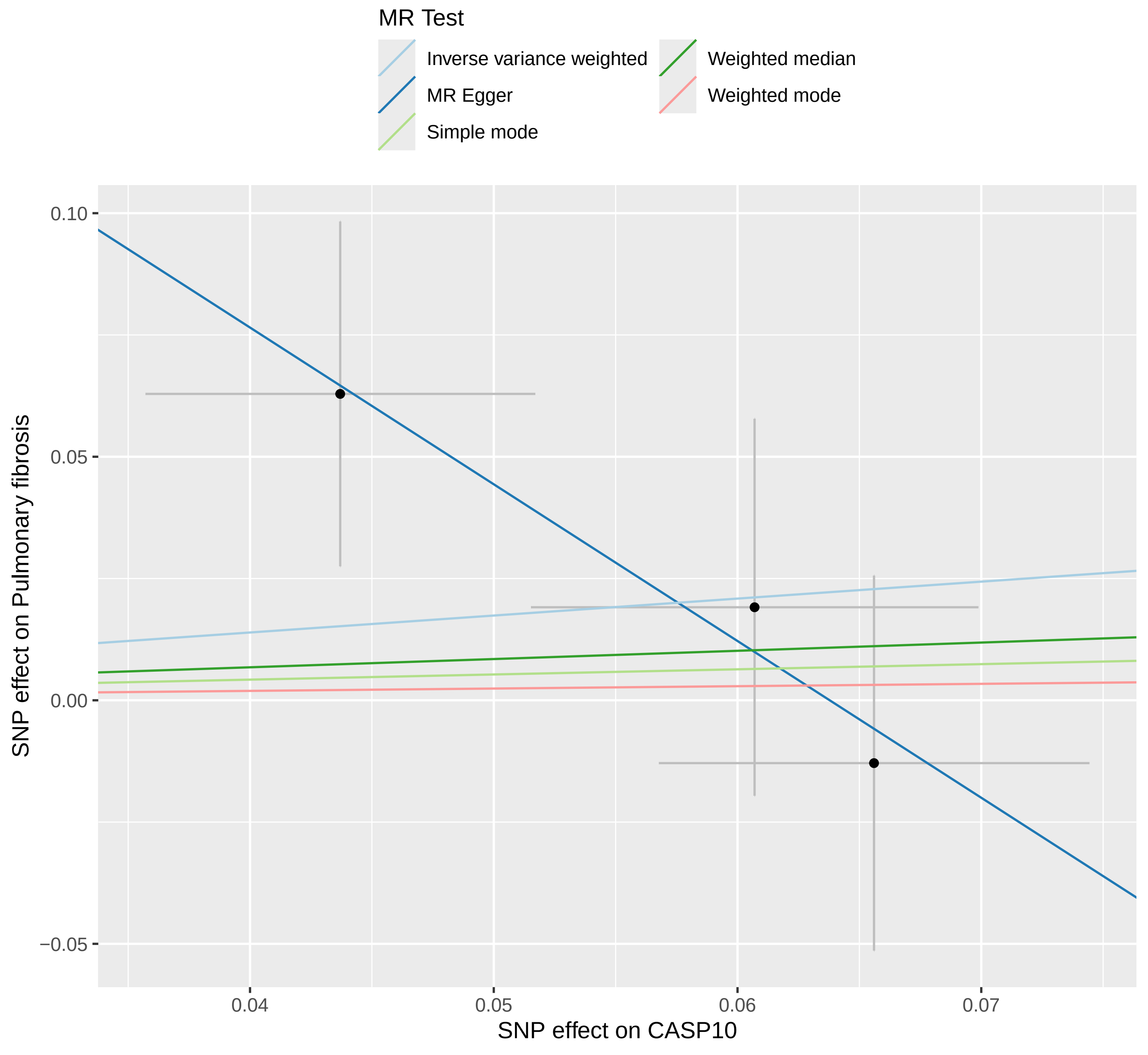

MR Test
Inverse variance weighted
MR Egger
Simple mode
Weighted median
Weighted mode
SNP effect on Pulmonary fibrosis
0.10
0.05
0.00
−0.05
0.04
0.05
0.06
0.07
SNP effect on CASP10

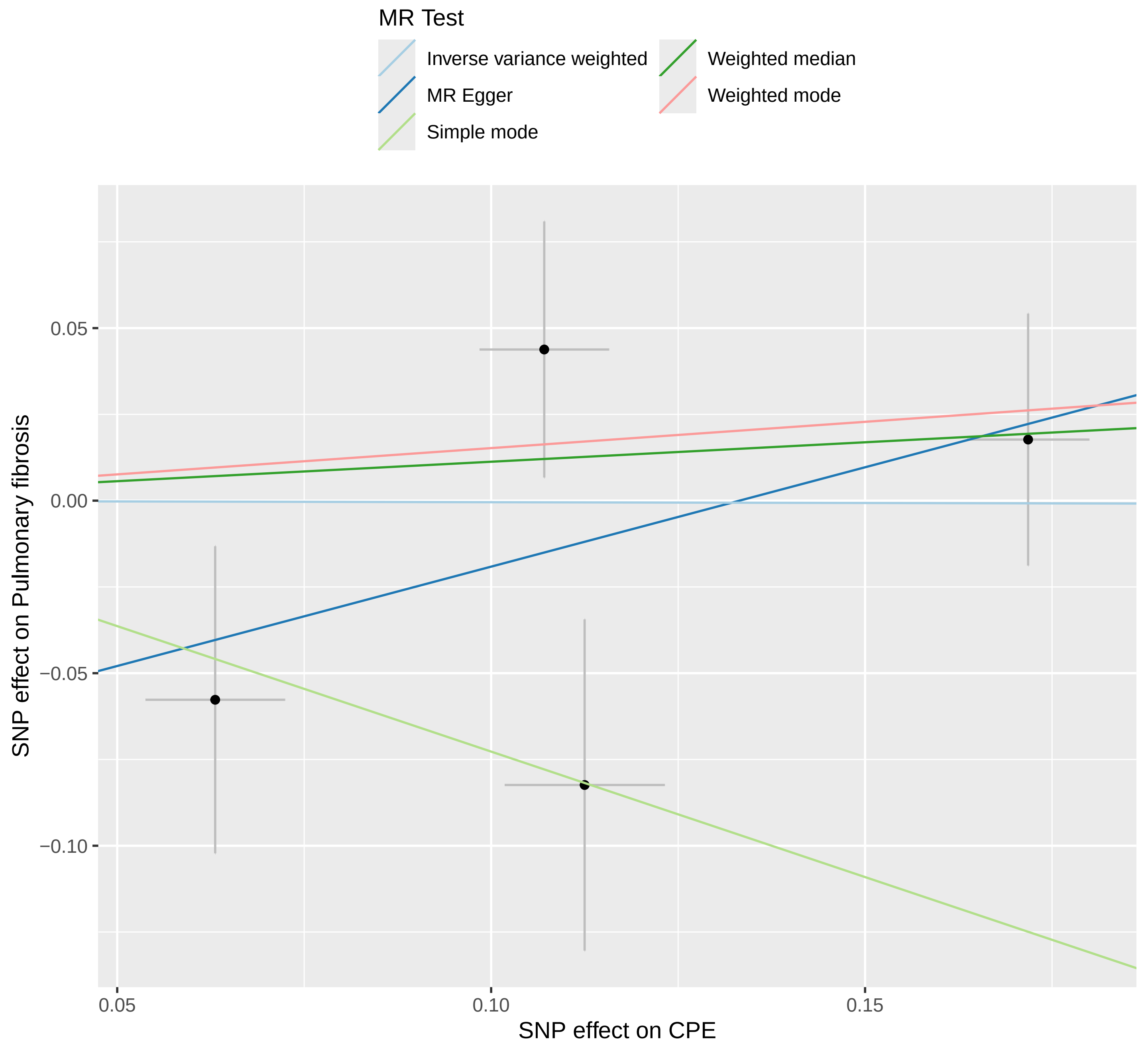

MR Test
Inverse variance weighted
MR Egger
Simple mode
Weighted median
Weighted mode
0.05
0.00
−0.05
−0.10
0.05
0.10
0.15
SNP effect on Pulmonary fibrosis
SNP effect on CPE

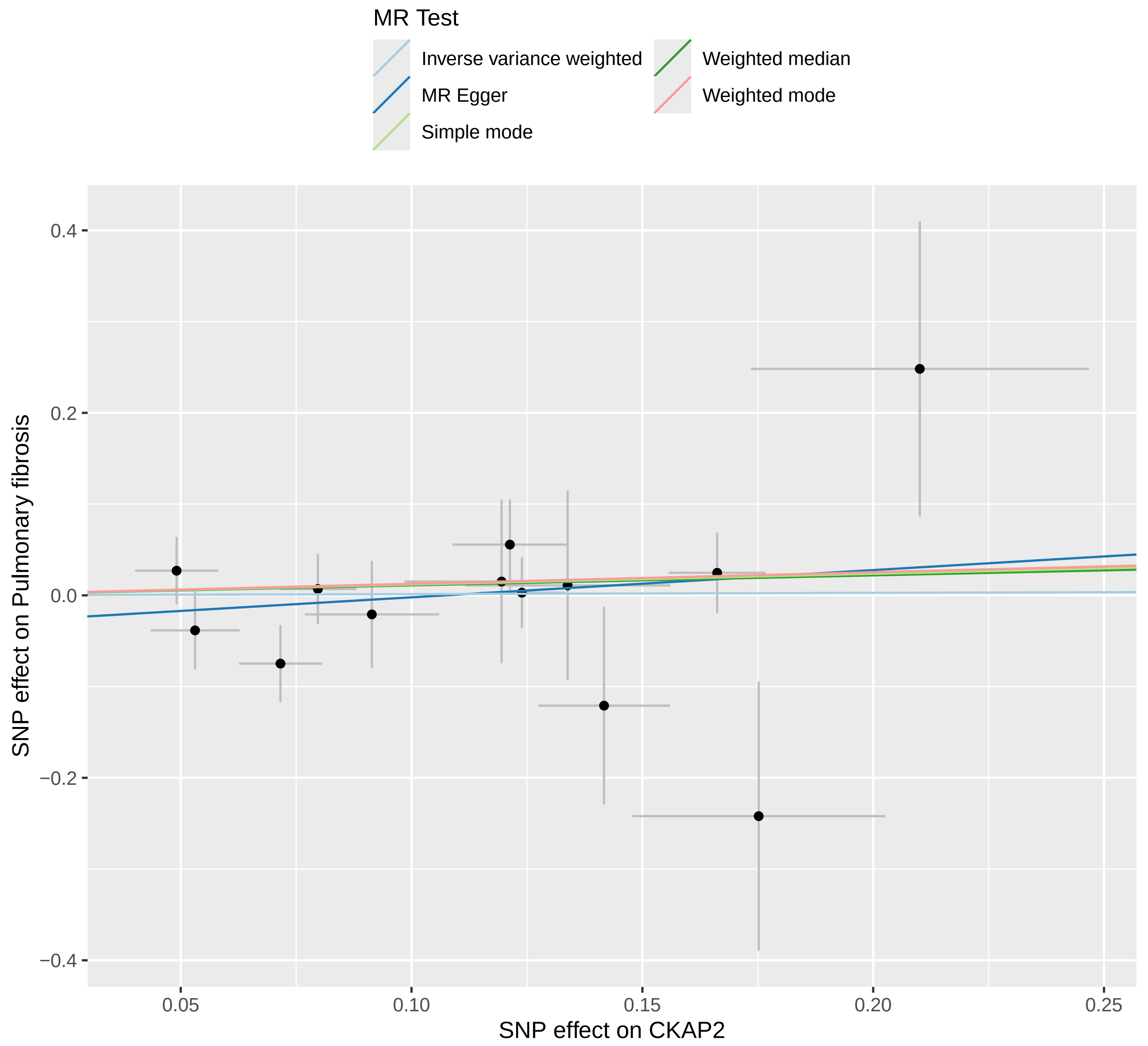

MR Test
Inverse variance weighted
MR Egger
Simple mode
Weighted median
Weighted mode
0.4
0.2
0.0
−0.2
−0.4
0.05
0.10
0.15
0.20
0.25
SNP effect on Pulmonary fibrosis
SNP effect on CKAP2

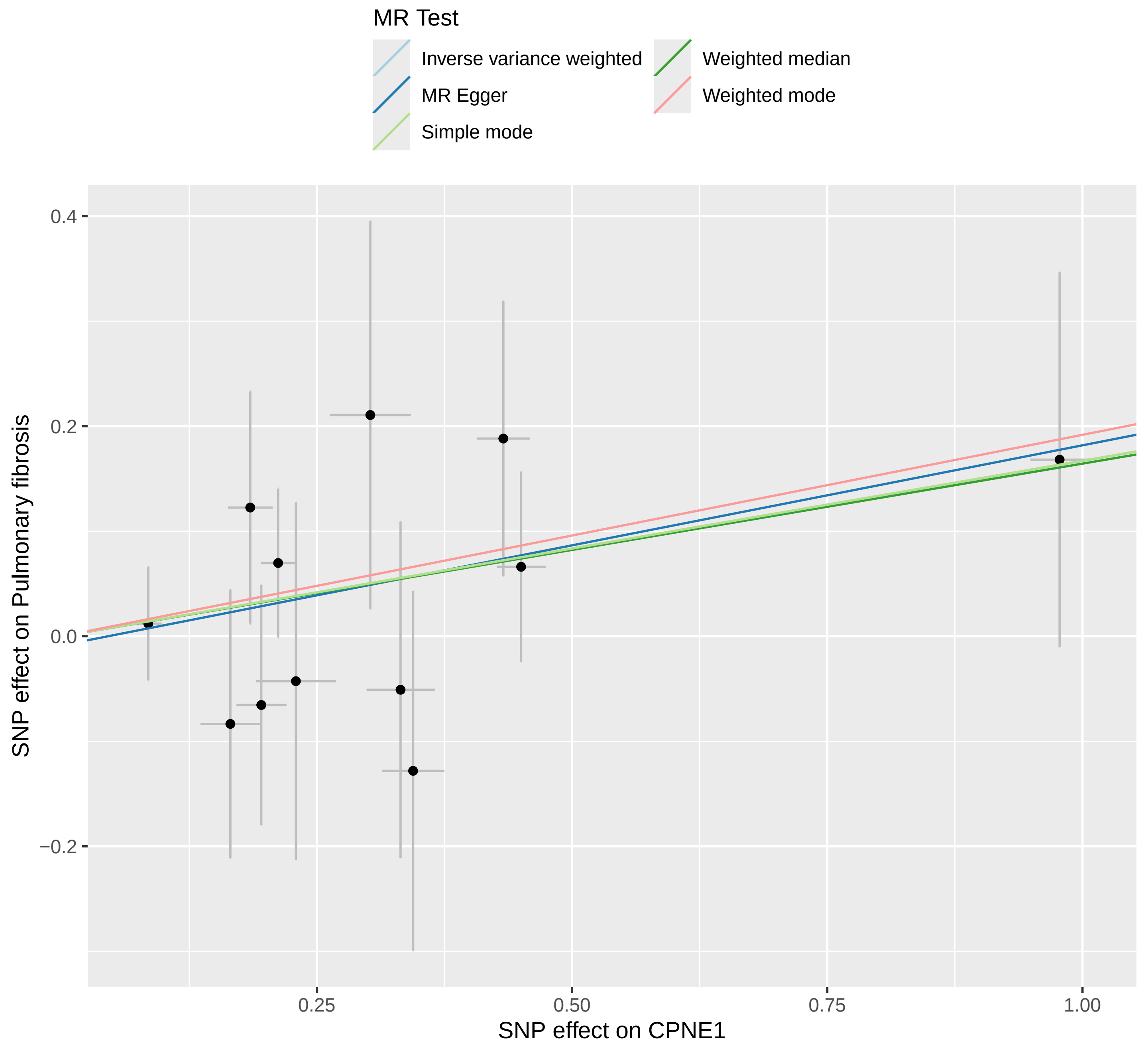

MR Test
Inverse variance weighted
MR Egger
Simple mode
Weighted median
Weighted mode
0.4
0.2
0.0
−0.2
0.25
0.50
0.75
1.00
SNP effect on CPNE1
SNP effect on Pulmonary fibrosis

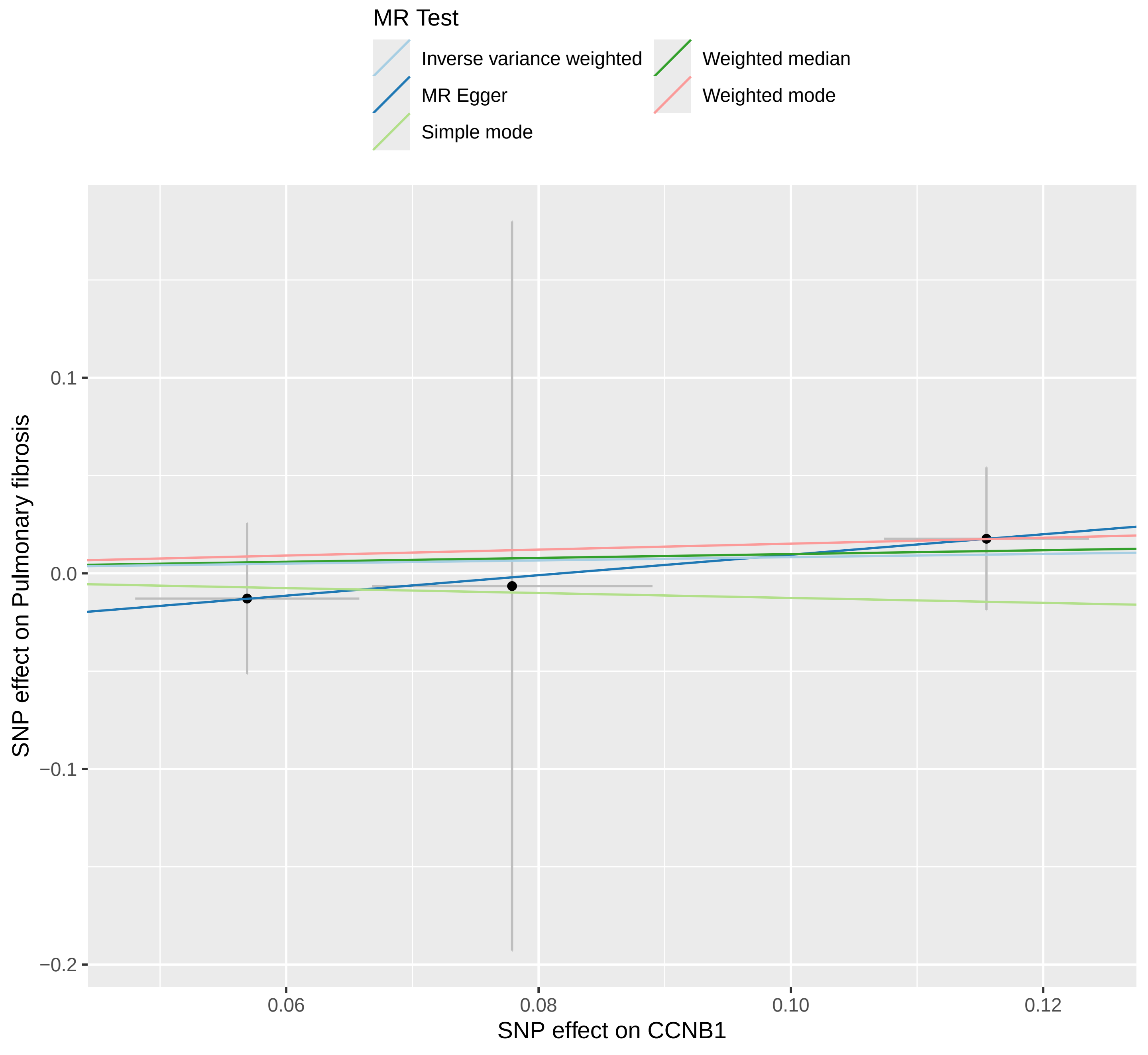
MR Test
Inverse variance weighted
MR Egger
Simple mode
Weighted median
Weighted mode
0.1
0.0
−0.1
−0.2
SNP effect on Pulmonary fibrosis
0.06
0.08
0.10
0.12
SNP effect on CCNB1

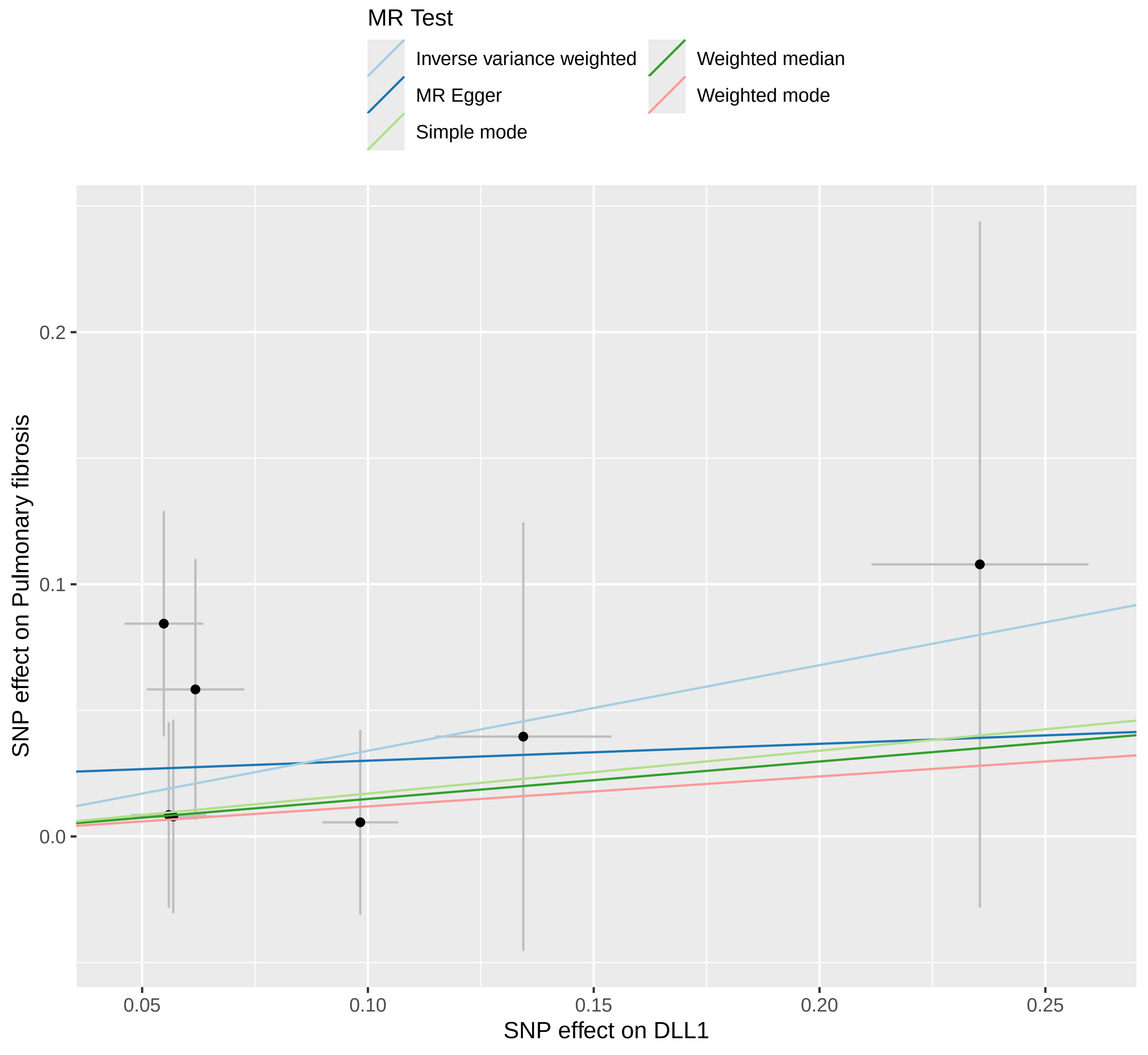

MR Test
Inverse variance weighted
MR Egger
Simple mode
Weighted median
Weighted mode
SNP effect on Pulmonary fibrosis
0.2
0.1
0.0
0.05
0.10
0.15
0.20
0.25
SNP effect on DLL1

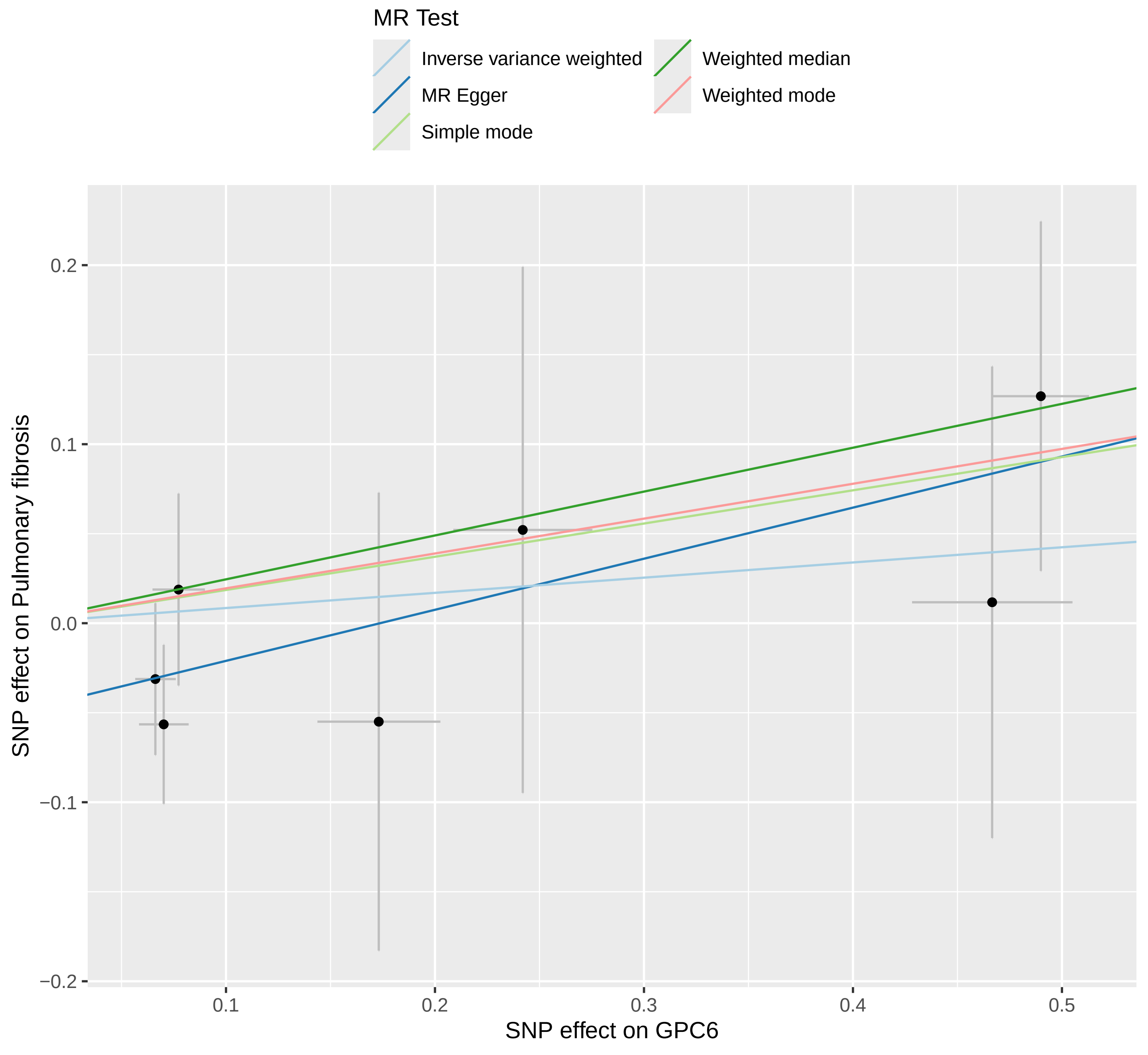
MR Test
Inverse variance weighted
MR Egger
Simple mode
Weighted median
Weighted mode
SNP effect on Pulmonary fibrosis
0.2
0.1
0.0
−0.1
−0.2
0.1
0.2
0.3
0.4
0.5
SNP effect on GPC6

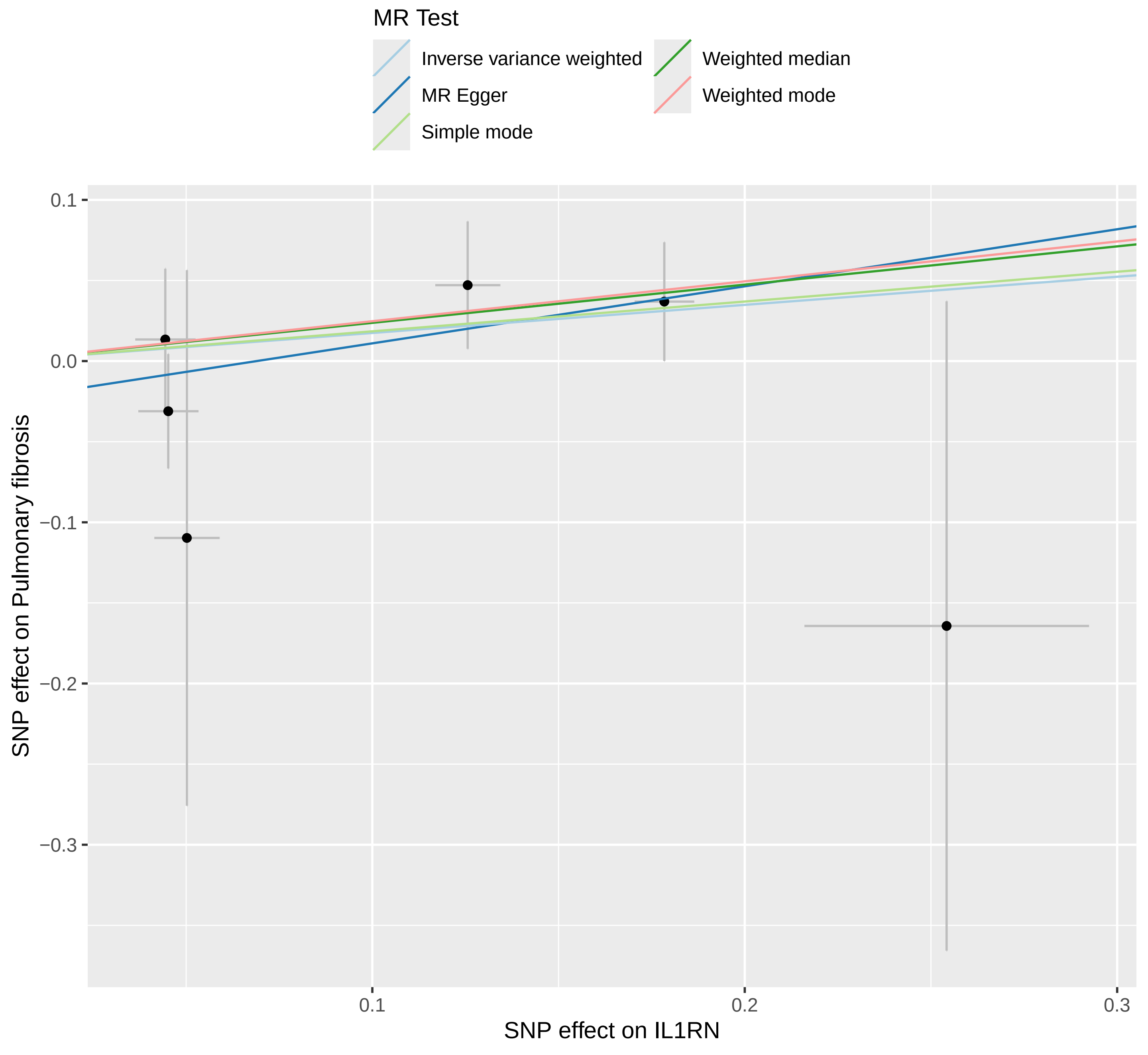
MR Test
Inverse variance weighted
MR Egger
Simple mode
Weighted median
Weighted mode
0.1
0.0
−0.1
−0.2
−0.3
0.1
0.2
0.3
SNP effect on Pulmonary fibrosis
SNP effect on IL1RN

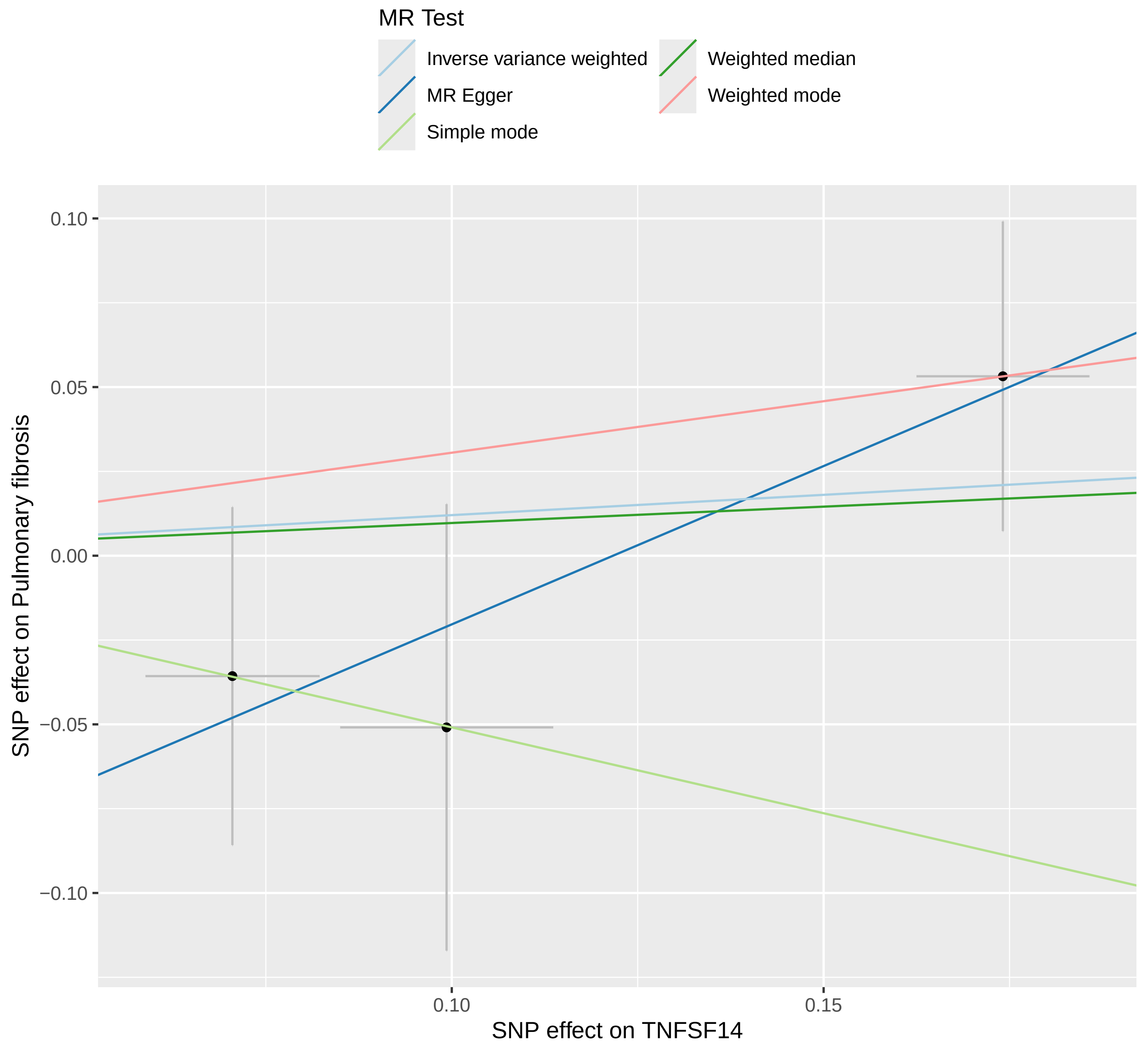

MR Test
Inverse variance weighted
MR Egger
Simple mode
Weighted median
Weighted mode
0.10
0.05
0.00
−0.05
−0.10
SNP effect on Pulmonary fibrosis
0.10
0.15
SNP effect on TNFSF14

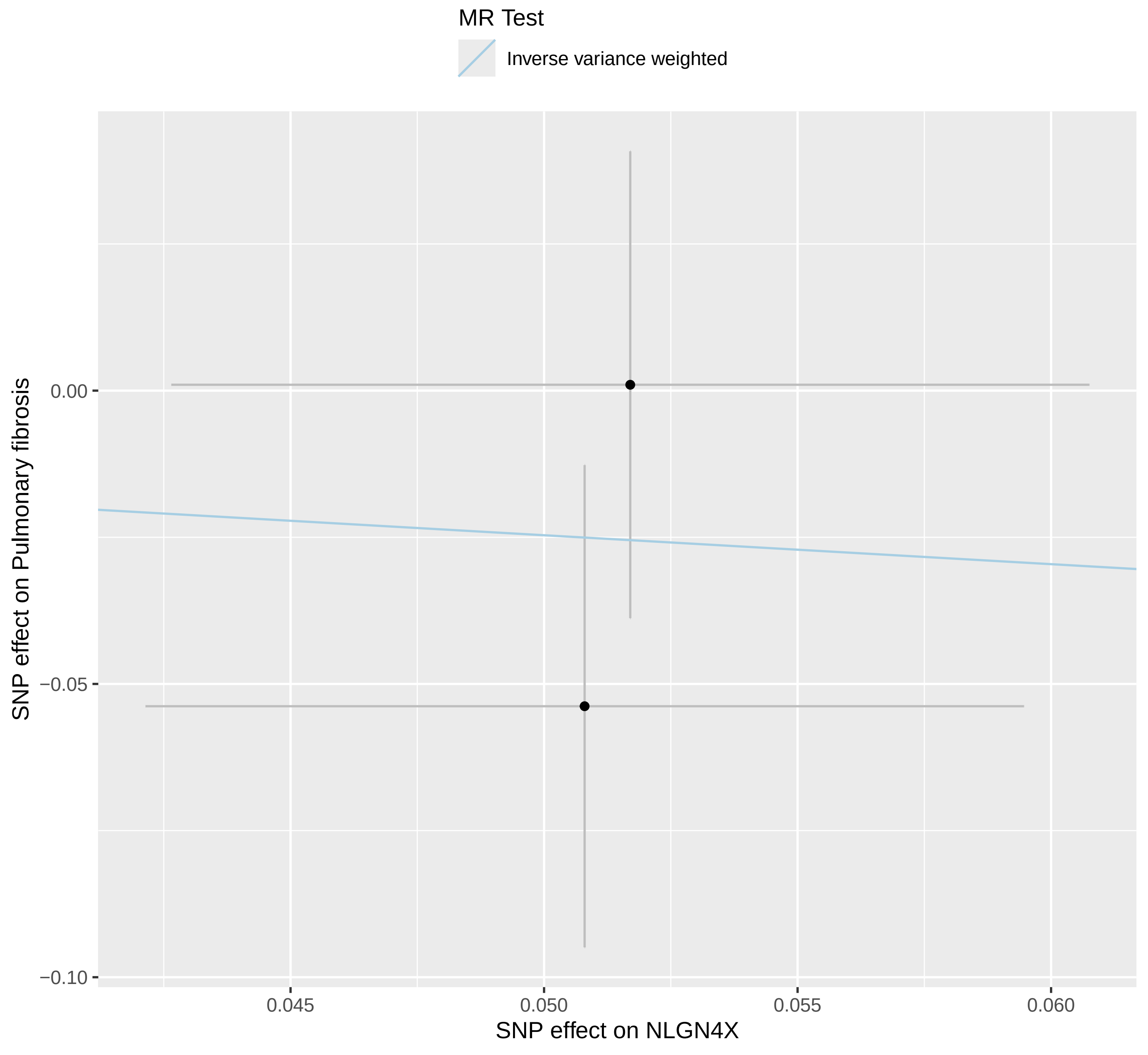

MR Test
Inverse variance weighted
SNP effect on Pulmonary fibrosis
0.00
−0.05
−0.10
0.045
0.050
0.055
0.060
SNP effect on NLGN4X

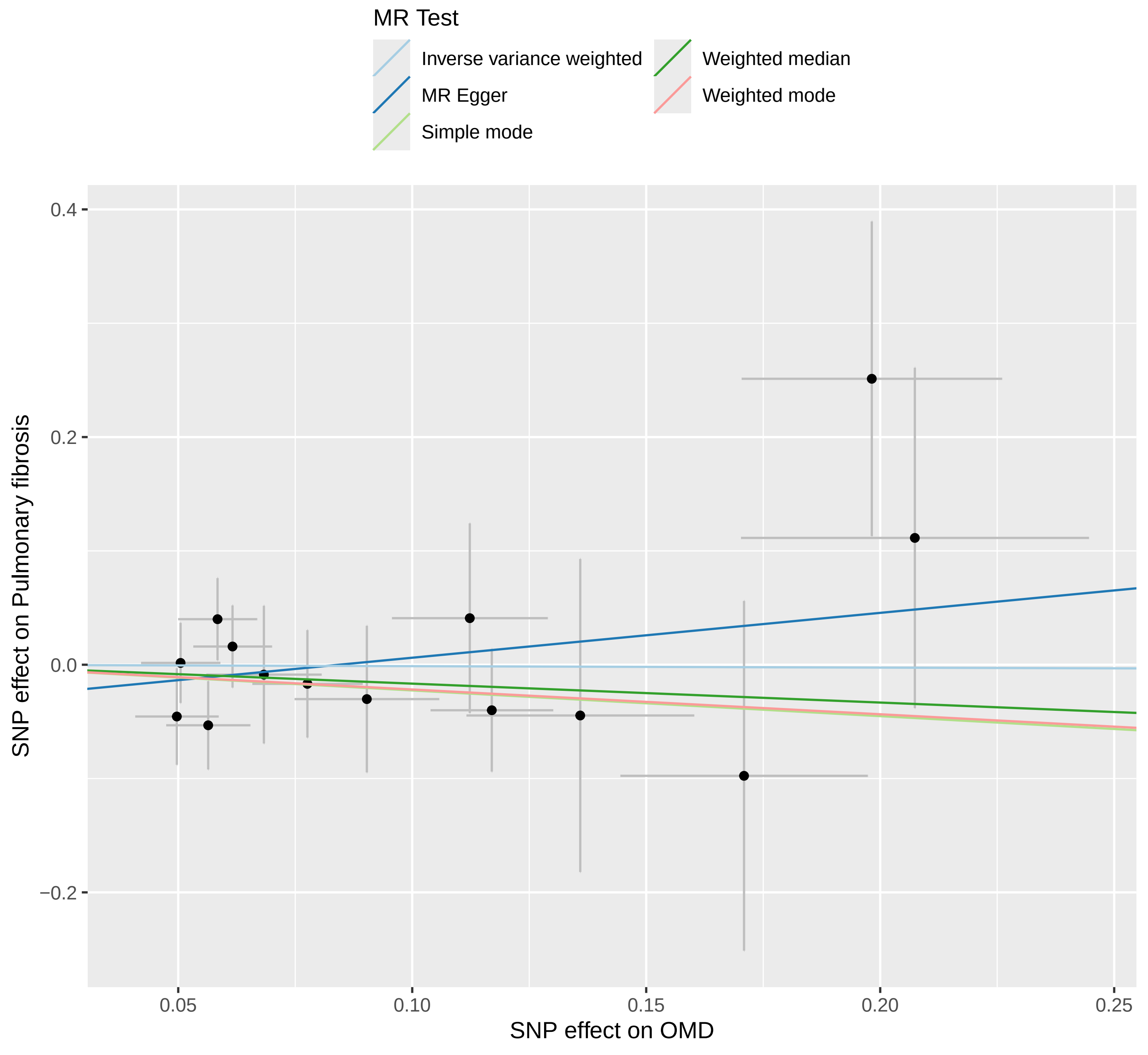

MR Test
Inverse variance weighted
MR Egger
Simple mode
Weighted median
Weighted mode
0.4
0.2
0.0
−0.2
0.05
0.10
0.15
0.20
0.25
SNP effect on OMD
SNP effect on Pulmonary fibrosis

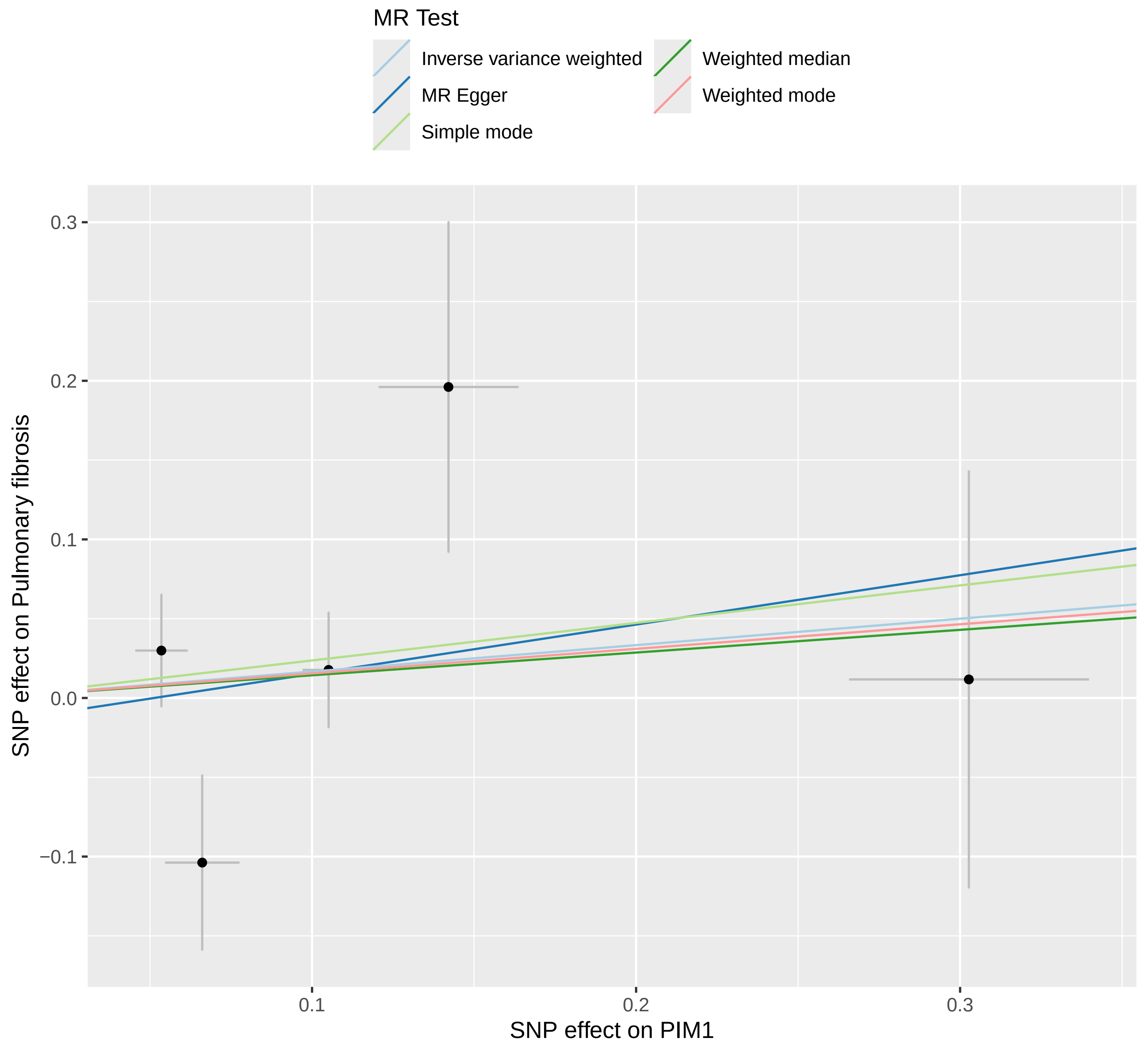

MR Test
Inverse variance weighted
MR Egger
Simple mode
Weighted median
Weighted mode
0.3
0.2
0.1
0.0
−0.1
0.1
0.2
0.3
SNP effect on Pulmonary fibrosis
SNP effect on PIM1

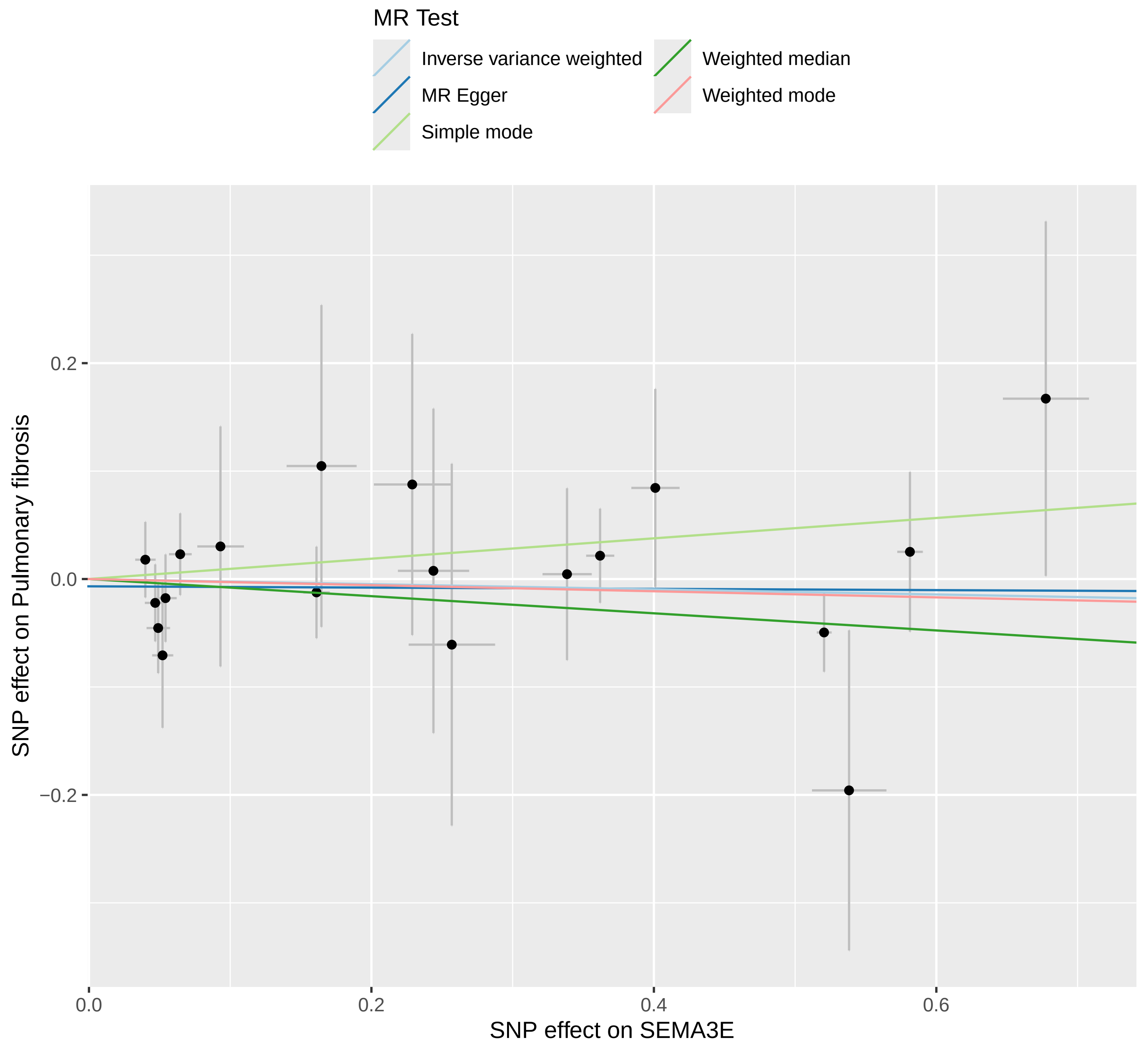
MR Test
Inverse variance weighted
MR Egger
Simple mode
Weighted median
Weighted mode
0.2
0.0
−0.2
SNP effect on Pulmonary fibrosis
0.0
0.2
0.4
0.6
SNP effect on SEMA3E

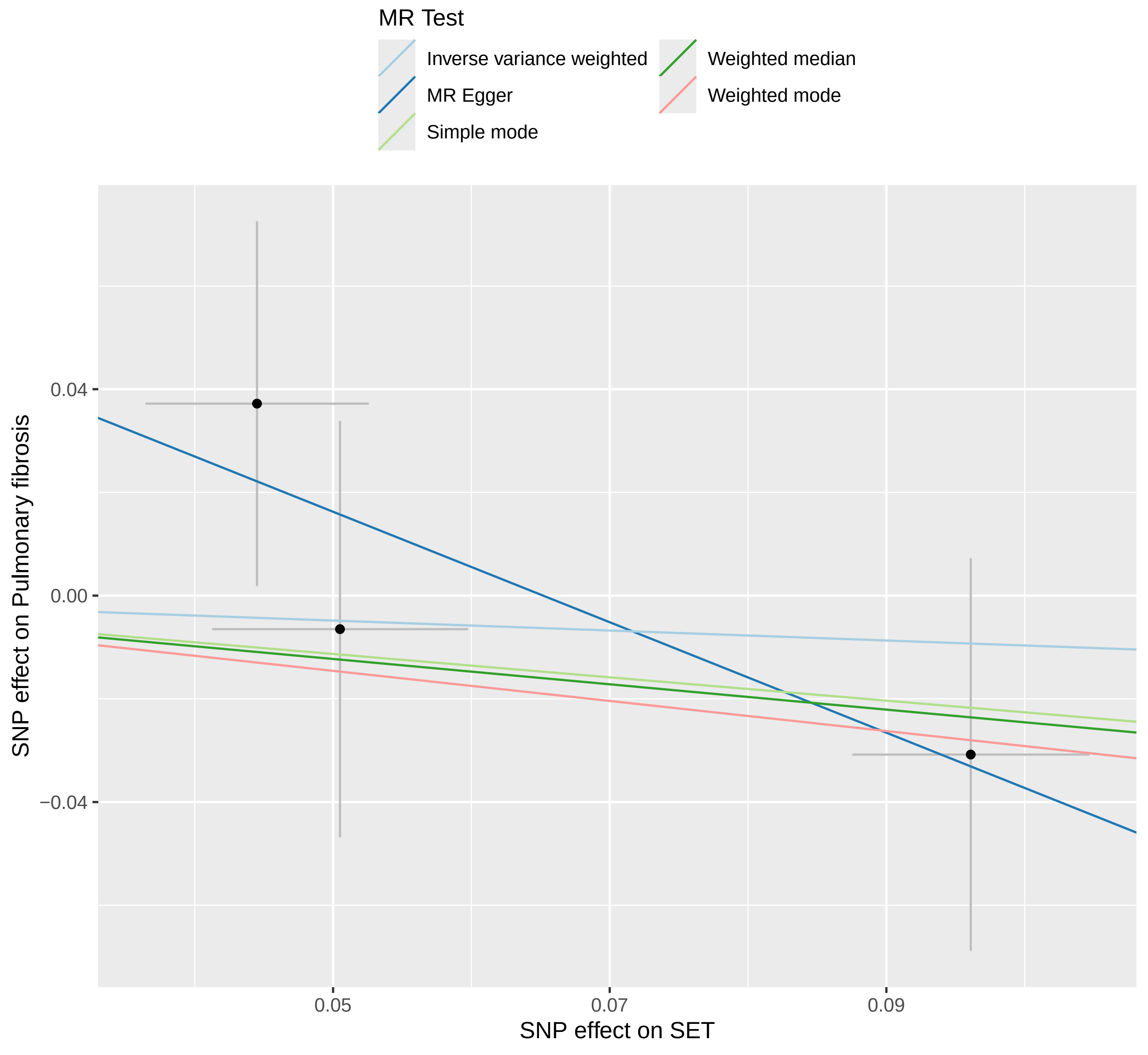

MR Test
Inverse variance weighted
MR Egger
Simple mode
Weighted median
Weighted mode
SNP effect on Pulmonary fibrosis
0.04
0.00
−0.04
0.05
0.07
0.09
SNP effect on SET

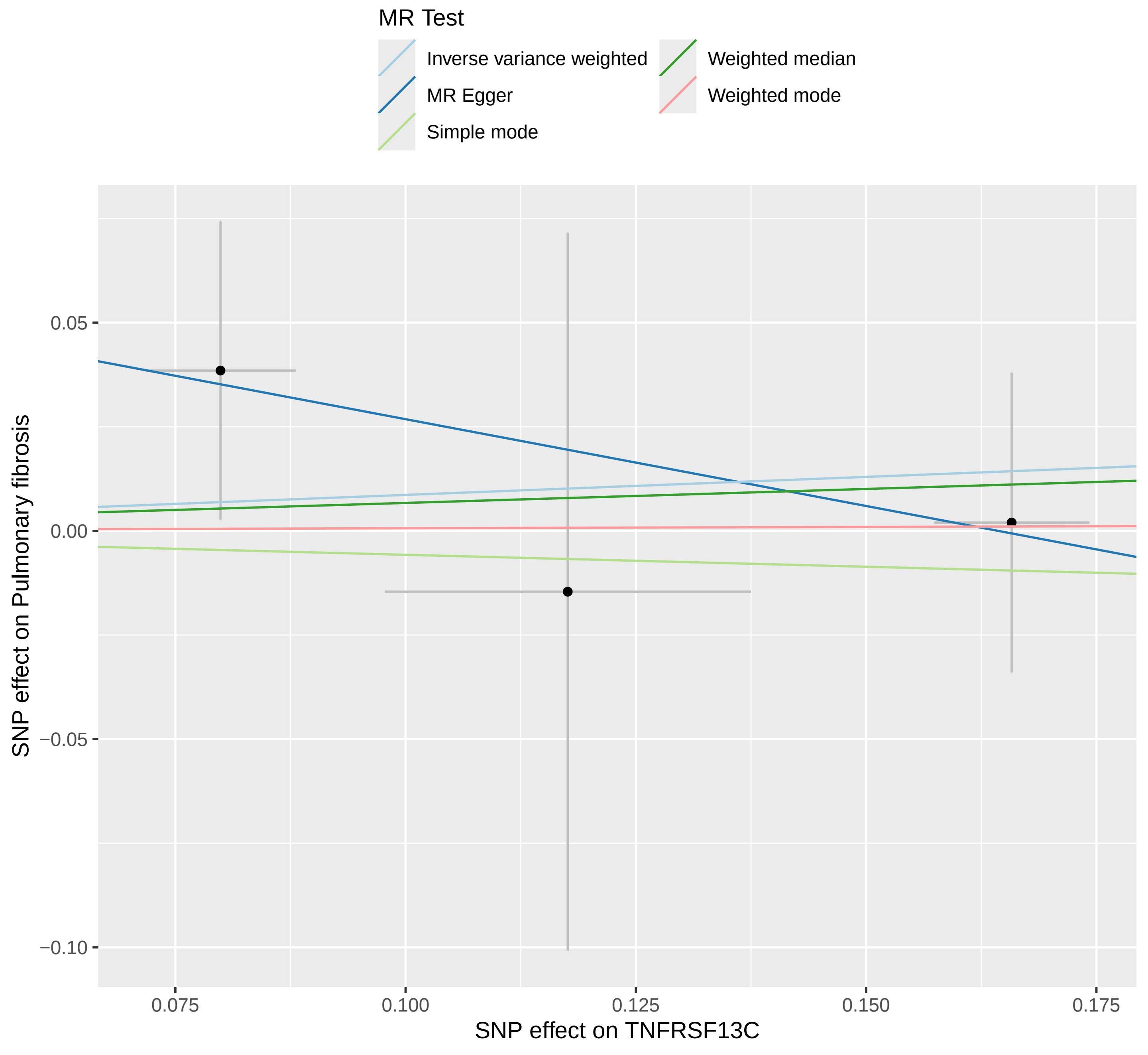

MR Test
Inverse variance weighted
MR Egger
Simple mode
Weighted median
Weighted mode
0.05
0.00
−0.05
−0.10
0.075
0.100
0.125
0.150
0.175
SNP effect on TNFRSF13C
SNP effect on Pulmonary fibrosis

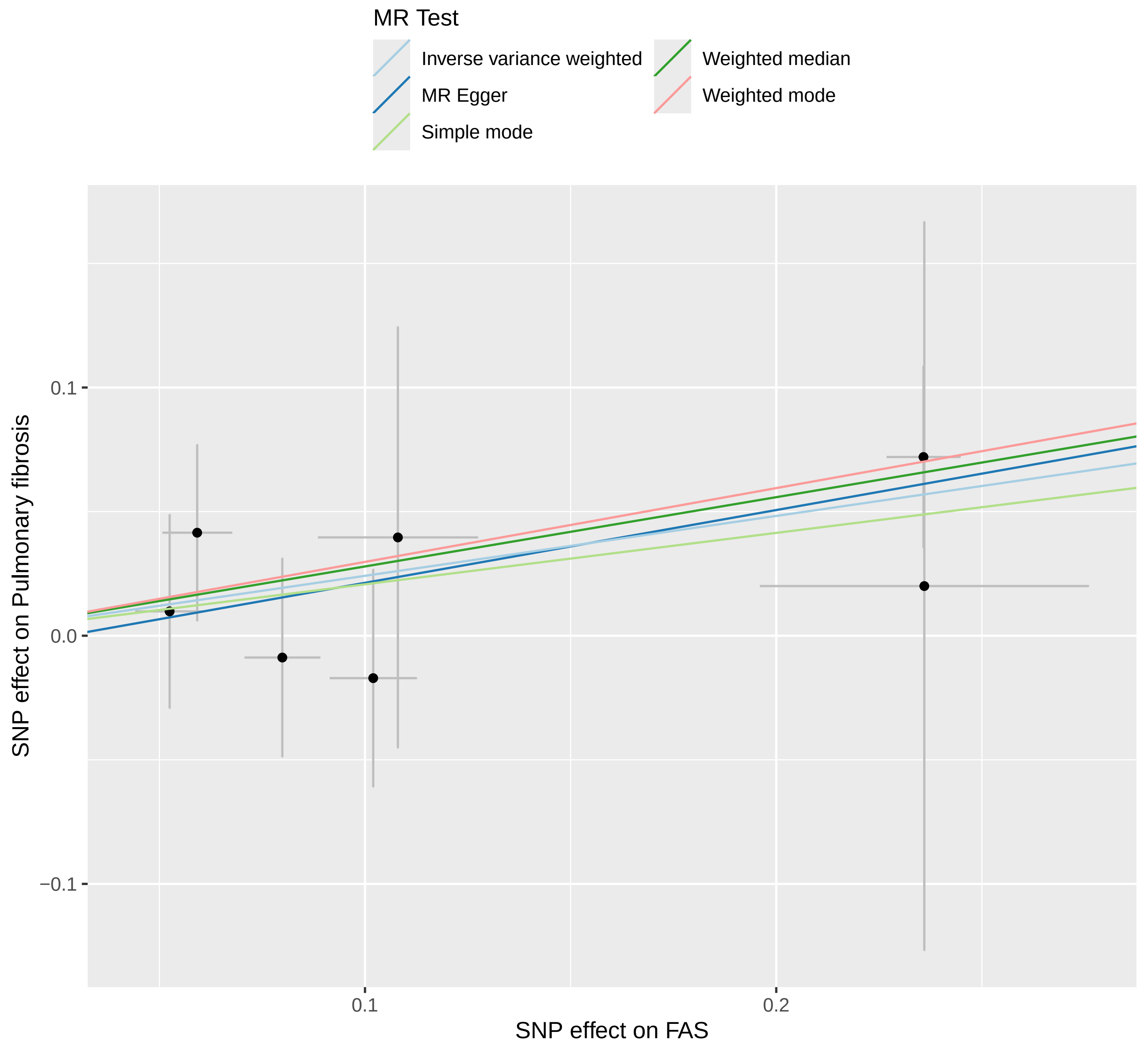

MR Test
Inverse variance weighted
MR Egger
Simple mode
Weighted median
Weighted mode
SNP effect on Pulmonary fibrosis
0.1
0.0
−0.1
0.1
0.2
SNP effect on FAS

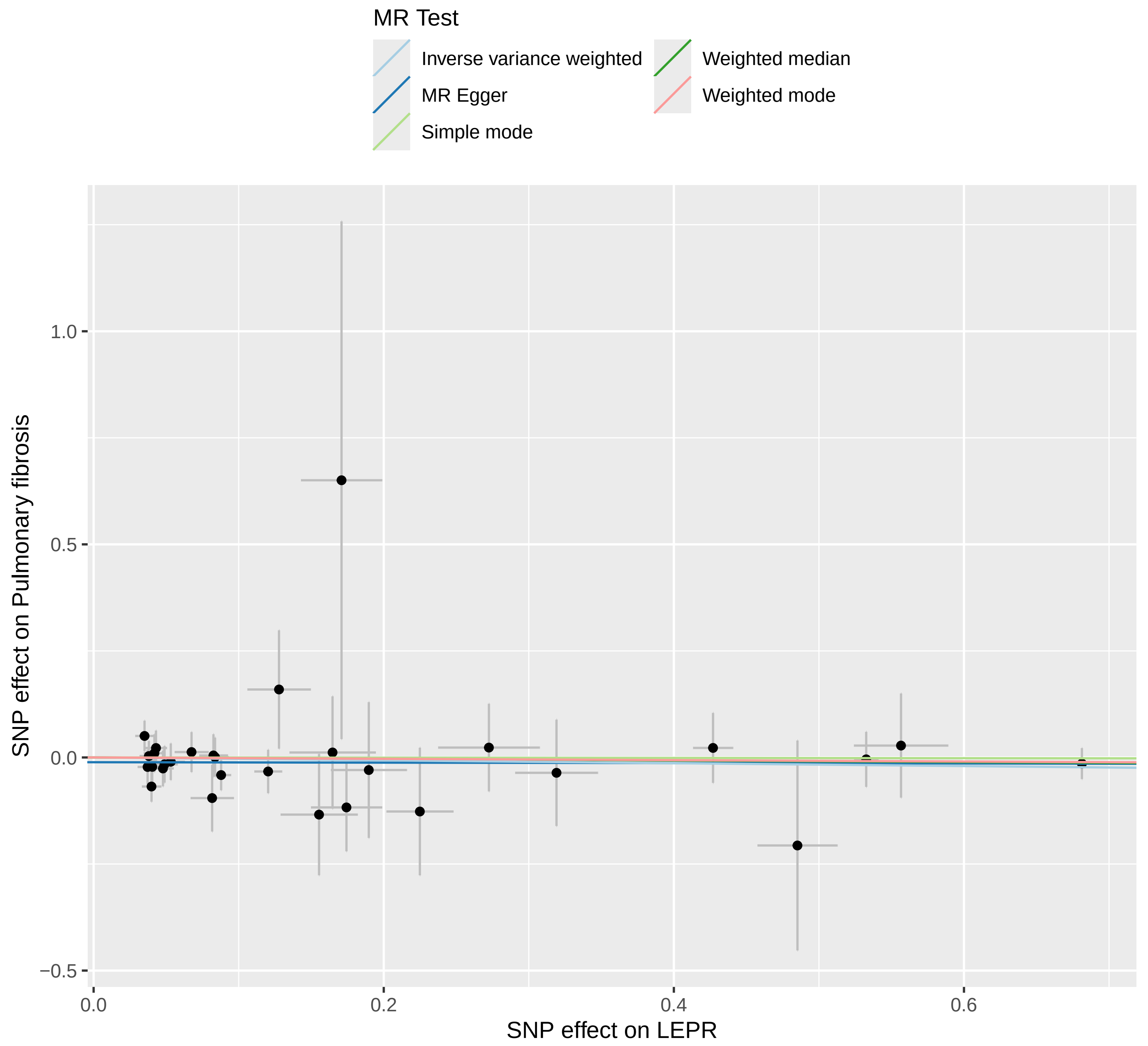
MR Test
Inverse variance weighted
MR Egger
Simple mode
Weighted median
Weighted mode
SNP effect on Pulmonary fibrosis
1.0
0.5
0.0
−0.5
0.0
0.2
0.4
0.6
SNP effect on LEPR

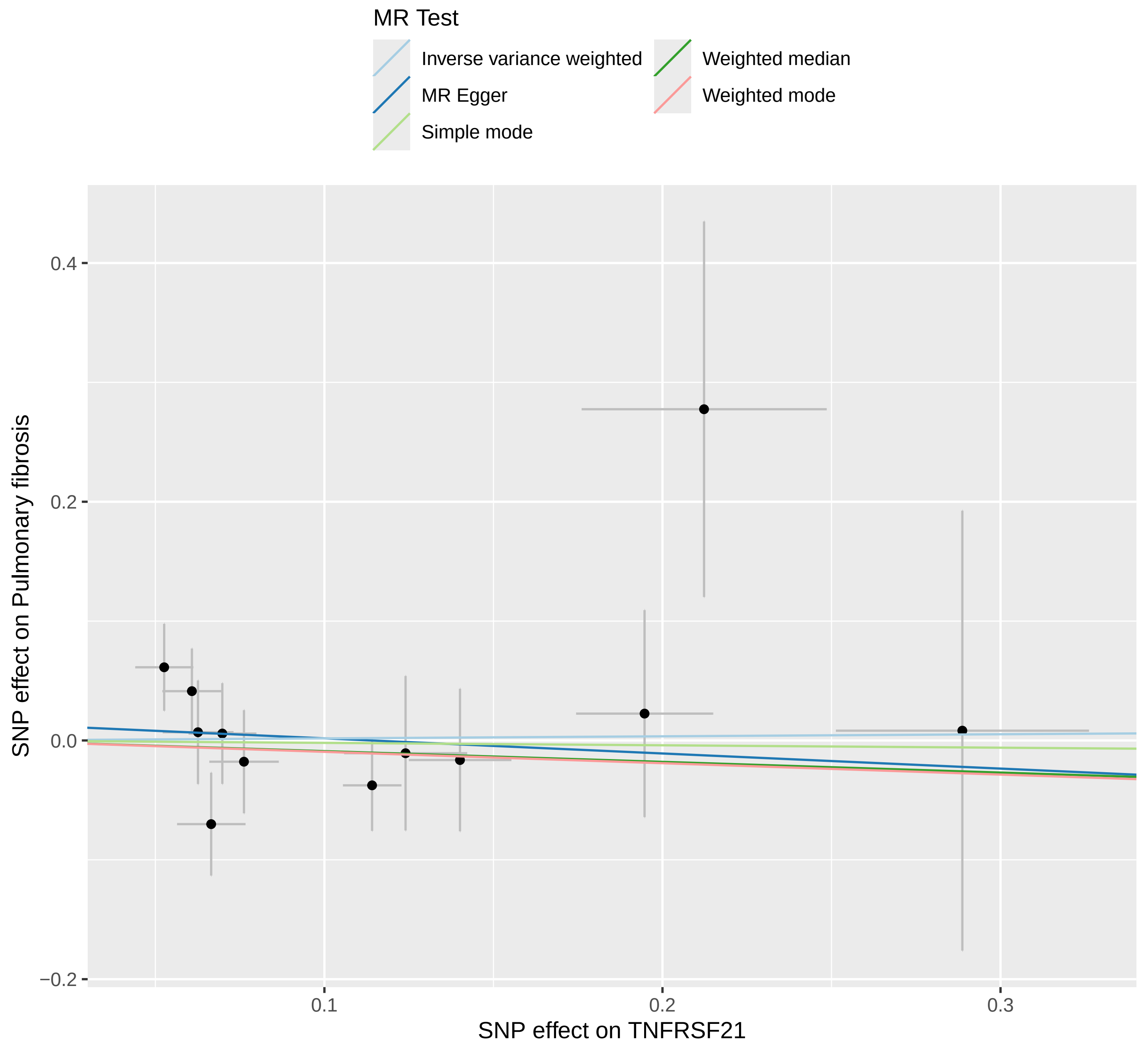
MR Test
Inverse variance weighted
MR Egger
Simple mode
Weighted median
Weighted mode
SNP effect on Pulmonary fibrosis
0.4
0.2
0.0
−0.2
0.1
0.2
0.3
SNP effect on TNFRSF21

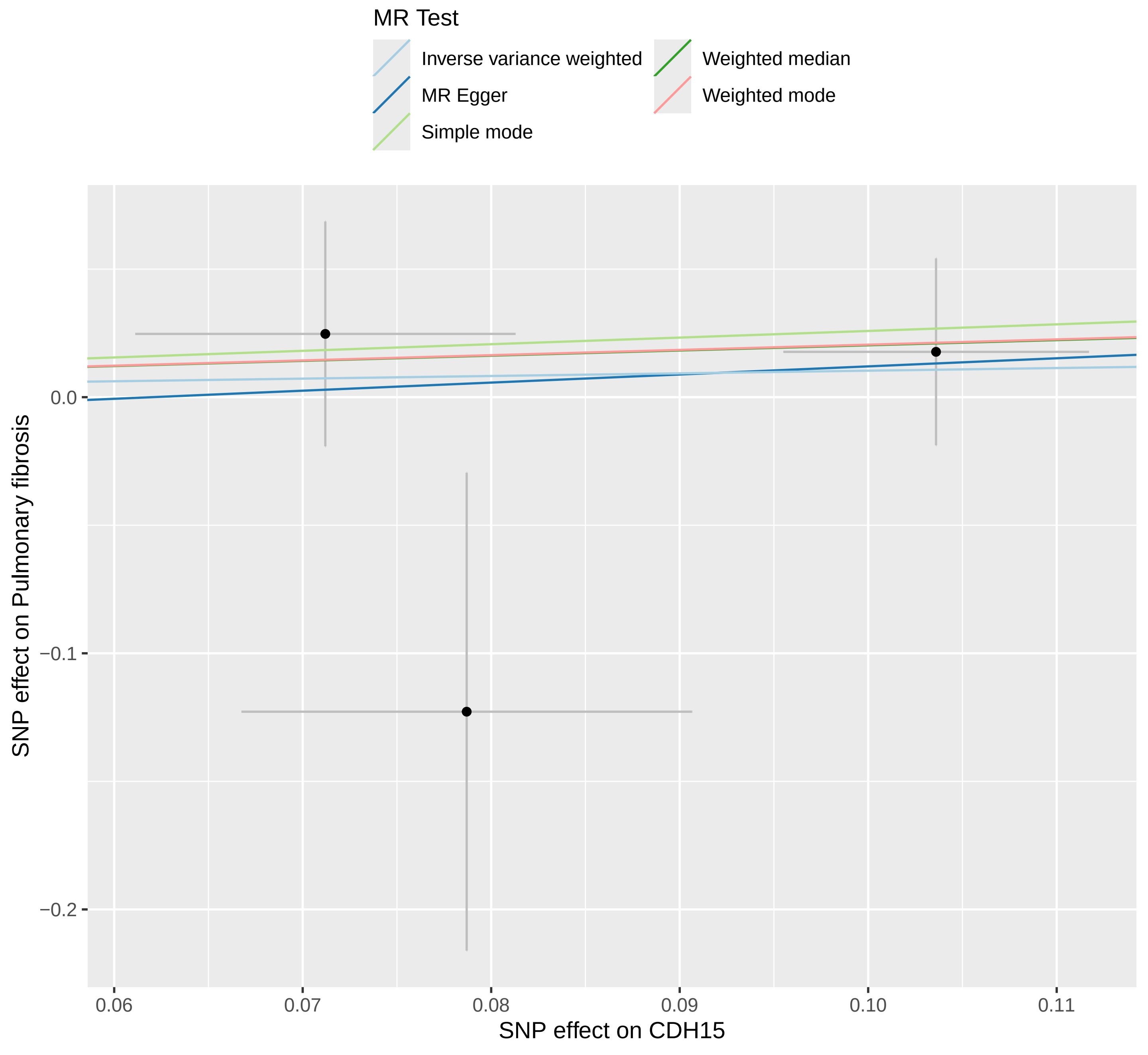

MR Test
Inverse variance weighted
MR Egger
Simple mode
Weighted median
Weighted mode
0.0
−0.1
−0.2
SNP effect on Pulmonary fibrosis
0.06
0.07
0.08
0.09
0.10
0.11
SNP effect on CDH15

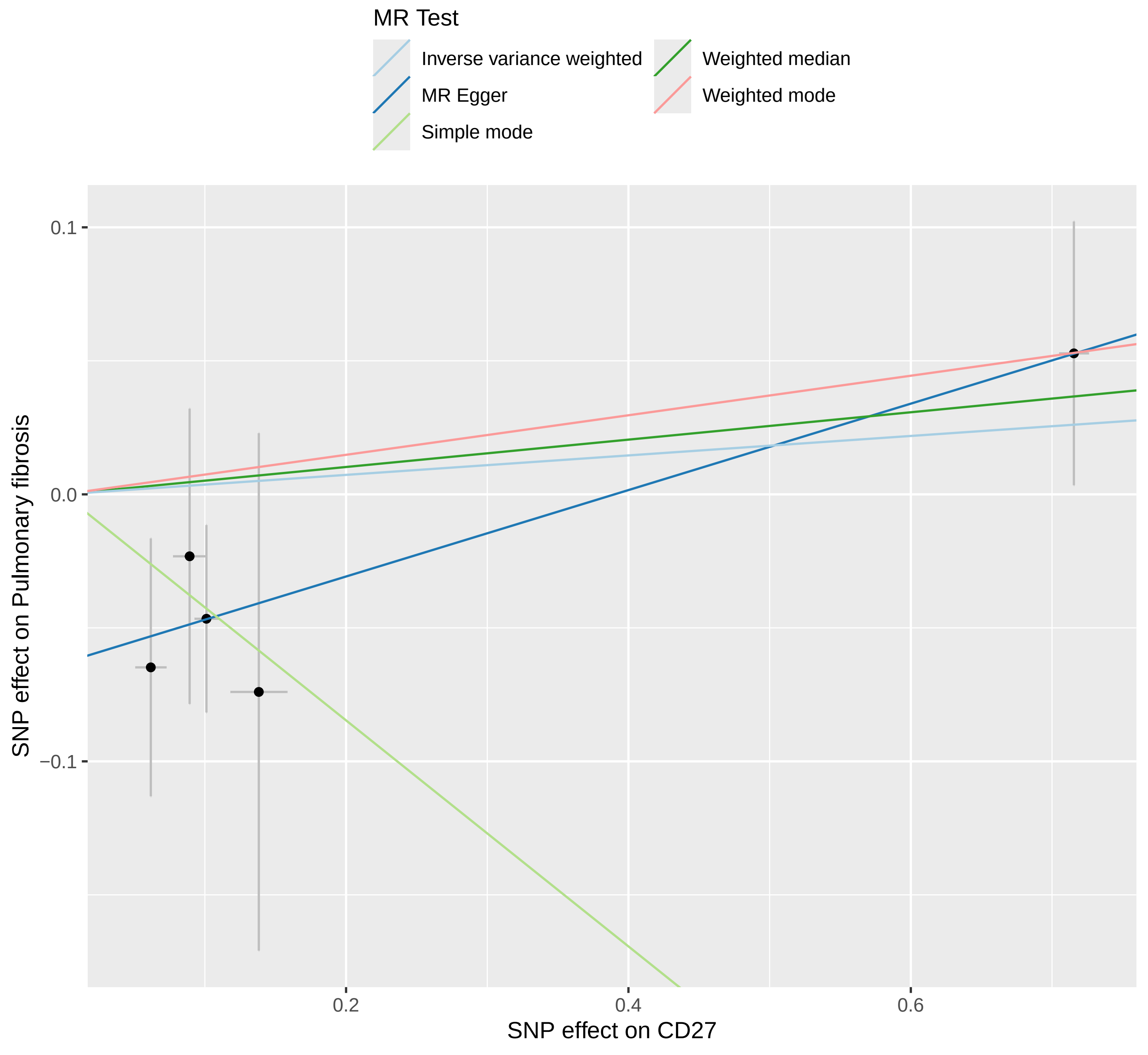

MR Test
Inverse variance weighted
MR Egger
Simple mode
Weighted median
Weighted mode
0.1
0.0
−0.1
0.2
0.4
0.6
SNP effect on Pulmonary fibrosis
SNP effect on CD27

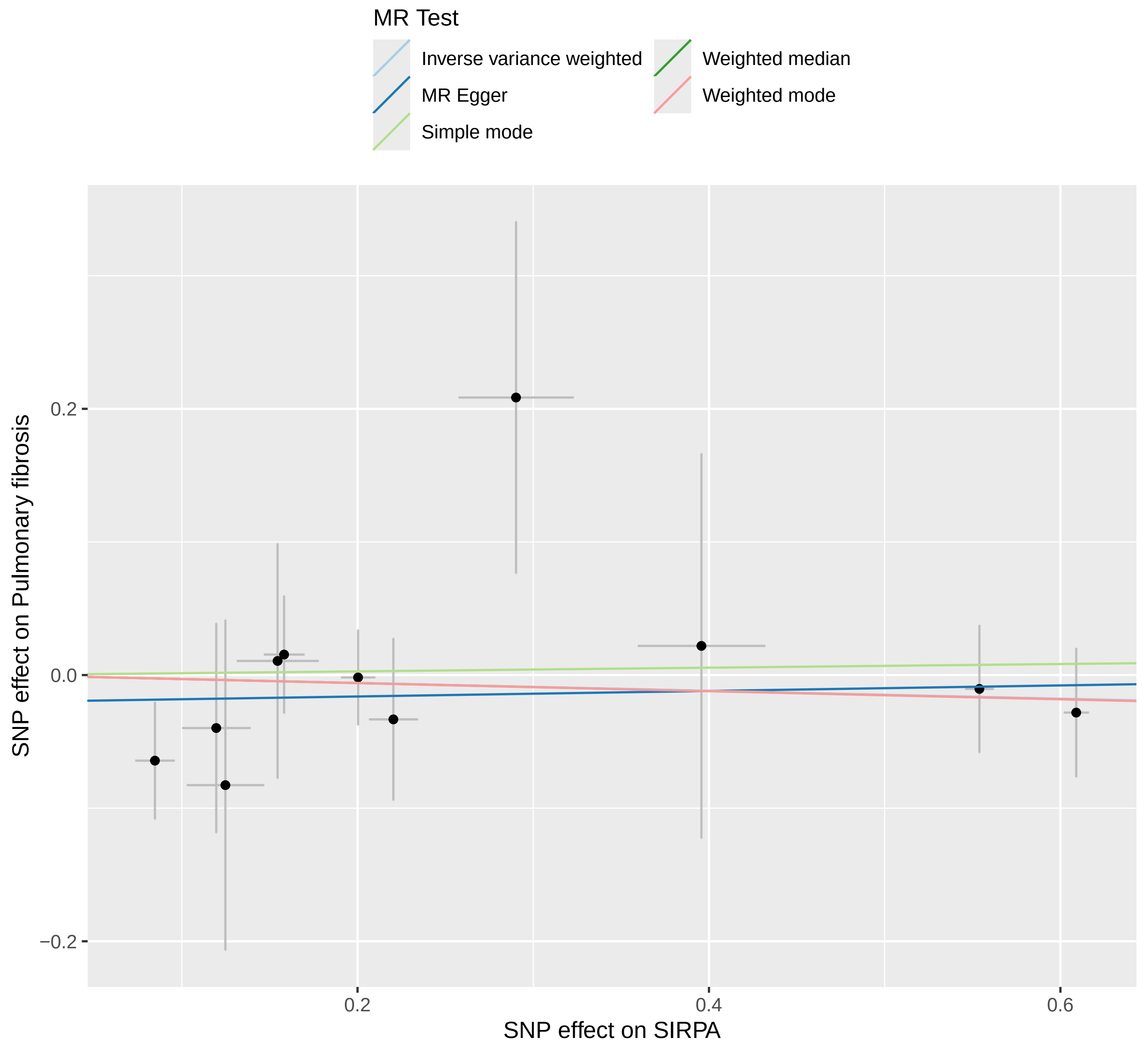
MR Test
Inverse variance weighted
MR Egger
Simple mode
Weighted median
Weighted mode
SNP effect on Pulmonary fibrosis
0.2
0.0
−0.2
0.2
0.4
0.6
SNP effect on SIRPA

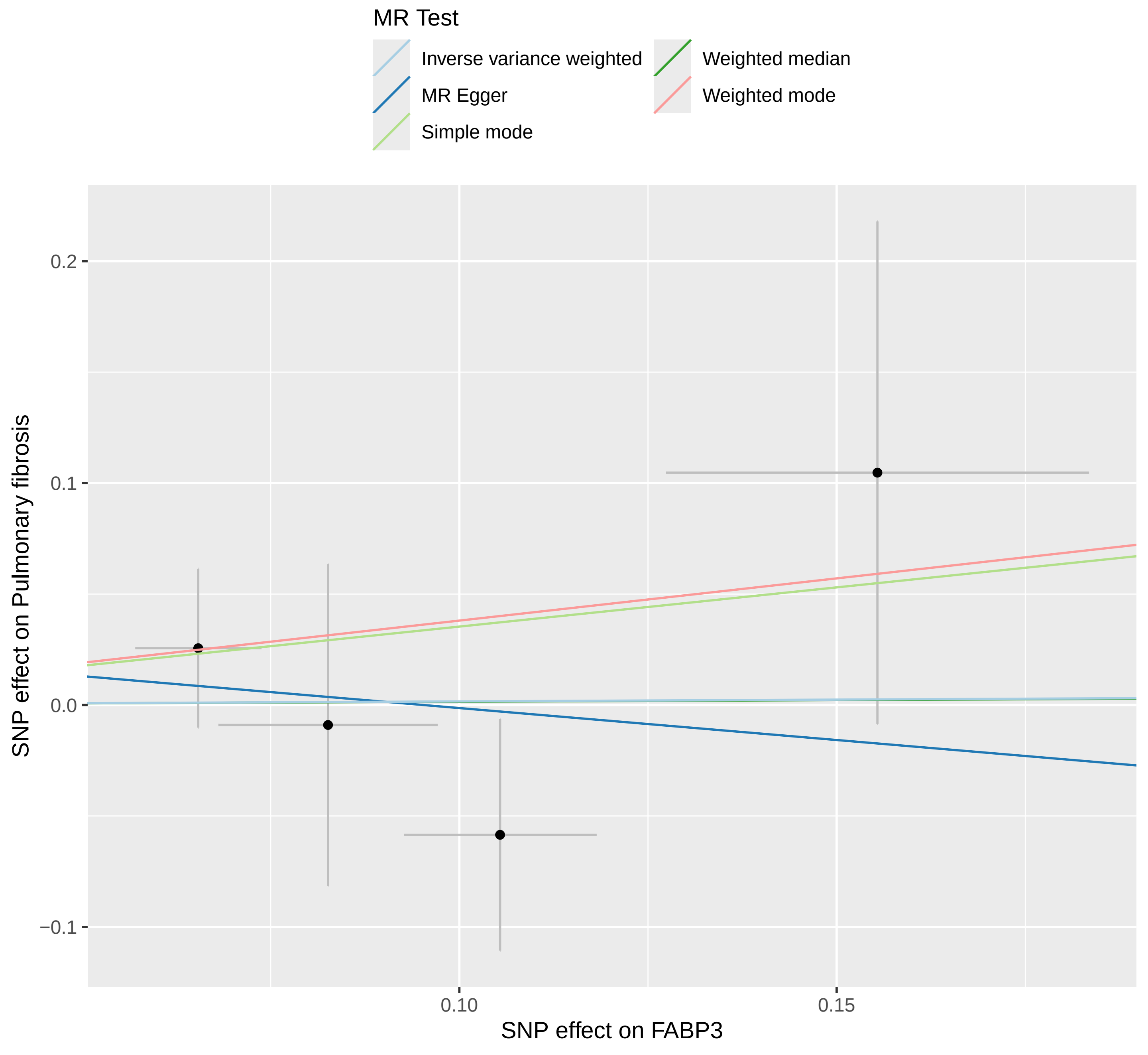
MR Test
Inverse variance weighted
MR Egger
Simple mode
Weighted median
Weighted mode
0.2
0.1
0.0
−0.1
0.10
0.15
SNP effect on FABP3
SNP effect on Pulmonary fibrosis

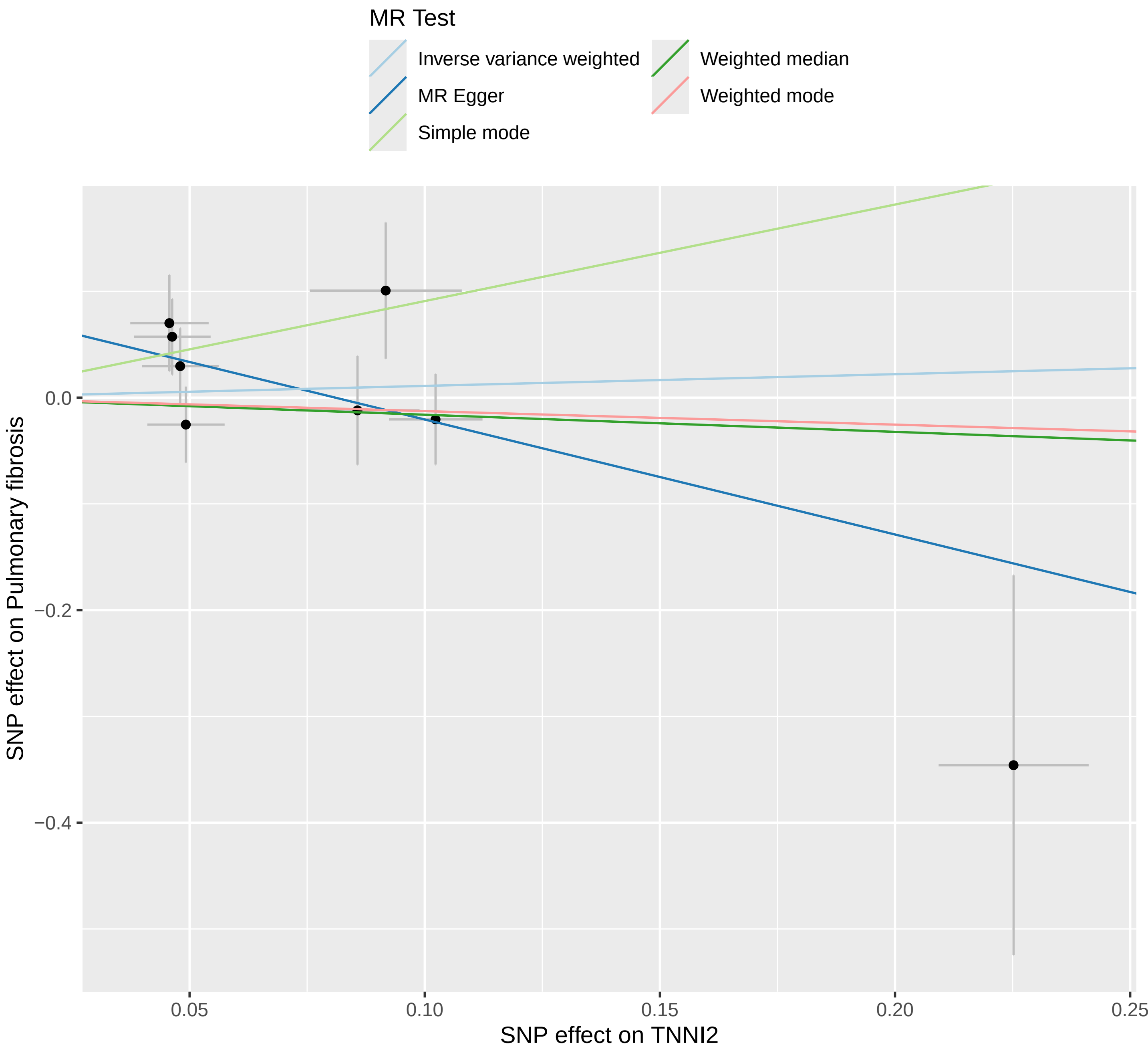

MR Test
Inverse variance weighted
MR Egger
Simple mode
Weighted median
Weighted mode
0.0
−0.2
−0.4
SNP effect on Pulmonary fibrosis
0.05
0.10
0.15
0.20
0.25
SNP effect on TNNI2

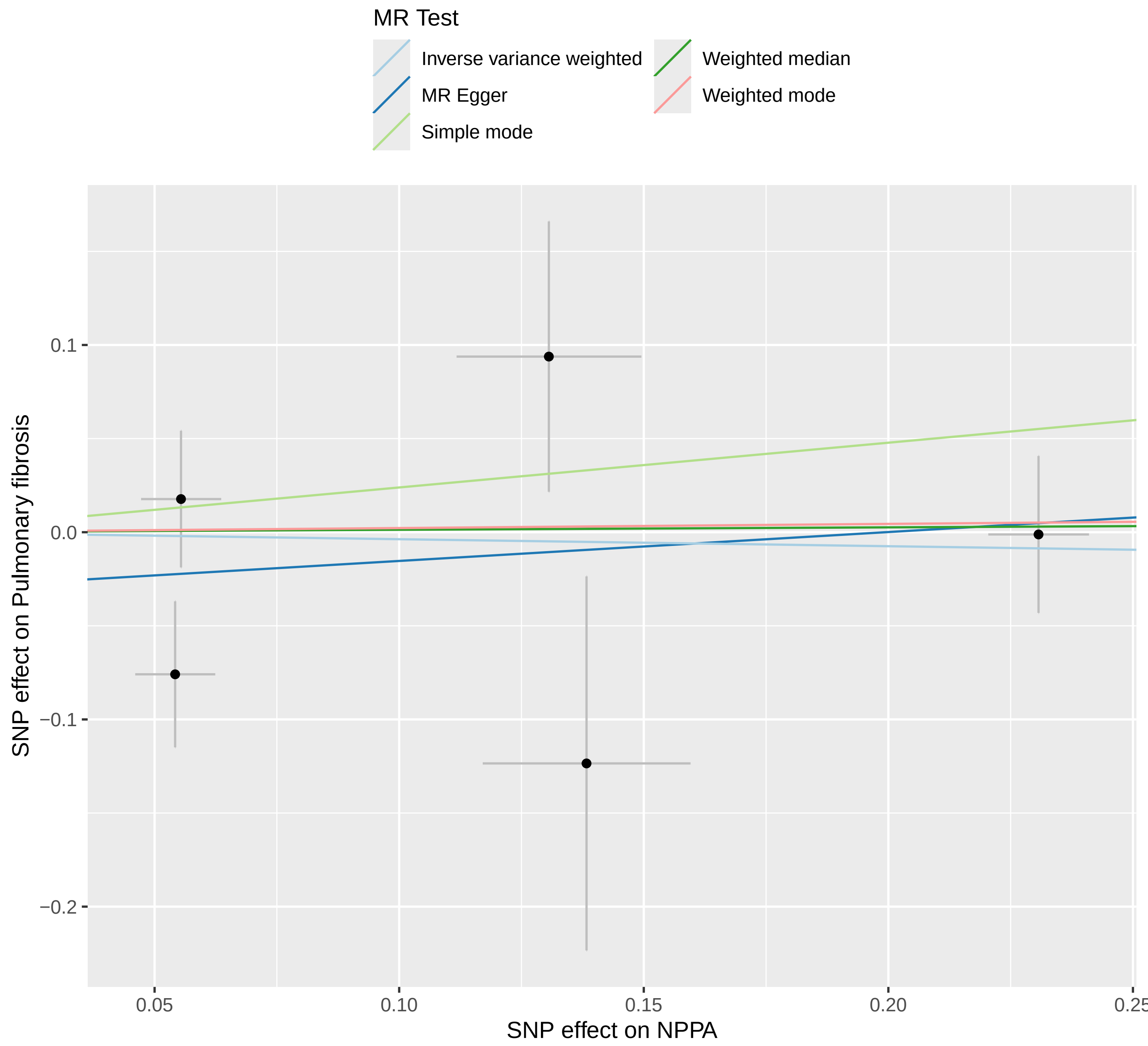
MR Test
Inverse variance weighted
MR Egger
Simple mode
Weighted median
Weighted mode
0.1
0.0
−0.1
−0.2
0.05
0.10
0.15
0.20
SNP effect on NPPA
SNP effect on Pulmonary fibrosis

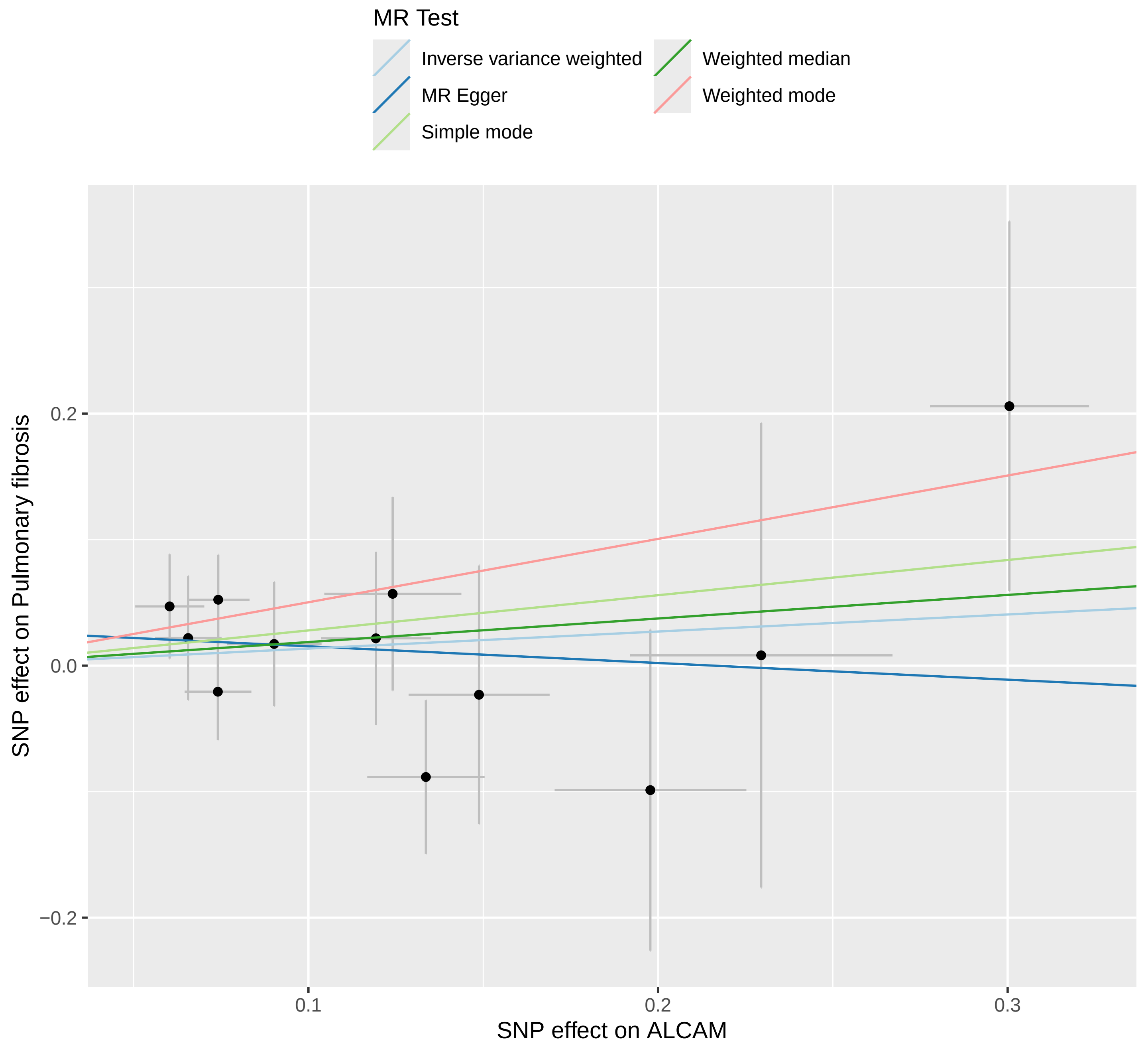

MR Test
Inverse variance weighted
MR Egger
Simple mode
Weighted median
Weighted mode
SNP effect on Pulmonary fibrosis
0.2
0.0
−0.2
0.1
0.2
0.3
SNP effect on ALCAM

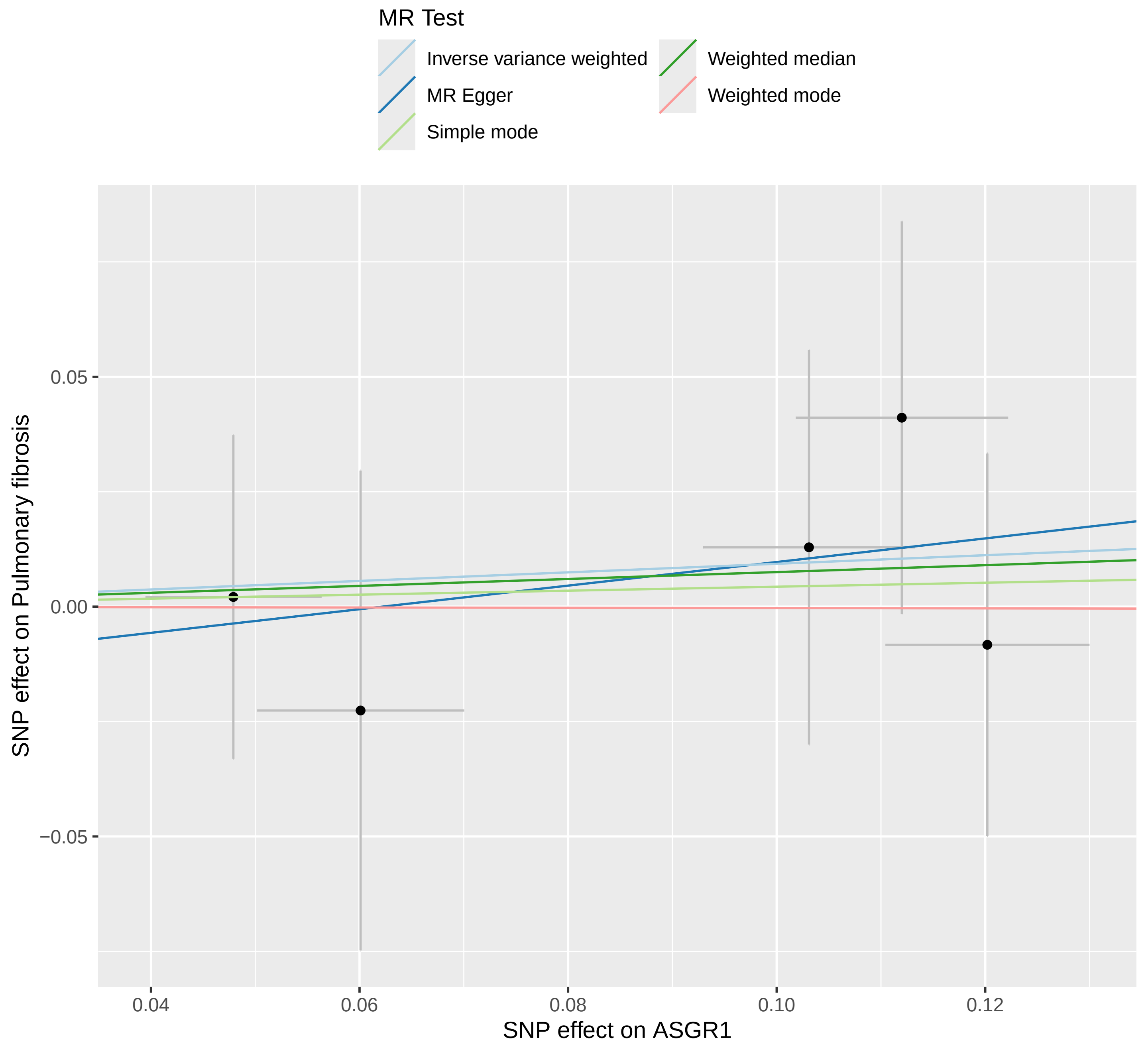

MR Test
Inverse variance weighted
MR Egger
Simple mode
Weighted median
Weighted mode
0.05
0.00
−0.05
SNP effect on Pulmonary fibrosis
0.04
0.06
0.08
0.10
0.12
SNP effect on ASGR1

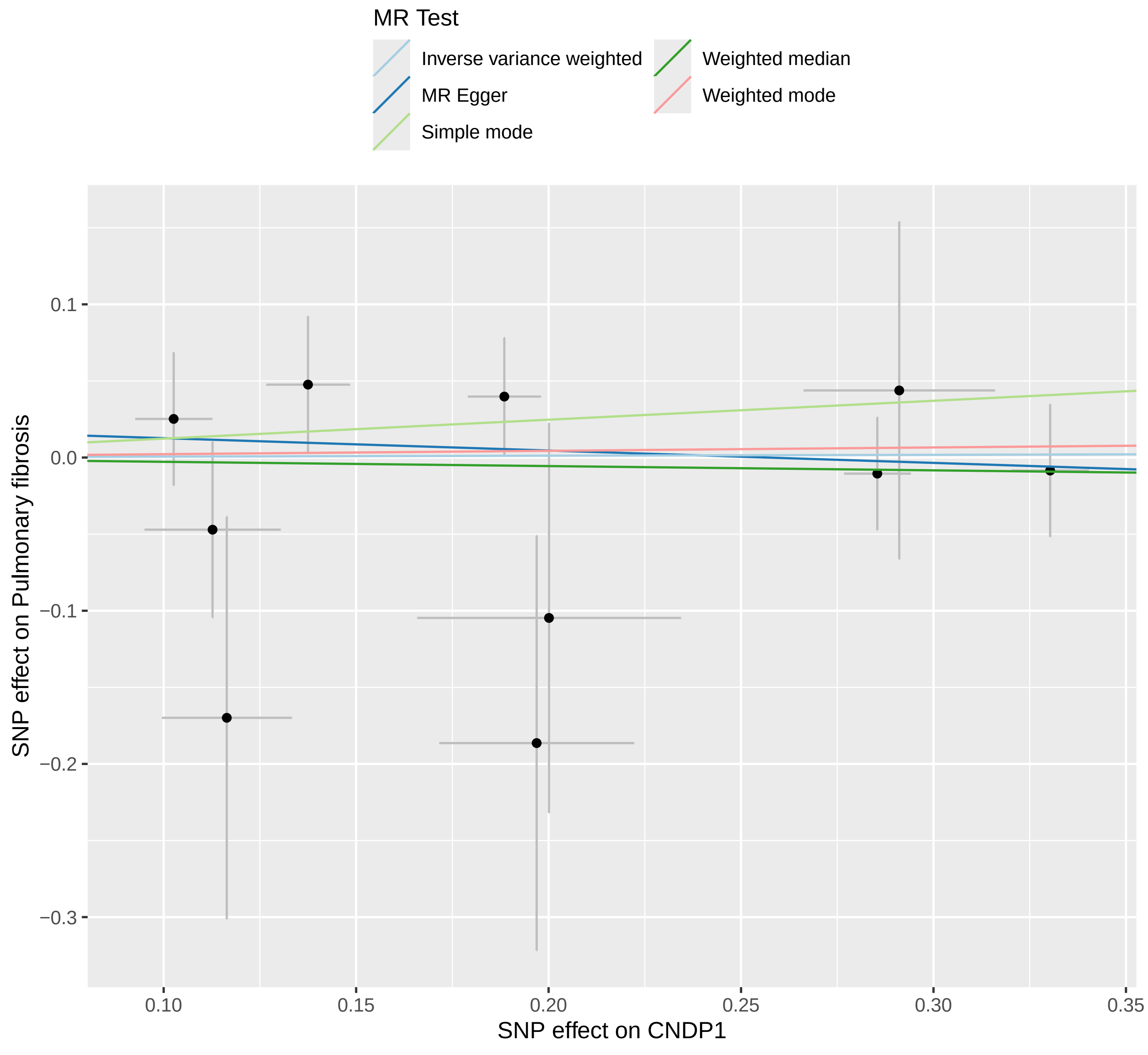

MR Test
Inverse variance weighted
MR Egger
Simple mode
Weighted median
Weighted mode
0.1
0.0
−0.1
−0.2
−0.3
SNP effect on Pulmonary fibrosis
0.10
0.15
0.20
0.25
0.30
0.35
SNP effect on CNDP1

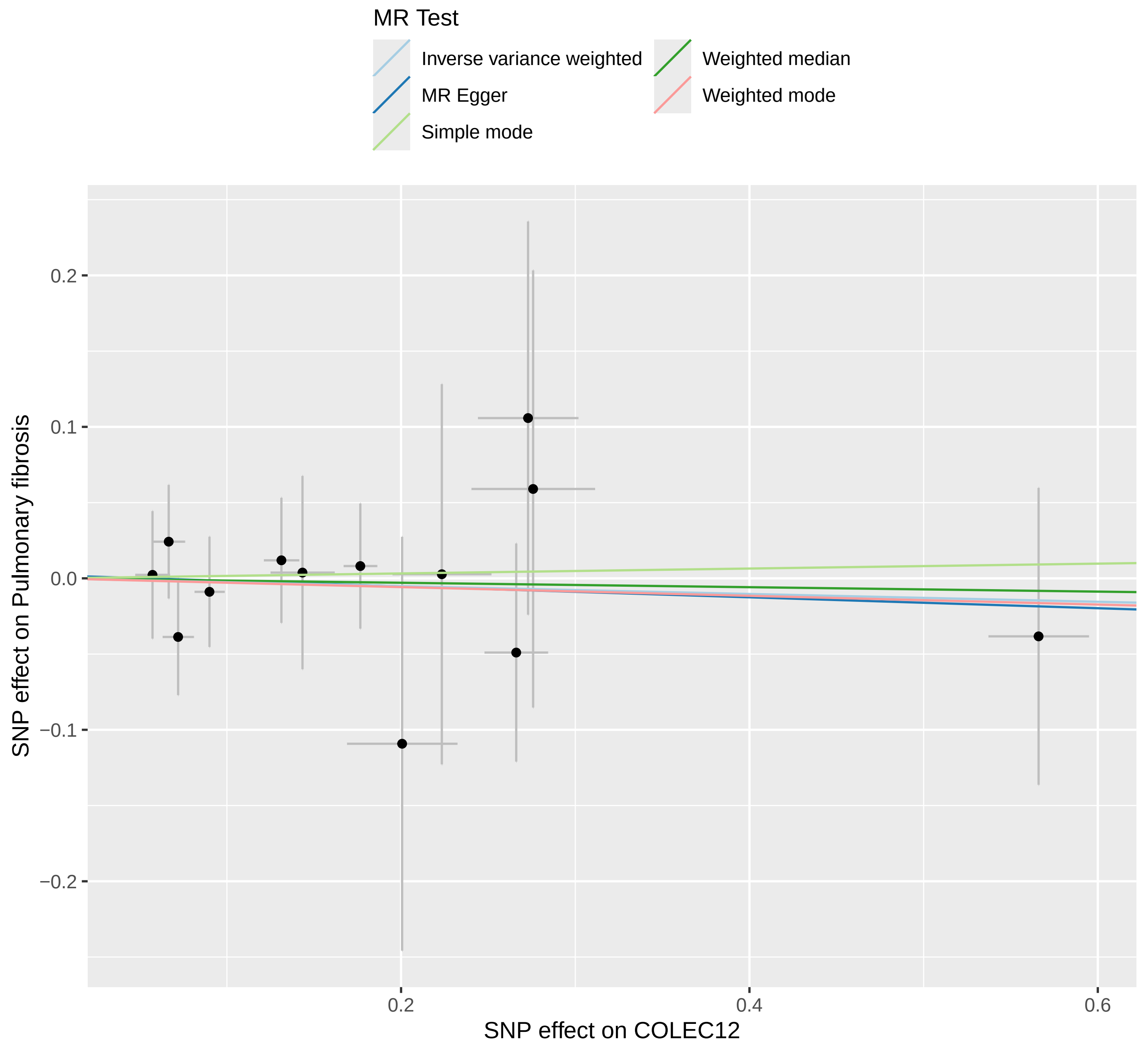
MR Test
Inverse variance weighted
MR Egger
Simple mode
Weighted median
Weighted mode
0.2
0.1
0.0
−0.1
−0.2
SNP effect on Pulmonary fibrosis
0.2
0.4
0.6
SNP effect on COLEC12

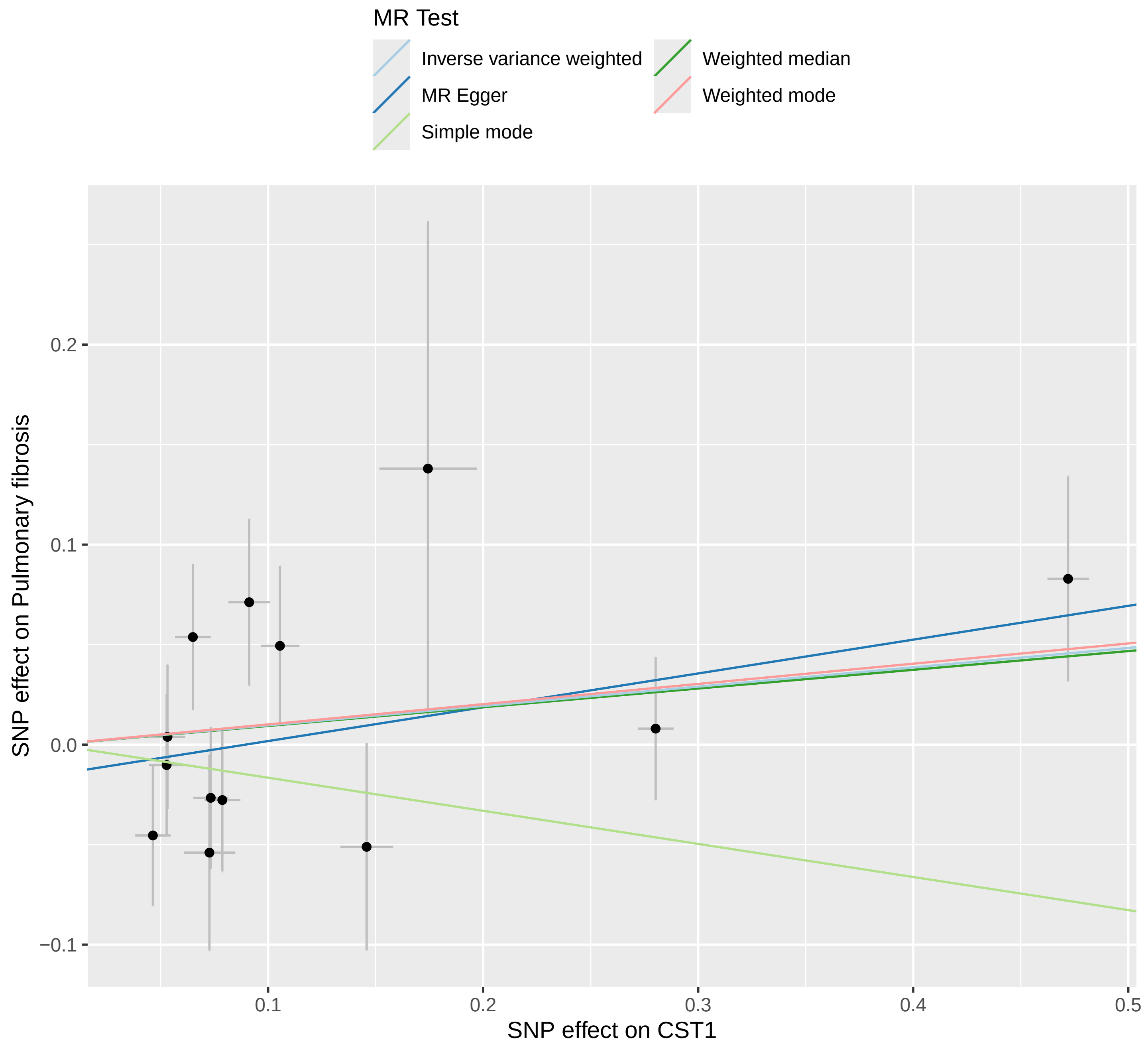

MR Test
Inverse variance weighted
MR Egger
Simple mode
Weighted median
Weighted mode
0.2
0.1
0.0
−0.1
0.1
0.2
0.3
0.4
0.5
SNP effect on CST1
SNP effect on Pulmonary fibrosis

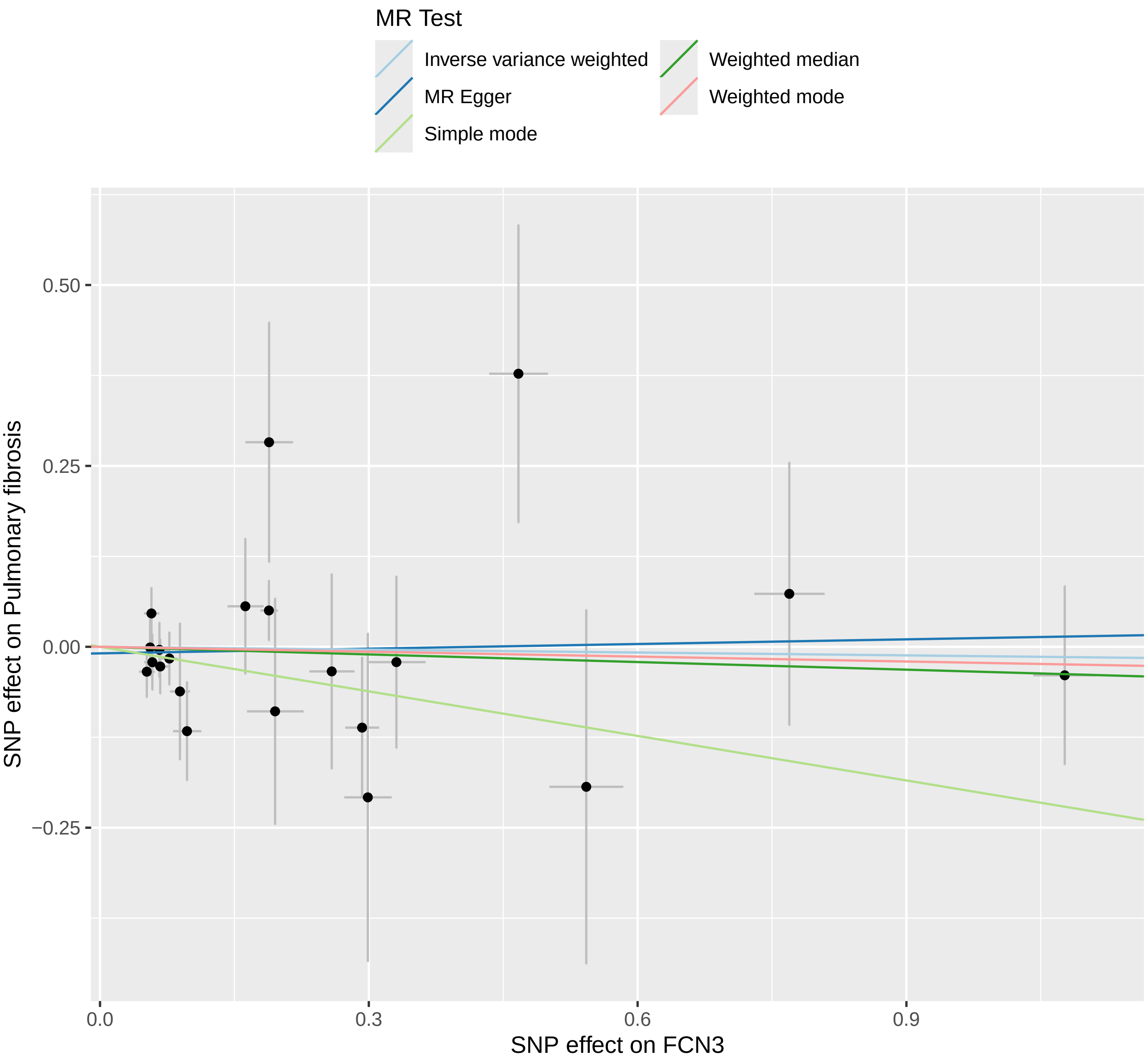

MR Test
Inverse variance weighted
MR Egger
Simple mode
Weighted median
Weighted mode
0.50
0.25
0.00
−0.25
0.0
0.3
0.6
0.9
SNP effect on Pulmonary fibrosis
SNP effect on FCN3

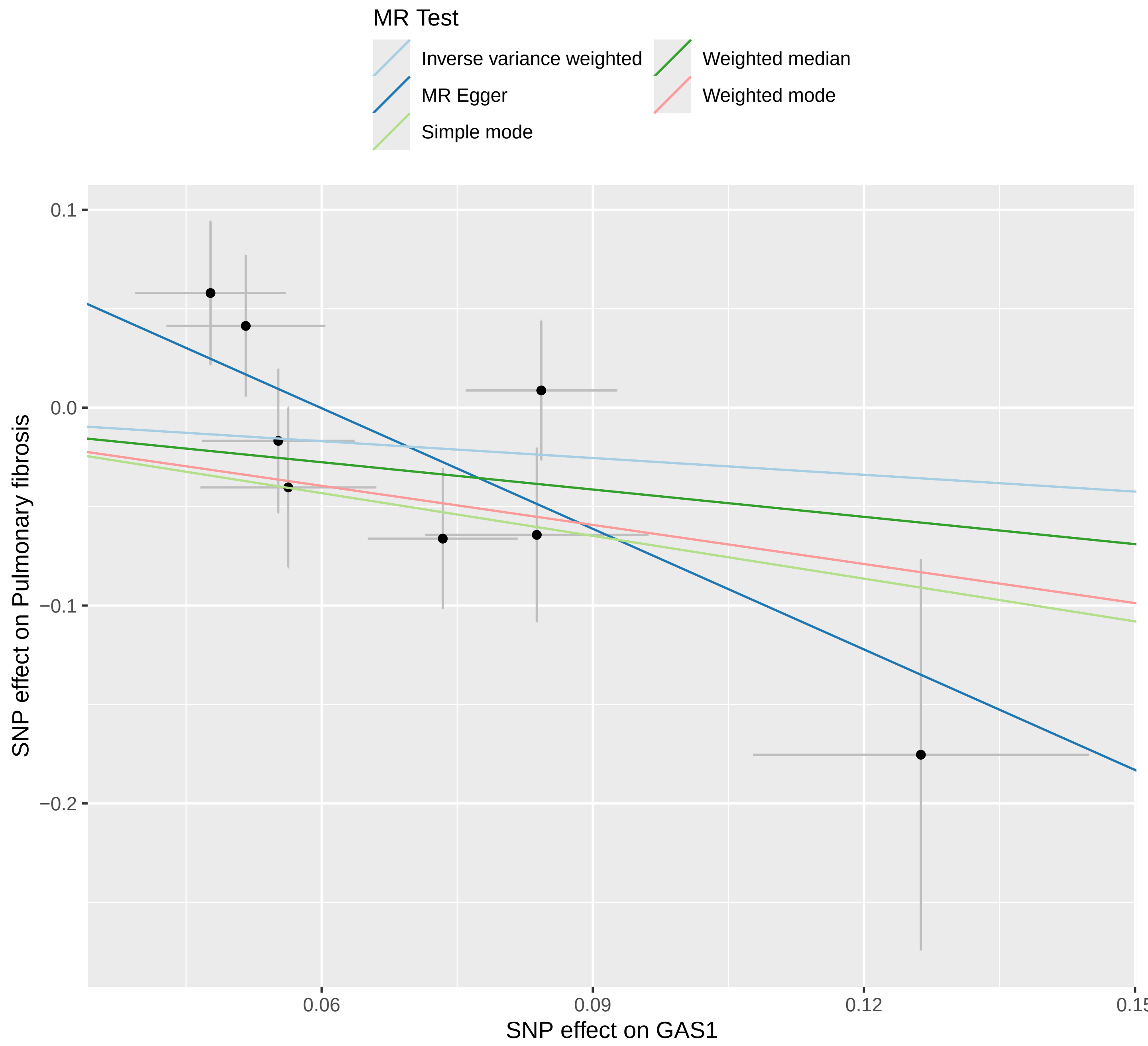
MR Test
Inverse variance weighted
MR Egger
Simple mode
Weighted median
Weighted mode
0.1
0.0
−0.1
−0.2
0.06
0.09
0.12
0.15
SNP effect on Pulmonary fibrosis
SNP effect on GAS1

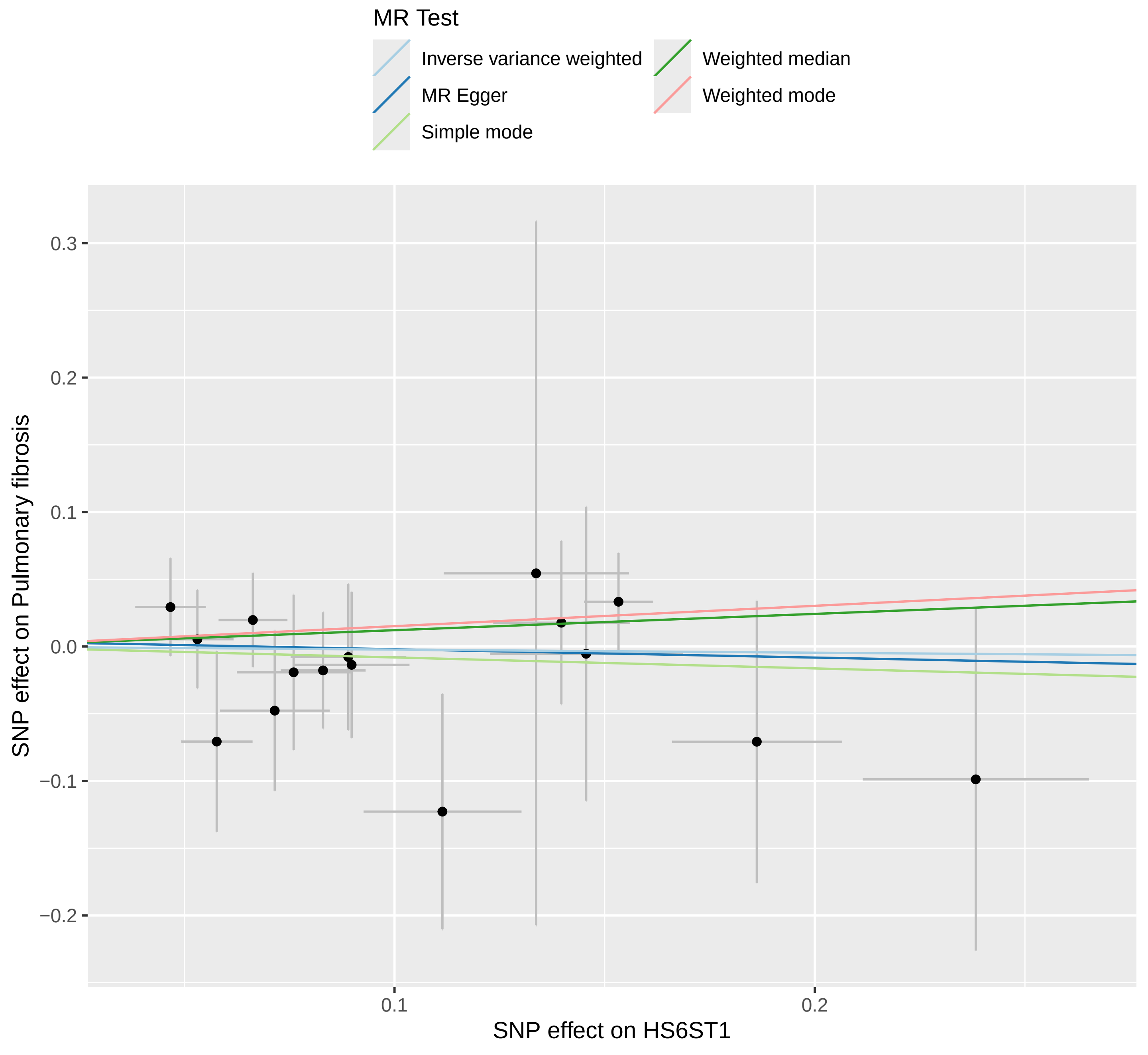

MR Test
Inverse variance weighted
MR Egger
Simple mode
Weighted median
Weighted mode
0.3
0.2
0.1
0.0
−0.1
−0.2
SNP effect on Pulmonary fibrosis
0.1
0.2
SNP effect on HS6ST1

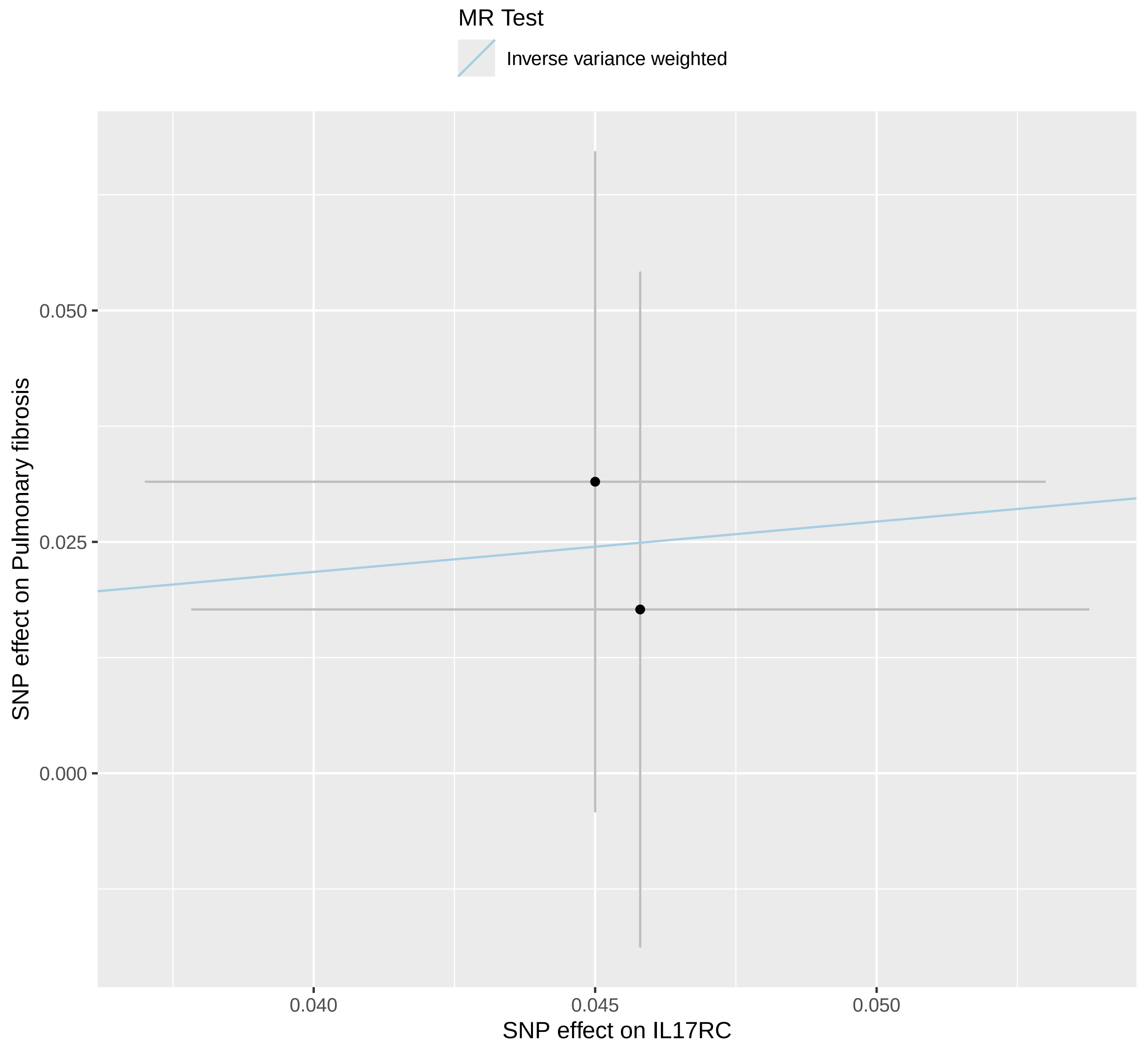

MR Test
Inverse variance weighted
0.050
0.025
0.000
0.040
0.045
0.050
SNP effect on Pulmonary fibrosis
SNP effect on IL17RC

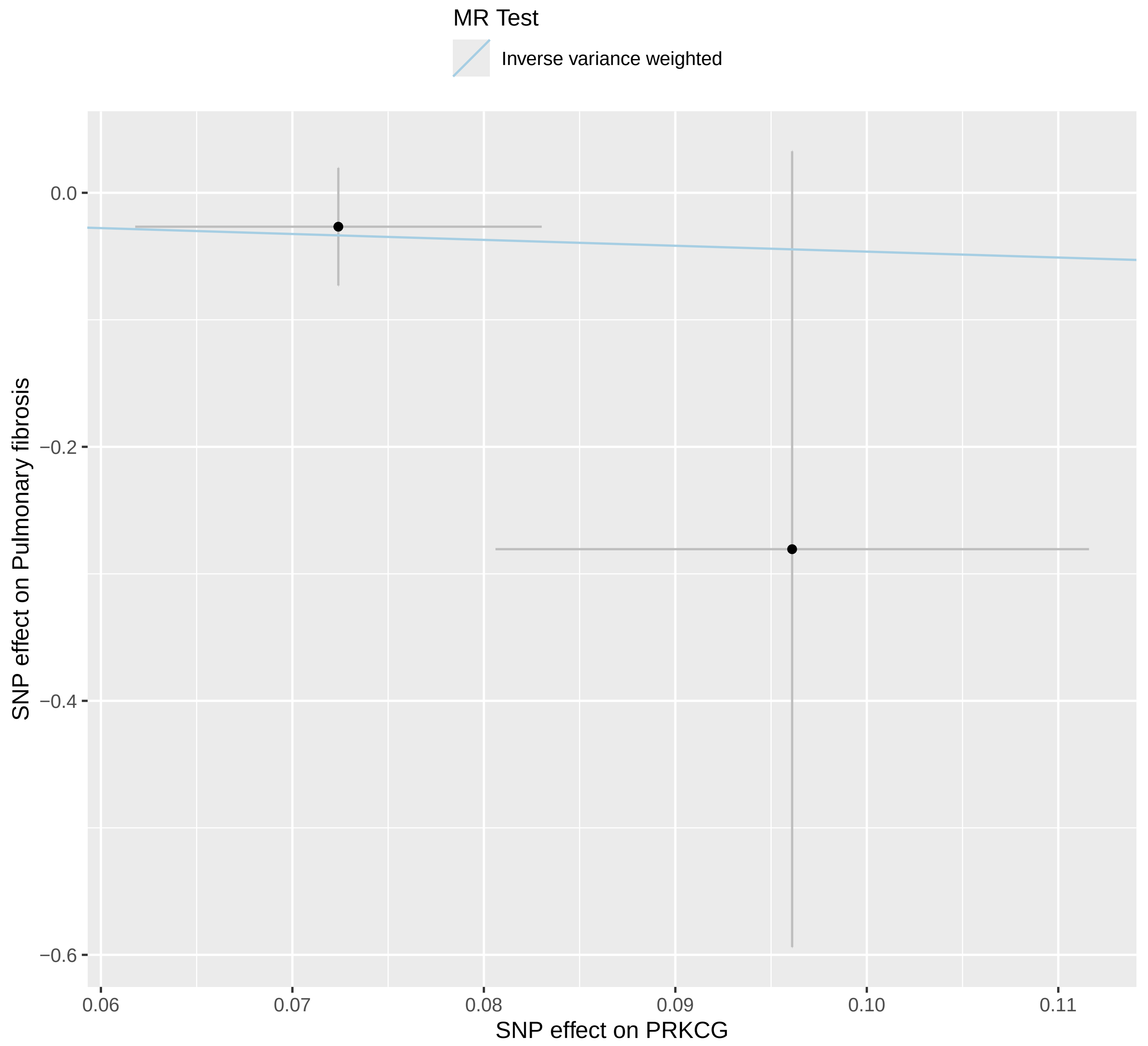

MR Test
Inverse variance weighted
0.0
−0.2
−0.4
−0.6
0.06
0.07
0.08
0.09
0.10
0.11
SNP effect on PRKCG
SNP effect on Pulmonary fibrosis

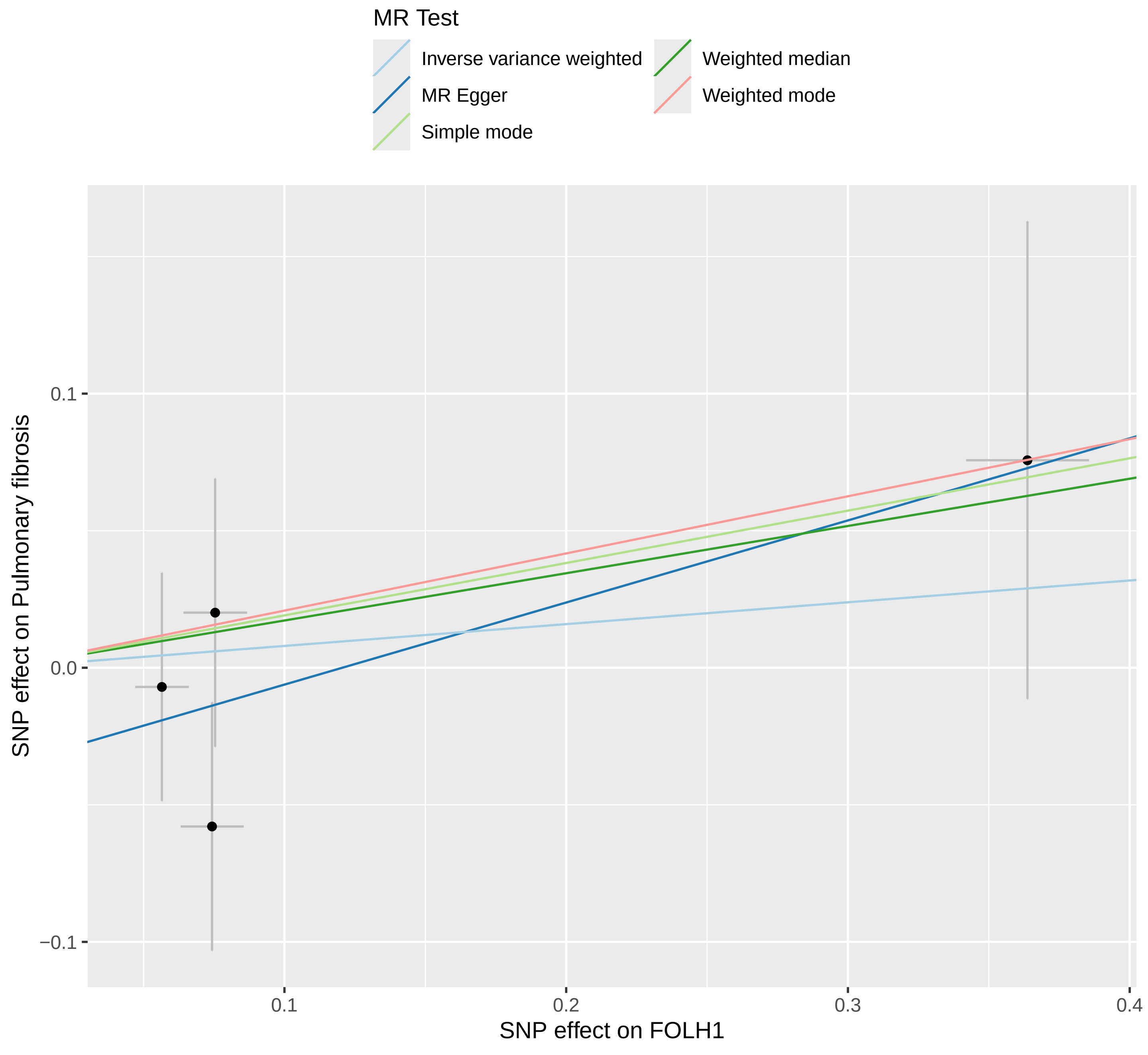

MR Test
Inverse variance weighted
MR Egger
Simple mode
Weighted median
Weighted mode
SNP effect on Pulmonary fibrosis
0.1
0.0
−0.1
0.1
0.2
0.3
0.4
SNP effect on FOLH1

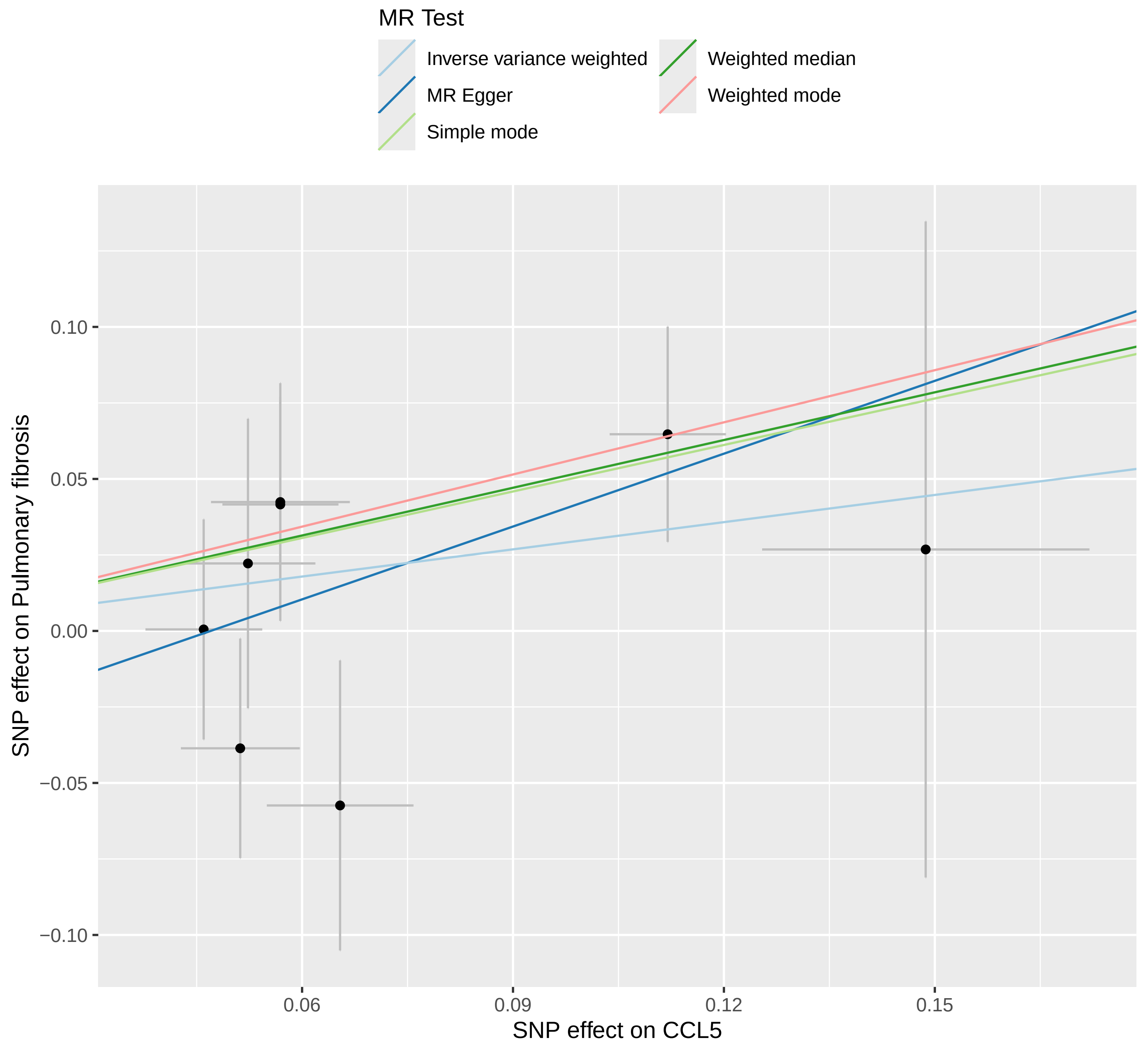

MR Test
Inverse variance weighted
MR Egger
Simple mode
Weighted median
Weighted mode
0.10
0.05
0.00
−0.05
−0.10
SNP effect on Pulmonary fibrosis
0.06
0.09
0.12
0.15
SNP effect on CCL5

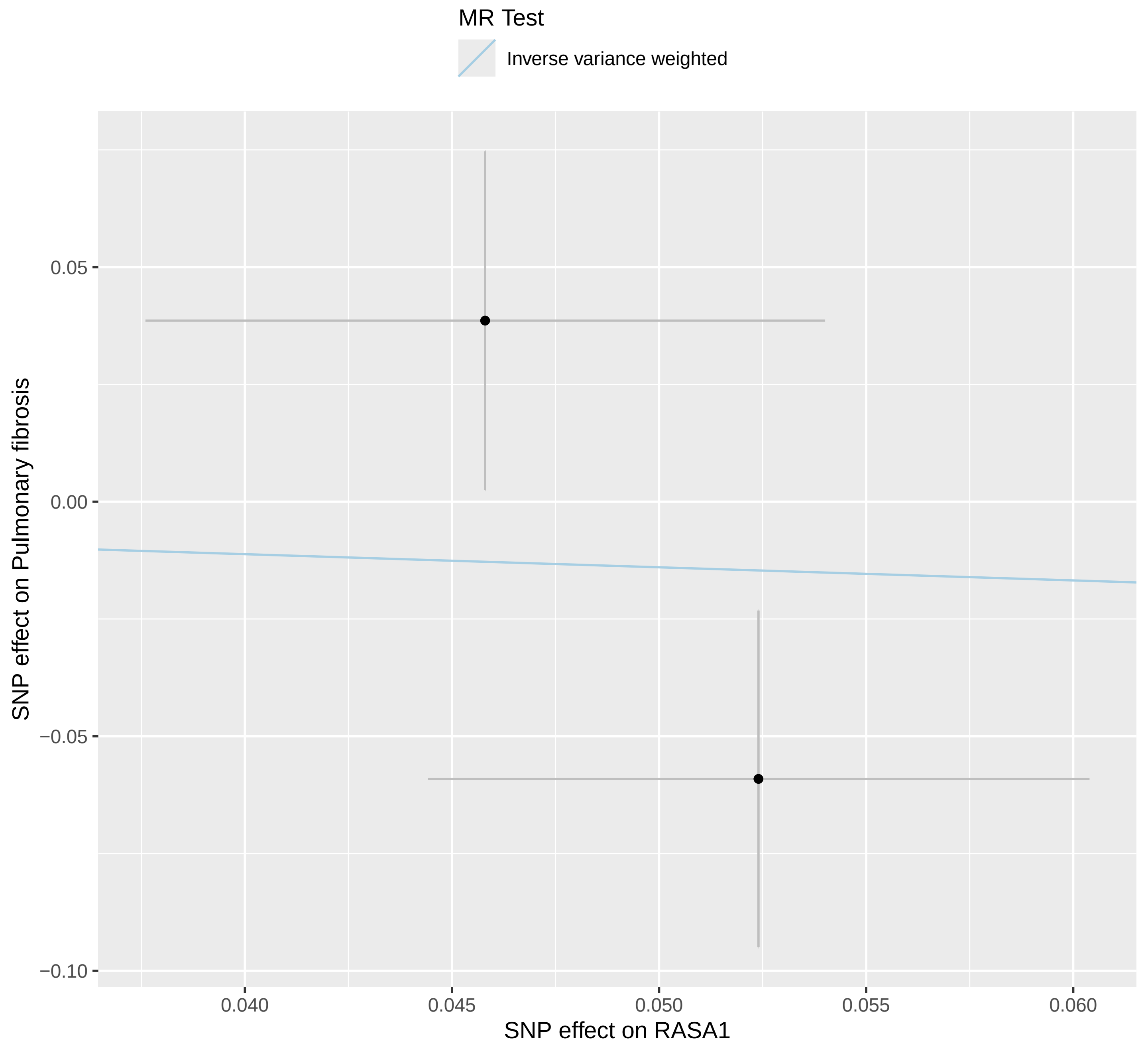

MR Test
Inverse variance weighted
0.05
0.00
−0.05
−0.10
SNP effect on Pulmonary fibrosis
0.040
0.045
0.050
0.055
0.060
SNP effect on RASA1

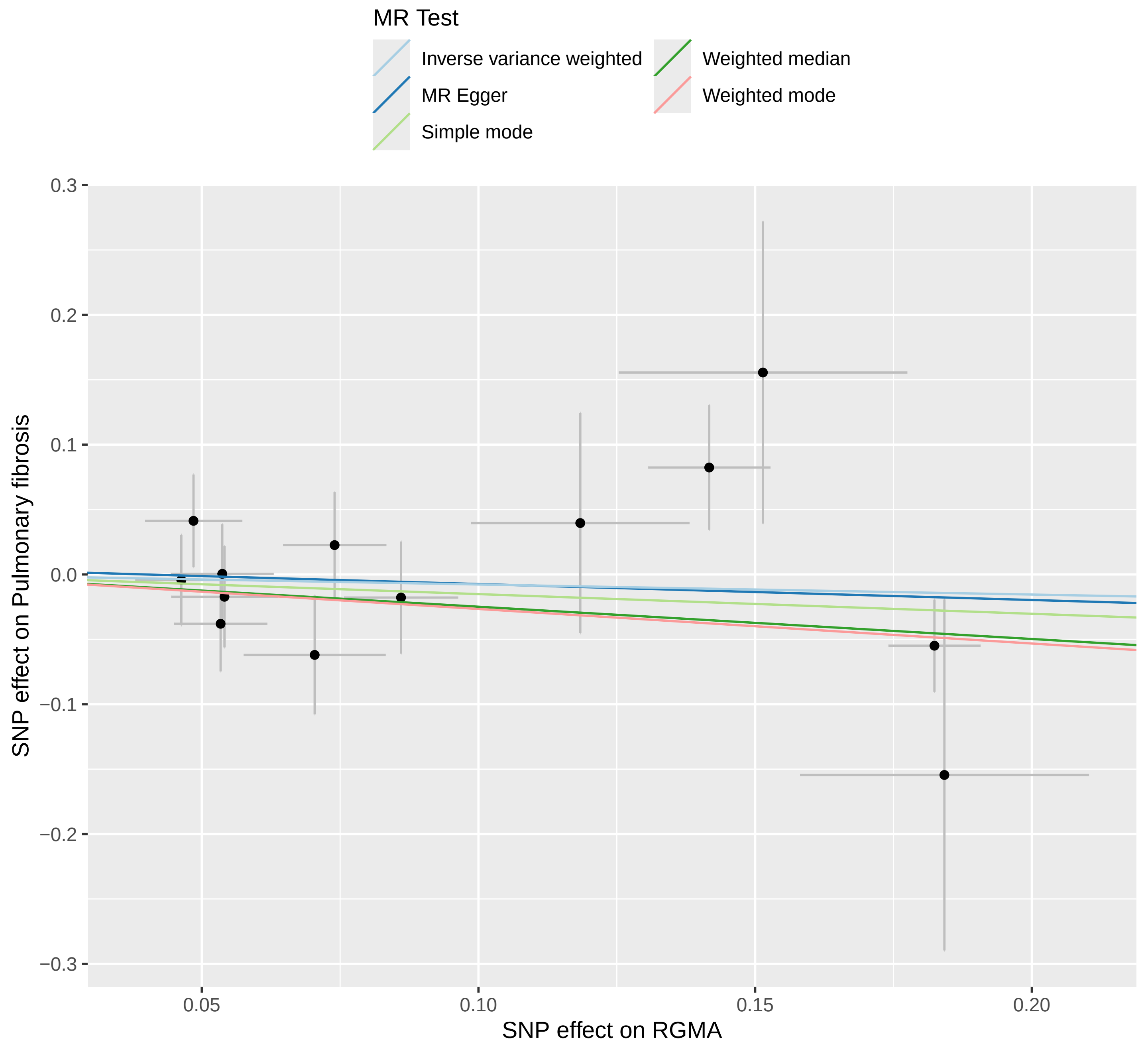

MR Test
Inverse variance weighted
MR Egger
Simple mode
Weighted median
Weighted mode
0.3
0.2
0.1
0.0
−0.1
−0.2
−0.3
SNP effect on Pulmonary fibrosis
0.05
0.10
0.15
0.20
SNP effect on RGMA

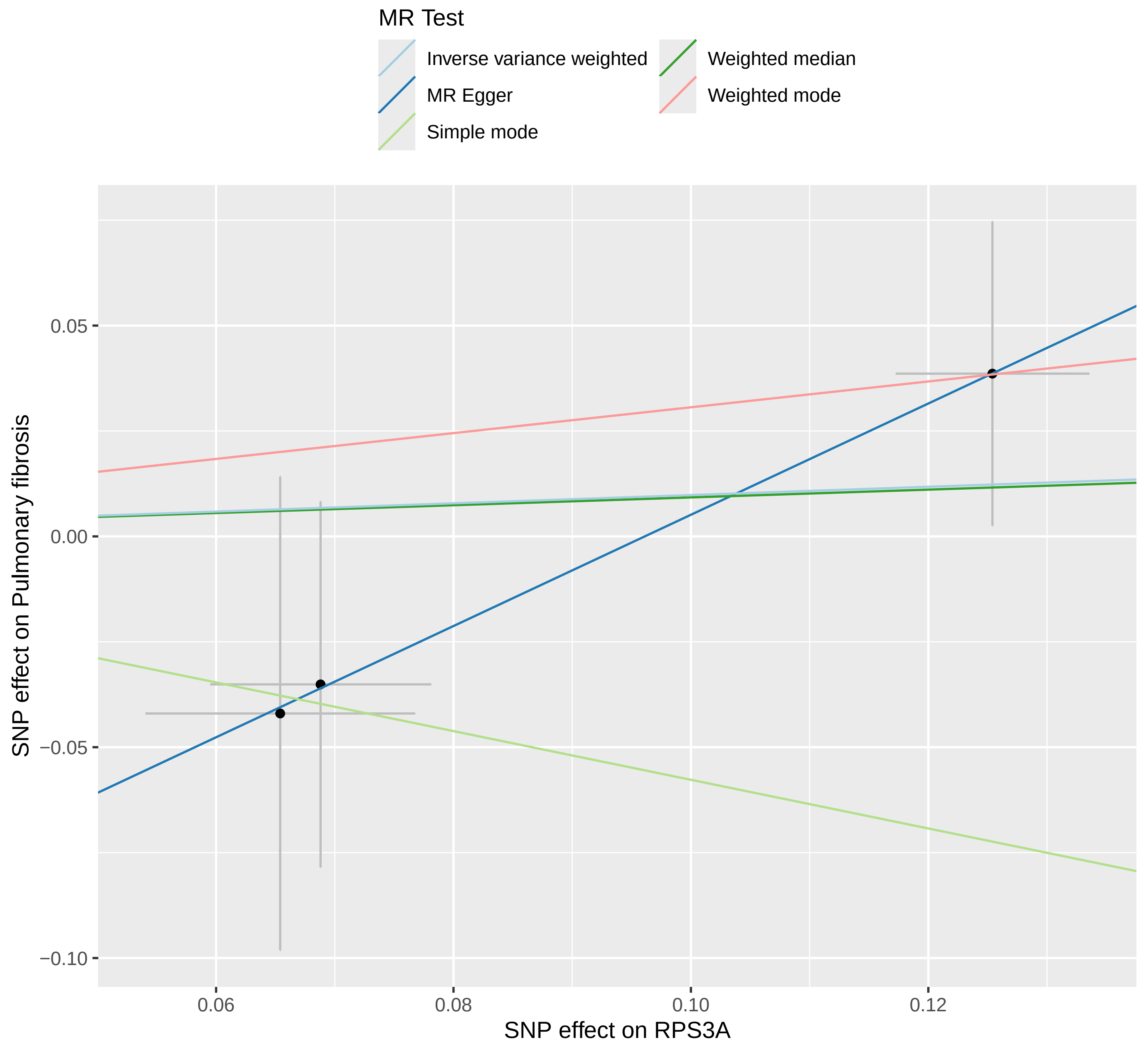

MR Test
Inverse variance weighted
MR Egger
Simple mode
Weighted median
Weighted mode
0.05
0.00
−0.05
−0.10
SNP effect on Pulmonary fibrosis
0.06
0.08
0.10
0.12
SNP effect on RPS3A

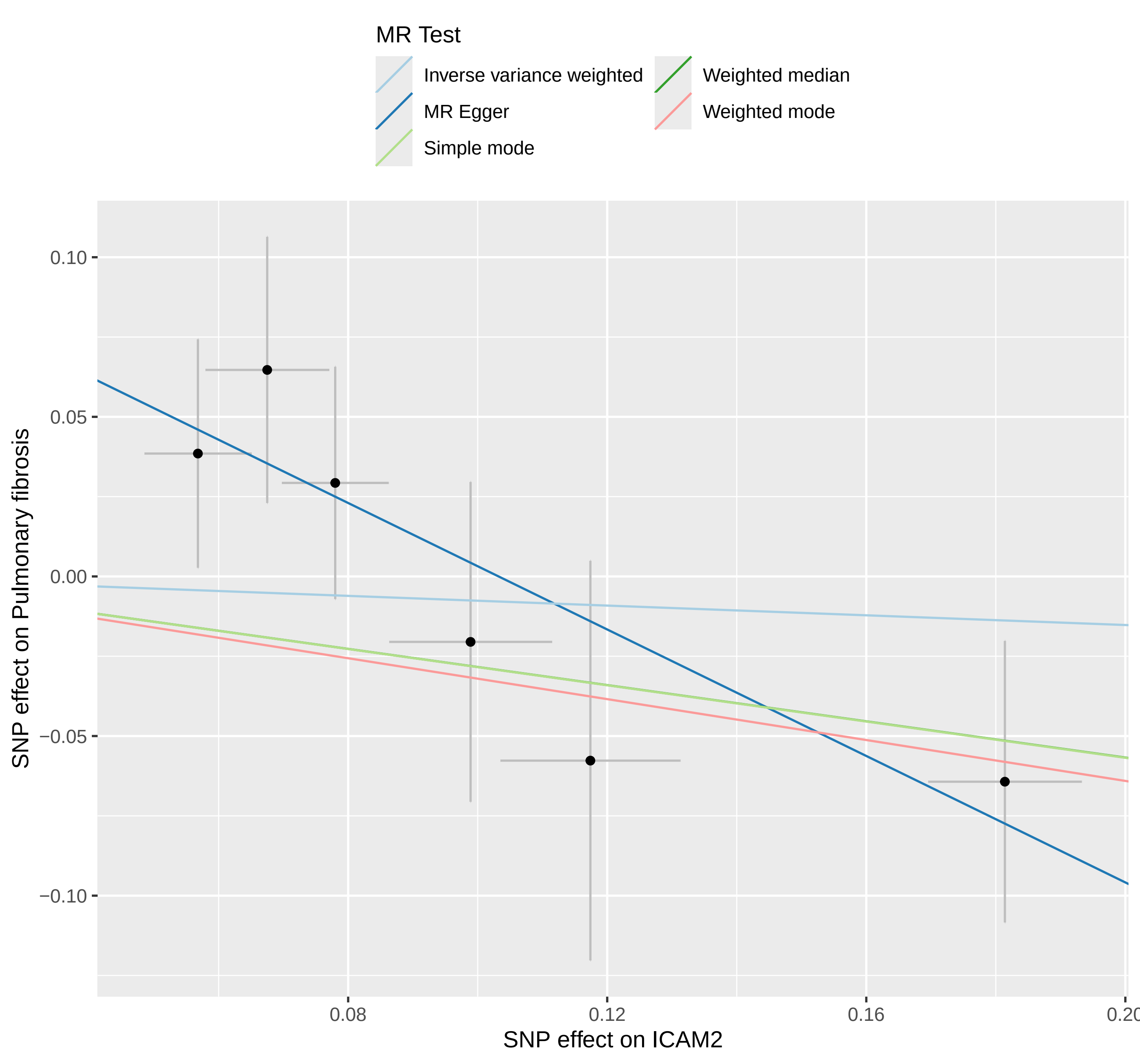

MR Test
Inverse variance weighted
MR Egger
Simple mode
Weighted median
Weighted mode
0.10
0.05
0.00
−0.05
−0.10
SNP effect on Pulmonary fibrosis
0.08
0.12
0.16
0.20
SNP effect on ICAM2

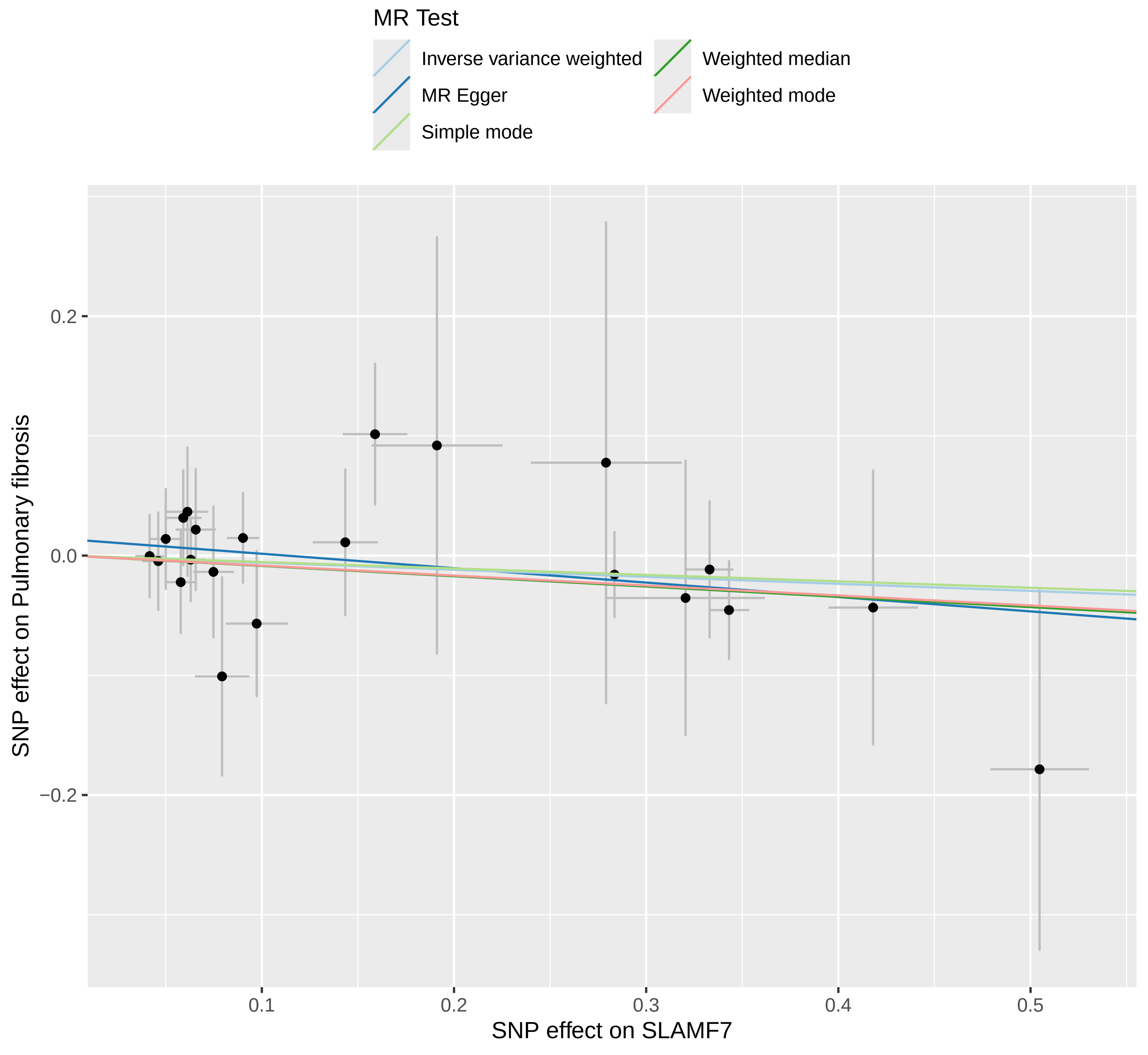

MR Test
Inverse variance weighted
Weighted median
MR Egger
Weighted mode
Simple mode
0.2
0.0
−0.2
0.1
0.2
0.3
0.4
0.5
SNP effect on Pulmonary fibrosis
SNP effect on SLAMF7

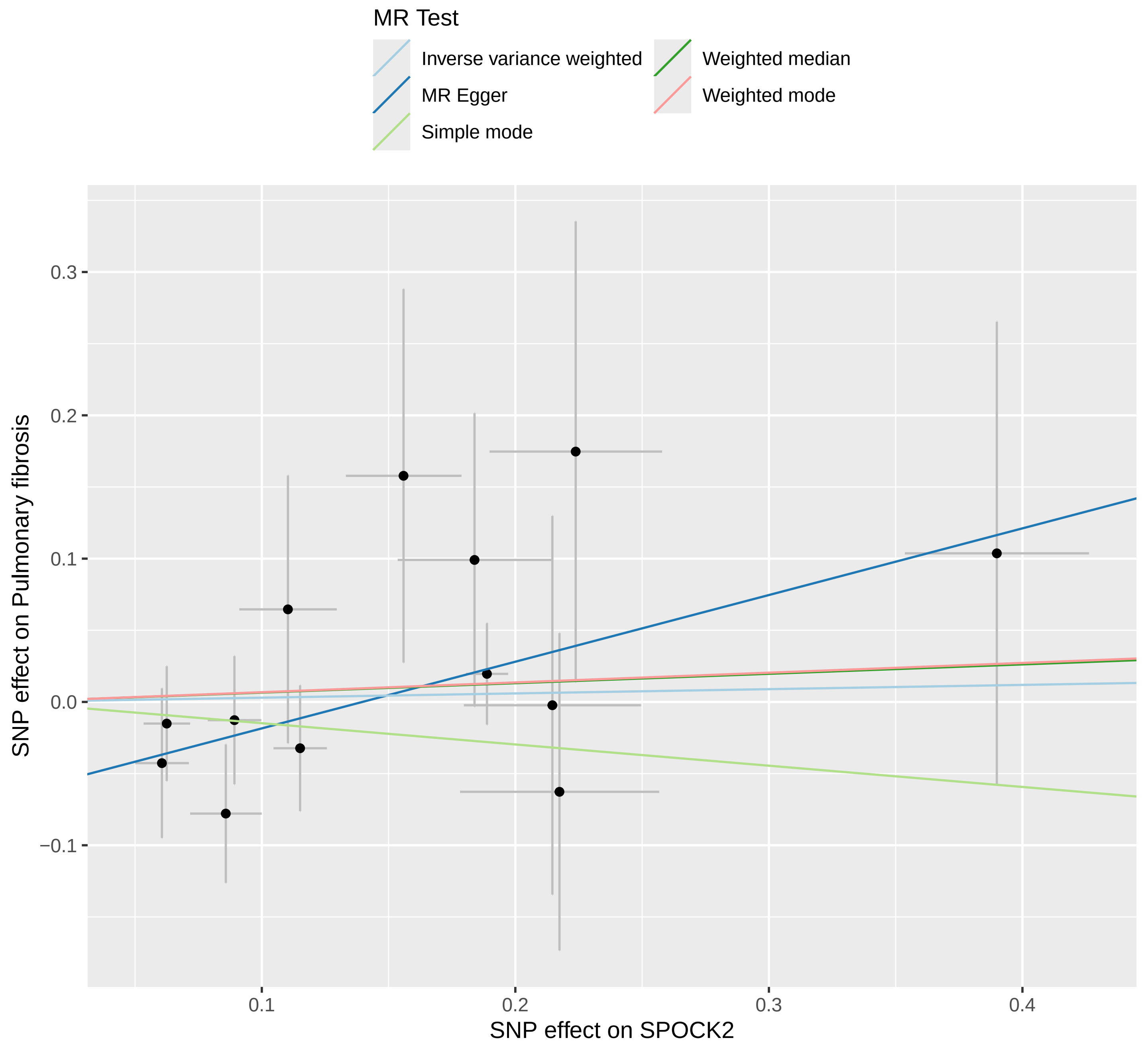
MR Test
Inverse variance weighted
MR Egger
Simple mode
Weighted median
Weighted mode
SNP effect on Pulmonary fibrosis
0.3
0.2
0.1
0.0
−0.1
0.1
0.2
0.3
0.4
SNP effect on SPOCK2

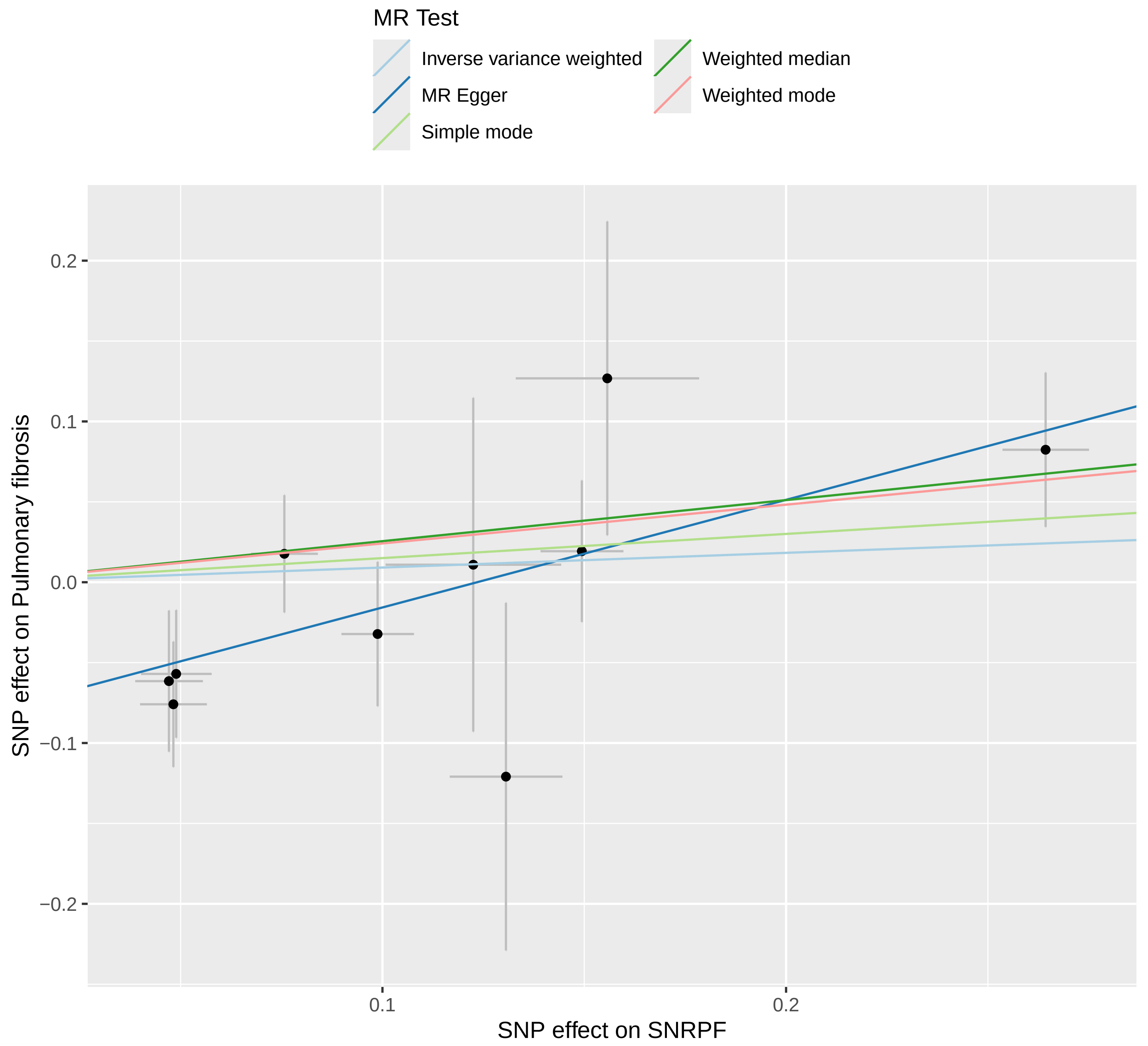
MR Test
Inverse variance weighted
MR Egger
Simple mode
Weighted median
Weighted mode
0.2
0.1
0.0
−0.1
−0.2
SNP effect on Pulmonary fibrosis
0.1
0.2
SNP effect on SNRPF

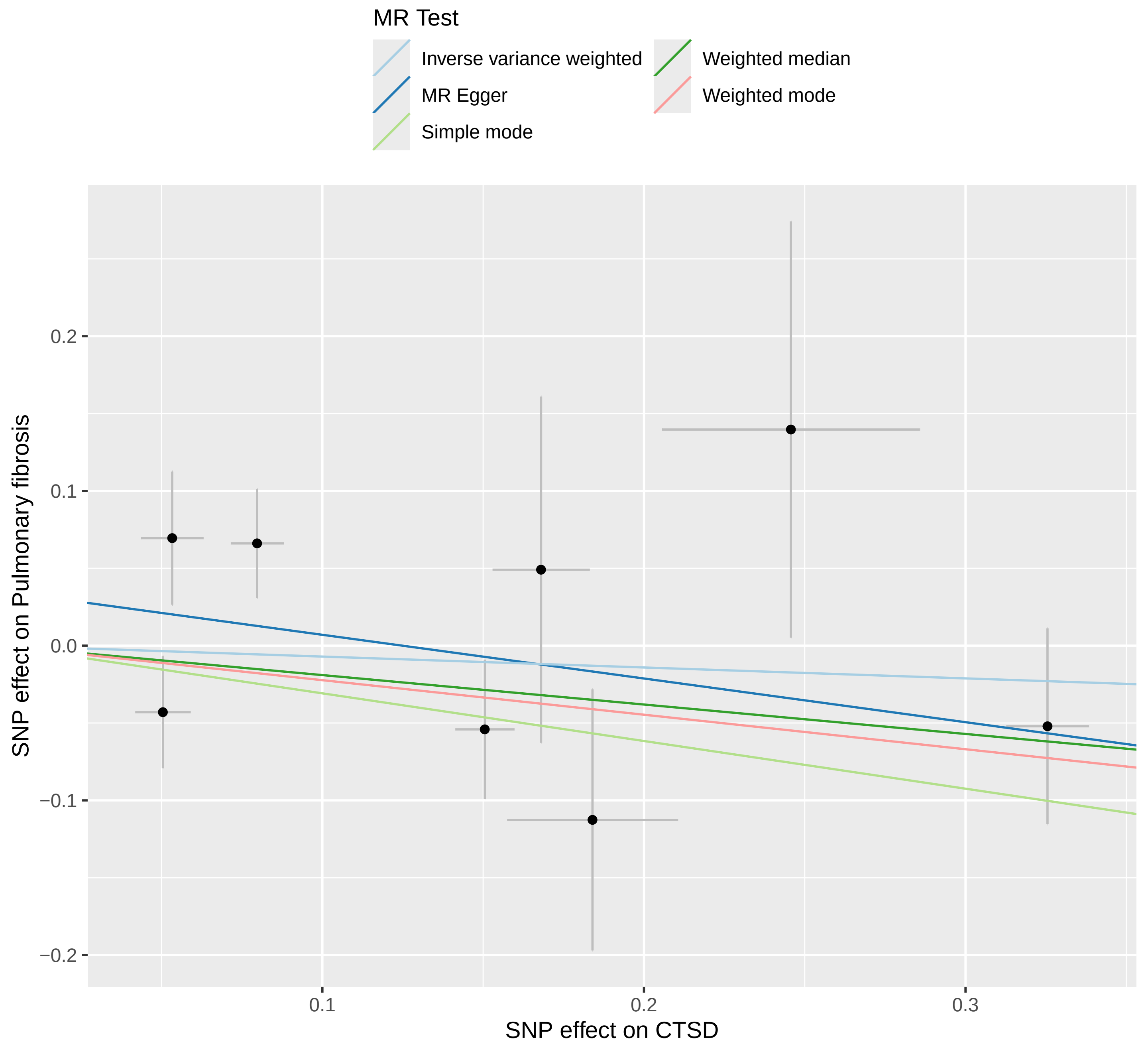

MR Test
Inverse variance weighted
MR Egger
Simple mode
Weighted median
Weighted mode
SNP effect on Pulmonary fibrosis
0.2
0.1
0.0
−0.1
−0.2
0.1
0.2
0.3
SNP effect on CTSD

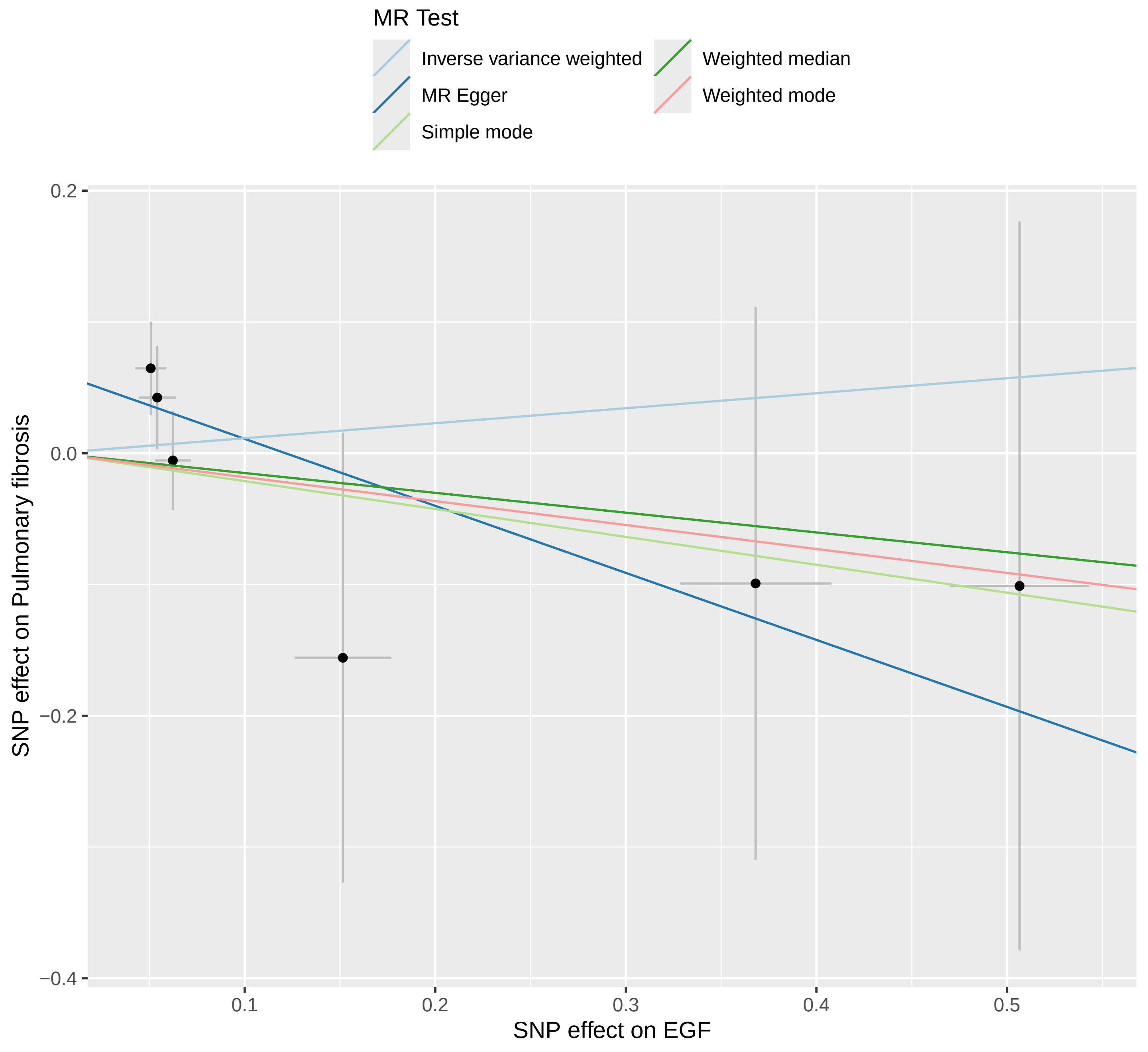
MR Test
Inverse variance weighted
MR Egger
Simple mode
Weighted median
Weighted mode
0.2
0.0
−0.2
−0.4
0.1
0.2
0.3
0.4
0.5
SNP effect on Pulmonary fibrosis
SNP effect on EGF

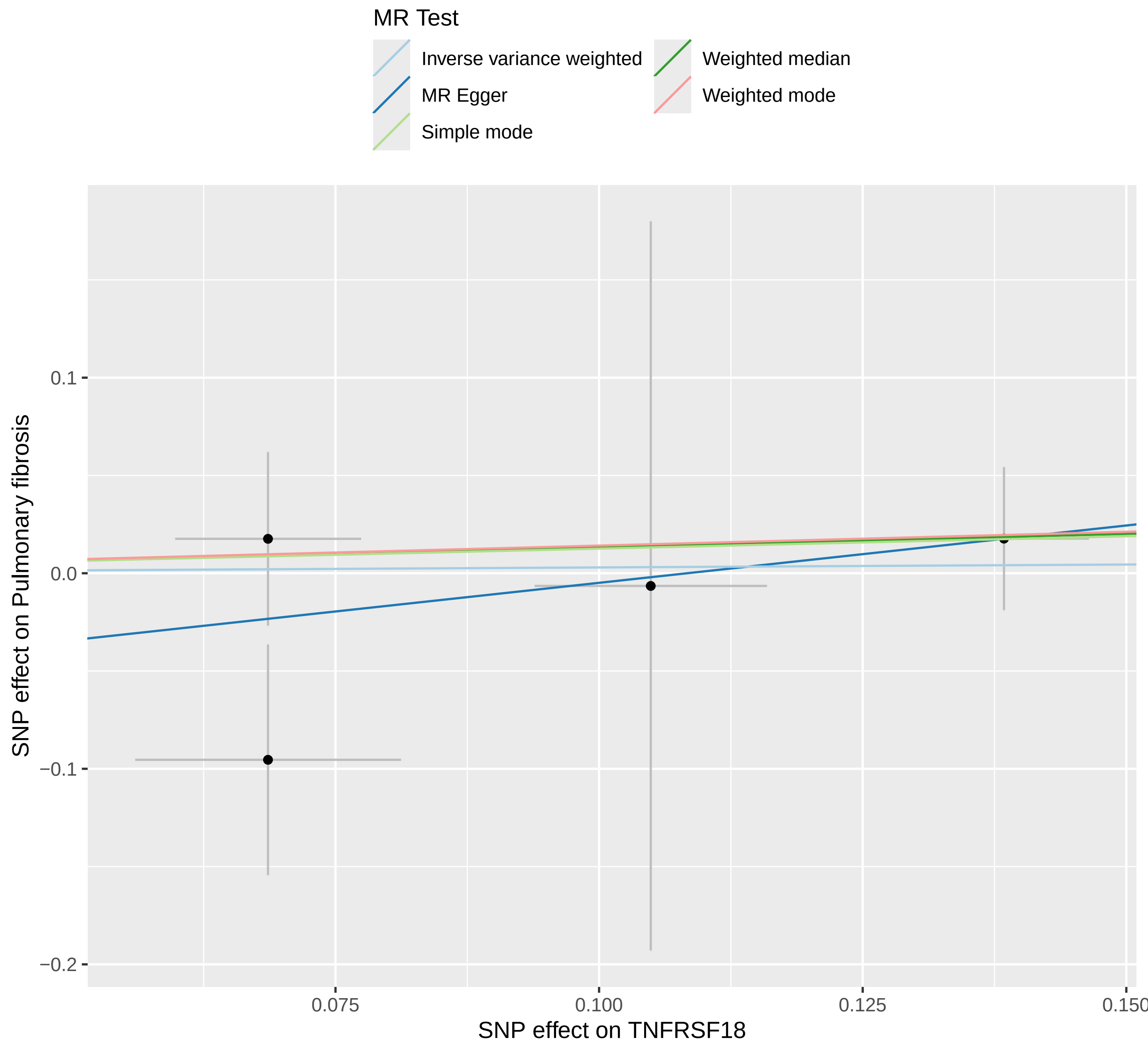

MR Test
Inverse variance weighted
MR Egger
Simple mode
Weighted median
Weighted mode
0.1
0.0
−0.1
−0.2
SNP effect on Pulmonary fibrosis
0.075
0.100
0.125
0.150
SNP effect on TNFRSF18

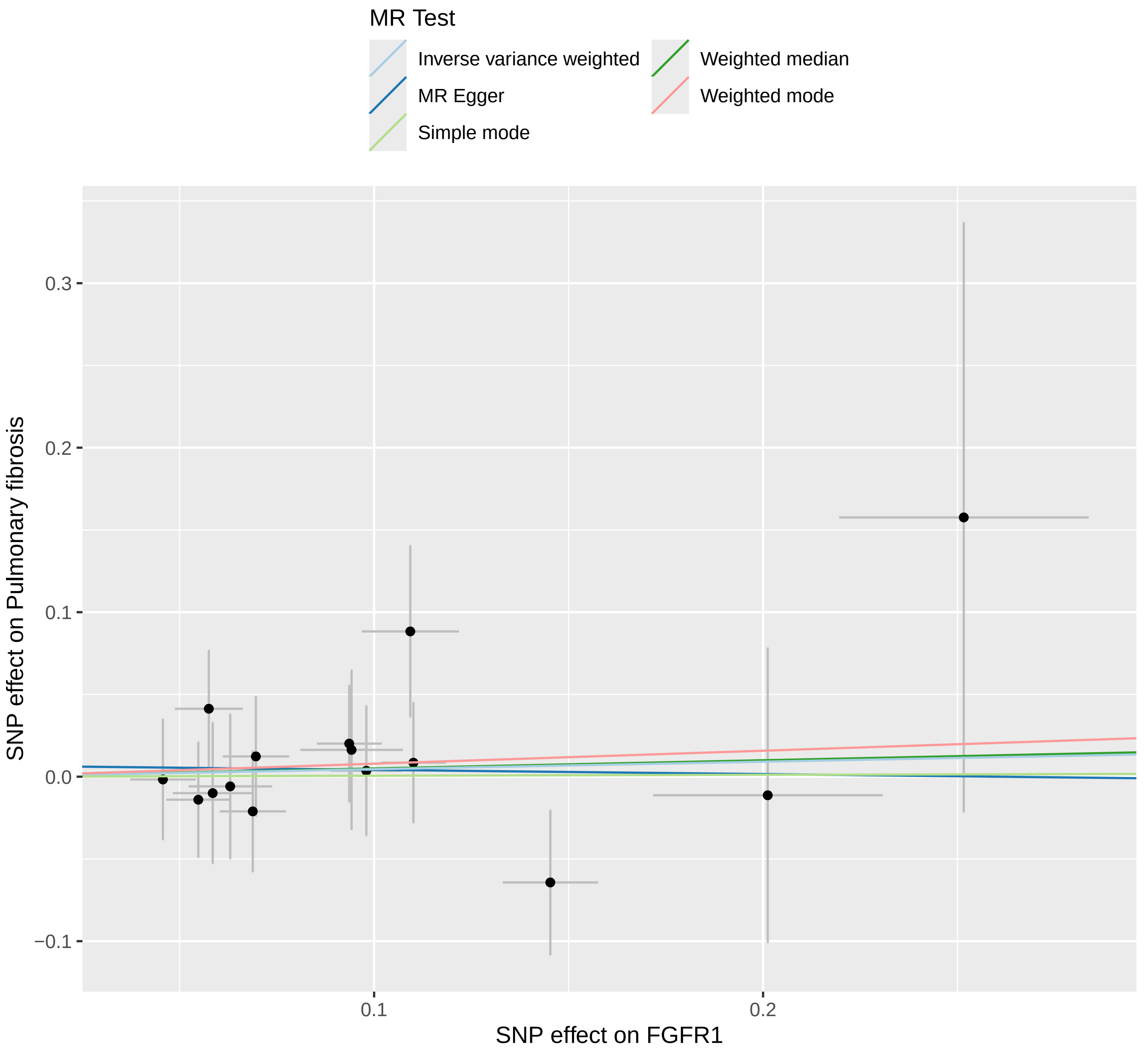

MR Test
Inverse variance weighted
MR Egger
Simple mode
Weighted median
Weighted mode
SNP effect on Pulmonary fibrosis
0.3
0.2
0.1
0.0
−0.1
0.1
0.2
SNP effect on FGFR1

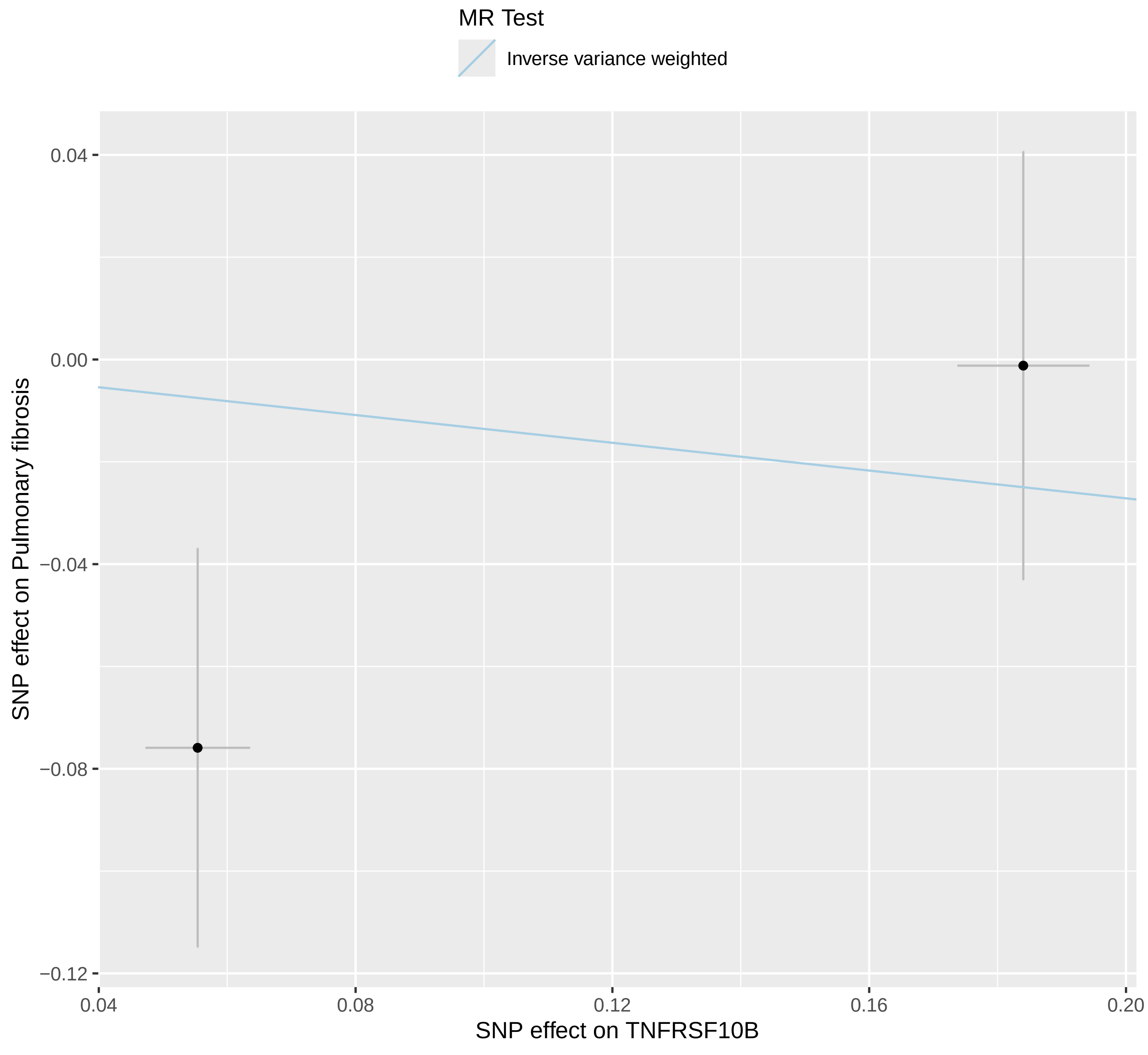

MR Test
Inverse variance weighted
0.04
0.00
−0.04
−0.08
−0.12
SNP effect on Pulmonary fibrosis
0.04
0.08
0.12
0.16
0.20
SNP effect on TNFRSF10B

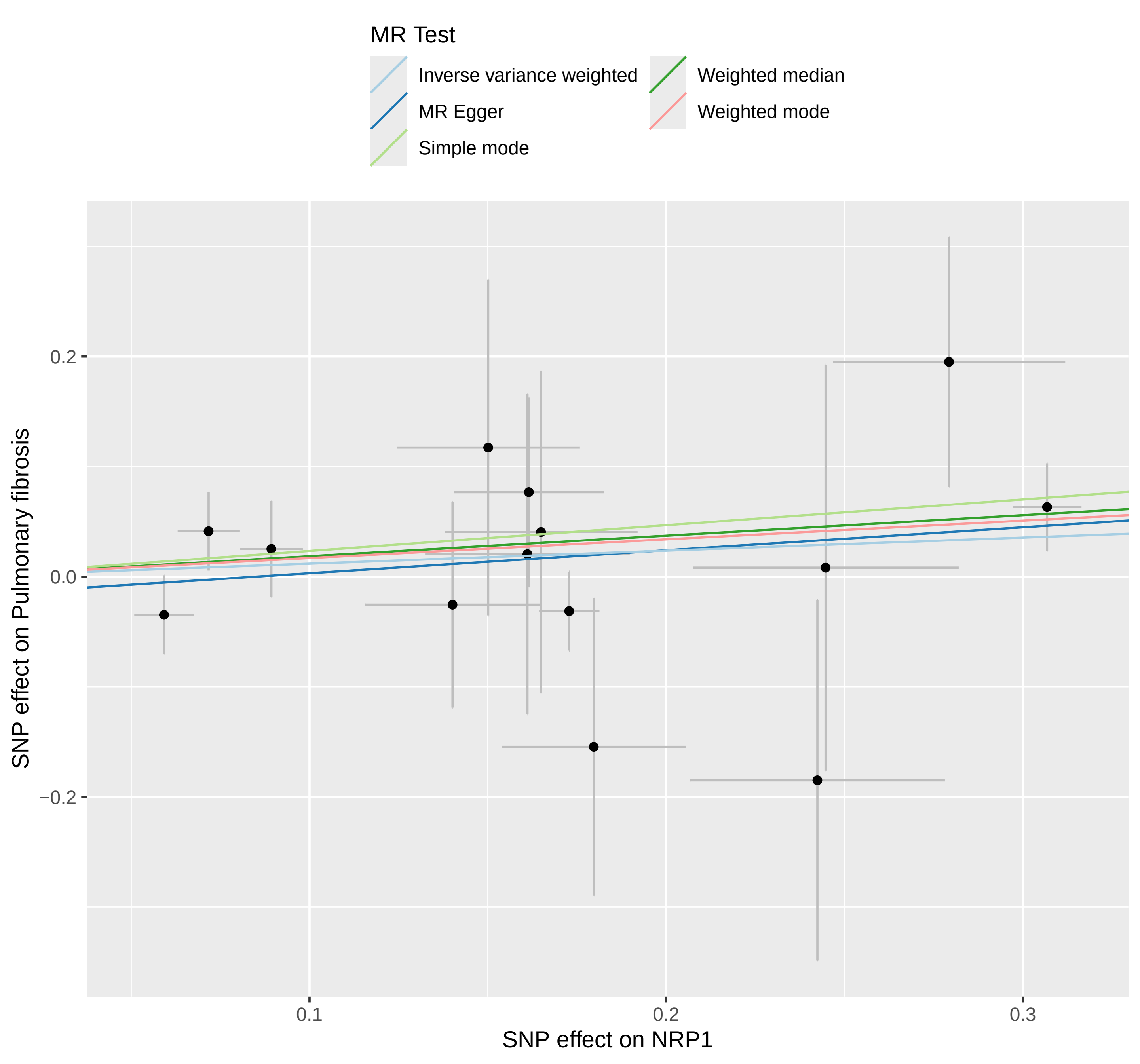

MR Test
Inverse variance weighted
MR Egger
Simple mode
Weighted median
Weighted mode
SNP effect on Pulmonary fibrosis
0.2
0.0
−0.2
0.1
0.2
0.3
SNP effect on NRP1

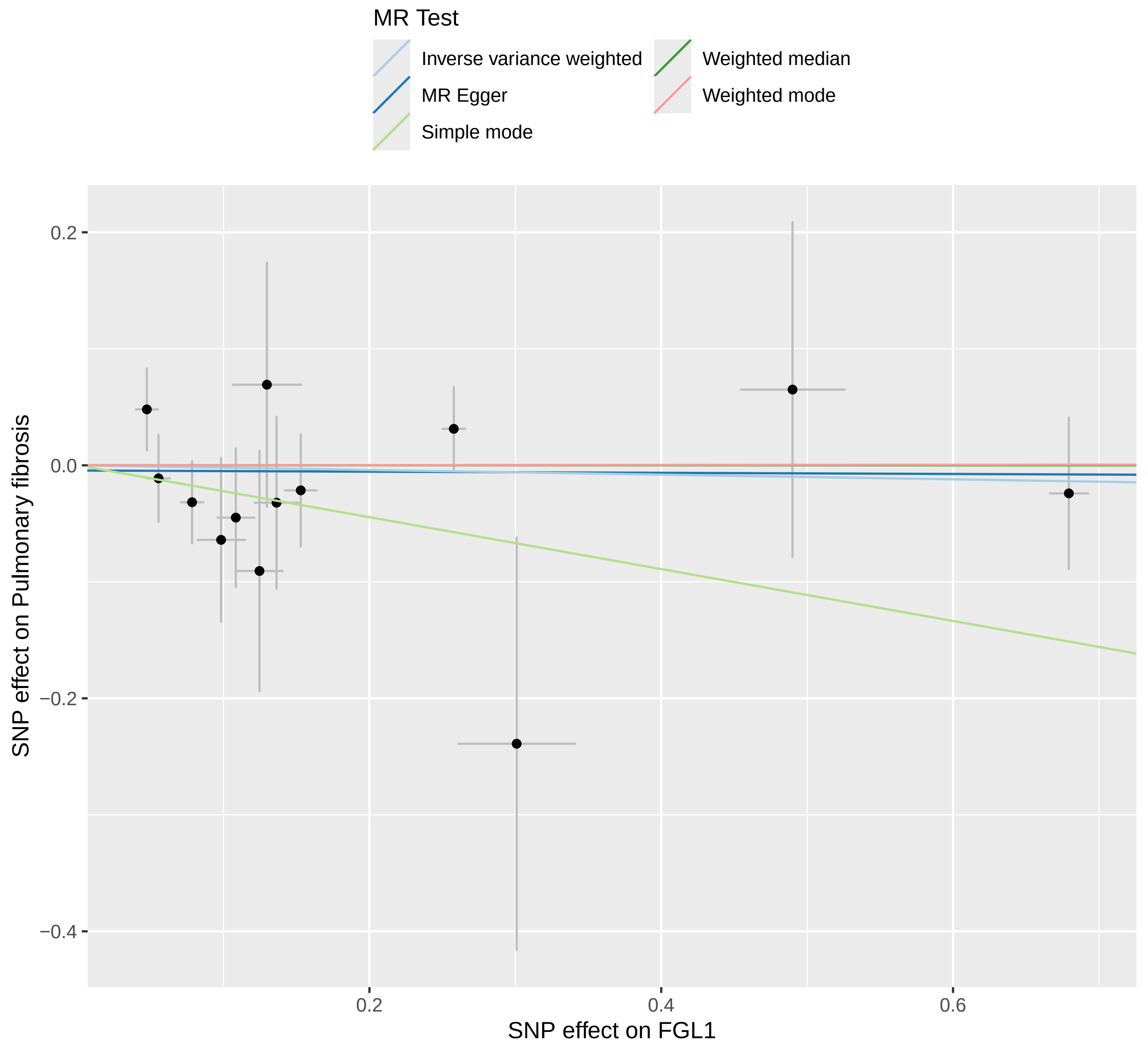

MR Test
Inverse variance weighted
MR Egger
Simple mode
Weighted median
Weighted mode
SNP effect on Pulmonary fibrosis
0.2
0.0
−0.2
−0.4
0.2
0.4
0.6
SNP effect on FGL1

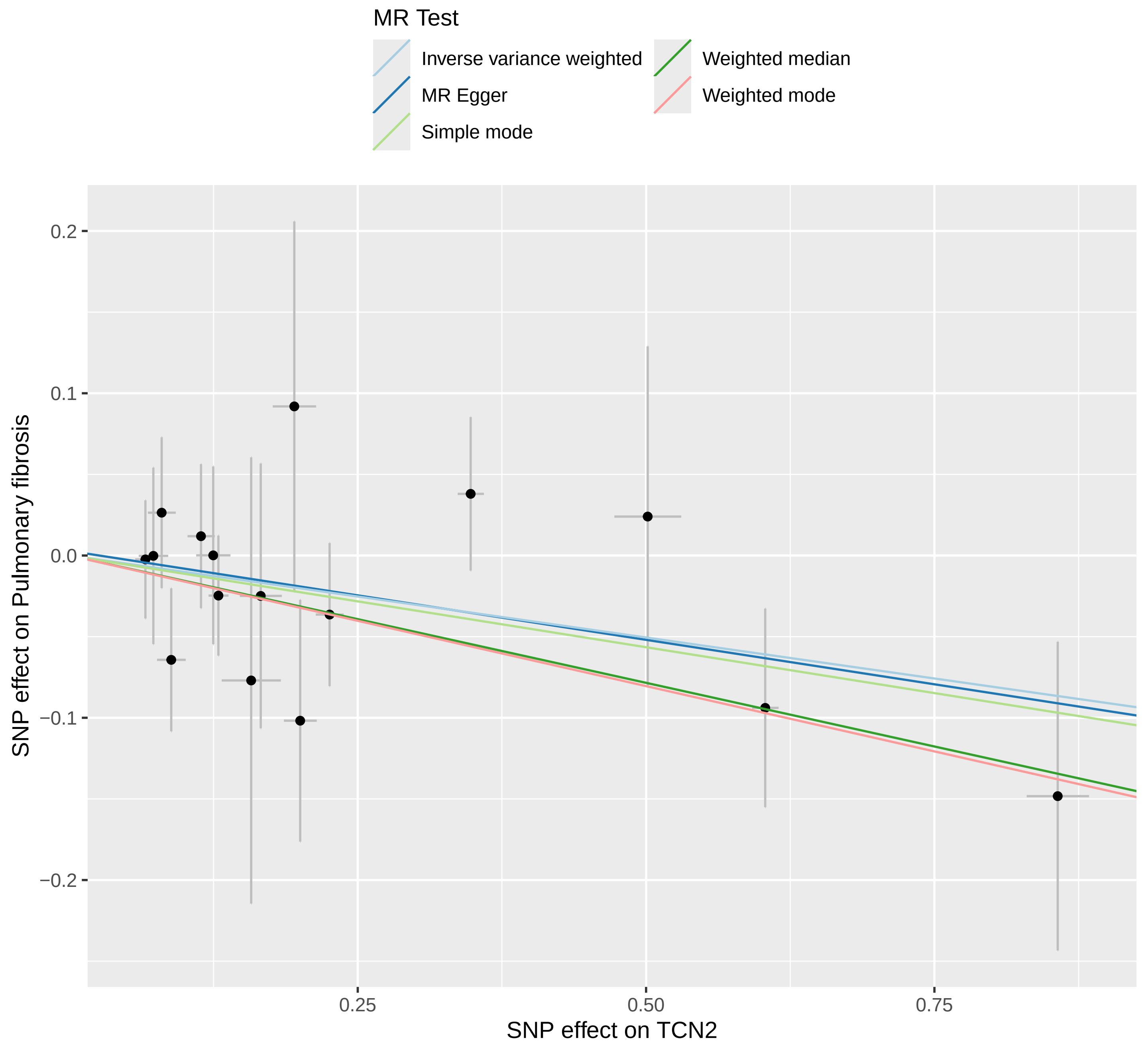

MR Test
Inverse variance weighted
MR Egger
Simple mode
Weighted median
Weighted mode
SNP effect on Pulmonary fibrosis
0.2
0.1
0.0
−0.1
−0.2
0.25
0.50
0.75
SNP effect on TCN2

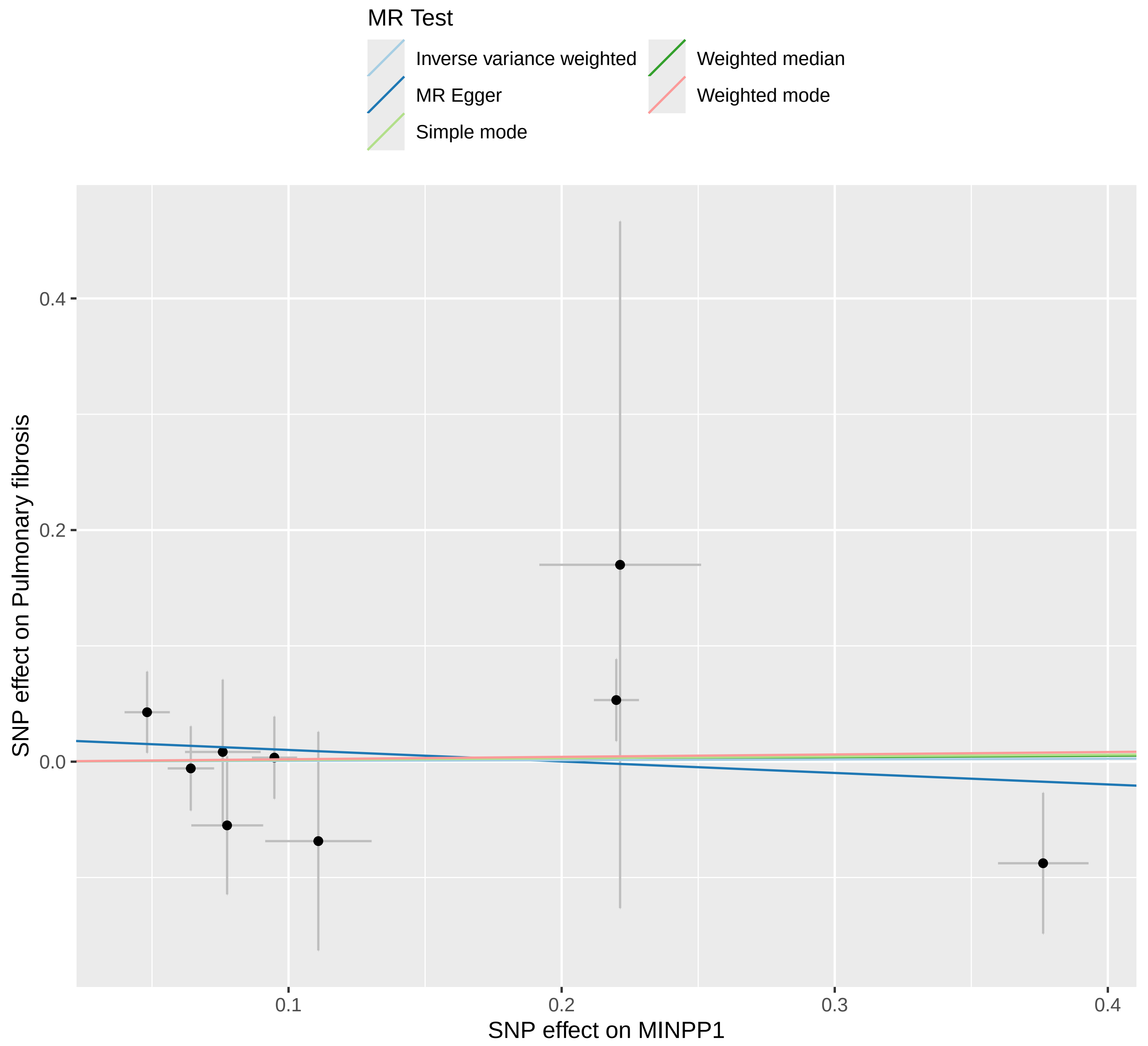

MR Test
Inverse variance weighted
MR Egger
Simple mode
Weighted median
Weighted mode
SNP effect on Pulmonary fibrosis
0.4
0.2
0.0
0.1
0.2
0.3
0.4
SNP effect on MINPP1

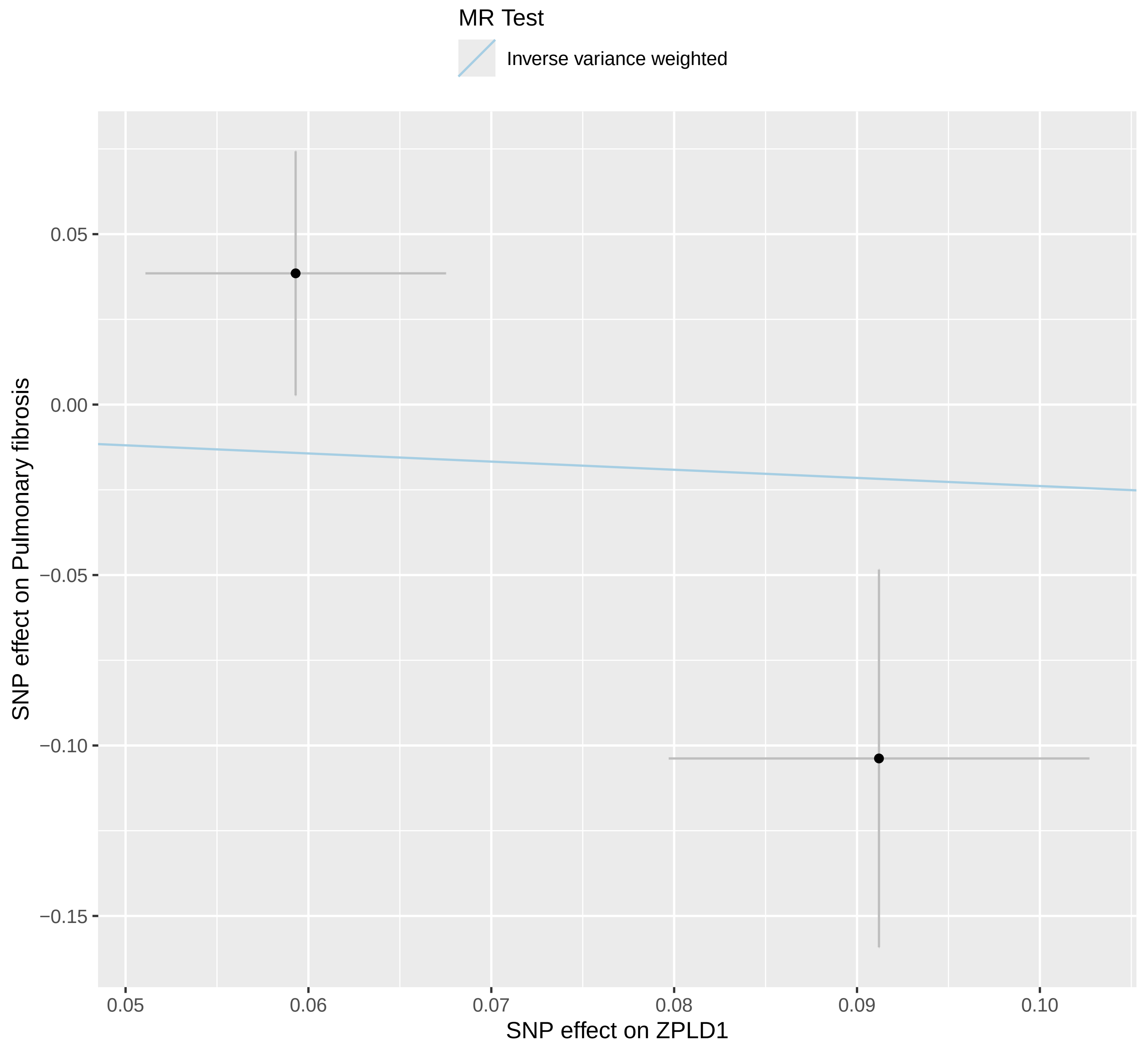
MR Test
Inverse variance weighted
SNP effect on Pulmonary fibrosis
0.05
0.00
−0.05
−0.10
−0.15
0.05
0.06
0.07
0.08
0.09
0.10
SNP effect on ZPLD1

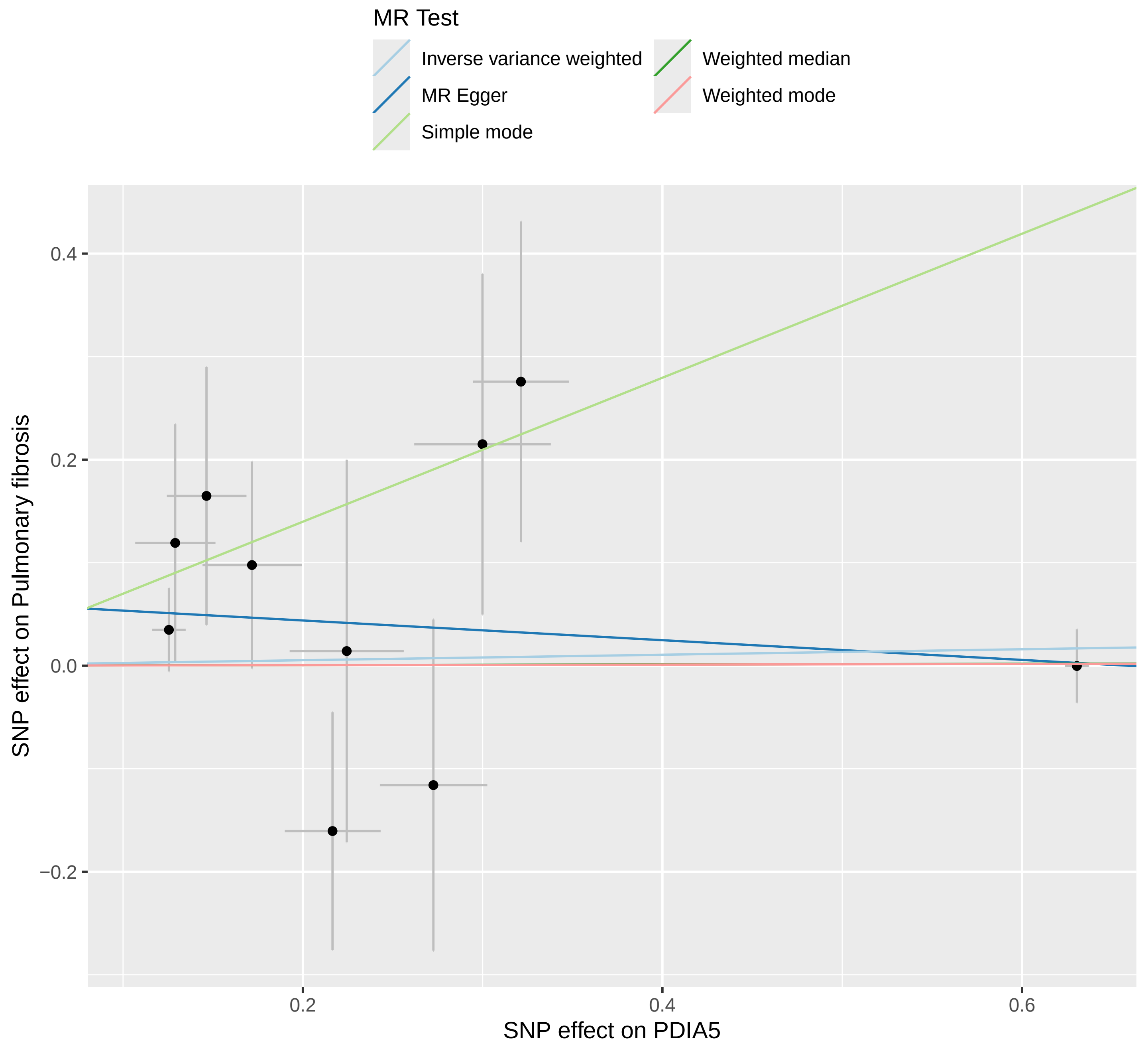
MR Test
Inverse variance weighted
MR Egger
Simple mode
Weighted median
Weighted mode
0.4
0.2
0.0
−0.2
0.2
0.4
0.6
SNP effect on Pulmonary fibrosis
SNP effect on PDIA5

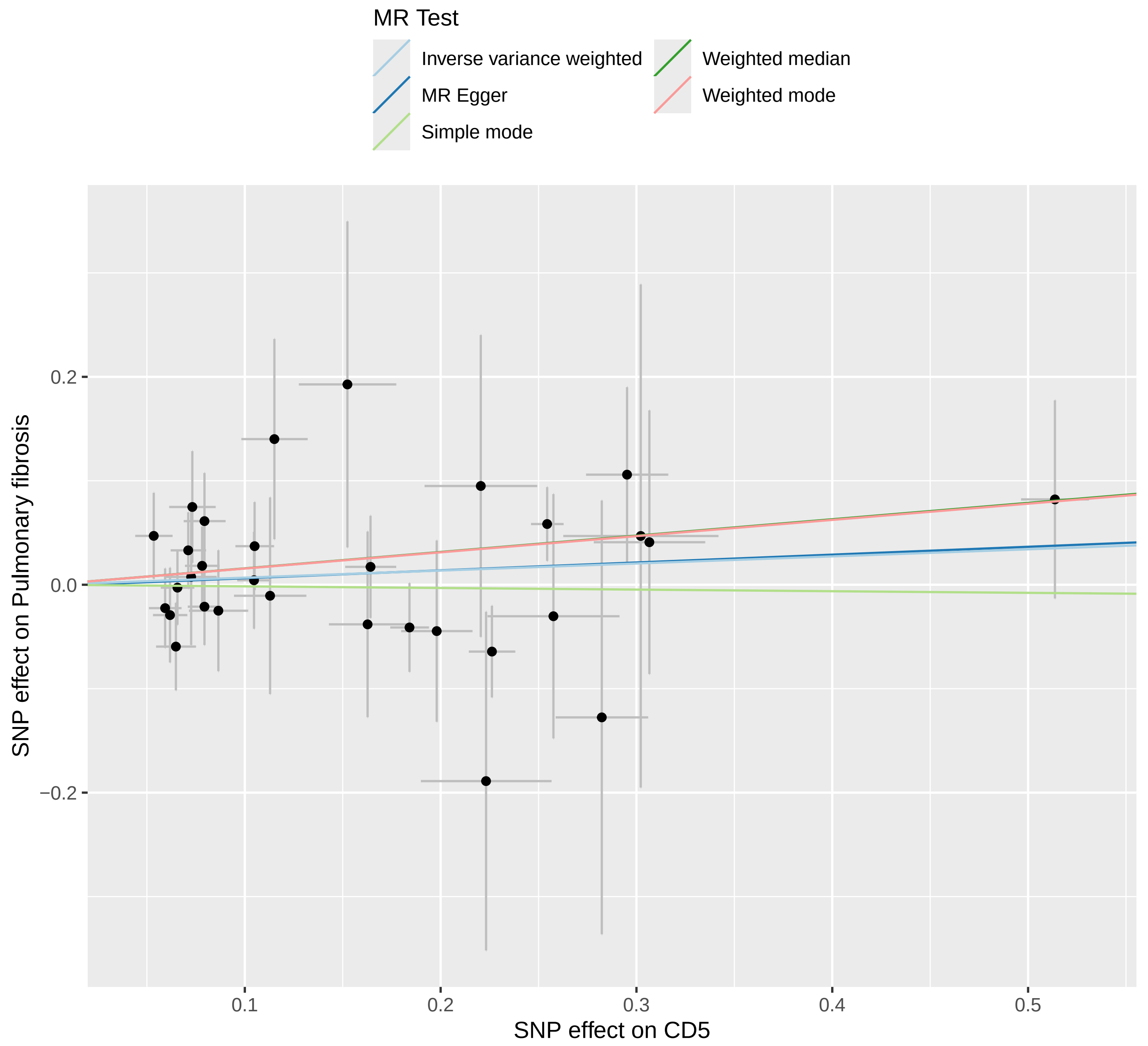
MR Test
Inverse variance weighted
MR Egger
Simple mode
Weighted median
Weighted mode
SNP effect on Pulmonary fibrosis
0.2
0.0
−0.2
0.1
0.2
0.3
0.4
0.5
SNP effect on CD5

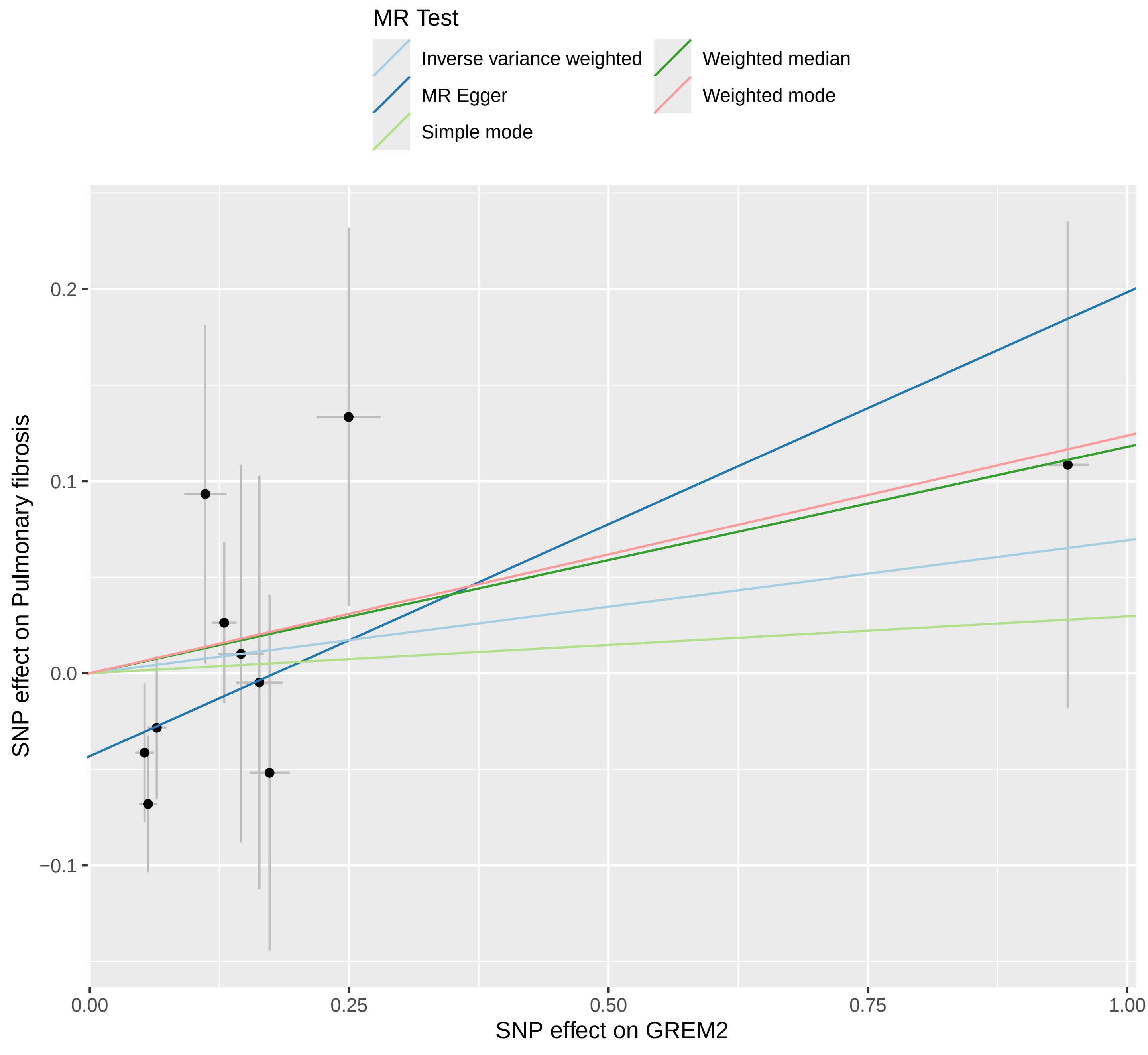

MR Test
Inverse variance weighted
MR Egger
Simple mode
Weighted median
Weighted mode
0.2
0.1
0.0
−0.1
SNP effect on Pulmonary fibrosis
0.00
0.25
0.50
0.75
1.00
SNP effect on GREM2

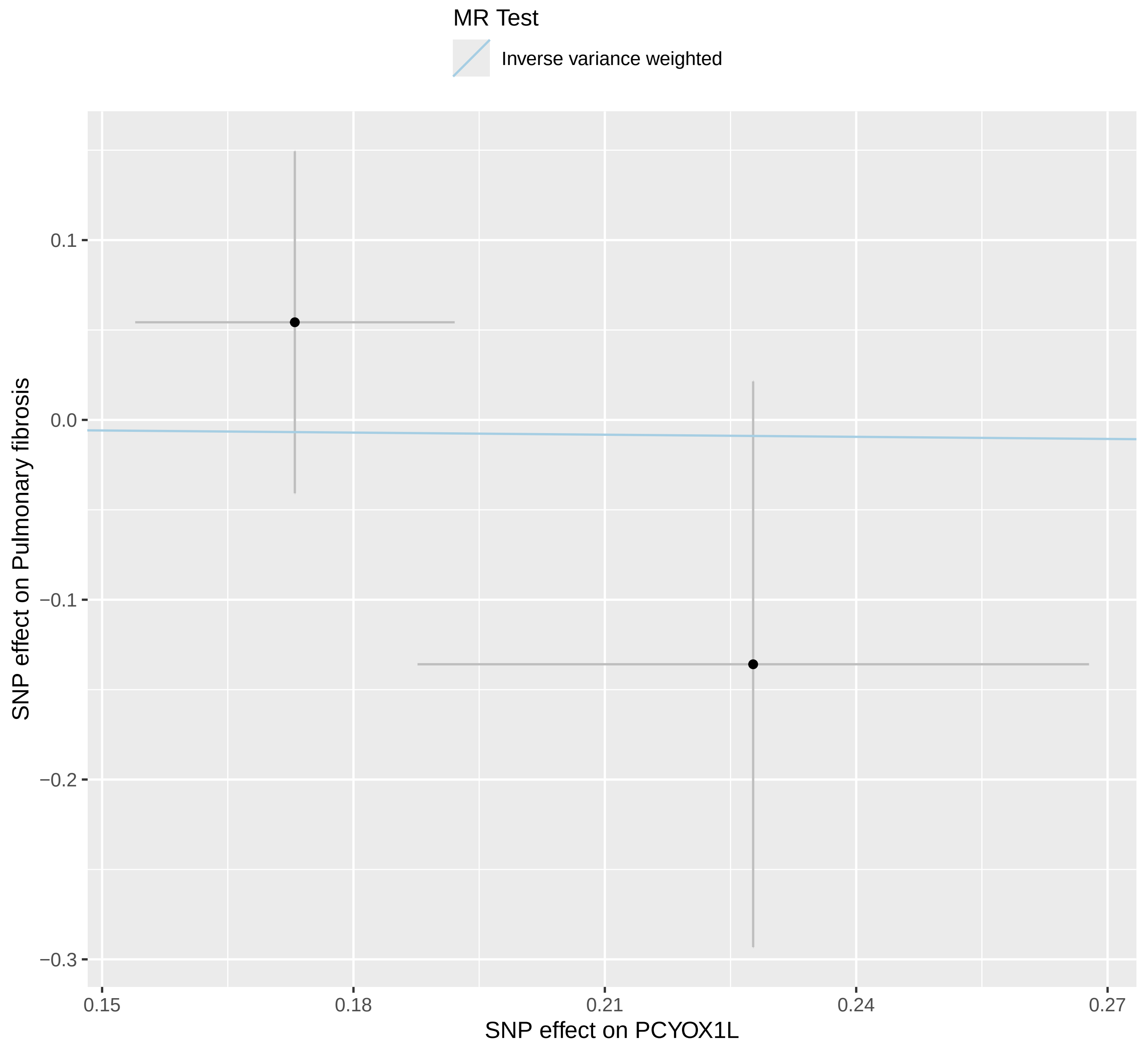

MR Test
Inverse variance weighted
0.1
0.0
−0.1
−0.2
−0.3
SNP effect on Pulmonary fibrosis
0.15
0.18
0.21
0.24
0.27
SNP effect on PCYOX1L

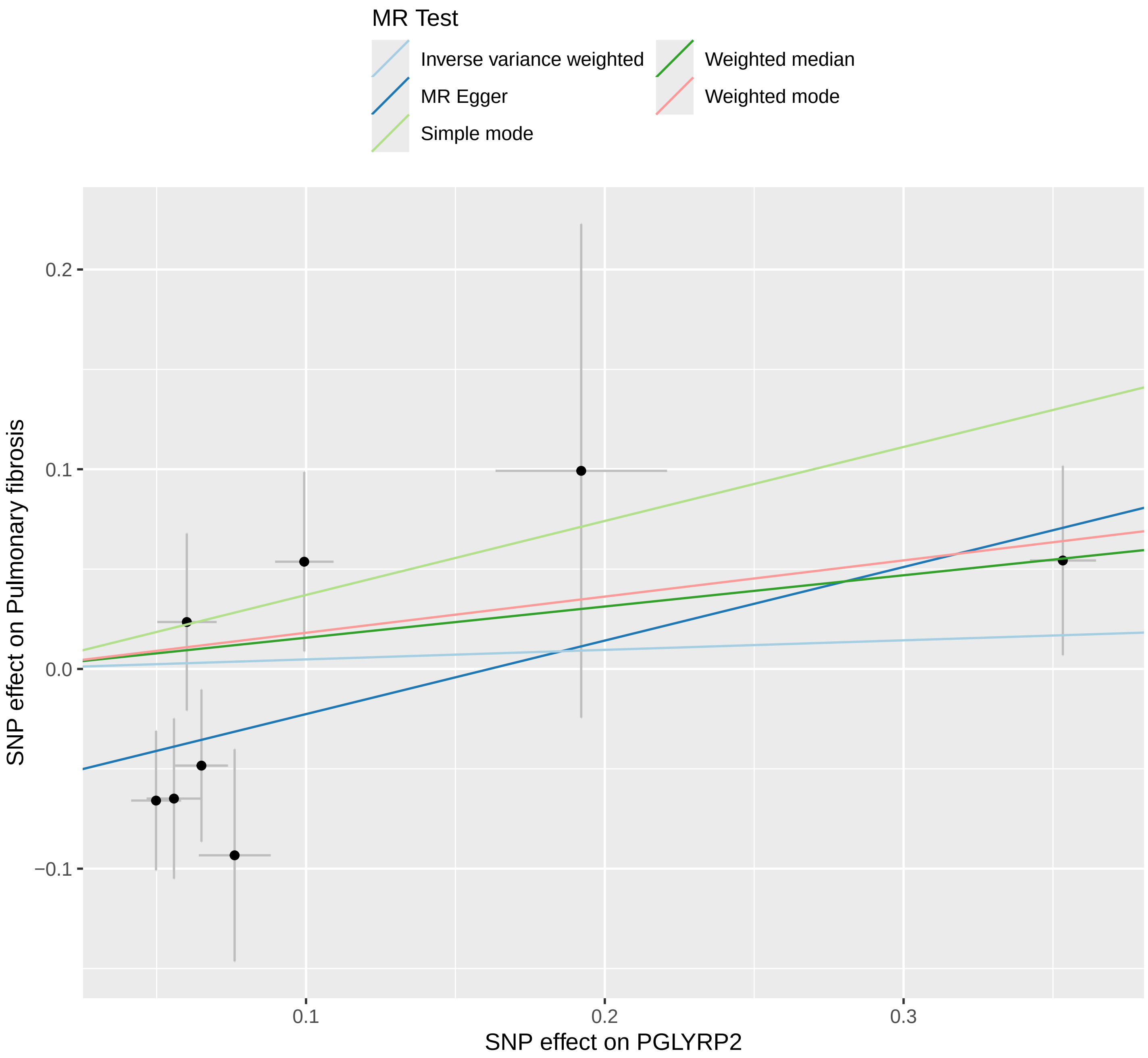
MR Test
Inverse variance weighted
MR Egger
Simple mode
Weighted median
Weighted mode
0.2
0.1
0.0
−0.1
SNP effect on Pulmonary fibrosis
0.1
0.2
0.3
SNP effect on PGLYRP2

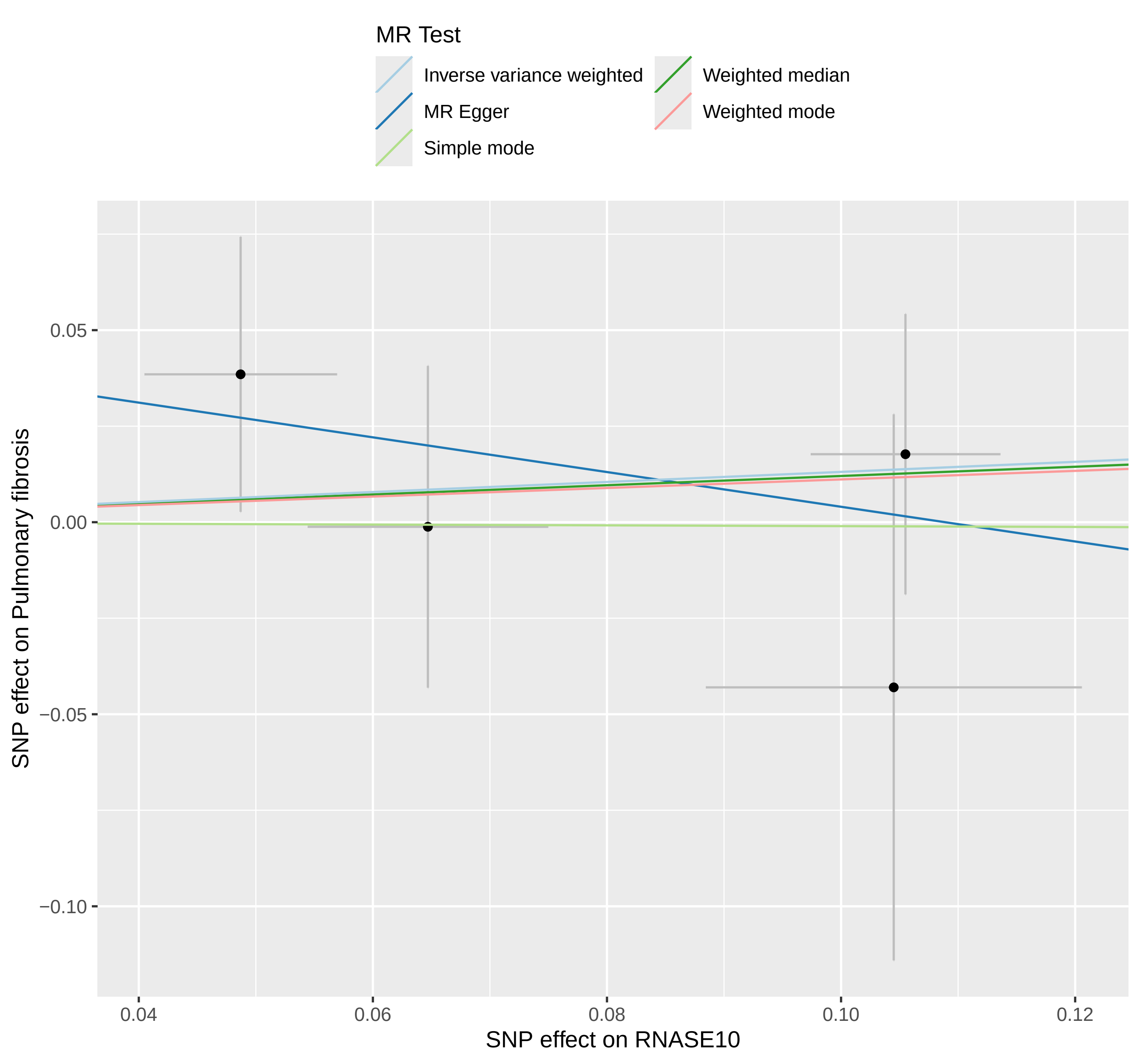

MR Test
Inverse variance weighted
MR Egger
Simple mode
Weighted median
Weighted mode
0.05
0.00
−0.05
−0.10
0.04
0.06
0.08
0.10
0.12
SNP effect on Pulmonary fibrosis
SNP effect on RNASE10

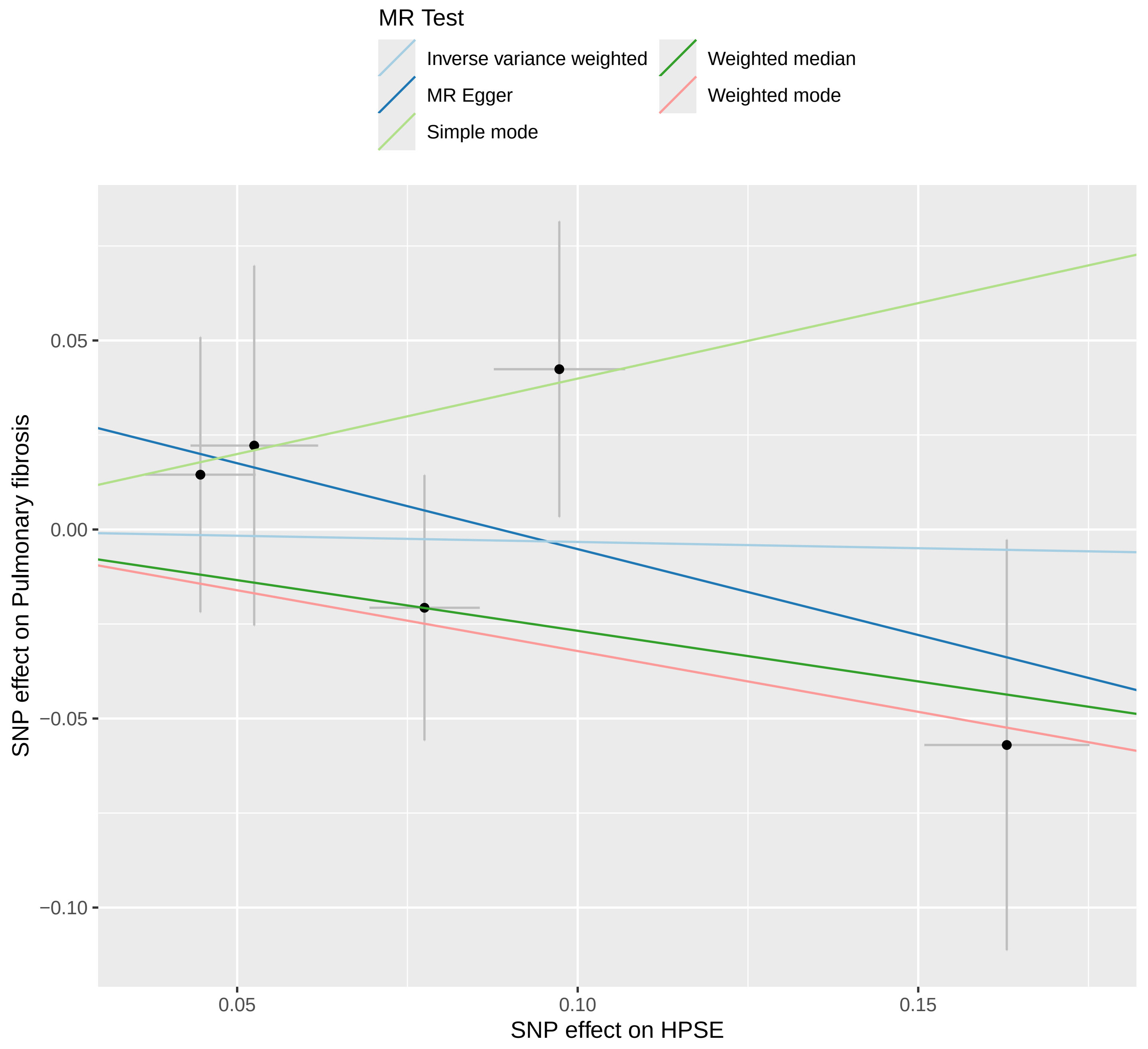

MR Test
Inverse variance weighted
MR Egger
Simple mode
Weighted median
Weighted mode
0.05
0.00
−0.05
−0.10
0.05
0.10
0.15
SNP effect on Pulmonary fibrosis
SNP effect on HPSE

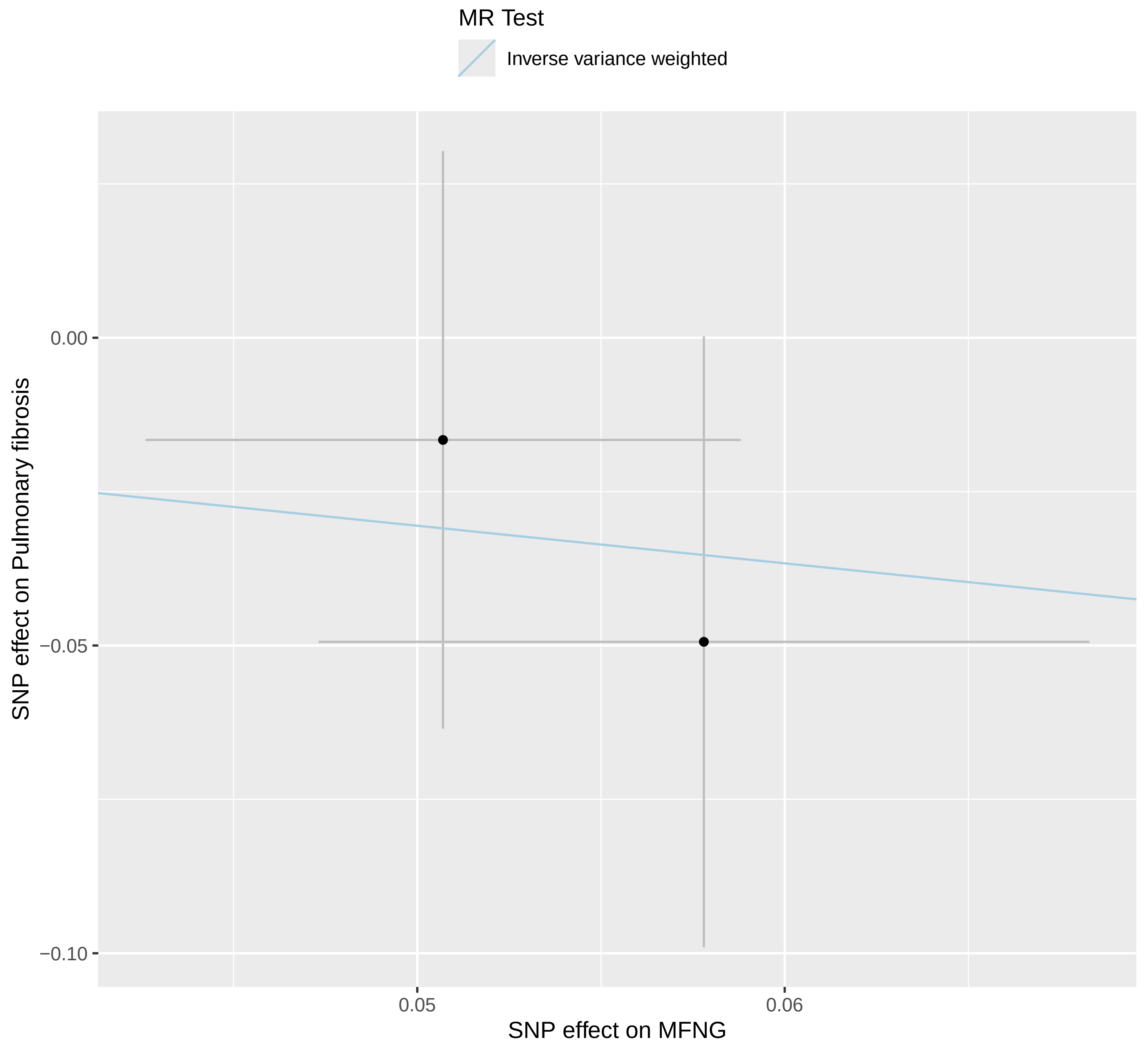

MR Test
Inverse variance weighted
SNP effect on Pulmonary fibrosis
0.00
−0.05
−0.10
0.05
0.06
SNP effect on MFNG

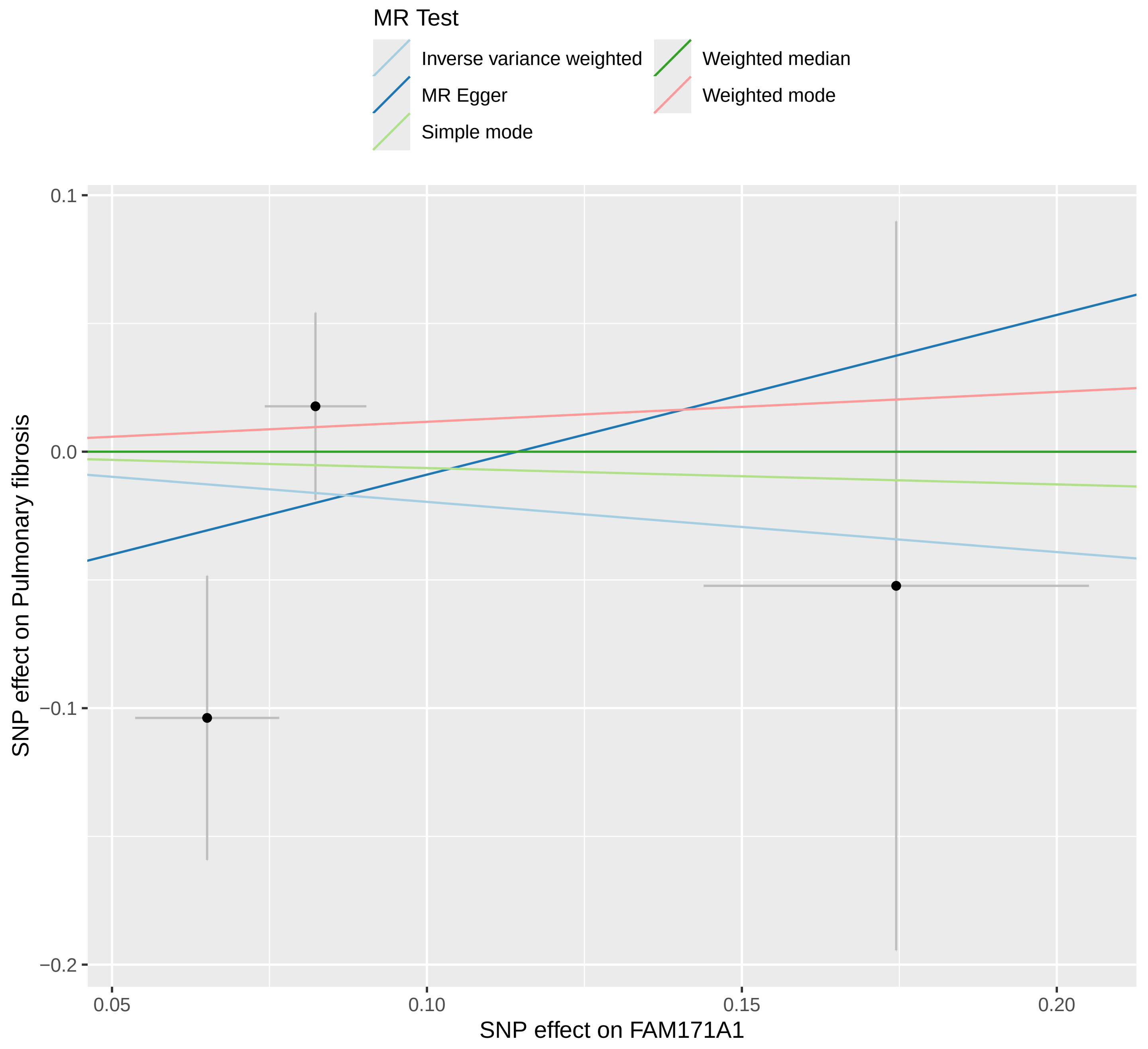
MR Test
Inverse variance weighted
MR Egger
Simple mode
Weighted median
Weighted mode
0.1
0.0
−0.1
−0.2
0.05
0.10
0.15
0.20
SNP effect on Pulmonary fibrosis
SNP effect on FAM171A1

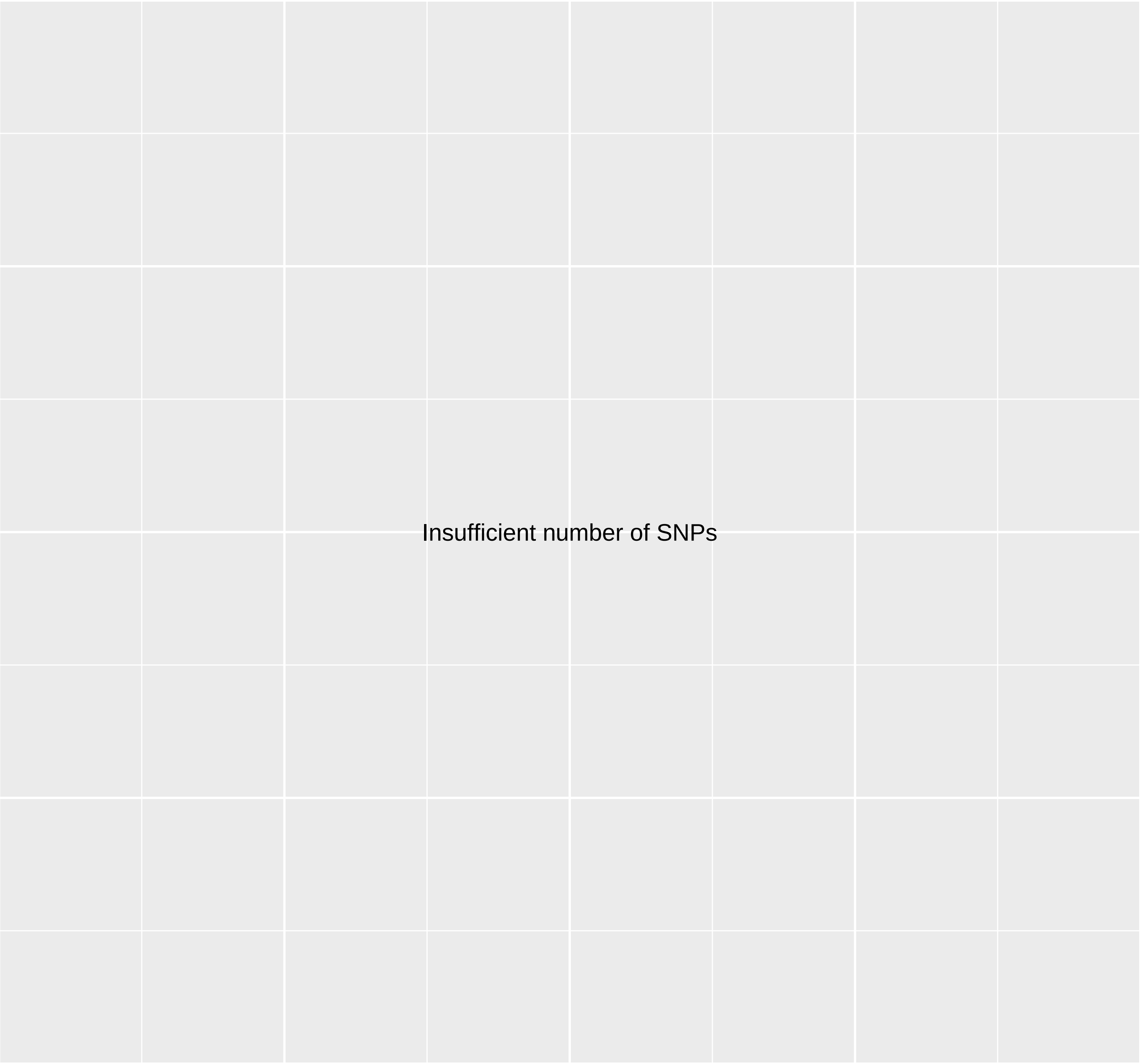
Insufficient number of SNPs

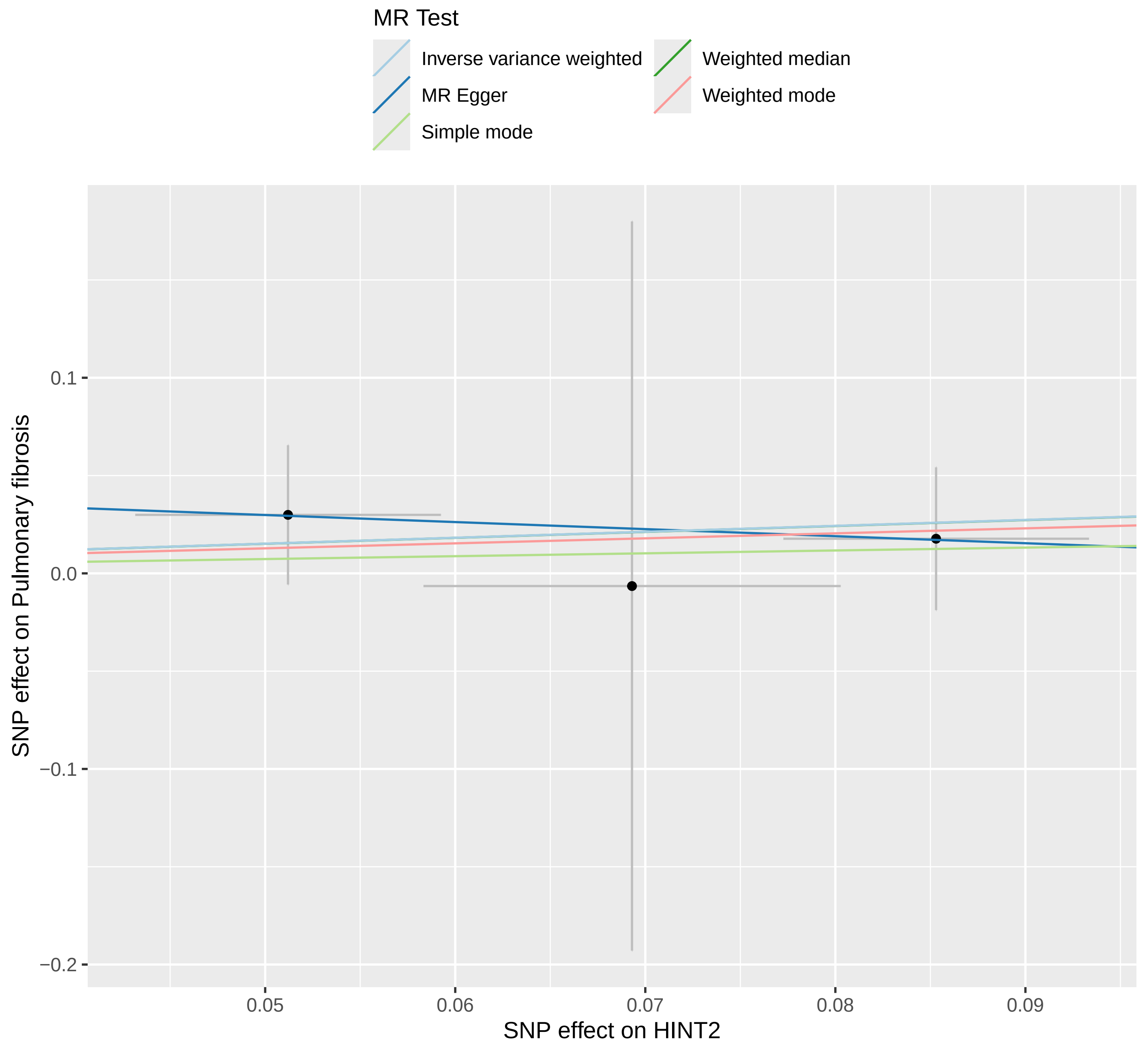
MR Test
Inverse variance weighted
MR Egger
Simple mode
Weighted median
Weighted mode
0.1
0.0
−0.1
−0.2
SNP effect on Pulmonary fibrosis
0.05
0.06
0.07
0.08
0.09
SNP effect on HINT2

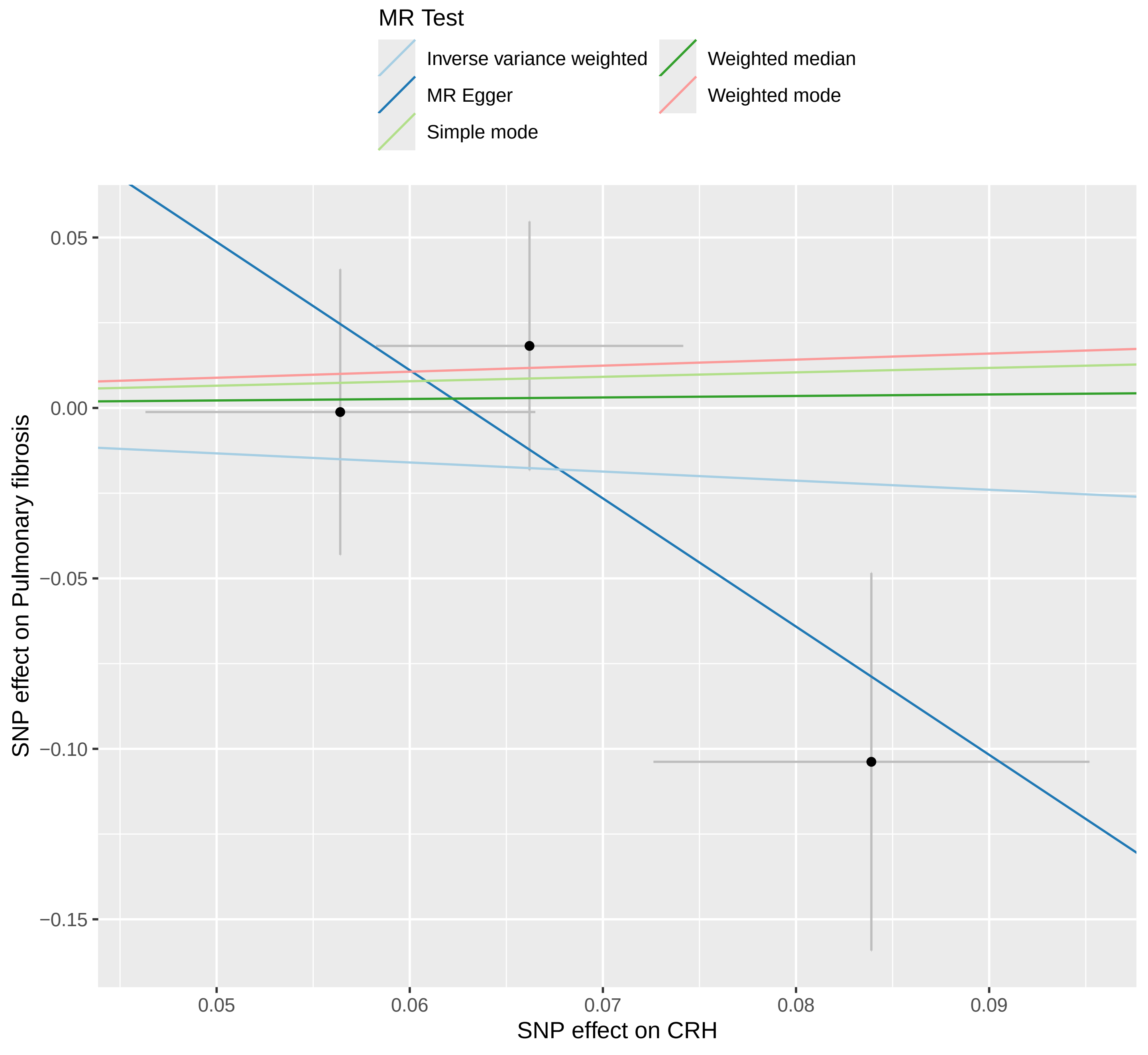

MR Test
Inverse variance weighted
MR Egger
Simple mode
Weighted median
Weighted mode
0.05
0.00
−0.05
−0.10
−0.15
SNP effect on Pulmonary fibrosis
0.05
0.06
0.07
0.08
0.09
SNP effect on CRH

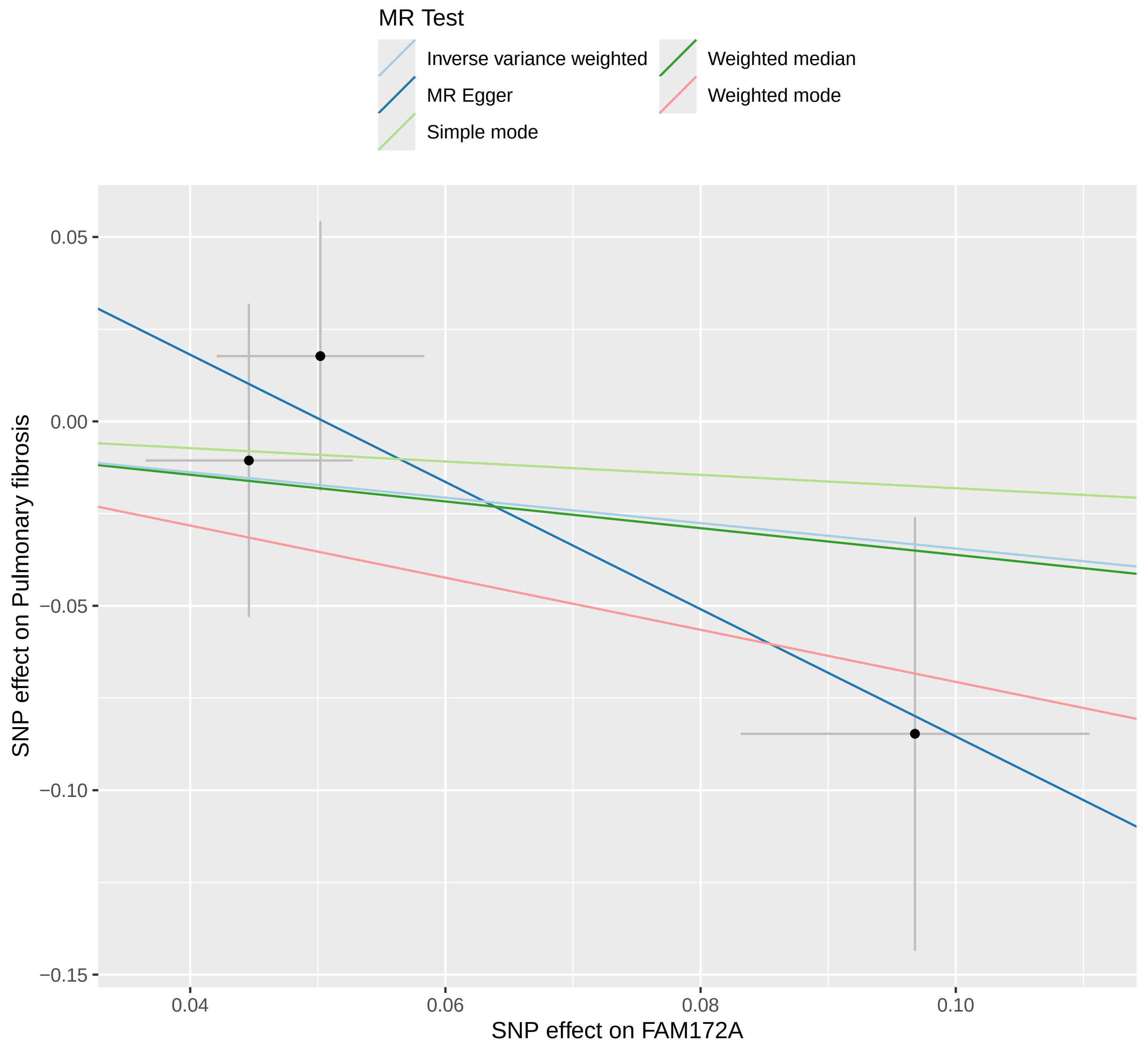

MR Test
Inverse variance weighted
MR Egger
Simple mode
Weighted median
Weighted mode
0.05
0.00
−0.05
−0.10
−0.15
0.04
0.06
0.08
0.10
SNP effect on Pulmonary fibrosis
SNP effect on FAM172A

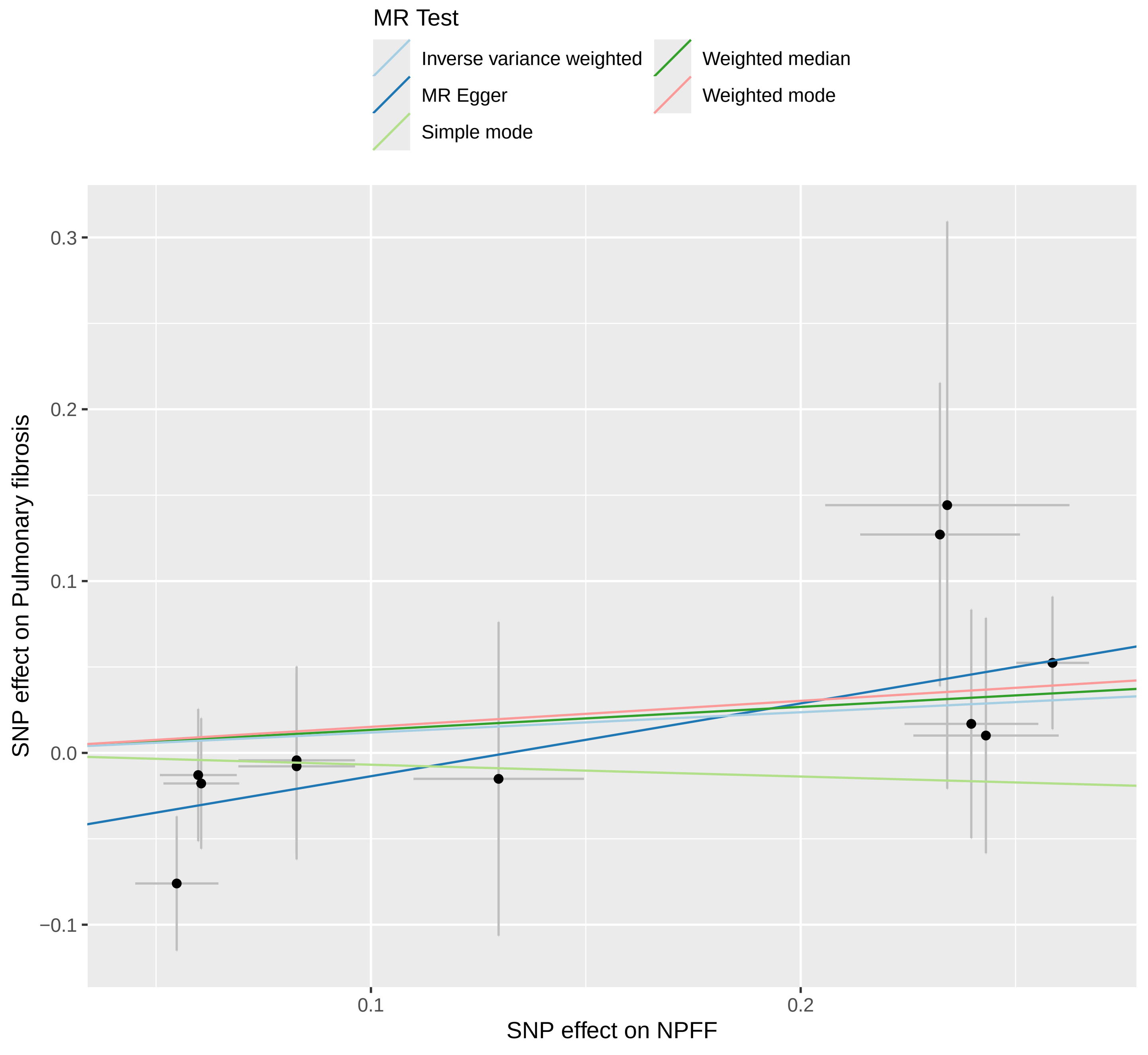

MR Test
Inverse variance weighted
MR Egger
Simple mode
Weighted median
Weighted mode
0.3
0.2
0.1
0.0
−0.1
SNP effect on Pulmonary fibrosis
0.1
0.2
SNP effect on NPFF

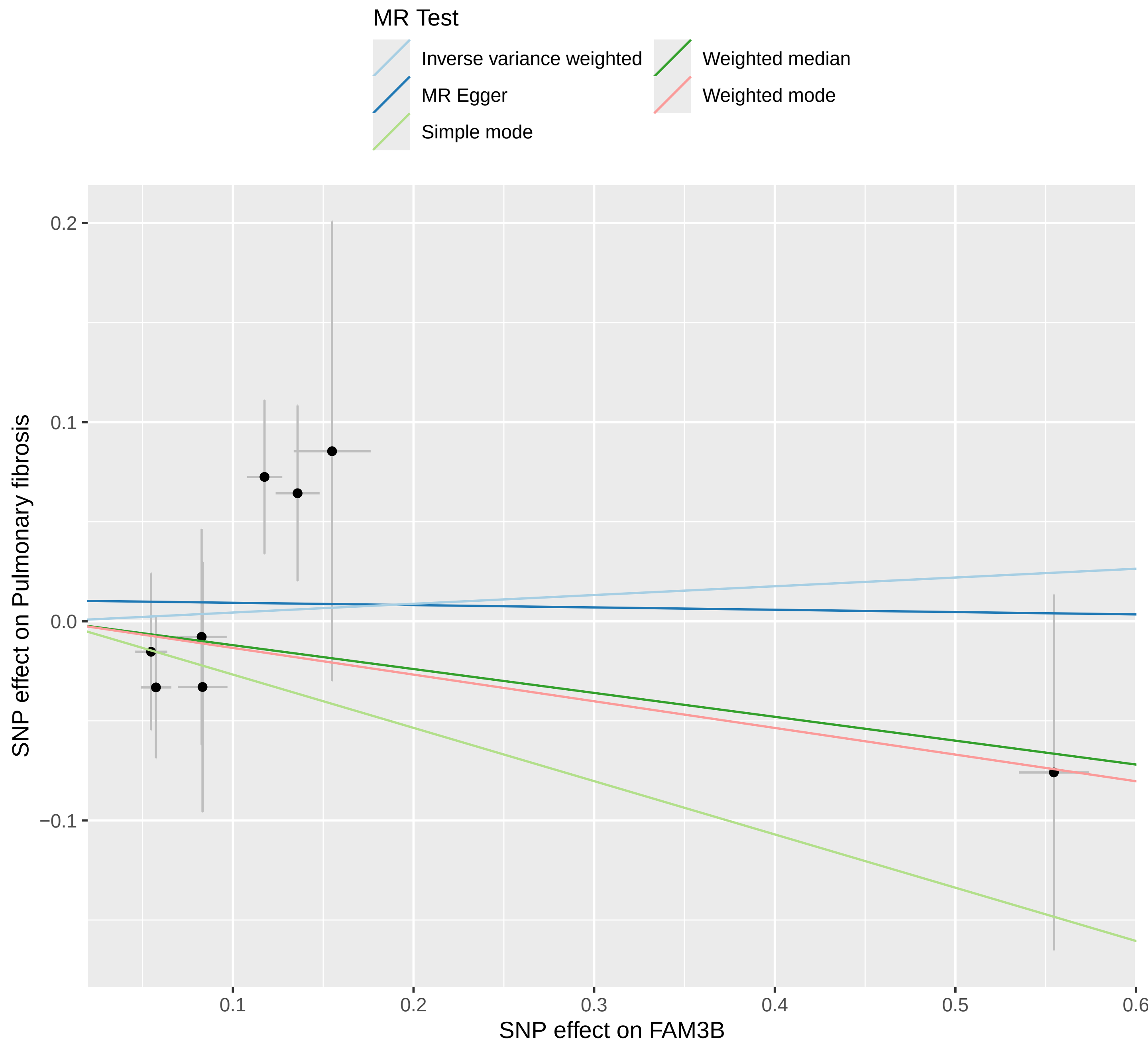
MR Test
Inverse variance weighted
MR Egger
Simple mode
Weighted median
Weighted mode
SNP effect on Pulmonary fibrosis
SNP effect on FAM3B
0.2
0.1
0.0
−0.1
0.1
0.2
0.3
0.4
0.5
0.6

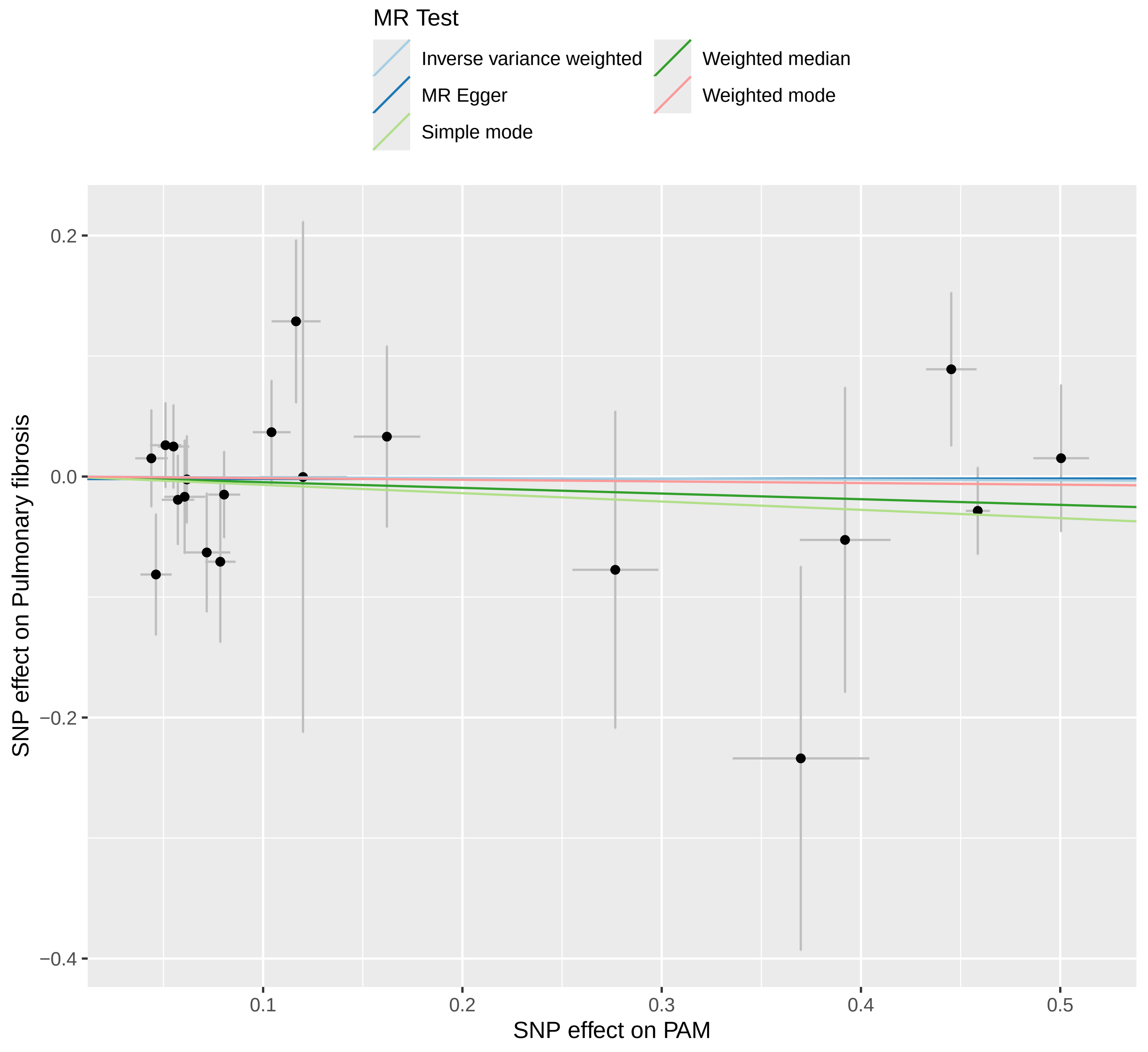

MR Test
Inverse variance weighted
MR Egger
Simple mode
Weighted median
Weighted mode
SNP effect on Pulmonary fibrosis
0.2
0.0
−0.2
−0.4
0.1
0.2
0.3
0.4
0.5
SNP effect on PAM

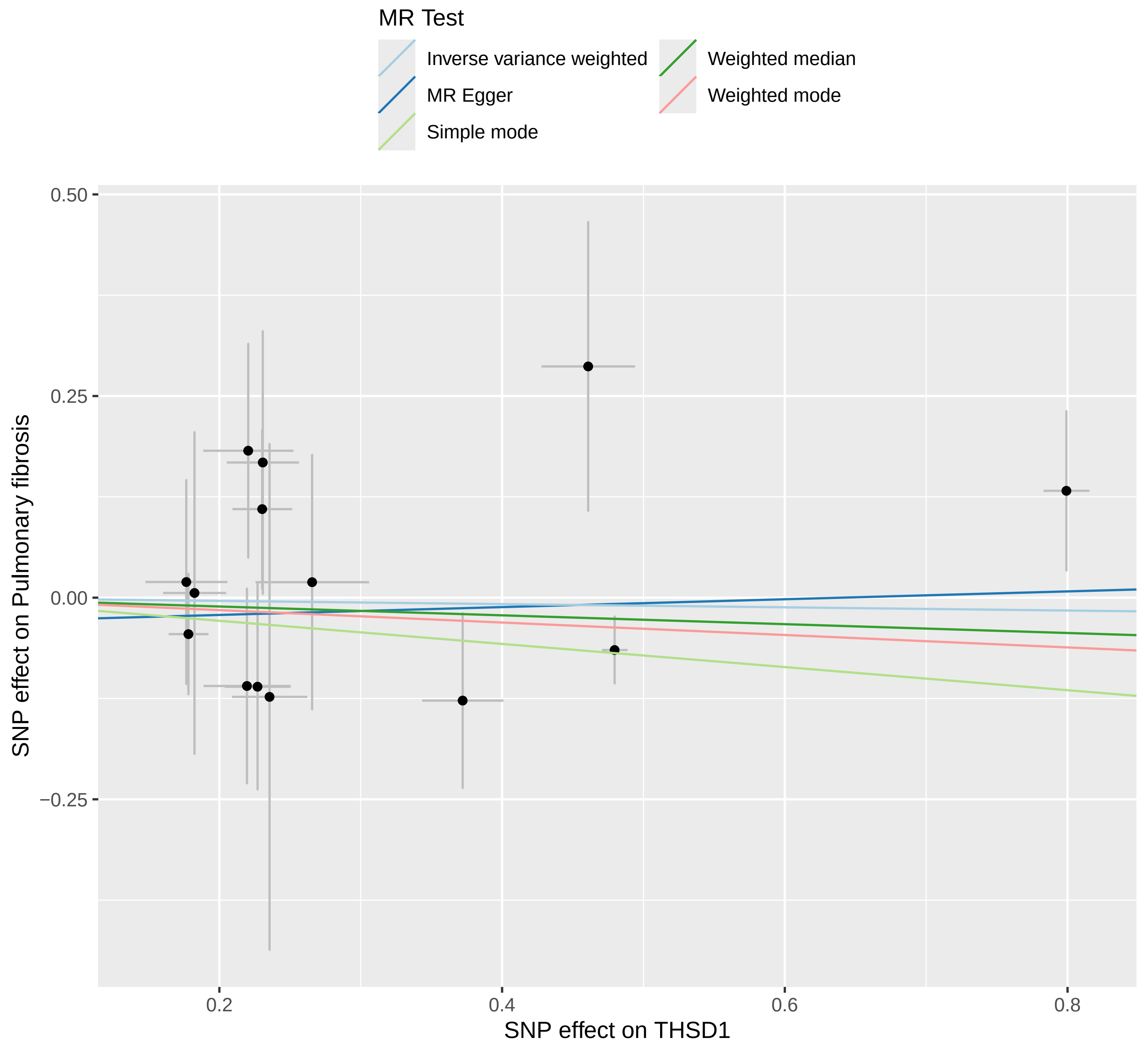
MR Test
Inverse variance weighted
MR Egger
Simple mode
Weighted median
Weighted mode
0.50
0.25
0.00
−0.25
SNP effect on Pulmonary fibrosis
0.2
0.4
0.6
0.8
SNP effect on THSD1

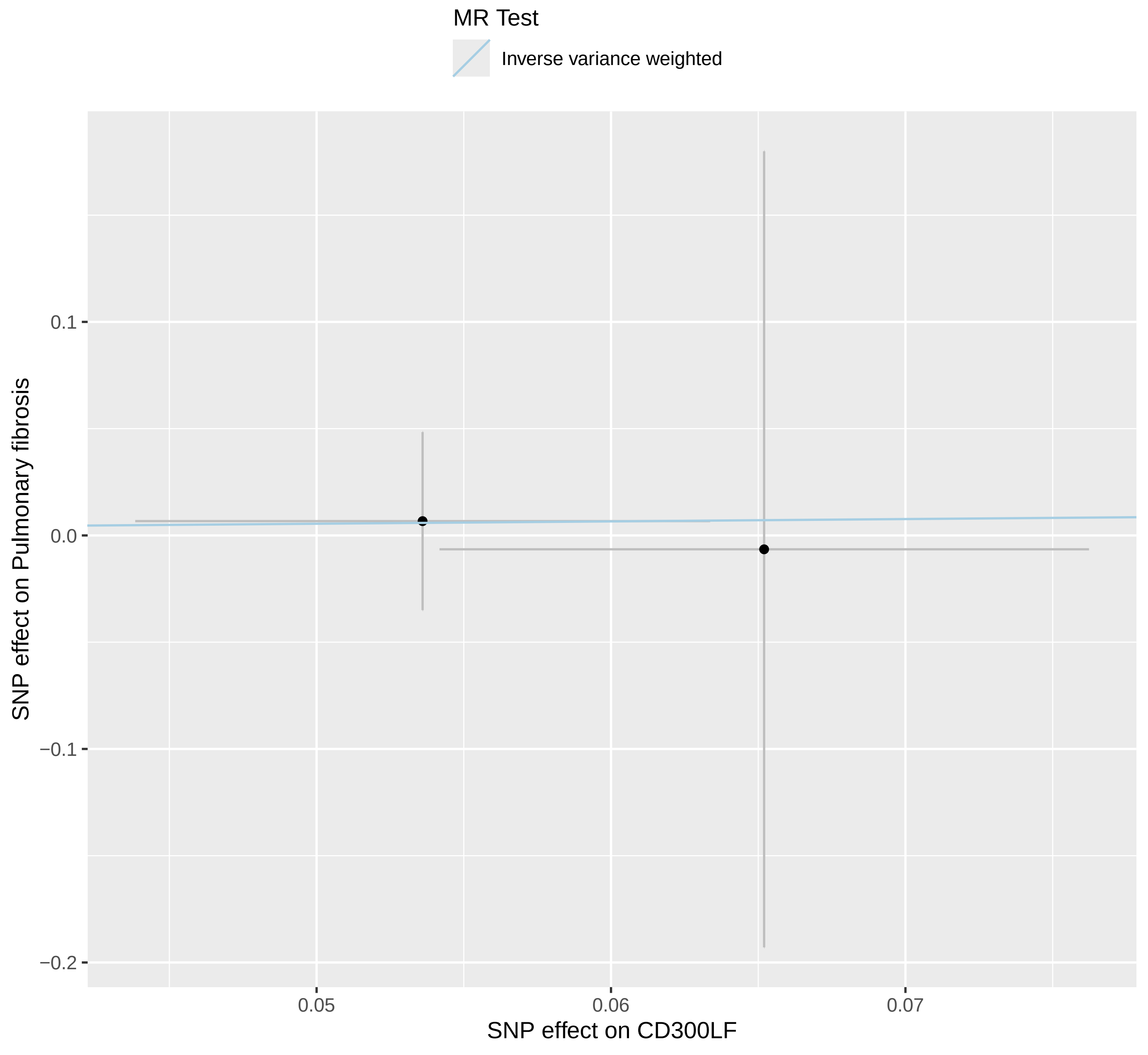

MR Test
Inverse variance weighted
0.1
0.0
−0.1
−0.2
0.05
0.06
0.07
SNP effect on CD300LF
SNP effect on Pulmonary fibrosis

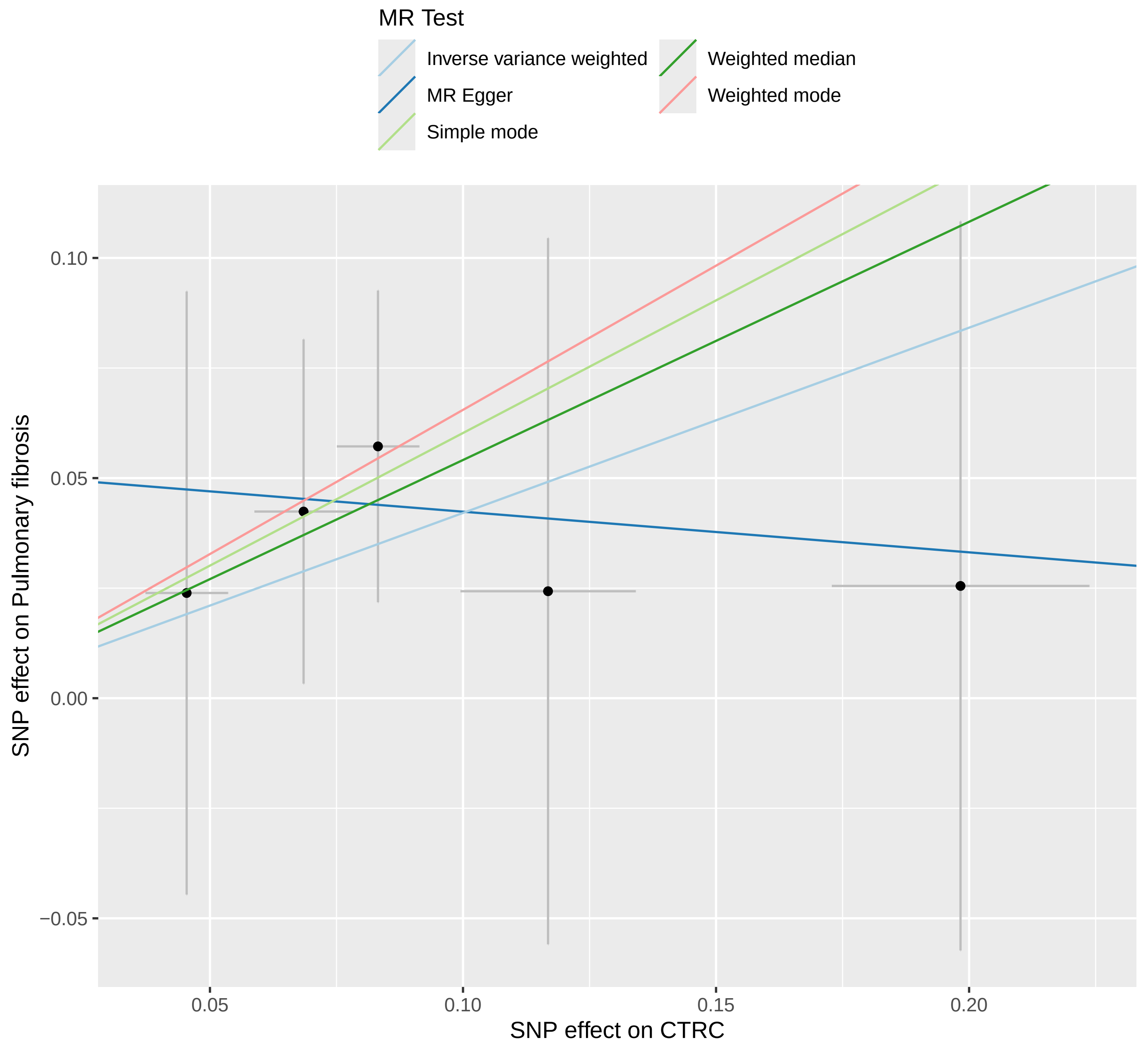
MR Test
Inverse variance weighted
MR Egger
Simple mode
Weighted median
Weighted mode
SNP effect on Pulmonary fibrosis
0.10
0.05
0.00
−0.05
0.05
0.10
0.15
0.20
SNP effect on CTRC

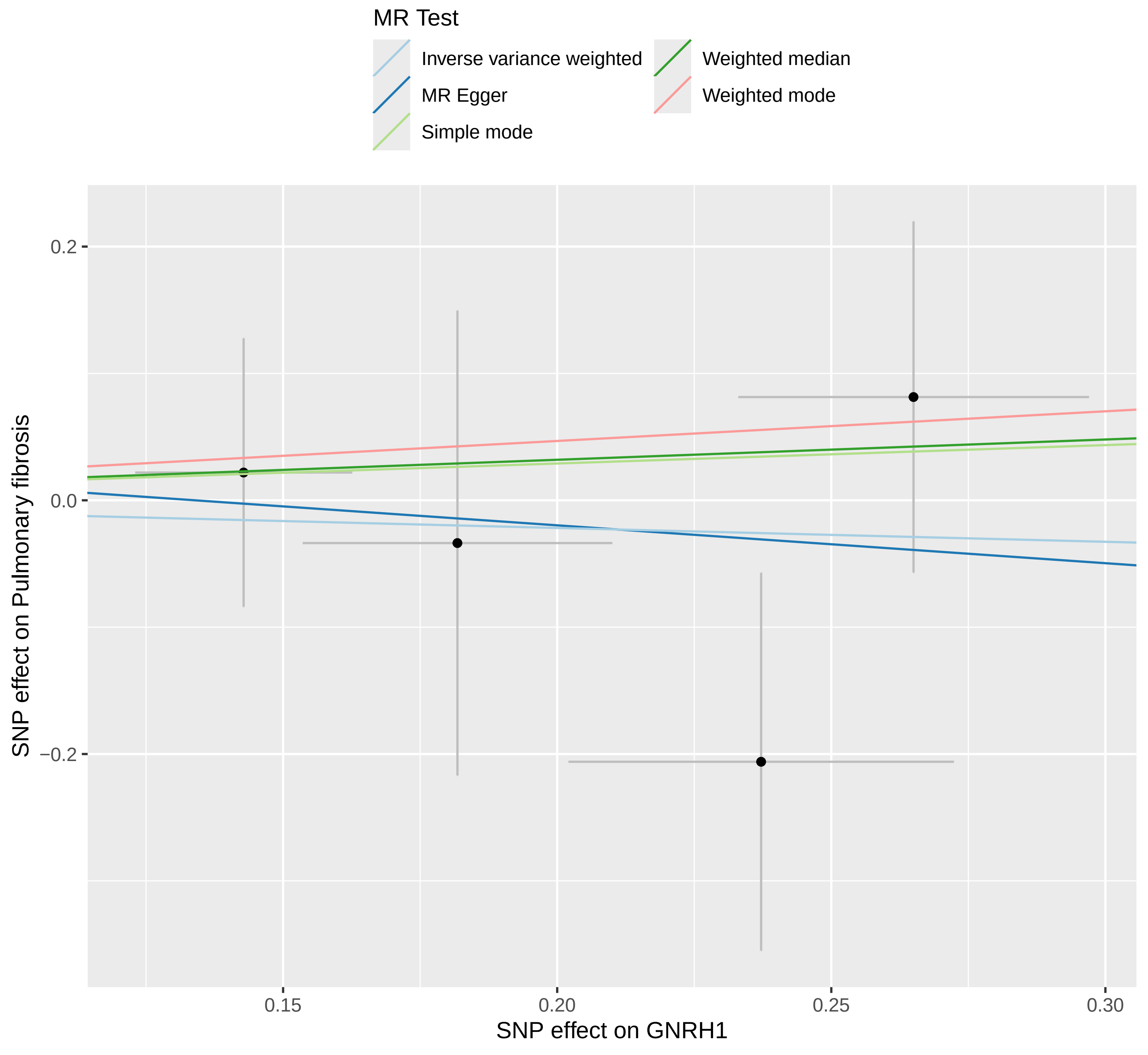

MR Test
Inverse variance weighted
MR Egger
Simple mode
Weighted median
Weighted mode
0.2
0.0
−0.2
0.15
0.20
0.25
0.30
SNP effect on Pulmonary fibrosis
SNP effect on GNRH1

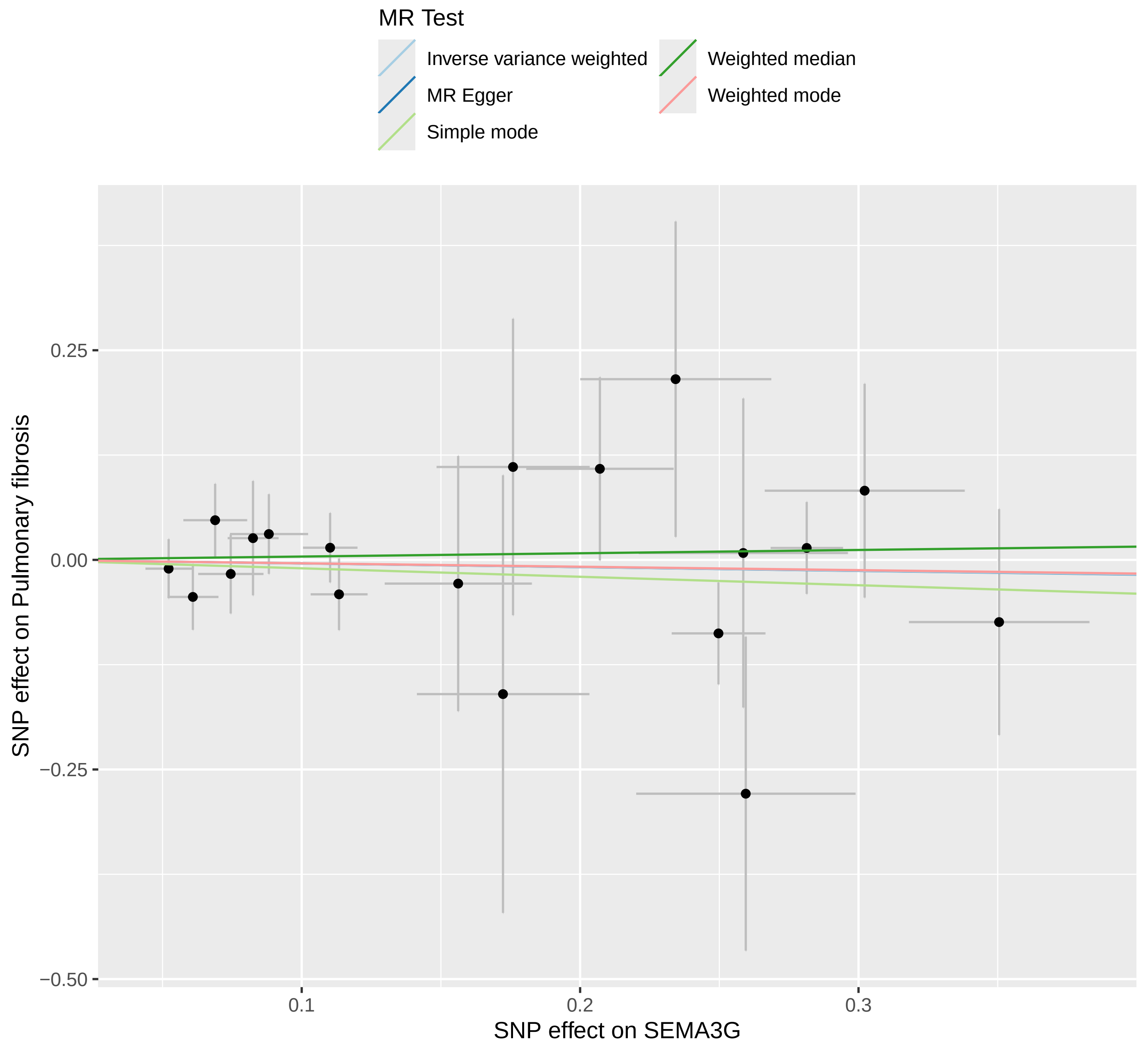

MR Test
Inverse variance weighted
MR Egger
Simple mode
Weighted median
Weighted mode
0.25
0.00
−0.25
−0.50
0.1
0.2
0.3
SNP effect on Pulmonary fibrosis
SNP effect on SEMA3G

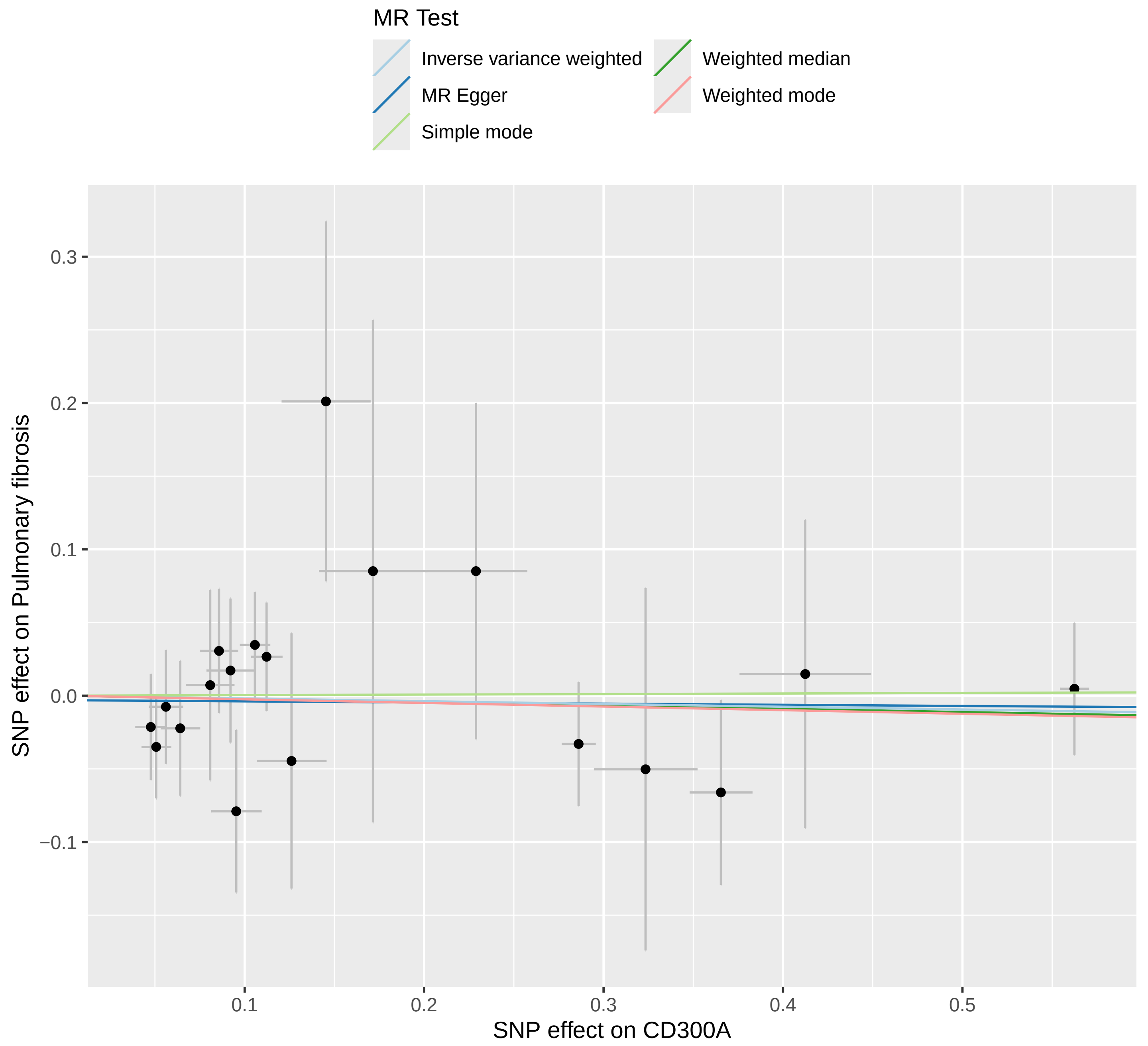
MR Test
Inverse variance weighted
MR Egger
Simple mode
Weighted median
Weighted mode
0.3
0.2
0.1
0.0
−0.1
SNP effect on Pulmonary fibrosis
0.1
0.2
0.3
0.4
0.5
SNP effect on CD300A

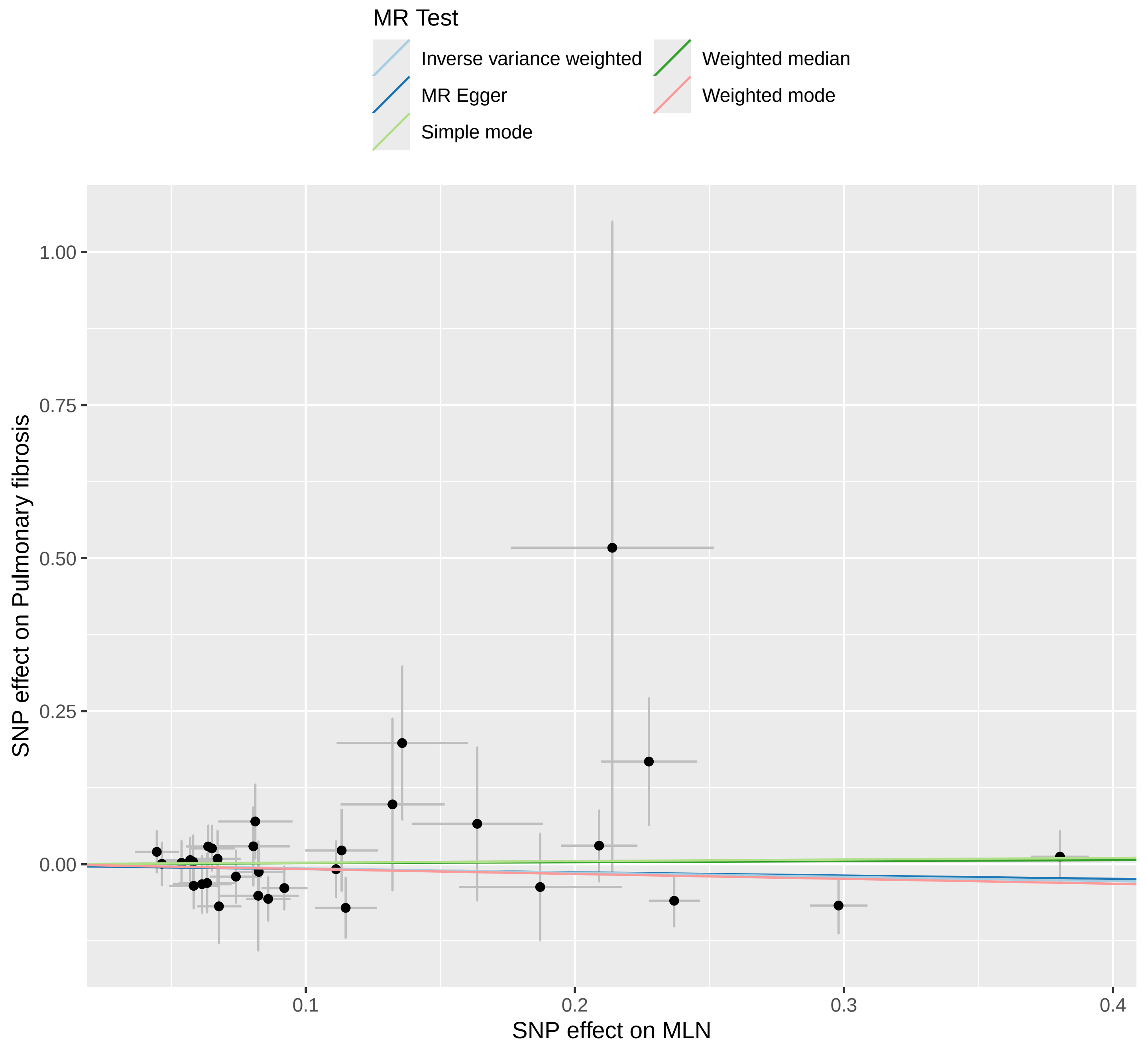

MR Test
Inverse variance weighted
MR Egger
Simple mode
Weighted median
Weighted mode
1.00
0.75
0.50
0.25
0.00
SNP effect on Pulmonary fibrosis
0.1
0.2
0.3
0.4
SNP effect on MLN

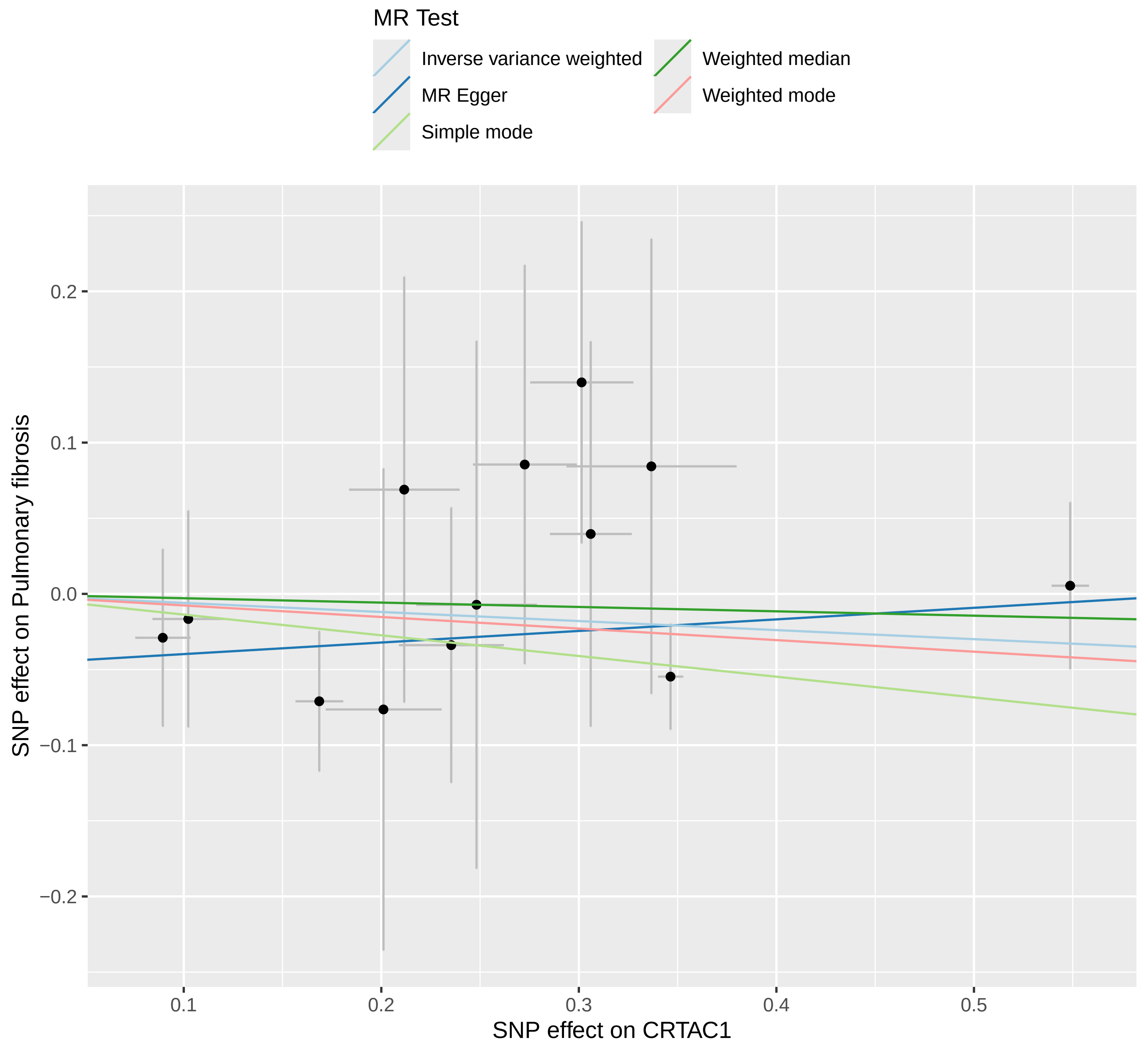
MR Test
Inverse variance weighted
MR Egger
Simple mode
Weighted median
Weighted mode
SNP effect on Pulmonary fibrosis
SNP effect on CRTAC1
0.2
0.1
0.0
−0.1
−0.2
0.1
0.2
0.3
0.4
0.5

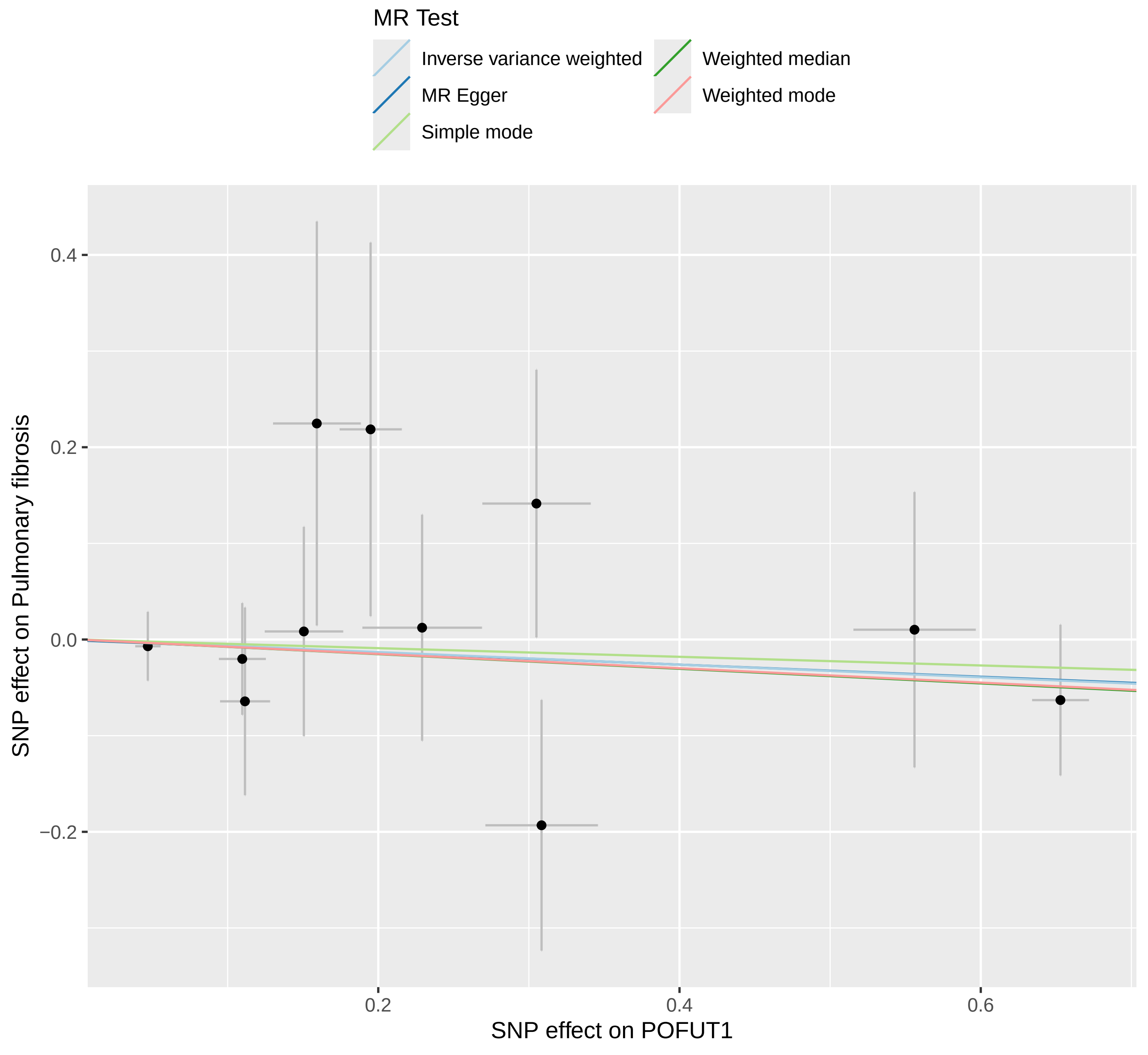
MR Test
Inverse variance weighted
MR Egger
Simple mode
Weighted median
Weighted mode
SNP effect on Pulmonary fibrosis
0.4
0.2
0.0
−0.2
0.2
0.4
0.6
SNP effect on POFUT1

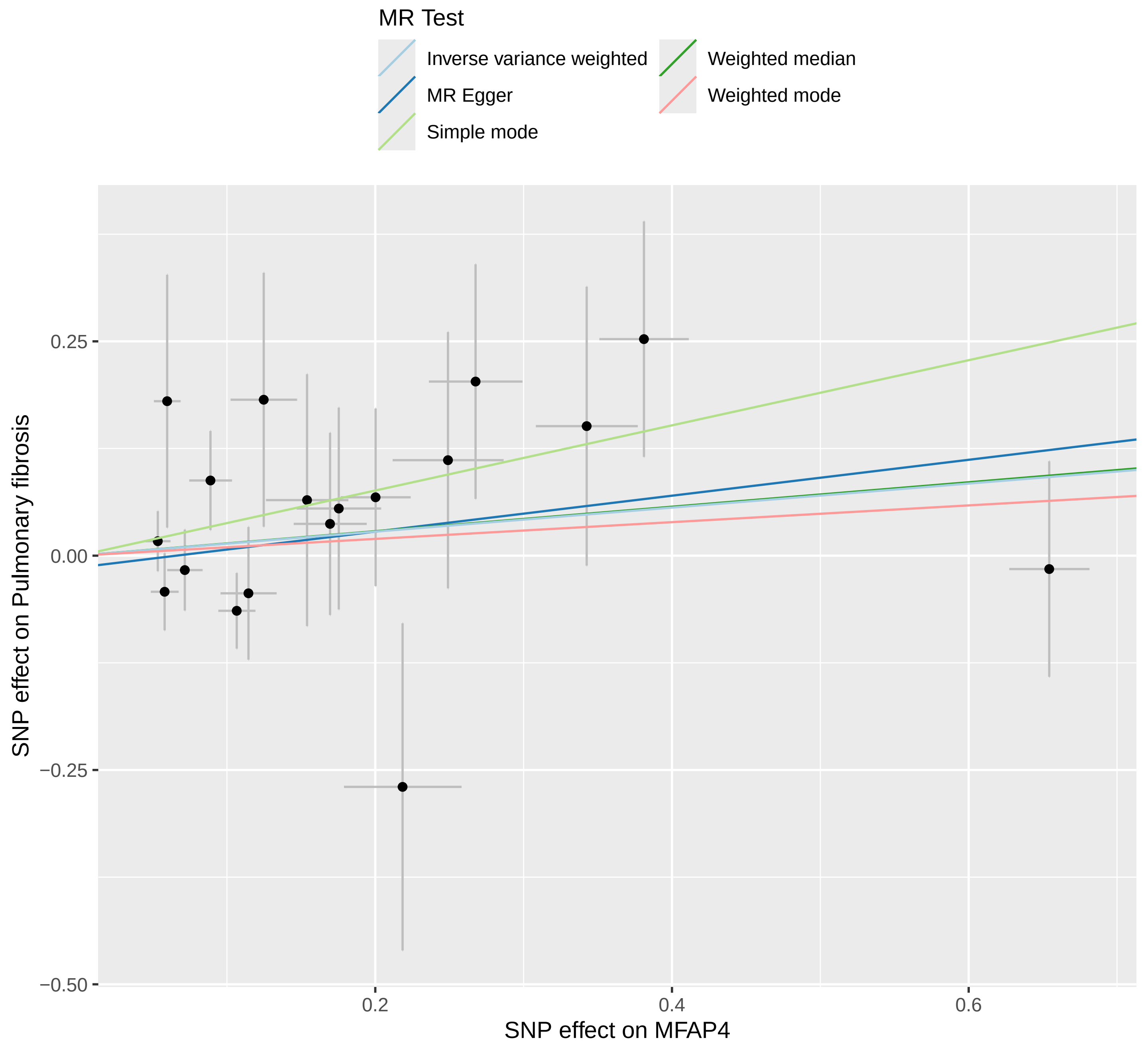

MR Test
Inverse variance weighted
MR Egger
Simple mode
Weighted median
Weighted mode
0.25
0.00
−0.25
−0.50
0.2
0.4
0.6
SNP effect on Pulmonary fibrosis
SNP effect on MFAP4

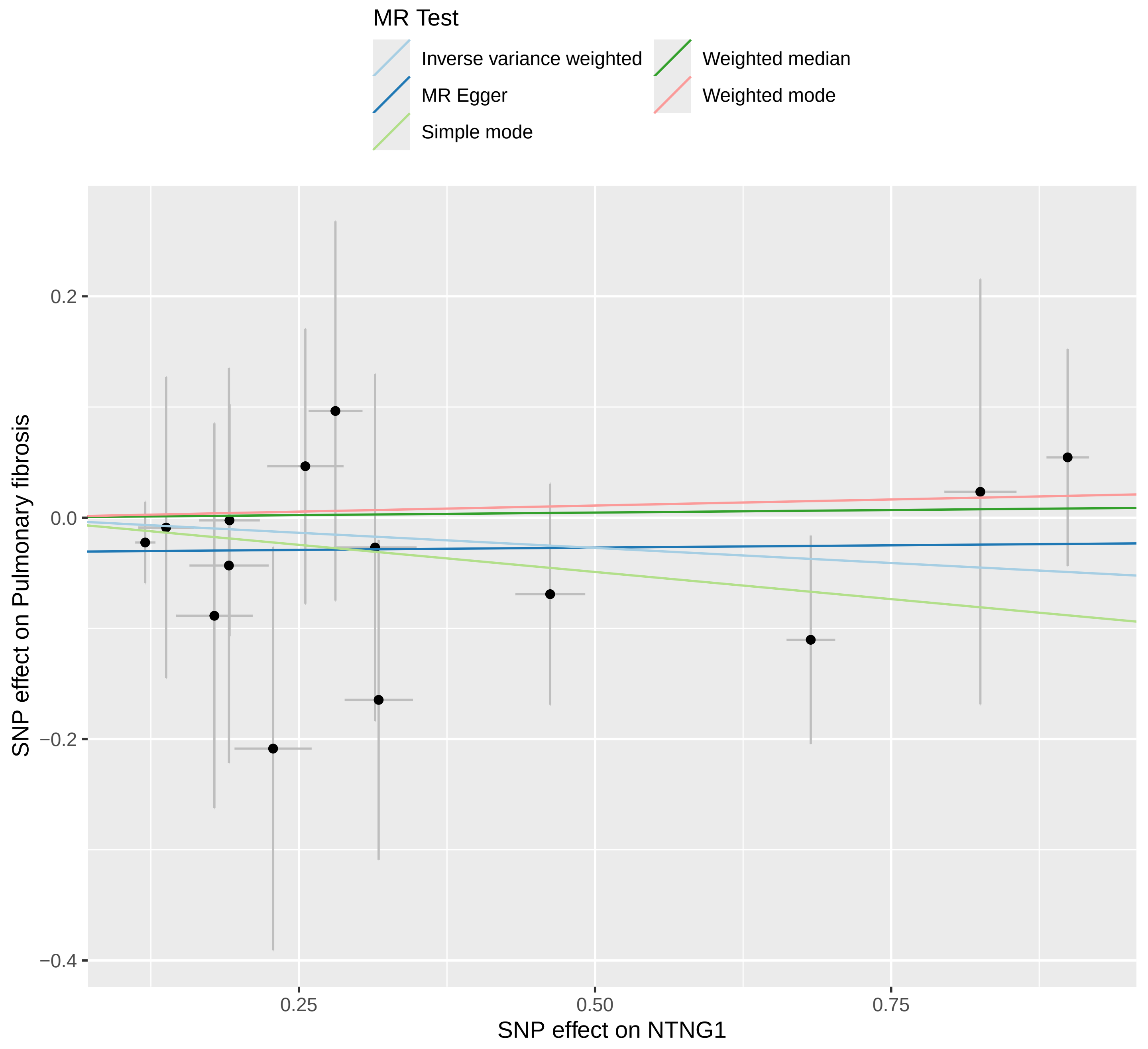
MR Test
Inverse variance weighted
MR Egger
Simple mode
Weighted median
Weighted mode
SNP effect on Pulmonary fibrosis
0.2
0.0
−0.2
−0.4
0.25
0.50
0.75
SNP effect on NTNG1

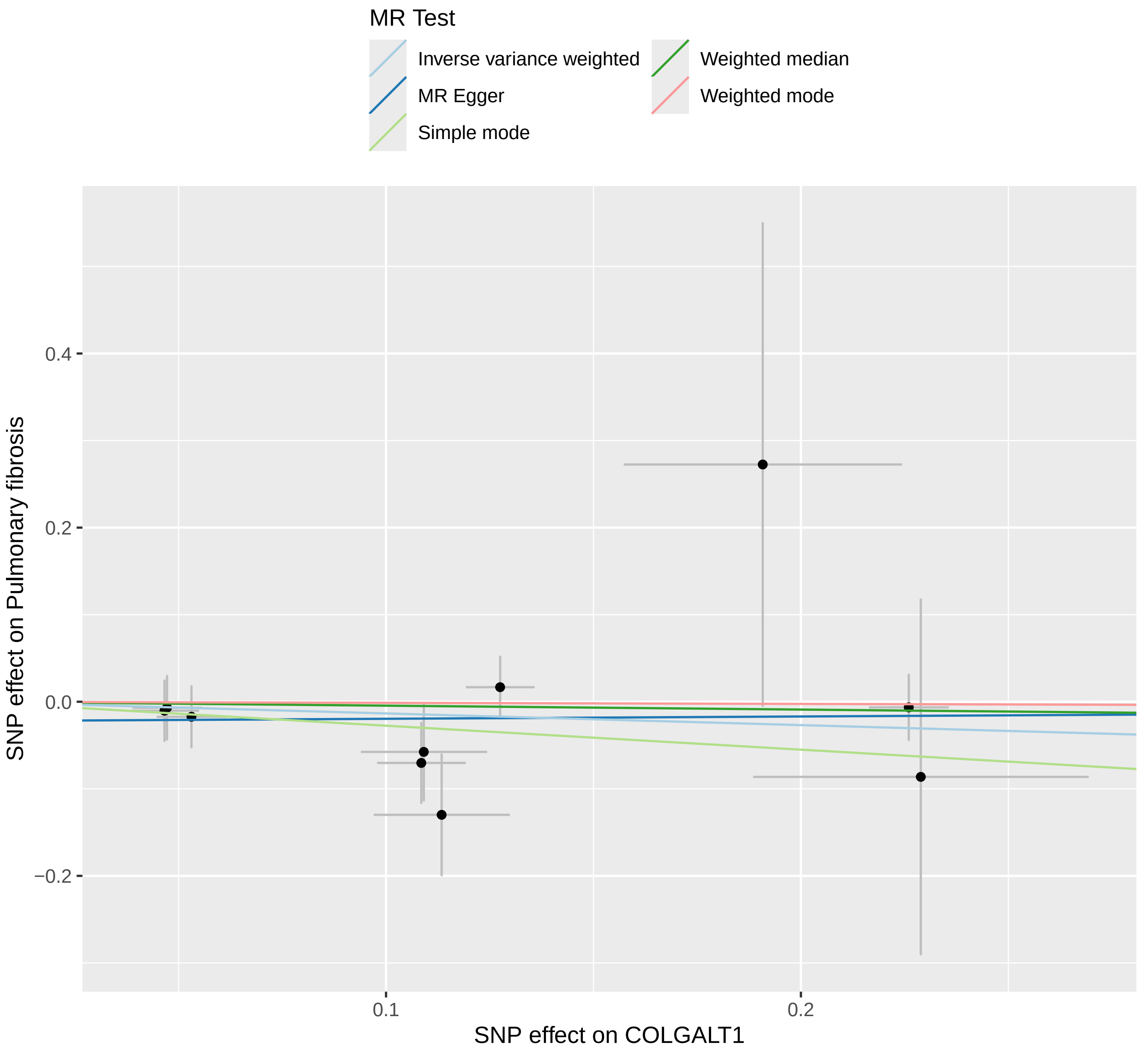

MR Test
Inverse variance weighted
MR Egger
Simple mode
Weighted median
Weighted mode
SNP effect on Pulmonary fibrosis
0.4
0.2
0.0
−0.2
0.1
0.2
SNP effect on COLGALT1

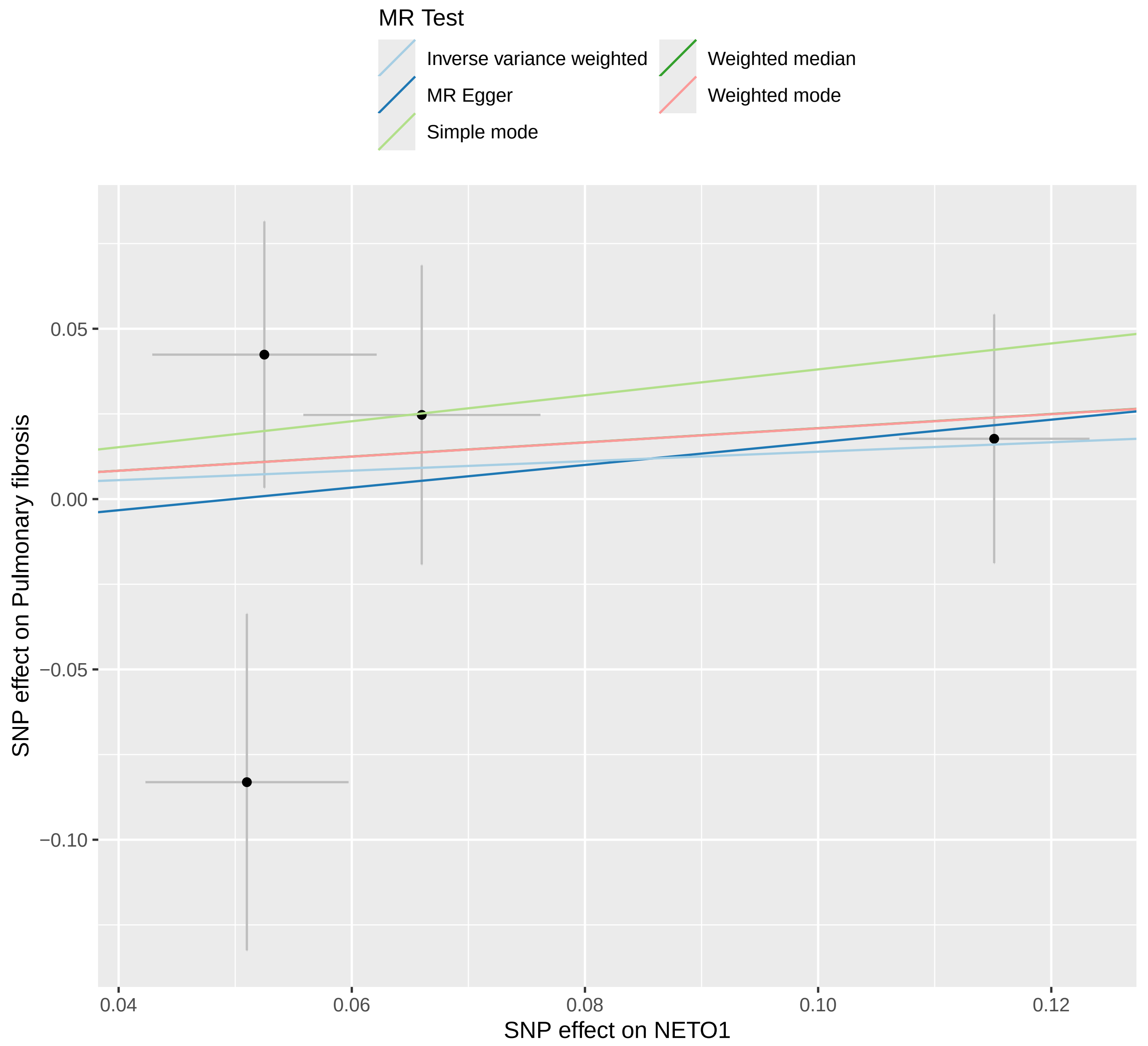

MR Test
Inverse variance weighted
MR Egger
Simple mode
Weighted median
Weighted mode
0.05
0.00
−0.05
−0.10
SNP effect on Pulmonary fibrosis
0.04
0.06
0.08
0.10
0.12
SNP effect on NETO1

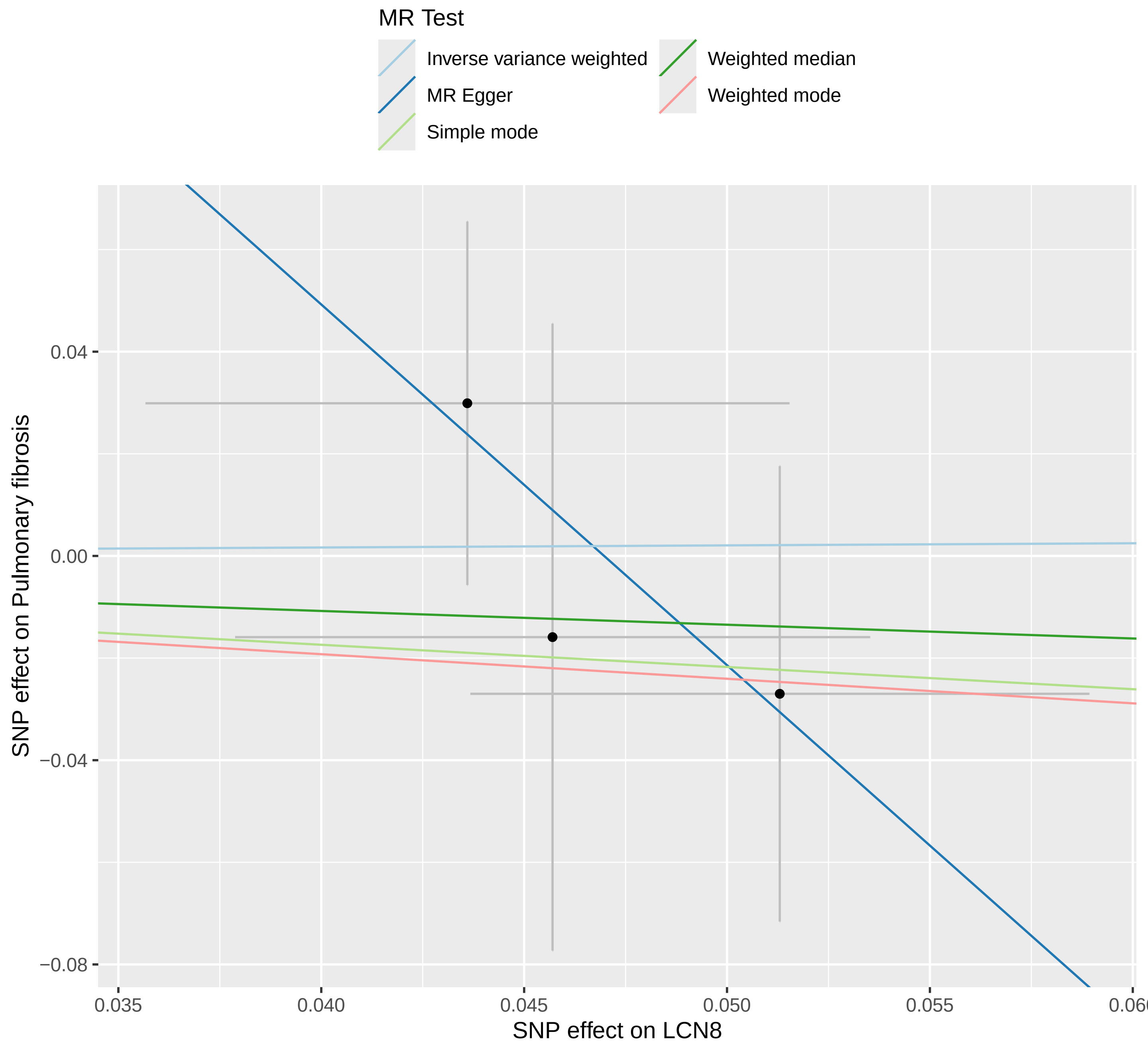
MR Test
Inverse variance weighted
MR Egger
Simple mode
Weighted median
Weighted mode
0.04
0.00
−0.04
−0.08
0.035
0.040
0.045
0.050
0.055
0.060
SNP effect on LCN8
SNP effect on Pulmonary fibrosis

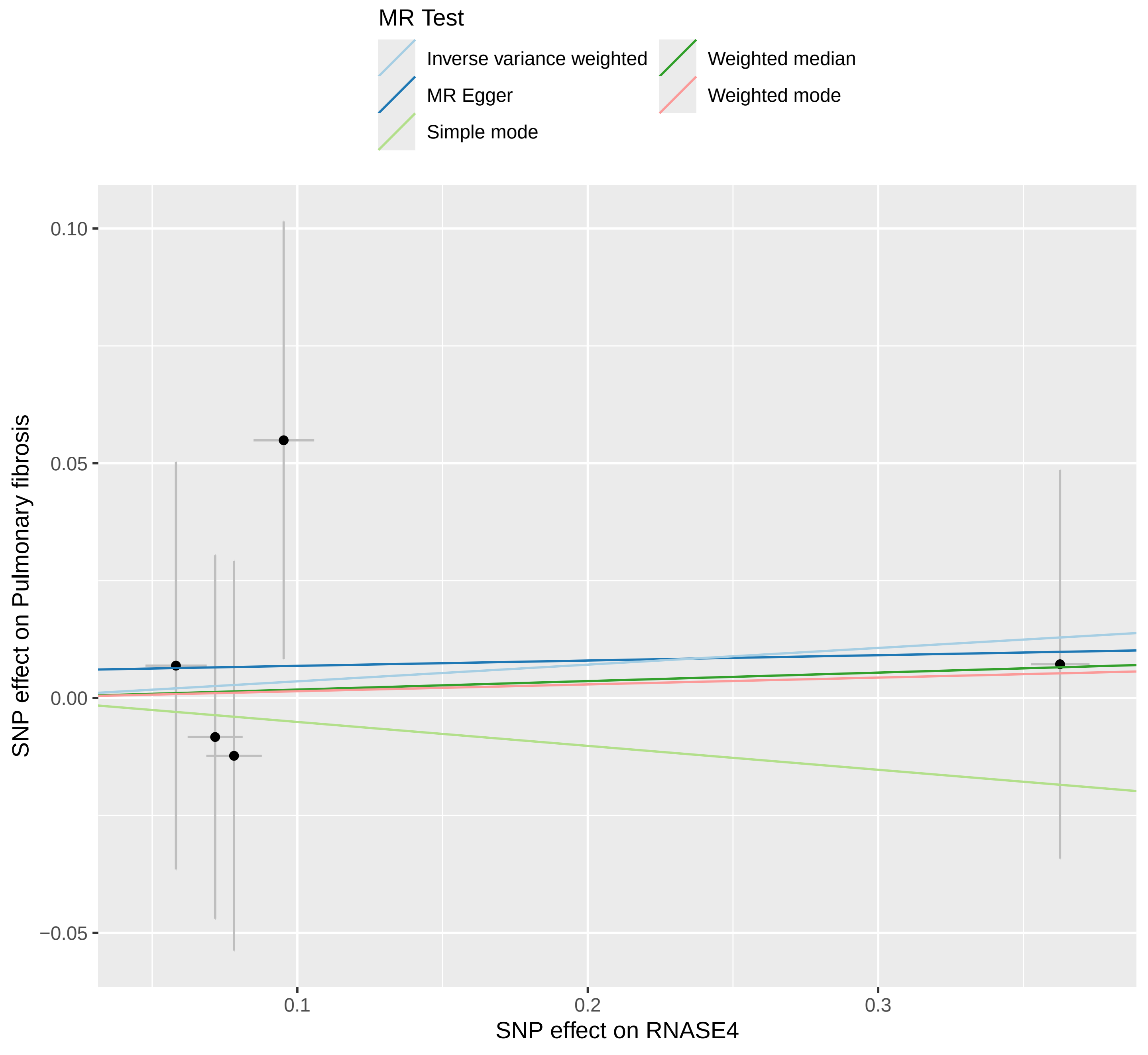

MR Test
Inverse variance weighted
MR Egger
Simple mode
Weighted median
Weighted mode
0.10
0.05
0.00
−0.05
SNP effect on Pulmonary fibrosis
0.1
0.2
0.3
SNP effect on RNASE4

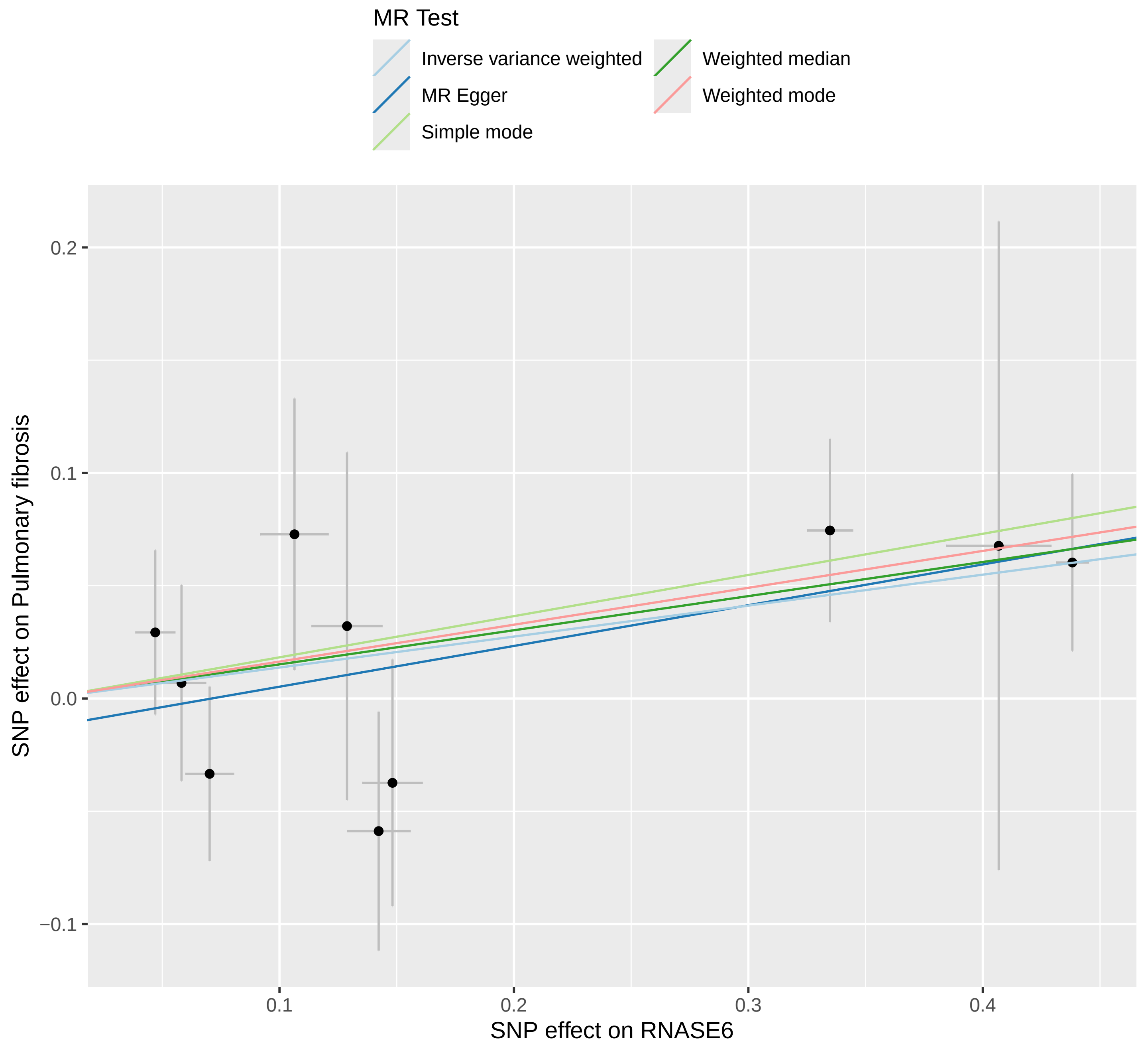
MR Test
Inverse variance weighted
MR Egger
Simple mode
Weighted median
Weighted mode
0.2
0.1
0.0
−0.1
SNP effect on Pulmonary fibrosis
0.1
0.2
0.3
0.4
SNP effect on RNASE6

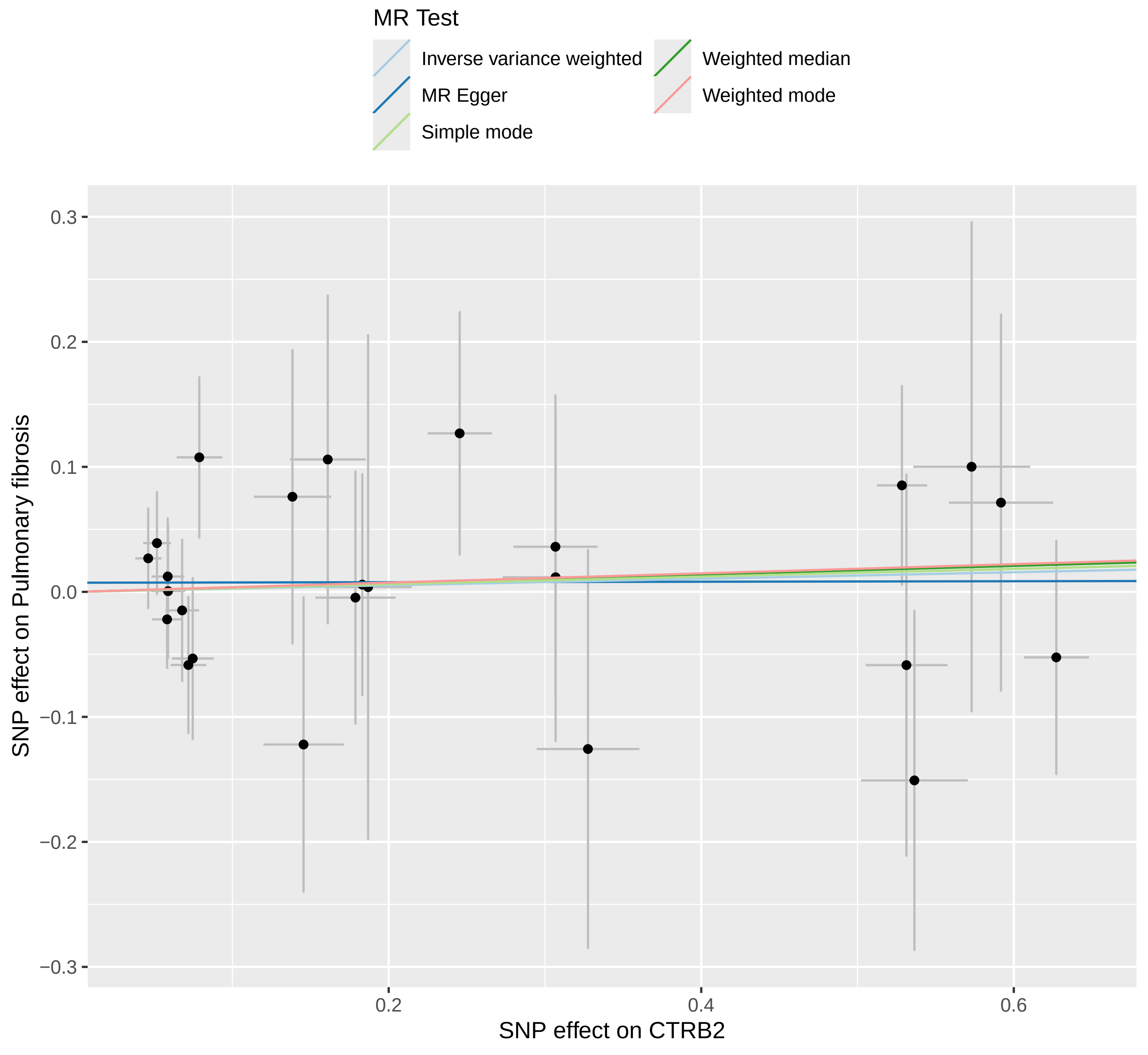
MR Test
Inverse variance weighted
MR Egger
Simple mode
Weighted median
Weighted mode
SNP effect on Pulmonary fibrosis
0.3
0.2
0.1
0.0
−0.1
−0.2
−0.3
0.2
0.4
0.6
SNP effect on CTRB2

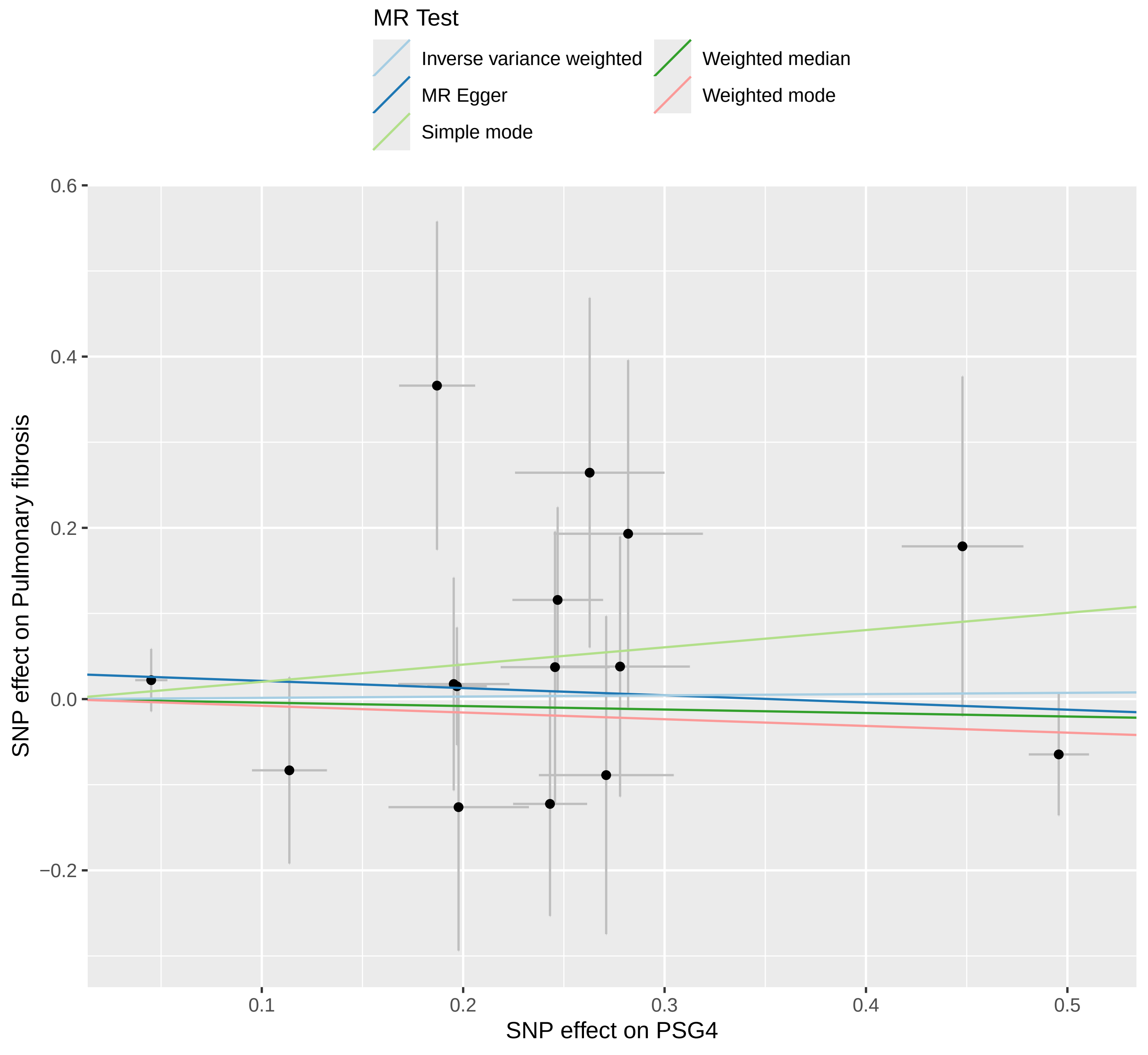
MR Test
Inverse variance weighted
MR Egger
Simple mode
Weighted median
Weighted mode
0.6
0.4
0.2
0.0
−0.2
0.1
0.2
0.3
0.4
0.5
SNP effect on Pulmonary fibrosis
SNP effect on PSG4

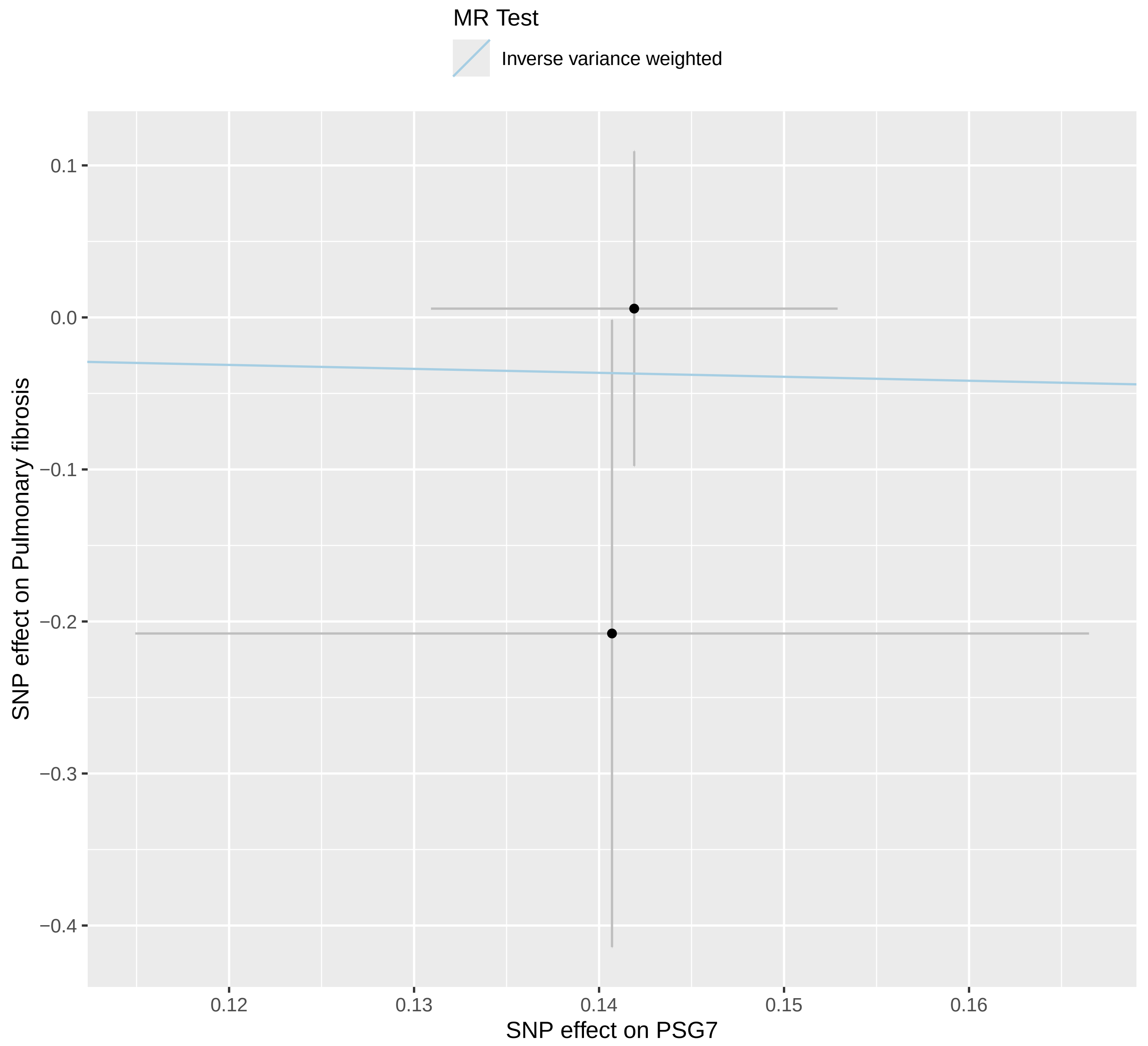

MR Test
Inverse variance weighted
0.1
0.0
−0.1
−0.2
−0.3
−0.4
SNP effect on Pulmonary fibrosis
0.12
0.13
0.14
0.15
0.16
SNP effect on PSG7

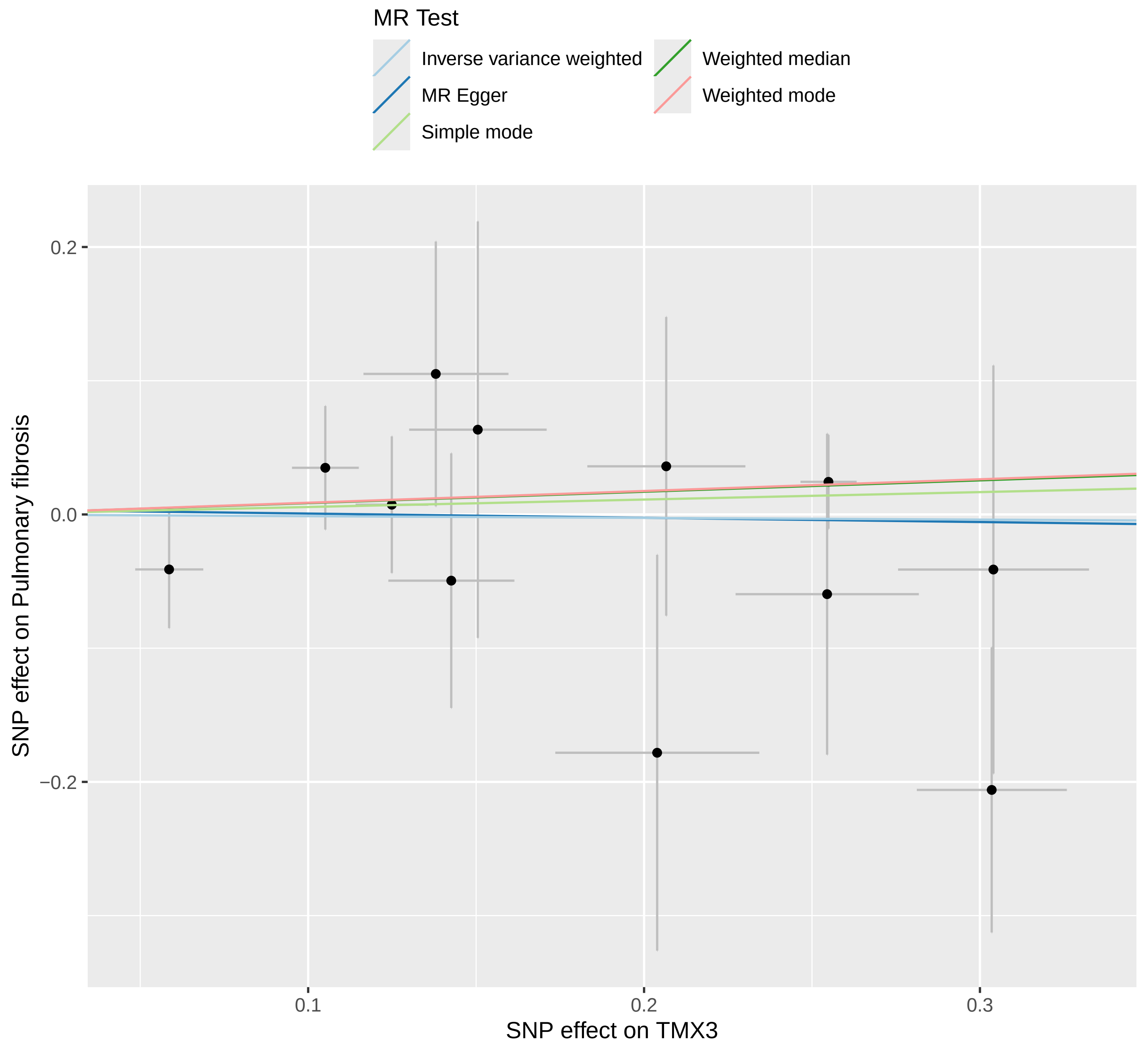
MR Test
Inverse variance weighted
MR Egger
Simple mode
Weighted median
Weighted mode
0.2
0.0
−0.2
0.1
0.2
0.3
SNP effect on Pulmonary fibrosis
SNP effect on TMX3

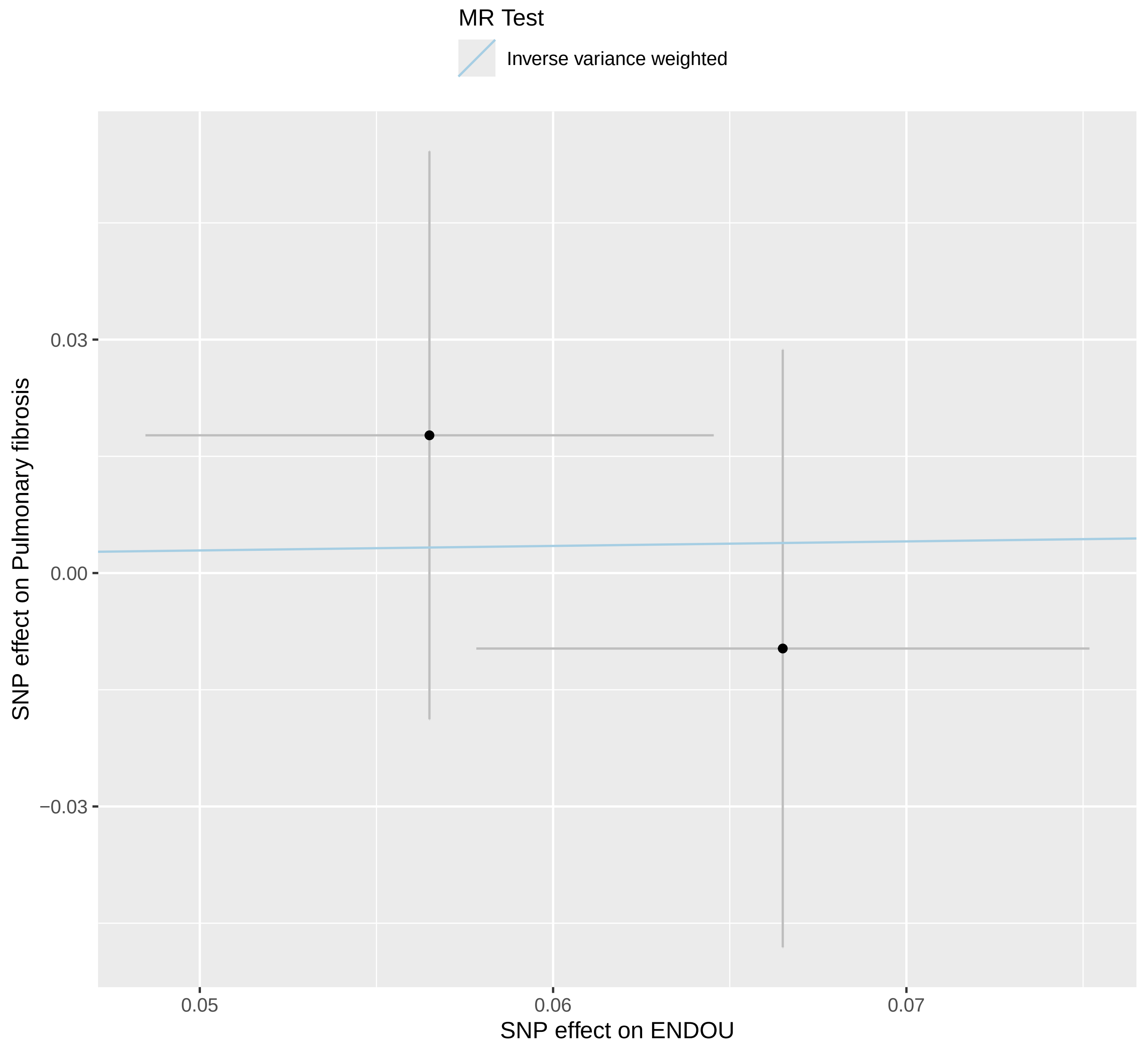

MR Test
Inverse variance weighted
0.03
0.00
−0.03
0.05
0.06
0.07
SNP effect on ENDOU
SNP effect on Pulmonary fibrosis

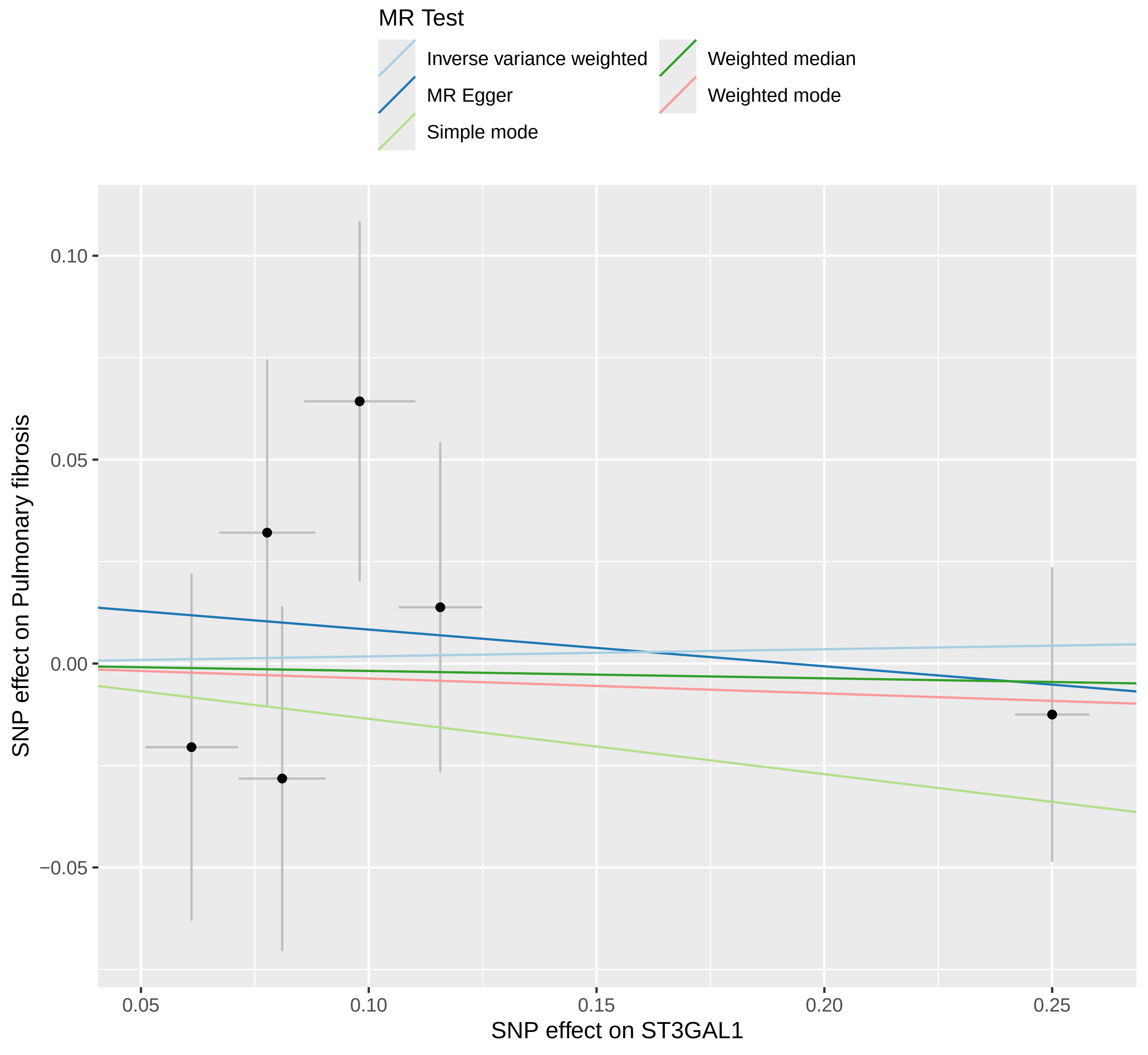
MR Test
Inverse variance weighted
MR Egger
Simple mode
Weighted median
Weighted mode
0.10
0.05
0.00
−0.05
0.05
0.10
0.15
0.20
0.25
SNP effect on ST3GAL1
SNP effect on Pulmonary fibrosis

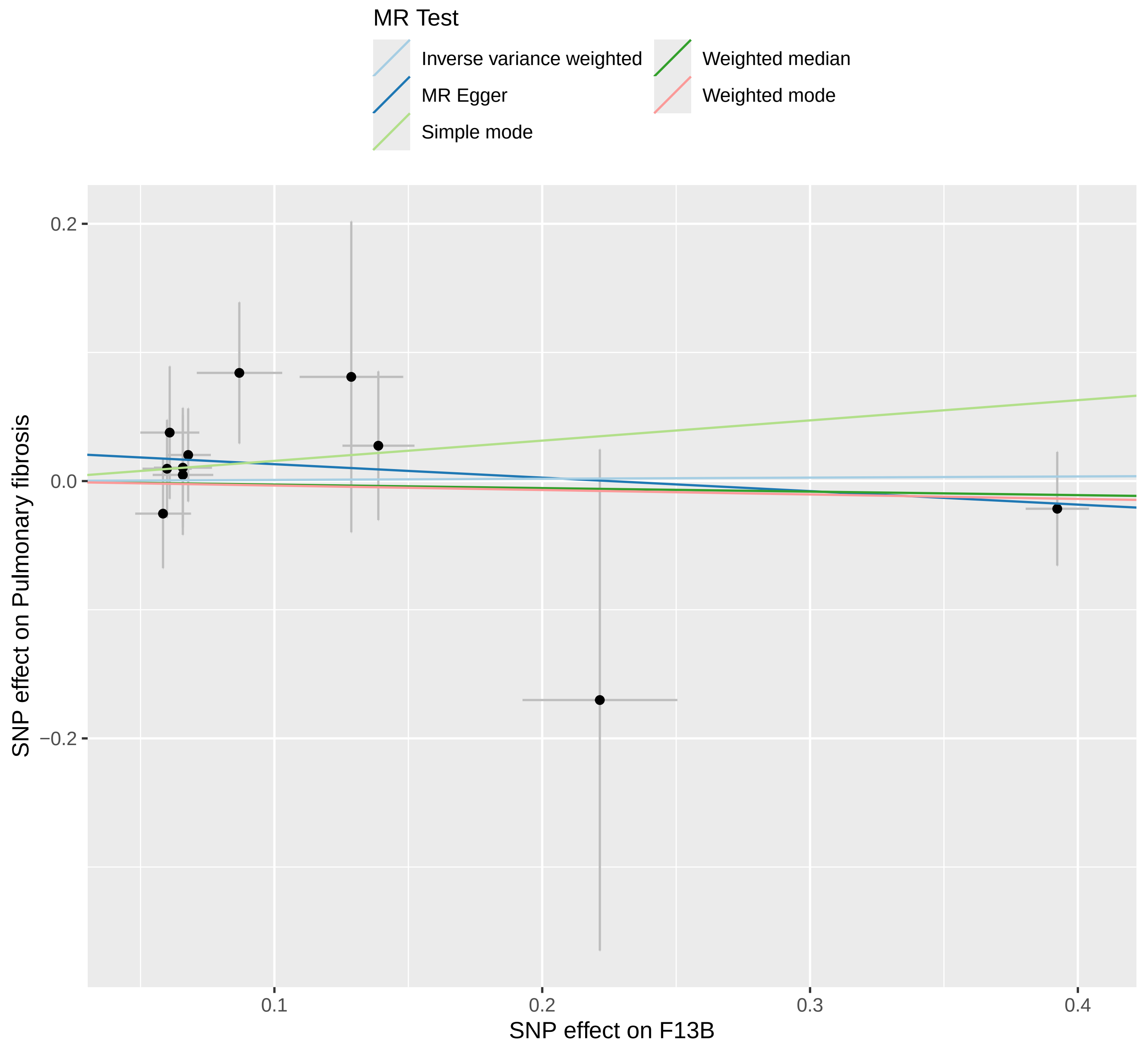

MR Test
Inverse variance weighted
MR Egger
Simple mode
Weighted median
Weighted mode
0.2
0.0
−0.2
SNP effect on Pulmonary fibrosis
0.1
0.2
0.3
0.4
SNP effect on F13B

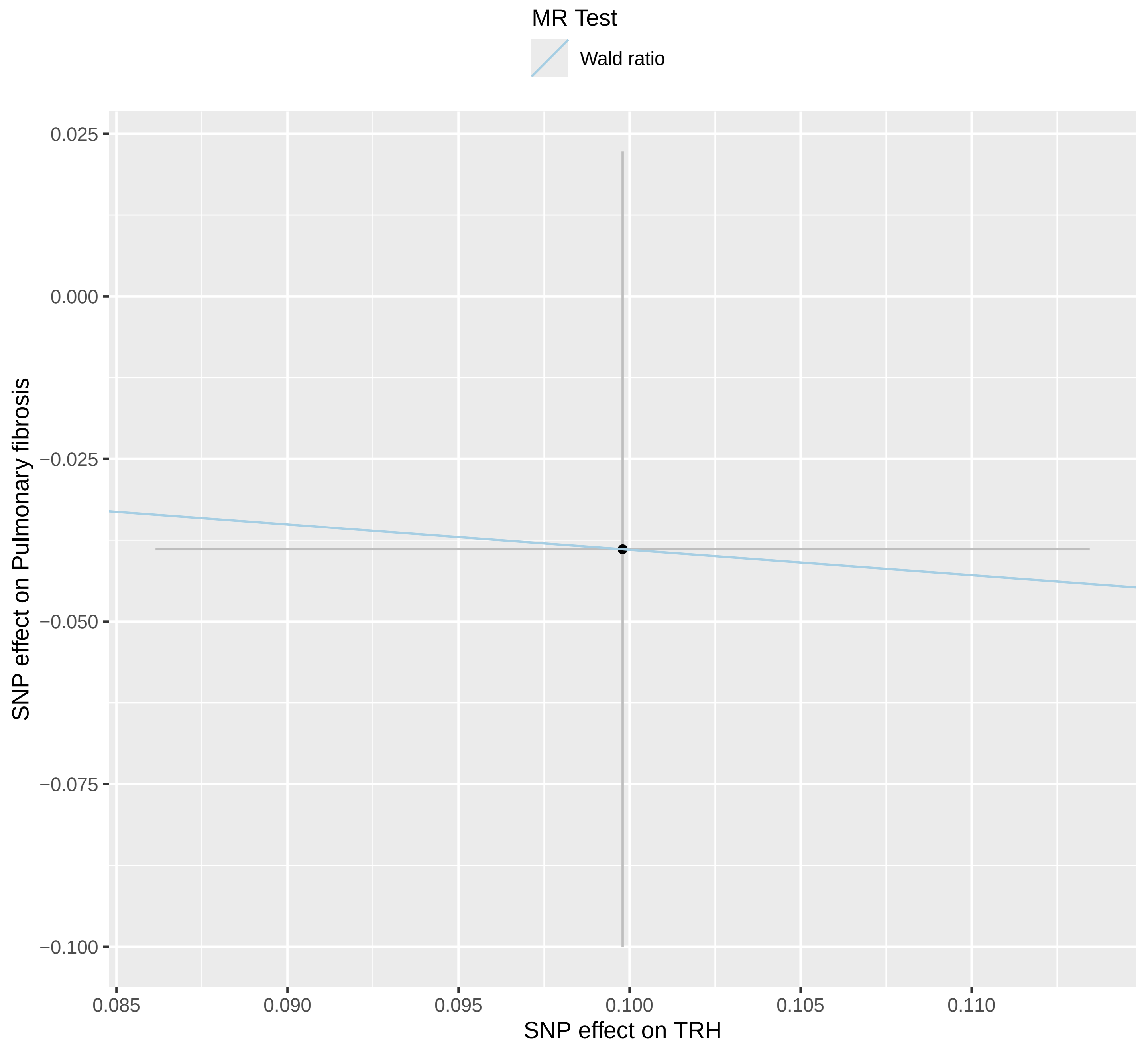
MR Test
Wald ratio
SNP effect on Pulmonary fibrosis
0.025
0.000
−0.025
−0.050
−0.075
−0.100
0.085
0.090
0.095
0.100
0.105
0.110
SNP effect on TRH

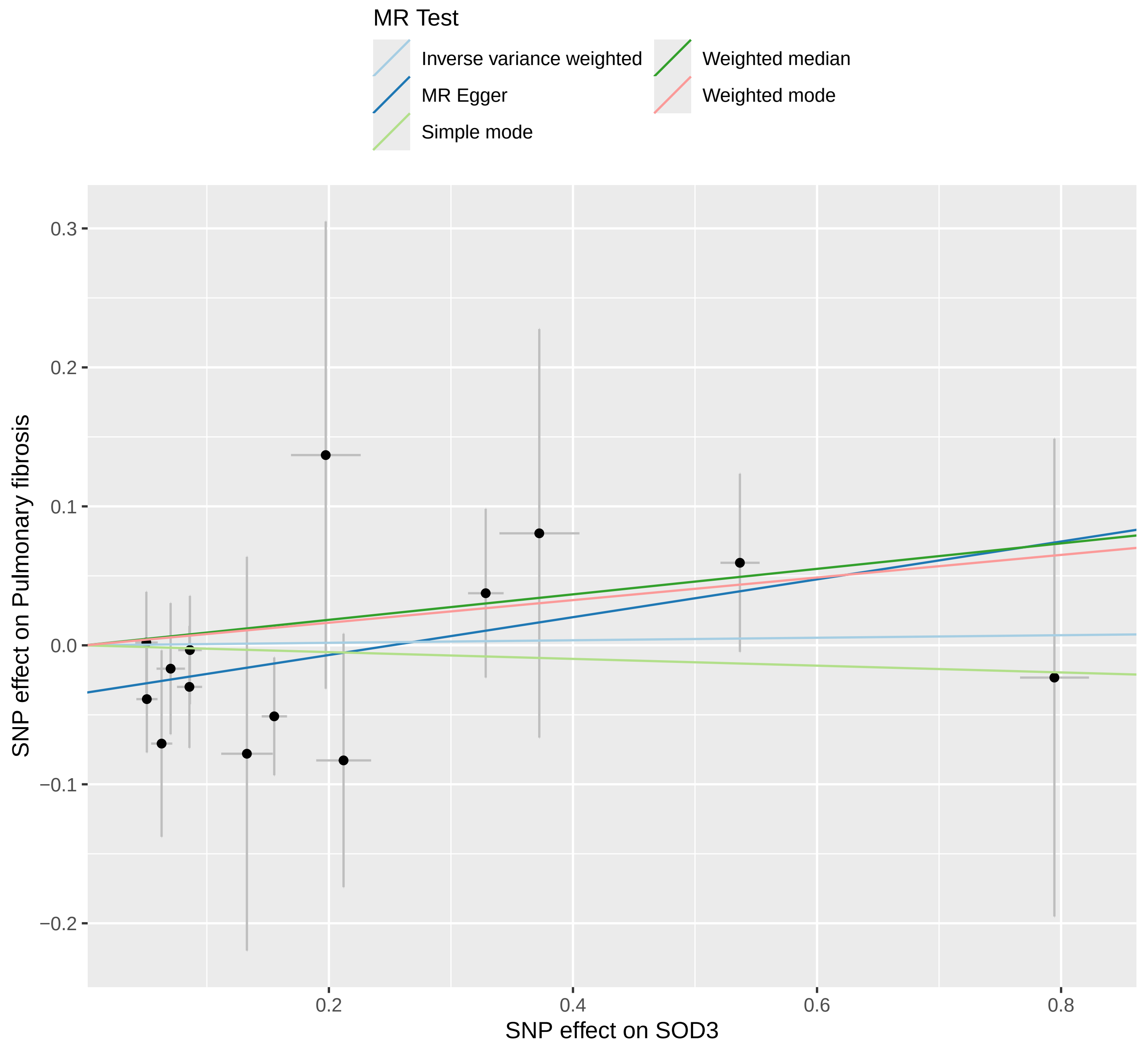

MR Test
Inverse variance weighted
MR Egger
Simple mode
Weighted median
Weighted mode
0.3
0.2
0.1
0.0
−0.1
−0.2
SNP effect on Pulmonary fibrosis
0.2
0.4
0.6
0.8
SNP effect on SOD3

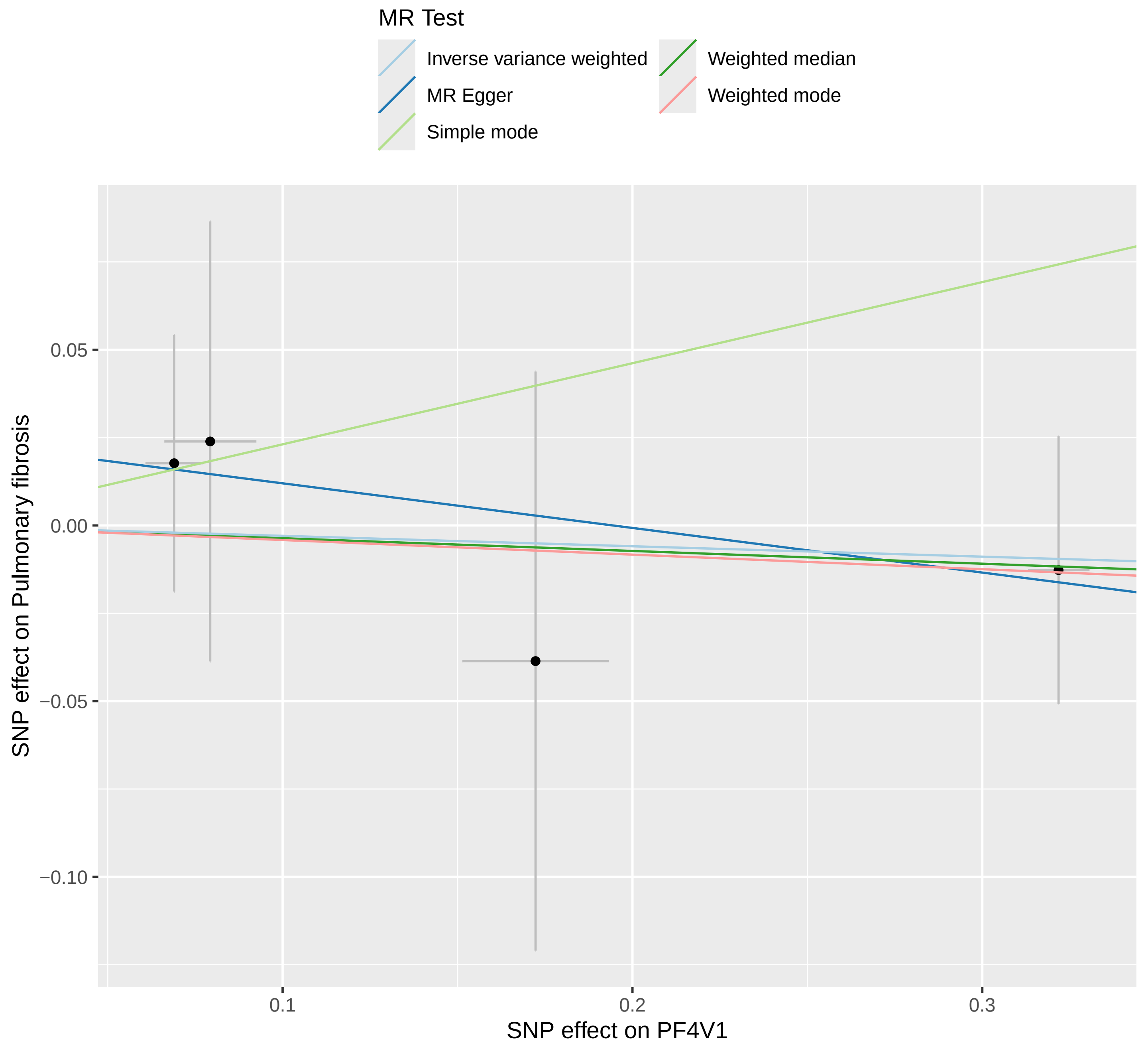

MR Test
Inverse variance weighted
MR Egger
Simple mode
Weighted median
Weighted mode
0.05
0.00
−0.05
−0.10
0.1
0.2
0.3
SNP effect on Pulmonary fibrosis
SNP effect on PF4V1

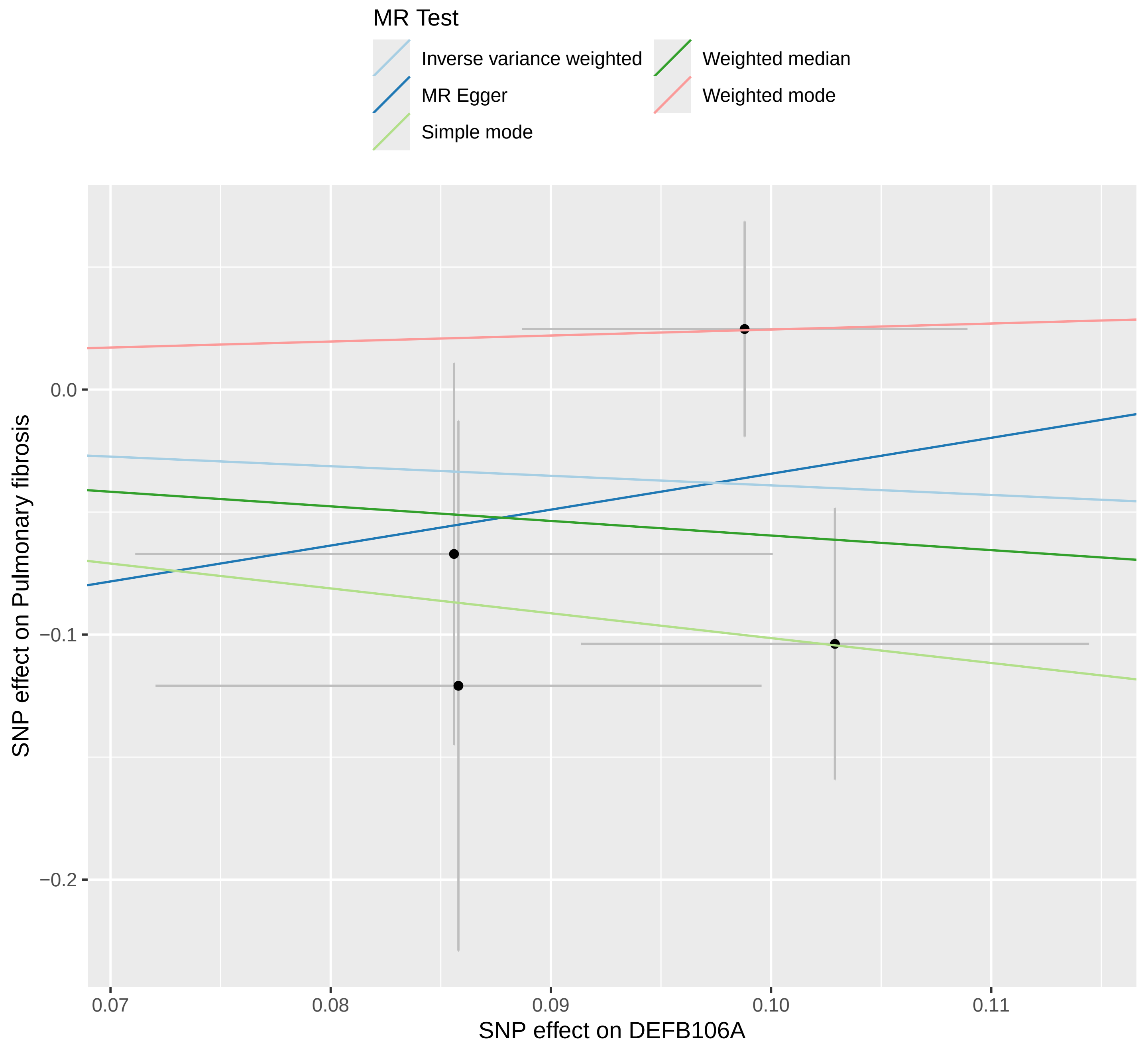
MR Test
Inverse variance weighted
MR Egger
Simple mode
Weighted median
Weighted mode
0.0
−0.1
−0.2
SNP effect on Pulmonary fibrosis
0.07
0.08
0.09
0.10
0.11
SNP effect on DEFB106A

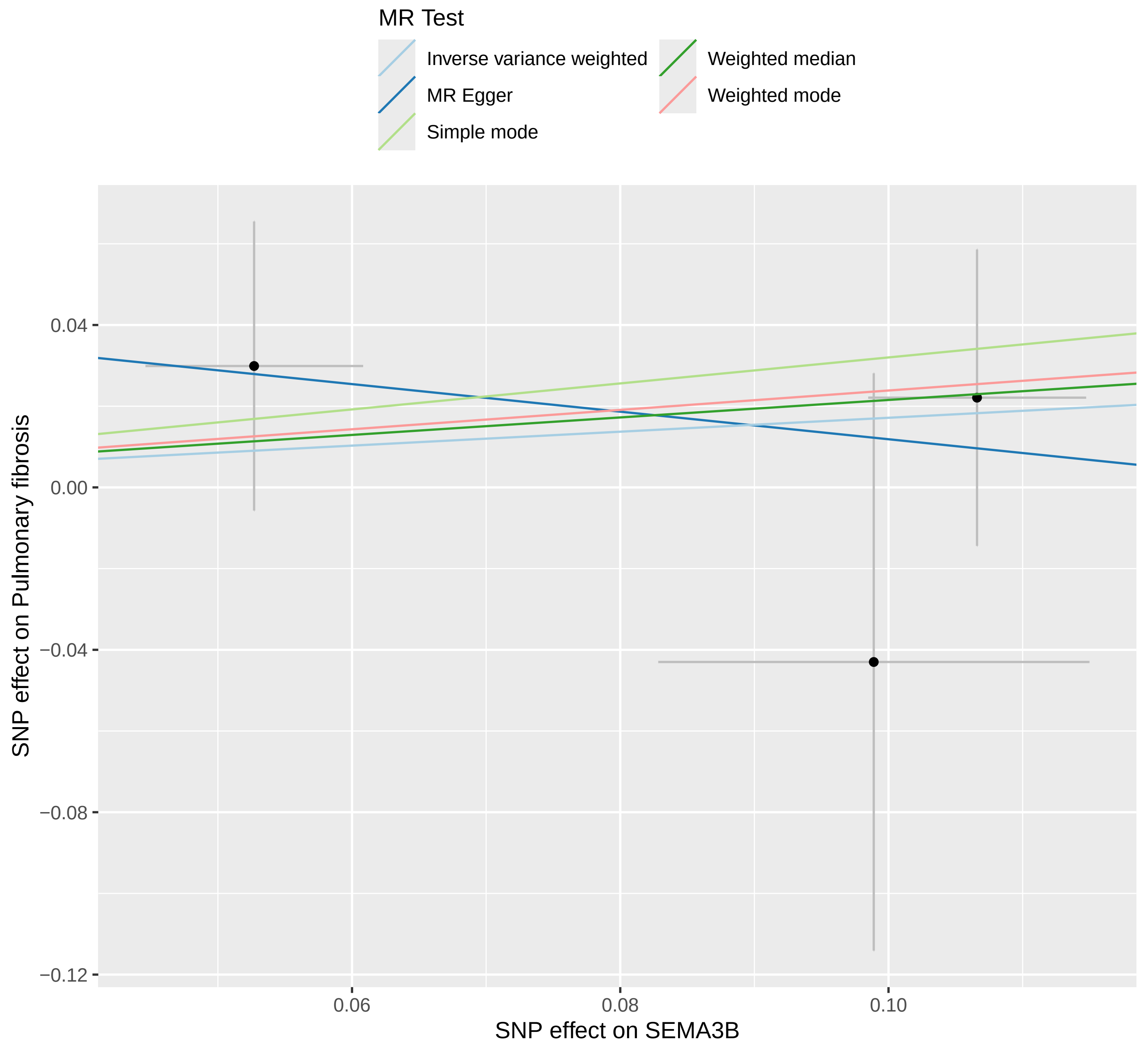
MR Test
Inverse variance weighted
MR Egger
Simple mode
Weighted median
Weighted mode
0.04
0.00
−0.04
−0.08
−0.12
SNP effect on Pulmonary fibrosis
0.06
0.08
0.10
SNP effect on SEMA3B

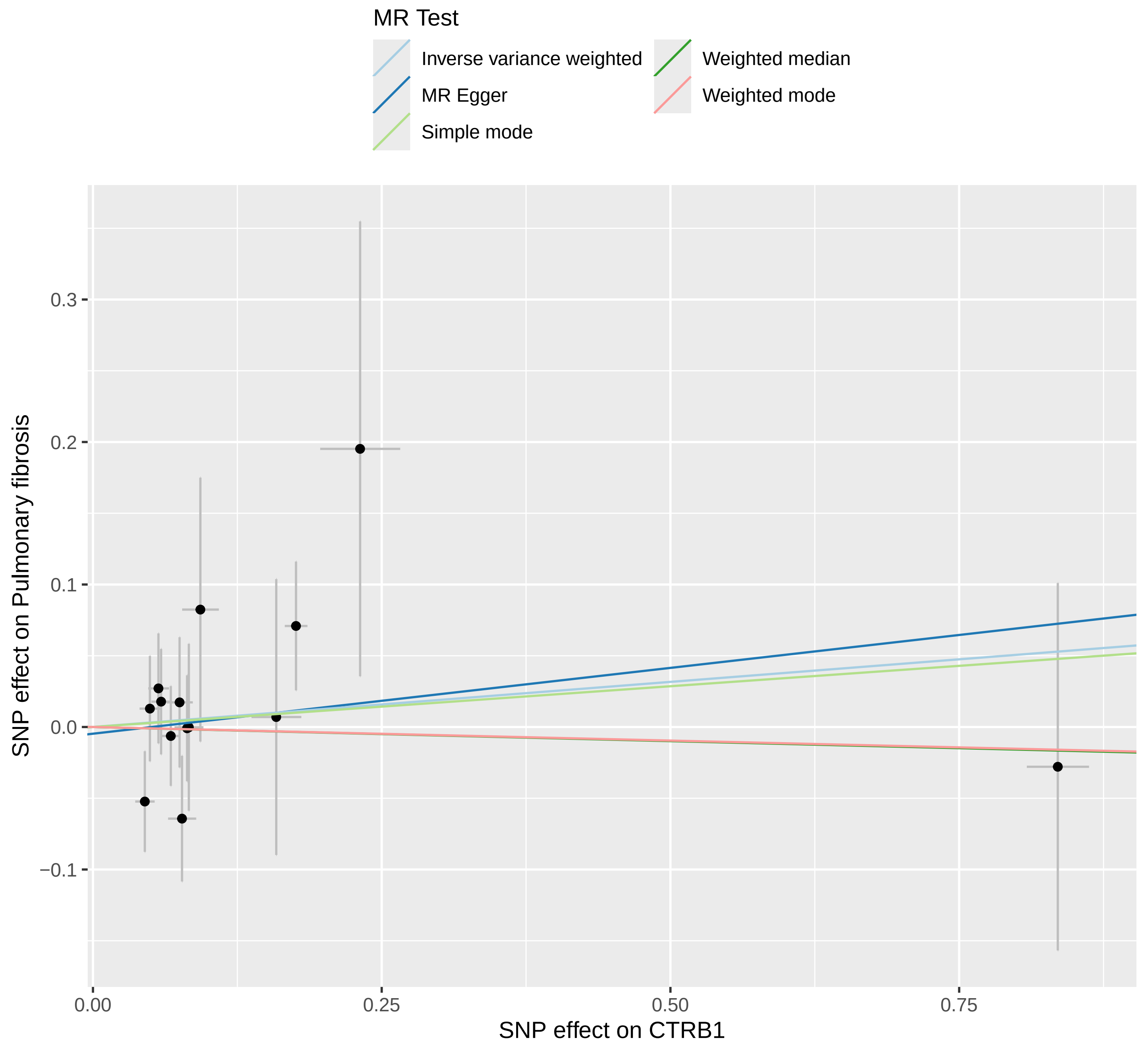

MR Test
Inverse variance weighted
MR Egger
Simple mode
Weighted median
Weighted mode
SNP effect on Pulmonary fibrosis
0.3
0.2
0.1
0.0
−0.1
0.00
0.25
0.50
0.75
SNP effect on CTRB1

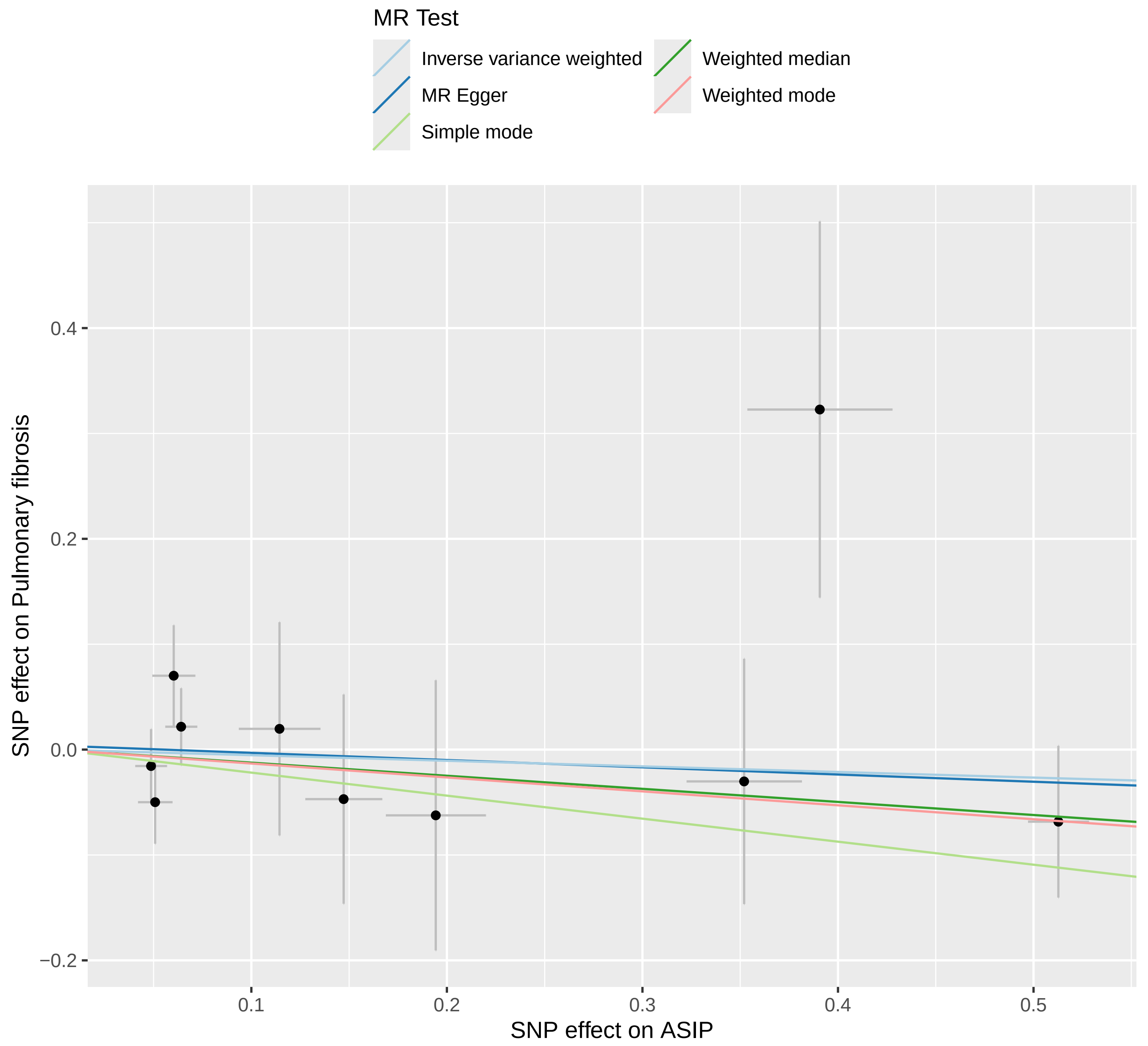
MR Test
Inverse variance weighted
MR Egger
Simple mode
Weighted median
Weighted mode
0.4
0.2
0.0
−0.2
0.1
0.2
0.3
0.4
0.5
SNP effect on Pulmonary fibrosis
SNP effect on ASIP

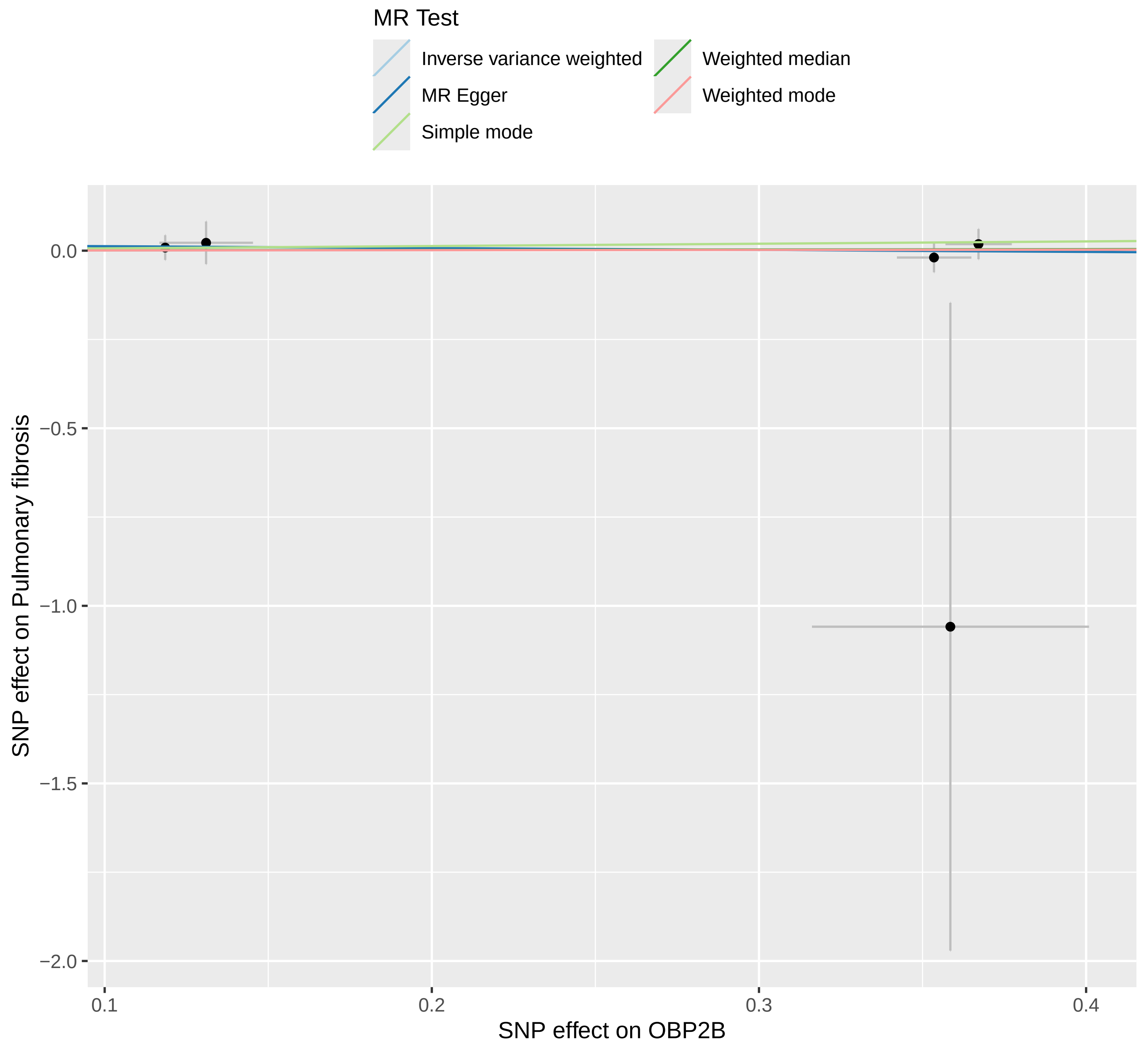

MR Test
Inverse variance weighted
MR Egger
Simple mode
Weighted median
Weighted mode
0.0
−0.5
−1.0
−1.5
−2.0
0.1
0.2
0.3
0.4
SNP effect on Pulmonary fibrosis
SNP effect on OBP2B

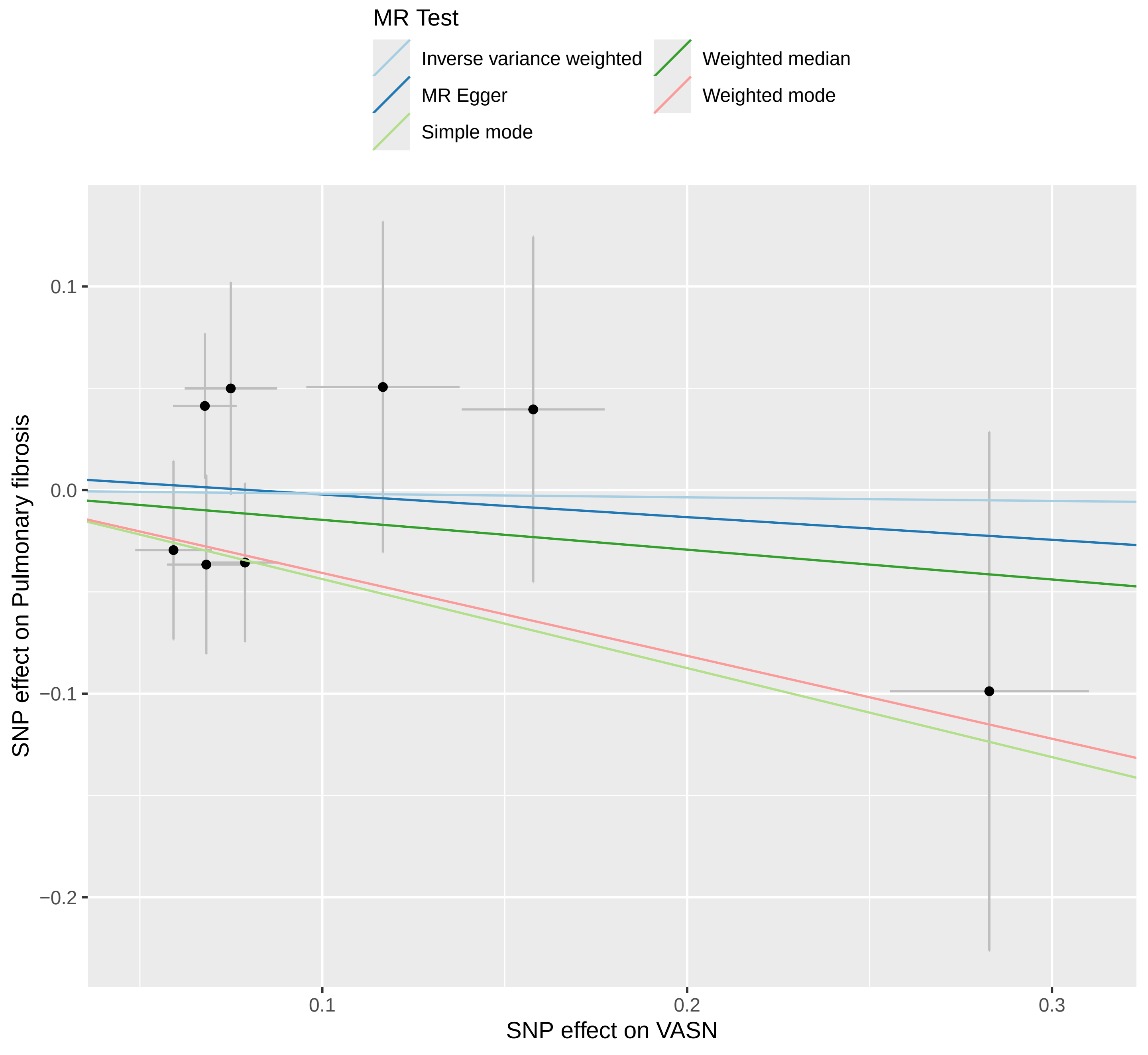
MR Test
Inverse variance weighted
MR Egger
Simple mode
Weighted median
Weighted mode
0.1
0.0
−0.1
−0.2
SNP effect on Pulmonary fibrosis
0.1
0.2
0.3
SNP effect on VASN

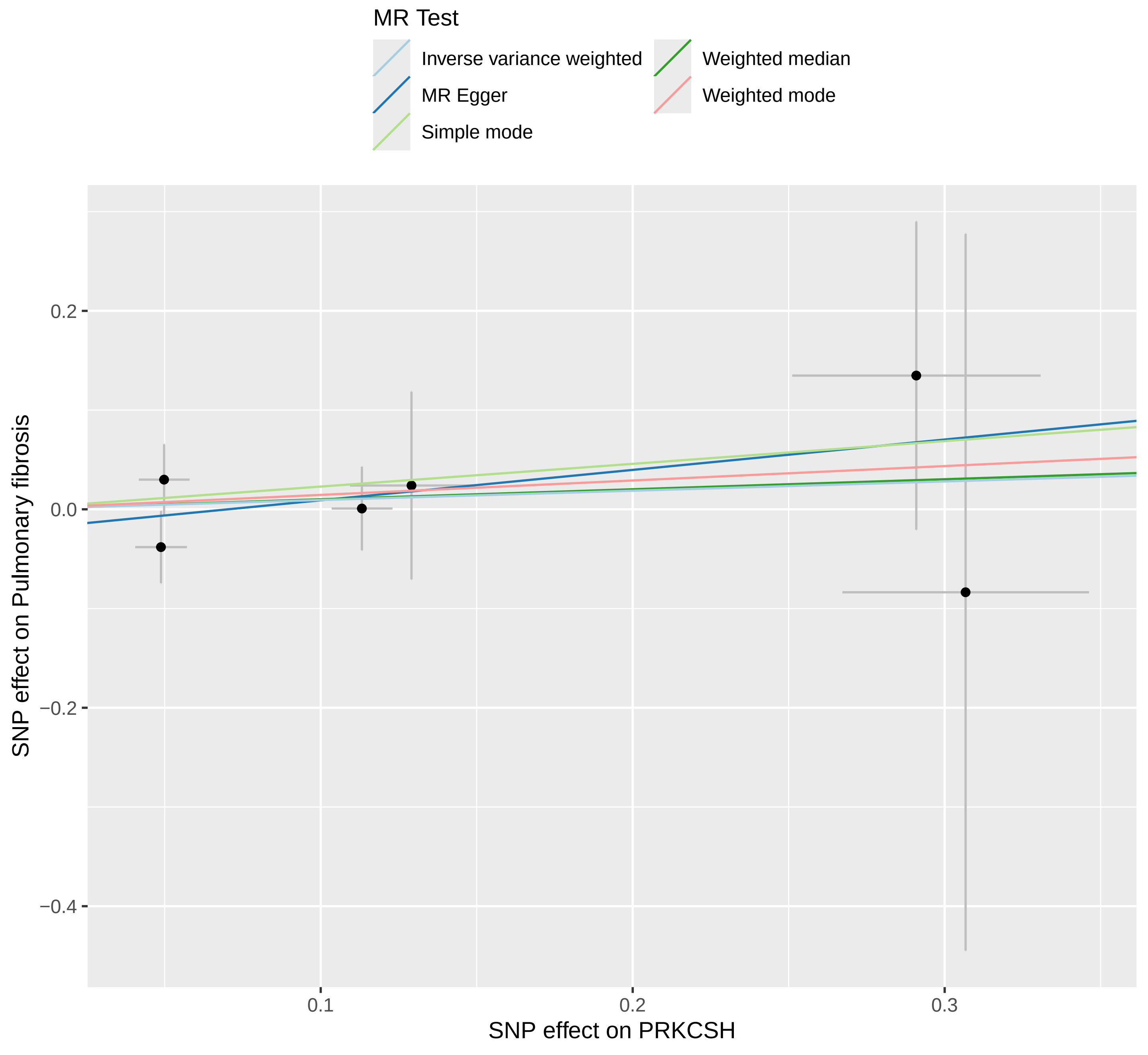

MR Test
Inverse variance weighted
MR Egger
Simple mode
Weighted median
Weighted mode
0.2
0.0
−0.2
−0.4
SNP effect on Pulmonary fibrosis
0.1
0.2
0.3
SNP effect on PRKCSH

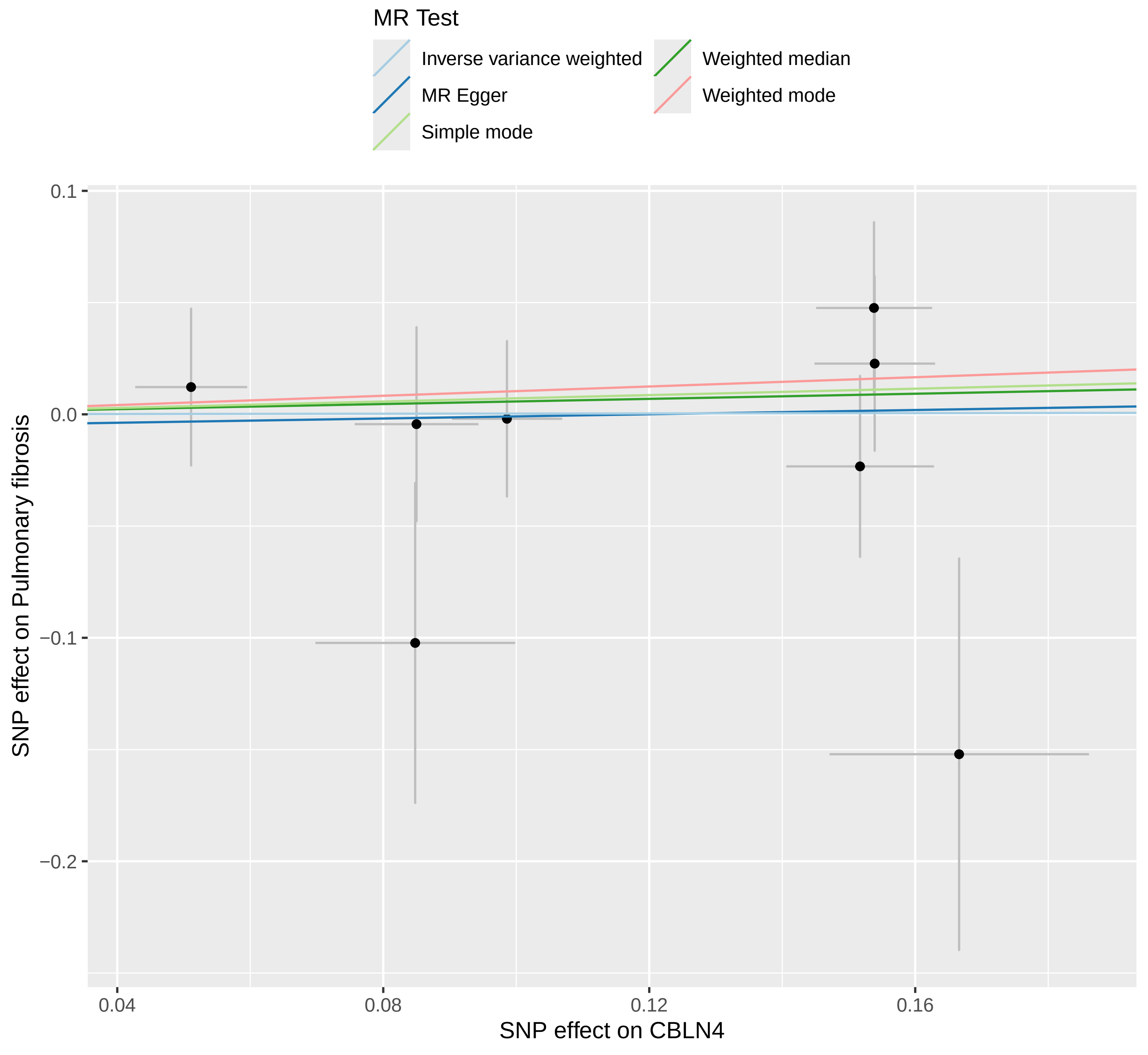

MR Test
Inverse variance weighted
MR Egger
Simple mode
Weighted median
Weighted mode
0.1
0.0
−0.1
−0.2
SNP effect on Pulmonary fibrosis
0.04
0.08
0.12
0.16
SNP effect on CBLN4

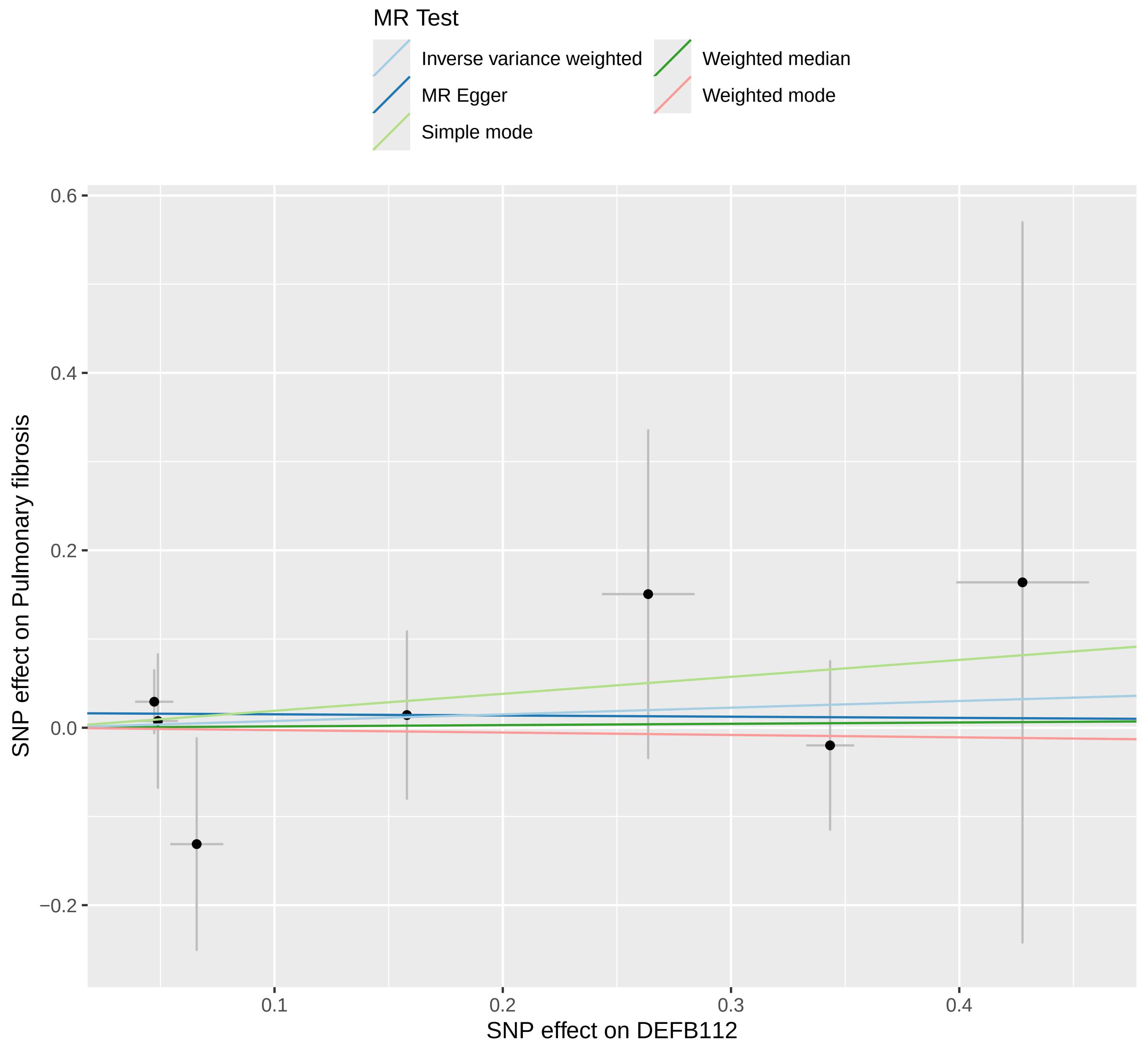
MR Test
Inverse variance weighted
MR Egger
Simple mode
Weighted median
Weighted mode
0.6
0.4
0.2
0.0
−0.2
0.1
0.2
0.3
0.4
SNP effect on Pulmonary fibrosis
SNP effect on DEFB112

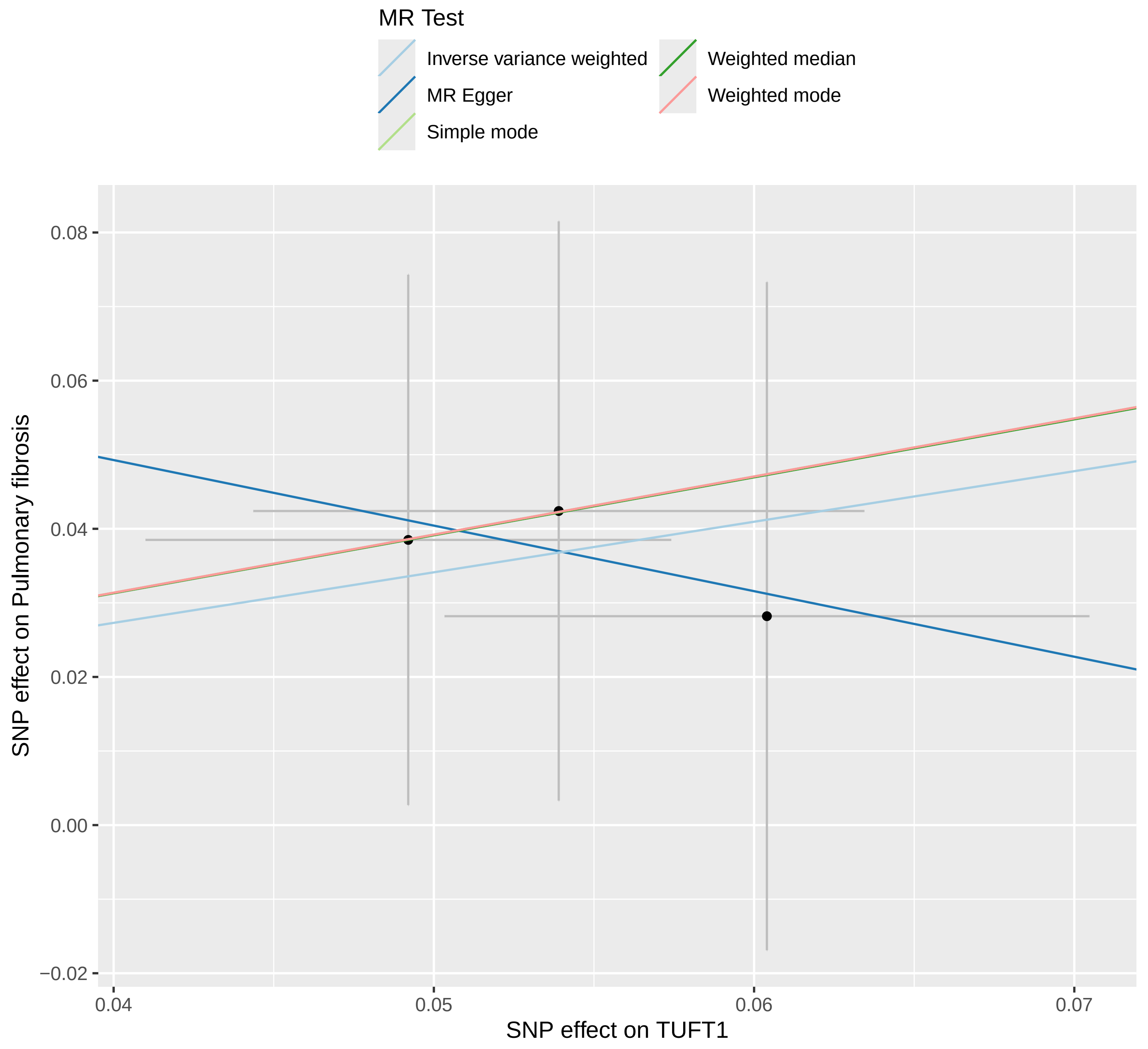

MR Test
Inverse variance weighted
MR Egger
Simple mode
Weighted median
Weighted mode
0.08
0.06
0.04
0.02
0.00
−0.02
0.04
0.05
0.06
0.07
SNP effect on Pulmonary fibrosis
SNP effect on TUFT1

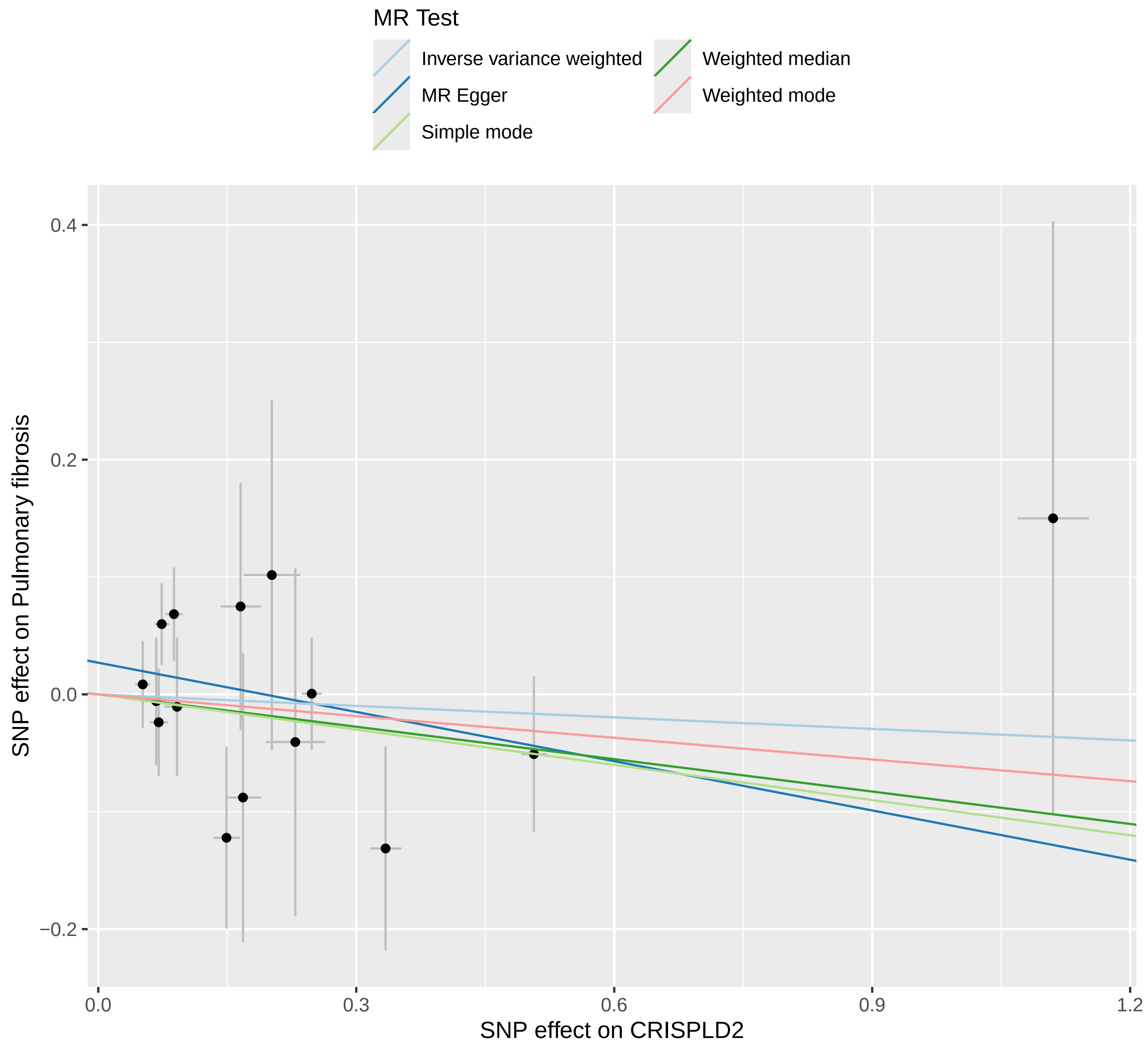

MR Test
Inverse variance weighted
MR Egger
Simple mode
Weighted median
Weighted mode
0.4
0.2
0.0
−0.2
0.0
0.3
0.6
0.9
1.2
SNP effect on CRISPLD2
SNP effect on Pulmonary fibrosis

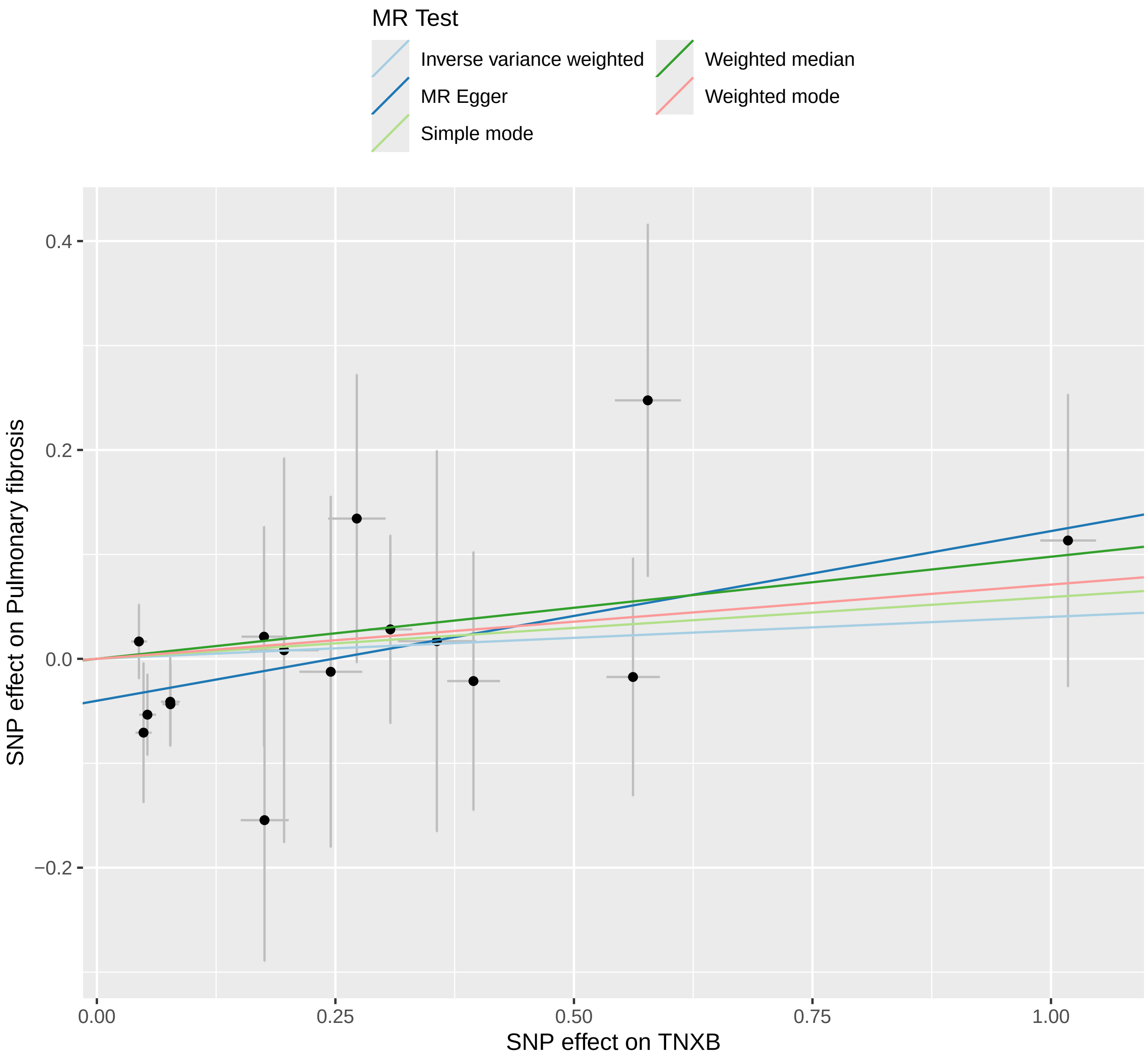

MR Test
Inverse variance weighted
MR Egger
Simple mode
Weighted median
Weighted mode
SNP effect on Pulmonary fibrosis
0.4
0.2
0.0
−0.2
0.00
0.25
0.50
0.75
1.00
SNP effect on TNXB

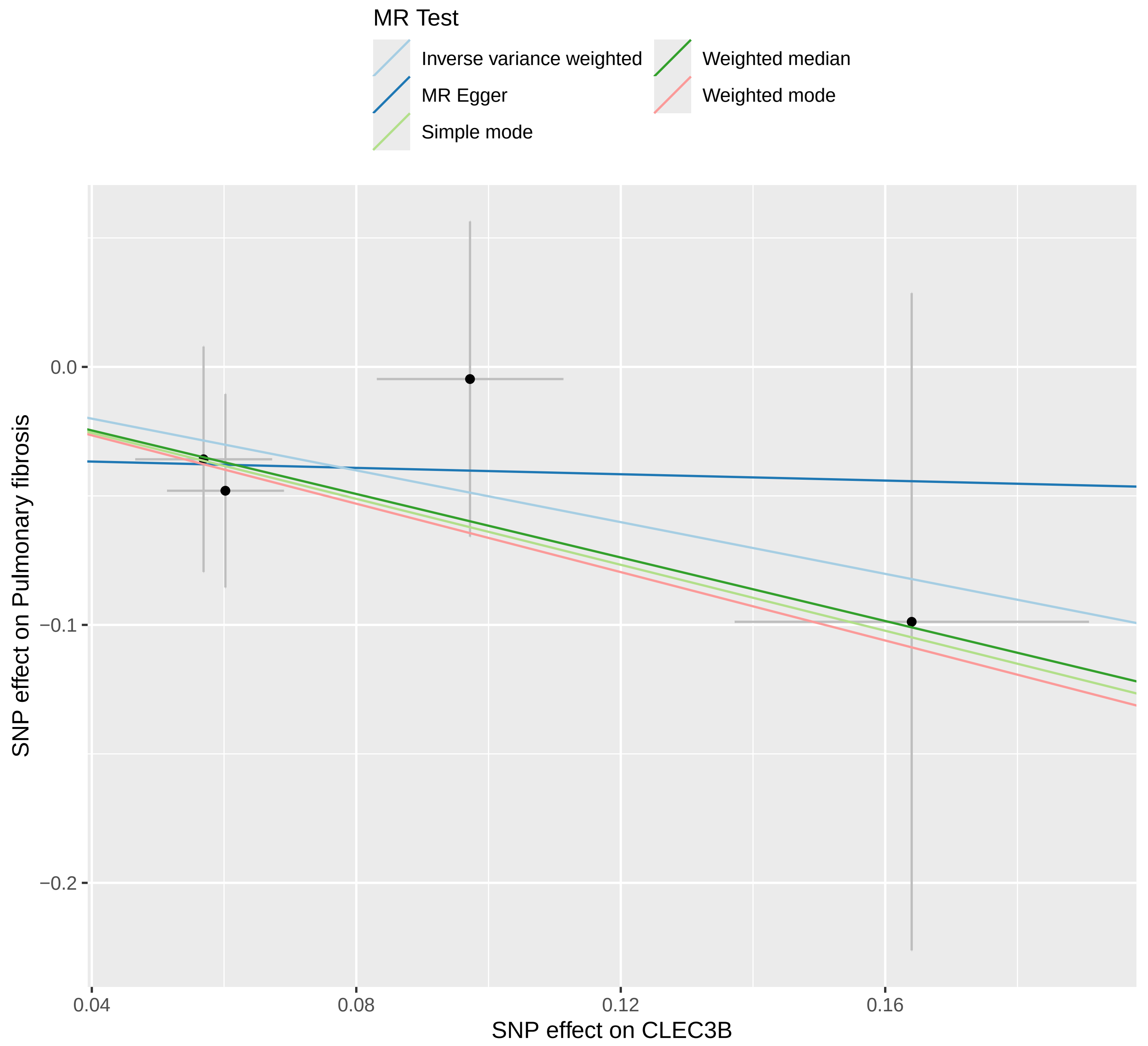

MR Test
Inverse variance weighted
MR Egger
Simple mode
Weighted median
Weighted mode
SNP effect on Pulmonary fibrosis
0.0
−0.1
−0.2
0.04
0.08
0.12
0.16
SNP effect on CLEC3B

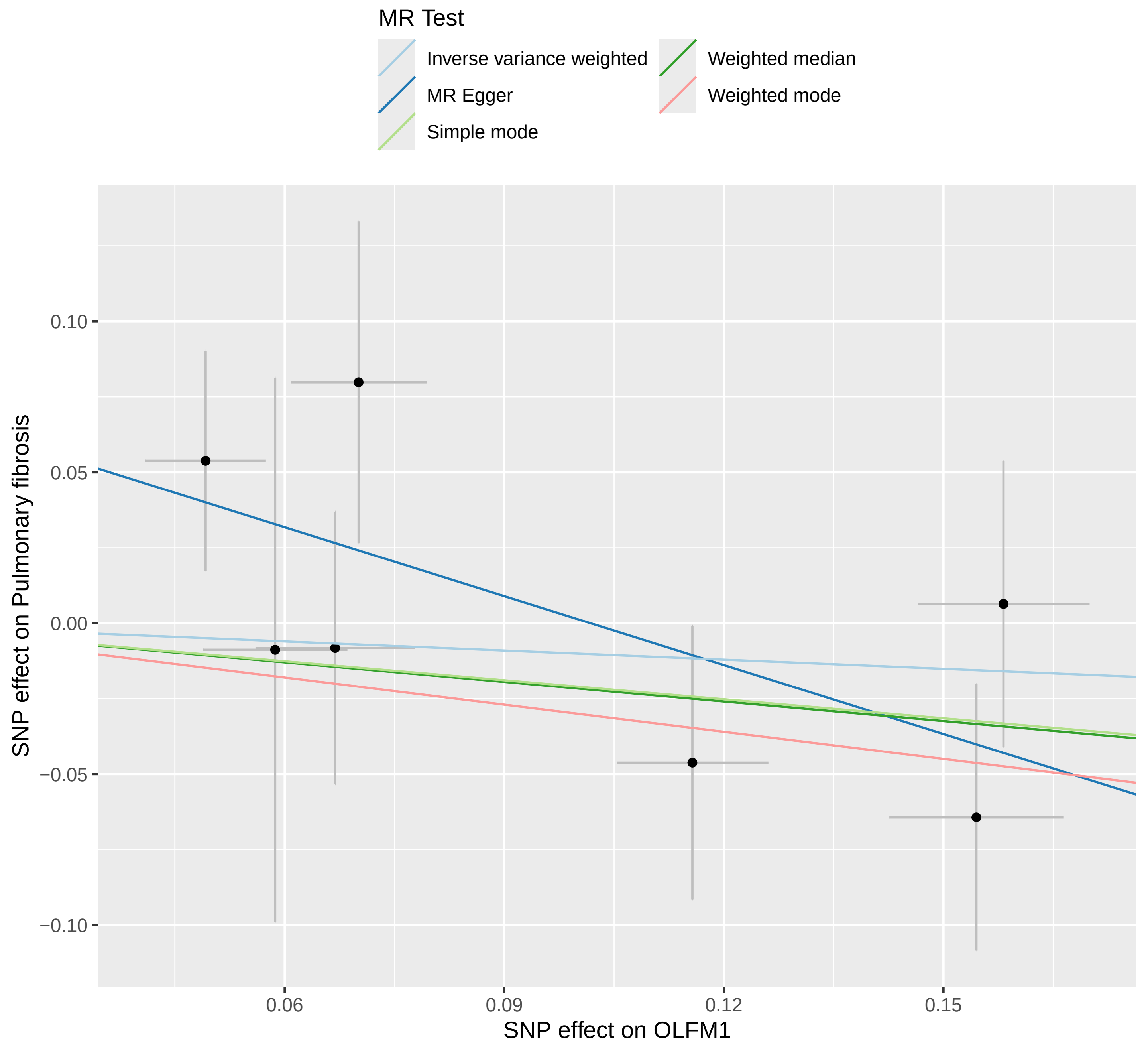

MR Test
Inverse variance weighted
MR Egger
Simple mode
Weighted median
Weighted mode
0.10
0.05
0.00
−0.05
−0.10
SNP effect on Pulmonary fibrosis
0.06
0.09
0.12
0.15
SNP effect on OLFM1

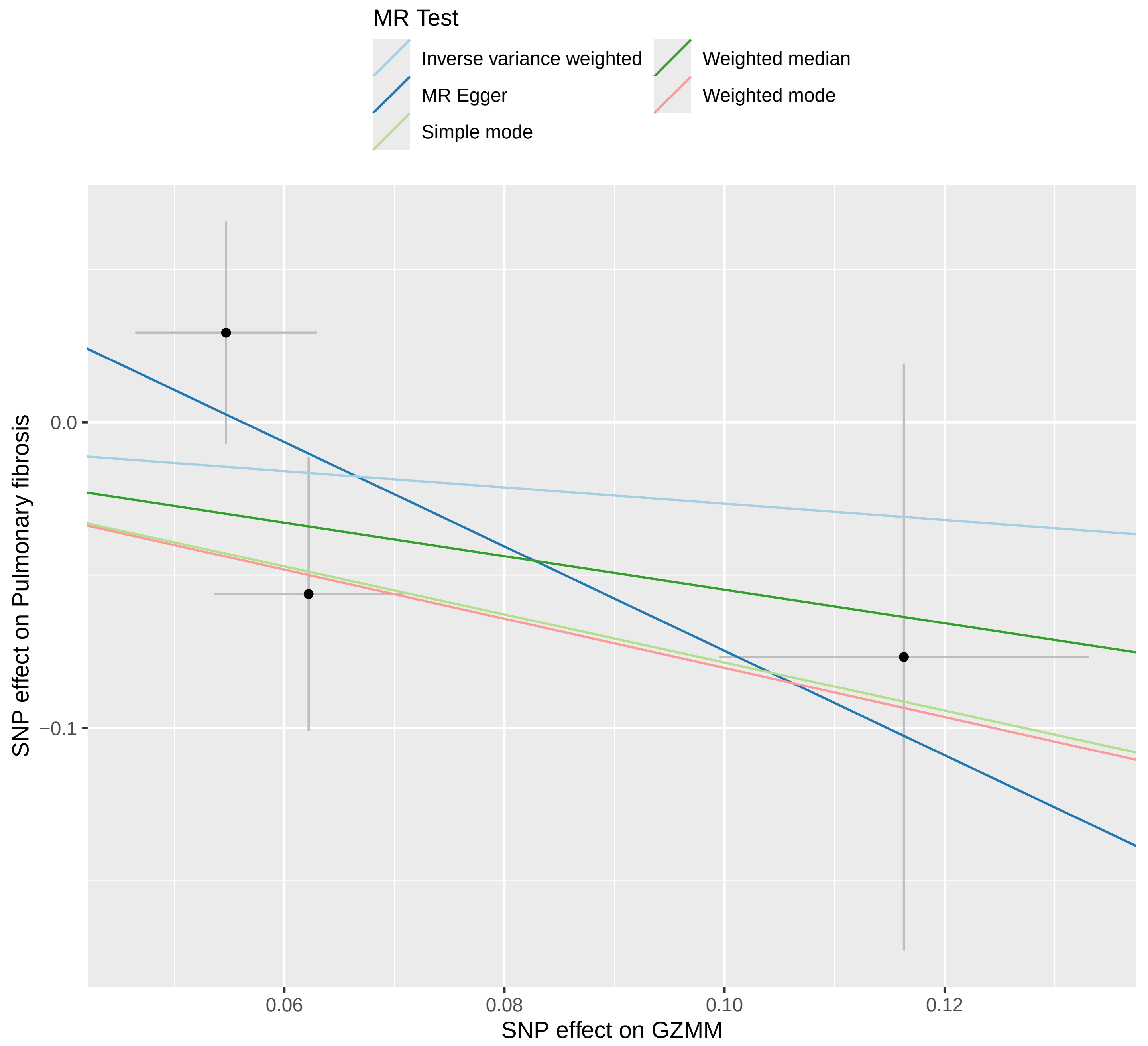

MR Test
Inverse variance weighted
MR Egger
Simple mode
Weighted median
Weighted mode
0.0
−0.1
SNP effect on Pulmonary fibrosis
0.06
0.08
0.10
0.12
SNP effect on GZMM

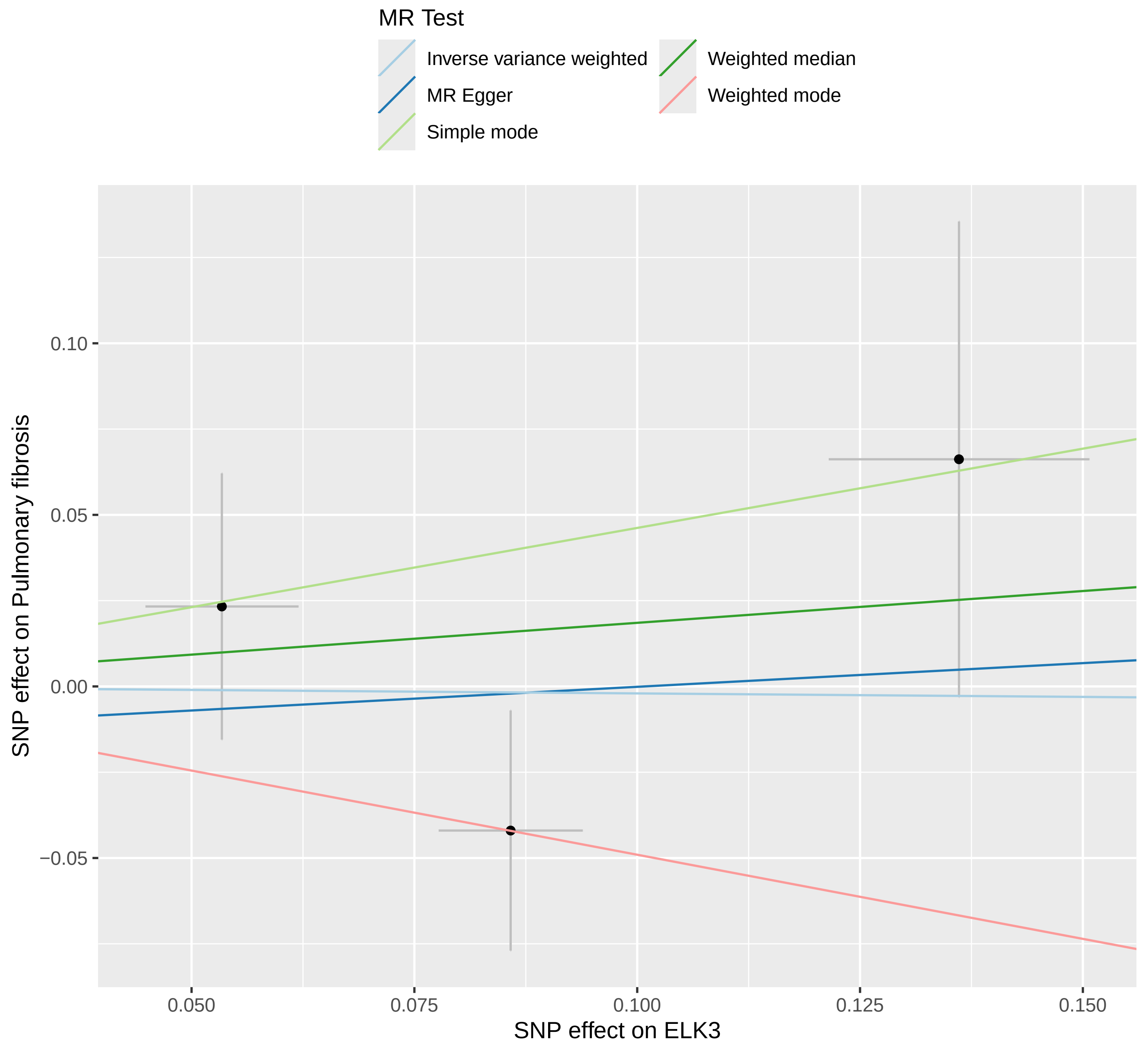

MR Test
Inverse variance weighted
MR Egger
Simple mode
Weighted median
Weighted mode
0.10
0.05
0.00
−0.05
SNP effect on Pulmonary fibrosis
0.050
0.075
0.100
0.125
0.150
SNP effect on ELK3

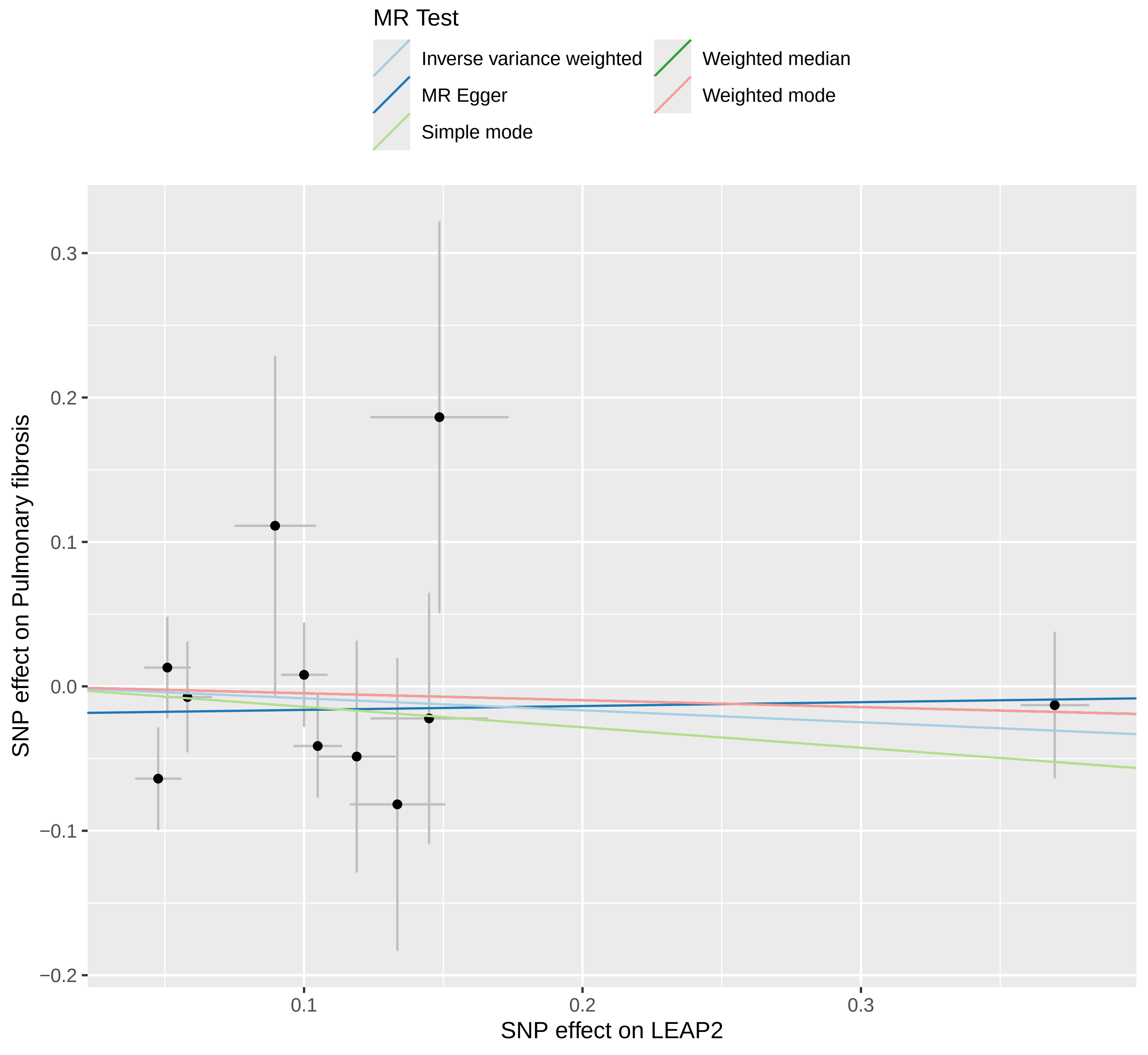

MR Test
Inverse variance weighted
MR Egger
Simple mode
Weighted median
Weighted mode
0.3
0.2
0.1
0.0
−0.1
−0.2
0.1
0.2
0.3
SNP effect on Pulmonary fibrosis
SNP effect on LEAP2

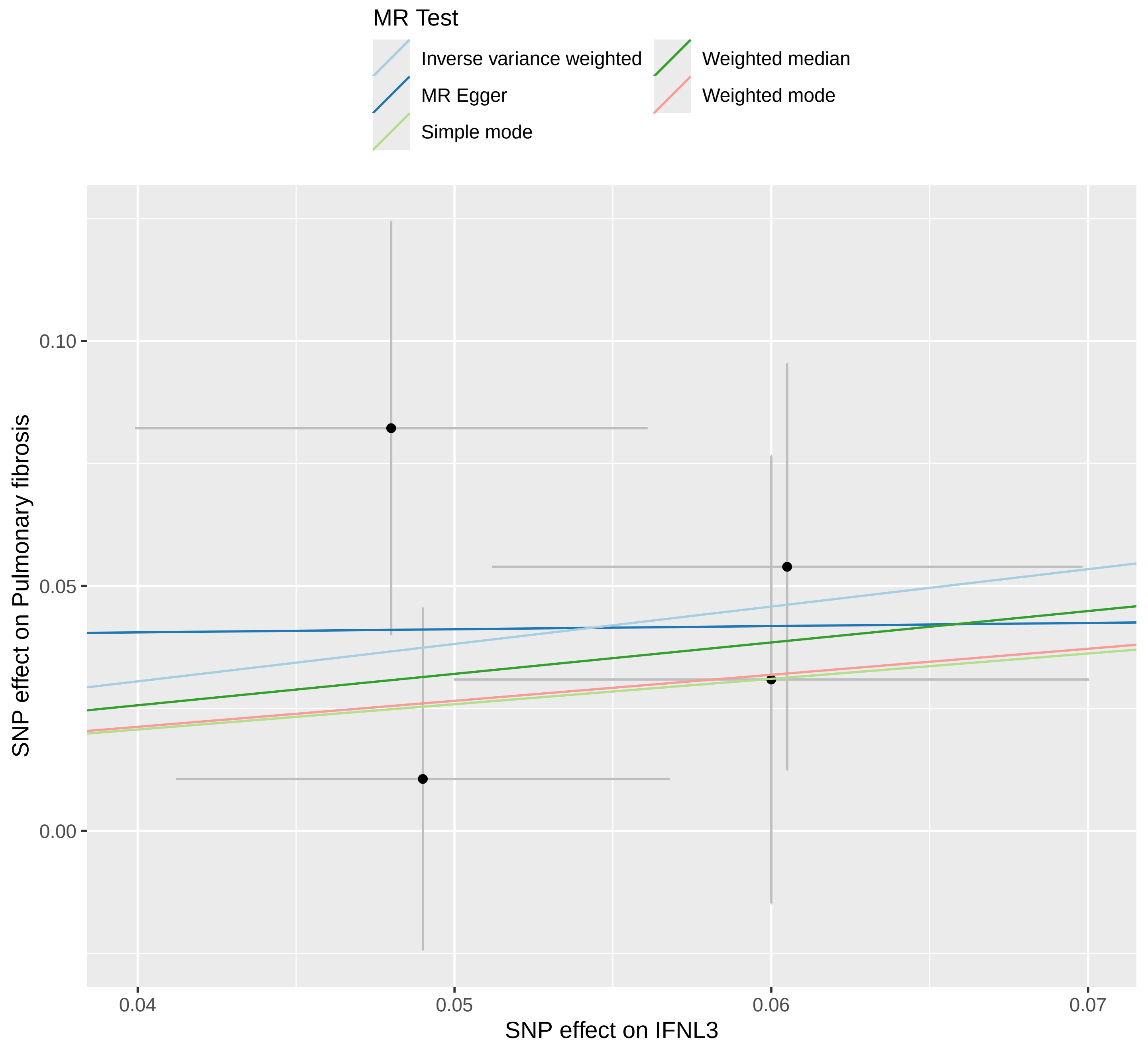

MR Test
Inverse variance weighted
MR Egger
Simple mode
Weighted median
Weighted mode
0.10
0.05
0.00
0.04
0.05
0.06
0.07
SNP effect on Pulmonary fibrosis
SNP effect on IFNL3

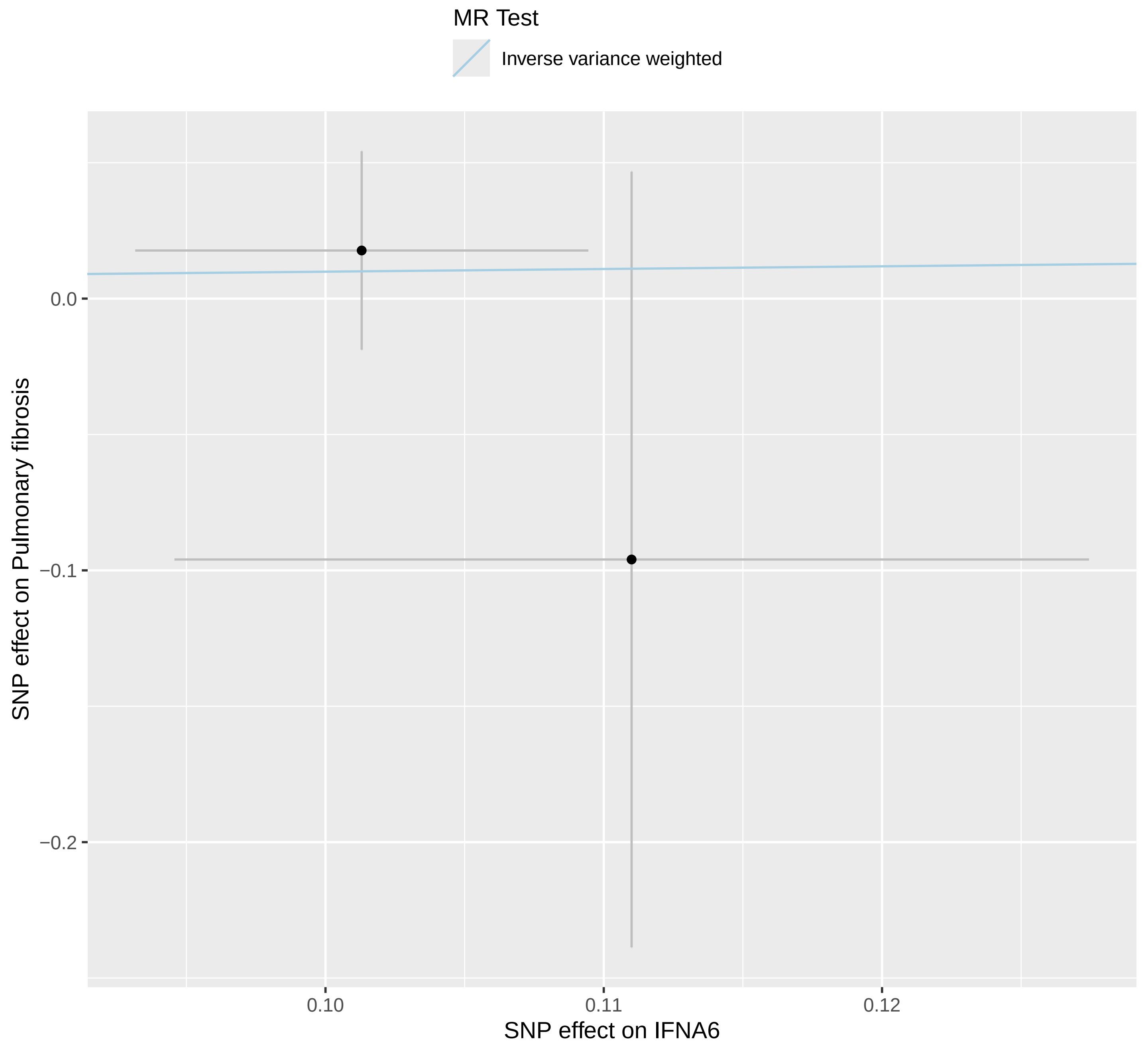

MR Test
Inverse variance weighted
SNP effect on Pulmonary fibrosis
0.0
−0.1
−0.2
0.10
0.11
0.12
SNP effect on IFNA6

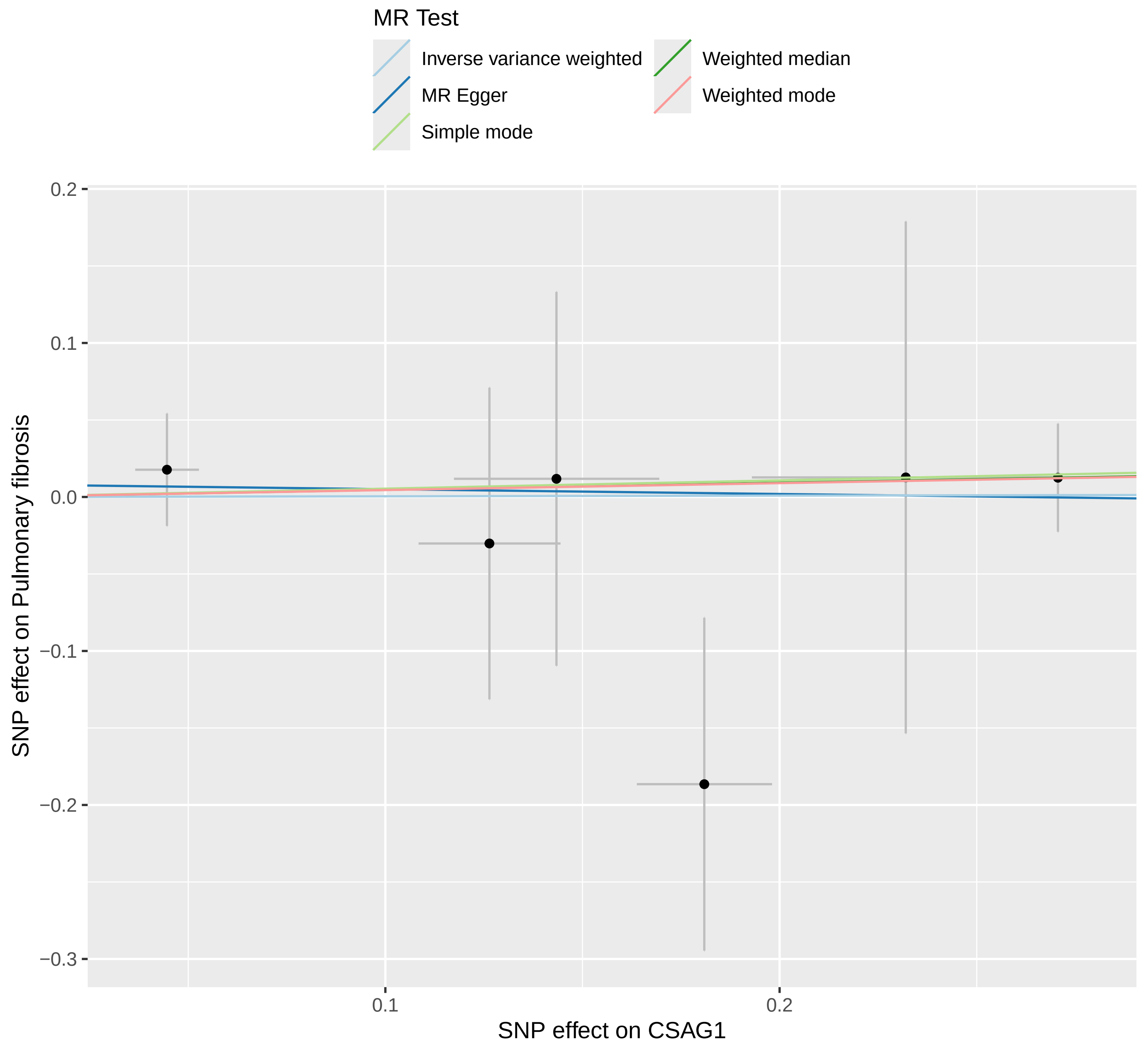

MR Test
Inverse variance weighted
MR Egger
Simple mode
Weighted median
Weighted mode
0.2
0.1
0.0
−0.1
−0.2
−0.3
SNP effect on Pulmonary fibrosis
0.1
0.2
SNP effect on CSAG1

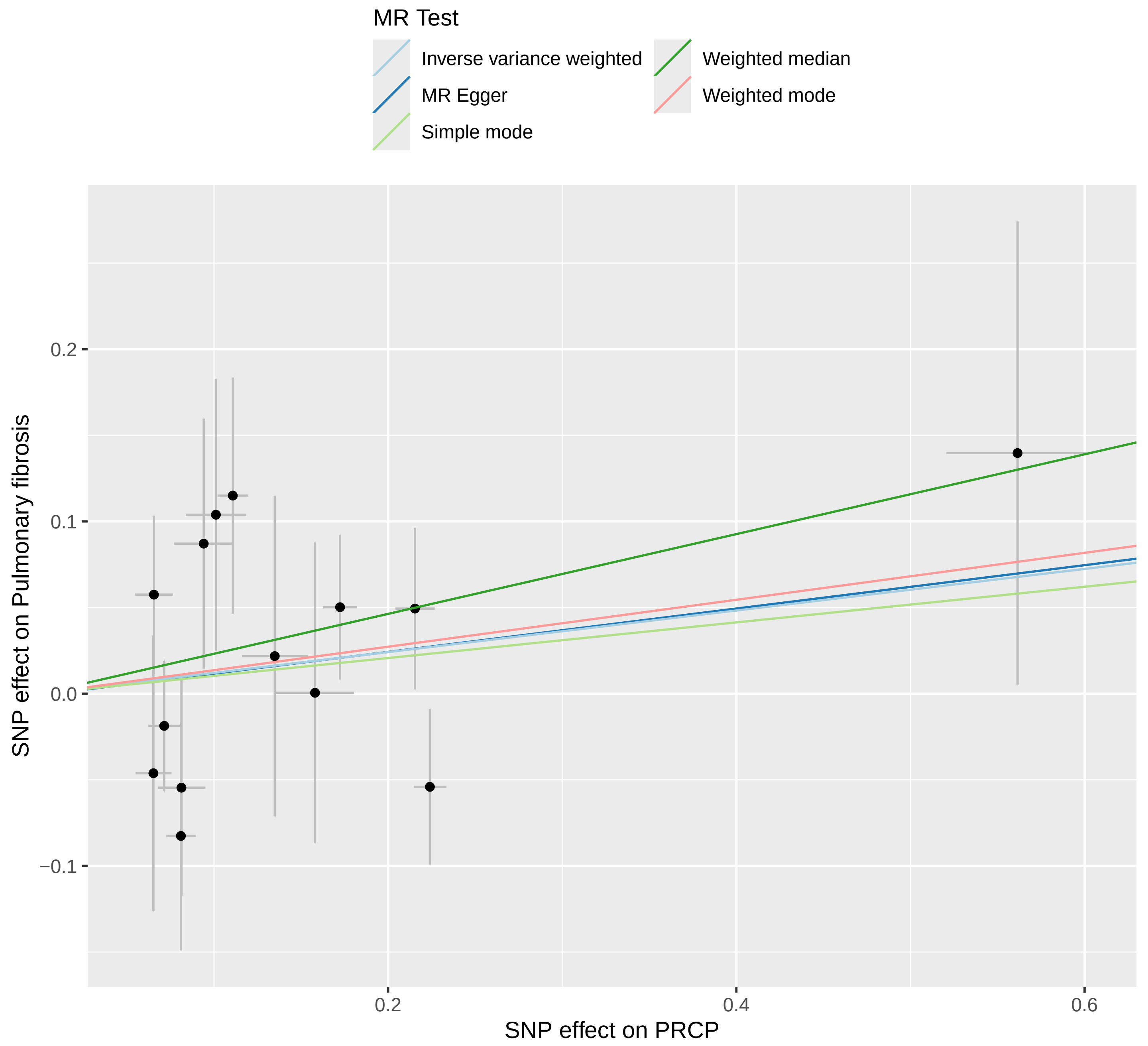

MR Test
Inverse variance weighted
MR Egger
Simple mode
Weighted median
Weighted mode
0.2
0.1
0.0
−0.1
SNP effect on Pulmonary fibrosis
0.2
0.4
0.6
SNP effect on PRCP

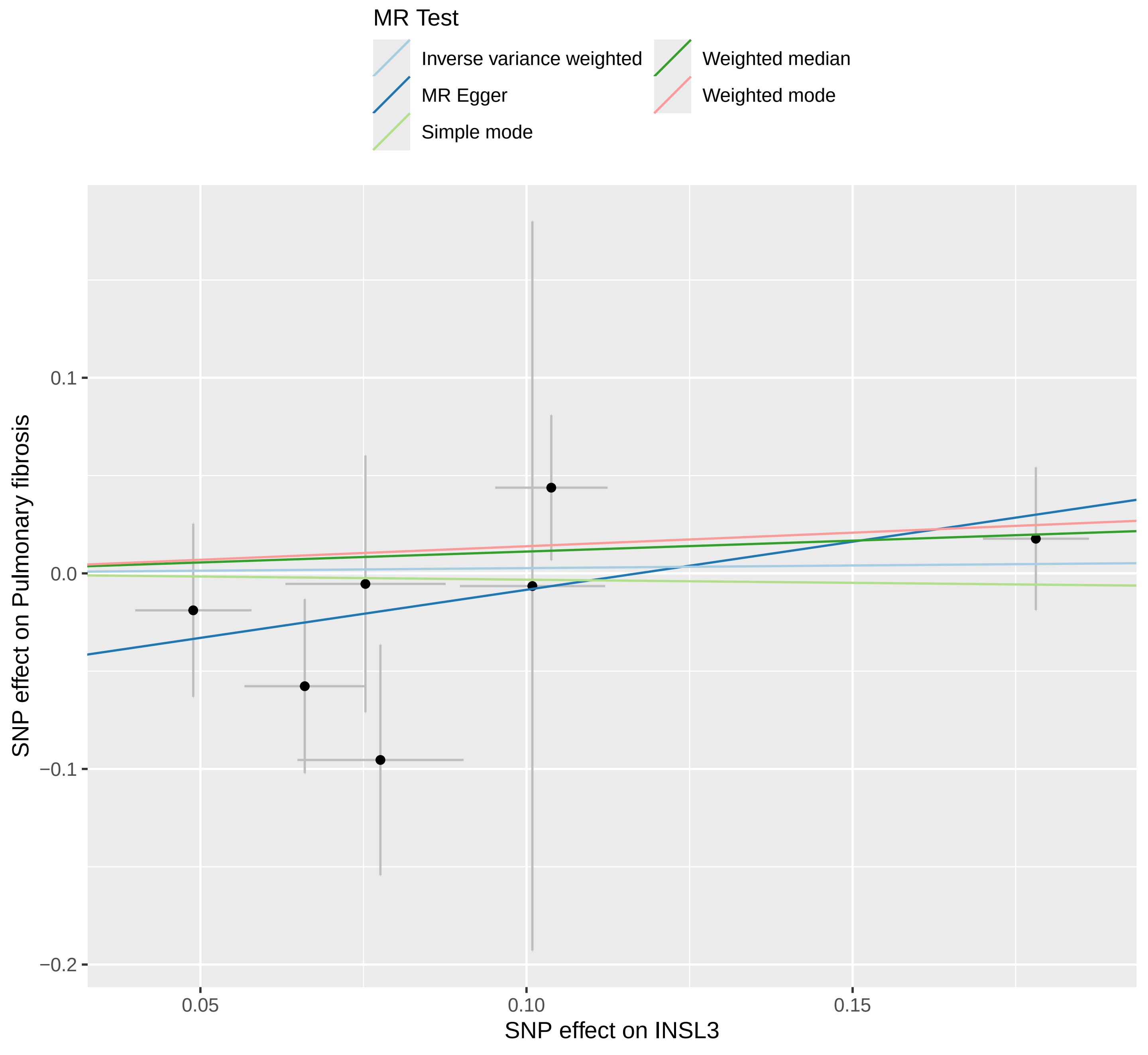

MR Test
Inverse variance weighted
MR Egger
Simple mode
Weighted median
Weighted mode
SNP effect on Pulmonary fibrosis
0.1
0.0
−0.1
−0.2
0.05
0.10
0.15
SNP effect on INSL3

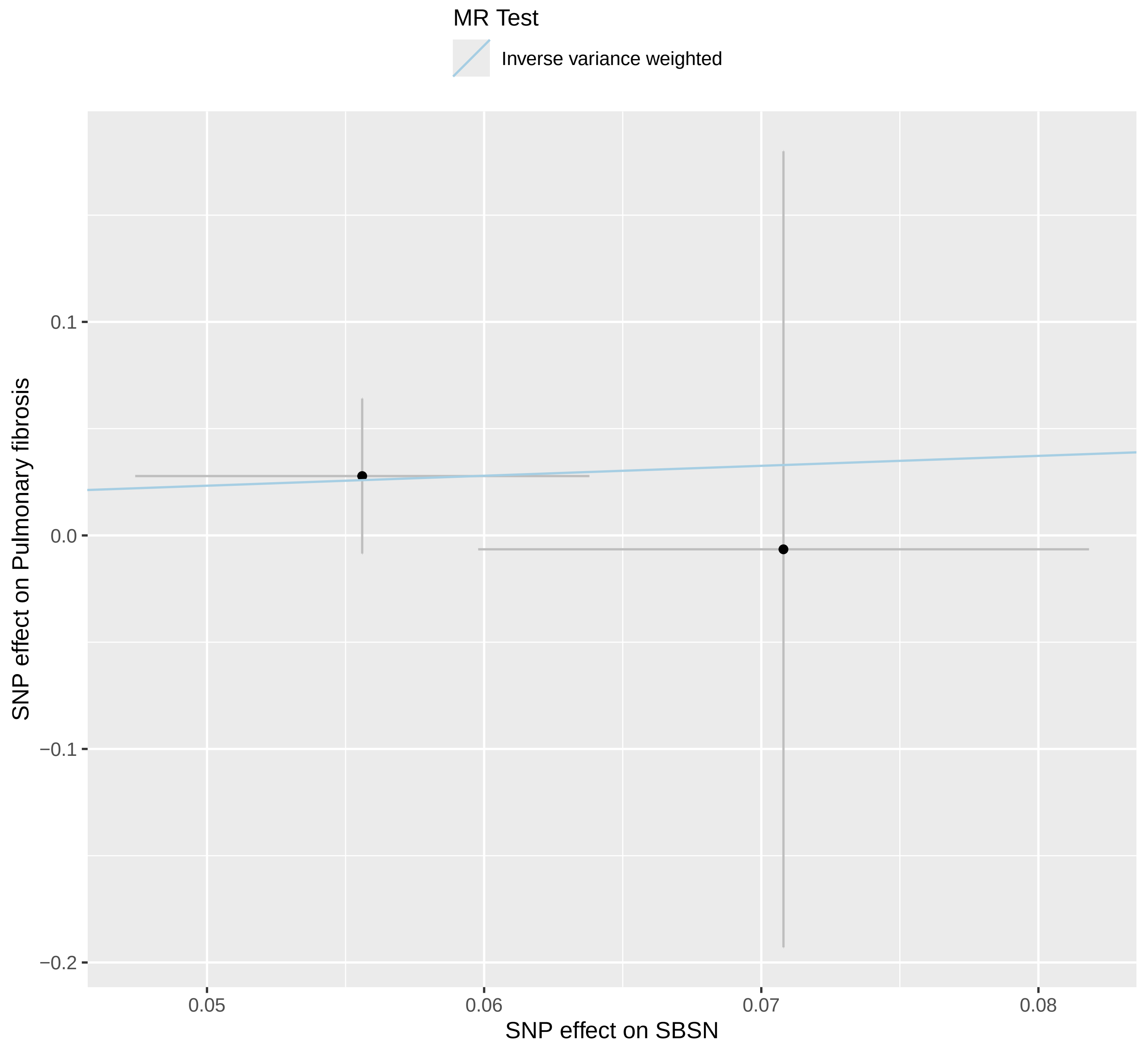
MR Test
Inverse variance weighted
0.1
0.0
−0.1
−0.2
SNP effect on Pulmonary fibrosis
0.05
0.06
0.07
0.08
SNP effect on SBSN

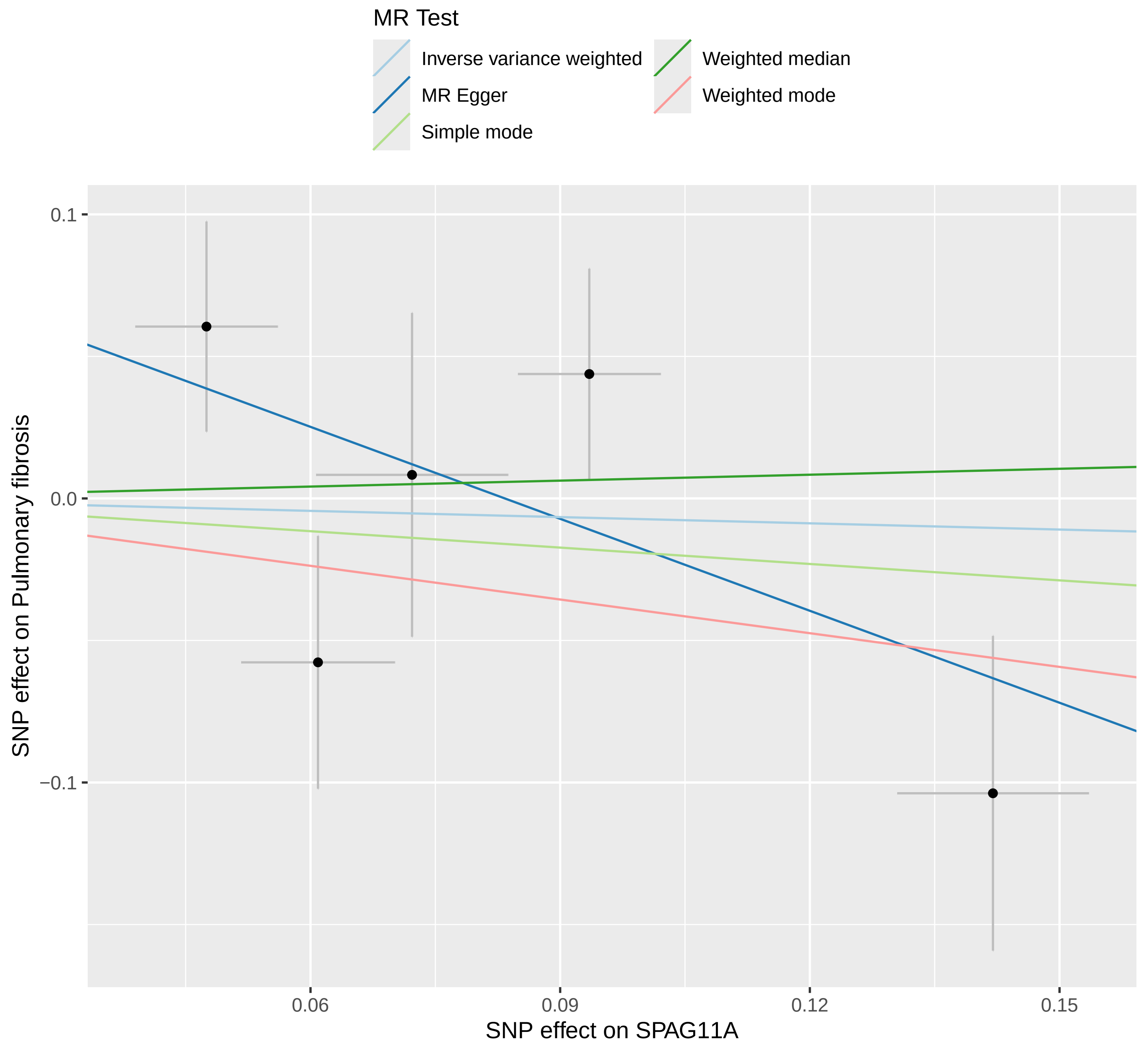
MR Test
Inverse variance weighted
MR Egger
Simple mode
Weighted median
Weighted mode
0.1
0.0
−0.1
0.06
0.09
0.12
0.15
SNP effect on Pulmonary fibrosis
SNP effect on SPAG11A

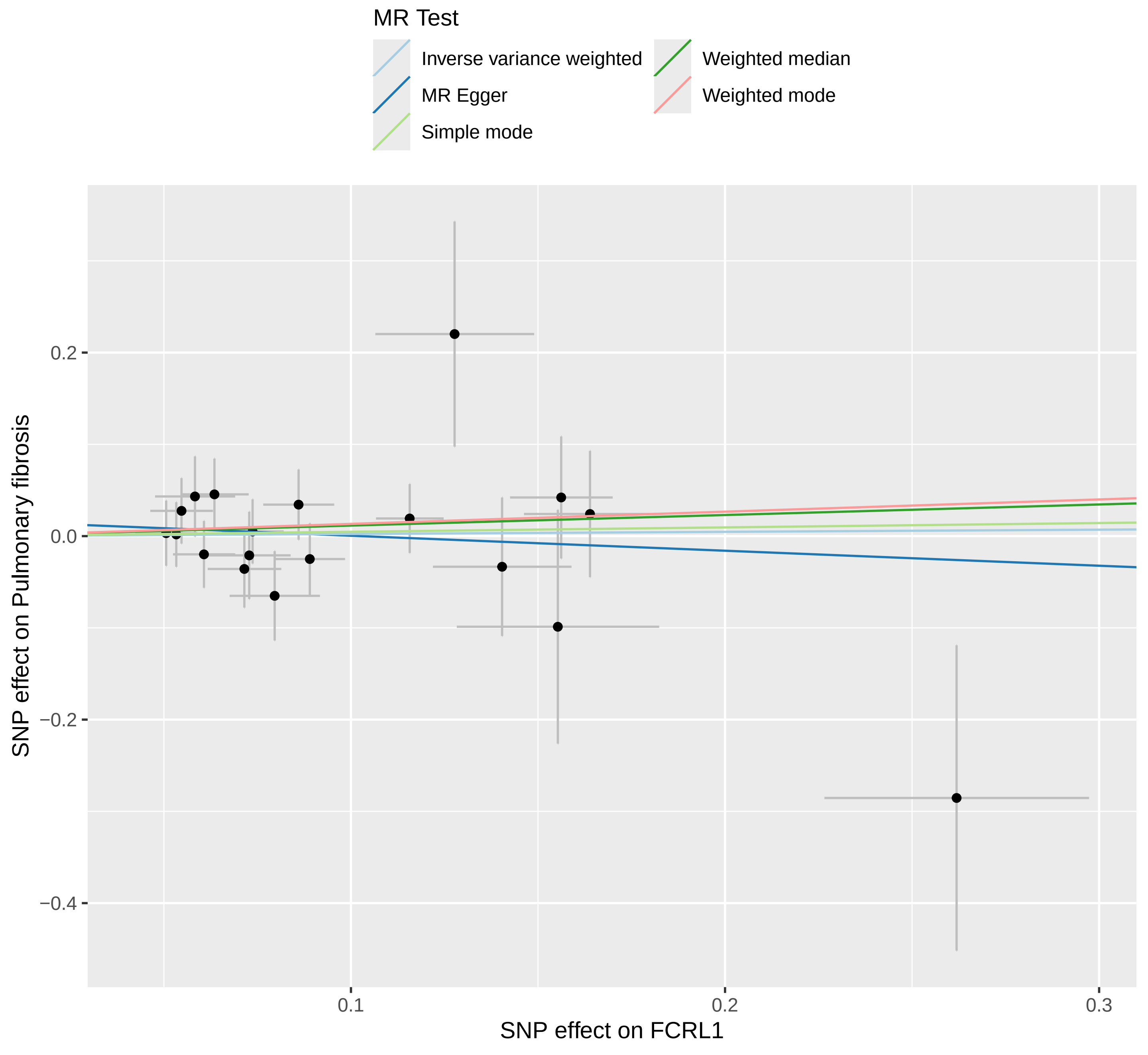
MR Test
Inverse variance weighted
MR Egger
Simple mode
Weighted median
Weighted mode
0.2
0.0
−0.2
−0.4
SNP effect on Pulmonary fibrosis
0.1
0.2
0.3
SNP effect on FCRL1

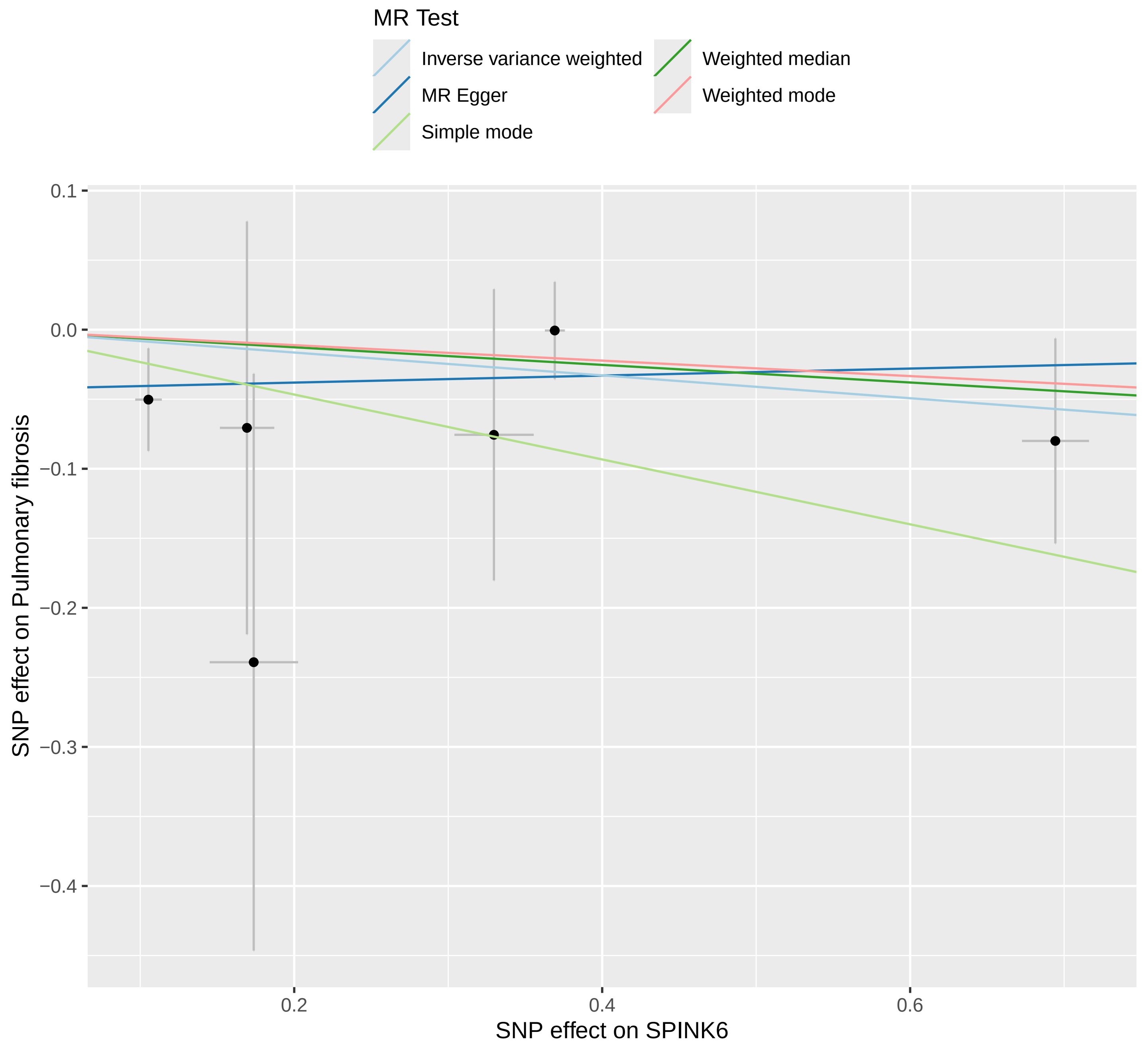

MR Test
Inverse variance weighted
MR Egger
Simple mode
Weighted median
Weighted mode
0.1
0.0
−0.1
−0.2
−0.3
−0.4
SNP effect on Pulmonary fibrosis
0.2
0.4
0.6
SNP effect on SPINK6

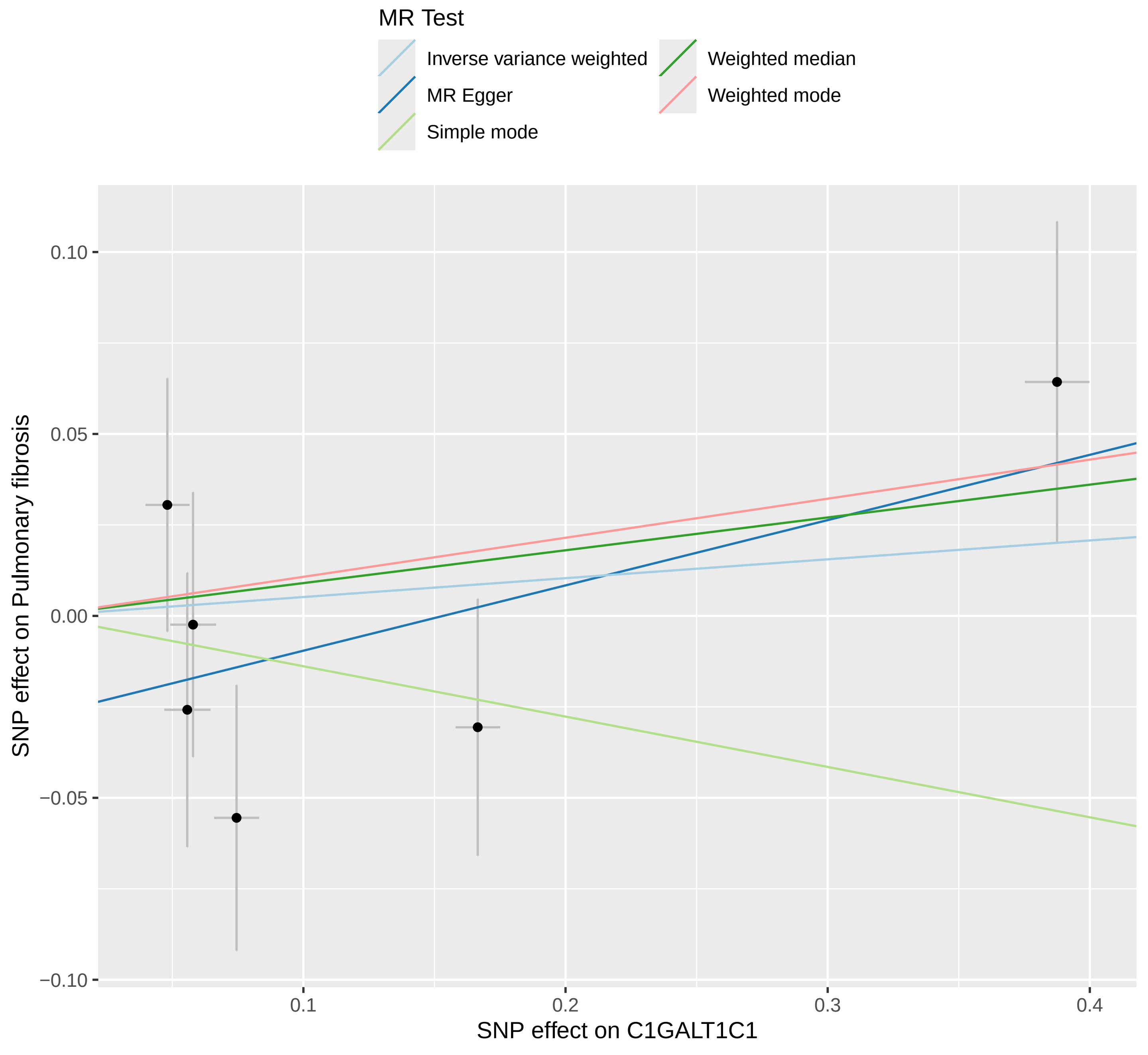

MR Test
Inverse variance weighted
MR Egger
Simple mode
Weighted median
Weighted mode
0.10
0.05
0.00
−0.05
−0.10
0.1
0.2
0.3
0.4
SNP effect on Pulmonary fibrosis
SNP effect on C1GALT1C1

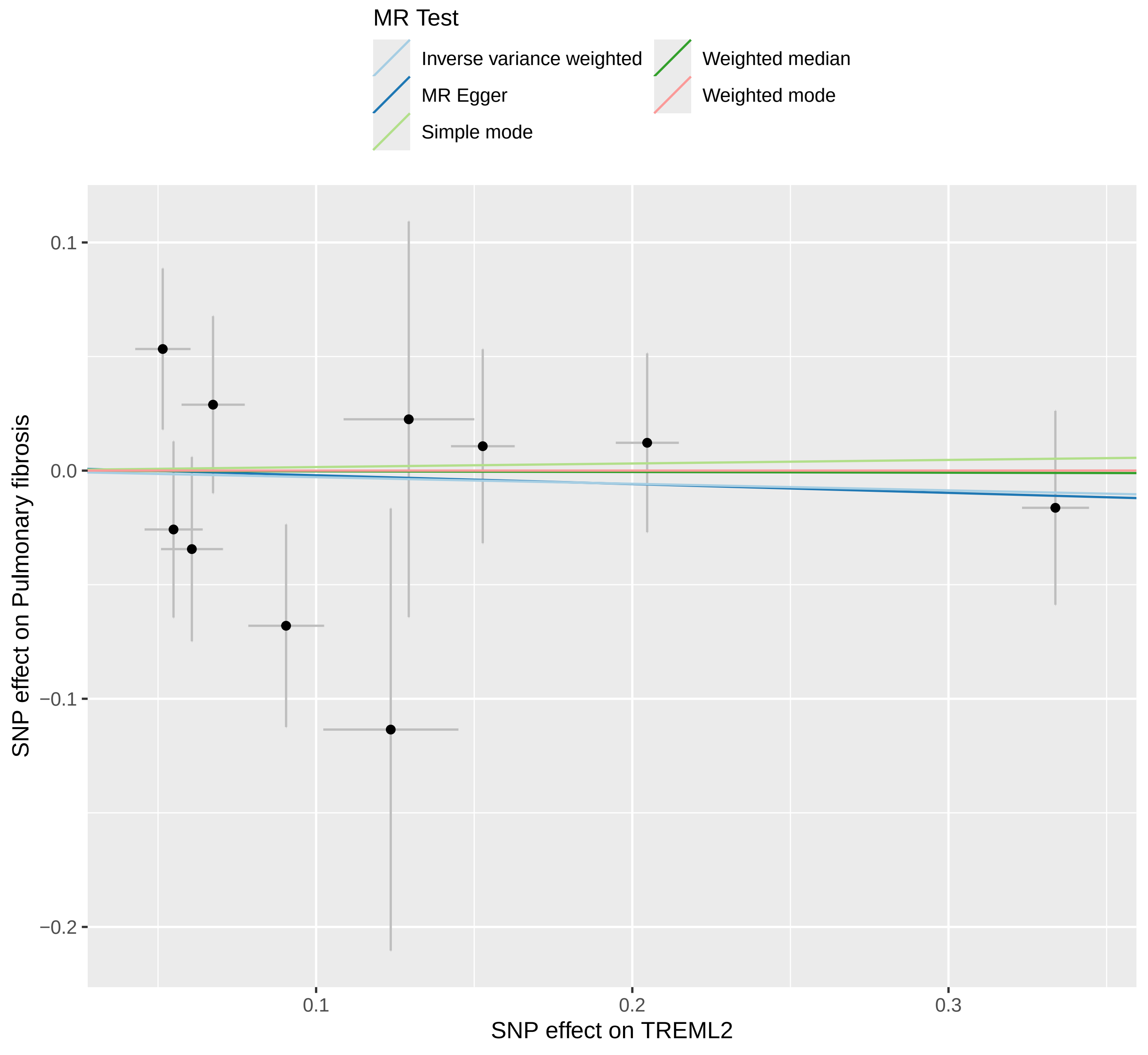
MR Test
Inverse variance weighted
MR Egger
Simple mode
Weighted median
Weighted mode
0.1
0.0
−0.1
−0.2
SNP effect on Pulmonary fibrosis
0.1
0.2
0.3
SNP effect on TREML2

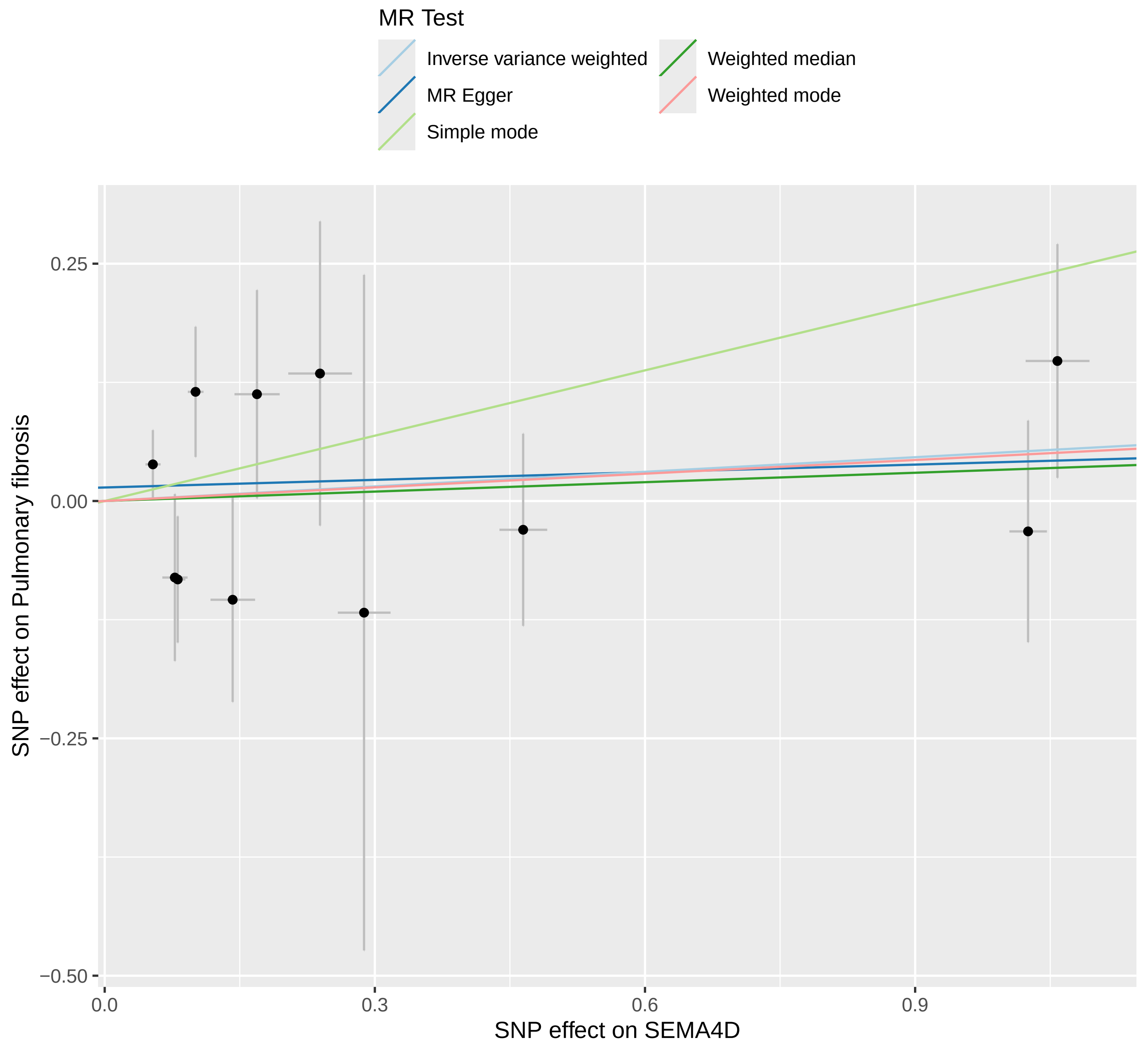

MR Test
Inverse variance weighted
MR Egger
Simple mode
Weighted median
Weighted mode
SNP effect on Pulmonary fibrosis
0.25
0.00
−0.25
−0.50
0.0
0.3
0.6
0.9
SNP effect on SEMA4D

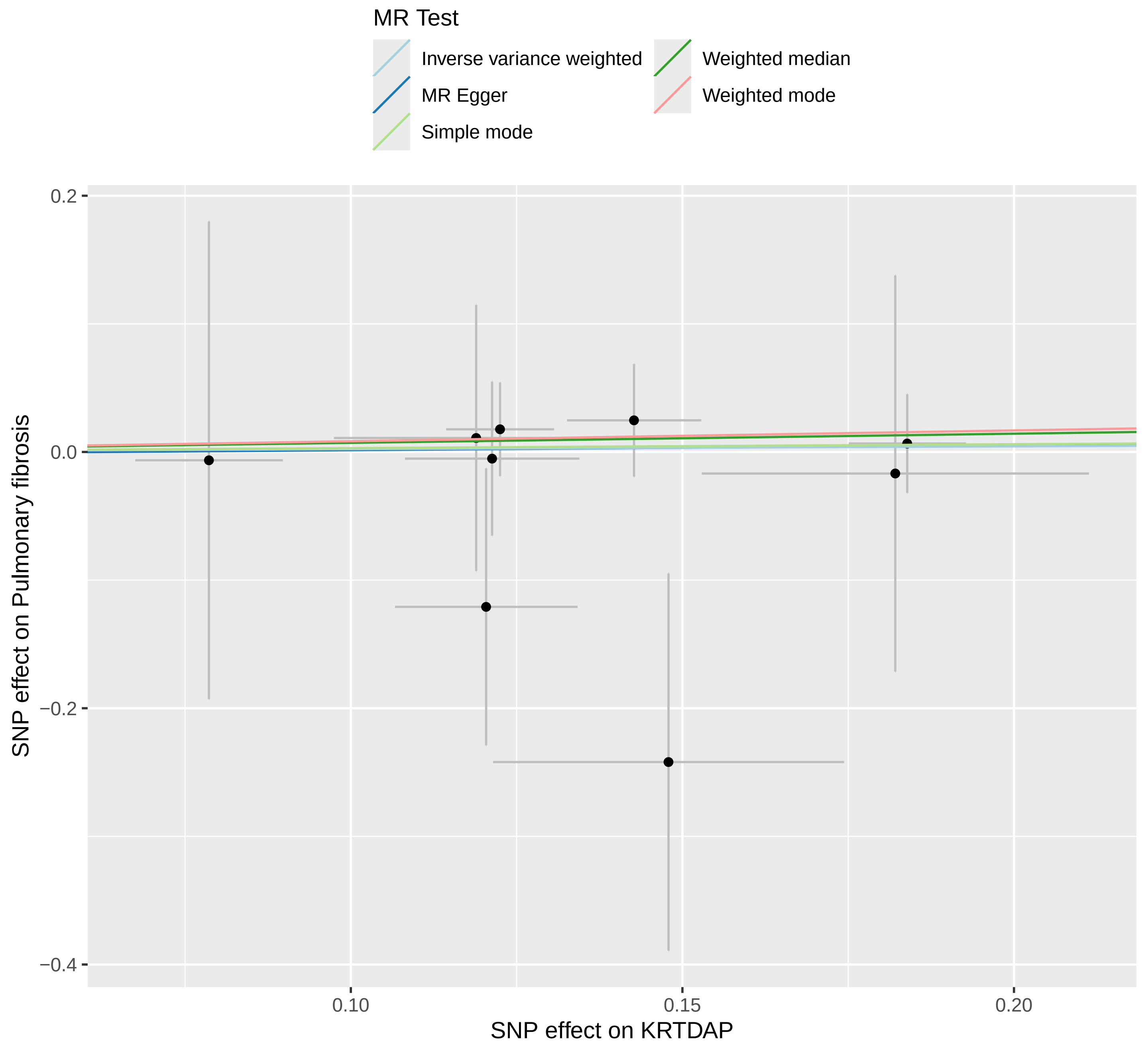
MR Test
Inverse variance weighted
MR Egger
Simple mode
Weighted median
Weighted mode
0.2
0.0
−0.2
−0.4
0.10
0.15
0.20
SNP effect on KRTDAP
SNP effect on Pulmonary fibrosis

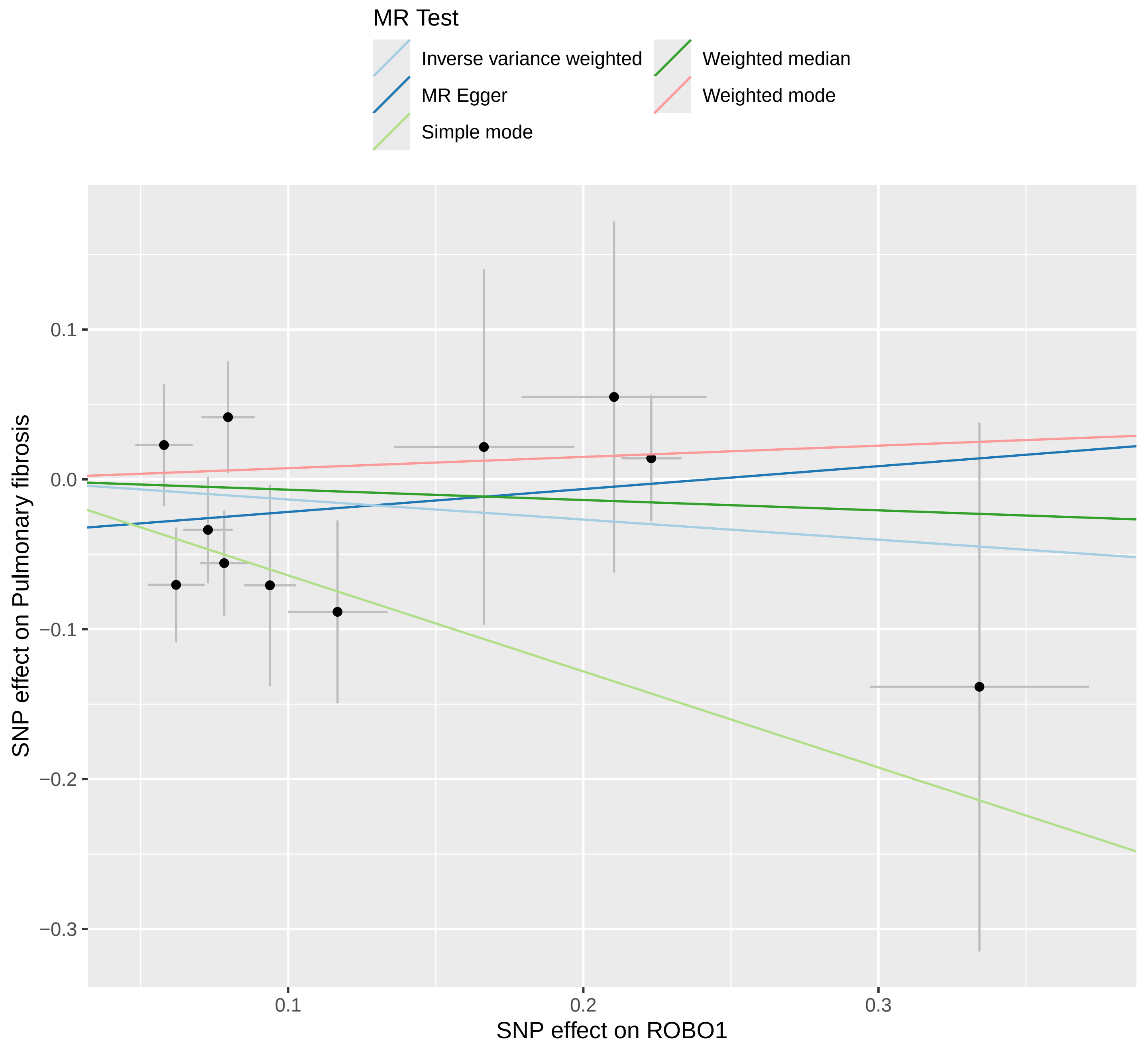
MR Test
Inverse variance weighted
MR Egger
Simple mode
Weighted median
Weighted mode
0.1
0.0
−0.1
−0.2
−0.3
SNP effect on Pulmonary fibrosis
0.1
0.2
0.3
SNP effect on ROBO1

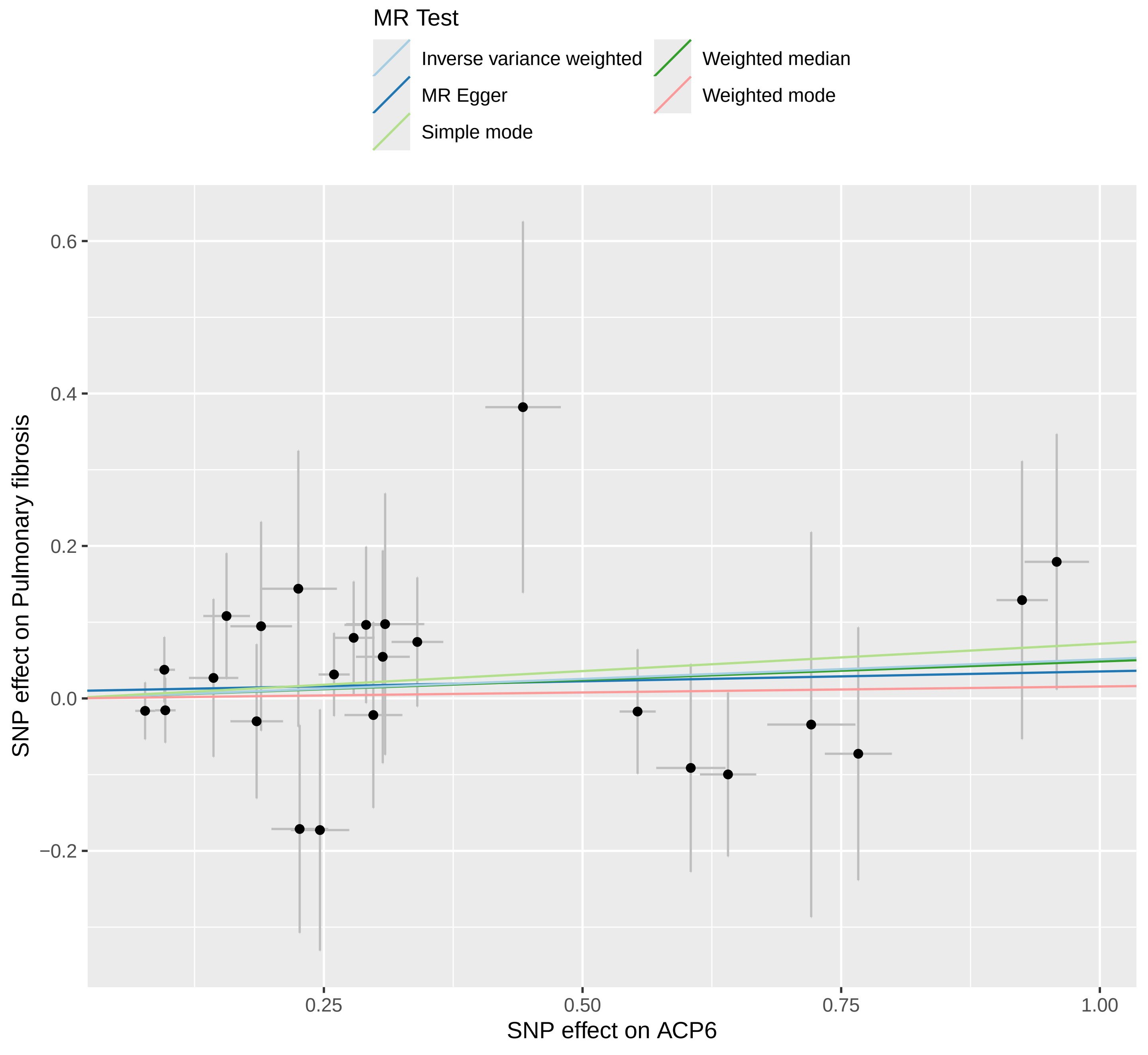
MR Test
Inverse variance weighted
MR Egger
Simple mode
Weighted median
Weighted mode
0.6
0.4
0.2
0.0
−0.2
0.25
0.50
0.75
1.00
SNP effect on Pulmonary fibrosis
SNP effect on ACP6

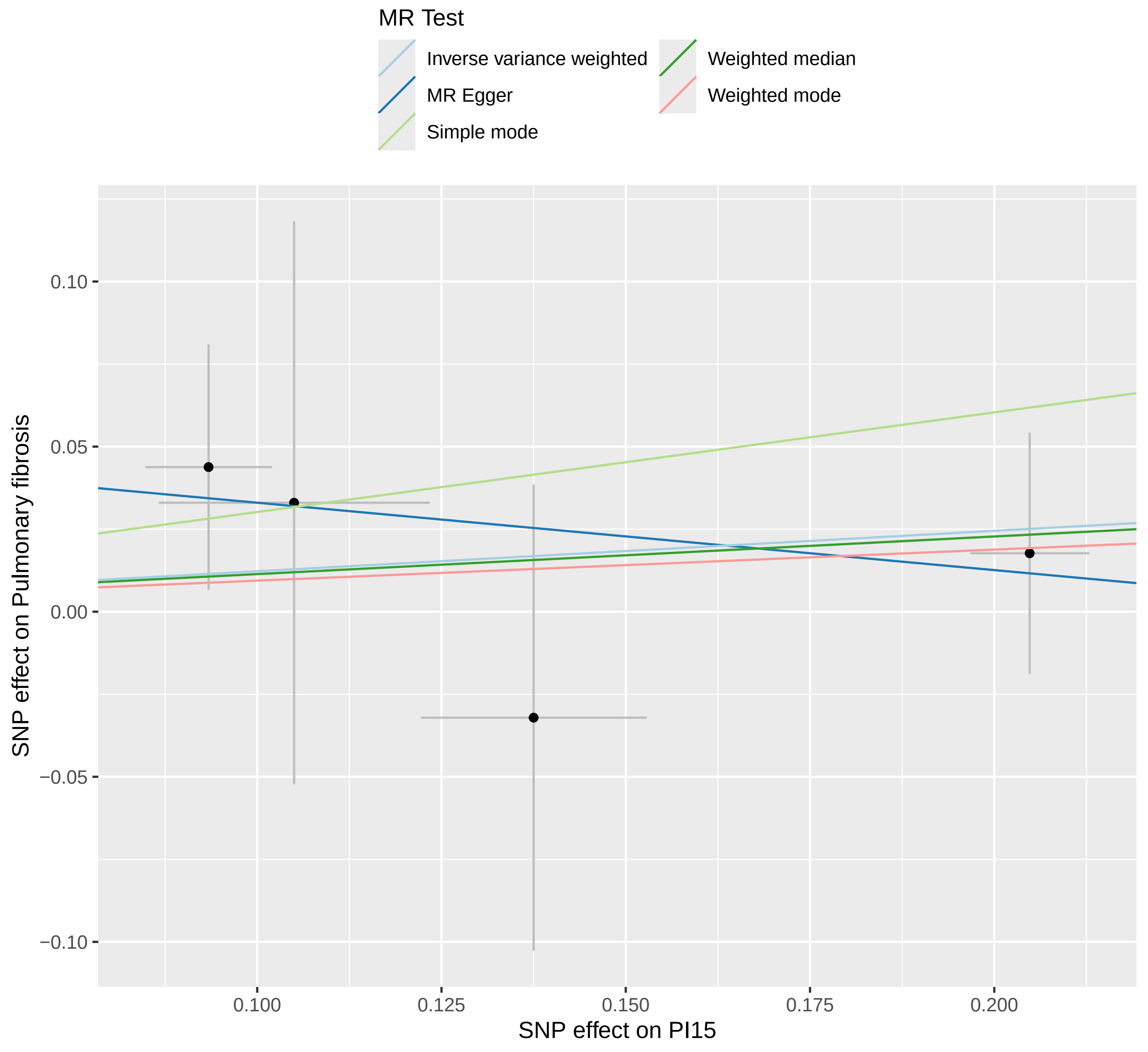
MR Test
Inverse variance weighted
MR Egger
Simple mode
Weighted median
Weighted mode
0.10
0.05
0.00
−0.05
−0.10
SNP effect on Pulmonary fibrosis
0.100
0.125
0.150
0.175
0.200
SNP effect on PI15

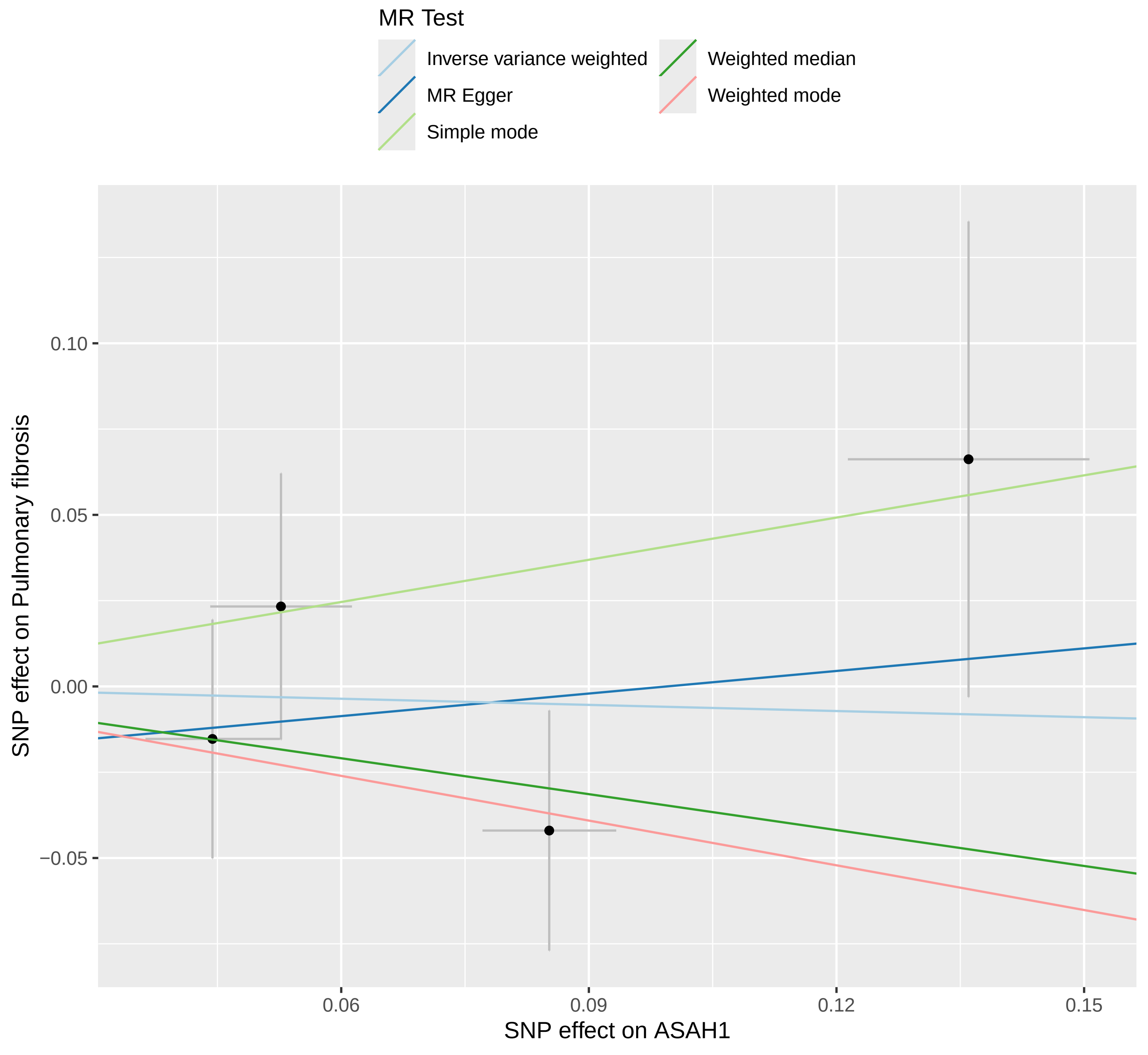

MR Test
Inverse variance weighted
MR Egger
Simple mode
Weighted median
Weighted mode
0.10
0.05
0.00
−0.05
SNP effect on Pulmonary fibrosis
0.06
0.09
0.12
0.15
SNP effect on ASAH1

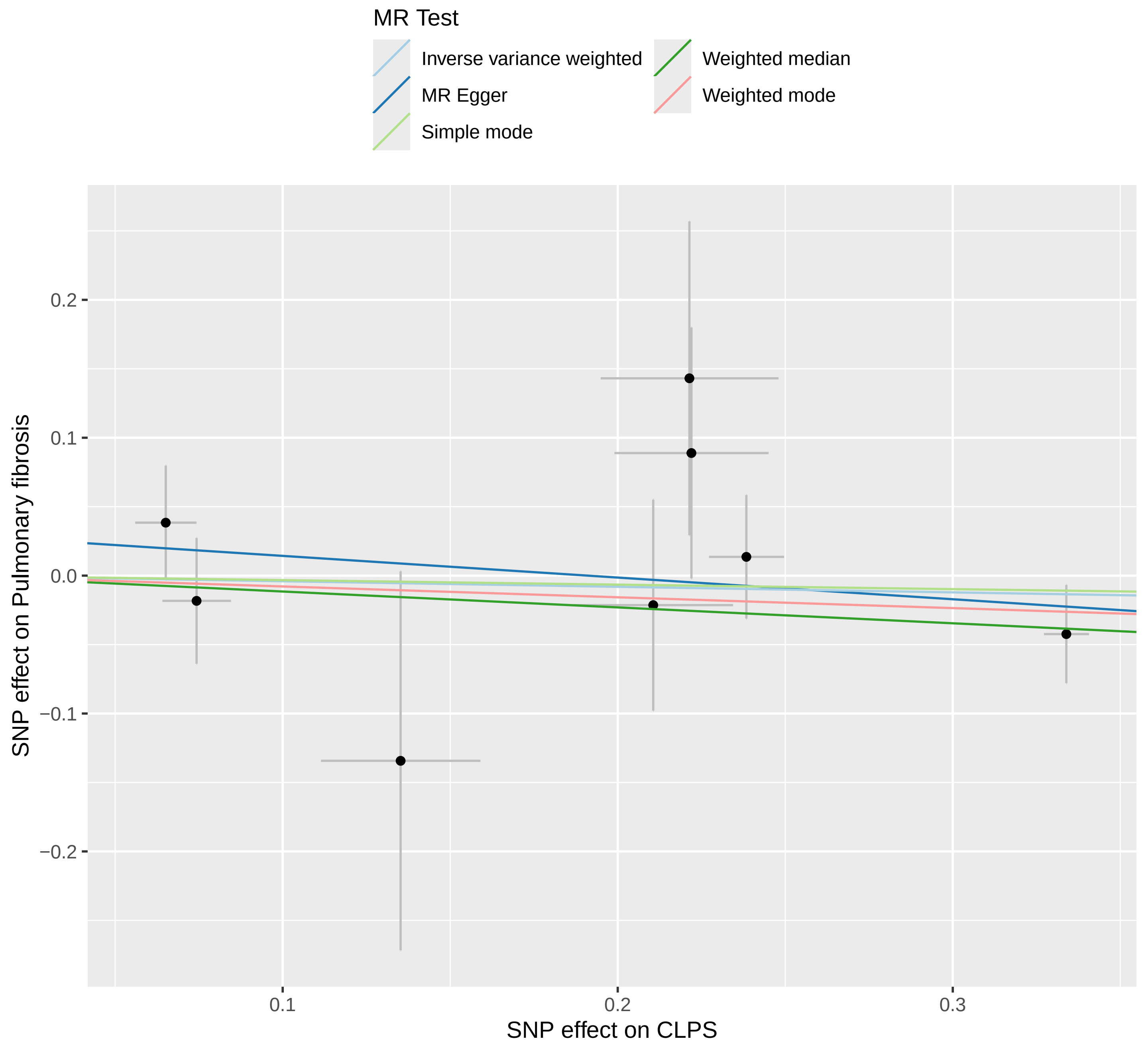

MR Test
Inverse variance weighted
MR Egger
Simple mode
Weighted median
Weighted mode
SNP effect on Pulmonary fibrosis
0.2
0.1
0.0
−0.1
−0.2
0.1
0.2
0.3
SNP effect on CLPS

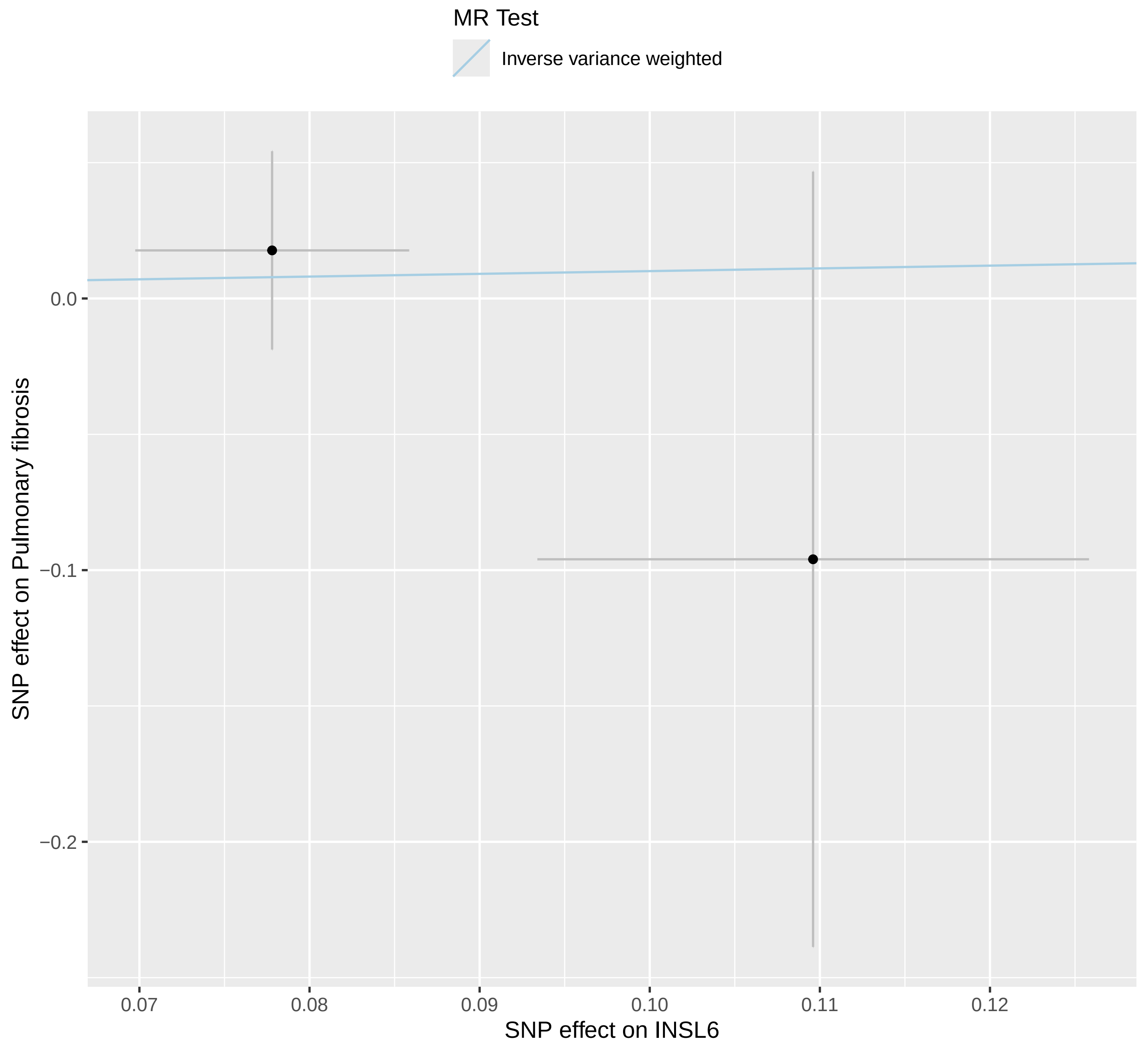
MR Test
Inverse variance weighted
0.0
−0.1
−0.2
SNP effect on Pulmonary fibrosis
0.07
0.08
0.09
0.10
0.11
0.12
SNP effect on INSL6

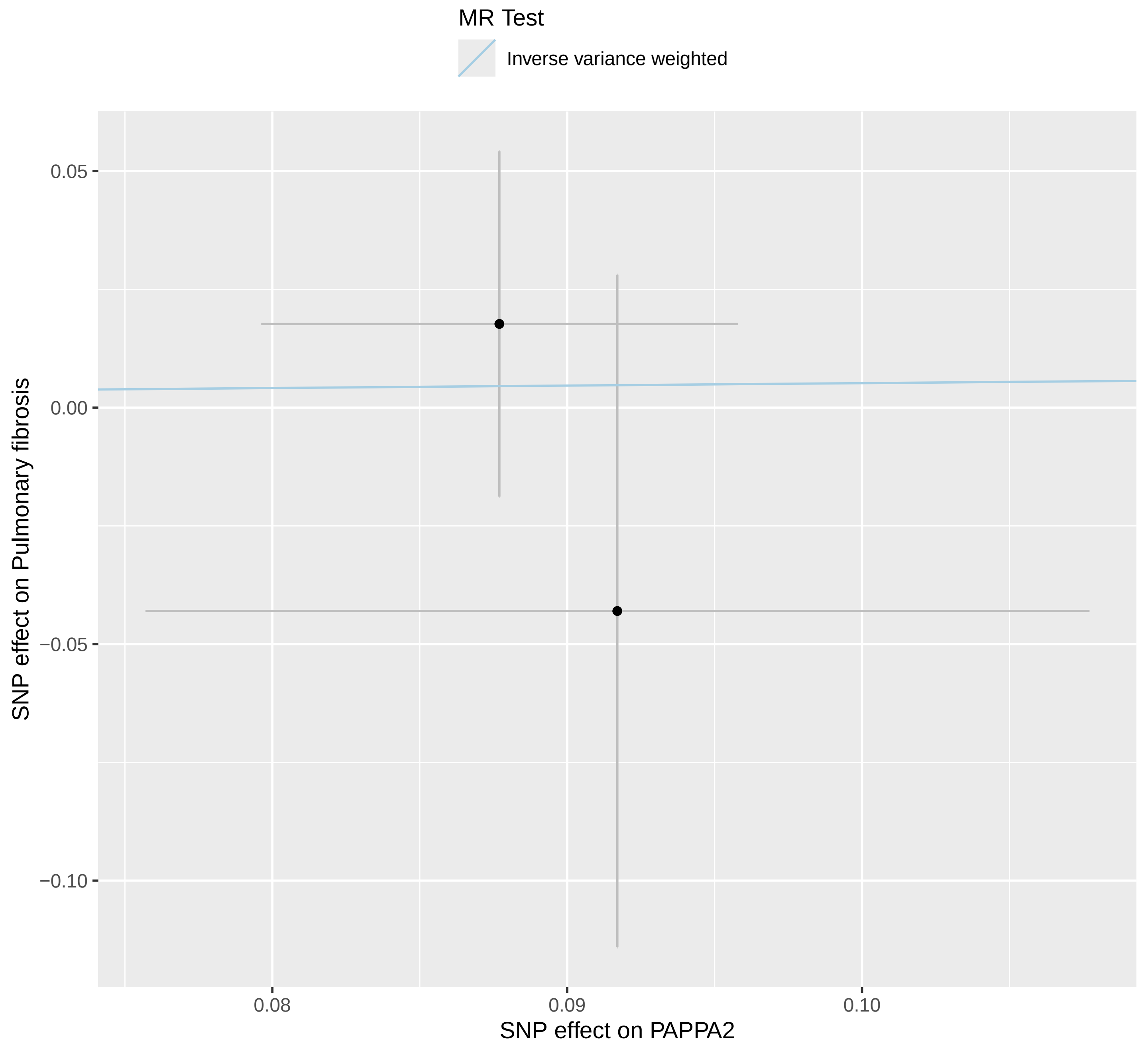
MR Test
Inverse variance weighted
0.05
0.00
−0.05
−0.10
0.08
0.09
0.10
SNP effect on PAPPA2
SNP effect on Pulmonary fibrosis

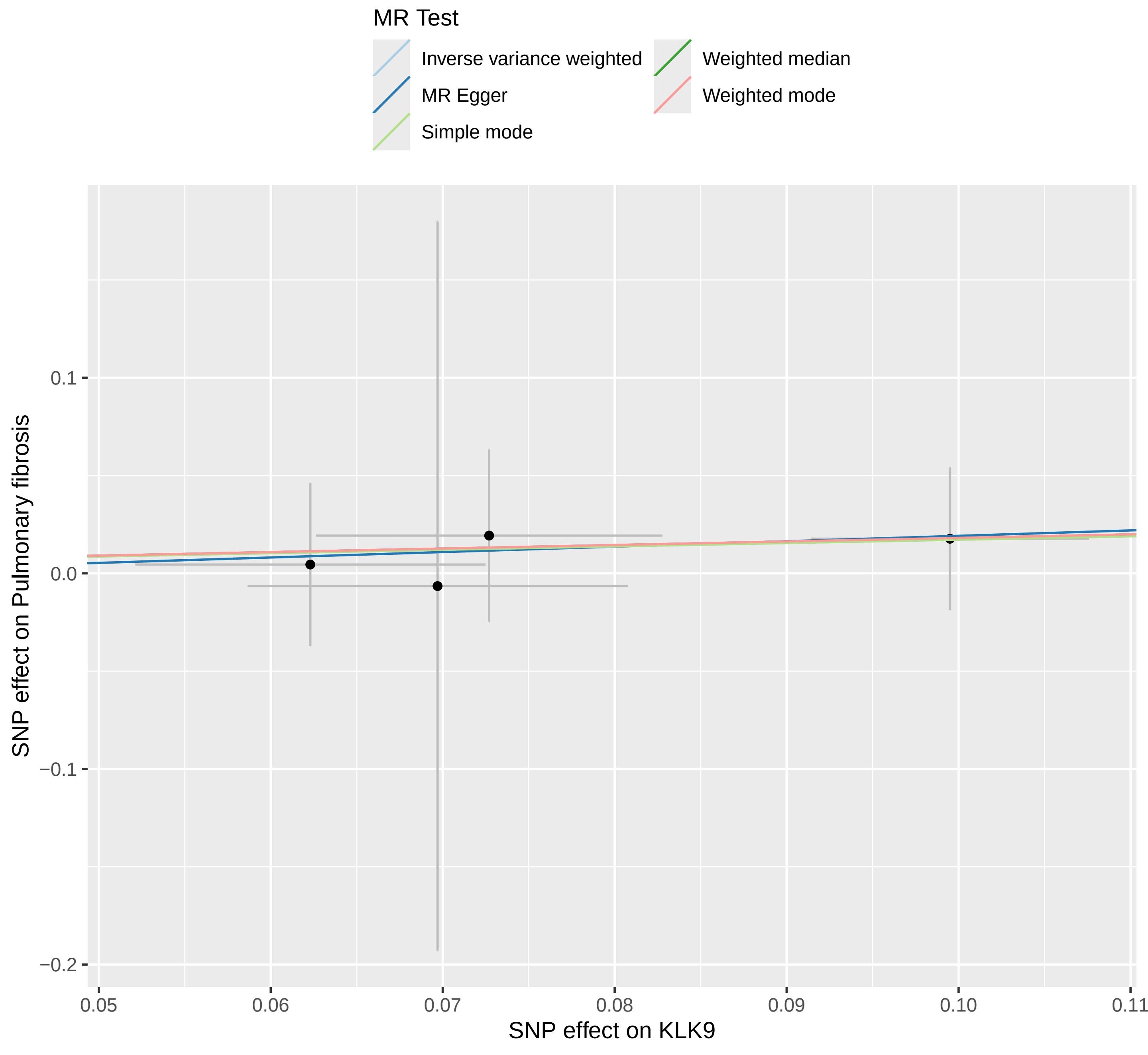

MR Test
Inverse variance weighted
MR Egger
Simple mode
Weighted median
Weighted mode
0.1
0.0
−0.1
−0.2
SNP effect on Pulmonary fibrosis
0.05
0.06
0.07
0.08
0.09
0.10
0.11
SNP effect on KLK9

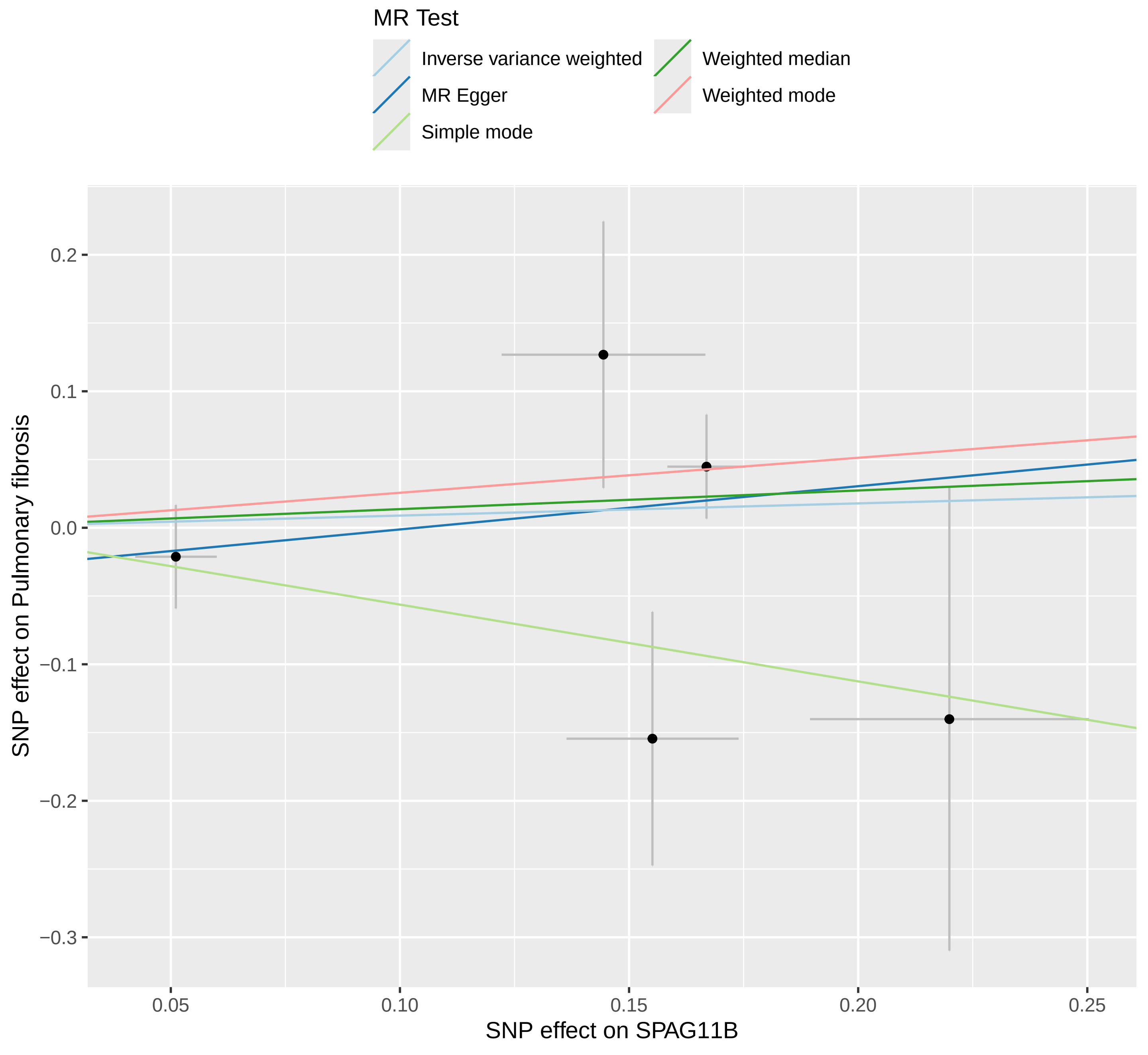
MR Test
Inverse variance weighted
MR Egger
Simple mode
Weighted median
Weighted mode
0.2
0.1
0.0
−0.1
−0.2
−0.3
0.05
0.10
0.15
0.20
0.25
SNP effect on SPAG11B
SNP effect on Pulmonary fibrosis

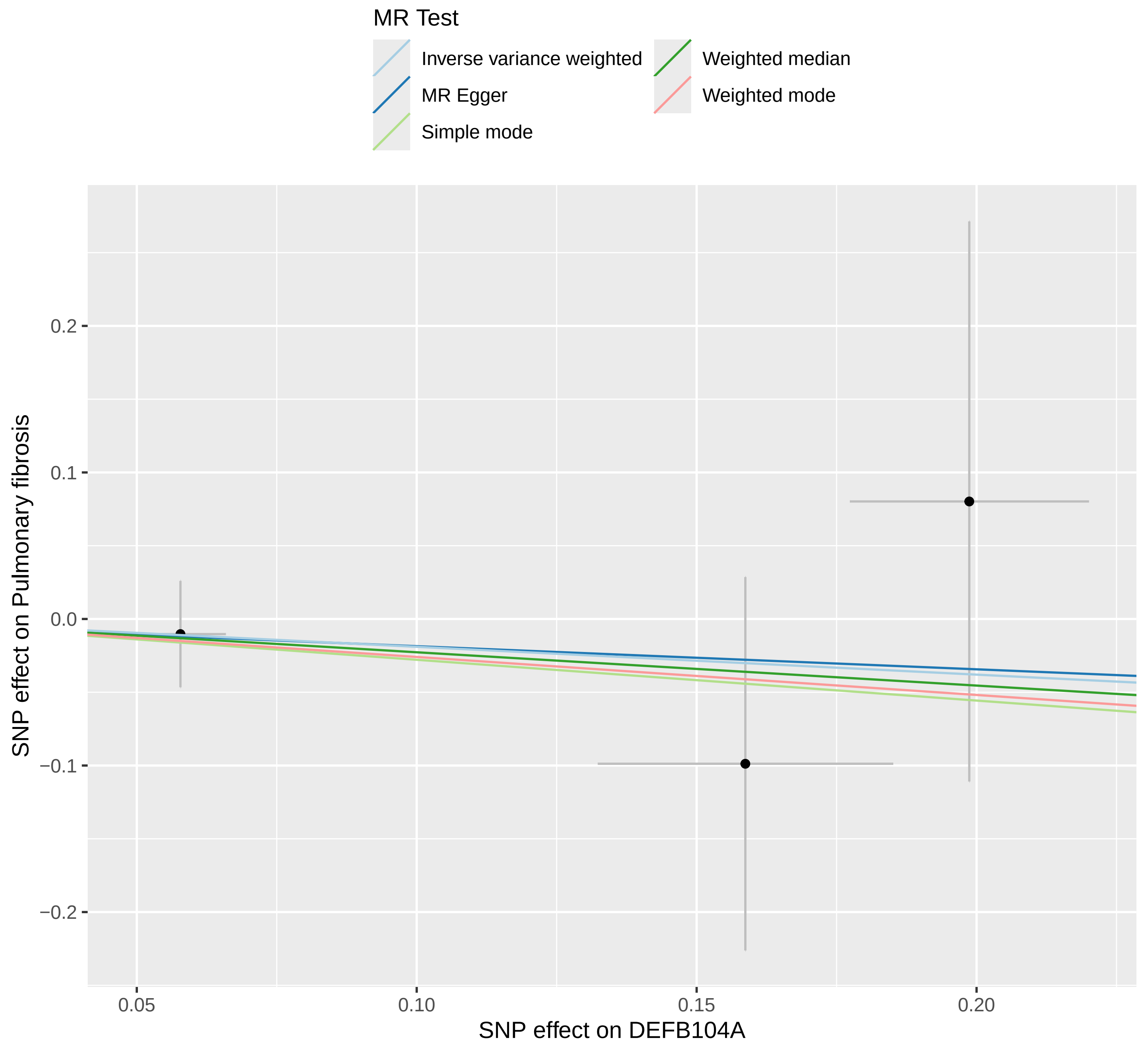
MR Test
Inverse variance weighted
MR Egger
Simple mode
Weighted median
Weighted mode
0.2
0.1
0.0
−0.1
−0.2
SNP effect on Pulmonary fibrosis
0.05
0.10
0.15
0.20
SNP effect on DEFB104A

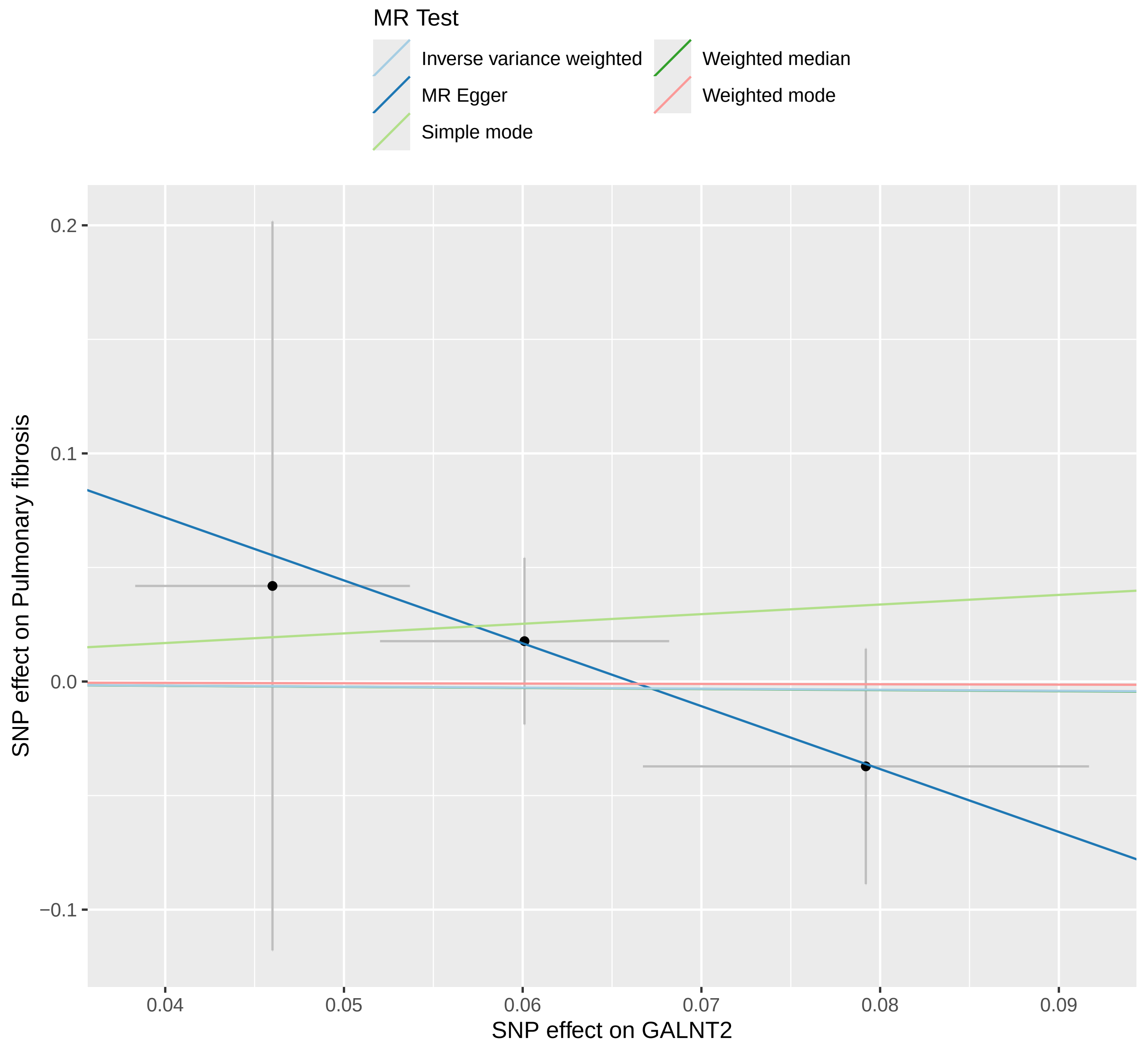
MR Test
Inverse variance weighted
MR Egger
Simple mode
Weighted median
Weighted mode
0.2
0.1
0.0
−0.1
SNP effect on Pulmonary fibrosis
0.04
0.05
0.06
0.07
0.08
0.09
SNP effect on GALNT2

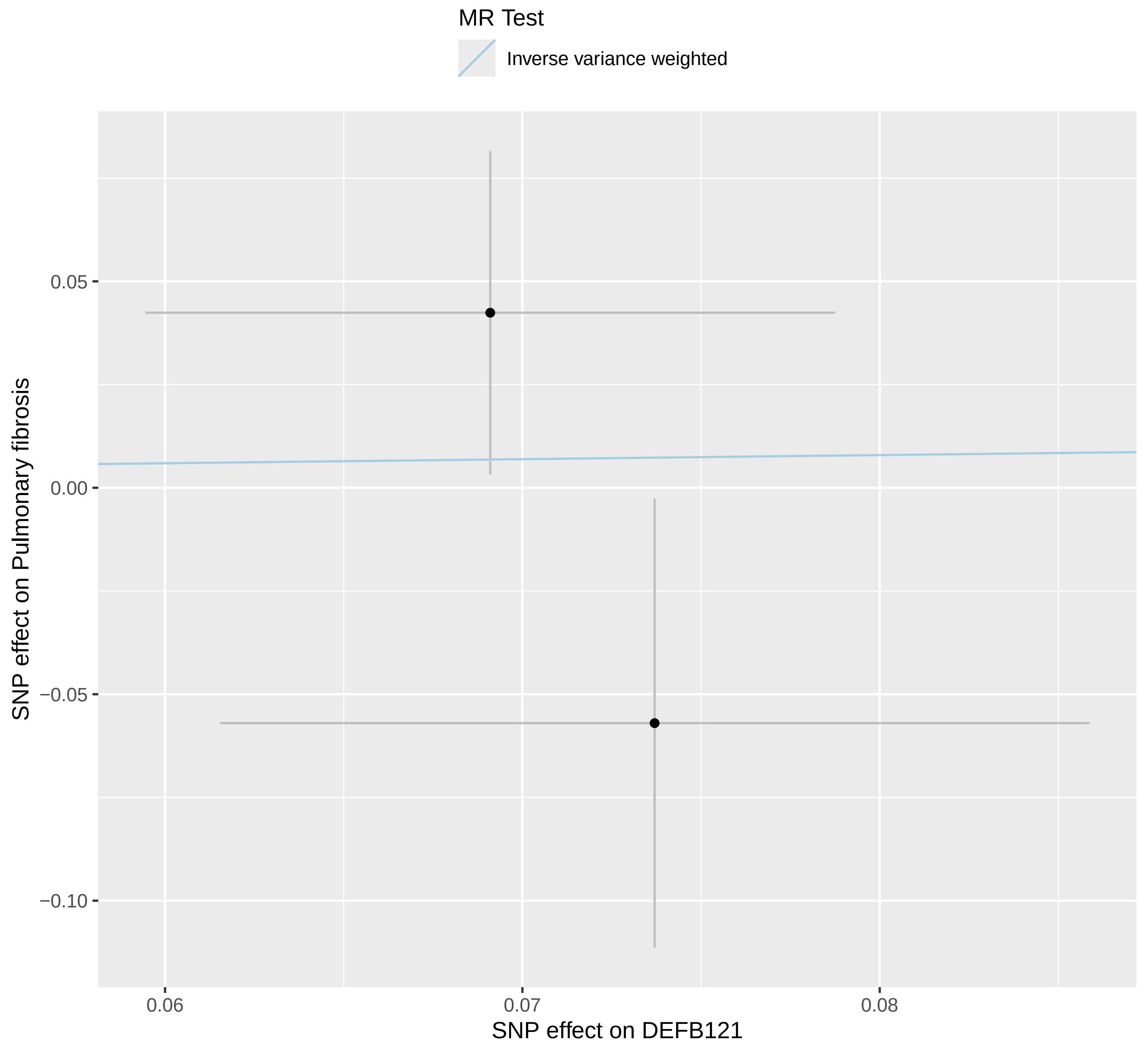

MR Test
Inverse variance weighted
0.05
0.00
−0.05
−0.10
SNP effect on Pulmonary fibrosis
0.06
0.07
0.08
SNP effect on DEFB121

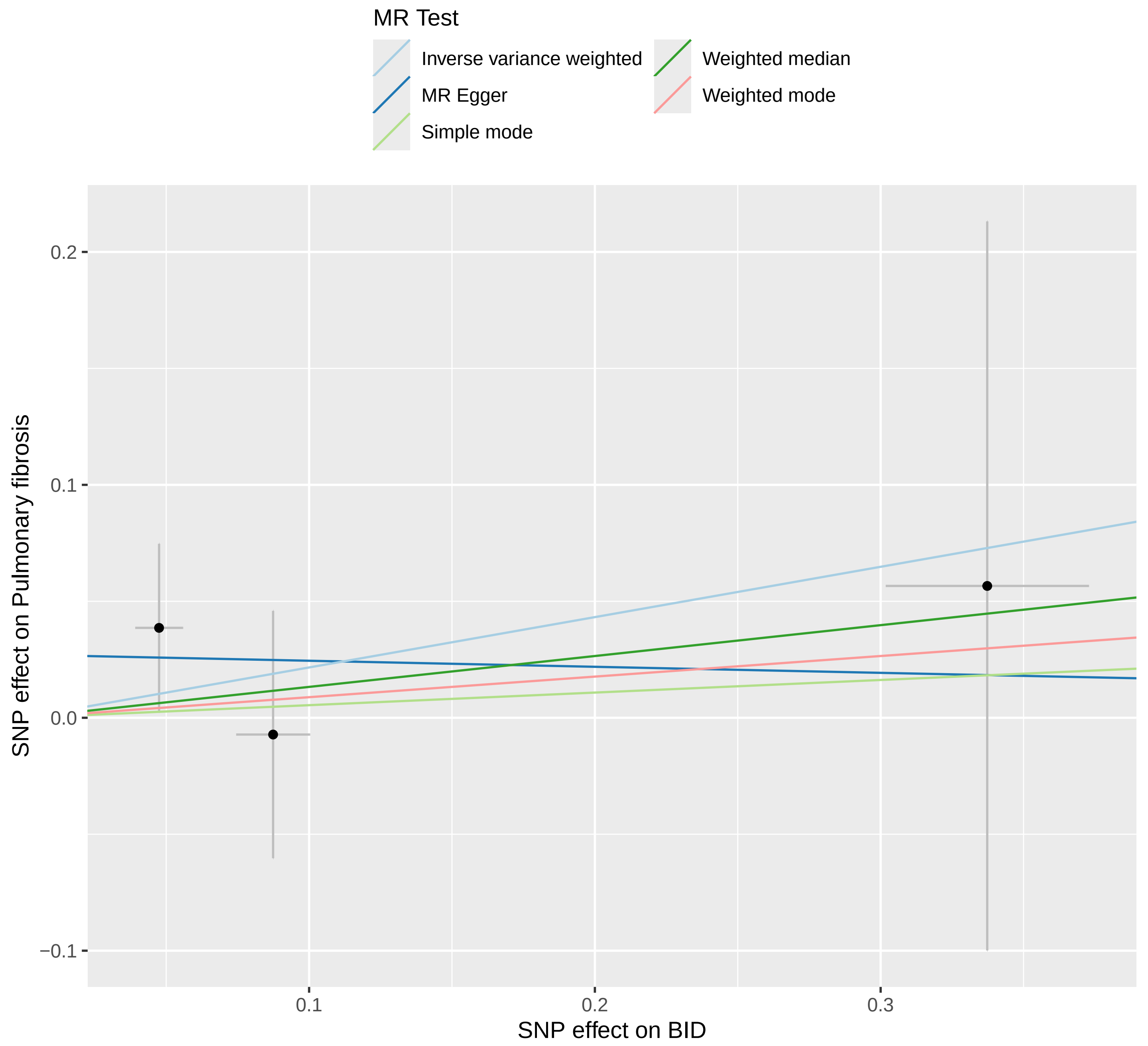
MR Test
Inverse variance weighted
MR Egger
Simple mode
Weighted median
Weighted mode
SNP effect on Pulmonary fibrosis
0.2
0.1
0.0
−0.1
0.1
0.2
0.3
SNP effect on BID

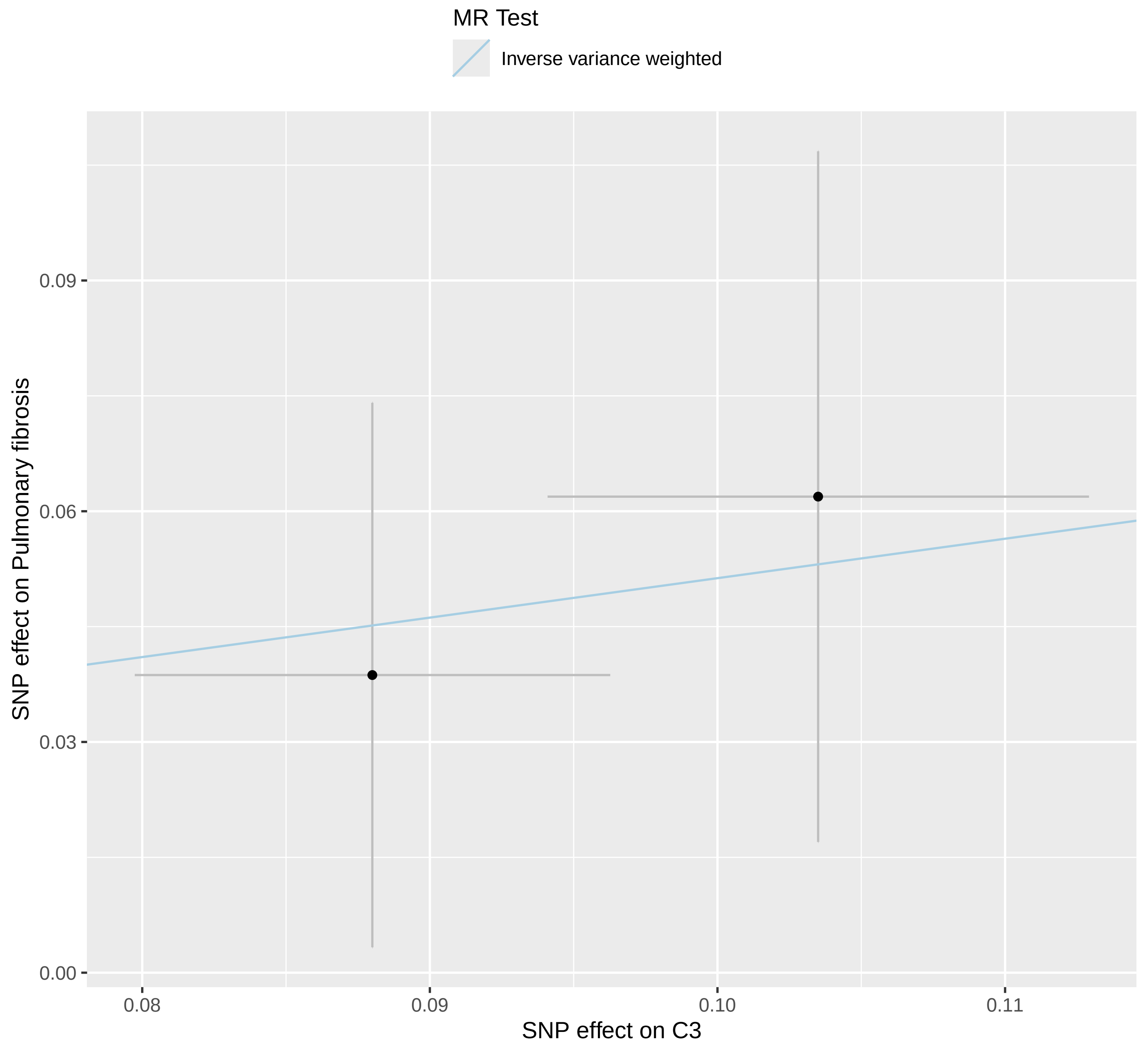

MR Test
Inverse variance weighted
0.09
0.06
0.03
0.00
SNP effect on Pulmonary fibrosis
0.08
0.09
0.10
0.11
SNP effect on C3

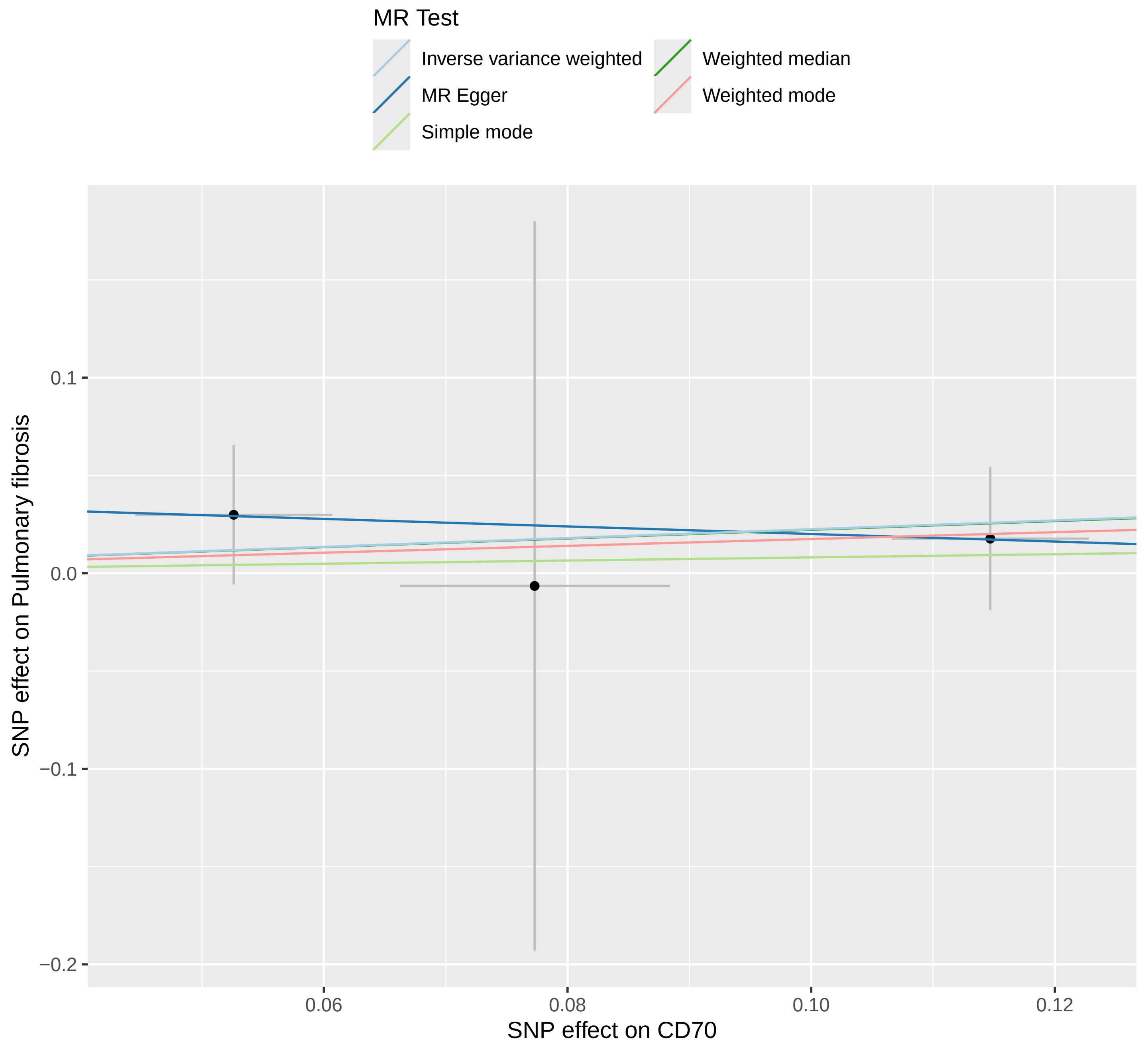

MR Test
Inverse variance weighted
MR Egger
Simple mode
Weighted median
Weighted mode
0.1
0.0
−0.1
−0.2
SNP effect on Pulmonary fibrosis
0.06
0.08
0.10
0.12
SNP effect on CD70

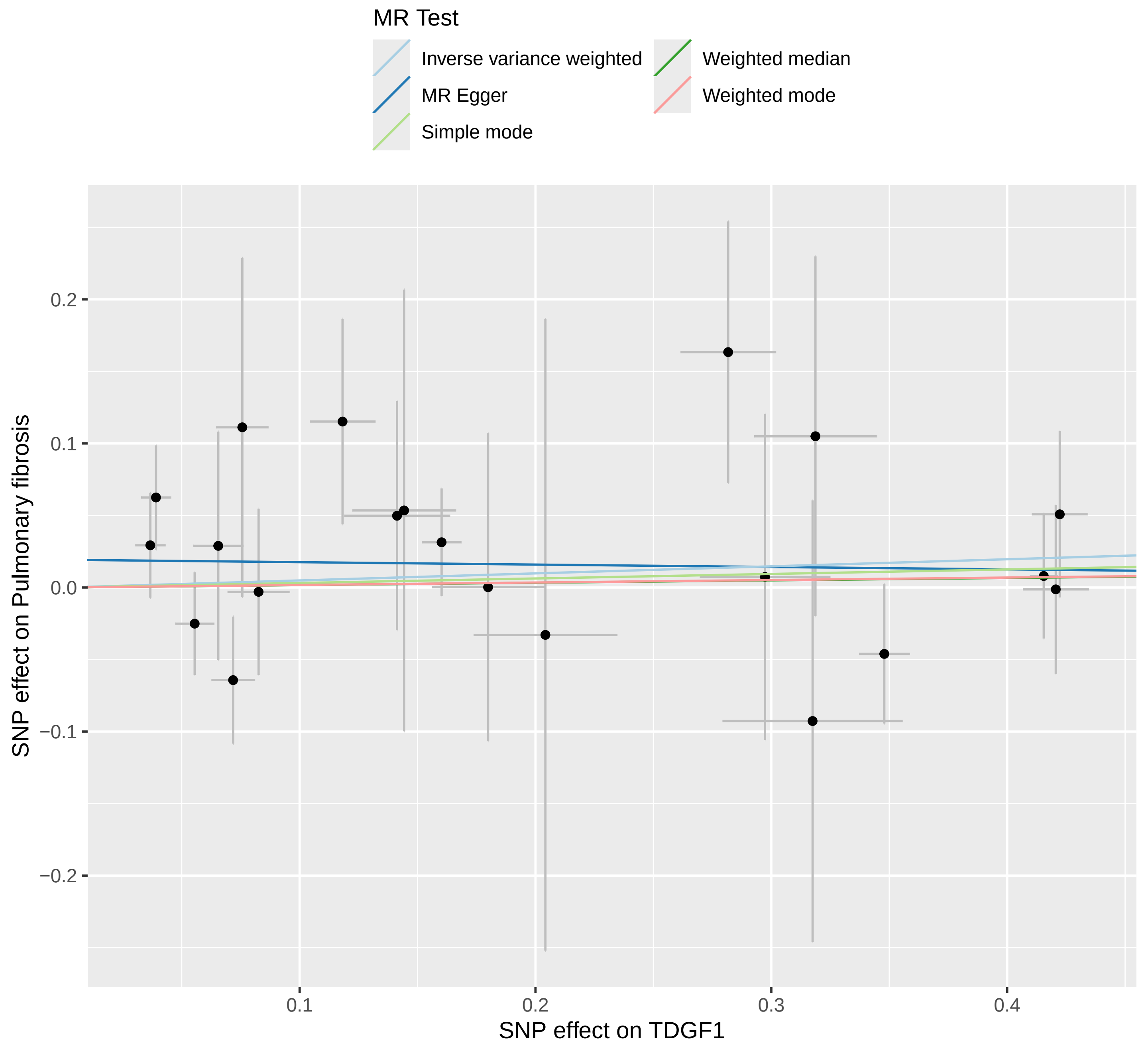
MR Test
Inverse variance weighted
MR Egger
Simple mode
Weighted median
Weighted mode
0.2
0.1
0.0
−0.1
−0.2
SNP effect on Pulmonary fibrosis
0.1
0.2
0.3
0.4
SNP effect on TDGF1

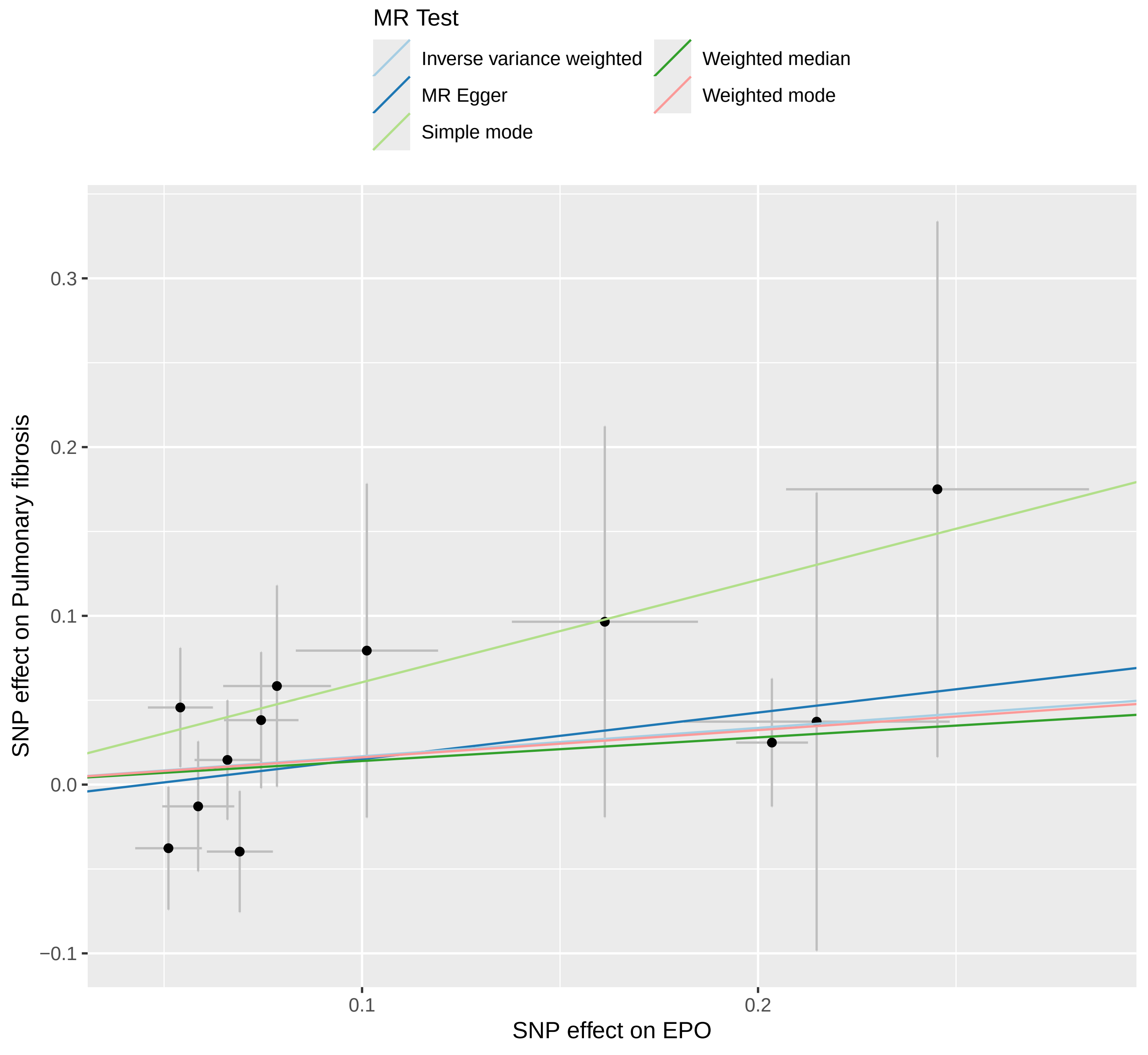

MR Test
Inverse variance weighted
MR Egger
Simple mode
Weighted median
Weighted mode
0.3
0.2
0.1
0.0
−0.1
SNP effect on Pulmonary fibrosis
0.1
0.2
SNP effect on EPO

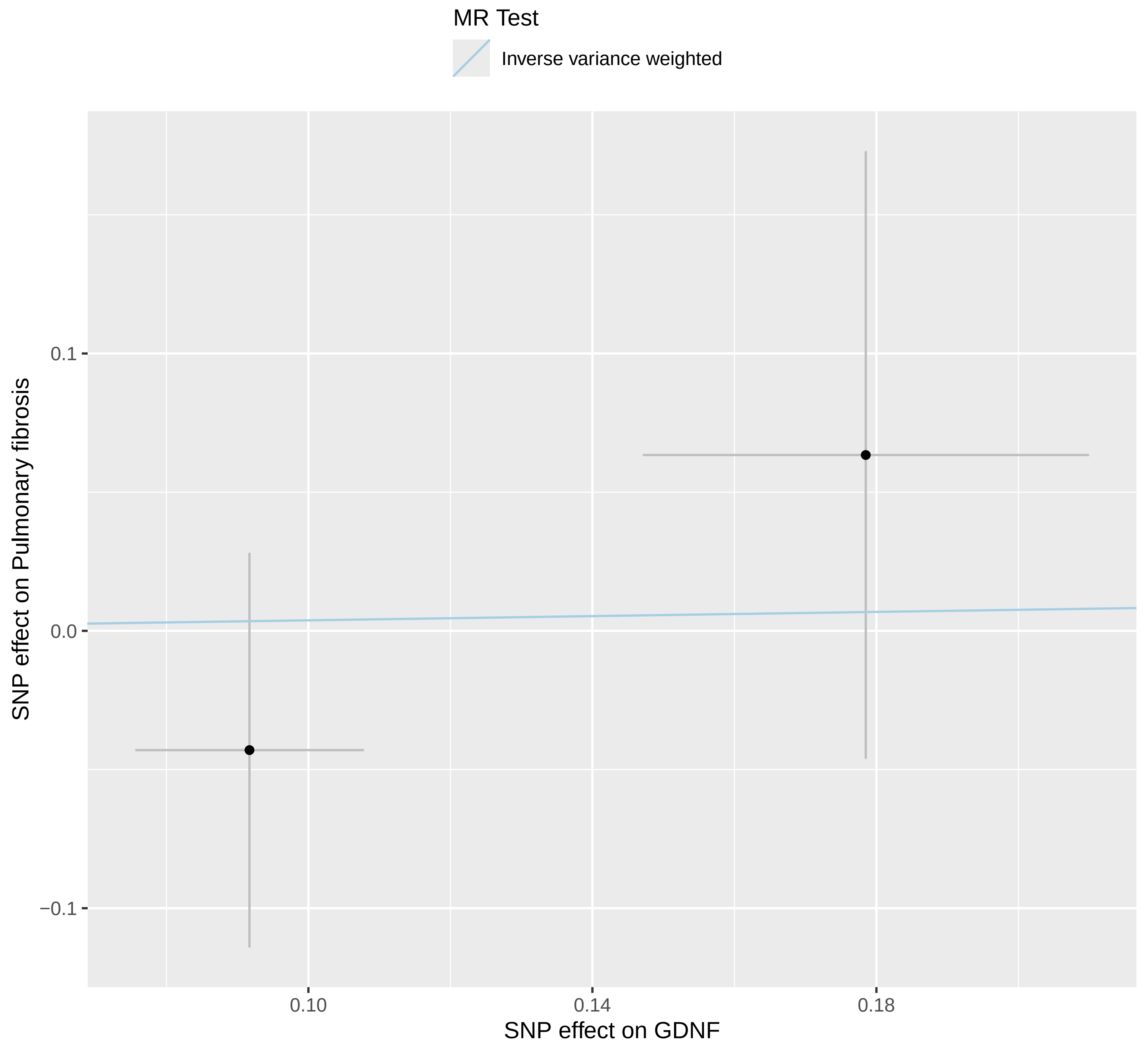

MR Test
Inverse variance weighted
SNP effect on Pulmonary fibrosis
0.1
0.0
−0.1
0.10
0.14
0.18
SNP effect on GDNF

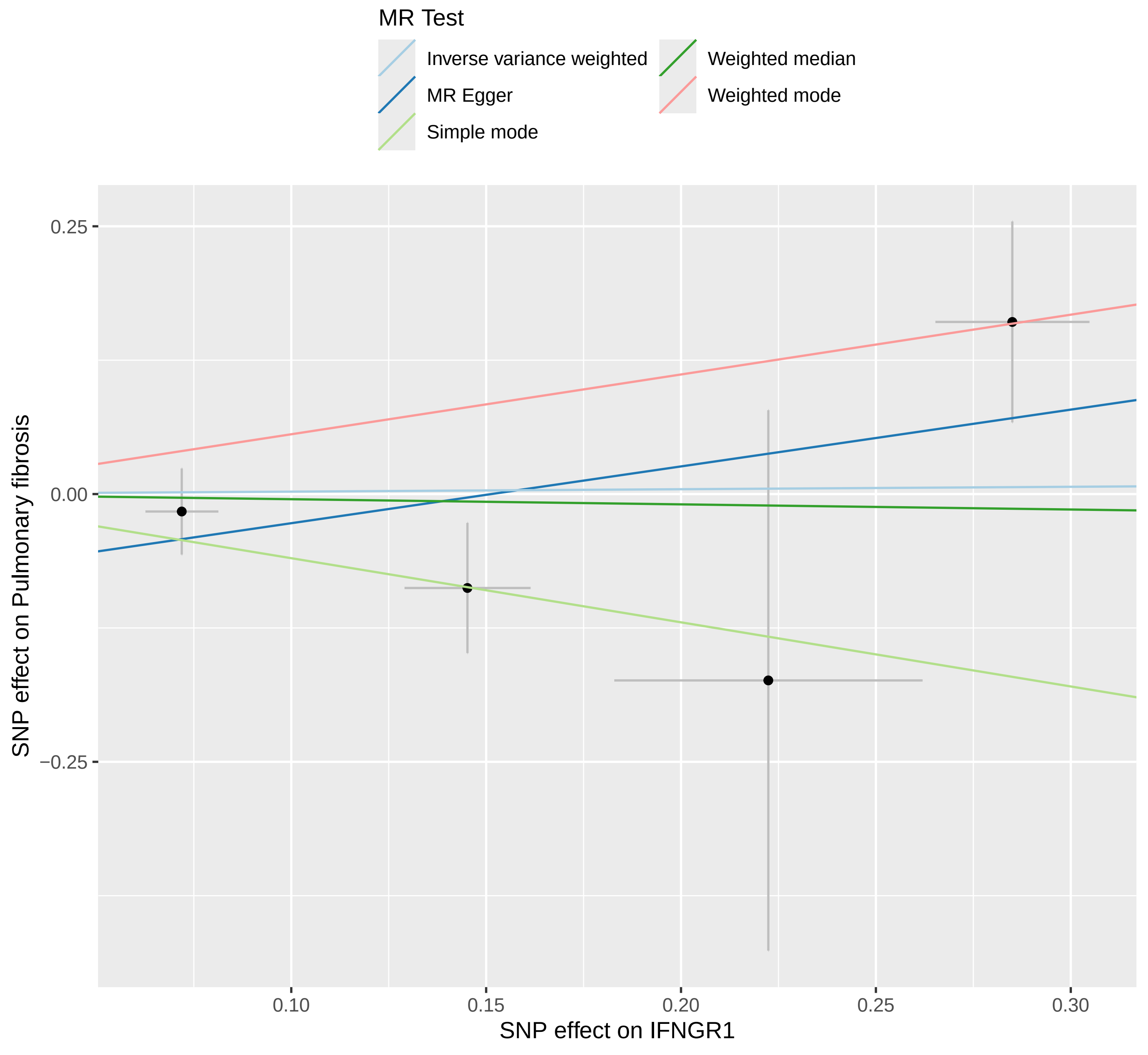
MR Test
Inverse variance weighted
MR Egger
Simple mode
Weighted median
Weighted mode
0.25
0.00
−0.25
SNP effect on Pulmonary fibrosis
0.10
0.15
0.20
0.25
0.30
SNP effect on IFNGR1

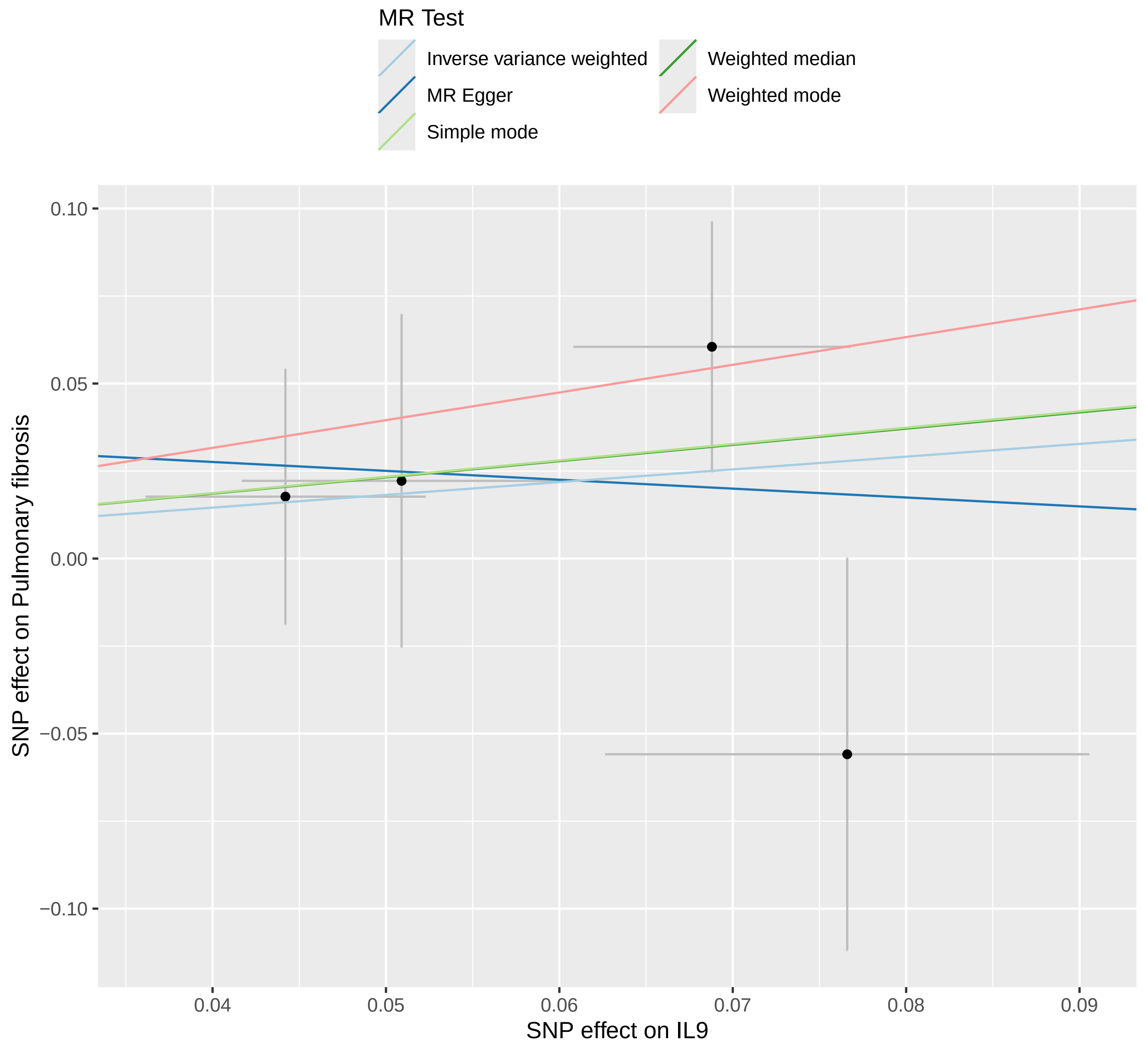

MR Test
Inverse variance weighted
MR Egger
Simple mode
Weighted median
Weighted mode
0.10
0.05
0.00
−0.05
−0.10
0.04
0.05
0.06
0.07
0.08
0.09
SNP effect on Pulmonary fibrosis
SNP effect on IL9

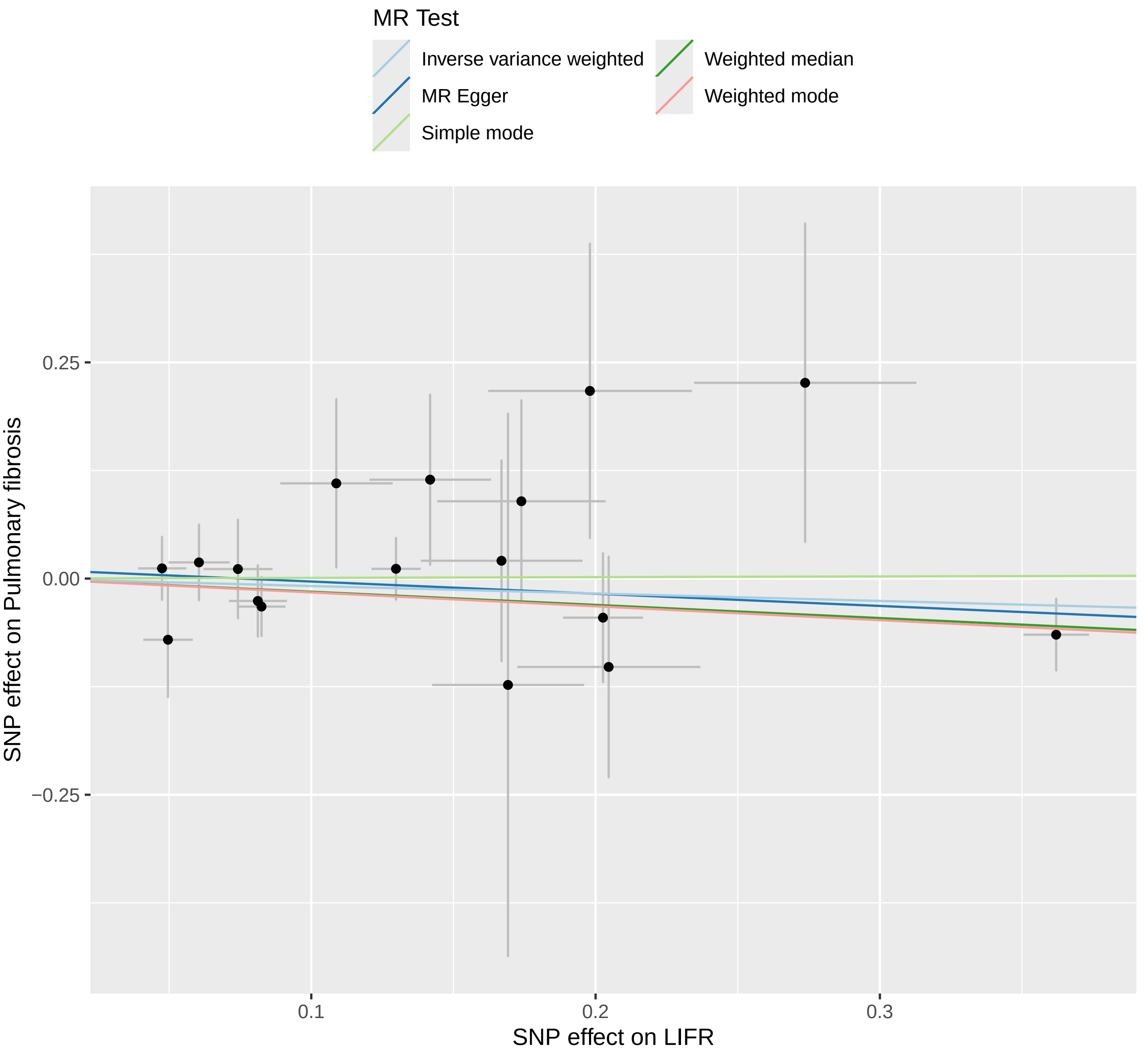
MR Test
Inverse variance weighted
MR Egger
Simple mode
Weighted median
Weighted mode
0.25
0.00
−0.25
SNP effect on Pulmonary fibrosis
0.1
0.2
0.3
SNP effect on LIFR

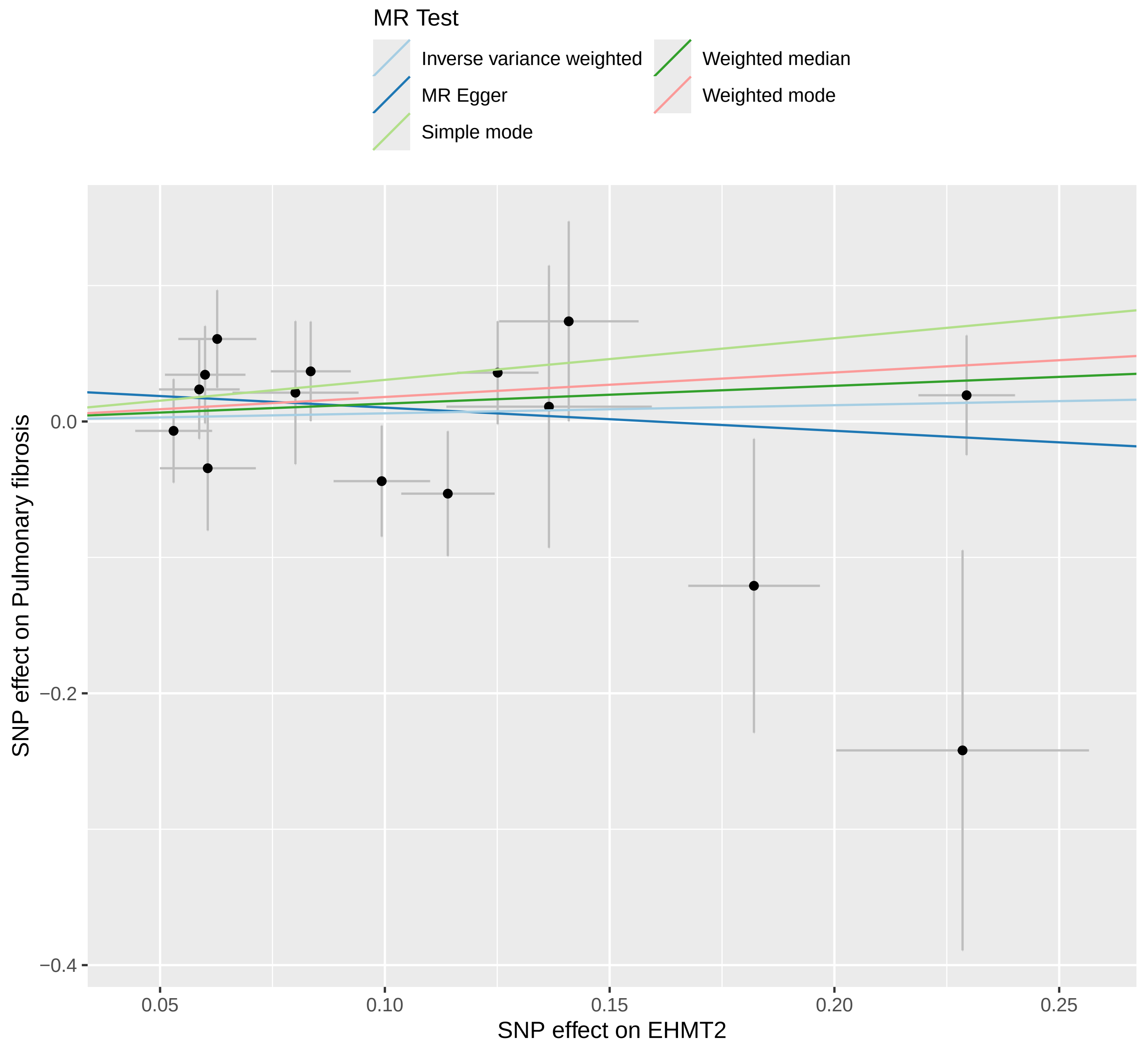

MR Test
Inverse variance weighted
MR Egger
Simple mode
Weighted median
Weighted mode
0.0
−0.2
−0.4
SNP effect on Pulmonary fibrosis
0.05
0.10
0.15
0.20
0.25
SNP effect on EHMT2

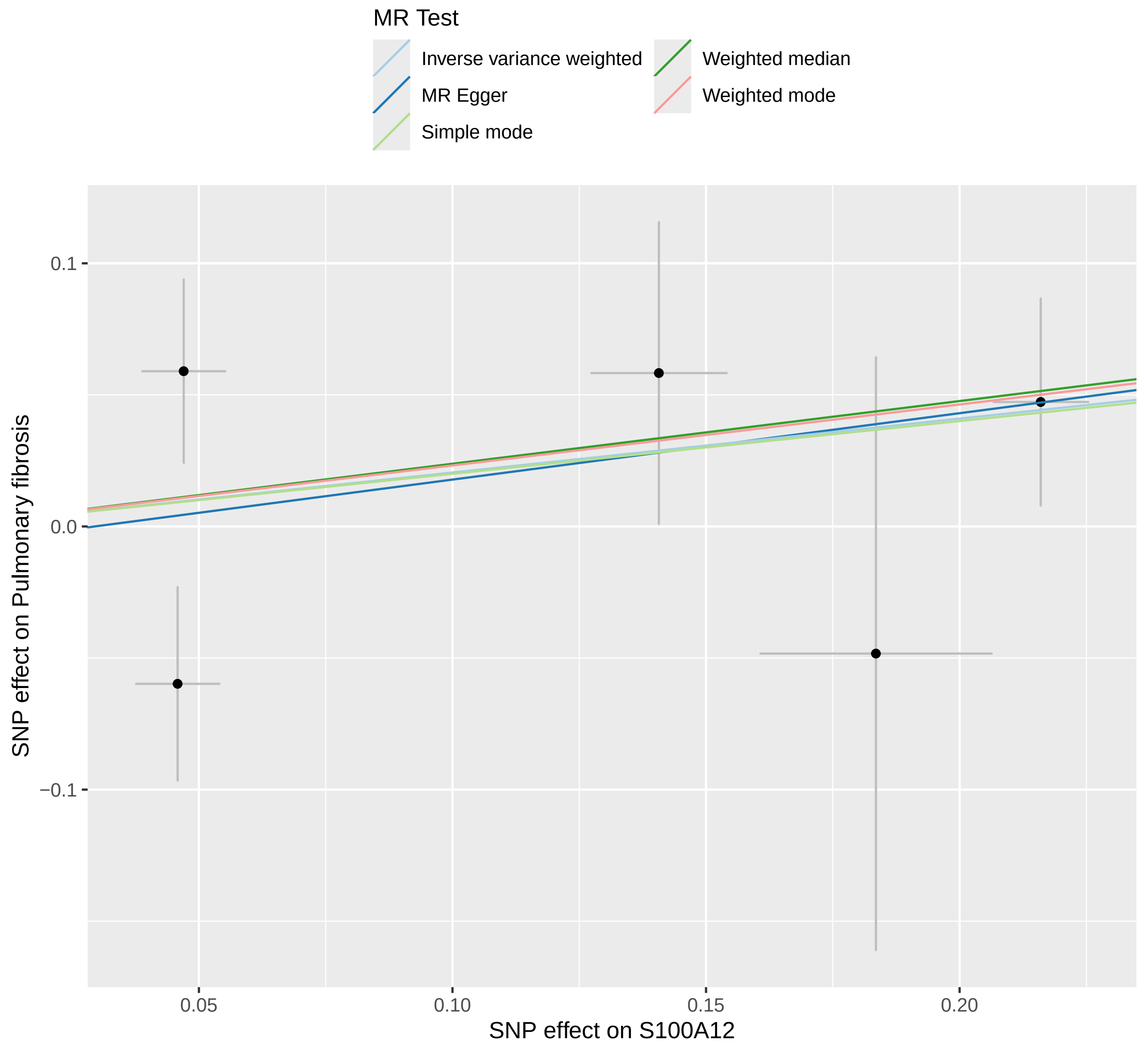

MR Test
Inverse variance weighted
MR Egger
Simple mode
Weighted median
Weighted mode
0.1
0.0
−0.1
0.05
0.10
0.15
0.20
SNP effect on Pulmonary fibrosis
SNP effect on S100A12

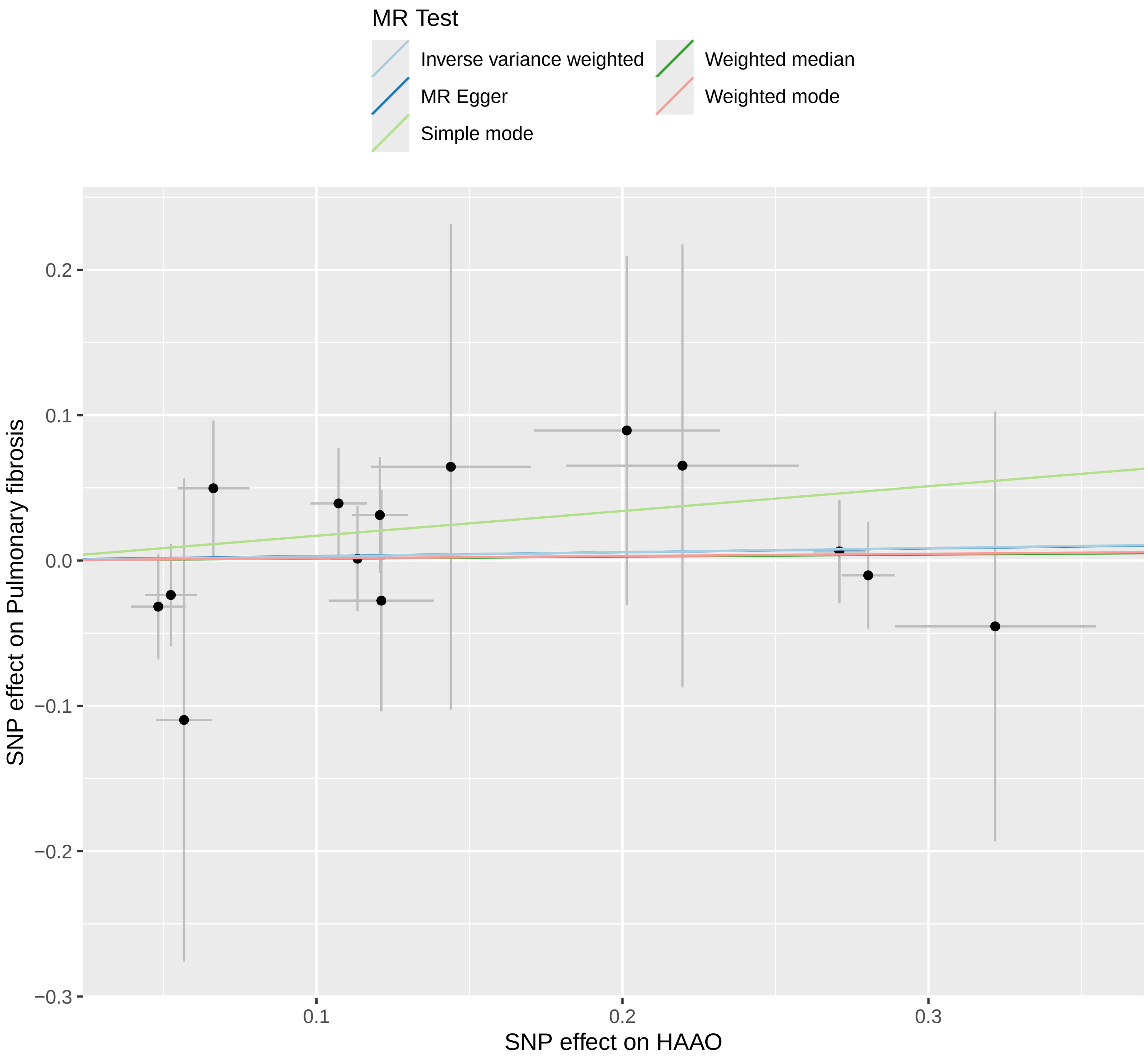
MR Test
Inverse variance weighted
MR Egger
Simple mode
Weighted median
Weighted mode
SNP effect on Pulmonary fibrosis
0.2
0.1
0.0
−0.1
−0.2
−0.3
0.1
0.2
0.3
SNP effect on HAAO

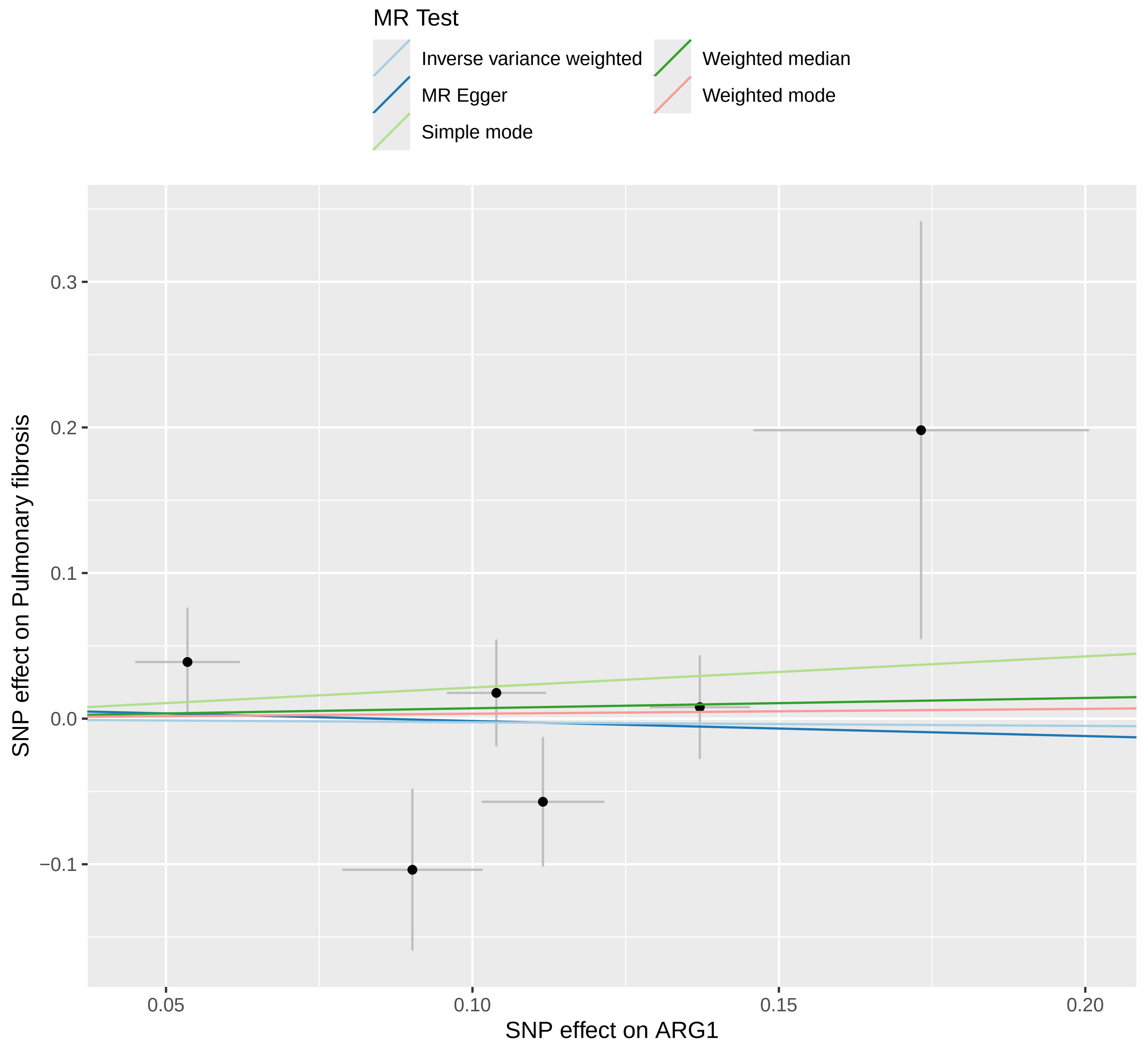

MR Test
Inverse variance weighted
MR Egger
Simple mode
Weighted median
Weighted mode
0.3
0.2
0.1
0.0
−0.1
0.05
0.10
0.15
0.20
SNP effect on ARG1
SNP effect on Pulmonary fibrosis

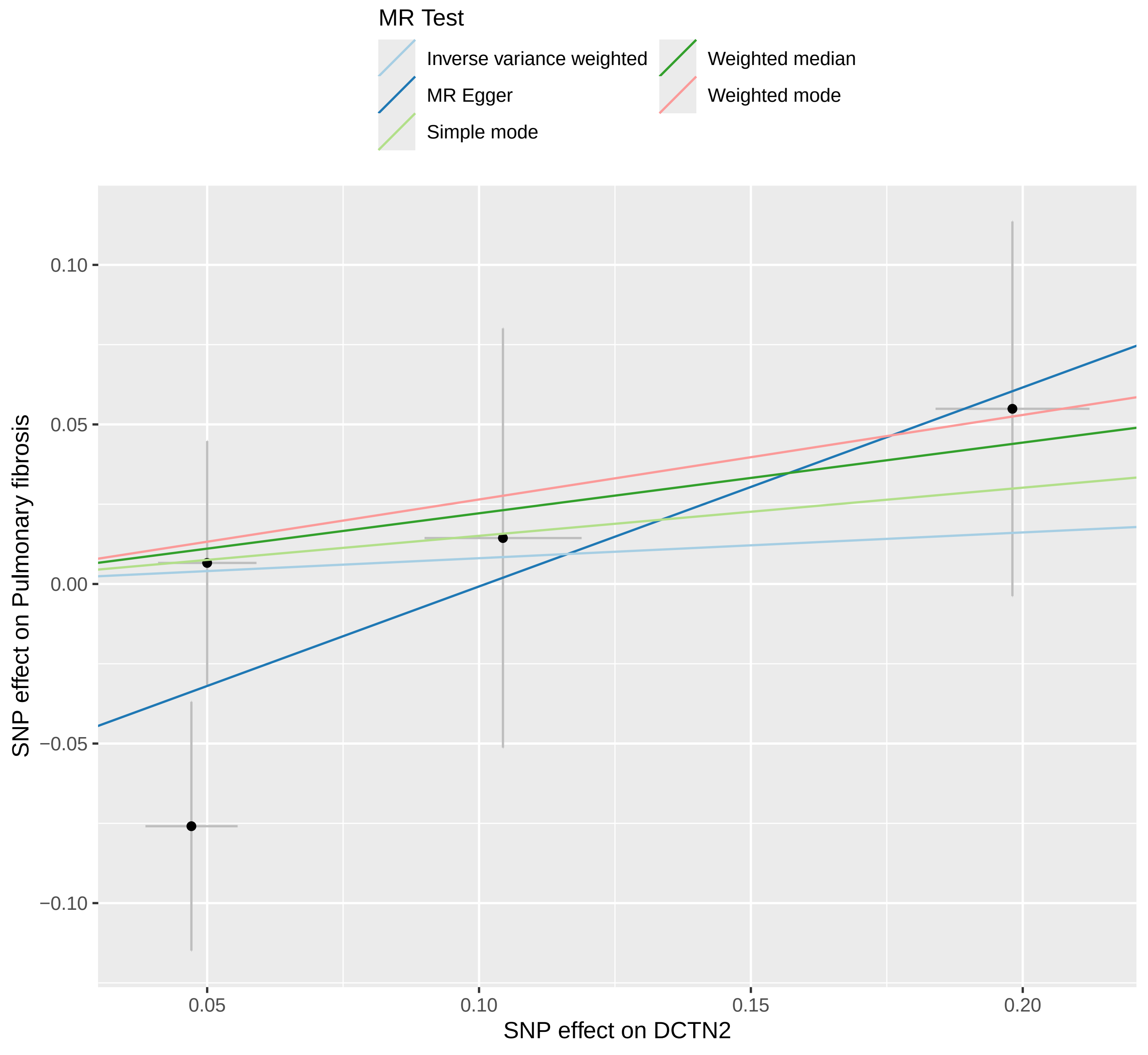

MR Test
Inverse variance weighted
MR Egger
Simple mode
Weighted median
Weighted mode
0.10
0.05
0.00
−0.05
−0.10
SNP effect on Pulmonary fibrosis
0.05
0.10
0.15
0.20
SNP effect on DCTN2

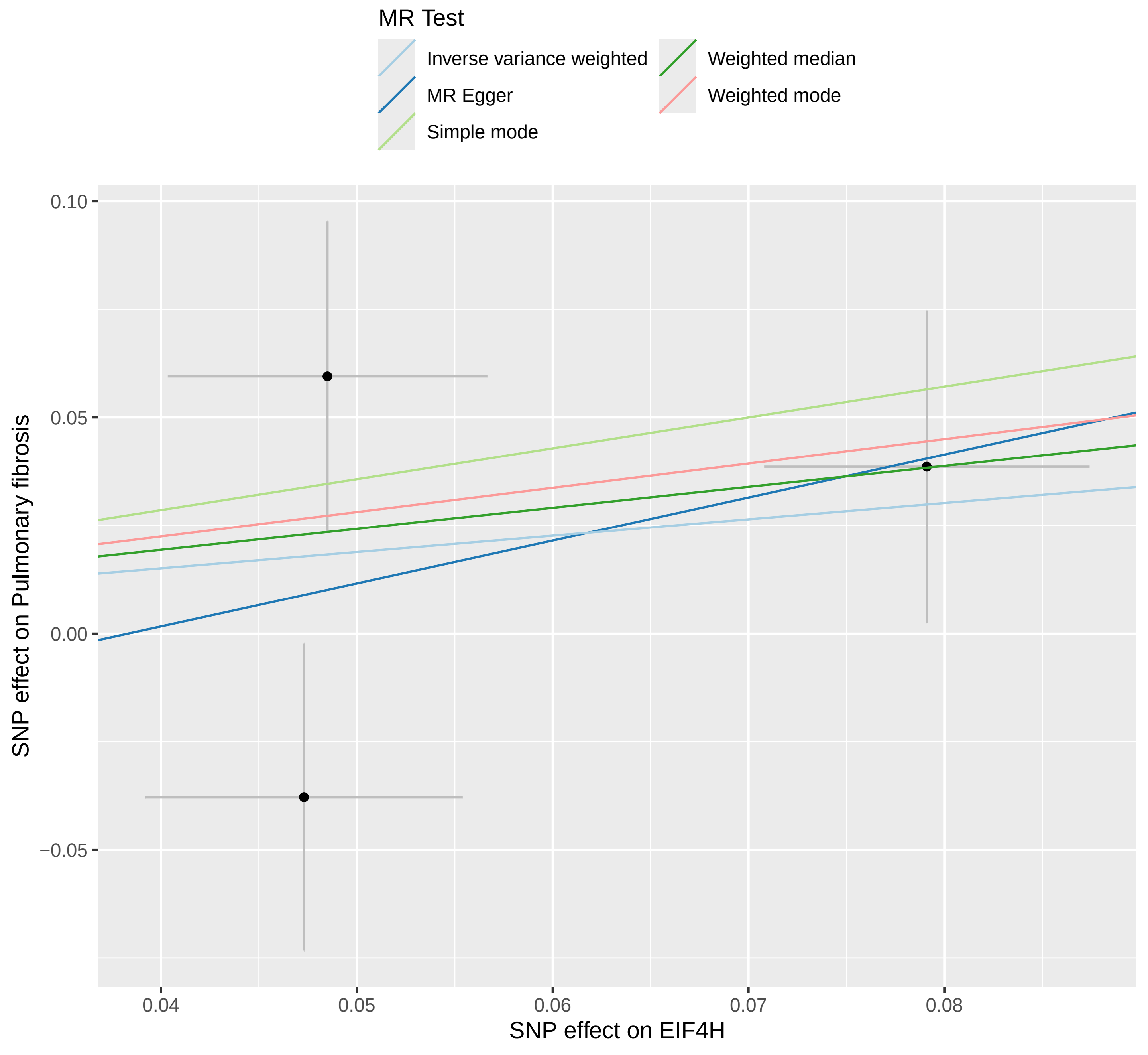
MR Test
Inverse variance weighted
MR Egger
Simple mode
Weighted median
Weighted mode
0.10
0.05
0.00
−0.05
SNP effect on Pulmonary fibrosis
0.04
0.05
0.06
0.07
0.08
SNP effect on EIF4H

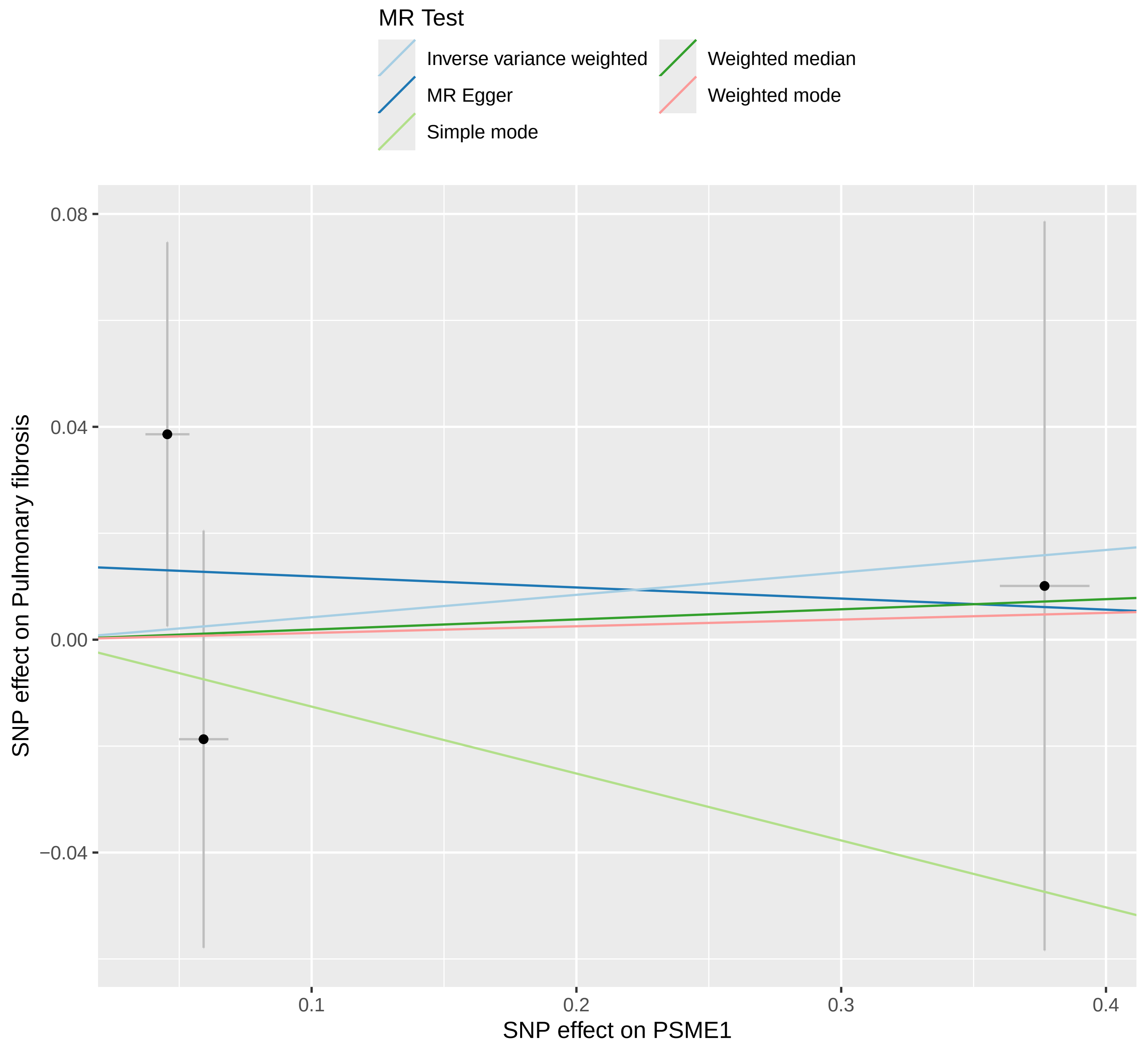
MR Test
Inverse variance weighted
MR Egger
Simple mode
Weighted median
Weighted mode
0.08
0.04
0.00
−0.04
SNP effect on Pulmonary fibrosis
0.1
0.2
0.3
0.4
SNP effect on PSME1

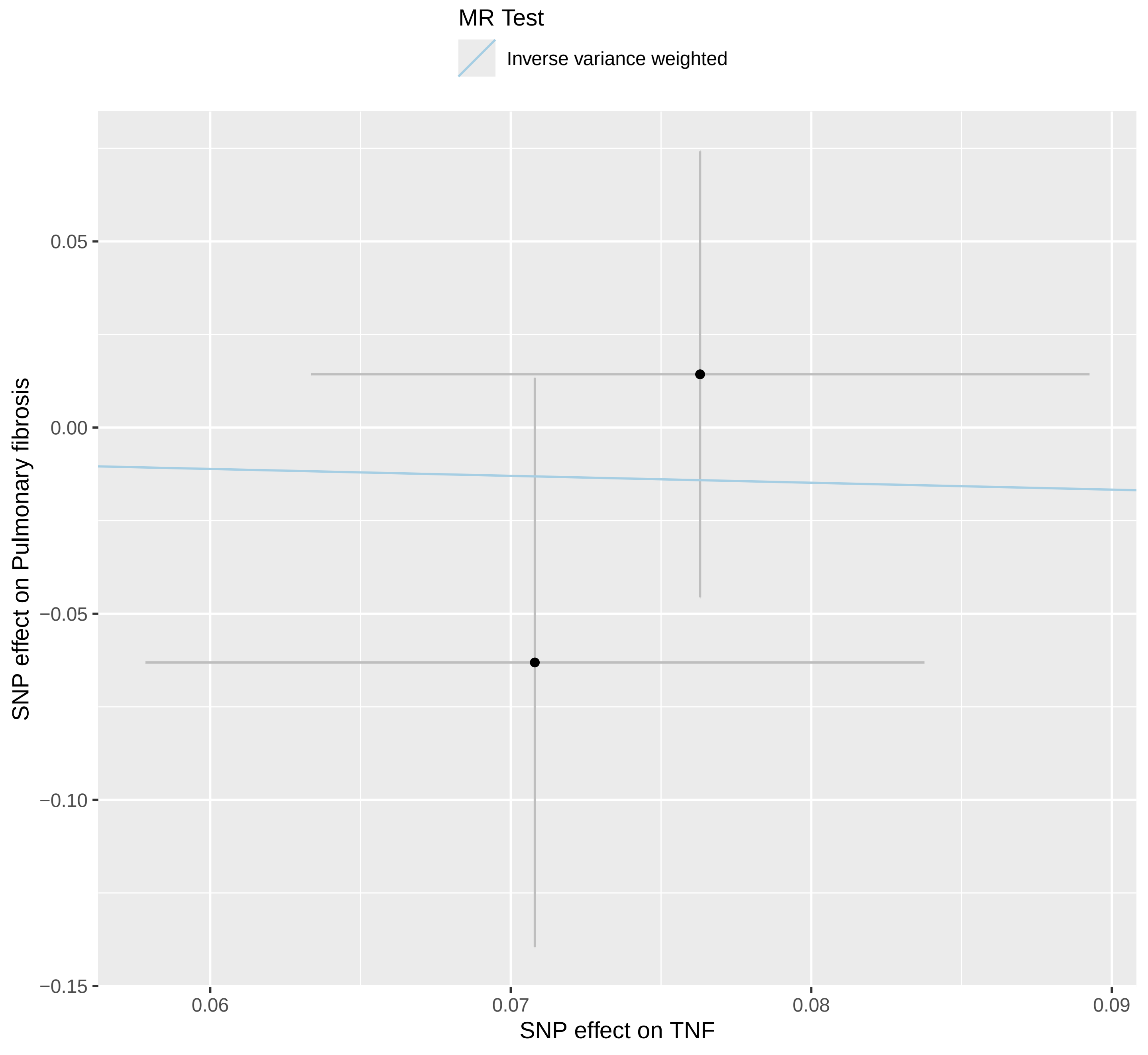
MR Test
Inverse variance weighted
0.05
0.00
−0.05
−0.10
−0.15
0.06
0.07
0.08
0.09
SNP effect on TNF
SNP effect on Pulmonary fibrosis

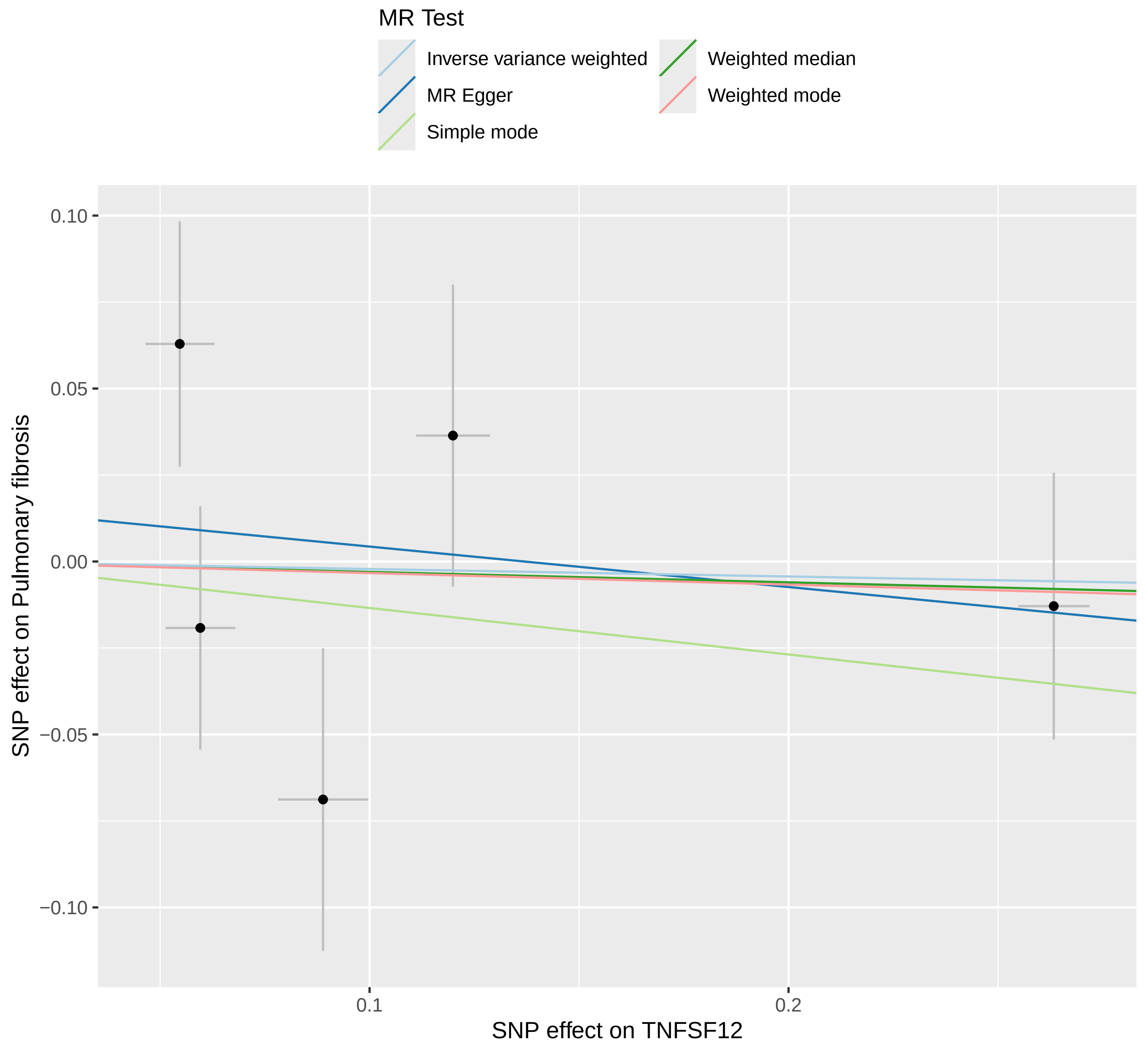

MR Test
Inverse variance weighted
MR Egger
Simple mode
Weighted median
Weighted mode
0.10
0.05
0.00
−0.05
−0.10
0.1
0.2
SNP effect on Pulmonary fibrosis
SNP effect on TNFSF12

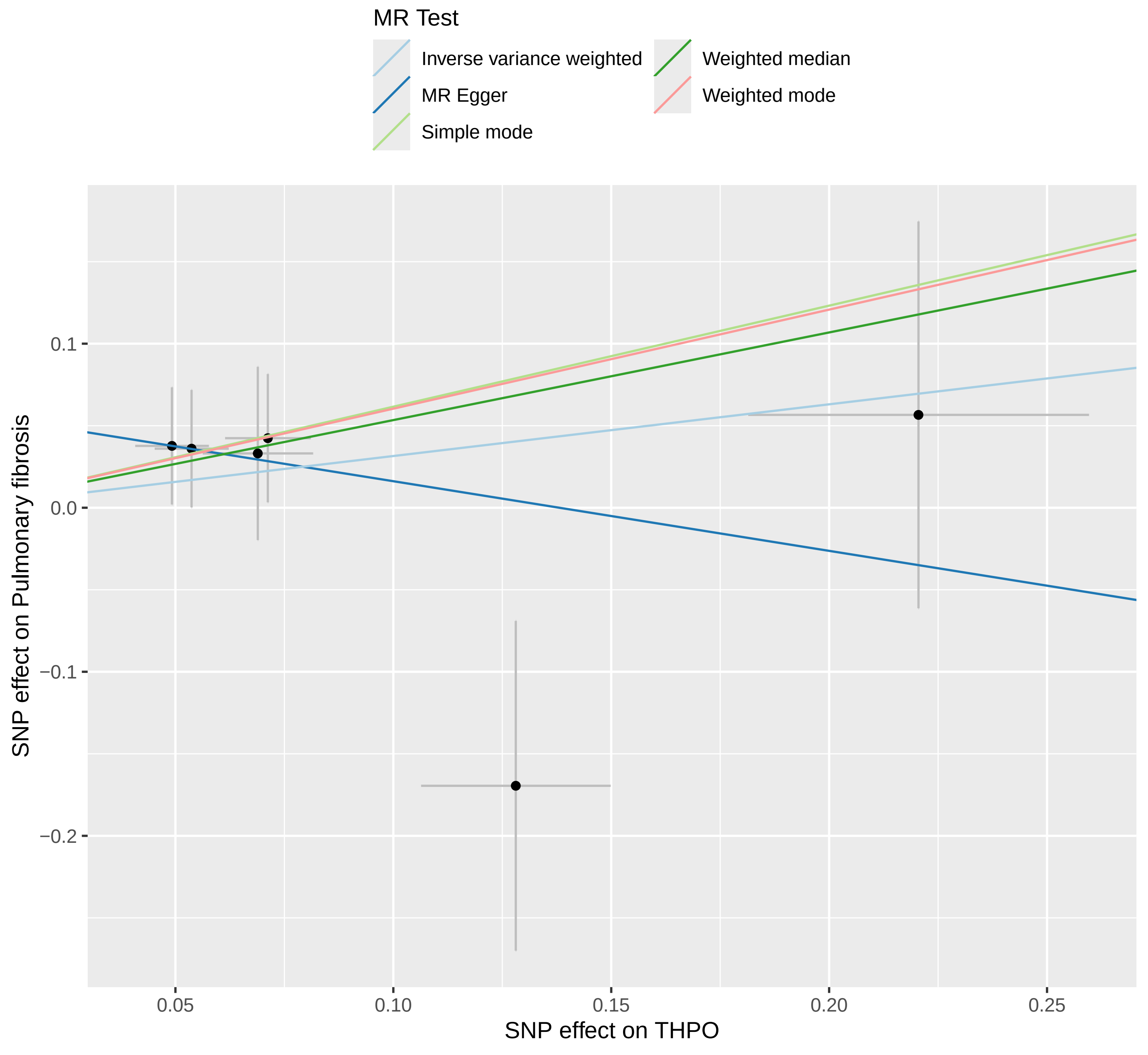
MR Test
Inverse variance weighted
MR Egger
Simple mode
Weighted median
Weighted mode
0.1
0.0
−0.1
−0.2
0.05
0.10
0.15
0.20
0.25
SNP effect on Pulmonary fibrosis
SNP effect on THPO

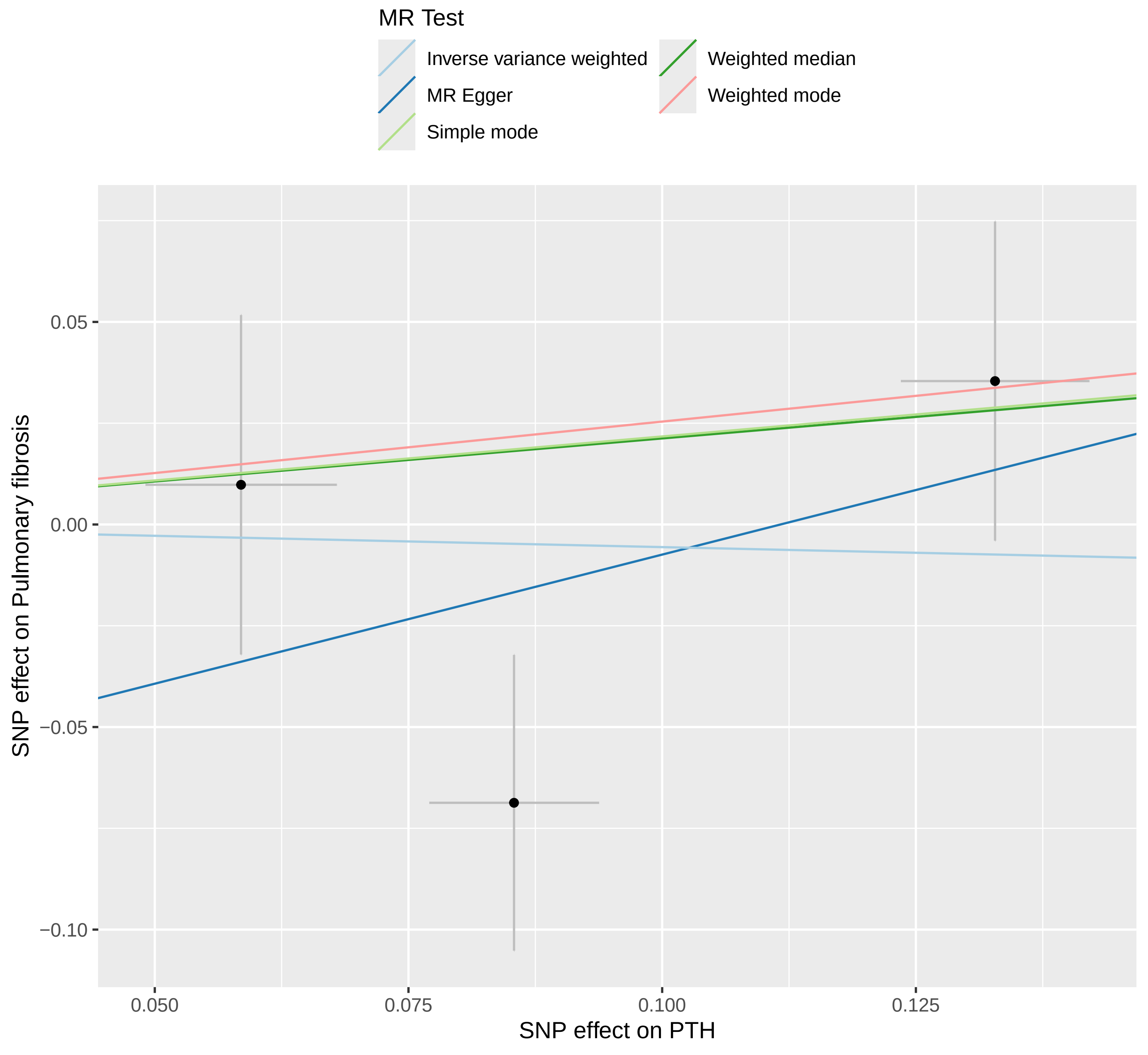

MR Test
Inverse variance weighted
MR Egger
Simple mode
Weighted median
Weighted mode
0.05
0.00
−0.05
−0.10
SNP effect on Pulmonary fibrosis
0.050
0.075
0.100
0.125
SNP effect on PTH

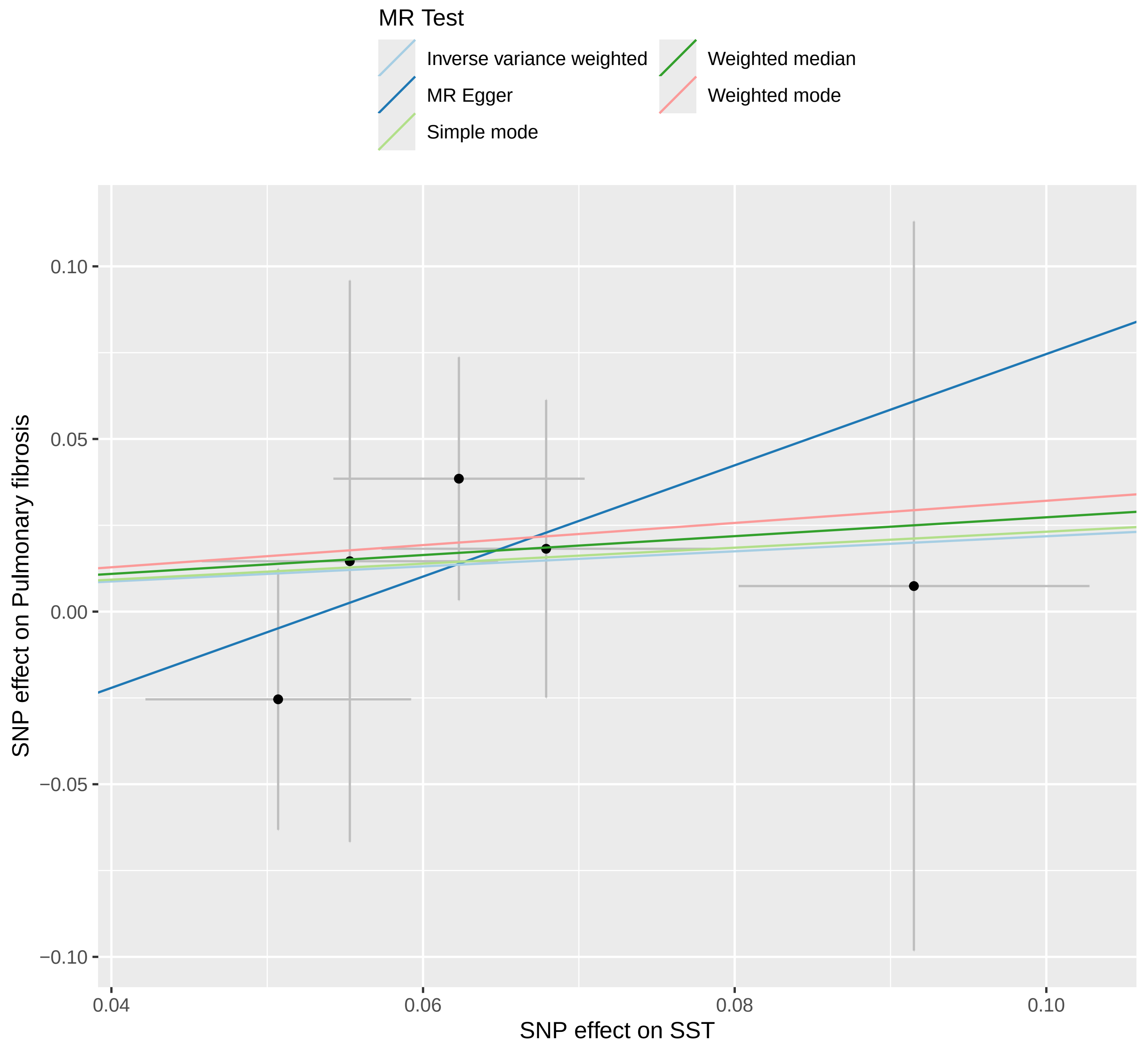

MR Test
Inverse variance weighted
MR Egger
Simple mode
Weighted median
Weighted mode
0.10
0.05
0.00
−0.05
−0.10
SNP effect on Pulmonary fibrosis
0.04
0.06
0.08
0.10
SNP effect on SST

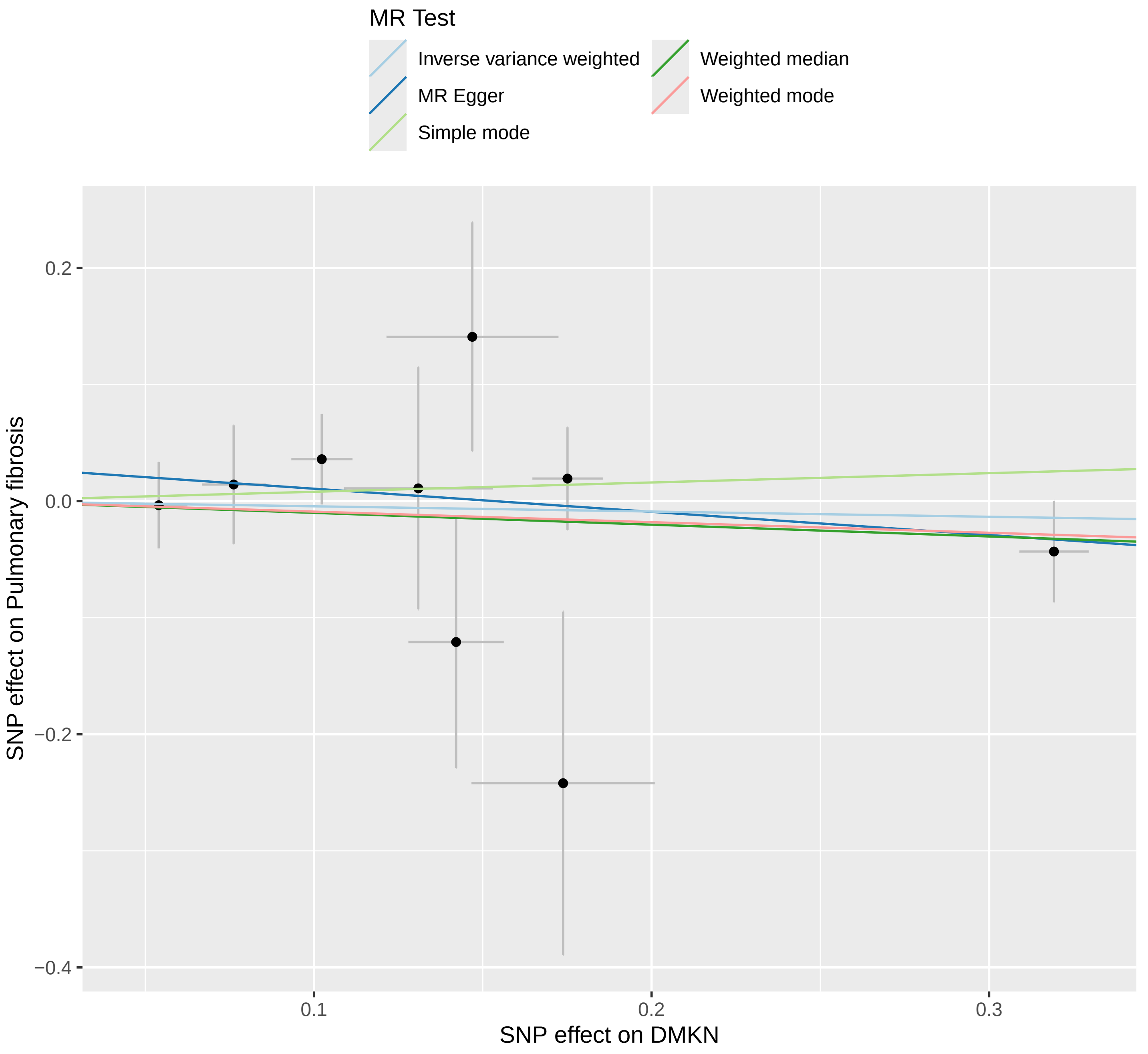
MR Test
Inverse variance weighted
MR Egger
Simple mode
Weighted median
Weighted mode
0.2
0.0
−0.2
−0.4
0.1
0.2
0.3
SNP effect on Pulmonary fibrosis
SNP effect on DMKN

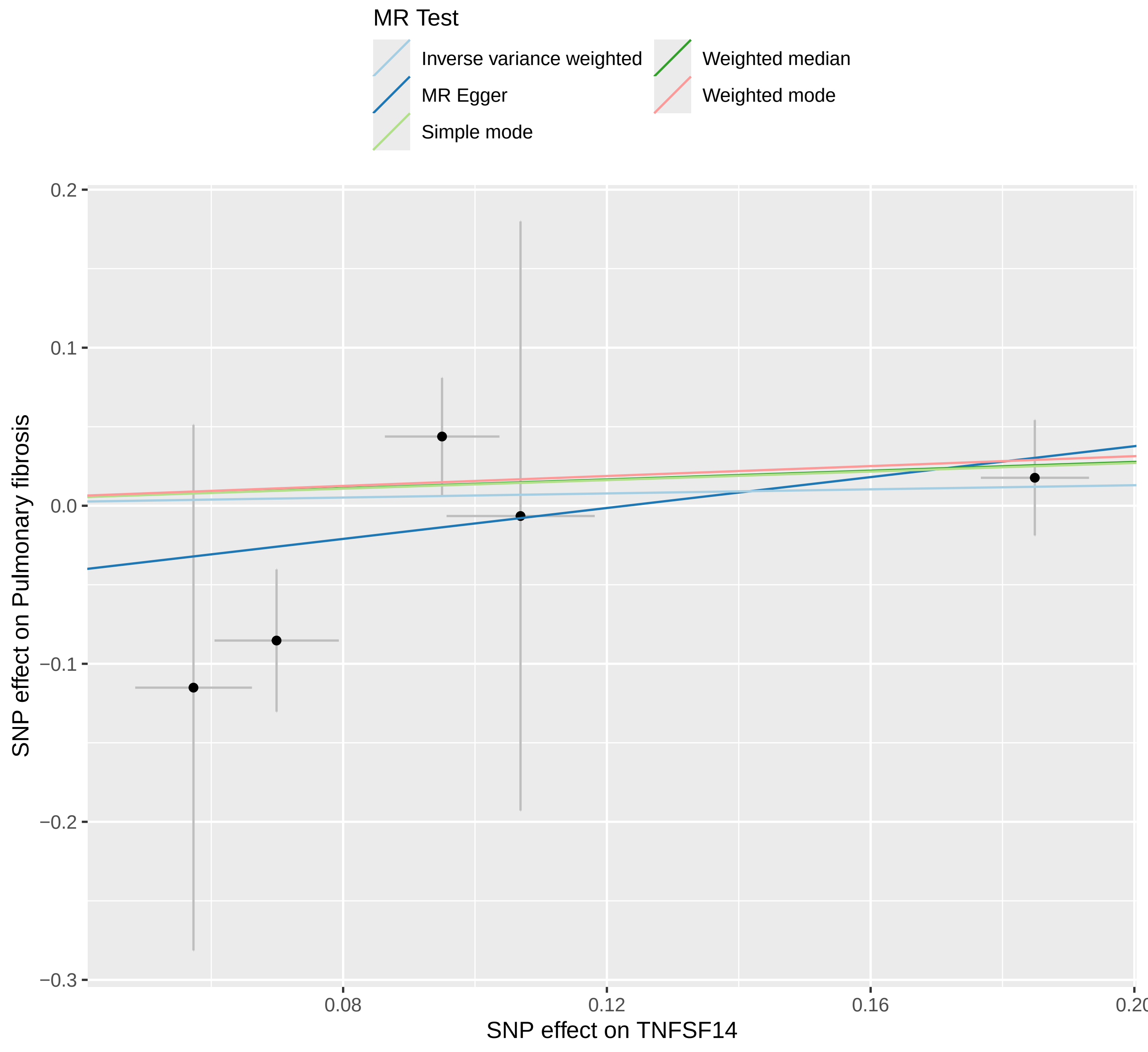

MR Test
Inverse variance weighted
MR Egger
Simple mode
Weighted median
Weighted mode
0.2
0.1
0.0
−0.1
−0.2
−0.3
0.08
0.12
0.16
0.20
SNP effect on TNFSF14
SNP effect on Pulmonary fibrosis

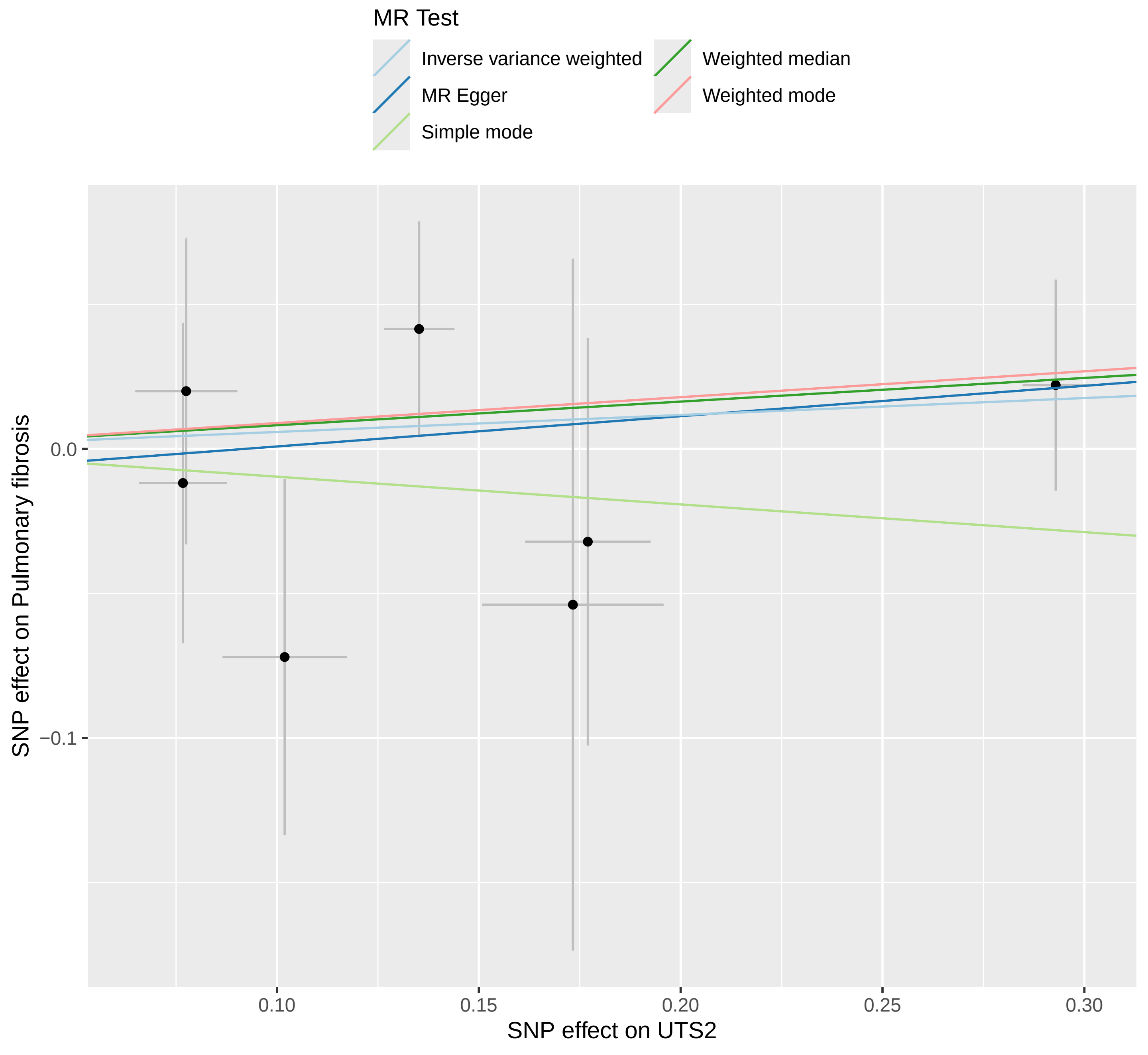

MR Test
Inverse variance weighted
MR Egger
Simple mode
Weighted median
Weighted mode
0.0
−0.1
0.10
0.15
0.20
0.25
0.30
SNP effect on UTS2
SNP effect on Pulmonary fibrosis

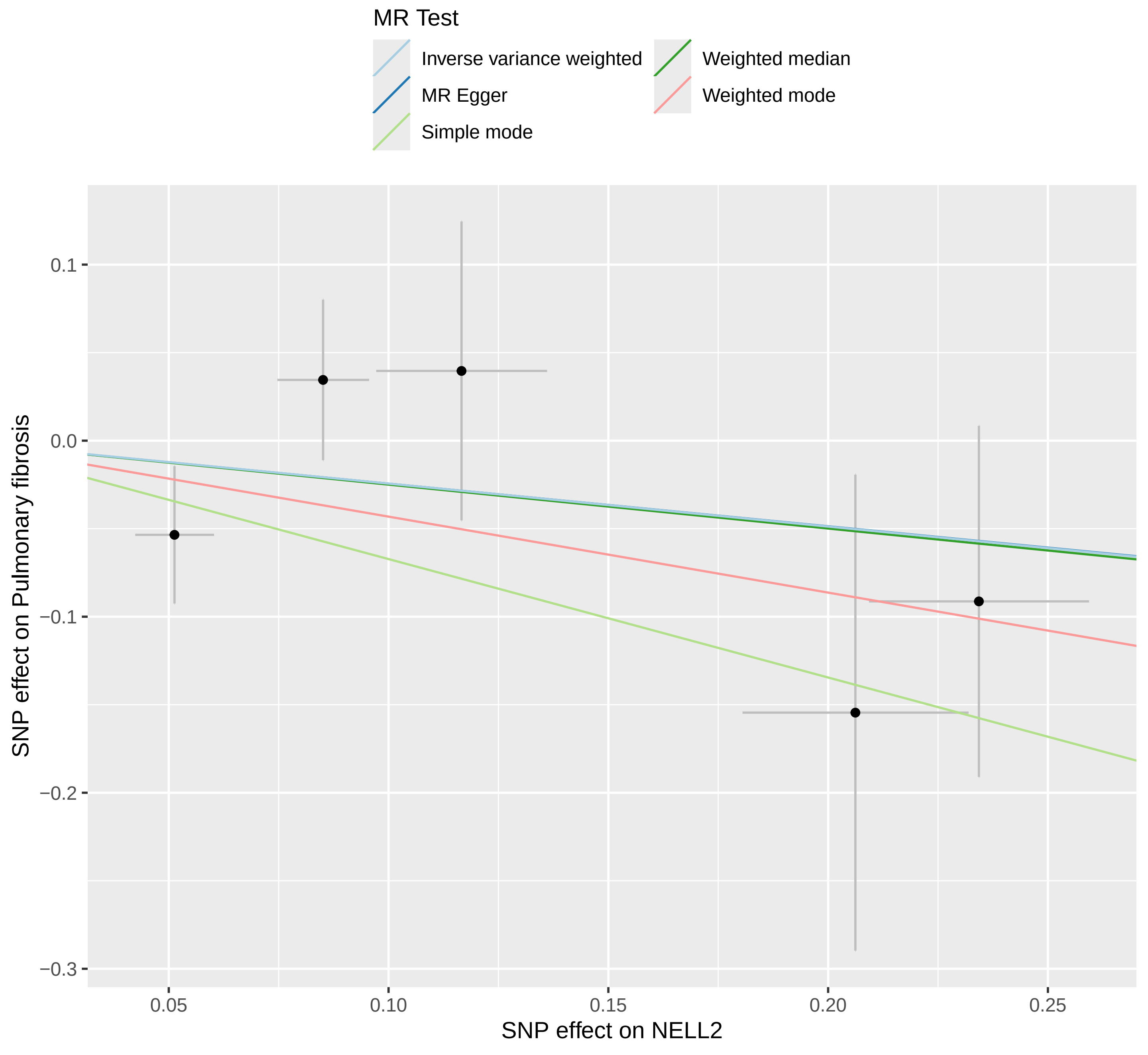

MR Test
Inverse variance weighted
MR Egger
Simple mode
Weighted median
Weighted mode
0.1
0.0
−0.1
−0.2
−0.3
SNP effect on Pulmonary fibrosis
0.05
0.10
0.15
0.20
0.25
SNP effect on NELL2

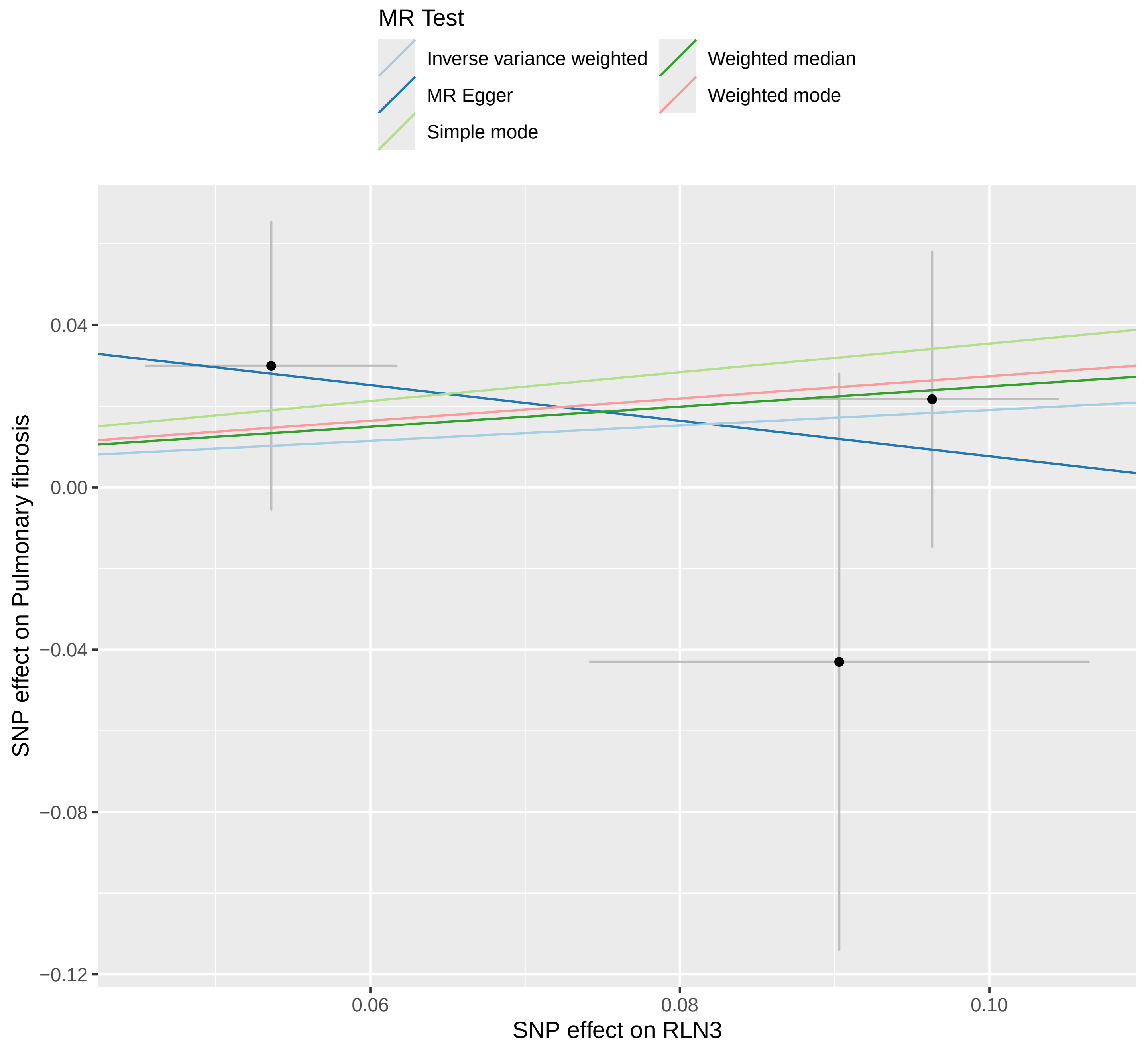
MR Test
Inverse variance weighted
MR Egger
Simple mode
Weighted median
Weighted mode
0.04
0.00
−0.04
−0.08
−0.12
SNP effect on Pulmonary fibrosis
0.06
0.08
0.10
SNP effect on RLN3

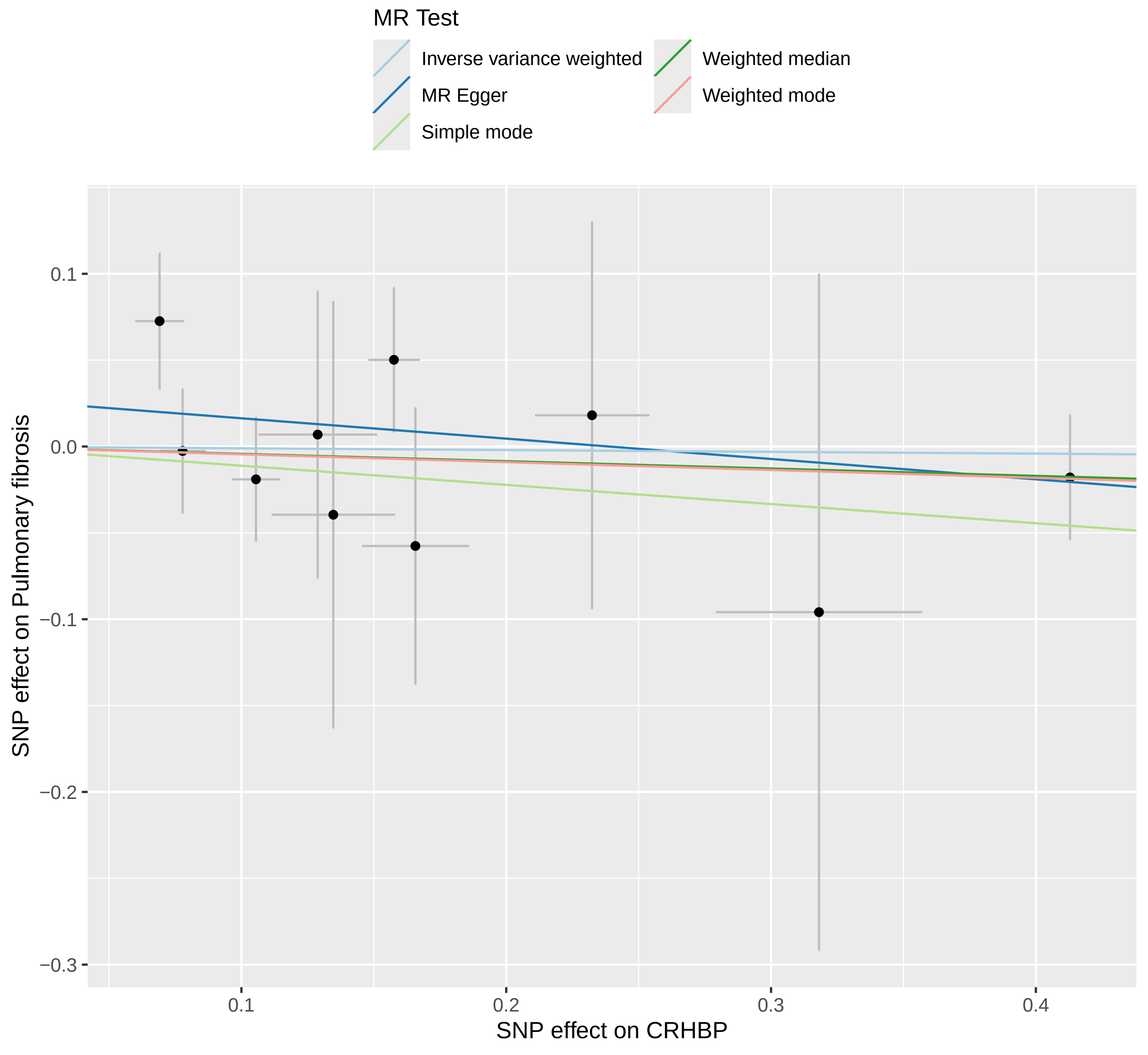
MR Test
Inverse variance weighted
MR Egger
Simple mode
Weighted median
Weighted mode
SNP effect on Pulmonary fibrosis
0.1
0.0
−0.1
−0.2
−0.3
0.1
0.2
0.3
0.4
SNP effect on CRHBP

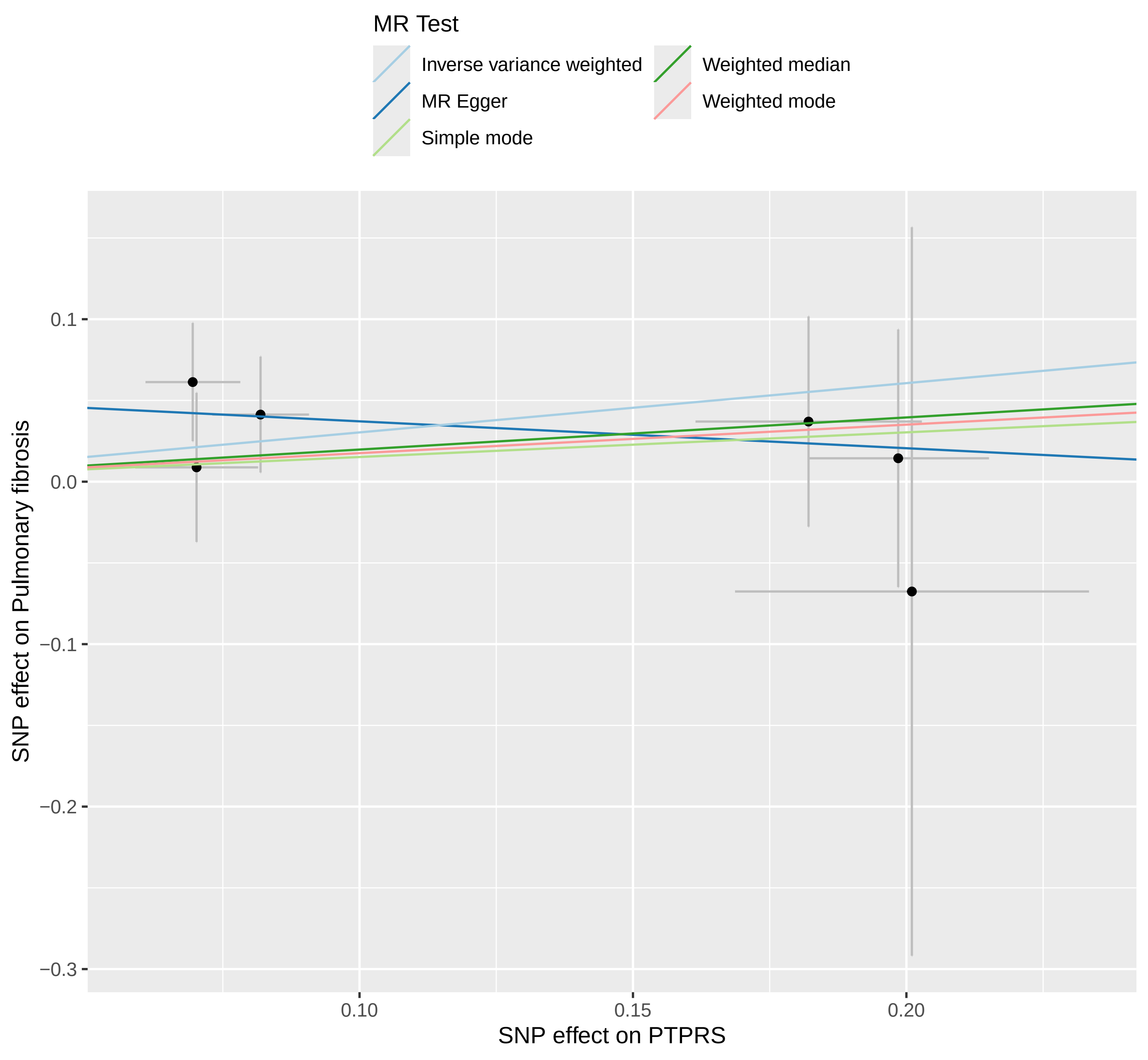
MR Test
Inverse variance weighted
MR Egger
Simple mode
Weighted median
Weighted mode
0.1
0.0
−0.1
−0.2
−0.3
0.10
0.15
0.20
SNP effect on PTPRS
SNP effect on Pulmonary fibrosis

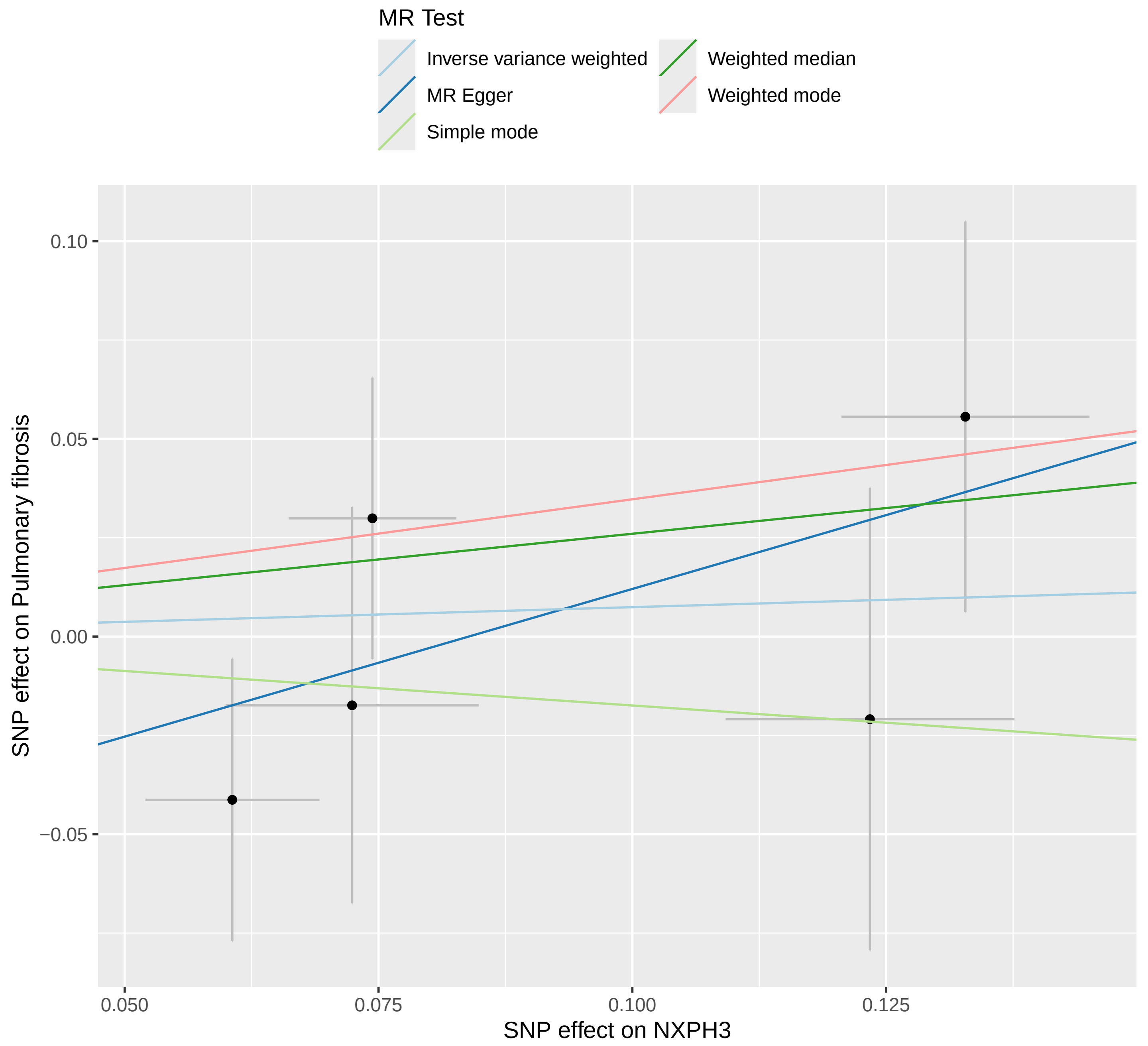
MR Test
Inverse variance weighted
MR Egger
Simple mode
Weighted median
Weighted mode
0.10
0.05
0.00
−0.05
0.050
0.075
0.100
0.125
SNP effect on NXPH3
SNP effect on Pulmonary fibrosis

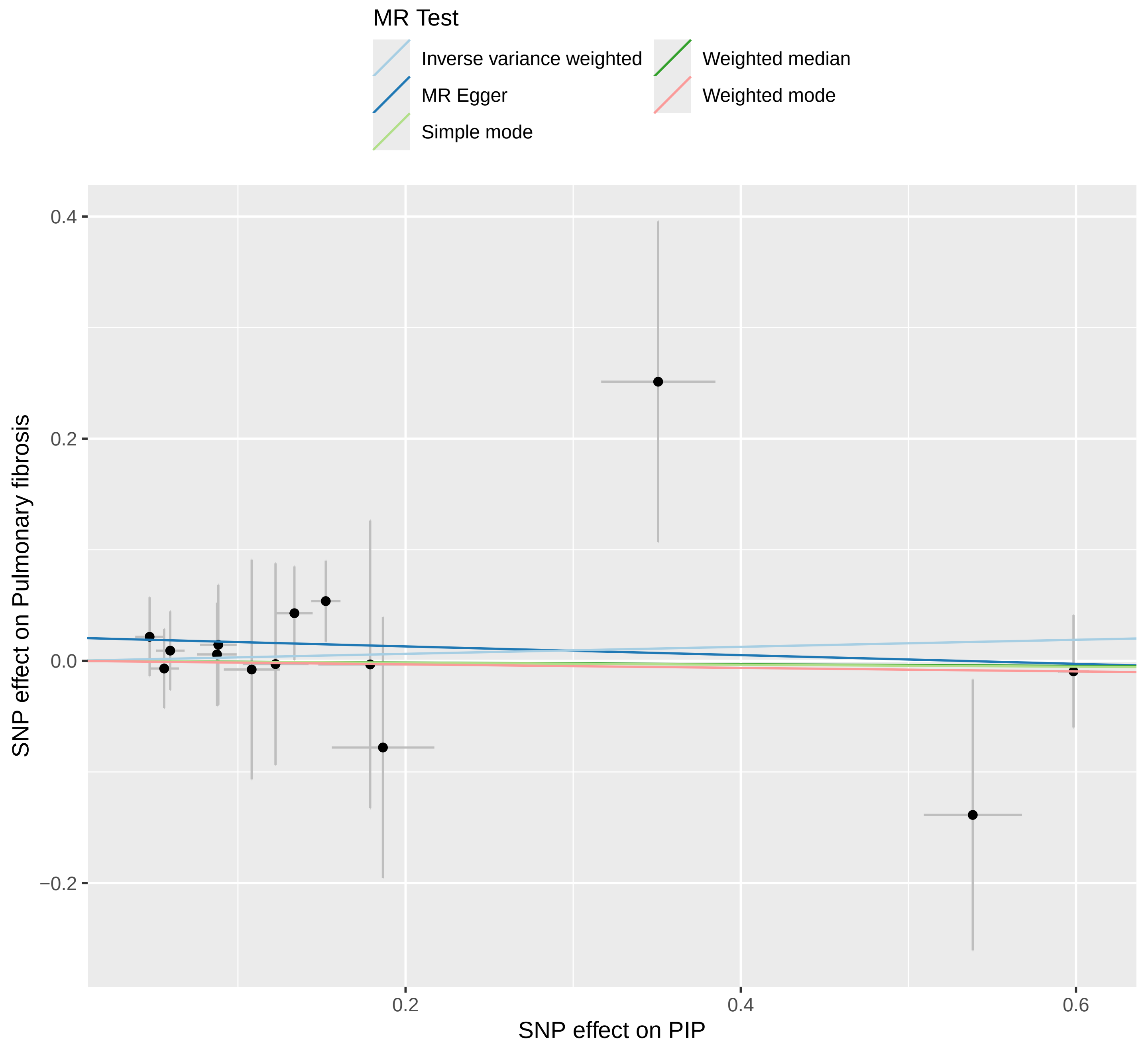

MR Test
Inverse variance weighted
MR Egger
Simple mode
Weighted median
Weighted mode
SNP effect on Pulmonary fibrosis
0.4
0.2
0.0
−0.2
0.2
0.4
0.6
SNP effect on PIP

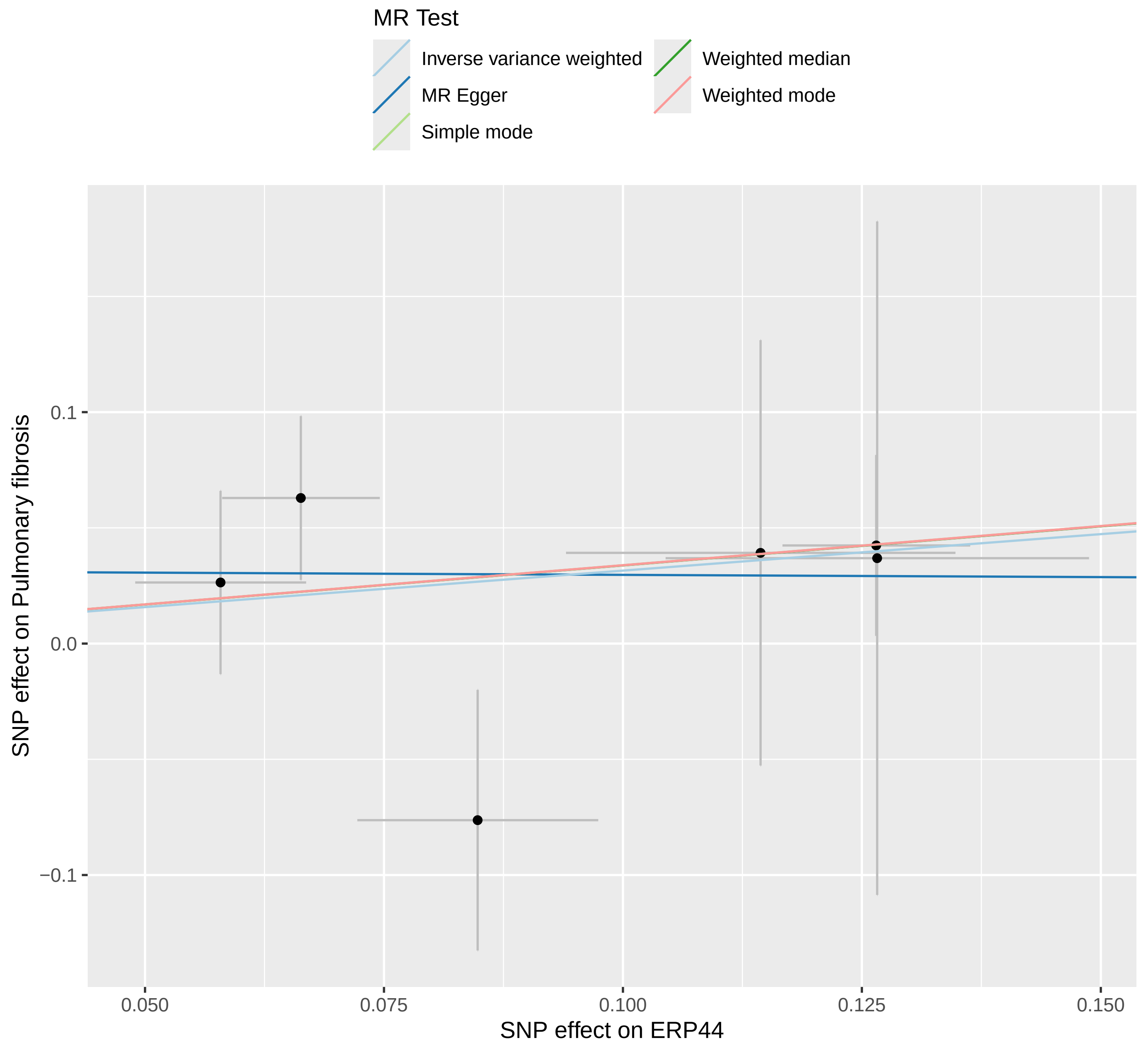
MR Test
Inverse variance weighted
MR Egger
Simple mode
Weighted median
Weighted mode
0.1
0.0
−0.1
SNP effect on Pulmonary fibrosis
0.050
0.075
0.100
0.125
0.150
SNP effect on ERP44

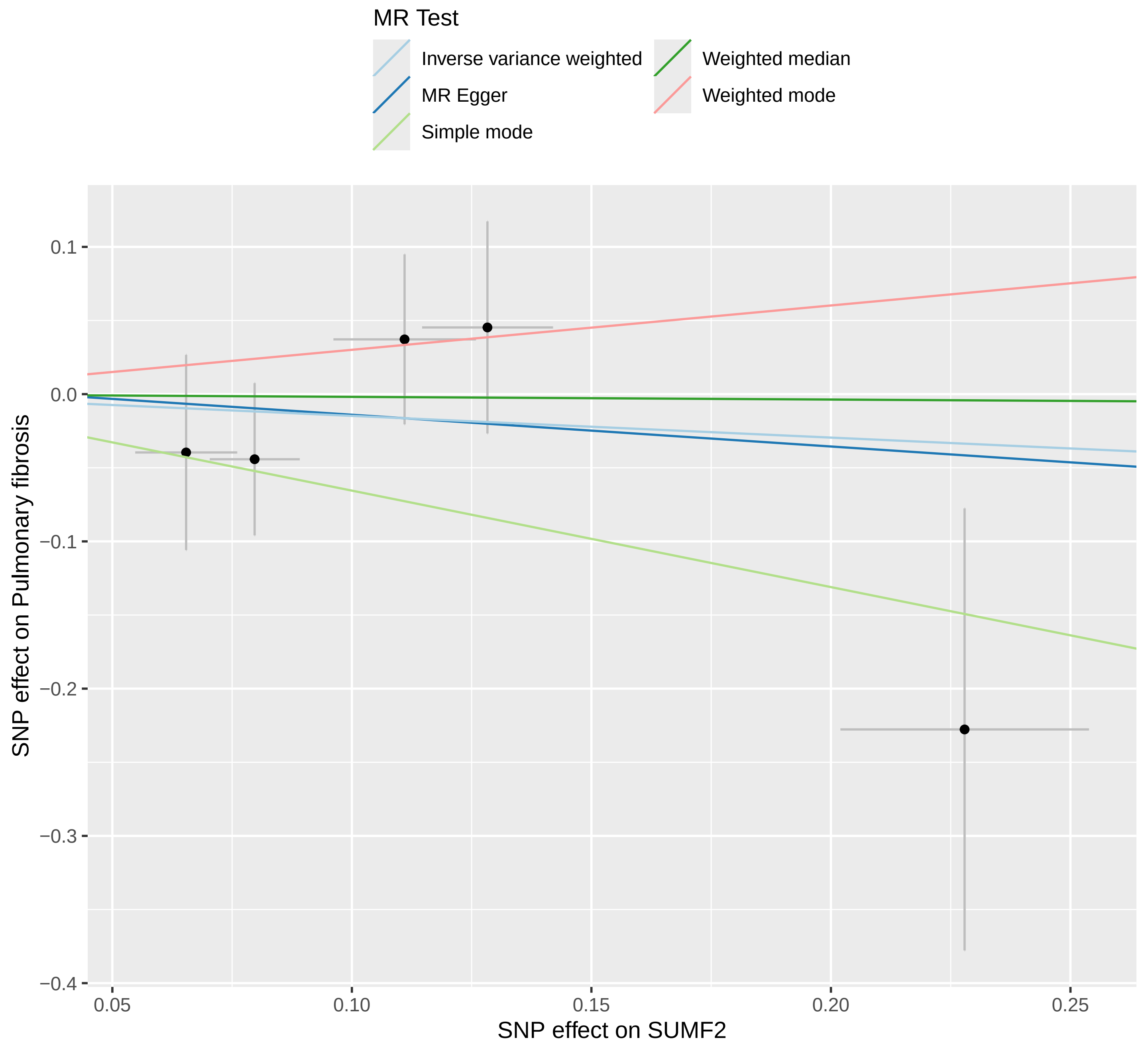

MR Test
Inverse variance weighted
MR Egger
Simple mode
Weighted median
Weighted mode
0.1
0.0
−0.1
−0.2
−0.3
−0.4
SNP effect on Pulmonary fibrosis
0.05
0.10
0.15
0.20
0.25
SNP effect on SUMF2

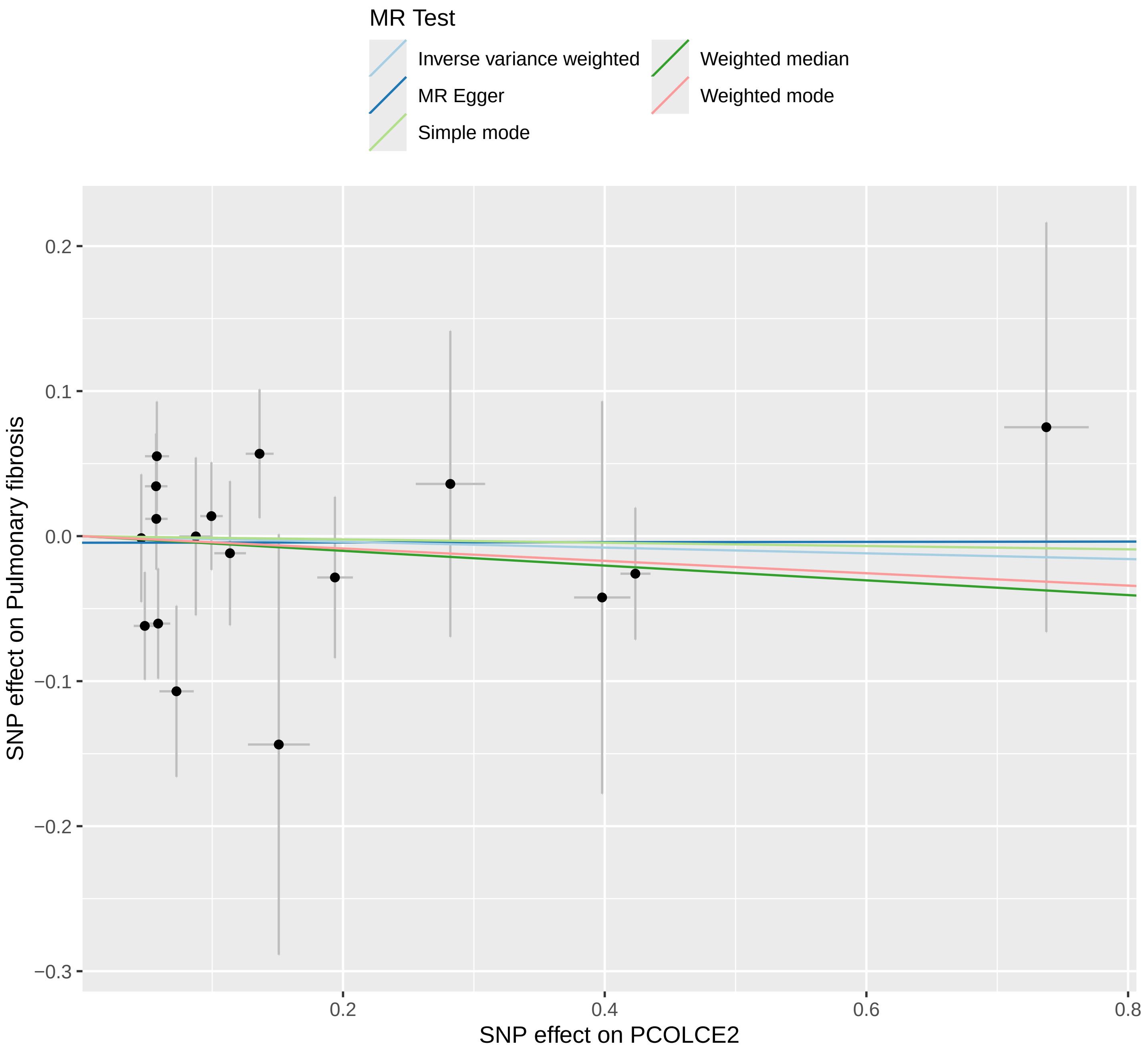
MR Test
Inverse variance weighted
MR Egger
Simple mode
Weighted median
Weighted mode
0.2
0.1
0.0
−0.1
−0.2
−0.3
0.2
0.4
0.6
0.8
SNP effect on Pulmonary fibrosis
SNP effect on PCOLCE2

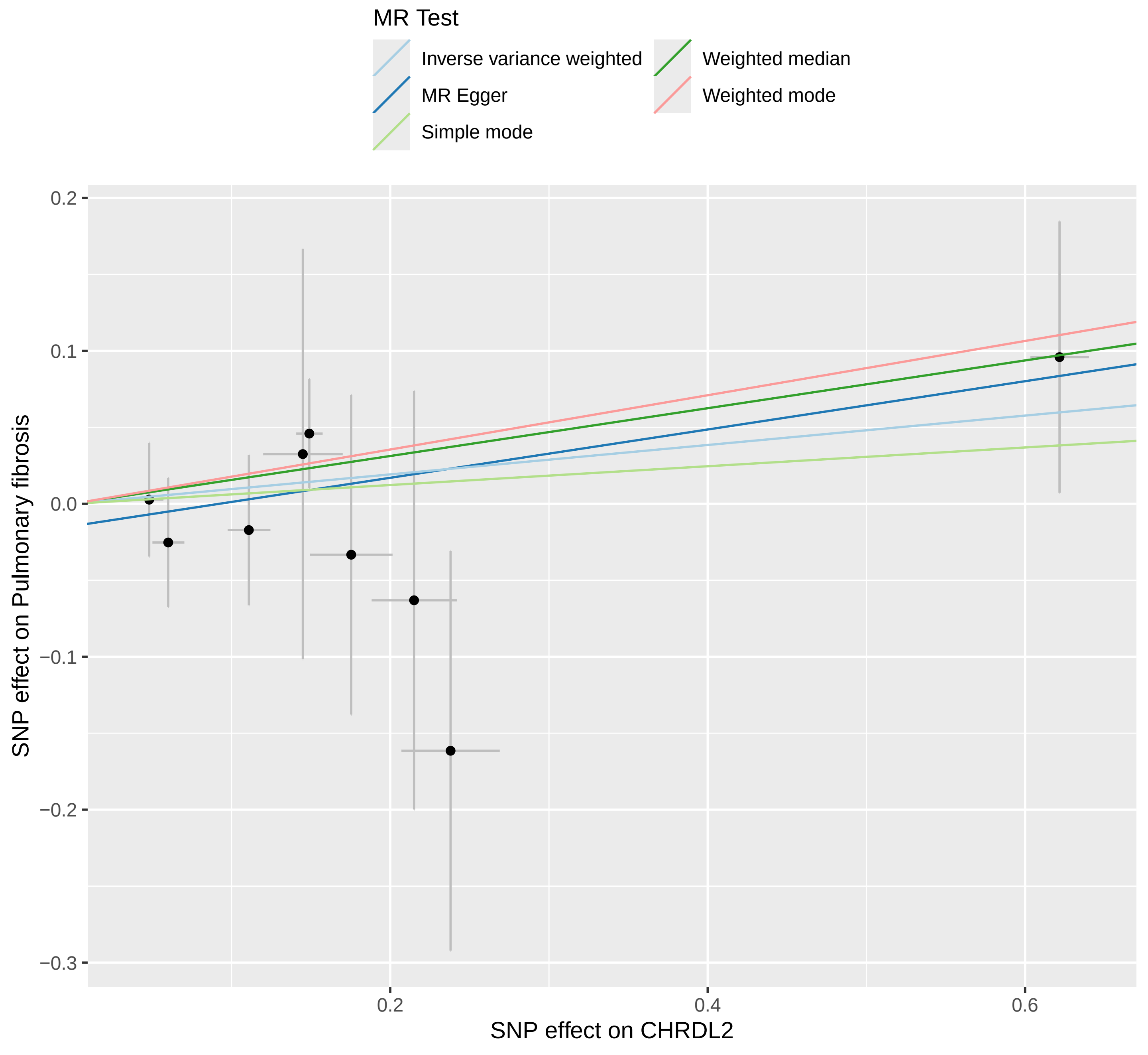

MR Test
Inverse variance weighted
MR Egger
Simple mode
Weighted median
Weighted mode
0.2
0.1
0.0
−0.1
−0.2
−0.3
SNP effect on Pulmonary fibrosis
0.2
0.4
0.6
SNP effect on CHRDL2

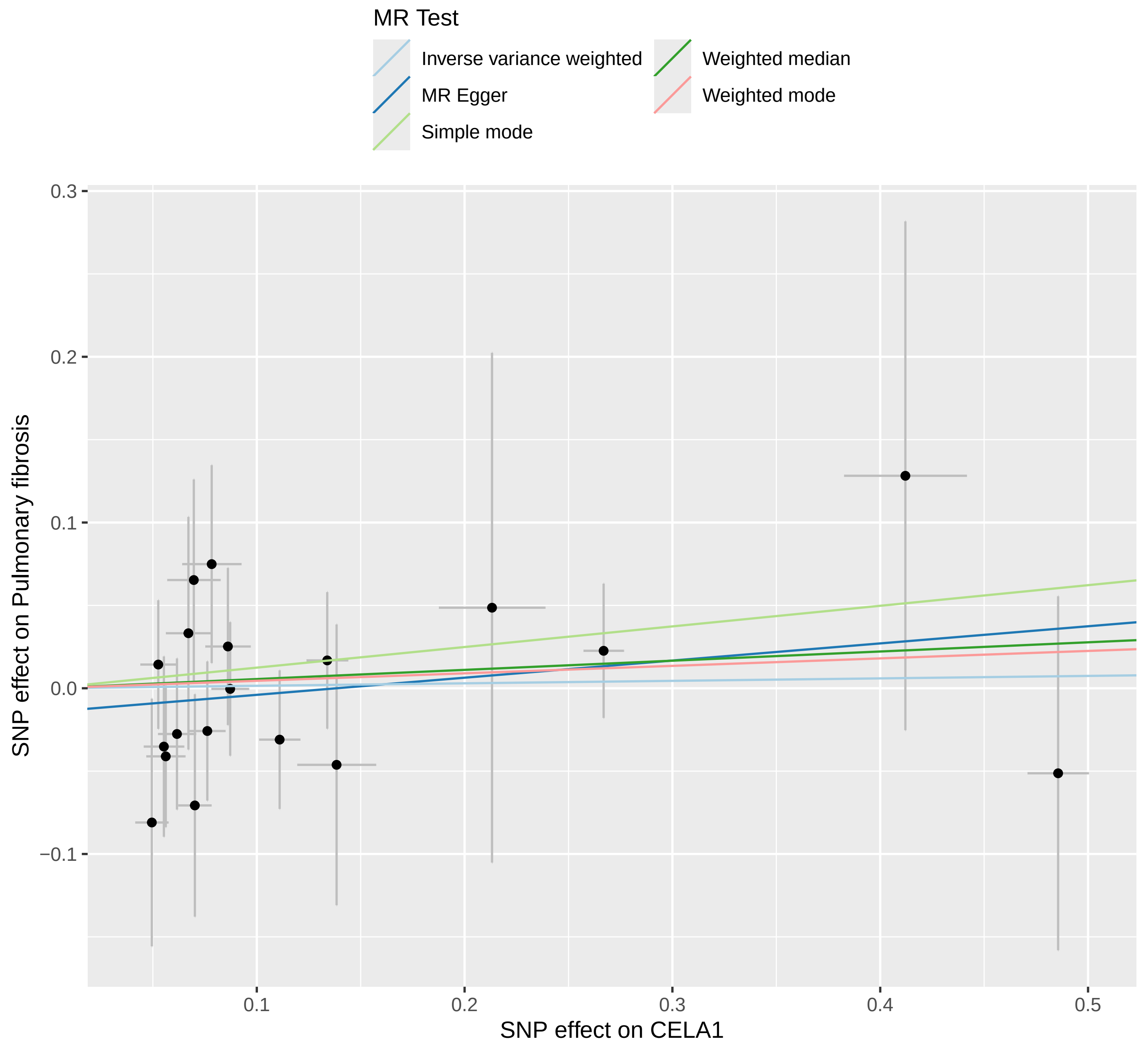

MR Test
Inverse variance weighted
MR Egger
Simple mode
Weighted median
Weighted mode
SNP effect on Pulmonary fibrosis
0.3
0.2
0.1
0.0
−0.1
0.1
0.2
0.3
0.4
0.5
SNP effect on CELA1

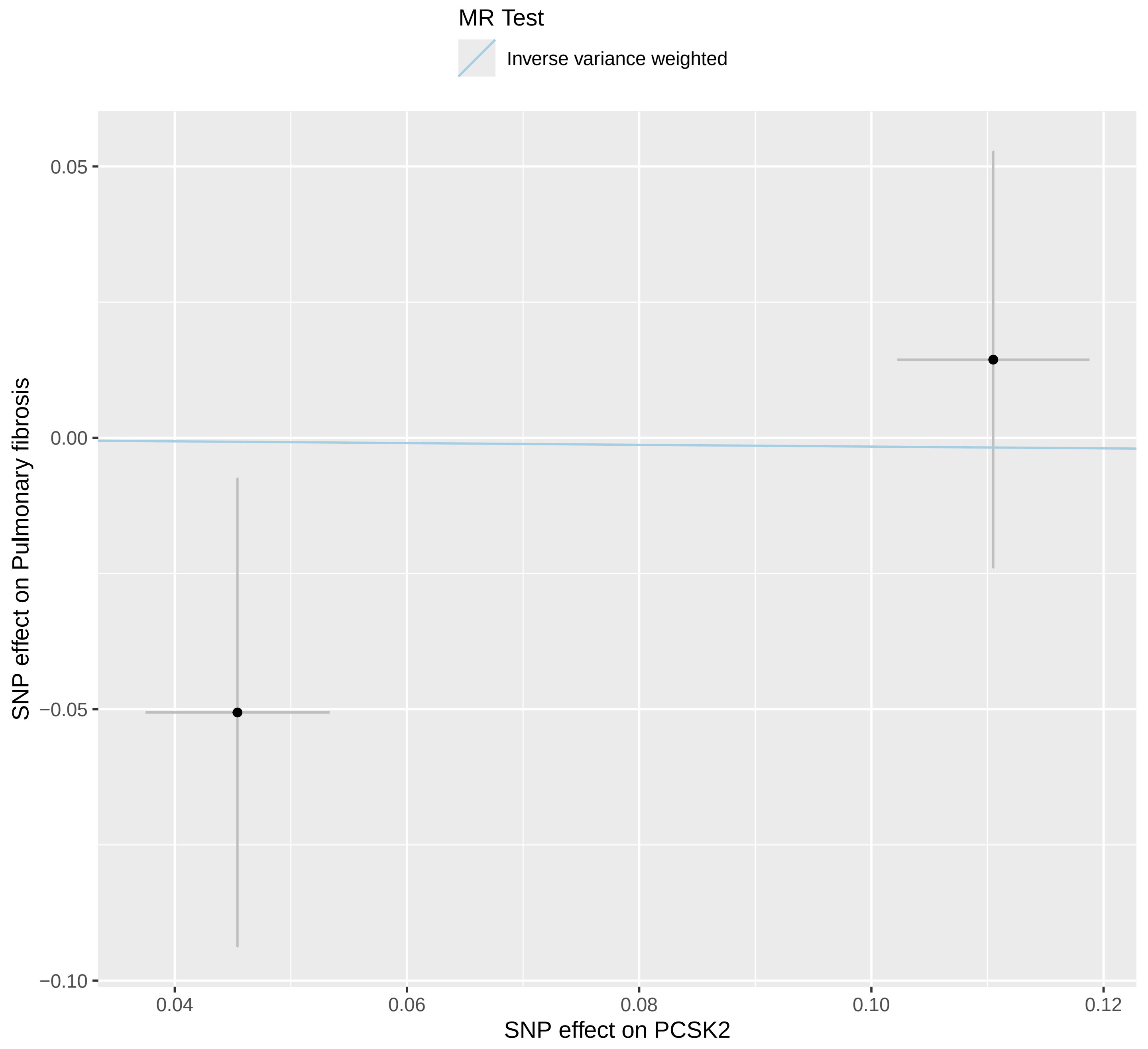
MR Test
Inverse variance weighted
0.05
0.00
−0.05
−0.10
0.04
0.06
0.08
0.10
0.12
SNP effect on PCSK2
SNP effect on Pulmonary fibrosis

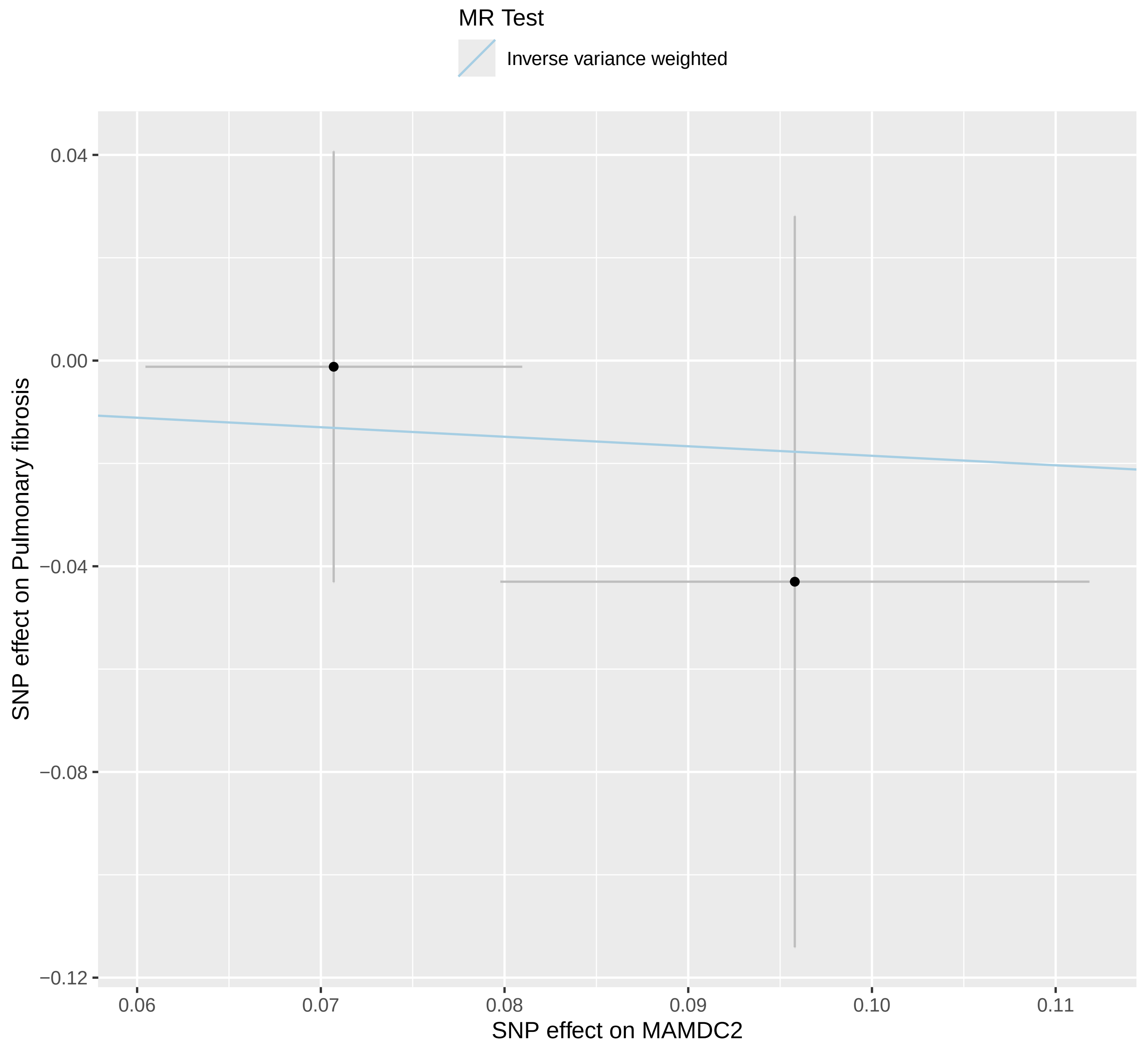

MR Test
Inverse variance weighted
0.04
0.00
−0.04
−0.08
−0.12
SNP effect on Pulmonary fibrosis
0.06
0.07
0.08
0.09
0.10
0.11
SNP effect on MAMDC2

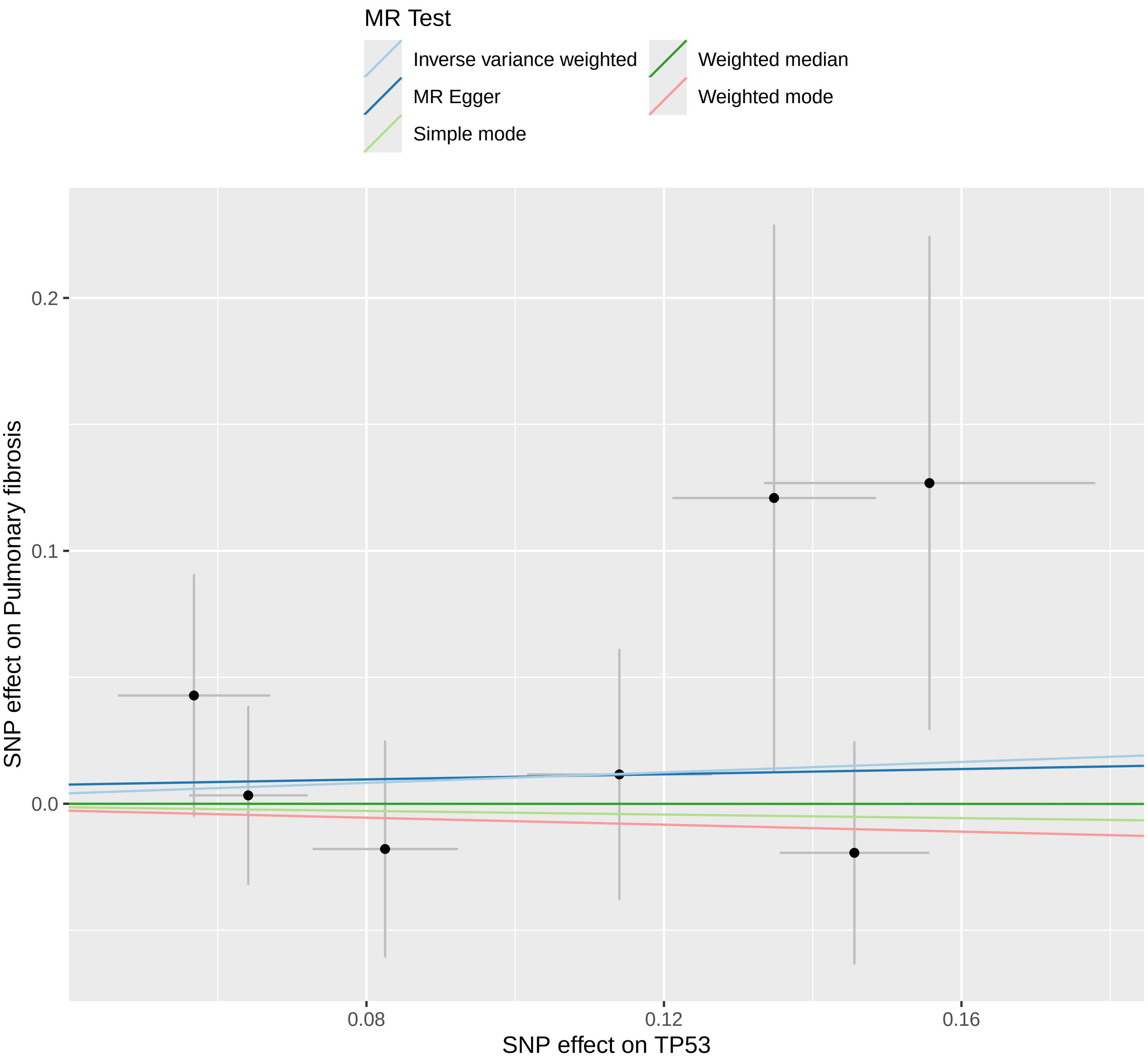

MR Test
Inverse variance weighted
MR Egger
Simple mode
Weighted median
Weighted mode
0.2
0.1
0.0
0.08
0.12
0.16
SNP effect on Pulmonary fibrosis
SNP effect on TP53

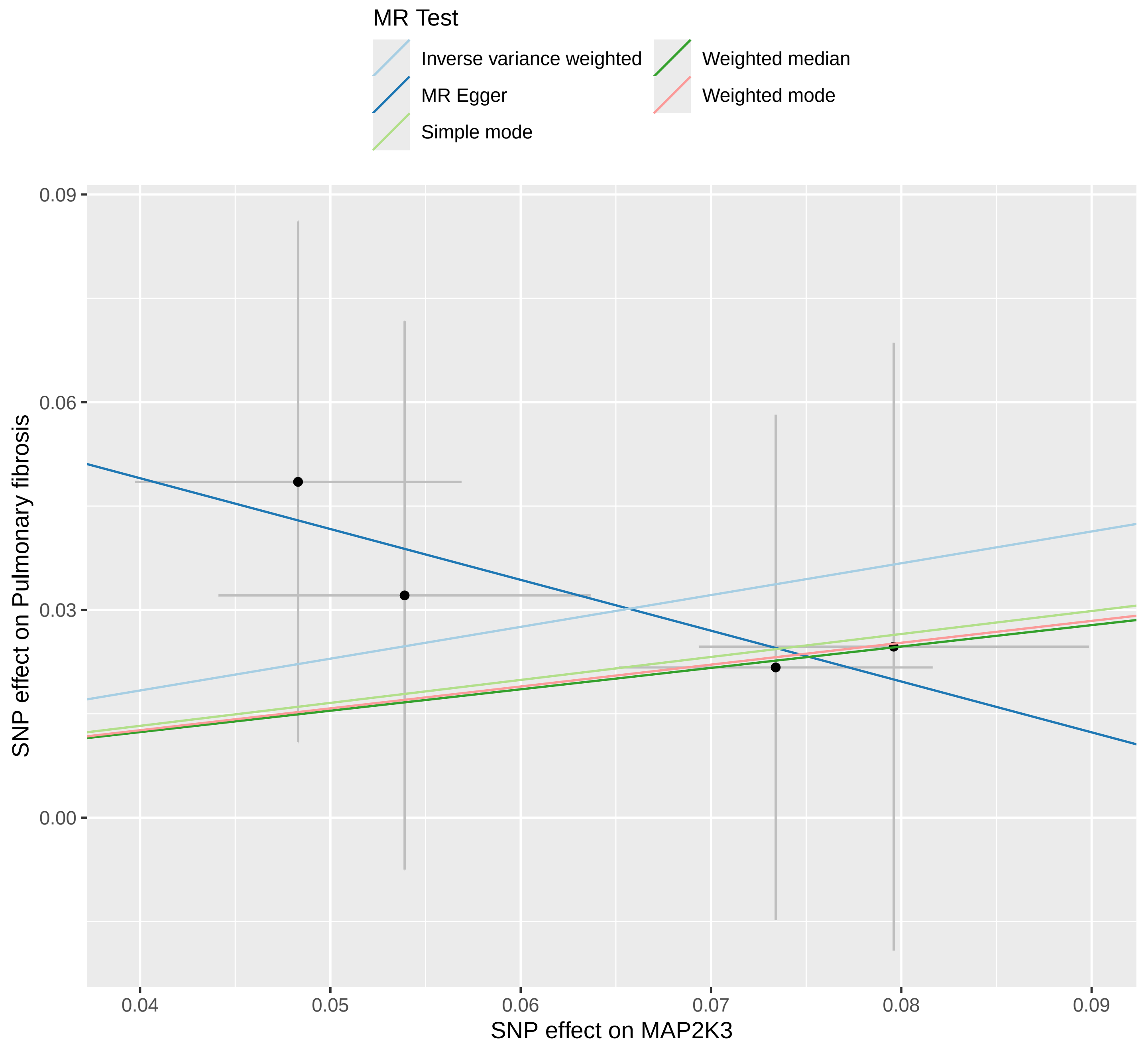

MR Test
Inverse variance weighted
MR Egger
Simple mode
Weighted median
Weighted mode
0.09
0.06
0.03
0.00
SNP effect on Pulmonary fibrosis
0.04
0.05
0.06
0.07
0.08
0.09
SNP effect on MAP2K3

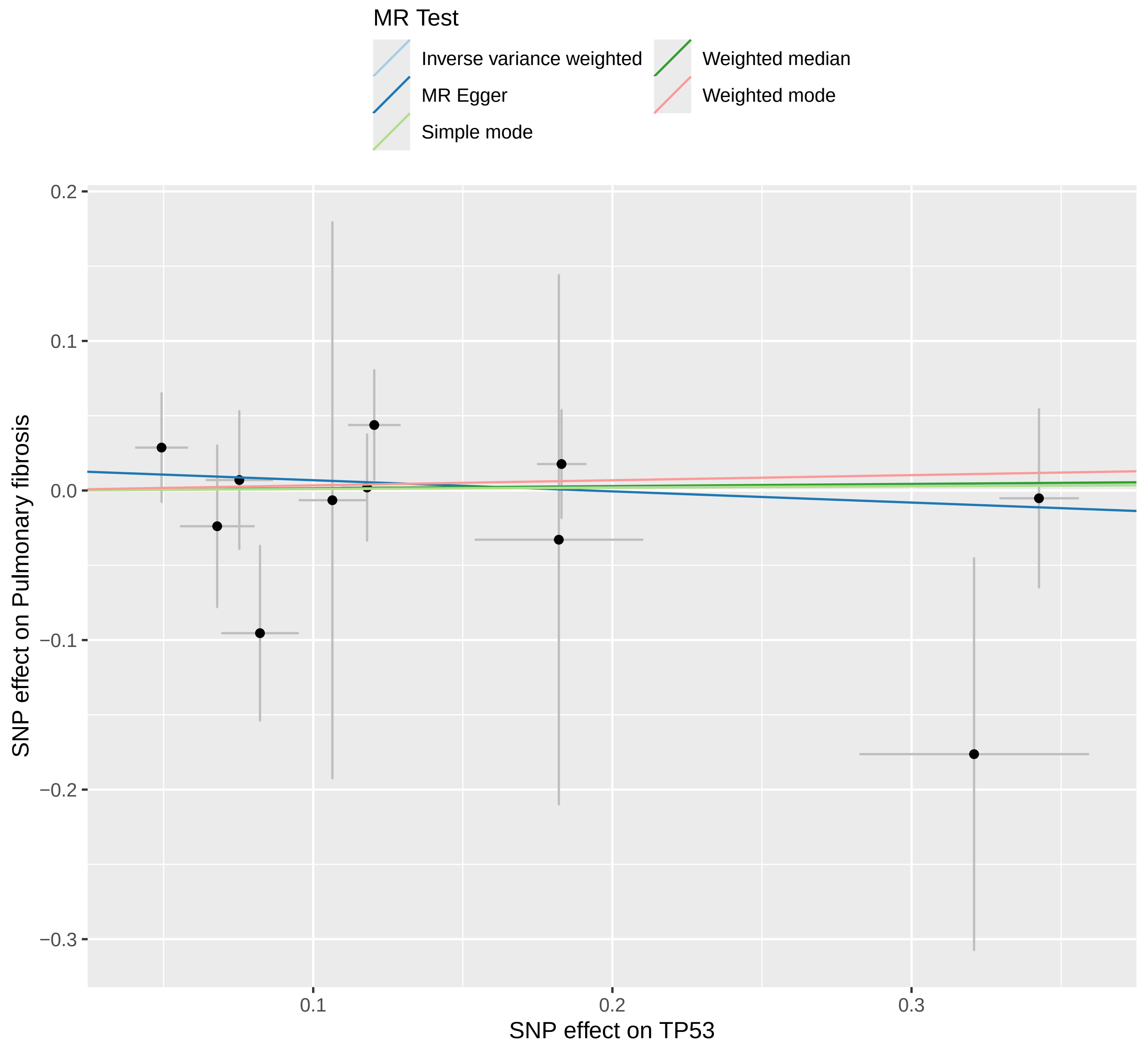

MR Test
Inverse variance weighted
MR Egger
Simple mode
Weighted median
Weighted mode
0.2
0.1
0.0
−0.1
−0.2
−0.3
SNP effect on Pulmonary fibrosis
0.1
0.2
0.3
SNP effect on TP53

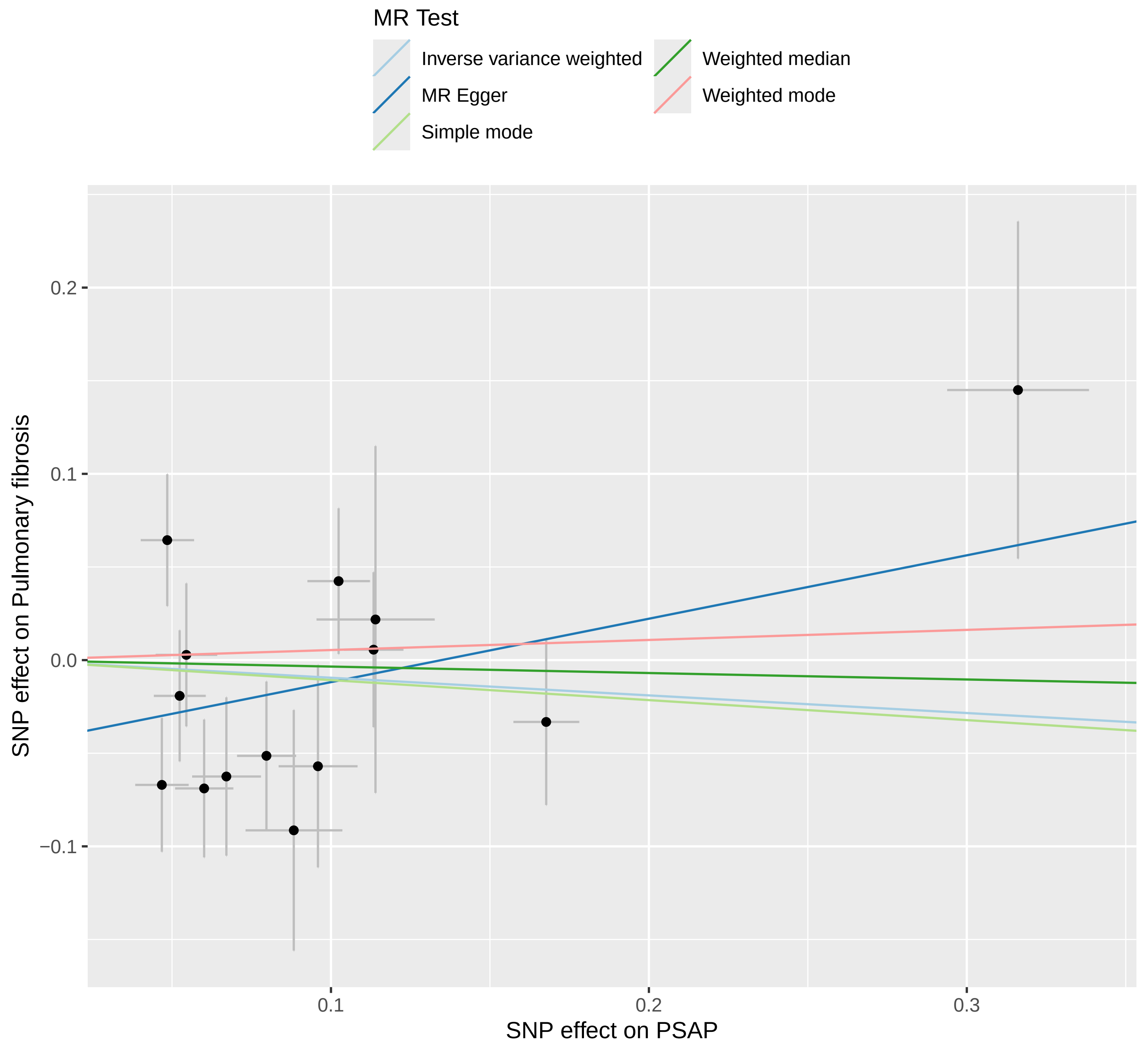
MR Test
Inverse variance weighted
MR Egger
Simple mode
Weighted median
Weighted mode
0.2
0.1
0.0
−0.1
0.1
0.2
0.3
SNP effect on Pulmonary fibrosis
SNP effect on PSAP

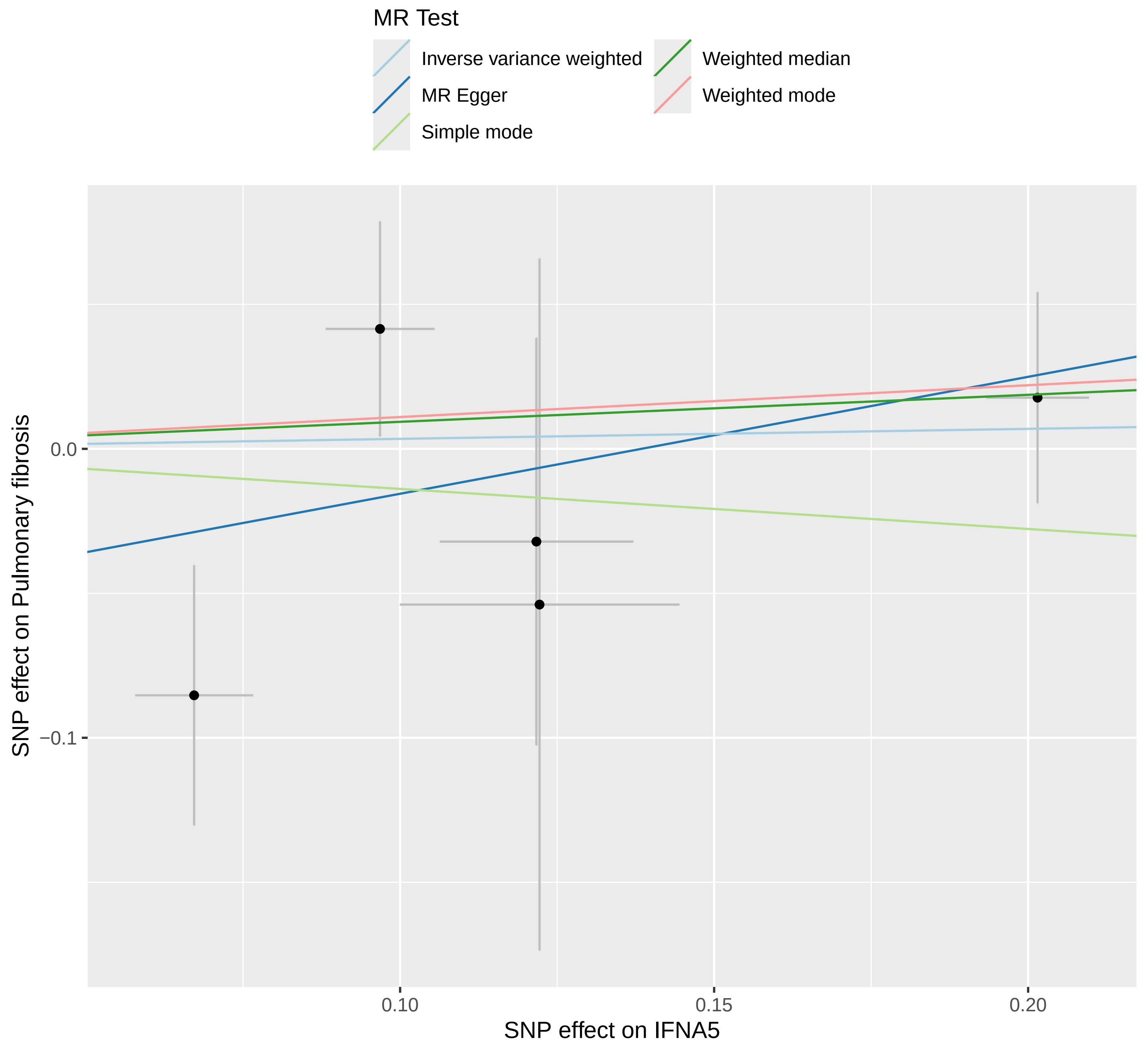
MR Test
Inverse variance weighted
MR Egger
Simple mode
Weighted median
Weighted mode
0.0
−0.1
0.10
0.15
0.20
SNP effect on Pulmonary fibrosis
SNP effect on IFNA5

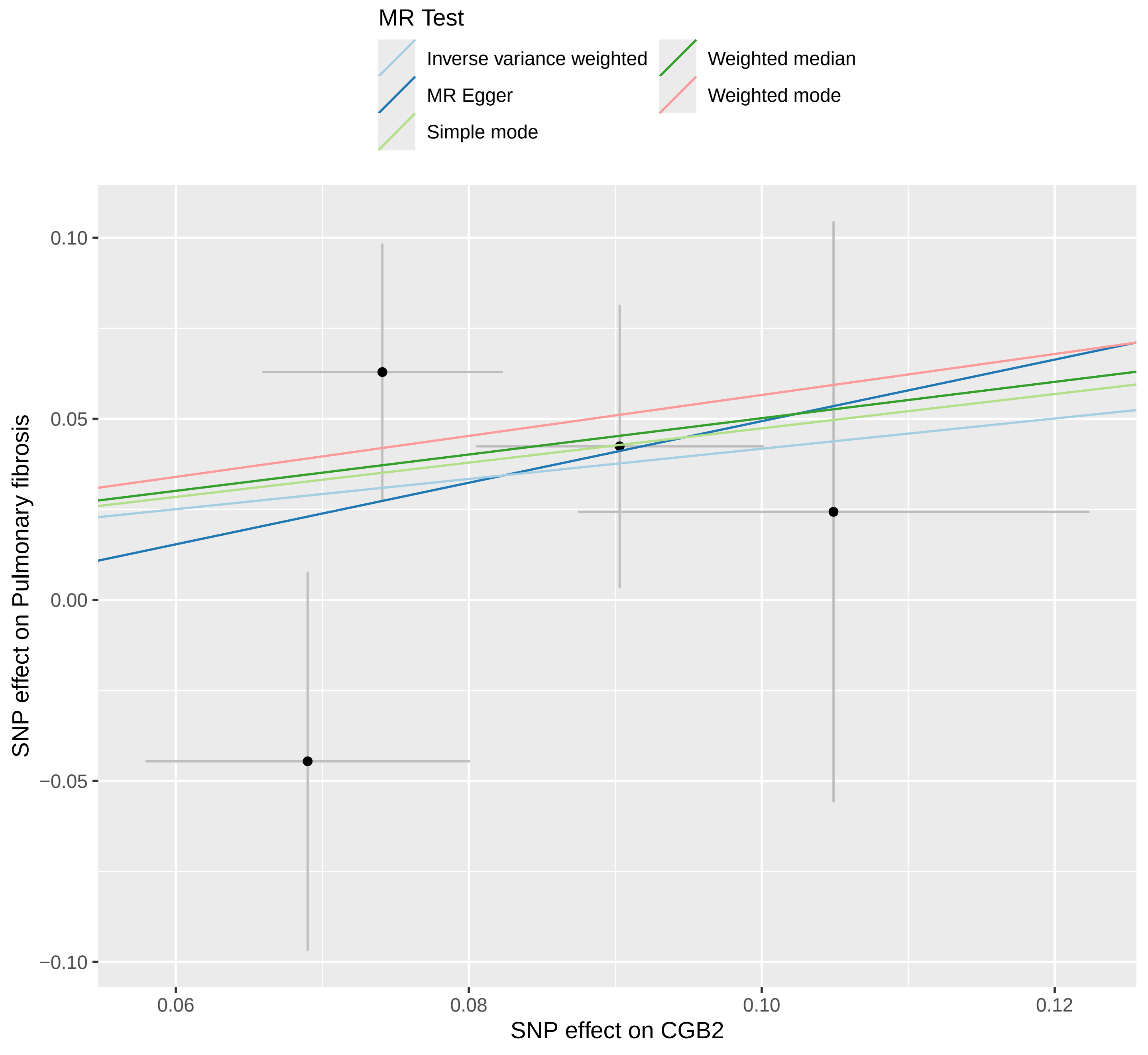

MR Test
Inverse variance weighted
MR Egger
Simple mode
Weighted median
Weighted mode
0.10
0.05
0.00
−0.05
−0.10
SNP effect on Pulmonary fibrosis
0.06
0.08
0.10
0.12
SNP effect on CGB2

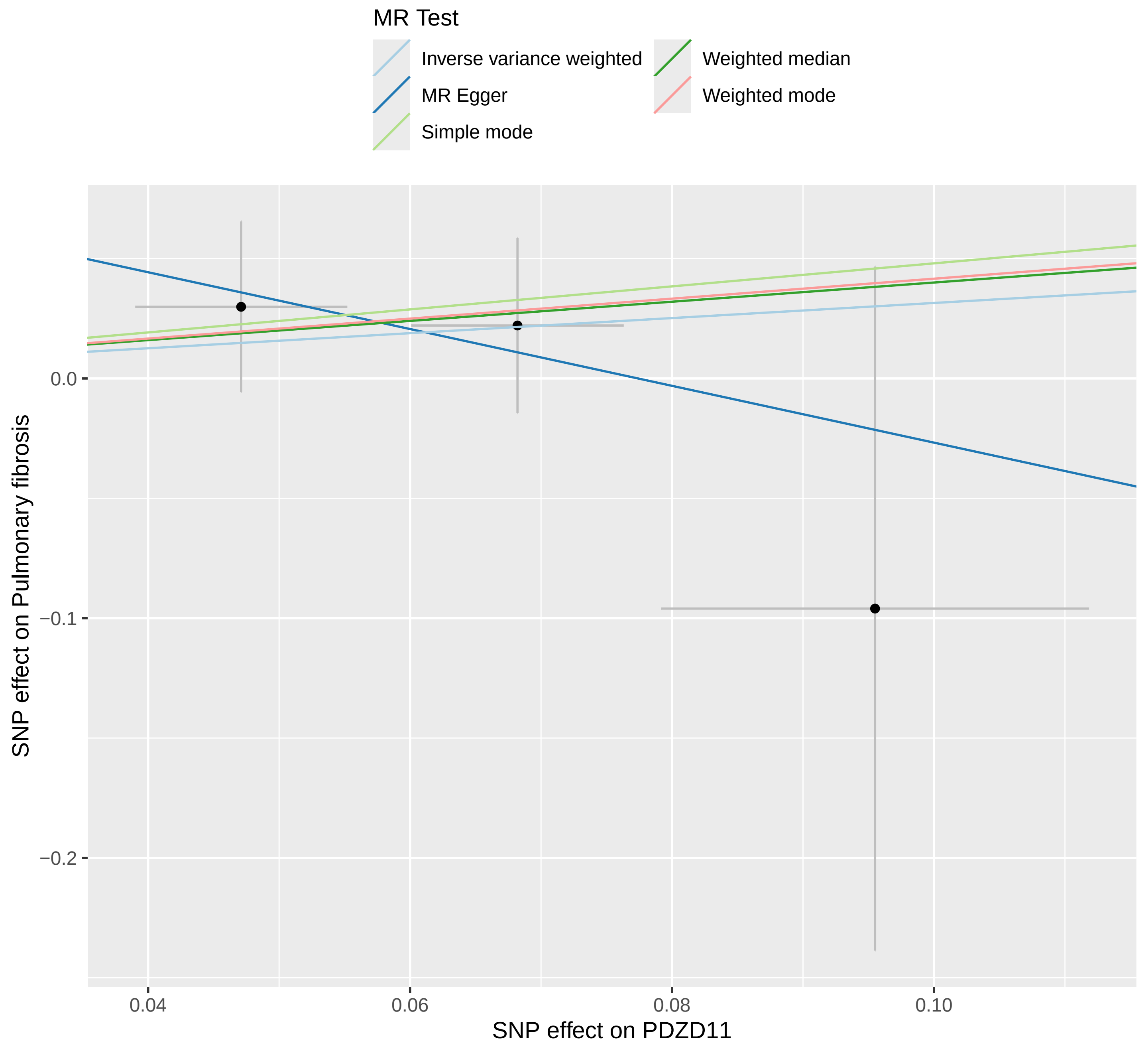

MR Test
Inverse variance weighted
MR Egger
Simple mode
Weighted median
Weighted mode
0.0
−0.1
−0.2
SNP effect on Pulmonary fibrosis
0.04
0.06
0.08
0.10
SNP effect on PDZD11

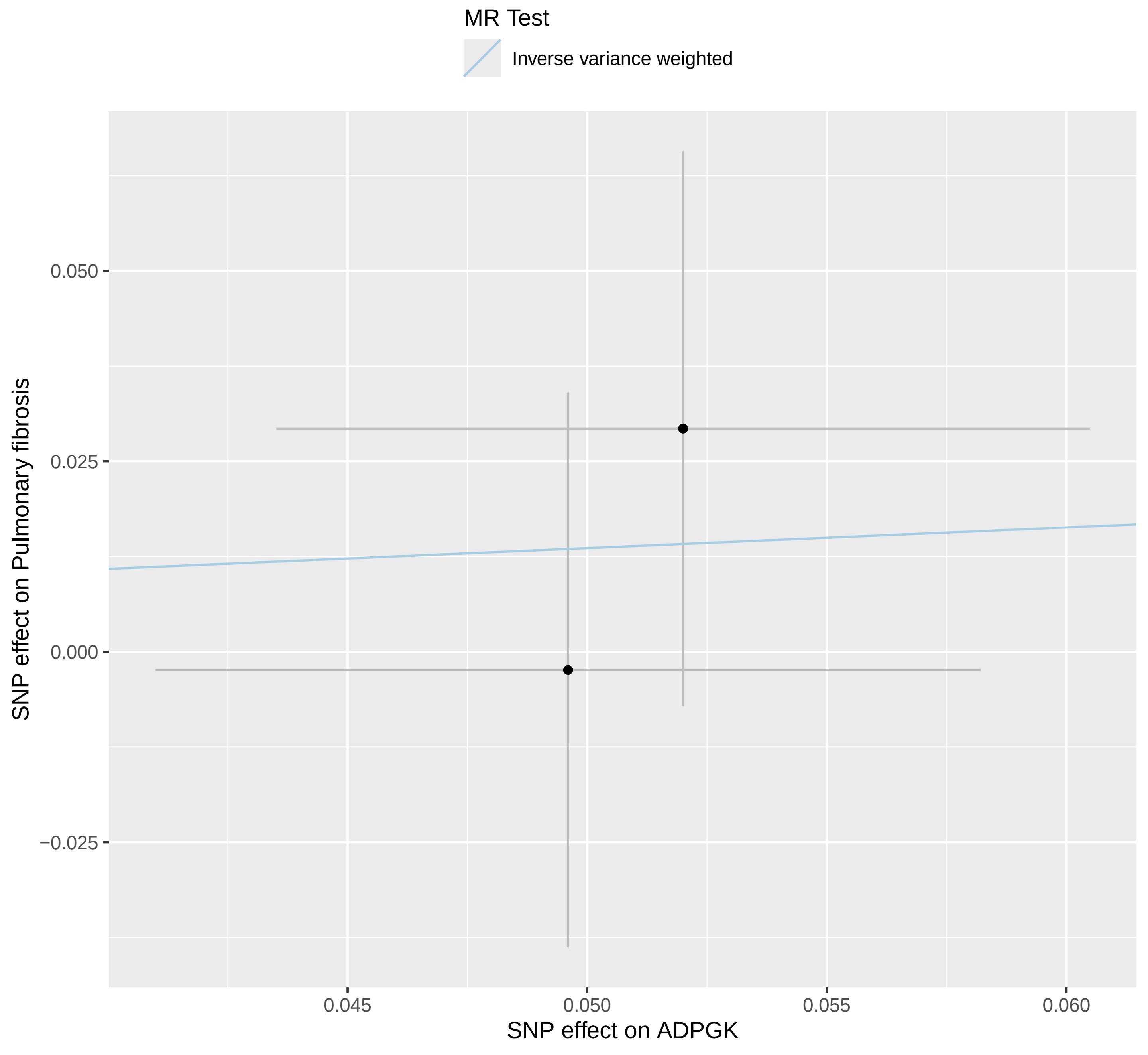

MR Test
Inverse variance weighted
0.050
0.025
0.000
−0.025
SNP effect on Pulmonary fibrosis
0.045
0.050
0.055
0.060
SNP effect on ADPGK

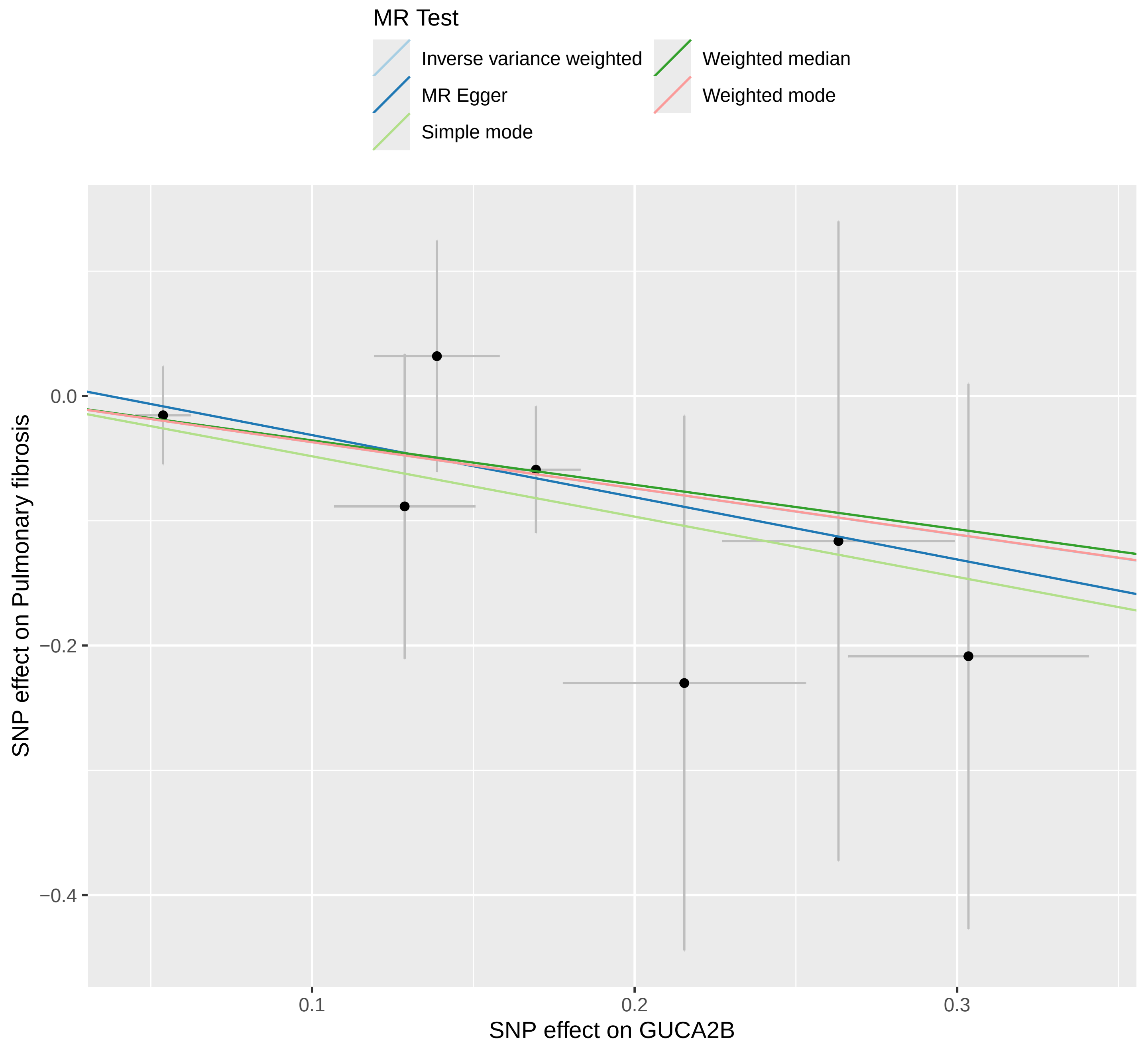
MR Test
Inverse variance weighted
MR Egger
Simple mode
Weighted median
Weighted mode
0.0
−0.2
−0.4
SNP effect on Pulmonary fibrosis
0.1
0.2
0.3
SNP effect on GUCA2B

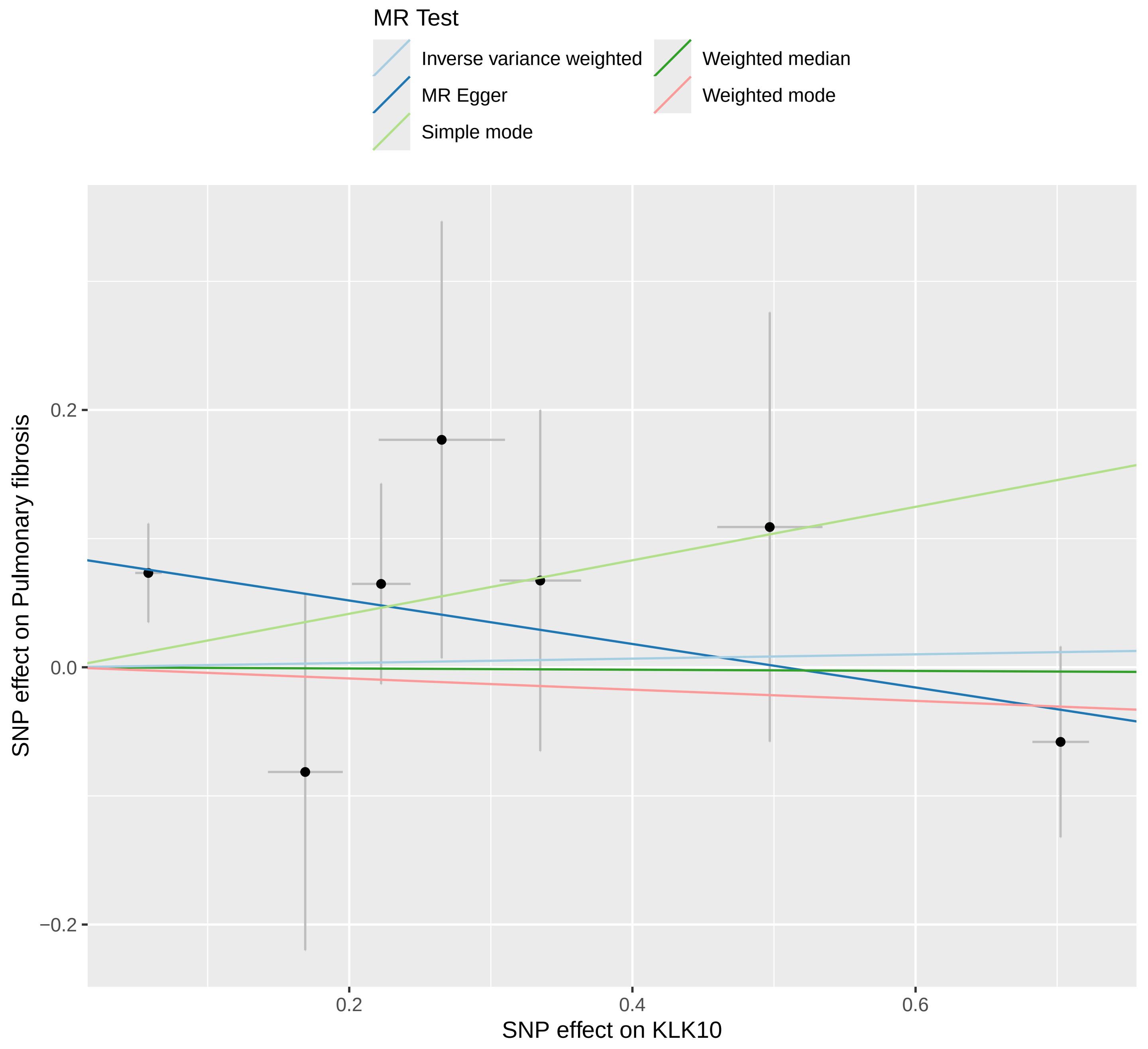

MR Test
Inverse variance weighted
Weighted median
MR Egger
Weighted mode
Simple mode
SNP effect on Pulmonary fibrosis
0.2
0.0
−0.2
0.2
0.4
0.6
SNP effect on KLK10

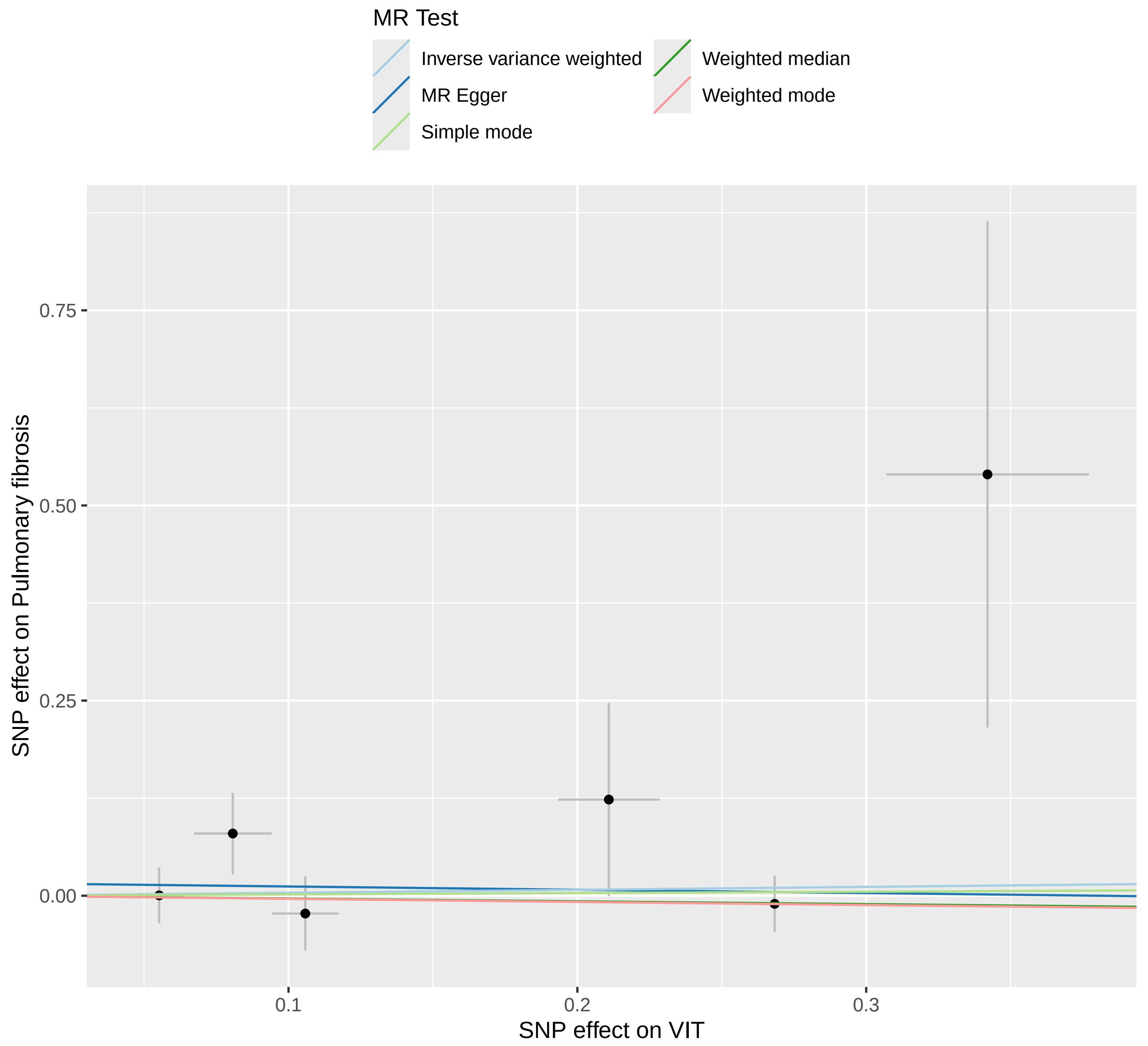

MR Test
Inverse variance weighted
MR Egger
Simple mode
Weighted median
Weighted mode
SNP effect on Pulmonary fibrosis
0.75
0.50
0.25
0.00
0.1
0.2
0.3
SNP effect on VIT

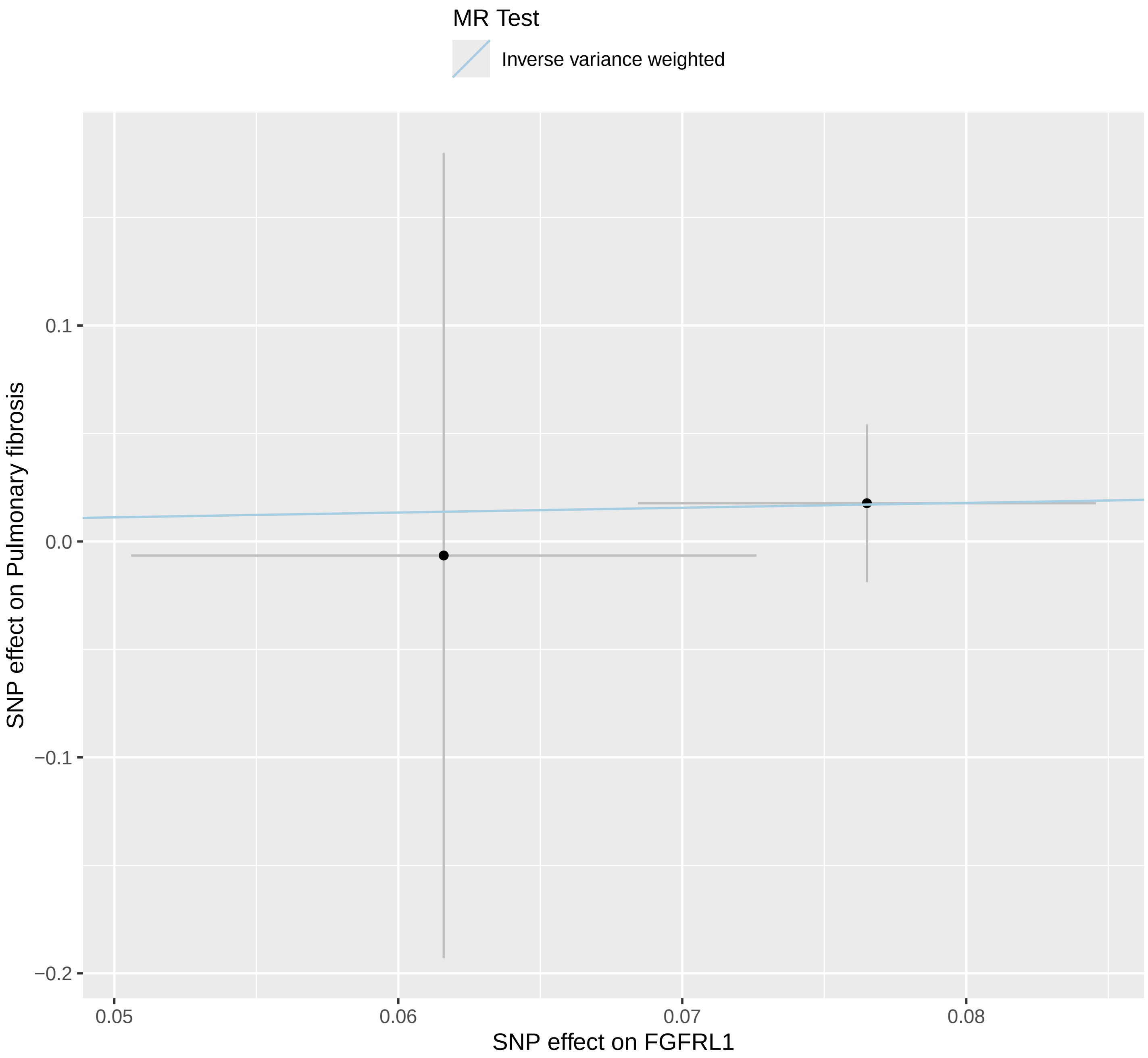
MR Test
Inverse variance weighted
0.1
0.0
−0.1
−0.2
SNP effect on Pulmonary fibrosis
0.05
0.06
0.07
0.08
SNP effect on FGFRL1

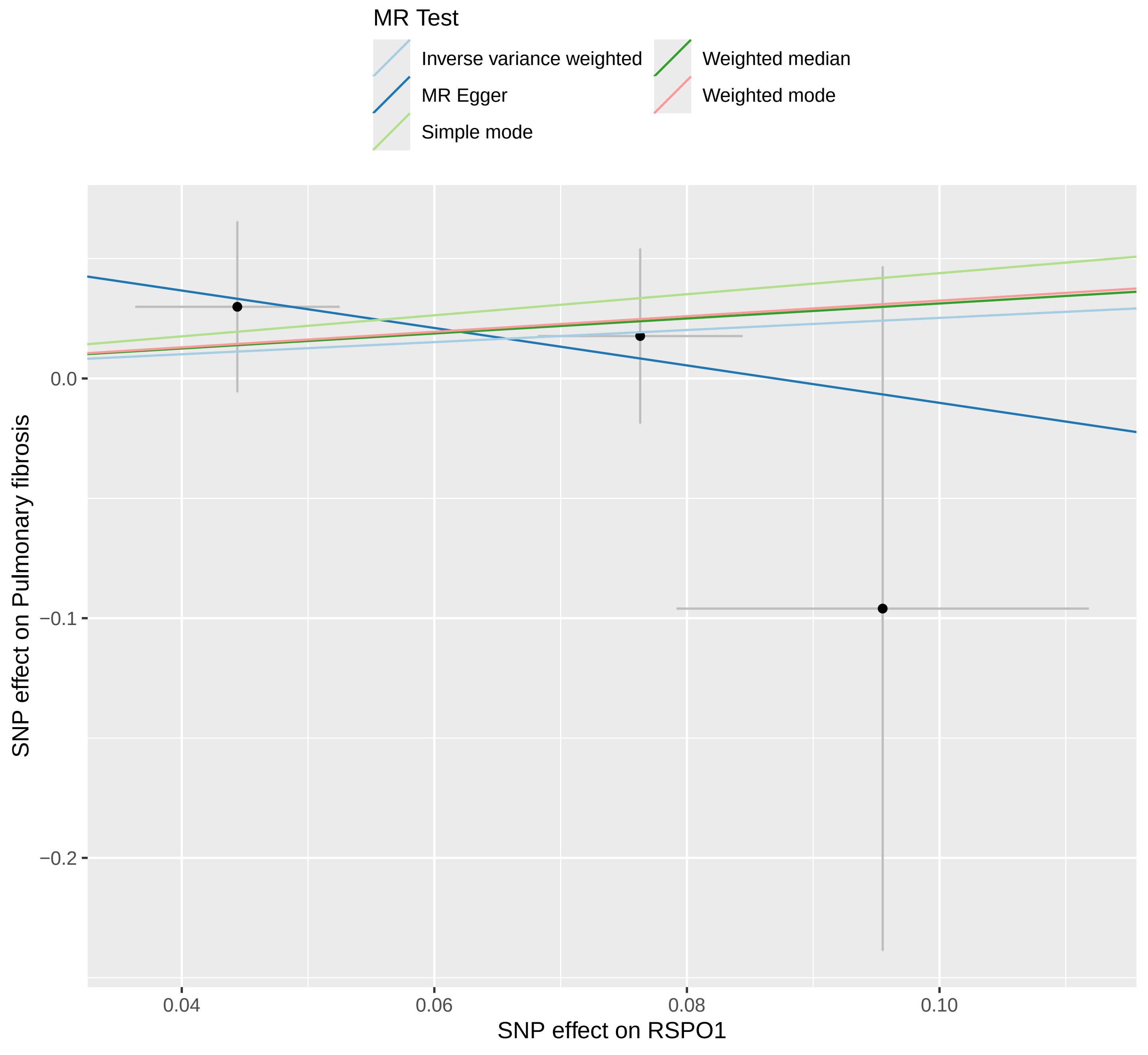

MR Test
Inverse variance weighted
MR Egger
Simple mode
Weighted median
Weighted mode
0.0
−0.1
−0.2
SNP effect on Pulmonary fibrosis
0.04
0.06
0.08
0.10
SNP effect on RSPO1

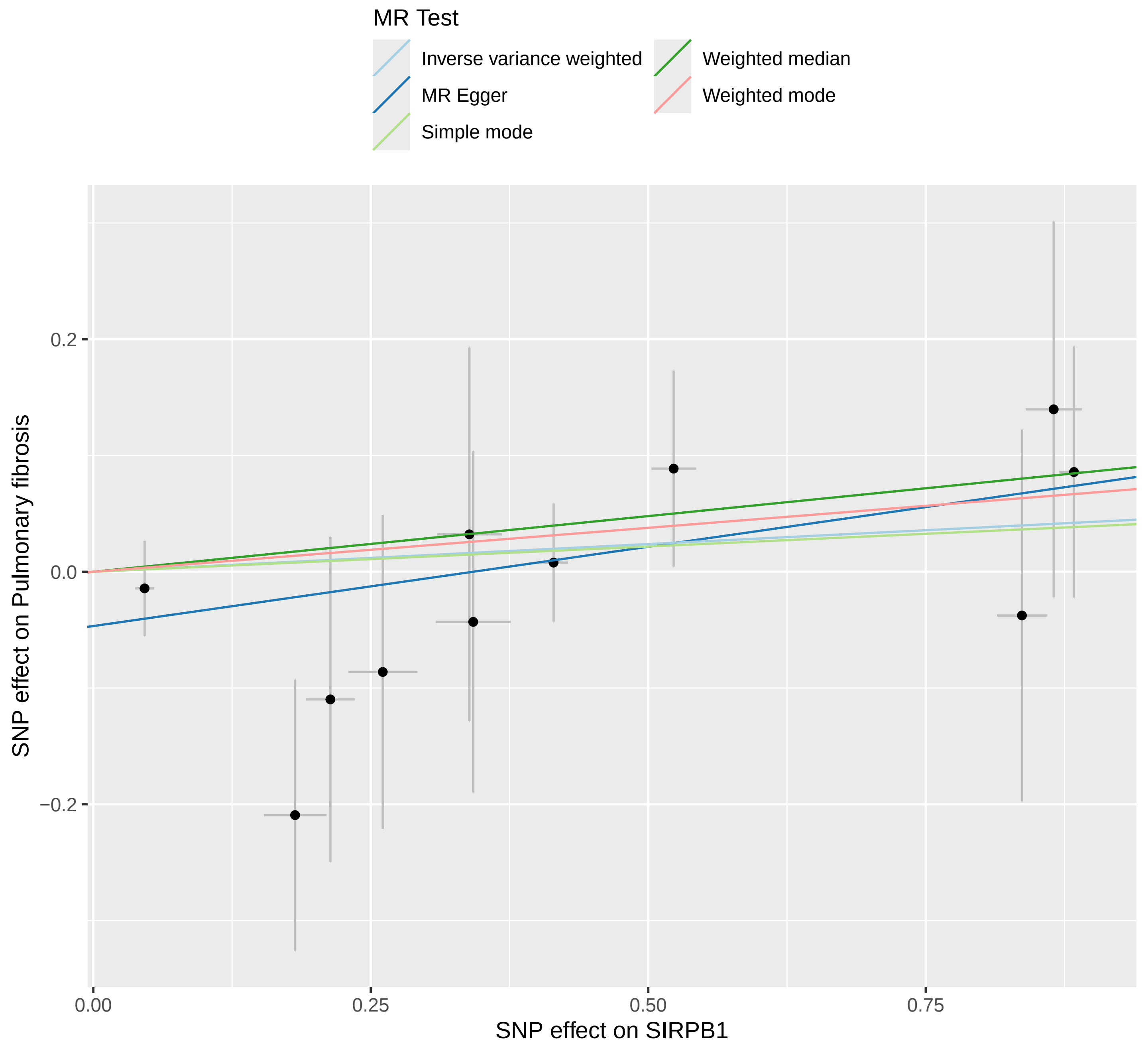
MR Test
Inverse variance weighted
MR Egger
Simple mode
Weighted median
Weighted mode
0.2
0.0
−0.2
0.00
0.25
0.50
0.75
SNP effect on SIRPB1
SNP effect on Pulmonary fibrosis

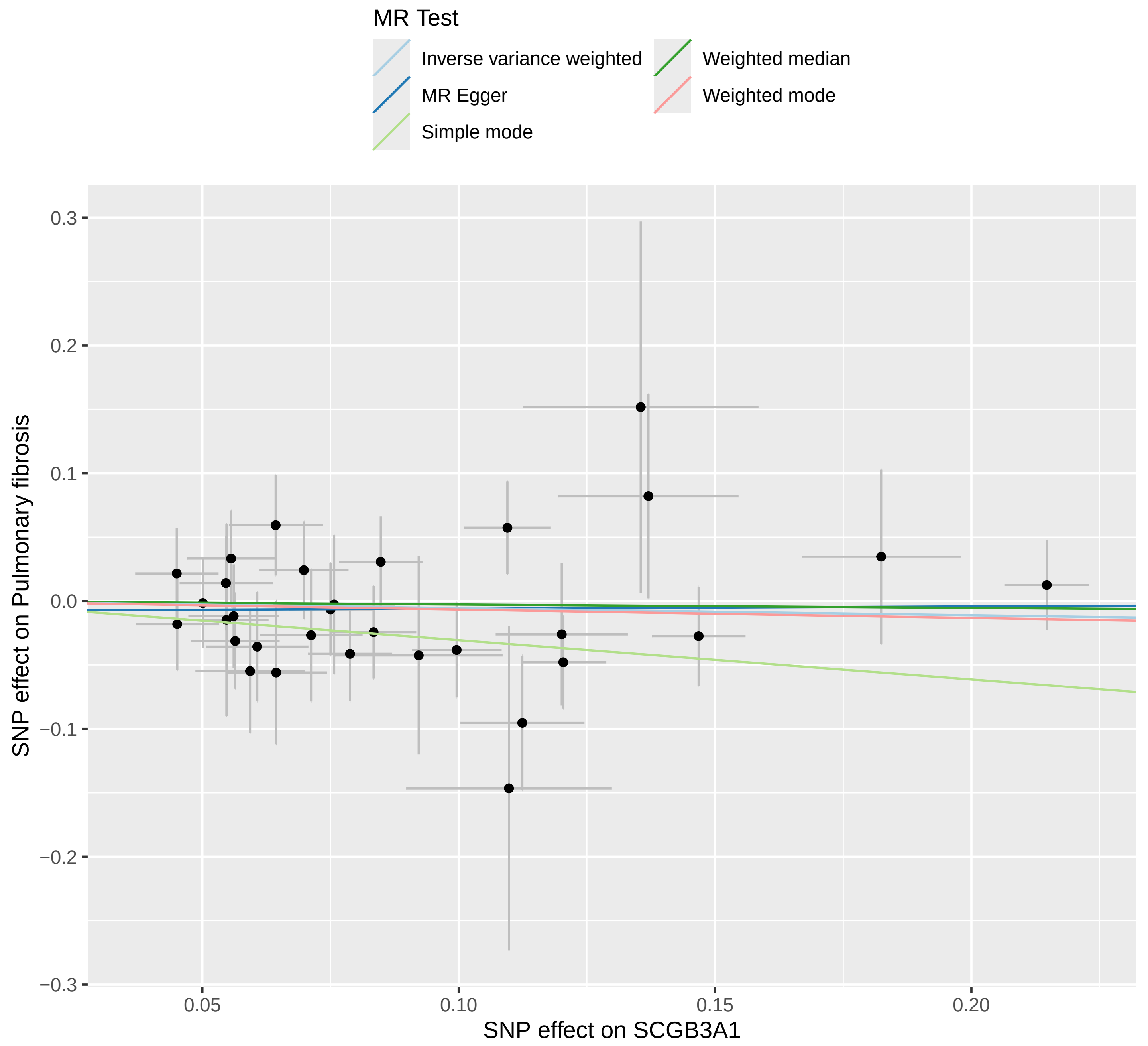

MR Test
Inverse variance weighted
MR Egger
Simple mode
Weighted median
Weighted mode
0.3
0.2
0.1
0.0
−0.1
−0.2
−0.3
0.05
0.10
0.15
0.20
SNP effect on Pulmonary fibrosis
SNP effect on SCGB3A1

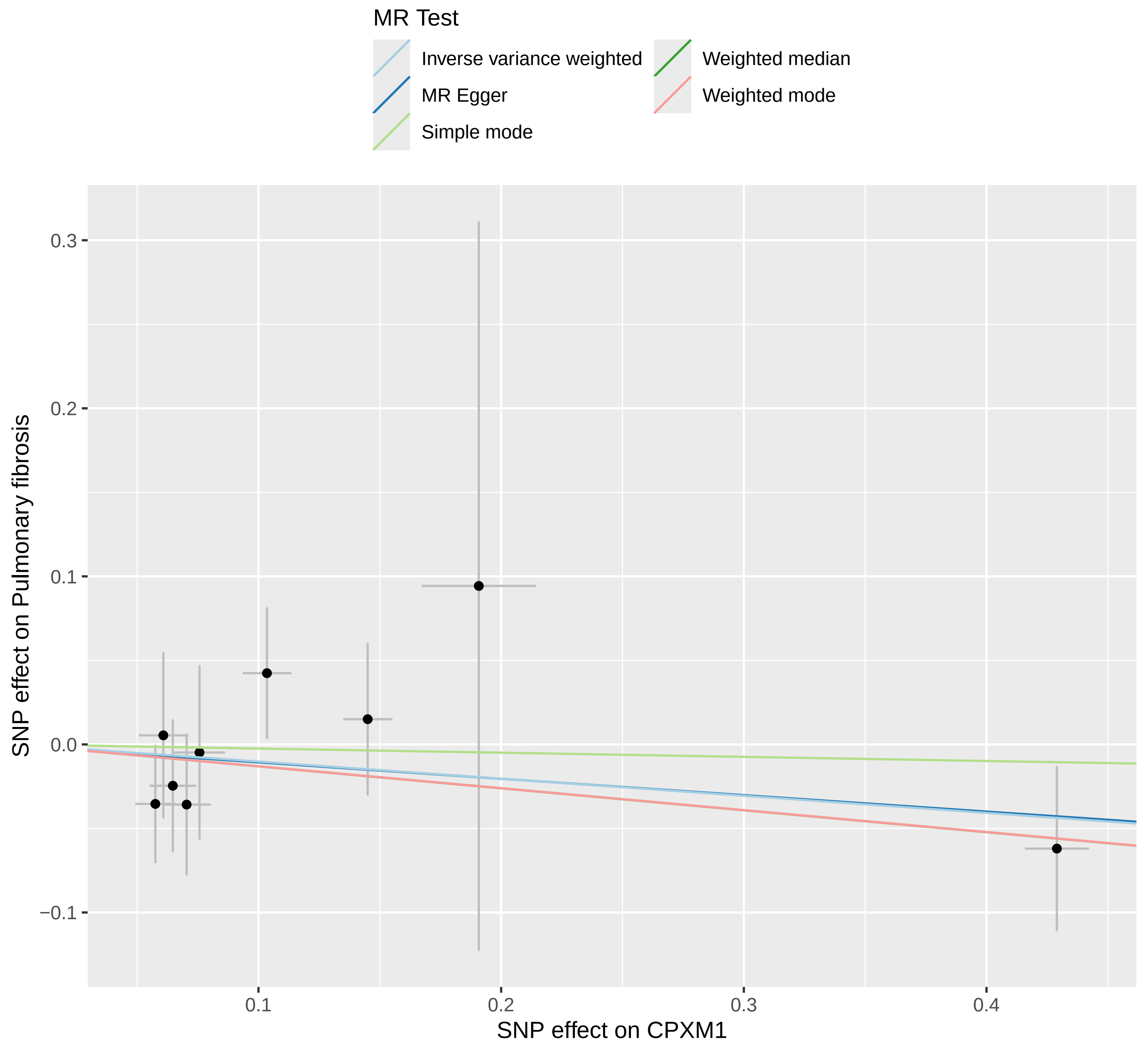
MR Test
Inverse variance weighted
MR Egger
Simple mode
Weighted median
Weighted mode
SNP effect on Pulmonary fibrosis
0.3
0.2
0.1
0.0
−0.1
0.1
0.2
0.3
0.4
SNP effect on CPXM1

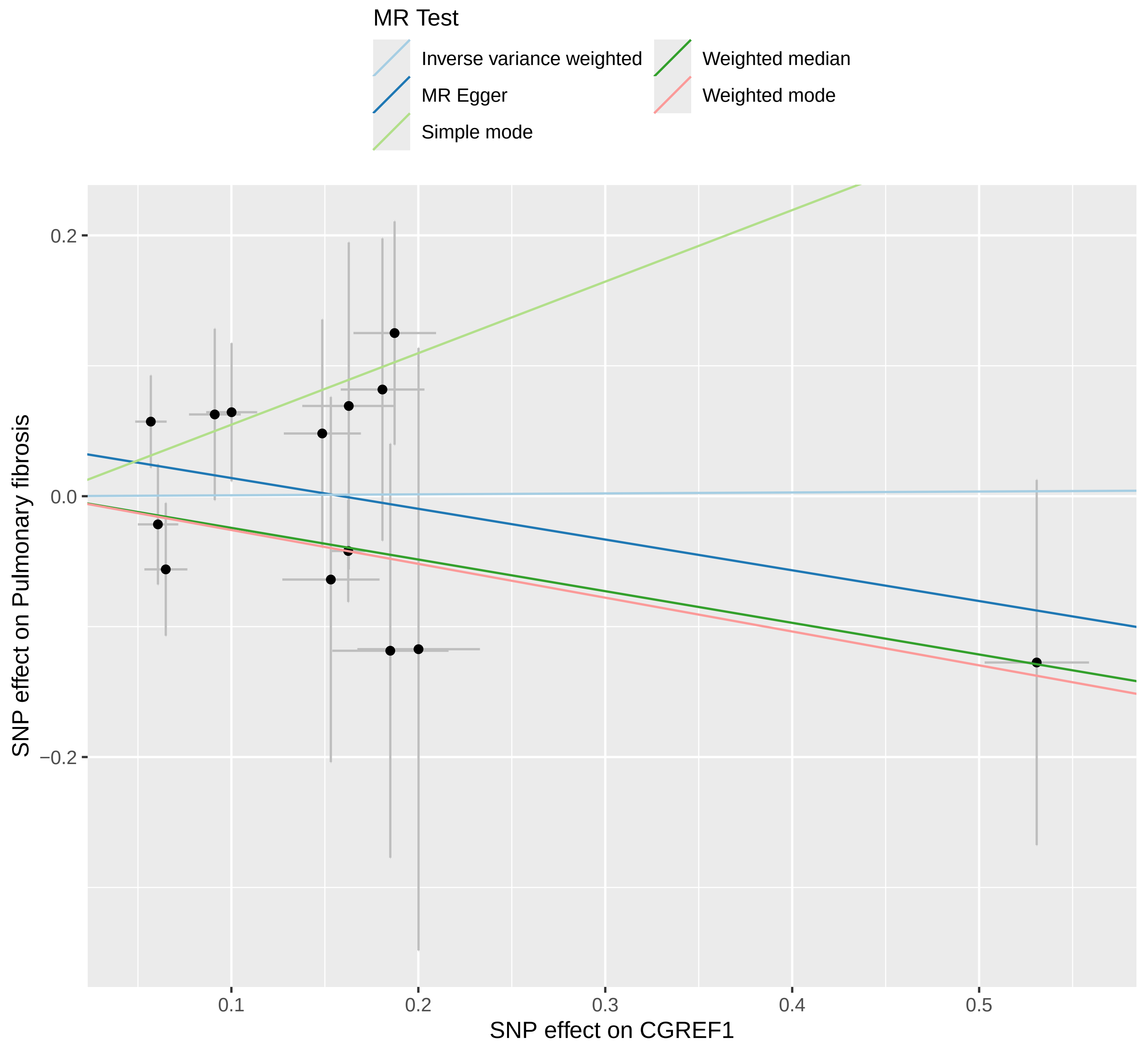
MR Test
Inverse variance weighted
MR Egger
Simple mode
Weighted median
Weighted mode
0.2
0.0
−0.2
0.1
0.2
0.3
0.4
0.5
SNP effect on Pulmonary fibrosis
SNP effect on CGREF1

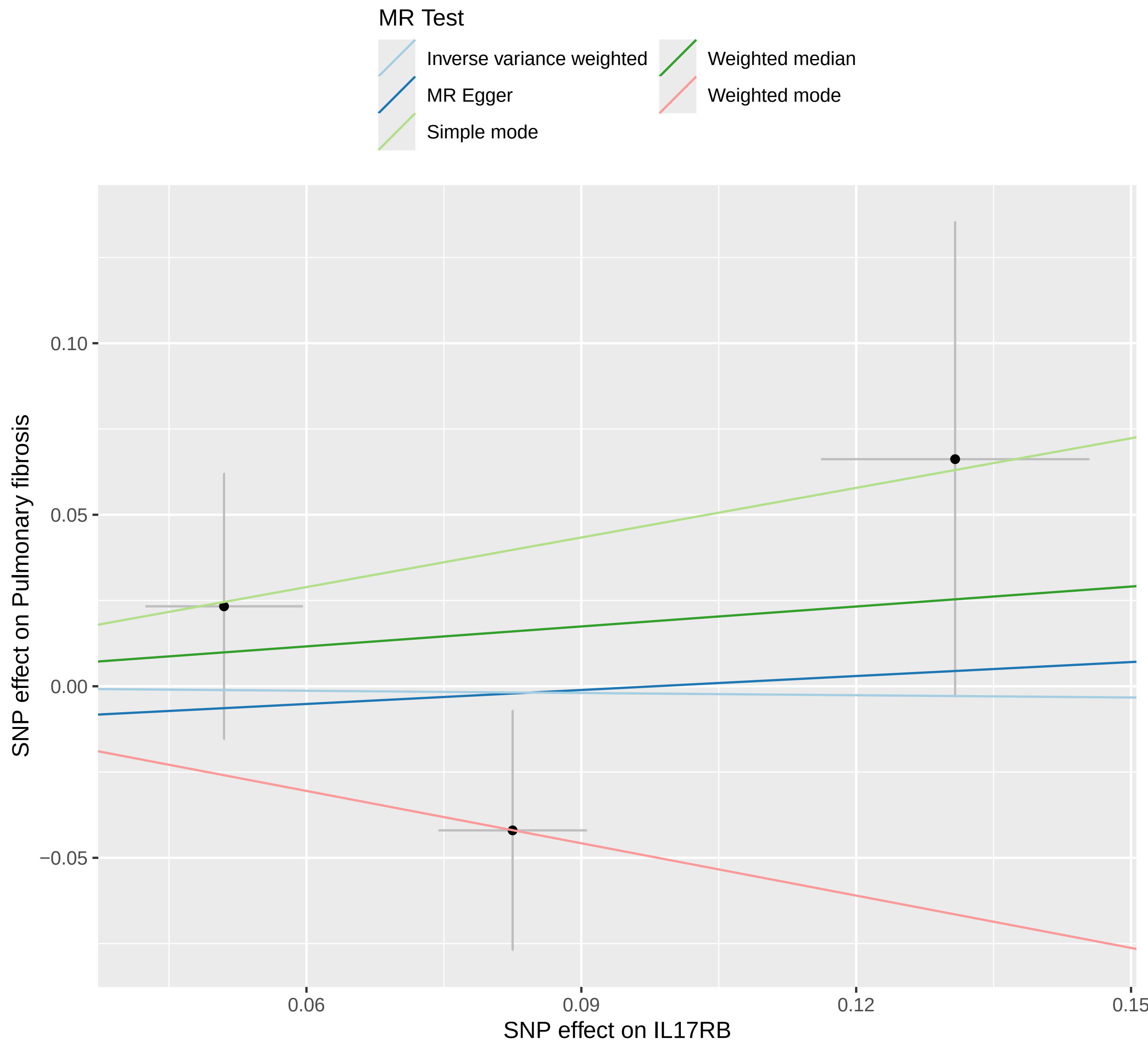

MR Test
Inverse variance weighted
MR Egger
Simple mode
Weighted median
Weighted mode
SNP effect on Pulmonary fibrosis
0.10
0.05
0.00
−0.05
0.06
0.09
0.12
0.15
SNP effect on IL17RB

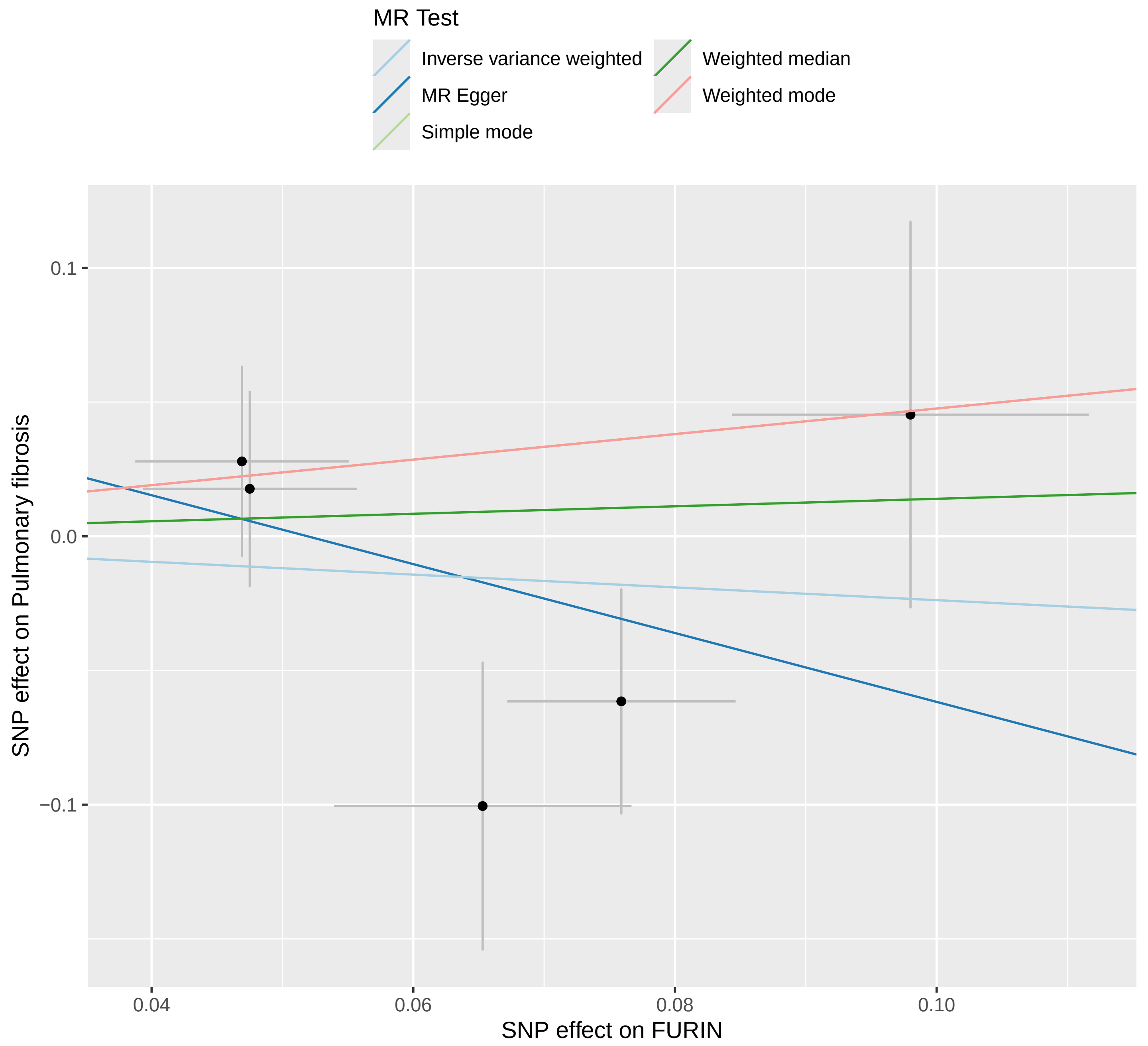
MR Test
Inverse variance weighted
MR Egger
Simple mode
Weighted median
Weighted mode
SNP effect on Pulmonary fibrosis
0.1
0.0
−0.1
0.04
0.06
0.08
0.10
SNP effect on FURIN

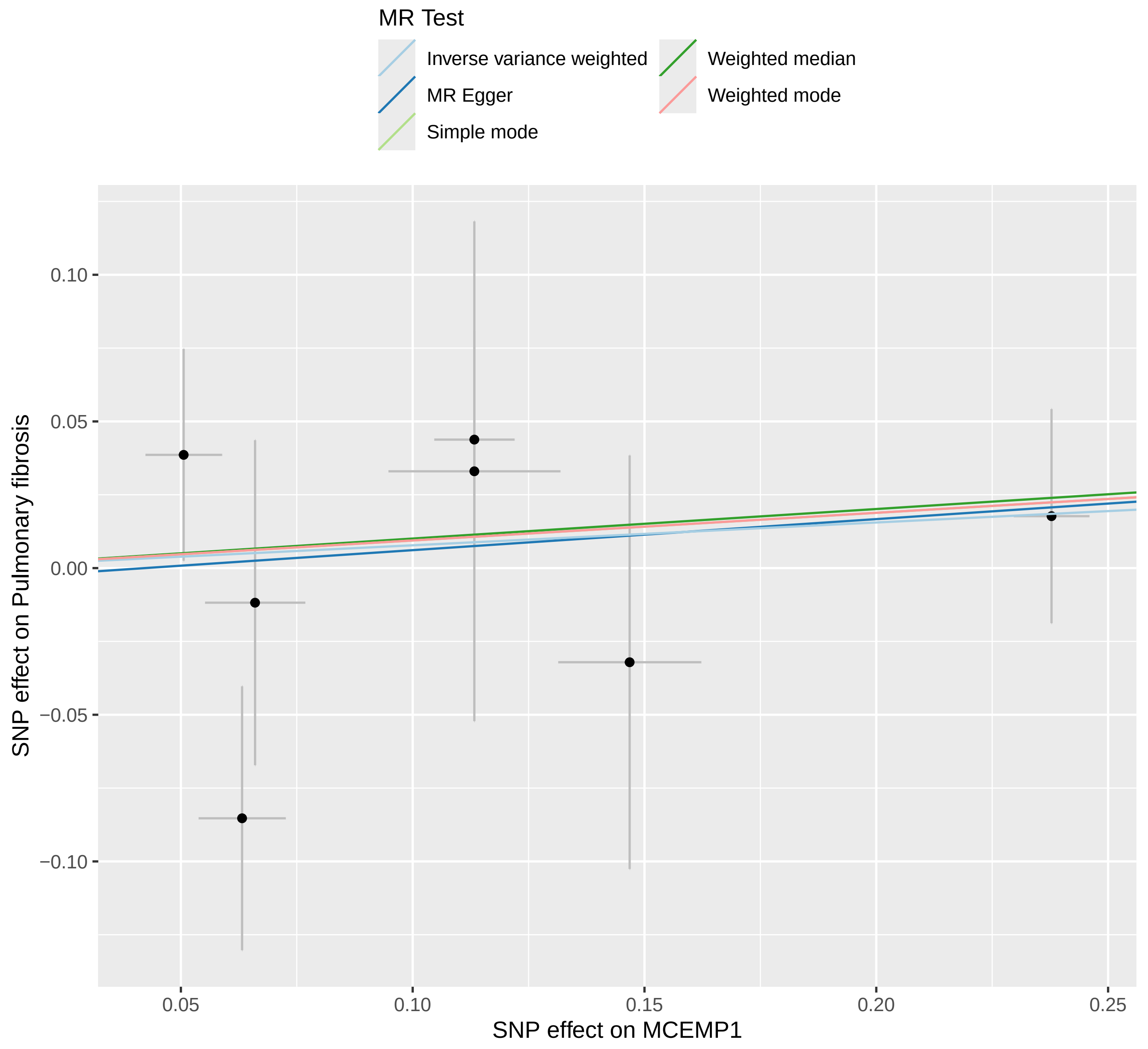

MR Test
Inverse variance weighted
MR Egger
Simple mode
Weighted median
Weighted mode
SNP effect on Pulmonary fibrosis
0.10
0.05
0.00
−0.05
−0.10
0.05
0.10
0.15
0.20
0.25
SNP effect on MCEMP1

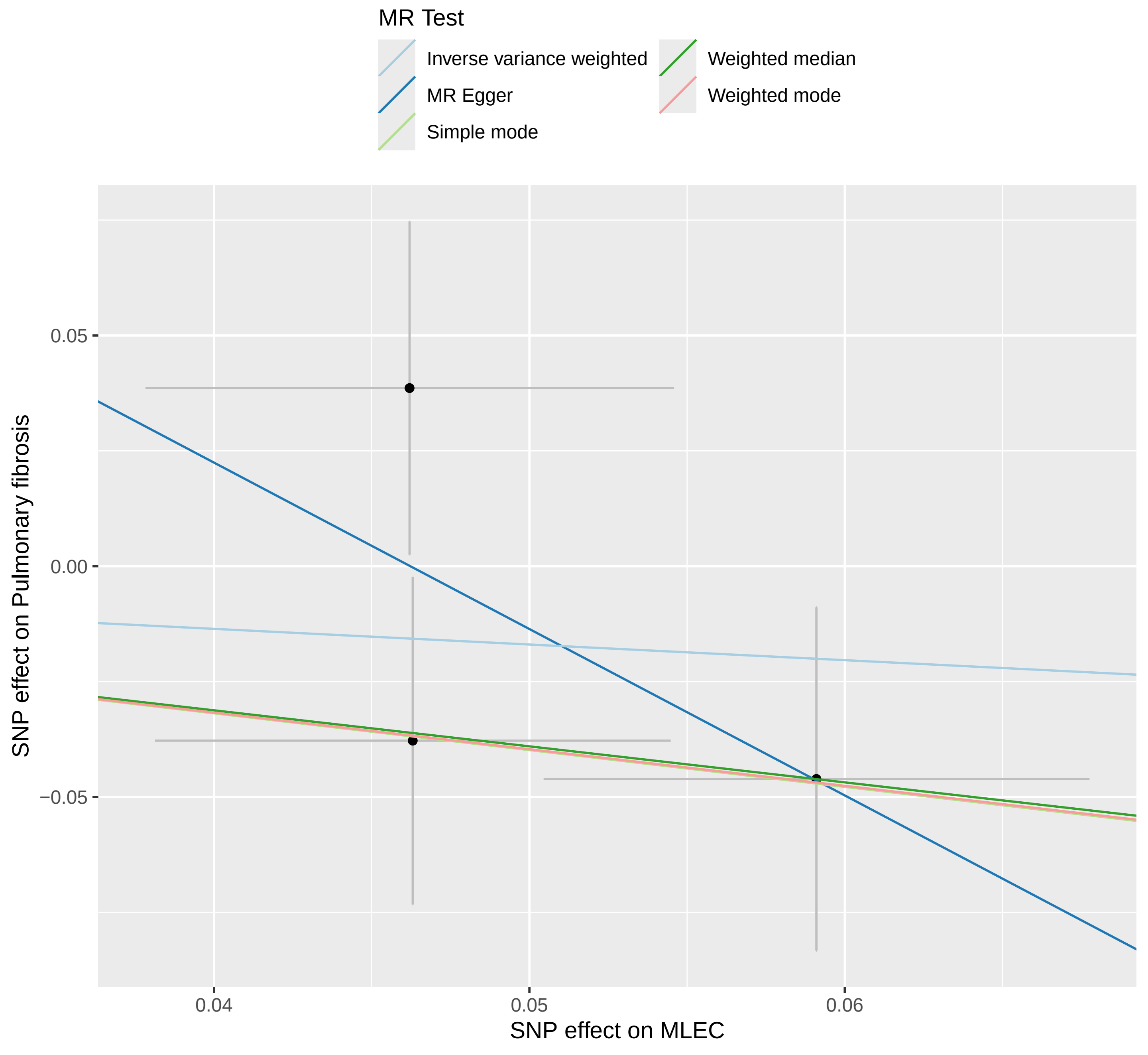
MR Test
Inverse variance weighted
MR Egger
Simple mode
Weighted median
Weighted mode
0.05
0.00
−0.05
SNP effect on Pulmonary fibrosis
0.04
0.05
0.06
SNP effect on MLEC

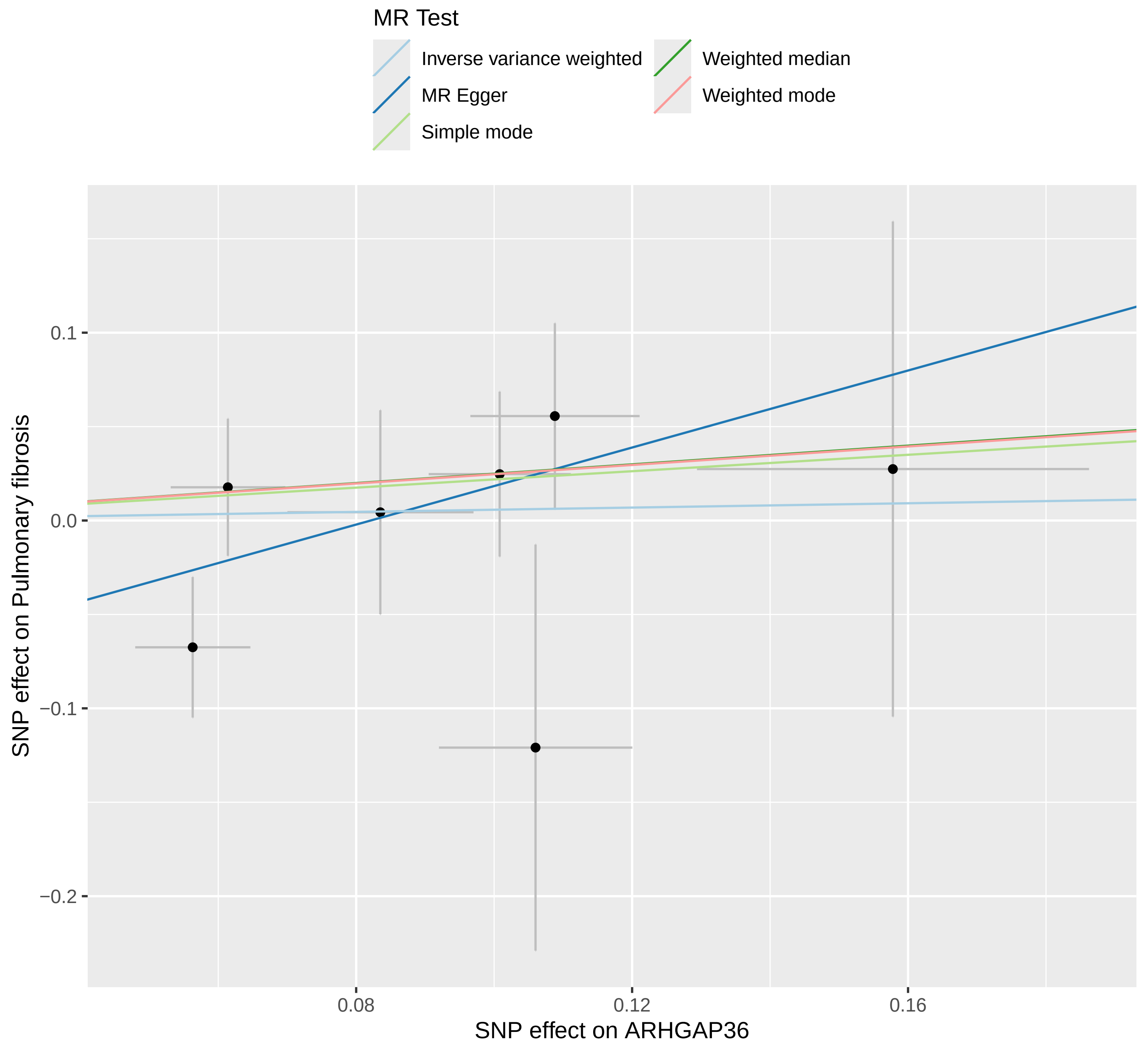

MR Test
Inverse variance weighted
MR Egger
Simple mode
Weighted median
Weighted mode
0.1
0.0
−0.1
−0.2
0.08
0.12
0.16
SNP effect on ARHGAP36
SNP effect on Pulmonary fibrosis

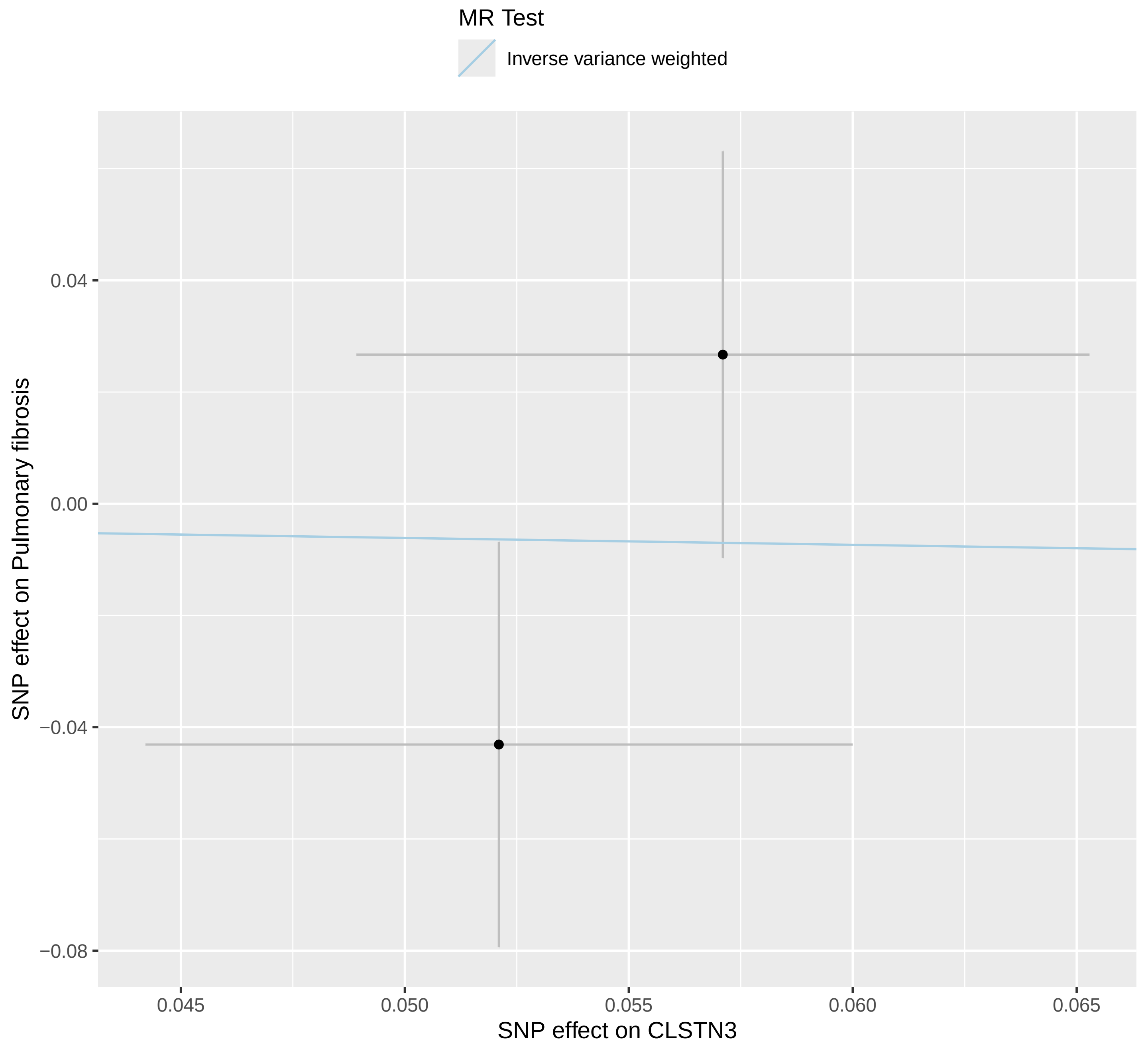
MR Test
Inverse variance weighted
SNP effect on Pulmonary fibrosis
0.04
0.00
−0.04
−0.08
0.045
0.050
0.055
0.060
0.065
SNP effect on CLSTN3

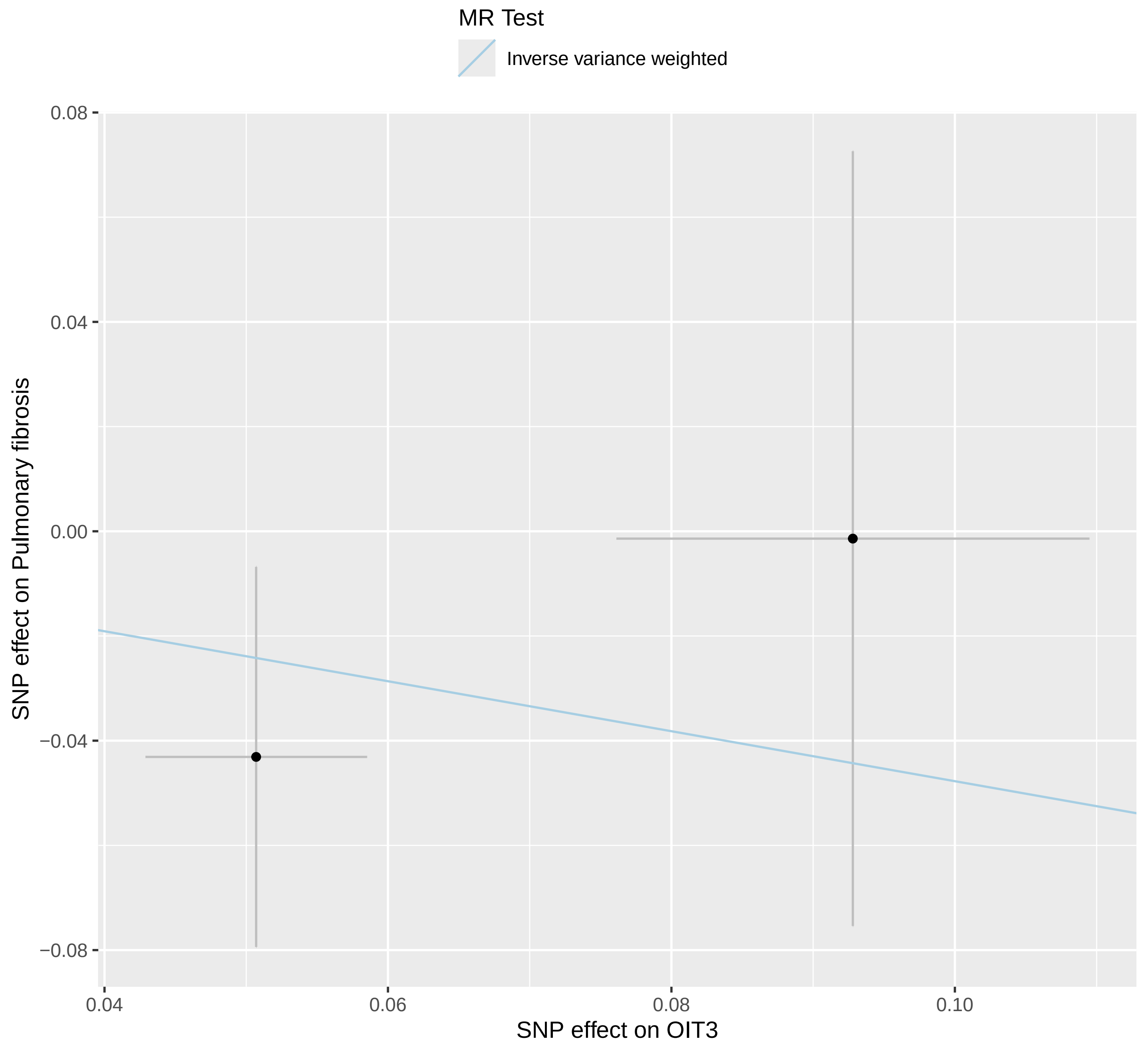
MR Test
Inverse variance weighted
0.08
0.04
0.00
−0.04
−0.08
SNP effect on Pulmonary fibrosis
0.04
0.06
0.08
0.10
SNP effect on OIT3

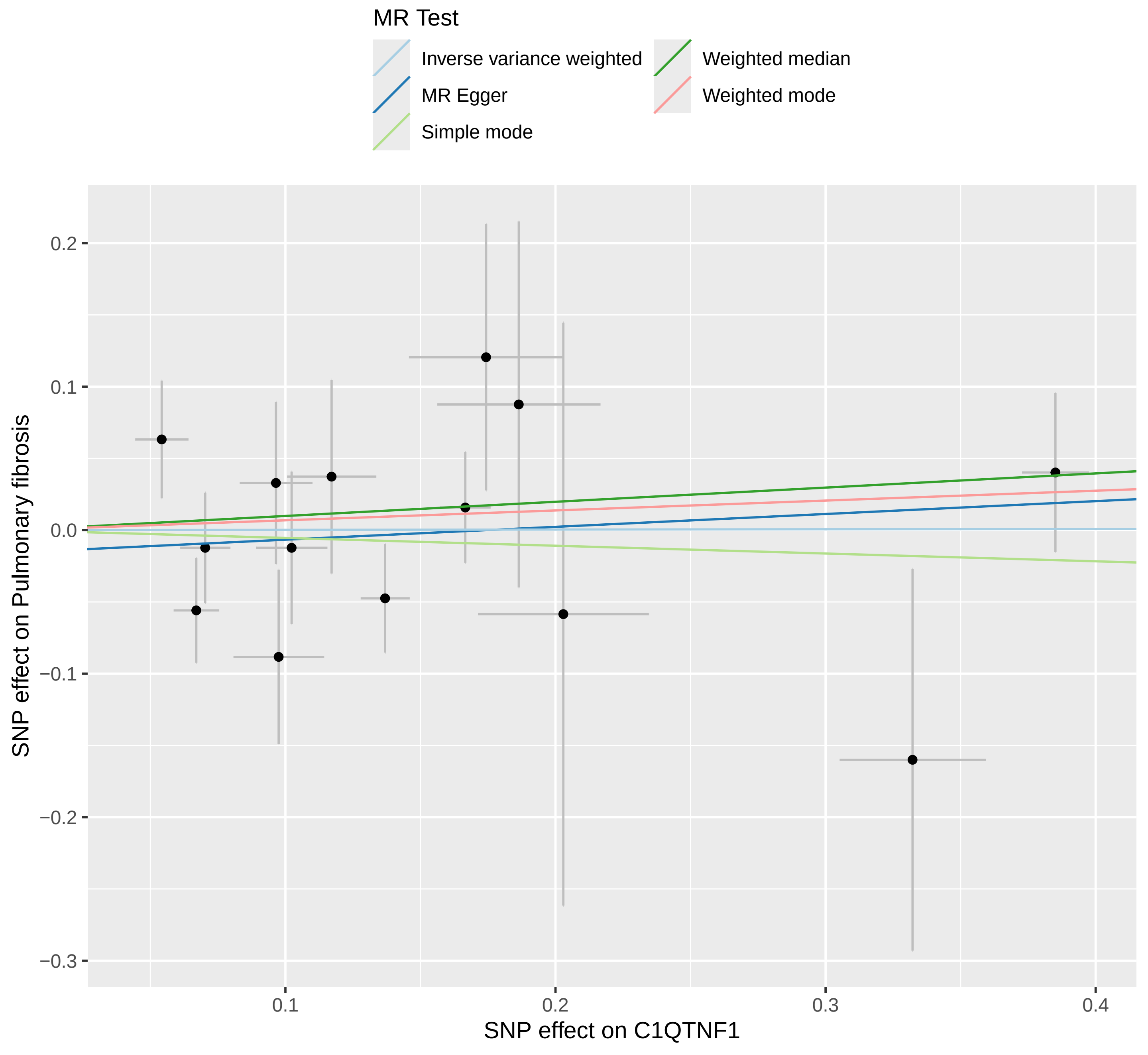

MR Test
Inverse variance weighted
MR Egger
Simple mode
Weighted median
Weighted mode
0.2
0.1
0.0
−0.1
−0.2
−0.3
SNP effect on Pulmonary fibrosis
0.1
0.2
0.3
0.4
SNP effect on C1QTNF1

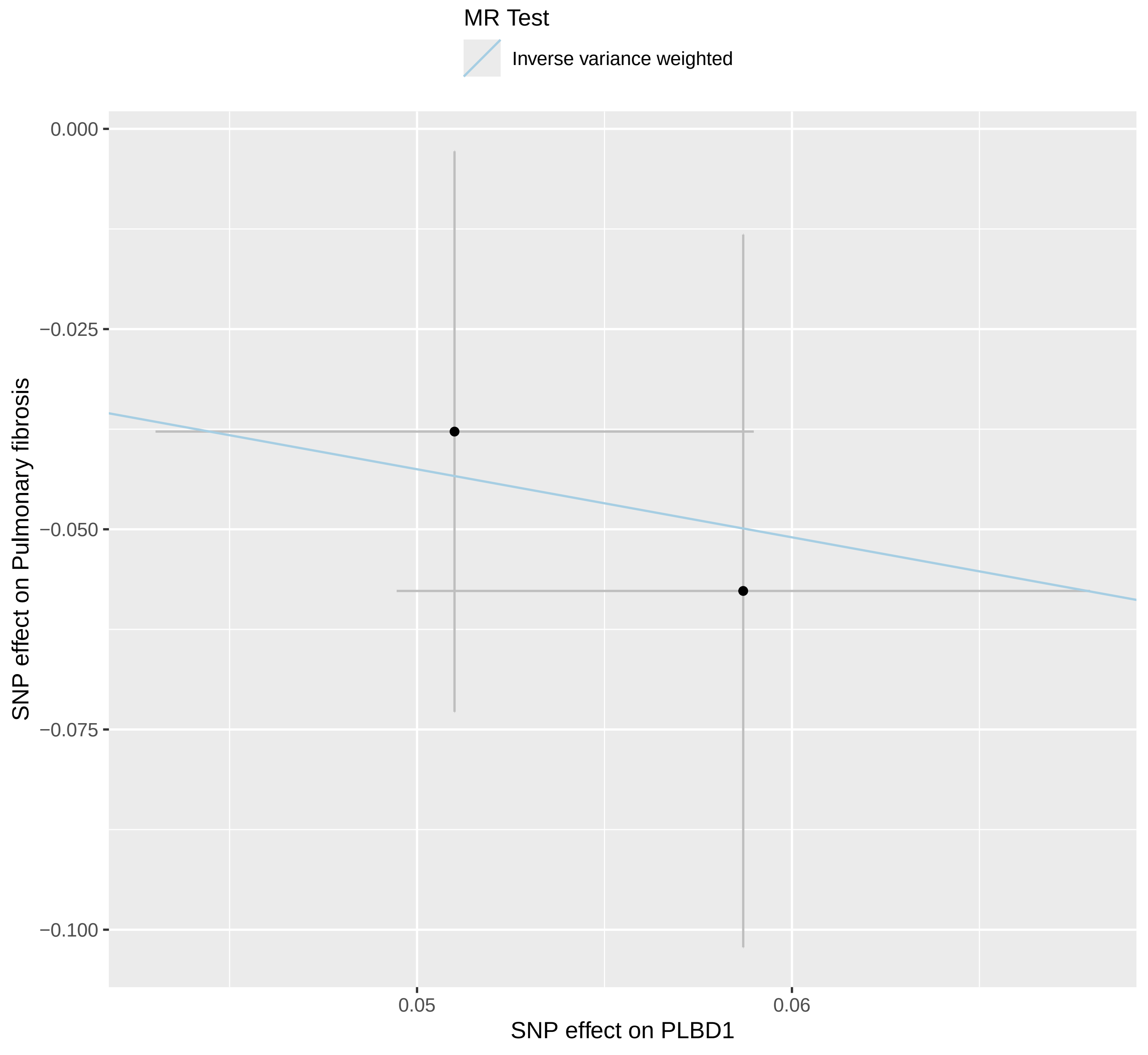
MR Test
Inverse variance weighted
0.000
−0.025
−0.050
−0.075
−0.100
SNP effect on Pulmonary fibrosis
0.05
0.06
SNP effect on PLBD1

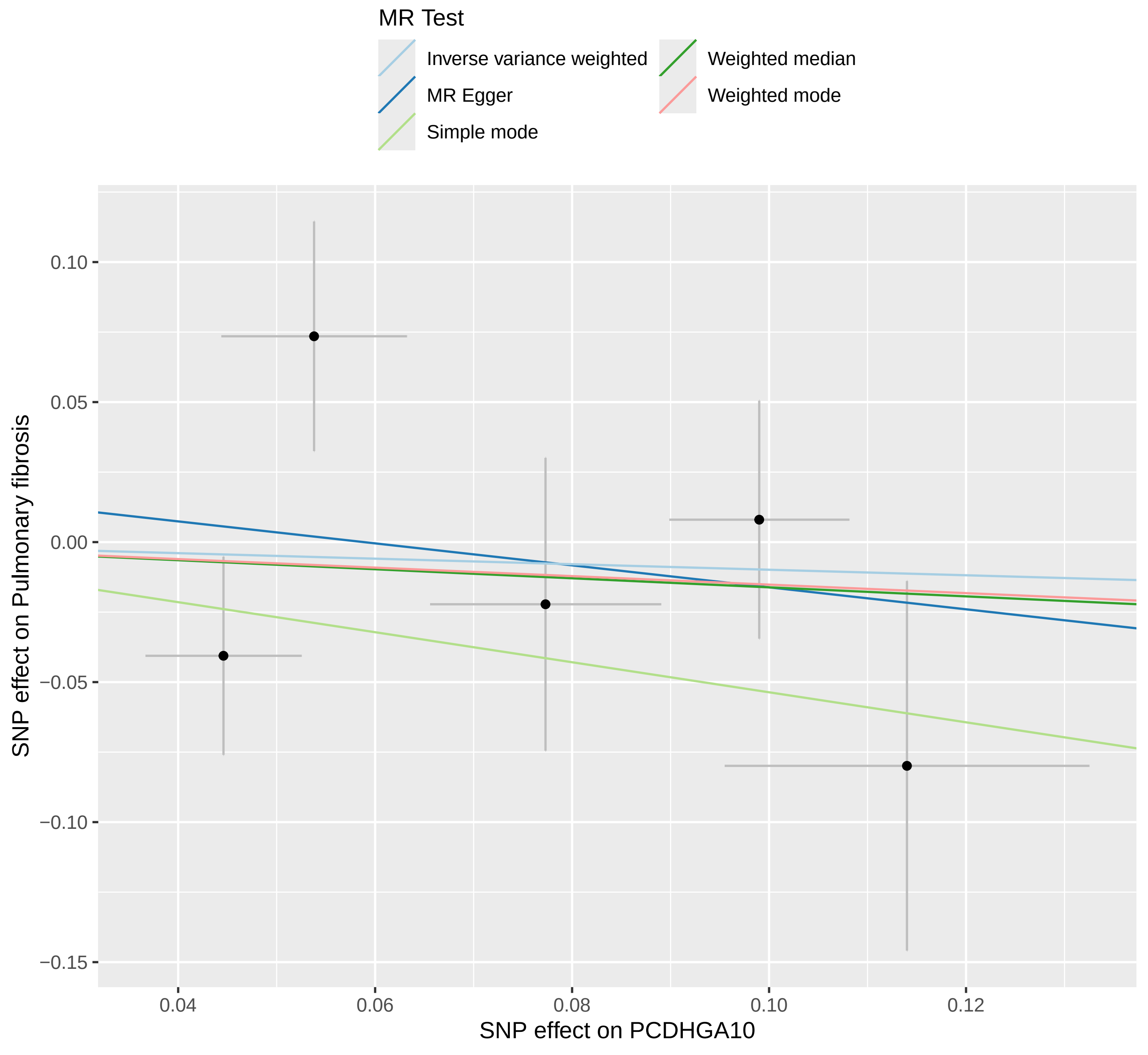

MR Test
Inverse variance weighted
MR Egger
Simple mode
Weighted median
Weighted mode
0.10
0.05
0.00
−0.05
−0.10
−0.15
SNP effect on Pulmonary fibrosis
0.04
0.06
0.08
0.10
0.12
SNP effect on PCDHGA10

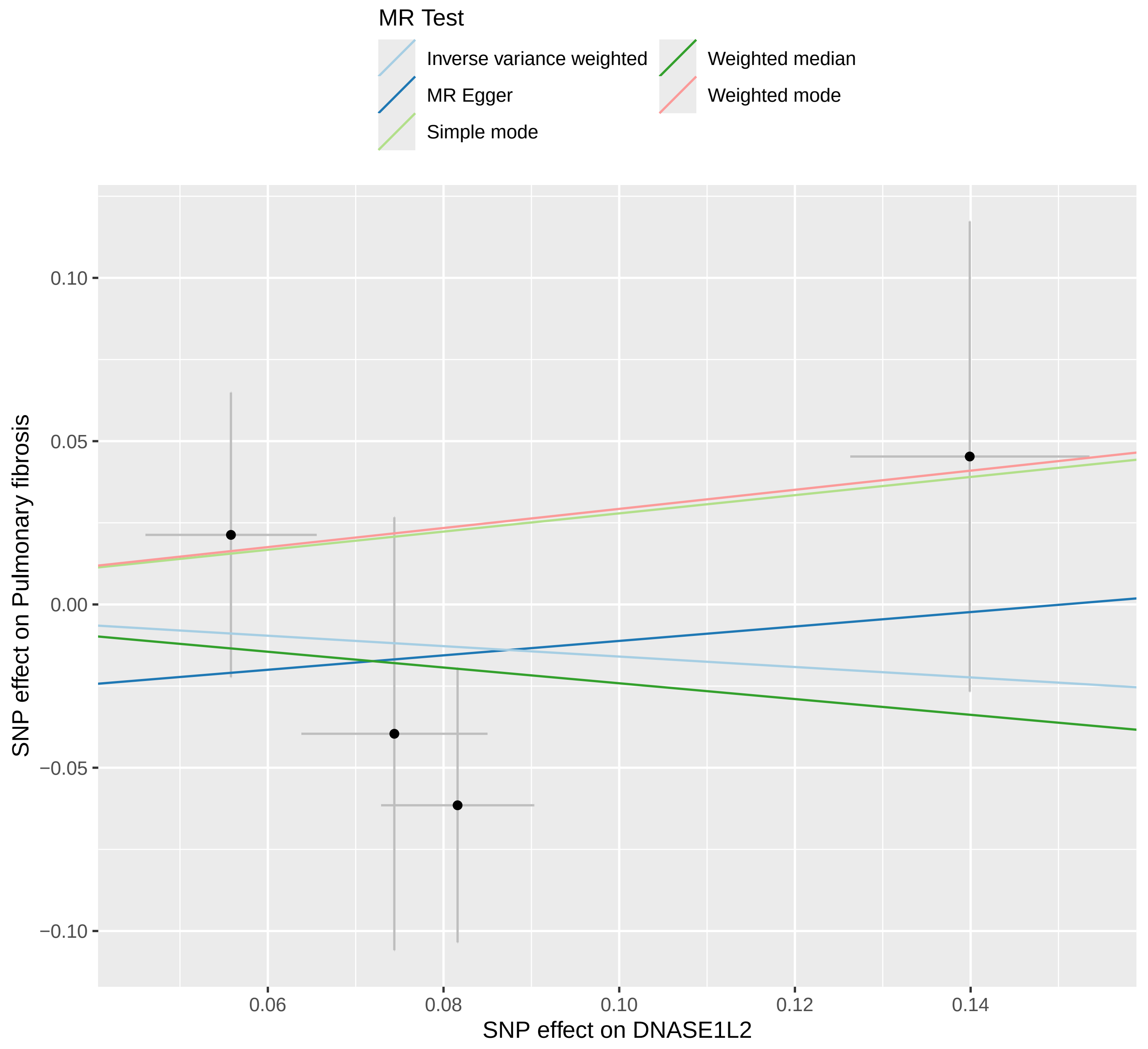
MR Test
Inverse variance weighted
MR Egger
Simple mode
Weighted median
Weighted mode
0.10
0.05
0.00
−0.05
−0.10
SNP effect on Pulmonary fibrosis
0.06
0.08
0.10
0.12
0.14
SNP effect on DNASE1L2

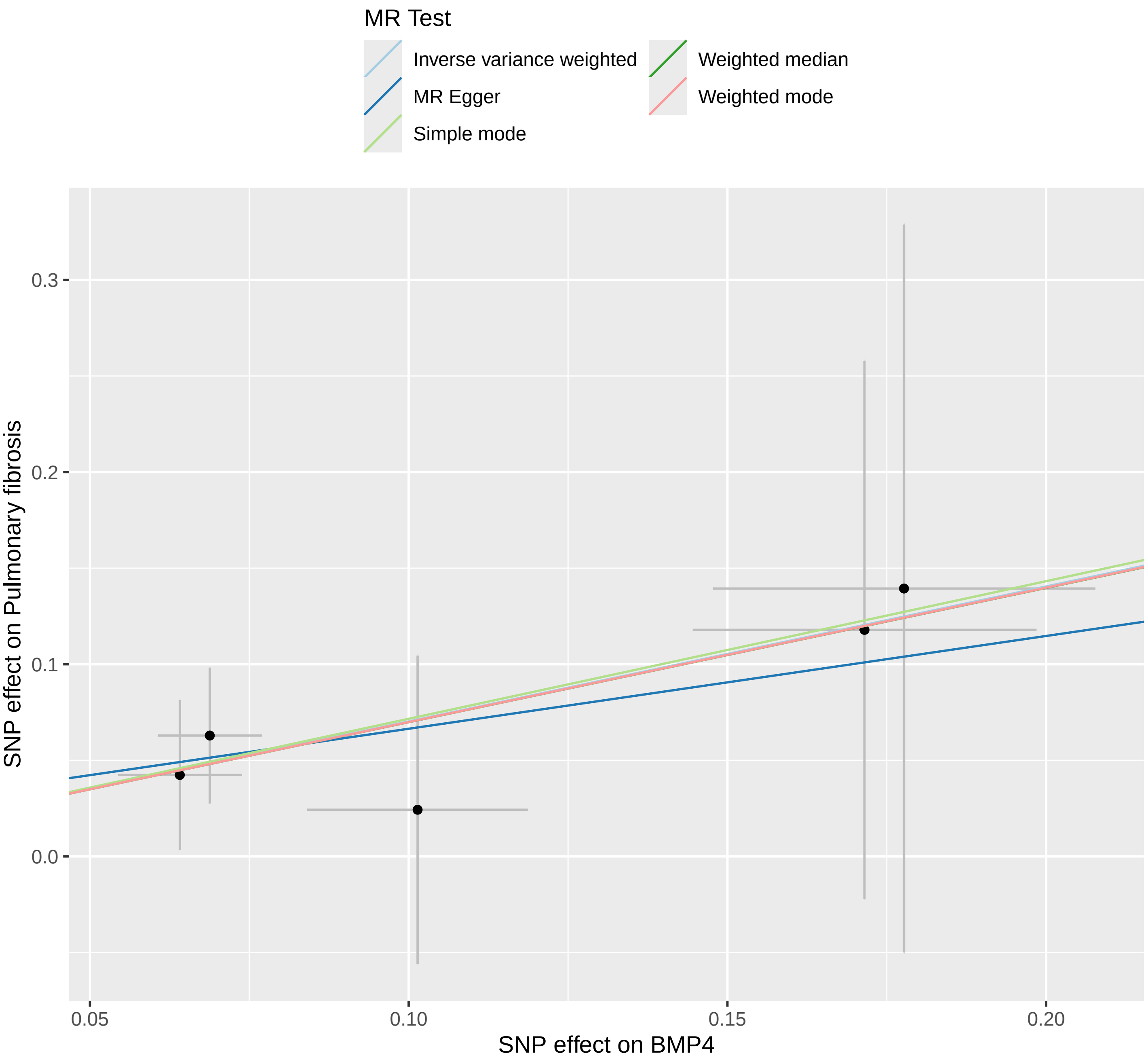

MR Test
Inverse variance weighted
MR Egger
Simple mode
Weighted median
Weighted mode
0.3
0.2
0.1
0.0
SNP effect on Pulmonary fibrosis
0.05
0.10
0.15
0.20
SNP effect on BMP4

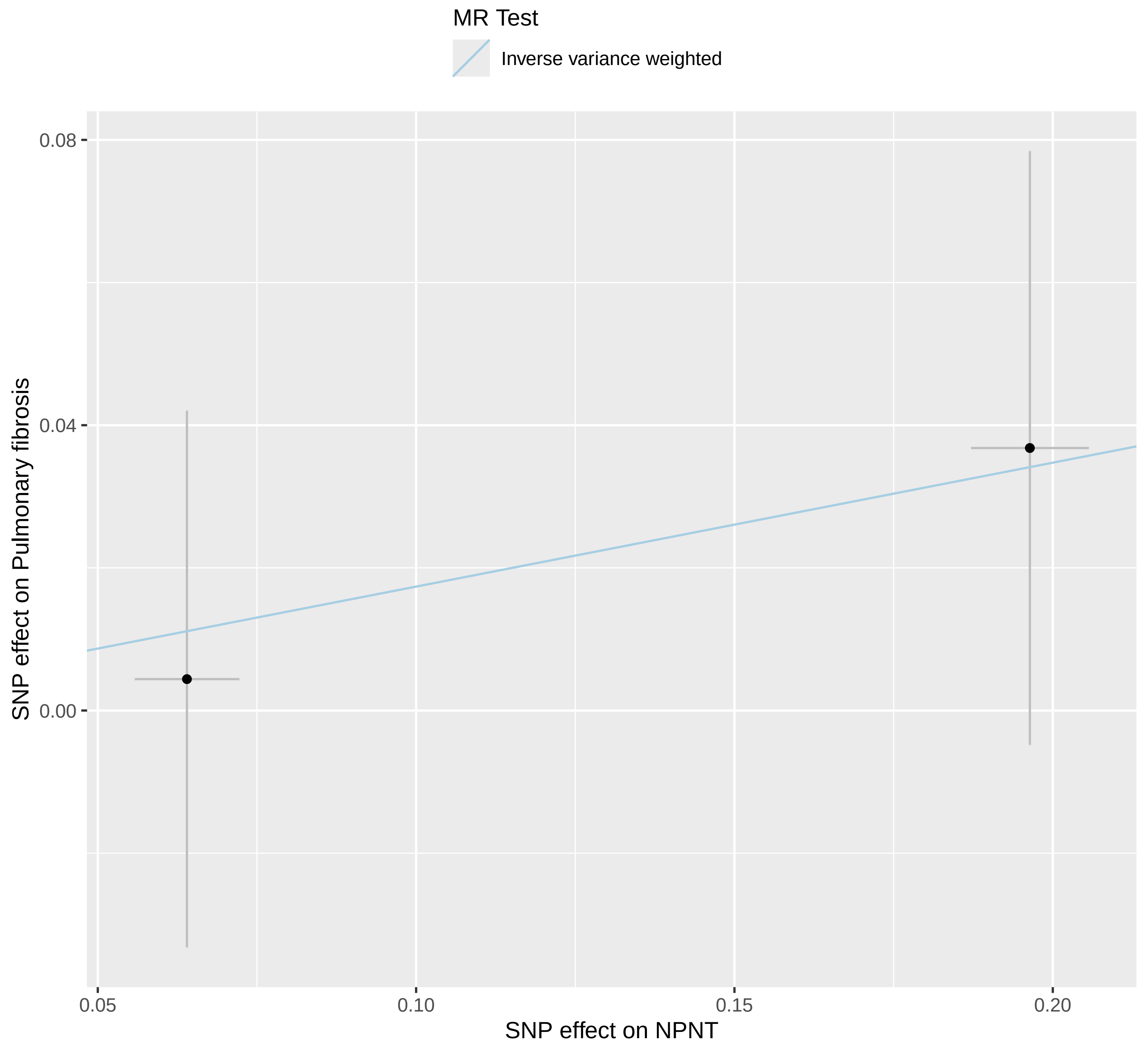
MR Test
Inverse variance weighted
0.08
0.04
0.00
0.05
0.10
0.15
0.20
SNP effect on Pulmonary fibrosis
SNP effect on NPNT

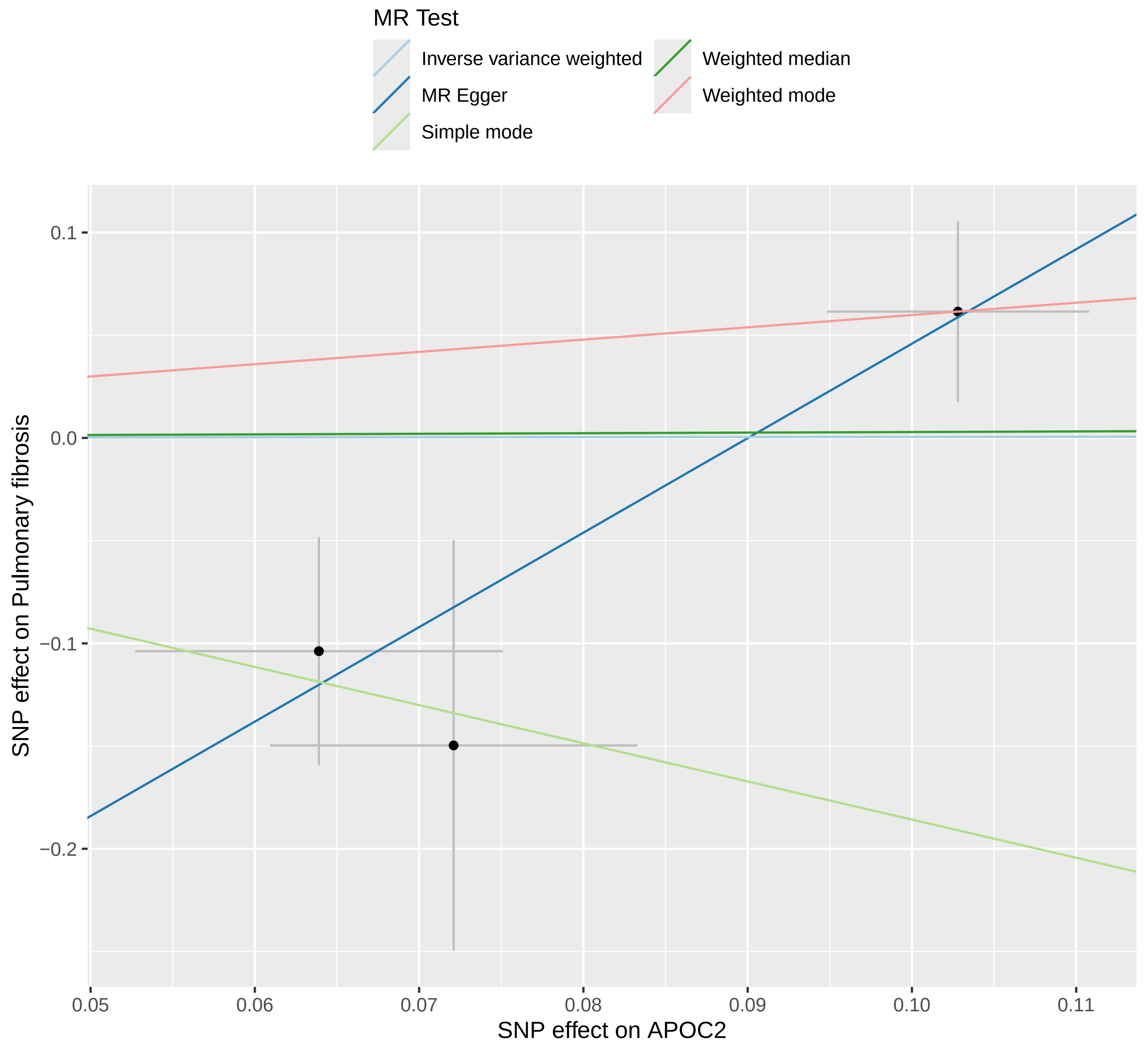
MR Test
Inverse variance weighted
MR Egger
Simple mode
Weighted median
Weighted mode
0.1
0.0
−0.1
−0.2
SNP effect on Pulmonary fibrosis
0.05
0.06
0.07
0.08
0.09
0.10
0.11
SNP effect on APOC2

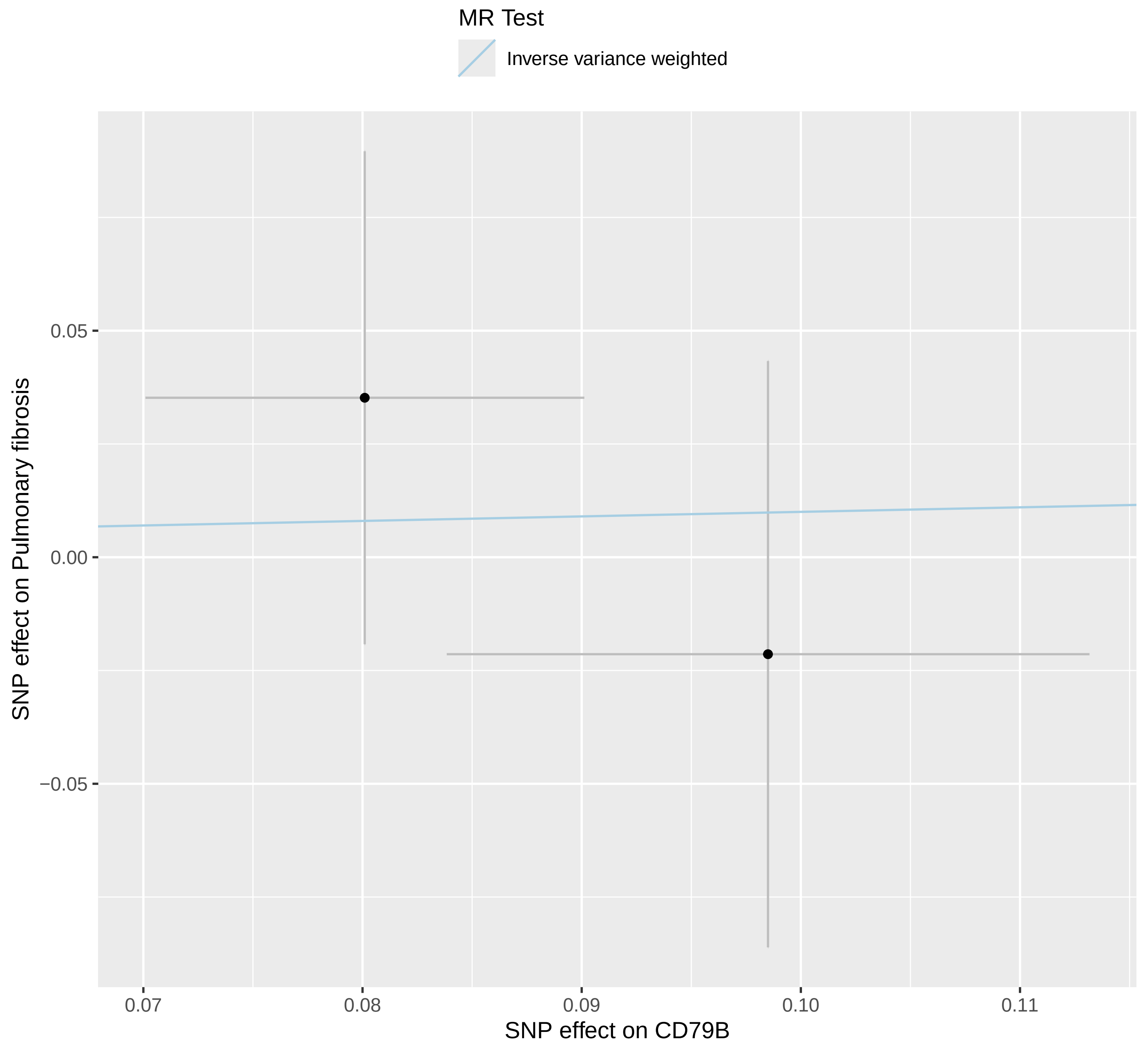

MR Test
Inverse variance weighted
0.05
0.00
−0.05
0.07
0.08
0.09
0.10
0.11
SNP effect on CD79B
SNP effect on Pulmonary fibrosis

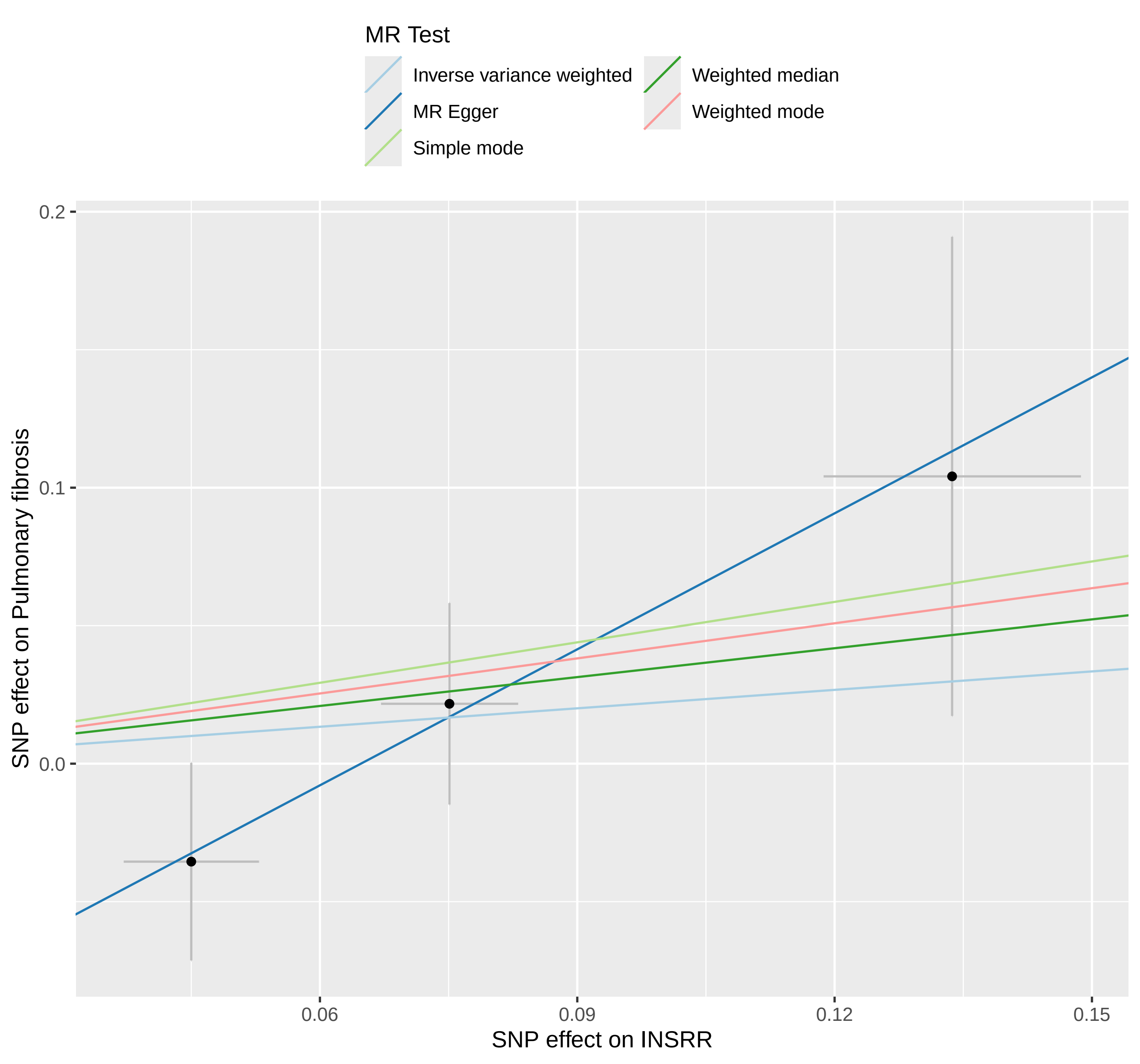
MR Test
Inverse variance weighted
MR Egger
Simple mode
Weighted median
Weighted mode
0.2
0.1
0.0
0.06
0.09
0.12
0.15
SNP effect on Pulmonary fibrosis
SNP effect on INSRR

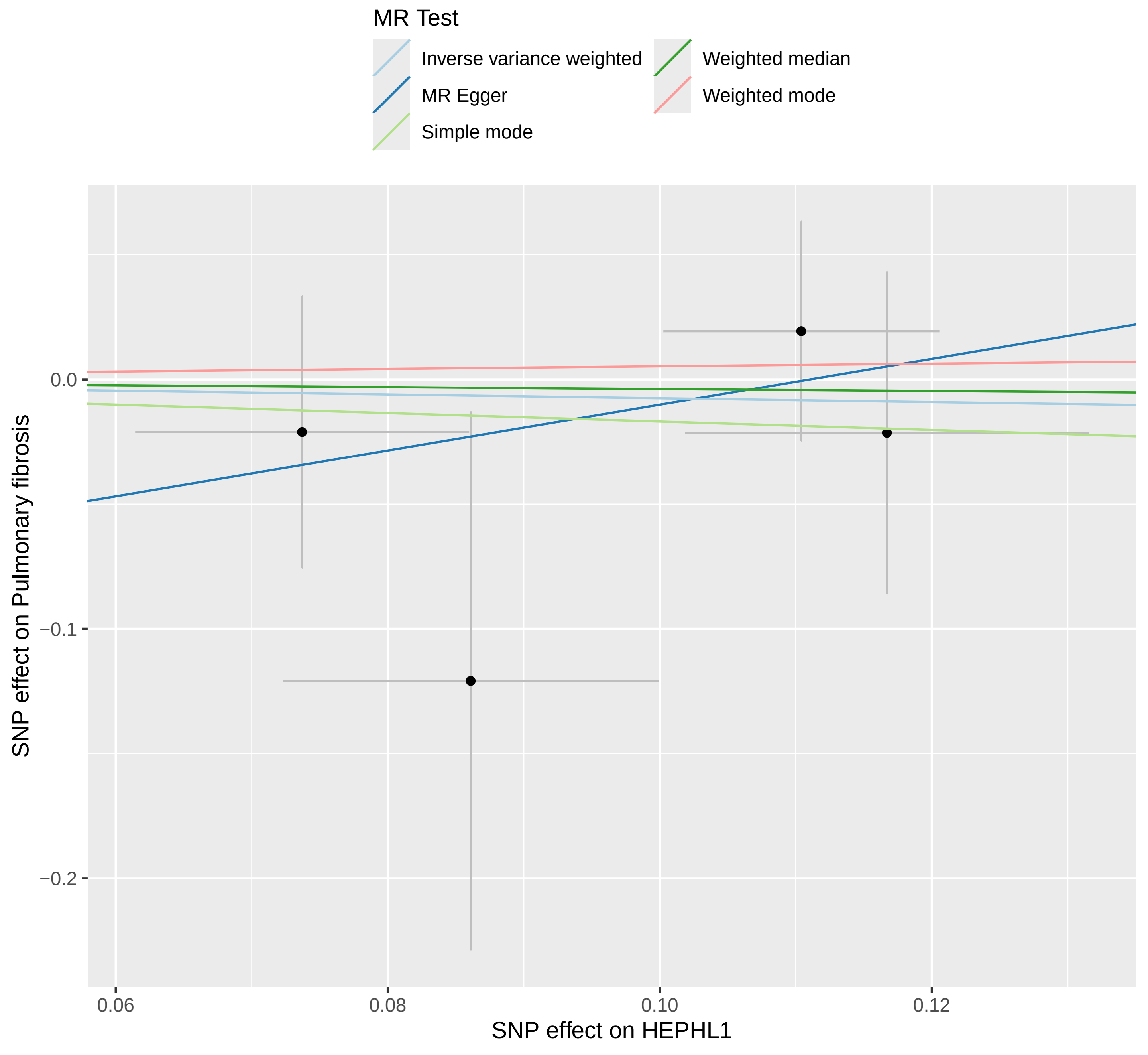

MR Test
Inverse variance weighted
MR Egger
Simple mode
Weighted median
Weighted mode
0.0
−0.1
−0.2
SNP effect on Pulmonary fibrosis
0.06
0.08
0.10
0.12
SNP effect on HEPHL1

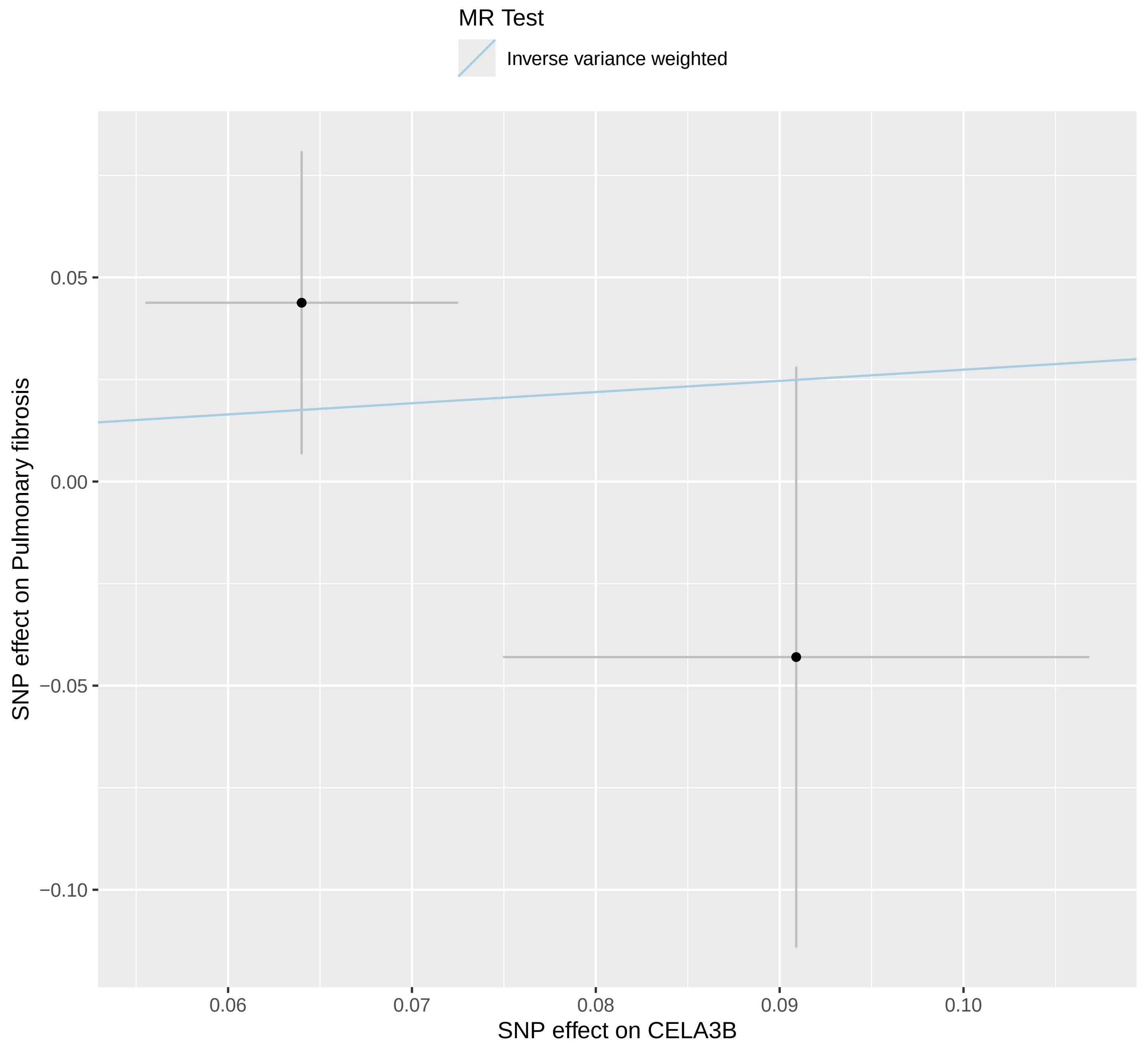
MR Test
Inverse variance weighted
SNP effect on Pulmonary fibrosis
0.05
0.00
−0.05
−0.10
0.06
0.07
0.08
0.09
0.10
SNP effect on CELA3B

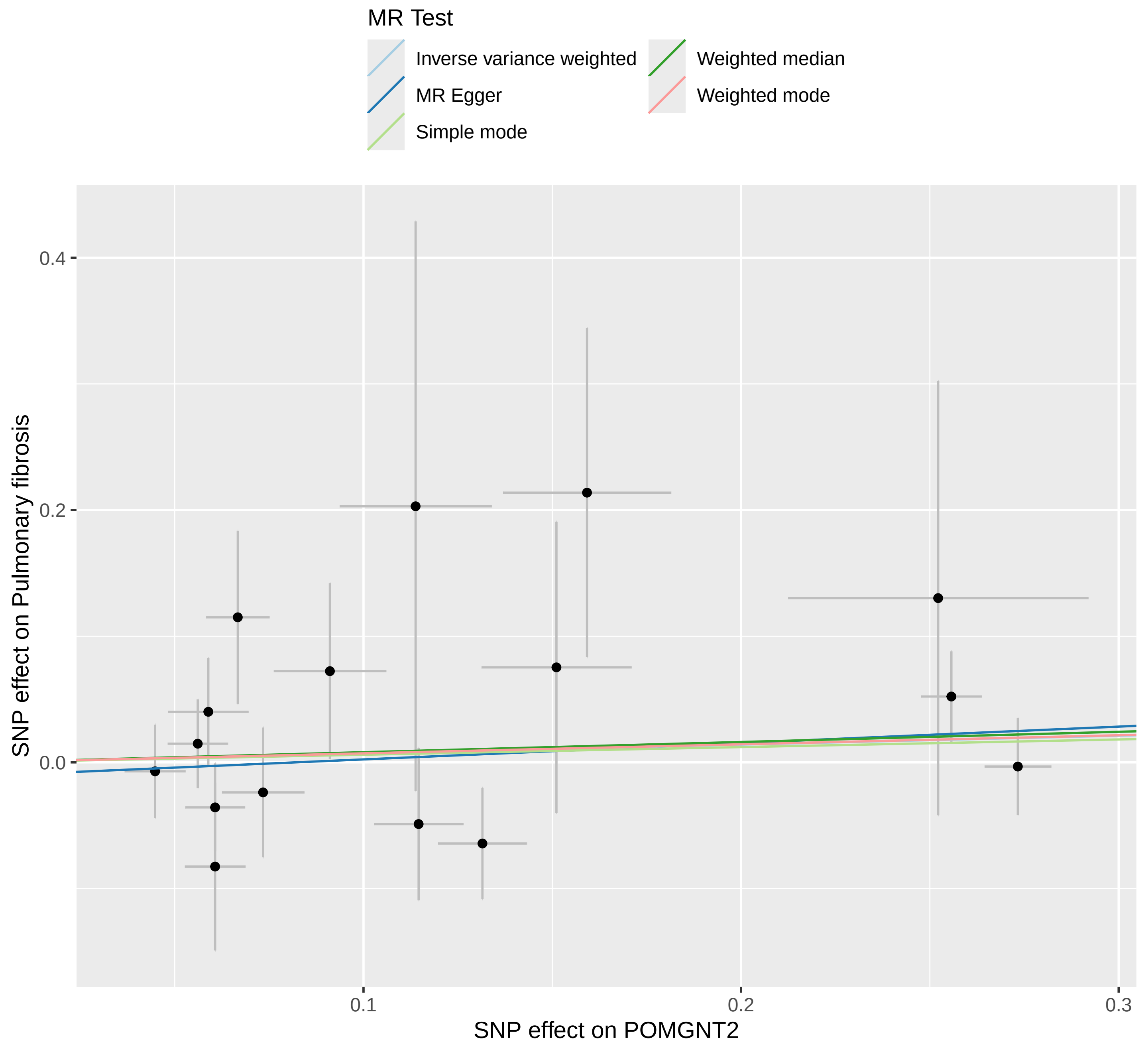
MR Test
Inverse variance weighted
MR Egger
Simple mode
Weighted median
Weighted mode
SNP effect on Pulmonary fibrosis
0.4
0.2
0.0
0.1
0.2
0.3
SNP effect on POMGNT2

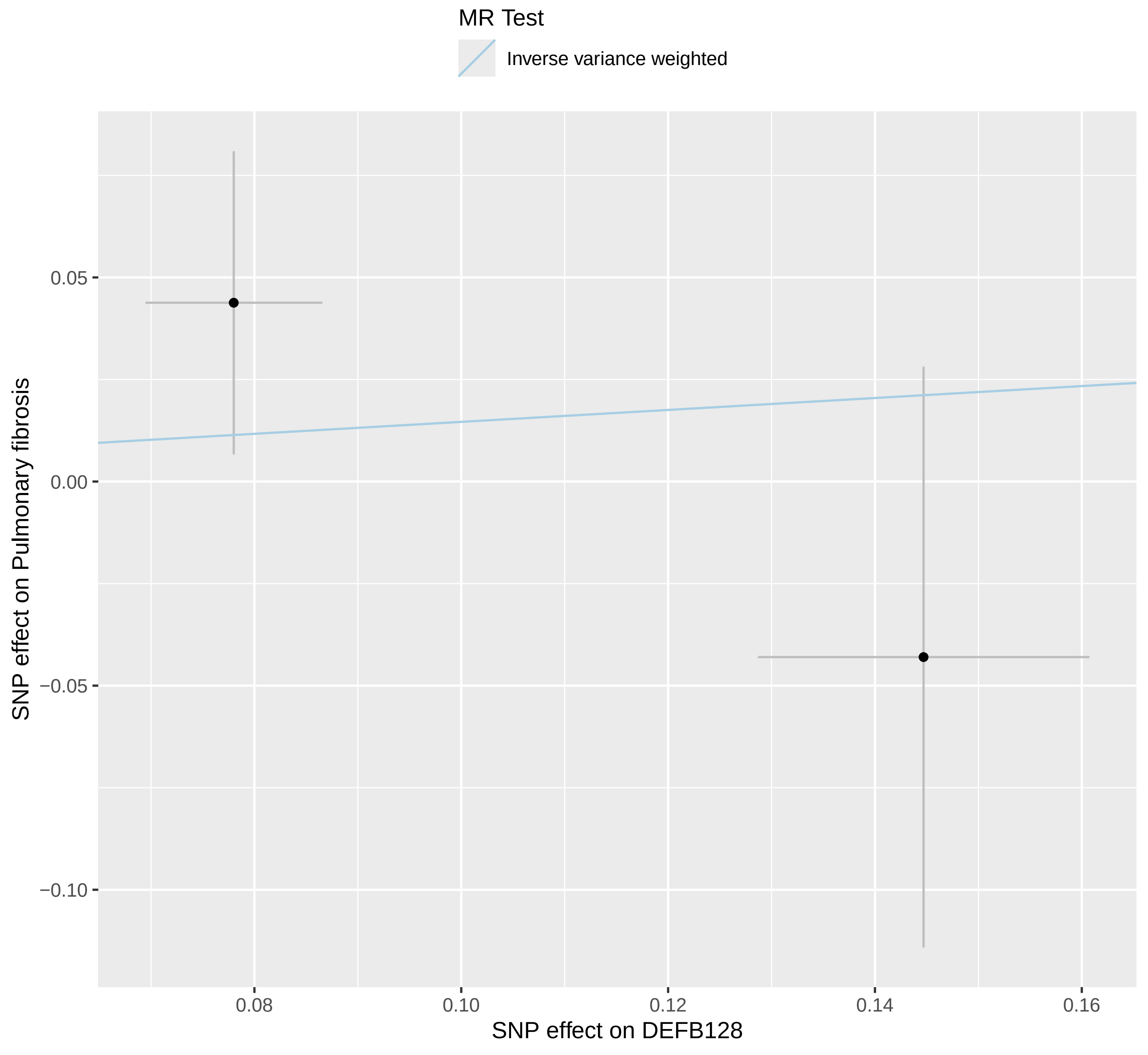

MR Test
Inverse variance weighted
0.05
0.00
−0.05
−0.10
SNP effect on Pulmonary fibrosis
0.08
0.10
0.12
0.14
0.16
SNP effect on DEFB128

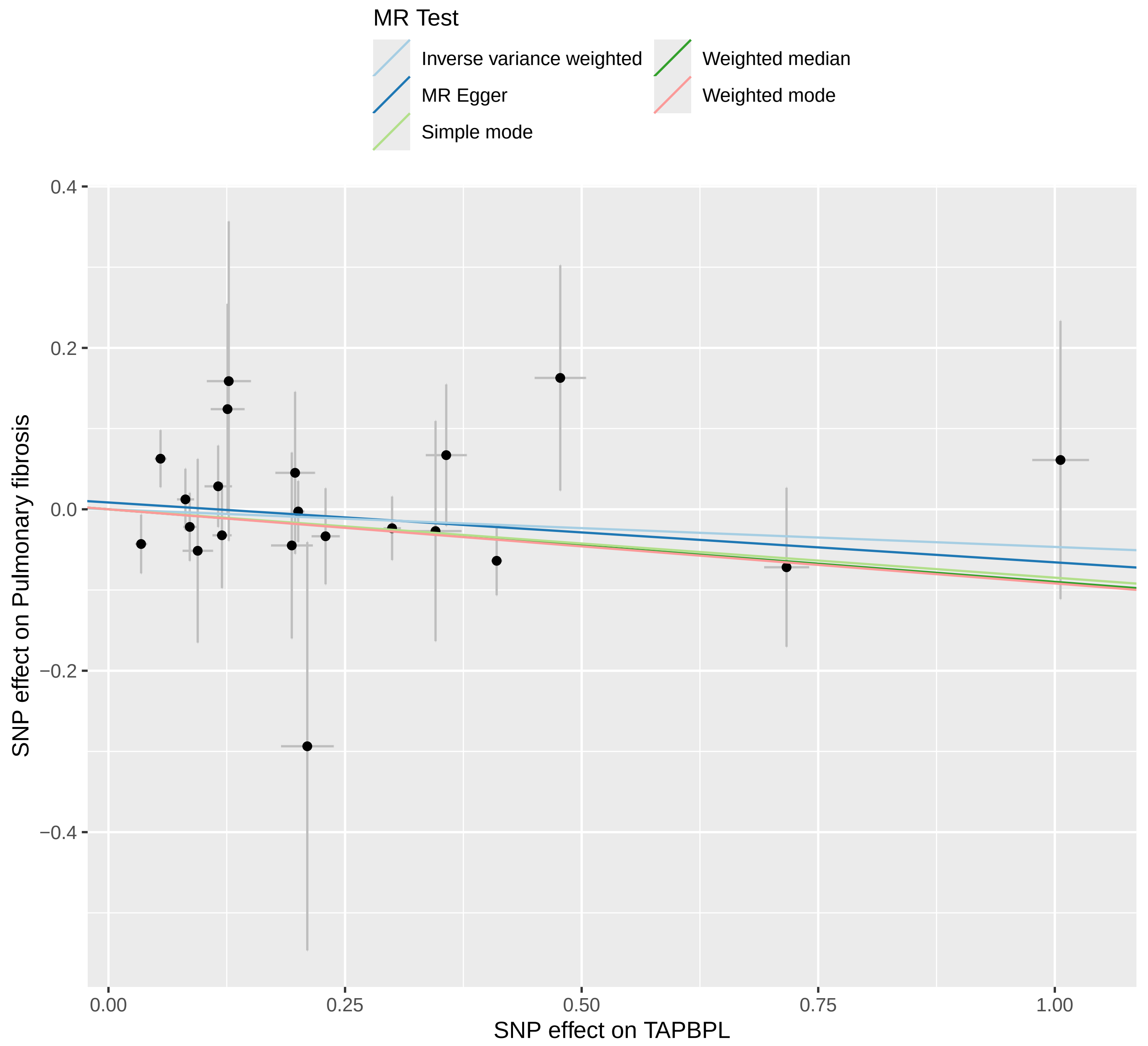

MR Test
Inverse variance weighted
MR Egger
Simple mode
Weighted median
Weighted mode
0.4
0.2
0.0
−0.2
−0.4
SNP effect on Pulmonary fibrosis
0.00
0.25
0.50
0.75
1.00
SNP effect on TAPBPL

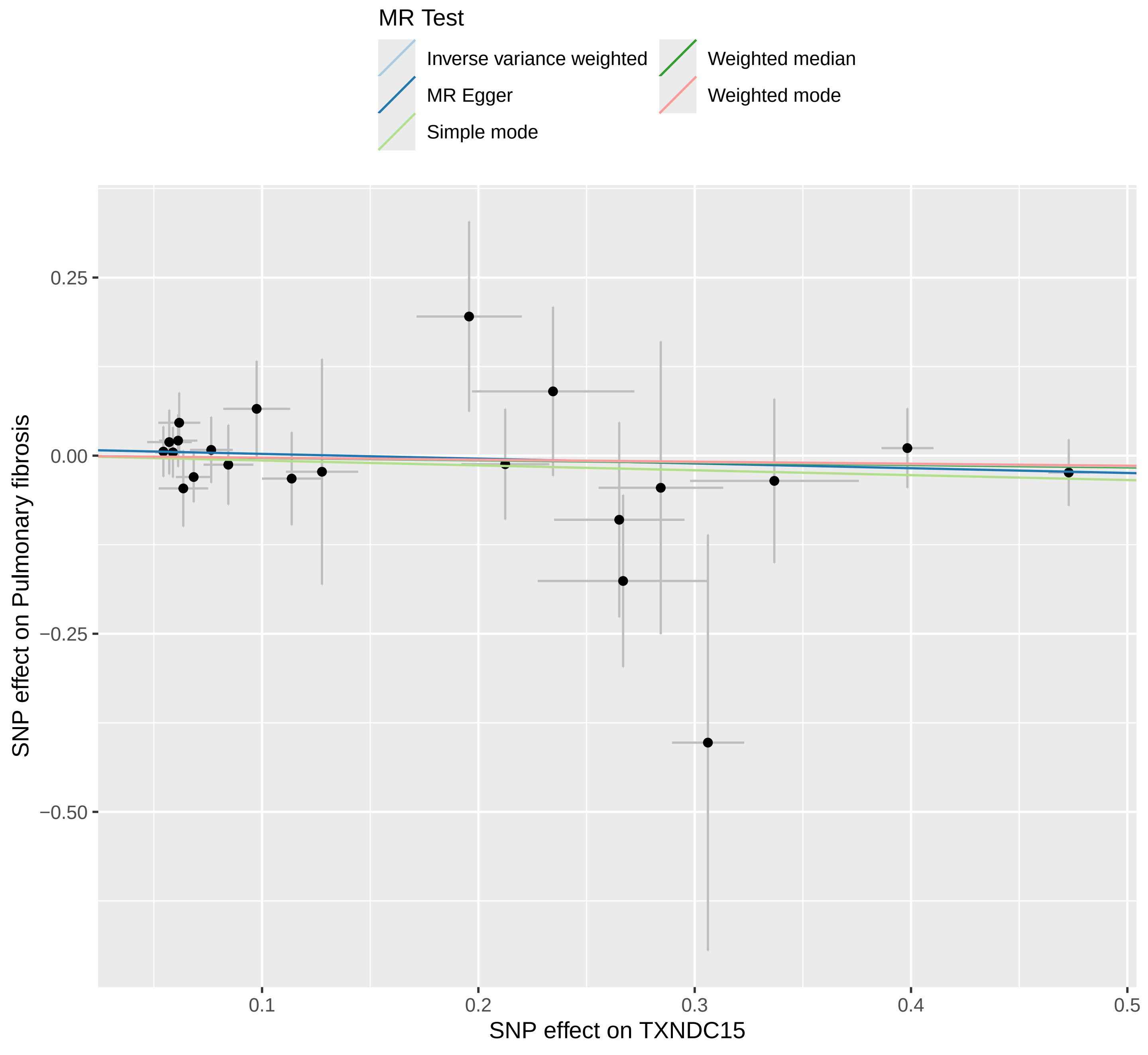

MR Test
Inverse variance weighted
MR Egger
Simple mode
Weighted median
Weighted mode
0.25
0.00
−0.25
−0.50
0.1
0.2
0.3
0.4
0.5
SNP effect on Pulmonary fibrosis
SNP effect on TXNDC15

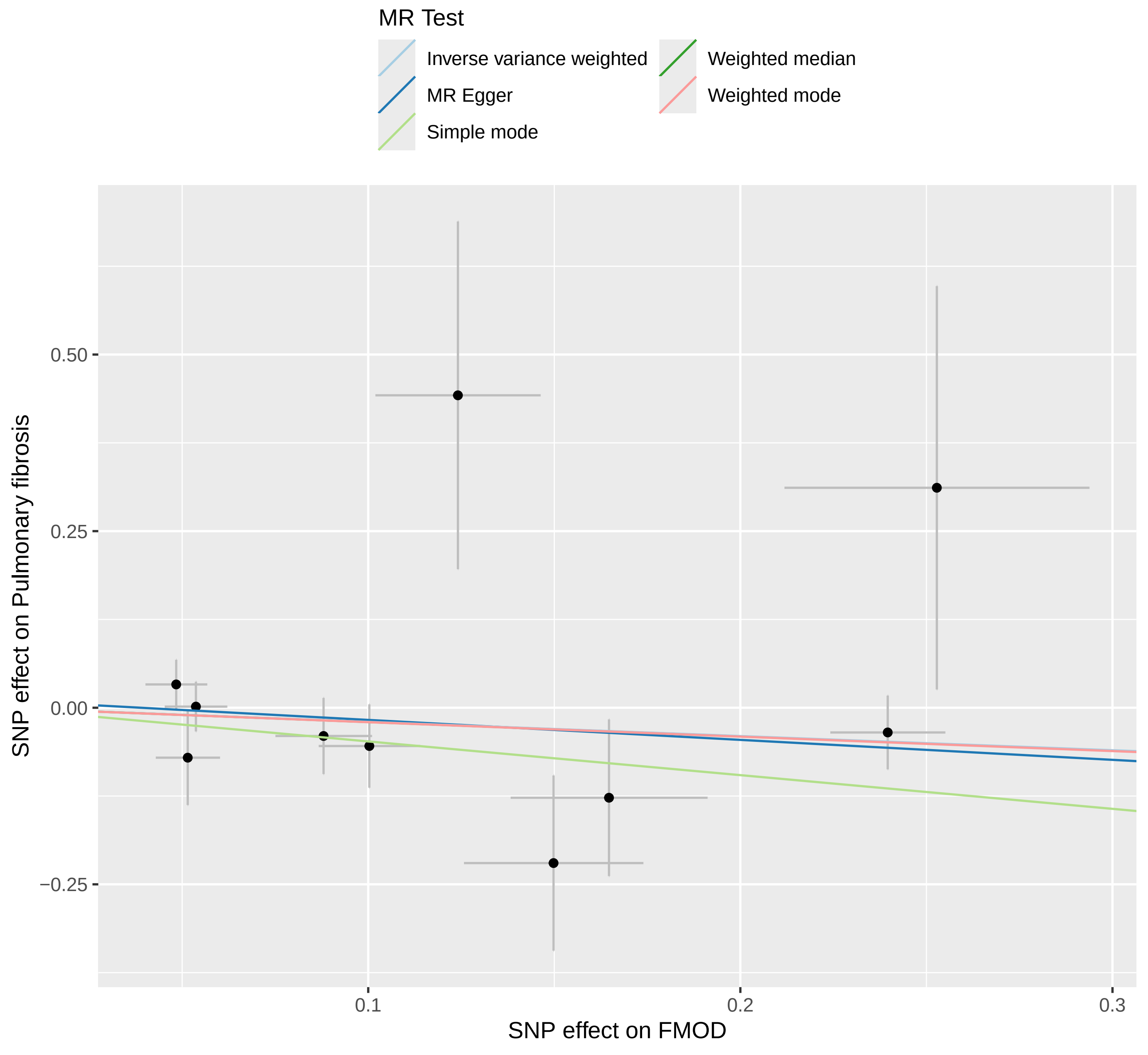
MR Test
Inverse variance weighted
MR Egger
Simple mode
Weighted median
Weighted mode
SNP effect on Pulmonary fibrosis
0.50
0.25
0.00
−0.25
0.1
0.2
0.3
SNP effect on FMOD

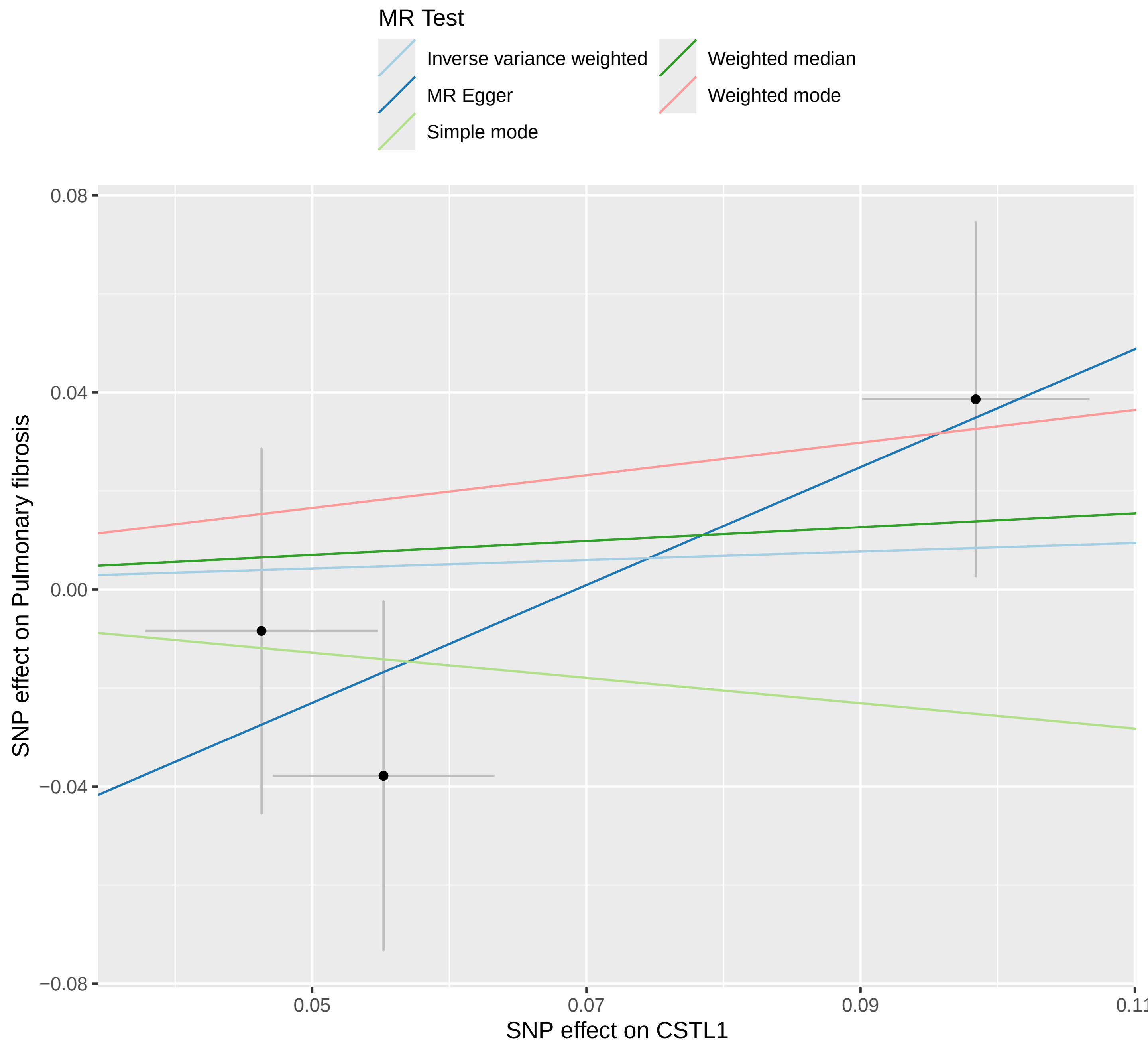
MR Test
Inverse variance weighted
MR Egger
Simple mode
Weighted median
Weighted mode
0.08
0.04
0.00
−0.04
−0.08
SNP effect on Pulmonary fibrosis
0.05
0.07
0.09
0.11
SNP effect on CSTL1

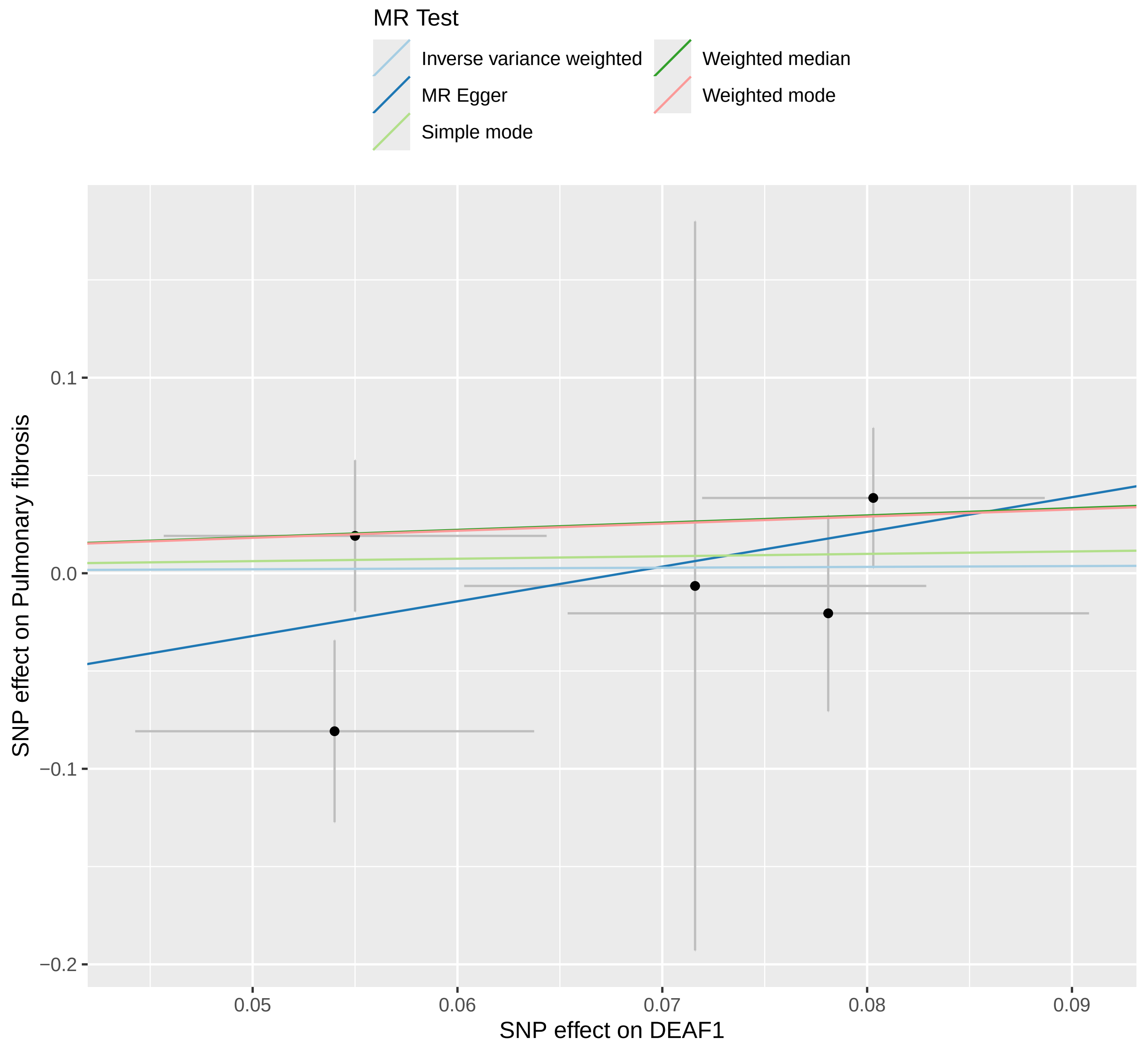

MR Test
Inverse variance weighted
MR Egger
Simple mode
Weighted median
Weighted mode
0.1
0.0
−0.1
−0.2
0.05
0.06
0.07
0.08
0.09
SNP effect on DEAF1
SNP effect on Pulmonary fibrosis

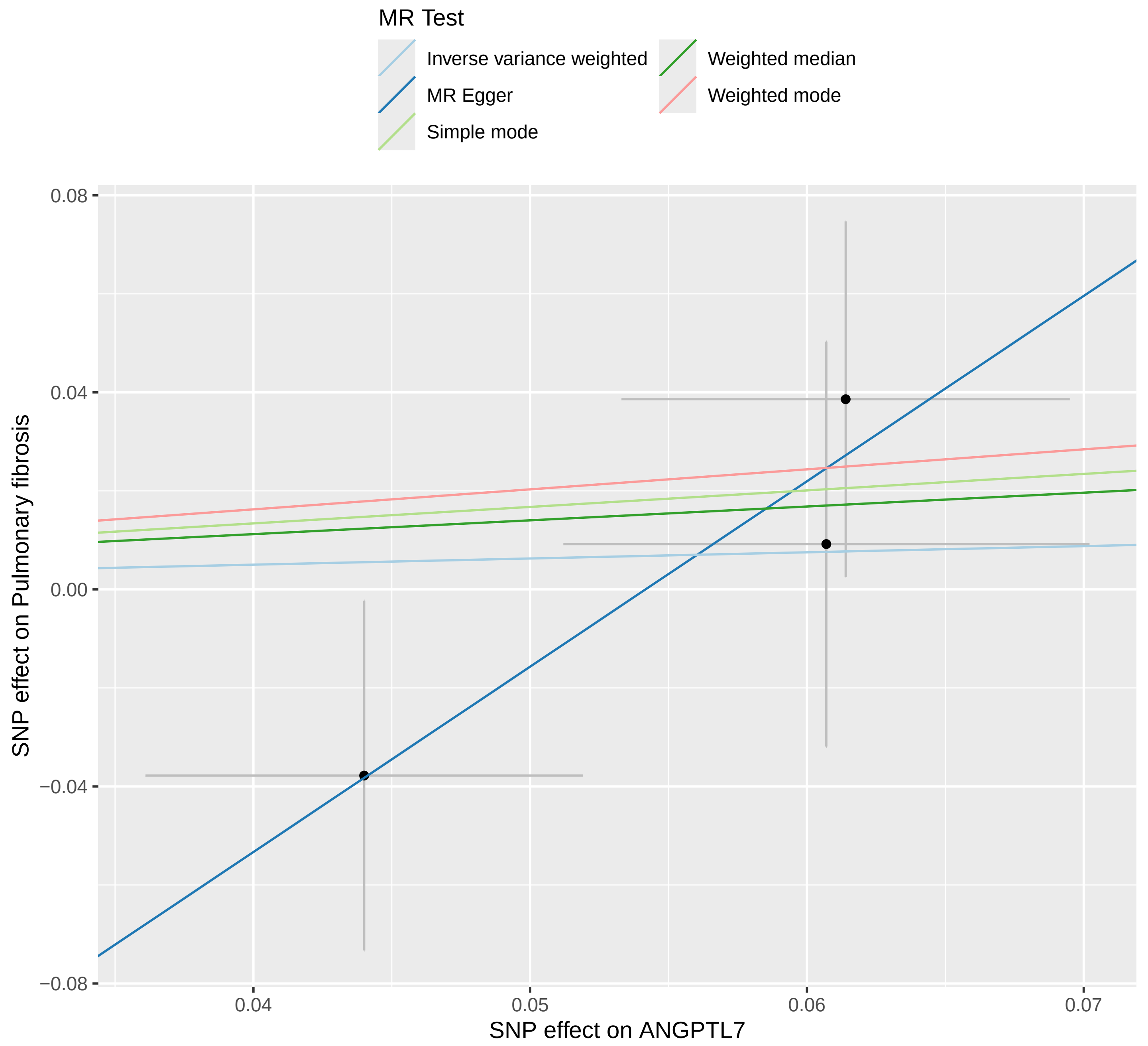

MR Test
Inverse variance weighted
MR Egger
Simple mode
Weighted median
Weighted mode
0.08
0.04
0.00
−0.04
−0.08
SNP effect on Pulmonary fibrosis
0.04
0.05
0.06
0.07
SNP effect on ANGPTL7

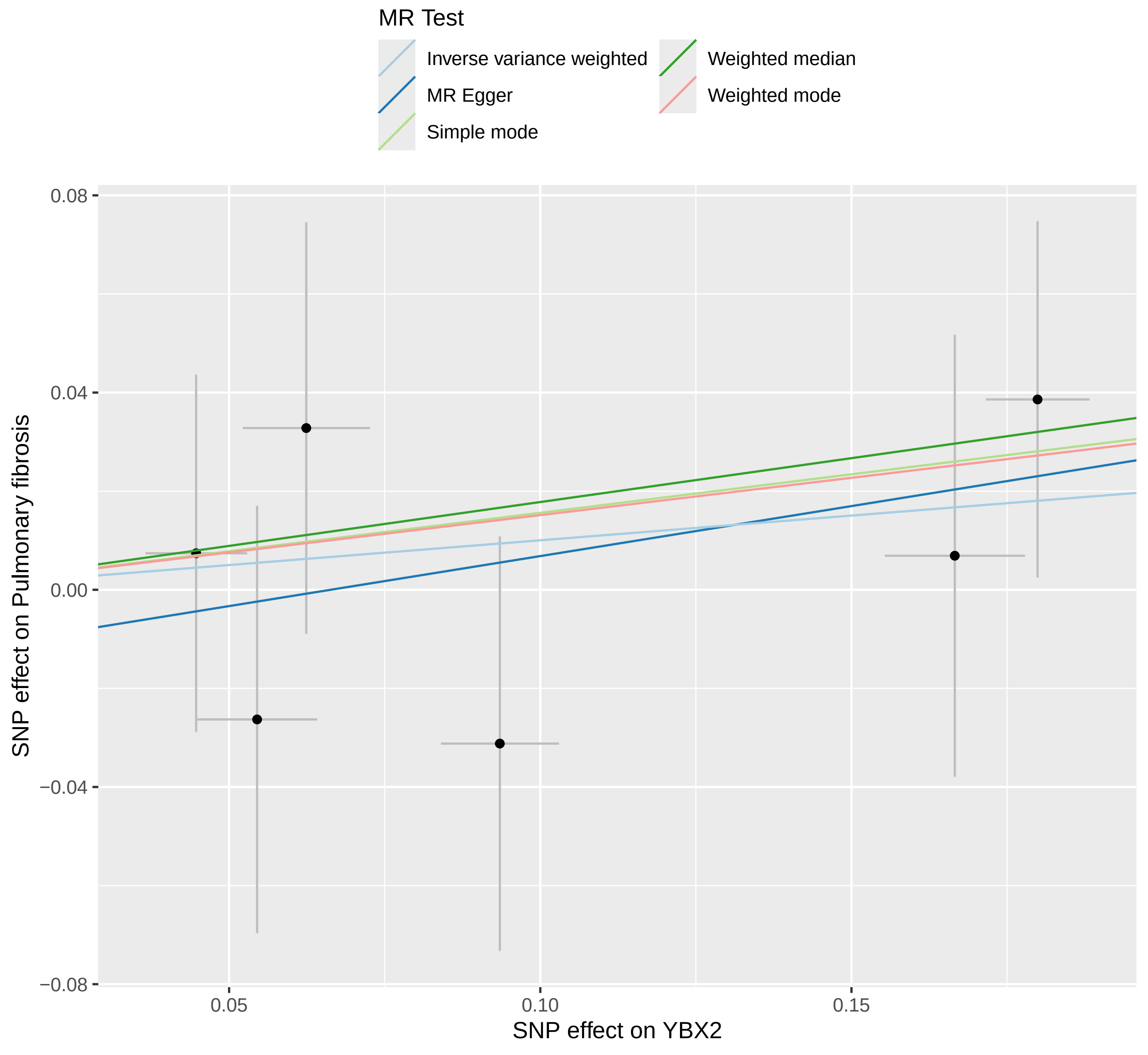

MR Test
Inverse variance weighted
MR Egger
Simple mode
Weighted median
Weighted mode
0.08
0.04
0.00
−0.04
−0.08
0.05
0.10
0.15
SNP effect on Pulmonary fibrosis
SNP effect on YBX2

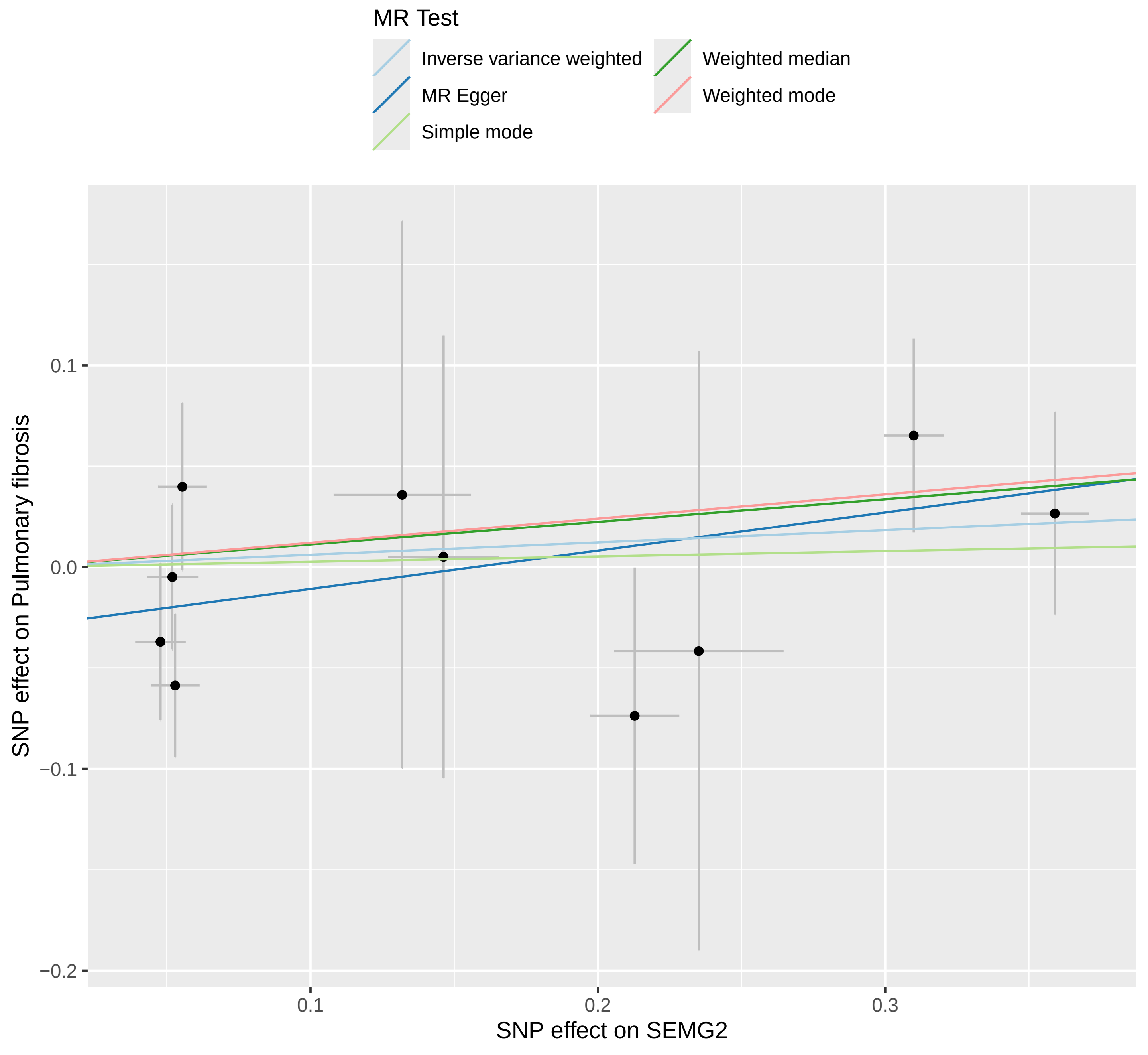
MR Test
Inverse variance weighted
MR Egger
Simple mode
Weighted median
Weighted mode
0.1
0.0
−0.1
−0.2
SNP effect on Pulmonary fibrosis
0.1
0.2
0.3
SNP effect on SEMG2

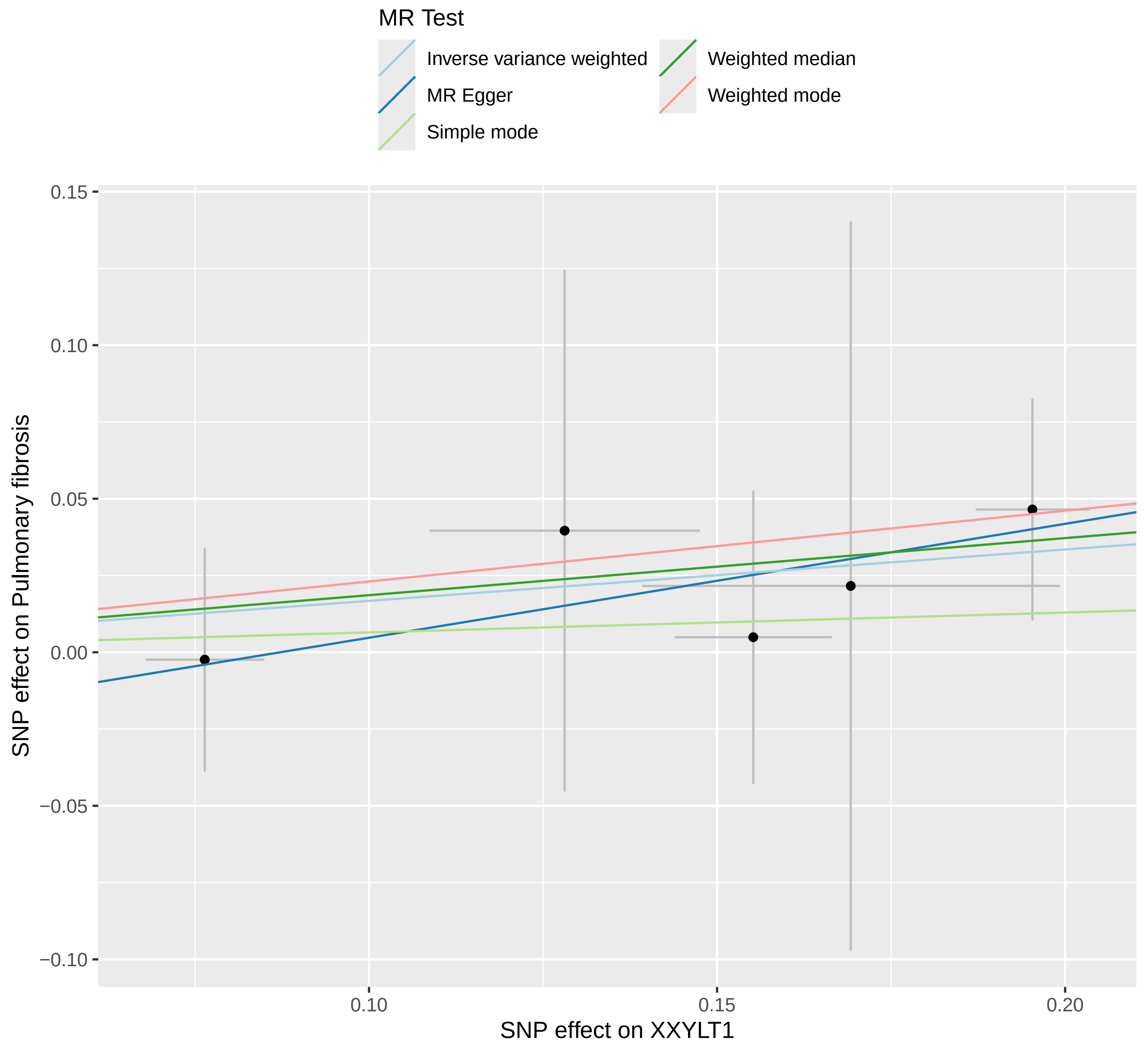

MR Test
Inverse variance weighted
MR Egger
Simple mode
Weighted median
Weighted mode
0.15
0.10
0.05
0.00
−0.05
−0.10
SNP effect on Pulmonary fibrosis
0.10
0.15
0.20
SNP effect on XXYLT1

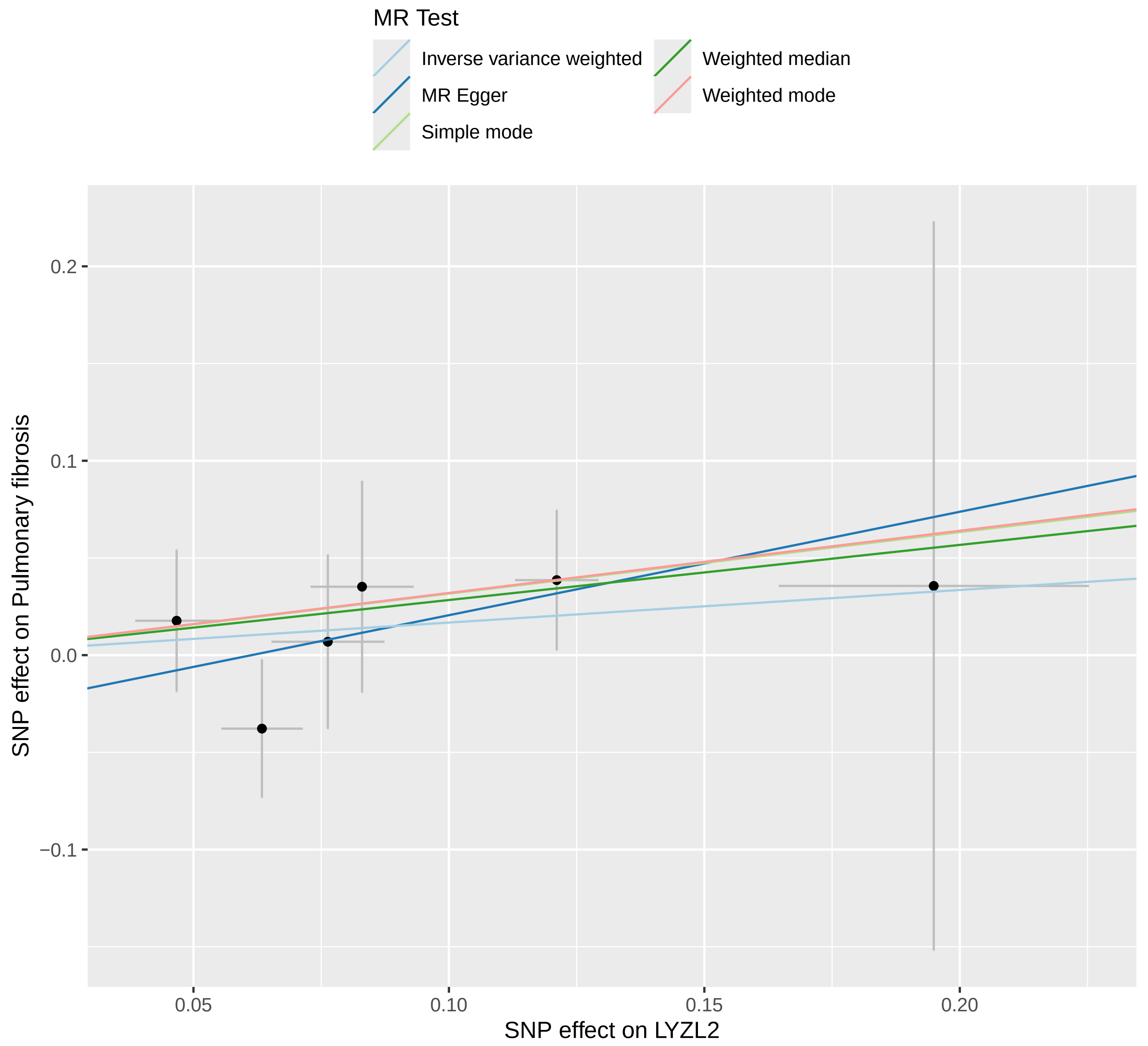

MR Test
Inverse variance weighted
MR Egger
Simple mode
Weighted median
Weighted mode
0.2
0.1
0.0
−0.1
0.05
0.10
0.15
0.20
SNP effect on Pulmonary fibrosis
SNP effect on LYZL2

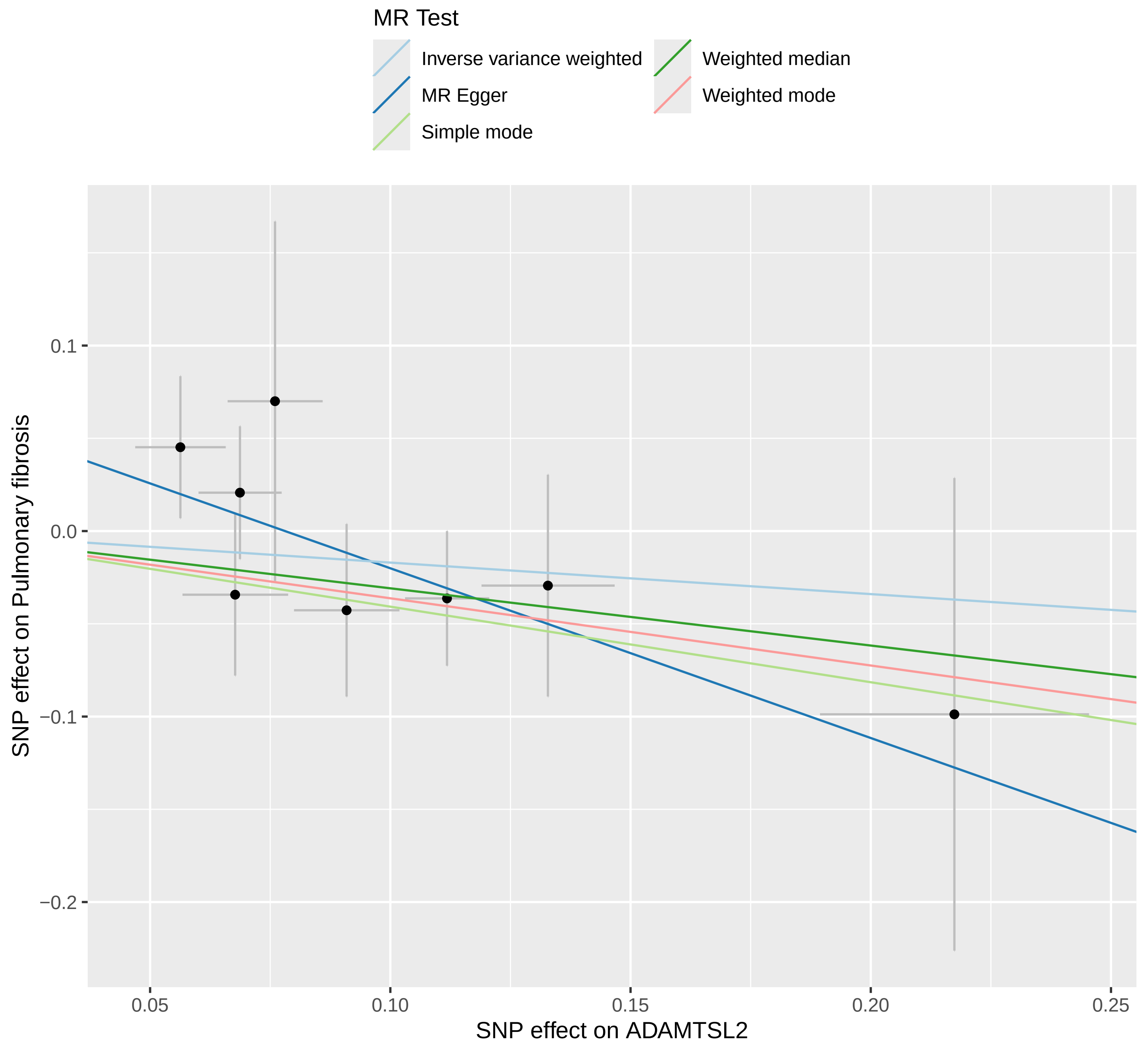
MR Test
Inverse variance weighted
MR Egger
Simple mode
Weighted median
Weighted mode
0.1
0.0
−0.1
−0.2
0.05
0.10
0.15
0.20
0.25
SNP effect on Pulmonary fibrosis
SNP effect on ADAMTSL2

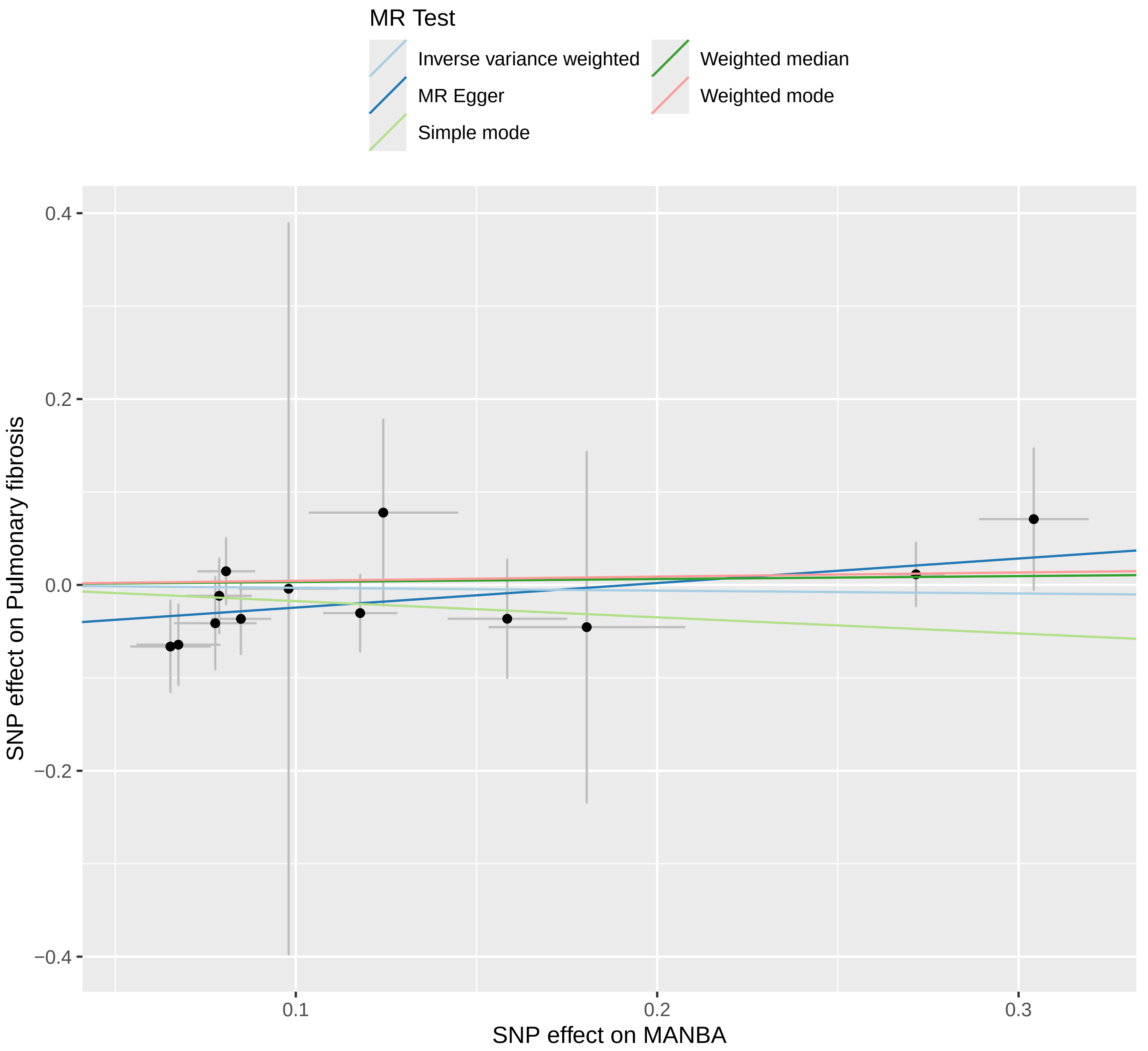
MR Test
Inverse variance weighted
MR Egger
Simple mode
Weighted median
Weighted mode
0.4
0.2
0.0
−0.2
−0.4
0.1
0.2
0.3
SNP effect on Pulmonary fibrosis
SNP effect on MANBA

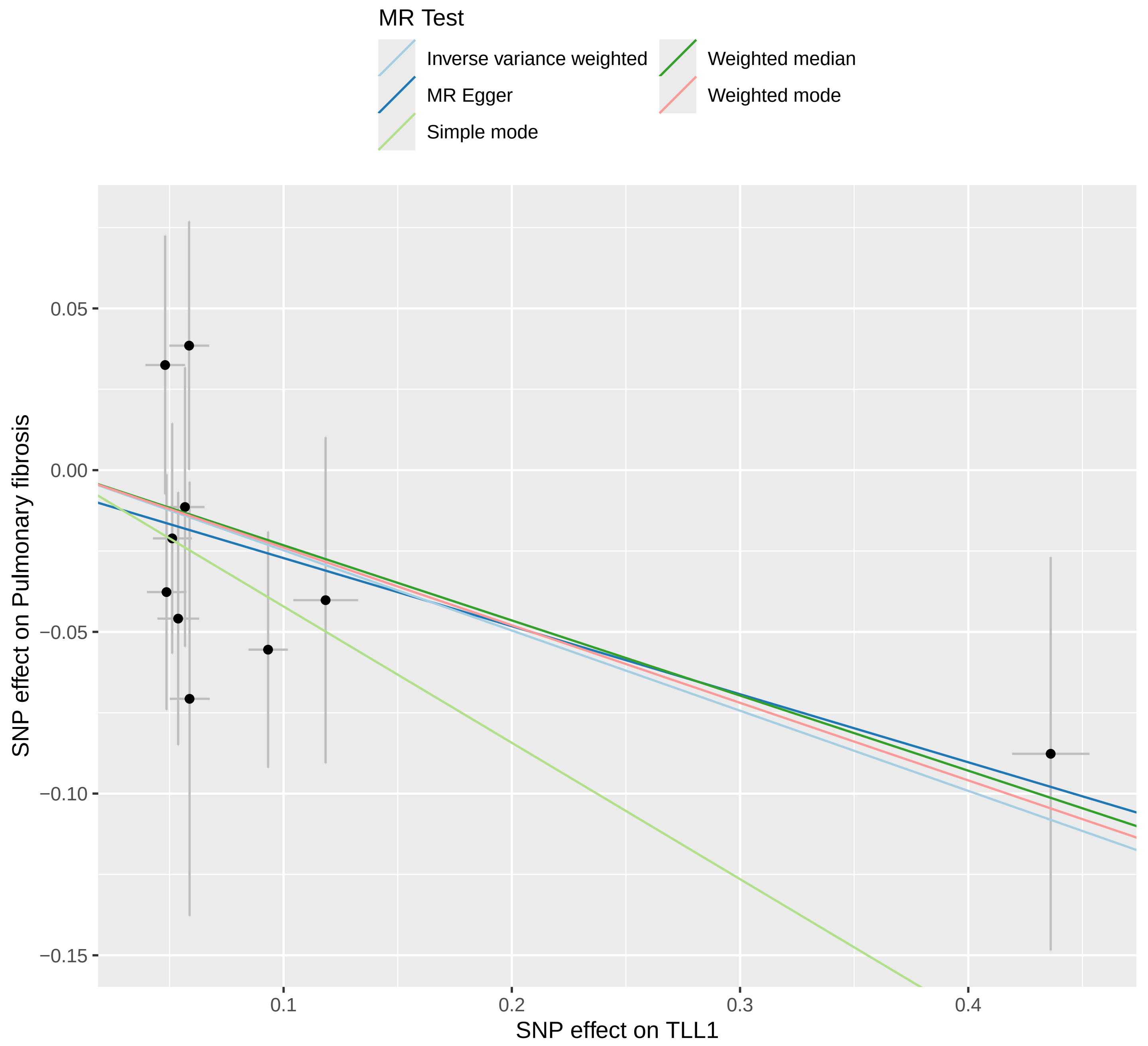
MR Test
Inverse variance weighted
MR Egger
Simple mode
Weighted median
Weighted mode
0.05
0.00
−0.05
−0.10
−0.15
SNP effect on Pulmonary fibrosis
0.1
0.2
0.3
0.4
SNP effect on TLL1

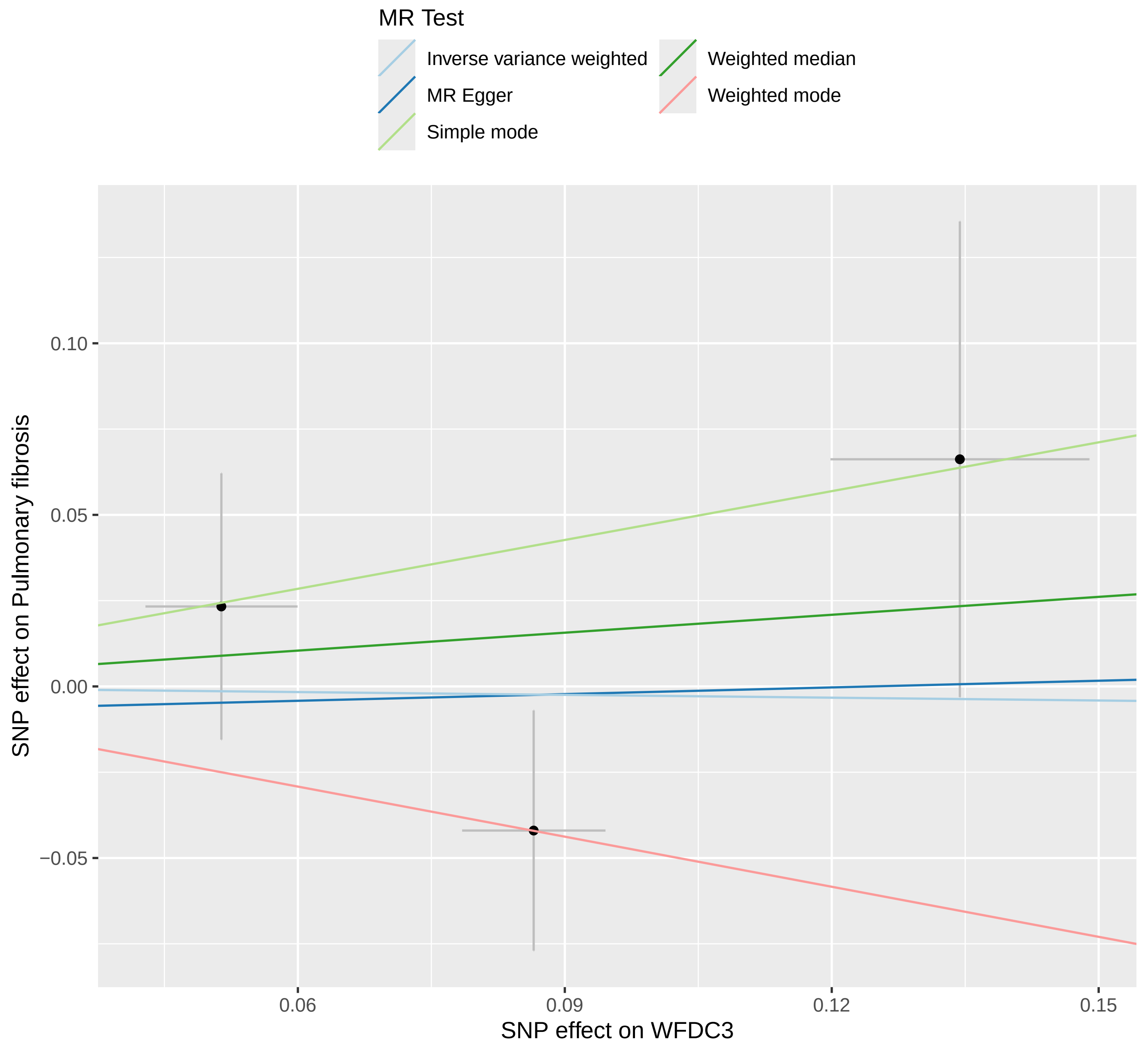
MR Test
Inverse variance weighted
MR Egger
Simple mode
Weighted median
Weighted mode
0.10
0.05
0.00
−0.05
SNP effect on Pulmonary fibrosis
0.06
0.09
0.12
0.15
SNP effect on WFDC3

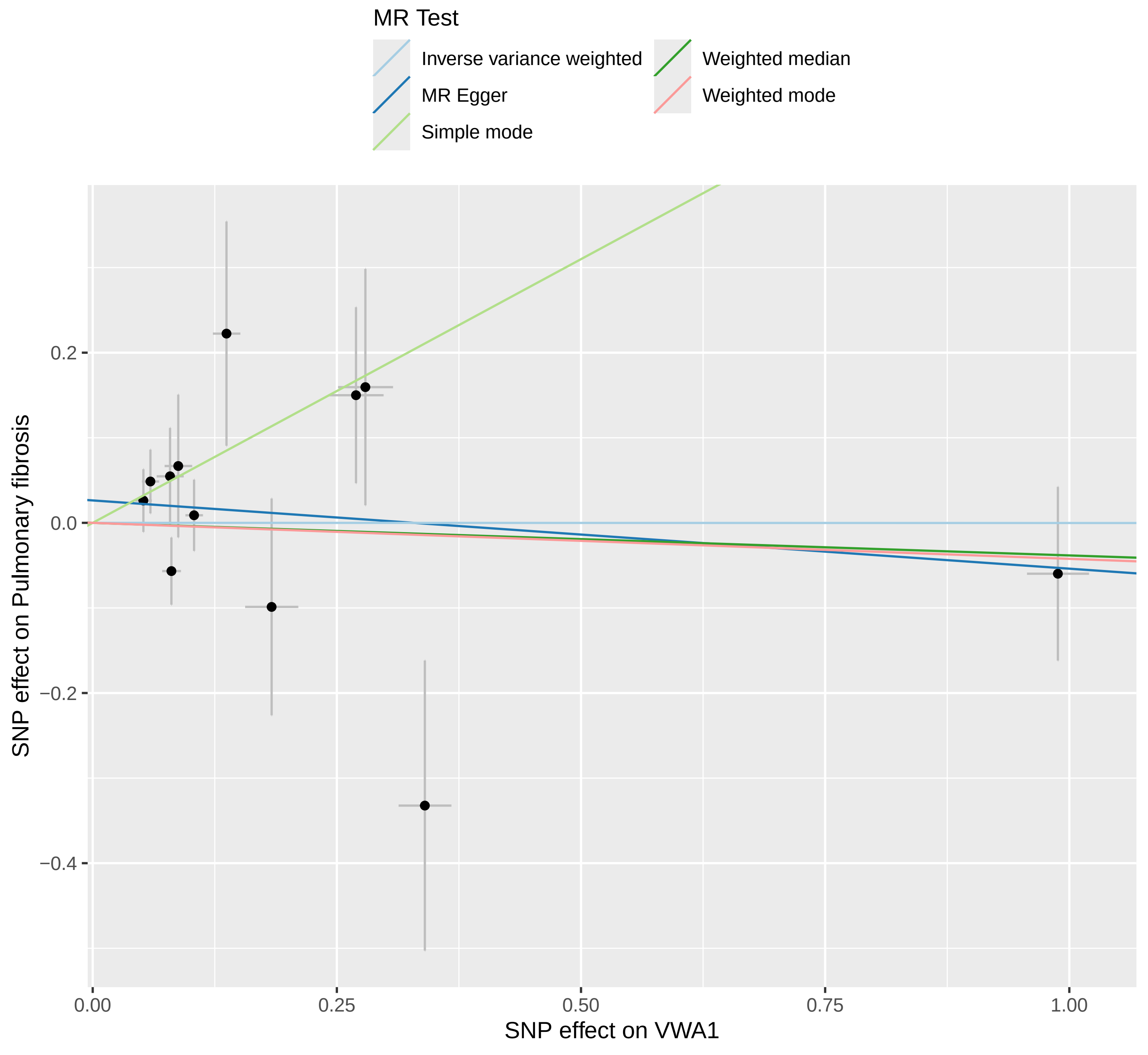
MR Test
Inverse variance weighted
MR Egger
Simple mode
Weighted median
Weighted mode
SNP effect on Pulmonary fibrosis
0.2
0.0
−0.2
−0.4
0.00
0.25
0.50
0.75
1.00
SNP effect on VWA1

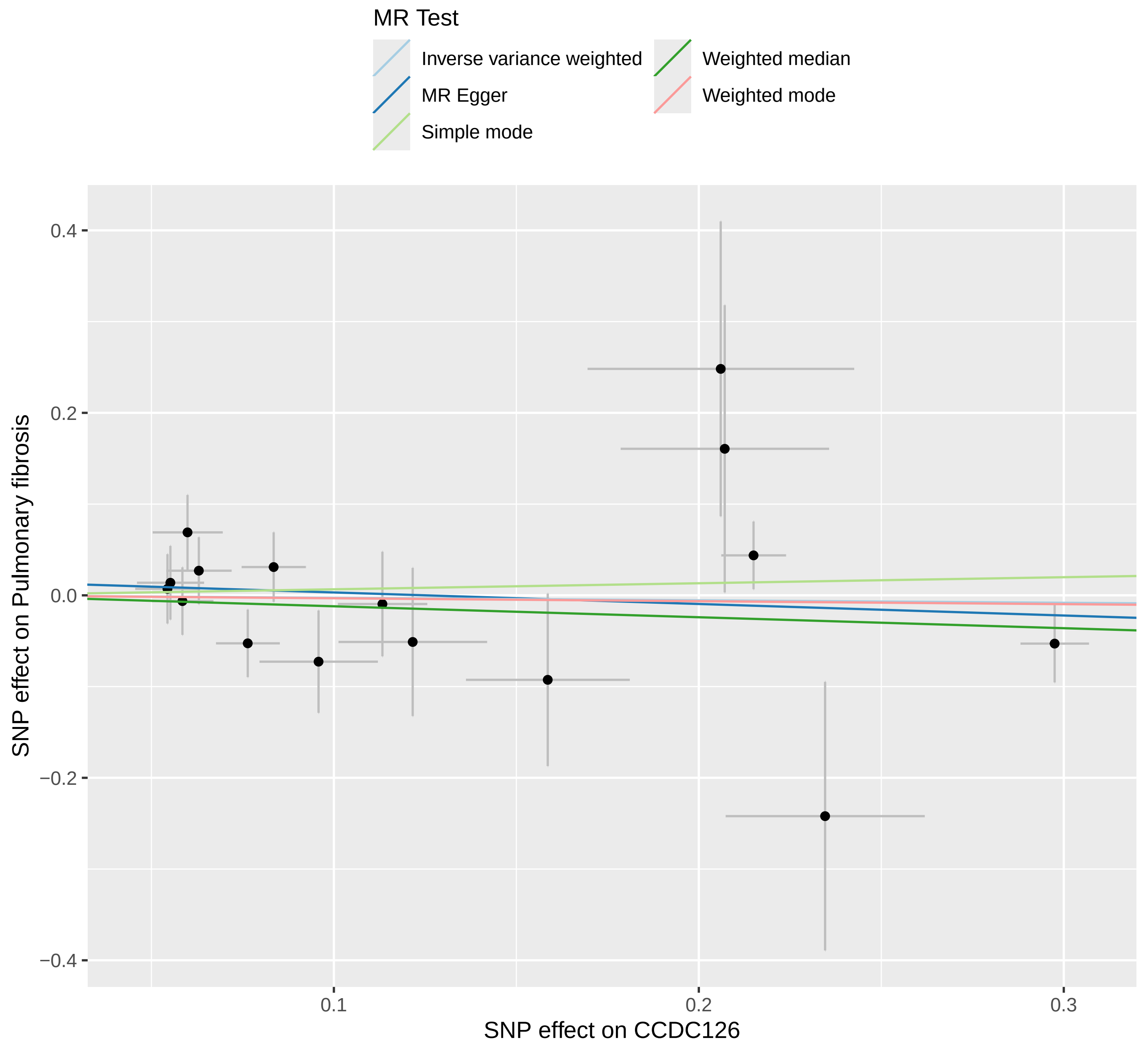
MR Test
Inverse variance weighted
MR Egger
Simple mode
Weighted median
Weighted mode
0.4
0.2
0.0
−0.2
−0.4
SNP effect on Pulmonary fibrosis
0.1
0.2
0.3
SNP effect on CCDC126

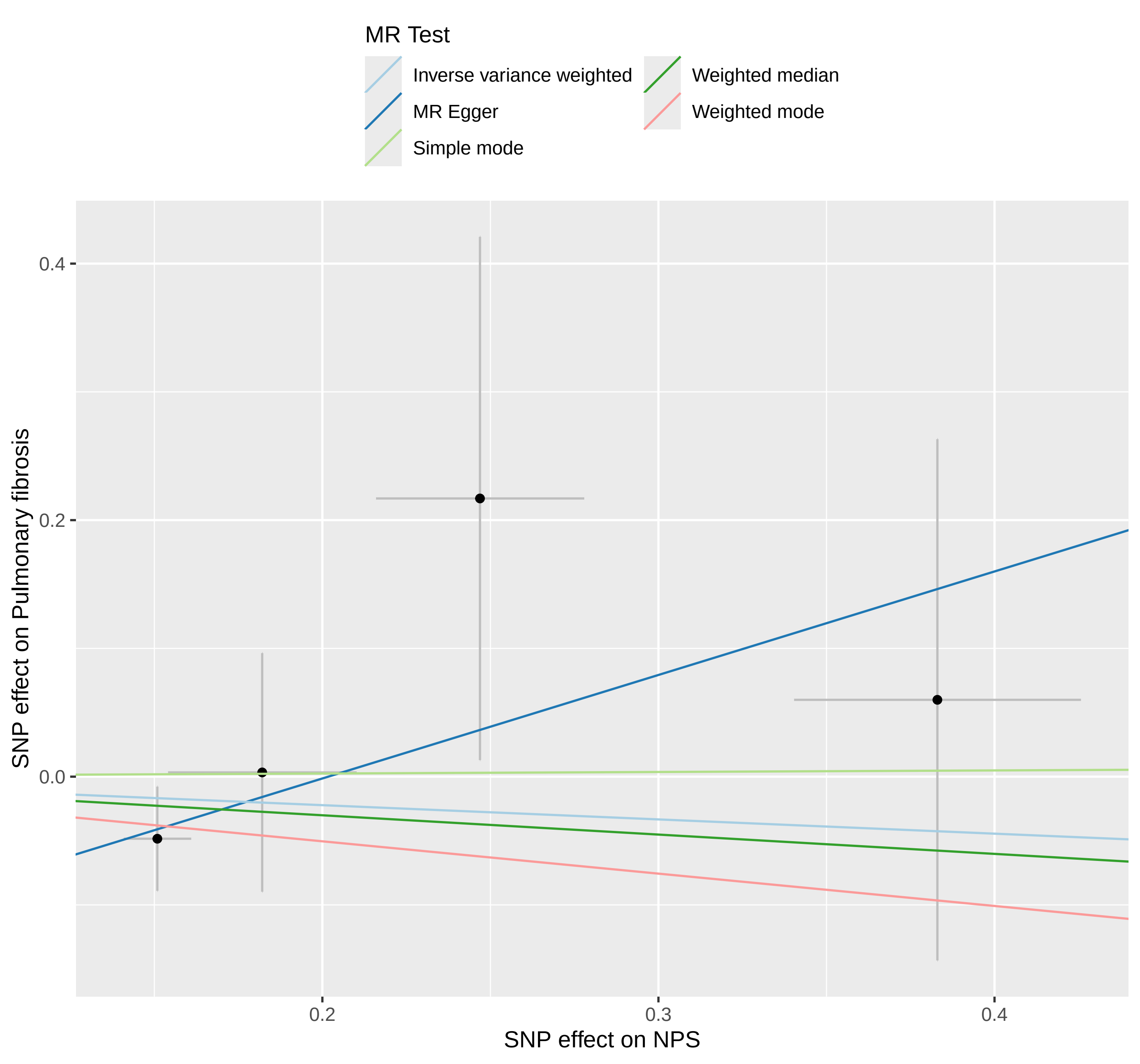

MR Test
Inverse variance weighted
MR Egger
Simple mode
Weighted median
Weighted mode
0.4
0.2
0.0
0.2
0.3
0.4
SNP effect on Pulmonary fibrosis
SNP effect on NPS

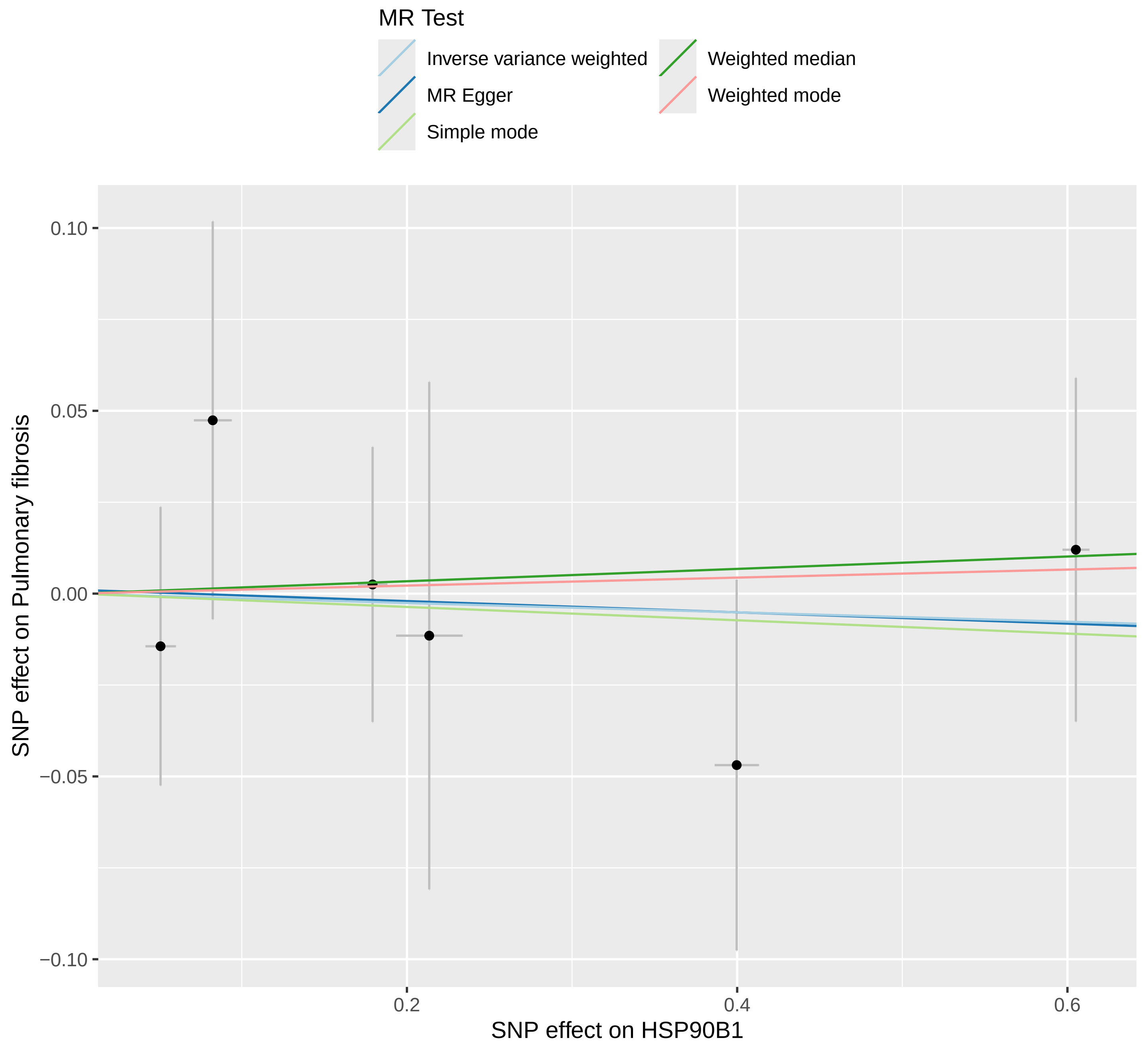

MR Test
Inverse variance weighted
MR Egger
Simple mode
Weighted median
Weighted mode
0.10
0.05
0.00
−0.05
−0.10
SNP effect on Pulmonary fibrosis
0.2
0.4
0.6
SNP effect on HSP90B1

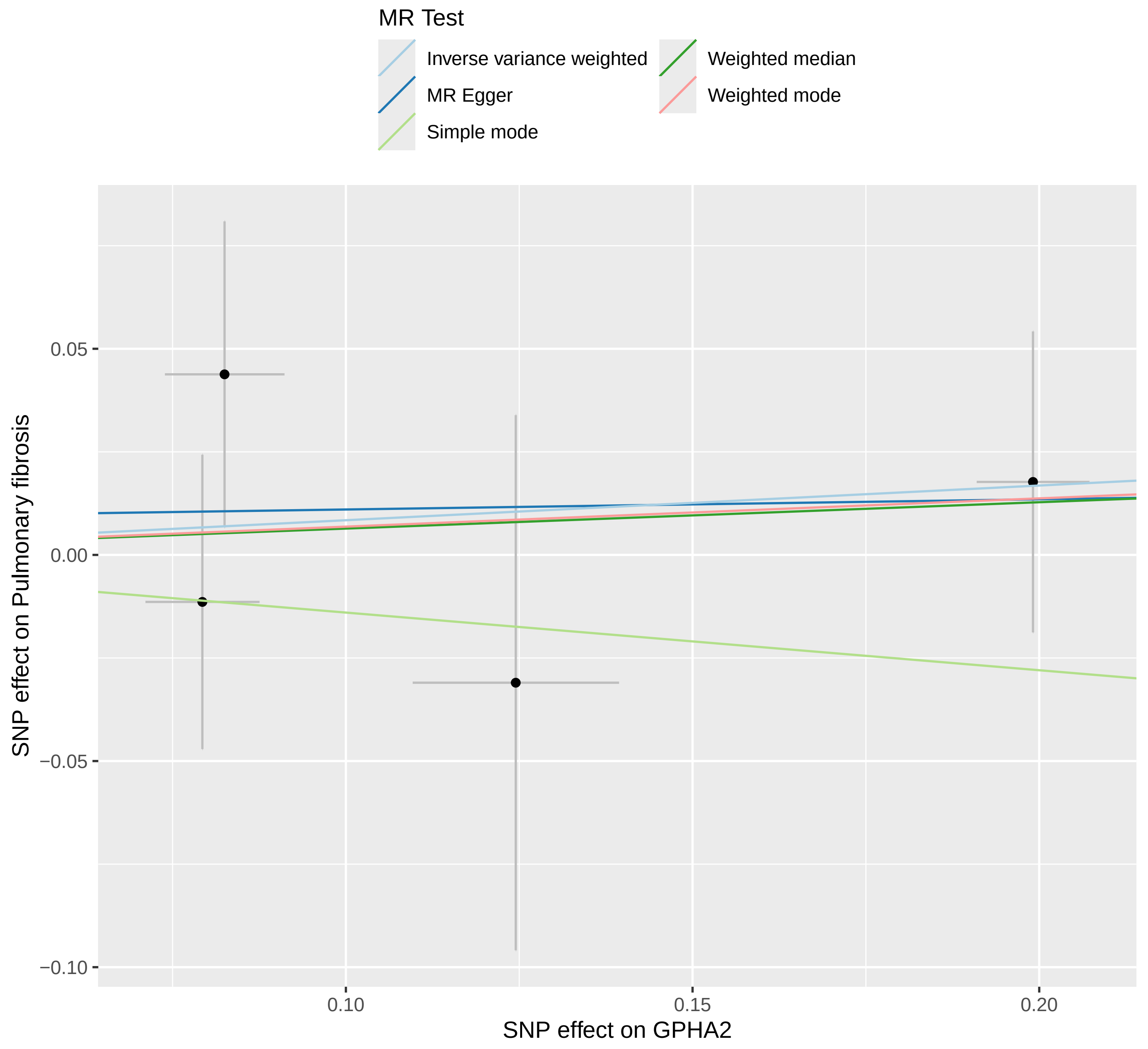
MR Test
Inverse variance weighted
MR Egger
Simple mode
Weighted median
Weighted mode
SNP effect on Pulmonary fibrosis
0.05
0.00
−0.05
−0.10
0.10
0.15
0.20
SNP effect on GPHA2

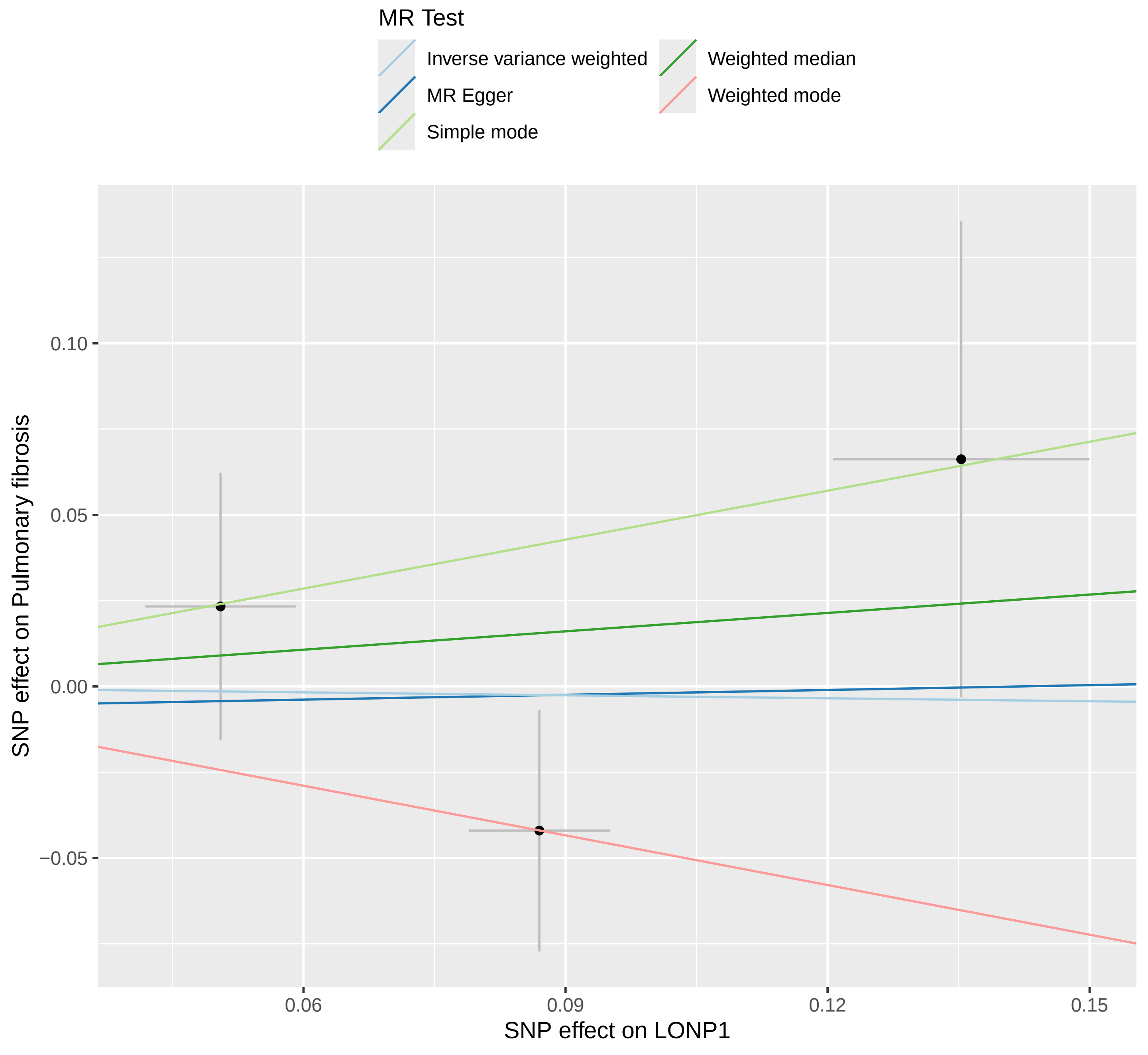
MR Test
Inverse variance weighted
MR Egger
Simple mode
Weighted median
Weighted mode
0.10
0.05
0.00
−0.05
0.06
0.09
0.12
0.15
SNP effect on LONP1
SNP effect on Pulmonary fibrosis

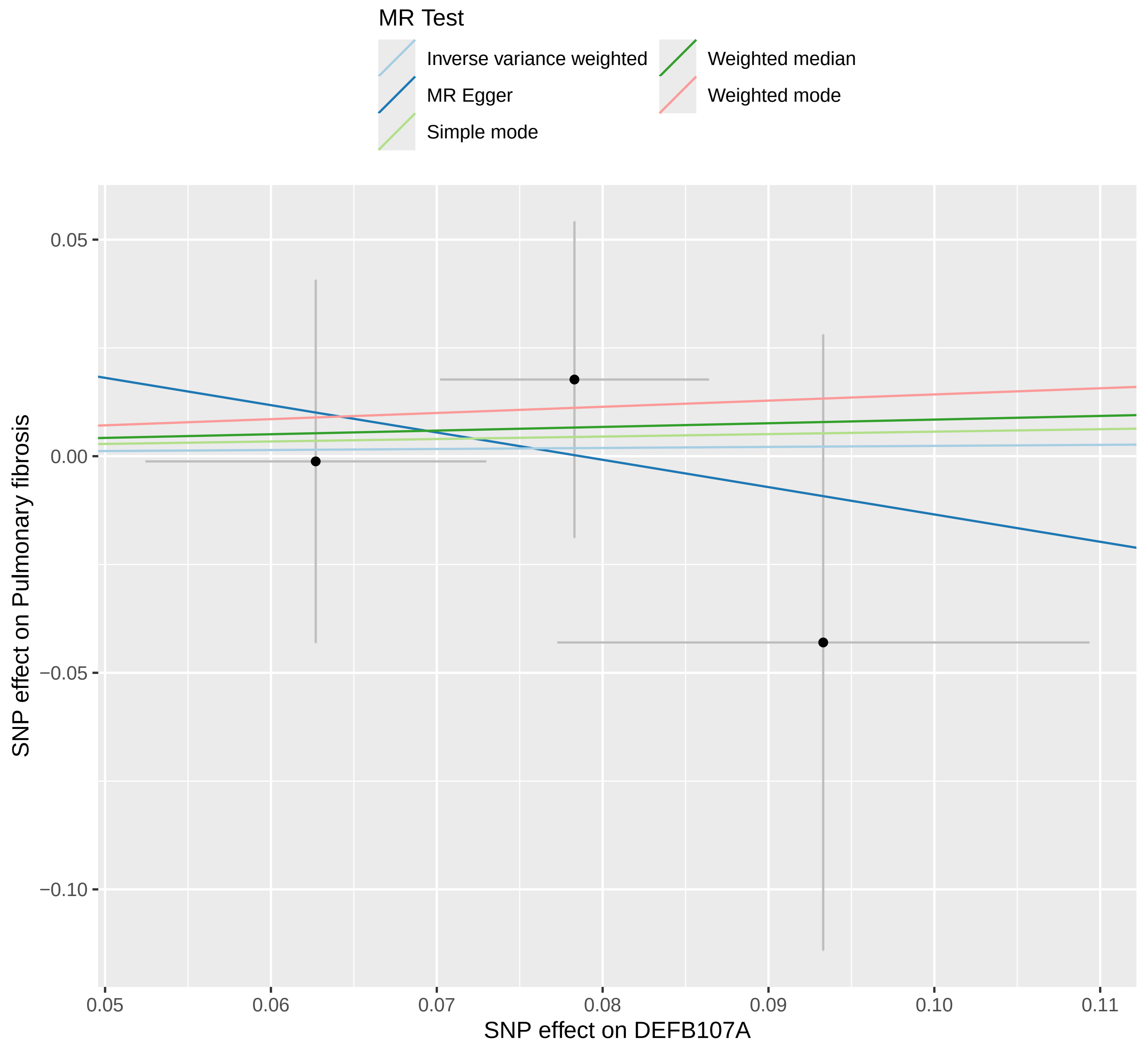
MR Test
Inverse variance weighted
MR Egger
Simple mode
Weighted median
Weighted mode
0.05
0.00
−0.05
−0.10
SNP effect on Pulmonary fibrosis
0.05
0.06
0.07
0.08
0.09
0.10
0.11
SNP effect on DEFB107A

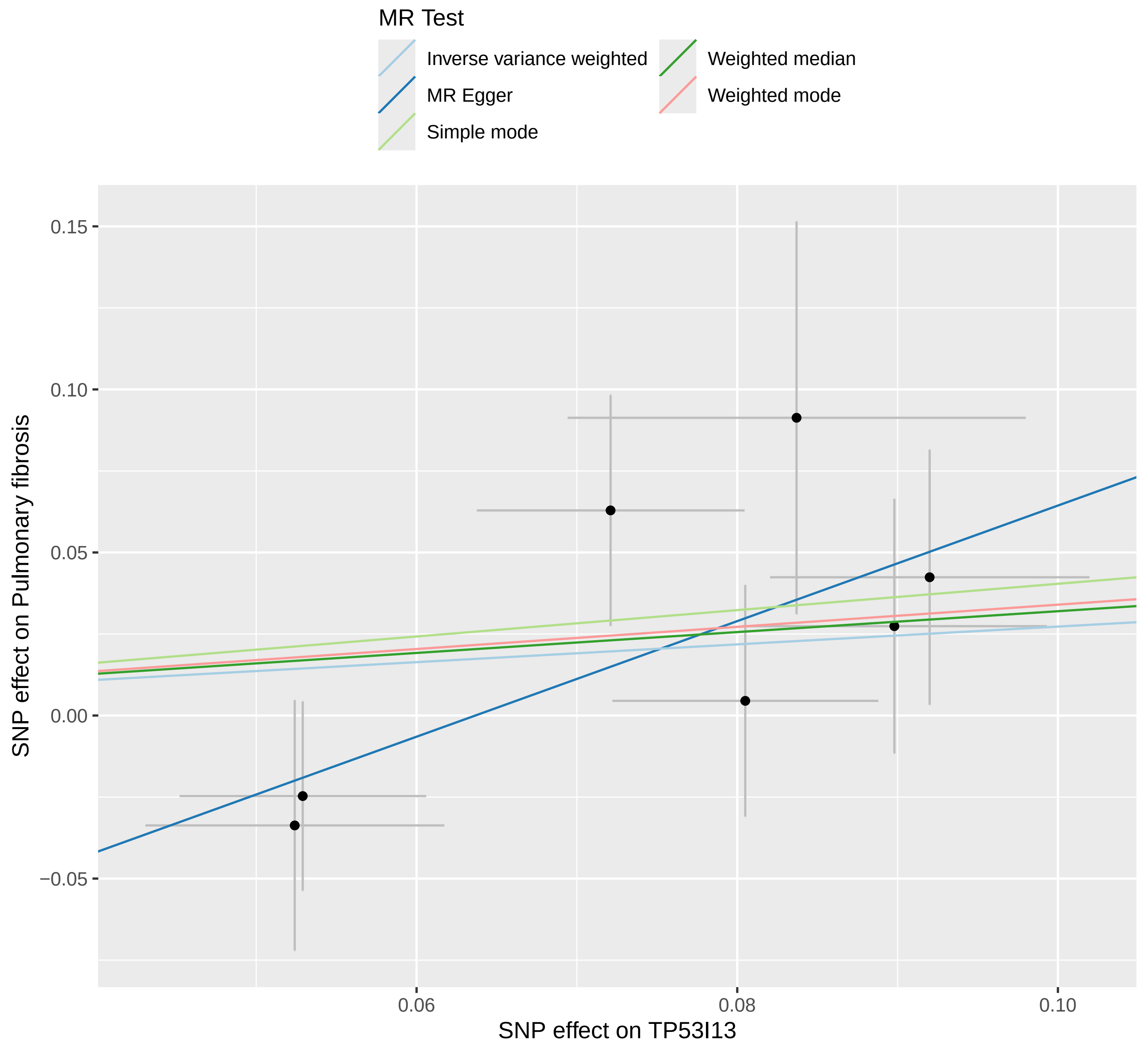

MR Test
Inverse variance weighted
MR Egger
Simple mode
Weighted median
Weighted mode
0.15
0.10
0.05
0.00
−0.05
0.06
0.08
0.10
SNP effect on Pulmonary fibrosis
SNP effect on TP53I13

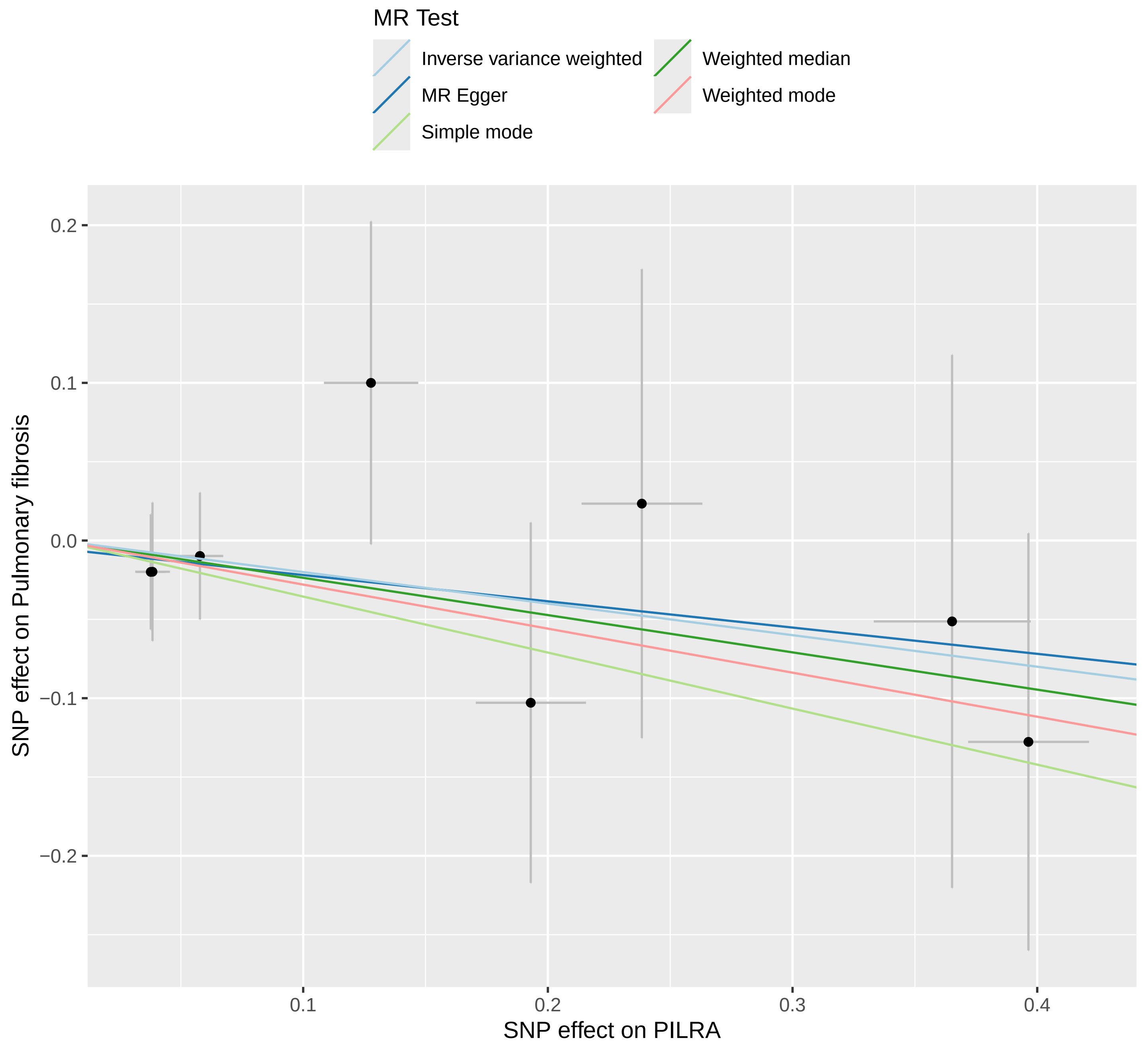

MR Test
Inverse variance weighted
MR Egger
Simple mode
Weighted median
Weighted mode
0.2
0.1
0.0
−0.1
−0.2
SNP effect on Pulmonary fibrosis
0.1
0.2
0.3
0.4
SNP effect on PILRA

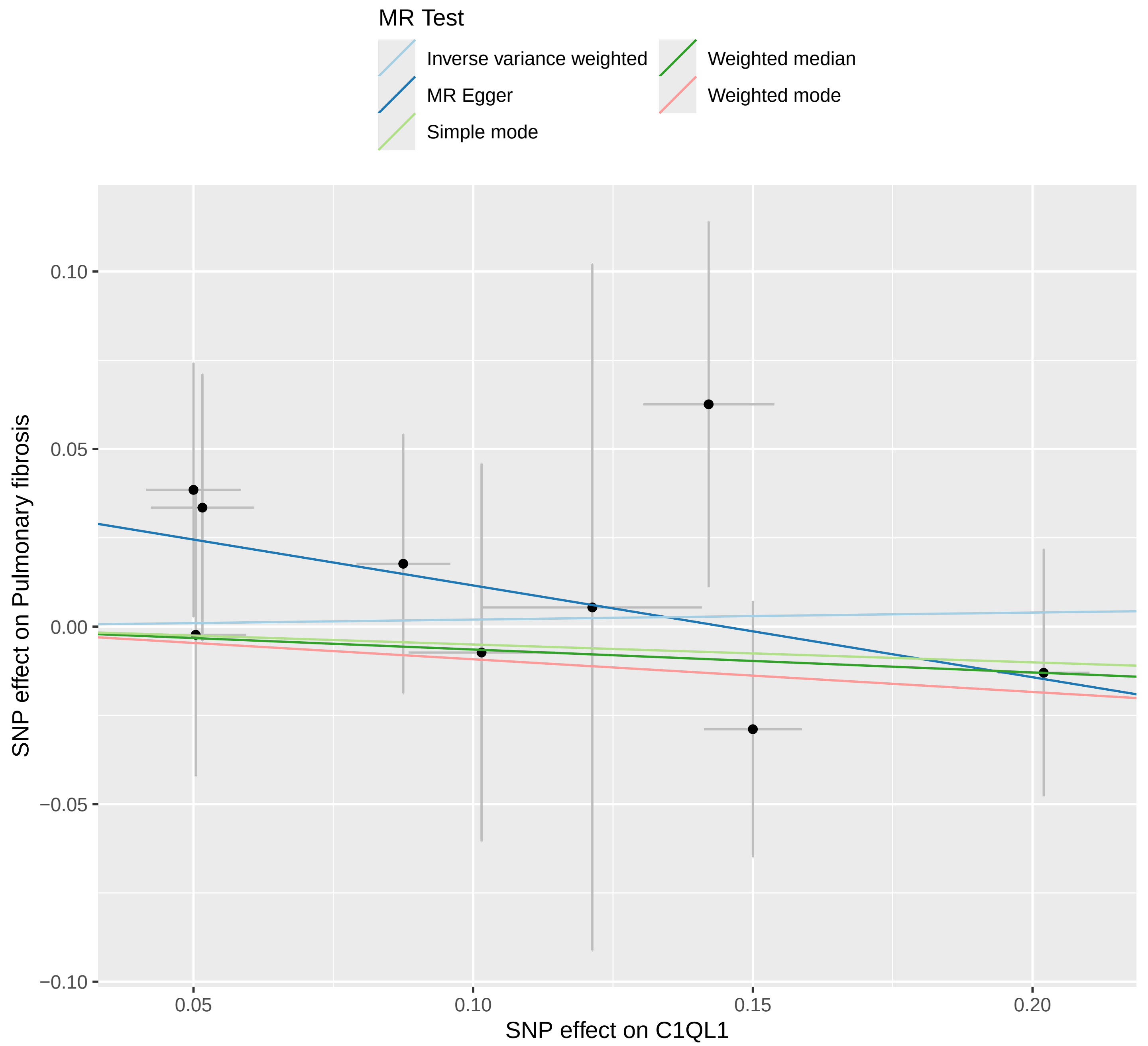

MR Test
Inverse variance weighted
MR Egger
Simple mode
Weighted median
Weighted mode
0.10
0.05
0.00
−0.05
−0.10
SNP effect on Pulmonary fibrosis
0.05
0.10
0.15
0.20
SNP effect on C1QL1

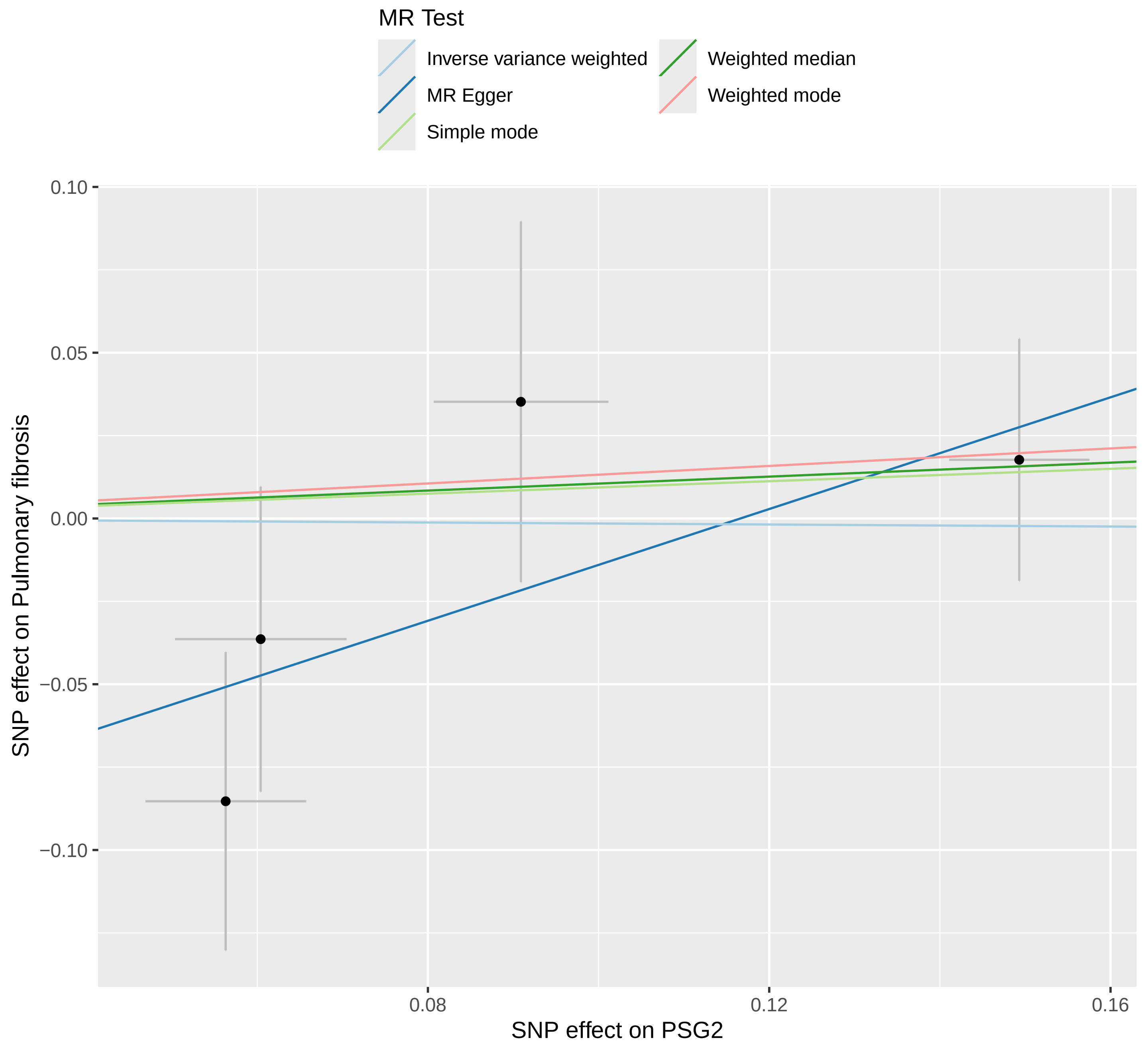

MR Test
Inverse variance weighted
MR Egger
Simple mode
Weighted median
Weighted mode
0.10
0.05
0.00
−0.05
−0.10
0.08
0.12
0.16
SNP effect on Pulmonary fibrosis
SNP effect on PSG2

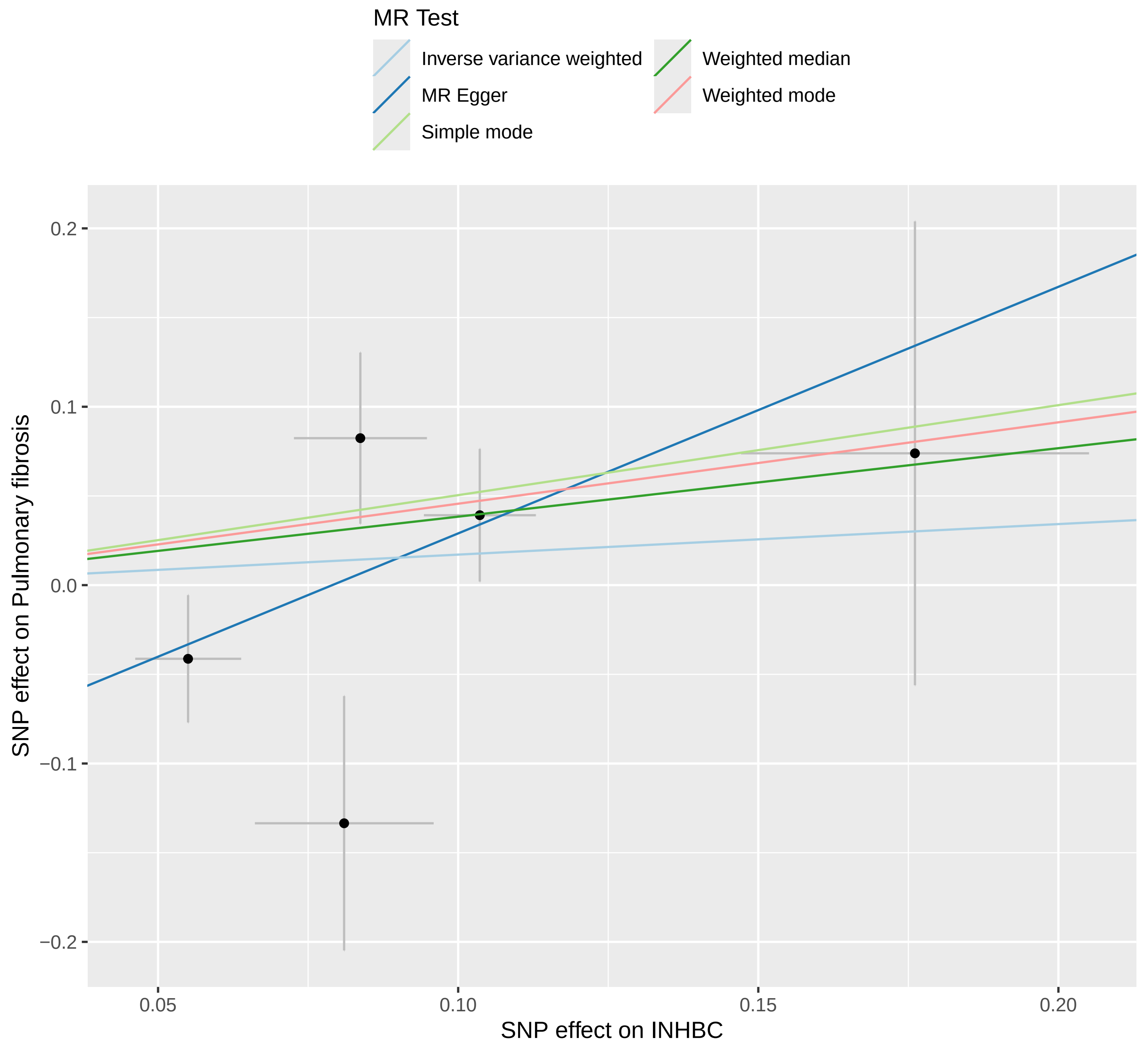
MR Test
Inverse variance weighted
MR Egger
Simple mode
Weighted median
Weighted mode
0.2
0.1
0.0
−0.1
−0.2
SNP effect on Pulmonary fibrosis
0.05
0.10
0.15
0.20
SNP effect on INHBC

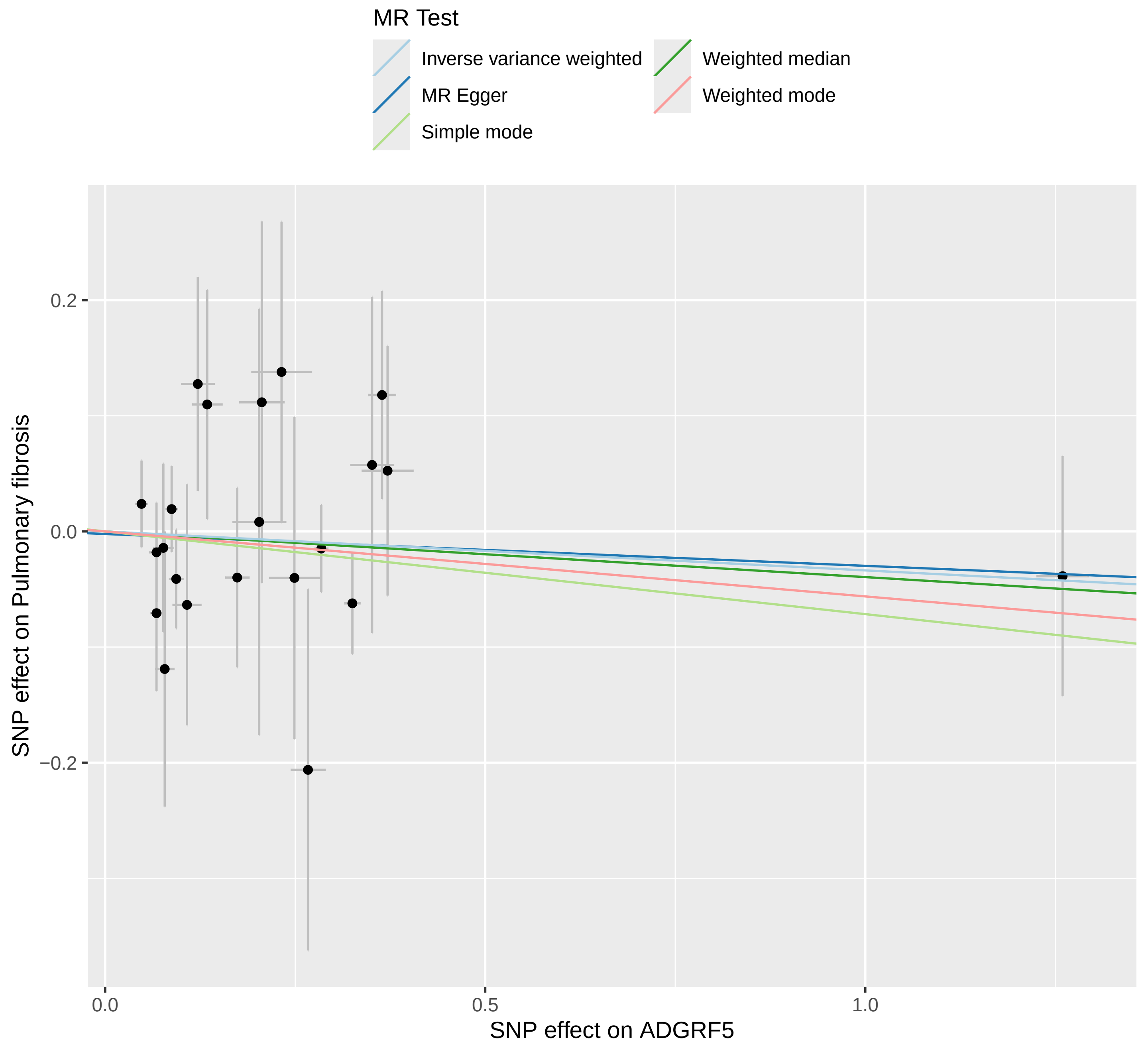

MR Test
Inverse variance weighted
MR Egger
Simple mode
Weighted median
Weighted mode
0.2
0.0
−0.2
0.0
0.5
1.0
SNP effect on ADGRF5
SNP effect on Pulmonary fibrosis

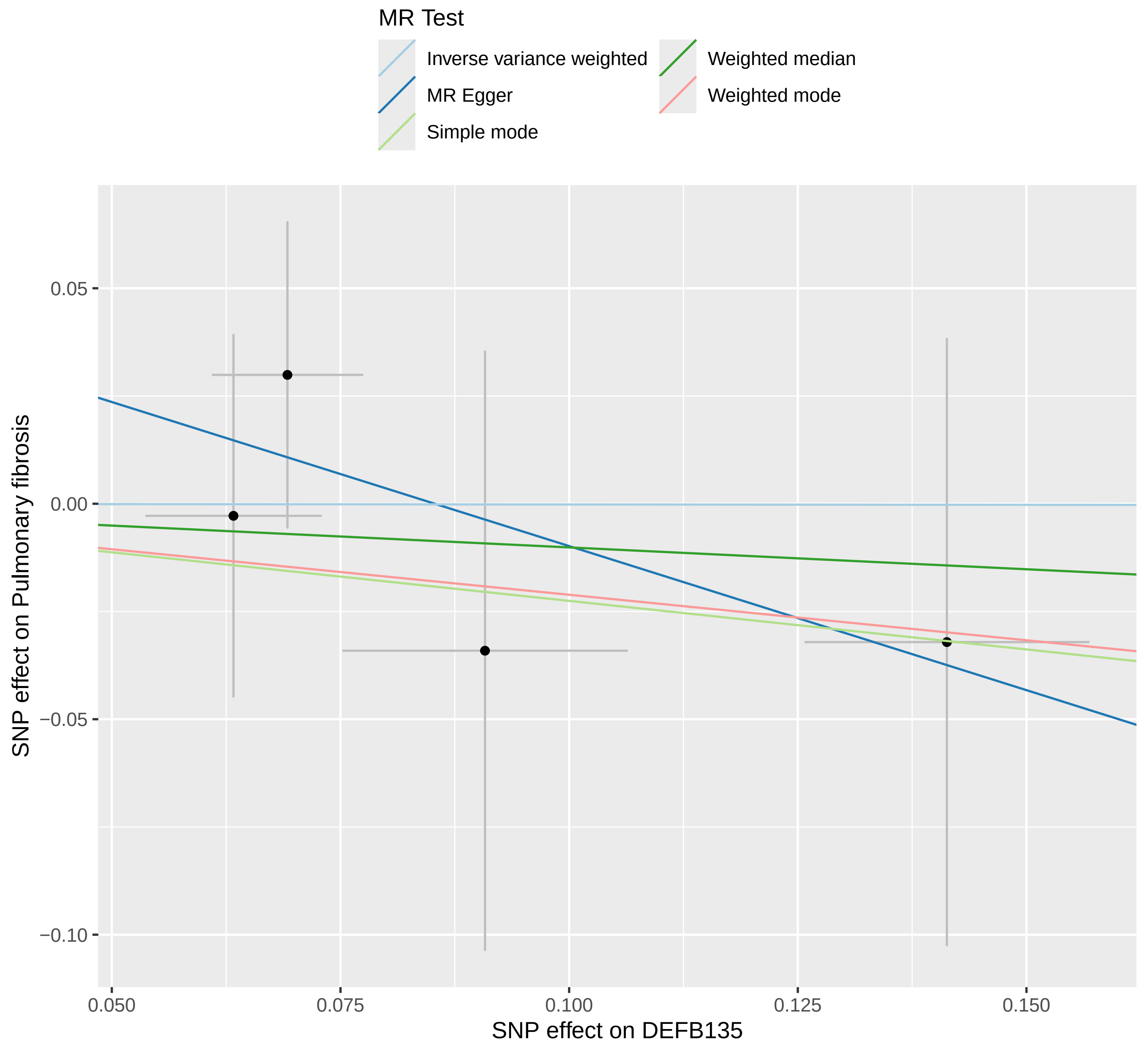

MR Test
Inverse variance weighted
MR Egger
Simple mode
Weighted median
Weighted mode
0.05
0.00
−0.05
−0.10
0.050
0.075
0.100
0.125
0.150
SNP effect on Pulmonary fibrosis
SNP effect on DEFB135

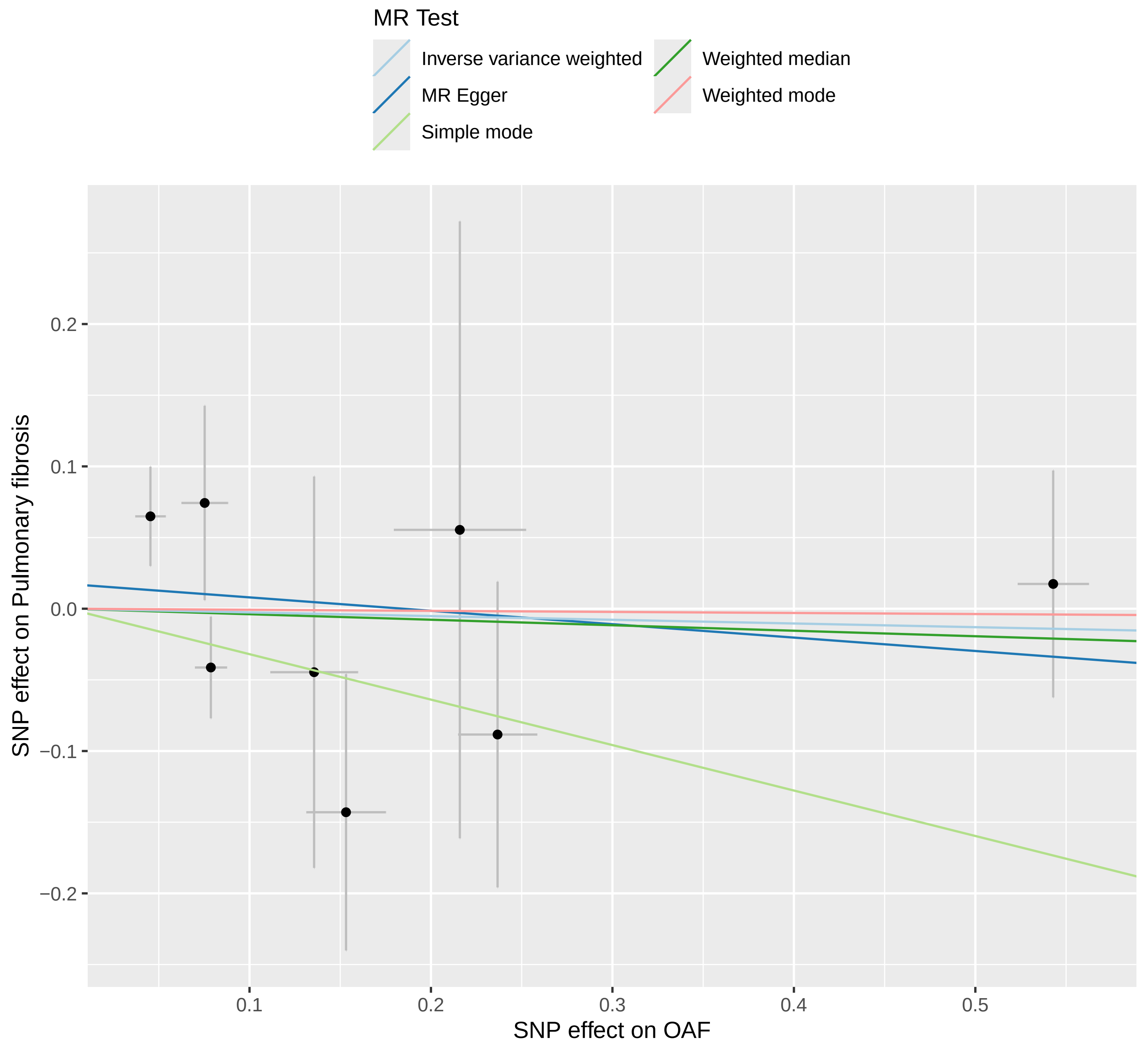
MR Test
Inverse variance weighted
MR Egger
Simple mode
Weighted median
Weighted mode
SNP effect on Pulmonary fibrosis
0.2
0.1
0.0
−0.1
−0.2
0.1
0.2
0.3
0.4
0.5
SNP effect on OAF

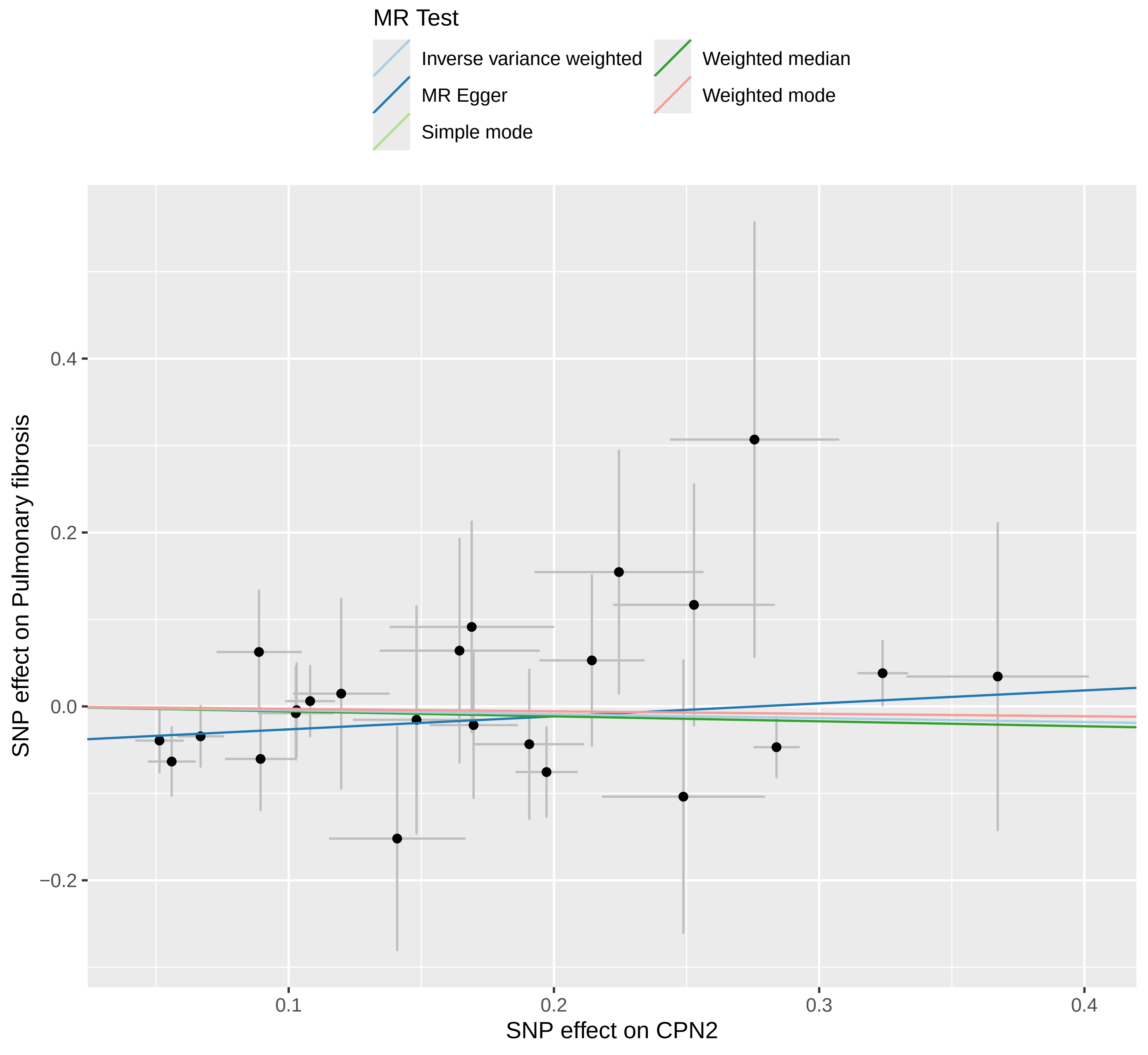

MR Test
Inverse variance weighted
MR Egger
Simple mode
Weighted median
Weighted mode
0.4
0.2
0.0
−0.2
SNP effect on Pulmonary fibrosis
0.1
0.2
0.3
0.4
SNP effect on CPN2

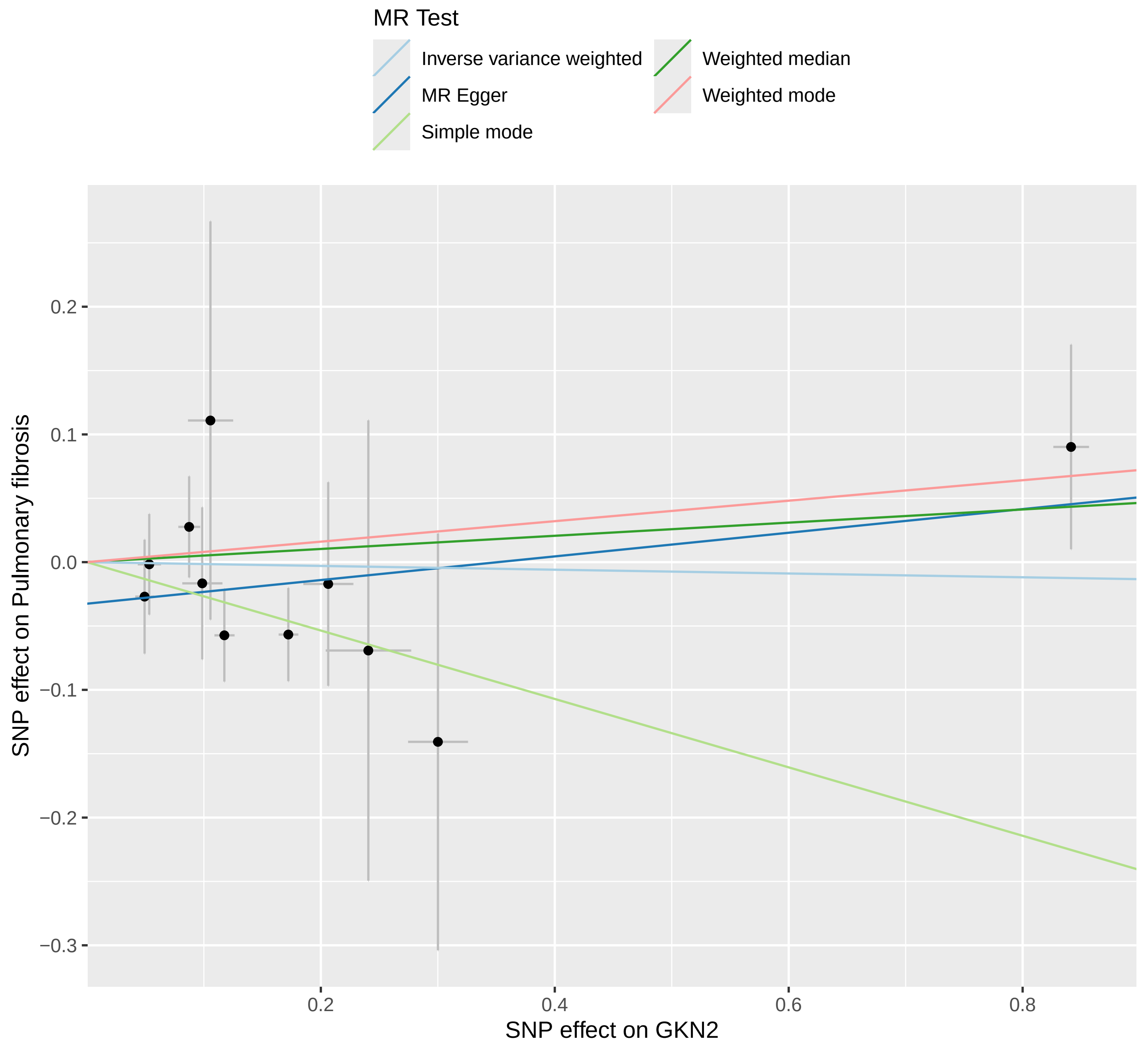
MR Test
Inverse variance weighted
MR Egger
Simple mode
Weighted median
Weighted mode
SNP effect on Pulmonary fibrosis
0.2
0.1
0.0
−0.1
−0.2
−0.3
0.2
0.4
0.6
0.8
SNP effect on GKN2

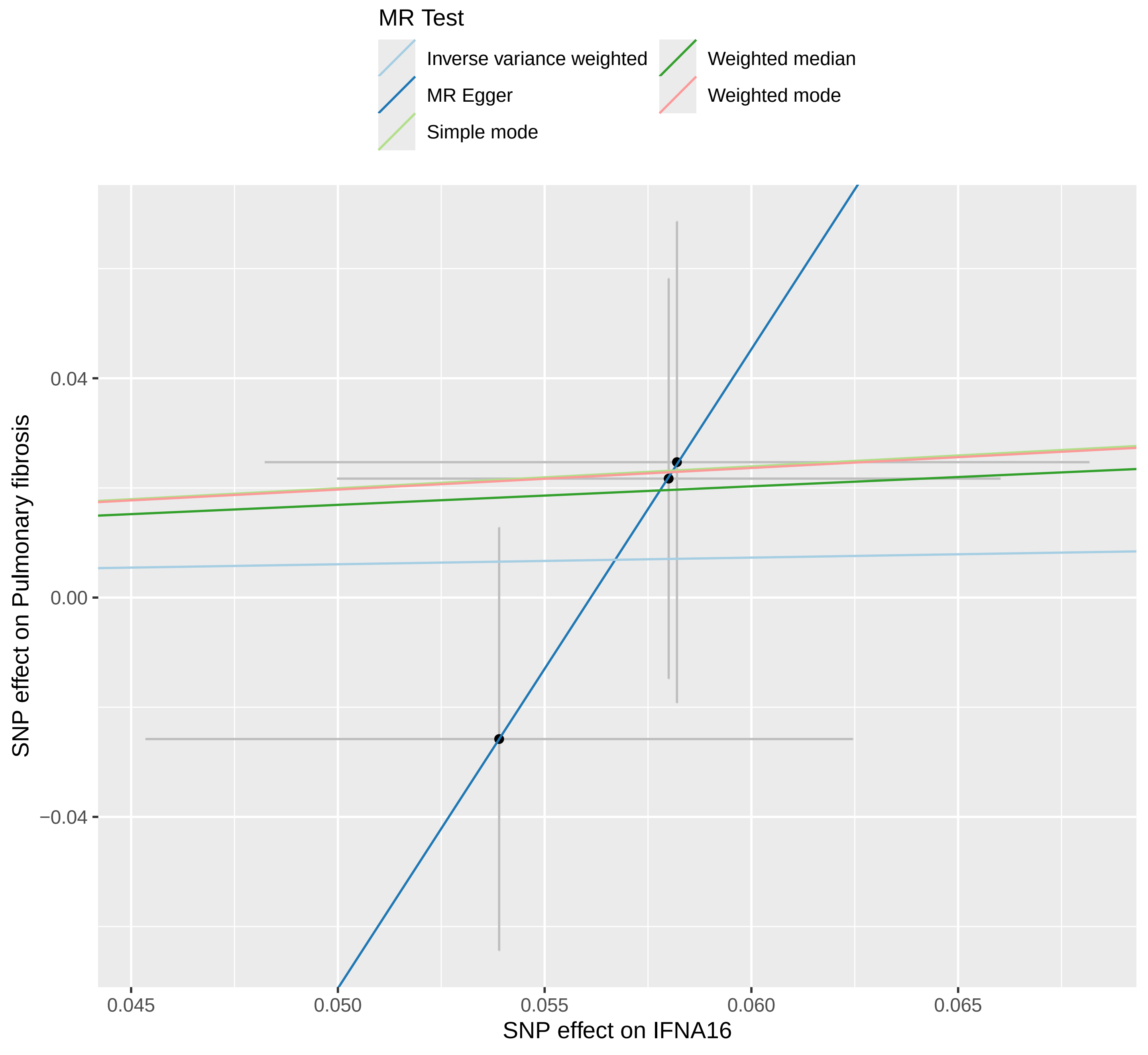
MR Test
Inverse variance weighted
MR Egger
Simple mode
Weighted median
Weighted mode
0.04
0.00
−0.04
0.045
0.050
0.055
0.060
0.065
SNP effect on IFNA16
SNP effect on Pulmonary fibrosis

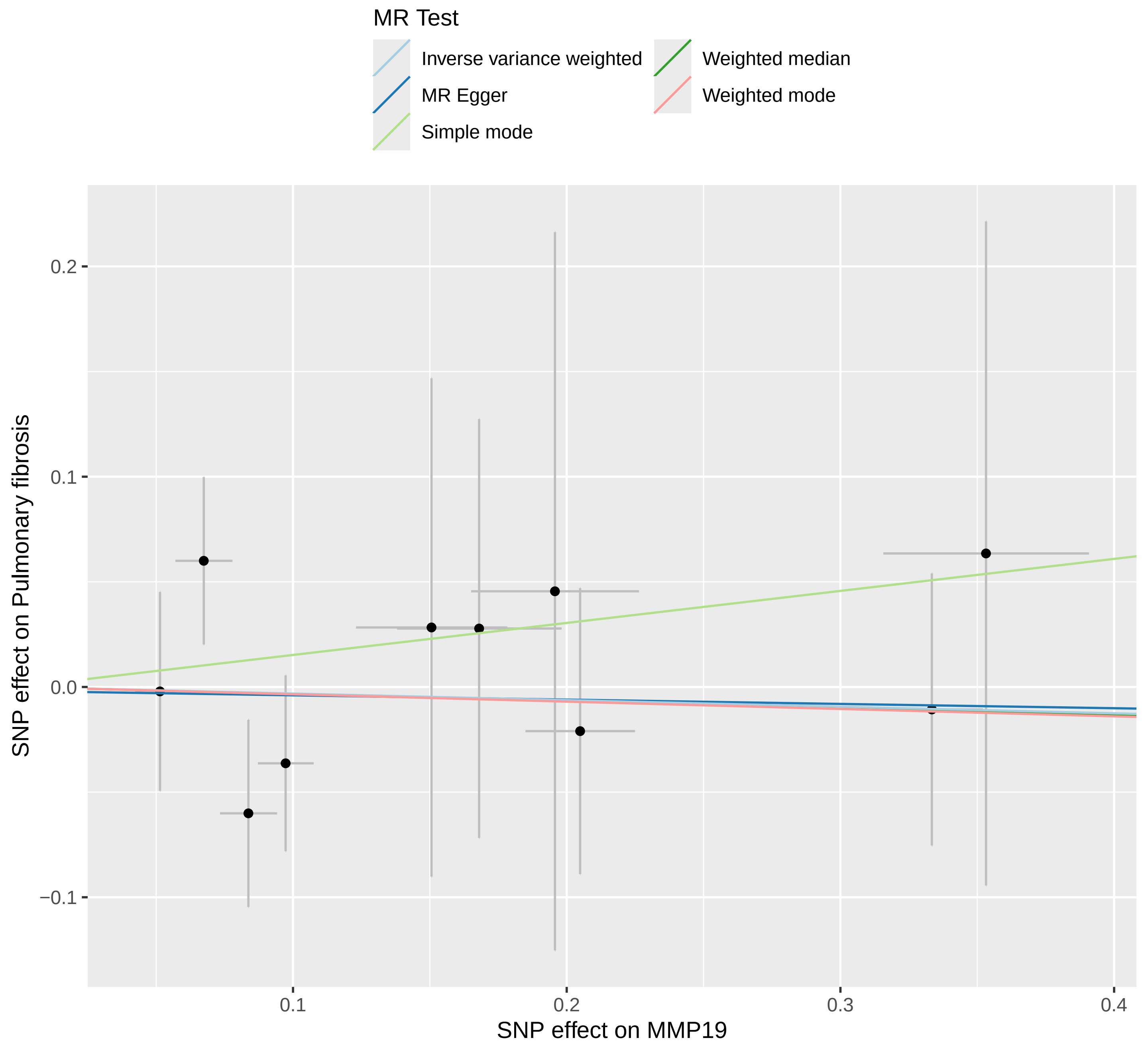
MR Test
Inverse variance weighted
MR Egger
Simple mode
Weighted median
Weighted mode
SNP effect on Pulmonary fibrosis
0.2
0.1
0.0
−0.1
0.1
0.2
0.3
0.4
SNP effect on MMP19

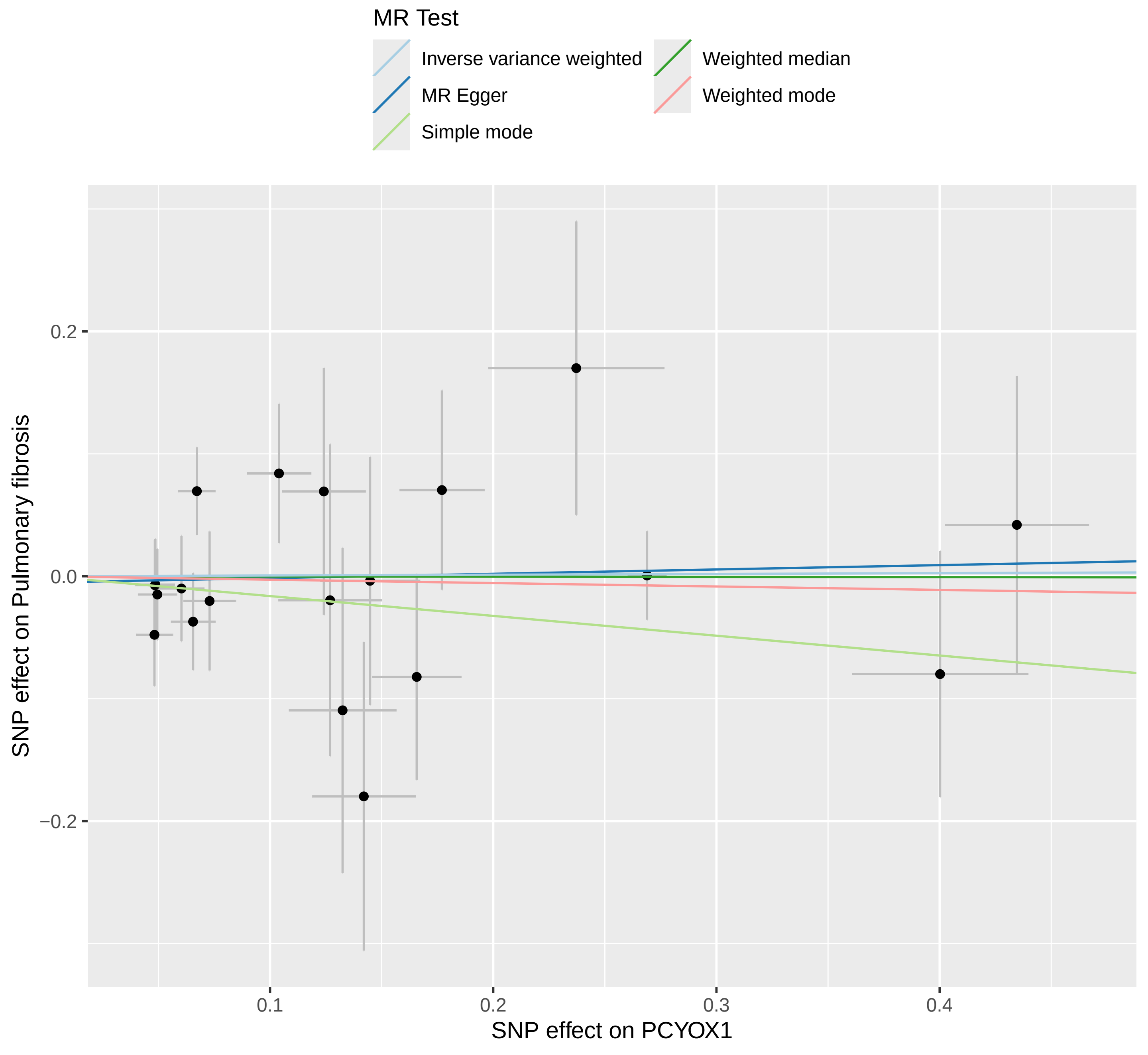
MR Test
Inverse variance weighted
MR Egger
Simple mode
Weighted median
Weighted mode
0.2
0.0
−0.2
0.1
0.2
0.3
0.4
SNP effect on Pulmonary fibrosis
SNP effect on PCYOX1

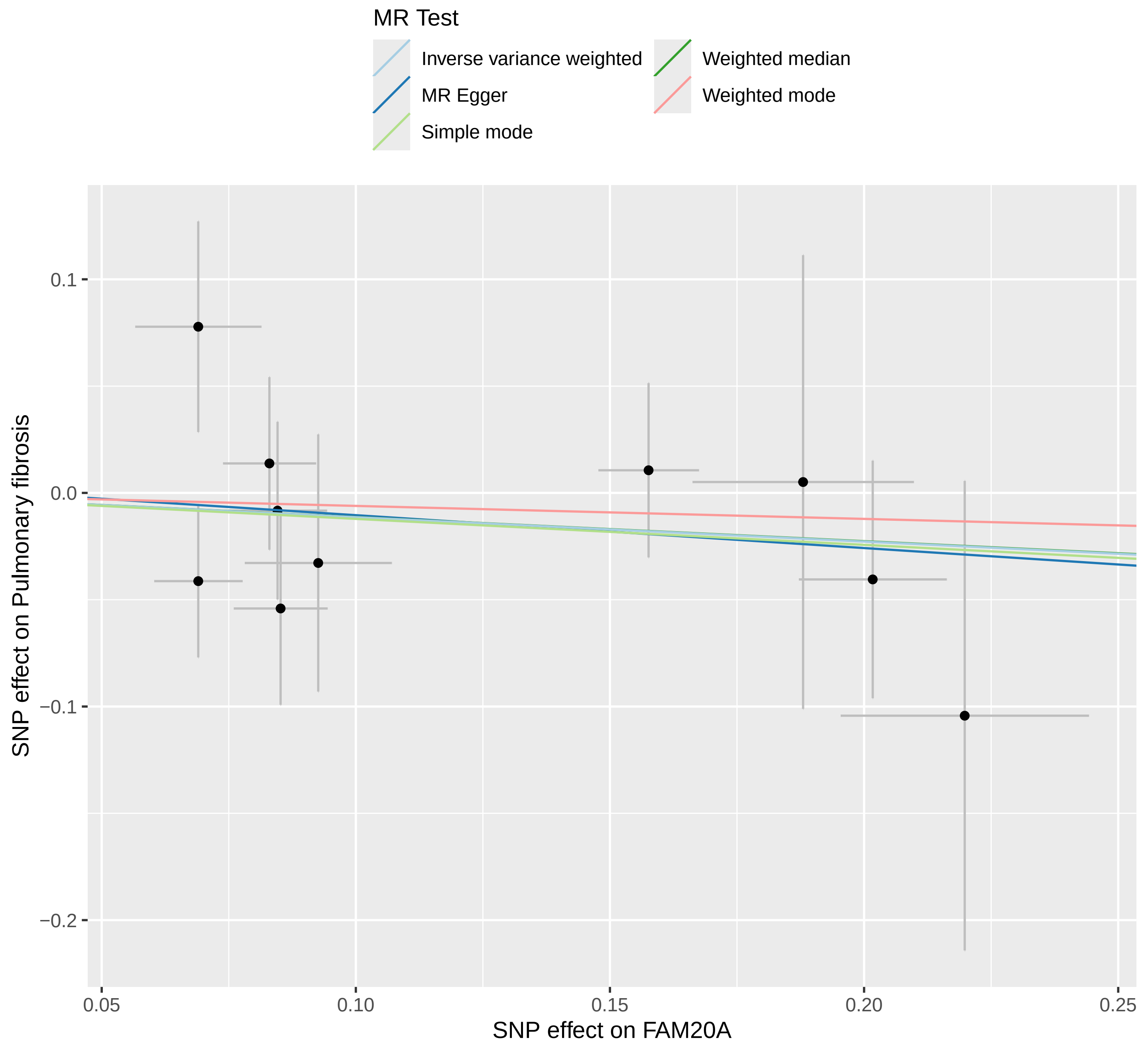

MR Test
Inverse variance weighted
MR Egger
Simple mode
Weighted median
Weighted mode
0.1
0.0
−0.1
−0.2
0.05
0.10
0.15
0.20
0.25
SNP effect on Pulmonary fibrosis
SNP effect on FAM20A

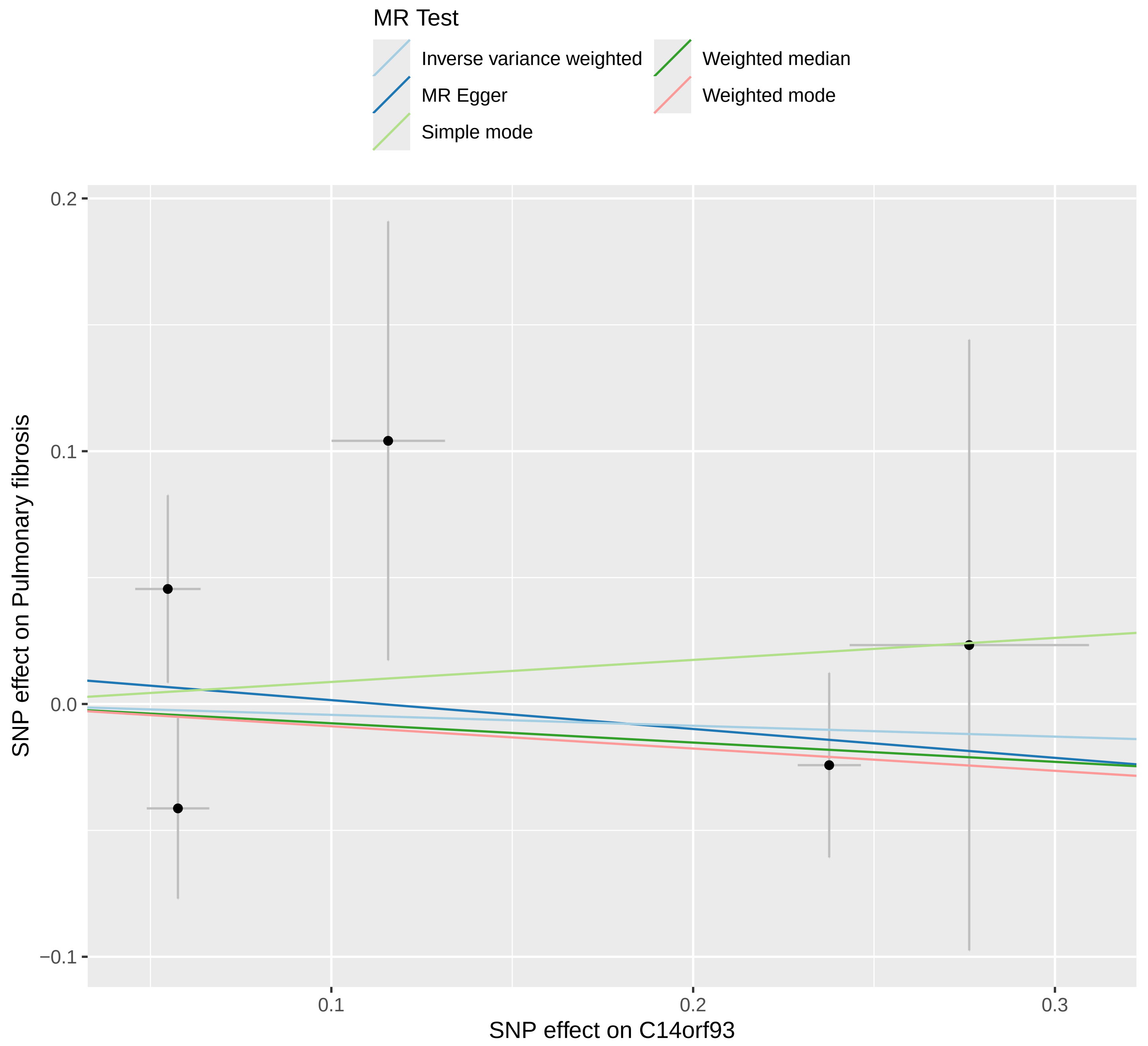

MR Test
Inverse variance weighted
MR Egger
Simple mode
Weighted median
Weighted mode
0.2
0.1
0.0
−0.1
SNP effect on Pulmonary fibrosis
0.1
0.2
0.3
SNP effect on C14orf93

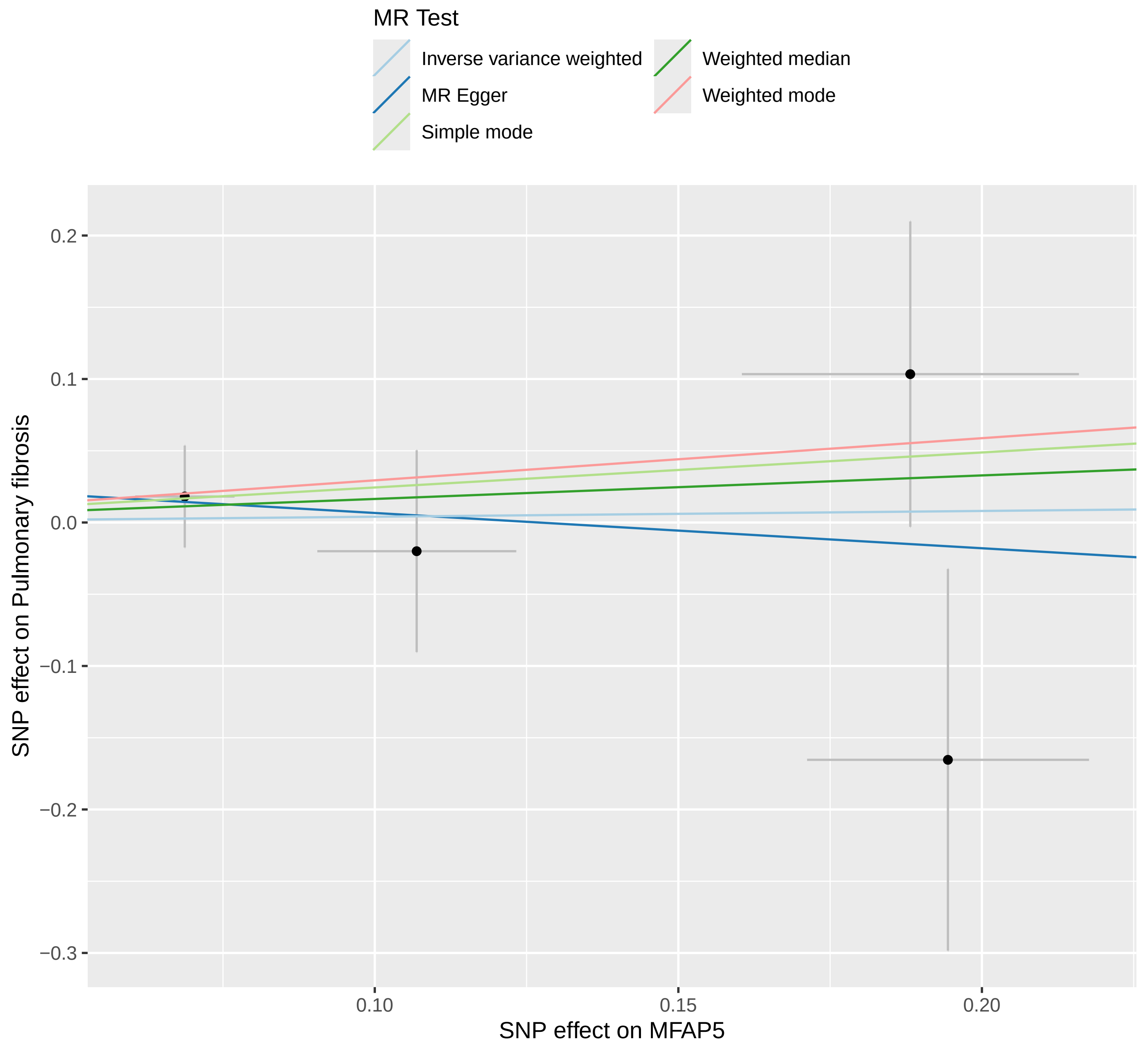
MR Test
Inverse variance weighted
MR Egger
Simple mode
Weighted median
Weighted mode
0.2
0.1
0.0
−0.1
−0.2
−0.3
0.10
0.15
0.20
SNP effect on Pulmonary fibrosis
SNP effect on MFAP5

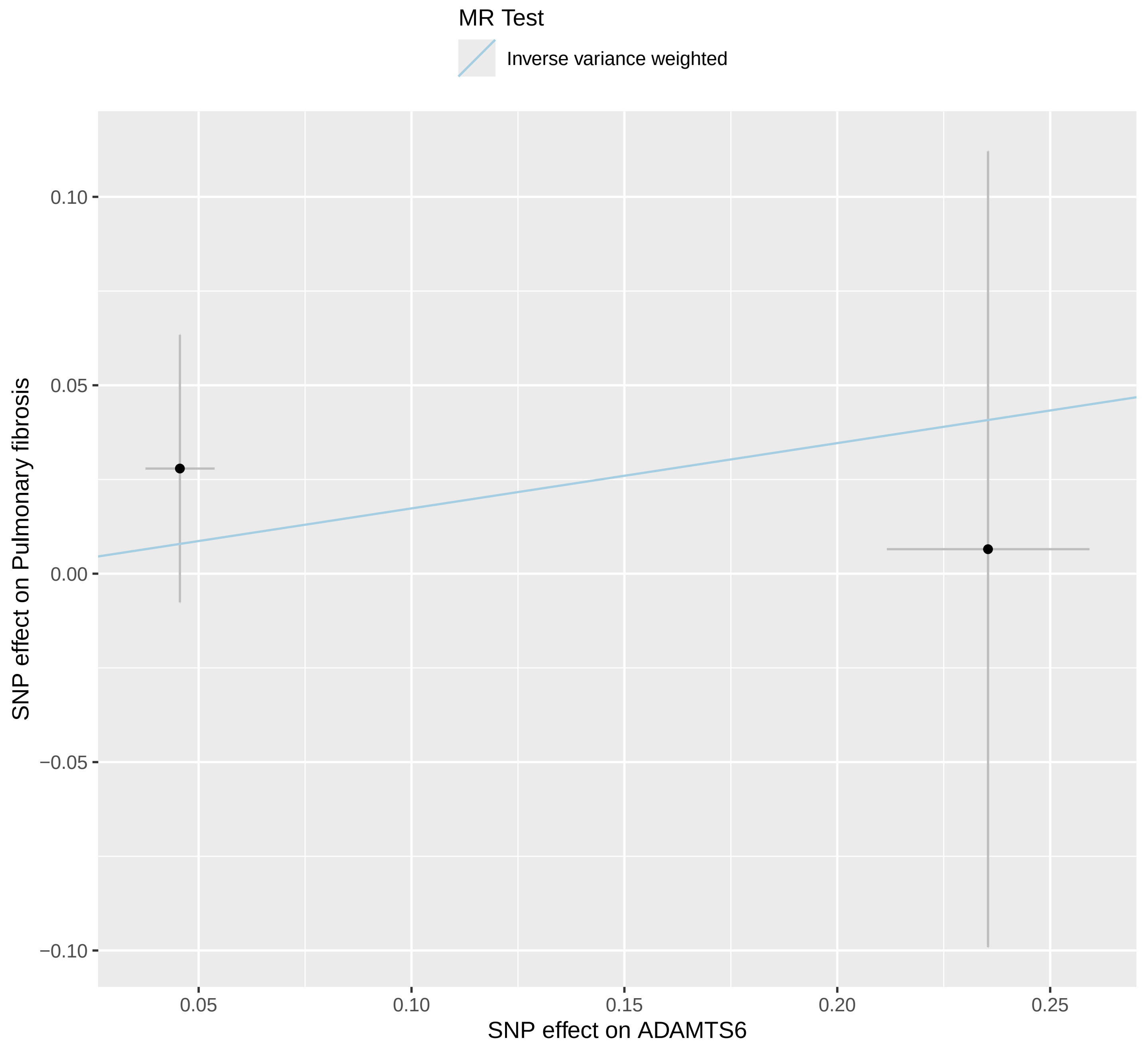
MR Test
Inverse variance weighted
0.10
0.05
0.00
−0.05
−0.10
0.05
0.10
0.15
0.20
0.25
SNP effect on Pulmonary fibrosis
SNP effect on ADAMTS6

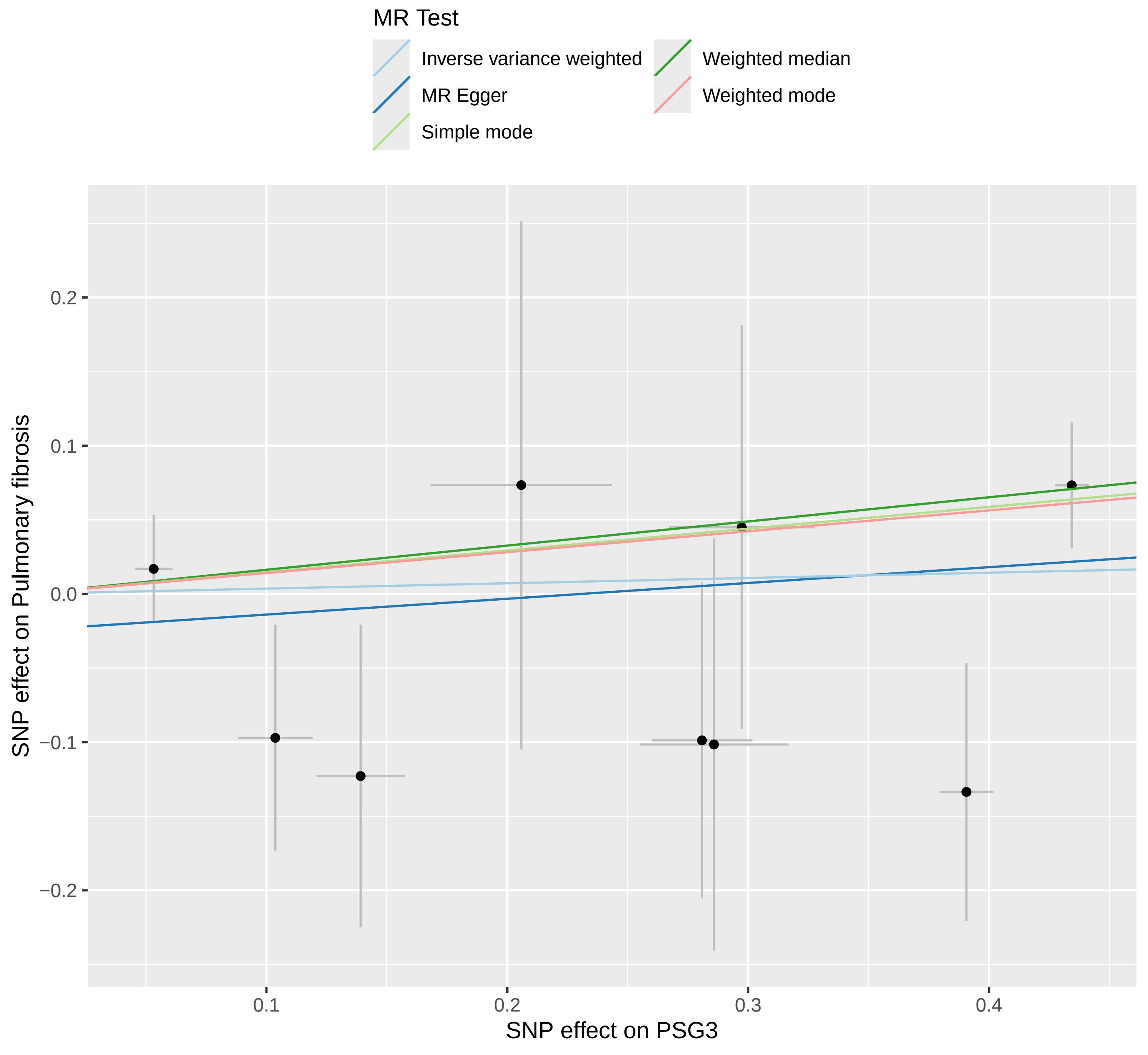
MR Test
Inverse variance weighted
MR Egger
Simple mode
Weighted median
Weighted mode
SNP effect on Pulmonary fibrosis
0.2
0.1
0.0
−0.1
−0.2
0.1
0.2
0.3
0.4
SNP effect on PSG3

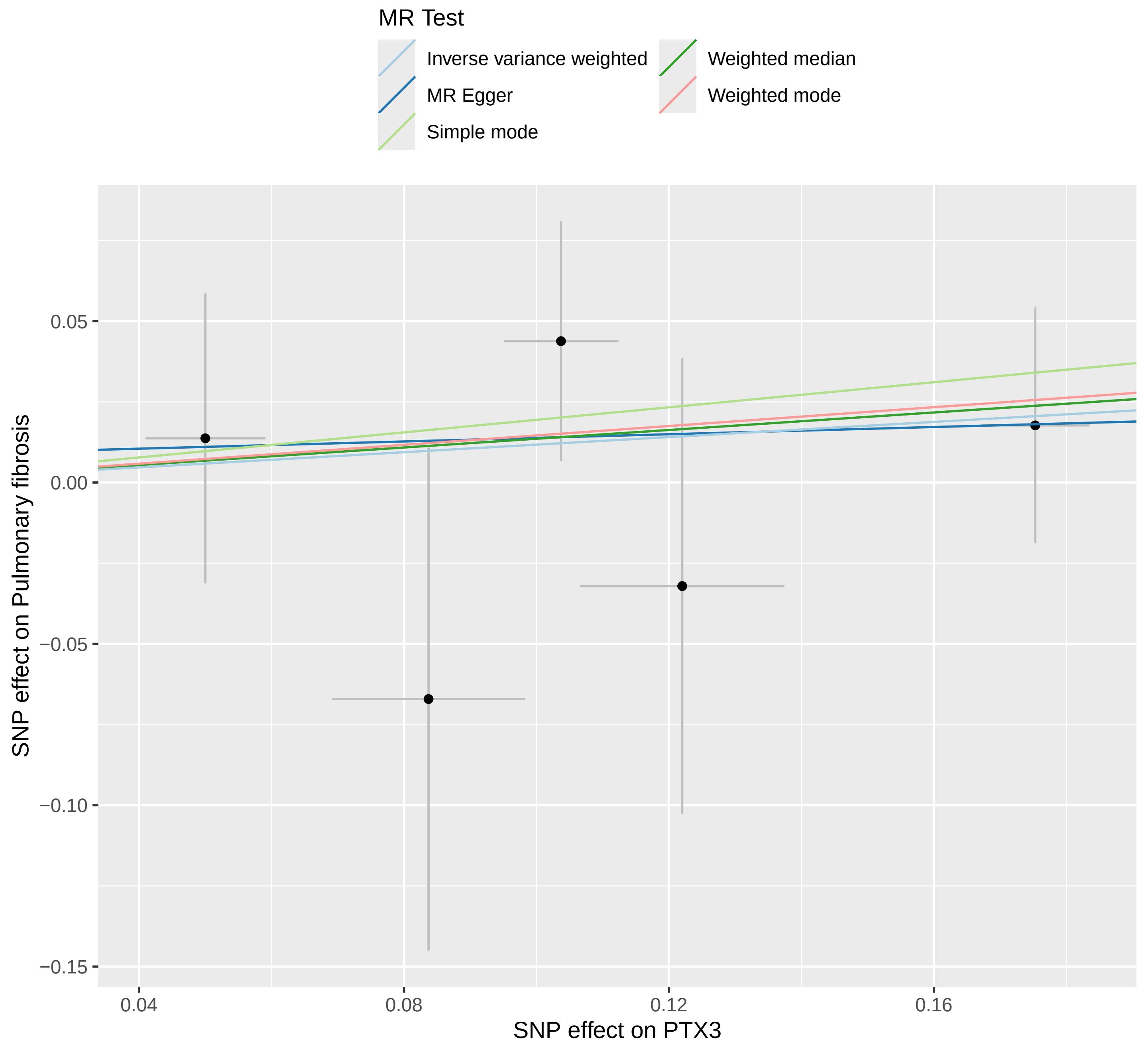

MR Test
Inverse variance weighted
MR Egger
Simple mode
Weighted median
Weighted mode
SNP effect on Pulmonary fibrosis
0.05
0.00
−0.05
−0.10
−0.15
0.04
0.08
0.12
0.16
SNP effect on PTX3

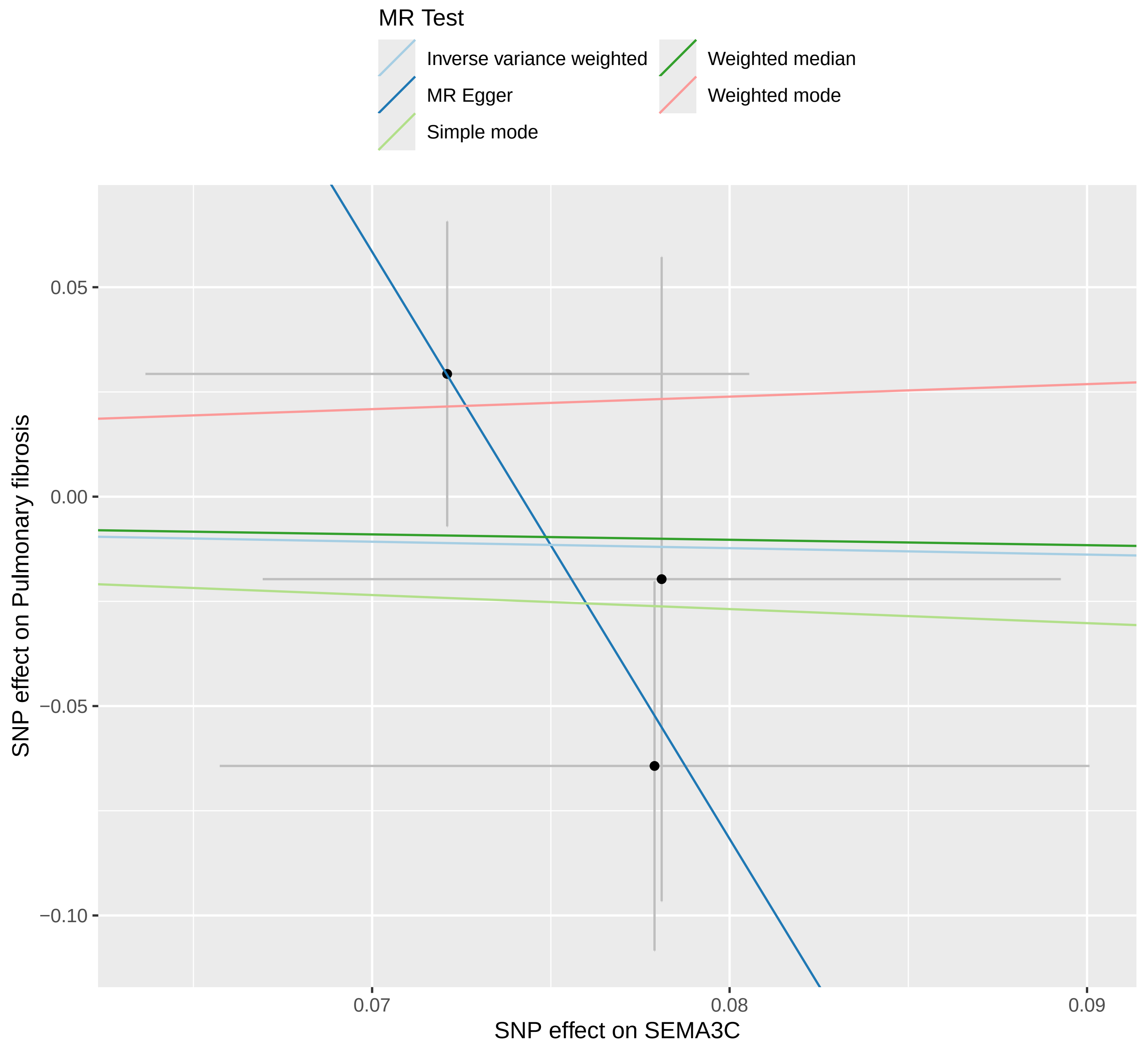
MR Test
Inverse variance weighted
MR Egger
Simple mode
Weighted median
Weighted mode
0.05
0.00
−0.05
−0.10
SNP effect on Pulmonary fibrosis
0.07
0.08
0.09
SNP effect on SEMA3C

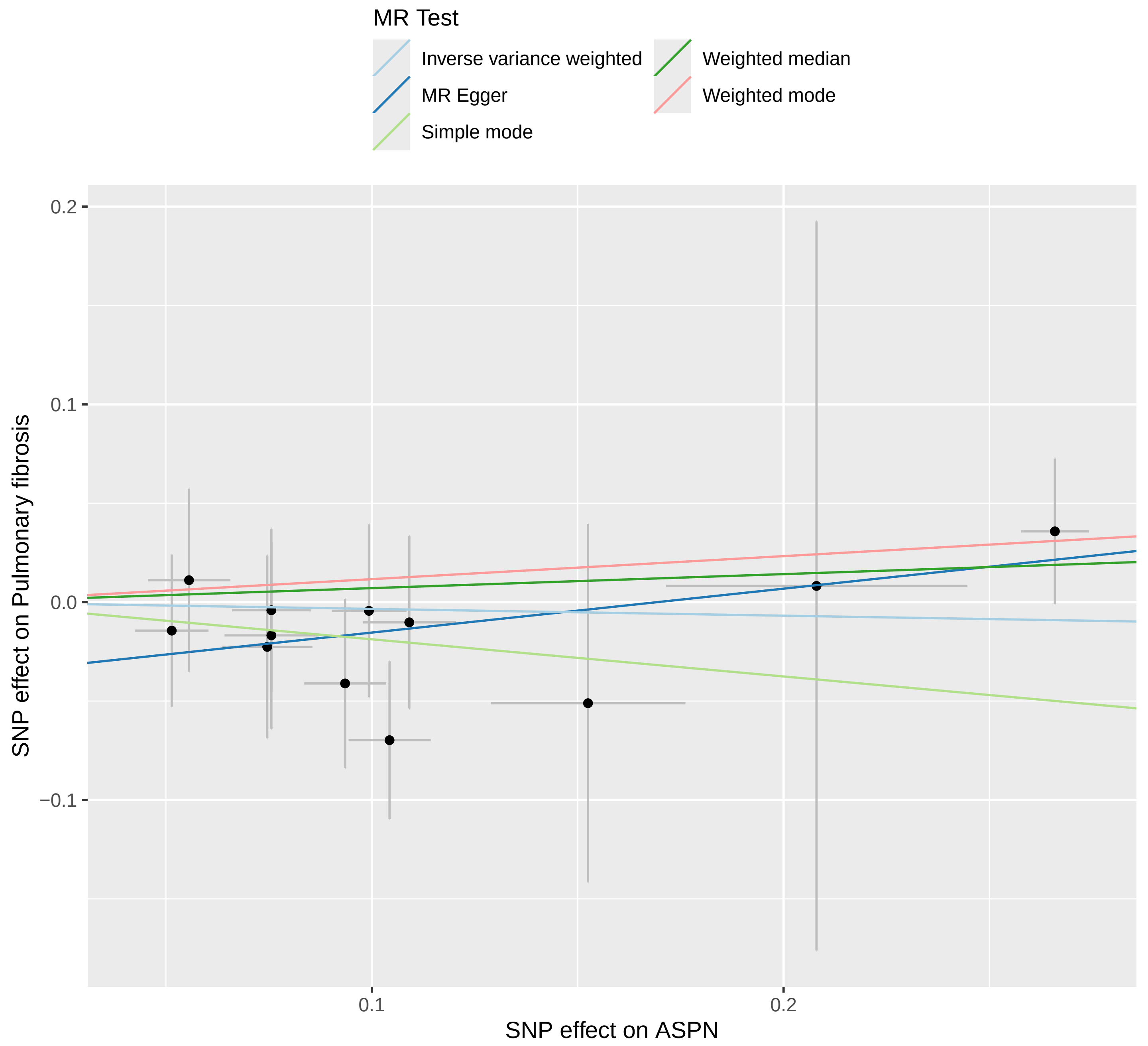
MR Test
Inverse variance weighted
MR Egger
Simple mode
Weighted median
Weighted mode
0.2
0.1
0.0
−0.1
0.1
0.2
SNP effect on ASPN
SNP effect on Pulmonary fibrosis

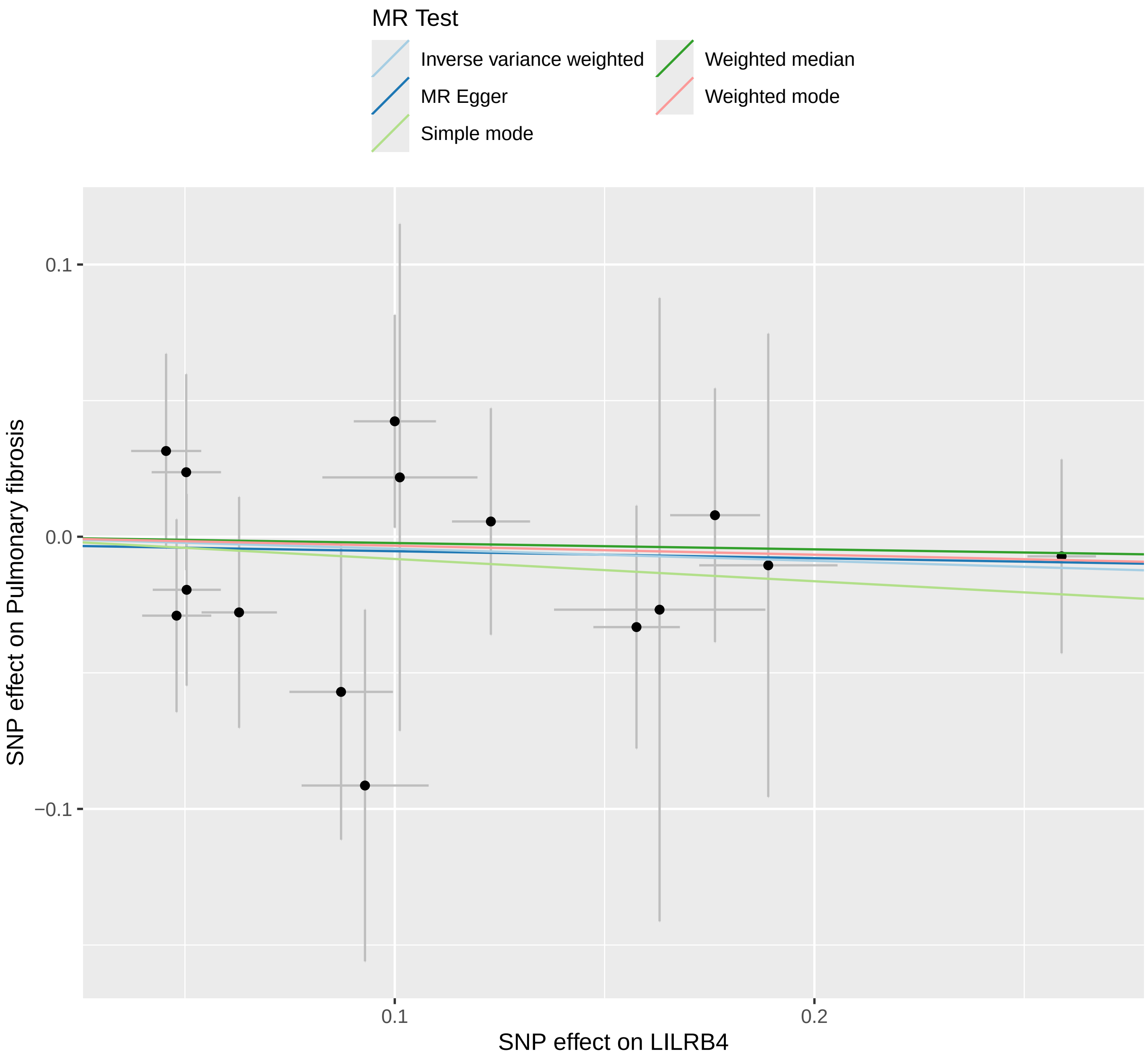
MR Test
Inverse variance weighted
MR Egger
Simple mode
Weighted median
Weighted mode
SNP effect on Pulmonary fibrosis
0.1
0.0
−0.1
0.1
0.2
SNP effect on LILRB4

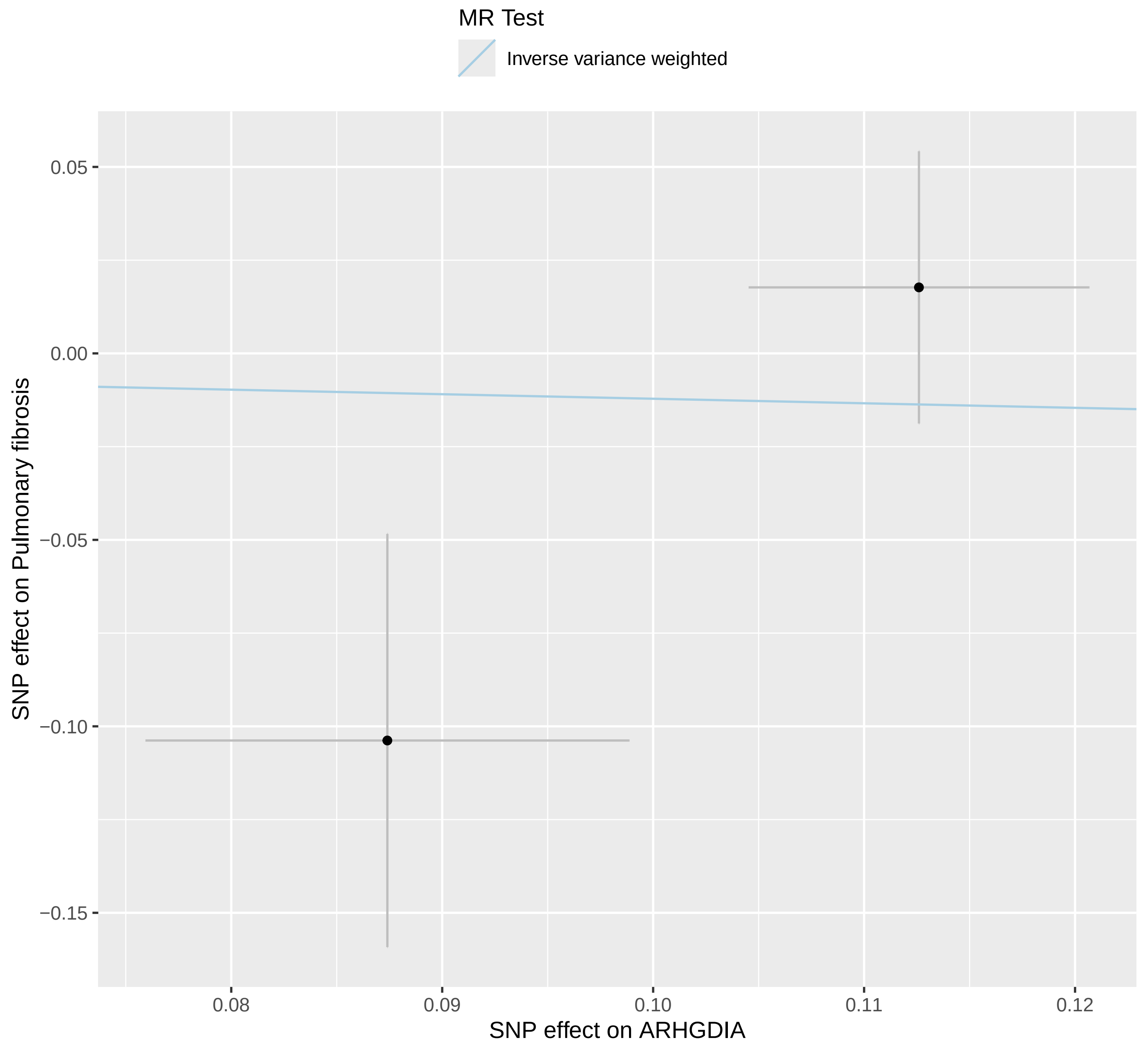

MR Test
Inverse variance weighted
0.05
0.00
−0.05
−0.10
−0.15
SNP effect on Pulmonary fibrosis
0.08
0.09
0.10
0.11
0.12
SNP effect on ARHGDIA

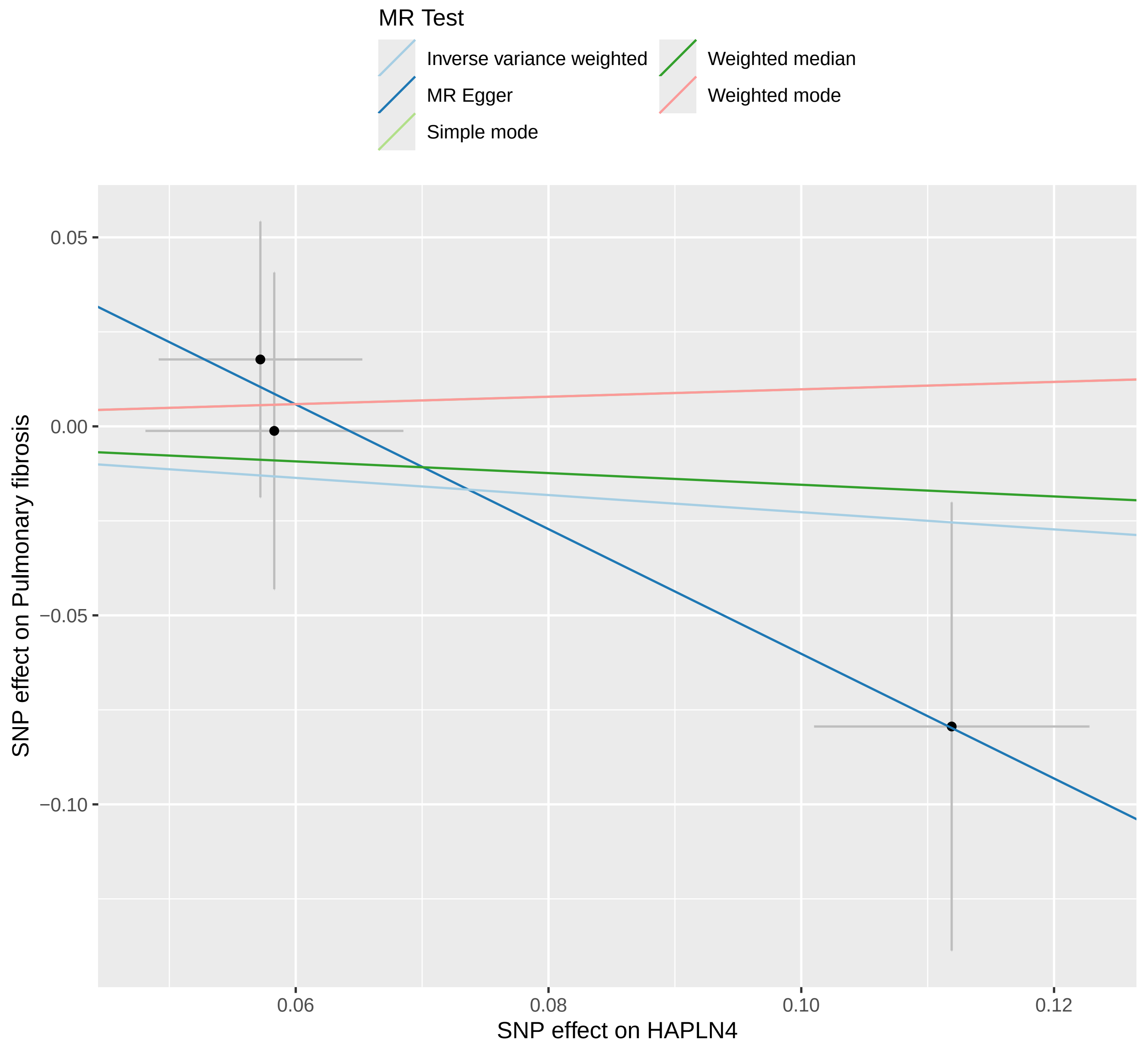
MR Test
Inverse variance weighted
MR Egger
Simple mode
Weighted median
Weighted mode
0.05
0.00
−0.05
−0.10
0.06
0.08
0.10
0.12
SNP effect on Pulmonary fibrosis
SNP effect on HAPLN4

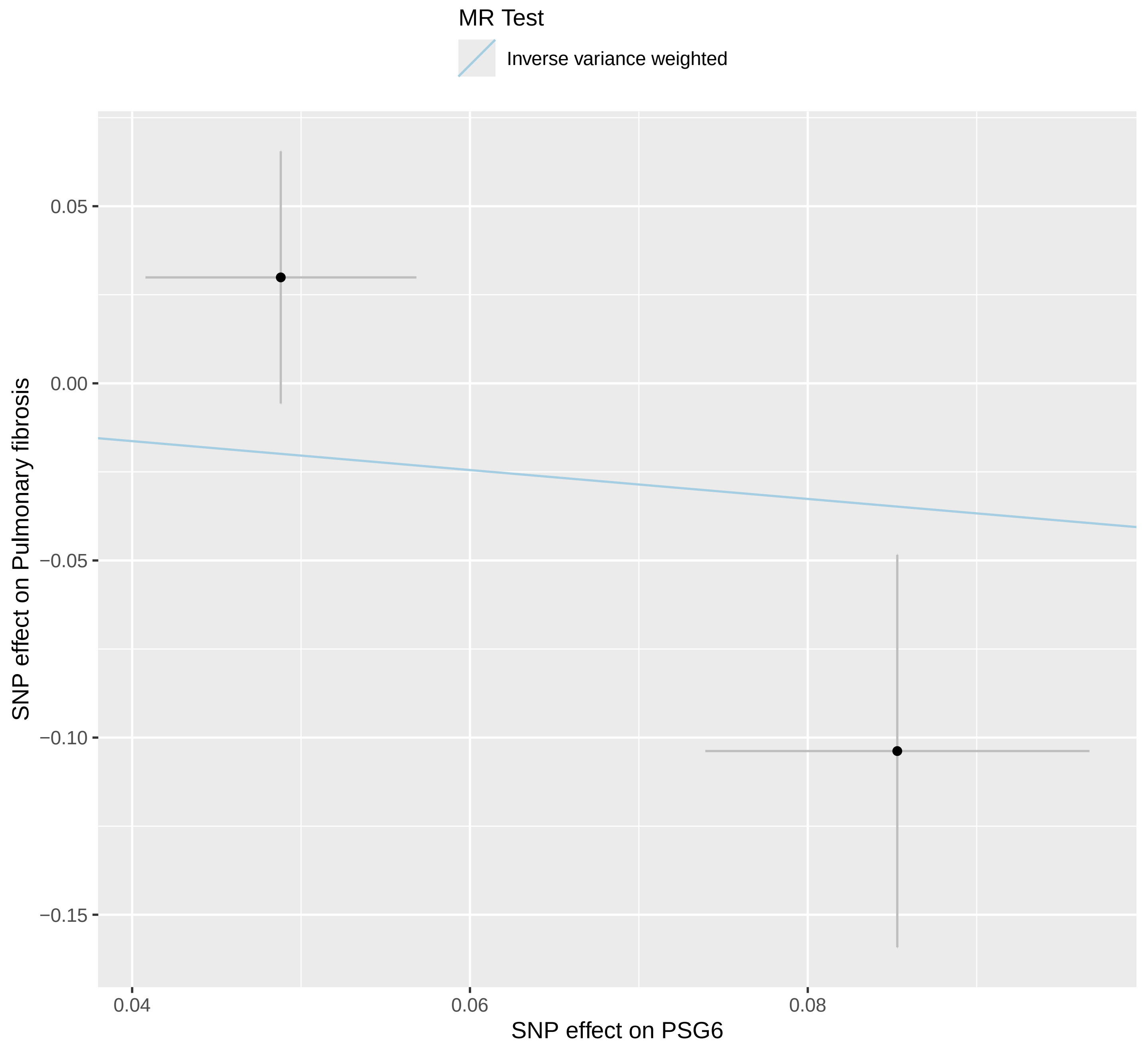

MR Test
Inverse variance weighted
0.05
0.00
−0.05
−0.10
−0.15
0.04
0.06
0.08
SNP effect on PSG6
SNP effect on Pulmonary fibrosis

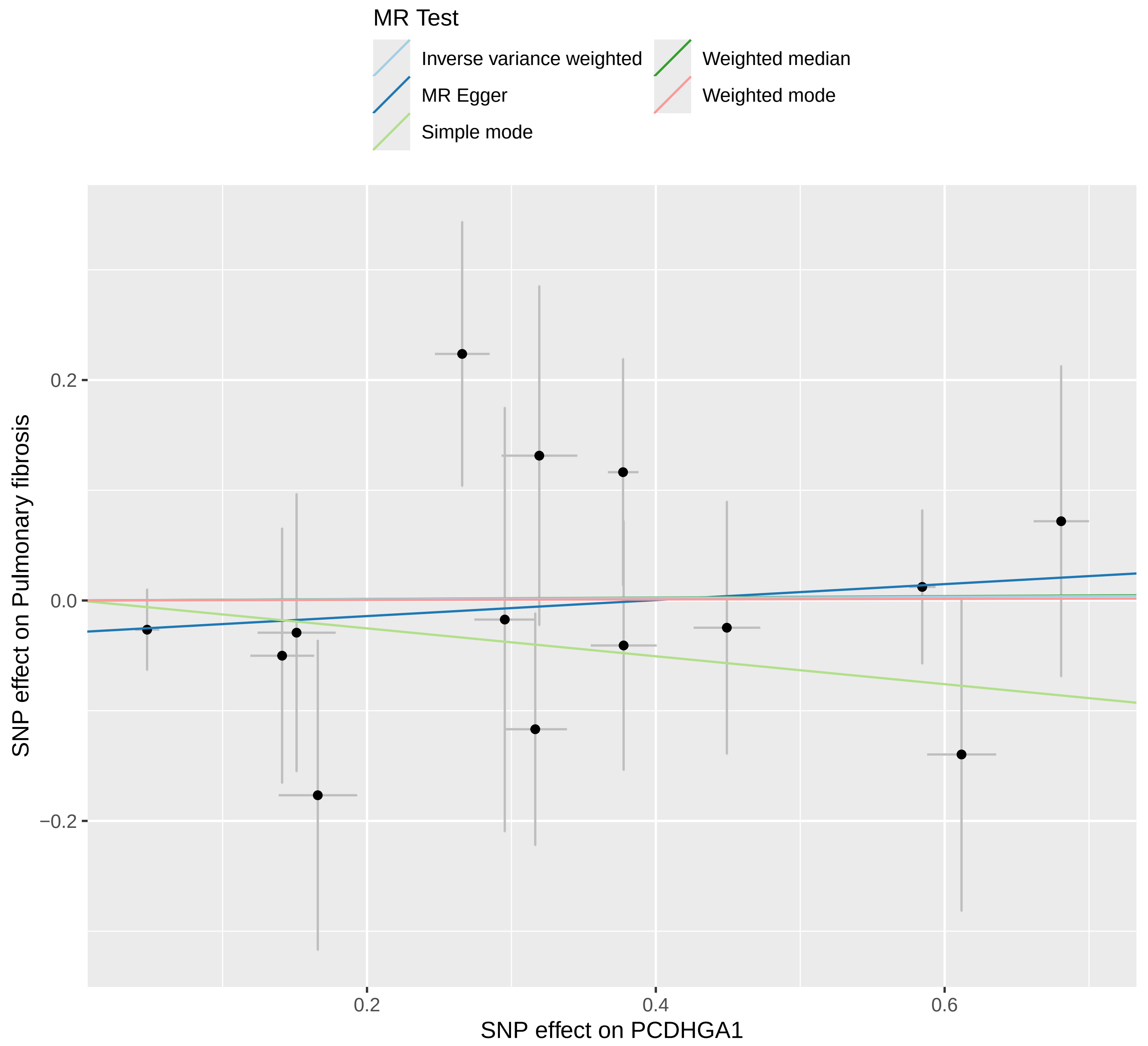

MR Test
Inverse variance weighted
MR Egger
Simple mode
Weighted median
Weighted mode
SNP effect on Pulmonary fibrosis
0.2
0.0
−0.2
0.2
0.4
0.6
SNP effect on PCDHGA1

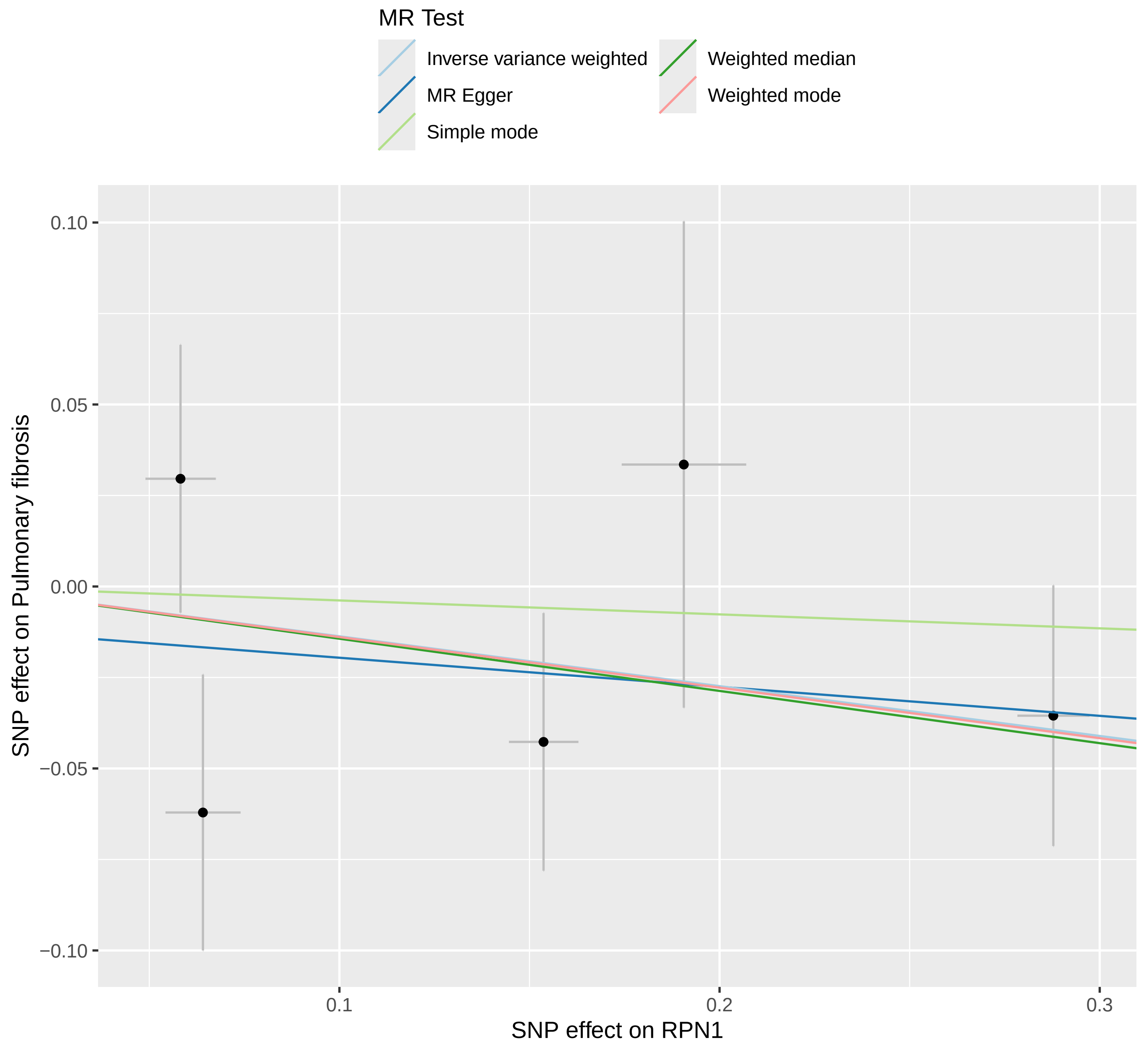

MR Test
Inverse variance weighted
MR Egger
Simple mode
Weighted median
Weighted mode
0.10
0.05
0.00
−0.05
−0.10
0.1
0.2
0.3
SNP effect on Pulmonary fibrosis
SNP effect on RPN1

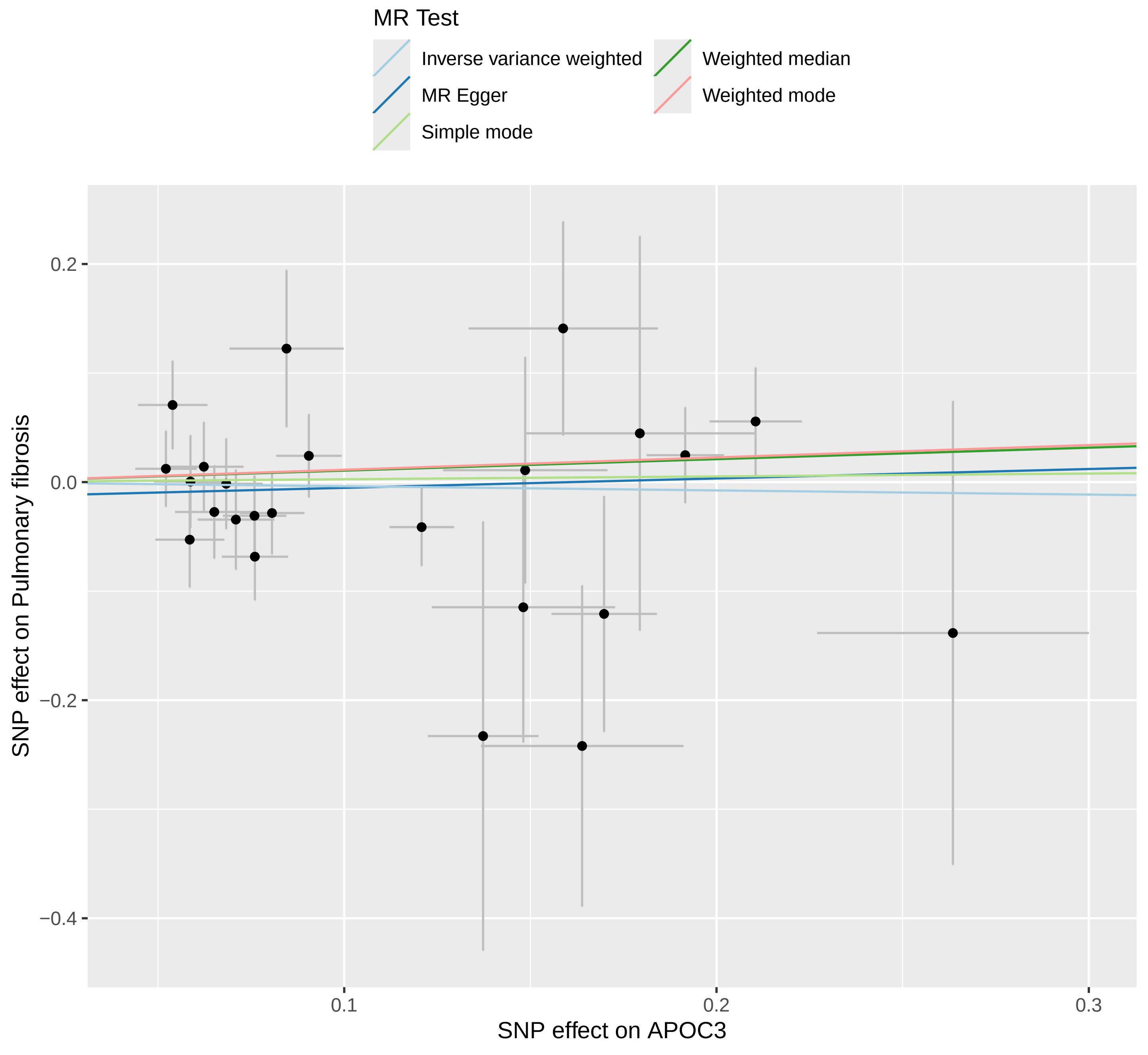
MR Test
Inverse variance weighted
MR Egger
Simple mode
Weighted median
Weighted mode
0.2
0.0
−0.2
−0.4
SNP effect on Pulmonary fibrosis
0.1
0.2
0.3
SNP effect on APOC3

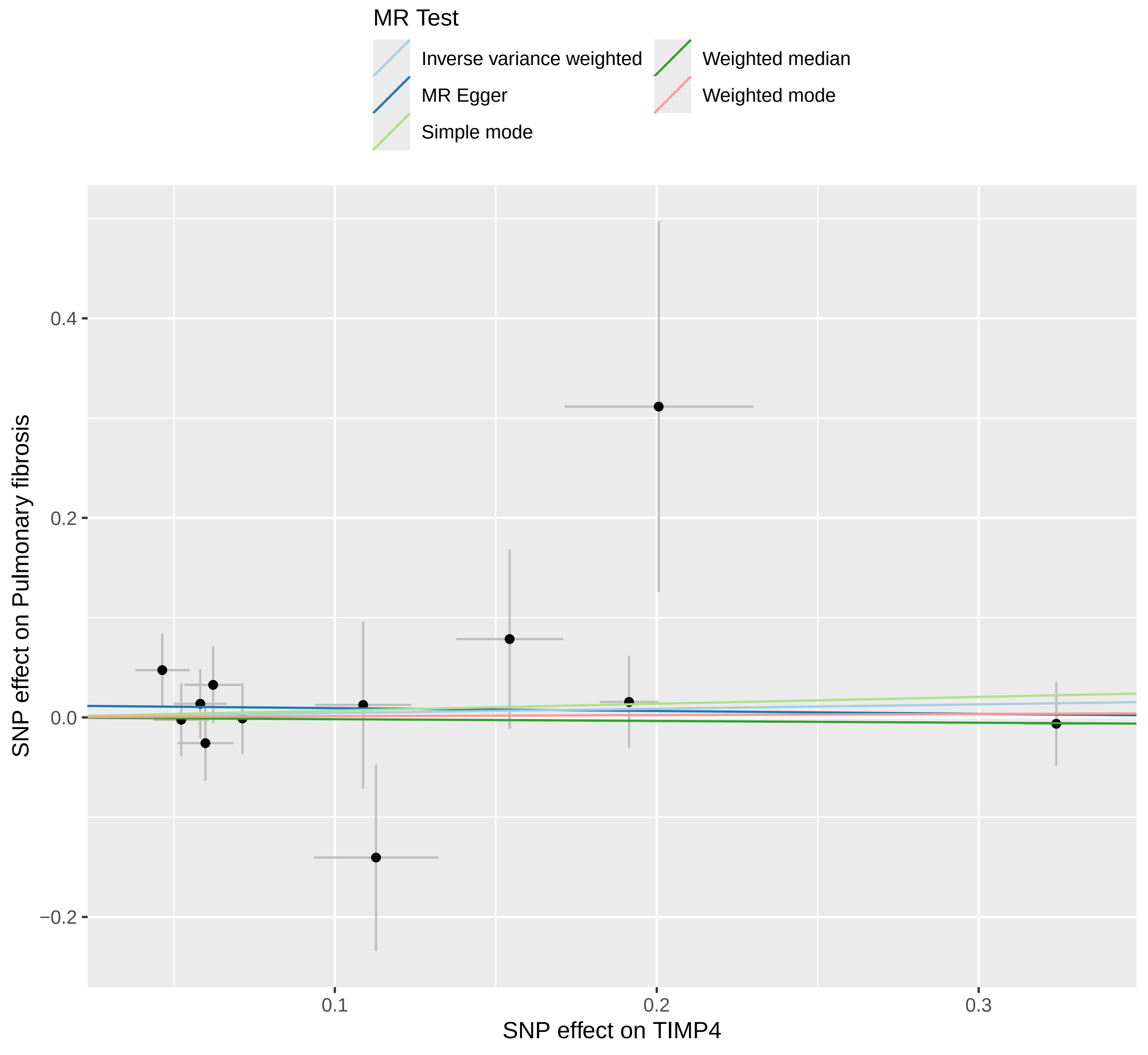
MR Test
Inverse variance weighted
MR Egger
Simple mode
Weighted median
Weighted mode
0.4
0.2
0.0
−0.2
0.1
0.2
0.3
SNP effect on Pulmonary fibrosis
SNP effect on TIMP4

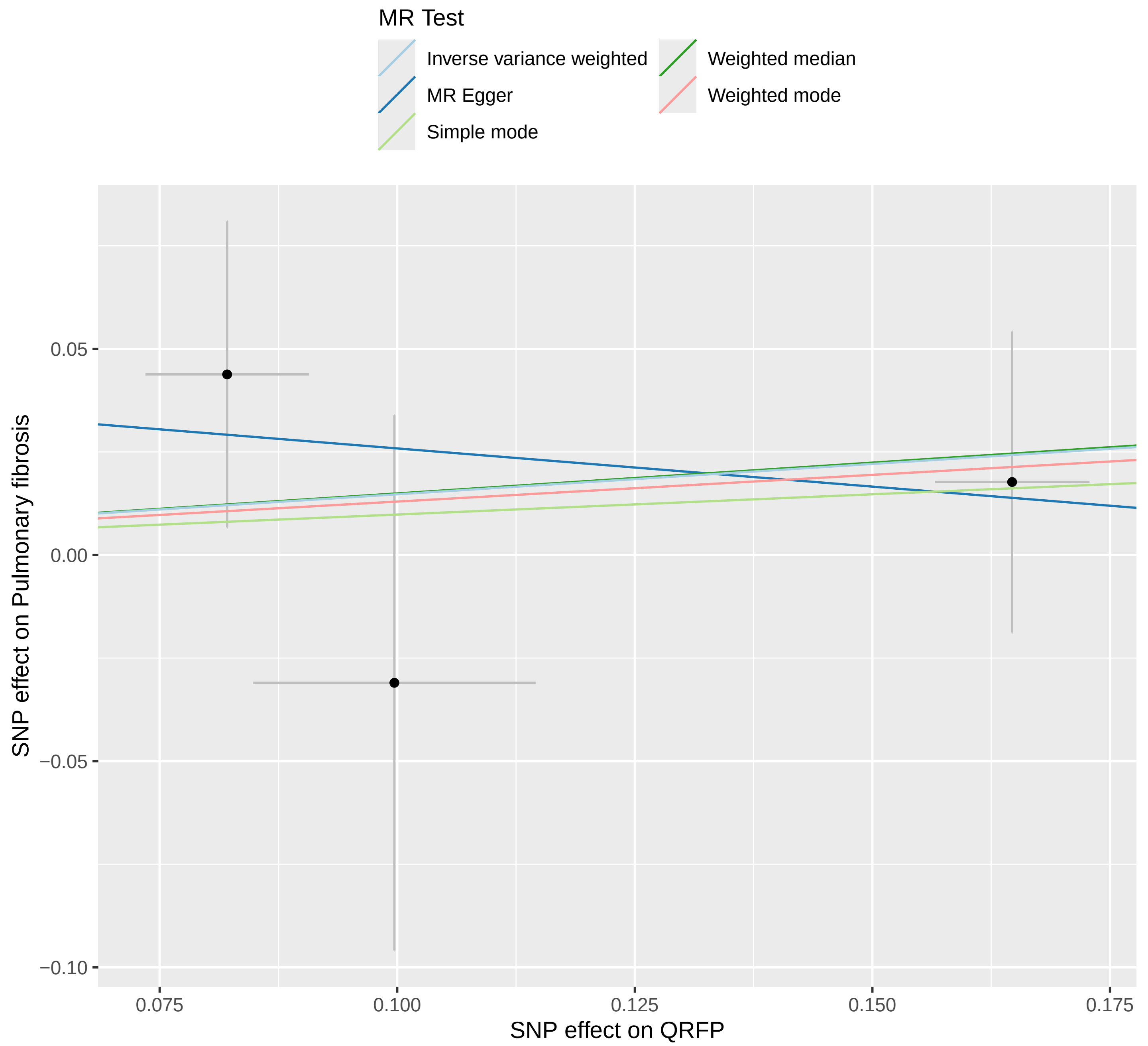

MR Test
Inverse variance weighted
MR Egger
Simple mode
Weighted median
Weighted mode
0.05
0.00
−0.05
−0.10
0.075
0.100
0.125
0.150
0.175
SNP effect on QRFP
SNP effect on Pulmonary fibrosis

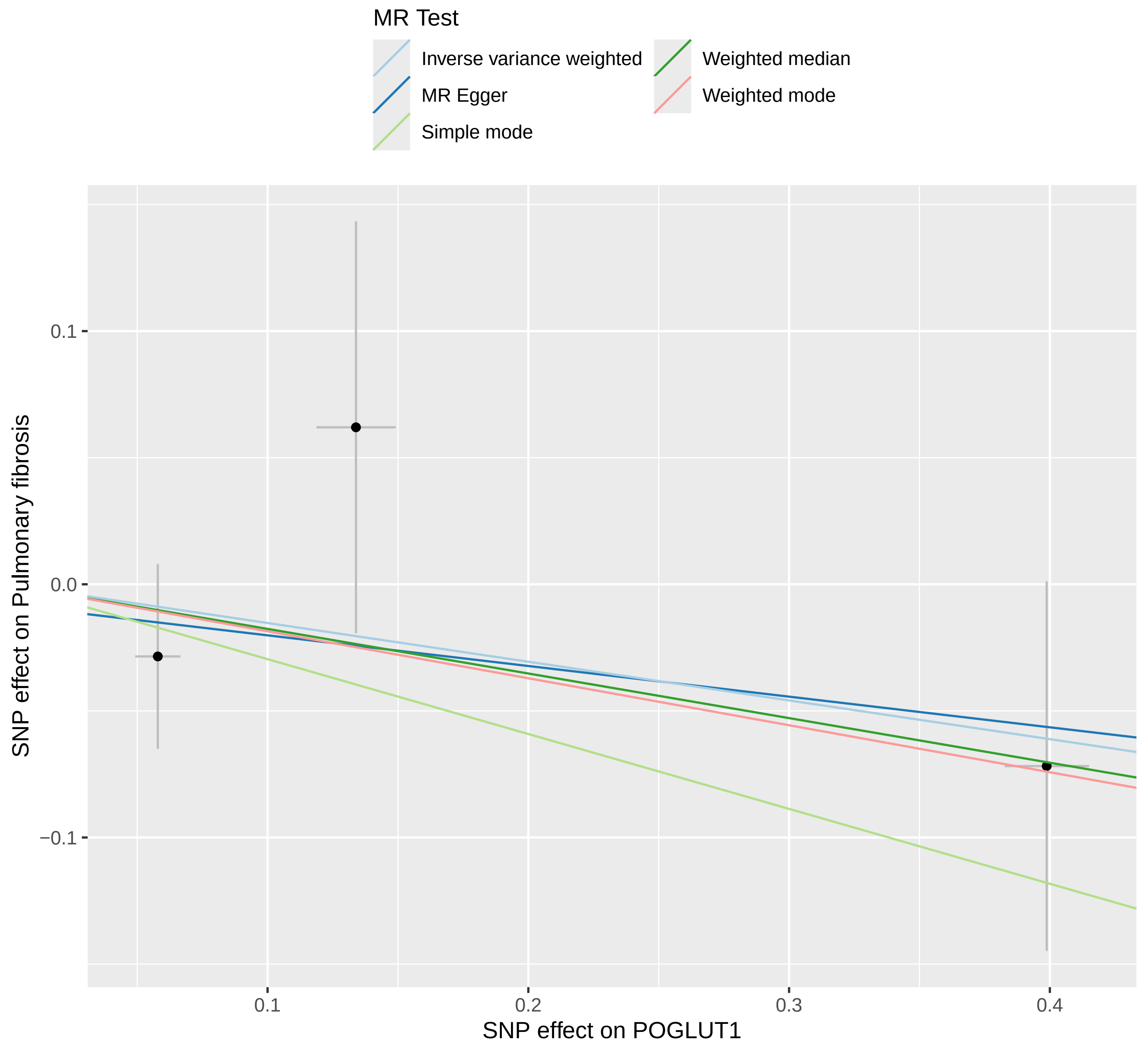
MR Test
Inverse variance weighted
MR Egger
Simple mode
Weighted median
Weighted mode
SNP effect on Pulmonary fibrosis
0.1
0.0
−0.1
0.1
0.2
0.3
0.4
SNP effect on POGLUT1

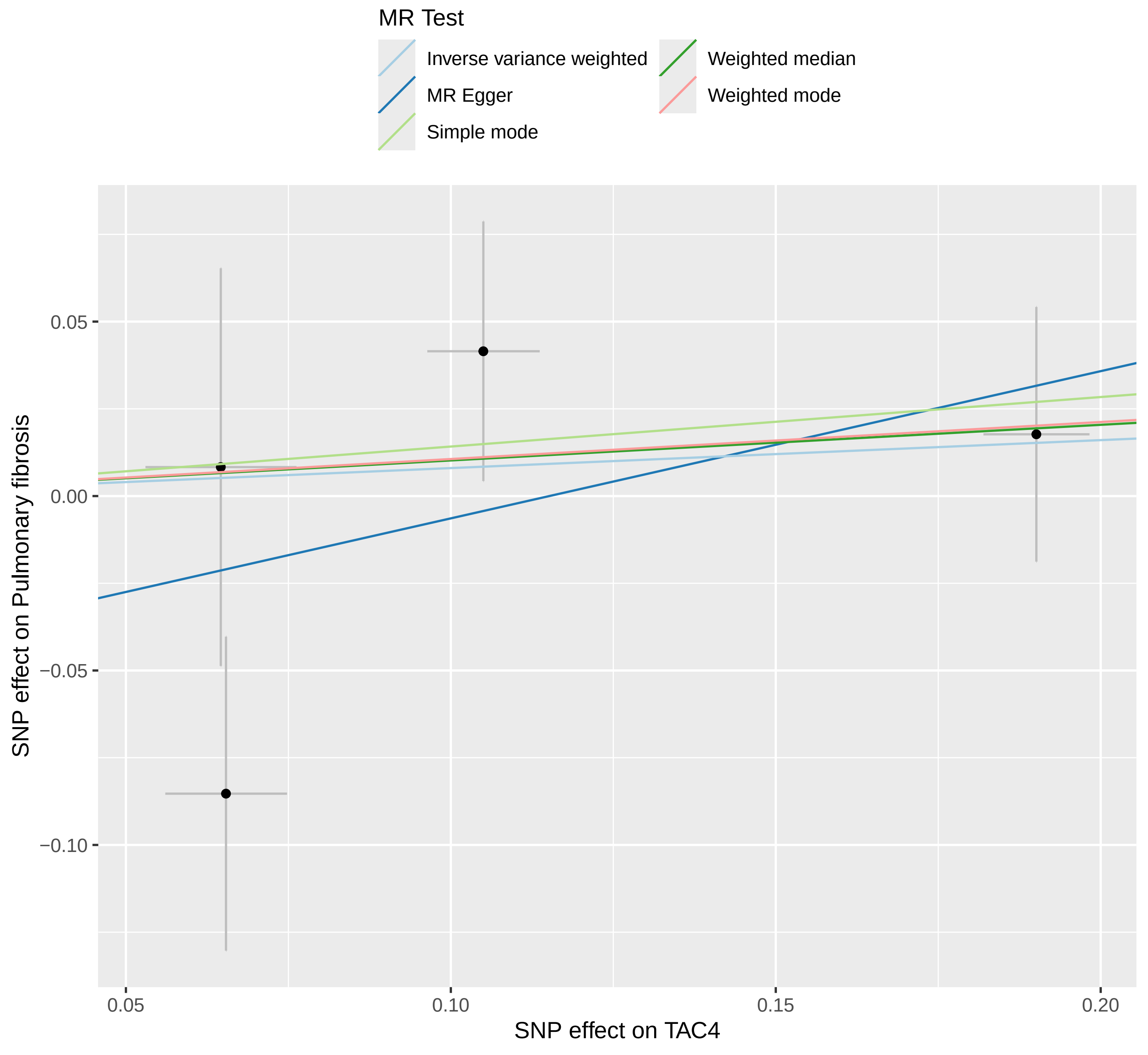
MR Test
Inverse variance weighted
MR Egger
Simple mode
Weighted median
Weighted mode
0.05
0.00
−0.05
−0.10
SNP effect on Pulmonary fibrosis
0.05
0.10
0.15
0.20
SNP effect on TAC4

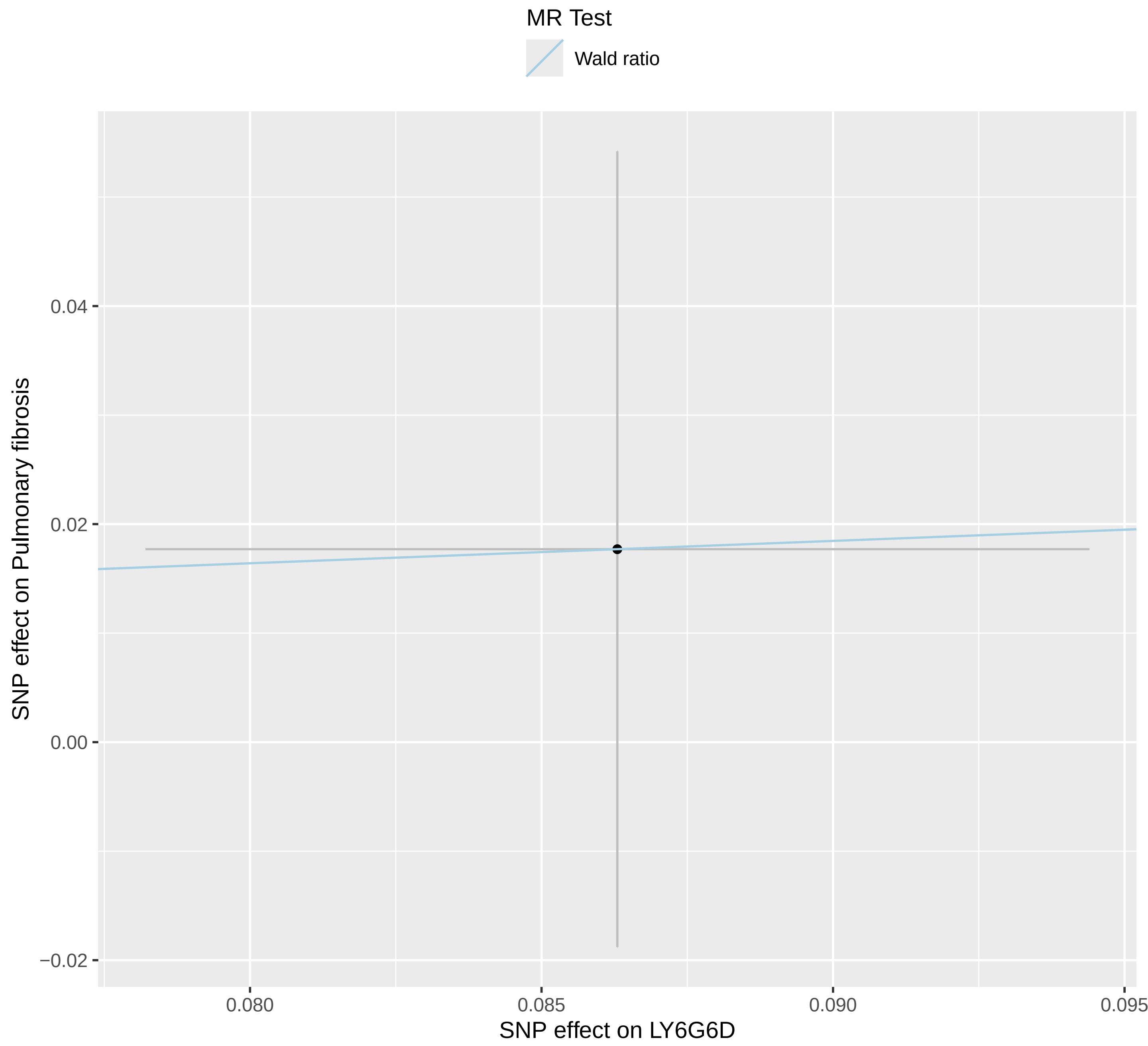

MR Test
Wald ratio
SNP effect on Pulmonary fibrosis
0.04
0.02
0.00
−0.02
0.080
0.085
0.090
0.095
SNP effect on LY6G6D

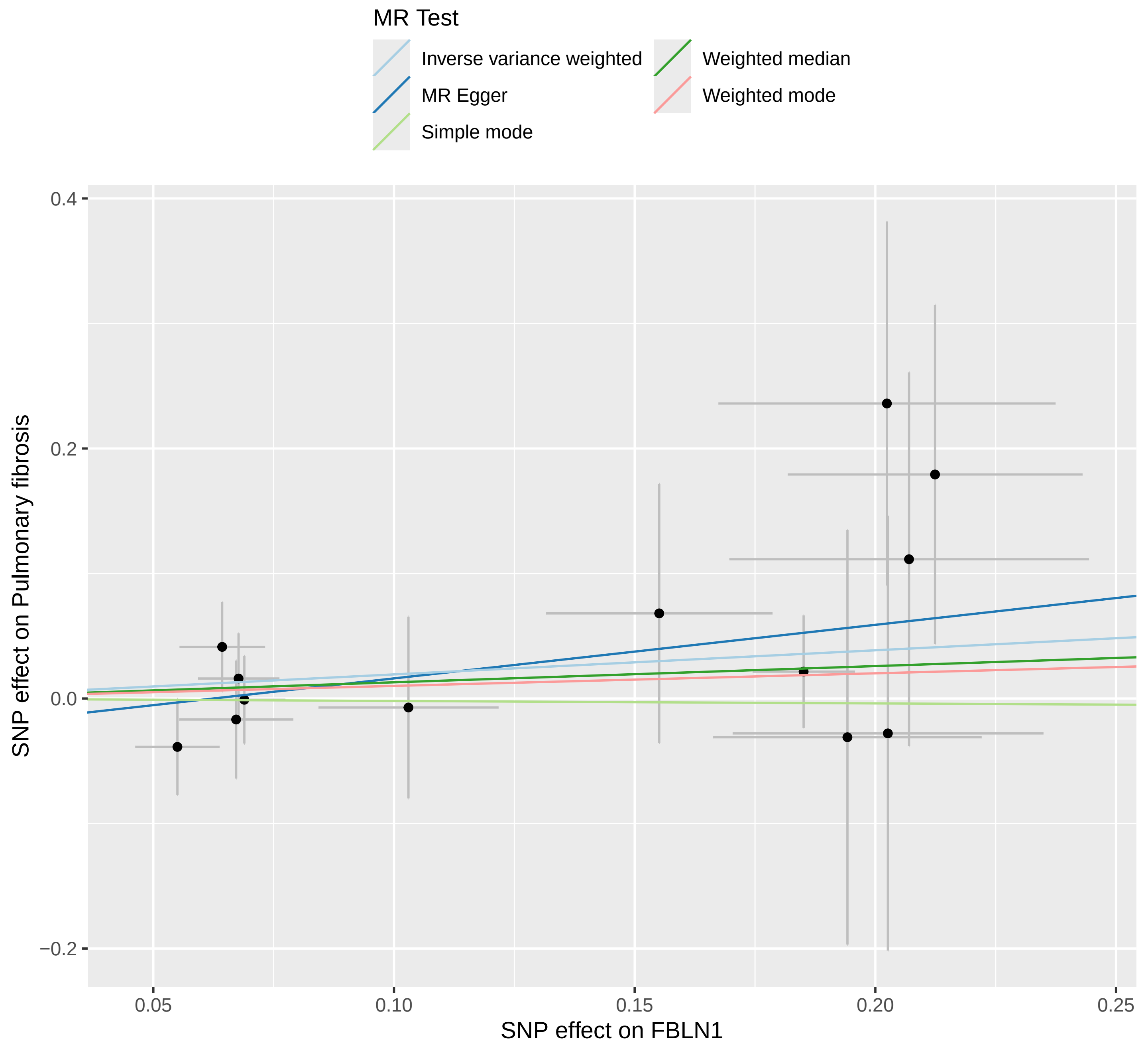

MR Test
Inverse variance weighted
MR Egger
Simple mode
Weighted median
Weighted mode
0.4
0.2
0.0
−0.2
0.05
0.10
0.15
0.20
0.25
SNP effect on Pulmonary fibrosis
SNP effect on FBLN1

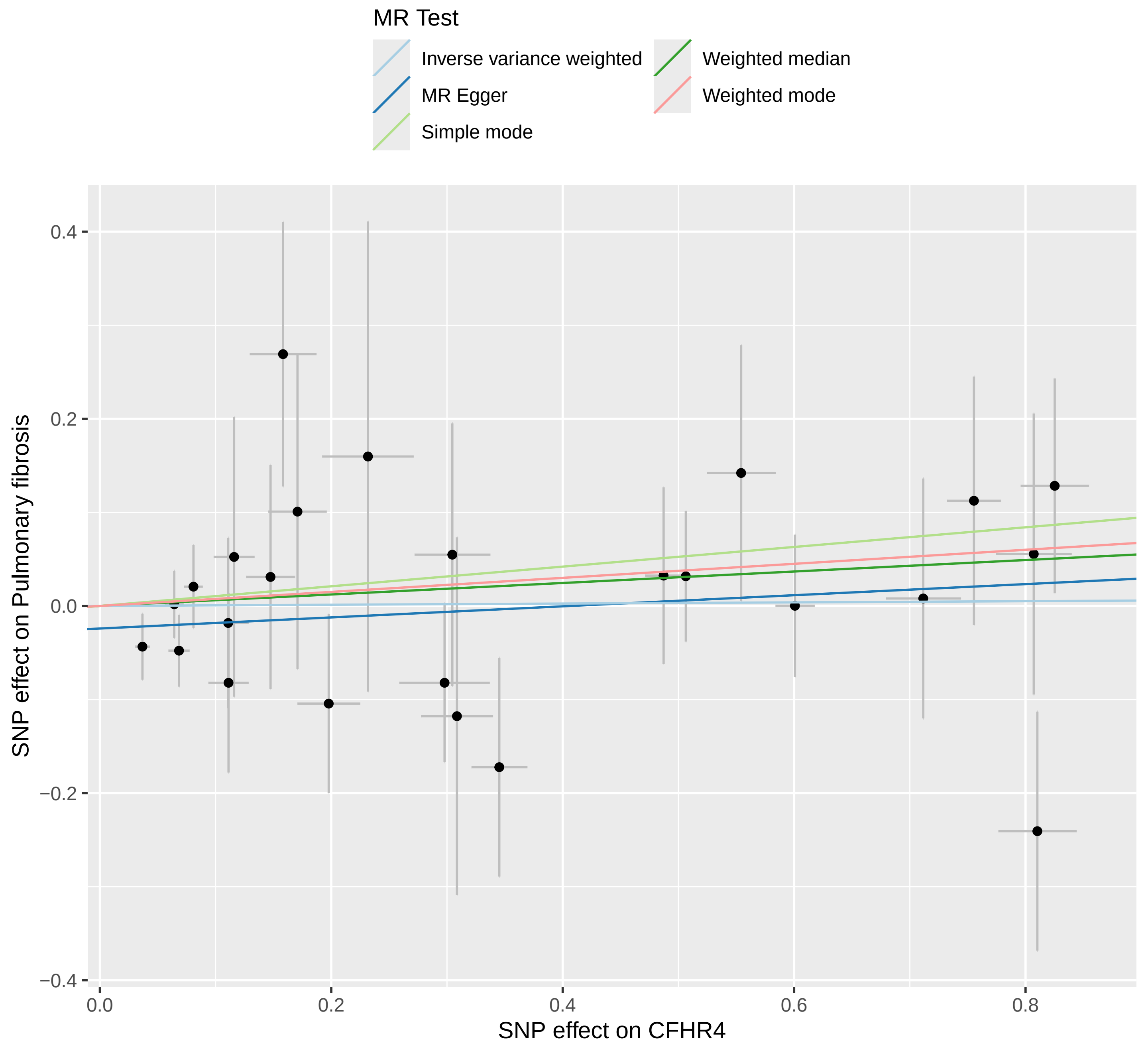

MR Test
Inverse variance weighted
MR Egger
Simple mode
Weighted median
Weighted mode
0.4
0.2
0.0
−0.2
−0.4
SNP effect on Pulmonary fibrosis
0.0
0.2
0.4
0.6
0.8
SNP effect on CFHR4

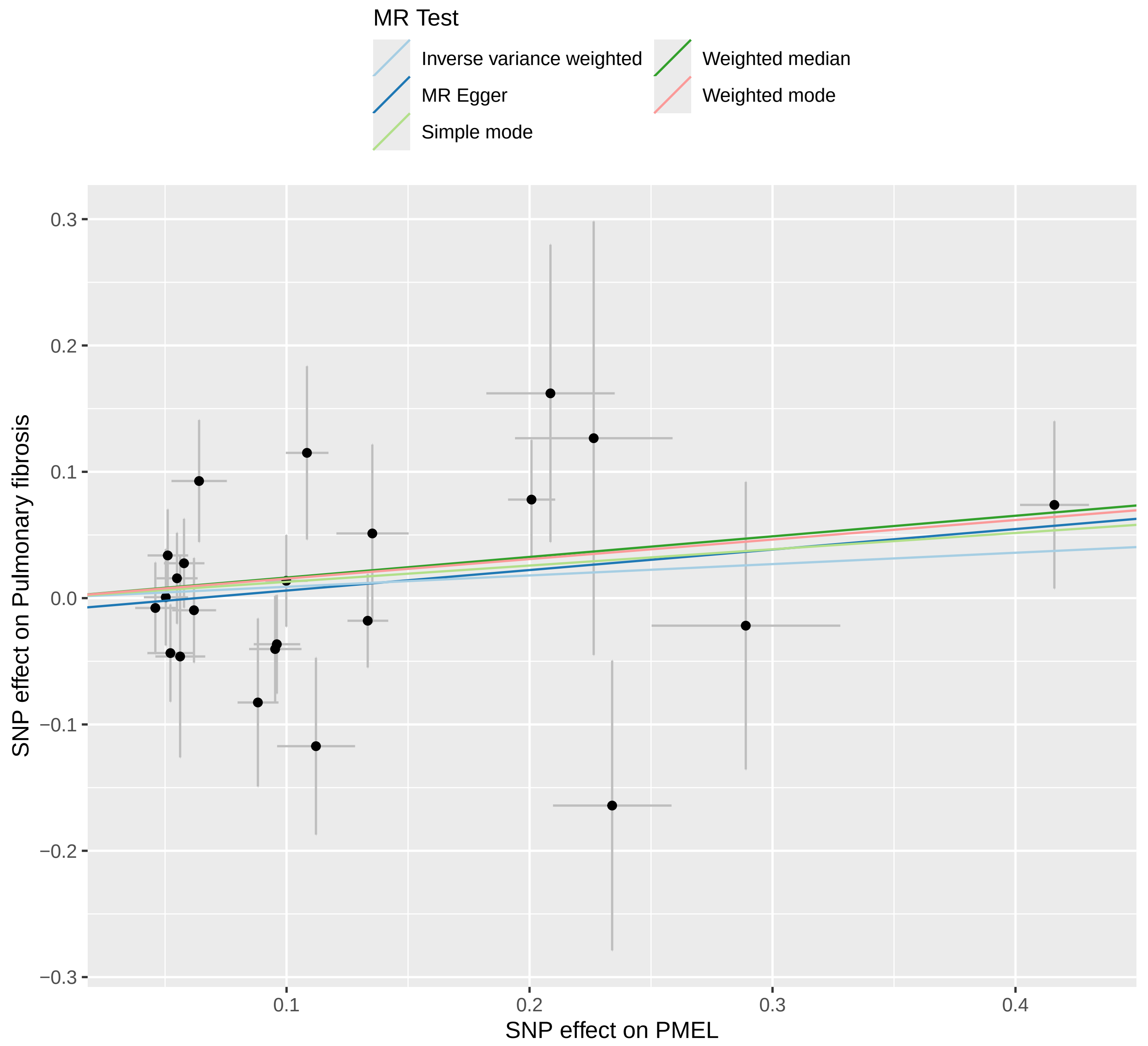

MR Test
Inverse variance weighted
MR Egger
Simple mode
Weighted median
Weighted mode
0.3
0.2
0.1
0.0
−0.1
−0.2
−0.3
0.1
0.2
0.3
0.4
SNP effect on Pulmonary fibrosis
SNP effect on PMEL

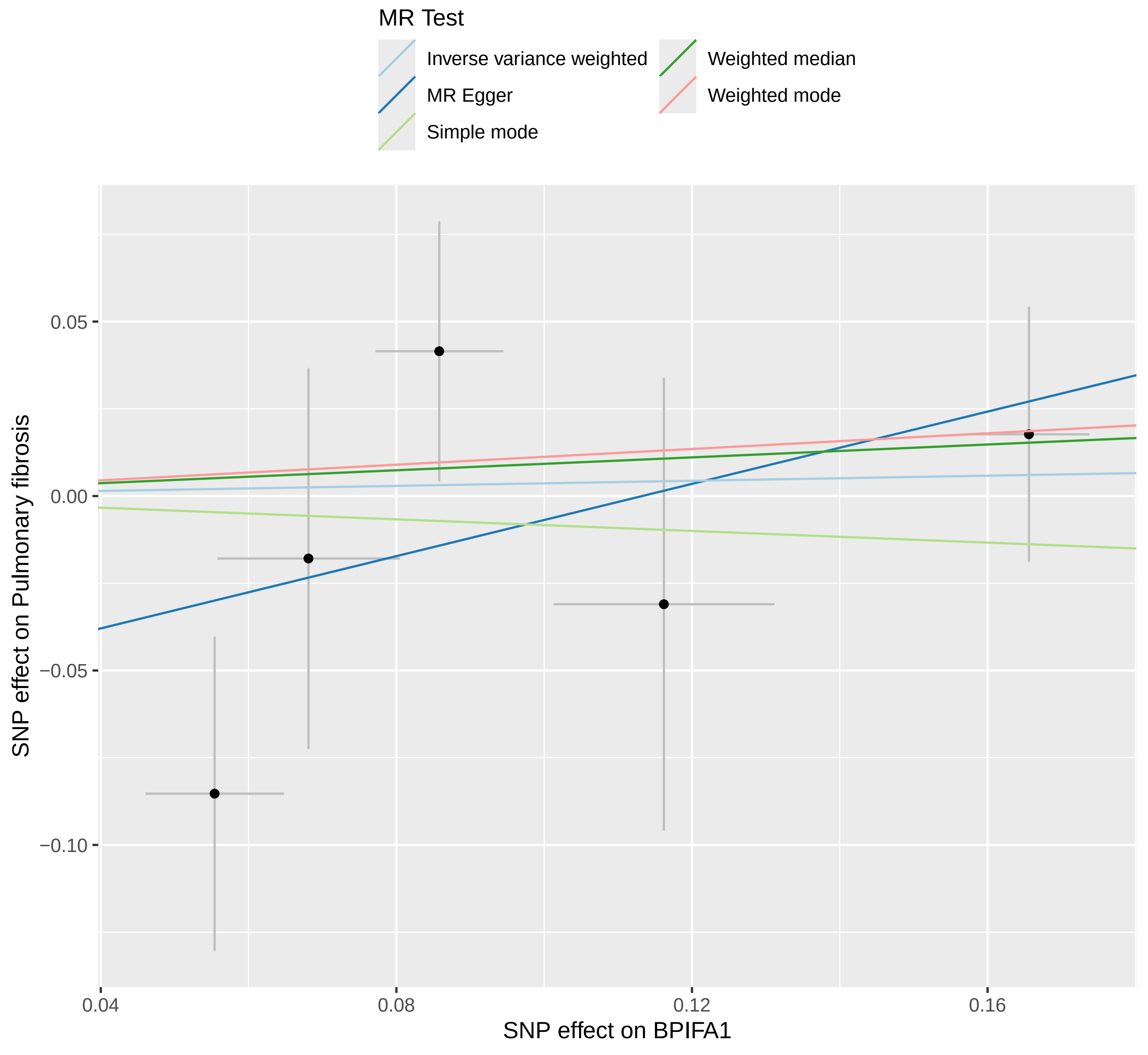
MR Test
Inverse variance weighted
MR Egger
Simple mode
Weighted median
Weighted mode
0.05
0.00
−0.05
−0.10
SNP effect on Pulmonary fibrosis
0.04
0.08
0.12
0.16
SNP effect on BPIFA1

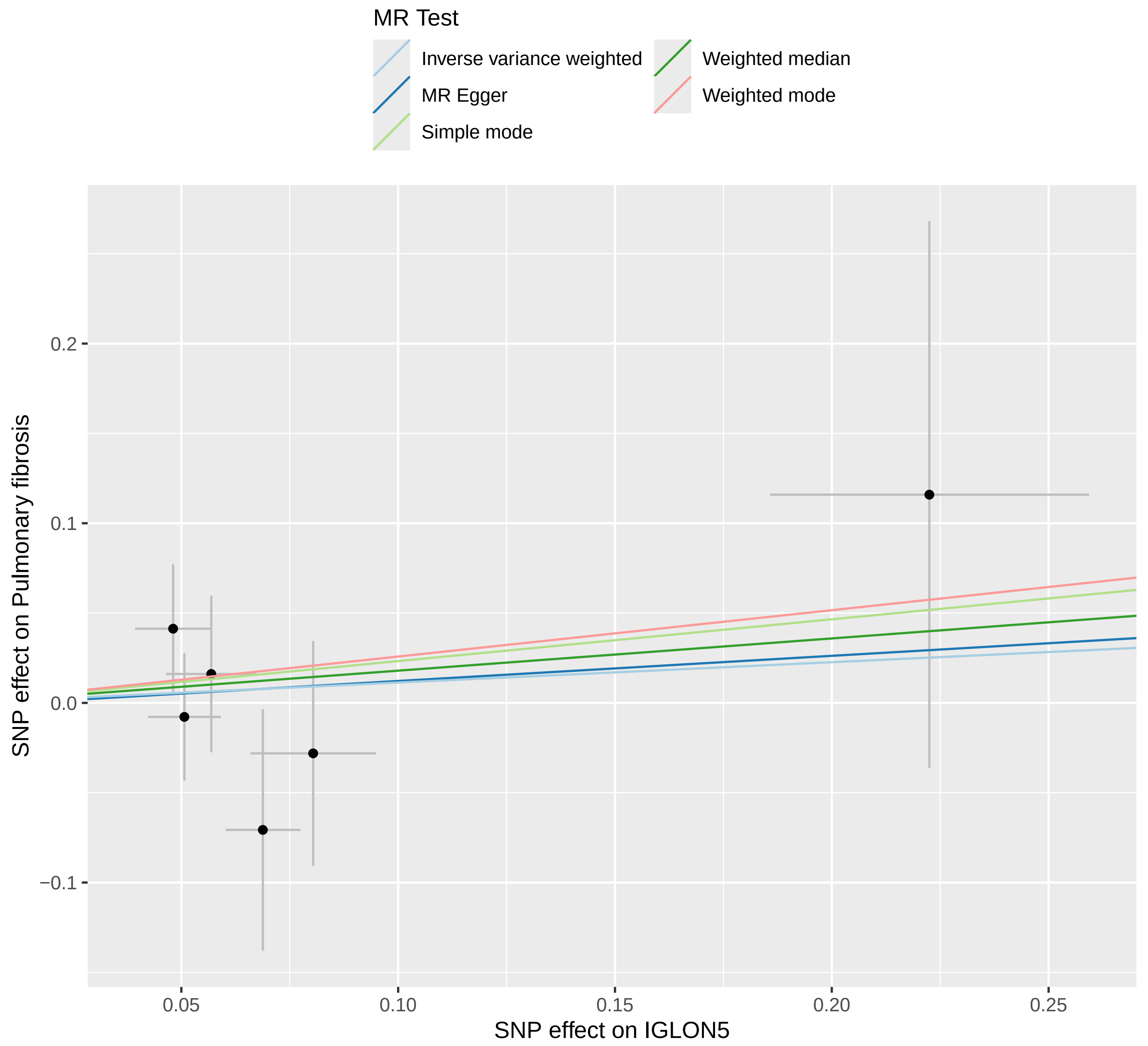

MR Test
Inverse variance weighted
MR Egger
Simple mode
Weighted median
Weighted mode
SNP effect on Pulmonary fibrosis
0.2
0.1
0.0
−0.1
0.05
0.10
0.15
0.20
0.25
SNP effect on IGLON5

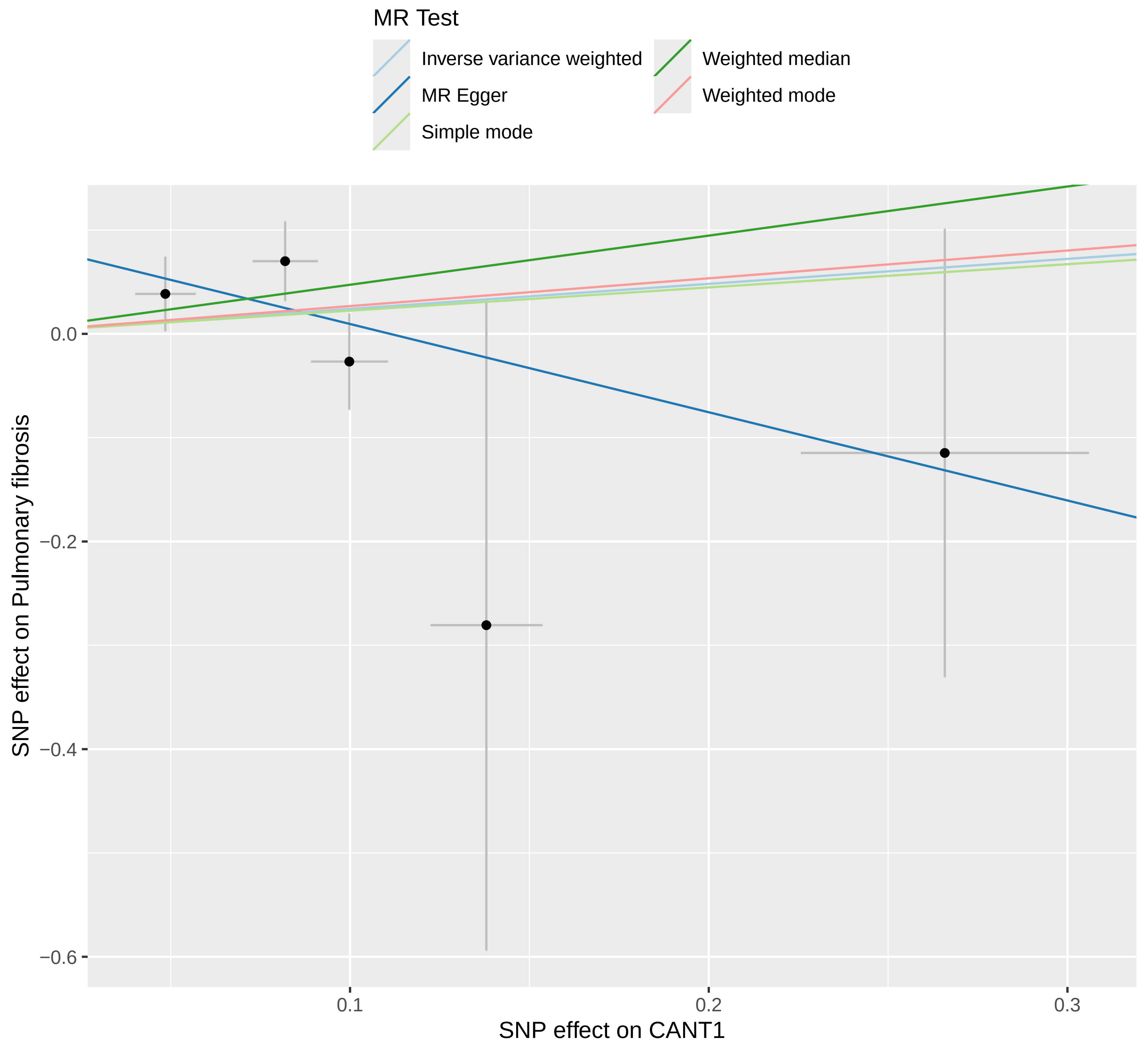

MR Test
Inverse variance weighted
MR Egger
Simple mode
Weighted median
Weighted mode
SNP effect on Pulmonary fibrosis
0.0
−0.2
−0.4
−0.6
0.1
0.2
0.3
SNP effect on CANT1

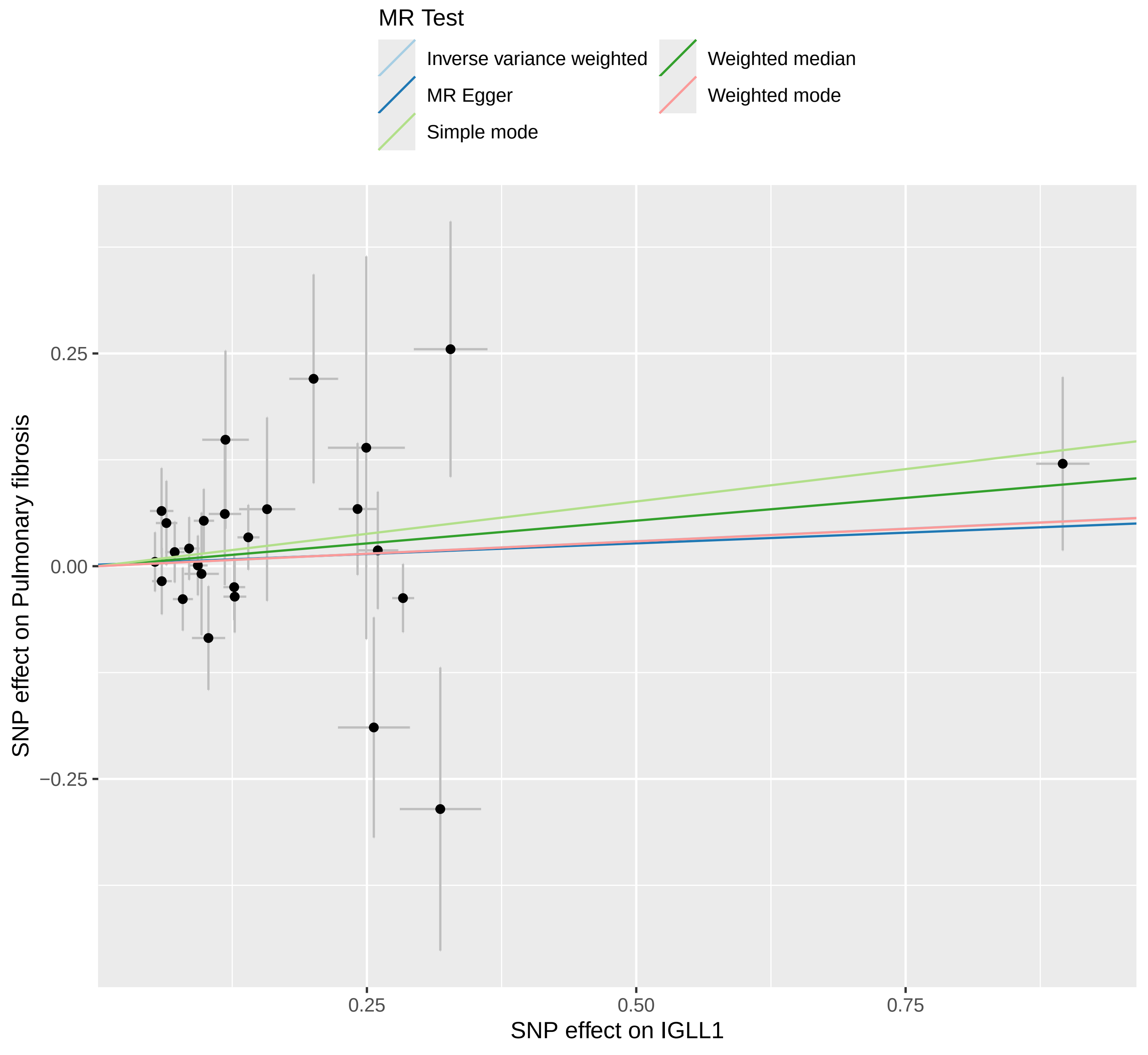

MR Test
Inverse variance weighted
MR Egger
Simple mode
Weighted median
Weighted mode
0.25
0.00
−0.25
SNP effect on Pulmonary fibrosis
0.25
0.50
0.75
SNP effect on IGLL1

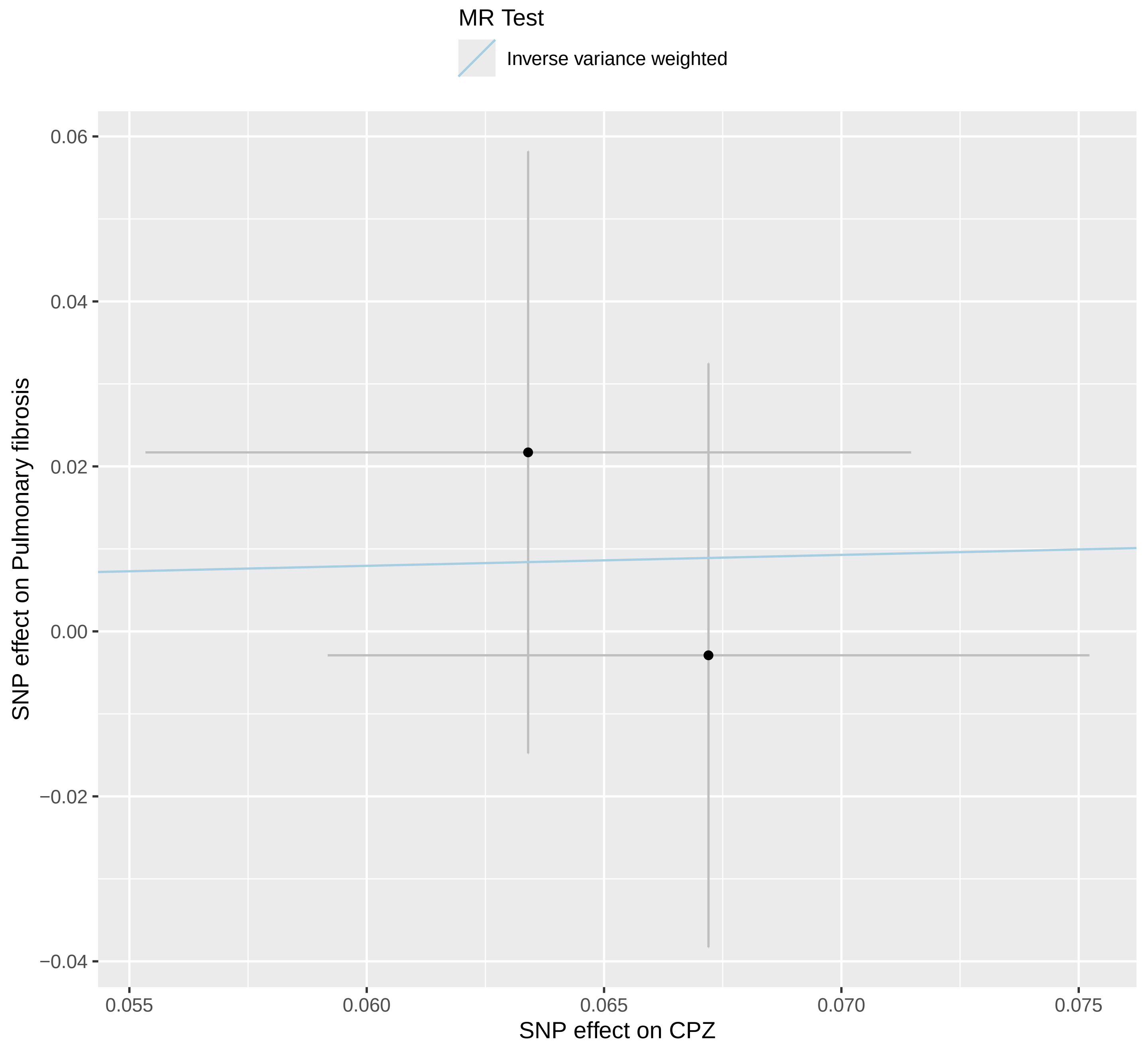

MR Test
Inverse variance weighted
0.06
0.04
0.02
0.00
−0.02
−0.04
SNP effect on Pulmonary fibrosis
0.055
0.060
0.065
0.070
0.075
SNP effect on CPZ

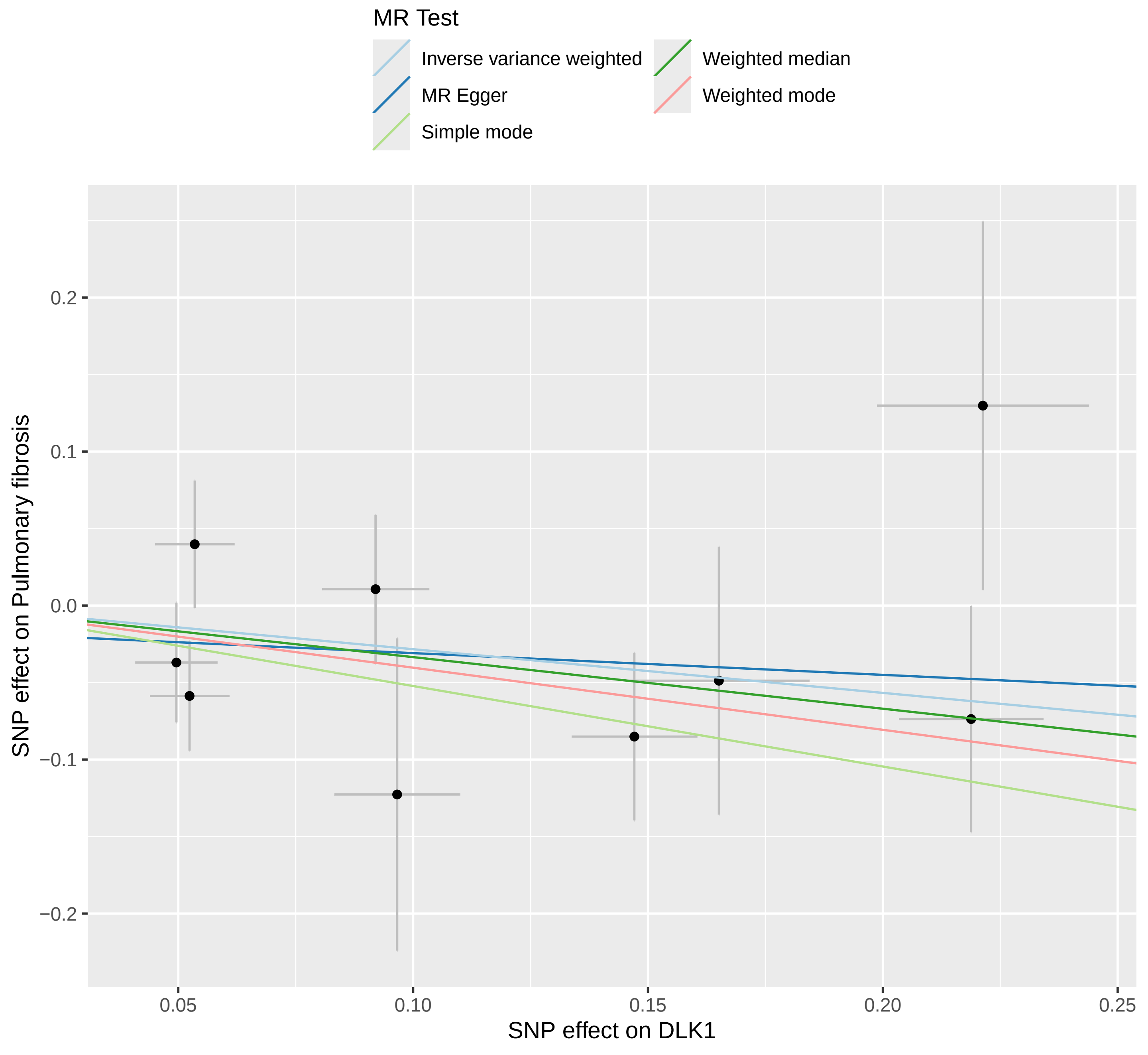
MR Test
Inverse variance weighted
MR Egger
Simple mode
Weighted median
Weighted mode
0.2
0.1
0.0
−0.1
−0.2
0.05
0.10
0.15
0.20
0.25
SNP effect on Pulmonary fibrosis
SNP effect on DLK1

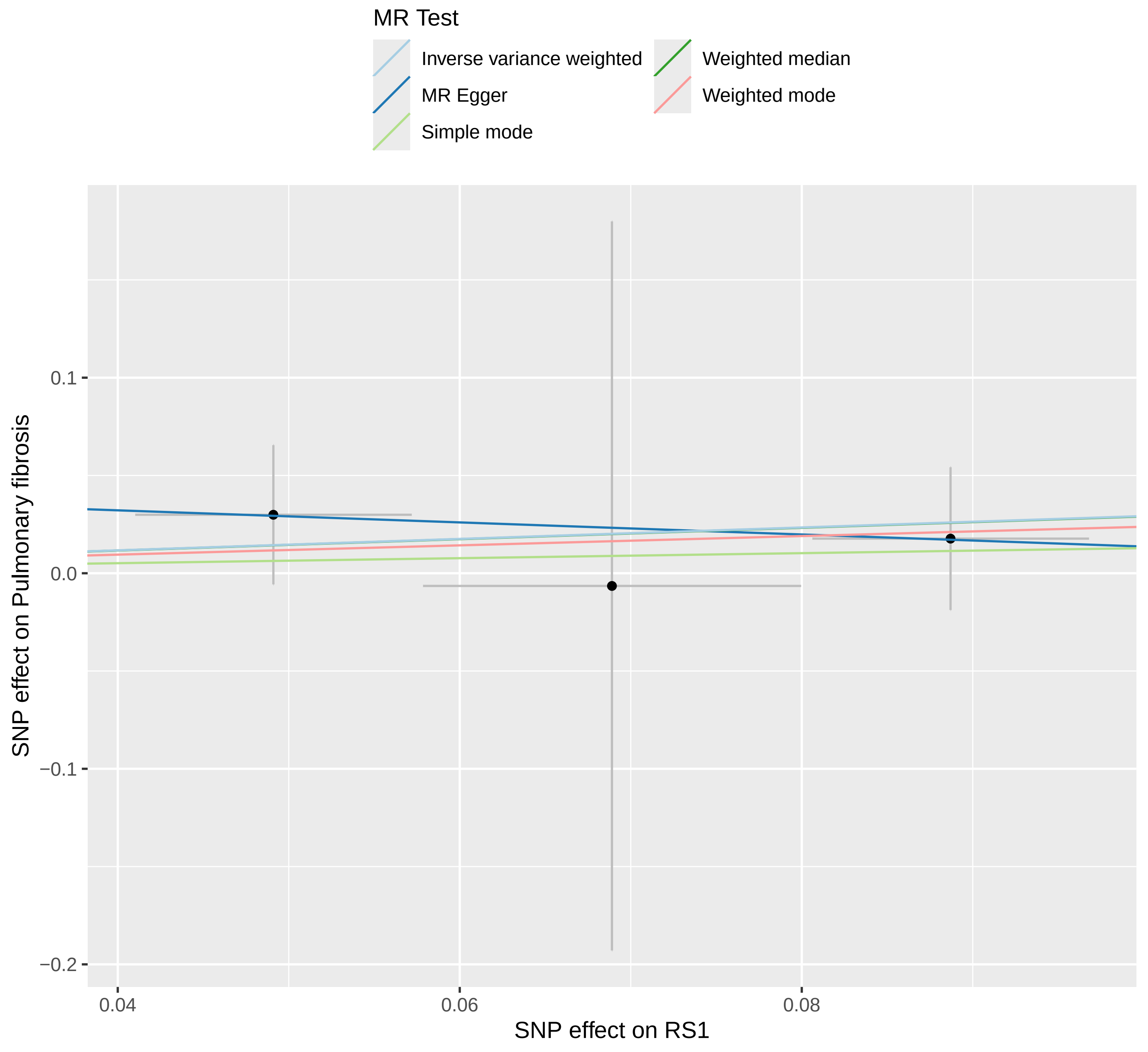

MR Test
Inverse variance weighted
MR Egger
Simple mode
Weighted median
Weighted mode
0.1
0.0
−0.1
−0.2
SNP effect on Pulmonary fibrosis
0.04
0.06
0.08
SNP effect on RS1

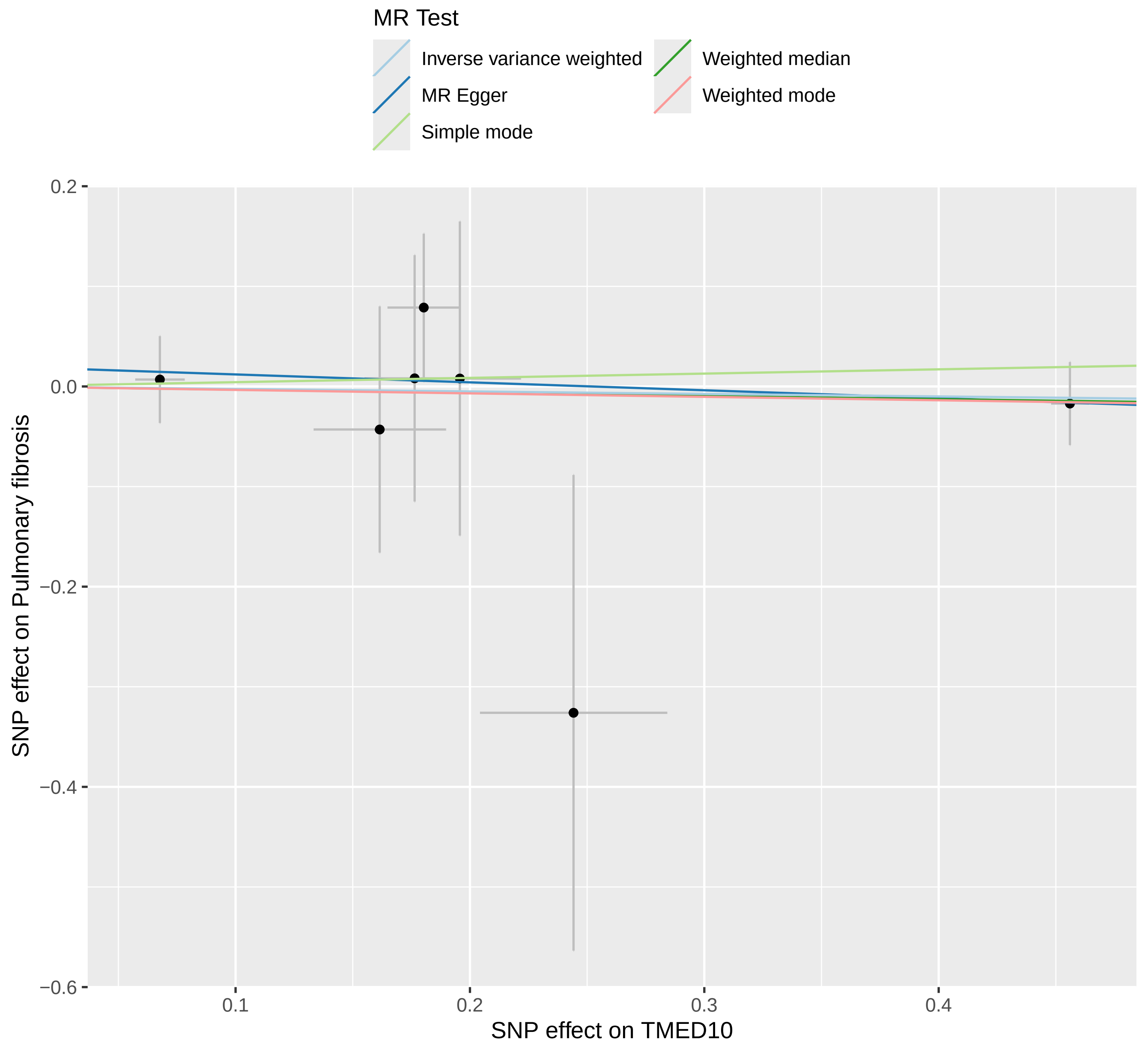

MR Test
Inverse variance weighted
MR Egger
Simple mode
Weighted median
Weighted mode
0.2
0.0
−0.2
−0.4
−0.6
0.1
0.2
0.3
0.4
SNP effect on Pulmonary fibrosis
SNP effect on TMED10

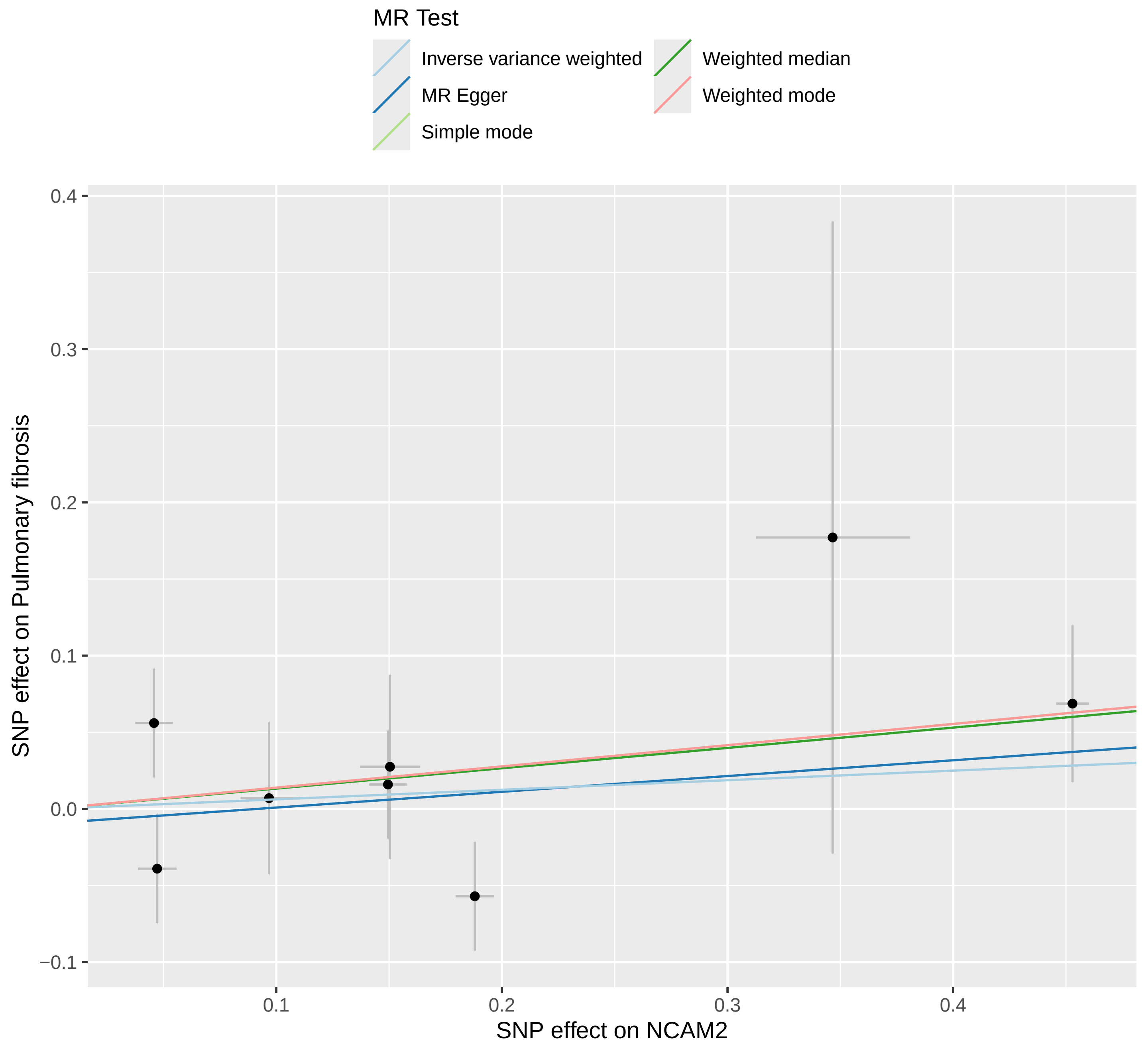
MR Test
Inverse variance weighted
MR Egger
Simple mode
Weighted median
Weighted mode
0.4
0.3
0.2
0.1
0.0
−0.1
0.1
0.2
0.3
0.4
SNP effect on Pulmonary fibrosis
SNP effect on NCAM2

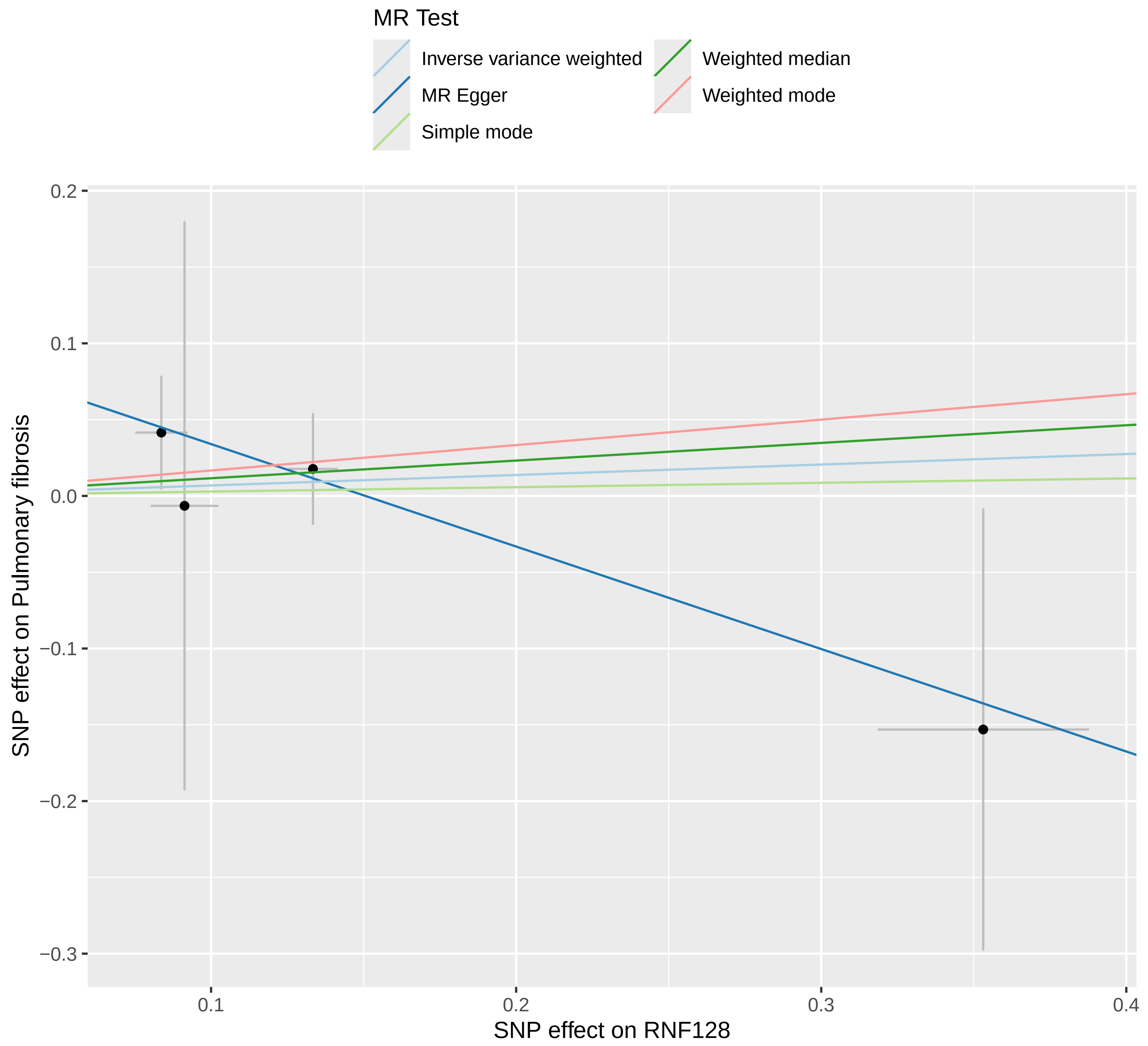
MR Test
Inverse variance weighted
MR Egger
Simple mode
Weighted median
Weighted mode
0.2
0.1
0.0
−0.1
−0.2
−0.3
0.1
0.2
0.3
0.4
SNP effect on RNF128
SNP effect on Pulmonary fibrosis

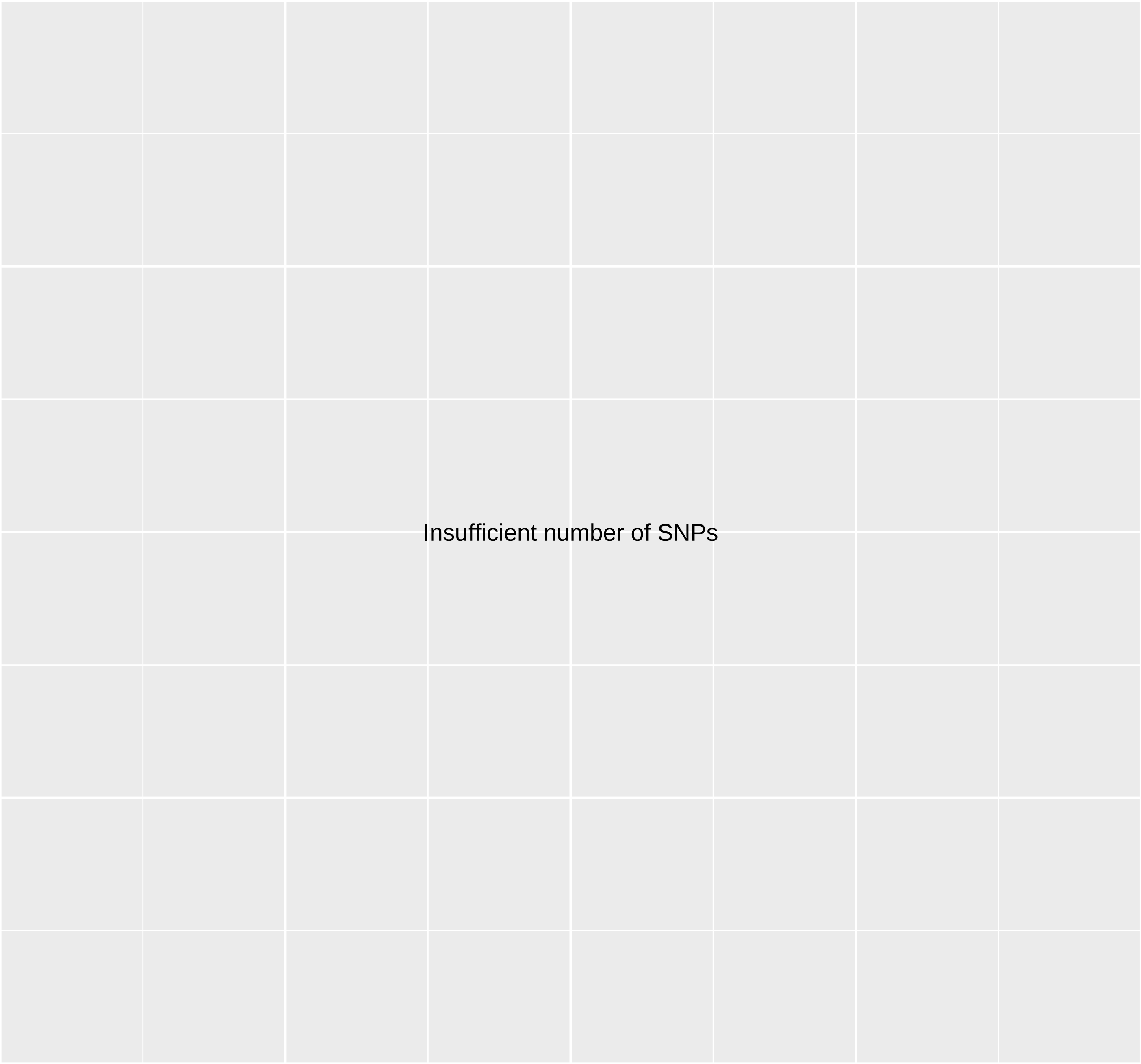
Insufficient number of SNPs

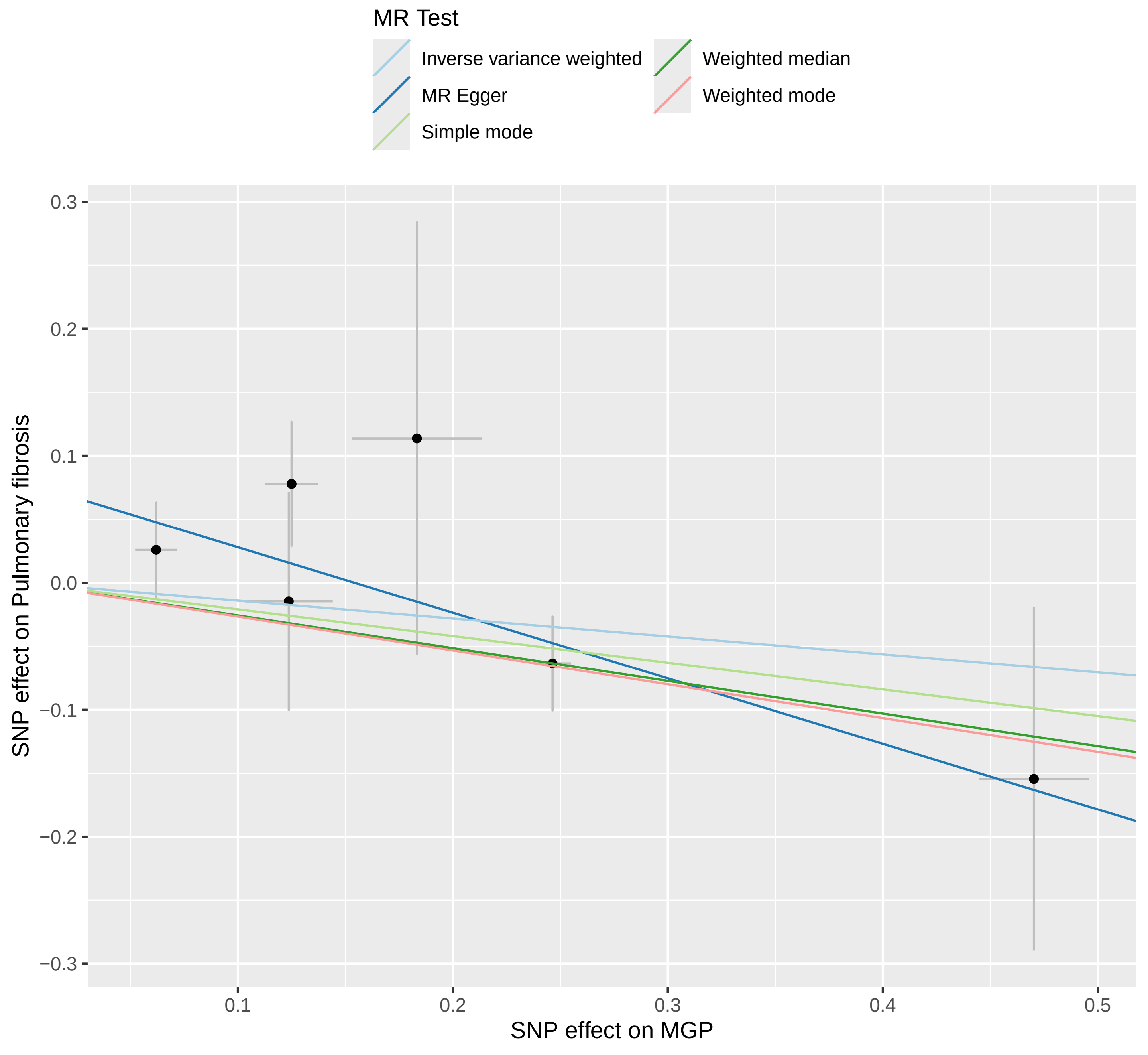

MR Test
Inverse variance weighted
MR Egger
Simple mode
Weighted median
Weighted mode
0.3
0.2
0.1
0.0
−0.1
−0.2
−0.3
0.1
0.2
0.3
0.4
0.5
SNP effect on Pulmonary fibrosis
SNP effect on MGP

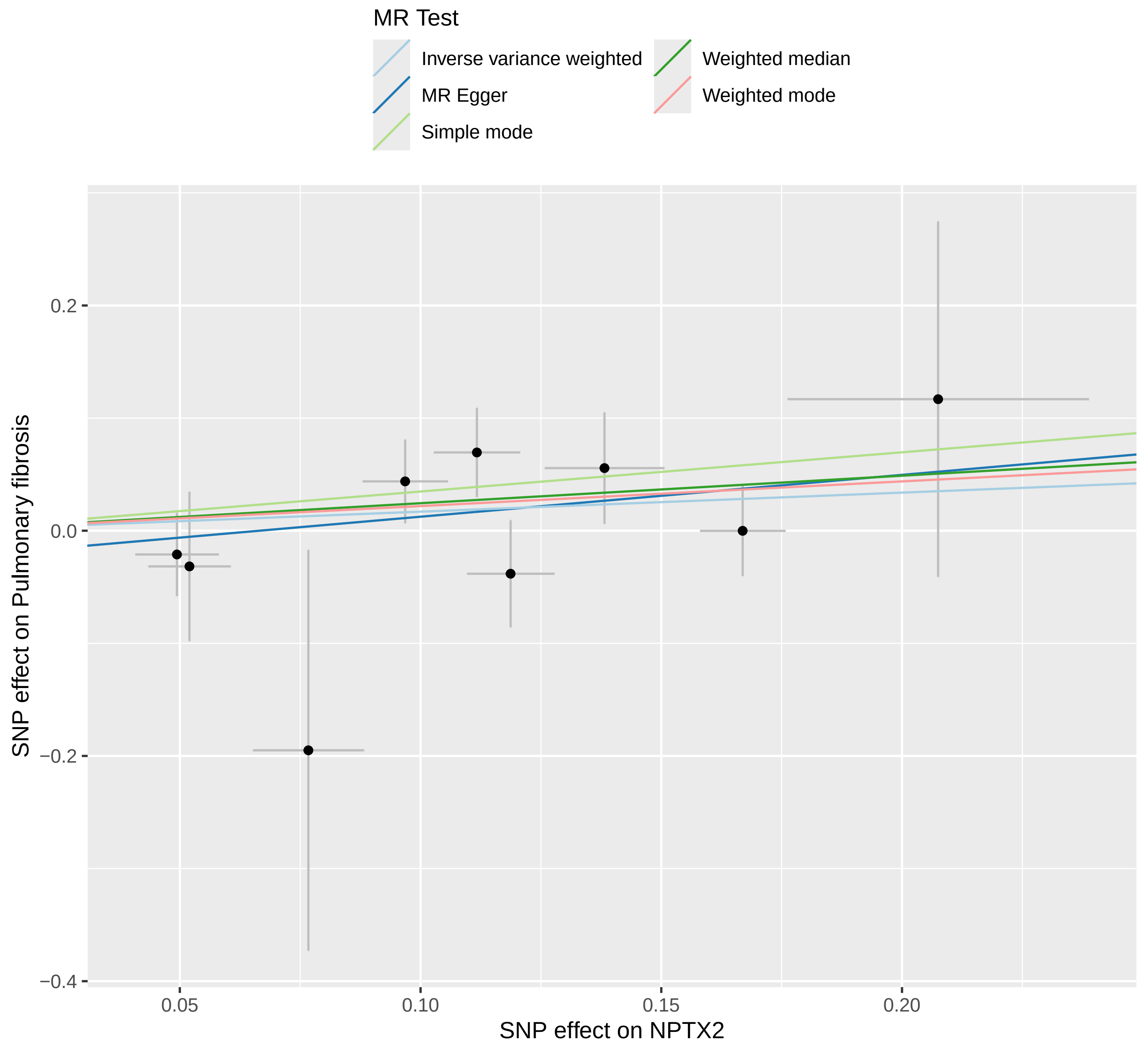
MR Test
Inverse variance weighted
MR Egger
Simple mode
Weighted median
Weighted mode
0.2
0.0
−0.2
−0.4
0.05
0.10
0.15
0.20
SNP effect on NPTX2
SNP effect on Pulmonary fibrosis

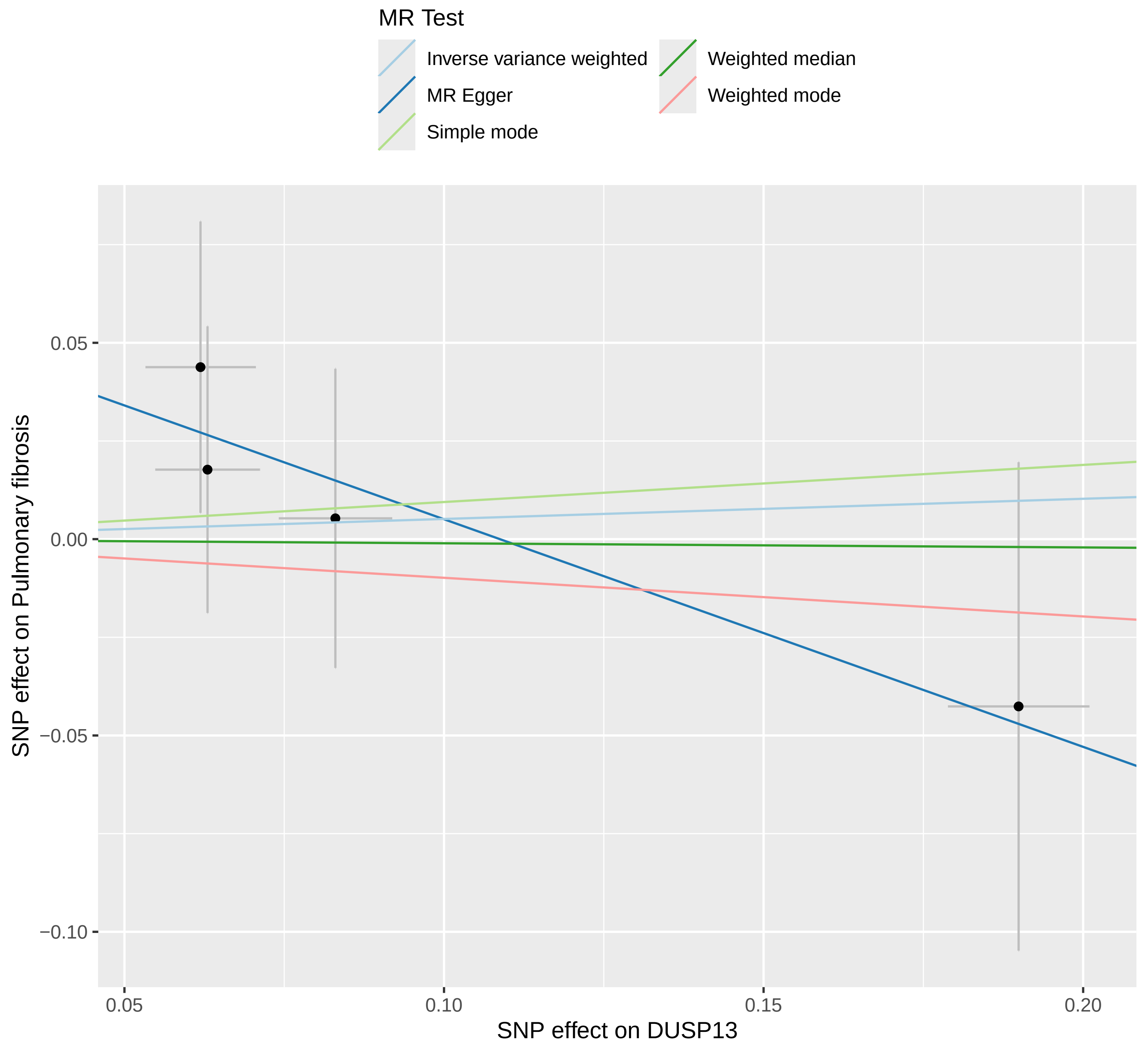
MR Test
Inverse variance weighted
MR Egger
Simple mode
Weighted median
Weighted mode
0.05
0.00
−0.05
−0.10
0.05
0.10
0.15
0.20
SNP effect on DUSP13
SNP effect on Pulmonary fibrosis

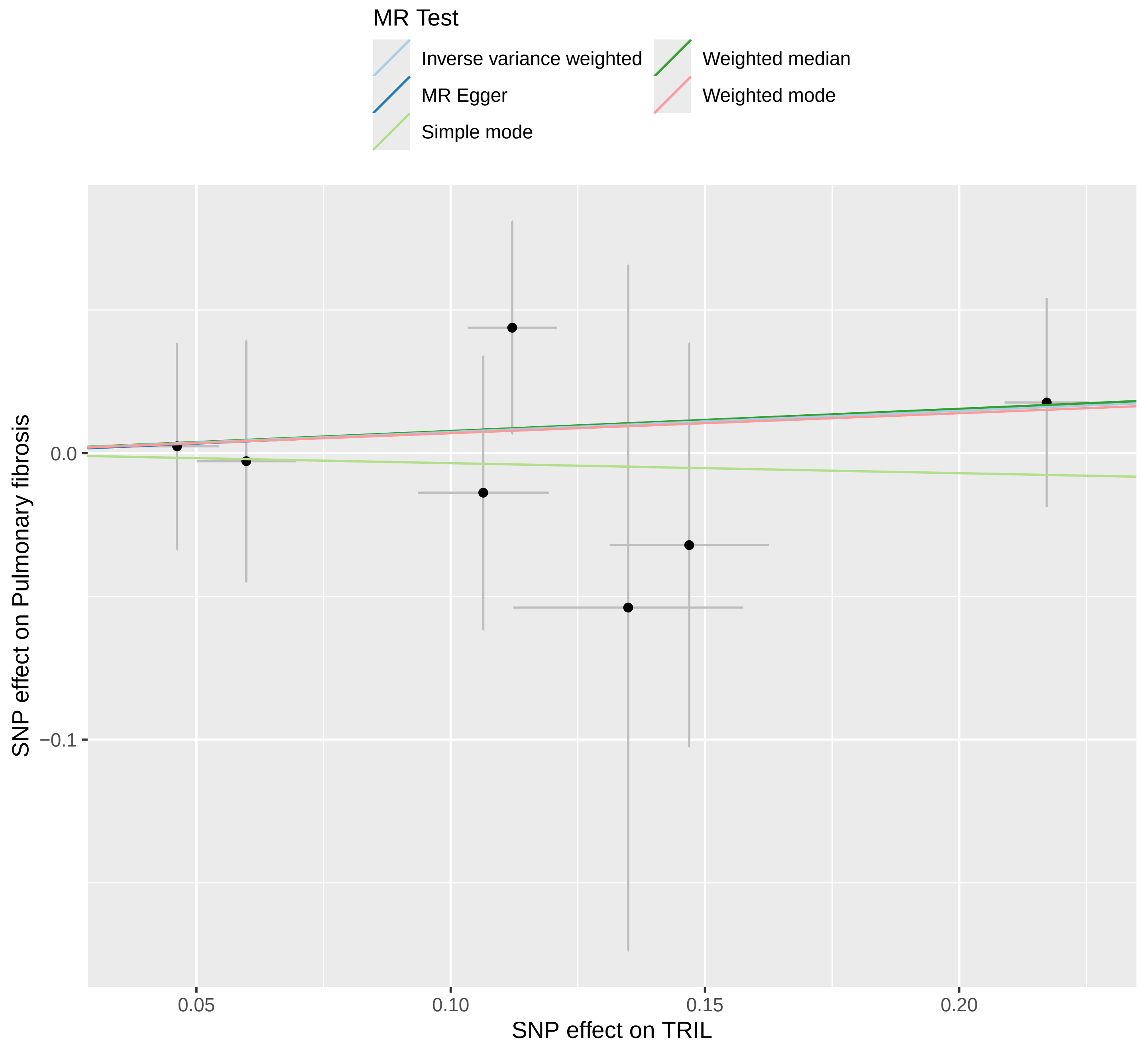
MR Test
Inverse variance weighted
MR Egger
Simple mode
Weighted median
Weighted mode
SNP effect on Pulmonary fibrosis
0.0
−0.1
0.05
0.10
0.15
0.20
SNP effect on TRIL

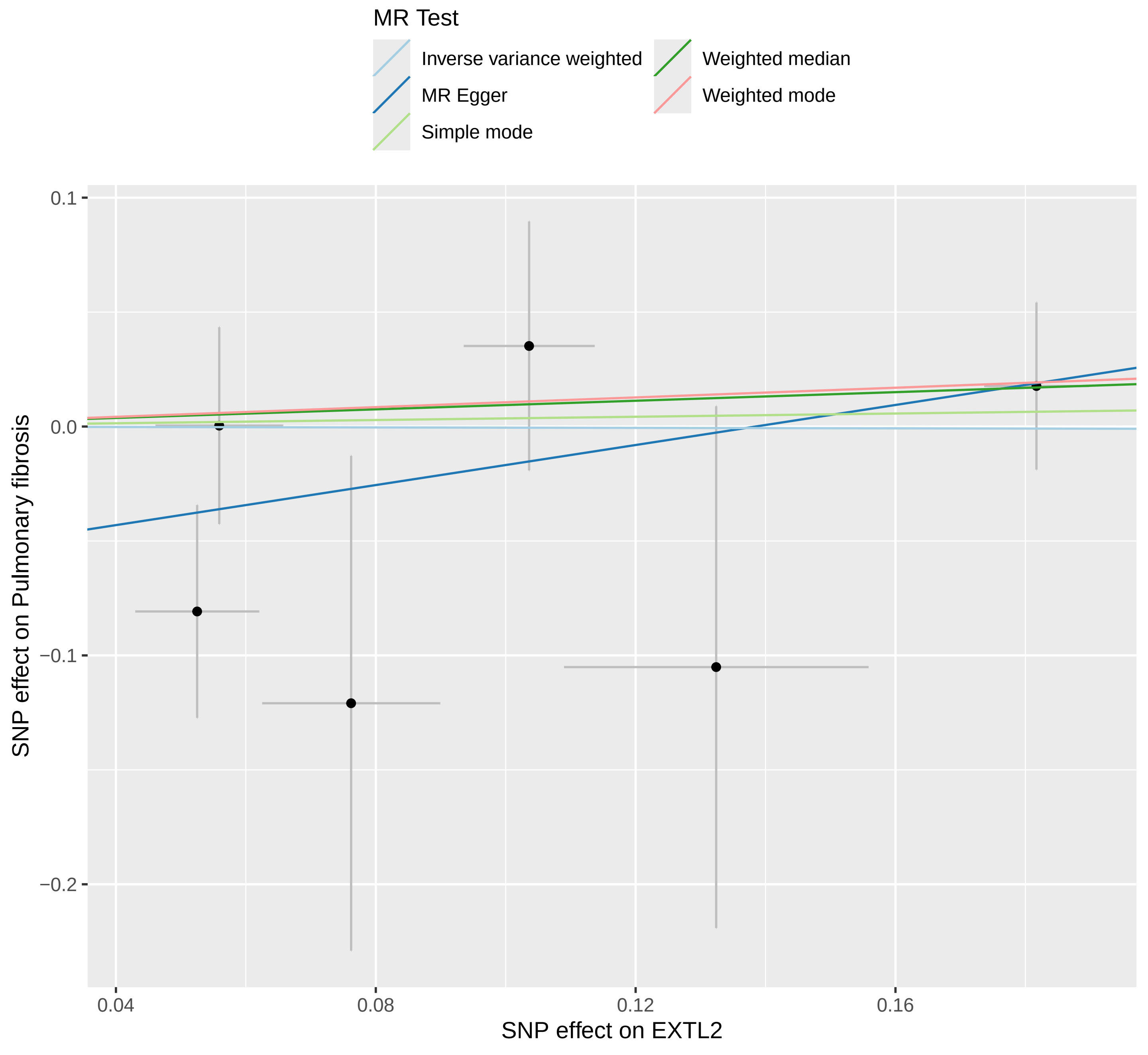
MR Test
Inverse variance weighted
MR Egger
Simple mode
Weighted median
Weighted mode
0.1
0.0
−0.1
−0.2
SNP effect on Pulmonary fibrosis
0.04
0.08
0.12
0.16
SNP effect on EXTL2

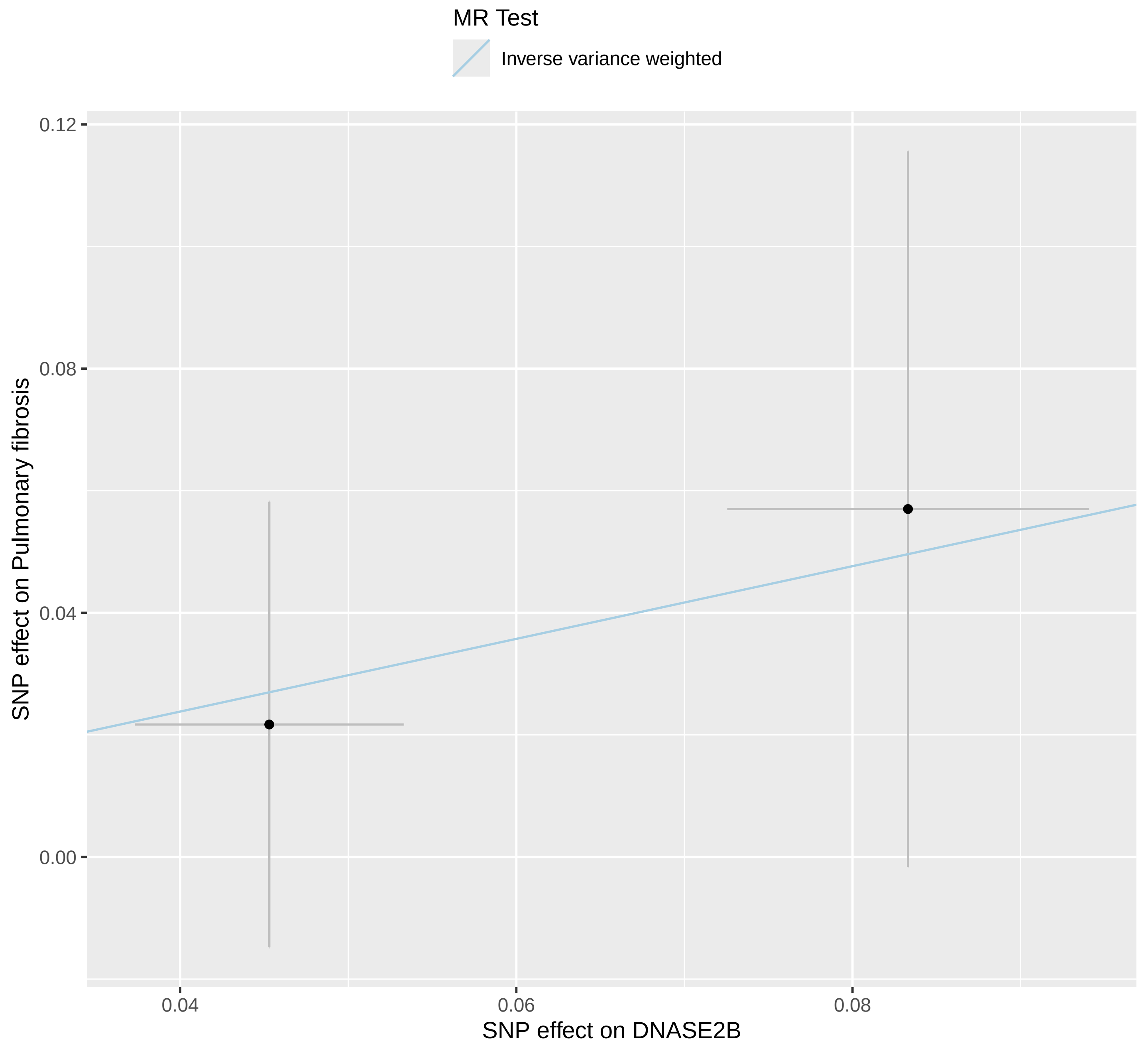
MR Test
Inverse variance weighted
0.12
0.08
0.04
0.00
SNP effect on Pulmonary fibrosis
0.04
0.06
0.08
SNP effect on DNASE2B

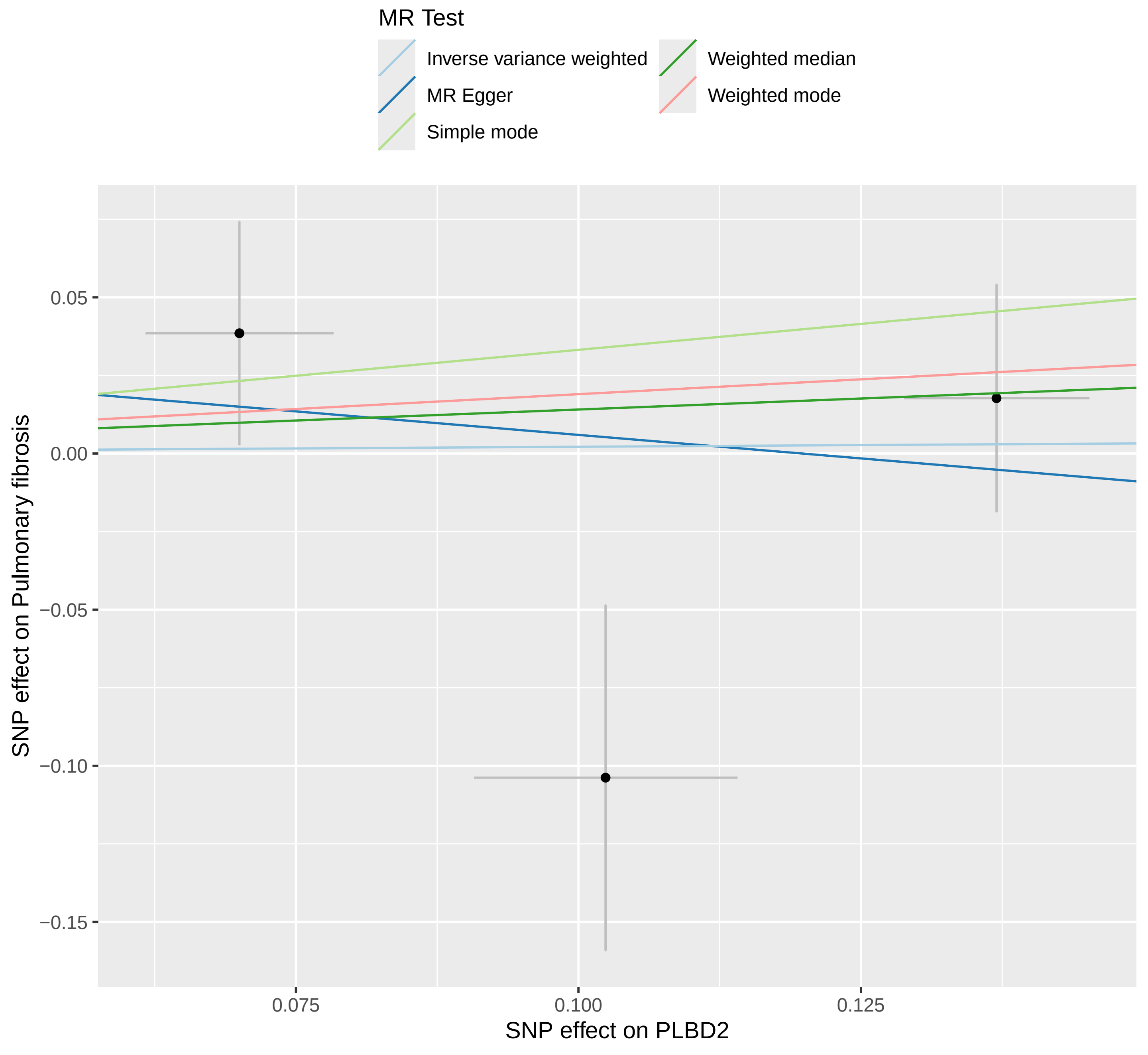

MR Test
Inverse variance weighted
MR Egger
Simple mode
Weighted median
Weighted mode
0.05
0.00
−0.05
−0.10
−0.15
SNP effect on Pulmonary fibrosis
0.075
0.100
0.125
SNP effect on PLBD2

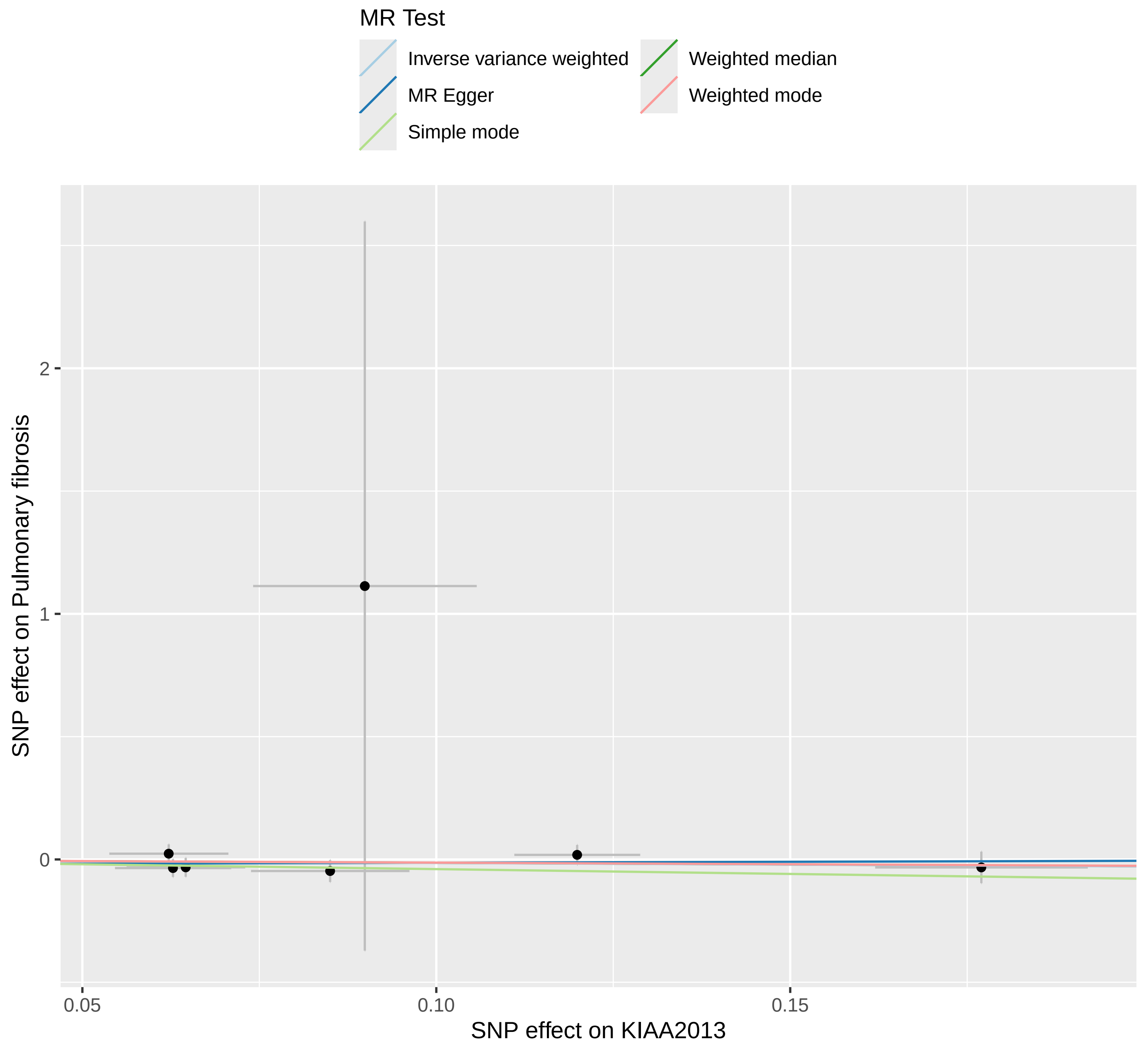

MR Test
Inverse variance weighted
MR Egger
Simple mode
Weighted median
Weighted mode
SNP effect on Pulmonary fibrosis
2
1
0
0.05
0.10
0.15
SNP effect on KIAA2013

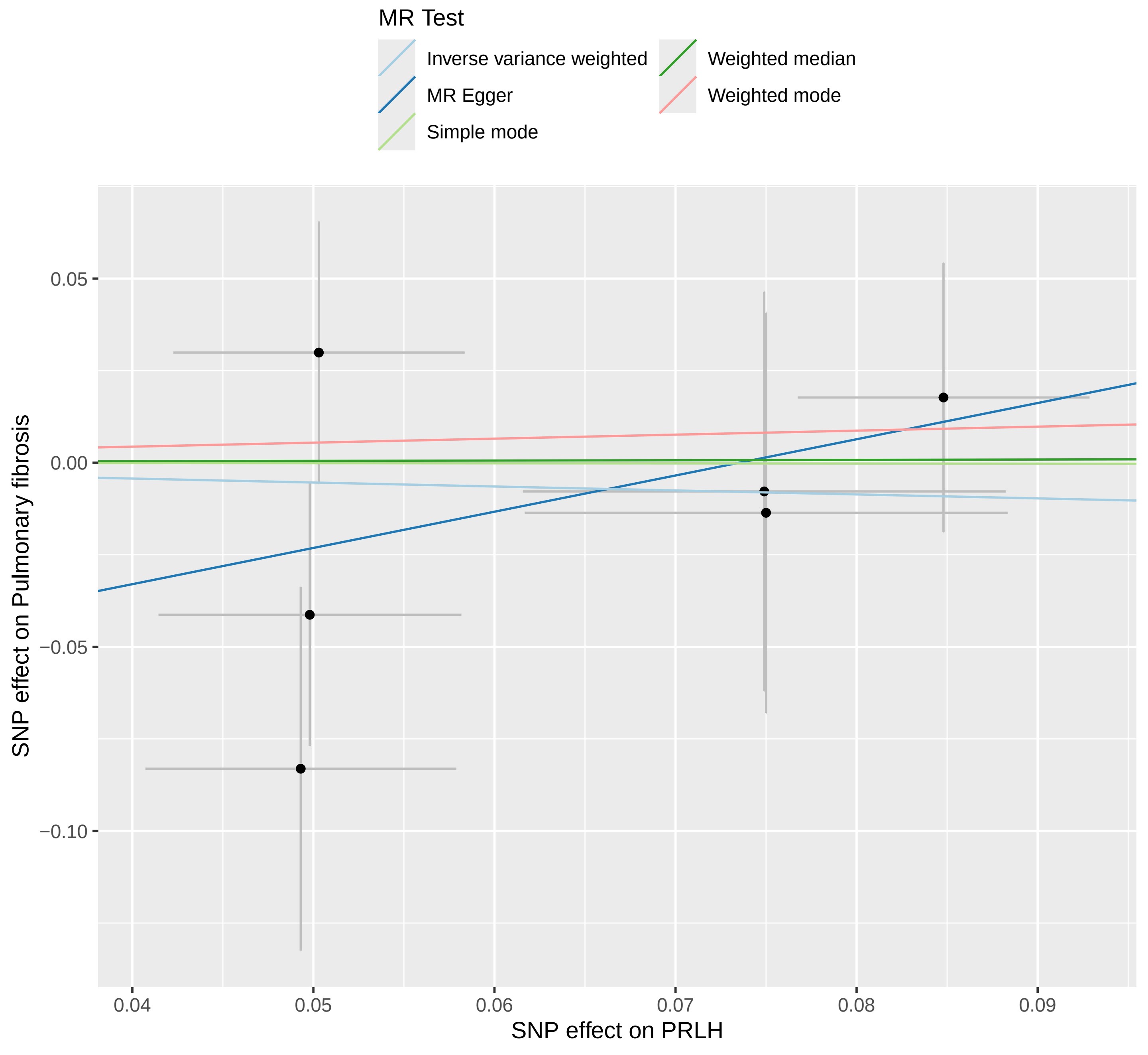

MR Test
Inverse variance weighted
MR Egger
Simple mode
Weighted median
Weighted mode
SNP effect on Pulmonary fibrosis
0.05
0.00
−0.05
−0.10
0.04
0.05
0.06
0.07
0.08
0.09
SNP effect on PRLH

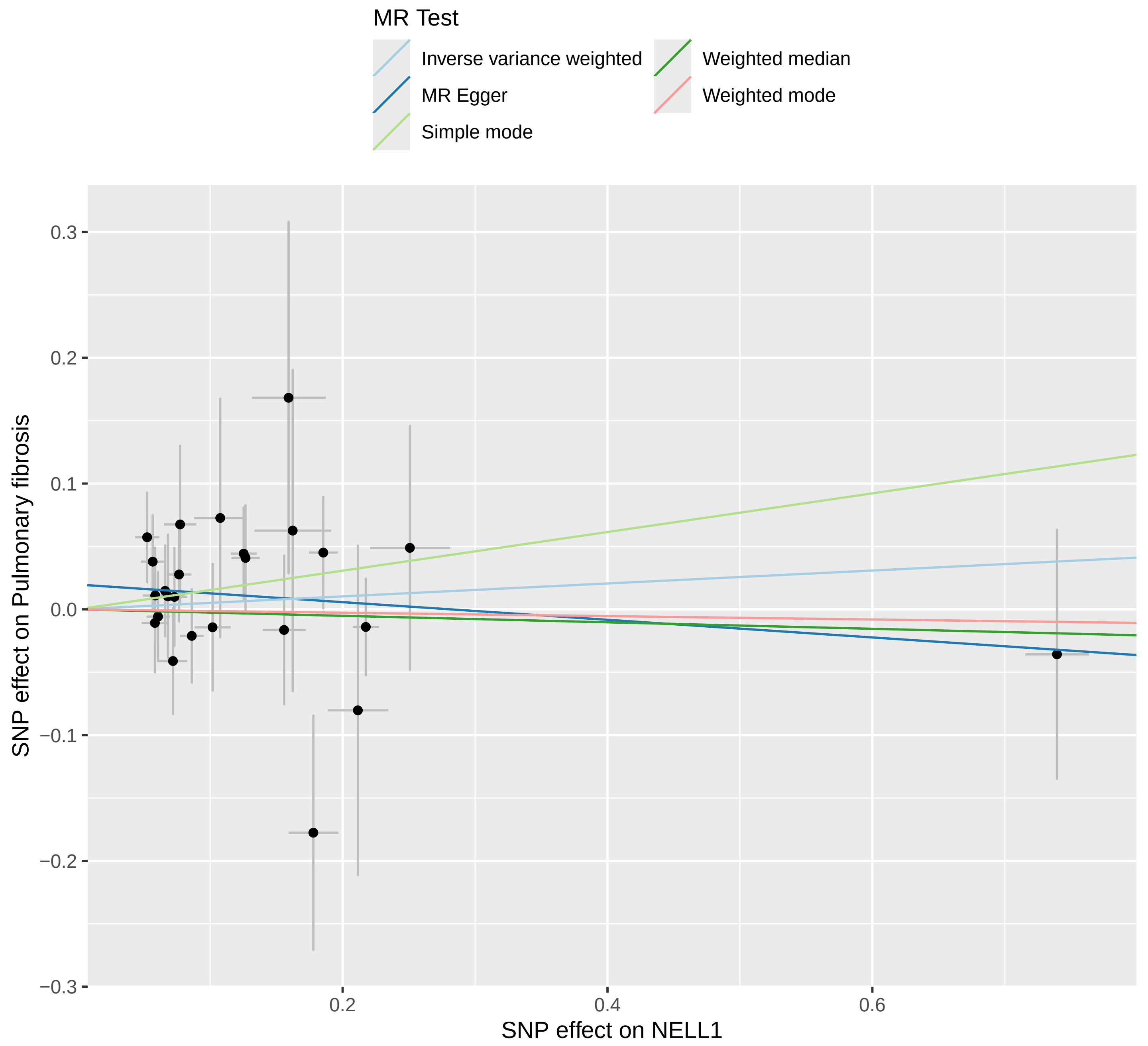

MR Test
Inverse variance weighted
MR Egger
Simple mode
Weighted median
Weighted mode
SNP effect on Pulmonary fibrosis
0.3
0.2
0.1
0.0
−0.1
−0.2
−0.3
0.2
0.4
0.6
SNP effect on NELL1

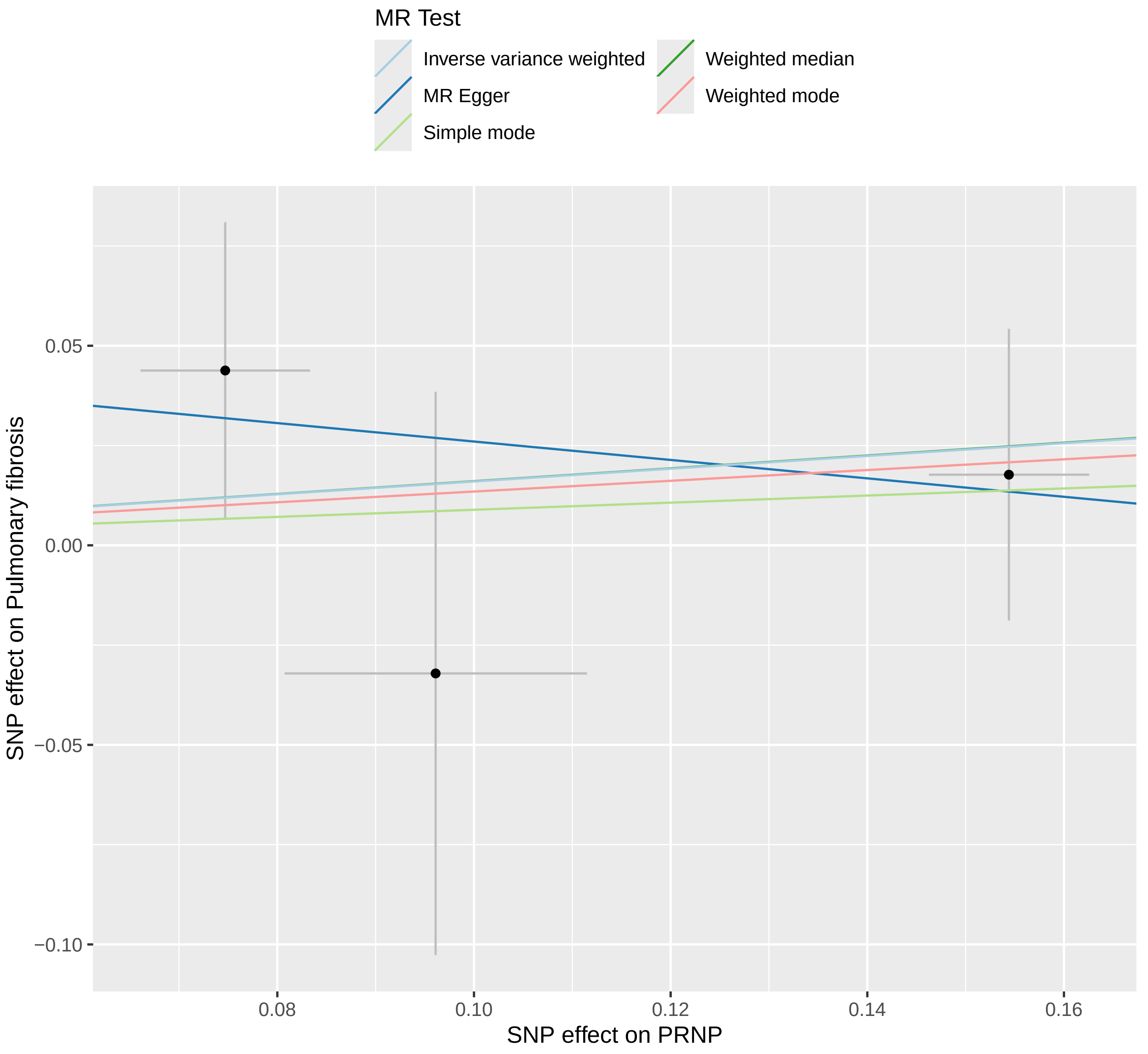
MR Test
Inverse variance weighted
MR Egger
Simple mode
Weighted median
Weighted mode
0.05
0.00
−0.05
−0.10
0.08
0.10
0.12
0.14
0.16
SNP effect on PRNP
SNP effect on Pulmonary fibrosis

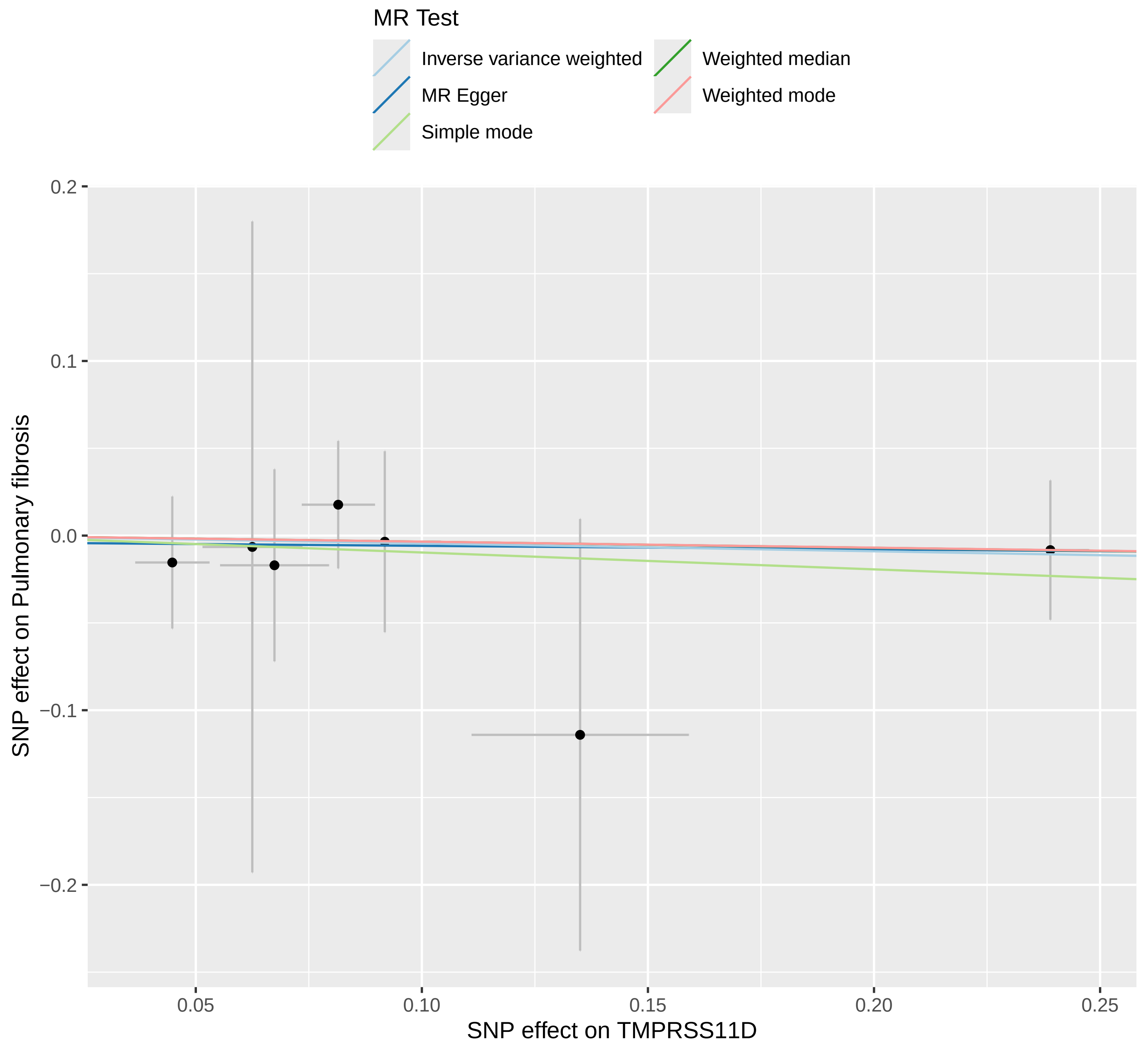

MR Test
Inverse variance weighted
MR Egger
Simple mode
Weighted median
Weighted mode
0.2
0.1
0.0
−0.1
−0.2
SNP effect on Pulmonary fibrosis
0.05
0.10
0.15
0.20
0.25
SNP effect on TMPRSS11D

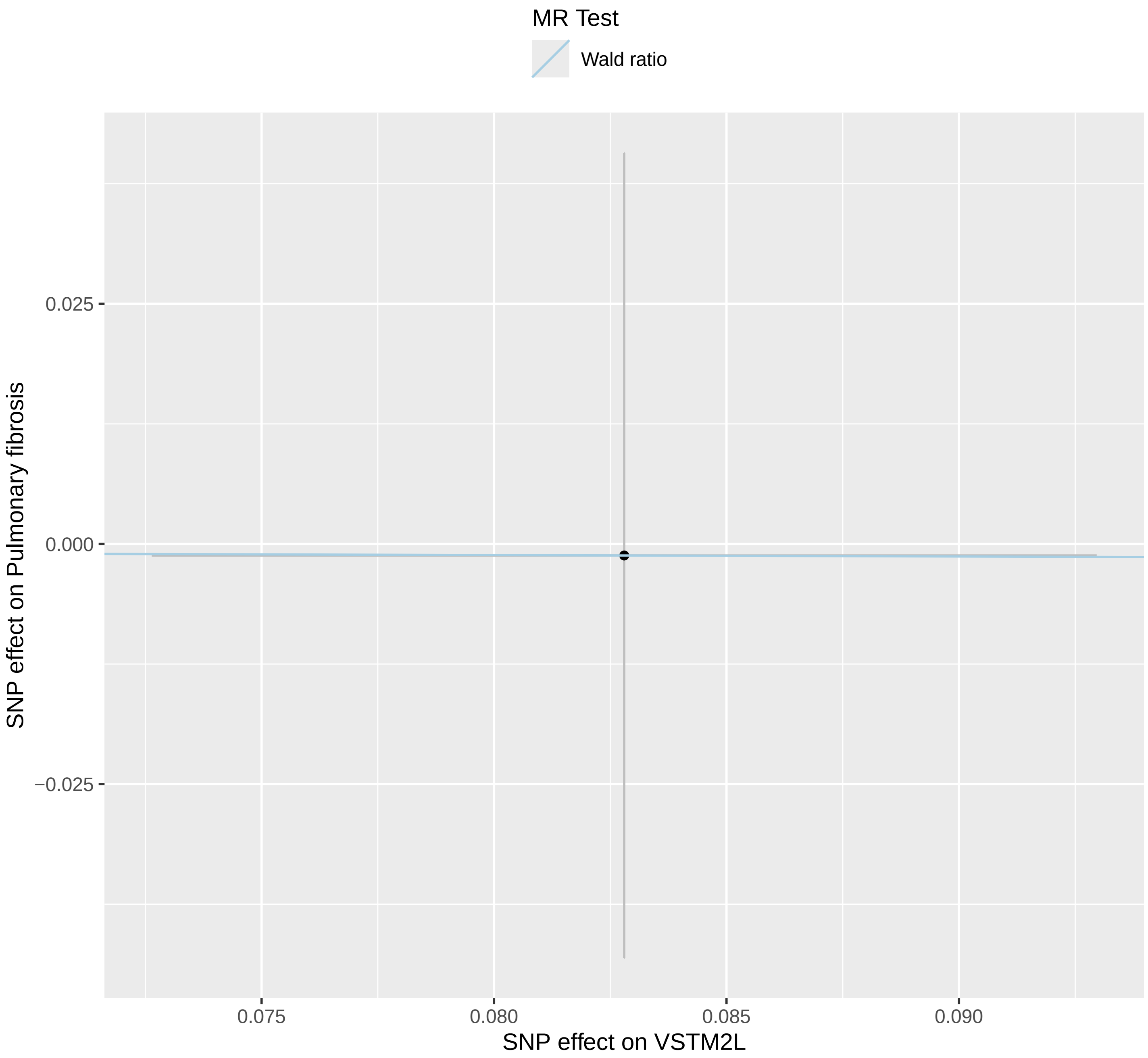
MR Test
Wald ratio
0.025
0.000
−0.025
0.075
0.080
0.085
0.090
SNP effect on Pulmonary fibrosis
SNP effect on VSTM2L

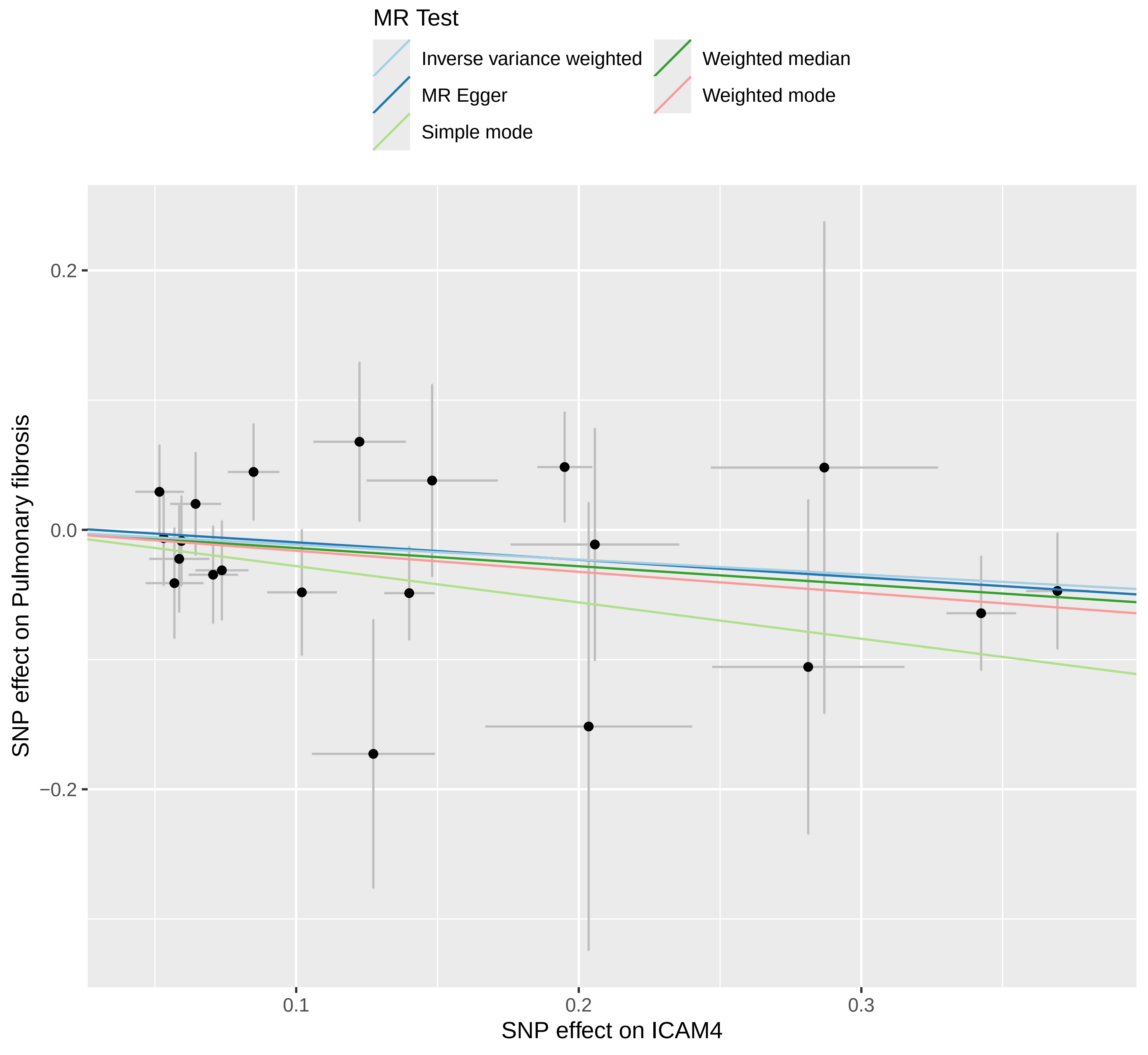

MR Test
Inverse variance weighted
MR Egger
Simple mode
Weighted median
Weighted mode
0.2
0.0
−0.2
0.1
0.2
0.3
SNP effect on Pulmonary fibrosis
SNP effect on ICAM4

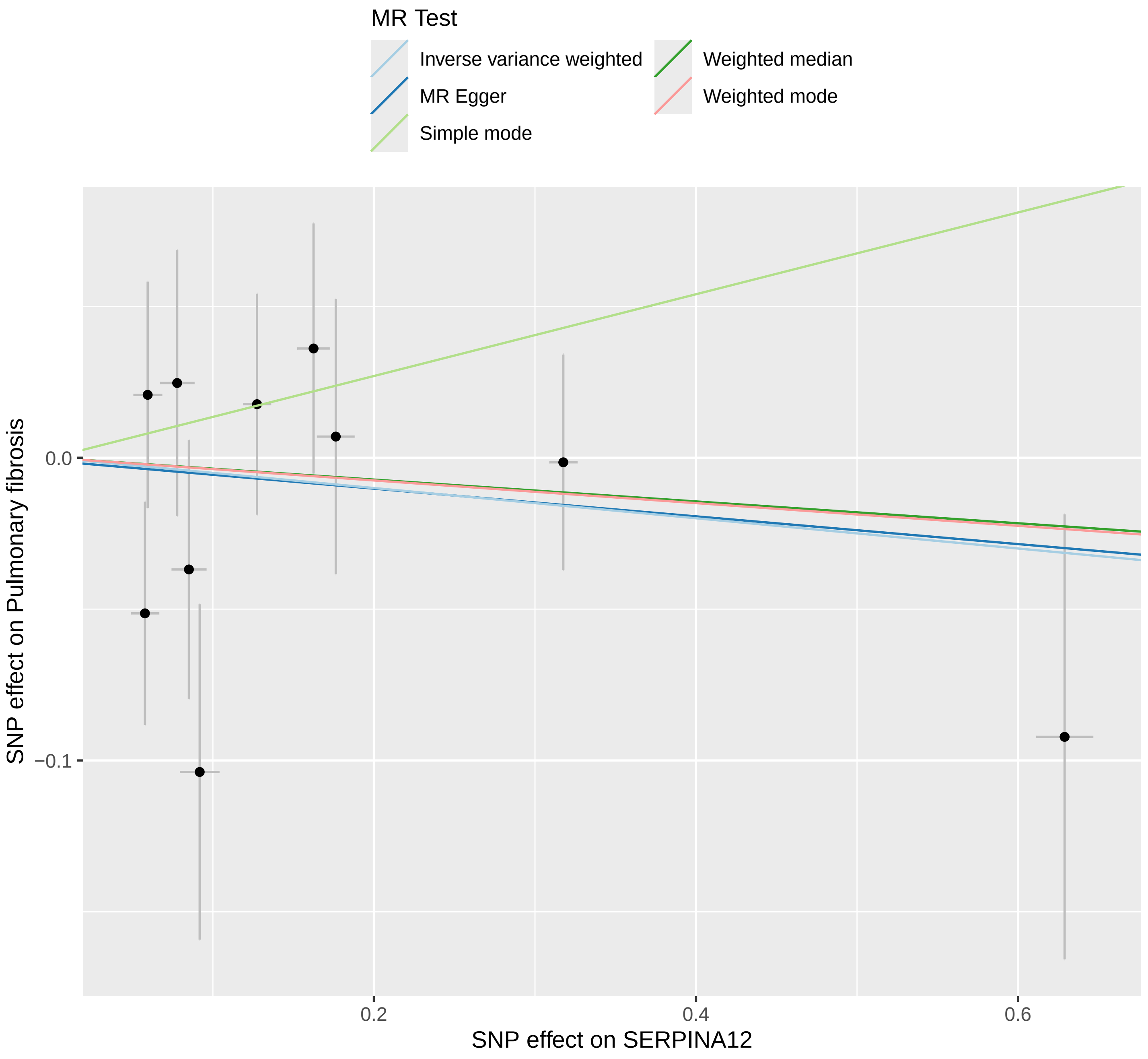
MR Test
Inverse variance weighted
MR Egger
Simple mode
Weighted median
Weighted mode
0.0
−0.1
0.2
0.4
0.6
SNP effect on Pulmonary fibrosis
SNP effect on SERPINA12

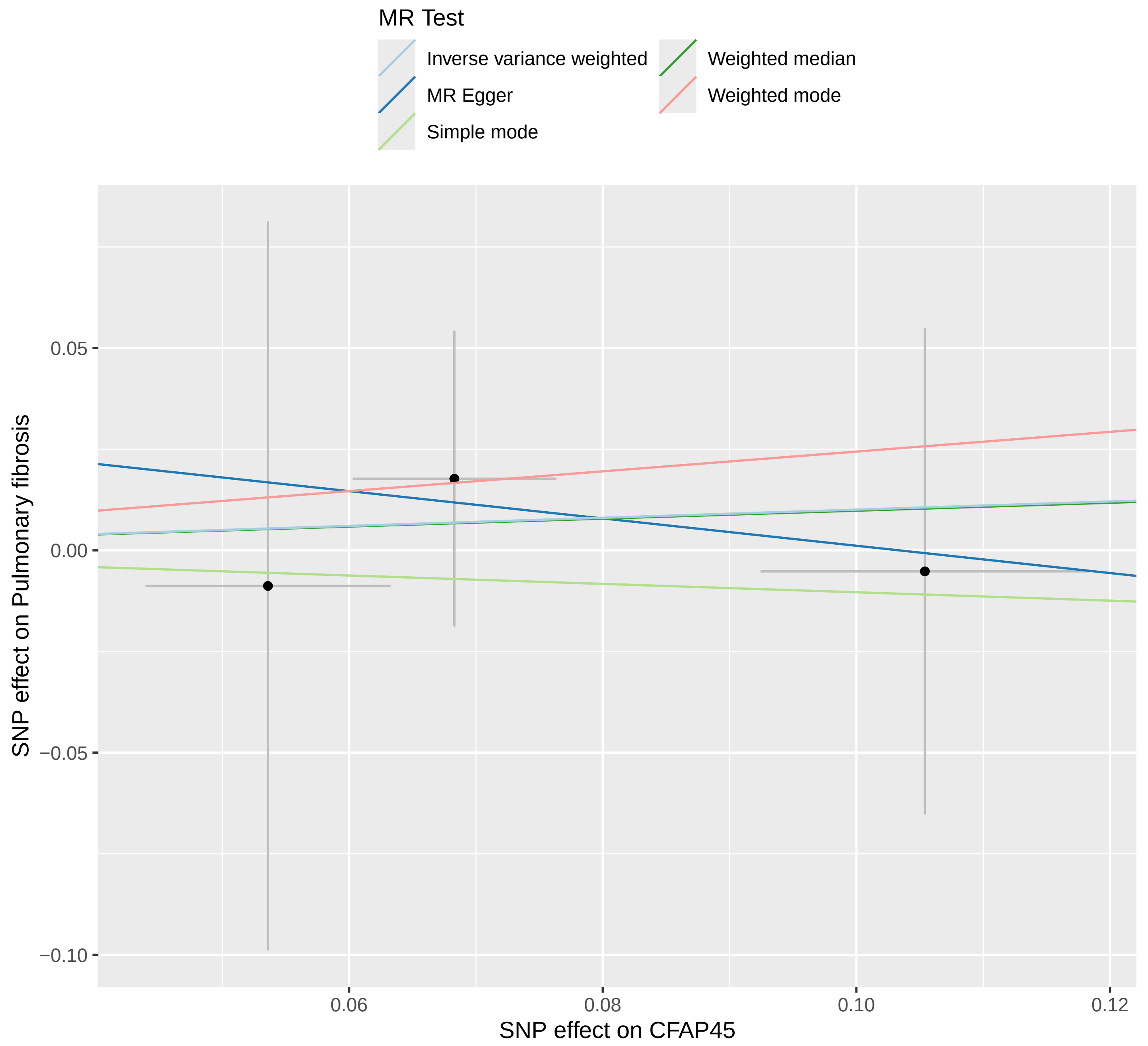

MR Test
Inverse variance weighted
MR Egger
Simple mode
Weighted median
Weighted mode
0.05
0.00
−0.05
−0.10
0.06
0.08
0.10
0.12
SNP effect on Pulmonary fibrosis
SNP effect on CFAP45

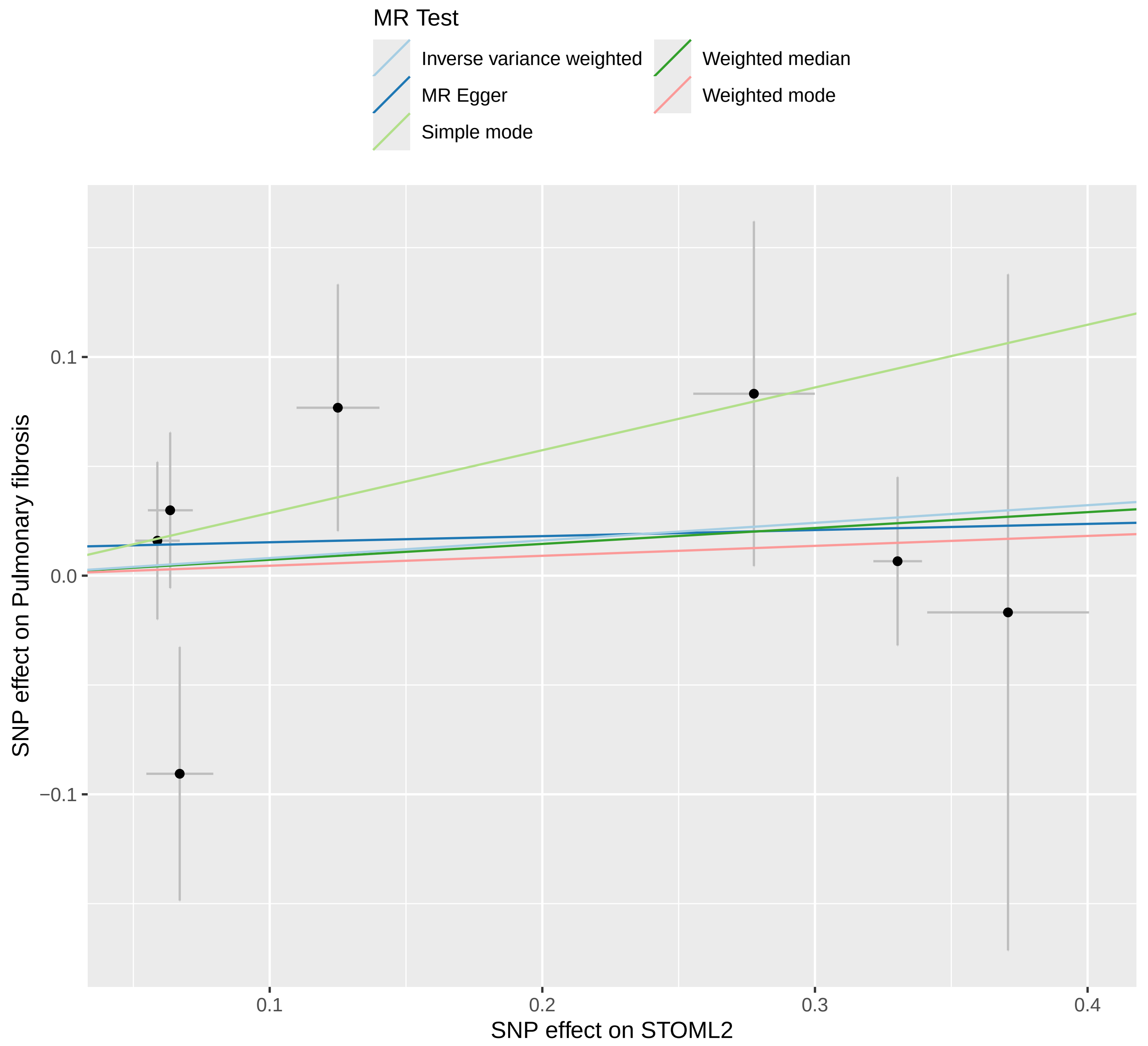

MR Test
Inverse variance weighted
MR Egger
Simple mode
Weighted median
Weighted mode
0.1
0.0
−0.1
SNP effect on Pulmonary fibrosis
0.1
0.2
0.3
0.4
SNP effect on STOML2

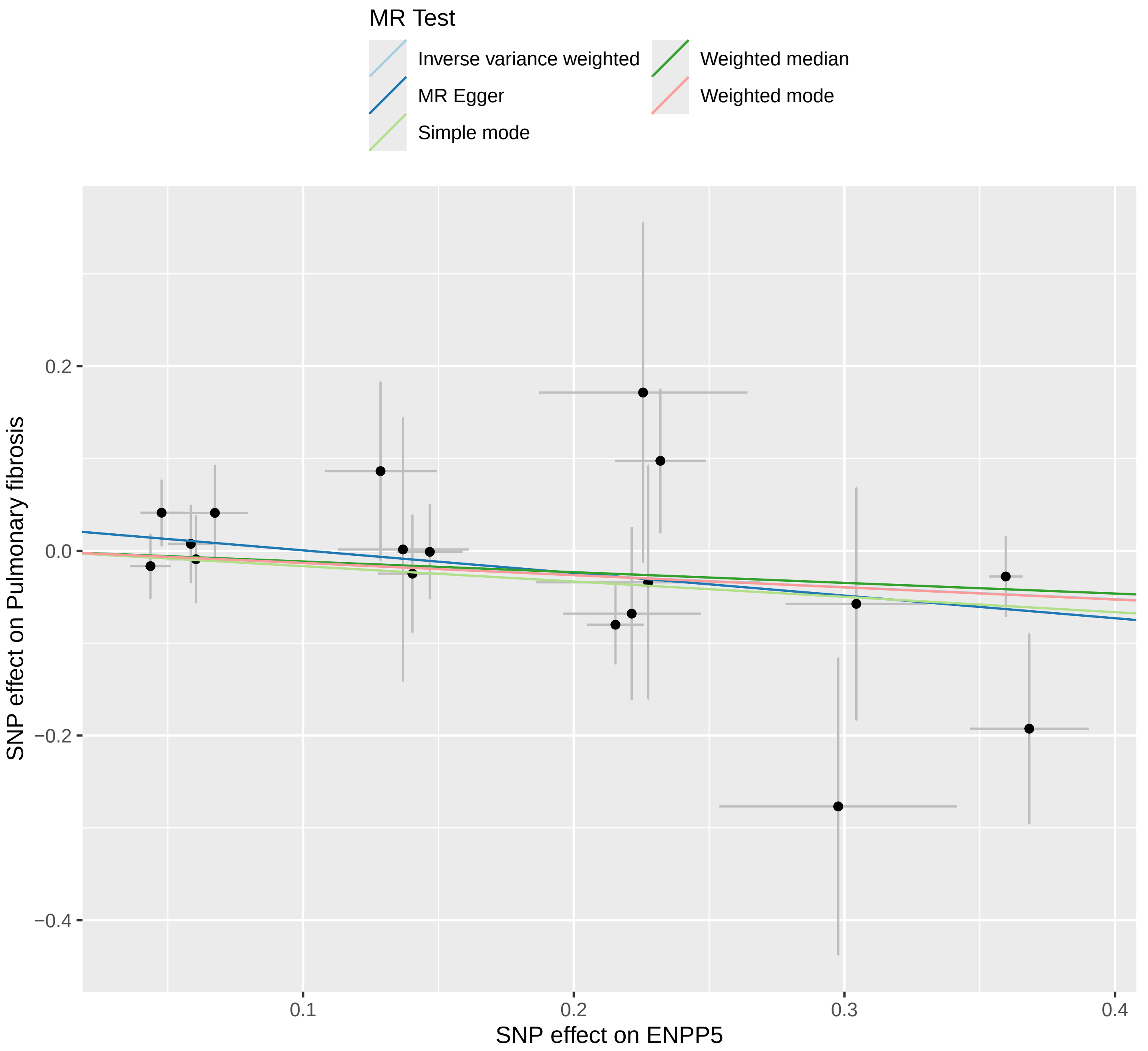

MR Test
Inverse variance weighted
MR Egger
Simple mode
Weighted median
Weighted mode
0.2
0.0
−0.2
−0.4
0.1
0.2
0.3
0.4
SNP effect on Pulmonary fibrosis
SNP effect on ENPP5

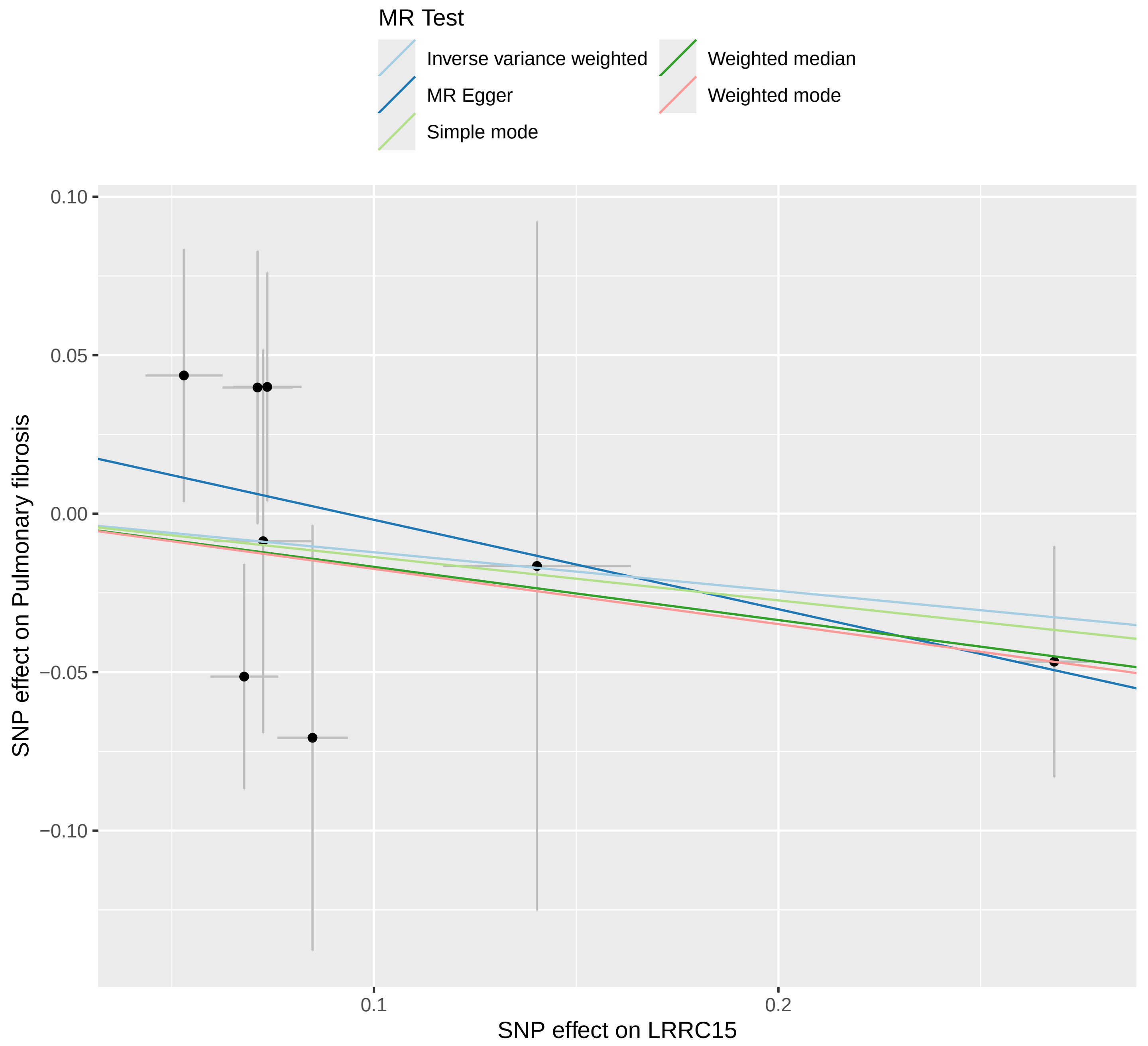

MR Test
Inverse variance weighted
MR Egger
Simple mode
Weighted median
Weighted mode
0.10
0.05
0.00
−0.05
−0.10
SNP effect on Pulmonary fibrosis
0.1
0.2
SNP effect on LRRC15

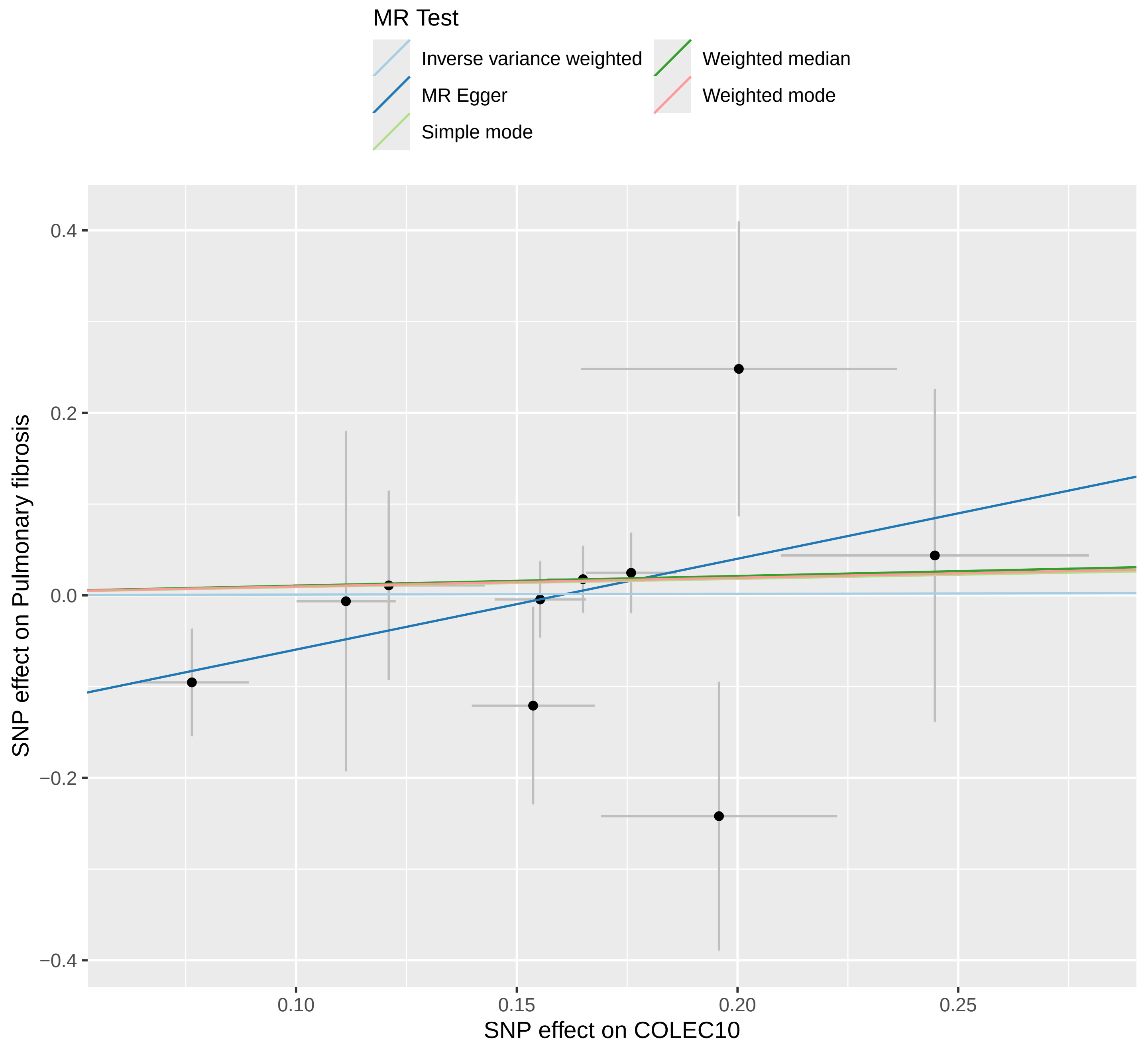

MR Test
Inverse variance weighted
MR Egger
Simple mode
Weighted median
Weighted mode
0.4
0.2
0.0
−0.2
−0.4
SNP effect on Pulmonary fibrosis
0.10
0.15
0.20
0.25
SNP effect on COLEC10

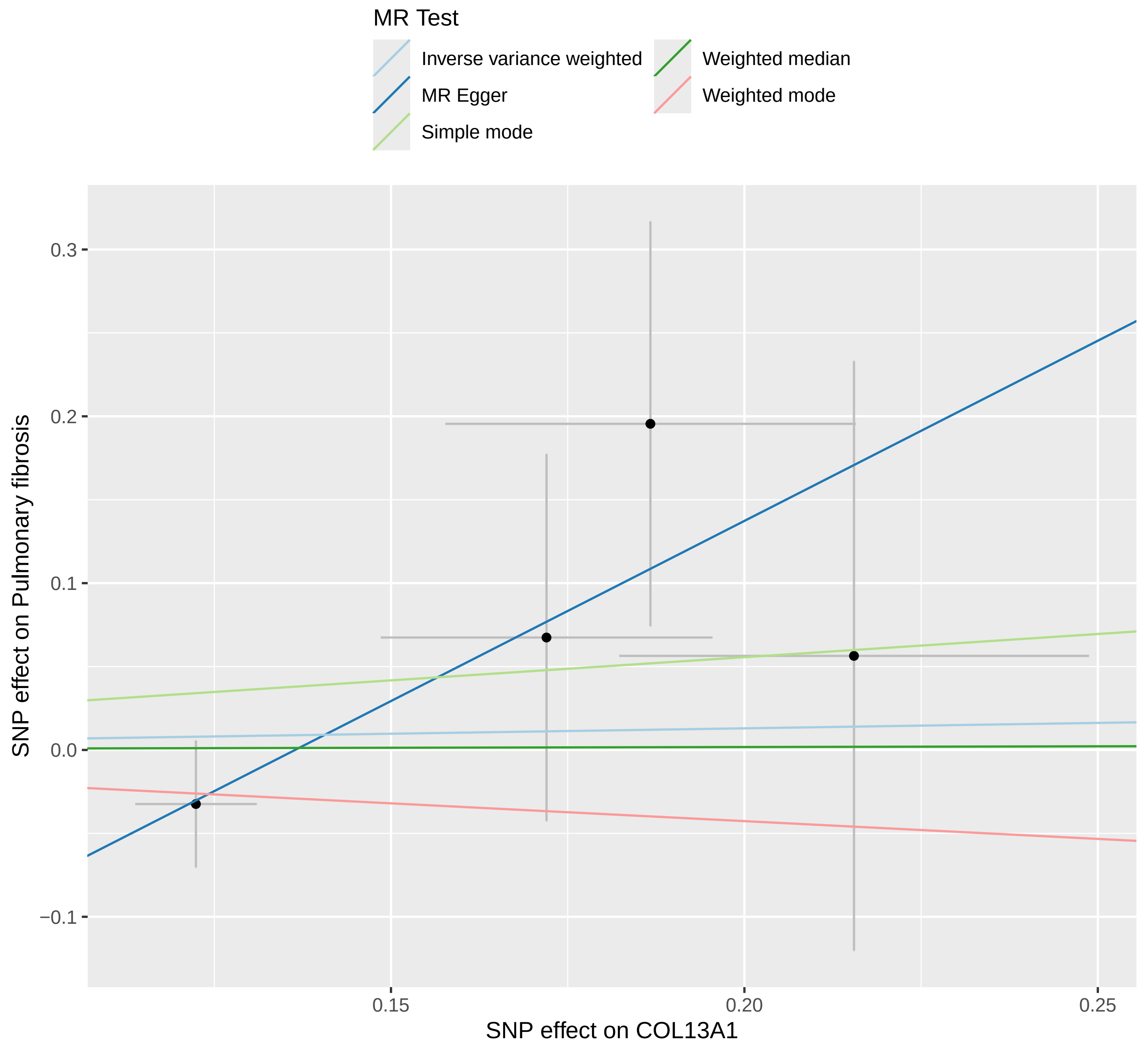

MR Test
Inverse variance weighted
MR Egger
Simple mode
Weighted median
Weighted mode
0.3
0.2
0.1
0.0
−0.1
0.15
0.20
0.25
SNP effect on Pulmonary fibrosis
SNP effect on COL13A1

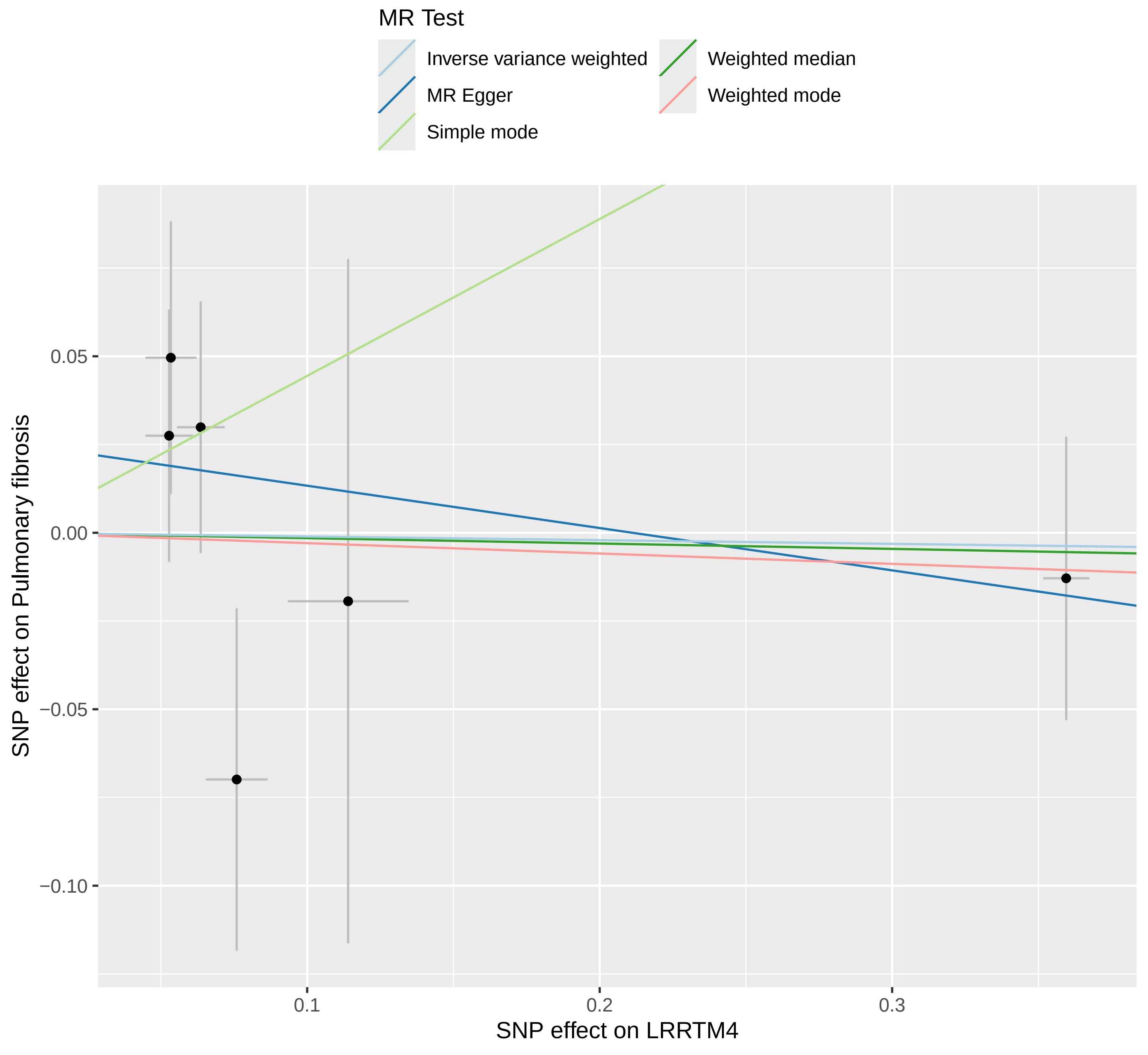

MR Test
Inverse variance weighted
MR Egger
Simple mode
Weighted median
Weighted mode
SNP effect on Pulmonary fibrosis
0.05
0.00
−0.05
−0.10
0.1
0.2
0.3
SNP effect on LRRTM4

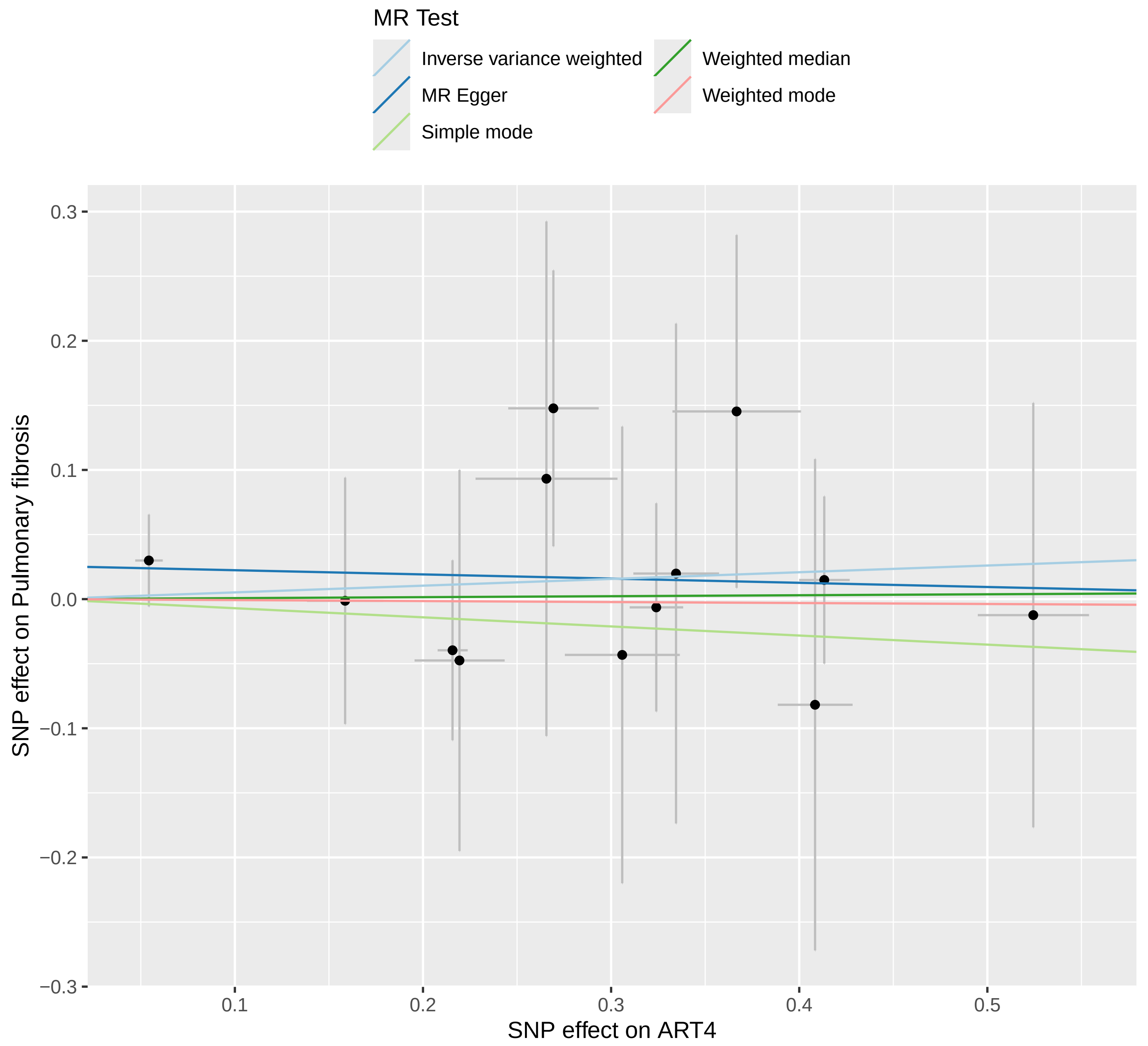
MR Test
Inverse variance weighted
MR Egger
Simple mode
Weighted median
Weighted mode
0.3
0.2
0.1
0.0
−0.1
−0.2
−0.3
0.1
0.2
0.3
0.4
0.5
SNP effect on Pulmonary fibrosis
SNP effect on ART4

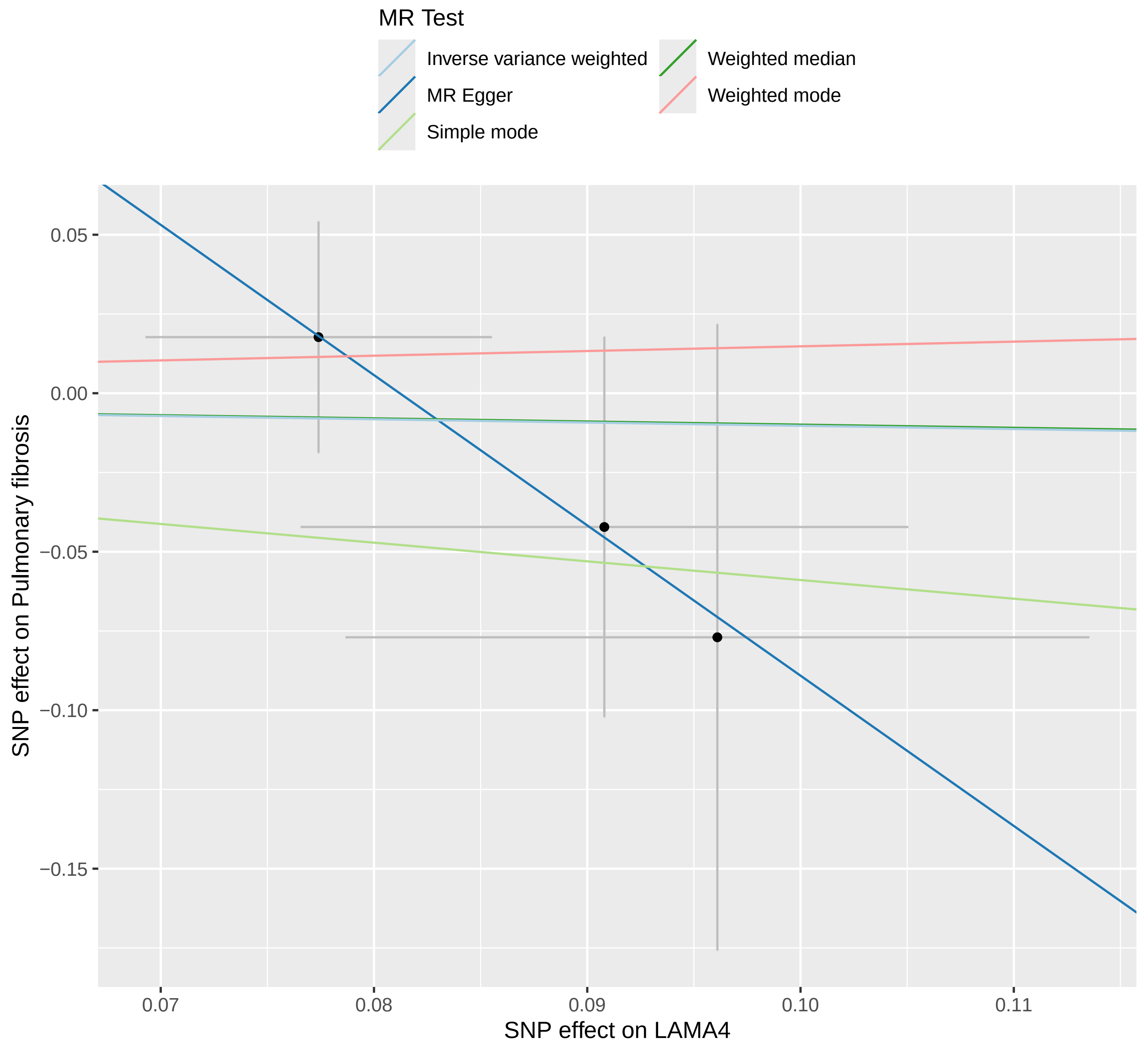
MR Test
Inverse variance weighted
MR Egger
Simple mode
Weighted median
Weighted mode
0.05
0.00
−0.05
−0.10
−0.15
SNP effect on Pulmonary fibrosis
0.07
0.08
0.09
0.10
0.11
SNP effect on LAMA4

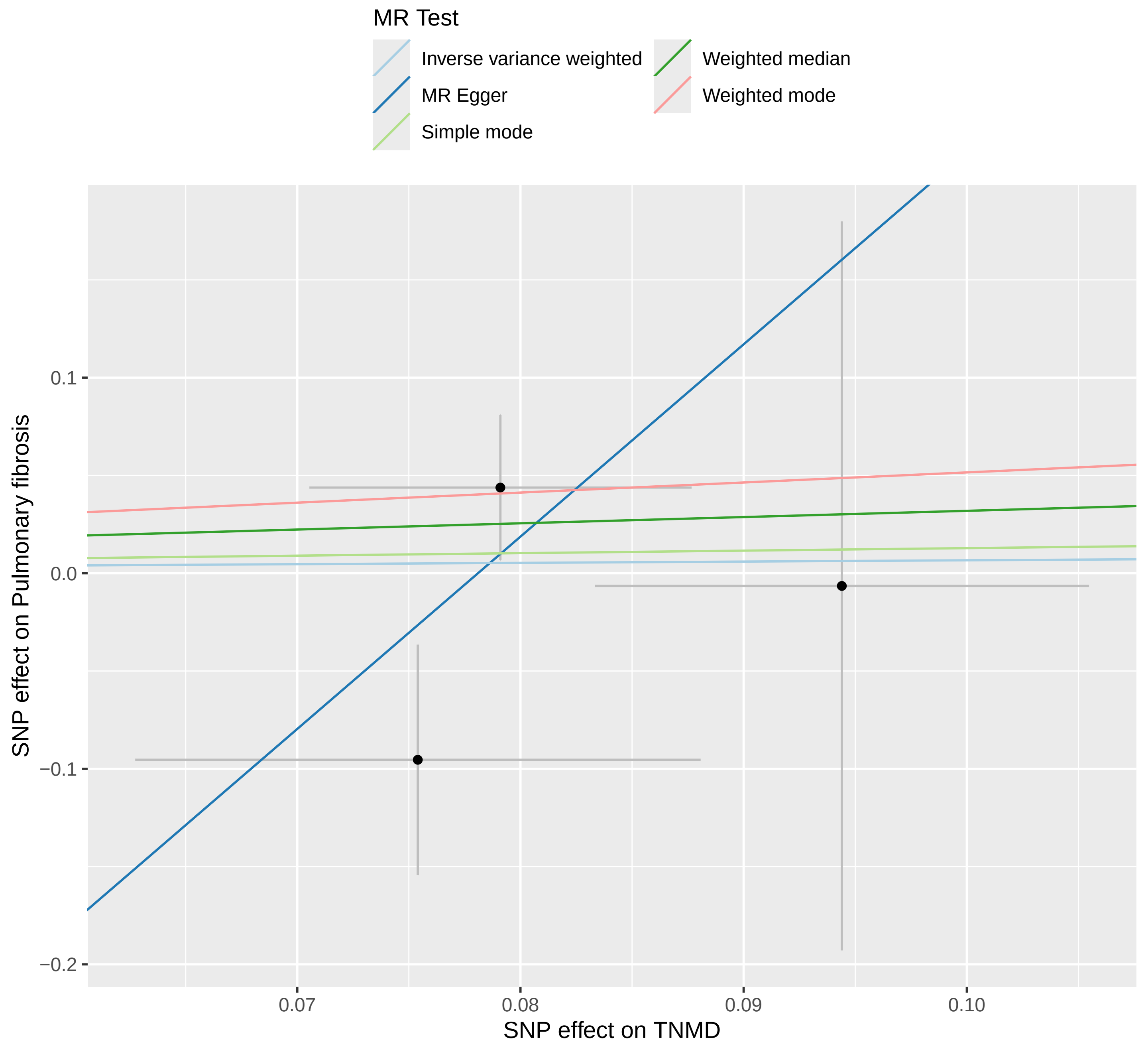

MR Test
Inverse variance weighted
MR Egger
Simple mode
Weighted median
Weighted mode
0.1
0.0
−0.1
−0.2
SNP effect on Pulmonary fibrosis
0.07
0.08
0.09
0.10
SNP effect on TNMD

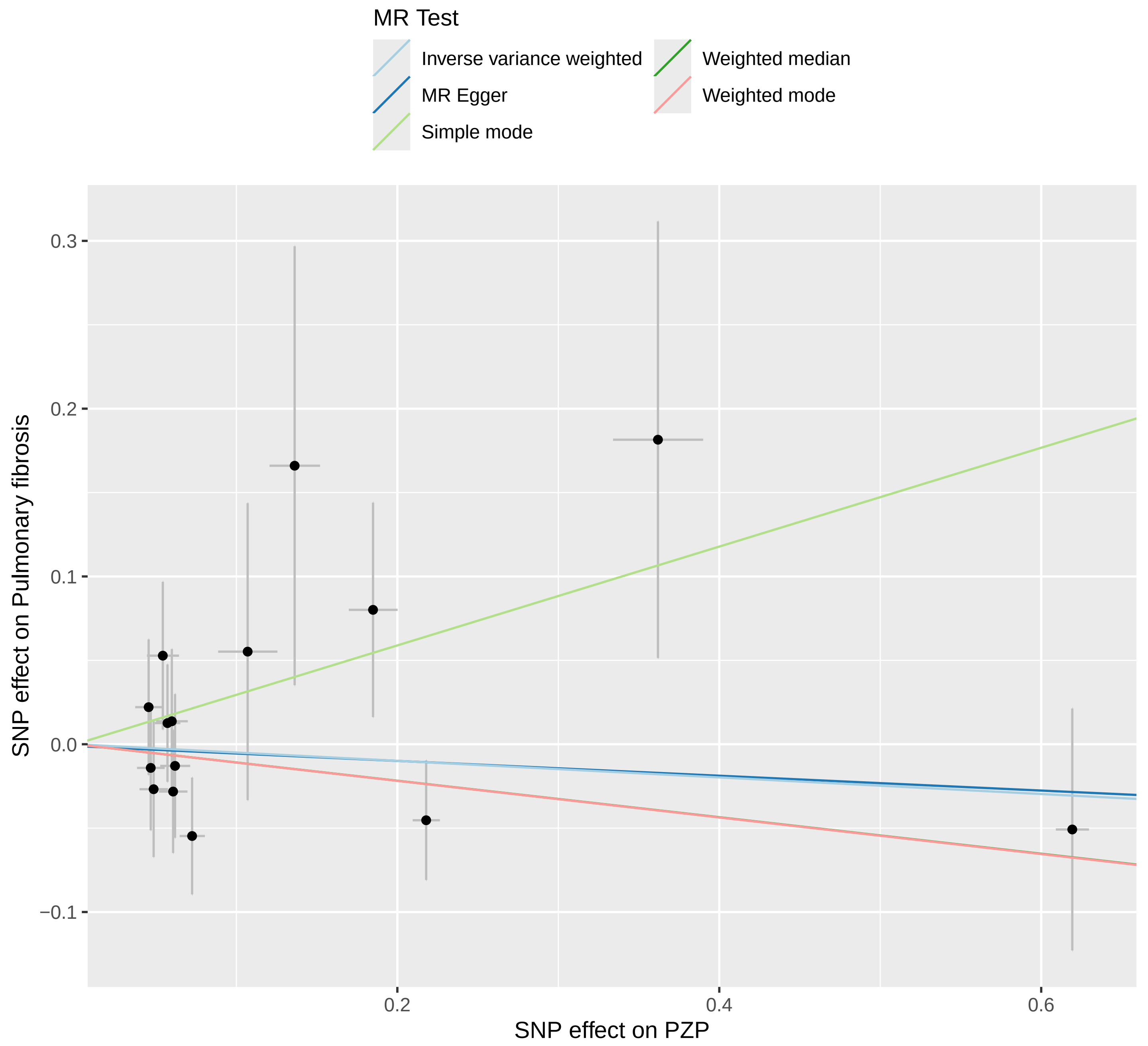

MR Test
Inverse variance weighted
MR Egger
Simple mode
Weighted median
Weighted mode
SNP effect on Pulmonary fibrosis
0.3
0.2
0.1
0.0
−0.1
0.2
0.4
0.6
SNP effect on PZP

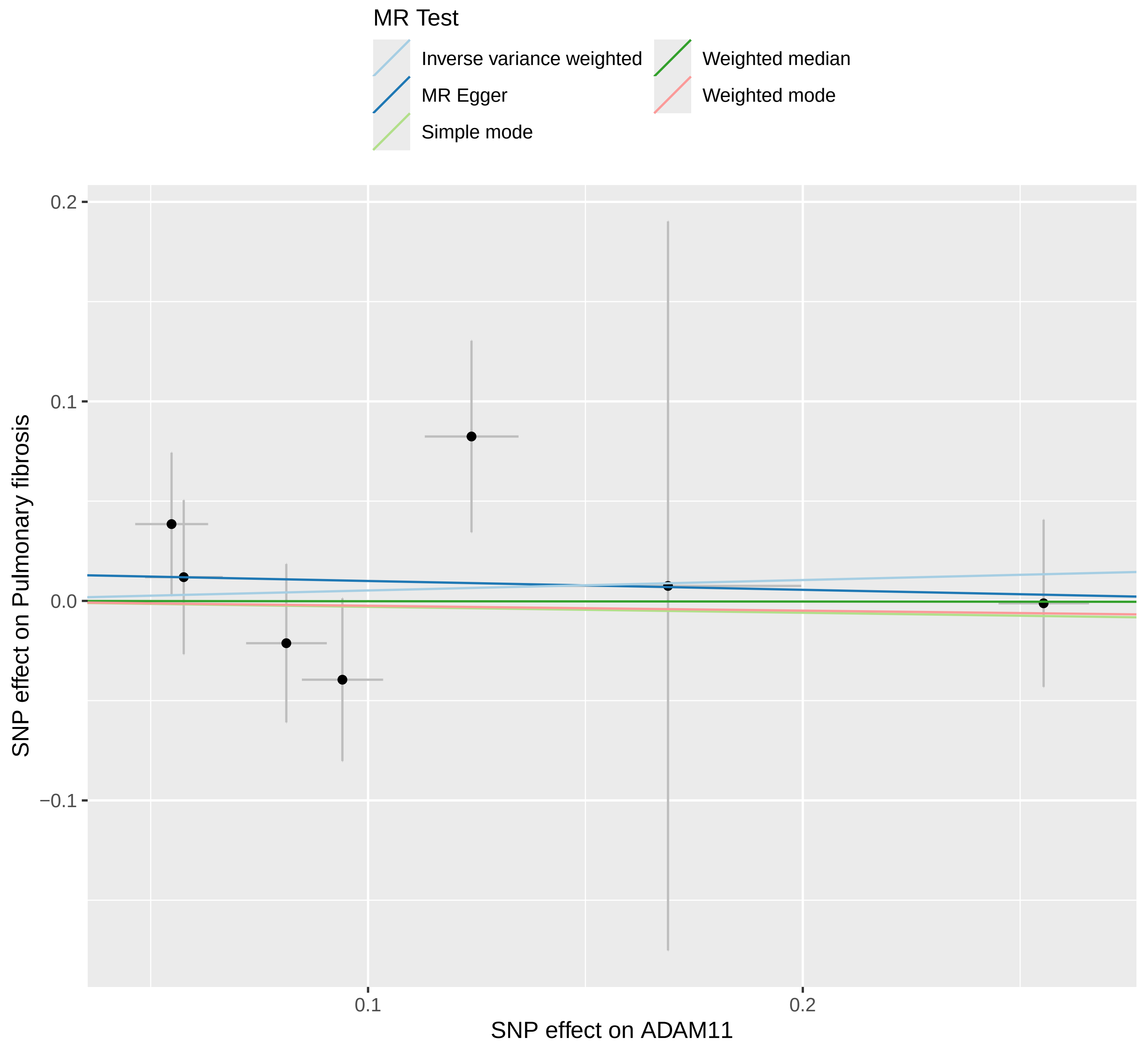
MR Test
Inverse variance weighted
Weighted median
MR Egger
Weighted mode
Simple mode
0.2
0.1
0.0
−0.1
0.1
0.2
SNP effect on Pulmonary fibrosis
SNP effect on ADAM11

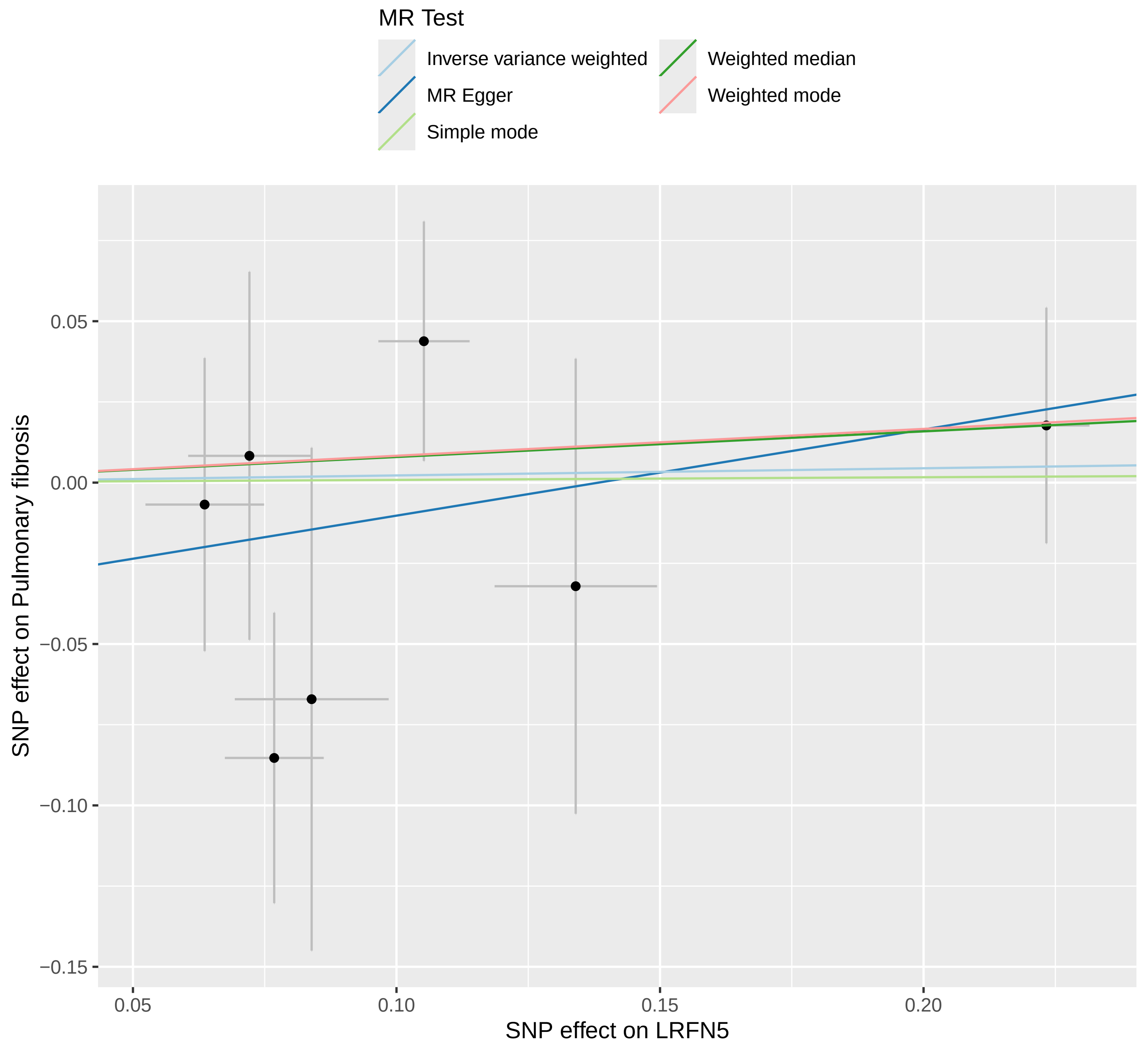

MR Test
Inverse variance weighted
MR Egger
Simple mode
Weighted median
Weighted mode
0.05
0.00
−0.05
−0.10
−0.15
SNP effect on Pulmonary fibrosis
0.05
0.10
0.15
0.20
SNP effect on LRFN5

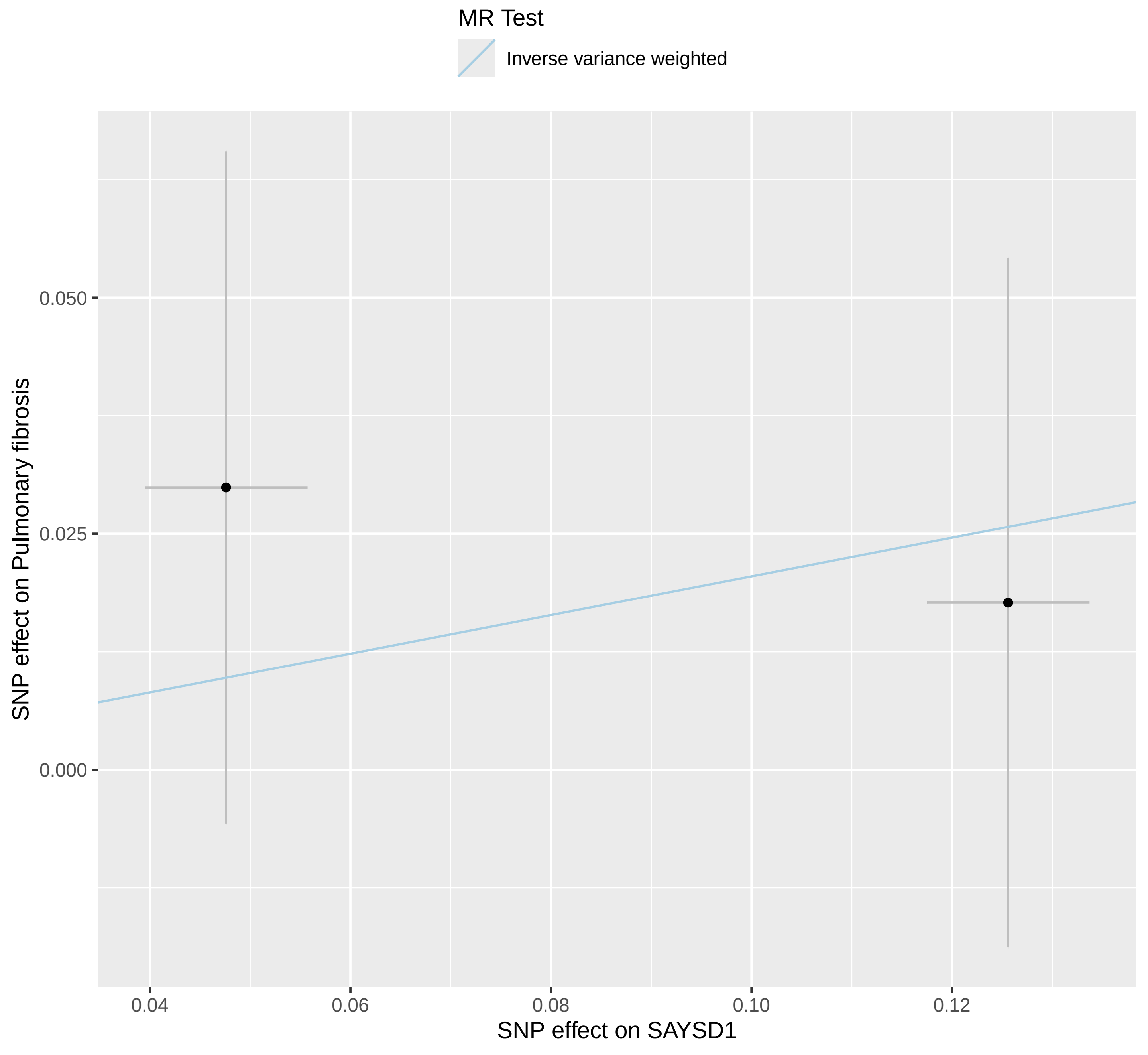

MR Test
Inverse variance weighted
0.050
0.025
0.000
SNP effect on Pulmonary fibrosis
0.04
0.06
0.08
0.10
0.12
SNP effect on SAYSD1

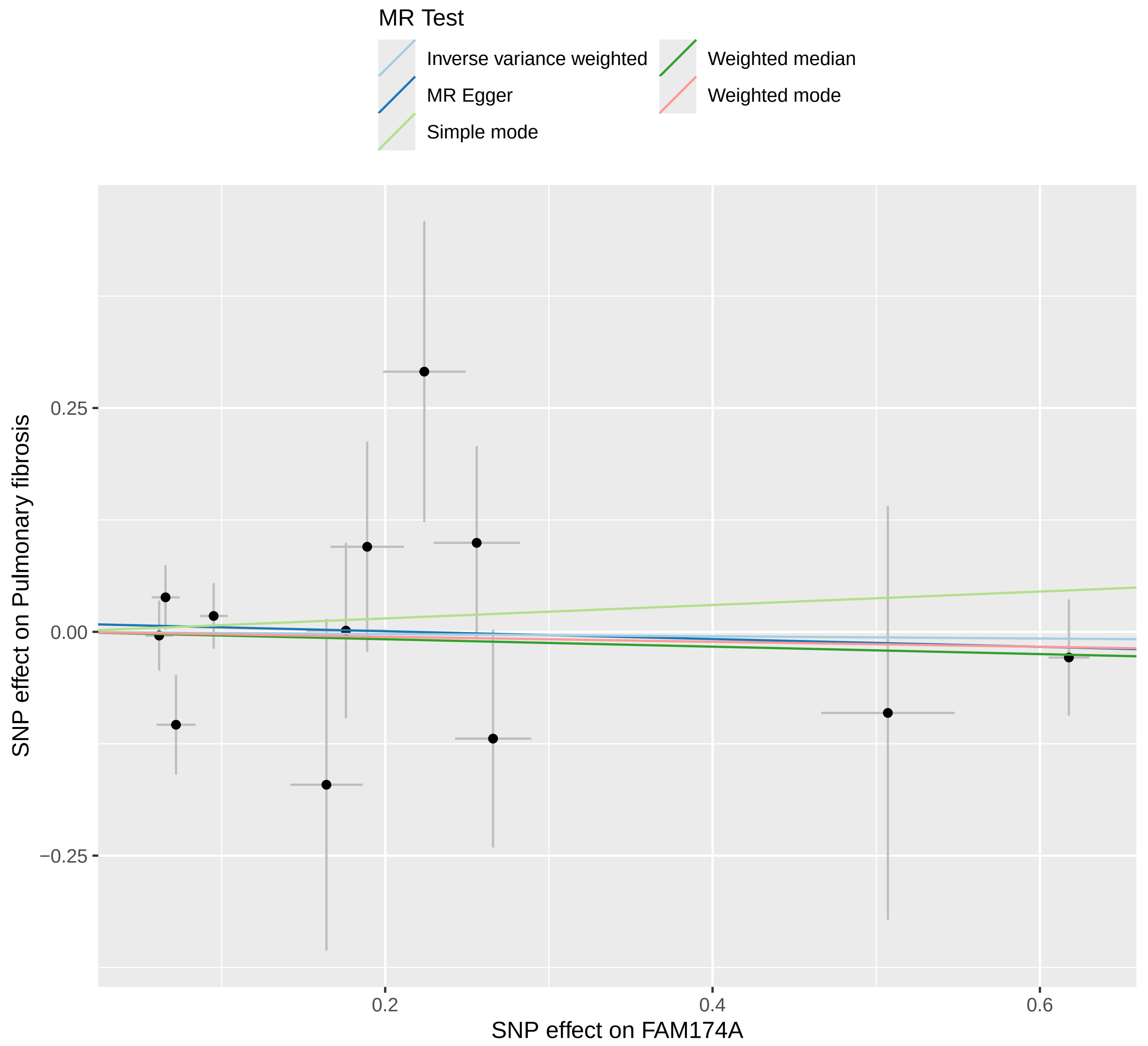

MR Test
Inverse variance weighted
MR Egger
Simple mode
Weighted median
Weighted mode
0.25
0.00
−0.25
0.2
0.4
0.6
SNP effect on Pulmonary fibrosis
SNP effect on FAM174A

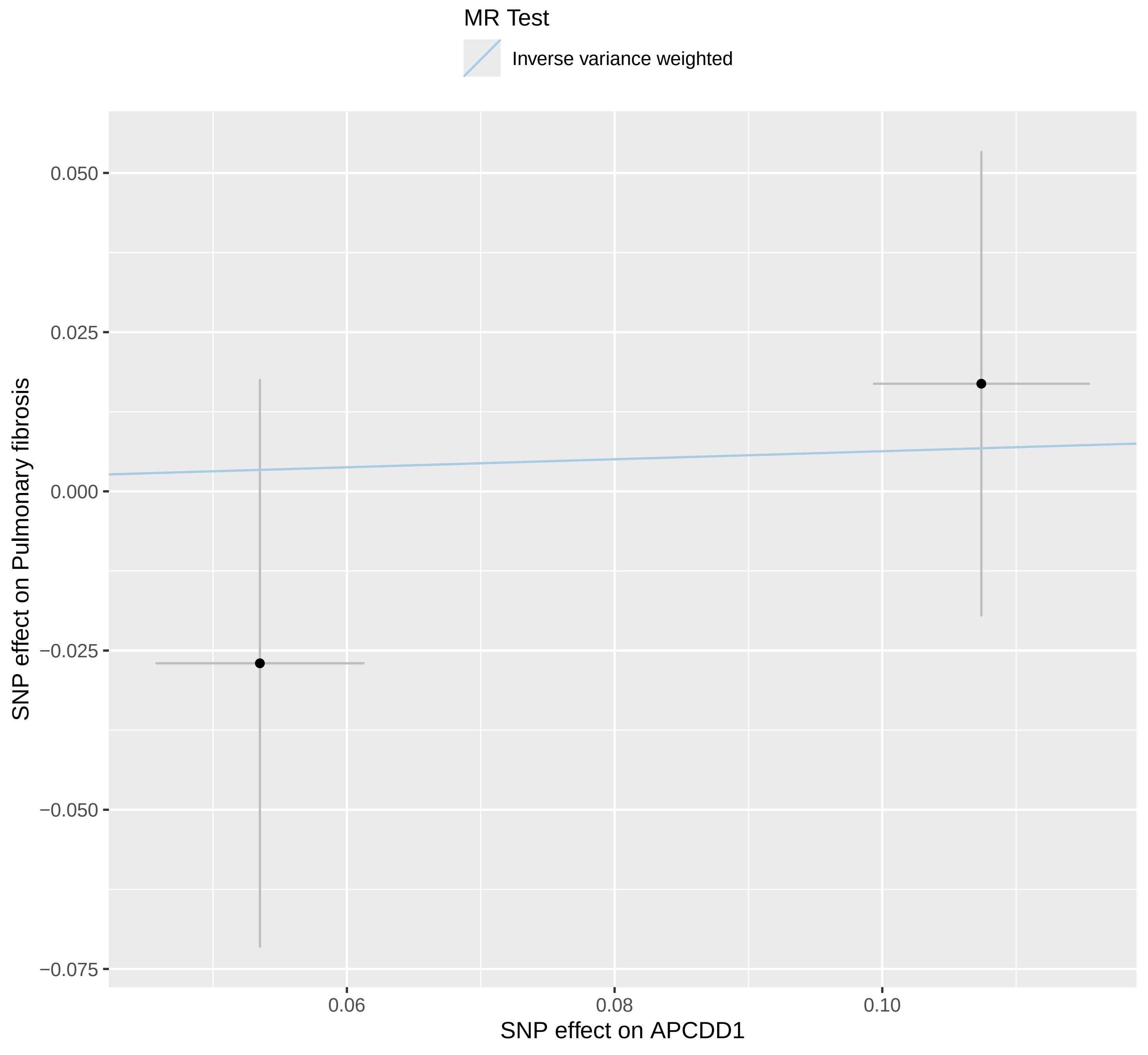

MR Test
Inverse variance weighted
0.050
0.025
0.000
−0.025
−0.050
−0.075
0.06
0.08
0.10
SNP effect on APCDD1
SNP effect on Pulmonary fibrosis

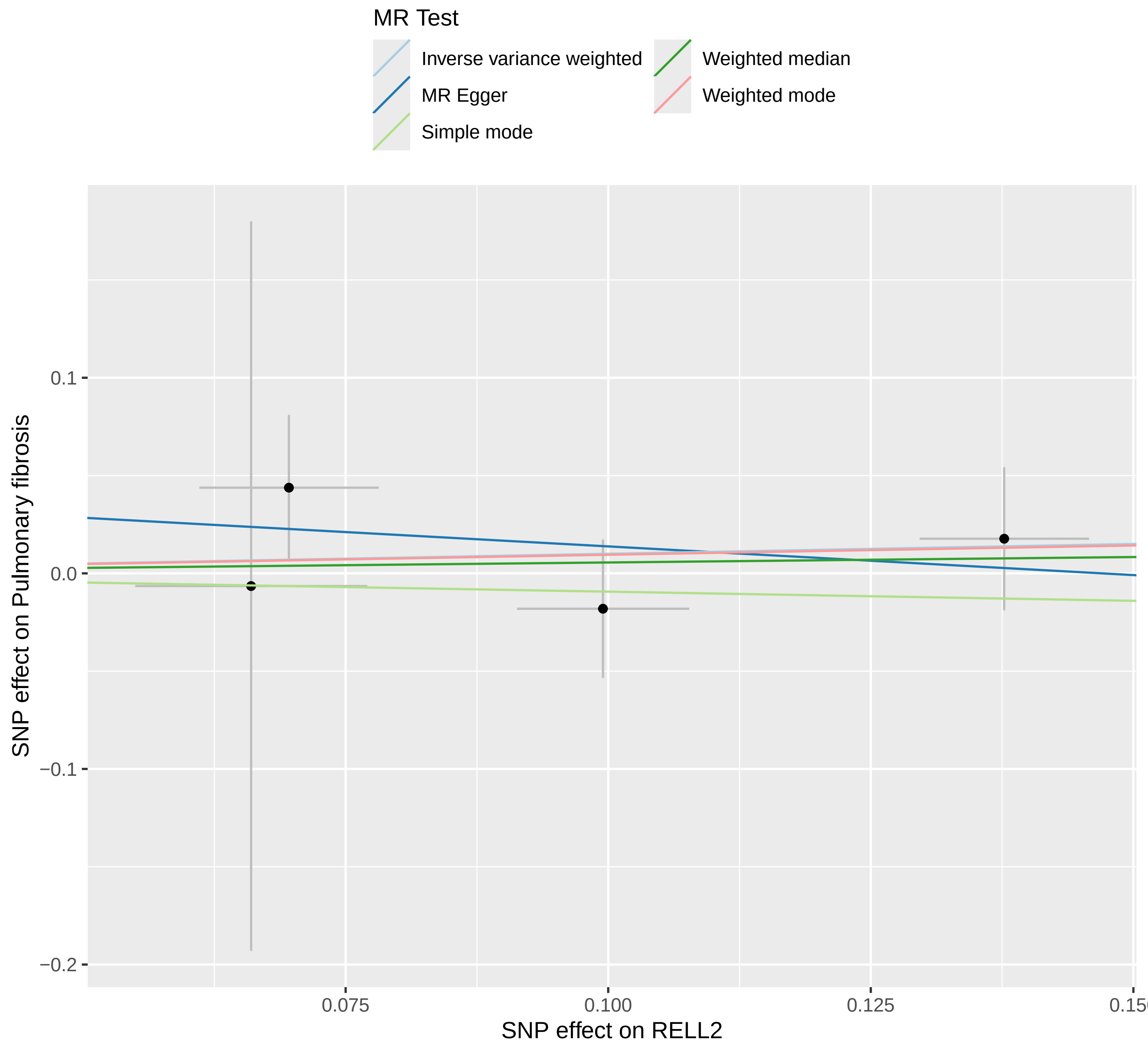

MR Test
Inverse variance weighted
MR Egger
Simple mode
Weighted median
Weighted mode
SNP effect on Pulmonary fibrosis
0.1
0.0
−0.1
−0.2
0.075
0.100
0.125
0.15
SNP effect on RELL2

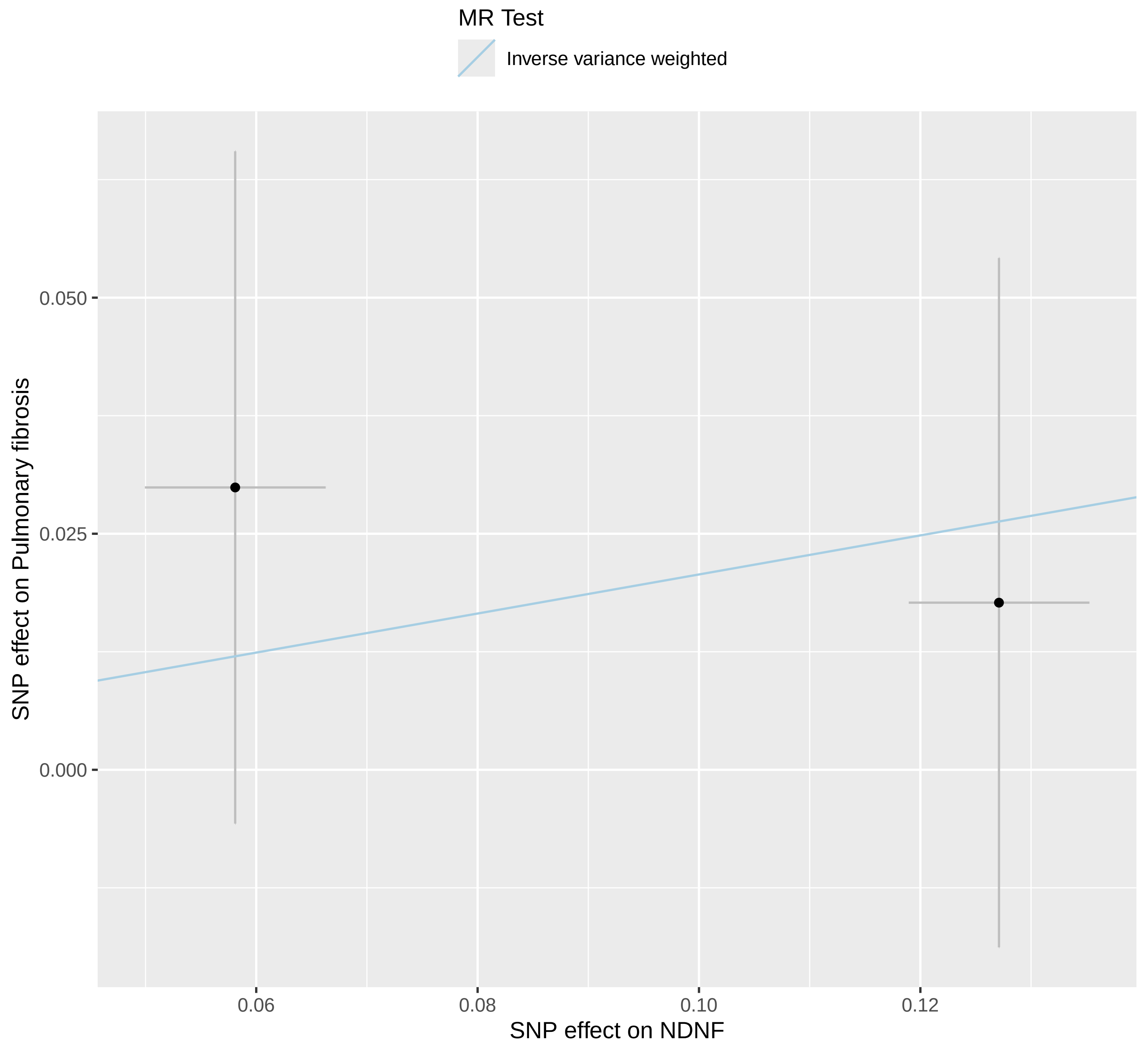
MR Test
Inverse variance weighted
0.050
0.025
0.000
0.06
0.08
0.10
0.12
SNP effect on Pulmonary fibrosis
SNP effect on NDNF

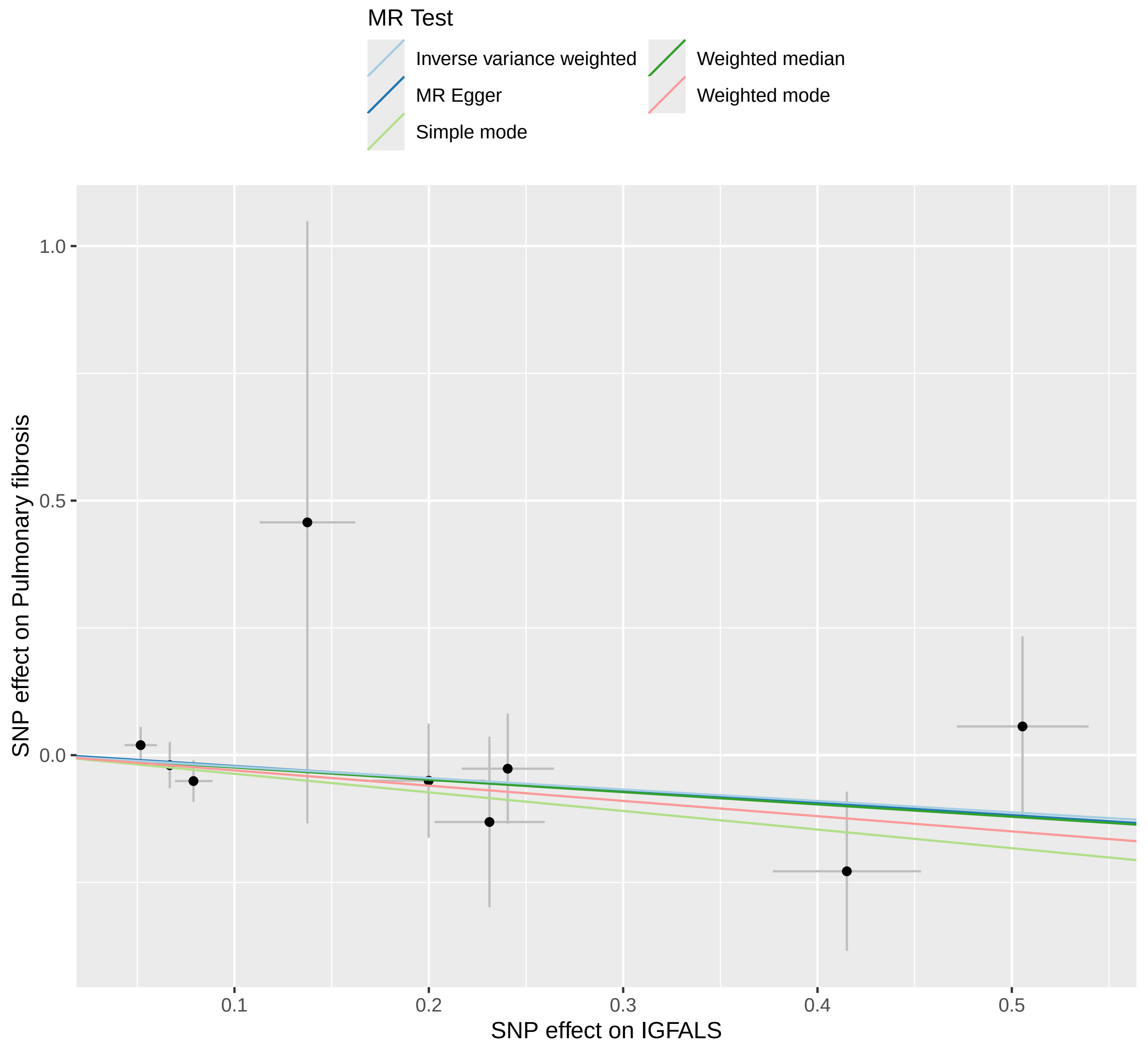
MR Test
Inverse variance weighted
MR Egger
Simple mode
Weighted median
Weighted mode
1.0
0.5
0.0
0.1
0.2
0.3
0.4
0.5
SNP effect on Pulmonary fibrosis
SNP effect on IGFALS

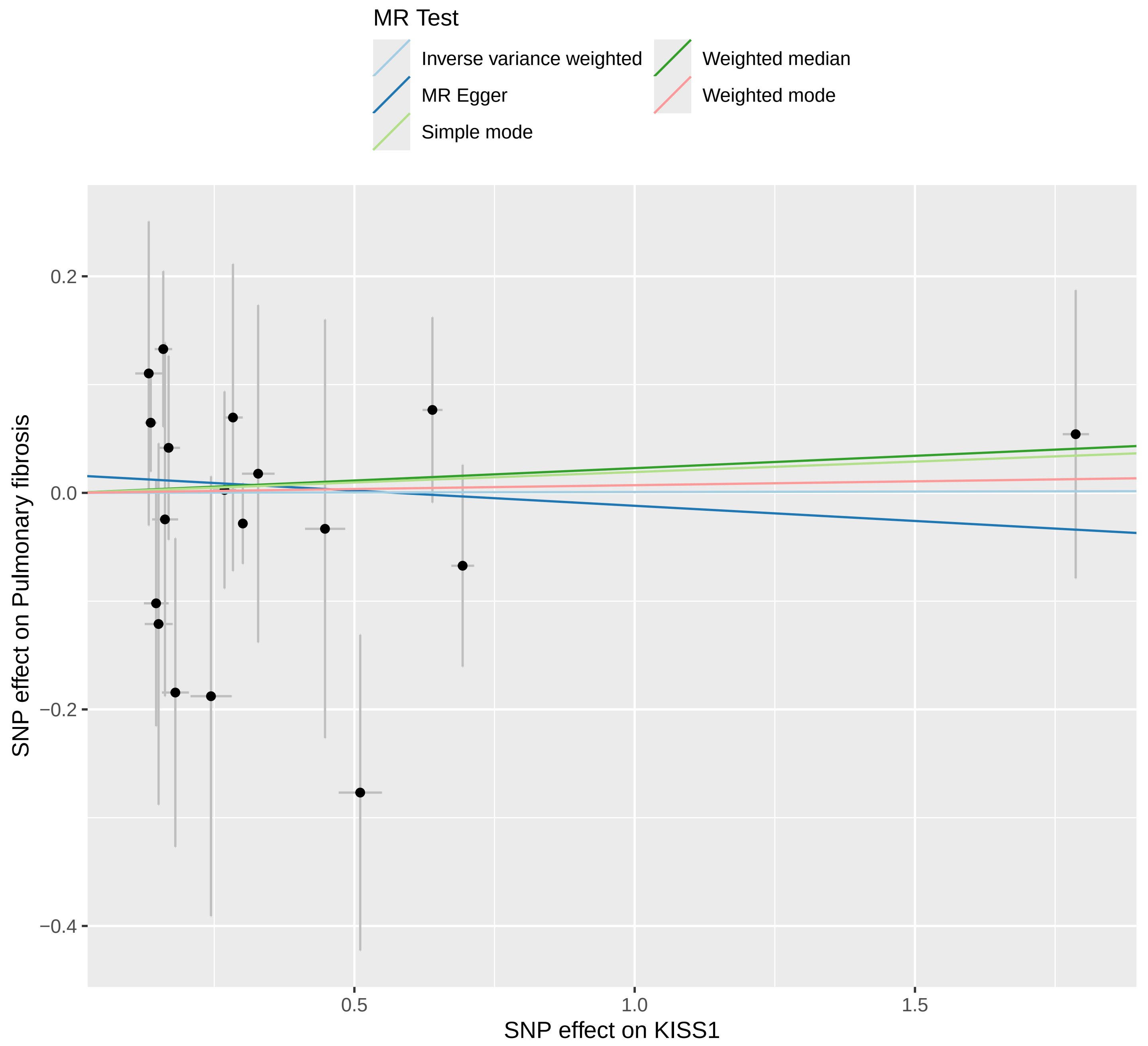

MR Test
Inverse variance weighted
MR Egger
Simple mode
Weighted median
Weighted mode
0.2
0.0
−0.2
−0.4
SNP effect on Pulmonary fibrosis
0.5
1.0
1.5
SNP effect on KISS1

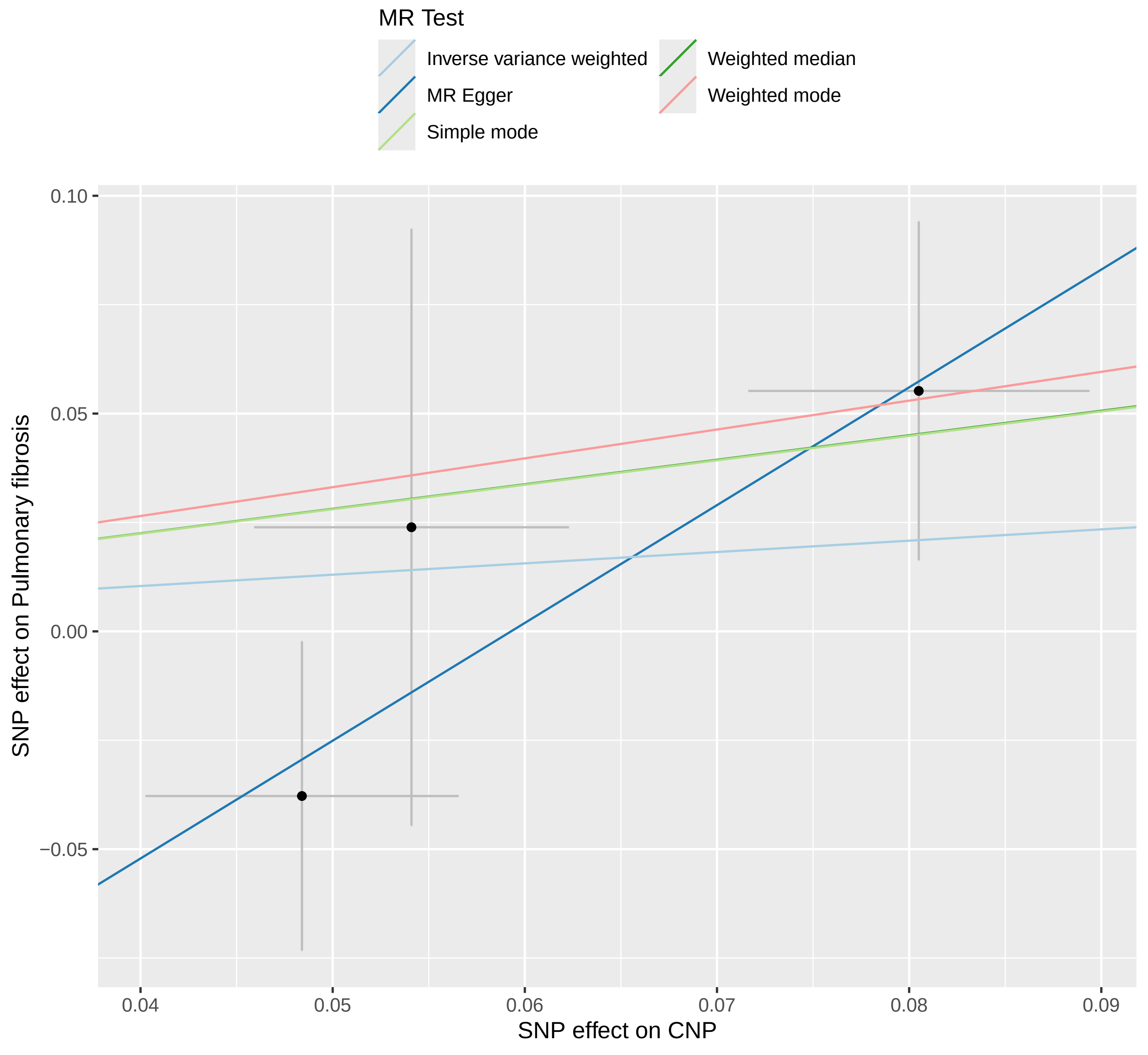
MR Test
Inverse variance weighted
MR Egger
Simple mode
Weighted median
Weighted mode
0.10
0.05
0.00
−0.05
SNP effect on Pulmonary fibrosis
0.04
0.05
0.06
0.07
0.08
0.09
SNP effect on CNP

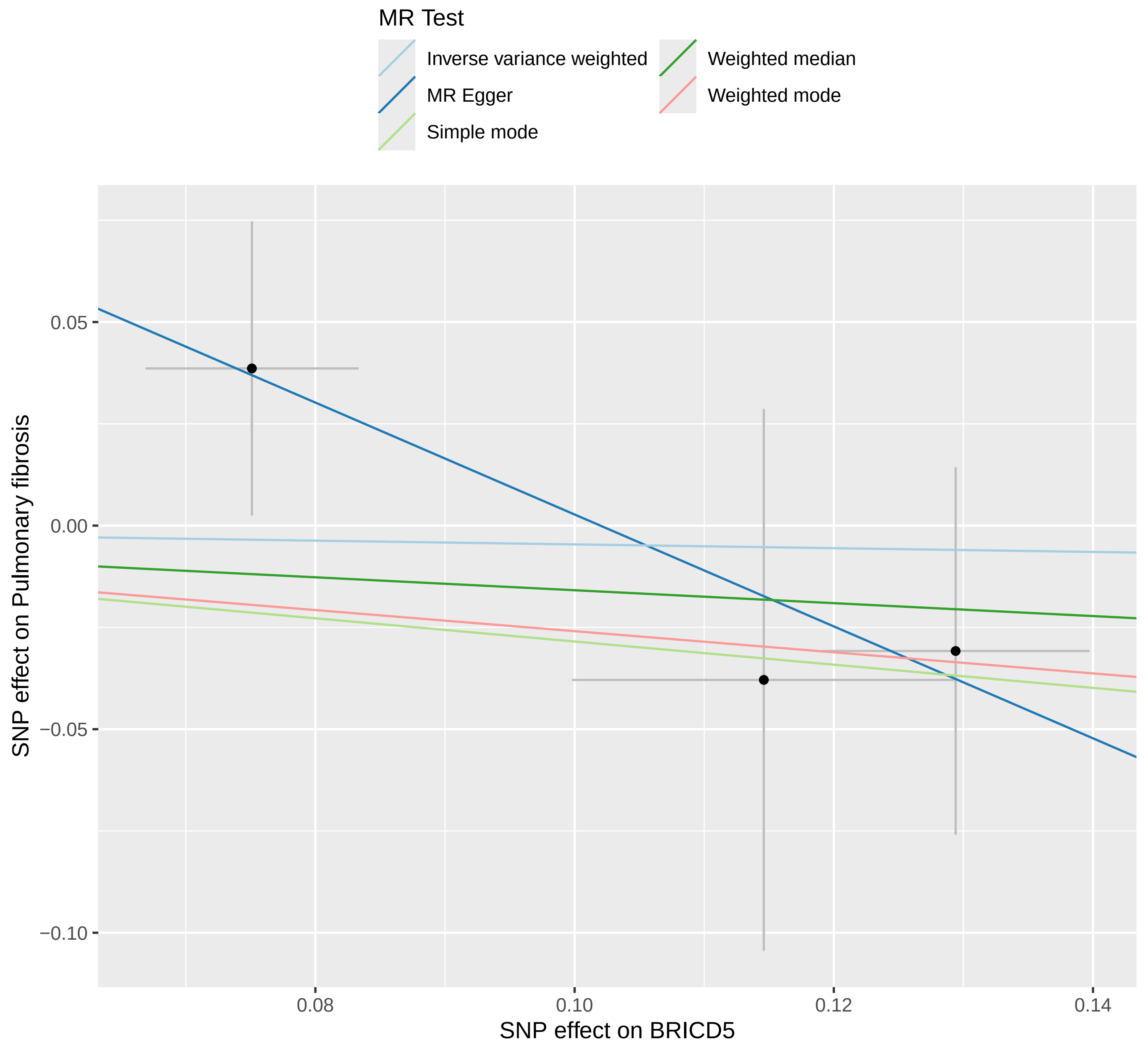
MR Test
Inverse variance weighted
MR Egger
Simple mode
Weighted median
Weighted mode
SNP effect on Pulmonary fibrosis
0.05
0.00
−0.05
−0.10
0.08
0.10
0.12
0.14
SNP effect on BRICD5

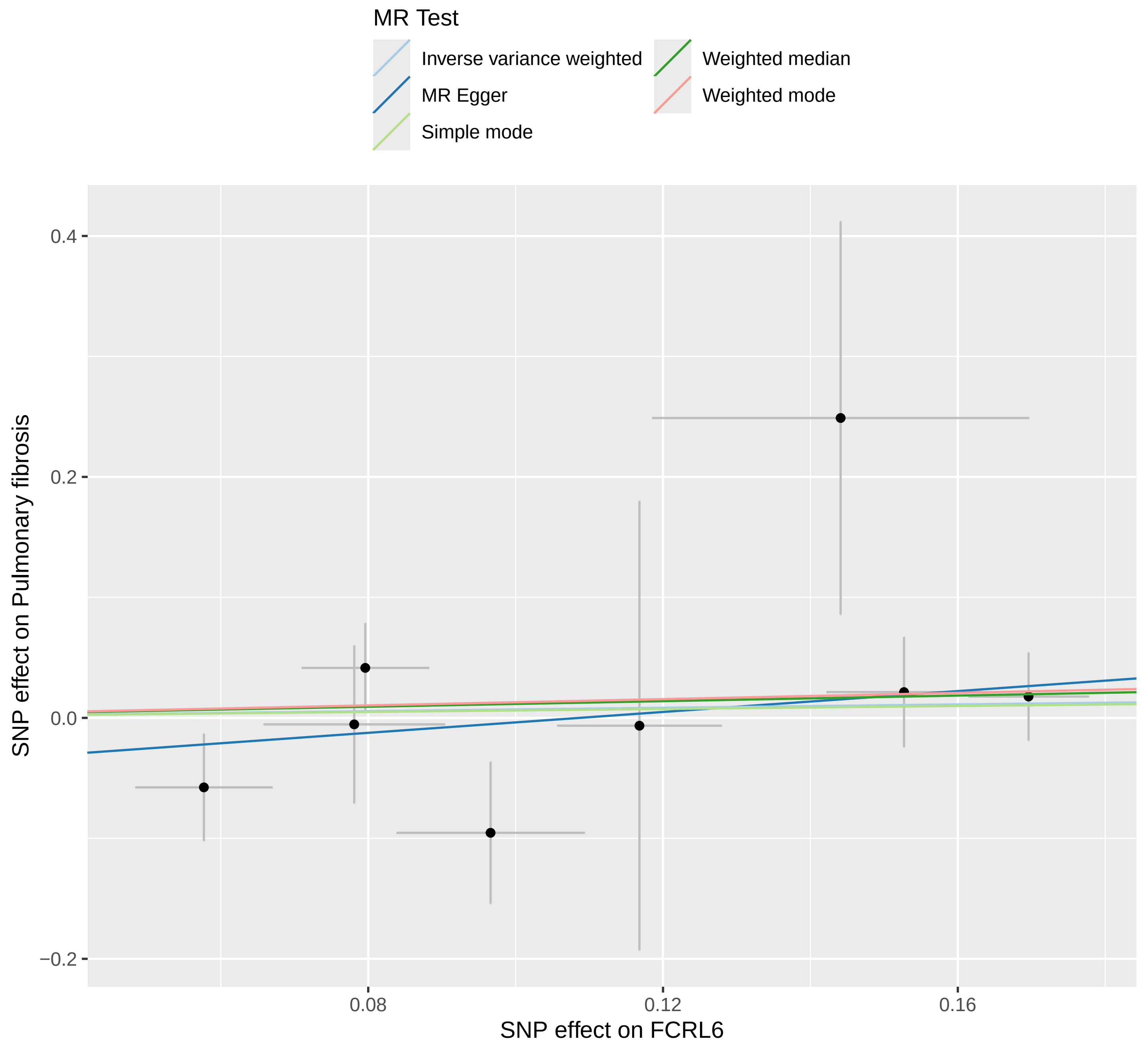
MR Test
Inverse variance weighted
MR Egger
Simple mode
Weighted median
Weighted mode
SNP effect on Pulmonary fibrosis
0.4
0.2
0.0
−0.2
0.08
0.12
0.16
SNP effect on FCRL6

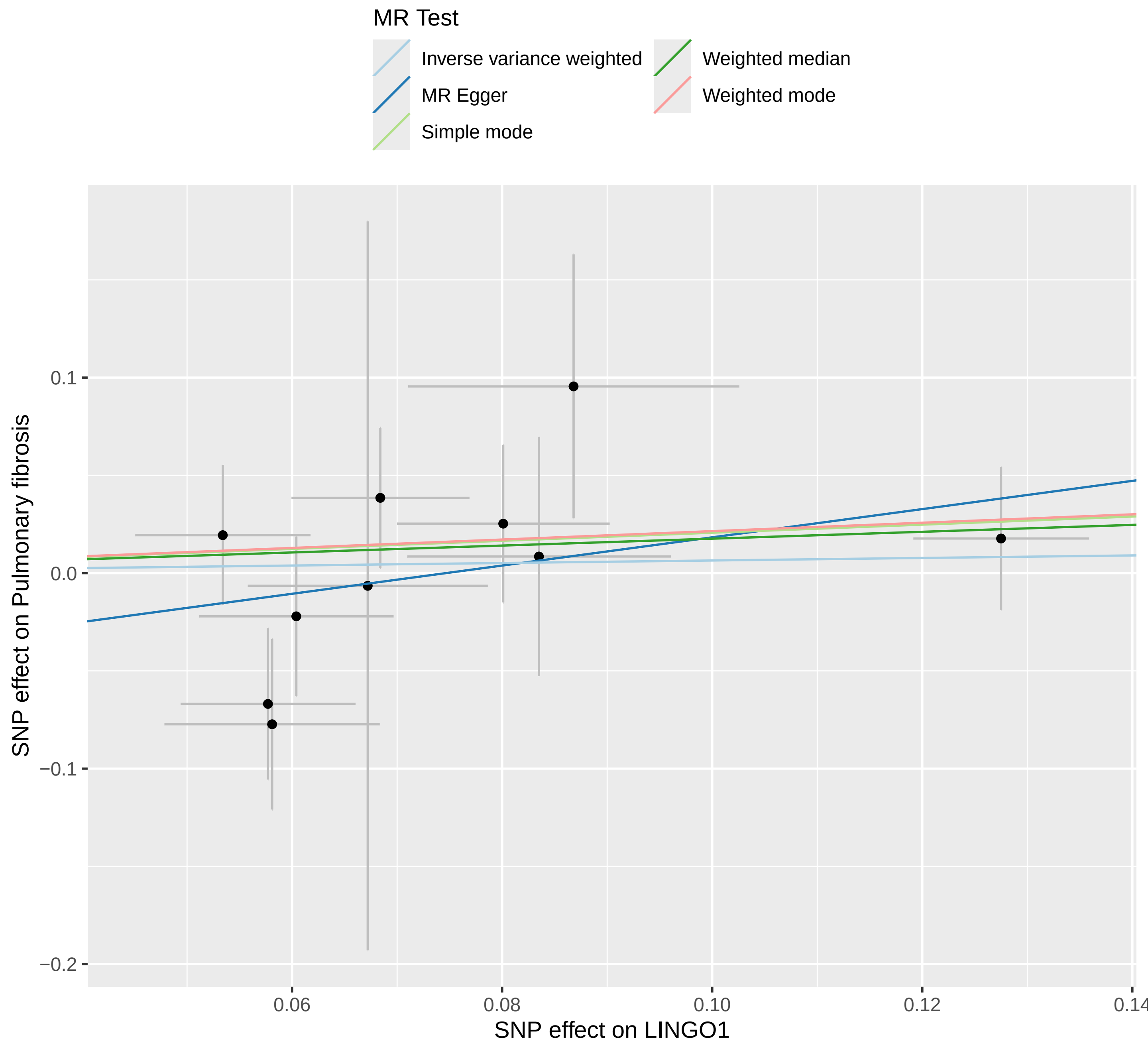
MR Test
Inverse variance weighted
MR Egger
Simple mode
Weighted median
Weighted mode
SNP effect on Pulmonary fibrosis
0.1
0.0
−0.1
−0.2
0.06
0.08
0.10
0.12
0.14
SNP effect on LINGO1

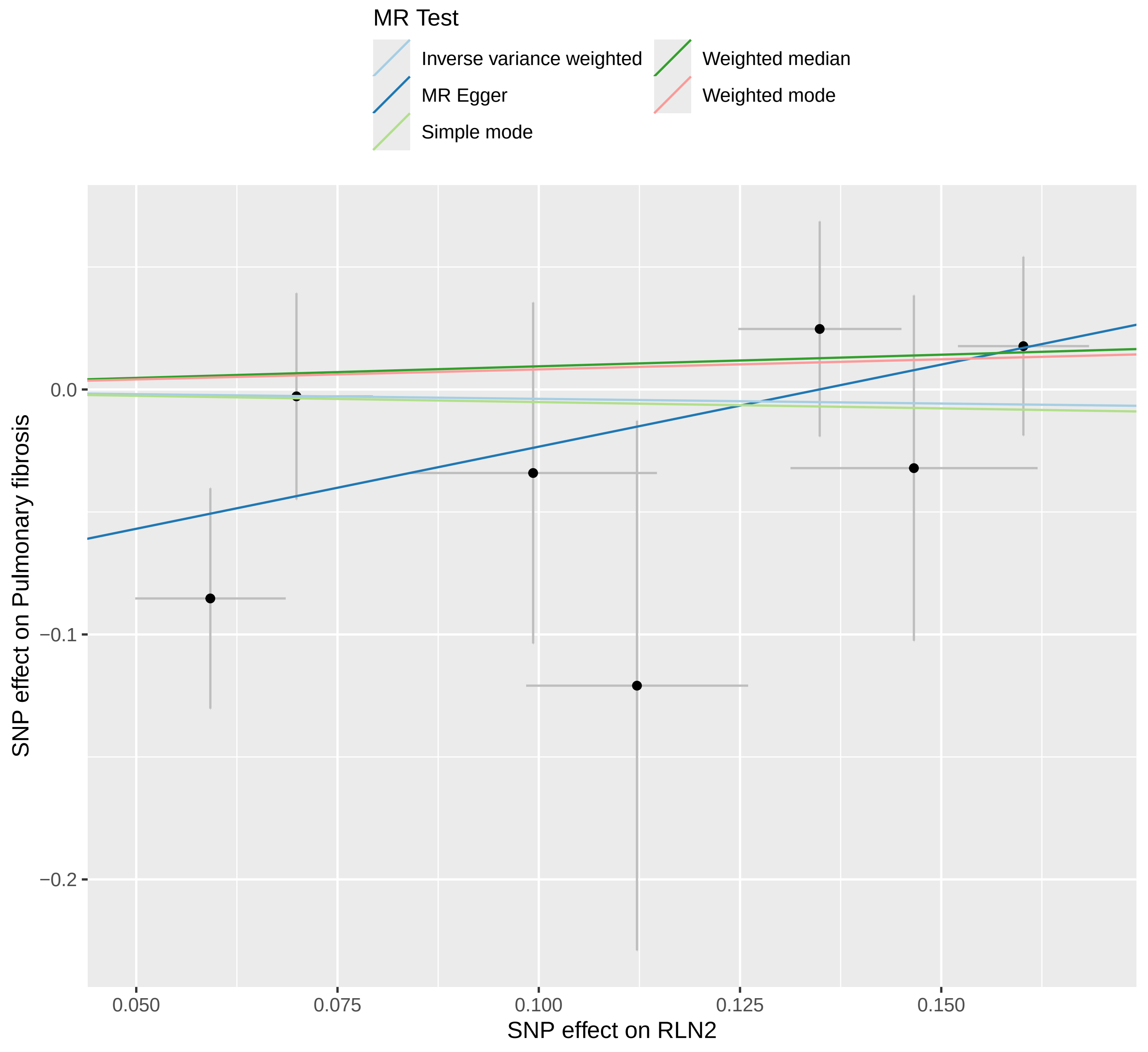
MR Test
Inverse variance weighted
MR Egger
Simple mode
Weighted median
Weighted mode
0.0
−0.1
−0.2
0.050
0.075
0.100
0.125
0.150
SNP effect on RLN2
SNP effect on Pulmonary fibrosis

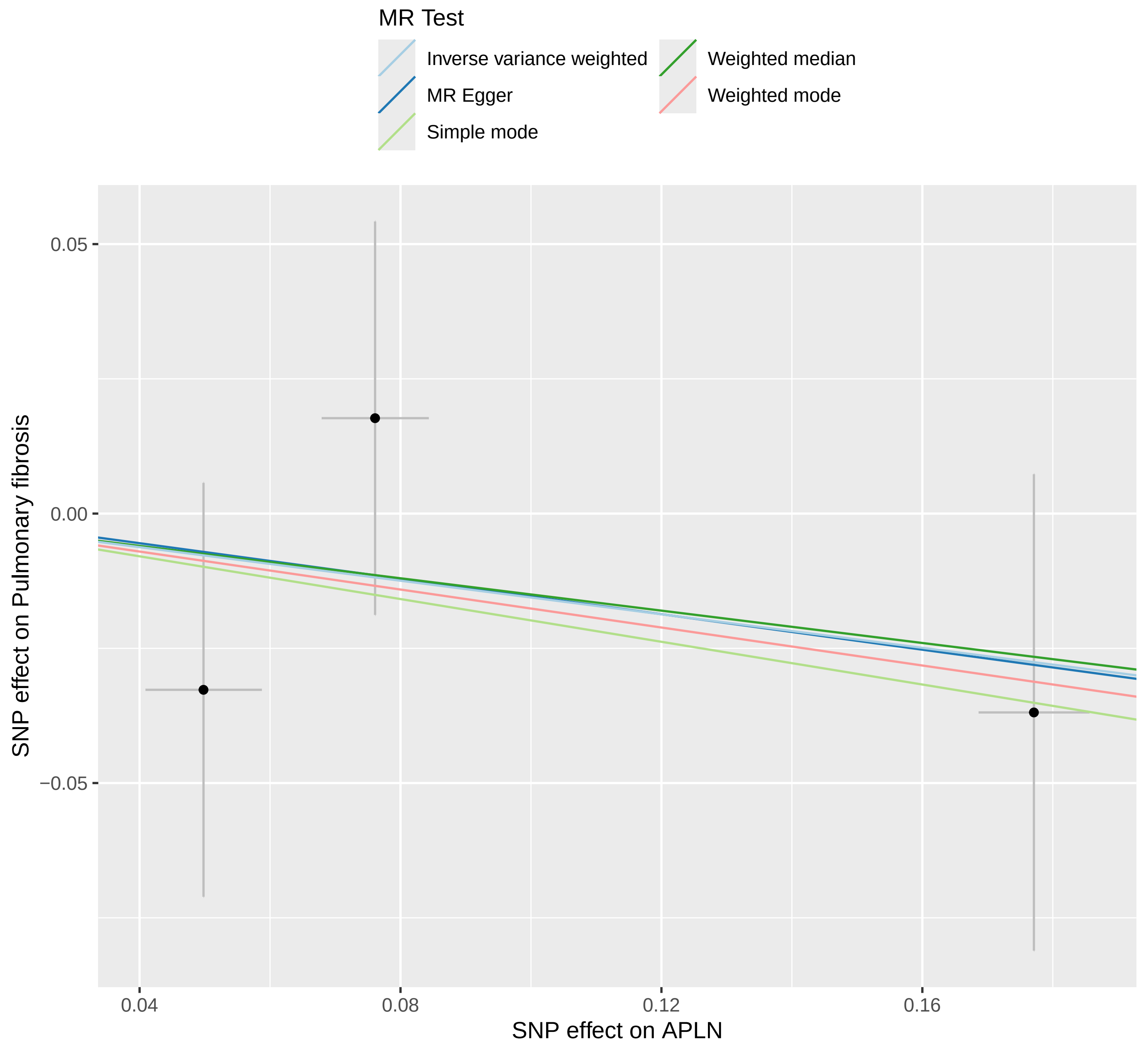

MR Test
Inverse variance weighted
MR Egger
Simple mode
Weighted median
Weighted mode
0.05
0.00
−0.05
SNP effect on Pulmonary fibrosis
0.04
0.08
0.12
0.16
SNP effect on APLN

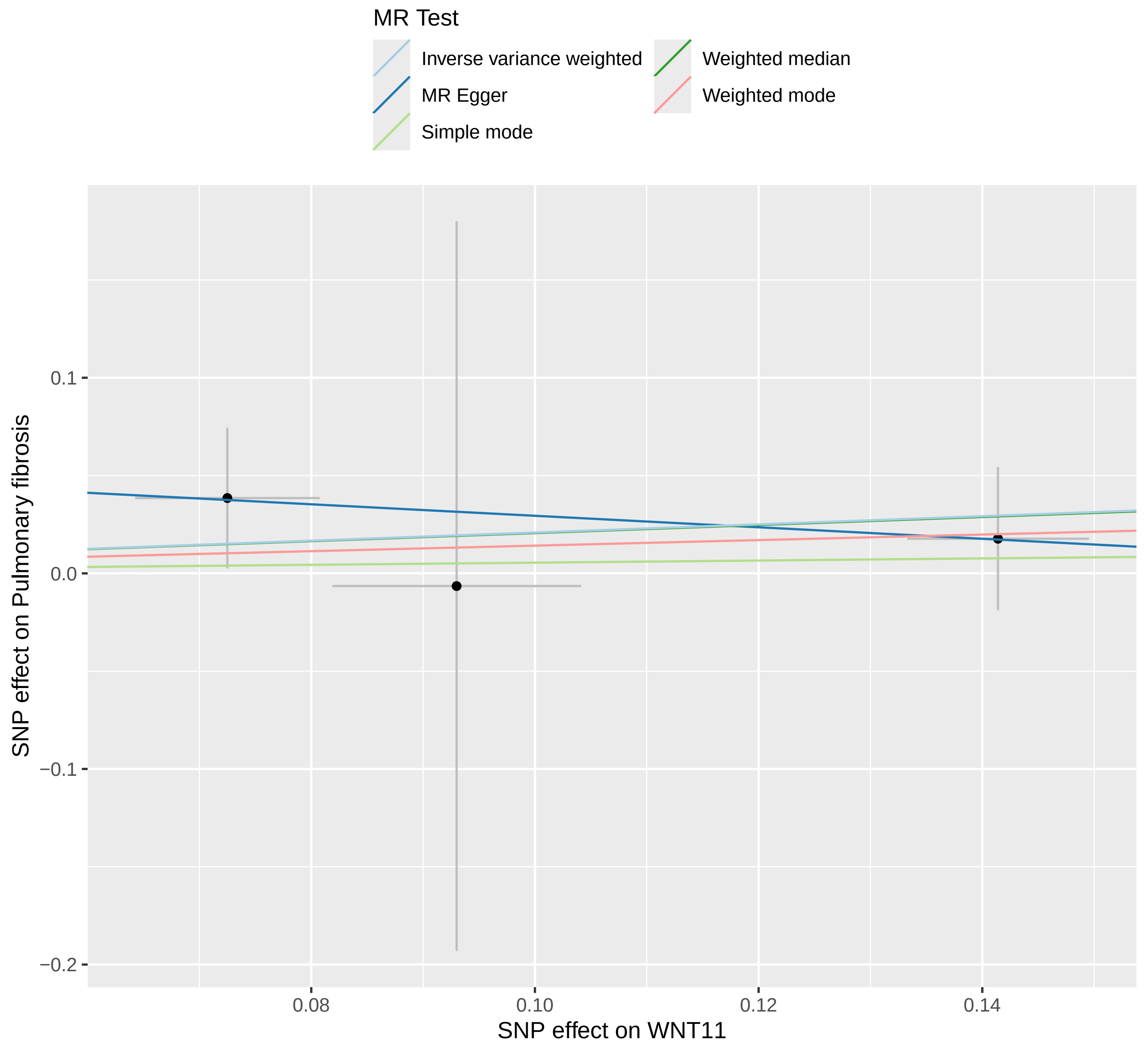
MR Test
Inverse variance weighted
MR Egger
Simple mode
Weighted median
Weighted mode
0.1
0.0
−0.1
−0.2
SNP effect on Pulmonary fibrosis
0.08
0.10
0.12
0.14
SNP effect on WNT11

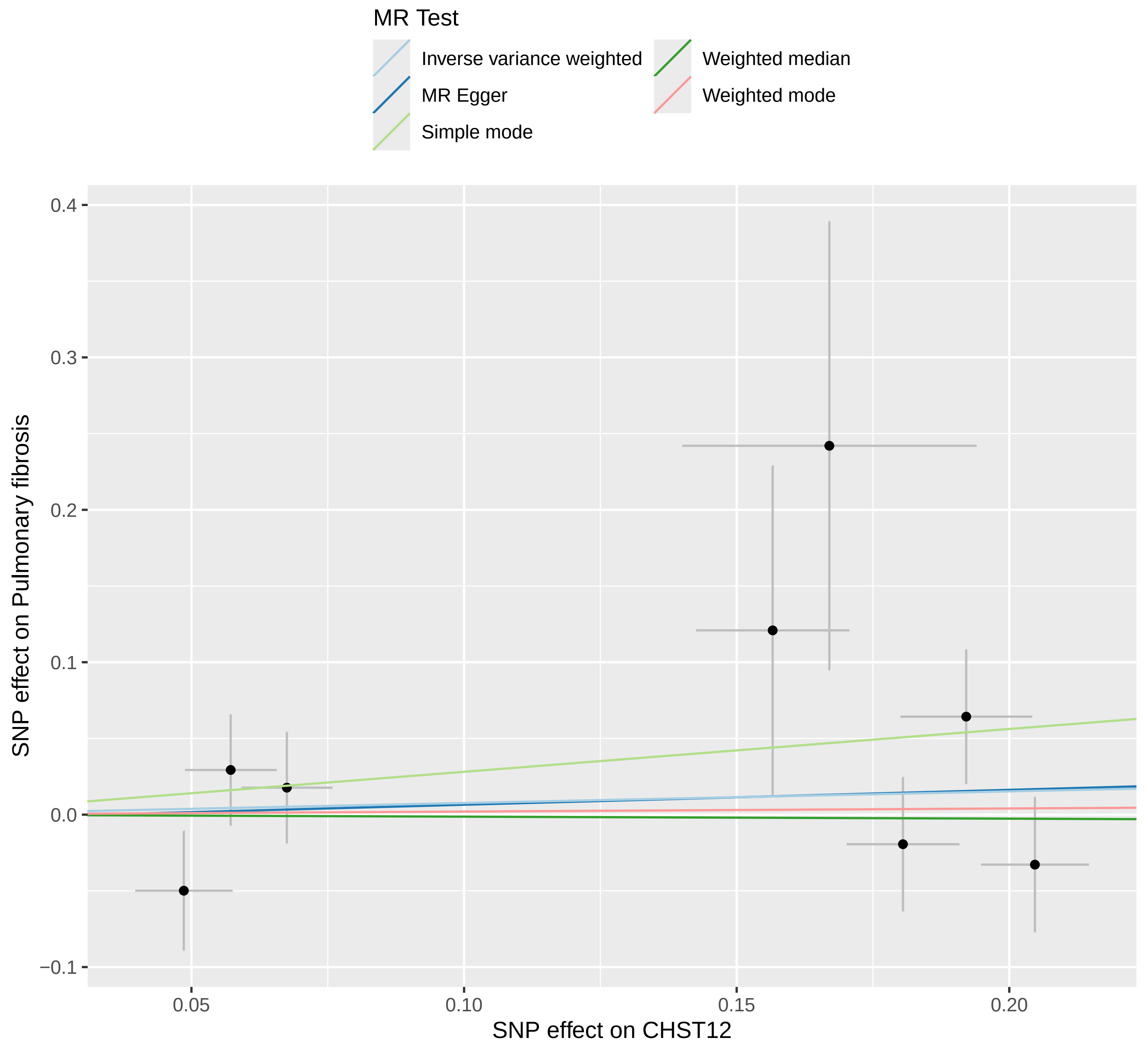
MR Test
Inverse variance weighted
MR Egger
Simple mode
Weighted median
Weighted mode
0.4
0.3
0.2
0.1
0.0
−0.1
0.05
0.10
0.15
0.20
SNP effect on CHST12
SNP effect on Pulmonary fibrosis

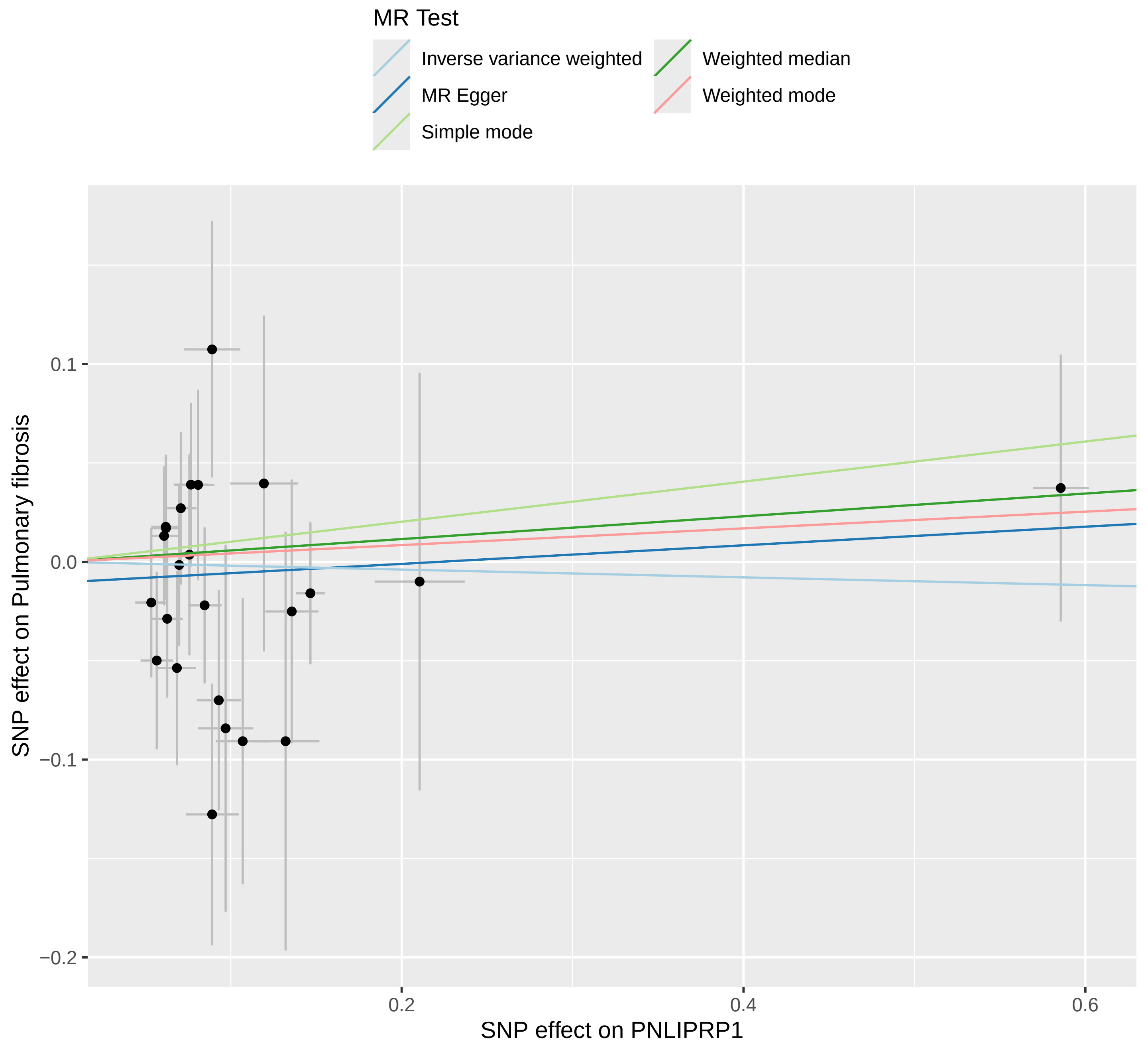
MR Test
Inverse variance weighted
MR Egger
Simple mode
Weighted median
Weighted mode
0.1
0.0
−0.1
−0.2
SNP effect on Pulmonary fibrosis
0.2
0.4
0.6
SNP effect on PNLIPRP1

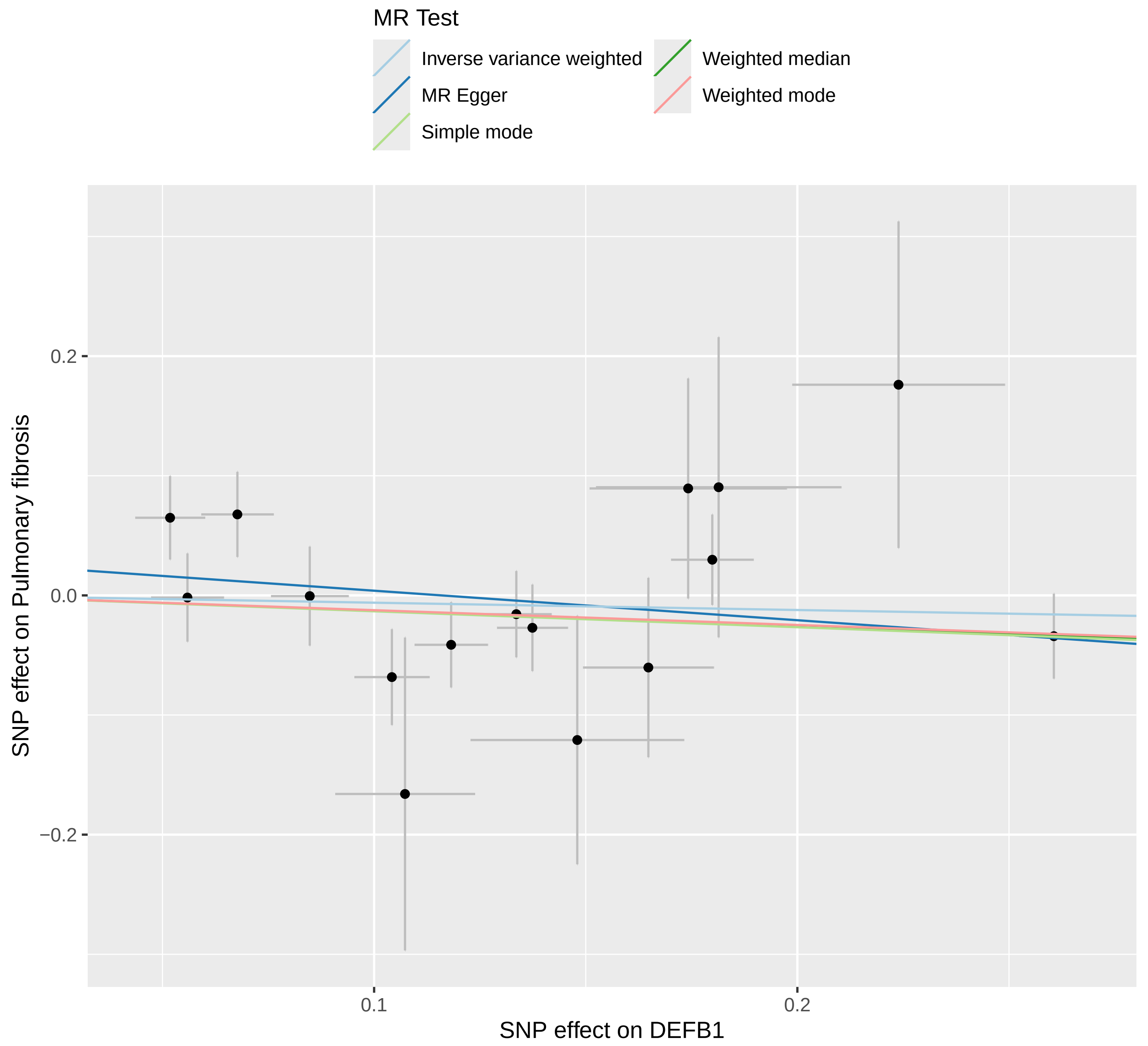

MR Test
Inverse variance weighted
MR Egger
Simple mode
Weighted median
Weighted mode
0.2
0.0
−0.2
SNP effect on Pulmonary fibrosis
0.1
0.2
SNP effect on DEFB1

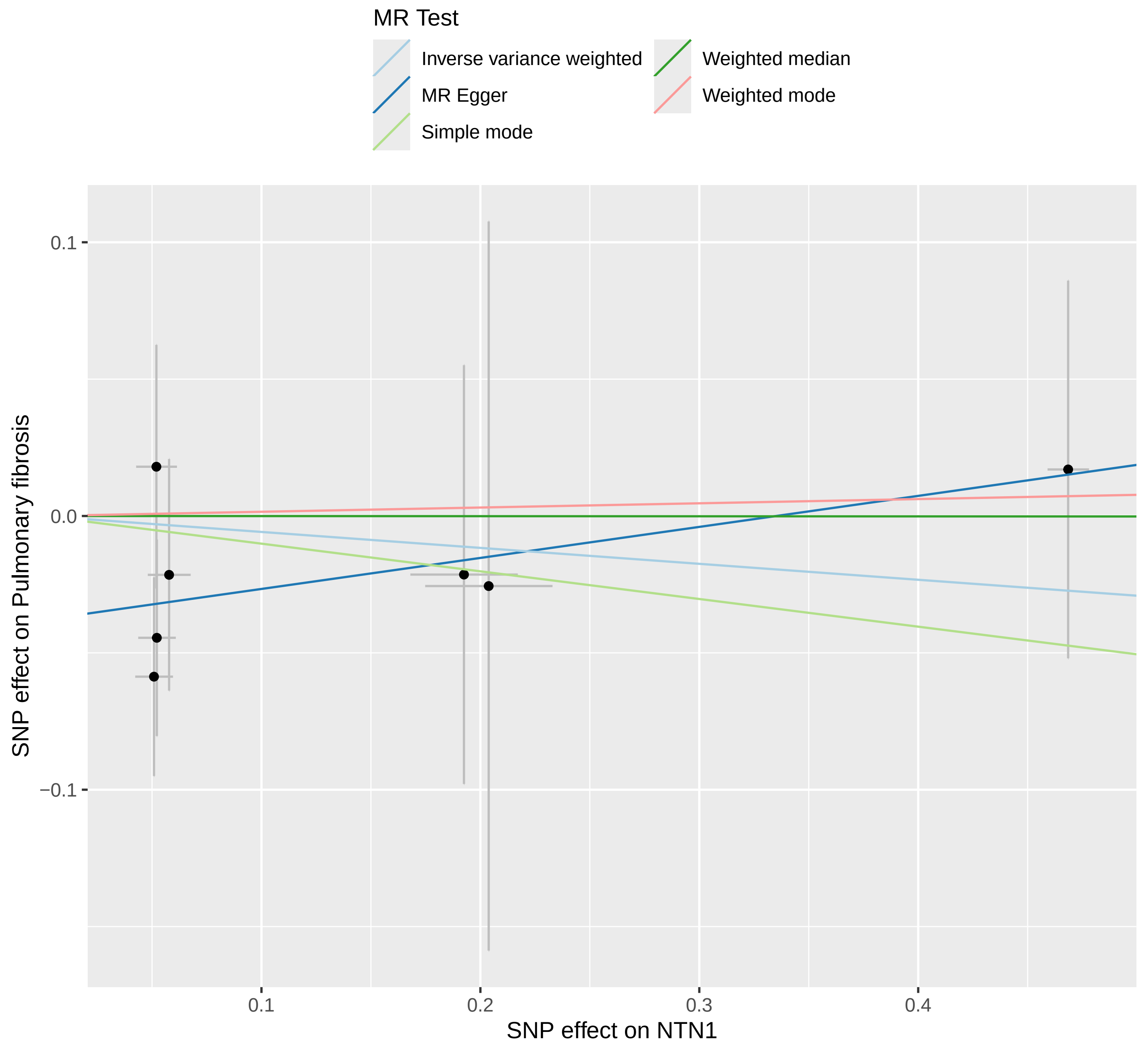

MR Test
Inverse variance weighted
MR Egger
Simple mode
Weighted median
Weighted mode
0.1
0.0
−0.1
SNP effect on Pulmonary fibrosis
0.1
0.2
0.3
0.4
SNP effect on NTN1

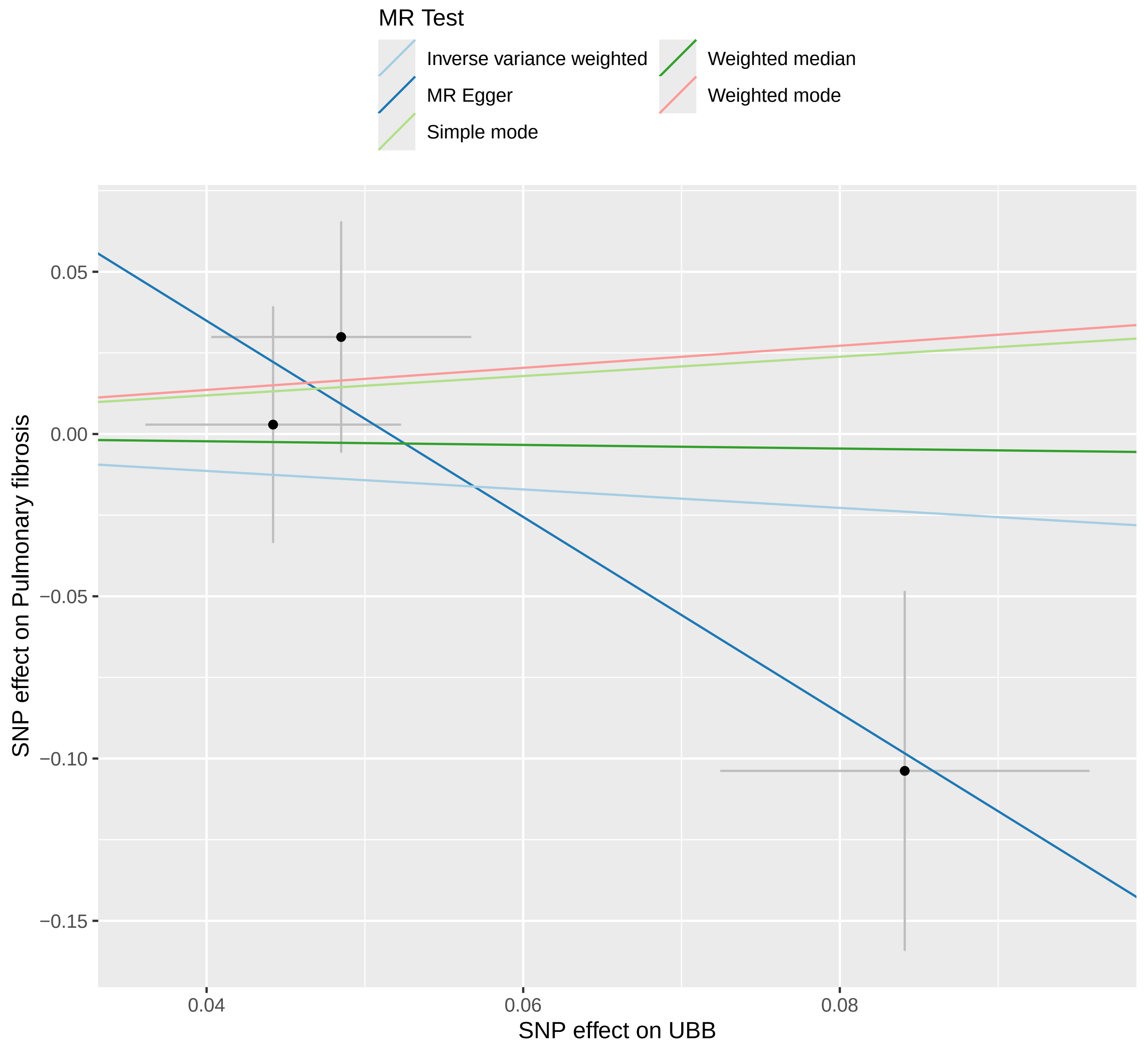
MR Test
Inverse variance weighted
MR Egger
Simple mode
Weighted median
Weighted mode
0.05
0.00
−0.05
−0.10
−0.15
0.04
0.06
0.08
SNP effect on Pulmonary fibrosis
SNP effect on UBB

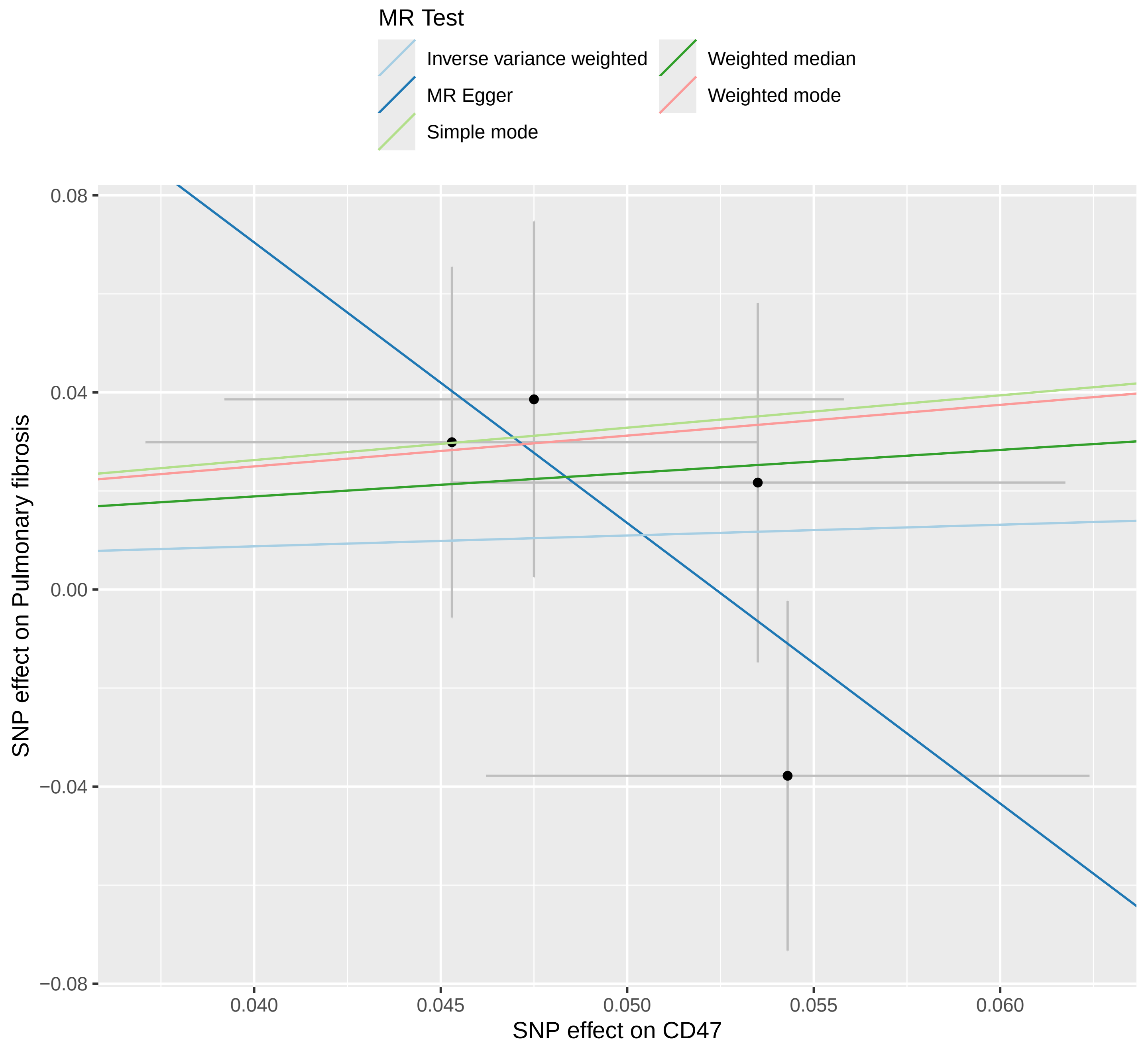
MR Test
Inverse variance weighted
MR Egger
Simple mode
Weighted median
Weighted mode
0.08
0.04
0.00
−0.04
−0.08
0.040
0.045
0.050
0.055
0.060
SNP effect on CD47
SNP effect on Pulmonary fibrosis

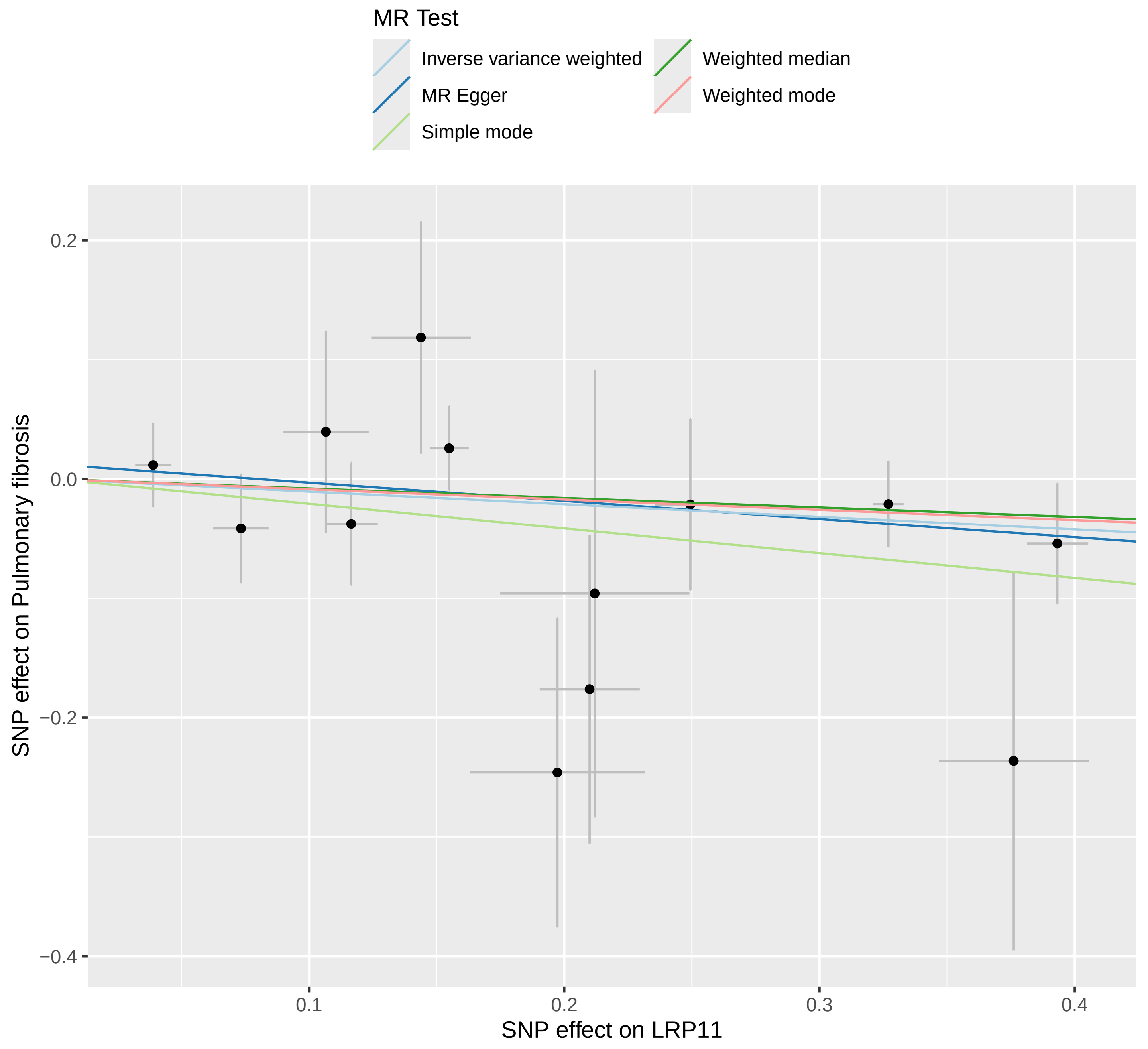
MR Test
Inverse variance weighted
MR Egger
Simple mode
Weighted median
Weighted mode
0.2
0.0
−0.2
−0.4
SNP effect on Pulmonary fibrosis
0.1
0.2
0.3
0.4
SNP effect on LRP11

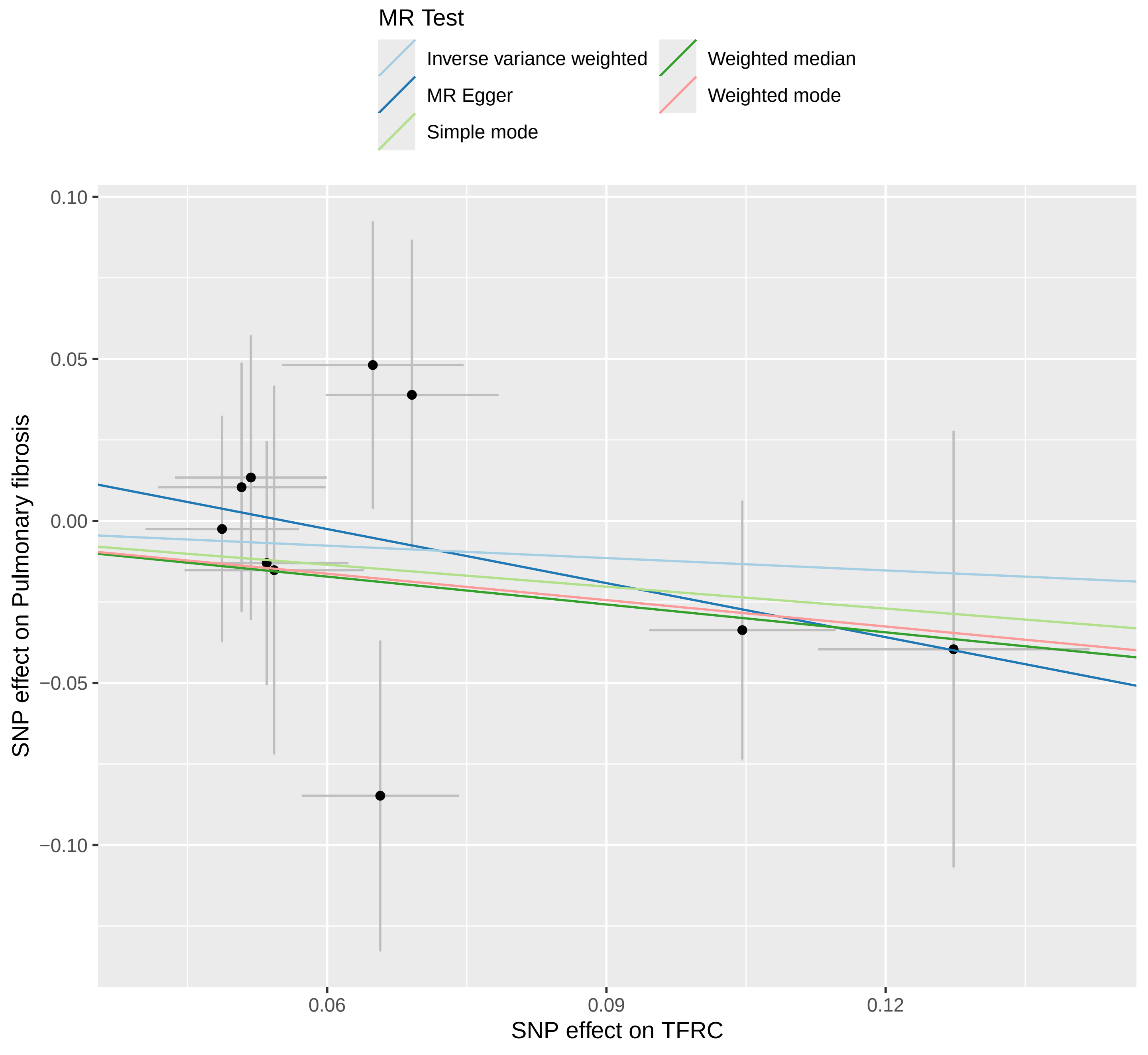

MR Test
Inverse variance weighted
MR Egger
Simple mode
Weighted median
Weighted mode
0.10
0.05
0.00
−0.05
−0.10
SNP effect on Pulmonary fibrosis
0.06
0.09
0.12
SNP effect on TFRC

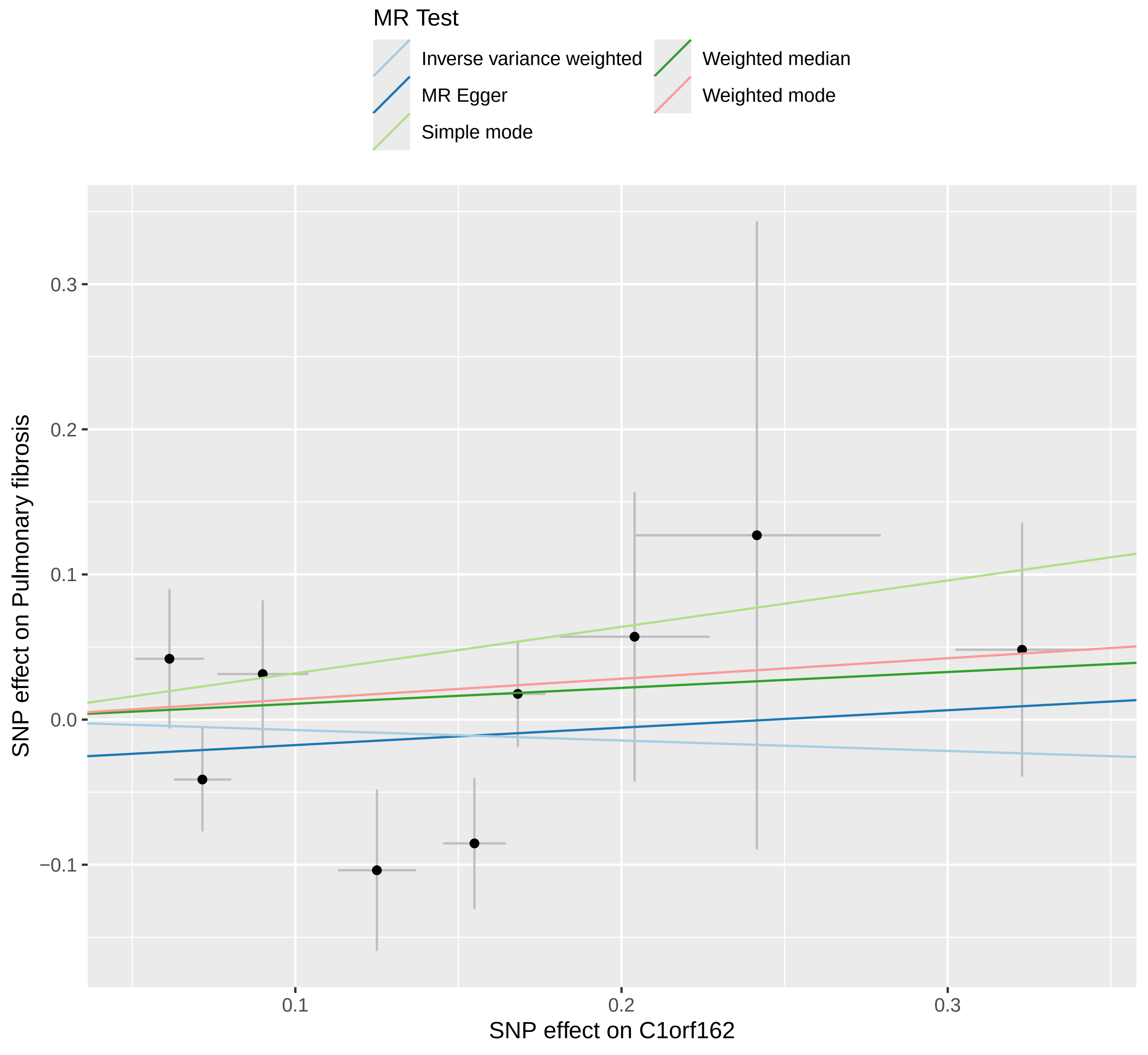
MR Test
Inverse variance weighted
MR Egger
Simple mode
Weighted median
Weighted mode
SNP effect on Pulmonary fibrosis
SNP effect on C1orf162
0.3
0.2
0.1
0.0
−0.1
0.1
0.2
0.3

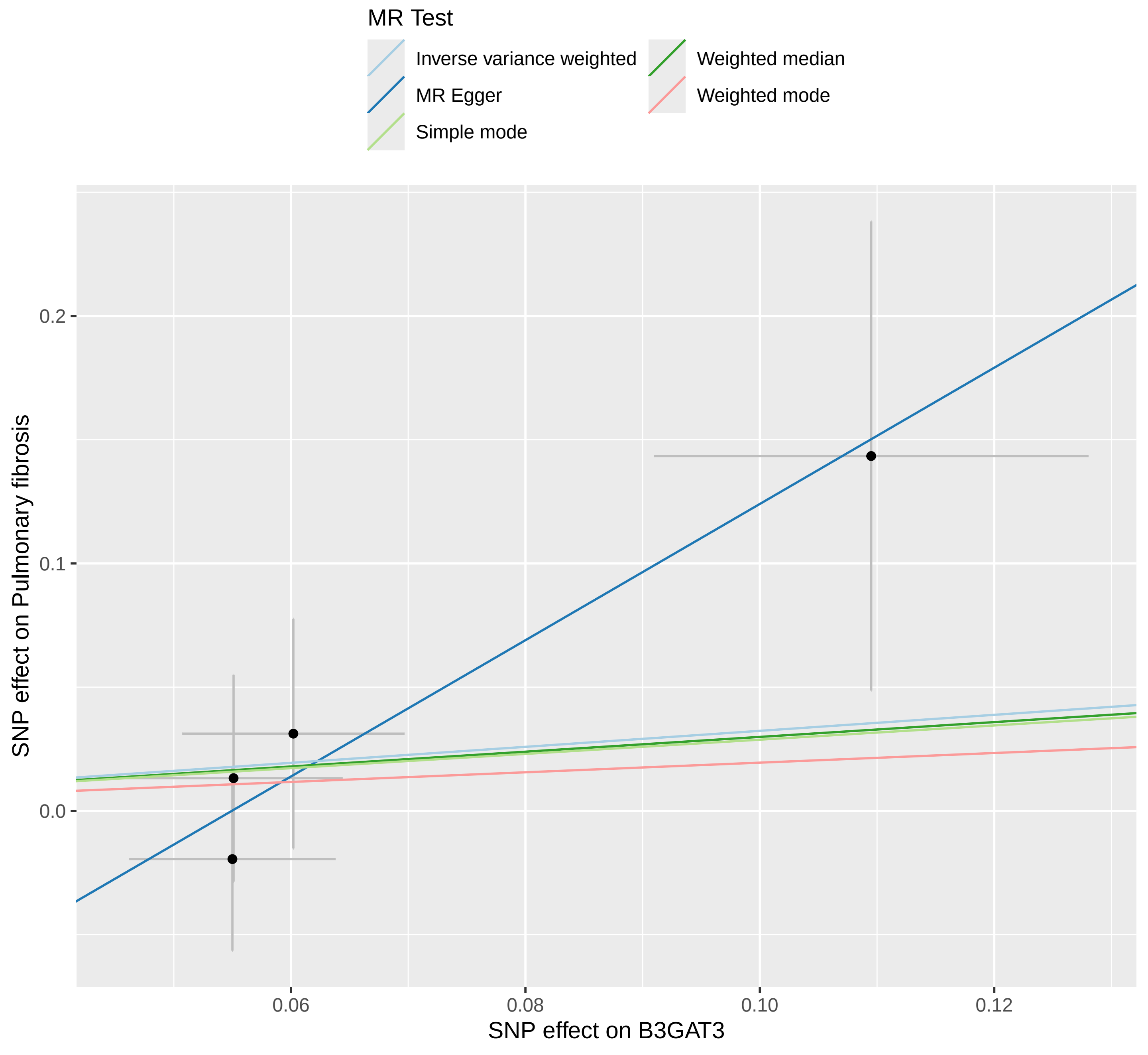

MR Test
Inverse variance weighted
MR Egger
Simple mode
Weighted median
Weighted mode
0.2
0.1
0.0
0.06
0.08
0.10
0.12
SNP effect on Pulmonary fibrosis
SNP effect on B3GAT3

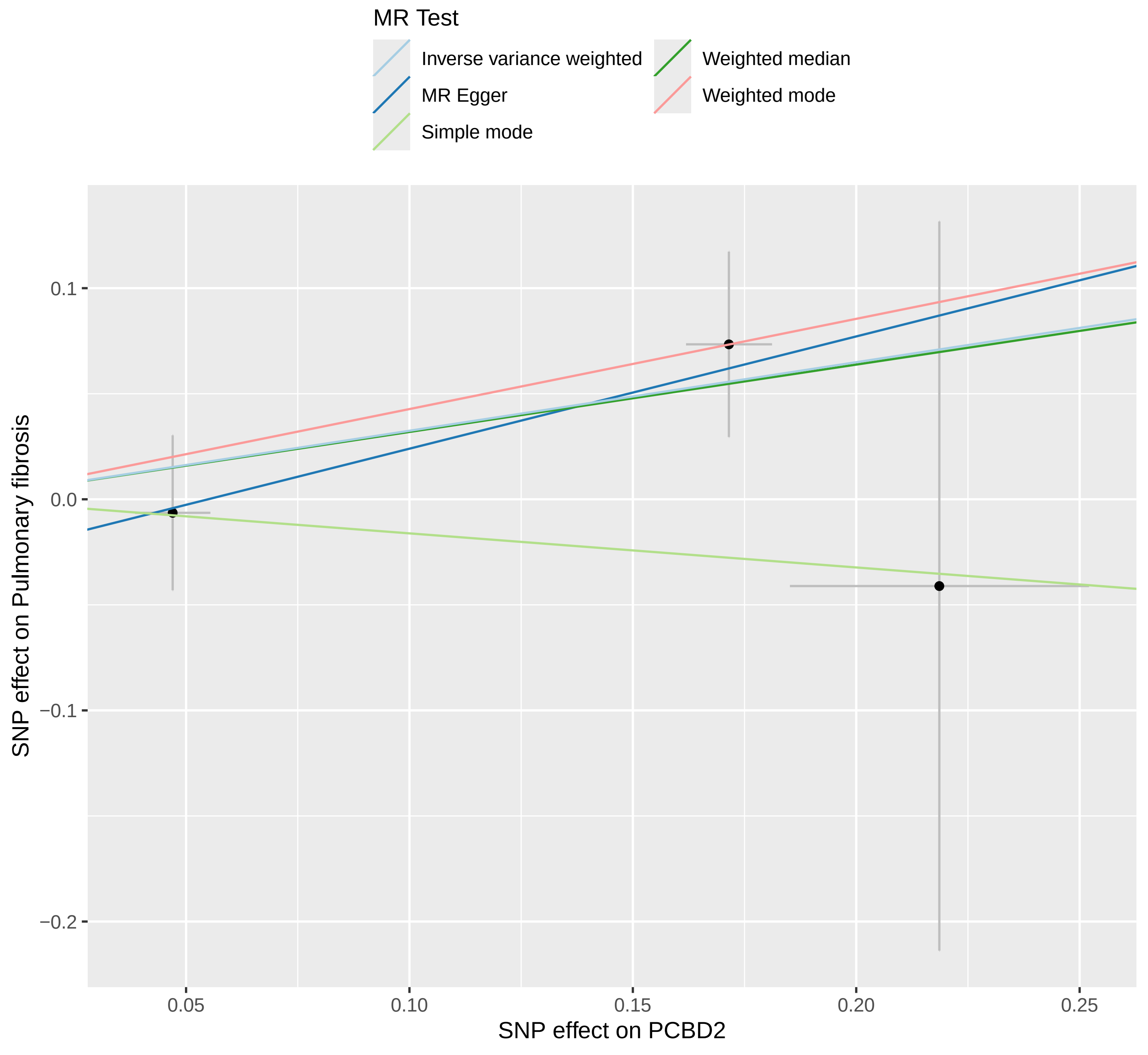
MR Test
Inverse variance weighted
MR Egger
Simple mode
Weighted median
Weighted mode
0.1
0.0
−0.1
−0.2
0.05
0.10
0.15
0.20
0.25
SNP effect on Pulmonary fibrosis
SNP effect on PCBD2

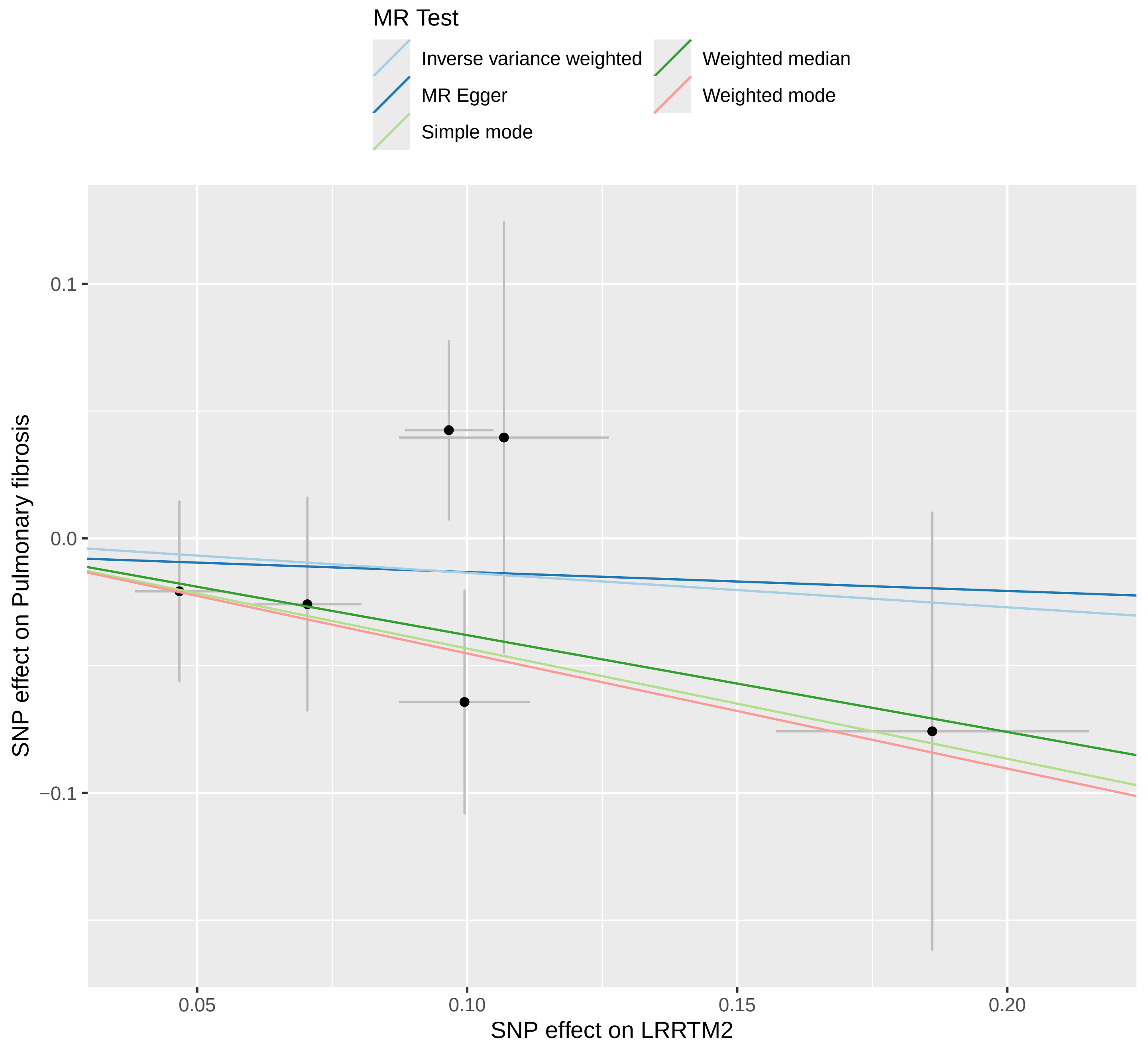

MR Test
Inverse variance weighted
MR Egger
Simple mode
Weighted median
Weighted mode
0.1
0.0
−0.1
0.05
0.10
0.15
0.20
SNP effect on Pulmonary fibrosis
SNP effect on LRRTM2

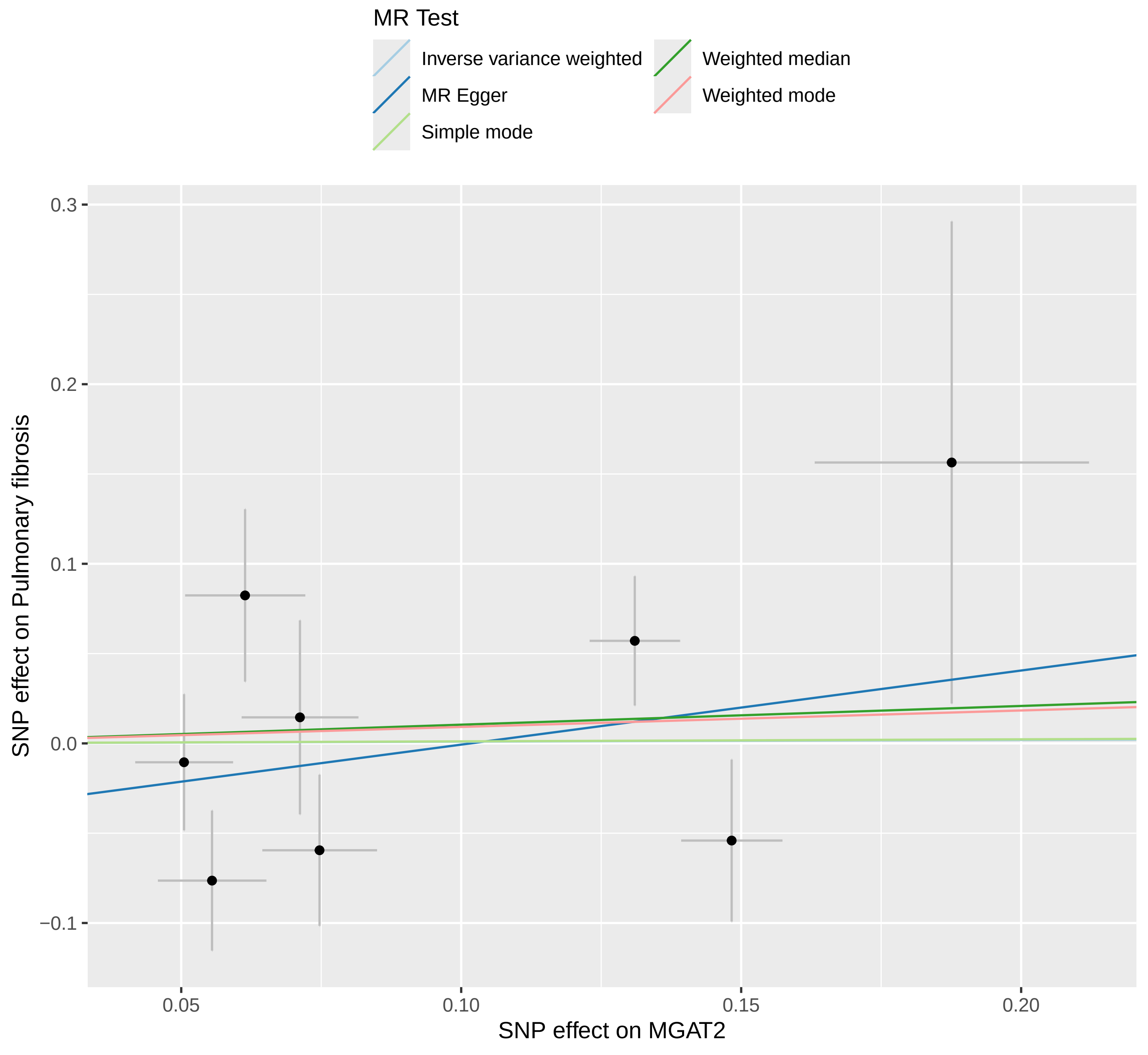

MR Test
Inverse variance weighted
MR Egger
Simple mode
Weighted median
Weighted mode
0.3
0.2
0.1
0.0
−0.1
SNP effect on Pulmonary fibrosis
0.05
0.10
0.15
0.20
SNP effect on MGAT2

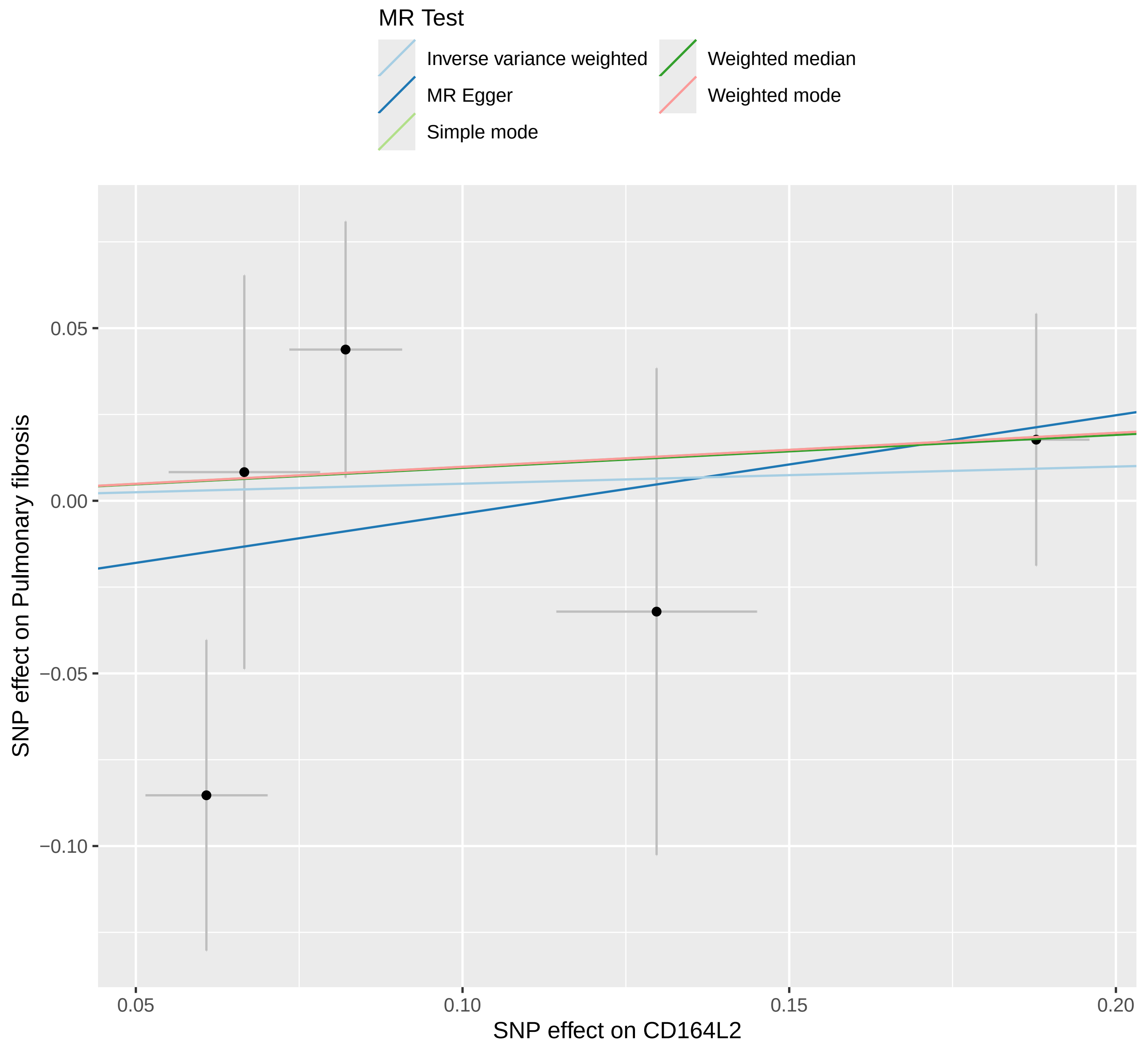

MR Test
Inverse variance weighted
MR Egger
Simple mode
Weighted median
Weighted mode
0.05
0.00
−0.05
−0.10
0.05
0.10
0.15
0.20
SNP effect on CD164L2
SNP effect on Pulmonary fibrosis

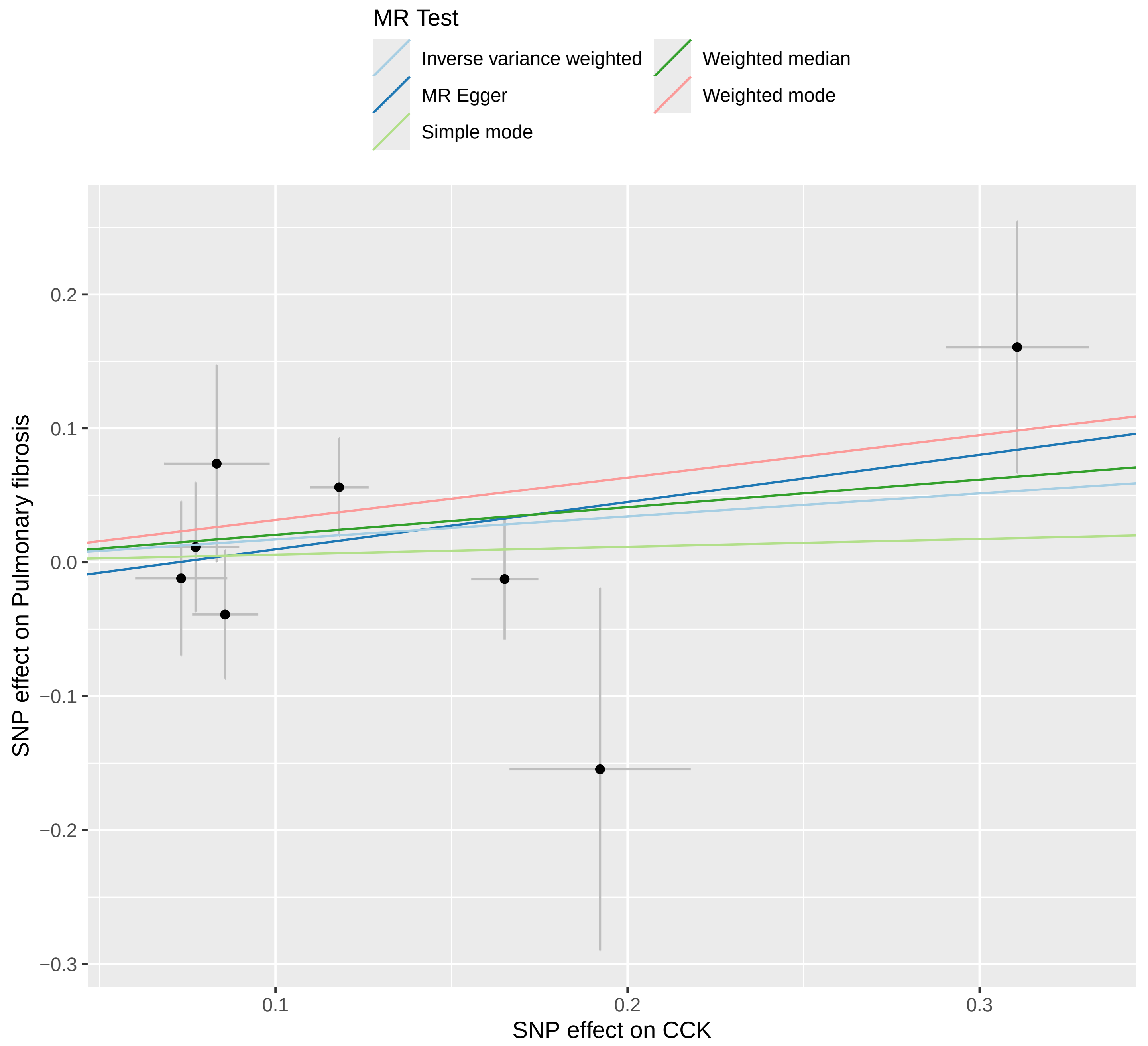
MR Test
Inverse variance weighted
MR Egger
Simple mode
Weighted median
Weighted mode
SNP effect on Pulmonary fibrosis
SNP effect on CCK
0.2
0.1
0.0
−0.1
−0.2
−0.3
0.1
0.2
0.3

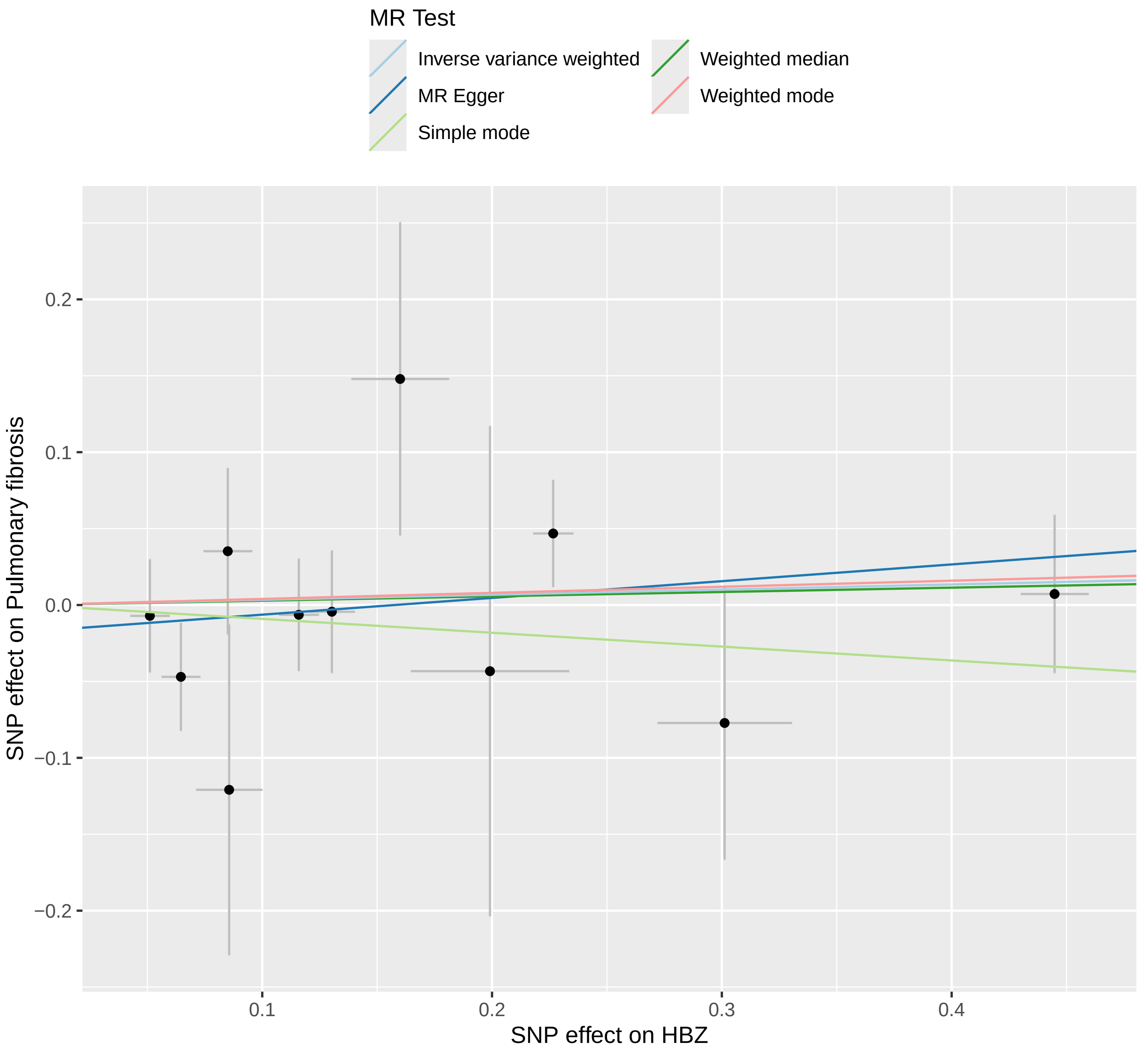
MR Test
Inverse variance weighted
MR Egger
Simple mode
Weighted median
Weighted mode
0.2
0.1
0.0
−0.1
−0.2
SNP effect on Pulmonary fibrosis
0.1
0.2
0.3
0.4
SNP effect on HBZ

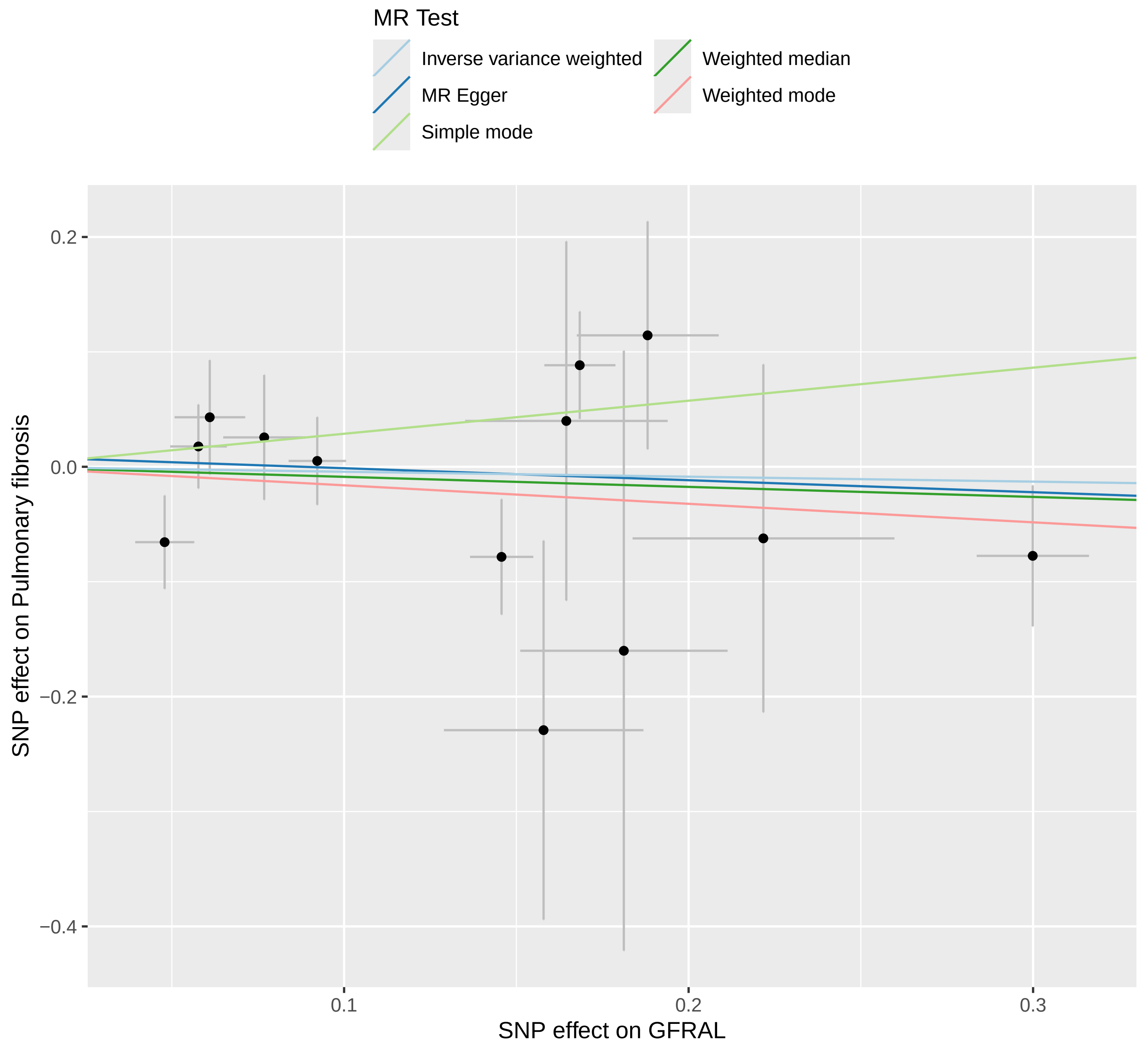

MR Test
Inverse variance weighted
MR Egger
Simple mode
Weighted median
Weighted mode
0.2
0.0
−0.2
−0.4
0.1
0.2
0.3
SNP effect on Pulmonary fibrosis
SNP effect on GFRAL

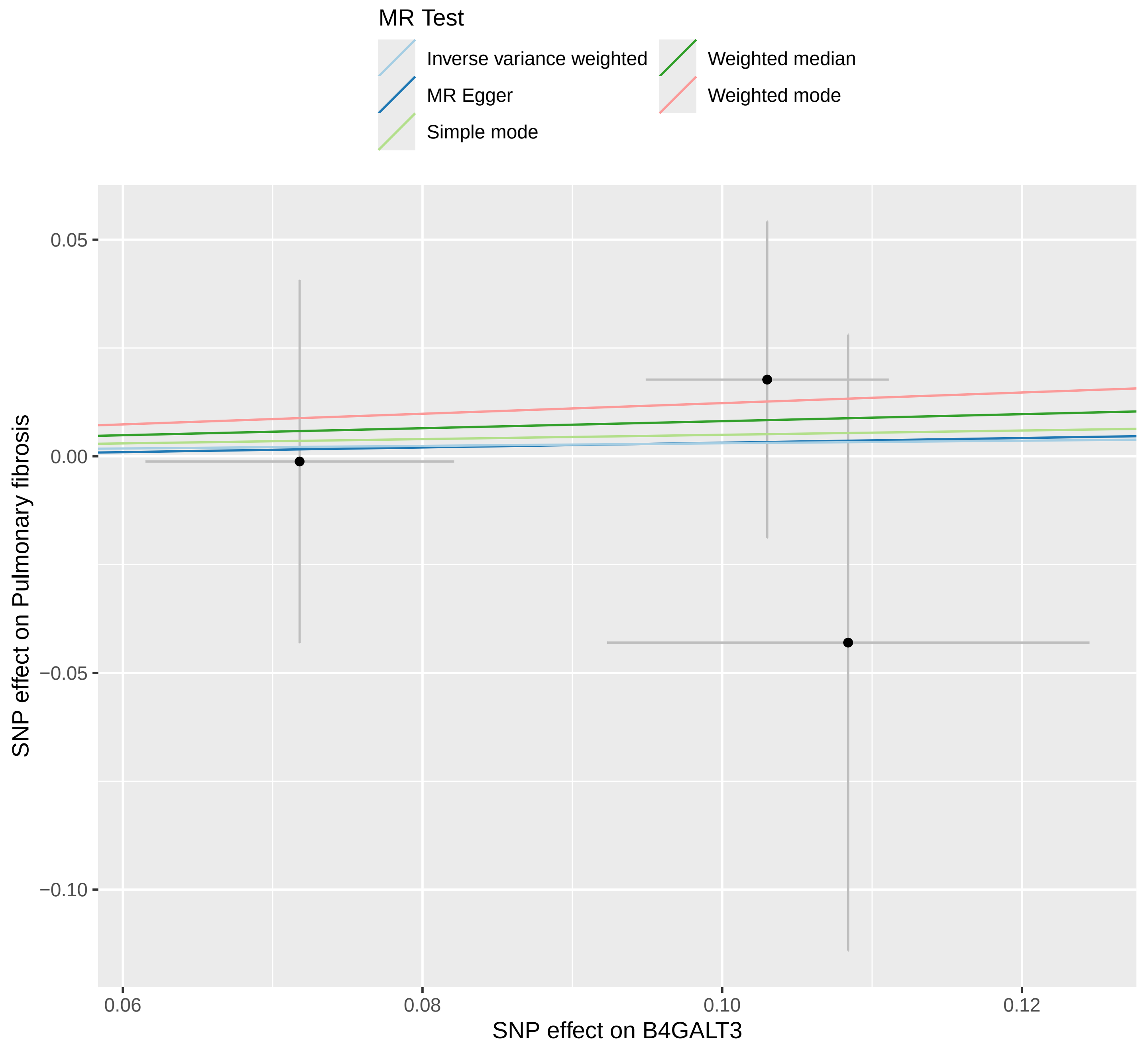
MR Test
Inverse variance weighted
MR Egger
Simple mode
Weighted median
Weighted mode
0.05
0.00
−0.05
−0.10
0.06
0.08
0.10
0.12
SNP effect on B4GALT3
SNP effect on Pulmonary fibrosis

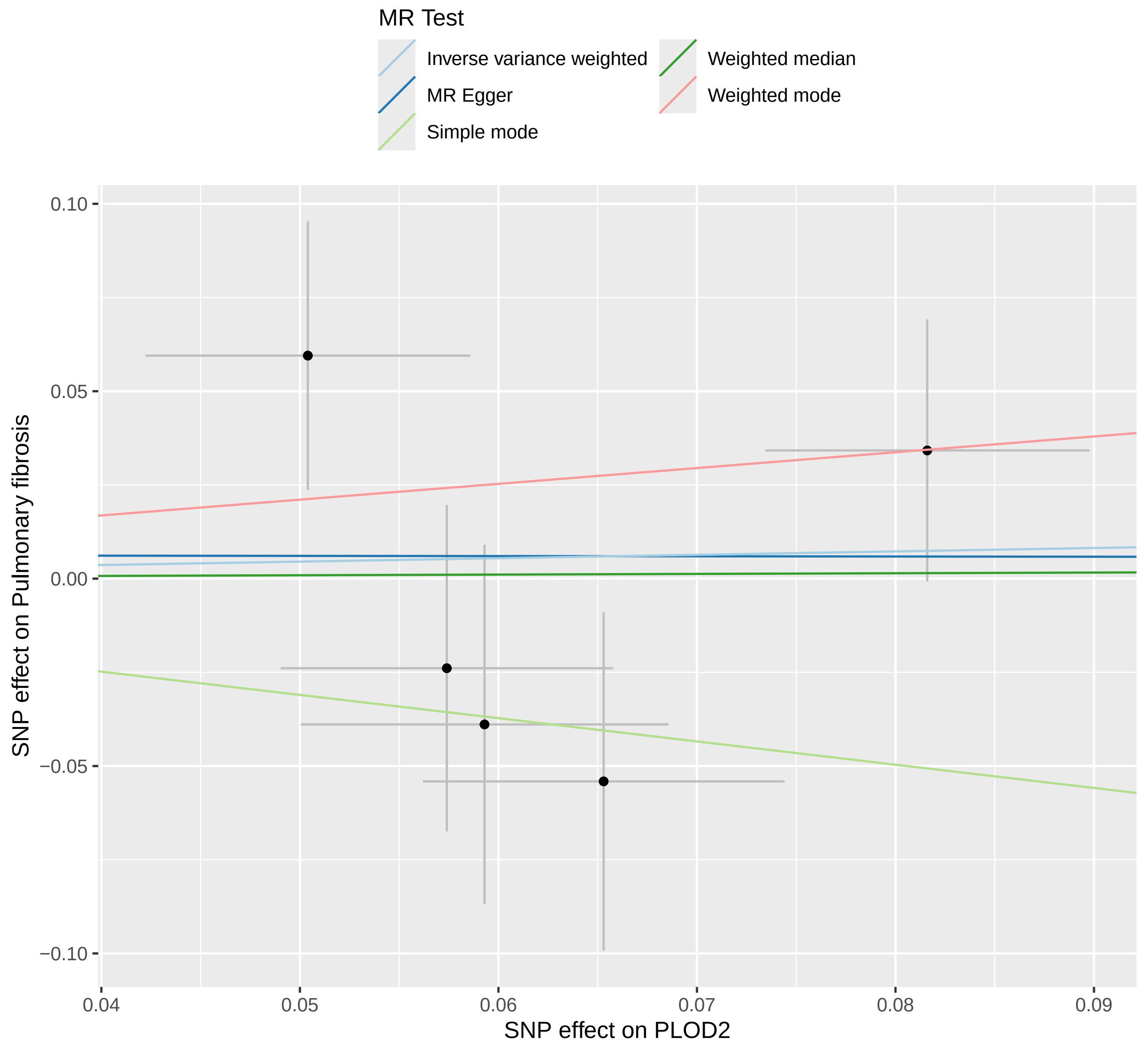

MR Test
Inverse variance weighted
MR Egger
Simple mode
Weighted median
Weighted mode
0.10
0.05
0.00
−0.05
−0.10
SNP effect on Pulmonary fibrosis
0.04
0.05
0.06
0.07
0.08
0.09
SNP effect on PLOD2

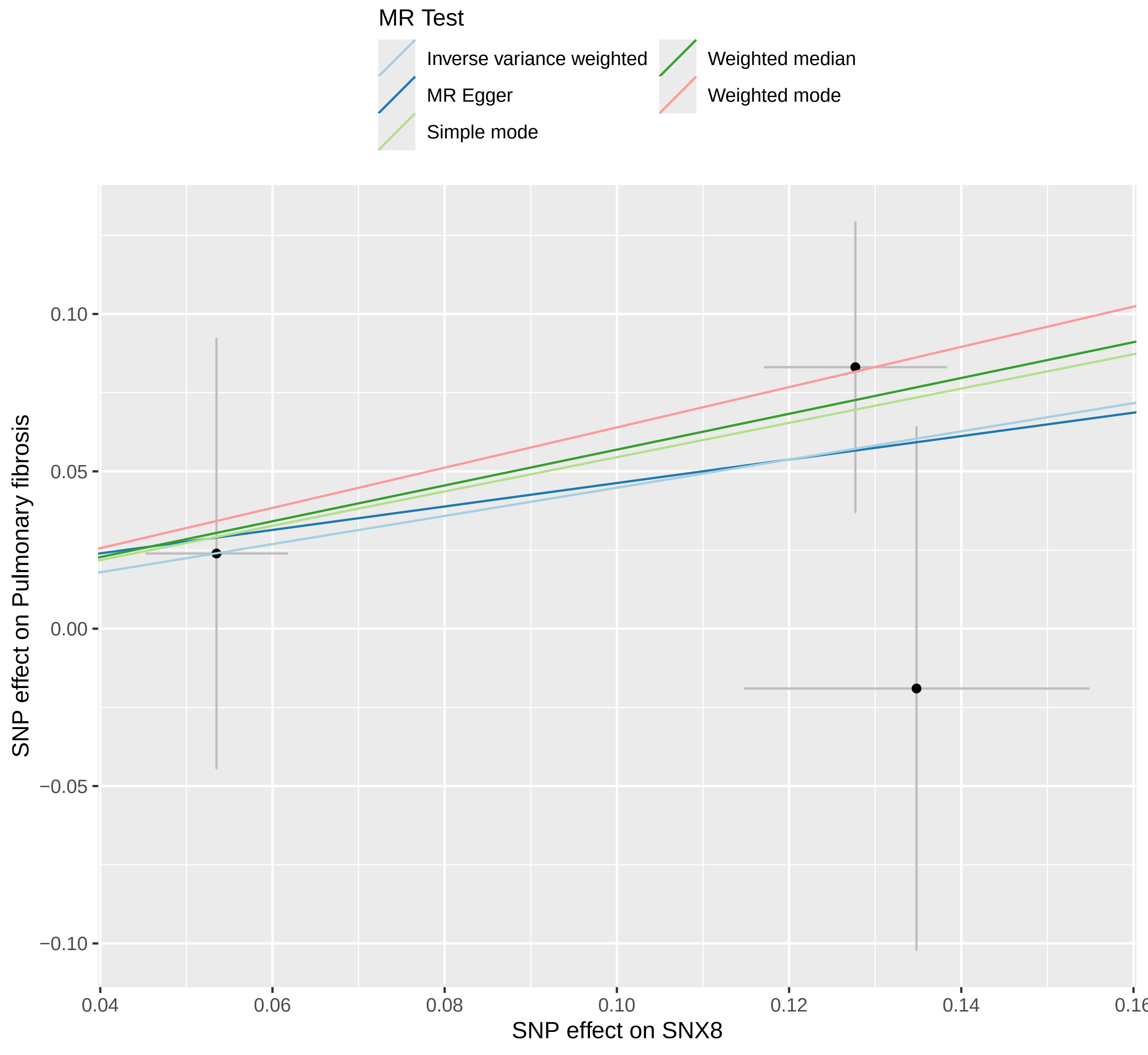

MR Test
Inverse variance weighted
MR Egger
Simple mode
Weighted median
Weighted mode
0.10
0.05
0.00
−0.05
−0.10
SNP effect on Pulmonary fibrosis
0.04
0.06
0.08
0.10
0.12
0.14
0.16
SNP effect on SNX8

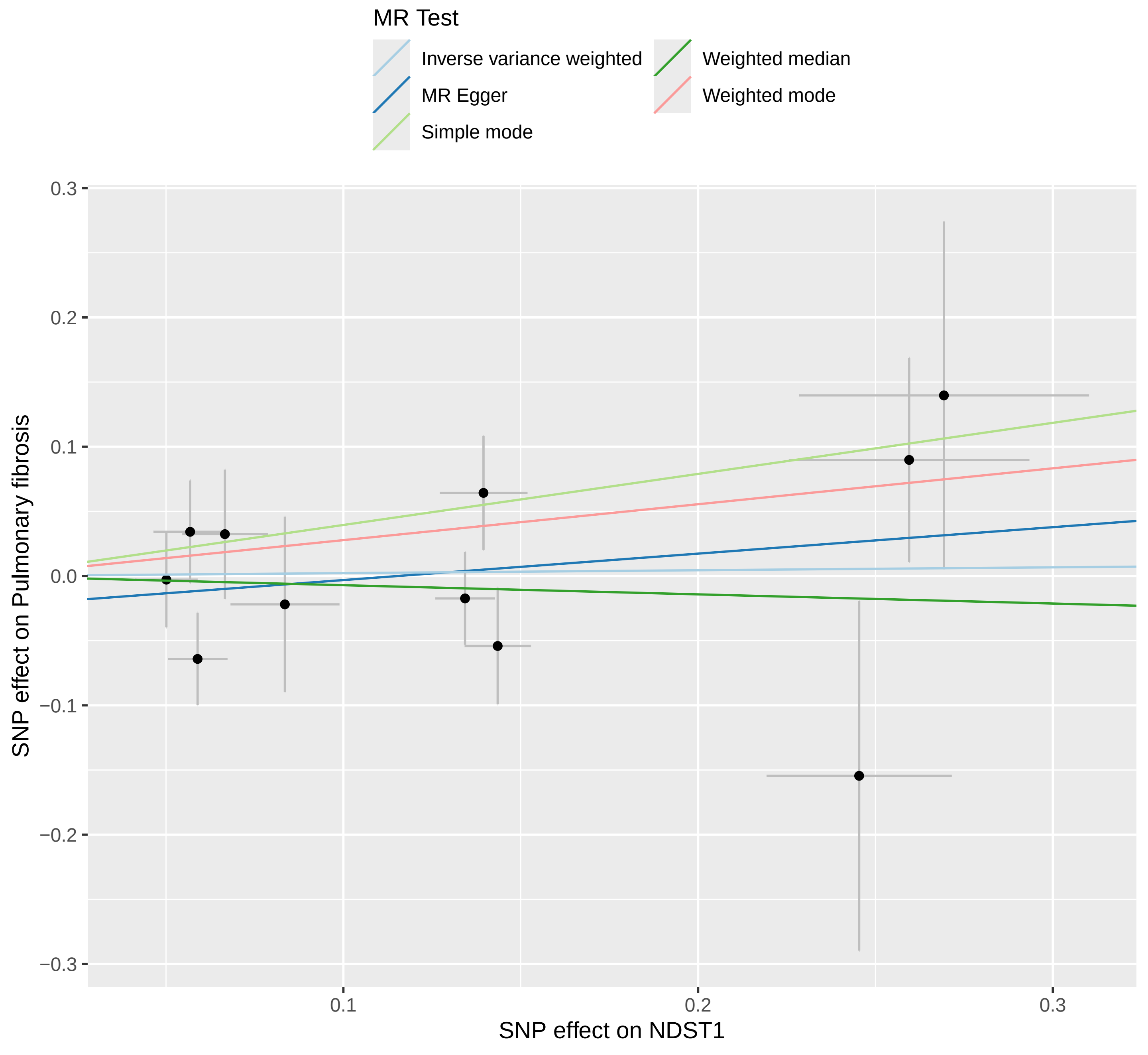
MR Test
Inverse variance weighted
MR Egger
Simple mode
Weighted median
Weighted mode
0.3
0.2
0.1
0.0
−0.1
−0.2
−0.3
SNP effect on Pulmonary fibrosis
0.1
0.2
0.3
SNP effect on NDST1

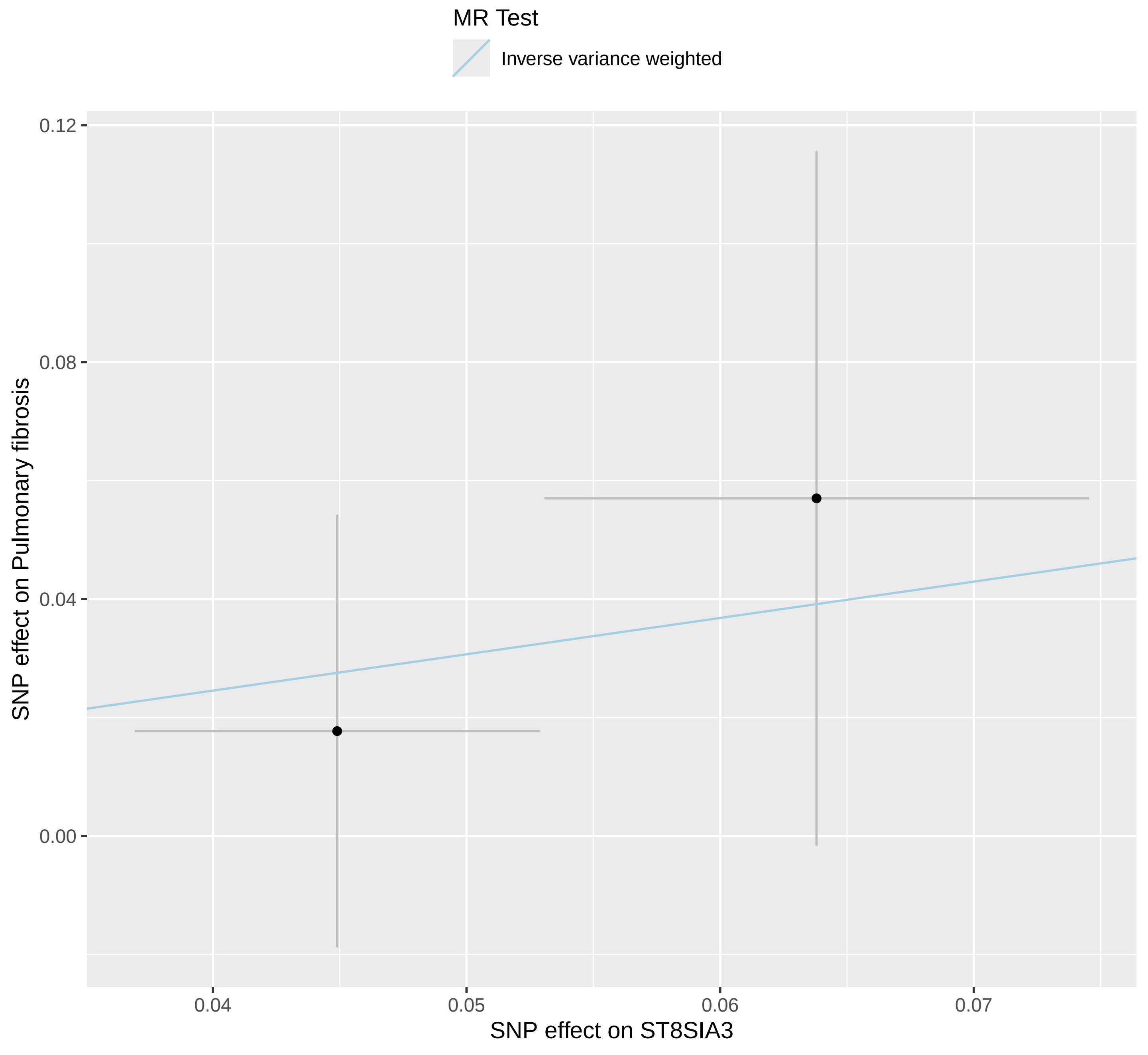

MR Test
Inverse variance weighted
0.12
0.08
0.04
0.00
SNP effect on Pulmonary fibrosis
0.04
0.05
0.06
0.07
SNP effect on ST8SIA3

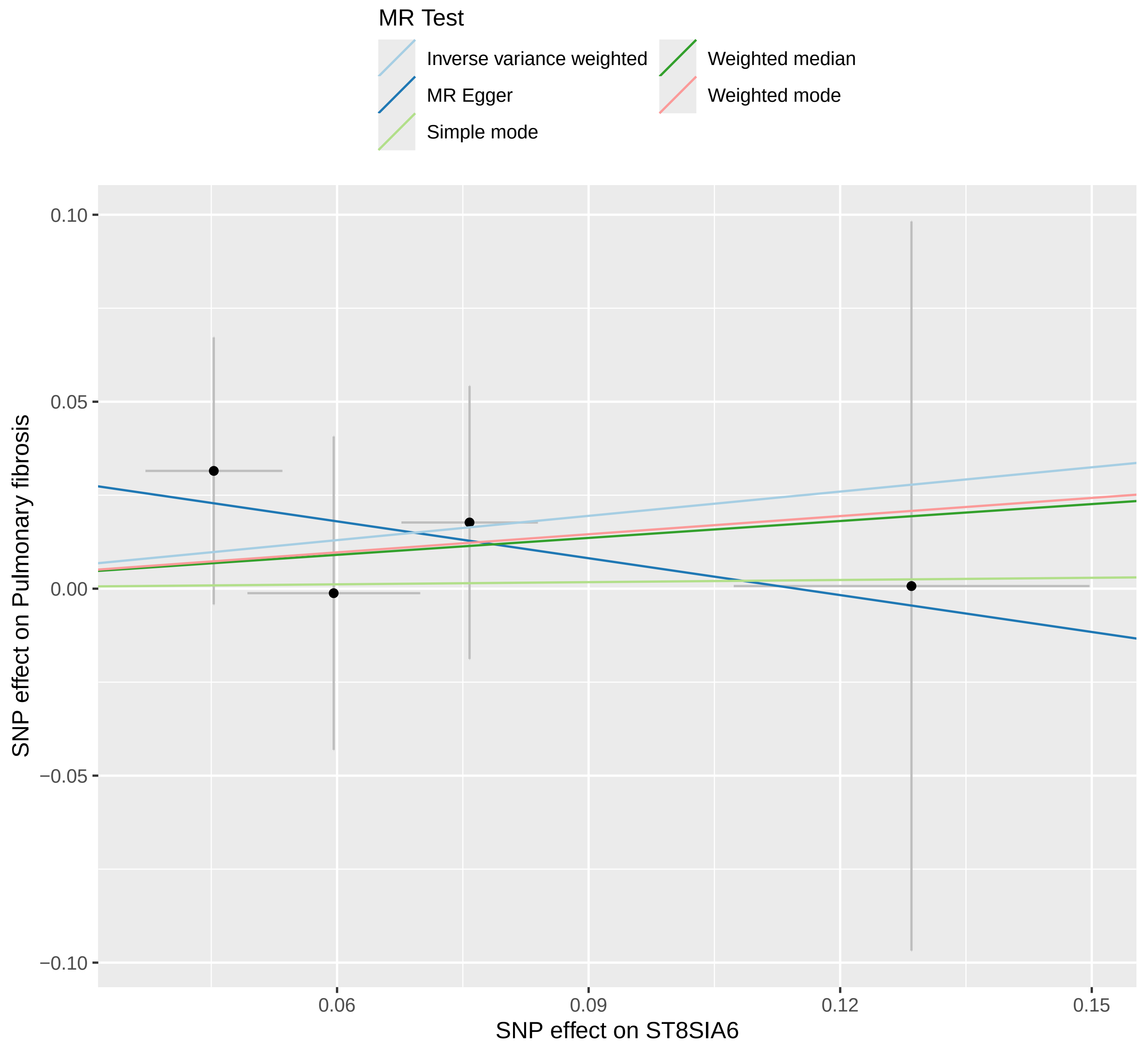

MR Test
Inverse variance weighted
MR Egger
Simple mode
Weighted median
Weighted mode
0.10
0.05
0.00
−0.05
−0.10
SNP effect on Pulmonary fibrosis
0.06
0.09
0.12
0.15
SNP effect on ST8SIA6

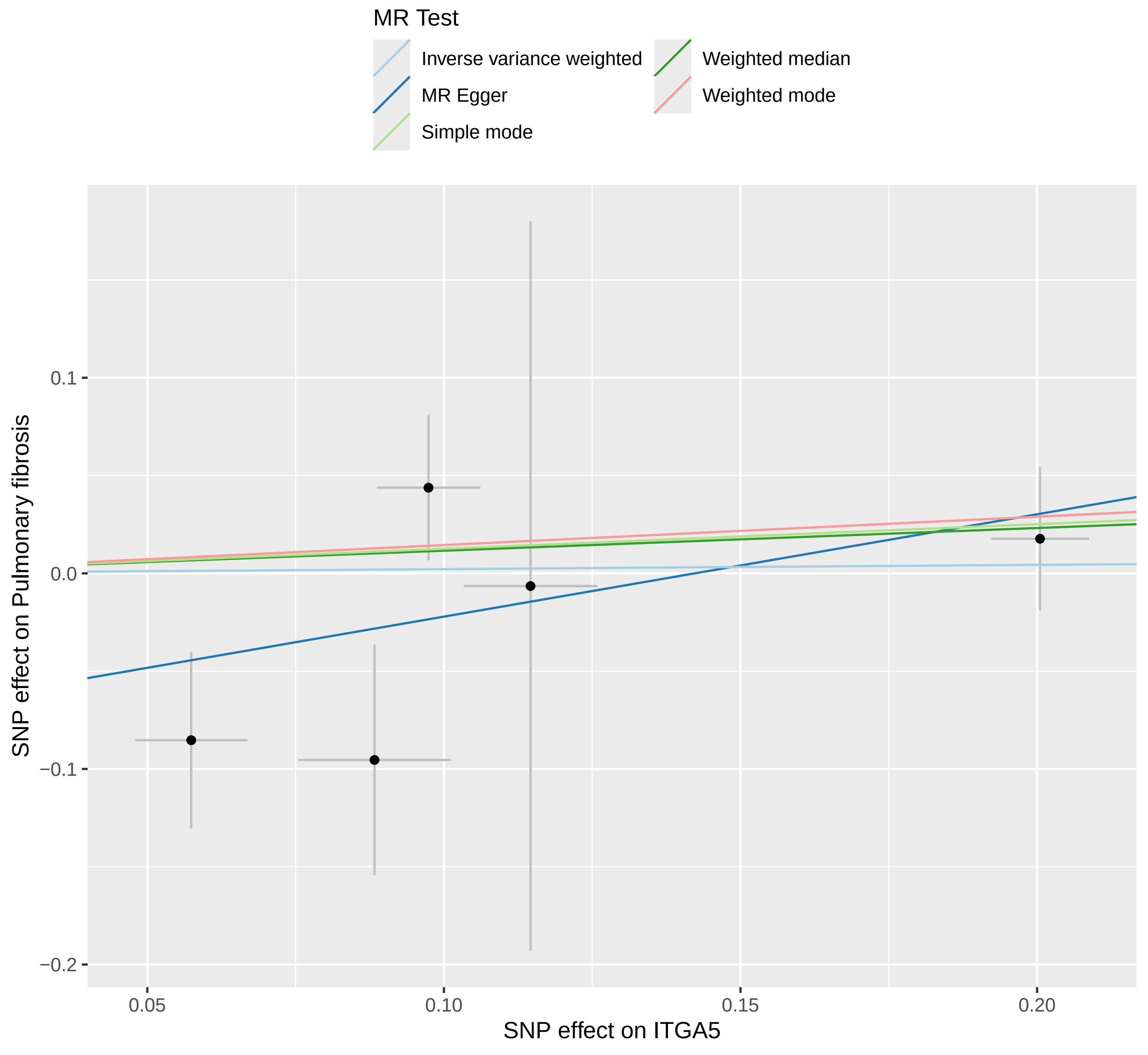

MR Test
Inverse variance weighted
MR Egger
Simple mode
Weighted median
Weighted mode
0.1
0.0
−0.1
−0.2
0.05
0.10
0.15
0.20
SNP effect on Pulmonary fibrosis
SNP effect on ITGA5

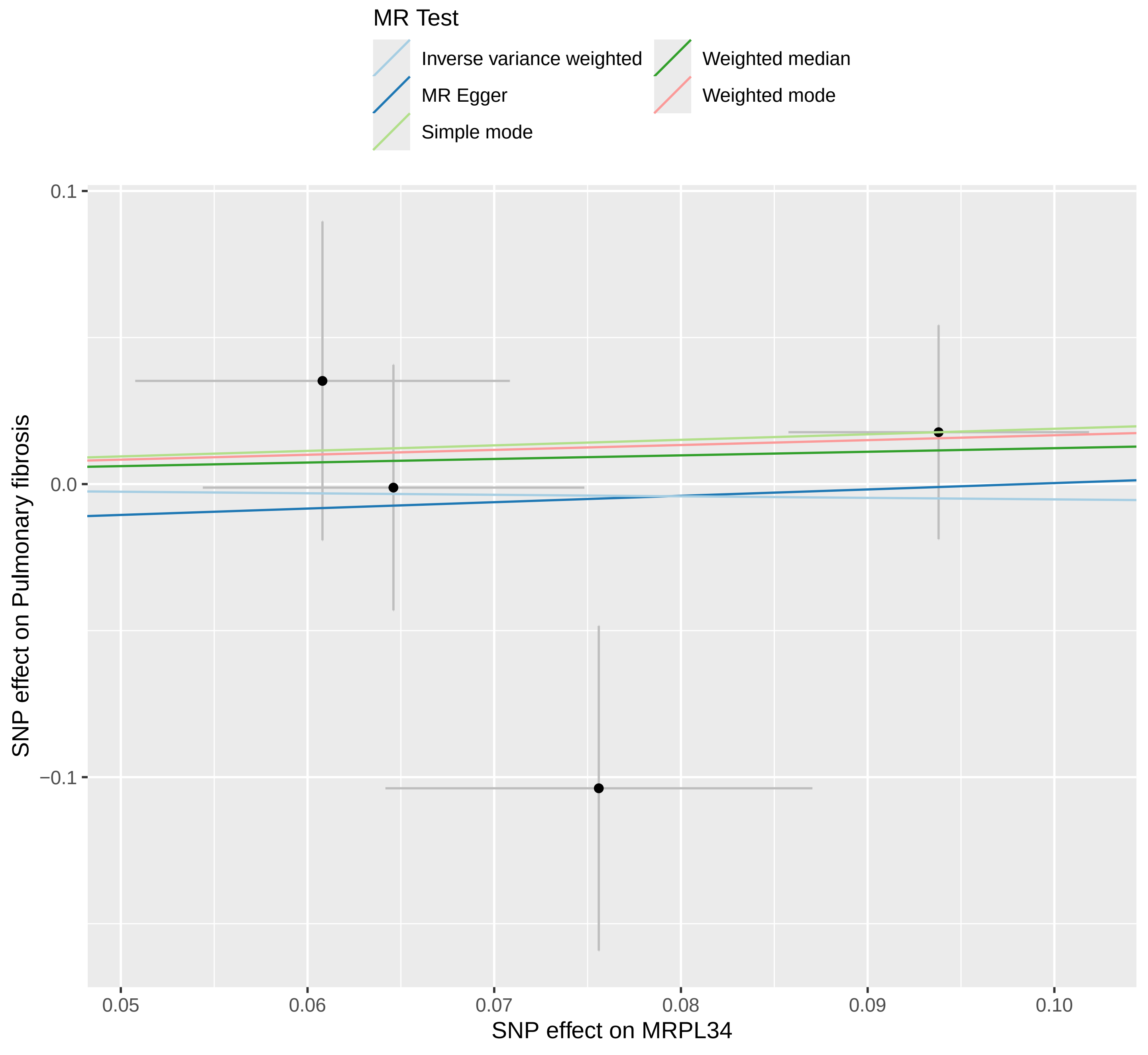

MR Test
Inverse variance weighted
MR Egger
Simple mode
Weighted median
Weighted mode
0.1
0.0
−0.1
SNP effect on Pulmonary fibrosis
0.05
0.06
0.07
0.08
0.09
0.10
SNP effect on MRPL34

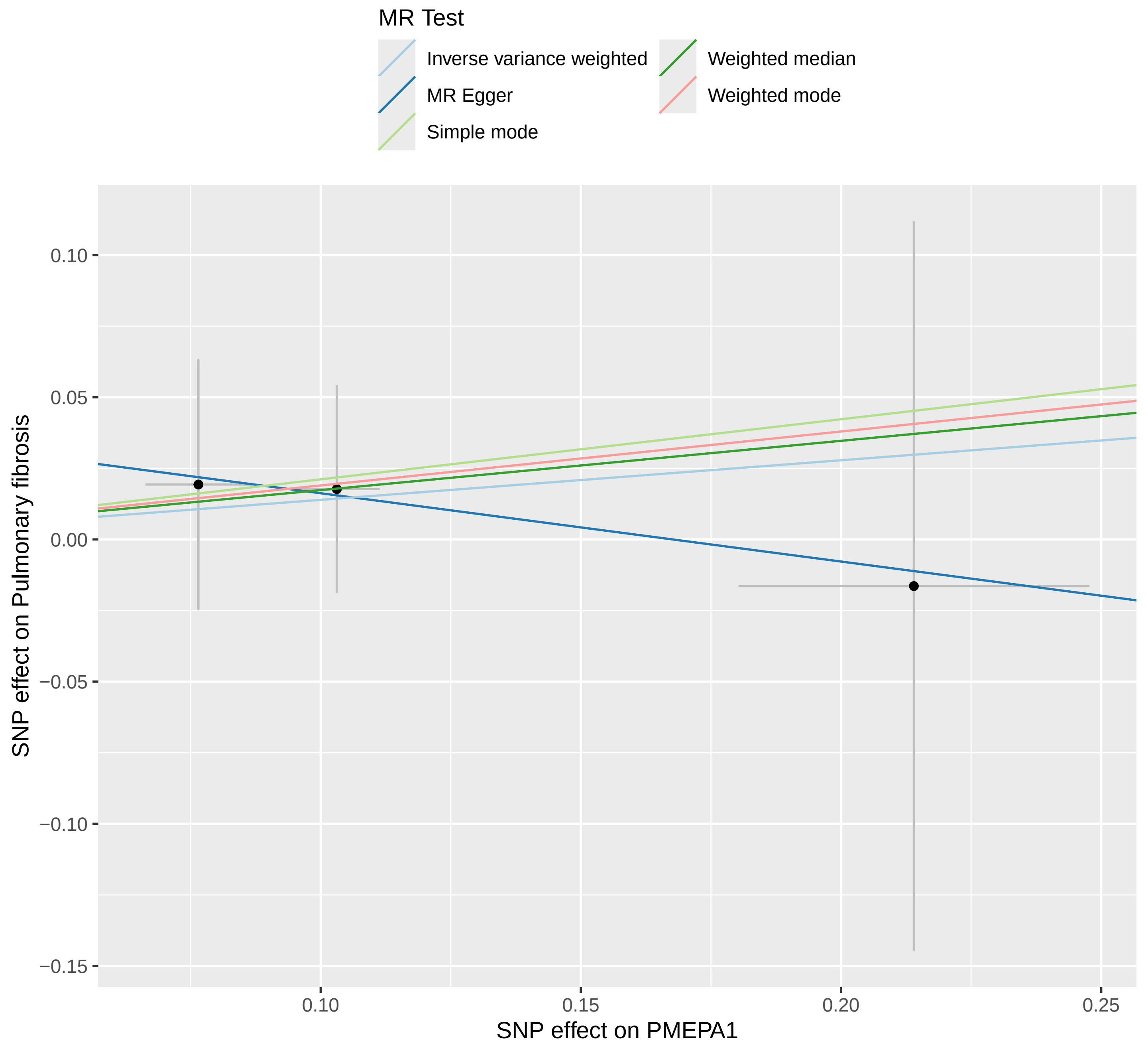

MR Test
Inverse variance weighted
MR Egger
Simple mode
Weighted median
Weighted mode
0.10
0.05
0.00
−0.05
−0.10
−0.15
SNP effect on Pulmonary fibrosis
0.10
0.15
0.20
0.25
SNP effect on PMEPA1

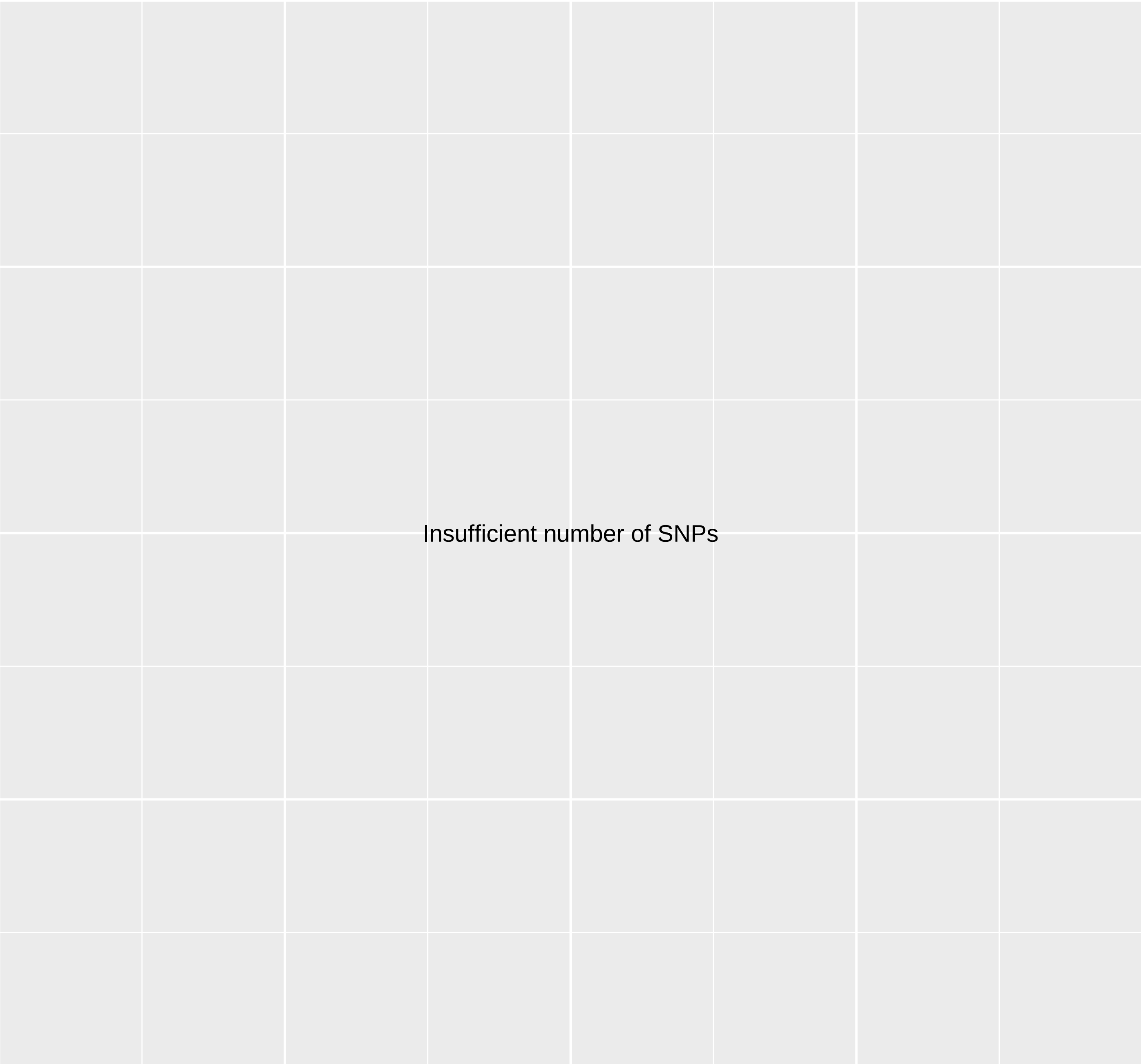
Insufficient number of SNPs

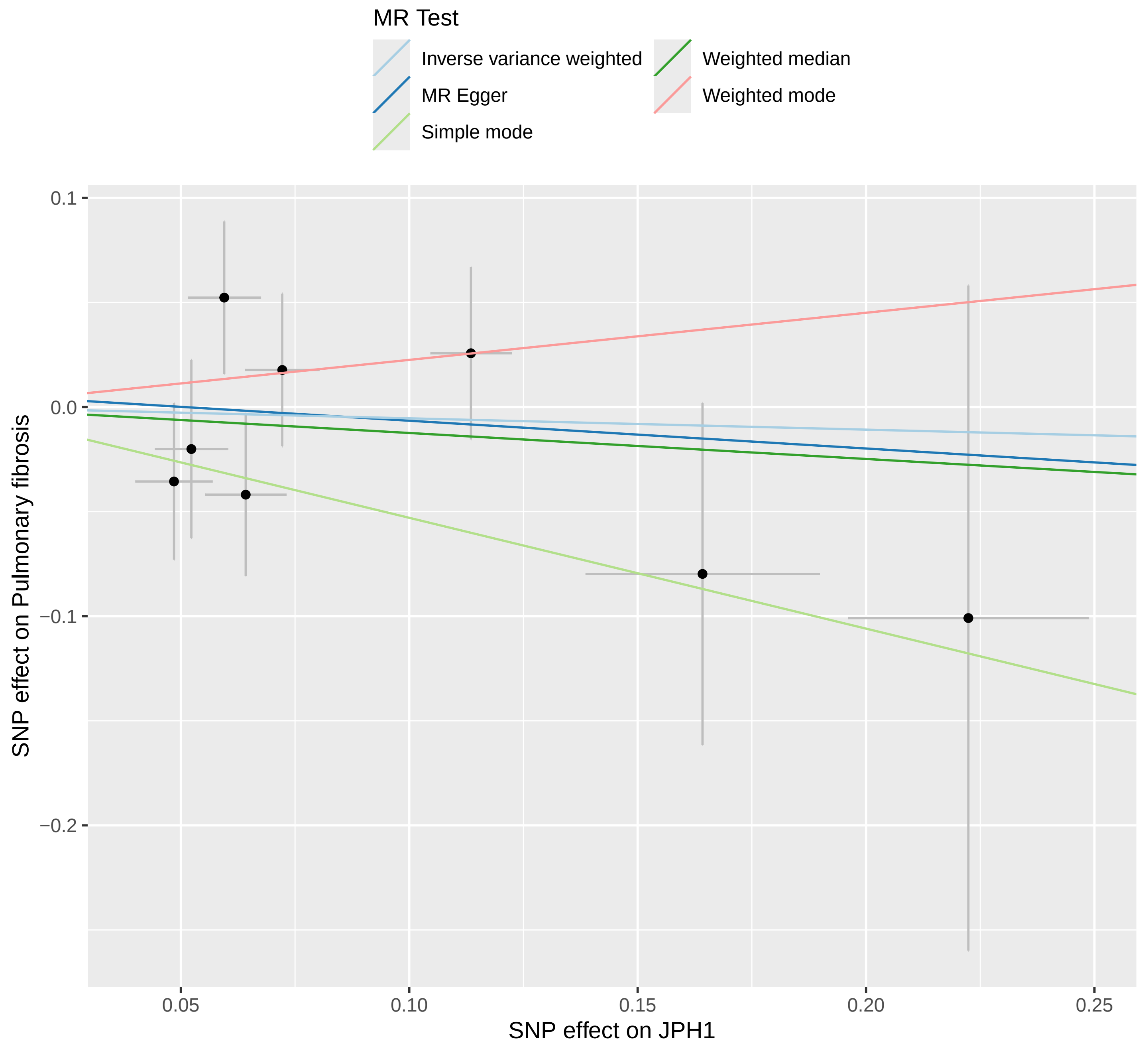

MR Test
Inverse variance weighted
MR Egger
Simple mode
Weighted median
Weighted mode
0.1
0.0
−0.1
−0.2
0.05
0.10
0.15
0.20
0.25
SNP effect on Pulmonary fibrosis
SNP effect on JPH1

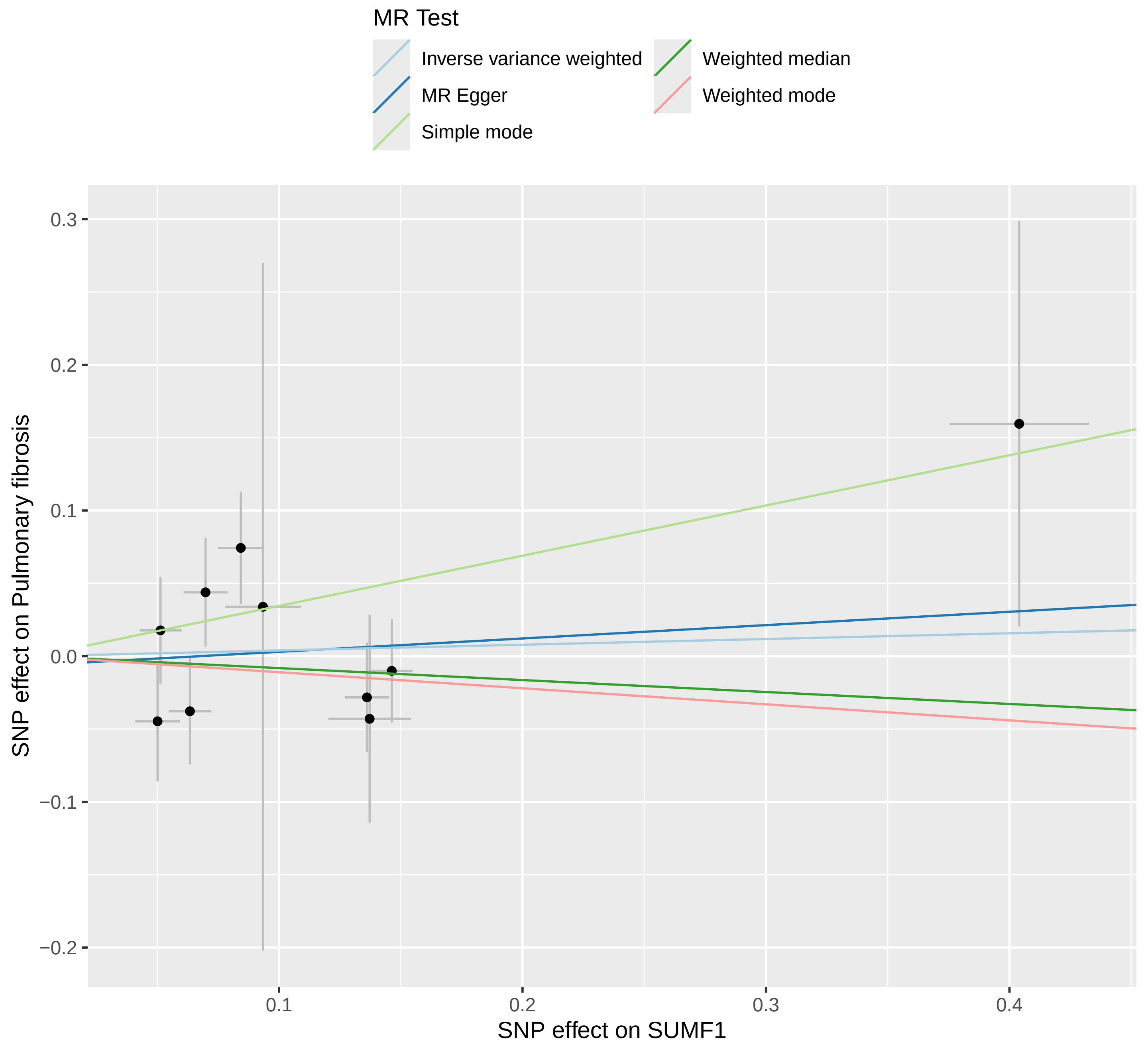

MR Test
Inverse variance weighted
MR Egger
Simple mode
Weighted median
Weighted mode
SNP effect on Pulmonary fibrosis
0.3
0.2
0.1
0.0
−0.1
−0.2
0.1
0.2
0.3
0.4
SNP effect on SUMF1

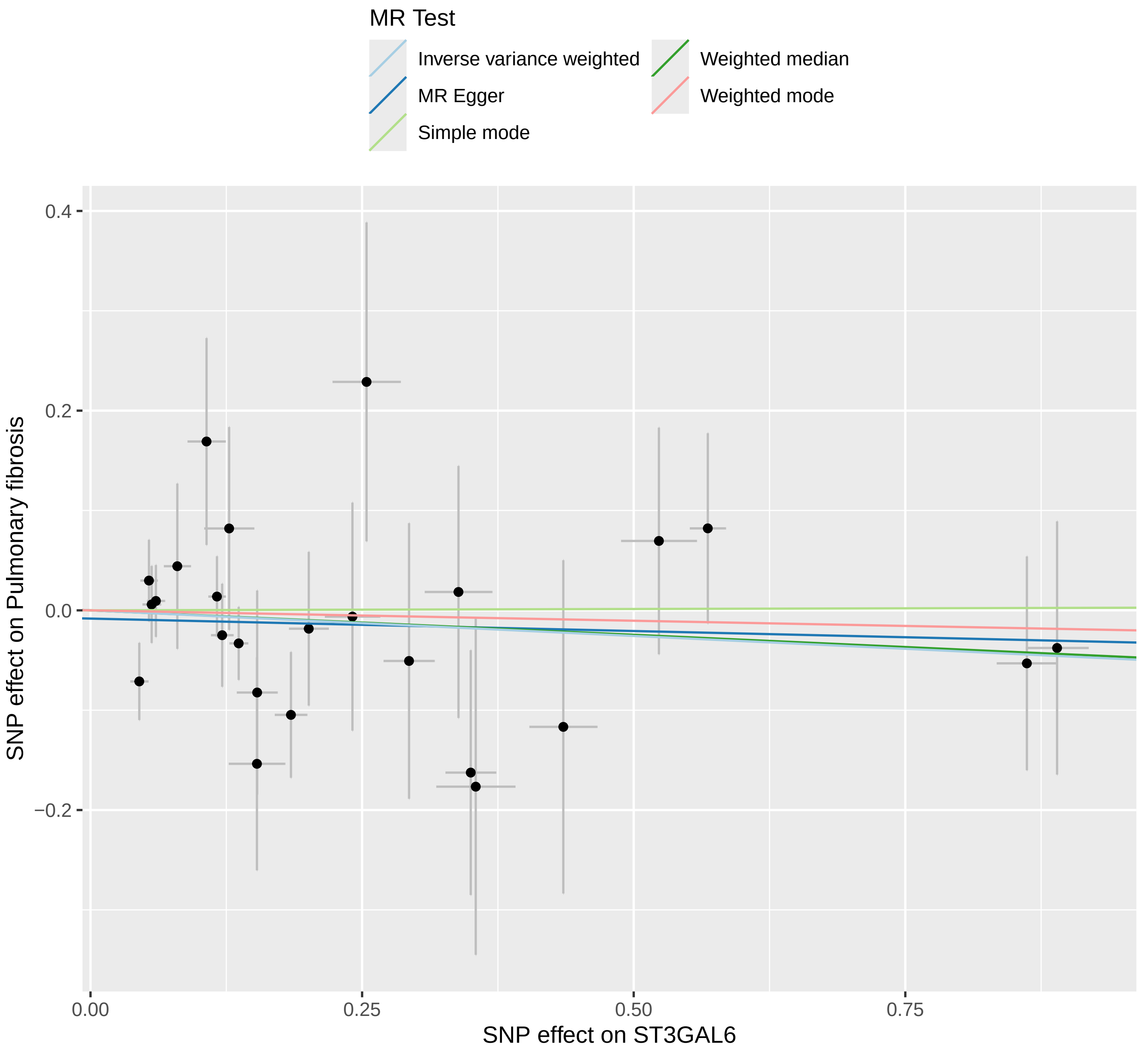
MR Test
Inverse variance weighted
MR Egger
Simple mode
Weighted median
Weighted mode
0.4
0.2
0.0
−0.2
SNP effect on Pulmonary fibrosis
0.00
0.25
0.50
0.75
SNP effect on ST3GAL6

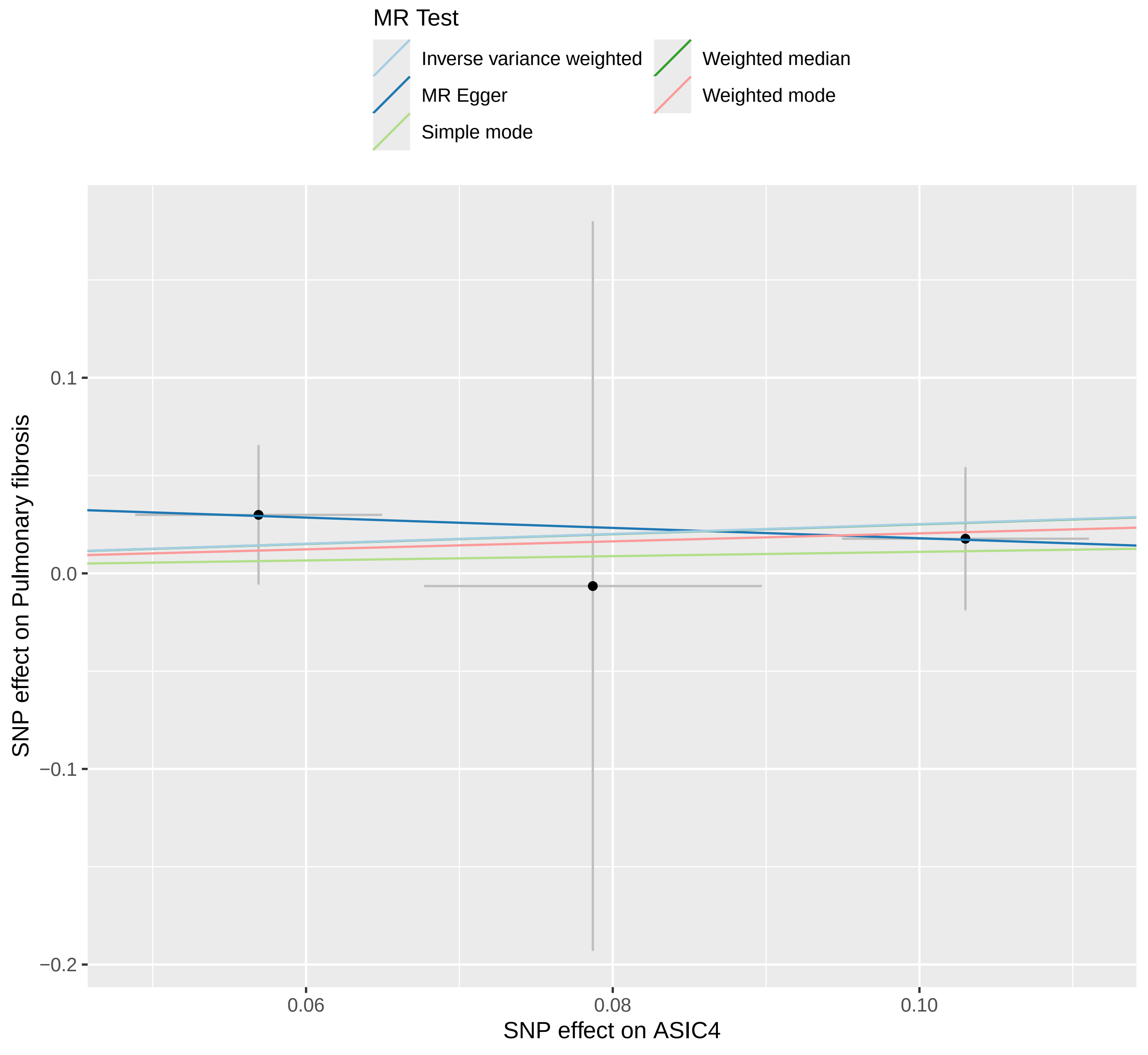
MR Test
Inverse variance weighted
MR Egger
Simple mode
Weighted median
Weighted mode
SNP effect on Pulmonary fibrosis
0.1
0.0
−0.1
−0.2
0.06
0.08
0.10
SNP effect on ASIC4

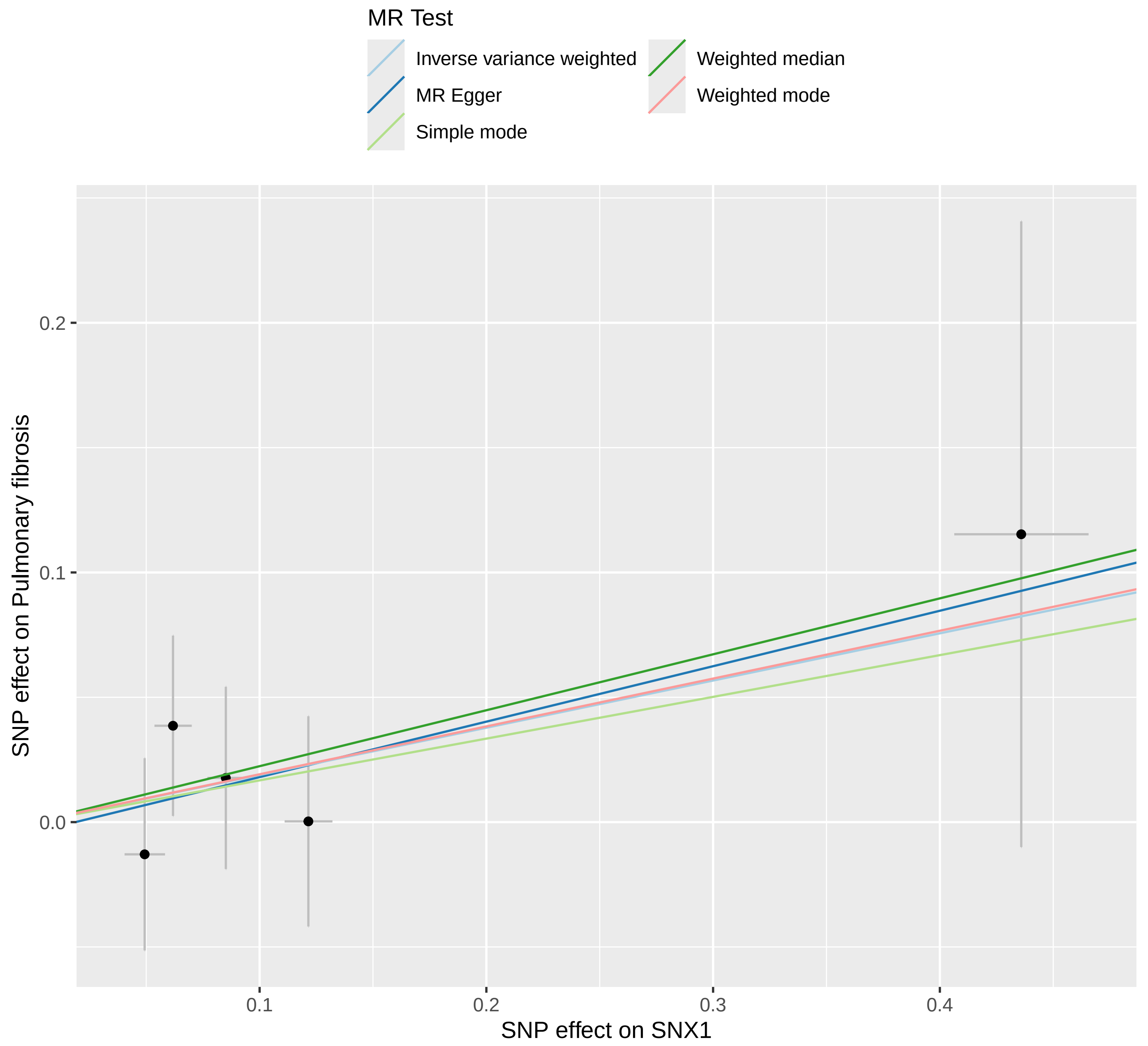
MR Test
Inverse variance weighted
MR Egger
Simple mode
Weighted median
Weighted mode
SNP effect on Pulmonary fibrosis
0.2
0.1
0.0
0.1
0.2
0.3
0.4
SNP effect on SNX1

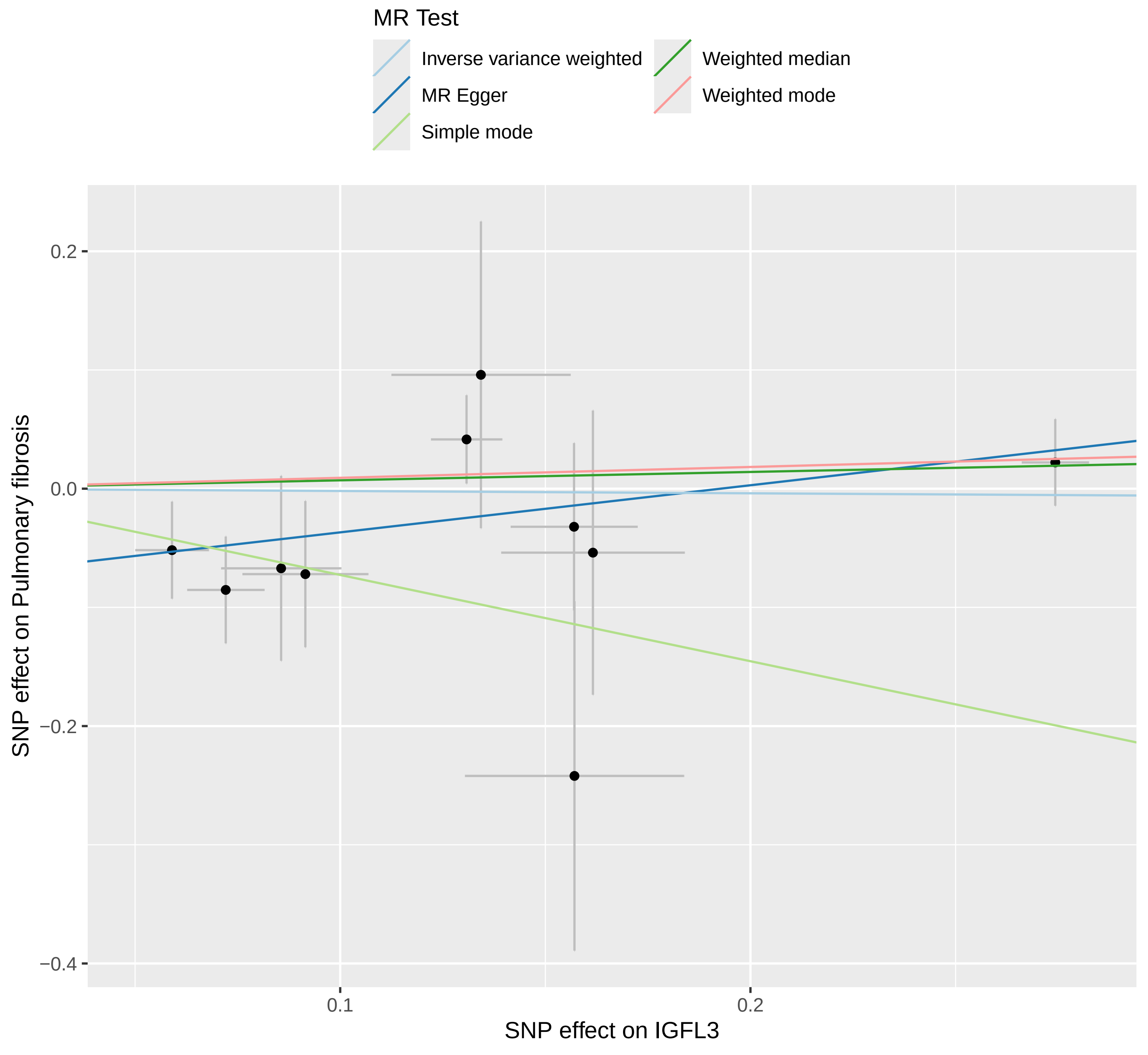
MR Test
Inverse variance weighted
MR Egger
Simple mode
Weighted median
Weighted mode
0.2
0.0
−0.2
−0.4
0.1
0.2
SNP effect on Pulmonary fibrosis
SNP effect on IGFL3

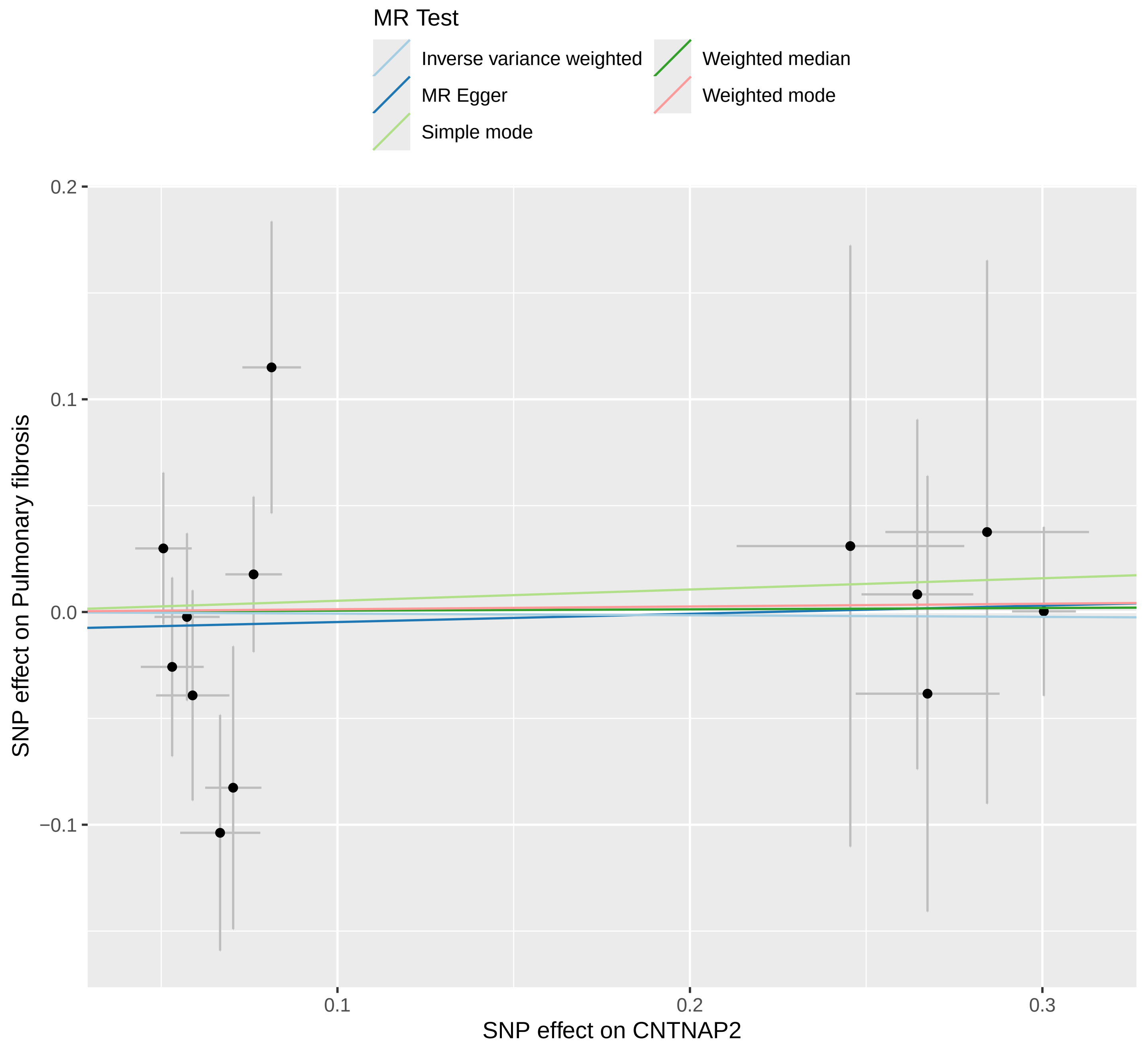

MR Test
Inverse variance weighted
MR Egger
Simple mode
Weighted median
Weighted mode
0.2
0.1
0.0
−0.1
0.1
0.2
0.3
SNP effect on Pulmonary fibrosis
SNP effect on CNTNAP2

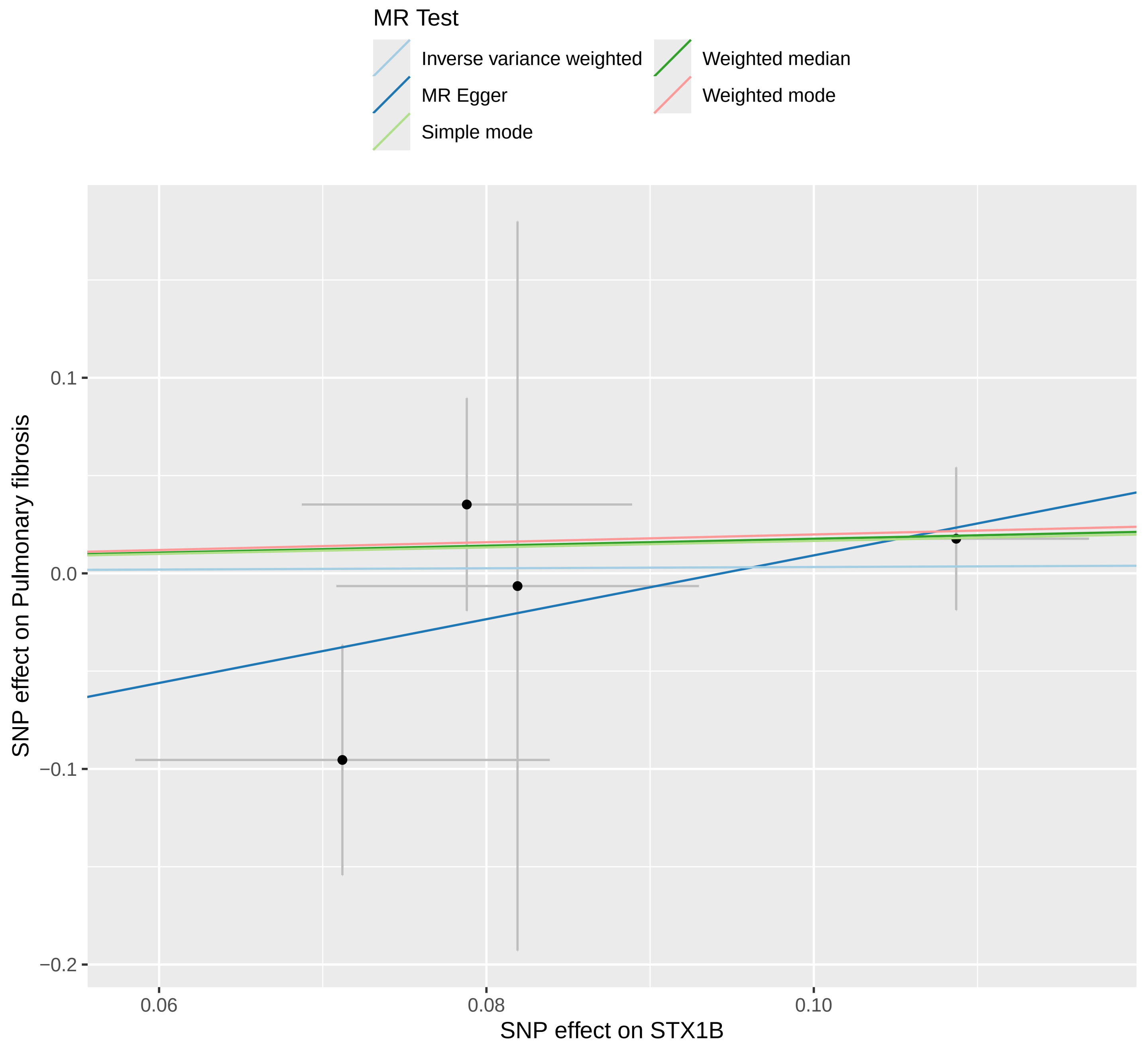

MR Test
Inverse variance weighted
MR Egger
Simple mode
Weighted median
Weighted mode
0.1
0.0
−0.1
−0.2
SNP effect on Pulmonary fibrosis
0.06
0.08
0.10
SNP effect on STX1B

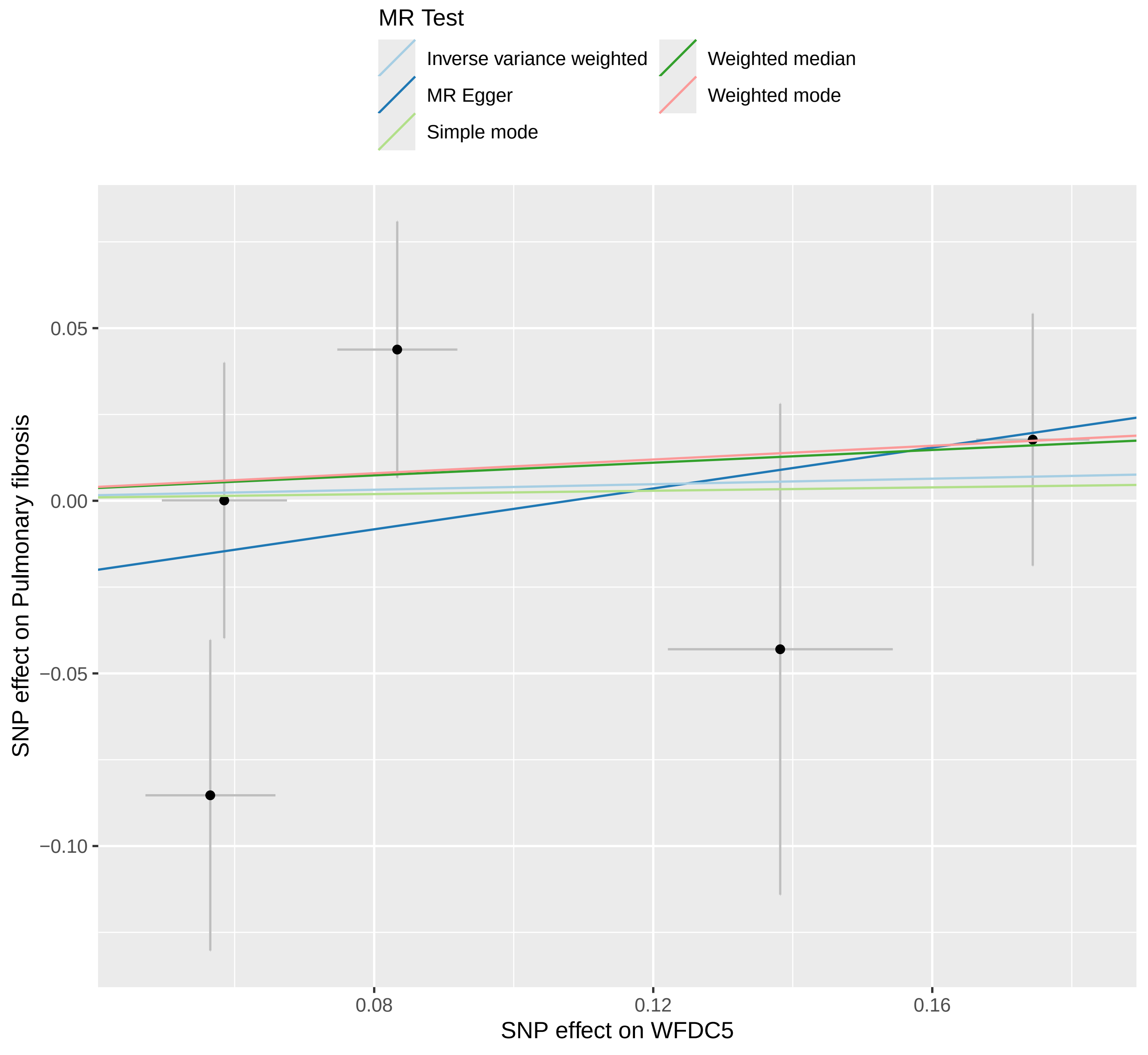
MR Test
Inverse variance weighted
MR Egger
Simple mode
Weighted median
Weighted mode
0.05
0.00
−0.05
−0.10
0.08
0.12
0.16
SNP effect on WFDC5
SNP effect on Pulmonary fibrosis

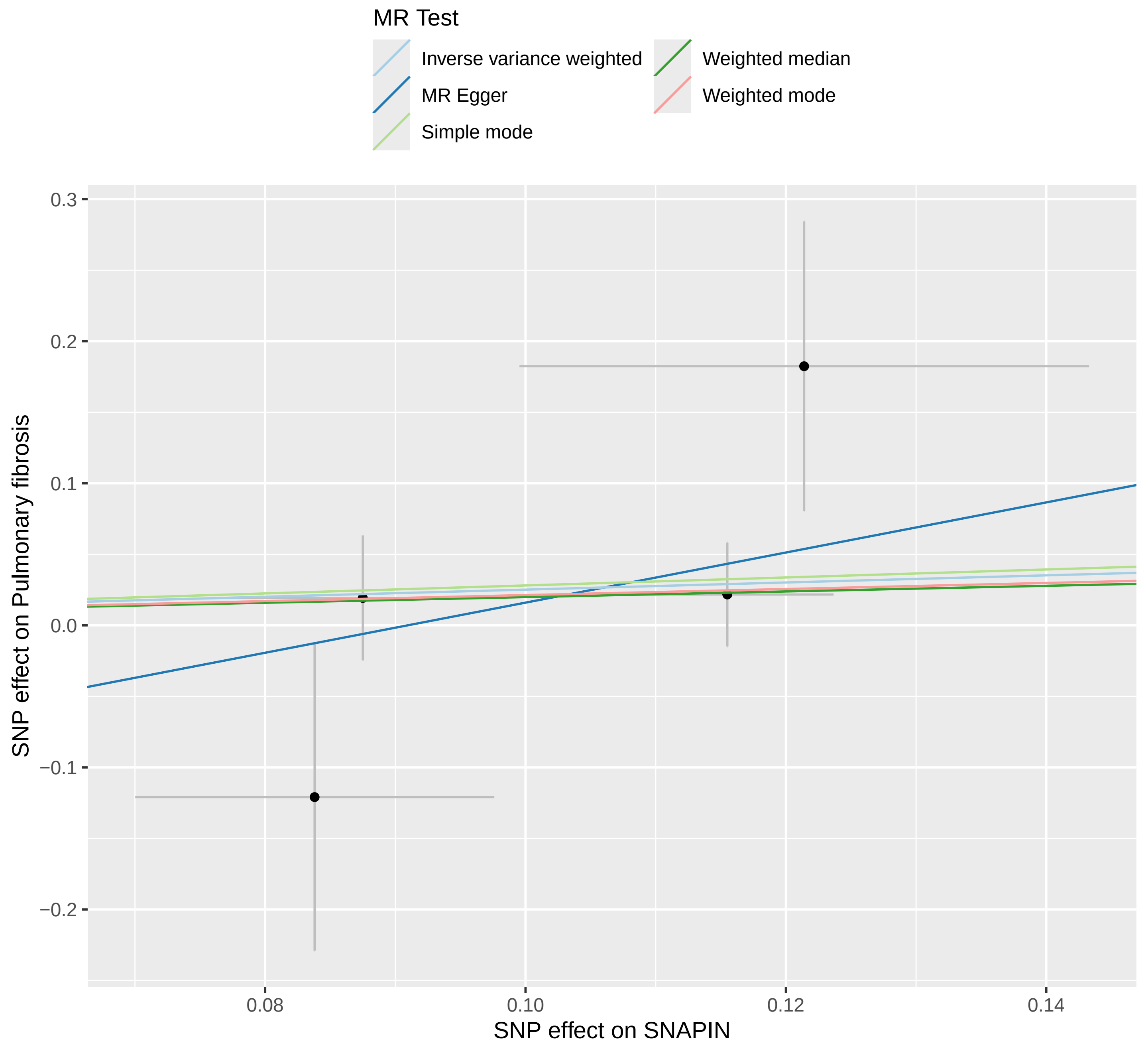

MR Test
Inverse variance weighted
MR Egger
Simple mode
Weighted median
Weighted mode
0.3
0.2
0.1
0.0
−0.1
−0.2
0.08
0.10
0.12
0.14
SNP effect on Pulmonary fibrosis
SNP effect on SNAPIN

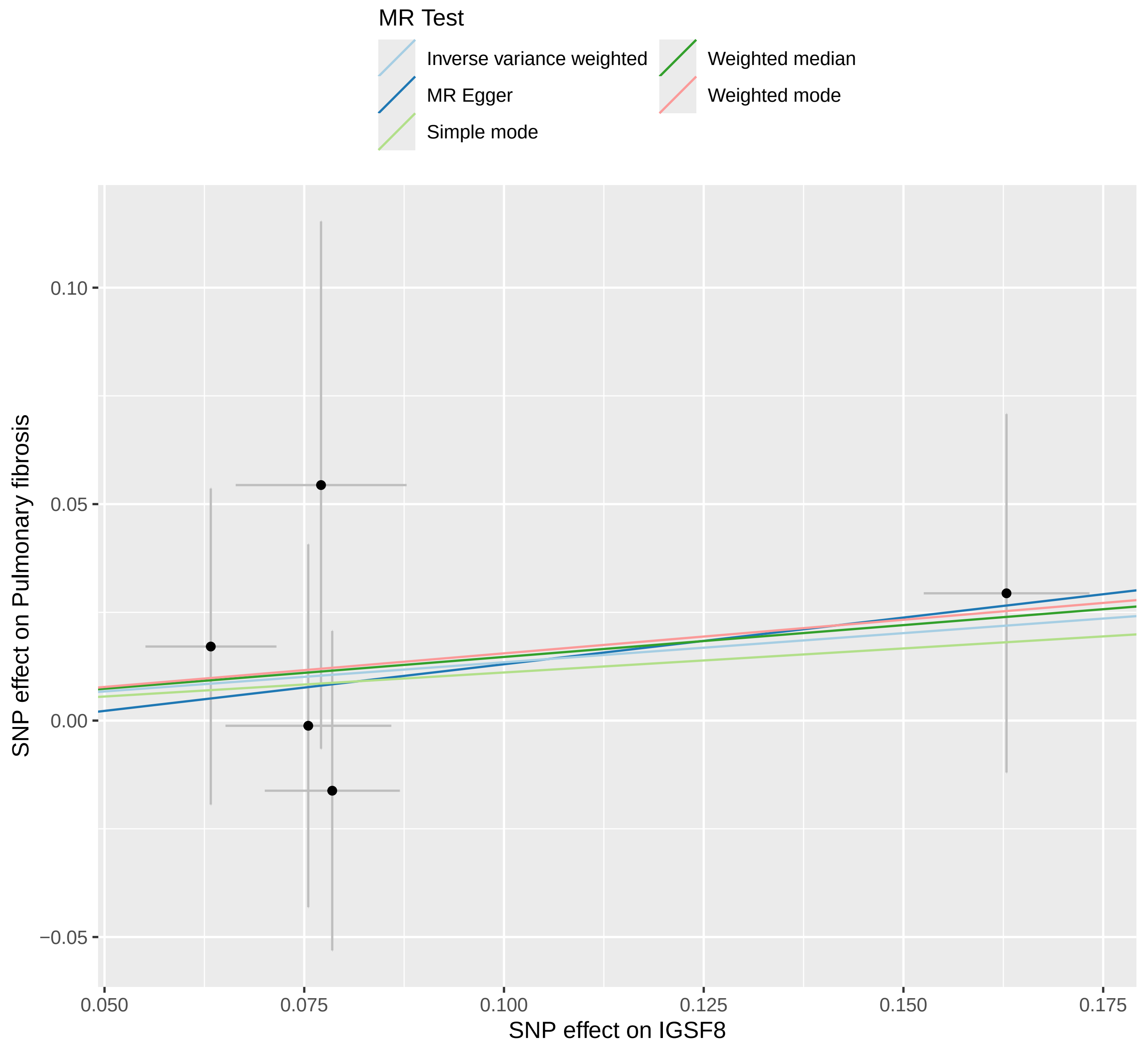
MR Test
Inverse variance weighted
MR Egger
Simple mode
Weighted median
Weighted mode
0.10
0.05
0.00
−0.05
SNP effect on Pulmonary fibrosis
0.050
0.075
0.100
0.125
0.150
0.175
SNP effect on IGSF8

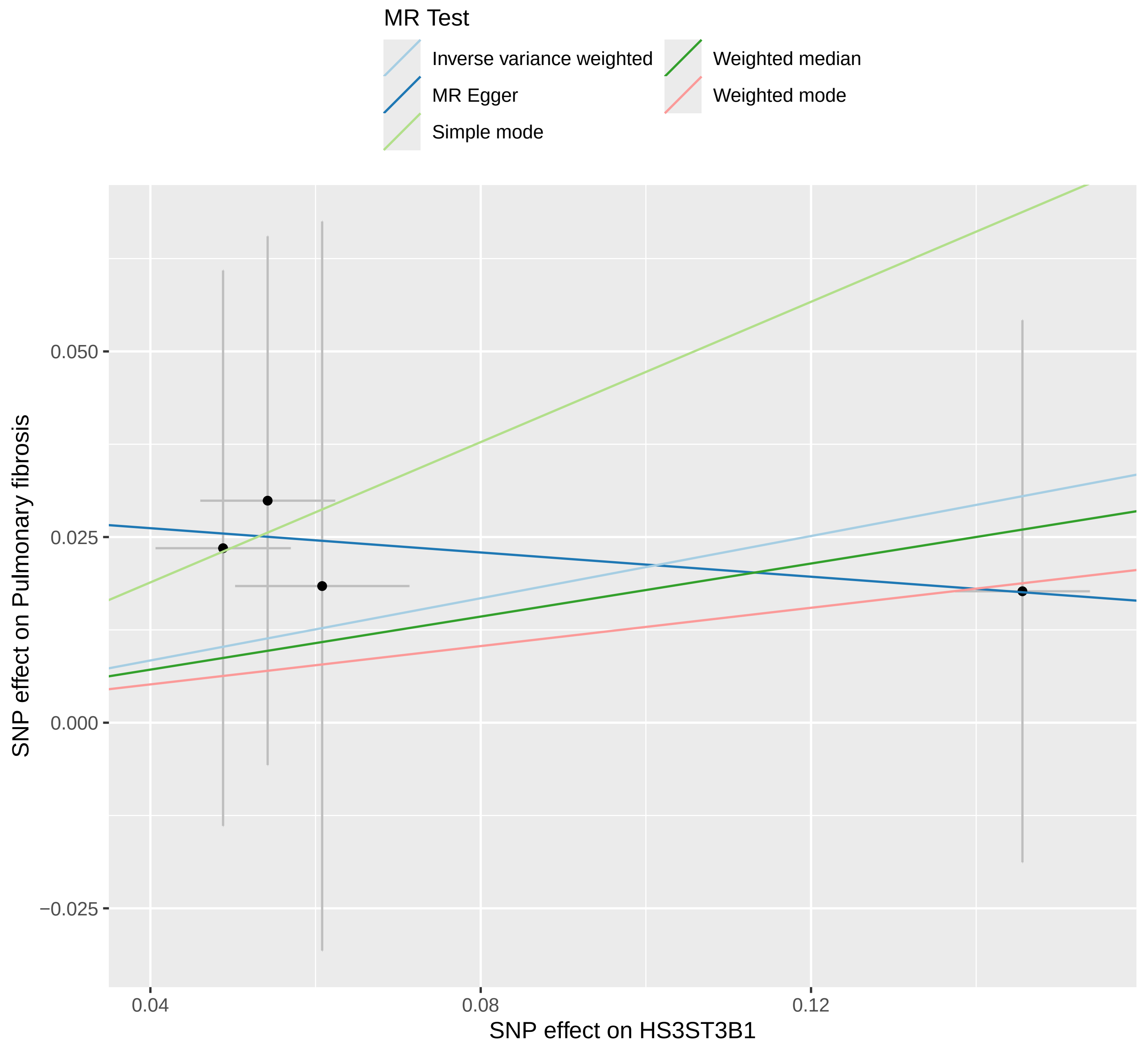
MR Test
Inverse variance weighted
MR Egger
Simple mode
Weighted median
Weighted mode
0.050
0.025
0.000
−0.025
0.04
0.08
0.12
SNP effect on HS3ST3B1
SNP effect on Pulmonary fibrosis

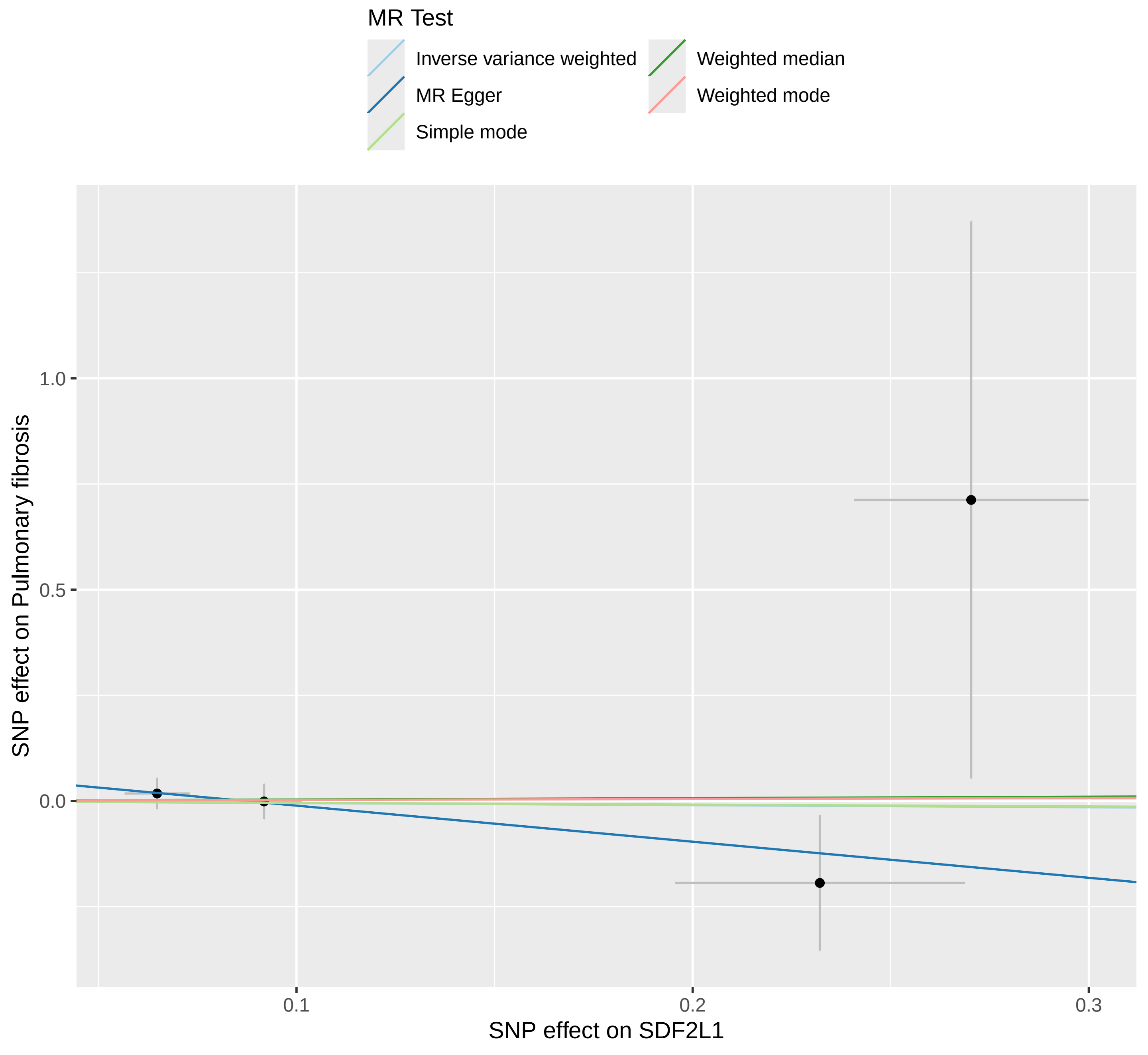

MR Test
Inverse variance weighted
MR Egger
Simple mode
Weighted median
Weighted mode
1.0
0.5
0.0
0.1
0.2
0.3
SNP effect on Pulmonary fibrosis
SNP effect on SDF2L1

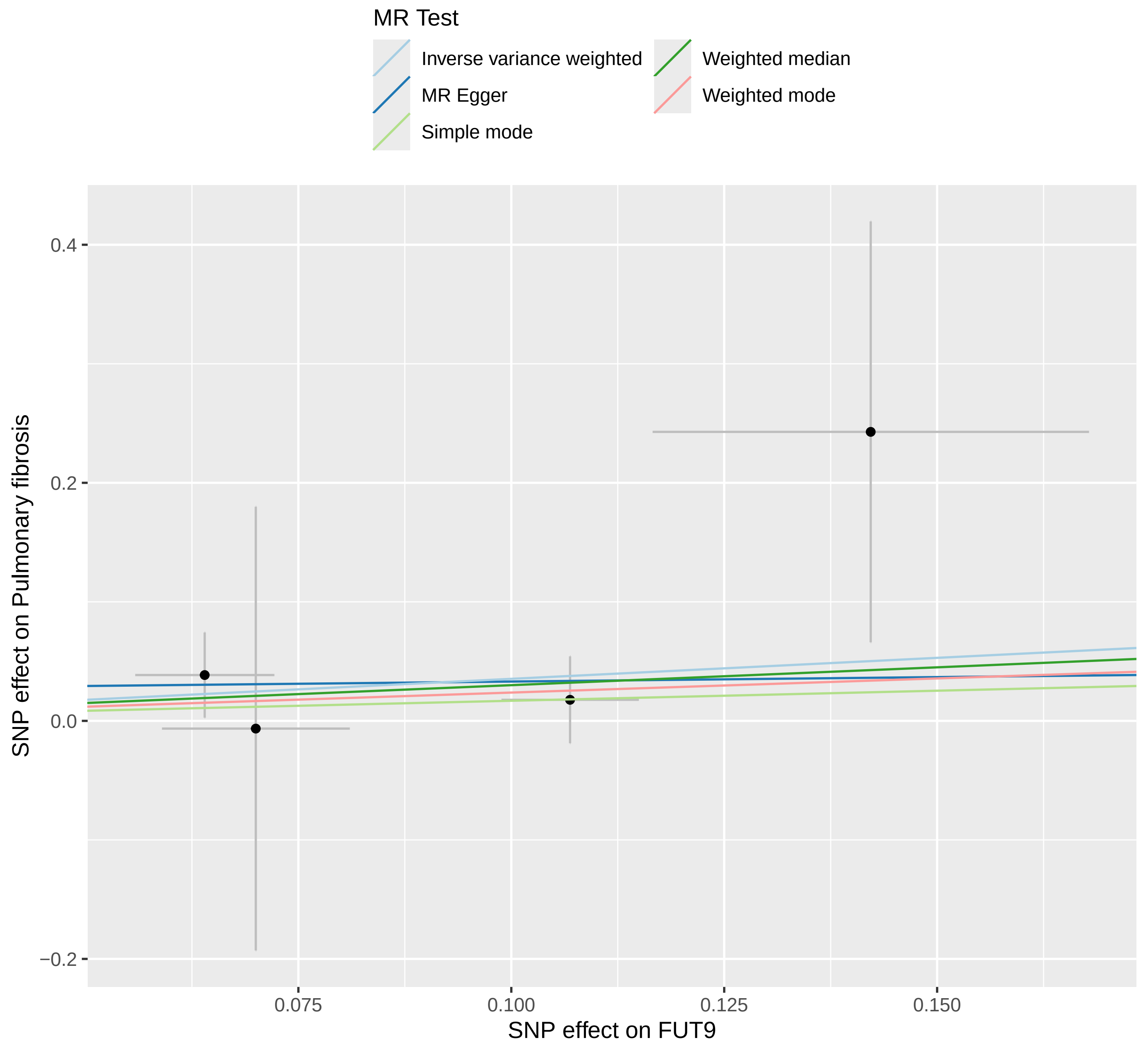

MR Test
Inverse variance weighted
MR Egger
Simple mode
Weighted median
Weighted mode
0.4
0.2
0.0
−0.2
0.075
0.100
0.125
0.150
SNP effect on Pulmonary fibrosis
SNP effect on FUT9

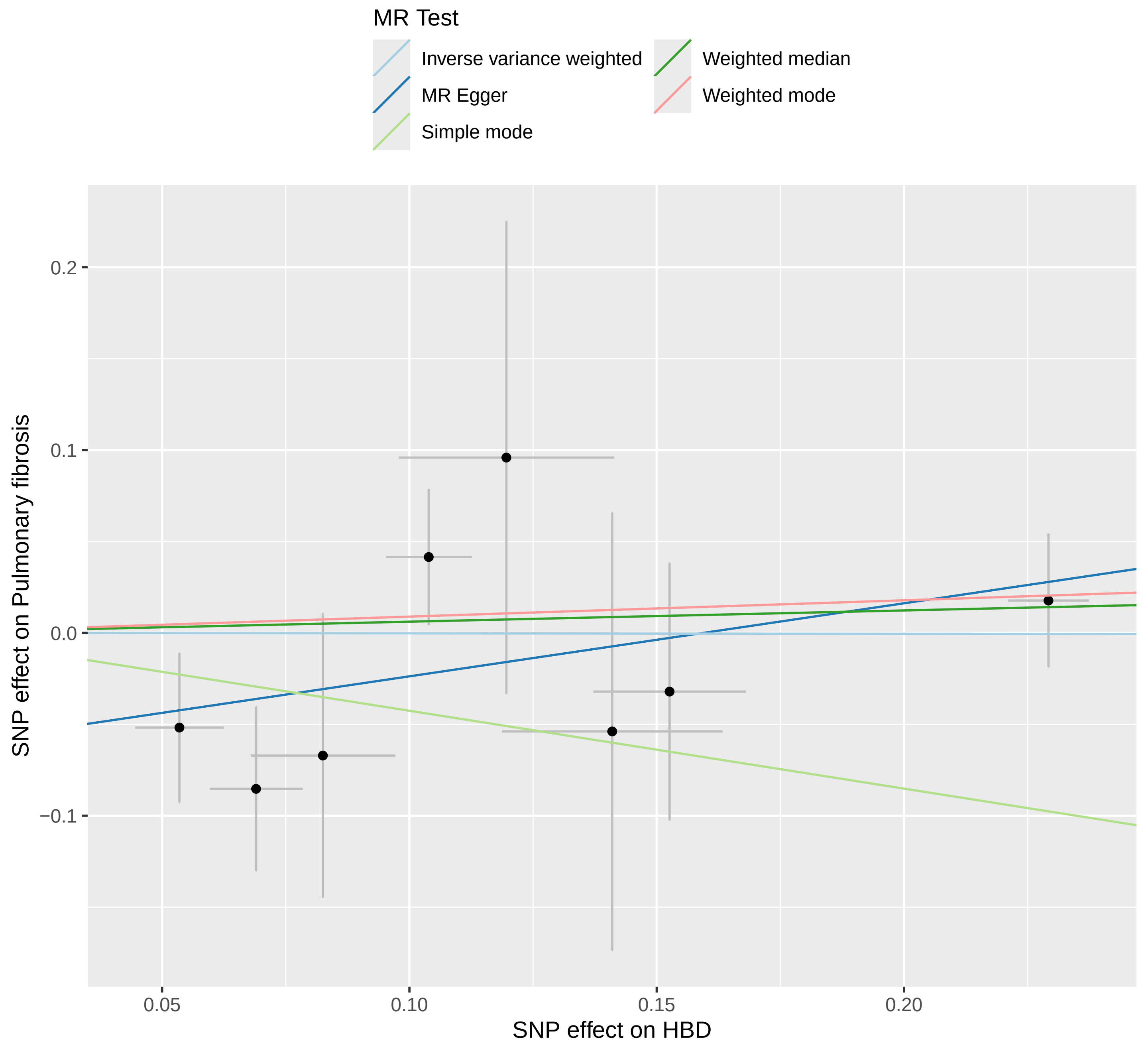

MR Test
Inverse variance weighted
MR Egger
Simple mode
Weighted median
Weighted mode
0.2
0.1
0.0
−0.1
SNP effect on Pulmonary fibrosis
0.05
0.10
0.15
0.20
SNP effect on HBD

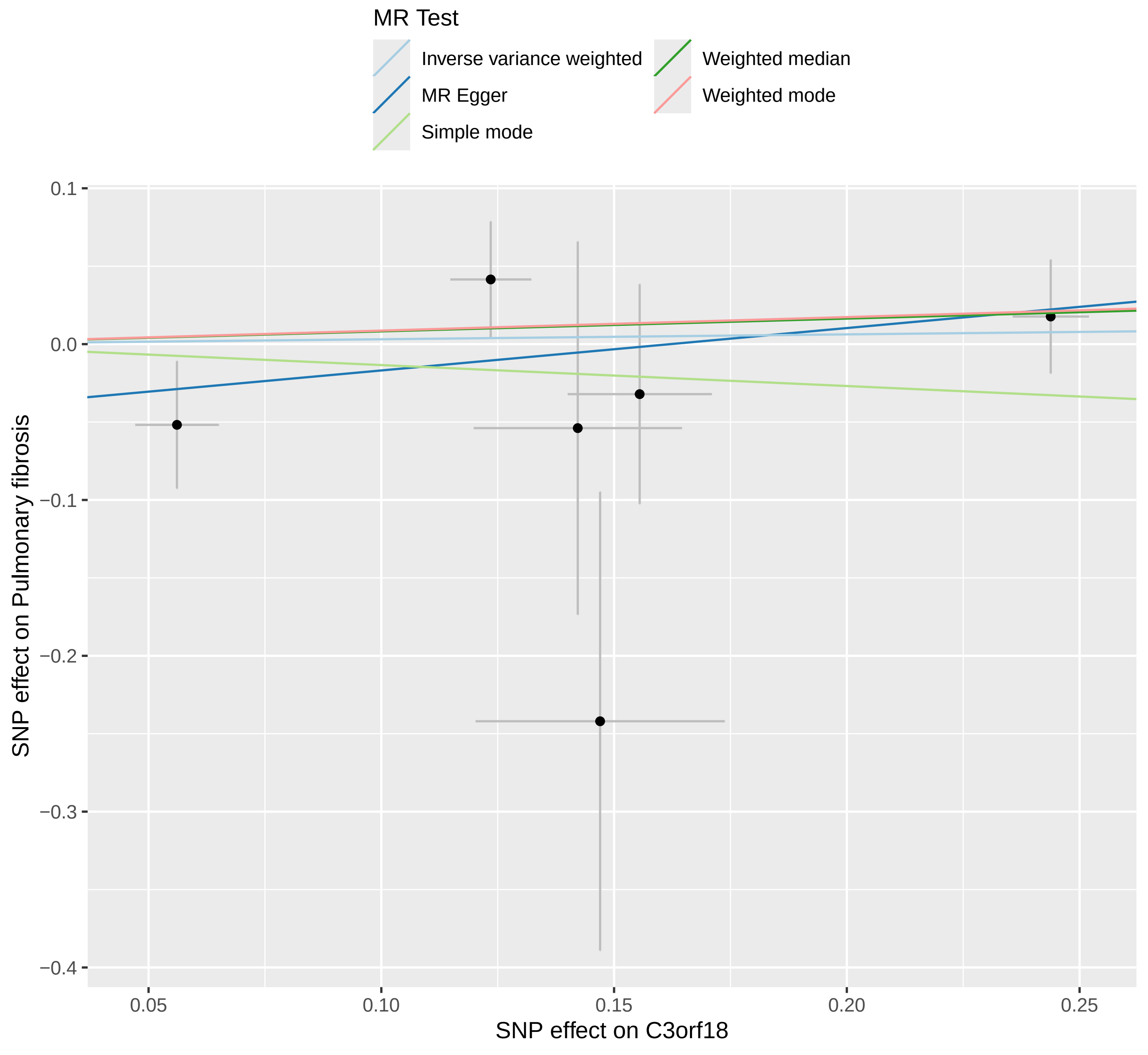
MR Test
Inverse variance weighted
MR Egger
Simple mode
Weighted median
Weighted mode
0.1
0.0
−0.1
−0.2
−0.3
−0.4
SNP effect on Pulmonary fibrosis
0.05
0.10
0.15
0.20
0.25
SNP effect on C3orf18

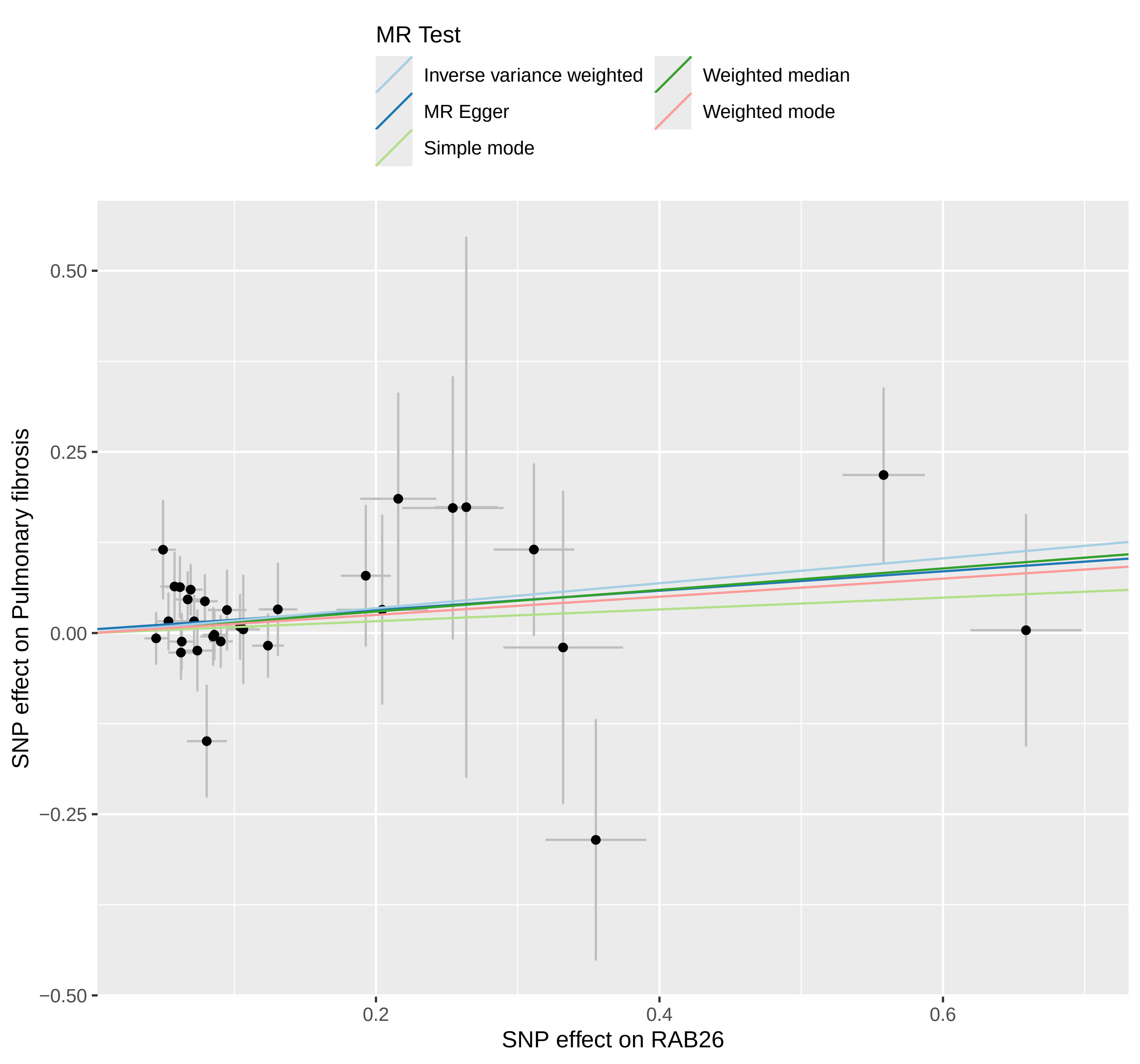
MR Test
Inverse variance weighted
MR Egger
Simple mode
Weighted median
Weighted mode
SNP effect on Pulmonary fibrosis
0.50
0.25
0.00
−0.25
−0.50
0.2
0.4
0.6
SNP effect on RAB26

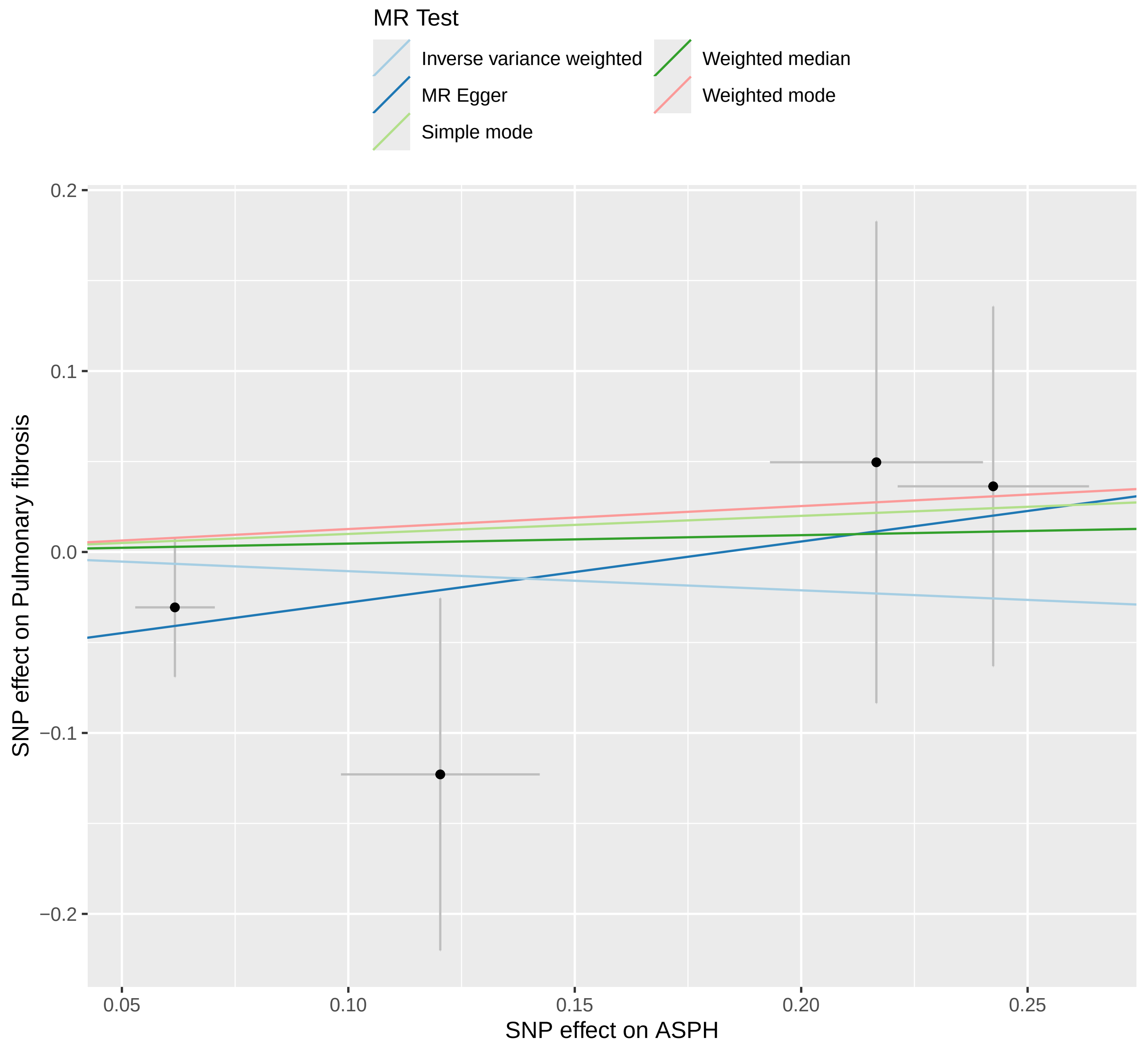

MR Test
Inverse variance weighted
MR Egger
Simple mode
Weighted median
Weighted mode
0.2
0.1
0.0
−0.1
−0.2
SNP effect on Pulmonary fibrosis
0.05
0.10
0.15
0.20
0.25
SNP effect on ASPH

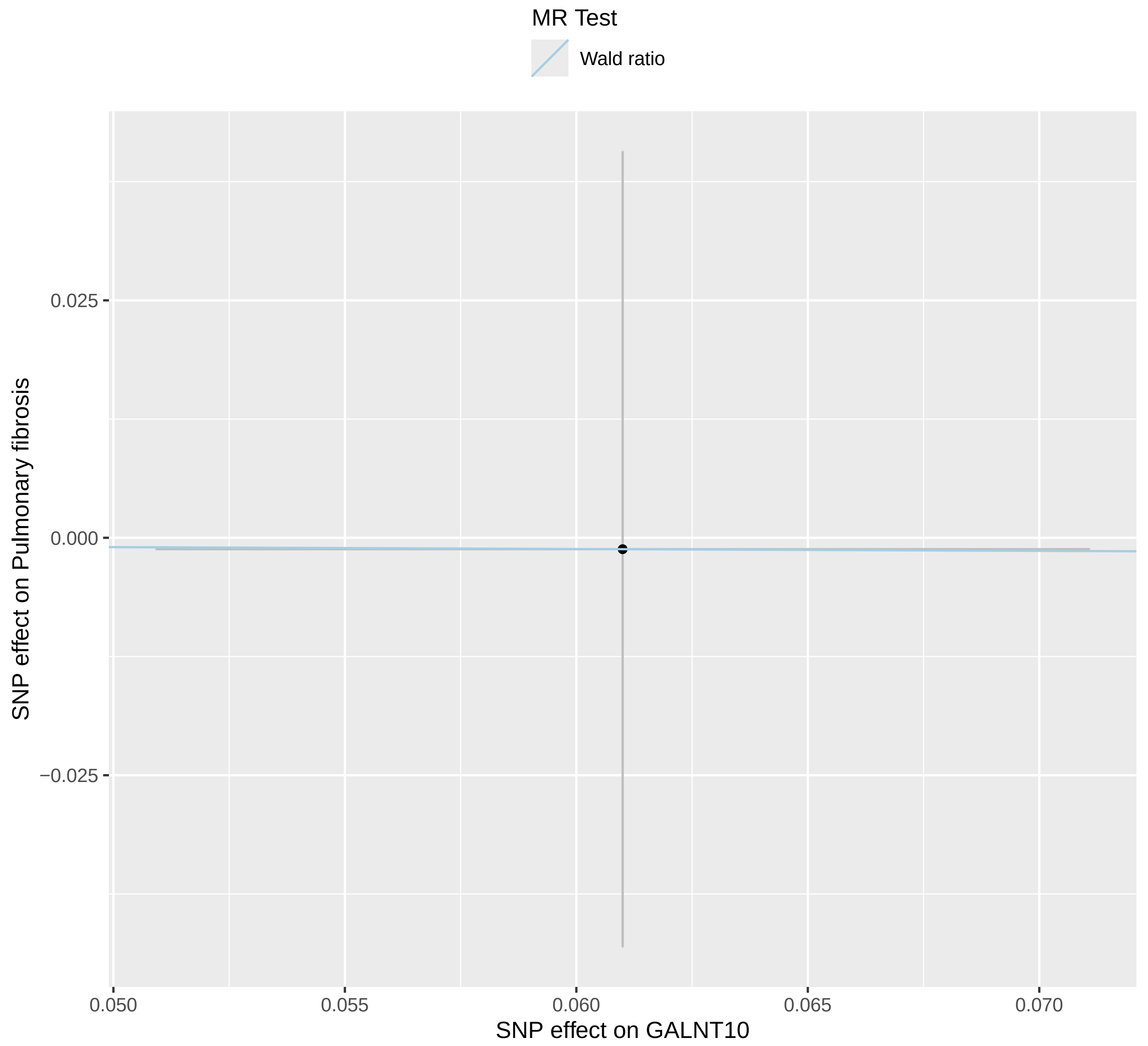
MR Test
Wald ratio
0.025
0.000
−0.025
0.050
0.055
0.060
0.065
0.070
SNP effect on Pulmonary fibrosis
SNP effect on GALNT10

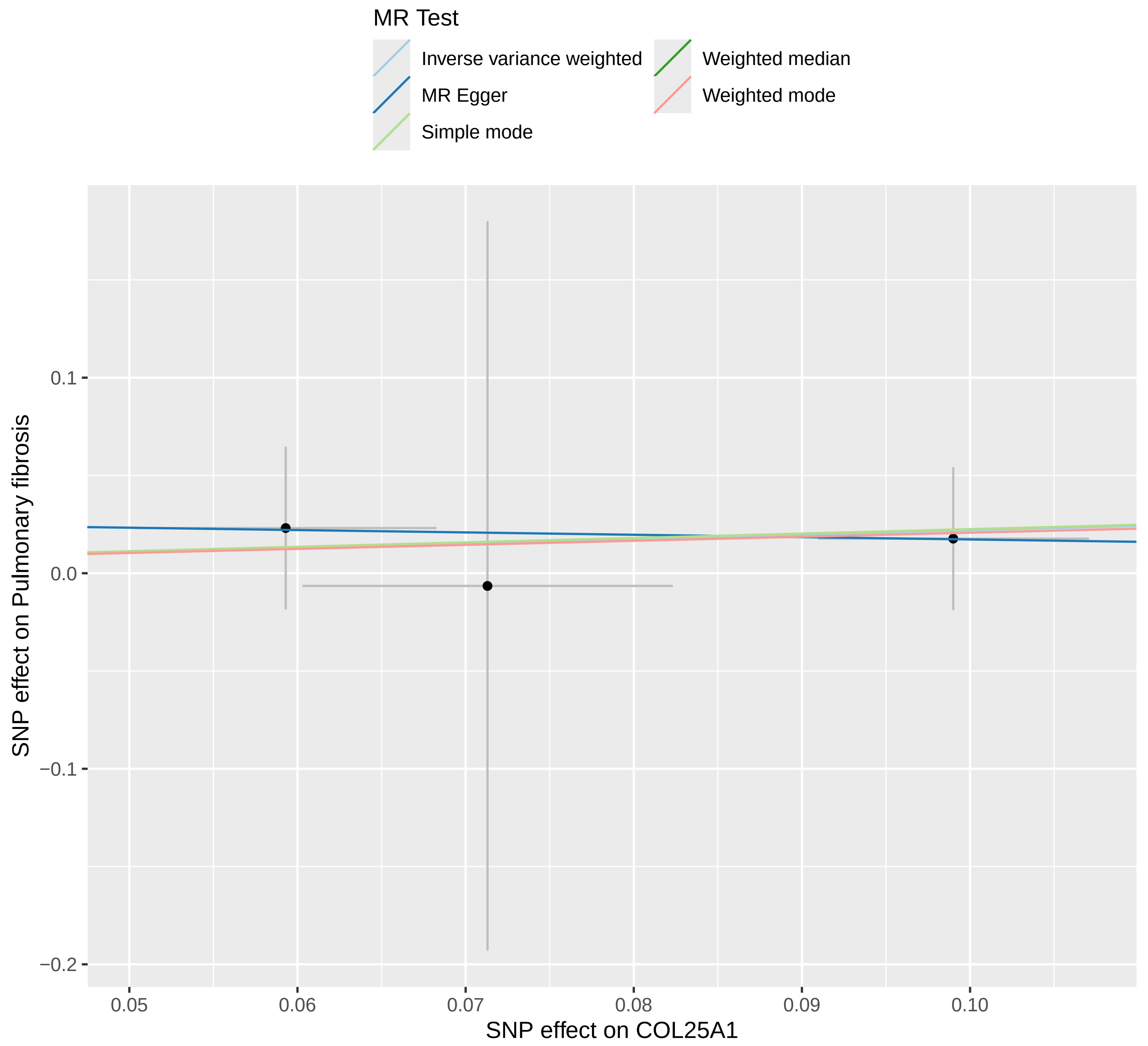

MR Test
Inverse variance weighted
MR Egger
Simple mode
Weighted median
Weighted mode
SNP effect on Pulmonary fibrosis
0.1
0.0
−0.1
−0.2
0.05
0.06
0.07
0.08
0.09
0.10
SNP effect on COL25A1

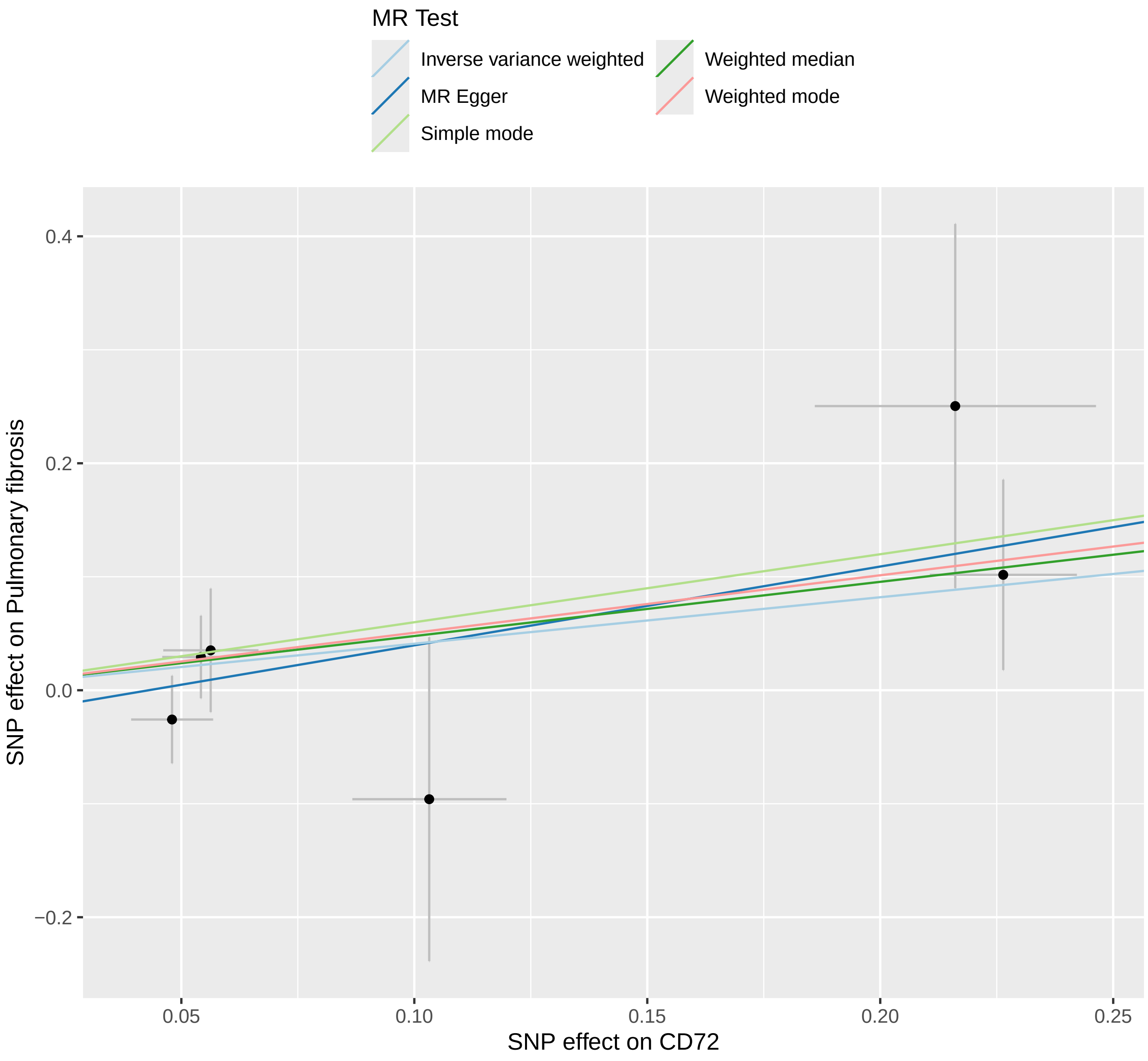
MR Test
Inverse variance weighted
MR Egger
Simple mode
Weighted median
Weighted mode
0.4
0.2
0.0
−0.2
0.05
0.10
0.15
0.20
0.25
SNP effect on Pulmonary fibrosis
SNP effect on CD72

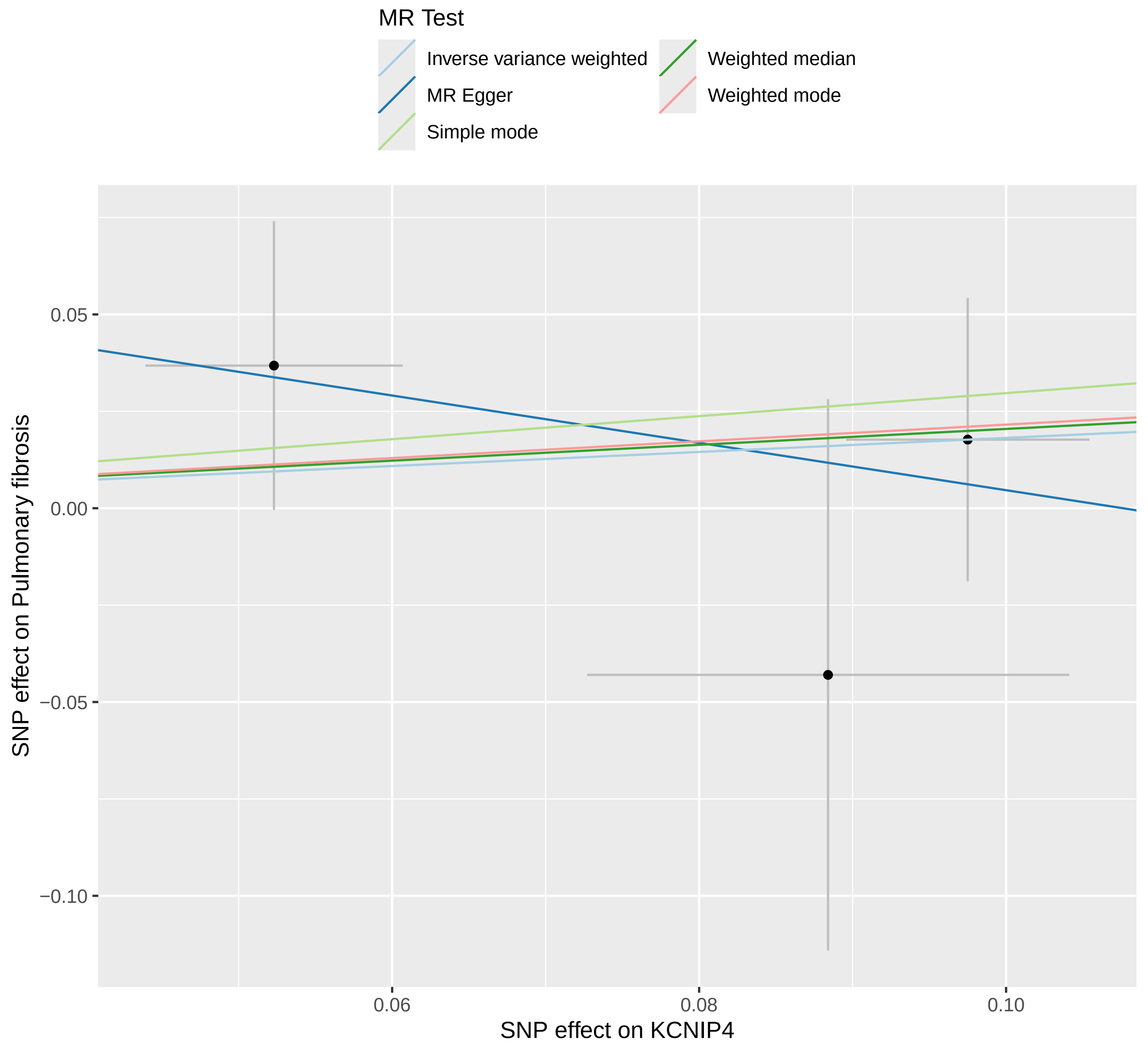

MR Test
Inverse variance weighted
MR Egger
Simple mode
Weighted median
Weighted mode
SNP effect on Pulmonary fibrosis
0.05
0.00
−0.05
−0.10
0.06
0.08
0.10
SNP effect on KCNIP4

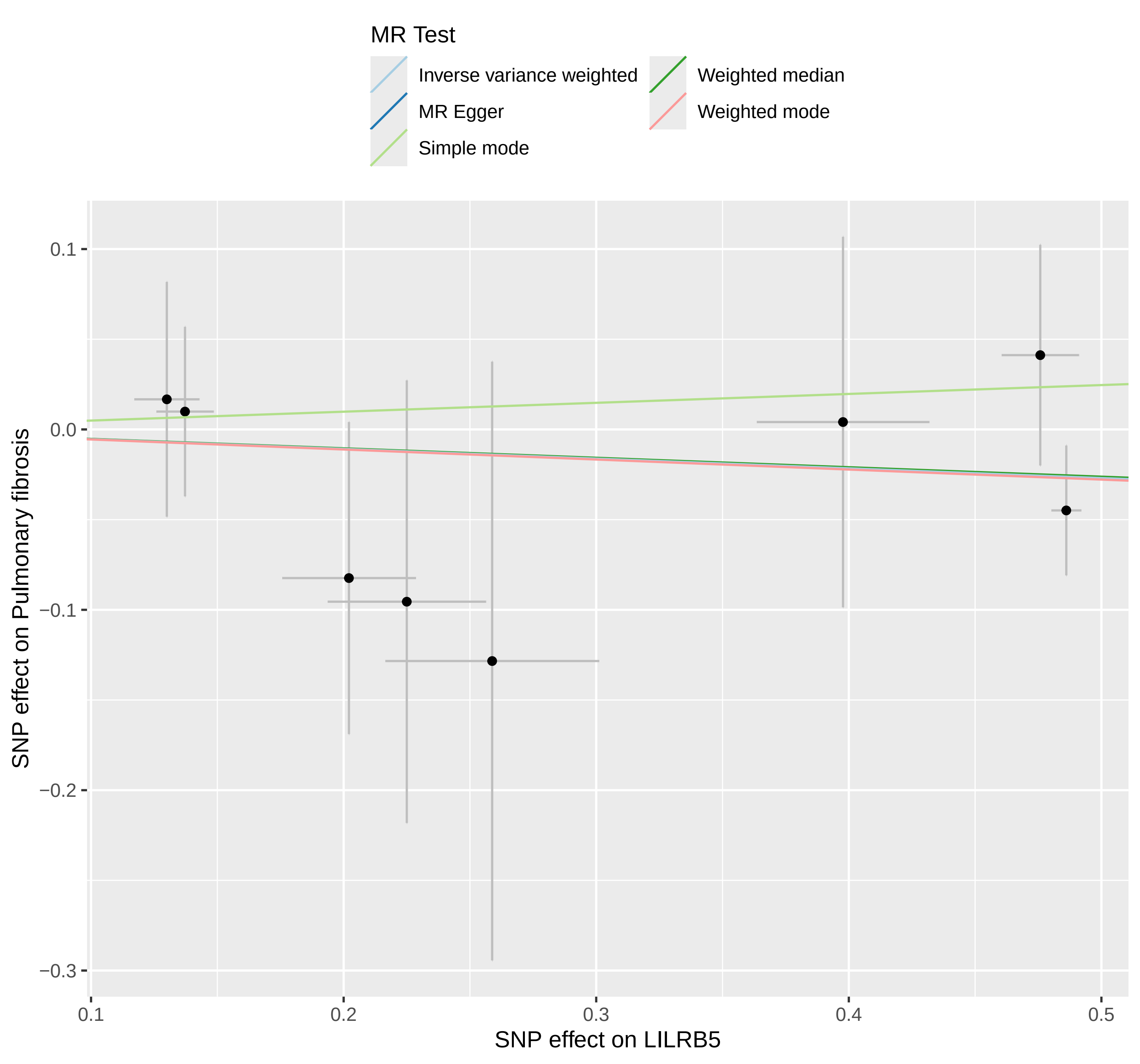

MR Test
Inverse variance weighted
MR Egger
Simple mode
Weighted median
Weighted mode
0.1
0.0
−0.1
−0.2
−0.3
SNP effect on Pulmonary fibrosis
0.1
0.2
0.3
0.4
0.5
SNP effect on LILRB5

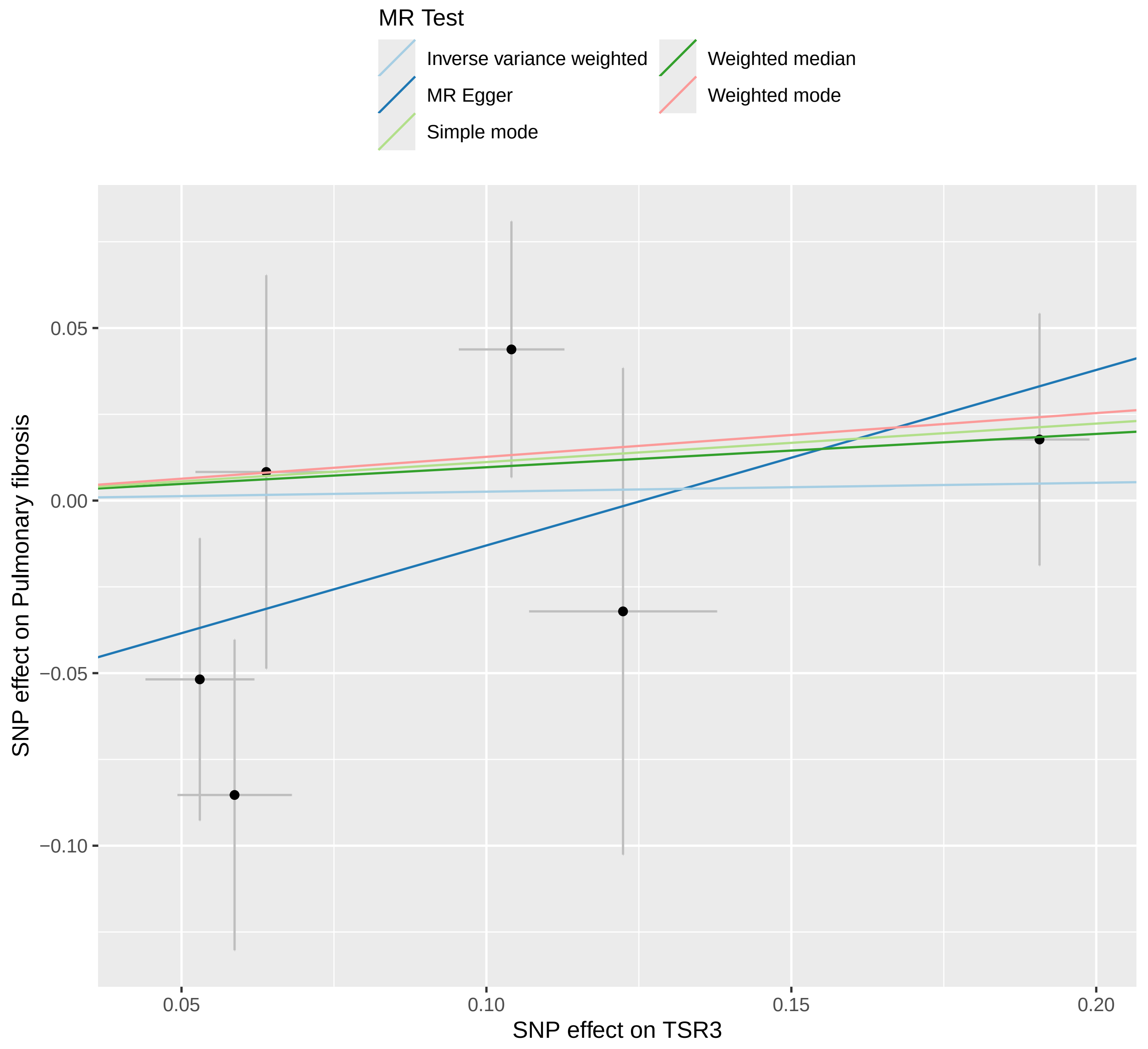
MR Test
Inverse variance weighted
MR Egger
Simple mode
Weighted median
Weighted mode
SNP effect on Pulmonary fibrosis
0.05
0.00
−0.05
−0.10
0.05
0.10
0.15
0.20
SNP effect on TSR3

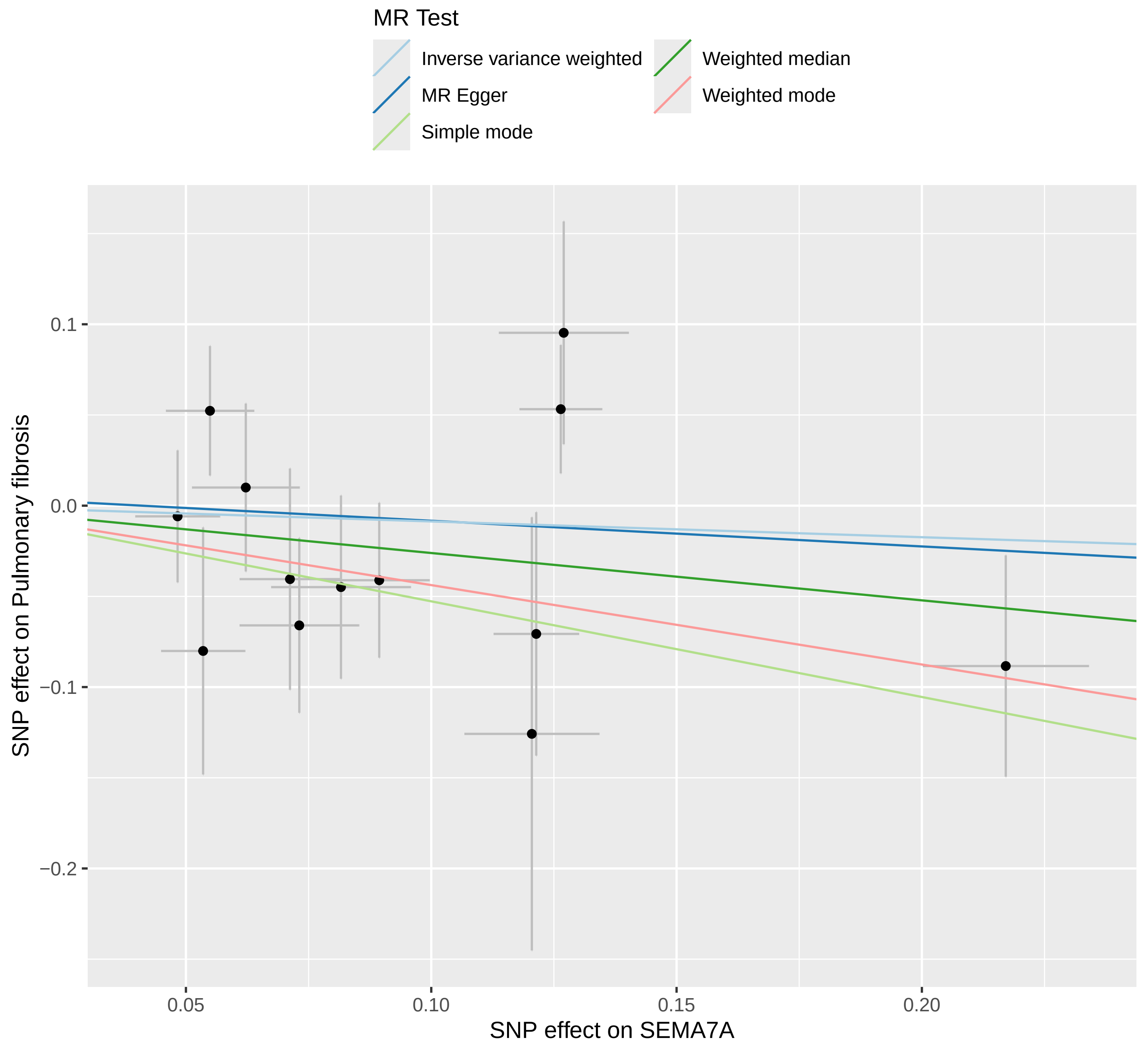

MR Test
Inverse variance weighted
MR Egger
Simple mode
Weighted median
Weighted mode
0.1
0.0
−0.1
−0.2
0.05
0.10
0.15
0.20
SNP effect on SEMA7A
SNP effect on Pulmonary fibrosis

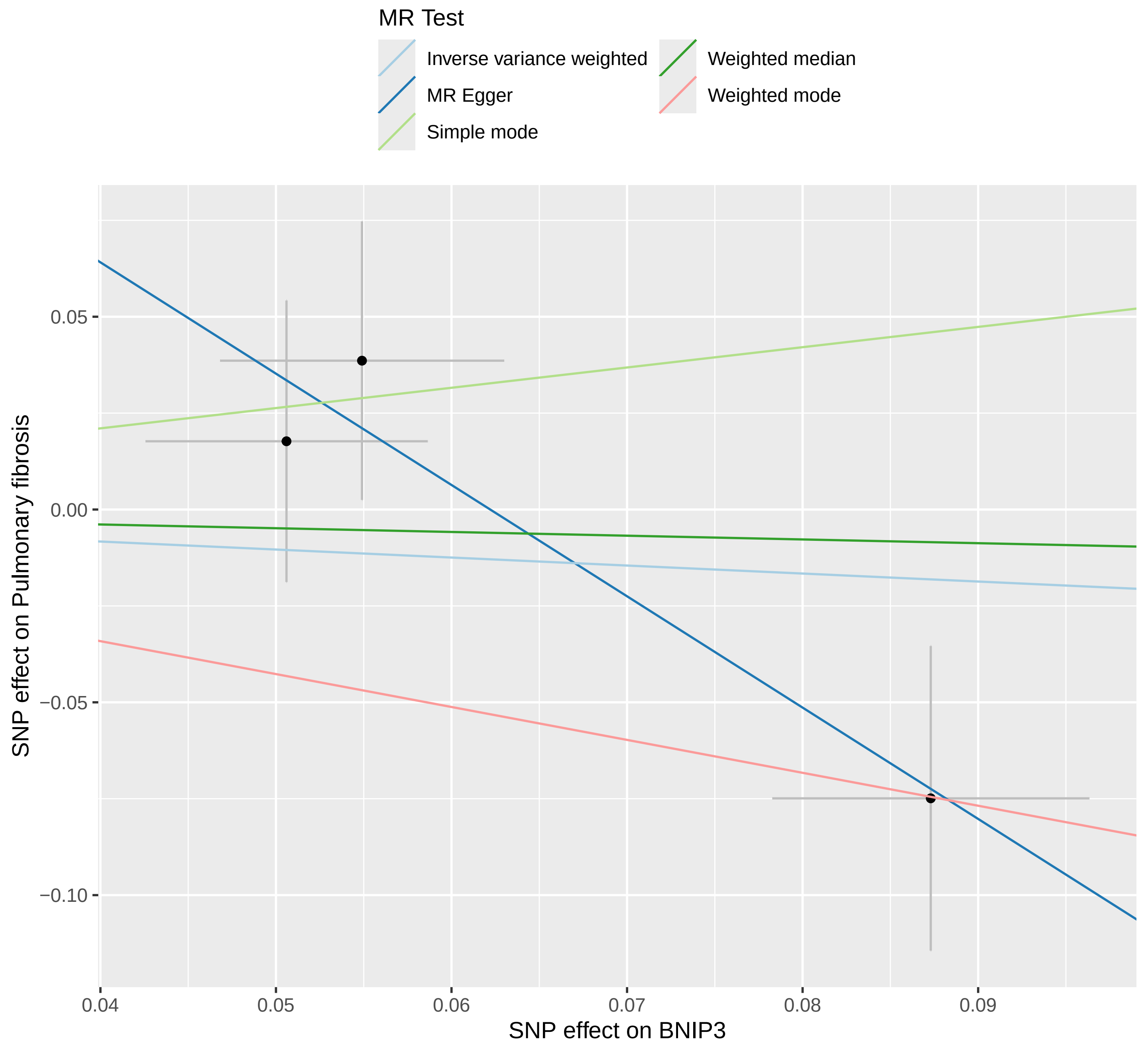
MR Test
Inverse variance weighted
MR Egger
Simple mode
Weighted median
Weighted mode
0.05
0.00
−0.05
−0.10
0.04
0.05
0.06
0.07
0.08
0.09
SNP effect on Pulmonary fibrosis
SNP effect on BNIP3

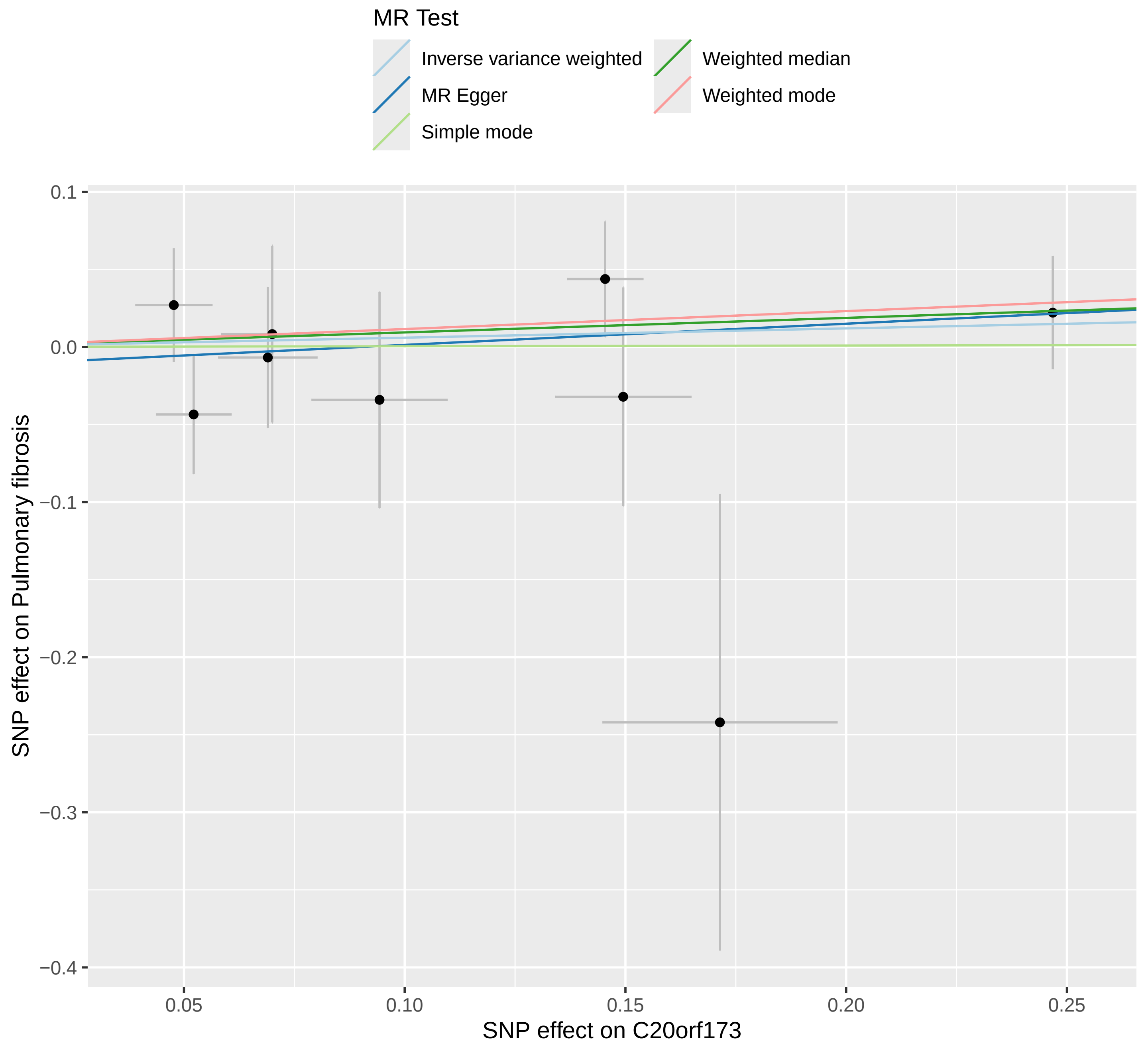
MR Test
Inverse variance weighted
MR Egger
Simple mode
Weighted median
Weighted mode
0.1
0.0
−0.1
−0.2
−0.3
−0.4
SNP effect on Pulmonary fibrosis
0.05
0.10
0.15
0.20
0.25
SNP effect on C20orf173

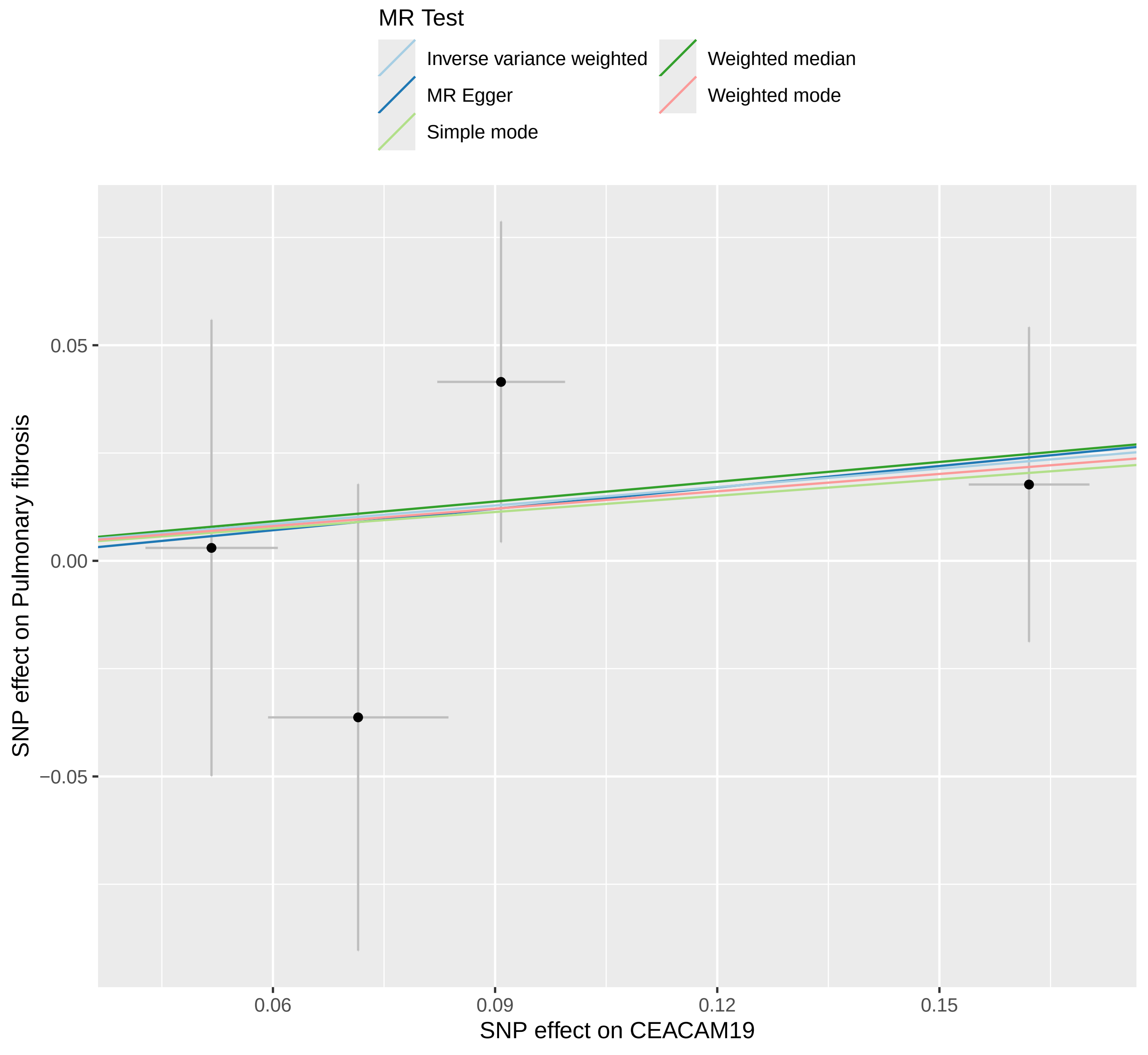
MR Test
Inverse variance weighted
MR Egger
Simple mode
Weighted median
Weighted mode
0.05
0.00
−0.05
0.06
0.09
0.12
0.15
SNP effect on Pulmonary fibrosis
SNP effect on CEACAM19

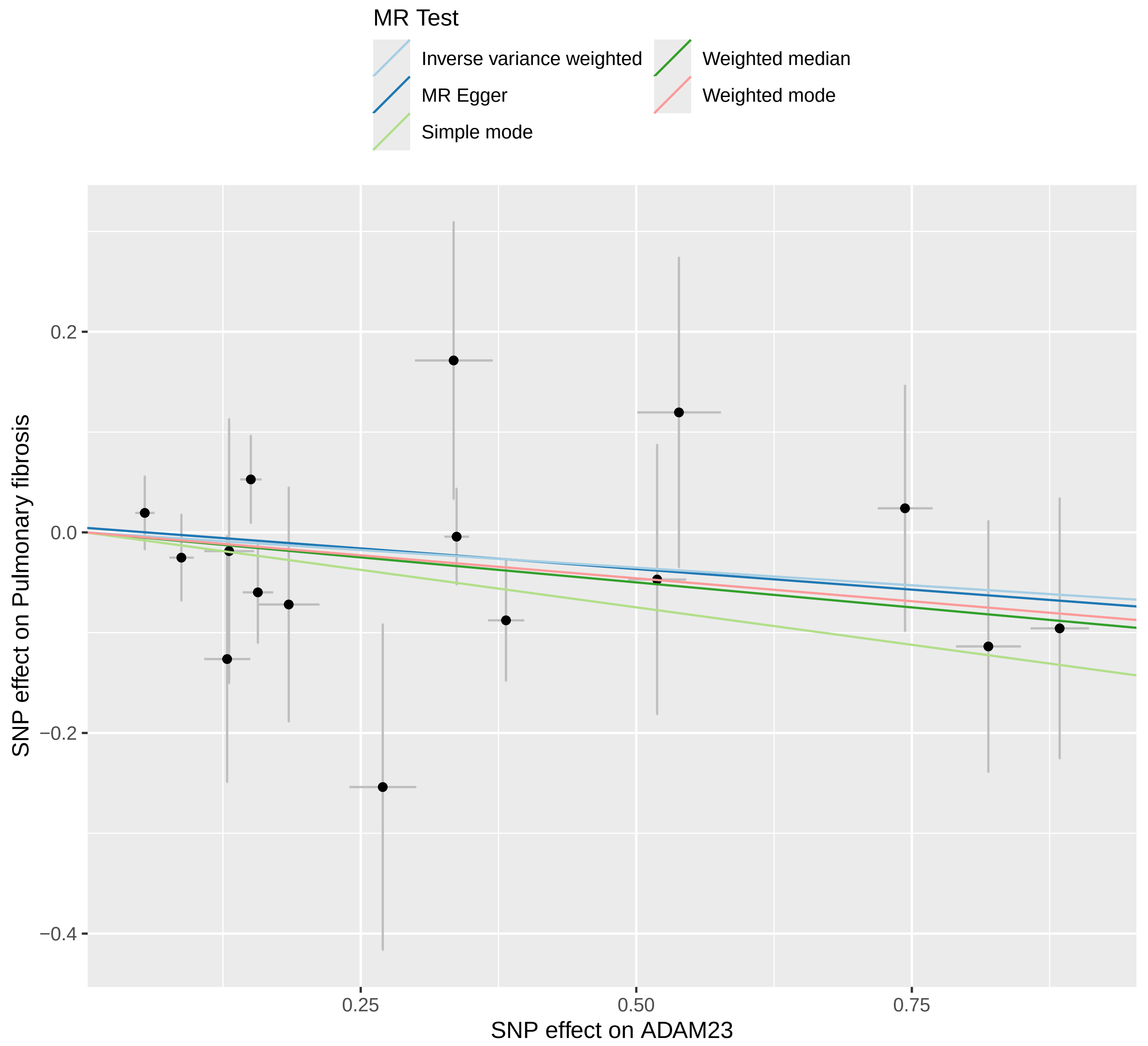

MR Test
Inverse variance weighted
MR Egger
Simple mode
Weighted median
Weighted mode
0.2
0.0
−0.2
−0.4
SNP effect on Pulmonary fibrosis
0.25
0.50
0.75
SNP effect on ADAM23

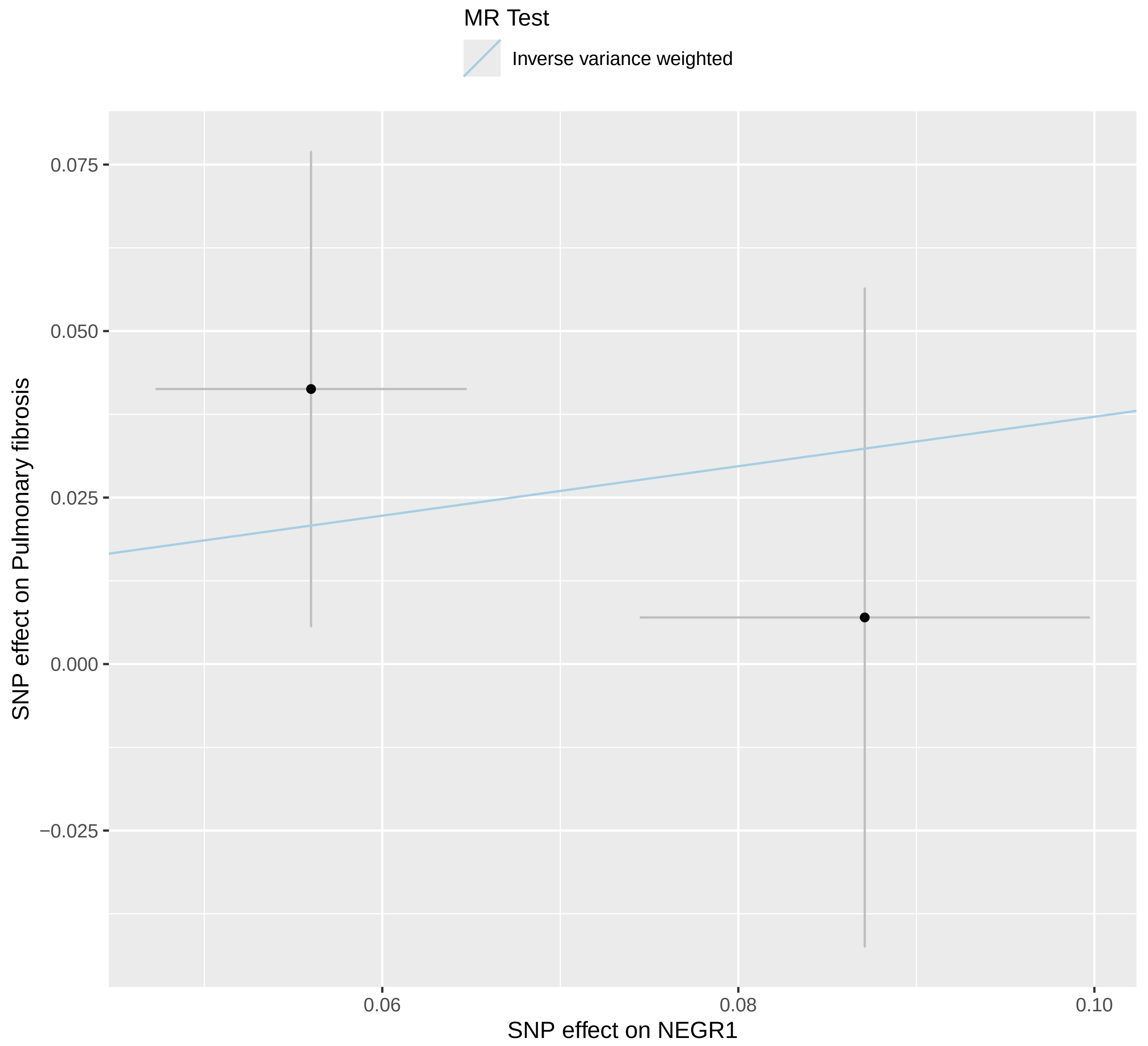

MR Test
Inverse variance weighted
0.075
0.050
0.025
0.000
−0.025
SNP effect on Pulmonary fibrosis
0.06
0.08
0.10
SNP effect on NEGR1

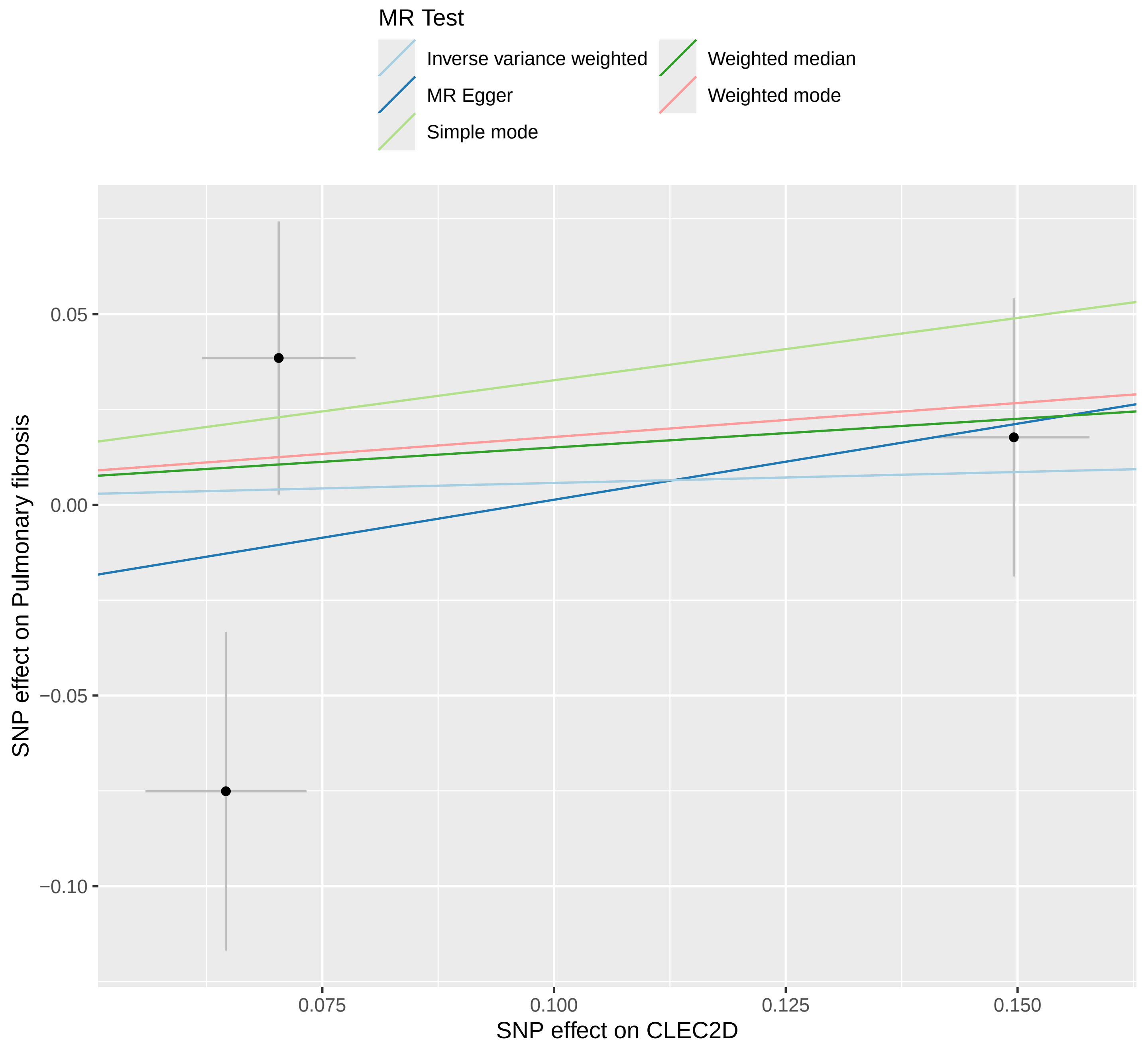
MR Test
Inverse variance weighted
MR Egger
Simple mode
Weighted median
Weighted mode
0.05
0.00
−0.05
−0.10
0.075
0.100
0.125
0.150
SNP effect on Pulmonary fibrosis
SNP effect on CLEC2D

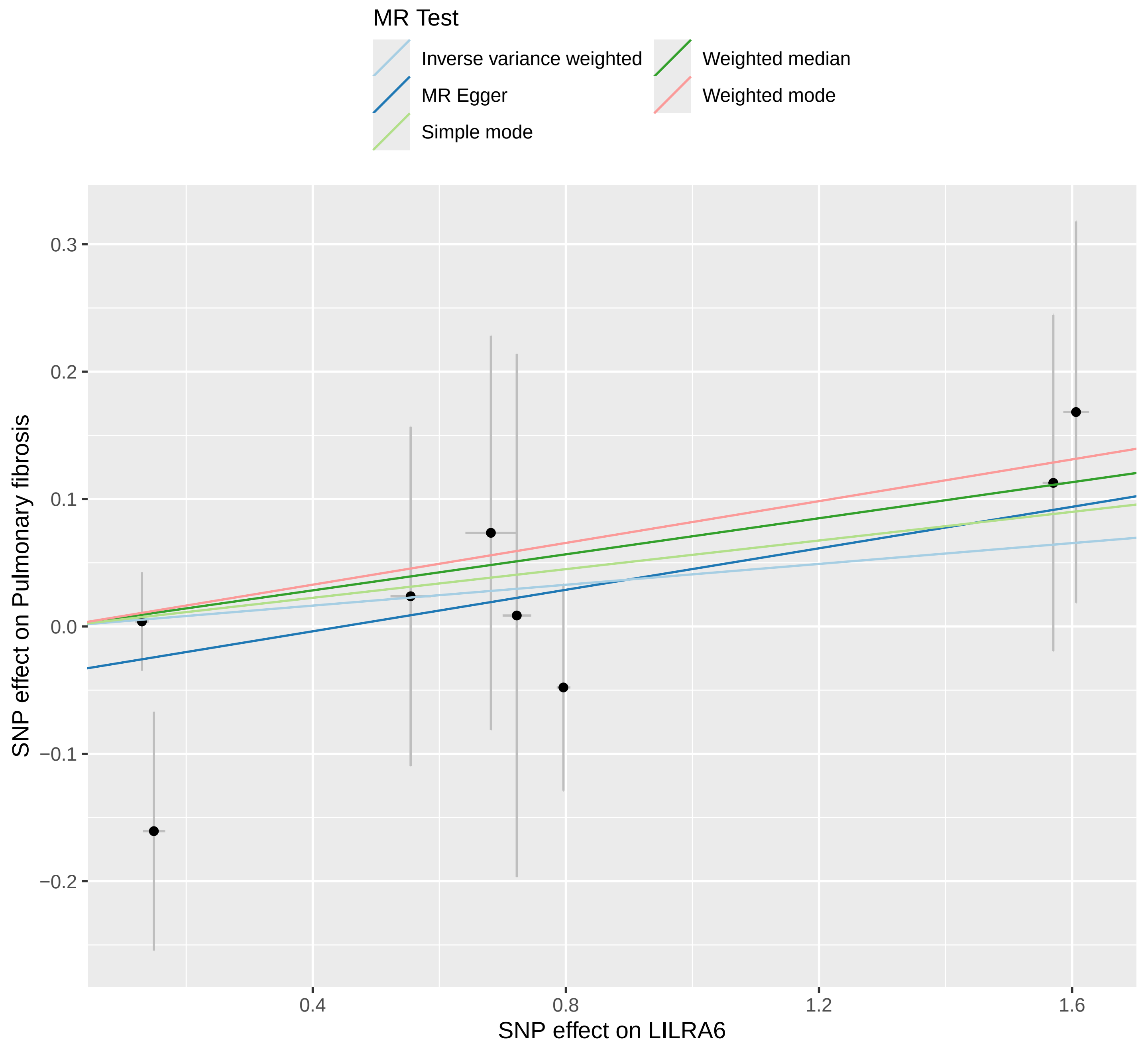
MR Test
Inverse variance weighted
MR Egger
Simple mode
Weighted median
Weighted mode
0.3
0.2
0.1
0.0
−0.1
−0.2
SNP effect on Pulmonary fibrosis
0.4
0.8
1.2
1.6
SNP effect on LILRA6

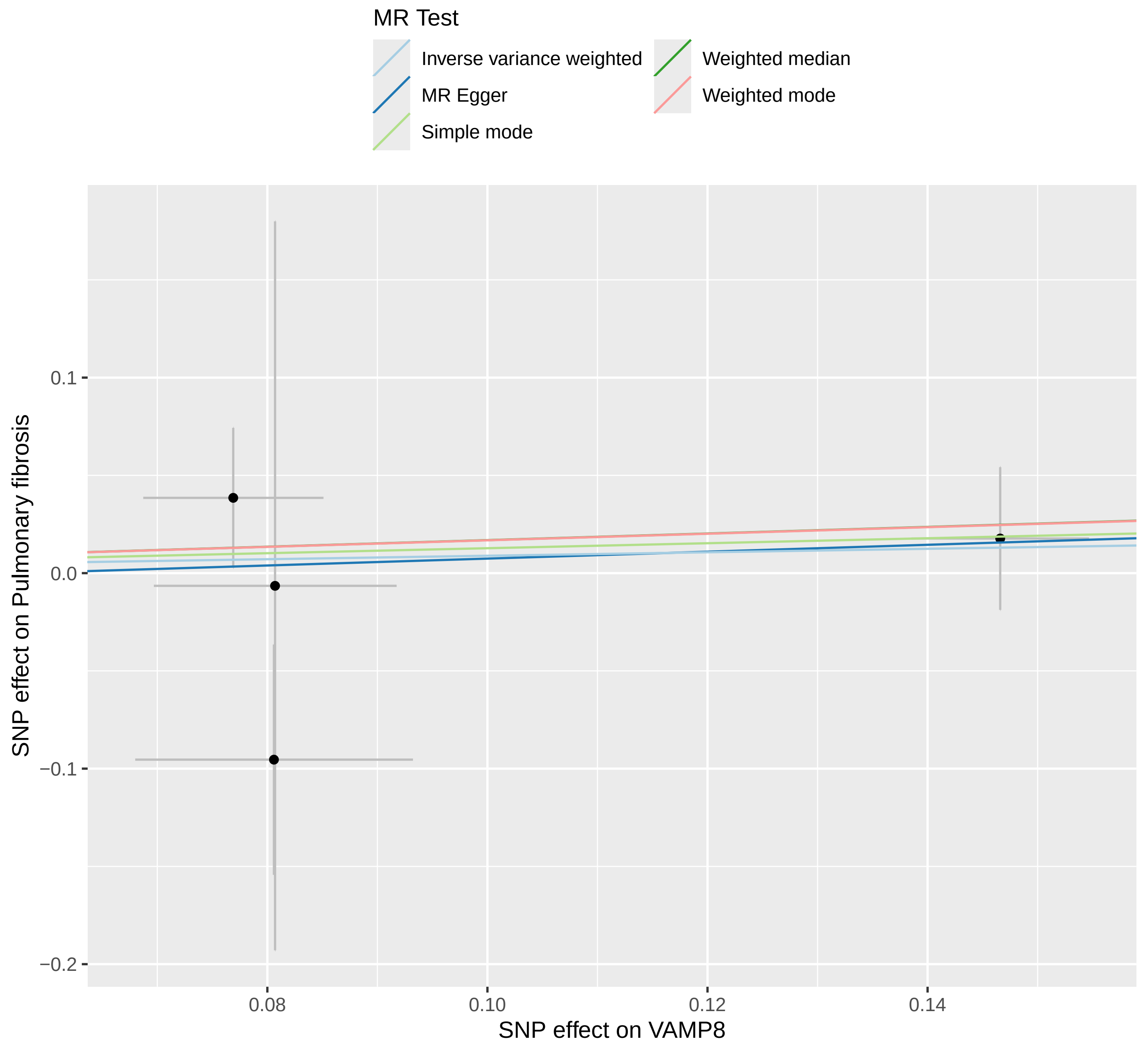

MR Test
Inverse variance weighted
MR Egger
Simple mode
Weighted median
Weighted mode
SNP effect on Pulmonary fibrosis
0.1
0.0
−0.1
−0.2
0.08
0.10
0.12
0.14
SNP effect on VAMP8

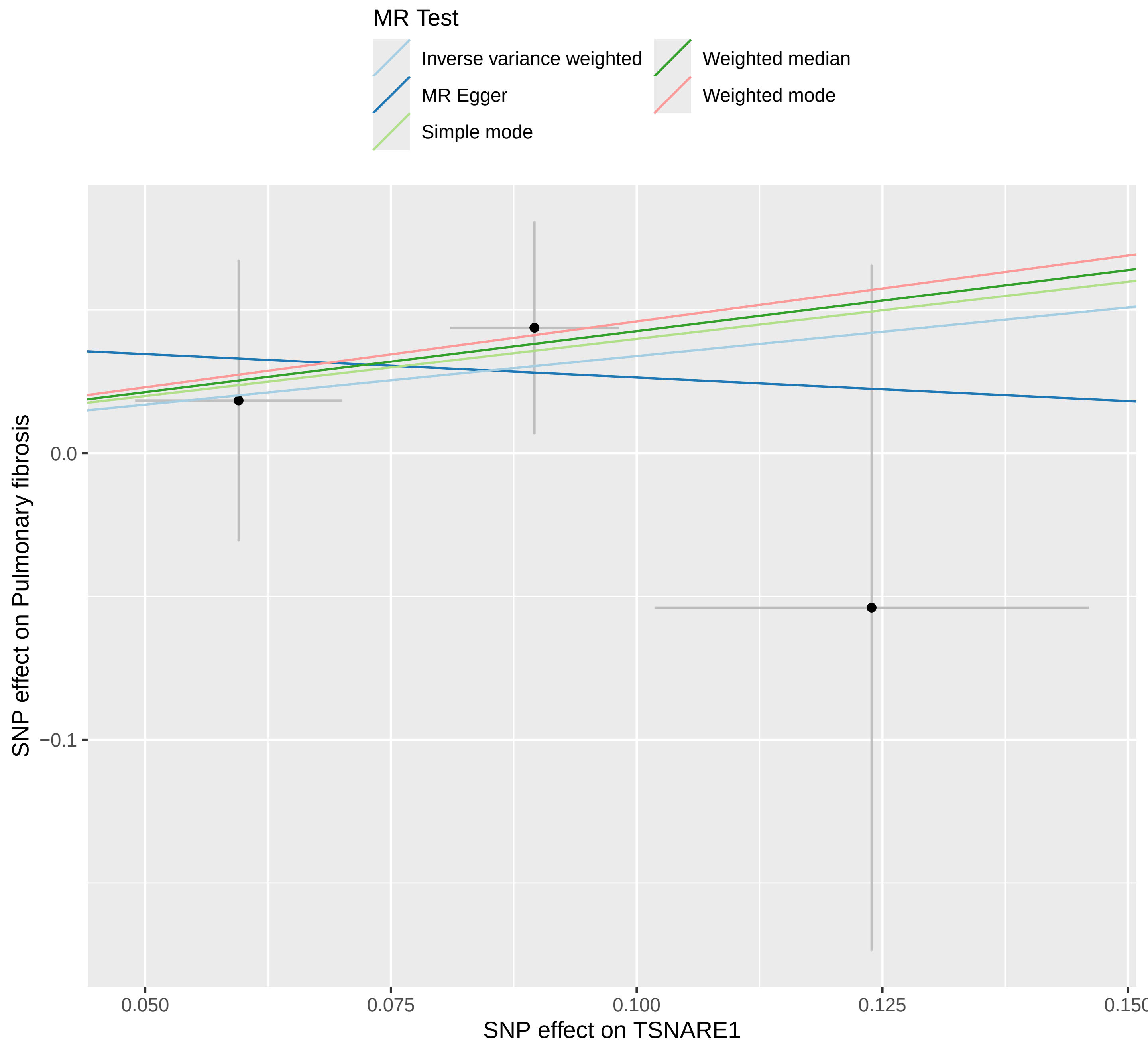
MR Test
Inverse variance weighted
MR Egger
Simple mode
Weighted median
Weighted mode
0.0
−0.1
0.050
0.075
0.100
0.125
0.150
SNP effect on TSNARE1
SNP effect on Pulmonary fibrosis

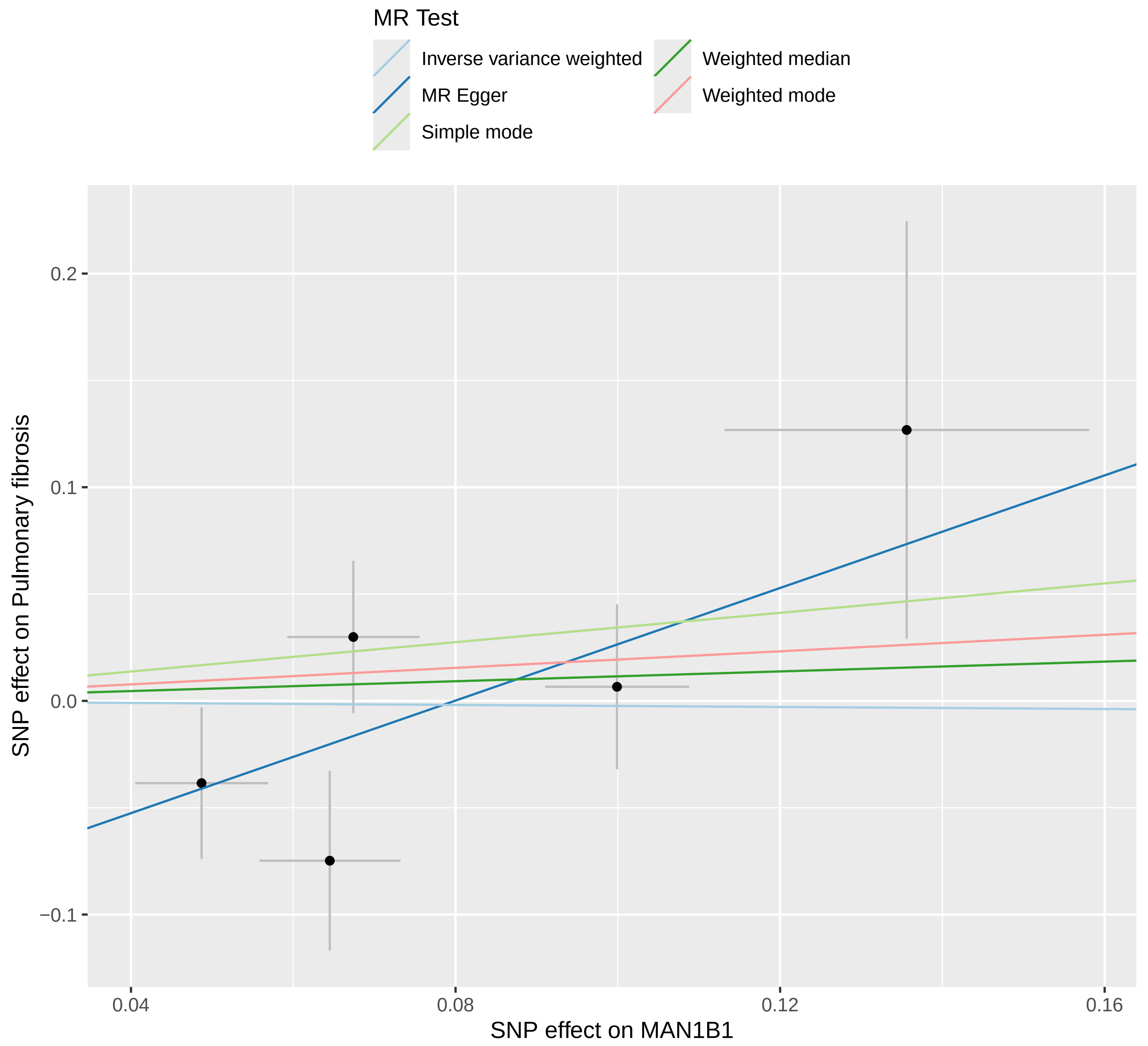

MR Test
Inverse variance weighted
MR Egger
Simple mode
Weighted median
Weighted mode
SNP effect on Pulmonary fibrosis
0.2
0.1
0.0
−0.1
0.04
0.08
0.12
0.16
SNP effect on MAN1B1

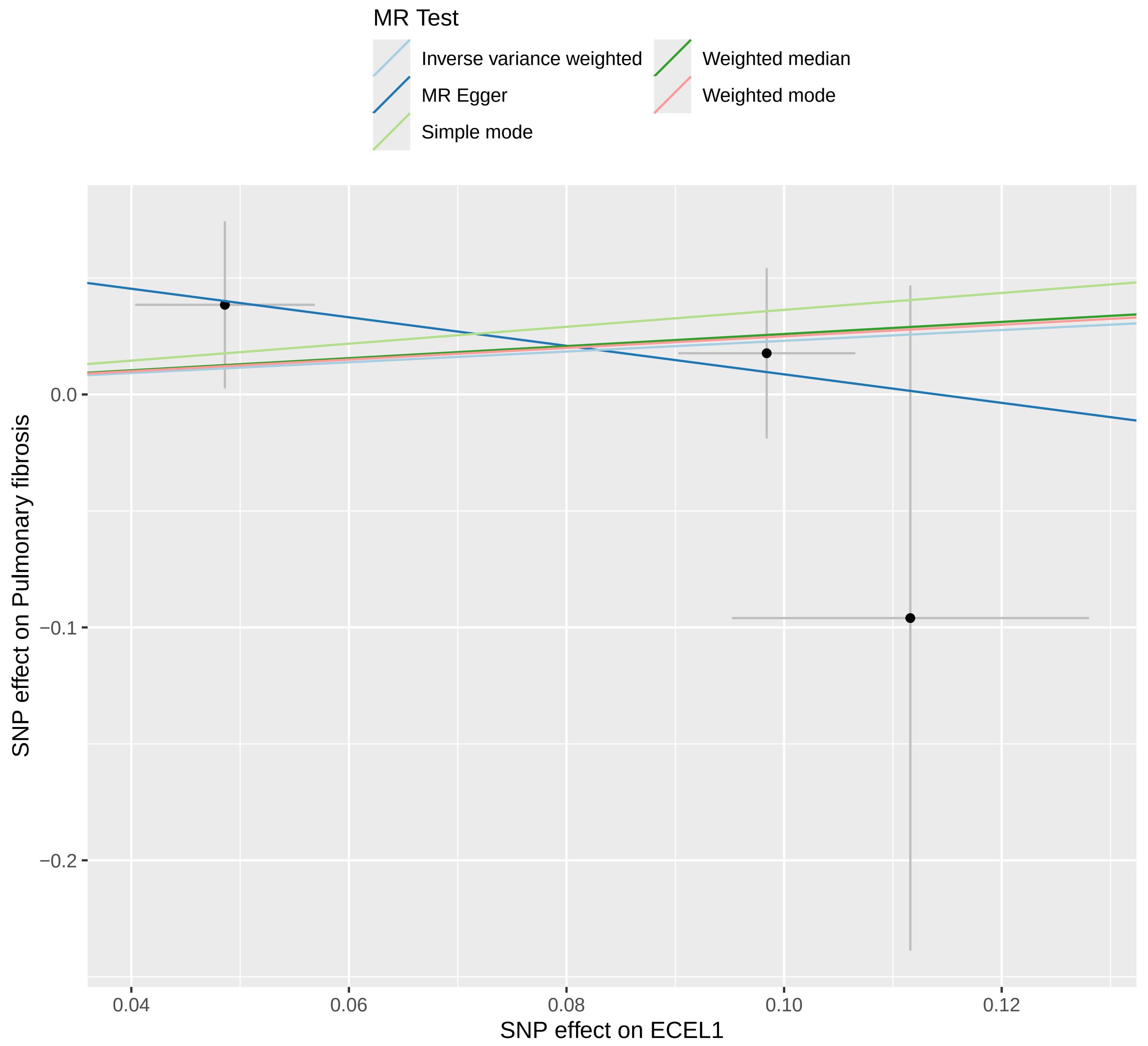
MR Test
Inverse variance weighted
MR Egger
Simple mode
Weighted median
Weighted mode
0.0
−0.1
−0.2
SNP effect on Pulmonary fibrosis
0.04
0.06
0.08
0.10
0.12
SNP effect on ECEL1

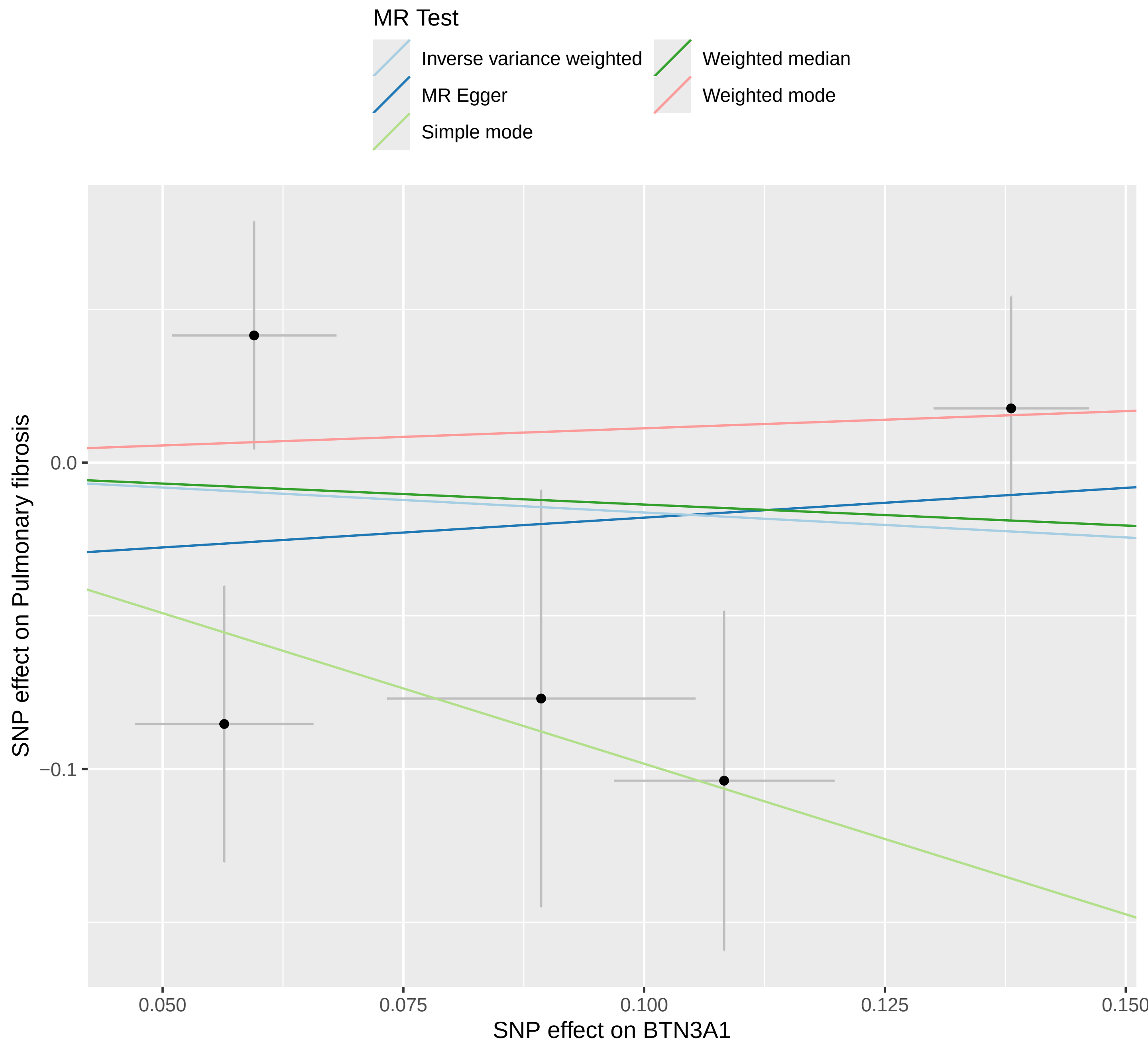

MR Test
Inverse variance weighted
MR Egger
Simple mode
Weighted median
Weighted mode
SNP effect on Pulmonary fibrosis
0.0
−0.1
0.050
0.075
0.100
0.125
0.150
SNP effect on BTN3A1

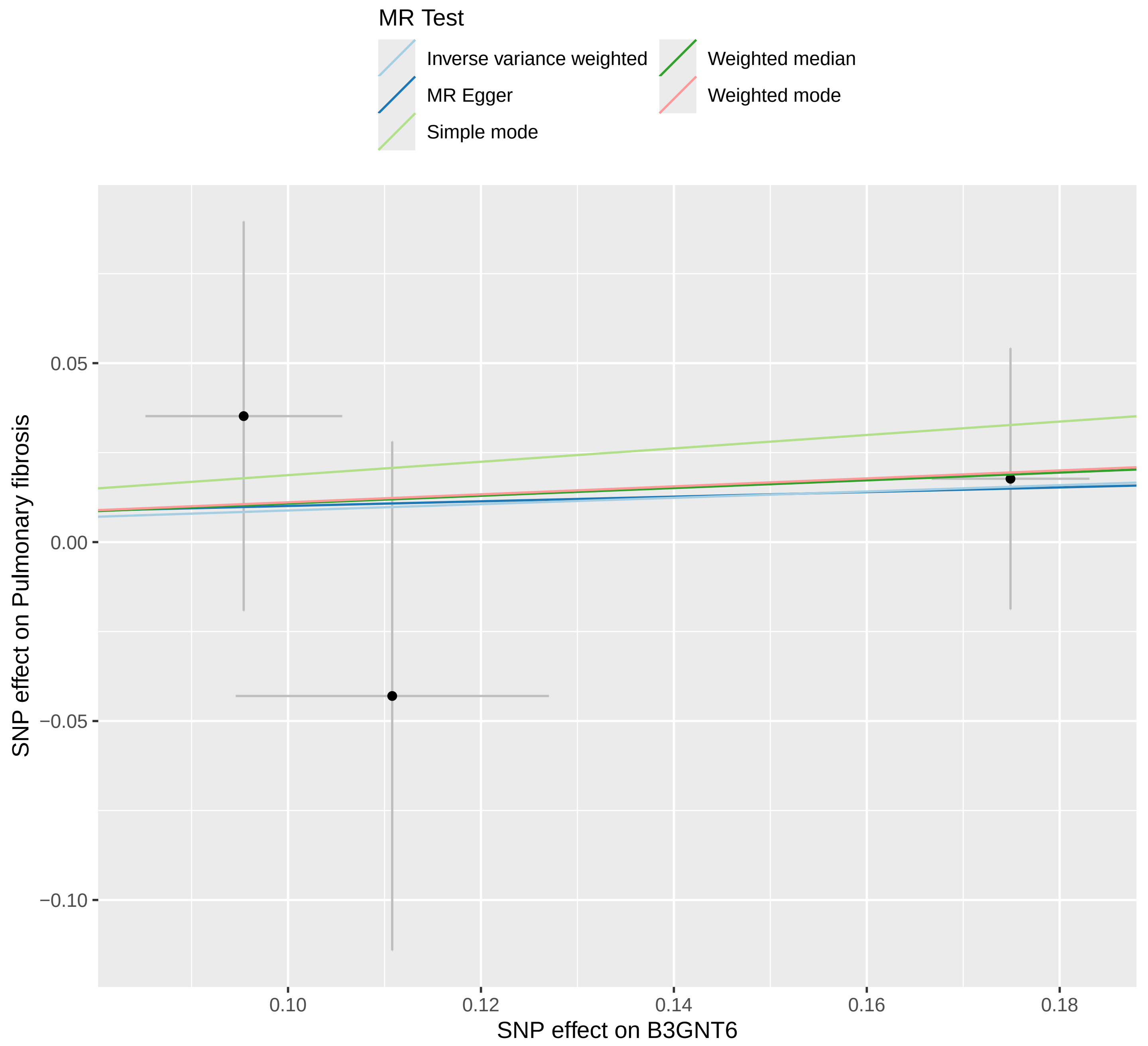
MR Test
Inverse variance weighted
MR Egger
Simple mode
Weighted median
Weighted mode
0.05
0.00
−0.05
−0.10
SNP effect on Pulmonary fibrosis
0.10
0.12
0.14
0.16
0.18
SNP effect on B3GNT6

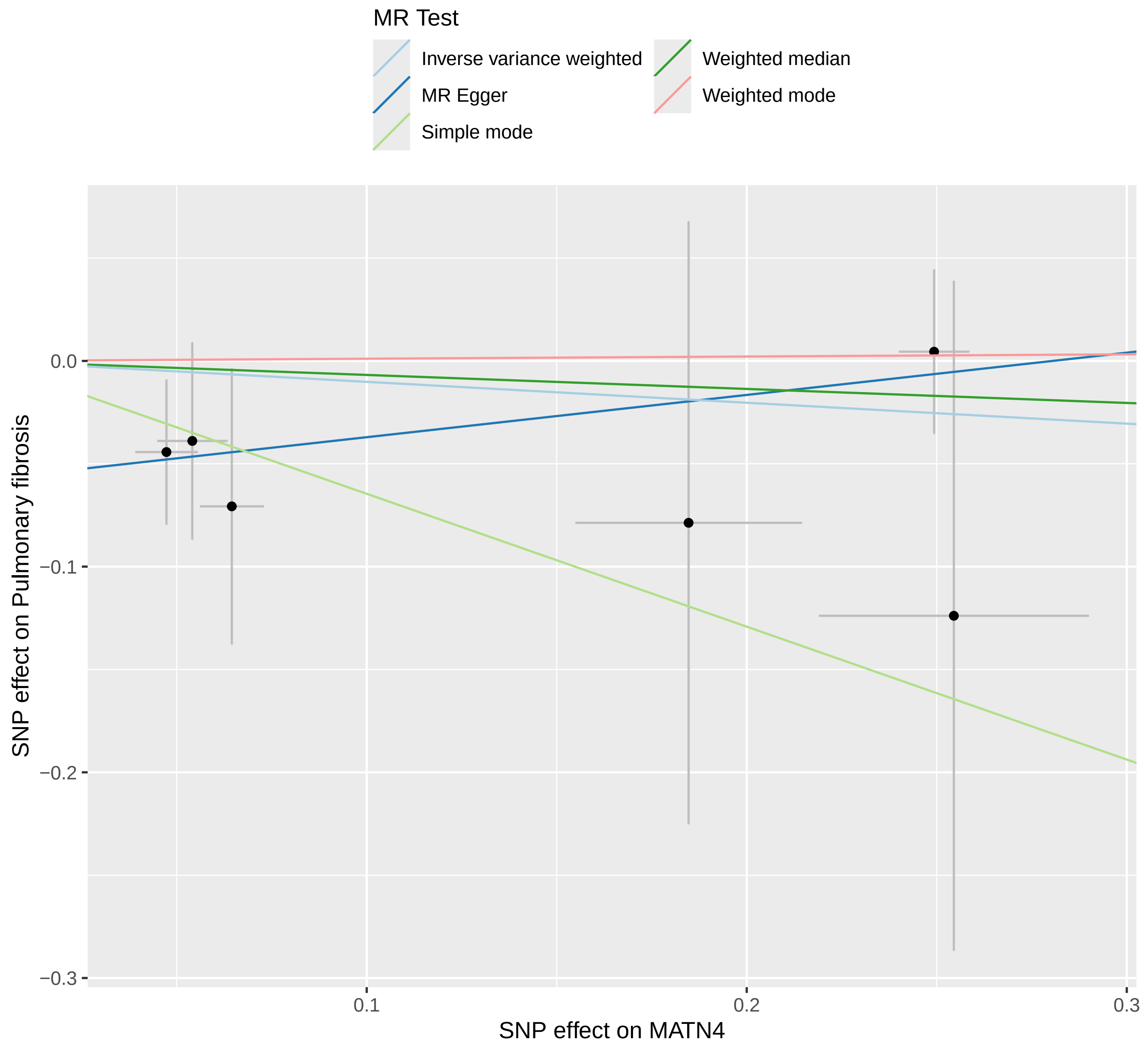

MR Test
Inverse variance weighted
MR Egger
Simple mode
Weighted median
Weighted mode
0.0
−0.1
−0.2
−0.3
SNP effect on Pulmonary fibrosis
0.1
0.2
0.3
SNP effect on MATN4

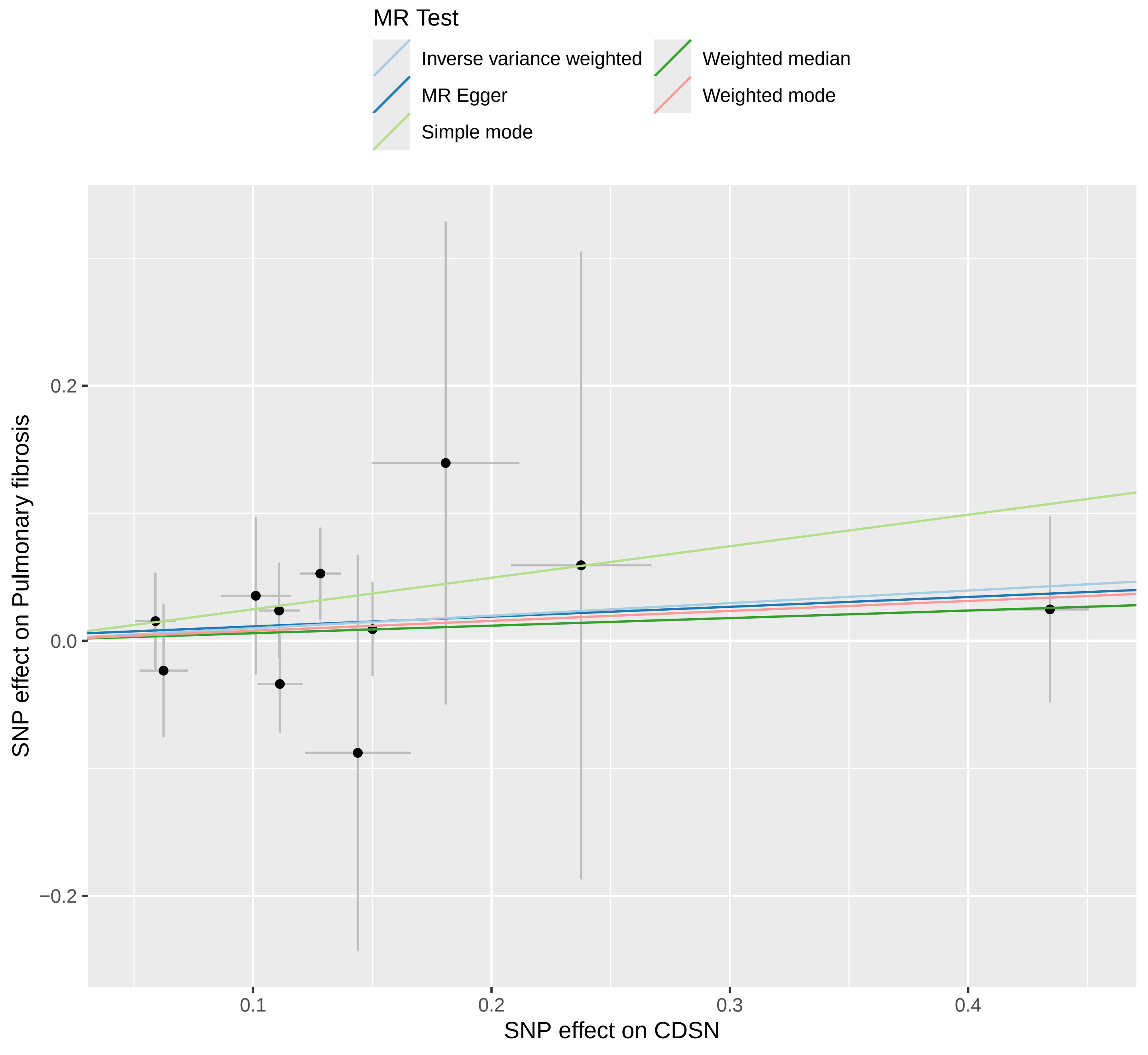

MR Test
Inverse variance weighted
MR Egger
Simple mode
Weighted median
Weighted mode
SNP effect on Pulmonary fibrosis
0.2
0.0
−0.2
0.1
0.2
0.3
0.4
SNP effect on CDSN

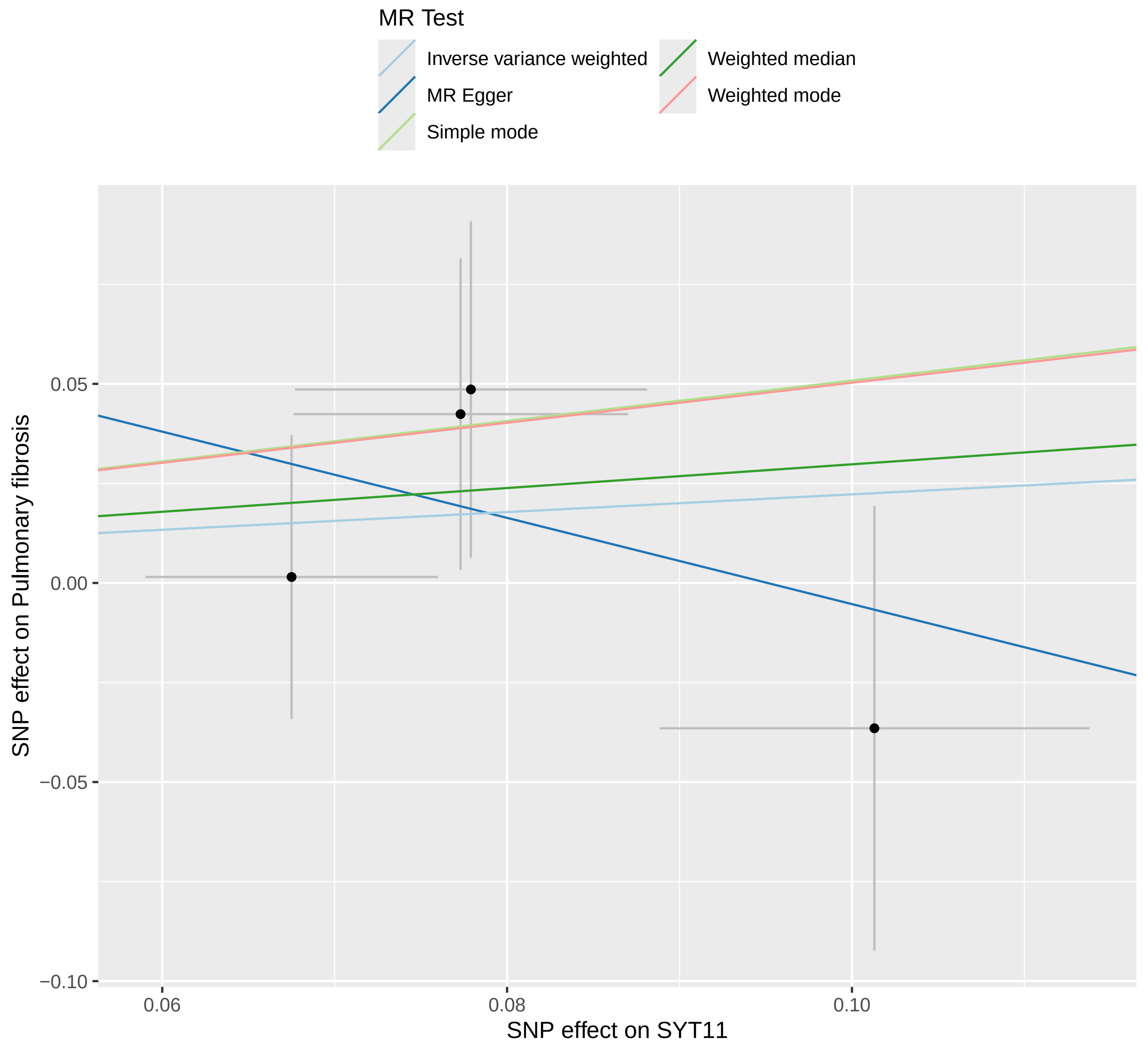
MR Test
Inverse variance weighted
MR Egger
Simple mode
Weighted median
Weighted mode
0.05
0.00
−0.05
−0.10
SNP effect on Pulmonary fibrosis
0.06
0.08
0.10
SNP effect on SYT11

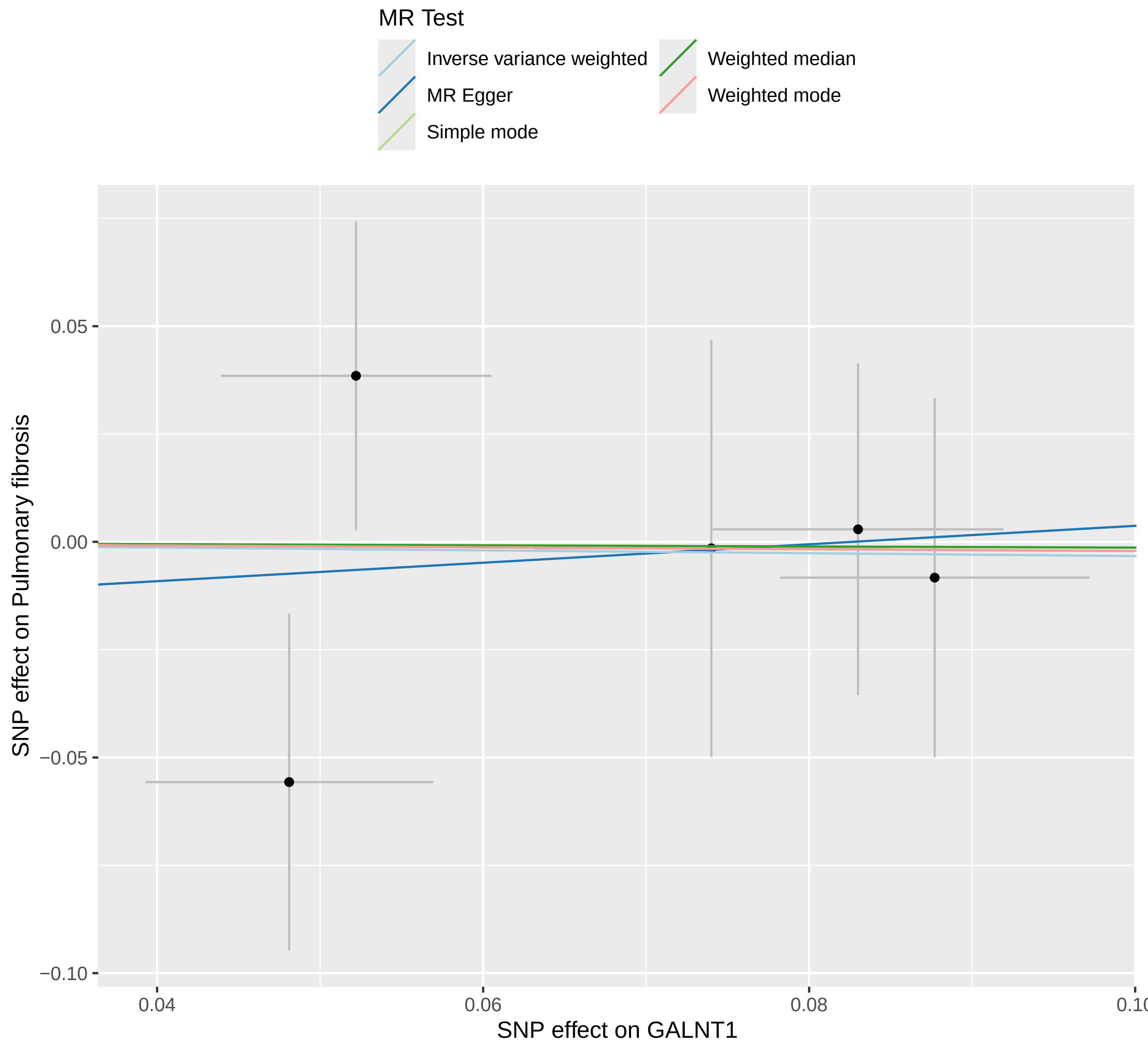
MR Test
Inverse variance weighted
MR Egger
Simple mode
Weighted median
Weighted mode
SNP effect on Pulmonary fibrosis
0.05
0.00
−0.05
−0.10
0.04
0.06
0.08
SNP effect on GALNT1

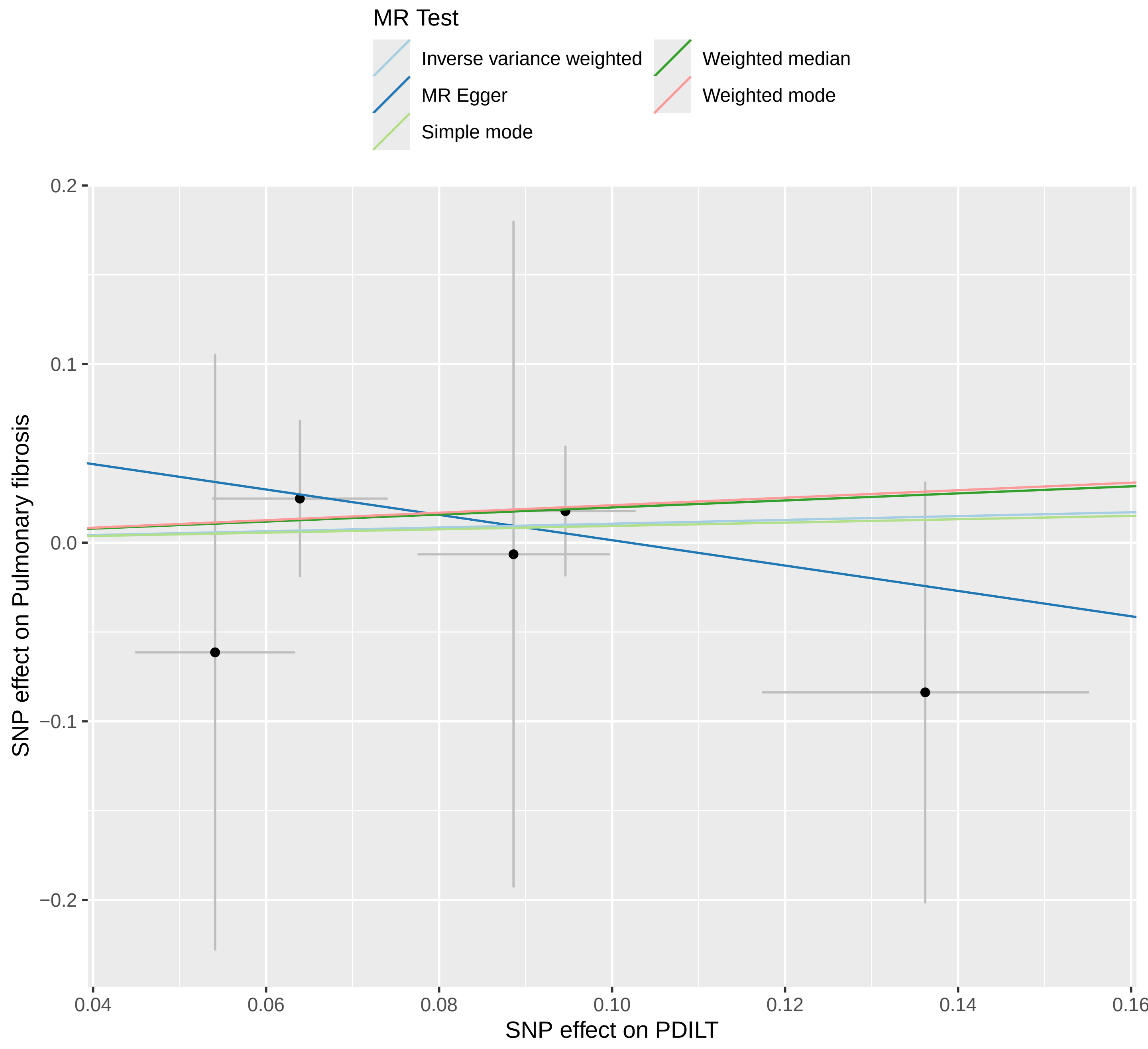

MR Test
Inverse variance weighted
MR Egger
Simple mode
Weighted median
Weighted mode
0.2
0.1
0.0
−0.1
−0.2
SNP effect on Pulmonary fibrosis
0.04
0.06
0.08
0.10
0.12
0.14
0.16
SNP effect on PDILT

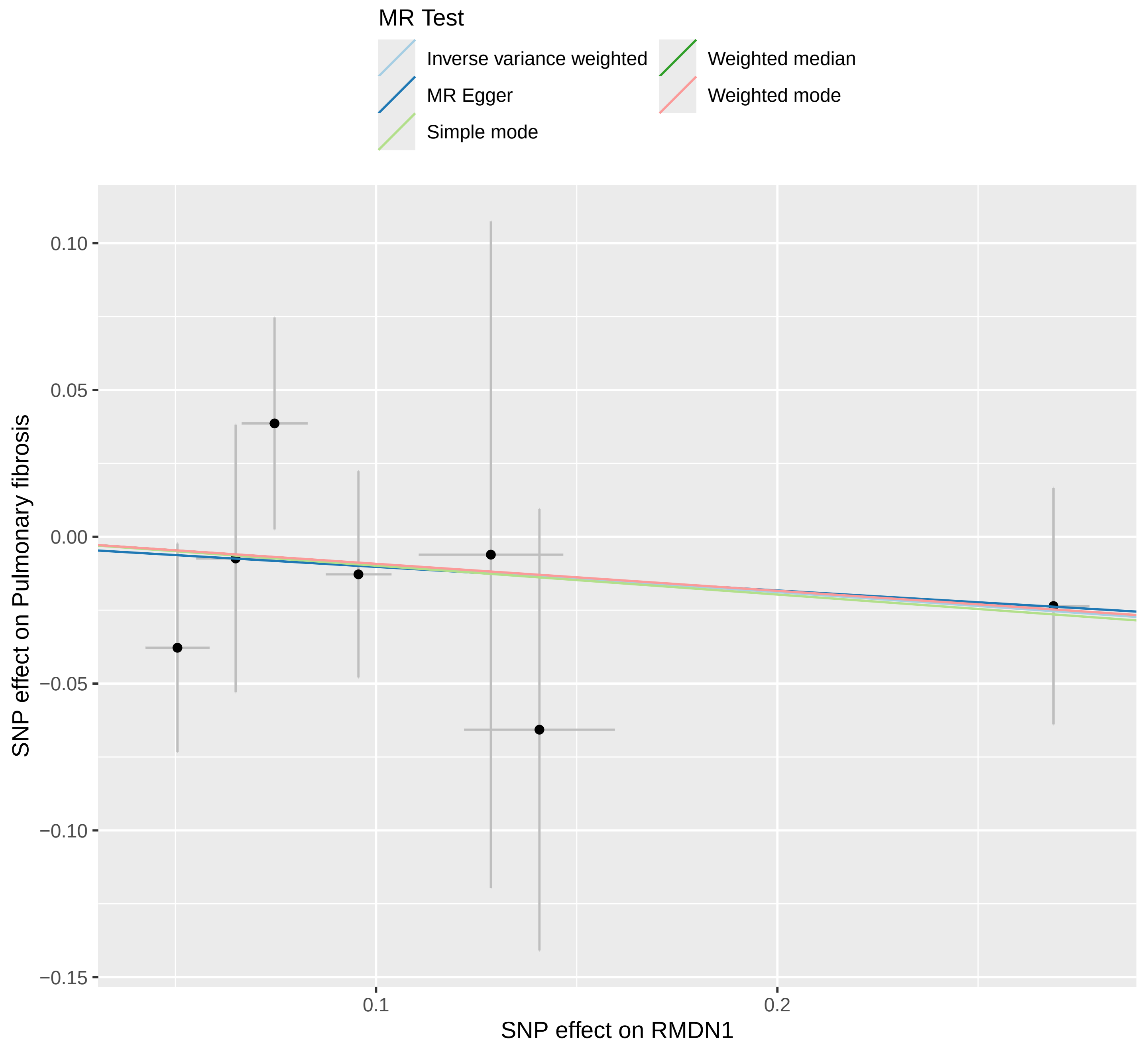

MR Test
Inverse variance weighted
MR Egger
Simple mode
Weighted median
Weighted mode
0.10
0.05
0.00
−0.05
−0.10
−0.15
SNP effect on Pulmonary fibrosis
0.1
0.2
SNP effect on RMDN1

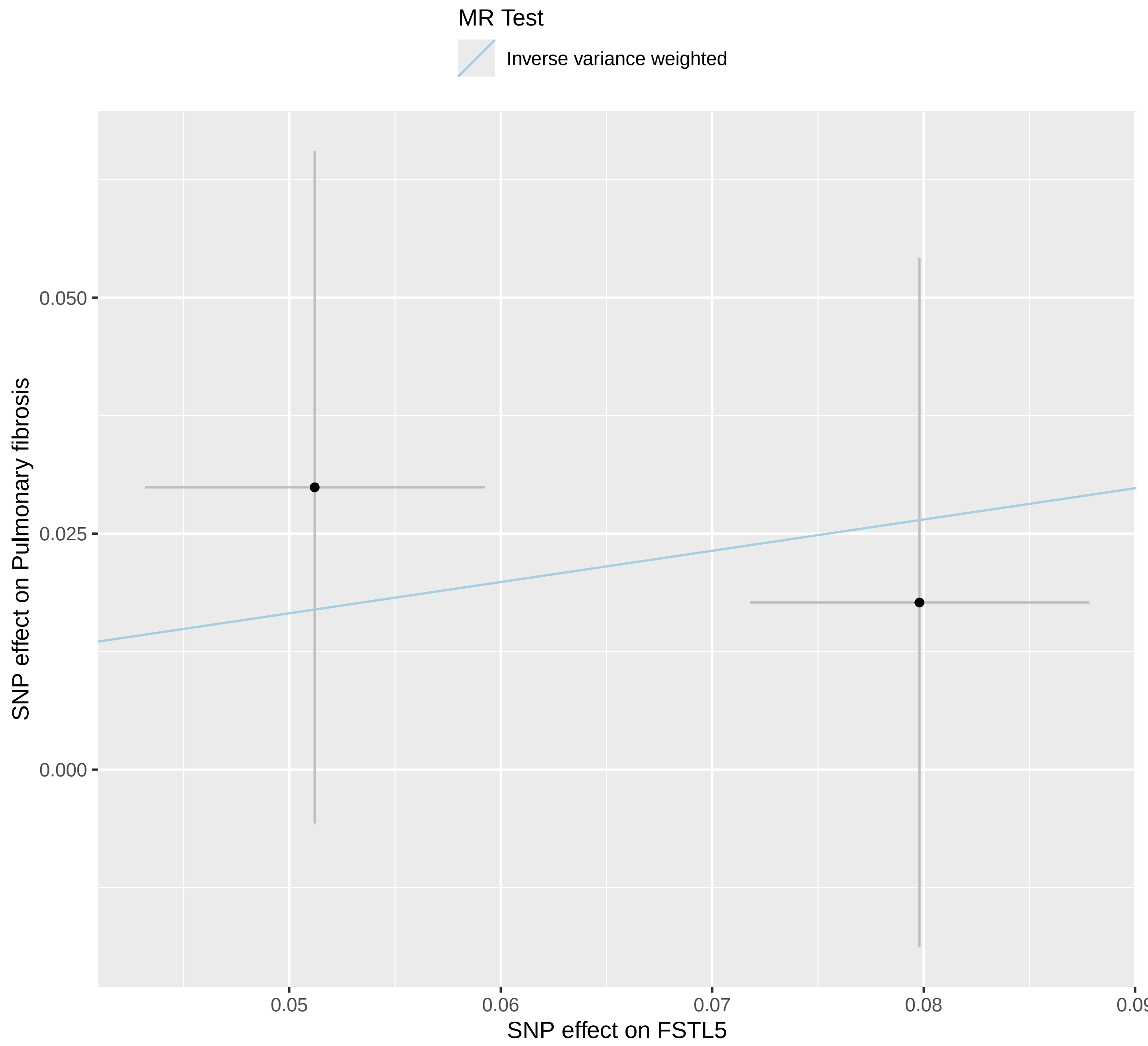
MR Test
Inverse variance weighted
SNP effect on Pulmonary fibrosis
0.050
0.025
0.000
0.05
0.06
0.07
0.08
0.09
SNP effect on FSTL5

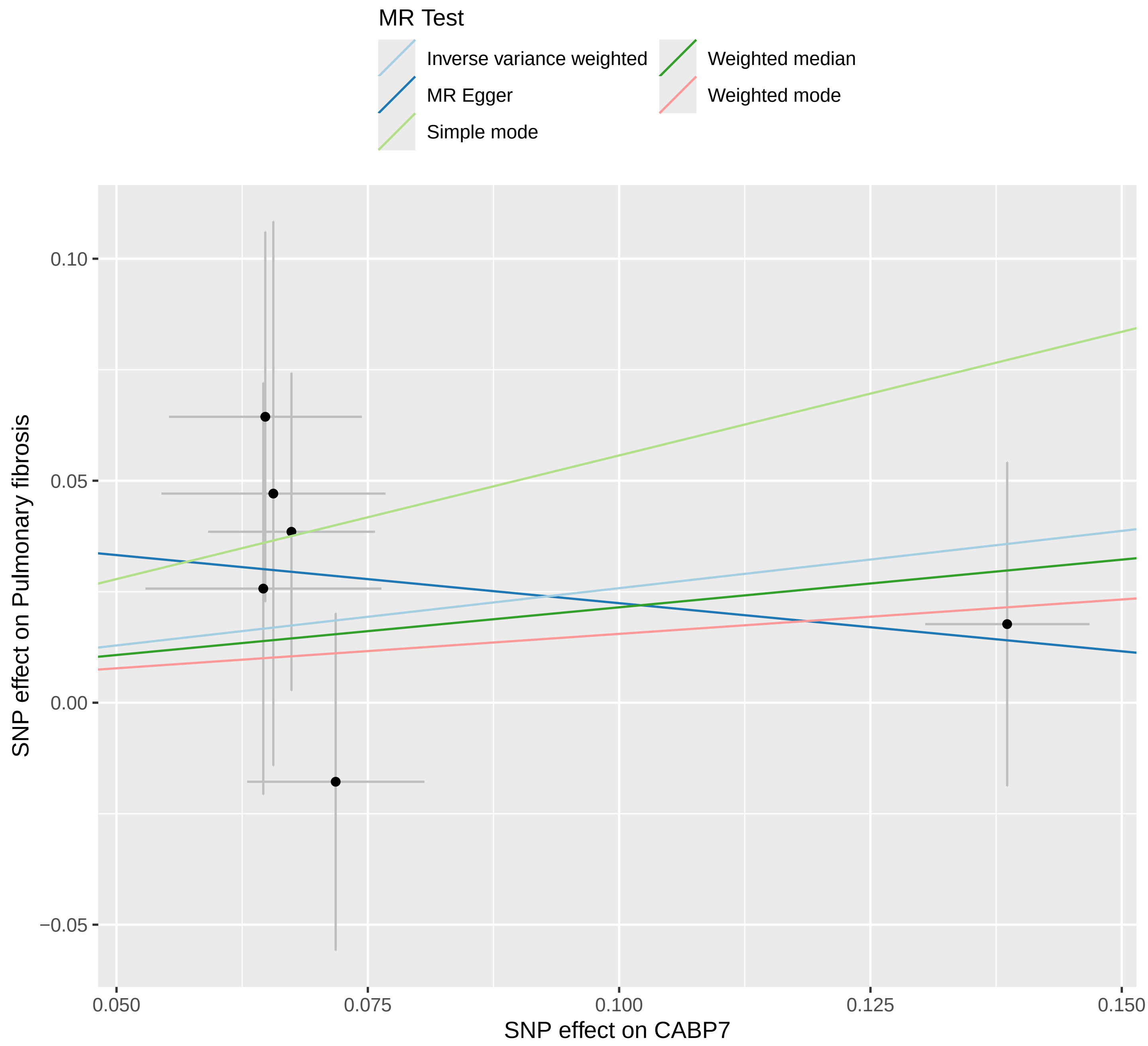
MR Test
Inverse variance weighted
MR Egger
Simple mode
Weighted median
Weighted mode
SNP effect on Pulmonary fibrosis
0.10
0.05
0.00
−0.05
0.050
0.075
0.100
0.125
0.150
SNP effect on CABP7

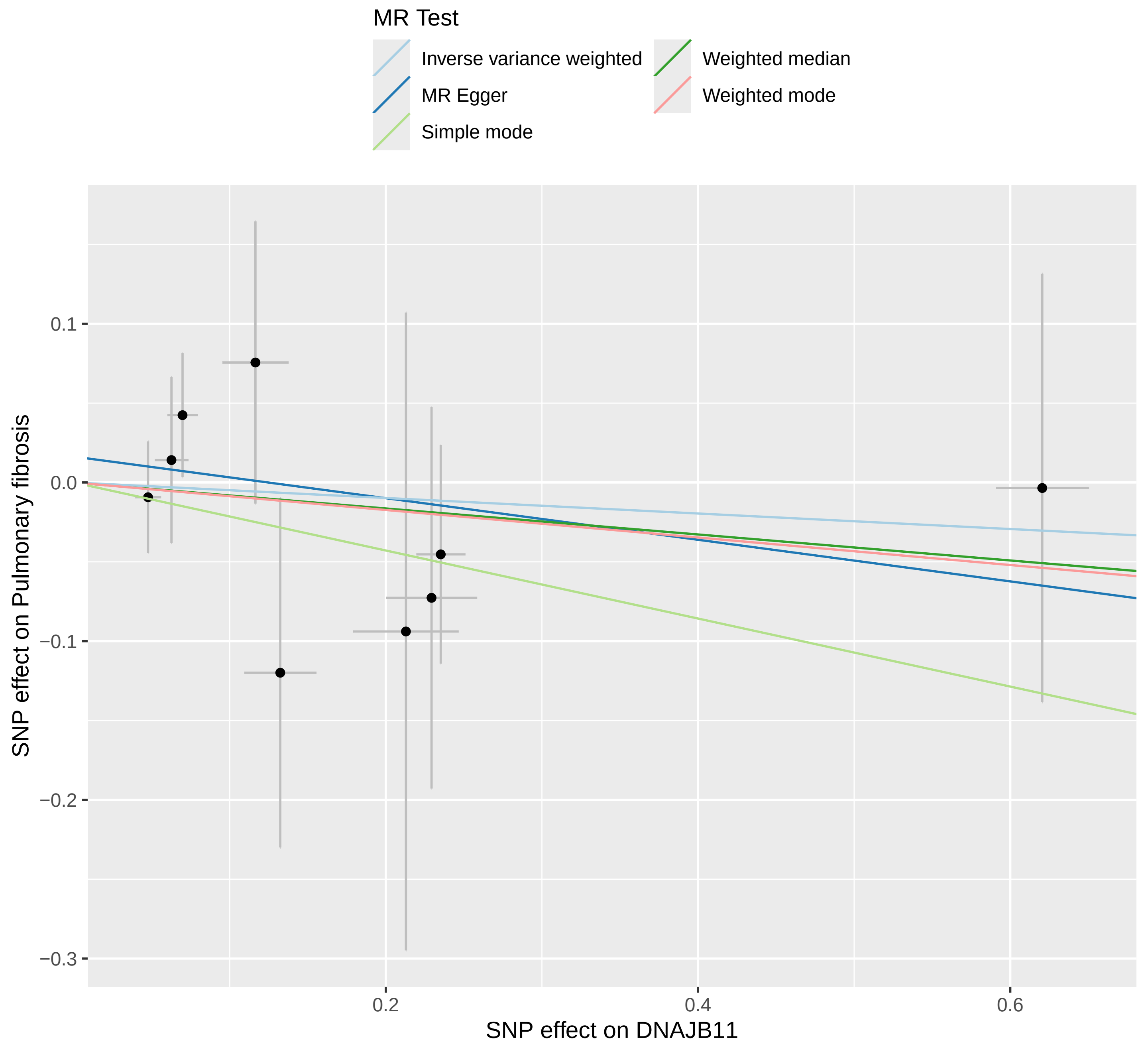
MR Test
Inverse variance weighted
Weighted median
MR Egger
Weighted mode
Simple mode
SNP effect on Pulmonary fibrosis
0.1
0.0
−0.1
−0.2
−0.3
0.2
0.4
0.6
SNP effect on DNAJB11

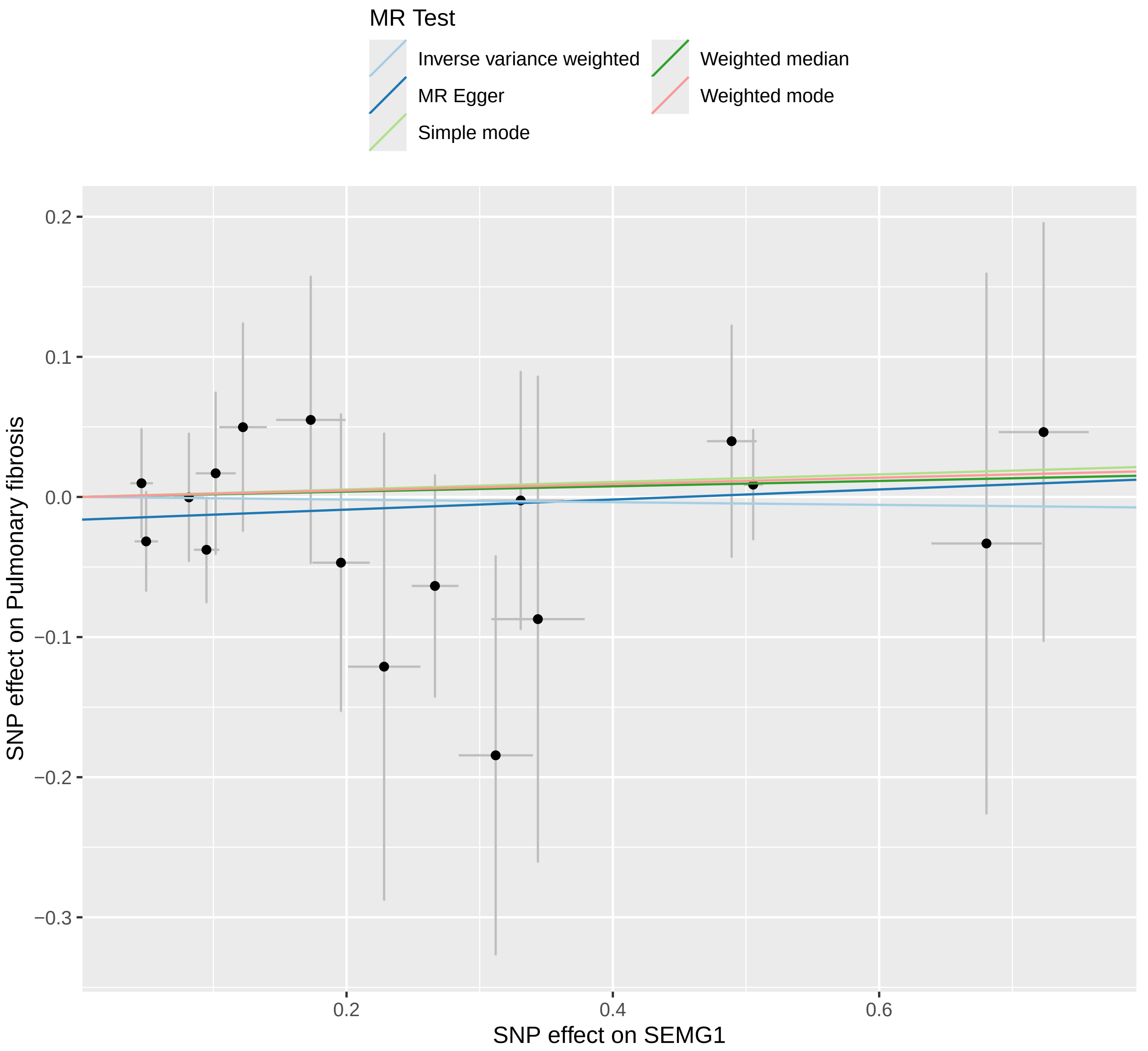

MR Test
Inverse variance weighted
MR Egger
Simple mode
Weighted median
Weighted mode
0.2
0.1
0.0
−0.1
−0.2
−0.3
SNP effect on Pulmonary fibrosis
0.2
0.4
0.6
SNP effect on SEMG1

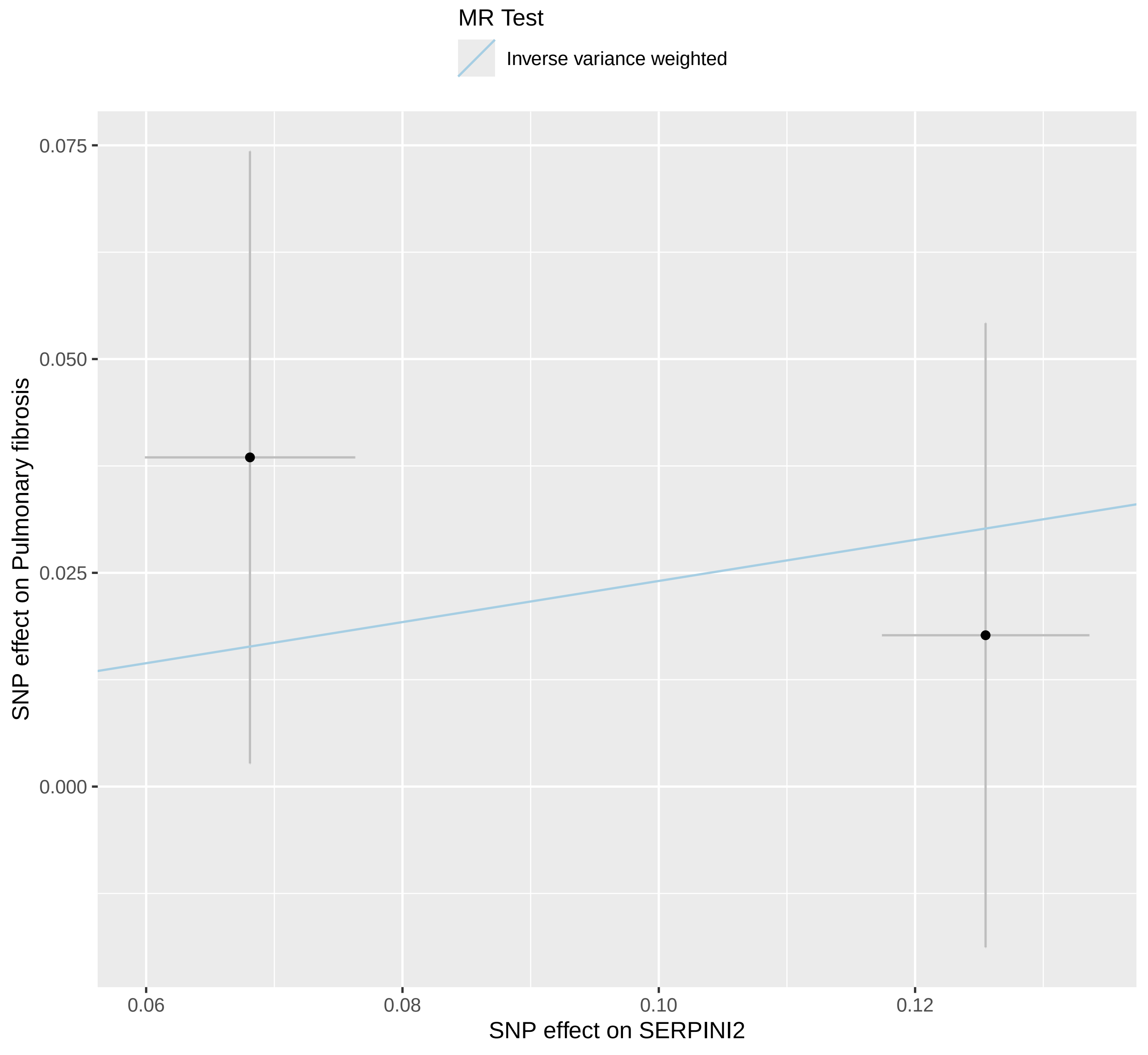
MR Test
Inverse variance weighted
0.075
0.050
0.025
0.000
SNP effect on Pulmonary fibrosis
0.06
0.08
0.10
0.12
SNP effect on SERPINI2

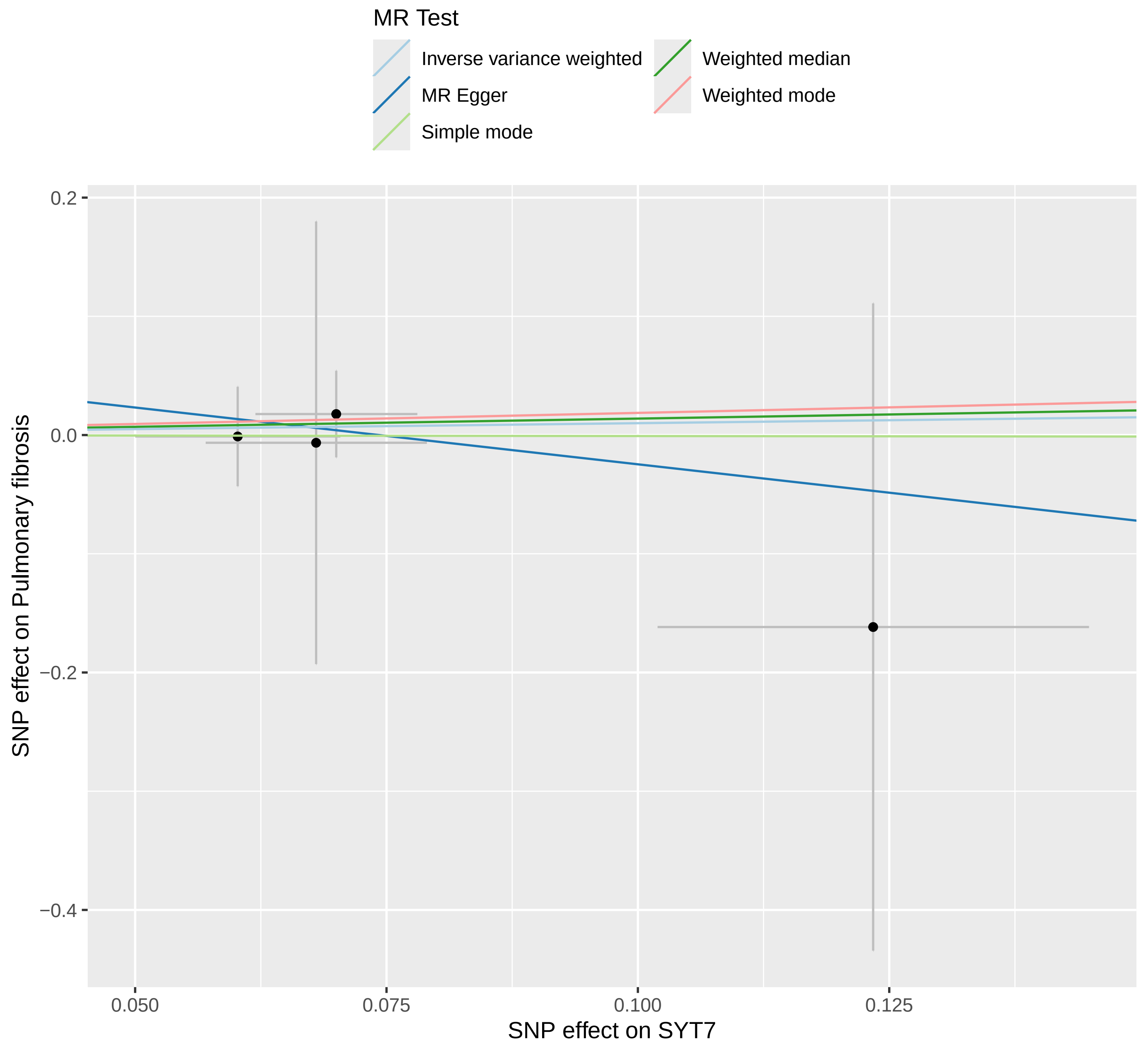
MR Test
Inverse variance weighted
MR Egger
Simple mode
Weighted median
Weighted mode
0.2
0.0
−0.2
−0.4
SNP effect on Pulmonary fibrosis
0.050
0.075
0.100
0.125
SNP effect on SYT7

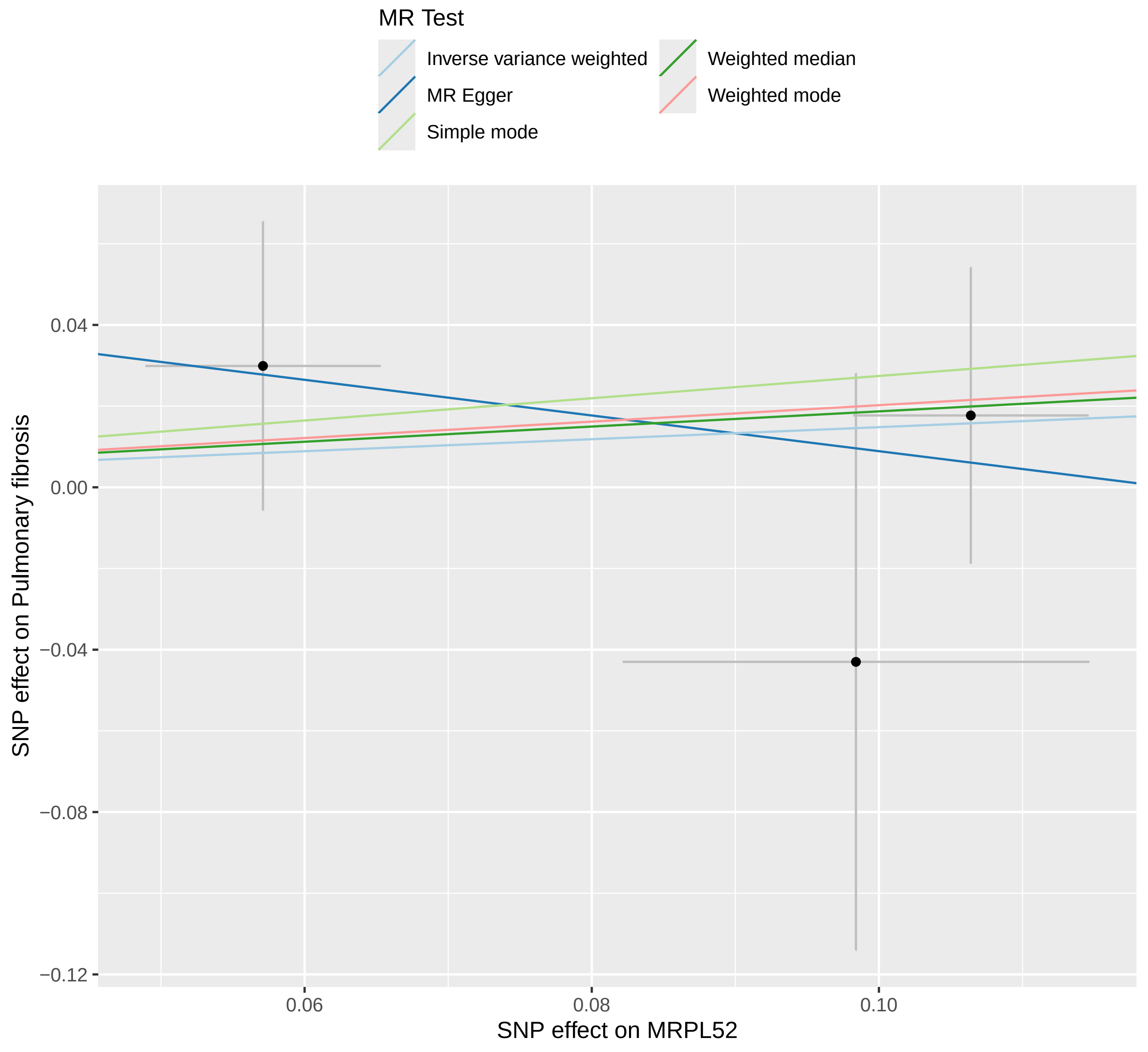

MR Test
Inverse variance weighted
MR Egger
Simple mode
Weighted median
Weighted mode
0.04
0.00
−0.04
−0.08
−0.12
SNP effect on Pulmonary fibrosis
0.06
0.08
0.10
SNP effect on MRPL52

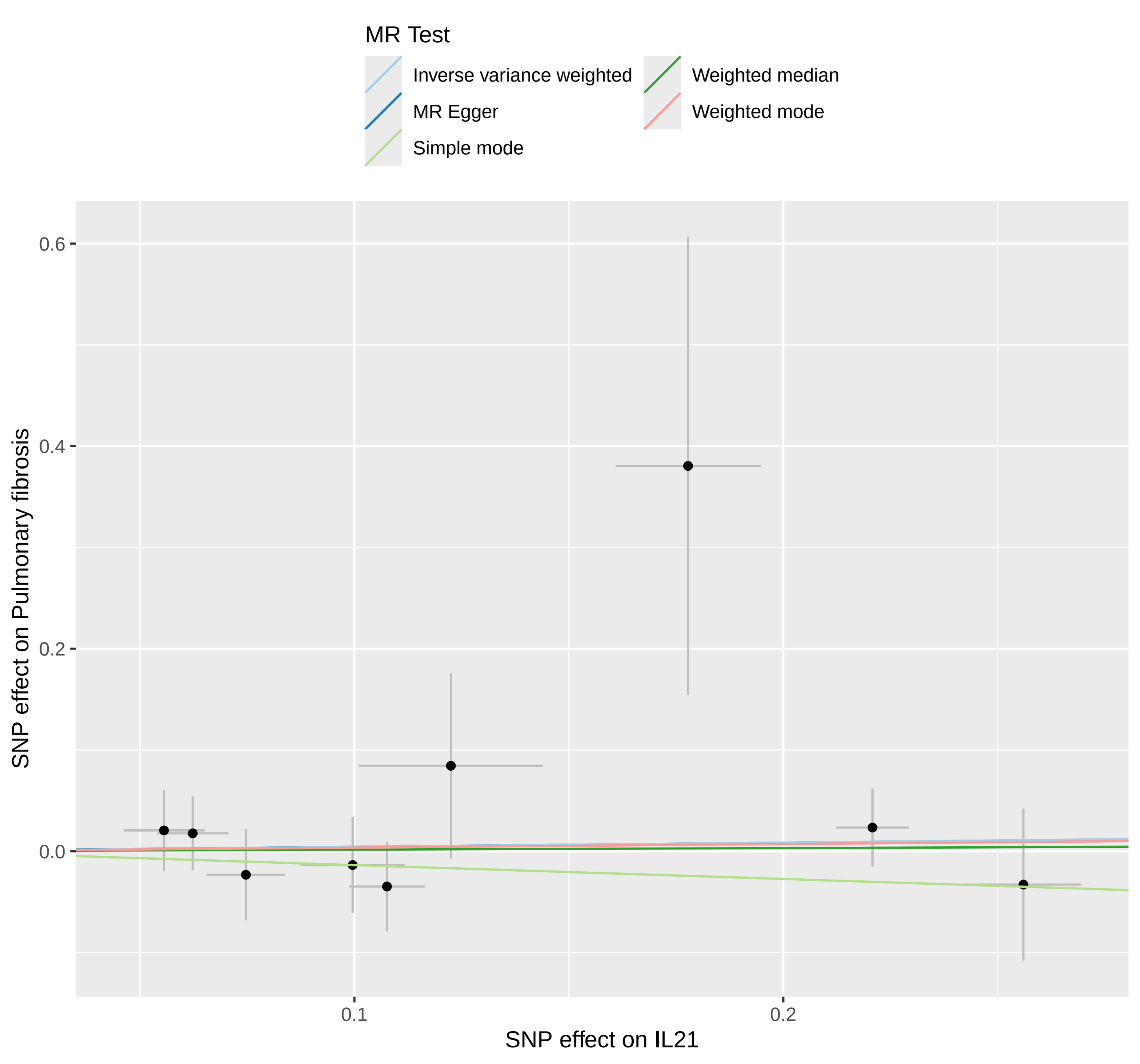
MR Test
Inverse variance weighted
MR Egger
Simple mode
Weighted median
Weighted mode
0.6
0.4
0.2
0.0
SNP effect on Pulmonary fibrosis
0.1
0.2
SNP effect on IL21

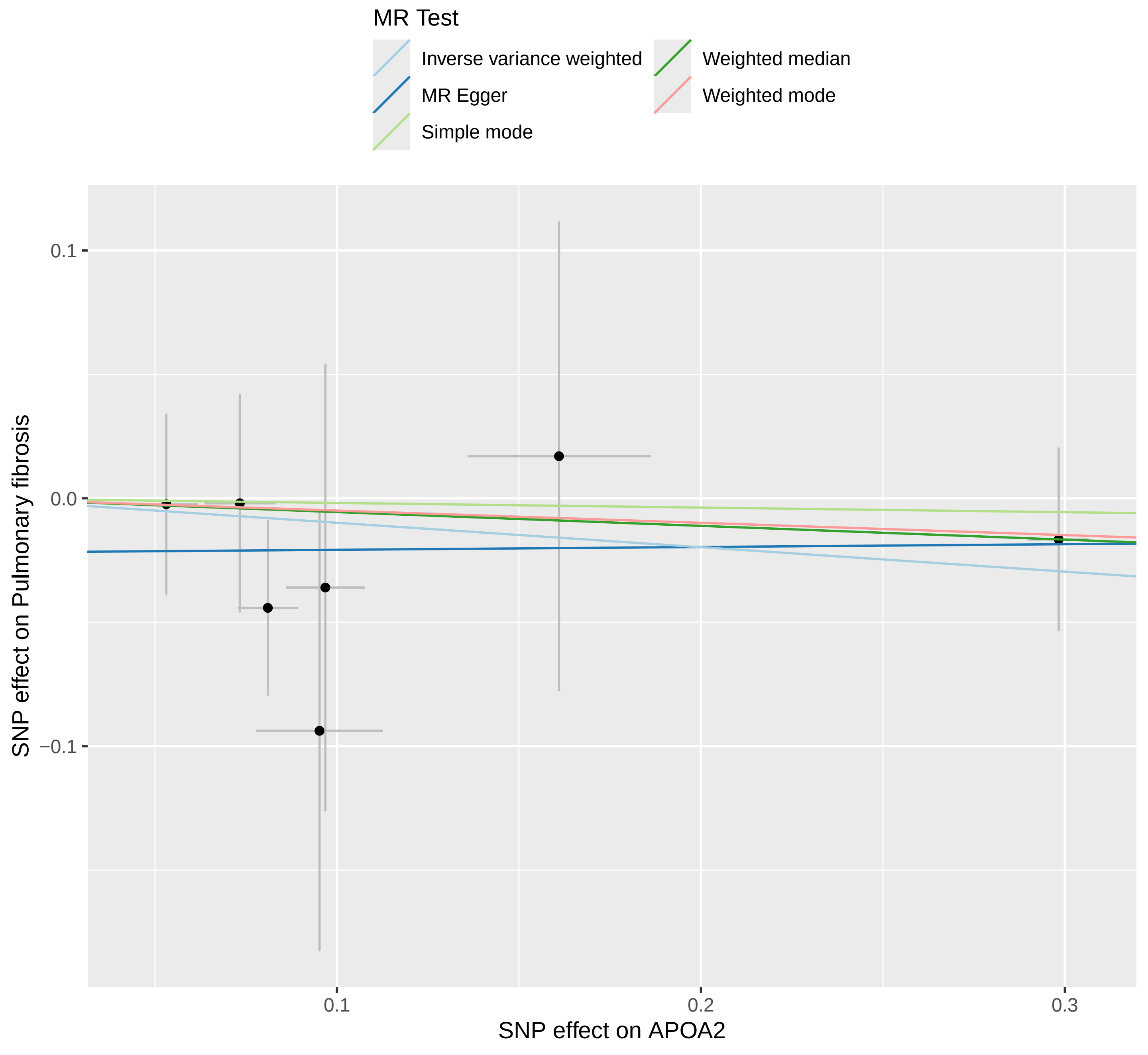
MR Test
Inverse variance weighted
MR Egger
Simple mode
Weighted median
Weighted mode
0.1
0.0
−0.1
SNP effect on Pulmonary fibrosis
0.1
0.2
0.3
SNP effect on APOA2

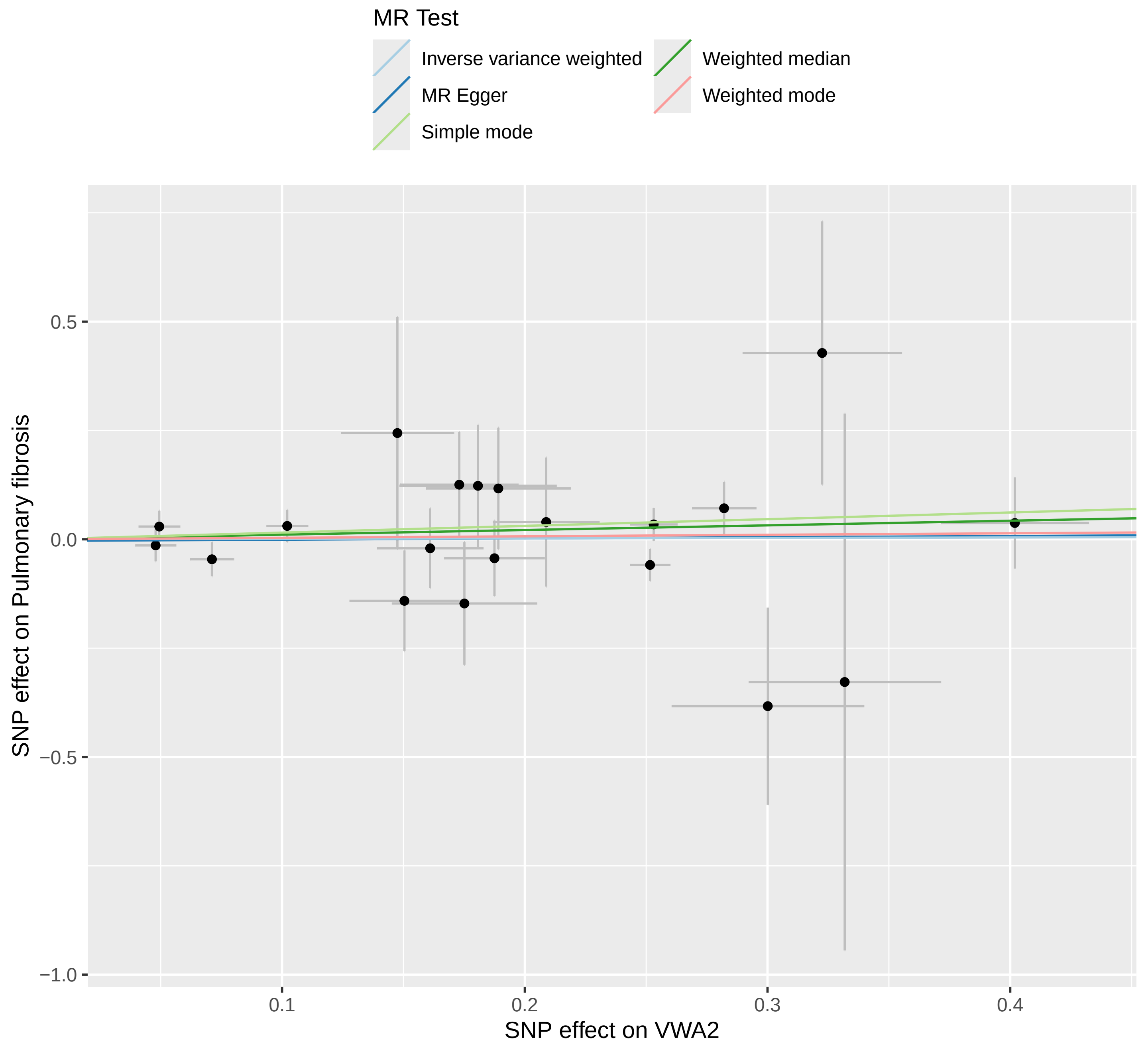

MR Test
Inverse variance weighted
MR Egger
Simple mode
Weighted median
Weighted mode
0.5
0.0
−0.5
−1.0
0.1
0.2
0.3
0.4
SNP effect on Pulmonary fibrosis
SNP effect on VWA2

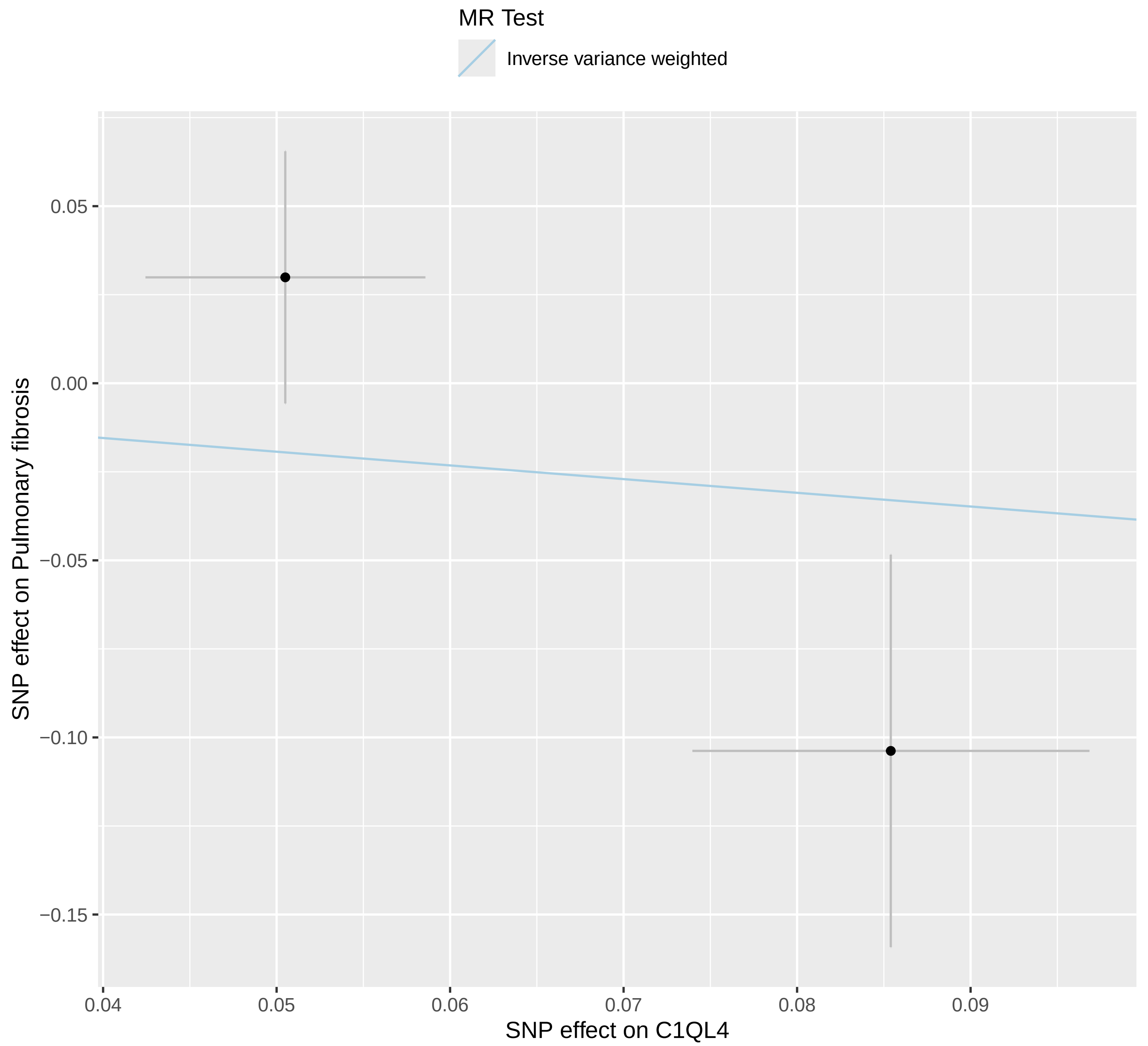
MR Test
Inverse variance weighted
SNP effect on Pulmonary fibrosis
0.05
0.00
−0.05
−0.10
−0.15
0.04
0.05
0.06
0.07
0.08
0.09
SNP effect on C1QL4

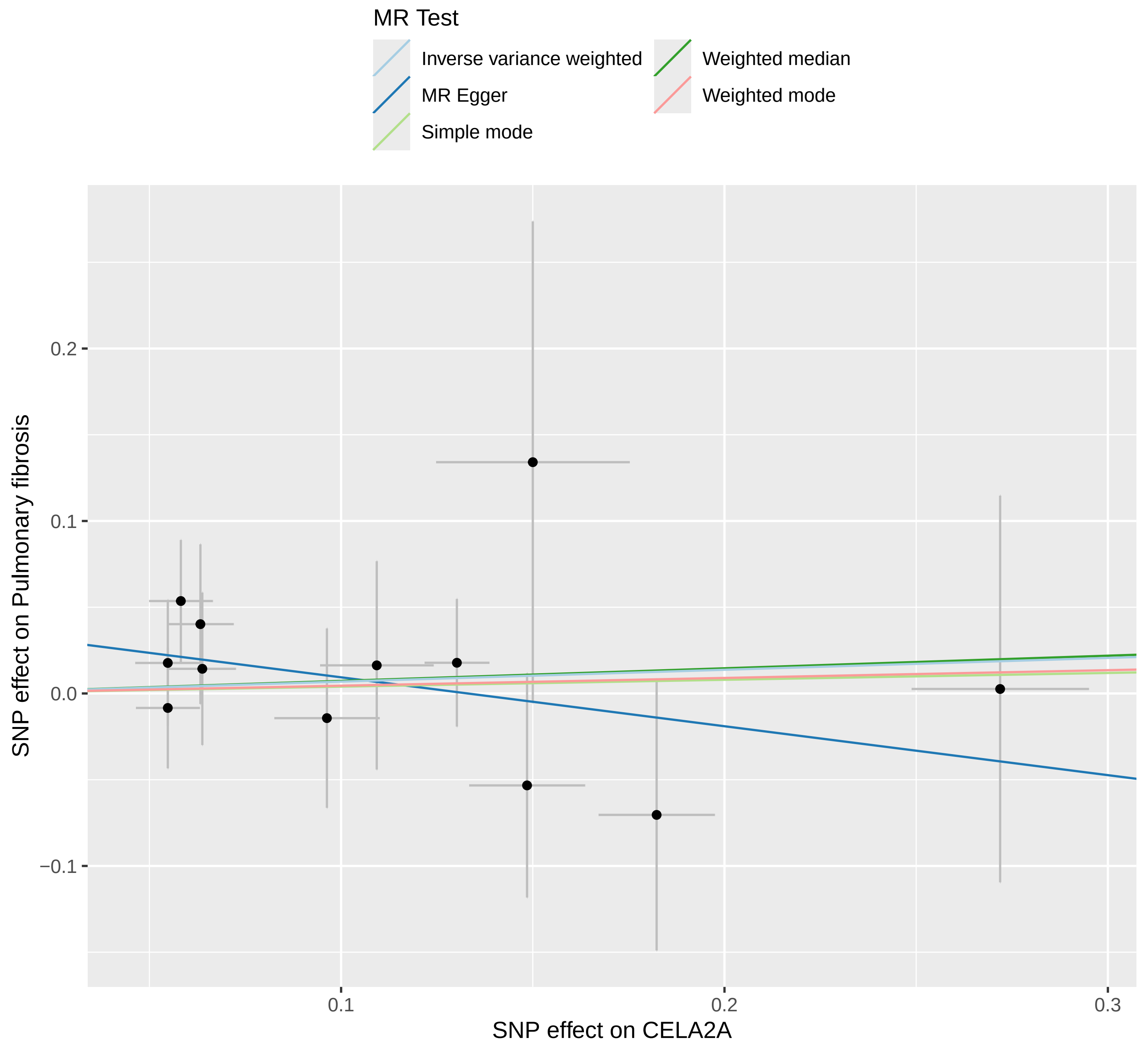
MR Test
Inverse variance weighted
MR Egger
Simple mode
Weighted median
Weighted mode
SNP effect on Pulmonary fibrosis
0.2
0.1
0.0
−0.1
SNP effect on CELA2A
0.1
0.2
0.3

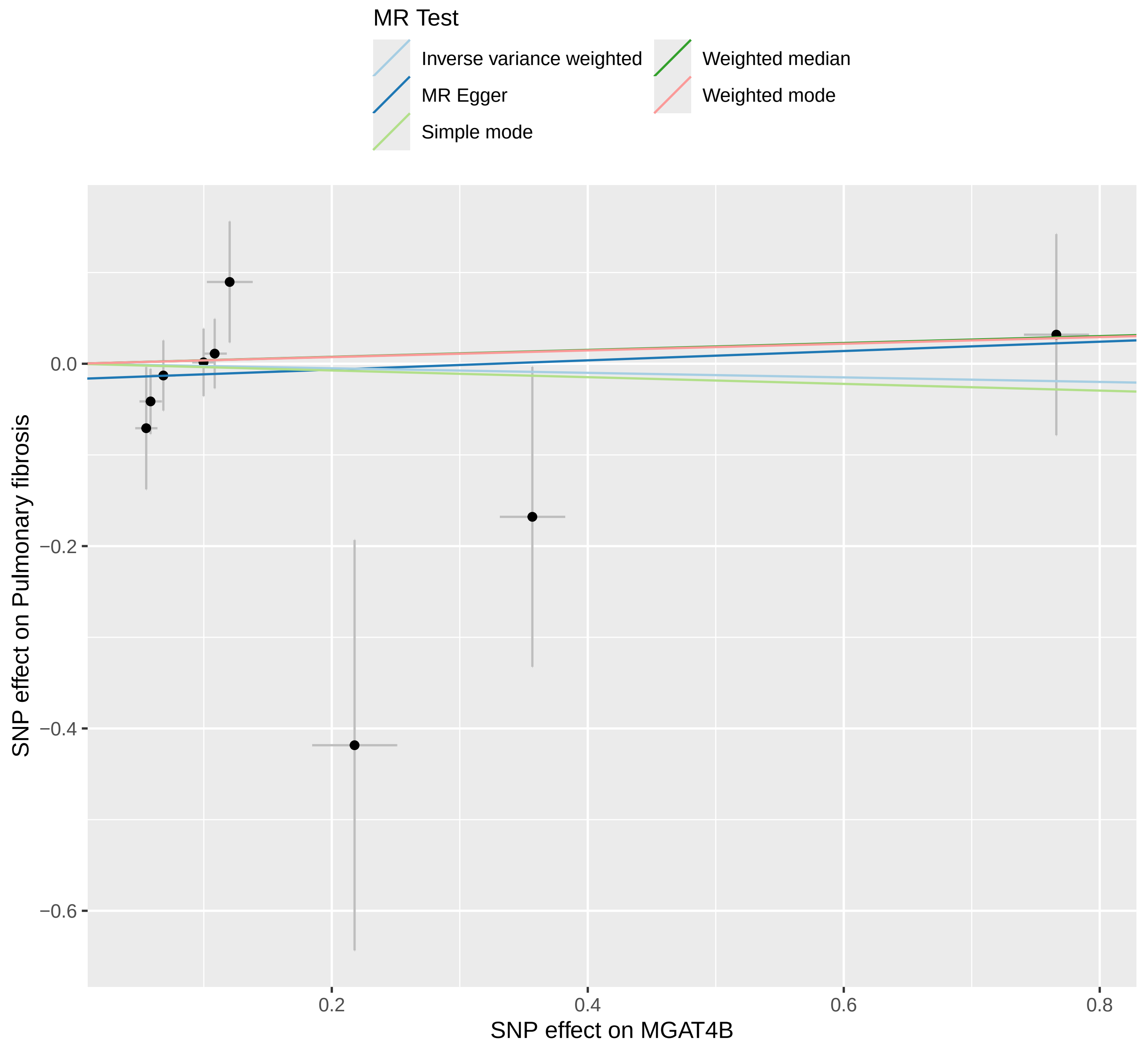
MR Test
Inverse variance weighted
MR Egger
Simple mode
Weighted median
Weighted mode
0.0
−0.2
−0.4
−0.6
0.2
0.4
0.6
0.8
SNP effect on Pulmonary fibrosis
SNP effect on MGAT4B

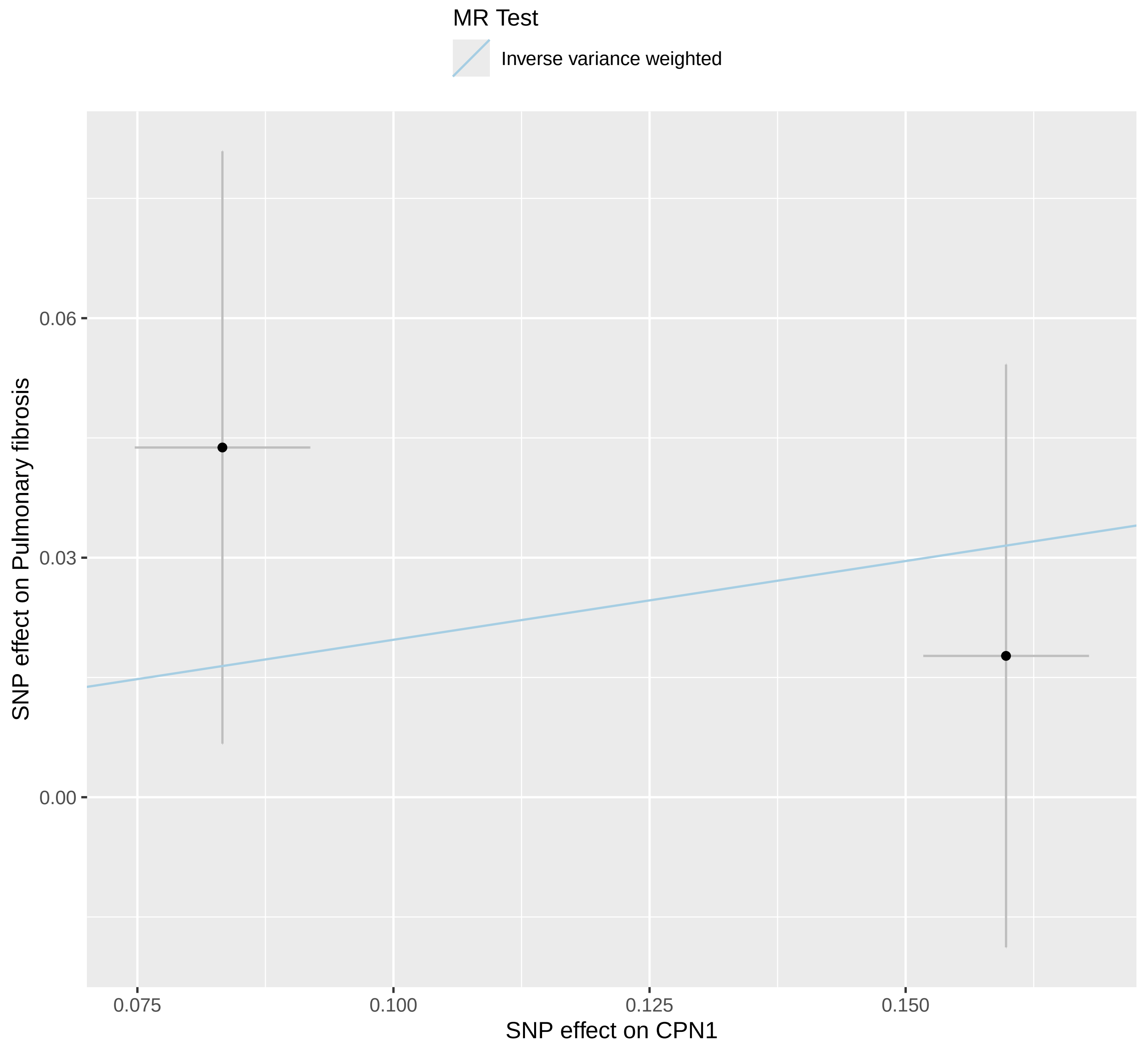
MR Test
Inverse variance weighted
SNP effect on Pulmonary fibrosis
0.06
0.03
0.00
0.075
0.100
0.125
0.150
SNP effect on CPN1

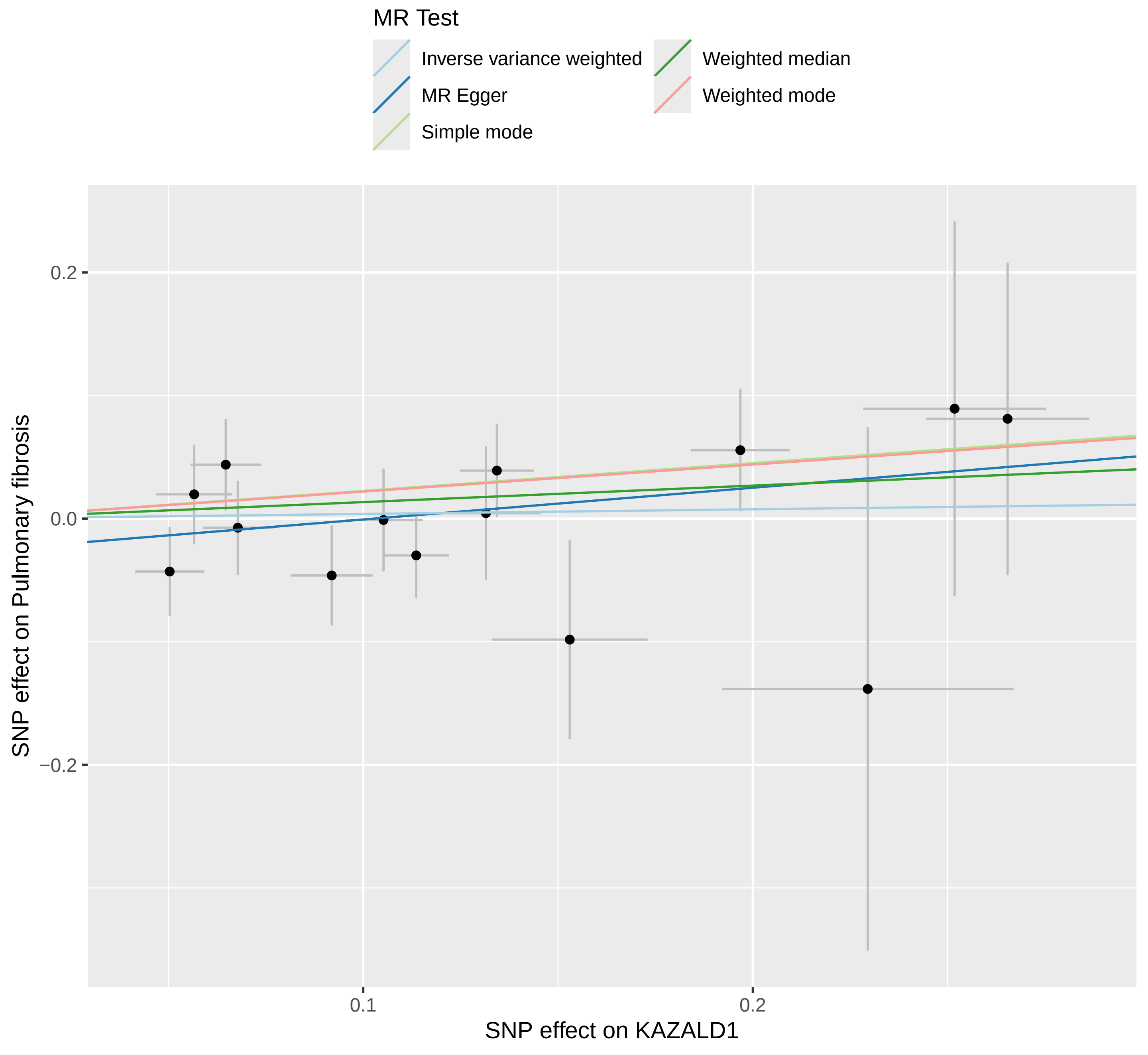

MR Test
Inverse variance weighted
MR Egger
Simple mode
Weighted median
Weighted mode
0.2
0.0
−0.2
0.1
0.2
SNP effect on Pulmonary fibrosis
SNP effect on KAZALD1

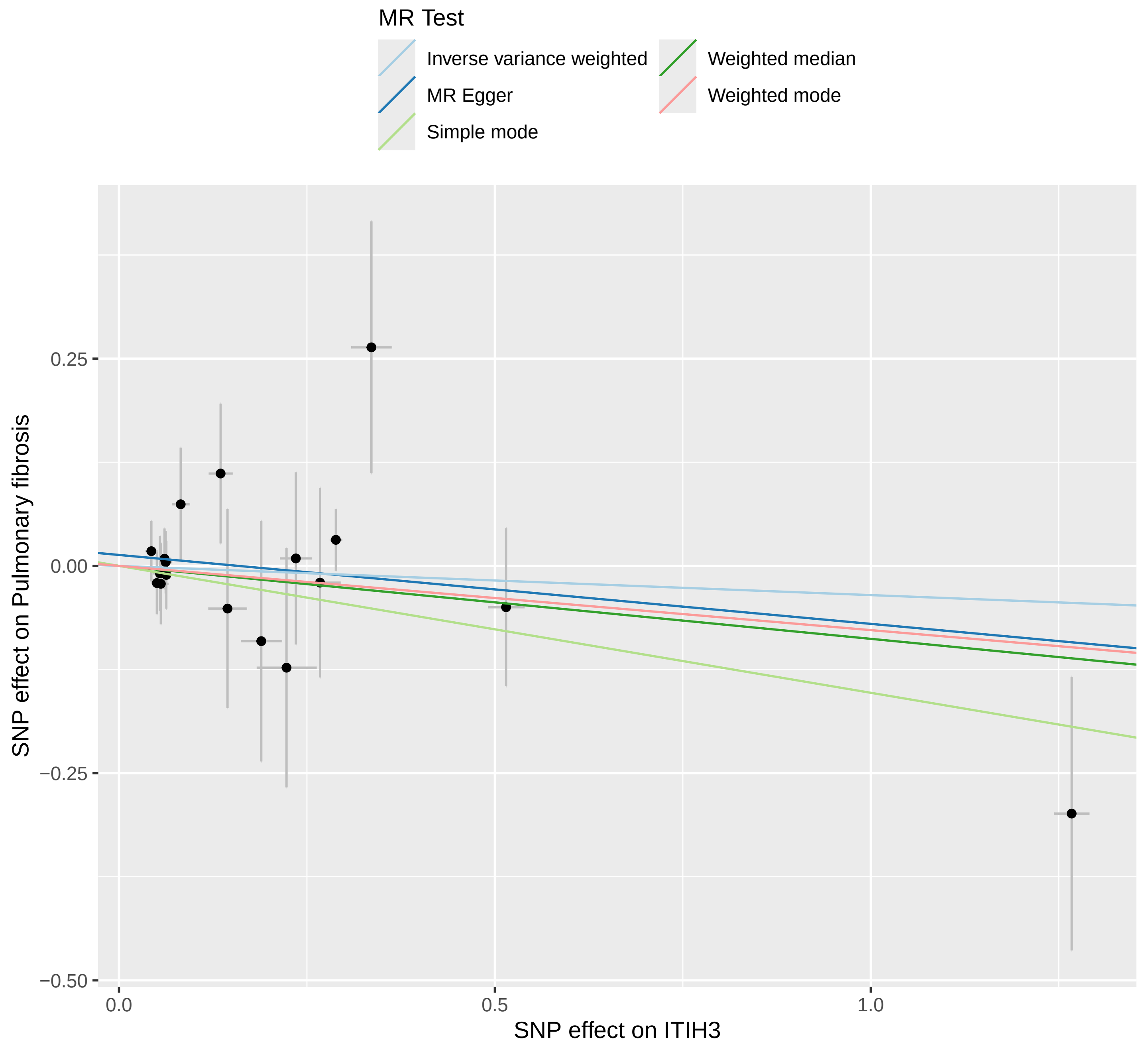

MR Test
Inverse variance weighted
MR Egger
Simple mode
Weighted median
Weighted mode
0.25
0.00
−0.25
−0.50
SNP effect on Pulmonary fibrosis
0.0
0.5
1.0
SNP effect on ITIH3

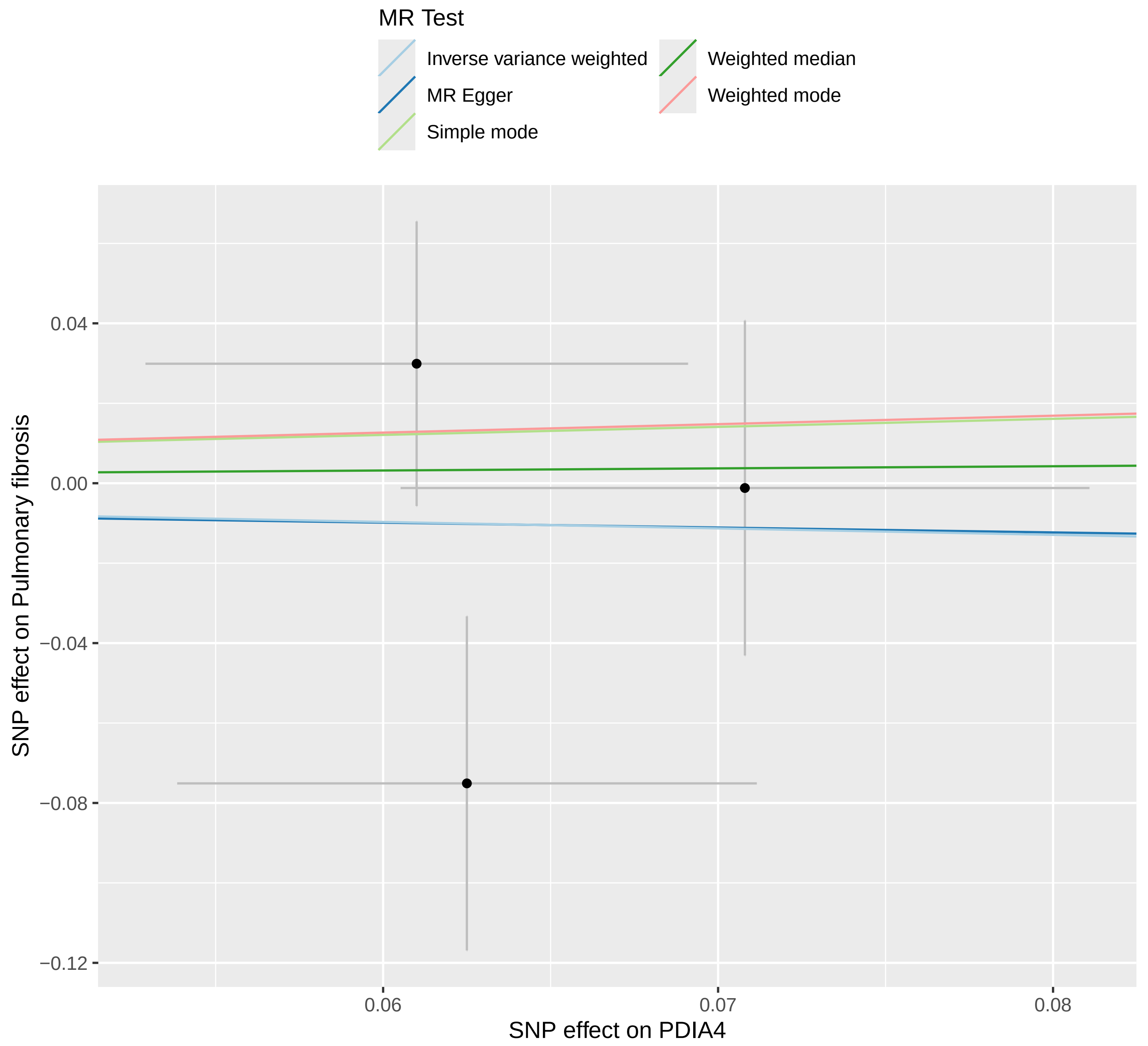
MR Test
Inverse variance weighted
MR Egger
Simple mode
Weighted median
Weighted mode
0.04
0.00
−0.04
−0.08
−0.12
0.06
0.07
0.08
SNP effect on Pulmonary fibrosis
SNP effect on PDIA4

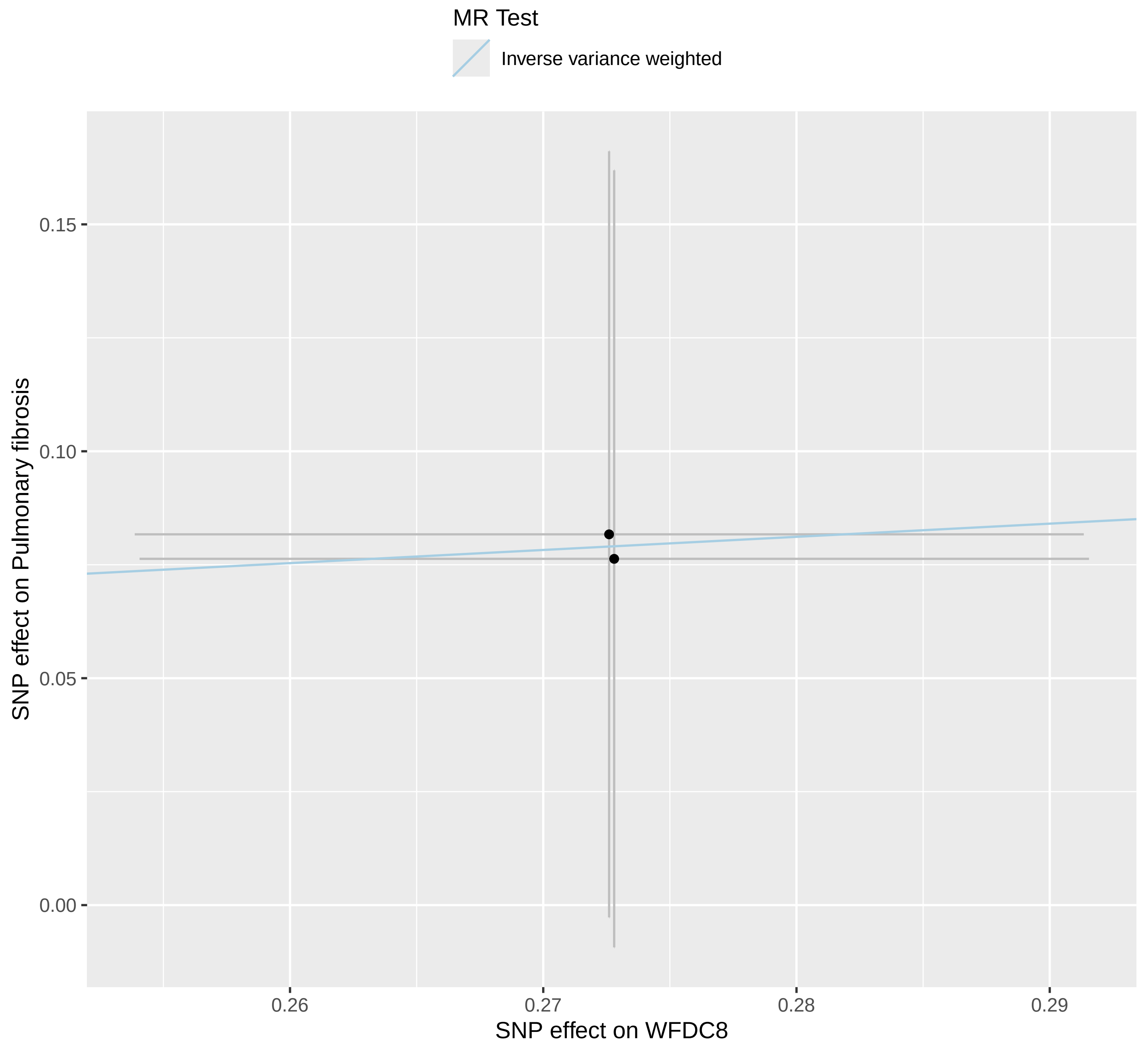
MR Test
Inverse variance weighted
0.15
0.10
0.05
0.00
SNP effect on Pulmonary fibrosis
0.26
0.27
0.28
0.29
SNP effect on WFDC8

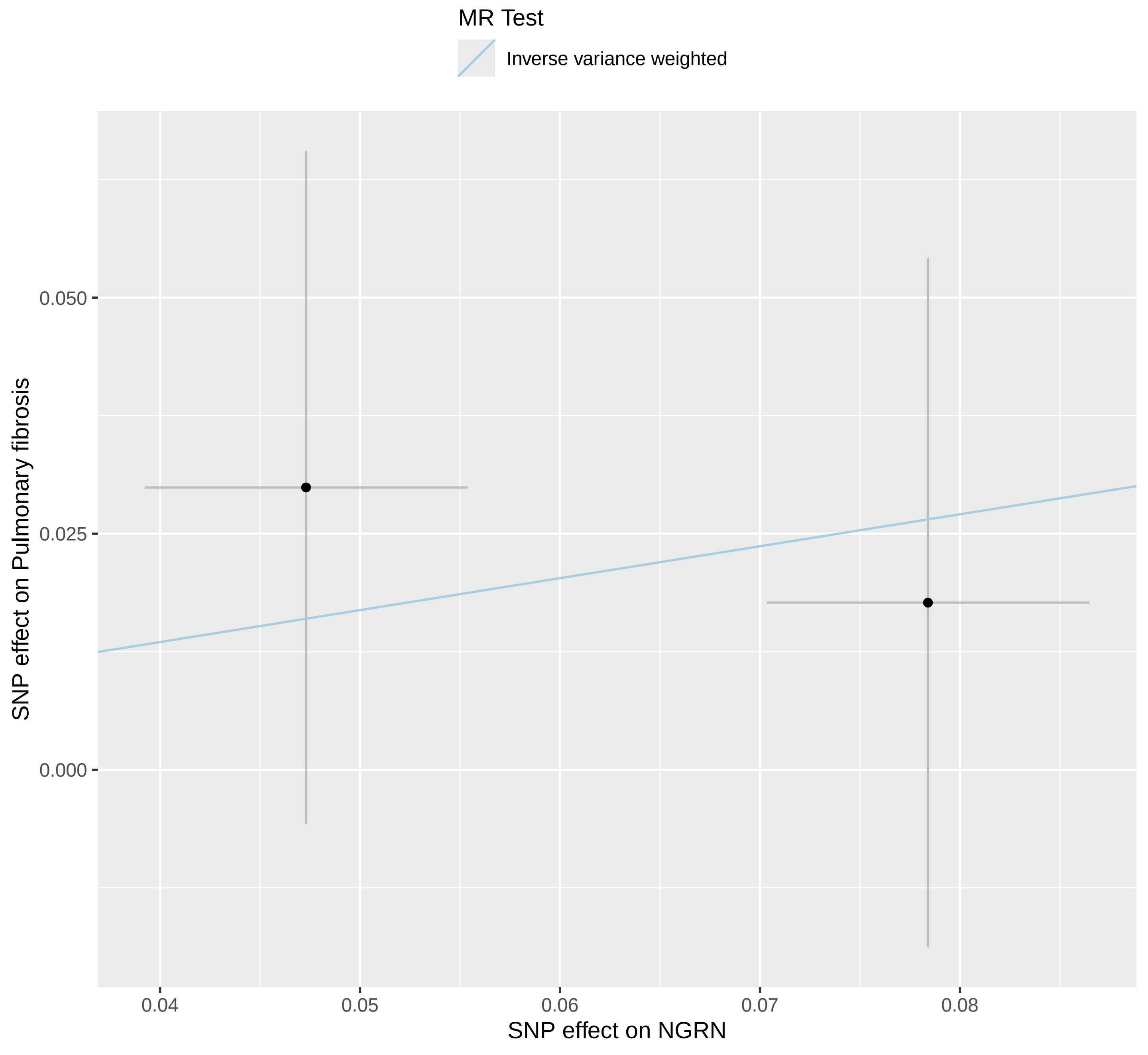
MR Test
Inverse variance weighted
0.050
0.025
0.000
0.04
0.05
0.06
0.07
0.08
SNP effect on Pulmonary fibrosis
SNP effect on NGRN

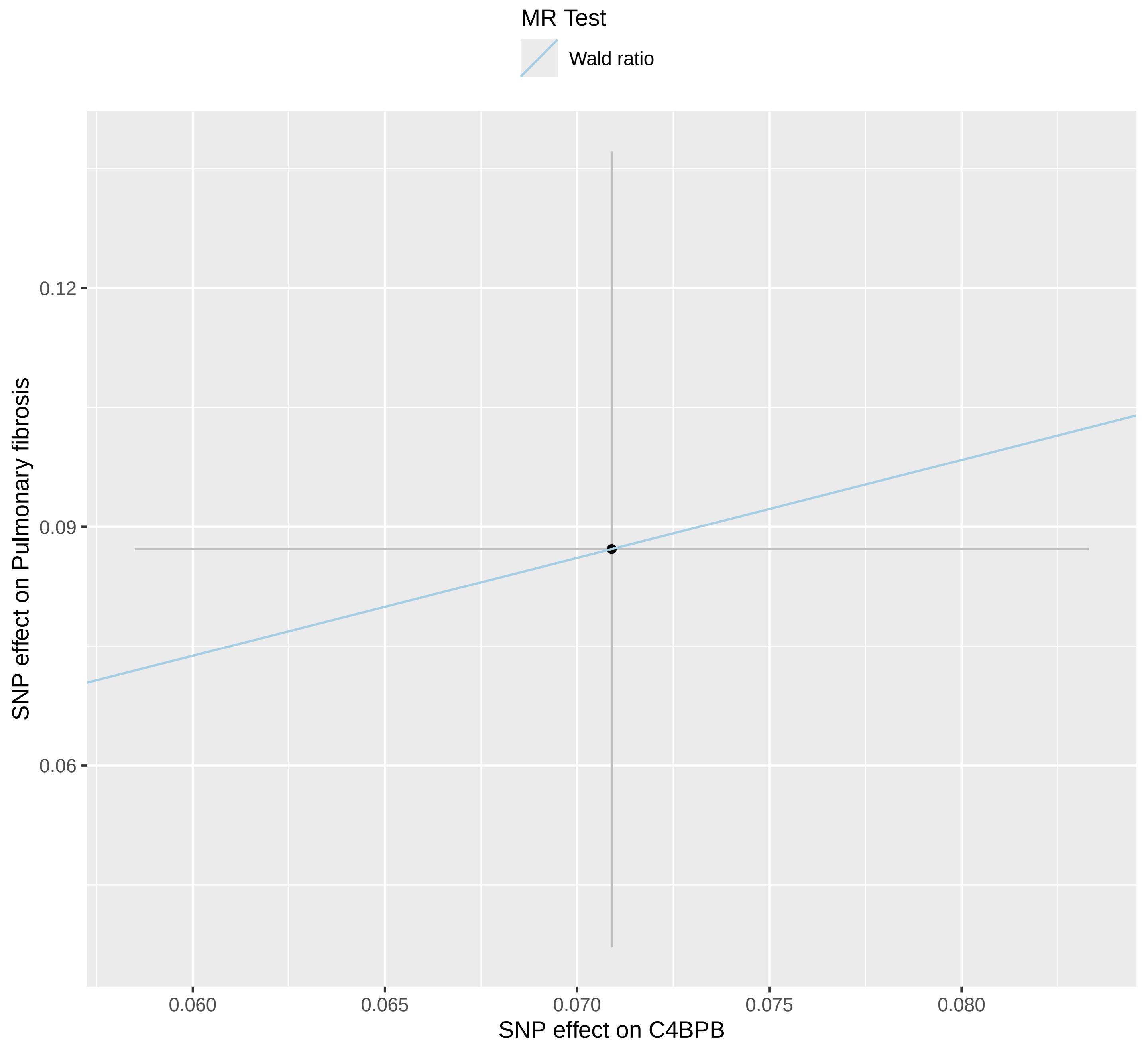
MR Test
Wald ratio
0.12
0.09
0.06
SNP effect on Pulmonary fibrosis
0.060
0.065
0.070
0.075
0.080
SNP effect on C4BPB

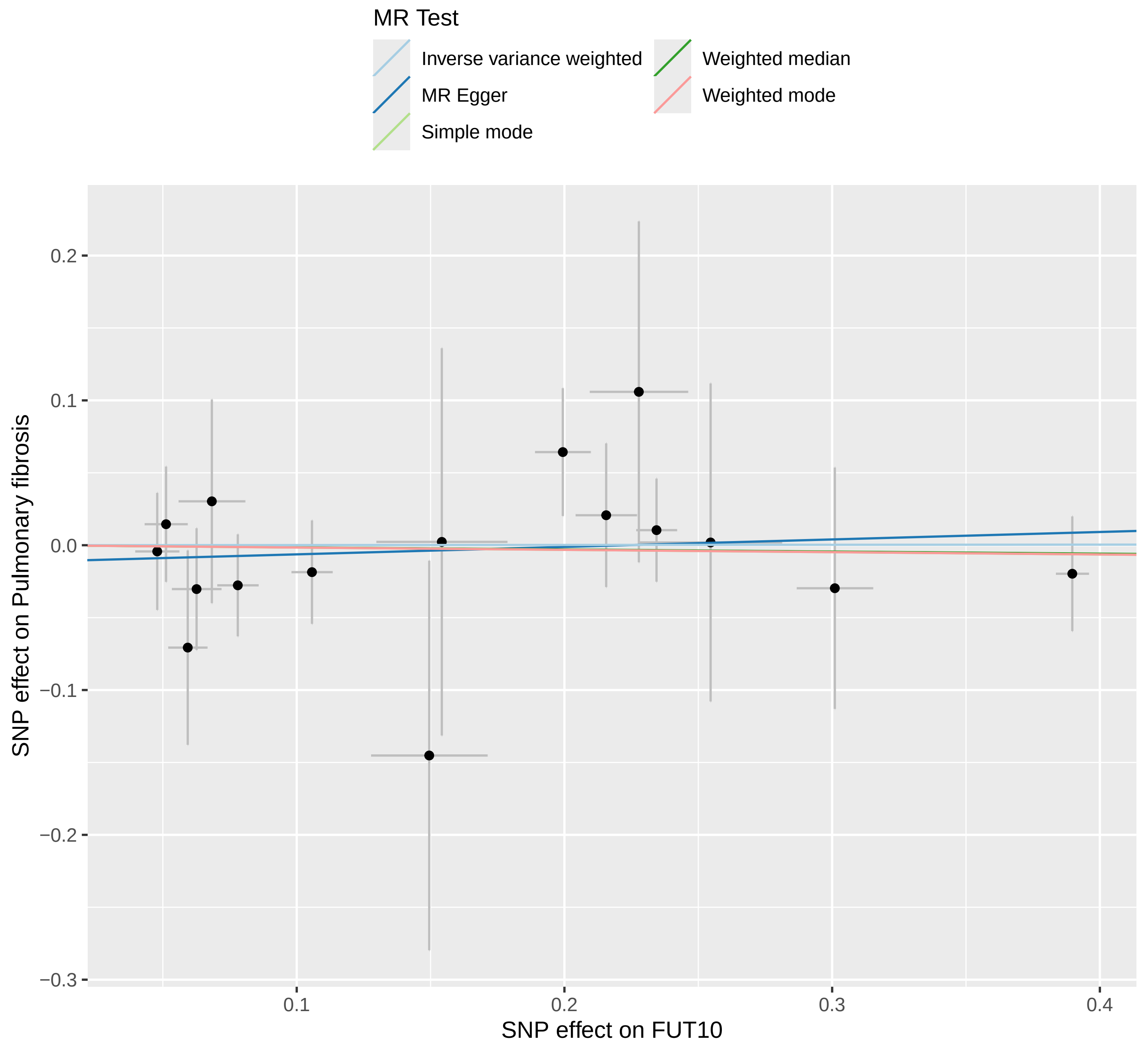

MR Test
Inverse variance weighted
MR Egger
Simple mode
Weighted median
Weighted mode
SNP effect on Pulmonary fibrosis
0.2
0.1
0.0
−0.1
−0.2
−0.3
0.1
0.2
0.3
0.4
SNP effect on FUT10

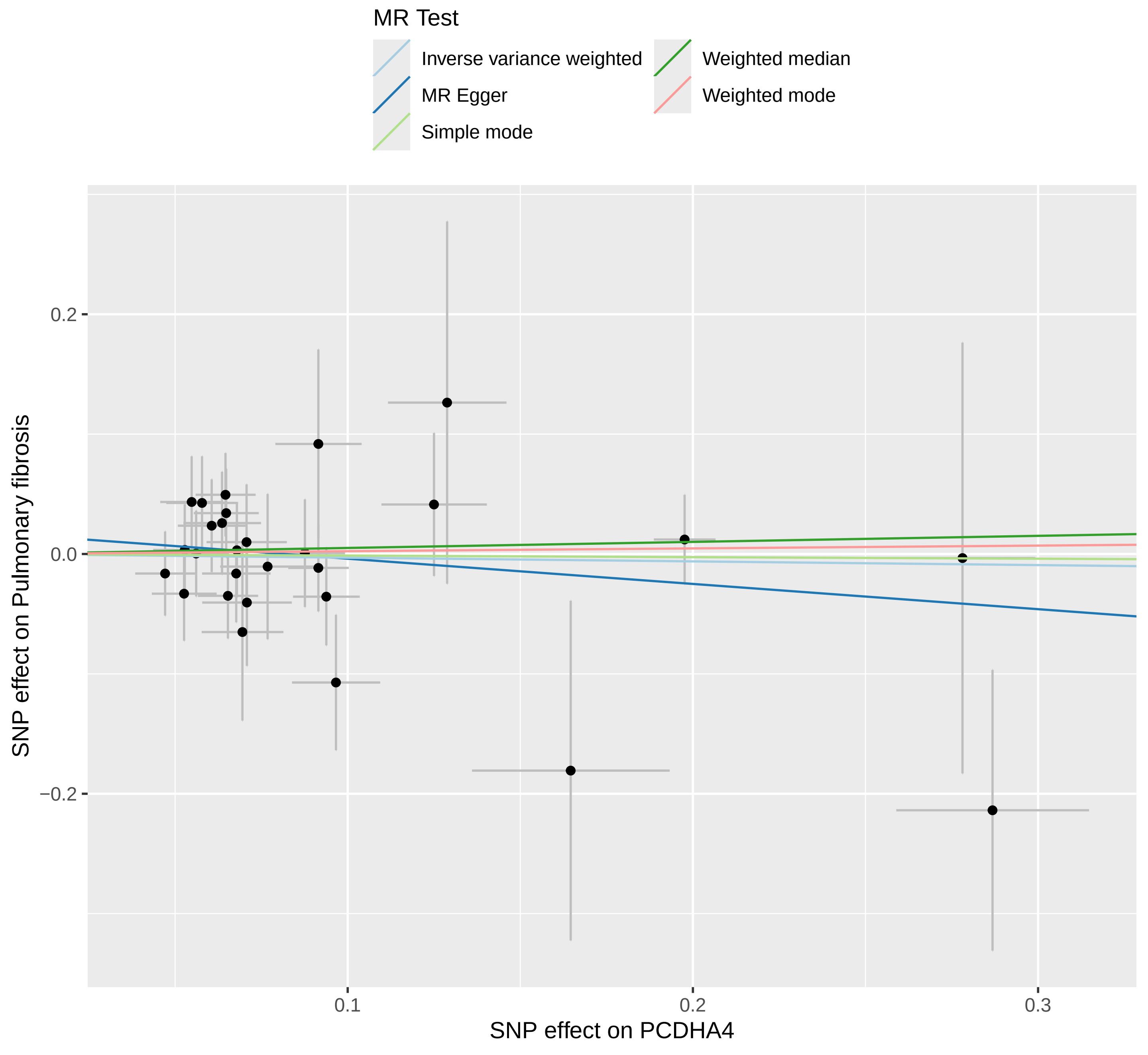

MR Test
Inverse variance weighted
MR Egger
Simple mode
Weighted median
Weighted mode
0.2
0.0
−0.2
0.1
0.2
0.3
SNP effect on Pulmonary fibrosis
SNP effect on PCDHA4

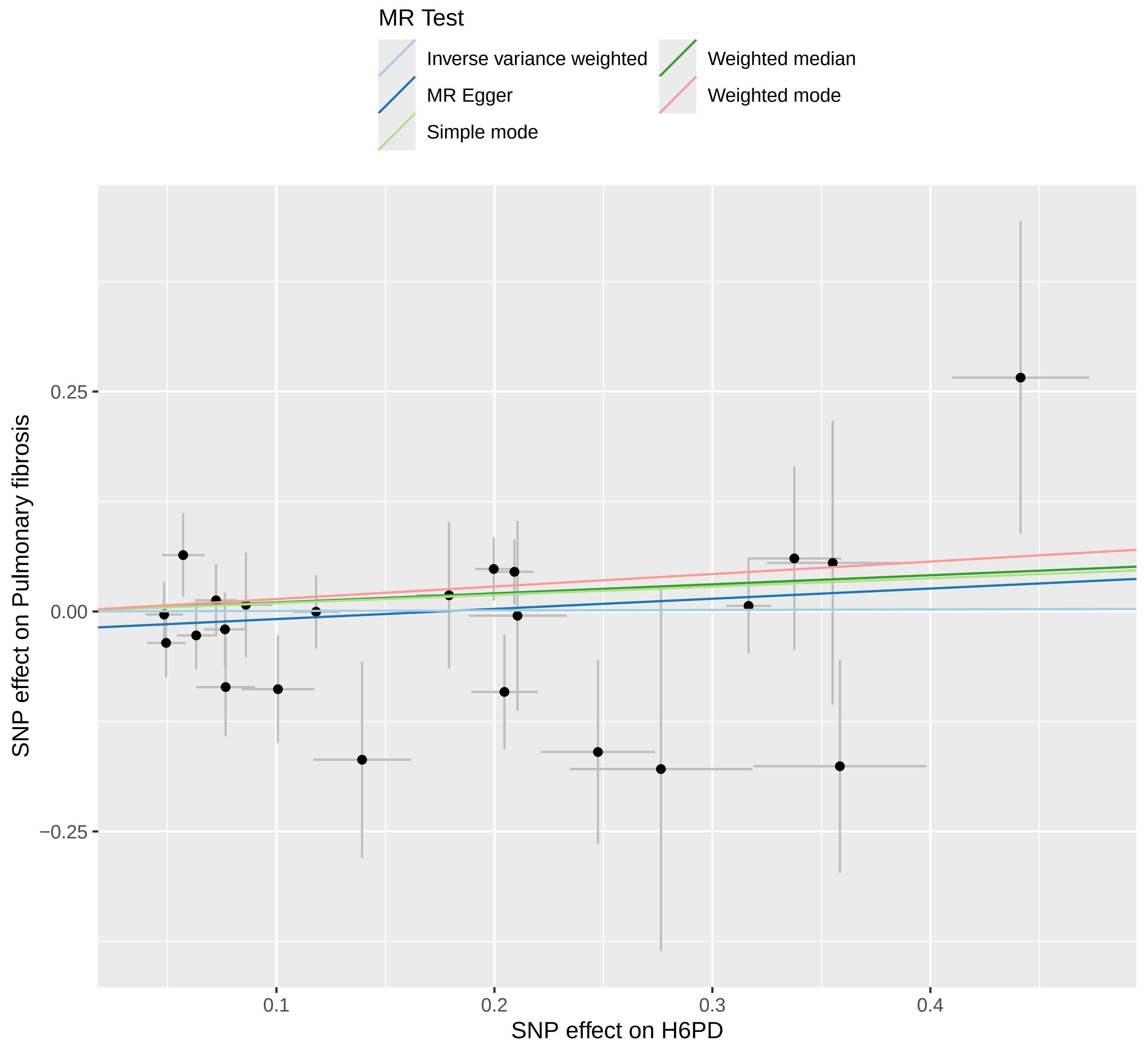

MR Test
Inverse variance weighted
MR Egger
Simple mode
Weighted median
Weighted mode
0.25
0.00
−0.25
0.1
0.2
0.3
0.4
SNP effect on Pulmonary fibrosis
SNP effect on H6PD

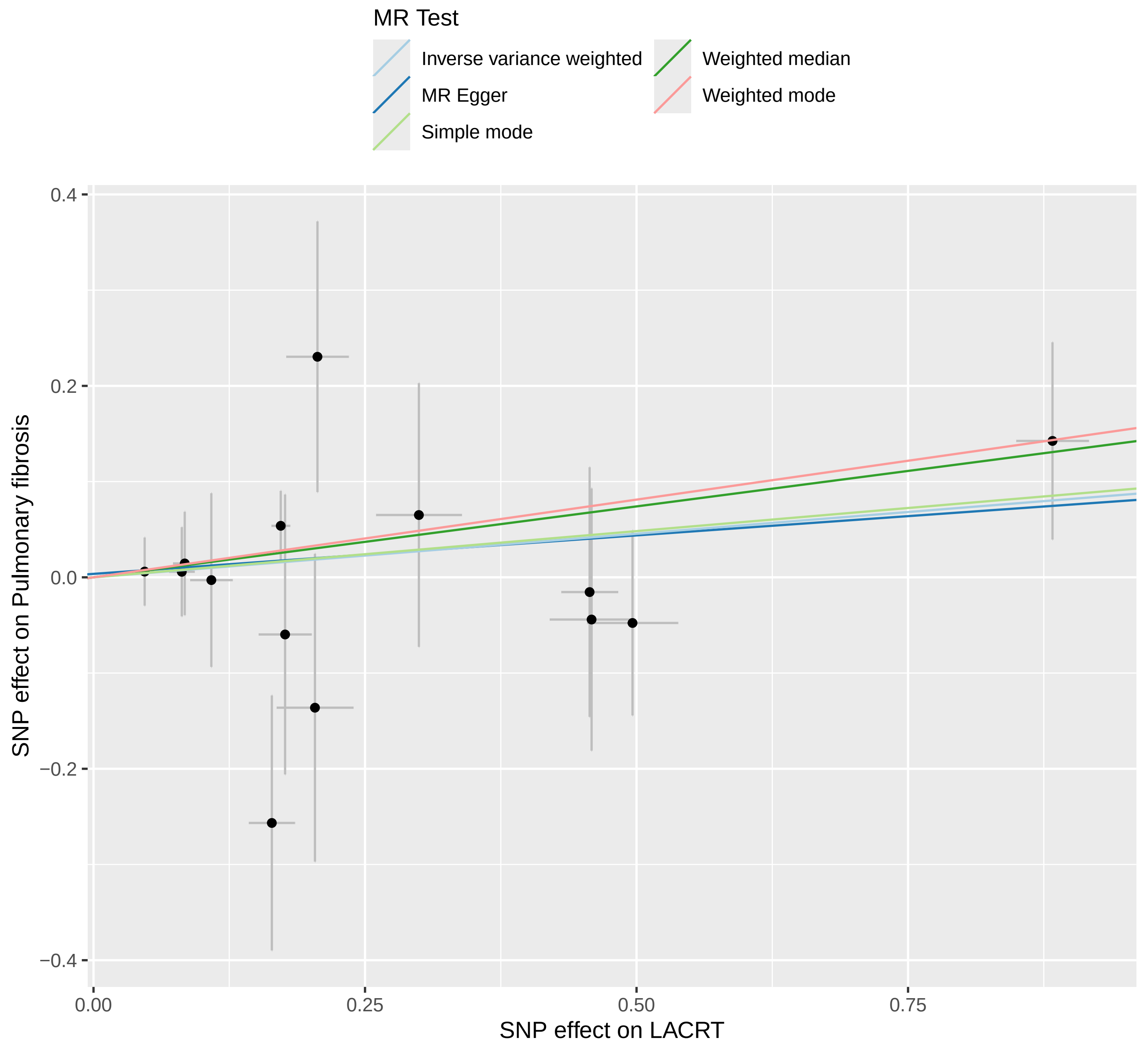
MR Test
Inverse variance weighted
MR Egger
Simple mode
Weighted median
Weighted mode
0.4
0.2
0.0
−0.2
−0.4
SNP effect on Pulmonary fibrosis
0.00
0.25
0.50
0.75
SNP effect on LACRT

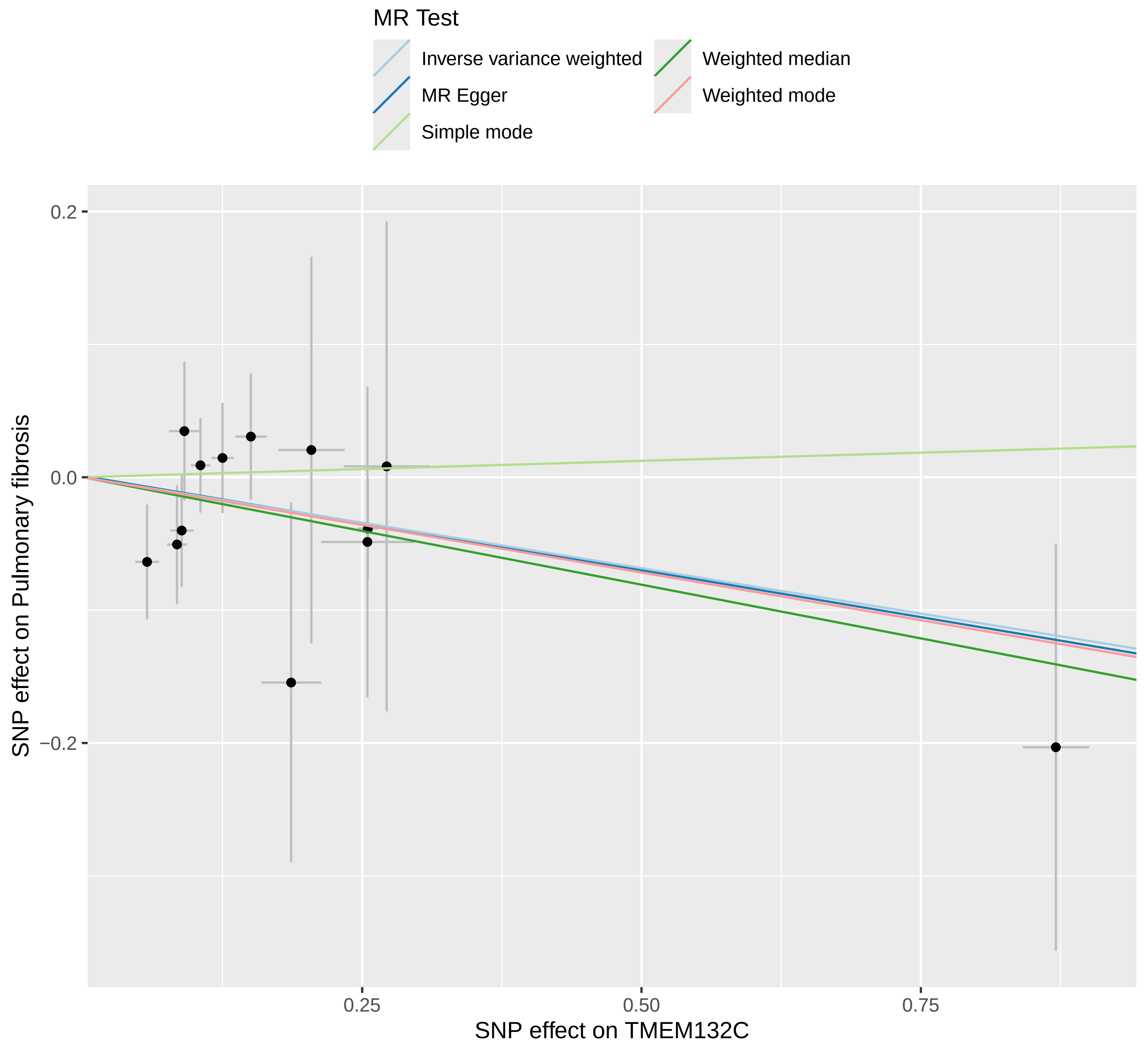
MR Test
Inverse variance weighted
MR Egger
Simple mode
Weighted median
Weighted mode
0.2
0.0
−0.2
0.25
0.50
0.75
SNP effect on Pulmonary fibrosis
SNP effect on TMEM132C

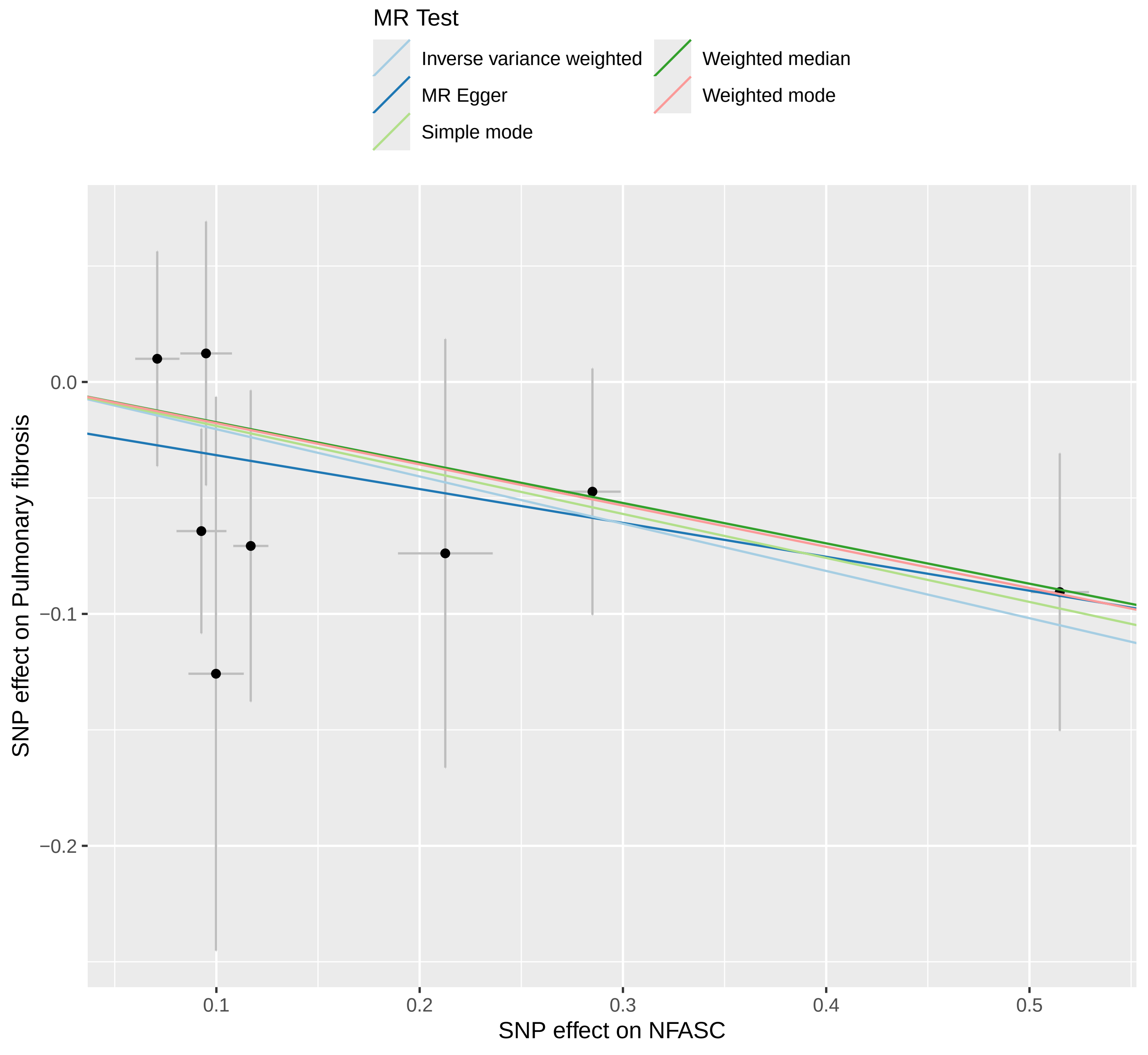

MR Test
Inverse variance weighted
MR Egger
Simple mode
Weighted median
Weighted mode
0.0
−0.1
−0.2
0.1
0.2
0.3
0.4
0.5
SNP effect on Pulmonary fibrosis
SNP effect on NFASC

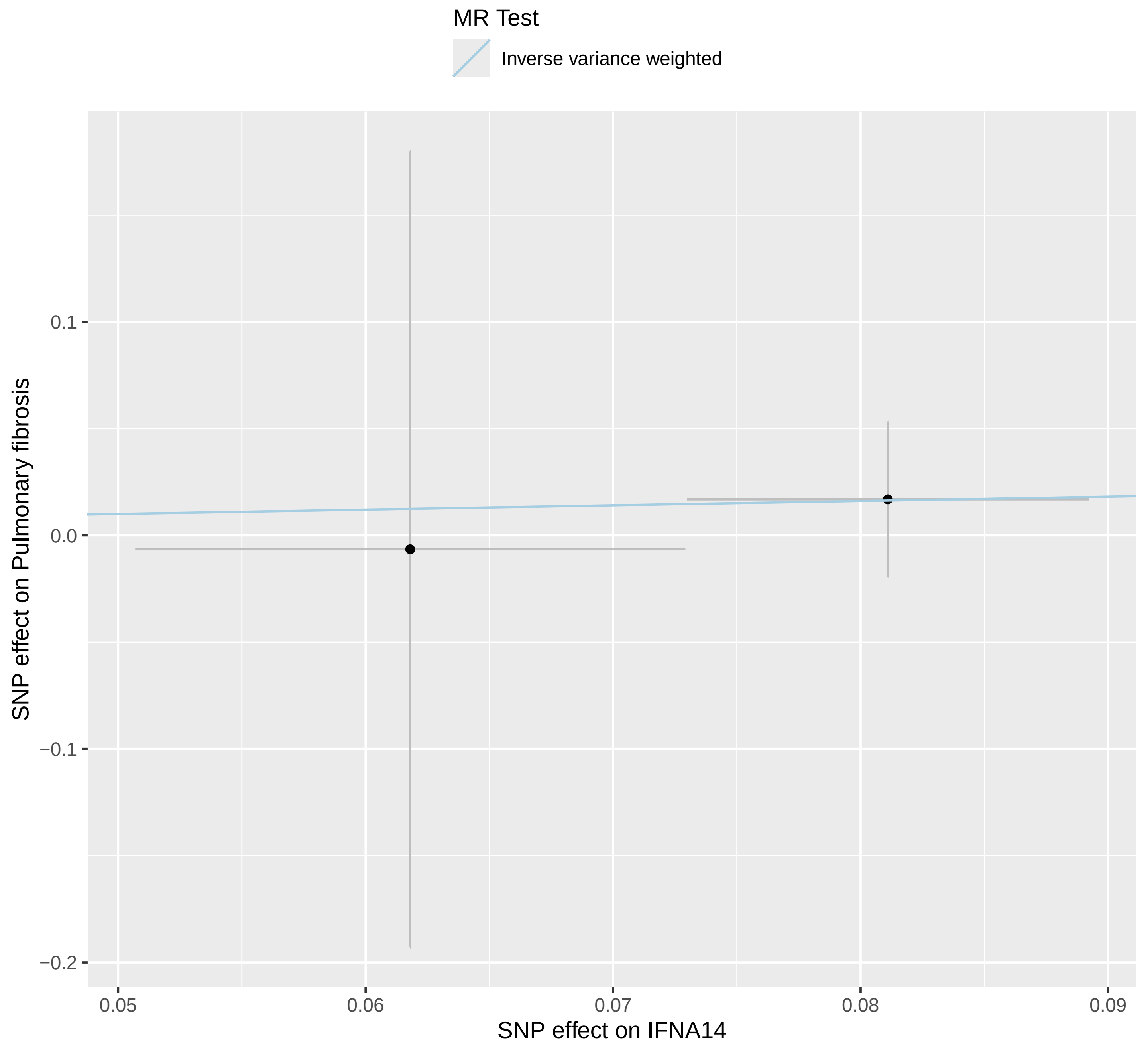

MR Test
Inverse variance weighted
0.1
0.0
−0.1
−0.2
SNP effect on Pulmonary fibrosis
0.05
0.06
0.07
0.08
0.09
SNP effect on IFNA14

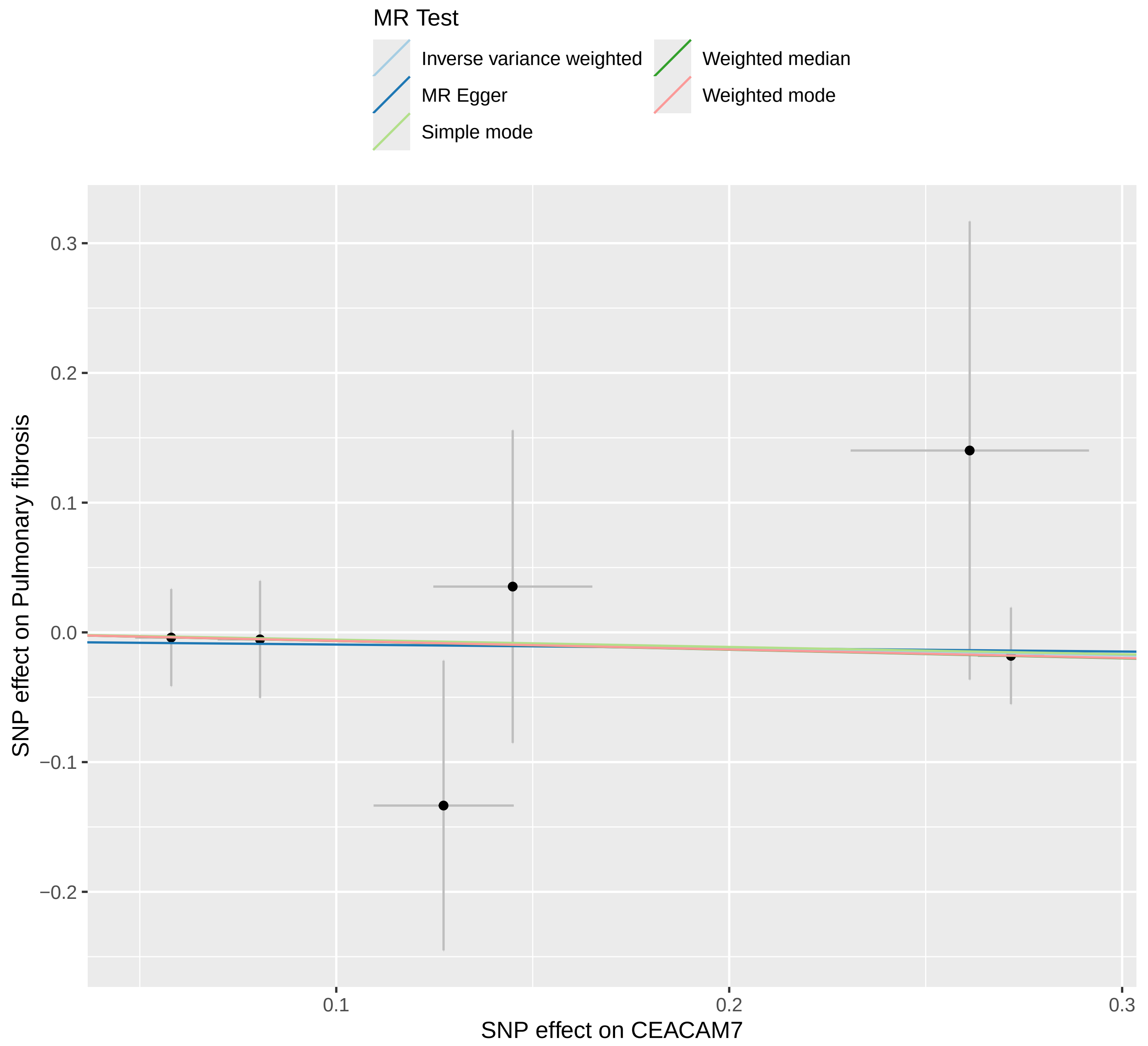

MR Test
Inverse variance weighted
MR Egger
Simple mode
Weighted median
Weighted mode
0.3
0.2
0.1
0.0
−0.1
−0.2
SNP effect on Pulmonary fibrosis
0.1
0.2
0.3
SNP effect on CEACAM7

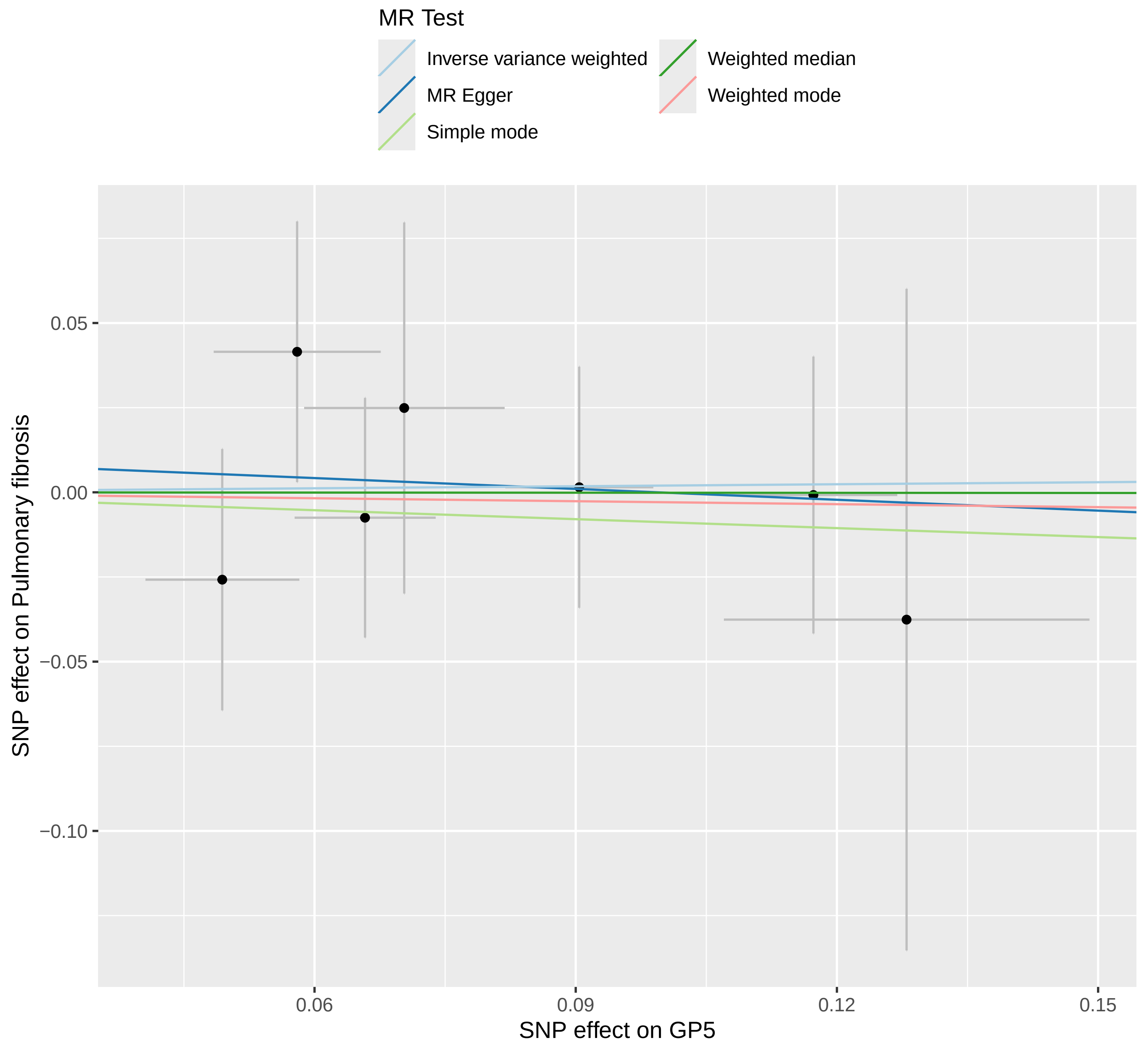
MR Test
Inverse variance weighted
MR Egger
Simple mode
Weighted median
Weighted mode
0.05
0.00
−0.05
−0.10
SNP effect on Pulmonary fibrosis
0.06
0.09
0.12
0.15
SNP effect on GP5

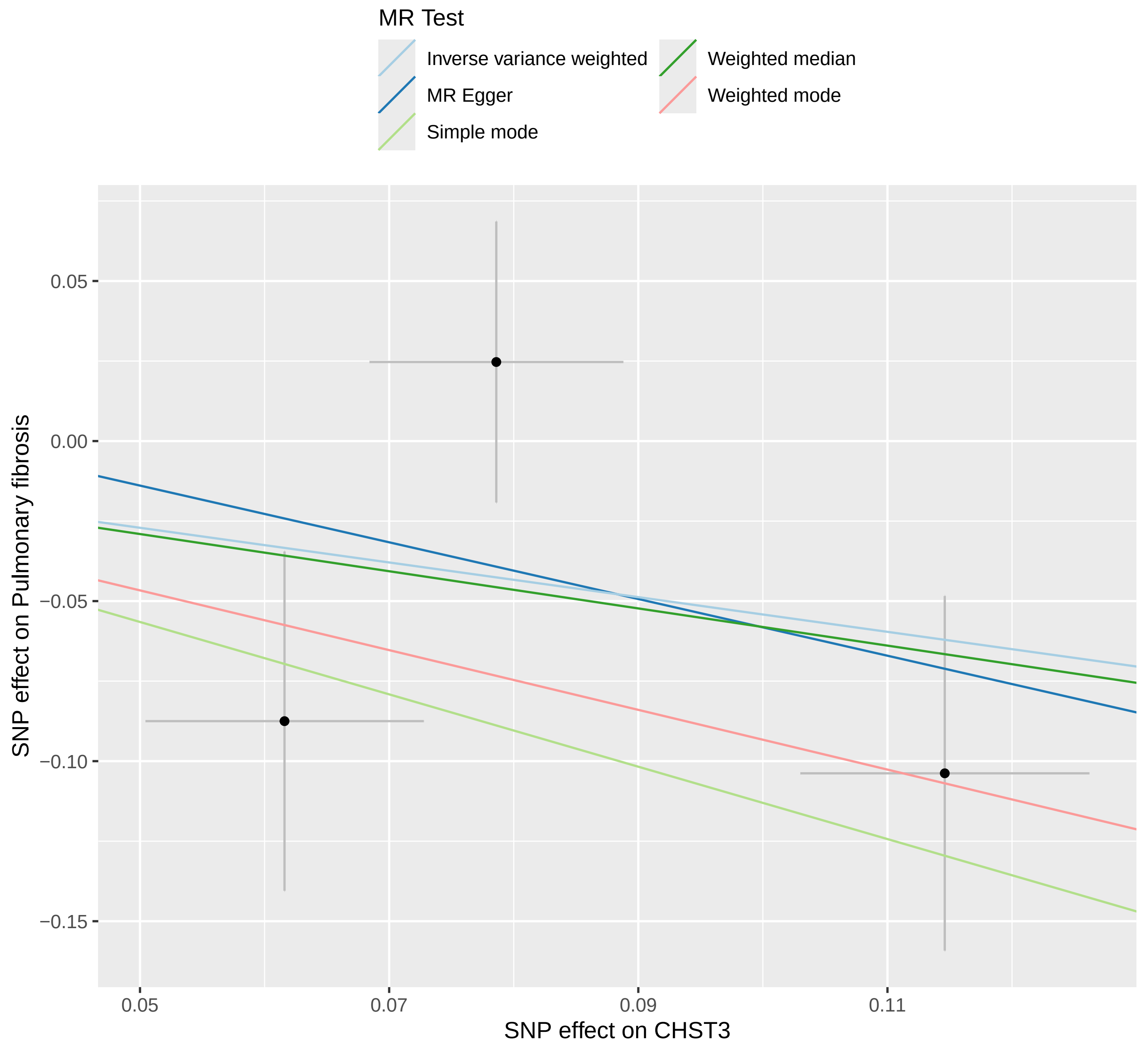
MR Test
Inverse variance weighted
MR Egger
Simple mode
Weighted median
Weighted mode
0.05
0.00
−0.05
−0.10
−0.15
SNP effect on Pulmonary fibrosis
0.05
0.07
0.09
0.11
SNP effect on CHST3

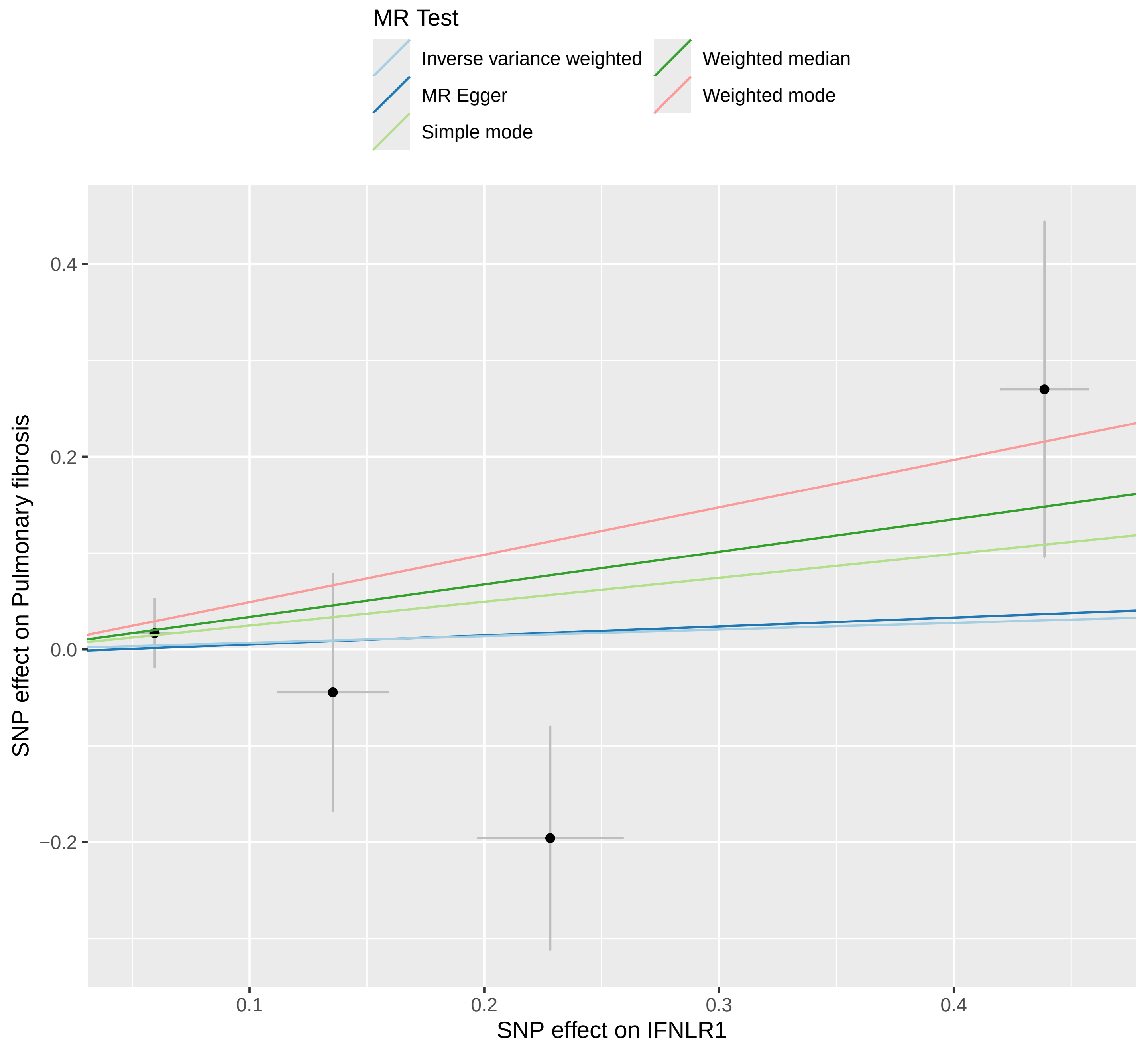
MR Test
Inverse variance weighted
MR Egger
Simple mode
Weighted median
Weighted mode
0.4
0.2
0.0
−0.2
0.1
0.2
0.3
0.4
SNP effect on Pulmonary fibrosis
SNP effect on IFNLR1

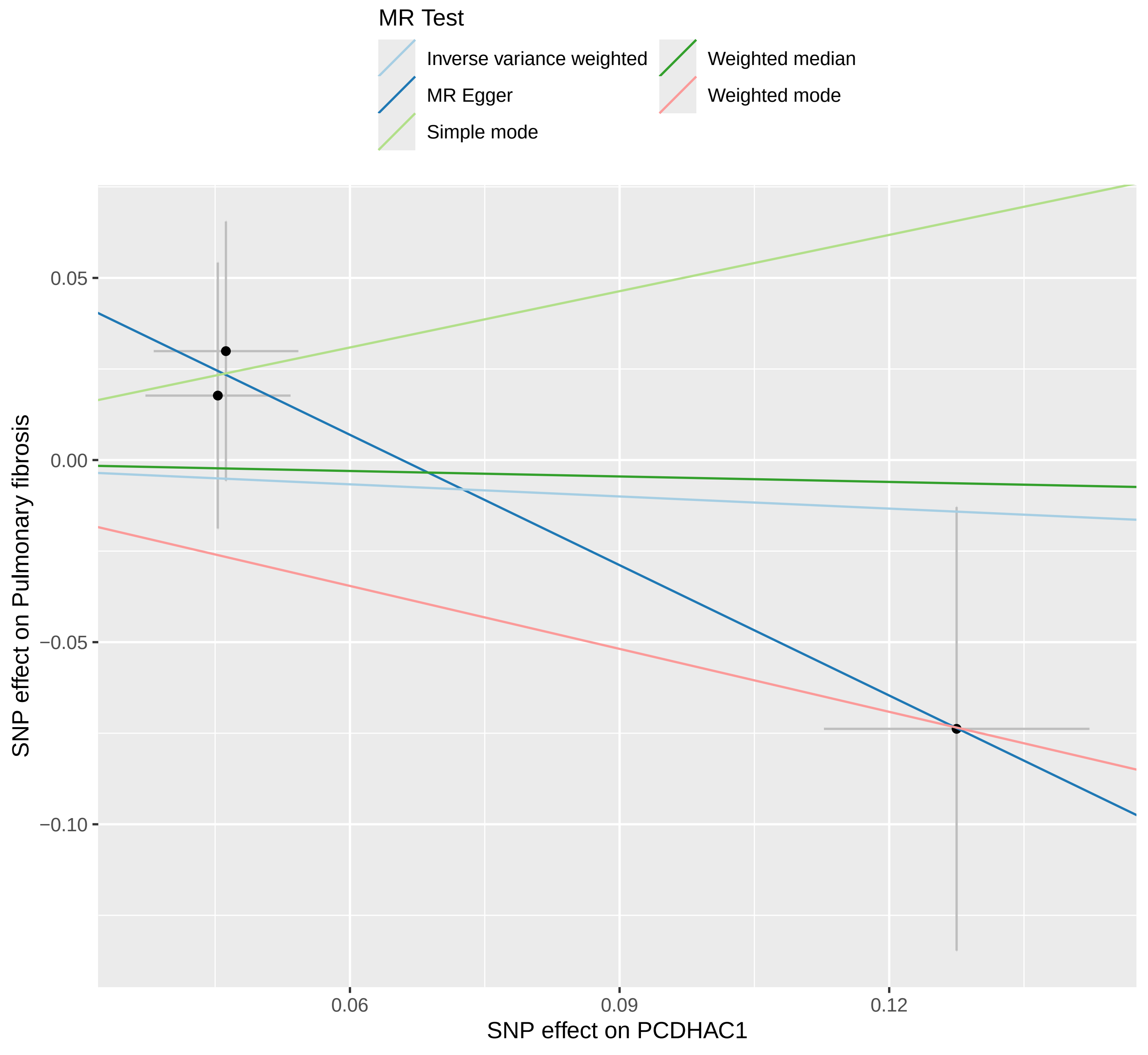
MR Test
Inverse variance weighted
MR Egger
Simple mode
Weighted median
Weighted mode
SNP effect on Pulmonary fibrosis
0.05
0.00
−0.05
−0.10
0.06
0.09
0.12
SNP effect on PCDHAC1

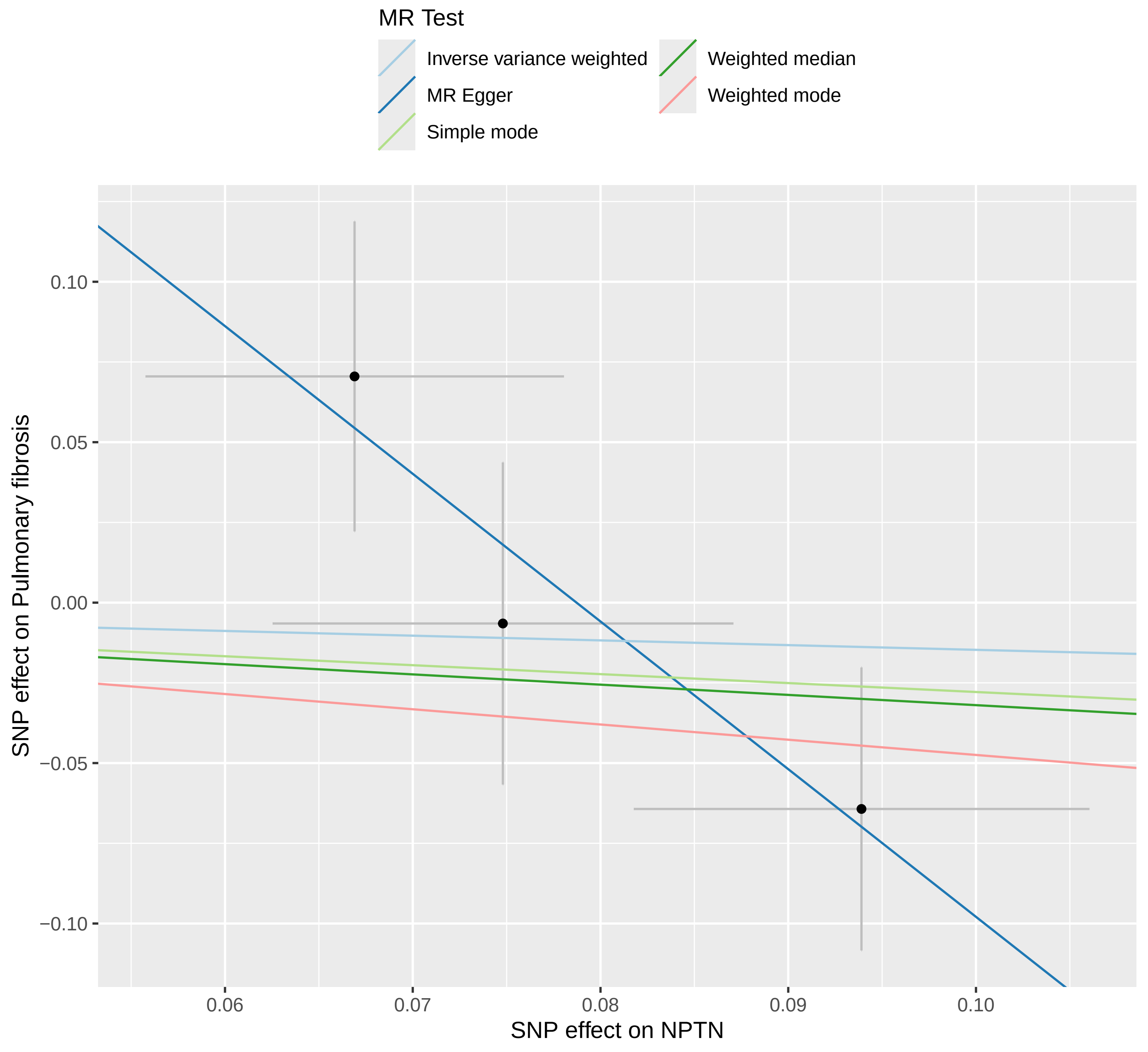

MR Test
Inverse variance weighted
MR Egger
Simple mode
Weighted median
Weighted mode
0.10
0.05
0.00
−0.05
−0.10
SNP effect on Pulmonary fibrosis
0.06
0.07
0.08
0.09
0.10
SNP effect on NPTN

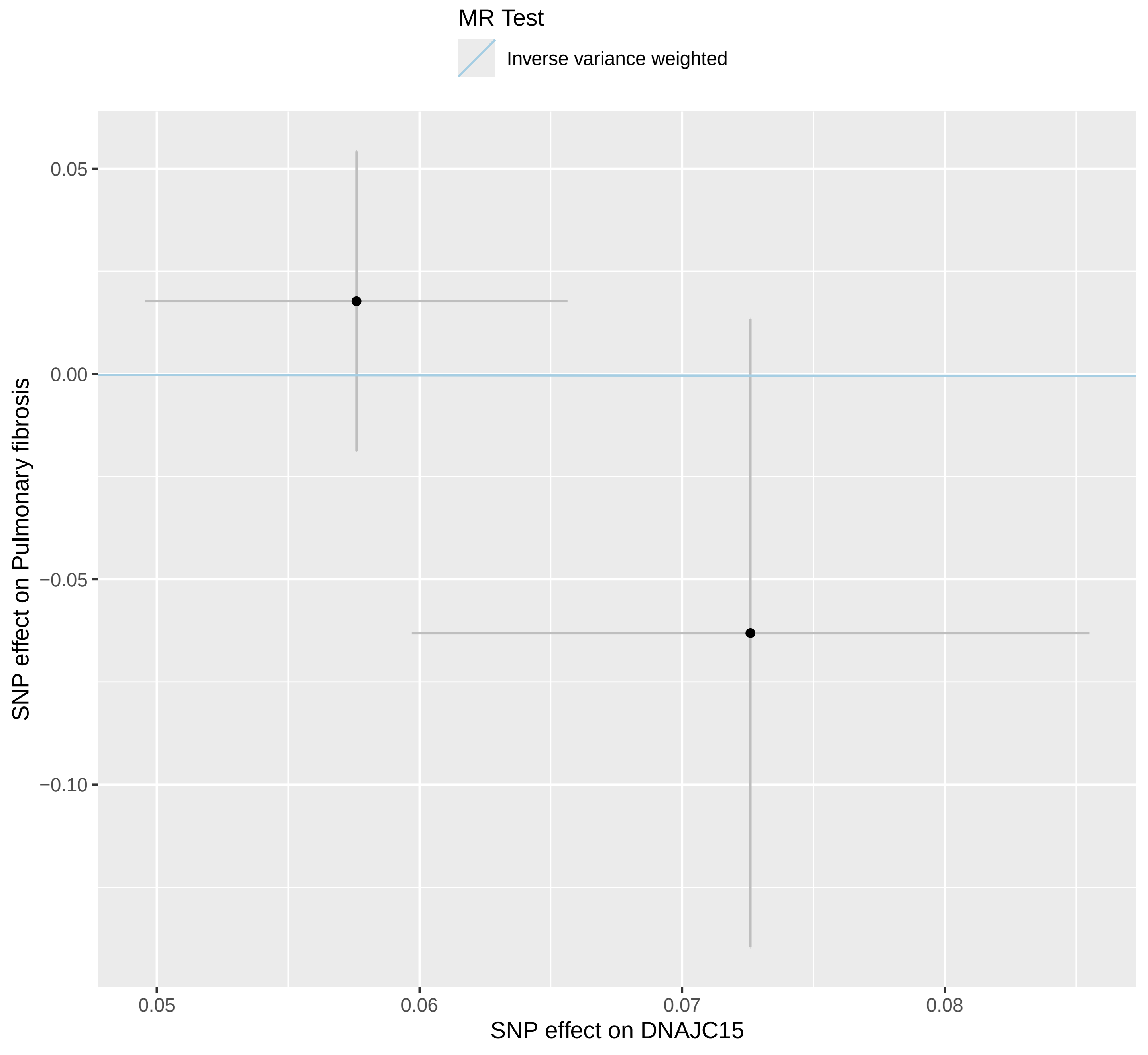

MR Test
Inverse variance weighted
0.05
0.00
−0.05
−0.10
SNP effect on Pulmonary fibrosis
0.05
0.06
0.07
0.08
SNP effect on DNAJC15

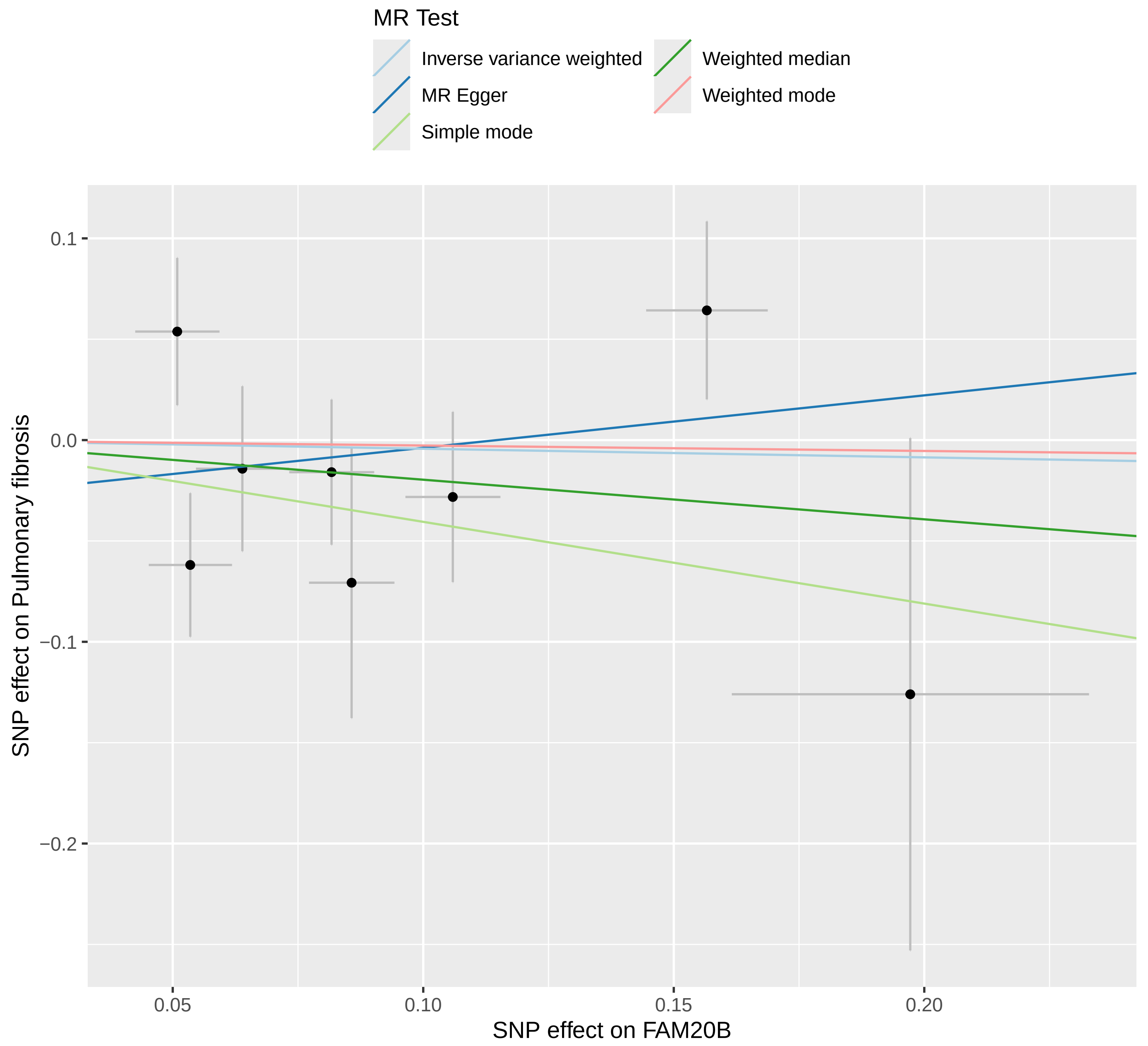

MR Test
Inverse variance weighted
MR Egger
Simple mode
Weighted median
Weighted mode
0.1
0.0
−0.1
−0.2
SNP effect on Pulmonary fibrosis
0.05
0.10
0.15
0.20
SNP effect on FAM20B

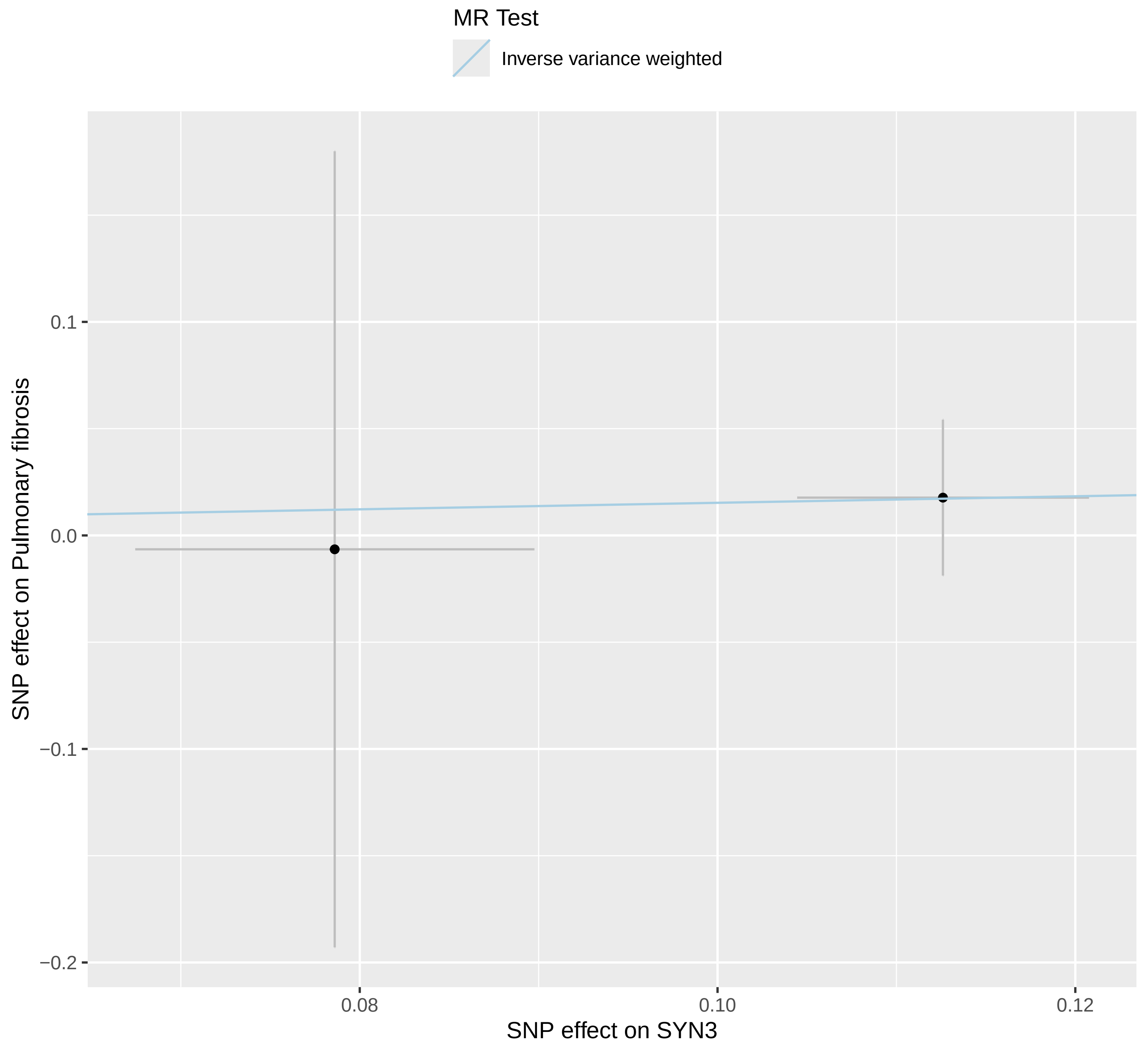

MR Test
Inverse variance weighted
0.1
0.0
−0.1
−0.2
SNP effect on Pulmonary fibrosis
0.08
0.10
0.12
SNP effect on SYN3

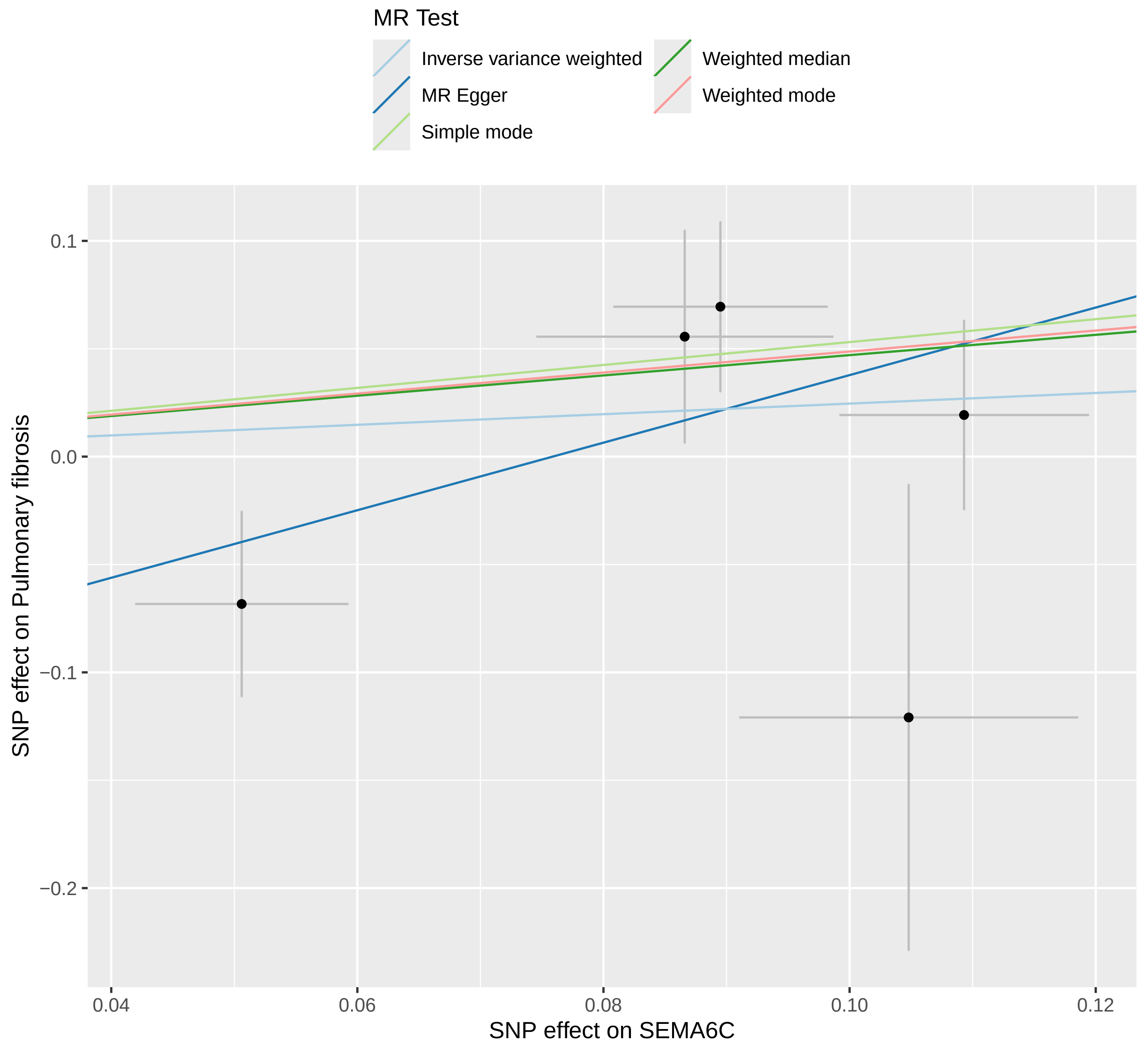
MR Test
Inverse variance weighted
MR Egger
Simple mode
Weighted median
Weighted mode
0.1
0.0
−0.1
−0.2
0.04
0.06
0.08
0.10
0.12
SNP effect on SEMA6C
SNP effect on Pulmonary fibrosis

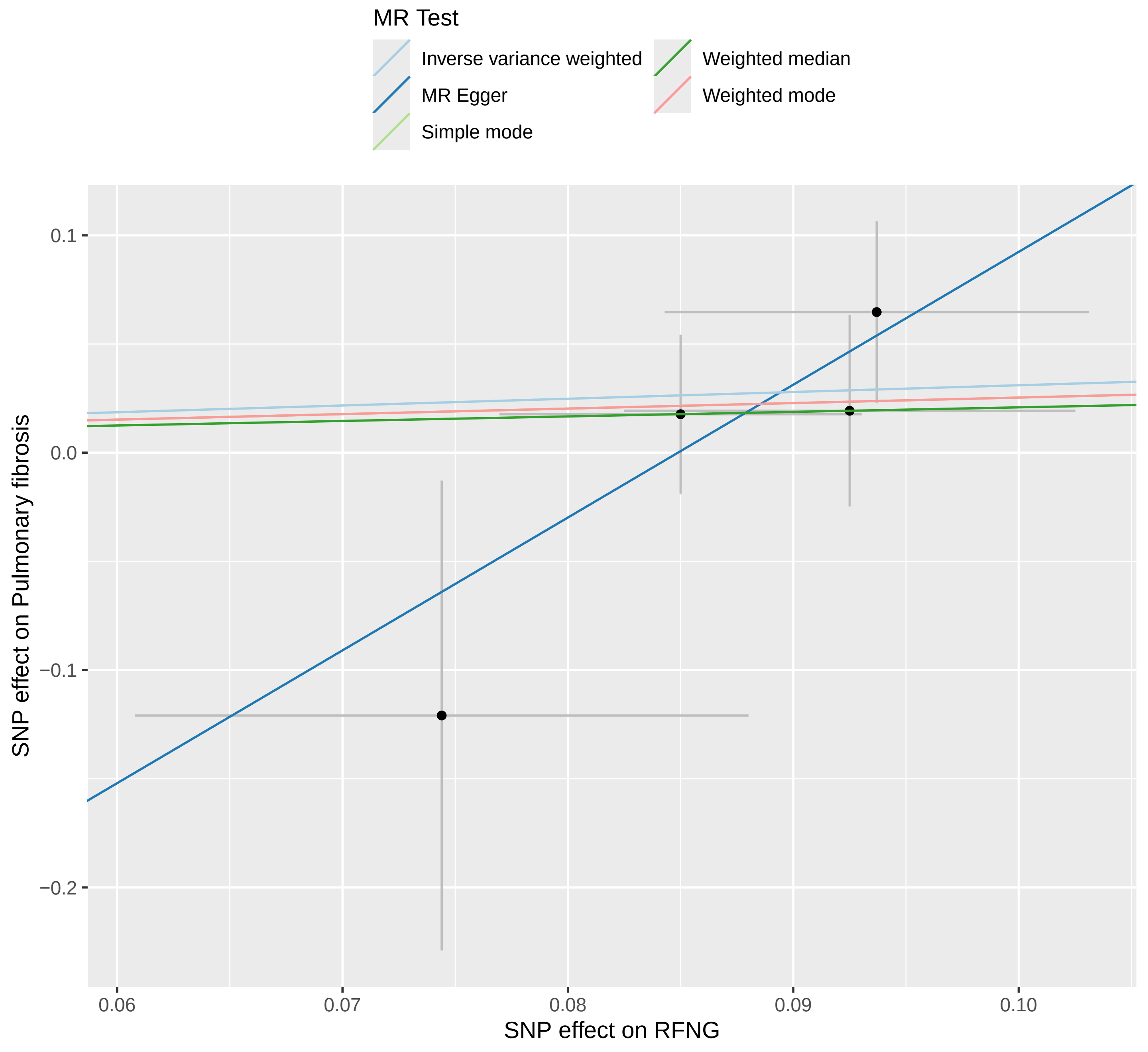
MR Test
Inverse variance weighted
MR Egger
Simple mode
Weighted median
Weighted mode
0.1
0.0
−0.1
−0.2
SNP effect on Pulmonary fibrosis
0.06
0.07
0.08
0.09
0.10
SNP effect on RFNG

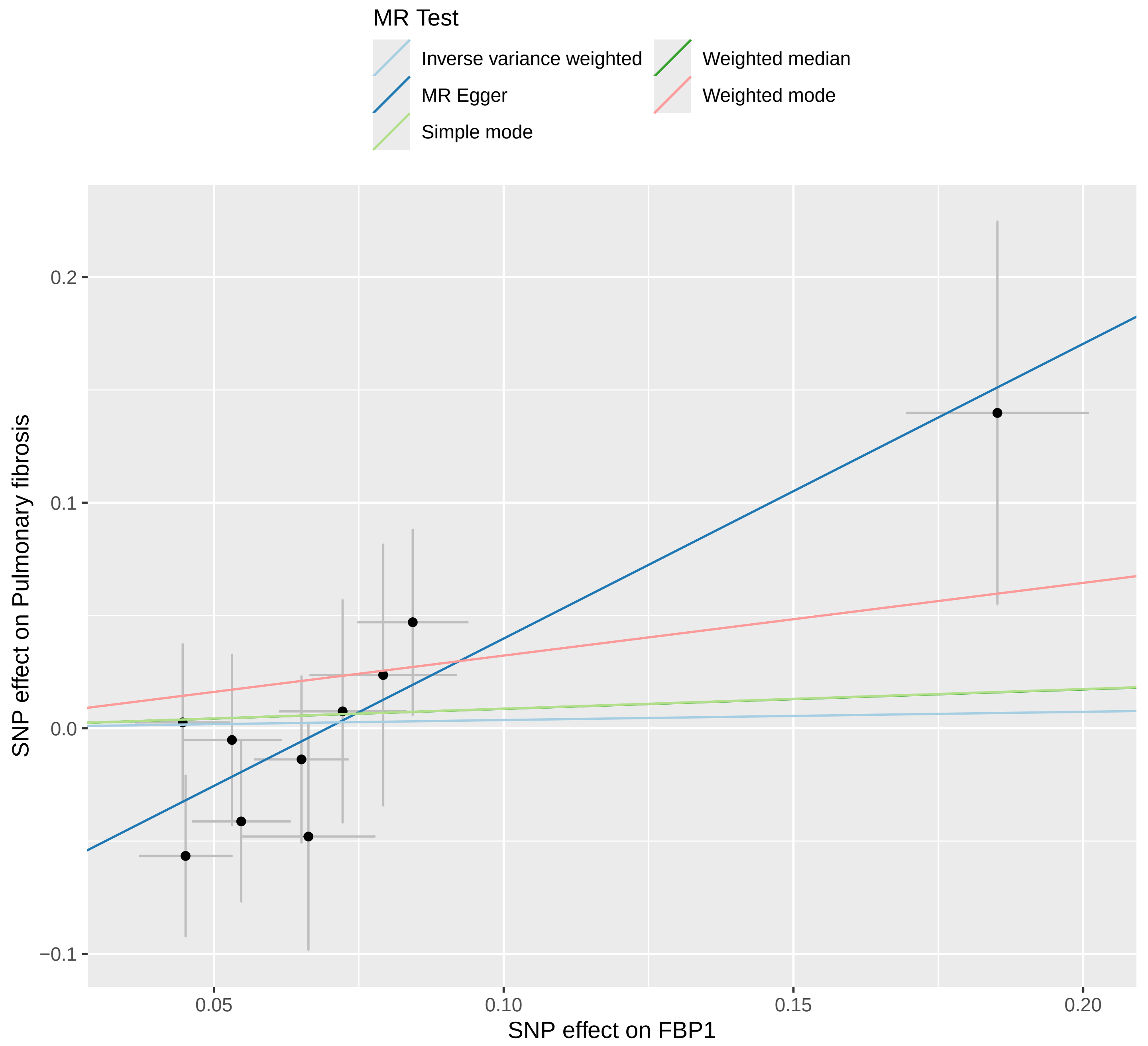
MR Test
Inverse variance weighted
MR Egger
Simple mode
Weighted median
Weighted mode
SNP effect on Pulmonary fibrosis
0.2
0.1
0.0
−0.1
0.05
0.10
0.15
0.20
SNP effect on FBP1

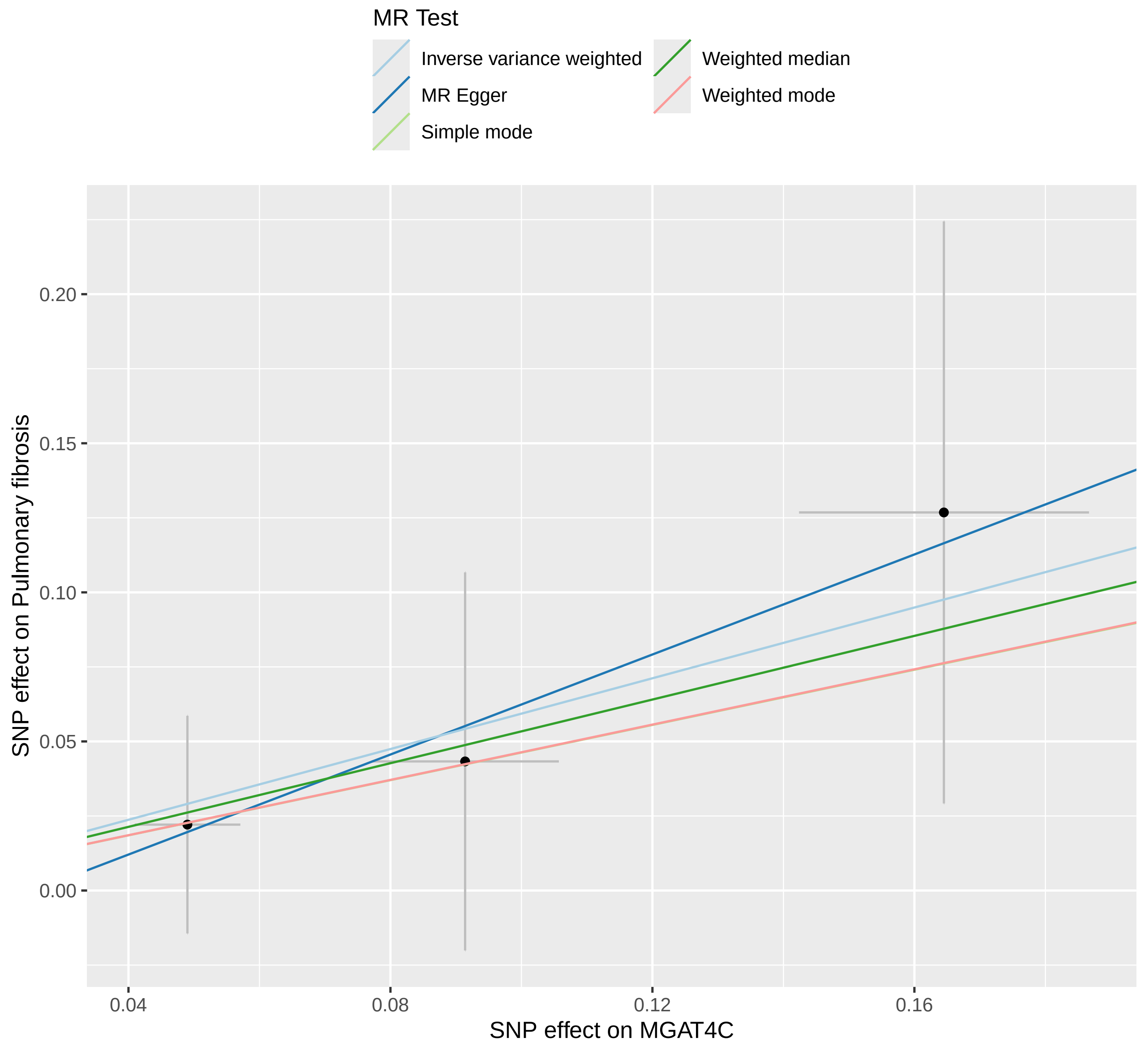
MR Test
Inverse variance weighted
MR Egger
Simple mode
Weighted median
Weighted mode
SNP effect on Pulmonary fibrosis
0.20
0.15
0.10
0.05
0.00
0.04
0.08
0.12
0.16
SNP effect on MGAT4C

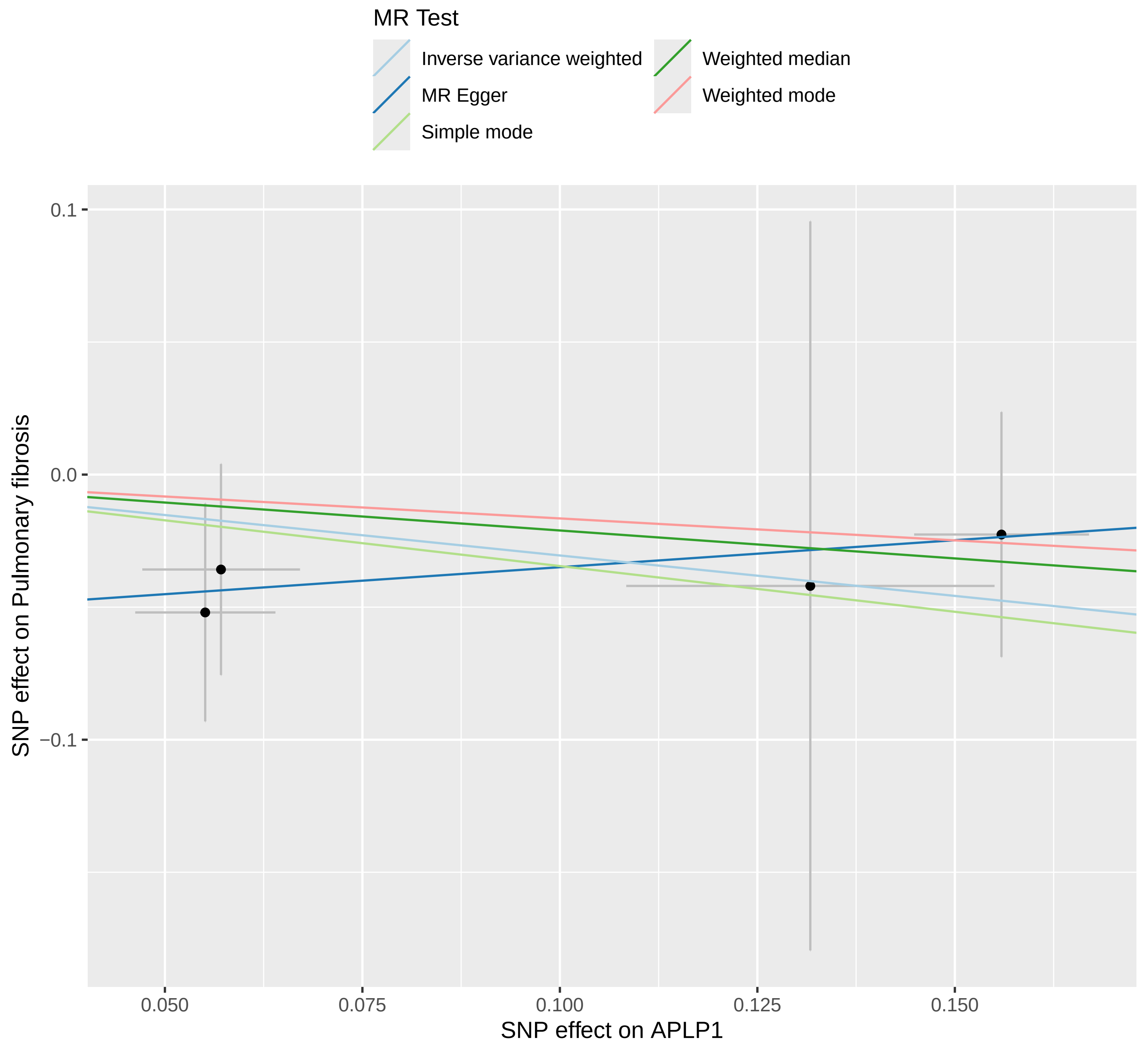
MR Test
Inverse variance weighted
MR Egger
Simple mode
Weighted median
Weighted mode
0.1
0.0
−0.1
SNP effect on Pulmonary fibrosis
0.050
0.075
0.100
0.125
0.150
SNP effect on APLP1

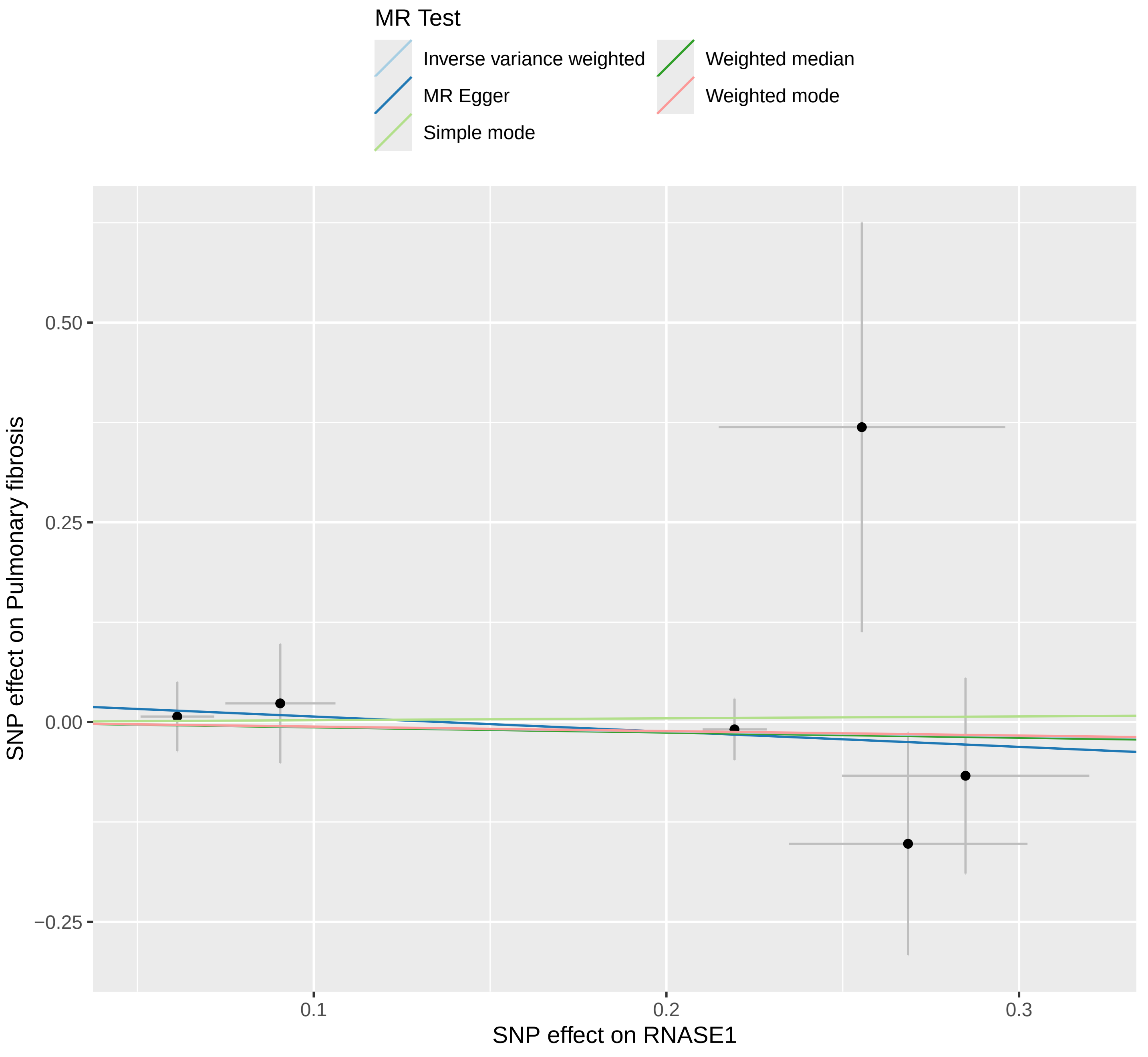

MR Test
Inverse variance weighted
MR Egger
Simple mode
Weighted median
Weighted mode
0.50
0.25
0.00
−0.25
0.1
0.2
0.3
SNP effect on RNASE1
SNP effect on Pulmonary fibrosis

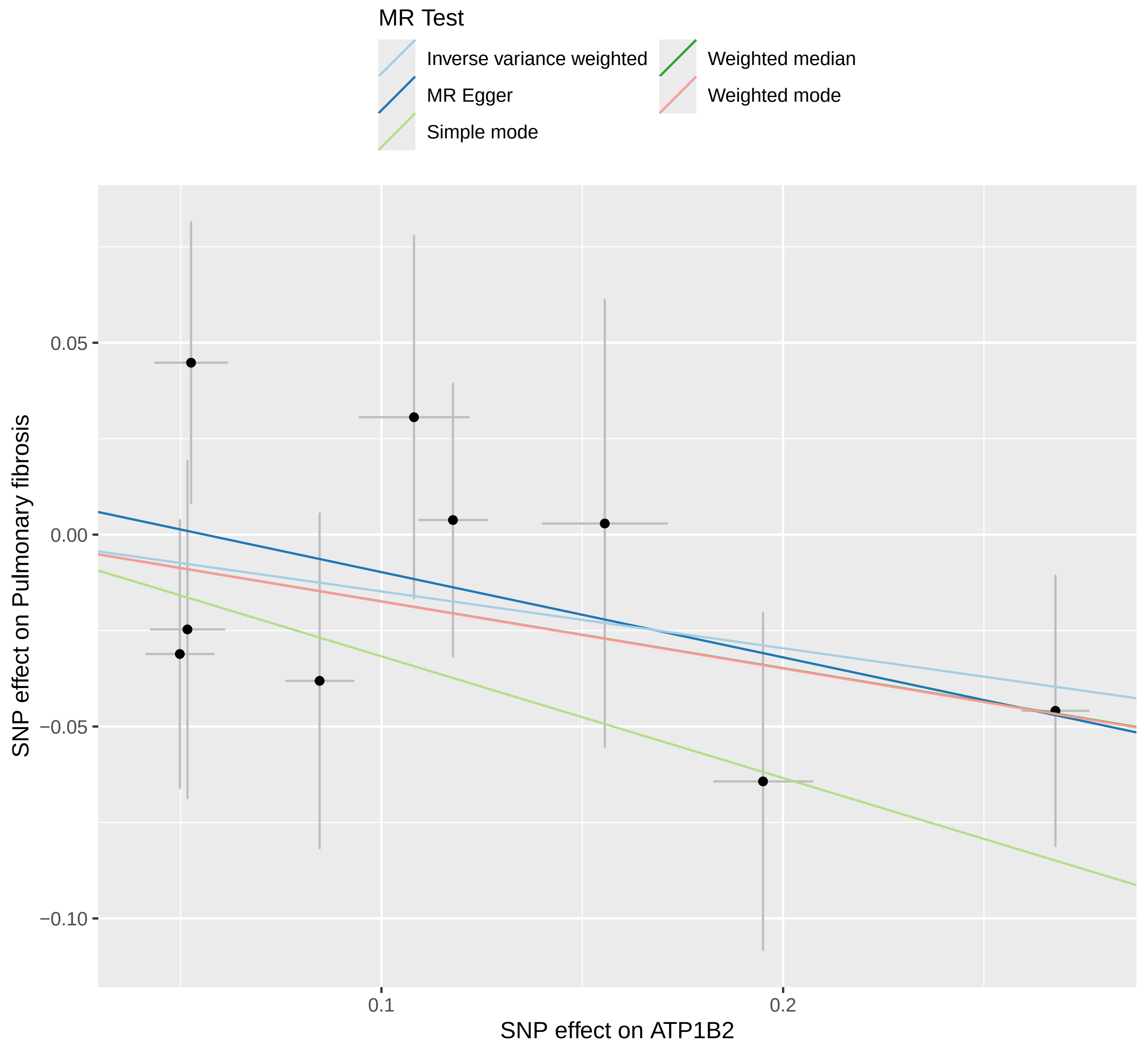
MR Test
Inverse variance weighted
MR Egger
Simple mode
Weighted median
Weighted mode
0.05
0.00
−0.05
−0.10
SNP effect on Pulmonary fibrosis
0.1
0.2
SNP effect on ATP1B2

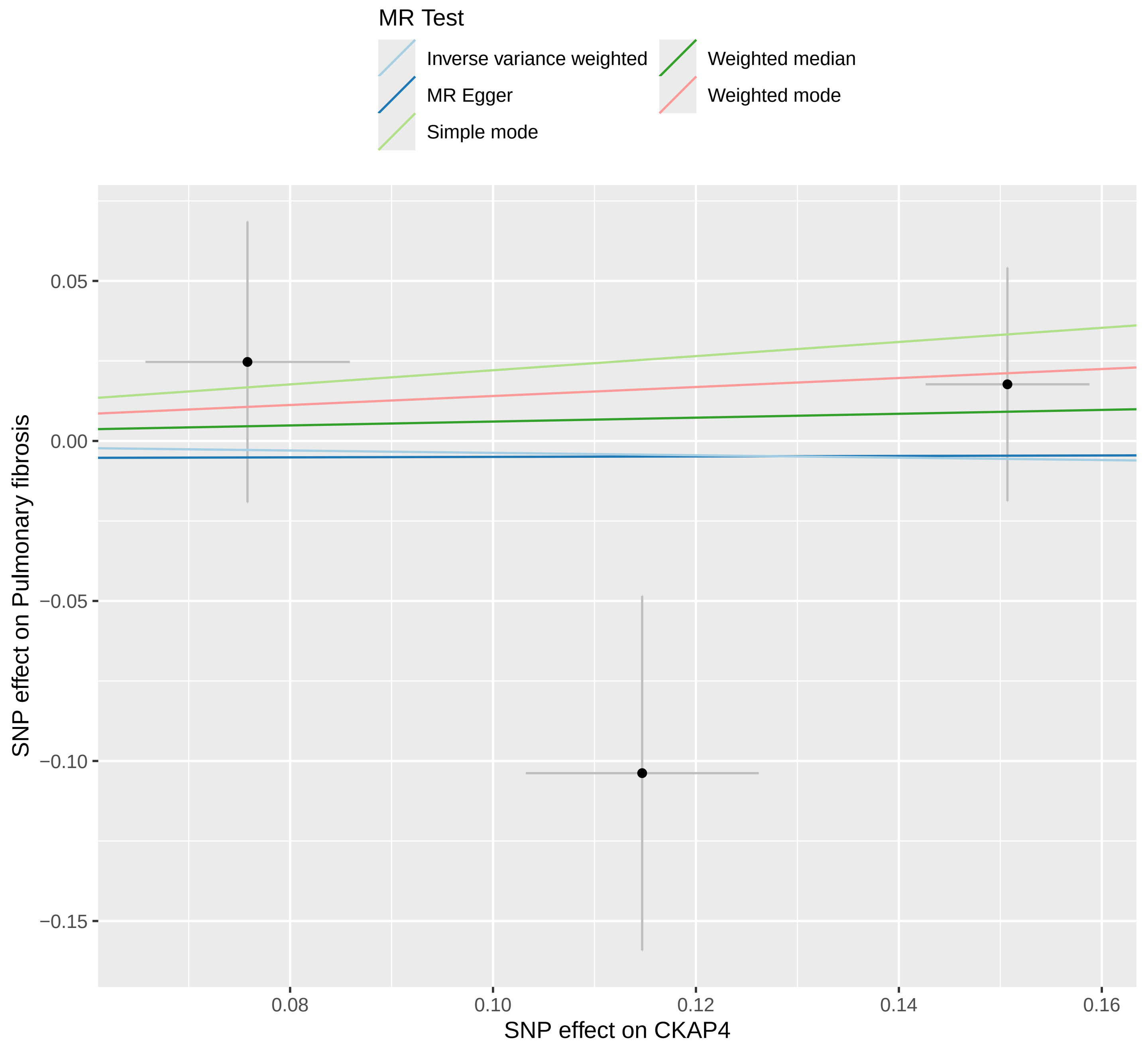

MR Test
Inverse variance weighted
MR Egger
Simple mode
Weighted median
Weighted mode
SNP effect on Pulmonary fibrosis
0.05
0.00
−0.05
−0.10
−0.15
0.08
0.10
0.12
0.14
0.16
SNP effect on CKAP4

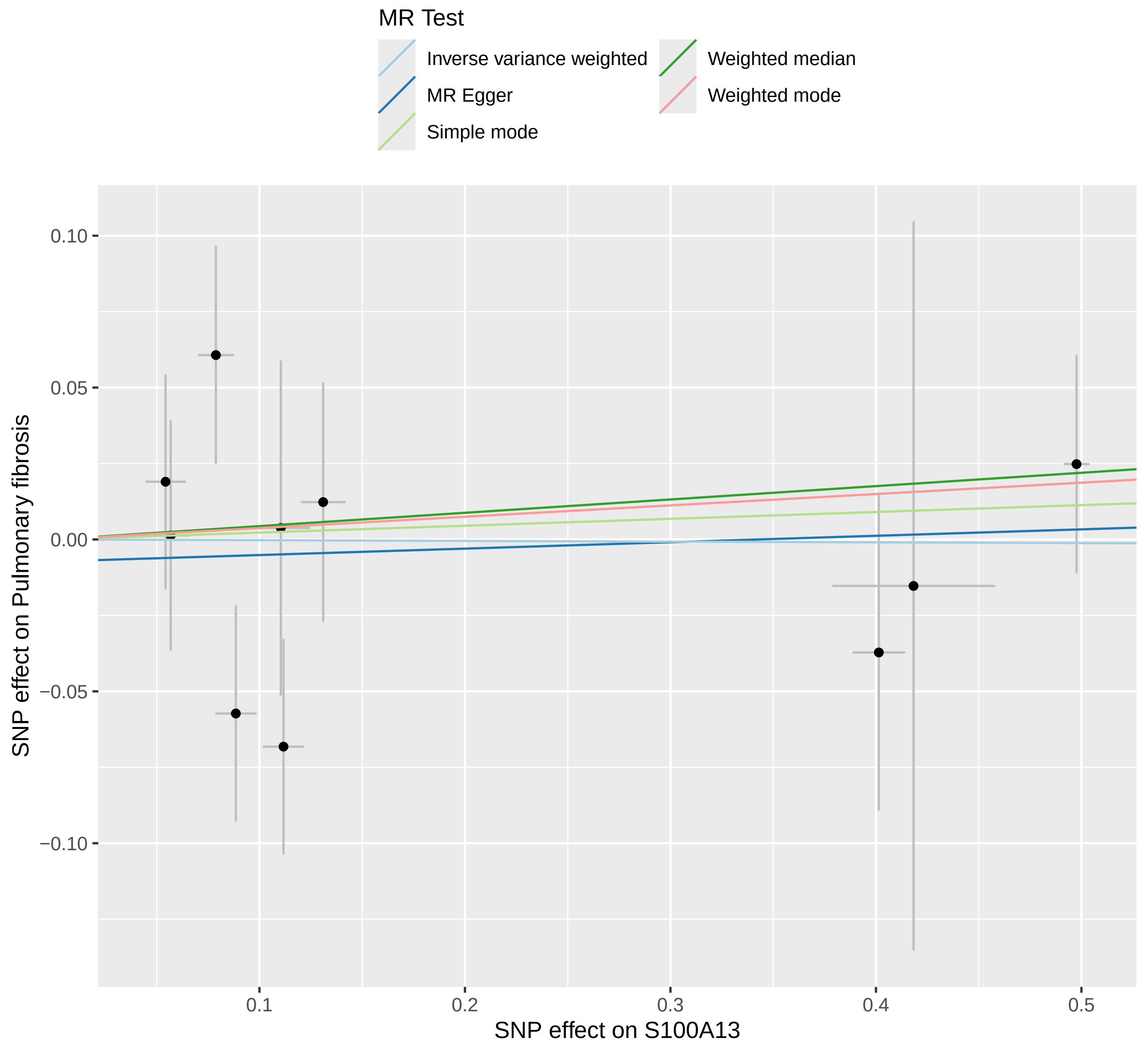
MR Test
Inverse variance weighted
MR Egger
Simple mode
Weighted median
Weighted mode
0.10
0.05
0.00
−0.05
−0.10
0.1
0.2
0.3
0.4
0.5
SNP effect on Pulmonary fibrosis
SNP effect on S100A13

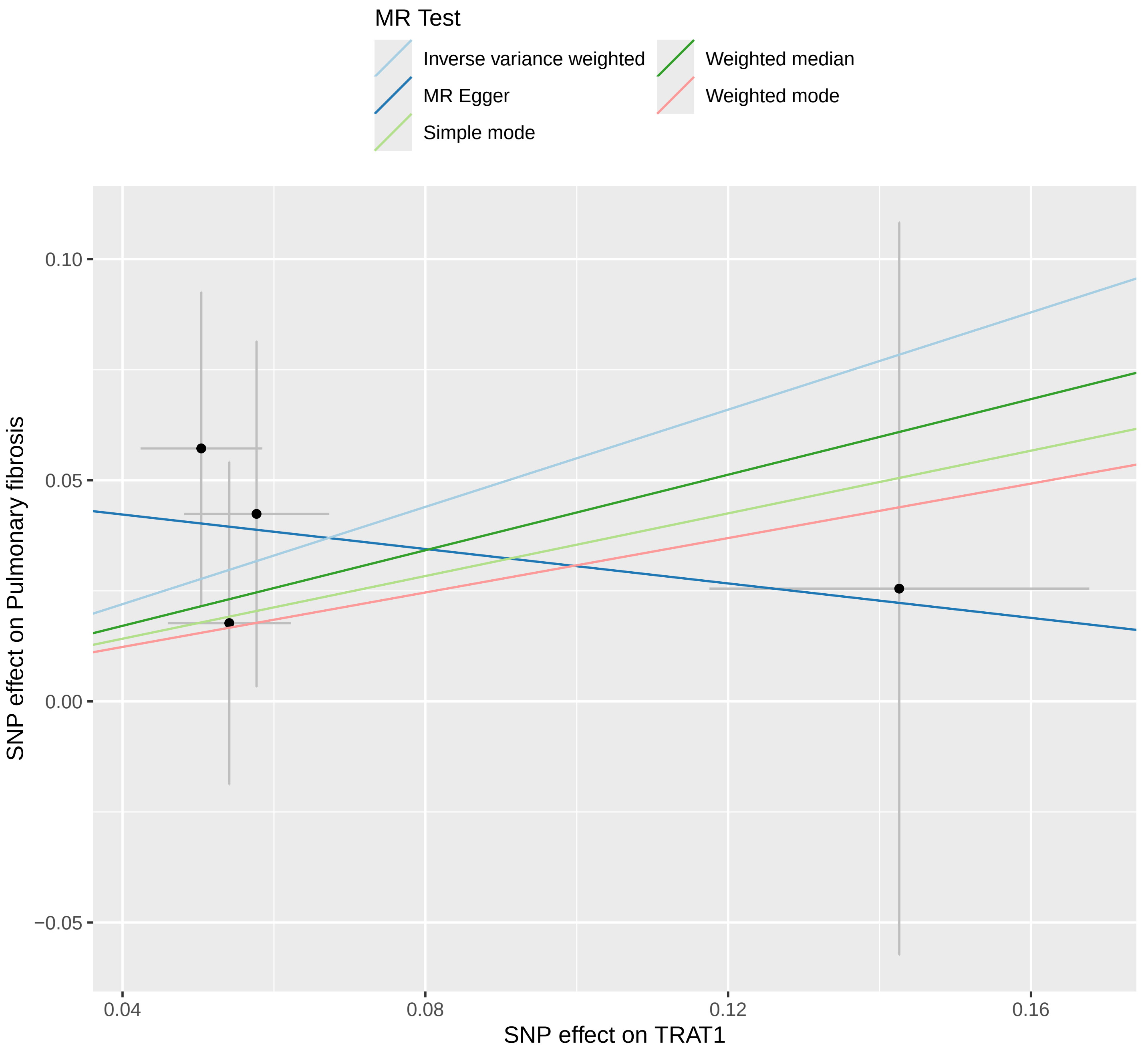
MR Test
Inverse variance weighted
MR Egger
Simple mode
Weighted median
Weighted mode
SNP effect on Pulmonary fibrosis
0.10
0.05
0.00
−0.05
0.04
0.08
0.12
0.16
SNP effect on TRAT1

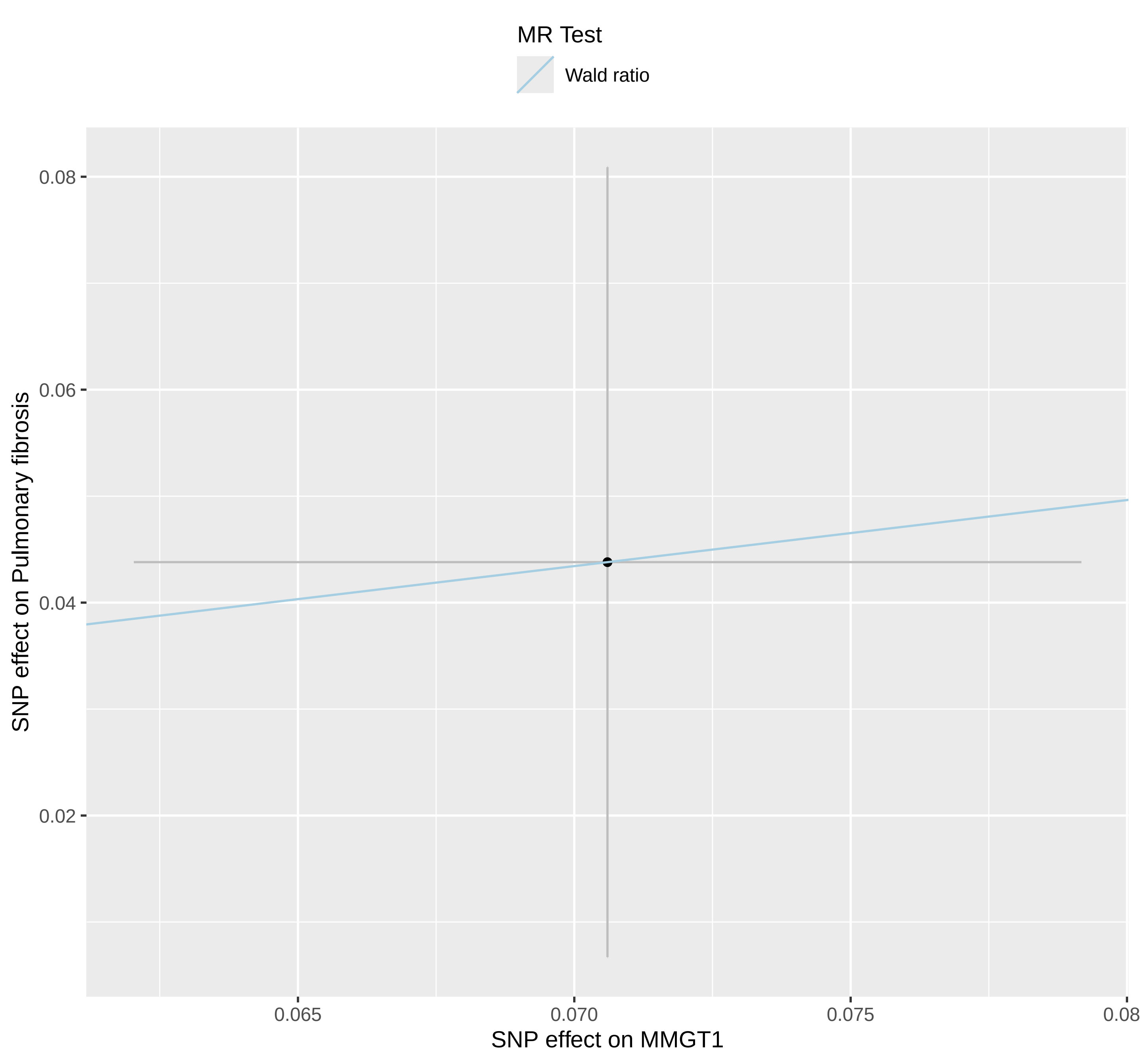

MR Test
Wald ratio
SNP effect on Pulmonary fibrosis
0.08
0.06
0.04
0.02
0.065
0.070
0.075
0.08
SNP effect on MMGT1

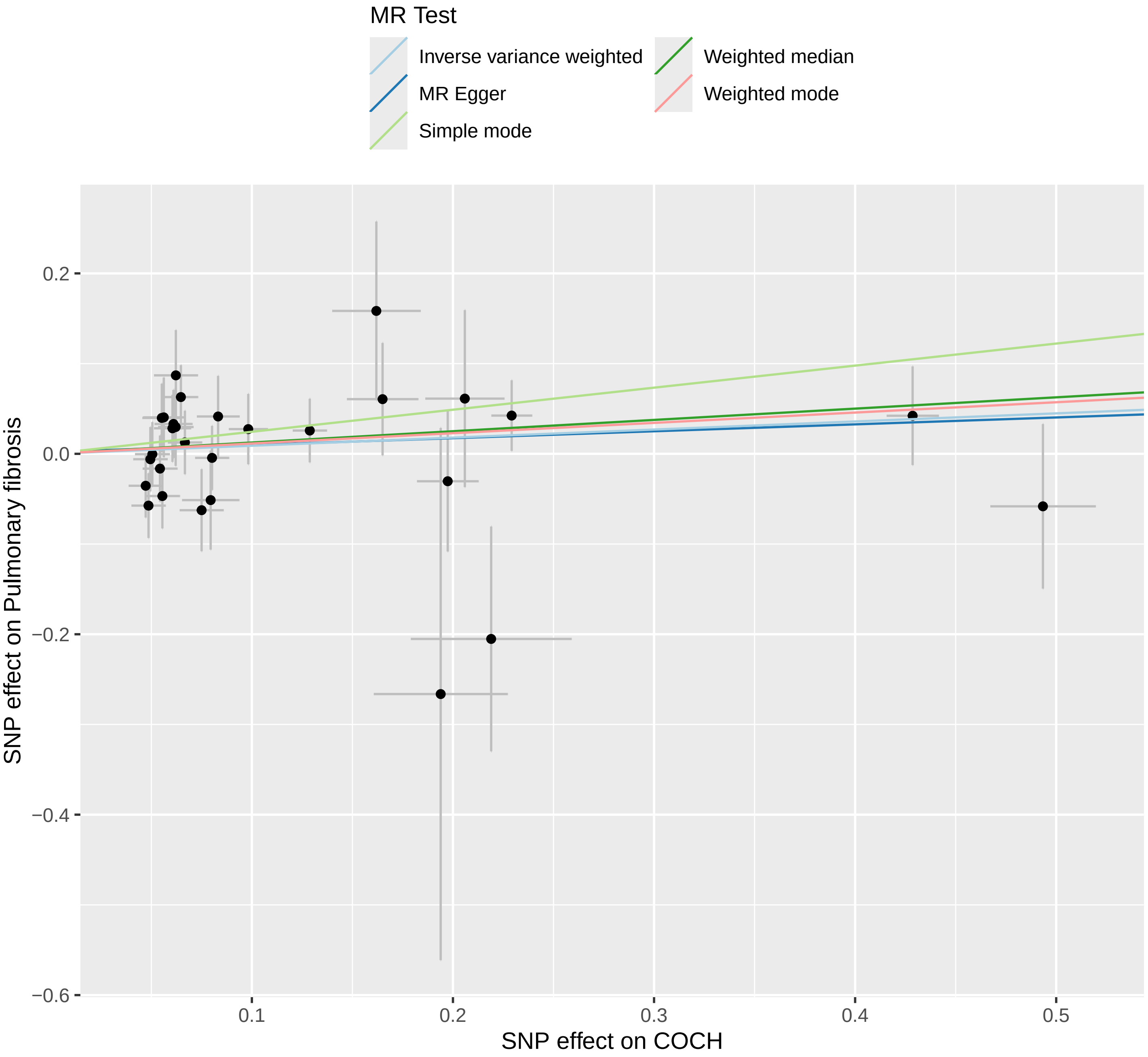
MR Test
Inverse variance weighted
MR Egger
Simple mode
Weighted median
Weighted mode
SNP effect on Pulmonary fibrosis
0.2
0.0
−0.2
−0.4
−0.6
0.1
0.2
0.3
0.4
0.5
SNP effect on COCH

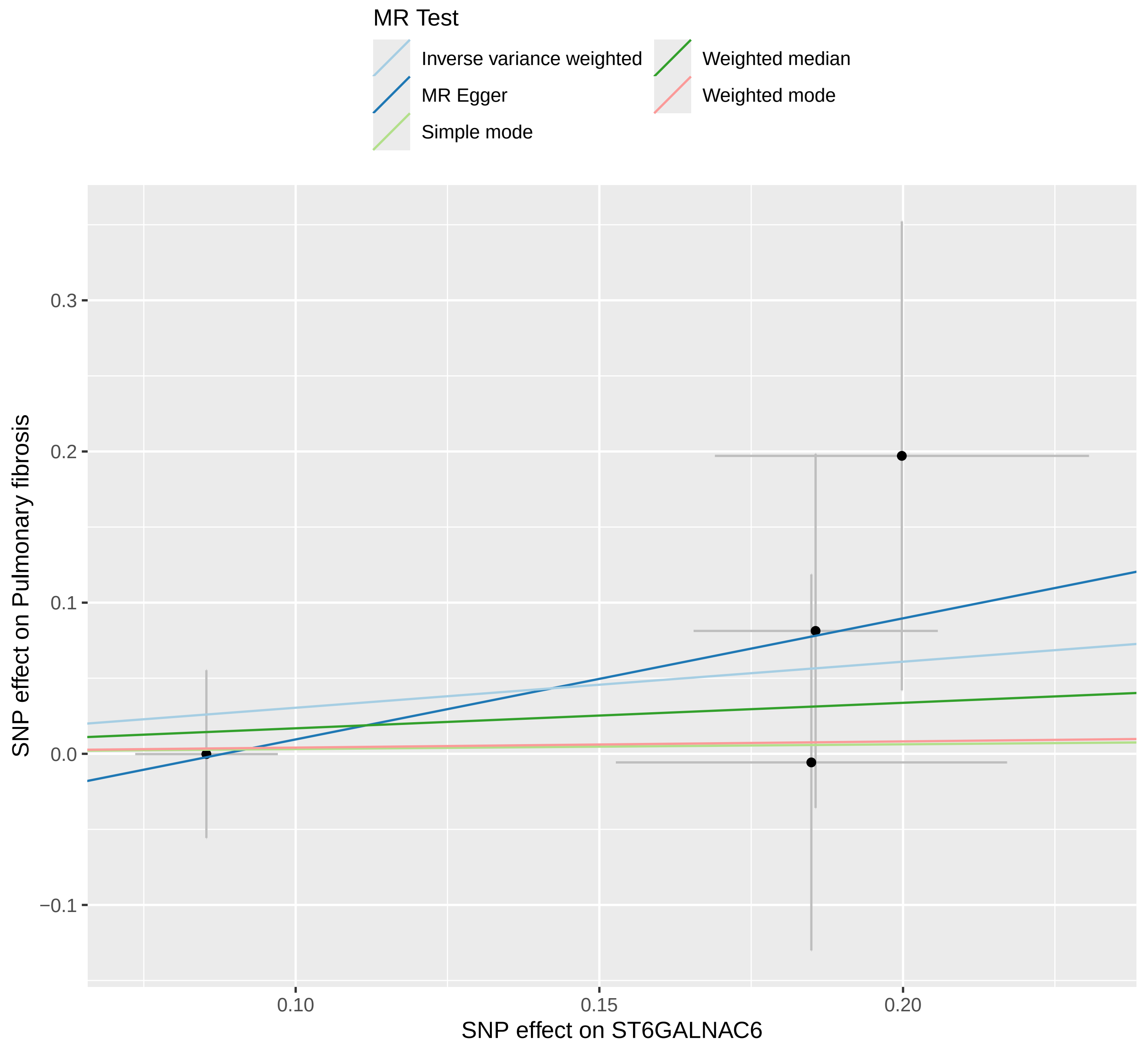

MR Test
Inverse variance weighted
MR Egger
Simple mode
Weighted median
Weighted mode
0.3
0.2
0.1
0.0
−0.1
SNP effect on Pulmonary fibrosis
0.10
0.15
0.20
SNP effect on ST6GALNAC6

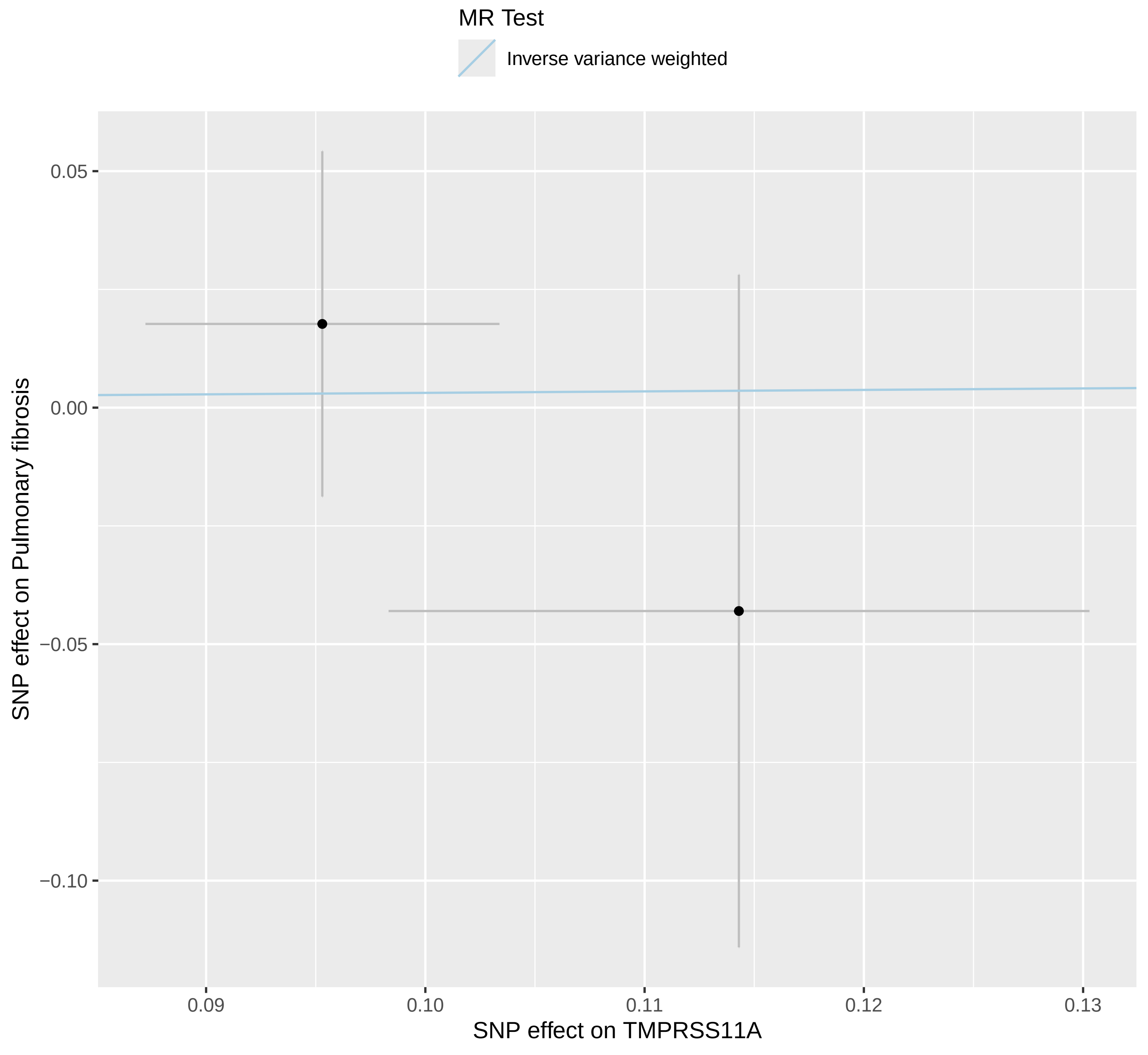
MR Test
Inverse variance weighted
0.05
0.00
−0.05
−0.10
0.09
0.10
0.11
0.12
0.13
SNP effect on Pulmonary fibrosis
SNP effect on TMPRSS11A

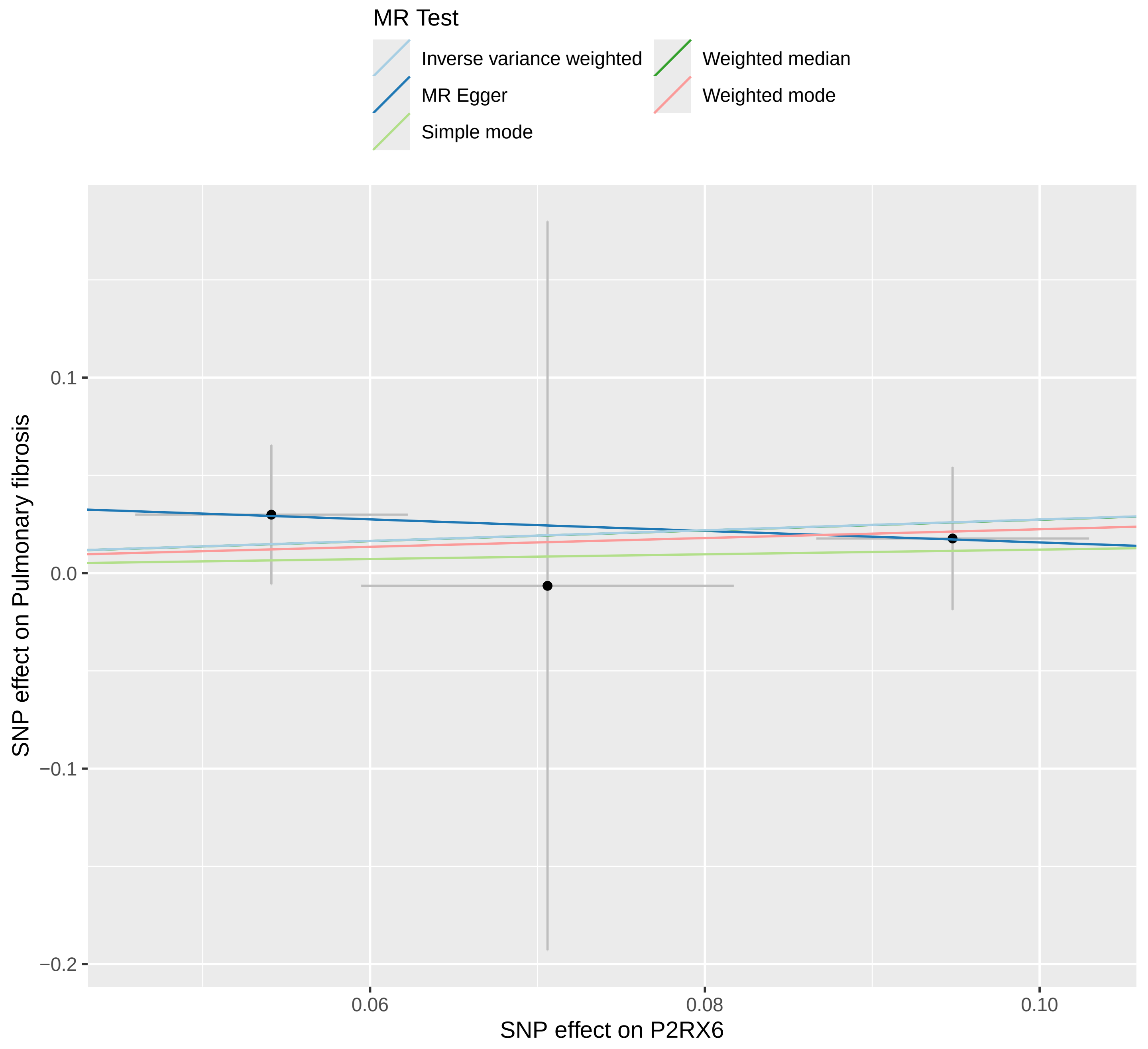
MR Test
Inverse variance weighted
MR Egger
Simple mode
Weighted median
Weighted mode
0.1
0.0
−0.1
−0.2
SNP effect on Pulmonary fibrosis
0.06
0.08
0.10
SNP effect on P2RX6

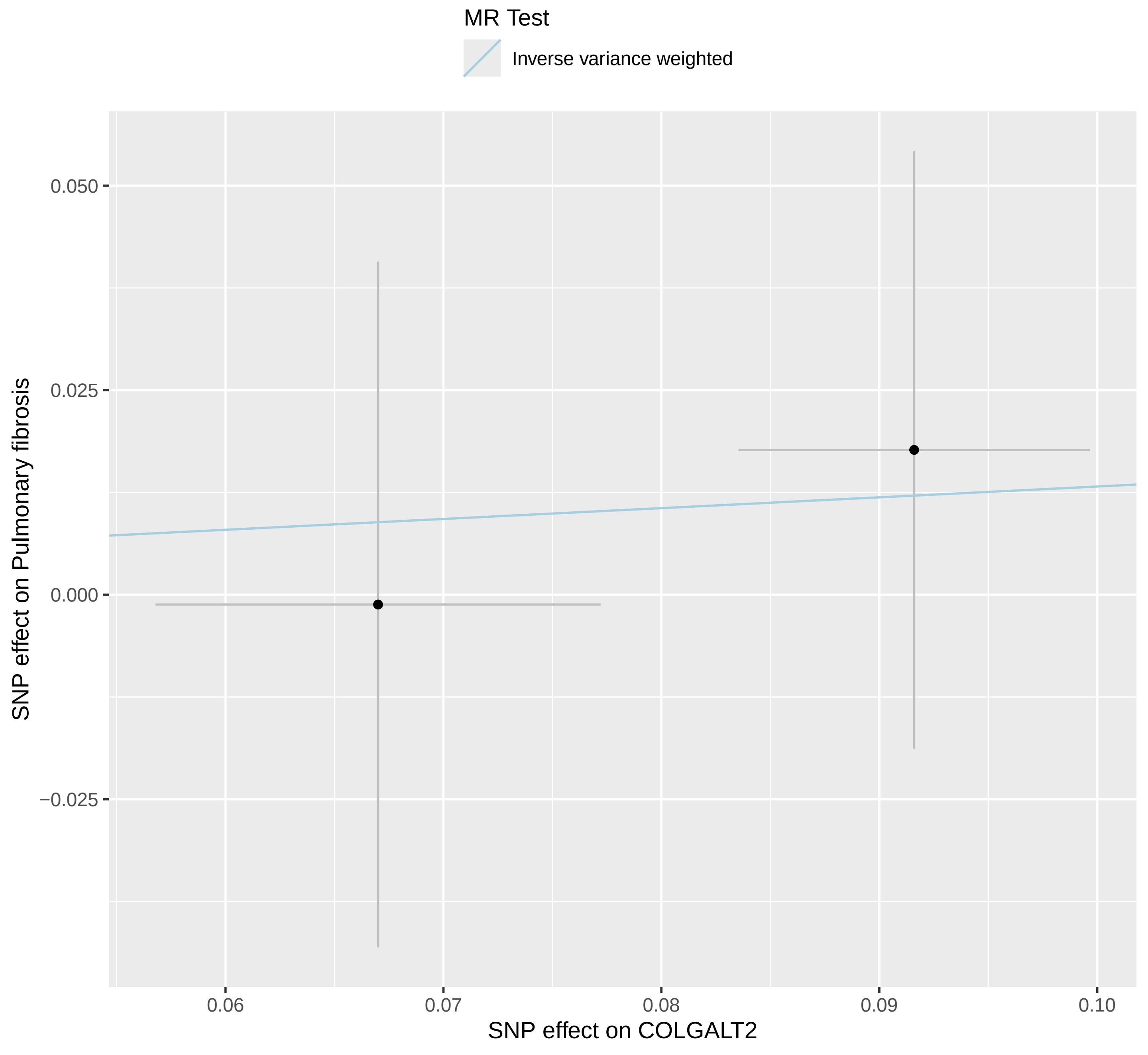

MR Test
Inverse variance weighted
0.050
0.025
0.000
−0.025
SNP effect on Pulmonary fibrosis
0.06
0.07
0.08
0.09
0.10
SNP effect on COLGALT2

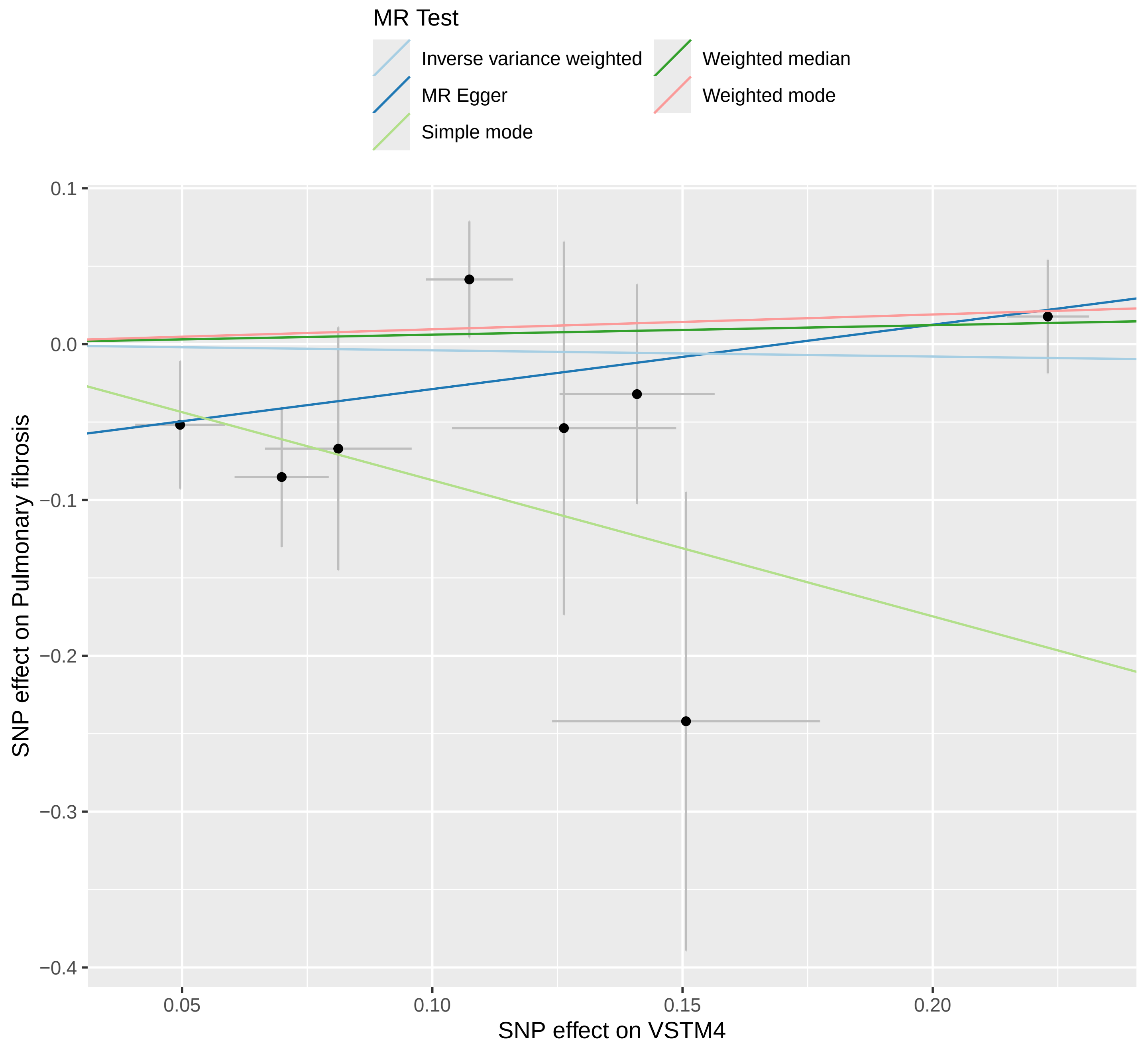
MR Test
Inverse variance weighted
MR Egger
Simple mode
Weighted median
Weighted mode
0.1
0.0
−0.1
−0.2
−0.3
−0.4
SNP effect on Pulmonary fibrosis
0.05
0.10
0.15
0.20
SNP effect on VSTM4

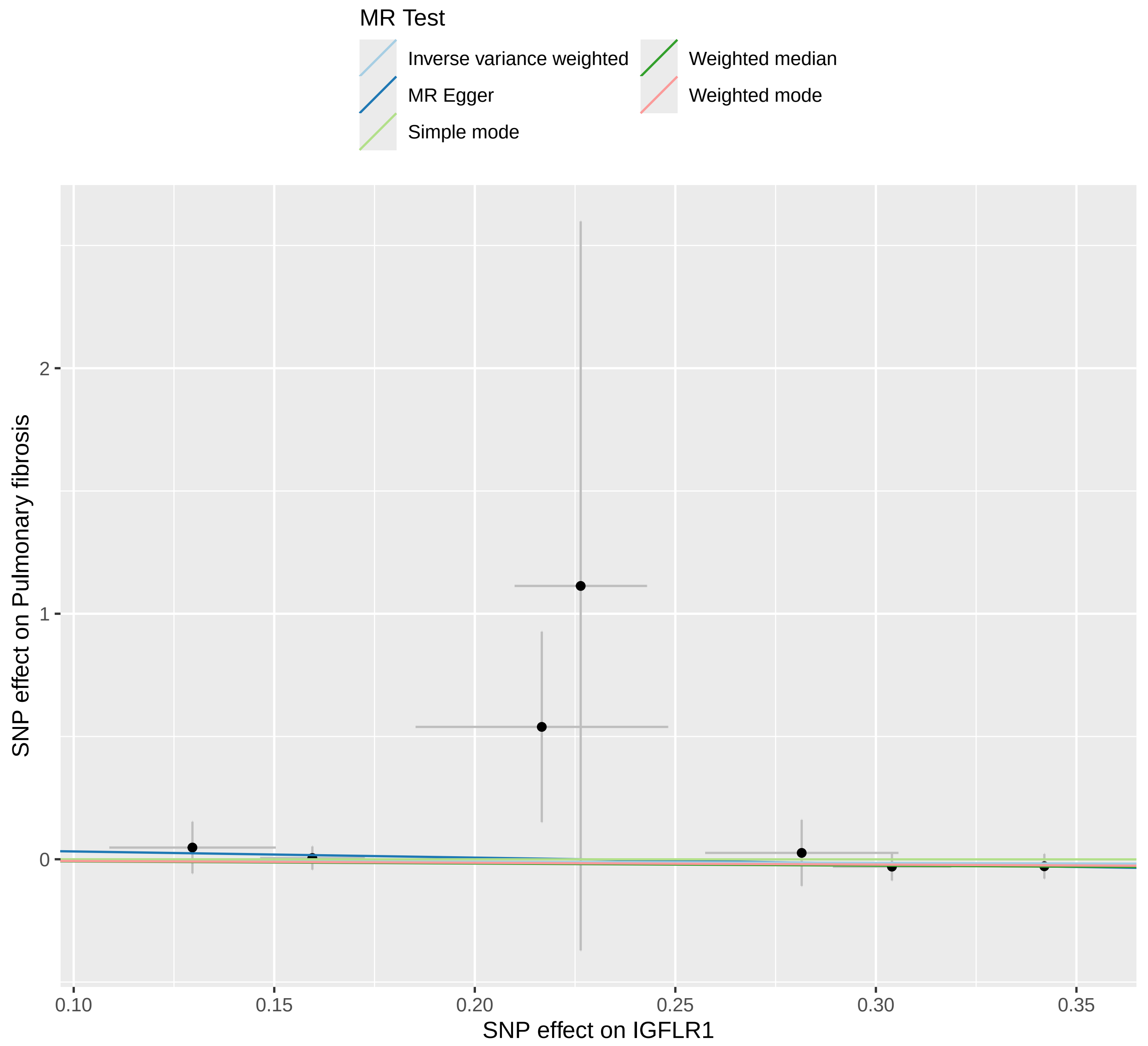
MR Test
Inverse variance weighted
MR Egger
Simple mode
Weighted median
Weighted mode
SNP effect on Pulmonary fibrosis
2
1
0
0.10
0.15
0.20
0.25
0.30
0.35
SNP effect on IGFLR1

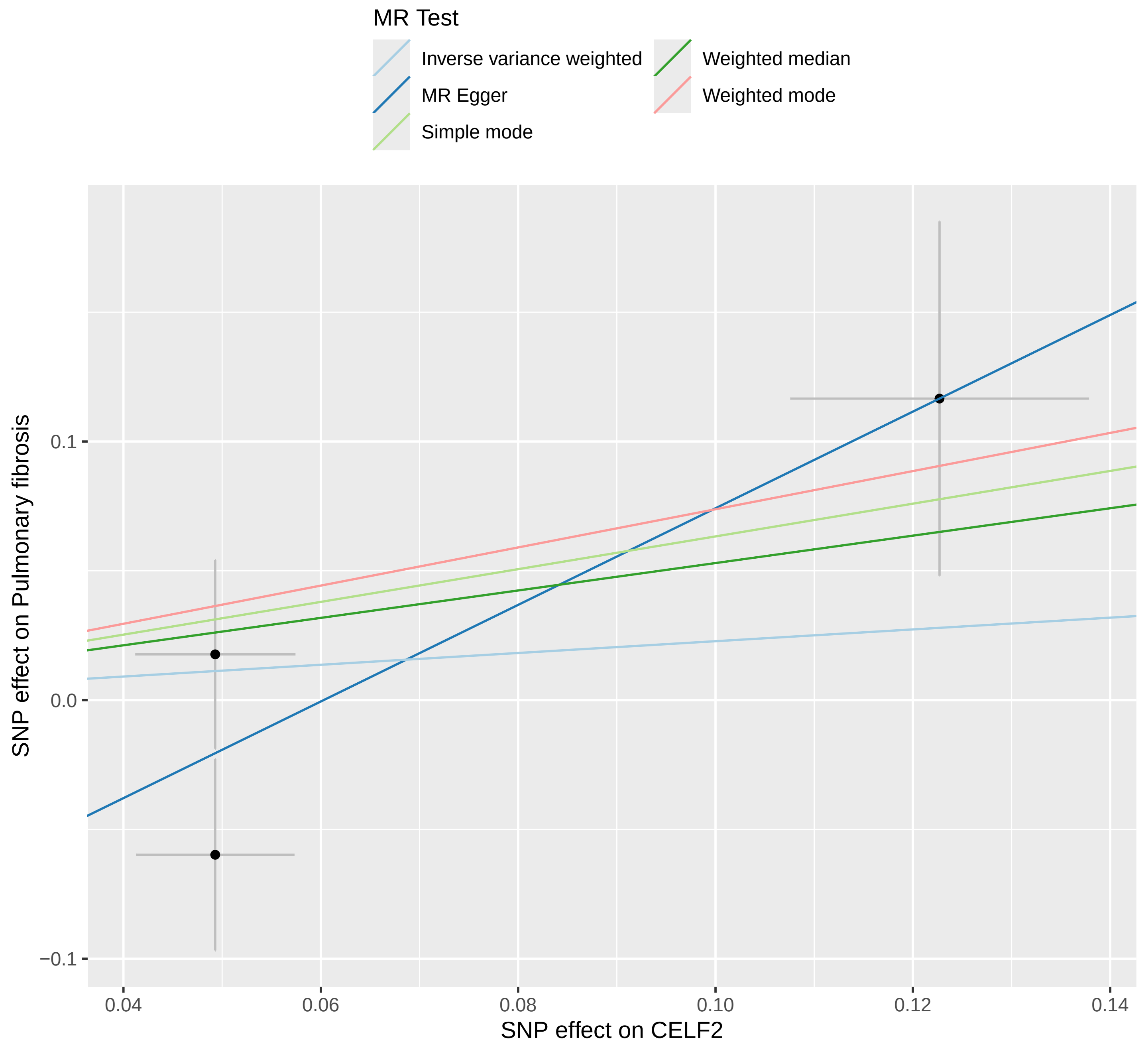

MR Test
Inverse variance weighted
MR Egger
Simple mode
Weighted median
Weighted mode
SNP effect on Pulmonary fibrosis
0.1
0.0
−0.1
0.04
0.06
0.08
0.10
0.12
0.14
SNP effect on CELF2

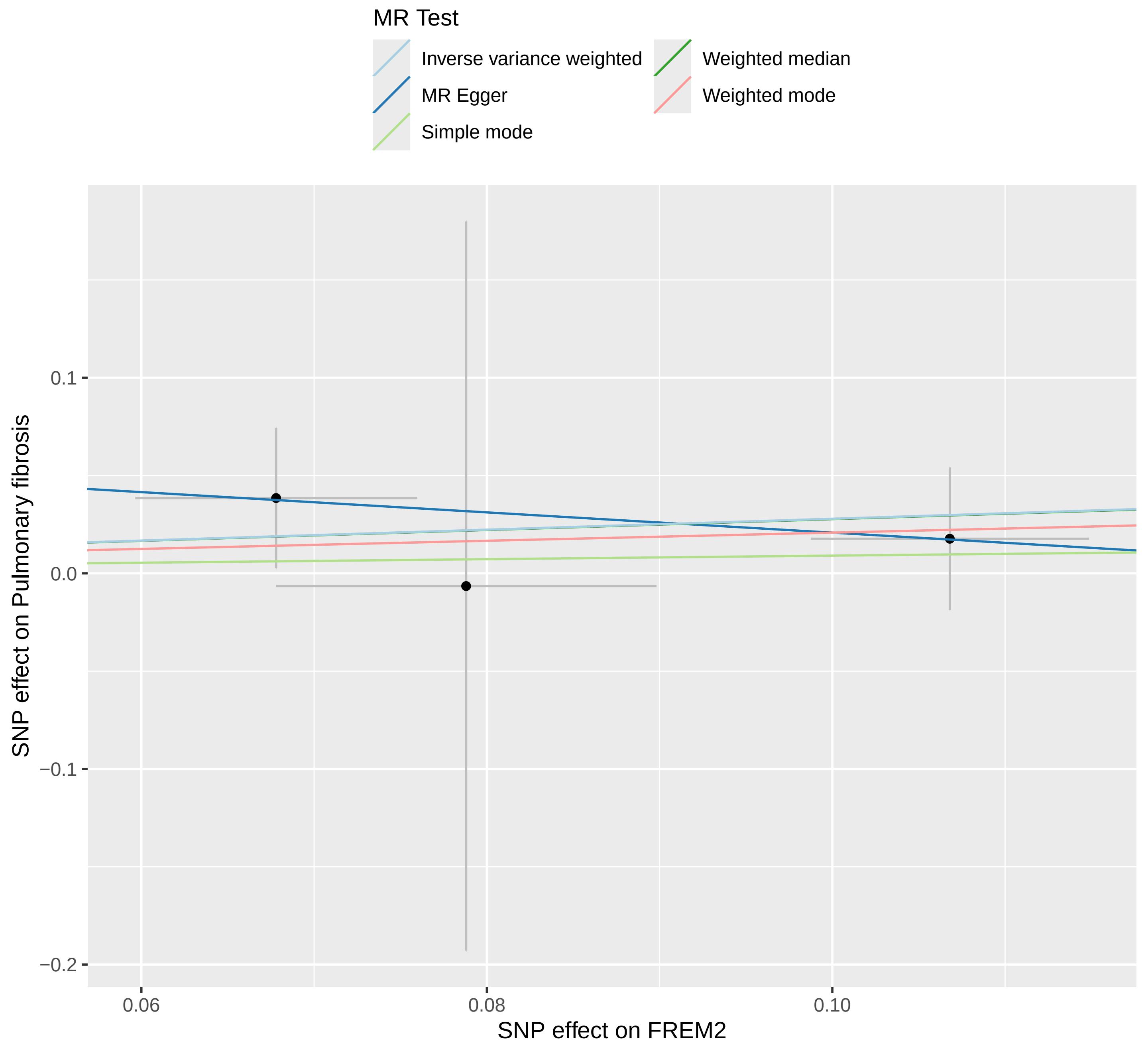
MR Test
Inverse variance weighted
MR Egger
Simple mode
Weighted median
Weighted mode
0.1
0.0
−0.1
−0.2
SNP effect on Pulmonary fibrosis
0.06
0.08
0.10
SNP effect on FREM2

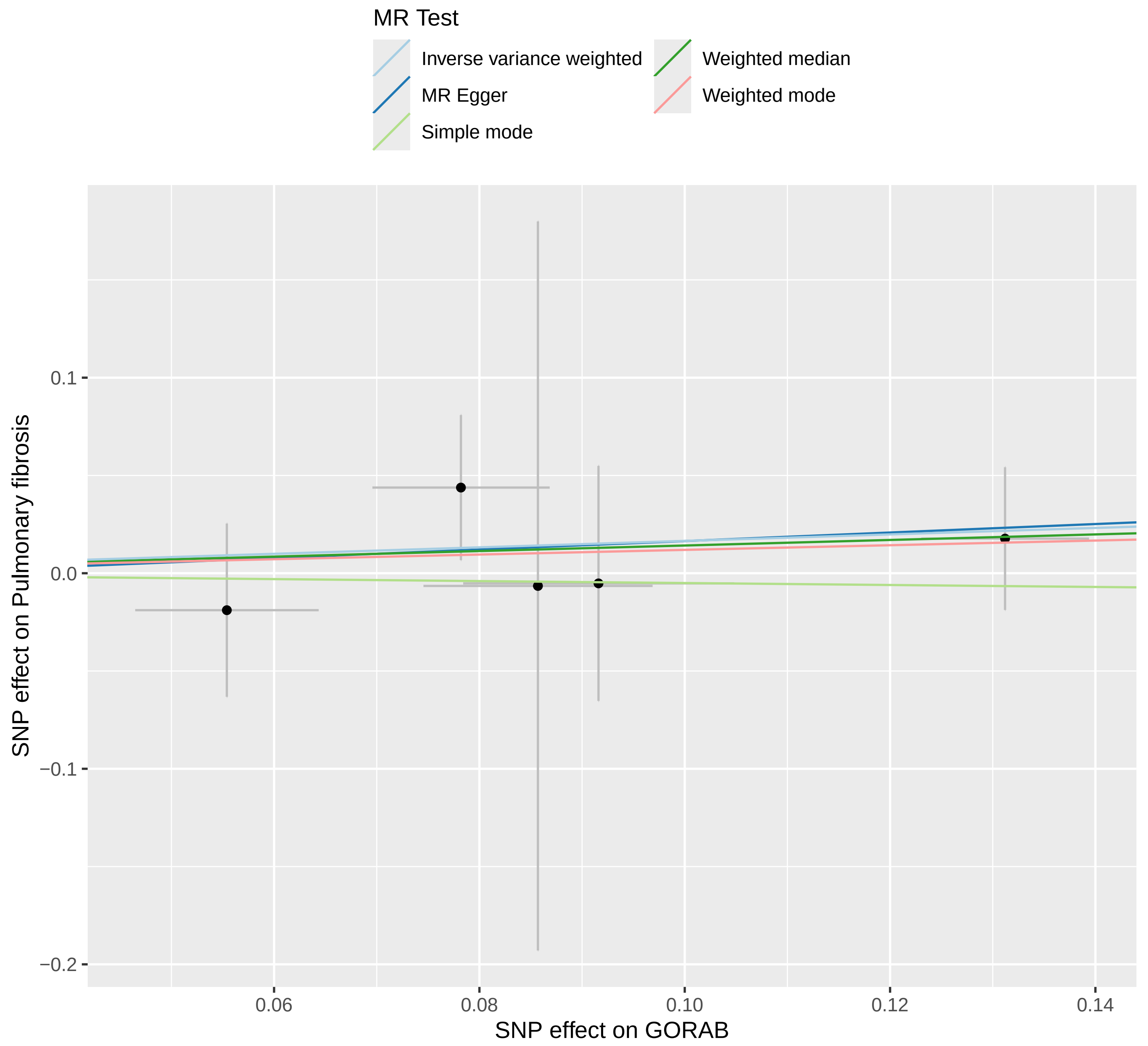

MR Test
Inverse variance weighted
MR Egger
Simple mode
Weighted median
Weighted mode
0.1
0.0
−0.1
−0.2
SNP effect on Pulmonary fibrosis
0.06
0.08
0.10
0.12
0.14
SNP effect on GORAB

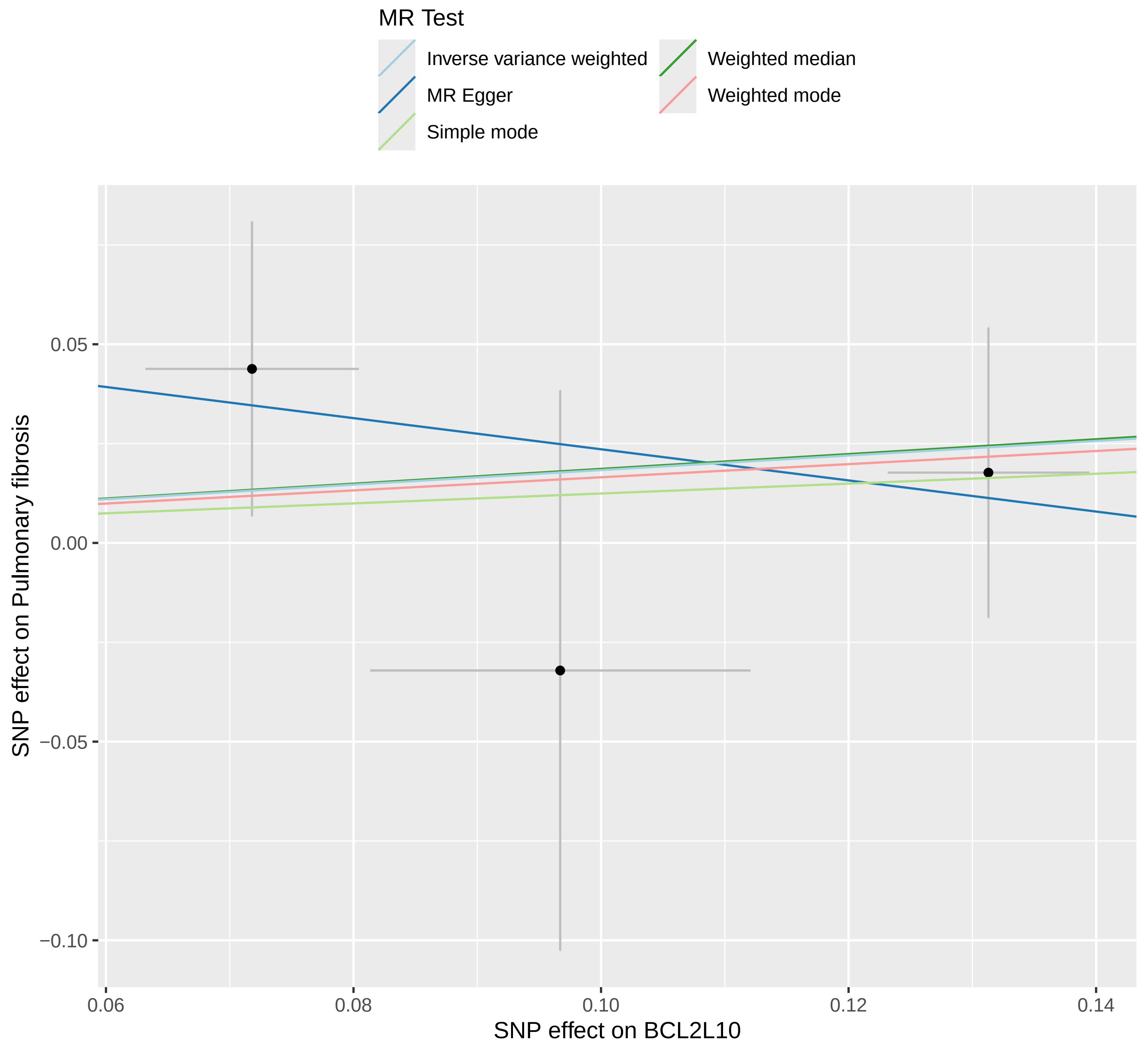

MR Test
Inverse variance weighted
MR Egger
Simple mode
Weighted median
Weighted mode
0.05
0.00
−0.05
−0.10
SNP effect on Pulmonary fibrosis
0.06
0.08
0.10
0.12
0.14
SNP effect on BCL2L10

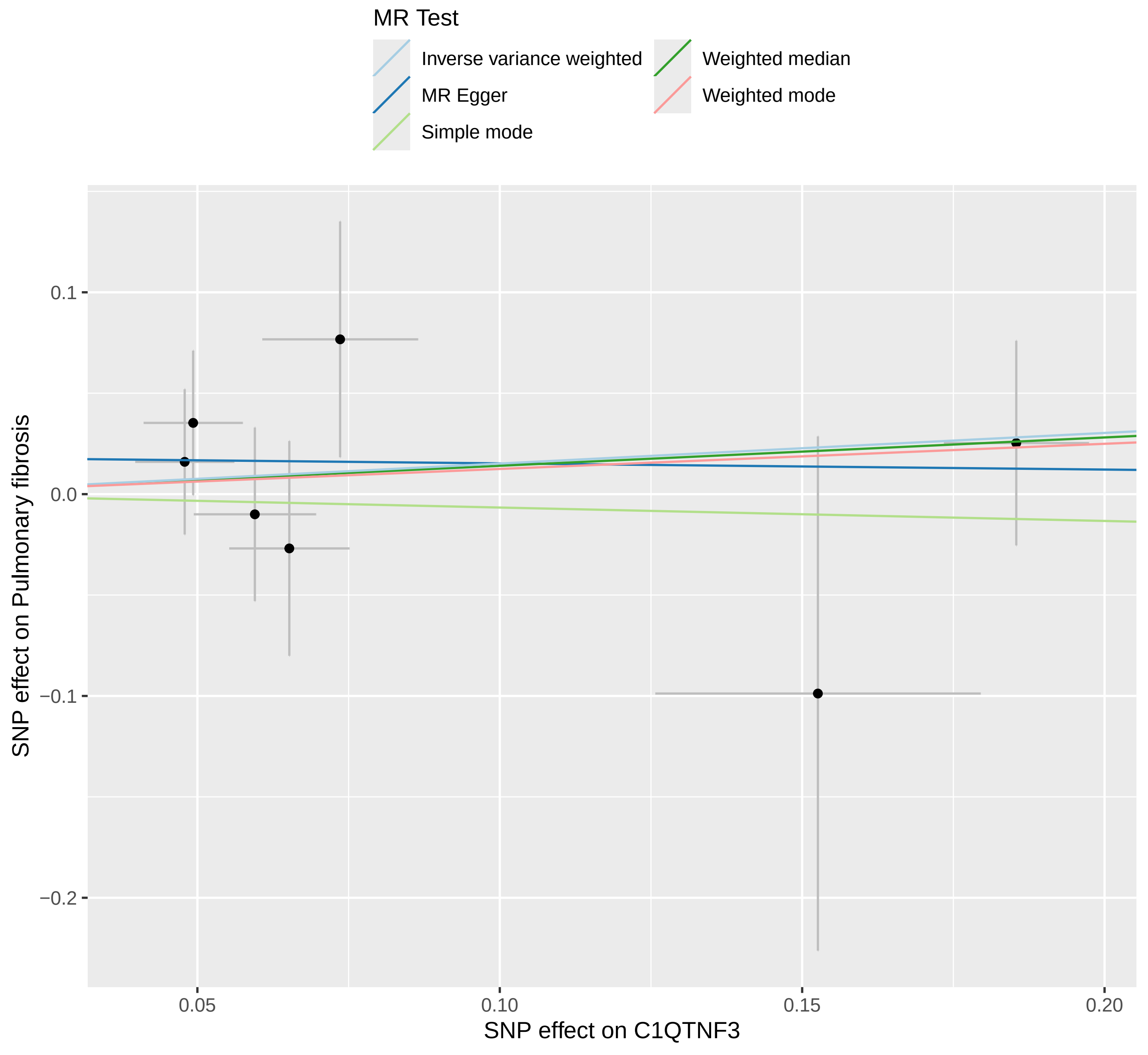

MR Test
Inverse variance weighted
MR Egger
Simple mode
Weighted median
Weighted mode
0.1
0.0
−0.1
−0.2
SNP effect on Pulmonary fibrosis
0.05
0.10
0.15
0.20
SNP effect on C1QTNF3

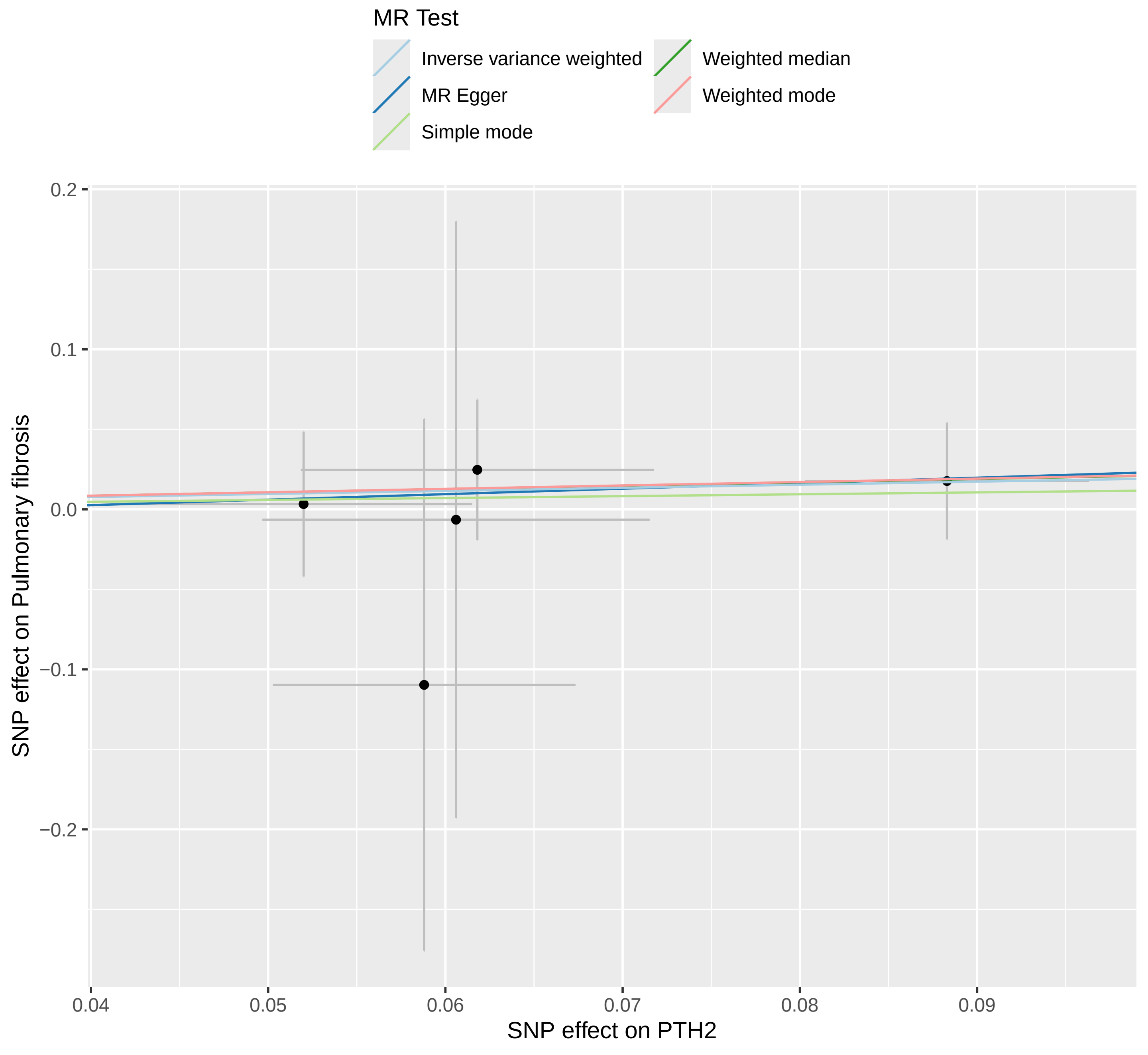
MR Test
Inverse variance weighted
MR Egger
Simple mode
Weighted median
Weighted mode
0.2
0.1
0.0
−0.1
−0.2
SNP effect on Pulmonary fibrosis
0.04
0.05
0.06
0.07
0.08
0.09
SNP effect on PTH2

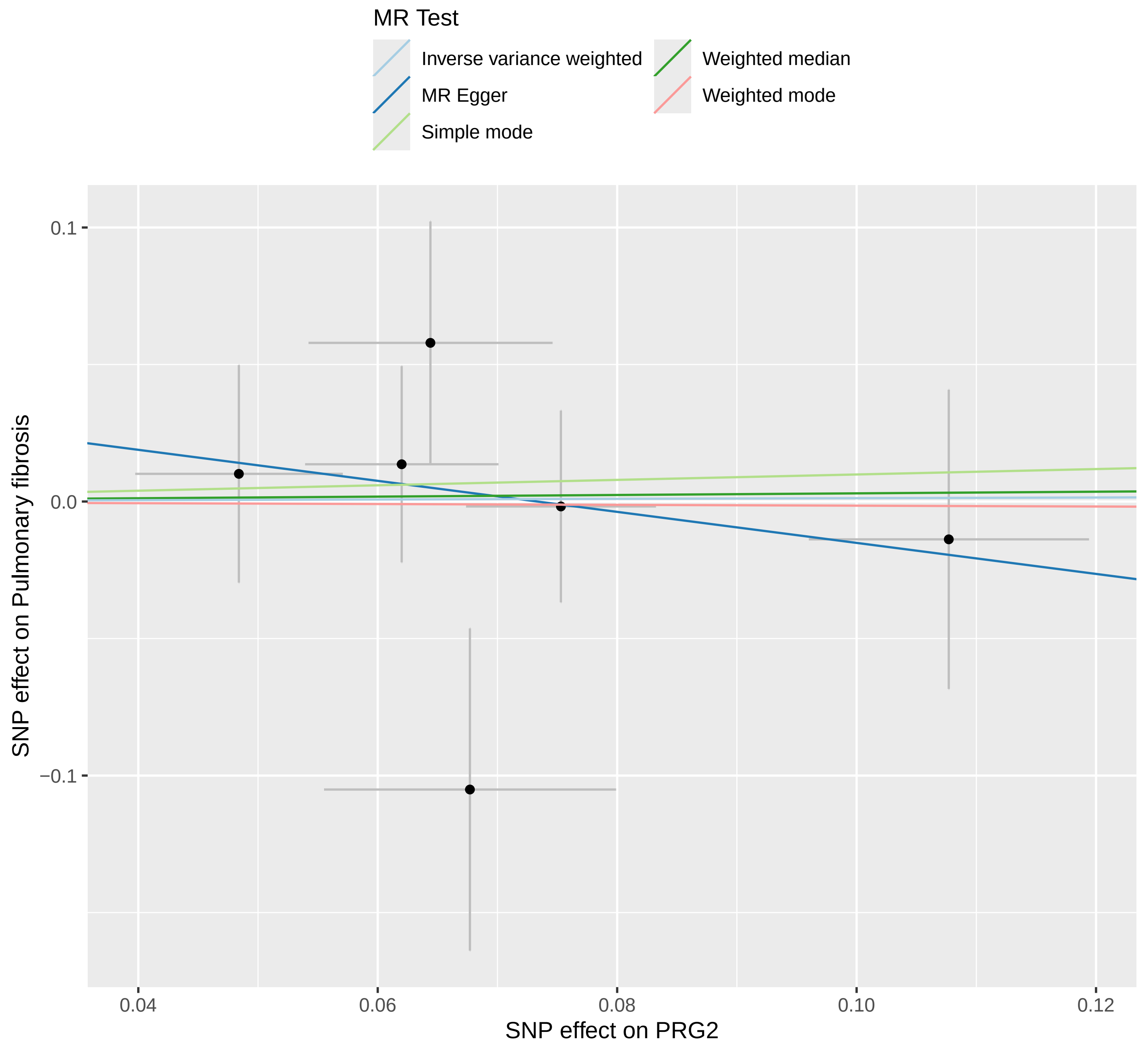
MR Test
Inverse variance weighted
MR Egger
Simple mode
Weighted median
Weighted mode
0.1
0.0
−0.1
0.04
0.06
0.08
0.10
0.12
SNP effect on PRG2
SNP effect on Pulmonary fibrosis

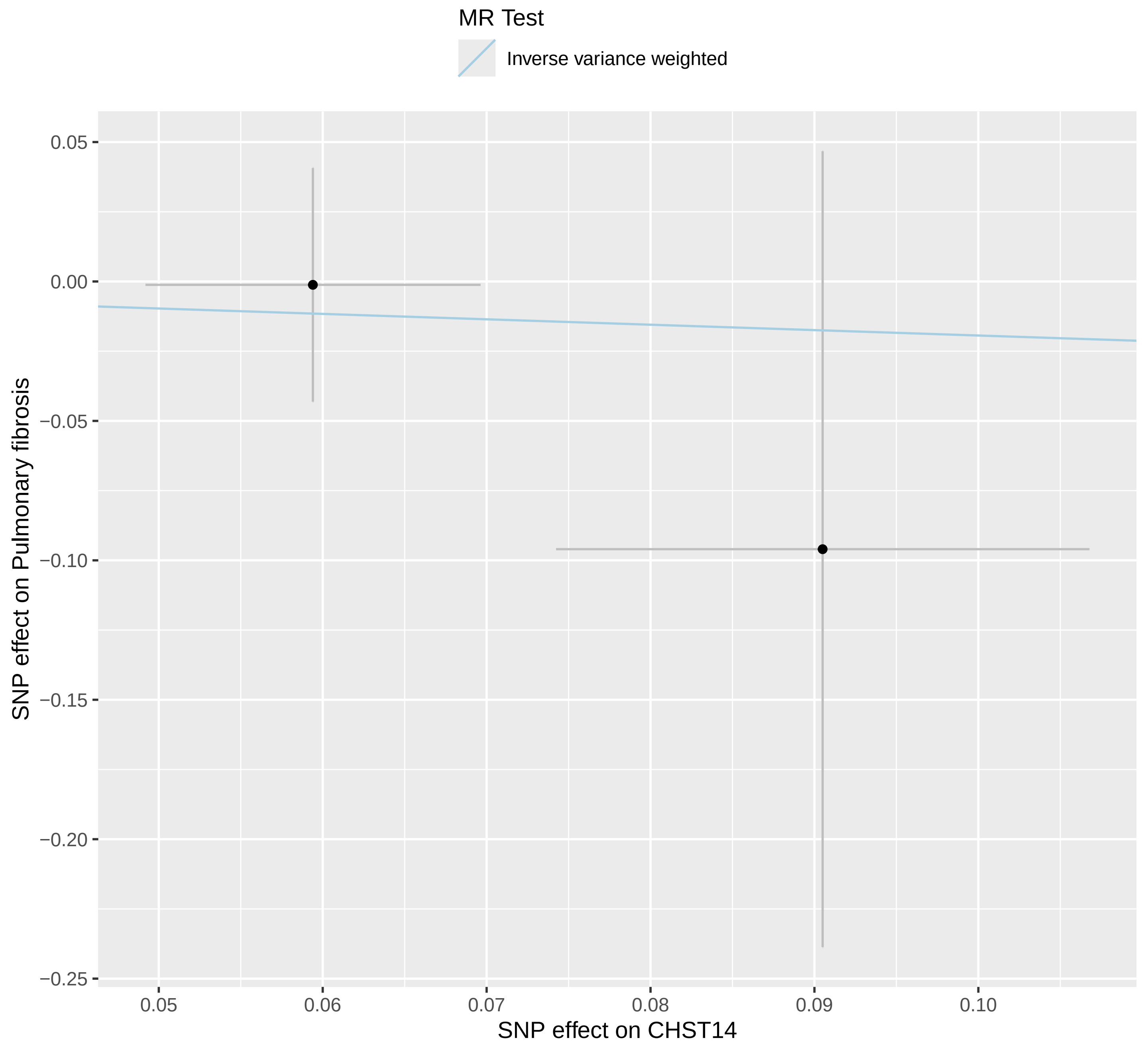

MR Test
Inverse variance weighted
0.05
0.00
−0.05
−0.10
−0.15
−0.20
−0.25
0.05
0.06
0.07
0.08
0.09
0.10
SNP effect on Pulmonary fibrosis
SNP effect on CHST14

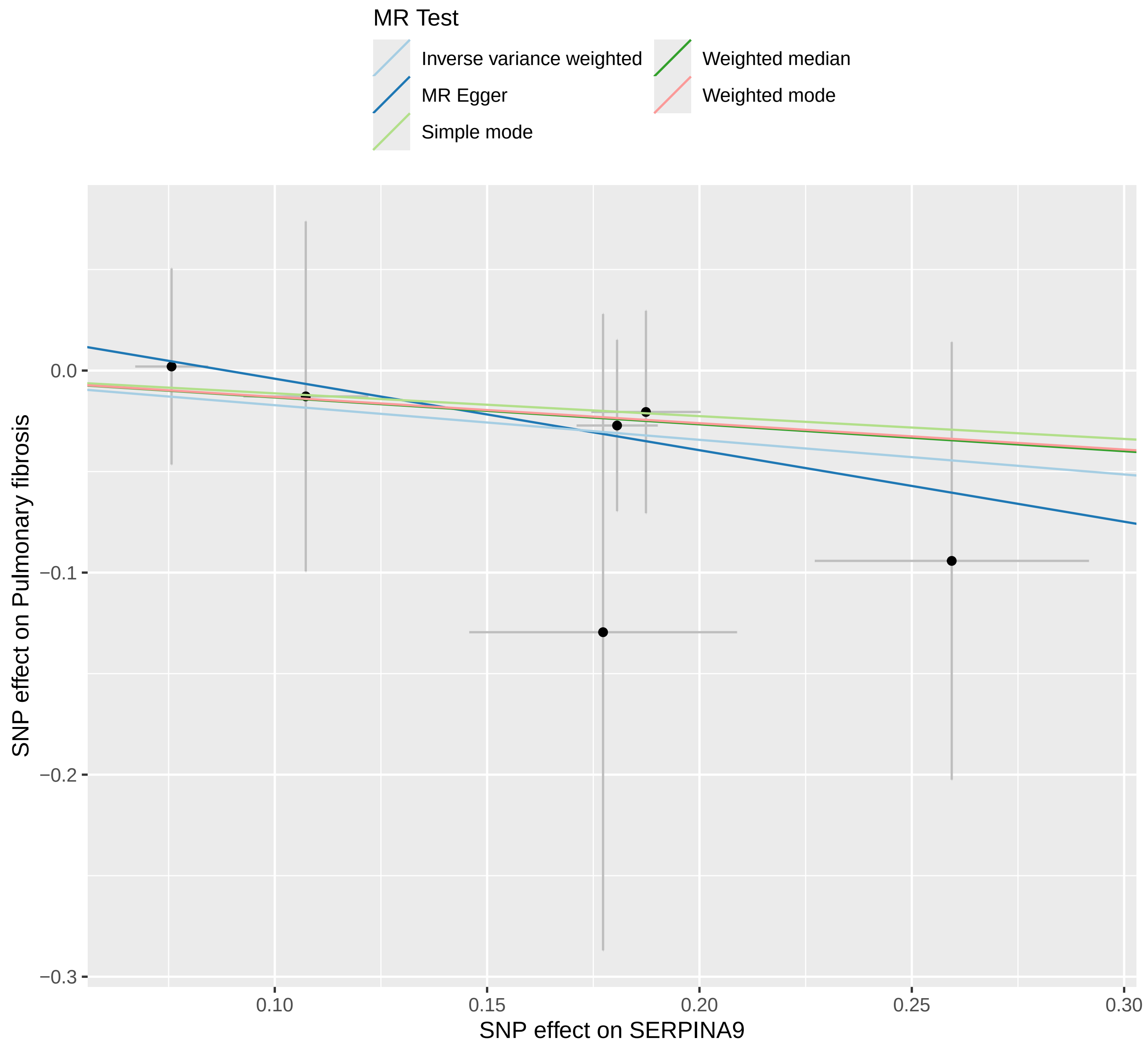

MR Test
Inverse variance weighted
MR Egger
Simple mode
Weighted median
Weighted mode
SNP effect on Pulmonary fibrosis
0.0
−0.1
−0.2
−0.3
0.10
0.15
0.20
0.25
0.30
SNP effect on SERPINA9

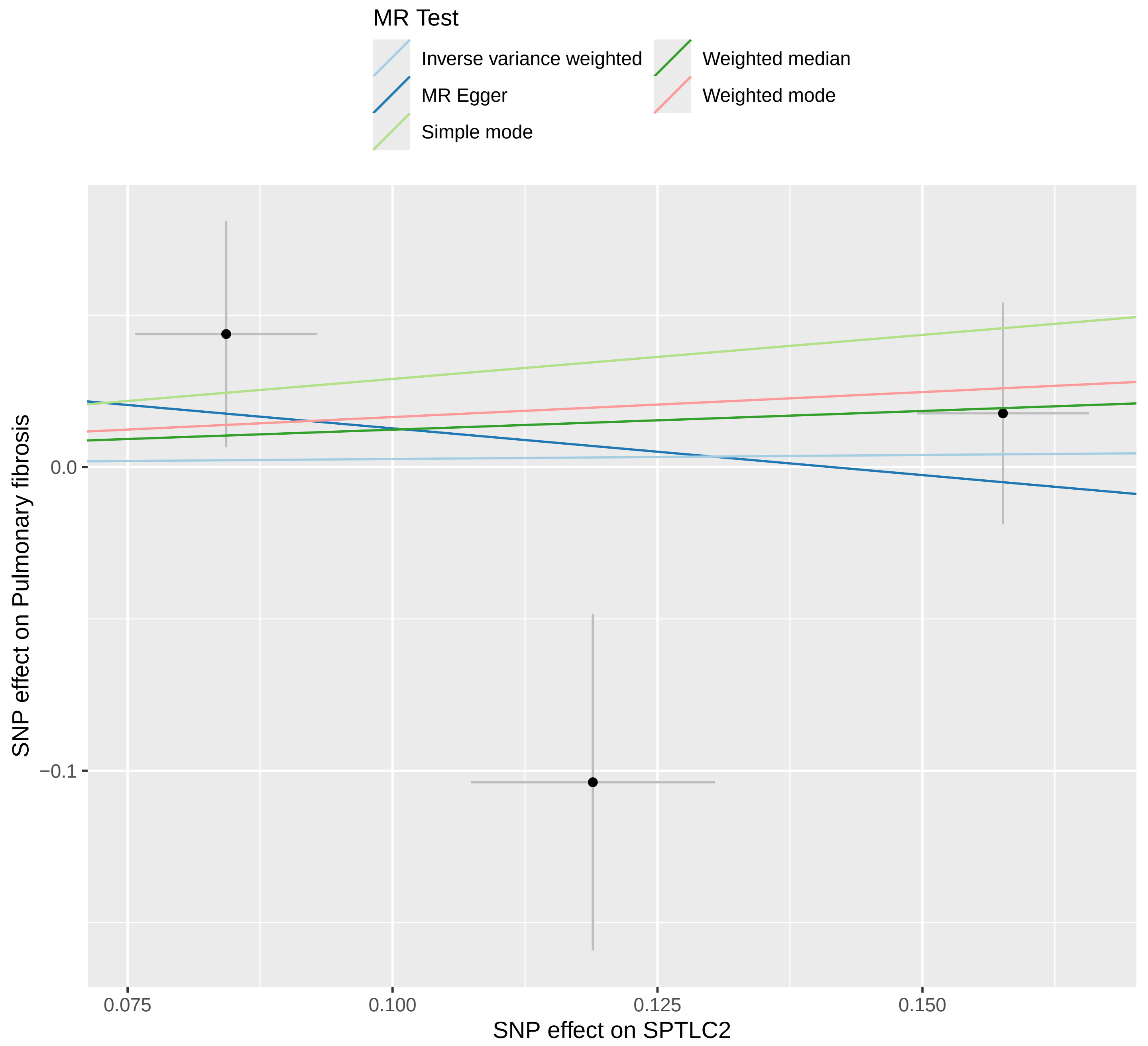

MR Test
Inverse variance weighted
MR Egger
Simple mode
Weighted median
Weighted mode
0.0
−0.1
SNP effect on Pulmonary fibrosis
0.075
0.100
0.125
0.150
SNP effect on SPTLC2

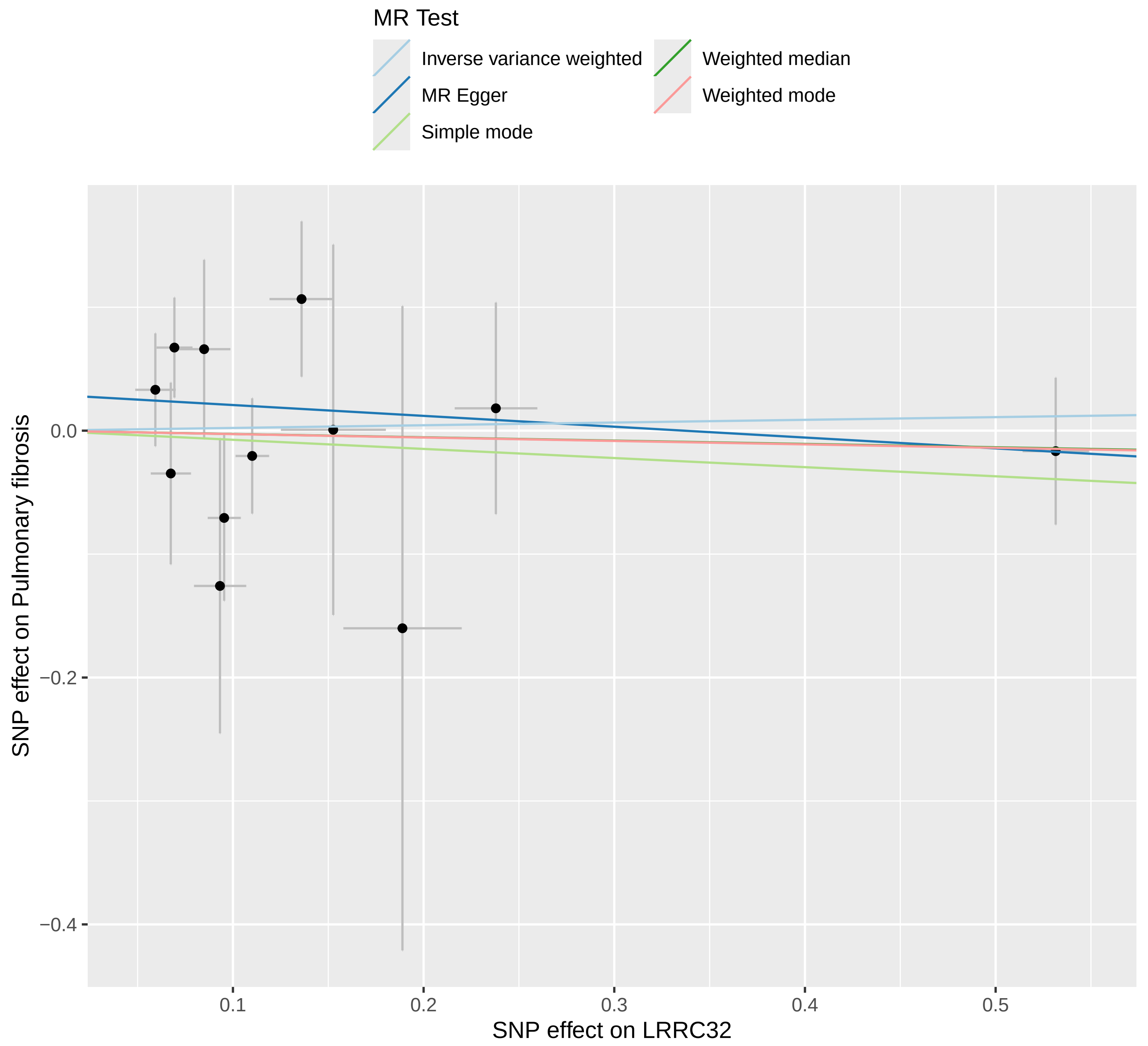
MR Test
Inverse variance weighted
MR Egger
Simple mode
Weighted median
Weighted mode
SNP effect on Pulmonary fibrosis
0.0
−0.2
−0.4
0.1
0.2
0.3
0.4
0.5
SNP effect on LRRC32

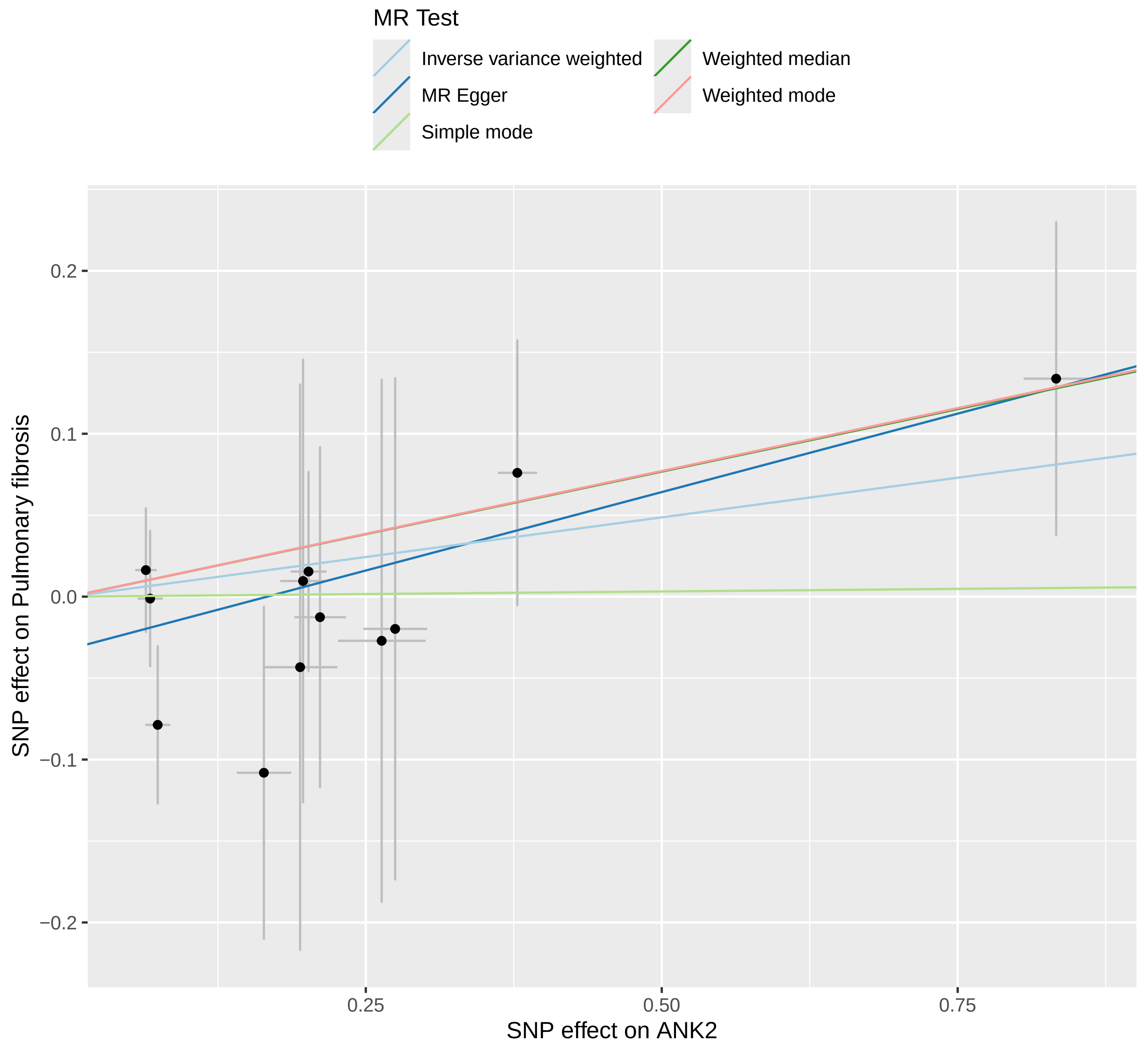
MR Test
Inverse variance weighted
MR Egger
Simple mode
Weighted median
Weighted mode
0.2
0.1
0.0
−0.1
−0.2
0.25
0.50
0.75
SNP effect on Pulmonary fibrosis
SNP effect on ANK2

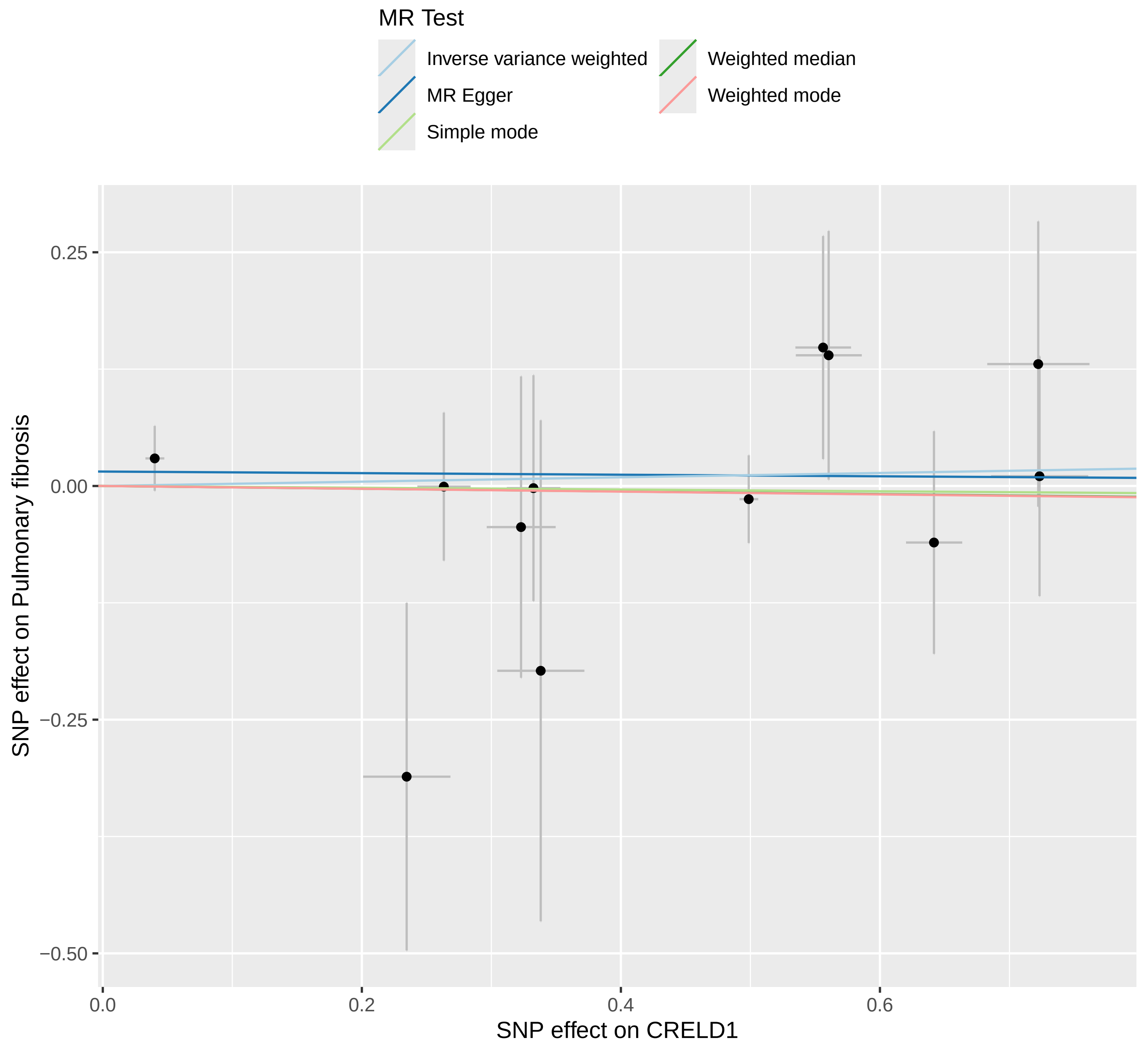
MR Test
Inverse variance weighted
MR Egger
Simple mode
Weighted median
Weighted mode
0.25
0.00
−0.25
−0.50
SNP effect on Pulmonary fibrosis
0.0
0.2
0.4
0.6
SNP effect on CRELD1

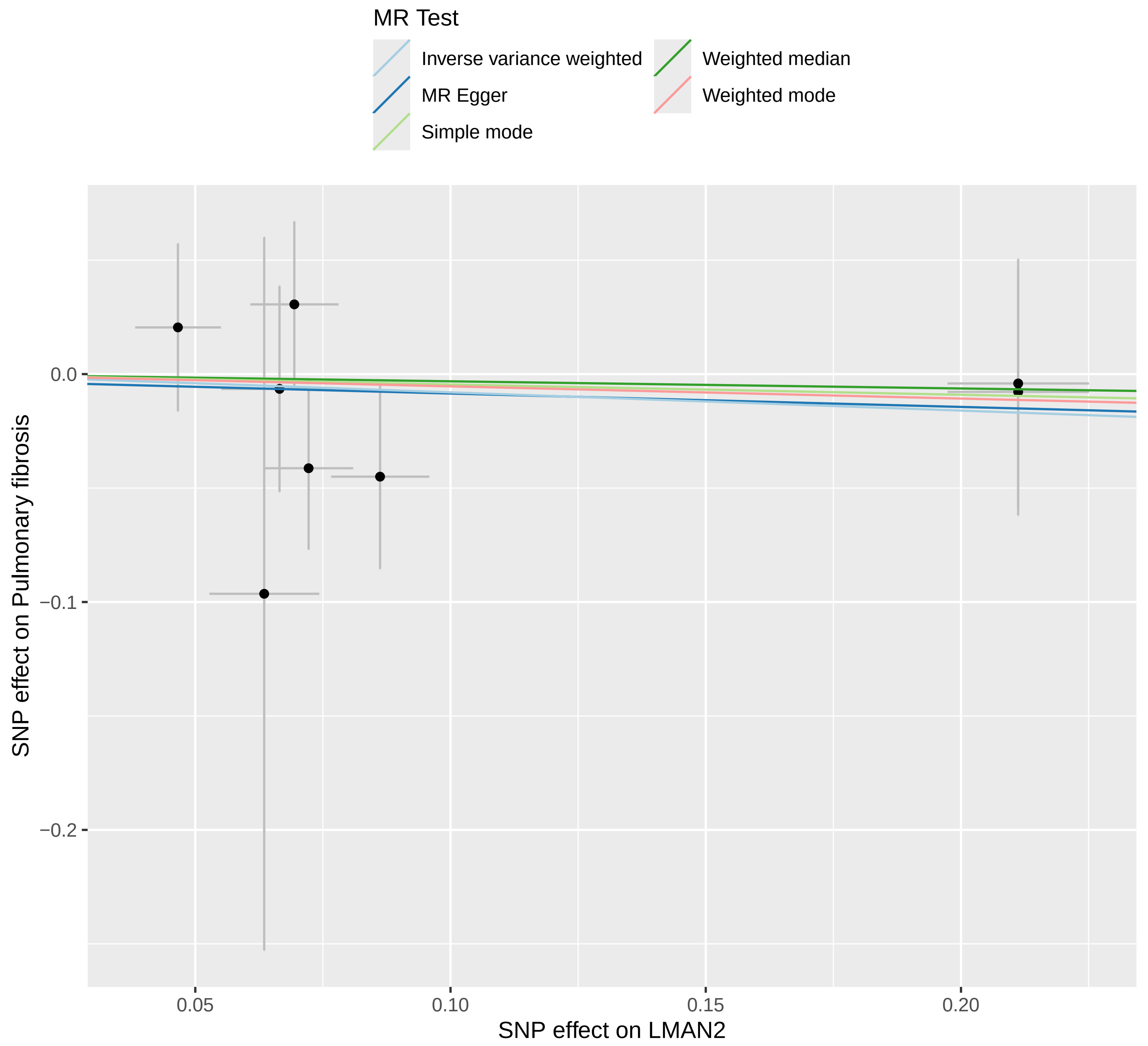

MR Test
Inverse variance weighted
MR Egger
Simple mode
Weighted median
Weighted mode
0.0
−0.1
−0.2
SNP effect on Pulmonary fibrosis
0.05
0.10
0.15
0.20
SNP effect on LMAN2

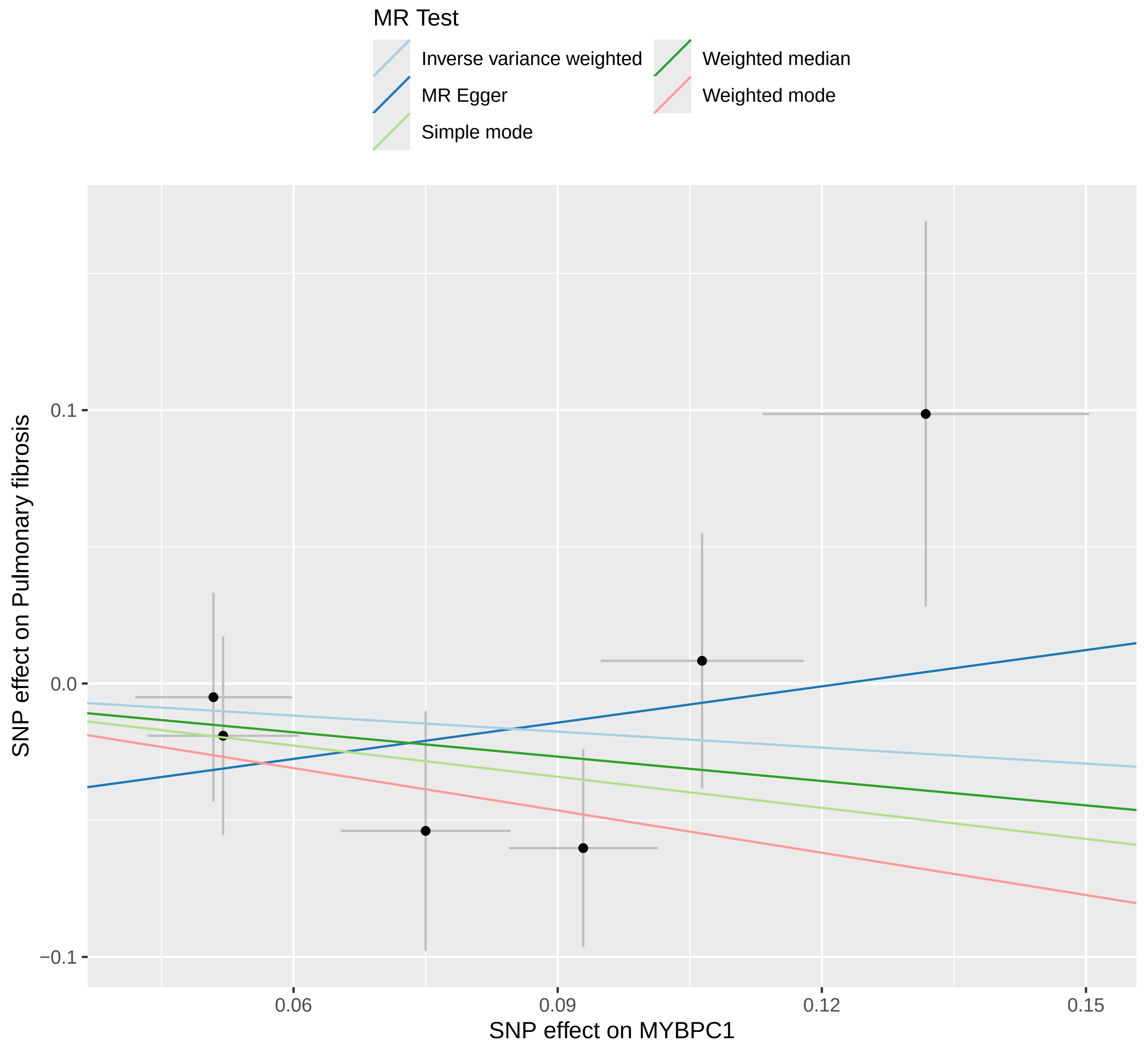
MR Test
Inverse variance weighted
MR Egger
Simple mode
Weighted median
Weighted mode
SNP effect on Pulmonary fibrosis
0.1
0.0
−0.1
0.06
0.09
0.12
0.15
SNP effect on MYBPC1

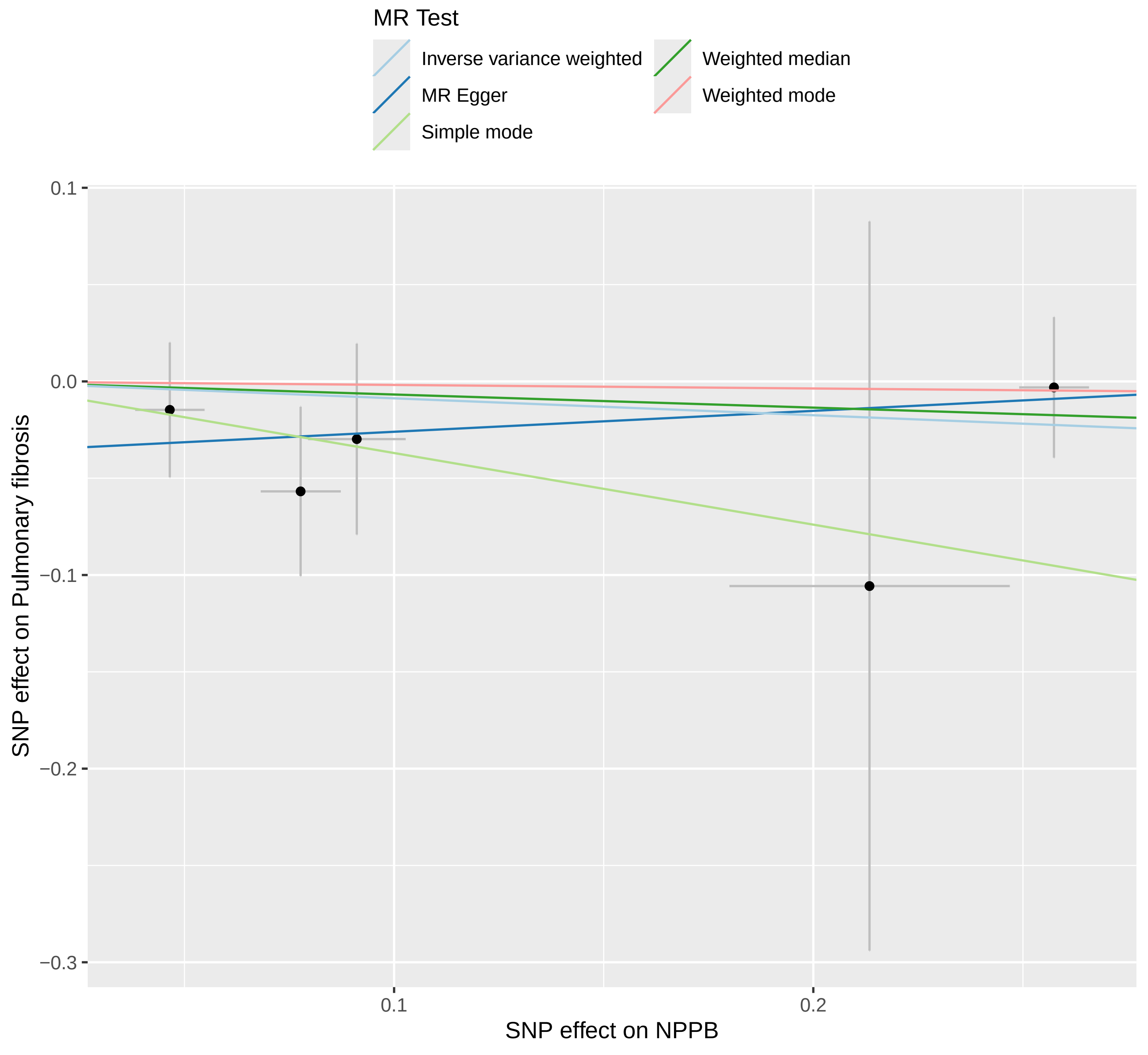

MR Test
Inverse variance weighted
MR Egger
Simple mode
Weighted median
Weighted mode
0.1
0.0
−0.1
−0.2
−0.3
SNP effect on Pulmonary fibrosis
0.1
0.2
SNP effect on NPPB

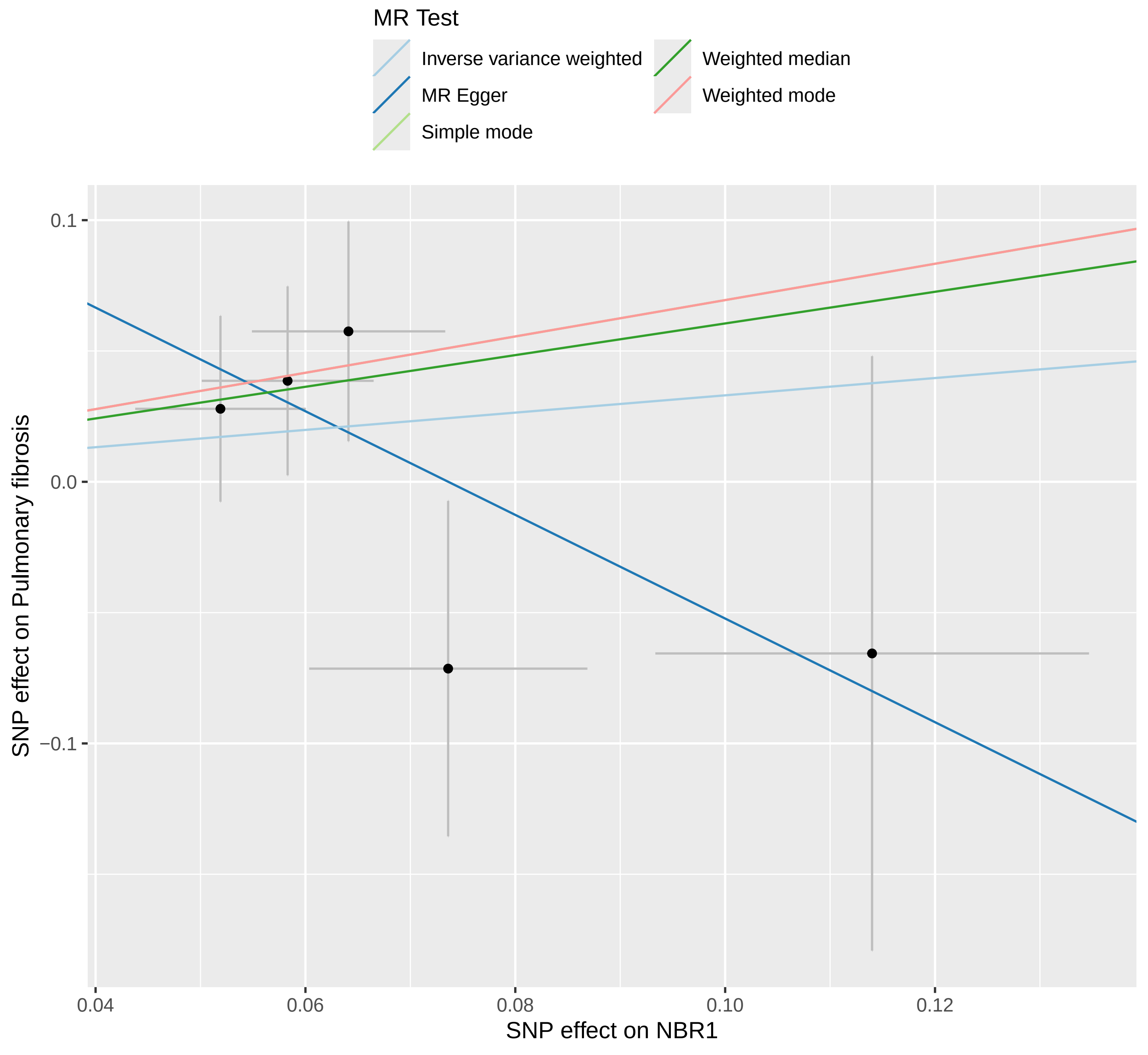

MR Test
Inverse variance weighted
MR Egger
Simple mode
Weighted median
Weighted mode
0.1
0.0
−0.1
0.04
0.06
0.08
0.10
0.12
SNP effect on NBR1
SNP effect on Pulmonary fibrosis

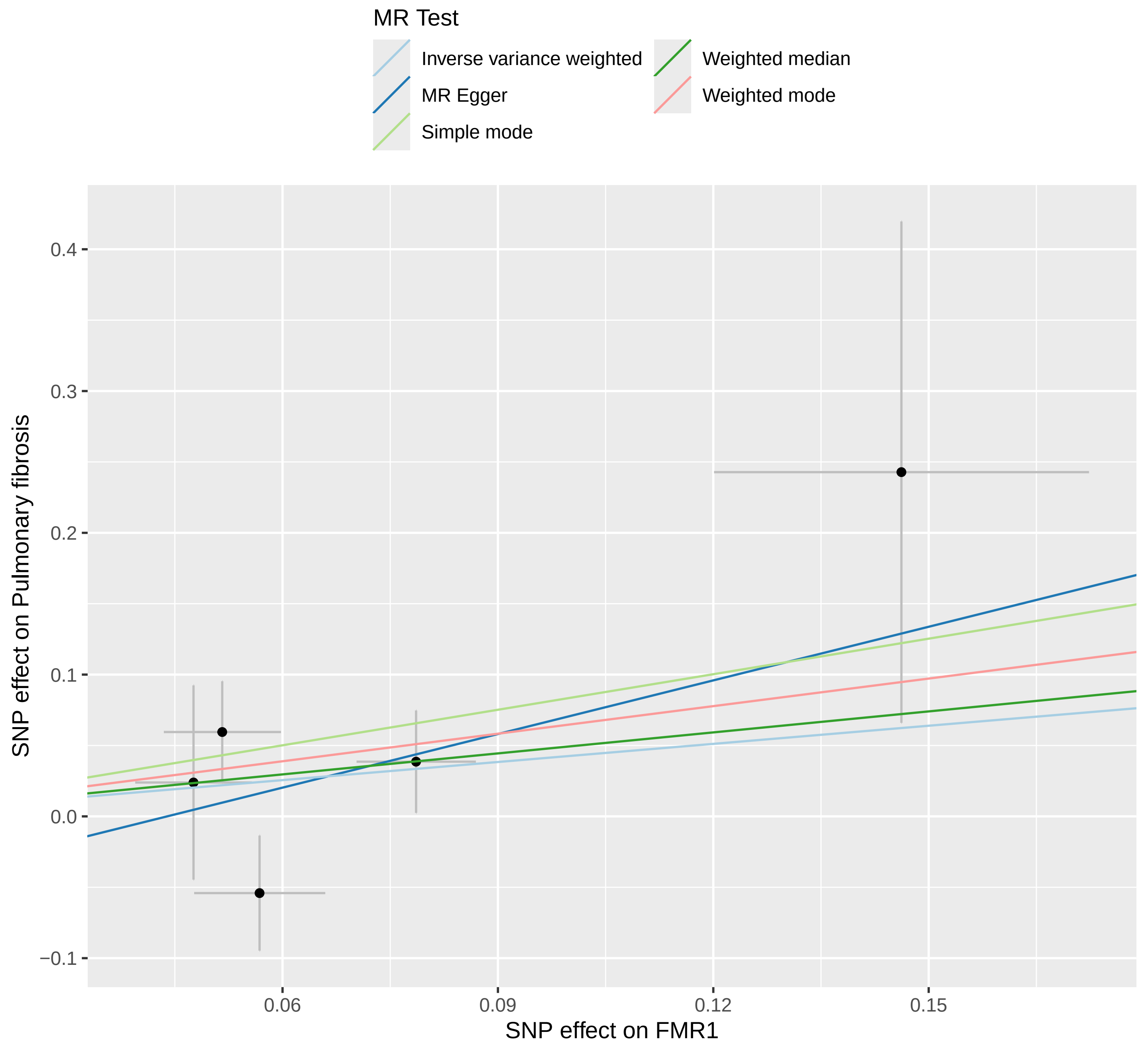
MR Test
Inverse variance weighted
MR Egger
Simple mode
Weighted median
Weighted mode
SNP effect on Pulmonary fibrosis
0.4
0.3
0.2
0.1
0.0
−0.1
0.06
0.09
0.12
0.15
SNP effect on FMR1

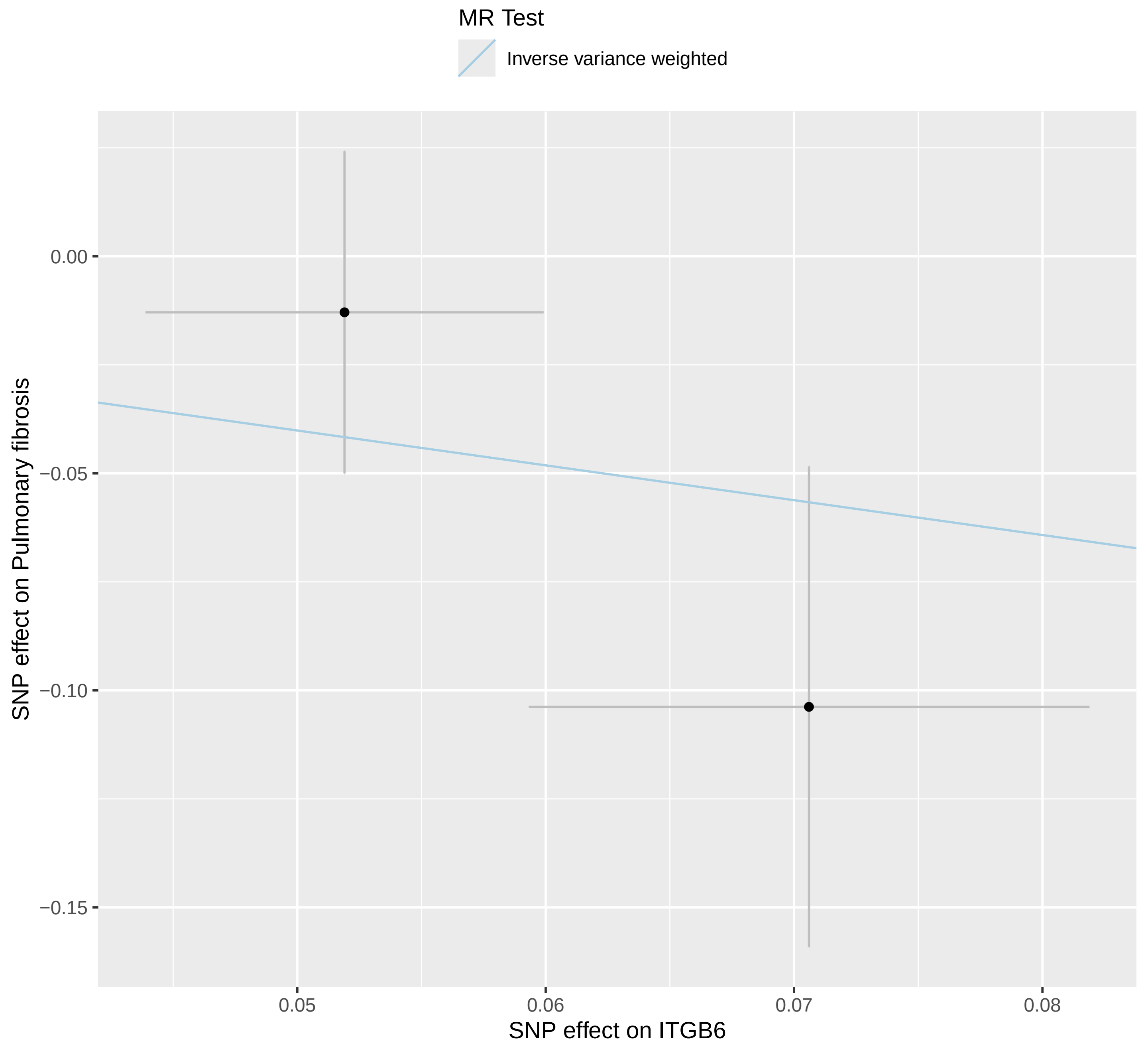

MR Test
Inverse variance weighted
0.00
−0.05
−0.10
−0.15
SNP effect on Pulmonary fibrosis
0.05
0.06
0.07
0.08
SNP effect on ITGB6

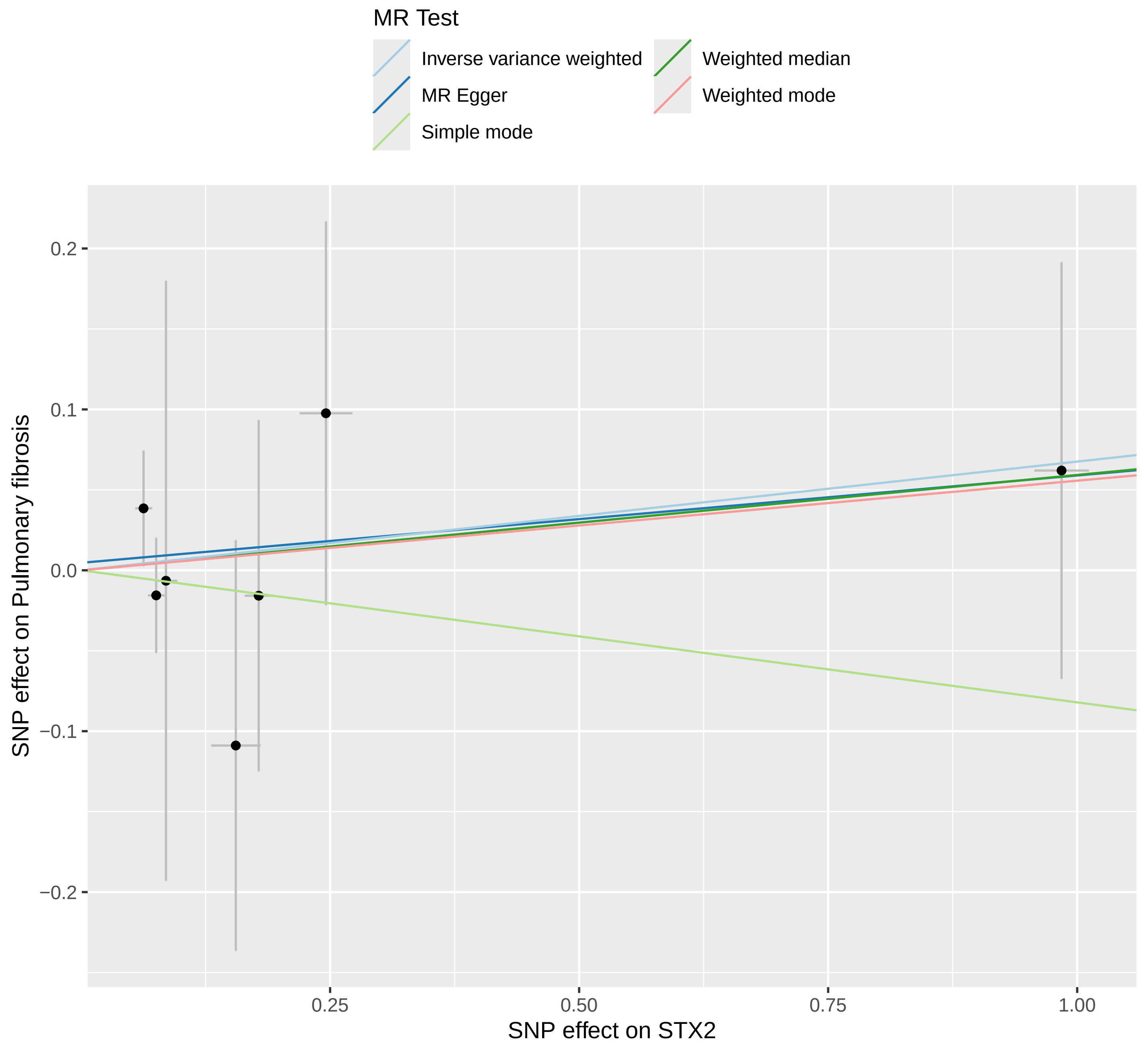

MR Test
Inverse variance weighted
MR Egger
Simple mode
Weighted median
Weighted mode
0.2
0.1
0.0
−0.1
−0.2
SNP effect on Pulmonary fibrosis
0.25
0.50
0.75
1.00
SNP effect on STX2

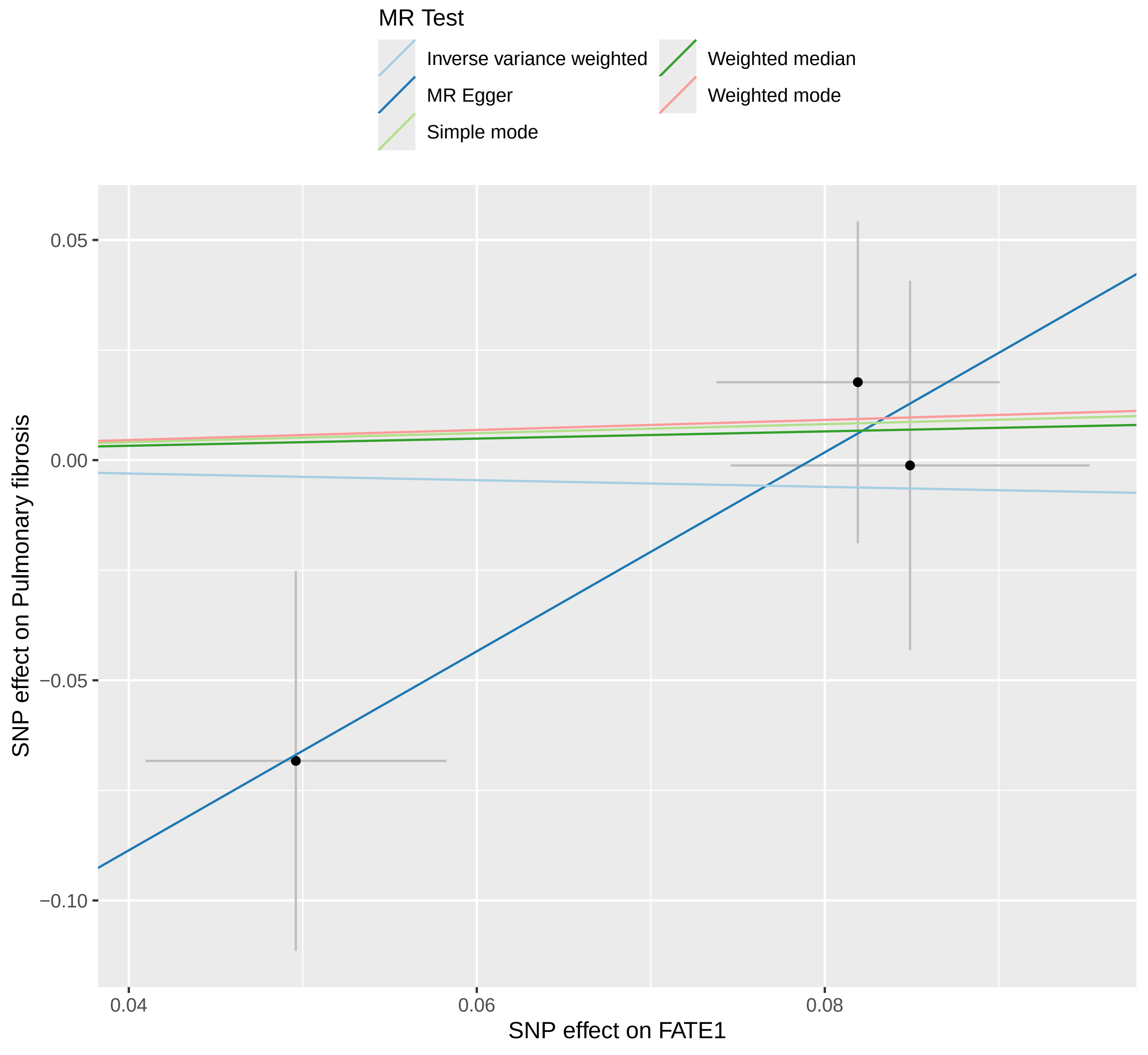
MR Test
Inverse variance weighted
MR Egger
Simple mode
Weighted median
Weighted mode
0.05
0.00
−0.05
−0.10
0.04
0.06
0.08
SNP effect on Pulmonary fibrosis
SNP effect on FATE1

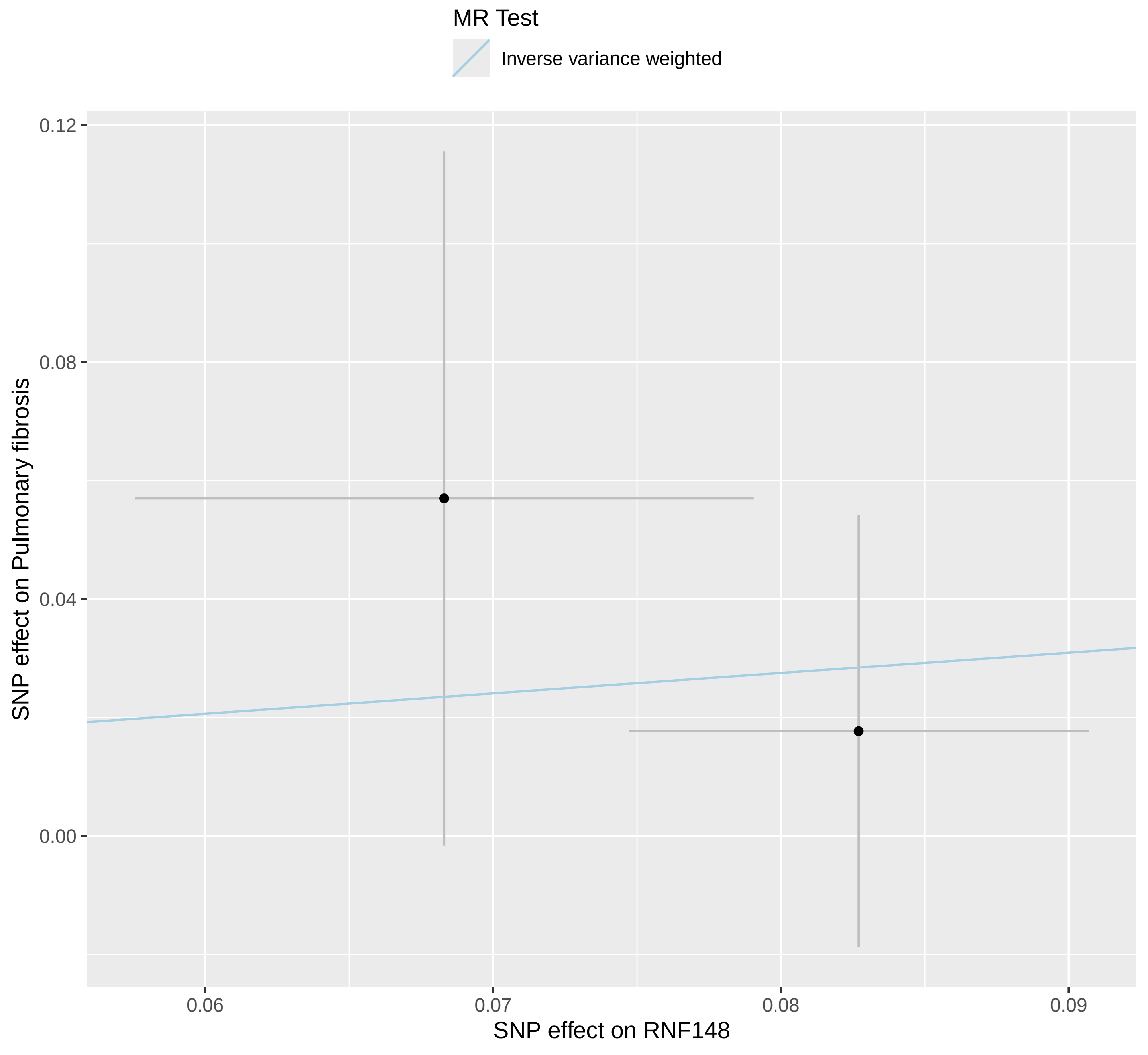

MR Test
Inverse variance weighted
0.12
0.08
0.04
0.00
SNP effect on Pulmonary fibrosis
0.06
0.07
0.08
0.09
SNP effect on RNF148

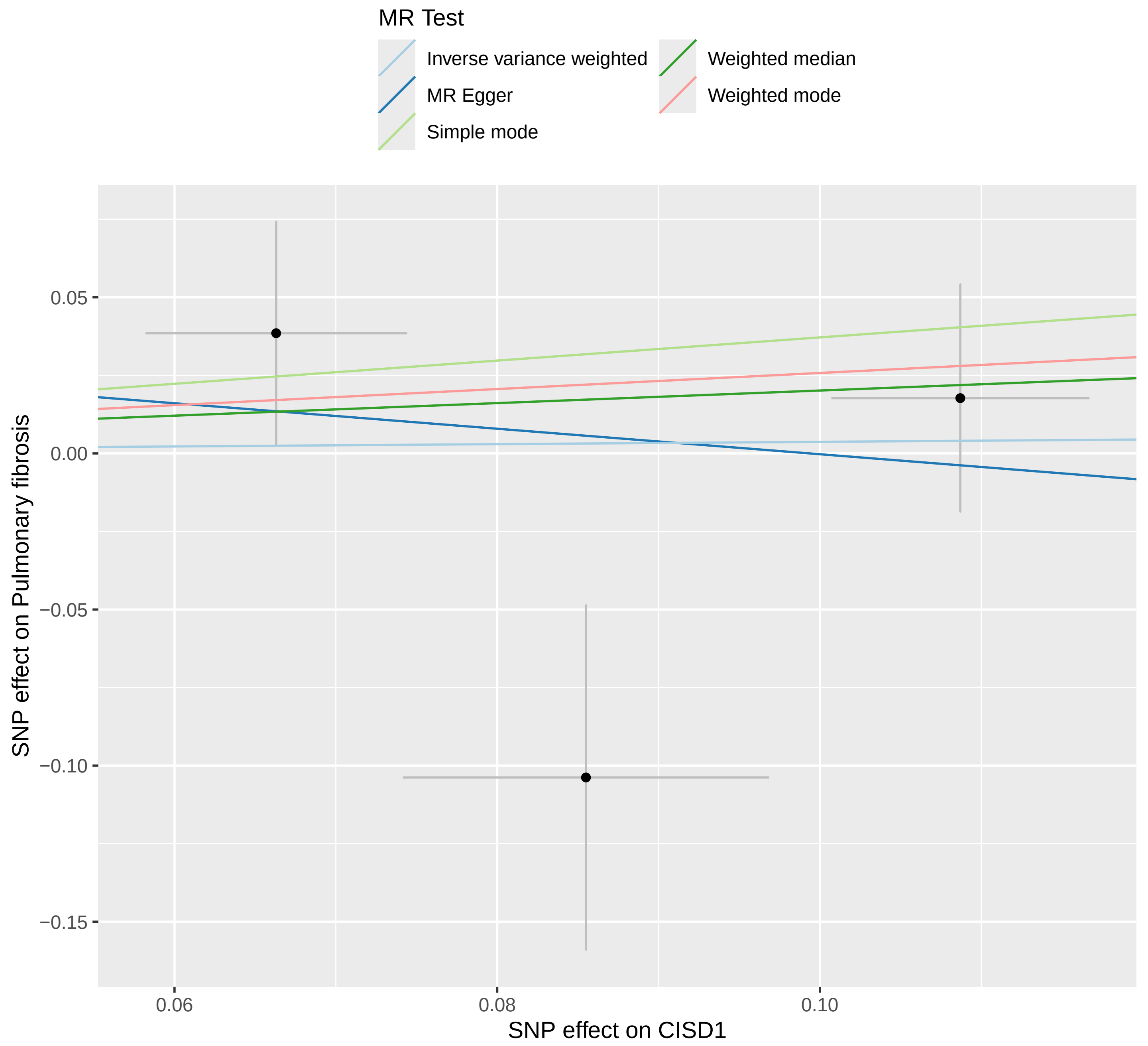
MR Test
Inverse variance weighted
MR Egger
Simple mode
Weighted median
Weighted mode
0.05
0.00
−0.05
−0.10
−0.15
SNP effect on Pulmonary fibrosis
0.06
0.08
0.10
SNP effect on CISD1

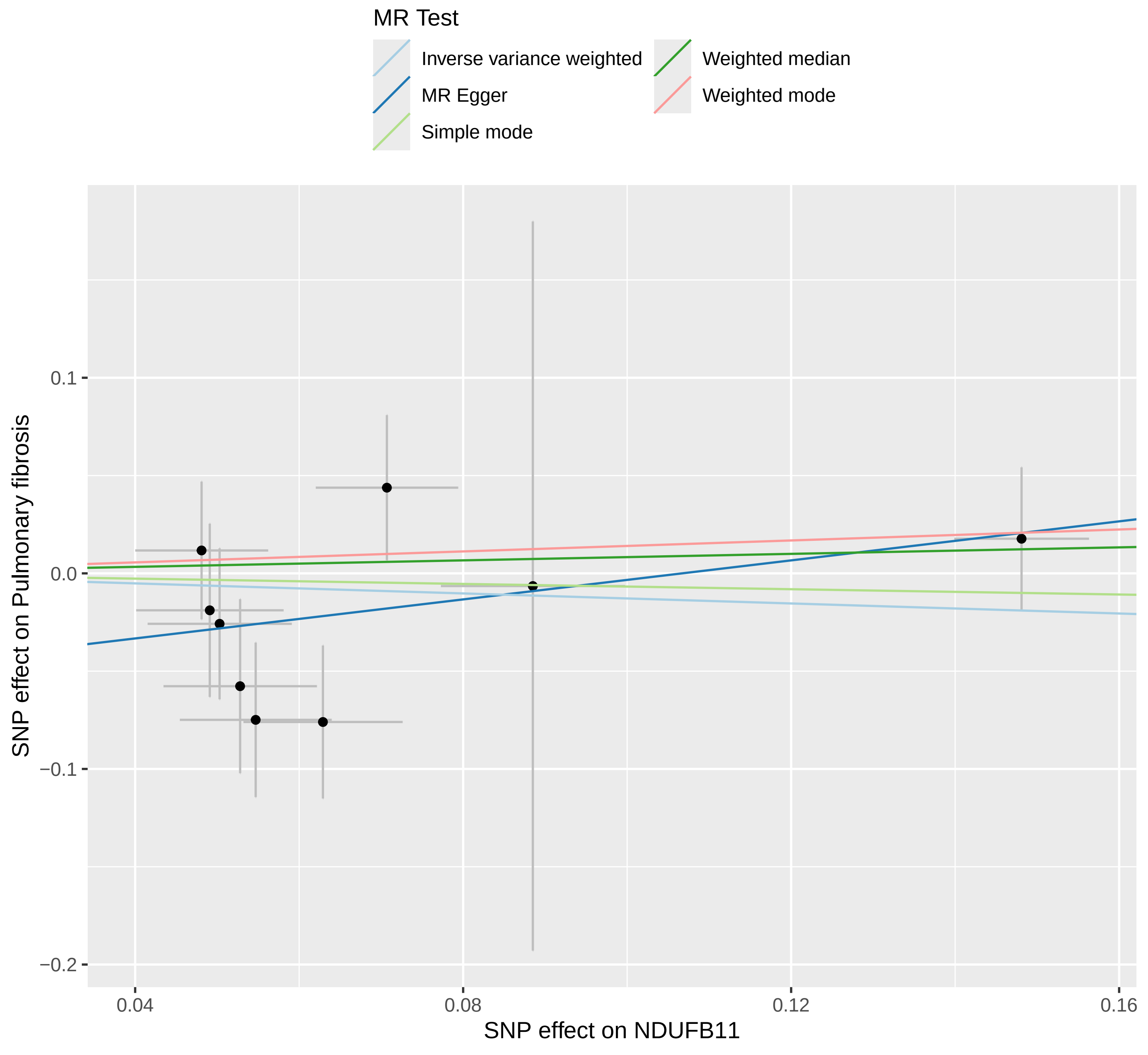

MR Test
Inverse variance weighted
MR Egger
Simple mode
Weighted median
Weighted mode
0.1
0.0
−0.1
−0.2
0.04
0.08
0.12
0.16
SNP effect on Pulmonary fibrosis
SNP effect on NDUFB11

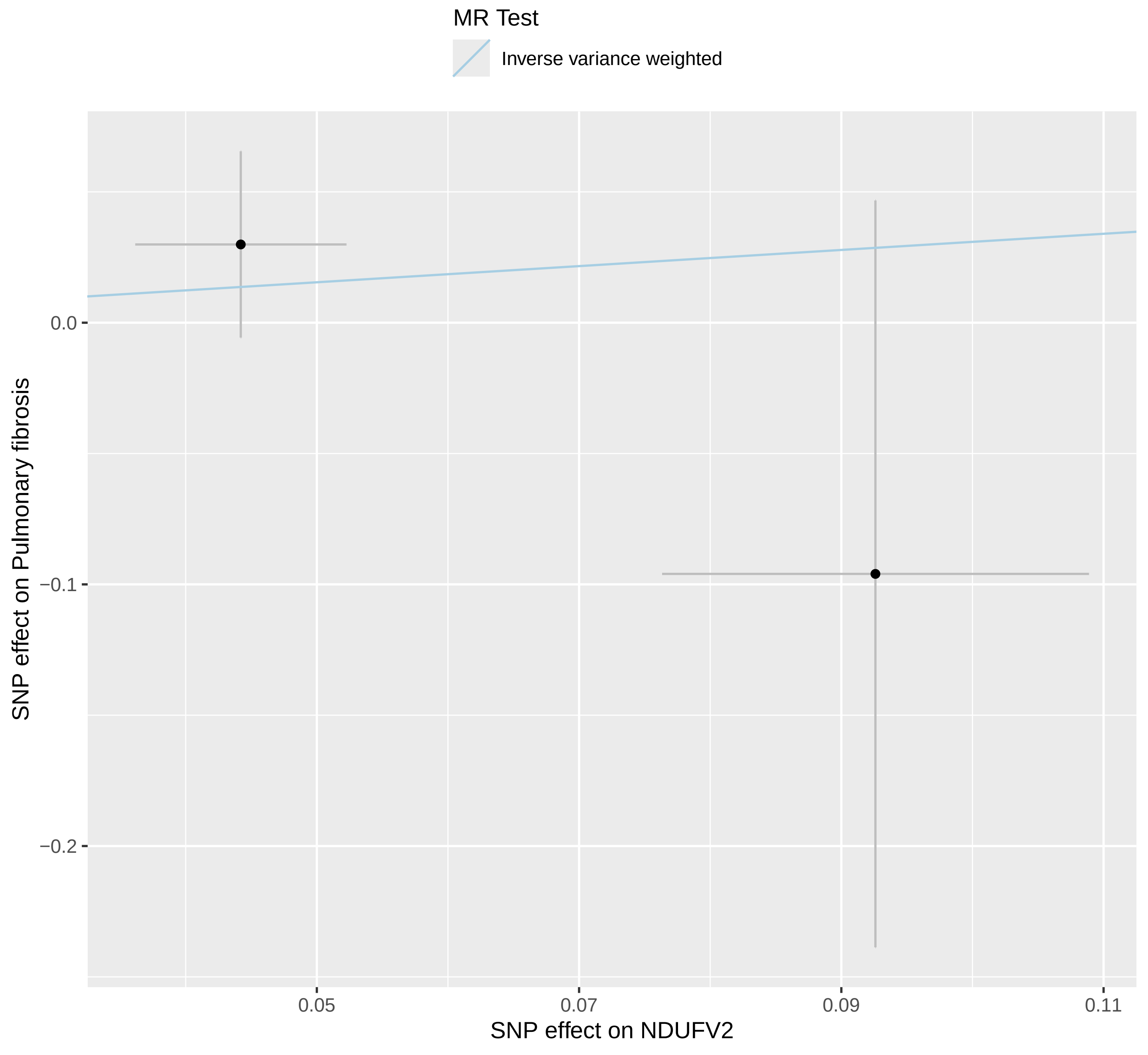
MR Test
Inverse variance weighted
0.0
−0.1
−0.2
SNP effect on Pulmonary fibrosis
0.05
0.07
0.09
0.11
SNP effect on NDUFV2

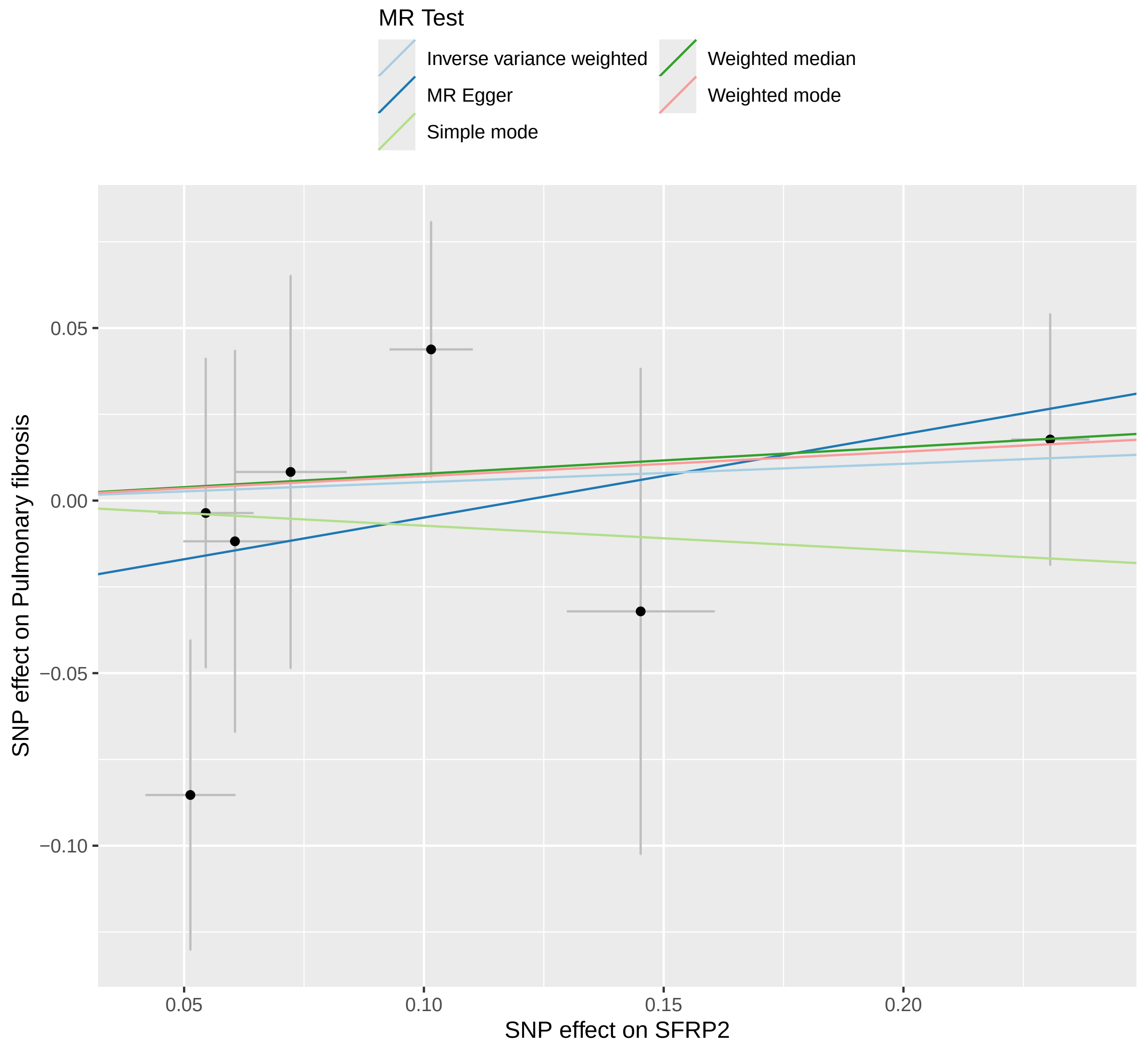
MR Test
Inverse variance weighted
MR Egger
Simple mode
Weighted median
Weighted mode
SNP effect on Pulmonary fibrosis
0.05
0.00
−0.05
−0.10
0.05
0.10
0.15
0.20
SNP effect on SFRP2

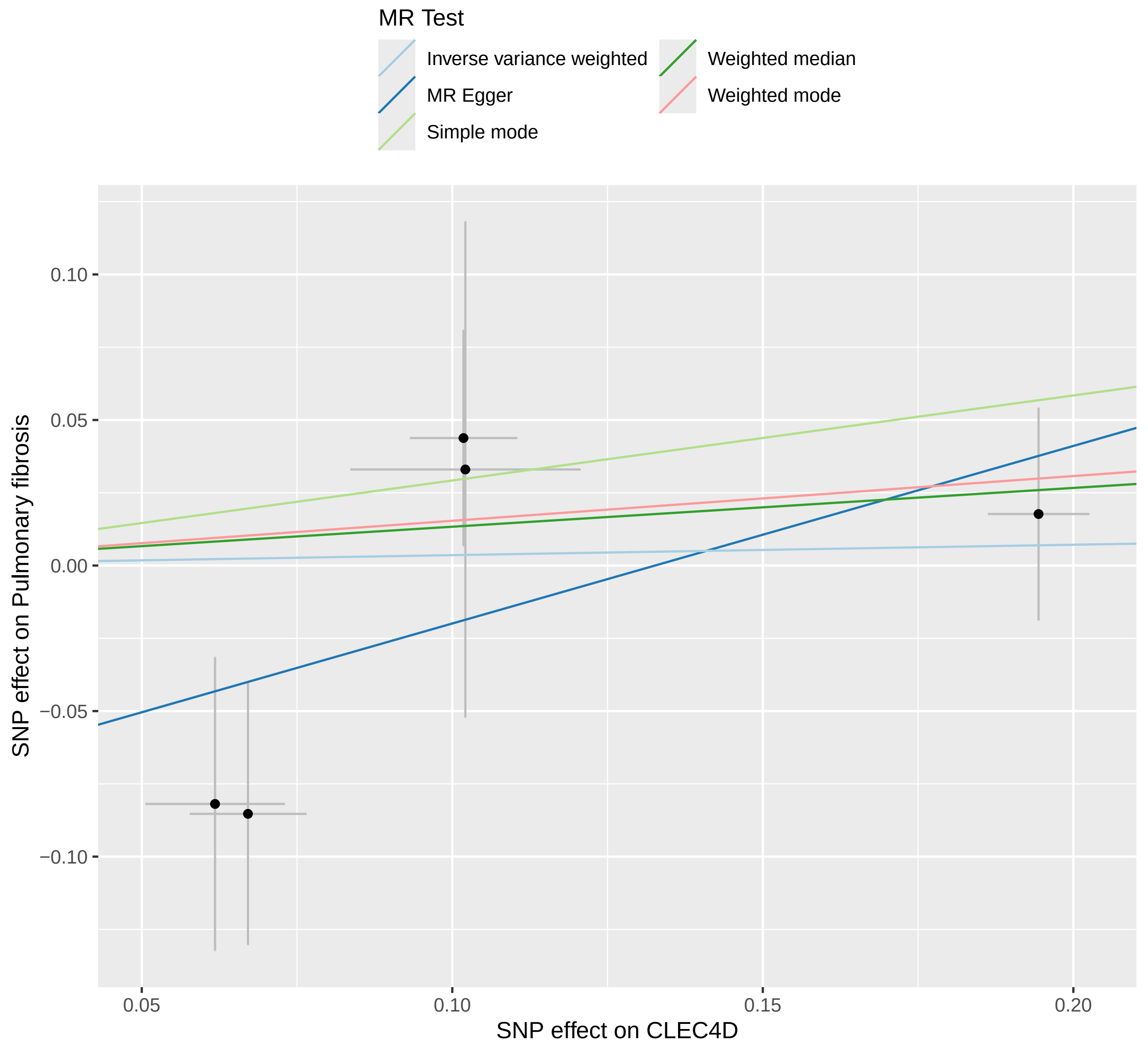

MR Test
Inverse variance weighted
MR Egger
Simple mode
Weighted median
Weighted mode
0.10
0.05
0.00
−0.05
−0.10
SNP effect on Pulmonary fibrosis
0.05
0.10
0.15
0.20
SNP effect on CLEC4D

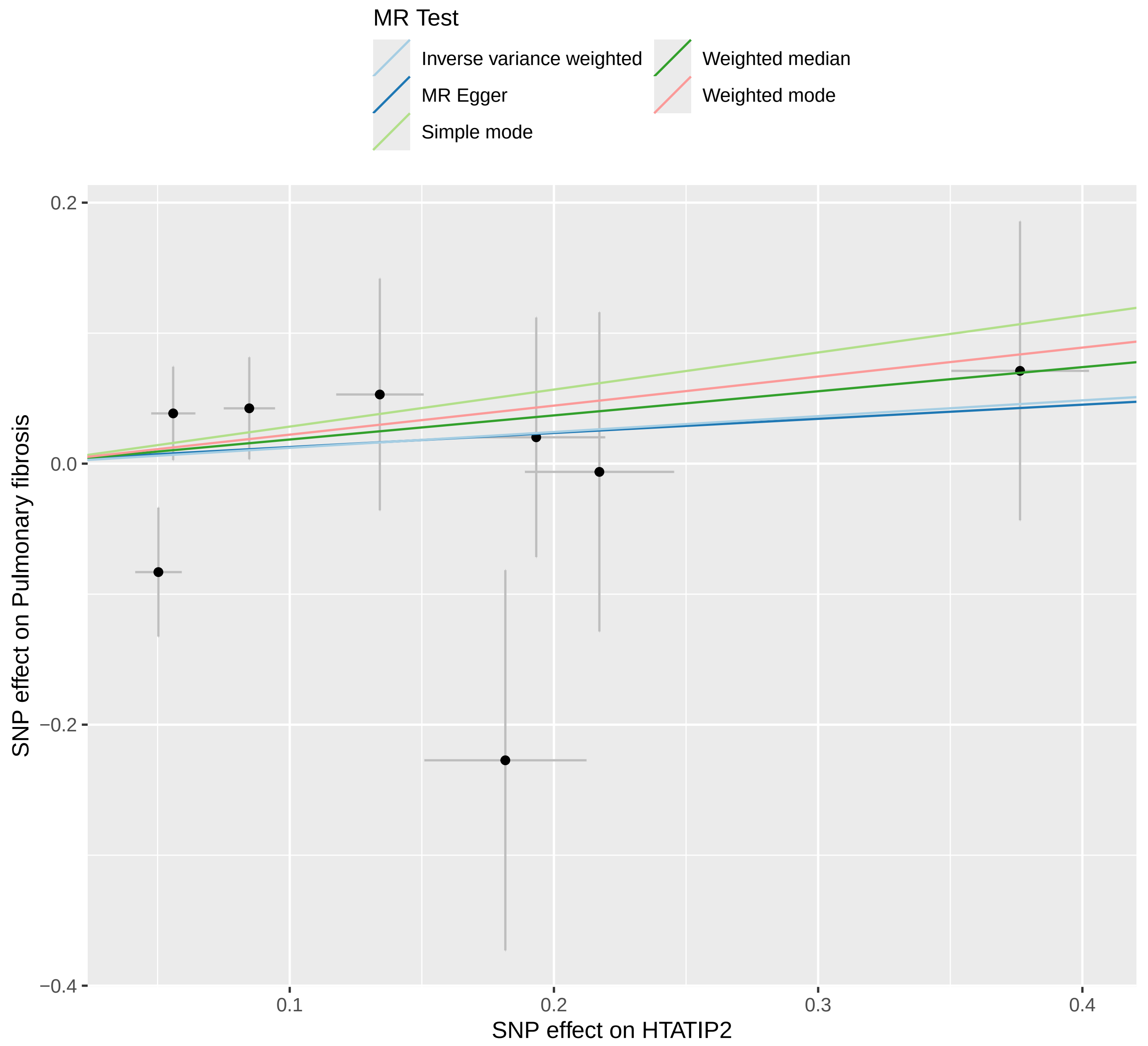

MR Test
Inverse variance weighted
MR Egger
Simple mode
Weighted median
Weighted mode
0.2
0.0
−0.2
−0.4
0.1
0.2
0.3
0.4
SNP effect on HTATIP2
SNP effect on Pulmonary fibrosis

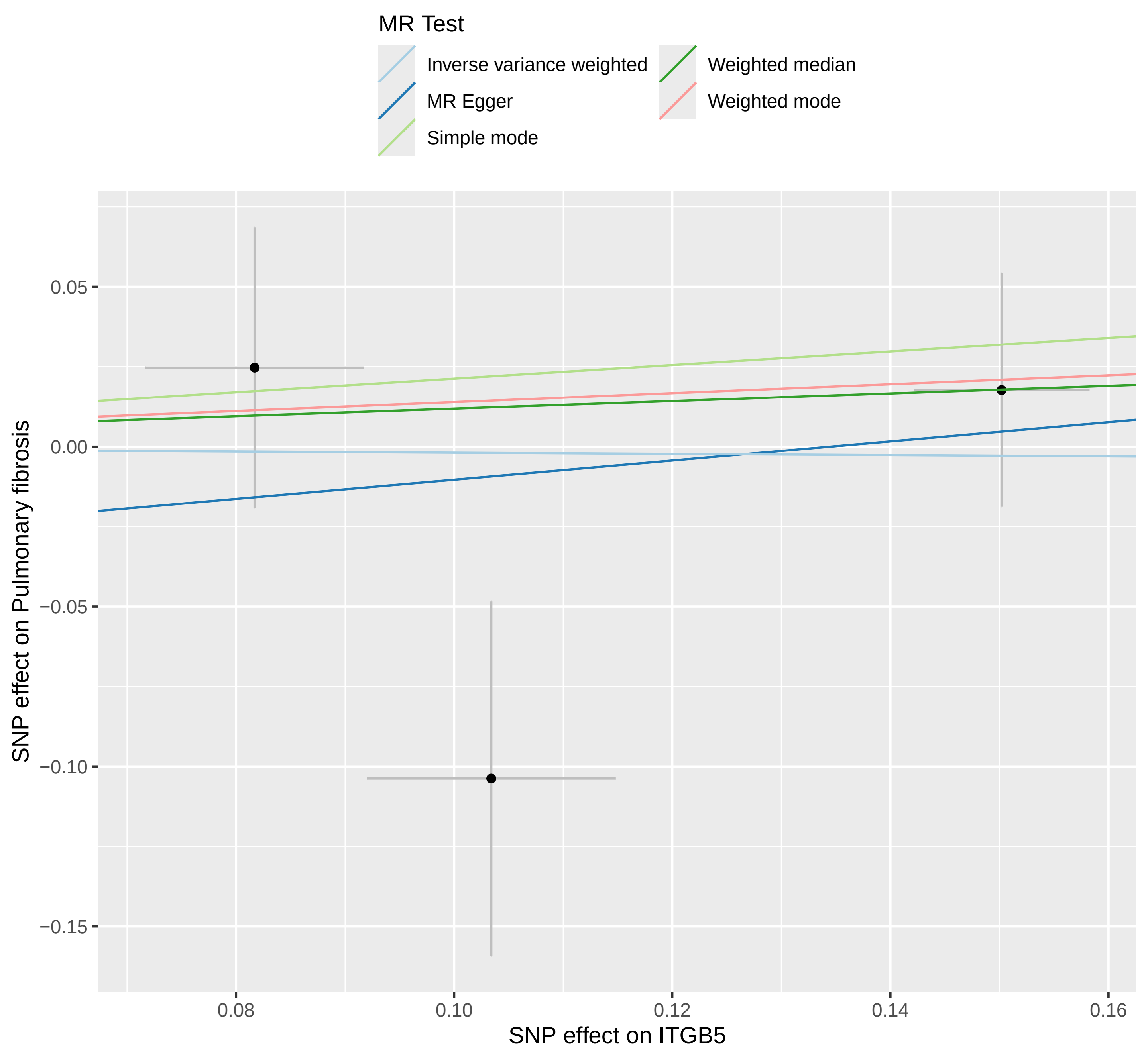
MR Test
Inverse variance weighted
MR Egger
Simple mode
Weighted median
Weighted mode
0.05
0.00
−0.05
−0.10
−0.15
0.08
0.10
0.12
0.14
0.16
SNP effect on Pulmonary fibrosis
SNP effect on ITGB5

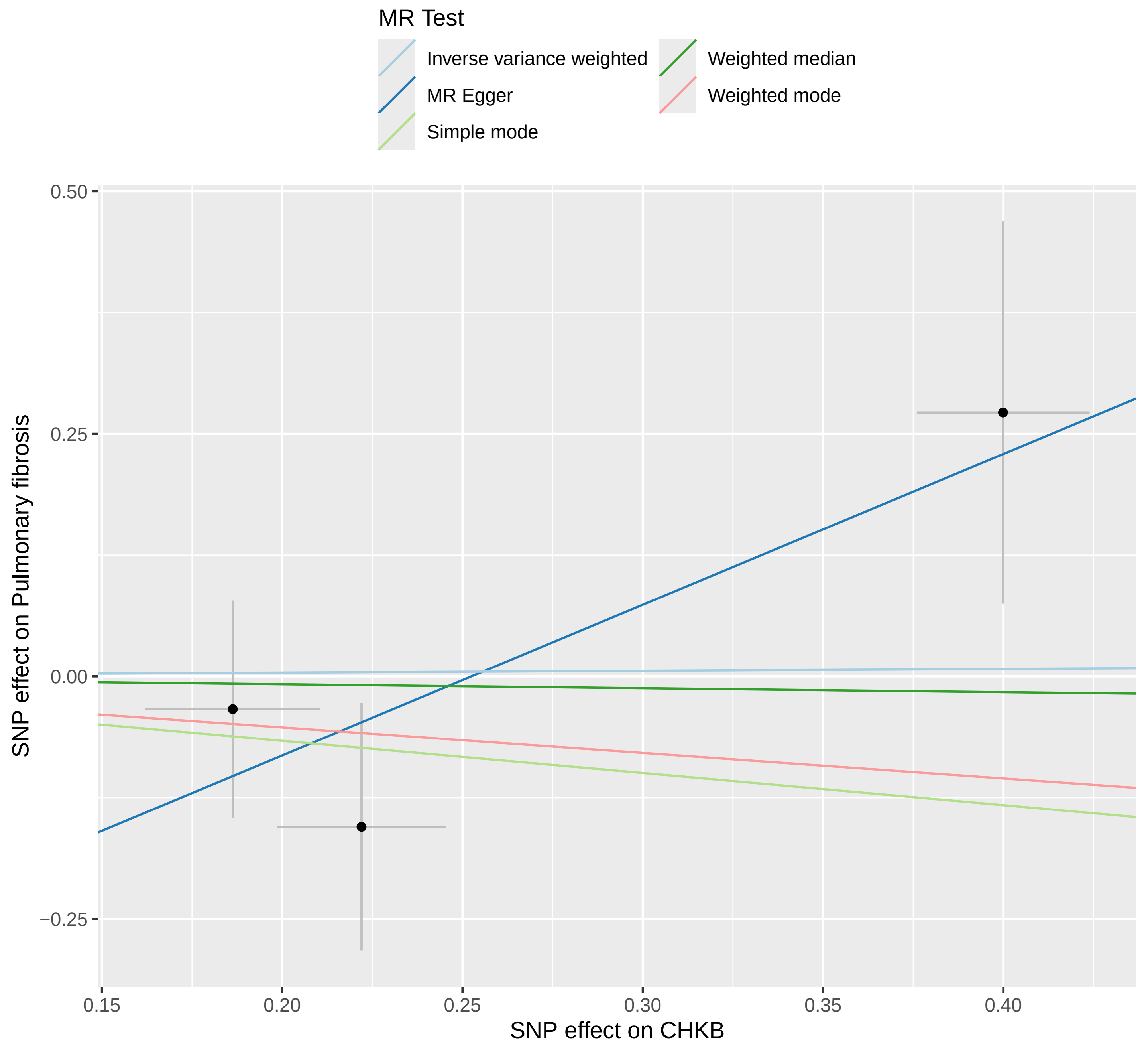
MR Test
Inverse variance weighted
MR Egger
Simple mode
Weighted median
Weighted mode
0.50
0.25
0.00
−0.25
SNP effect on Pulmonary fibrosis
0.15
0.20
0.25
0.30
0.35
0.40
SNP effect on CHKB

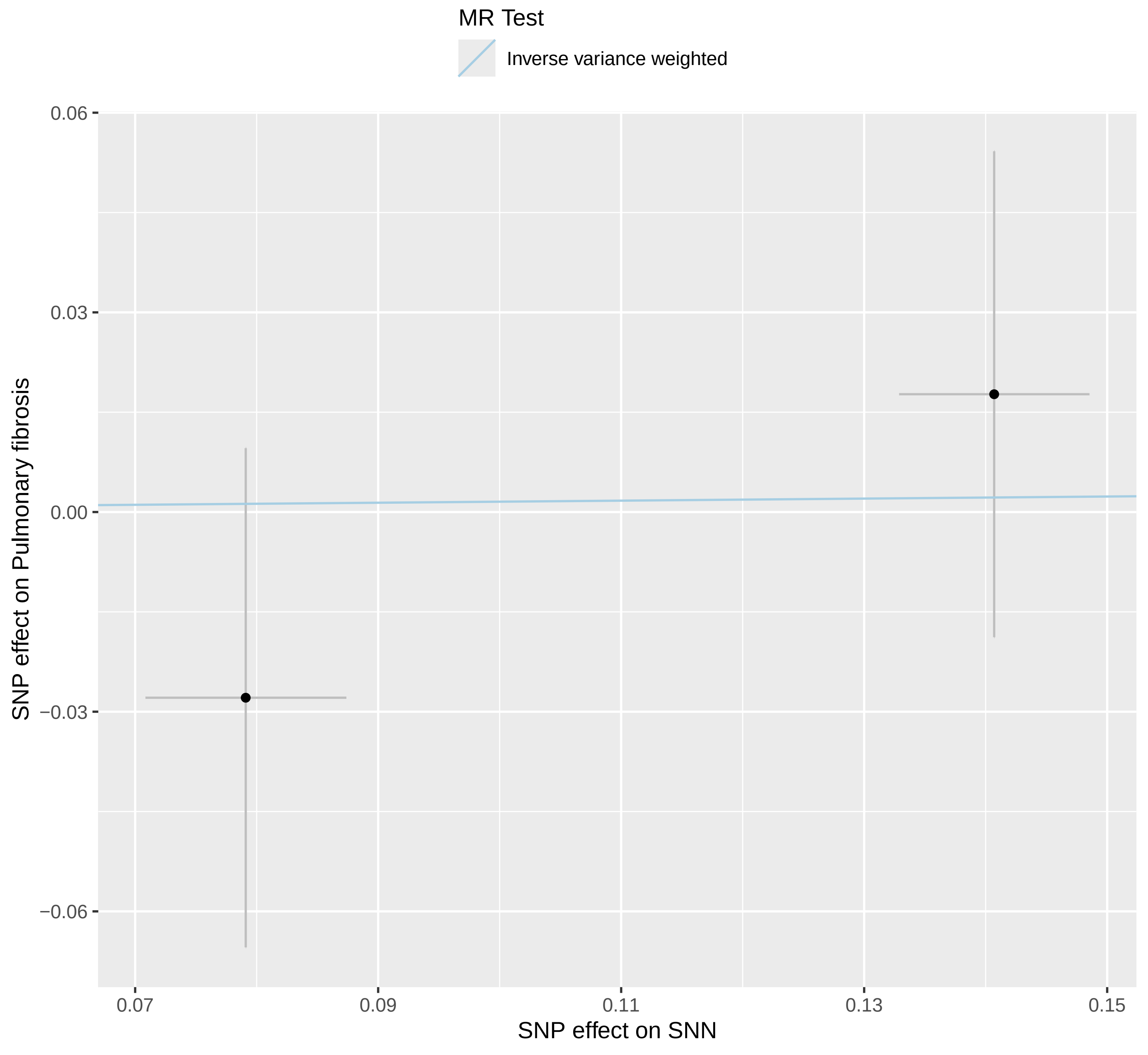

MR Test
Inverse variance weighted
0.06
0.03
0.00
−0.03
−0.06
SNP effect on Pulmonary fibrosis
0.07
0.09
0.11
0.13
0.15
SNP effect on SNN

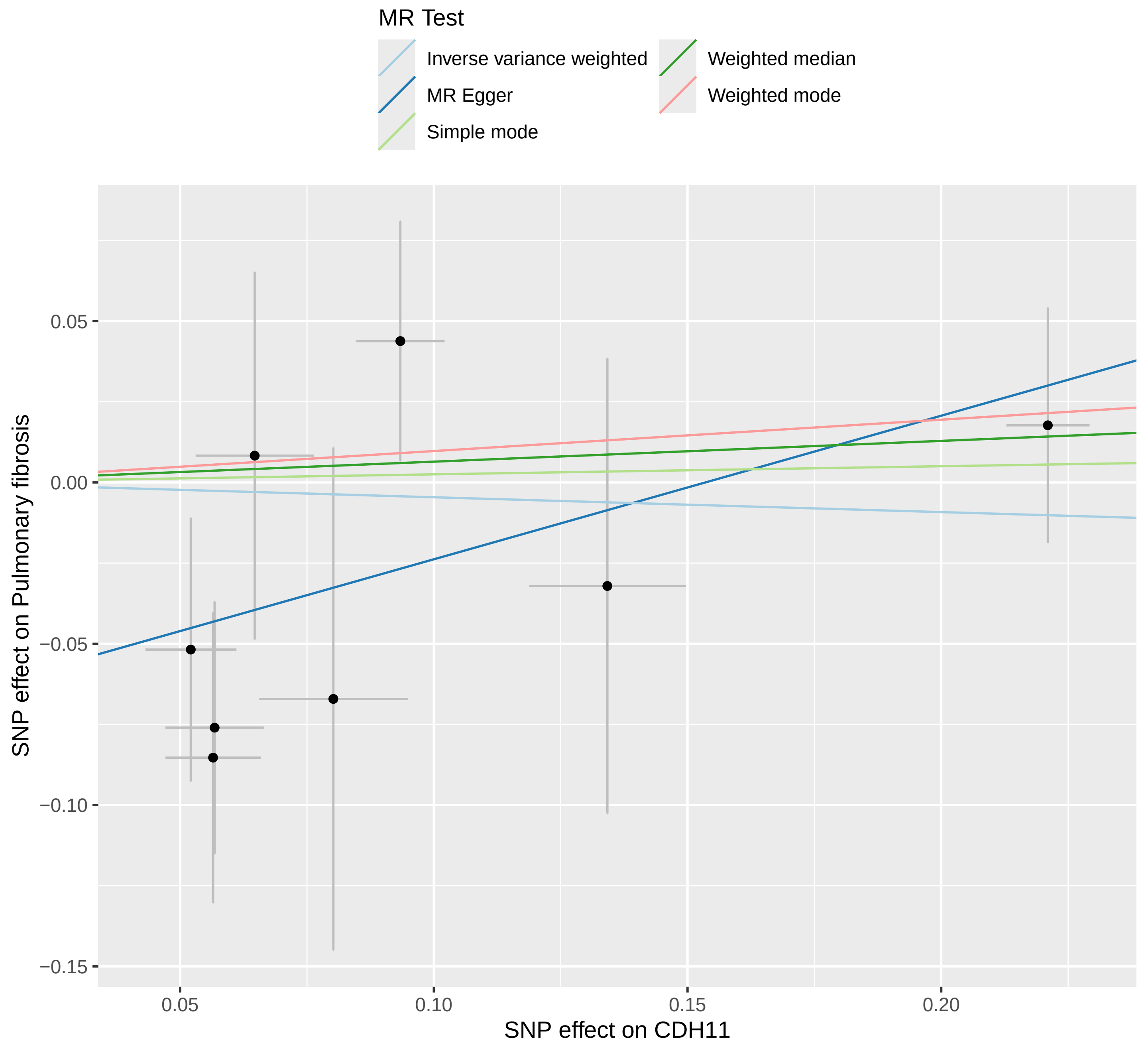
MR Test
Inverse variance weighted
MR Egger
Simple mode
Weighted median
Weighted mode
0.05
0.00
−0.05
−0.10
−0.15
SNP effect on Pulmonary fibrosis
0.05
0.10
0.15
0.20
SNP effect on CDH11

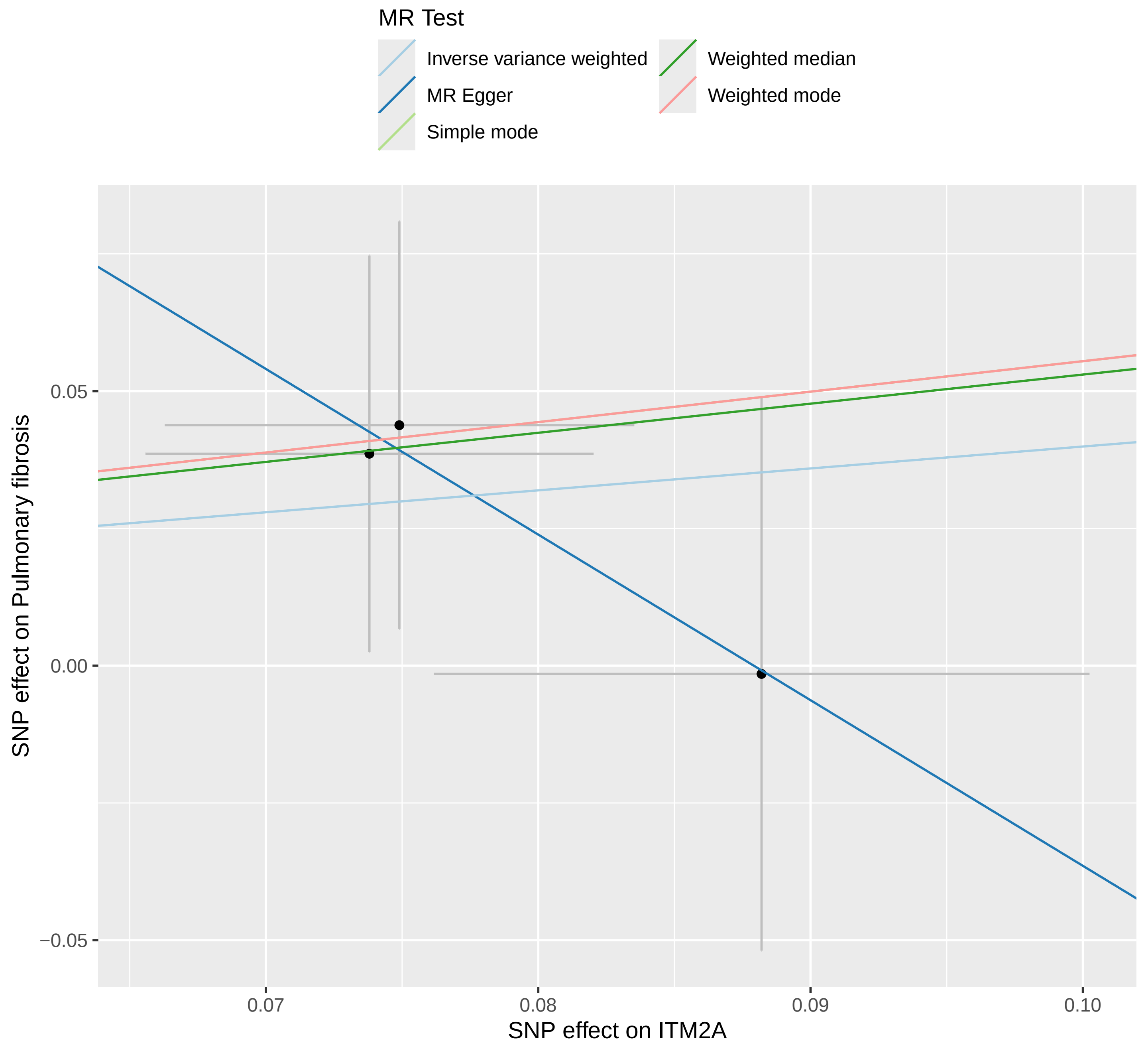

MR Test
Inverse variance weighted
MR Egger
Simple mode
Weighted median
Weighted mode
0.05
0.00
−0.05
SNP effect on Pulmonary fibrosis
0.07
0.08
0.09
0.10
SNP effect on ITM2A

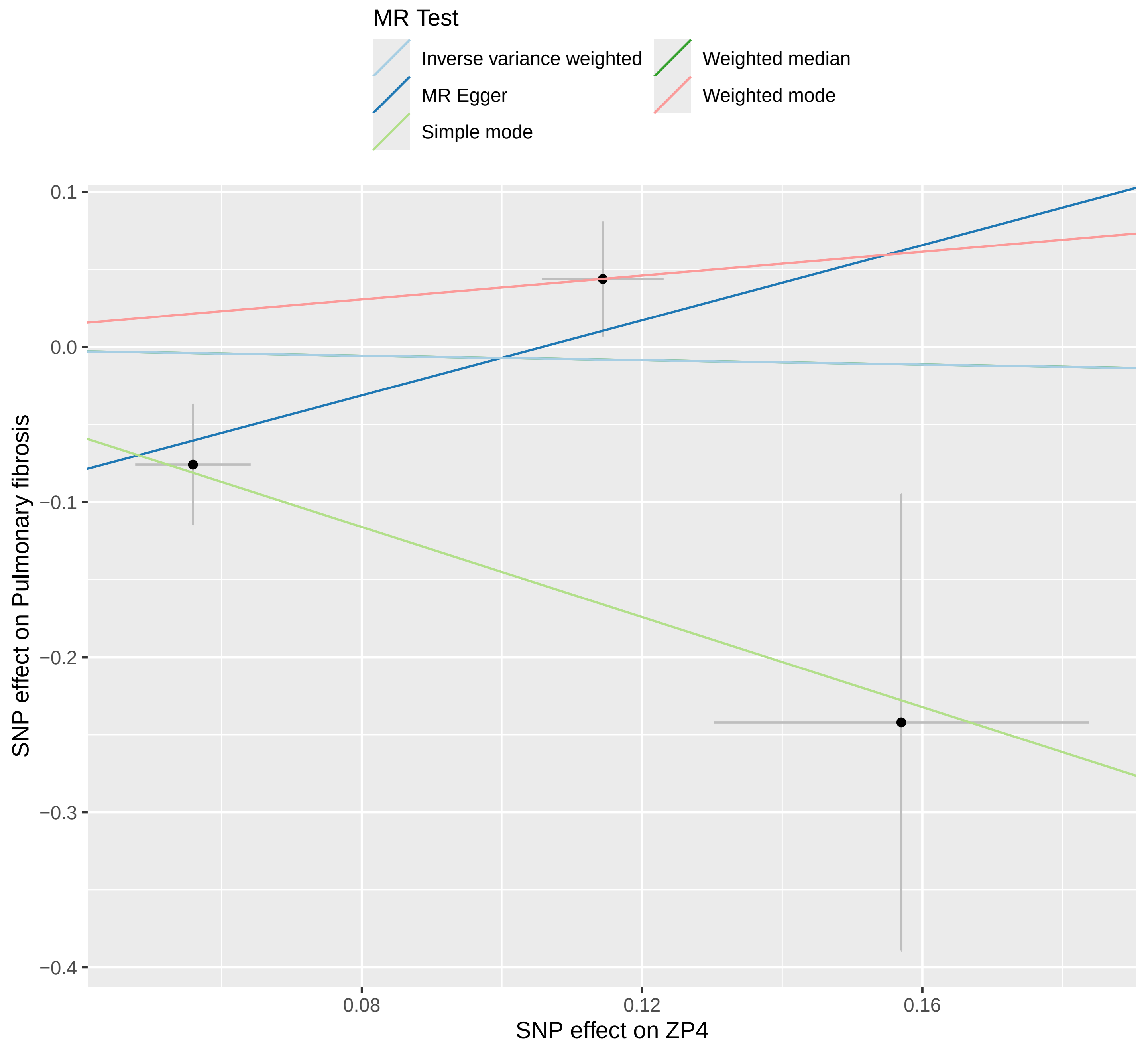
MR Test
Inverse variance weighted
MR Egger
Simple mode
Weighted median
Weighted mode
0.1
0.0
−0.1
−0.2
−0.3
−0.4
0.08
0.12
0.16
SNP effect on Pulmonary fibrosis
SNP effect on ZP4

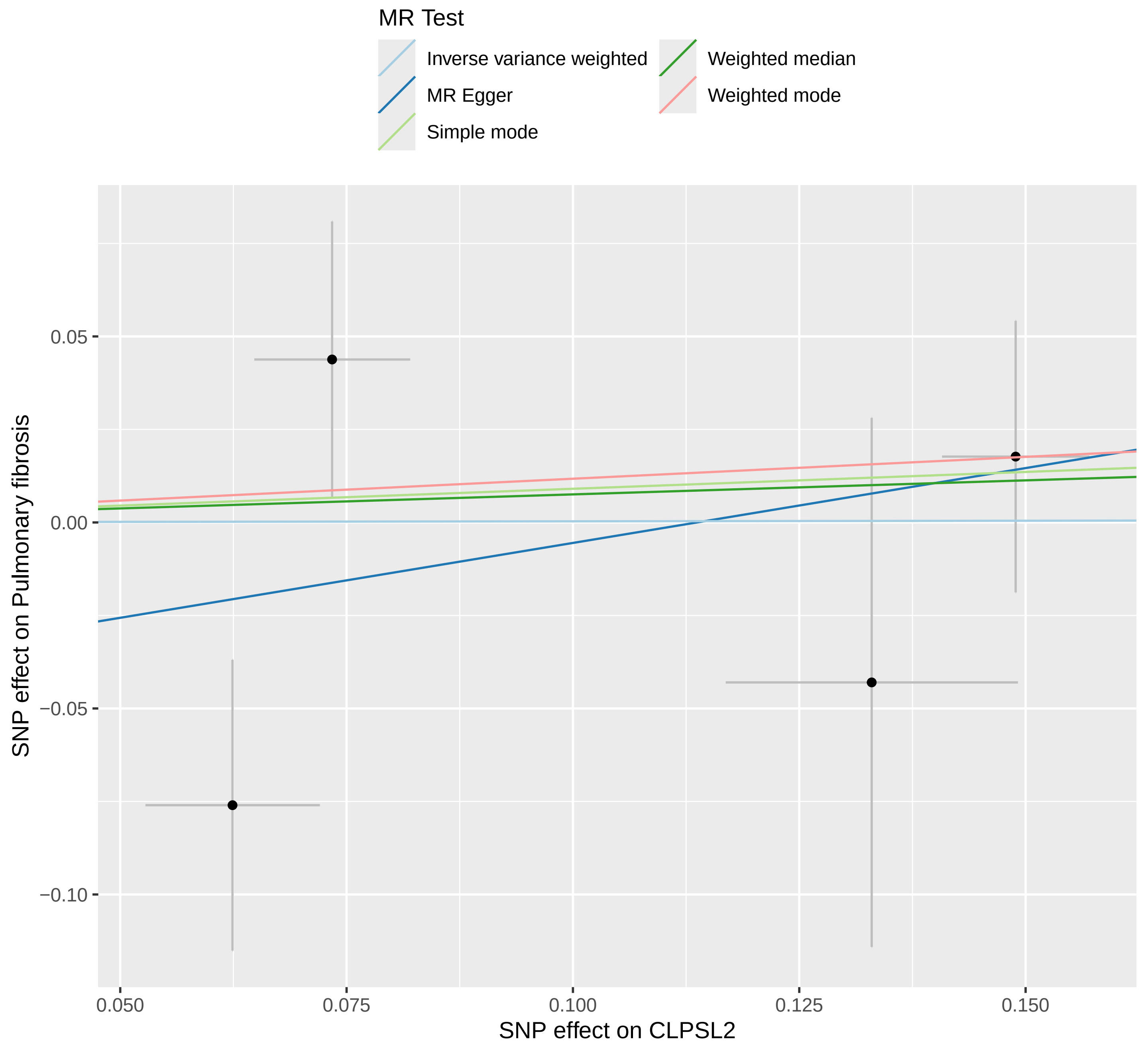

MR Test
Inverse variance weighted
MR Egger
Simple mode
Weighted median
Weighted mode
0.05
0.00
−0.05
−0.10
SNP effect on Pulmonary fibrosis
0.050
0.075
0.100
0.125
0.150
SNP effect on CLPSL2

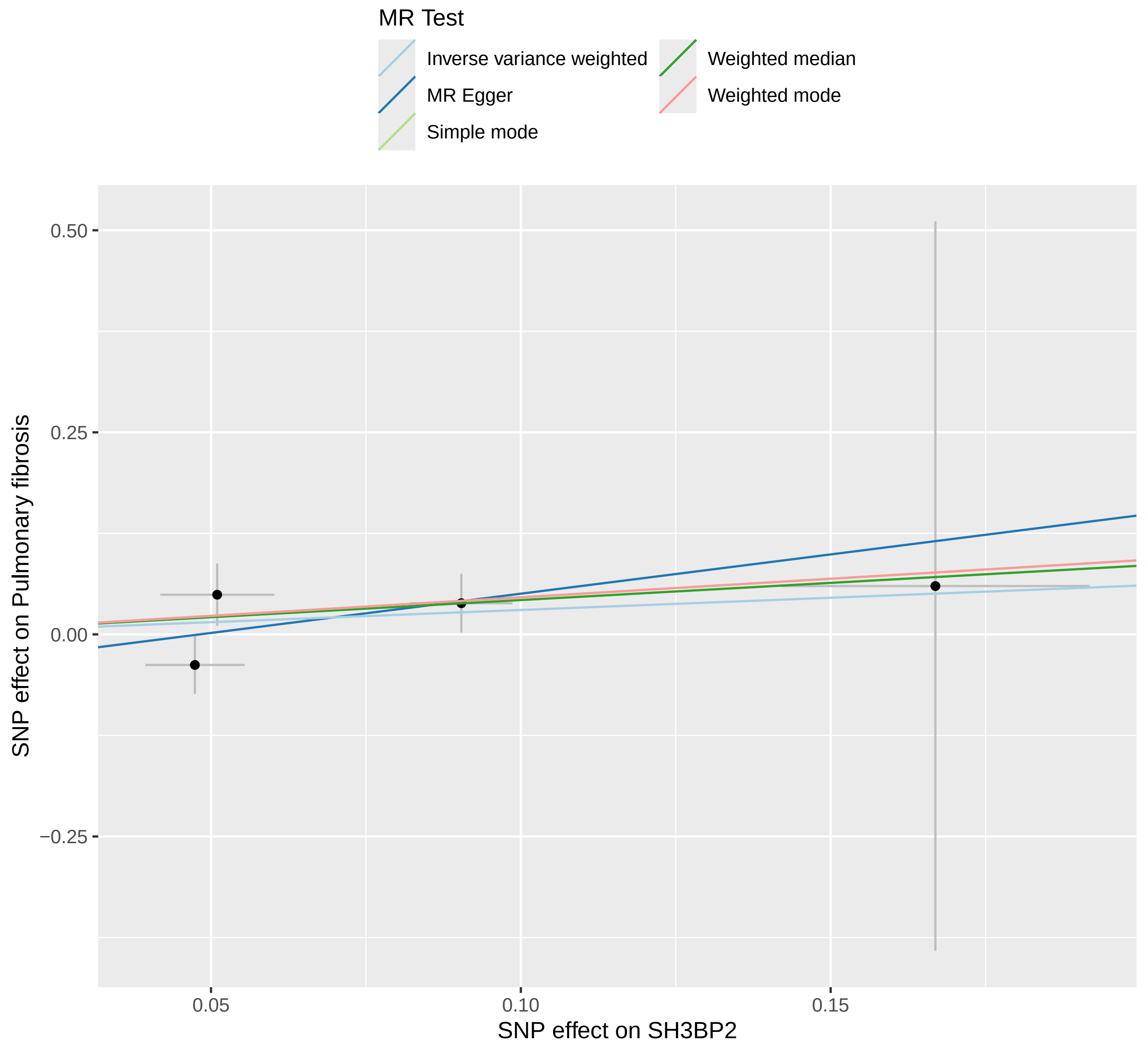

MR Test
Inverse variance weighted
MR Egger
Simple mode
Weighted median
Weighted mode
0.50
0.25
0.00
−0.25
0.05
0.10
0.15
SNP effect on Pulmonary fibrosis
SNP effect on SH3BP2

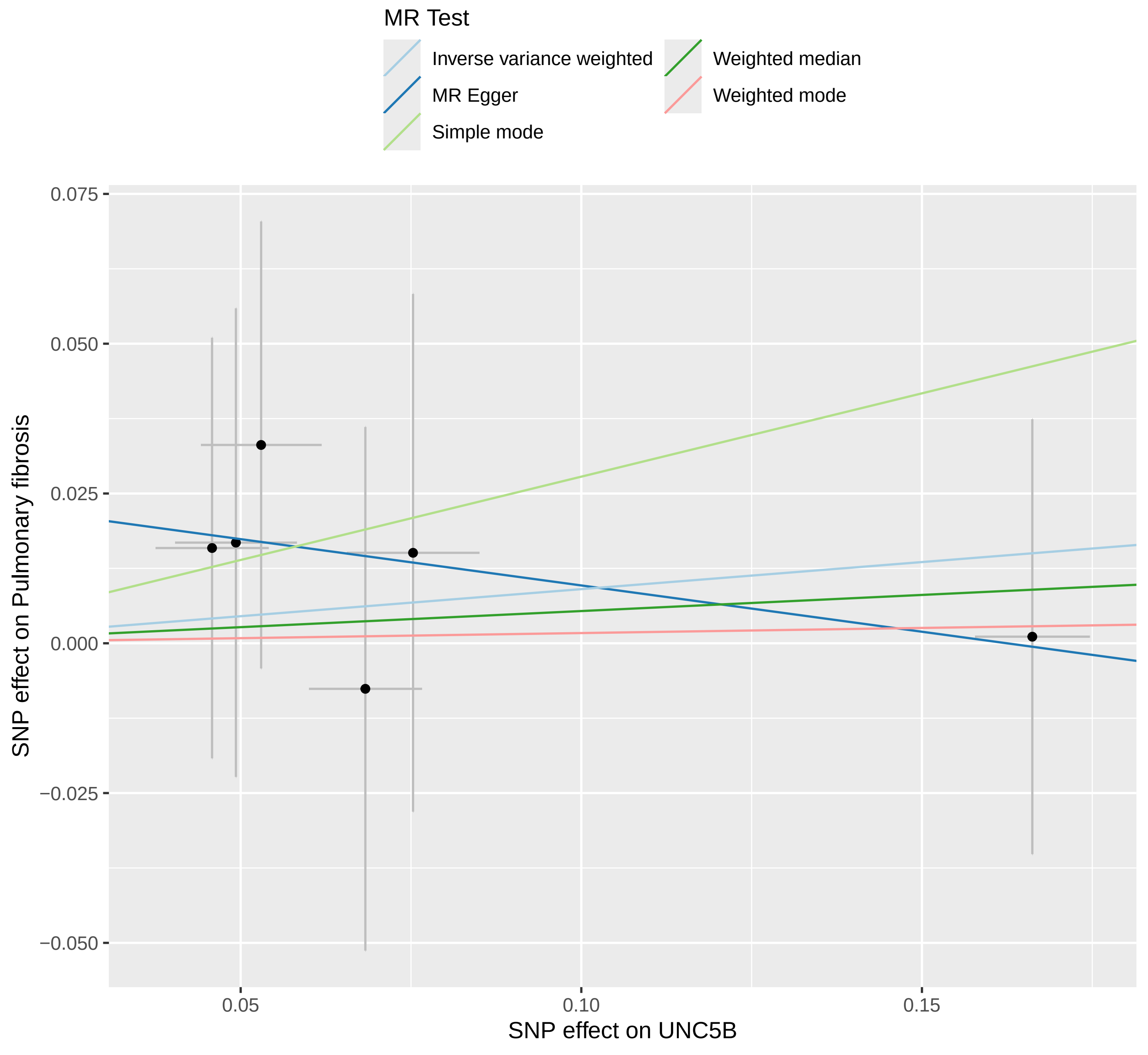
MR Test
Inverse variance weighted
MR Egger
Simple mode
Weighted median
Weighted mode
0.075
0.050
0.025
0.000
−0.025
−0.050
SNP effect on Pulmonary fibrosis
0.05
0.10
0.15
SNP effect on UNC5B

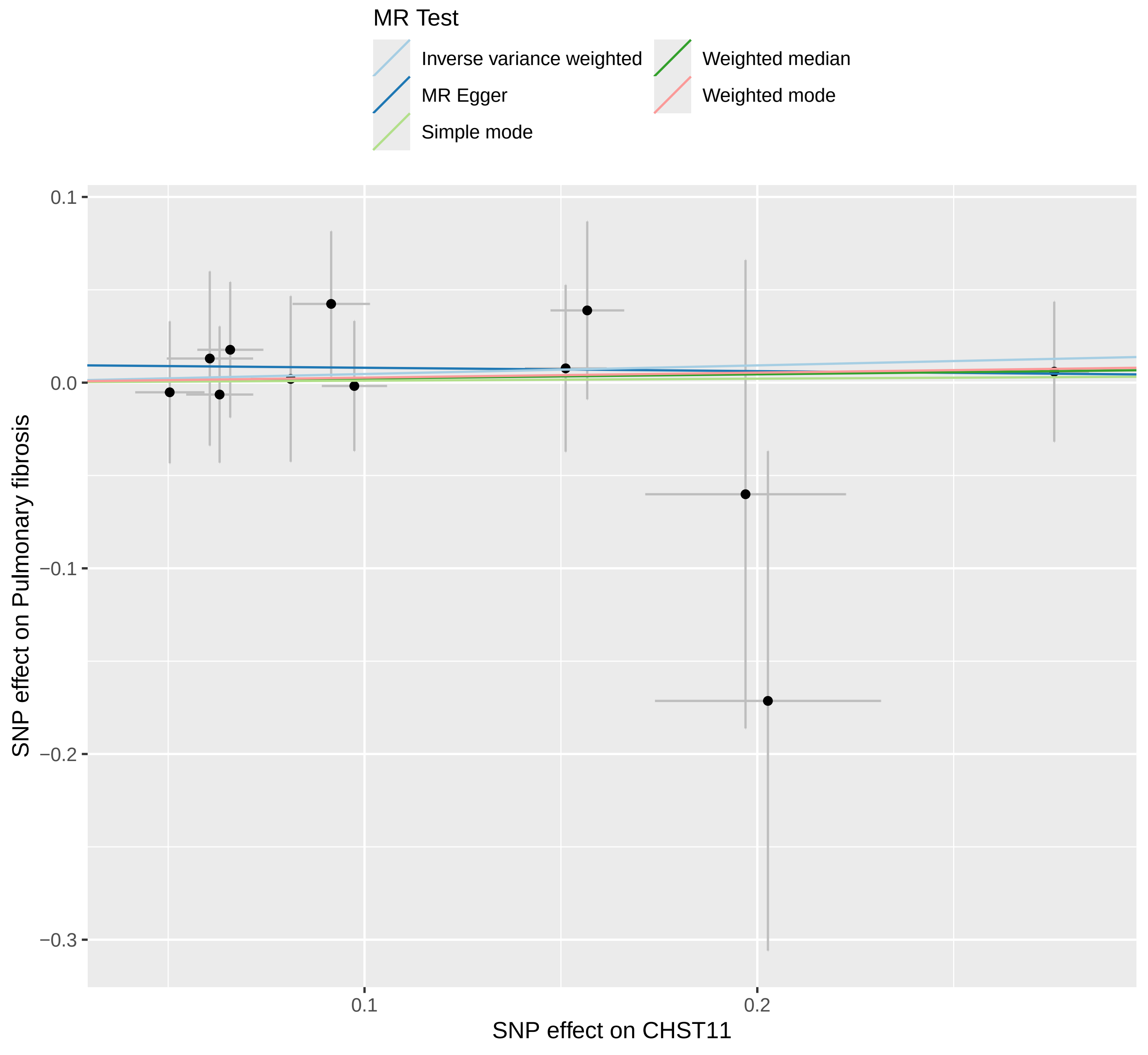

MR Test
Inverse variance weighted
MR Egger
Simple mode
Weighted median
Weighted mode
0.1
0.0
−0.1
−0.2
−0.3
SNP effect on Pulmonary fibrosis
0.1
0.2
SNP effect on CHST11

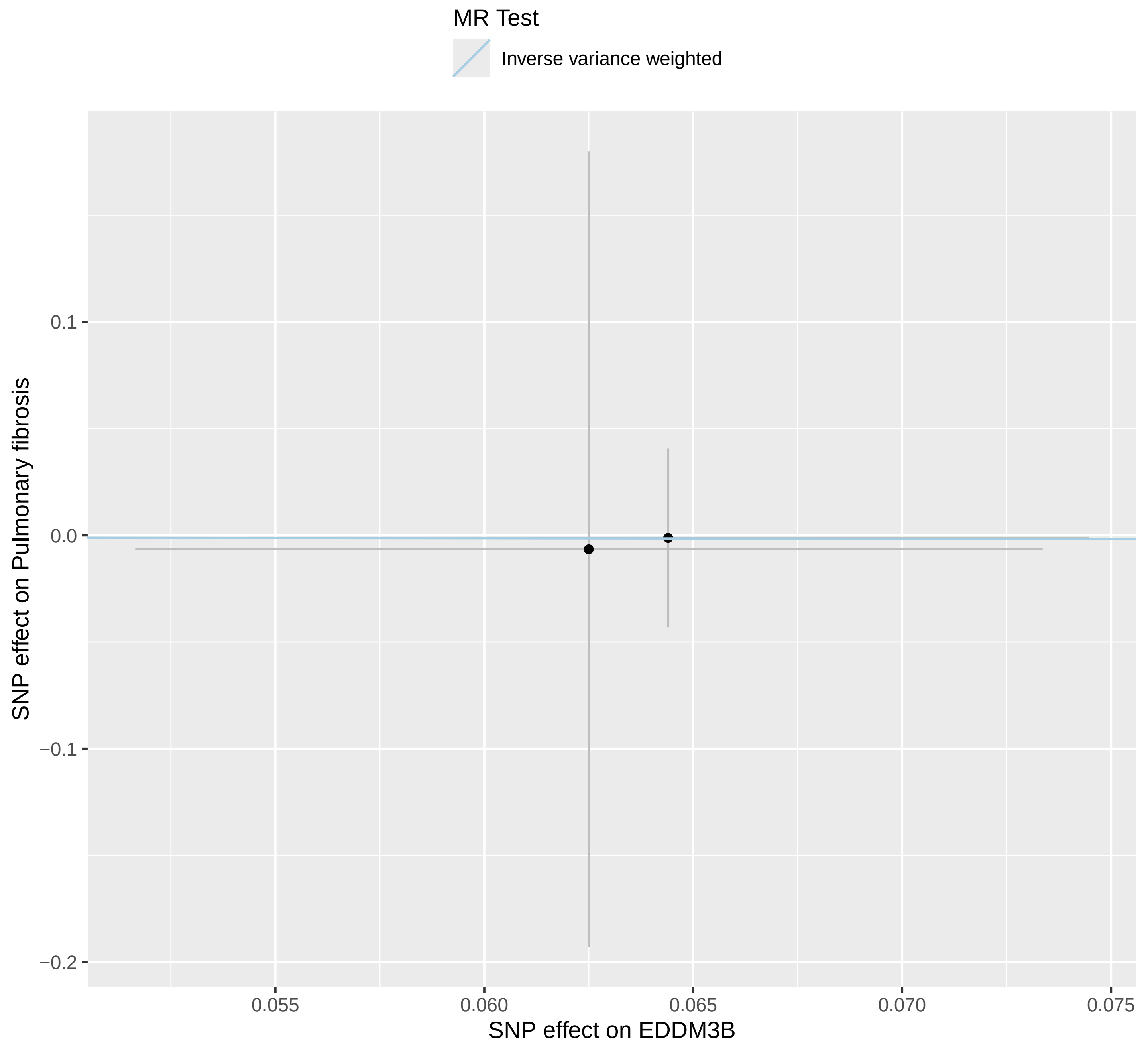
MR Test
Inverse variance weighted
SNP effect on Pulmonary fibrosis
0.1
0.0
−0.1
−0.2
0.055
0.060
0.065
0.070
0.075
SNP effect on EDDM3B

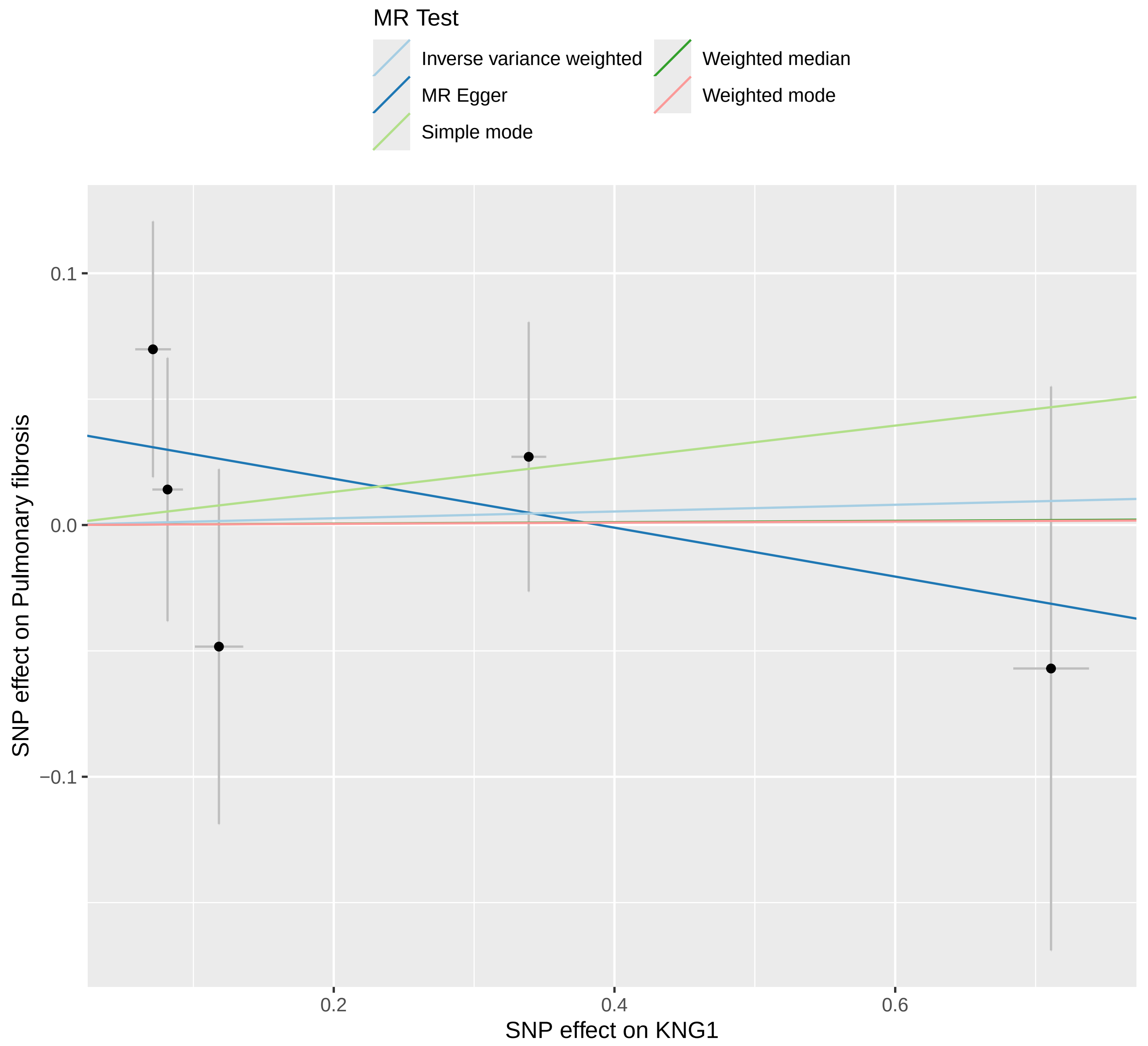

MR Test
Inverse variance weighted
MR Egger
Simple mode
Weighted median
Weighted mode
0.1
0.0
−0.1
SNP effect on Pulmonary fibrosis
0.2
0.4
0.6
SNP effect on KNG1

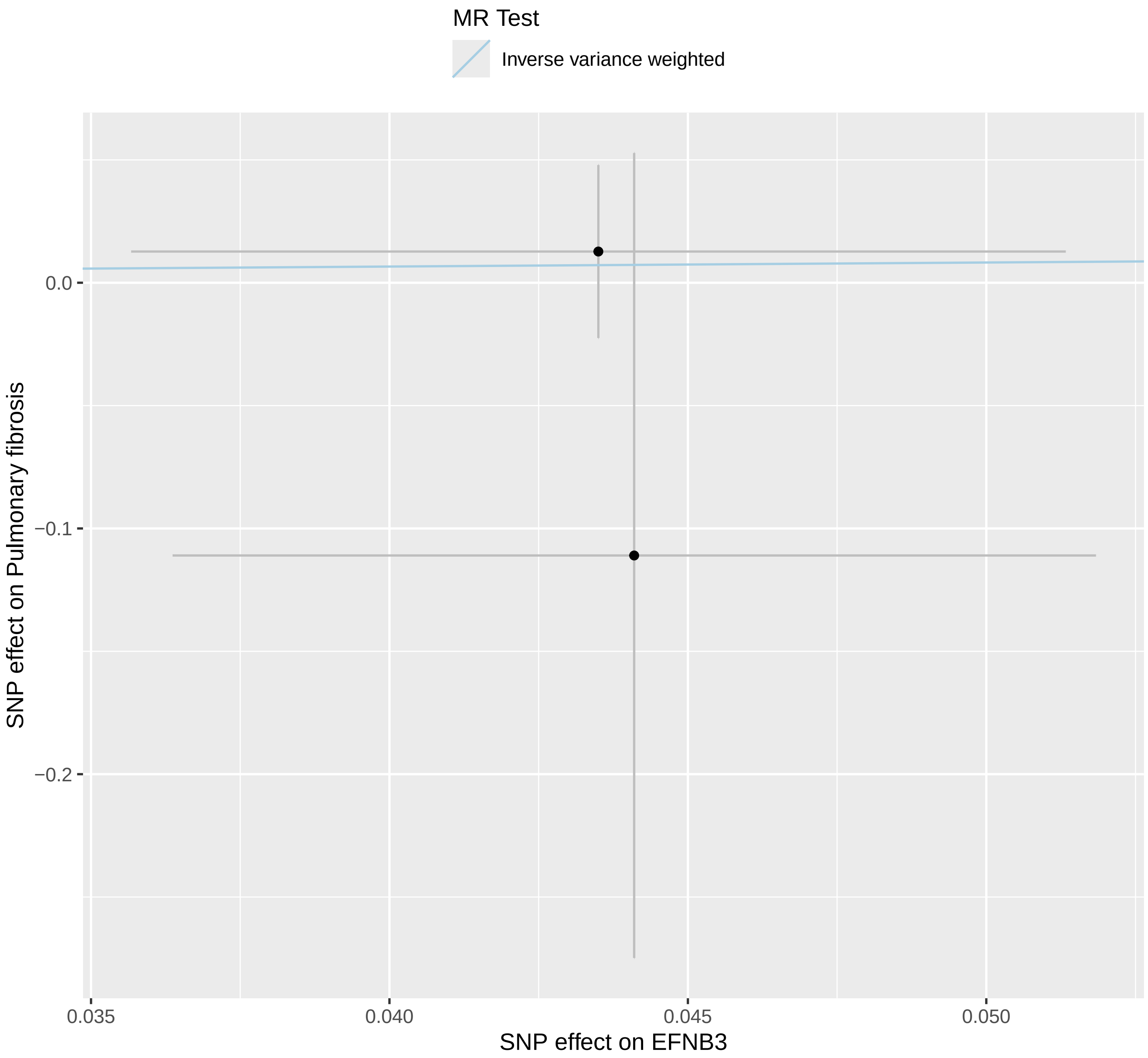

MR Test
Inverse variance weighted
0.0
−0.1
−0.2
SNP effect on Pulmonary fibrosis
0.035
0.040
0.045
0.050
SNP effect on EFNB3

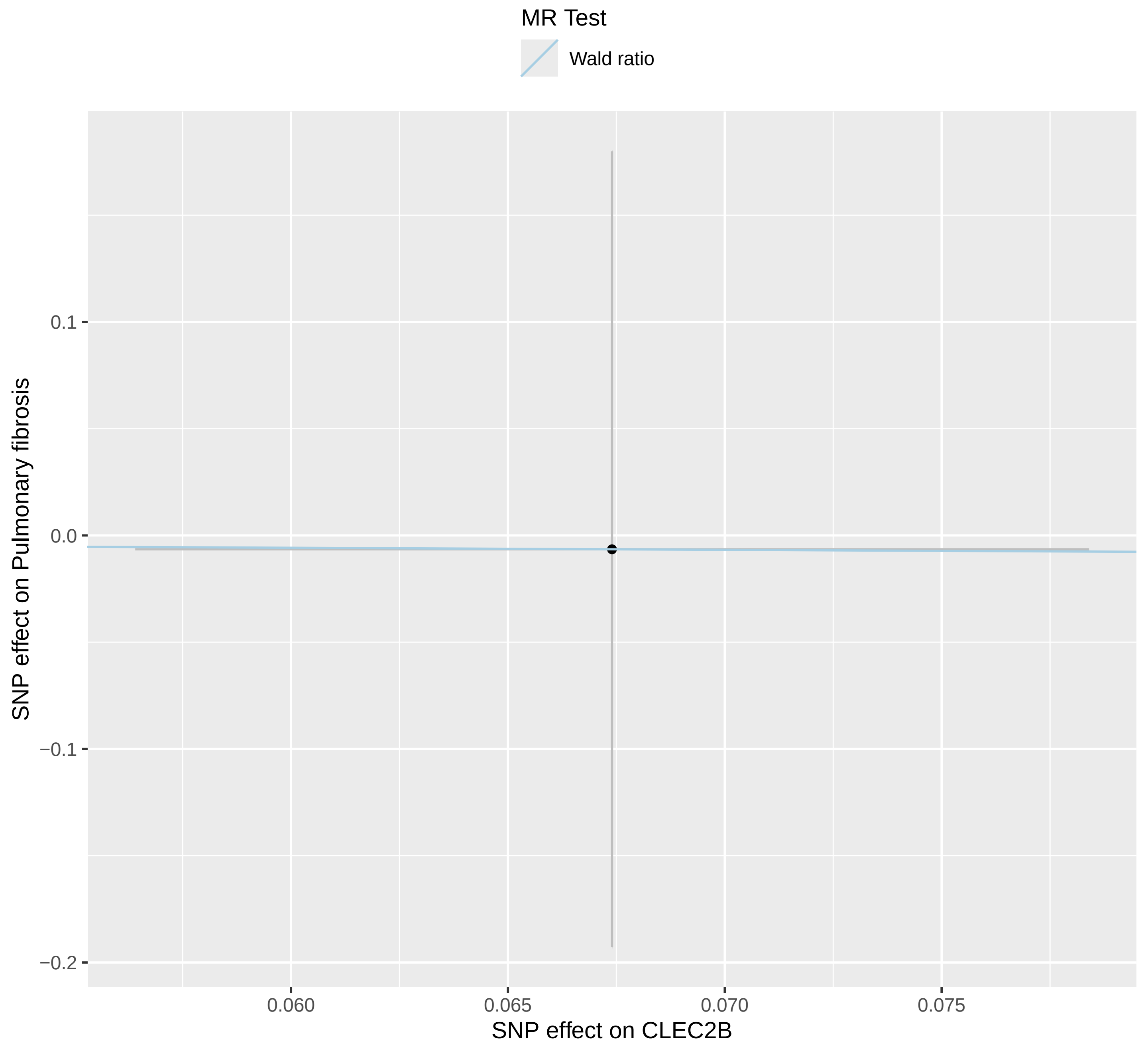

MR Test
Wald ratio
0.1
0.0
−0.1
−0.2
0.060
0.065
0.070
0.075
SNP effect on CLEC2B
SNP effect on Pulmonary fibrosis

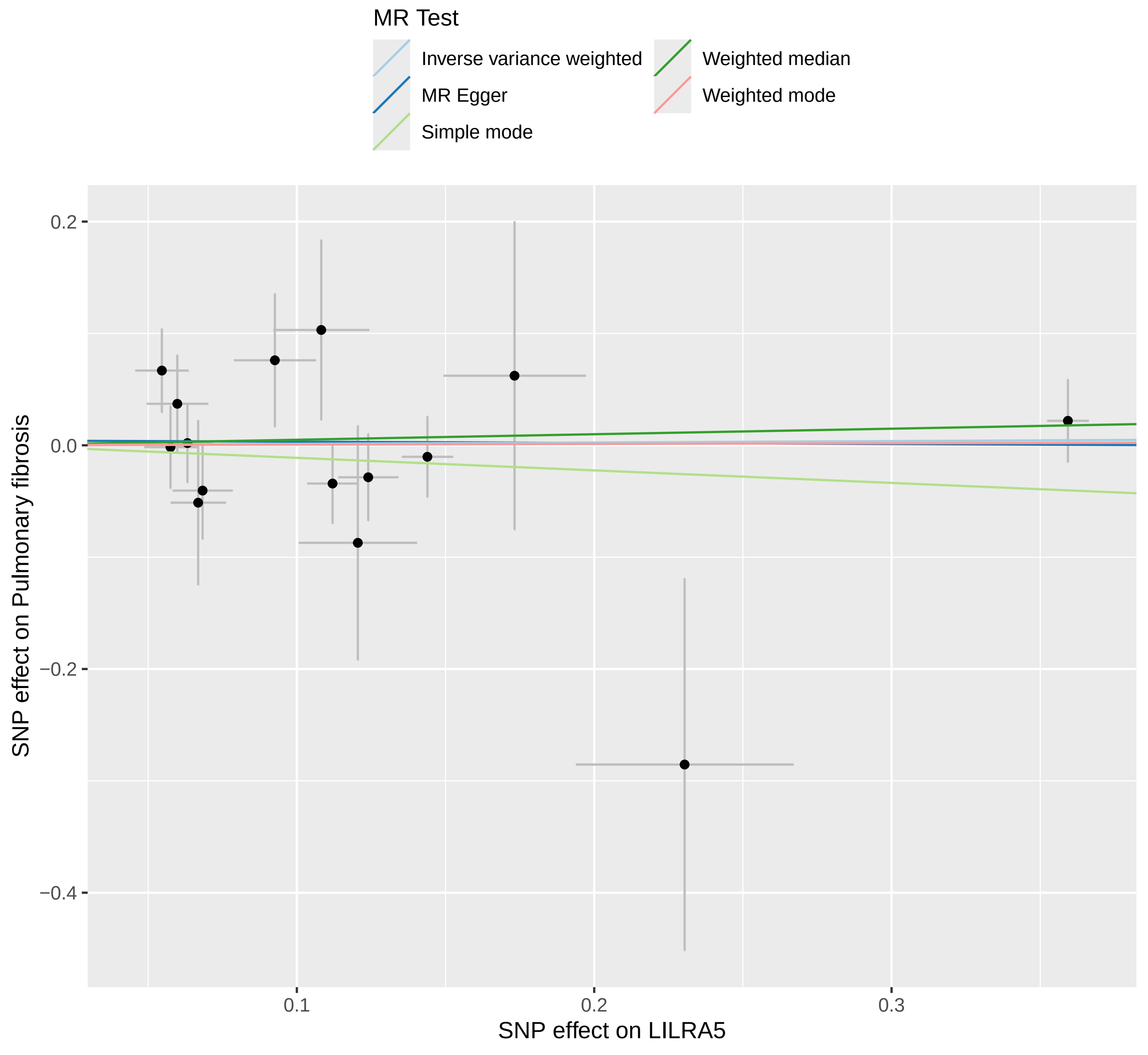

MR Test
Inverse variance weighted
MR Egger
Simple mode
Weighted median
Weighted mode
0.2
0.0
−0.2
−0.4
0.1
0.2
0.3
SNP effect on Pulmonary fibrosis
SNP effect on LILRA5

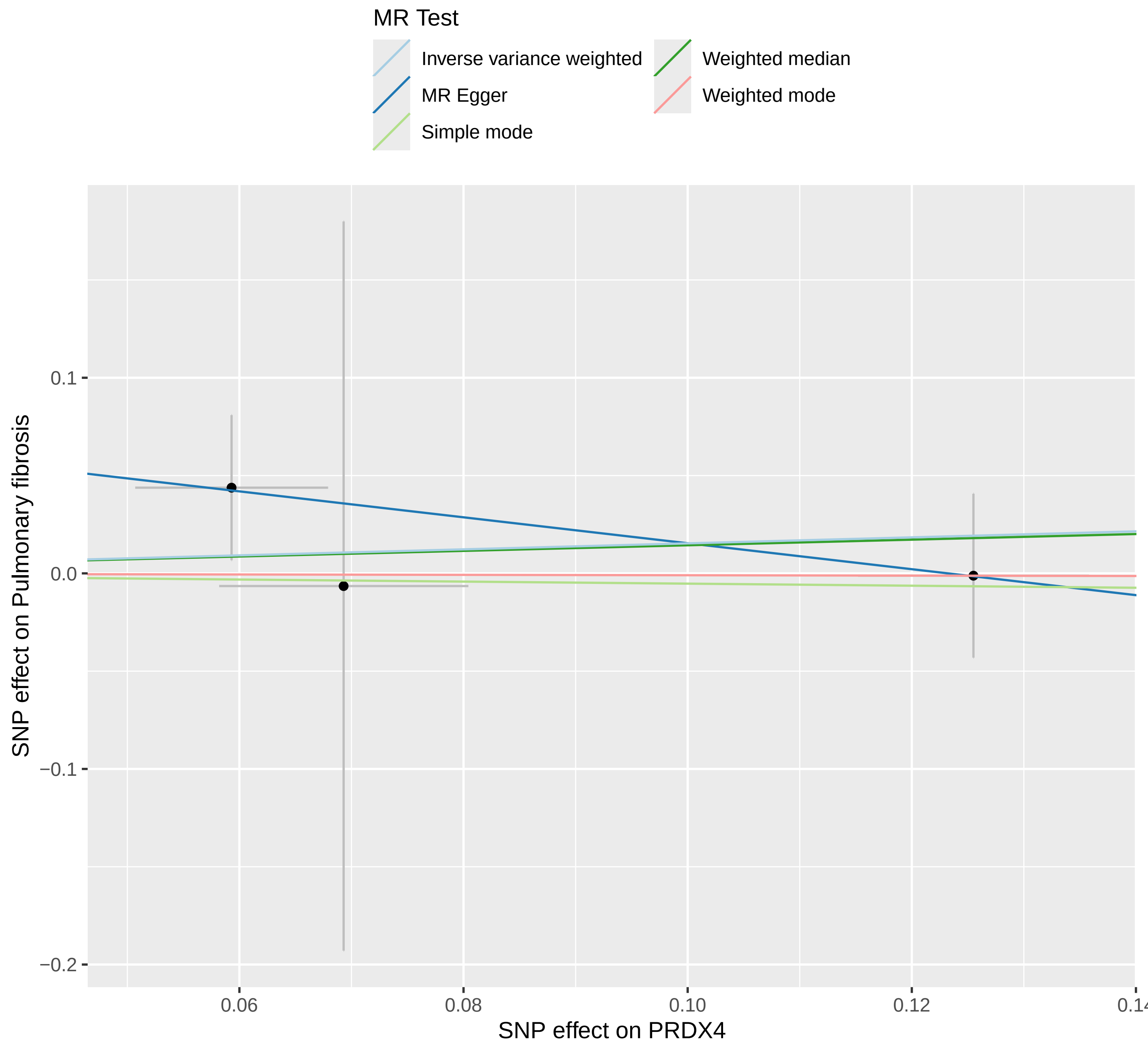
MR Test
Inverse variance weighted
MR Egger
Simple mode
Weighted median
Weighted mode
0.1
0.0
−0.1
−0.2
SNP effect on Pulmonary fibrosis
0.06
0.08
0.10
0.12
SNP effect on PRDX4

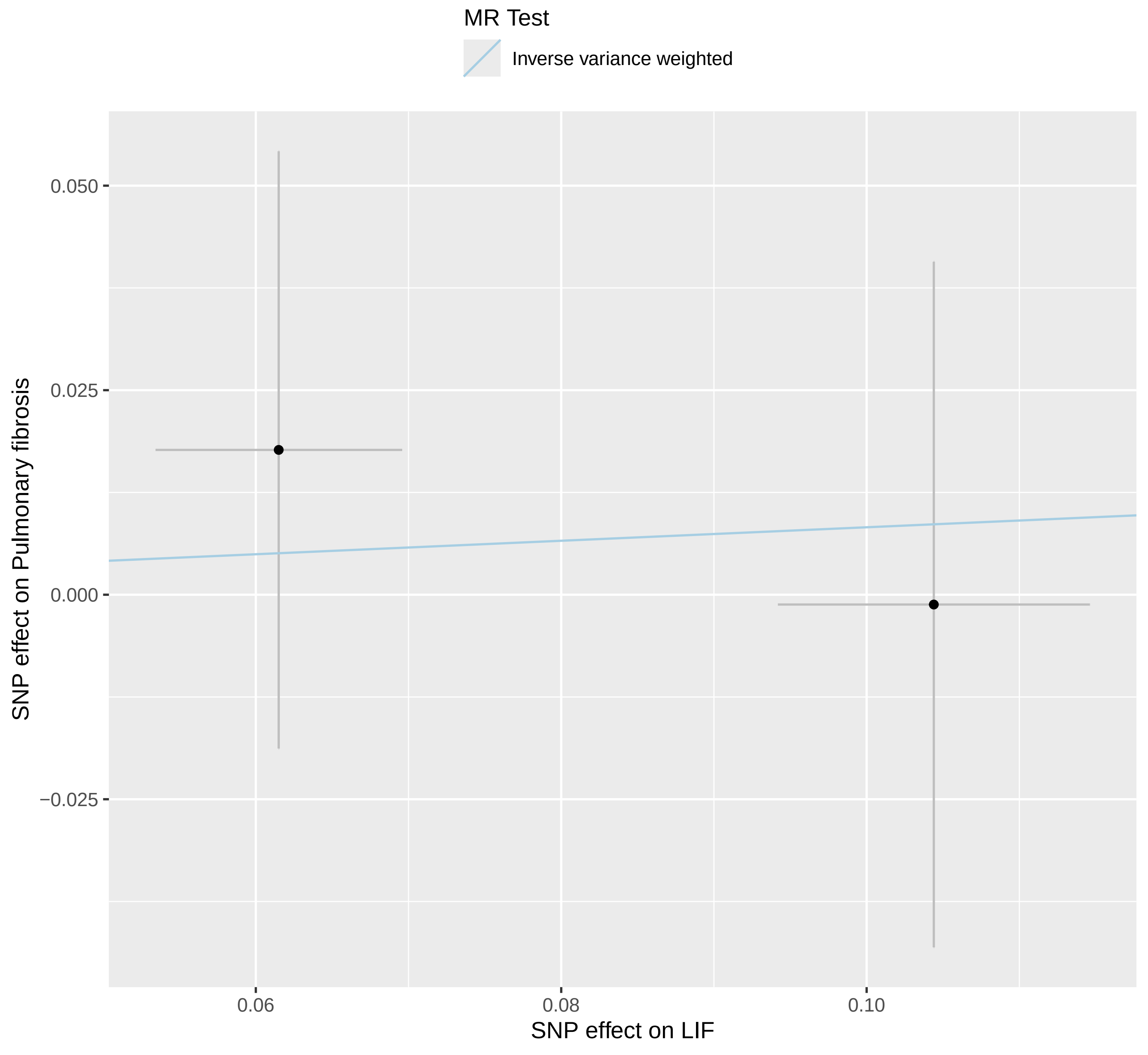
MR Test
Inverse variance weighted
0.050
0.025
0.000
−0.025
SNP effect on Pulmonary fibrosis
0.06
0.08
0.10
SNP effect on LIF

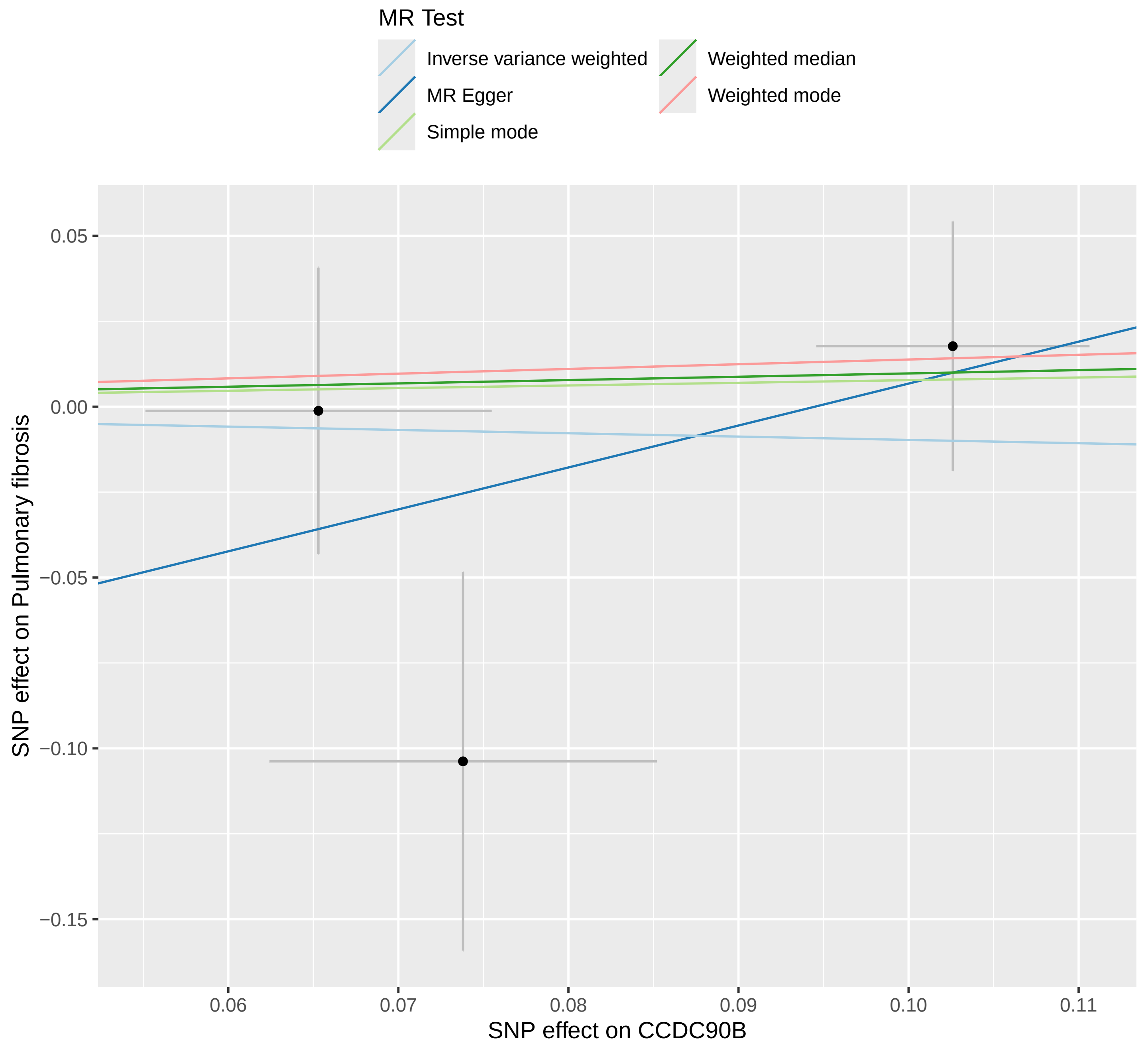

MR Test
Inverse variance weighted
MR Egger
Simple mode
Weighted median
Weighted mode
SNP effect on Pulmonary fibrosis
0.05
0.00
−0.05
−0.10
−0.15
0.06
0.07
0.08
0.09
0.10
0.11
SNP effect on CCDC90B

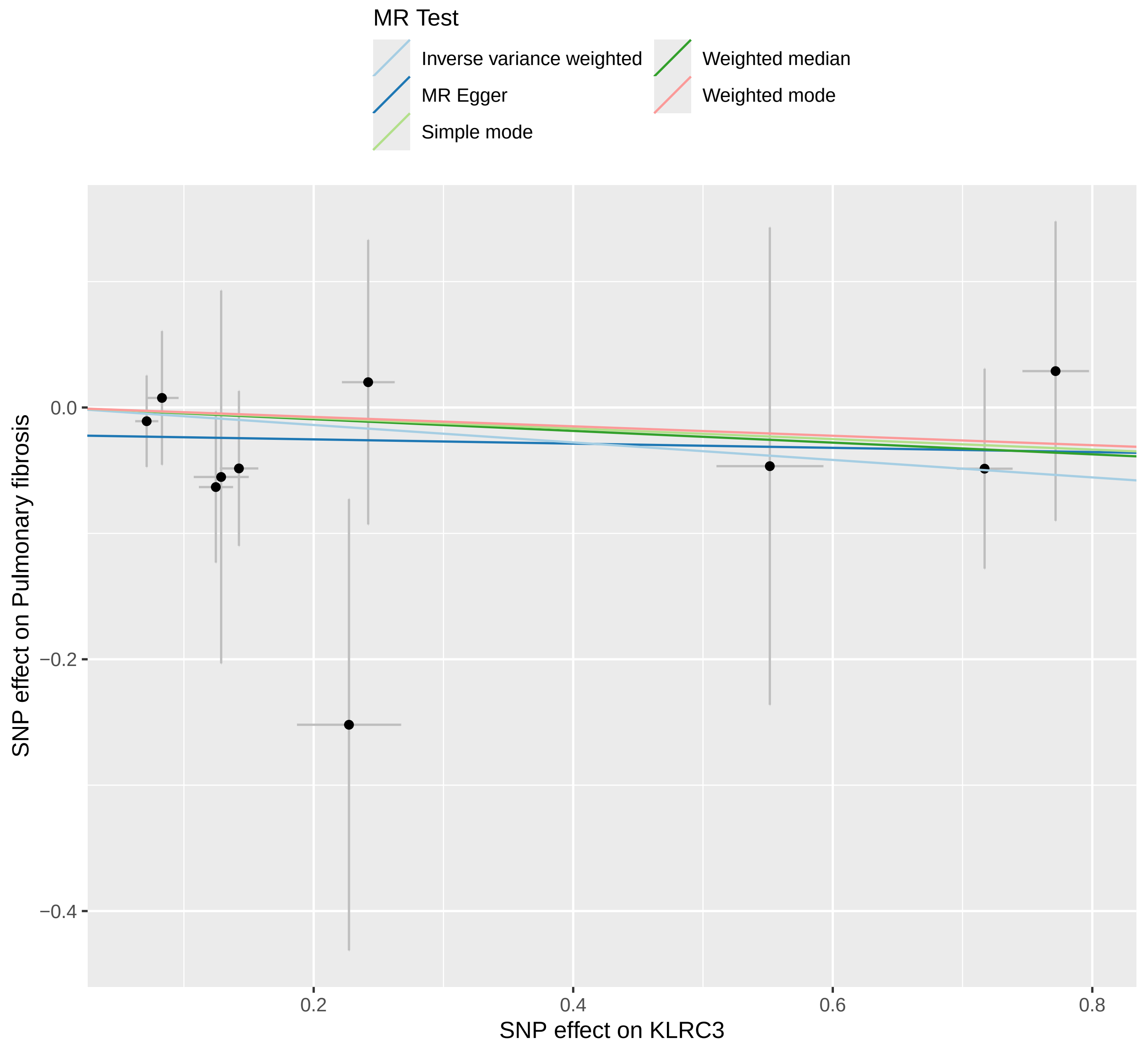

MR Test
Inverse variance weighted
MR Egger
Simple mode
Weighted median
Weighted mode
SNP effect on Pulmonary fibrosis
0.0
−0.2
−0.4
0.2
0.4
0.6
0.8
SNP effect on KLRC3

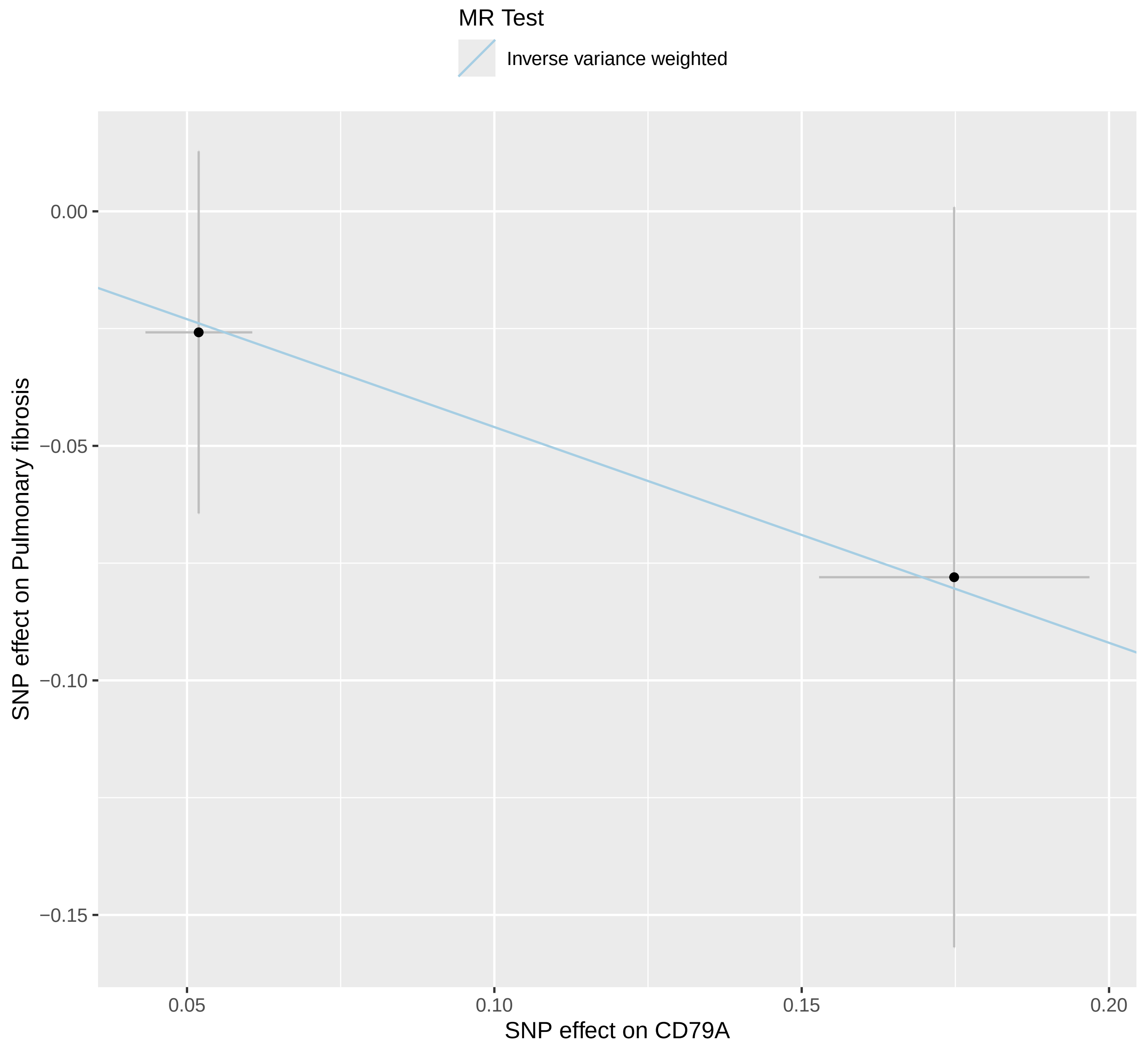

MR Test
Inverse variance weighted
0.00
−0.05
−0.10
−0.15
SNP effect on Pulmonary fibrosis
0.05
0.10
0.15
0.20
SNP effect on CD79A

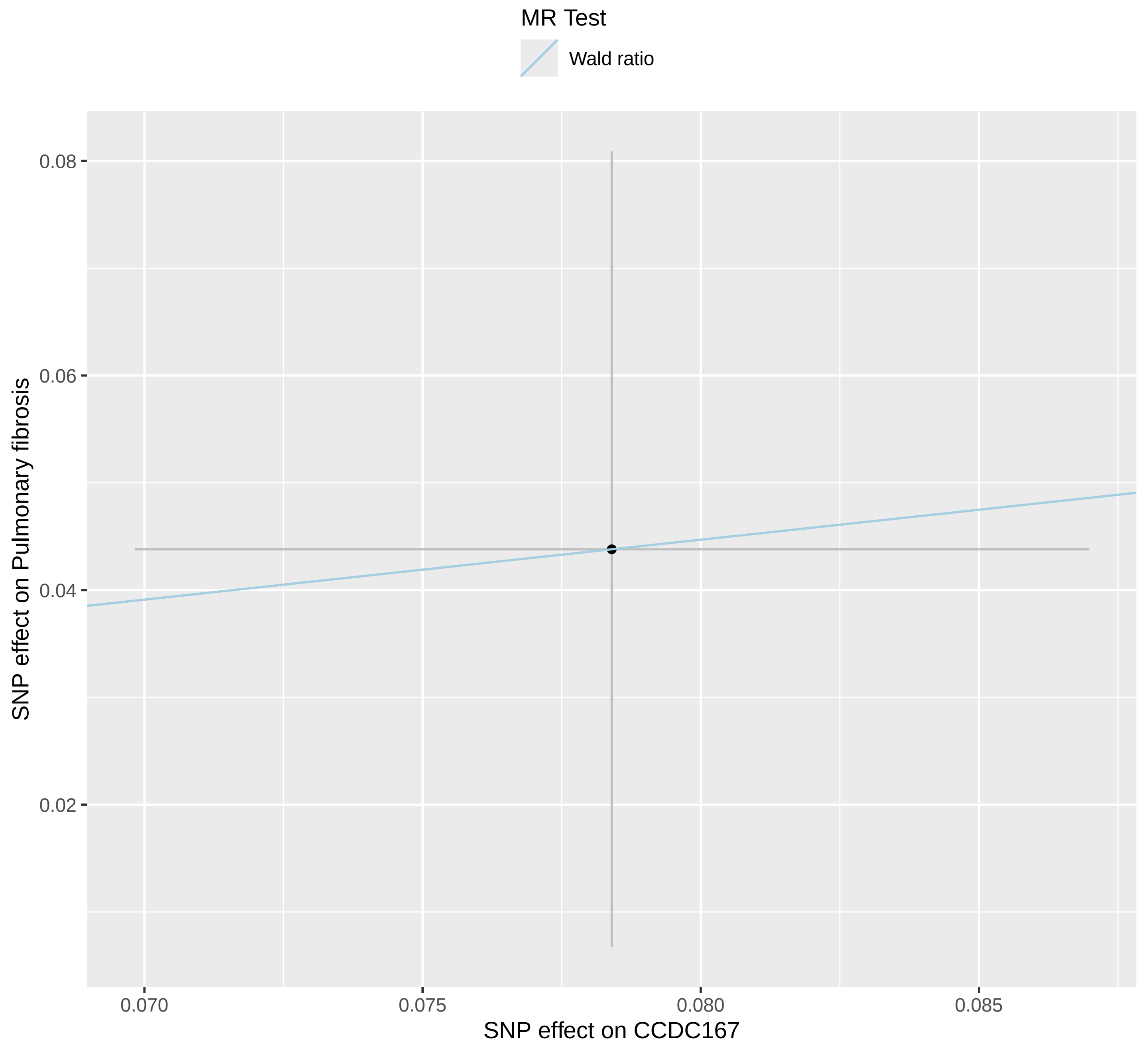

MR Test
Wald ratio
SNP effect on Pulmonary fibrosis
0.08
0.06
0.04
0.02
0.070
0.075
0.080
0.085
SNP effect on CCDC167

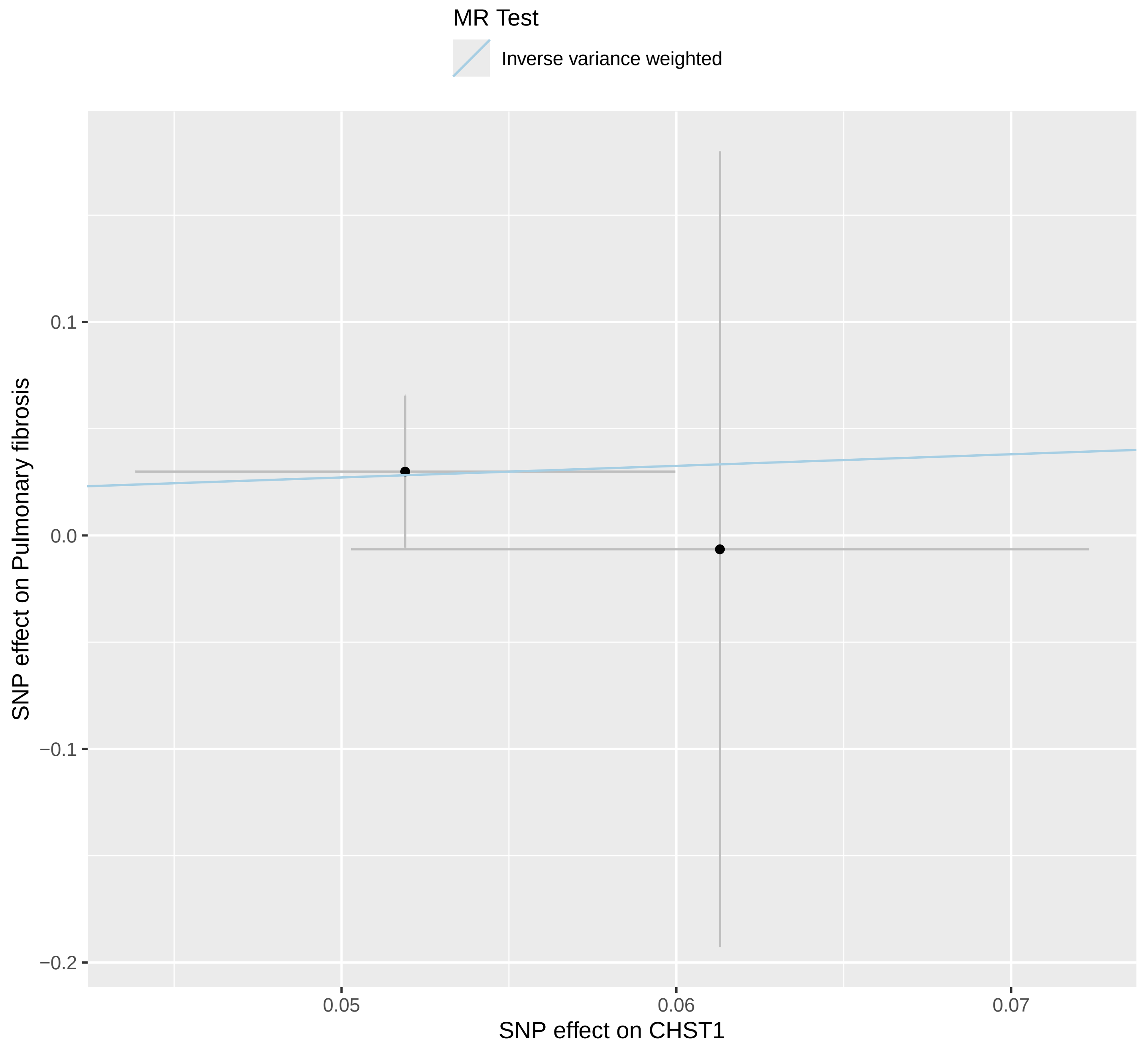
MR Test
Inverse variance weighted
0.1
0.0
−0.1
−0.2
SNP effect on Pulmonary fibrosis
0.05
0.06
0.07
SNP effect on CHST1

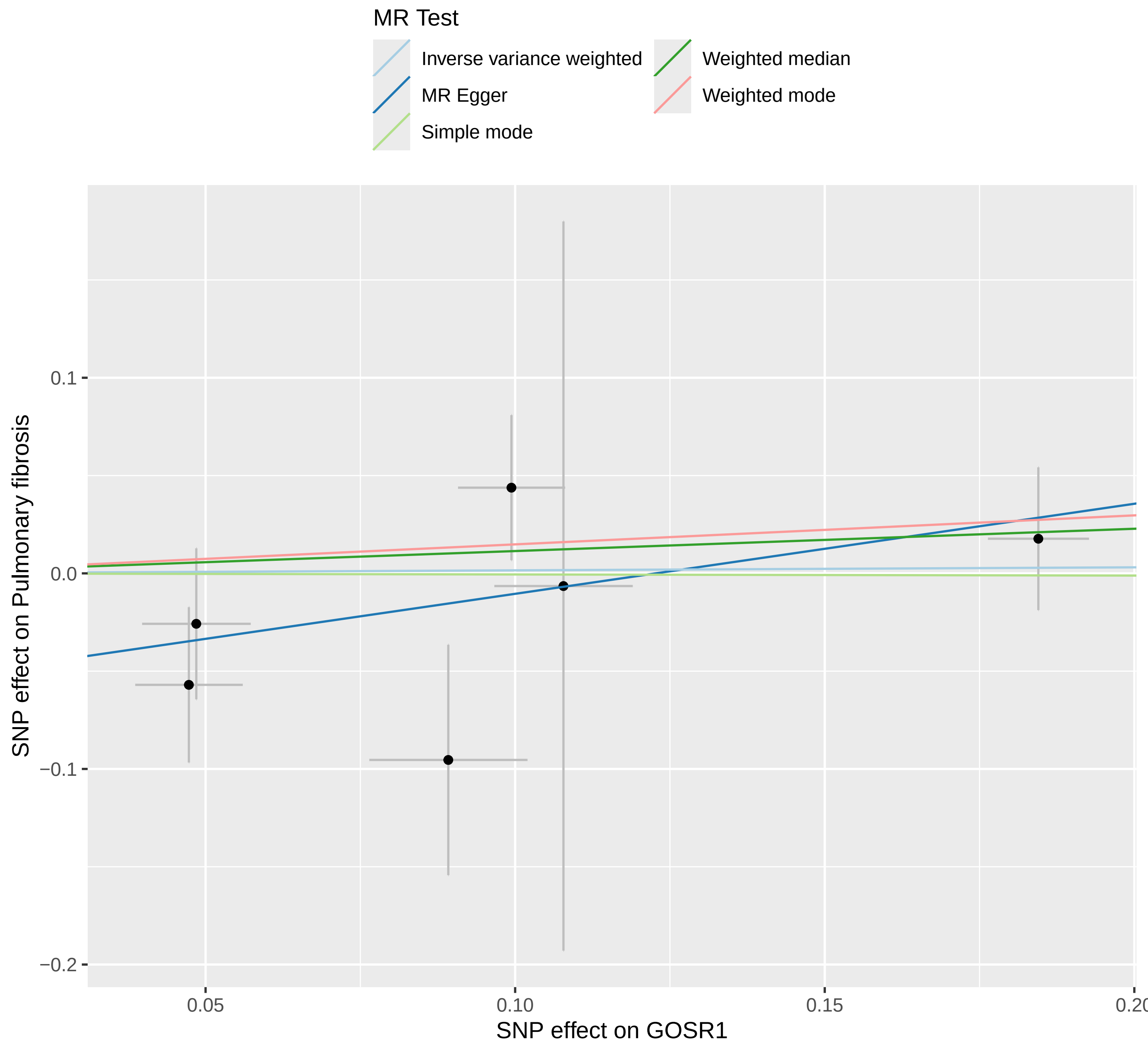

MR Test
Inverse variance weighted
MR Egger
Simple mode
Weighted median
Weighted mode
0.1
0.0
−0.1
−0.2
0.05
0.10
0.15
SNP effect on Pulmonary fibrosis
SNP effect on GOSR1

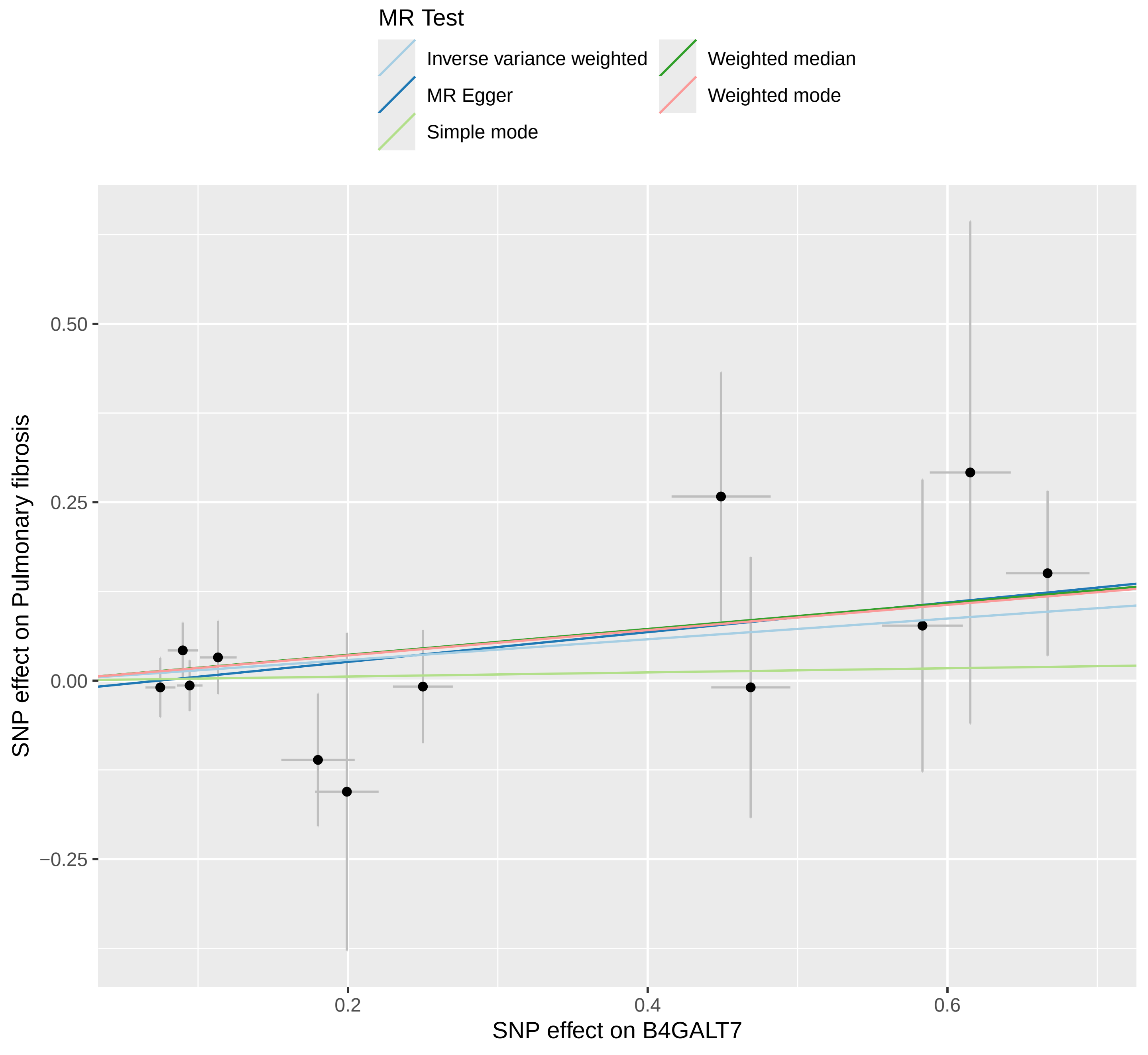
MR Test
Inverse variance weighted
MR Egger
Simple mode
Weighted median
Weighted mode
0.50
0.25
0.00
−0.25
0.2
0.4
0.6
SNP effect on Pulmonary fibrosis
SNP effect on B4GALT7

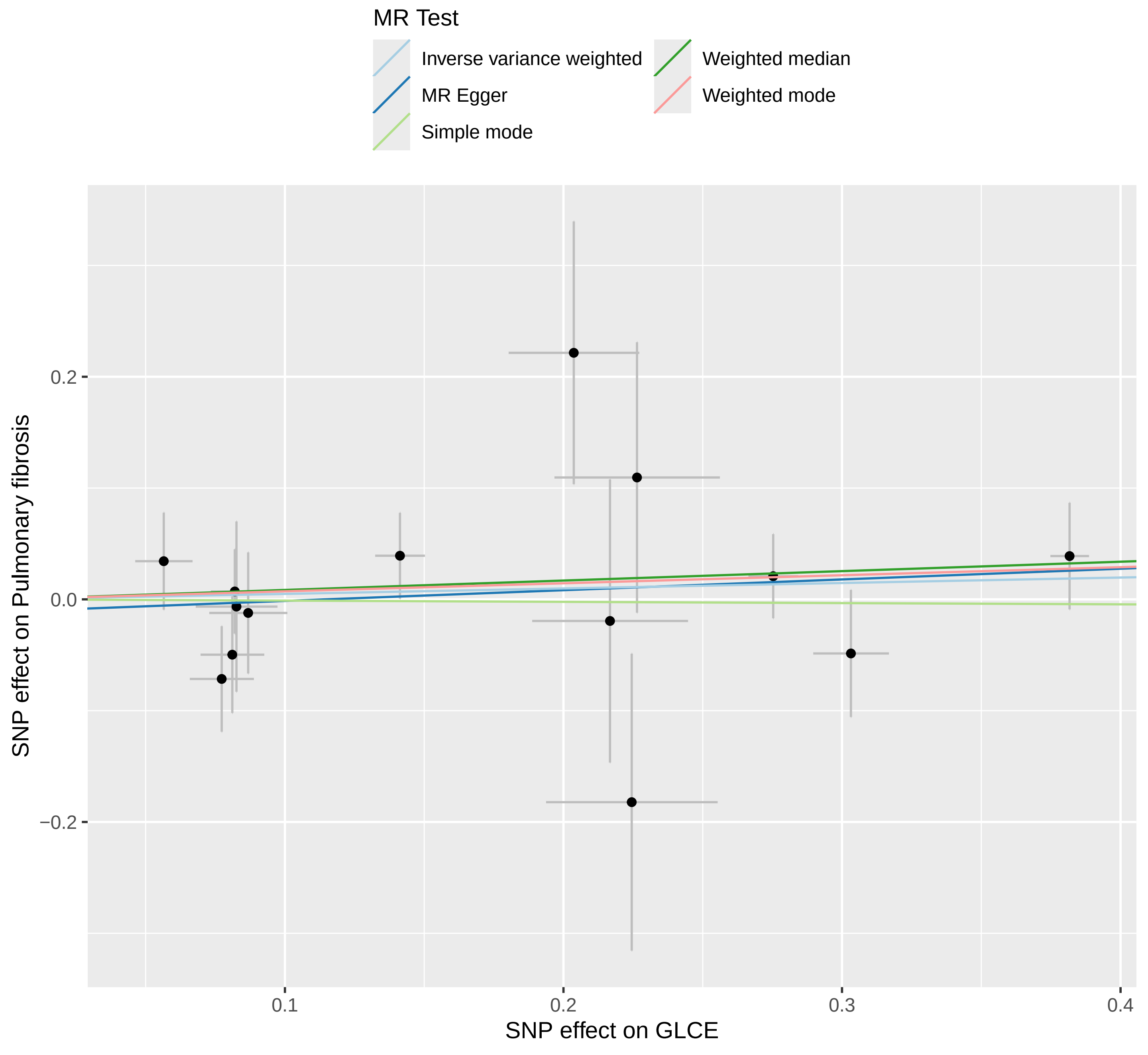

MR Test
Inverse variance weighted
MR Egger
Simple mode
Weighted median
Weighted mode
0.2
0.0
−0.2
SNP effect on Pulmonary fibrosis
0.1
0.2
0.3
0.4
SNP effect on GLCE

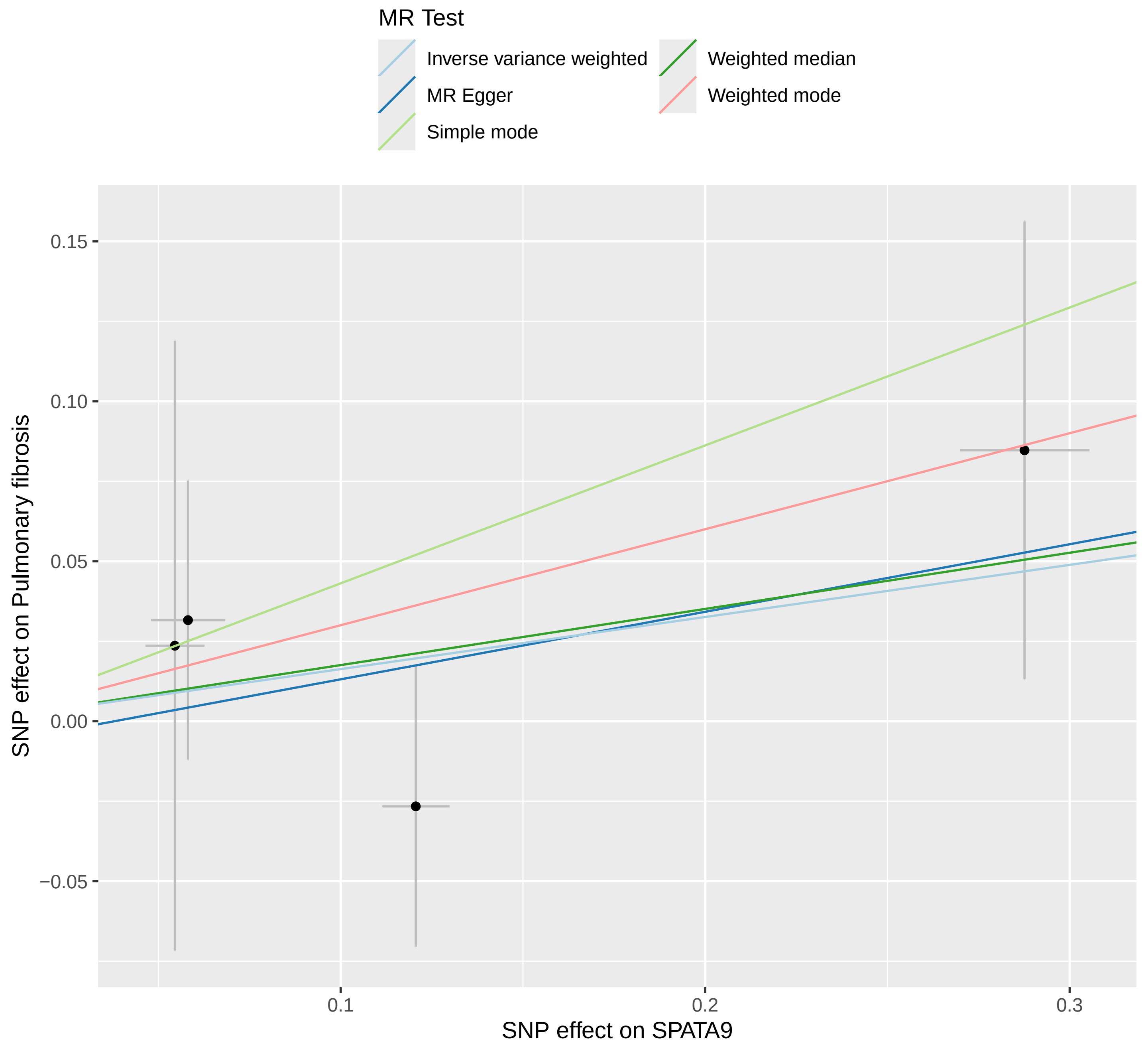
MR Test
Inverse variance weighted
MR Egger
Simple mode
Weighted median
Weighted mode
SNP effect on Pulmonary fibrosis
0.15
0.10
0.05
0.00
−0.05
0.1
0.2
0.3
SNP effect on SPATA9

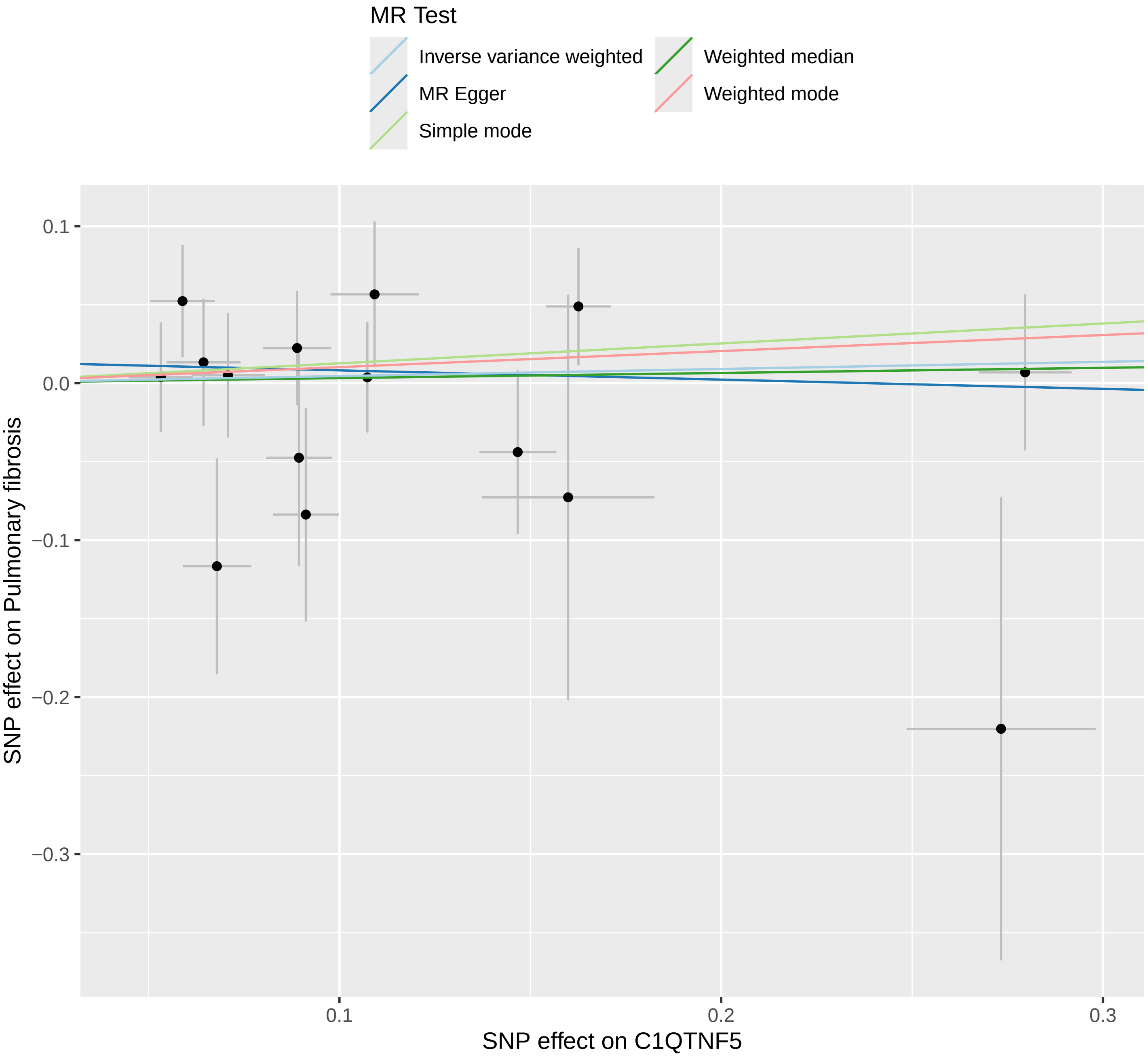
MR Test
Inverse variance weighted
MR Egger
Simple mode
Weighted median
Weighted mode
0.1
0.0
−0.1
−0.2
−0.3
SNP effect on Pulmonary fibrosis
0.1
0.2
0.3
SNP effect on C1QTNF5

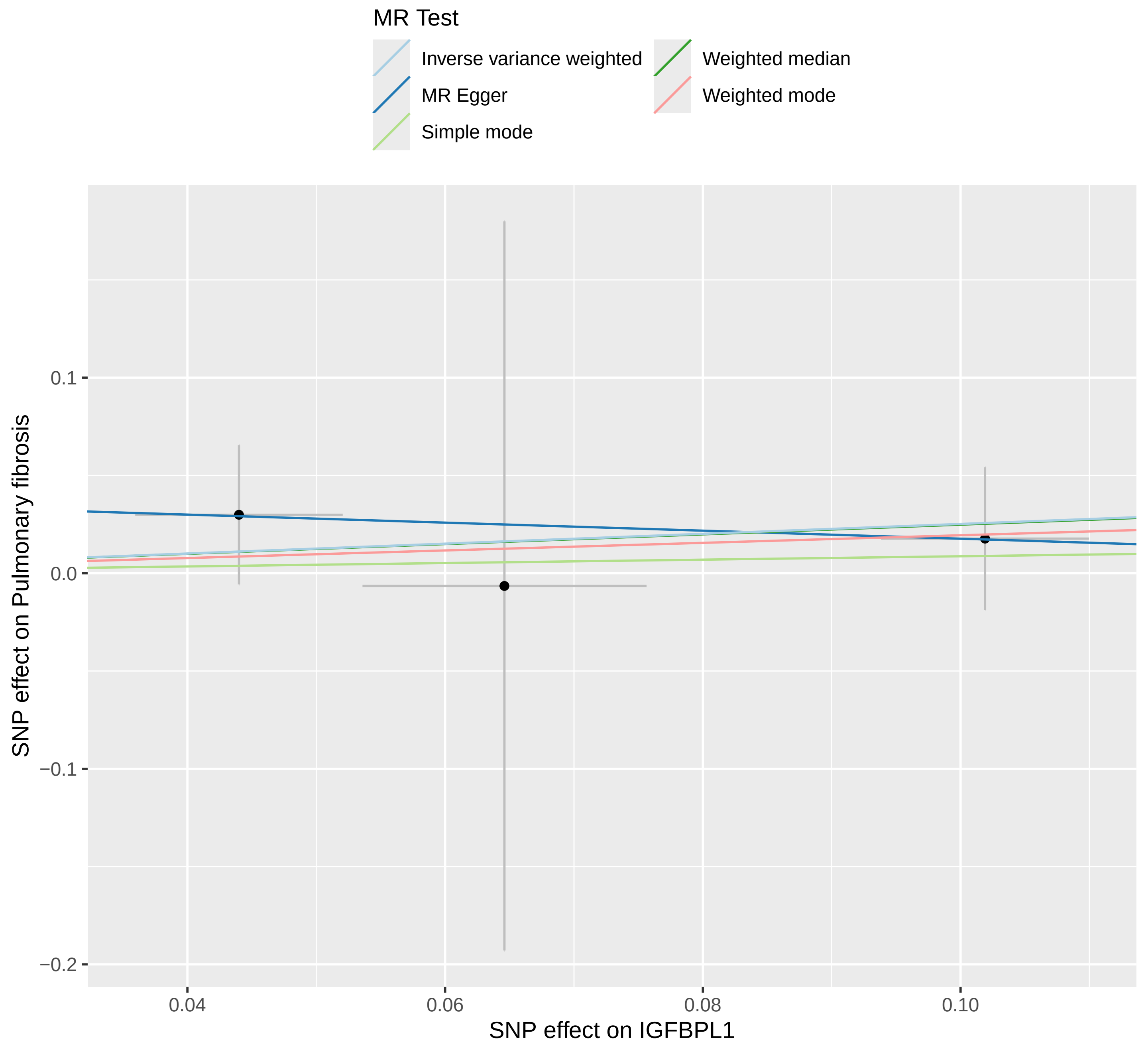
MR Test
Inverse variance weighted
MR Egger
Simple mode
Weighted median
Weighted mode
0.1
0.0
−0.1
−0.2
0.04
0.06
0.08
0.10
SNP effect on Pulmonary fibrosis
SNP effect on IGFBPL1

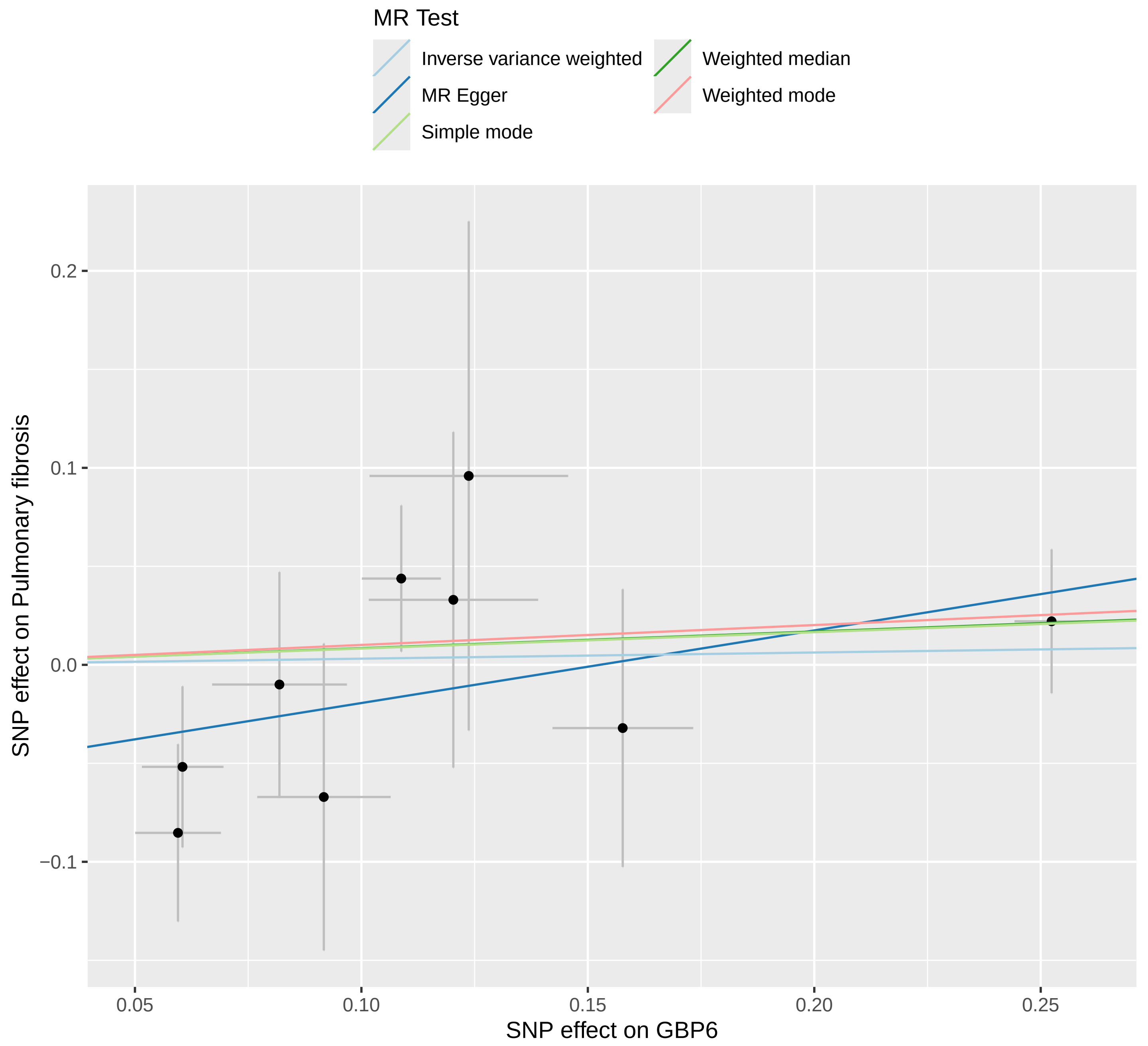
MR Test
Inverse variance weighted
MR Egger
Simple mode
Weighted median
Weighted mode
0.2
0.1
0.0
−0.1
0.05
0.10
0.15
0.20
0.25
SNP effect on GBP6
SNP effect on Pulmonary fibrosis

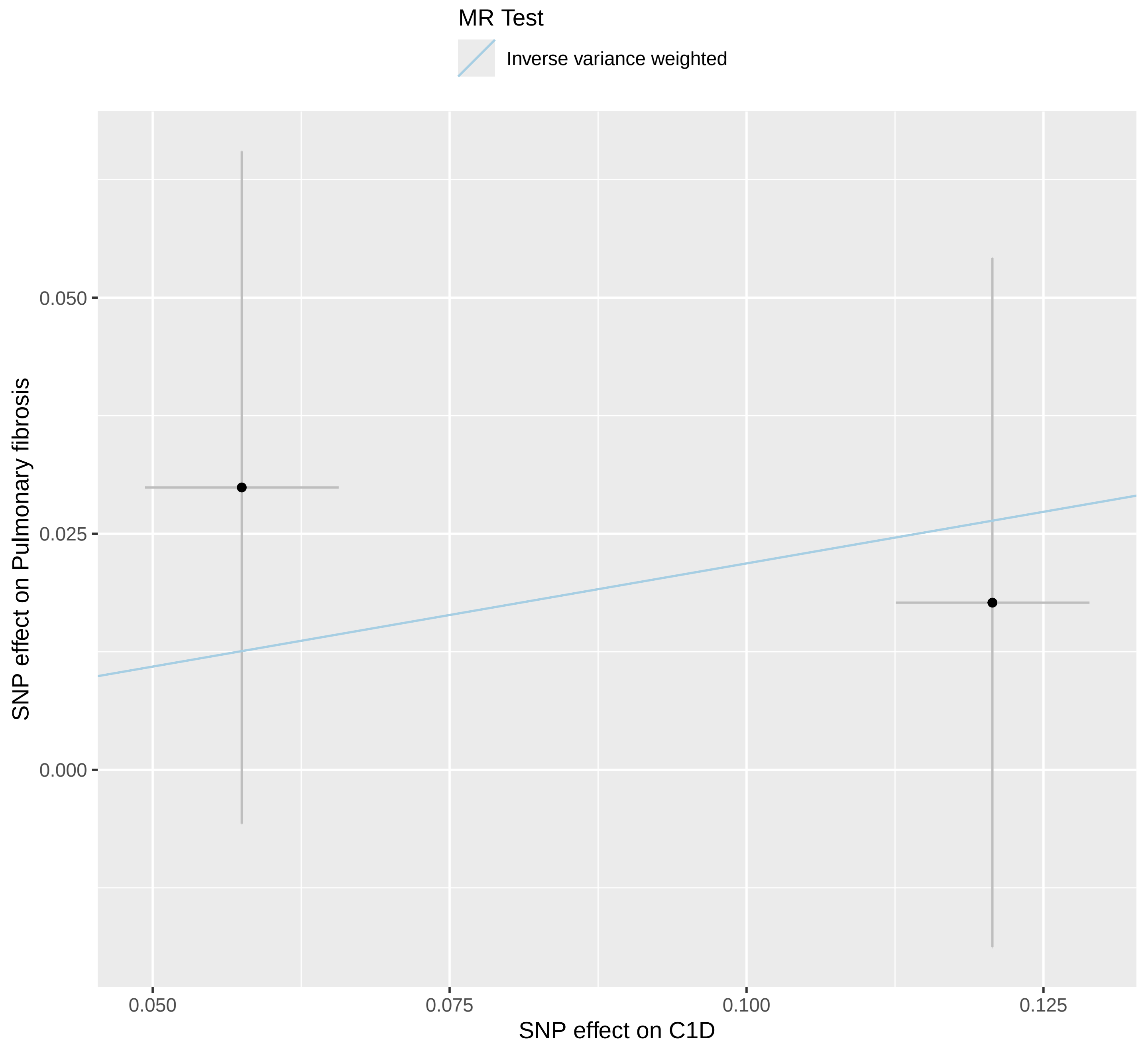

MR Test
Inverse variance weighted
SNP effect on Pulmonary fibrosis
0.050
0.025
0.000
0.050
0.075
0.100
0.125
SNP effect on C1D

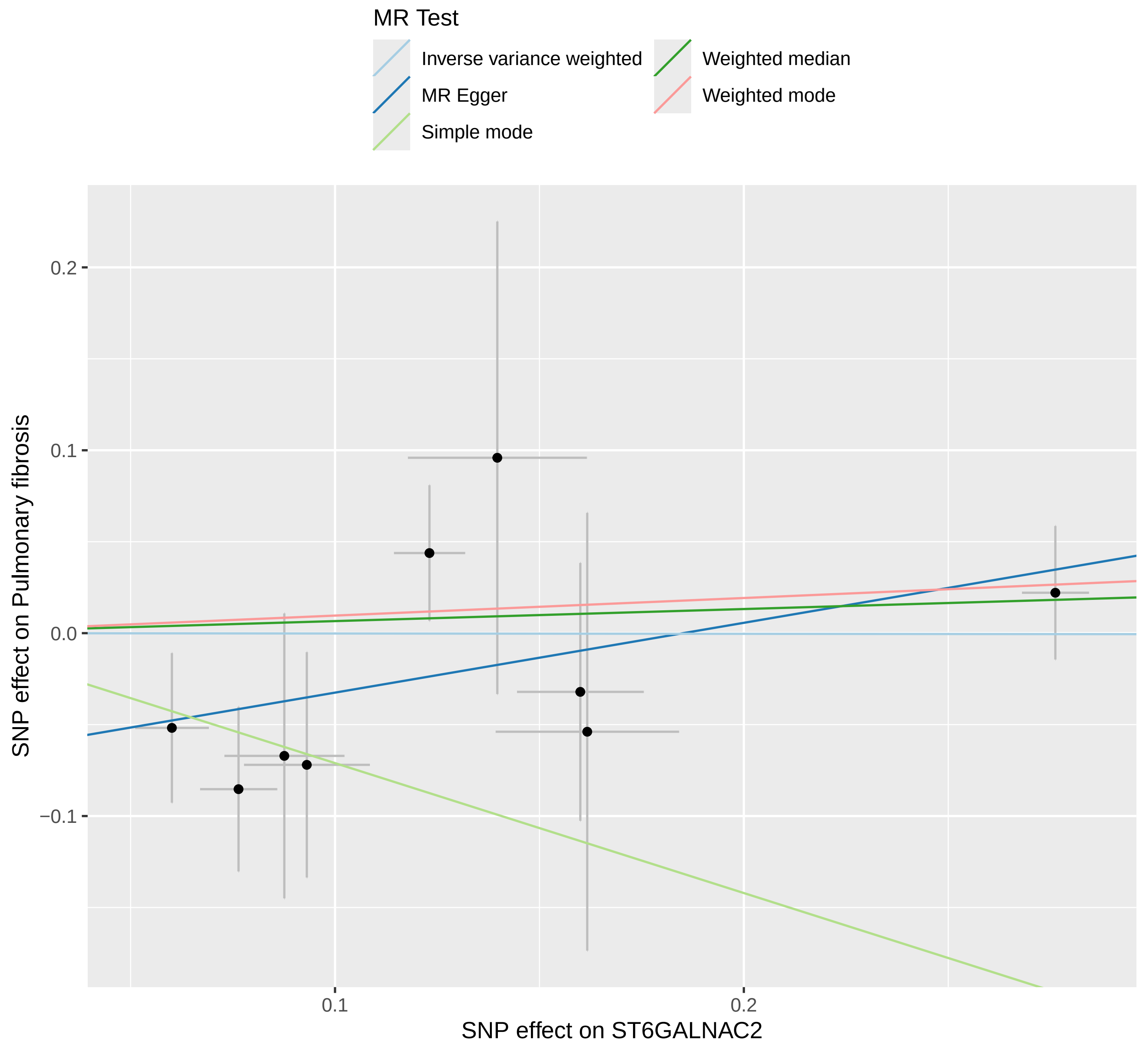
MR Test
Inverse variance weighted
MR Egger
Simple mode
Weighted median
Weighted mode
0.2
0.1
0.0
−0.1
0.1
0.2
SNP effect on Pulmonary fibrosis
SNP effect on ST6GALNAC2

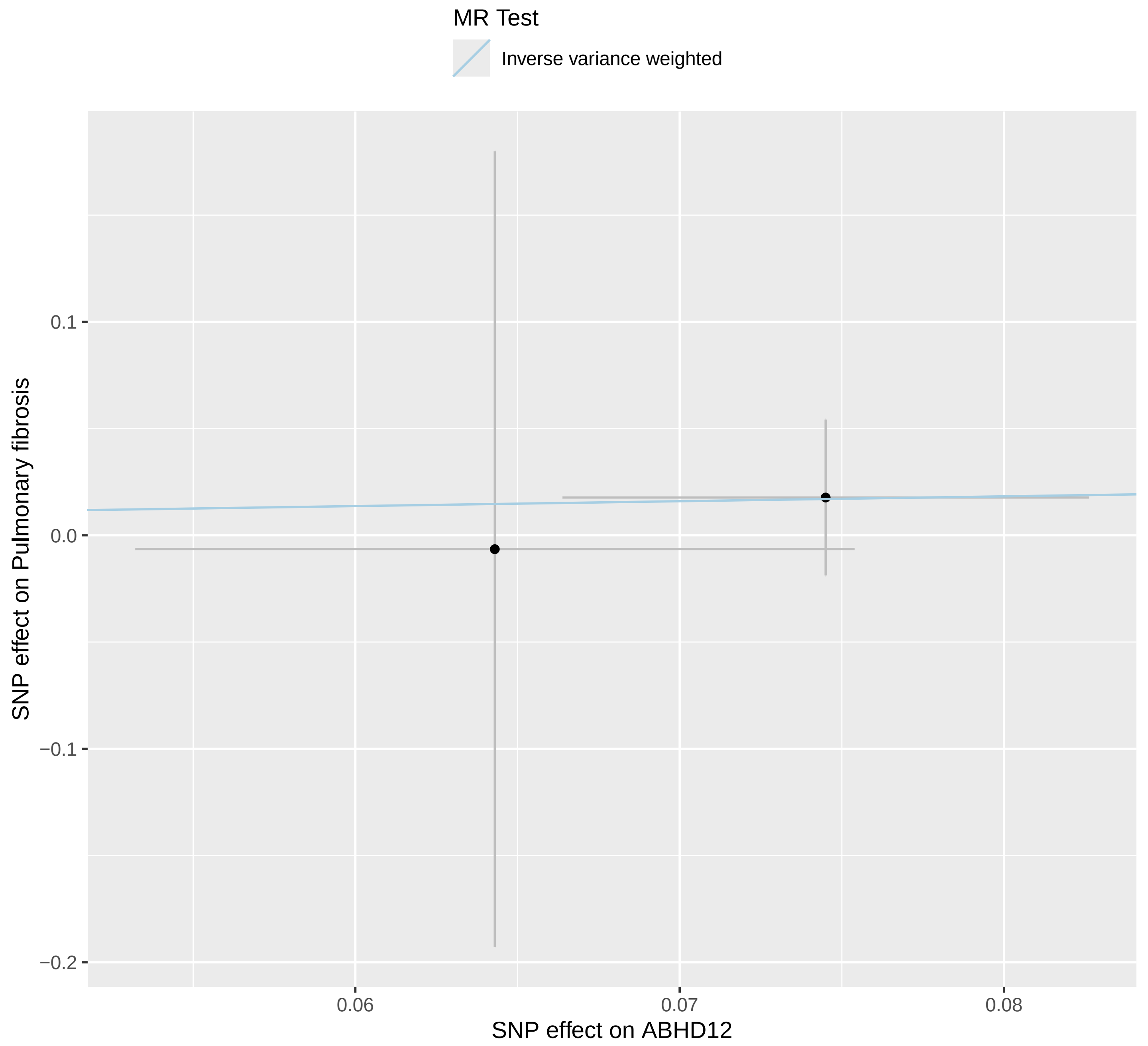
MR Test
Inverse variance weighted
SNP effect on Pulmonary fibrosis
0.1
0.0
−0.1
−0.2
0.06
0.07
0.08
SNP effect on ABHD12

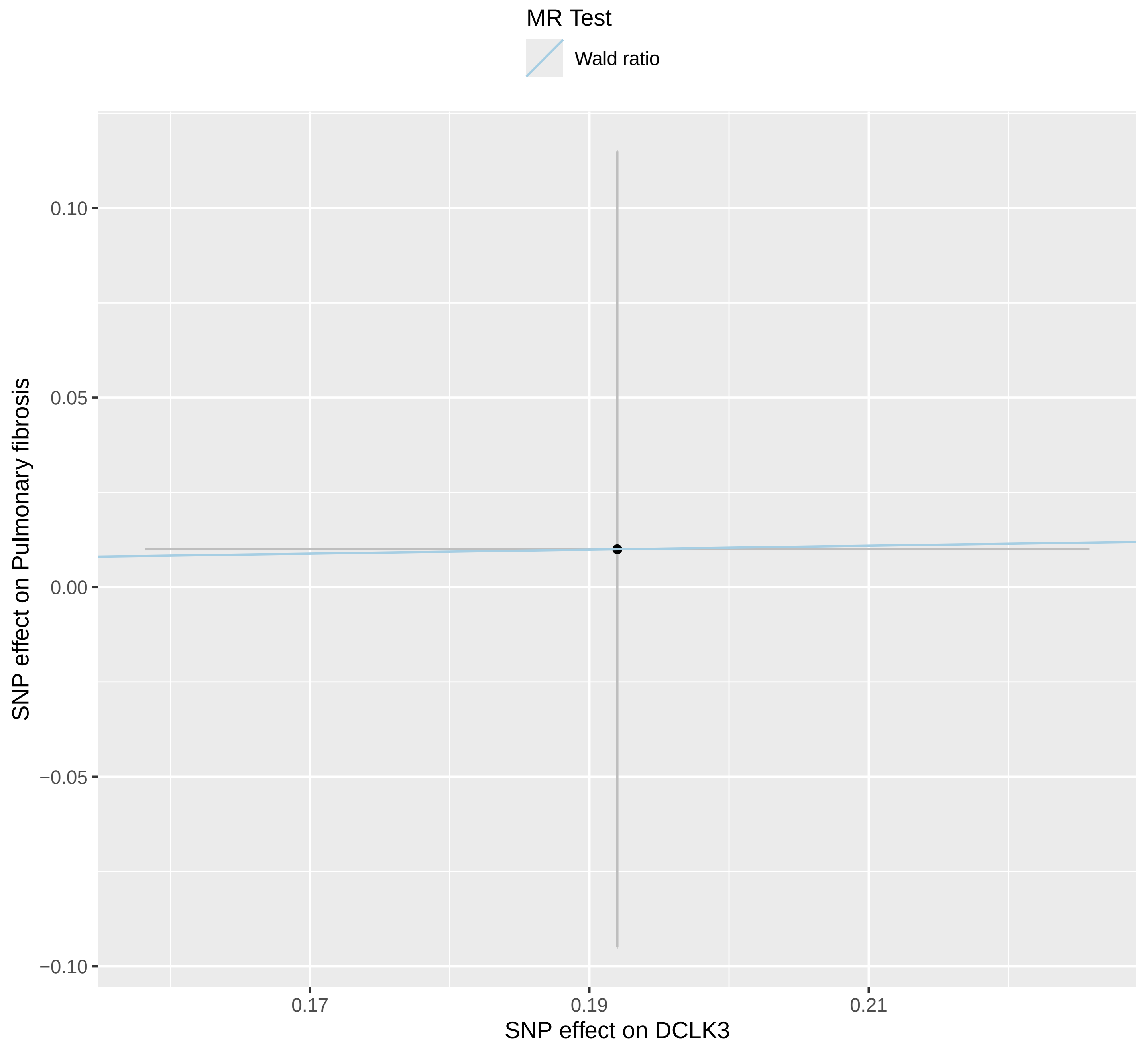
MR Test
Wald ratio
SNP effect on Pulmonary fibrosis
0.10
0.05
0.00
−0.05
−0.10
0.17
0.19
0.21
SNP effect on DCLK3

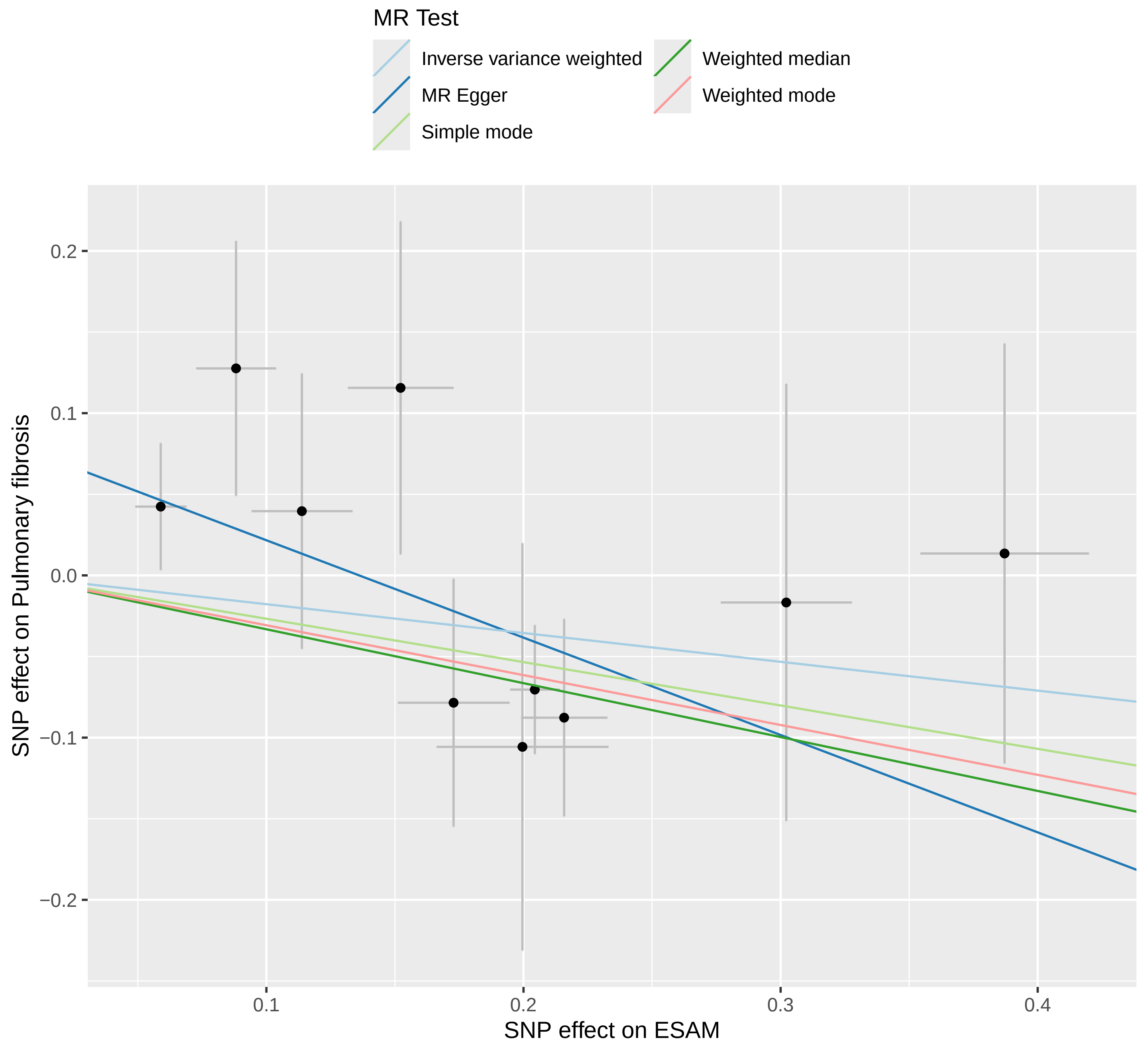

MR Test
Inverse variance weighted
MR Egger
Simple mode
Weighted median
Weighted mode
SNP effect on Pulmonary fibrosis
0.2
0.1
0.0
−0.1
−0.2
0.1
0.2
0.3
0.4
SNP effect on ESAM

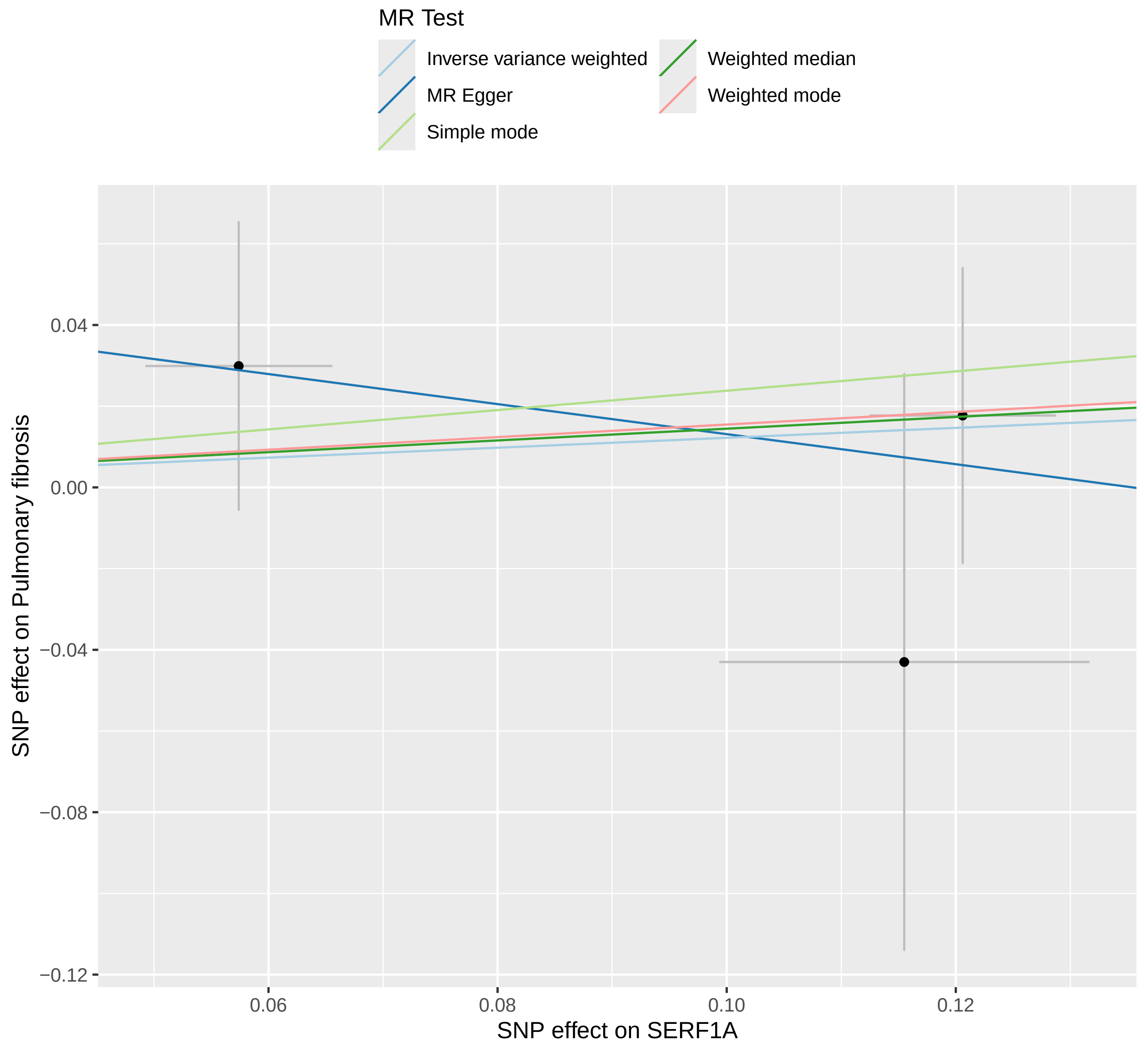
MR Test
Inverse variance weighted
MR Egger
Simple mode
Weighted median
Weighted mode
0.04
0.00
−0.04
−0.08
−0.12
SNP effect on Pulmonary fibrosis
0.06
0.08
0.10
0.12
SNP effect on SERF1A

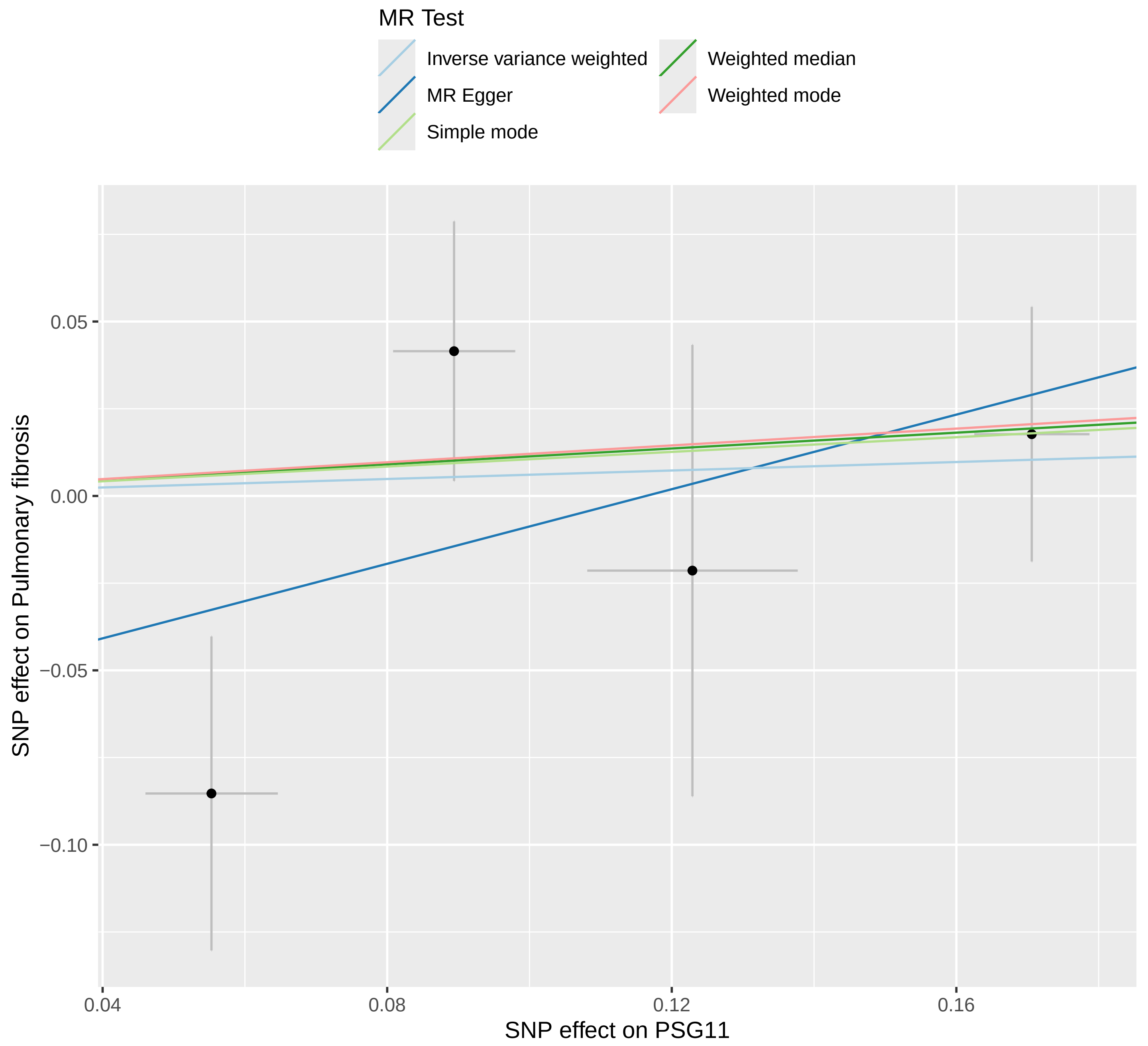
MR Test
Inverse variance weighted
MR Egger
Simple mode
Weighted median
Weighted mode
0.05
0.00
−0.05
−0.10
0.04
0.08
0.12
0.16
SNP effect on Pulmonary fibrosis
SNP effect on PSG11

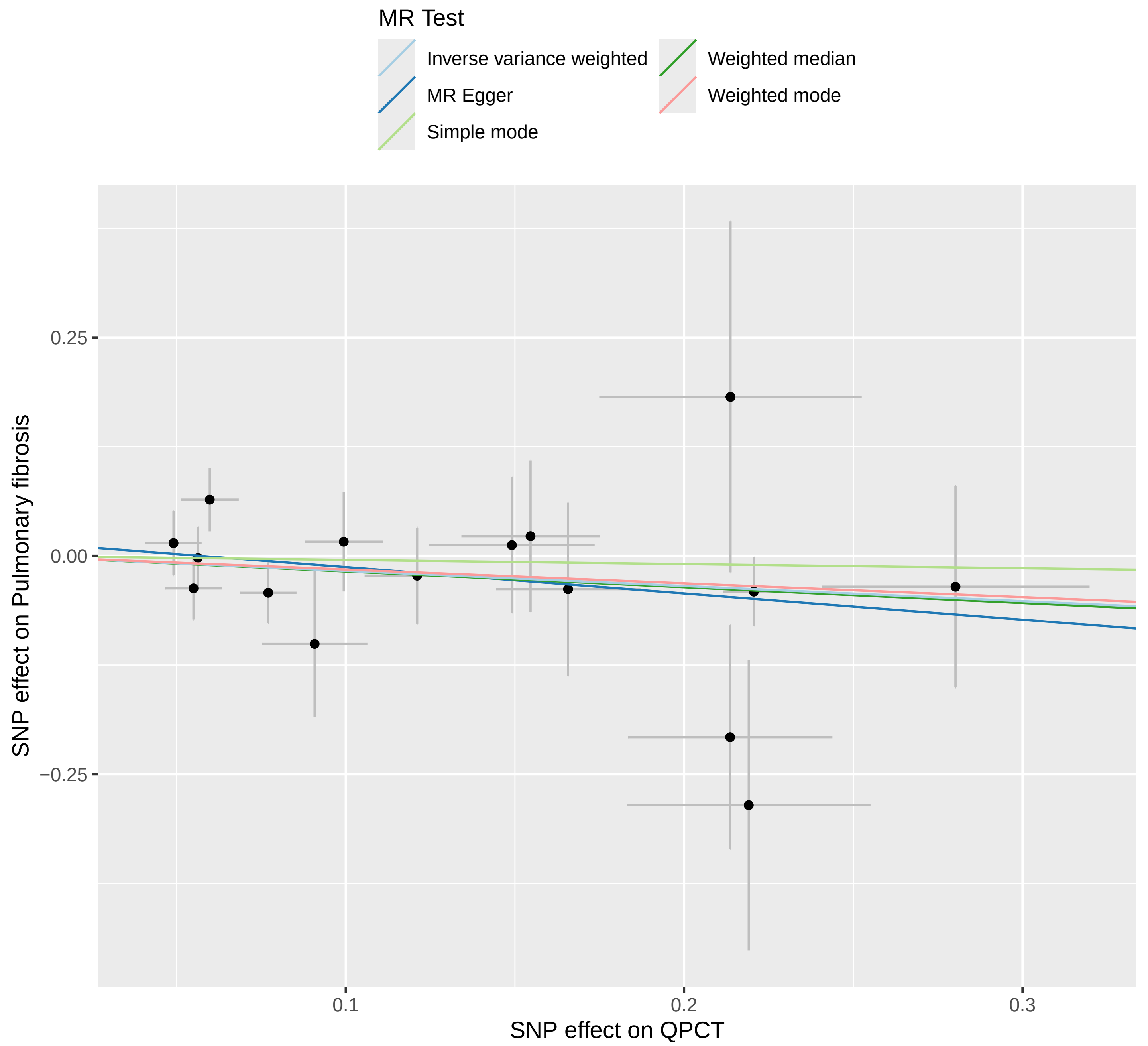

MR Test
Inverse variance weighted
MR Egger
Simple mode
Weighted median
Weighted mode
0.25
0.00
−0.25
SNP effect on Pulmonary fibrosis
0.1
0.2
0.3
SNP effect on QPCT

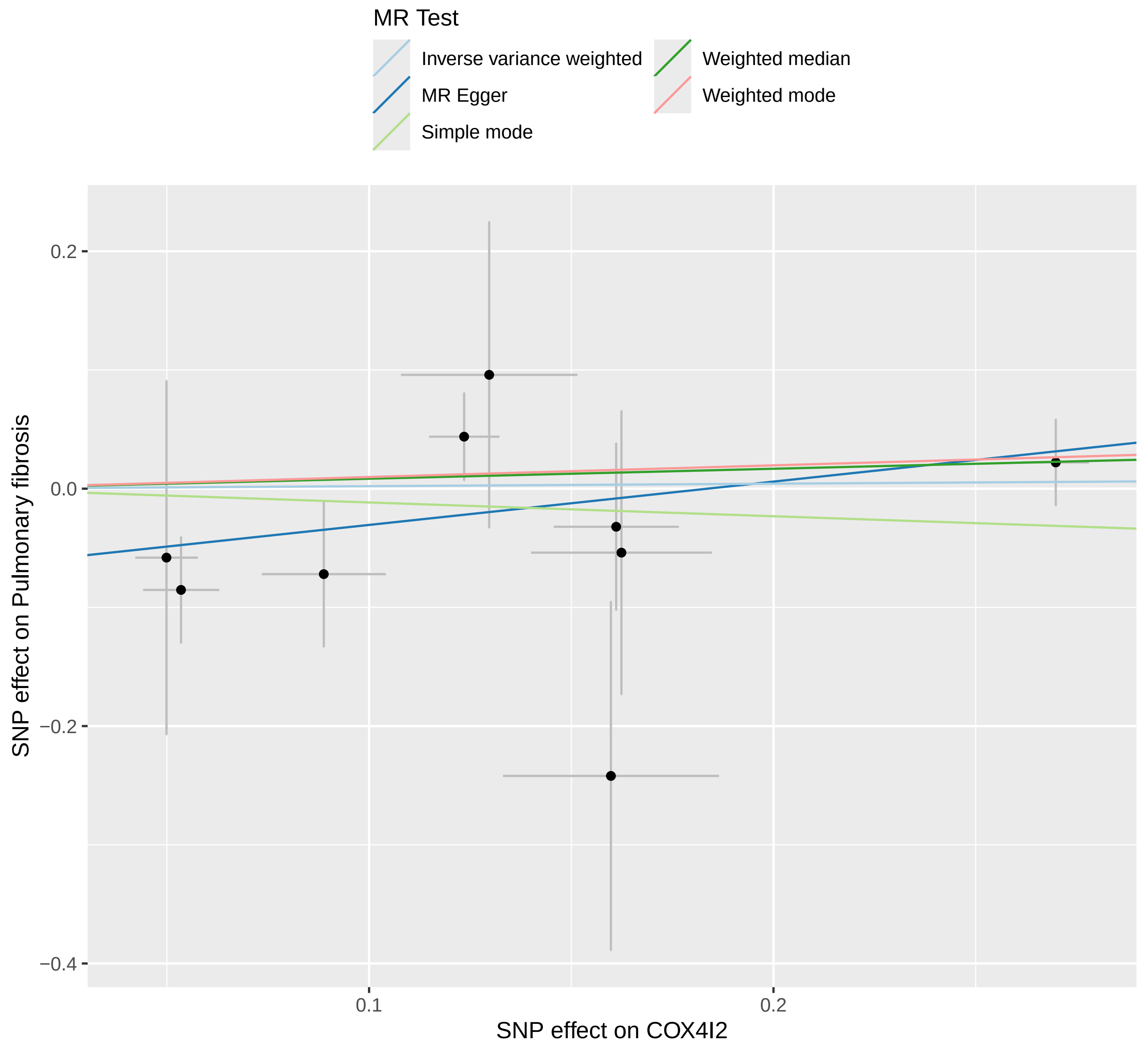
MR Test
Inverse variance weighted
MR Egger
Simple mode
Weighted median
Weighted mode
0.2
0.0
−0.2
−0.4
0.1
0.2
SNP effect on Pulmonary fibrosis
SNP effect on COX4I2

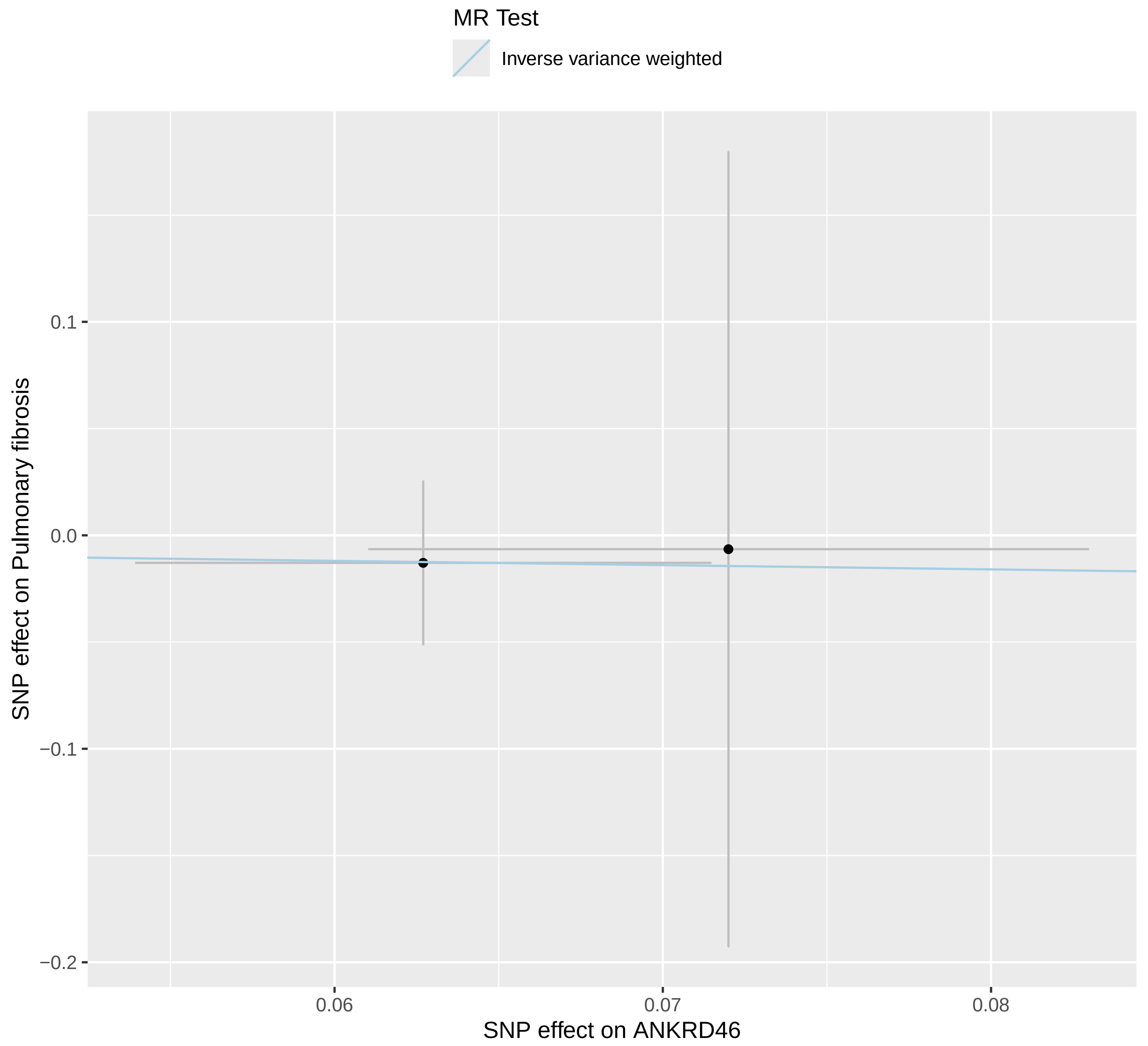

MR Test
Inverse variance weighted
SNP effect on Pulmonary fibrosis
0.1
0.0
−0.1
−0.2
0.06
0.07
0.08
SNP effect on ANKRD46

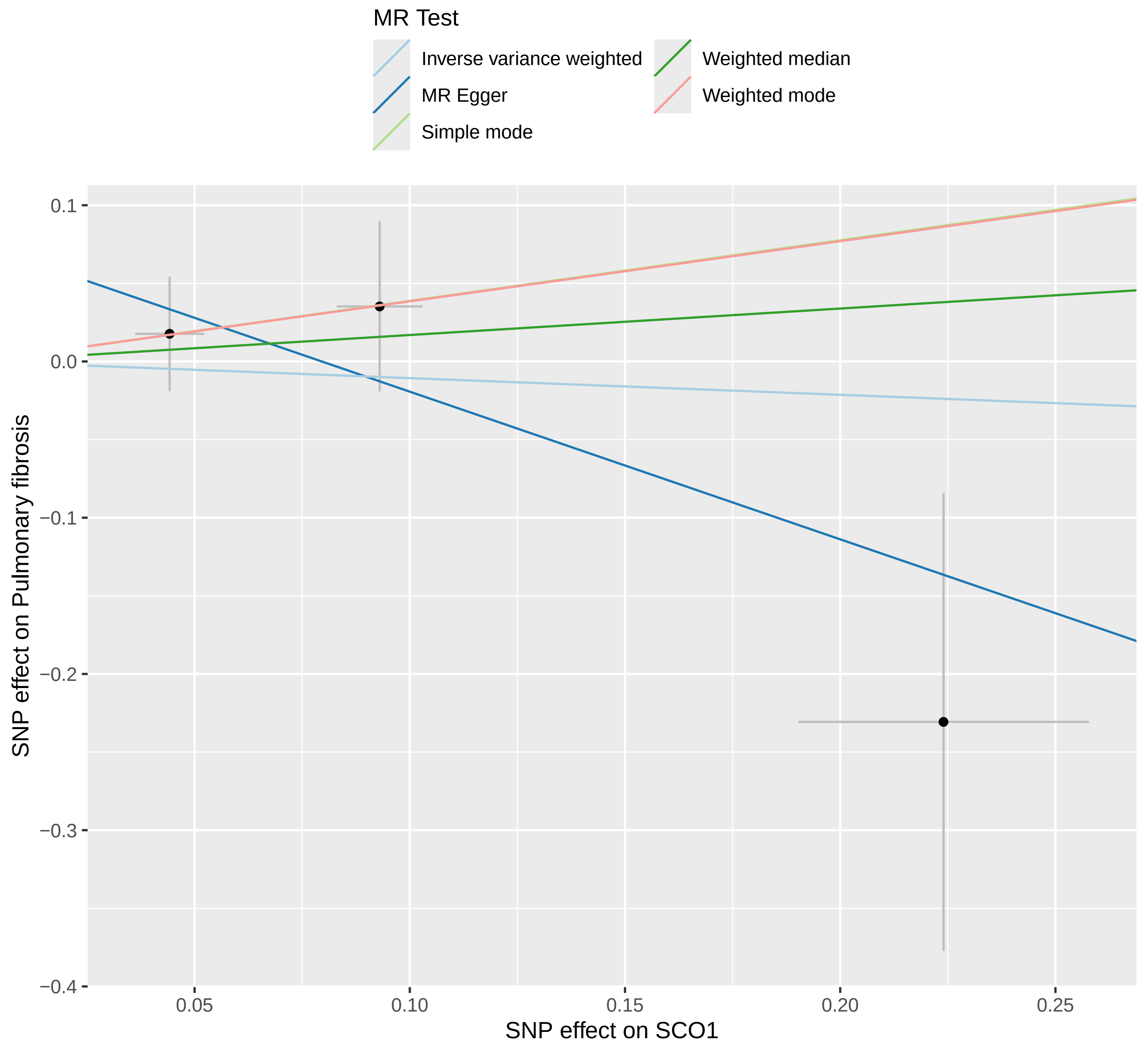

MR Test
Inverse variance weighted
MR Egger
Simple mode
Weighted median
Weighted mode
0.1
0.0
−0.1
−0.2
−0.3
−0.4
0.05
0.10
0.15
0.20
0.25
SNP effect on Pulmonary fibrosis
SNP effect on SCO1

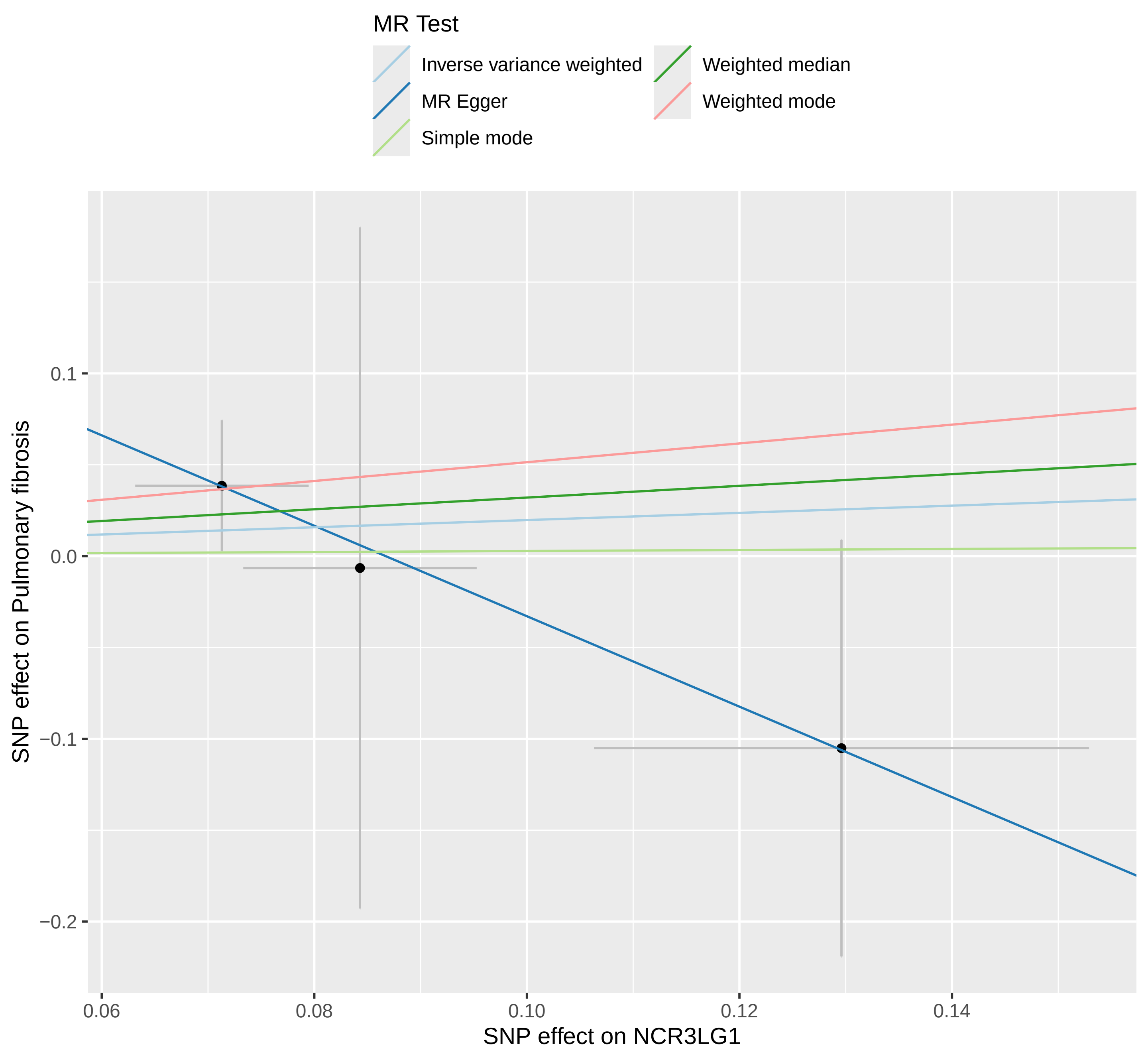

MR Test
Inverse variance weighted
MR Egger
Simple mode
Weighted median
Weighted mode
0.1
0.0
−0.1
−0.2
0.06
0.08
0.10
0.12
0.14
SNP effect on NCR3LG1
SNP effect on Pulmonary fibrosis

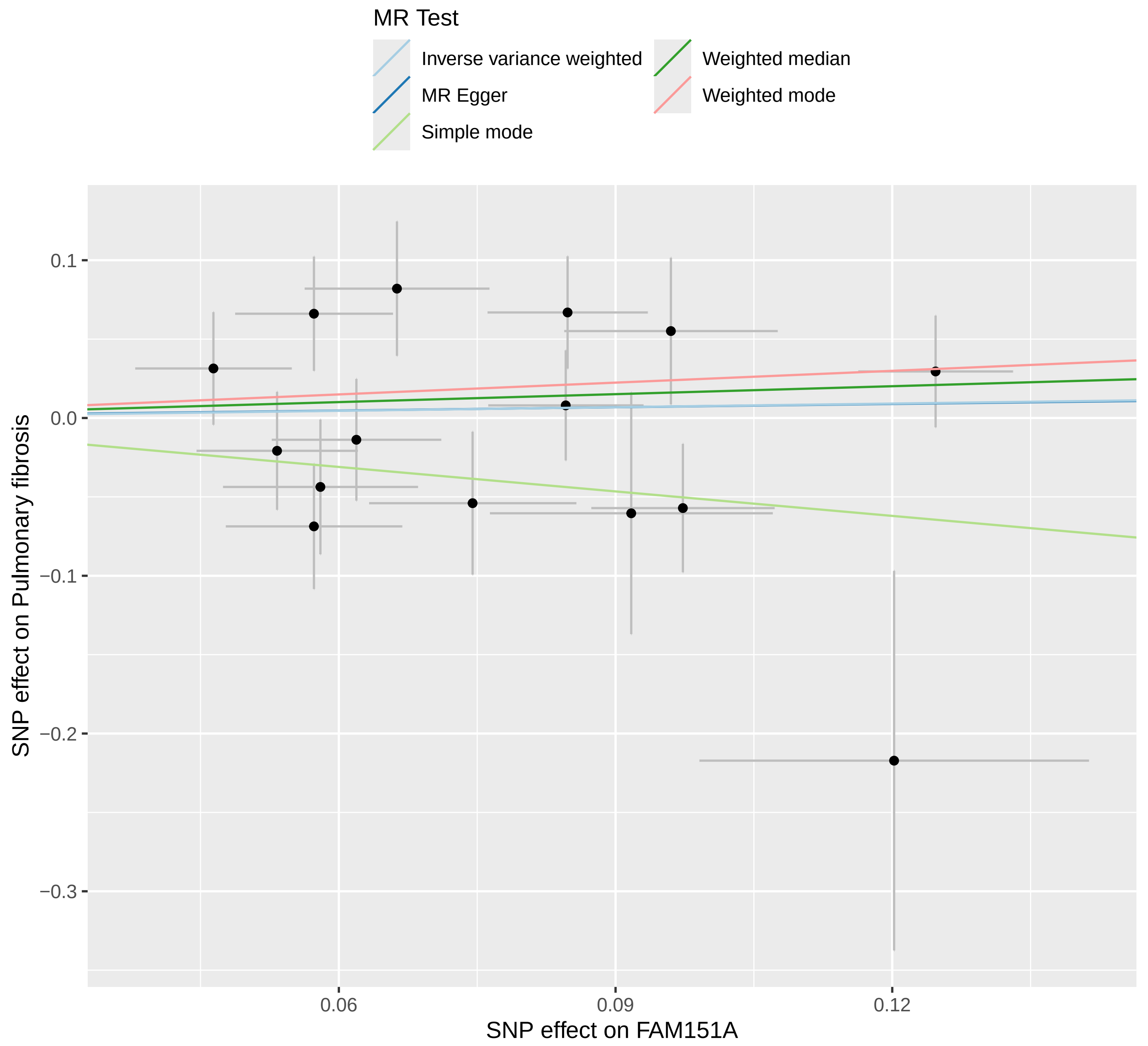

MR Test
Inverse variance weighted
MR Egger
Simple mode
Weighted median
Weighted mode
0.1
0.0
−0.1
−0.2
−0.3
SNP effect on Pulmonary fibrosis
0.06
0.09
0.12
SNP effect on FAM151A

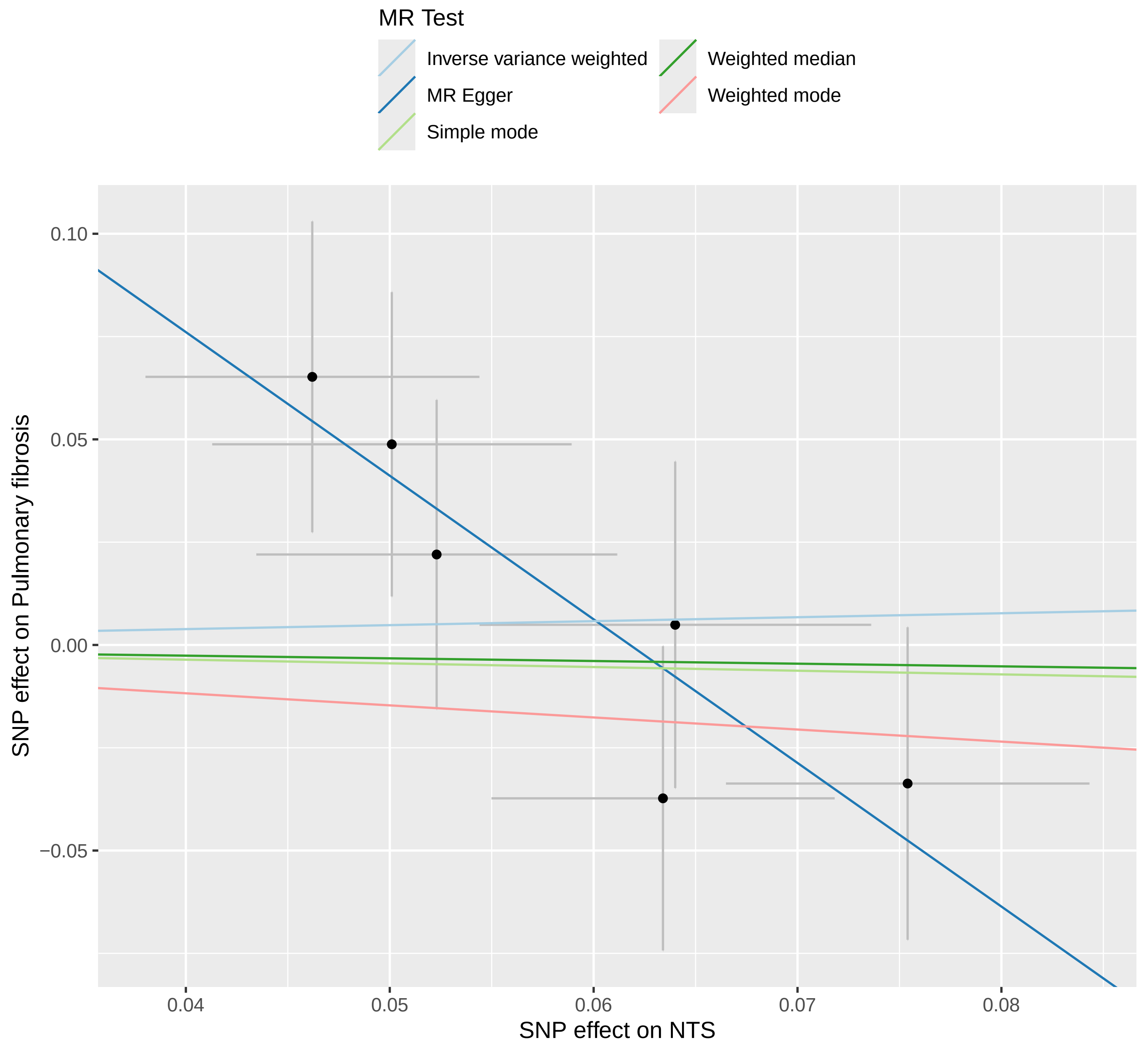
MR Test
Inverse variance weighted
MR Egger
Simple mode
Weighted median
Weighted mode
0.10
0.05
0.00
−0.05
SNP effect on Pulmonary fibrosis
0.04
0.05
0.06
0.07
0.08
SNP effect on NTS

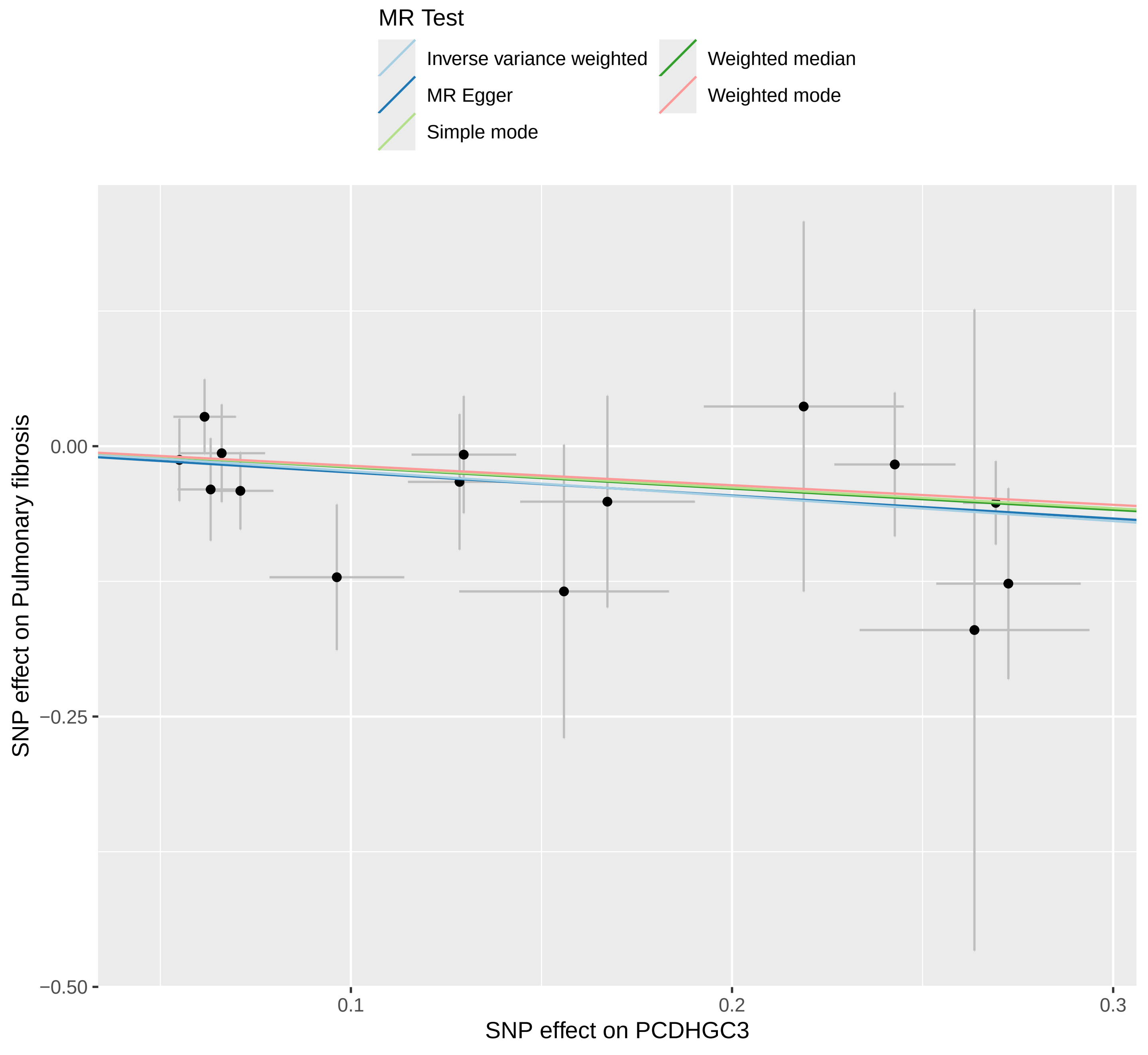

MR Test
Inverse variance weighted
MR Egger
Simple mode
Weighted median
Weighted mode
SNP effect on Pulmonary fibrosis
0.00
−0.25
−0.50
0.1
0.2
0.3
SNP effect on PCDHGC3

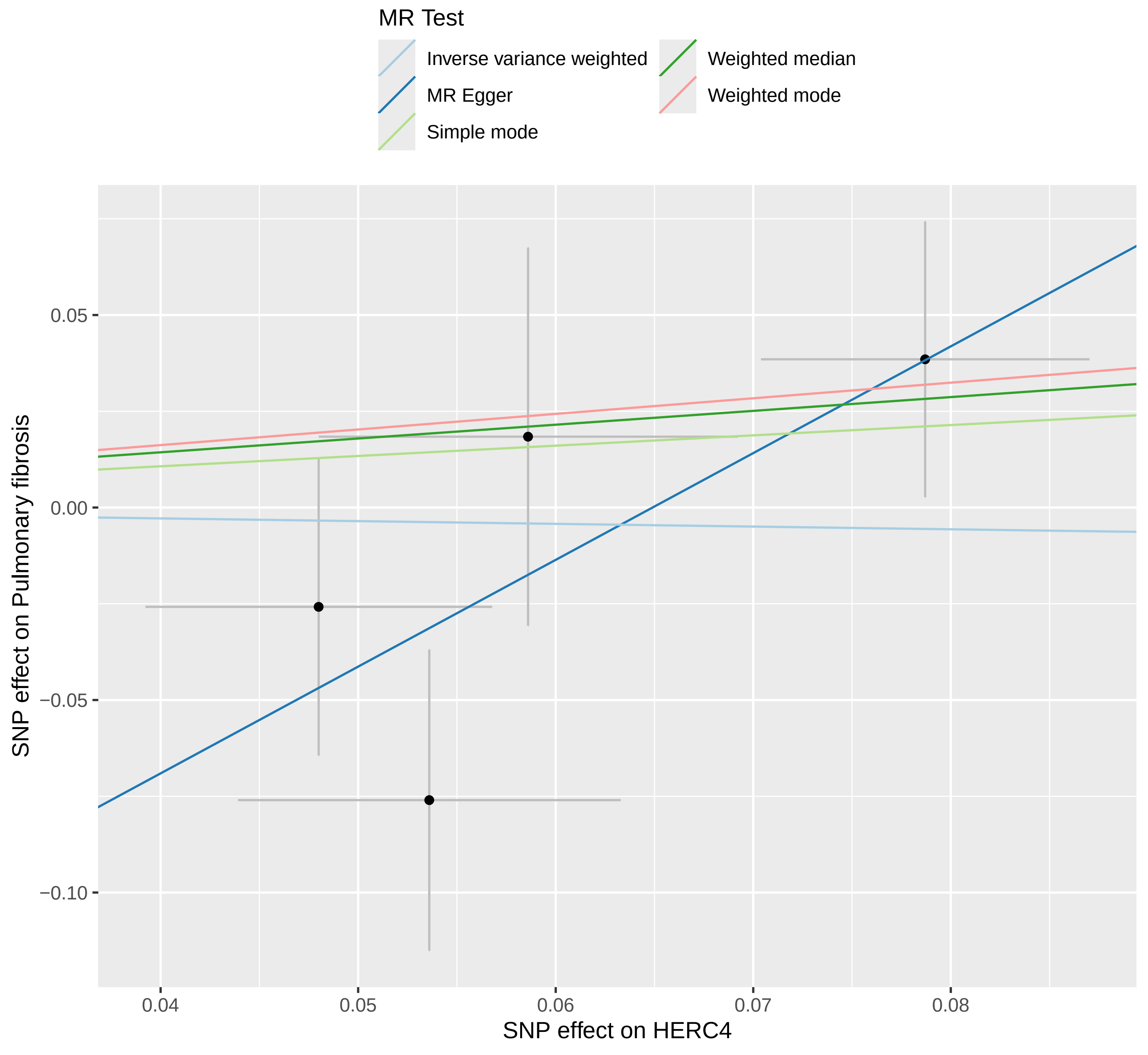
MR Test
Inverse variance weighted
MR Egger
Simple mode
Weighted median
Weighted mode
0.05
0.00
−0.05
−0.10
0.04
0.05
0.06
0.07
0.08
SNP effect on HERC4
SNP effect on Pulmonary fibrosis

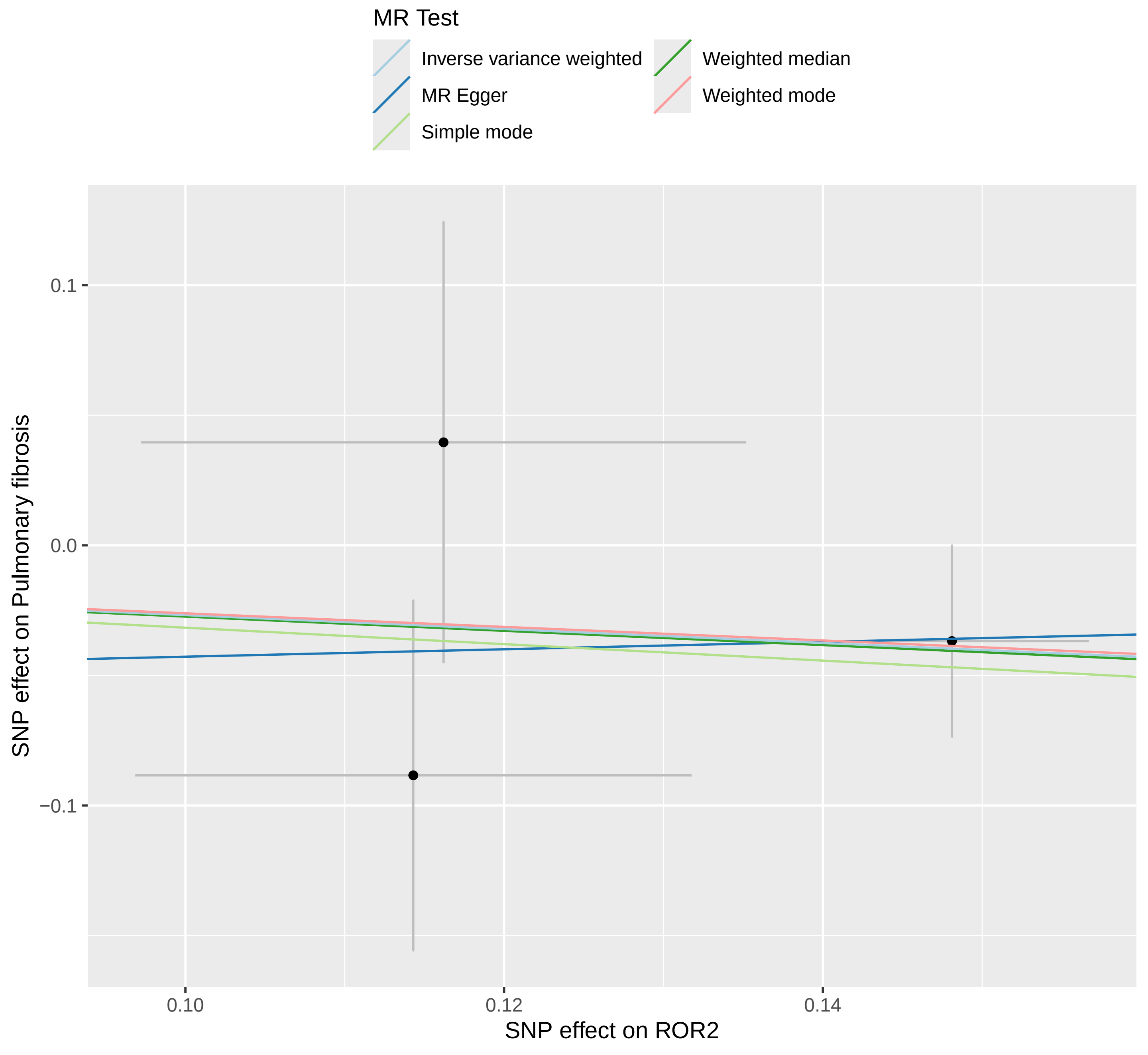

MR Test
Inverse variance weighted
MR Egger
Simple mode
Weighted median
Weighted mode
SNP effect on Pulmonary fibrosis
0.1
0.0
−0.1
0.10
0.12
0.14
SNP effect on ROR2

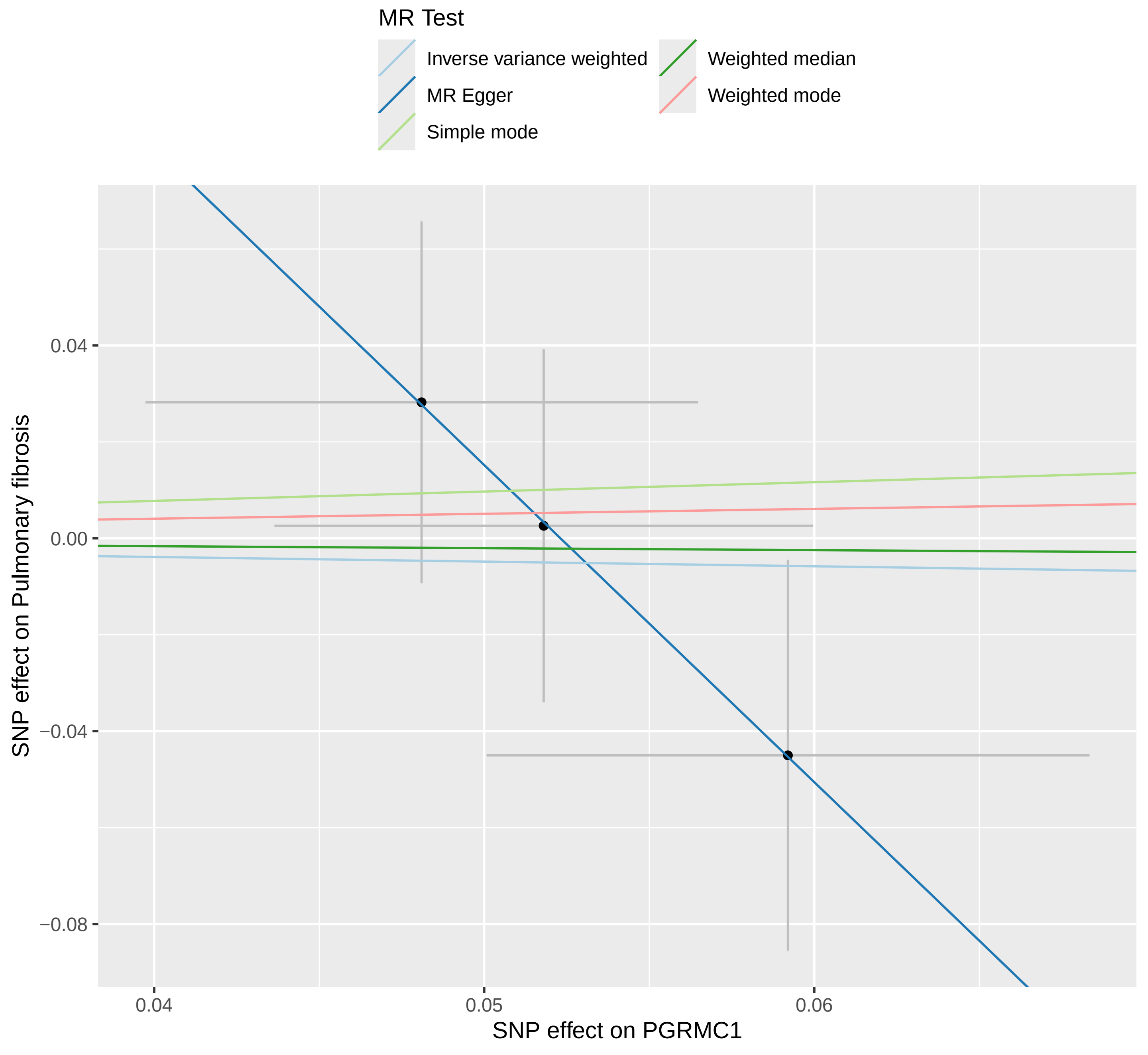
MR Test
Inverse variance weighted
MR Egger
Simple mode
Weighted median
Weighted mode
0.04
0.00
−0.04
−0.08
SNP effect on Pulmonary fibrosis
0.04
0.05
0.06
SNP effect on PGRMC1

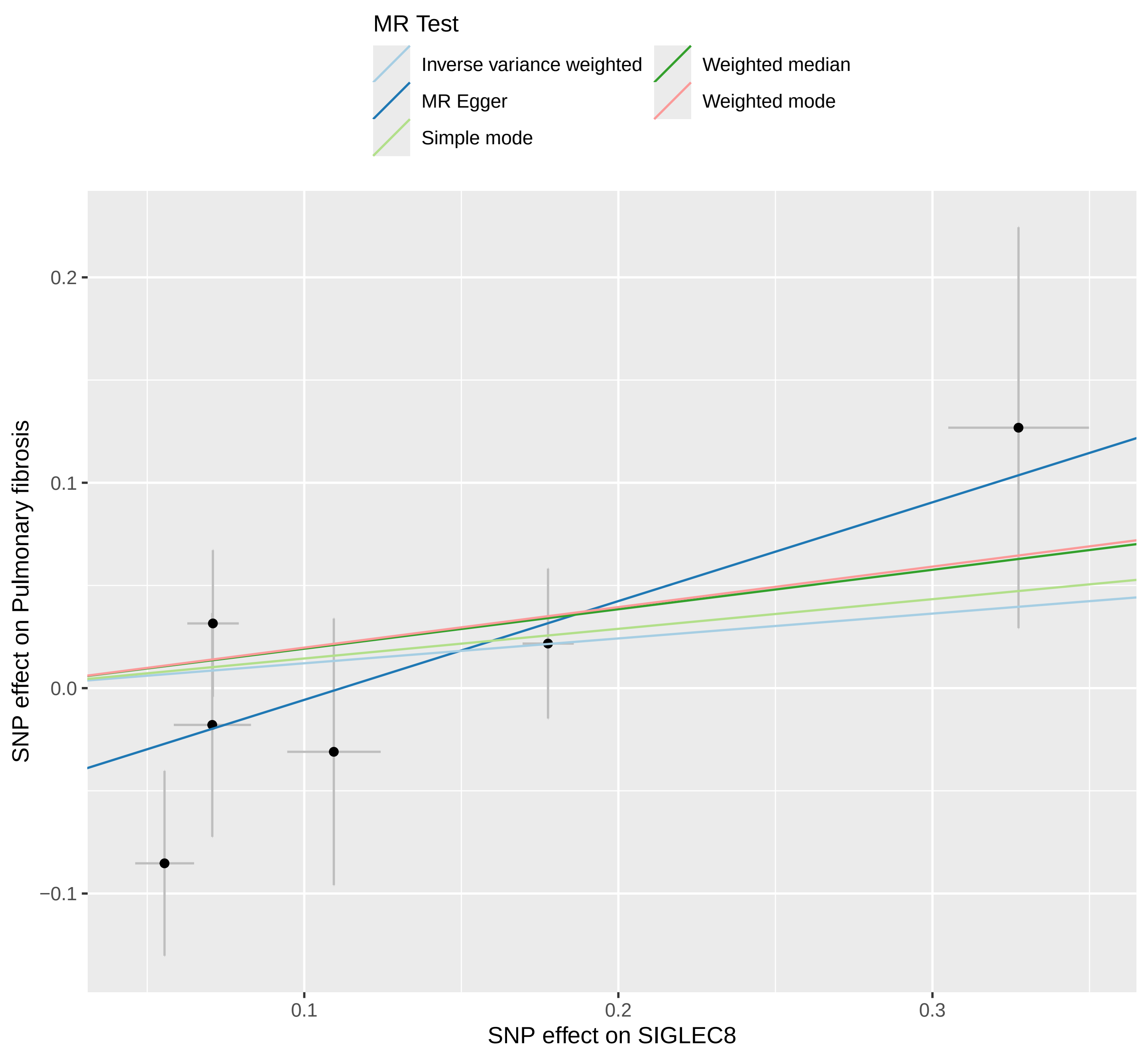
MR Test
Inverse variance weighted
MR Egger
Simple mode
Weighted median
Weighted mode
0.2
0.1
0.0
−0.1
SNP effect on Pulmonary fibrosis
0.1
0.2
0.3
SNP effect on SIGLEC8

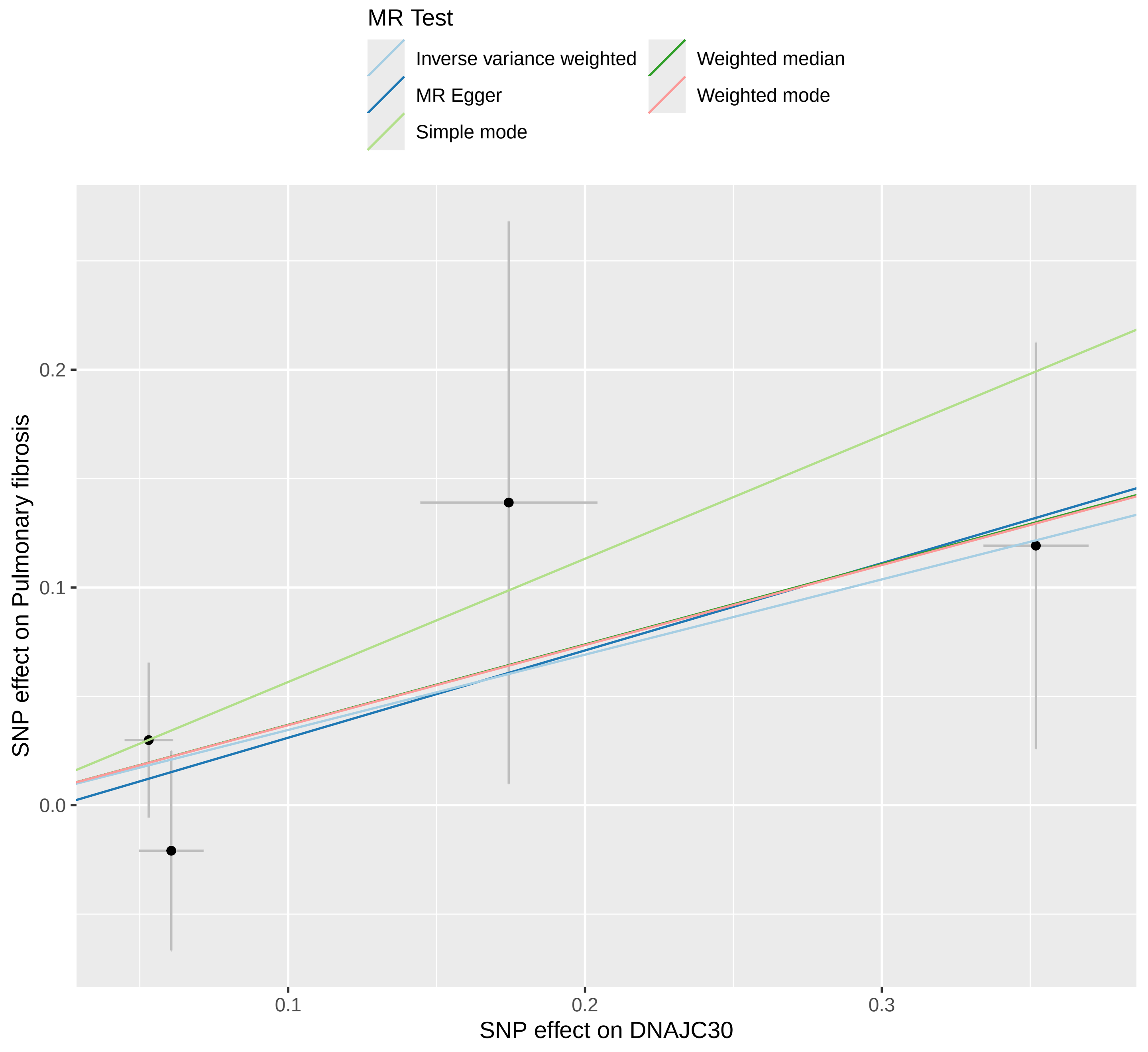
MR Test
Inverse variance weighted
MR Egger
Simple mode
Weighted median
Weighted mode
0.2
0.1
0.0
SNP effect on Pulmonary fibrosis
0.1
0.2
0.3
SNP effect on DNAJC30

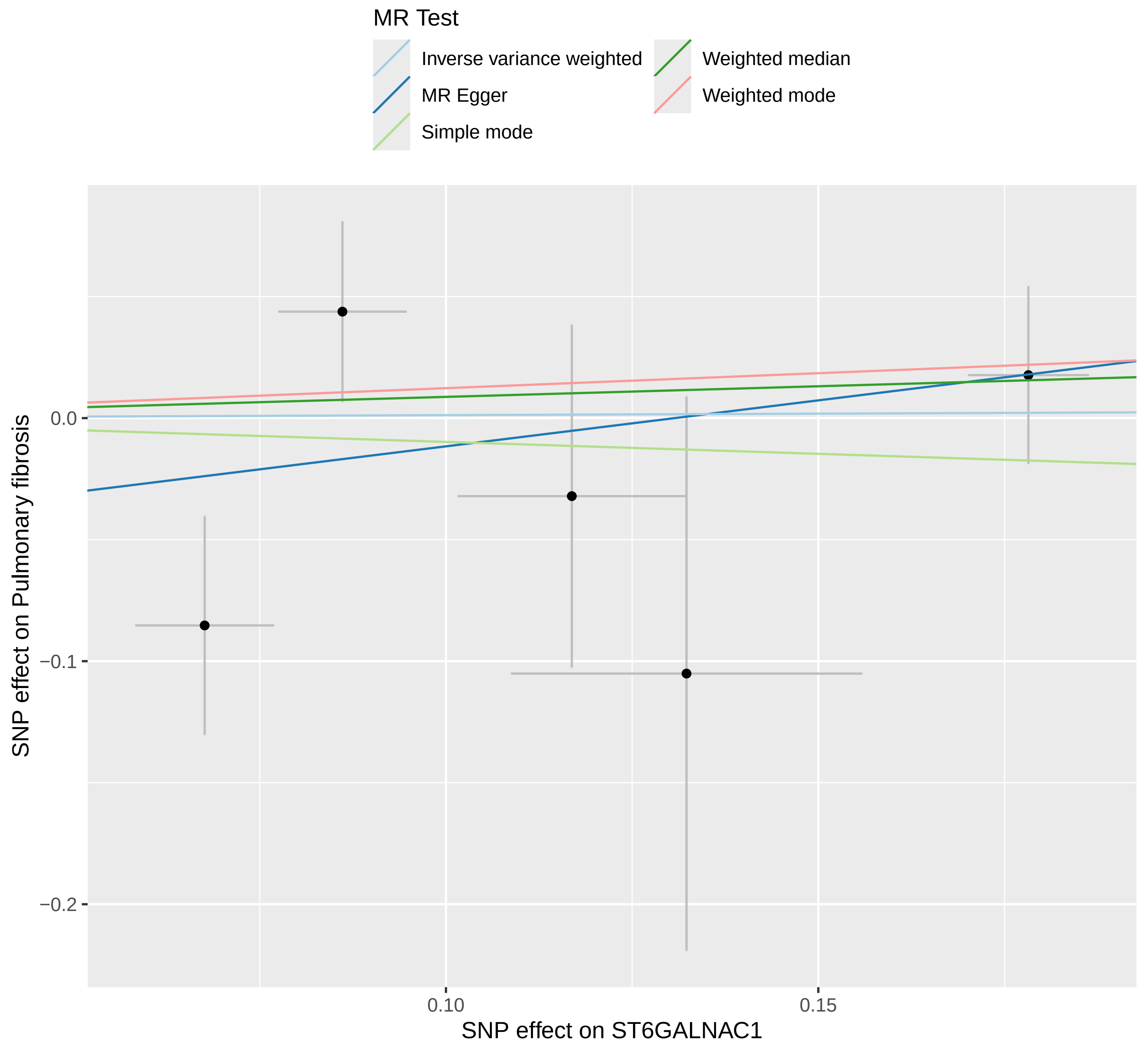

MR Test
Inverse variance weighted
MR Egger
Simple mode
Weighted median
Weighted mode
SNP effect on Pulmonary fibrosis
0.0
−0.1
−0.2
0.10
0.15
SNP effect on ST6GALNAC1

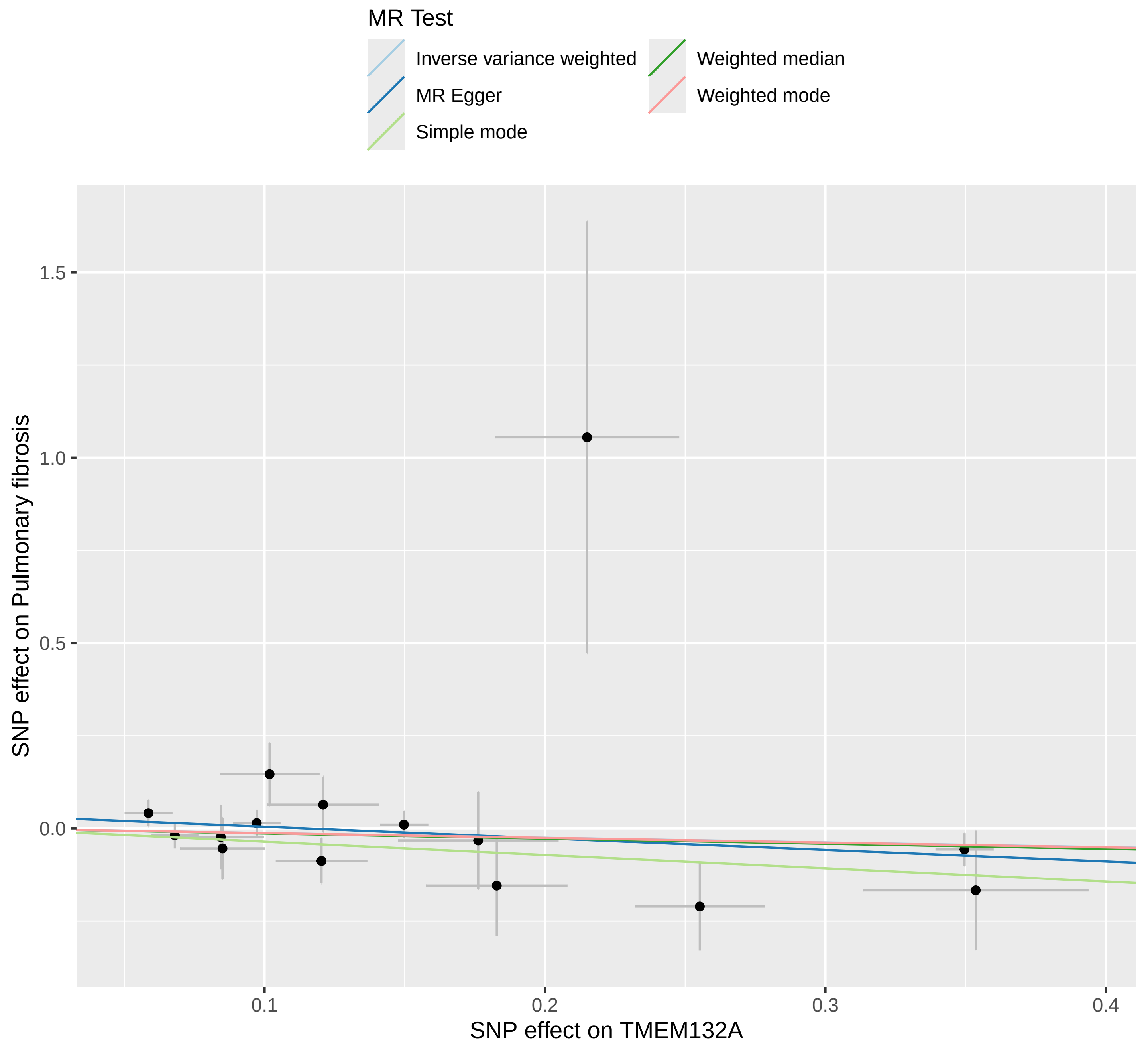
MR Test
Inverse variance weighted
MR Egger
Simple mode
Weighted median
Weighted mode
1.5
1.0
0.5
0.0
SNP effect on Pulmonary fibrosis
0.1
0.2
0.3
0.4
SNP effect on TMEM132A

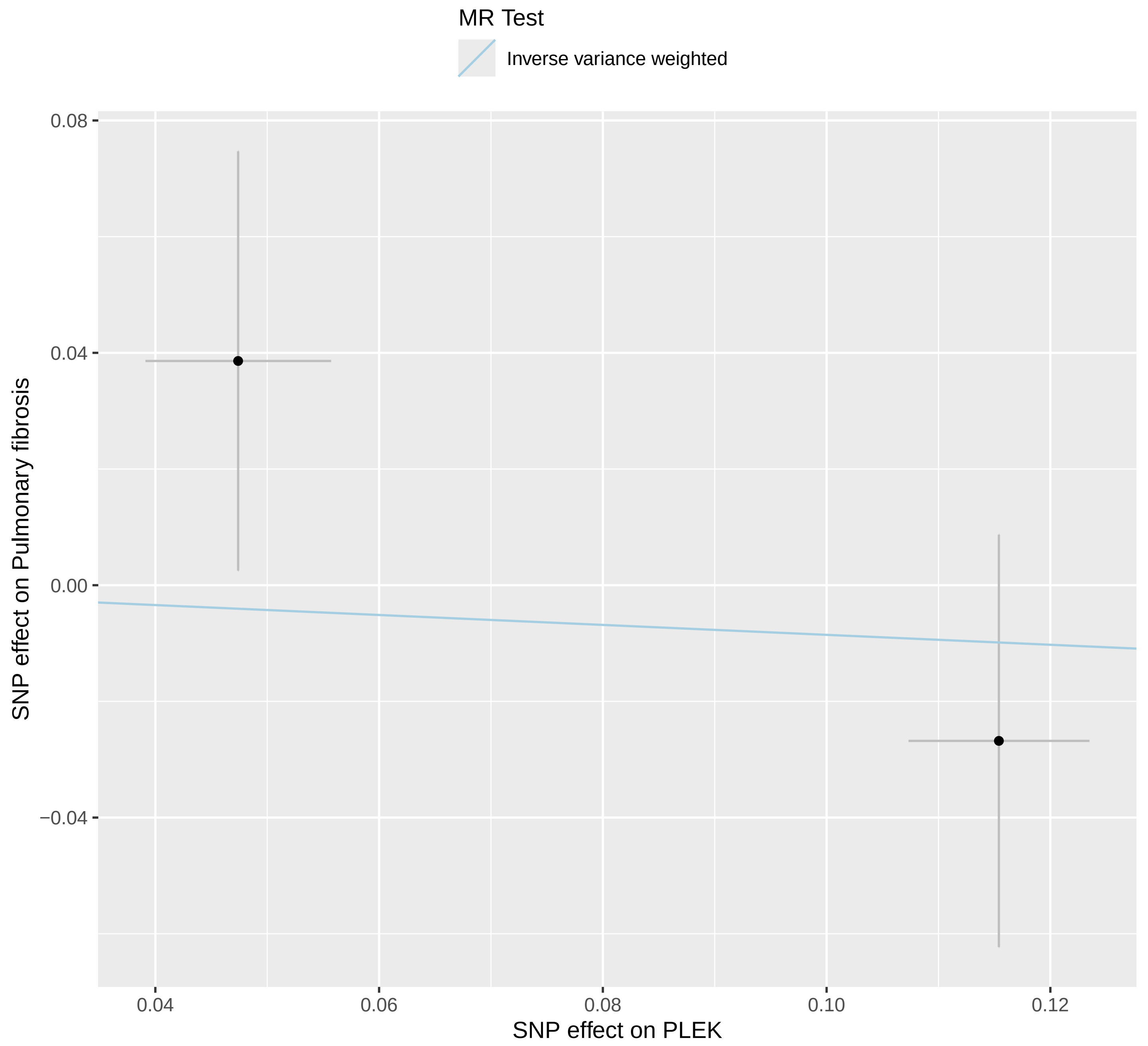
MR Test
Inverse variance weighted
0.08
0.04
0.00
−0.04
SNP effect on Pulmonary fibrosis
0.04
0.06
0.08
0.10
0.12
SNP effect on PLEK

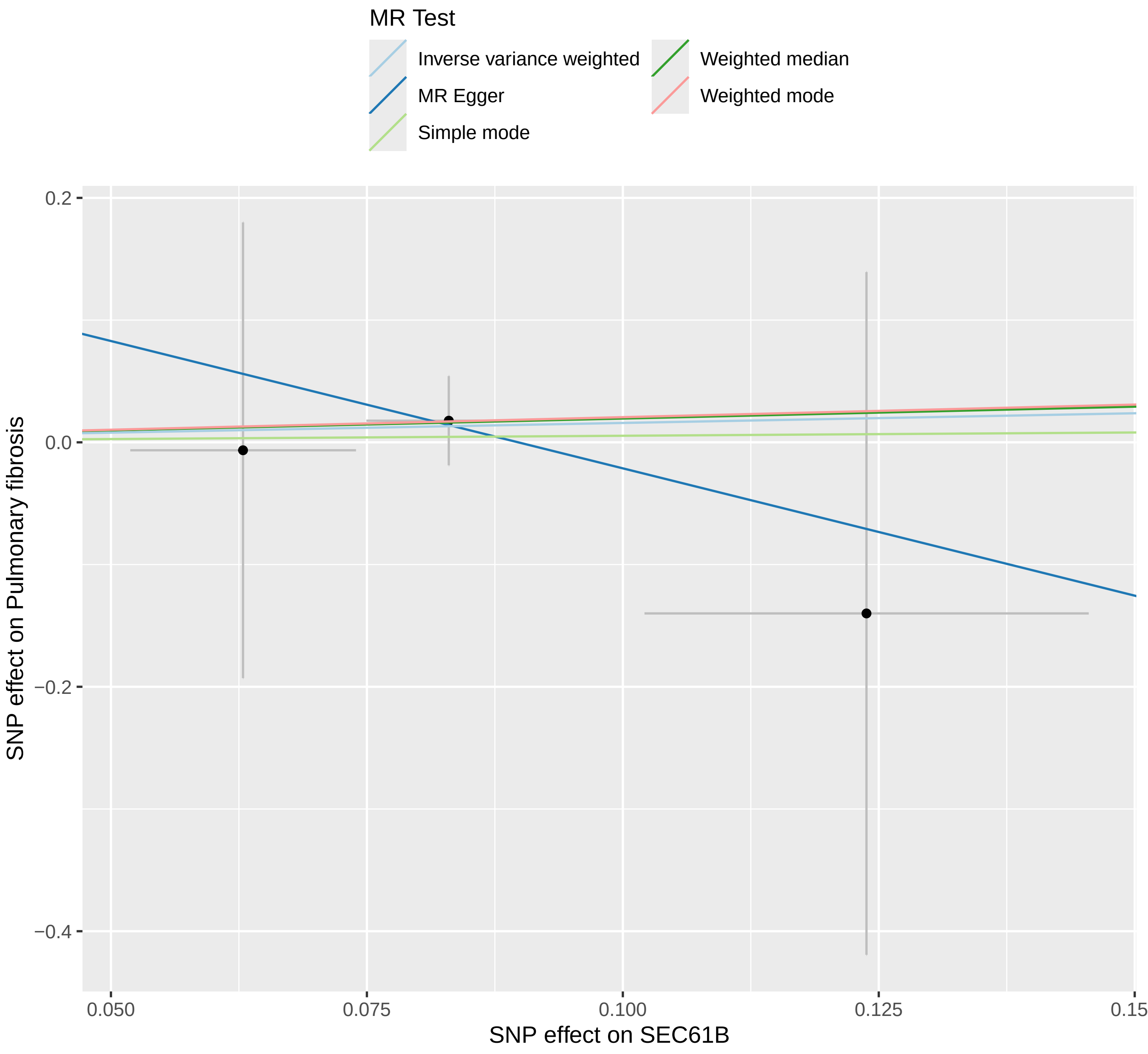
MR Test
Inverse variance weighted
MR Egger
Simple mode
Weighted median
Weighted mode
0.2
0.0
−0.2
−0.4
0.050
0.075
0.100
0.125
0.150
SNP effect on SEC61B
SNP effect on Pulmonary fibrosis

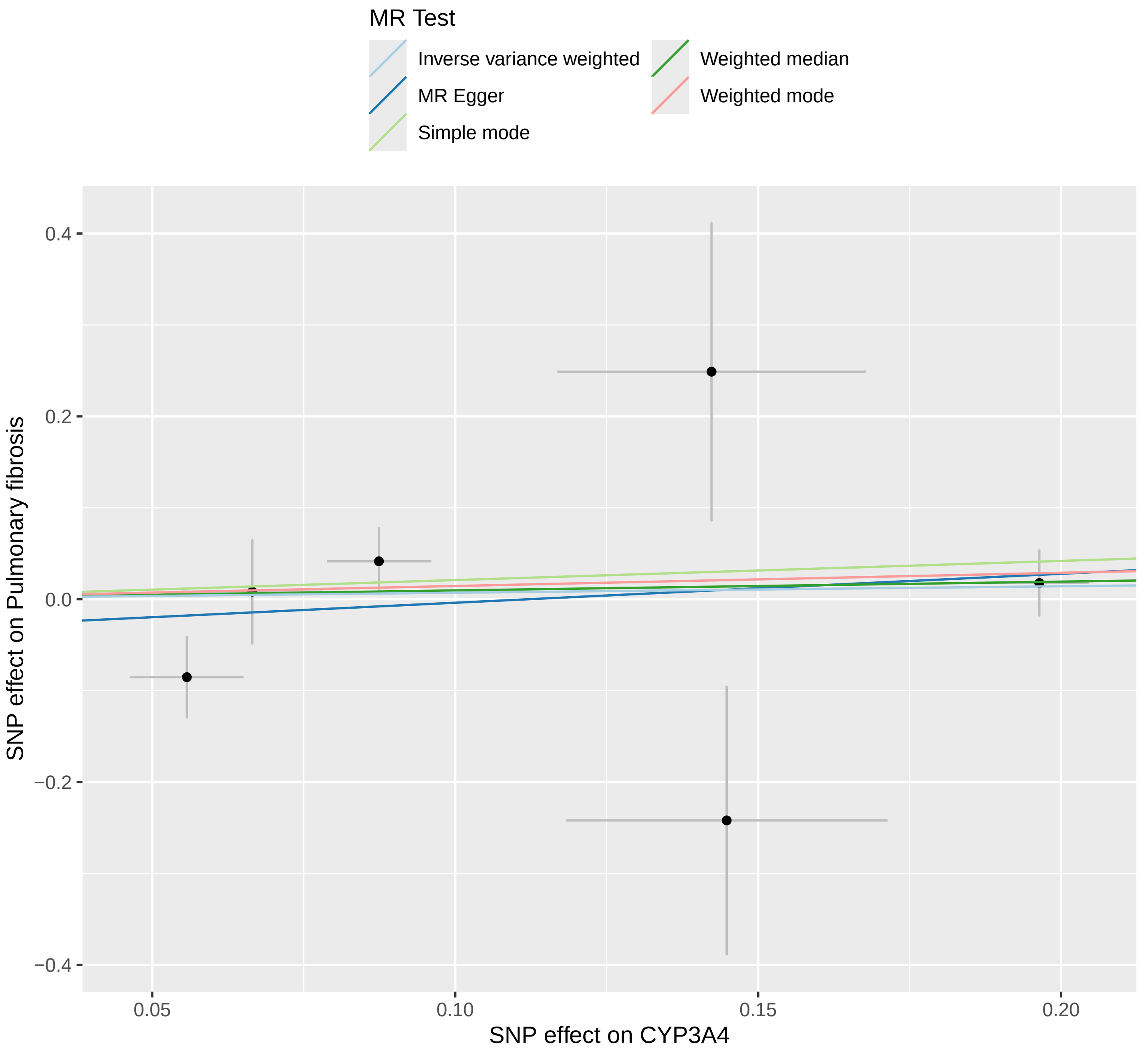
MR Test
Inverse variance weighted
MR Egger
Simple mode
Weighted median
Weighted mode
SNP effect on Pulmonary fibrosis
0.4
0.2
0.0
−0.2
−0.4
0.05
0.10
0.15
0.20
SNP effect on CYP3A4

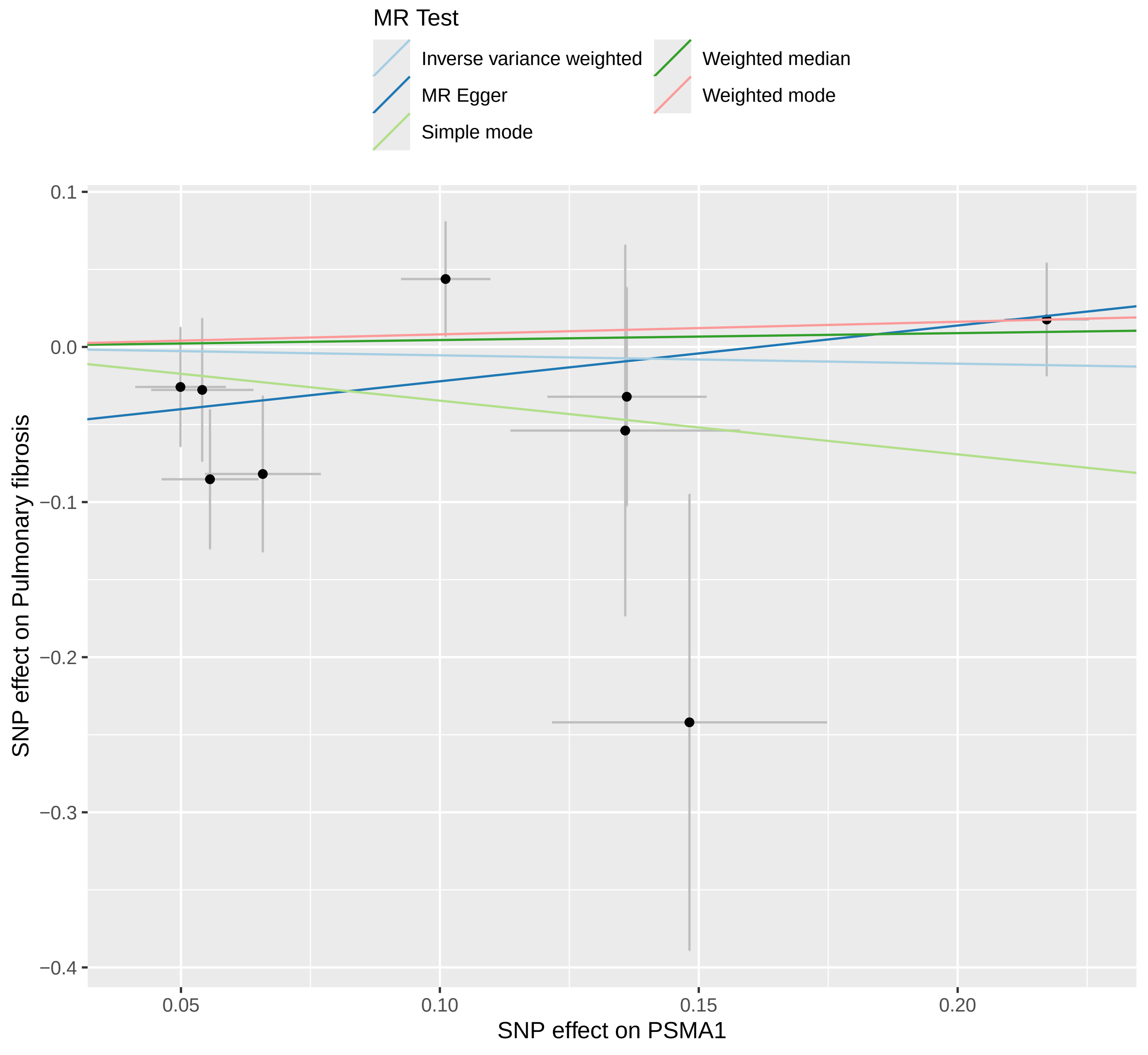

MR Test
Inverse variance weighted
MR Egger
Simple mode
Weighted median
Weighted mode
0.1
0.0
−0.1
−0.2
−0.3
−0.4
SNP effect on Pulmonary fibrosis
0.05
0.10
0.15
0.20
SNP effect on PSMA1

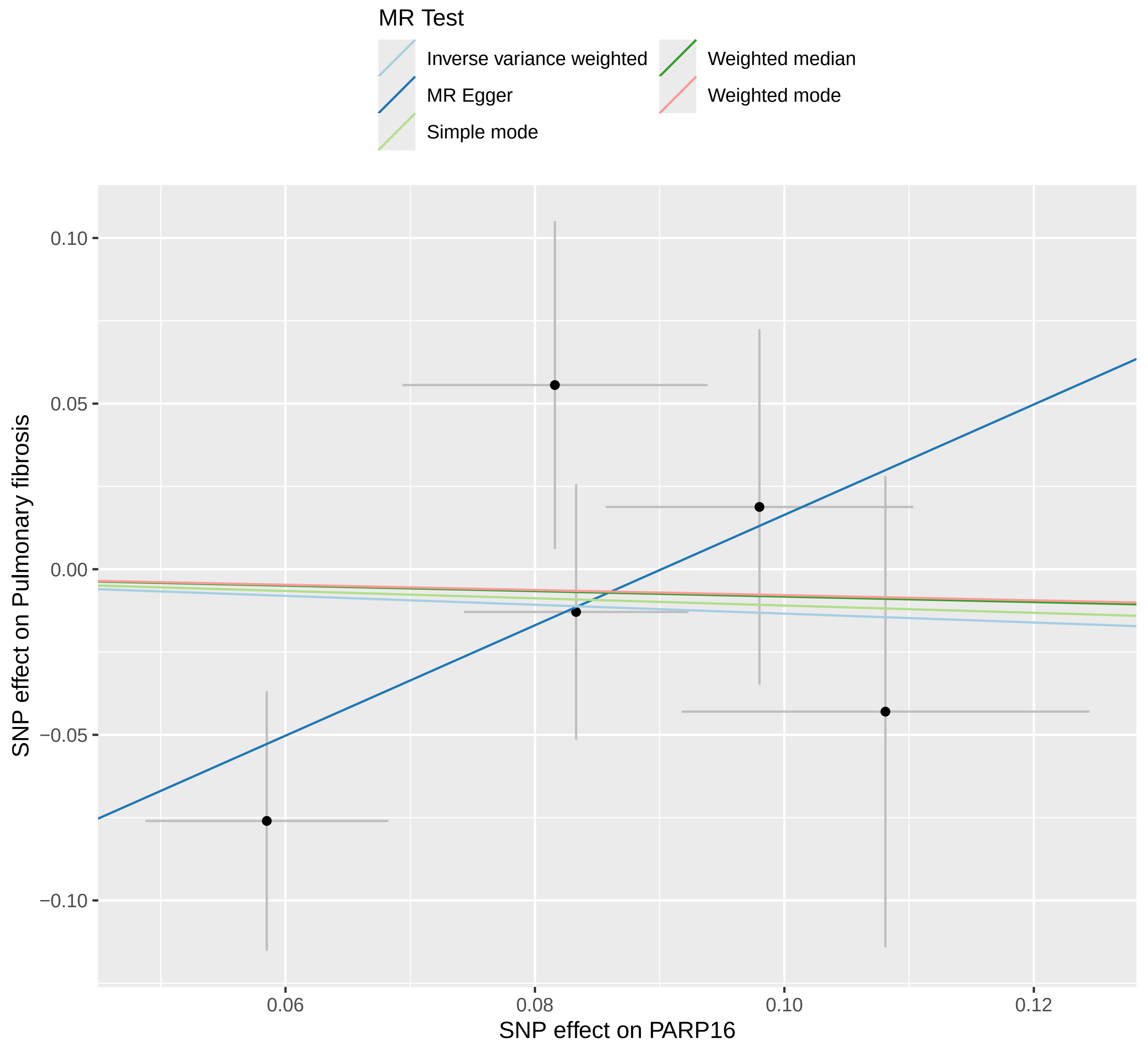

MR Test
Inverse variance weighted
MR Egger
Simple mode
Weighted median
Weighted mode
0.10
0.05
0.00
−0.05
−0.10
SNP effect on Pulmonary fibrosis
0.06
0.08
0.10
0.12
SNP effect on PARP16

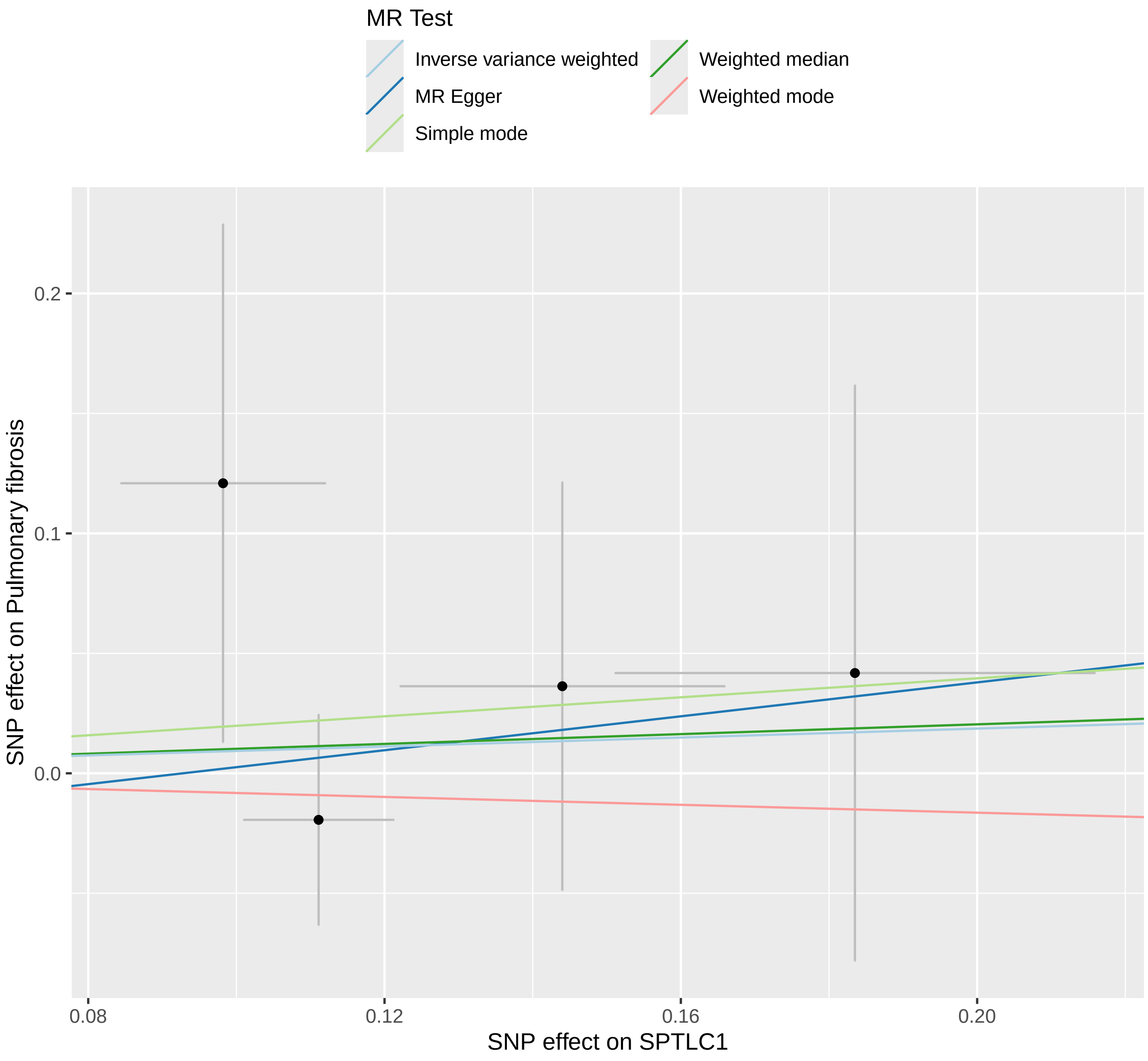

MR Test
Inverse variance weighted
MR Egger
Simple mode
Weighted median
Weighted mode
0.2
0.1
0.0
0.08
0.12
0.16
0.20
SNP effect on SPTLC1
SNP effect on Pulmonary fibrosis

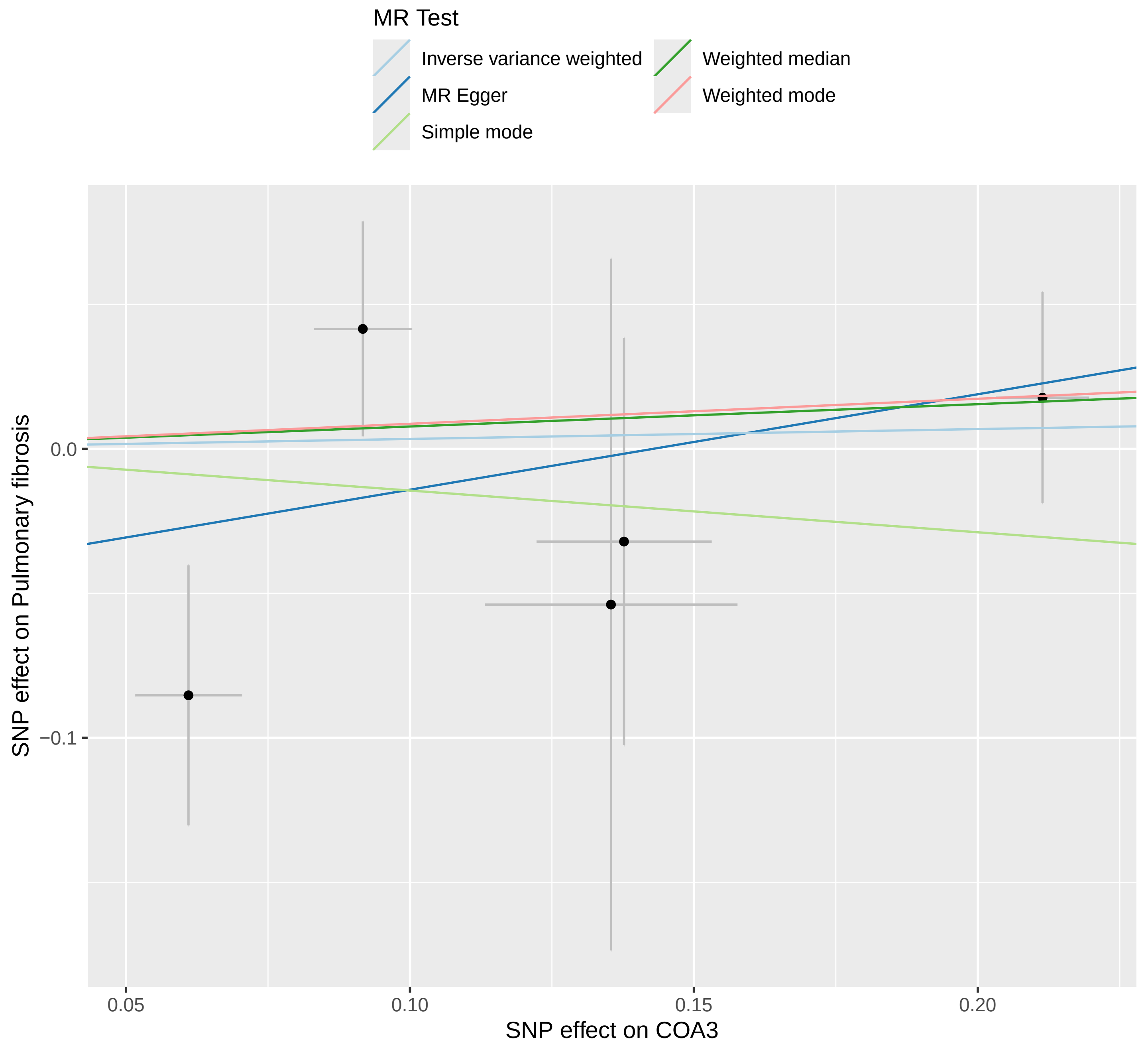
MR Test
Inverse variance weighted
MR Egger
Simple mode
Weighted median
Weighted mode
SNP effect on Pulmonary fibrosis
0.0
−0.1
0.05
0.10
0.15
0.20
SNP effect on COA3

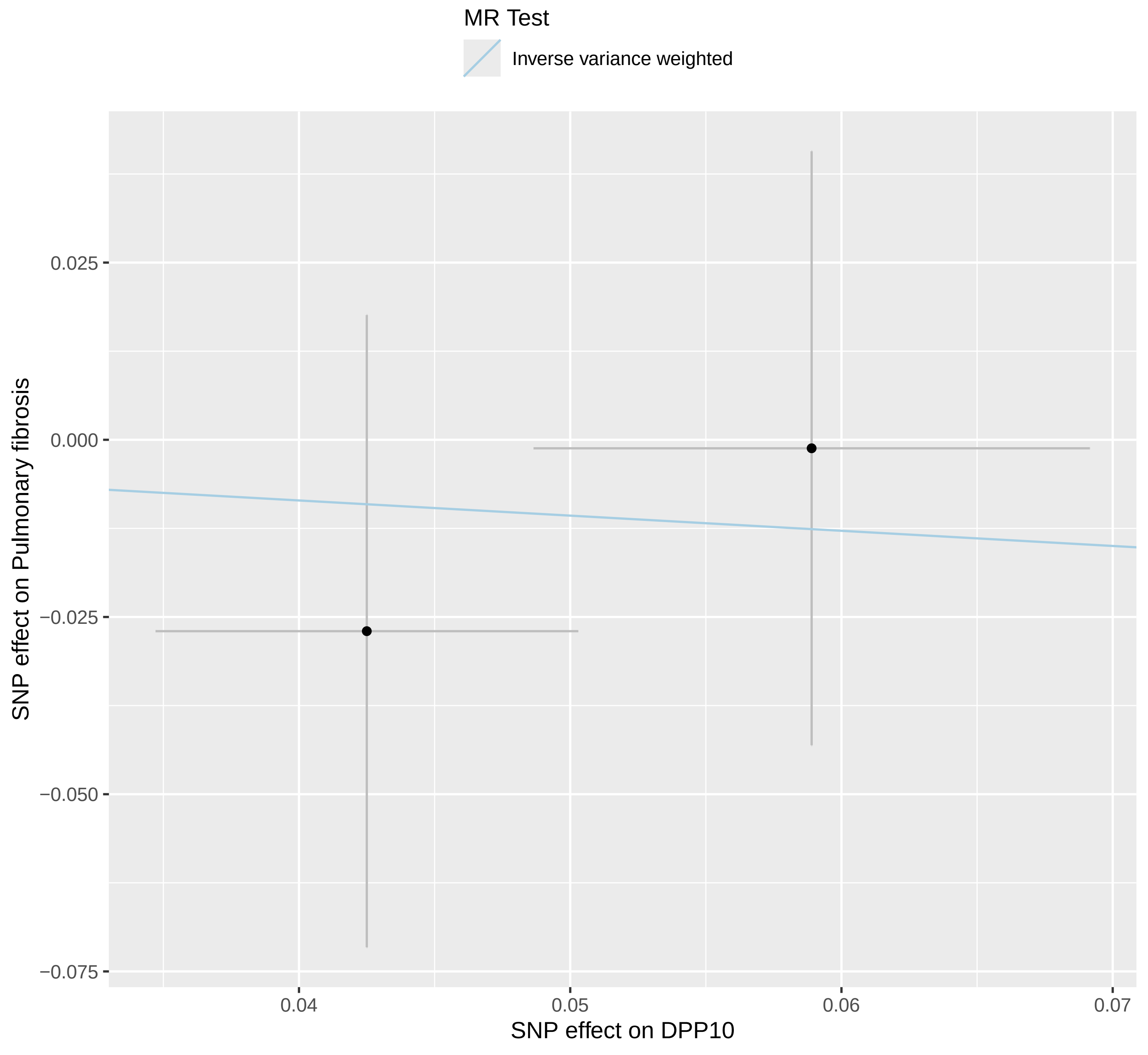

MR Test
Inverse variance weighted
0.025
0.000
−0.025
−0.050
−0.075
0.04
0.05
0.06
0.07
SNP effect on Pulmonary fibrosis
SNP effect on DPP10

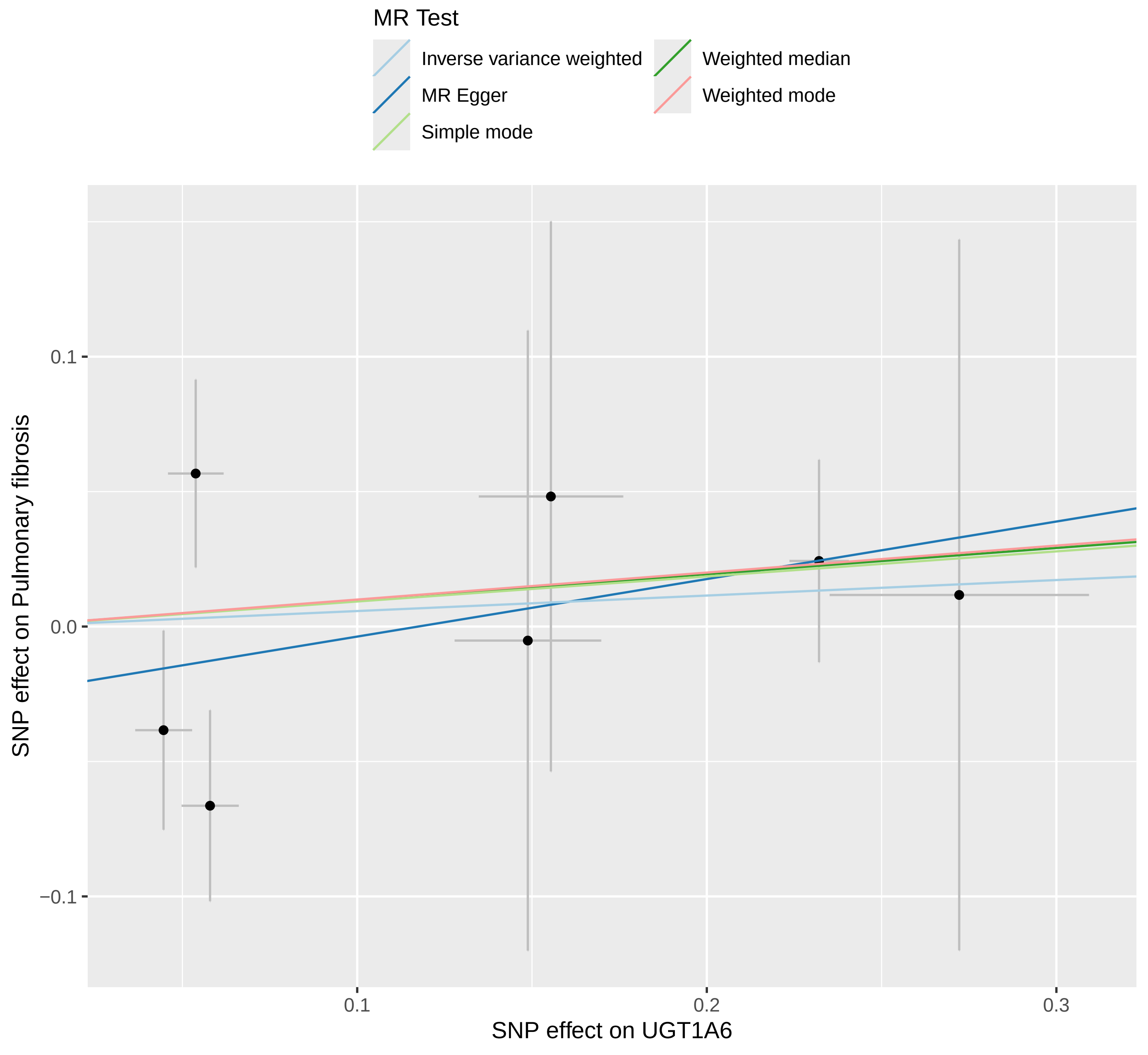
MR Test
Inverse variance weighted
MR Egger
Simple mode
Weighted median
Weighted mode
0.1
0.0
−0.1
0.1
0.2
0.3
SNP effect on Pulmonary fibrosis
SNP effect on UGT1A6

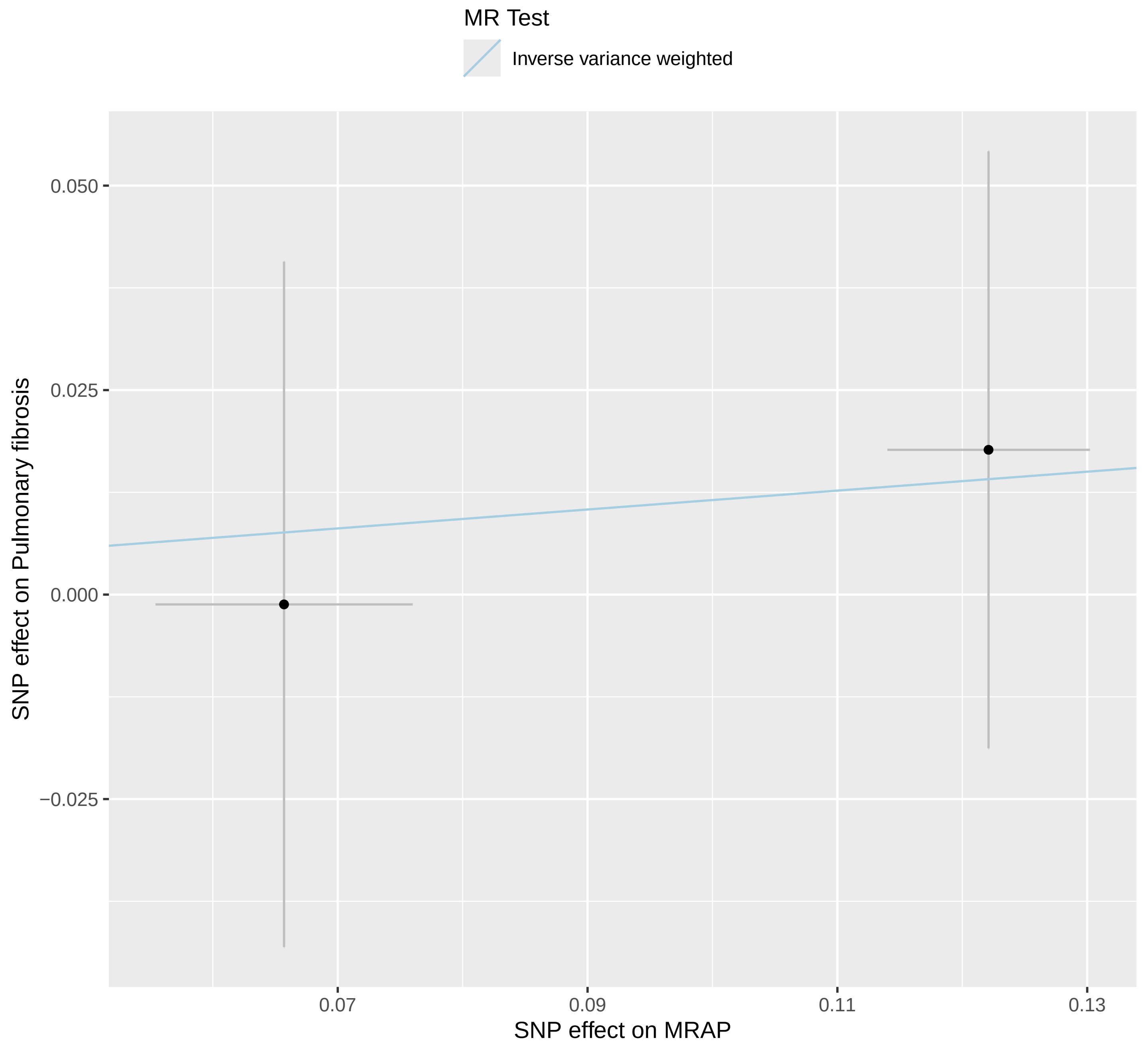

MR Test
Inverse variance weighted
0.050
0.025
0.000
−0.025
SNP effect on Pulmonary fibrosis
0.07
0.09
0.11
0.13
SNP effect on MRAP

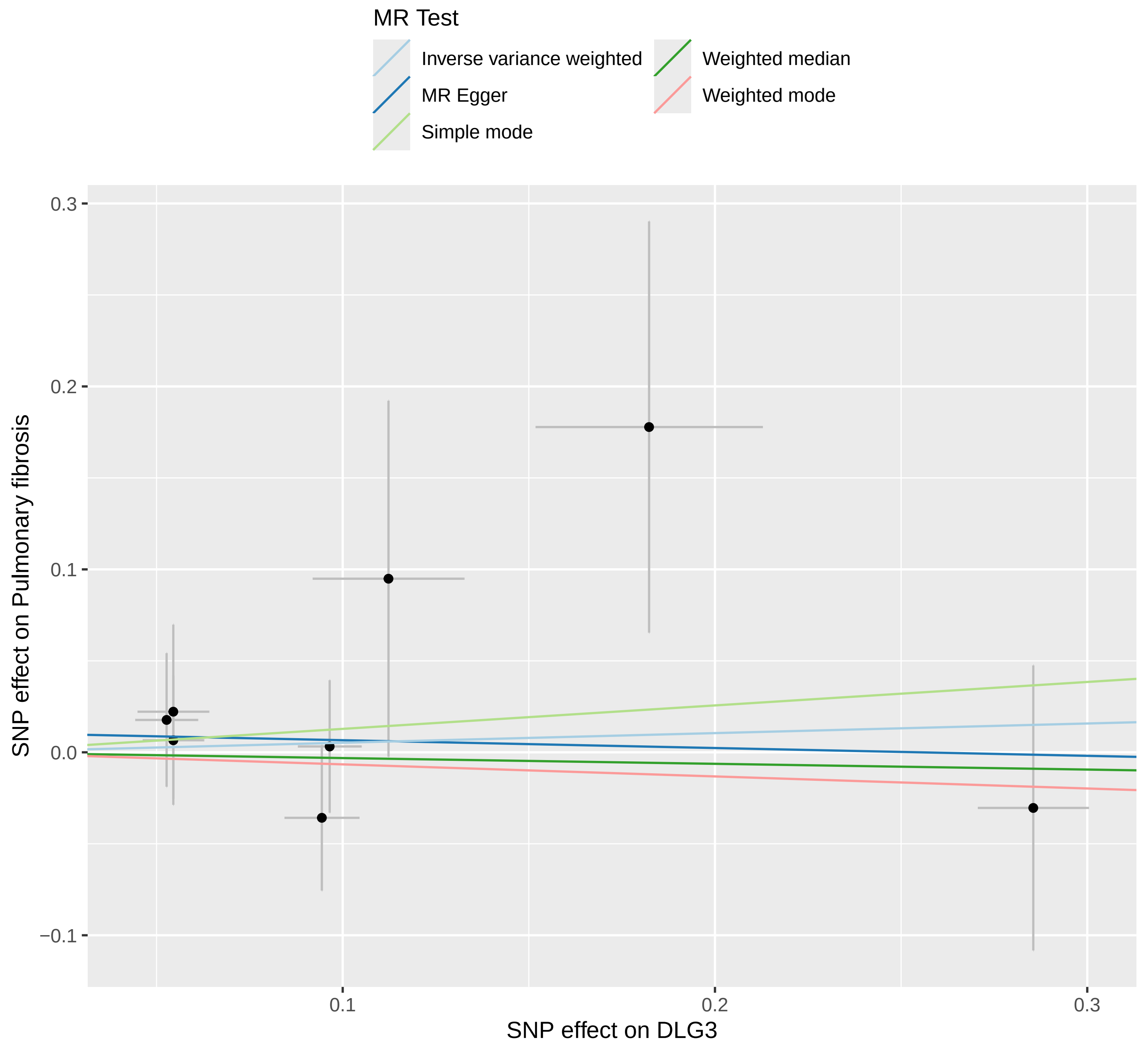

MR Test
Inverse variance weighted
MR Egger
Simple mode
Weighted median
Weighted mode
0.3
0.2
0.1
0.0
−0.1
SNP effect on Pulmonary fibrosis
0.1
0.2
0.3
SNP effect on DLG3

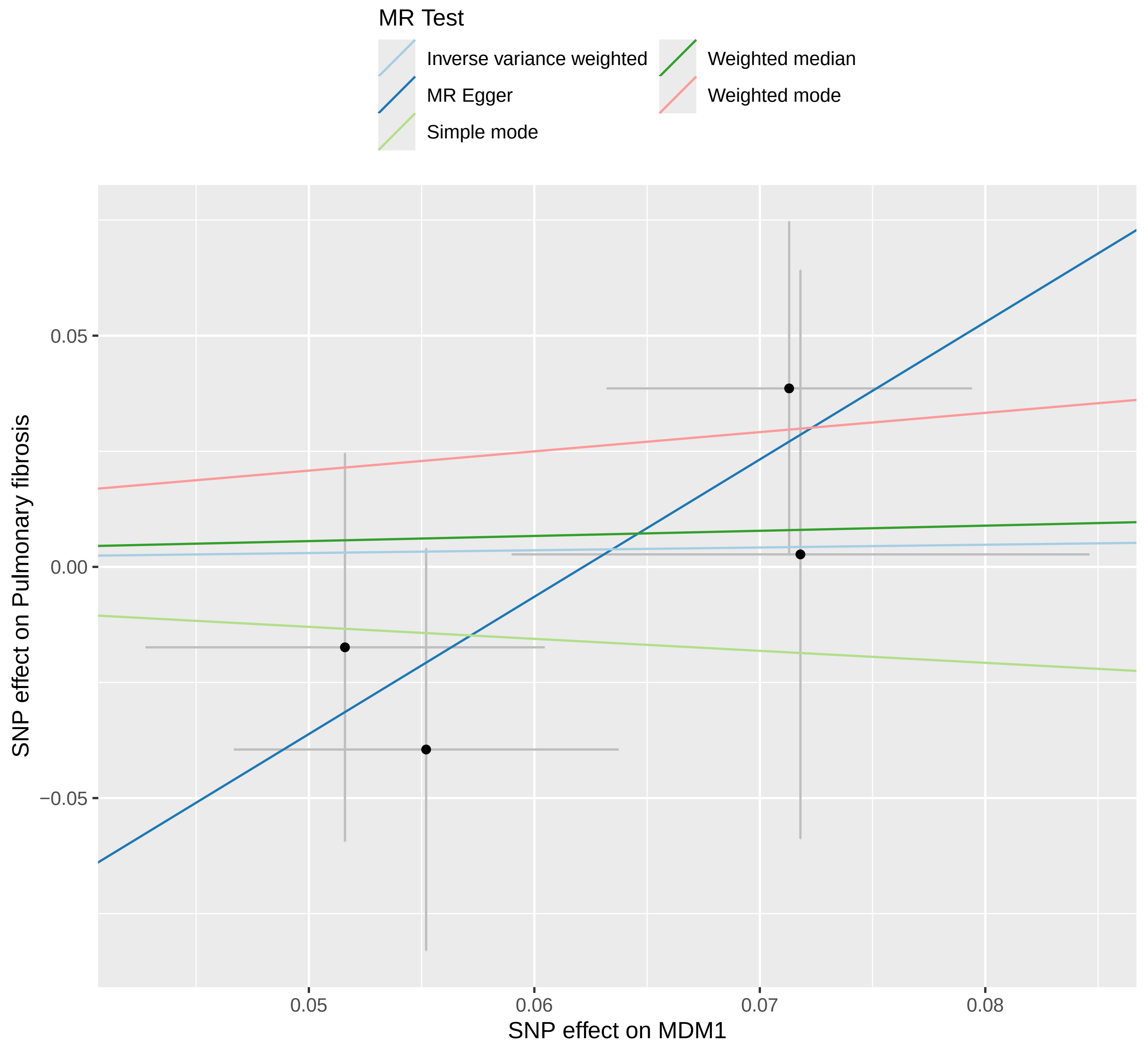

MR Test
Inverse variance weighted
MR Egger
Simple mode
Weighted median
Weighted mode
0.05
0.00
−0.05
0.05
0.06
0.07
0.08
SNP effect on MDM1
SNP effect on Pulmonary fibrosis

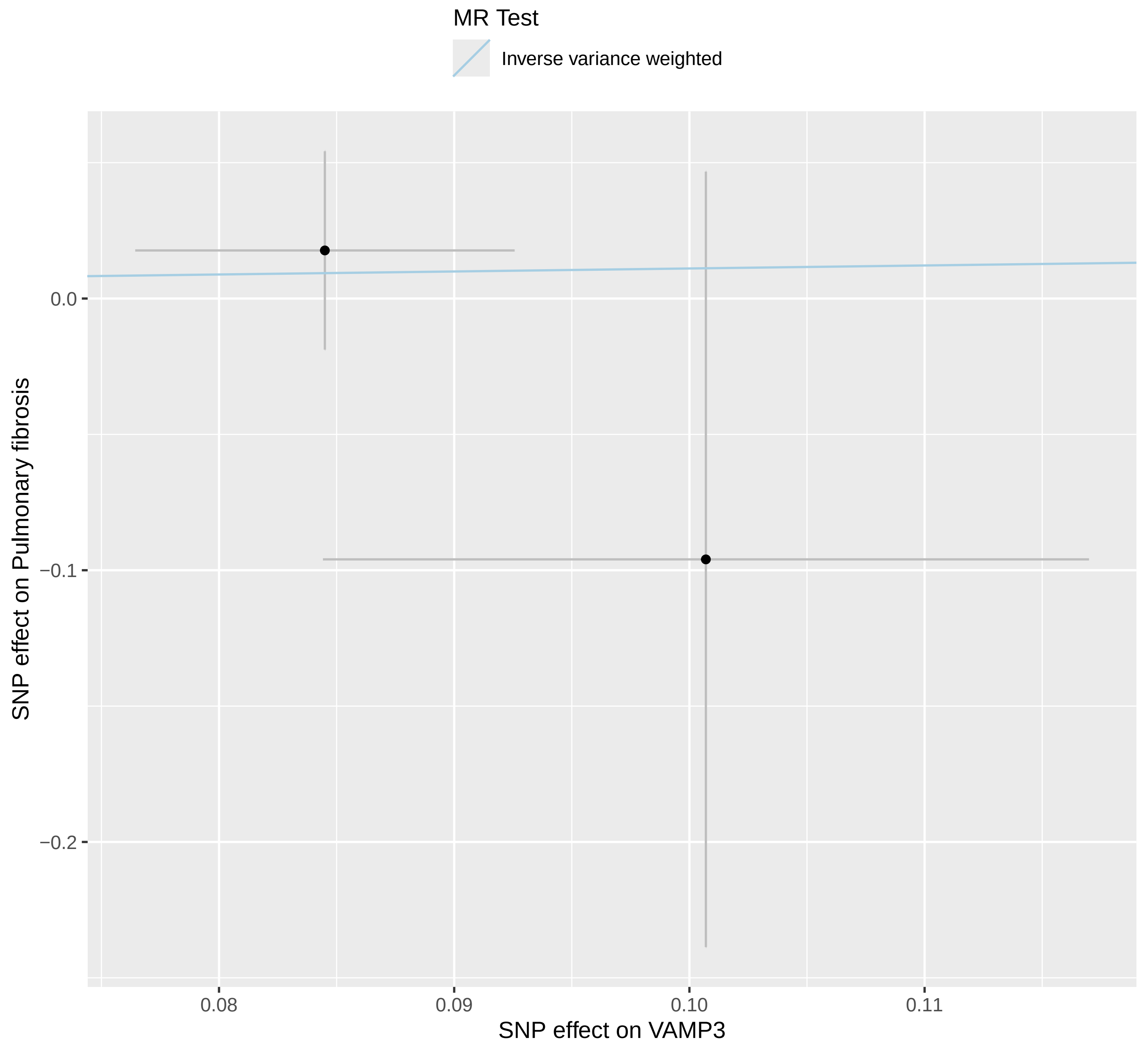

MR Test
Inverse variance weighted
0.0
−0.1
−0.2
0.08
0.09
0.10
0.11
SNP effect on VAMP3
SNP effect on Pulmonary fibrosis

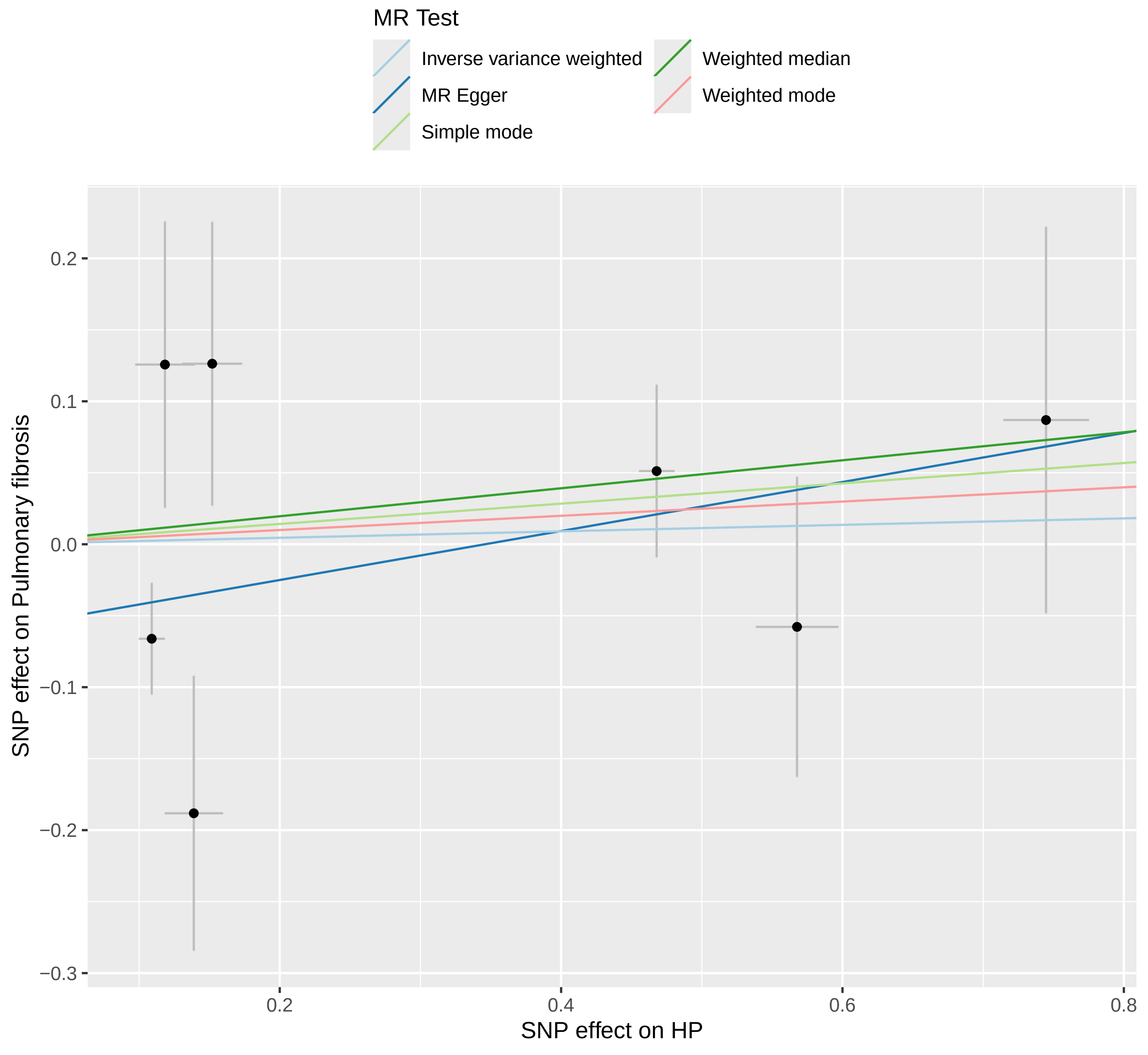

MR Test
Inverse variance weighted
MR Egger
Simple mode
Weighted median
Weighted mode
SNP effect on Pulmonary fibrosis
0.2
0.1
0.0
−0.1
−0.2
−0.3
0.2
0.4
0.6
0.8
SNP effect on HP

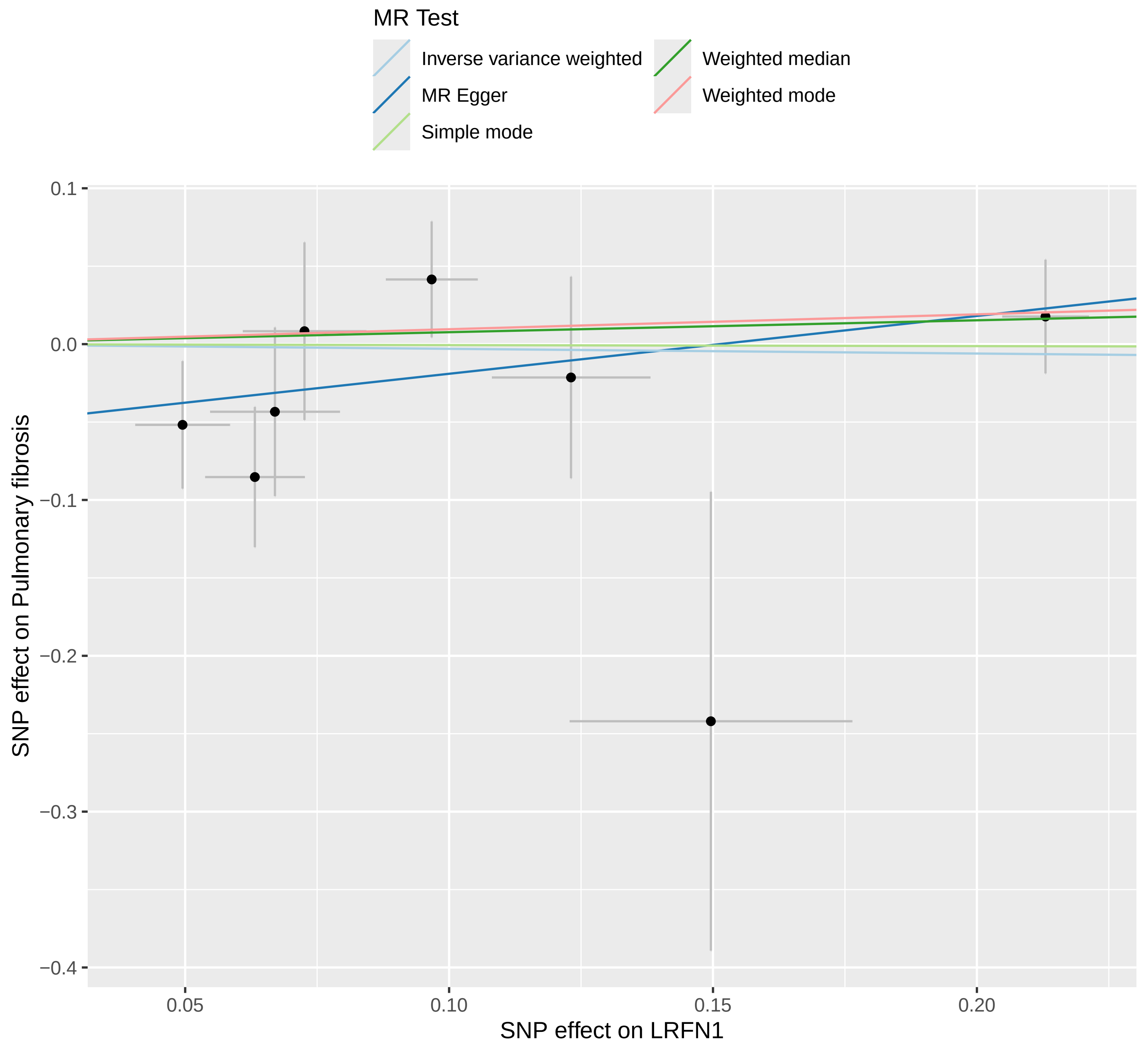

MR Test
Inverse variance weighted
MR Egger
Simple mode
Weighted median
Weighted mode
0.1
0.0
−0.1
−0.2
−0.3
−0.4
SNP effect on Pulmonary fibrosis
0.05
0.10
0.15
0.20
SNP effect on LRFN1

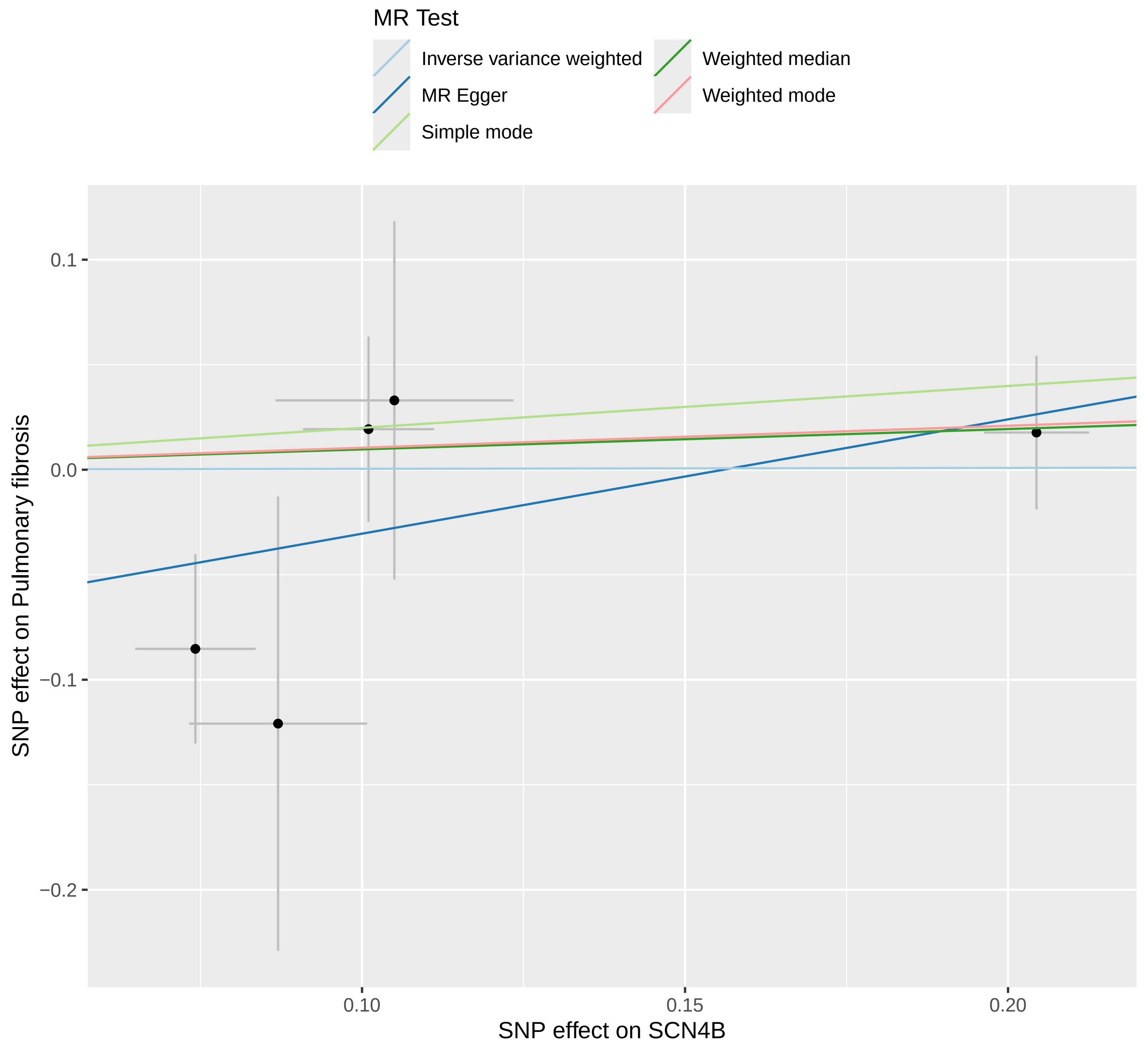

MR Test
Inverse variance weighted
MR Egger
Simple mode
Weighted median
Weighted mode
0.1
0.0
−0.1
−0.2
SNP effect on Pulmonary fibrosis
0.10
0.15
0.20
SNP effect on SCN4B

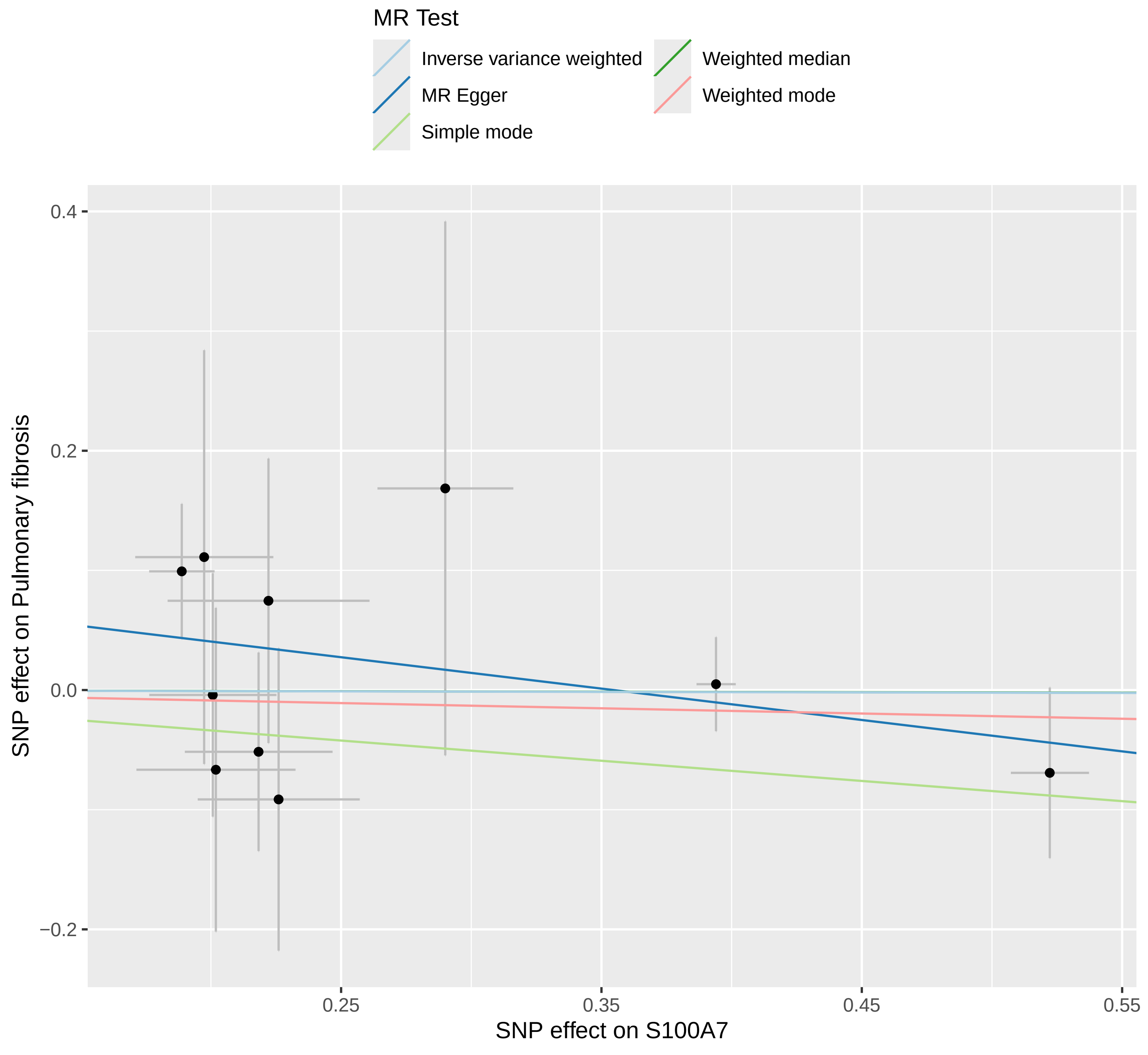

MR Test
Inverse variance weighted
MR Egger
Simple mode
Weighted median
Weighted mode
0.4
0.2
0.0
−0.2
0.25
0.35
0.45
0.55
SNP effect on Pulmonary fibrosis
SNP effect on S100A7

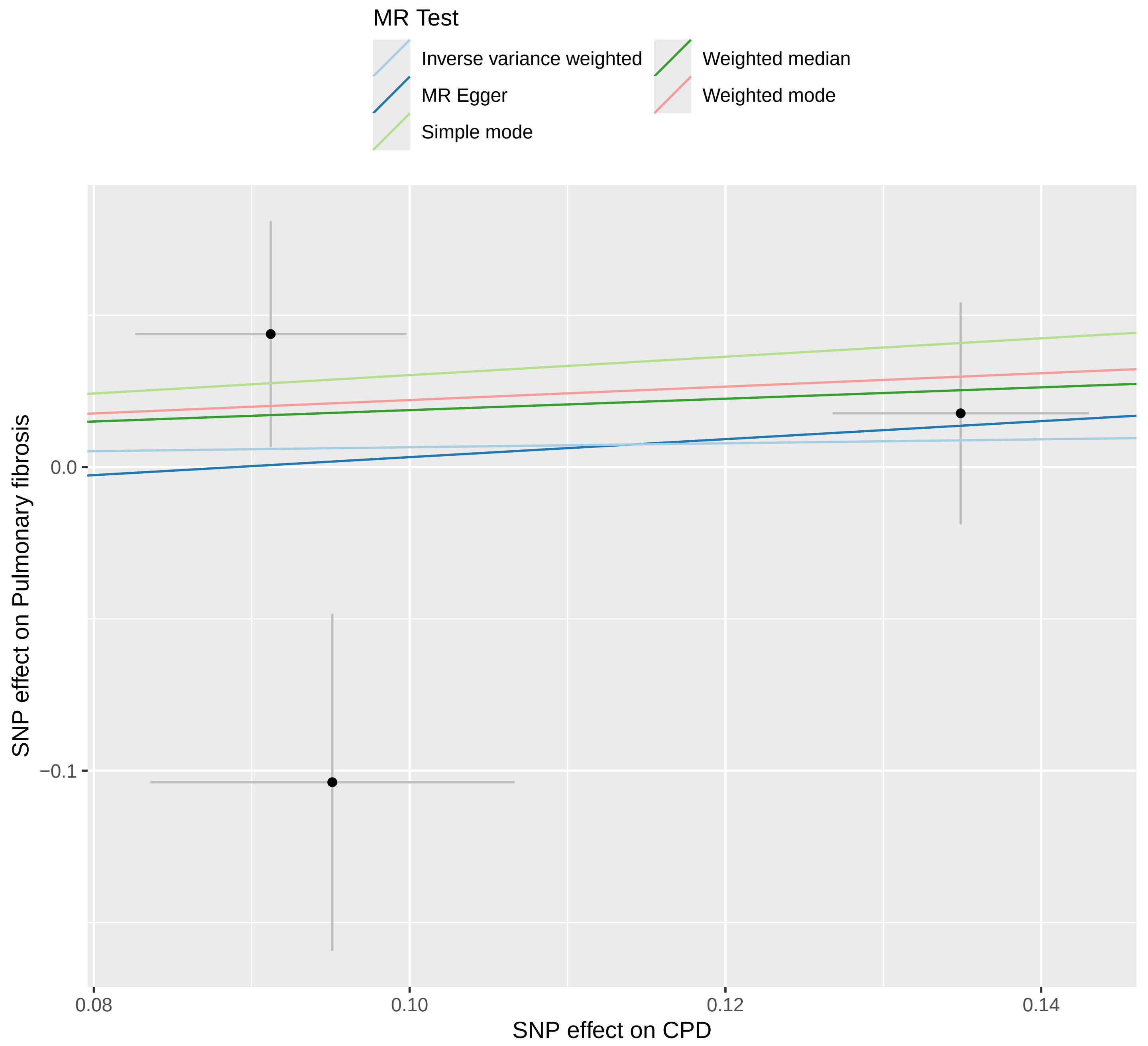

MR Test
Inverse variance weighted
MR Egger
Simple mode
Weighted median
Weighted mode
SNP effect on Pulmonary fibrosis
0.0
−0.1
0.08
0.10
0.12
0.14
SNP effect on CPD

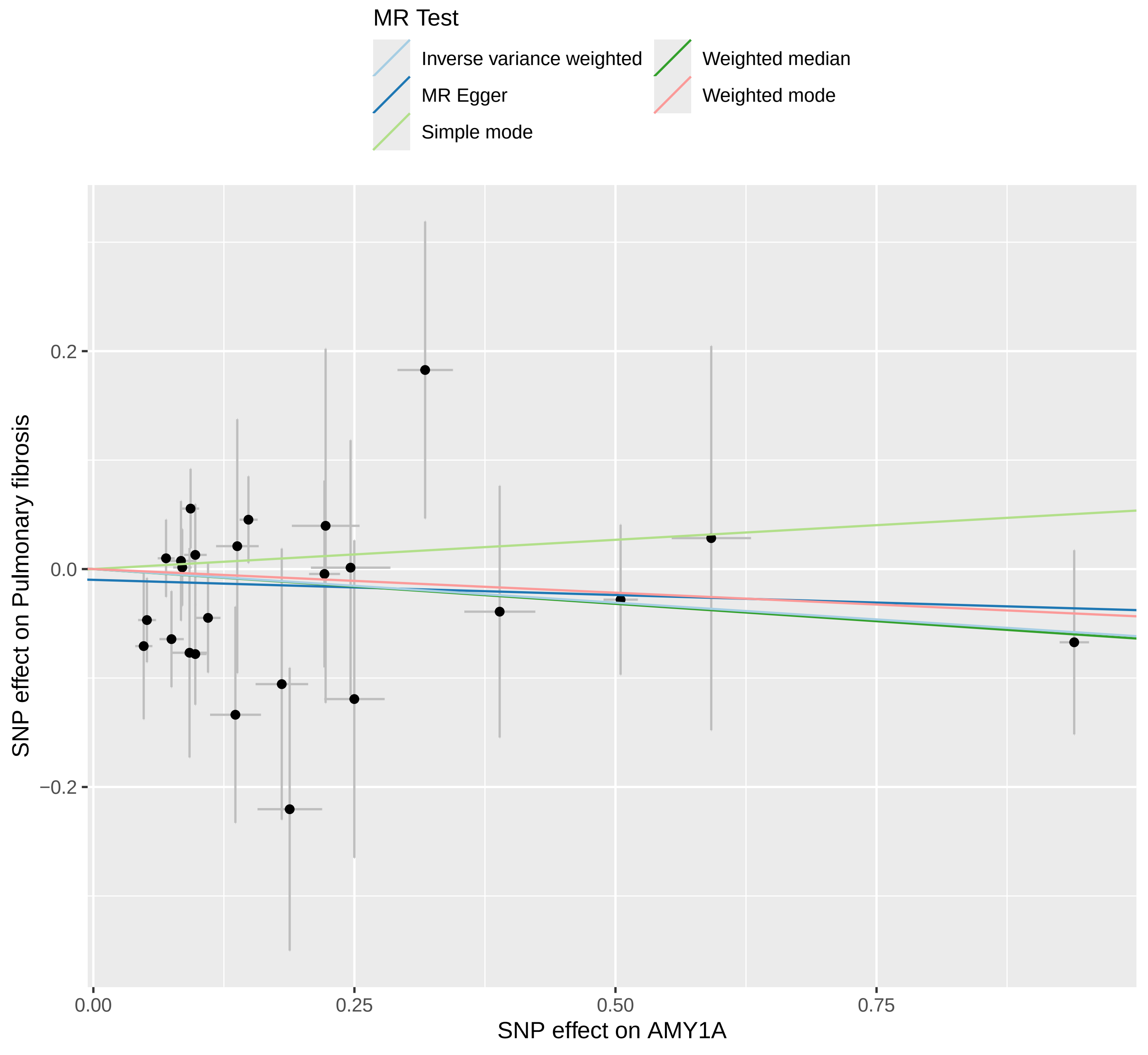

MR Test
Inverse variance weighted
MR Egger
Simple mode
Weighted median
Weighted mode
0.2
0.0
−0.2
0.00
0.25
0.50
0.75
SNP effect on Pulmonary fibrosis
SNP effect on AMY1A

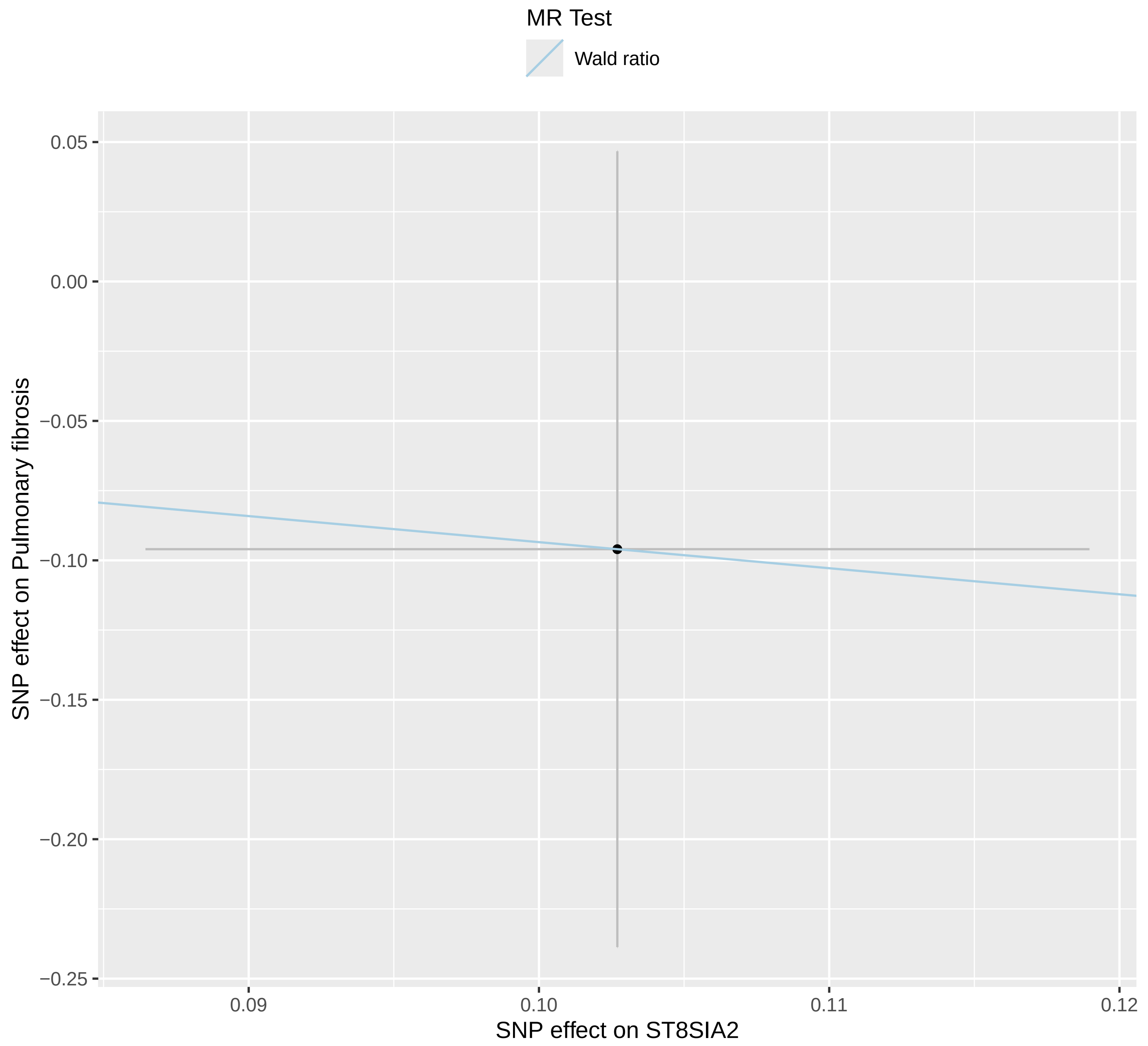

MR Test
Wald ratio
SNP effect on Pulmonary fibrosis
SNP effect on ST8SIA2
0.05
0.00
−0.05
−0.10
−0.15
−0.20
−0.25
0.09
0.10
0.11
0.12

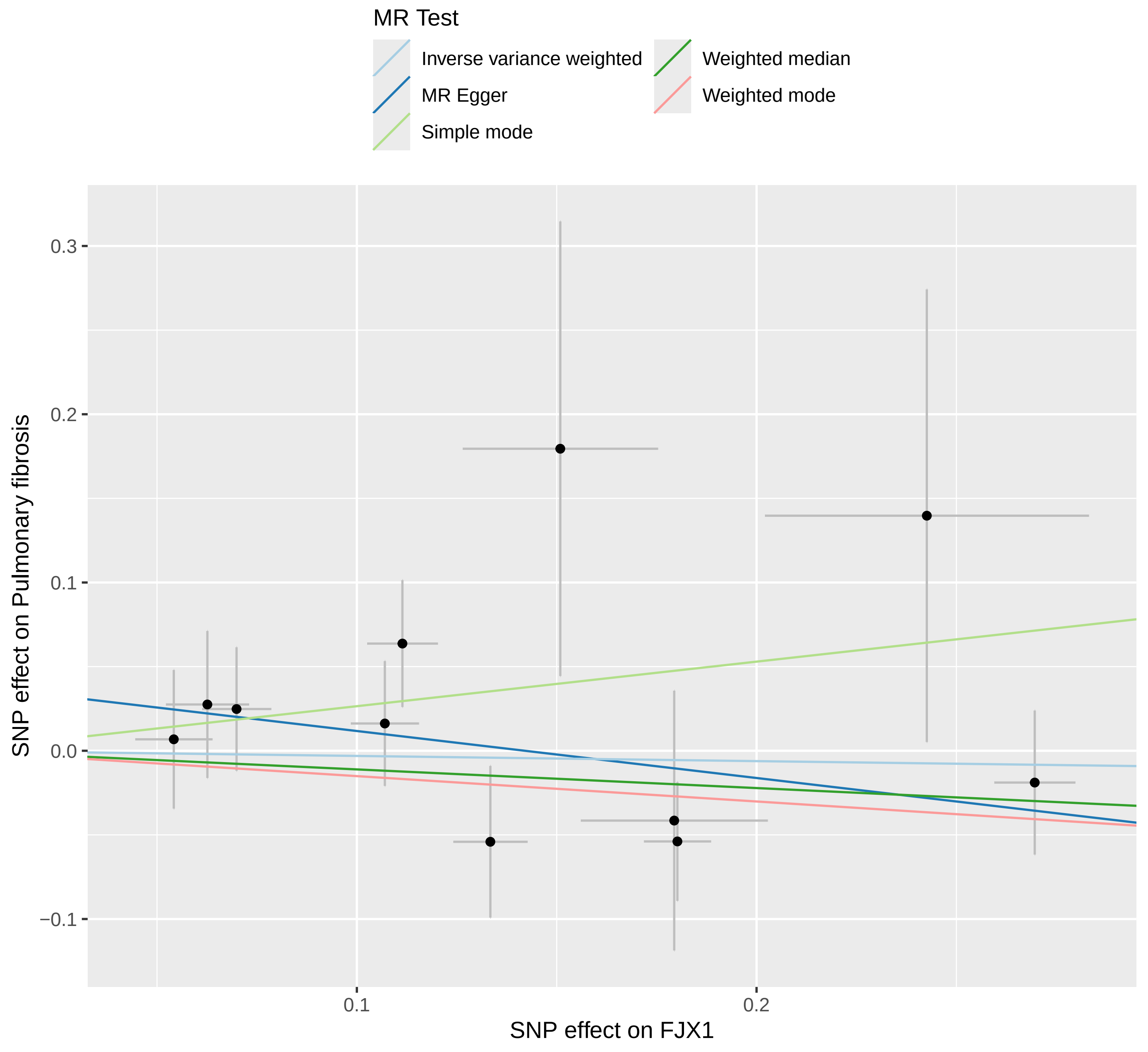
MR Test
Inverse variance weighted
MR Egger
Simple mode
Weighted median
Weighted mode
SNP effect on Pulmonary fibrosis
0.3
0.2
0.1
0.0
−0.1
0.1
0.2
SNP effect on FJX1

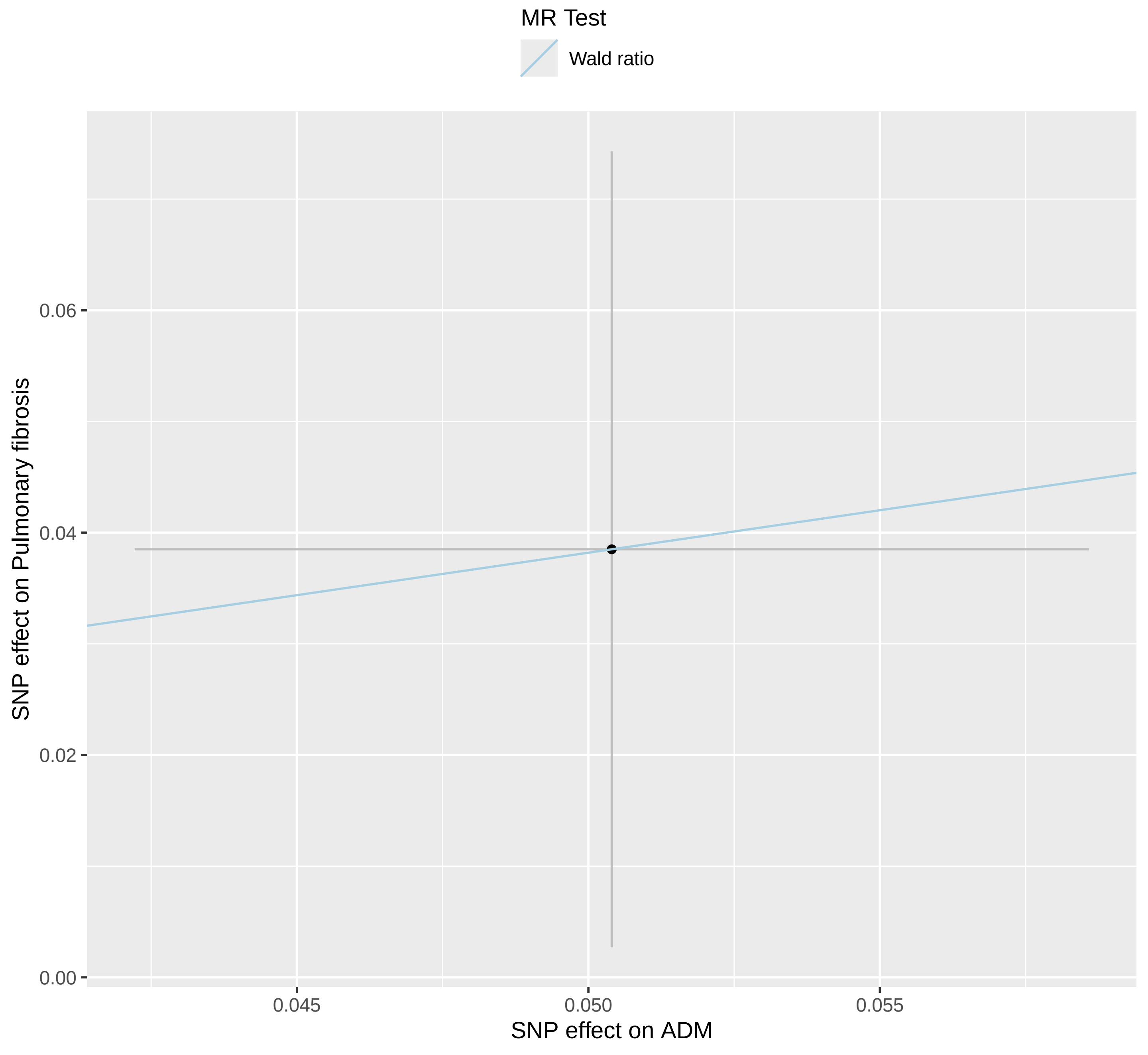

MR Test
Wald ratio
0.06
0.04
0.02
0.00
0.045
0.050
0.055
SNP effect on ADM
SNP effect on Pulmonary fibrosis

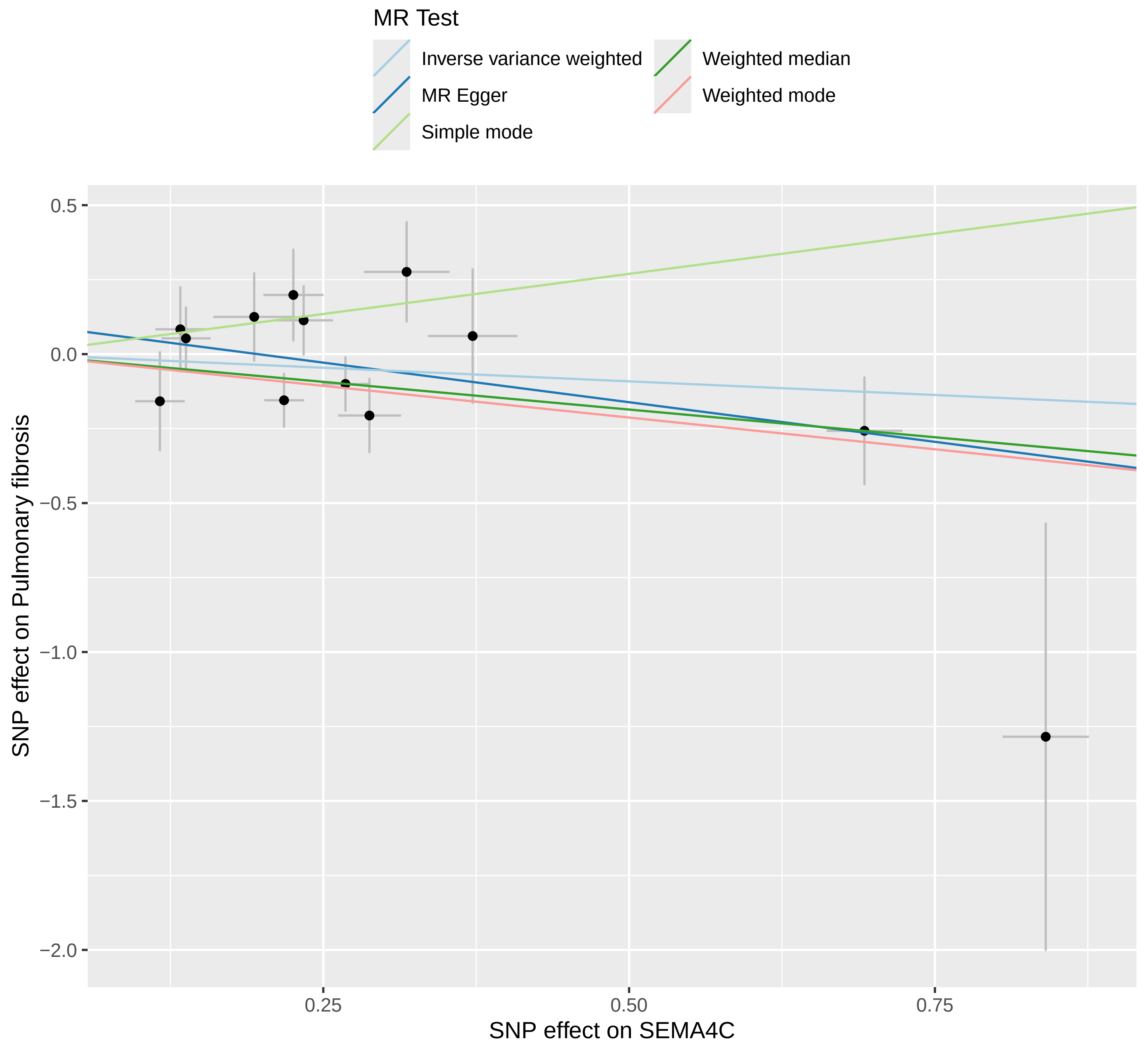

MR Test
Inverse variance weighted
MR Egger
Simple mode
Weighted median
Weighted mode
0.5
0.0
−0.5
−1.0
−1.5
−2.0
0.25
0.50
0.75
SNP effect on Pulmonary fibrosis
SNP effect on SEMA4C

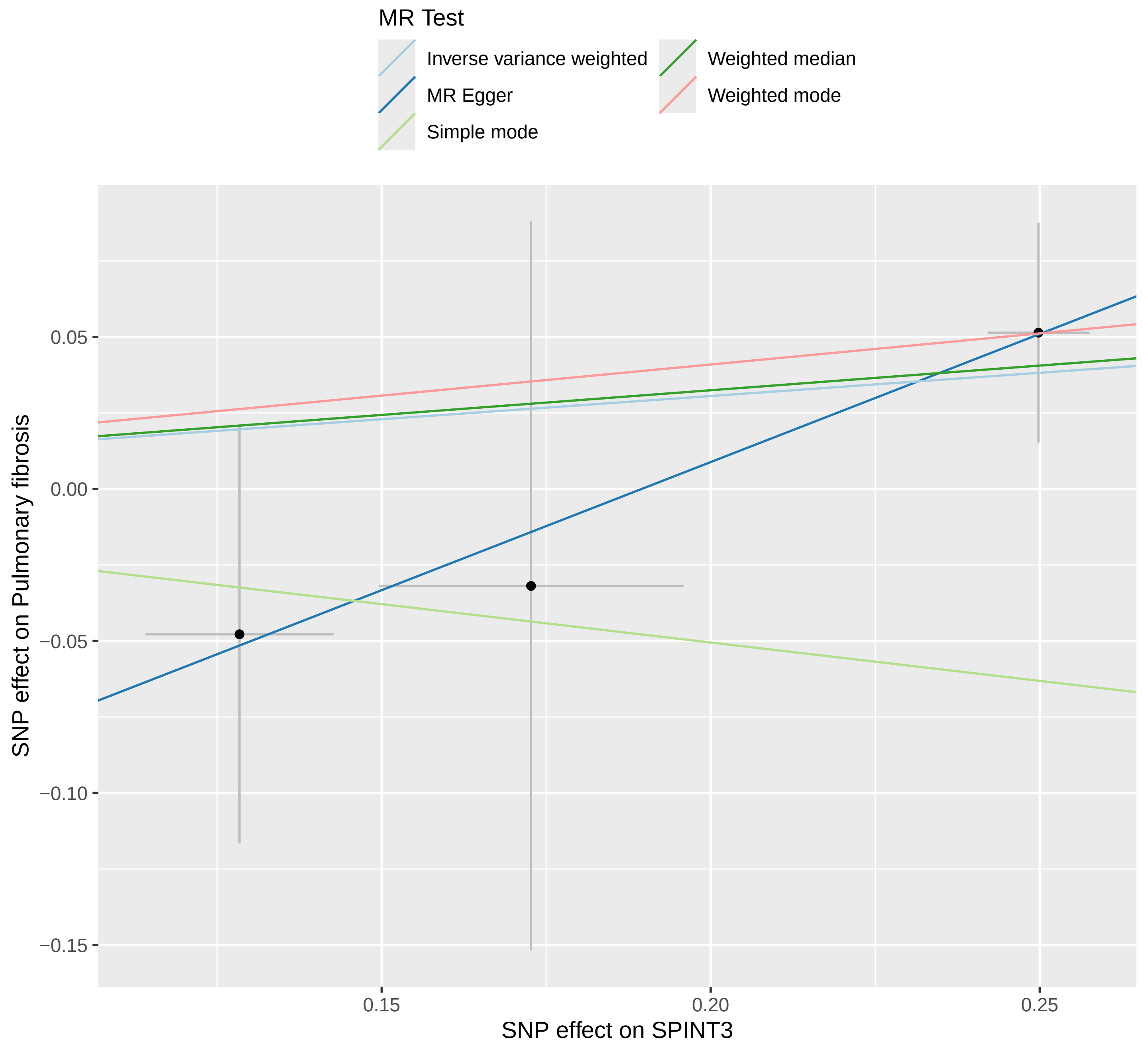

MR Test
Inverse variance weighted
MR Egger
Simple mode
Weighted median
Weighted mode
0.05
0.00
−0.05
−0.10
−0.15
SNP effect on Pulmonary fibrosis
0.15
0.20
0.25
SNP effect on SPINT3

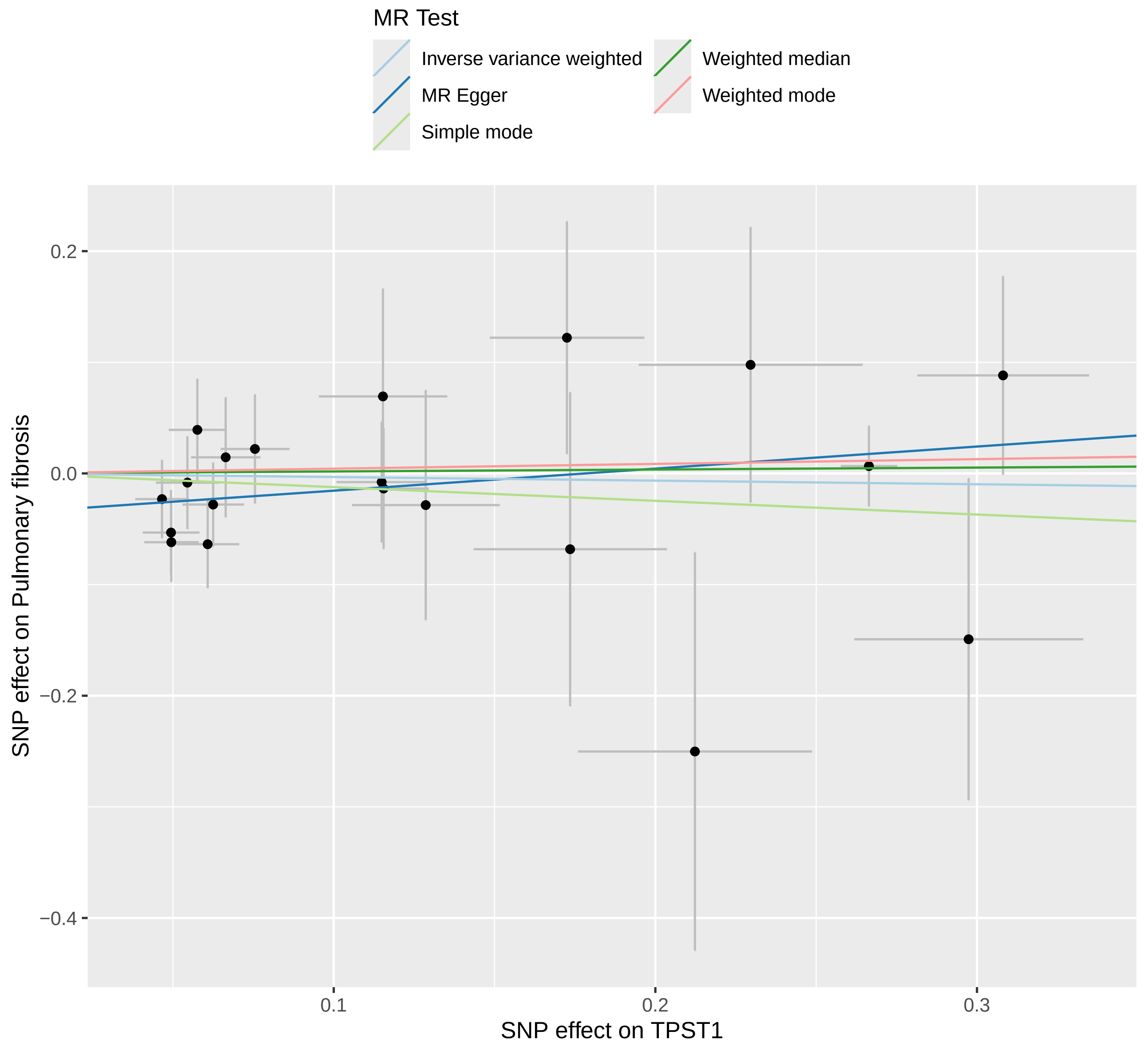
MR Test
Inverse variance weighted
MR Egger
Simple mode
Weighted median
Weighted mode
0.2
0.0
−0.2
−0.4
0.1
0.2
0.3
SNP effect on Pulmonary fibrosis
SNP effect on TPST1

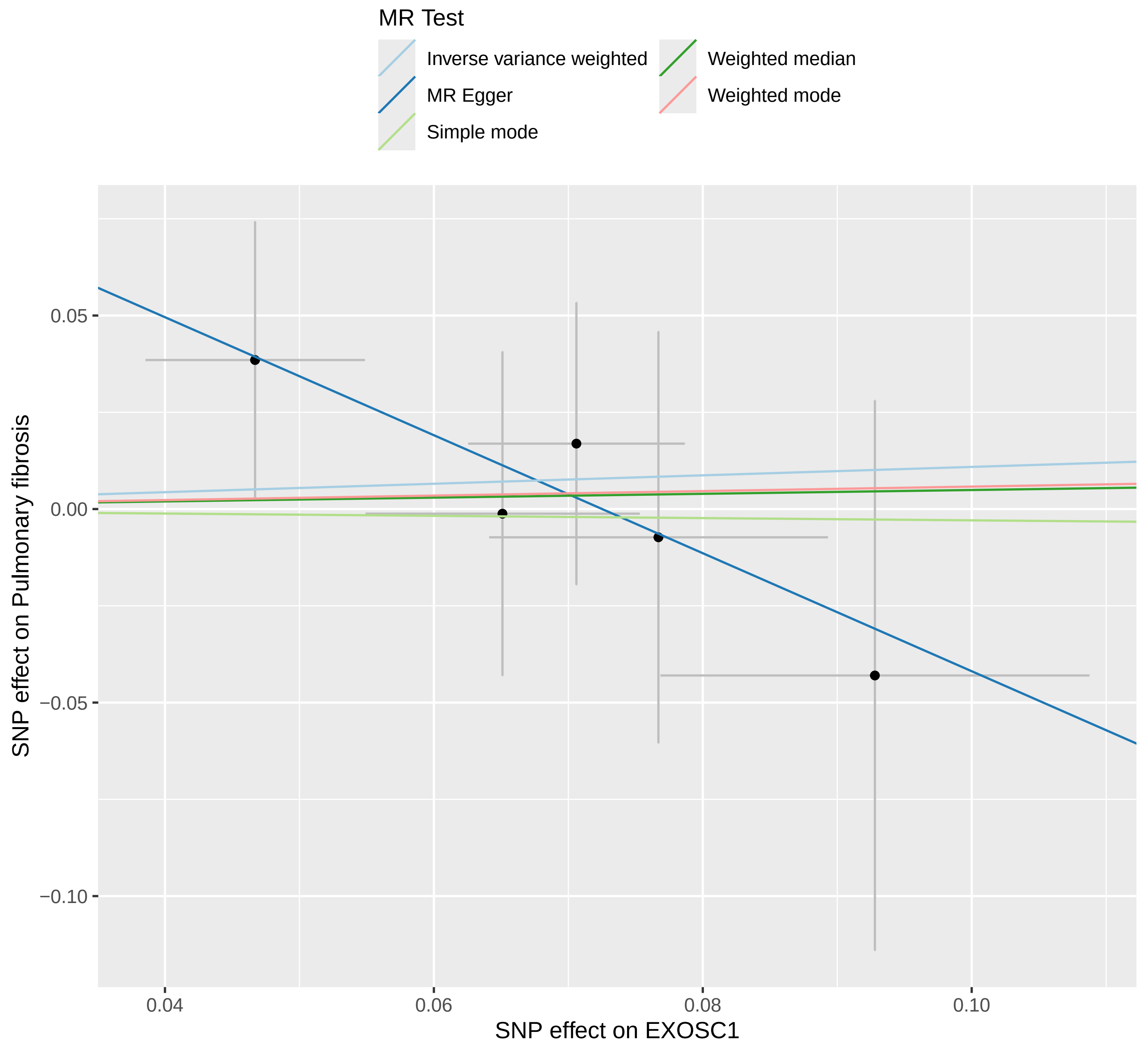

MR Test
Inverse variance weighted
MR Egger
Simple mode
Weighted median
Weighted mode
0.05
0.00
−0.05
−0.10
0.04
0.06
0.08
0.10
SNP effect on Pulmonary fibrosis
SNP effect on EXOSC1

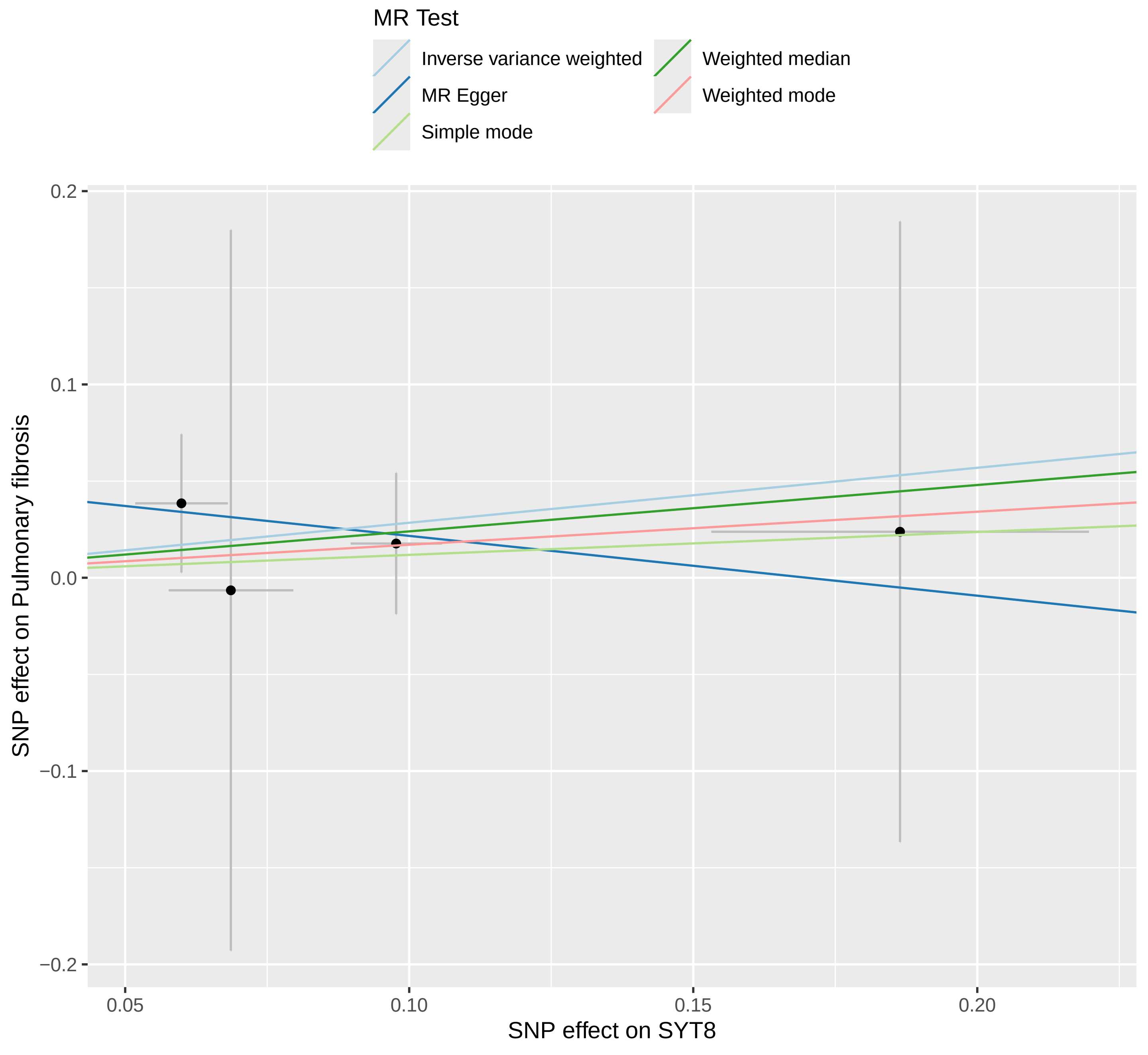

MR Test
Inverse variance weighted
MR Egger
Simple mode
Weighted median
Weighted mode
0.2
0.1
0.0
−0.1
−0.2
0.05
0.10
0.15
0.20
SNP effect on Pulmonary fibrosis
SNP effect on SYT8

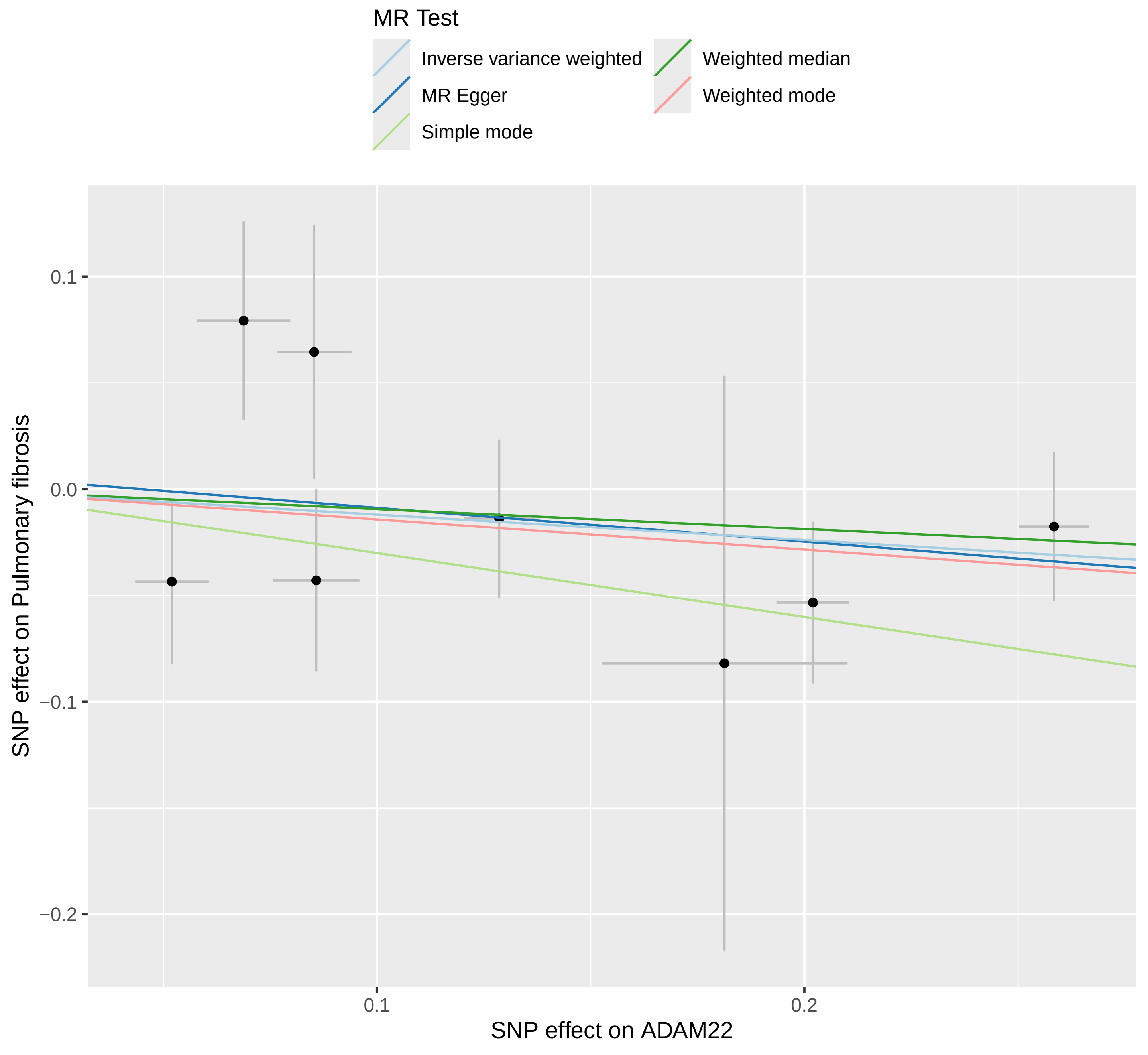
MR Test
Inverse variance weighted
MR Egger
Simple mode
Weighted median
Weighted mode
SNP effect on Pulmonary fibrosis
0.1
0.0
−0.1
−0.2
0.1
0.2
SNP effect on ADAM22

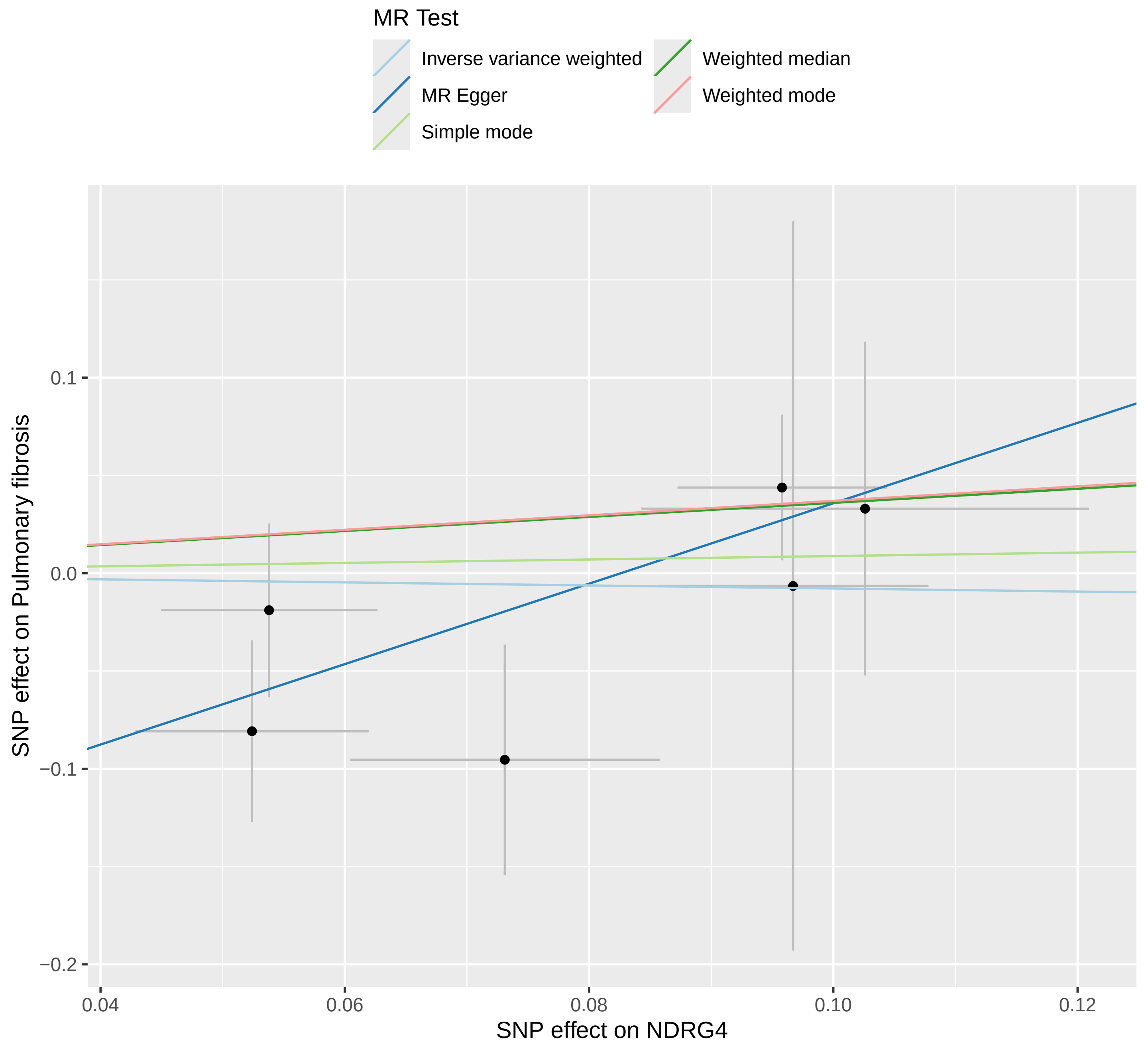
MR Test
Inverse variance weighted
MR Egger
Simple mode
Weighted median
Weighted mode
0.1
0.0
−0.1
−0.2
0.04
0.06
0.08
0.10
0.12
SNP effect on NDRG4
SNP effect on Pulmonary fibrosis

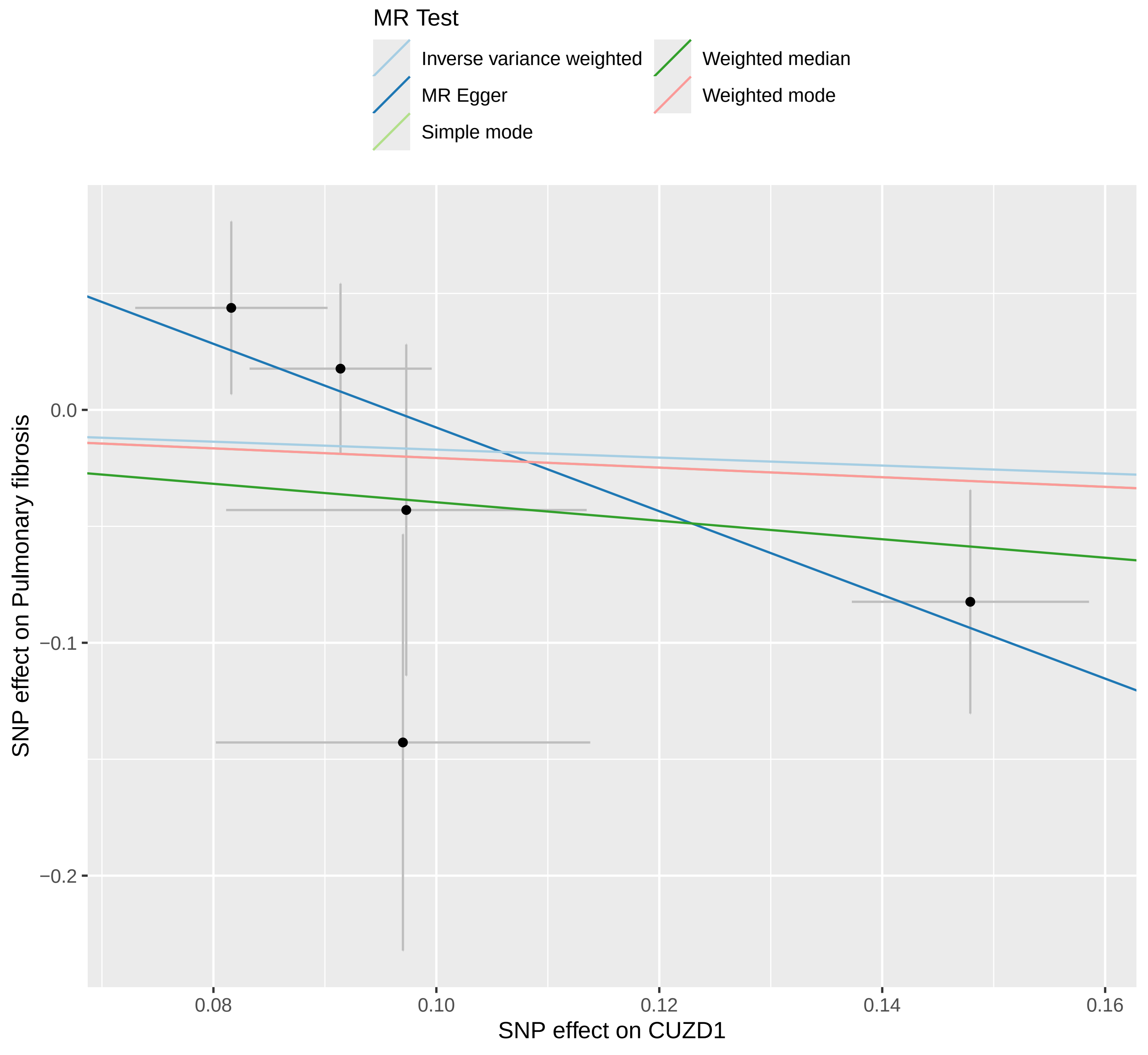

MR Test
Inverse variance weighted
MR Egger
Simple mode
Weighted median
Weighted mode
0.0
−0.1
−0.2
0.08
0.10
0.12
0.14
0.16
SNP effect on Pulmonary fibrosis
SNP effect on CUZD1

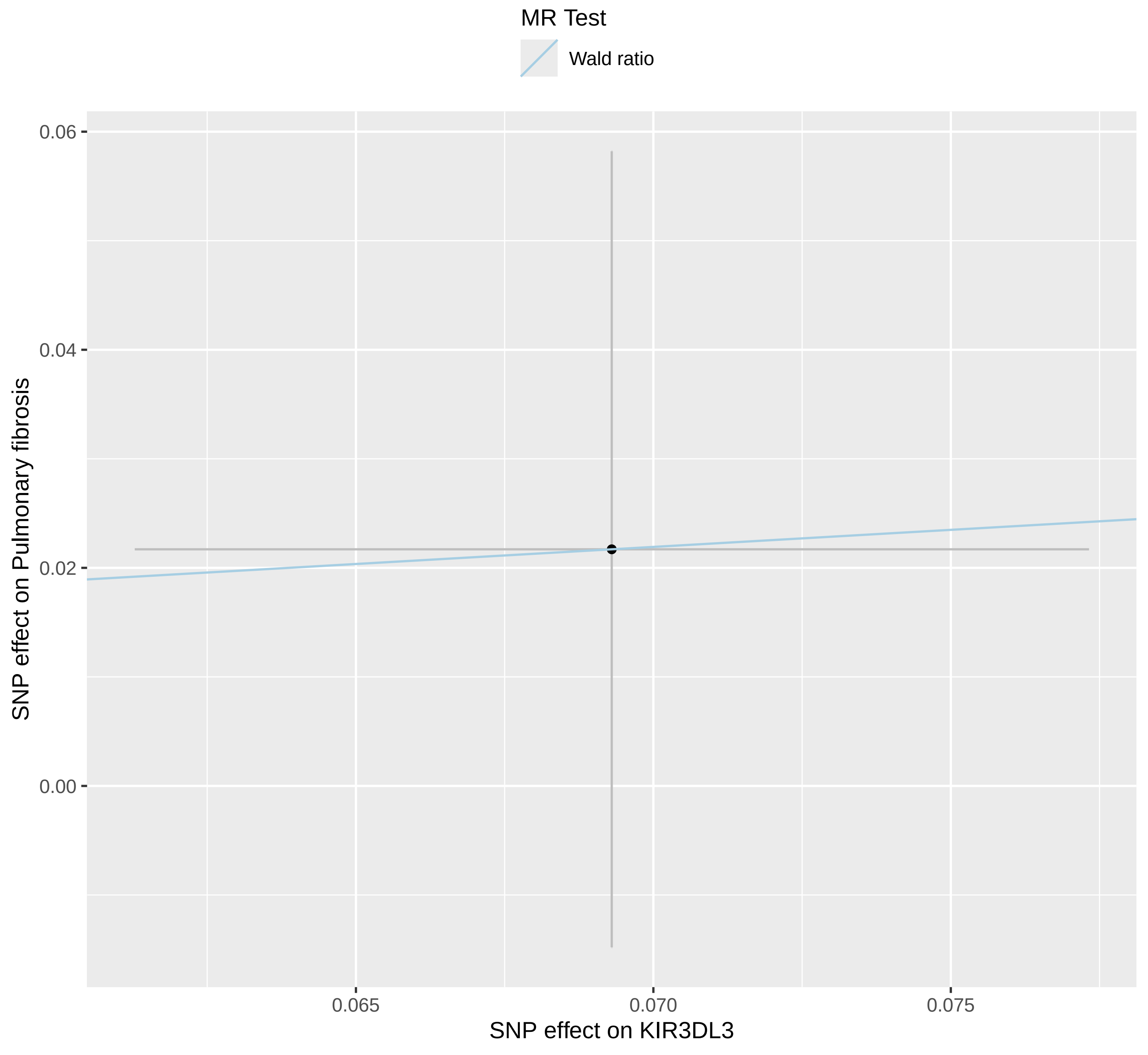
MR Test
Wald ratio
0.06
0.04
0.02
0.00
0.065
0.070
0.075
SNP effect on Pulmonary fibrosis
SNP effect on KIR3DL3

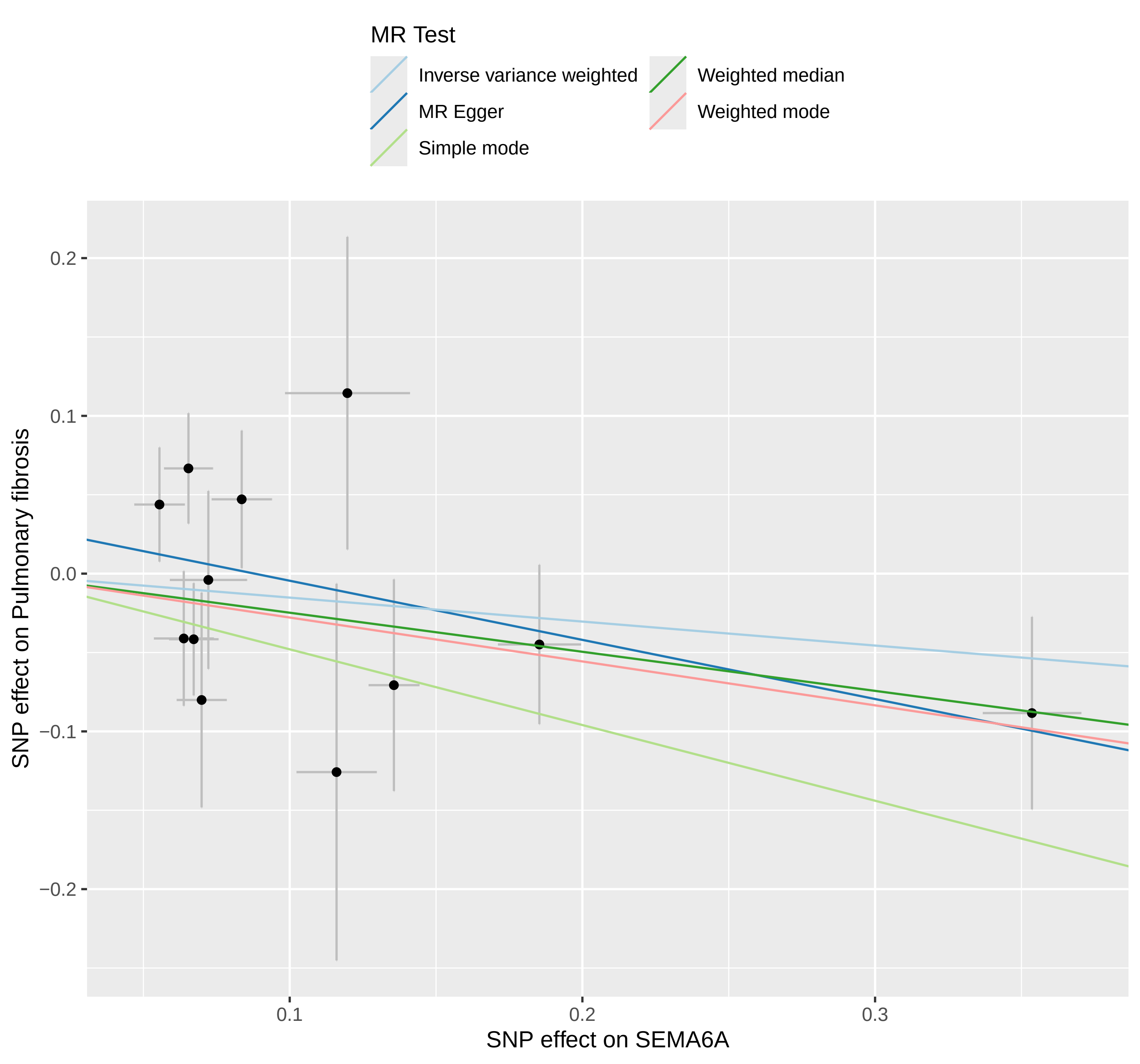
MR Test
Inverse variance weighted
MR Egger
Simple mode
Weighted median
Weighted mode
0.2
0.1
0.0
−0.1
−0.2
SNP effect on Pulmonary fibrosis
0.1
0.2
0.3
SNP effect on SEMA6A

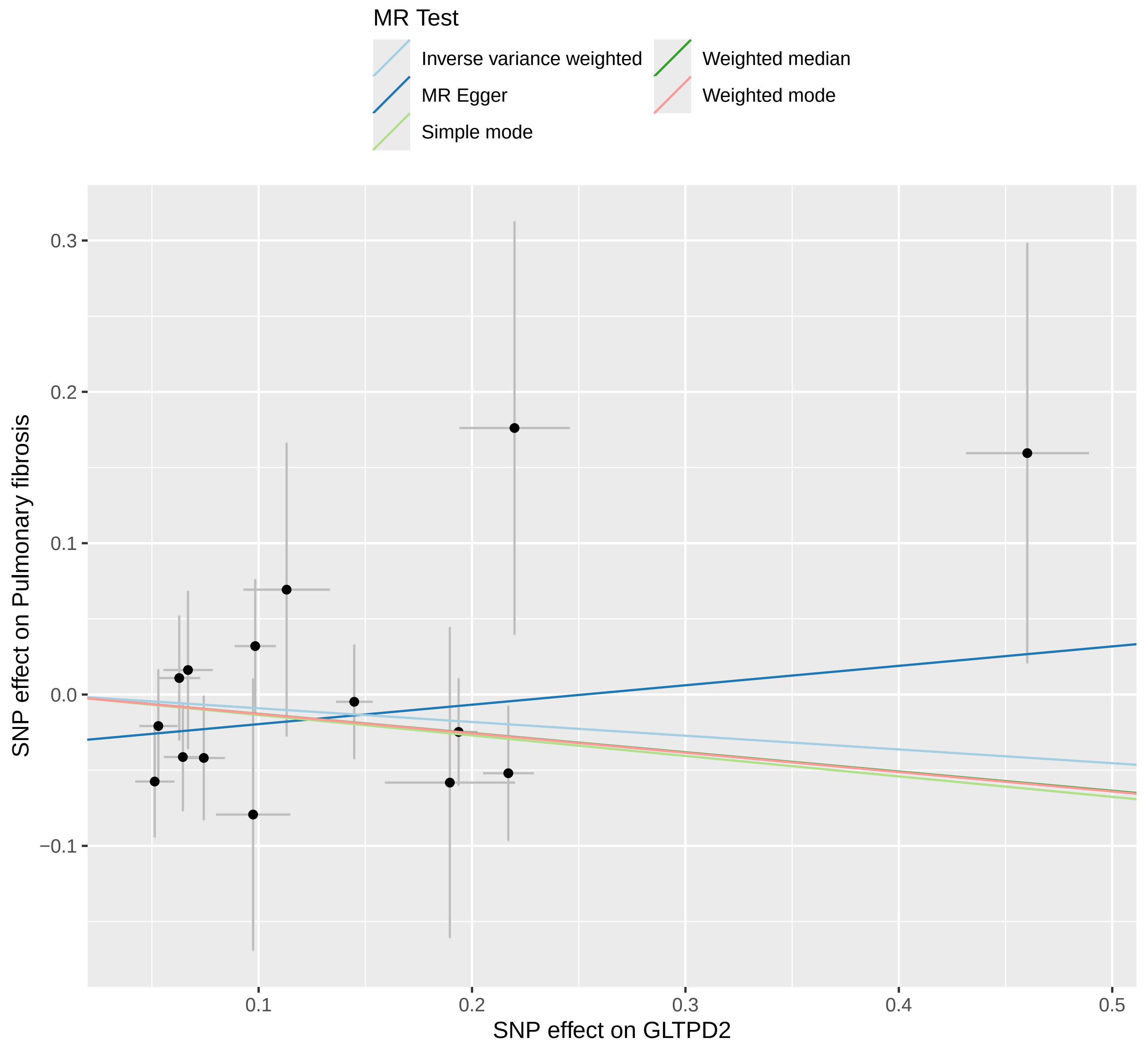

MR Test
Inverse variance weighted
MR Egger
Simple mode
Weighted median
Weighted mode
SNP effect on Pulmonary fibrosis
0.3
0.2
0.1
0.0
−0.1
0.1
0.2
0.3
0.4
0.5
SNP effect on GLTPD2

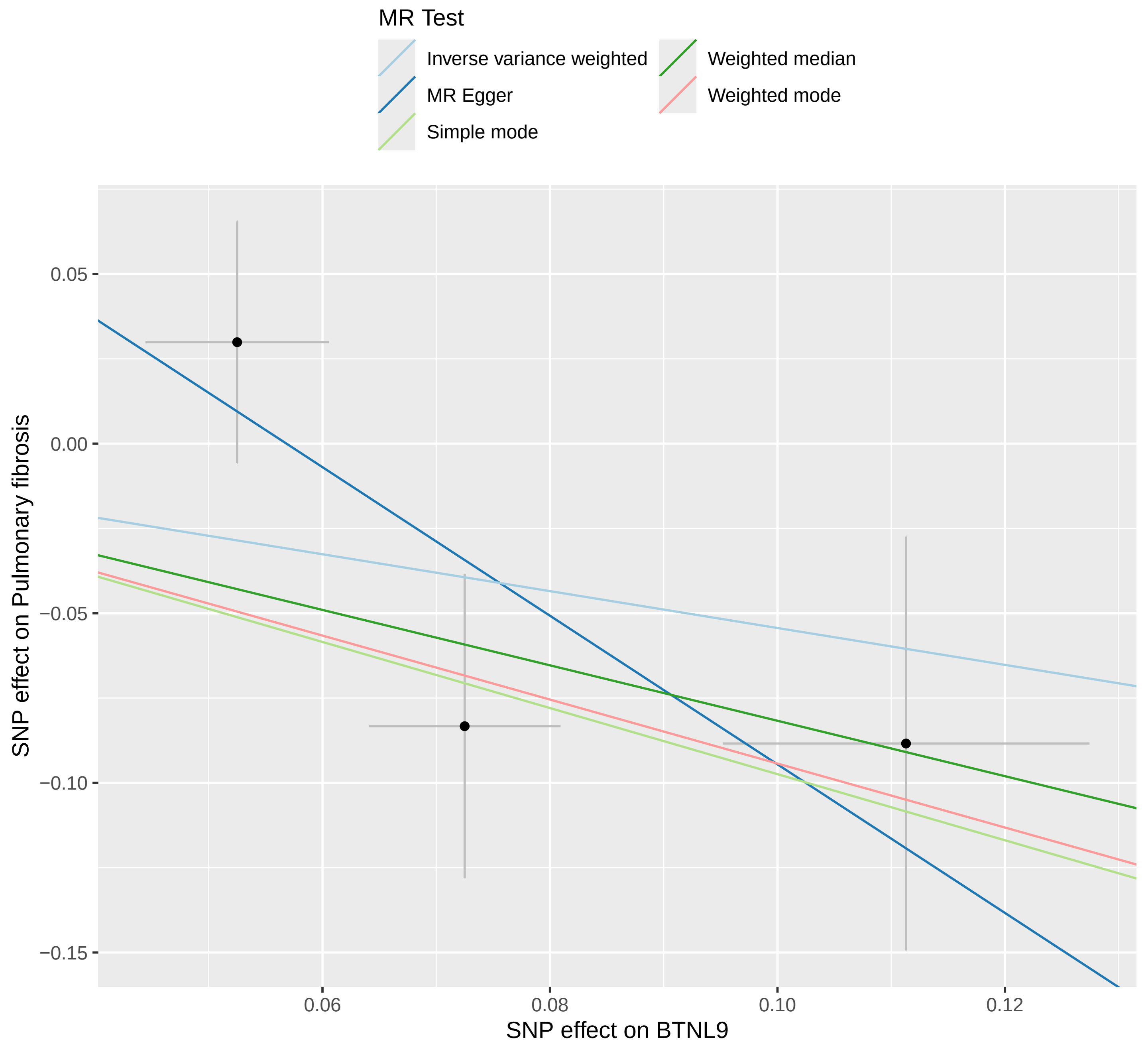
MR Test
Inverse variance weighted
MR Egger
Simple mode
Weighted median
Weighted mode
0.05
0.00
−0.05
−0.10
−0.15
0.06
0.08
0.10
0.12
SNP effect on BTNL9
SNP effect on Pulmonary fibrosis

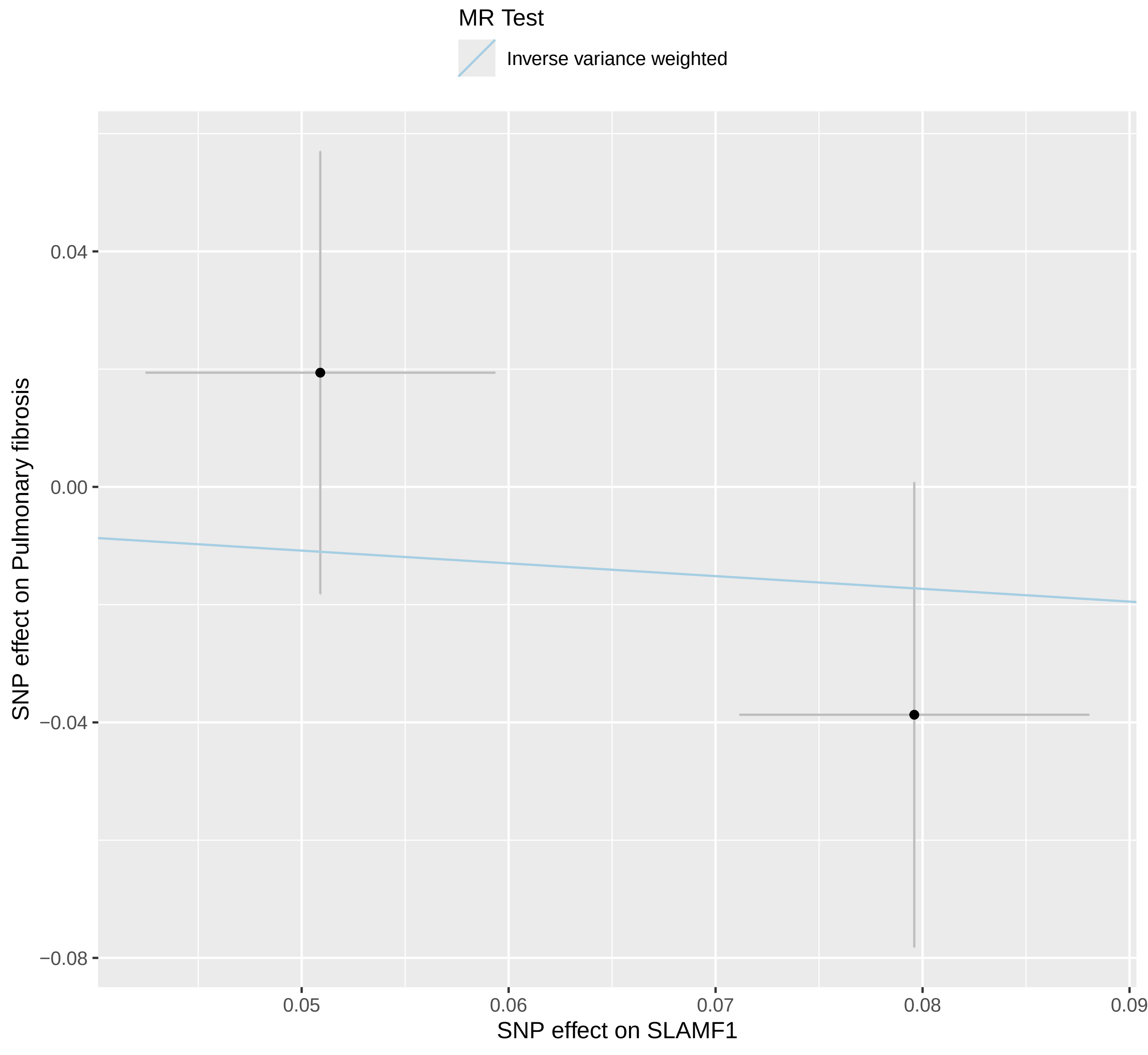
MR Test
Inverse variance weighted
0.04
0.00
−0.04
−0.08
SNP effect on Pulmonary fibrosis
0.05
0.06
0.07
0.08
0.09
SNP effect on SLAMF1

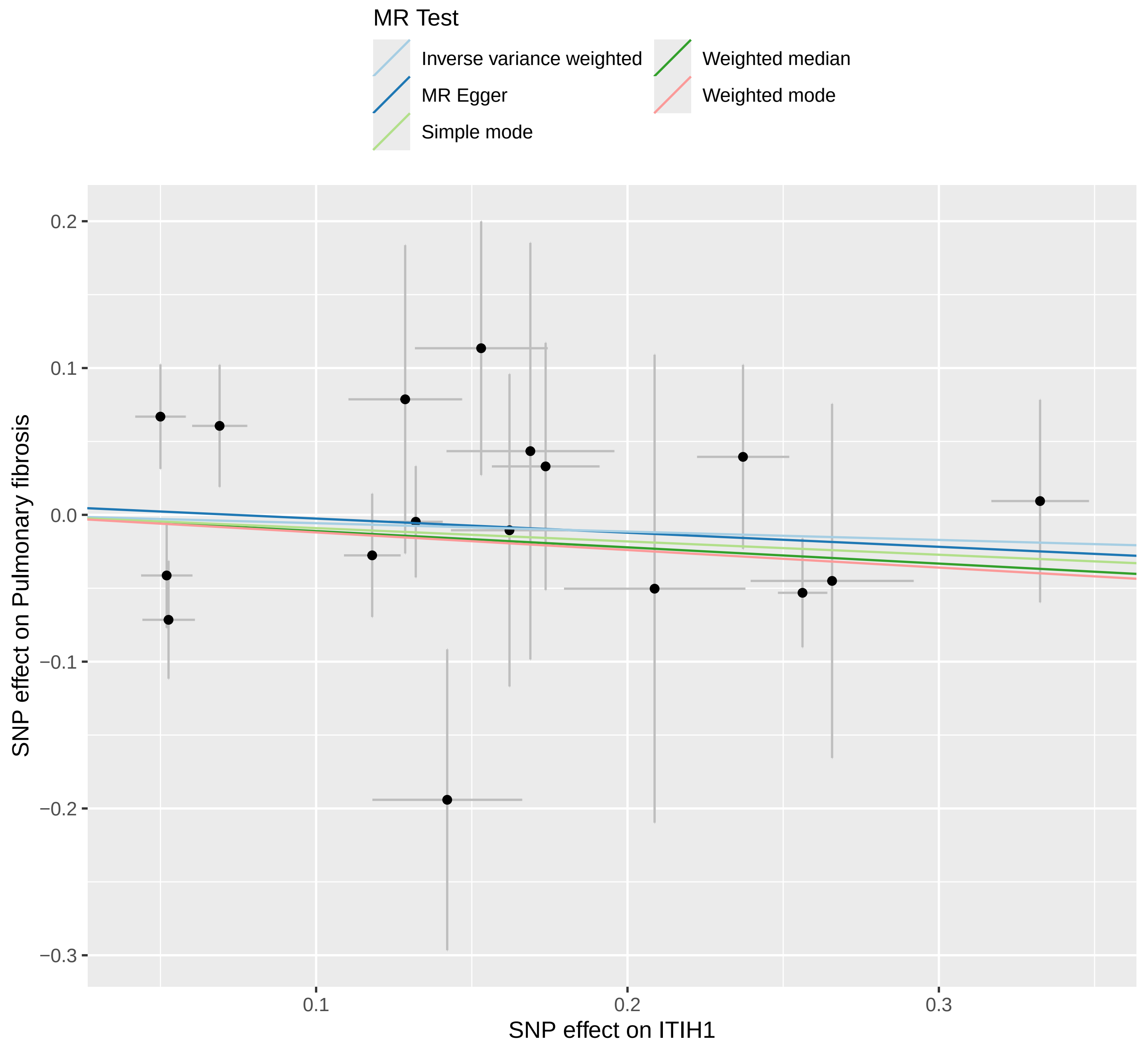

MR Test
Inverse variance weighted
MR Egger
Simple mode
Weighted median
Weighted mode
0.2
0.1
0.0
−0.1
−0.2
−0.3
SNP effect on Pulmonary fibrosis
0.1
0.2
0.3
SNP effect on ITIH1

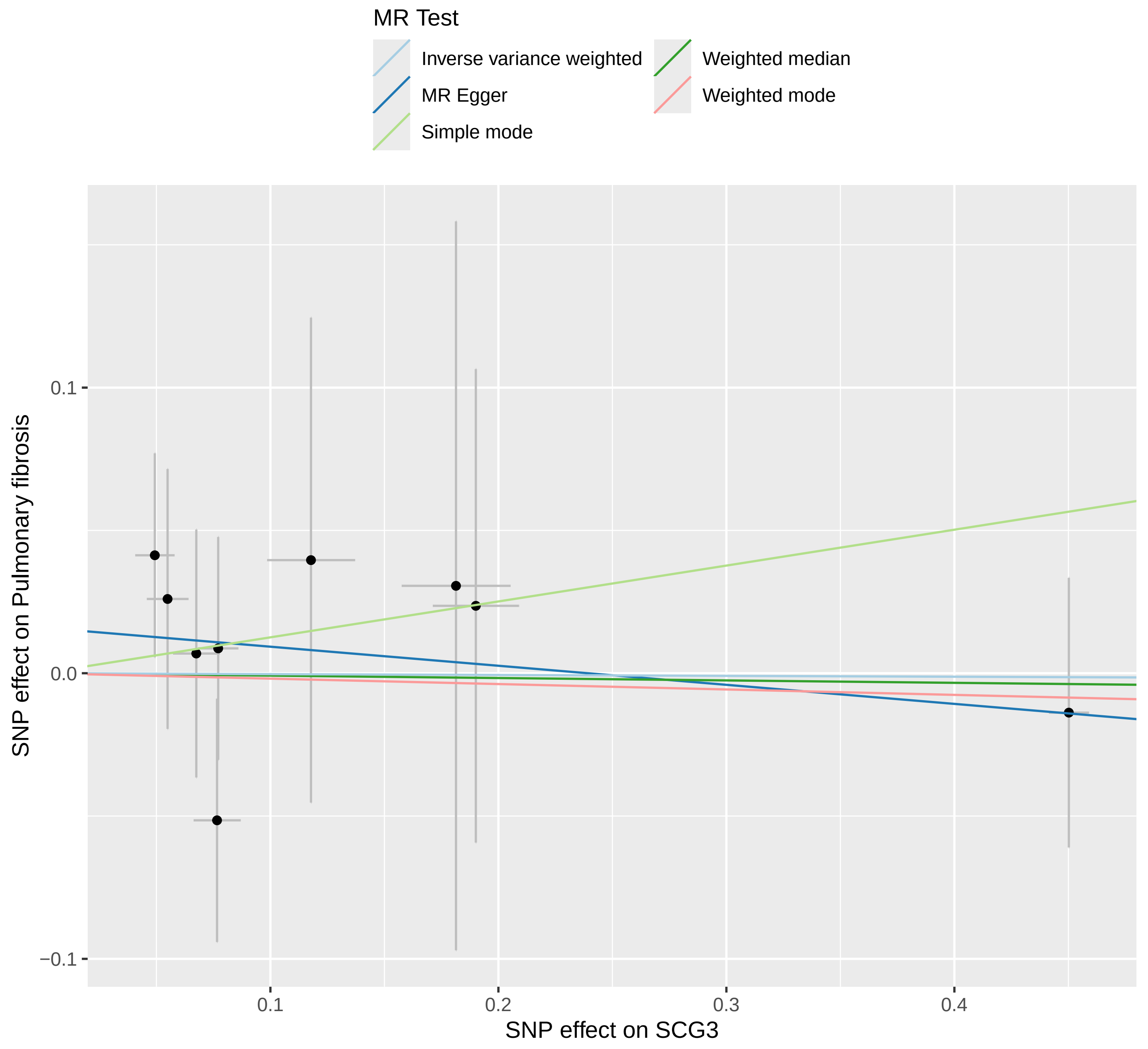
MR Test
Inverse variance weighted
MR Egger
Simple mode
Weighted median
Weighted mode
0.1
0.0
−0.1
SNP effect on Pulmonary fibrosis
0.1
0.2
0.3
0.4
SNP effect on SCG3

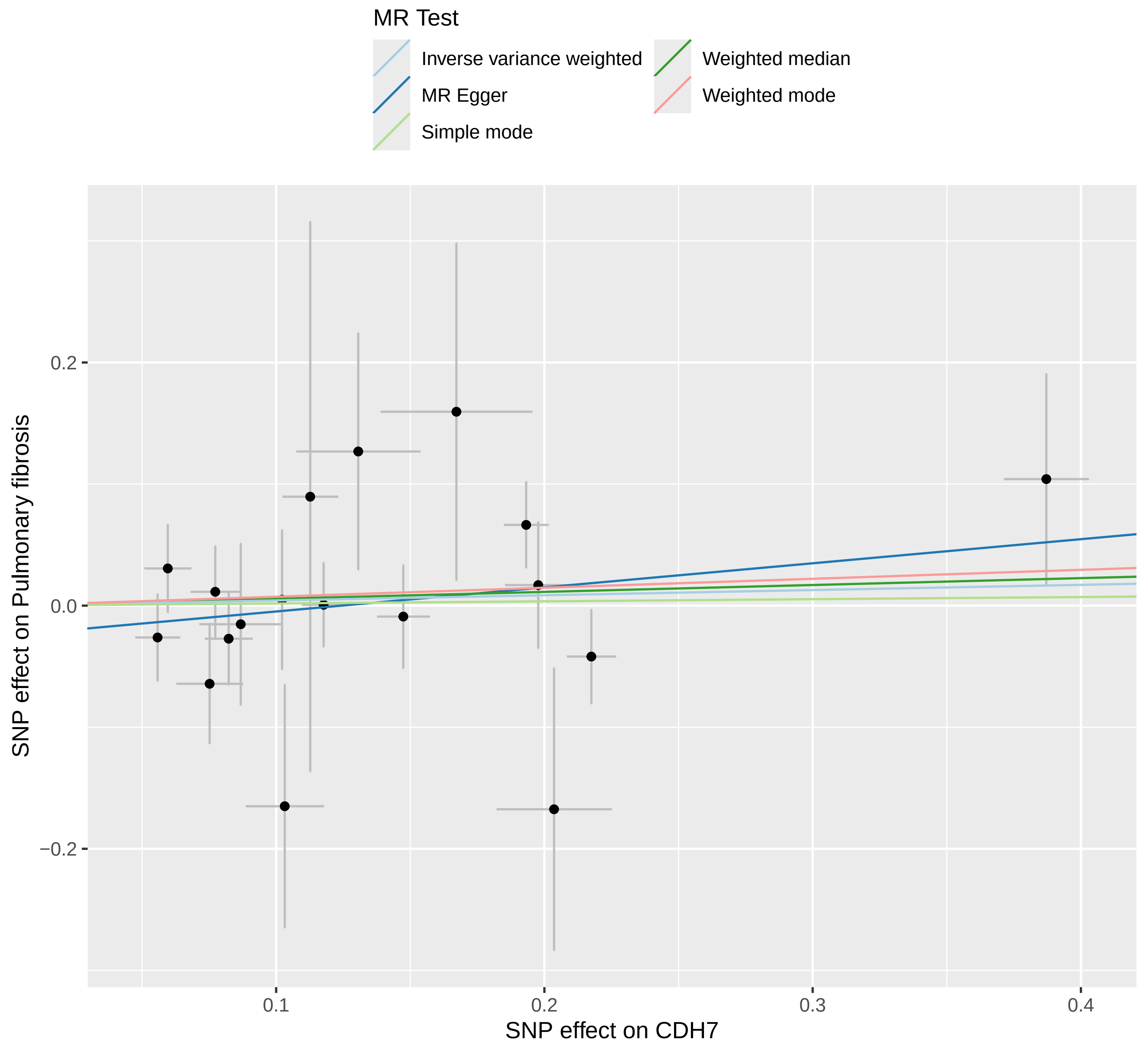
MR Test
Inverse variance weighted
MR Egger
Simple mode
Weighted median
Weighted mode
0.2
0.0
−0.2
0.1
0.2
0.3
0.4
SNP effect on CDH7
SNP effect on Pulmonary fibrosis

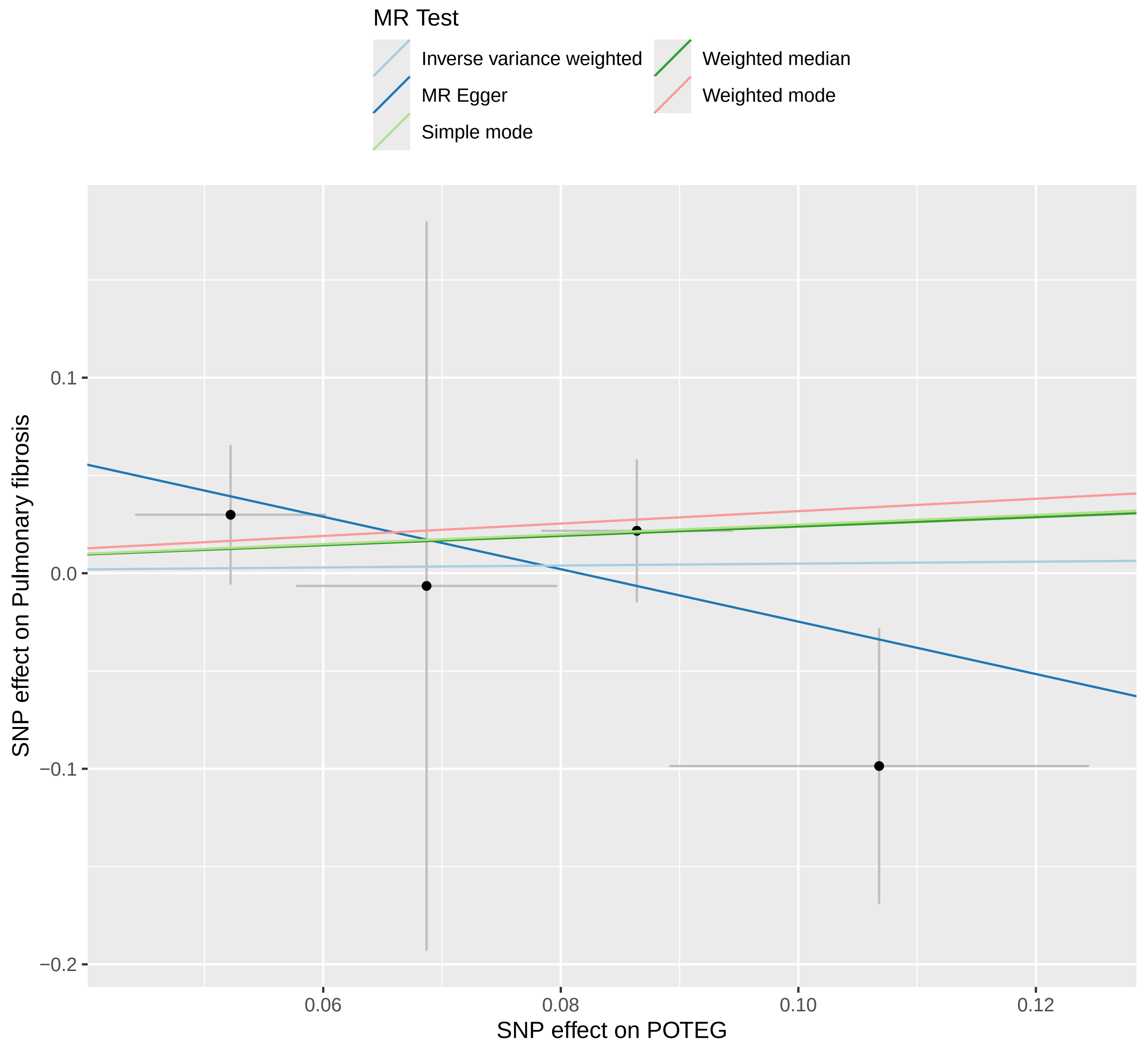

MR Test
Inverse variance weighted
MR Egger
Simple mode
Weighted median
Weighted mode
SNP effect on Pulmonary fibrosis
0.1
0.0
−0.1
−0.2
0.06
0.08
0.10
0.12
SNP effect on POTEG

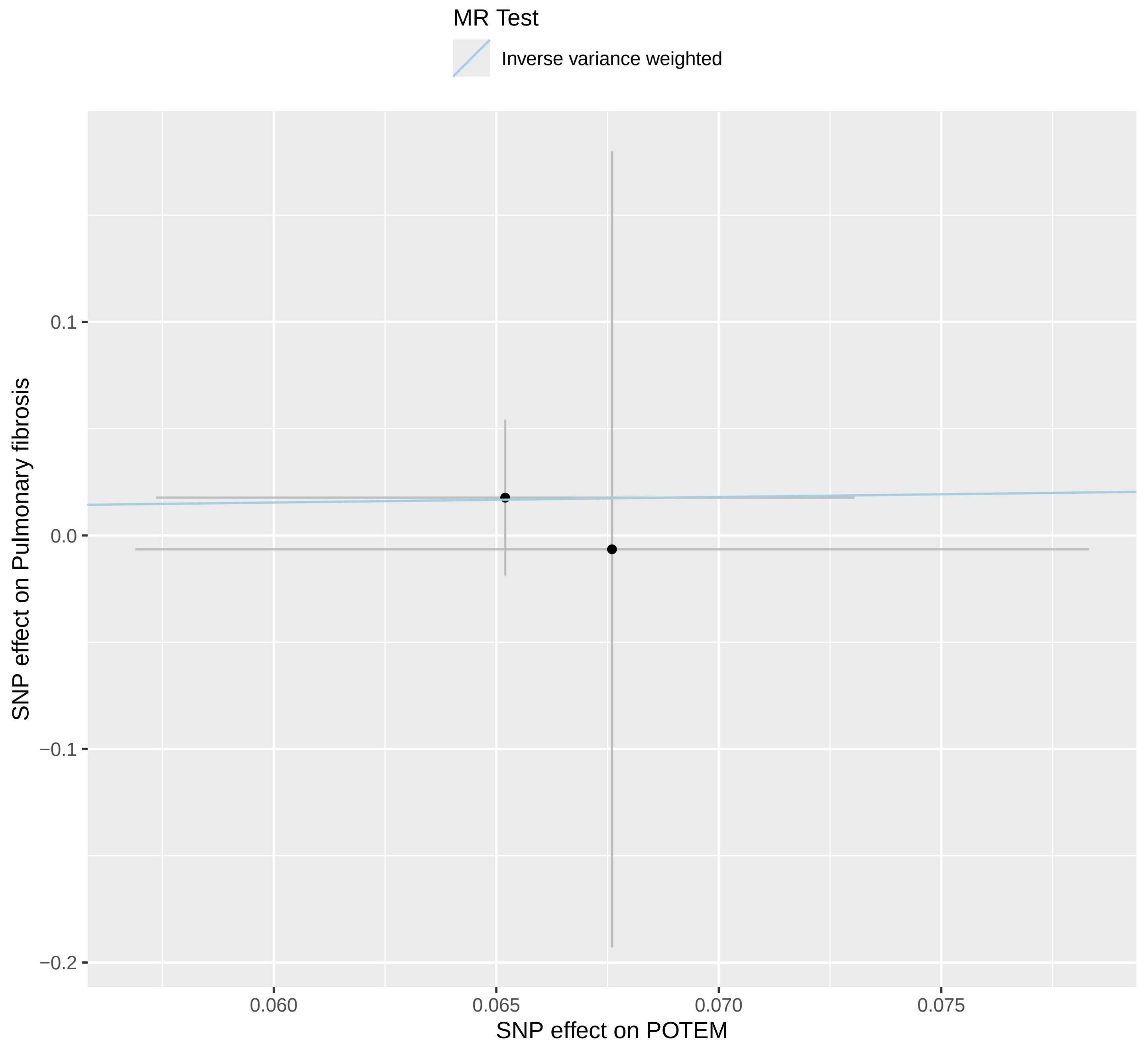
MR Test
Inverse variance weighted
0.1
0.0
−0.1
−0.2
0.060
0.065
0.070
0.075
SNP effect on POTEM
SNP effect on Pulmonary fibrosis

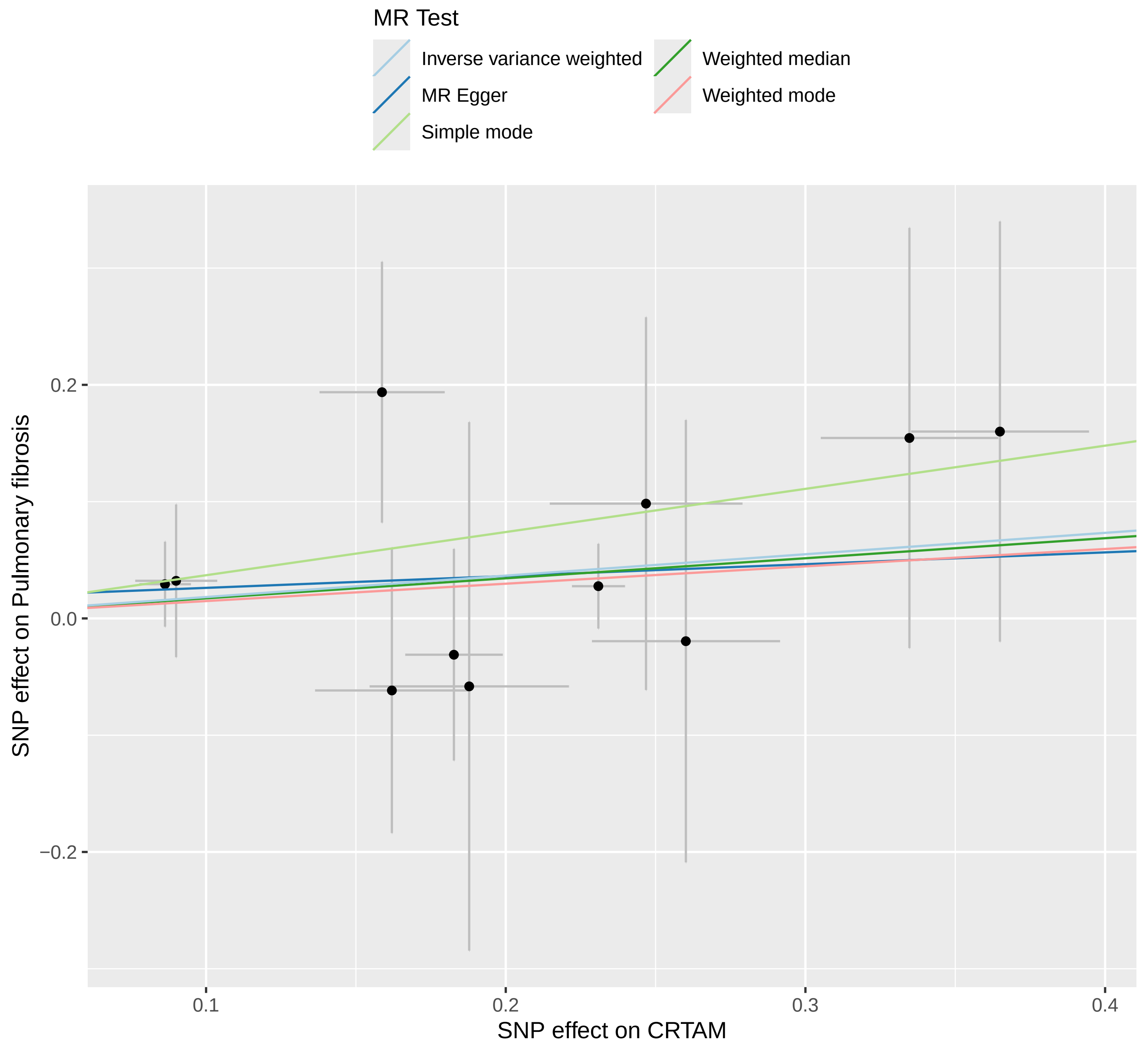

MR Test
Inverse variance weighted
MR Egger
Simple mode
Weighted median
Weighted mode
SNP effect on Pulmonary fibrosis
0.2
0.0
−0.2
0.1
0.2
0.3
0.4
SNP effect on CRTAM

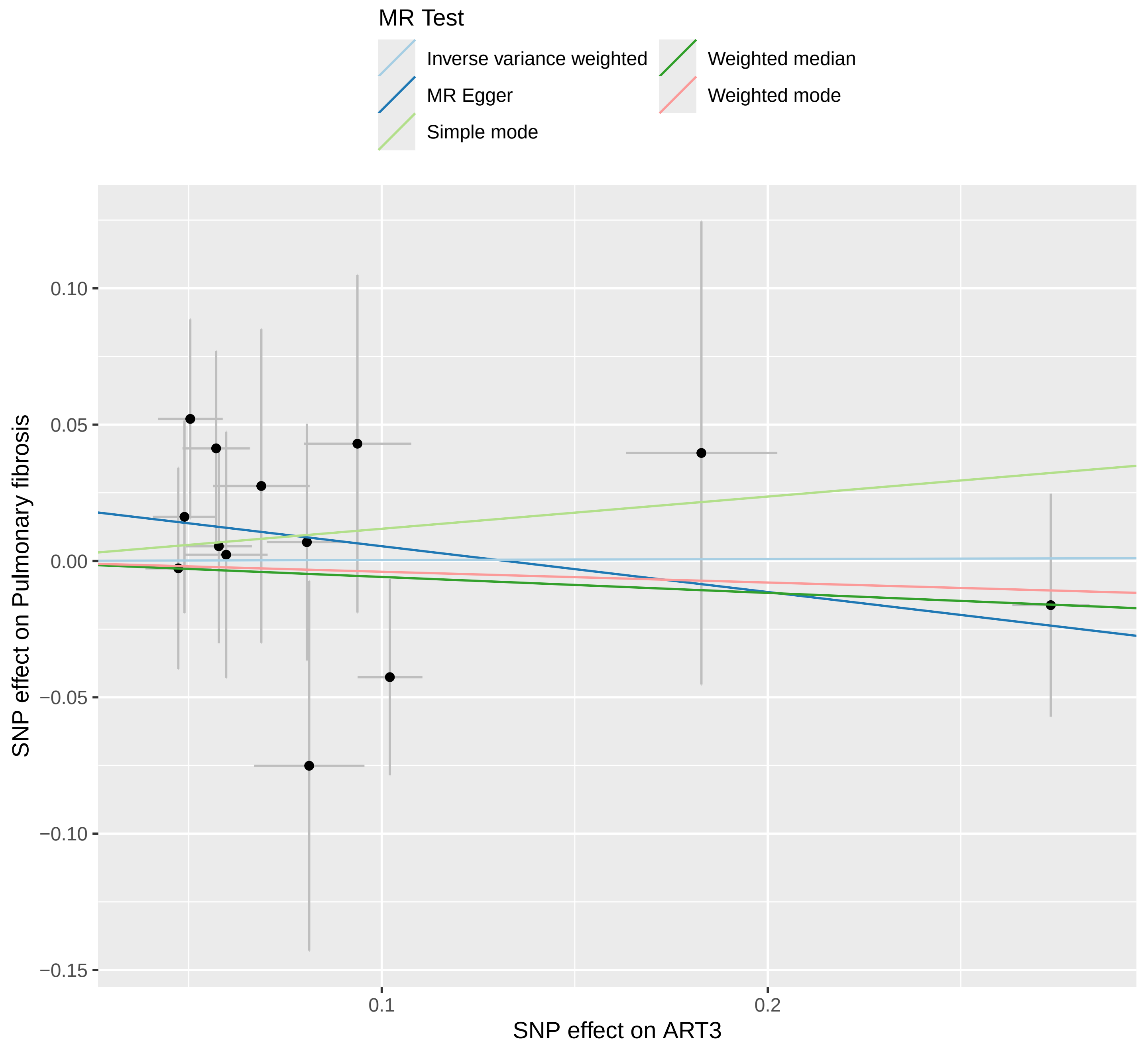

MR Test
Inverse variance weighted
MR Egger
Simple mode
Weighted median
Weighted mode
SNP effect on Pulmonary fibrosis
0.10
0.05
0.00
−0.05
−0.10
−0.15
0.1
0.2
SNP effect on ART3

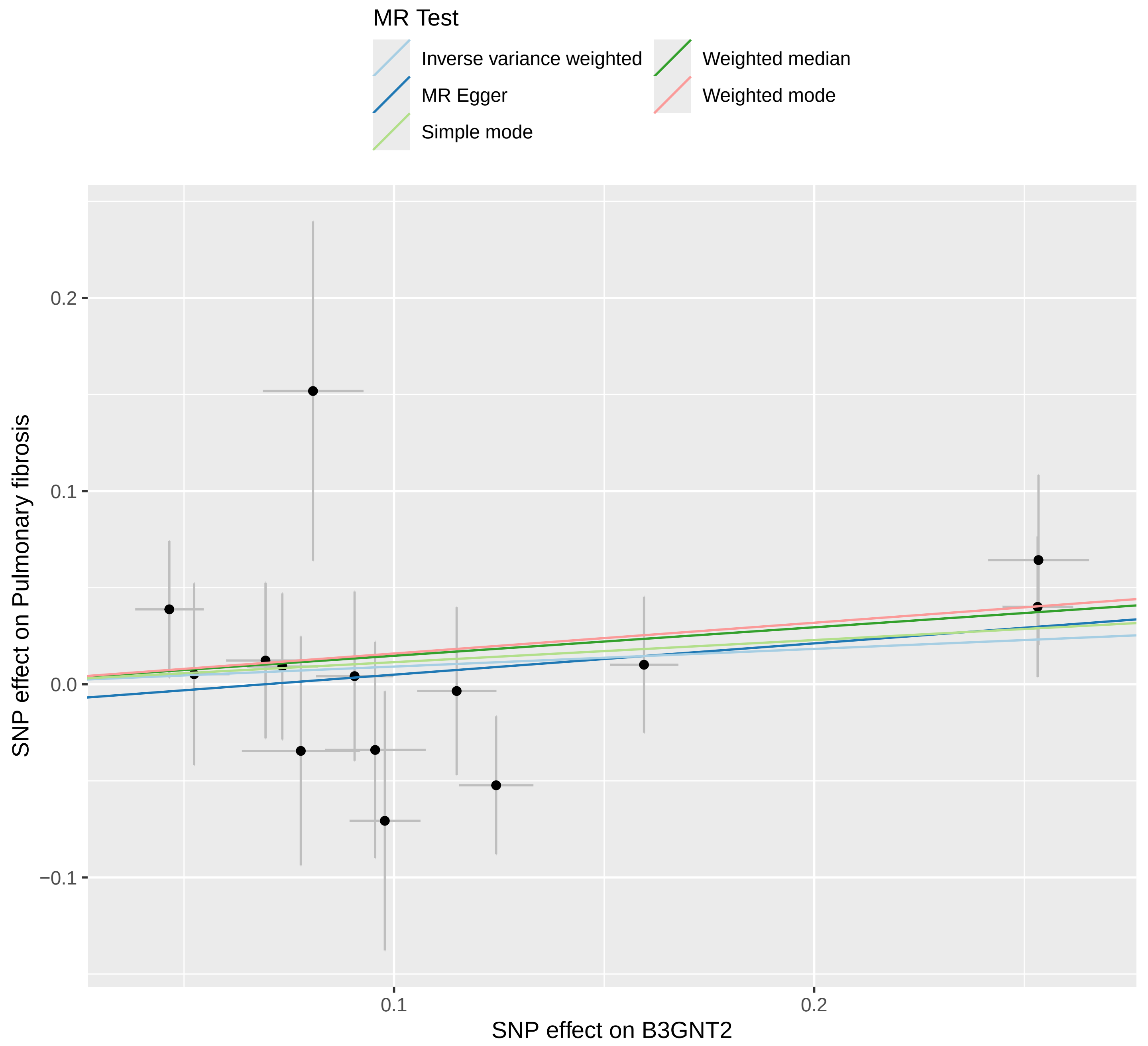
MR Test
Inverse variance weighted
MR Egger
Simple mode
Weighted median
Weighted mode
SNP effect on Pulmonary fibrosis
0.2
0.1
0.0
−0.1
0.1
0.2
SNP effect on B3GNT2

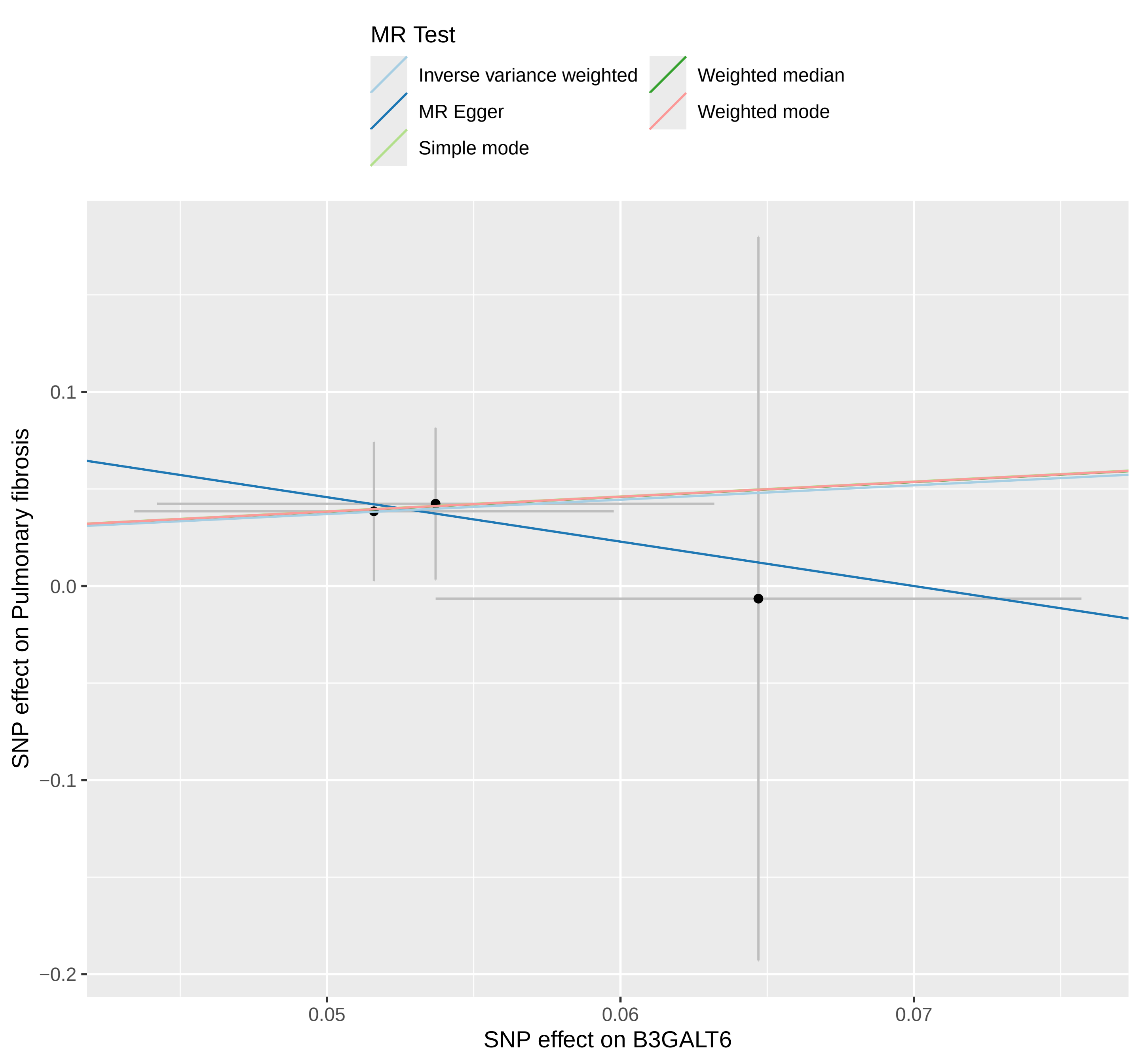

MR Test
Inverse variance weighted
MR Egger
Simple mode
Weighted median
Weighted mode
0.1
0.0
−0.1
−0.2
SNP effect on Pulmonary fibrosis
0.05
0.06
0.07
SNP effect on B3GALT6

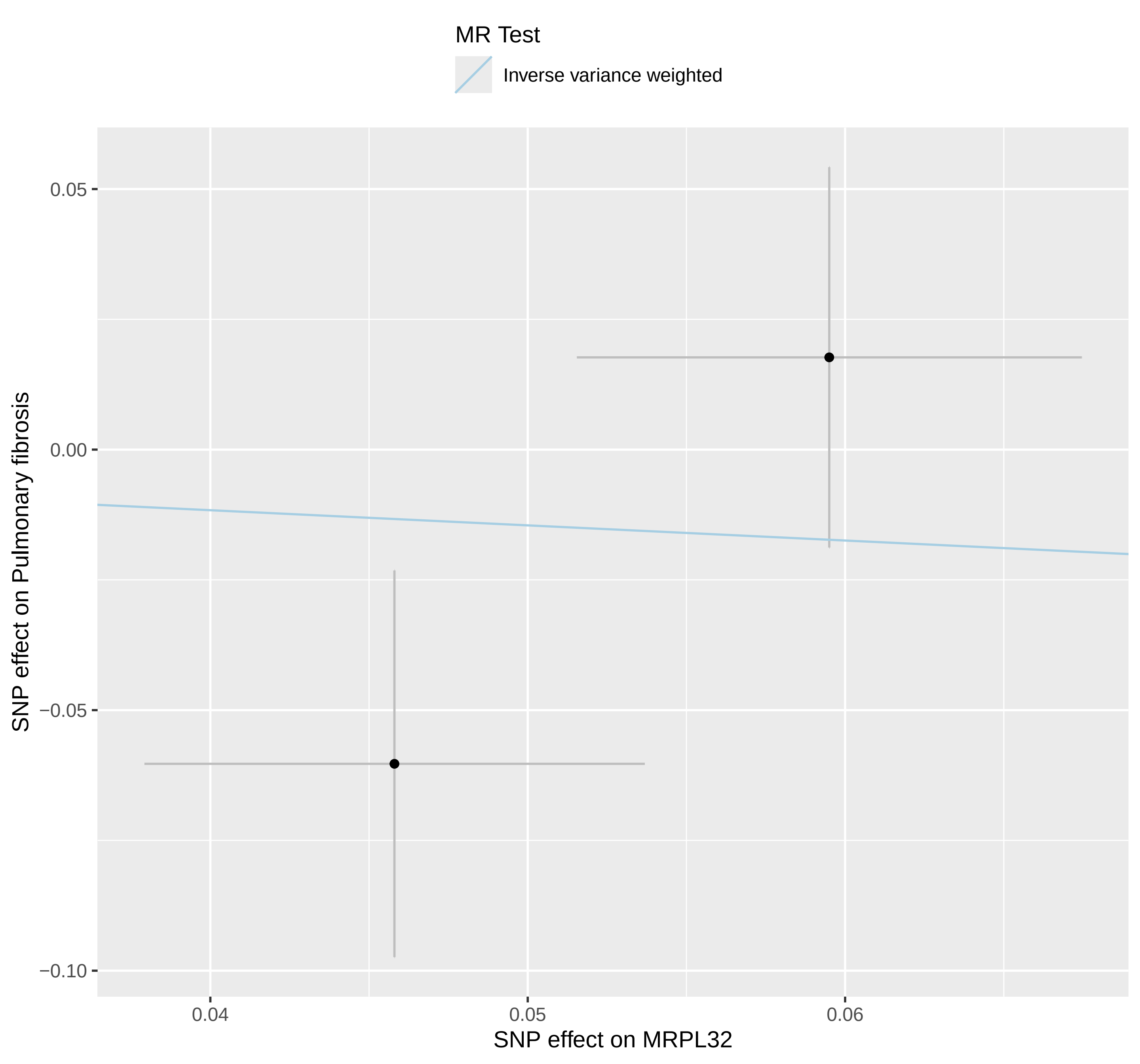

MR Test
Inverse variance weighted
0.05
0.00
−0.05
−0.10
0.04
0.05
0.06
SNP effect on MRPL32
SNP effect on Pulmonary fibrosis

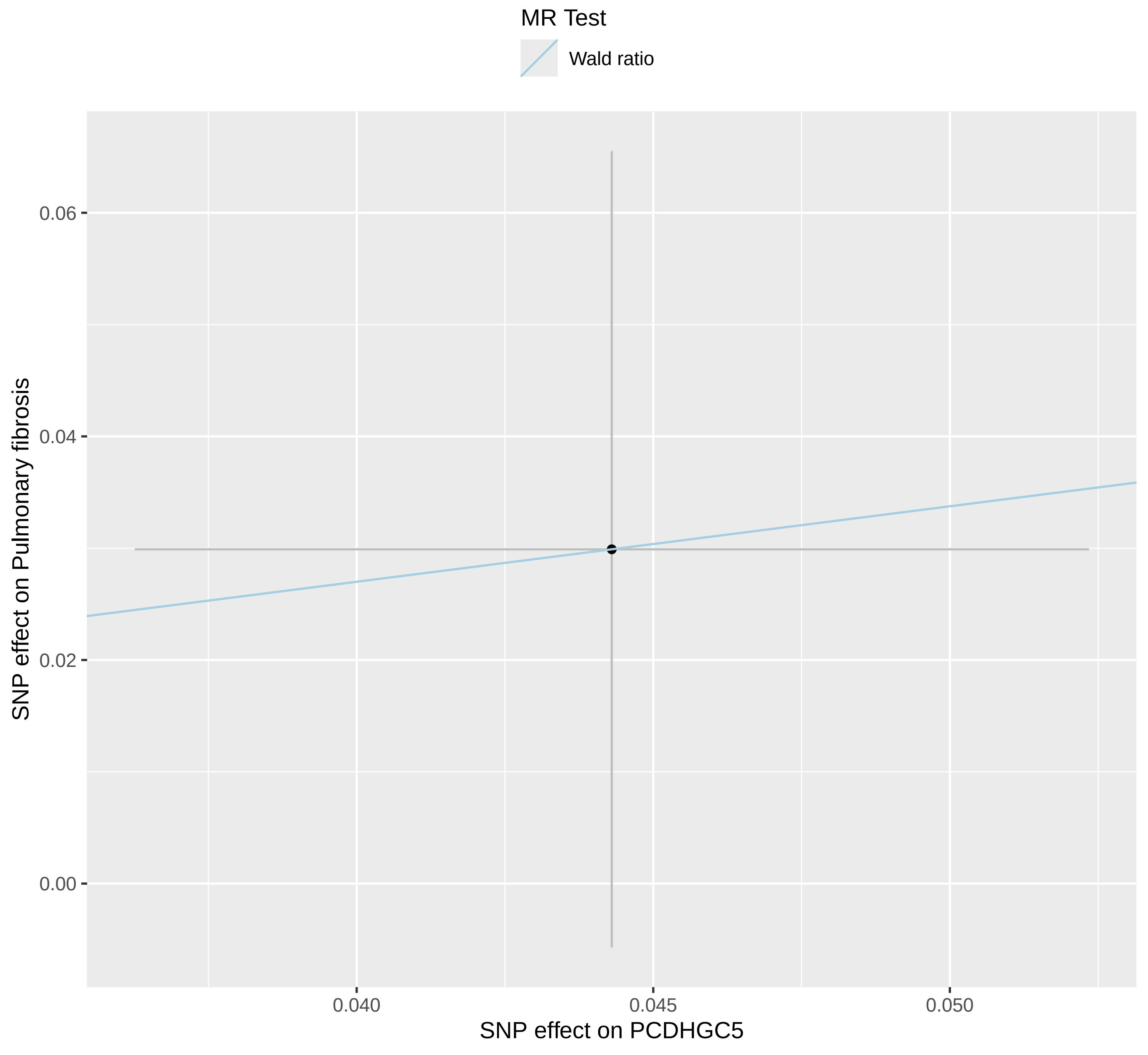
MR Test
Wald ratio
0.06
0.04
0.02
0.00
0.040
0.045
0.050
SNP effect on Pulmonary fibrosis
SNP effect on PCDHGC5

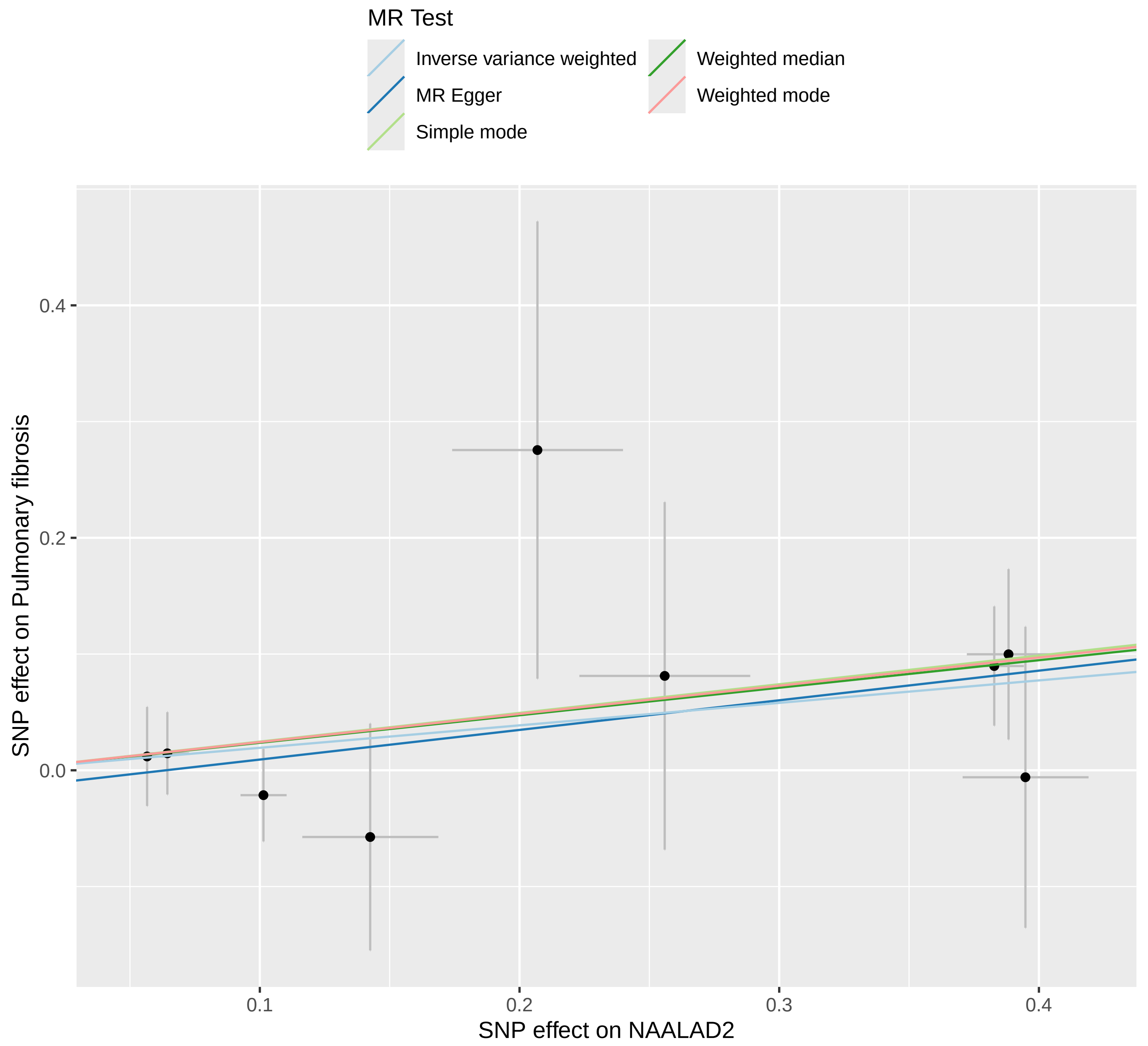
MR Test
Inverse variance weighted
MR Egger
Simple mode
Weighted median
Weighted mode
0.4
0.2
0.0
SNP effect on Pulmonary fibrosis
0.1
0.2
0.3
0.4
SNP effect on NAALAD2

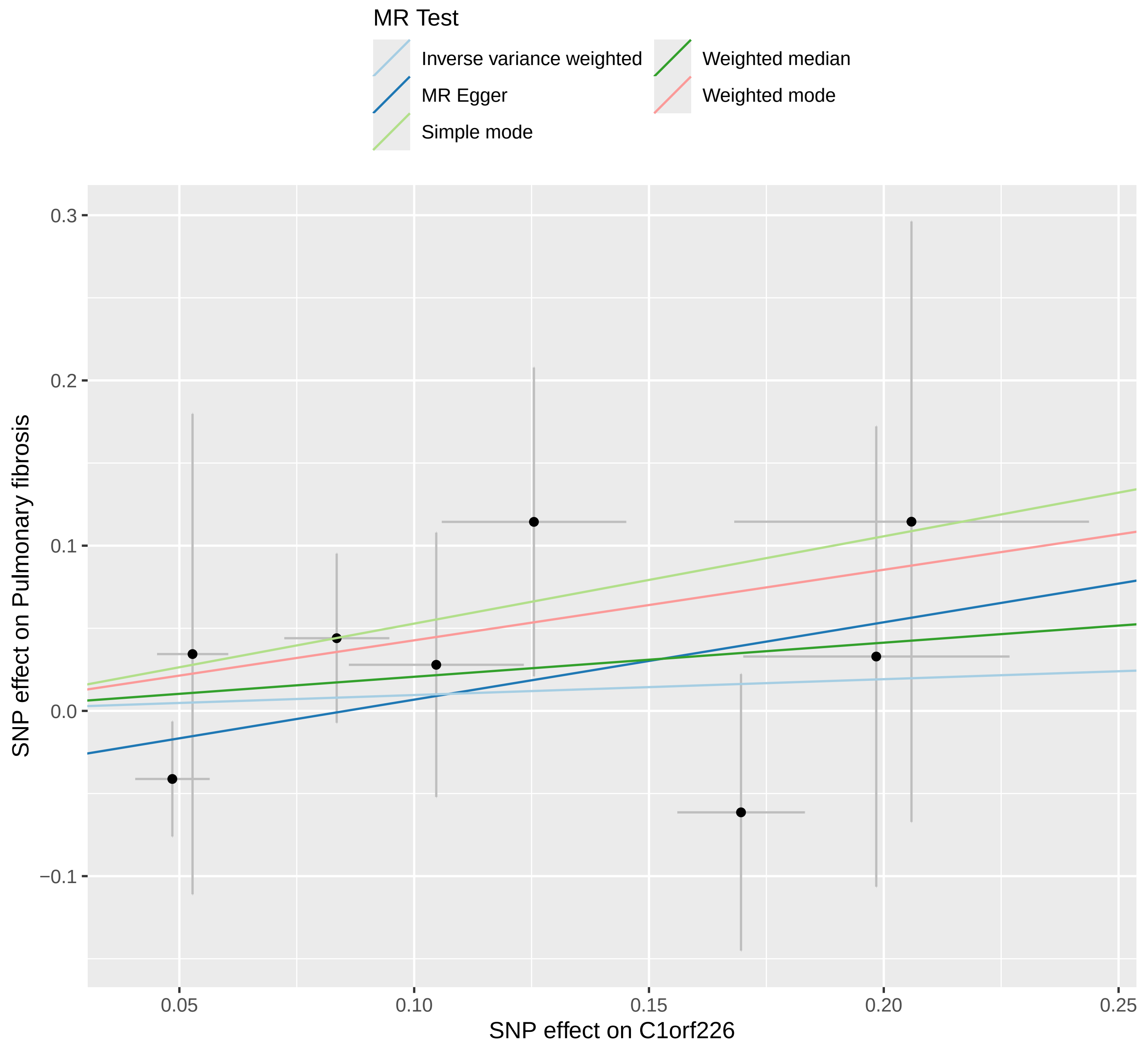
MR Test
Inverse variance weighted
MR Egger
Simple mode
Weighted median
Weighted mode
0.3
0.2
0.1
0.0
−0.1
0.05
0.10
0.15
0.20
0.25
SNP effect on Pulmonary fibrosis
SNP effect on C1orf226

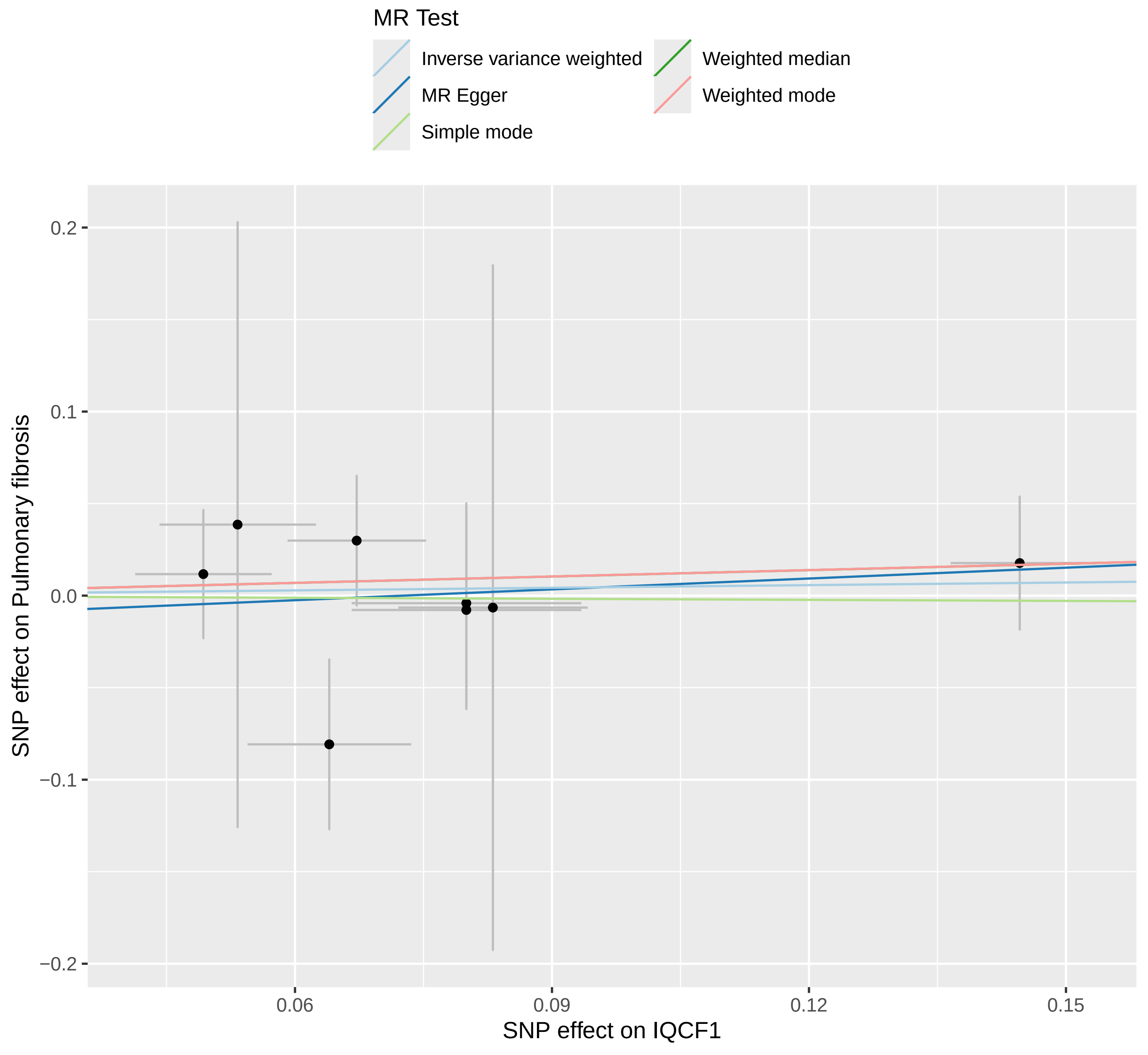

MR Test
Inverse variance weighted
MR Egger
Simple mode
Weighted median
Weighted mode
SNP effect on Pulmonary fibrosis
0.2
0.1
0.0
−0.1
−0.2
0.06
0.09
0.12
0.15
SNP effect on IQCF1

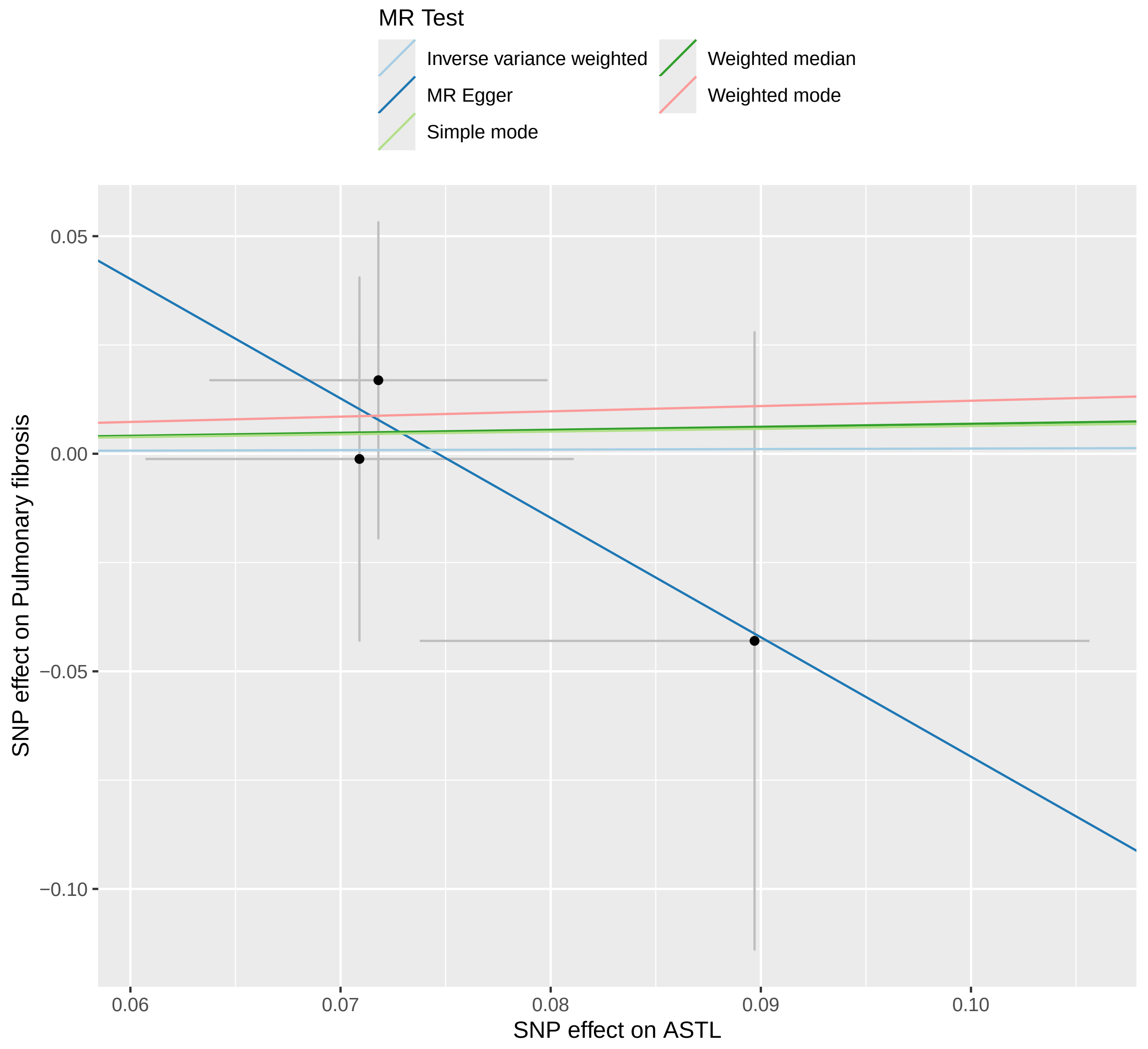
MR Test
Inverse variance weighted
MR Egger
Simple mode
Weighted median
Weighted mode
0.05
0.00
−0.05
−0.10
SNP effect on Pulmonary fibrosis
0.06
0.07
0.08
0.09
0.10
SNP effect on ASTL

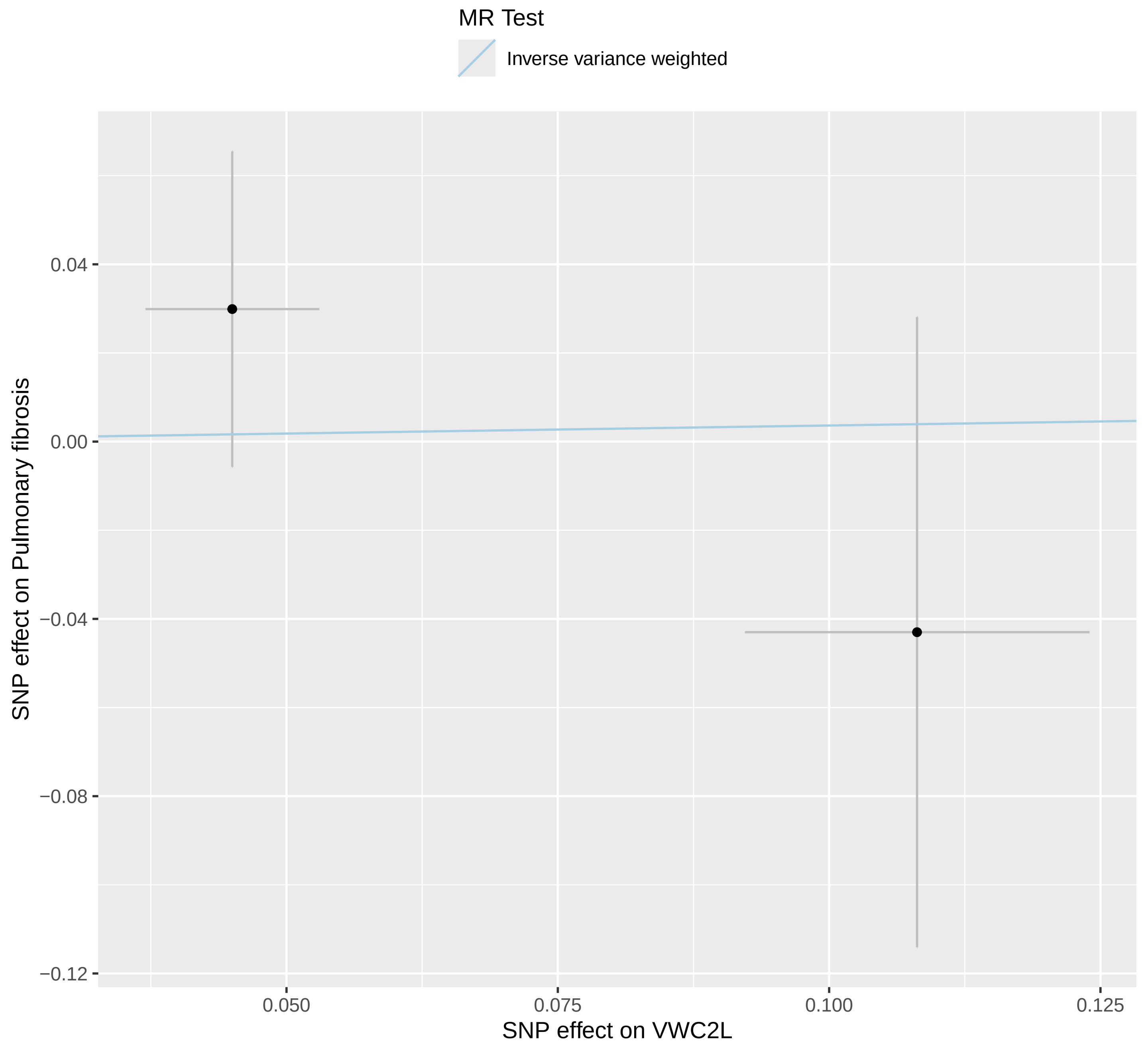

MR Test
Inverse variance weighted
0.04
0.00
−0.04
−0.08
−0.12
0.050
0.075
0.100
0.125
SNP effect on Pulmonary fibrosis
SNP effect on VWC2L

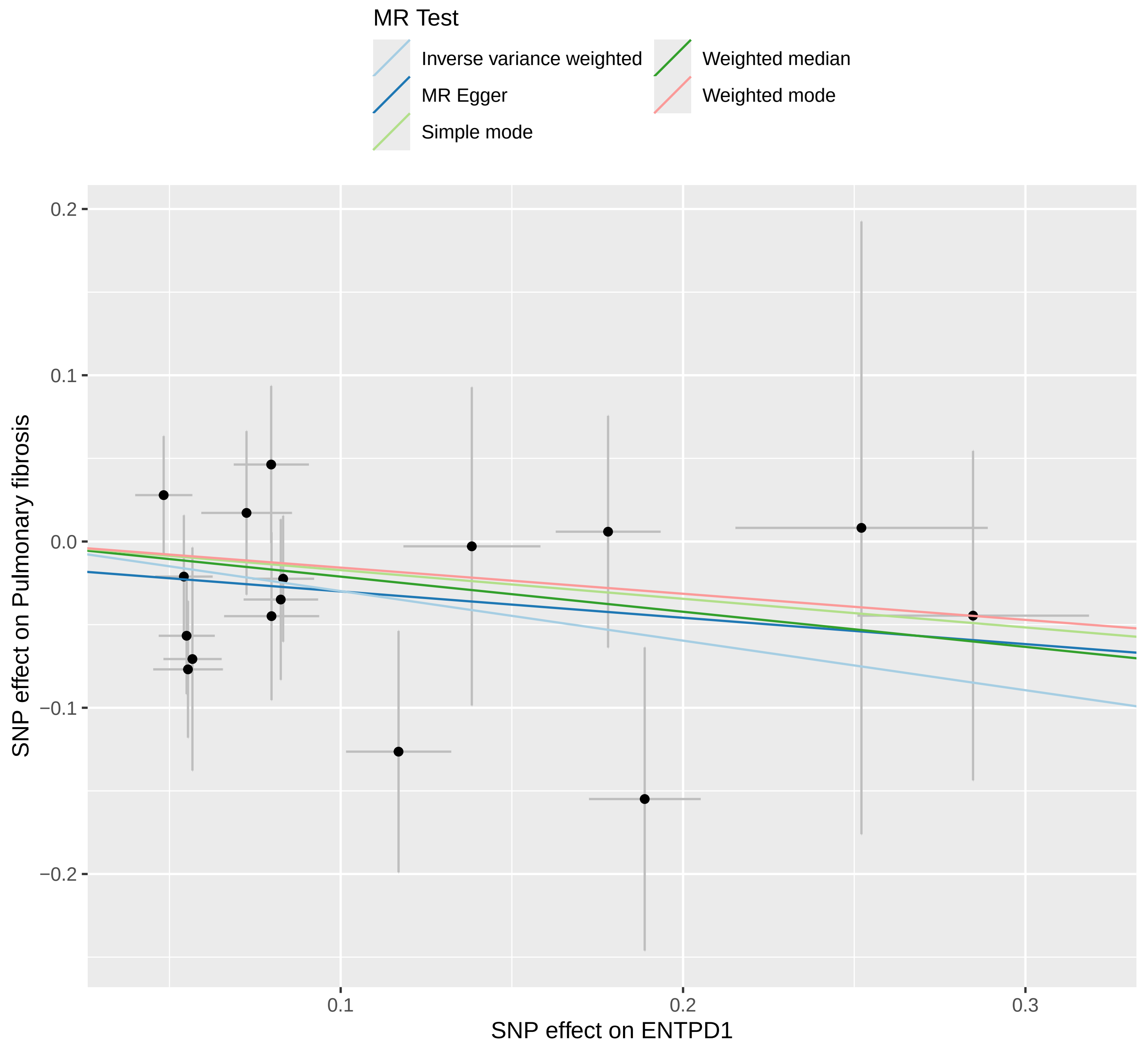
MR Test
Inverse variance weighted
MR Egger
Simple mode
Weighted median
Weighted mode
0.2
0.1
0.0
−0.1
−0.2
SNP effect on Pulmonary fibrosis
0.1
0.2
0.3
SNP effect on ENTPD1

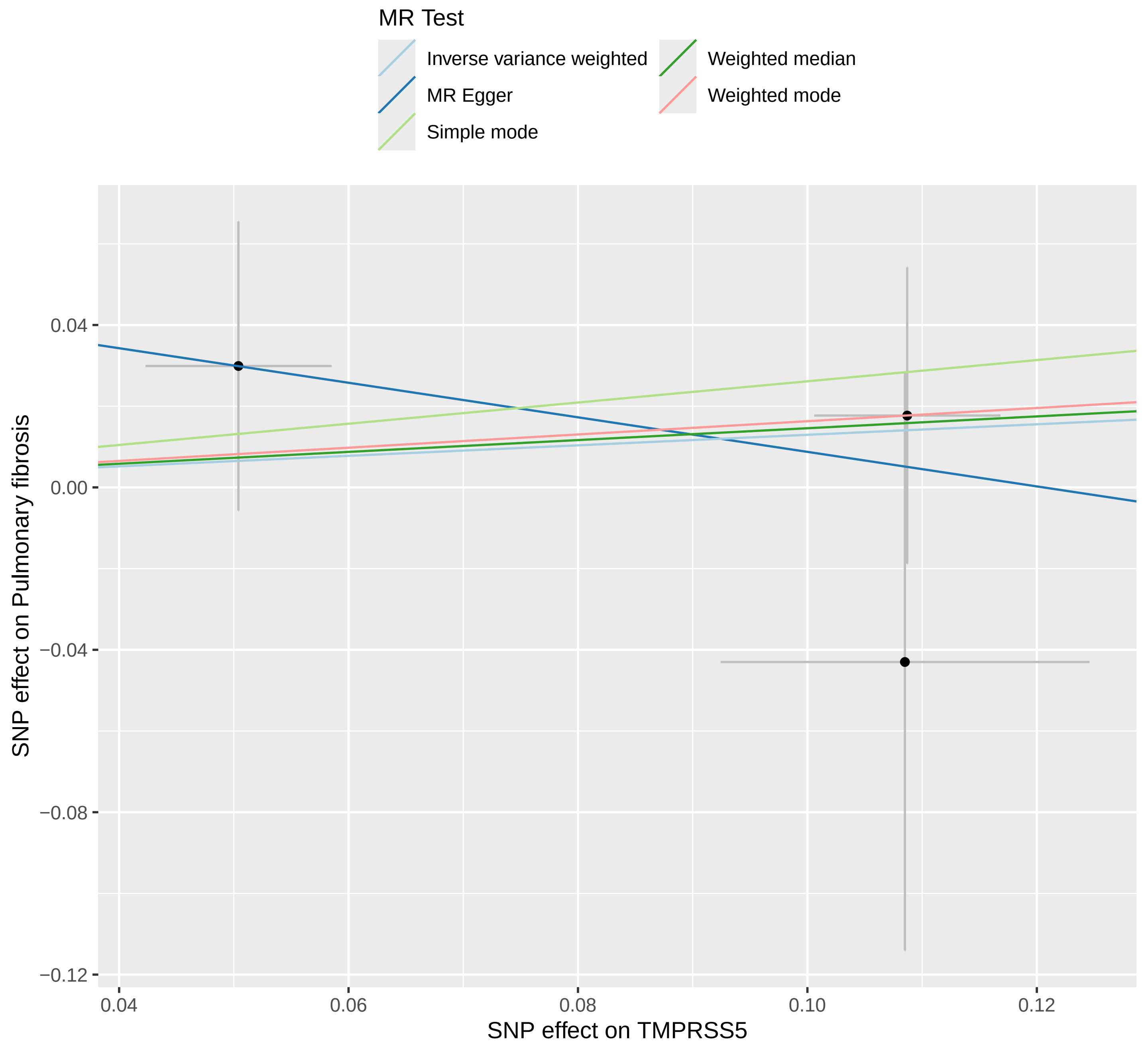

MR Test
Inverse variance weighted
MR Egger
Simple mode
Weighted median
Weighted mode
0.04
0.00
−0.04
−0.08
−0.12
SNP effect on Pulmonary fibrosis
0.04
0.06
0.08
0.10
0.12
SNP effect on TMPRSS5

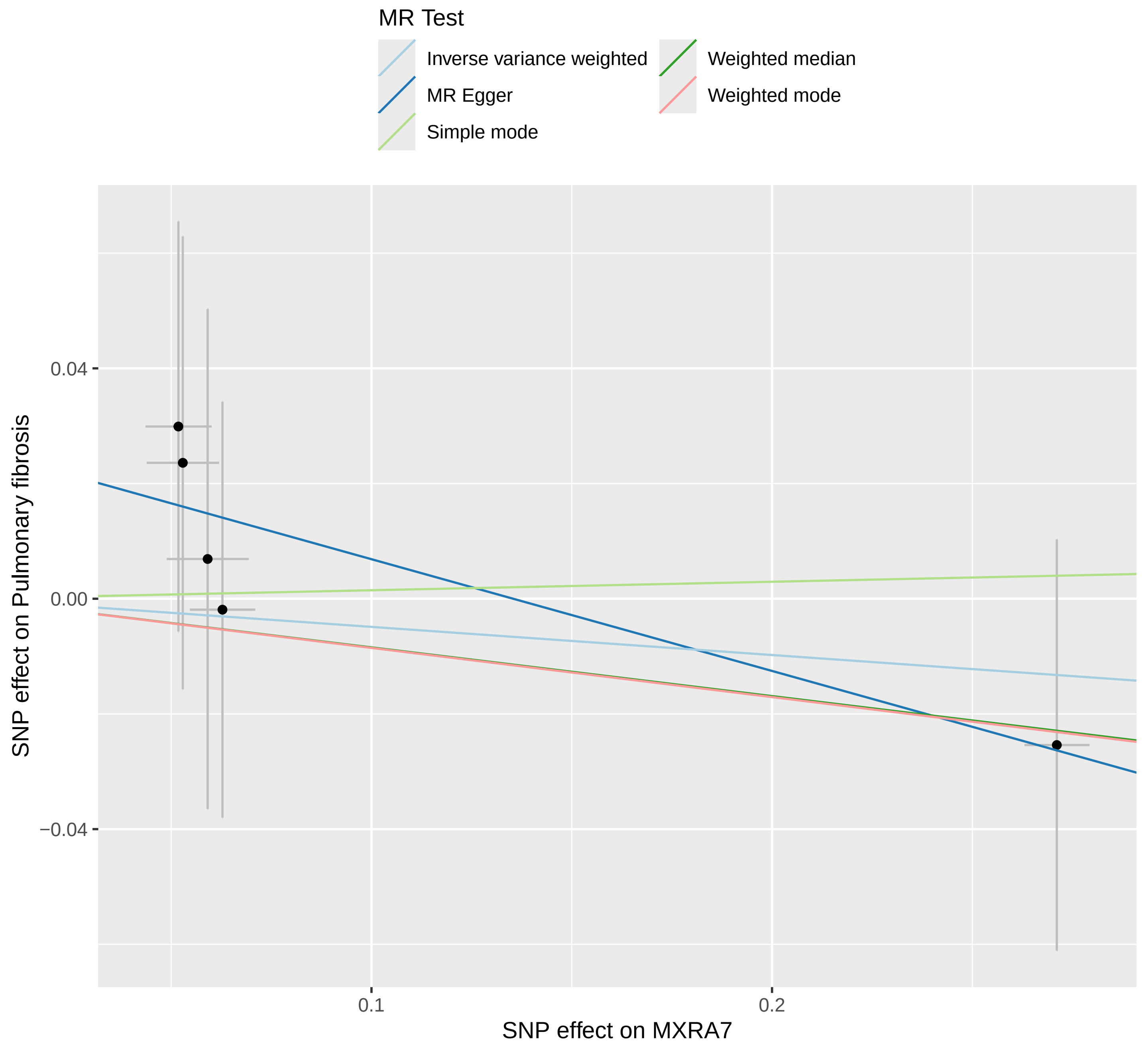
MR Test
Inverse variance weighted
MR Egger
Simple mode
Weighted median
Weighted mode
0.04
0.00
−0.04
0.1
0.2
SNP effect on Pulmonary fibrosis
SNP effect on MXRA7

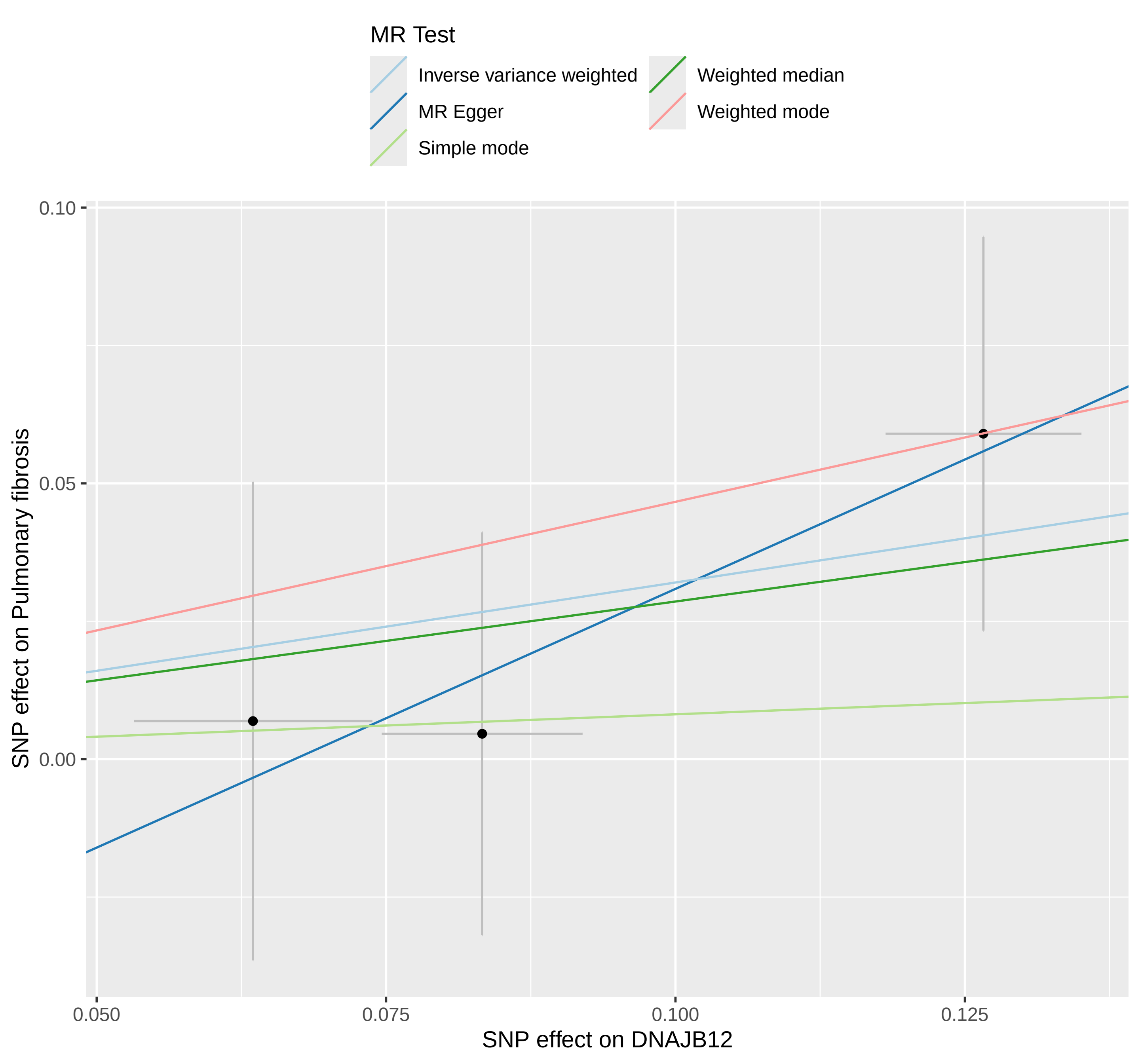
MR Test
Inverse variance weighted
MR Egger
Simple mode
Weighted median
Weighted mode
0.10
0.05
0.00
0.050
0.075
0.100
0.125
SNP effect on Pulmonary fibrosis
SNP effect on DNAJB12

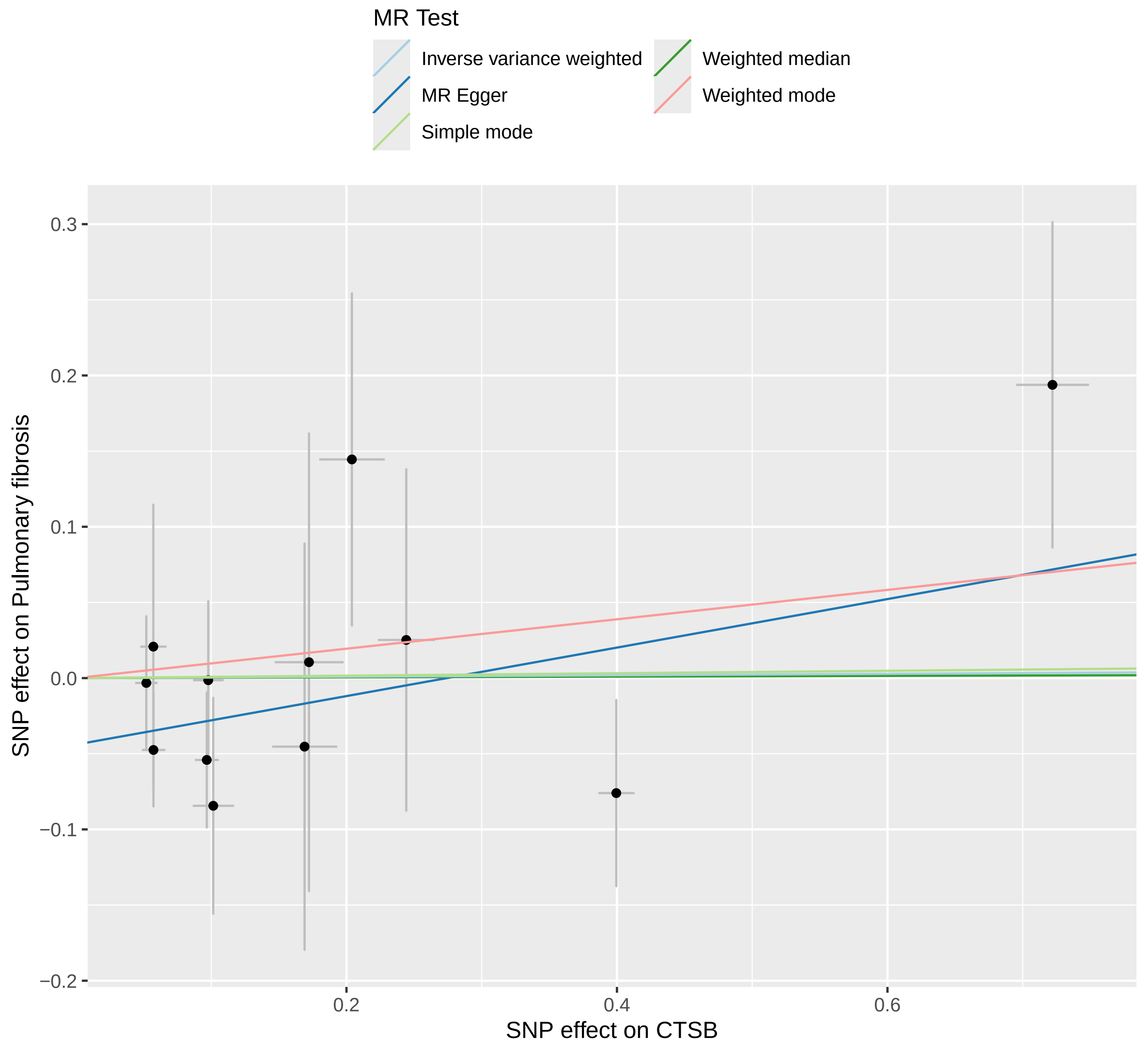

MR Test
Inverse variance weighted
MR Egger
Simple mode
Weighted median
Weighted mode
0.3
0.2
0.1
0.0
−0.1
−0.2
SNP effect on Pulmonary fibrosis
0.2
0.4
0.6
SNP effect on CTSB

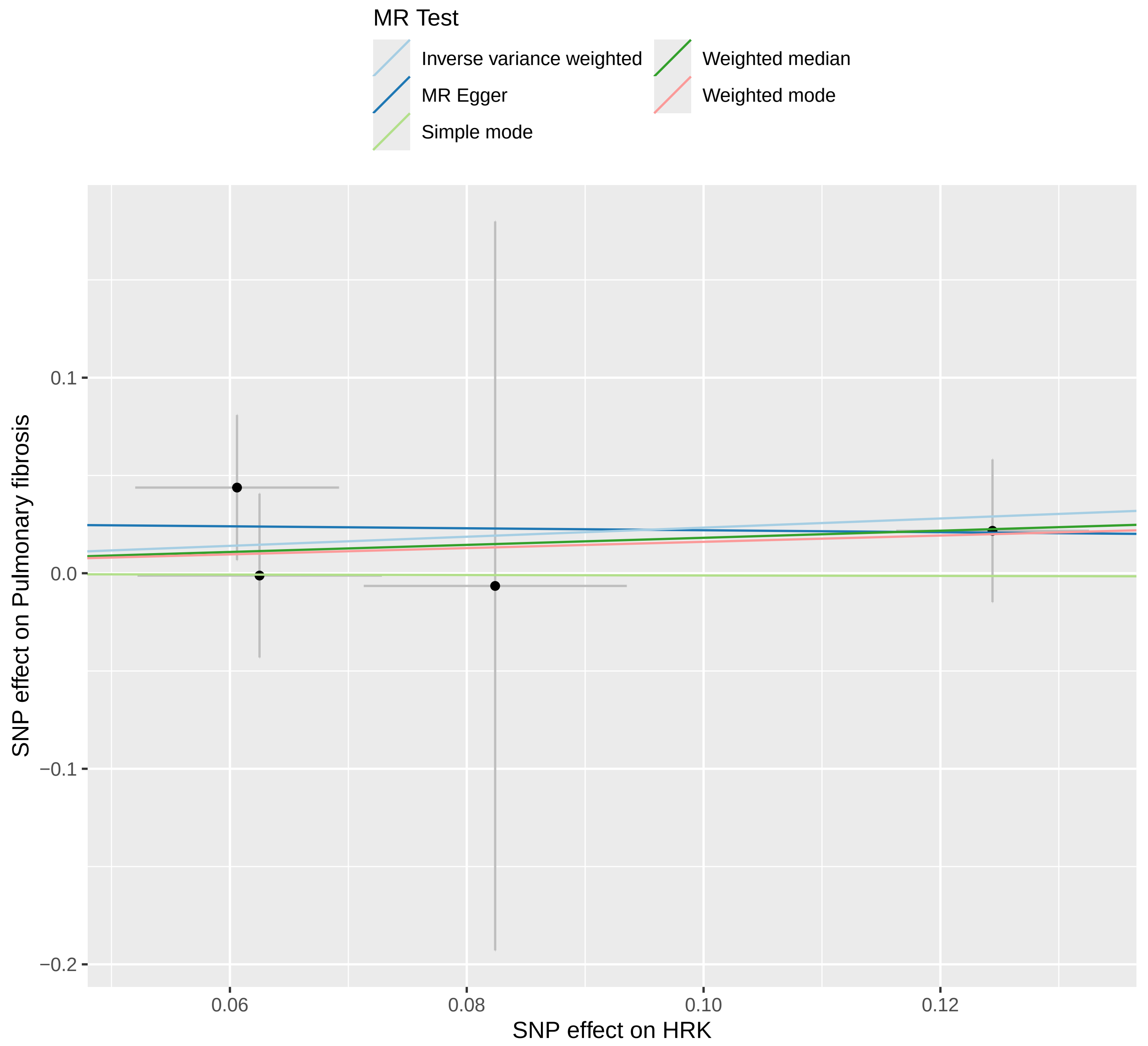

MR Test
Inverse variance weighted
MR Egger
Simple mode
Weighted median
Weighted mode
0.1
0.0
−0.1
−0.2
SNP effect on Pulmonary fibrosis
0.06
0.08
0.10
0.12
SNP effect on HRK

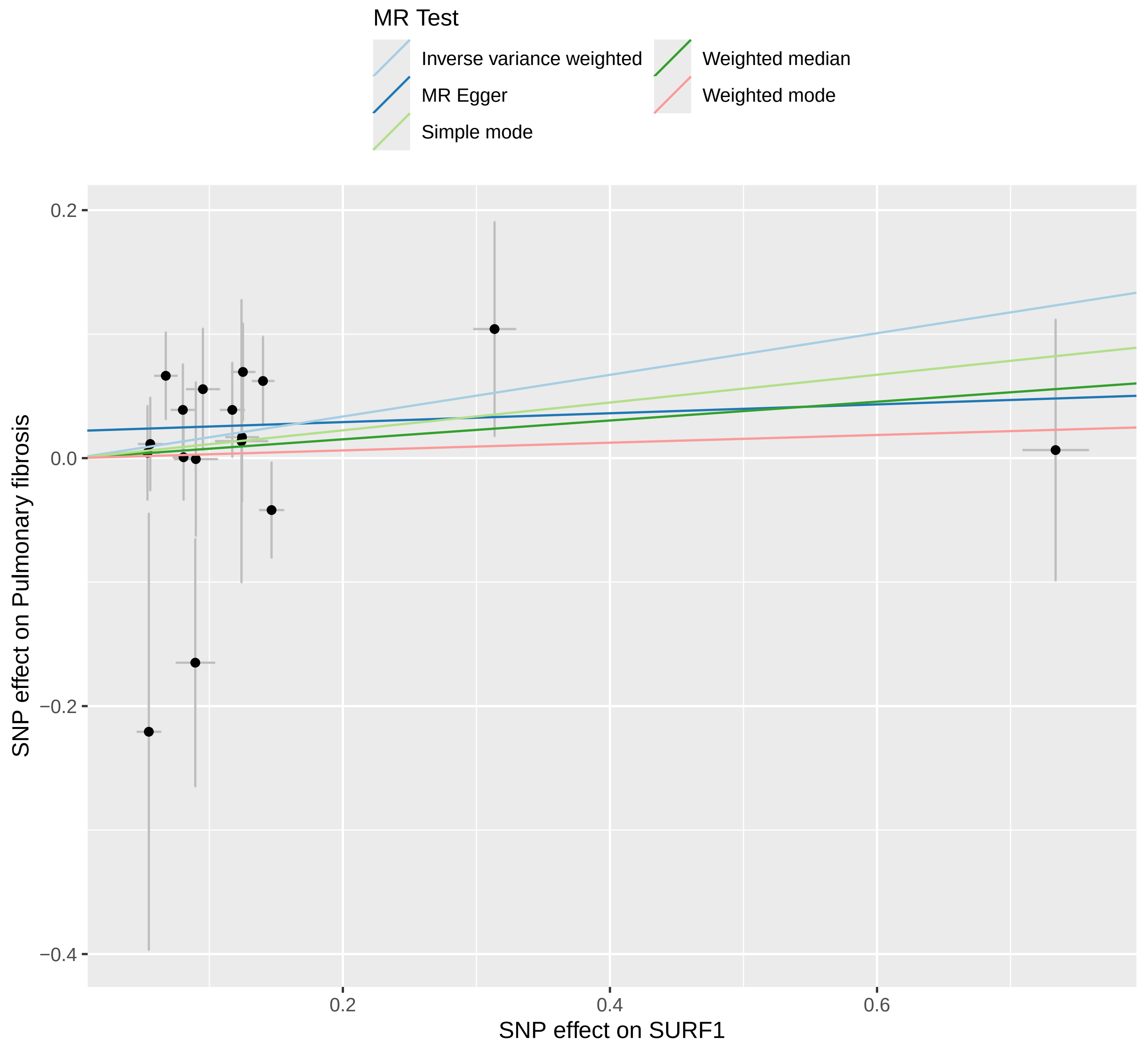

MR Test
Inverse variance weighted
MR Egger
Simple mode
Weighted median
Weighted mode
0.2
0.0
−0.2
−0.4
SNP effect on Pulmonary fibrosis
0.2
0.4
0.6
SNP effect on SURF1

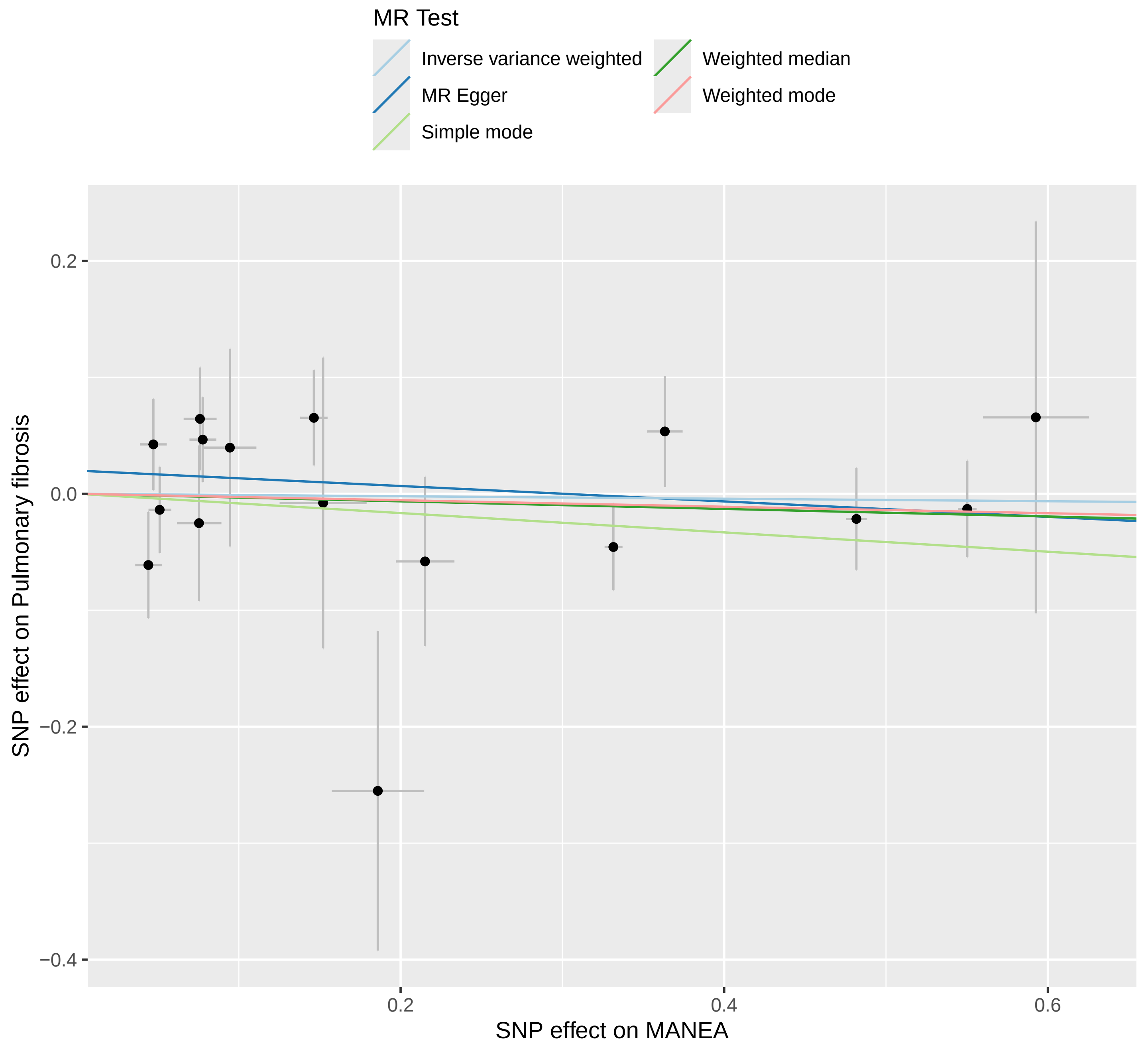
MR Test
Inverse variance weighted
MR Egger
Simple mode
Weighted median
Weighted mode
0.2
0.0
−0.2
−0.4
SNP effect on Pulmonary fibrosis
0.2
0.4
0.6
SNP effect on MANEA

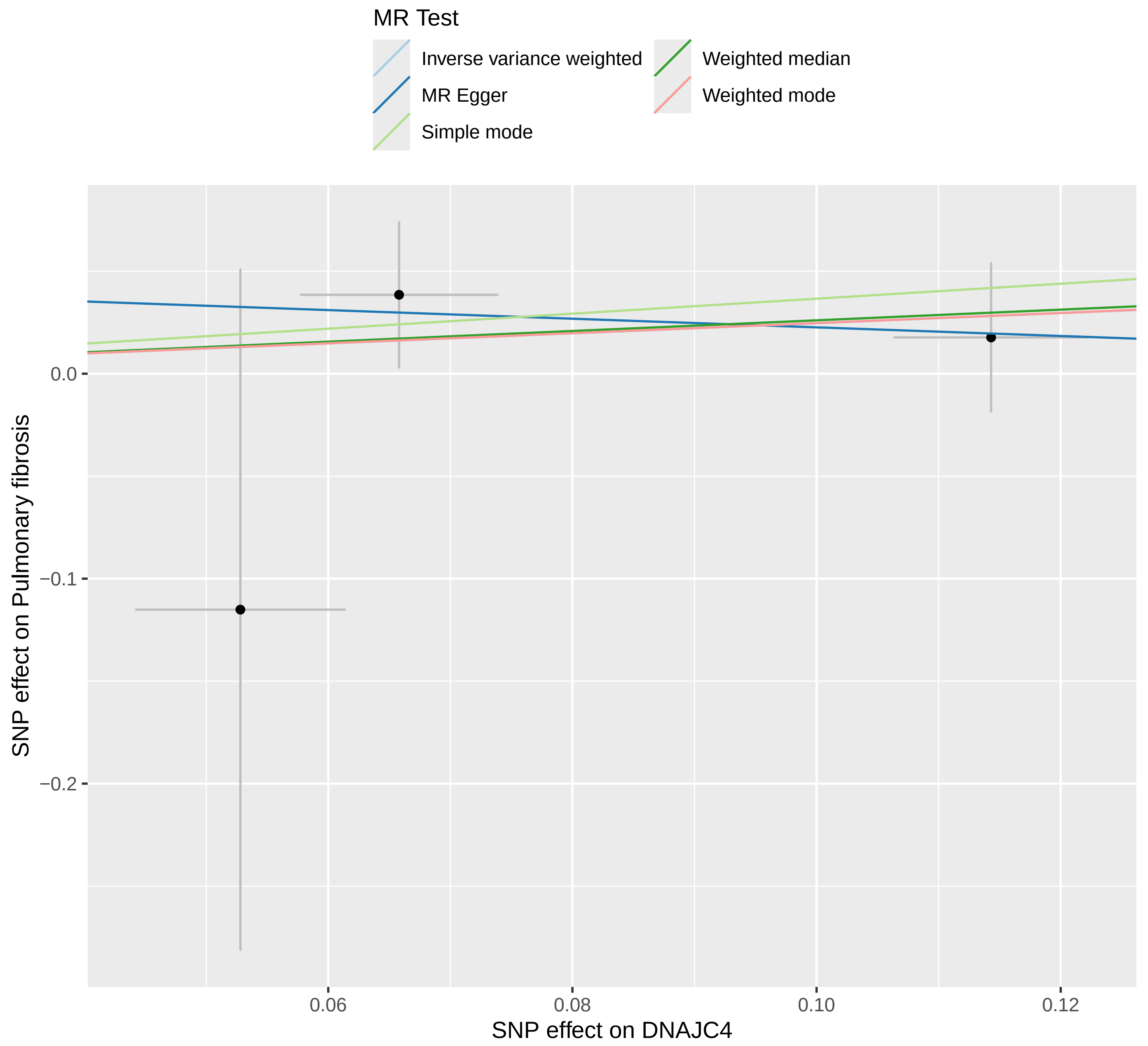

MR Test
Inverse variance weighted
MR Egger
Simple mode
Weighted median
Weighted mode
0.0
−0.1
−0.2
0.06
0.08
0.10
0.12
SNP effect on Pulmonary fibrosis
SNP effect on DNAJC4

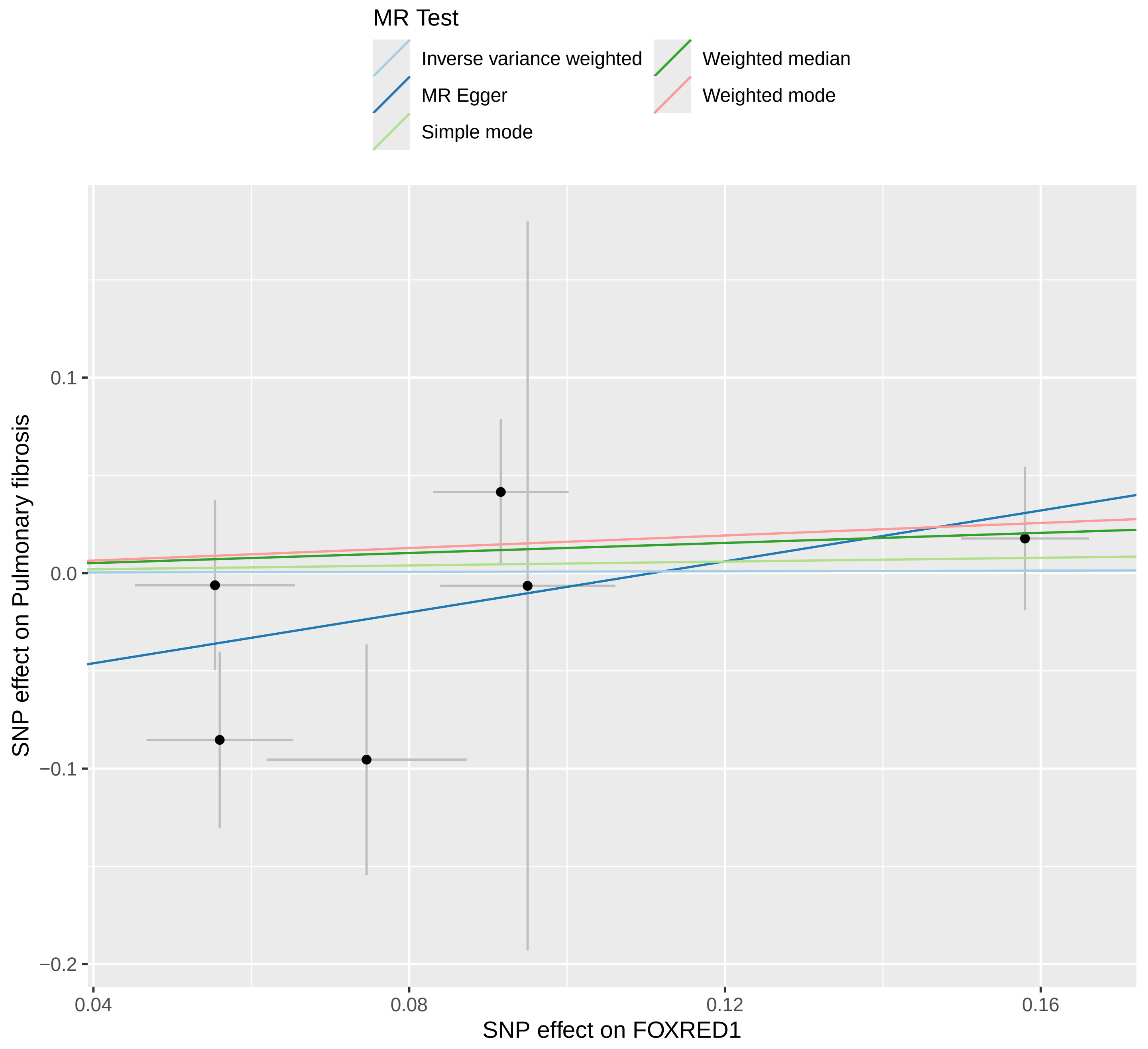

MR Test
Inverse variance weighted
MR Egger
Simple mode
Weighted median
Weighted mode
SNP effect on Pulmonary fibrosis
0.1
0.0
−0.1
−0.2
0.04
0.08
0.12
0.16
SNP effect on FOXRED1

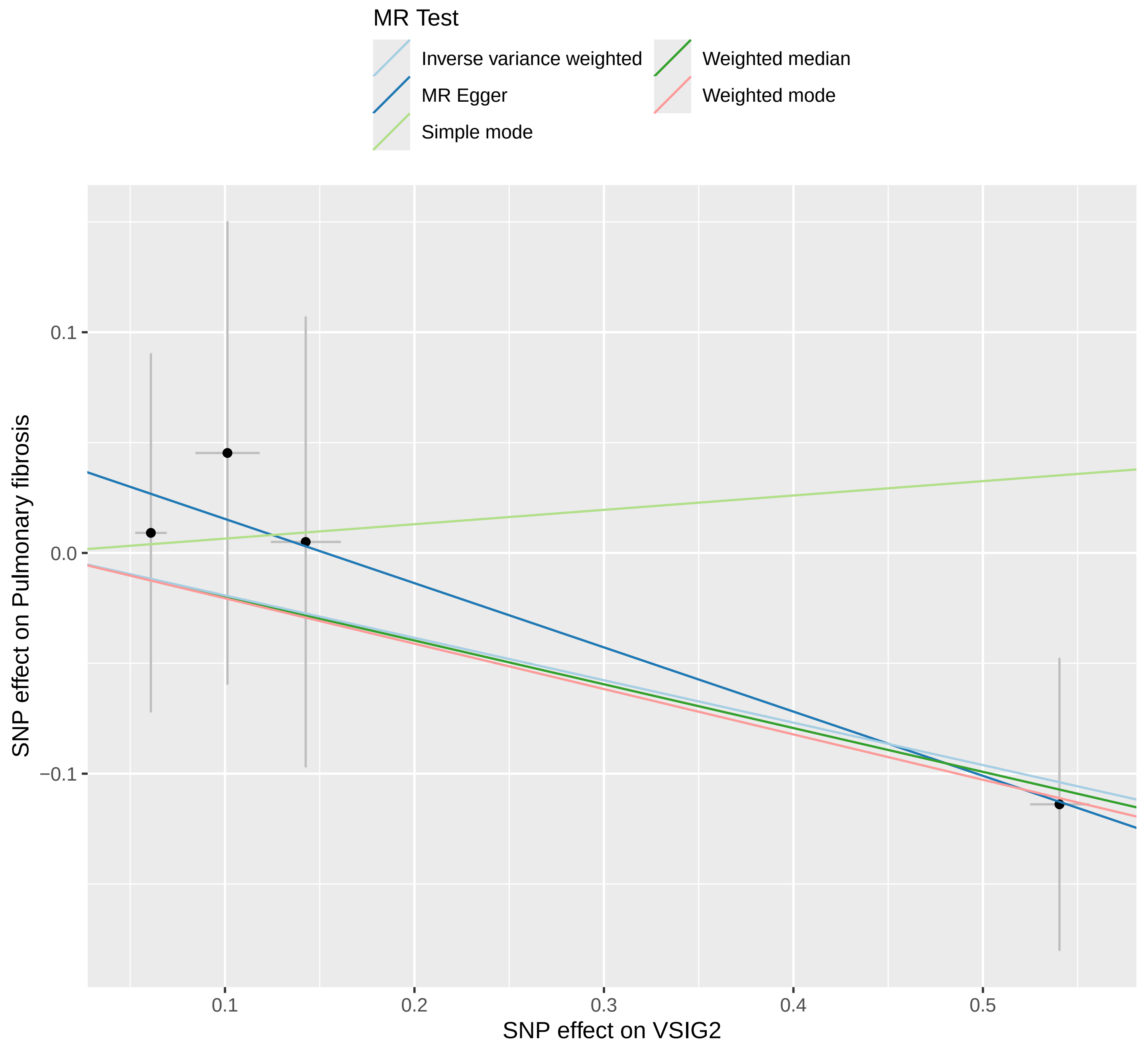

MR Test
Inverse variance weighted
MR Egger
Simple mode
Weighted median
Weighted mode
0.1
0.0
−0.1
0.1
0.2
0.3
0.4
0.5
SNP effect on VSIG2
SNP effect on Pulmonary fibrosis

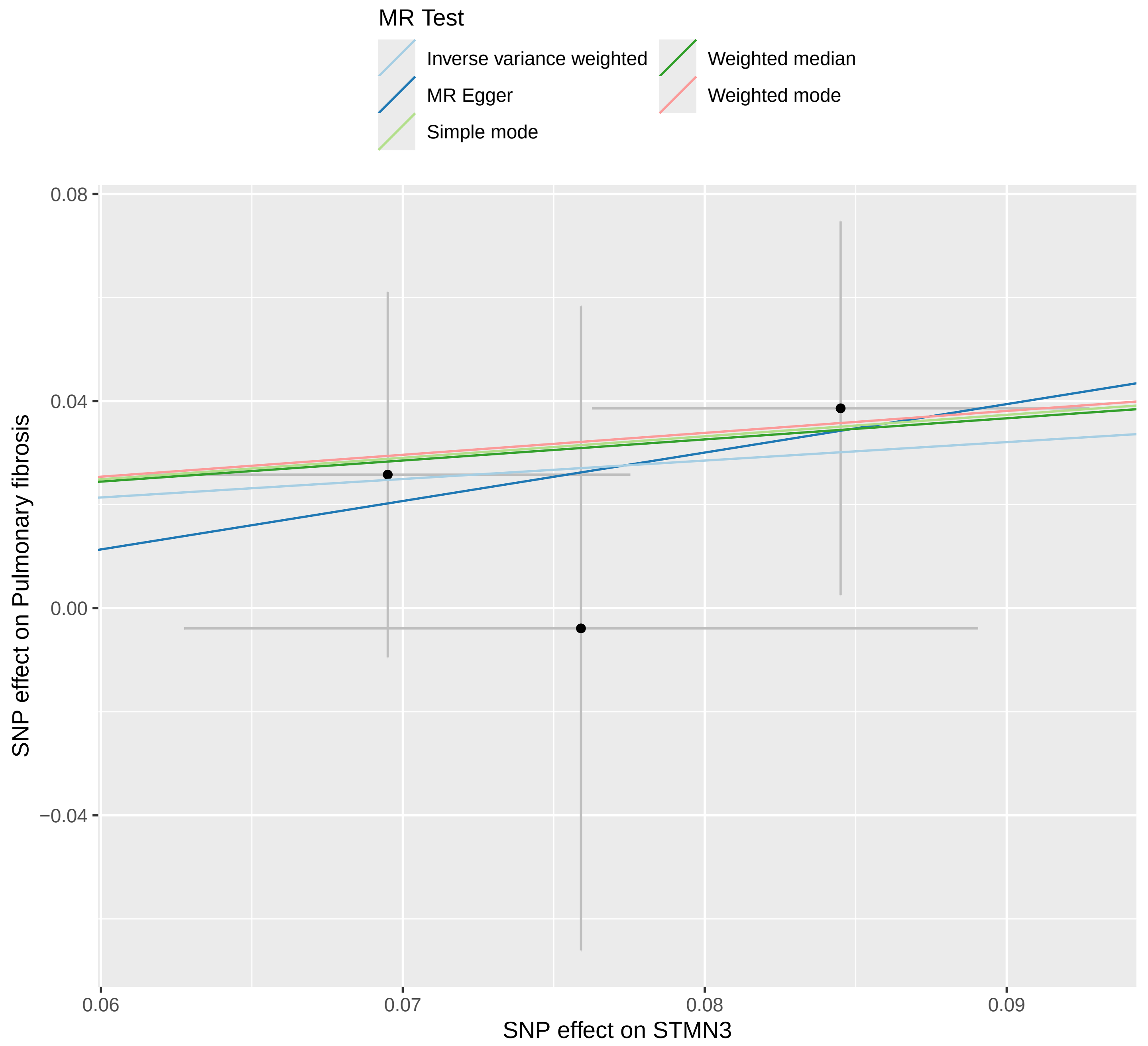
MR Test
Inverse variance weighted
MR Egger
Simple mode
Weighted median
Weighted mode
0.08
0.04
0.00
−0.04
SNP effect on Pulmonary fibrosis
0.06
0.07
0.08
0.09
SNP effect on STMN3

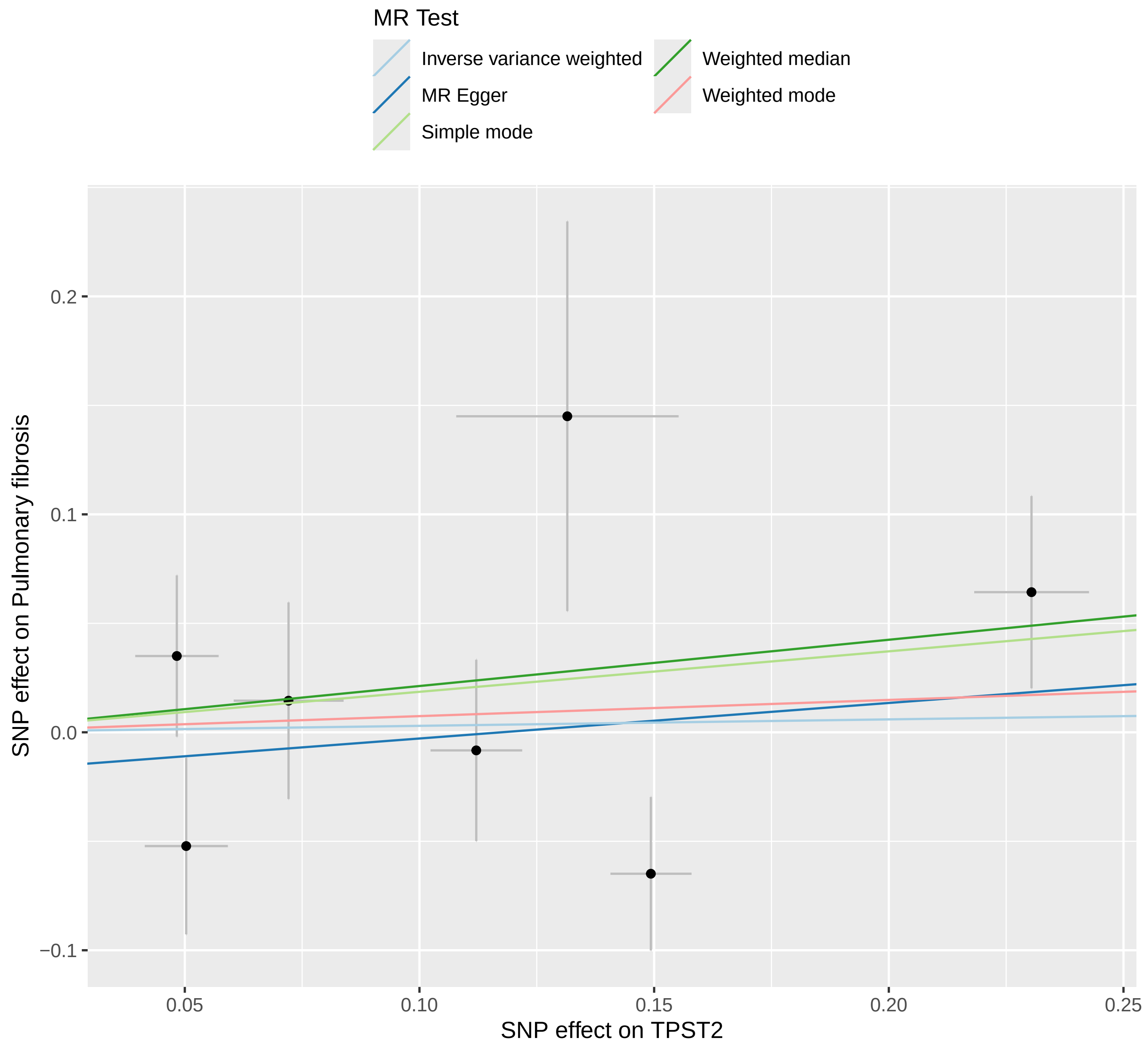

MR Test
Inverse variance weighted
MR Egger
Simple mode
Weighted median
Weighted mode
0.2
0.1
0.0
−0.1
0.05
0.10
0.15
0.20
0.25
SNP effect on TPST2
SNP effect on Pulmonary fibrosis

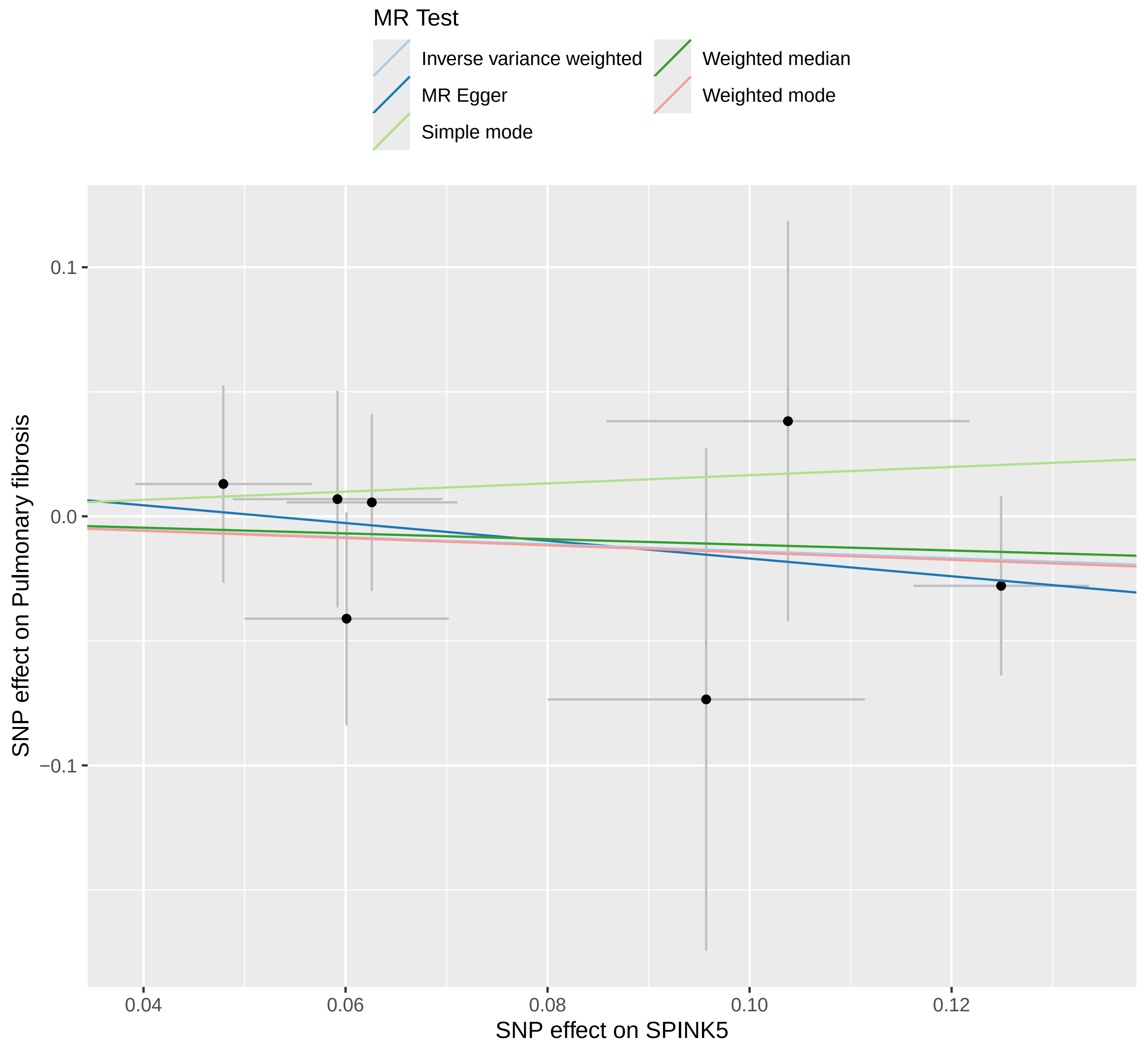
MR Test
Inverse variance weighted
MR Egger
Simple mode
Weighted median
Weighted mode
0.1
0.0
−0.1
0.04
0.06
0.08
0.10
0.12
SNP effect on SPINK5
SNP effect on Pulmonary fibrosis

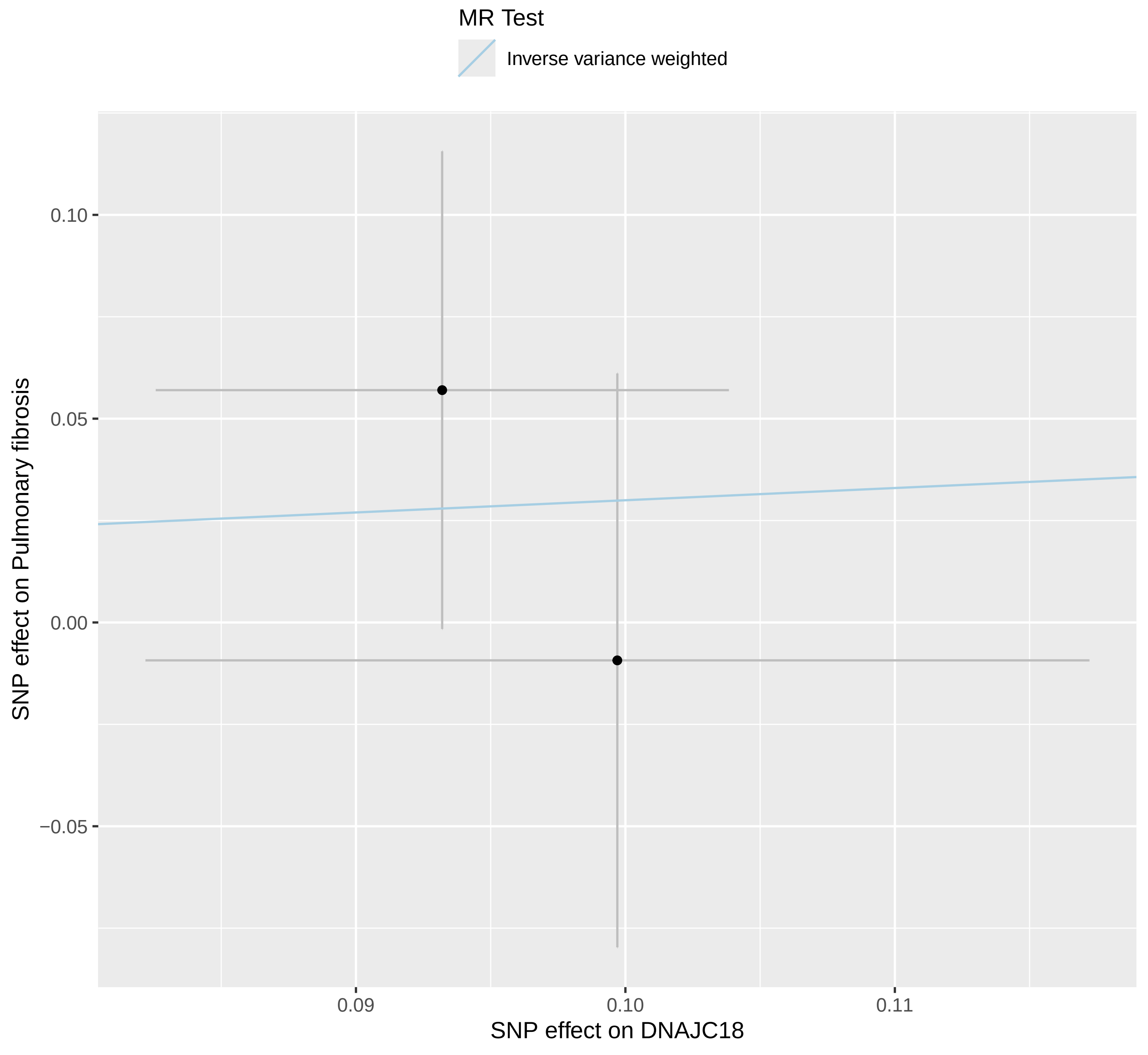
MR Test
Inverse variance weighted
0.10
0.05
0.00
−0.05
SNP effect on Pulmonary fibrosis
0.09
0.10
0.11
SNP effect on DNAJC18

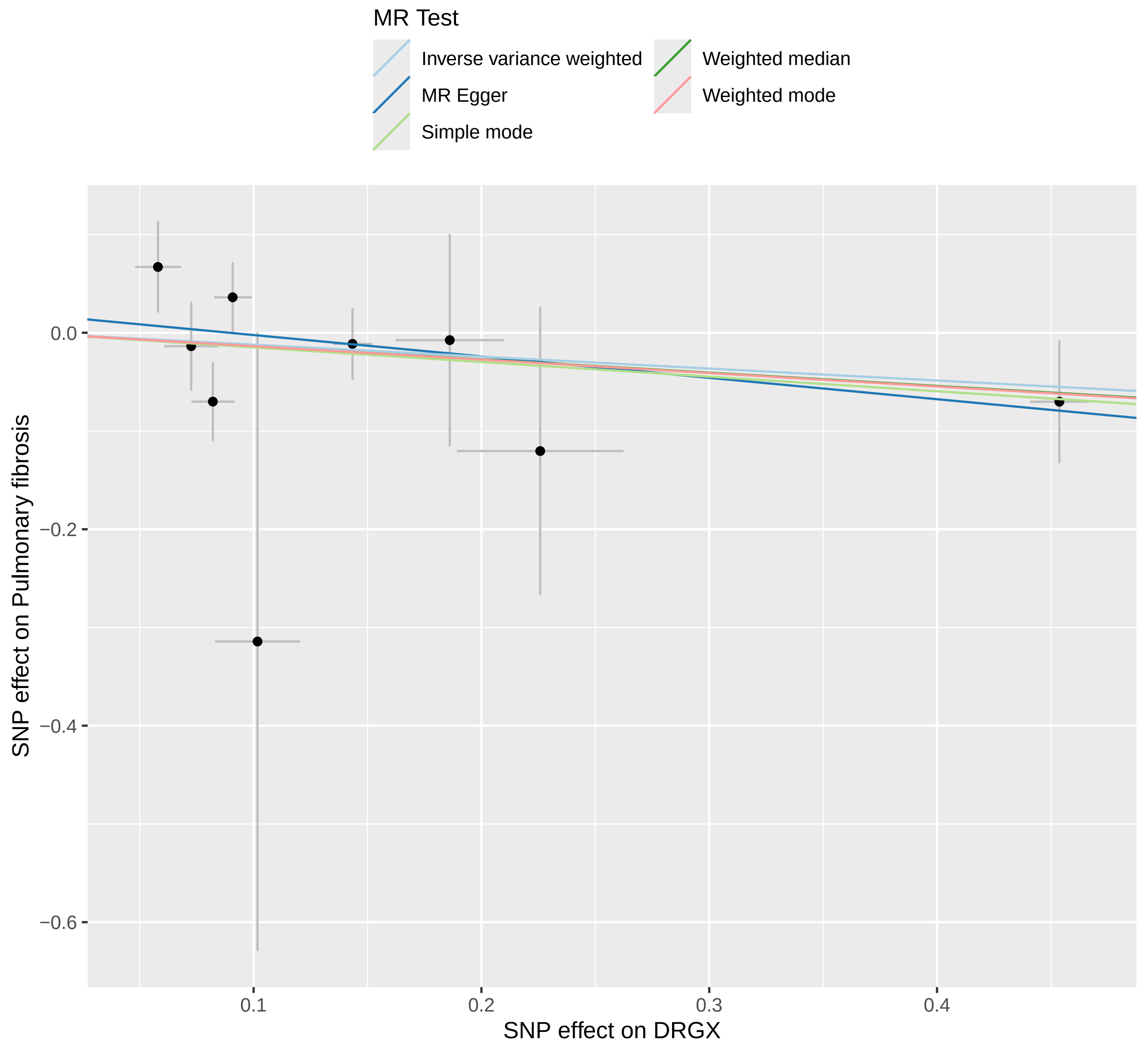

MR Test
Inverse variance weighted
MR Egger
Simple mode
Weighted median
Weighted mode
SNP effect on Pulmonary fibrosis
0.0
−0.2
−0.4
−0.6
0.1
0.2
0.3
0.4
SNP effect on DRGX

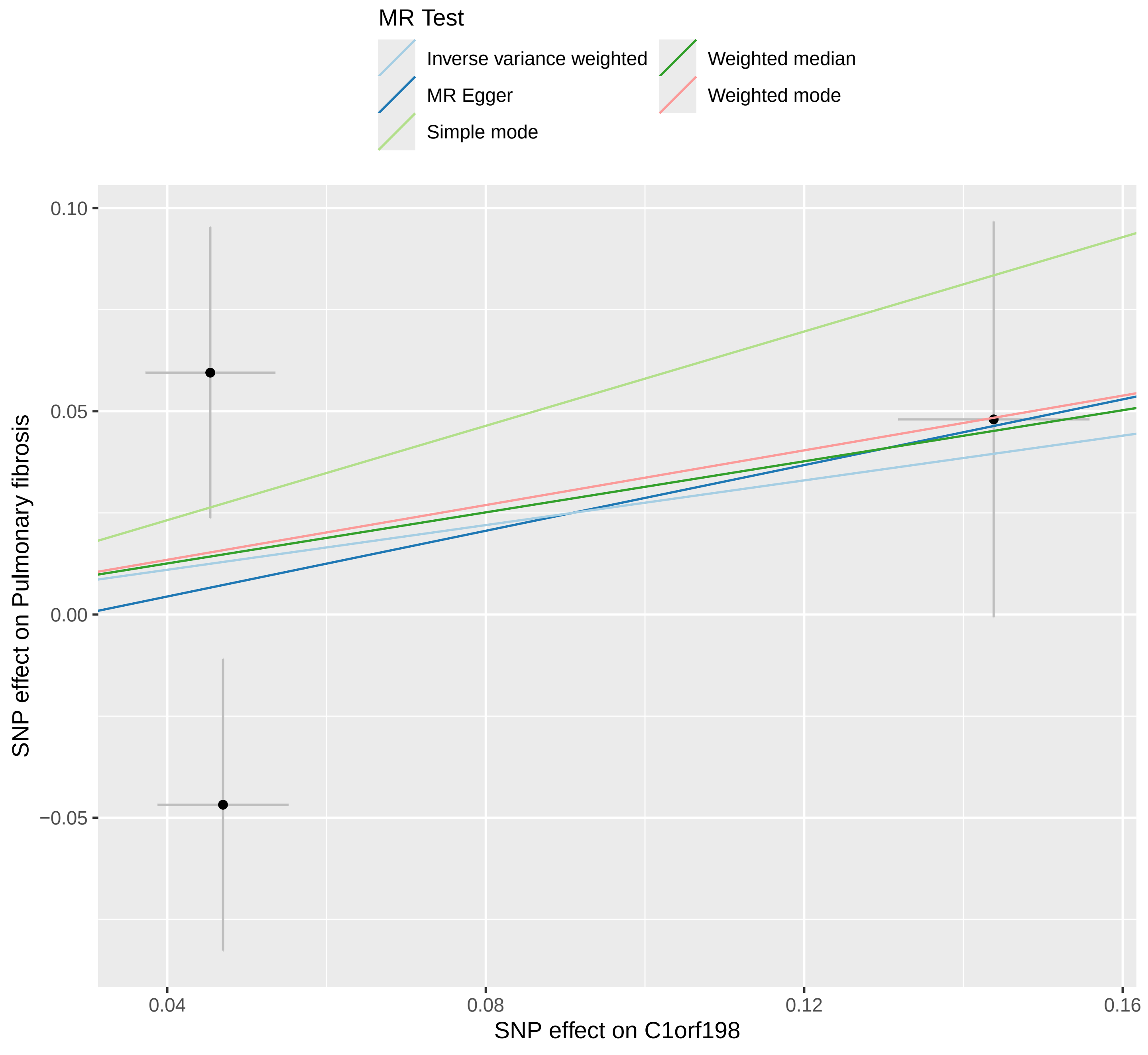

MR Test
Inverse variance weighted
MR Egger
Simple mode
Weighted median
Weighted mode
0.10
0.05
0.00
−0.05
SNP effect on Pulmonary fibrosis
0.04
0.08
0.12
0.16
SNP effect on C1orf198

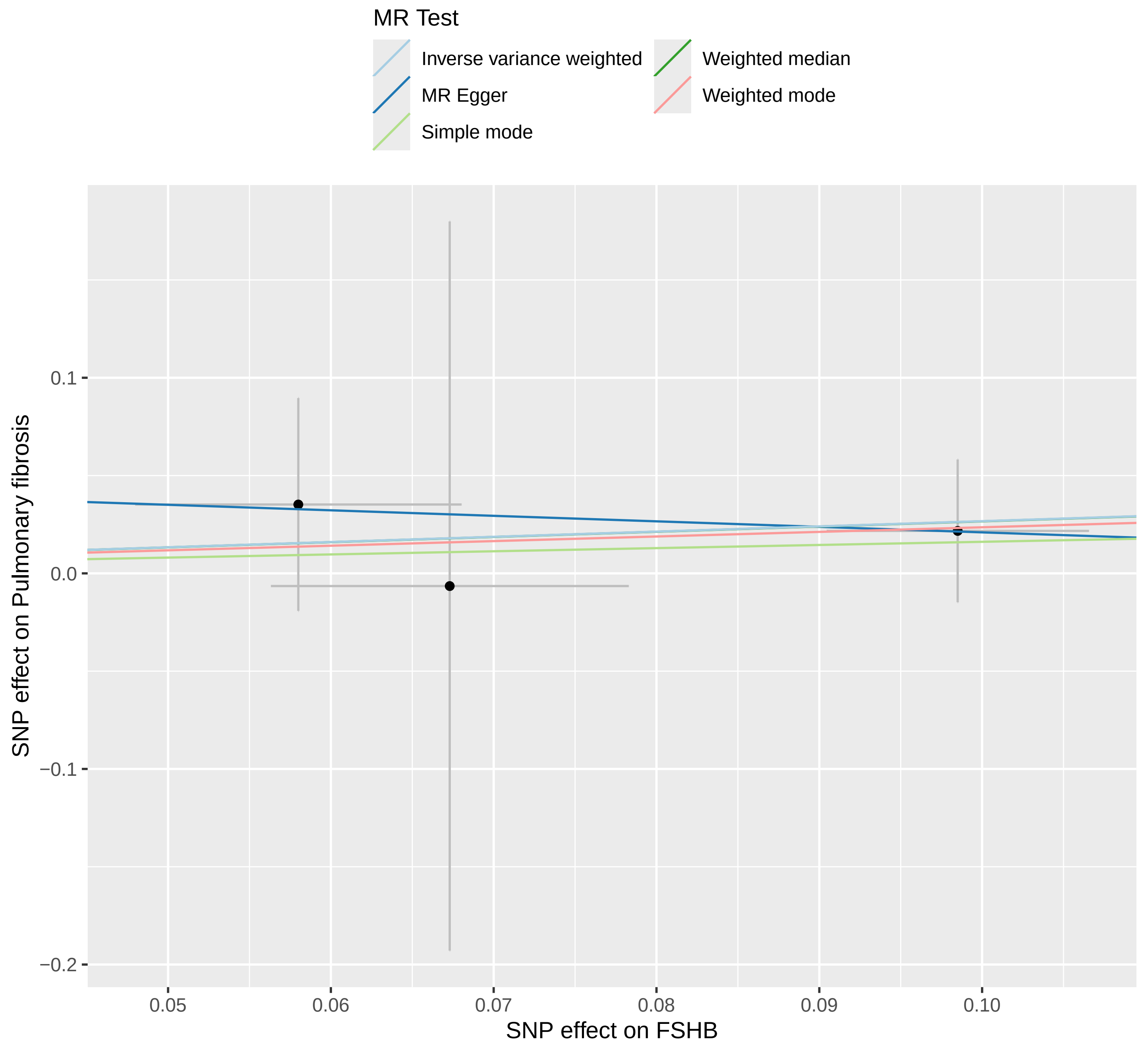

MR Test
Inverse variance weighted
MR Egger
Simple mode
Weighted median
Weighted mode
0.1
0.0
−0.1
−0.2
0.05
0.06
0.07
0.08
0.09
0.10
SNP effect on FSHB
SNP effect on Pulmonary fibrosis

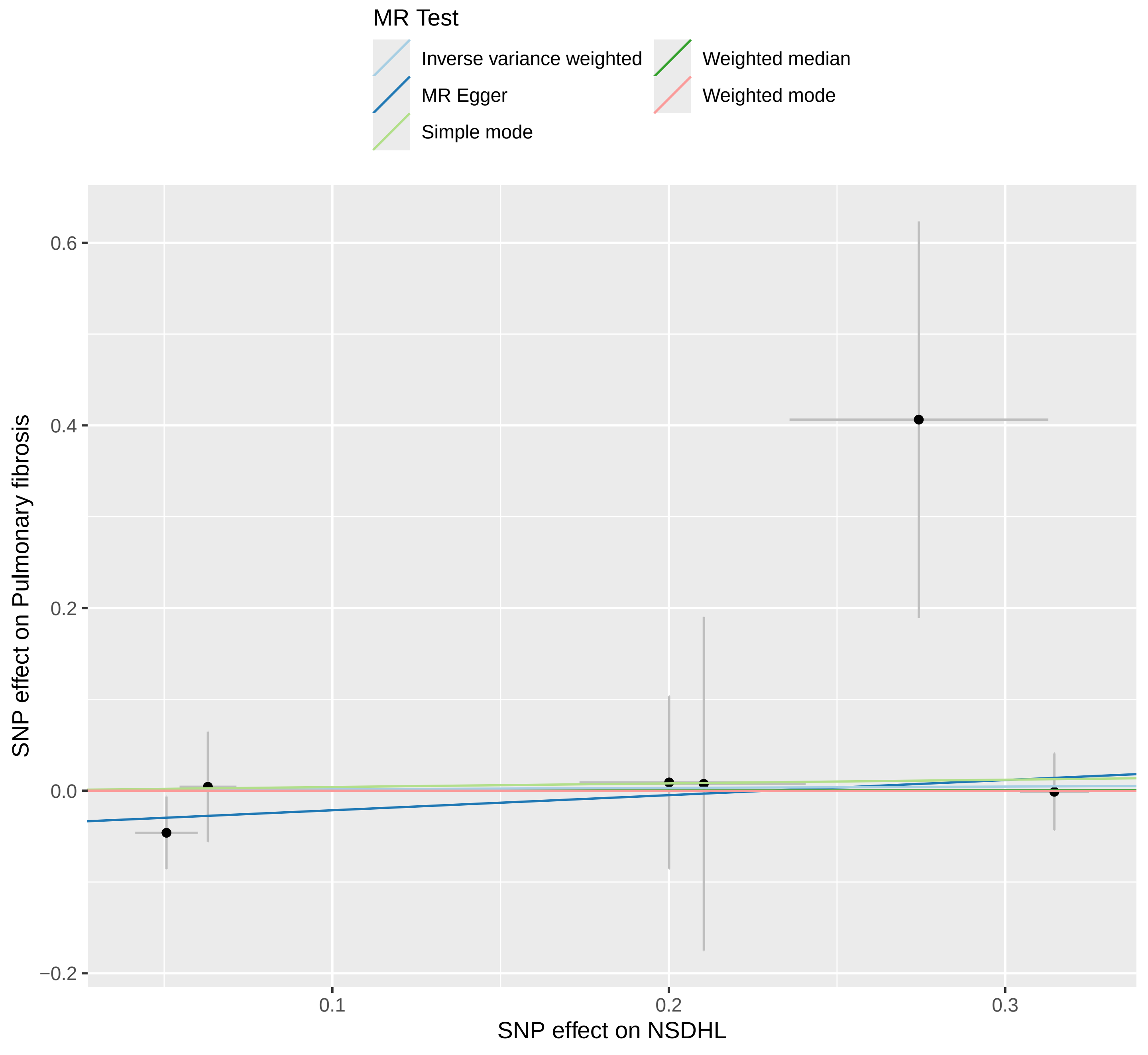
MR Test
Inverse variance weighted
MR Egger
Simple mode
Weighted median
Weighted mode
0.6
0.4
0.2
0.0
−0.2
0.1
0.2
0.3
SNP effect on Pulmonary fibrosis
SNP effect on NSDHL

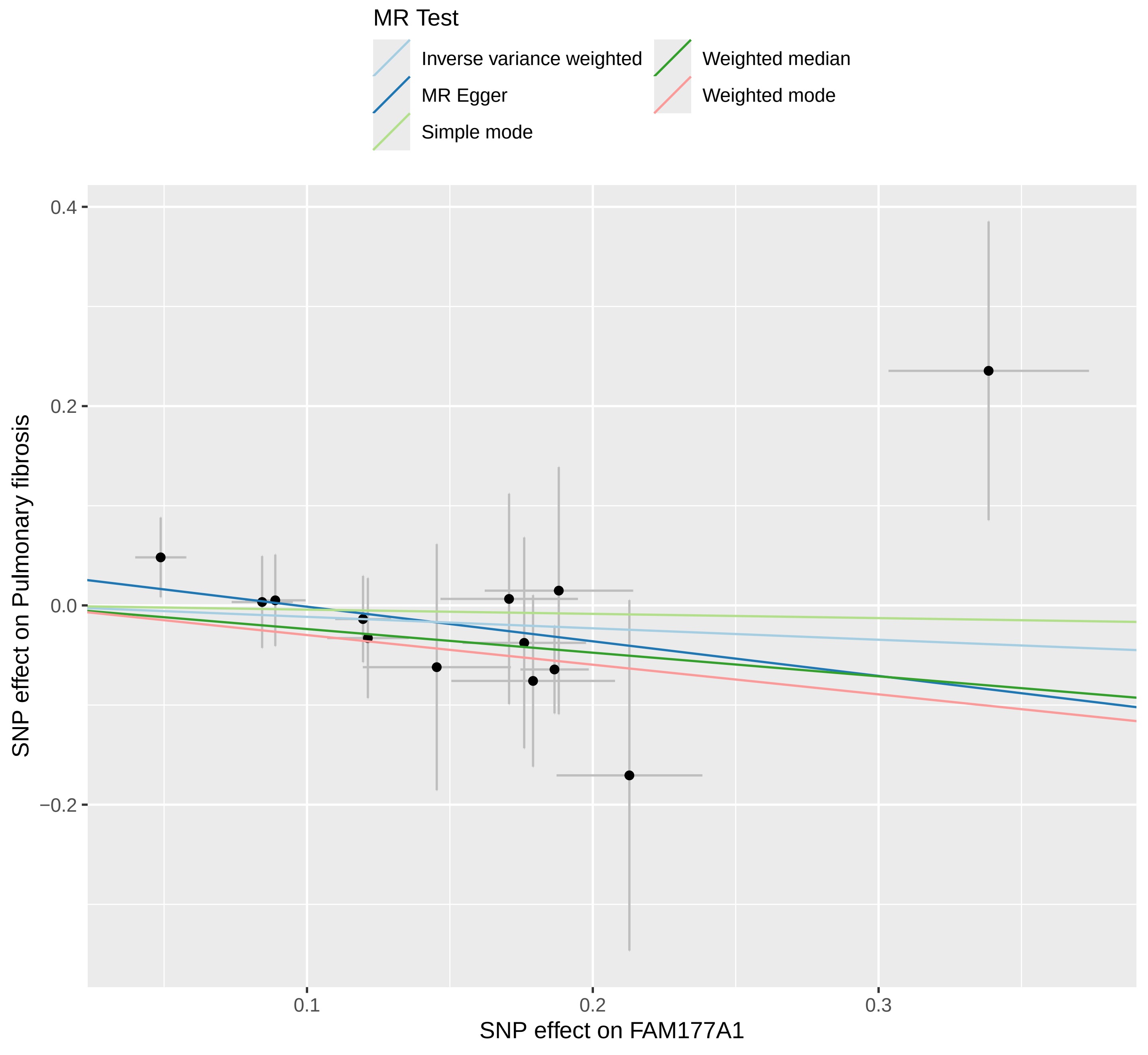
MR Test
Inverse variance weighted
MR Egger
Simple mode
Weighted median
Weighted mode
0.4
0.2
0.0
−0.2
SNP effect on Pulmonary fibrosis
0.1
0.2
0.3
SNP effect on FAM177A1

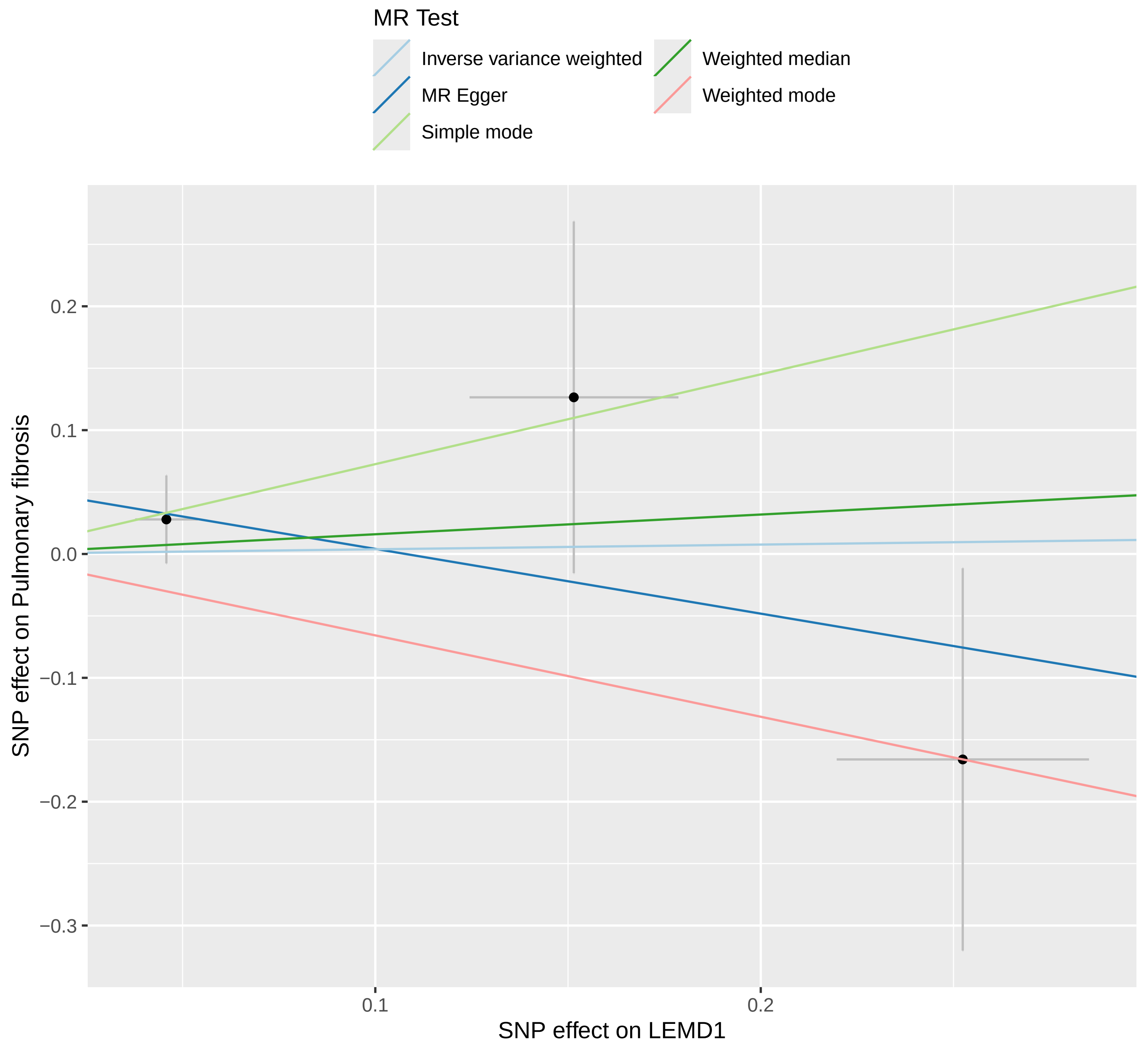
MR Test
Inverse variance weighted
MR Egger
Simple mode
Weighted median
Weighted mode
SNP effect on Pulmonary fibrosis
0.2
0.1
0.0
−0.1
−0.2
−0.3
0.1
0.2
SNP effect on LEMD1

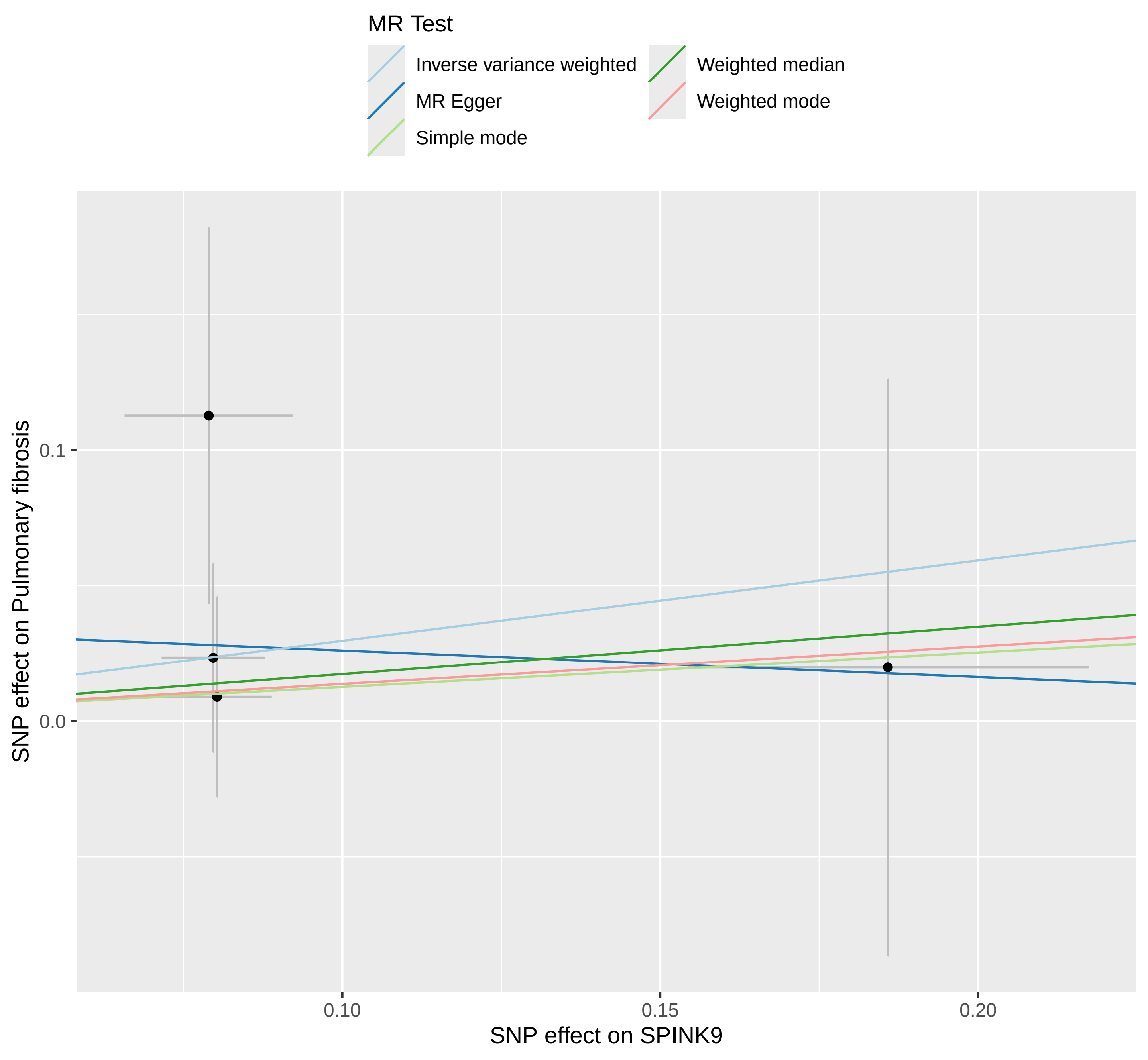

MR Test
Inverse variance weighted
MR Egger
Simple mode
Weighted median
Weighted mode
SNP effect on Pulmonary fibrosis
0.1
0.0
0.10
0.15
0.20
SNP effect on SPINK9

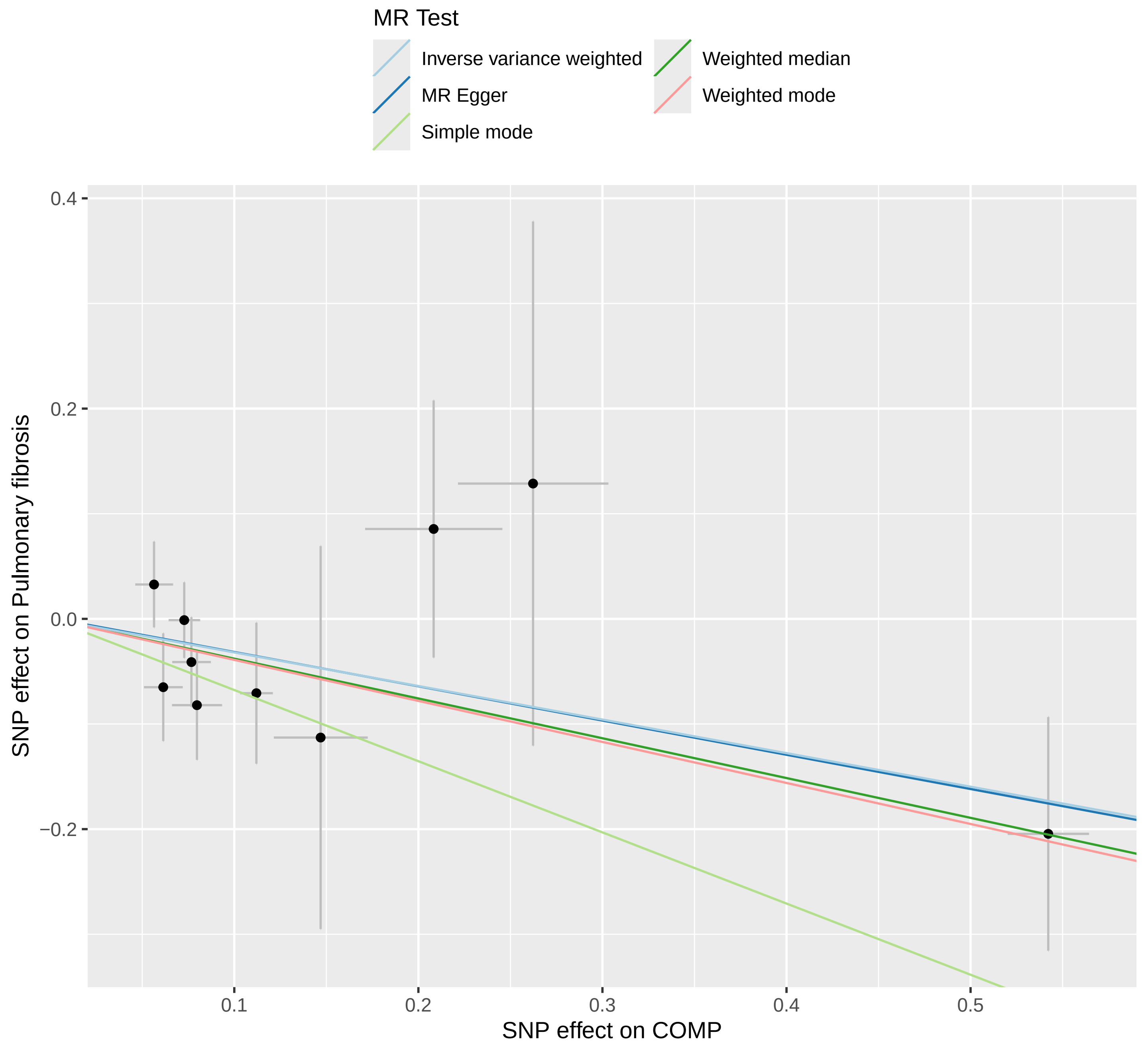
MR Test
Inverse variance weighted
MR Egger
Simple mode
Weighted median
Weighted mode
0.4
0.2
0.0
−0.2
0.1
0.2
0.3
0.4
0.5
SNP effect on COMP
SNP effect on Pulmonary fibrosis

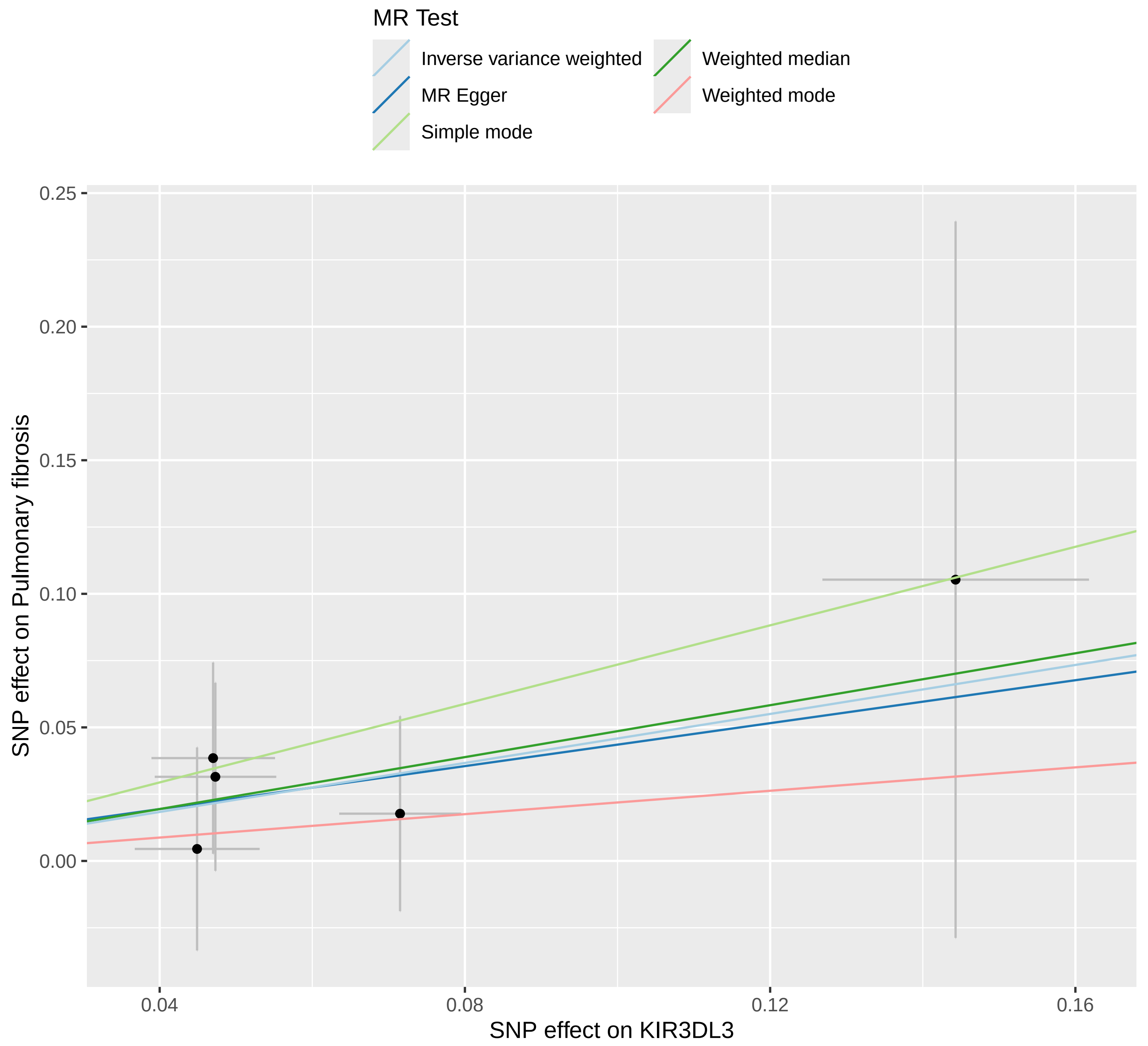
MR Test
Inverse variance weighted
MR Egger
Simple mode
Weighted median
Weighted mode
0.25
0.20
0.15
0.10
0.05
0.00
0.04
0.08
0.12
0.16
SNP effect on Pulmonary fibrosis
SNP effect on KIR3DL3

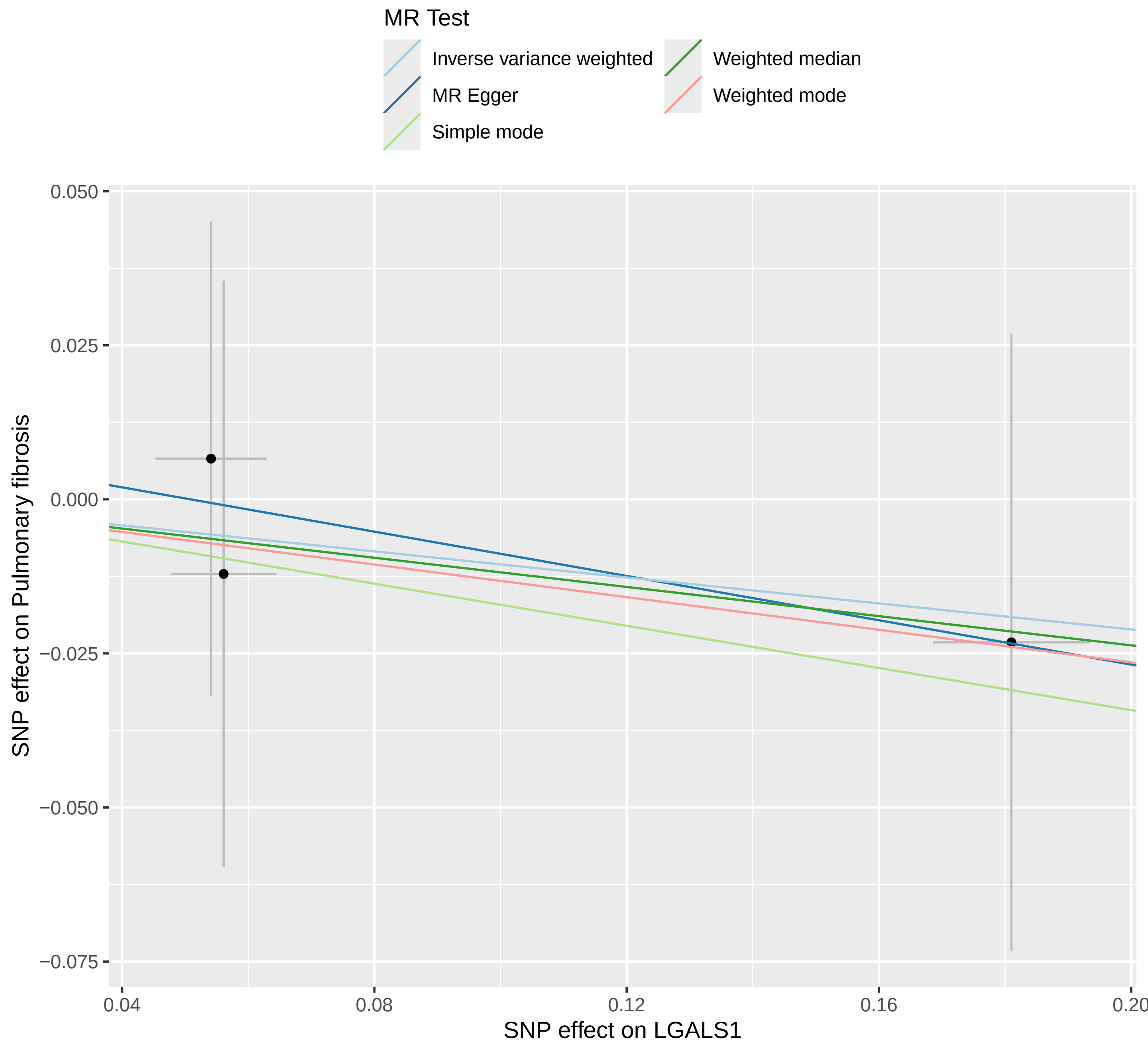
MR Test
Inverse variance weighted
MR Egger
Simple mode
Weighted median
Weighted mode
0.050
0.025
0.000
−0.025
−0.050
−0.075
SNP effect on Pulmonary fibrosis
0.04
0.08
0.12
0.16
0.20
SNP effect on LGALS1

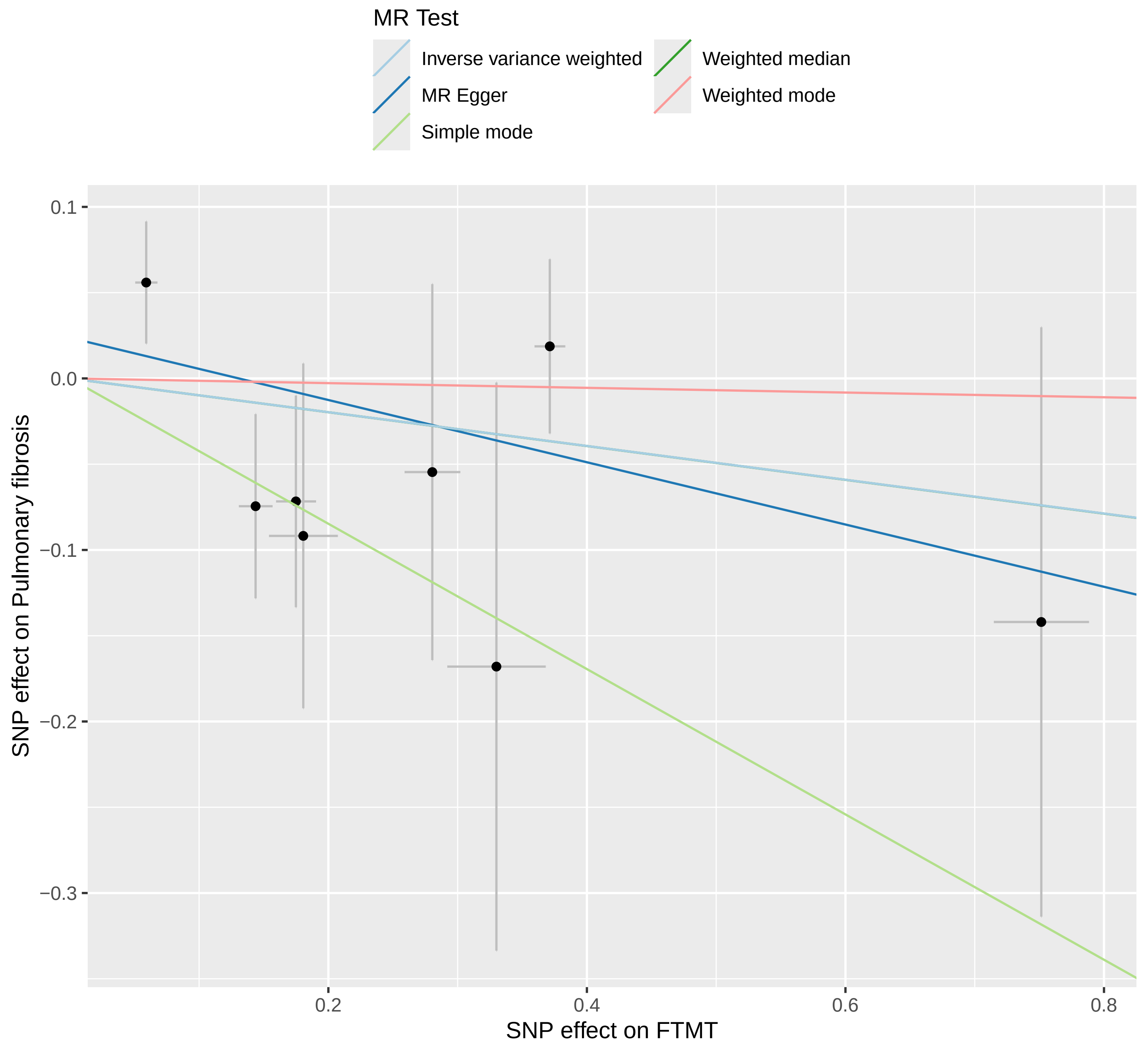
MR Test
Inverse variance weighted
MR Egger
Simple mode
Weighted median
Weighted mode
0.1
0.0
−0.1
−0.2
−0.3
0.2
0.4
0.6
0.8
SNP effect on Pulmonary fibrosis
SNP effect on FTMT

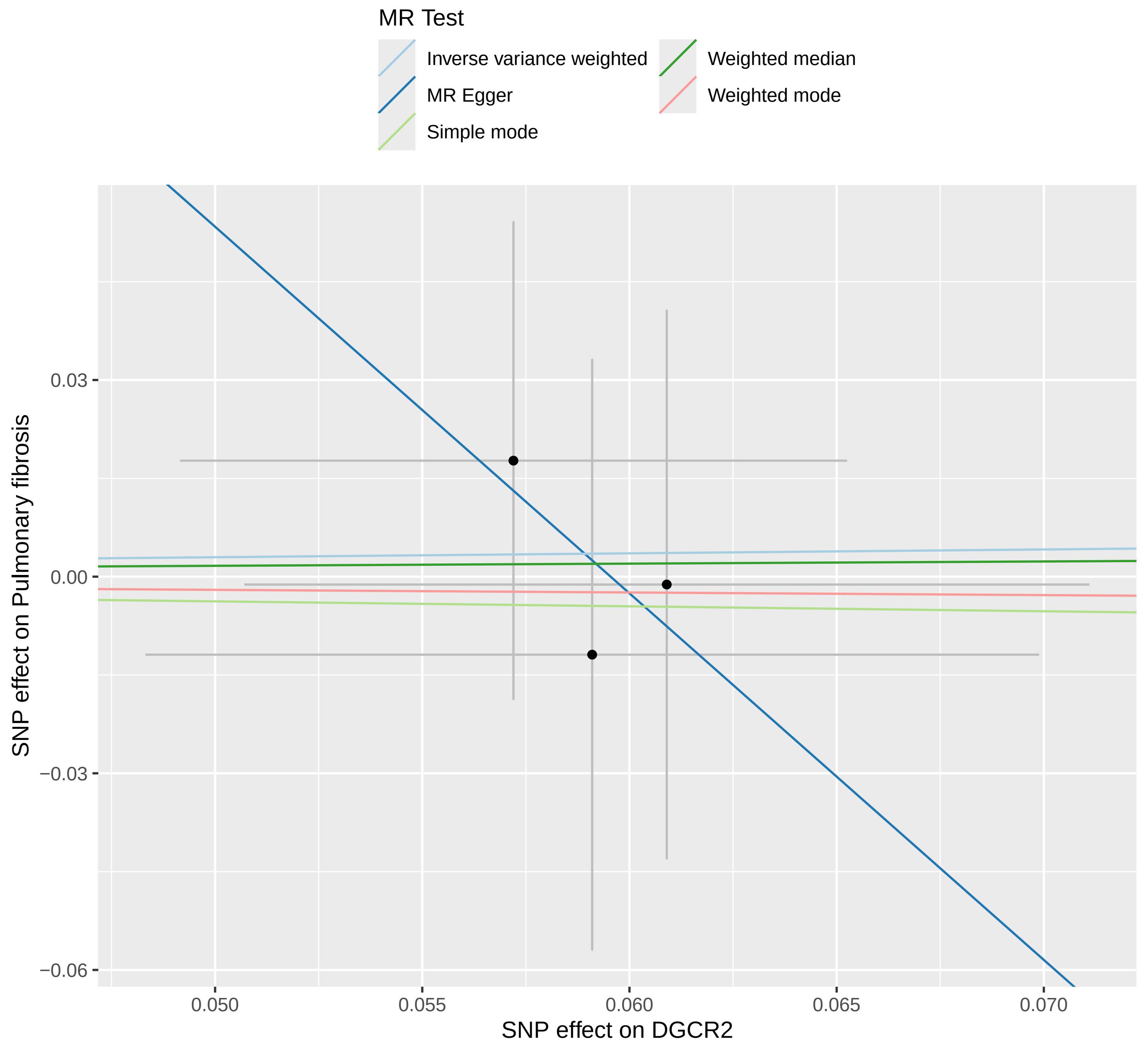
MR Test
Inverse variance weighted
MR Egger
Simple mode
Weighted median
Weighted mode
0.03
0.00
−0.03
−0.06
SNP effect on Pulmonary fibrosis
0.050
0.055
0.060
0.065
0.070
SNP effect on DGCR2

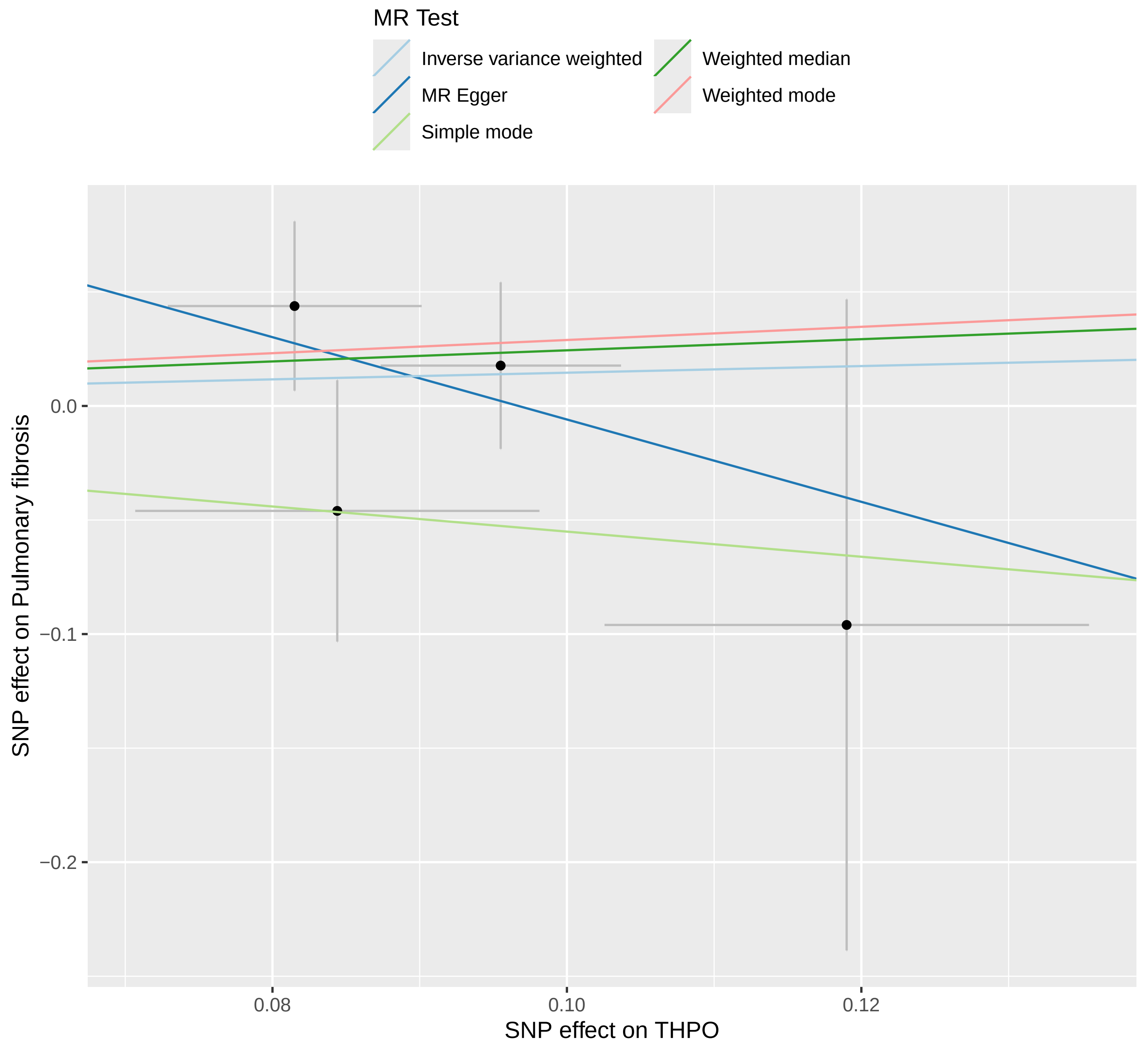
MR Test
Inverse variance weighted
MR Egger
Simple mode
Weighted median
Weighted mode
0.0
−0.1
−0.2
SNP effect on Pulmonary fibrosis
0.08
0.10
0.12
SNP effect on THPO

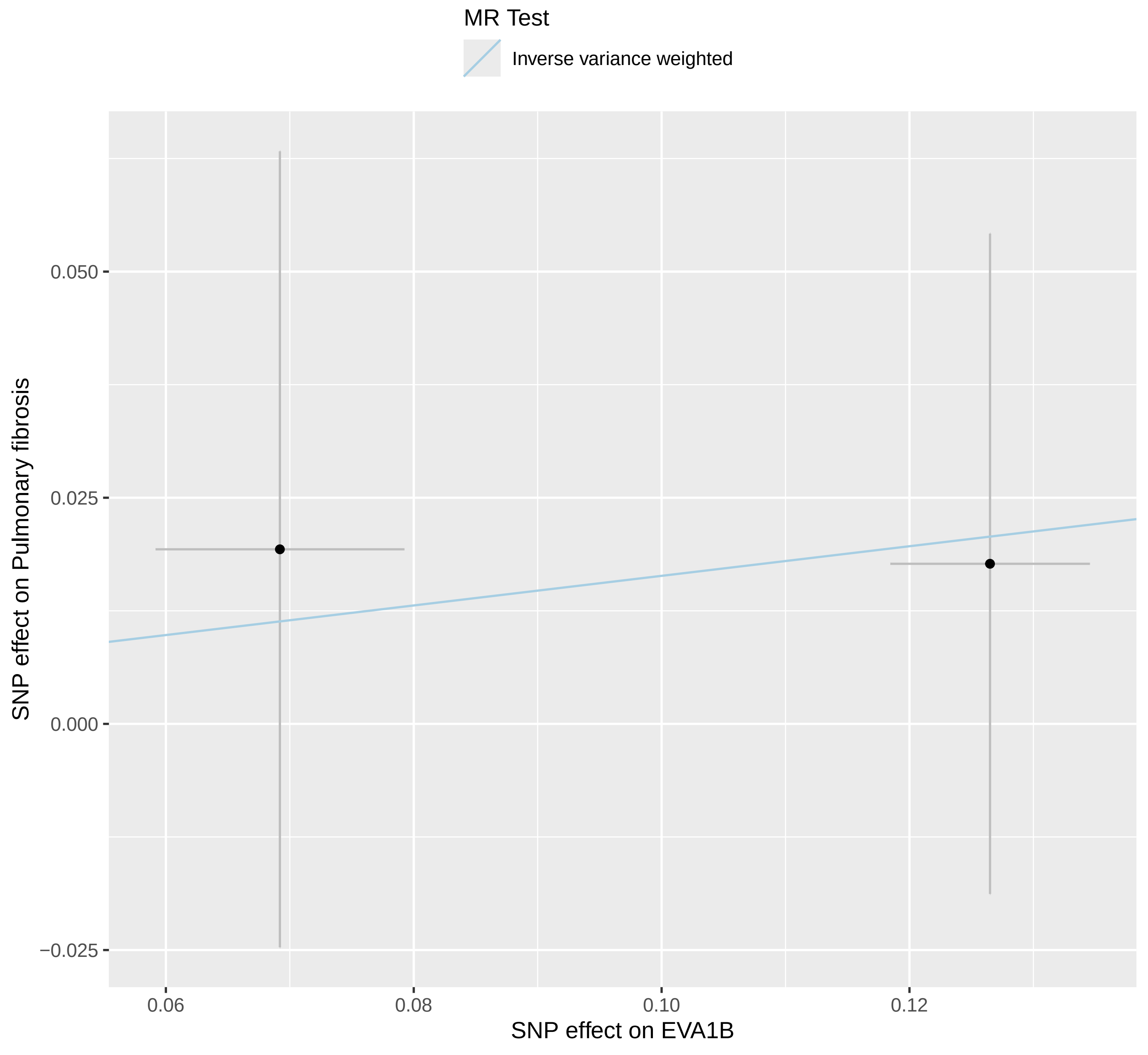
MR Test
Inverse variance weighted
0.050
0.025
0.000
−0.025
SNP effect on Pulmonary fibrosis
0.06
0.08
0.10
0.12
SNP effect on EVA1B

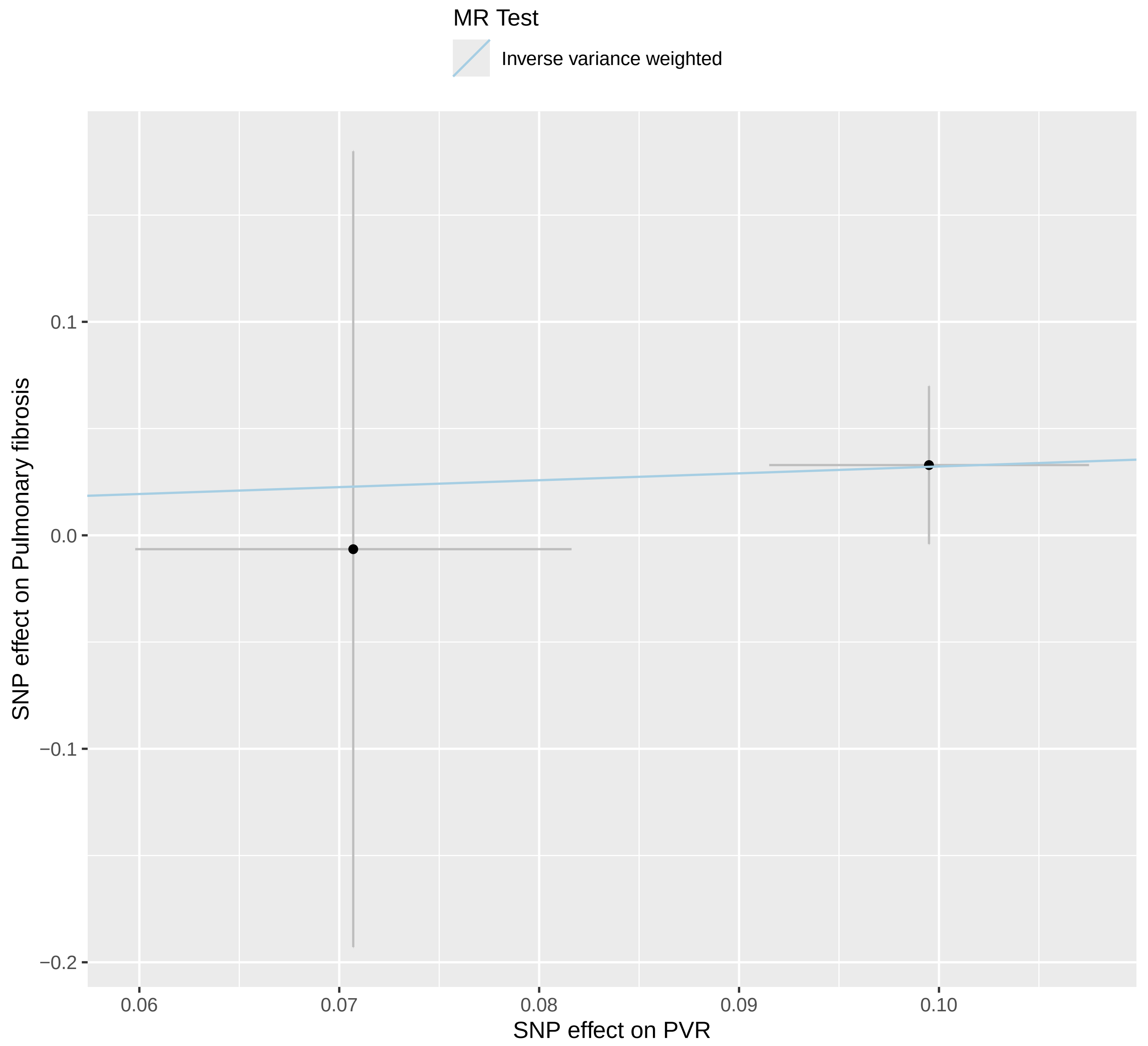
MR Test
Inverse variance weighted
0.1
0.0
−0.1
−0.2
SNP effect on Pulmonary fibrosis
0.06
0.07
0.08
0.09
0.10
SNP effect on PVR

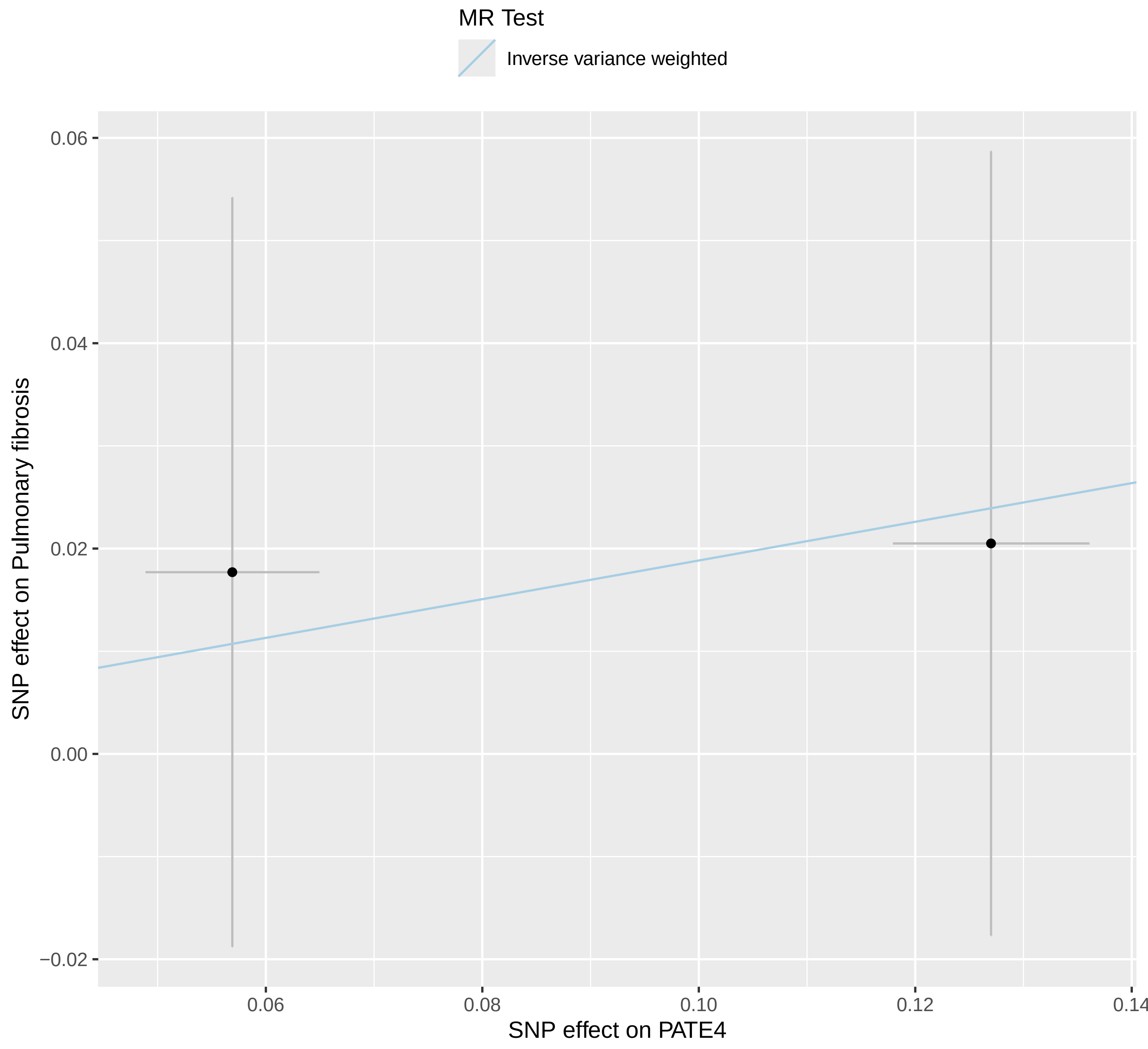
MR Test
Inverse variance weighted
0.06
0.04
0.02
0.00
−0.02
0.06
0.08
0.10
0.12
0.14
SNP effect on Pulmonary fibrosis
SNP effect on PATE4

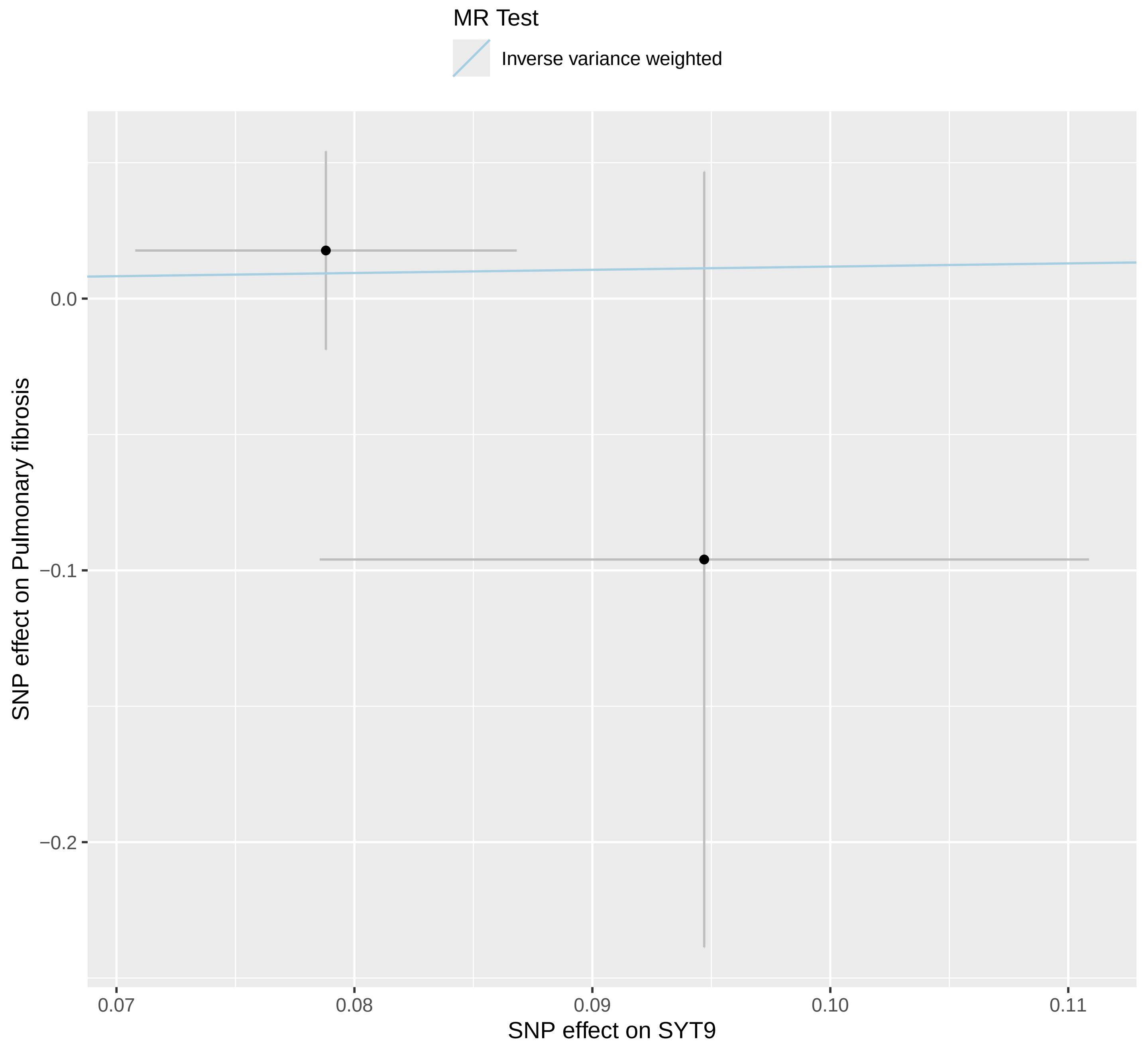
MR Test
Inverse variance weighted
0.0
−0.1
−0.2
SNP effect on Pulmonary fibrosis
0.07
0.08
0.09
0.10
0.11
SNP effect on SYT9

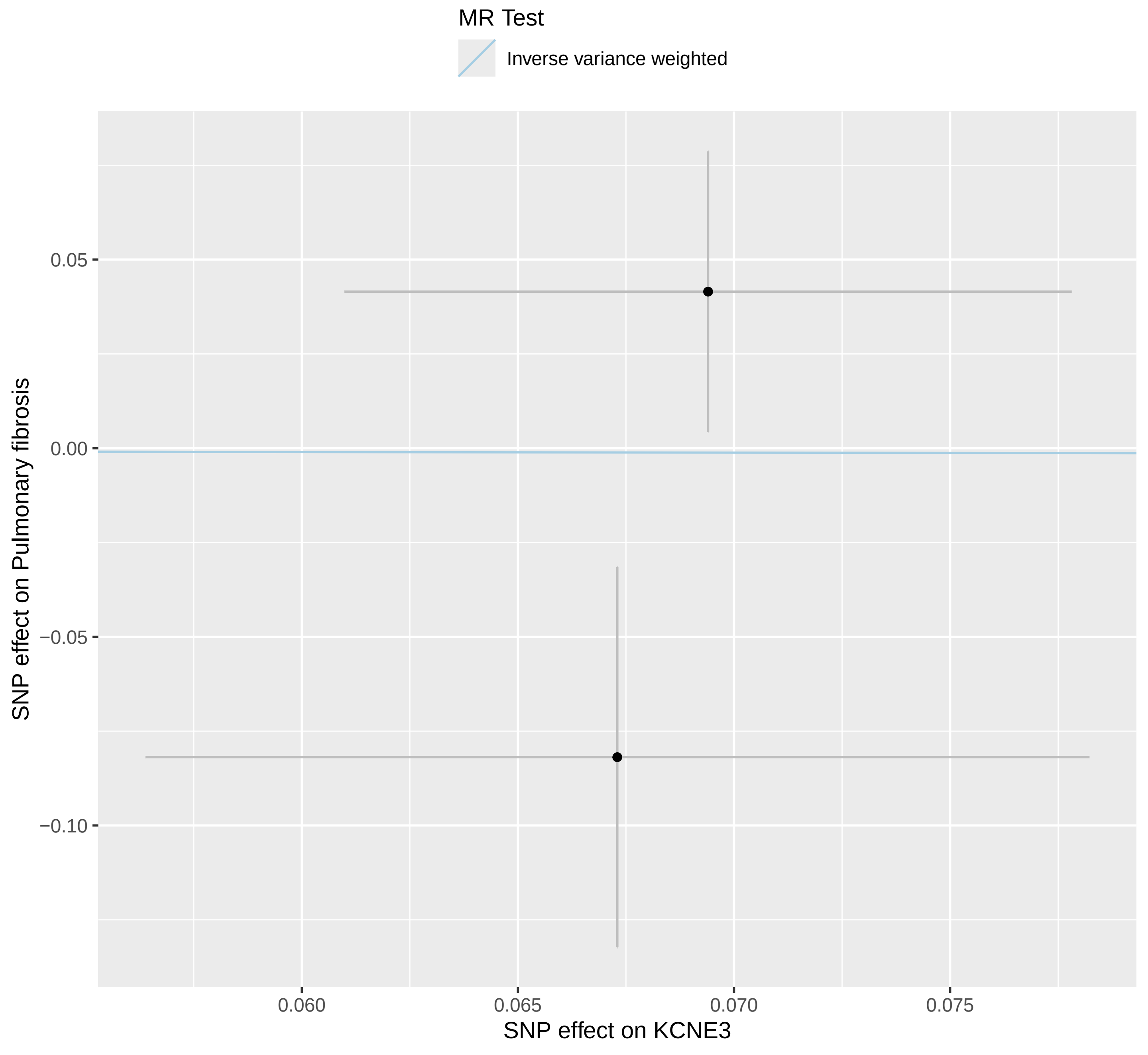

MR Test
Inverse variance weighted
0.05
0.00
−0.05
−0.10
SNP effect on Pulmonary fibrosis
0.060
0.065
0.070
0.075
SNP effect on KCNE3

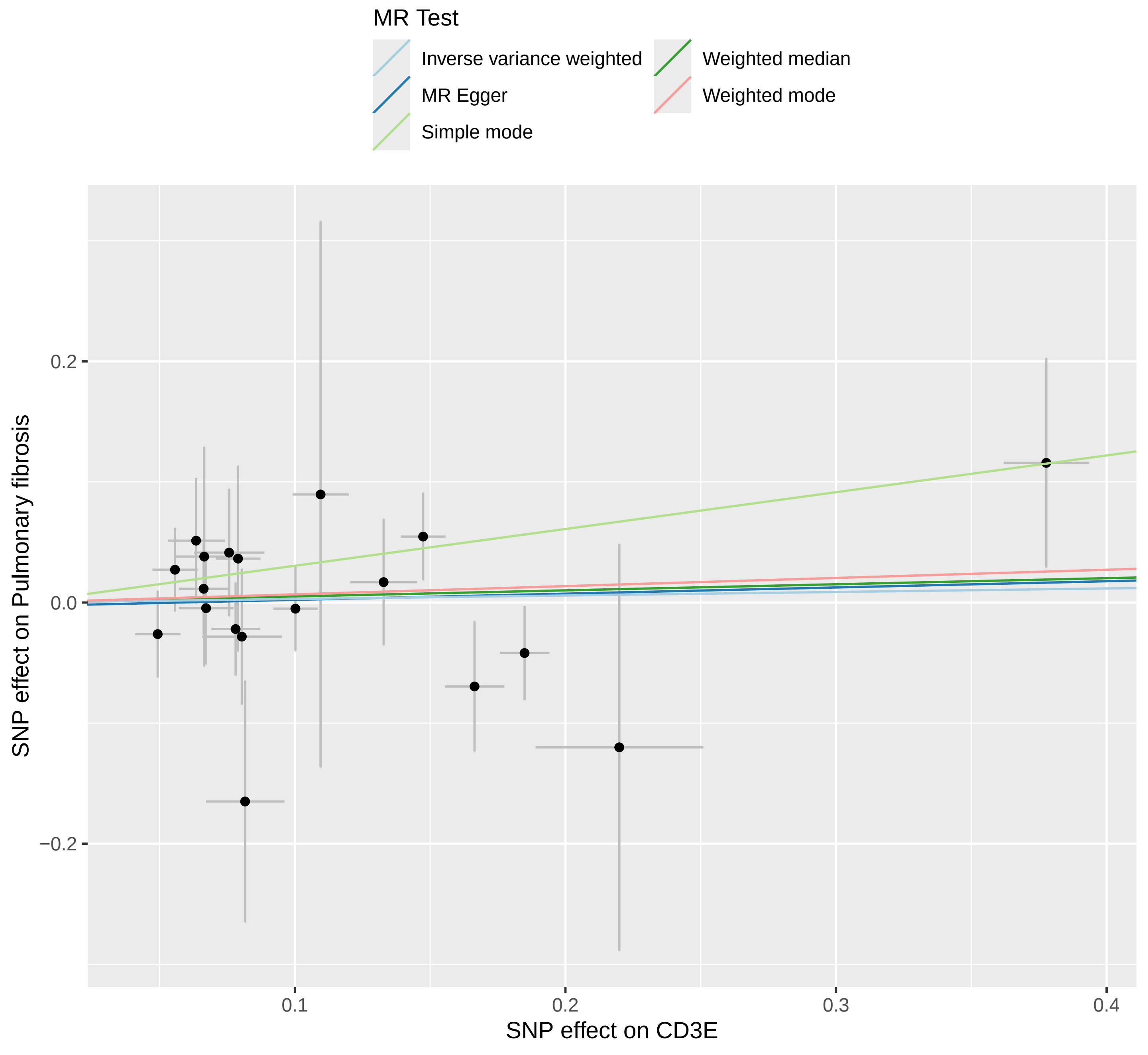
MR Test
Inverse variance weighted
MR Egger
Simple mode
Weighted median
Weighted mode
0.2
0.0
−0.2
0.1
0.2
0.3
0.4
SNP effect on Pulmonary fibrosis
SNP effect on CD3E

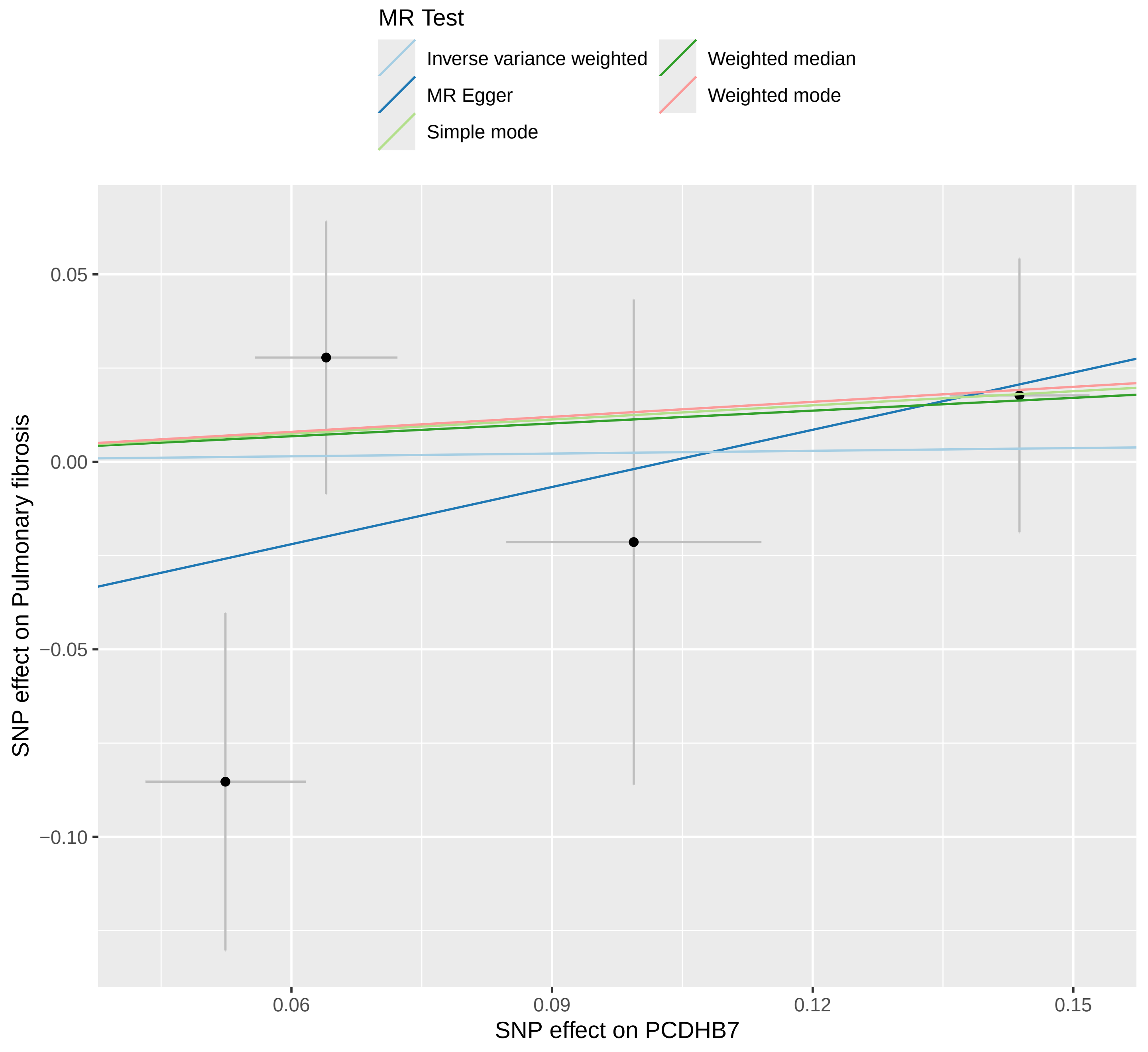
MR Test
Inverse variance weighted
MR Egger
Simple mode
Weighted median
Weighted mode
0.05
0.00
−0.05
−0.10
0.06
0.09
0.12
0.15
SNP effect on PCDHB7
SNP effect on Pulmonary fibrosis

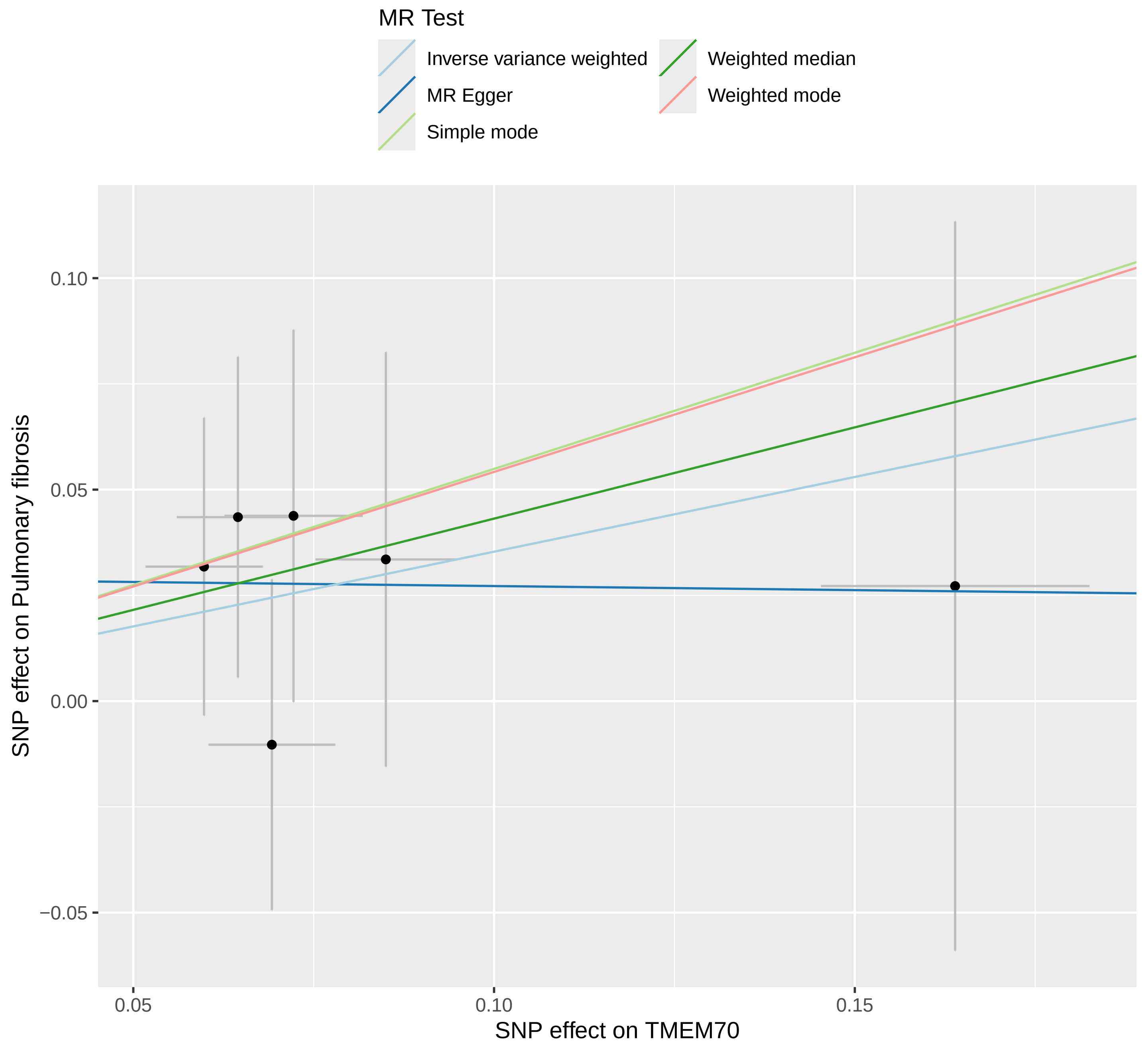

MR Test
Inverse variance weighted
MR Egger
Simple mode
Weighted median
Weighted mode
SNP effect on Pulmonary fibrosis
0.10
0.05
0.00
−0.05
0.05
0.10
0.15
SNP effect on TMEM70

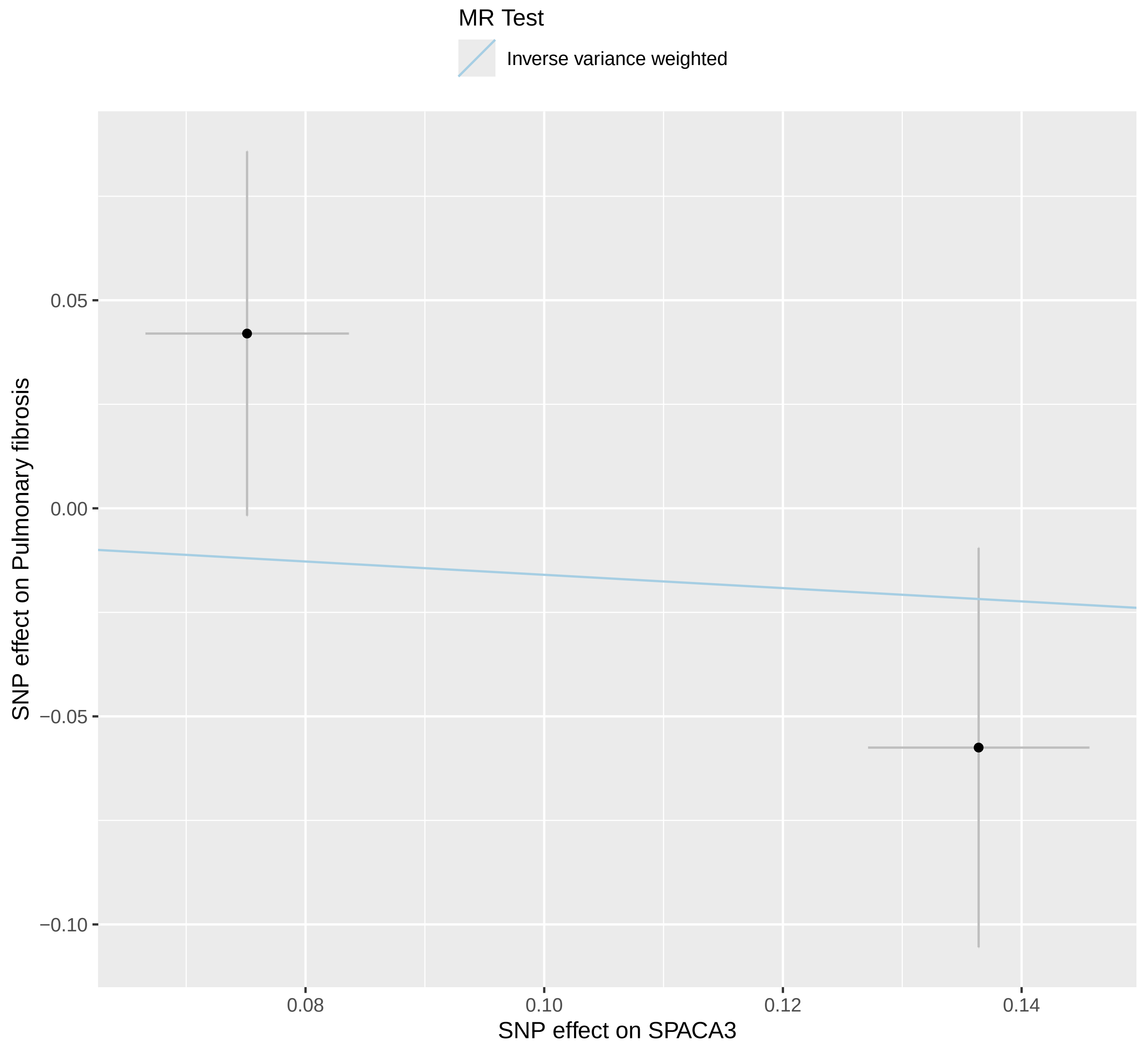
MR Test
Inverse variance weighted
0.05
0.00
−0.05
−0.10
SNP effect on Pulmonary fibrosis
0.08
0.10
0.12
0.14
SNP effect on SPACA3

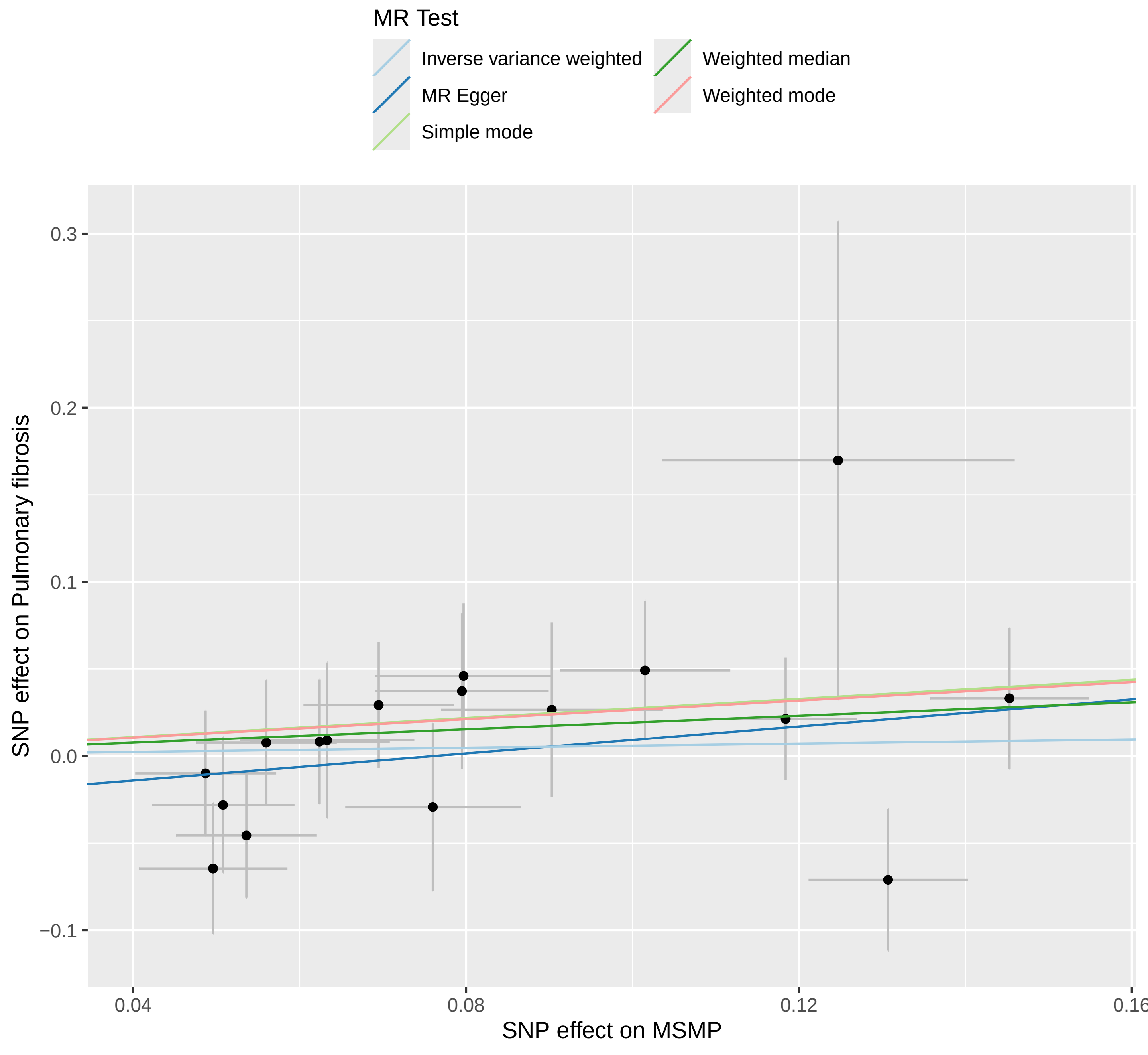

MR Test
Inverse variance weighted
MR Egger
Simple mode
Weighted median
Weighted mode
0.3
0.2
0.1
0.0
−0.1
0.04
0.08
0.12
0.16
SNP effect on MSMP
SNP effect on Pulmonary fibrosis

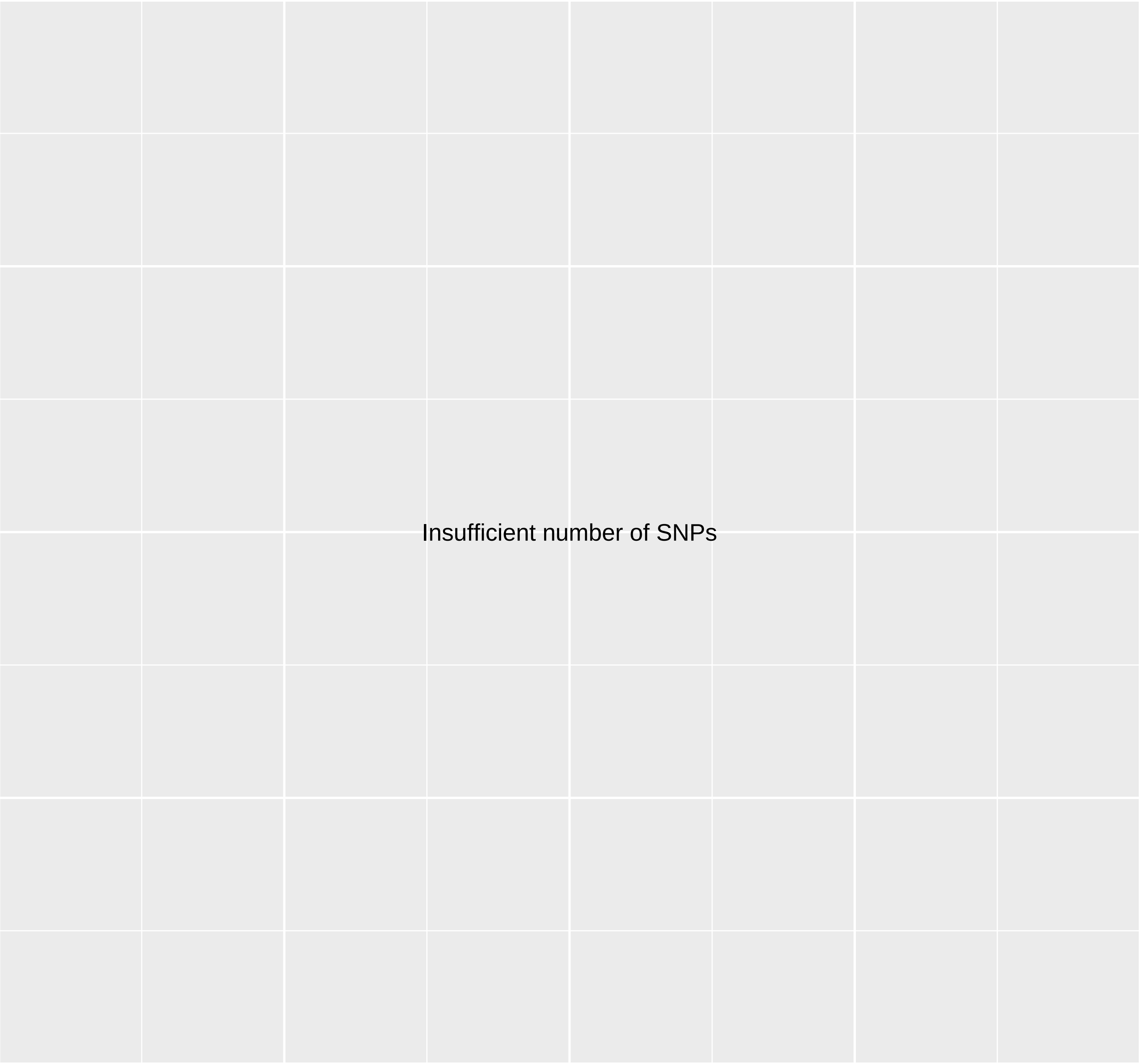
Insufficient number of SNPs

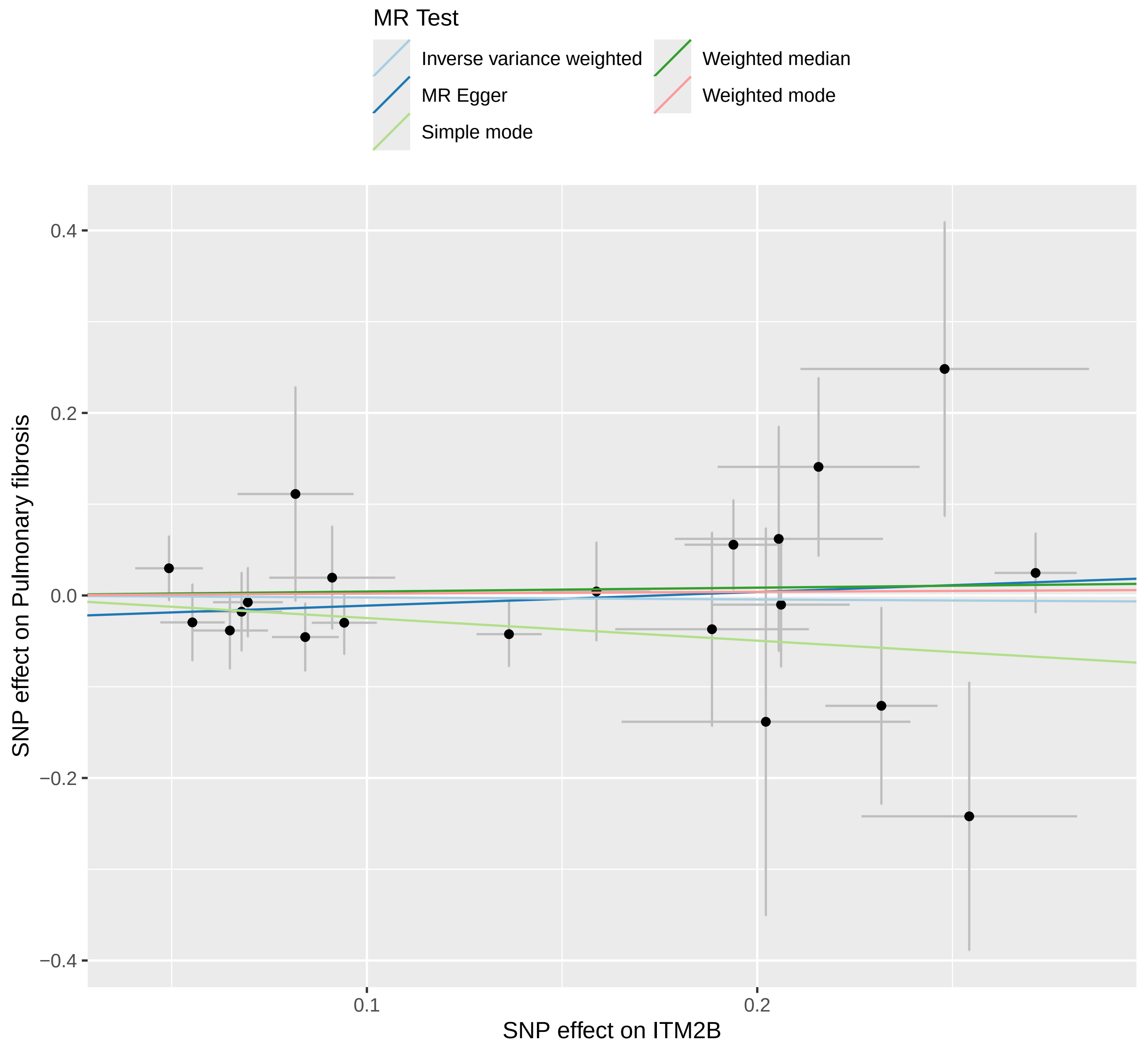

MR Test
Inverse variance weighted
MR Egger
Simple mode
Weighted median
Weighted mode
0.4
0.2
0.0
−0.2
−0.4
SNP effect on Pulmonary fibrosis
0.1
0.2
SNP effect on ITM2B

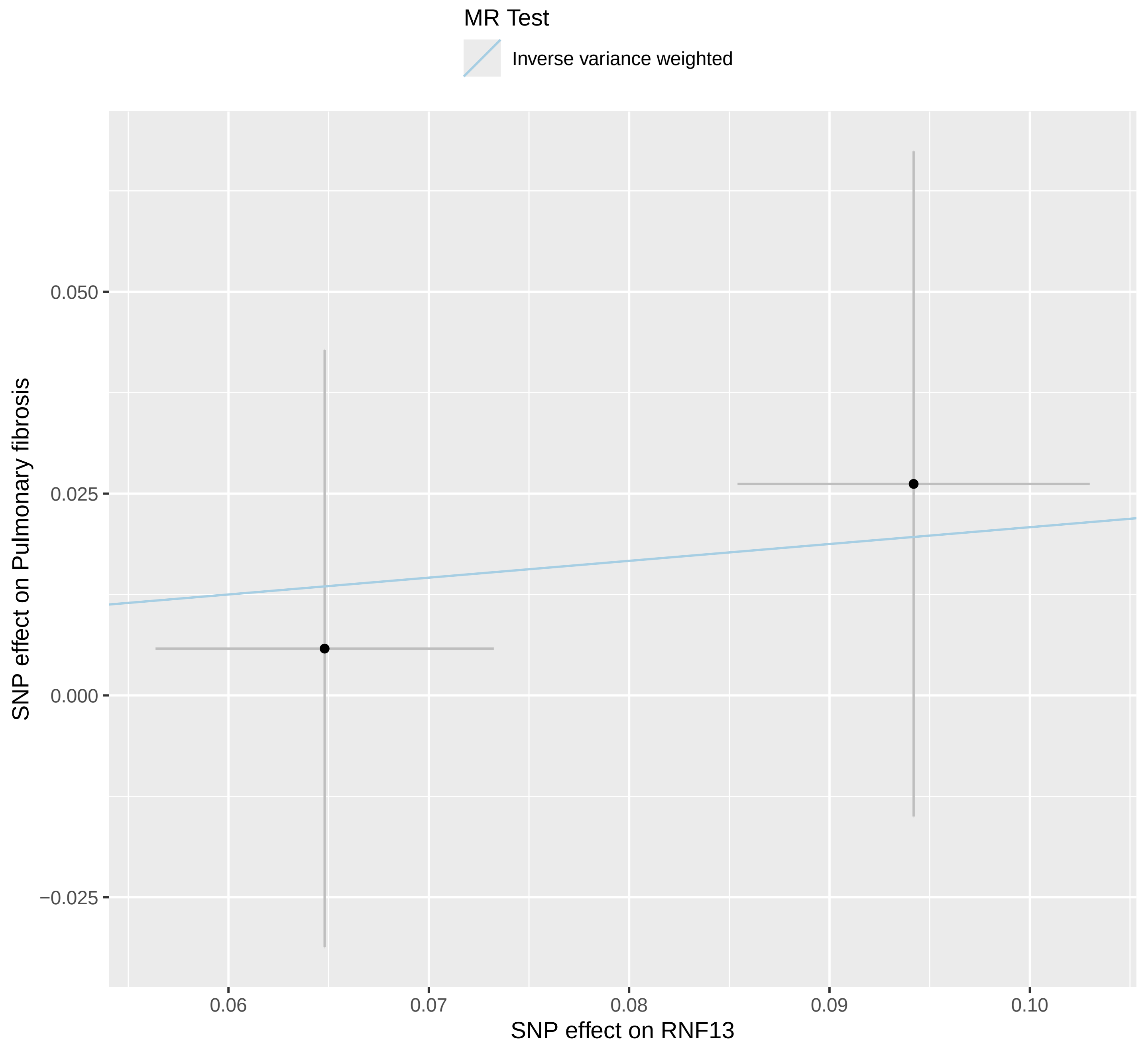
MR Test
Inverse variance weighted
0.050
0.025
0.000
−0.025
SNP effect on Pulmonary fibrosis
0.06
0.07
0.08
0.09
0.10
SNP effect on RNF13

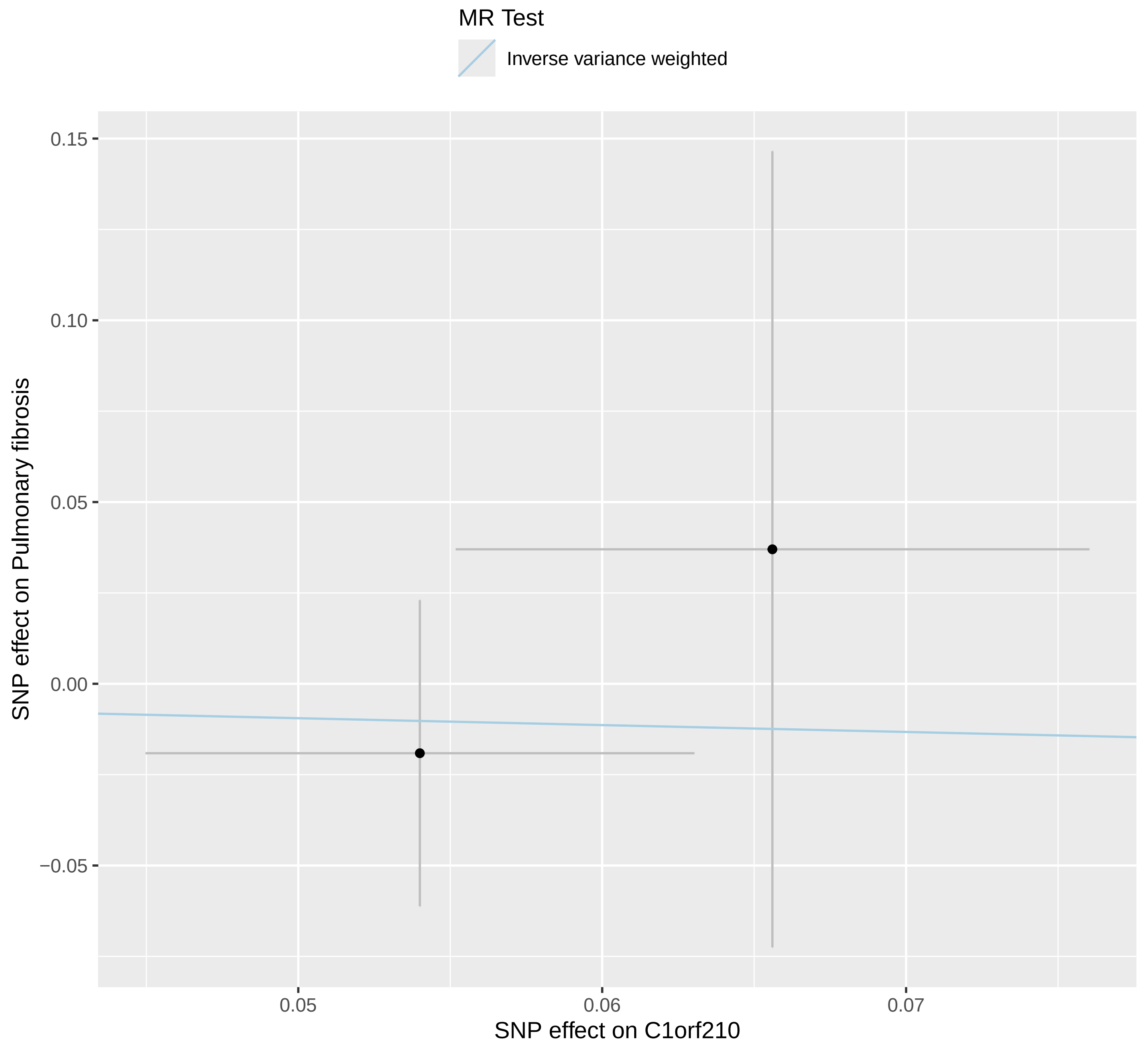

MR Test
Inverse variance weighted
0.15
0.10
0.05
0.00
−0.05
SNP effect on Pulmonary fibrosis
0.05
0.06
0.07
SNP effect on C1orf210

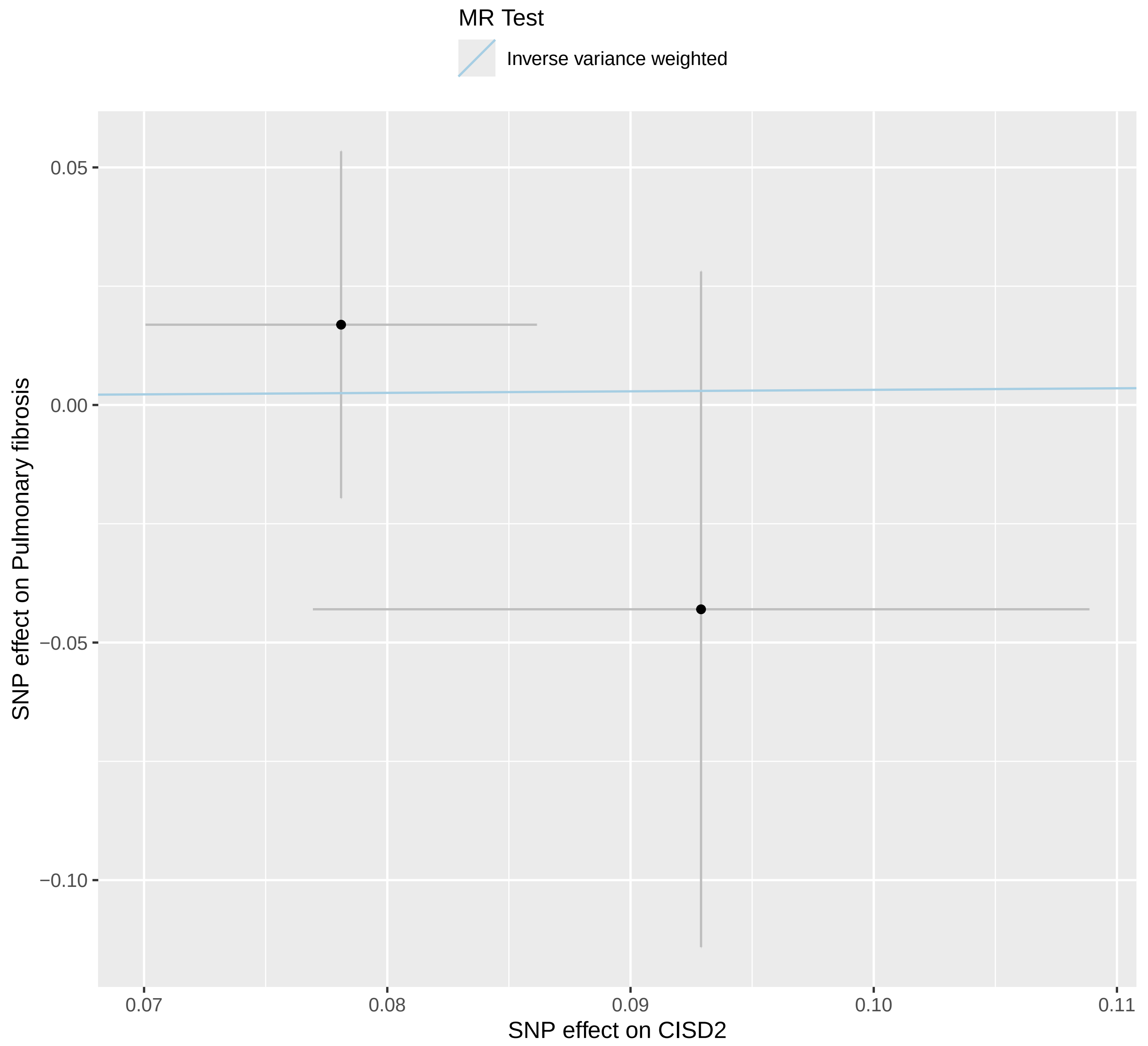

MR Test
Inverse variance weighted
0.05
0.00
−0.05
−0.10
SNP effect on Pulmonary fibrosis
0.07
0.08
0.09
0.10
0.11
SNP effect on CISD2

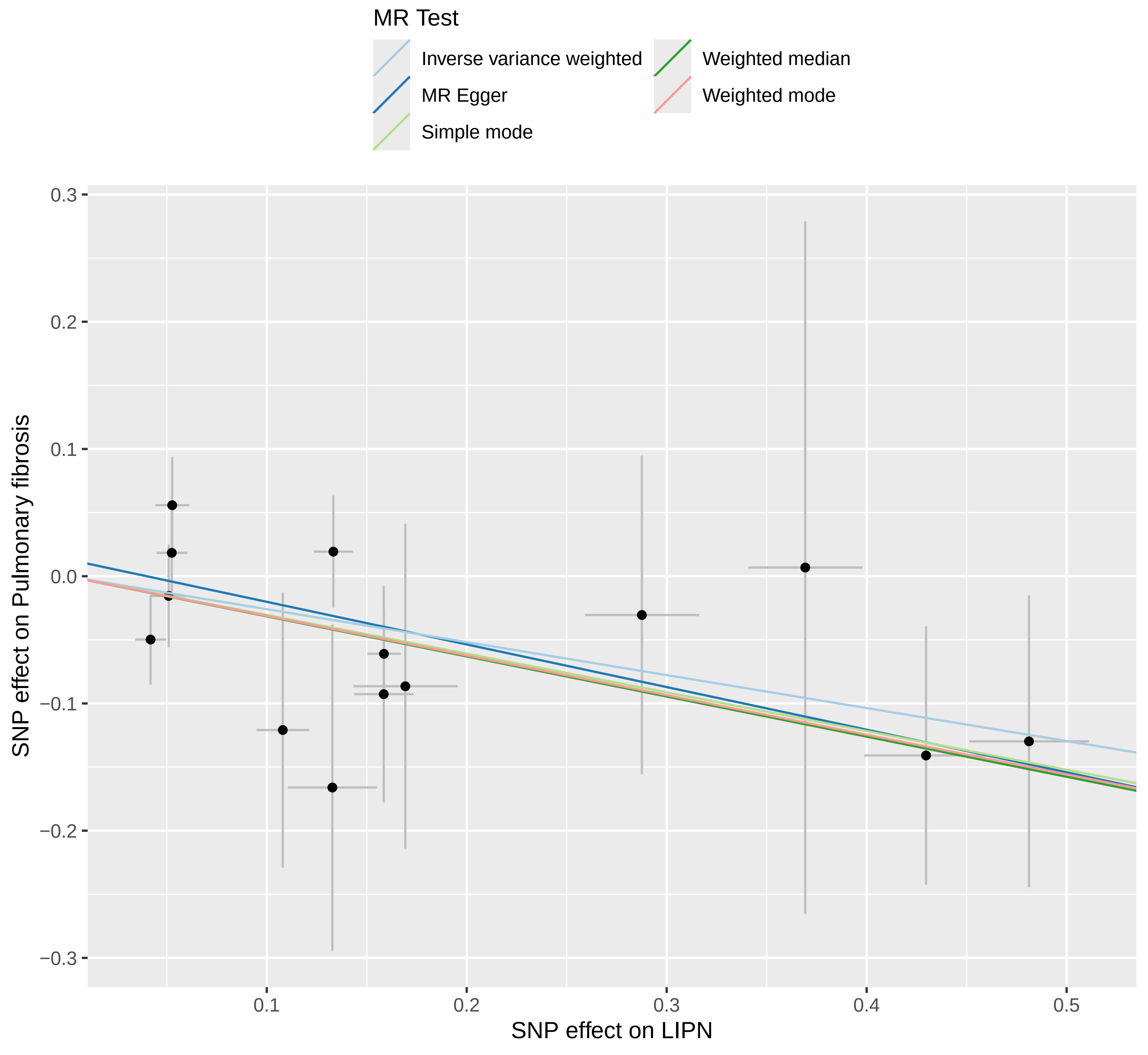
MR Test
Inverse variance weighted
MR Egger
Simple mode
Weighted median
Weighted mode
SNP effect on Pulmonary fibrosis
0.3
0.2
0.1
0.0
−0.1
−0.2
−0.3
0.1
0.2
0.3
0.4
0.5
SNP effect on LIPN

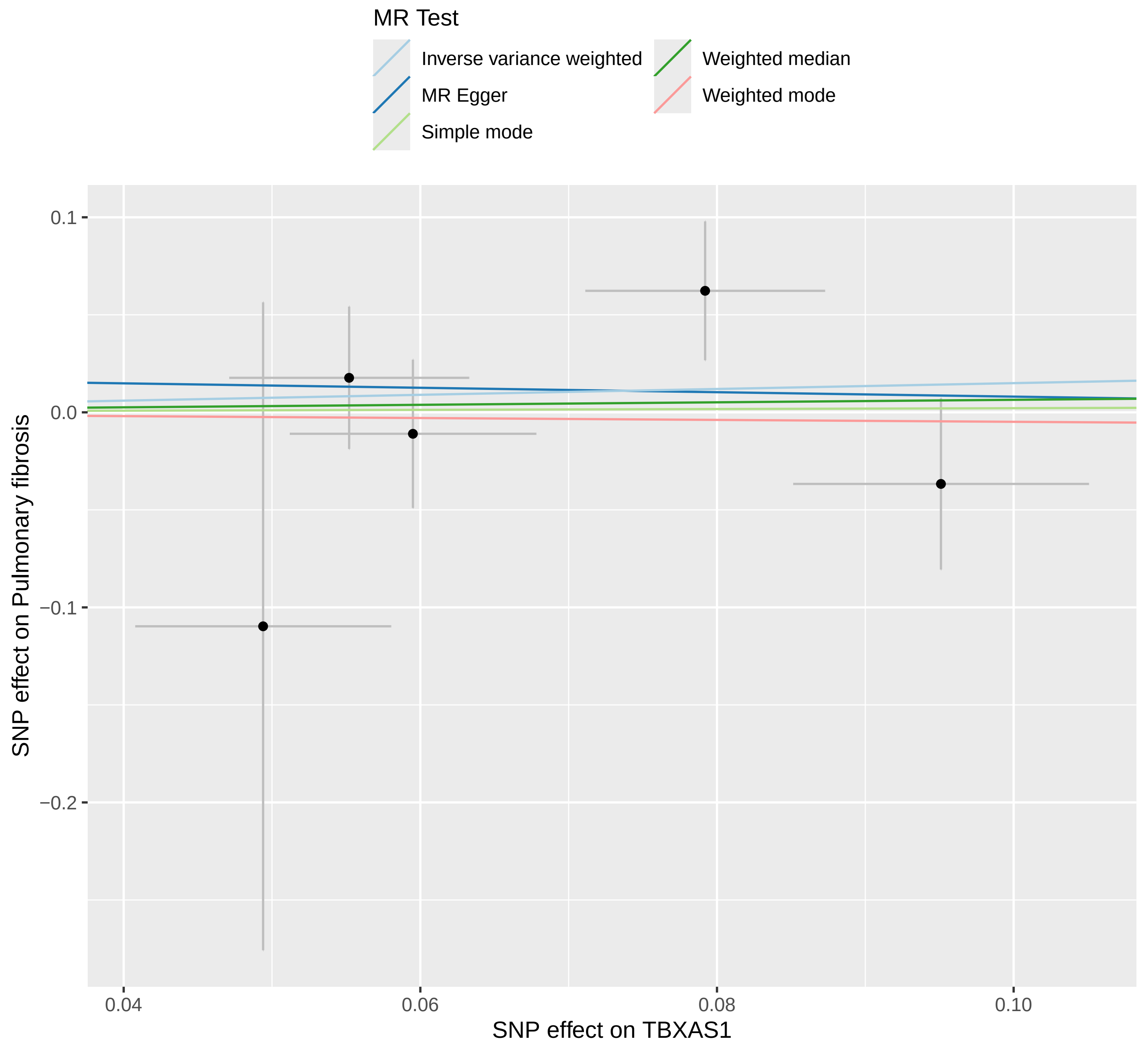

MR Test
Inverse variance weighted
MR Egger
Simple mode
Weighted median
Weighted mode
0.1
0.0
−0.1
−0.2
SNP effect on Pulmonary fibrosis
0.04
0.06
0.08
0.10
SNP effect on TBXAS1

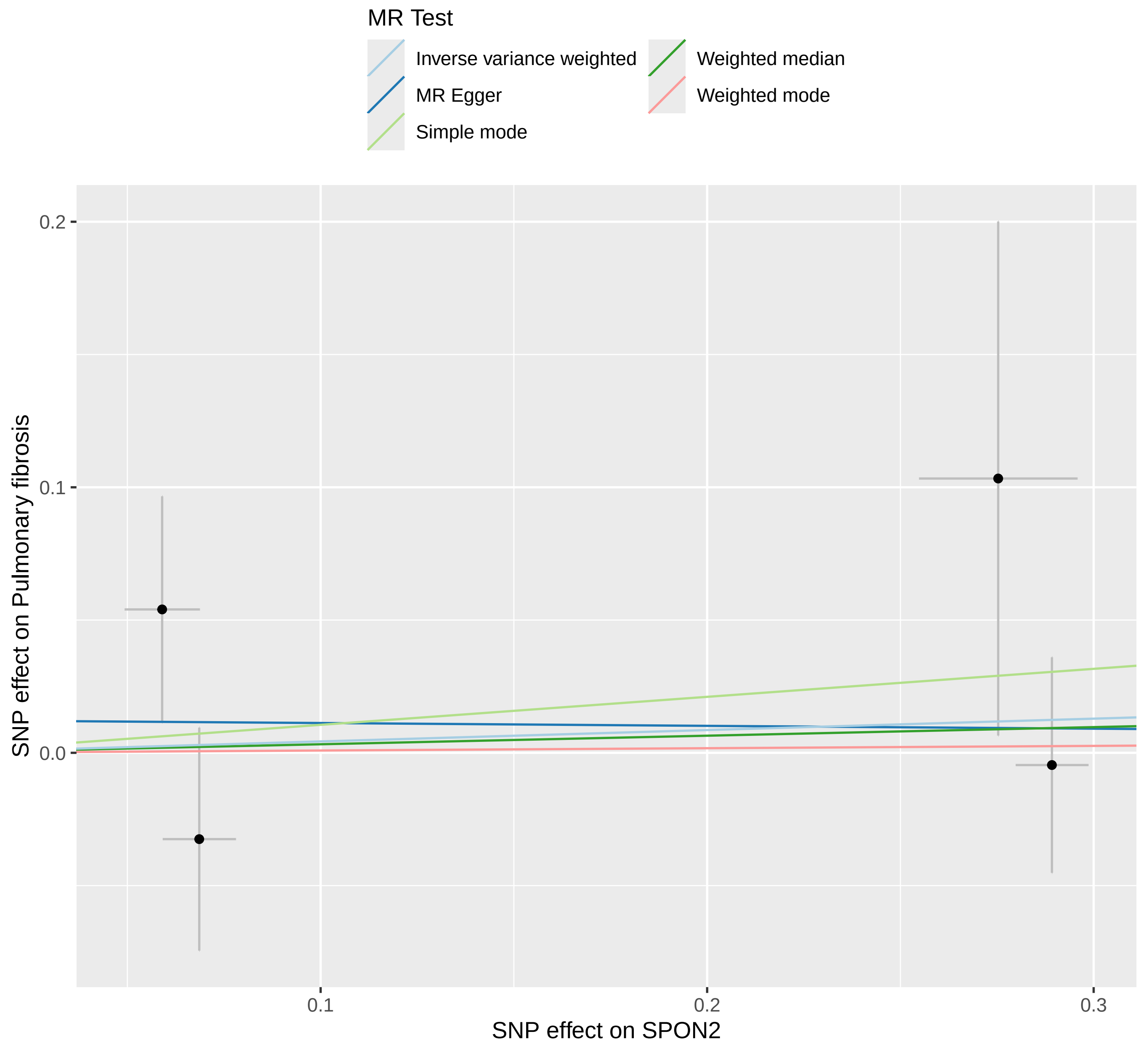
MR Test
Inverse variance weighted
MR Egger
Simple mode
Weighted median
Weighted mode
0.2
0.1
0.0
SNP effect on Pulmonary fibrosis
0.1
0.2
0.3
SNP effect on SPON2

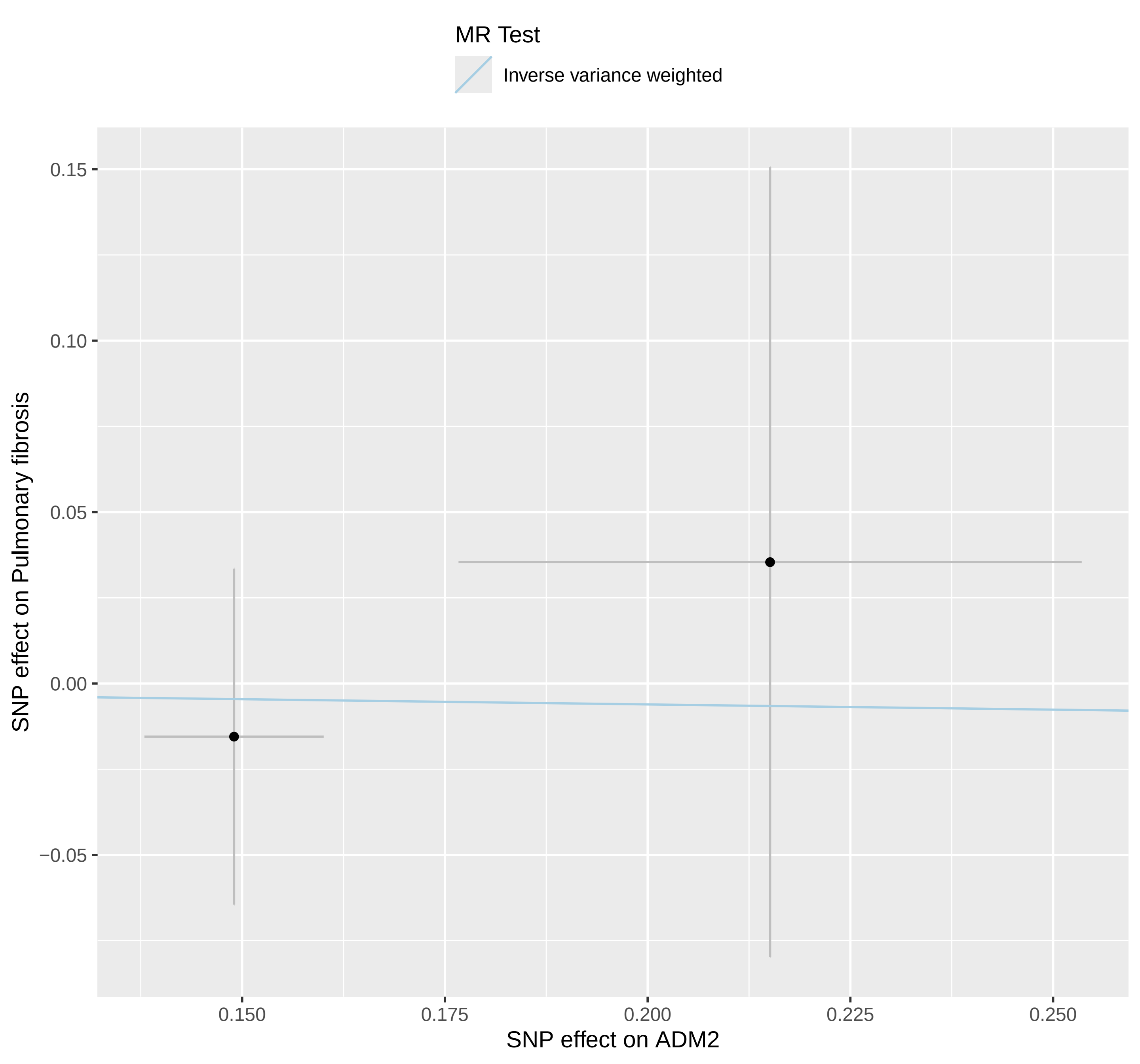
MR Test
Inverse variance weighted
0.15
0.10
0.05
0.00
−0.05
SNP effect on Pulmonary fibrosis
0.150
0.175
0.200
0.225
0.250
SNP effect on ADM2

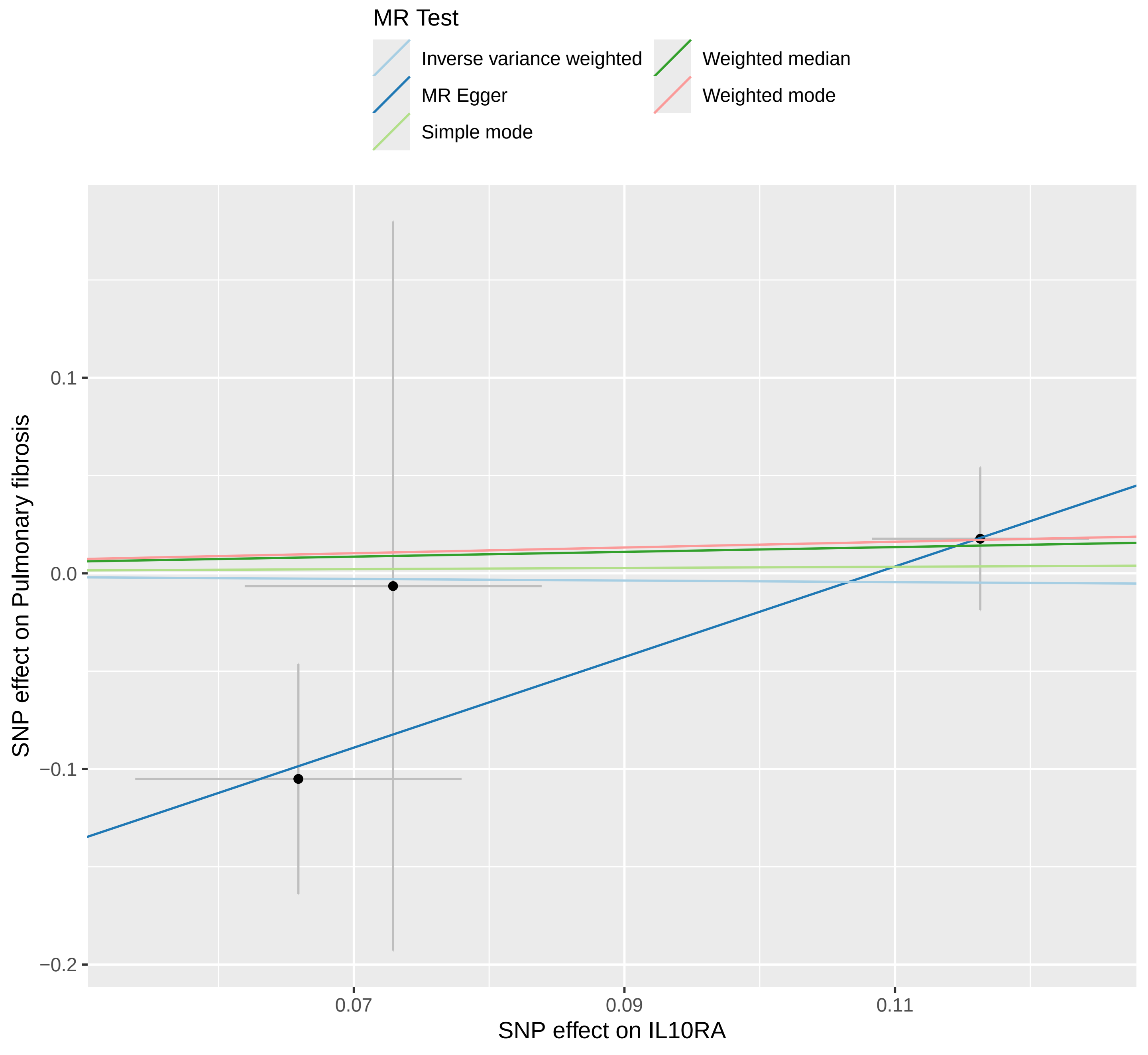
MR Test
Inverse variance weighted
MR Egger
Simple mode
Weighted median
Weighted mode
0.1
0.0
−0.1
−0.2
SNP effect on Pulmonary fibrosis
0.07
0.09
0.11
SNP effect on IL10RA

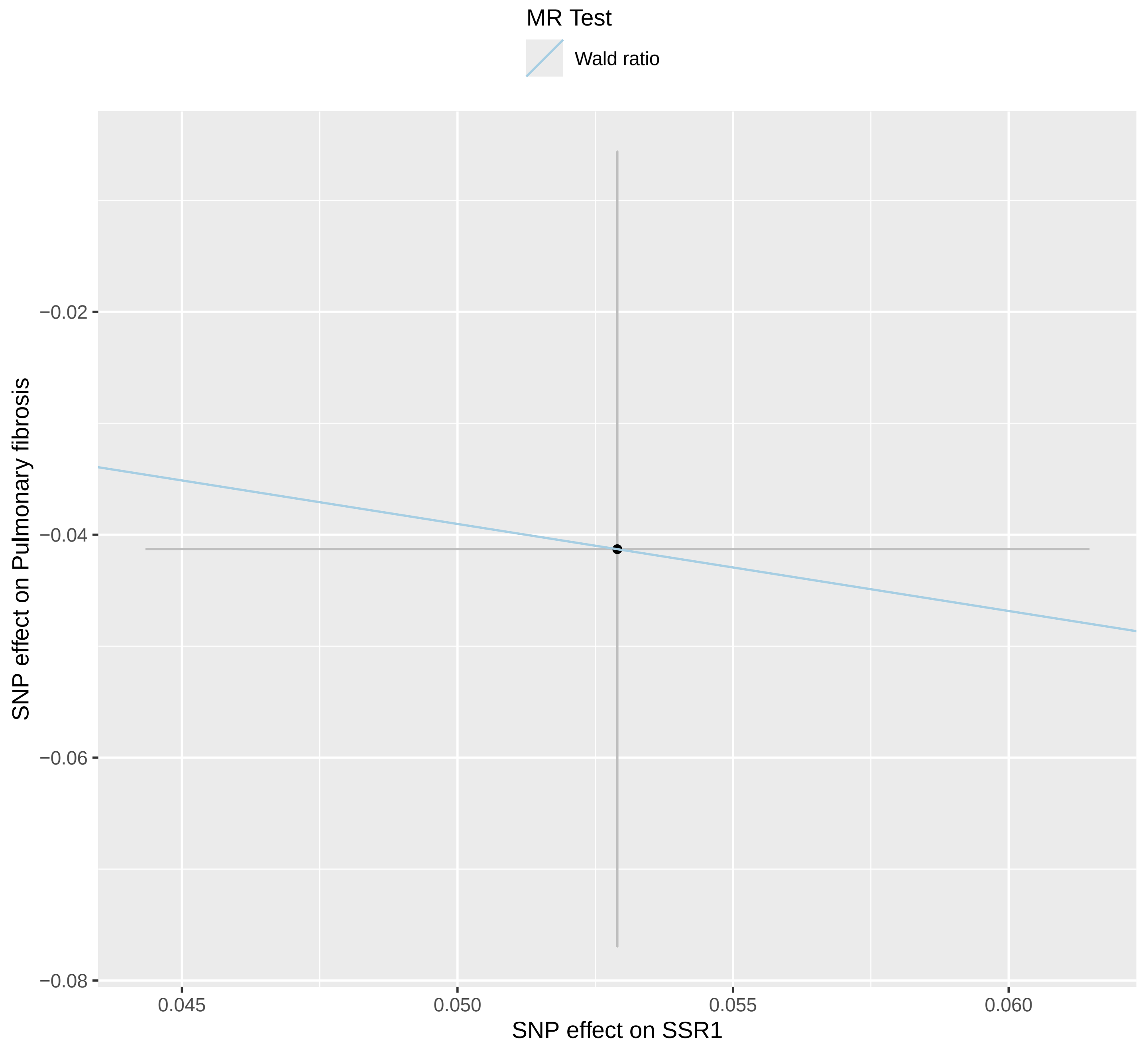
MR Test
Wald ratio
SNP effect on Pulmonary fibrosis
−0.02
−0.04
−0.06
−0.08
0.045
0.050
0.055
0.060
SNP effect on SSR1

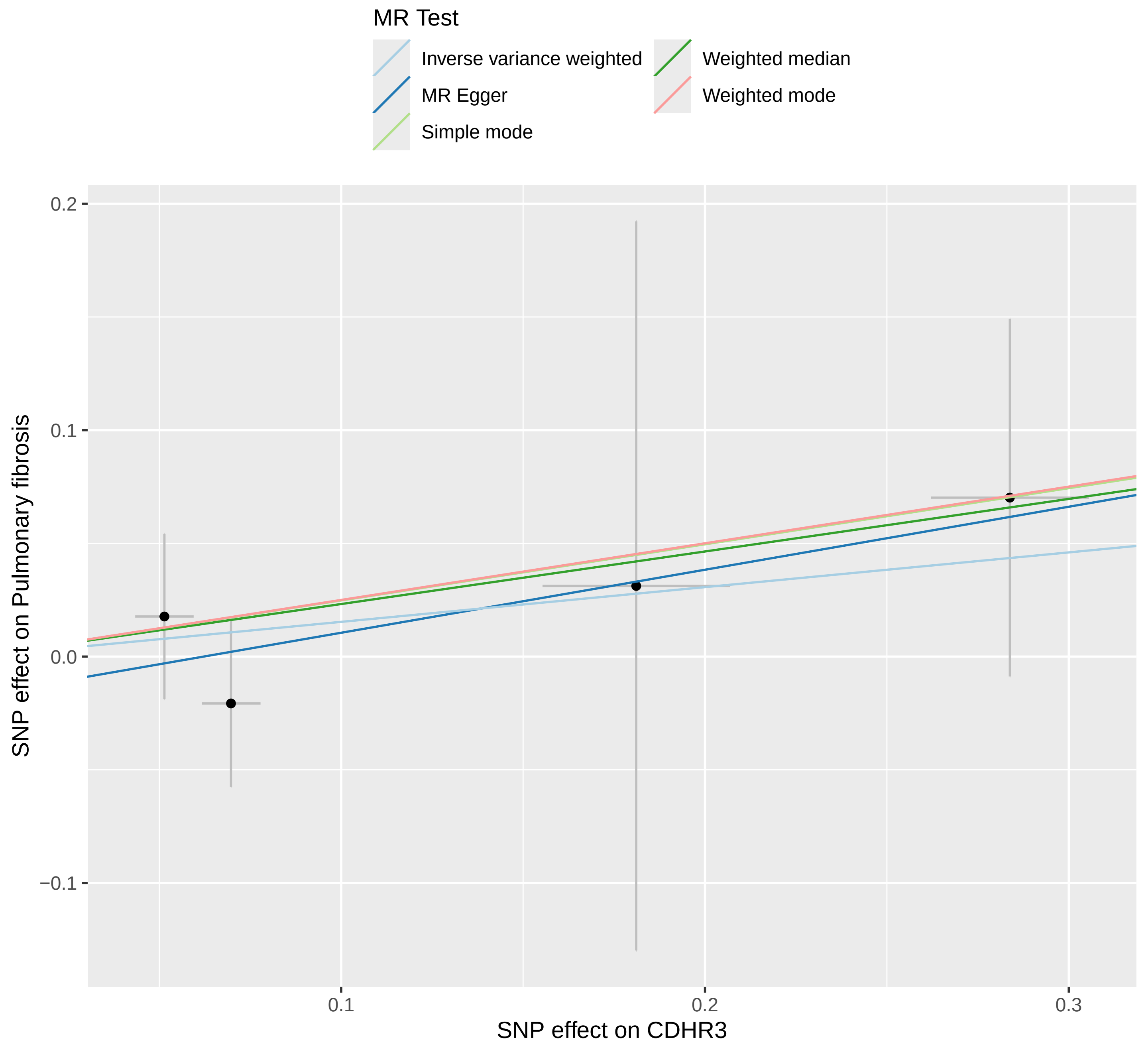

MR Test
Inverse variance weighted
MR Egger
Simple mode
Weighted median
Weighted mode
0.2
0.1
0.0
−0.1
SNP effect on Pulmonary fibrosis
0.1
0.2
0.3
SNP effect on CDHR3

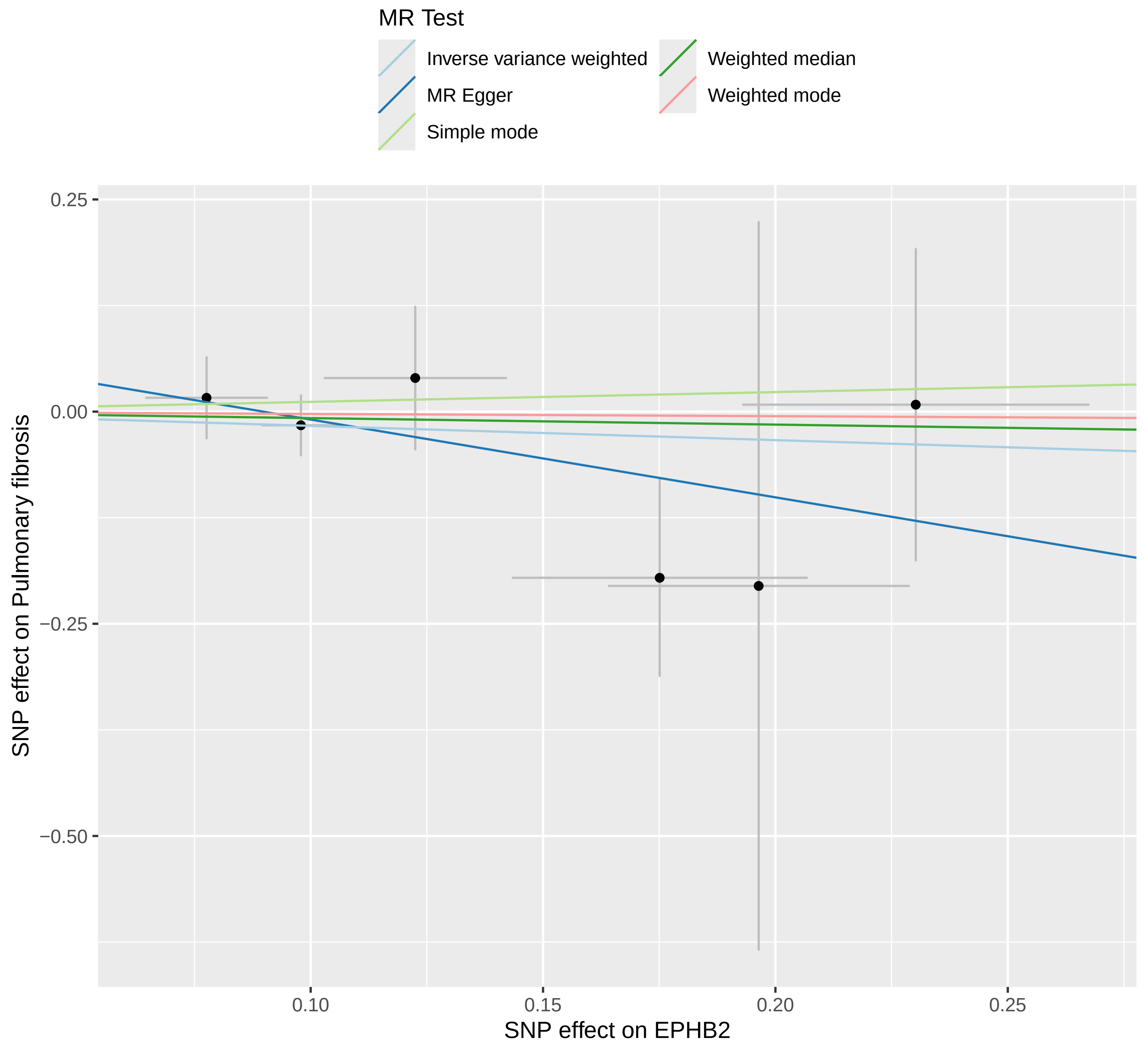

MR Test
Inverse variance weighted
MR Egger
Simple mode
Weighted median
Weighted mode
0.25
0.00
−0.25
−0.50
0.10
0.15
0.20
0.25
SNP effect on Pulmonary fibrosis
SNP effect on EPHB2

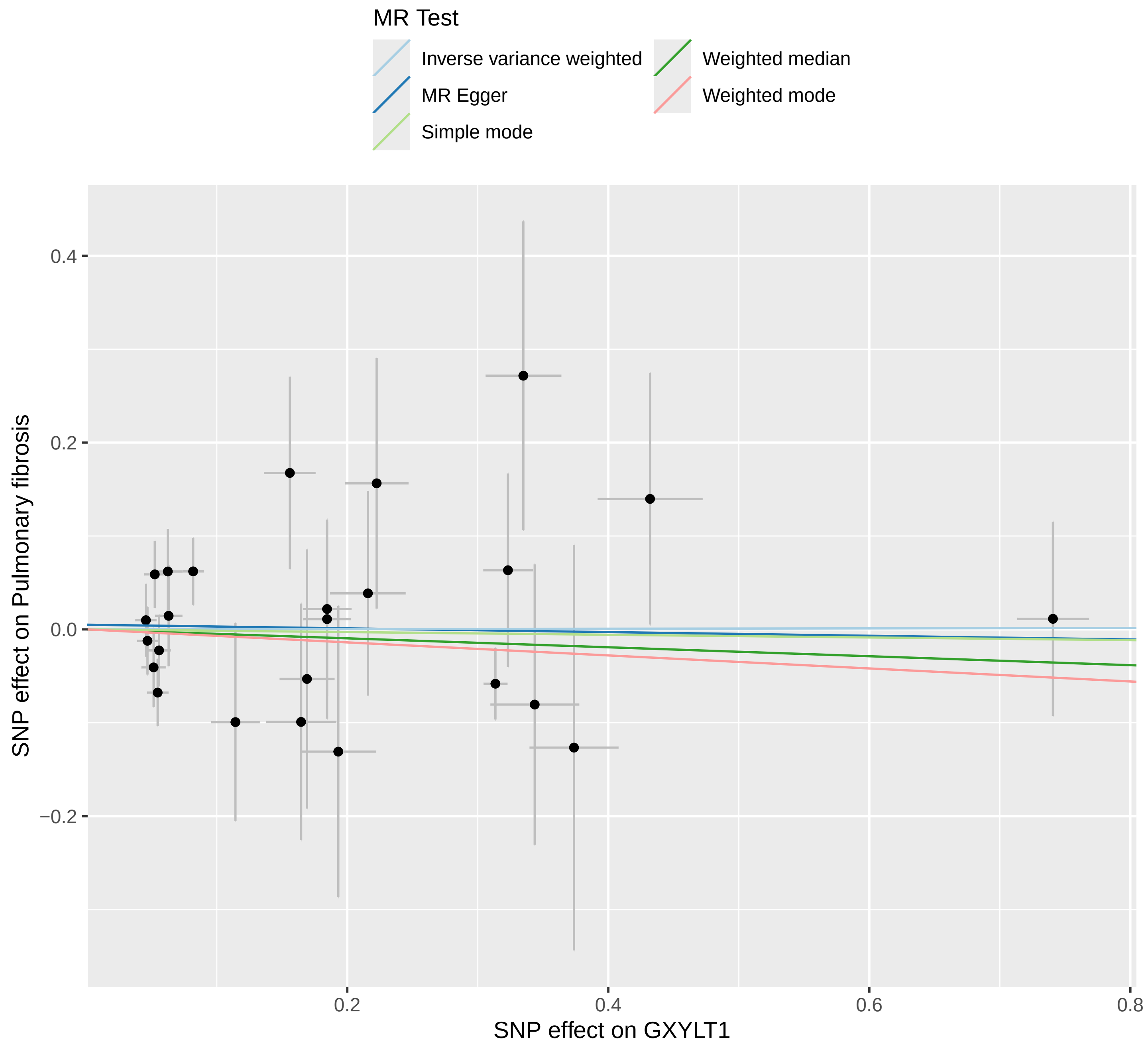

MR Test
Inverse variance weighted
MR Egger
Simple mode
Weighted median
Weighted mode
0.4
0.2
0.0
−0.2
SNP effect on Pulmonary fibrosis
0.2
0.4
0.6
0.8
SNP effect on GXYLT1

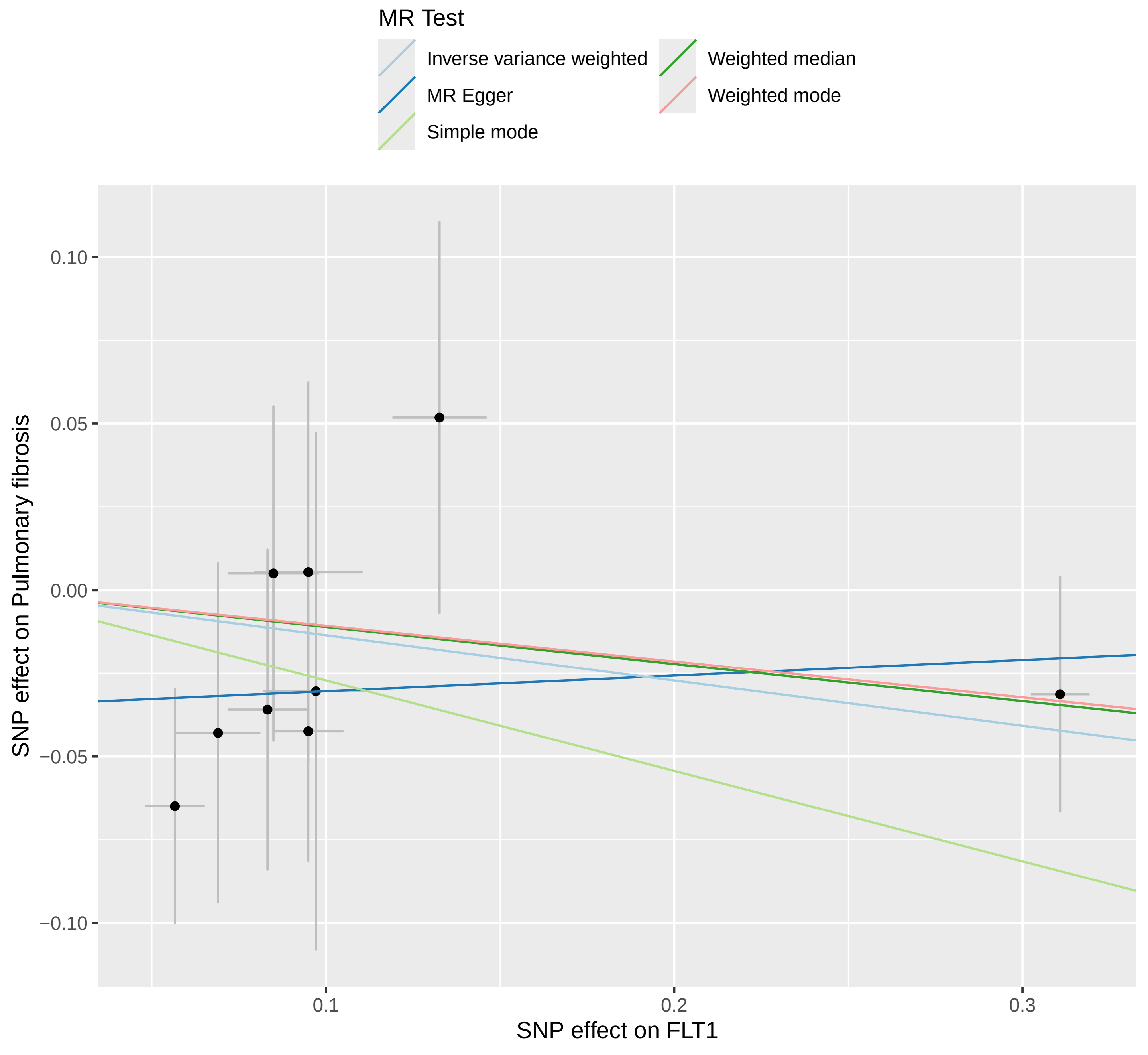
MR Test
Inverse variance weighted
MR Egger
Simple mode
Weighted median
Weighted mode
SNP effect on Pulmonary fibrosis
0.10
0.05
0.00
−0.05
−0.10
0.1
0.2
0.3
SNP effect on FLT1

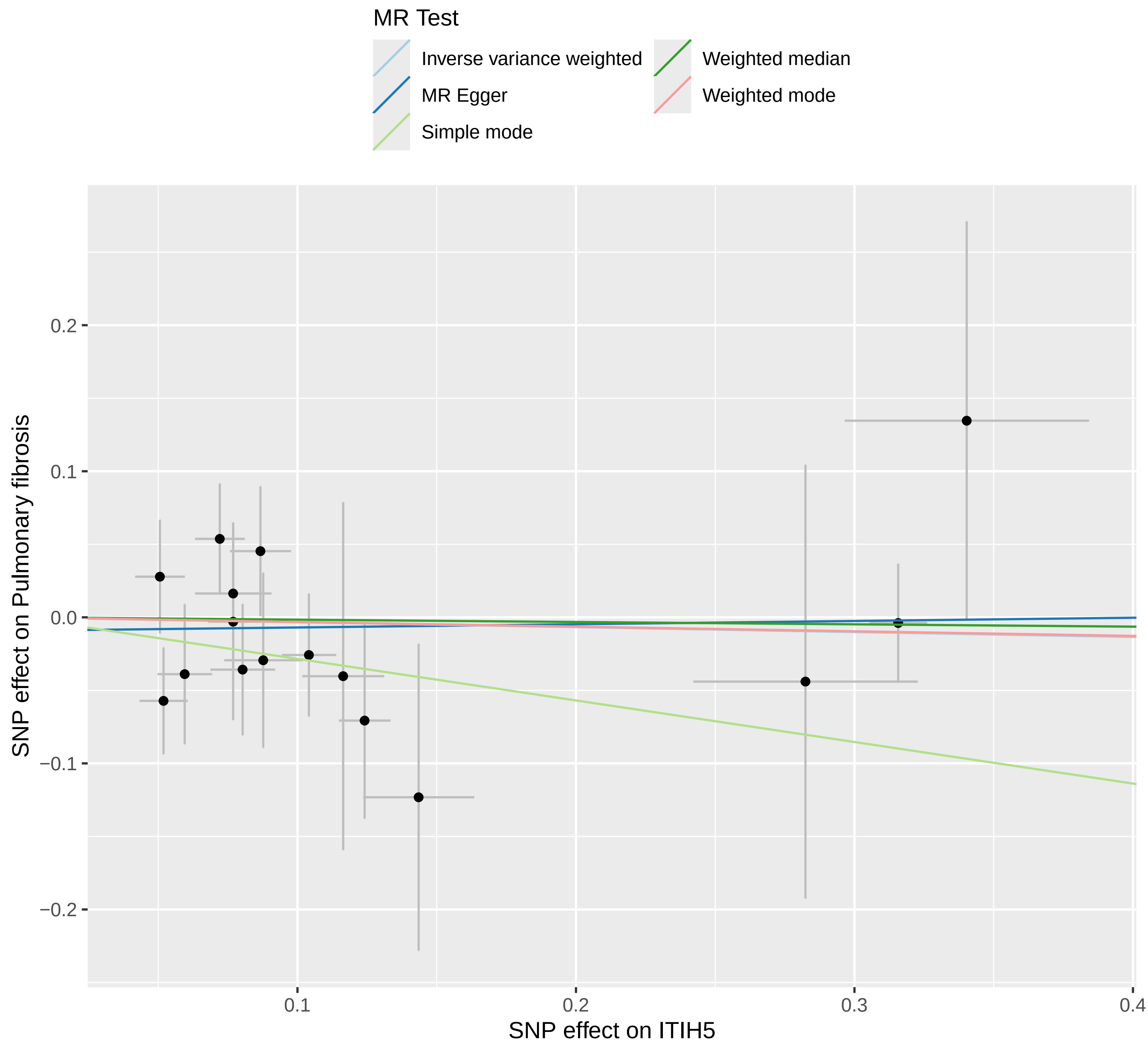
MR Test
Inverse variance weighted
MR Egger
Simple mode
Weighted median
Weighted mode
SNP effect on Pulmonary fibrosis
0.2
0.1
0.0
−0.1
−0.2
0.1
0.2
0.3
0.4
SNP effect on ITIH5

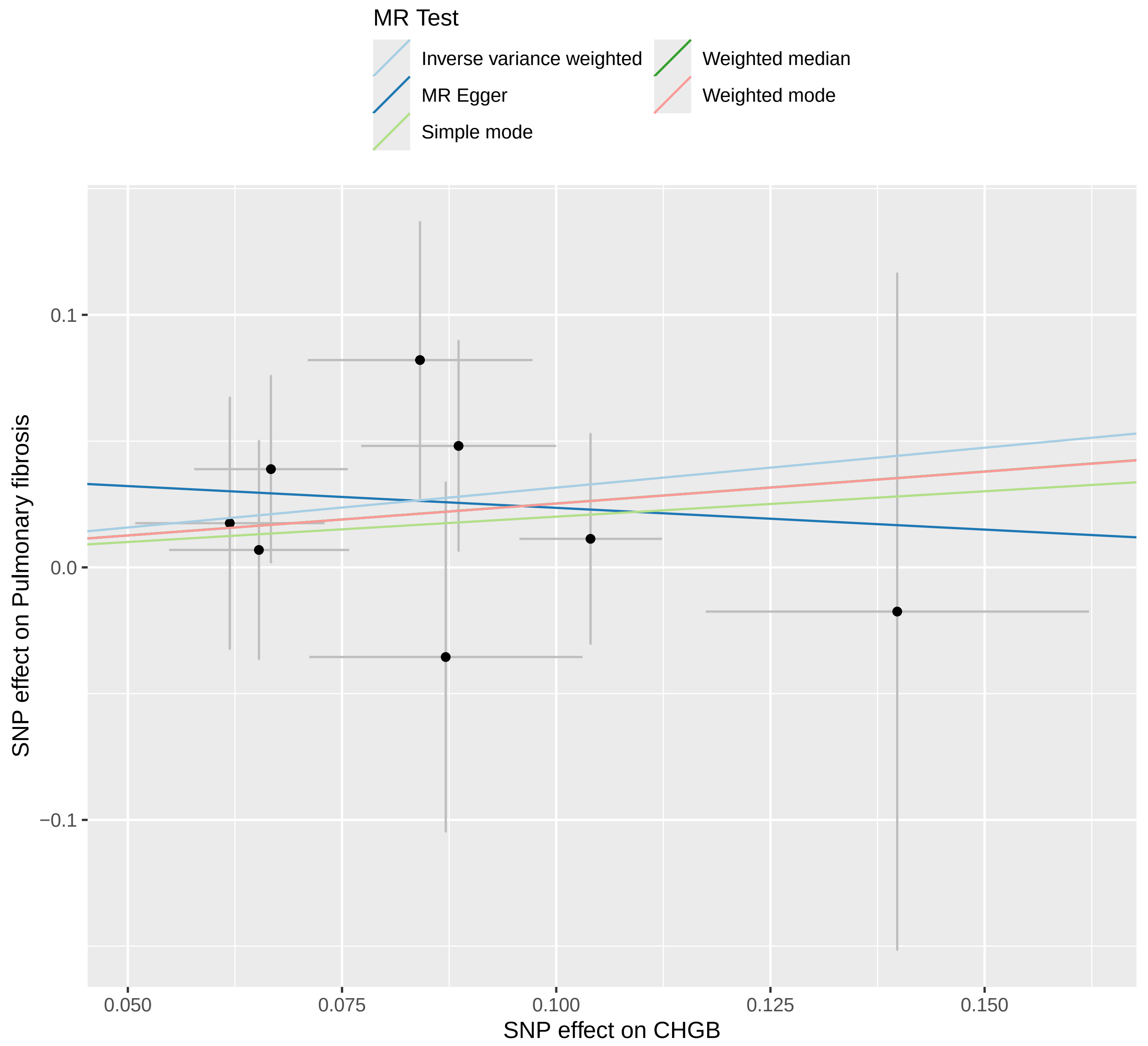

MR Test
Inverse variance weighted
MR Egger
Simple mode
Weighted median
Weighted mode
0.1
0.0
−0.1
0.050
0.075
0.100
0.125
0.150
SNP effect on CHGB
SNP effect on Pulmonary fibrosis

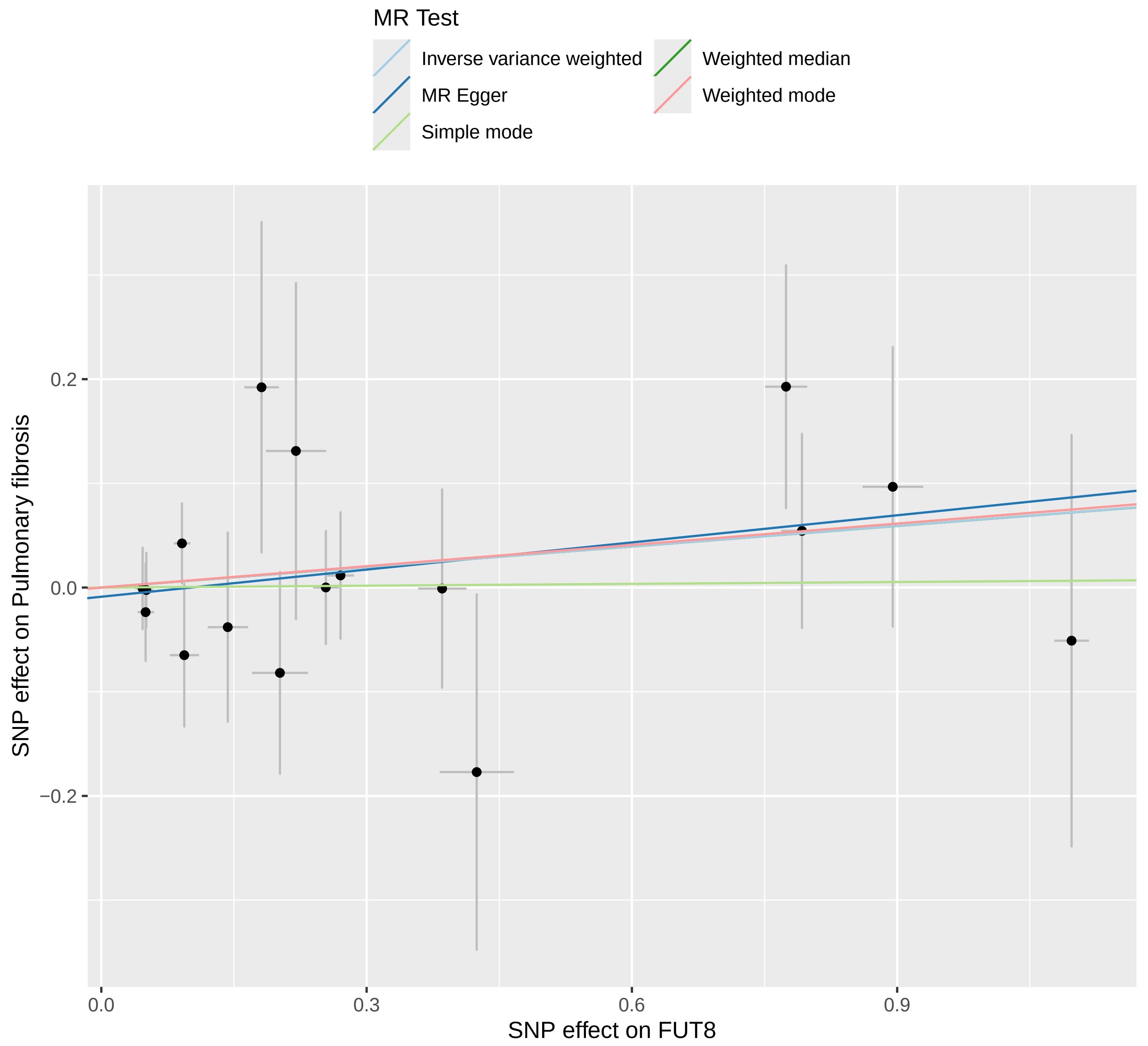

MR Test
Inverse variance weighted
MR Egger
Simple mode
Weighted median
Weighted mode
0.2
0.0
−0.2
0.0
0.3
0.6
0.9
SNP effect on FUT8
SNP effect on Pulmonary fibrosis

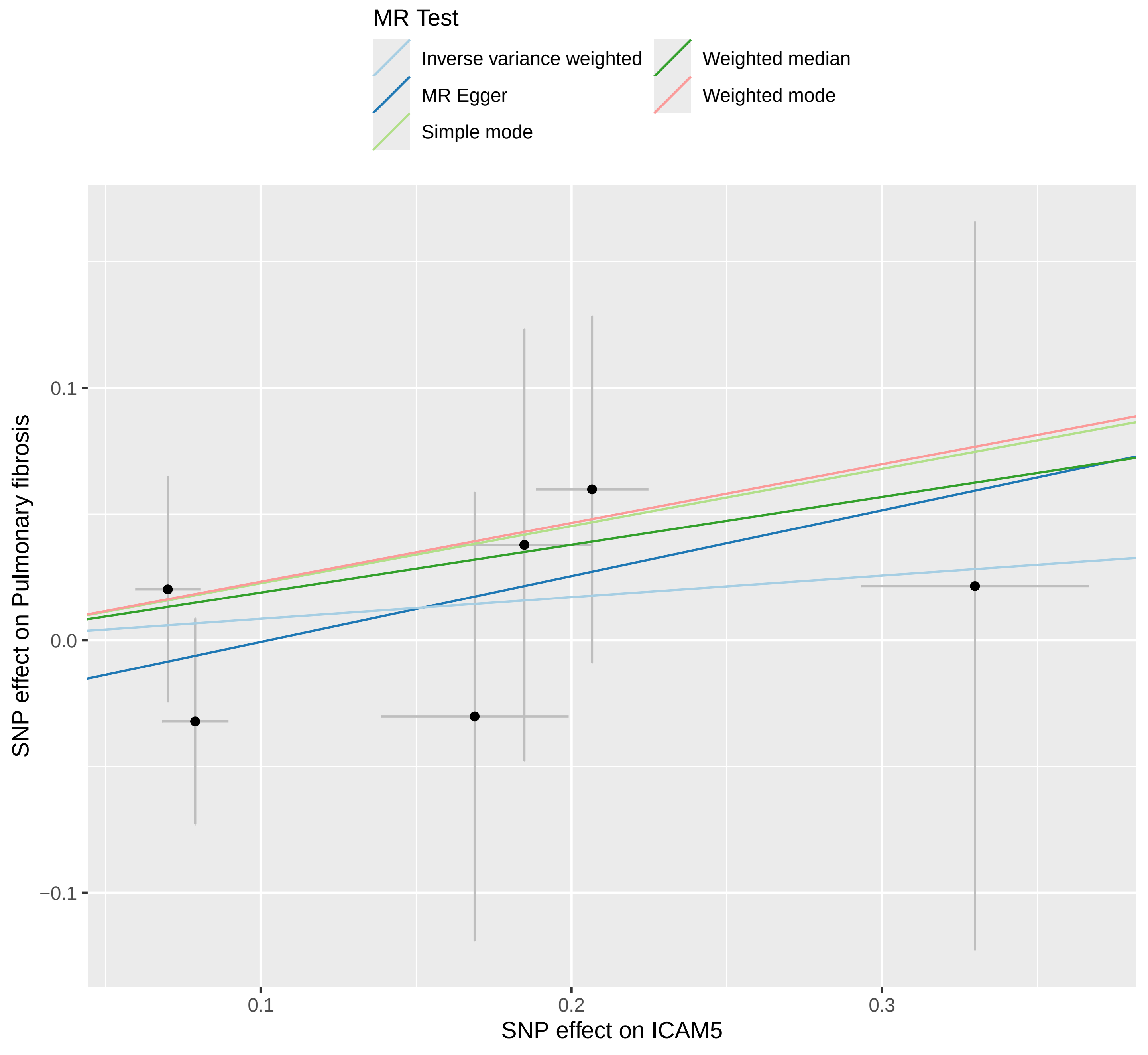
MR Test
Inverse variance weighted
MR Egger
Simple mode
Weighted median
Weighted mode
SNP effect on Pulmonary fibrosis
0.1
0.0
−0.1
0.1
0.2
0.3
SNP effect on ICAM5

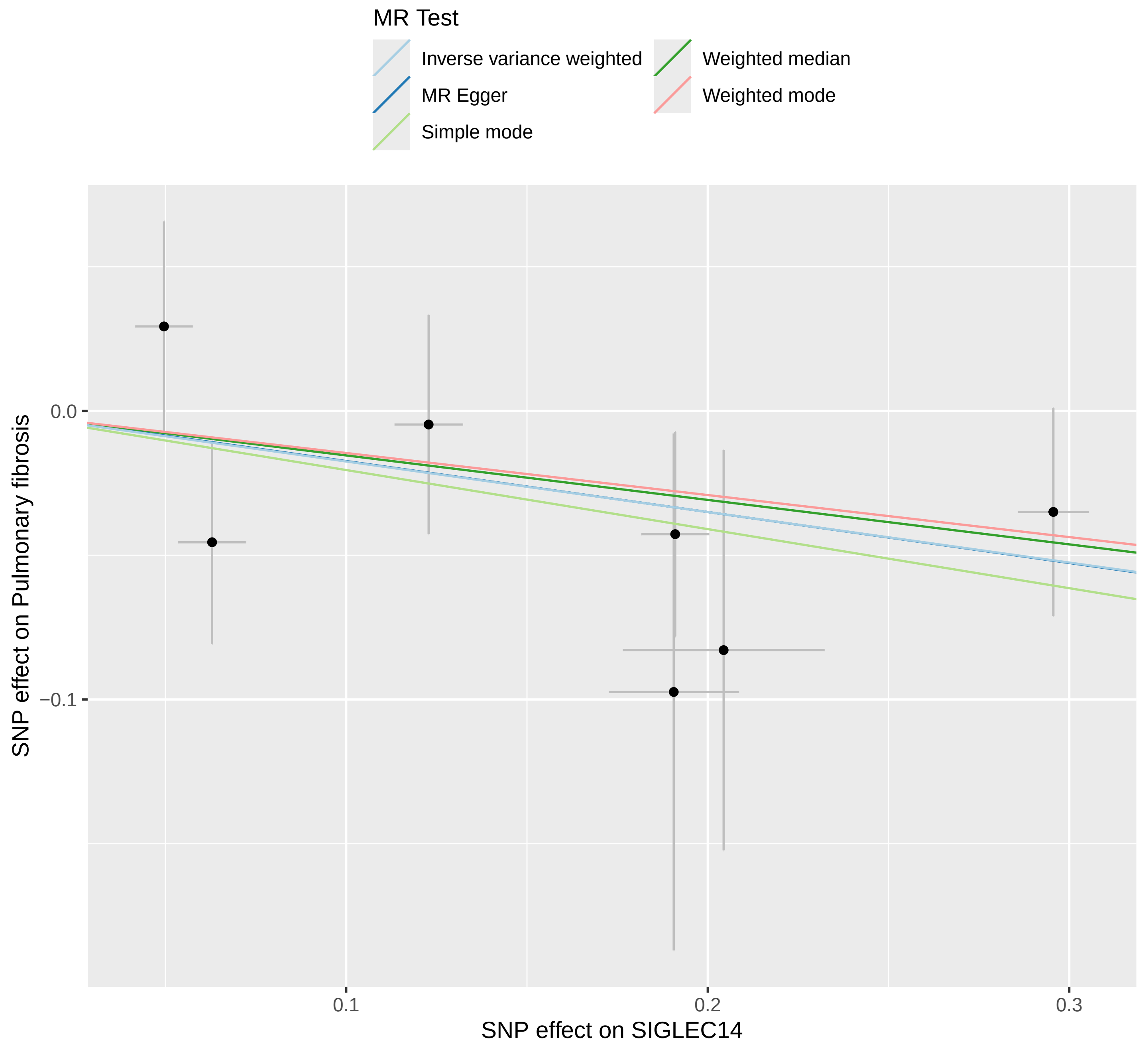
MR Test
Inverse variance weighted
MR Egger
Simple mode
Weighted median
Weighted mode
0.0
−0.1
0.1
0.2
0.3
SNP effect on Pulmonary fibrosis
SNP effect on SIGLEC14

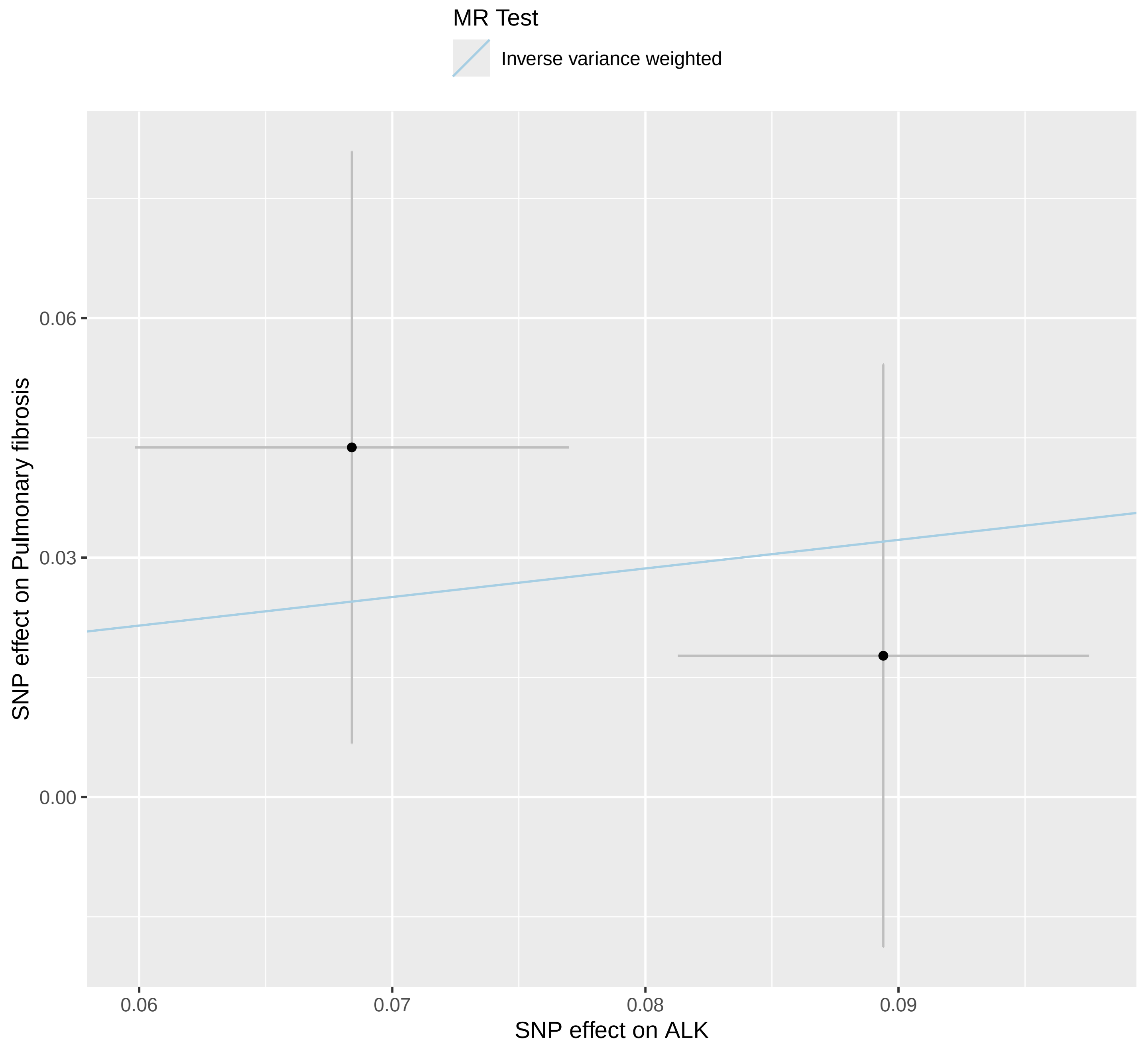
MR Test
Inverse variance weighted
0.06
0.03
0.00
0.06
0.07
0.08
0.09
SNP effect on Pulmonary fibrosis
SNP effect on ALK

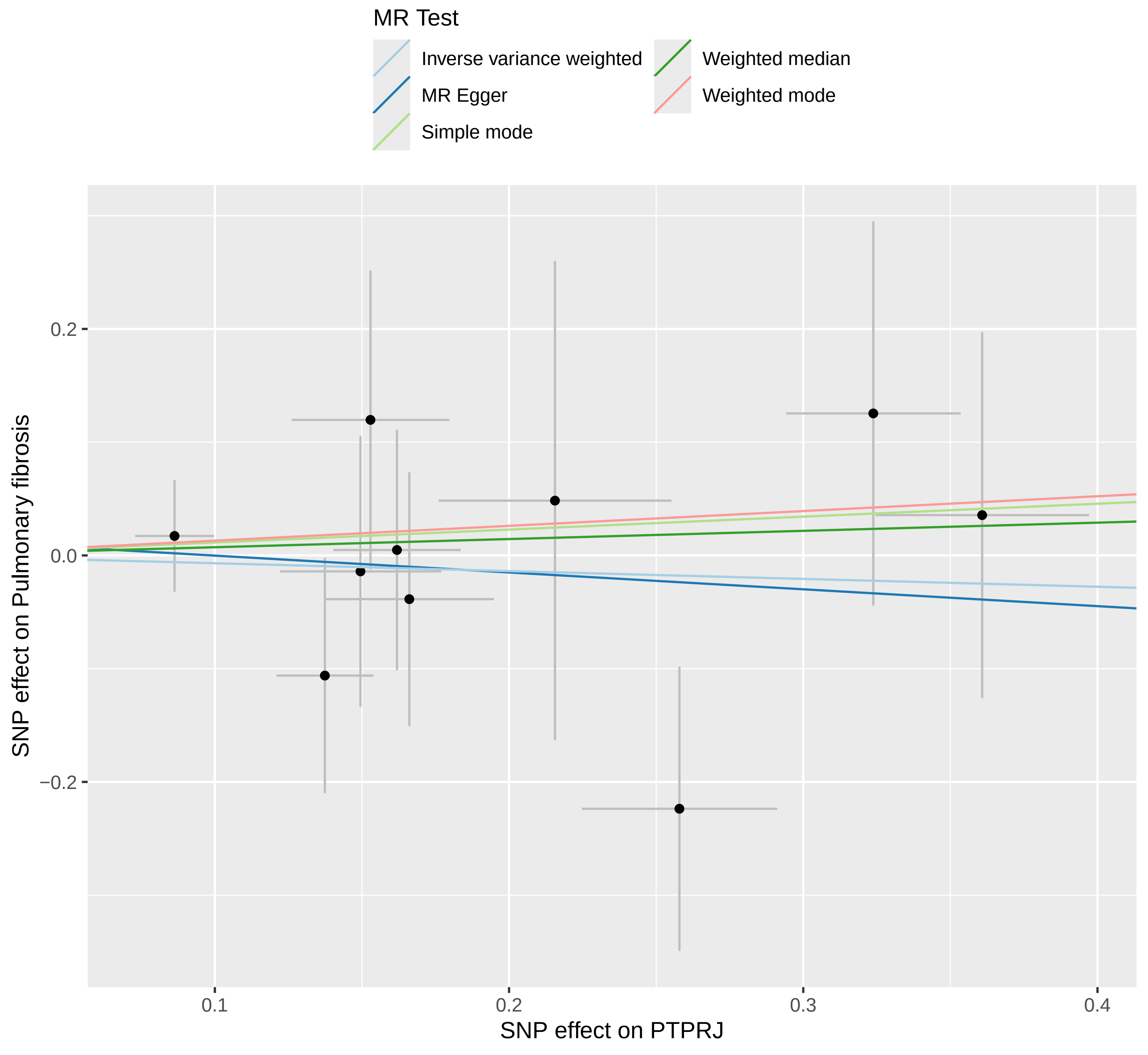

MR Test
Inverse variance weighted
MR Egger
Simple mode
Weighted median
Weighted mode
0.2
0.0
−0.2
SNP effect on Pulmonary fibrosis
0.1
0.2
0.3
0.4
SNP effect on PTPRJ

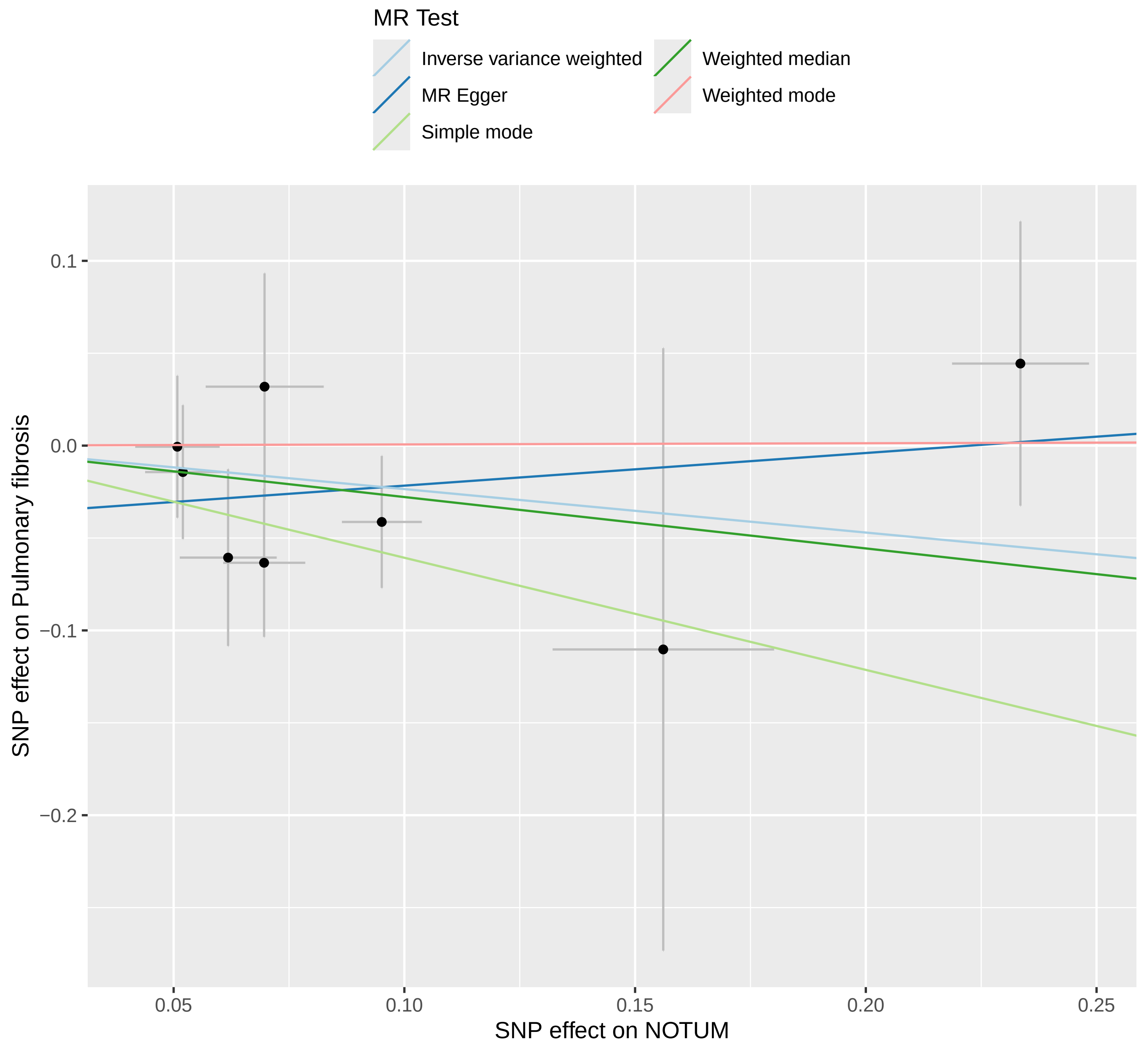
MR Test
Inverse variance weighted
MR Egger
Simple mode
Weighted median
Weighted mode
0.1
0.0
−0.1
−0.2
SNP effect on Pulmonary fibrosis
0.05
0.10
0.15
0.20
0.25
SNP effect on NOTUM

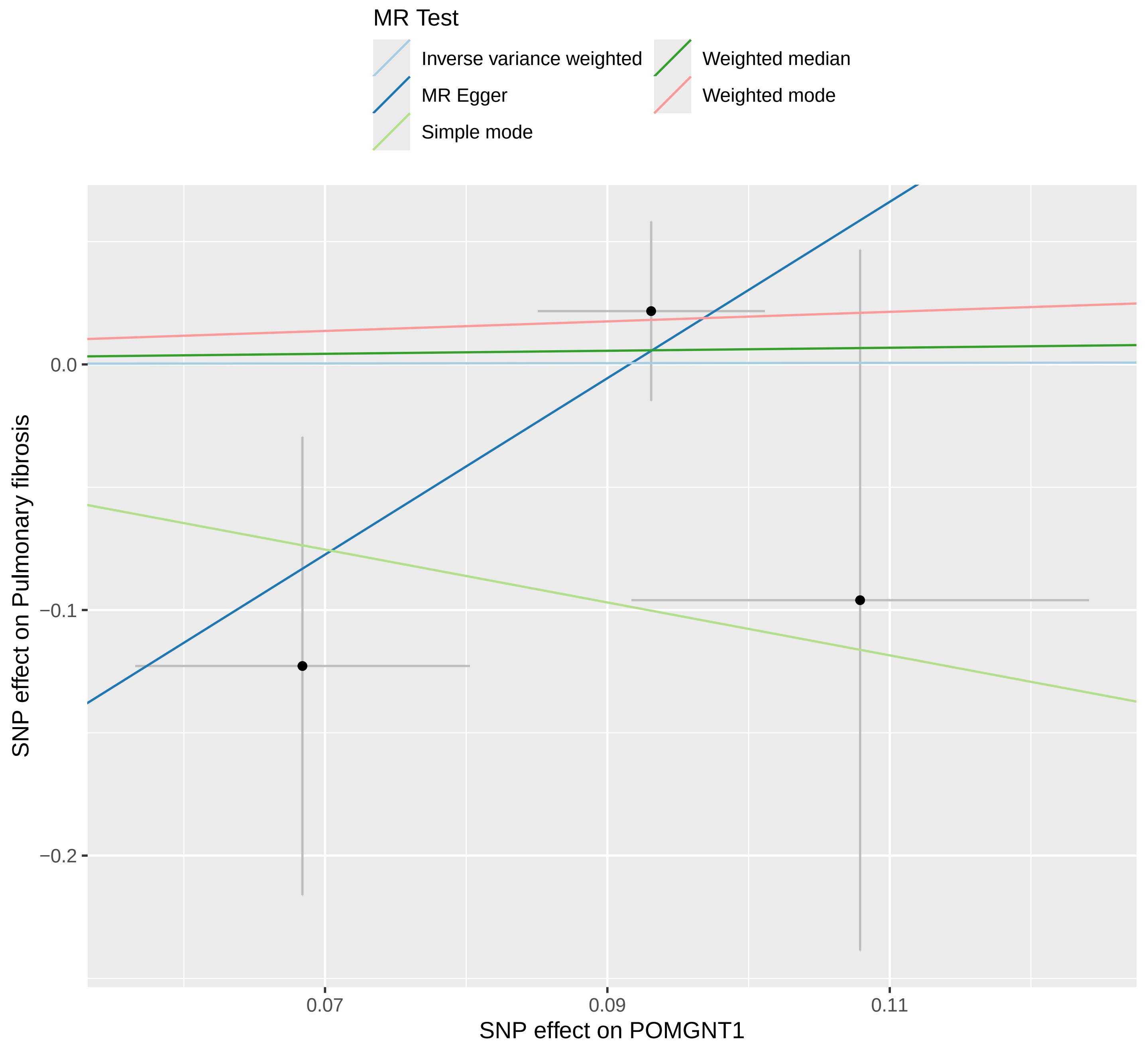

MR Test
Inverse variance weighted
MR Egger
Simple mode
Weighted median
Weighted mode
0.0
−0.1
−0.2
0.07
0.09
0.11
SNP effect on Pulmonary fibrosis
SNP effect on POMGNT1

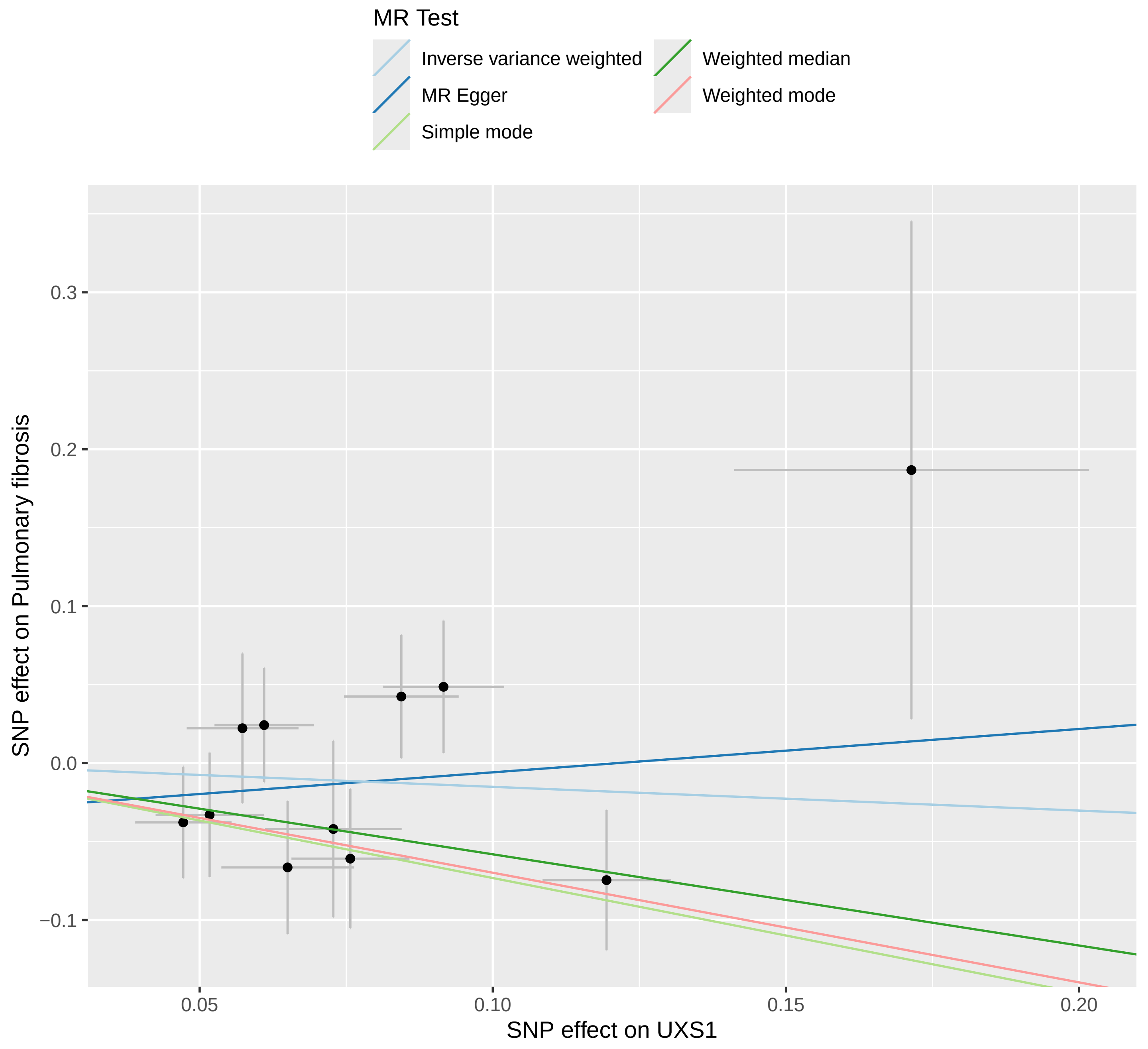

MR Test
Inverse variance weighted
MR Egger
Simple mode
Weighted median
Weighted mode
0.3
0.2
0.1
0.0
−0.1
0.05
0.10
0.15
0.20
SNP effect on UXS1
SNP effect on Pulmonary fibrosis

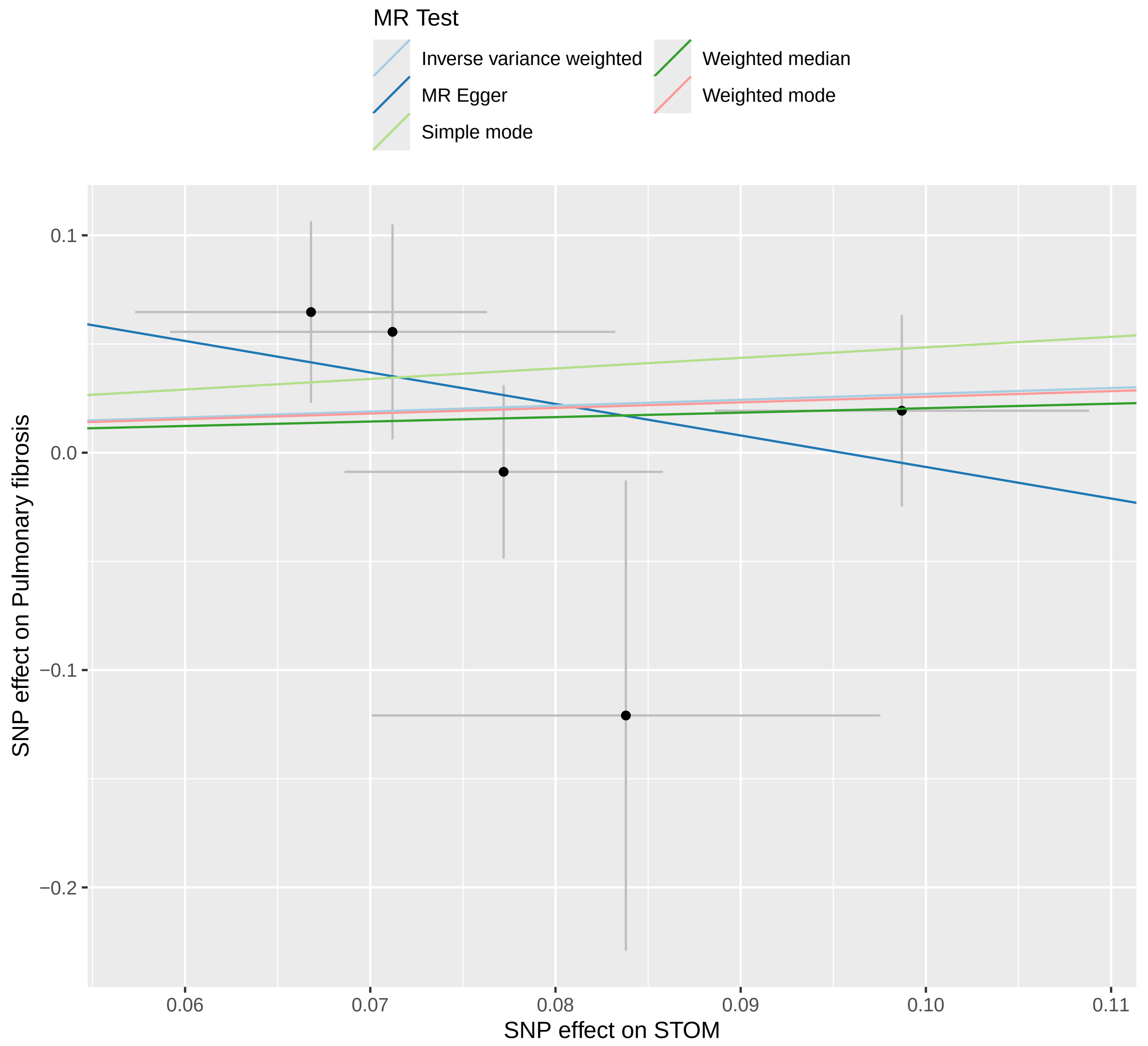

MR Test
Inverse variance weighted
MR Egger
Simple mode
Weighted median
Weighted mode
0.1
0.0
−0.1
−0.2
SNP effect on Pulmonary fibrosis
0.06
0.07
0.08
0.09
0.10
0.11
SNP effect on STOM

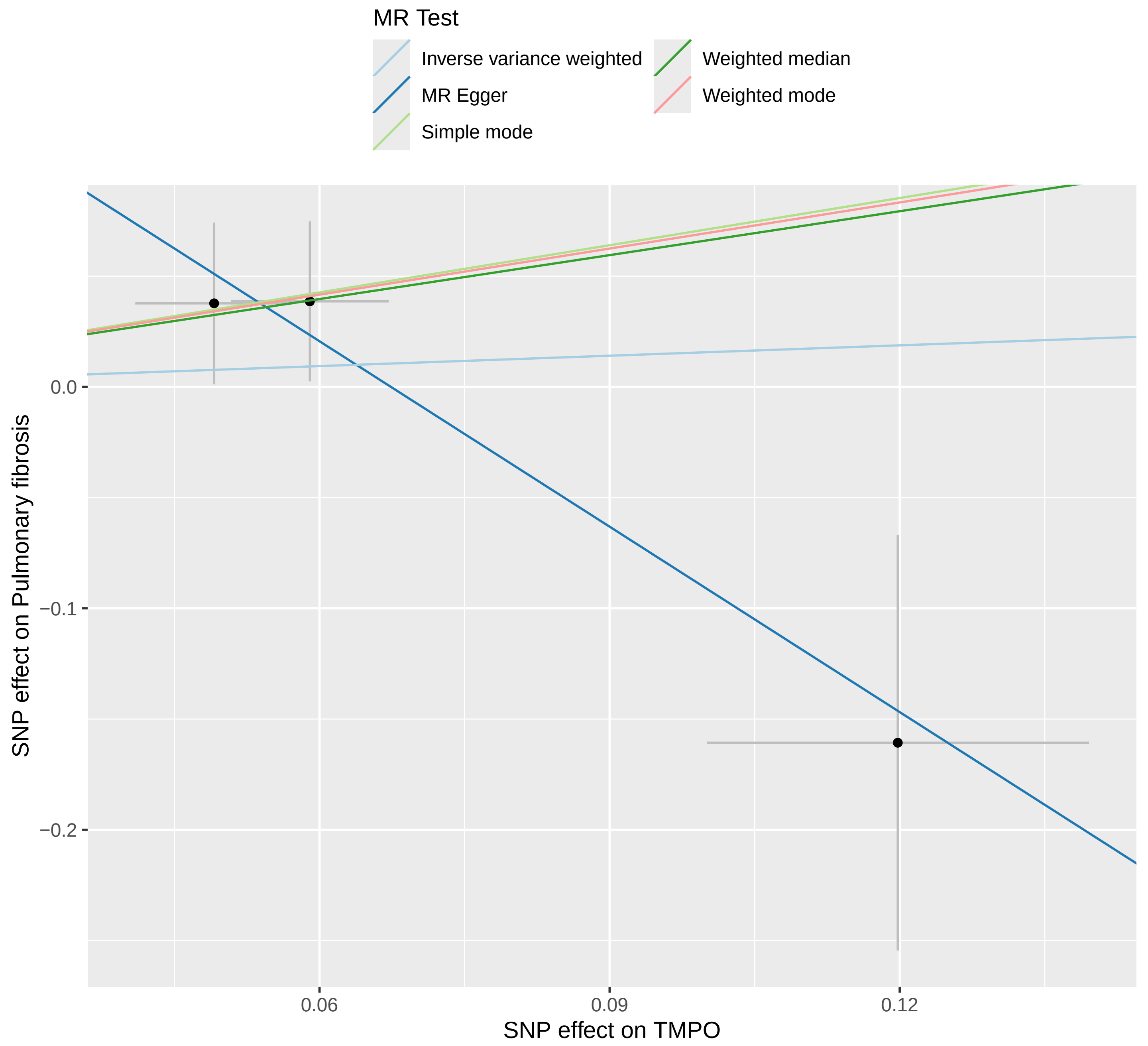
MR Test
Inverse variance weighted
MR Egger
Simple mode
Weighted median
Weighted mode
0.0
−0.1
−0.2
SNP effect on Pulmonary fibrosis
0.06
0.09
0.12
SNP effect on TMPO

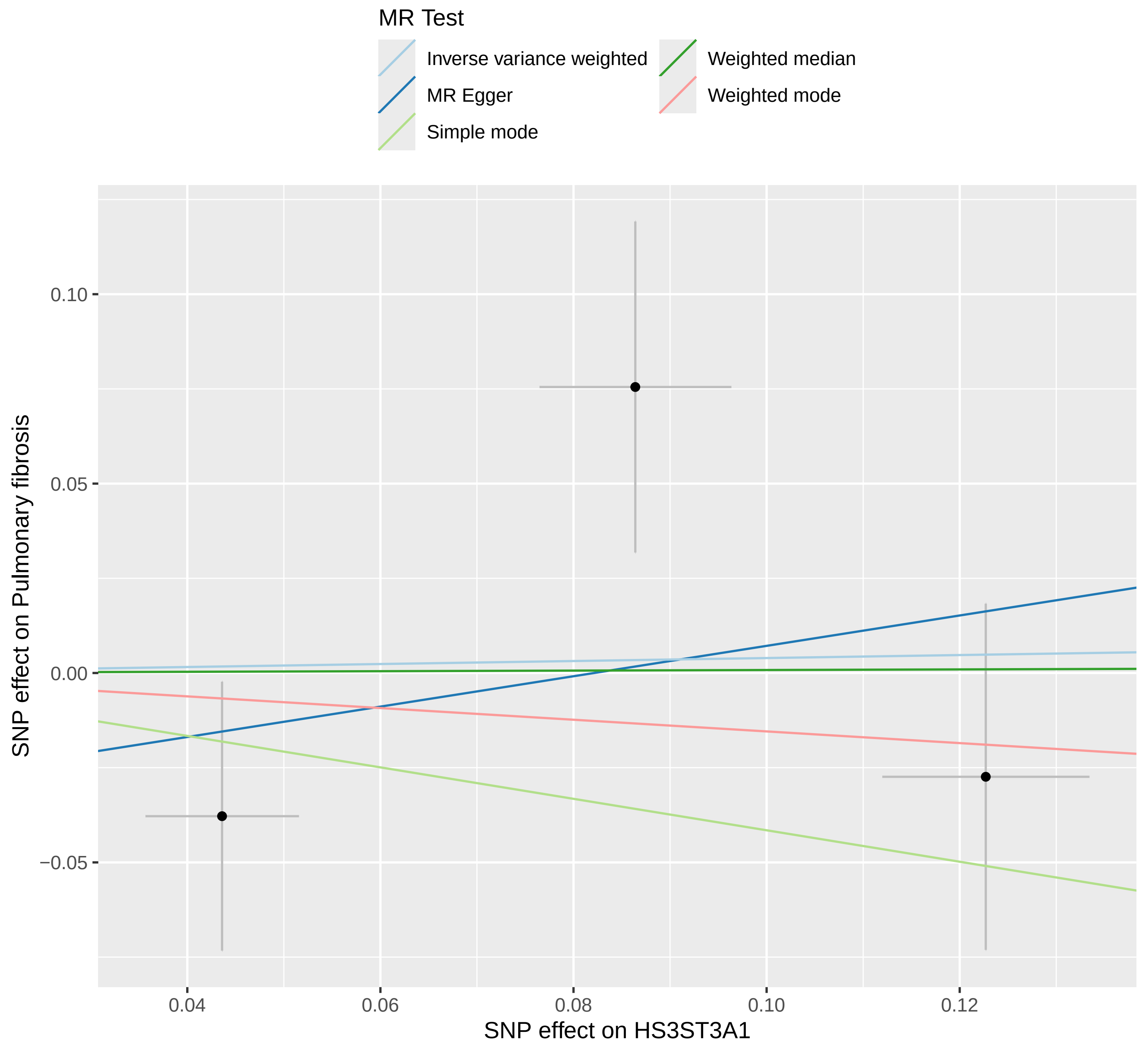
MR Test
Inverse variance weighted
MR Egger
Simple mode
Weighted median
Weighted mode
0.10
0.05
0.00
−0.05
SNP effect on Pulmonary fibrosis
0.04
0.06
0.08
0.10
0.12
SNP effect on HS3ST3A1

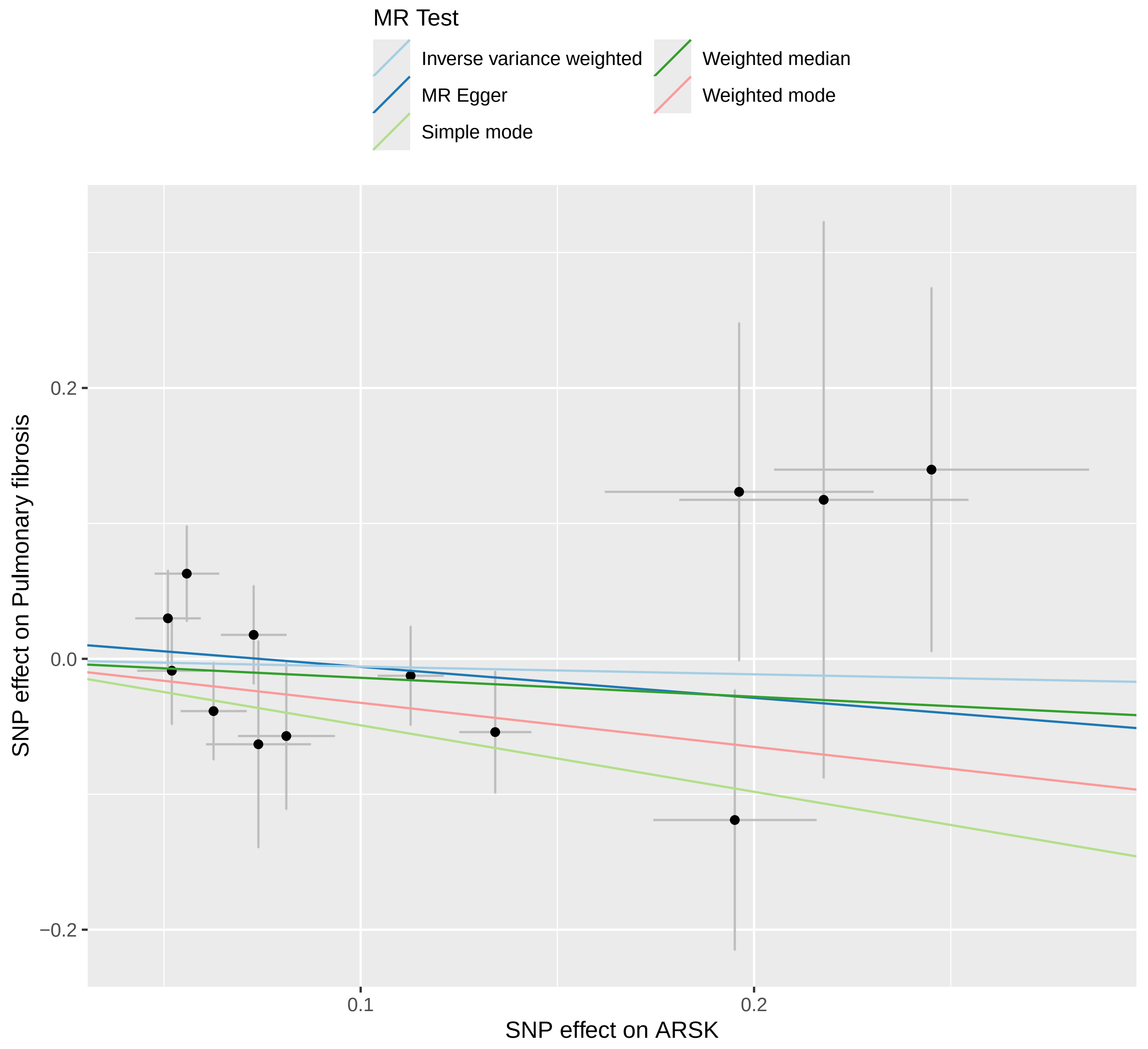
MR Test
Inverse variance weighted
MR Egger
Simple mode
Weighted median
Weighted mode
0.2
0.0
−0.2
0.1
0.2
SNP effect on Pulmonary fibrosis
SNP effect on ARSK

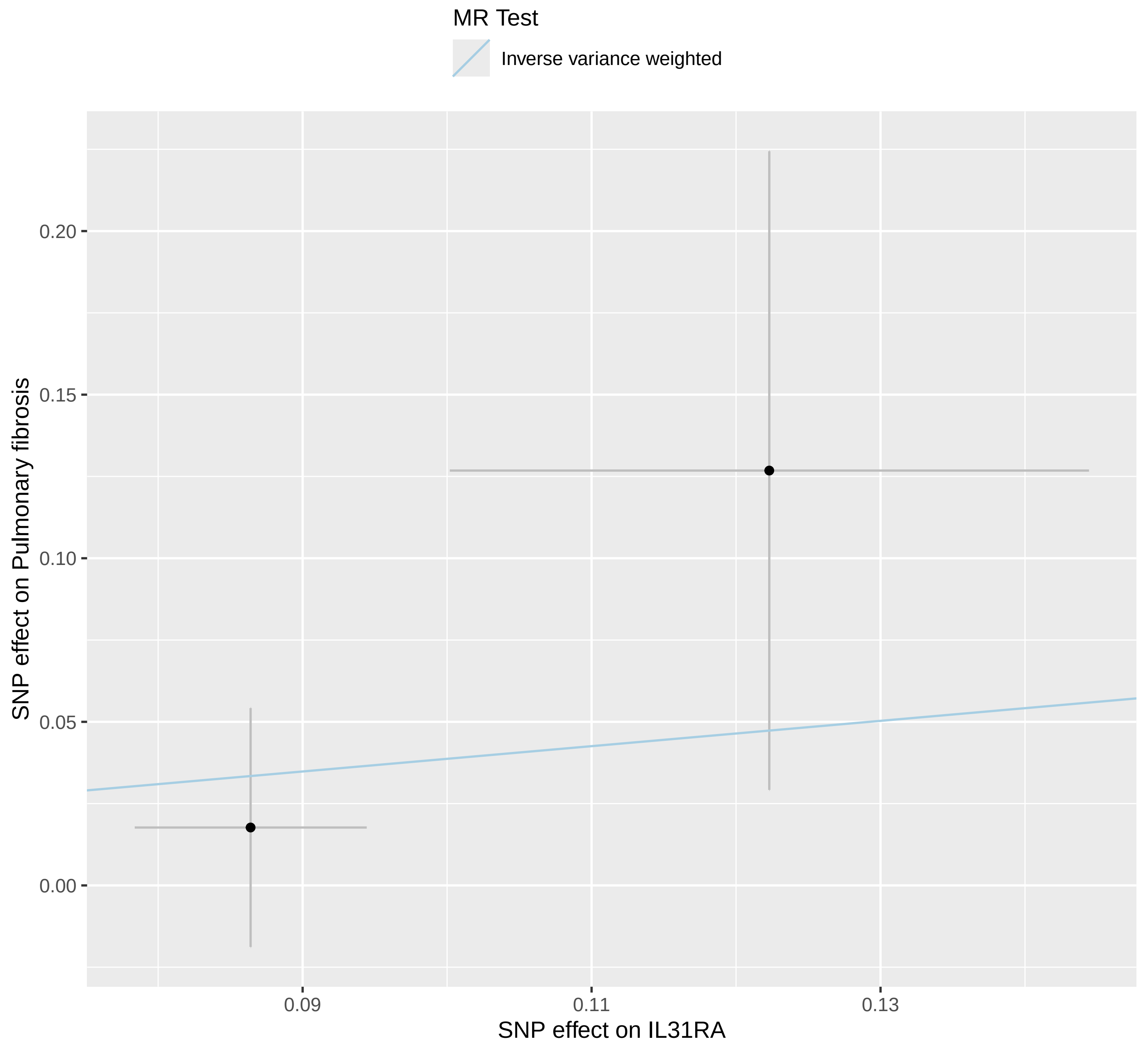

MR Test
Inverse variance weighted
SNP effect on Pulmonary fibrosis
0.20
0.15
0.10
0.05
0.00
0.09
0.11
0.13
SNP effect on IL31RA

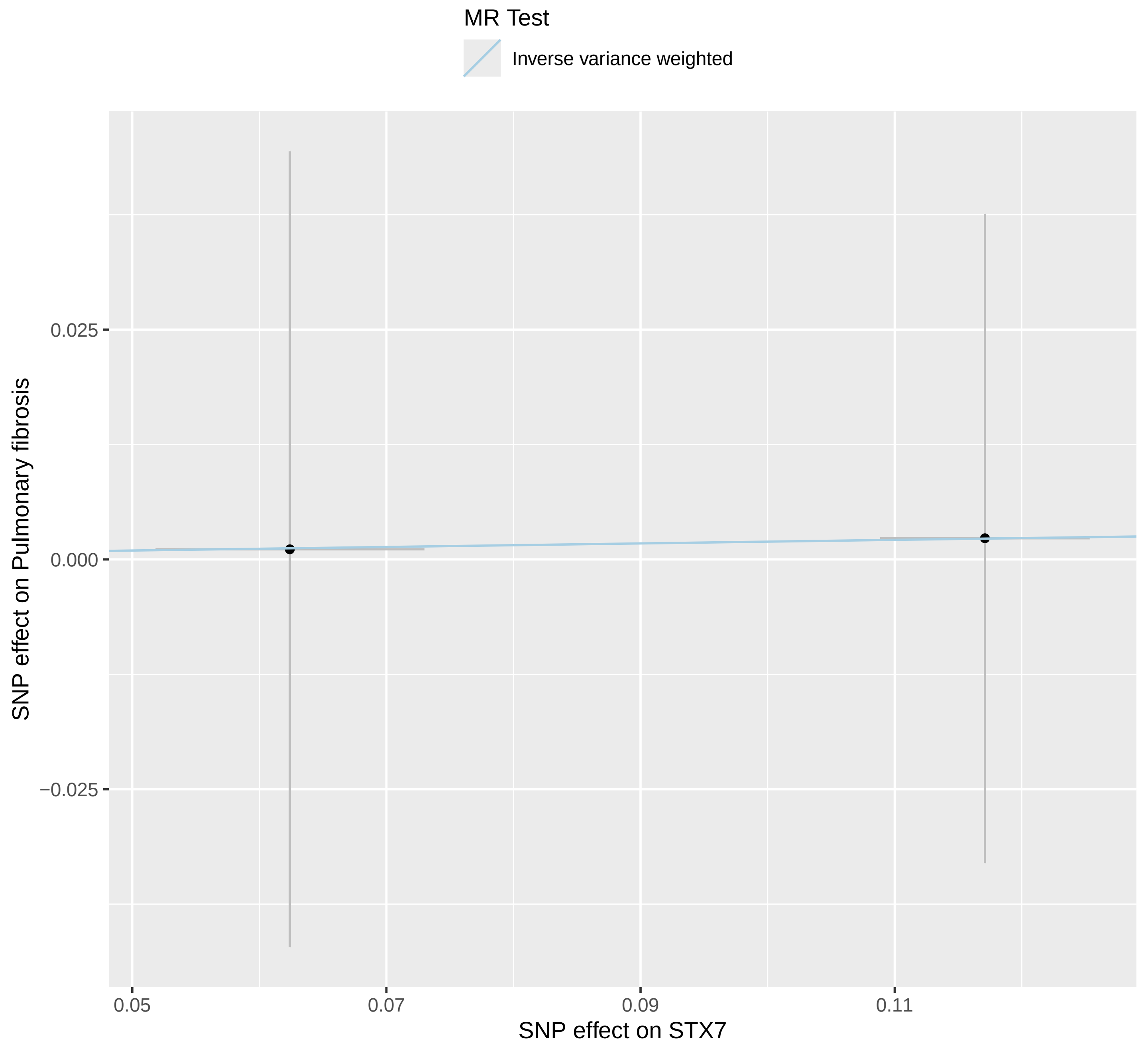

MR Test
Inverse variance weighted
SNP effect on Pulmonary fibrosis
0.025
0.000
−0.025
0.05
0.07
0.09
0.11
SNP effect on STX7

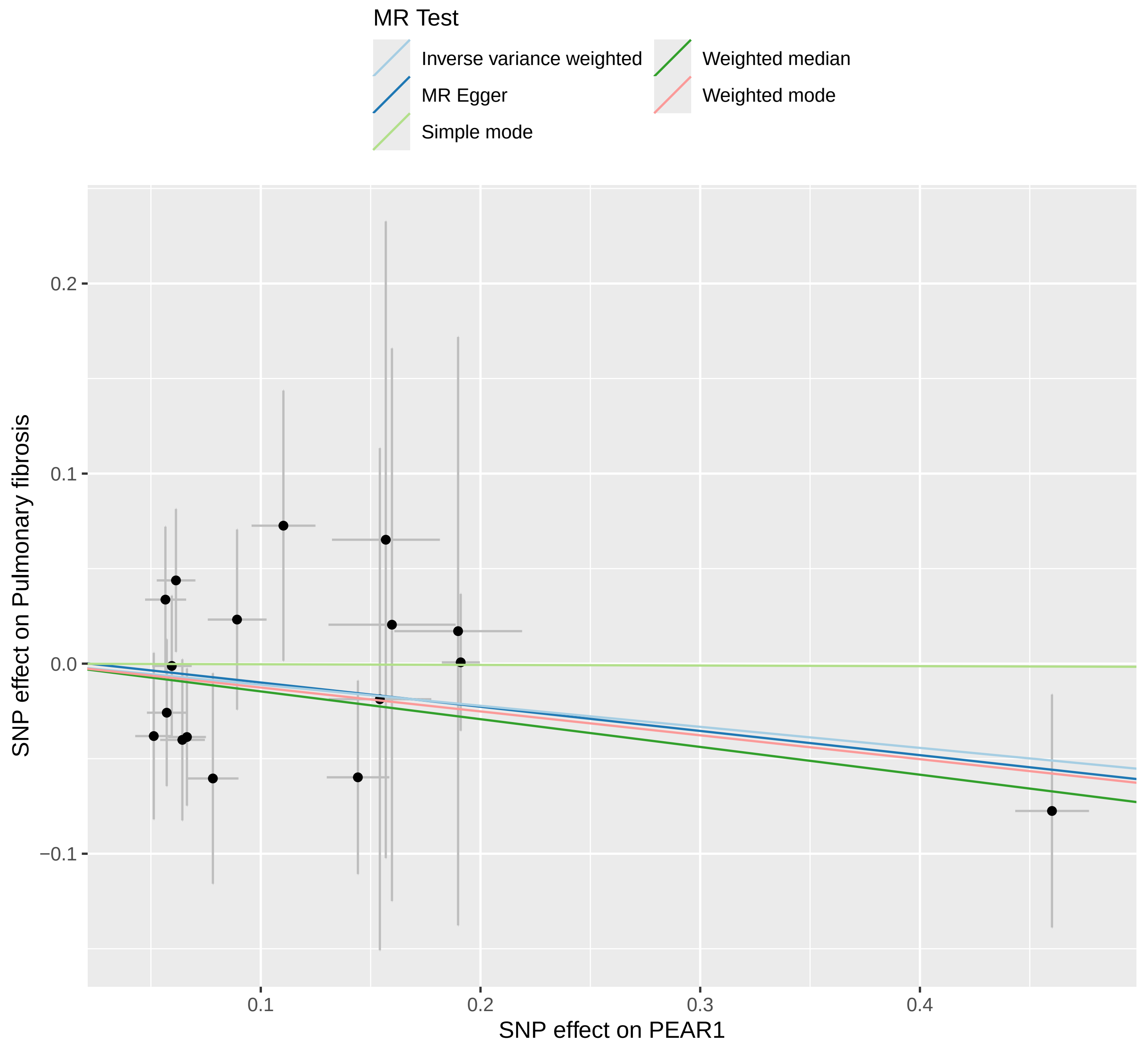

MR Test
Inverse variance weighted
MR Egger
Simple mode
Weighted median
Weighted mode
0.2
0.1
0.0
−0.1
0.1
0.2
0.3
0.4
SNP effect on Pulmonary fibrosis
SNP effect on PEAR1

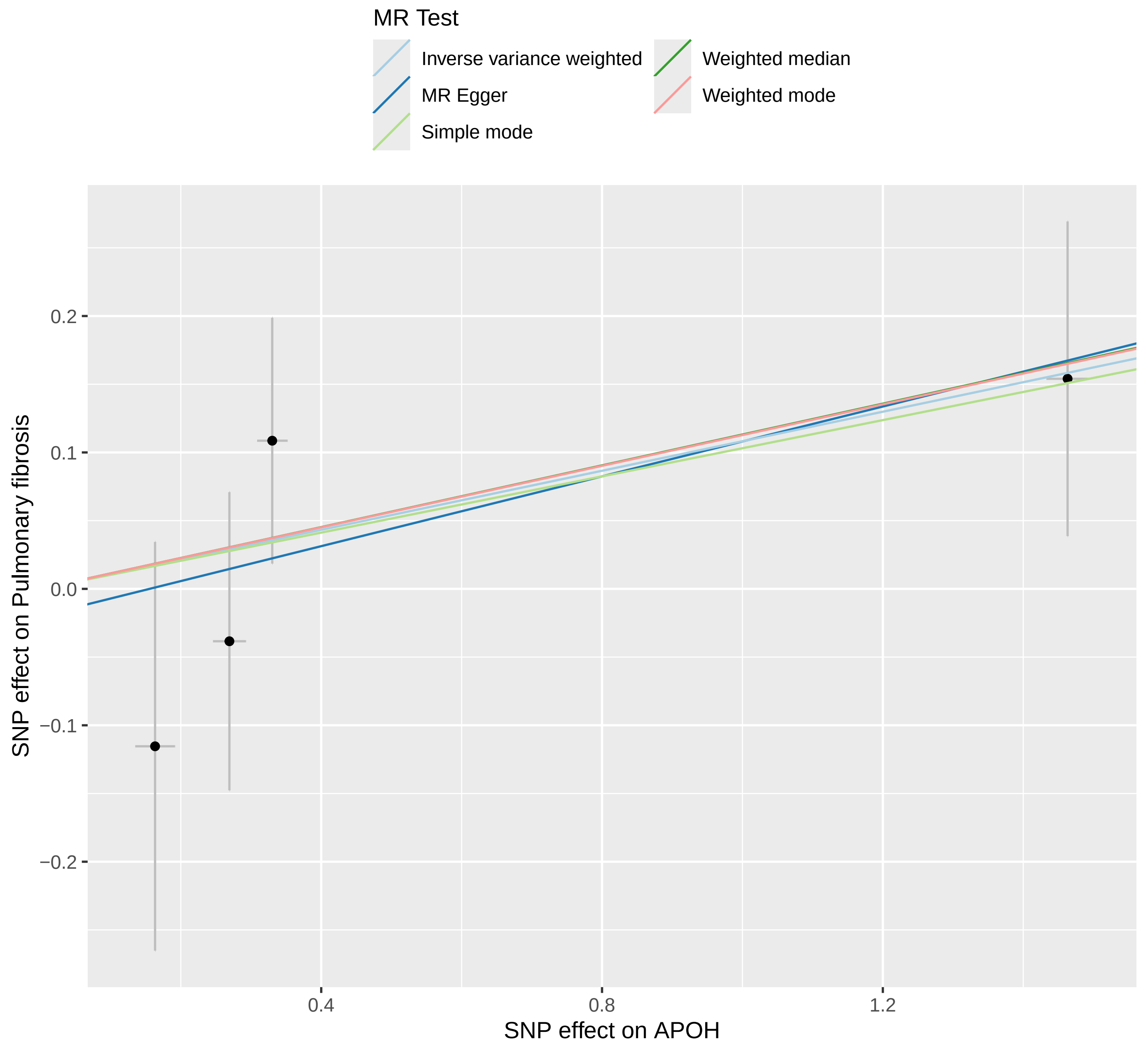

MR Test
Inverse variance weighted
MR Egger
Simple mode
Weighted median
Weighted mode
0.2
0.1
0.0
−0.1
−0.2
0.4
0.8
1.2
SNP effect on Pulmonary fibrosis
SNP effect on APOH

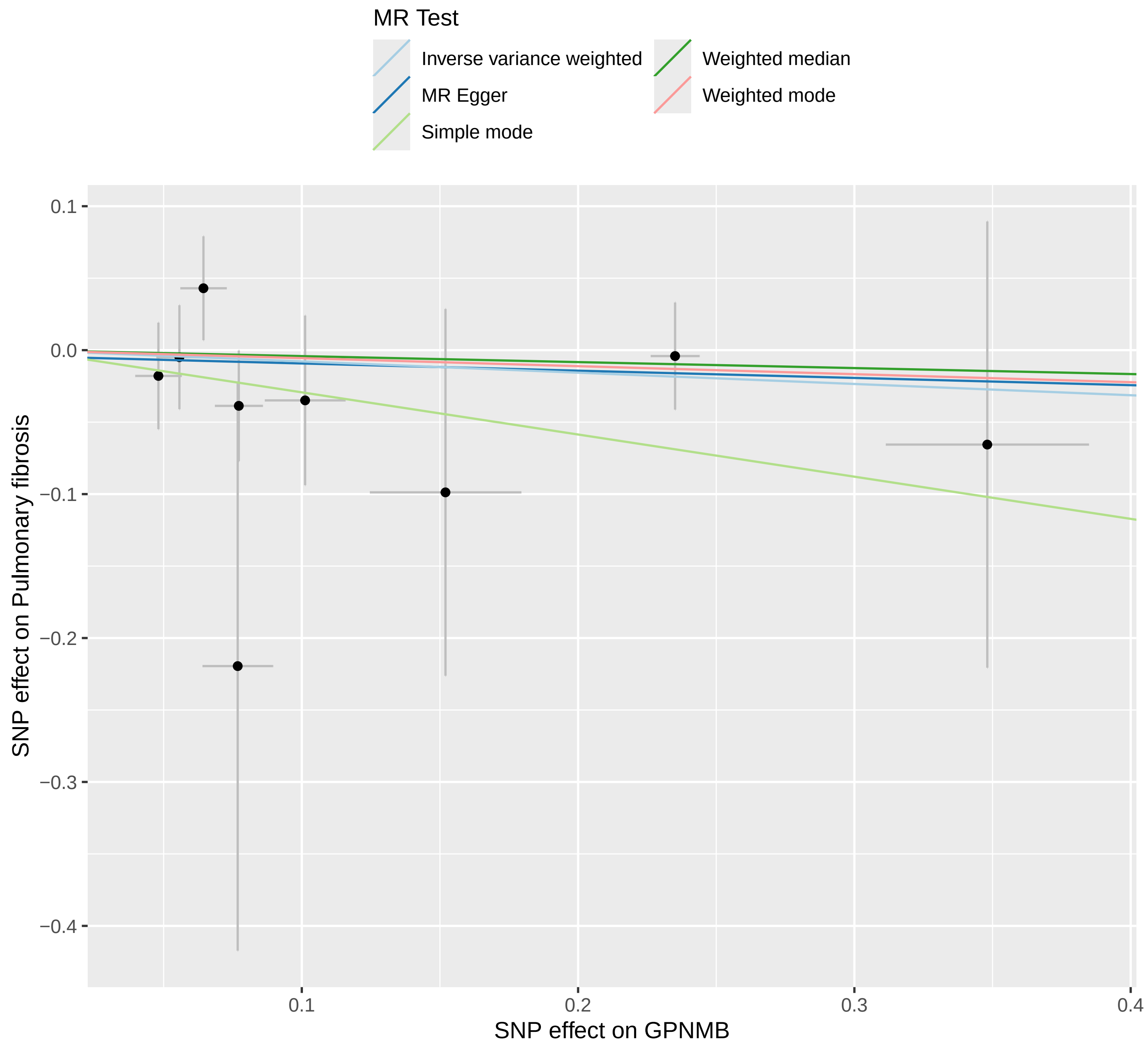

MR Test
Inverse variance weighted
MR Egger
Simple mode
Weighted median
Weighted mode
0.1
0.0
−0.1
−0.2
−0.3
−0.4
SNP effect on Pulmonary fibrosis
0.1
0.2
0.3
0.4
SNP effect on GPNMB

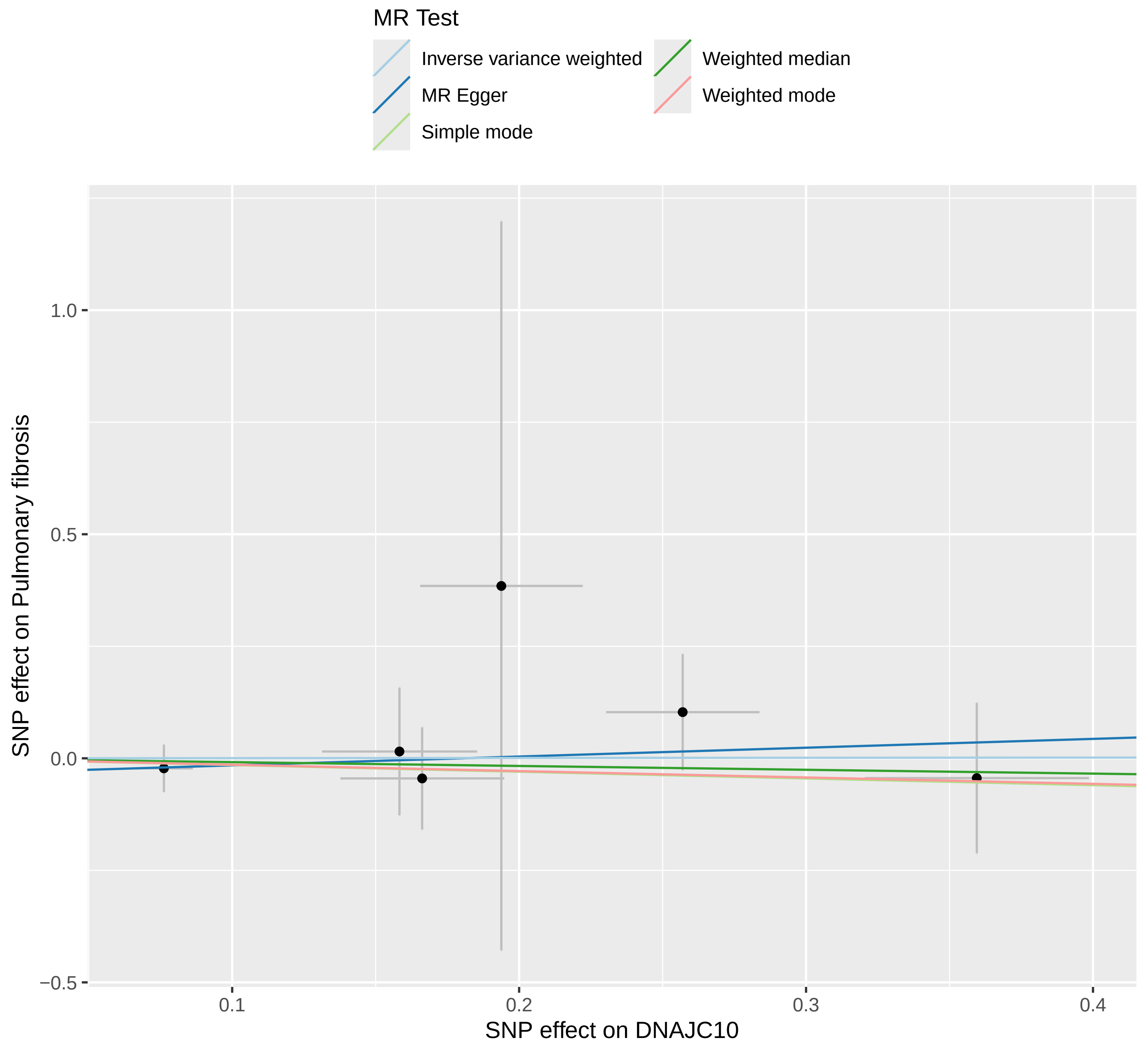
MR Test
Inverse variance weighted
MR Egger
Simple mode
Weighted median
Weighted mode
1.0
0.5
0.0
−0.5
0.1
0.2
0.3
0.4
SNP effect on Pulmonary fibrosis
SNP effect on DNAJC10

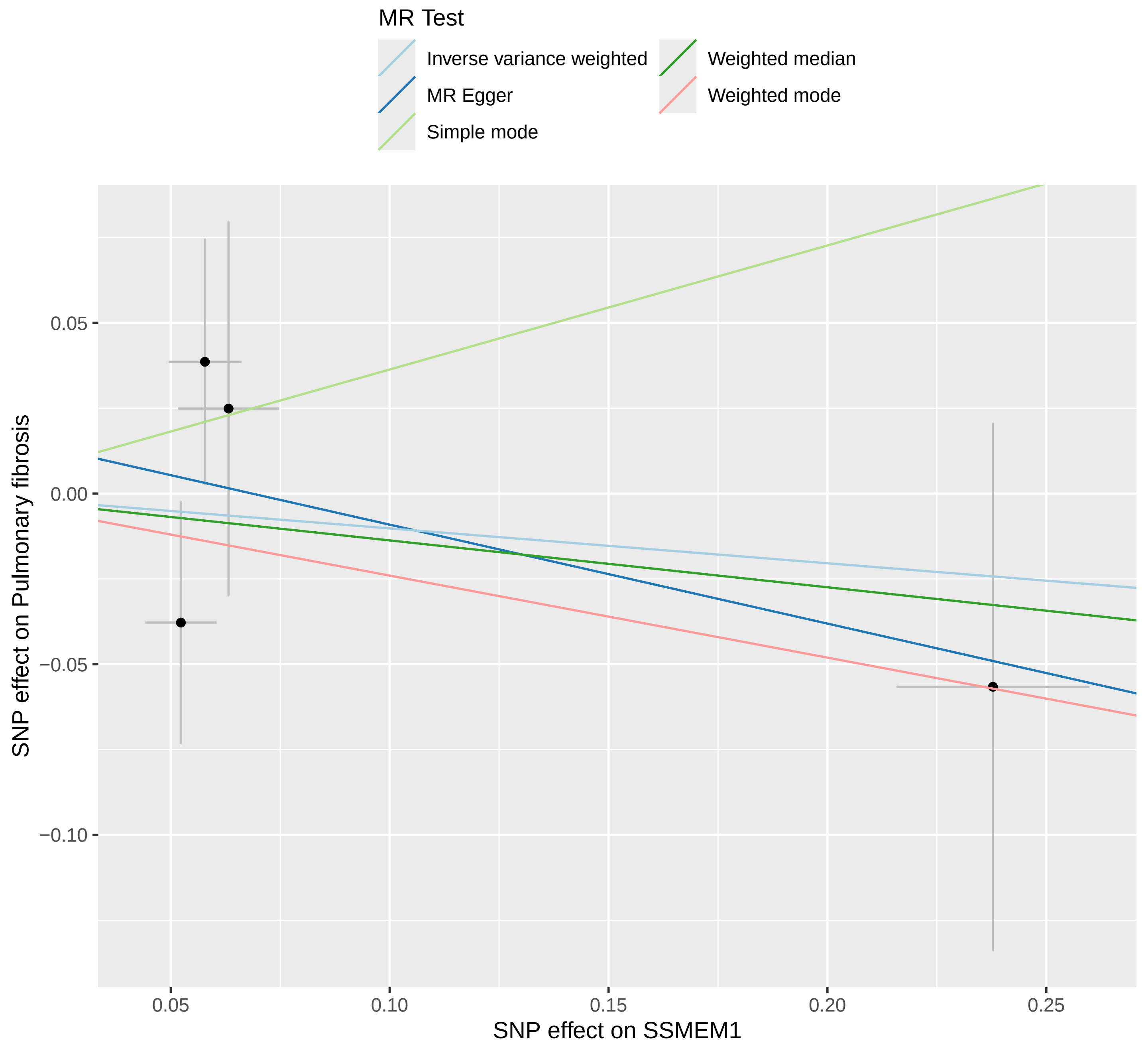
MR Test
Inverse variance weighted
MR Egger
Simple mode
Weighted median
Weighted mode
0.05
0.00
−0.05
−0.10
0.05
0.10
0.15
0.20
0.25
SNP effect on Pulmonary fibrosis
SNP effect on SSMEM1

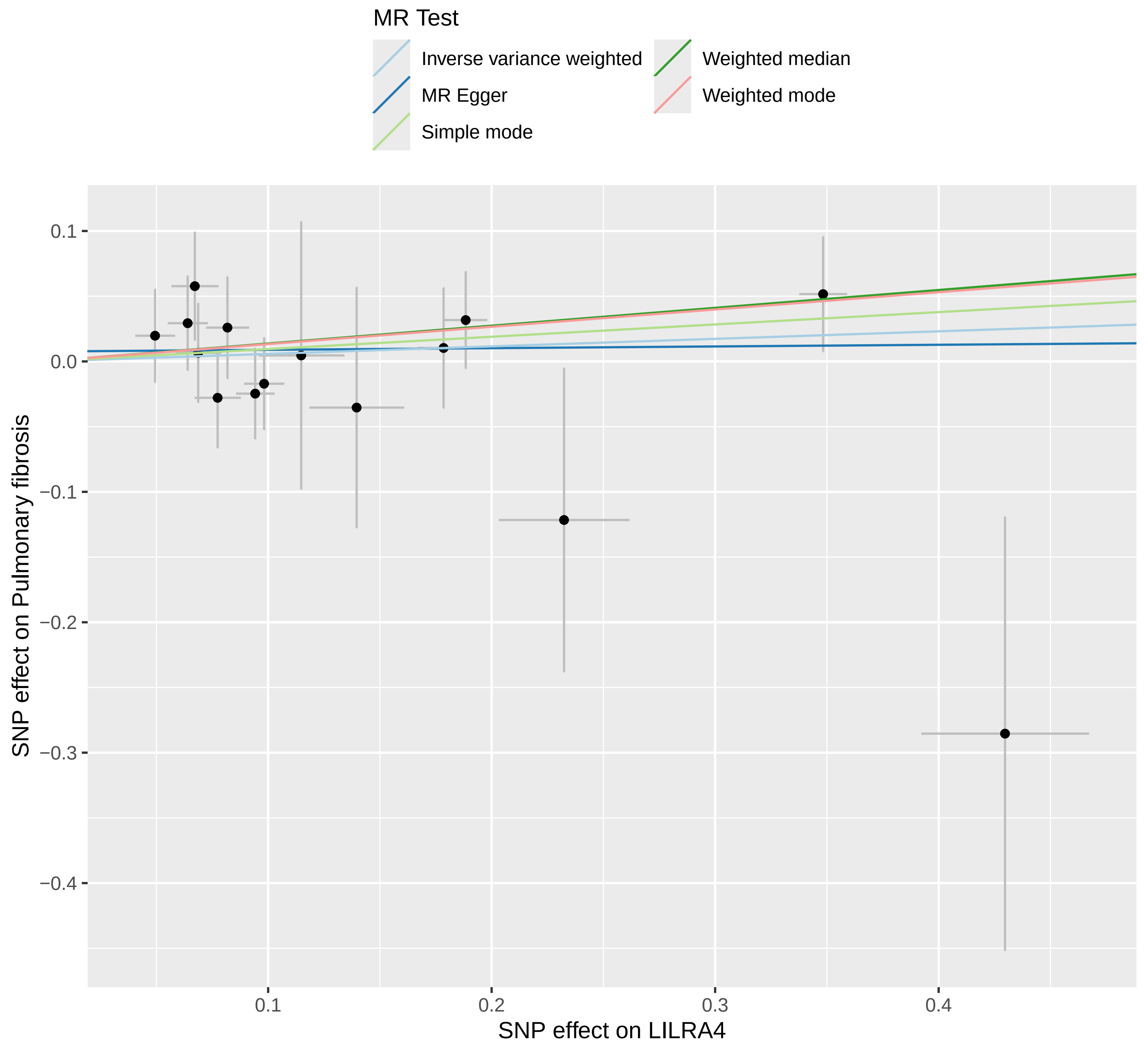
MR Test
Inverse variance weighted
MR Egger
Simple mode
Weighted median
Weighted mode
0.1
0.0
−0.1
−0.2
−0.3
−0.4
SNP effect on Pulmonary fibrosis
0.1
0.2
0.3
0.4
SNP effect on LILRA4

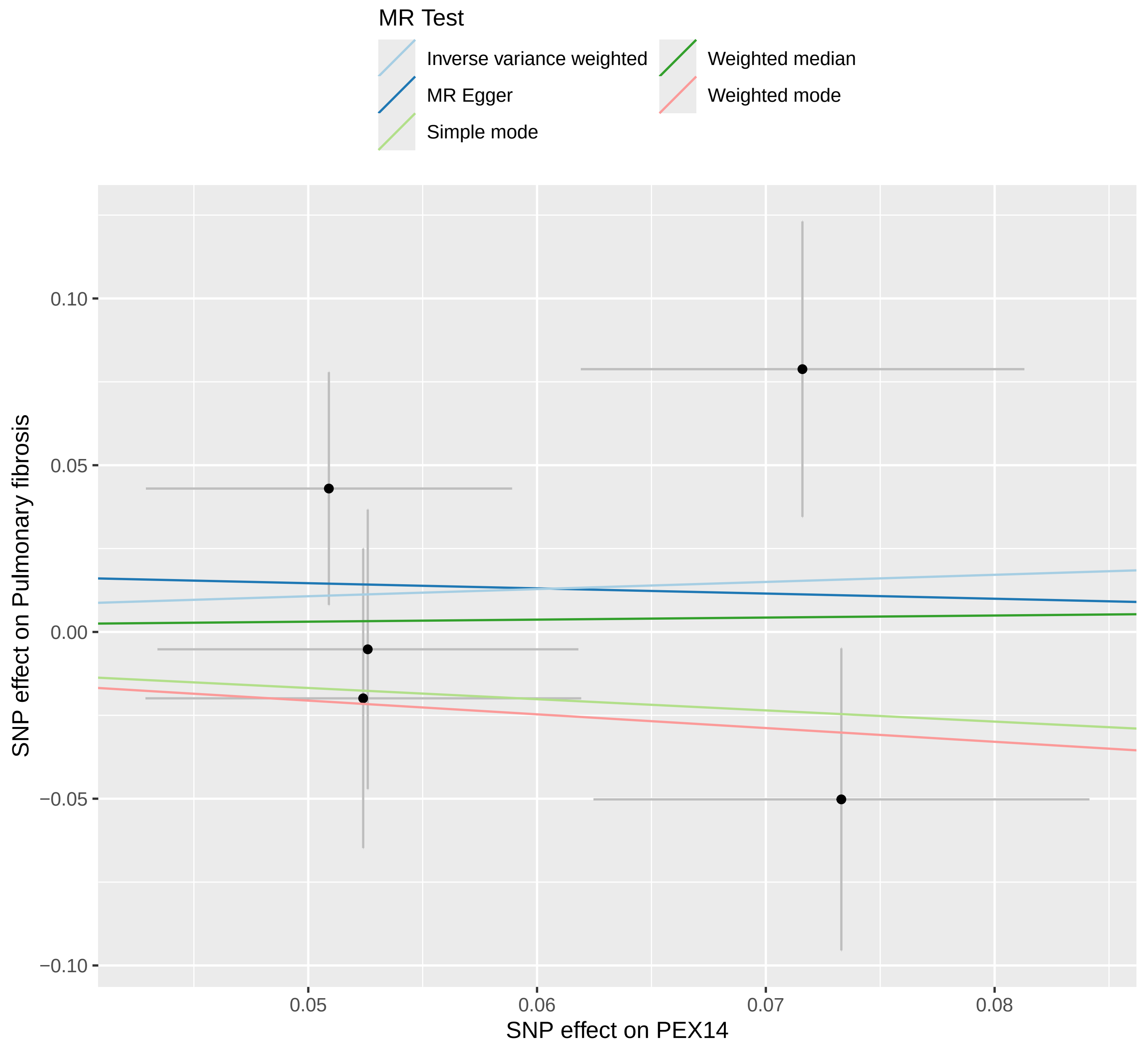

MR Test
Inverse variance weighted
MR Egger
Simple mode
Weighted median
Weighted mode
0.10
0.05
0.00
−0.05
−0.10
SNP effect on Pulmonary fibrosis
0.05
0.06
0.07
0.08
SNP effect on PEX14

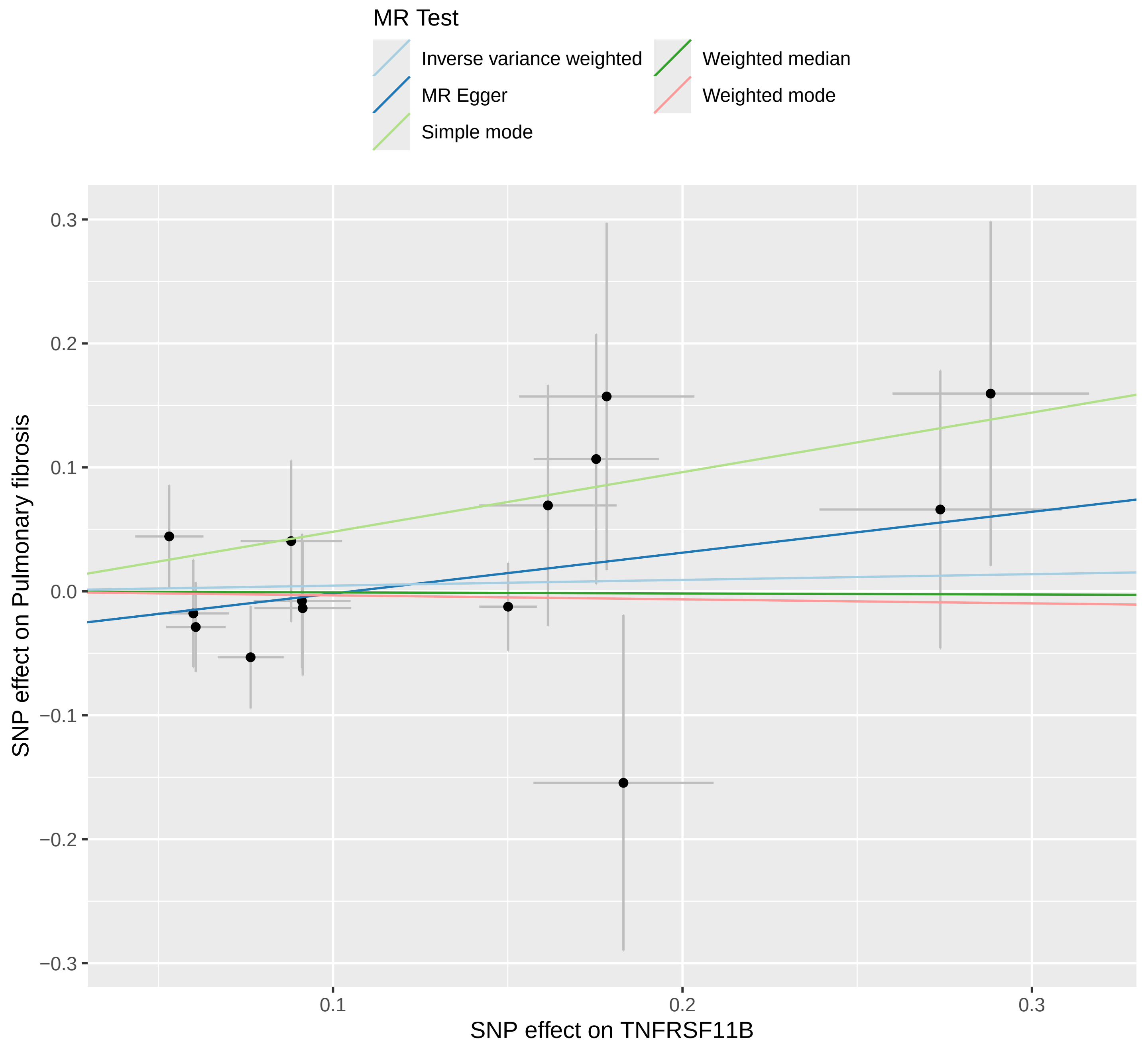

MR Test
Inverse variance weighted
MR Egger
Simple mode
Weighted median
Weighted mode
SNP effect on Pulmonary fibrosis
0.3
0.2
0.1
0.0
−0.1
−0.2
−0.3
0.1
0.2
0.3
SNP effect on TNFRSF11B

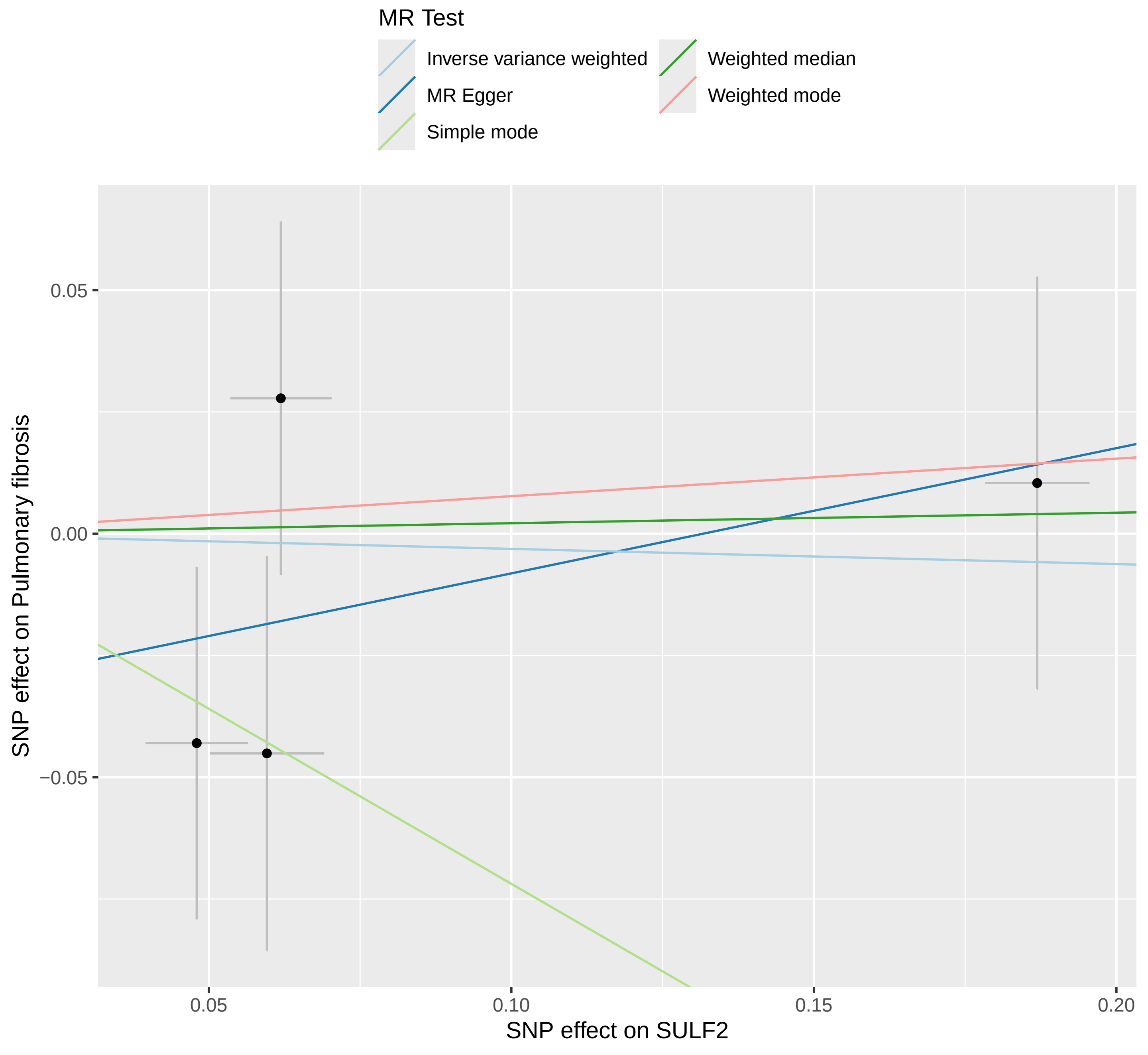

MR Test
Inverse variance weighted
MR Egger
Simple mode
Weighted median
Weighted mode
0.05
0.00
−0.05
SNP effect on Pulmonary fibrosis
0.05
0.10
0.15
0.20
SNP effect on SULF2

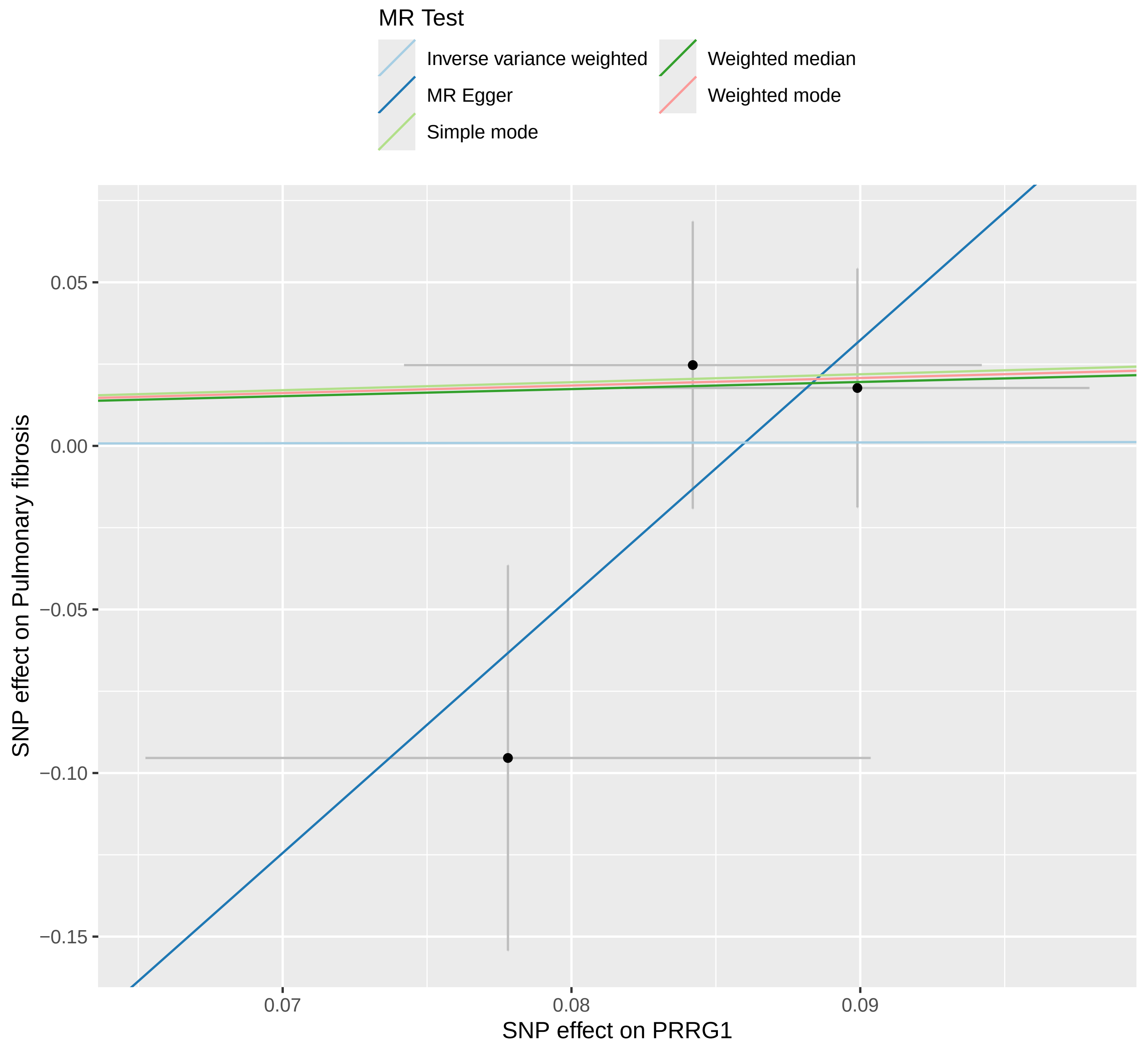
MR Test
Inverse variance weighted
MR Egger
Simple mode
Weighted median
Weighted mode
0.05
0.00
−0.05
−0.10
−0.15
0.07
0.08
0.09
SNP effect on Pulmonary fibrosis
SNP effect on PRRG1

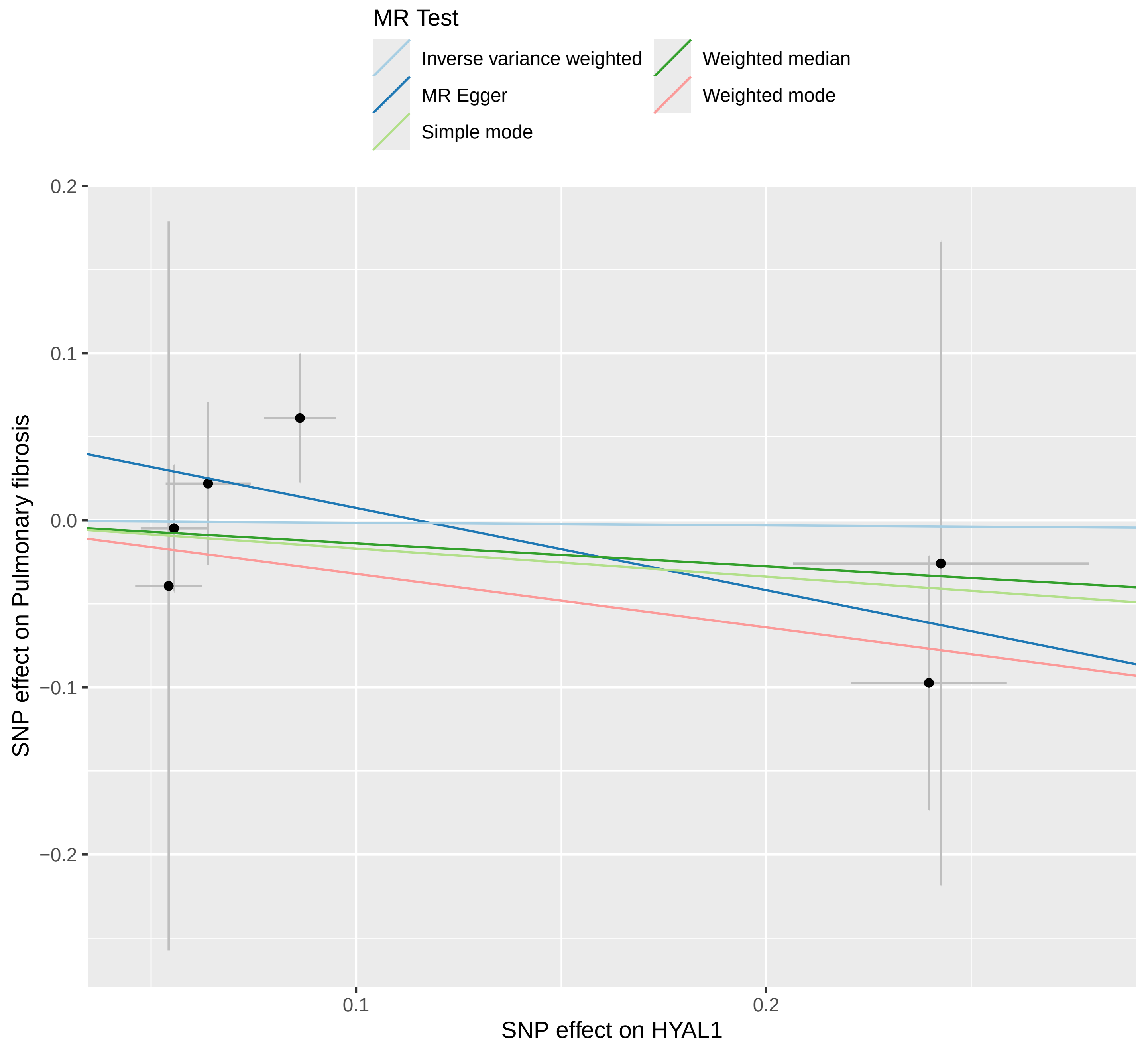
MR Test
Inverse variance weighted
MR Egger
Simple mode
Weighted median
Weighted mode
0.2
0.1
0.0
−0.1
−0.2
SNP effect on Pulmonary fibrosis
0.1
0.2
SNP effect on HYAL1

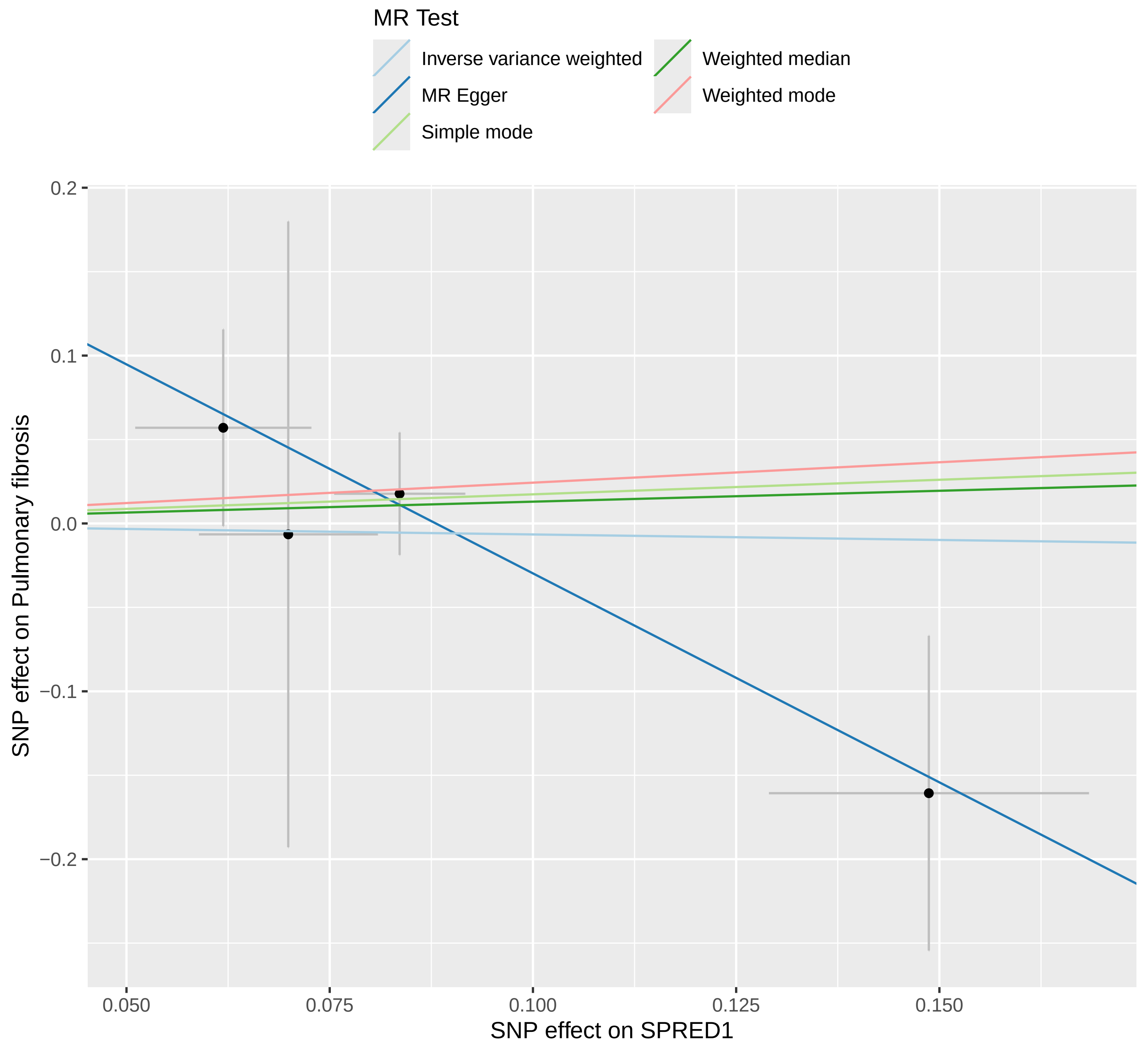

MR Test
Inverse variance weighted
MR Egger
Simple mode
Weighted median
Weighted mode
0.2
0.1
0.0
−0.1
−0.2
0.050
0.075
0.100
0.125
0.150
SNP effect on Pulmonary fibrosis
SNP effect on SPRED1

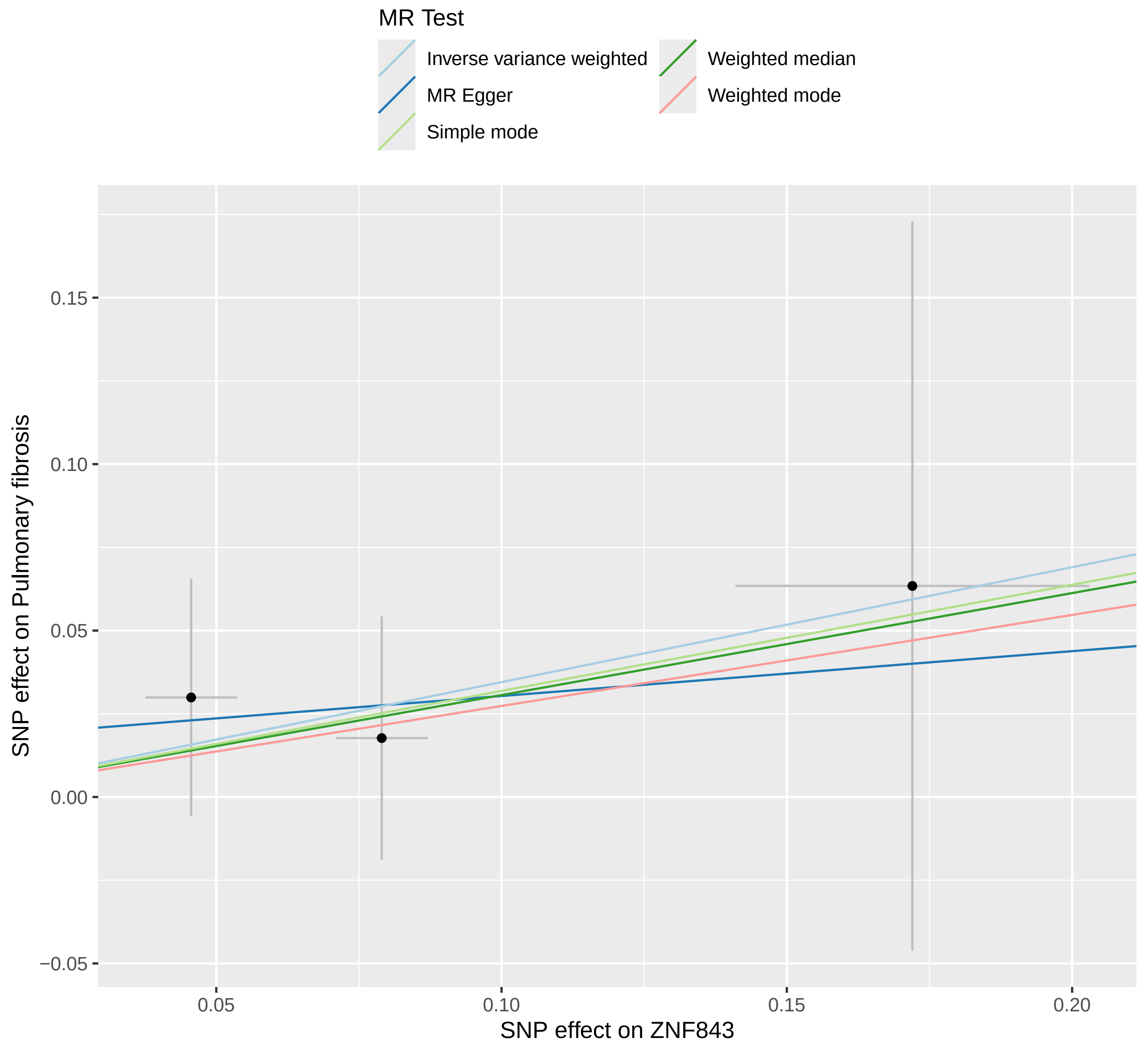
MR Test
Inverse variance weighted
MR Egger
Simple mode
Weighted median
Weighted mode
0.15
0.10
0.05
0.00
−0.05
0.05
0.10
0.15
0.20
SNP effect on Pulmonary fibrosis
SNP effect on ZNF843

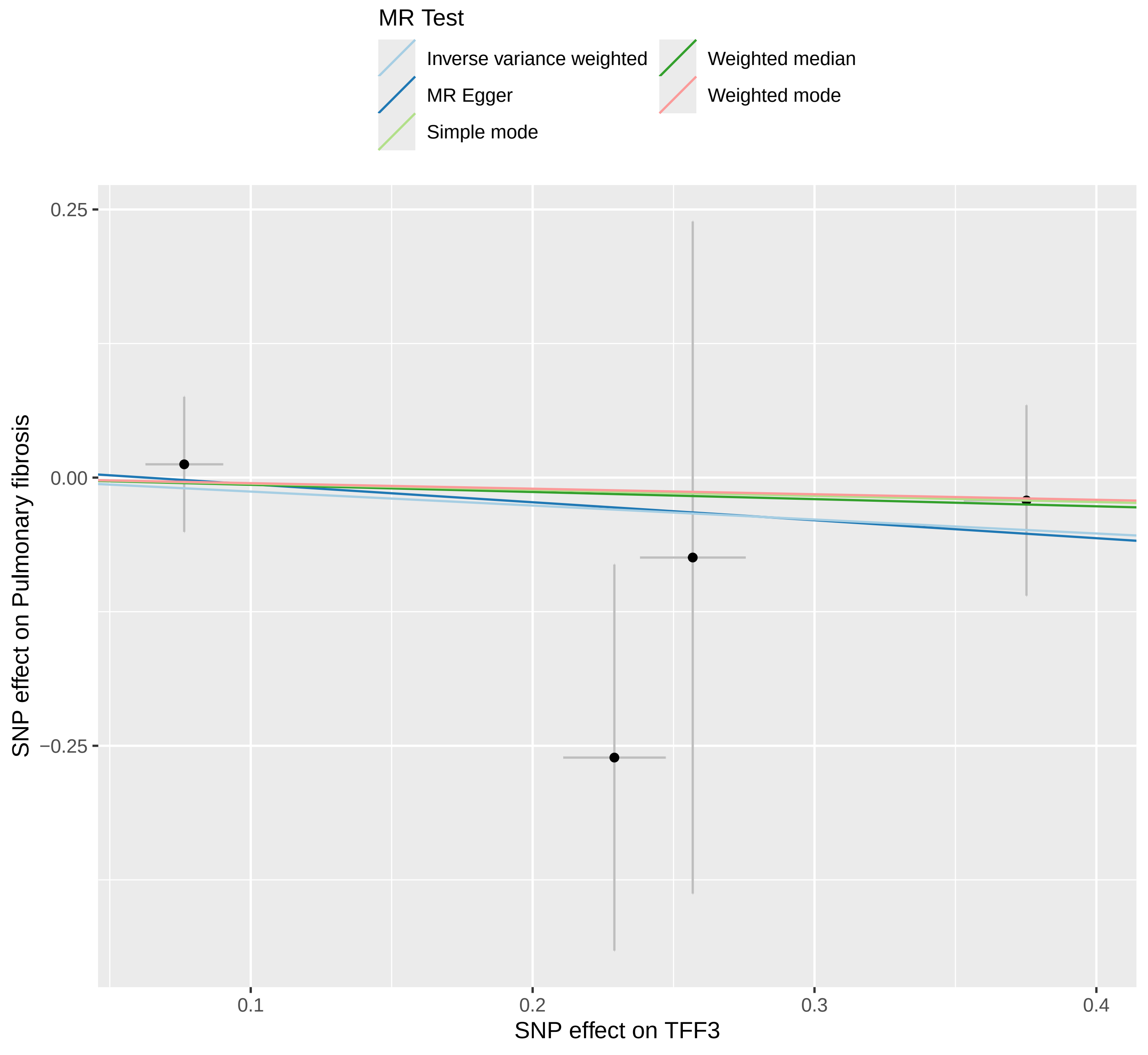
MR Test
Inverse variance weighted
MR Egger
Simple mode
Weighted median
Weighted mode
0.25
0.00
−0.25
SNP effect on Pulmonary fibrosis
0.1
0.2
0.3
0.4
SNP effect on TFF3

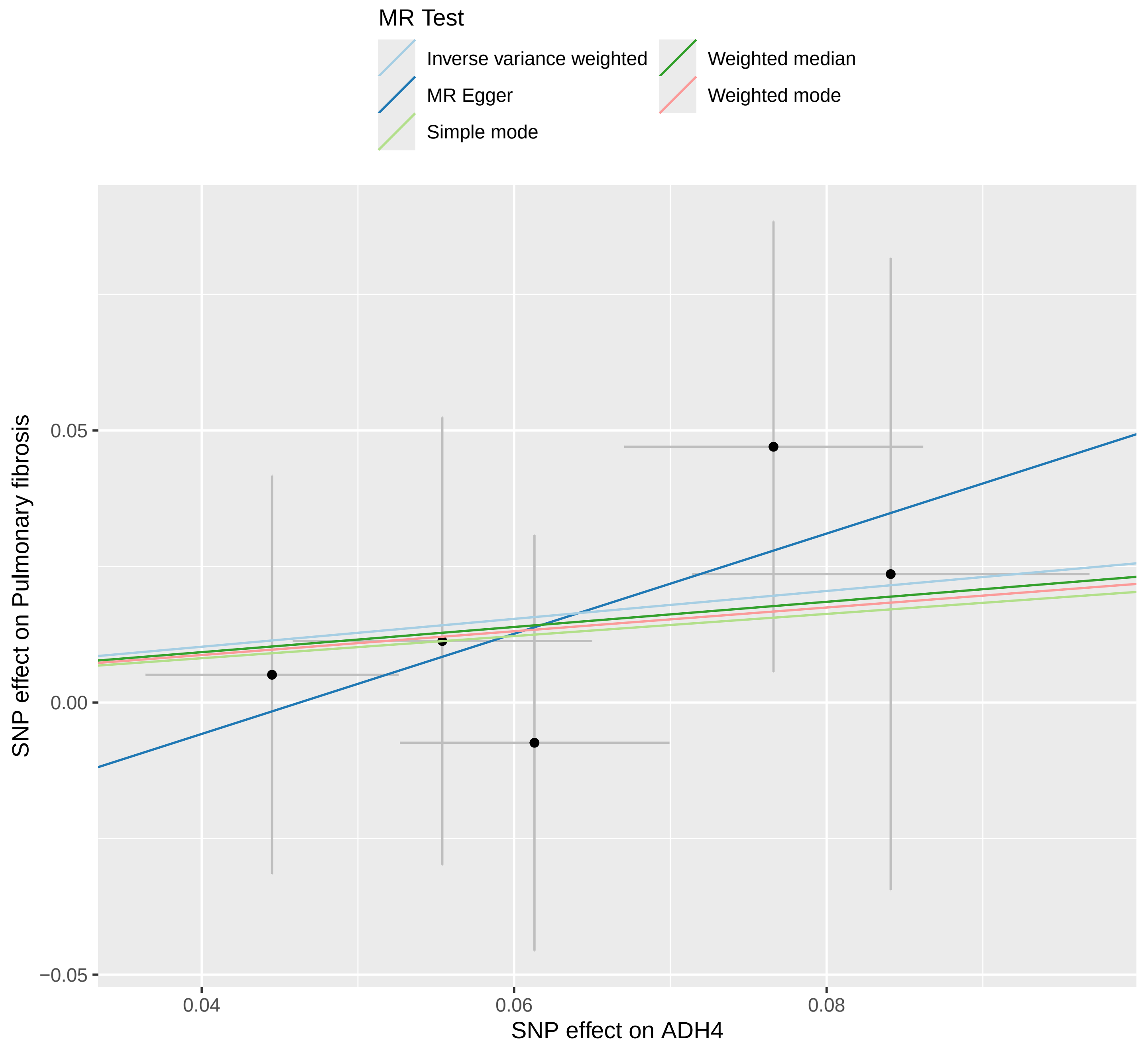
MR Test
Inverse variance weighted
MR Egger
Simple mode
Weighted median
Weighted mode
SNP effect on Pulmonary fibrosis
0.05
0.00
−0.05
0.04
0.06
0.08
SNP effect on ADH4

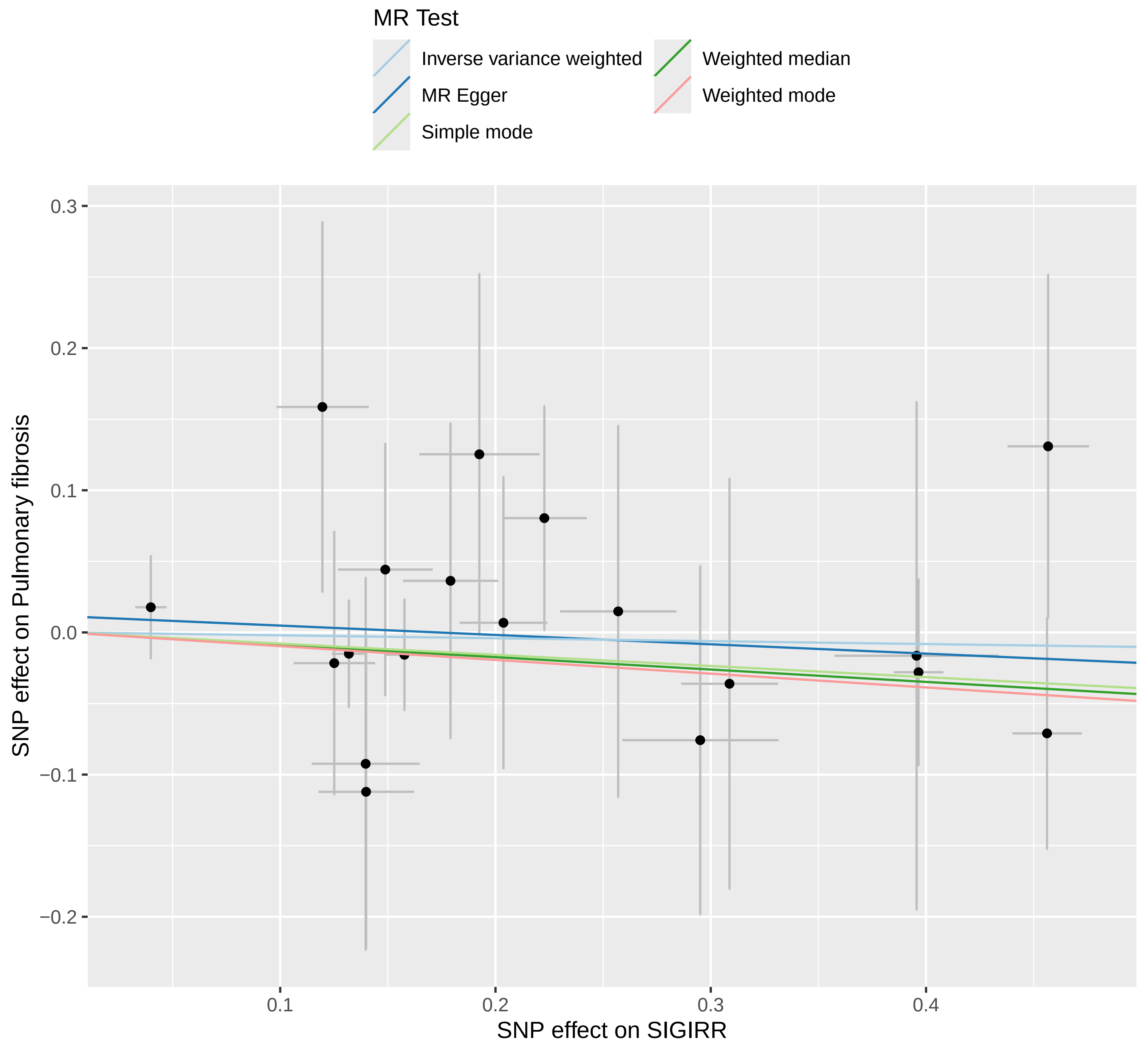

MR Test
Inverse variance weighted
MR Egger
Simple mode
Weighted median
Weighted mode
0.3
0.2
0.1
0.0
−0.1
−0.2
SNP effect on Pulmonary fibrosis
0.1
0.2
0.3
0.4
SNP effect on SIGIRR

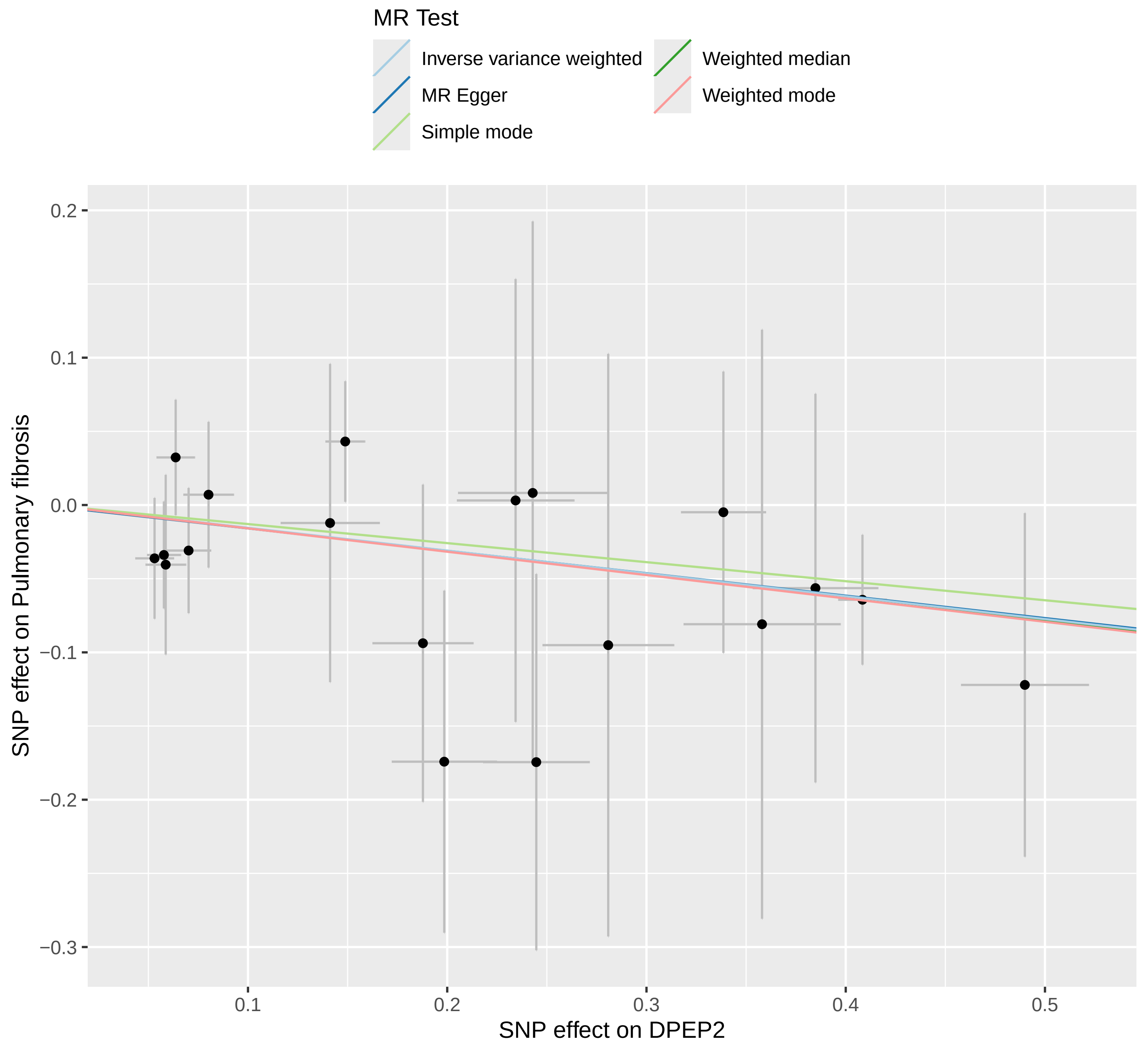

MR Test
Inverse variance weighted
MR Egger
Simple mode
Weighted median
Weighted mode
SNP effect on Pulmonary fibrosis
0.2
0.1
0.0
−0.1
−0.2
−0.3
0.1
0.2
0.3
0.4
0.5
SNP effect on DPEP2

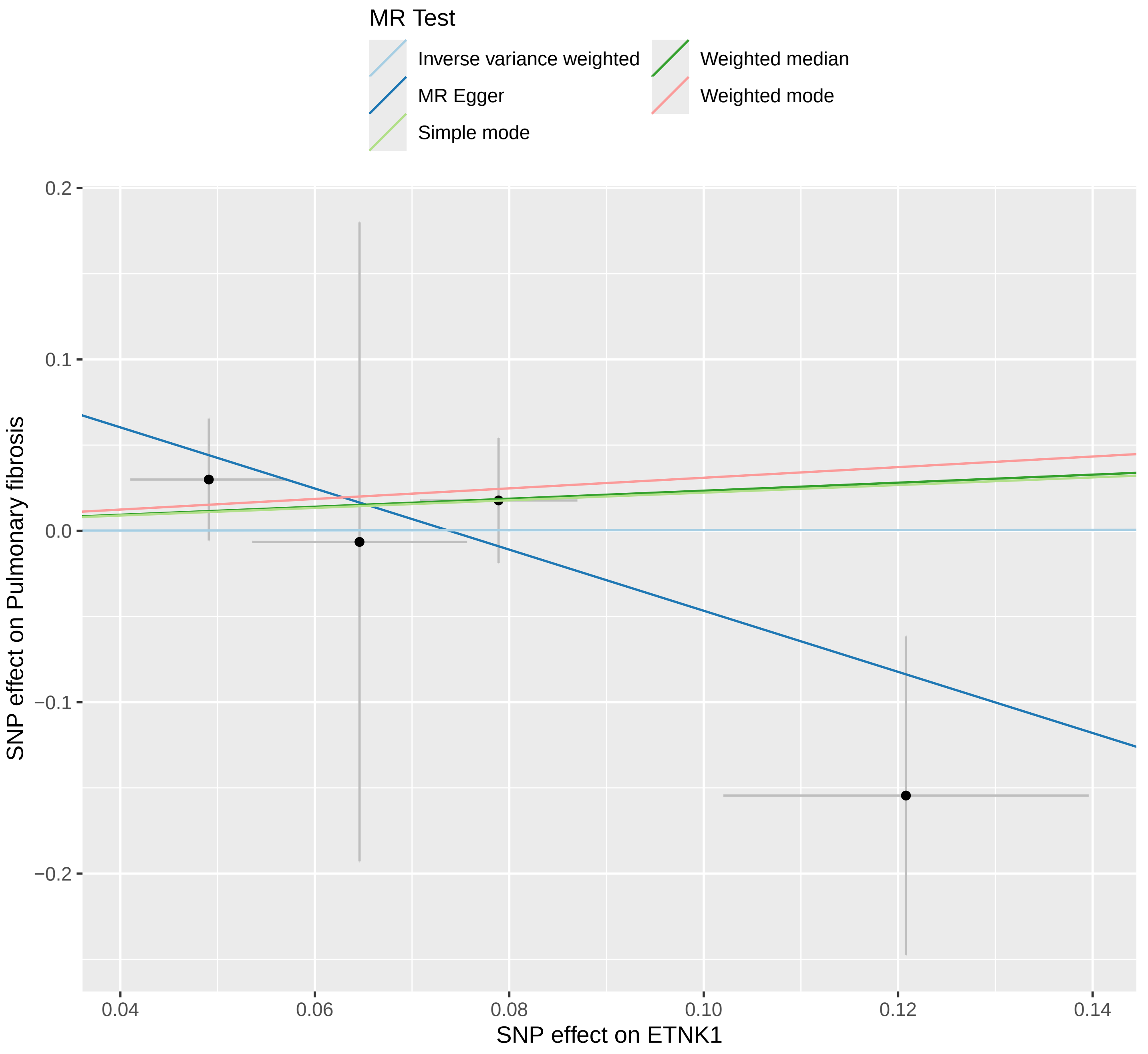

MR Test
Inverse variance weighted
MR Egger
Simple mode
Weighted median
Weighted mode
SNP effect on Pulmonary fibrosis
0.2
0.1
0.0
−0.1
−0.2
0.04
0.06
0.08
0.10
0.12
0.14
SNP effect on ETNK1

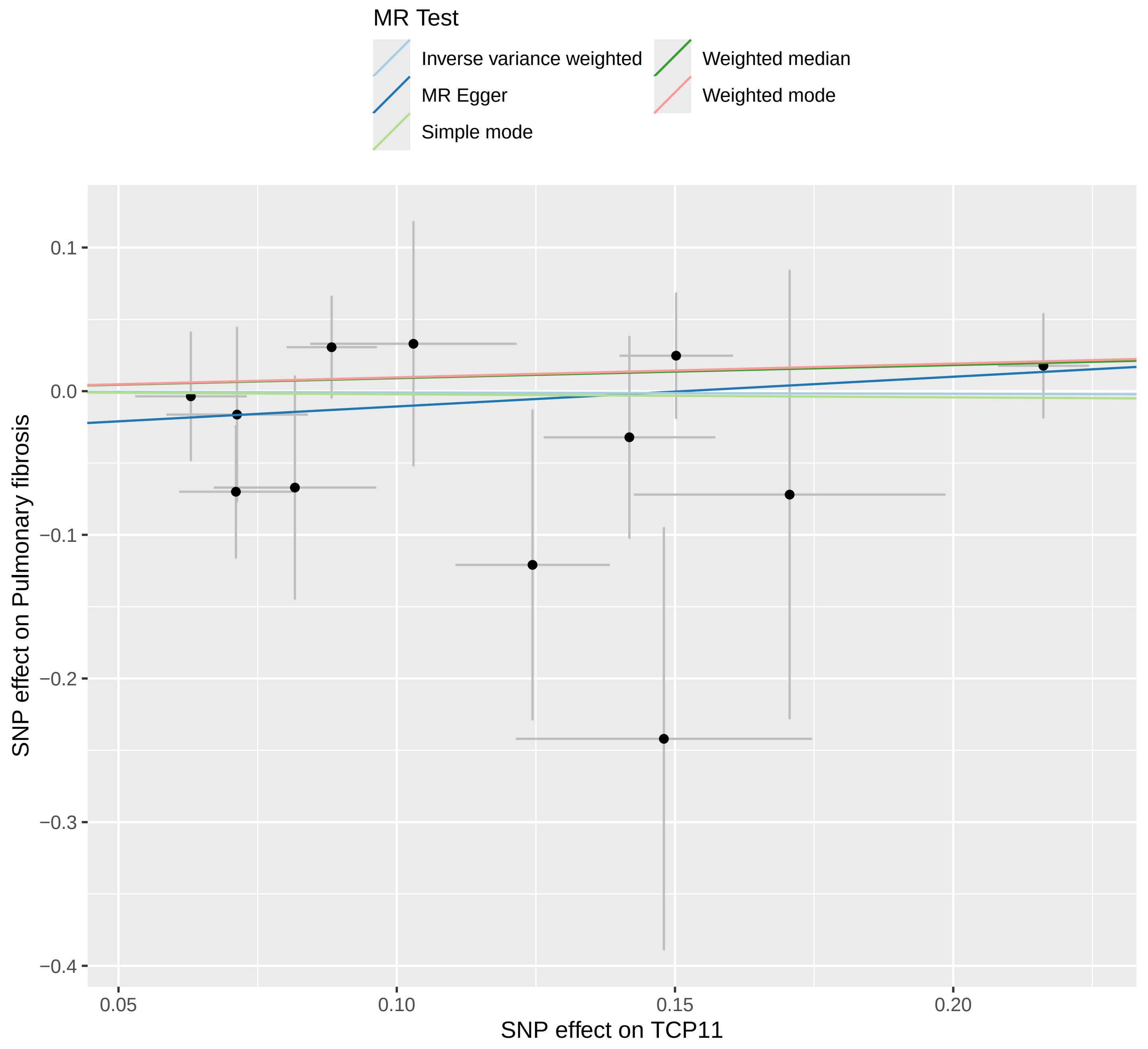
MR Test
Inverse variance weighted
Weighted median
MR Egger
Weighted mode
Simple mode
0.1
0.0
−0.1
−0.2
−0.3
−0.4
SNP effect on Pulmonary fibrosis
0.05
0.10
0.15
0.20
SNP effect on TCP11

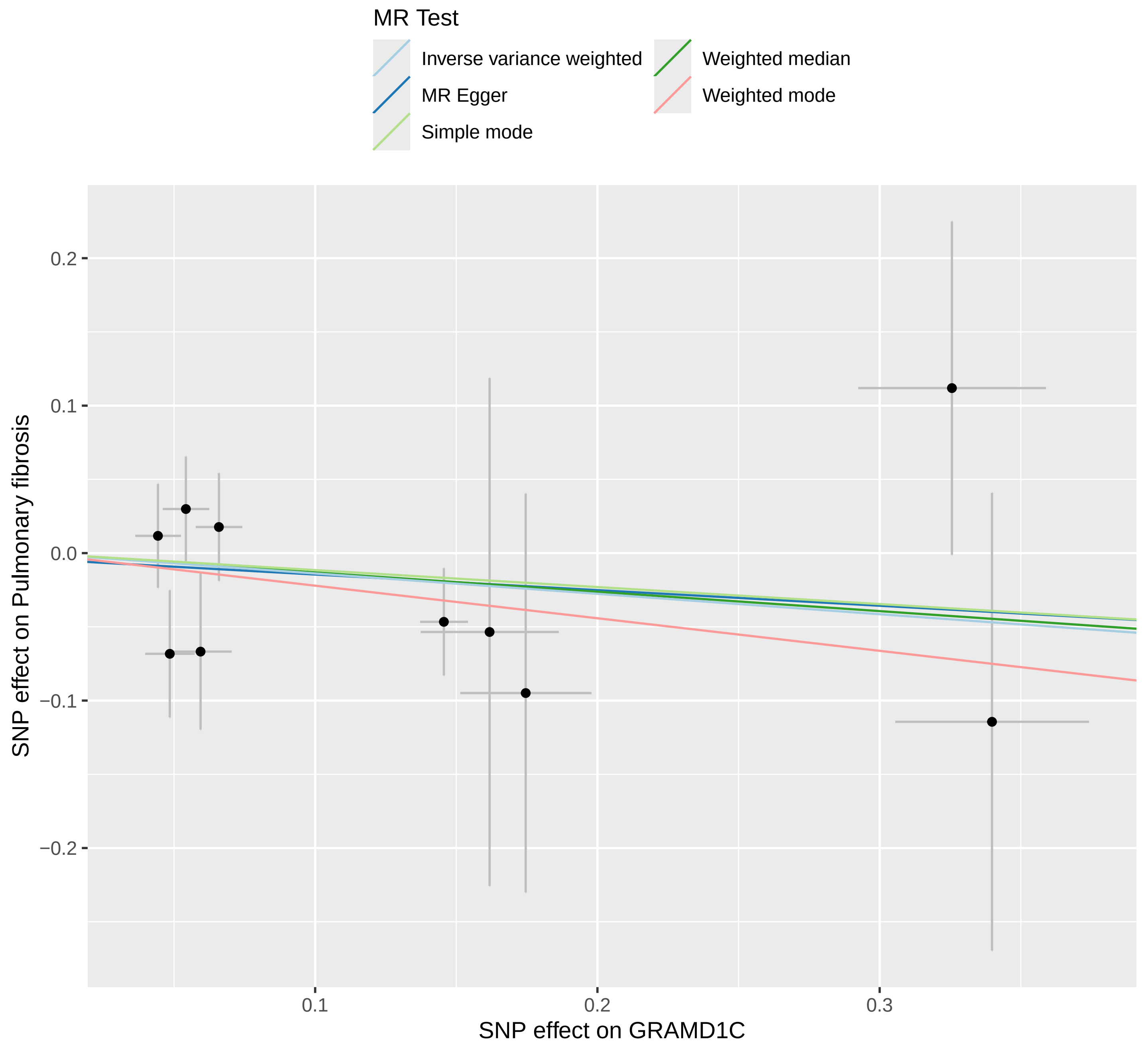

MR Test
Inverse variance weighted
MR Egger
Simple mode
Weighted median
Weighted mode
0.2
0.1
0.0
−0.1
−0.2
0.1
0.2
0.3
SNP effect on GRAMD1C
SNP effect on Pulmonary fibrosis

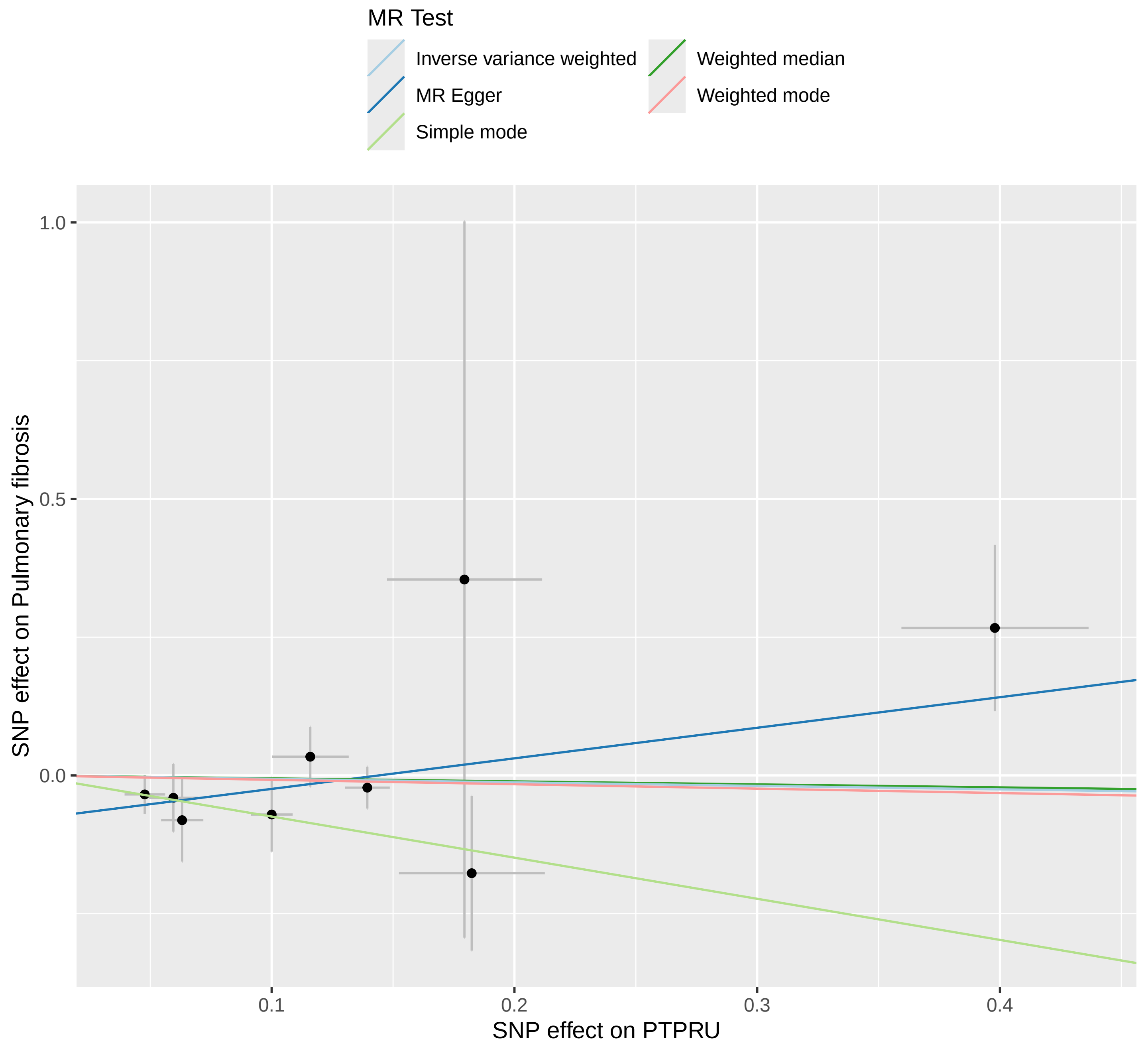

MR Test
Inverse variance weighted
MR Egger
Simple mode
Weighted median
Weighted mode
SNP effect on Pulmonary fibrosis
1.0
0.5
0.0
0.1
0.2
0.3
0.4
SNP effect on PTPRU

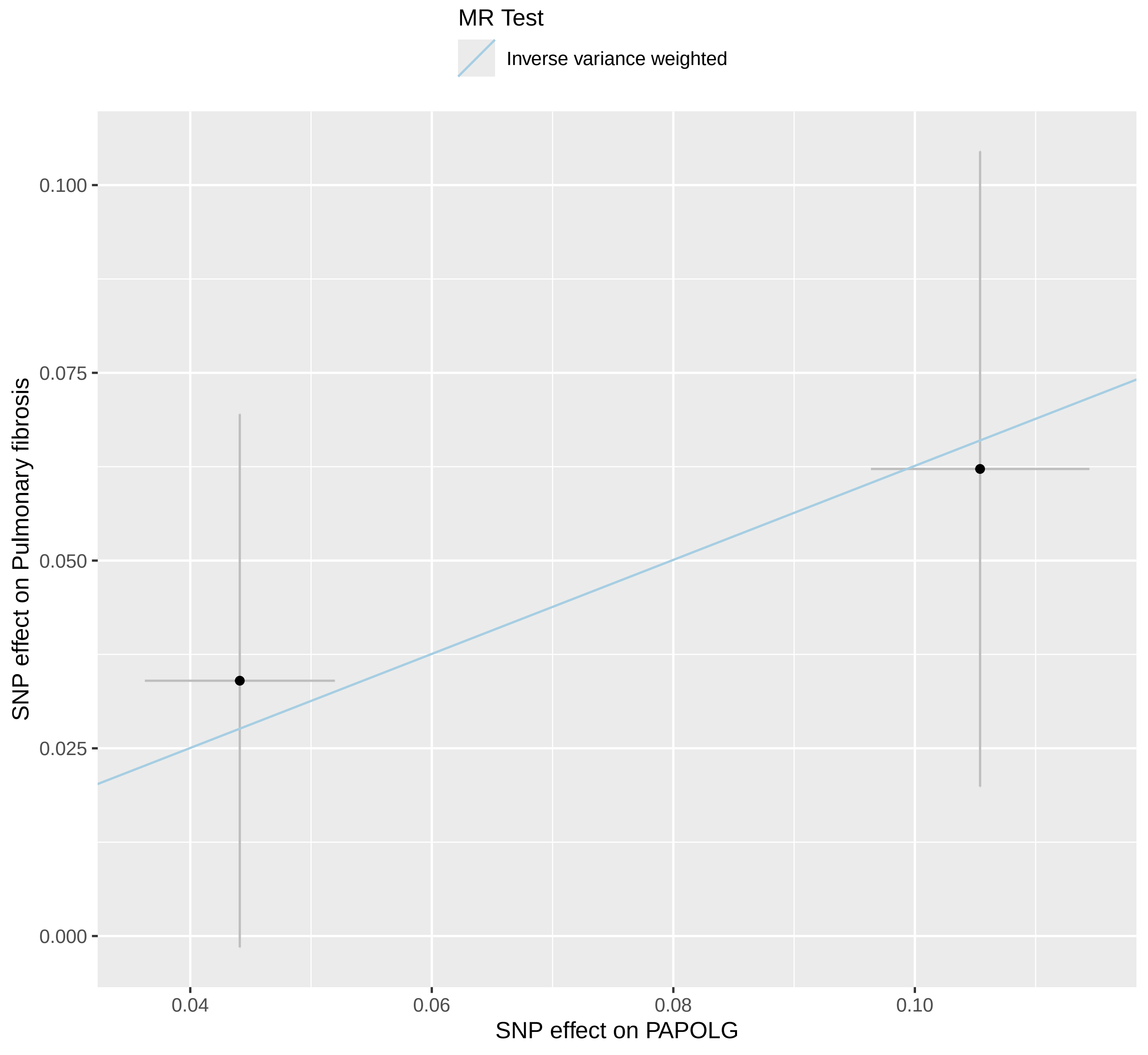
MR Test
Inverse variance weighted
0.100
0.075
0.050
0.025
0.000
SNP effect on Pulmonary fibrosis
0.04
0.06
0.08
0.10
SNP effect on PAPOLG

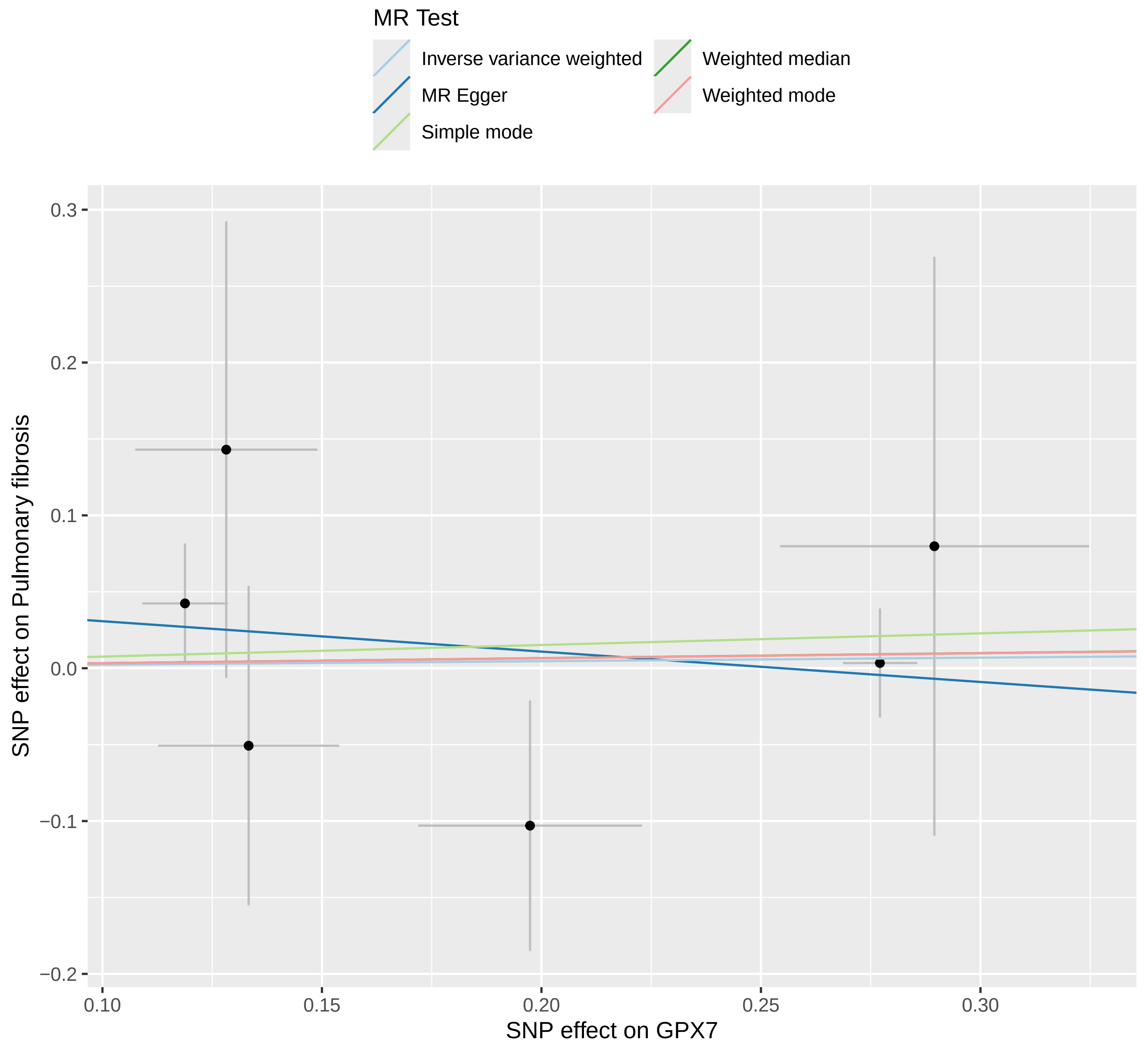
MR Test
Inverse variance weighted
MR Egger
Simple mode
Weighted median
Weighted mode
0.3
0.2
0.1
0.0
−0.1
−0.2
SNP effect on Pulmonary fibrosis
0.10
0.15
0.20
0.25
0.30
SNP effect on GPX7

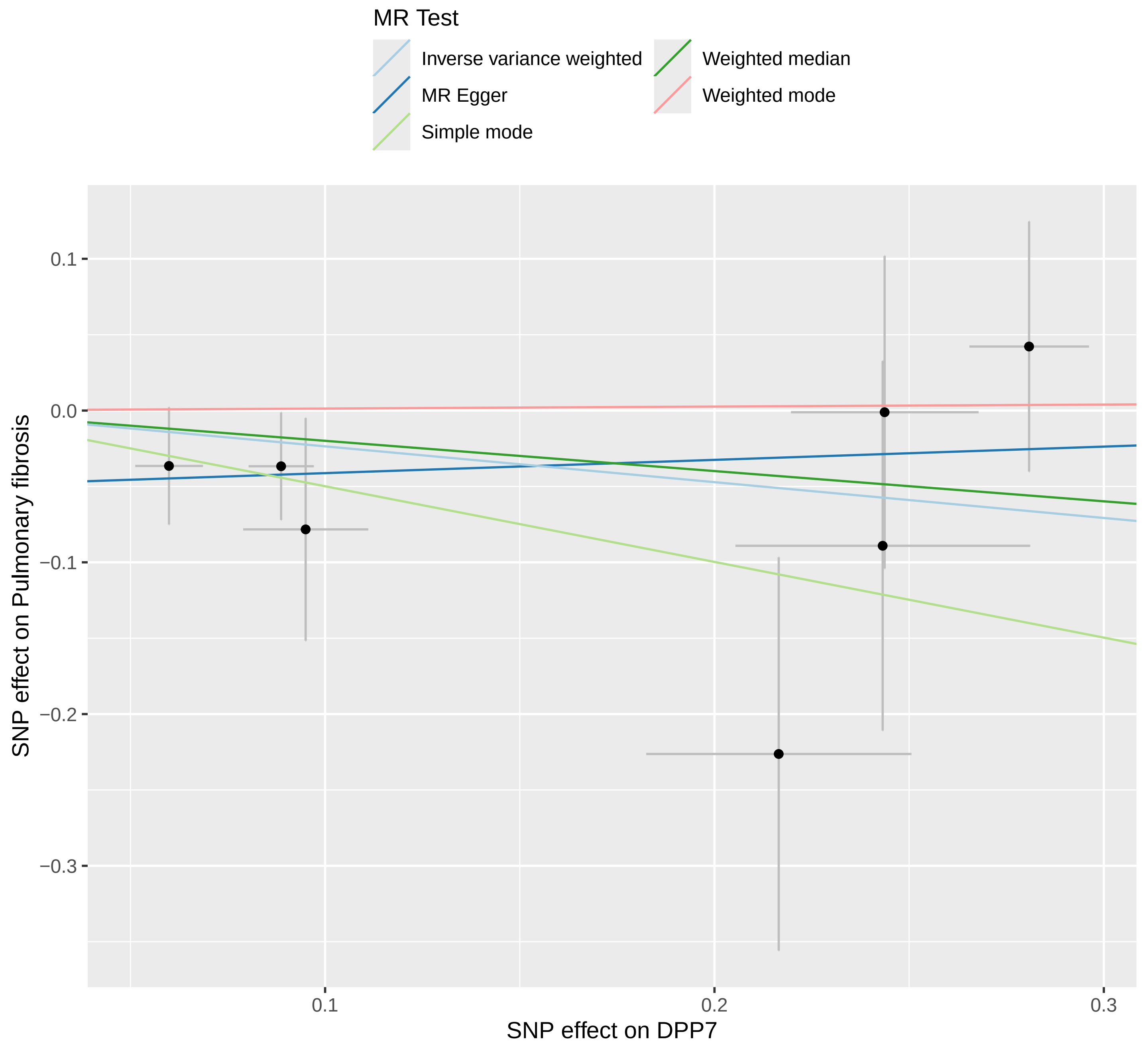

MR Test
Inverse variance weighted
MR Egger
Simple mode
Weighted median
Weighted mode
0.1
0.0
−0.1
−0.2
−0.3
SNP effect on Pulmonary fibrosis
0.1
0.2
0.3
SNP effect on DPP7

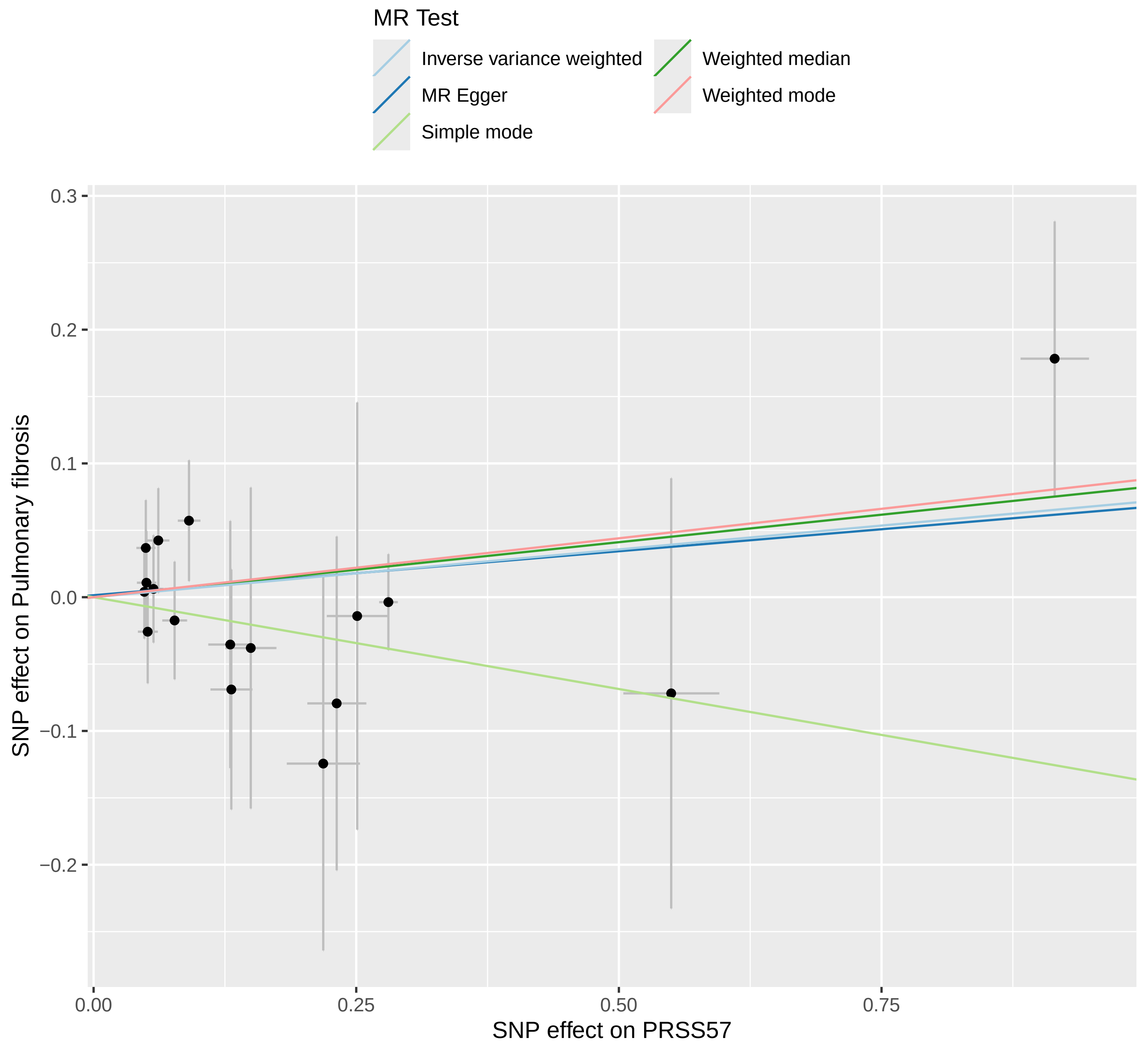
MR Test
Inverse variance weighted
MR Egger
Simple mode
Weighted median
Weighted mode
0.3
0.2
0.1
0.0
−0.1
−0.2
SNP effect on Pulmonary fibrosis
0.00
0.25
0.50
0.75
SNP effect on PRSS57

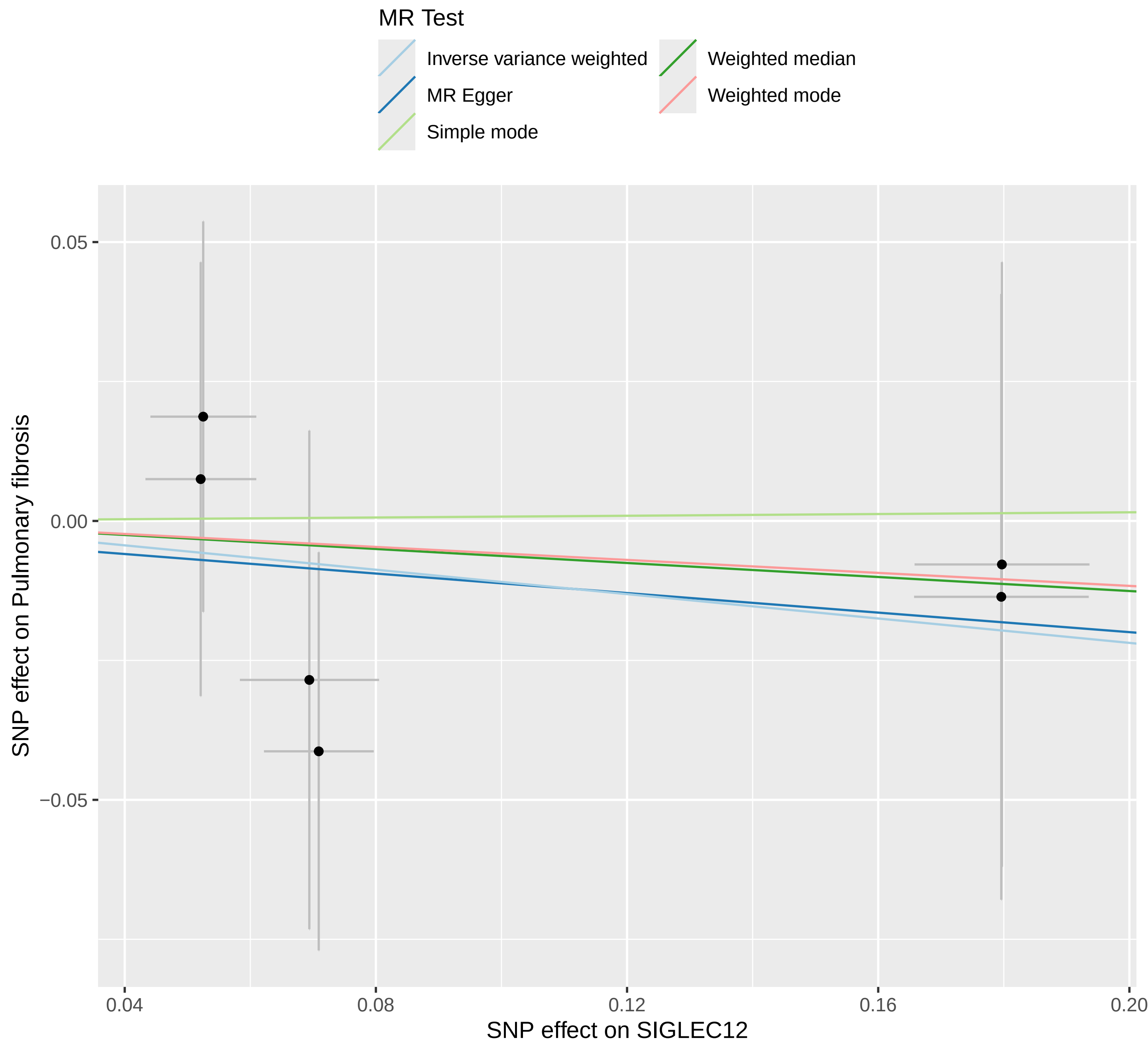

MR Test
Inverse variance weighted
MR Egger
Simple mode
Weighted median
Weighted mode
0.05
0.00
−0.05
0.04
0.08
0.12
0.16
0.20
SNP effect on SIGLEC12
SNP effect on Pulmonary fibrosis

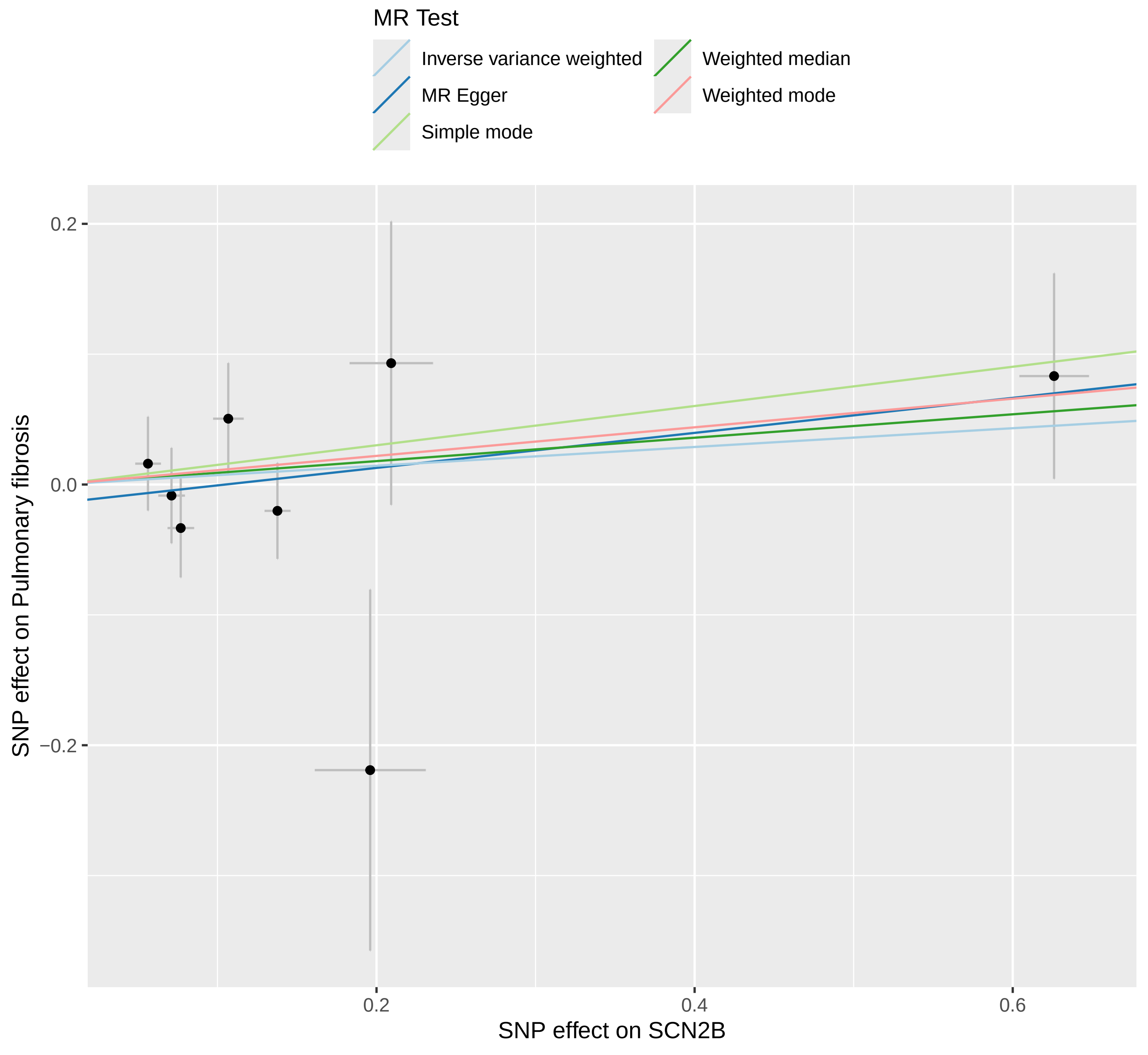
MR Test
Inverse variance weighted
MR Egger
Simple mode
Weighted median
Weighted mode
0.2
0.0
−0.2
0.2
0.4
0.6
SNP effect on Pulmonary fibrosis
SNP effect on SCN2B

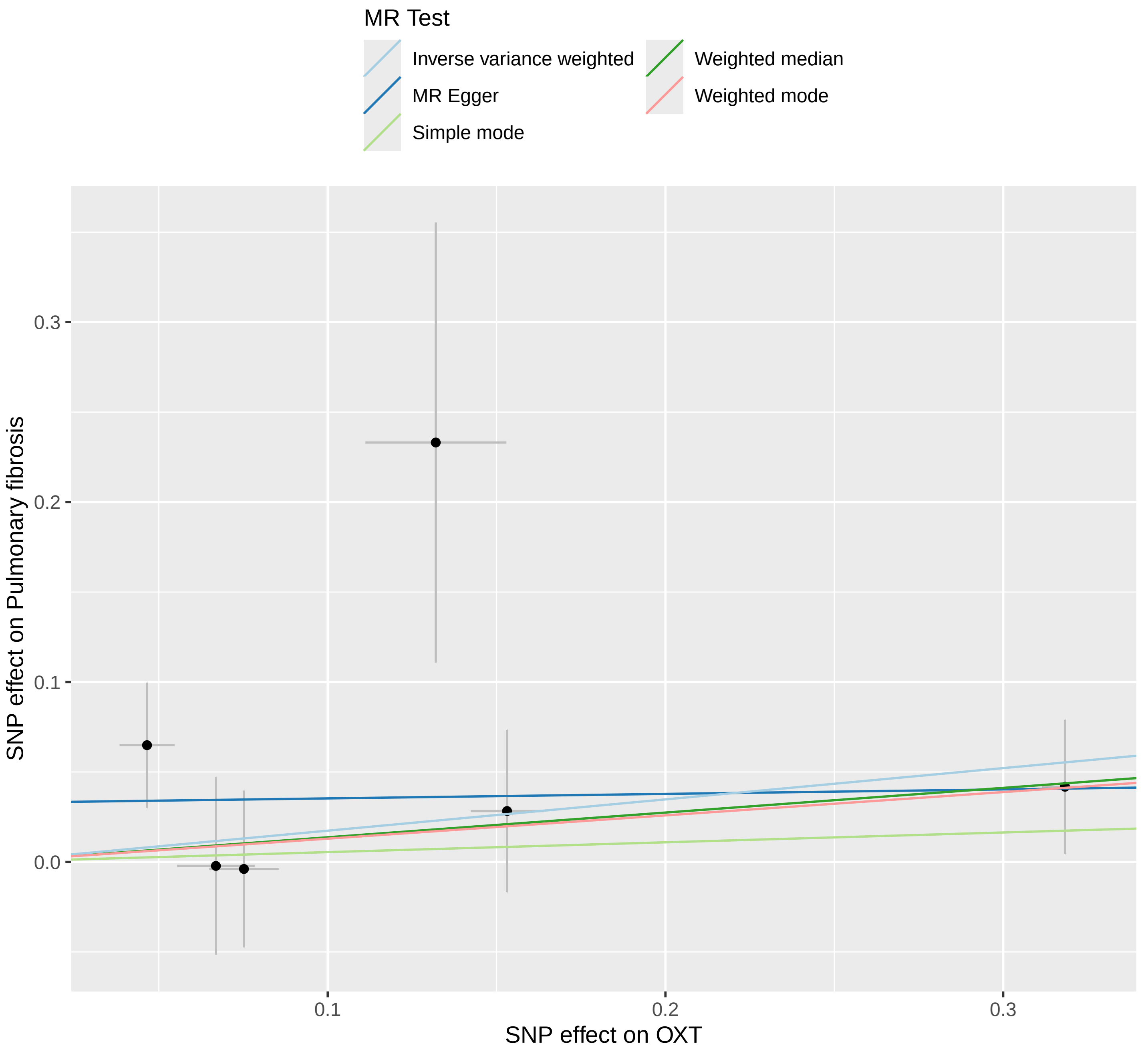
MR Test
Inverse variance weighted
MR Egger
Simple mode
Weighted median
Weighted mode
SNP effect on Pulmonary fibrosis
0.3
0.2
0.1
0.0
0.1
0.2
0.3
SNP effect on OXT

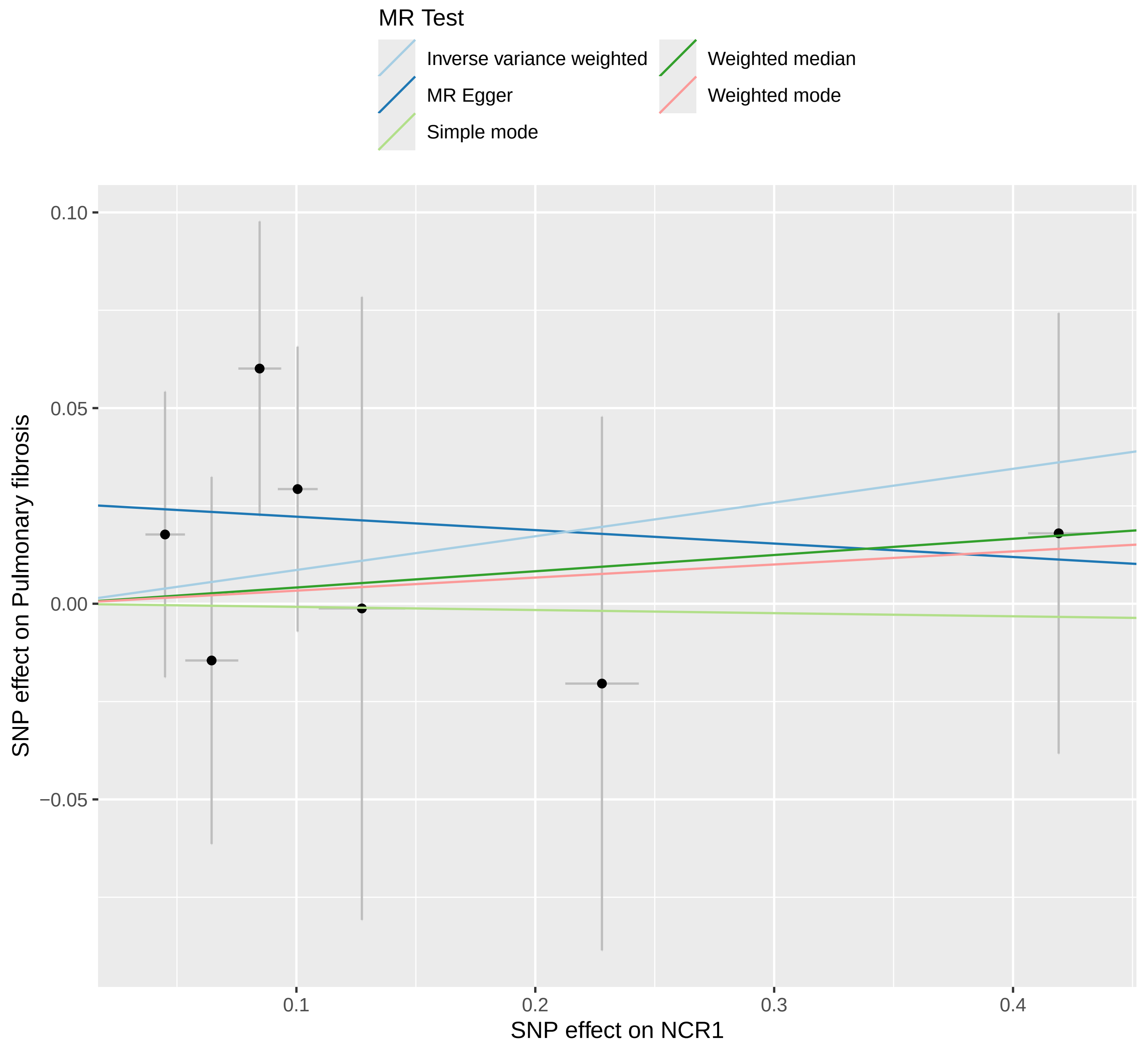
MR Test
Inverse variance weighted
MR Egger
Simple mode
Weighted median
Weighted mode
0.10
0.05
0.00
−0.05
SNP effect on Pulmonary fibrosis
0.1
0.2
0.3
0.4
SNP effect on NCR1

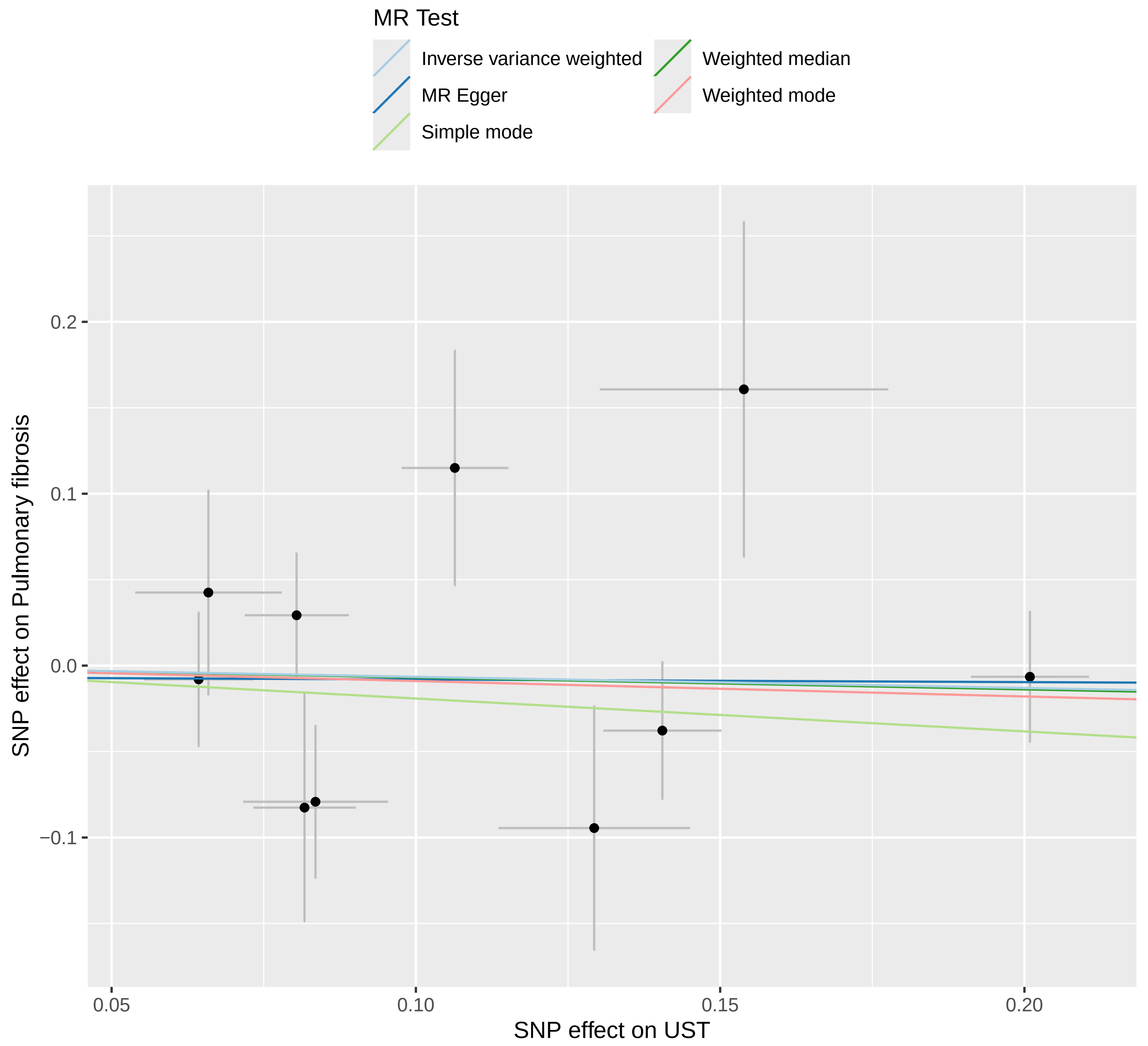

MR Test
Inverse variance weighted
MR Egger
Simple mode
Weighted median
Weighted mode
0.2
0.1
0.0
−0.1
0.05
0.10
0.15
0.20
SNP effect on Pulmonary fibrosis
SNP effect on UST

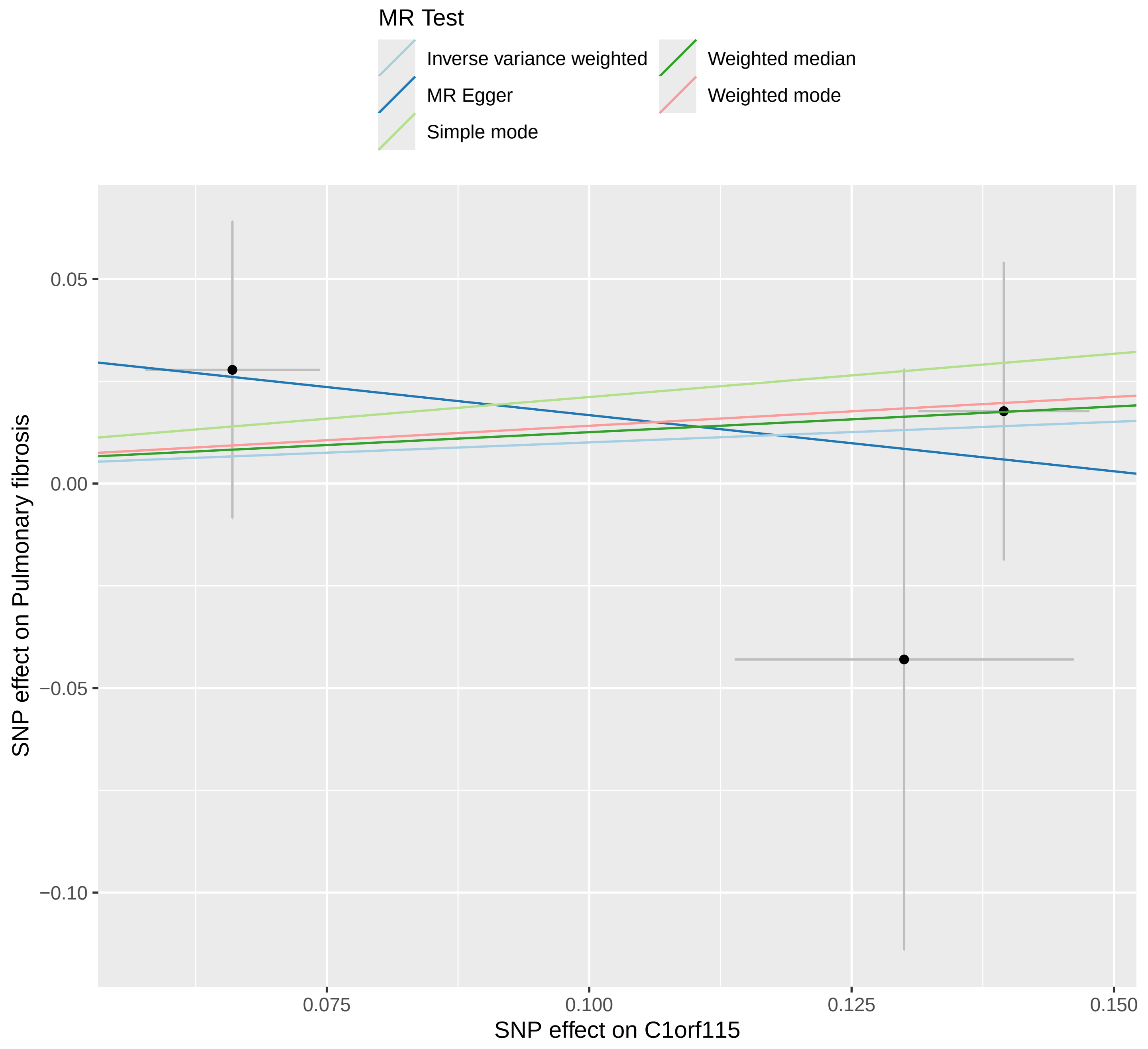

MR Test
Inverse variance weighted
MR Egger
Simple mode
Weighted median
Weighted mode
0.05
0.00
−0.05
−0.10
0.075
0.100
0.125
0.150
SNP effect on Pulmonary fibrosis
SNP effect on C1orf115

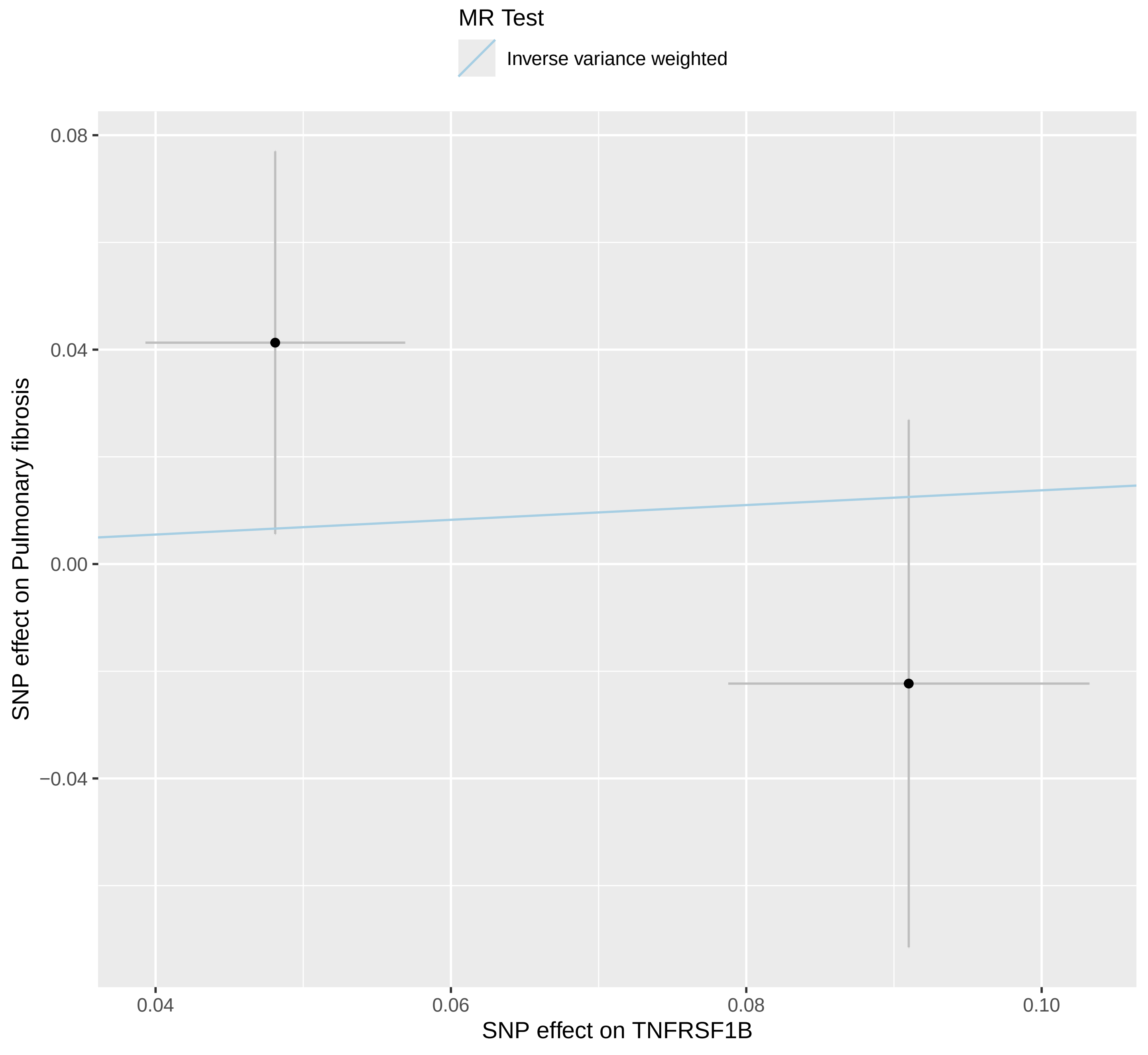
MR Test
Inverse variance weighted
0.08
0.04
0.00
−0.04
SNP effect on Pulmonary fibrosis
0.04
0.06
0.08
0.10
SNP effect on TNFRSF1B

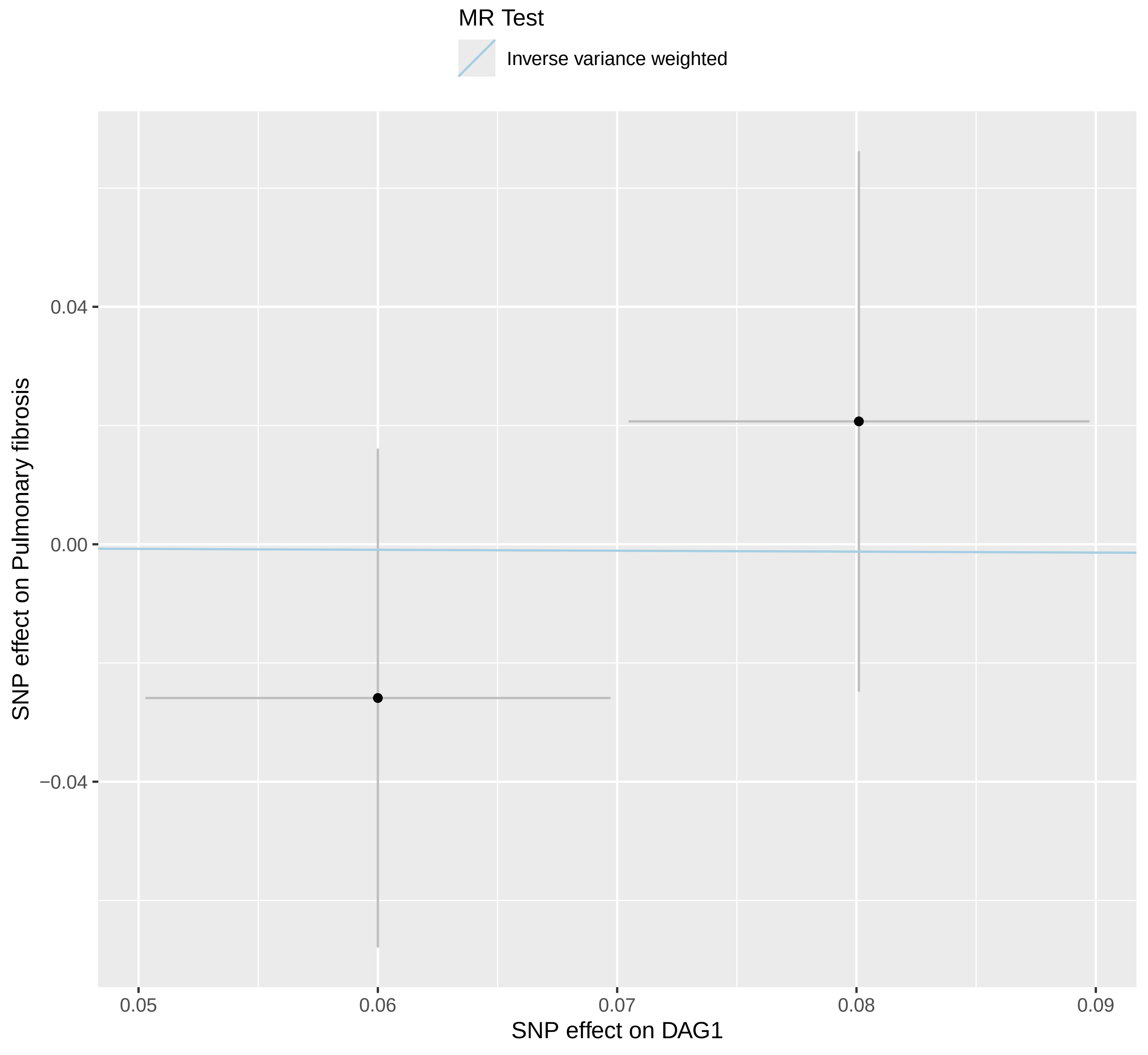

MR Test
Inverse variance weighted
SNP effect on Pulmonary fibrosis
0.04
0.00
−0.04
0.05
0.06
0.07
0.08
0.09
SNP effect on DAG1

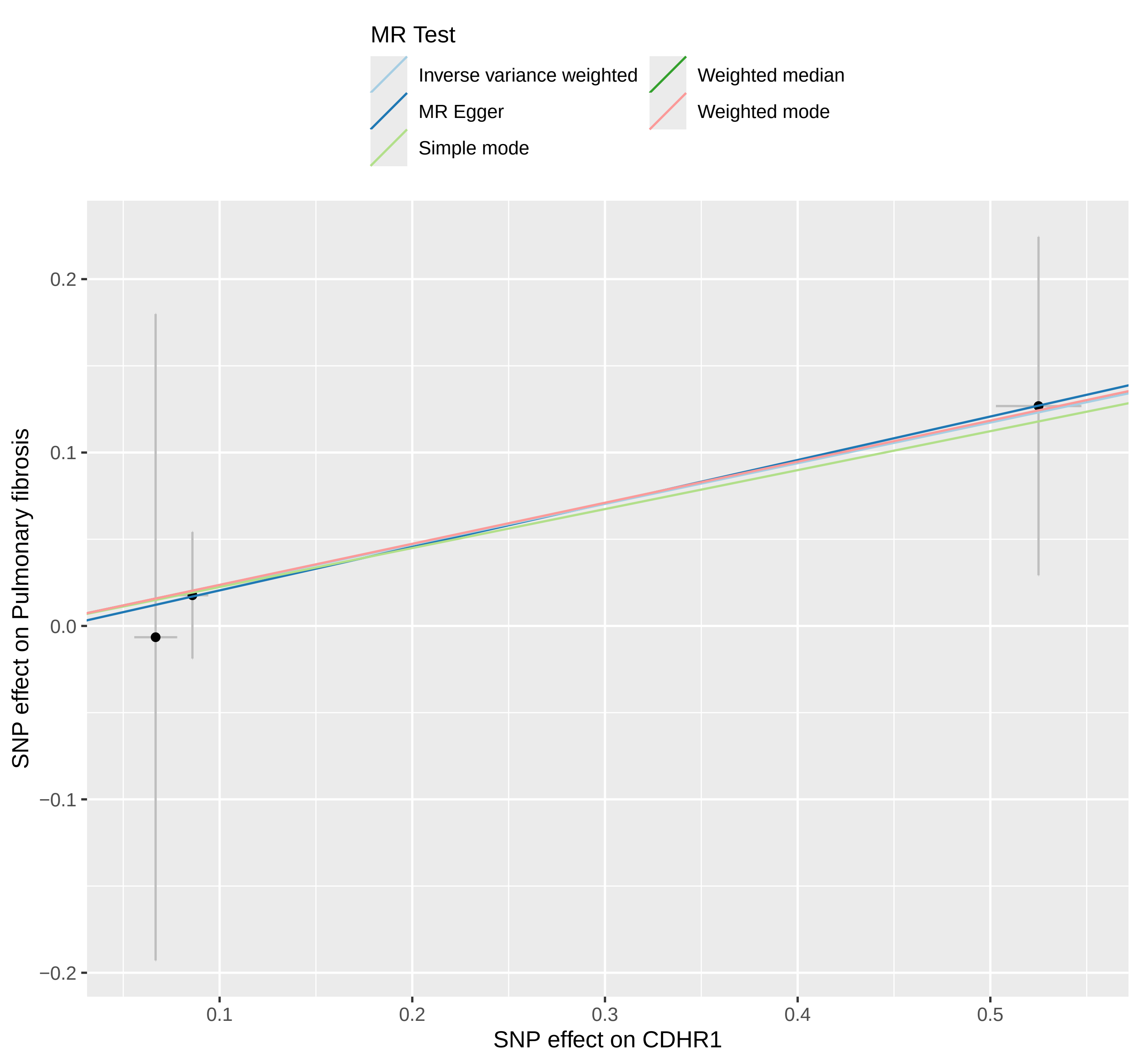

MR Test
Inverse variance weighted
MR Egger
Simple mode
Weighted median
Weighted mode
0.2
0.1
0.0
−0.1
−0.2
0.1
0.2
0.3
0.4
0.5
SNP effect on Pulmonary fibrosis
SNP effect on CDHR1

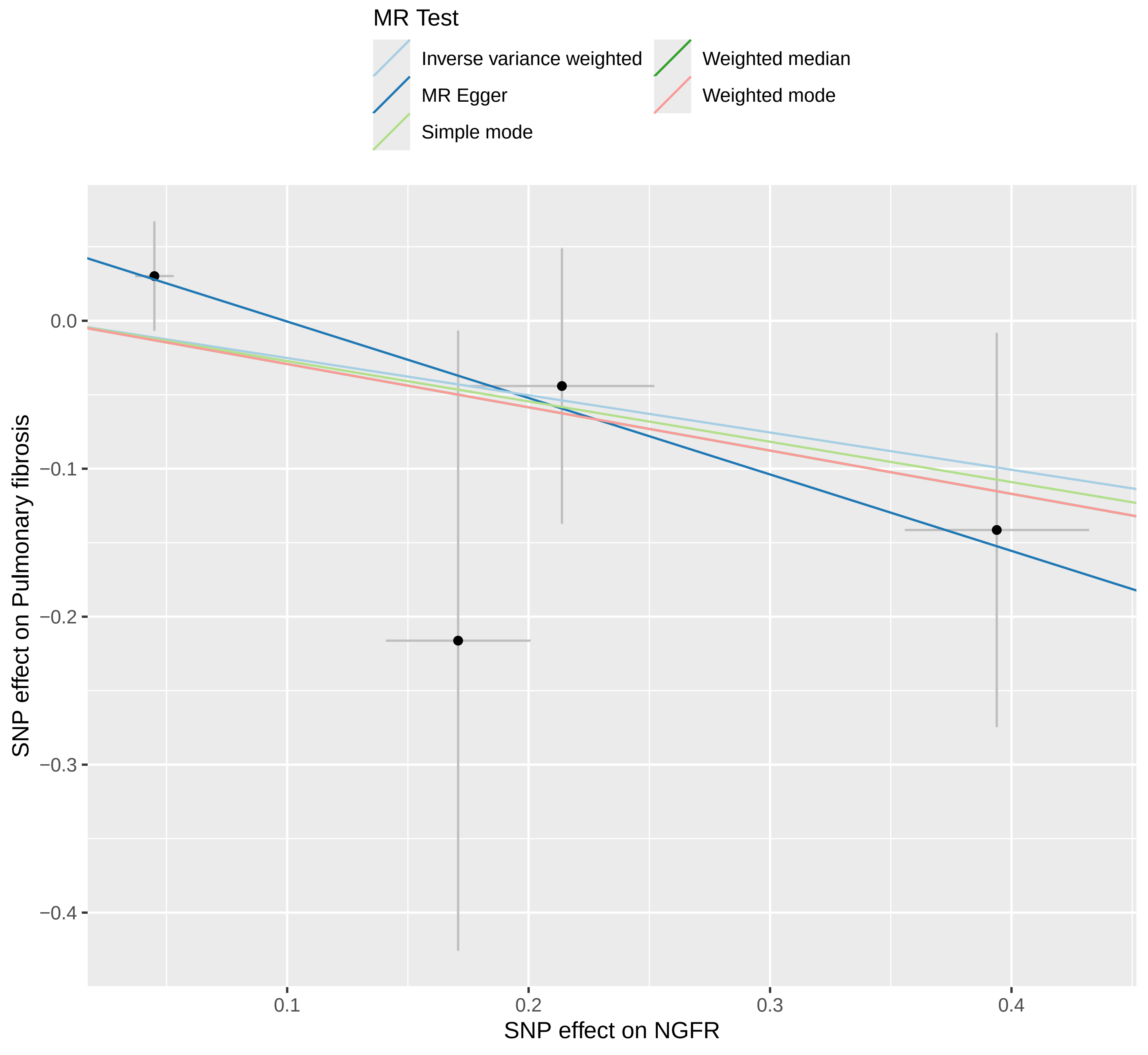
MR Test
Inverse variance weighted
MR Egger
Simple mode
Weighted median
Weighted mode
0.0
−0.1
−0.2
−0.3
−0.4
SNP effect on Pulmonary fibrosis
0.1
0.2
0.3
0.4
SNP effect on NGFR

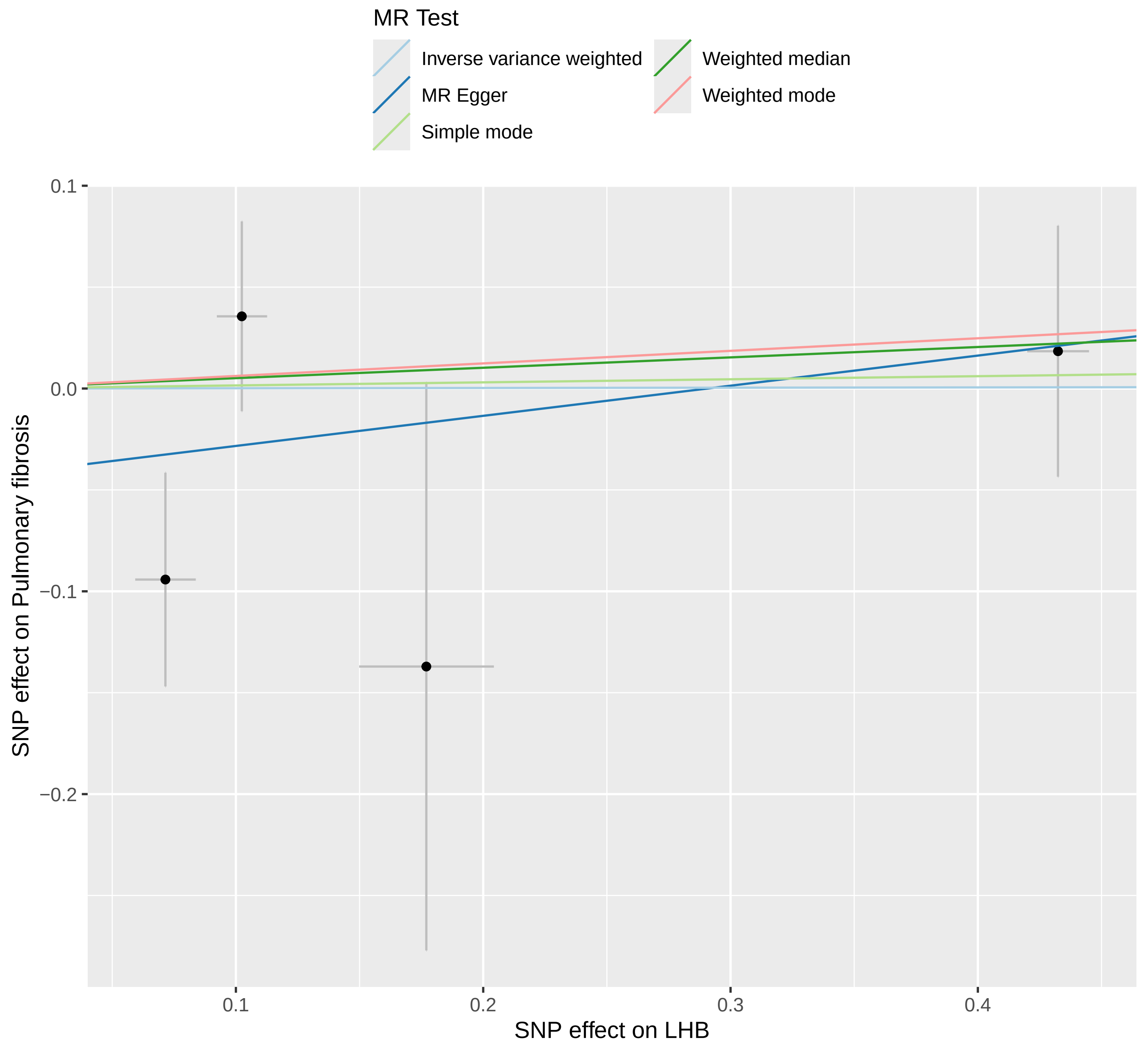

MR Test
Inverse variance weighted
MR Egger
Simple mode
Weighted median
Weighted mode
0.1
0.0
−0.1
−0.2
SNP effect on Pulmonary fibrosis
0.1
0.2
0.3
0.4
SNP effect on LHB

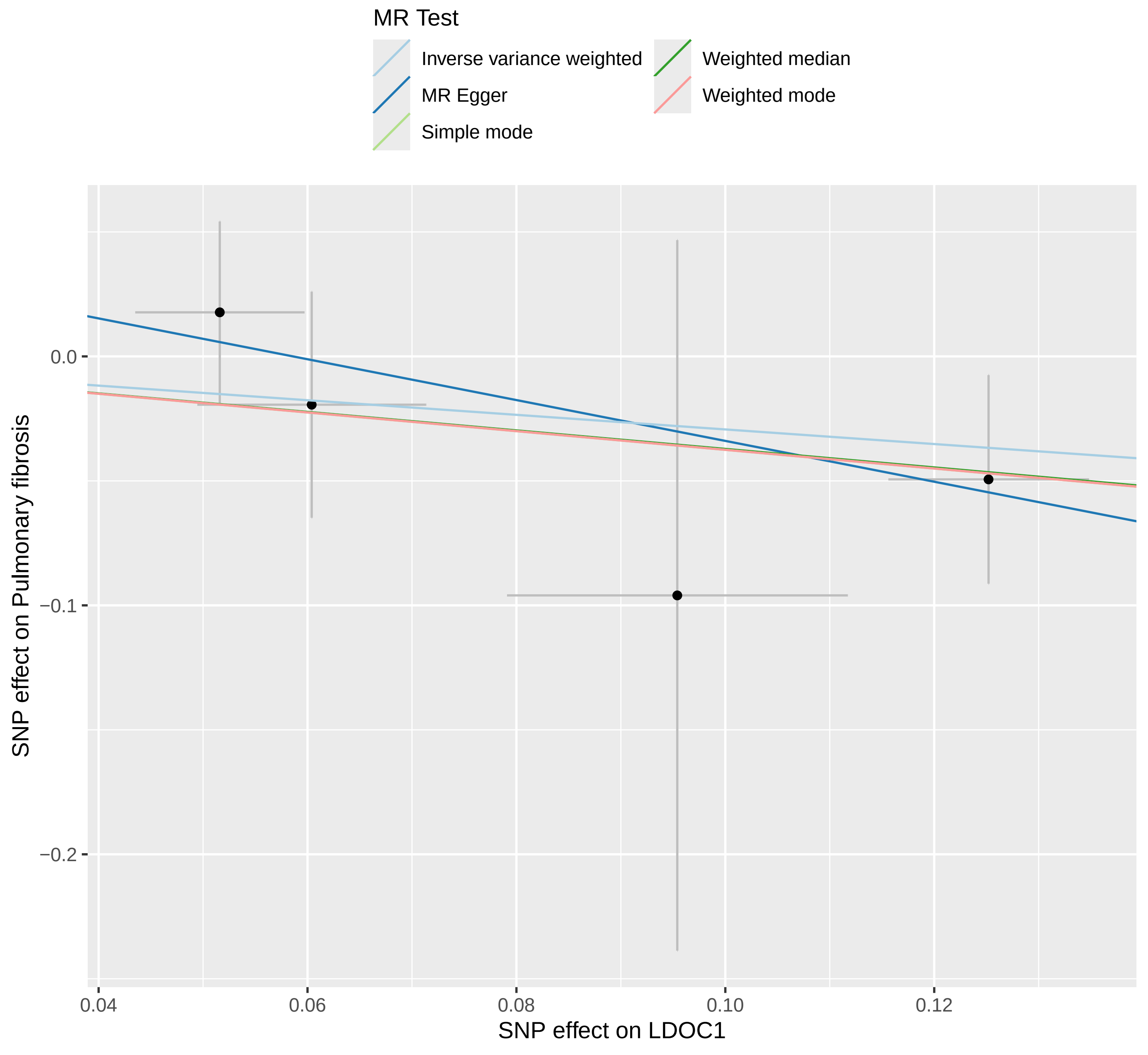

MR Test
Inverse variance weighted
MR Egger
Simple mode
Weighted median
Weighted mode
0.0
−0.1
−0.2
0.04
0.06
0.08
0.10
0.12
SNP effect on LDOC1
SNP effect on Pulmonary fibrosis

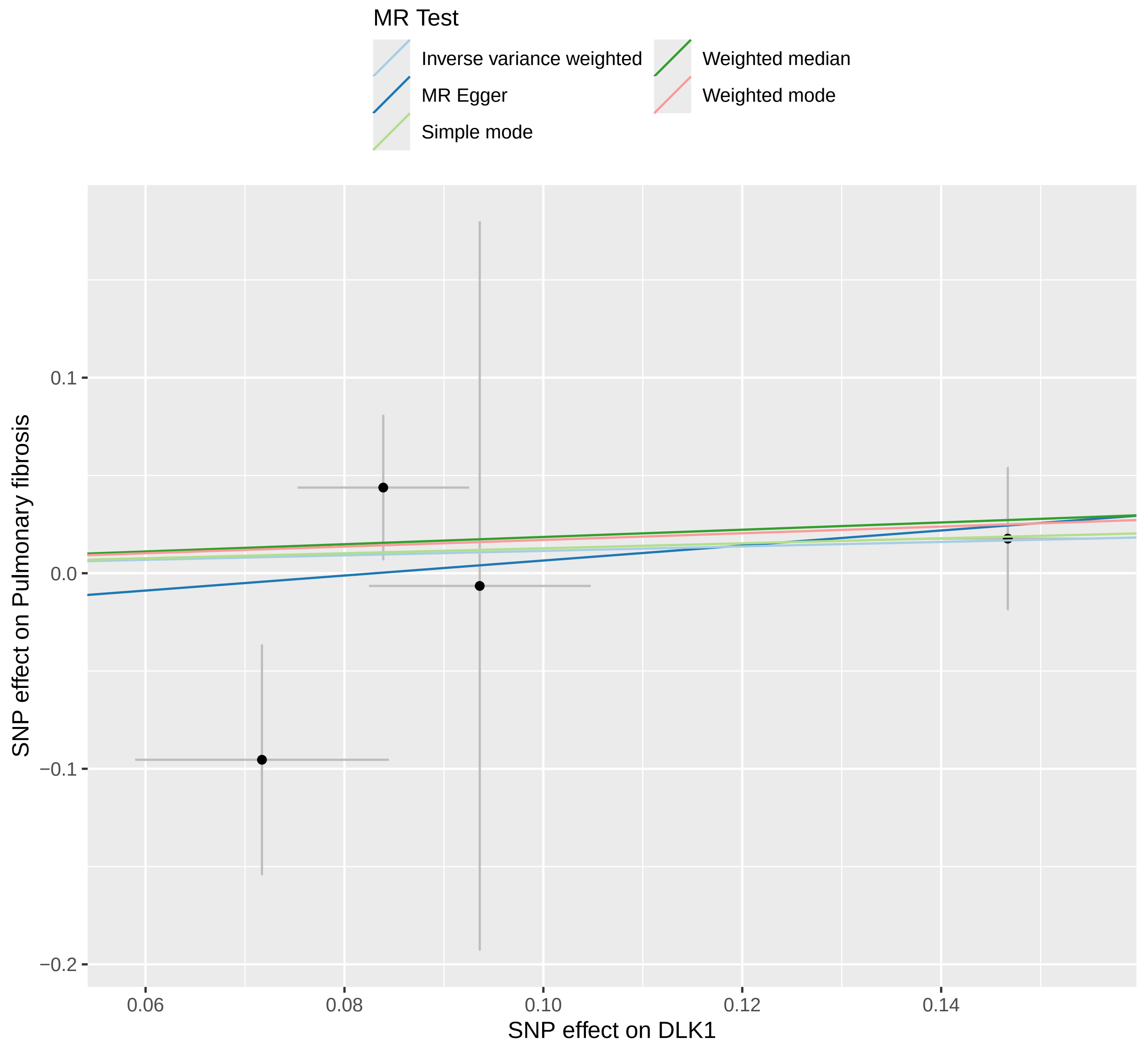

MR Test
Inverse variance weighted
MR Egger
Simple mode
Weighted median
Weighted mode
0.1
0.0
−0.1
−0.2
SNP effect on Pulmonary fibrosis
0.06
0.08
0.10
0.12
0.14
SNP effect on DLK1

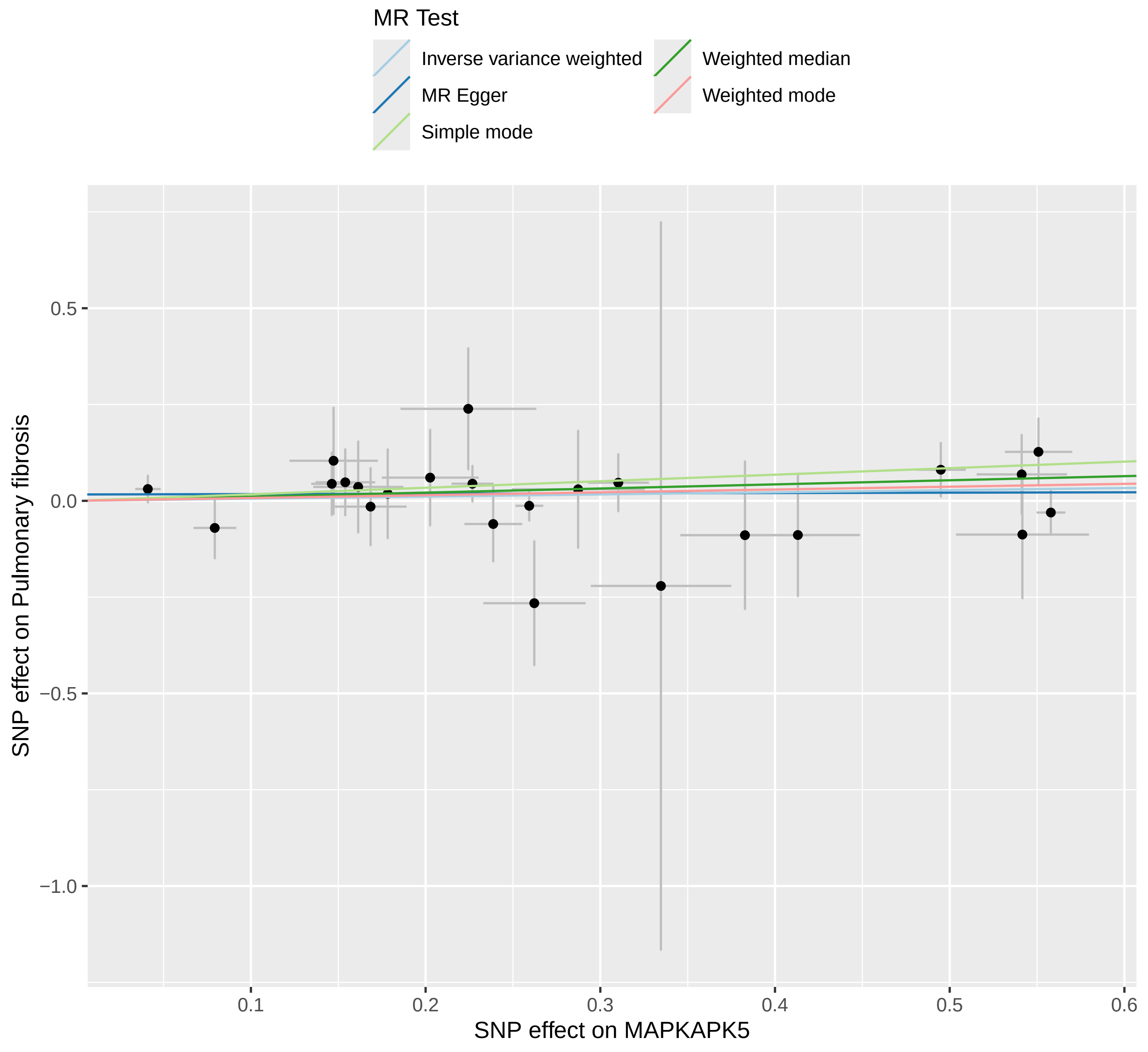

MR Test
Inverse variance weighted
MR Egger
Simple mode
Weighted median
Weighted mode
SNP effect on Pulmonary fibrosis
0.5
0.0
−0.5
−1.0
0.1
0.2
0.3
0.4
0.5
0.6
SNP effect on MAPKAPK5

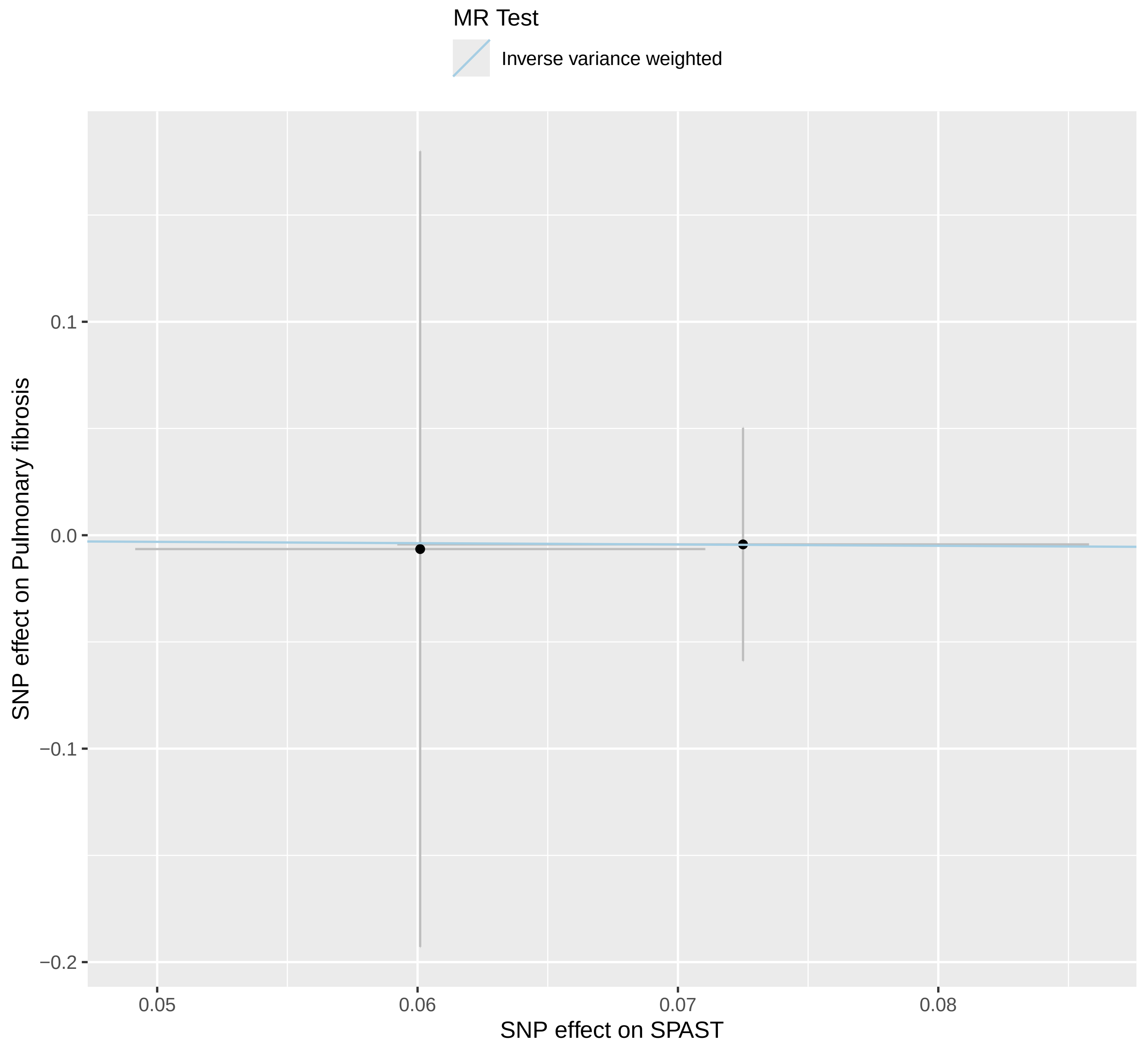

MR Test
Inverse variance weighted
0.1
0.0
−0.1
−0.2
0.05
0.06
0.07
0.08
SNP effect on Pulmonary fibrosis
SNP effect on SPAST

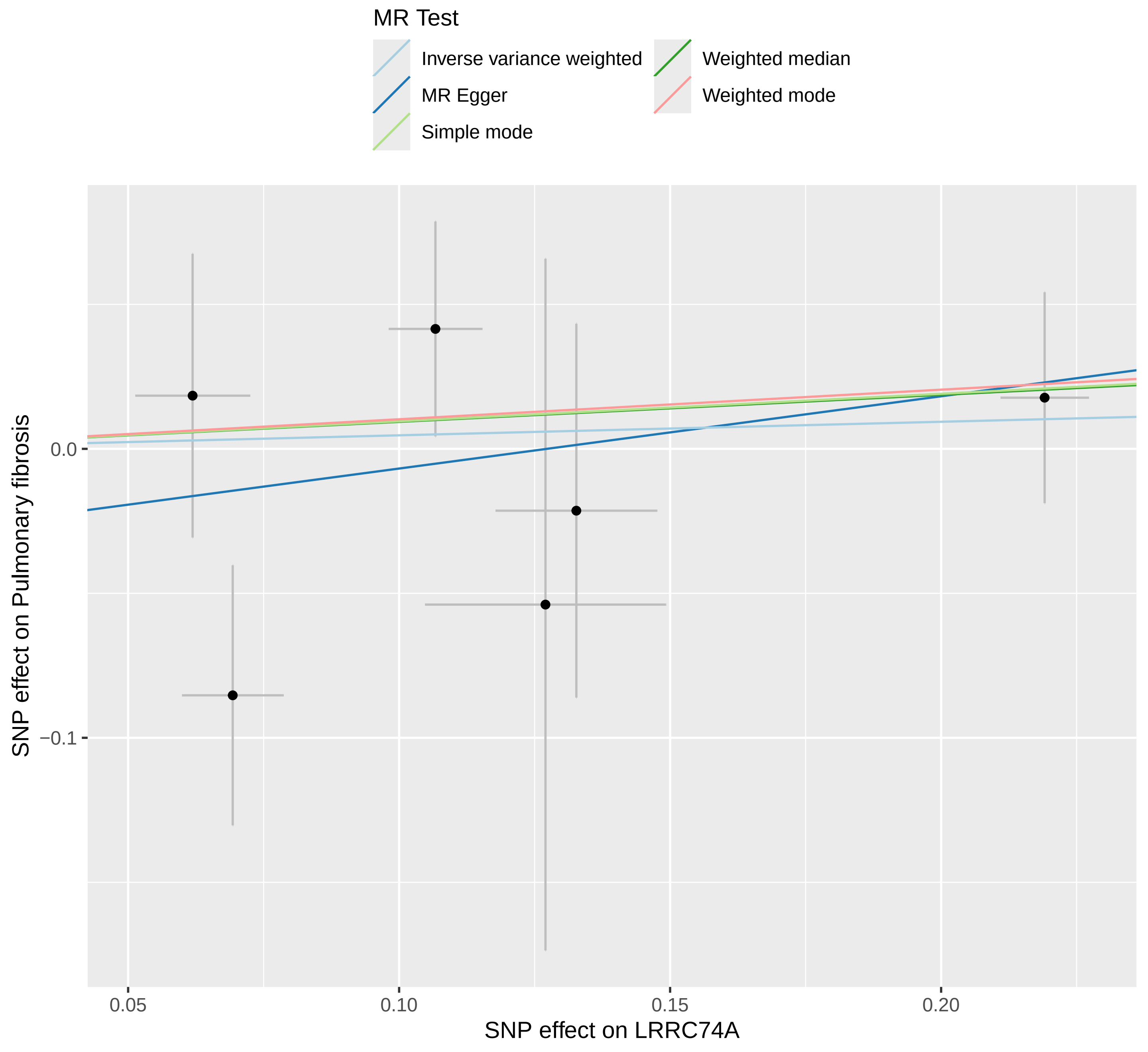

MR Test
Inverse variance weighted
MR Egger
Simple mode
Weighted median
Weighted mode
SNP effect on Pulmonary fibrosis
0.0
−0.1
0.05
0.10
0.15
0.20
SNP effect on LRRC74A

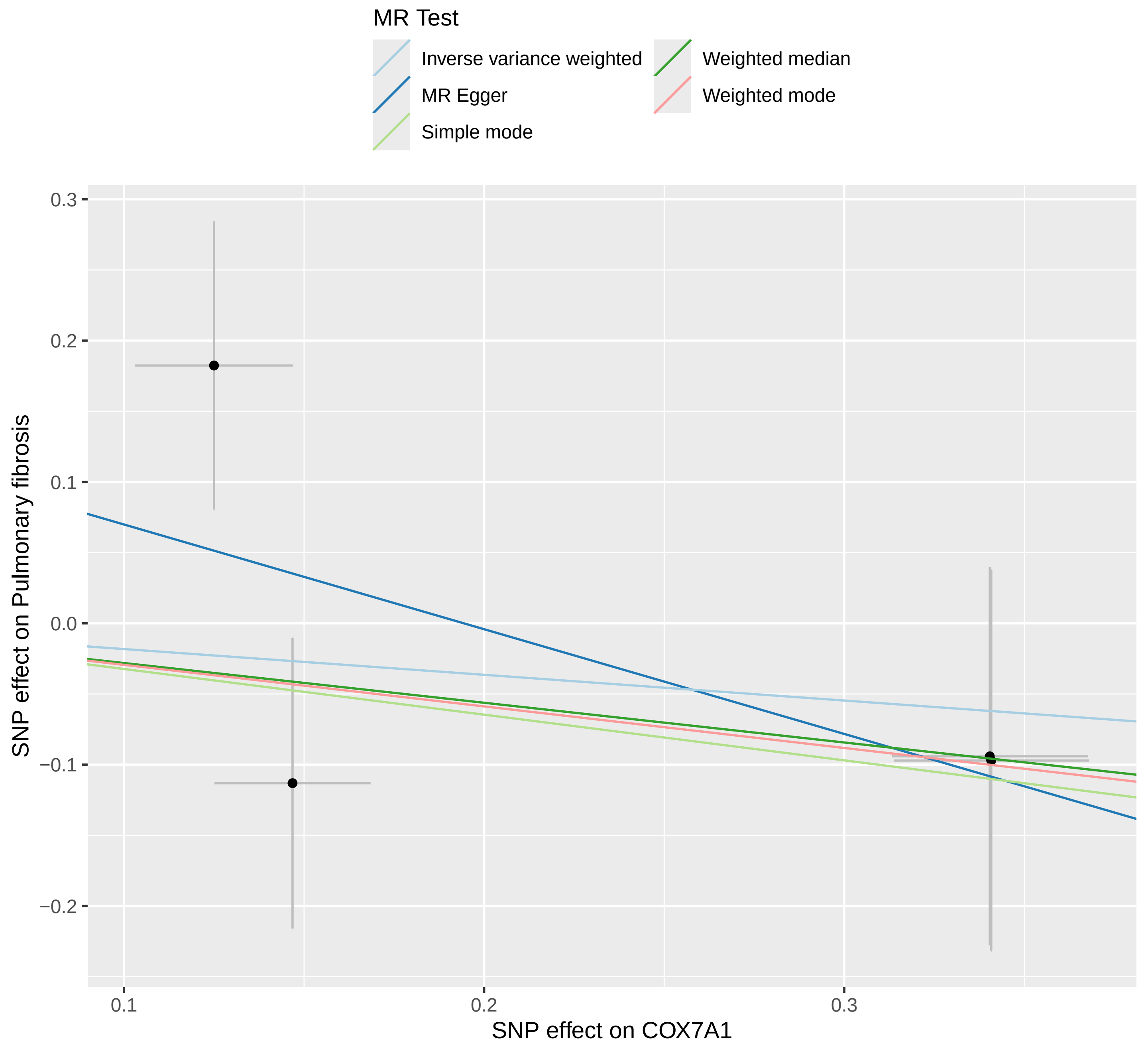
MR Test
Inverse variance weighted
MR Egger
Simple mode
Weighted median
Weighted mode
0.3
0.2
0.1
0.0
−0.1
−0.2
SNP effect on Pulmonary fibrosis
0.1
0.2
0.3
SNP effect on COX7A1

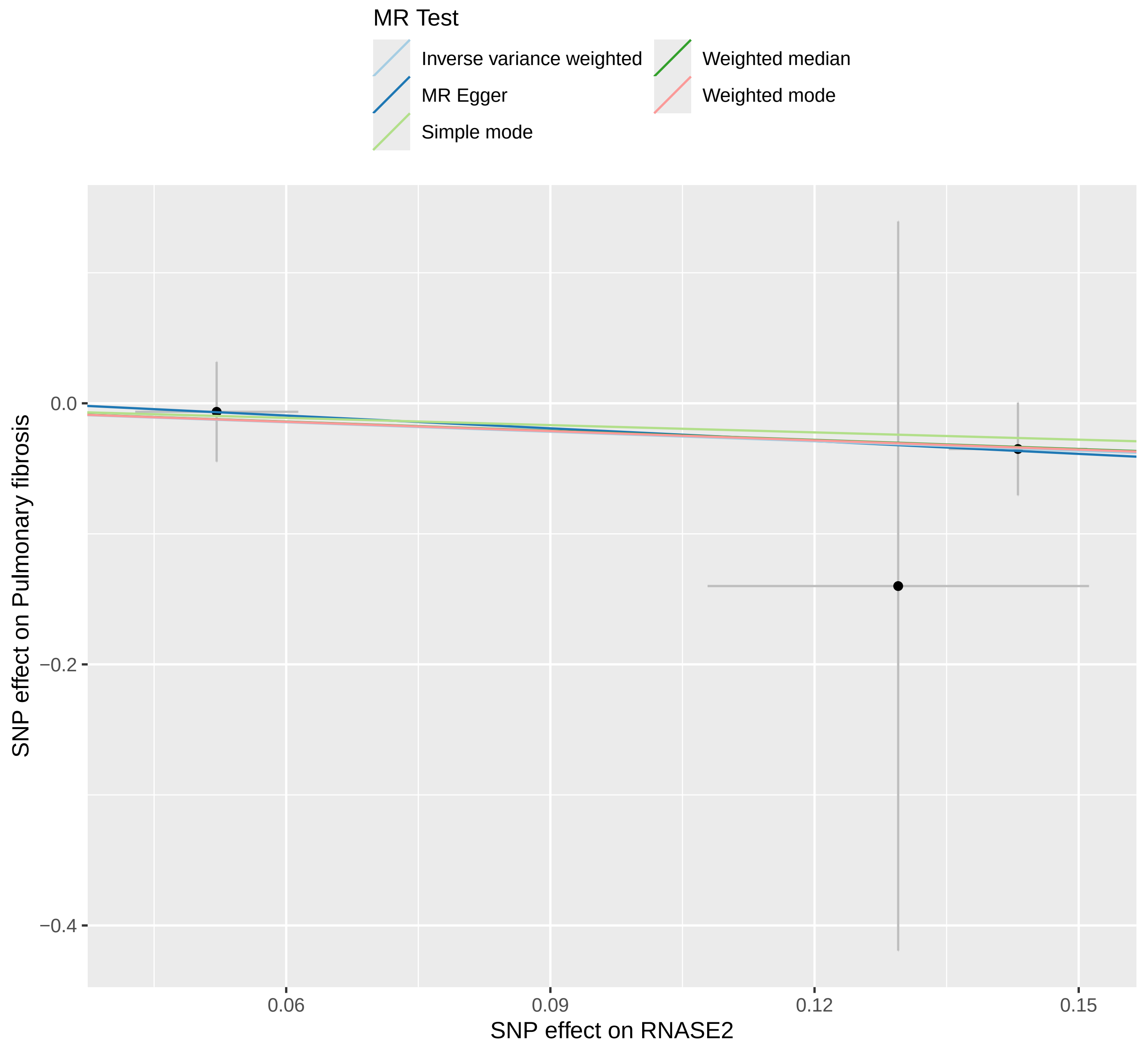

MR Test
Inverse variance weighted
MR Egger
Simple mode
Weighted median
Weighted mode
SNP effect on Pulmonary fibrosis
0.0
−0.2
−0.4
0.06
0.09
0.12
0.15
SNP effect on RNASE2

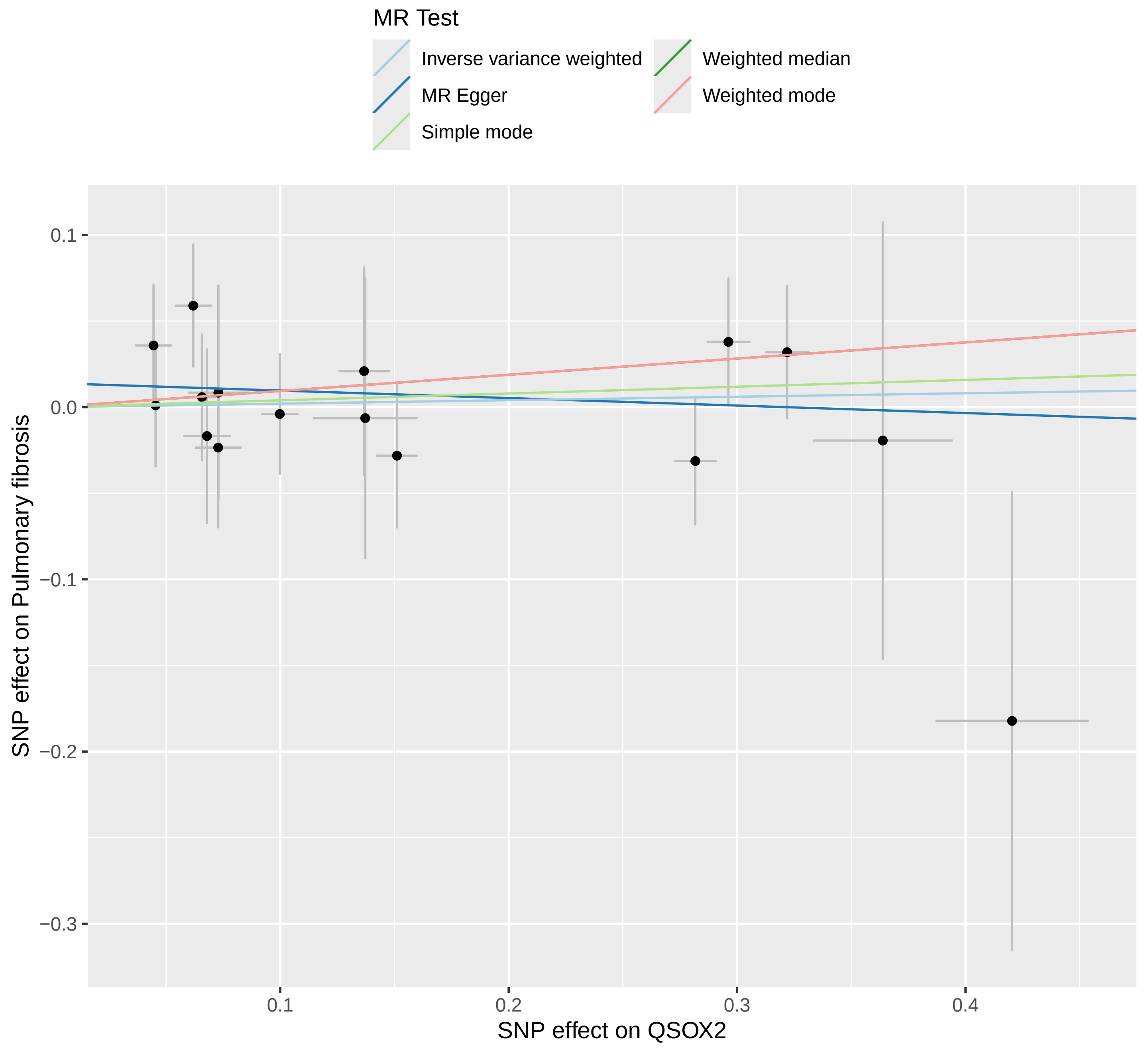
MR Test
Inverse variance weighted
MR Egger
Simple mode
Weighted median
Weighted mode
SNP effect on Pulmonary fibrosis
0.1
0.0
−0.1
−0.2
−0.3
0.1
0.2
0.3
0.4
SNP effect on QSOX2

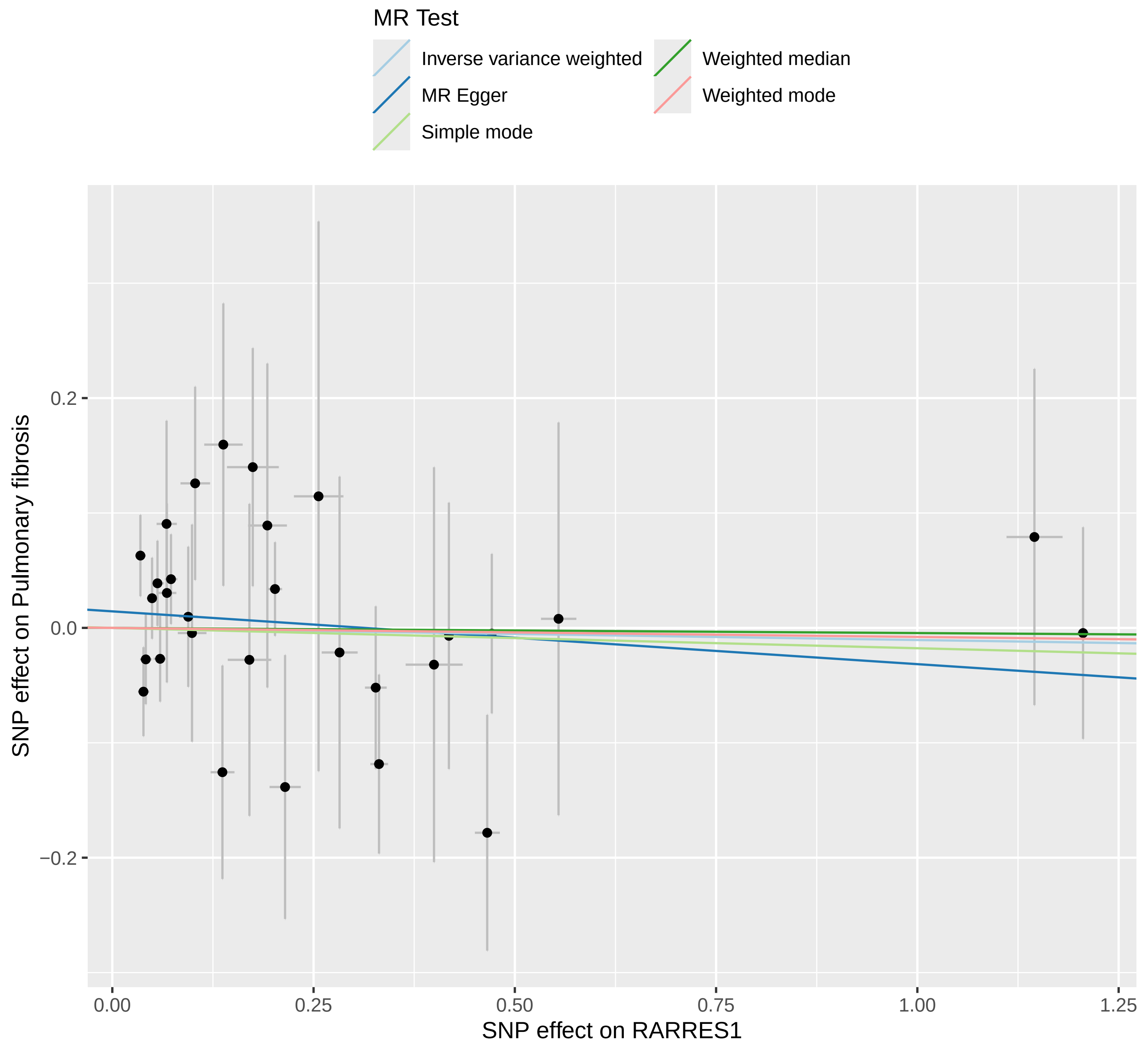
MR Test
Inverse variance weighted
MR Egger
Simple mode
Weighted median
Weighted mode
0.2
0.0
−0.2
SNP effect on Pulmonary fibrosis
0.00
0.25
0.50
0.75
1.00
1.25
SNP effect on RARRES1

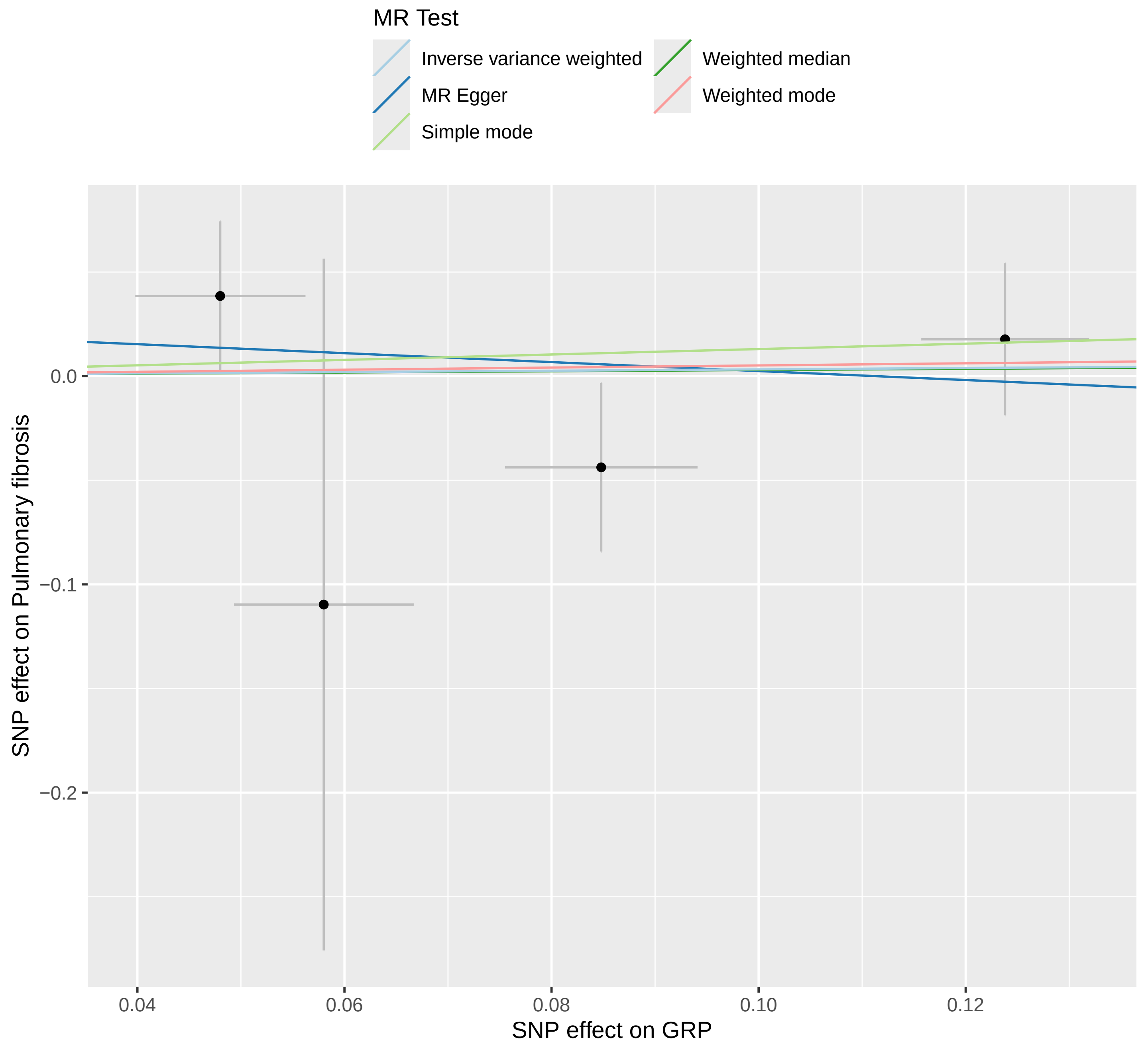

MR Test
Inverse variance weighted
MR Egger
Simple mode
Weighted median
Weighted mode
SNP effect on Pulmonary fibrosis
0.0
−0.1
−0.2
0.04
0.06
0.08
0.10
0.12
SNP effect on GRP

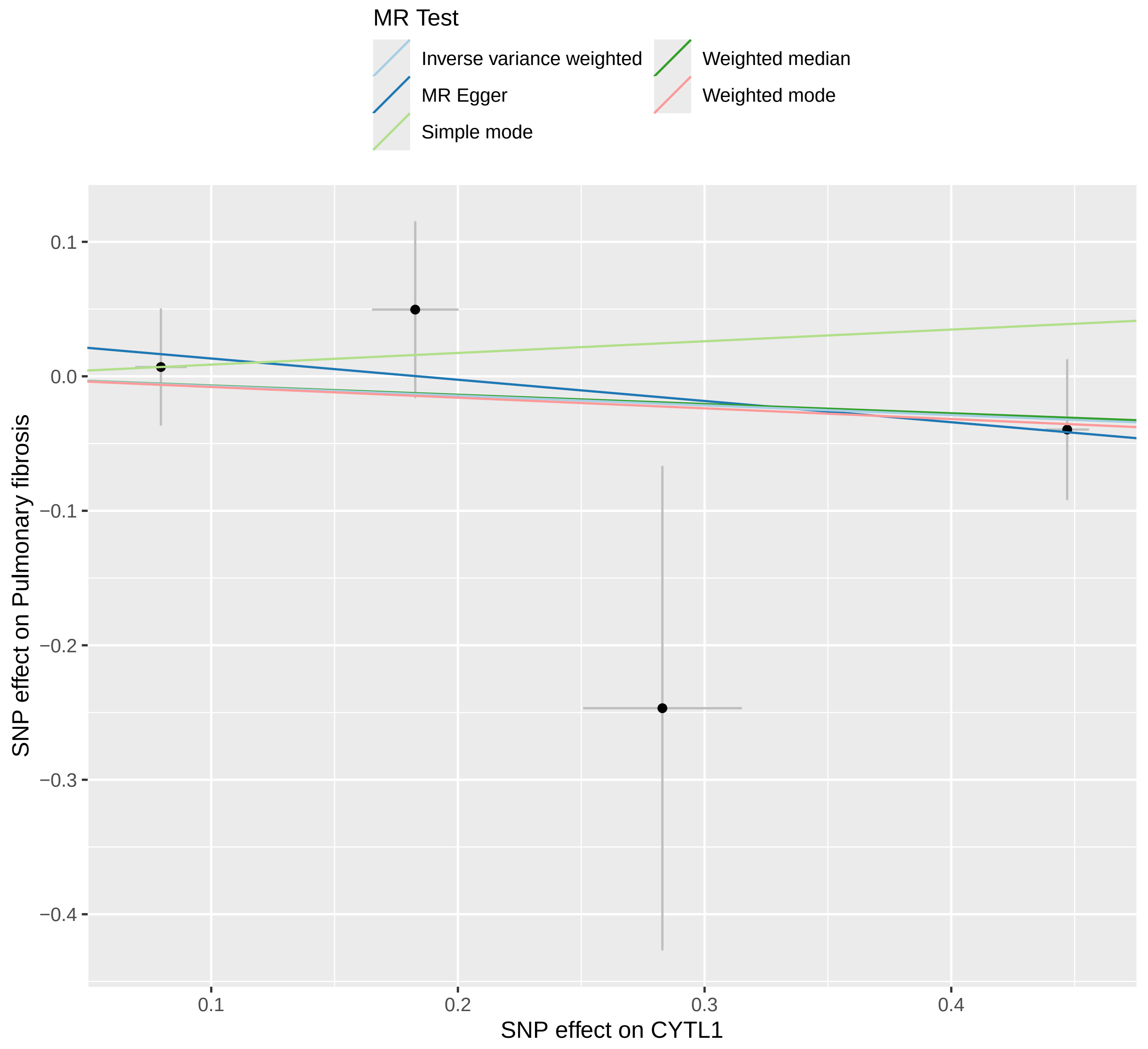
MR Test
Inverse variance weighted
MR Egger
Simple mode
Weighted median
Weighted mode
0.1
0.0
−0.1
−0.2
−0.3
−0.4
0.1
0.2
0.3
0.4
SNP effect on Pulmonary fibrosis
SNP effect on CYTL1

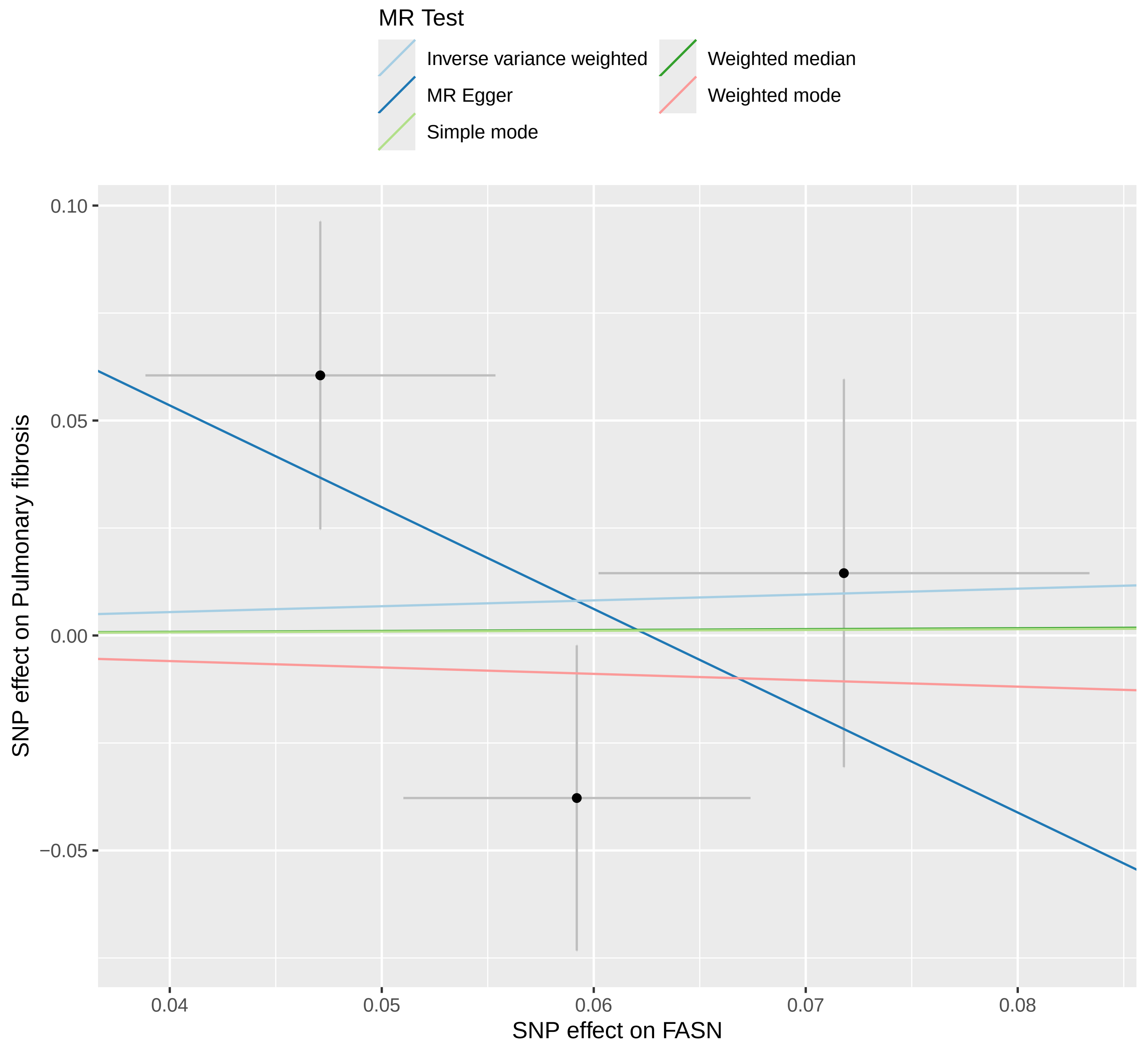

MR Test
Inverse variance weighted
MR Egger
Simple mode
Weighted median
Weighted mode
0.10
0.05
0.00
−0.05
SNP effect on Pulmonary fibrosis
0.04
0.05
0.06
0.07
0.08
SNP effect on FASN

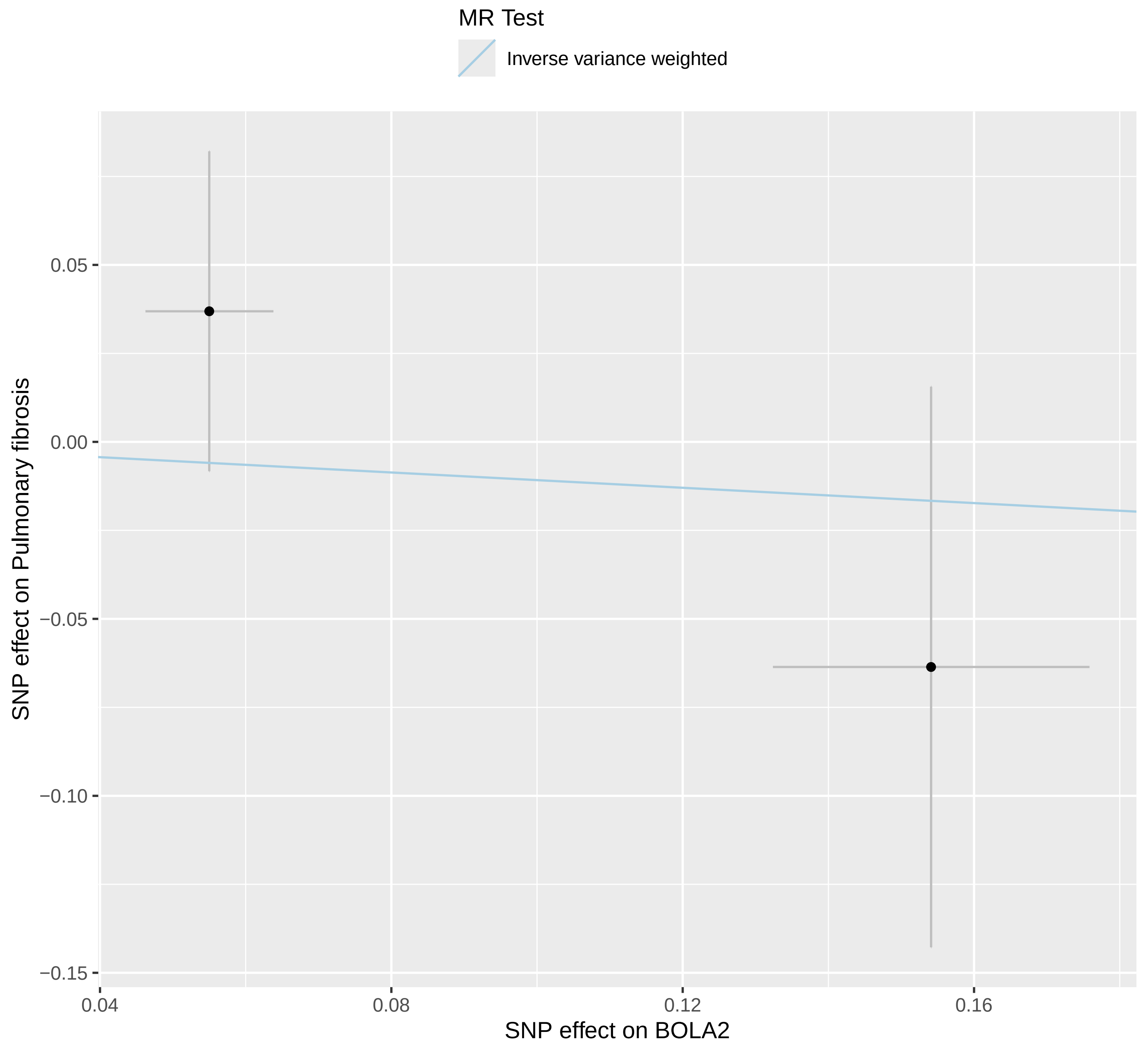
MR Test
Inverse variance weighted
0.05
0.00
−0.05
−0.10
−0.15
0.04
0.08
0.12
0.16
SNP effect on BOLA2
SNP effect on Pulmonary fibrosis

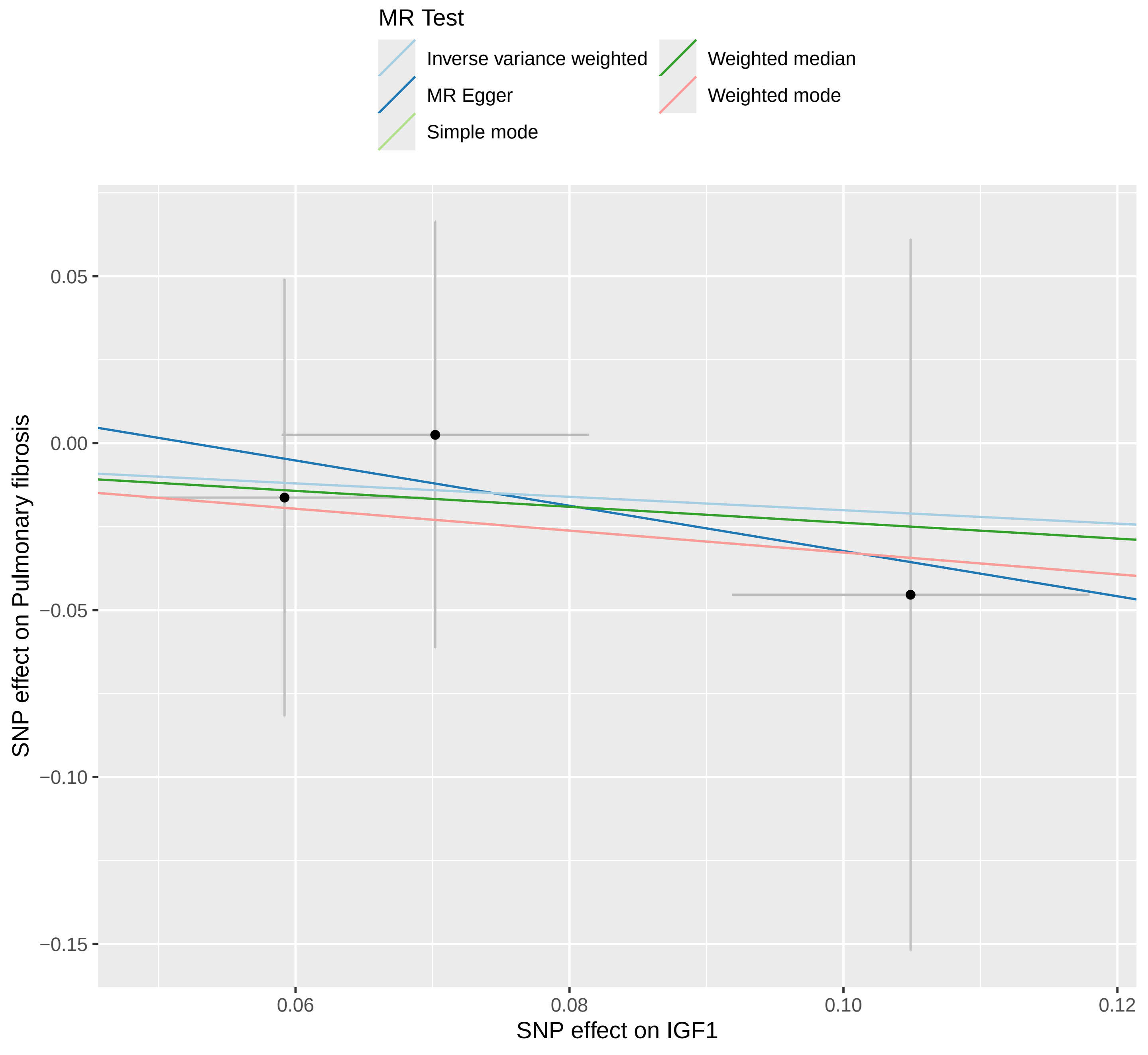

MR Test
Inverse variance weighted
MR Egger
Simple mode
Weighted median
Weighted mode
0.05
0.00
−0.05
−0.10
−0.15
SNP effect on Pulmonary fibrosis
0.06
0.08
0.10
0.12
SNP effect on IGF1

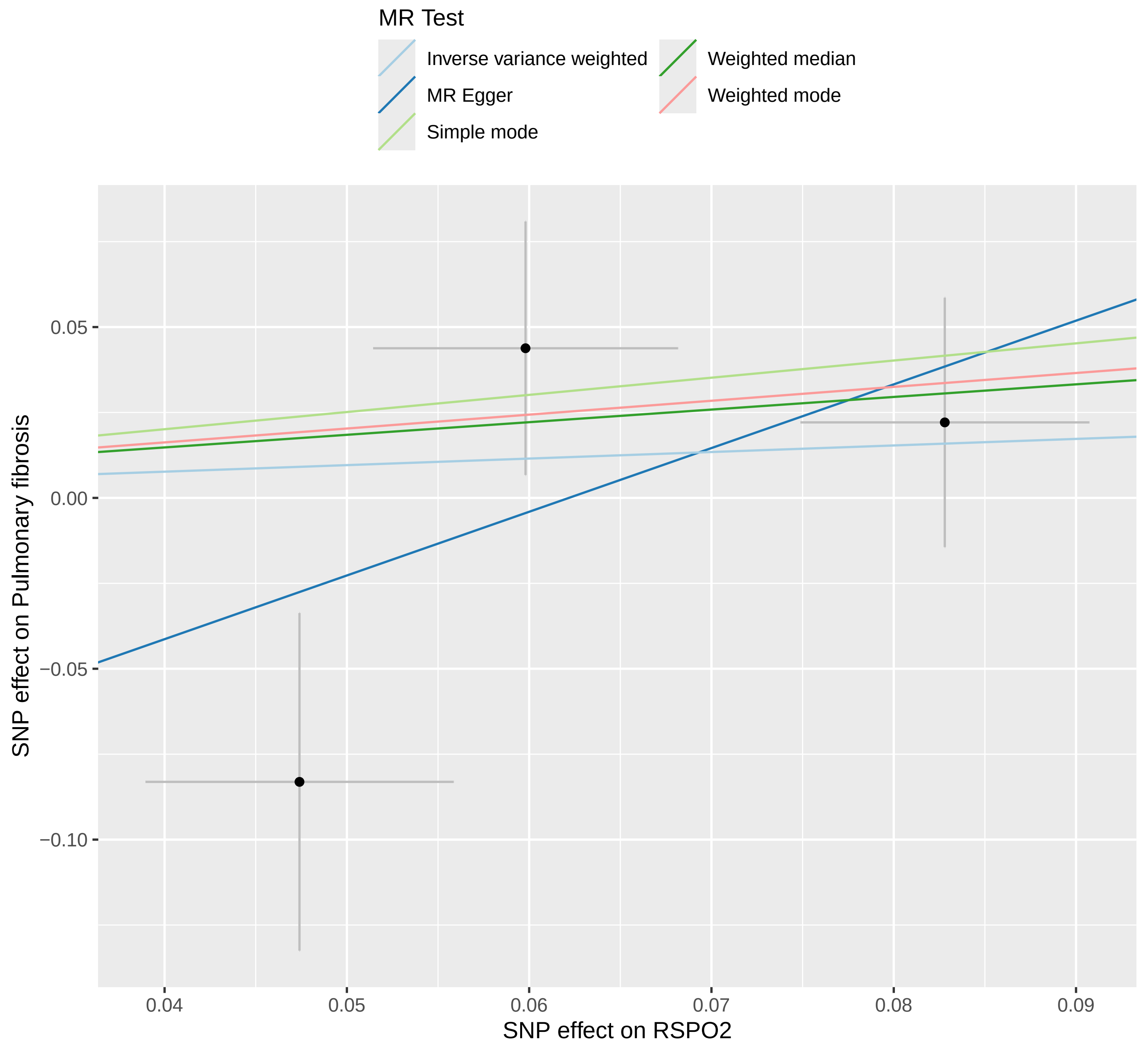
MR Test
Inverse variance weighted
MR Egger
Simple mode
Weighted median
Weighted mode
0.05
0.00
−0.05
−0.10
SNP effect on Pulmonary fibrosis
0.04
0.05
0.06
0.07
0.08
0.09
SNP effect on RSPO2

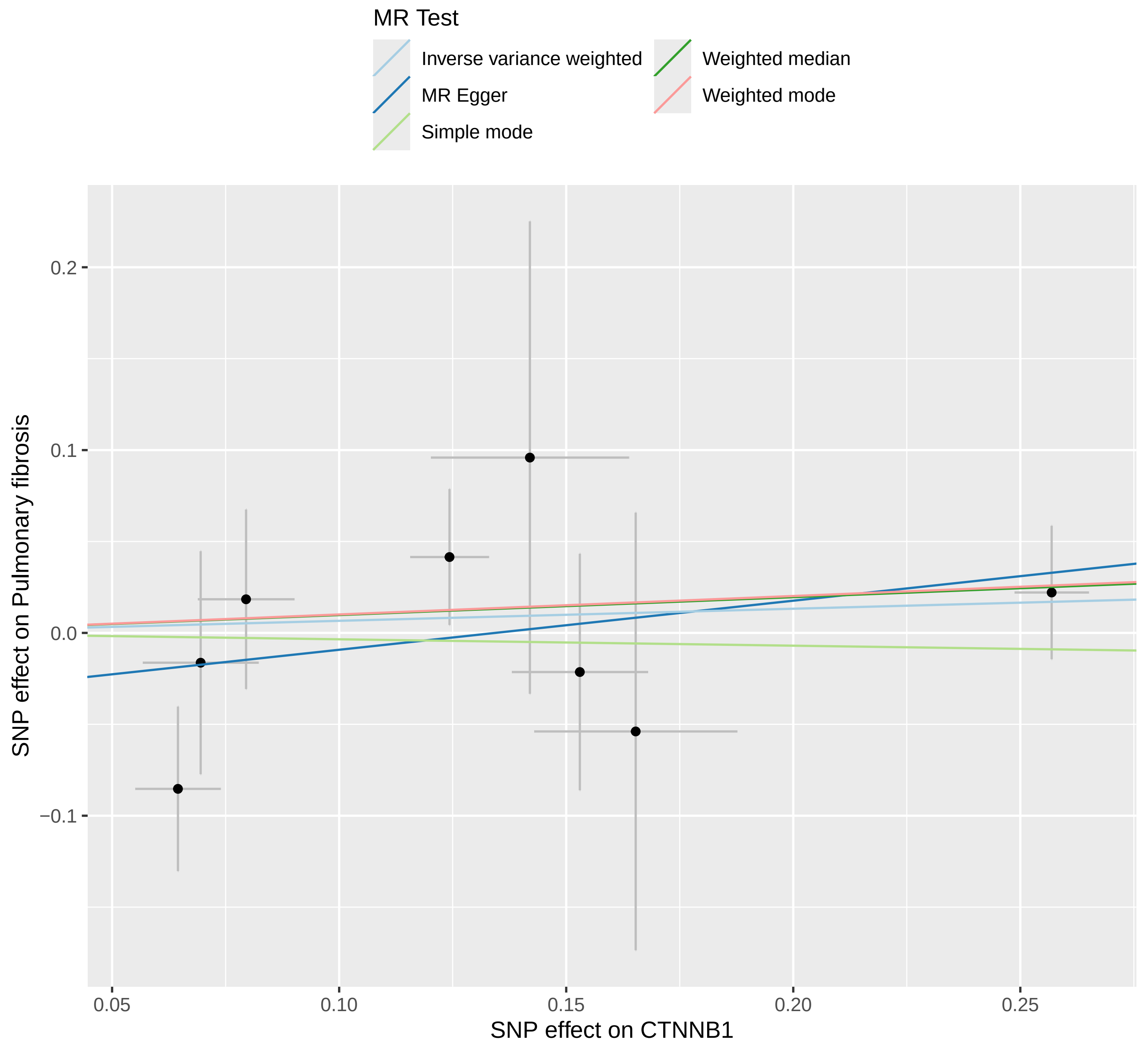

MR Test
Inverse variance weighted
MR Egger
Simple mode
Weighted median
Weighted mode
0.2
0.1
0.0
−0.1
SNP effect on Pulmonary fibrosis
0.05
0.10
0.15
0.20
0.25
SNP effect on CTNNB1

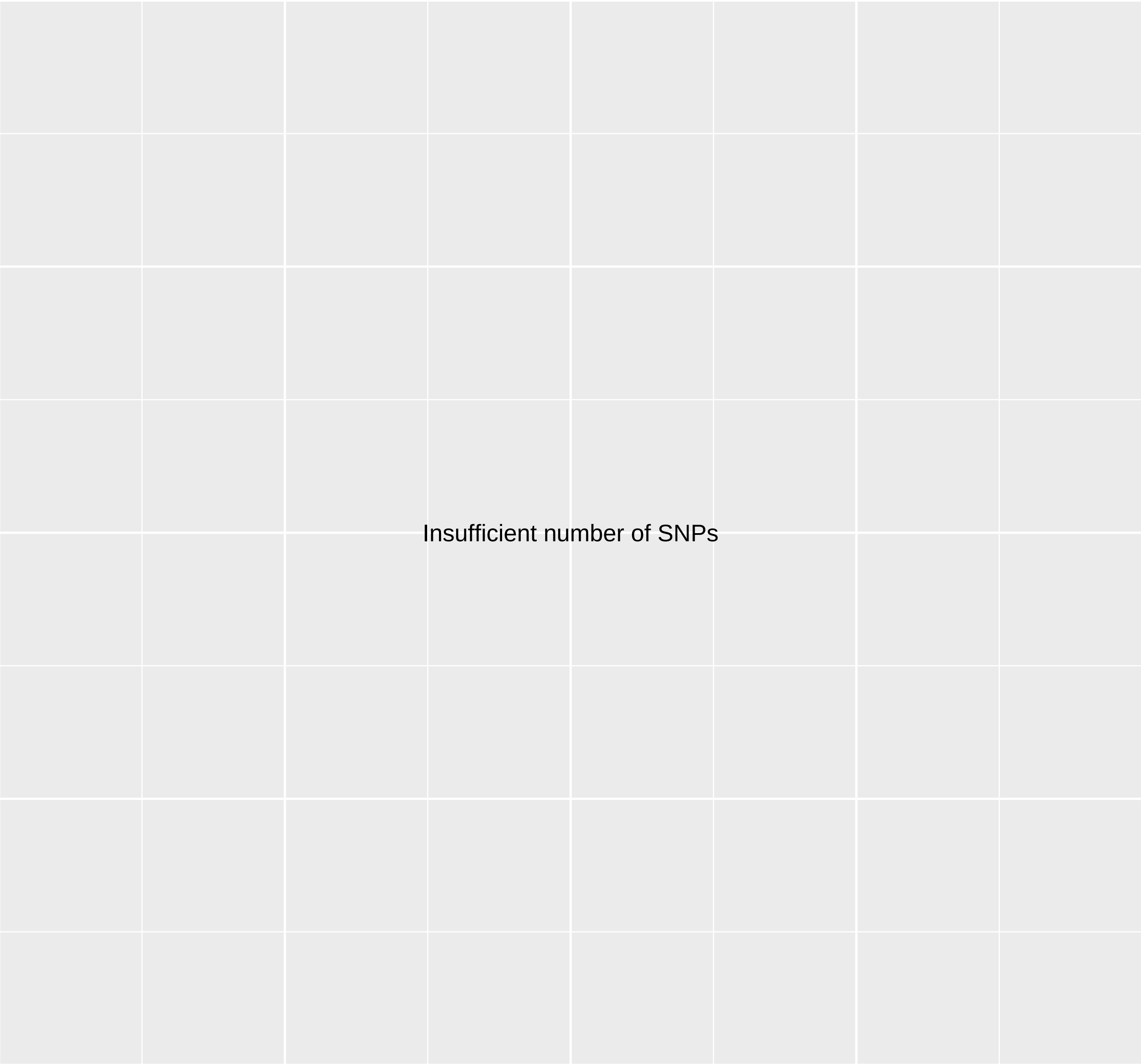
Insufficient number of SNPs

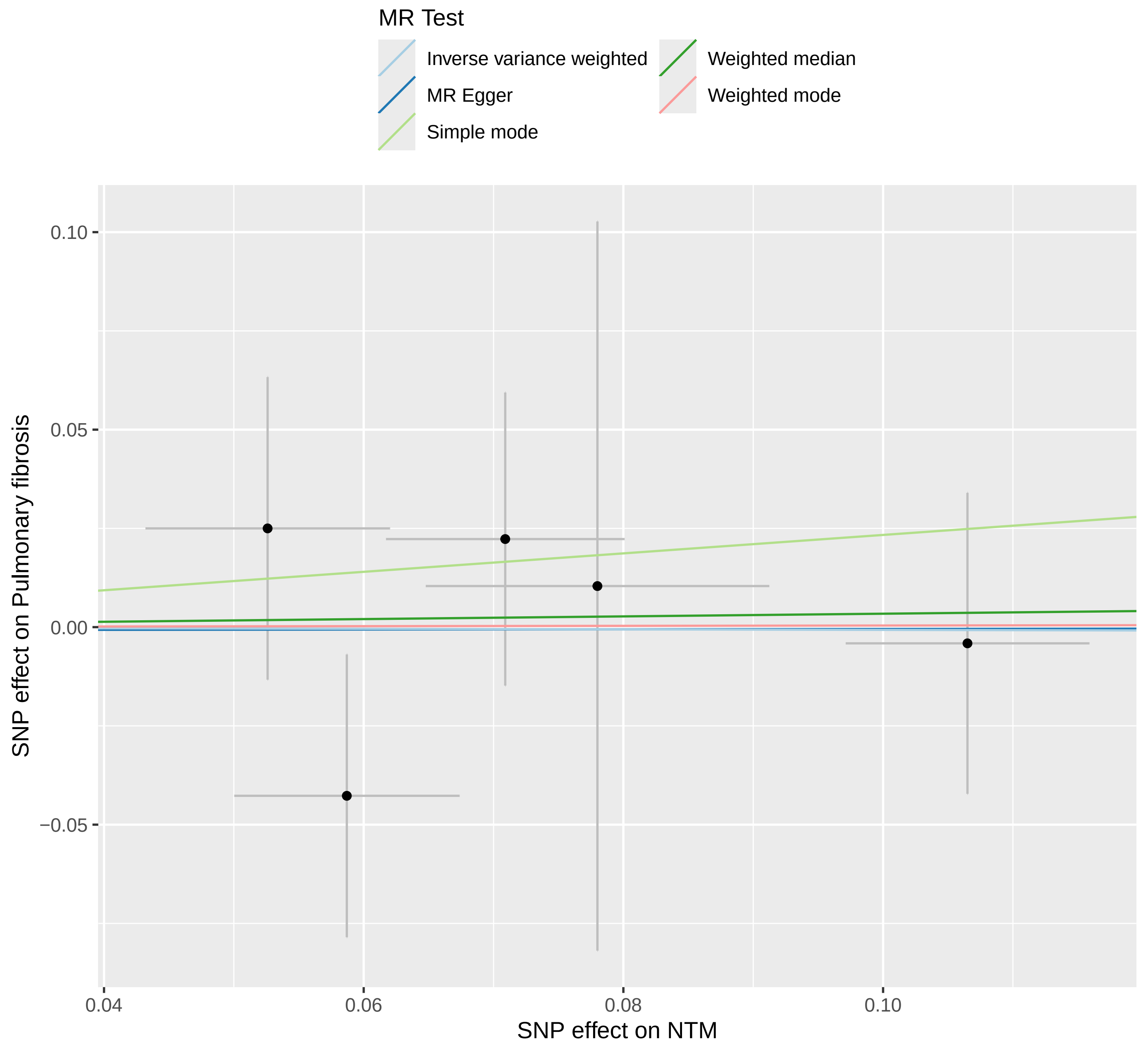

MR Test
Inverse variance weighted
MR Egger
Simple mode
Weighted median
Weighted mode
0.10
0.05
0.00
−0.05
SNP effect on Pulmonary fibrosis
0.04
0.06
0.08
0.10
SNP effect on NTM

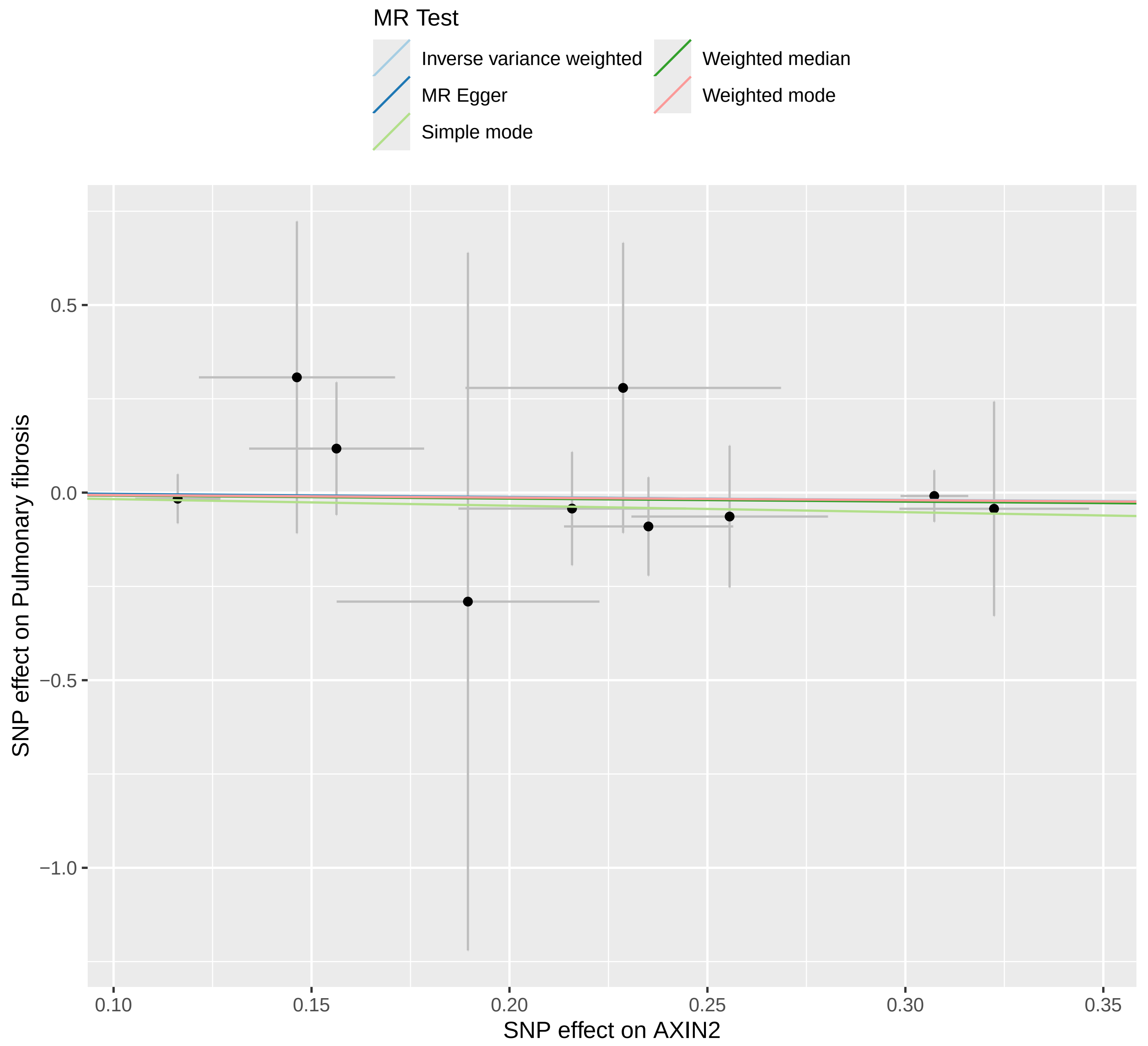

MR Test
Inverse variance weighted
MR Egger
Simple mode
Weighted median
Weighted mode
SNP effect on Pulmonary fibrosis
0.5
0.0
−0.5
−1.0
0.10
0.15
0.20
0.25
0.30
0.35
SNP effect on AXIN2

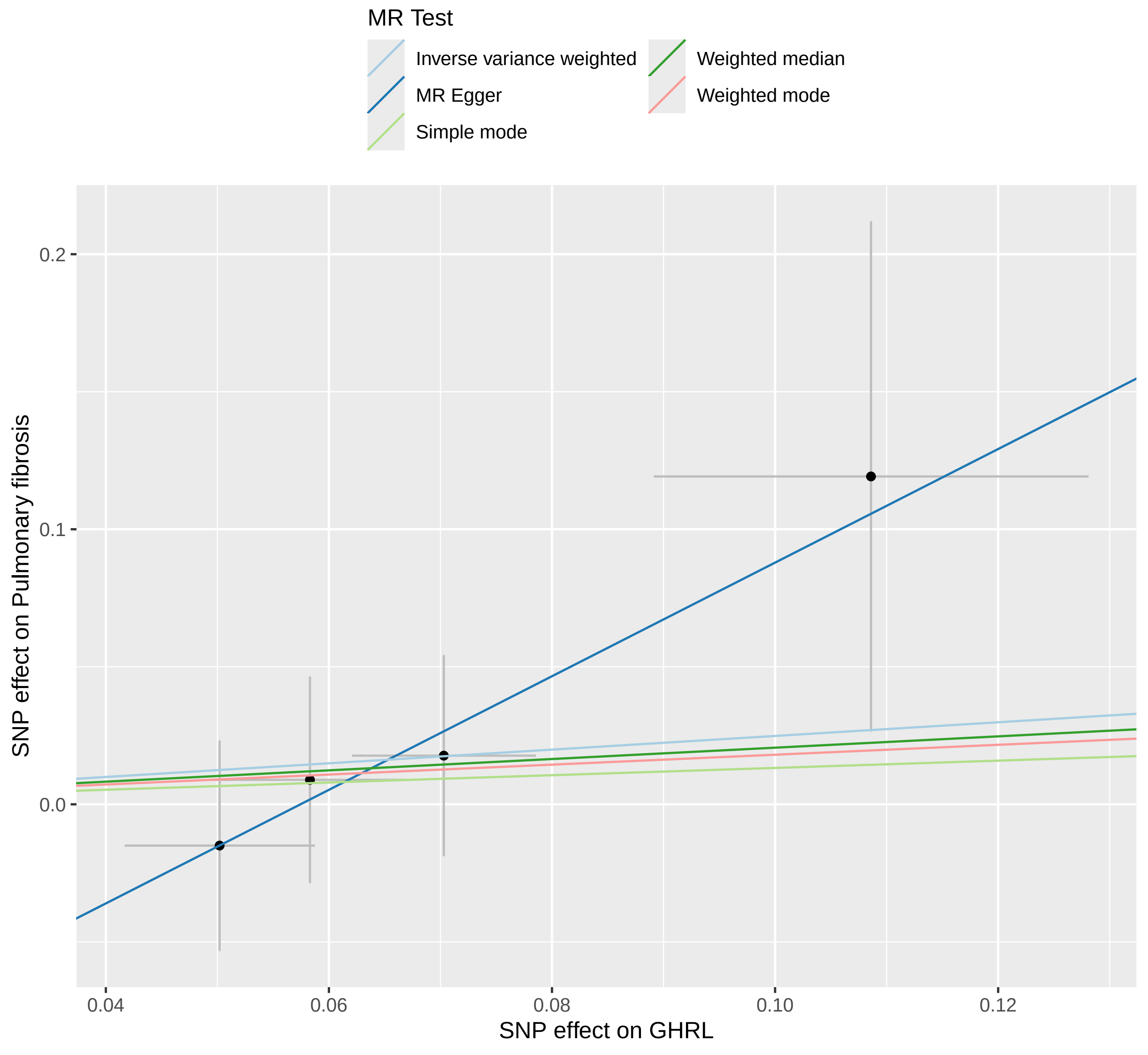

MR Test
Inverse variance weighted
MR Egger
Simple mode
Weighted median
Weighted mode
0.2
0.1
0.0
0.04
0.06
0.08
0.10
0.12
SNP effect on Pulmonary fibrosis
SNP effect on GHRL

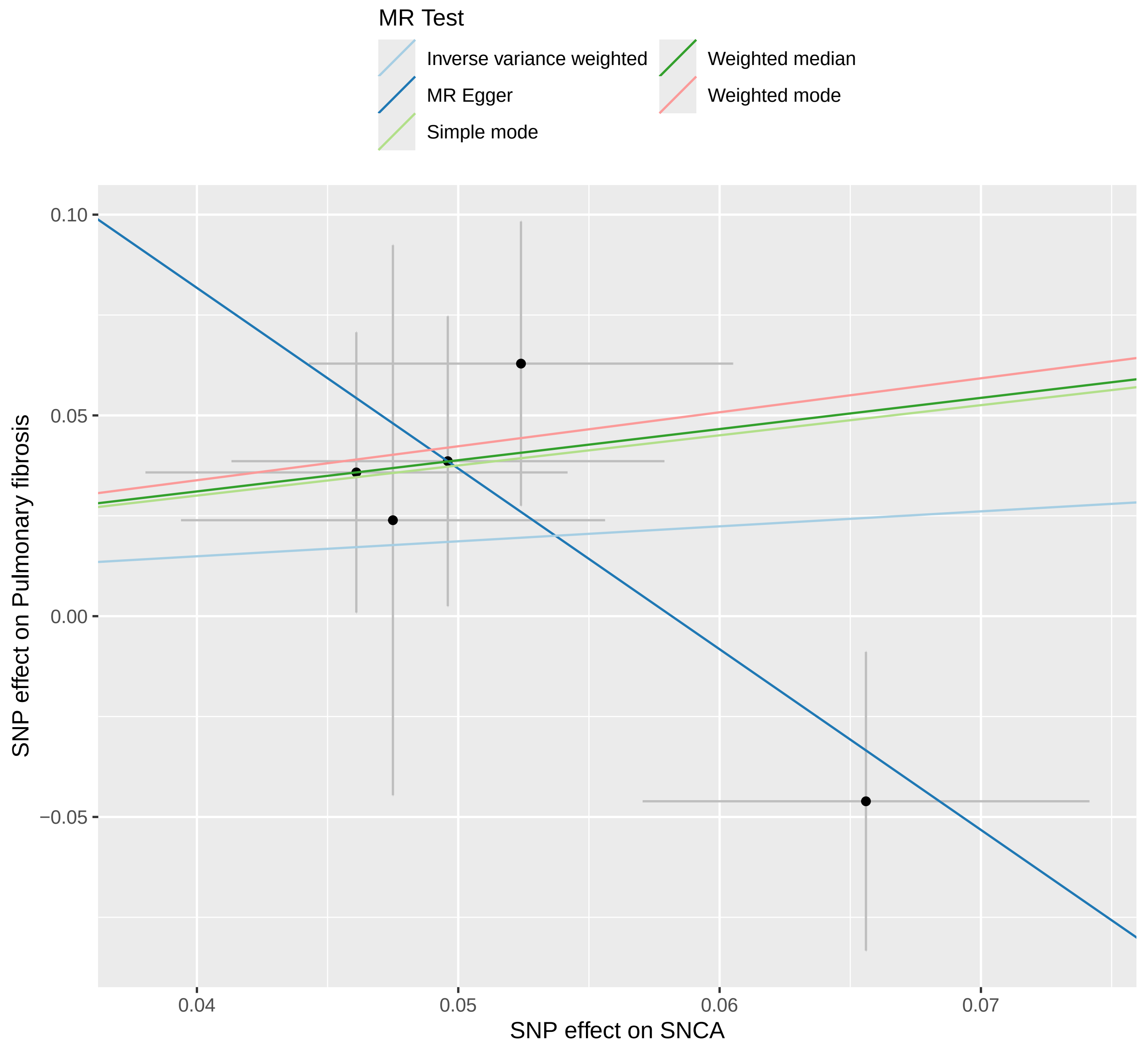
MR Test
Inverse variance weighted
MR Egger
Simple mode
Weighted median
Weighted mode
SNP effect on Pulmonary fibrosis
0.10
0.05
0.00
−0.05
0.04
0.05
0.06
0.07
SNP effect on SNCA

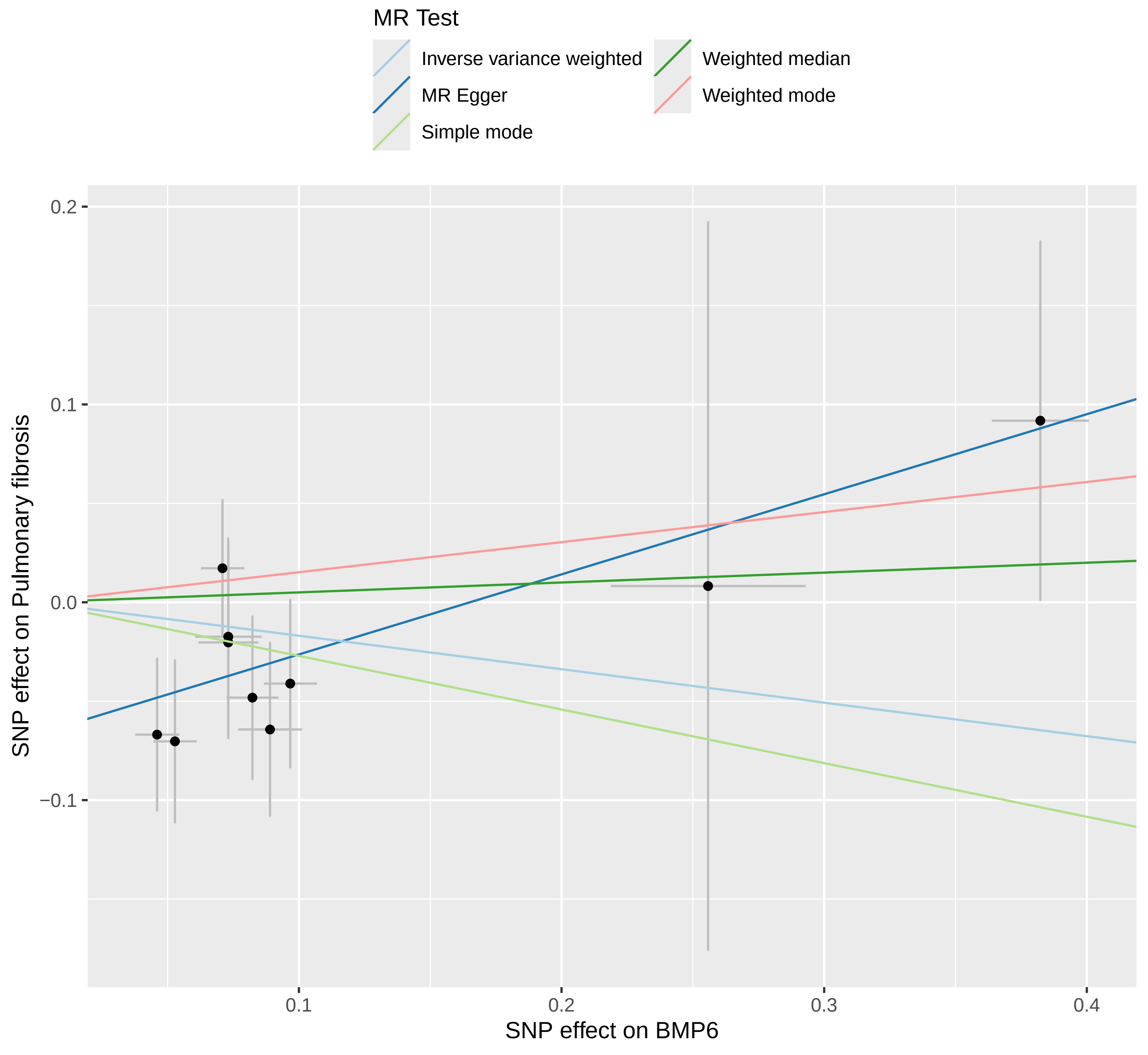
MR Test
Inverse variance weighted
MR Egger
Simple mode
Weighted median
Weighted mode
0.2
0.1
0.0
−0.1
0.1
0.2
0.3
0.4
SNP effect on Pulmonary fibrosis
SNP effect on BMP6

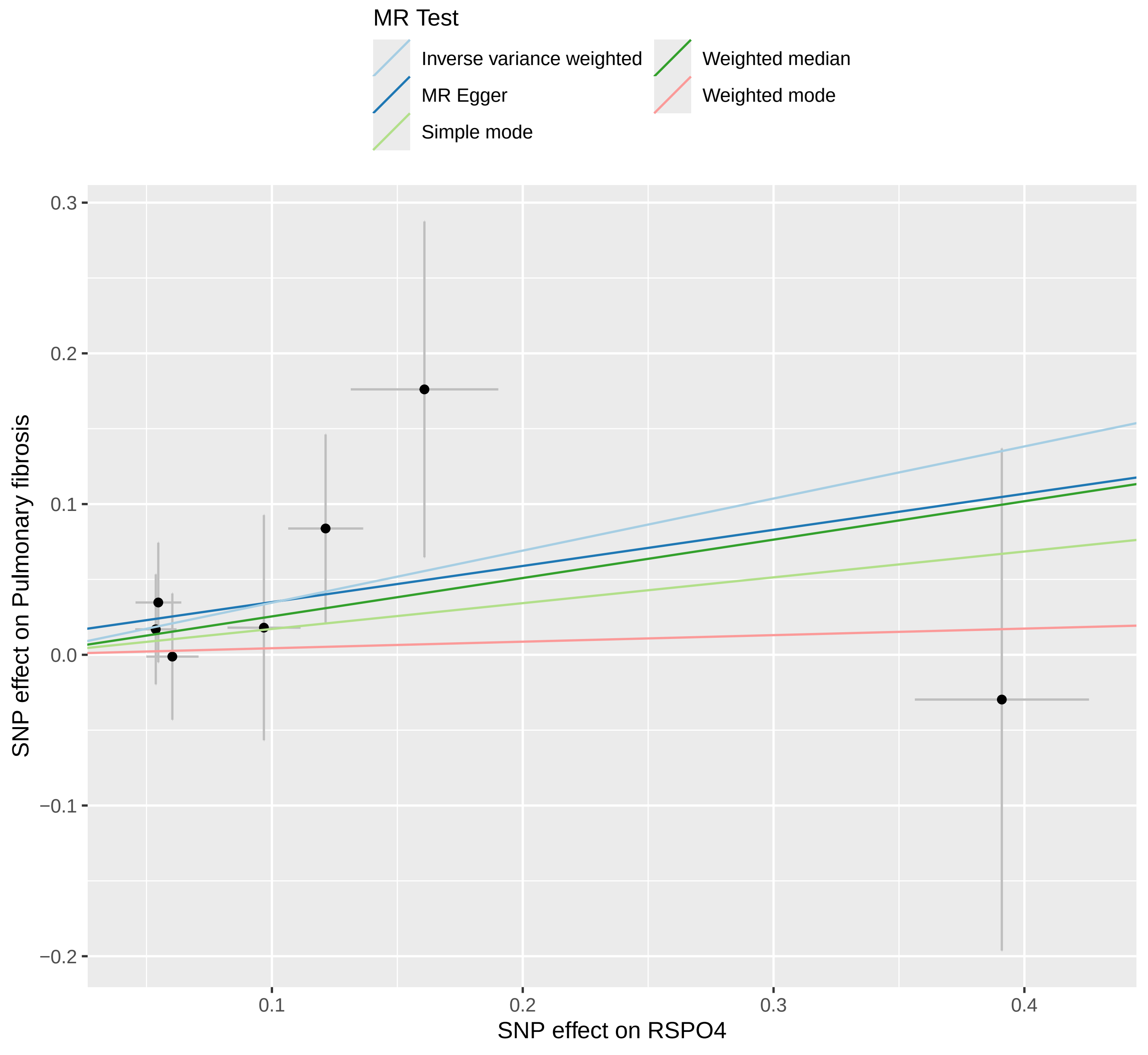

MR Test
Inverse variance weighted
MR Egger
Simple mode
Weighted median
Weighted mode
0.3
0.2
0.1
0.0
−0.1
−0.2
SNP effect on Pulmonary fibrosis
0.1
0.2
0.3
0.4
SNP effect on RSPO4

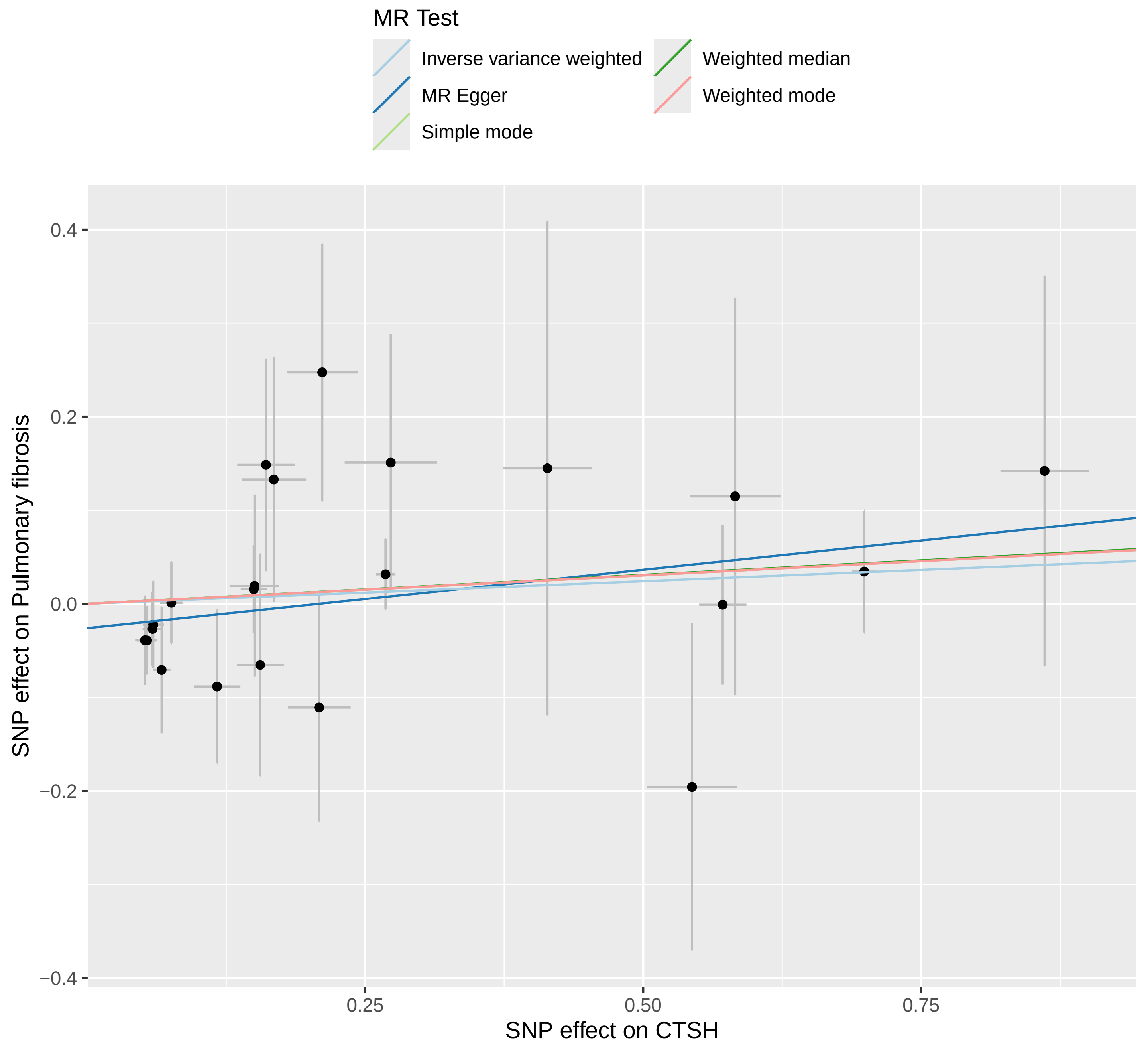
MR Test
Inverse variance weighted
MR Egger
Simple mode
Weighted median
Weighted mode
0.4
0.2
0.0
−0.2
−0.4
0.25
0.50
0.75
SNP effect on Pulmonary fibrosis
SNP effect on CTSH

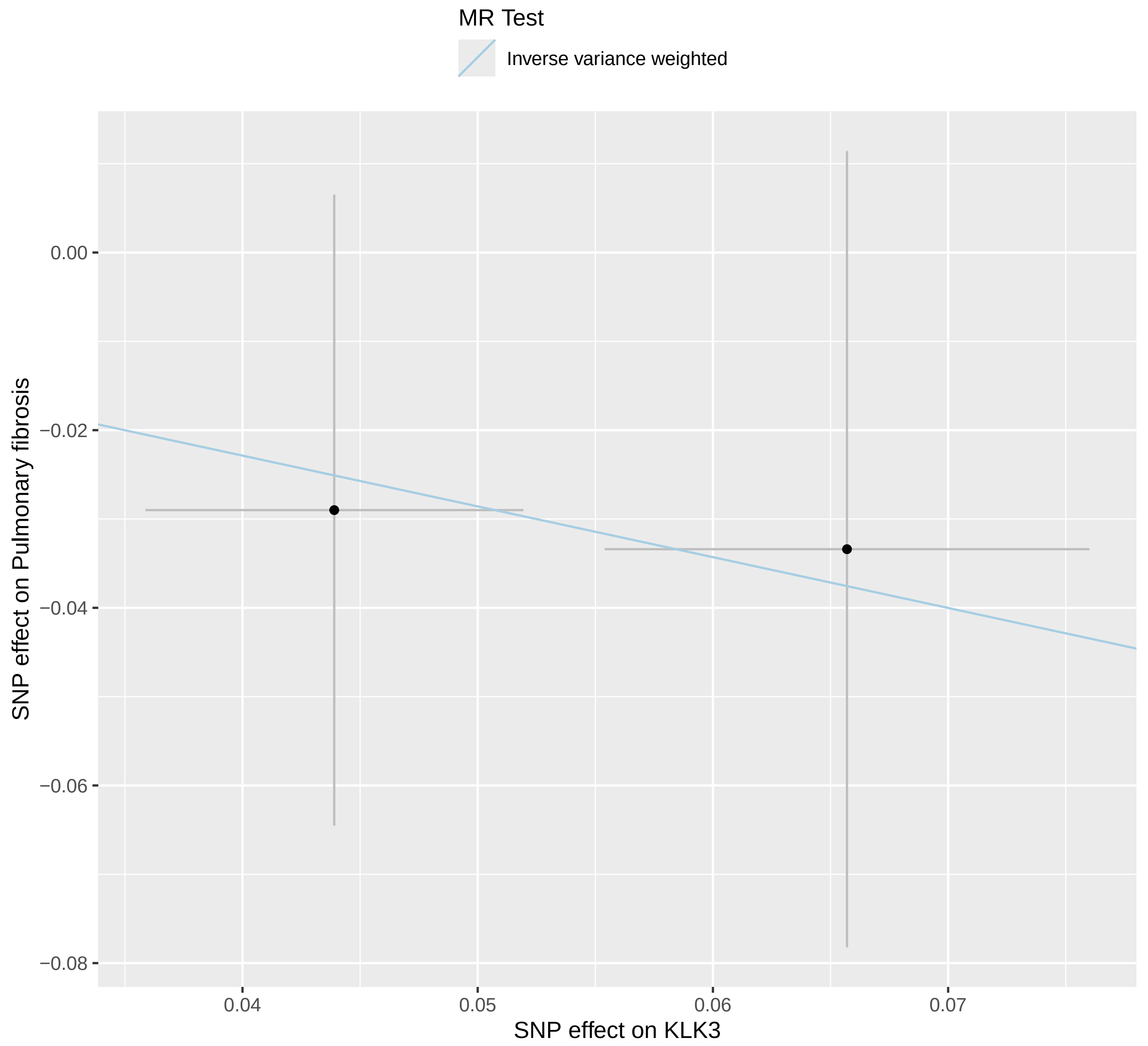

MR Test
Inverse variance weighted
0.00
−0.02
−0.04
−0.06
−0.08
SNP effect on Pulmonary fibrosis
0.04
0.05
0.06
0.07
SNP effect on KLK3

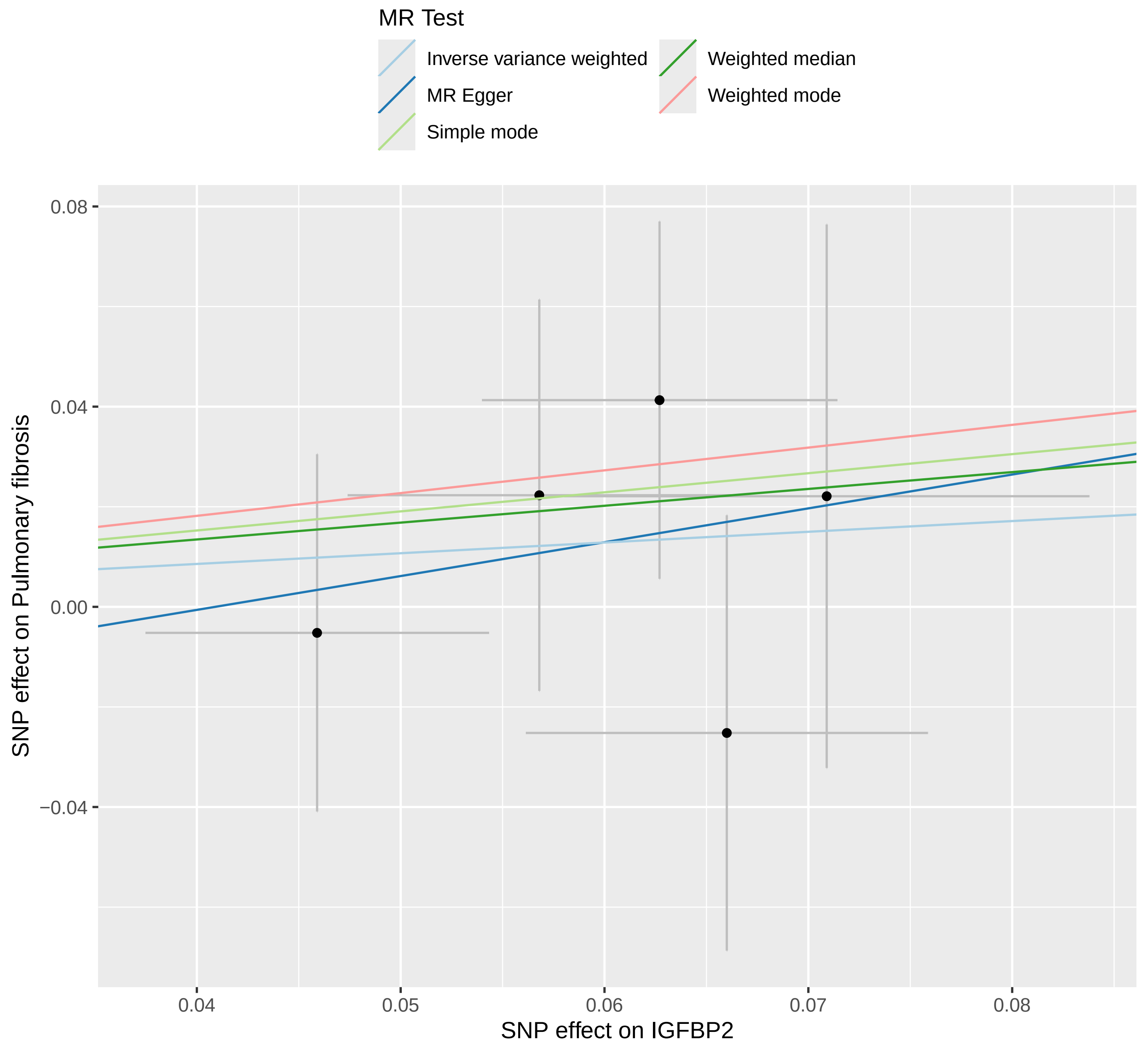

MR Test
Inverse variance weighted
MR Egger
Simple mode
Weighted median
Weighted mode
0.08
0.04
0.00
−0.04
SNP effect on Pulmonary fibrosis
0.04
0.05
0.06
0.07
0.08
SNP effect on IGFBP2

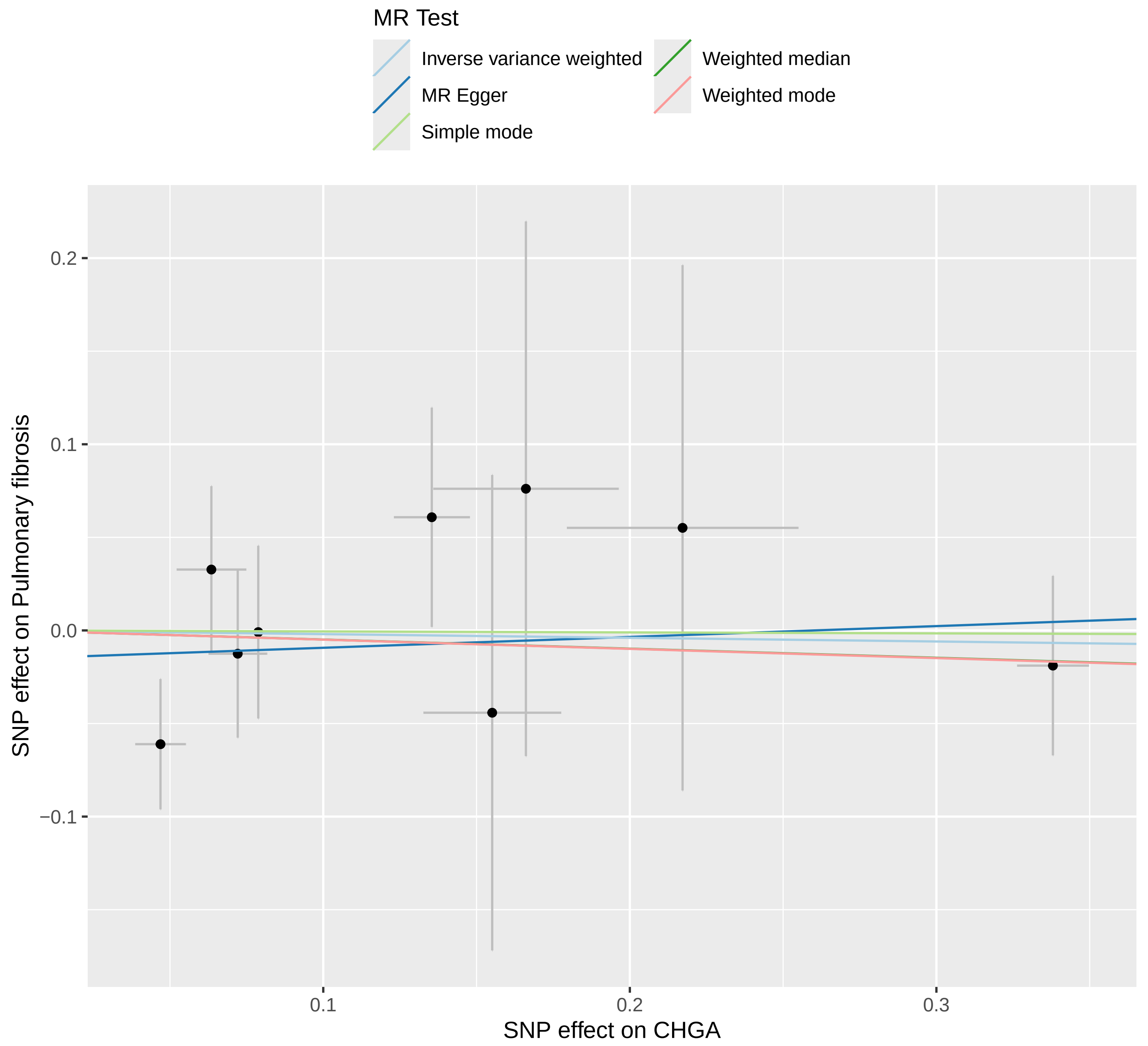

MR Test
Inverse variance weighted
MR Egger
Simple mode
Weighted median
Weighted mode
0.2
0.1
0.0
−0.1
SNP effect on Pulmonary fibrosis
0.1
0.2
0.3
SNP effect on CHGA

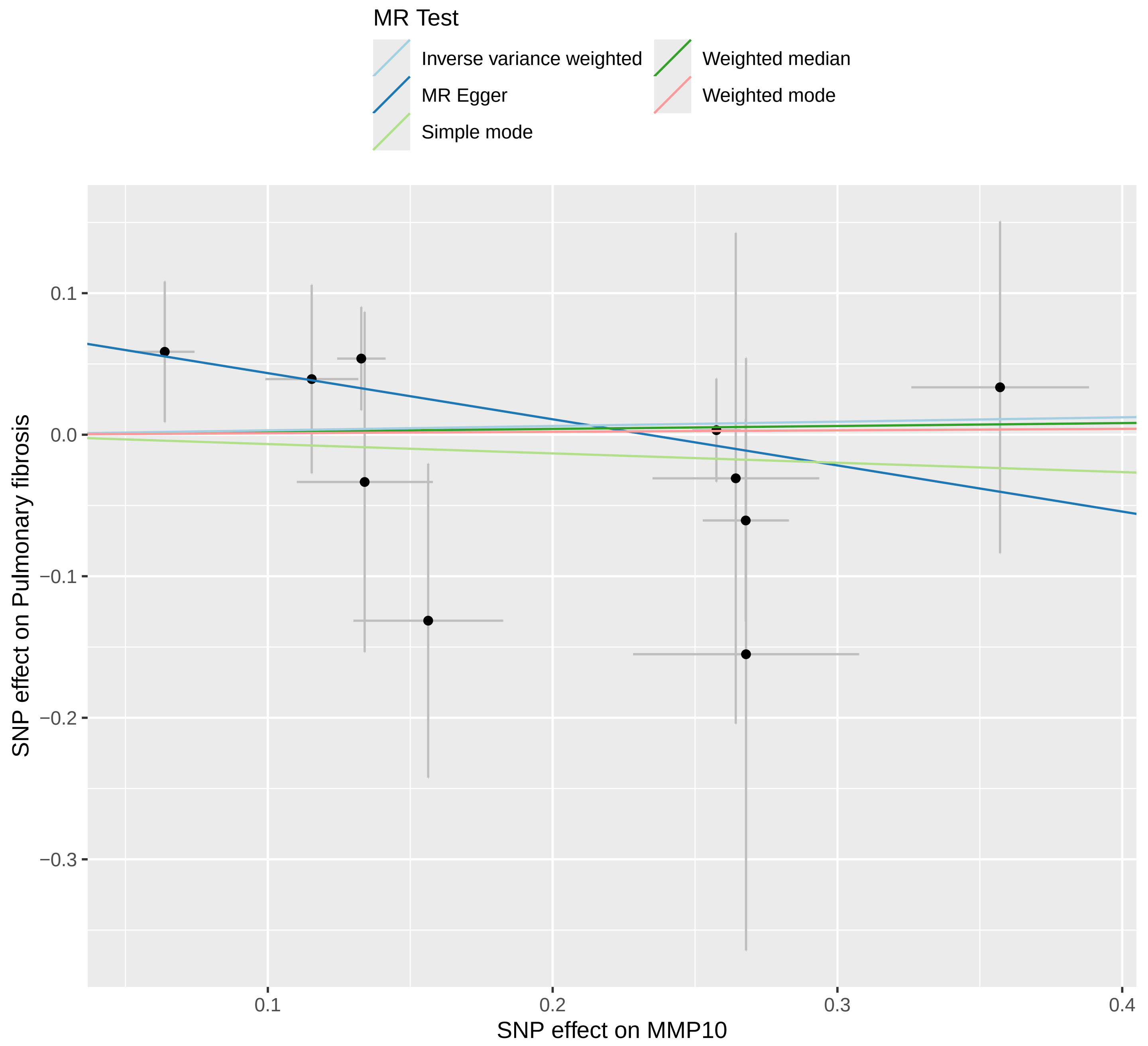

MR Test
Inverse variance weighted
MR Egger
Simple mode
Weighted median
Weighted mode
0.1
0.0
−0.1
−0.2
−0.3
SNP effect on Pulmonary fibrosis
0.1
0.2
0.3
0.4
SNP effect on MMP10

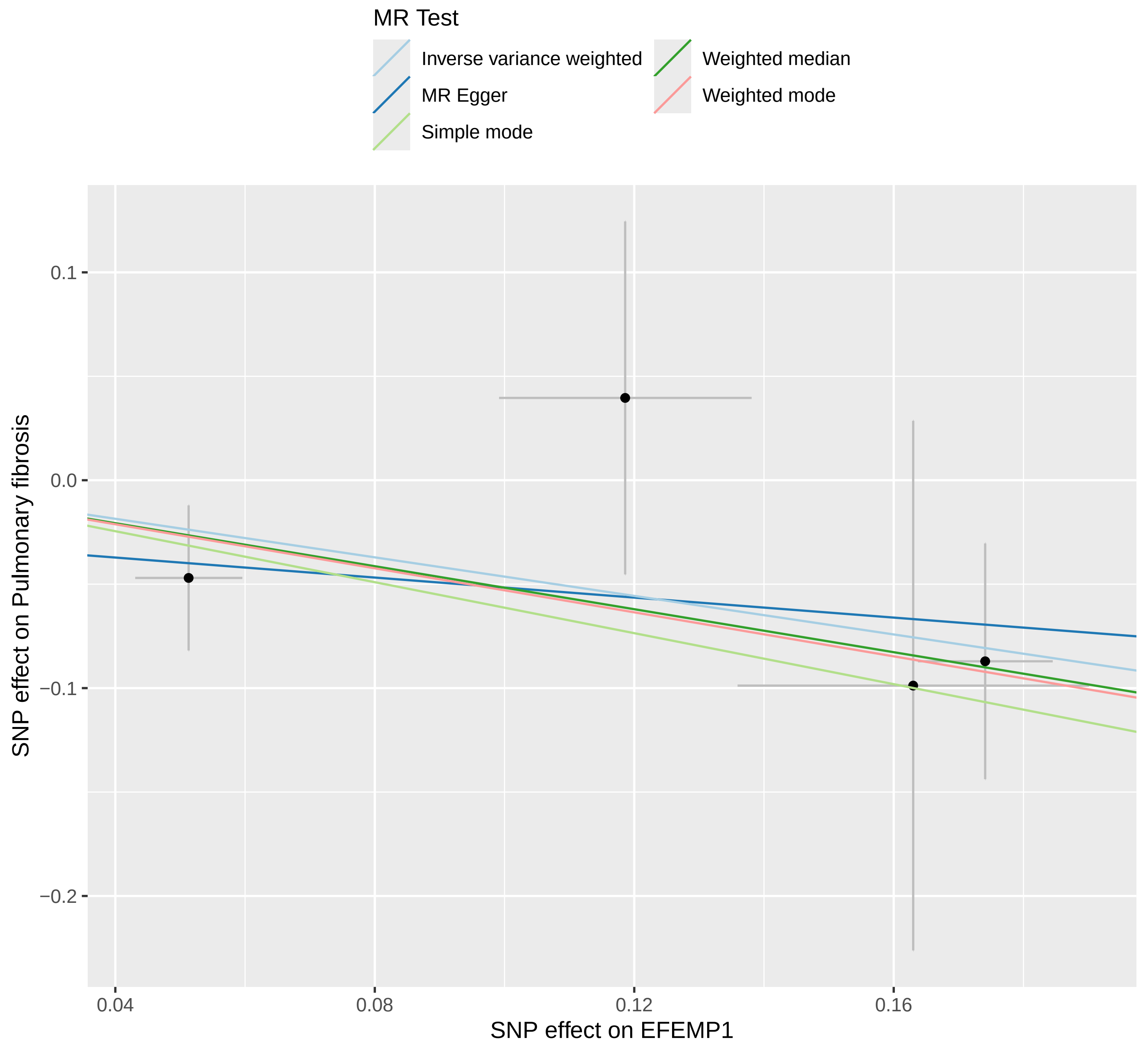
MR Test
Inverse variance weighted
MR Egger
Simple mode
Weighted median
Weighted mode
0.1
0.0
−0.1
−0.2
SNP effect on Pulmonary fibrosis
0.04
0.08
0.12
0.16
SNP effect on EFEMP1

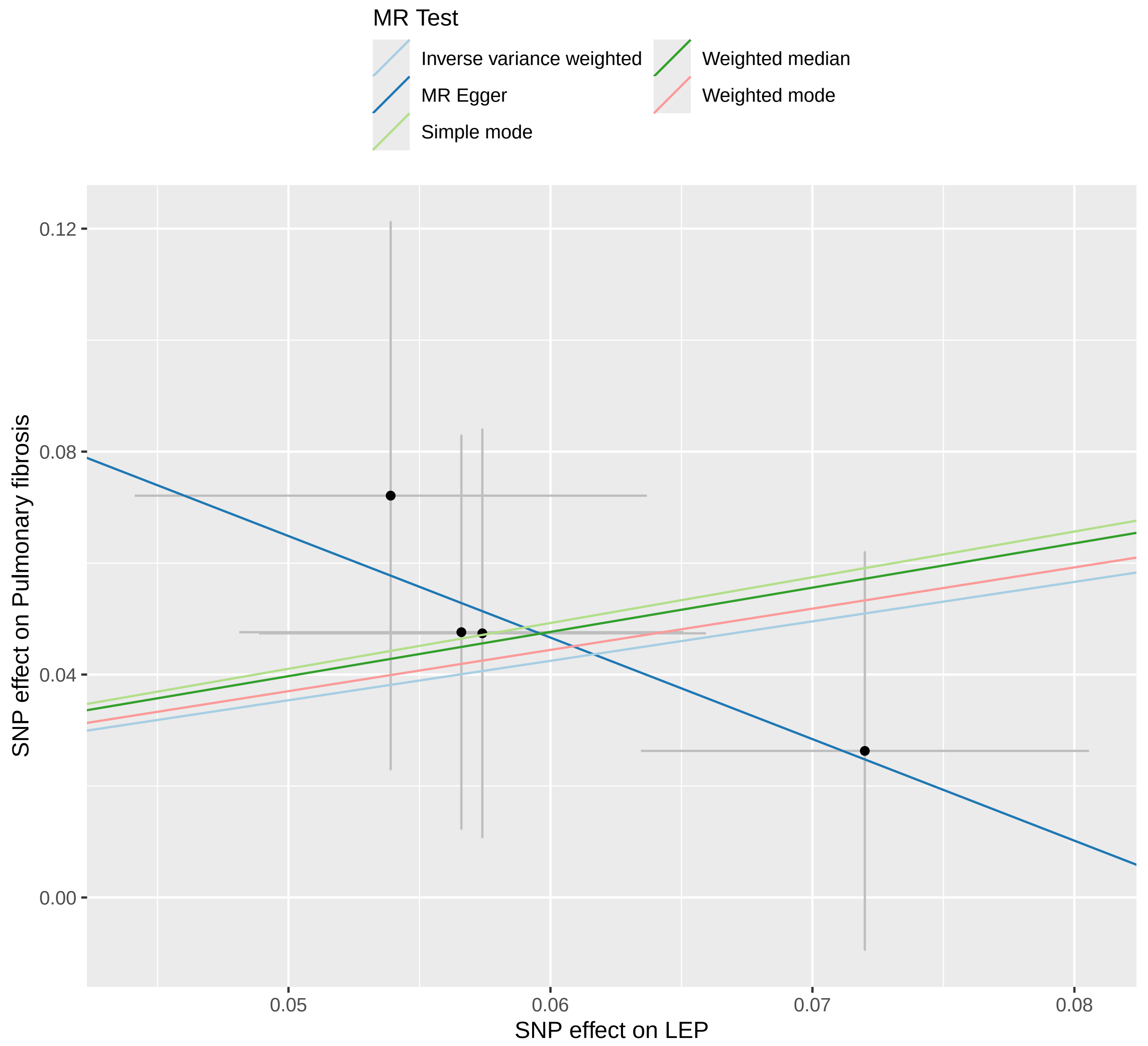

MR Test
Inverse variance weighted
MR Egger
Simple mode
Weighted median
Weighted mode
0.12
0.08
0.04
0.00
SNP effect on Pulmonary fibrosis
0.05
0.06
0.07
0.08
SNP effect on LEP

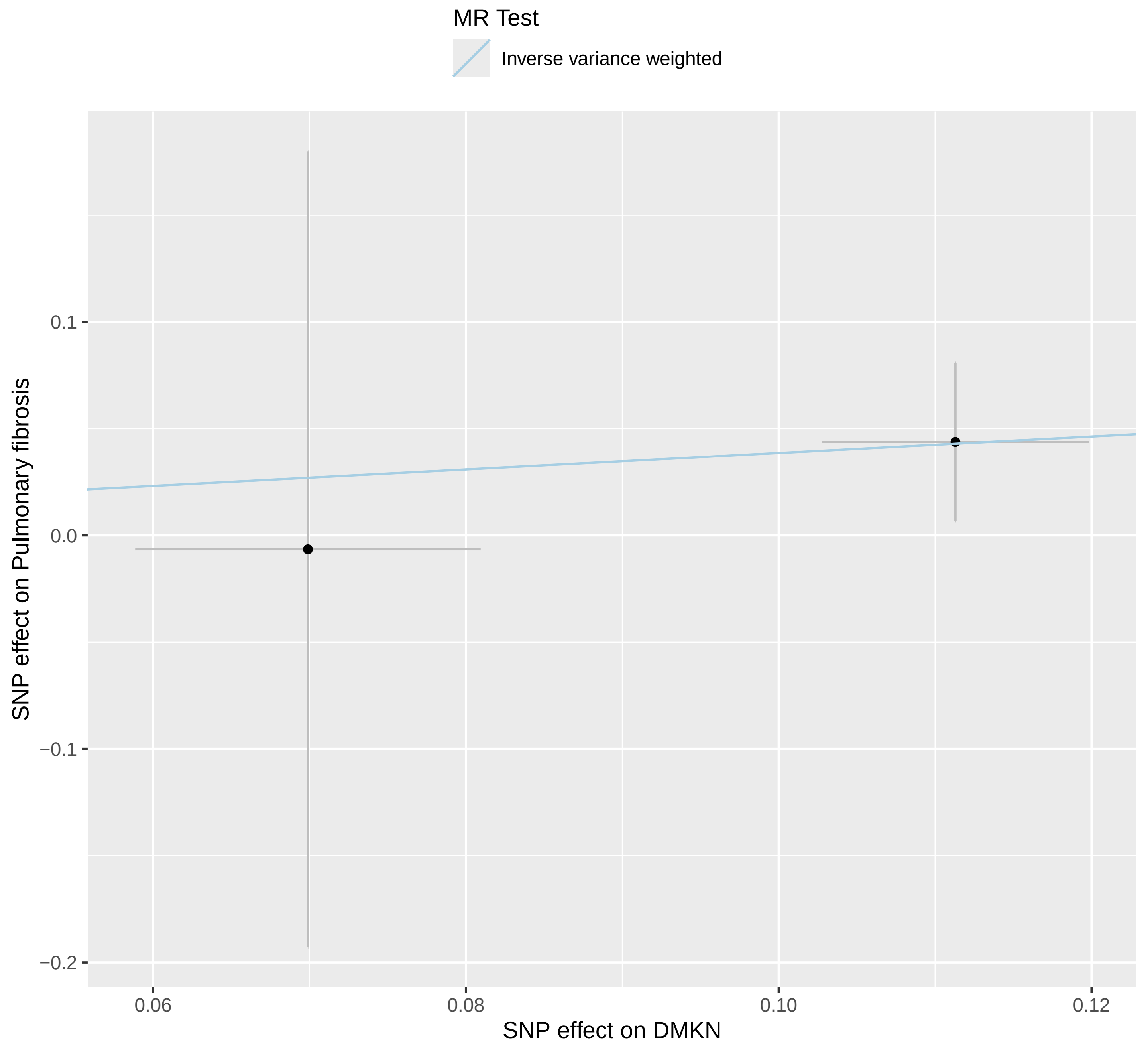
MR Test
Inverse variance weighted
0.1
0.0
−0.1
−0.2
SNP effect on Pulmonary fibrosis
0.06
0.08
0.10
0.12
SNP effect on DMKN

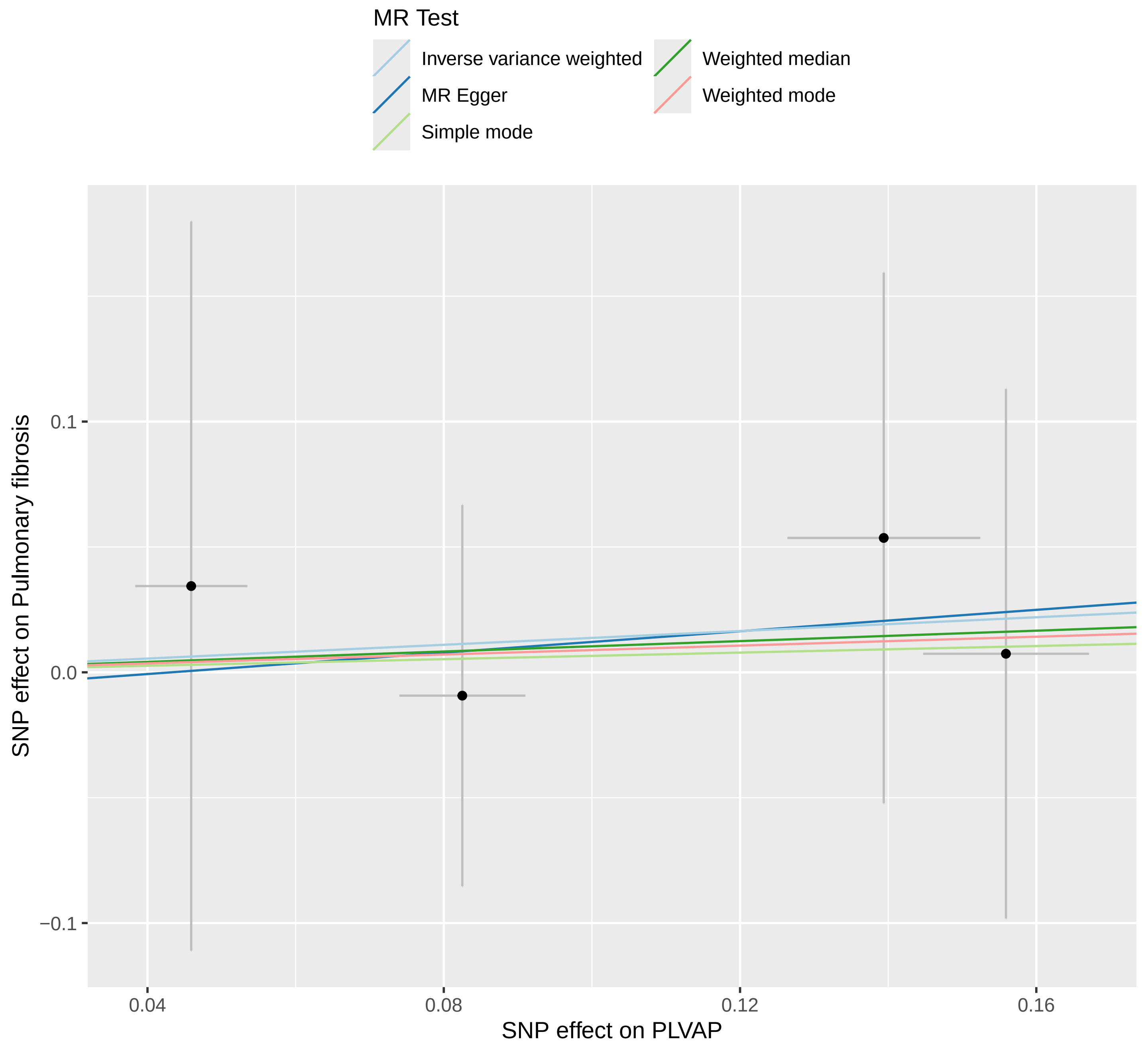

MR Test
Inverse variance weighted
MR Egger
Simple mode
Weighted median
Weighted mode
SNP effect on Pulmonary fibrosis
0.1
0.0
−0.1
0.04
0.08
0.12
0.16
SNP effect on PLVAP

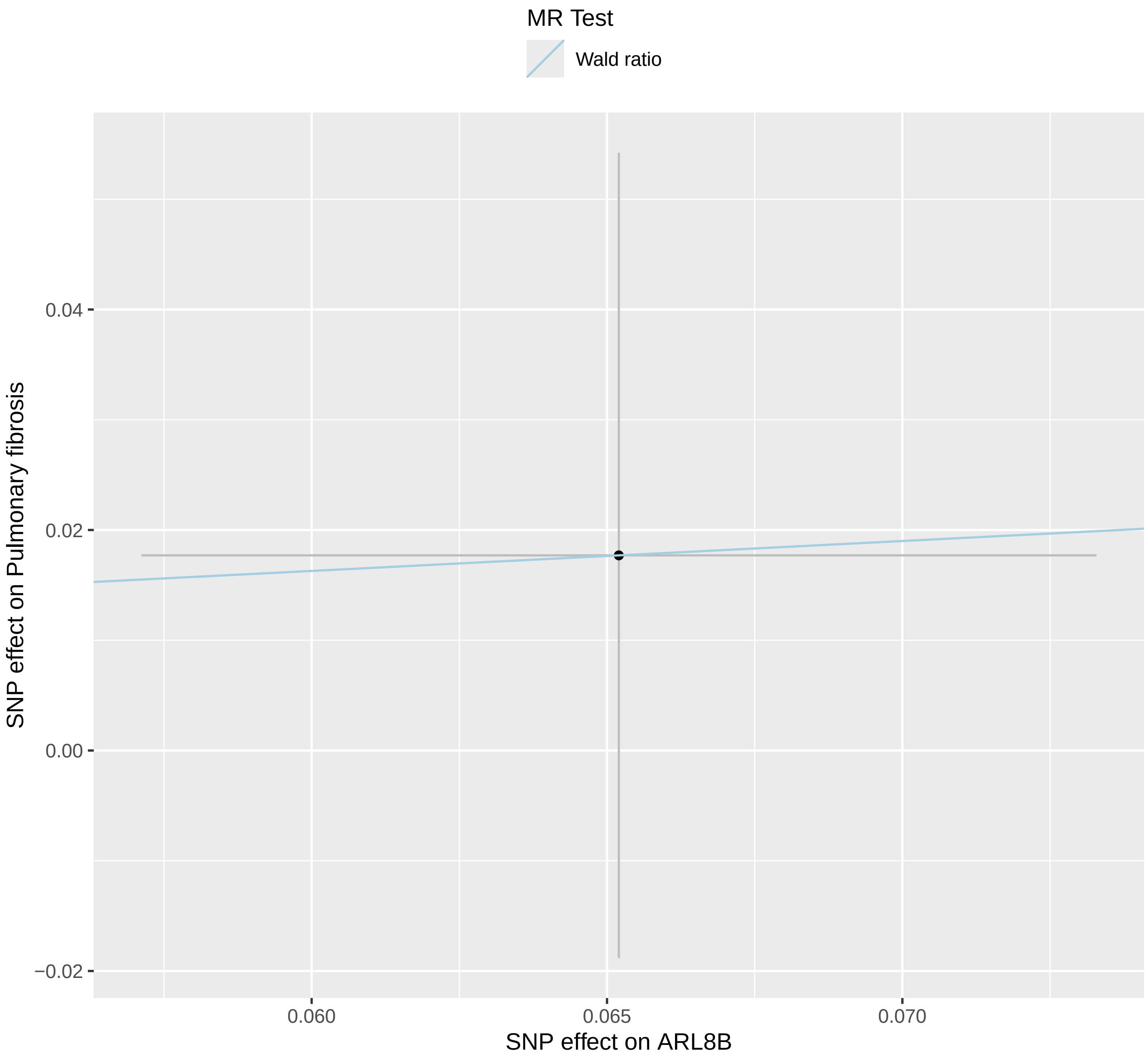

MR Test
Wald ratio
0.04
0.02
0.00
−0.02
0.060
0.065
0.070
SNP effect on Pulmonary fibrosis
SNP effect on ARL8B

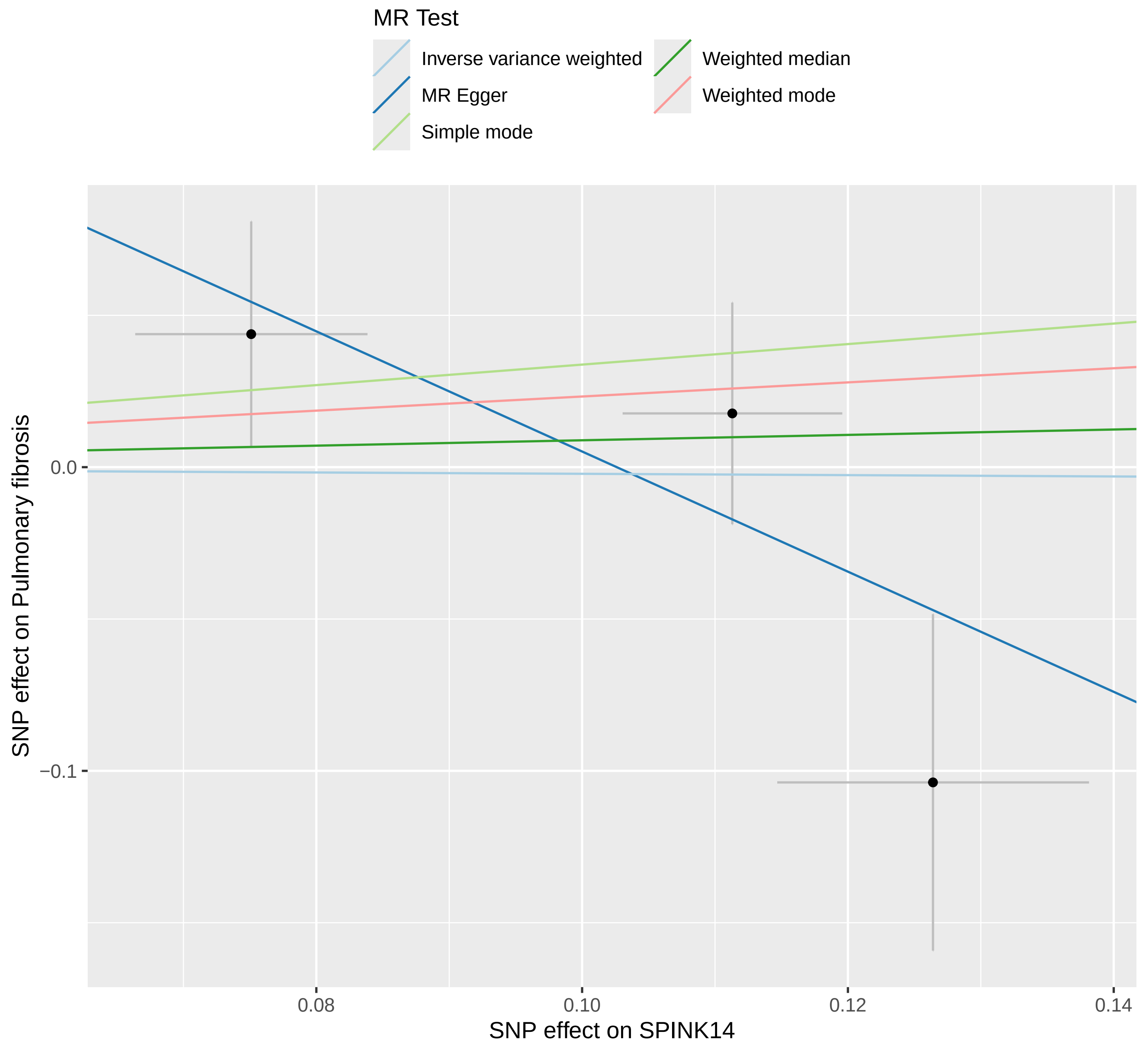

MR Test
Inverse variance weighted
MR Egger
Simple mode
Weighted median
Weighted mode
0.0
−0.1
0.08
0.10
0.12
0.14
SNP effect on Pulmonary fibrosis
SNP effect on SPINK14

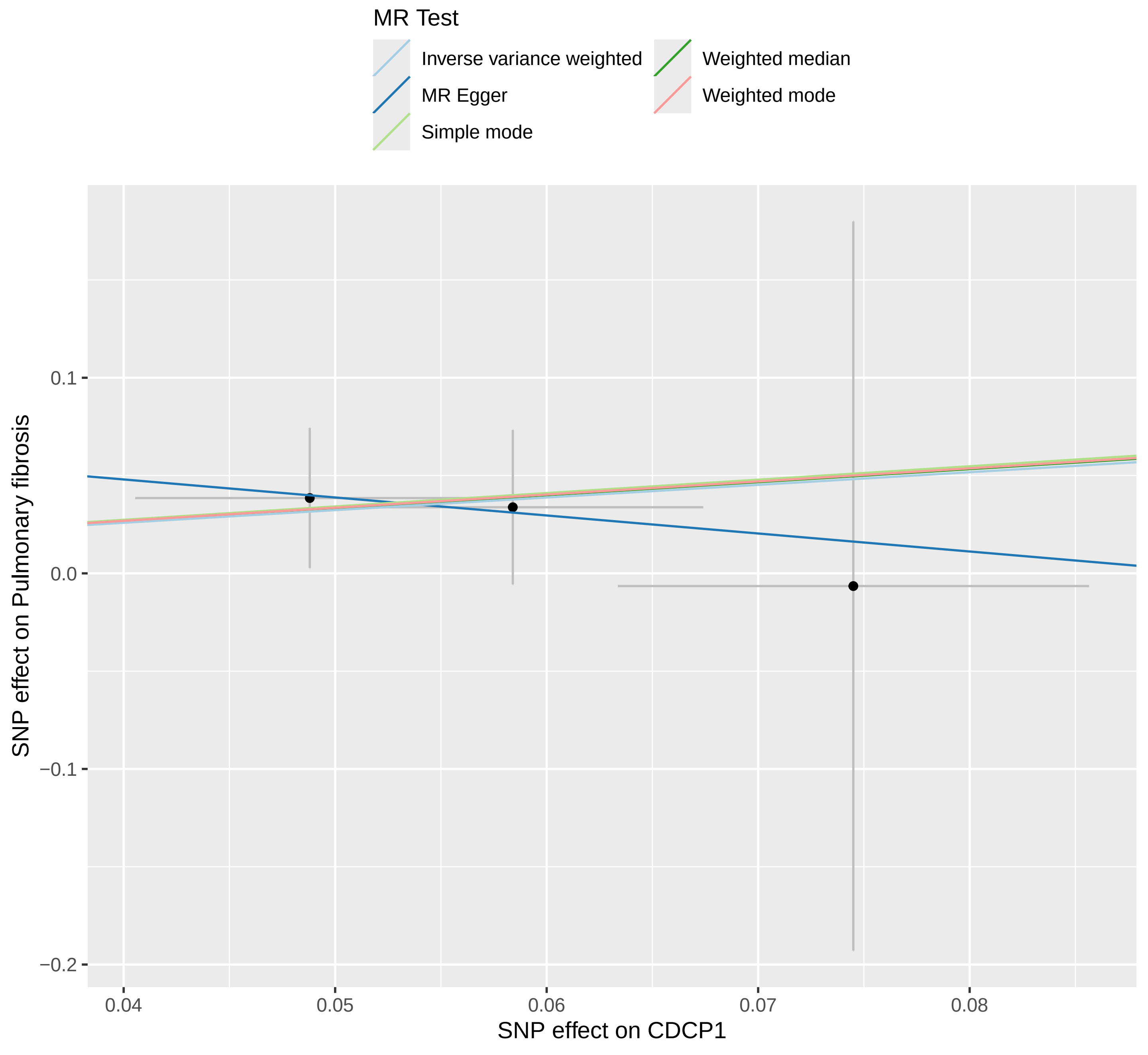

MR Test
Inverse variance weighted
MR Egger
Simple mode
Weighted median
Weighted mode
0.1
0.0
−0.1
−0.2
0.04
0.05
0.06
0.07
0.08
SNP effect on Pulmonary fibrosis
SNP effect on CDCP1

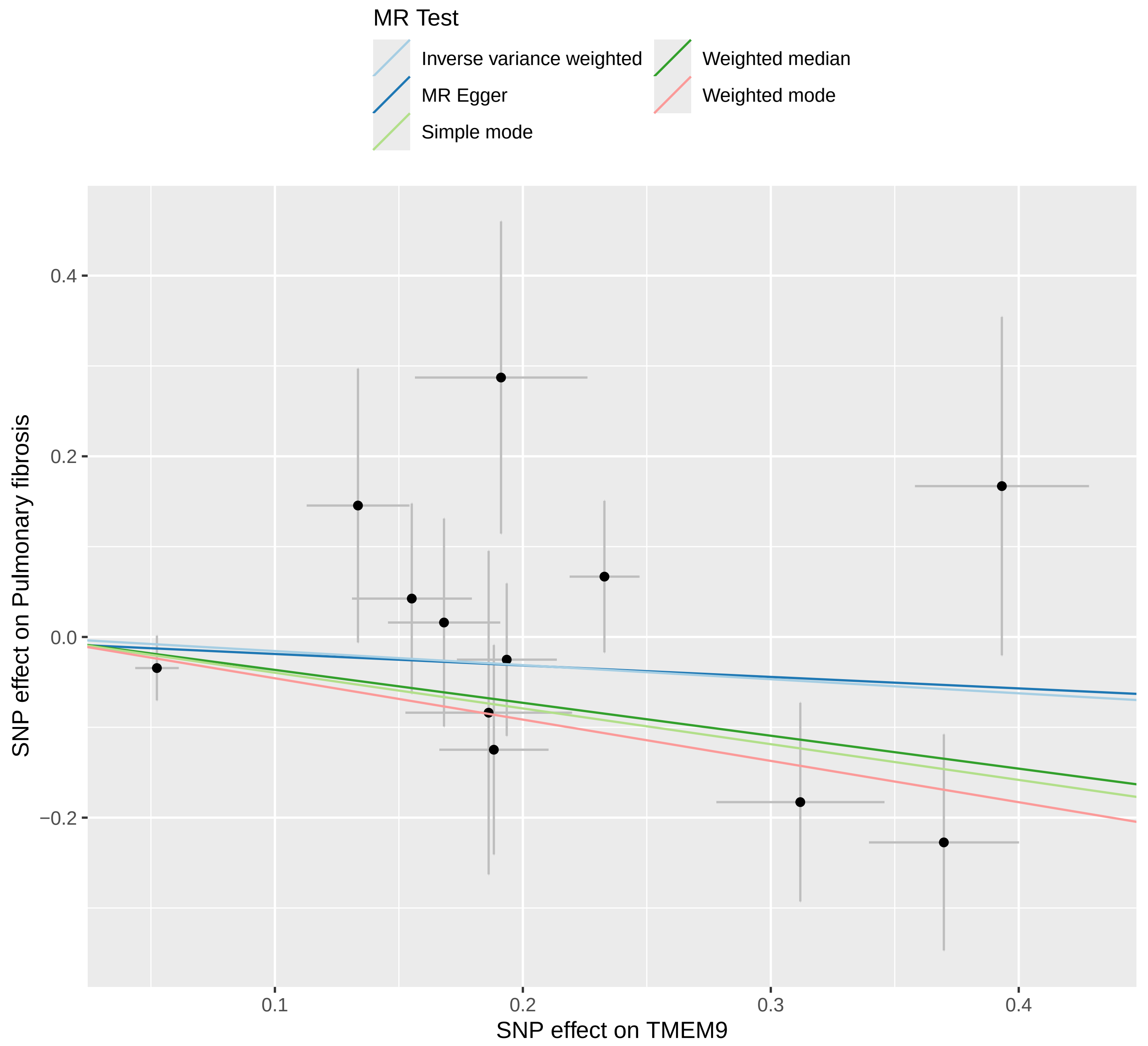

MR Test
Inverse variance weighted
MR Egger
Simple mode
Weighted median
Weighted mode
0.4
0.2
0.0
−0.2
0.1
0.2
0.3
0.4
SNP effect on TMEM9
SNP effect on Pulmonary fibrosis

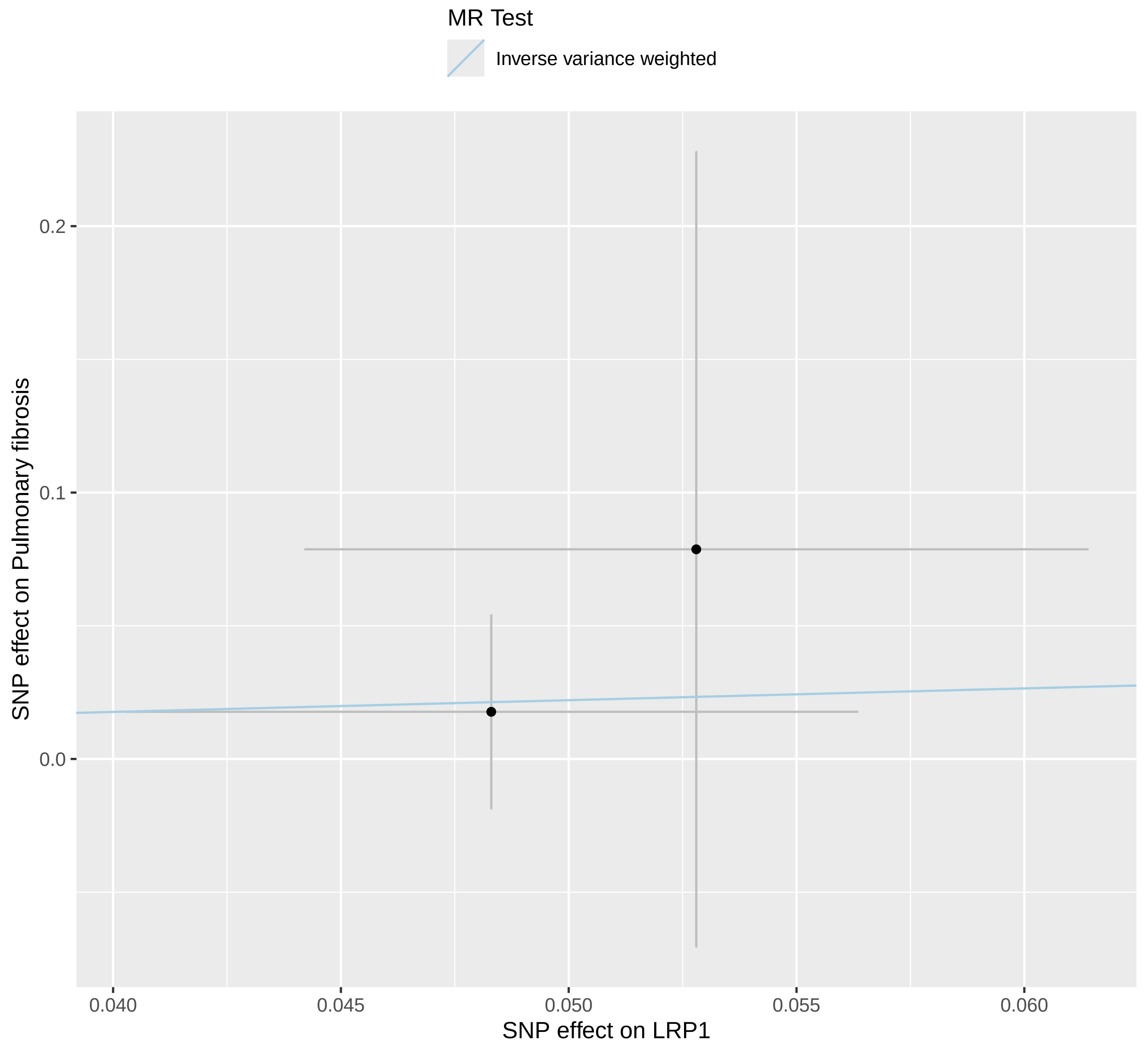
MR Test
Inverse variance weighted
0.2
0.1
0.0
0.040
0.045
0.050
0.055
0.060
SNP effect on LRP1
SNP effect on Pulmonary fibrosis

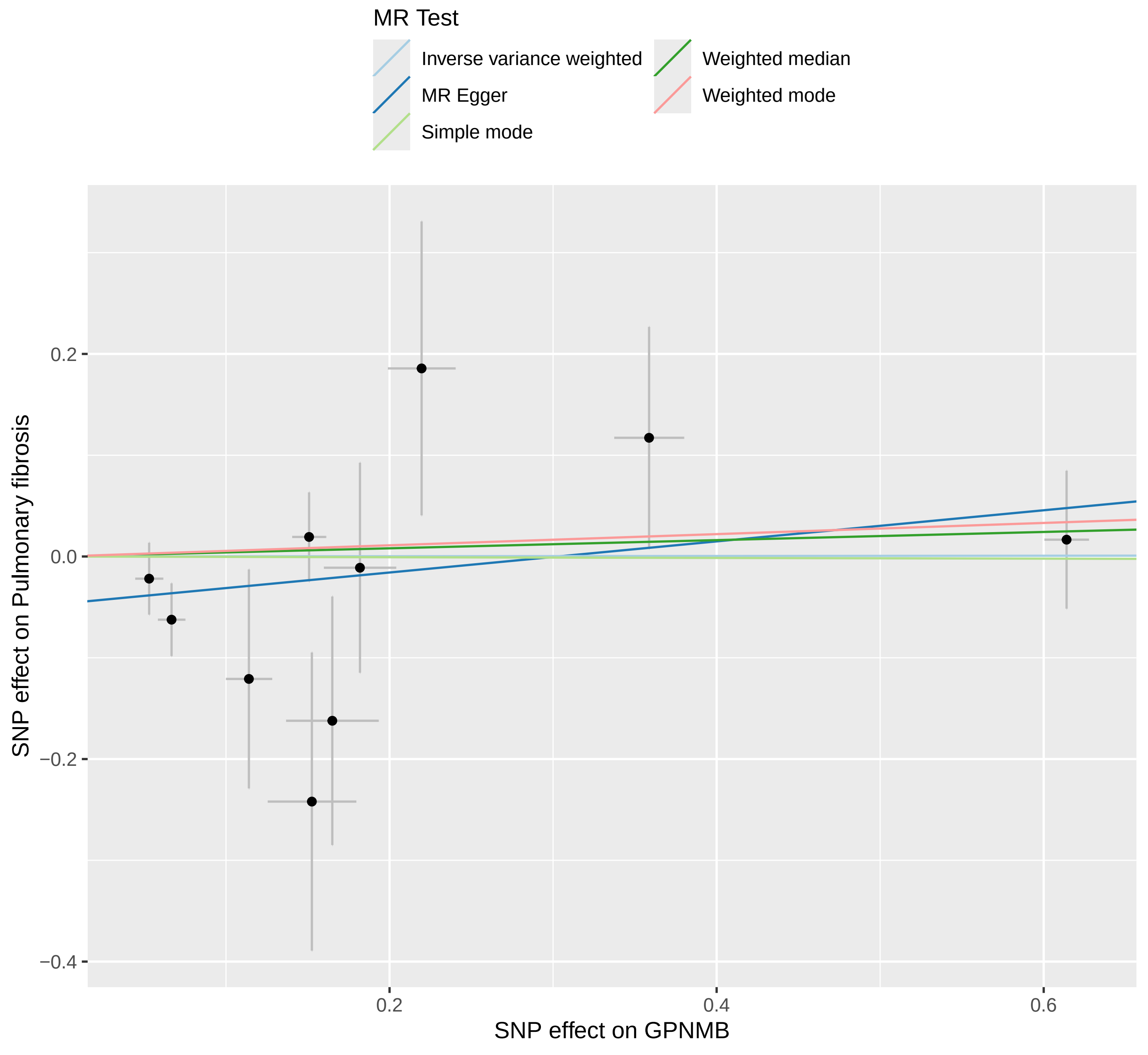

MR Test
Inverse variance weighted
MR Egger
Simple mode
Weighted median
Weighted mode
SNP effect on Pulmonary fibrosis
0.2
0.0
−0.2
−0.4
0.2
0.4
0.6
SNP effect on GPNMB

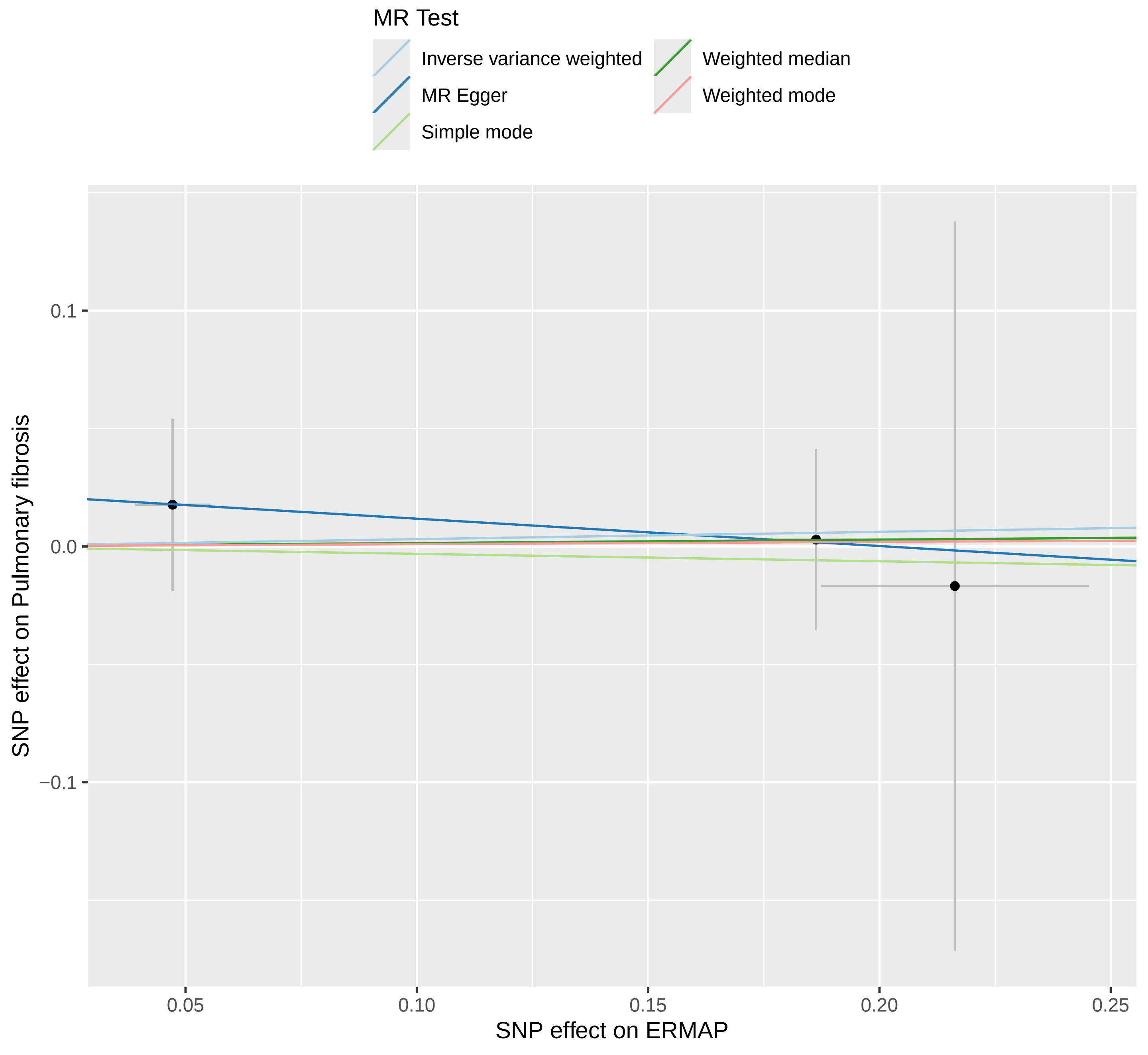

MR Test
Inverse variance weighted
MR Egger
Simple mode
Weighted median
Weighted mode
0.1
0.0
−0.1
0.05
0.10
0.15
0.20
0.25
SNP effect on ERMAP
SNP effect on Pulmonary fibrosis

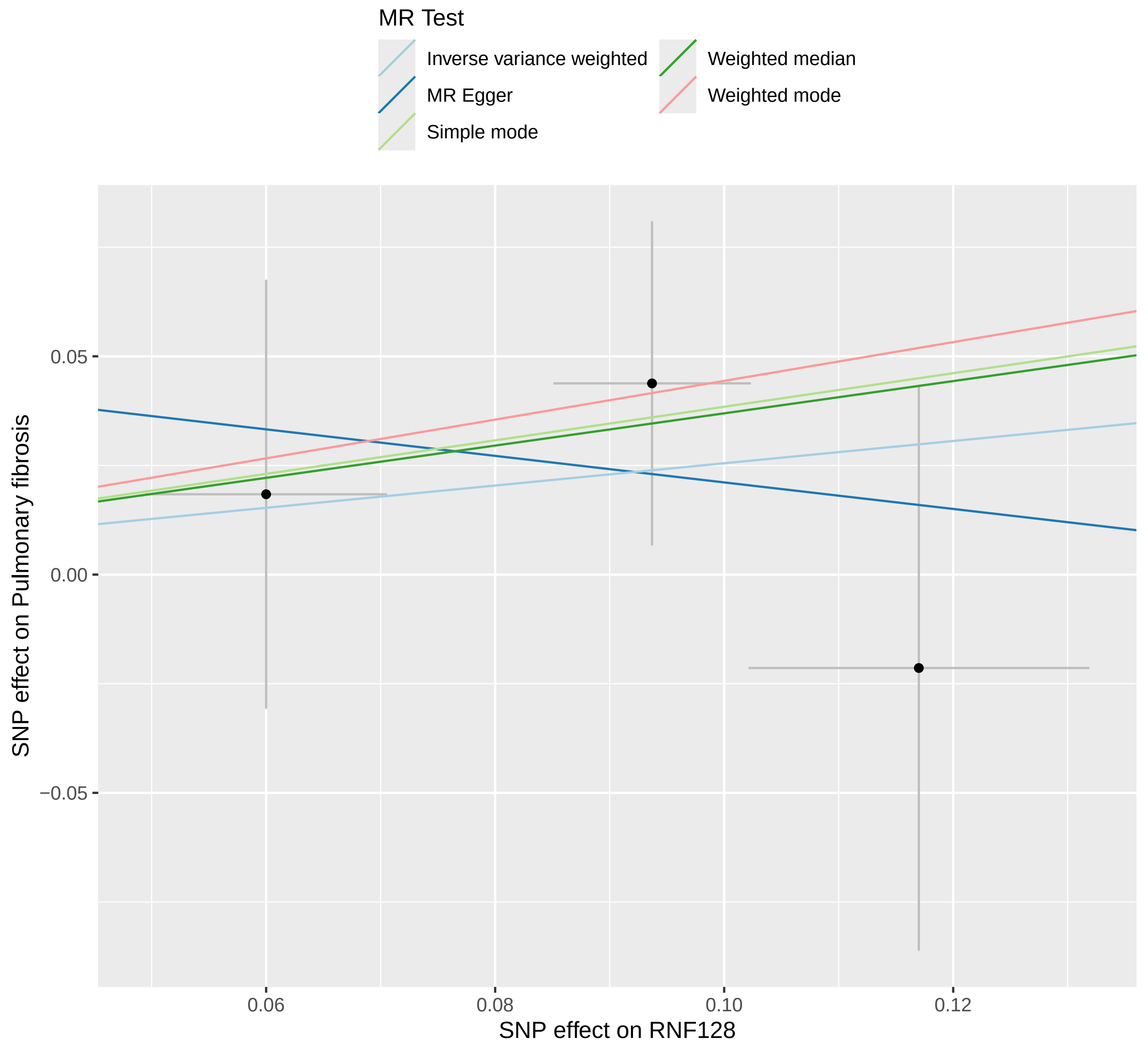
MR Test
Inverse variance weighted
MR Egger
Simple mode
Weighted median
Weighted mode
0.05
0.00
−0.05
SNP effect on Pulmonary fibrosis
0.06
0.08
0.10
0.12
SNP effect on RNF128

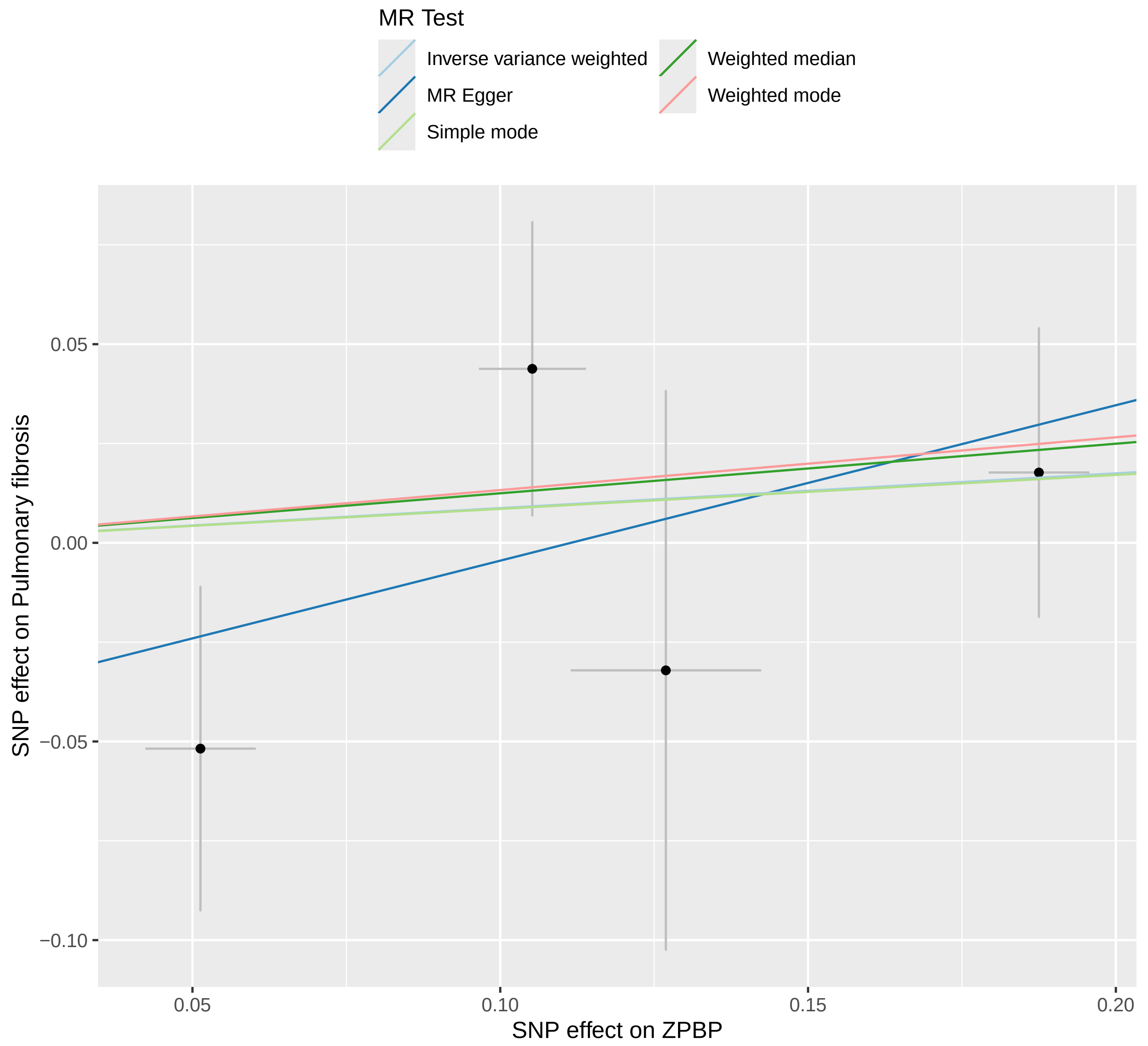

MR Test
Inverse variance weighted
MR Egger
Simple mode
Weighted median
Weighted mode
0.05
0.00
−0.05
−0.10
0.05
0.10
0.15
0.20
SNP effect on Pulmonary fibrosis
SNP effect on ZPBP

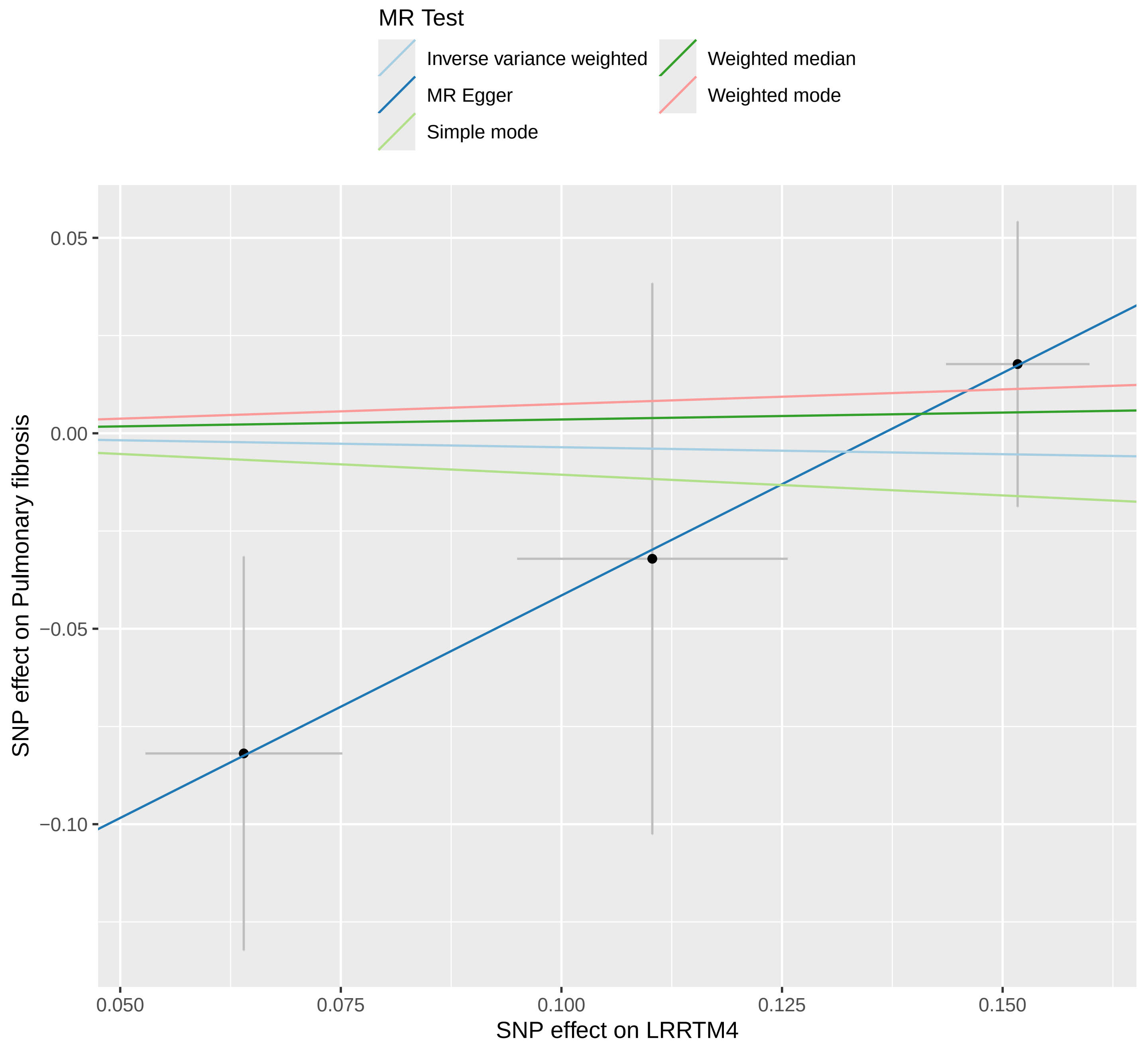
MR Test
Inverse variance weighted
MR Egger
Simple mode
Weighted median
Weighted mode
SNP effect on Pulmonary fibrosis
0.05
0.00
−0.05
−0.10
0.050
0.075
0.100
0.125
0.150
SNP effect on LRRTM4

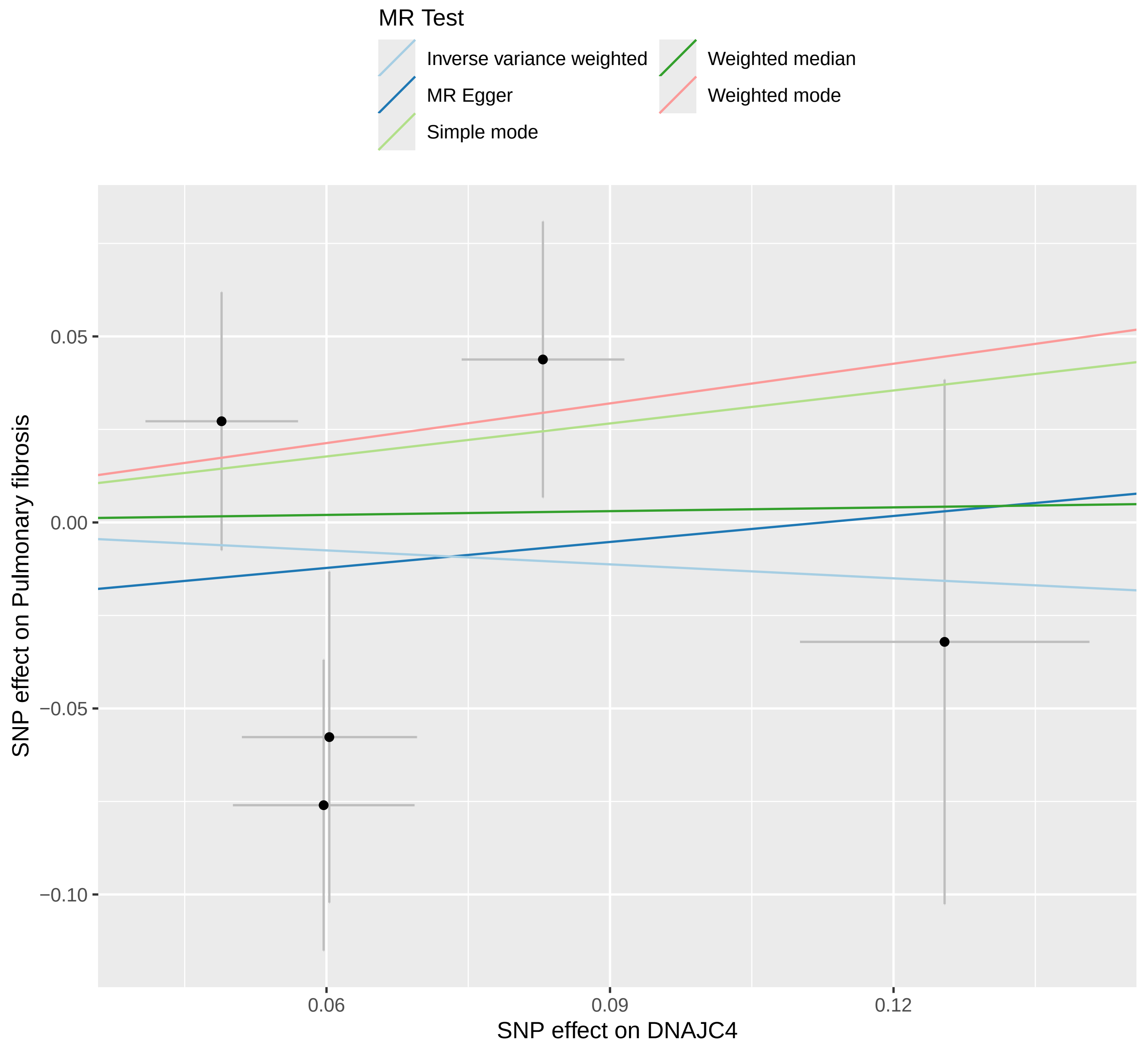

MR Test
Inverse variance weighted
MR Egger
Simple mode
Weighted median
Weighted mode
SNP effect on Pulmonary fibrosis
0.05
0.00
−0.05
−0.10
0.06
0.09
0.12
SNP effect on DNAJC4

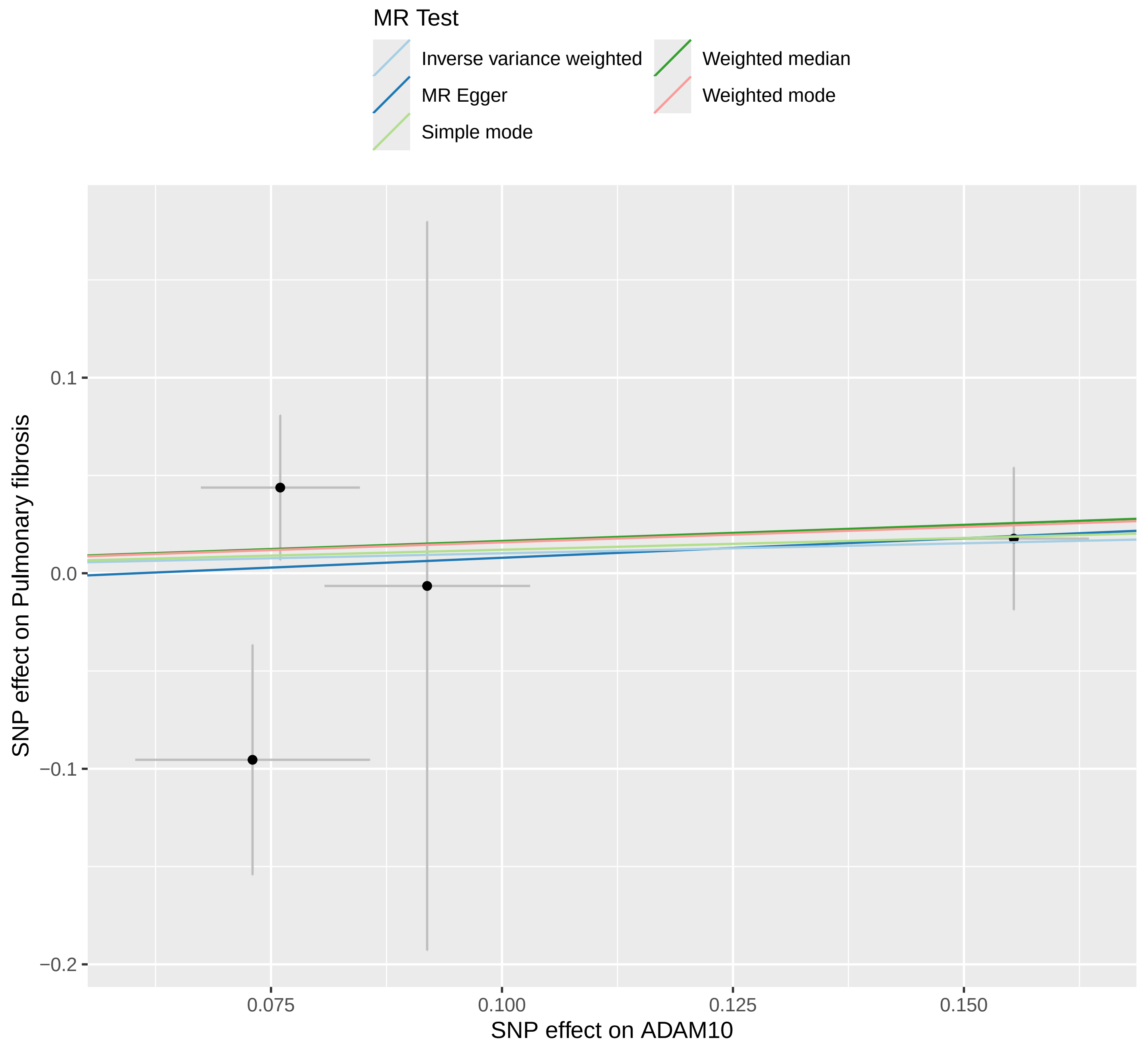

MR Test
Inverse variance weighted
MR Egger
Simple mode
Weighted median
Weighted mode
SNP effect on Pulmonary fibrosis
0.1
0.0
−0.1
−0.2
0.075
0.100
0.125
0.150
SNP effect on ADAM10

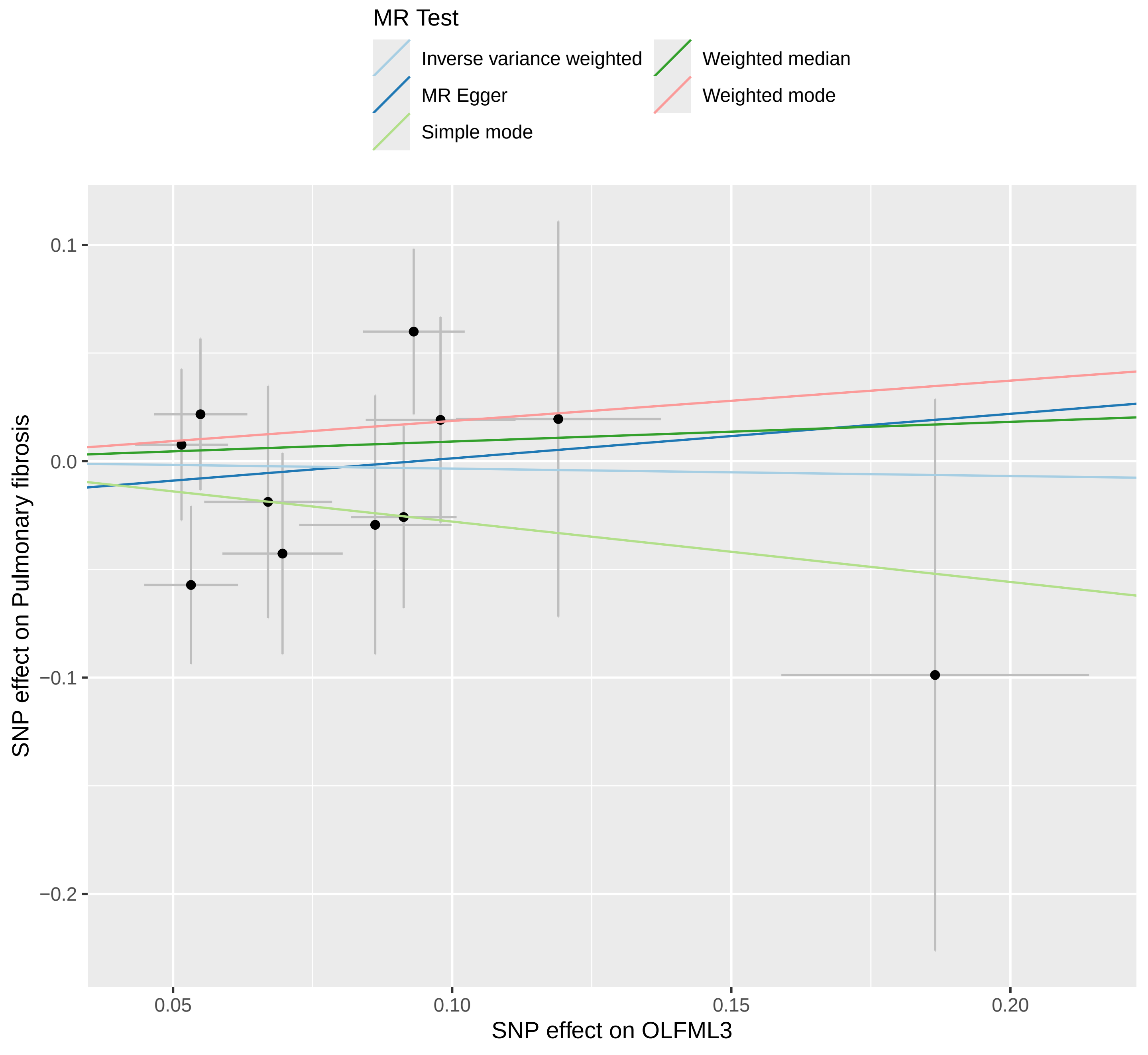
MR Test
Inverse variance weighted
Weighted median
MR Egger
Weighted mode
Simple mode
0.1
0.0
−0.1
−0.2
SNP effect on Pulmonary fibrosis
0.05
0.10
0.15
0.20
SNP effect on OLFML3

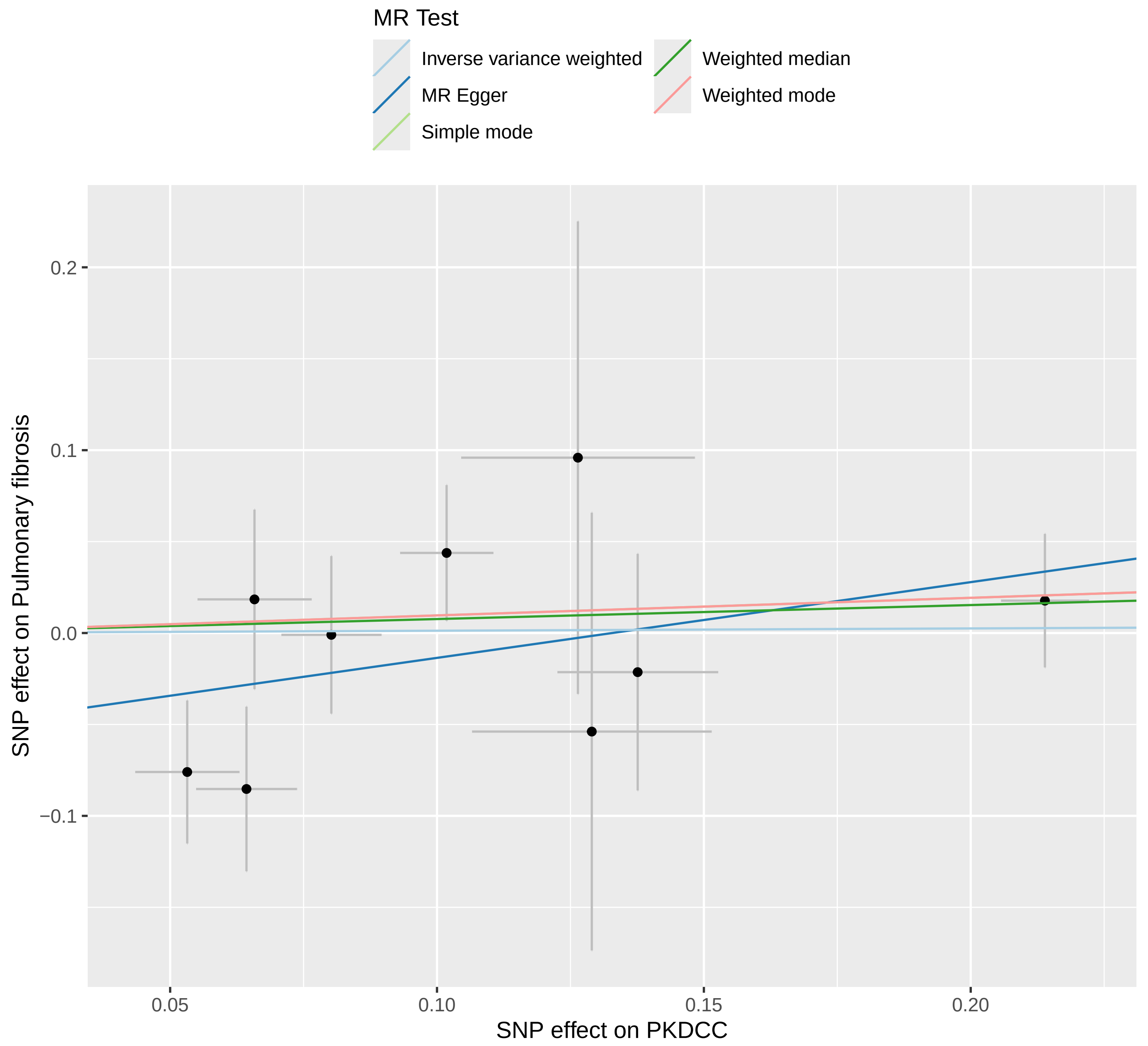

MR Test
Inverse variance weighted
MR Egger
Simple mode
Weighted median
Weighted mode
0.2
0.1
0.0
−0.1
SNP effect on Pulmonary fibrosis
0.05
0.10
0.15
0.20
SNP effect on PKDCC

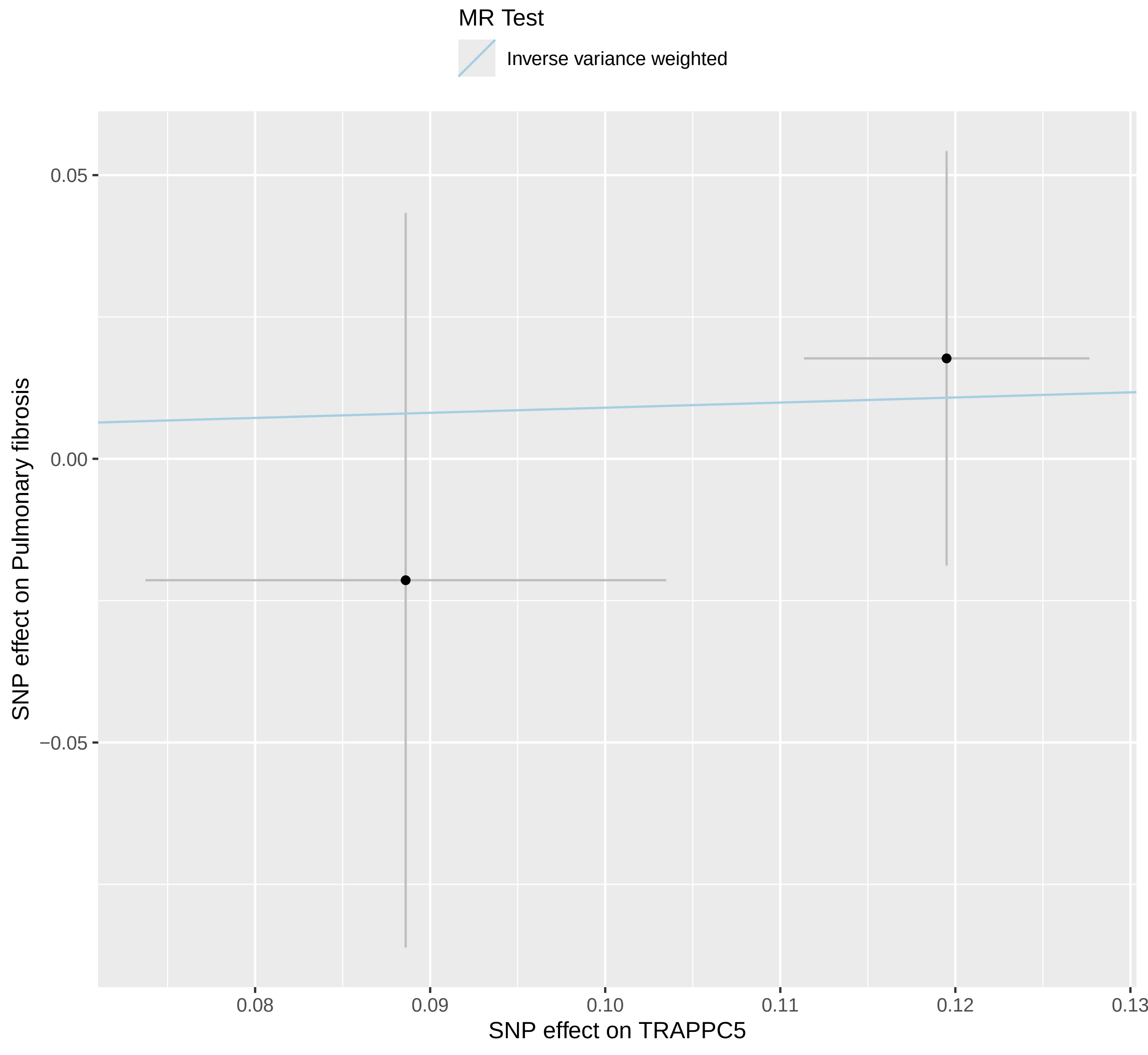

MR Test
Inverse variance weighted
0.05
0.00
−0.05
SNP effect on Pulmonary fibrosis
0.08
0.09
0.10
0.11
0.12
0.13
SNP effect on TRAPPC5

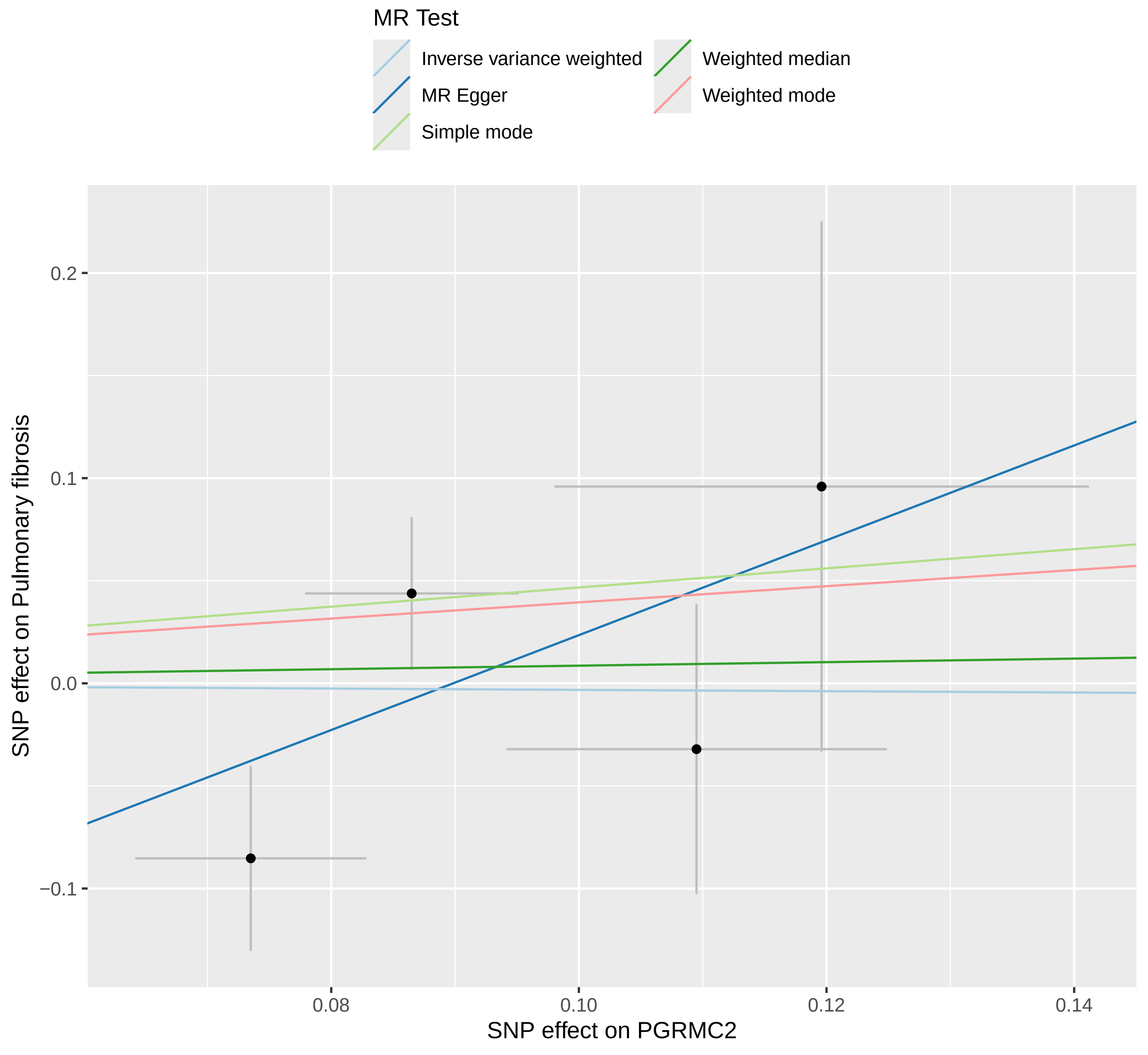
MR Test
Inverse variance weighted
MR Egger
Simple mode
Weighted median
Weighted mode
0.2
0.1
0.0
−0.1
0.08
0.10
0.12
0.14
SNP effect on PGRMC2
SNP effect on Pulmonary fibrosis

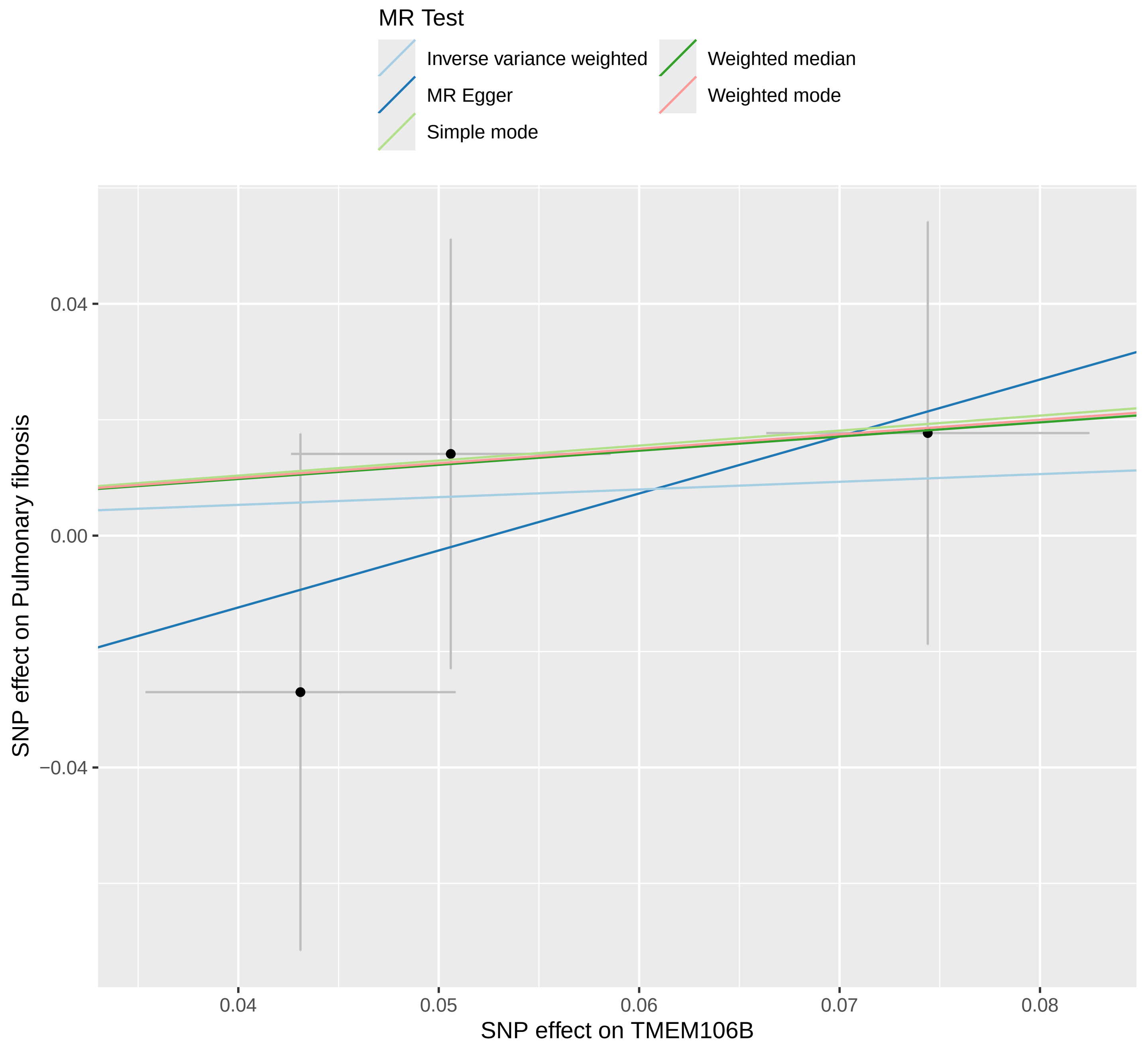
MR Test
Inverse variance weighted
MR Egger
Simple mode
Weighted median
Weighted mode
0.04
0.00
−0.04
0.04
0.05
0.06
0.07
0.08
SNP effect on Pulmonary fibrosis
SNP effect on TMEM106B

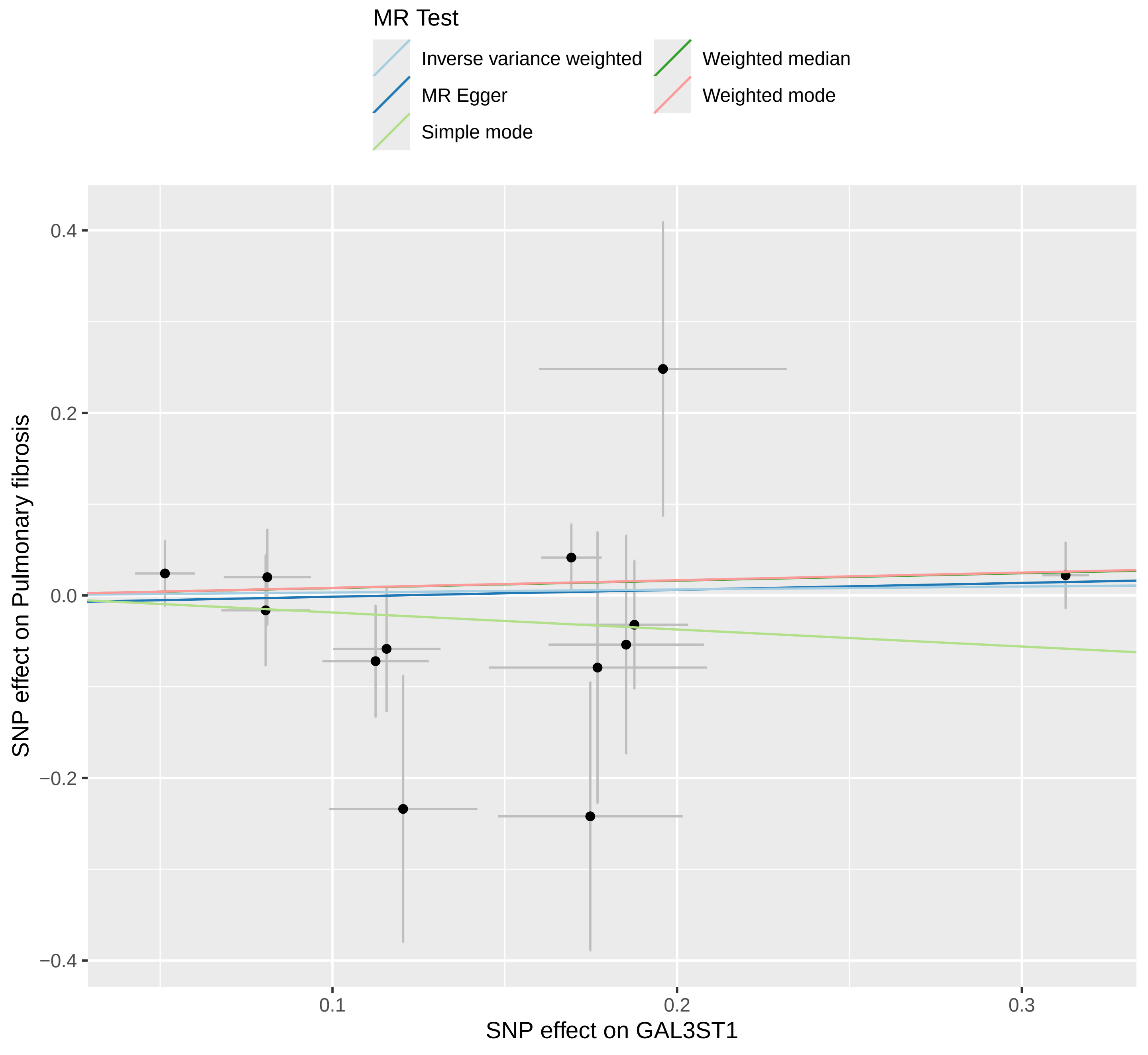

MR Test
Inverse variance weighted
MR Egger
Simple mode
Weighted median
Weighted mode
0.4
0.2
0.0
−0.2
−0.4
SNP effect on Pulmonary fibrosis
0.1
0.2
0.3
SNP effect on GAL3ST1

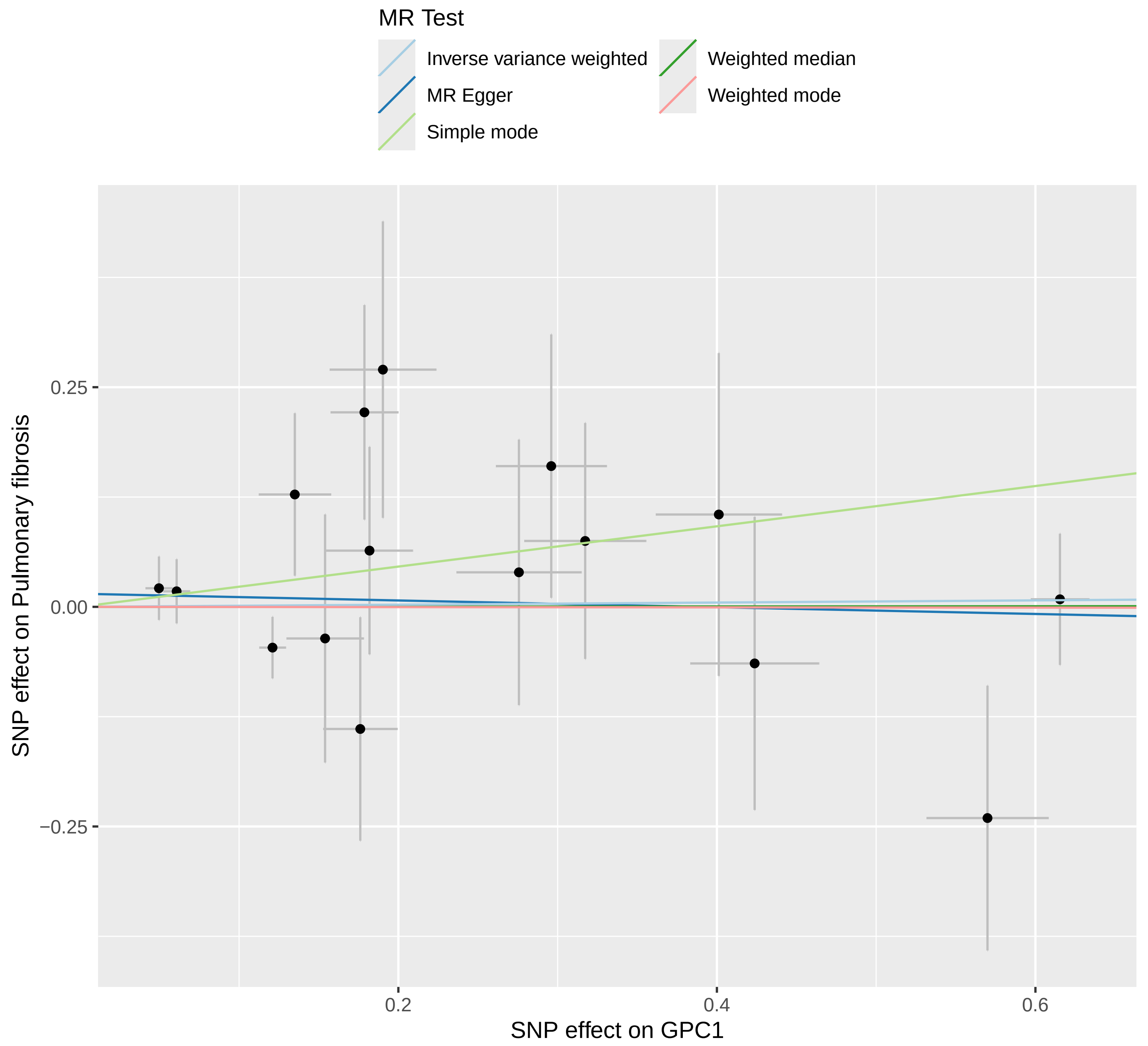

MR Test
Inverse variance weighted
MR Egger
Simple mode
Weighted median
Weighted mode
0.25
0.00
−0.25
0.2
0.4
0.6
SNP effect on Pulmonary fibrosis
SNP effect on GPC1

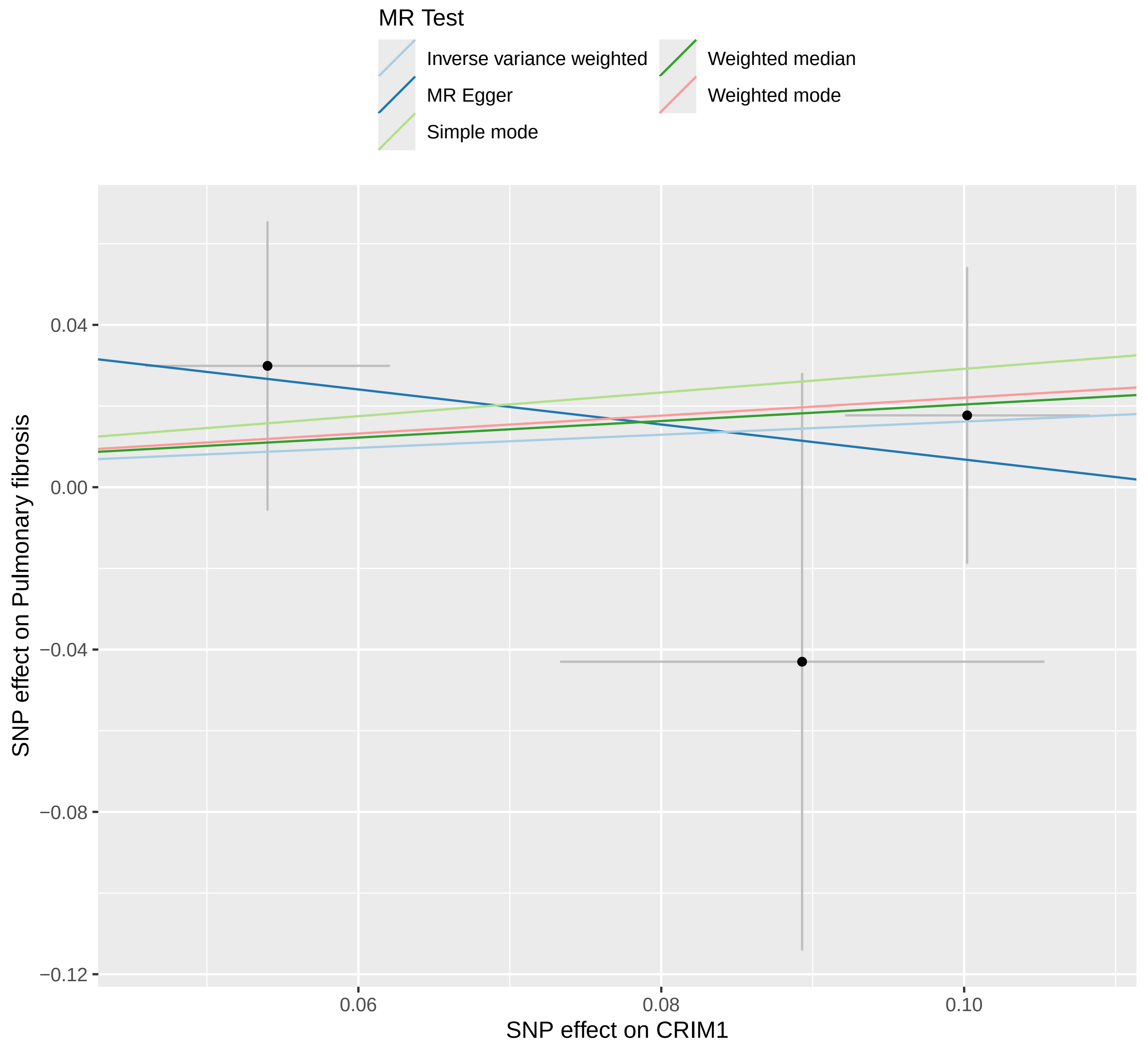

MR Test
Inverse variance weighted
MR Egger
Simple mode
Weighted median
Weighted mode
0.04
0.00
−0.04
−0.08
−0.12
0.06
0.08
0.10
SNP effect on Pulmonary fibrosis
SNP effect on CRIM1

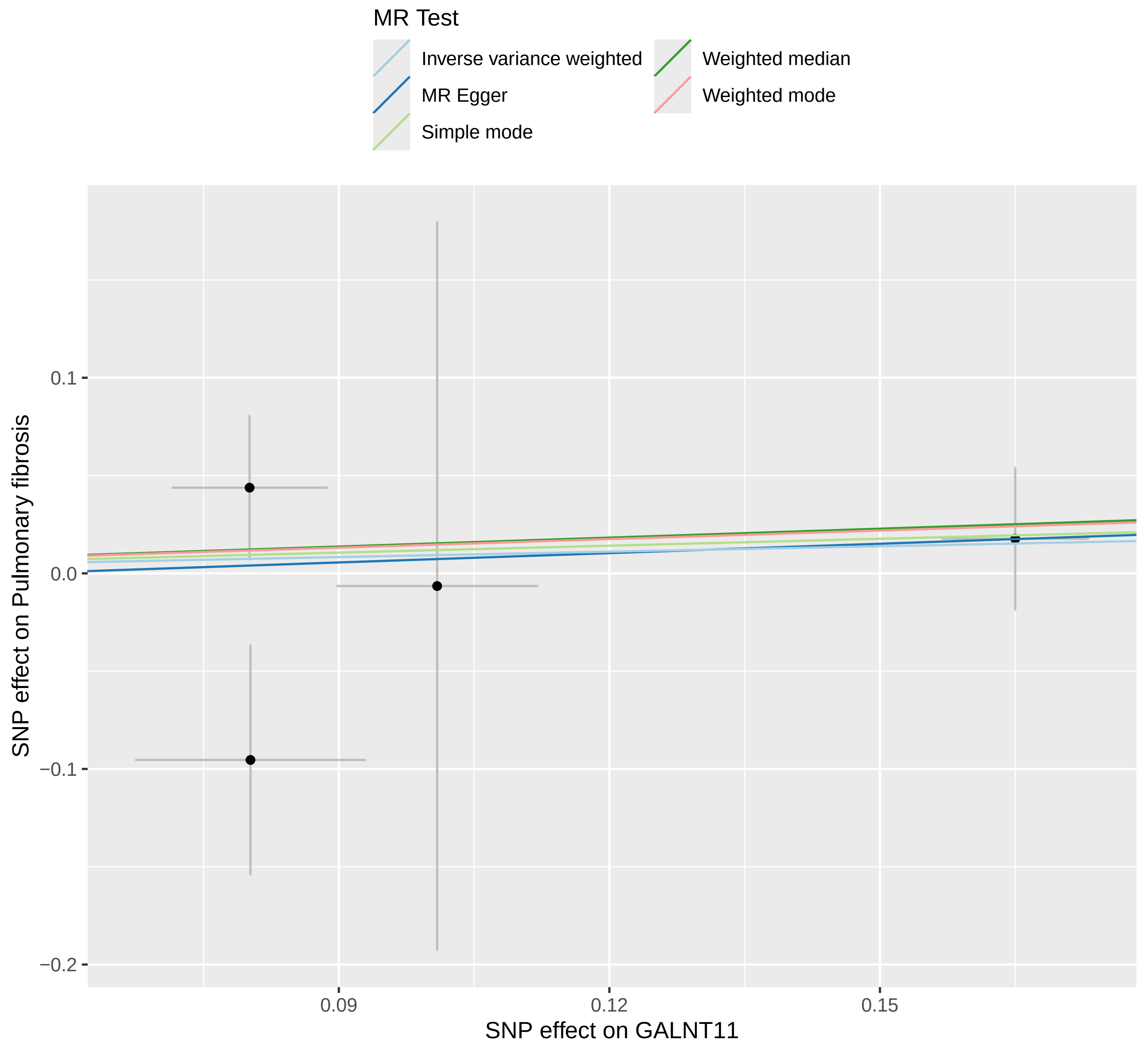

MR Test
Inverse variance weighted
MR Egger
Simple mode
Weighted median
Weighted mode
0.1
0.0
−0.1
−0.2
0.09
0.12
0.15
SNP effect on Pulmonary fibrosis
SNP effect on GALNT11

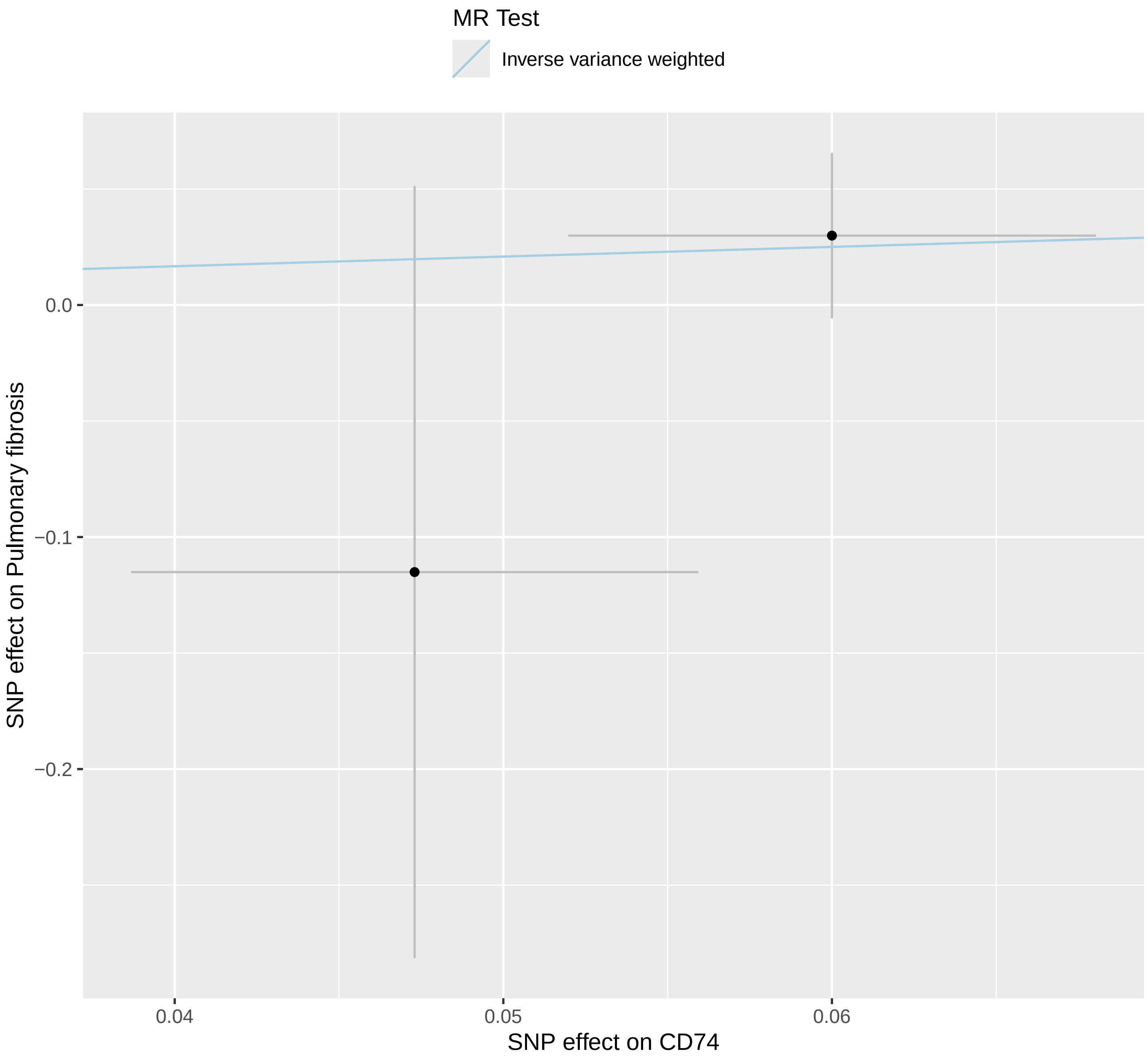
MR Test
Inverse variance weighted
0.0
−0.1
−0.2
SNP effect on Pulmonary fibrosis
0.04
0.05
0.06
SNP effect on CD74

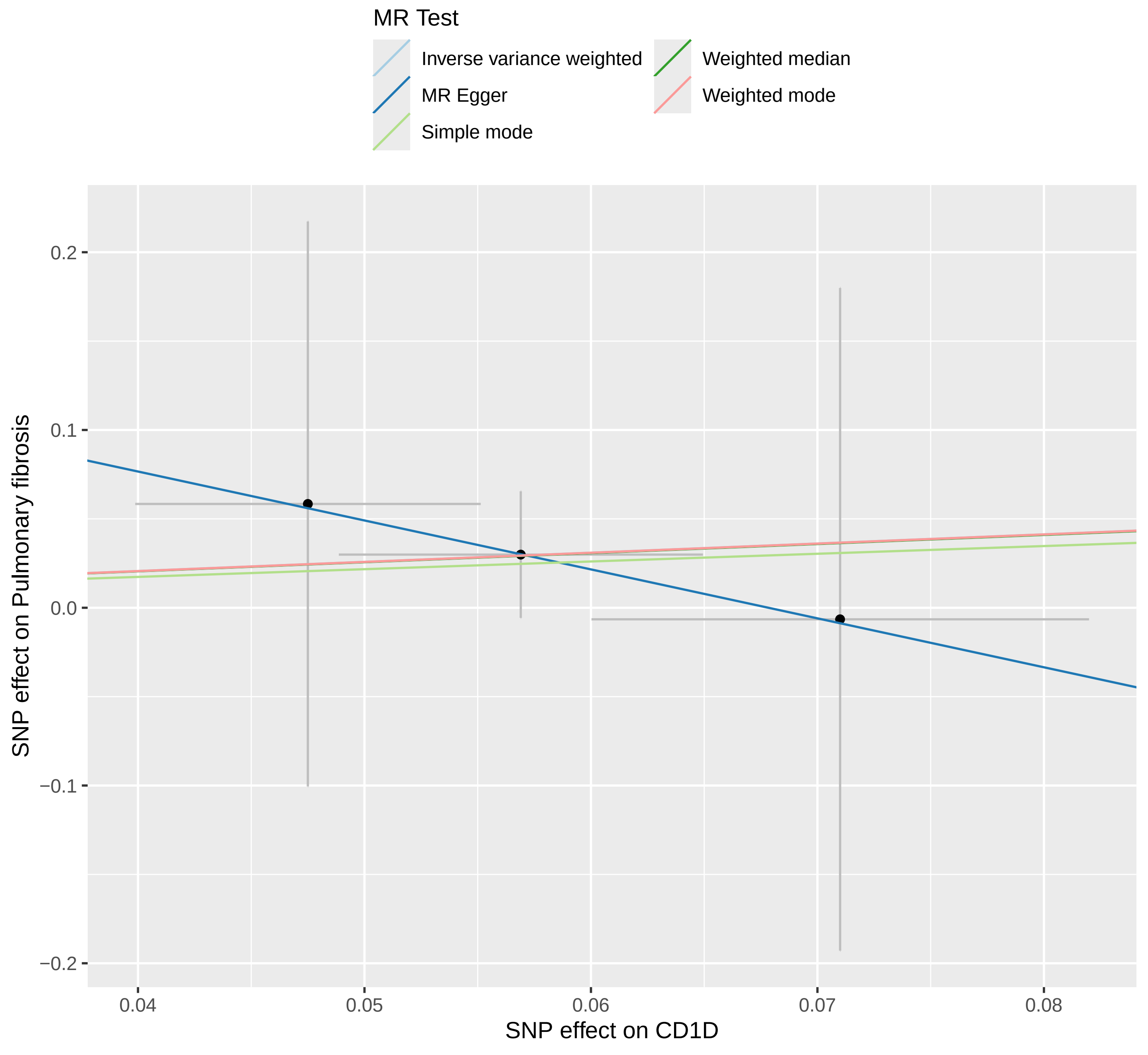
MR Test
Inverse variance weighted
MR Egger
Simple mode
Weighted median
Weighted mode
0.2
0.1
0.0
−0.1
−0.2
SNP effect on Pulmonary fibrosis
0.04
0.05
0.06
0.07
0.08
SNP effect on CD1D

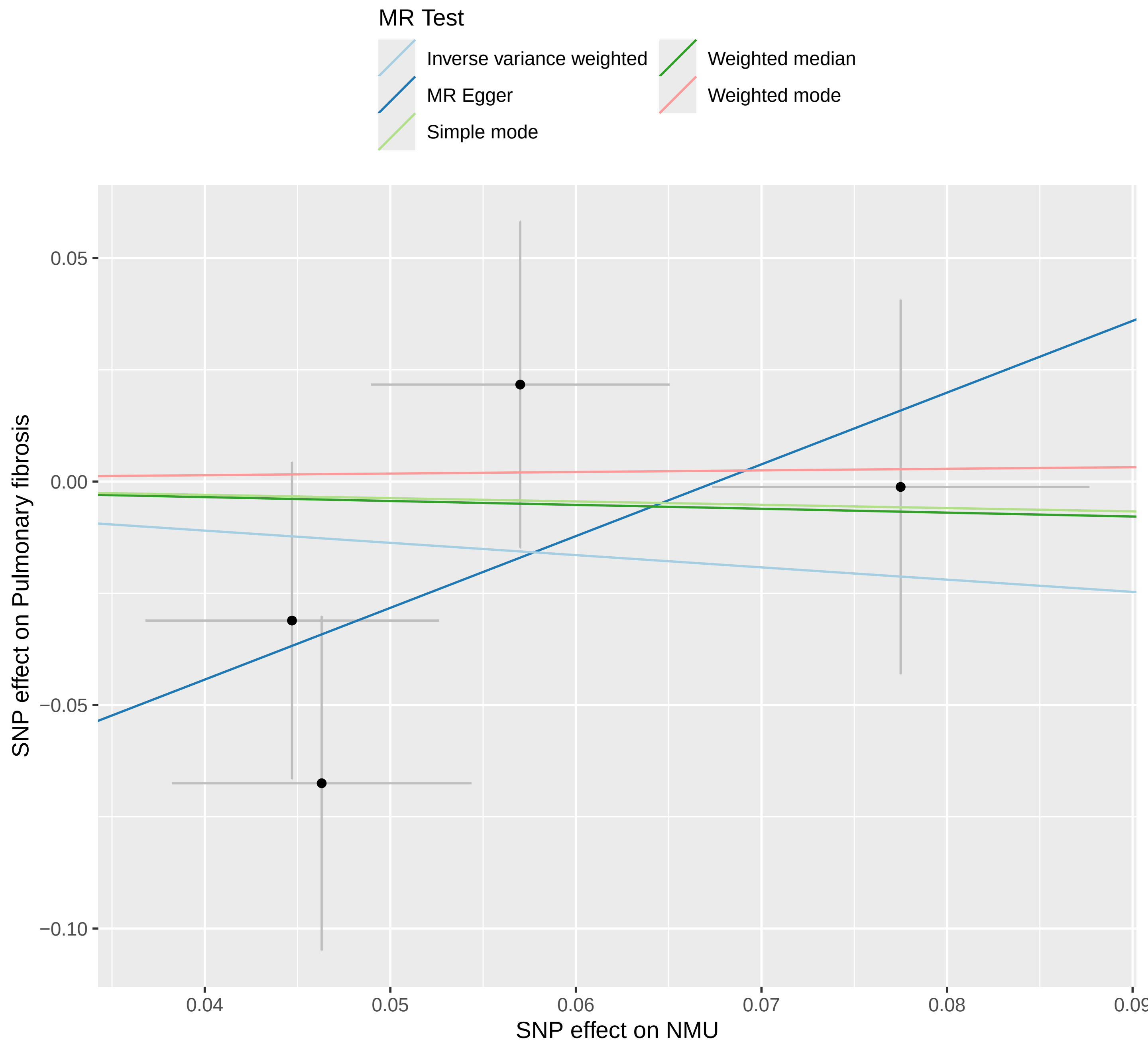

MR Test
Inverse variance weighted
MR Egger
Simple mode
Weighted median
Weighted mode
0.05
0.00
−0.05
−0.10
SNP effect on Pulmonary fibrosis
0.04
0.05
0.06
0.07
0.08
SNP effect on NMU

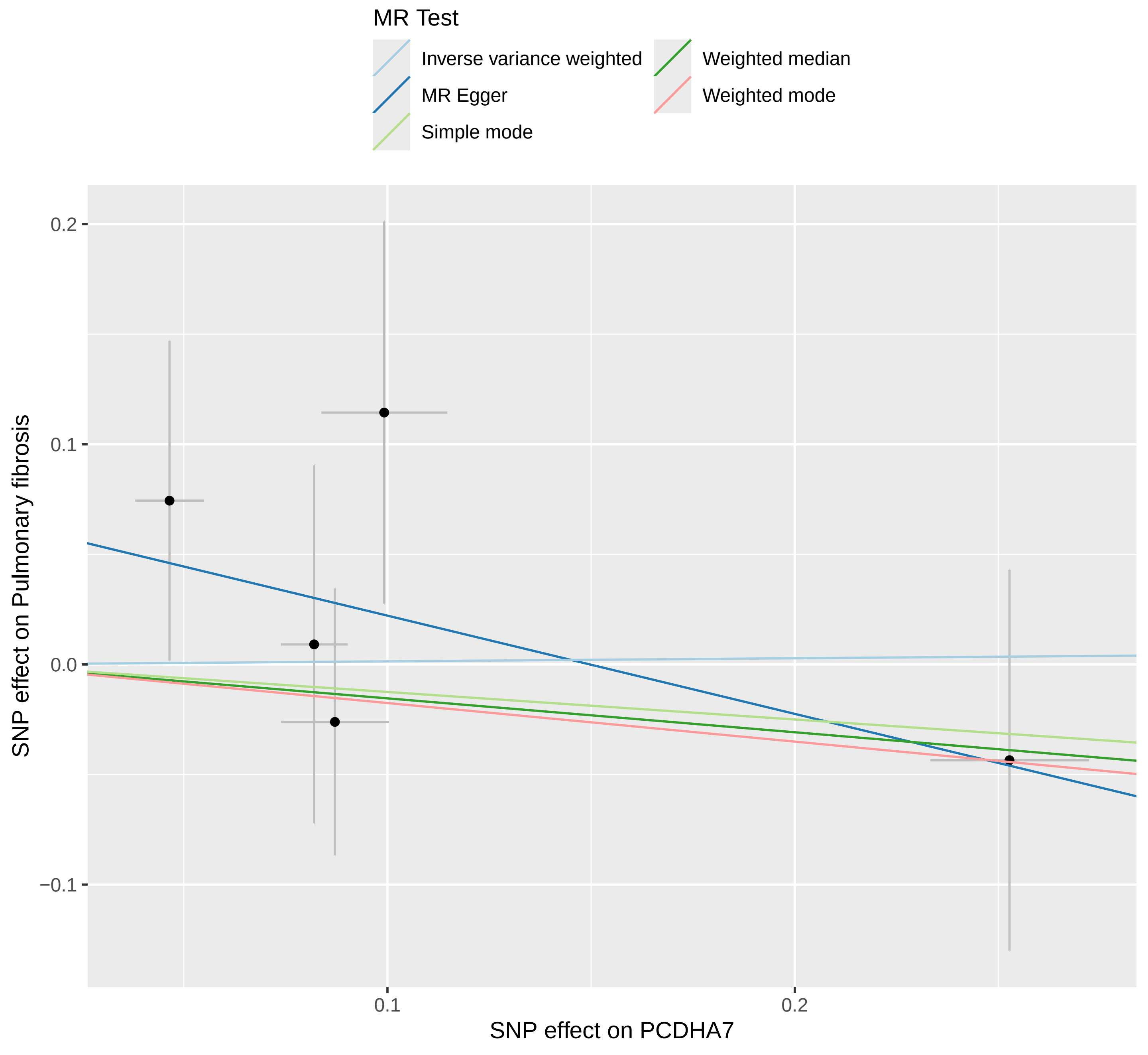
MR Test
Inverse variance weighted
MR Egger
Simple mode
Weighted median
Weighted mode
0.2
0.1
0.0
−0.1
0.1
0.2
SNP effect on PCDHA7
SNP effect on Pulmonary fibrosis

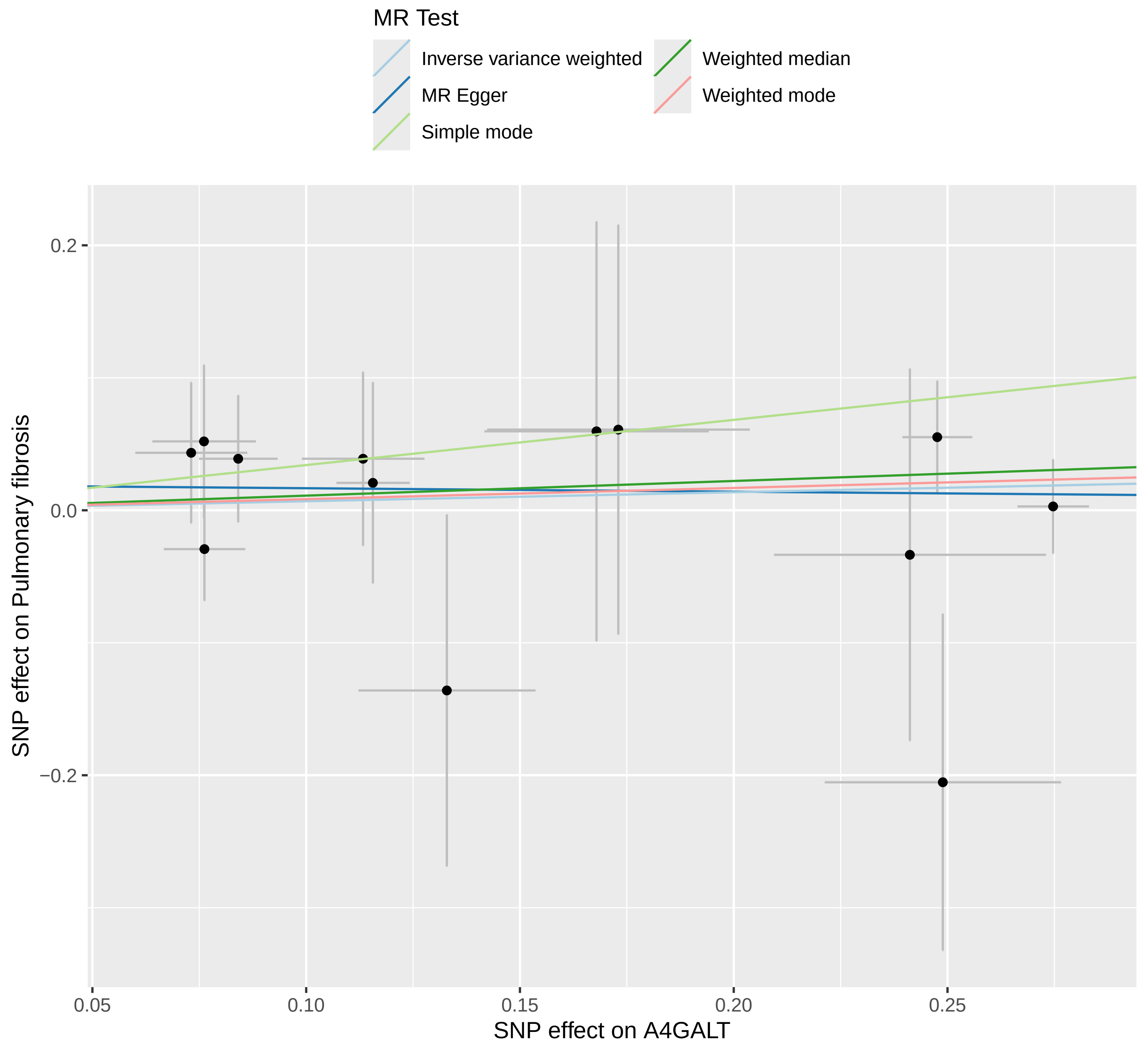
MR Test
Inverse variance weighted
MR Egger
Simple mode
Weighted median
Weighted mode
0.2
0.0
−0.2
SNP effect on Pulmonary fibrosis
0.05
0.10
0.15
0.20
0.25
SNP effect on A4GALT

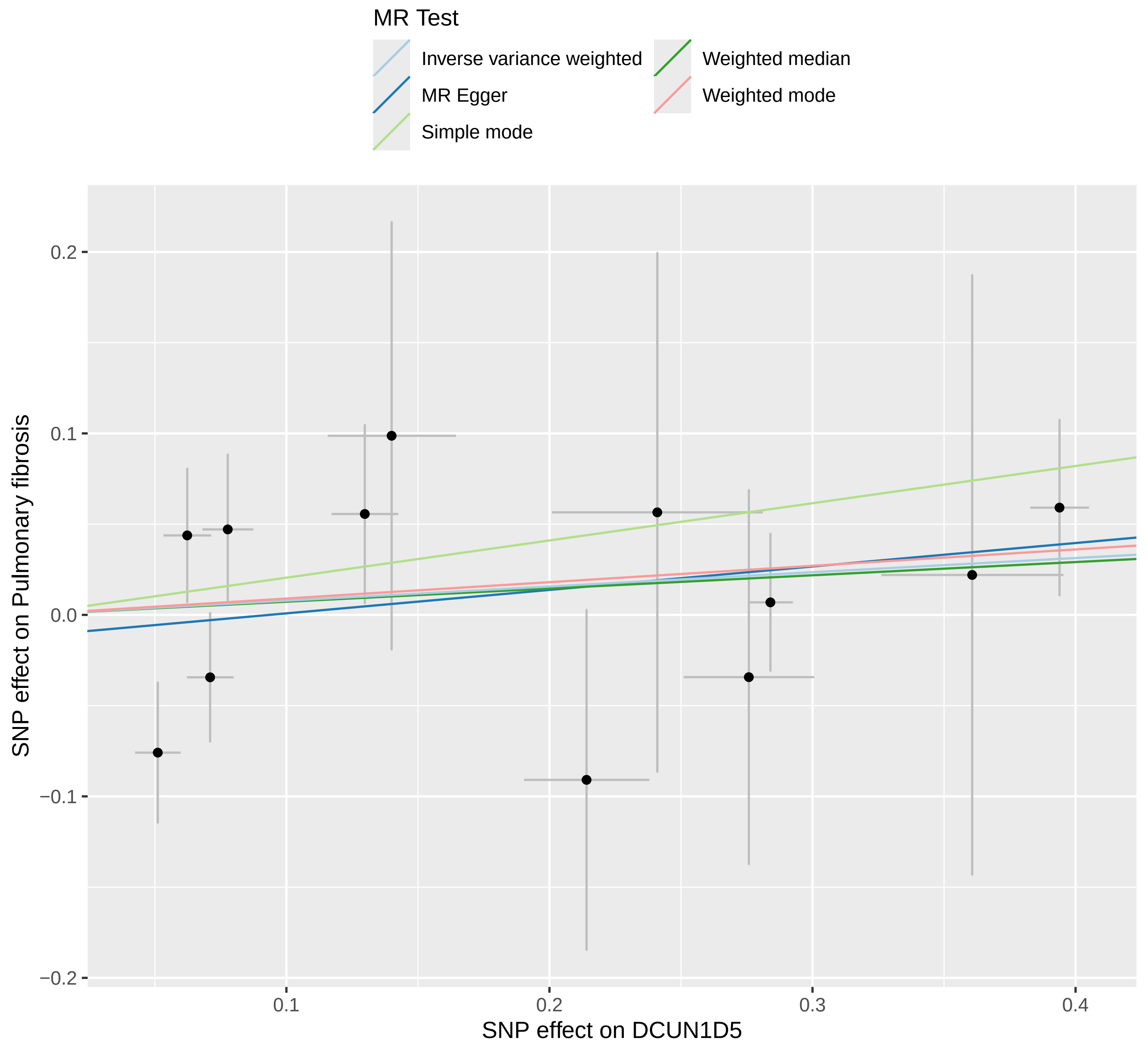

MR Test
Inverse variance weighted
MR Egger
Simple mode
Weighted median
Weighted mode
0.2
0.1
0.0
−0.1
−0.2
SNP effect on Pulmonary fibrosis
0.1
0.2
0.3
0.4
SNP effect on DCUN1D5

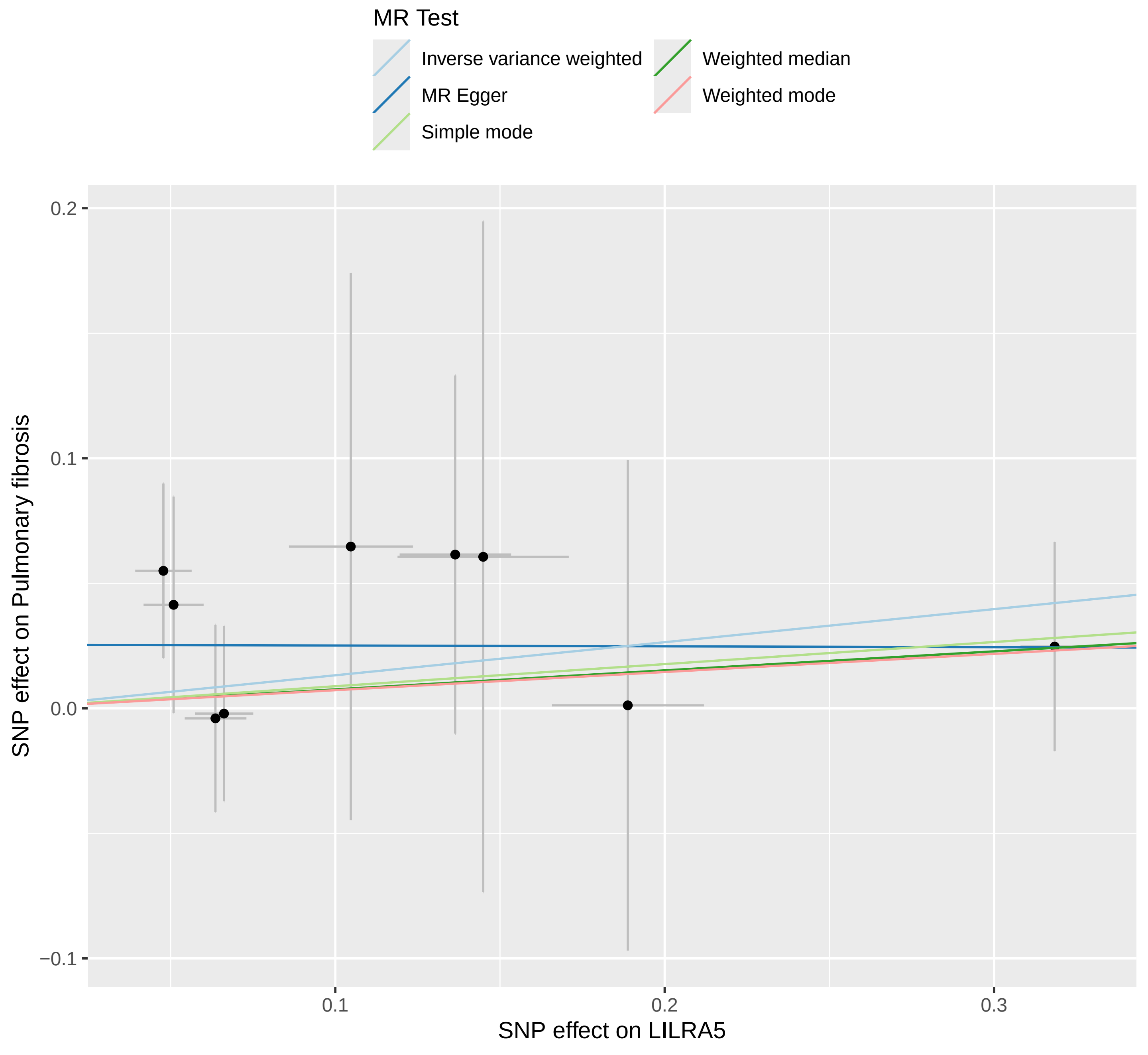

MR Test
Inverse variance weighted
MR Egger
Simple mode
Weighted median
Weighted mode
0.2
0.1
0.0
−0.1
0.1
0.2
0.3
SNP effect on Pulmonary fibrosis
SNP effect on LILRA5

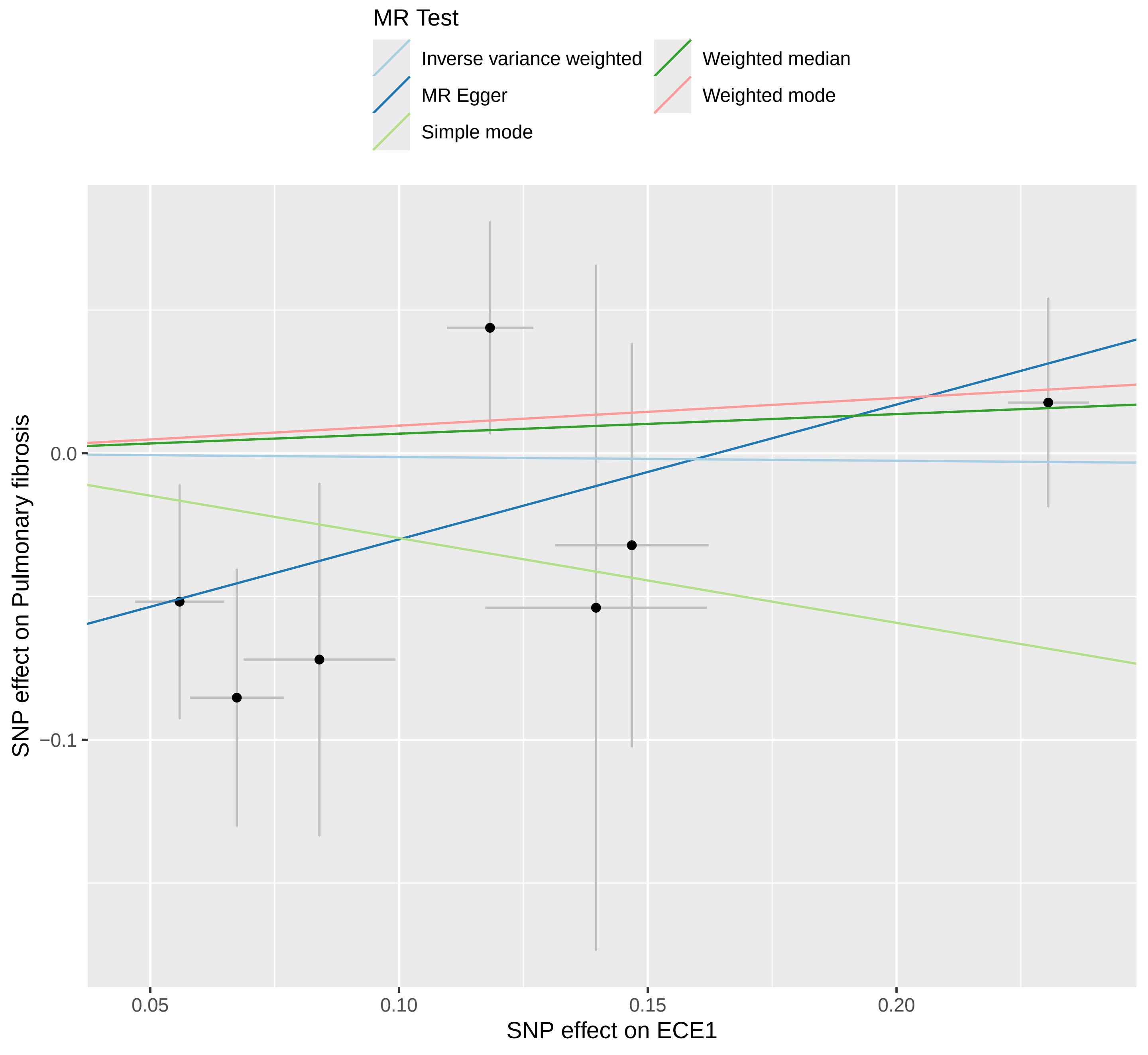

MR Test
Inverse variance weighted
MR Egger
Simple mode
Weighted median
Weighted mode
SNP effect on Pulmonary fibrosis
0.0
−0.1
0.05
0.10
0.15
0.20
SNP effect on ECE1

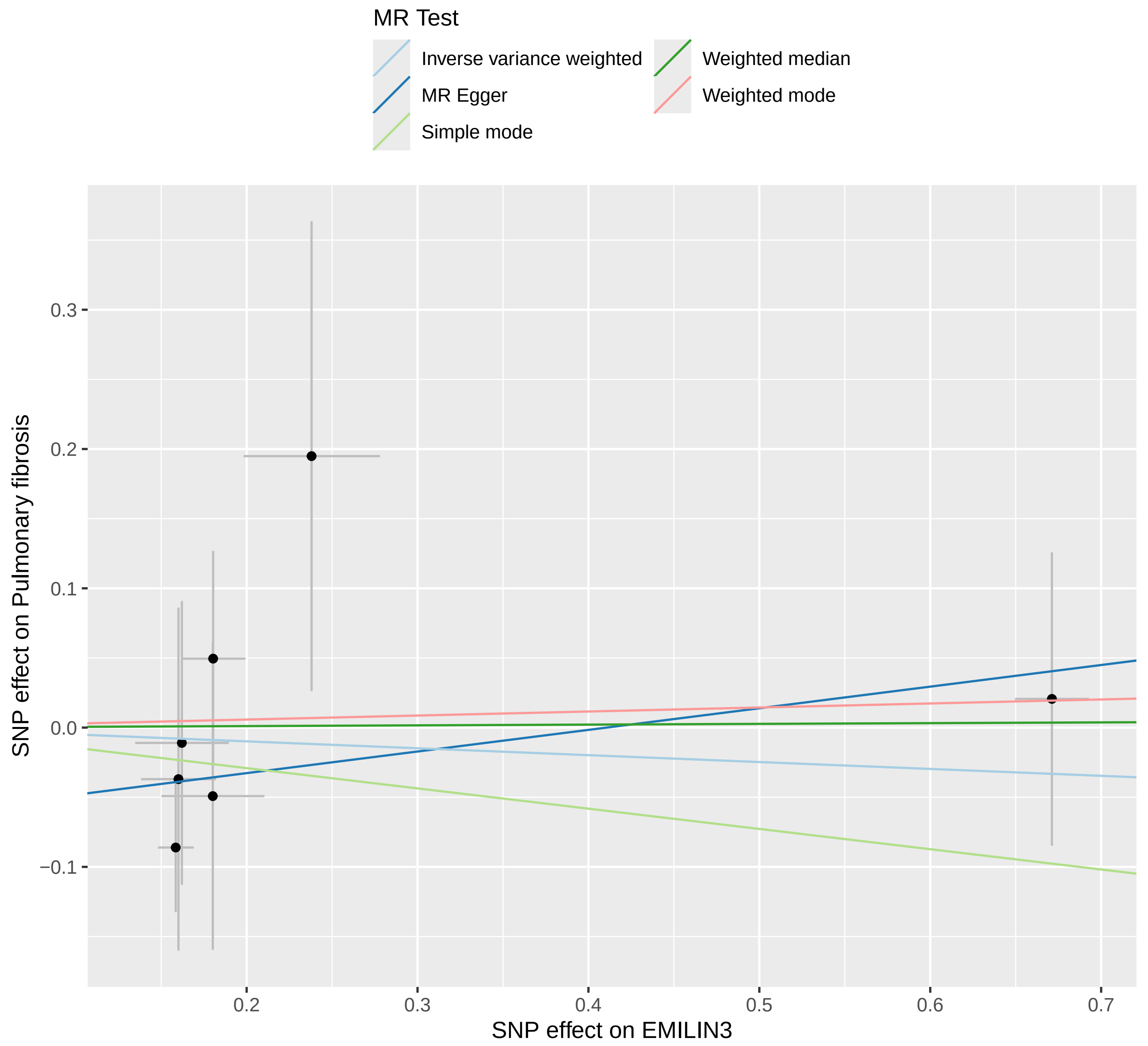

MR Test
Inverse variance weighted
MR Egger
Simple mode
Weighted median
Weighted mode
SNP effect on Pulmonary fibrosis
0.3
0.2
0.1
0.0
−0.1
0.2
0.3
0.4
0.5
0.6
0.7
SNP effect on EMILIN3

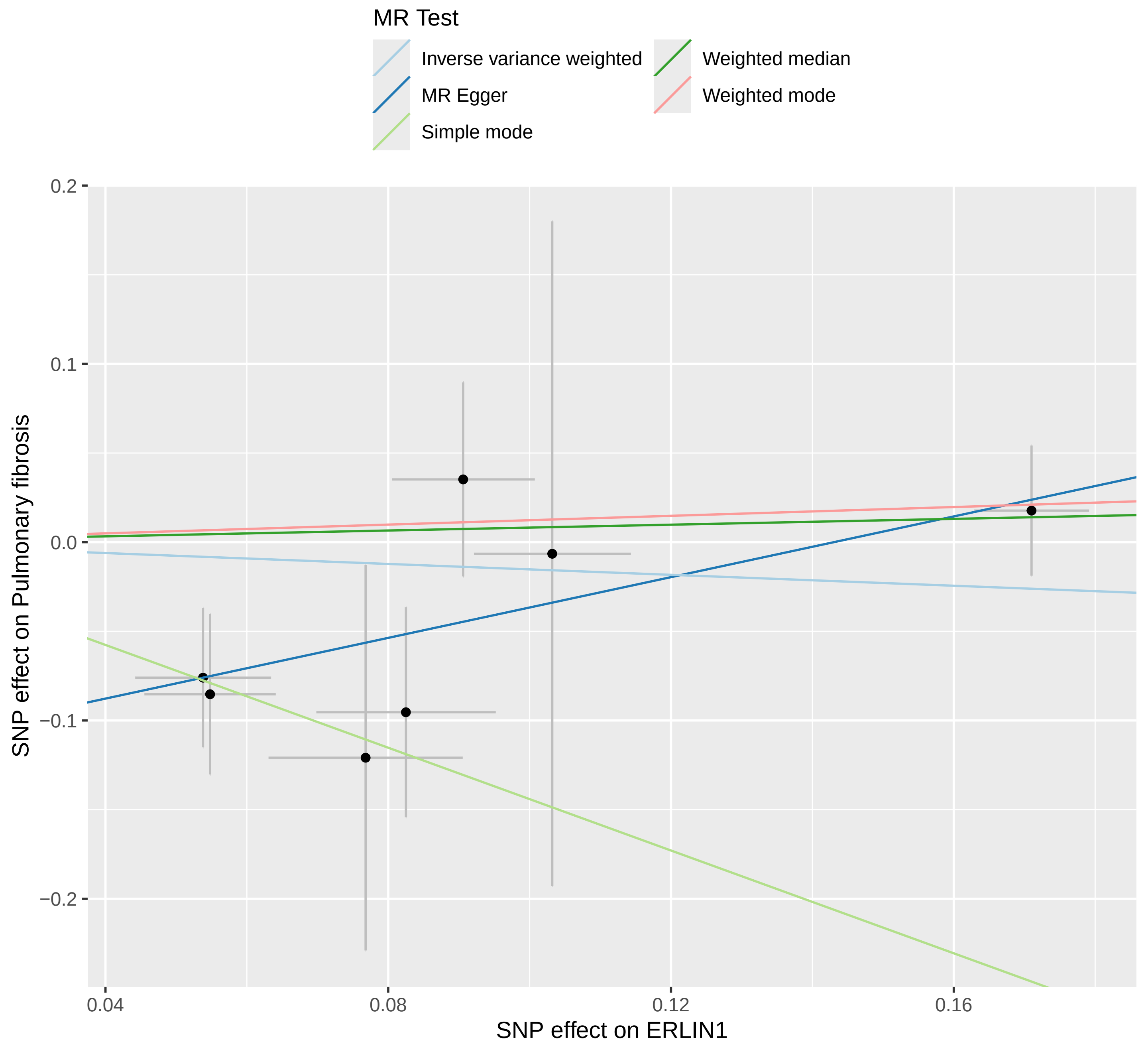
MR Test
Inverse variance weighted
MR Egger
Simple mode
Weighted median
Weighted mode
0.2
0.1
0.0
−0.1
−0.2
SNP effect on Pulmonary fibrosis
0.04
0.08
0.12
0.16
SNP effect on ERLIN1

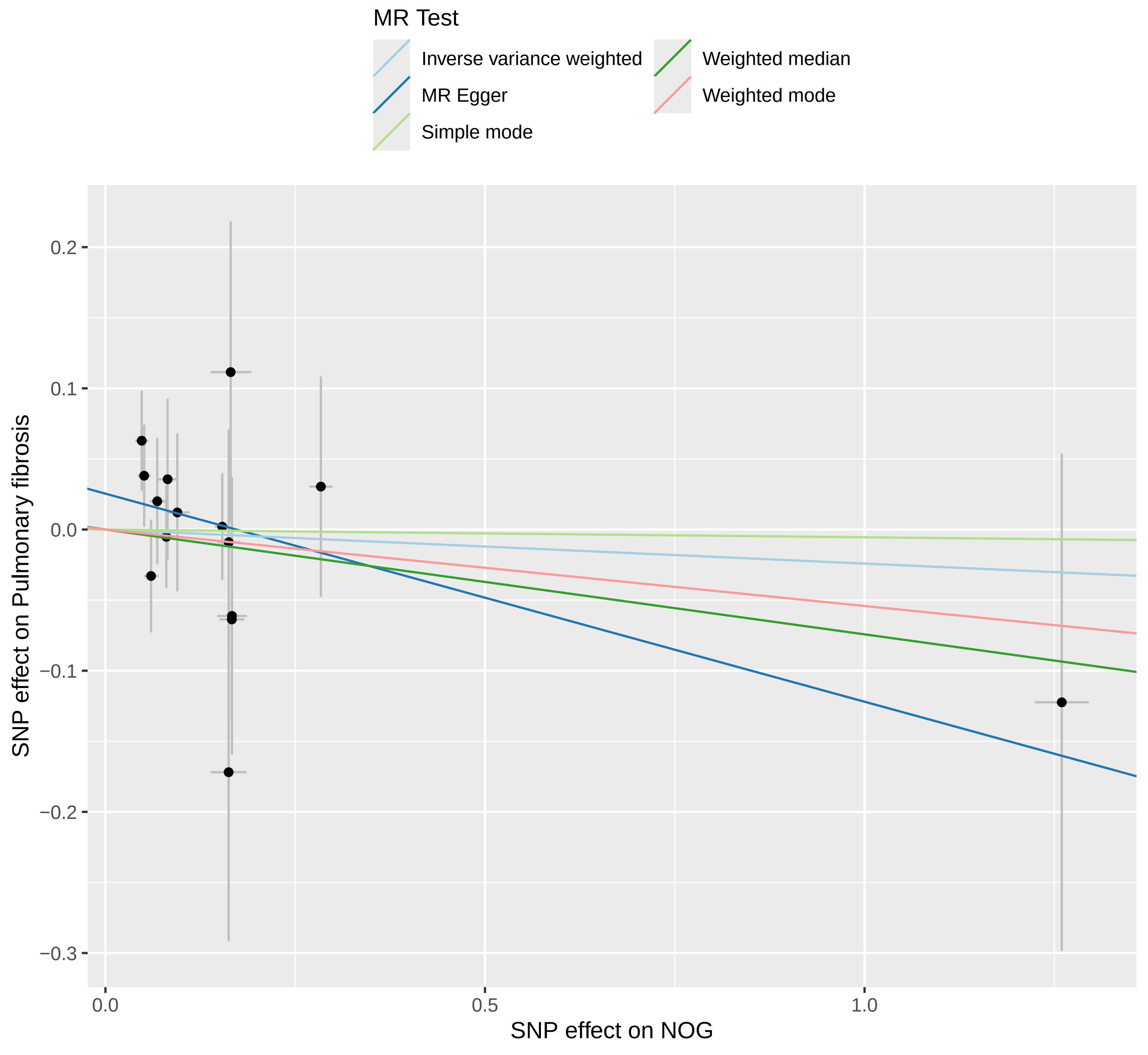
MR Test
Inverse variance weighted
MR Egger
Simple mode
Weighted median
Weighted mode
0.2
0.1
0.0
−0.1
−0.2
−0.3
SNP effect on Pulmonary fibrosis
0.0
0.5
1.0
SNP effect on NOG

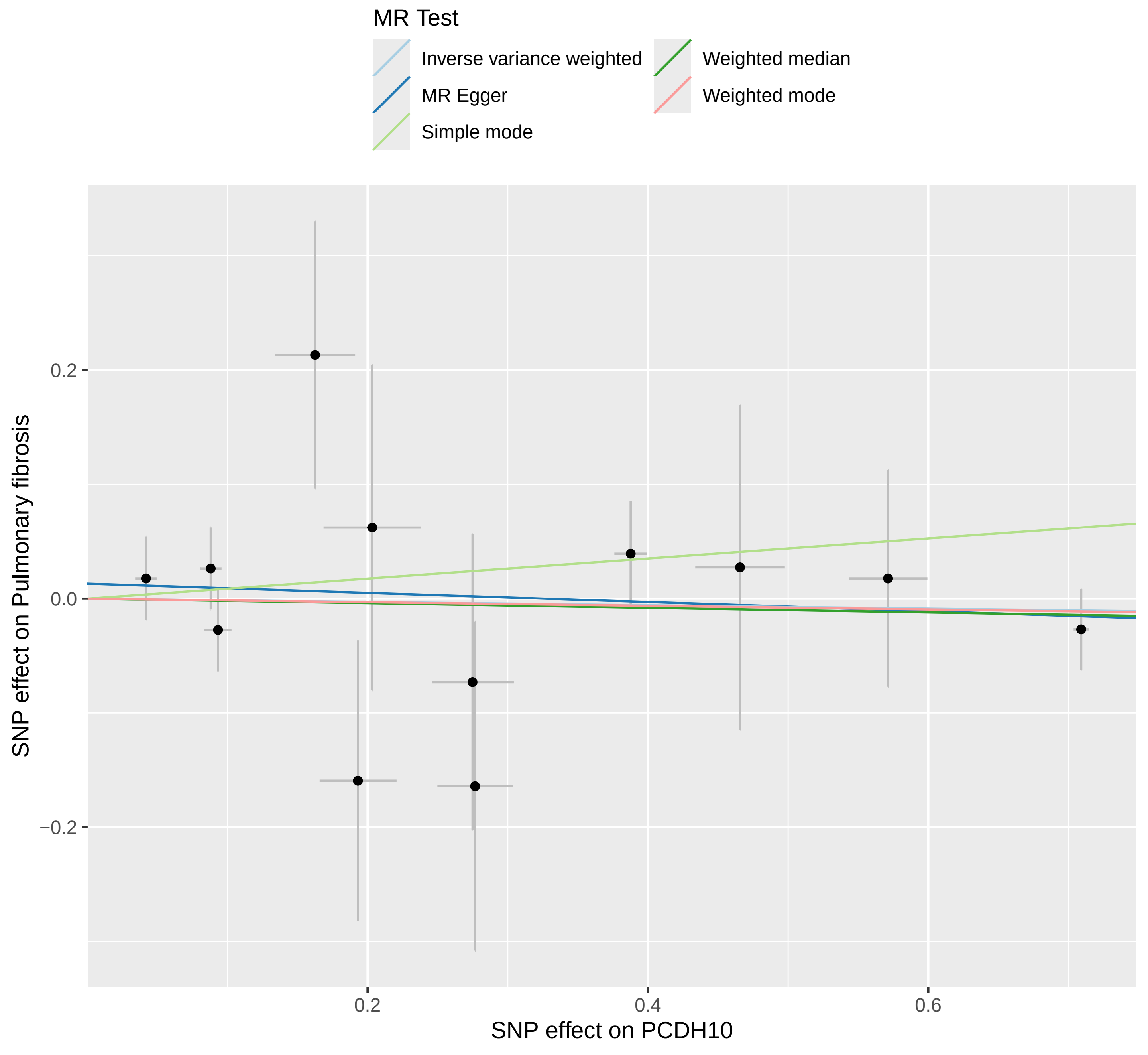
MR Test
Inverse variance weighted
MR Egger
Simple mode
Weighted median
Weighted mode
0.2
0.0
−0.2
SNP effect on Pulmonary fibrosis
0.2
0.4
0.6
SNP effect on PCDH10

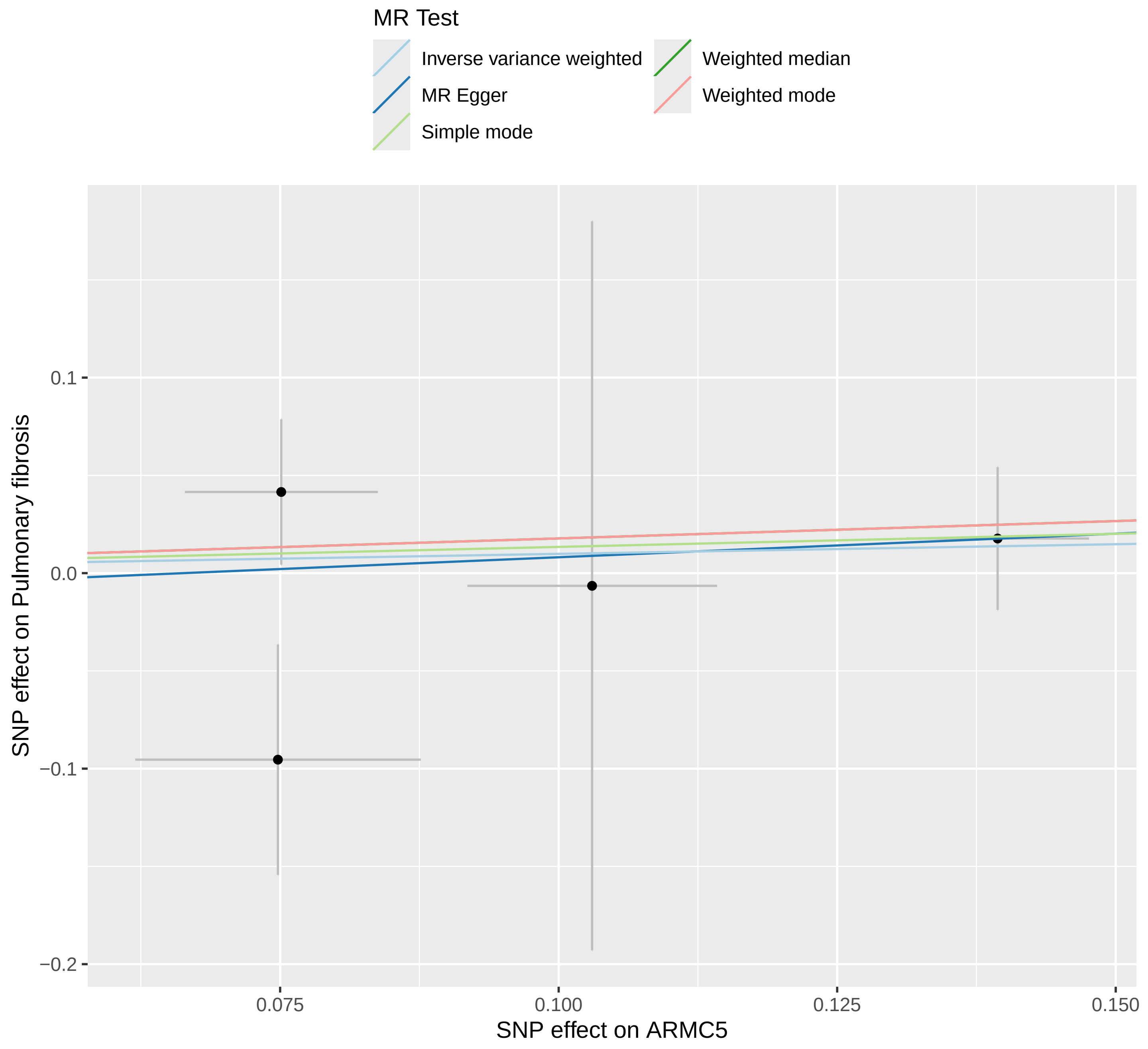

MR Test
Inverse variance weighted
MR Egger
Simple mode
Weighted median
Weighted mode
0.1
0.0
−0.1
−0.2
0.075
0.100
0.125
0.150
SNP effect on Pulmonary fibrosis
SNP effect on ARMC5

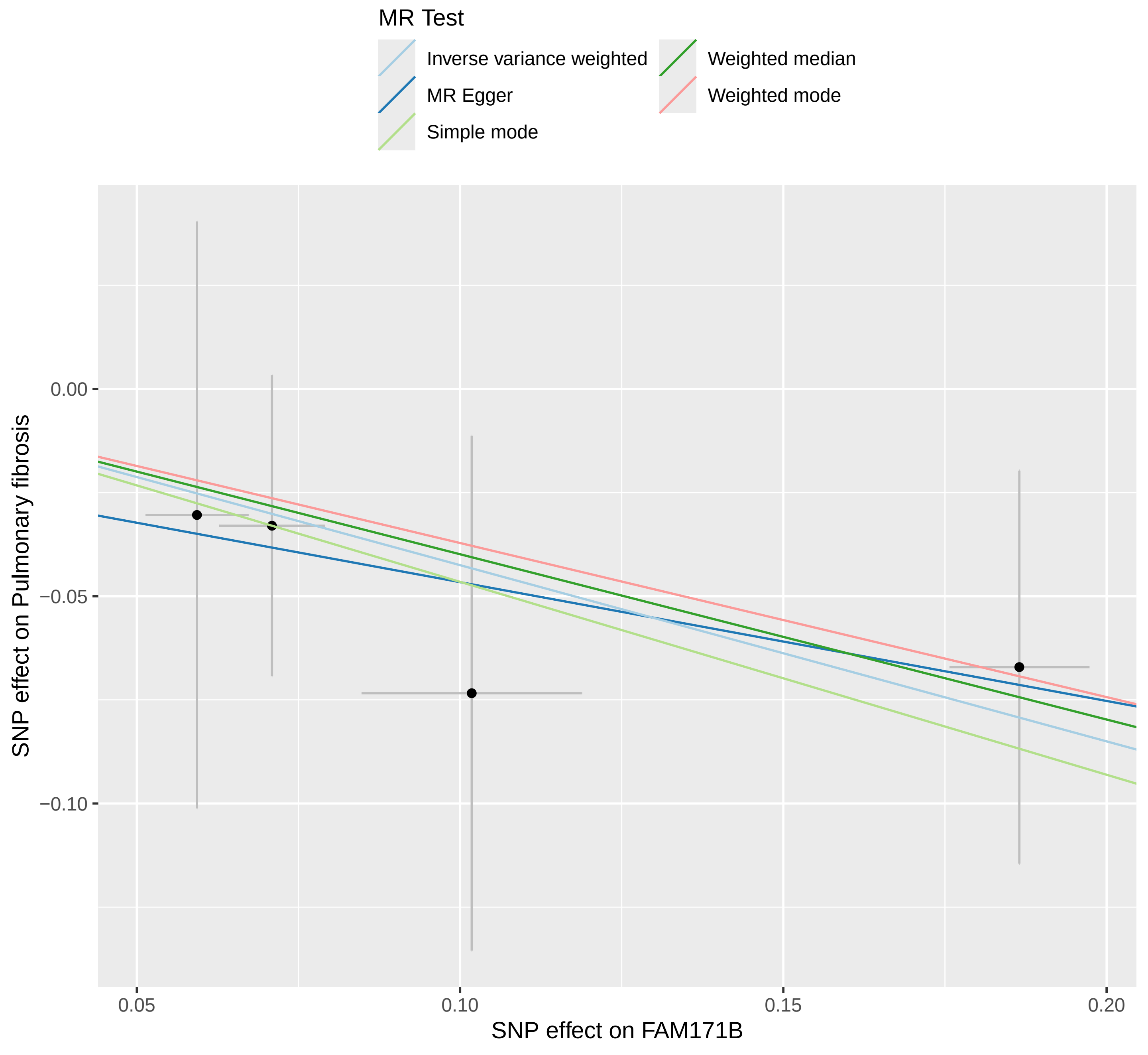

MR Test
Inverse variance weighted
MR Egger
Simple mode
Weighted median
Weighted mode
0.00
−0.05
−0.10
0.05
0.10
0.15
0.20
SNP effect on Pulmonary fibrosis
SNP effect on FAM171B

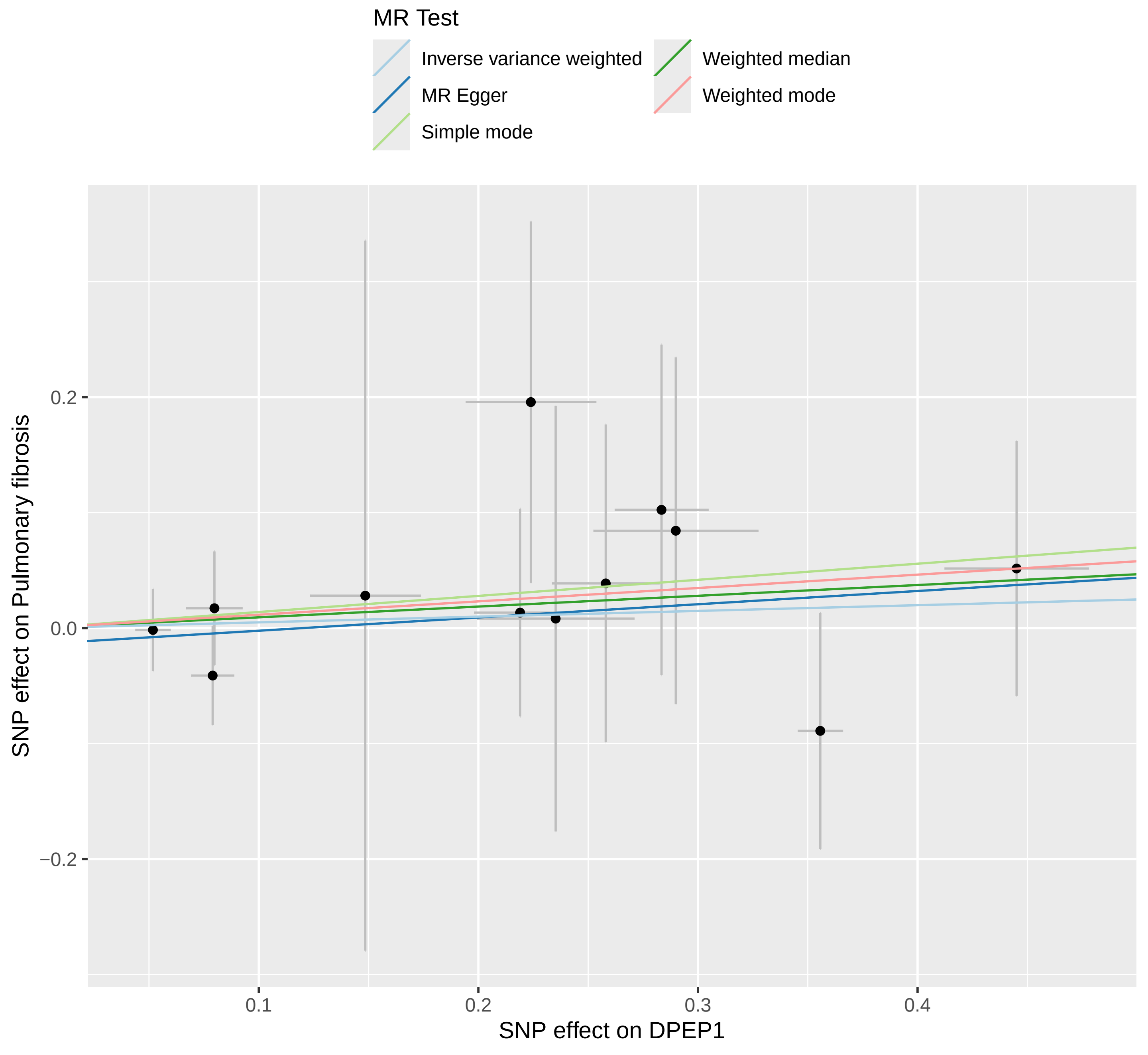
MR Test
Inverse variance weighted
MR Egger
Simple mode
Weighted median
Weighted mode
0.2
0.0
−0.2
0.1
0.2
0.3
0.4
SNP effect on Pulmonary fibrosis
SNP effect on DPEP1

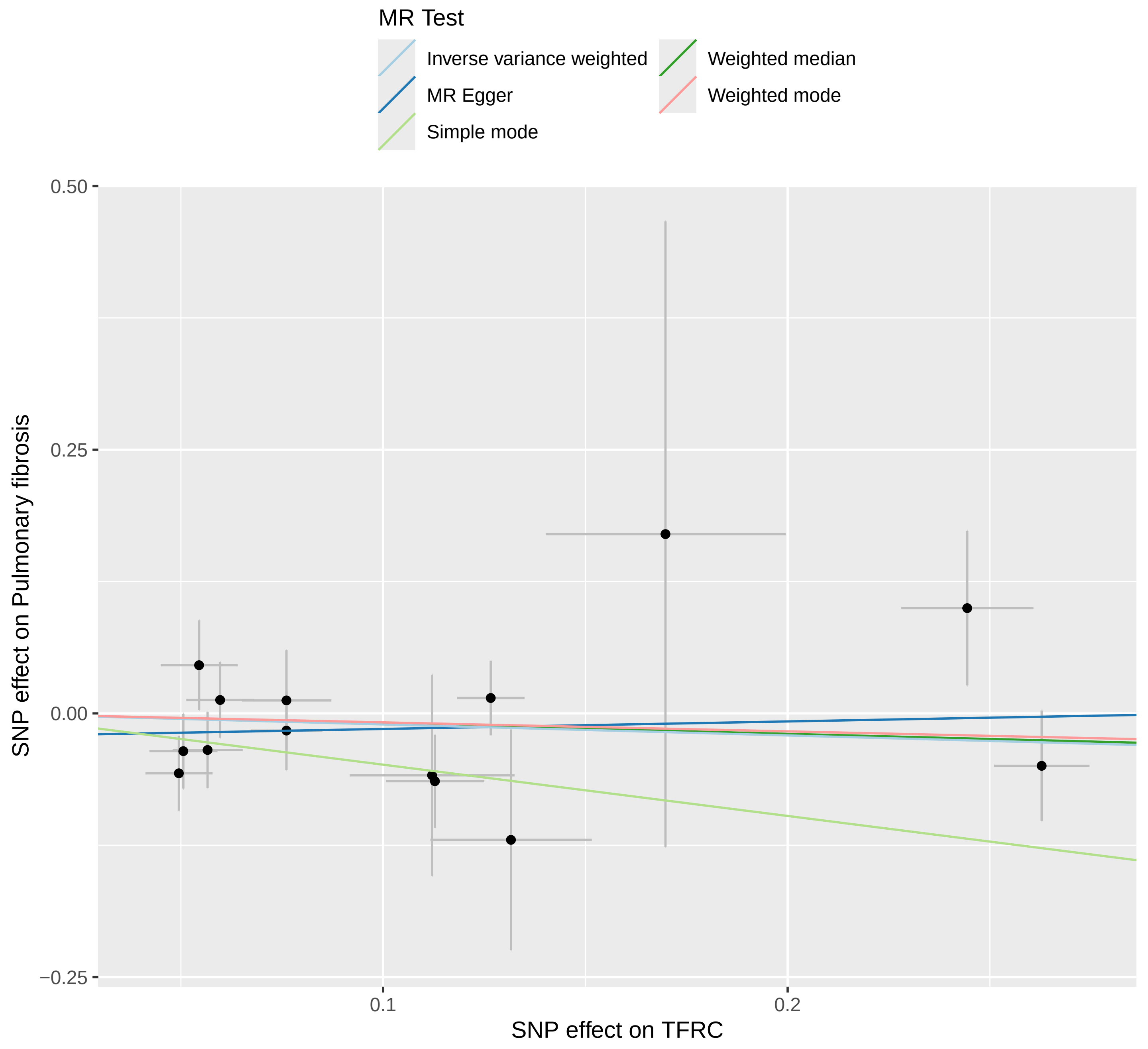
MR Test
Inverse variance weighted
MR Egger
Simple mode
Weighted median
Weighted mode
0.50
0.25
0.00
−0.25
0.1
0.2
SNP effect on Pulmonary fibrosis
SNP effect on TFRC

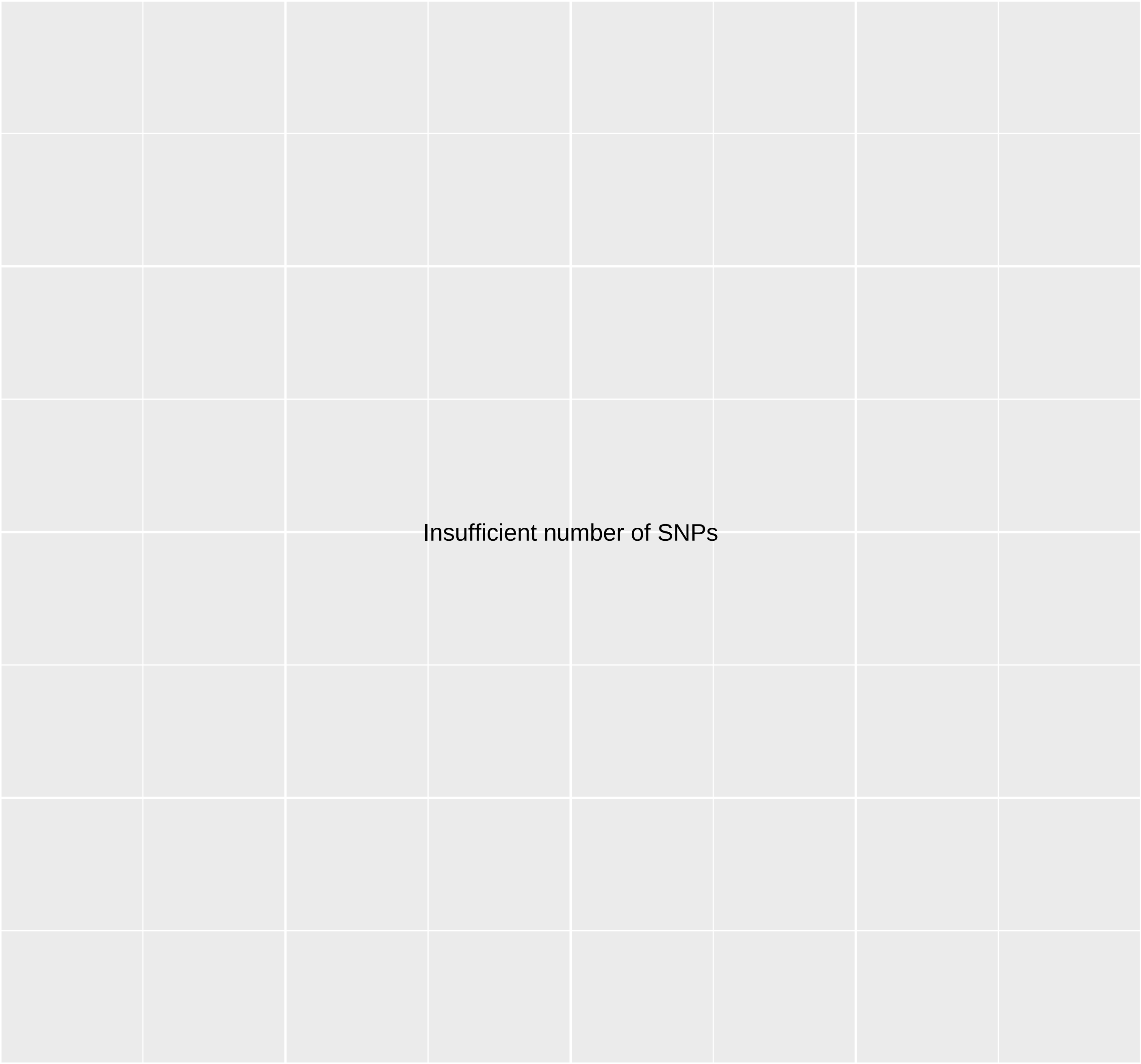
Insufficient number of SNPs

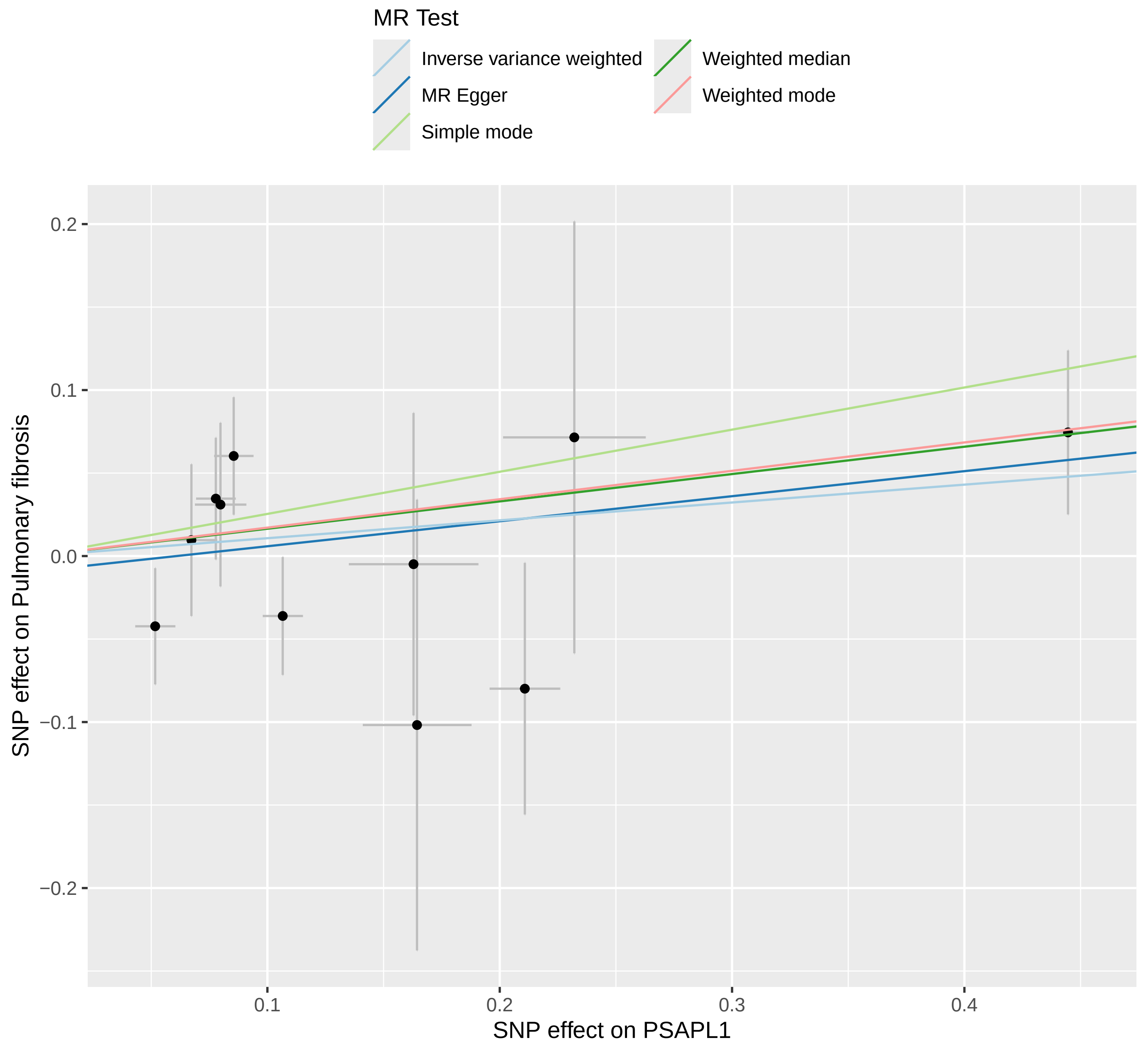
MR Test
Inverse variance weighted
MR Egger
Simple mode
Weighted median
Weighted mode
0.2
0.1
0.0
−0.1
−0.2
SNP effect on Pulmonary fibrosis
0.1
0.2
0.3
0.4
SNP effect on PSAPL1

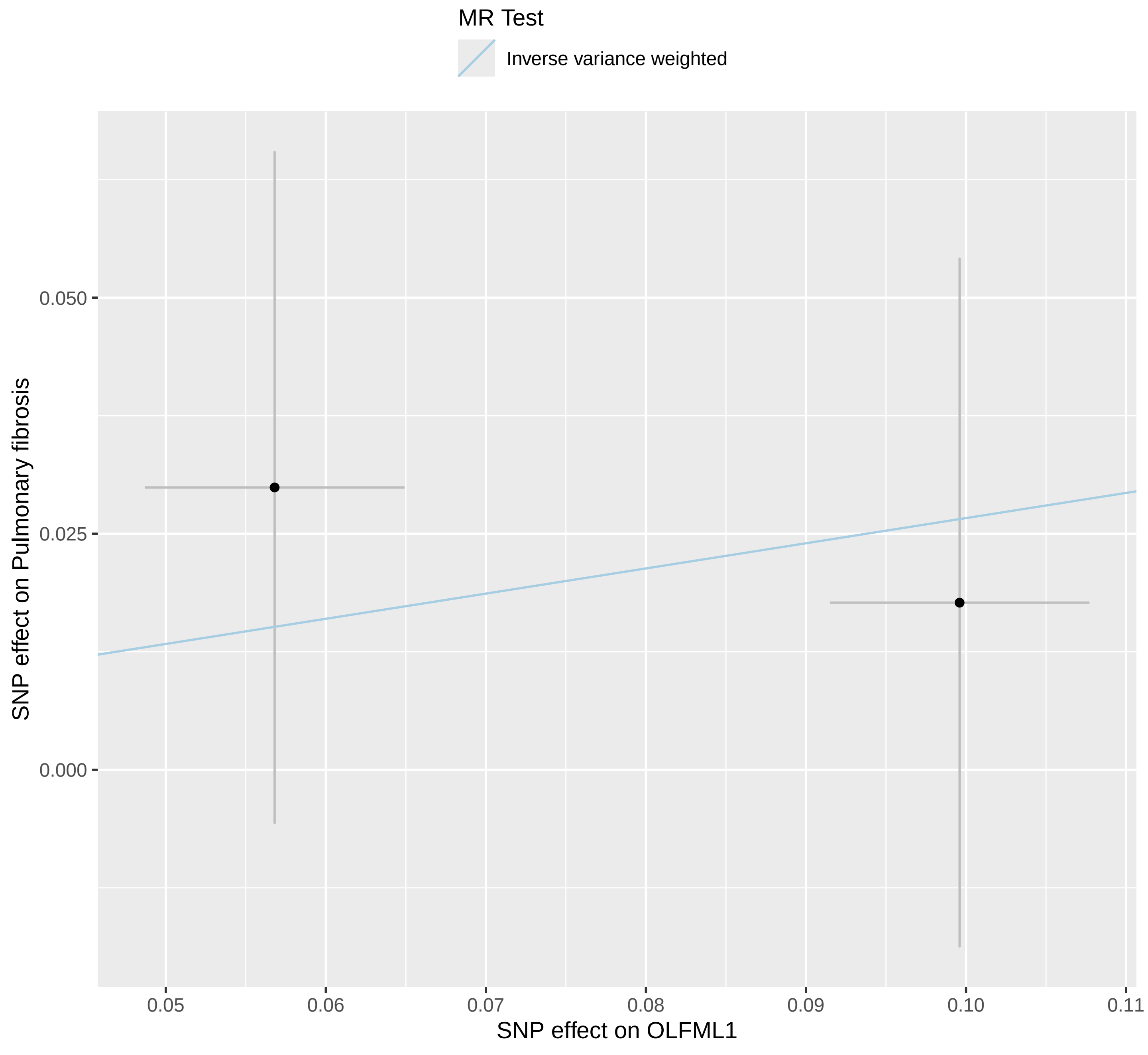

MR Test
Inverse variance weighted
0.050
0.025
0.000
SNP effect on Pulmonary fibrosis
0.05
0.06
0.07
0.08
0.09
0.10
0.11
SNP effect on OLFML1

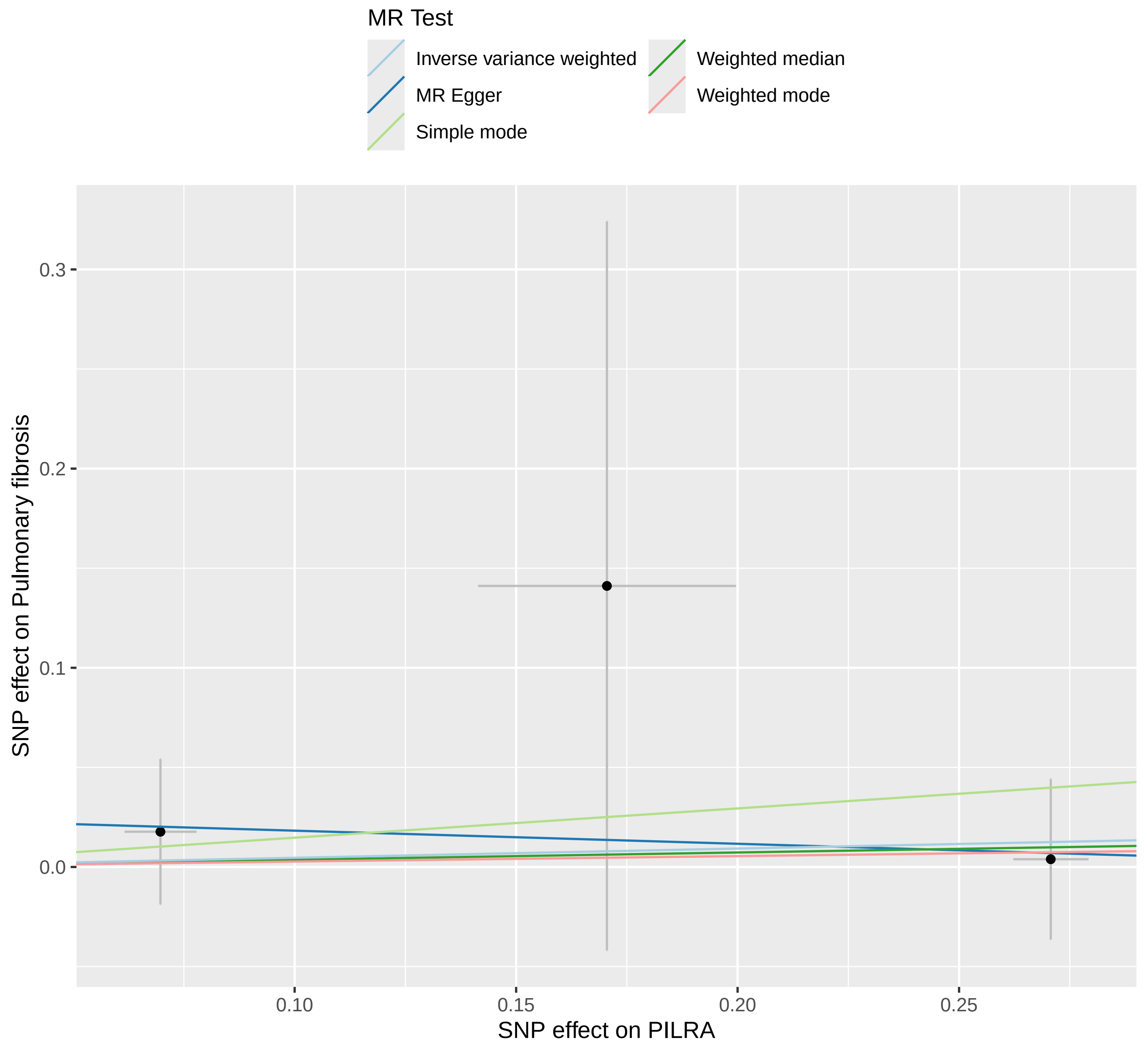
MR Test
Inverse variance weighted
MR Egger
Simple mode
Weighted median
Weighted mode
0.3
0.2
0.1
0.0
0.10
0.15
0.20
0.25
SNP effect on Pulmonary fibrosis
SNP effect on PILRA

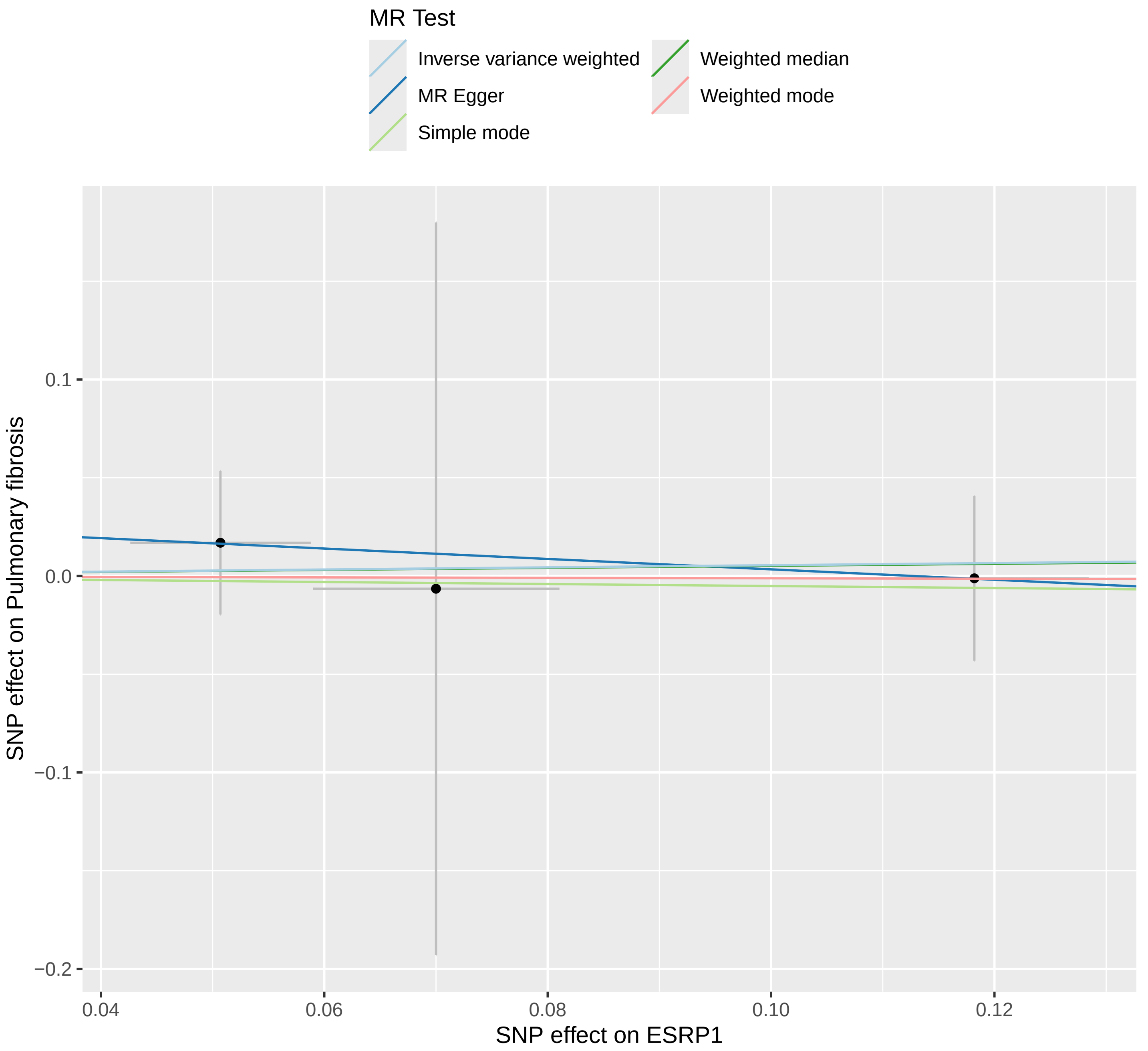

MR Test
Inverse variance weighted
MR Egger
Simple mode
Weighted median
Weighted mode
0.1
0.0
−0.1
−0.2
SNP effect on Pulmonary fibrosis
0.04
0.06
0.08
0.10
0.12
SNP effect on ESRP1

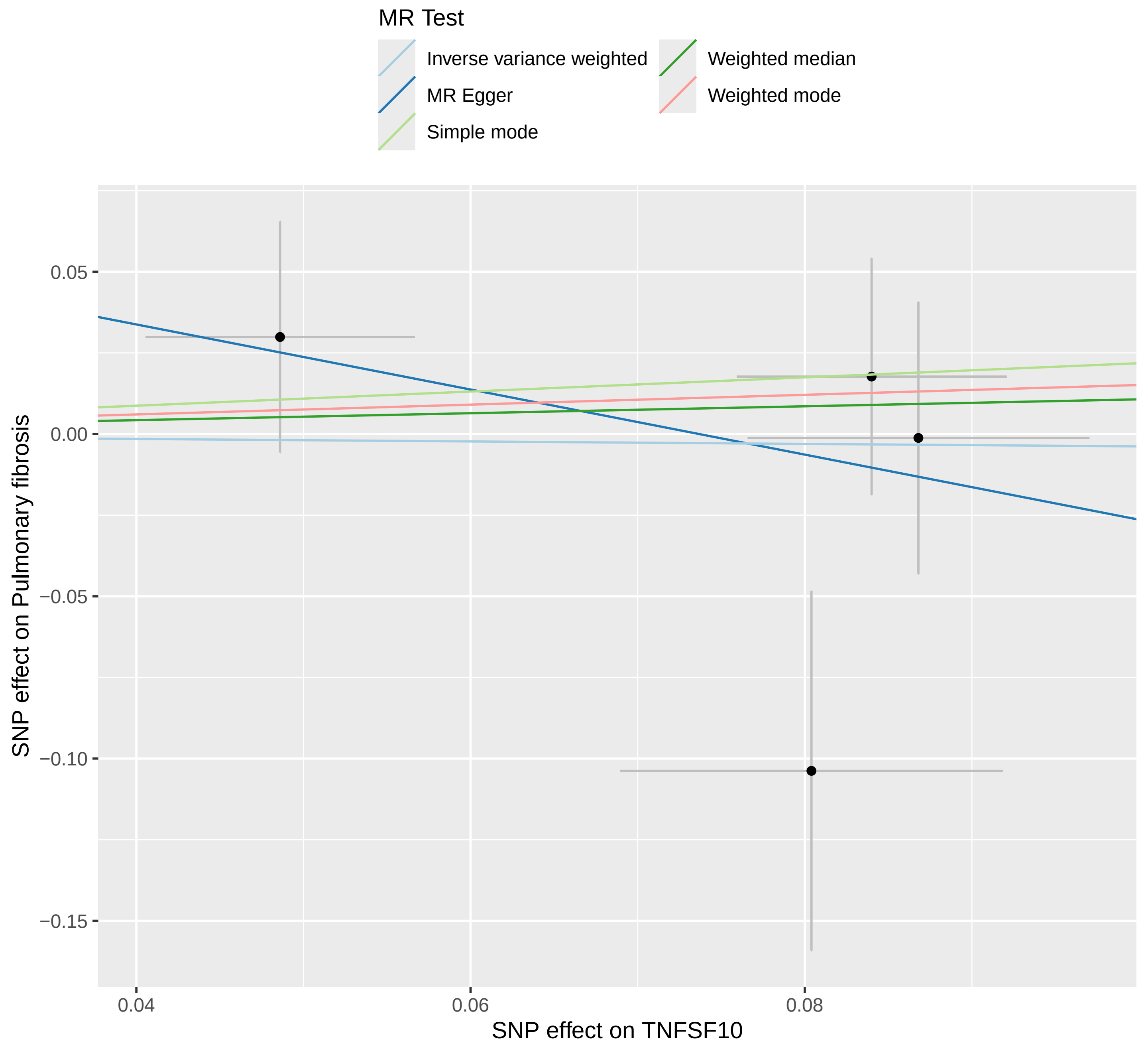
MR Test
Inverse variance weighted
MR Egger
Simple mode
Weighted median
Weighted mode
0.05
0.00
−0.05
−0.10
−0.15
SNP effect on Pulmonary fibrosis
0.04
0.06
0.08
SNP effect on TNFSF10

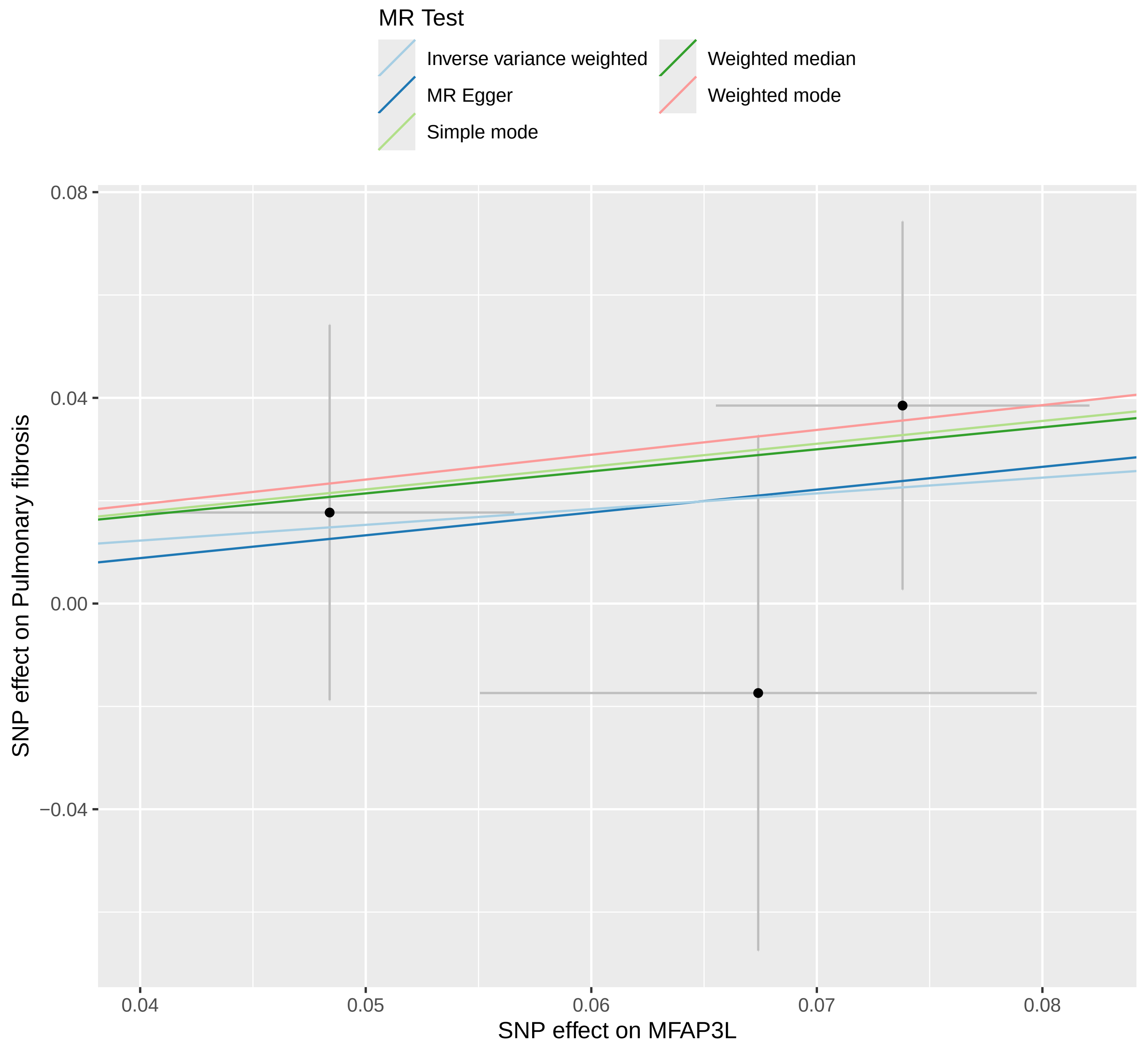
MR Test
Inverse variance weighted
MR Egger
Simple mode
Weighted median
Weighted mode
0.08
0.04
0.00
−0.04
0.04
0.05
0.06
0.07
0.08
SNP effect on MFAP3L
SNP effect on Pulmonary fibrosis

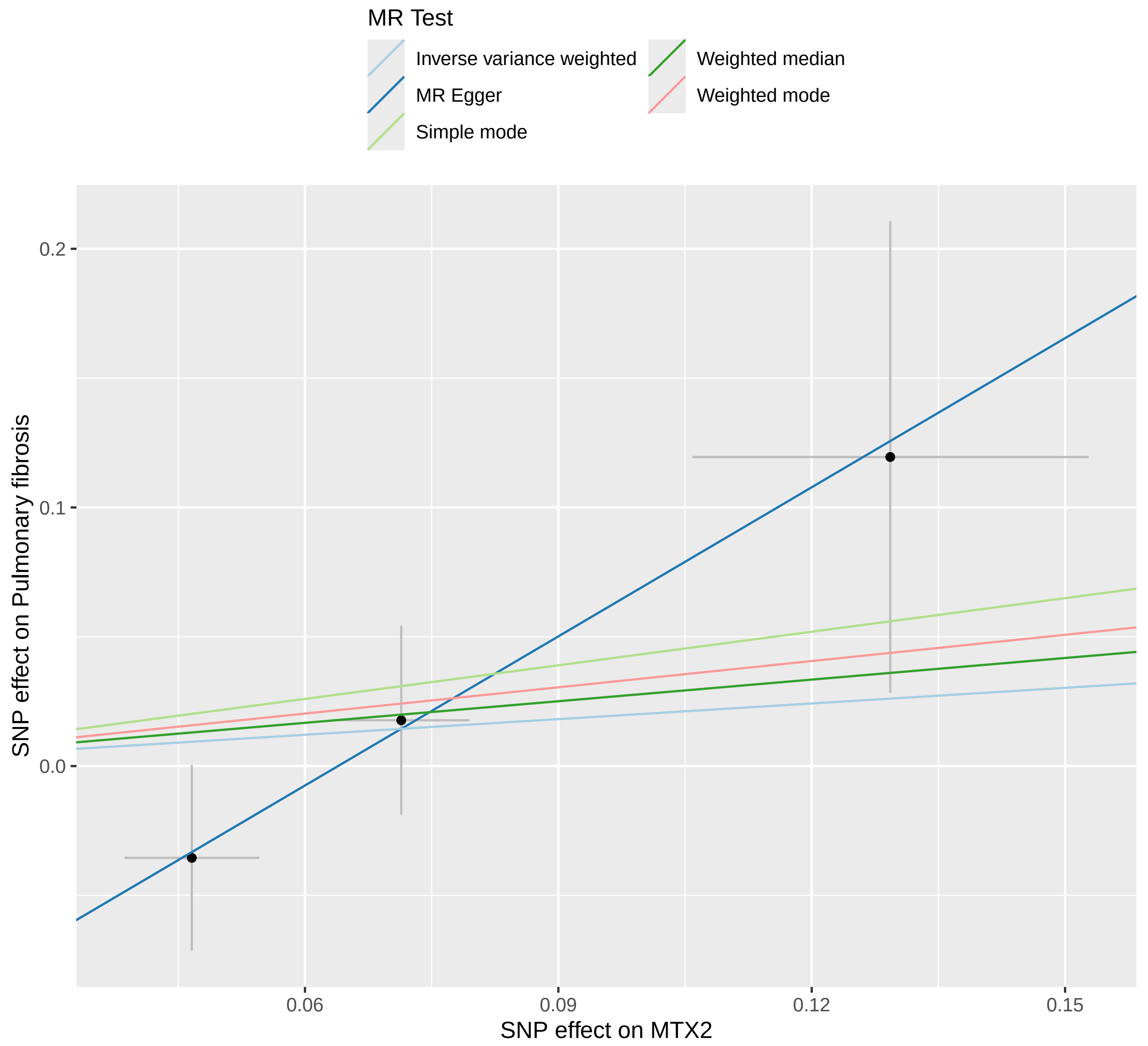

MR Test
Inverse variance weighted
MR Egger
Simple mode
Weighted median
Weighted mode
SNP effect on Pulmonary fibrosis
0.2
0.1
0.0
0.06
0.09
0.12
0.15
SNP effect on MTX2

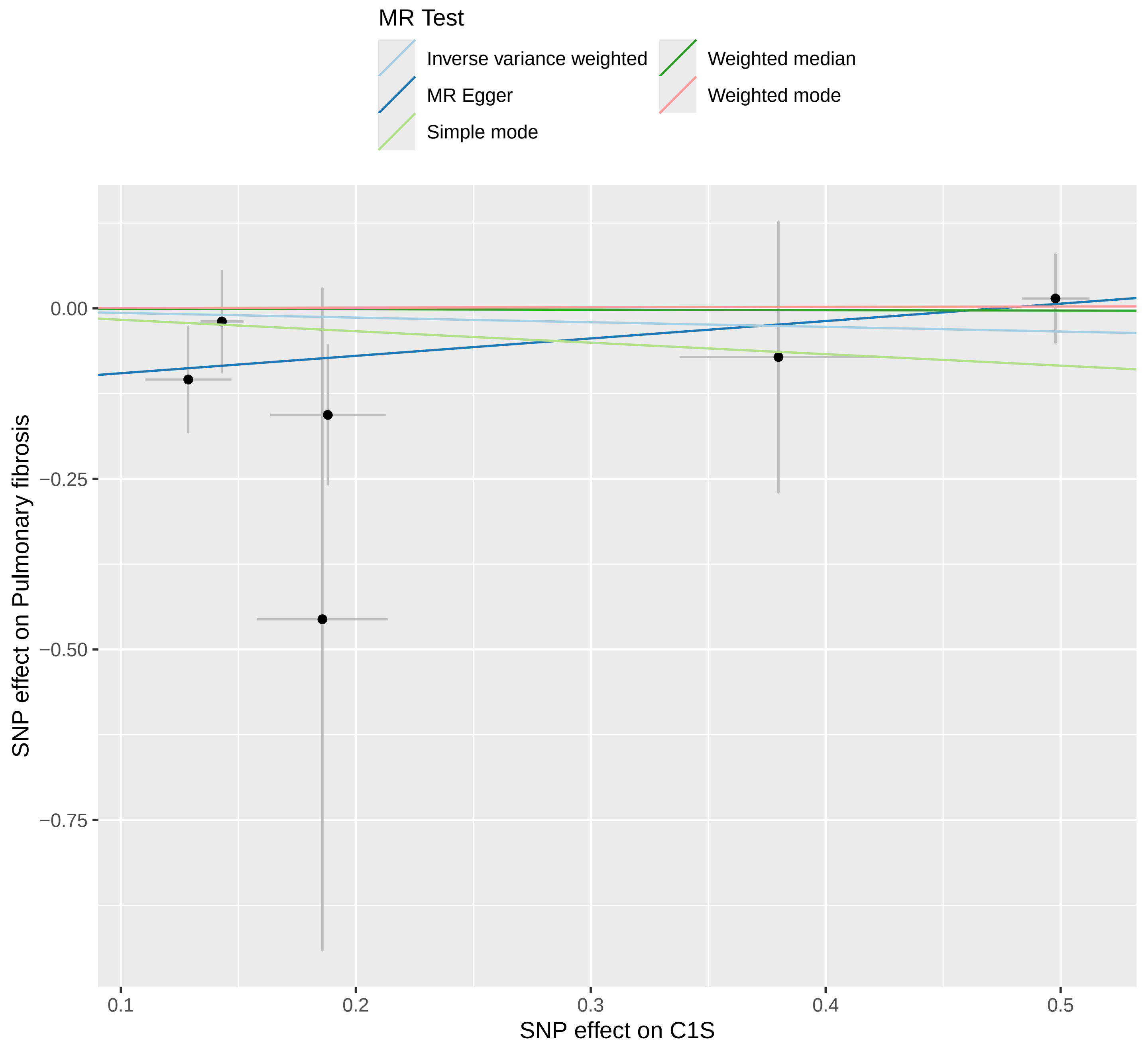

MR Test
Inverse variance weighted
MR Egger
Simple mode
Weighted median
Weighted mode
0.00
−0.25
−0.50
−0.75
SNP effect on Pulmonary fibrosis
0.1
0.2
0.3
0.4
0.5
SNP effect on C1S

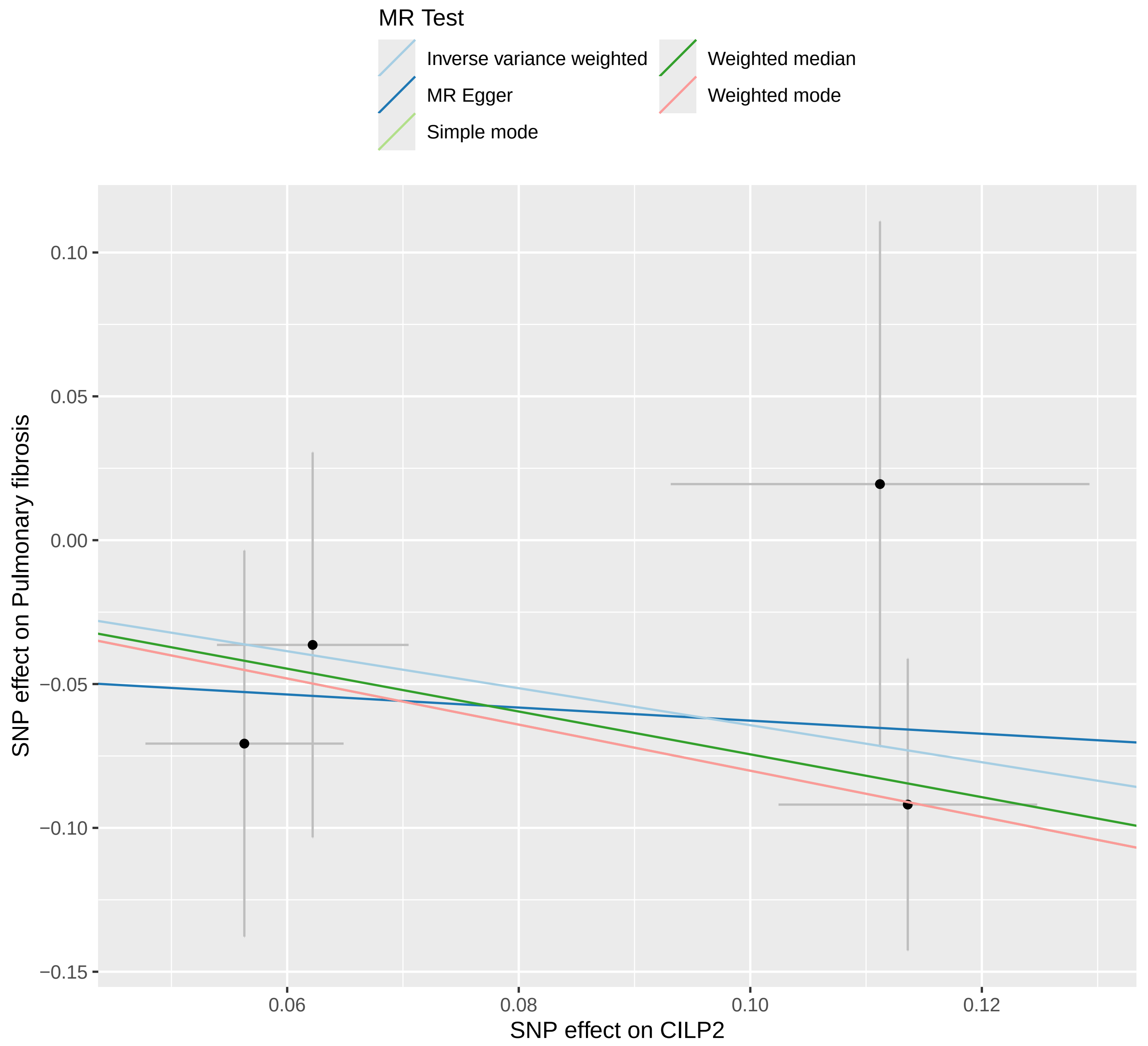
MR Test
Inverse variance weighted
MR Egger
Simple mode
Weighted median
Weighted mode
0.10
0.05
0.00
−0.05
−0.10
−0.15
SNP effect on Pulmonary fibrosis
0.06
0.08
0.10
0.12
SNP effect on CILP2

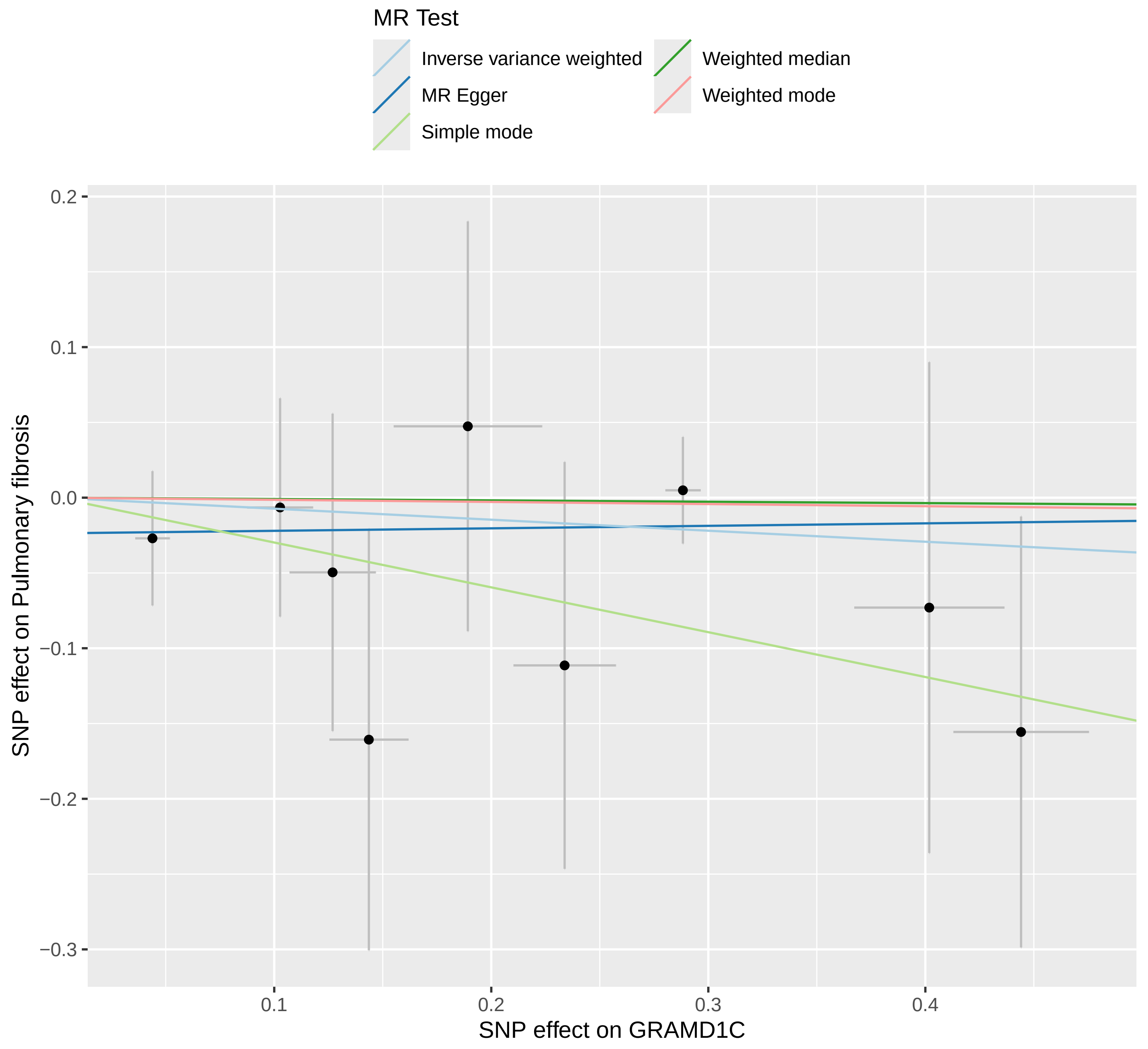
MR Test
Inverse variance weighted
MR Egger
Simple mode
Weighted median
Weighted mode
0.2
0.1
0.0
−0.1
−0.2
−0.3
0.1
0.2
0.3
0.4
SNP effect on Pulmonary fibrosis
SNP effect on GRAMD1C

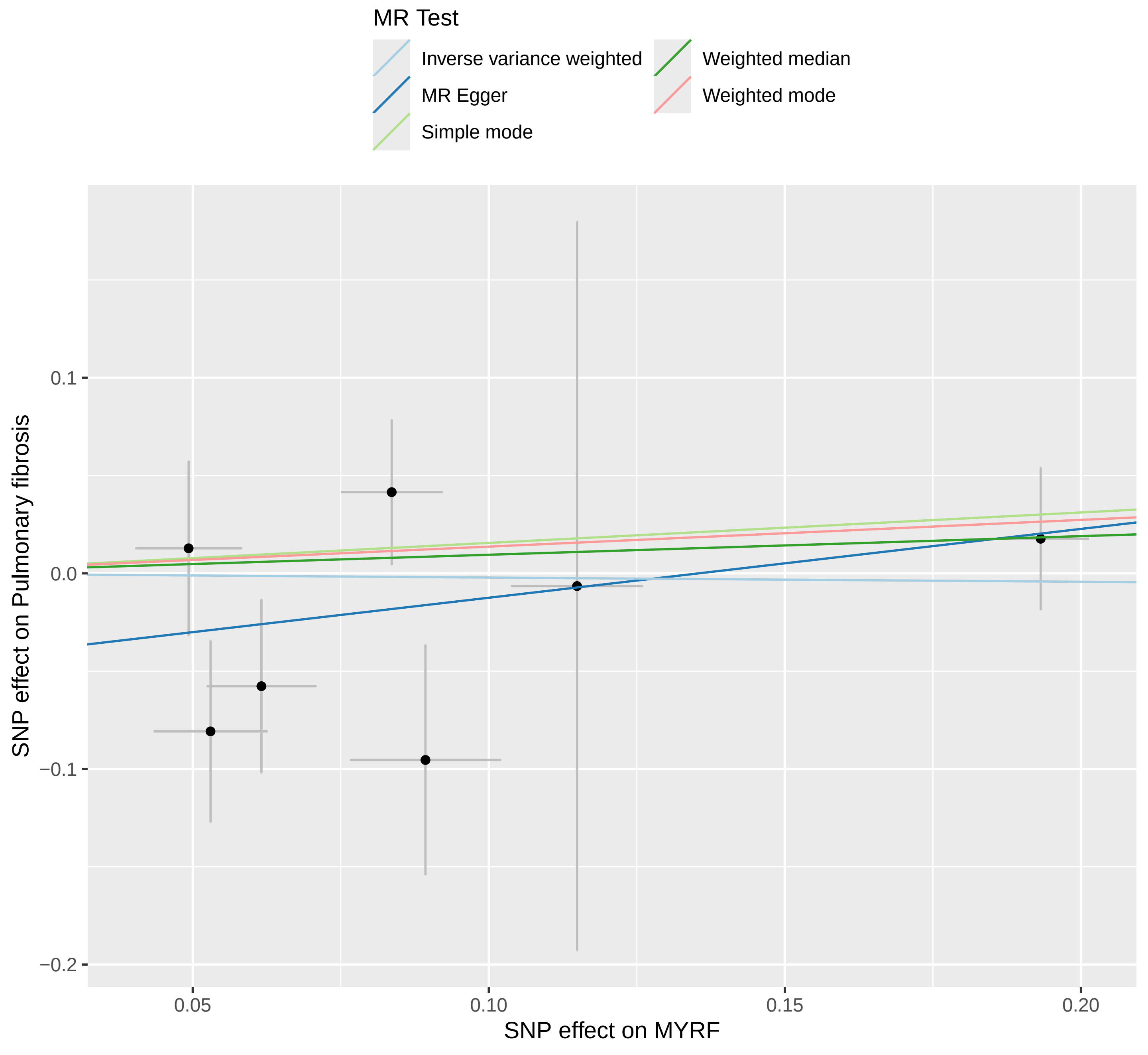

MR Test
Inverse variance weighted
MR Egger
Simple mode
Weighted median
Weighted mode
0.1
0.0
−0.1
−0.2
0.05
0.10
0.15
0.20
SNP effect on Pulmonary fibrosis
SNP effect on MYRF

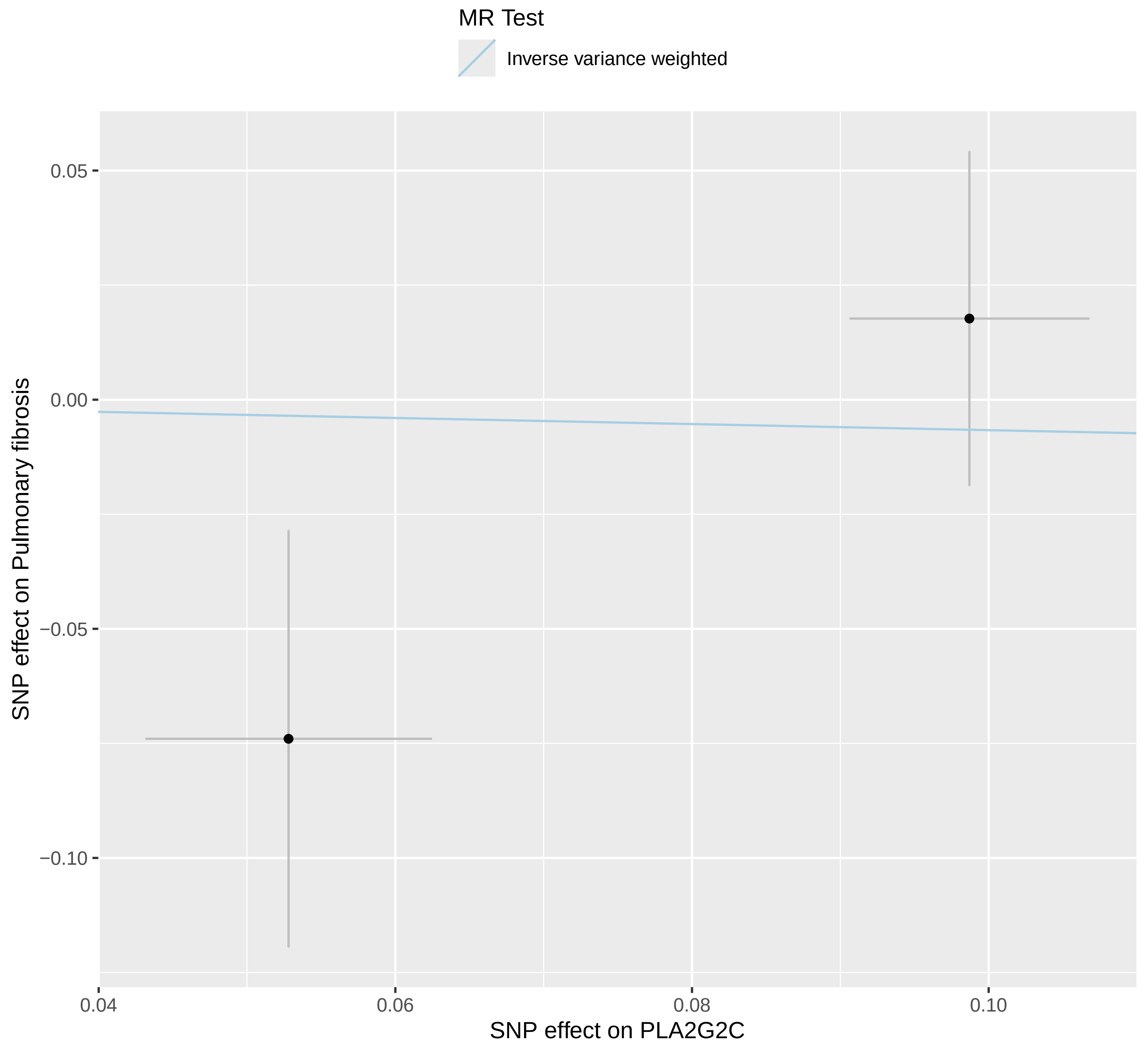
MR Test
Inverse variance weighted
0.05
0.00
−0.05
−0.10
SNP effect on Pulmonary fibrosis
0.04
0.06
0.08
0.10
SNP effect on PLA2G2C

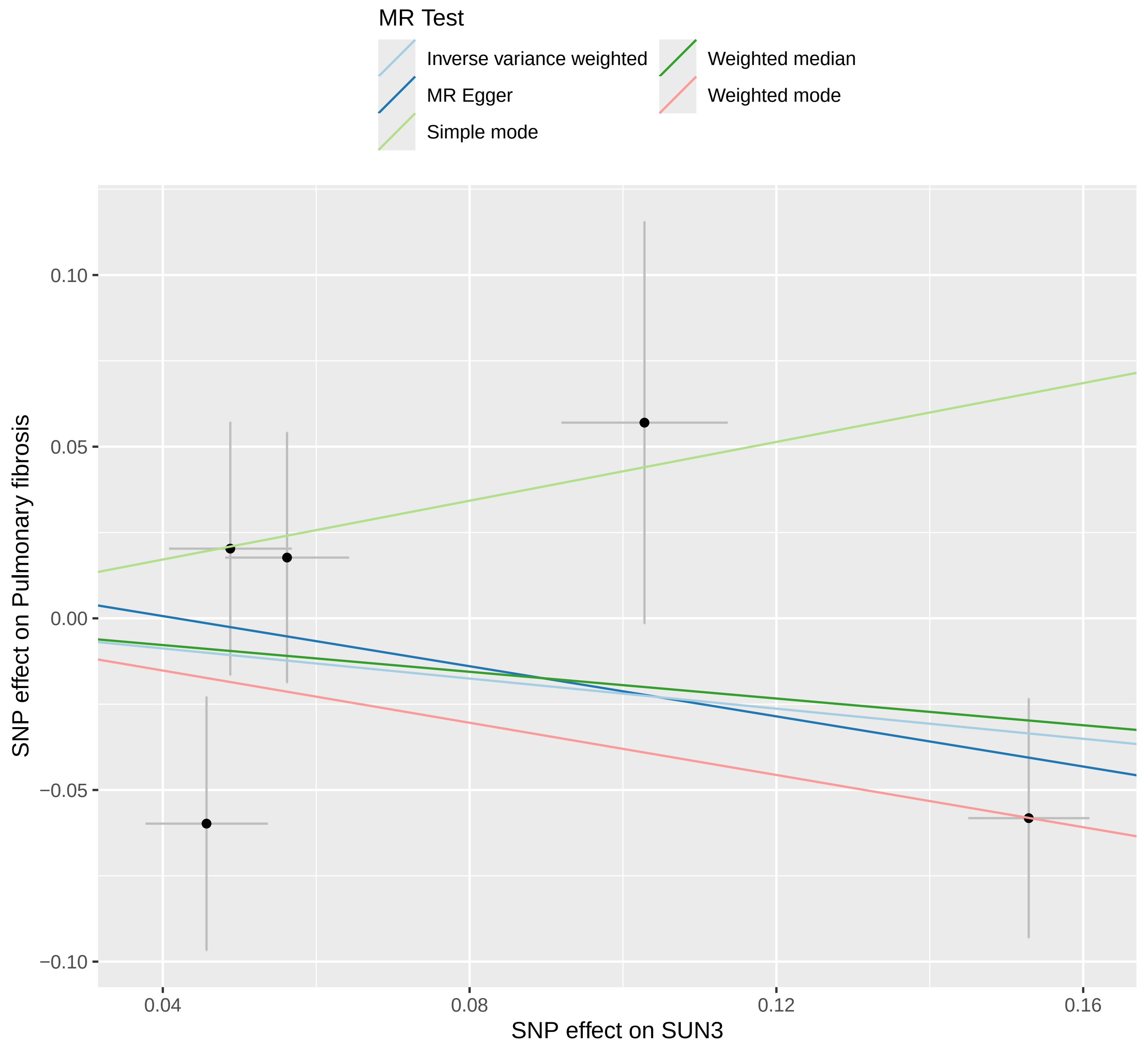

MR Test
Inverse variance weighted
MR Egger
Simple mode
Weighted median
Weighted mode
SNP effect on Pulmonary fibrosis
0.10
0.05
0.00
−0.05
−0.10
0.04
0.08
0.12
0.16
SNP effect on SUN3

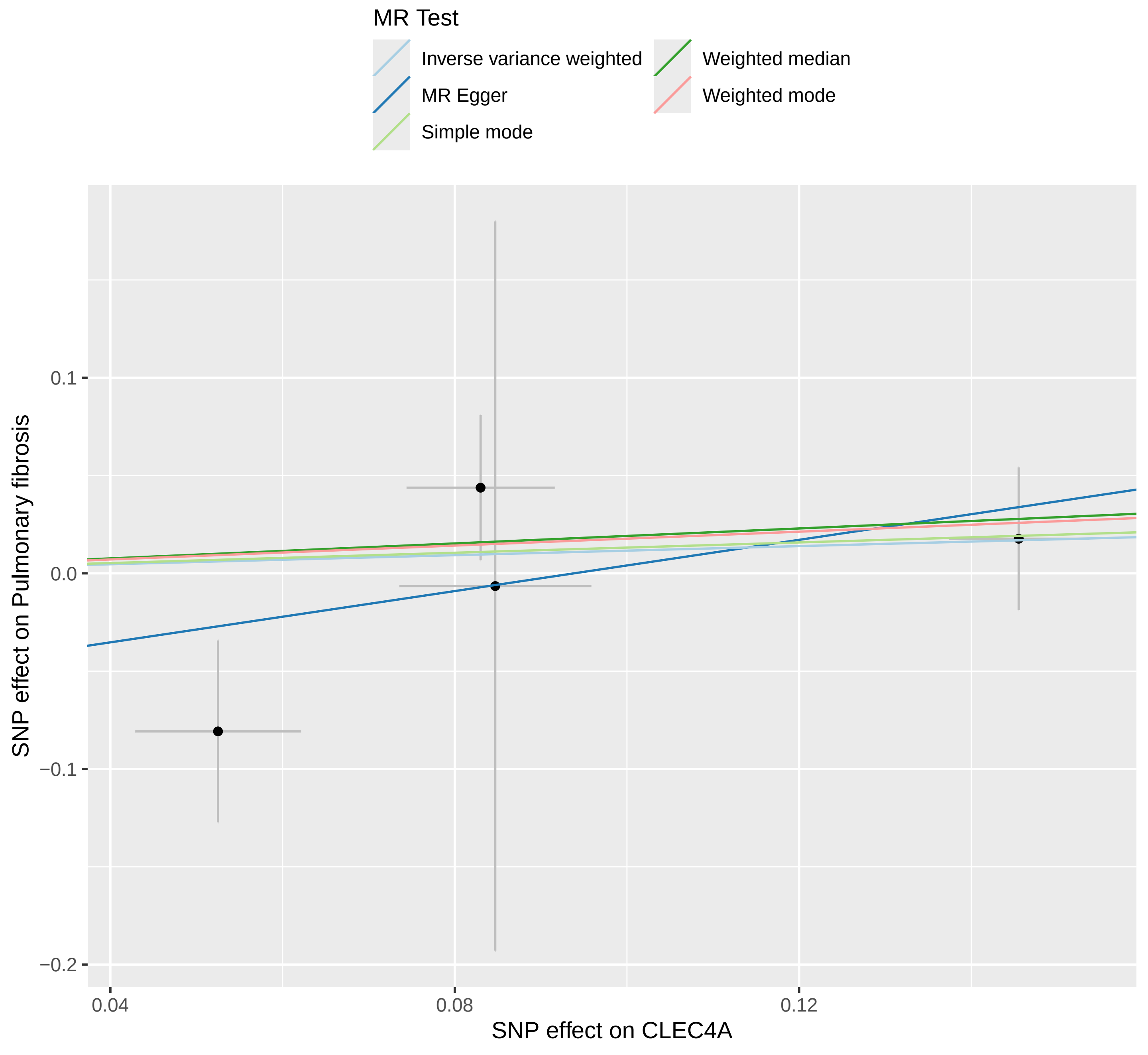
MR Test
Inverse variance weighted
Weighted median
MR Egger
Weighted mode
Simple mode
0.1
0.0
−0.1
−0.2
SNP effect on Pulmonary fibrosis
0.04
0.08
0.12
SNP effect on CLEC4A

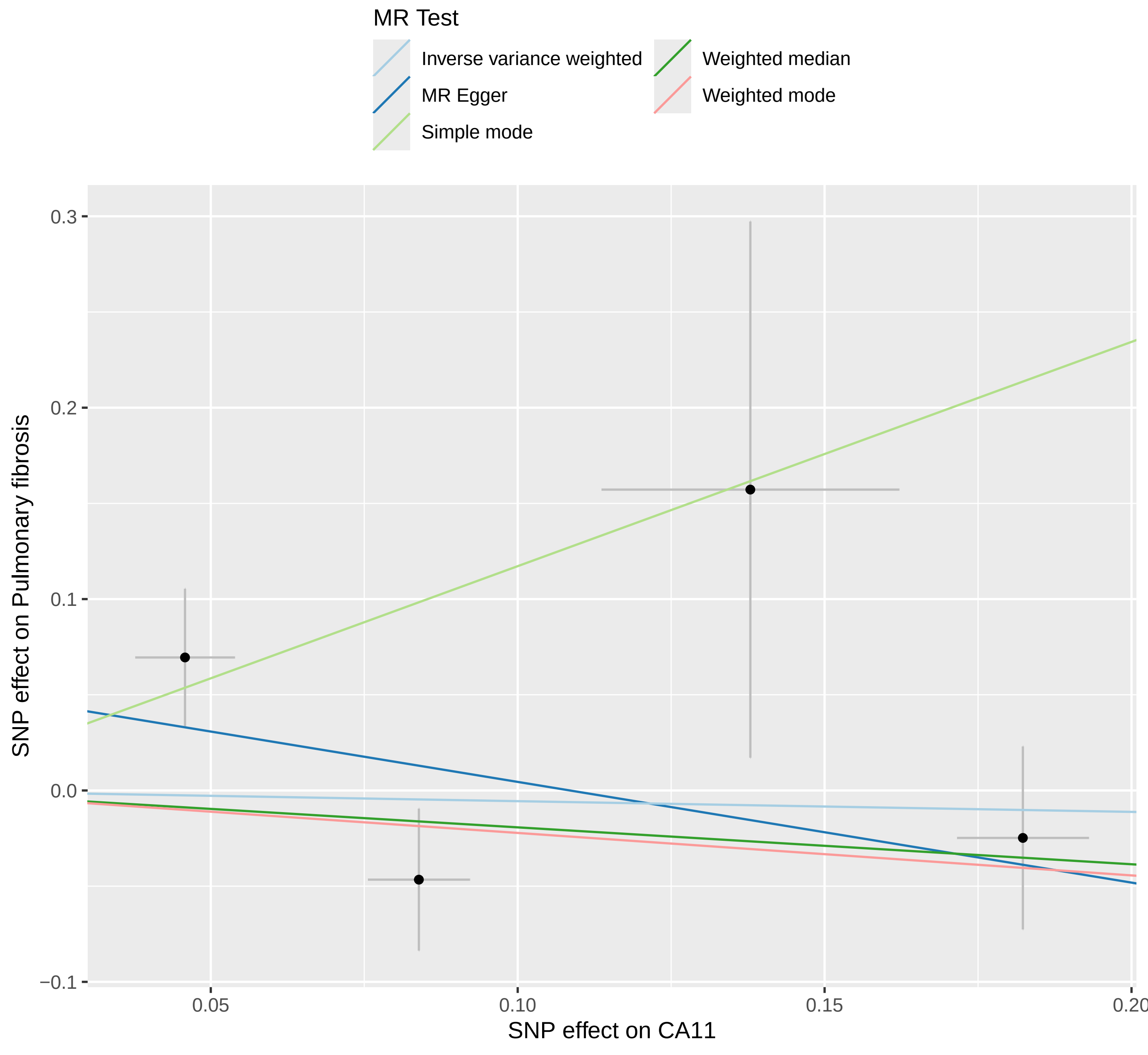
MR Test
Inverse variance weighted
MR Egger
Simple mode
Weighted median
Weighted mode
0.3
0.2
0.1
0.0
−0.1
0.05
0.10
0.15
0.20
SNP effect on CA11
SNP effect on Pulmonary fibrosis

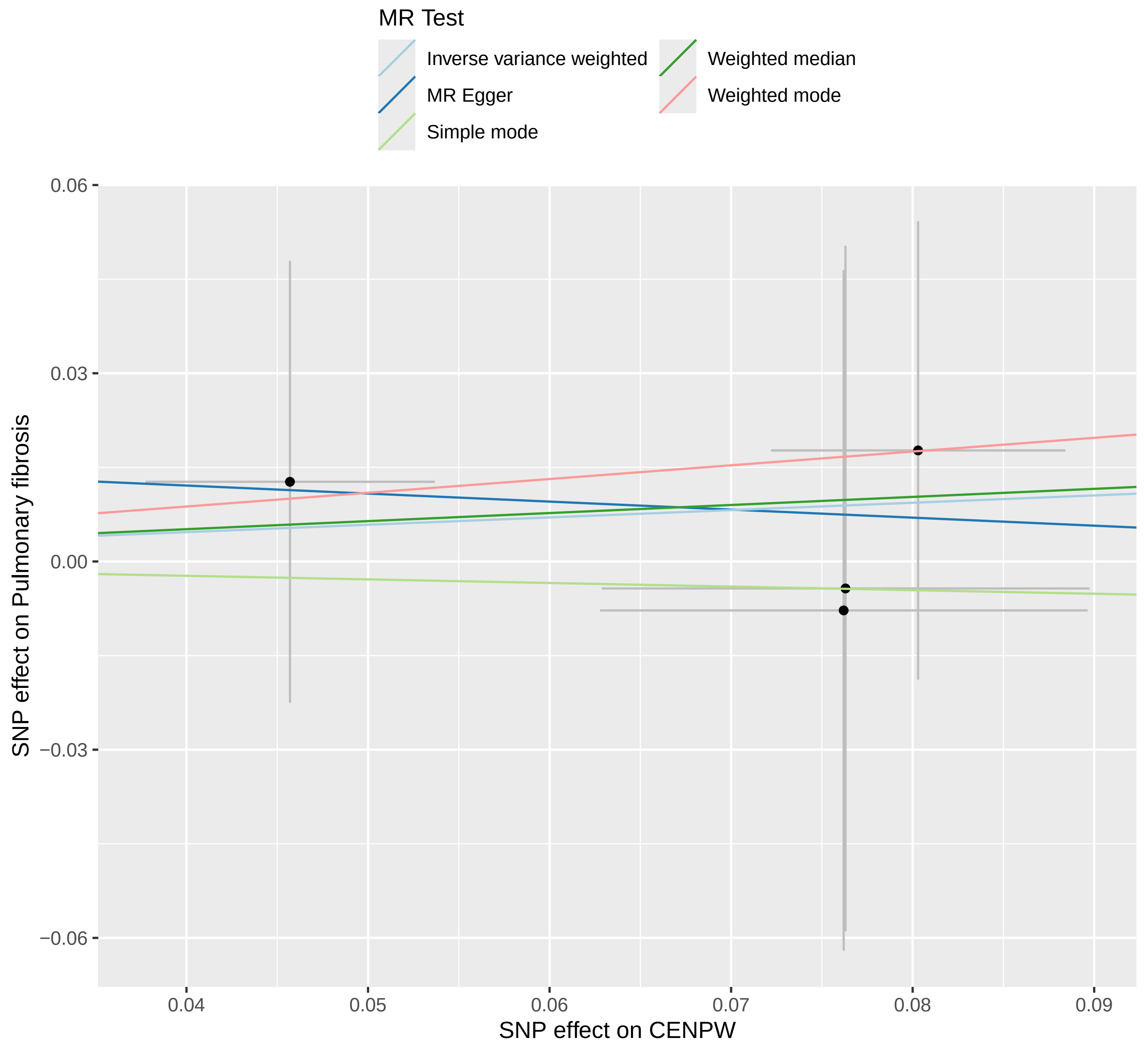
MR Test
Inverse variance weighted
MR Egger
Simple mode
Weighted median
Weighted mode
0.06
0.03
0.00
−0.03
−0.06
SNP effect on Pulmonary fibrosis
0.04
0.05
0.06
0.07
0.08
0.09
SNP effect on CENPW

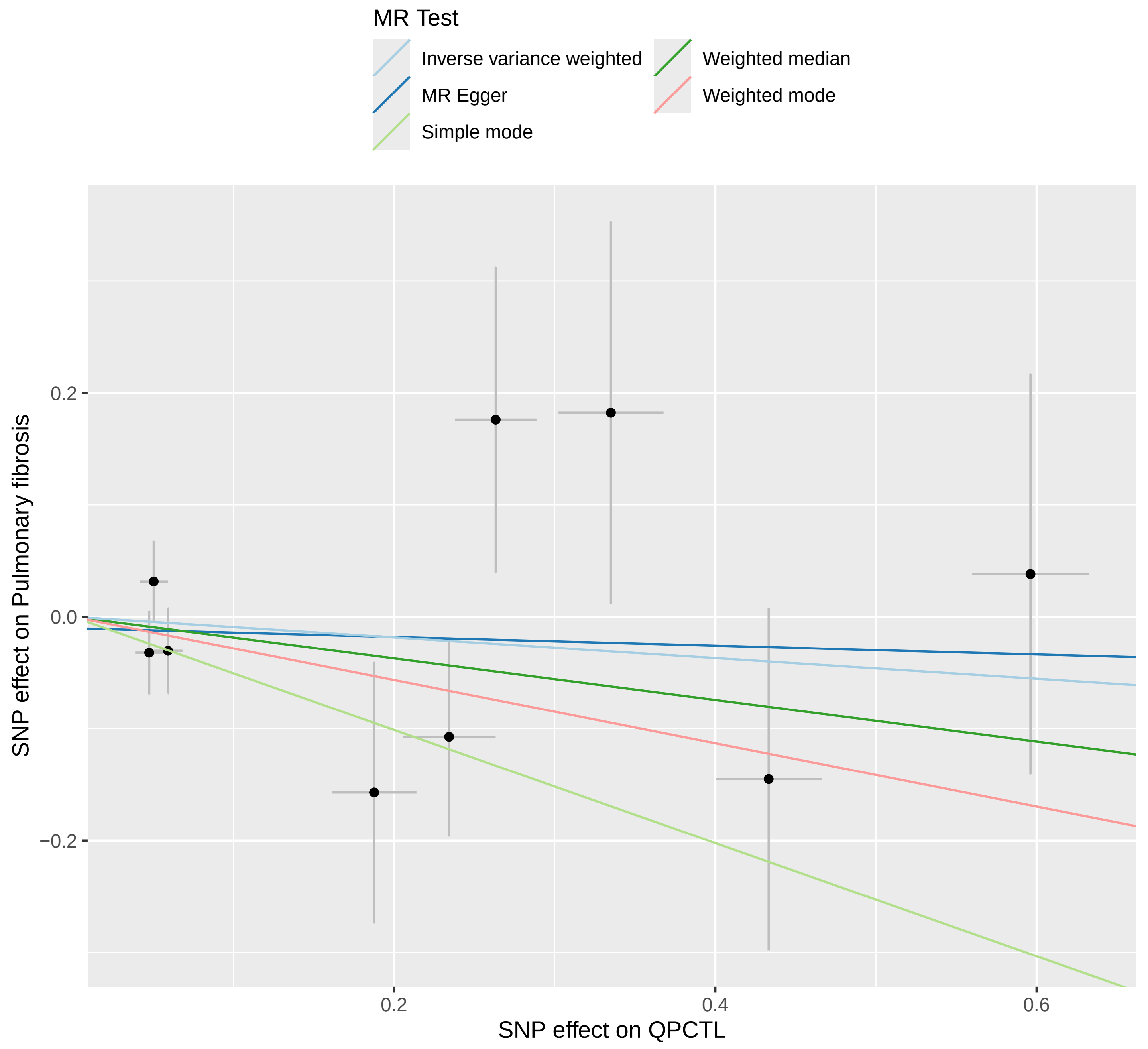

MR Test
Inverse variance weighted
MR Egger
Simple mode
Weighted median
Weighted mode
0.2
0.0
−0.2
SNP effect on Pulmonary fibrosis
0.2
0.4
0.6
SNP effect on QPCTL

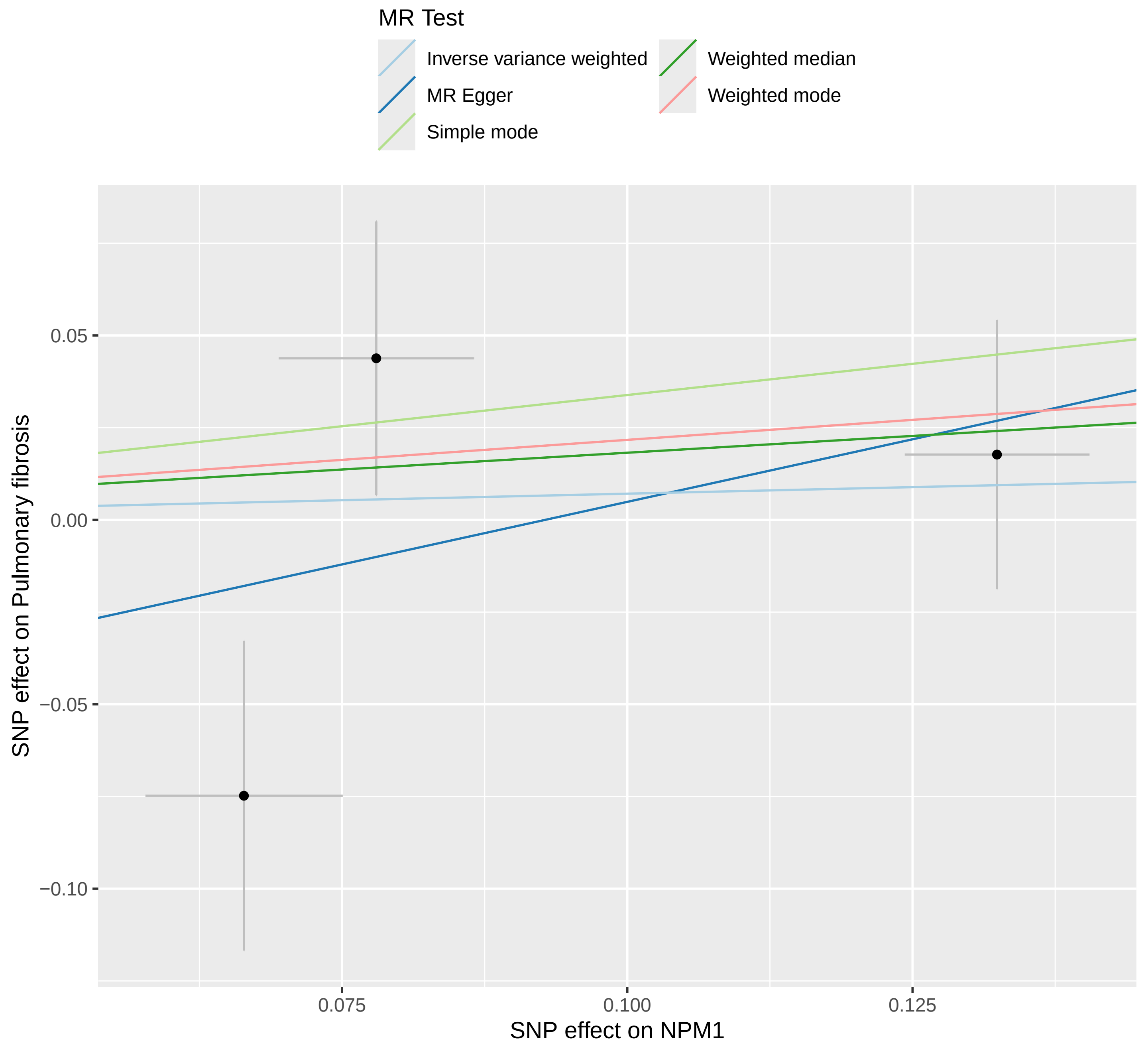

MR Test
Inverse variance weighted
MR Egger
Simple mode
Weighted median
Weighted mode
0.05
0.00
−0.05
−0.10
SNP effect on Pulmonary fibrosis
0.075
0.100
0.125
SNP effect on NPM1

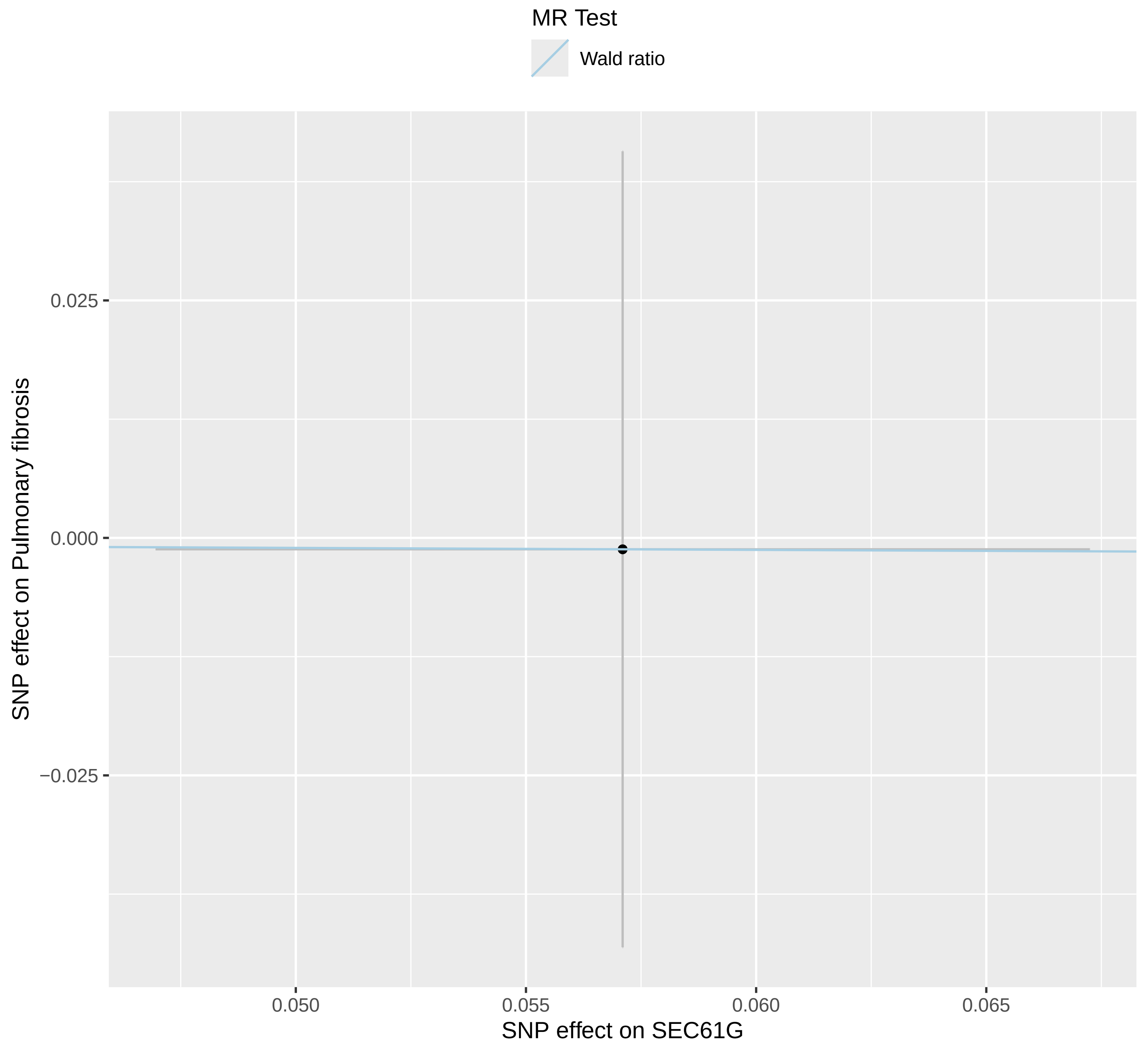
MR Test
Wald ratio
0.025
0.000
−0.025
0.050
0.055
0.060
0.065
SNP effect on Pulmonary fibrosis
SNP effect on SEC61G

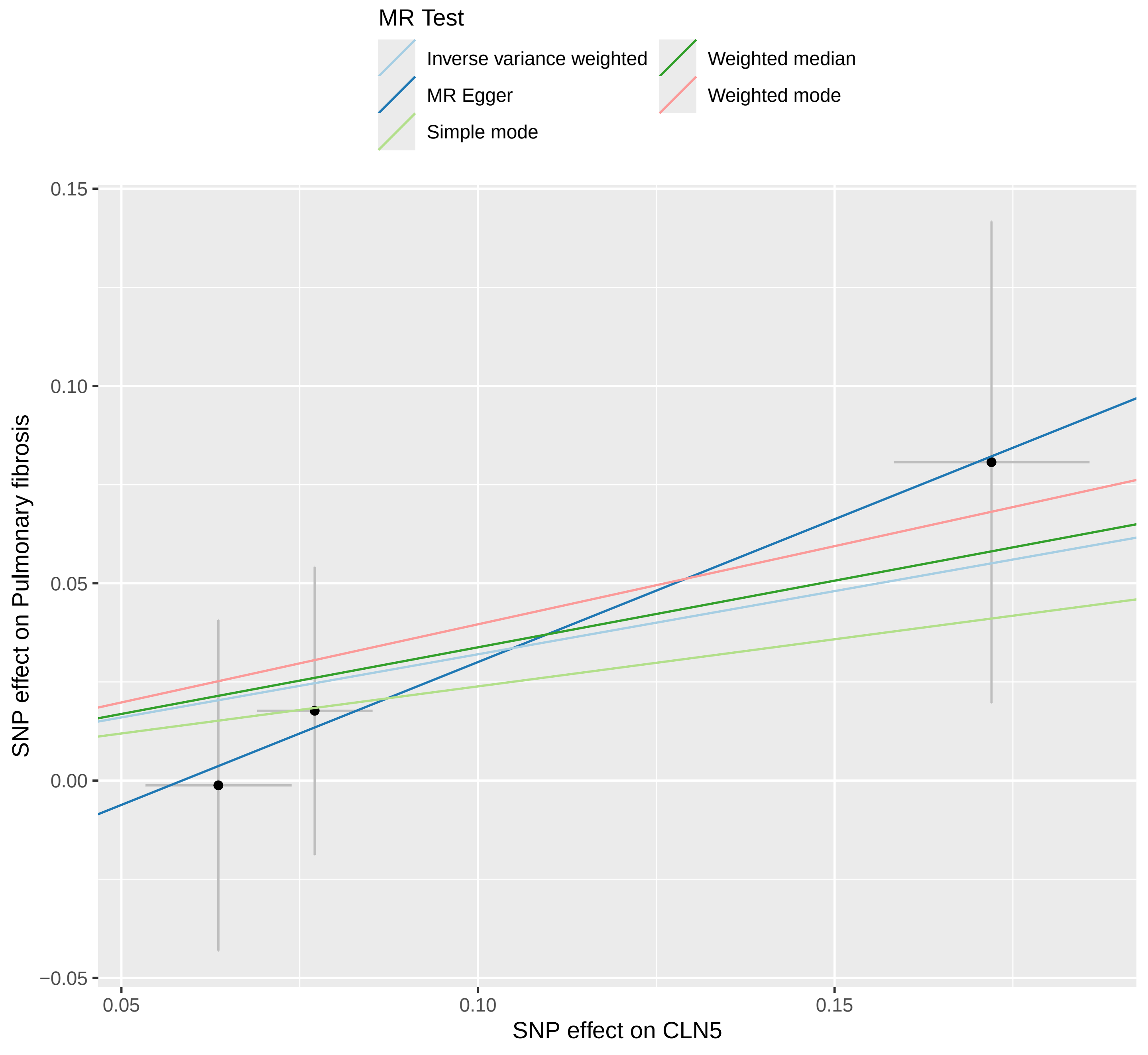

MR Test
Inverse variance weighted
MR Egger
Simple mode
Weighted median
Weighted mode
0.15
0.10
0.05
0.00
−0.05
SNP effect on Pulmonary fibrosis
0.05
0.10
0.15
SNP effect on CLN5

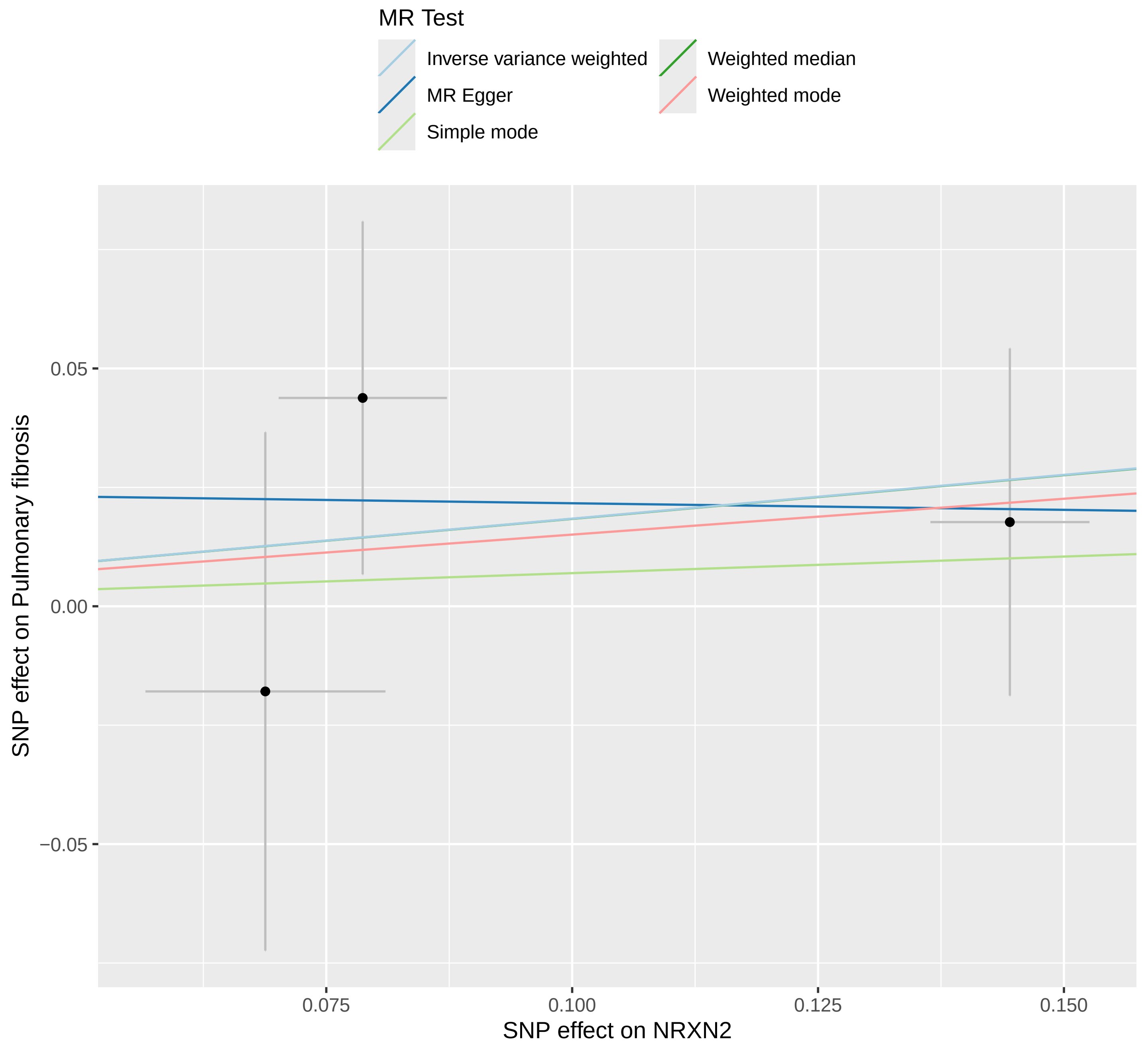

MR Test
Inverse variance weighted
MR Egger
Simple mode
Weighted median
Weighted mode
0.05
0.00
−0.05
0.075
0.100
0.125
0.150
SNP effect on NRXN2
SNP effect on Pulmonary fibrosis

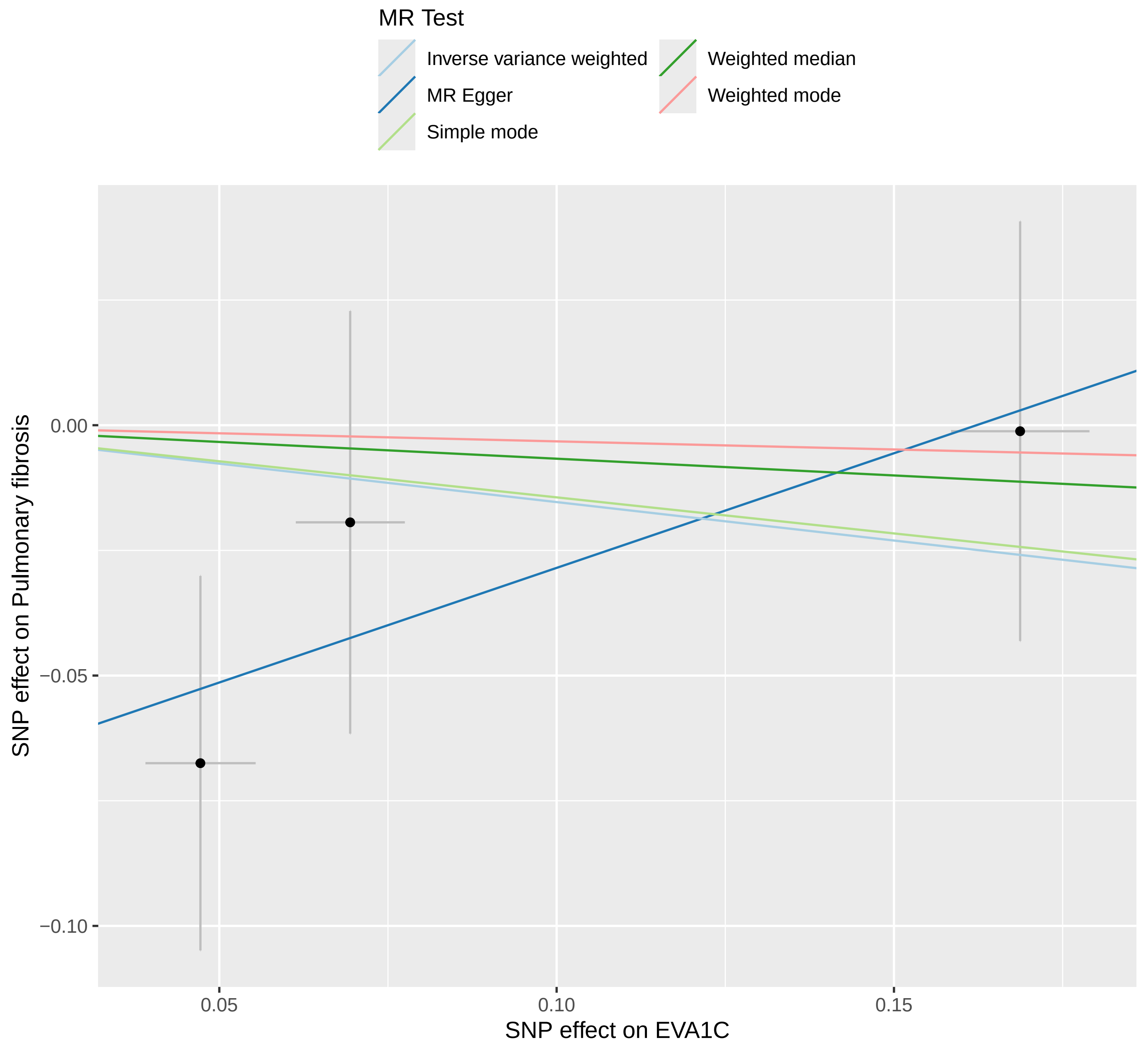

MR Test
Inverse variance weighted
MR Egger
Simple mode
Weighted median
Weighted mode
0.00
−0.05
−0.10
0.05
0.10
0.15
SNP effect on EVA1C
SNP effect on Pulmonary fibrosis

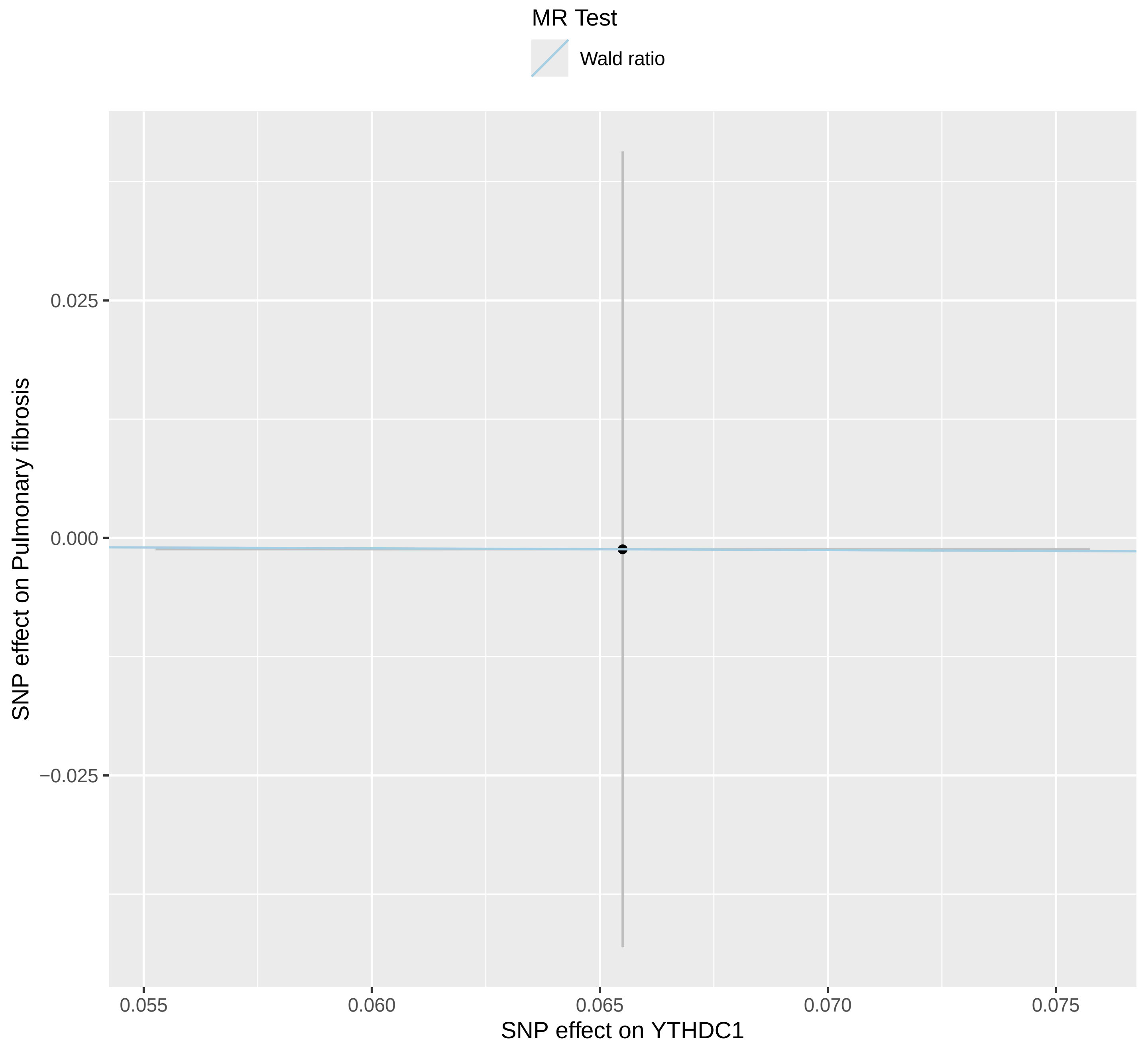

MR Test
Wald ratio
0.025
0.000
−0.025
0.055
0.060
0.065
0.070
0.075
SNP effect on Pulmonary fibrosis
SNP effect on YTHDC1

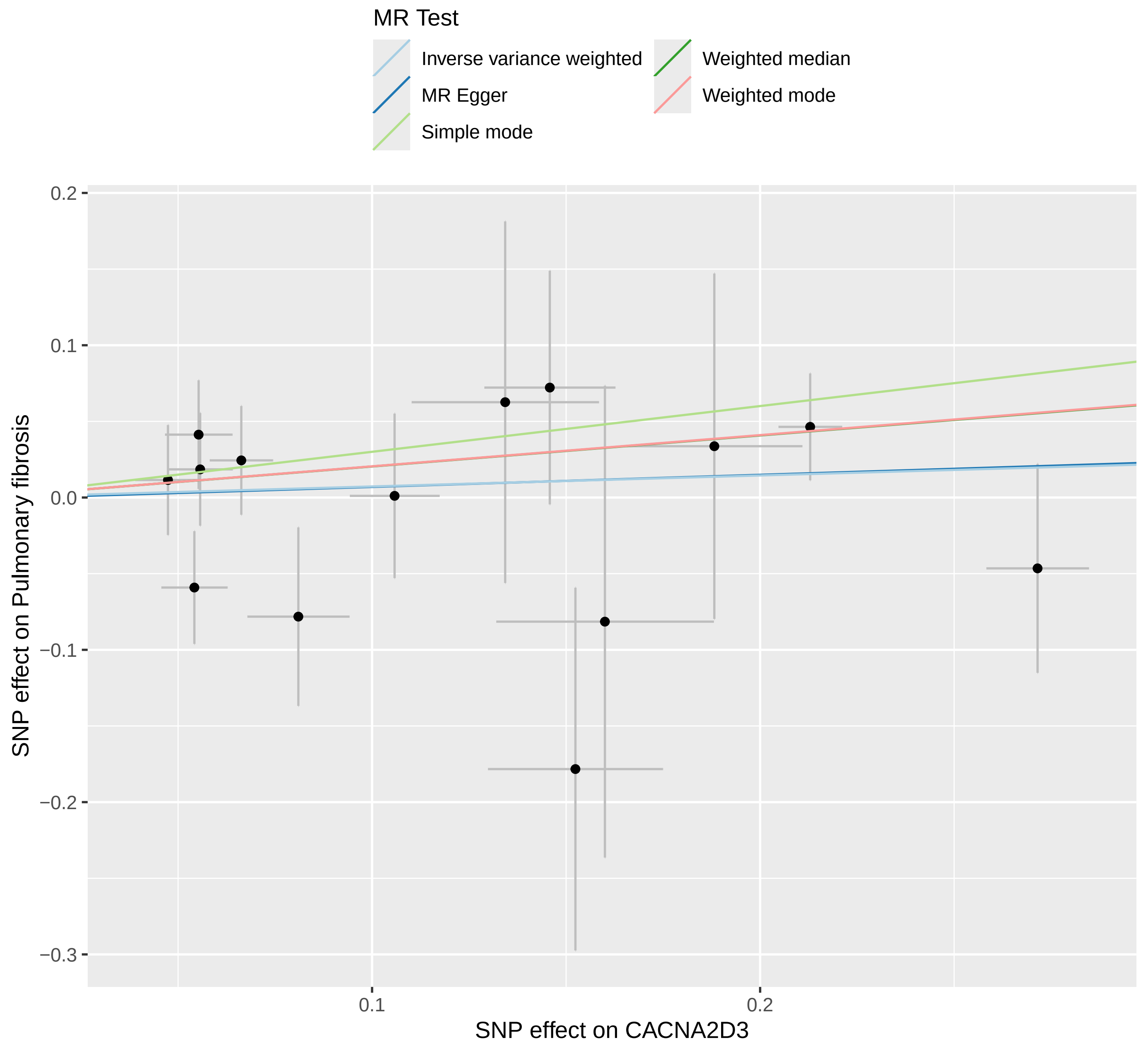

MR Test
Inverse variance weighted
MR Egger
Simple mode
Weighted median
Weighted mode
0.2
0.1
0.0
−0.1
−0.2
−0.3
SNP effect on Pulmonary fibrosis
0.1
0.2
SNP effect on CACNA2D3

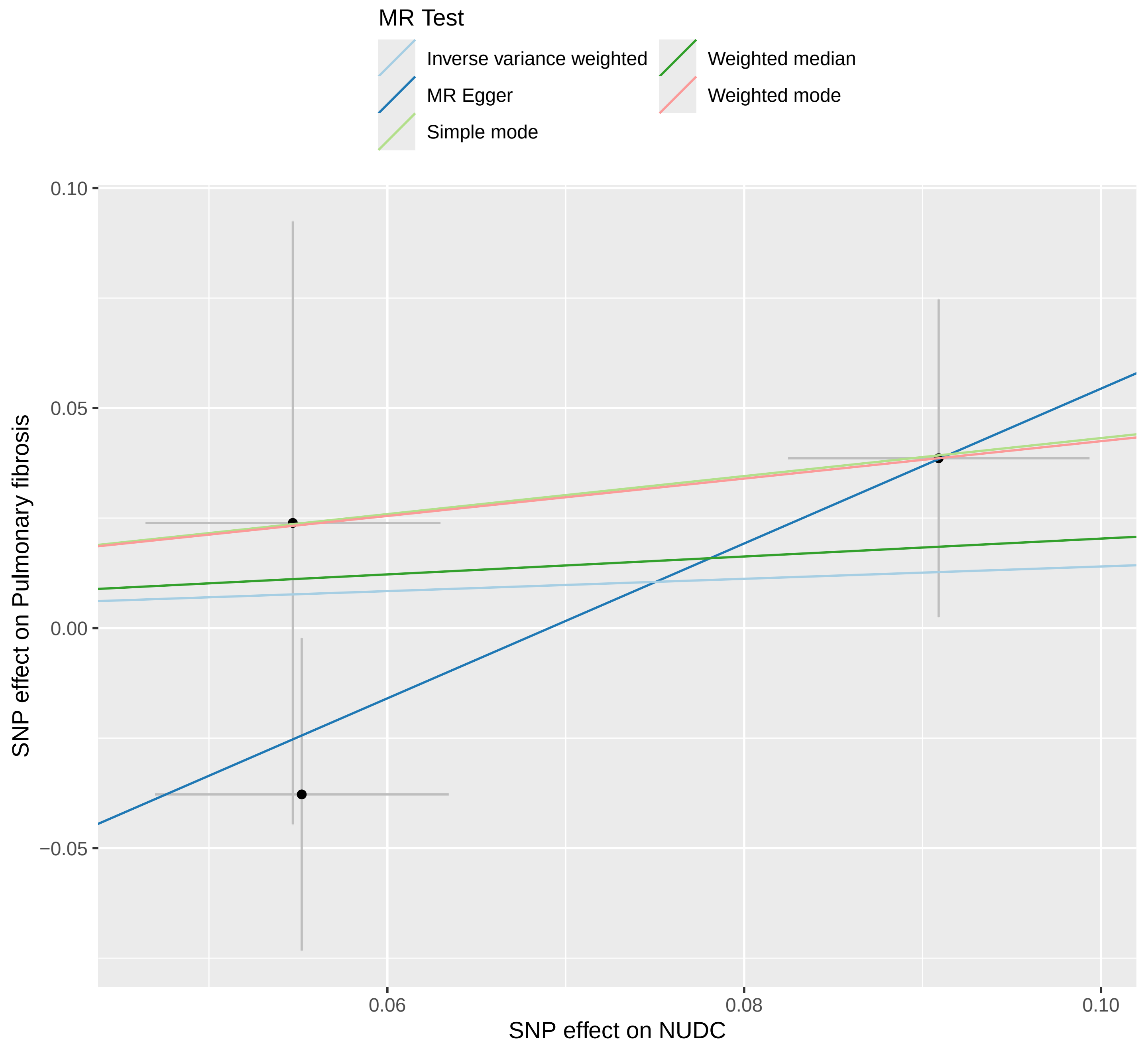
MR Test
Inverse variance weighted
MR Egger
Simple mode
Weighted median
Weighted mode
0.10
0.05
0.00
−0.05
0.06
0.08
0.10
SNP effect on Pulmonary fibrosis
SNP effect on NUDC

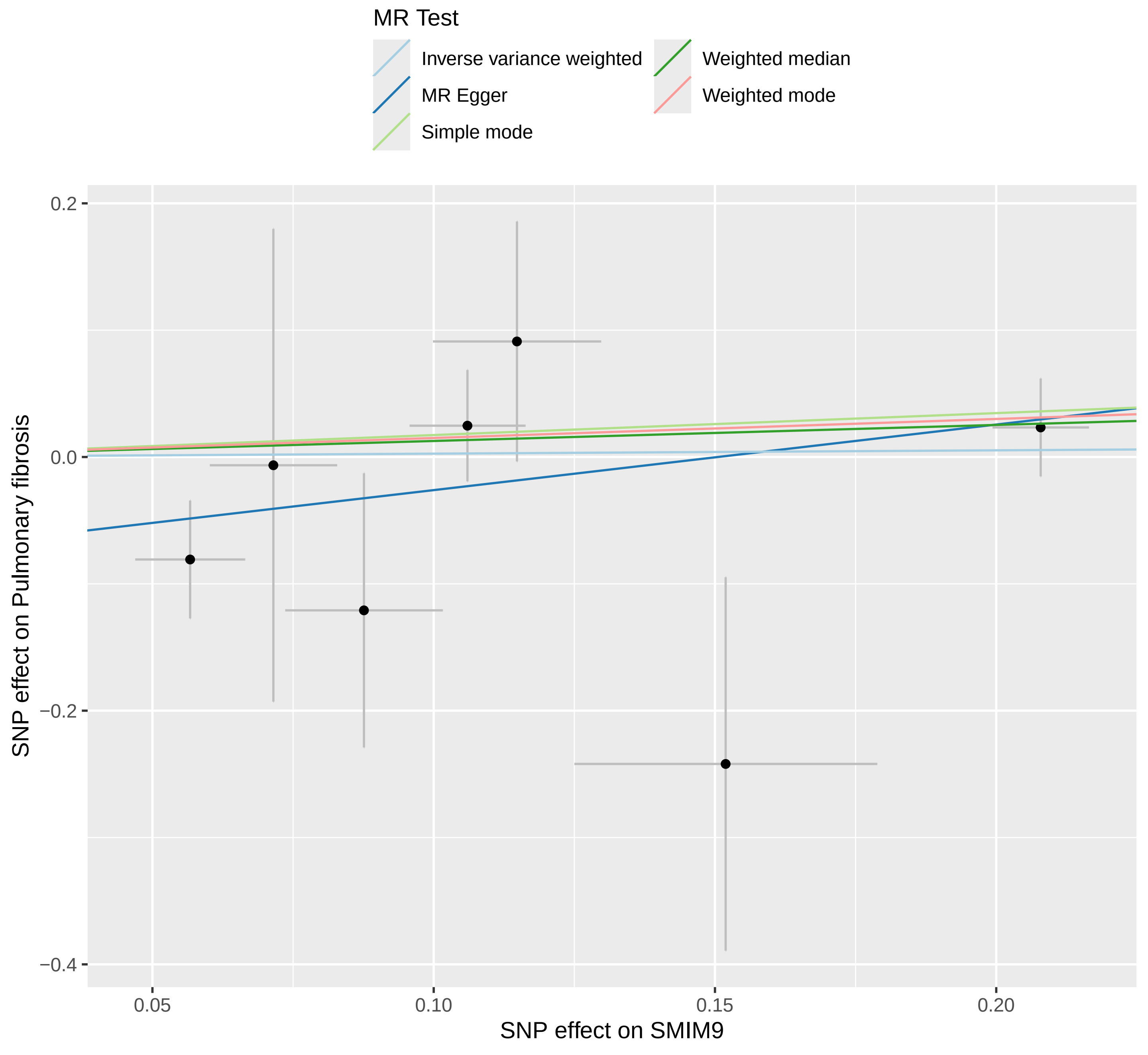
MR Test
Inverse variance weighted
MR Egger
Simple mode
Weighted median
Weighted mode
0.2
0.0
−0.2
−0.4
0.05
0.10
0.15
0.20
SNP effect on Pulmonary fibrosis
SNP effect on SMIM9

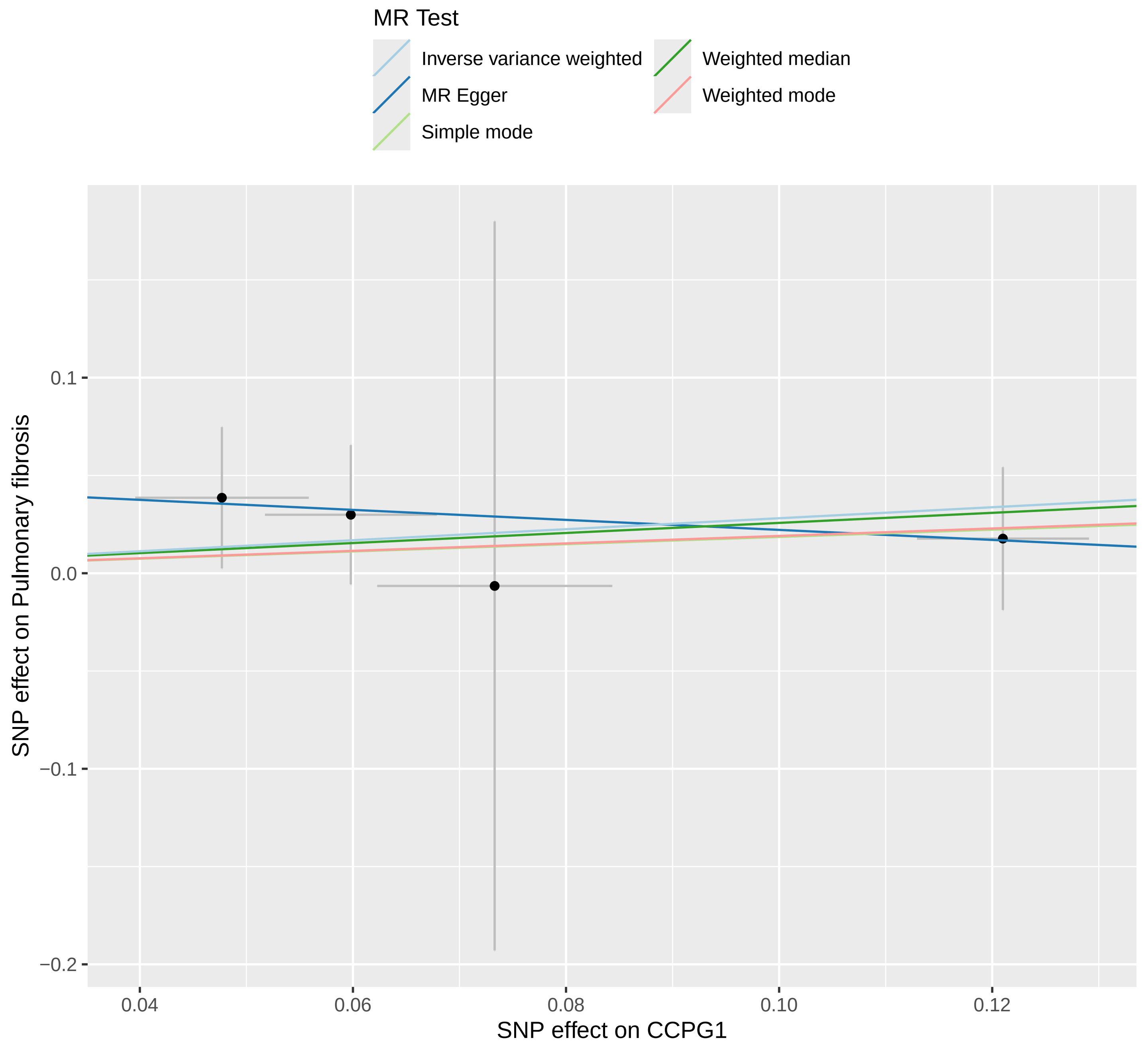

MR Test
Inverse variance weighted
MR Egger
Simple mode
Weighted median
Weighted mode
0.1
0.0
−0.1
−0.2
0.04
0.06
0.08
0.10
0.12
SNP effect on CCPG1
SNP effect on Pulmonary fibrosis

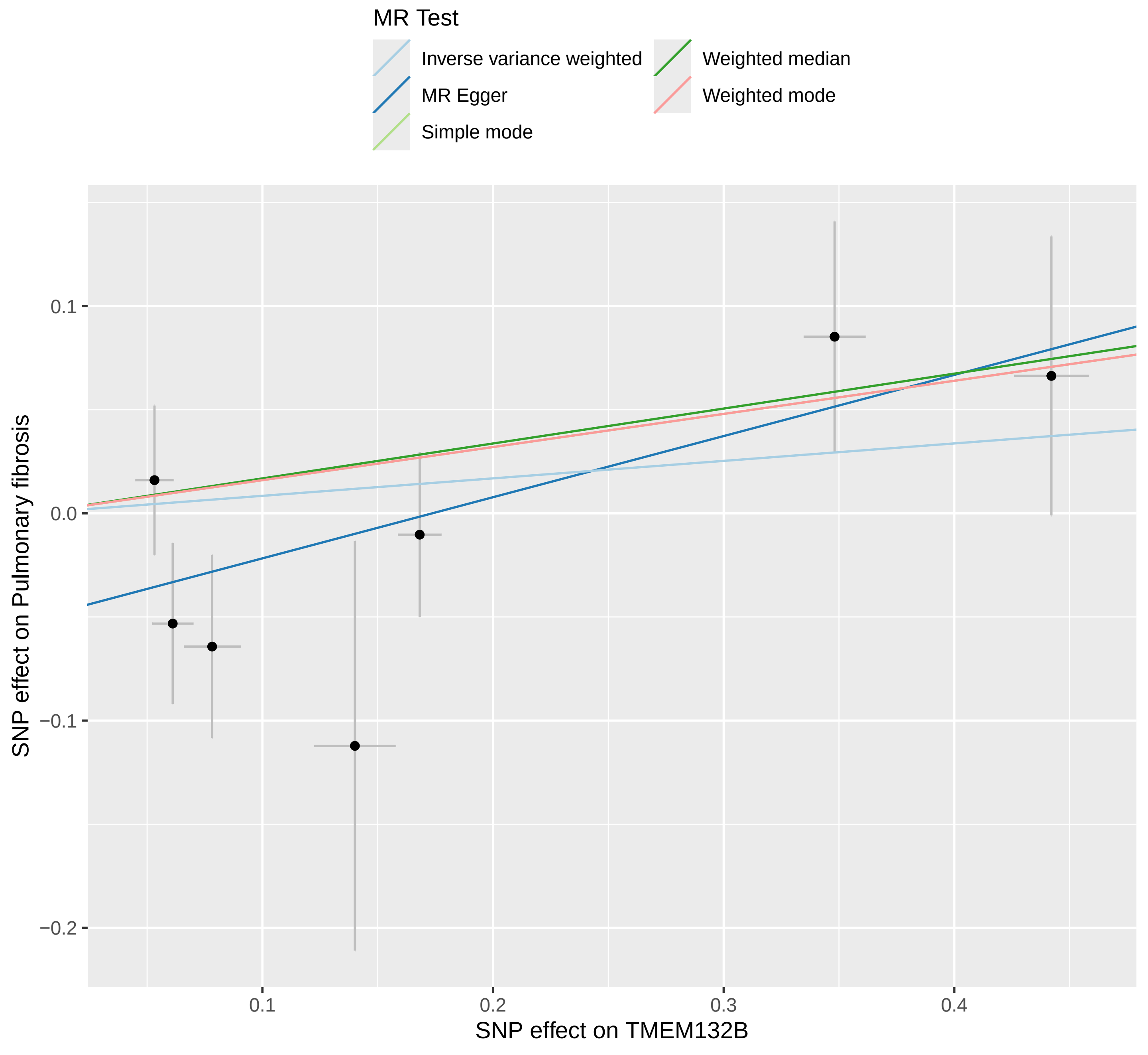
MR Test
Inverse variance weighted
MR Egger
Simple mode
Weighted median
Weighted mode
0.1
0.0
−0.1
−0.2
SNP effect on Pulmonary fibrosis
0.1
0.2
0.3
0.4
SNP effect on TMEM132B

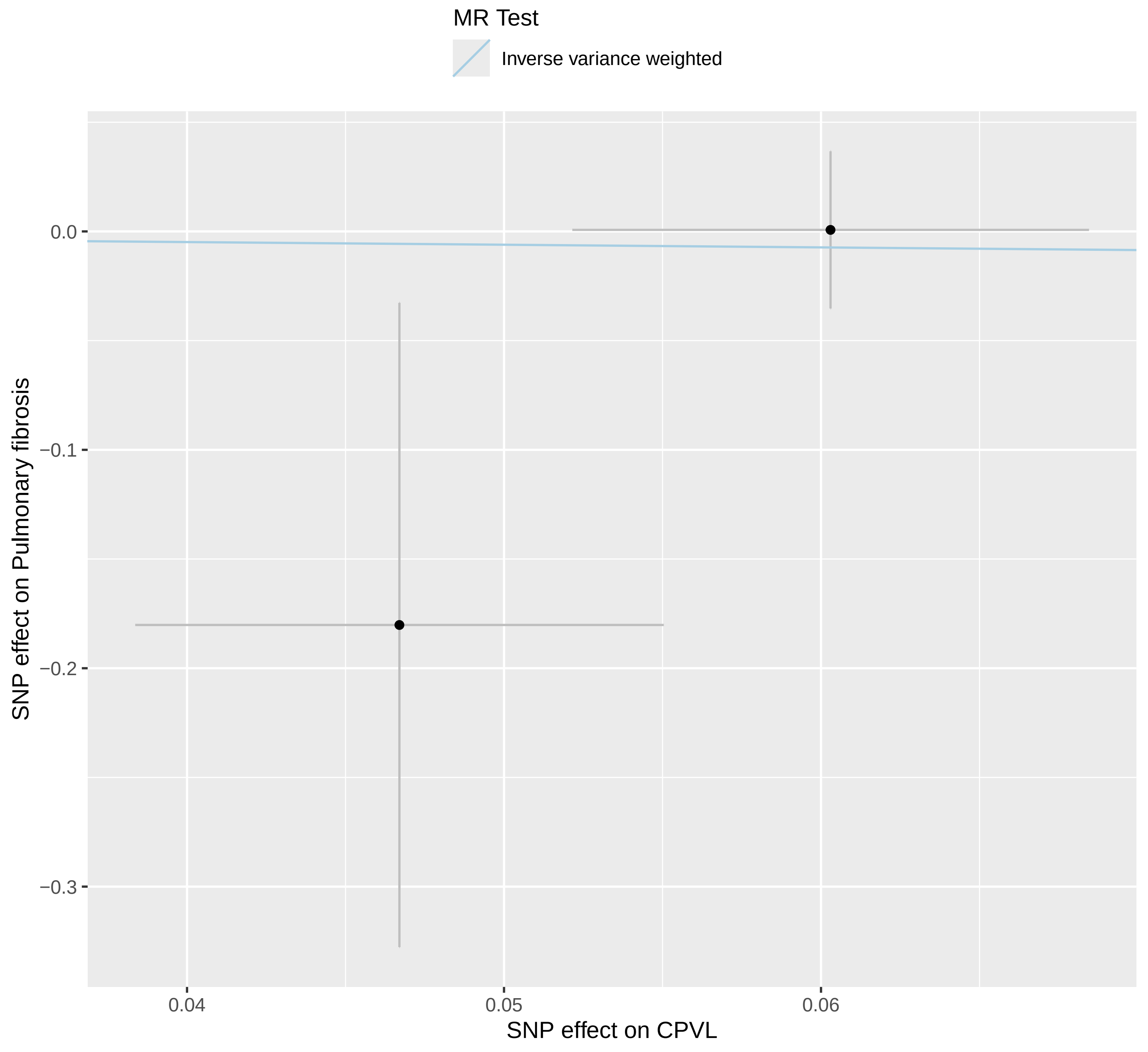
MR Test
Inverse variance weighted
0.0
−0.1
−0.2
−0.3
SNP effect on Pulmonary fibrosis
0.04
0.05
0.06
SNP effect on CPVL

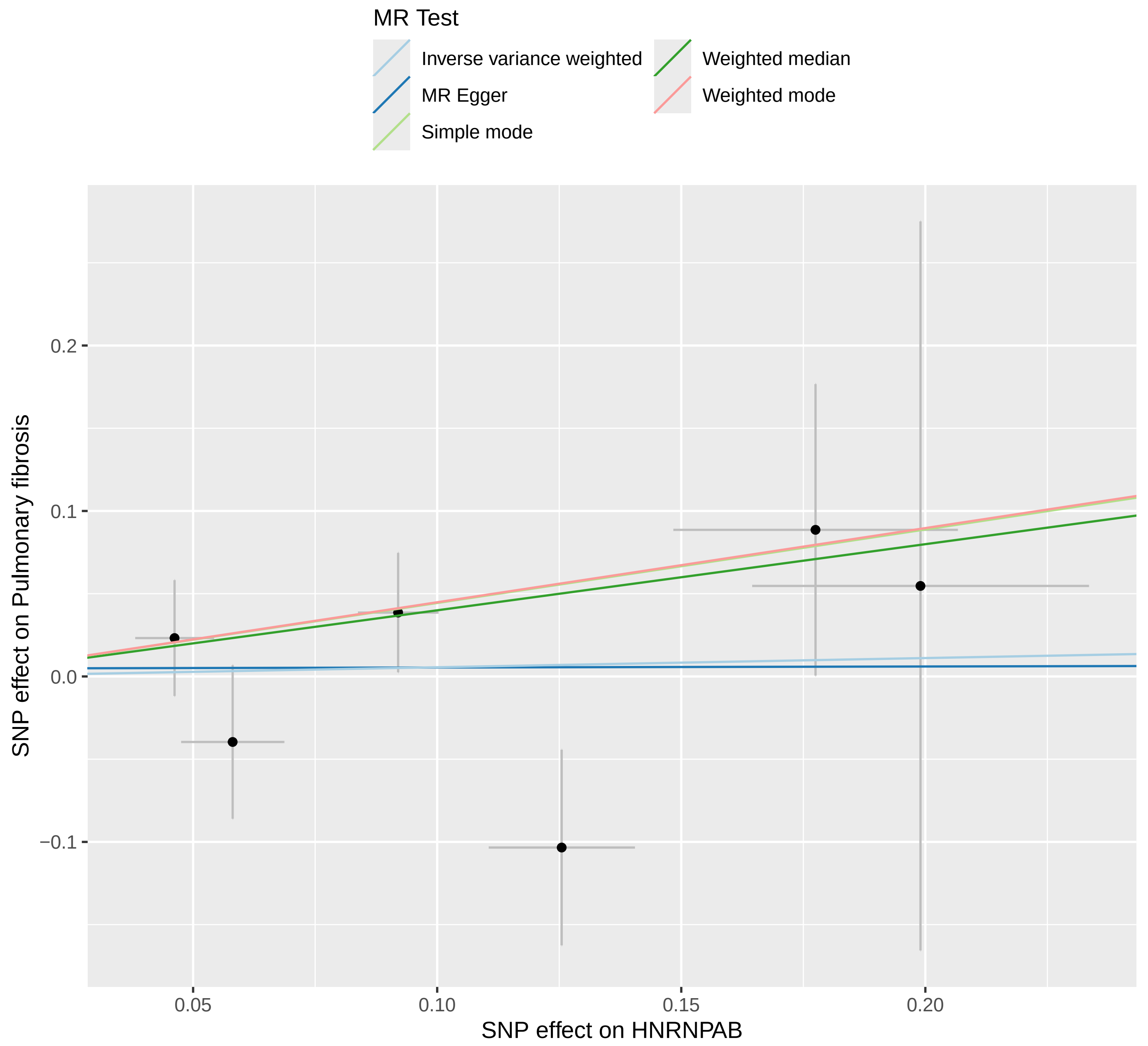
MR Test
Inverse variance weighted
MR Egger
Simple mode
Weighted median
Weighted mode
0.2
0.1
0.0
−0.1
0.05
0.10
0.15
0.20
SNP effect on HNRNPAB
SNP effect on Pulmonary fibrosis

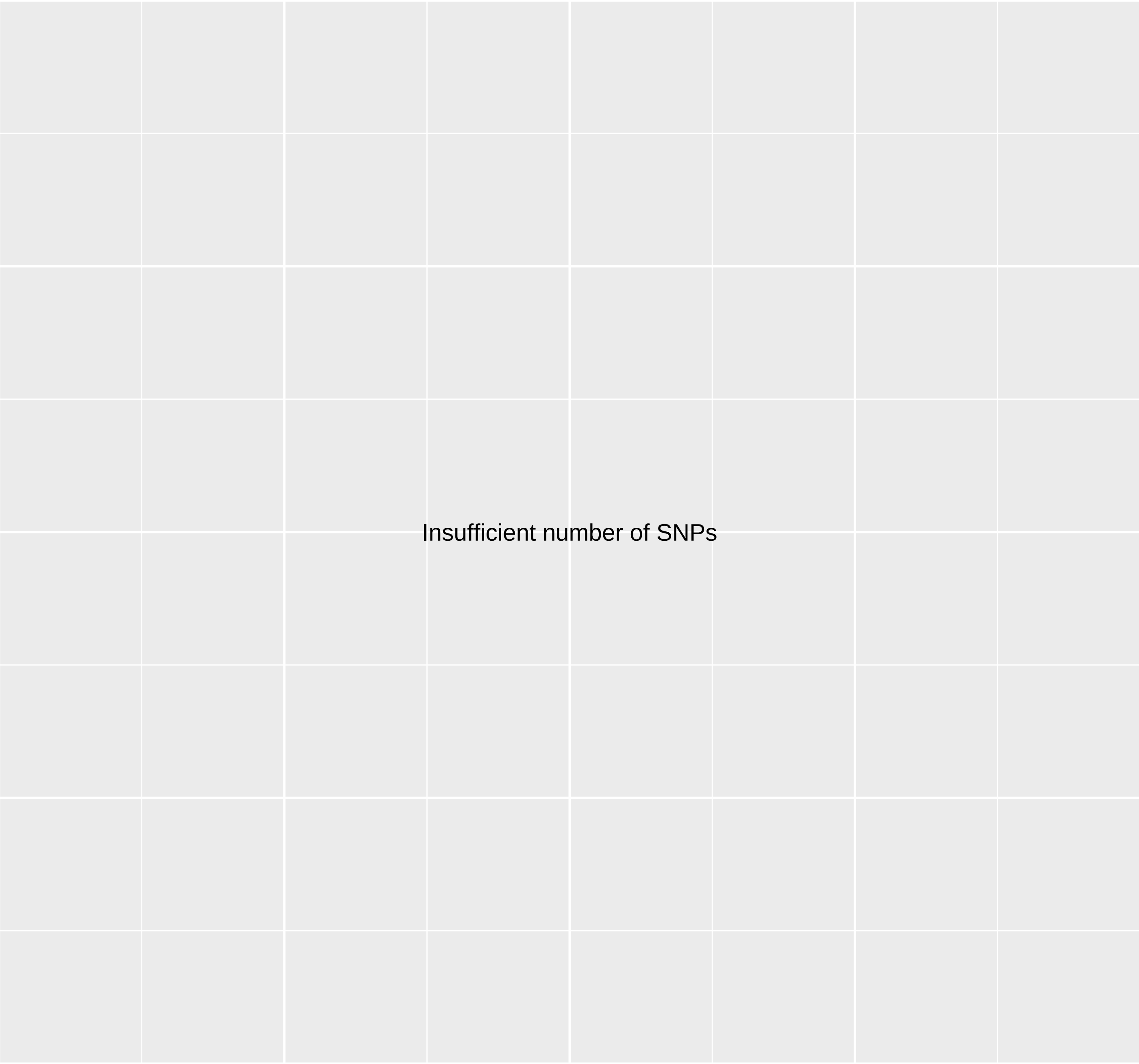
Insufficient number of SNPs

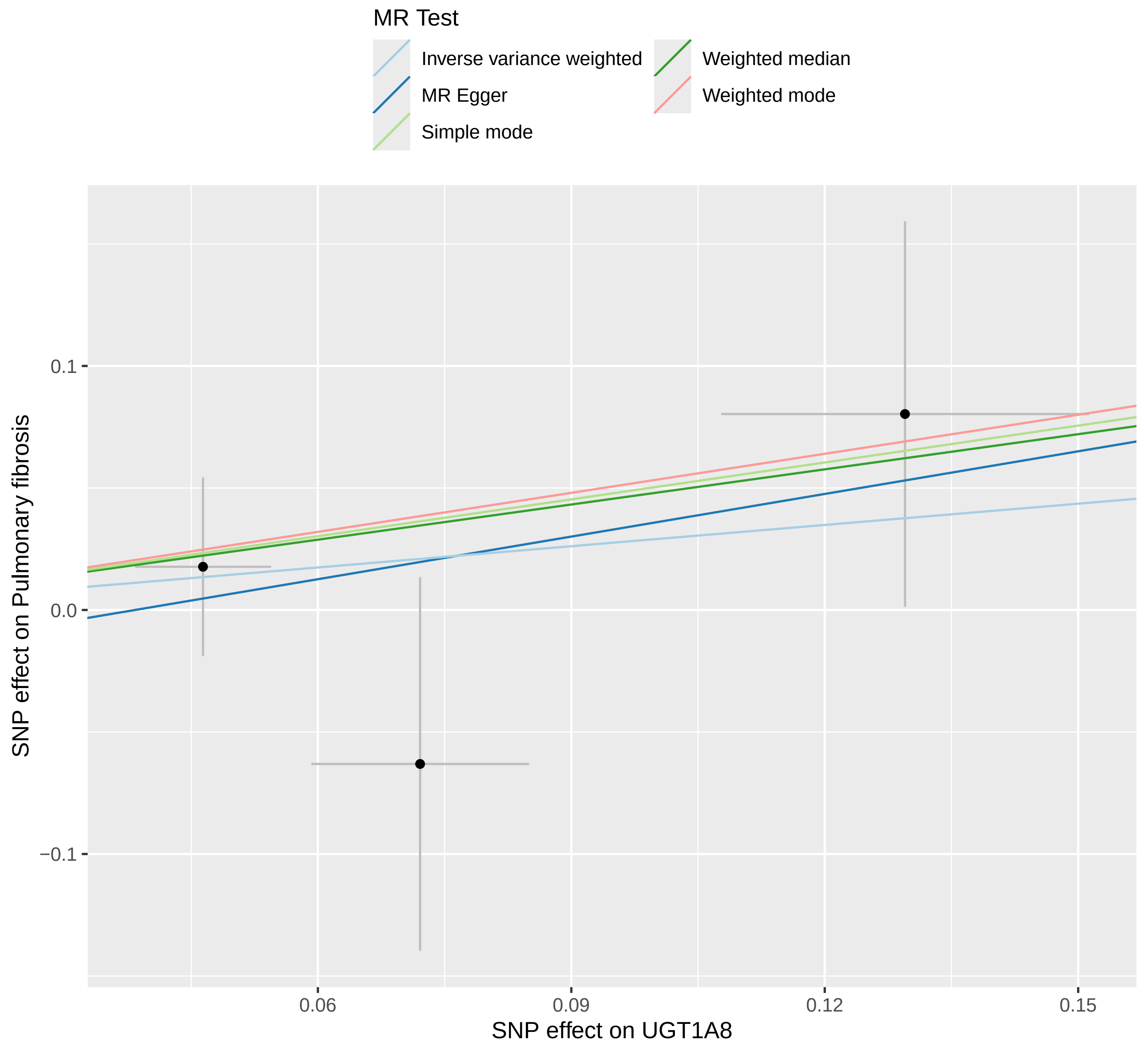
MR Test
Inverse variance weighted
MR Egger
Simple mode
Weighted median
Weighted mode
SNP effect on Pulmonary fibrosis
0.1
0.0
−0.1
0.06
0.09
0.12
0.15
SNP effect on UGT1A8

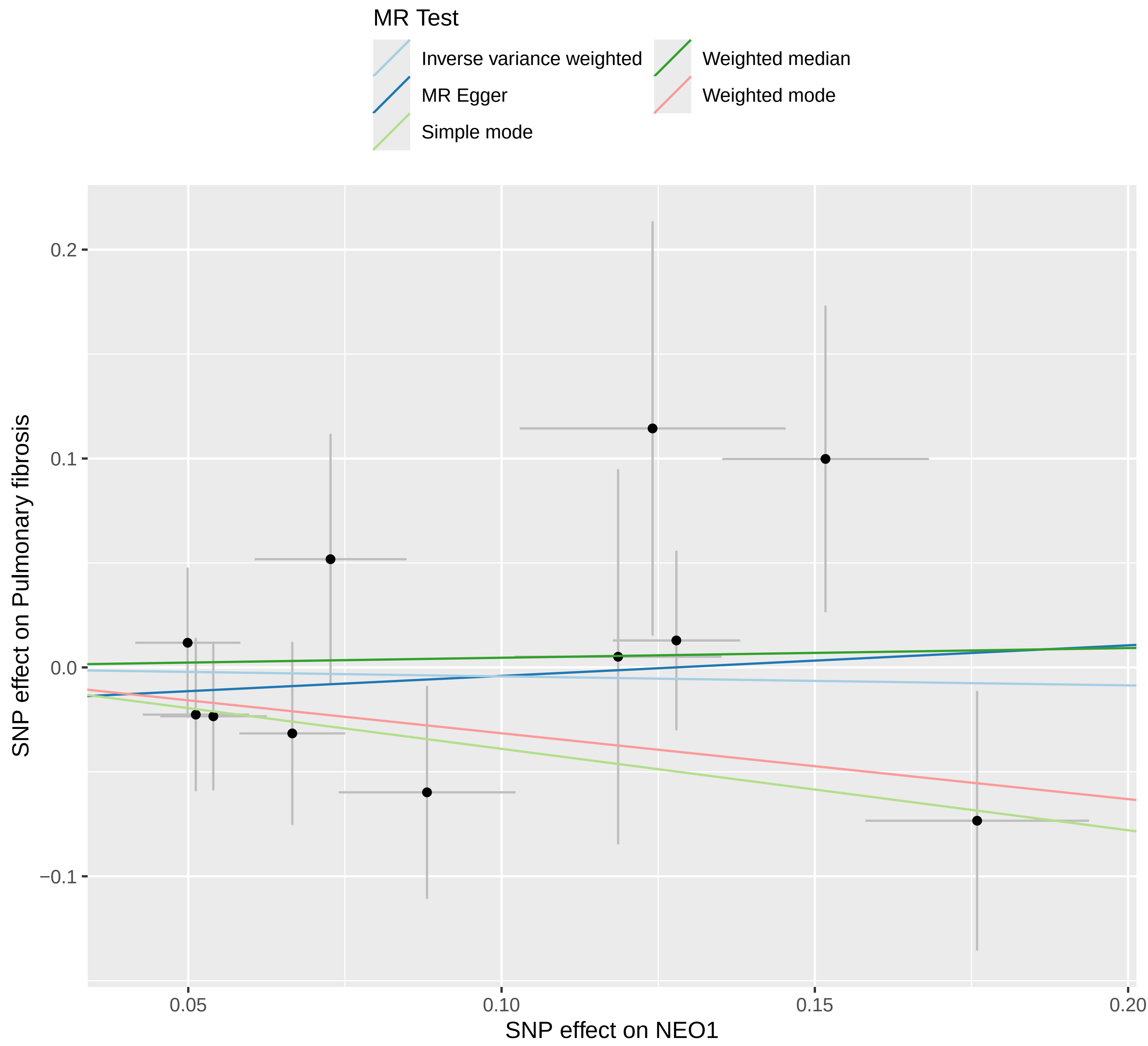

MR Test
Inverse variance weighted
MR Egger
Simple mode
Weighted median
Weighted mode
SNP effect on Pulmonary fibrosis
0.2
0.1
0.0
−0.1
0.05
0.10
0.15
0.20
SNP effect on NEO1

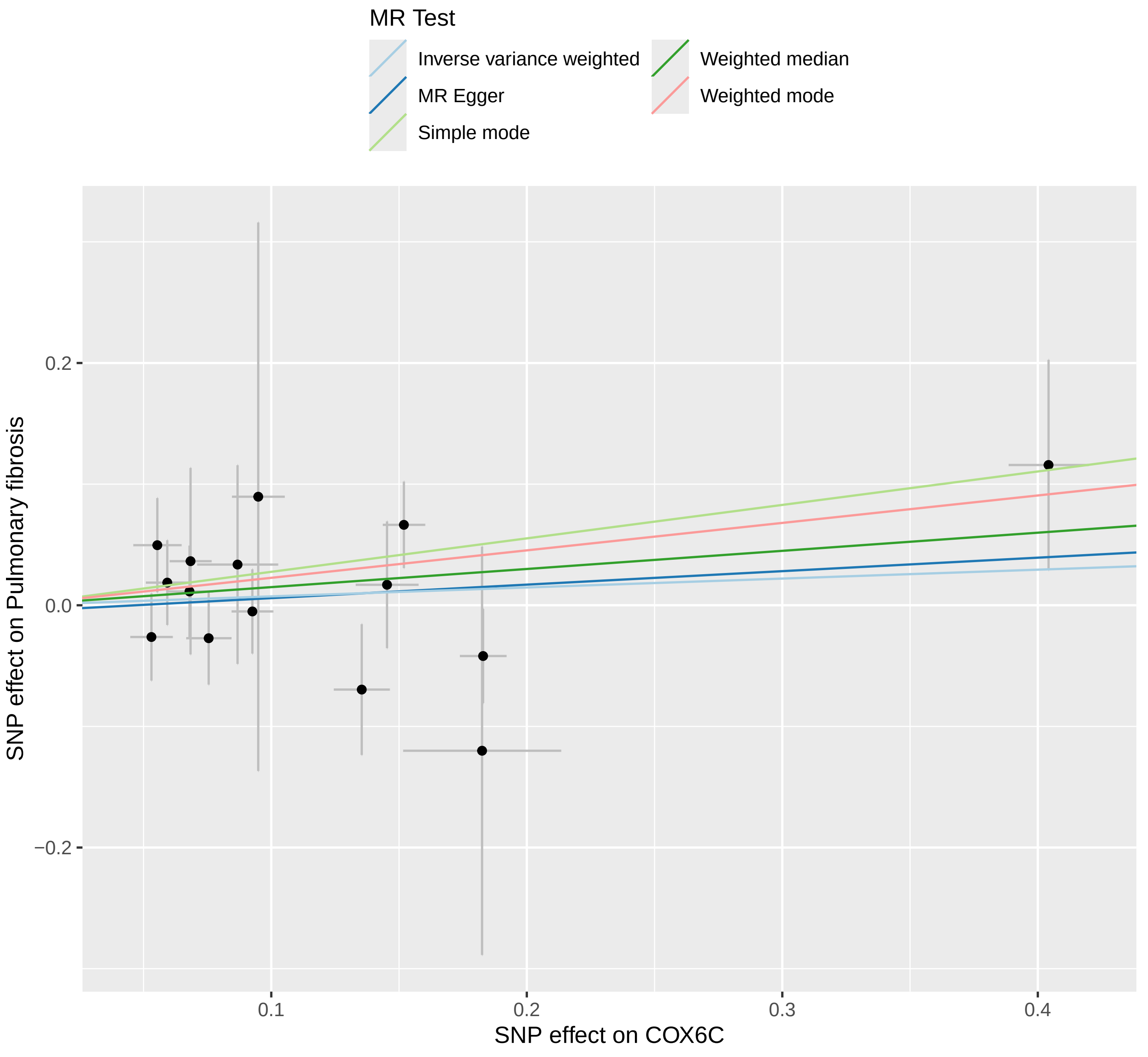
MR Test
Inverse variance weighted
MR Egger
Simple mode
Weighted median
Weighted mode
0.2
0.0
−0.2
0.1
0.2
0.3
0.4
SNP effect on Pulmonary fibrosis
SNP effect on COX6C

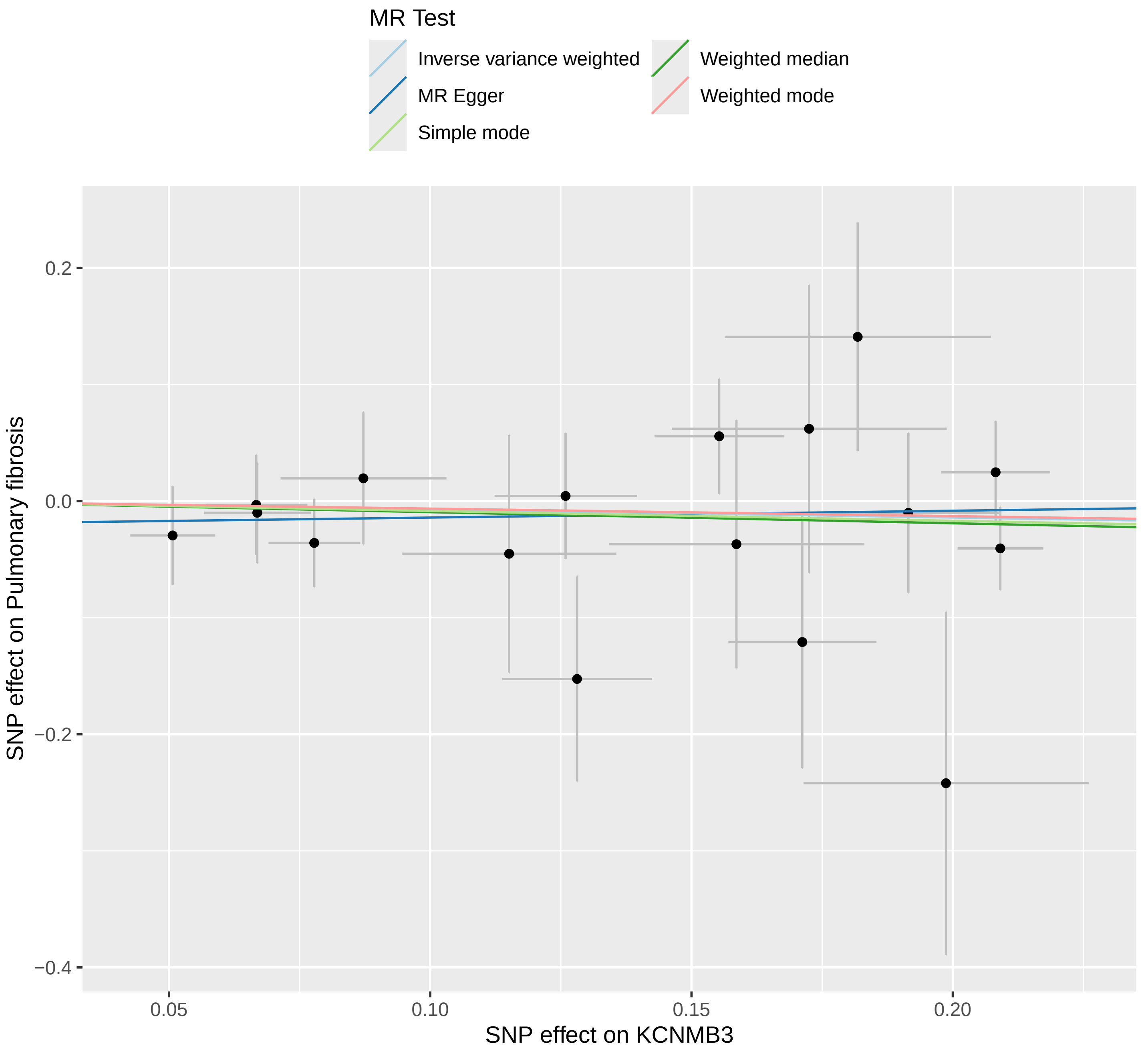
MR Test
Inverse variance weighted
MR Egger
Simple mode
Weighted median
Weighted mode
SNP effect on Pulmonary fibrosis
0.2
0.0
−0.2
−0.4
0.05
0.10
0.15
0.20
SNP effect on KCNMB3

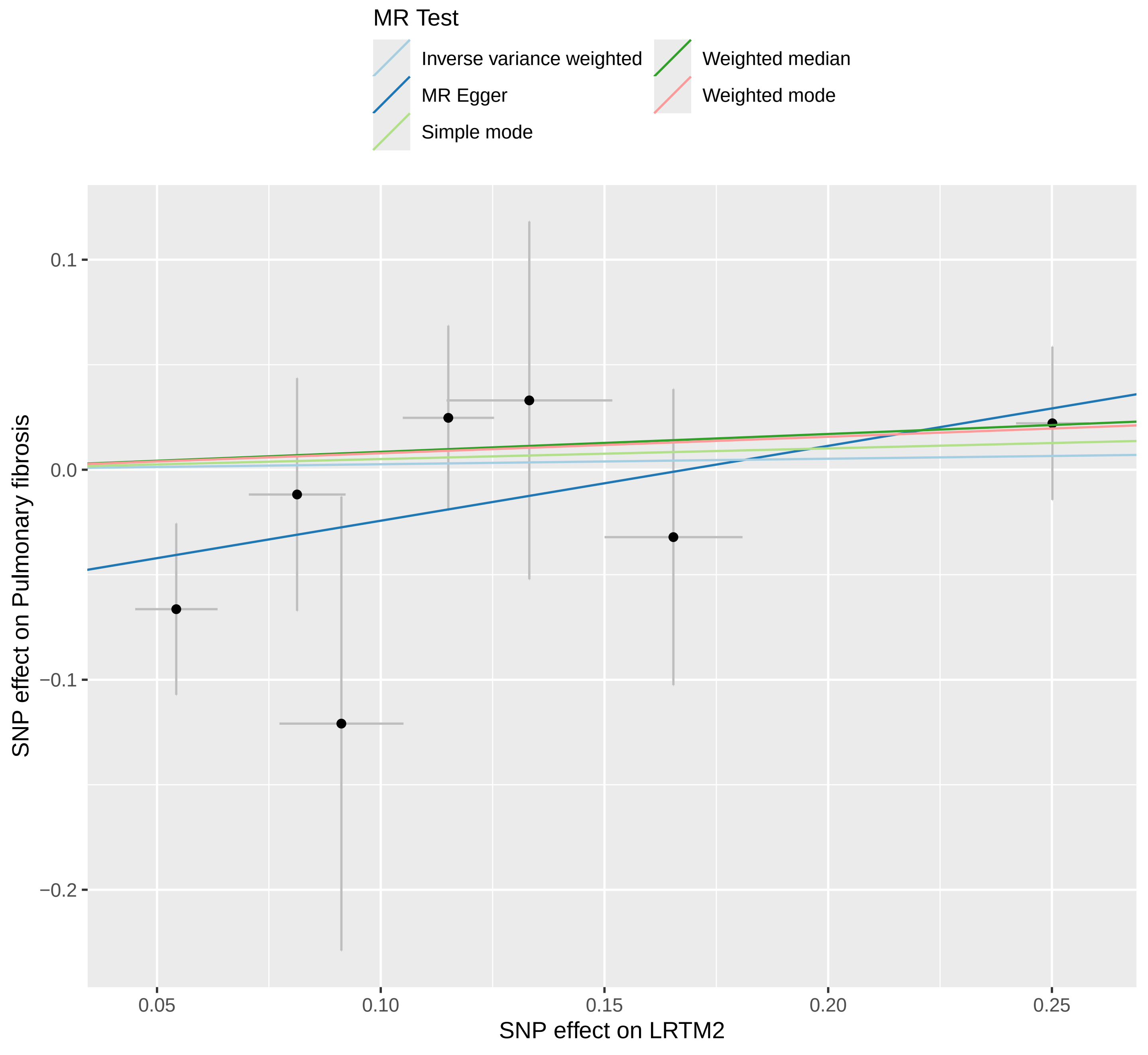

MR Test
Inverse variance weighted
MR Egger
Simple mode
Weighted median
Weighted mode
0.1
0.0
−0.1
−0.2
0.05
0.10
0.15
0.20
0.25
SNP effect on Pulmonary fibrosis
SNP effect on LRTM2

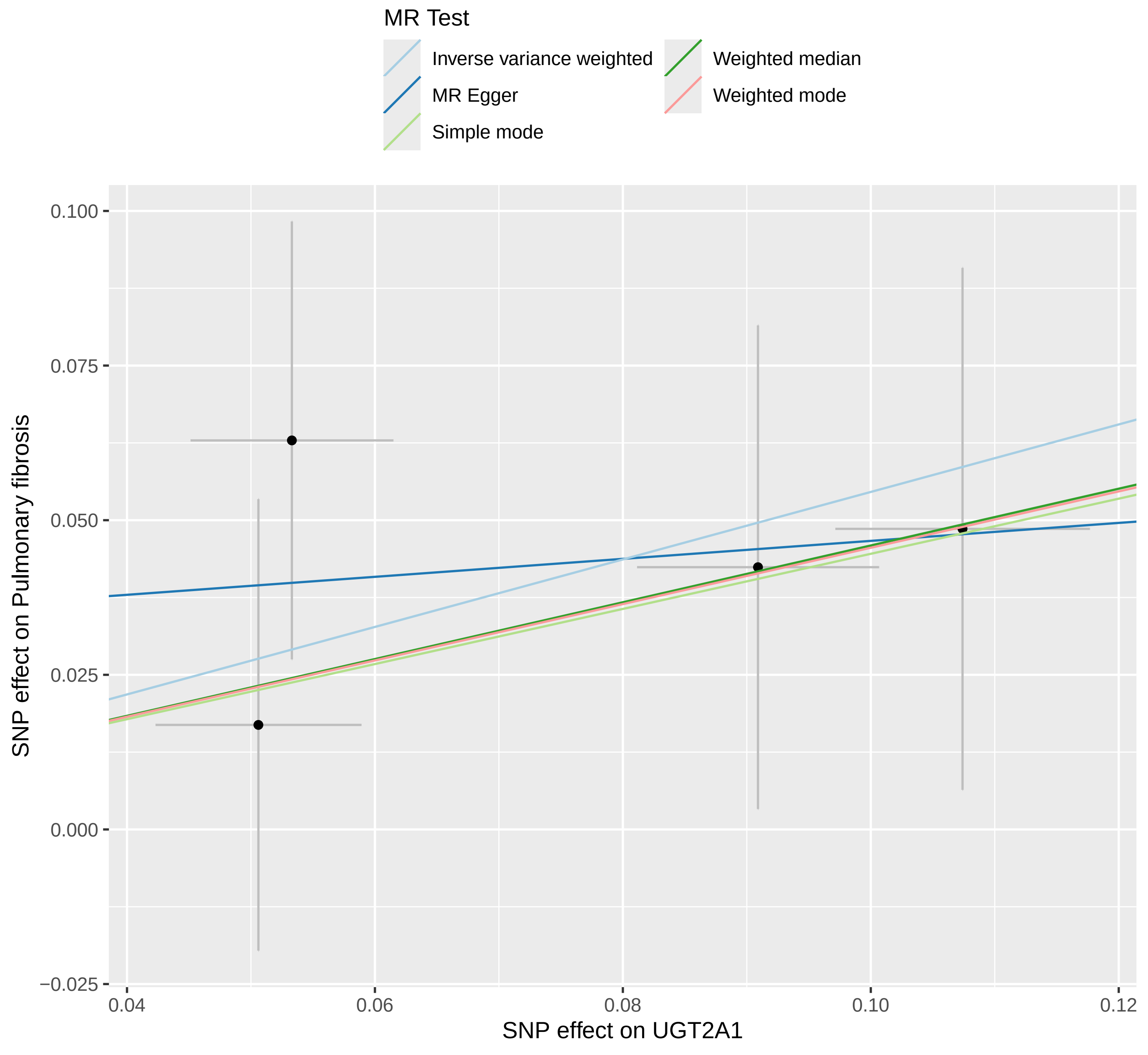

MR Test
Inverse variance weighted
MR Egger
Simple mode
Weighted median
Weighted mode
0.100
0.075
0.050
0.025
0.000
−0.025
0.04
0.06
0.08
0.10
0.12
SNP effect on Pulmonary fibrosis
SNP effect on UGT2A1

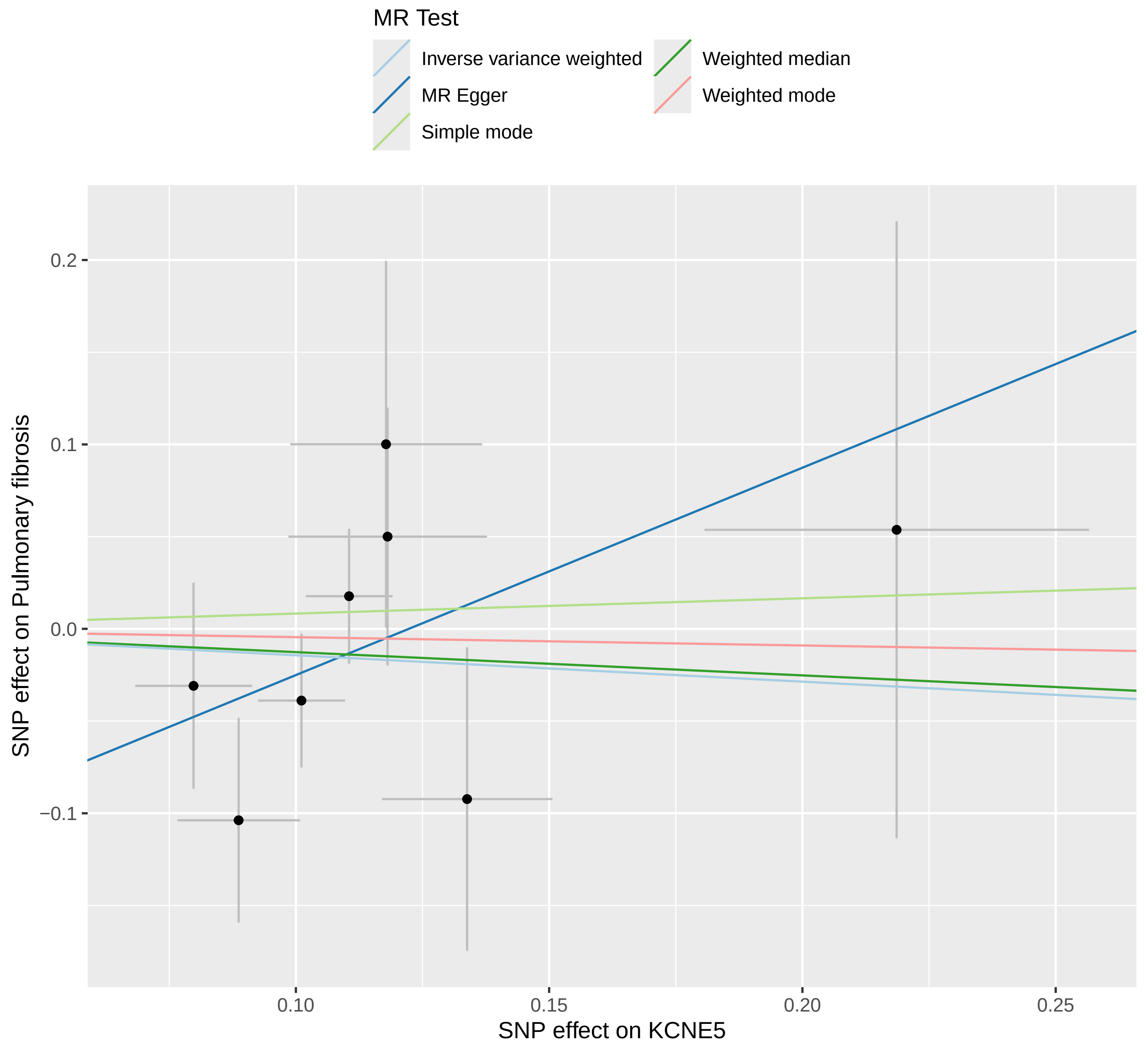

MR Test
Inverse variance weighted
MR Egger
Simple mode
Weighted median
Weighted mode
SNP effect on Pulmonary fibrosis
0.2
0.1
0.0
−0.1
0.10
0.15
0.20
0.25
SNP effect on KCNE5

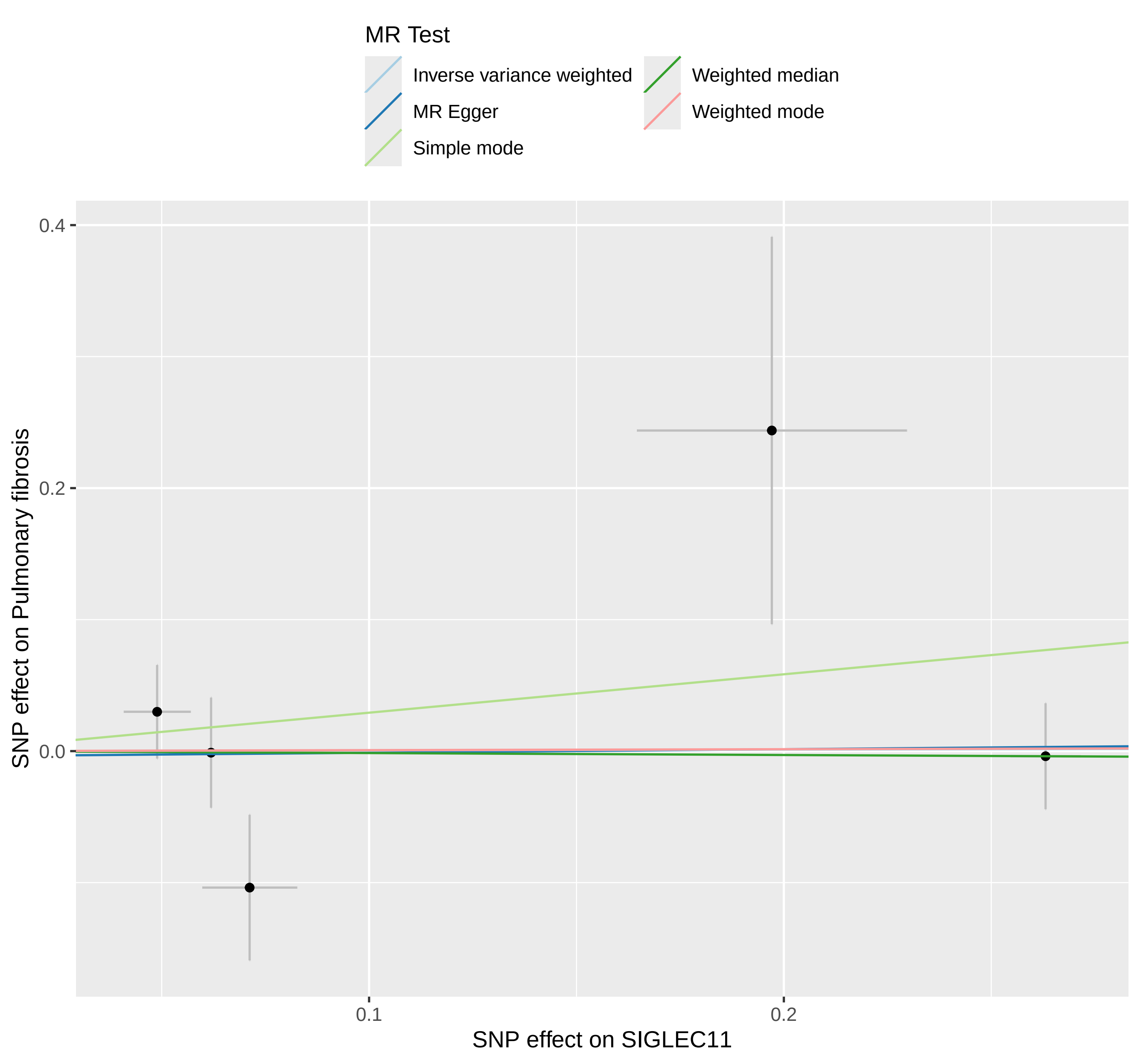
MR Test
Inverse variance weighted
MR Egger
Simple mode
Weighted median
Weighted mode
0.4
0.2
0.0
0.1
0.2
SNP effect on SIGLEC11
SNP effect on Pulmonary fibrosis

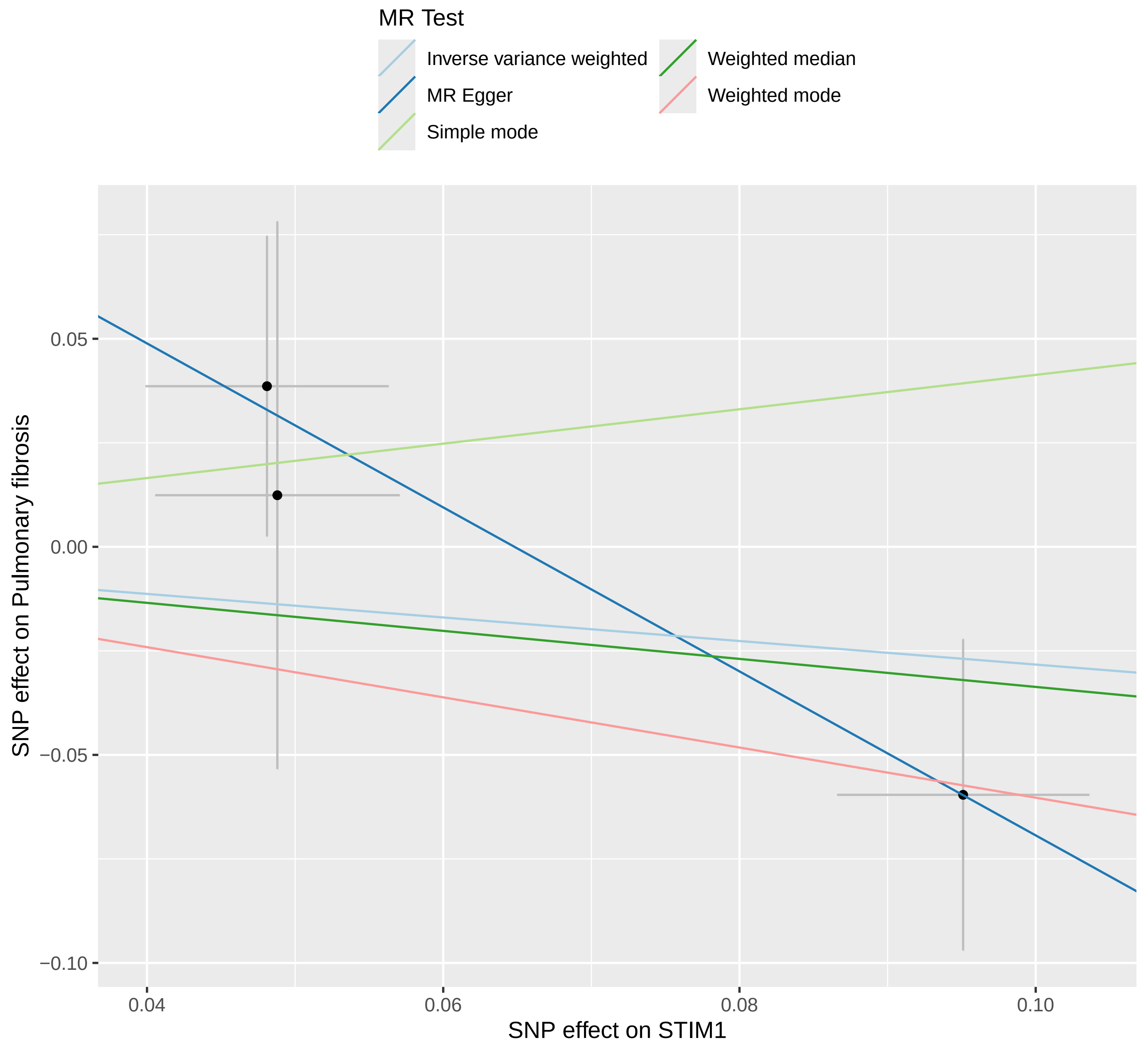
MR Test
Inverse variance weighted
MR Egger
Simple mode
Weighted median
Weighted mode
0.05
0.00
−0.05
−0.10
SNP effect on Pulmonary fibrosis
0.04
0.06
0.08
0.10
SNP effect on STIM1

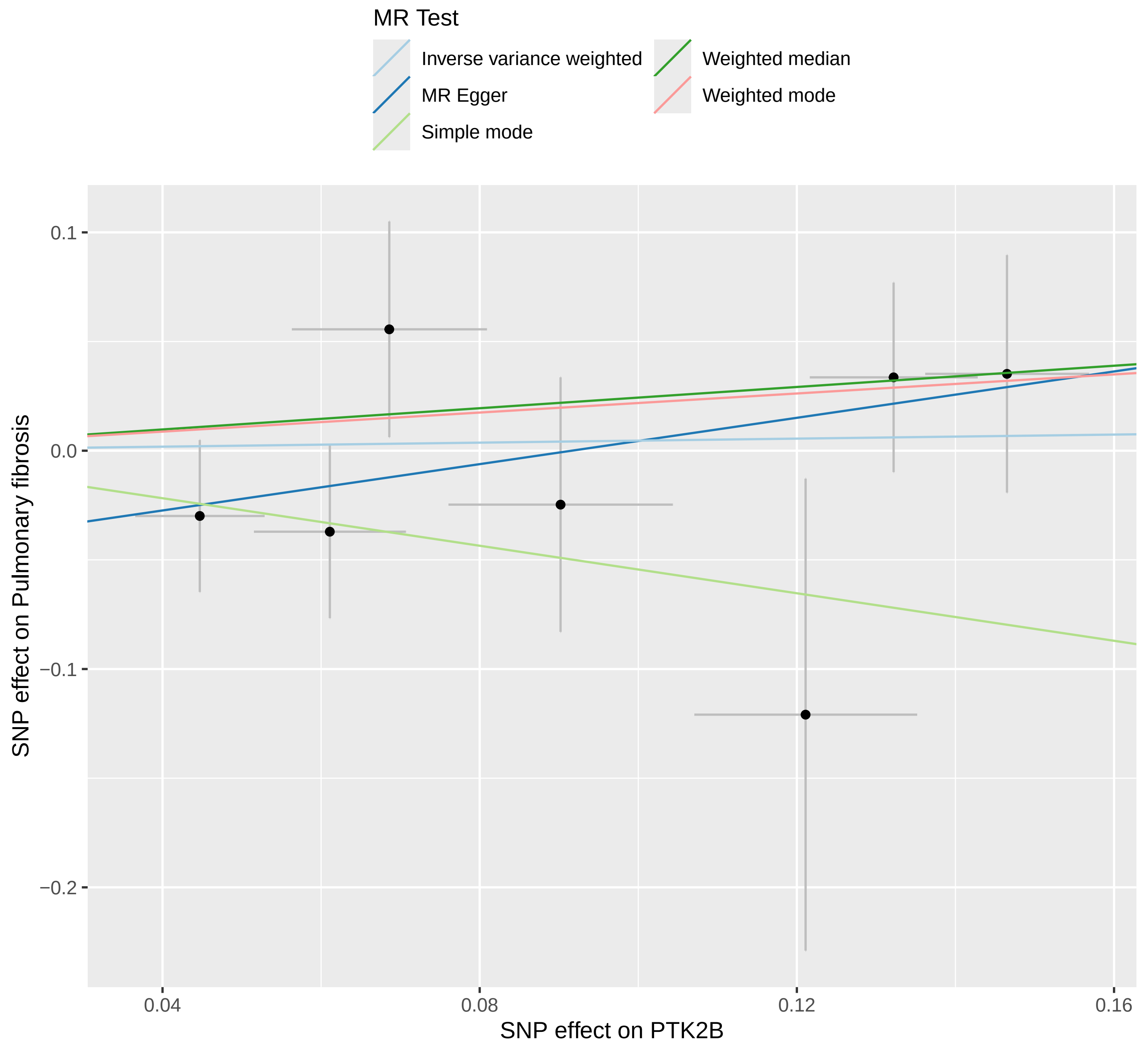
MR Test
Inverse variance weighted
MR Egger
Simple mode
Weighted median
Weighted mode
0.1
0.0
−0.1
−0.2
SNP effect on Pulmonary fibrosis
0.04
0.08
0.12
0.16
SNP effect on PTK2B

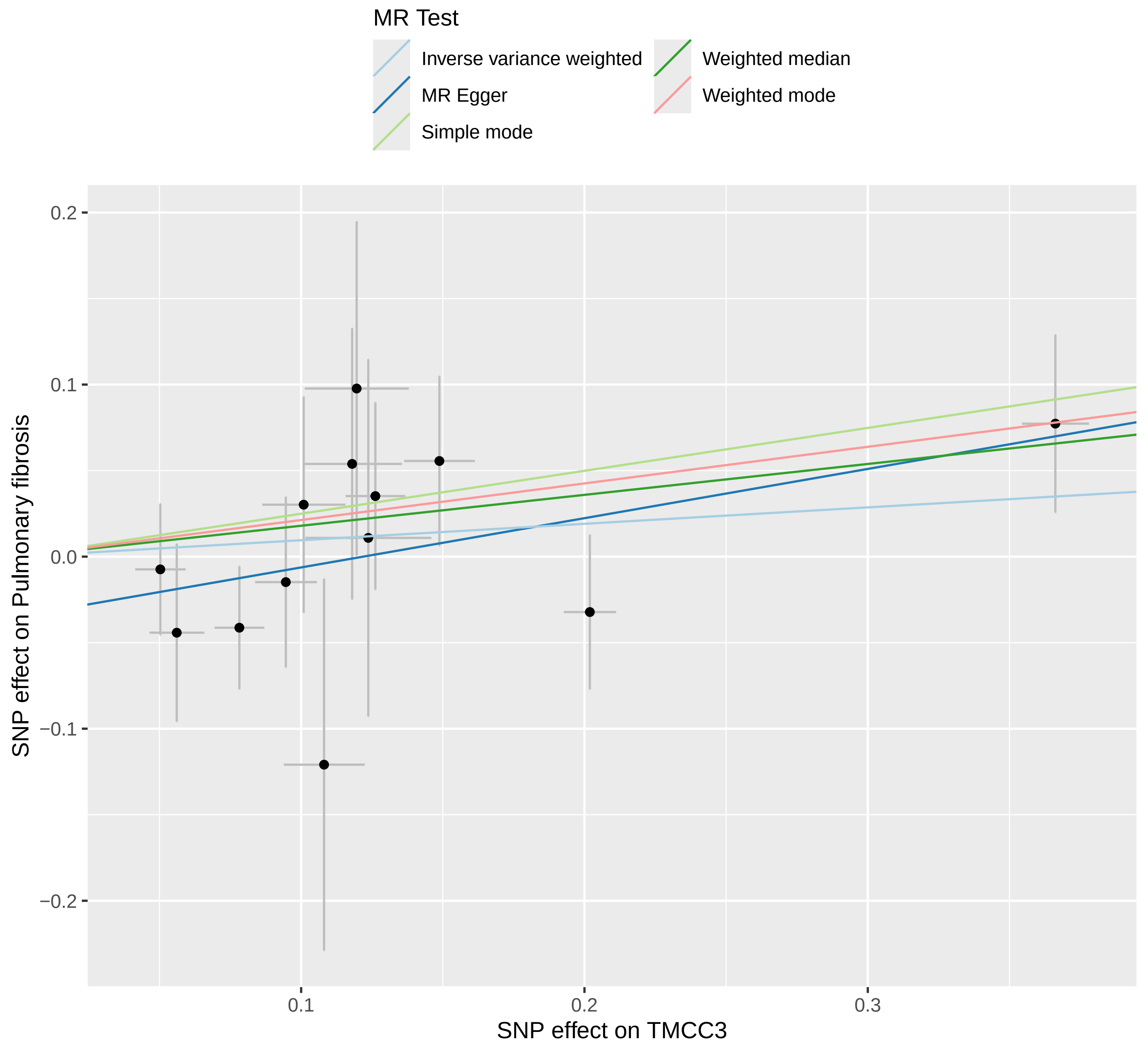
MR Test
Inverse variance weighted
MR Egger
Simple mode
Weighted median
Weighted mode
0.2
0.1
0.0
−0.1
−0.2
SNP effect on Pulmonary fibrosis
0.1
0.2
0.3
SNP effect on TMCC3

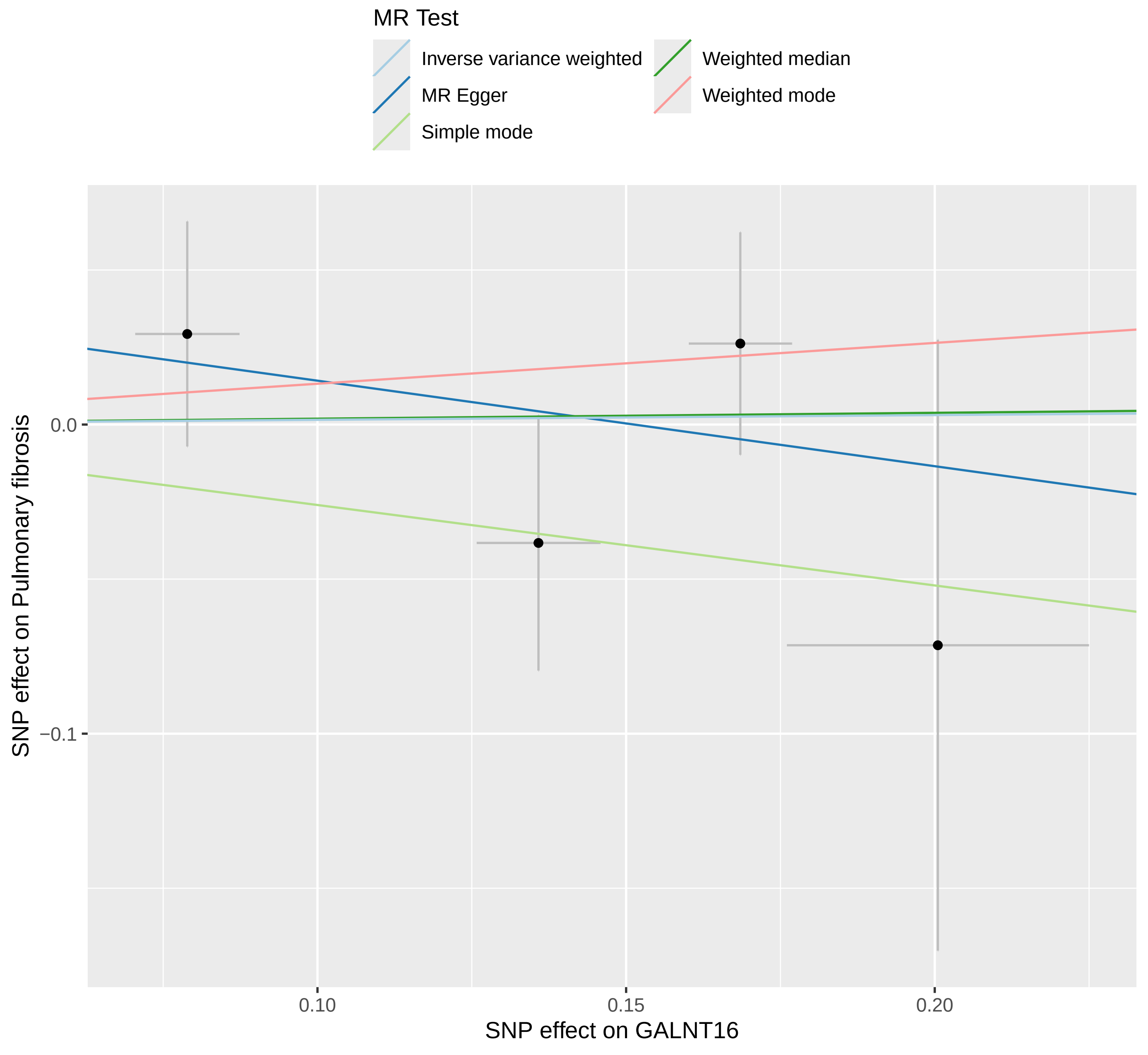
MR Test
Inverse variance weighted
MR Egger
Simple mode
Weighted median
Weighted mode
0.0
−0.1
SNP effect on Pulmonary fibrosis
0.10
0.15
0.20
SNP effect on GALNT16

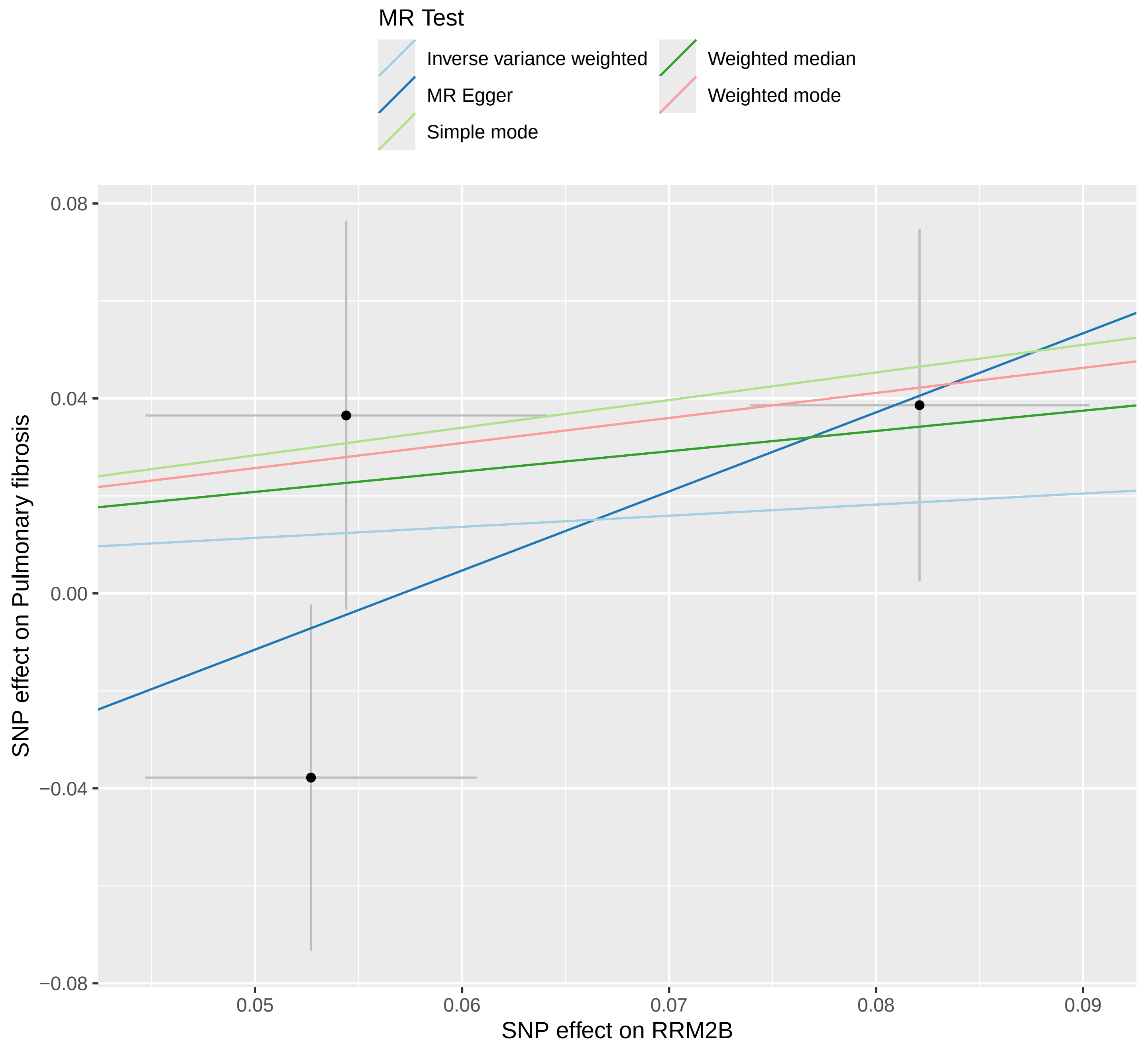

MR Test
Inverse variance weighted
MR Egger
Simple mode
Weighted median
Weighted mode
0.08
0.04
0.00
−0.04
−0.08
0.05
0.06
0.07
0.08
0.09
SNP effect on Pulmonary fibrosis
SNP effect on RRM2B

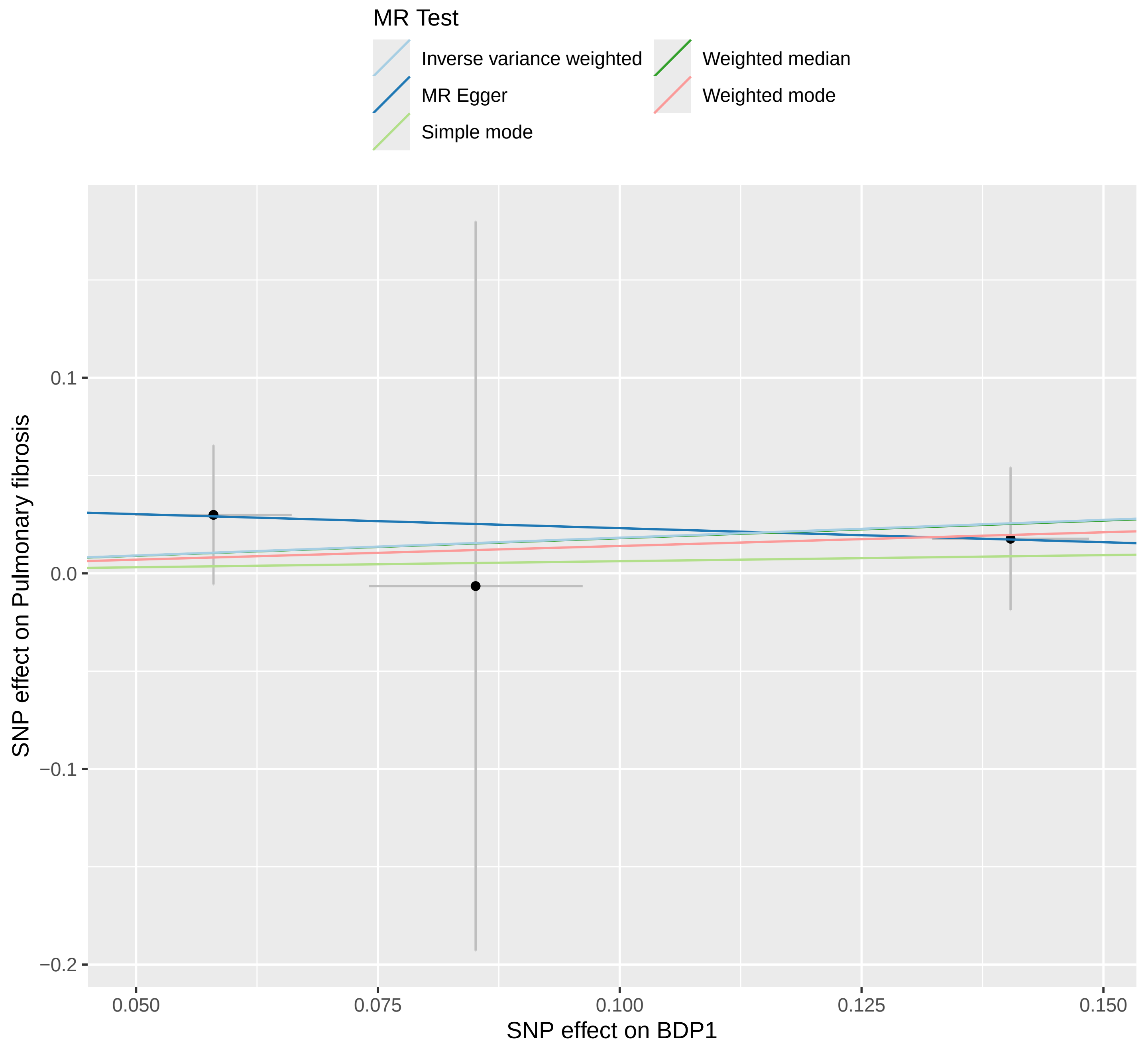
MR Test
Inverse variance weighted
MR Egger
Simple mode
Weighted median
Weighted mode
0.1
0.0
−0.1
−0.2
SNP effect on Pulmonary fibrosis
0.050
0.075
0.100
0.125
0.150
SNP effect on BDP1

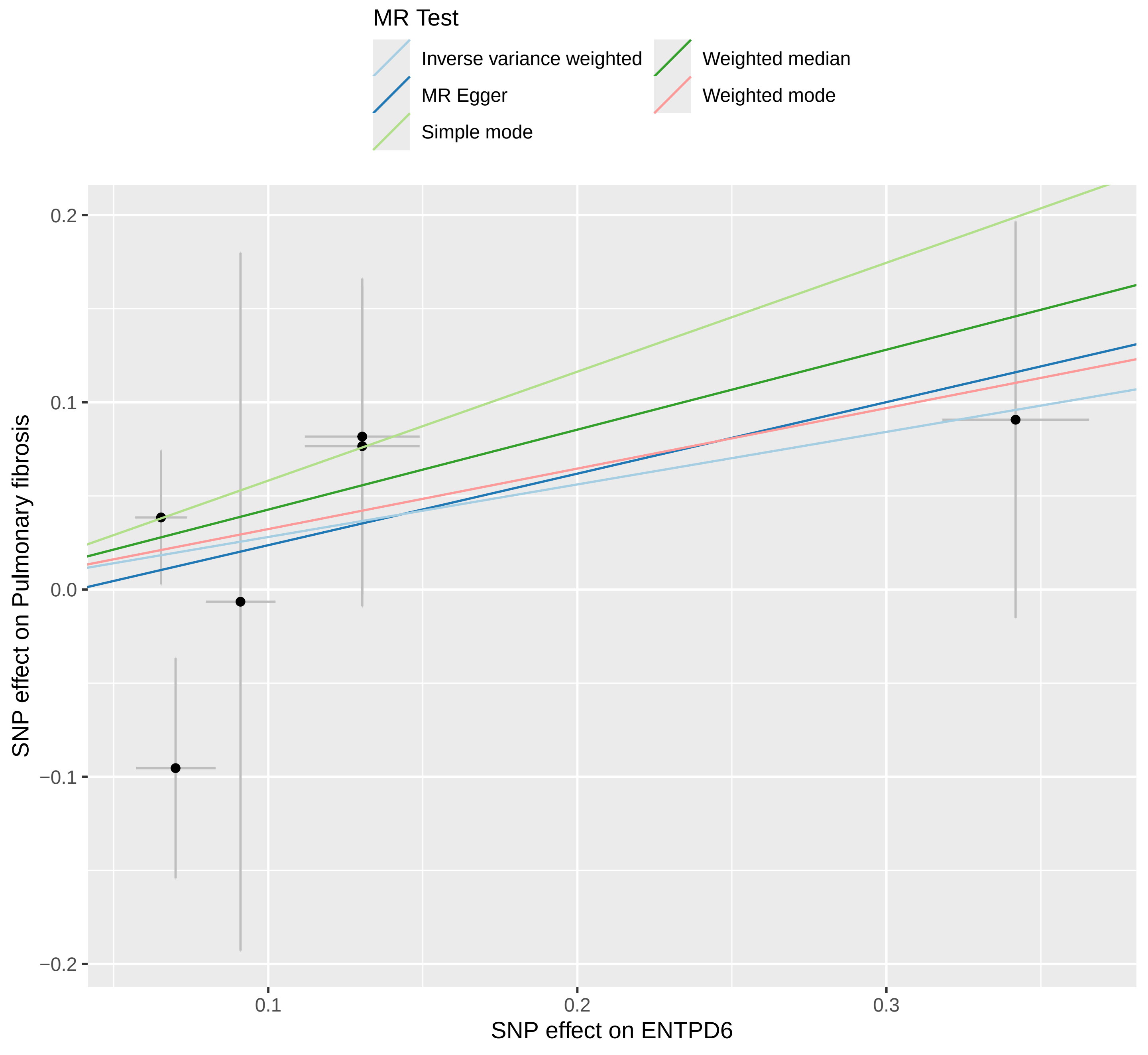

MR Test
Inverse variance weighted
MR Egger
Simple mode
Weighted median
Weighted mode
0.2
0.1
0.0
−0.1
−0.2
0.1
0.2
0.3
SNP effect on Pulmonary fibrosis
SNP effect on ENTPD6

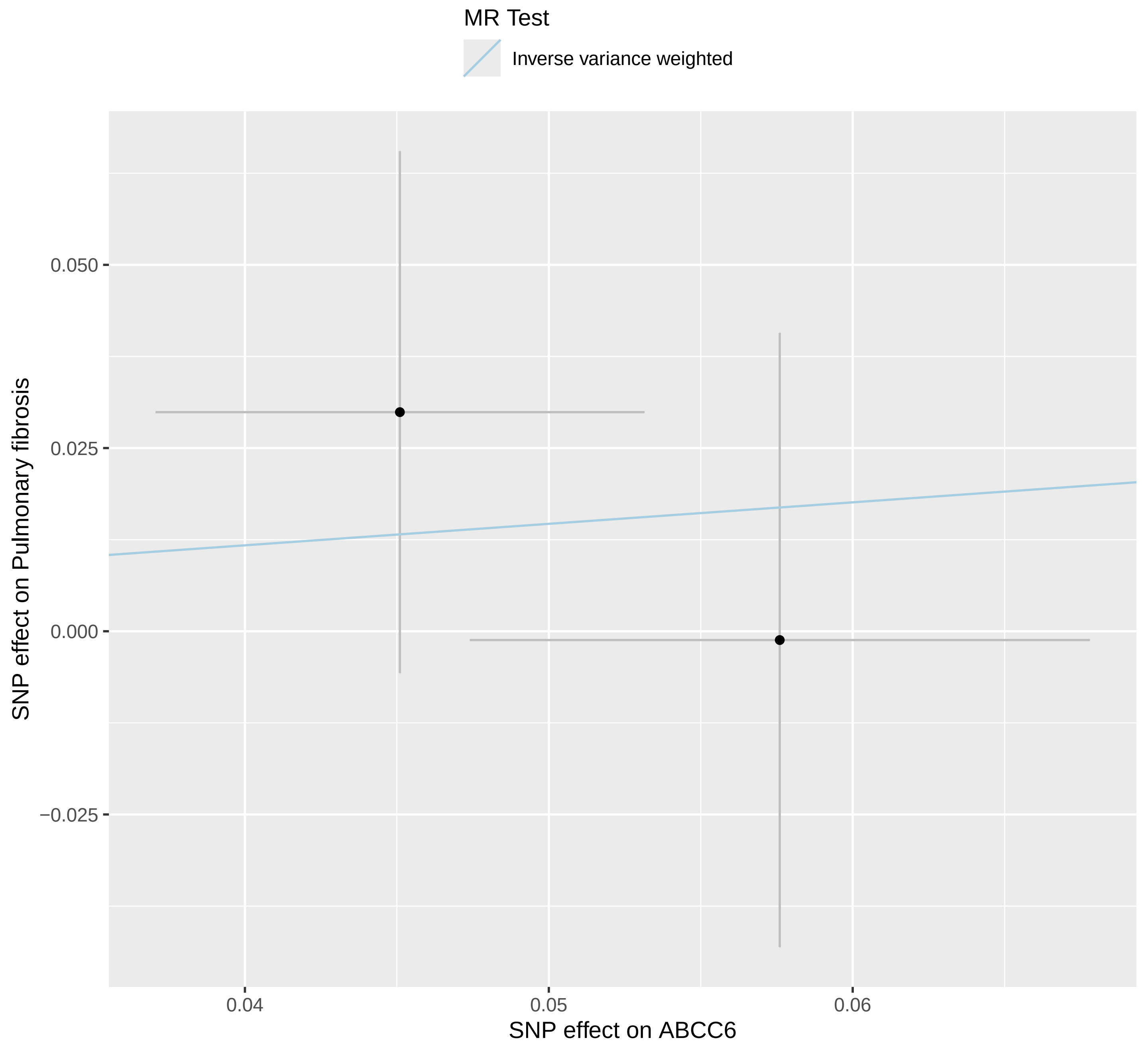
MR Test
Inverse variance weighted
0.050
0.025
0.000
−0.025
SNP effect on Pulmonary fibrosis
0.04
0.05
0.06
SNP effect on ABCC6

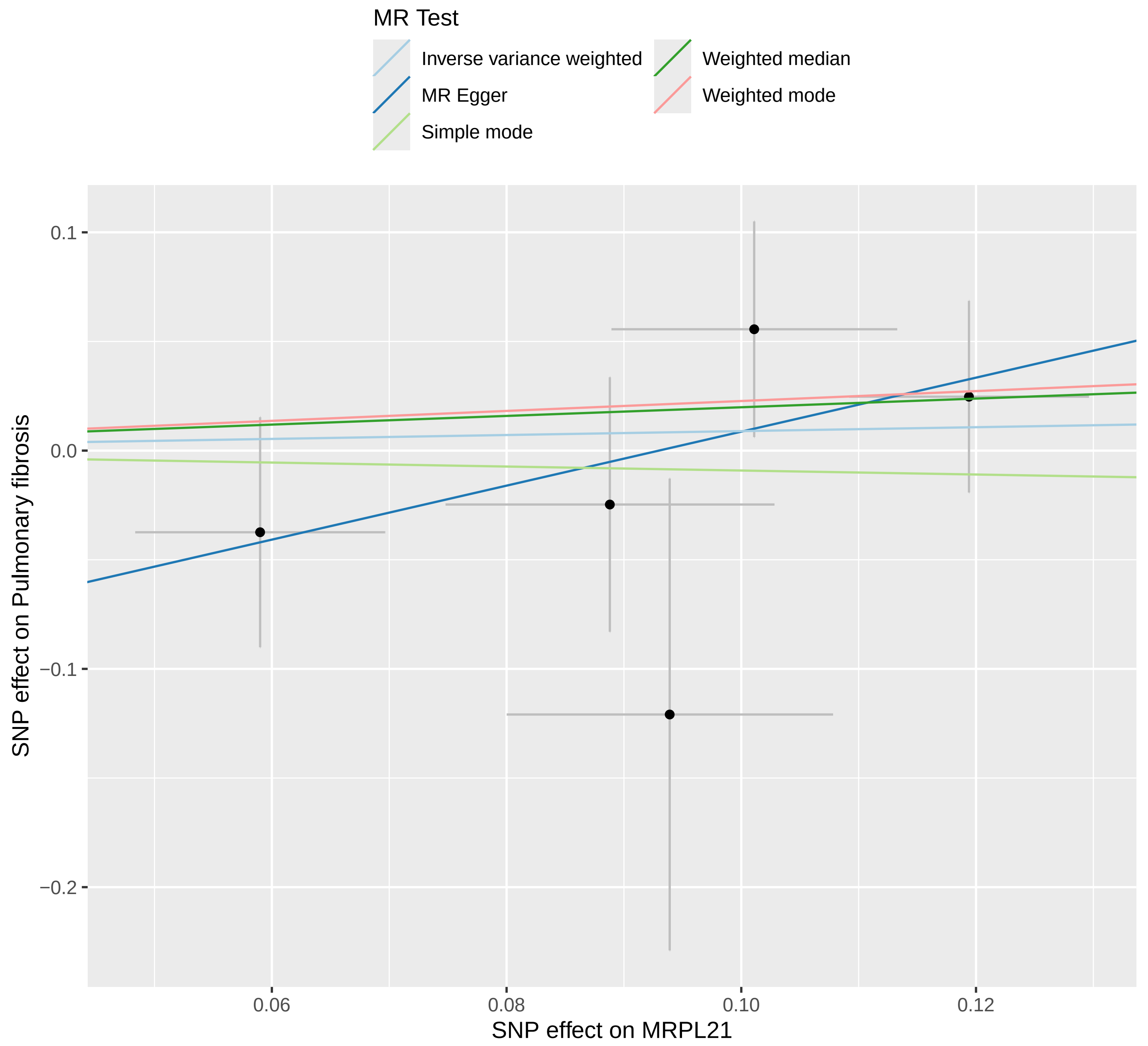

MR Test
Inverse variance weighted
MR Egger
Simple mode
Weighted median
Weighted mode
0.1
0.0
−0.1
−0.2
0.06
0.08
0.10
0.12
SNP effect on Pulmonary fibrosis
SNP effect on MRPL21

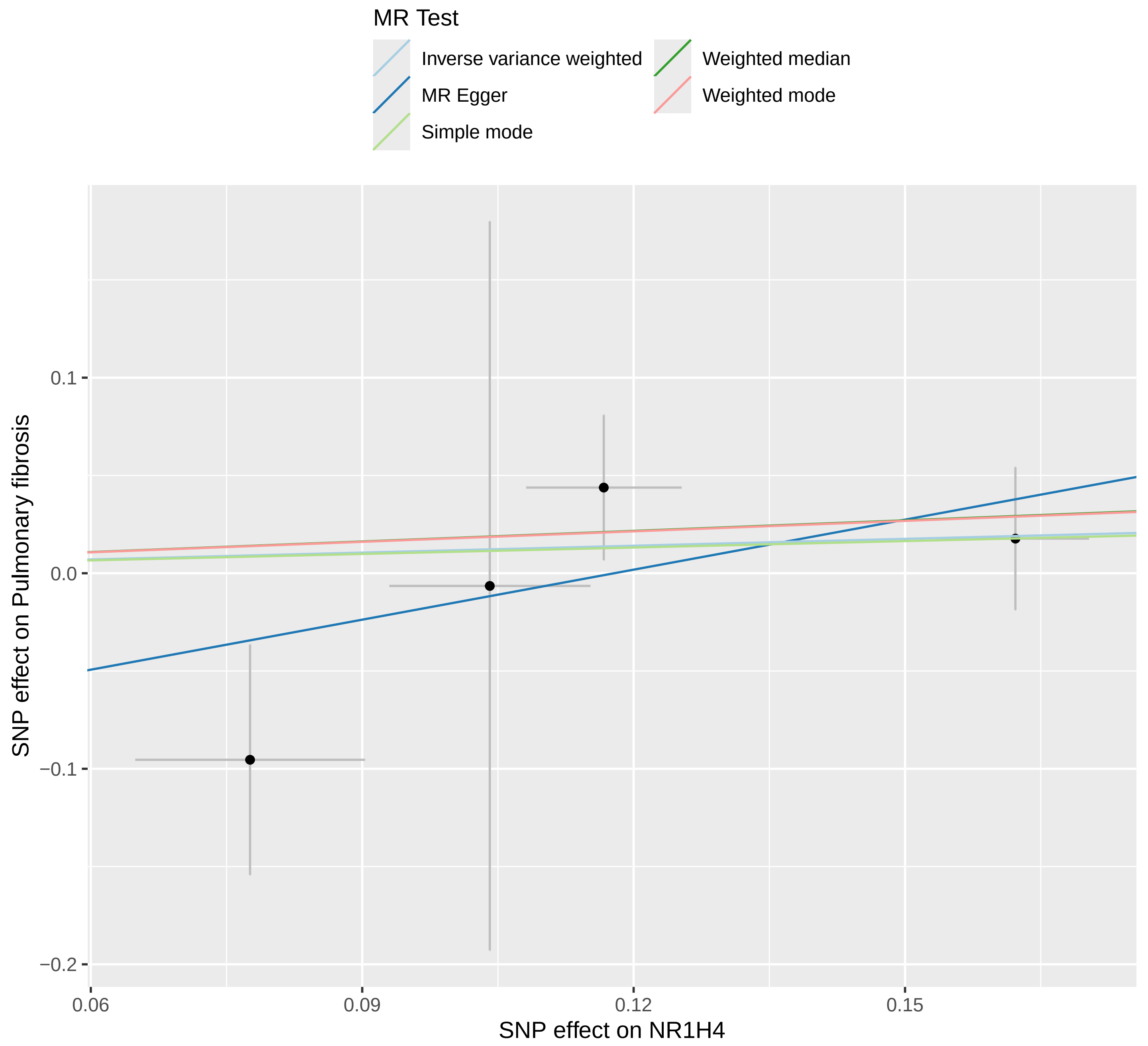

MR Test
Inverse variance weighted
MR Egger
Simple mode
Weighted median
Weighted mode
SNP effect on Pulmonary fibrosis
0.1
0.0
−0.1
−0.2
0.06
0.09
0.12
0.15
SNP effect on NR1H4

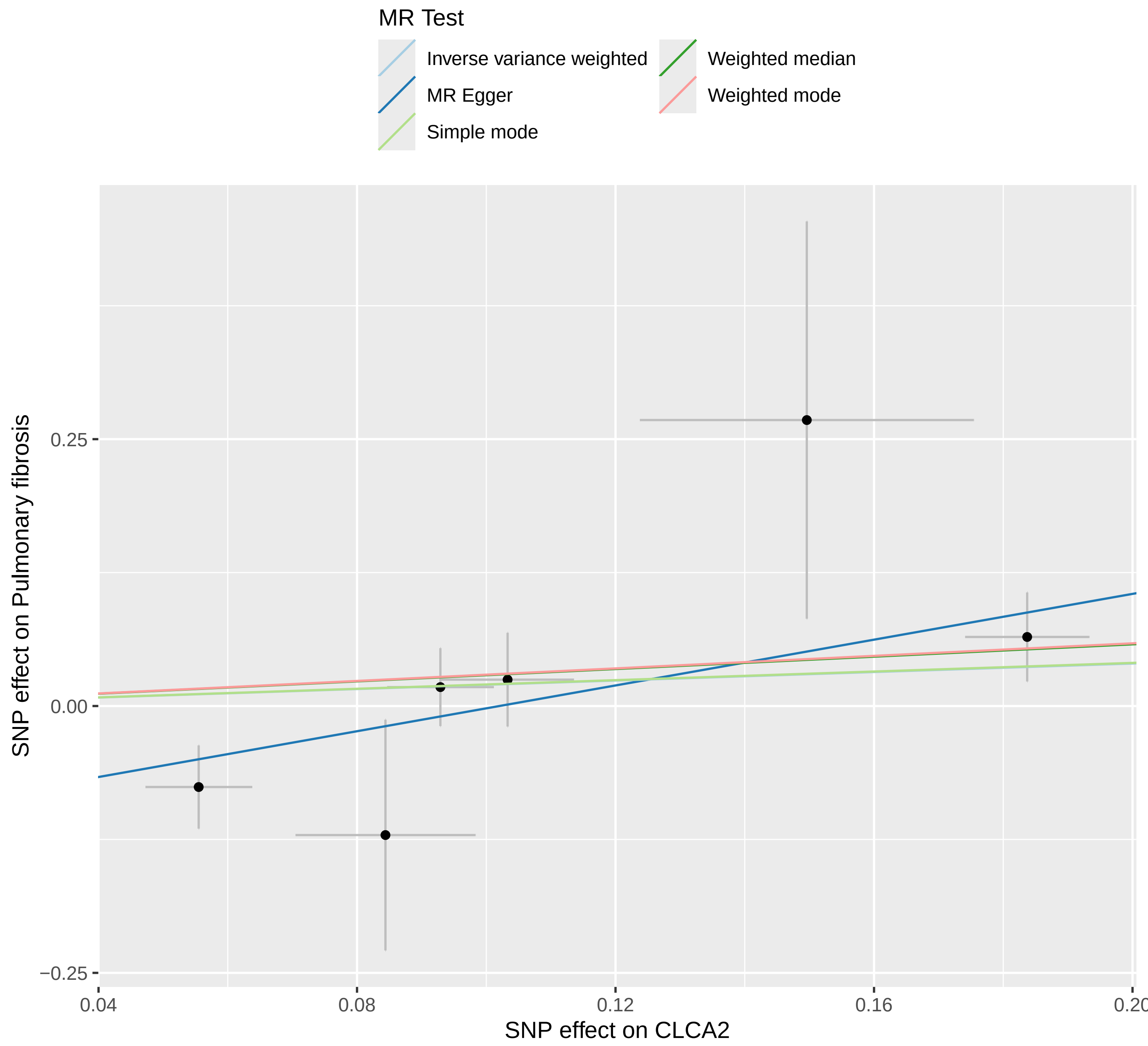

MR Test
Inverse variance weighted
MR Egger
Simple mode
Weighted median
Weighted mode
SNP effect on Pulmonary fibrosis
0.25
0.00
−0.25
0.04
0.08
0.12
0.16
0.20
SNP effect on CLCA2

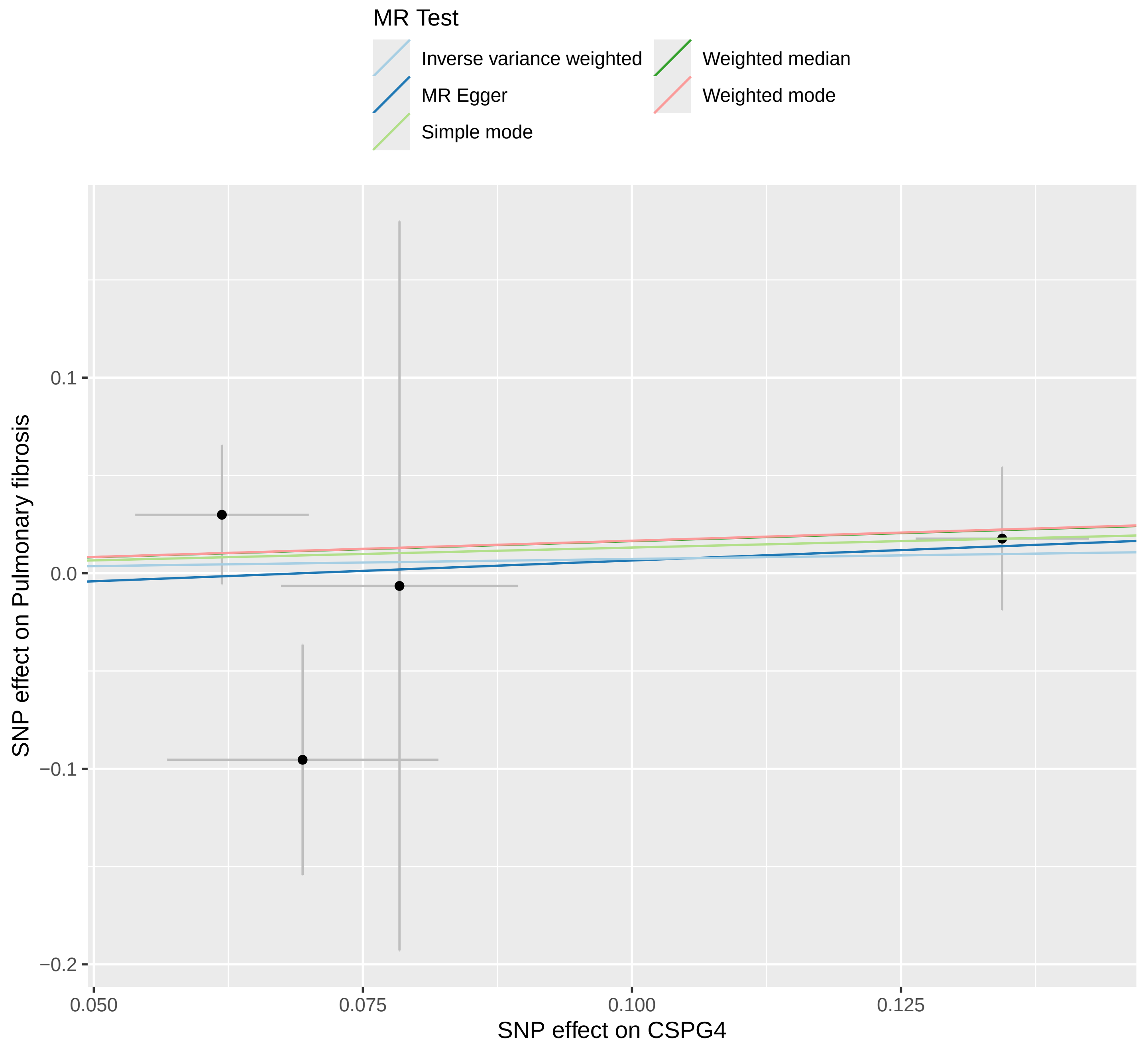

MR Test
Inverse variance weighted
MR Egger
Simple mode
Weighted median
Weighted mode
SNP effect on Pulmonary fibrosis
0.1
0.0
−0.1
−0.2
0.050
0.075
0.100
0.125
SNP effect on CSPG4

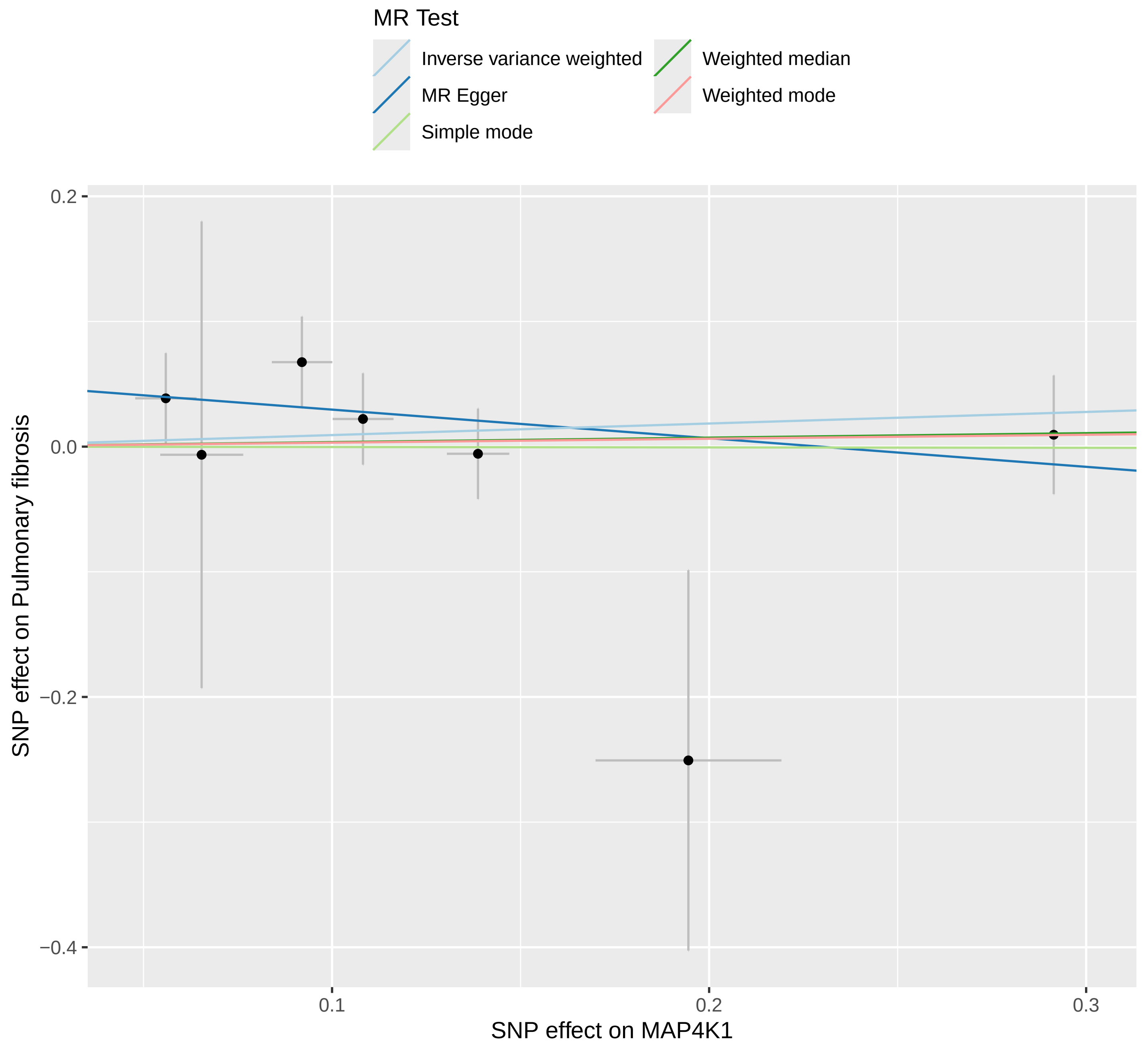
MR Test
Inverse variance weighted
MR Egger
Simple mode
Weighted median
Weighted mode
0.2
0.0
−0.2
−0.4
0.1
0.2
0.3
SNP effect on Pulmonary fibrosis
SNP effect on MAP4K1

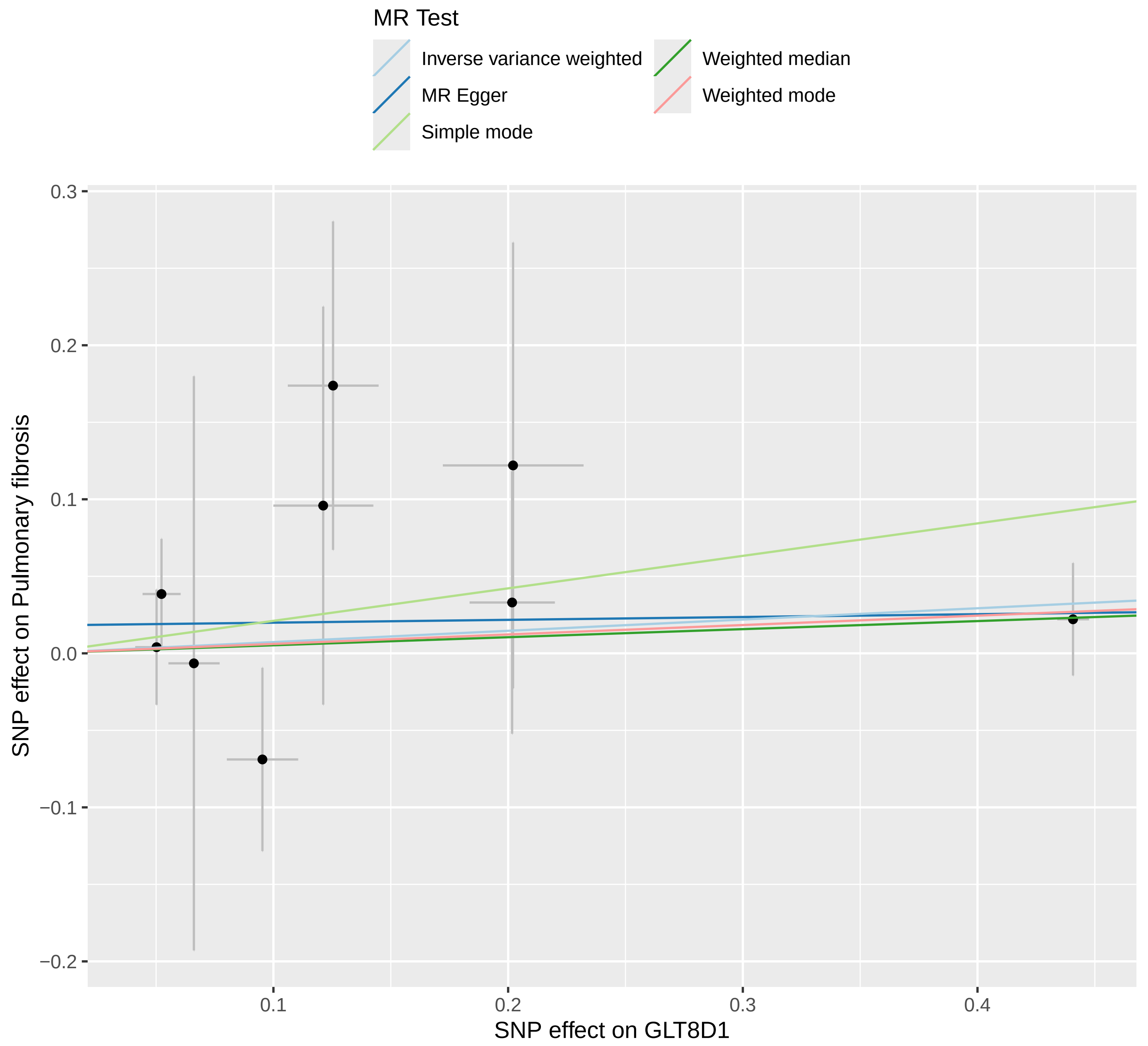

MR Test
Inverse variance weighted
MR Egger
Simple mode
Weighted median
Weighted mode
0.3
0.2
0.1
0.0
−0.1
−0.2
0.1
0.2
0.3
0.4
SNP effect on Pulmonary fibrosis
SNP effect on GLT8D1

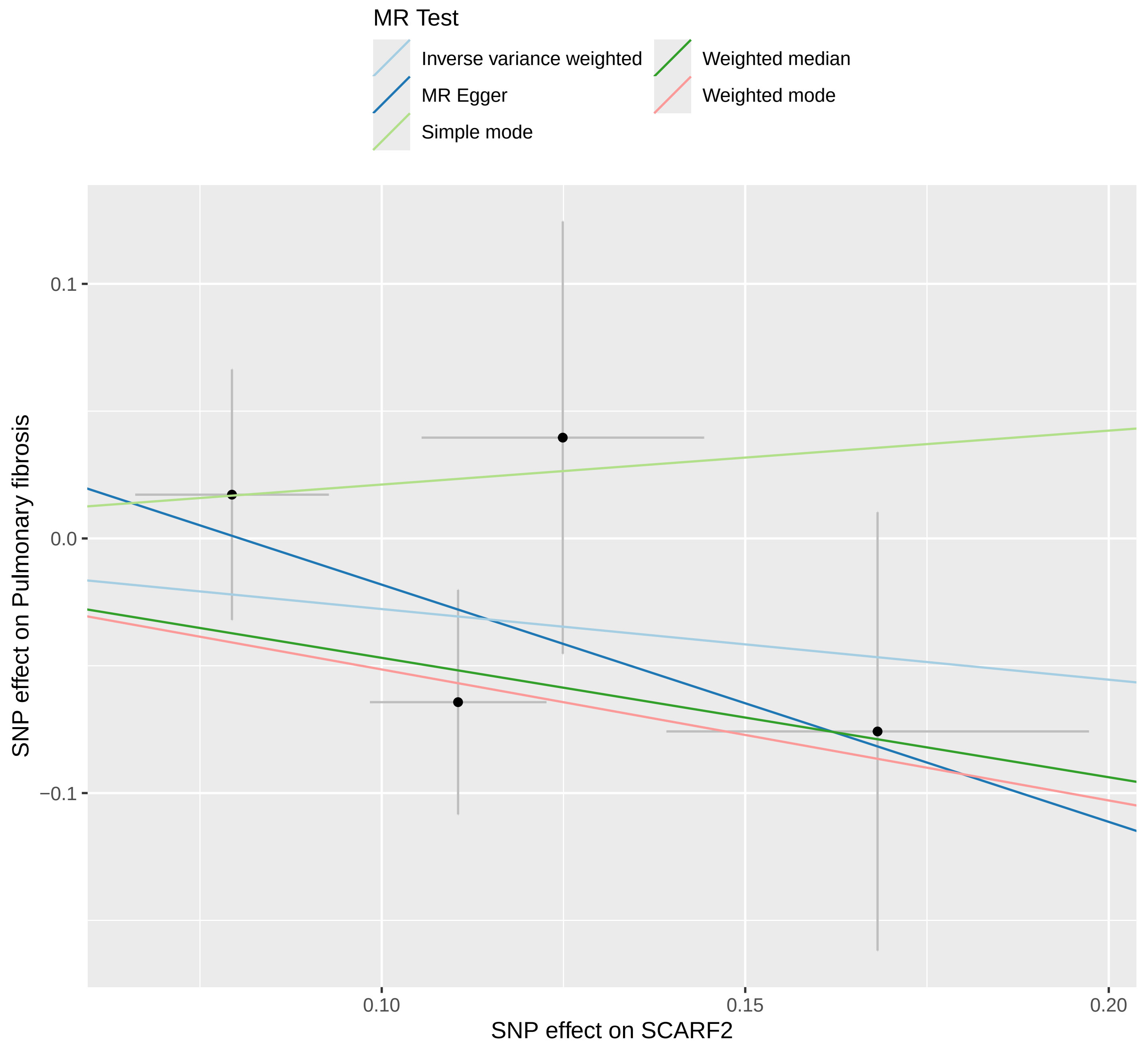

MR Test
Inverse variance weighted
MR Egger
Simple mode
Weighted median
Weighted mode
0.1
0.0
−0.1
0.10
0.15
0.20
SNP effect on Pulmonary fibrosis
SNP effect on SCARF2

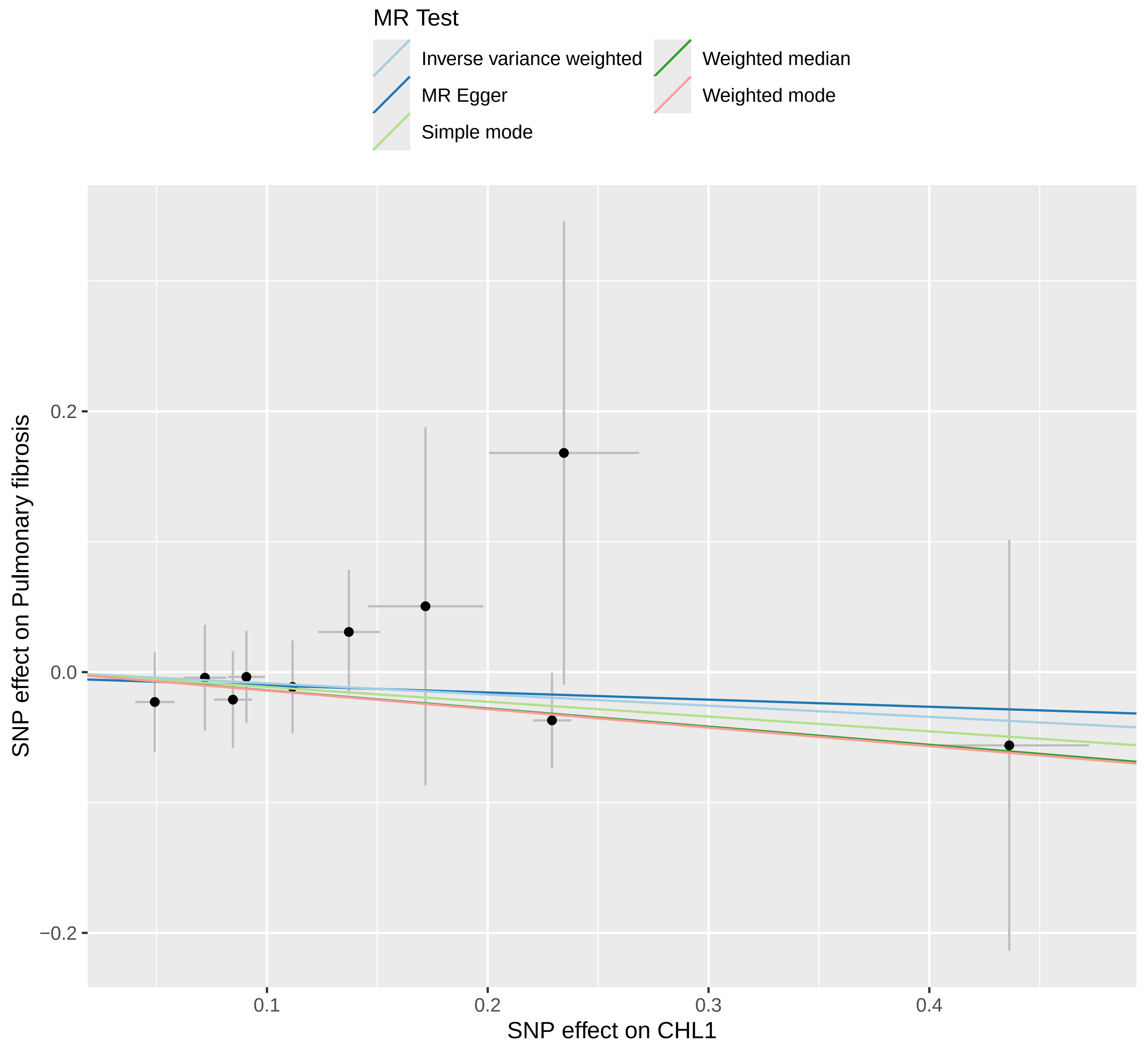
MR Test
Inverse variance weighted
MR Egger
Simple mode
Weighted median
Weighted mode
0.2
0.0
−0.2
0.1
0.2
0.3
0.4
SNP effect on CHL1
SNP effect on Pulmonary fibrosis

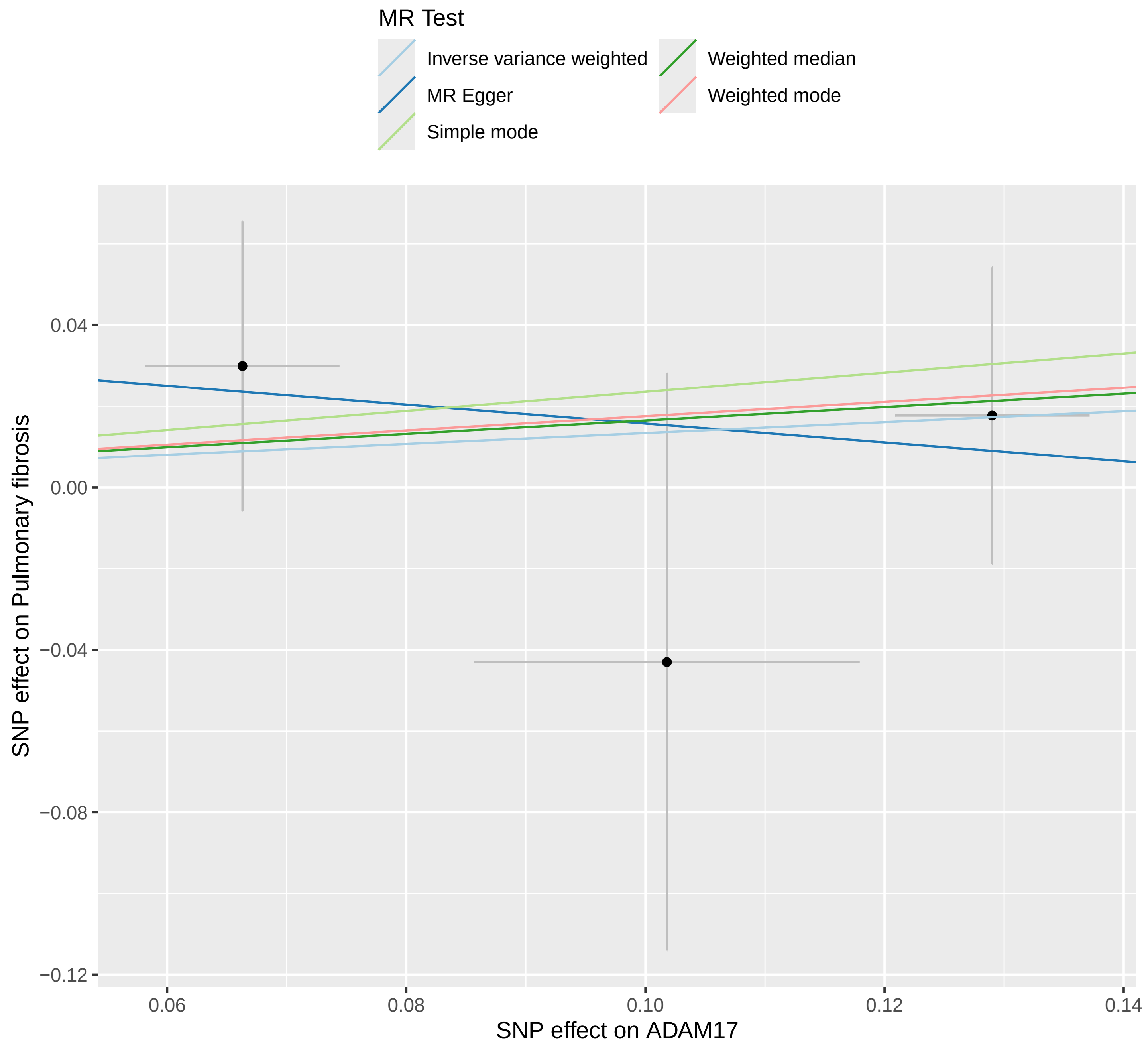
MR Test
Inverse variance weighted
MR Egger
Simple mode
Weighted median
Weighted mode
SNP effect on Pulmonary fibrosis
0.04
0.00
−0.04
−0.08
−0.12
0.06
0.08
0.10
0.12
0.14
SNP effect on ADAM17

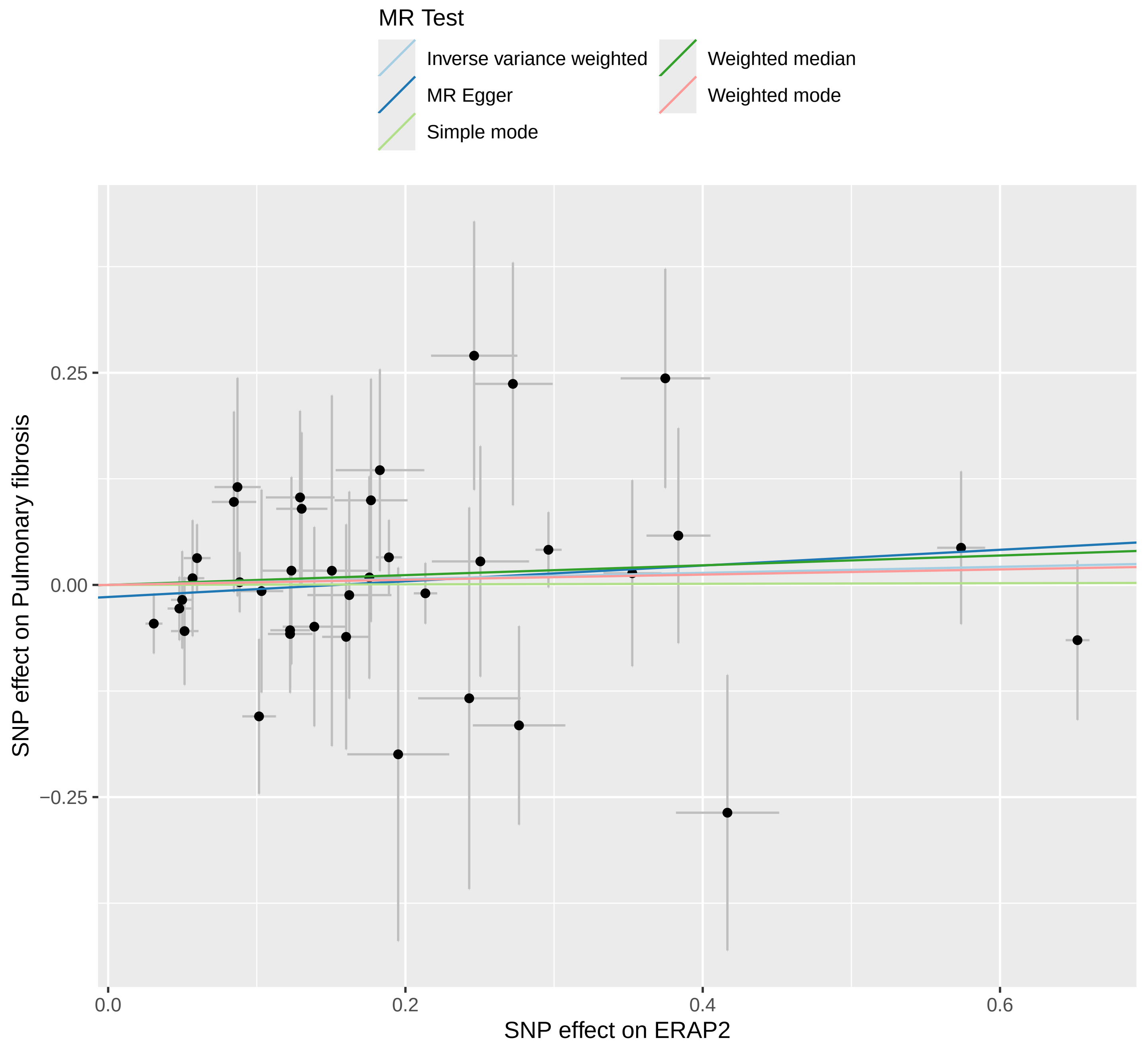
MR Test
Inverse variance weighted
MR Egger
Simple mode
Weighted median
Weighted mode
0.25
0.00
−0.25
0.0
0.2
0.4
0.6
SNP effect on ERAP2
SNP effect on Pulmonary fibrosis

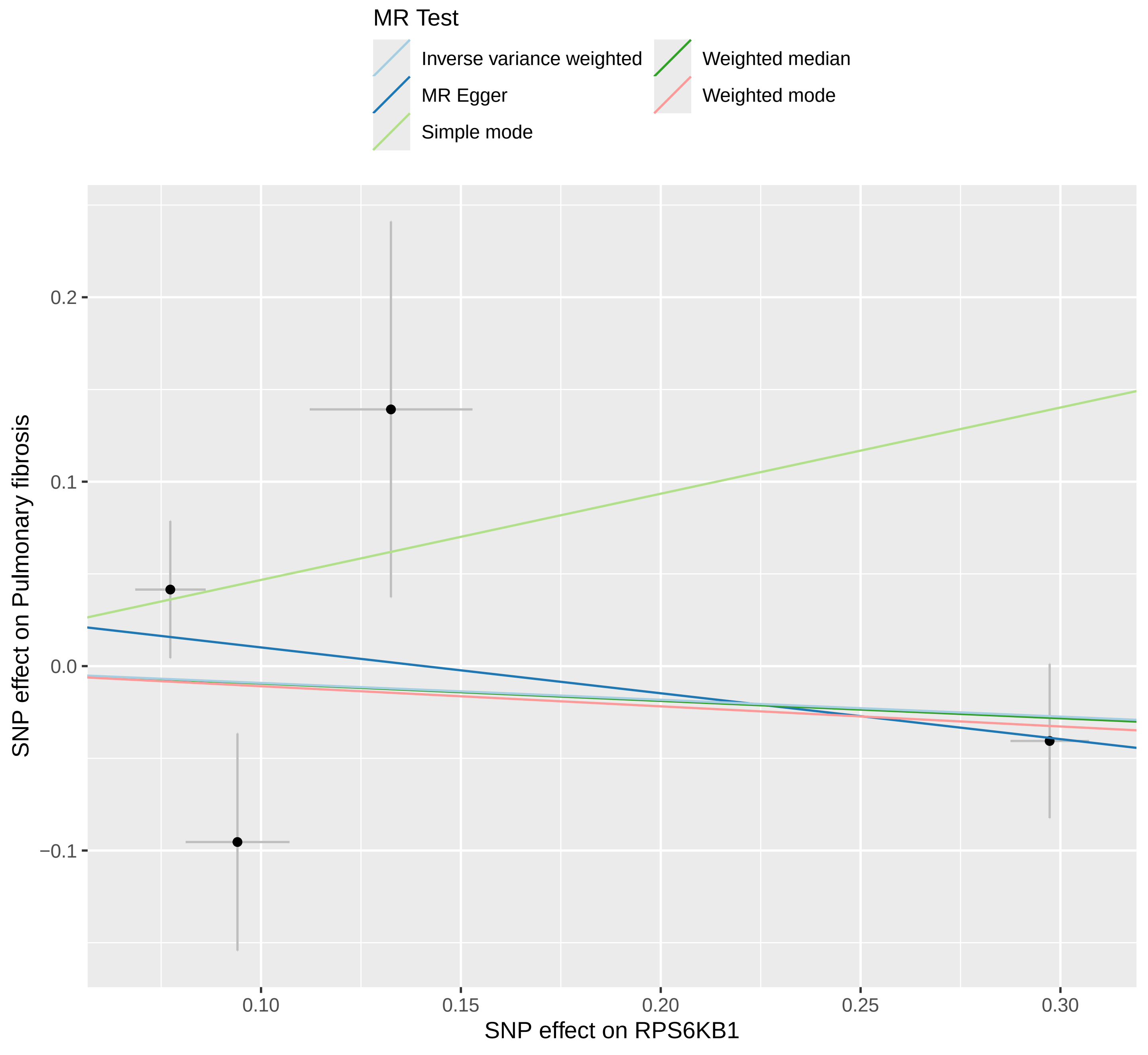

MR Test
Inverse variance weighted
MR Egger
Simple mode
Weighted median
Weighted mode
0.2
0.1
0.0
−0.1
SNP effect on Pulmonary fibrosis
0.10
0.15
0.20
0.25
0.30
SNP effect on RPS6KB1

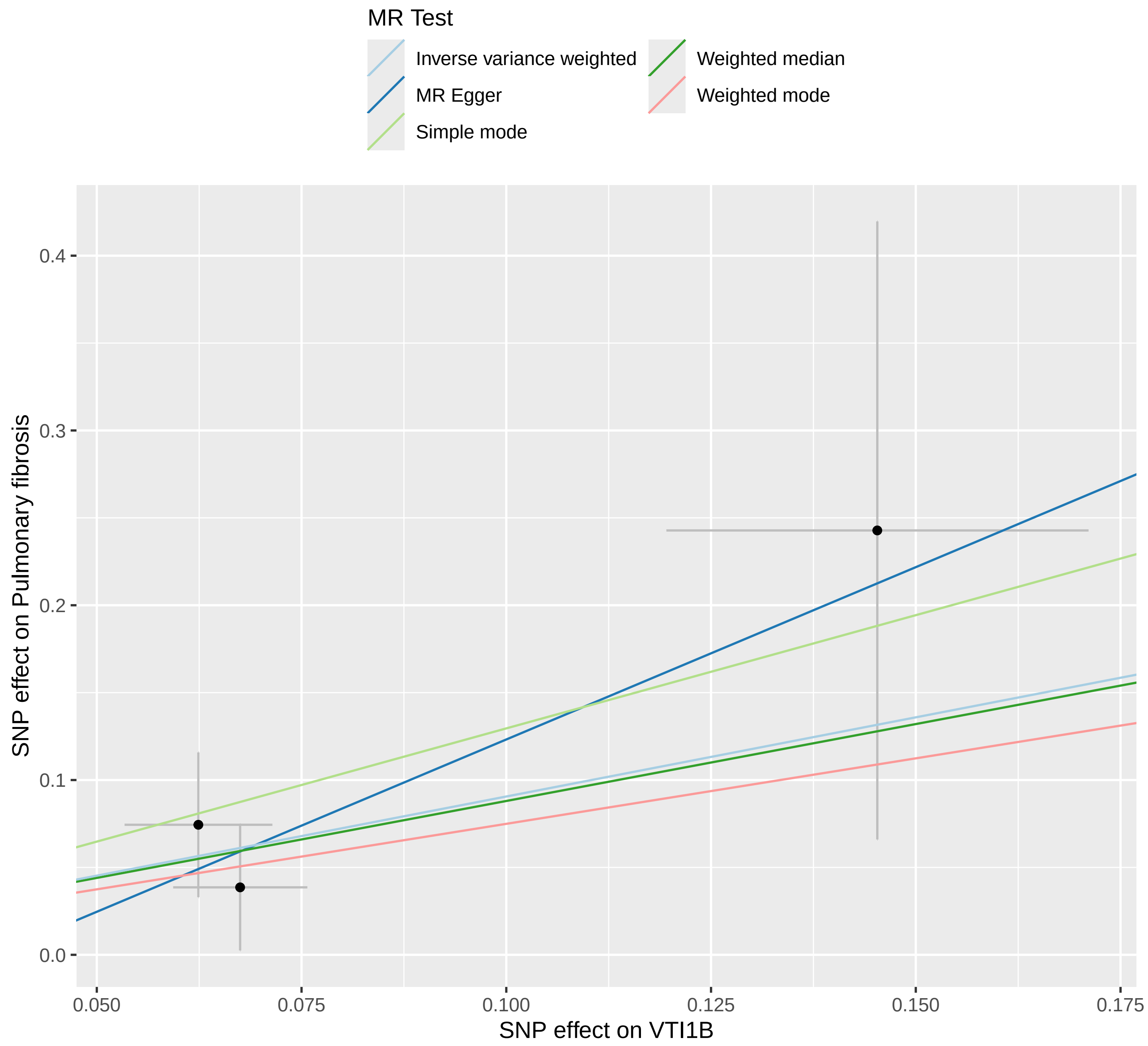

MR Test
Inverse variance weighted
MR Egger
Simple mode
Weighted median
Weighted mode
SNP effect on Pulmonary fibrosis
0.0
0.1
0.2
0.3
0.4
0.050
0.075
0.100
0.125
0.150
0.175
SNP effect on VTI1B

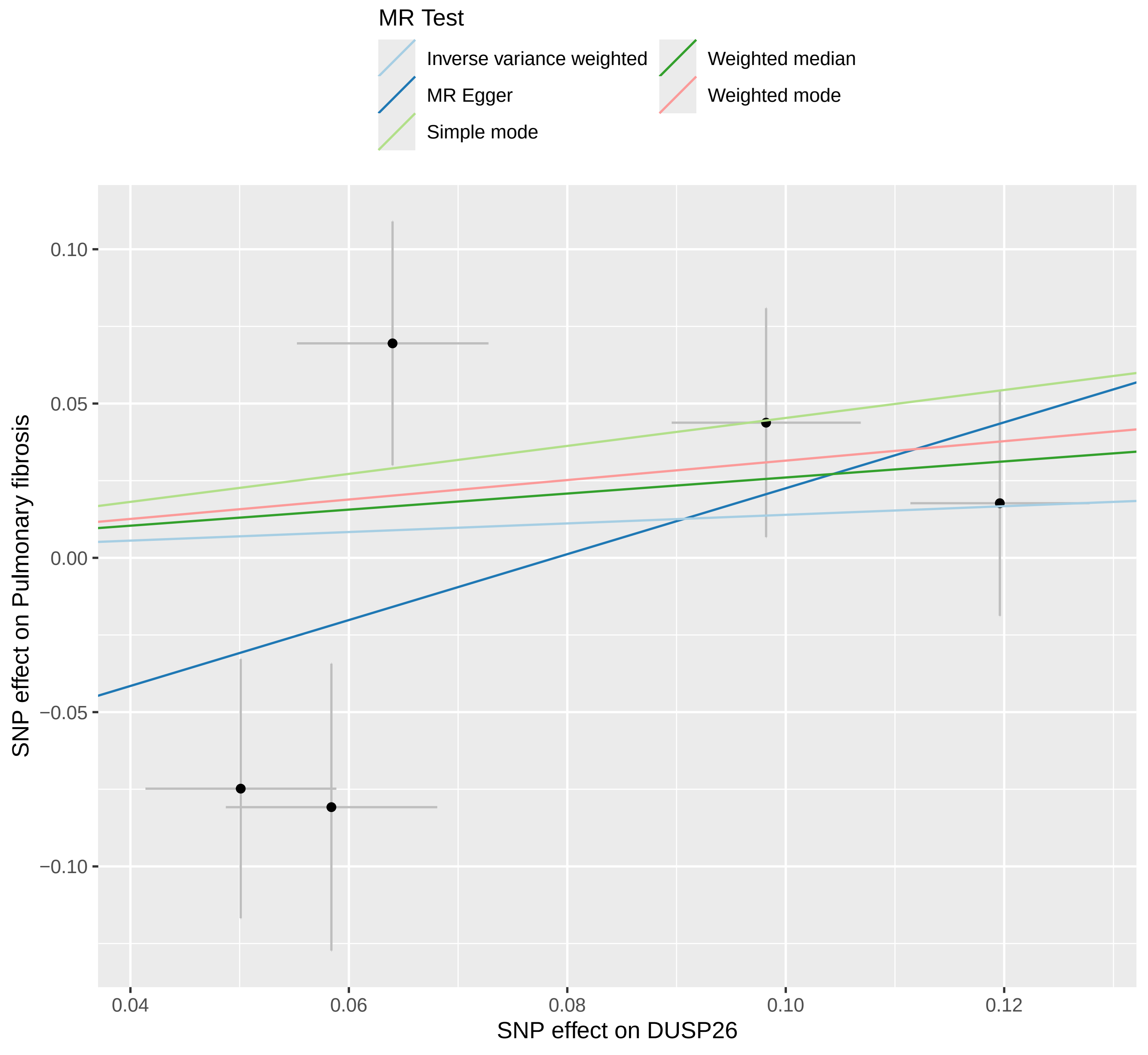
MR Test
Inverse variance weighted
MR Egger
Simple mode
Weighted median
Weighted mode
0.10
0.05
0.00
−0.05
−0.10
SNP effect on Pulmonary fibrosis
0.04
0.06
0.08
0.10
0.12
SNP effect on DUSP26

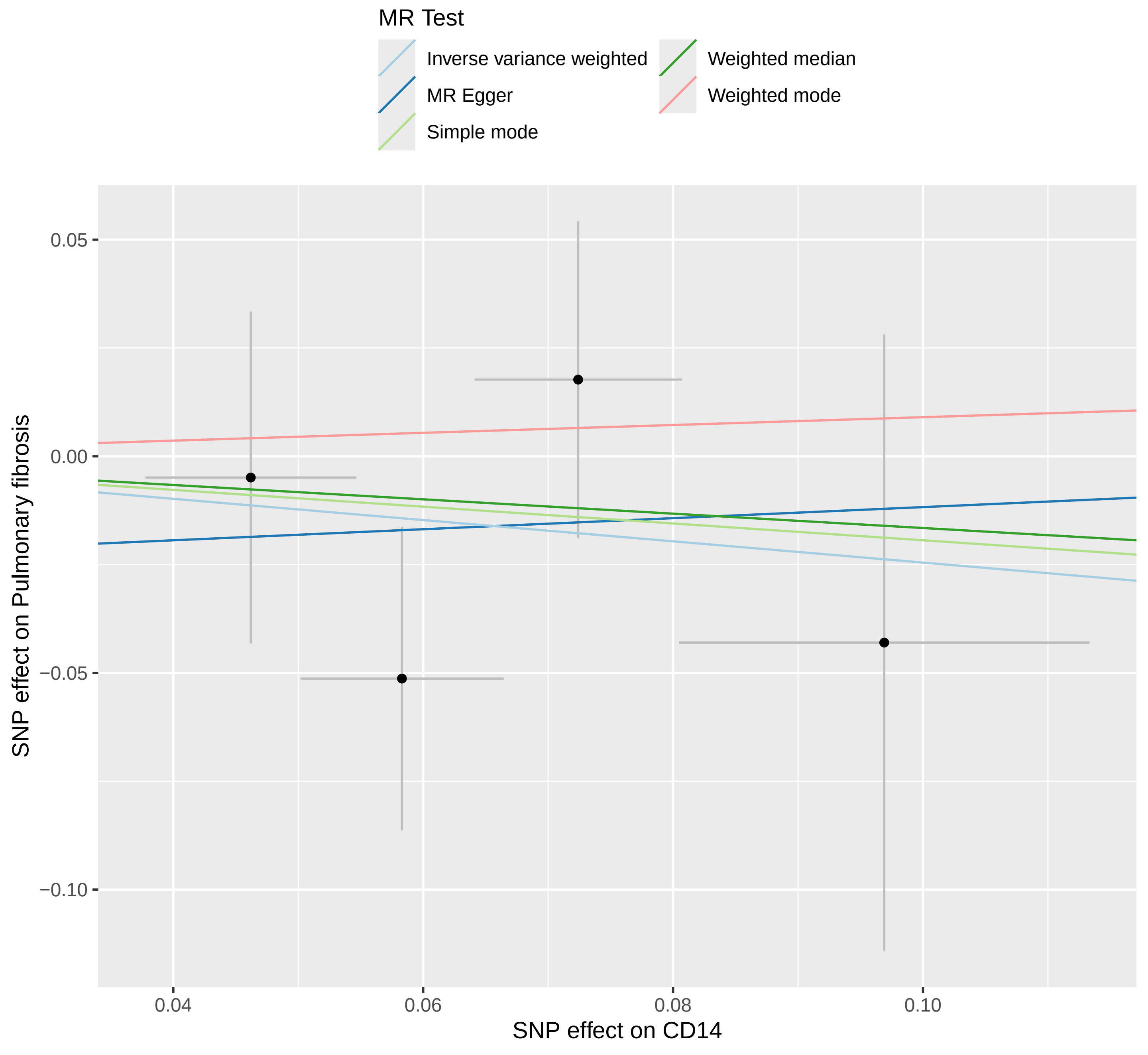

MR Test
Inverse variance weighted
MR Egger
Simple mode
Weighted median
Weighted mode
SNP effect on Pulmonary fibrosis
0.05
0.00
−0.05
−0.10
0.04
0.06
0.08
0.10
SNP effect on CD14

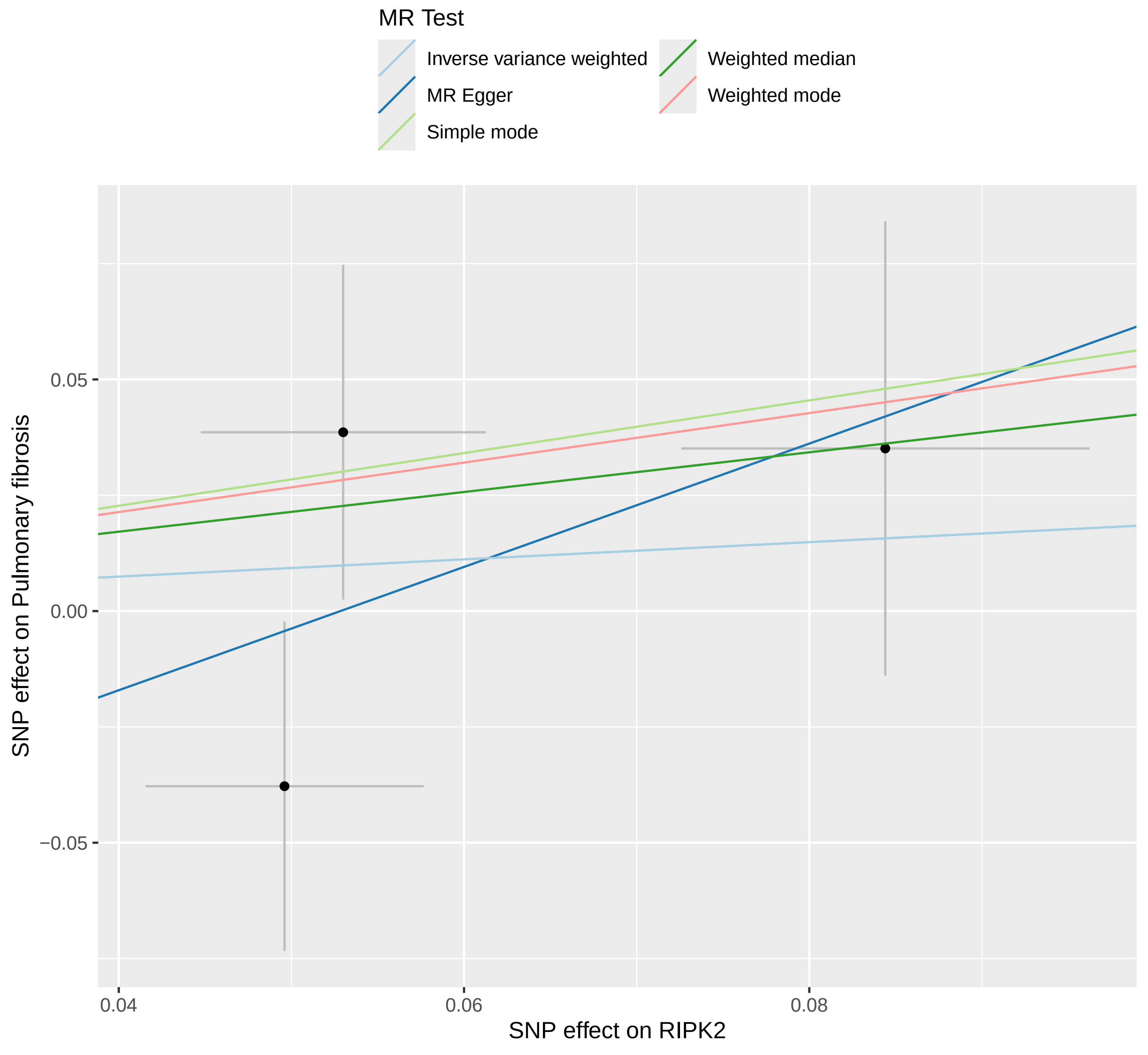

MR Test
Inverse variance weighted
MR Egger
Simple mode
Weighted median
Weighted mode
0.05
0.00
−0.05
SNP effect on Pulmonary fibrosis
0.04
0.06
0.08
SNP effect on RIPK2

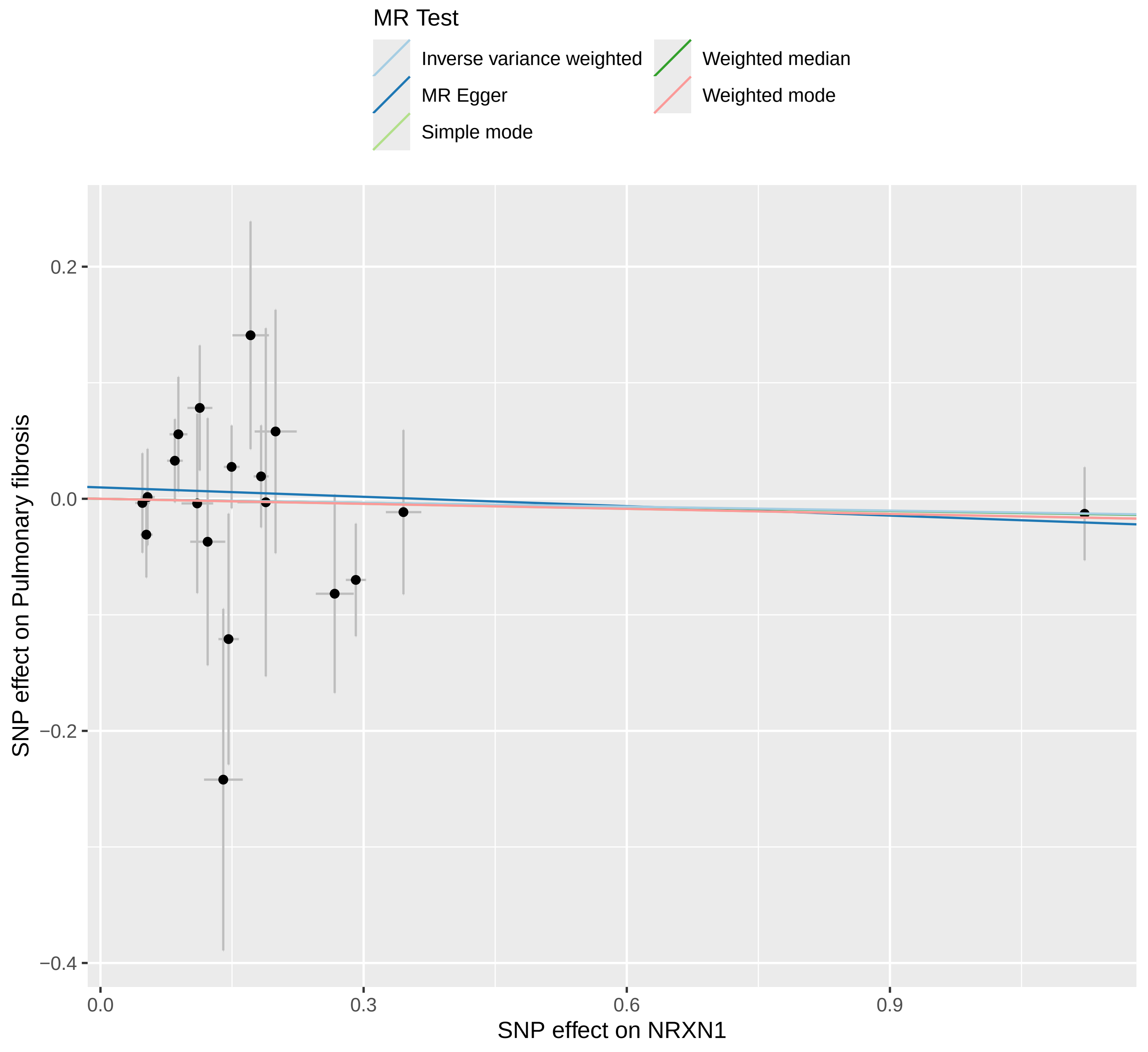
MR Test
Inverse variance weighted
MR Egger
Simple mode
Weighted median
Weighted mode
0.2
0.0
−0.2
−0.4
SNP effect on Pulmonary fibrosis
0.0
0.3
0.6
0.9
SNP effect on NRXN1

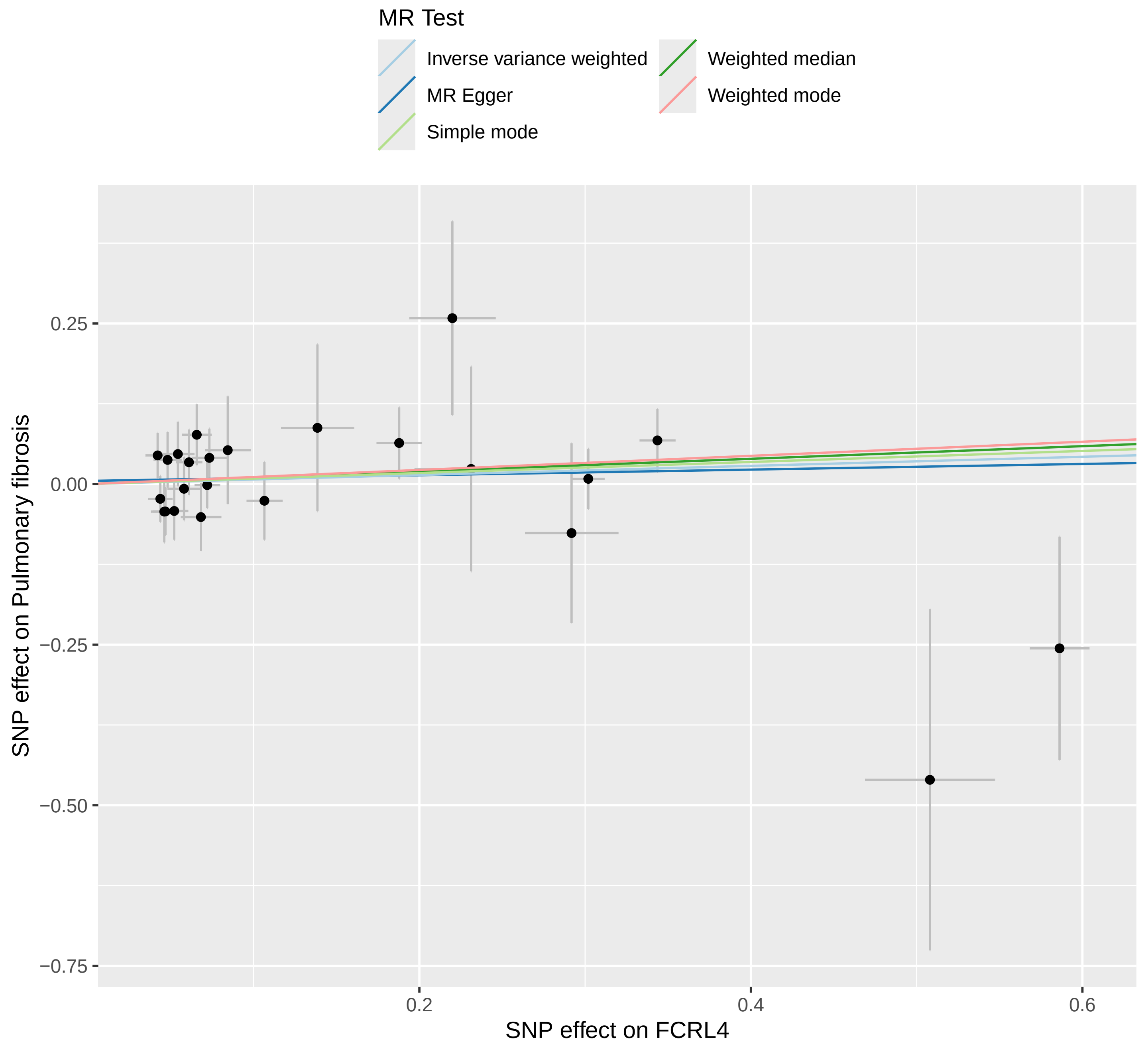
MR Test
Inverse variance weighted
MR Egger
Simple mode
Weighted median
Weighted mode
SNP effect on Pulmonary fibrosis
0.25
0.00
−0.25
−0.50
−0.75
0.2
0.4
0.6
SNP effect on FCRL4

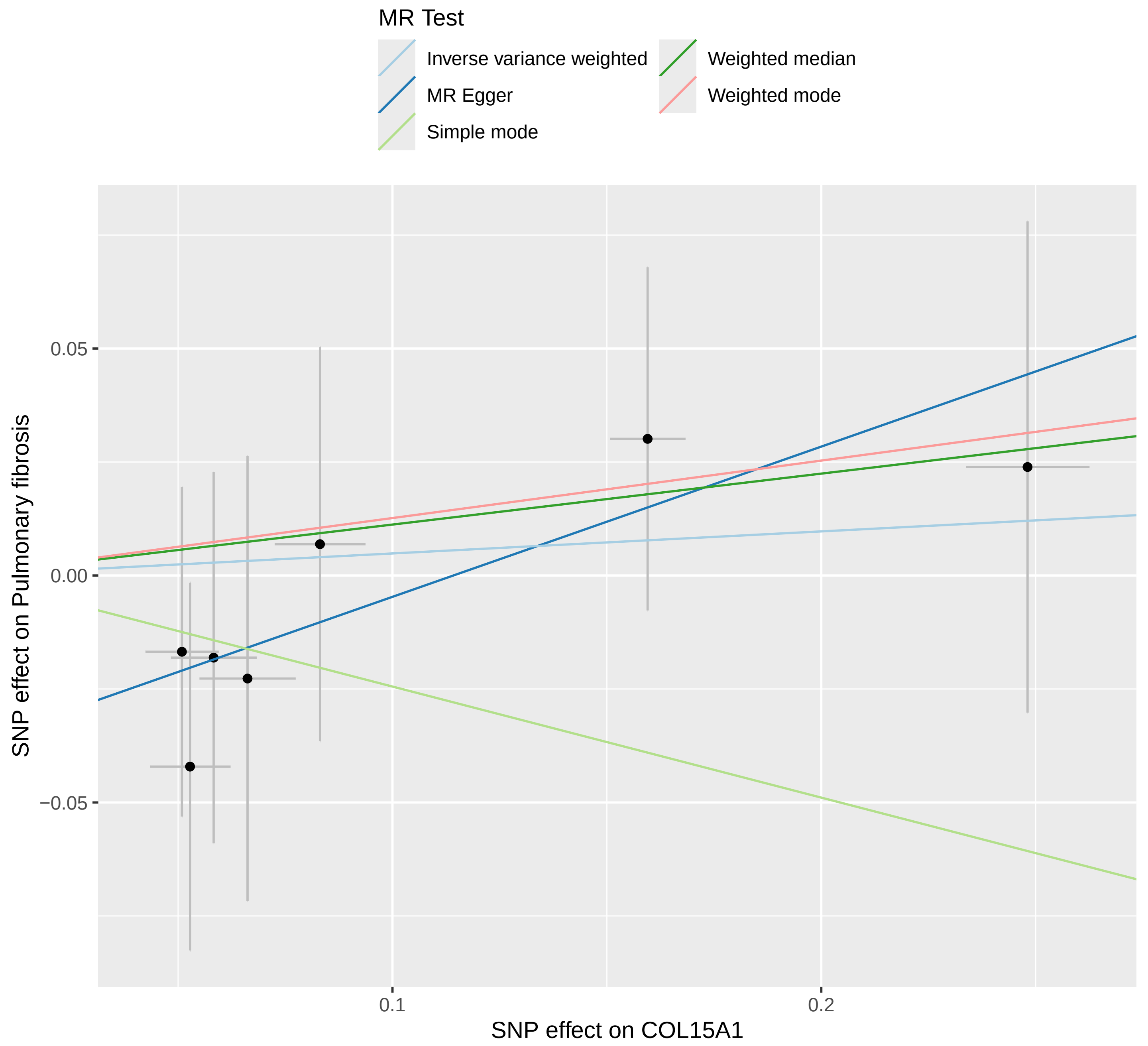
MR Test
Inverse variance weighted
MR Egger
Simple mode
Weighted median
Weighted mode
0.05
0.00
−0.05
0.1
0.2
SNP effect on Pulmonary fibrosis
SNP effect on COL15A1

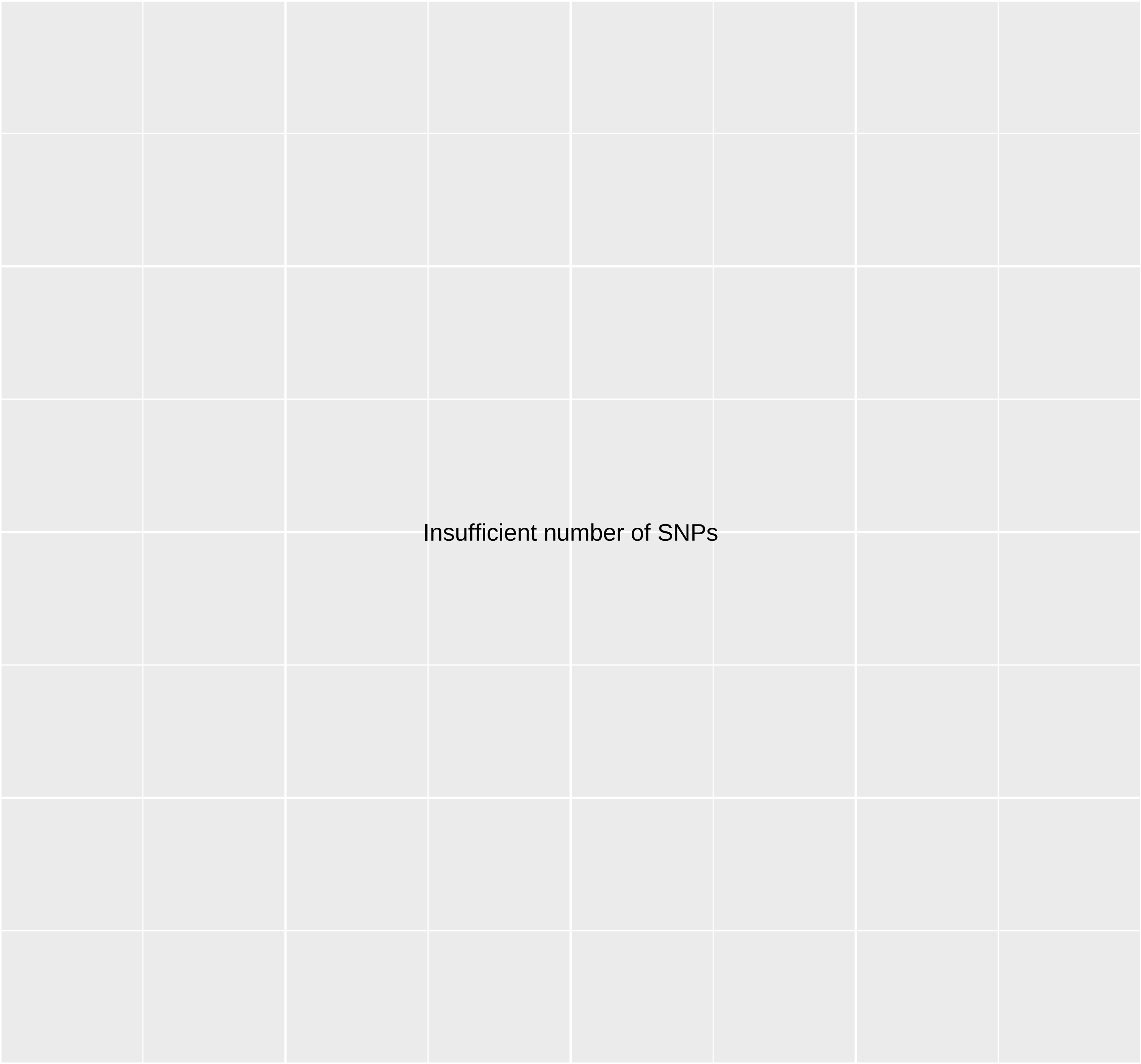
Insufficient number of SNPs

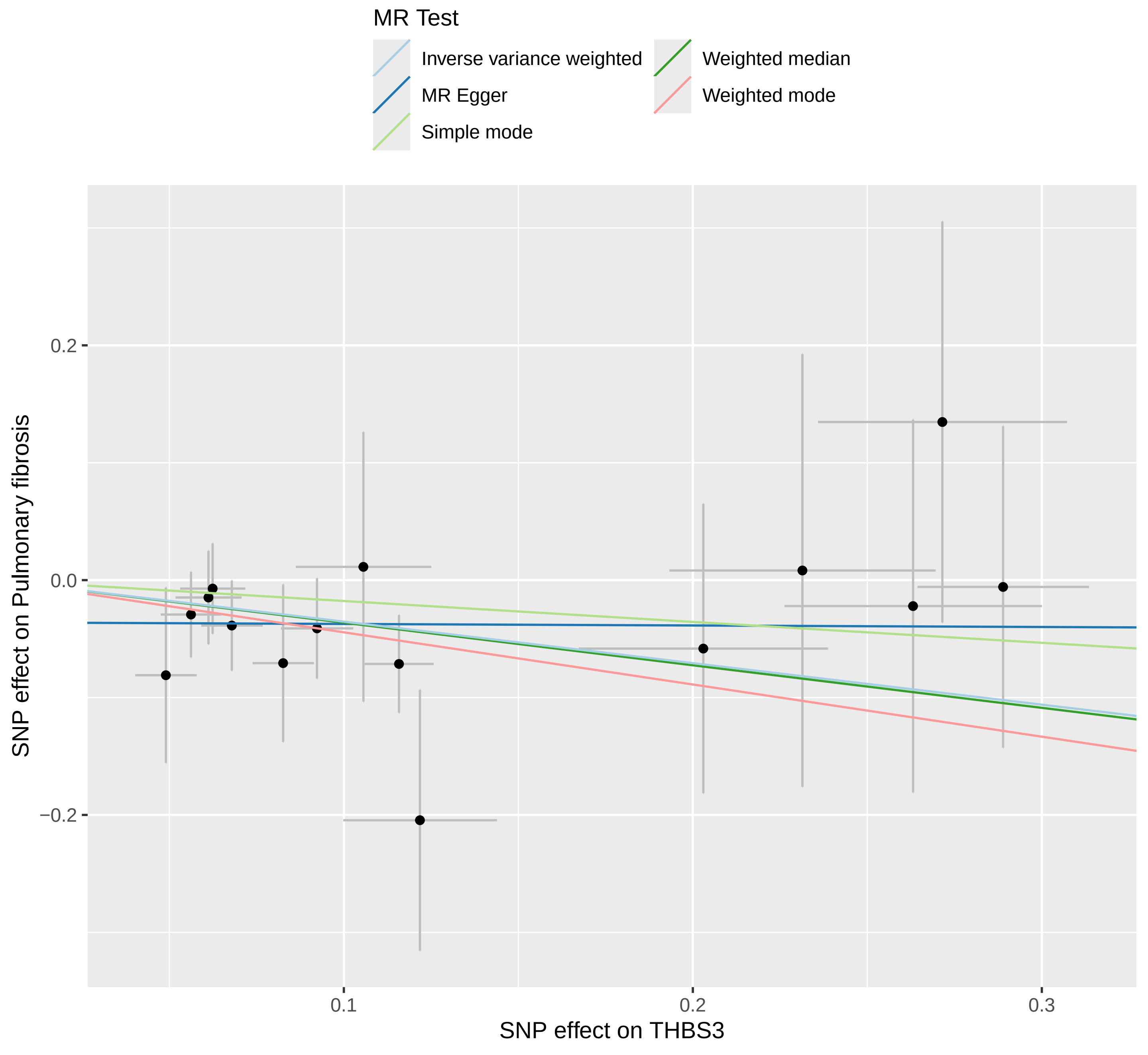

MR Test
Inverse variance weighted
MR Egger
Simple mode
Weighted median
Weighted mode
0.2
0.0
−0.2
0.1
0.2
0.3
SNP effect on THBS3
SNP effect on Pulmonary fibrosis

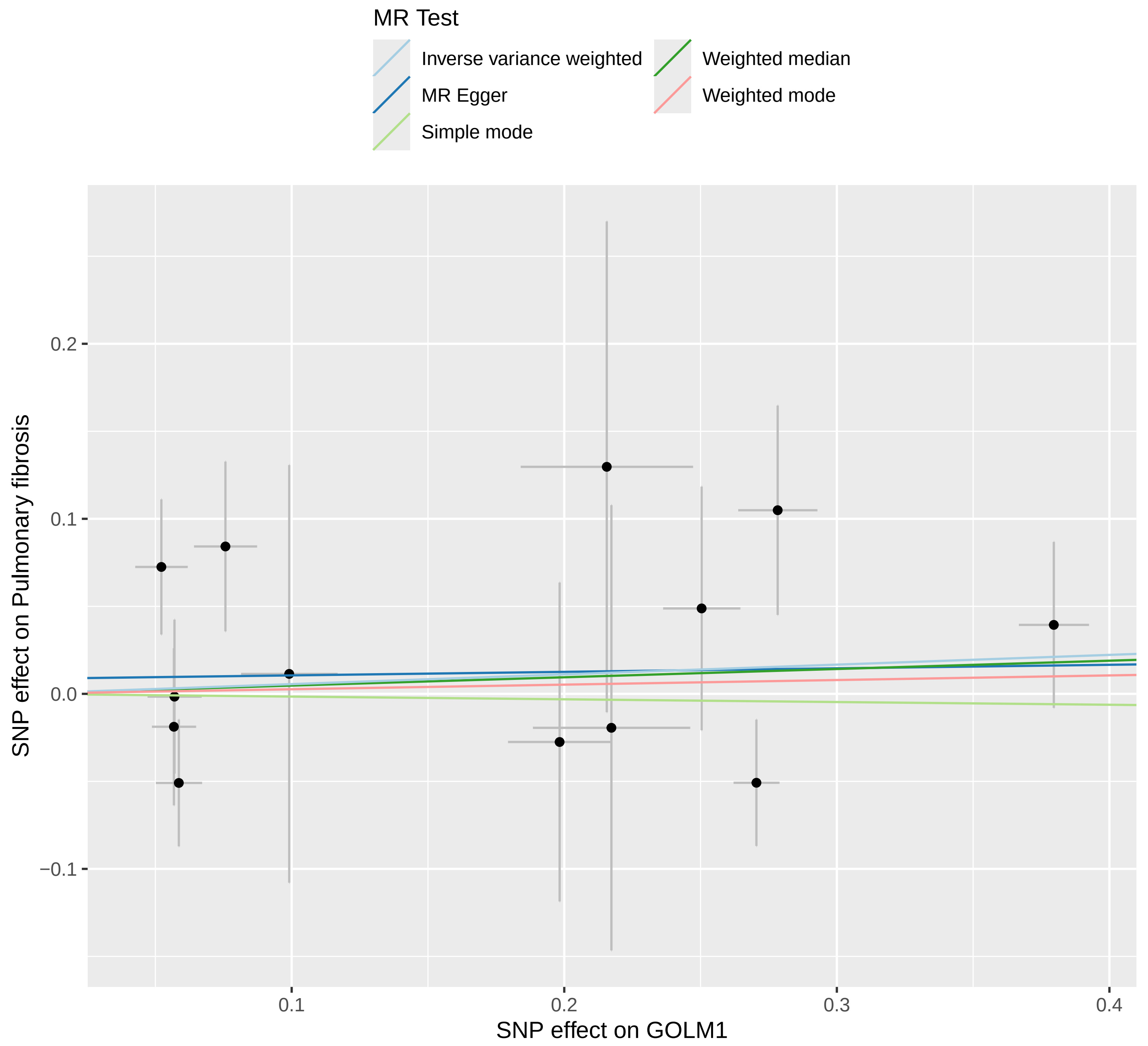

MR Test
Inverse variance weighted
MR Egger
Simple mode
Weighted median
Weighted mode
SNP effect on Pulmonary fibrosis
0.2
0.1
0.0
−0.1
0.1
0.2
0.3
0.4
SNP effect on GOLM1

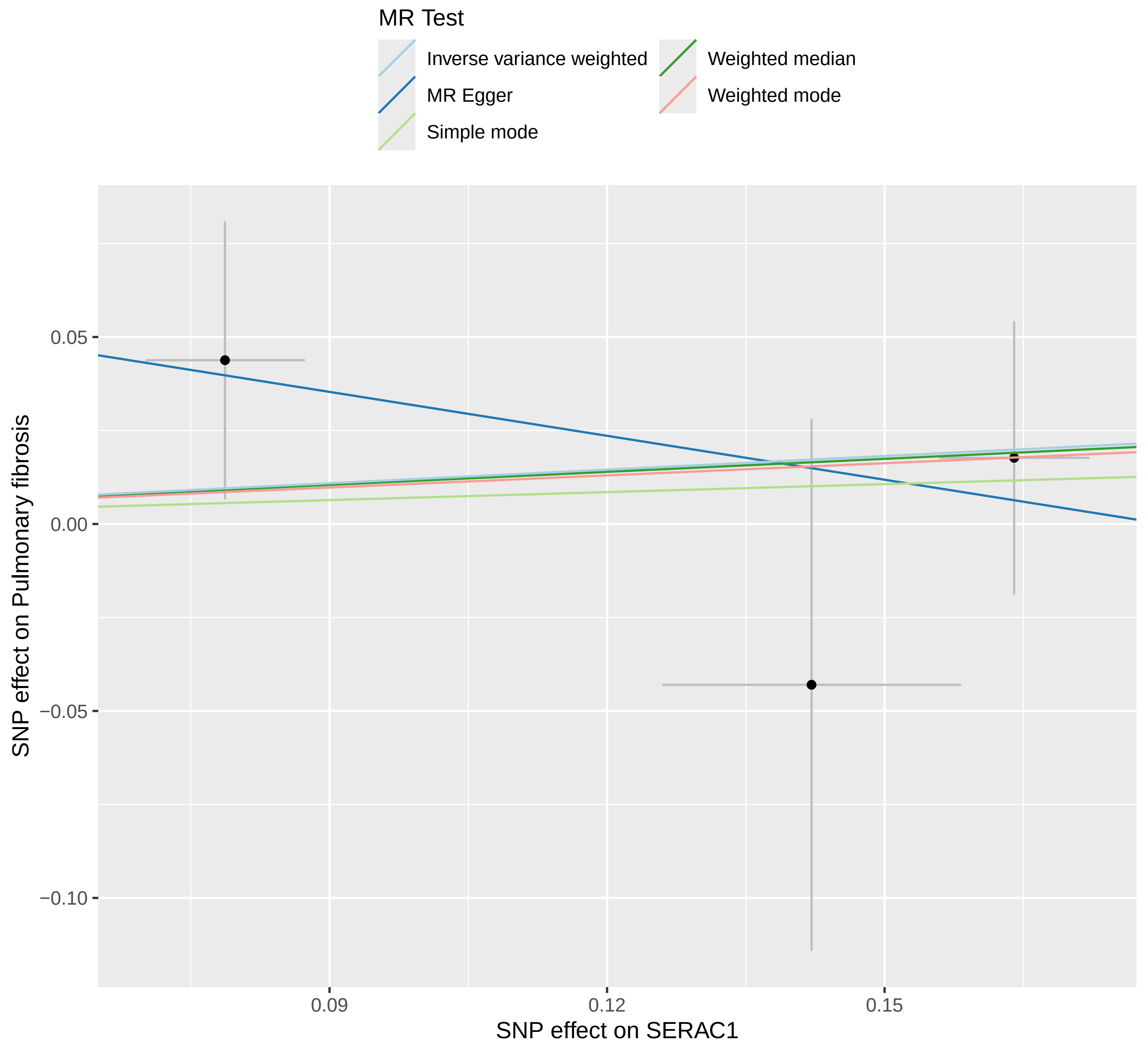

MR Test
Inverse variance weighted
MR Egger
Simple mode
Weighted median
Weighted mode
0.05
0.00
−0.05
−0.10
SNP effect on Pulmonary fibrosis
0.09
0.12
0.15
SNP effect on SERAC1

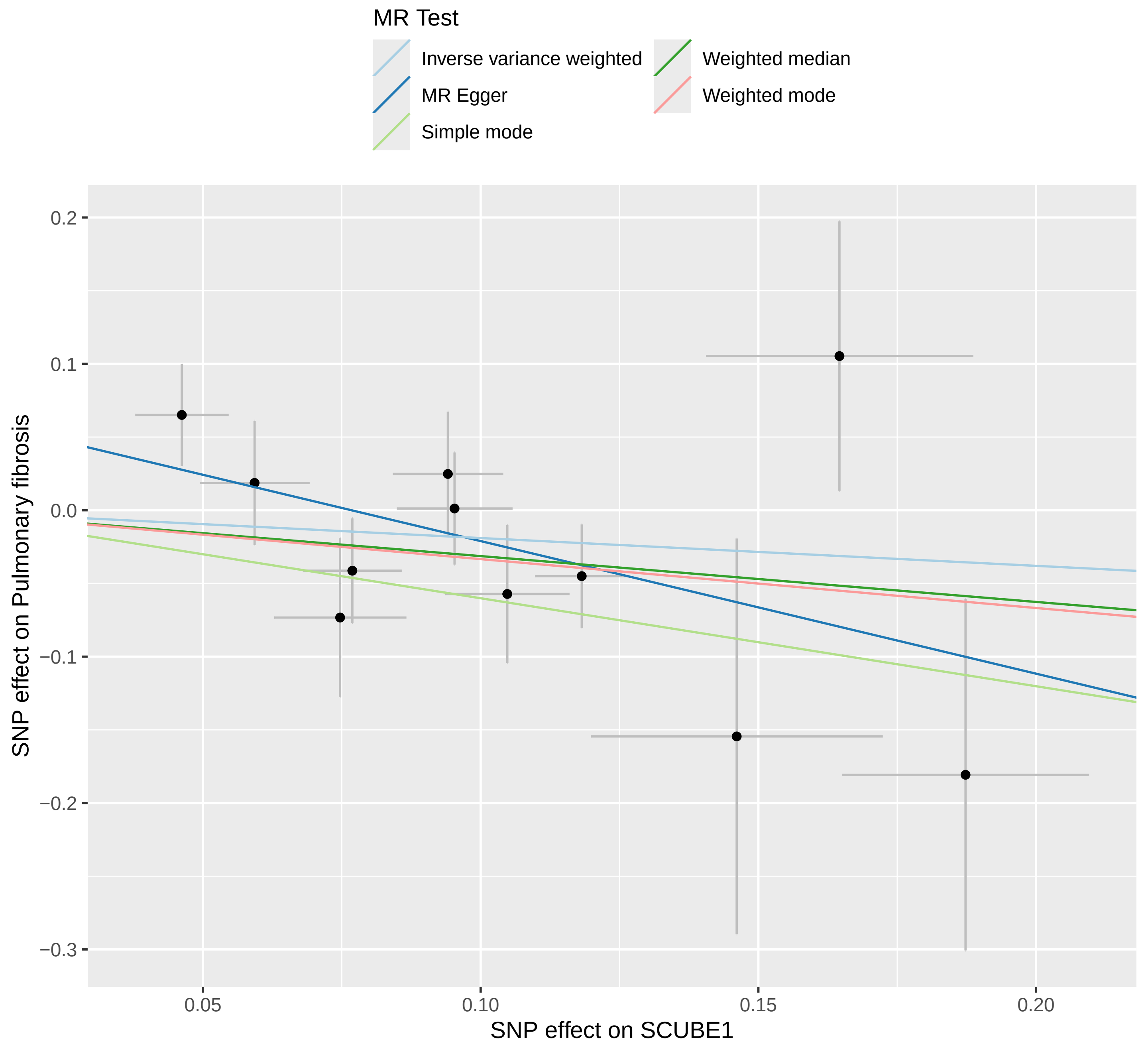

MR Test
Inverse variance weighted
MR Egger
Simple mode
Weighted median
Weighted mode
0.2
0.1
0.0
−0.1
−0.2
−0.3
SNP effect on Pulmonary fibrosis
0.05
0.10
0.15
0.20
SNP effect on SCUBE1

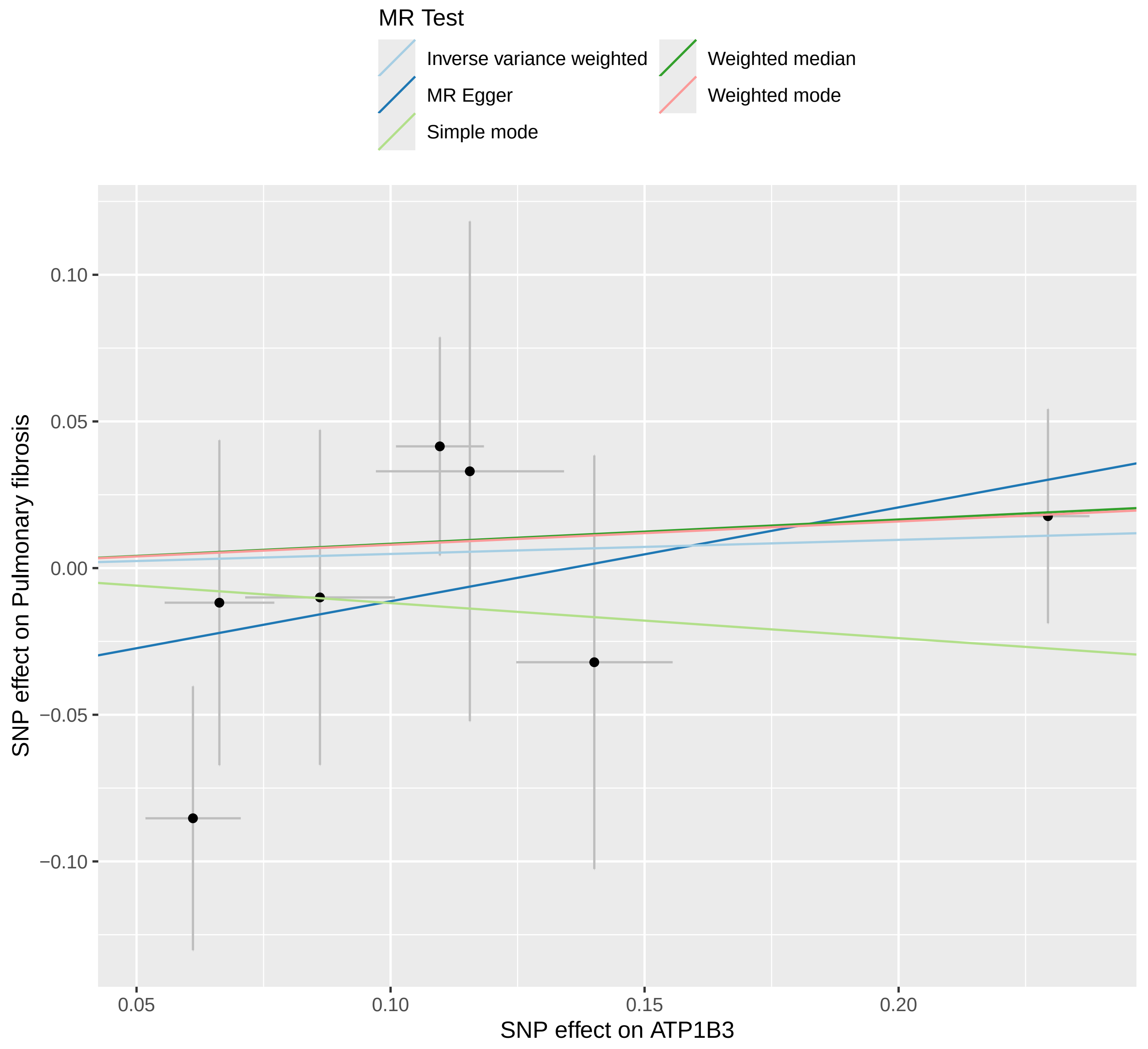
MR Test
Inverse variance weighted
MR Egger
Simple mode
Weighted median
Weighted mode
0.10
0.05
0.00
−0.05
−0.10
SNP effect on Pulmonary fibrosis
0.05
0.10
0.15
0.20
SNP effect on ATP1B3

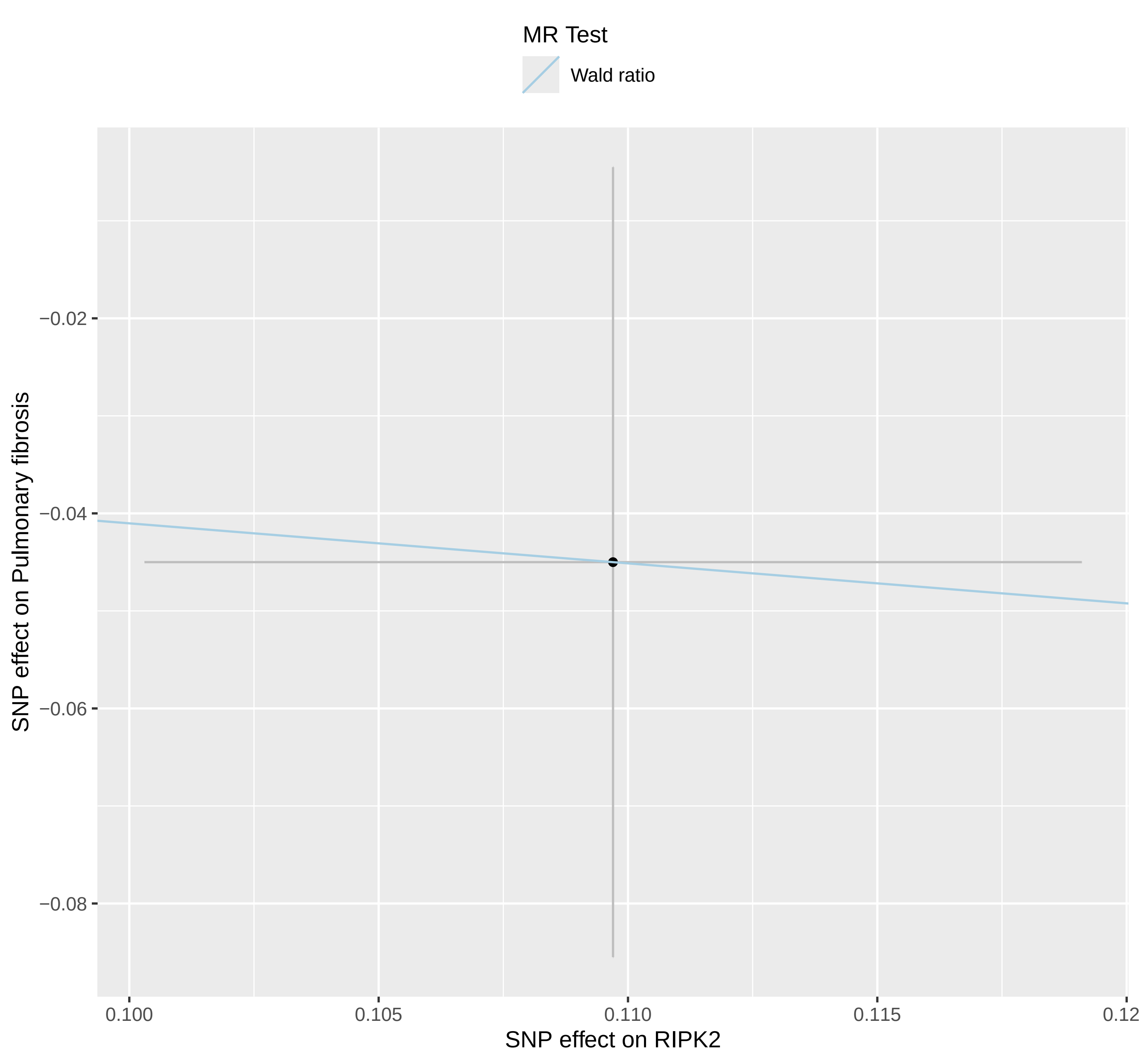

MR Test
Wald ratio
−0.02
−0.04
−0.06
−0.08
SNP effect on Pulmonary fibrosis
0.100
0.105
0.110
0.115
0.12
SNP effect on RIPK2

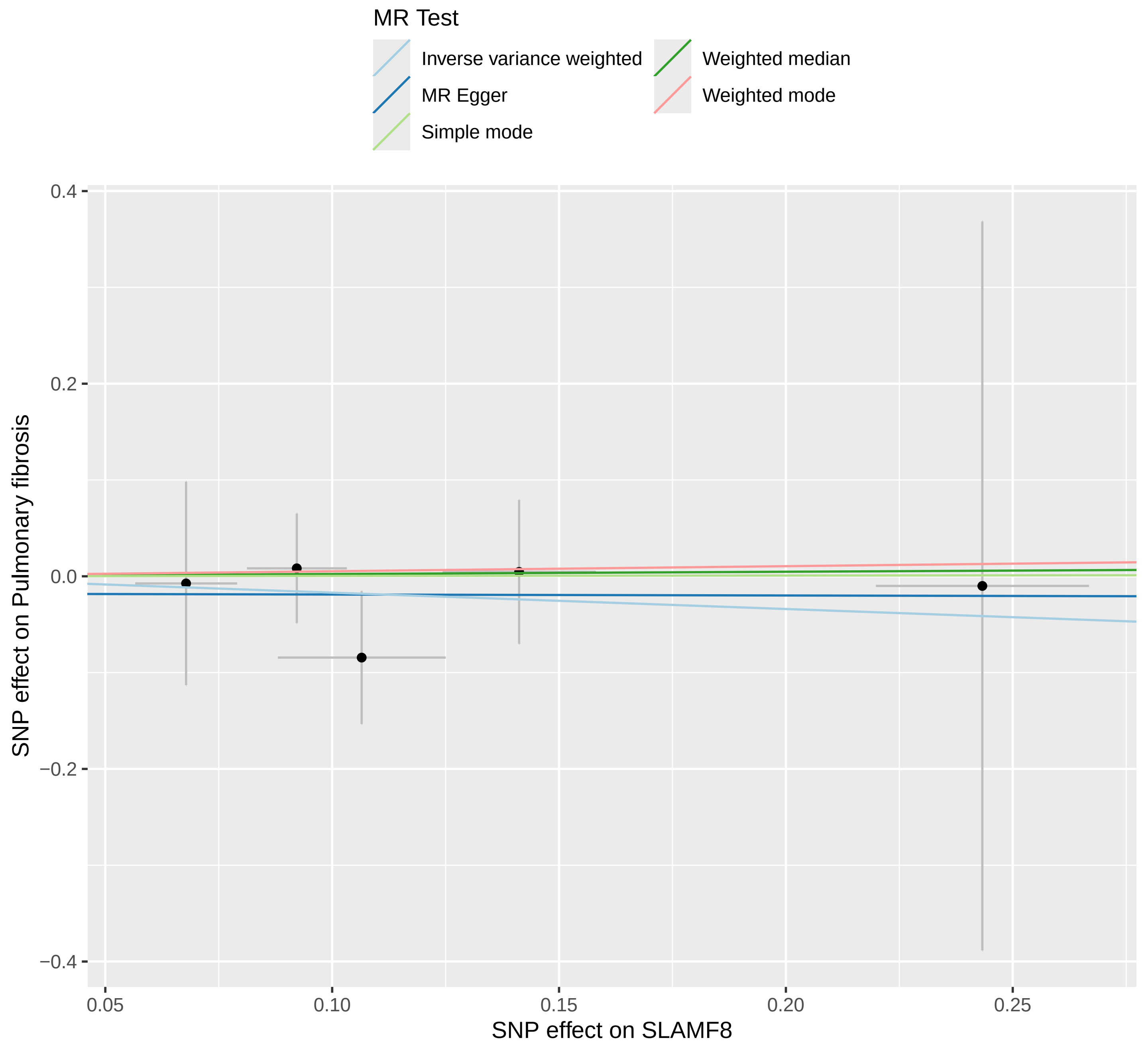

MR Test
Inverse variance weighted
MR Egger
Simple mode
Weighted median
Weighted mode
0.4
0.2
0.0
−0.2
−0.4
SNP effect on Pulmonary fibrosis
0.05
0.10
0.15
0.20
0.25
SNP effect on SLAMF8

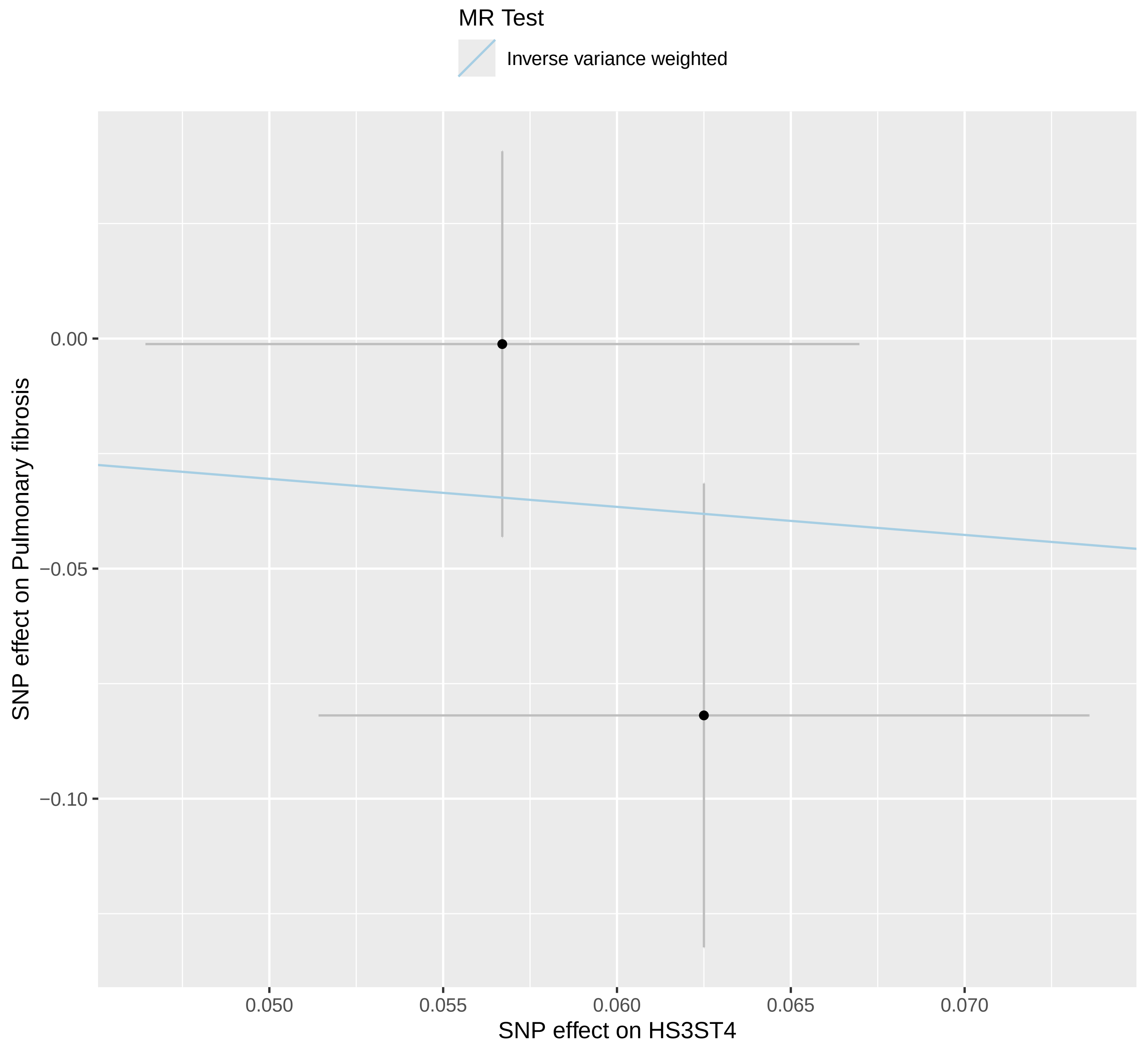

MR Test
Inverse variance weighted
0.00
−0.05
−0.10
0.050
0.055
0.060
0.065
0.070
SNP effect on HS3ST4
SNP effect on Pulmonary fibrosis

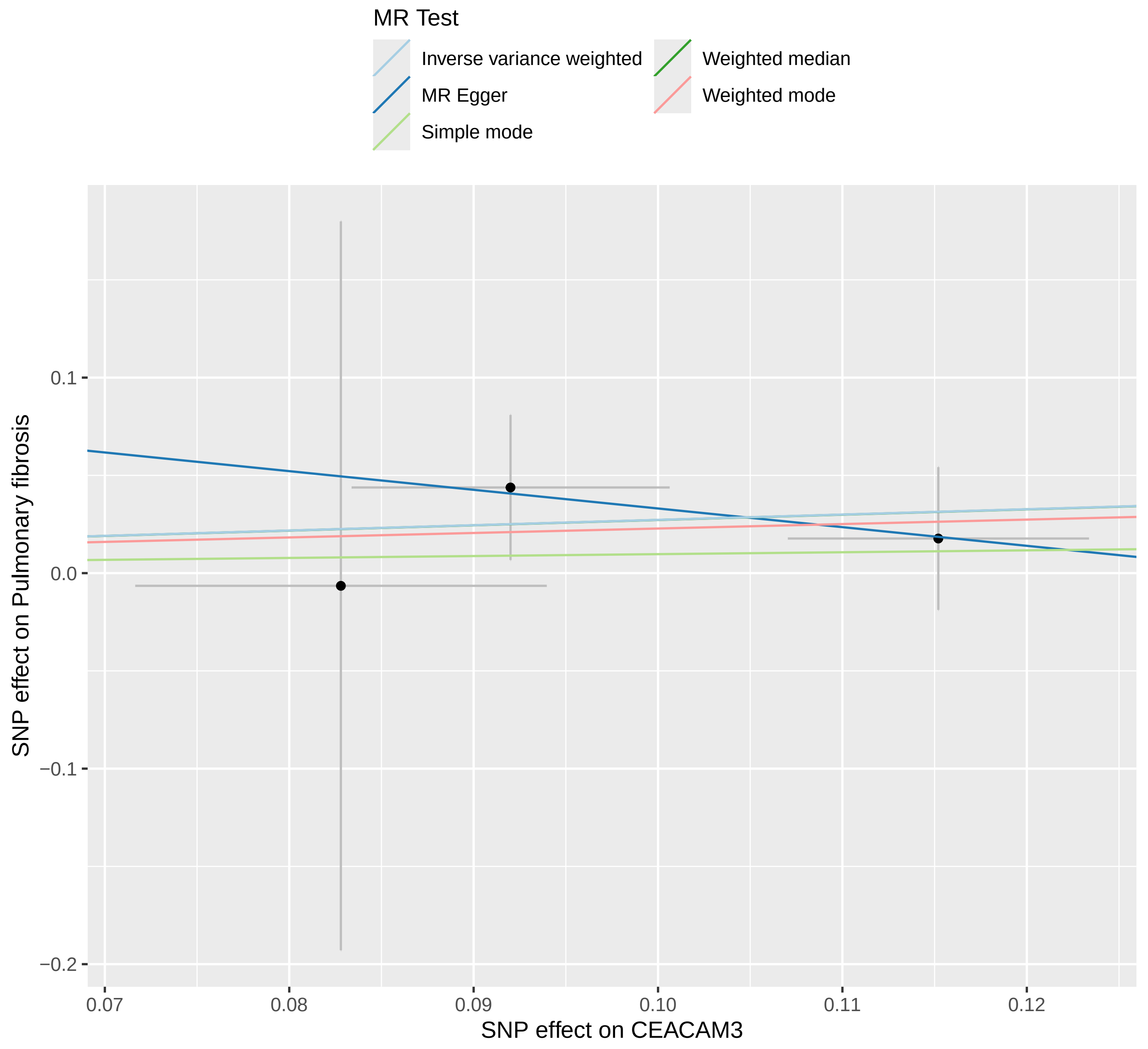

MR Test
Inverse variance weighted
MR Egger
Simple mode
Weighted median
Weighted mode
0.1
0.0
−0.1
−0.2
SNP effect on Pulmonary fibrosis
0.07
0.08
0.09
0.10
0.11
0.12
SNP effect on CEACAM3

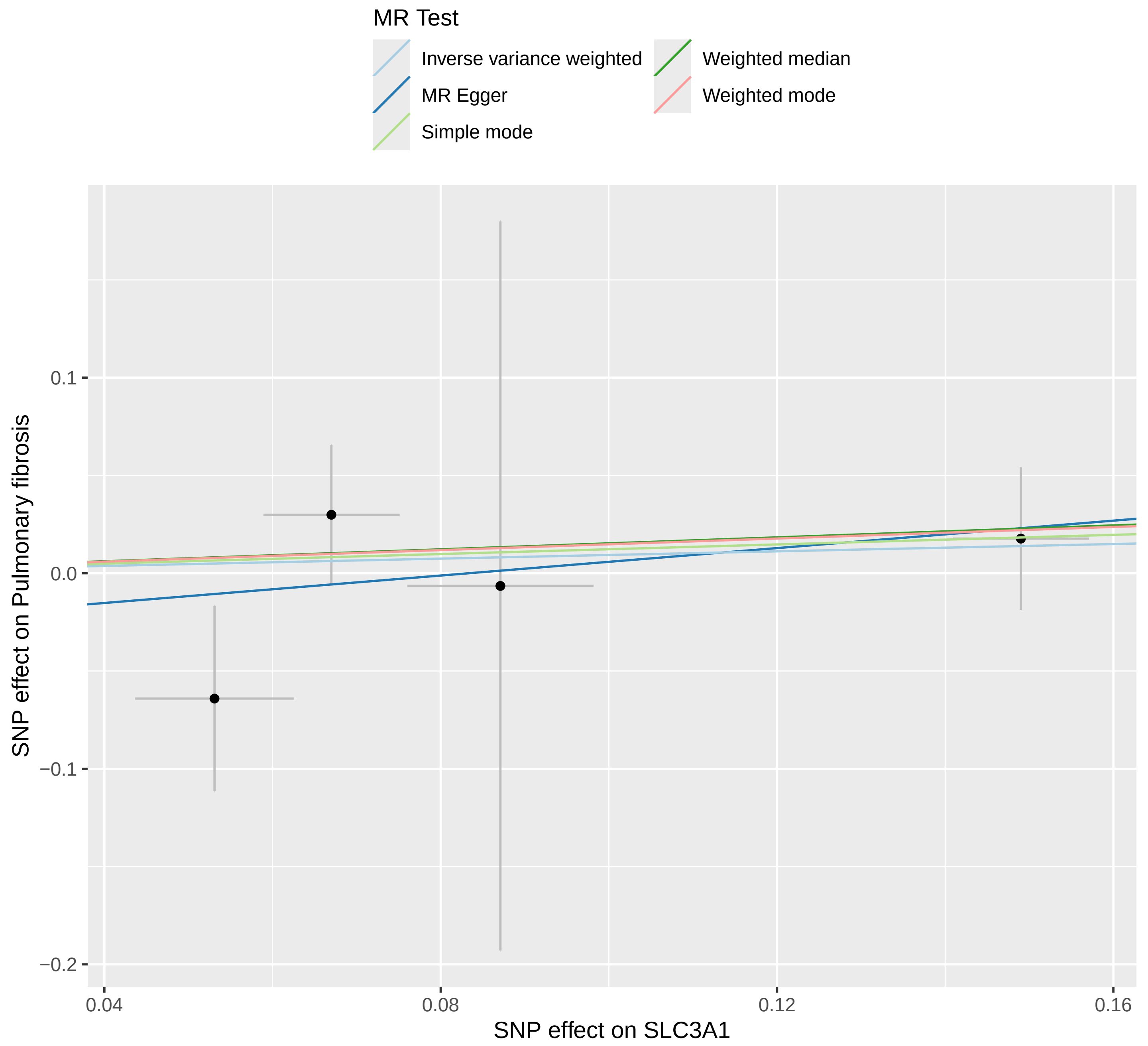

MR Test
Inverse variance weighted
MR Egger
Simple mode
Weighted median
Weighted mode
0.1
0.0
−0.1
−0.2
SNP effect on Pulmonary fibrosis
0.04
0.08
0.12
0.16
SNP effect on SLC3A1

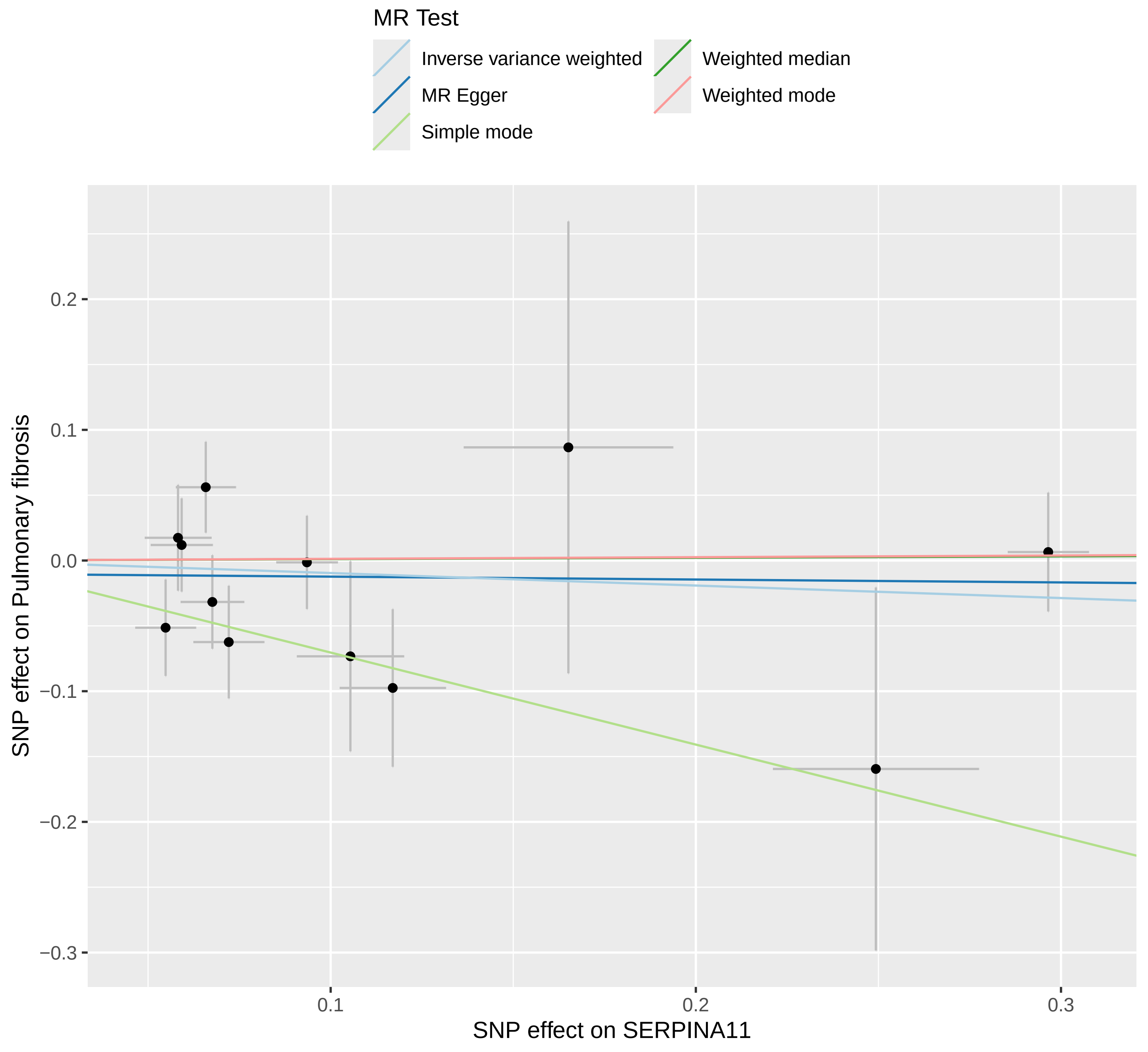

MR Test
Inverse variance weighted
MR Egger
Simple mode
Weighted median
Weighted mode
SNP effect on Pulmonary fibrosis
0.2
0.1
0.0
−0.1
−0.2
−0.3
0.1
0.2
0.3
SNP effect on SERPINA11

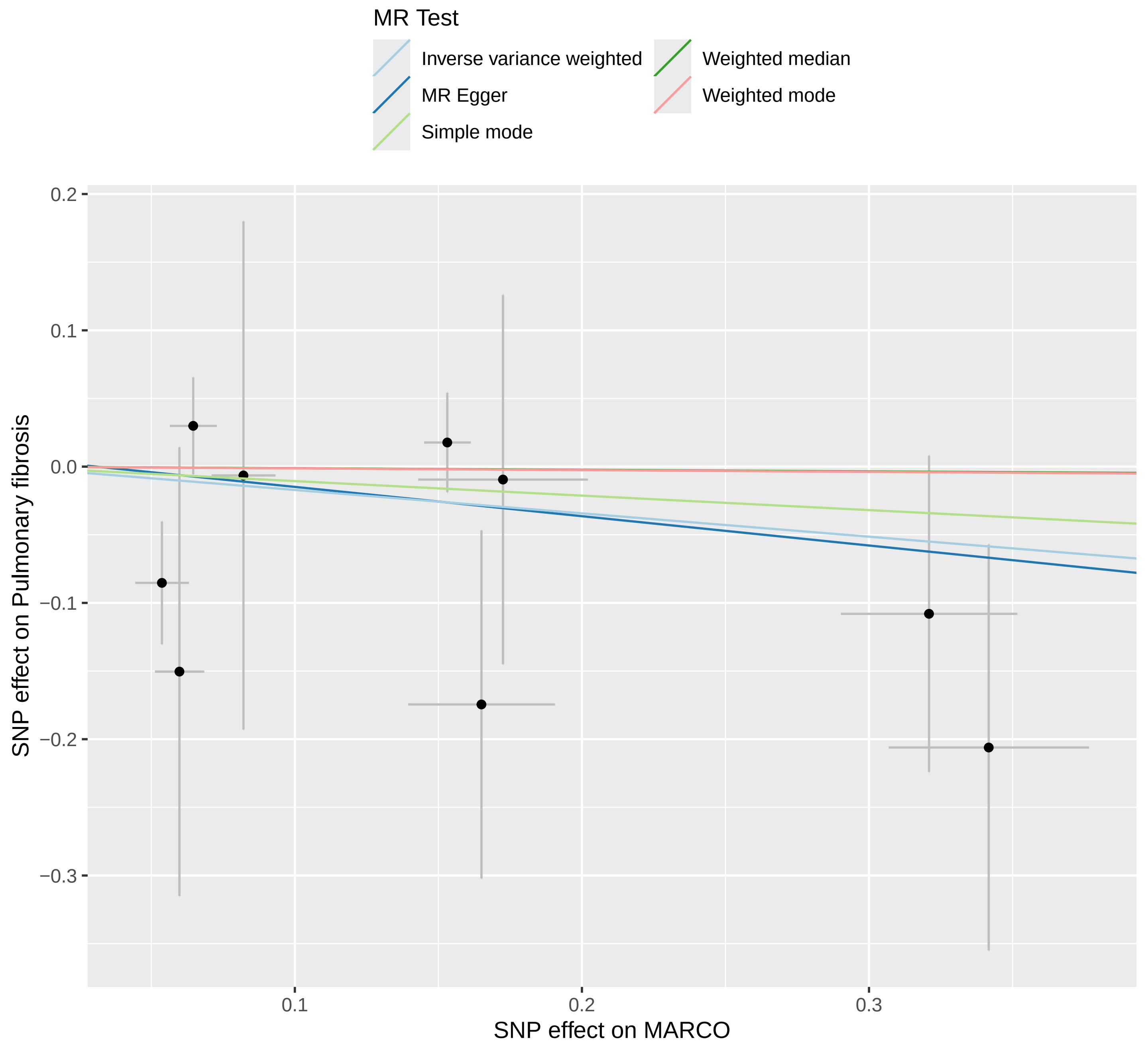
MR Test
Inverse variance weighted
MR Egger
Simple mode
Weighted median
Weighted mode
0.2
0.1
0.0
−0.1
−0.2
−0.3
SNP effect on Pulmonary fibrosis
0.1
0.2
0.3
SNP effect on MARCO

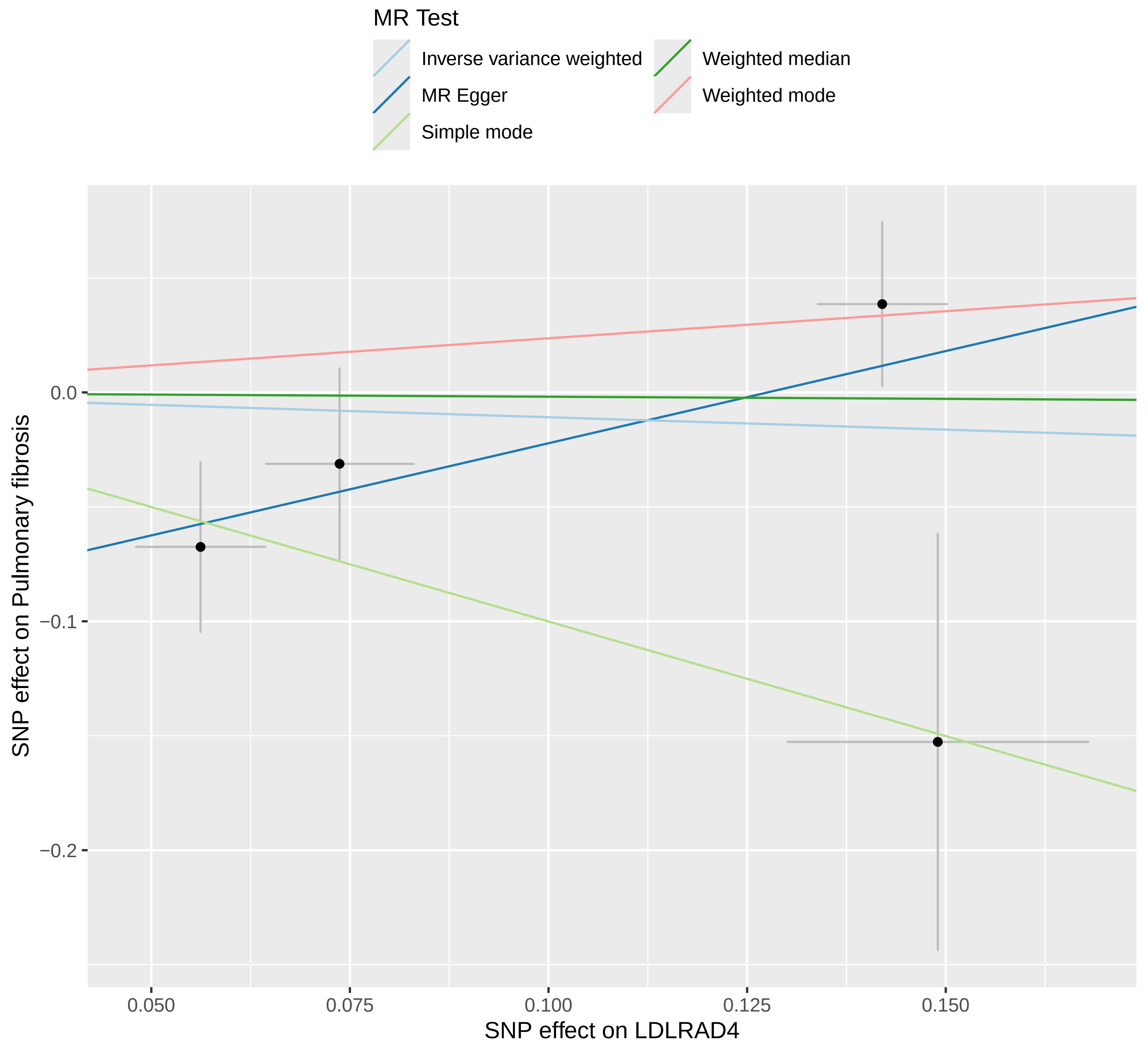
MR Test
Inverse variance weighted
MR Egger
Simple mode
Weighted median
Weighted mode
SNP effect on Pulmonary fibrosis
0.0
−0.1
−0.2
0.050
0.075
0.100
0.125
0.150
SNP effect on LDLRAD4

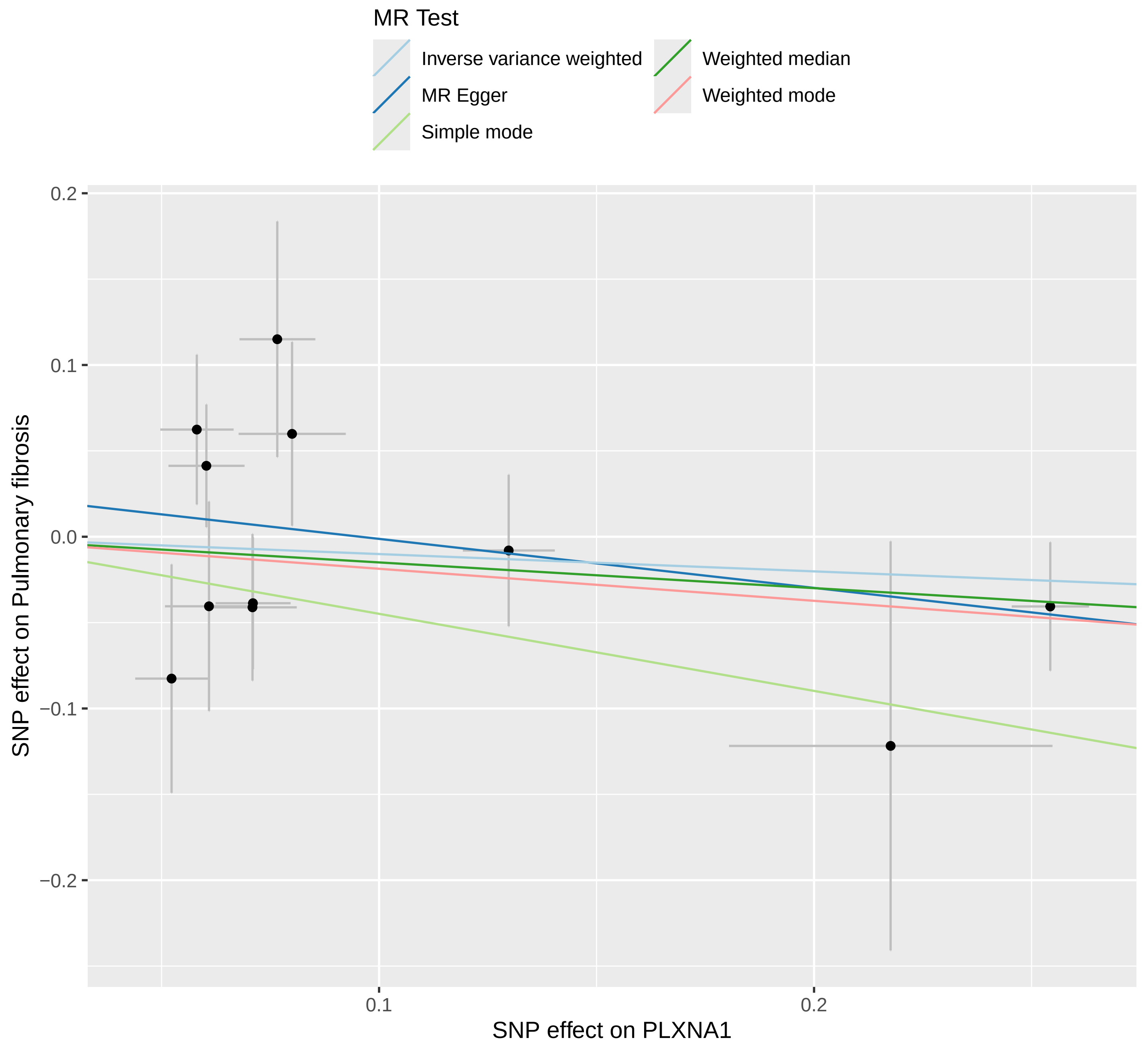

MR Test
Inverse variance weighted
MR Egger
Simple mode
Weighted median
Weighted mode
0.2
0.1
0.0
−0.1
−0.2
SNP effect on Pulmonary fibrosis
0.1
0.2
SNP effect on PLXNA1

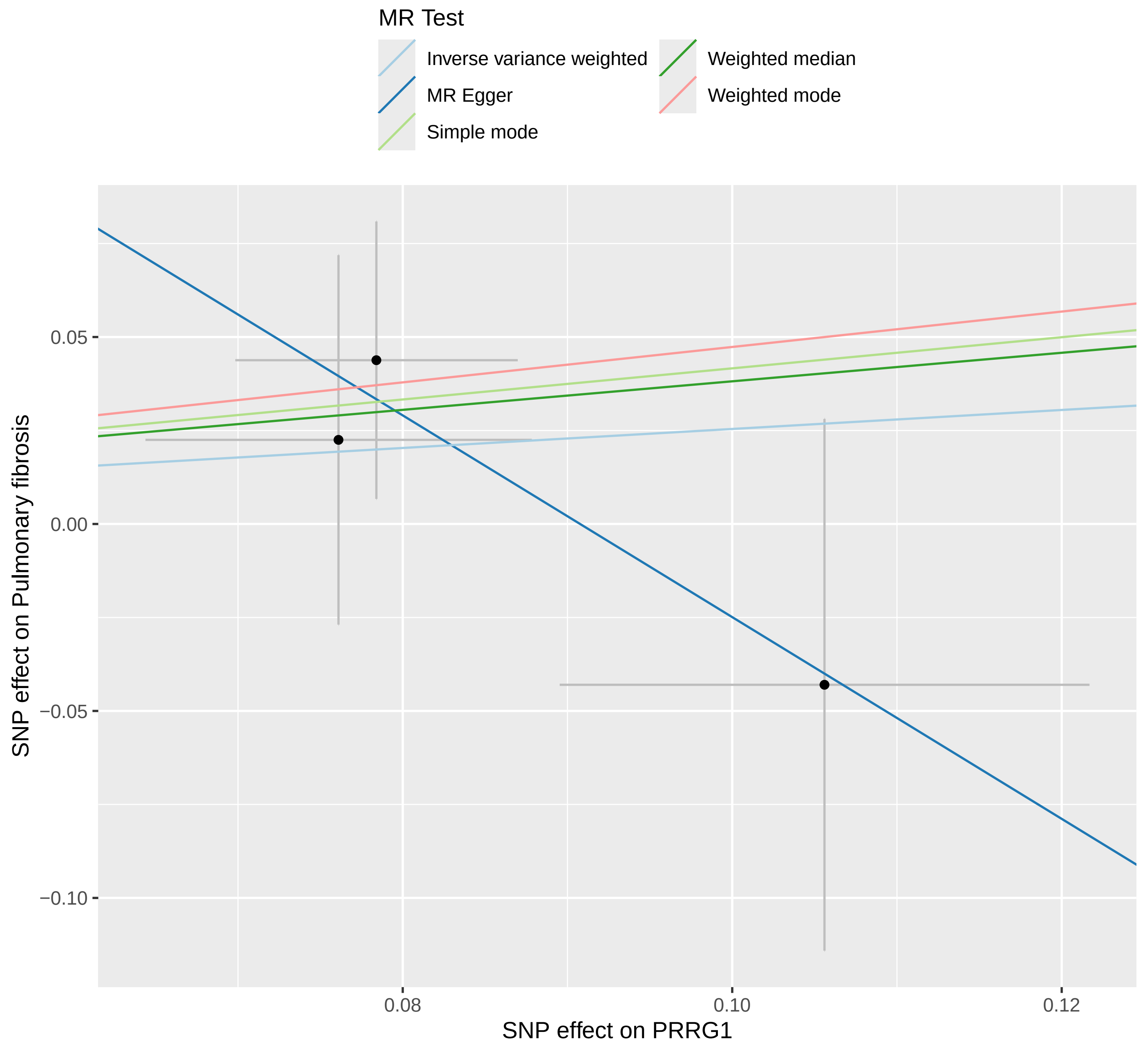
MR Test
Inverse variance weighted
MR Egger
Simple mode
Weighted median
Weighted mode
0.05
0.00
−0.05
−0.10
SNP effect on Pulmonary fibrosis
0.08
0.10
0.12
SNP effect on PRRG1

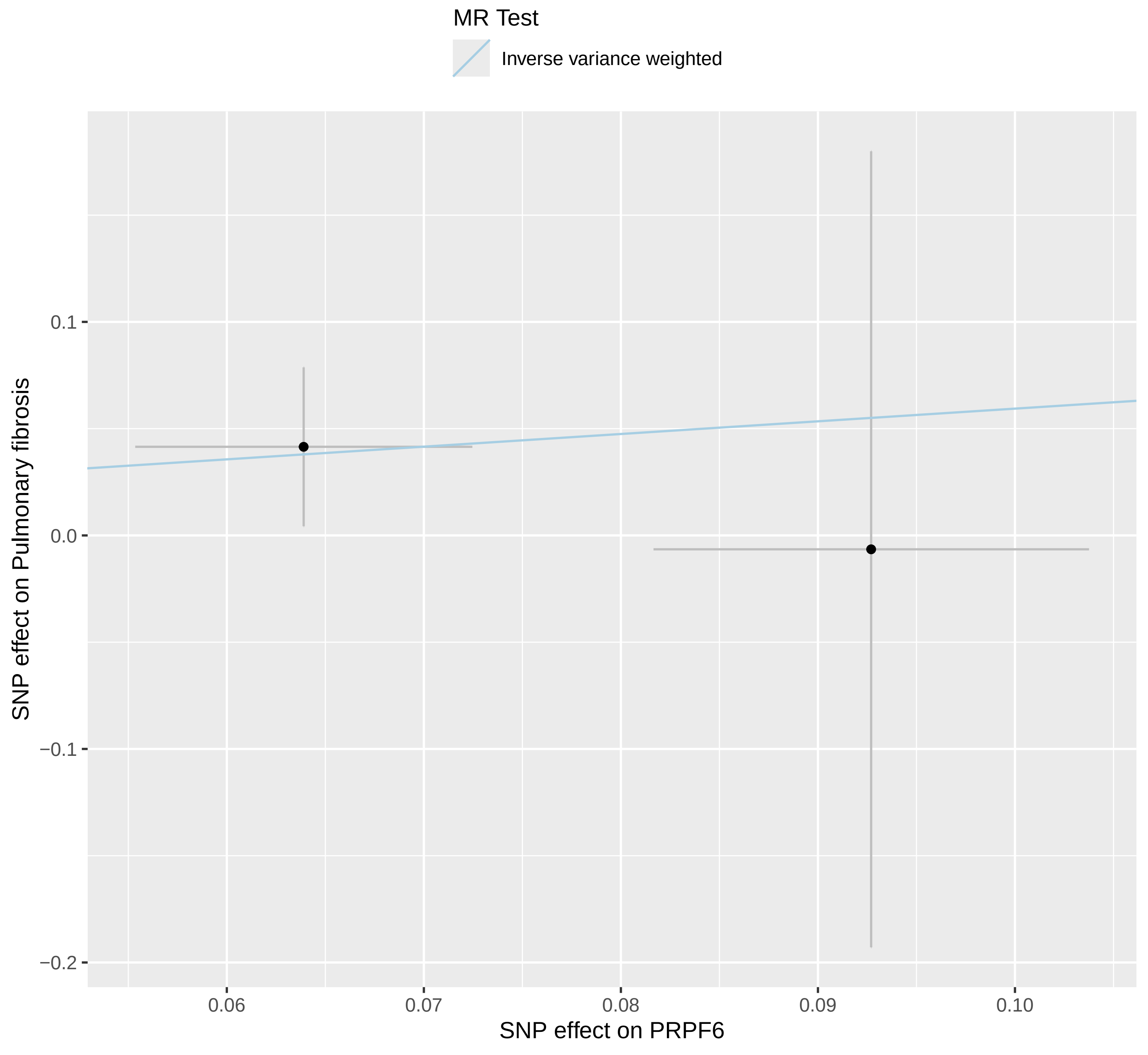

MR Test
Inverse variance weighted
SNP effect on Pulmonary fibrosis
0.1
0.0
−0.1
−0.2
0.06
0.07
0.08
0.09
0.10
SNP effect on PRPF6

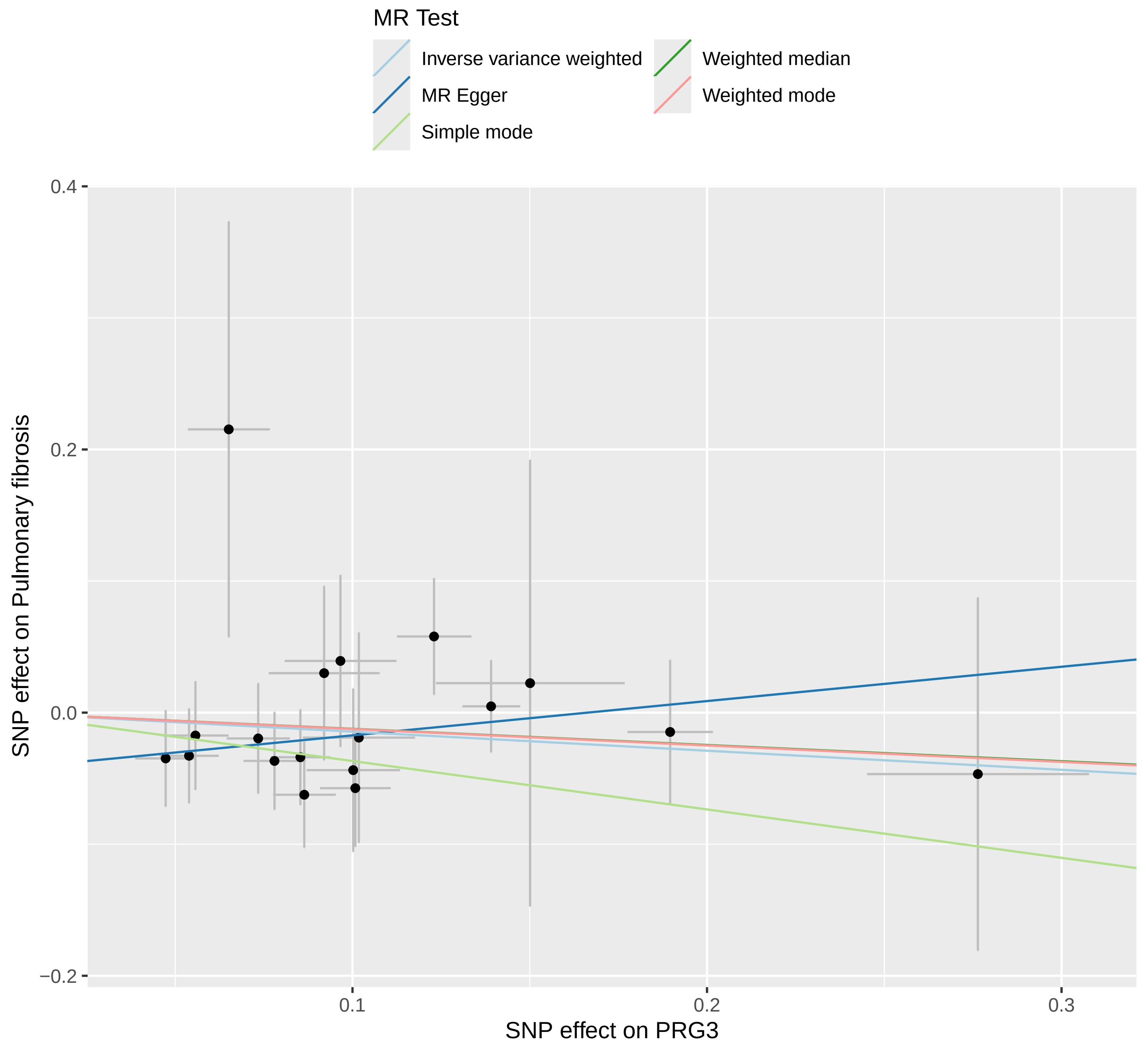

MR Test
Inverse variance weighted
MR Egger
Simple mode
Weighted median
Weighted mode
0.4
0.2
0.0
−0.2
0.1
0.2
0.3
SNP effect on Pulmonary fibrosis
SNP effect on PRG3

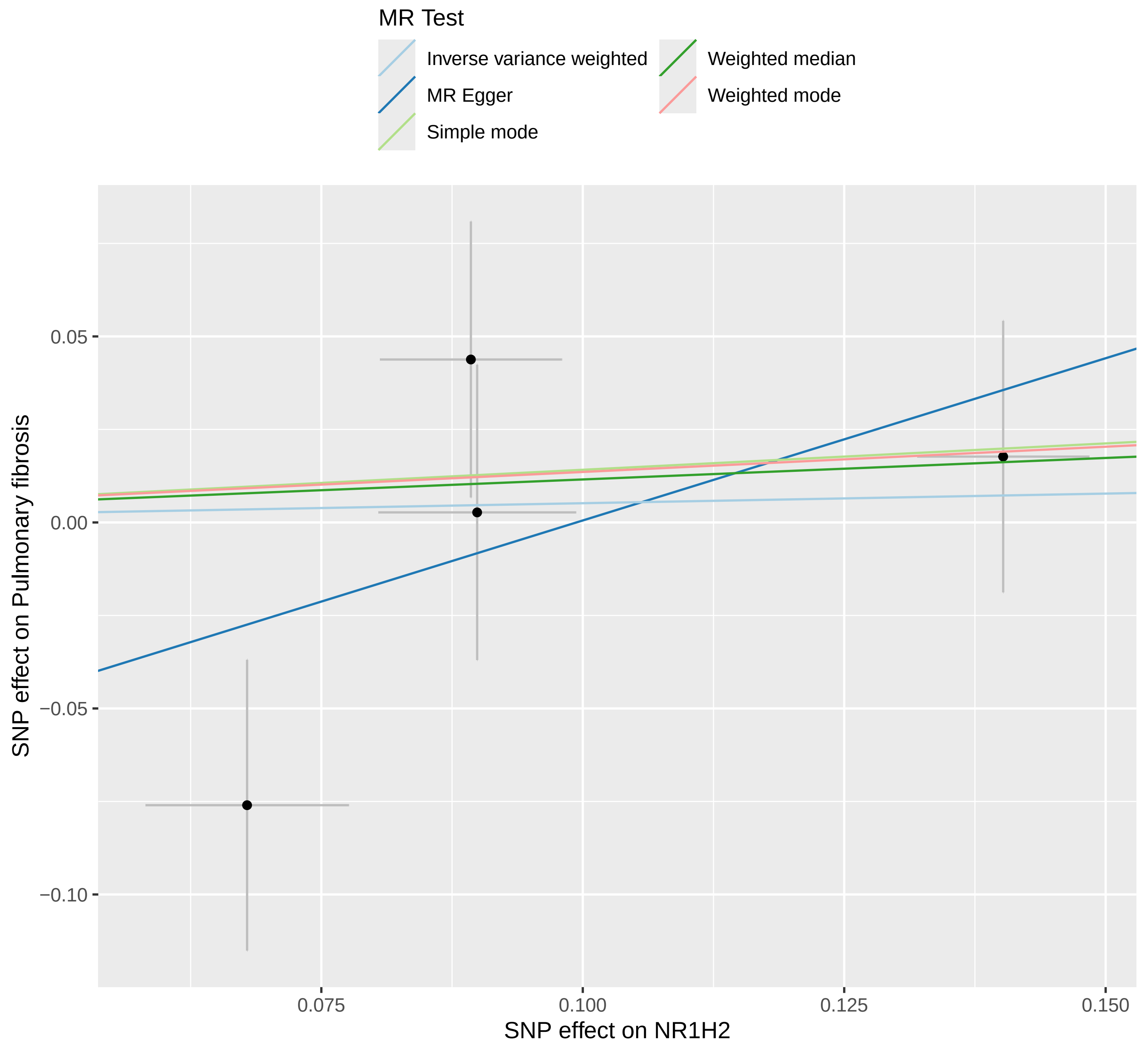

MR Test
Inverse variance weighted
MR Egger
Simple mode
Weighted median
Weighted mode
0.05
0.00
−0.05
−0.10
0.075
0.100
0.125
0.150
SNP effect on NR1H2
SNP effect on Pulmonary fibrosis

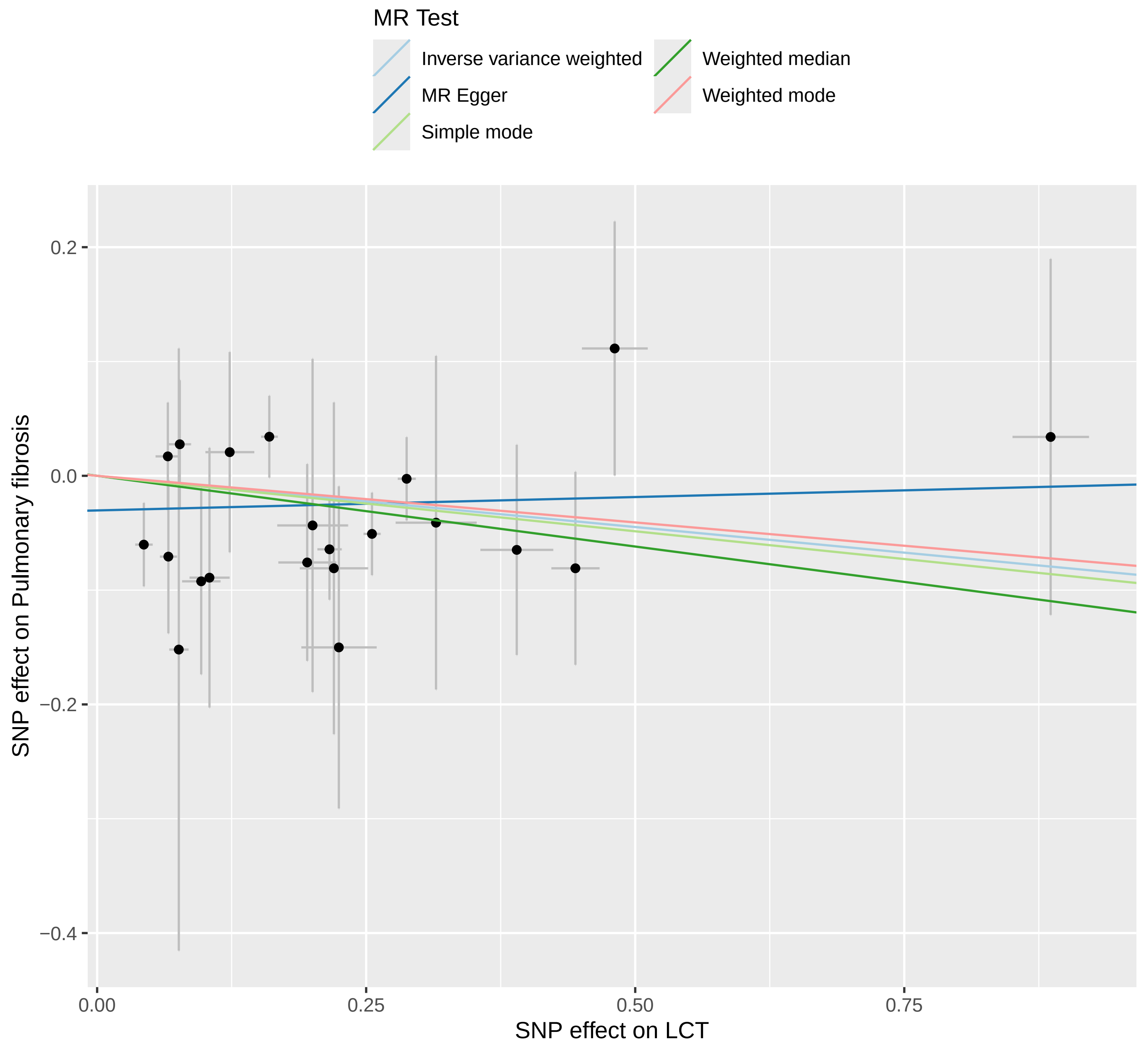

MR Test
Inverse variance weighted
MR Egger
Simple mode
Weighted median
Weighted mode
0.2
0.0
−0.2
−0.4
SNP effect on Pulmonary fibrosis
0.00
0.25
0.50
0.75
SNP effect on LCT

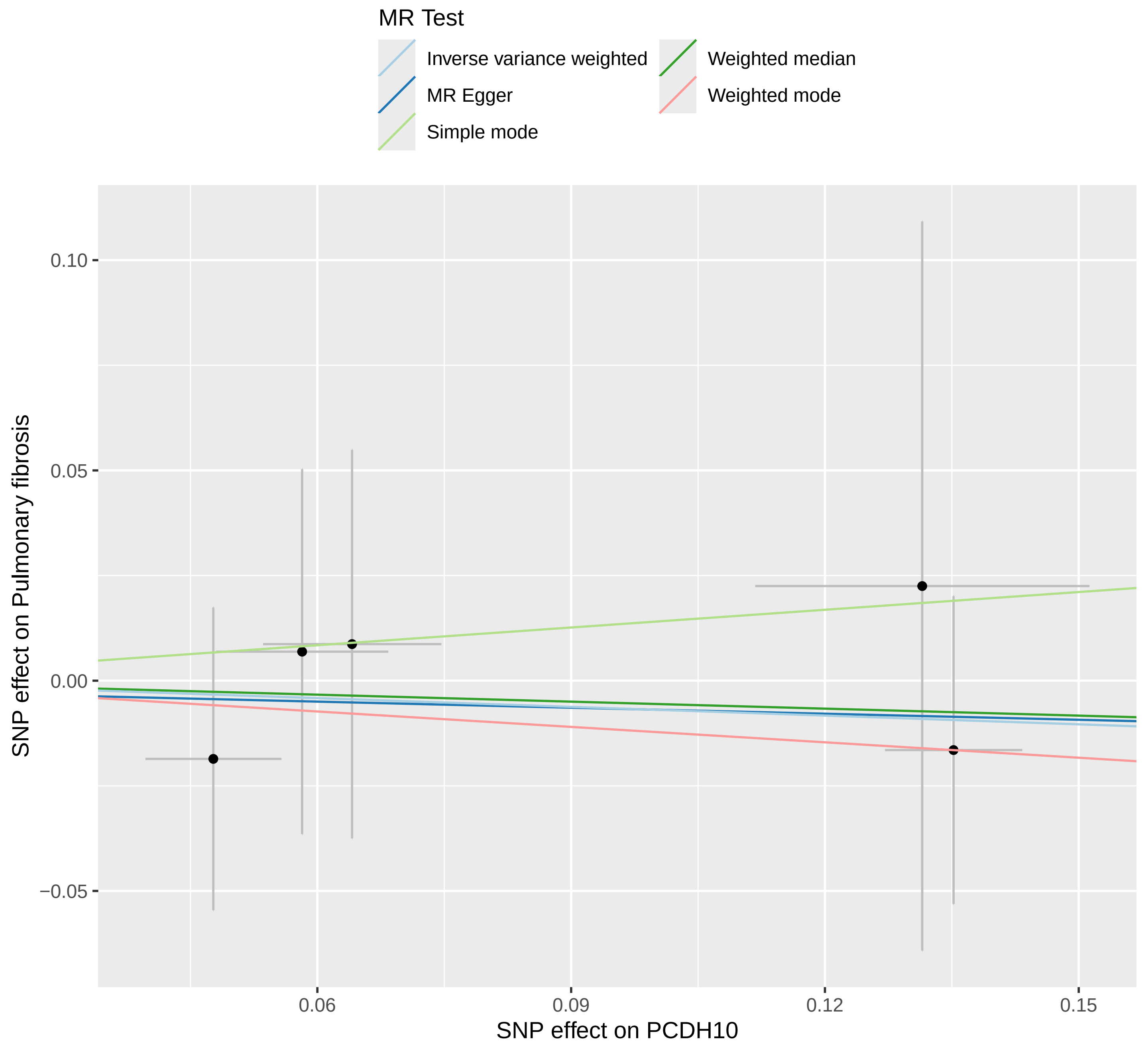

MR Test
Inverse variance weighted
MR Egger
Simple mode
Weighted median
Weighted mode
0.10
0.05
0.00
−0.05
0.06
0.09
0.12
0.15
SNP effect on Pulmonary fibrosis
SNP effect on PCDH10

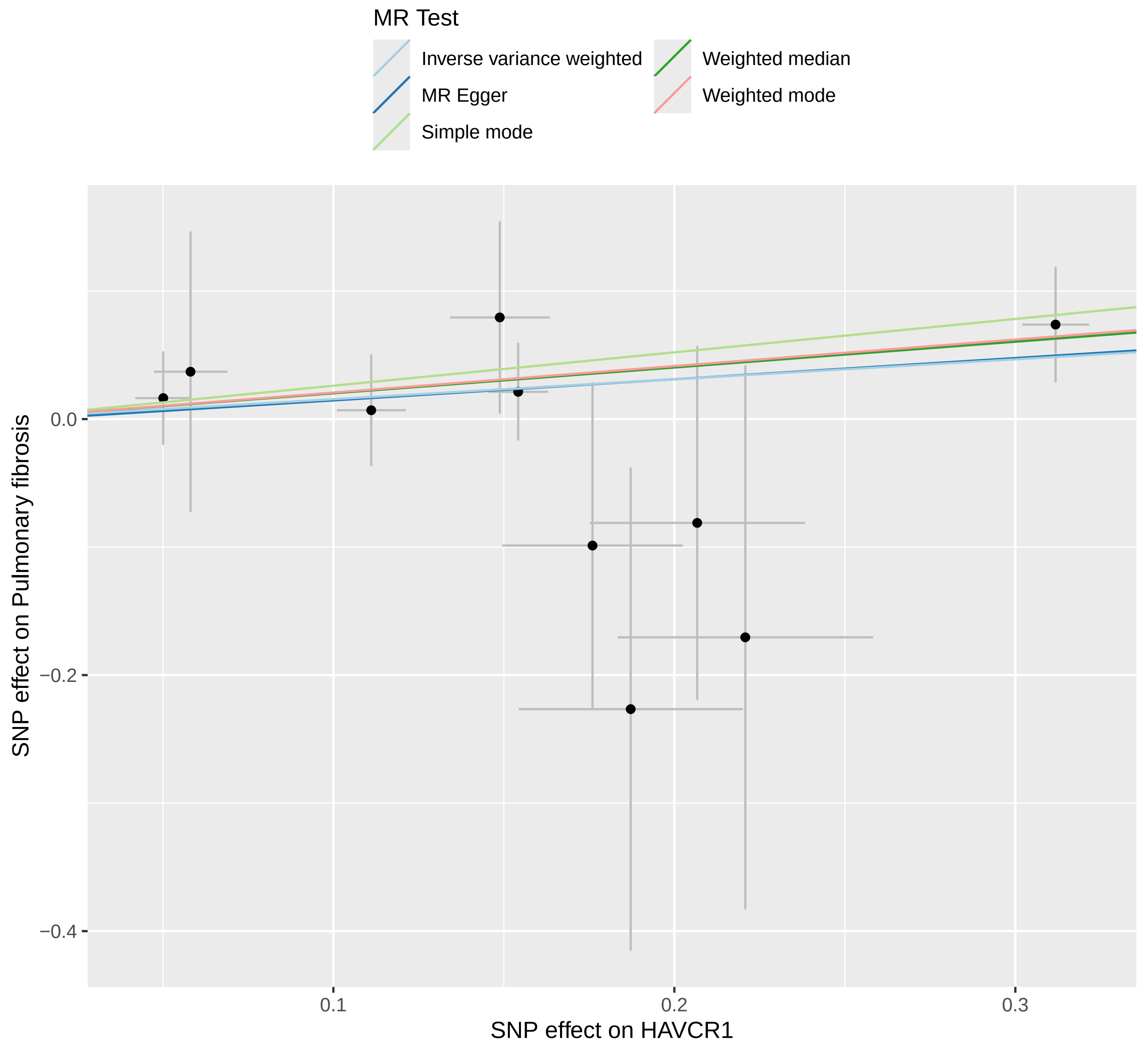
MR Test
Inverse variance weighted
MR Egger
Simple mode
Weighted median
Weighted mode
SNP effect on Pulmonary fibrosis
0.0
−0.2
−0.4
0.1
0.2
0.3
SNP effect on HAVCR1

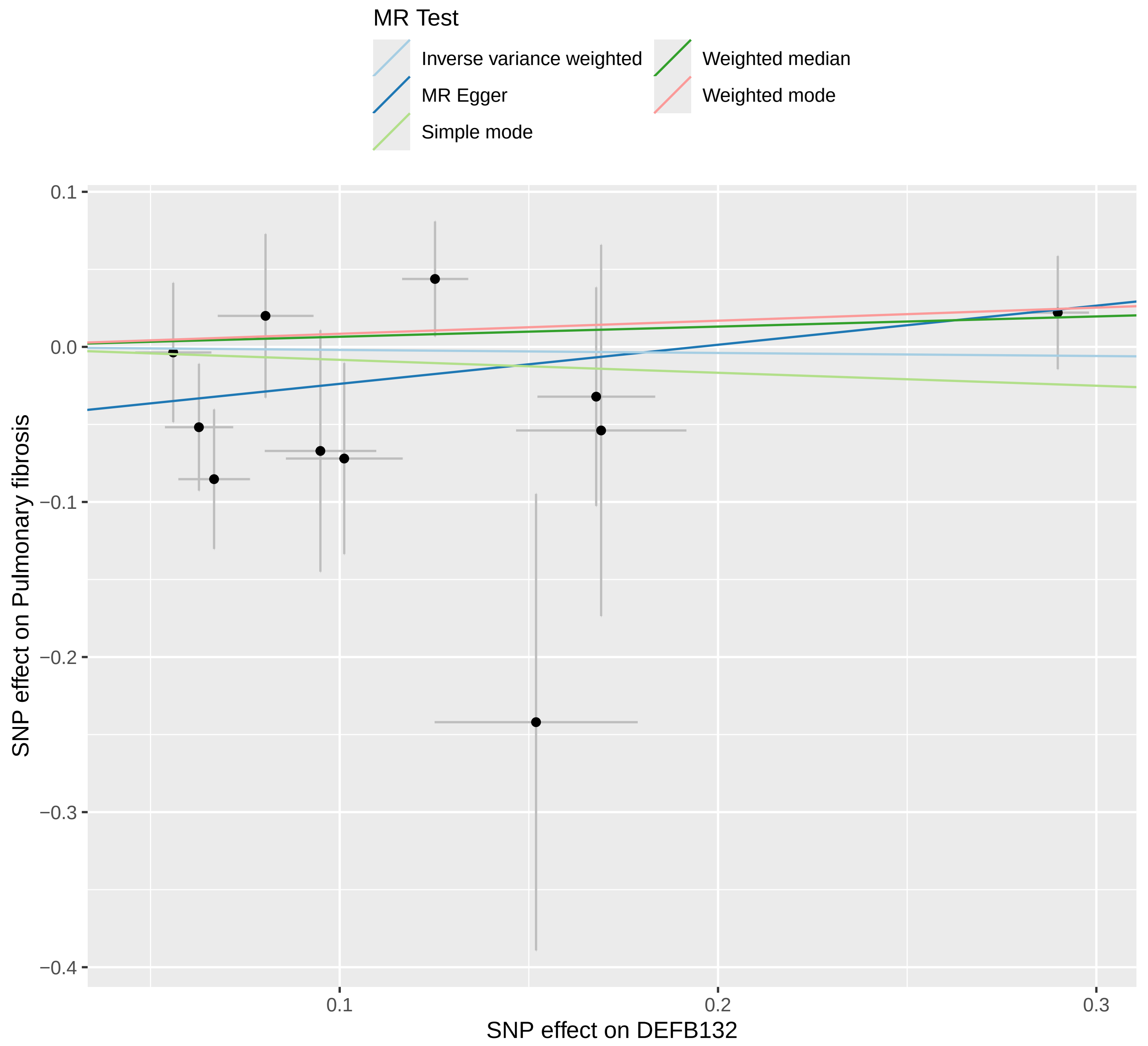

MR Test
Inverse variance weighted
MR Egger
Simple mode
Weighted median
Weighted mode
0.1
0.0
−0.1
−0.2
−0.3
−0.4
SNP effect on Pulmonary fibrosis
0.1
0.2
0.3
SNP effect on DEFB132

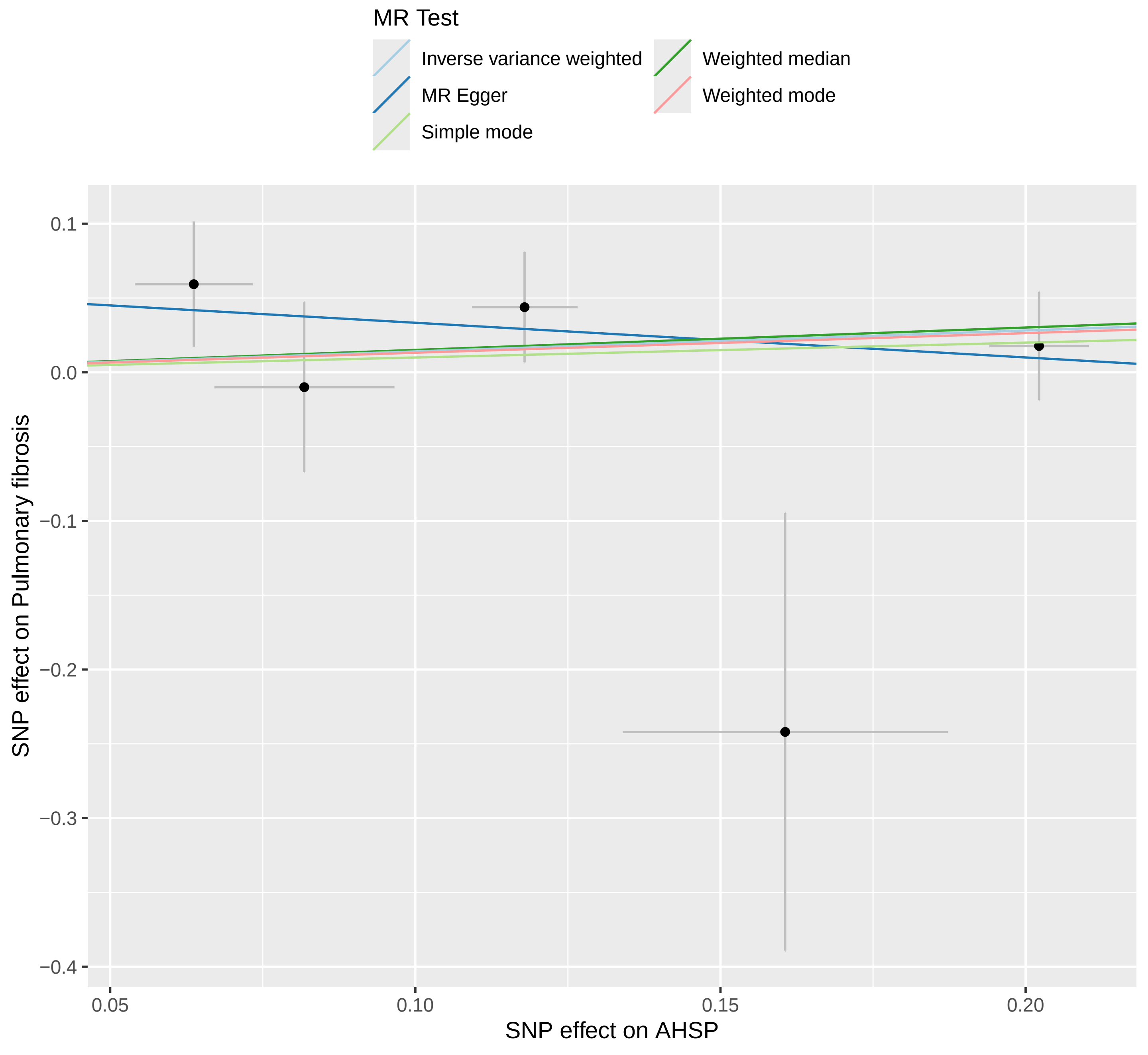

MR Test
Inverse variance weighted
MR Egger
Simple mode
Weighted median
Weighted mode
0.1
0.0
−0.1
−0.2
−0.3
−0.4
SNP effect on Pulmonary fibrosis
0.05
0.10
0.15
0.20
SNP effect on AHSP

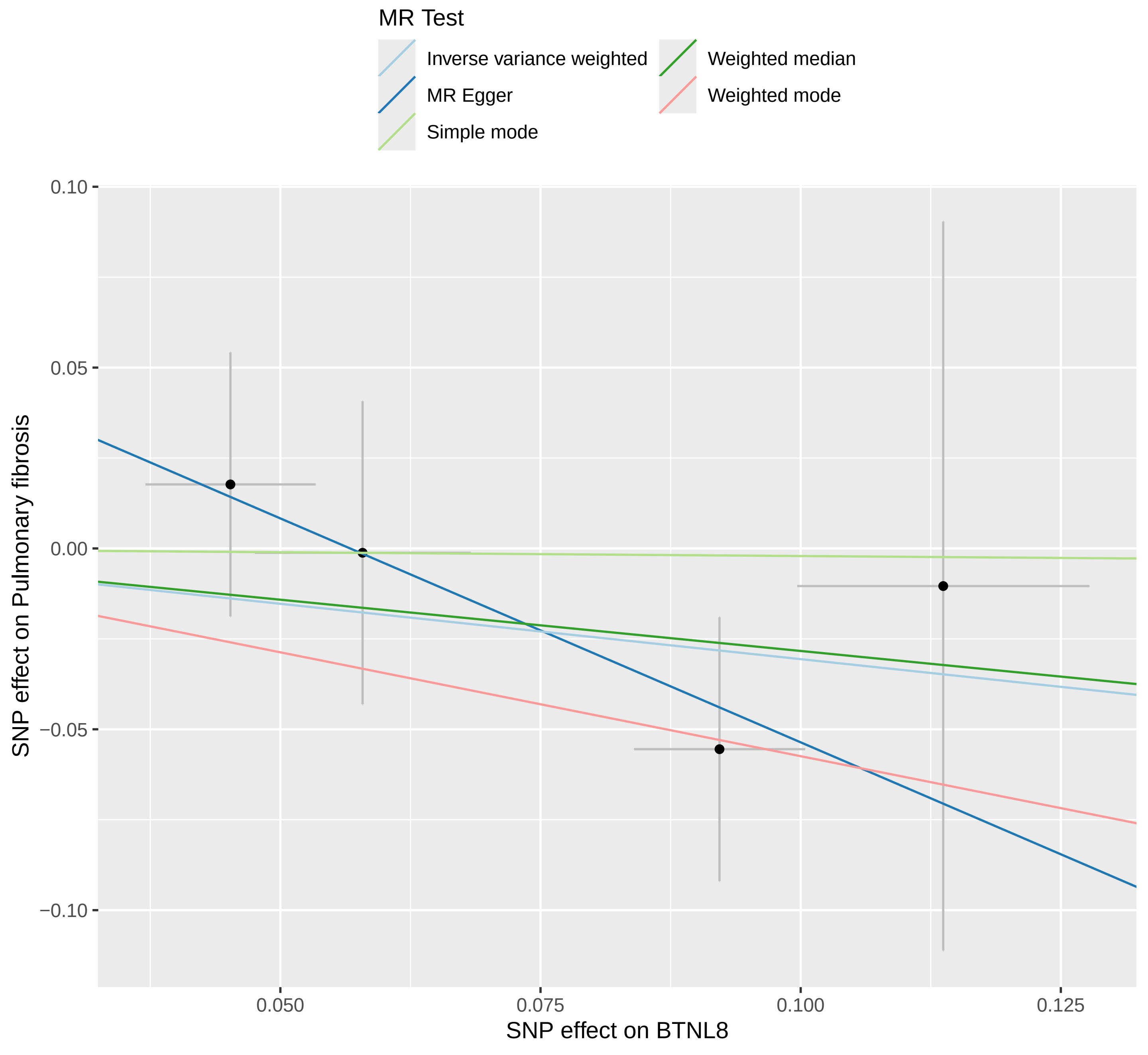
MR Test
Inverse variance weighted
MR Egger
Simple mode
Weighted median
Weighted mode
0.10
0.05
0.00
−0.05
−0.10
0.050
0.075
0.100
0.125
SNP effect on Pulmonary fibrosis
SNP effect on BTNL8

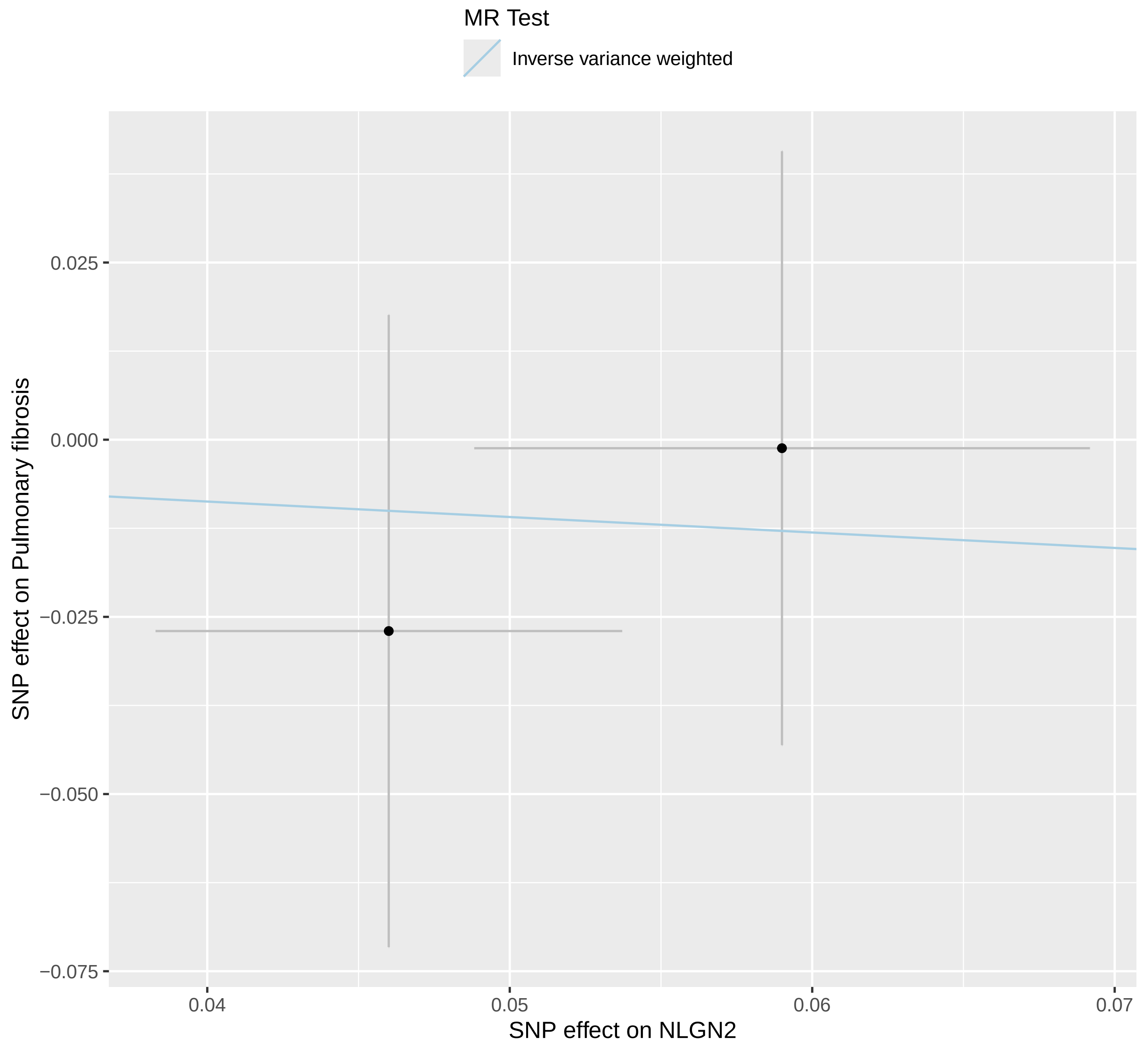
MR Test
Inverse variance weighted
0.025
0.000
−0.025
−0.050
−0.075
0.04
0.05
0.06
0.07
SNP effect on Pulmonary fibrosis
SNP effect on NLGN2

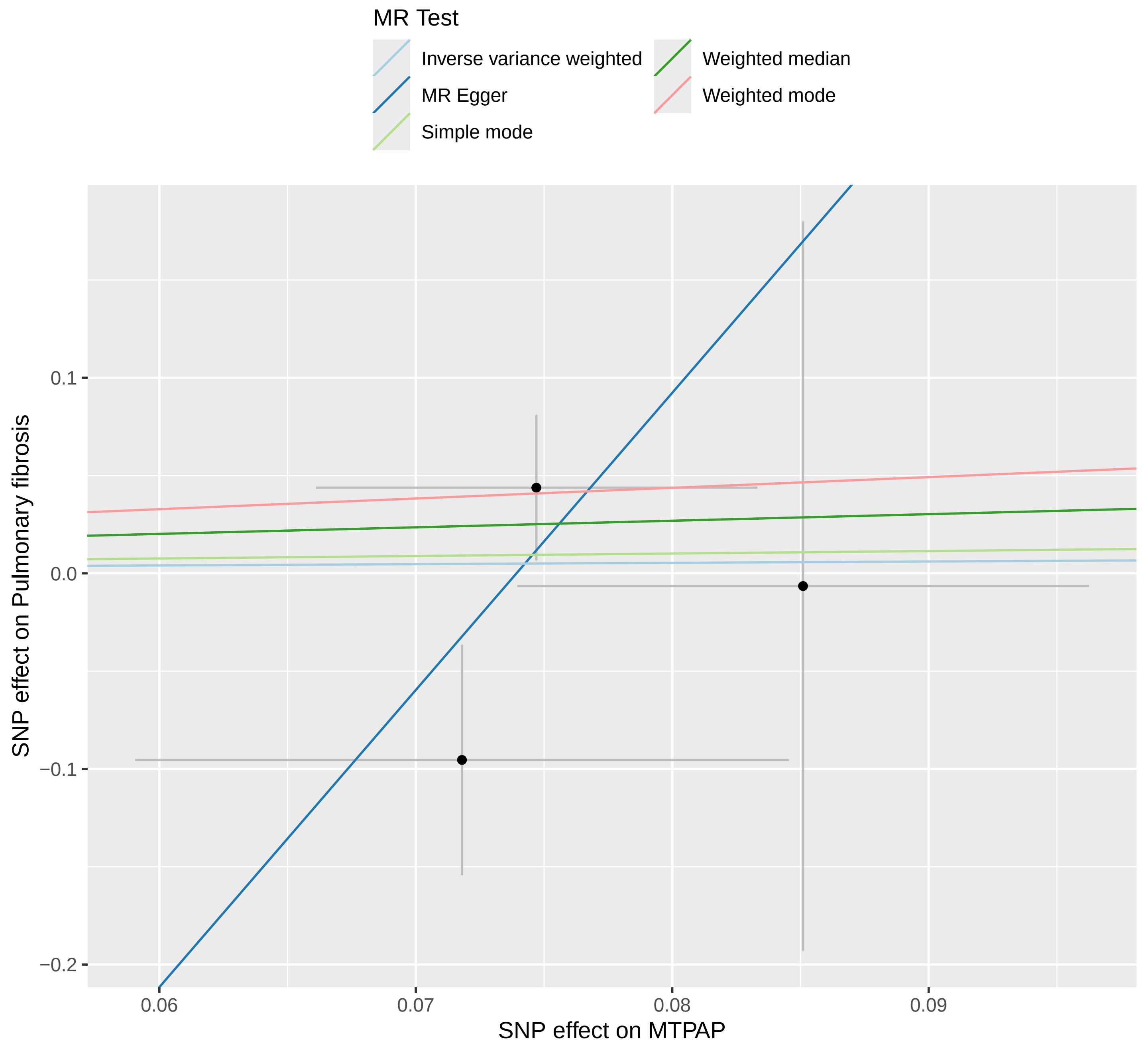

MR Test
Inverse variance weighted
MR Egger
Simple mode
Weighted median
Weighted mode
SNP effect on Pulmonary fibrosis
SNP effect on MTPAP
0.1
0.0
−0.1
−0.2
0.06
0.07
0.08
0.09

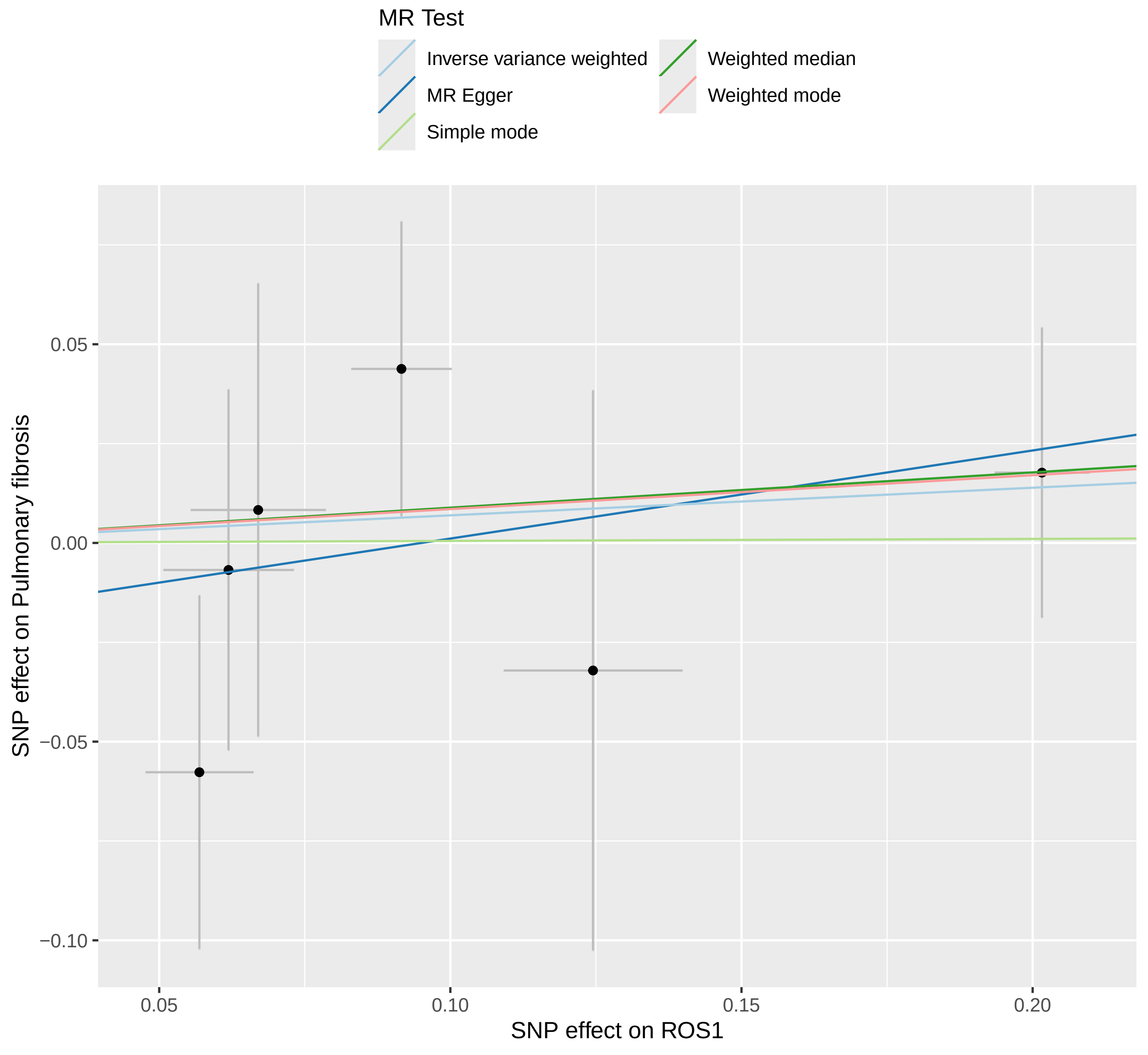

MR Test
Inverse variance weighted
MR Egger
Simple mode
Weighted median
Weighted mode
SNP effect on Pulmonary fibrosis
0.05
0.00
−0.05
−0.10
0.05
0.10
0.15
0.20
SNP effect on ROS1

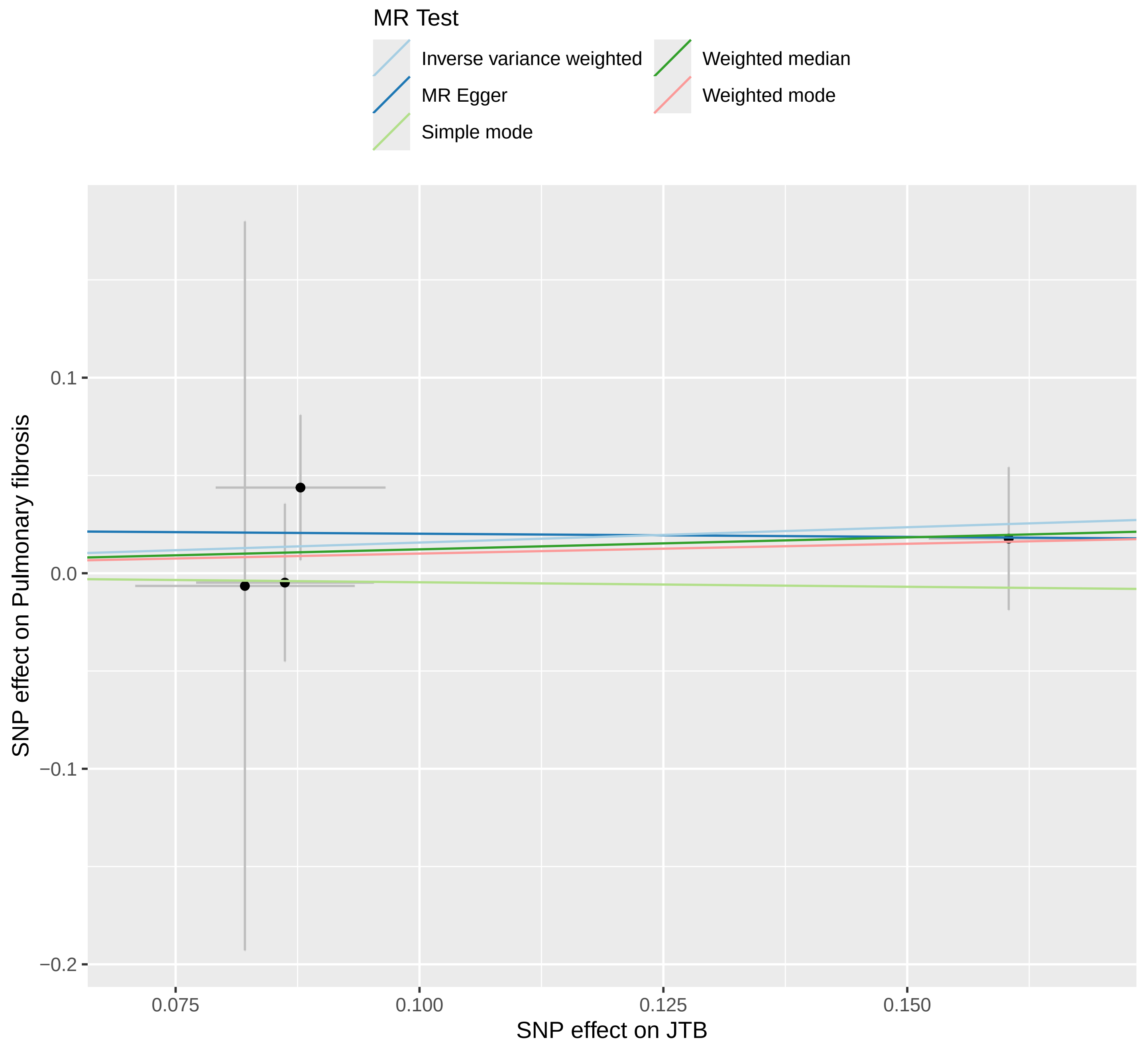
MR Test
Inverse variance weighted
MR Egger
Simple mode
Weighted median
Weighted mode
0.1
0.0
−0.1
−0.2
SNP effect on Pulmonary fibrosis
0.075
0.100
0.125
0.150
SNP effect on JTB

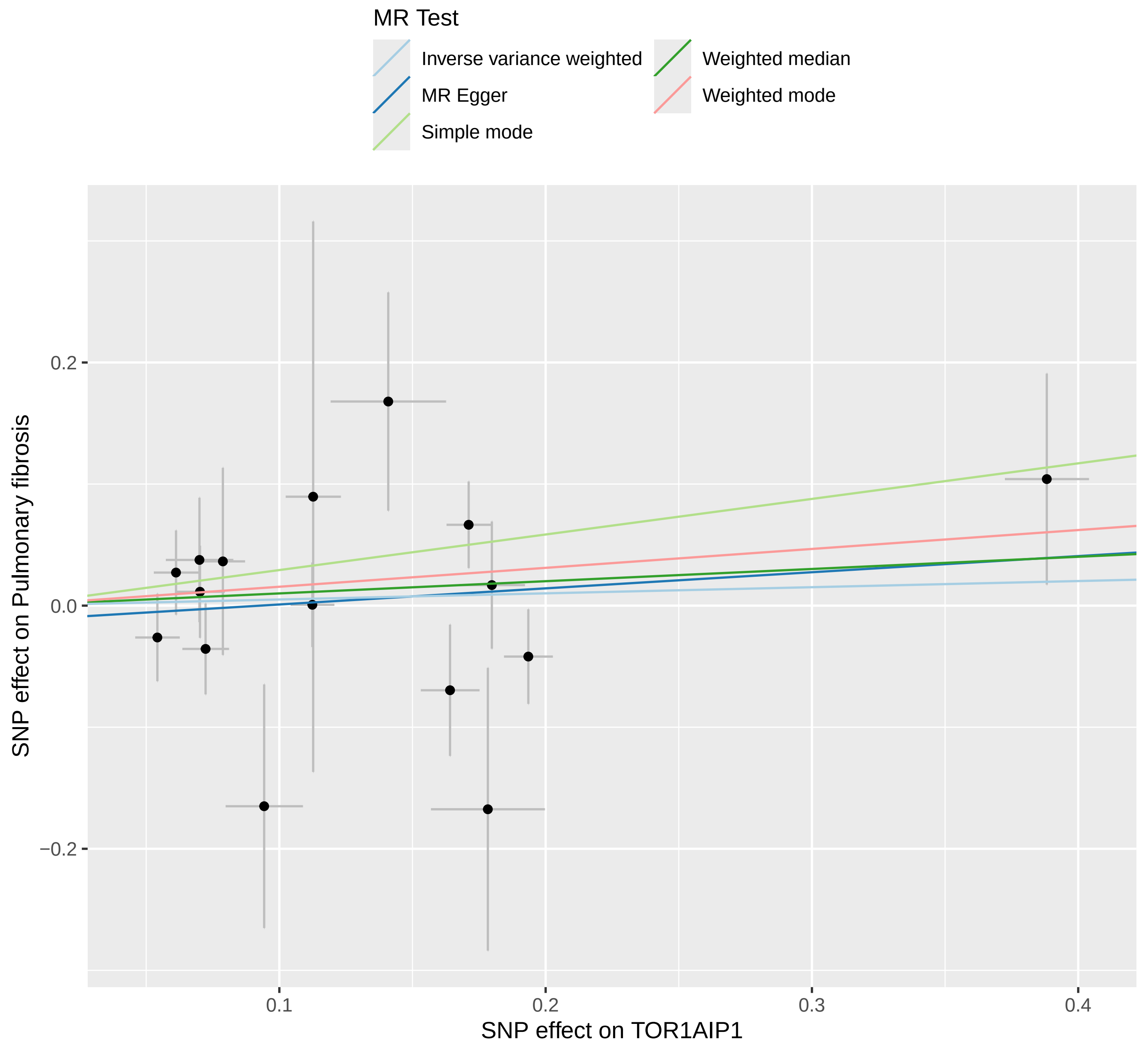
MR Test
Inverse variance weighted
MR Egger
Simple mode
Weighted median
Weighted mode
0.2
0.0
−0.2
0.1
0.2
0.3
0.4
SNP effect on Pulmonary fibrosis
SNP effect on TOR1AIP1

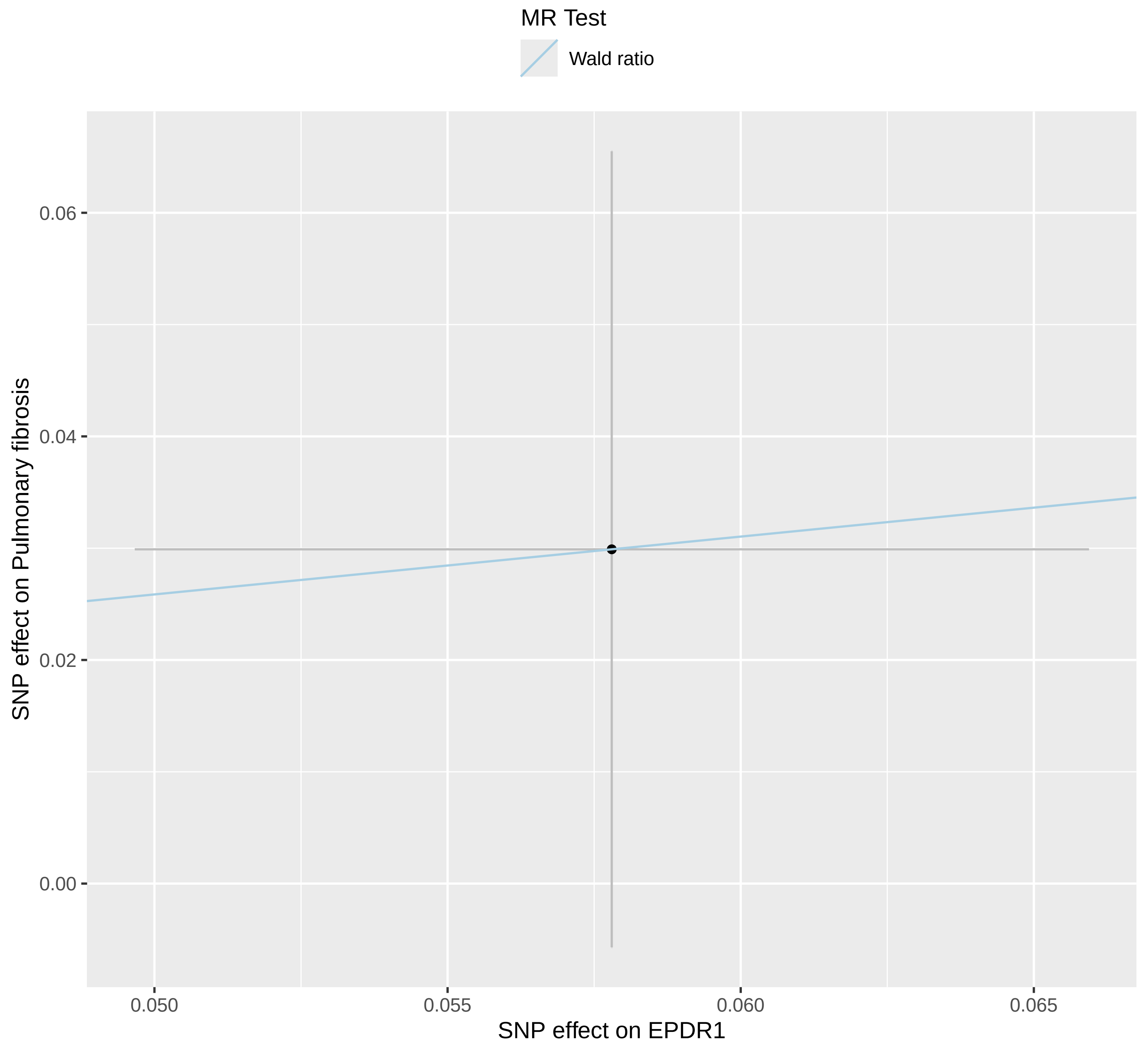

MR Test
Wald ratio
0.06
0.04
0.02
0.00
0.050
0.055
0.060
0.065
SNP effect on EPDR1
SNP effect on Pulmonary fibrosis

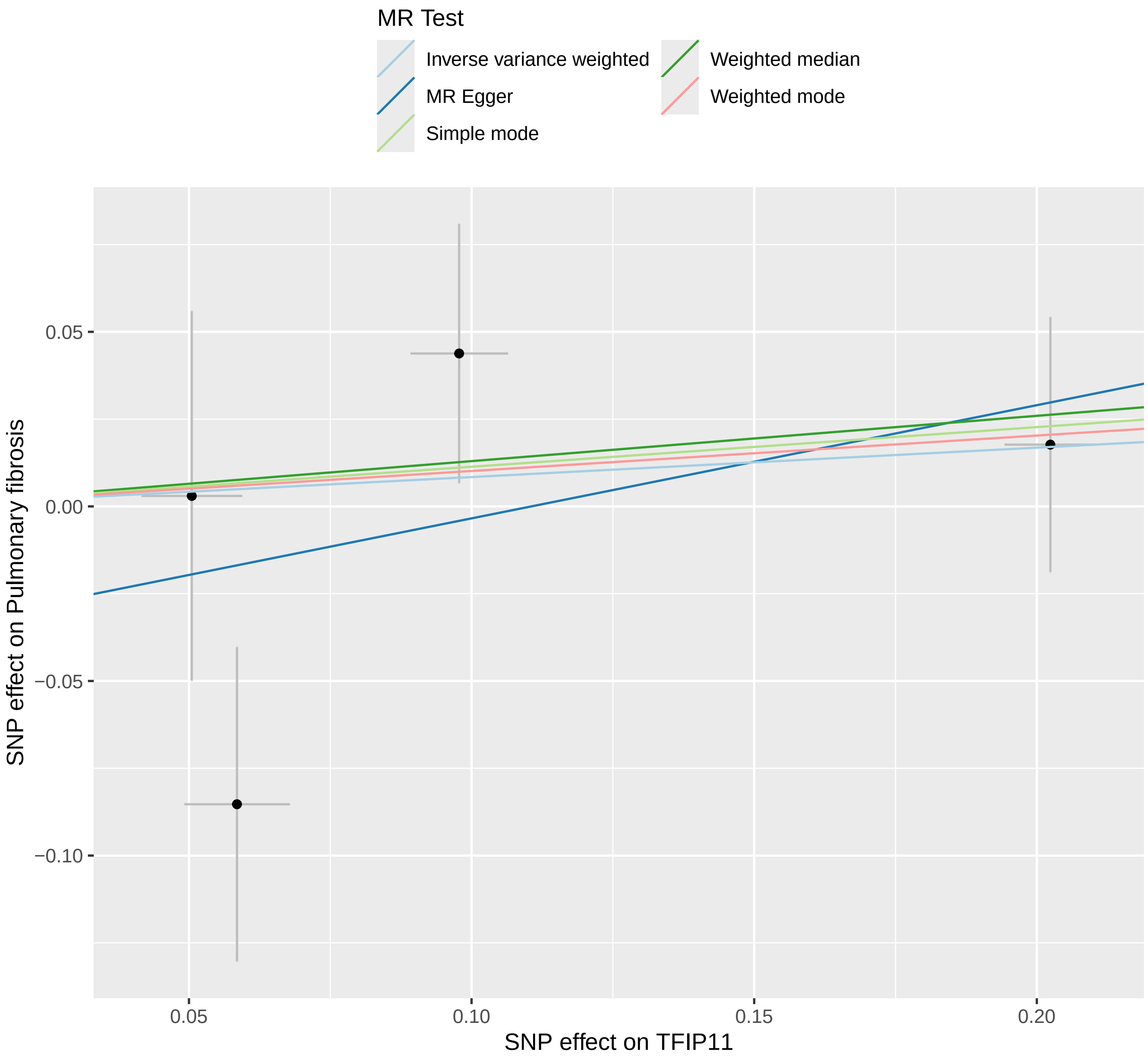
MR Test
Inverse variance weighted
MR Egger
Simple mode
Weighted median
Weighted mode
SNP effect on Pulmonary fibrosis
0.05
0.00
−0.05
−0.10
0.05
0.10
0.15
0.20
SNP effect on TFIP11

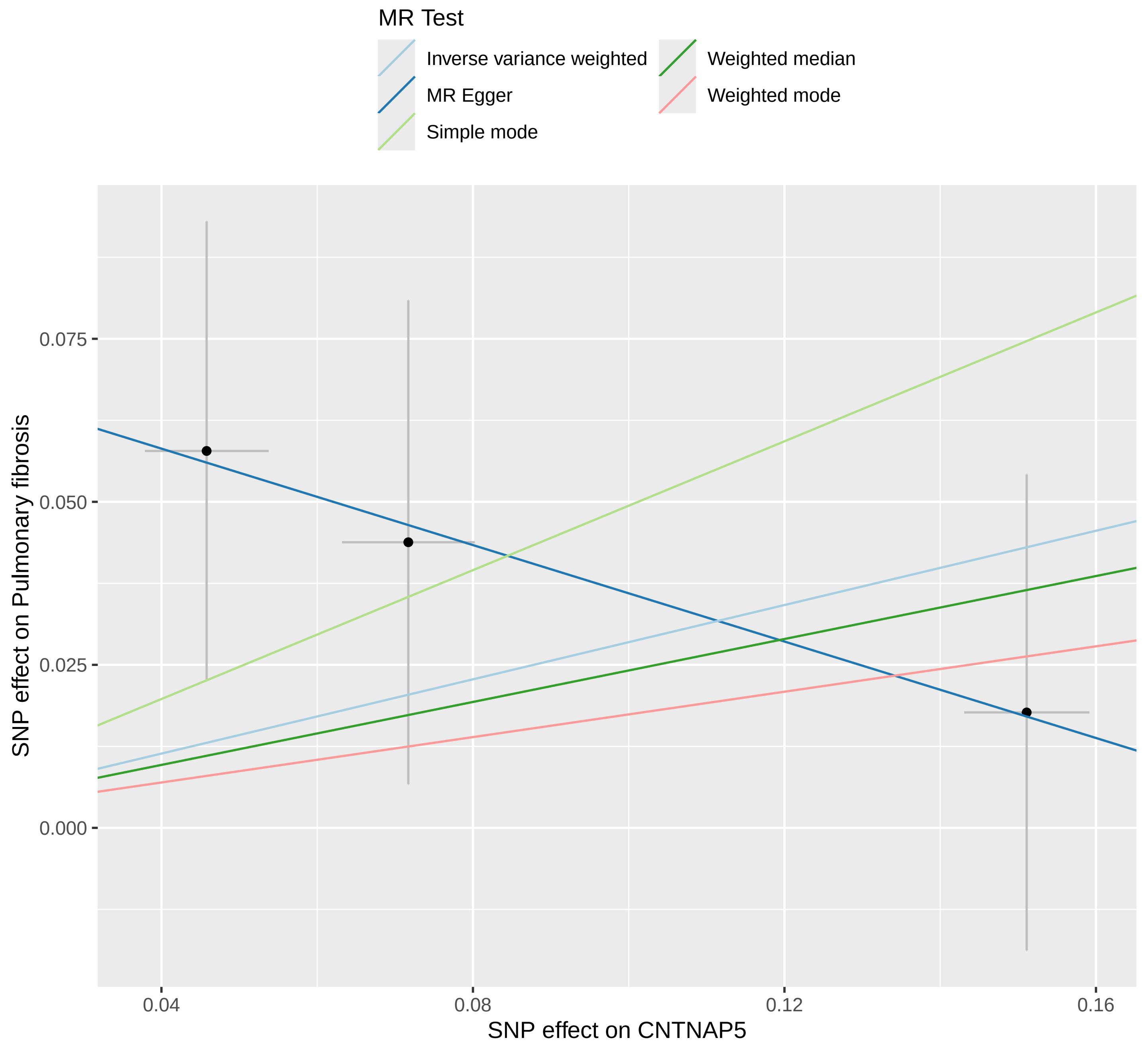
MR Test
Inverse variance weighted
MR Egger
Simple mode
Weighted median
Weighted mode
SNP effect on Pulmonary fibrosis
0.075
0.050
0.025
0.000
0.04
0.08
0.12
0.16
SNP effect on CNTNAP5

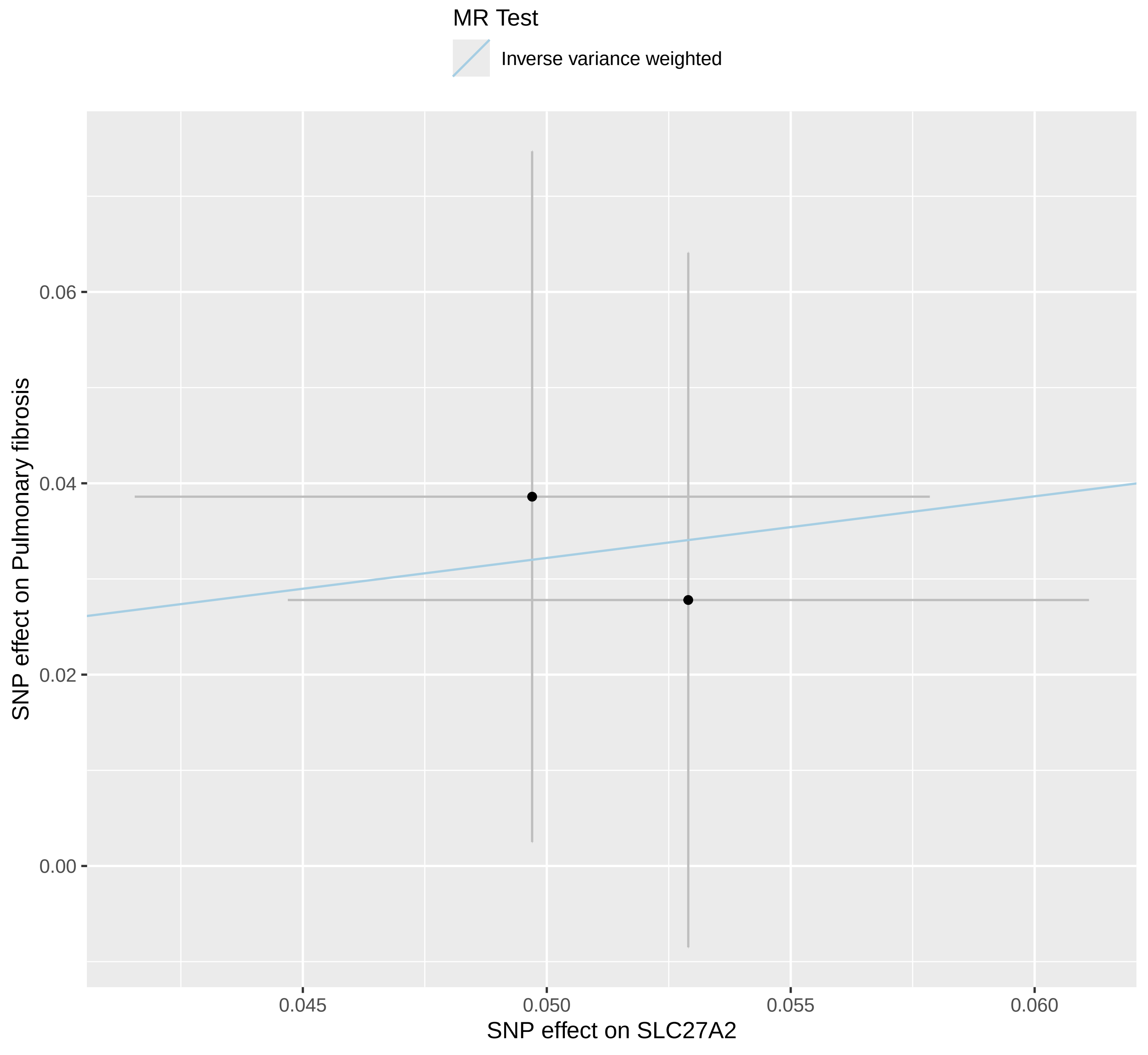
MR Test
Inverse variance weighted
0.06
0.04
0.02
0.00
SNP effect on Pulmonary fibrosis
0.045
0.050
0.055
0.060
SNP effect on SLC27A2

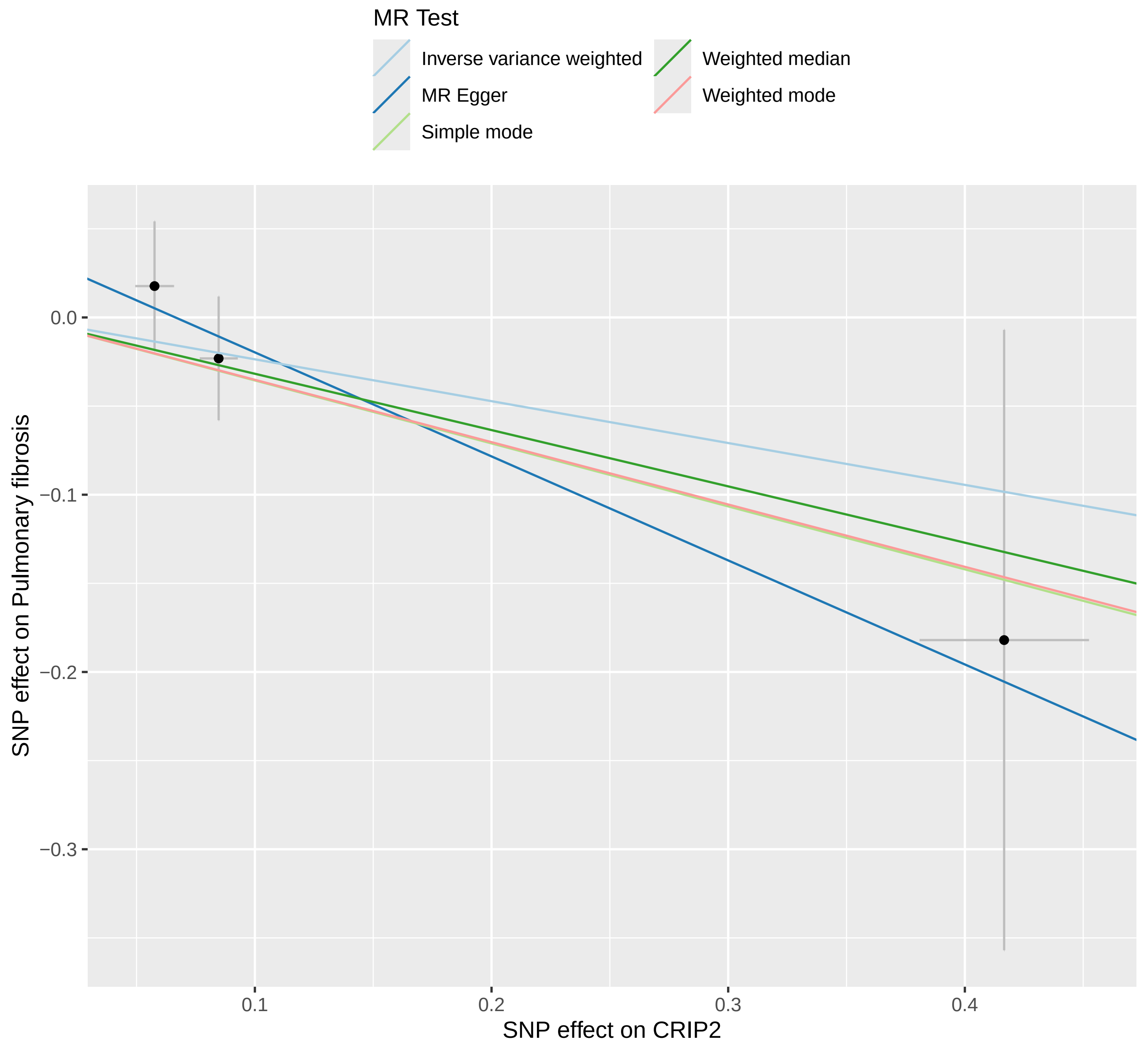
MR Test
Inverse variance weighted
MR Egger
Simple mode
Weighted median
Weighted mode
0.0
−0.1
−0.2
−0.3
0.1
0.2
0.3
0.4
SNP effect on Pulmonary fibrosis
SNP effect on CRIP2

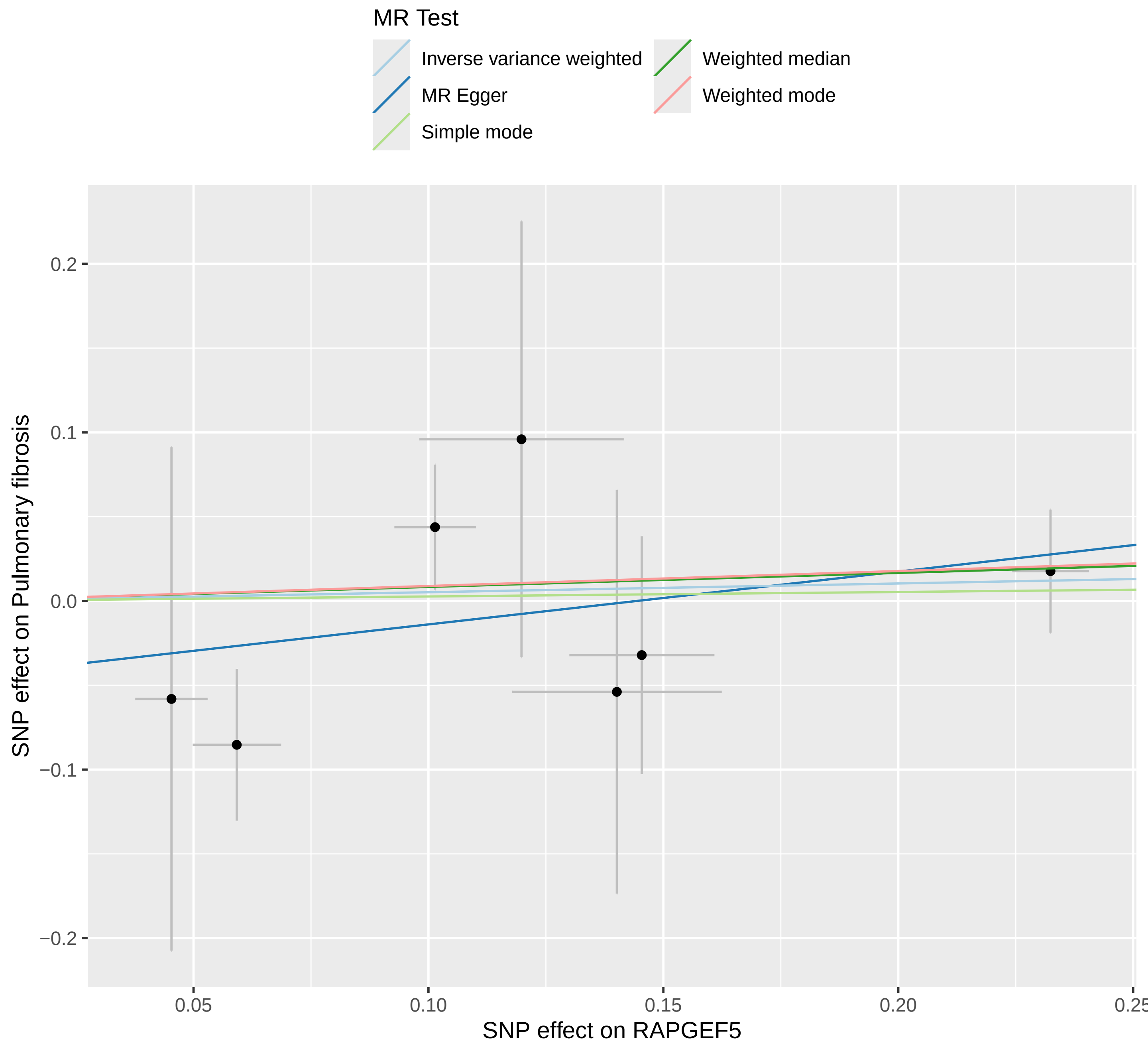

MR Test
Inverse variance weighted
MR Egger
Simple mode
Weighted median
Weighted mode
0.2
0.1
0.0
−0.1
−0.2
0.05
0.10
0.15
0.20
0.25
SNP effect on RAPGEF5
SNP effect on Pulmonary fibrosis

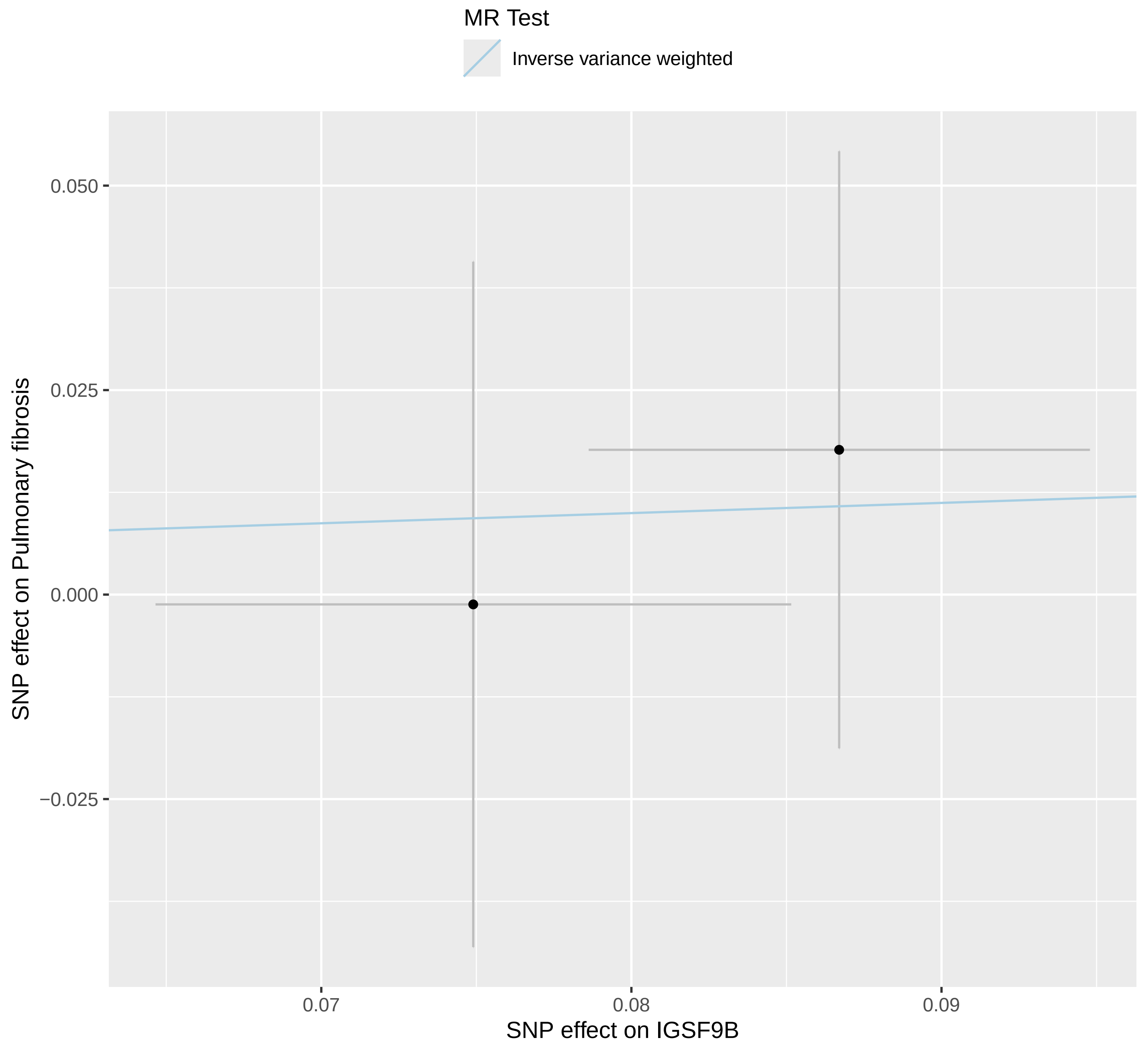

MR Test
Inverse variance weighted
0.050
0.025
0.000
−0.025
SNP effect on Pulmonary fibrosis
0.07
0.08
0.09
SNP effect on IGSF9B

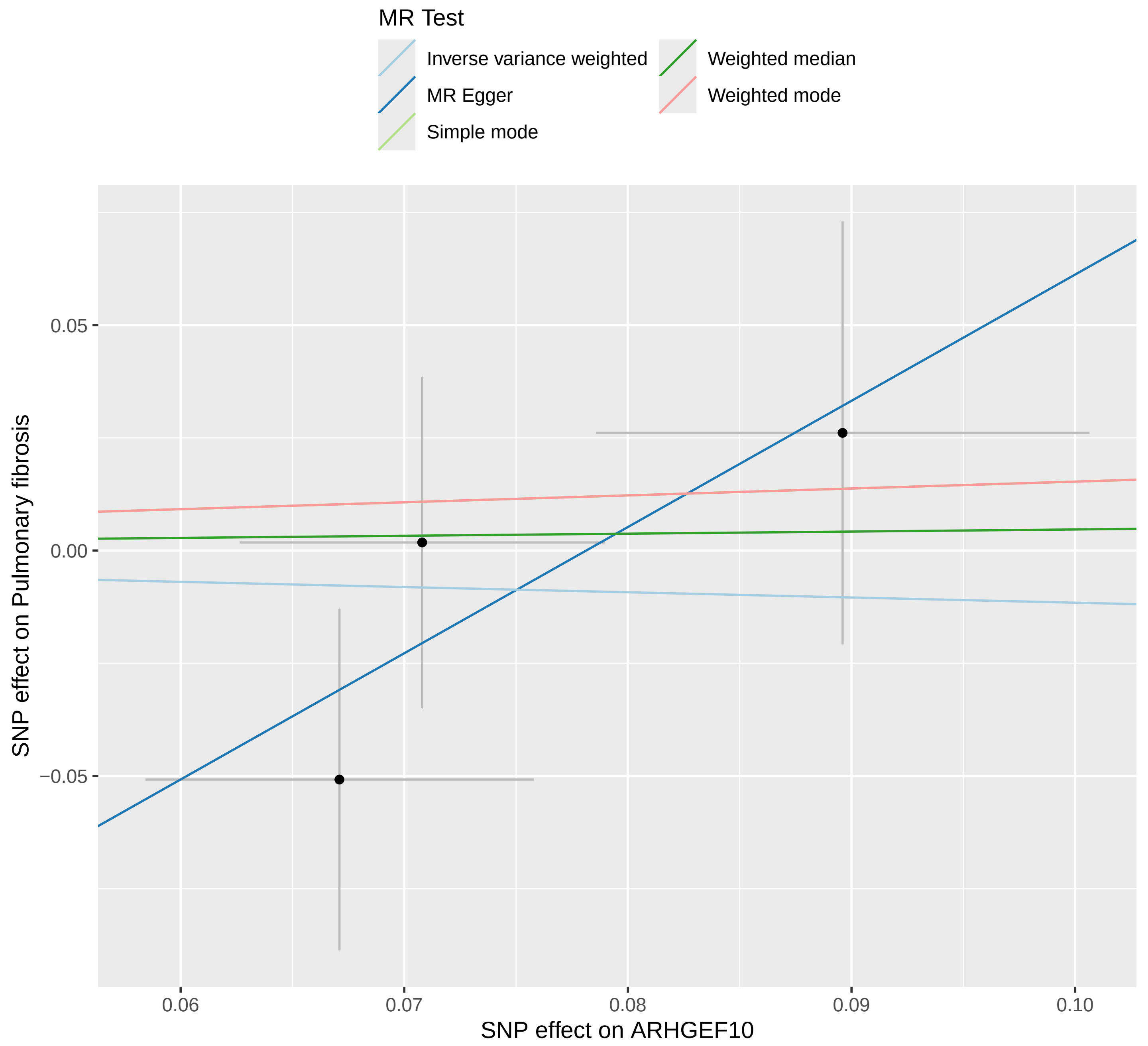

MR Test
Inverse variance weighted
MR Egger
Simple mode
Weighted median
Weighted mode
0.05
0.00
−0.05
0.06
0.07
0.08
0.09
0.10
SNP effect on ARHGEF10
SNP effect on Pulmonary fibrosis

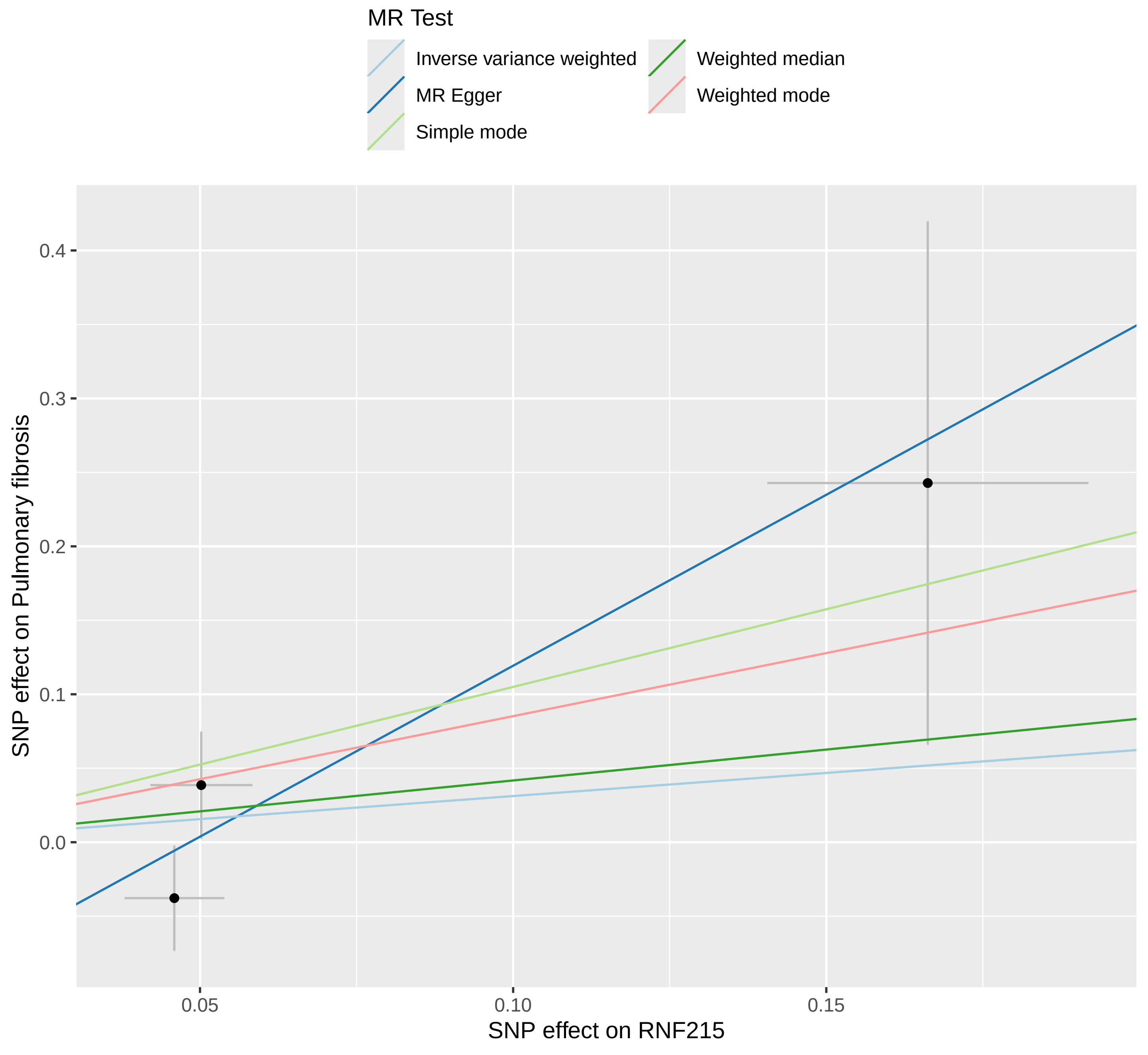
MR Test
Inverse variance weighted
MR Egger
Simple mode
Weighted median
Weighted mode
0.4
0.3
0.2
0.1
0.0
0.05
0.10
0.15
SNP effect on Pulmonary fibrosis
SNP effect on RNF215

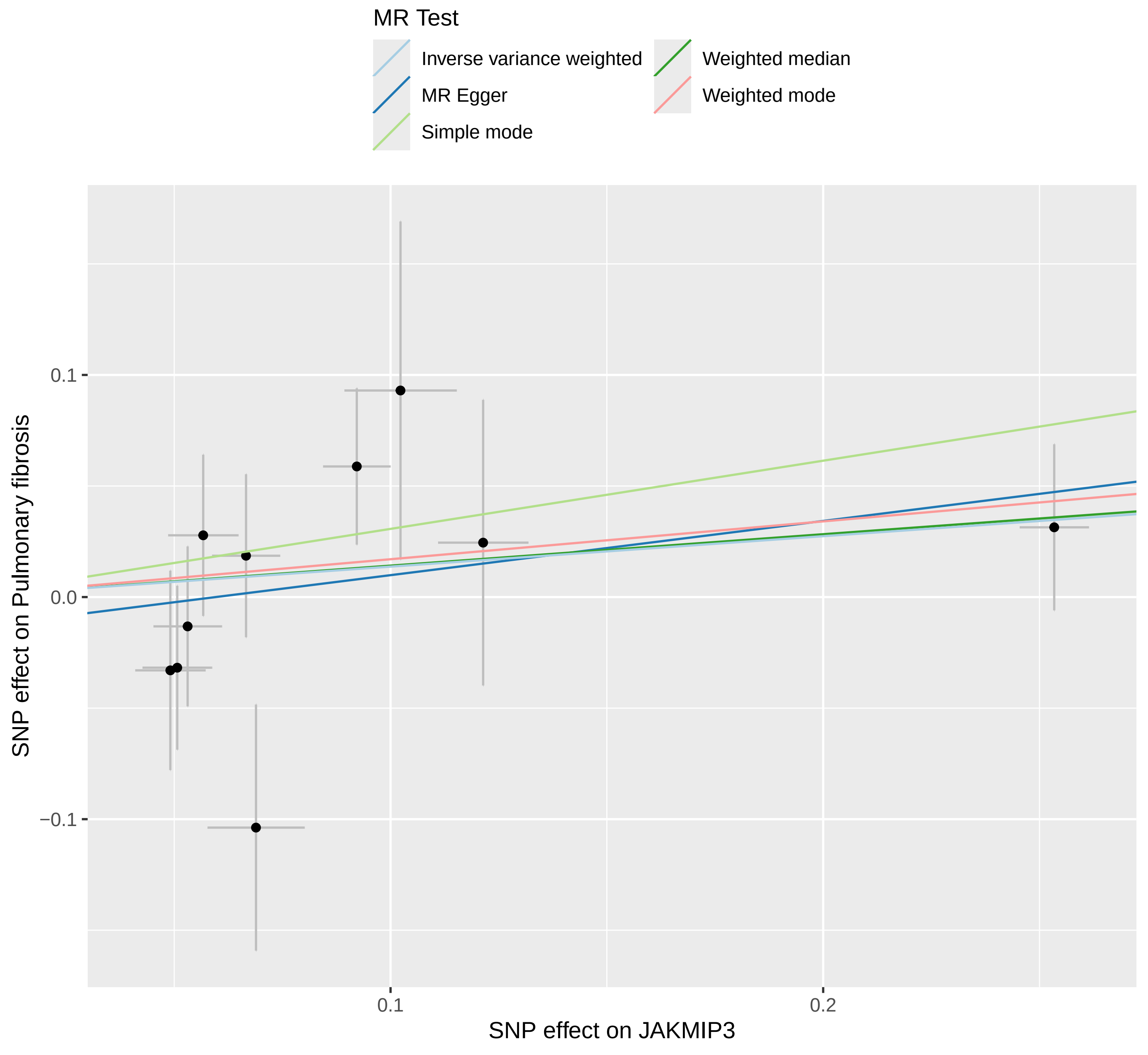
MR Test
Inverse variance weighted
MR Egger
Simple mode
Weighted median
Weighted mode
0.1
0.0
−0.1
SNP effect on Pulmonary fibrosis
0.1
0.2
SNP effect on JAKMIP3

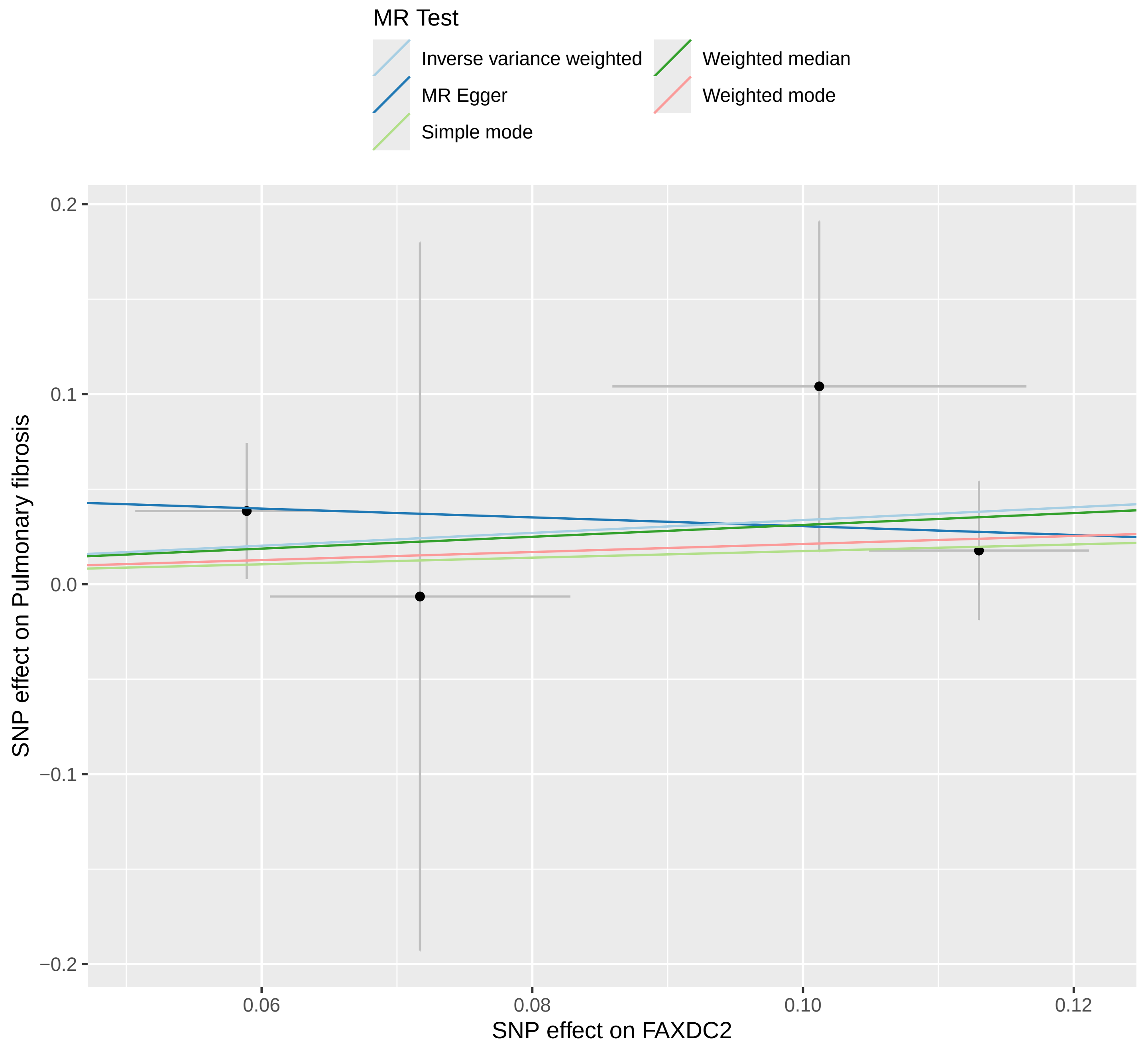
MR Test
Inverse variance weighted
MR Egger
Simple mode
Weighted median
Weighted mode
SNP effect on Pulmonary fibrosis
SNP effect on FAXDC2
0.2
0.1
0.0
−0.1
−0.2
0.06
0.08
0.10
0.12

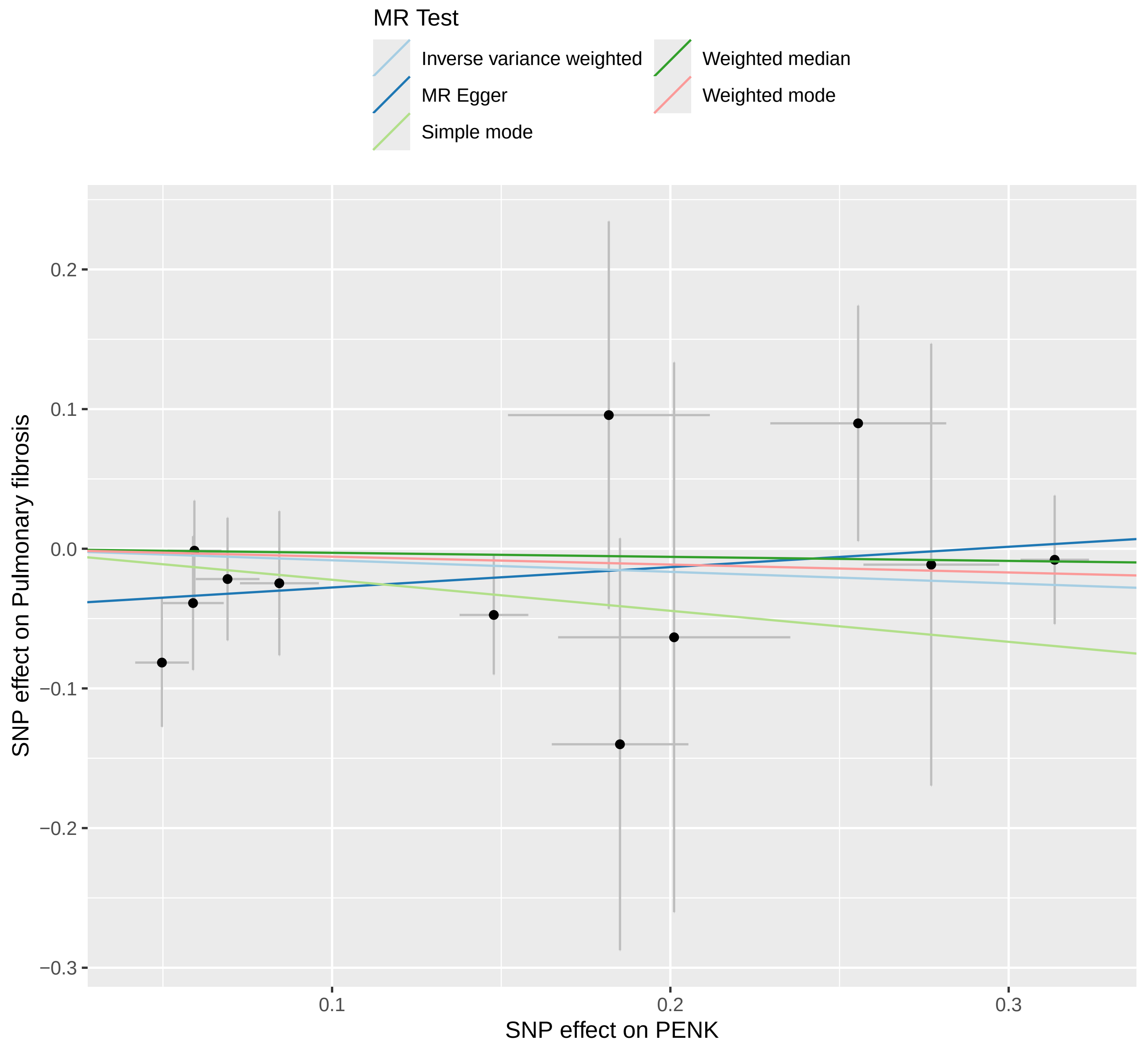

MR Test
Inverse variance weighted
MR Egger
Simple mode
Weighted median
Weighted mode
0.2
0.1
0.0
−0.1
−0.2
−0.3
SNP effect on Pulmonary fibrosis
0.1
0.2
0.3
SNP effect on PENK

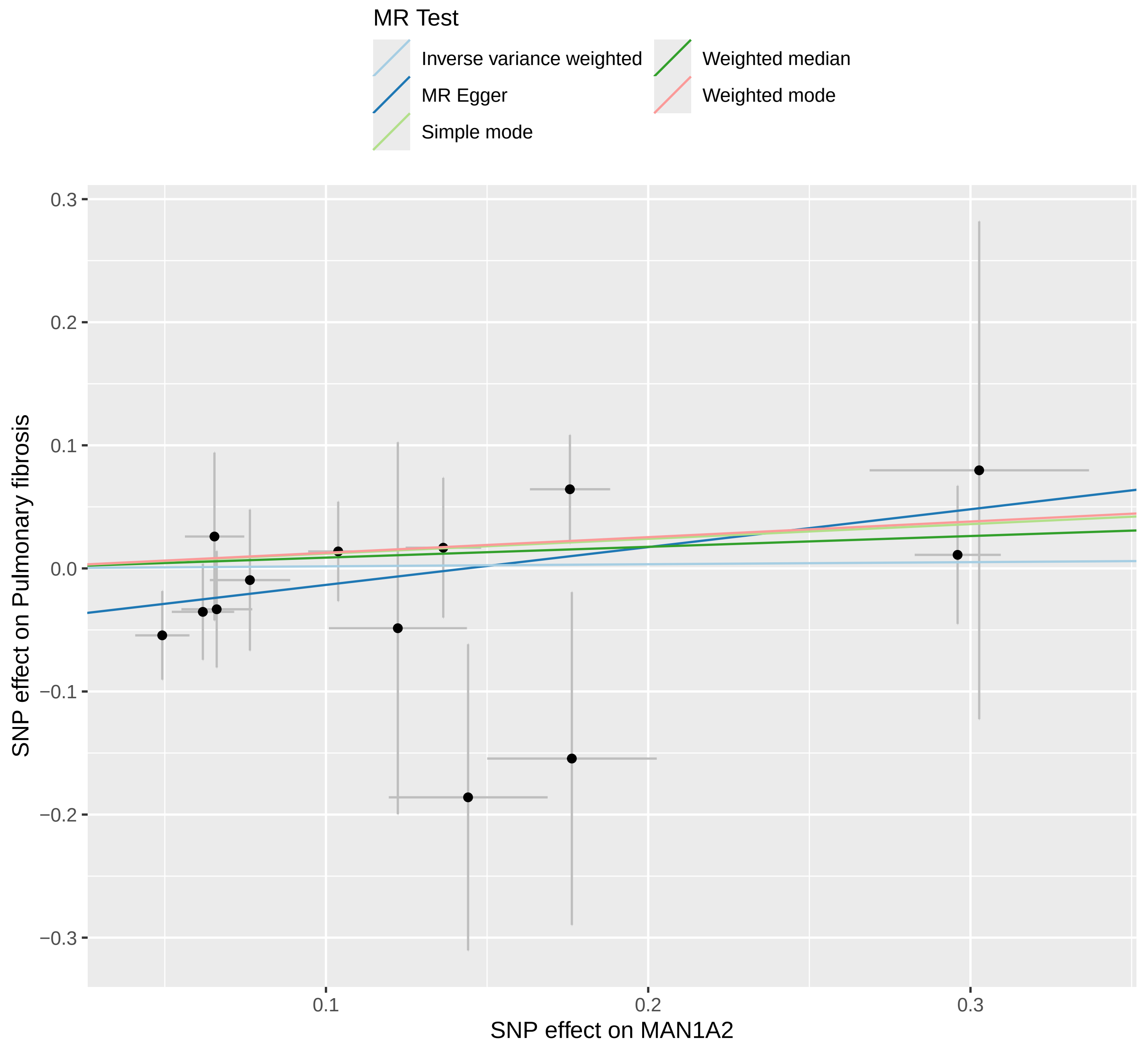
MR Test
Inverse variance weighted
MR Egger
Simple mode
Weighted median
Weighted mode
0.3
0.2
0.1
0.0
−0.1
−0.2
−0.3
SNP effect on Pulmonary fibrosis
0.1
0.2
0.3
SNP effect on MAN1A2

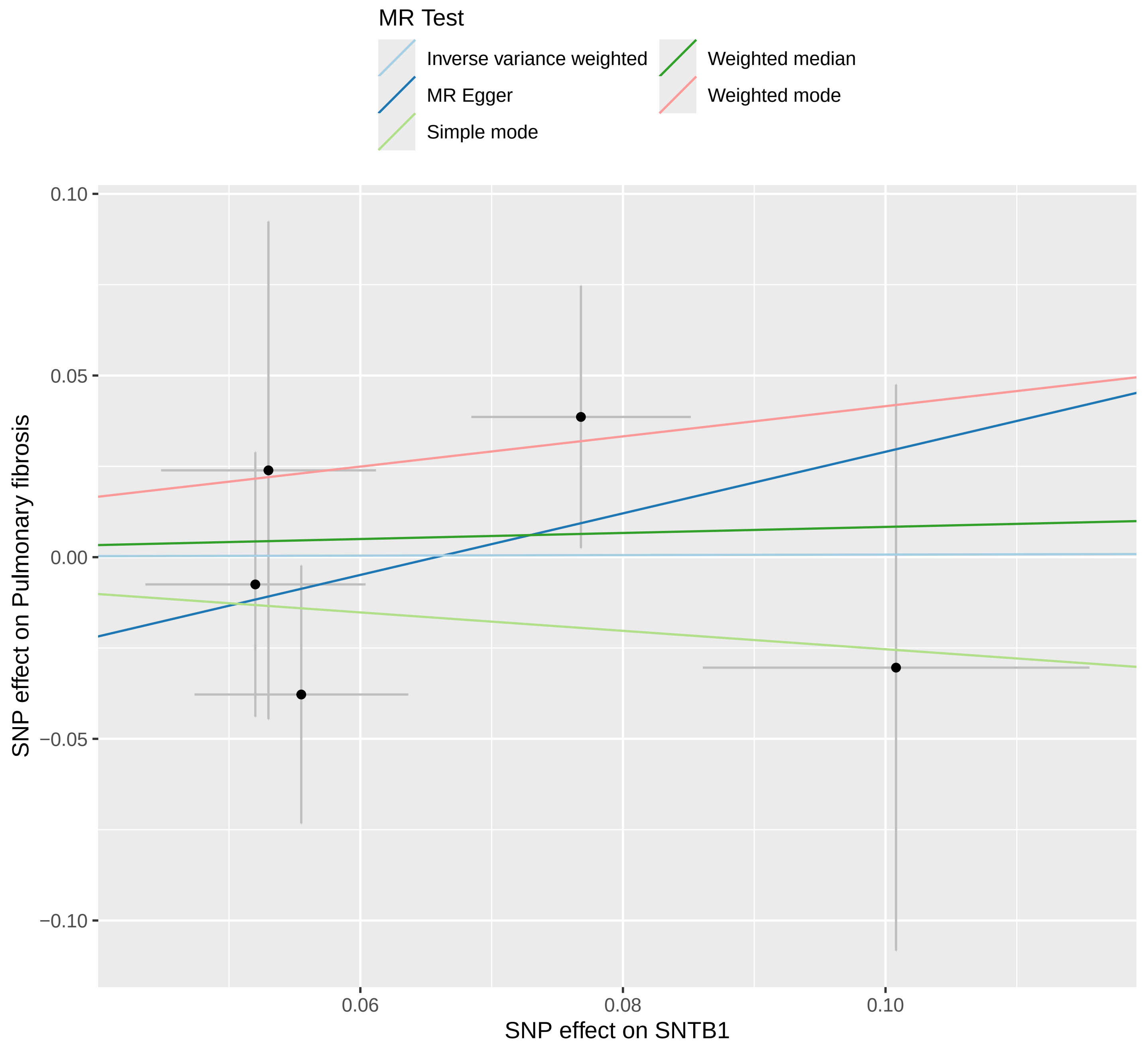
MR Test
Inverse variance weighted
MR Egger
Simple mode
Weighted median
Weighted mode
0.10
0.05
0.00
−0.05
−0.10
SNP effect on Pulmonary fibrosis
0.06
0.08
0.10
SNP effect on SNTB1

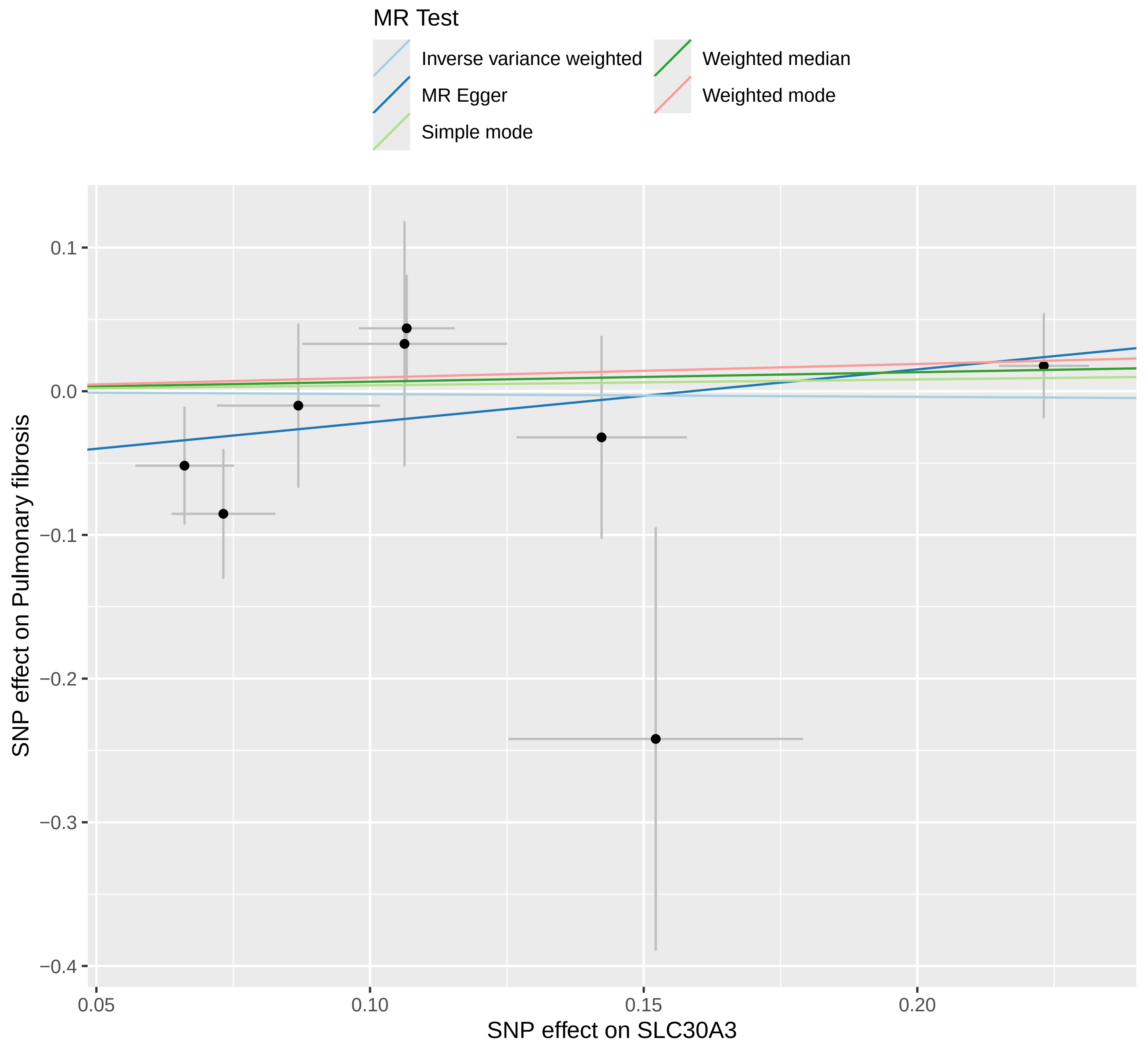

MR Test
Inverse variance weighted
MR Egger
Simple mode
Weighted median
Weighted mode
0.1
0.0
−0.1
−0.2
−0.3
−0.4
0.05
0.10
0.15
0.20
SNP effect on Pulmonary fibrosis
SNP effect on SLC30A3

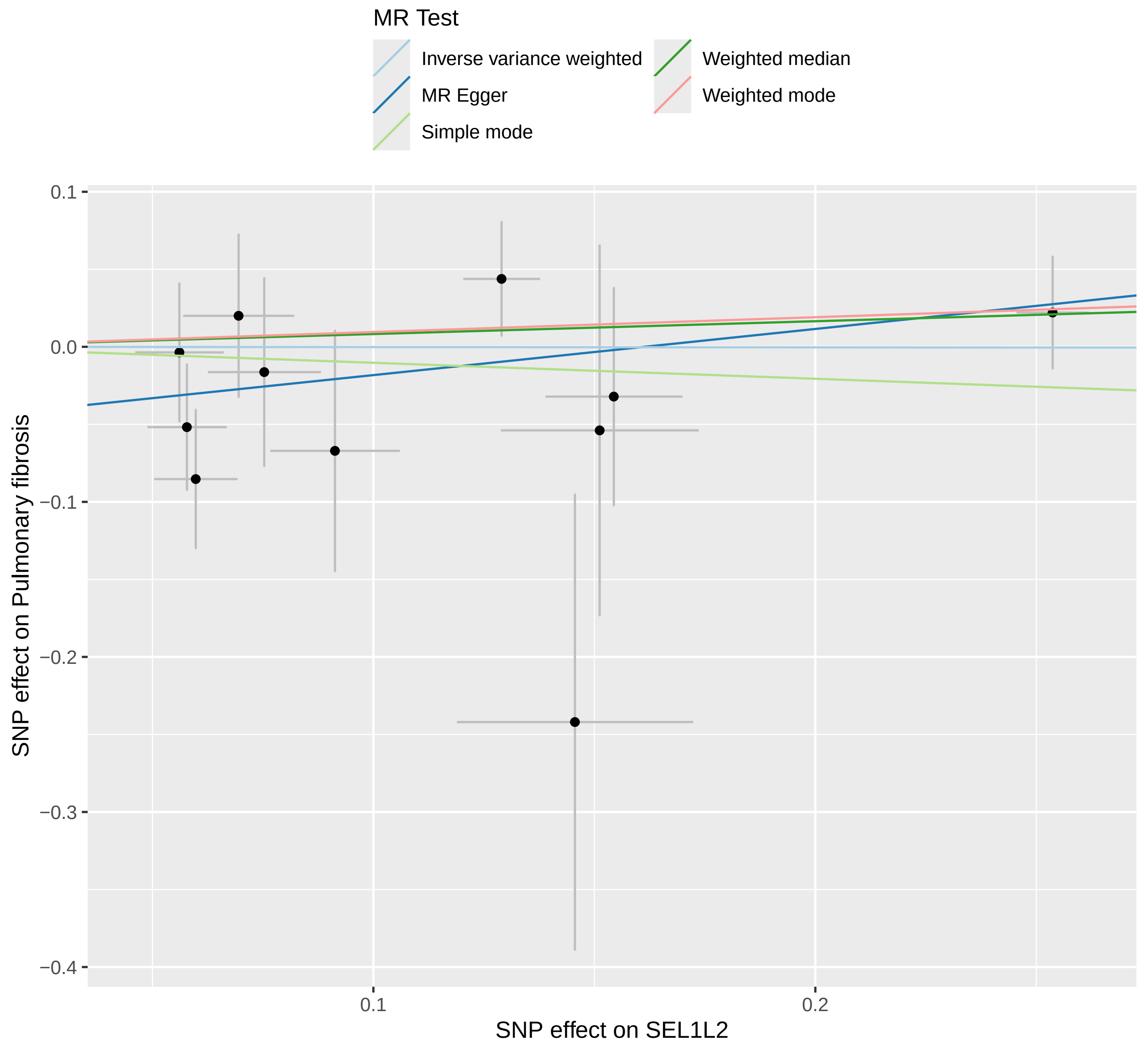

MR Test
Inverse variance weighted
MR Egger
Simple mode
Weighted median
Weighted mode
0.1
0.0
−0.1
−0.2
−0.3
−0.4
SNP effect on Pulmonary fibrosis
0.1
0.2
SNP effect on SEL1L2

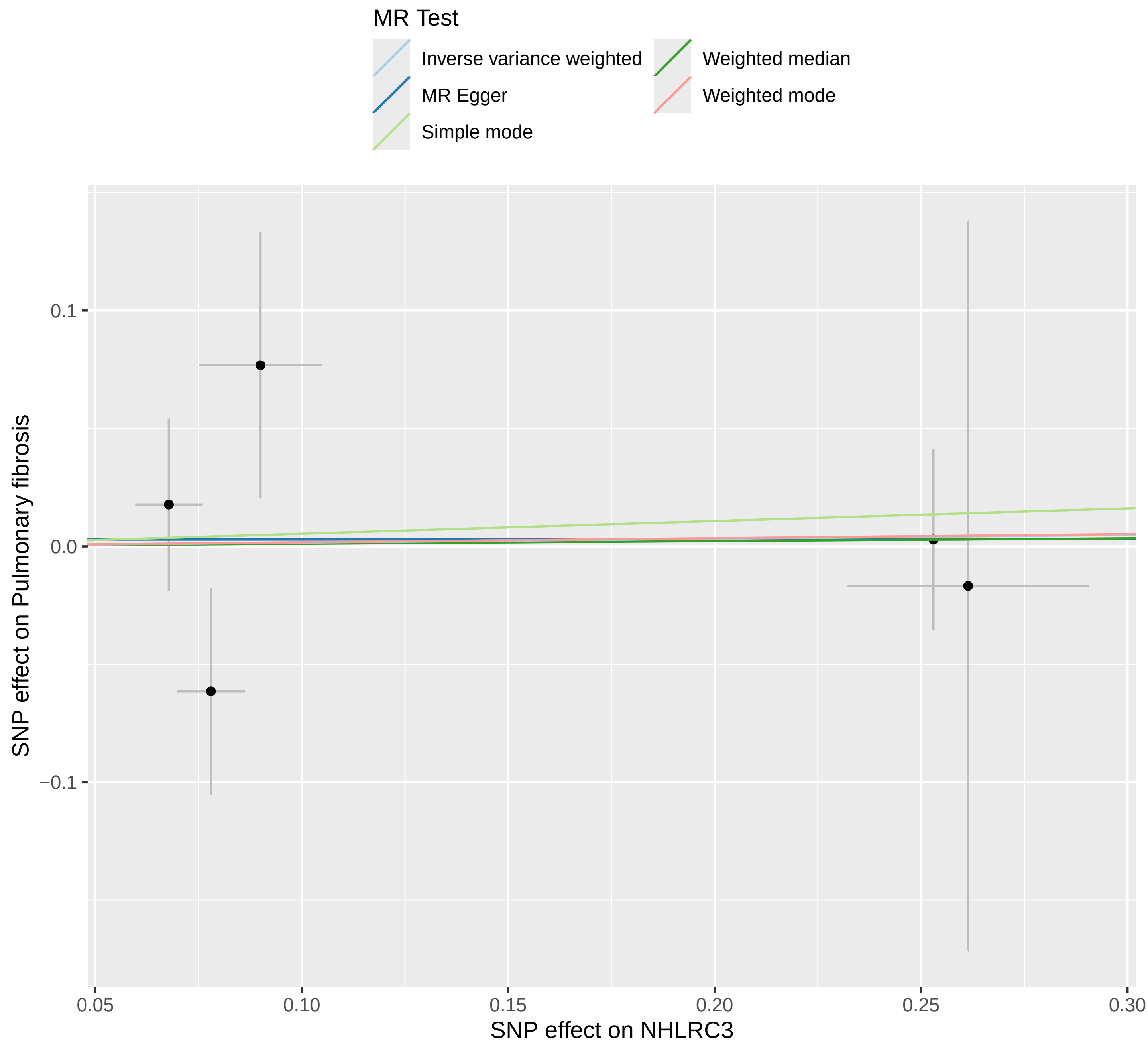

MR Test
Inverse variance weighted
MR Egger
Simple mode
Weighted median
Weighted mode
0.1
0.0
−0.1
SNP effect on Pulmonary fibrosis
0.05
0.10
0.15
0.20
0.25
0.30
SNP effect on NHLRC3

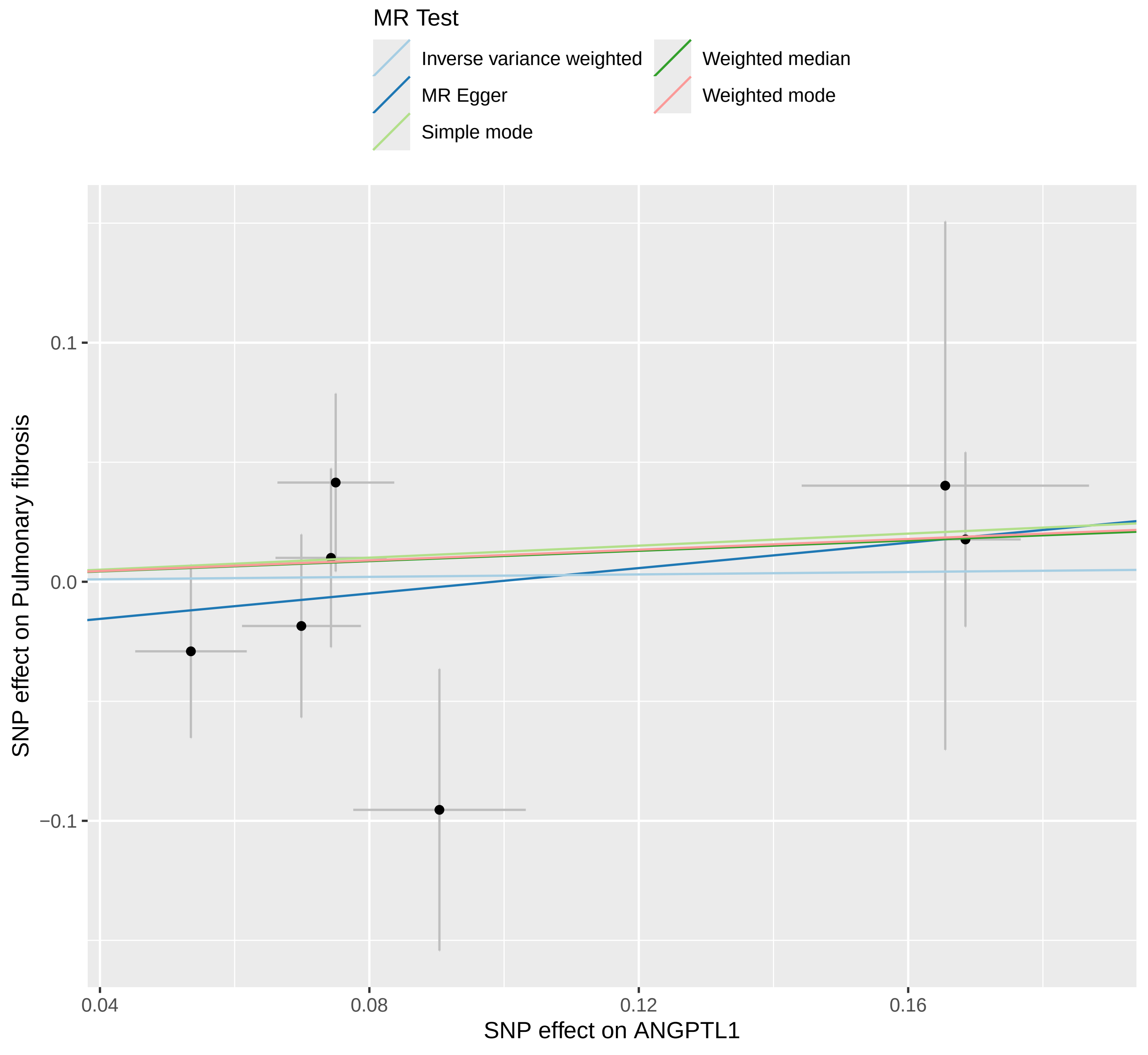
MR Test
Inverse variance weighted
MR Egger
Simple mode
Weighted median
Weighted mode
SNP effect on Pulmonary fibrosis
0.1
0.0
−0.1
0.04
0.08
0.12
0.16
SNP effect on ANGPTL1

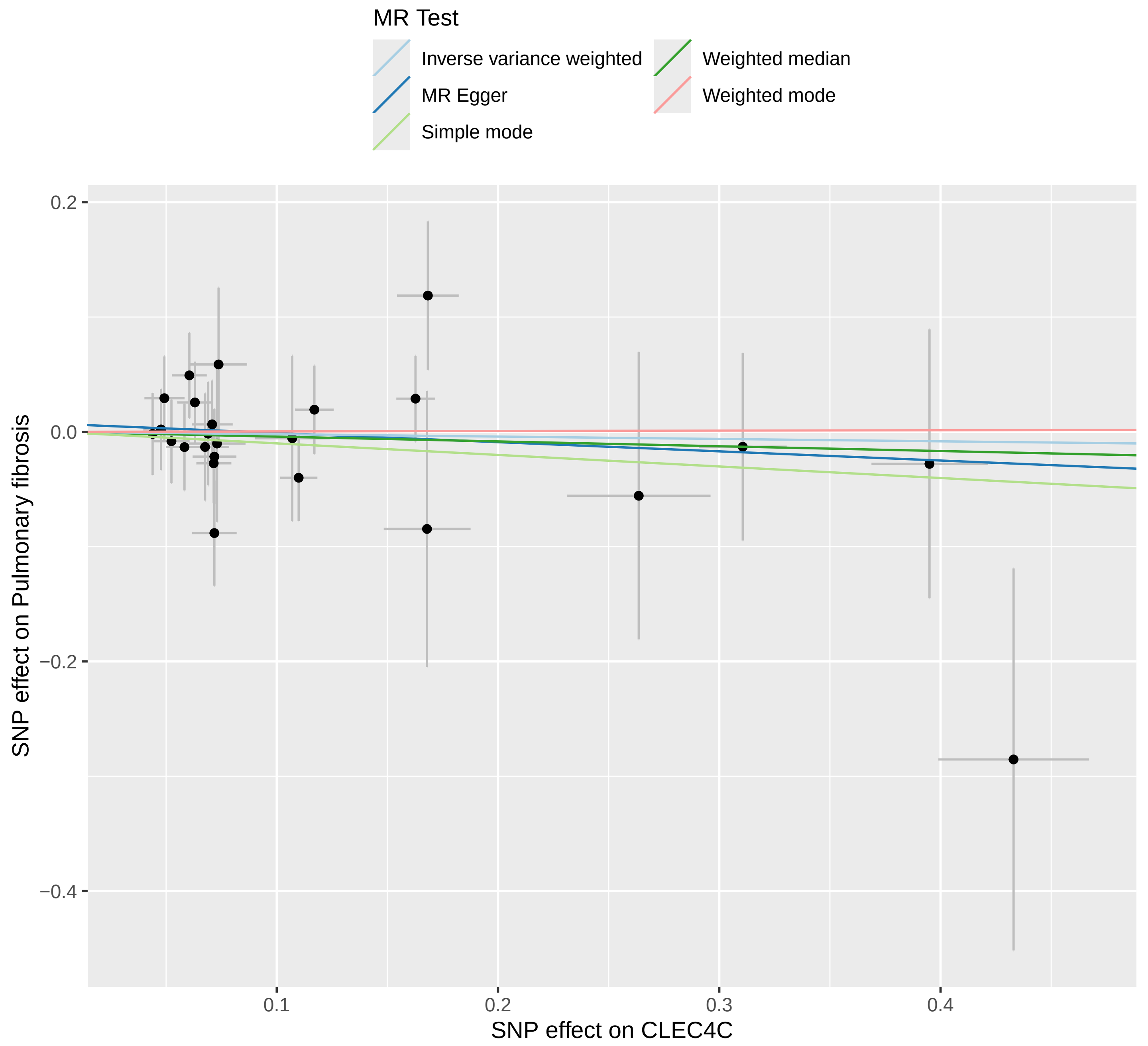
MR Test
Inverse variance weighted
MR Egger
Simple mode
Weighted median
Weighted mode
0.2
0.0
−0.2
−0.4
0.1
0.2
0.3
0.4
SNP effect on CLEC4C
SNP effect on Pulmonary fibrosis

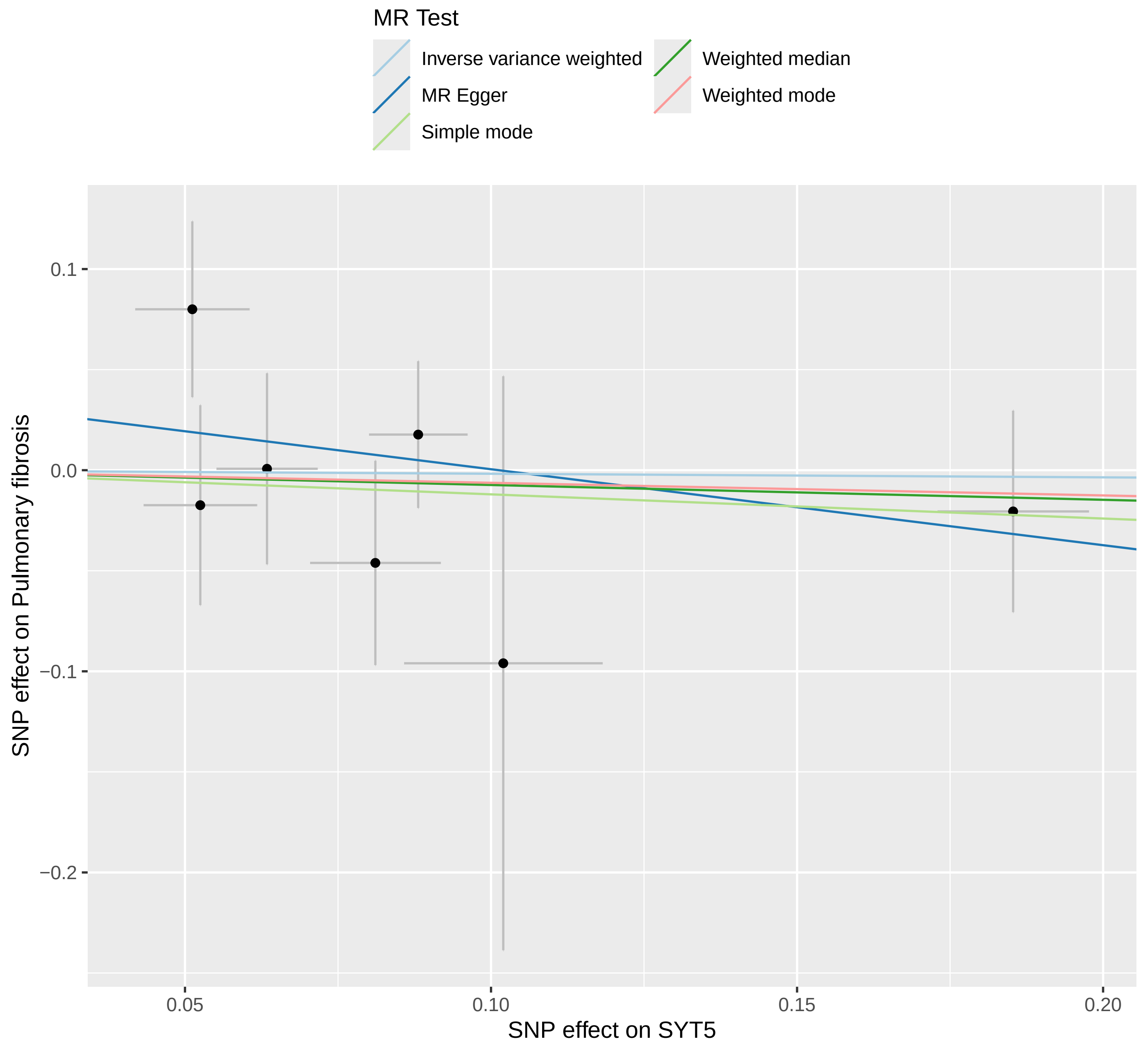
MR Test
Inverse variance weighted
MR Egger
Simple mode
Weighted median
Weighted mode
SNP effect on Pulmonary fibrosis
0.1
0.0
−0.1
−0.2
0.05
0.10
0.15
0.20
SNP effect on SYT5

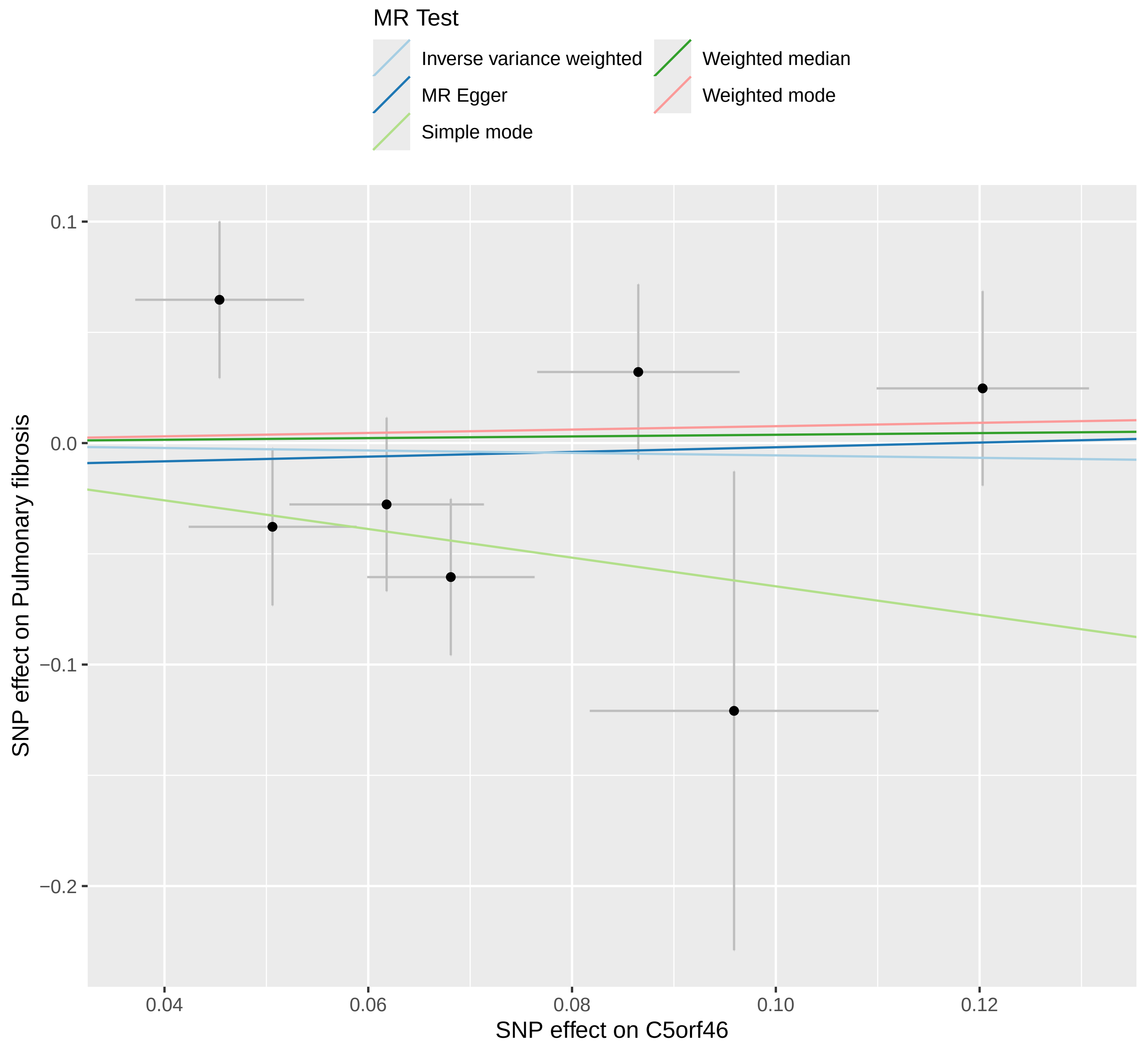

MR Test
Inverse variance weighted
MR Egger
Simple mode
Weighted median
Weighted mode
0.1
0.0
−0.1
−0.2
SNP effect on Pulmonary fibrosis
0.04
0.06
0.08
0.10
0.12
SNP effect on C5orf46

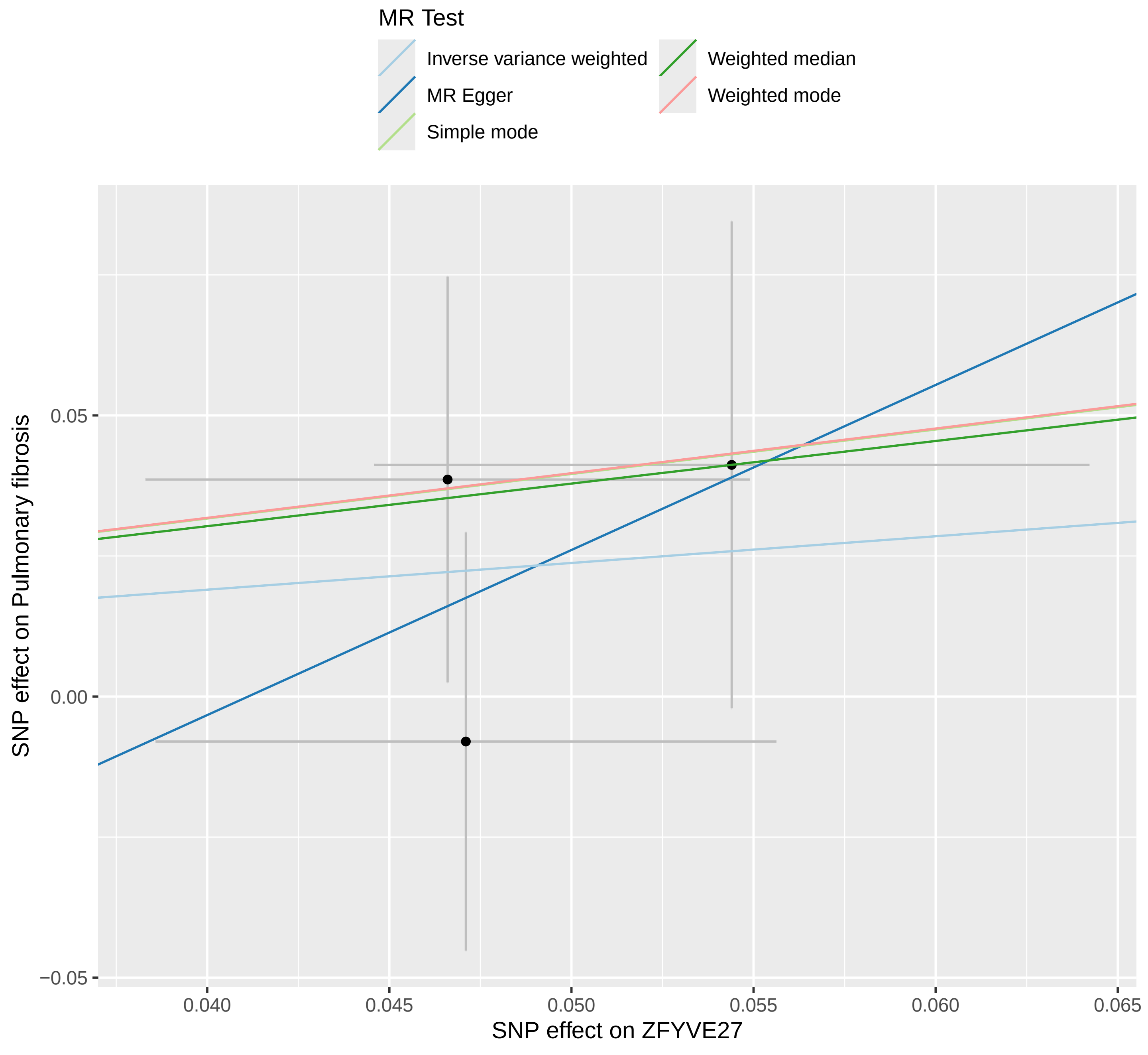

MR Test
Inverse variance weighted
MR Egger
Simple mode
Weighted median
Weighted mode
SNP effect on Pulmonary fibrosis
0.05
0.00
−0.05
0.040
0.045
0.050
0.055
0.060
0.065
SNP effect on ZFYVE27

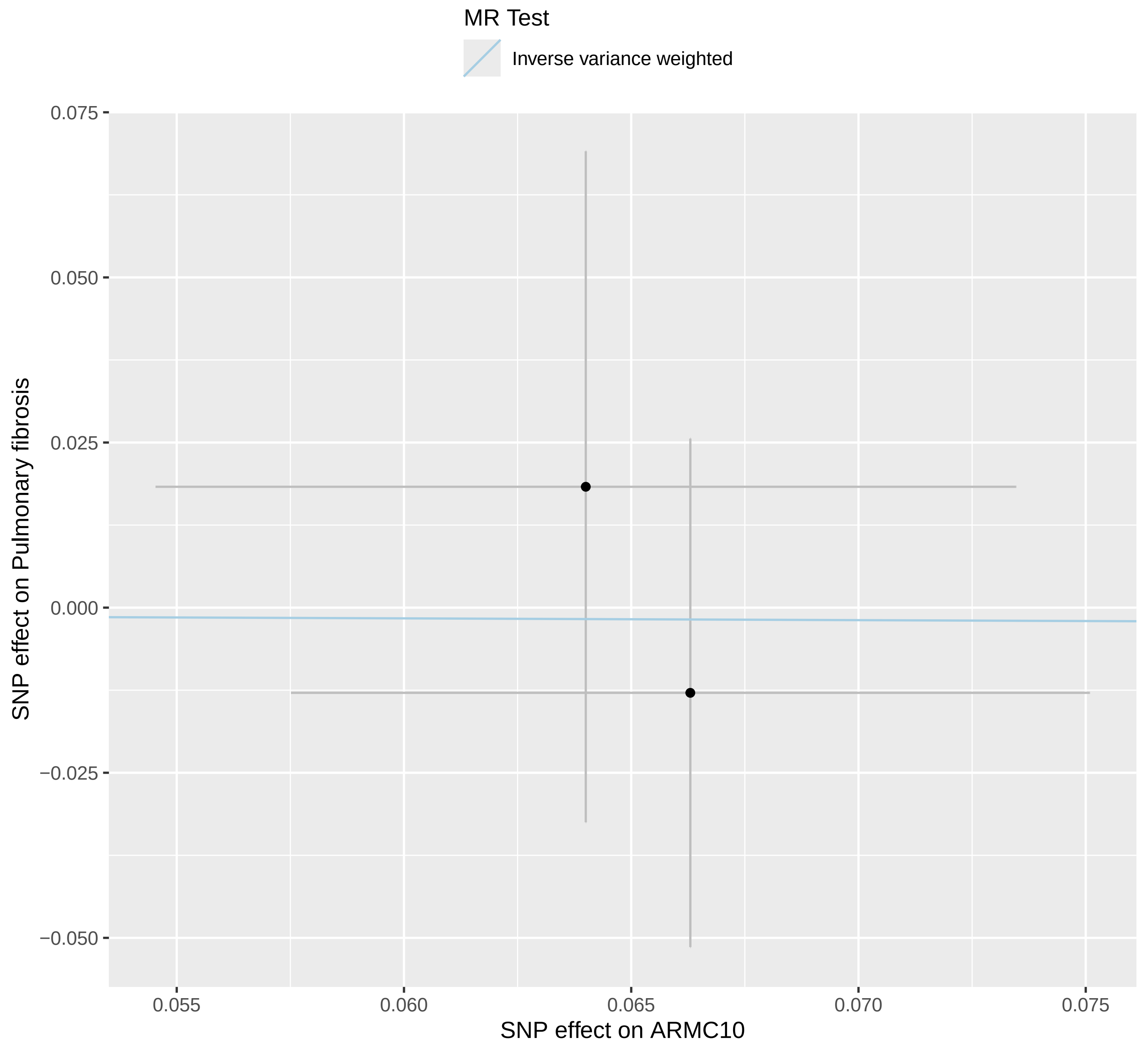
MR Test
Inverse variance weighted
0.075
0.050
0.025
0.000
−0.025
−0.050
SNP effect on Pulmonary fibrosis
0.055
0.060
0.065
0.070
0.075
SNP effect on ARMC10

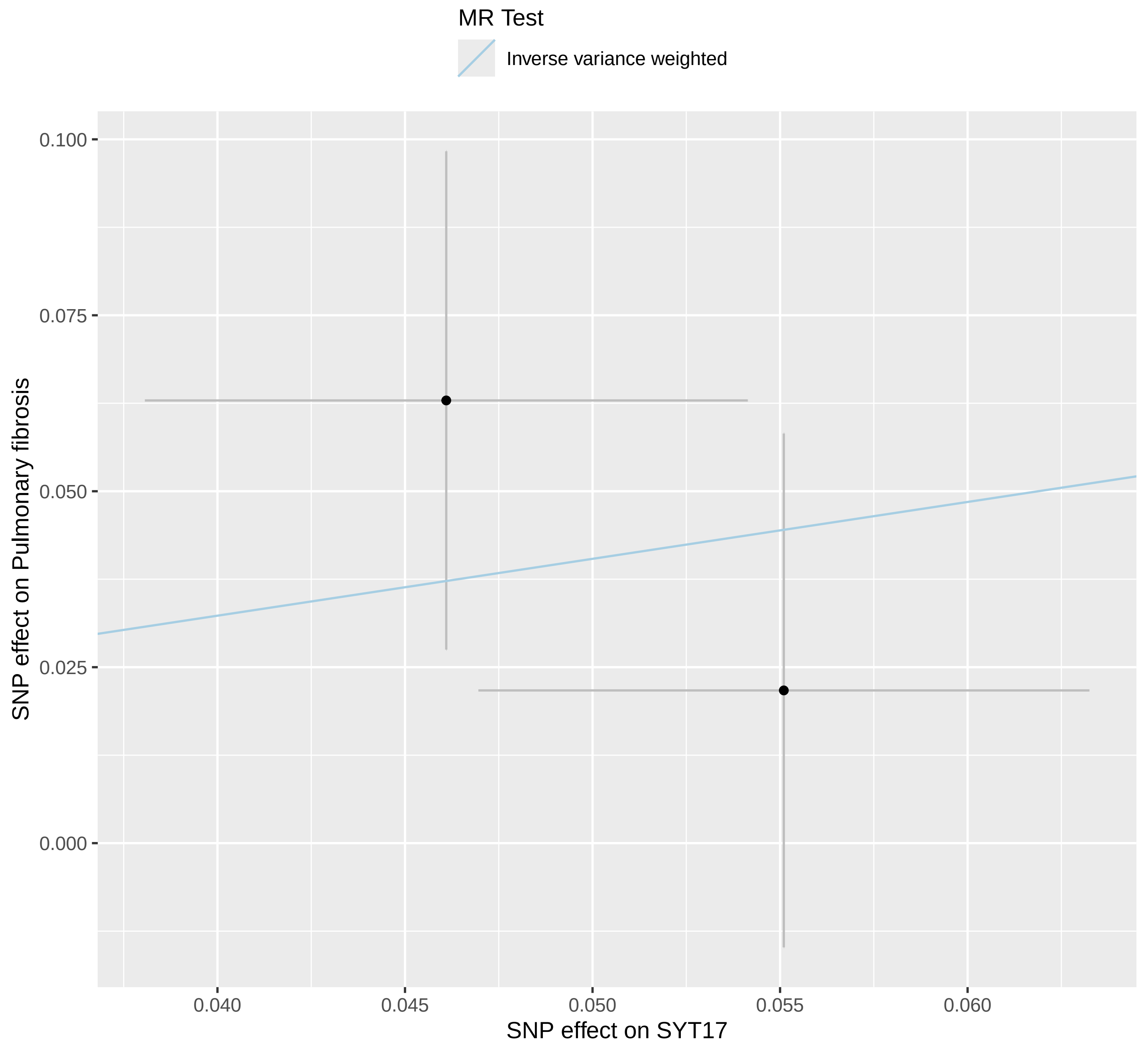
MR Test
Inverse variance weighted
0.100
0.075
0.050
0.025
0.000
0.040
0.045
0.050
0.055
0.060
SNP effect on SYT17
SNP effect on Pulmonary fibrosis

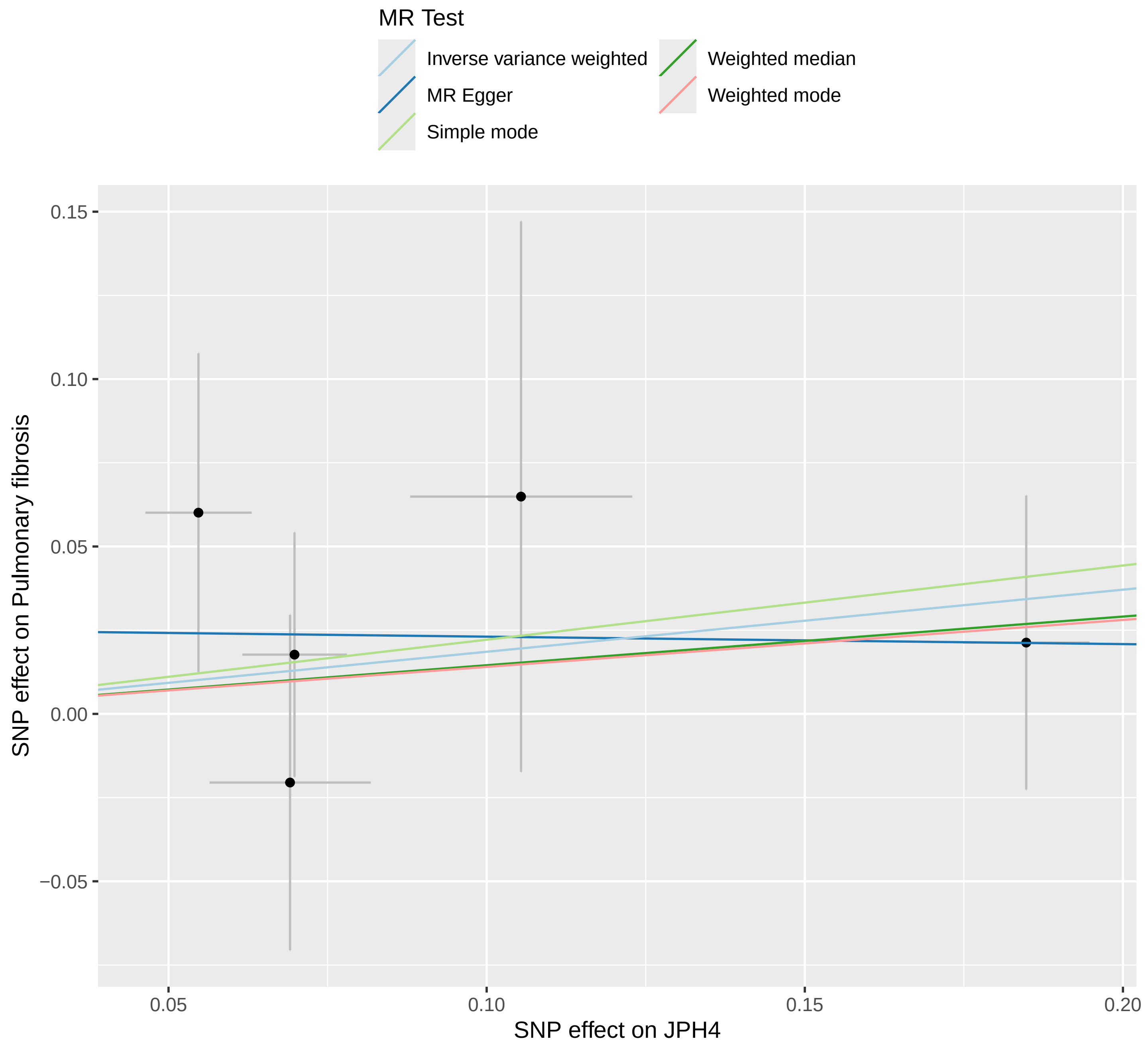
MR Test
Inverse variance weighted
MR Egger
Simple mode
Weighted median
Weighted mode
0.15
0.10
0.05
0.00
−0.05
SNP effect on Pulmonary fibrosis
0.05
0.10
0.15
0.20
SNP effect on JPH4

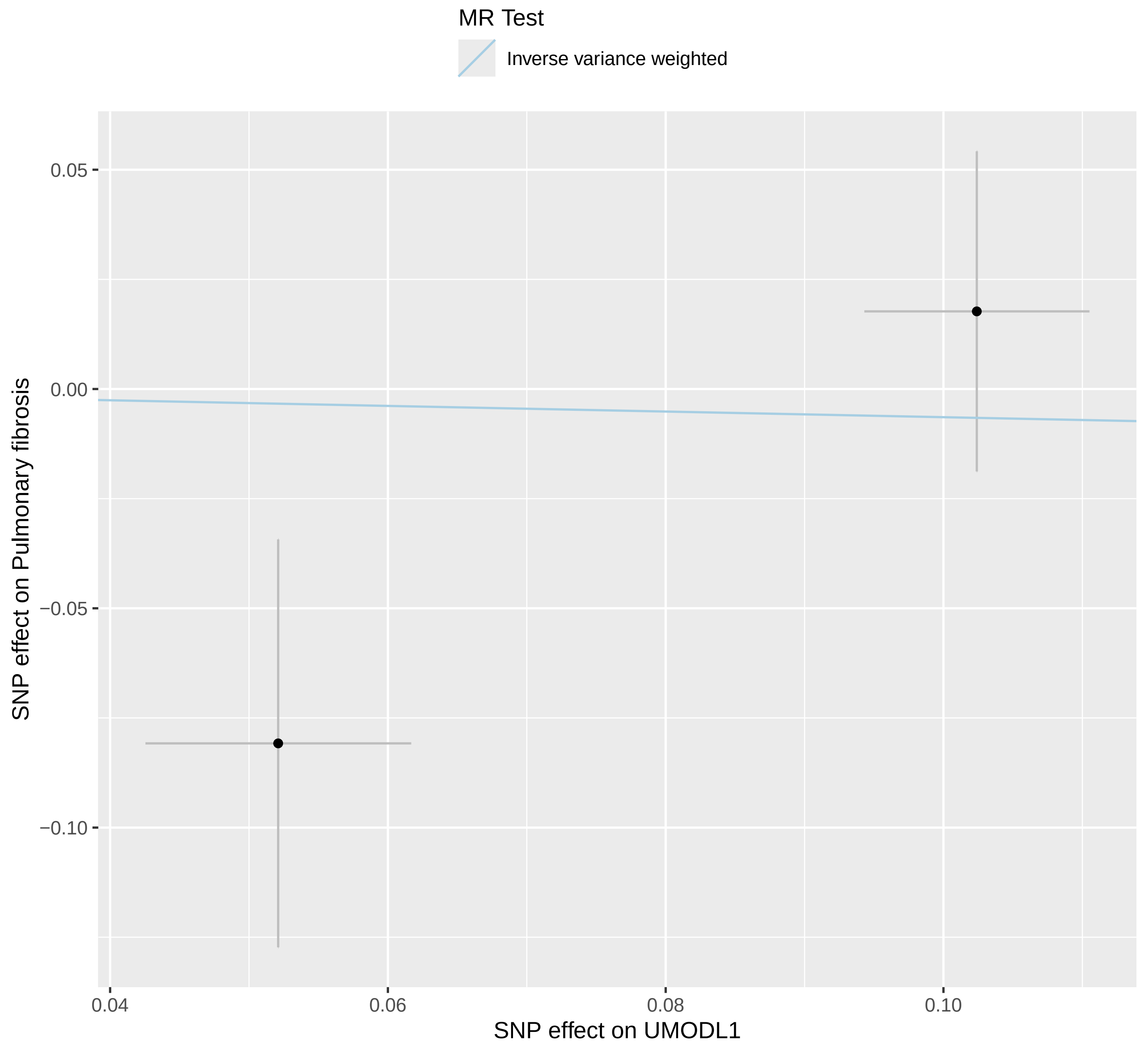
MR Test
Inverse variance weighted
0.05
0.00
−0.05
−0.10
SNP effect on Pulmonary fibrosis
0.04
0.06
0.08
0.10
SNP effect on UMODL1

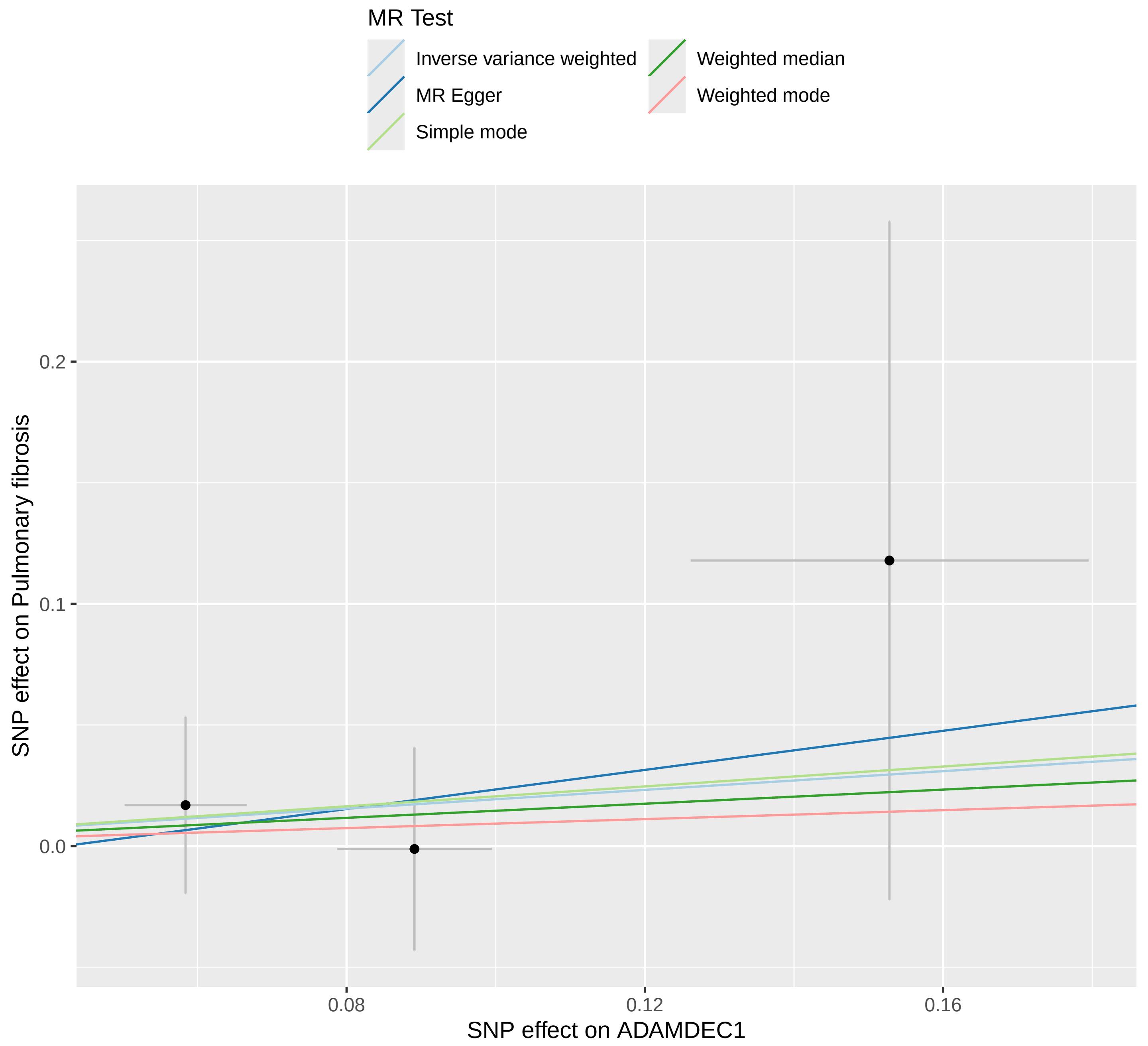
MR Test
Inverse variance weighted
MR Egger
Simple mode
Weighted median
Weighted mode
0.2
0.1
0.0
0.08
0.12
0.16
SNP effect on ADAMDEC1
SNP effect on Pulmonary fibrosis

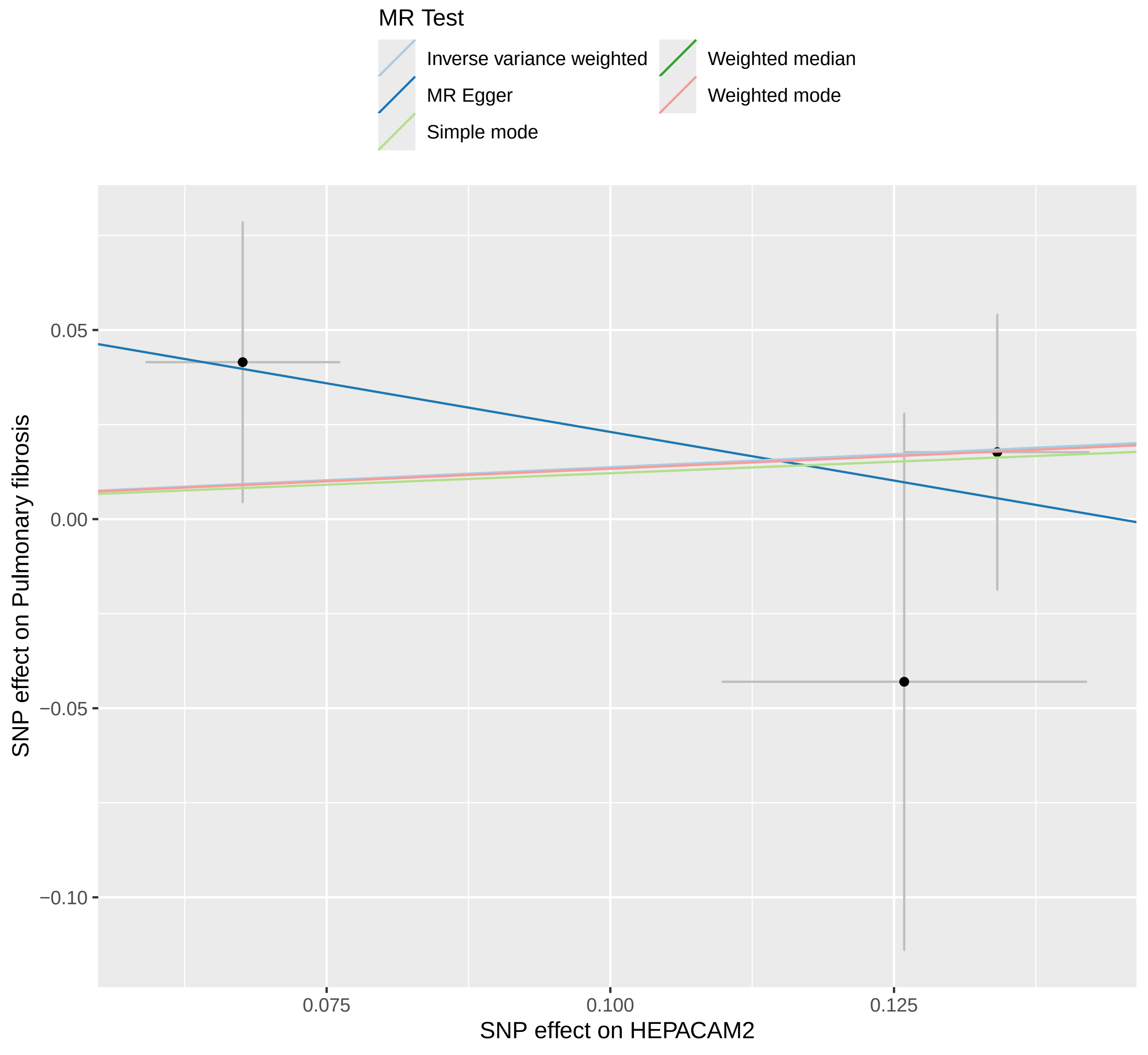

MR Test
Inverse variance weighted
MR Egger
Simple mode
Weighted median
Weighted mode
0.05
0.00
−0.05
−0.10
0.075
0.100
0.125
SNP effect on Pulmonary fibrosis
SNP effect on HEPACAM2

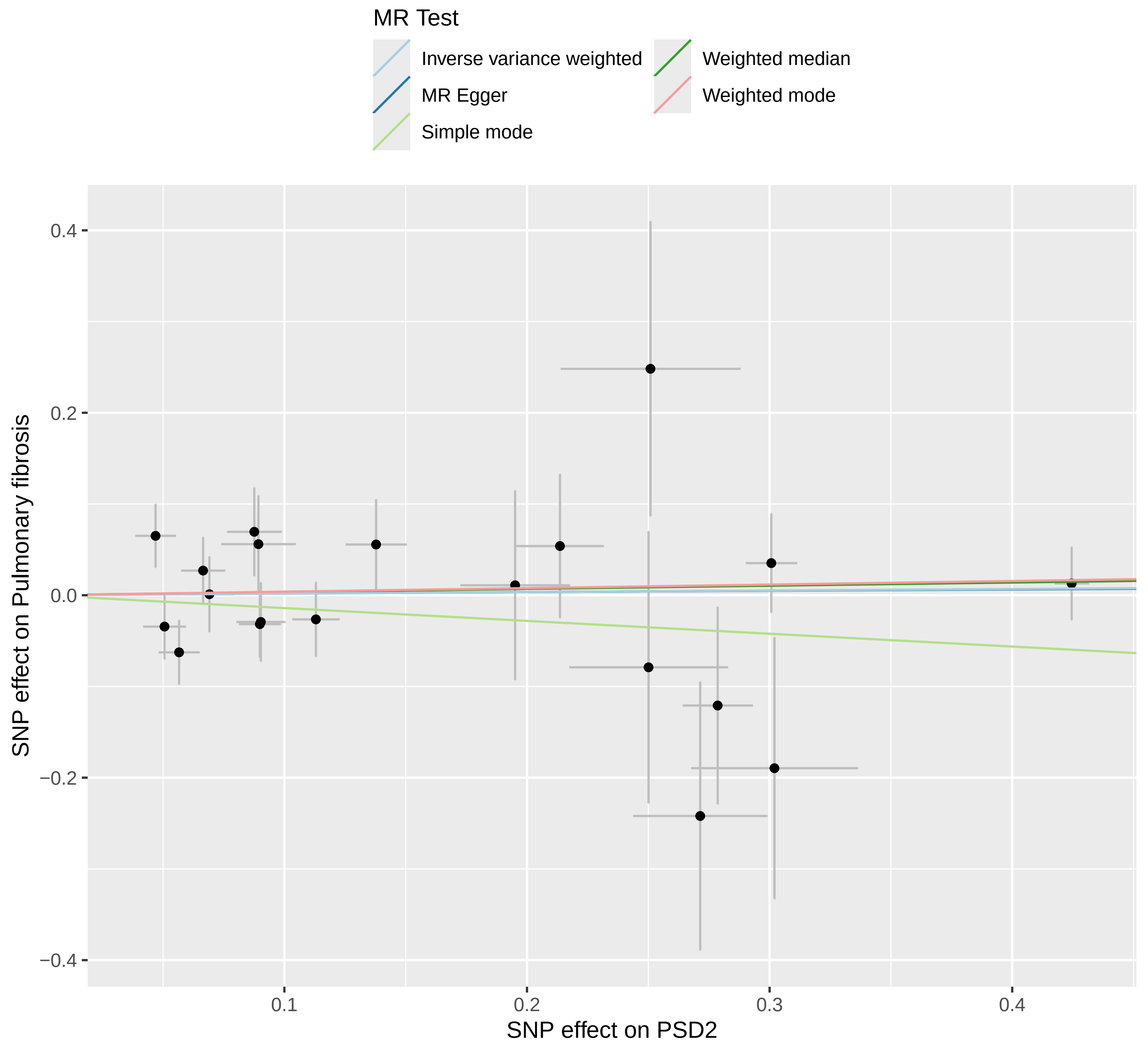
MR Test
Inverse variance weighted
MR Egger
Simple mode
Weighted median
Weighted mode
SNP effect on Pulmonary fibrosis
0.4
0.2
0.0
−0.2
−0.4
0.1
0.2
0.3
0.4
SNP effect on PSD2

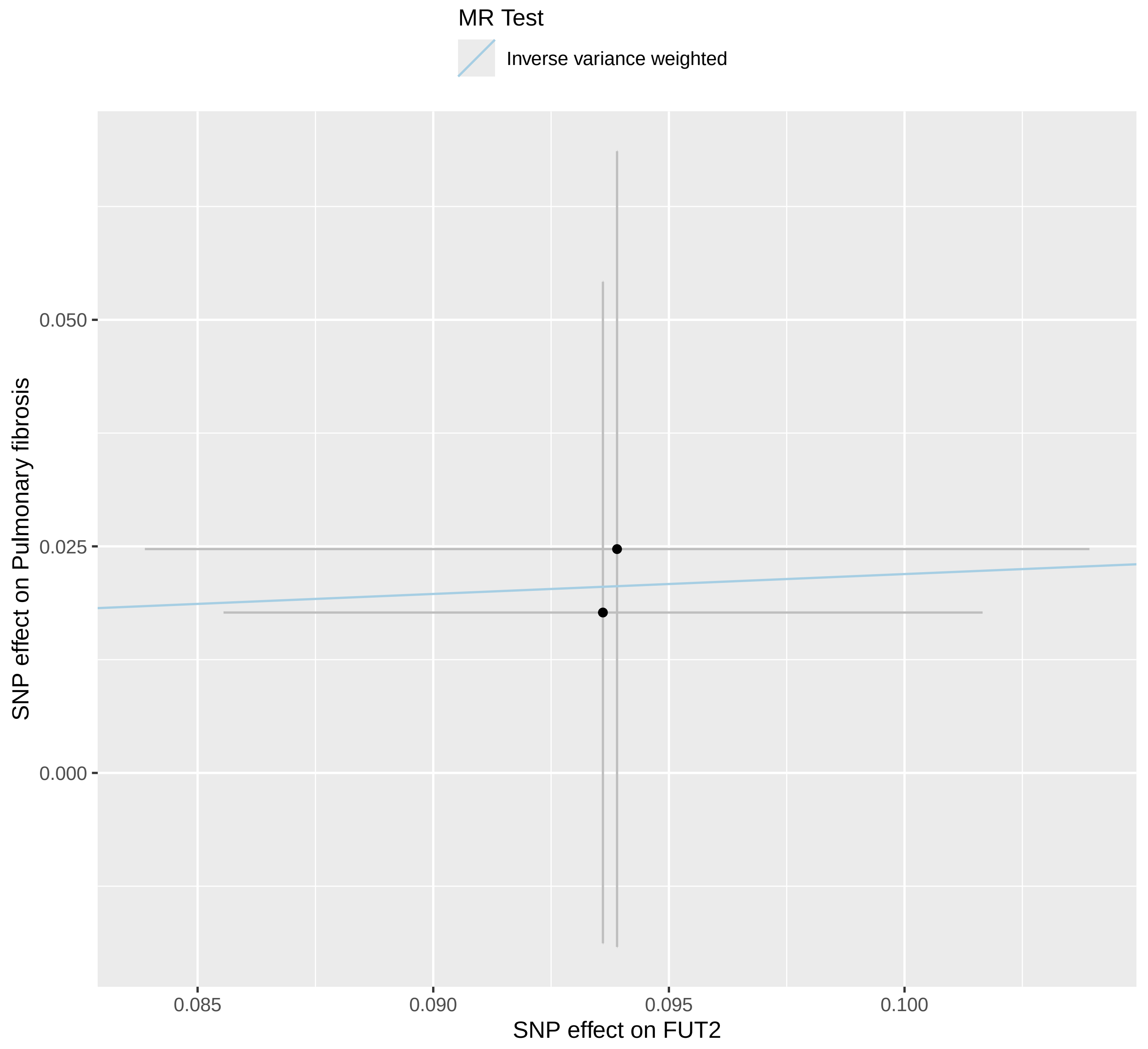

MR Test
Inverse variance weighted
0.050
0.025
0.000
0.085
0.090
0.095
0.100
SNP effect on FUT2
SNP effect on Pulmonary fibrosis

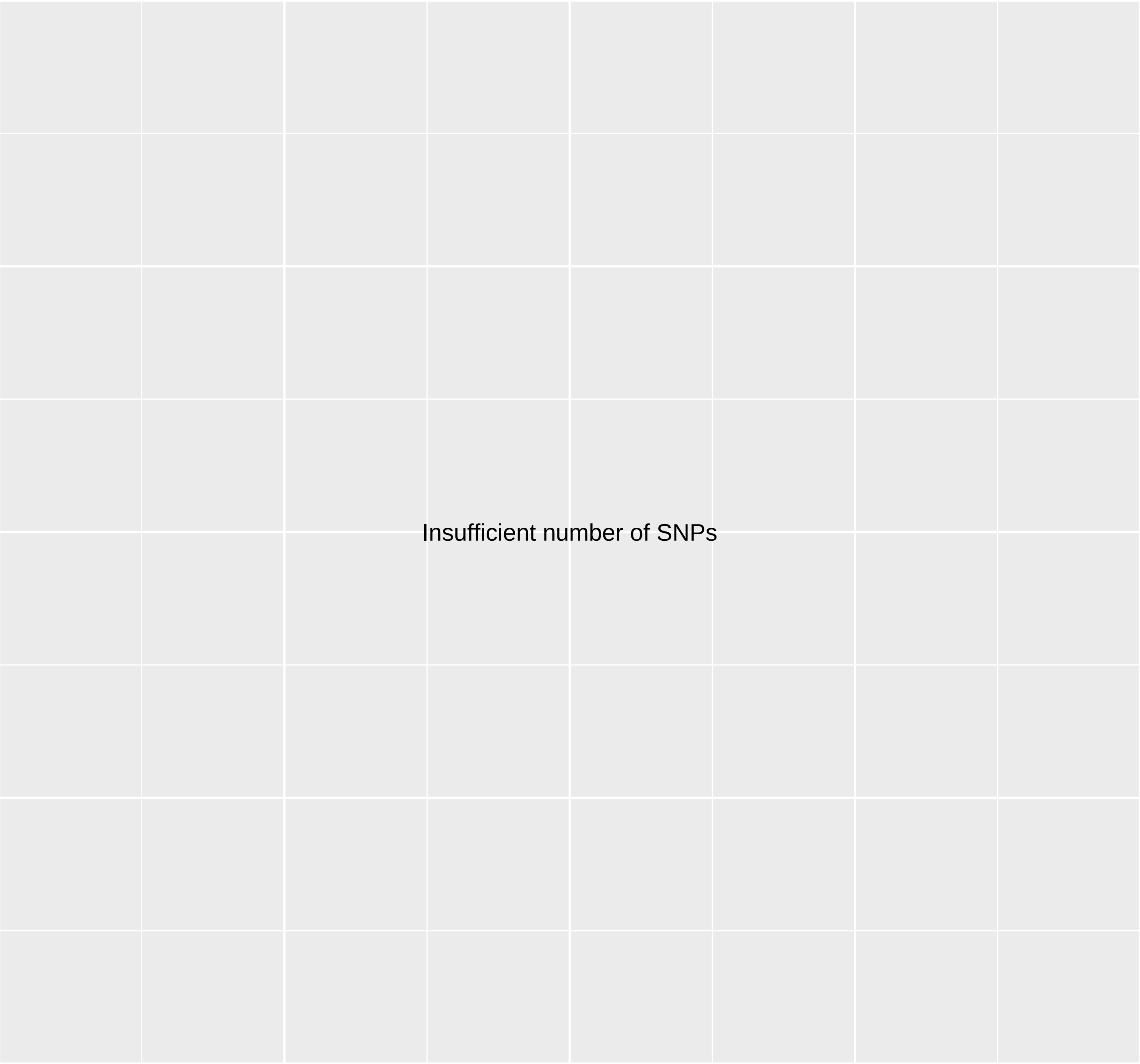
Insufficient number of SNPs

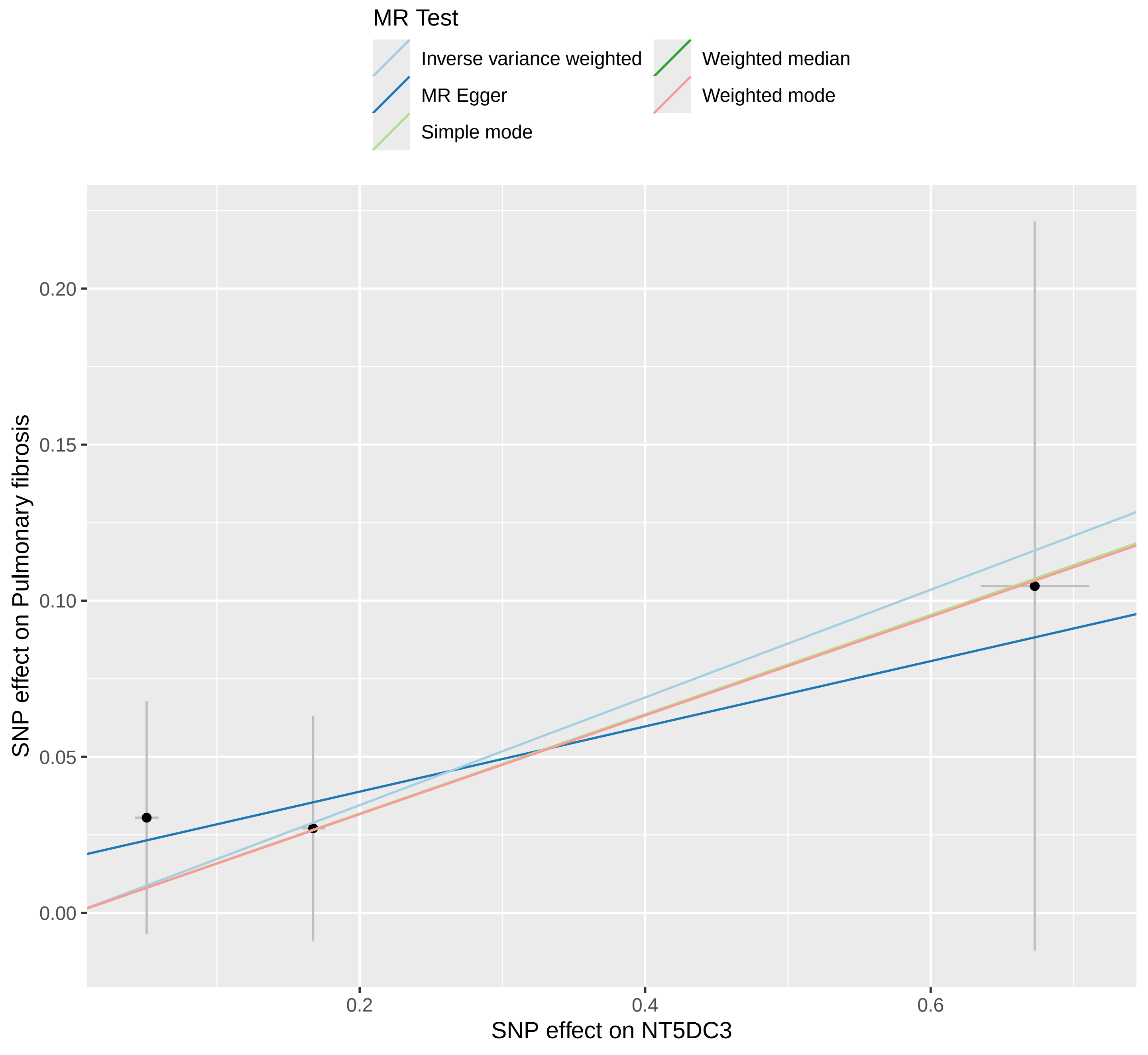

MR Test
Inverse variance weighted
MR Egger
Simple mode
Weighted median
Weighted mode
0.20
0.15
0.10
0.05
0.00
0.2
0.4
0.6
SNP effect on Pulmonary fibrosis
SNP effect on NT5DC3

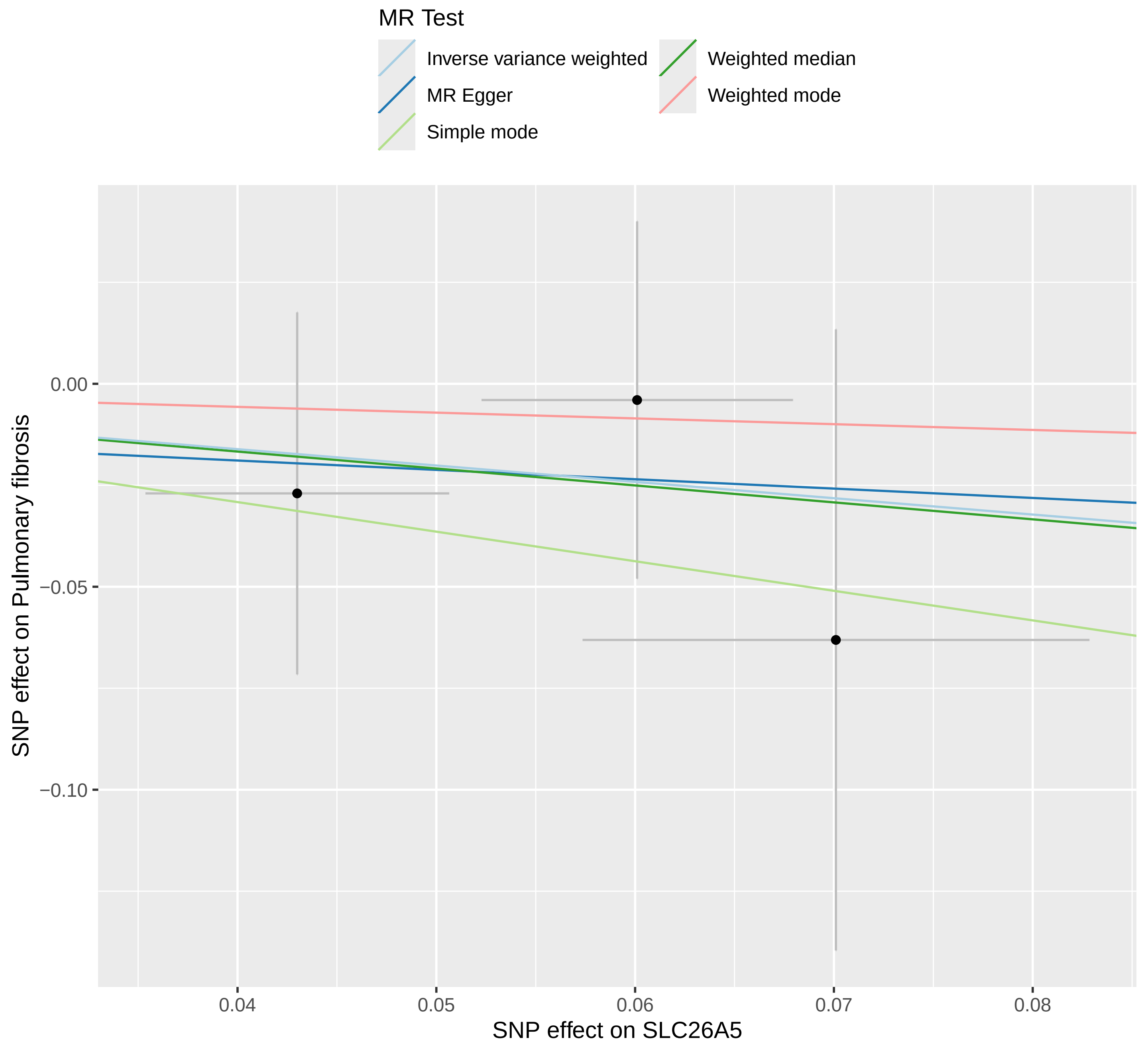

MR Test
Inverse variance weighted
MR Egger
Simple mode
Weighted median
Weighted mode
0.00
−0.05
−0.10
SNP effect on Pulmonary fibrosis
0.04
0.05
0.06
0.07
0.08
SNP effect on SLC26A5

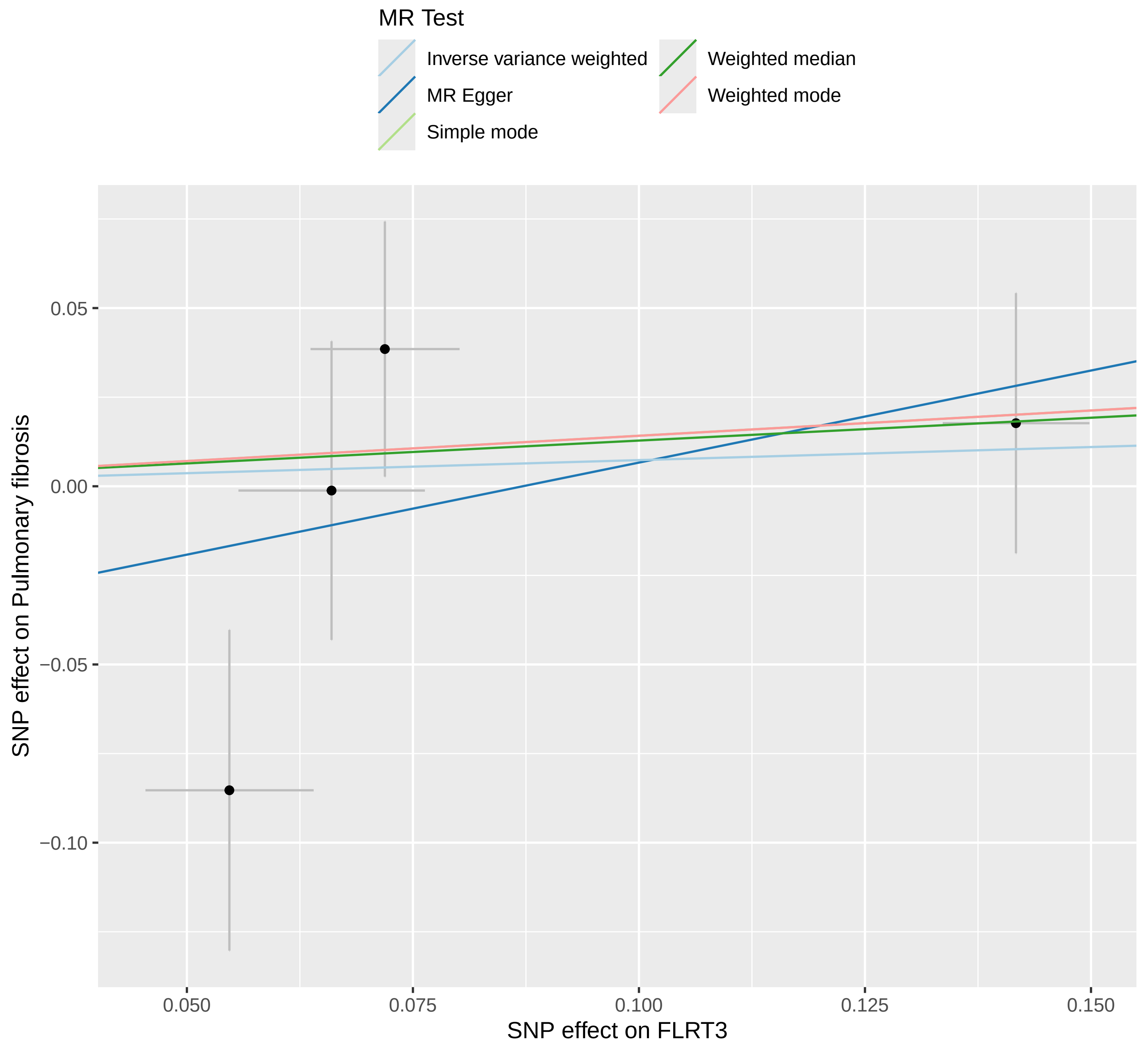

MR Test
Inverse variance weighted
MR Egger
Simple mode
Weighted median
Weighted mode
0.05
0.00
−0.05
−0.10
0.050
0.075
0.100
0.125
0.150
SNP effect on Pulmonary fibrosis
SNP effect on FLRT3

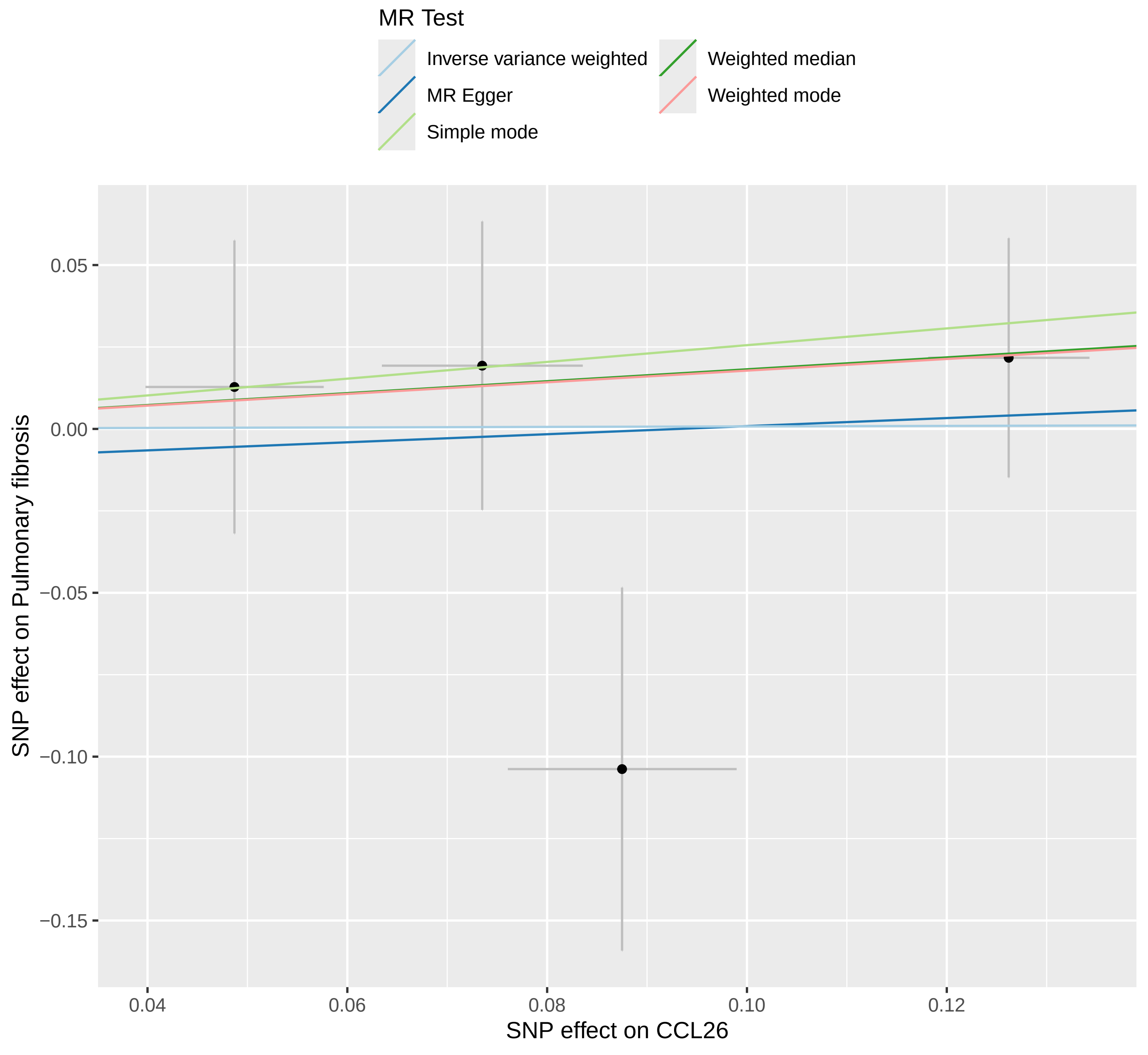

MR Test
Inverse variance weighted
MR Egger
Simple mode
Weighted median
Weighted mode
0.05
0.00
−0.05
−0.10
−0.15
SNP effect on Pulmonary fibrosis
0.04
0.06
0.08
0.10
0.12
SNP effect on CCL26

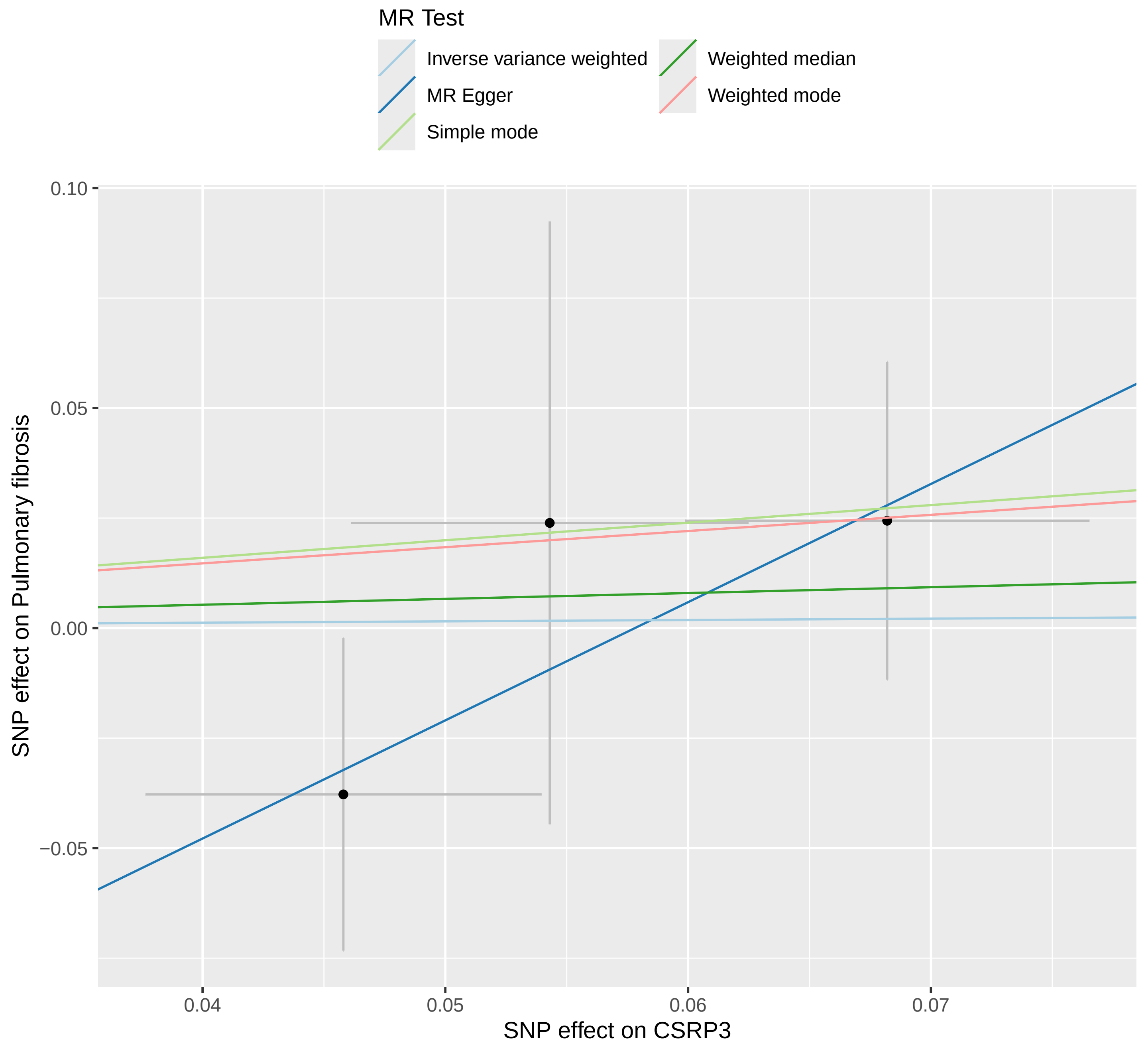

MR Test
Inverse variance weighted
MR Egger
Simple mode
Weighted median
Weighted mode
0.10
0.05
0.00
−0.05
SNP effect on Pulmonary fibrosis
0.04
0.05
0.06
0.07
SNP effect on CSRP3

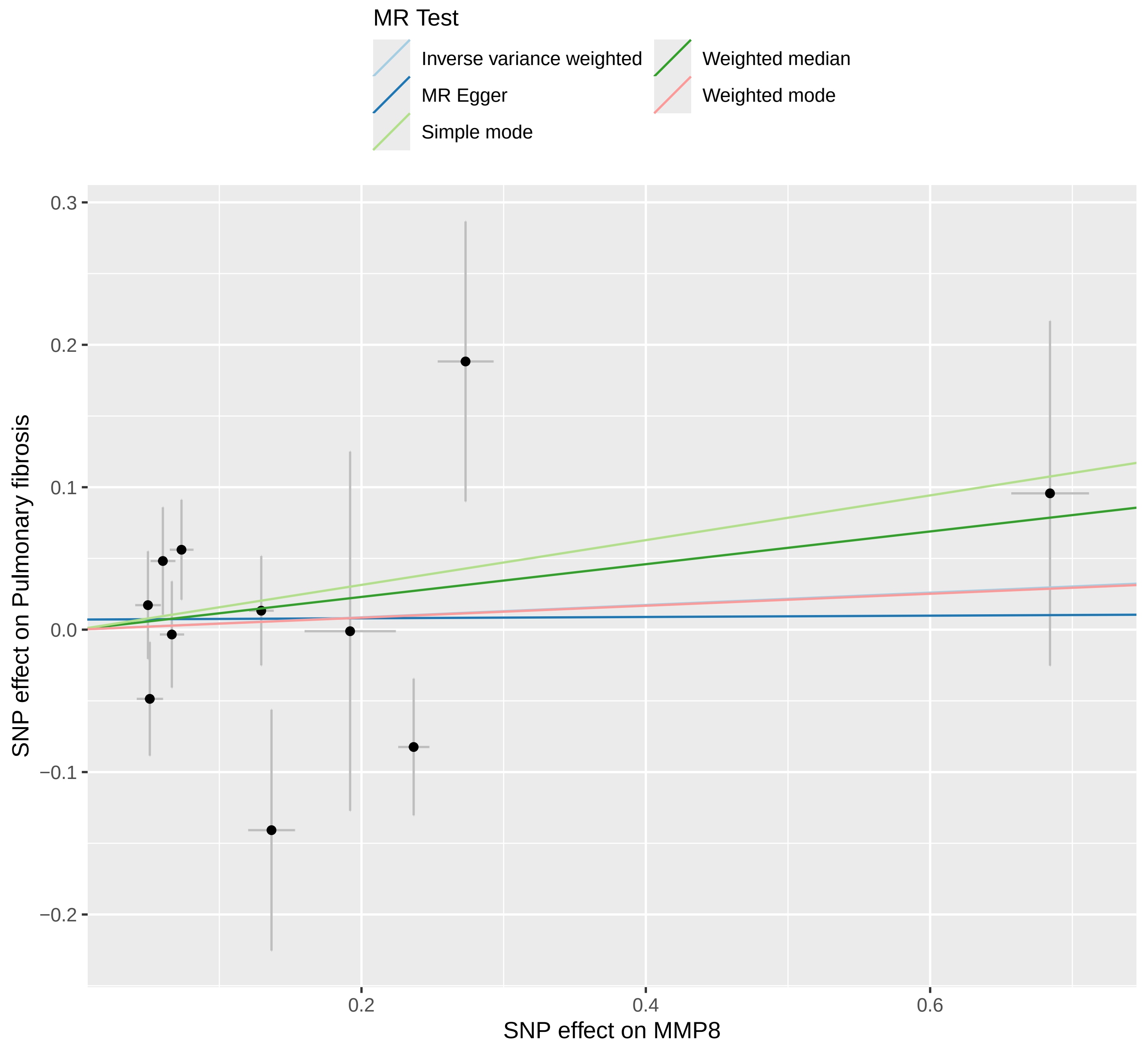
MR Test
Inverse variance weighted
MR Egger
Simple mode
Weighted median
Weighted mode
SNP effect on Pulmonary fibrosis
0.3
0.2
0.1
0.0
−0.1
−0.2
0.2
0.4
0.6
SNP effect on MMP8

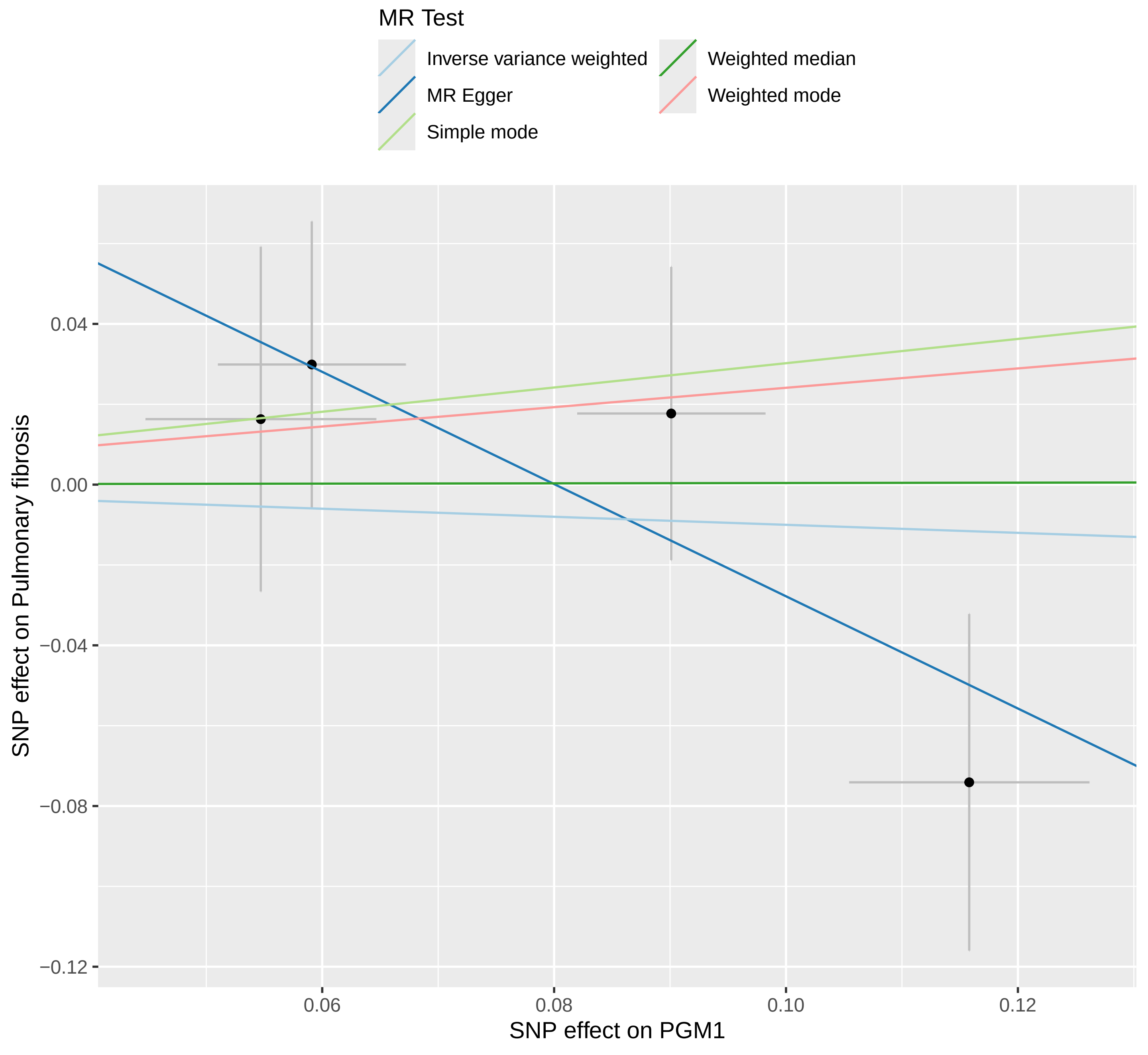
MR Test
Inverse variance weighted
MR Egger
Simple mode
Weighted median
Weighted mode
0.04
0.00
−0.04
−0.08
−0.12
SNP effect on Pulmonary fibrosis
0.06
0.08
0.10
0.12
SNP effect on PGM1

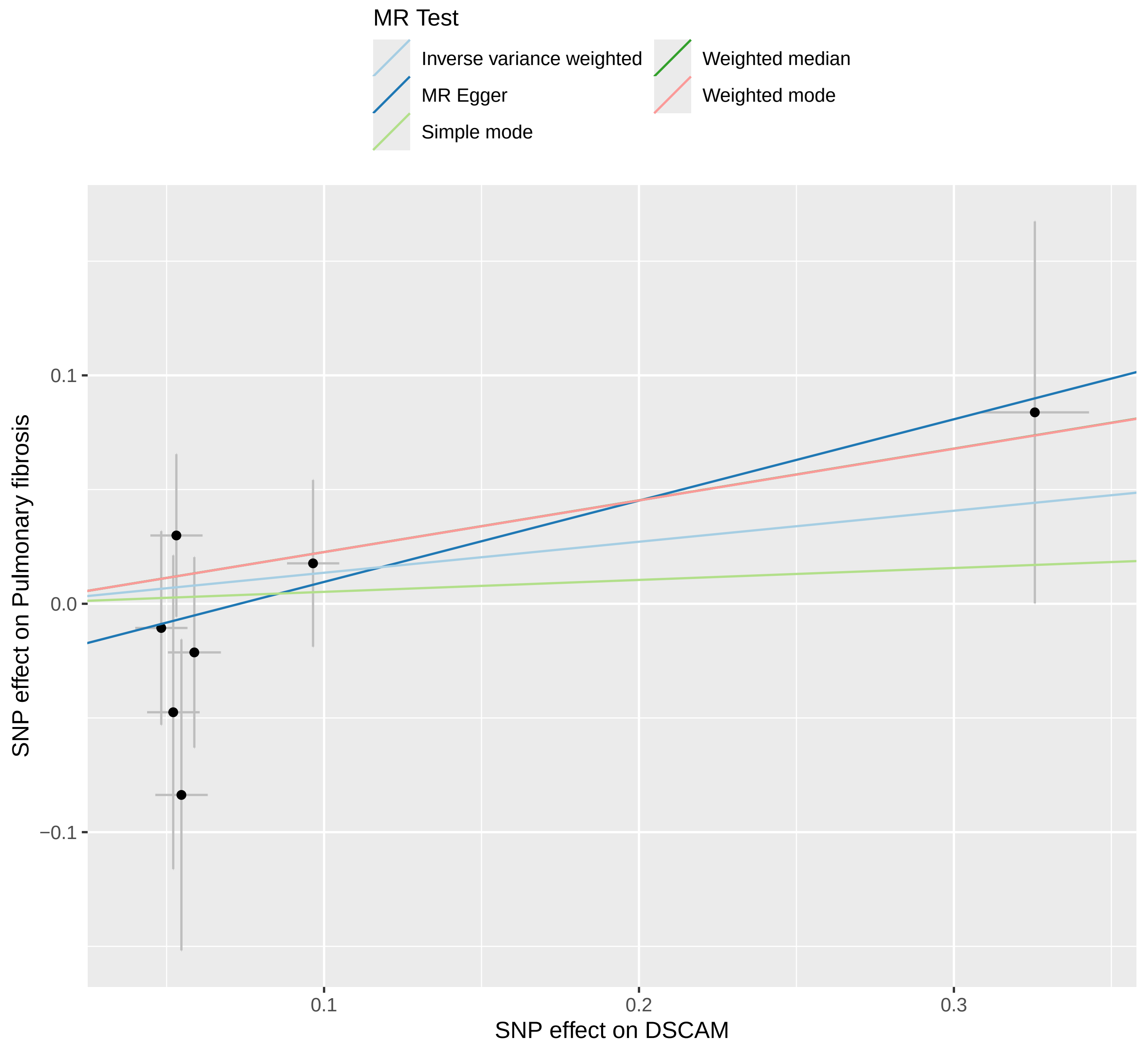

MR Test
Inverse variance weighted
MR Egger
Simple mode
Weighted median
Weighted mode
0.1
0.0
−0.1
SNP effect on Pulmonary fibrosis
0.1
0.2
0.3
SNP effect on DSCAM

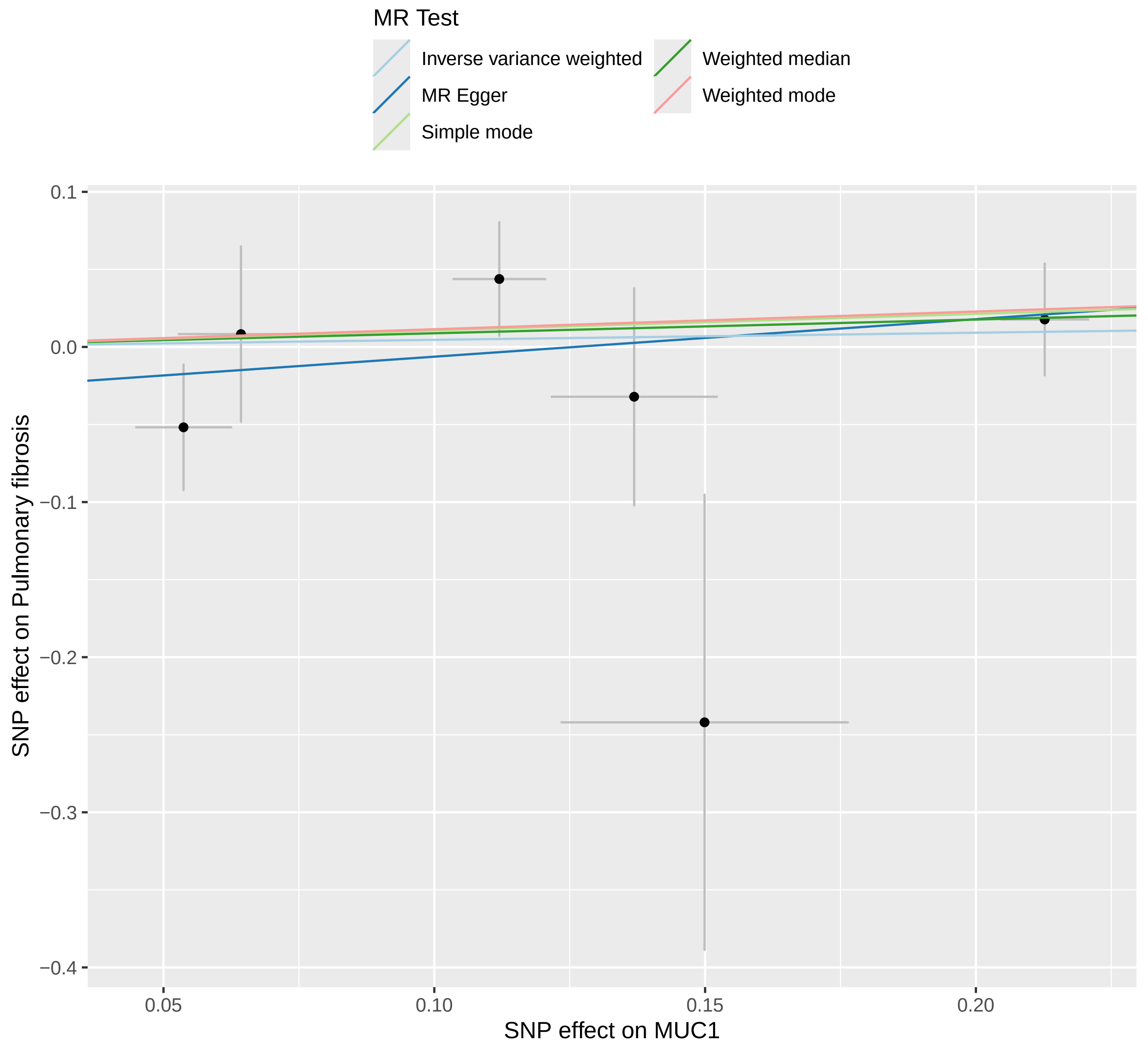
MR Test
Inverse variance weighted
MR Egger
Simple mode
Weighted median
Weighted mode
0.1
0.0
−0.1
−0.2
−0.3
−0.4
SNP effect on Pulmonary fibrosis
0.05
0.10
0.15
0.20
SNP effect on MUC1

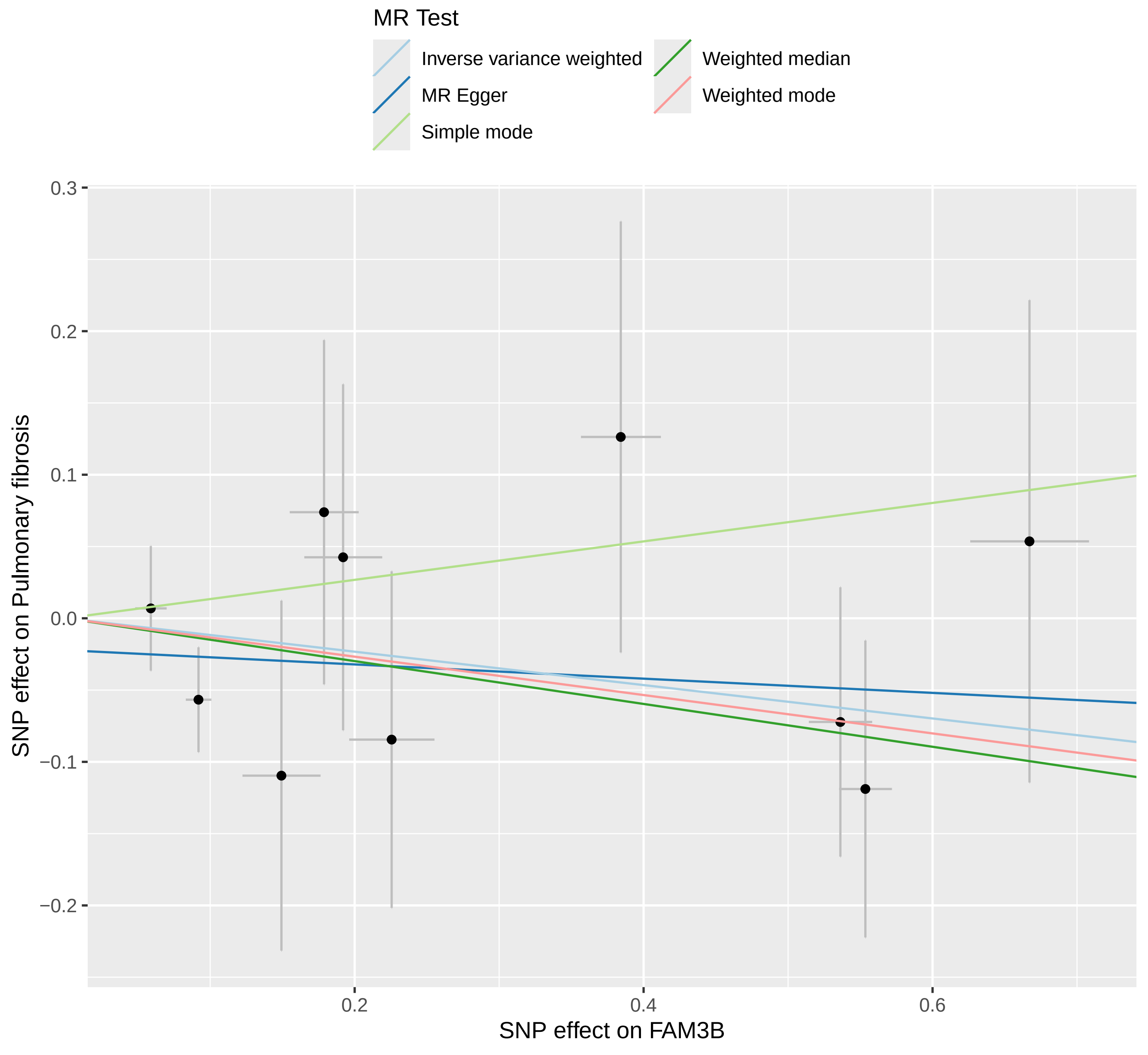

MR Test
Inverse variance weighted
MR Egger
Simple mode
Weighted median
Weighted mode
0.3
0.2
0.1
0.0
−0.1
−0.2
0.2
0.4
0.6
SNP effect on FAM3B
SNP effect on Pulmonary fibrosis

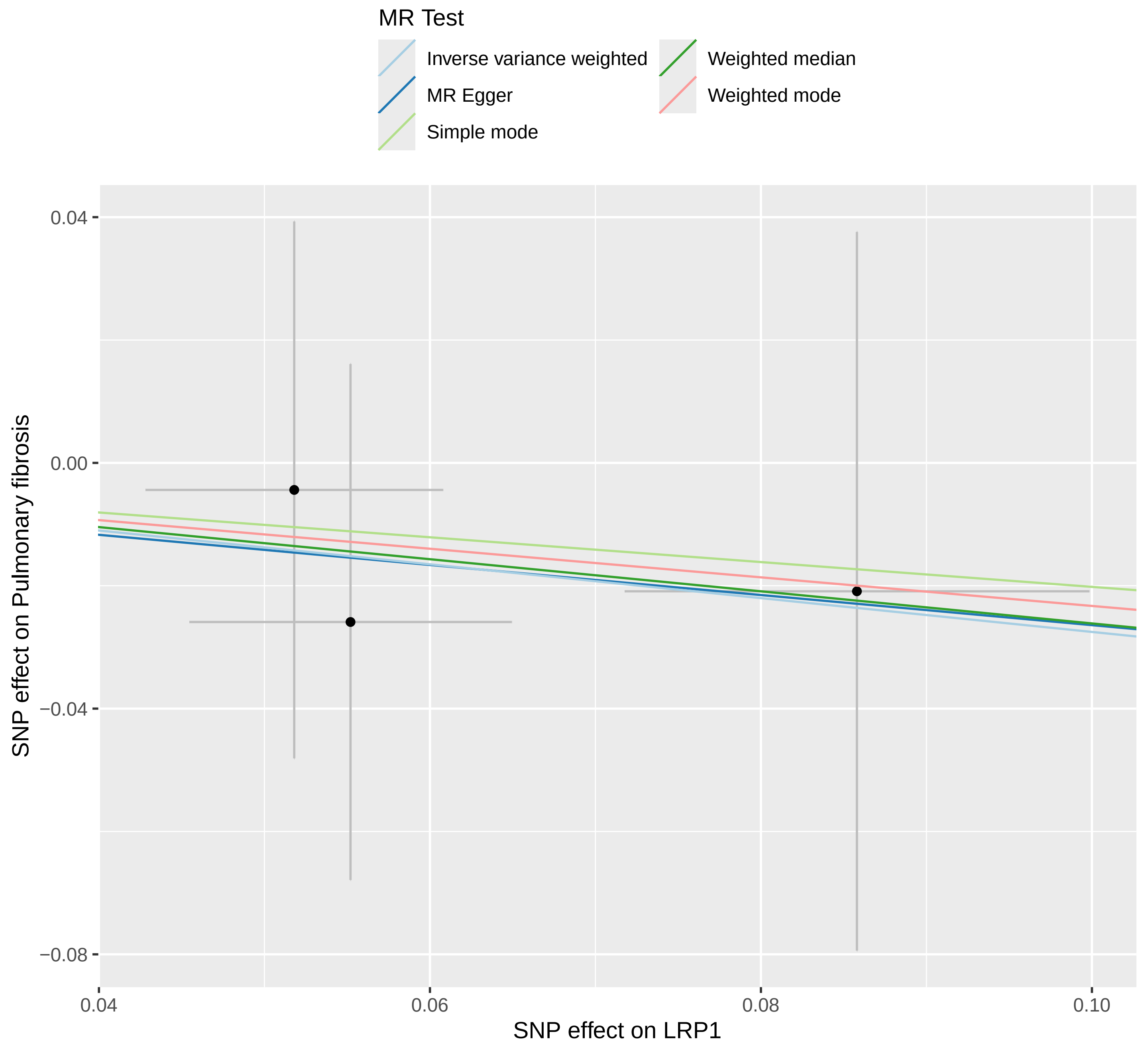
MR Test
Inverse variance weighted
MR Egger
Simple mode
Weighted median
Weighted mode
0.04
0.00
−0.04
−0.08
SNP effect on Pulmonary fibrosis
0.04
0.06
0.08
0.10
SNP effect on LRP1

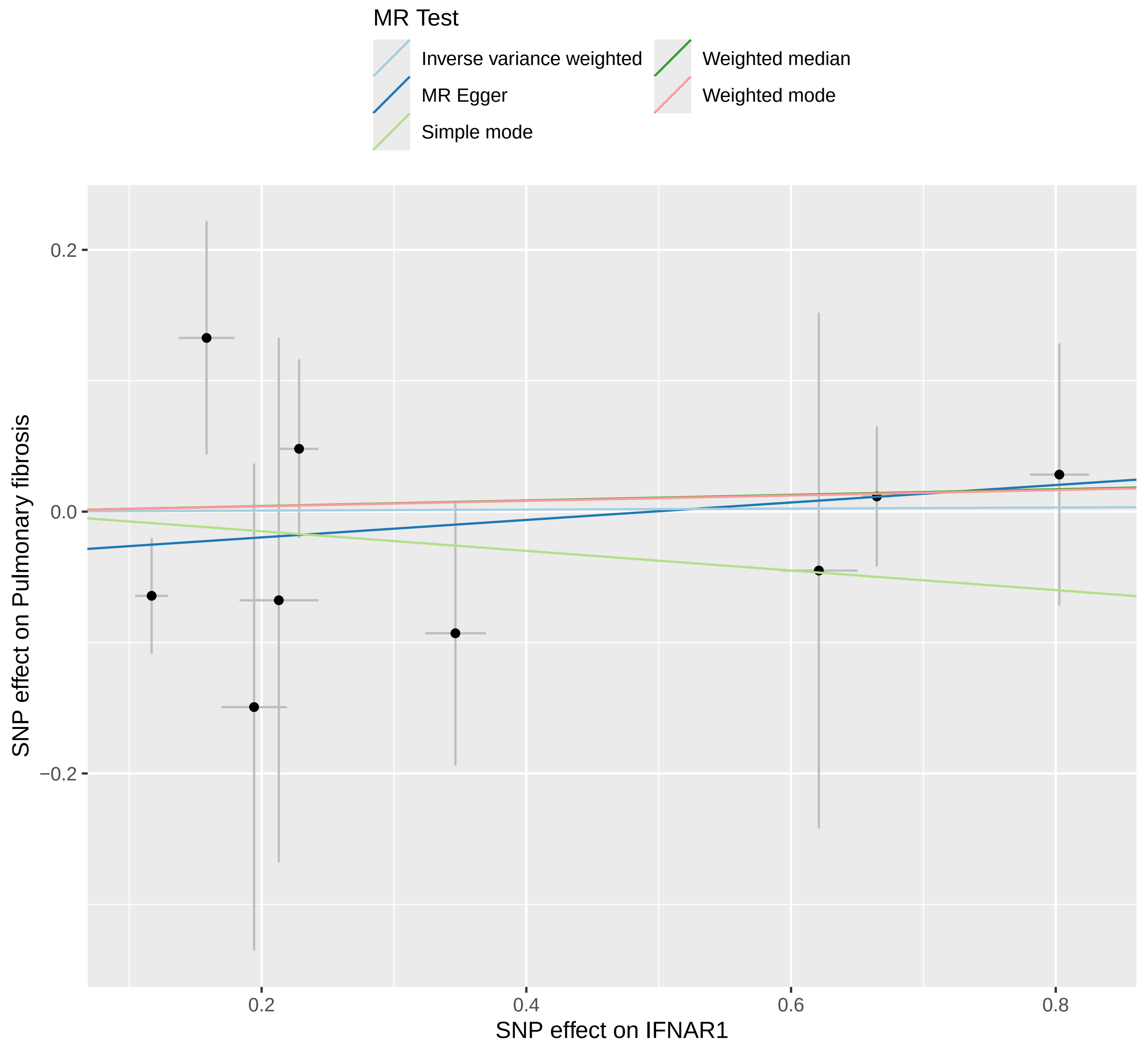

MR Test
Inverse variance weighted
MR Egger
Simple mode
Weighted median
Weighted mode
0.2
0.0
−0.2
0.2
0.4
0.6
0.8
SNP effect on Pulmonary fibrosis
SNP effect on IFNAR1

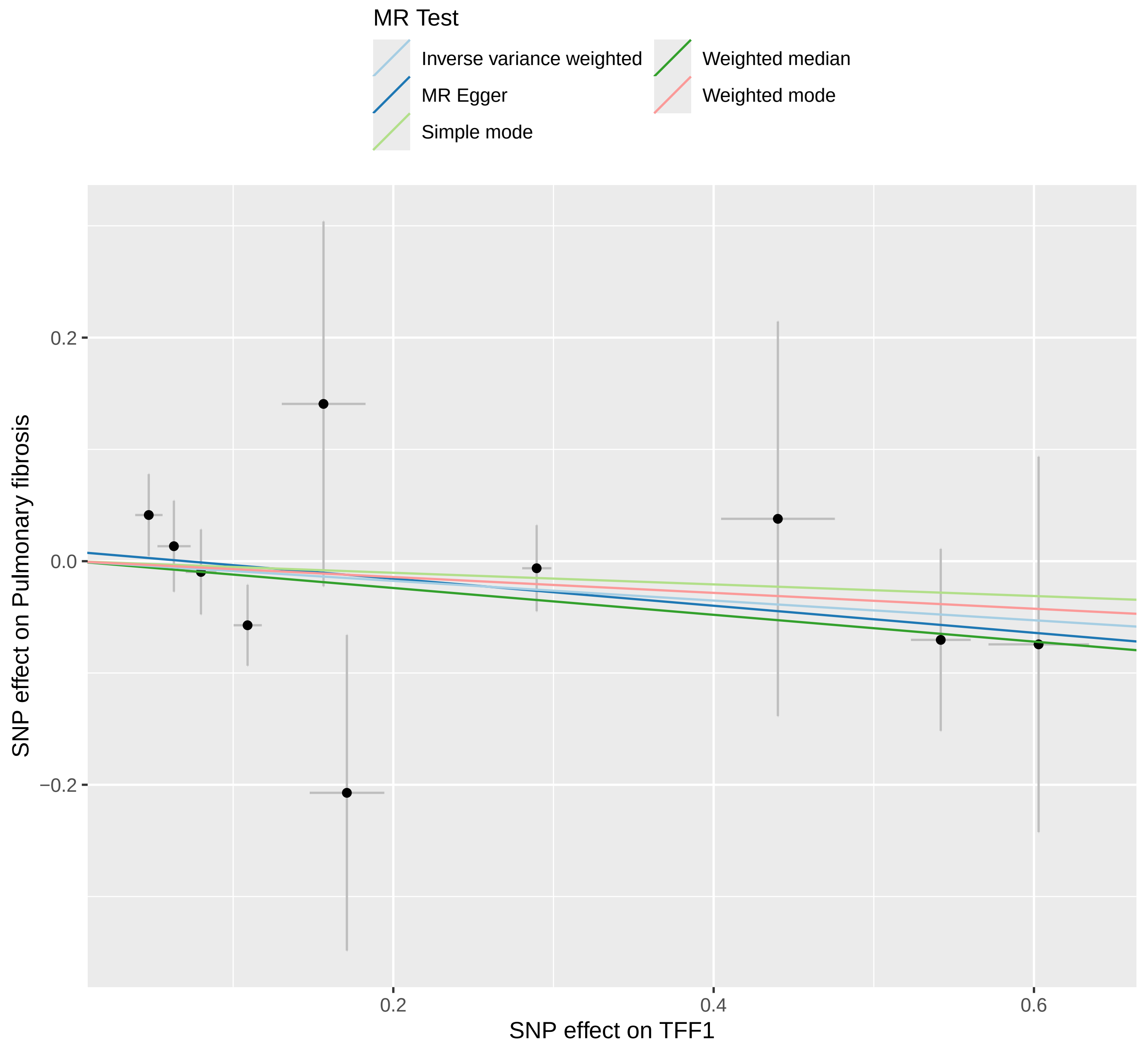
MR Test
Inverse variance weighted
MR Egger
Simple mode
Weighted median
Weighted mode
0.2
0.0
−0.2
SNP effect on Pulmonary fibrosis
0.2
0.4
0.6
SNP effect on TFF1

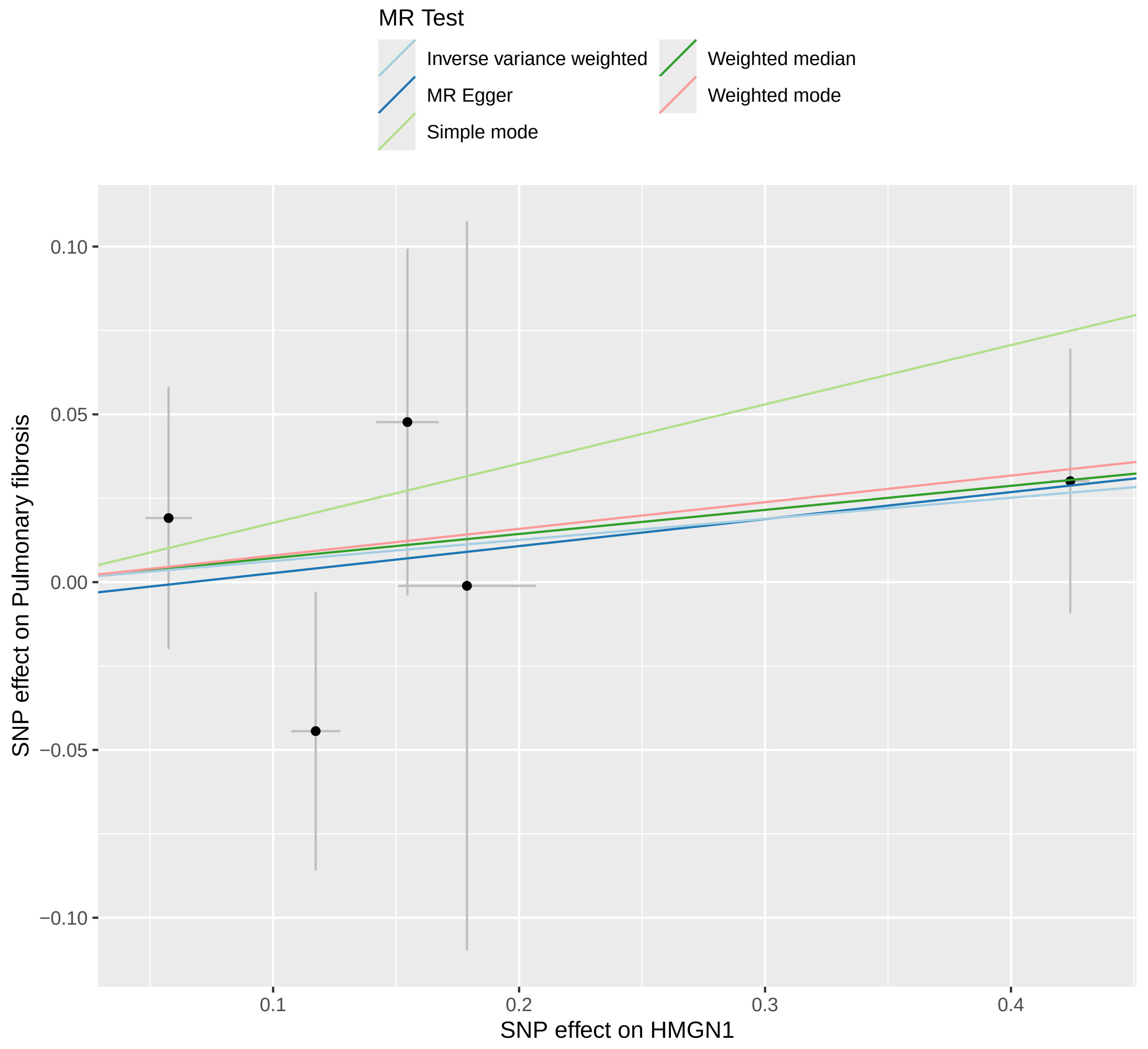
MR Test
Inverse variance weighted
MR Egger
Simple mode
Weighted median
Weighted mode
0.10
0.05
0.00
−0.05
−0.10
SNP effect on Pulmonary fibrosis
0.1
0.2
0.3
0.4
SNP effect on HMGN1

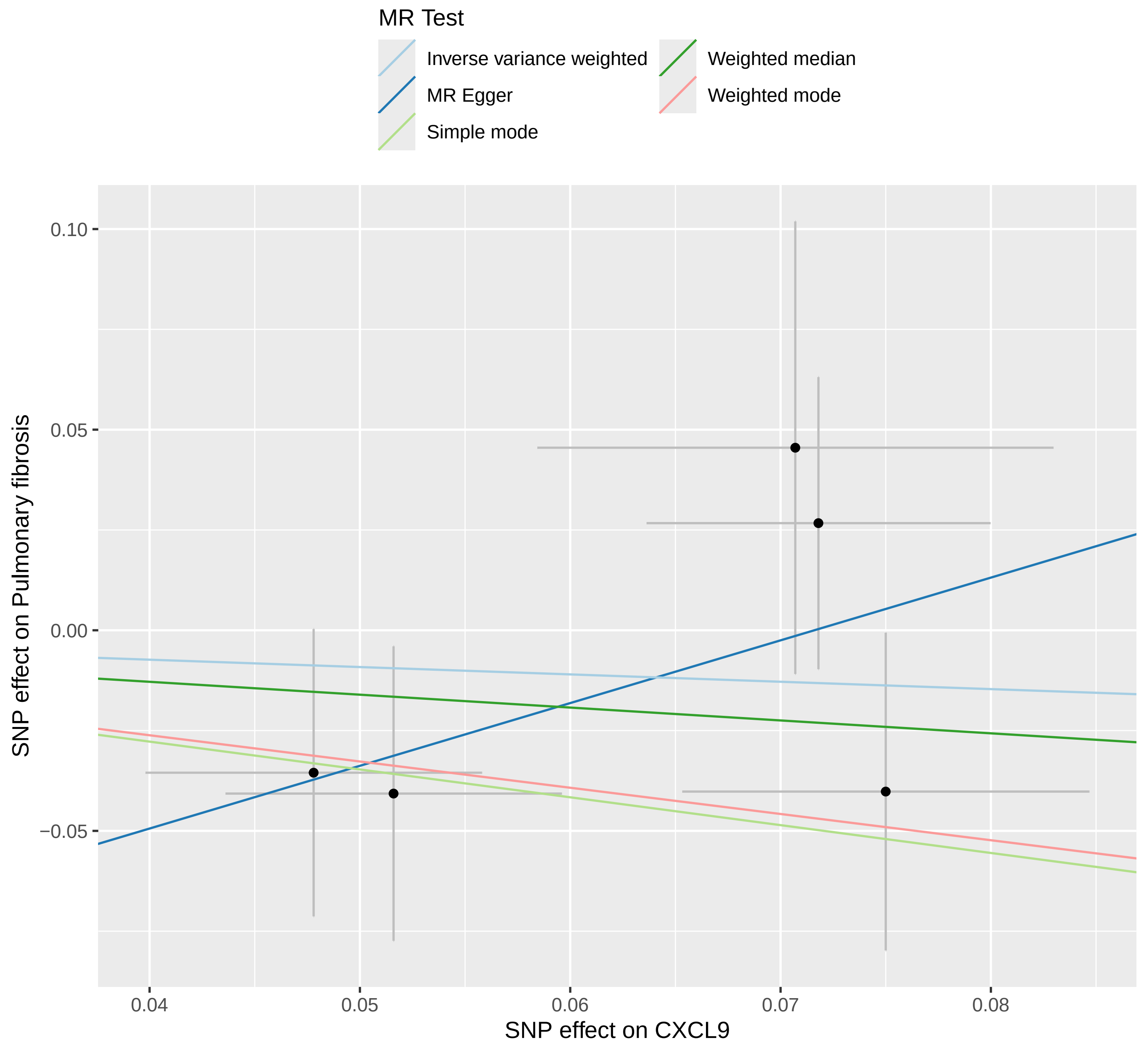
MR Test
Inverse variance weighted
MR Egger
Simple mode
Weighted median
Weighted mode
0.10
0.05
0.00
−0.05
SNP effect on Pulmonary fibrosis
0.04
0.05
0.06
0.07
0.08
SNP effect on CXCL9

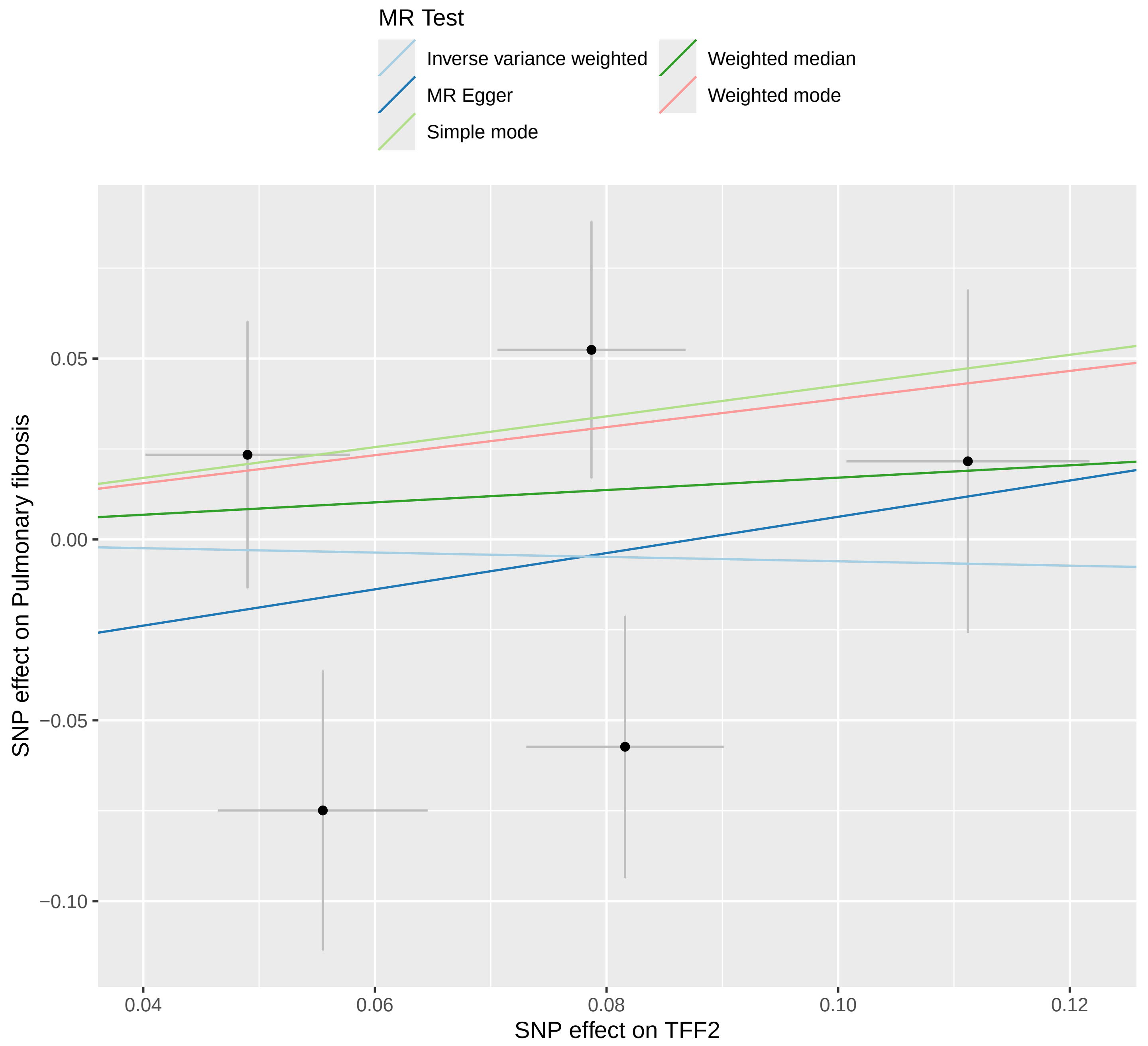

MR Test
Inverse variance weighted
MR Egger
Simple mode
Weighted median
Weighted mode
0.05
0.00
−0.05
−0.10
SNP effect on Pulmonary fibrosis
0.04
0.06
0.08
0.10
0.12
SNP effect on TFF2

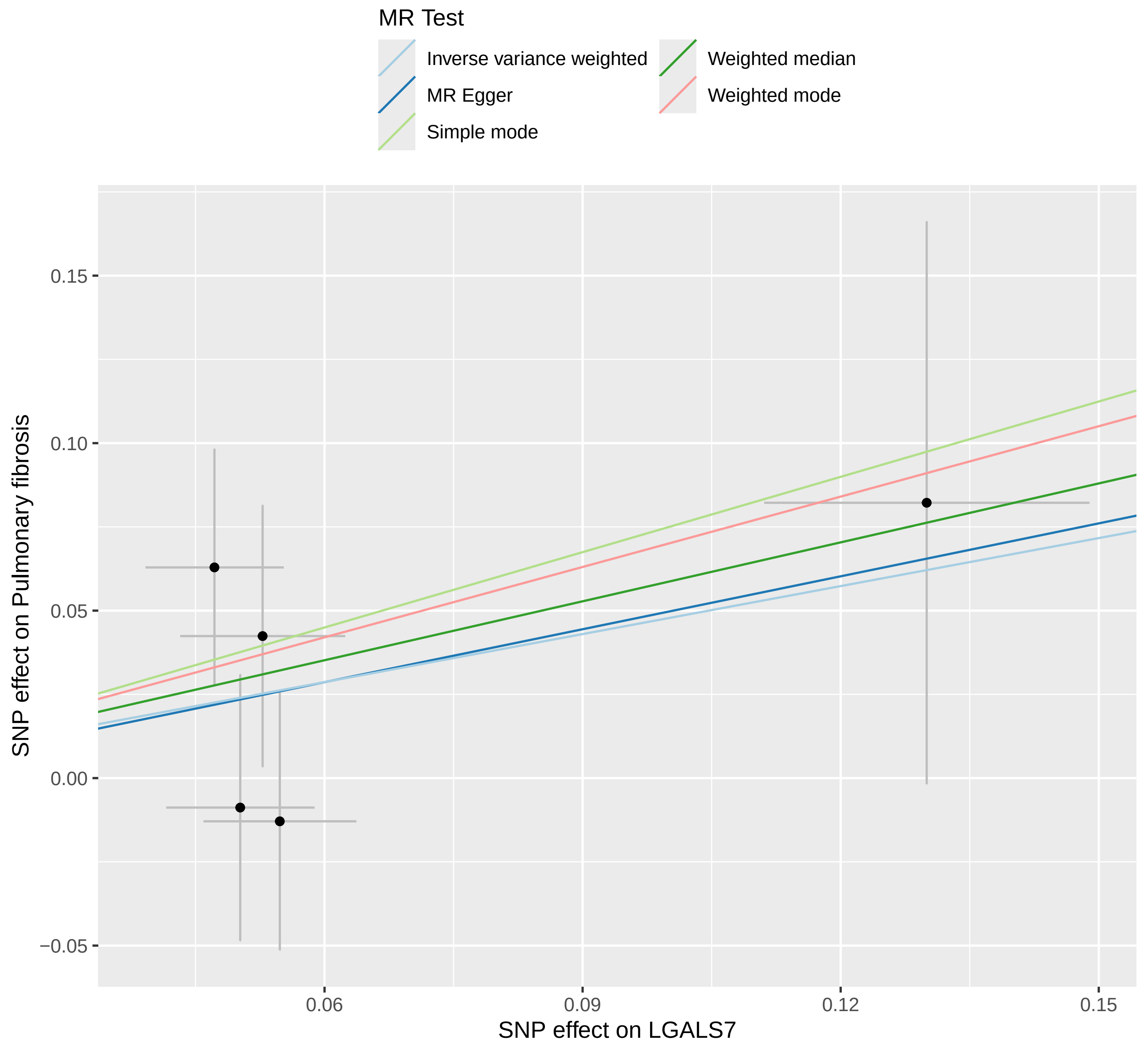
MR Test
Inverse variance weighted
MR Egger
Simple mode
Weighted median
Weighted mode
0.15
0.10
0.05
0.00
−0.05
0.06
0.09
0.12
0.15
SNP effect on Pulmonary fibrosis
SNP effect on LGALS7

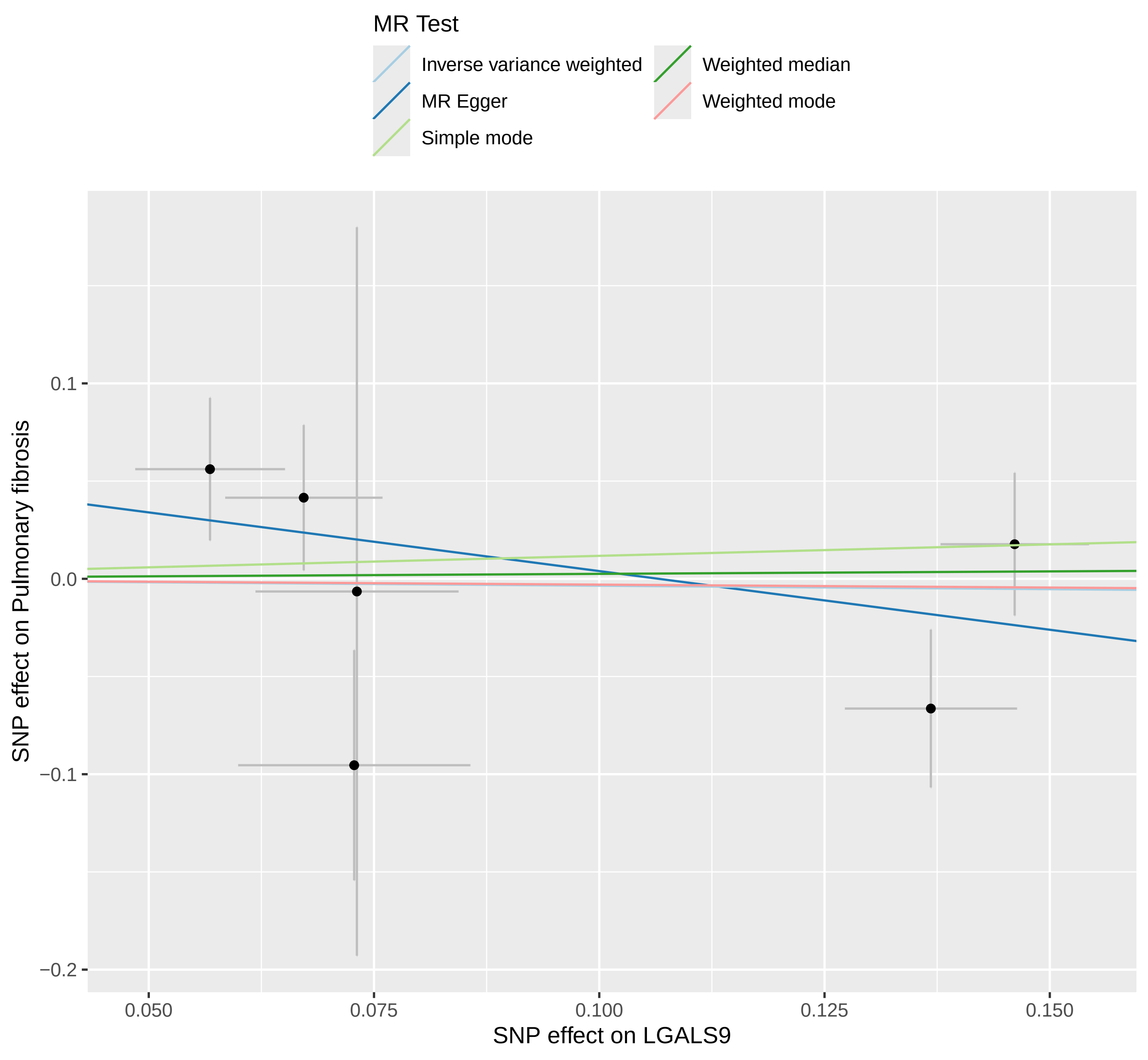
MR Test
Inverse variance weighted
MR Egger
Simple mode
Weighted median
Weighted mode
0.1
0.0
−0.1
−0.2
0.050
0.075
0.100
0.125
0.150
SNP effect on Pulmonary fibrosis
SNP effect on LGALS9

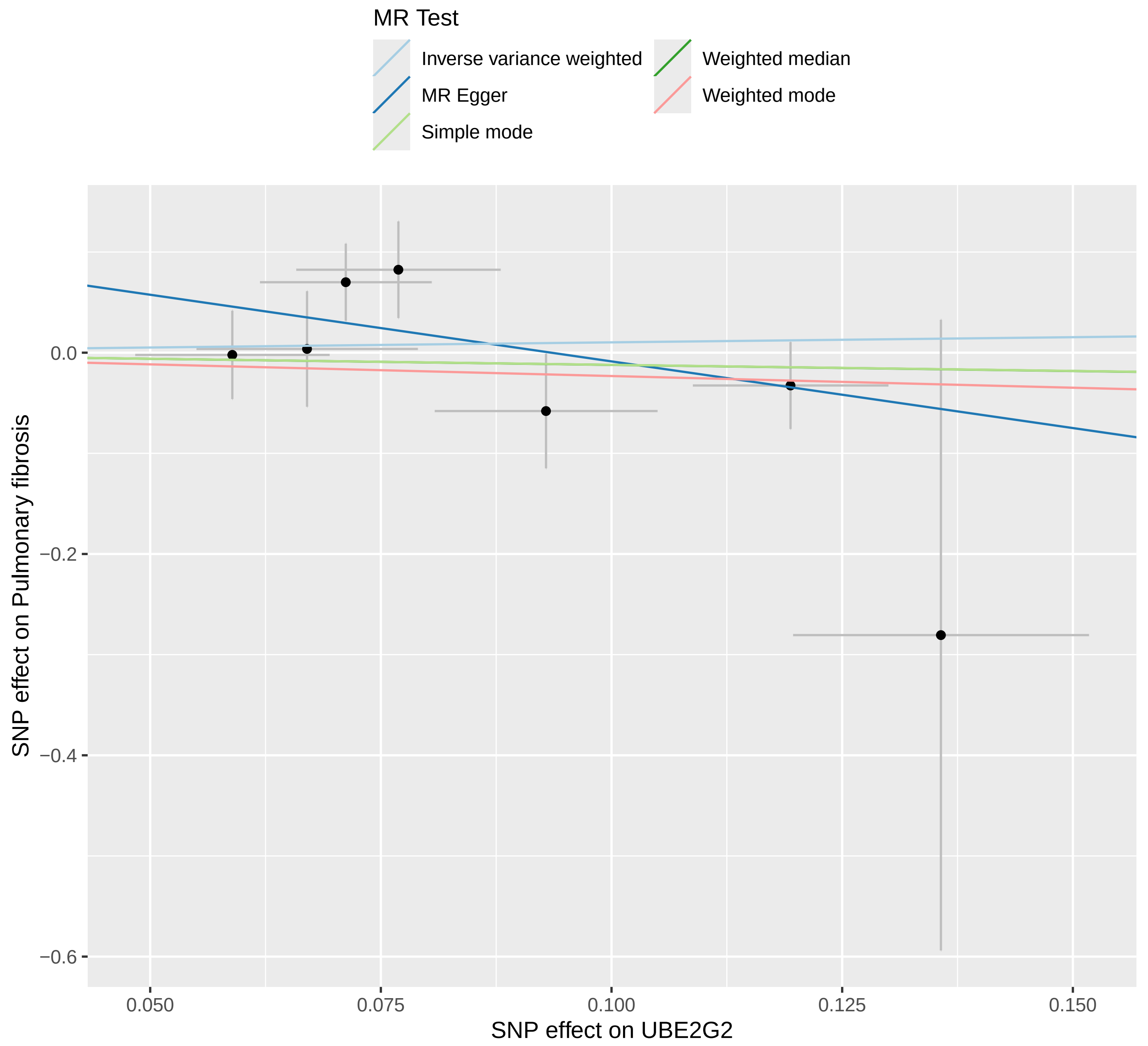
MR Test
Inverse variance weighted
MR Egger
Simple mode
Weighted median
Weighted mode
SNP effect on Pulmonary fibrosis
0.0
−0.2
−0.4
−0.6
0.050
0.075
0.100
0.125
0.150
SNP effect on UBE2G2

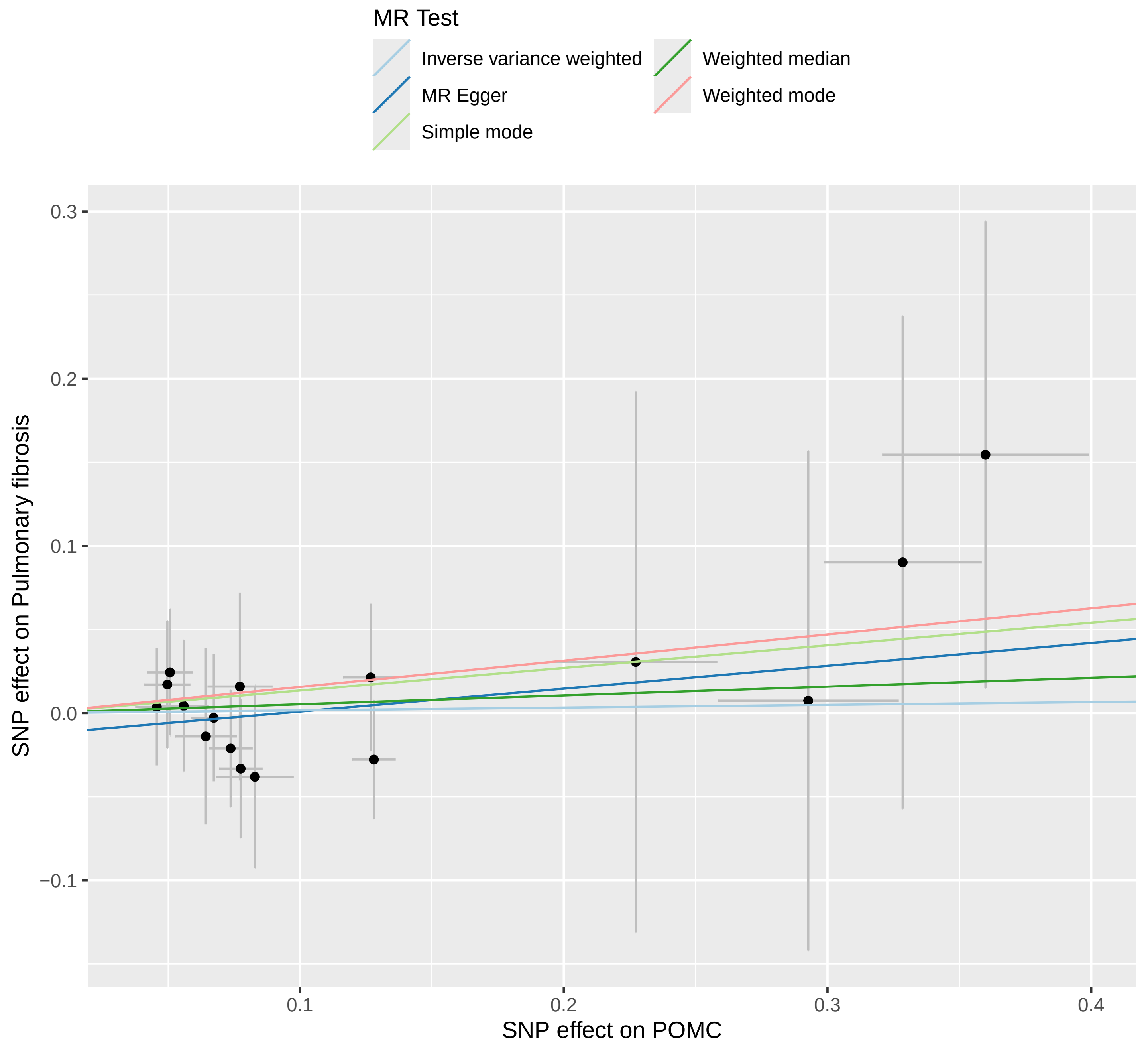

MR Test
Inverse variance weighted
MR Egger
Simple mode
Weighted median
Weighted mode
0.3
0.2
0.1
0.0
−0.1
0.1
0.2
0.3
0.4
SNP effect on Pulmonary fibrosis
SNP effect on POMC

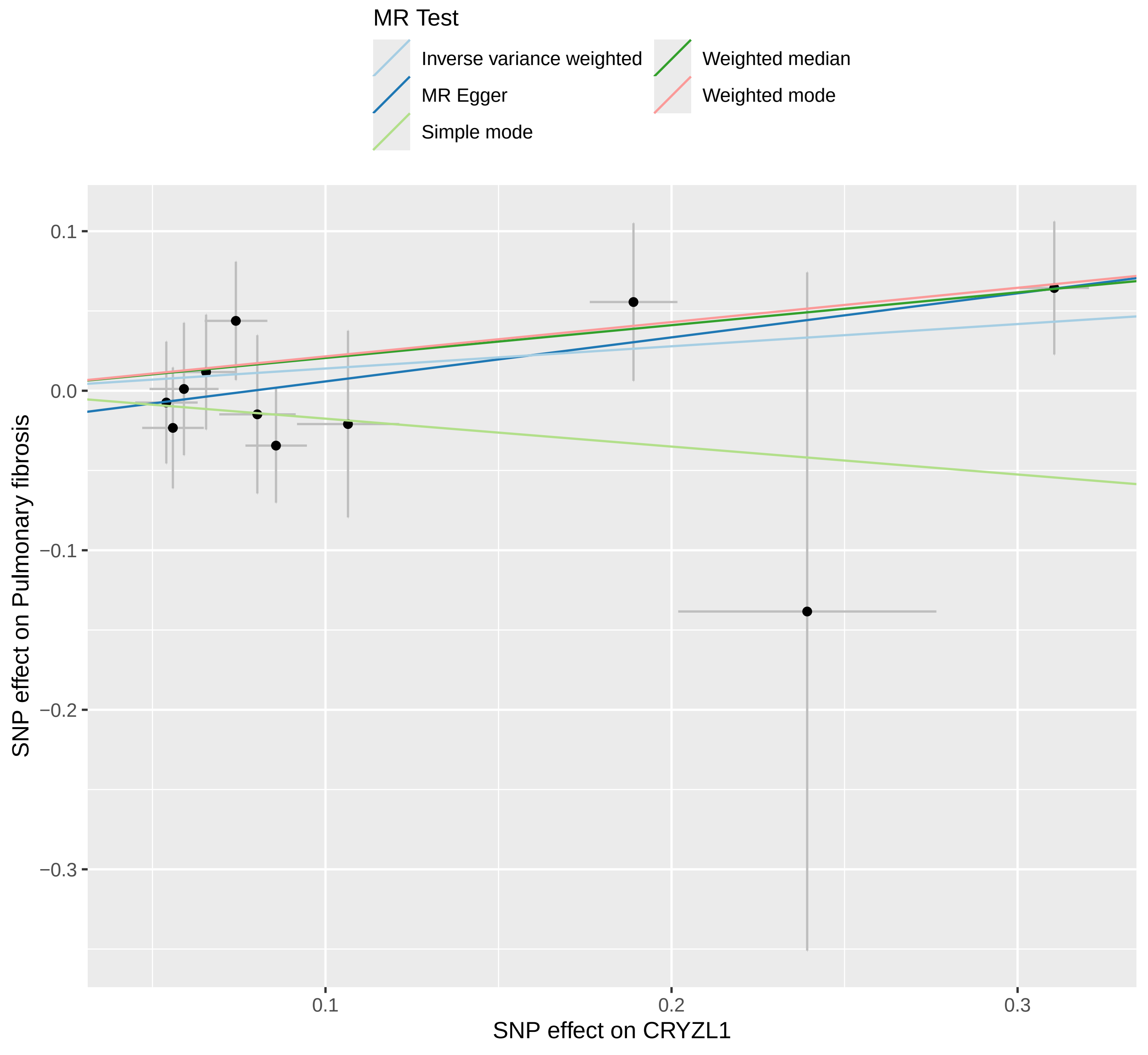

MR Test
Inverse variance weighted
MR Egger
Simple mode
Weighted median
Weighted mode
0.1
0.0
−0.1
−0.2
−0.3
SNP effect on Pulmonary fibrosis
0.1
0.2
0.3
SNP effect on CRYZL1

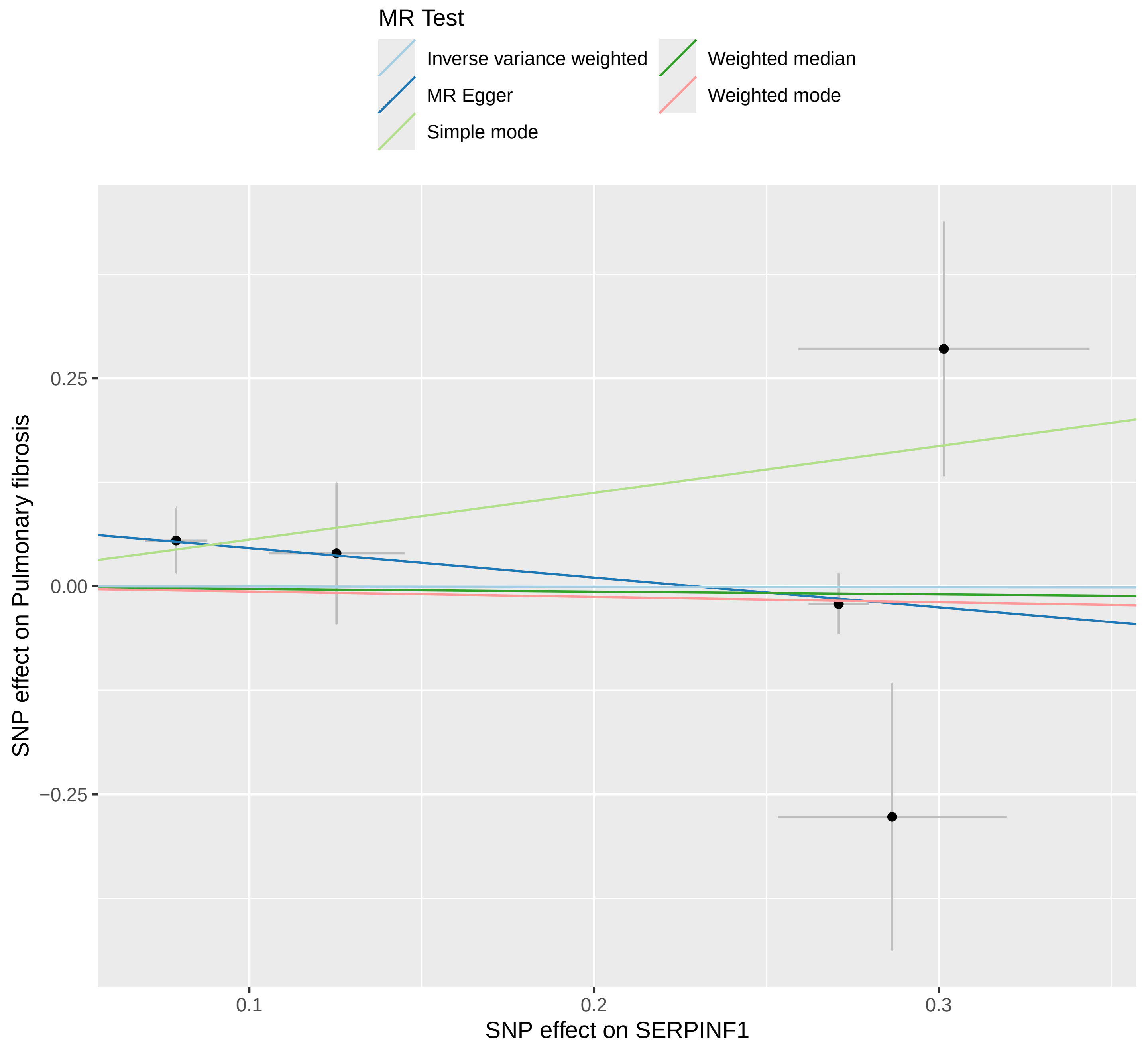
MR Test
Inverse variance weighted
MR Egger
Simple mode
Weighted median
Weighted mode
0.25
0.00
−0.25
0.1
0.2
0.3
SNP effect on Pulmonary fibrosis
SNP effect on SERPINF1

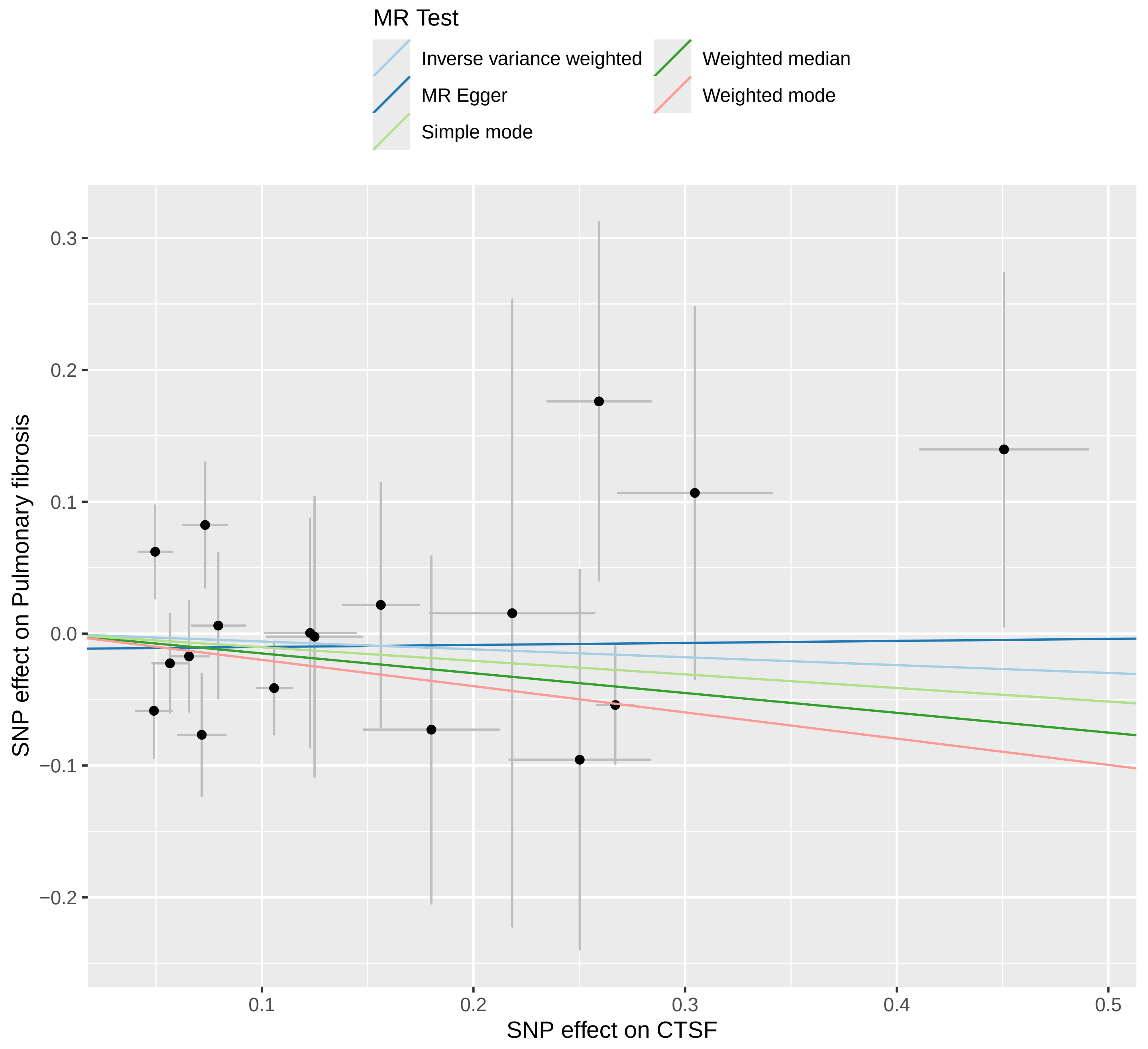

MR Test
Inverse variance weighted
MR Egger
Simple mode
Weighted median
Weighted mode
0.3
0.2
0.1
0.0
−0.1
−0.2
SNP effect on Pulmonary fibrosis
0.1
0.2
0.3
0.4
0.5
SNP effect on CTSF

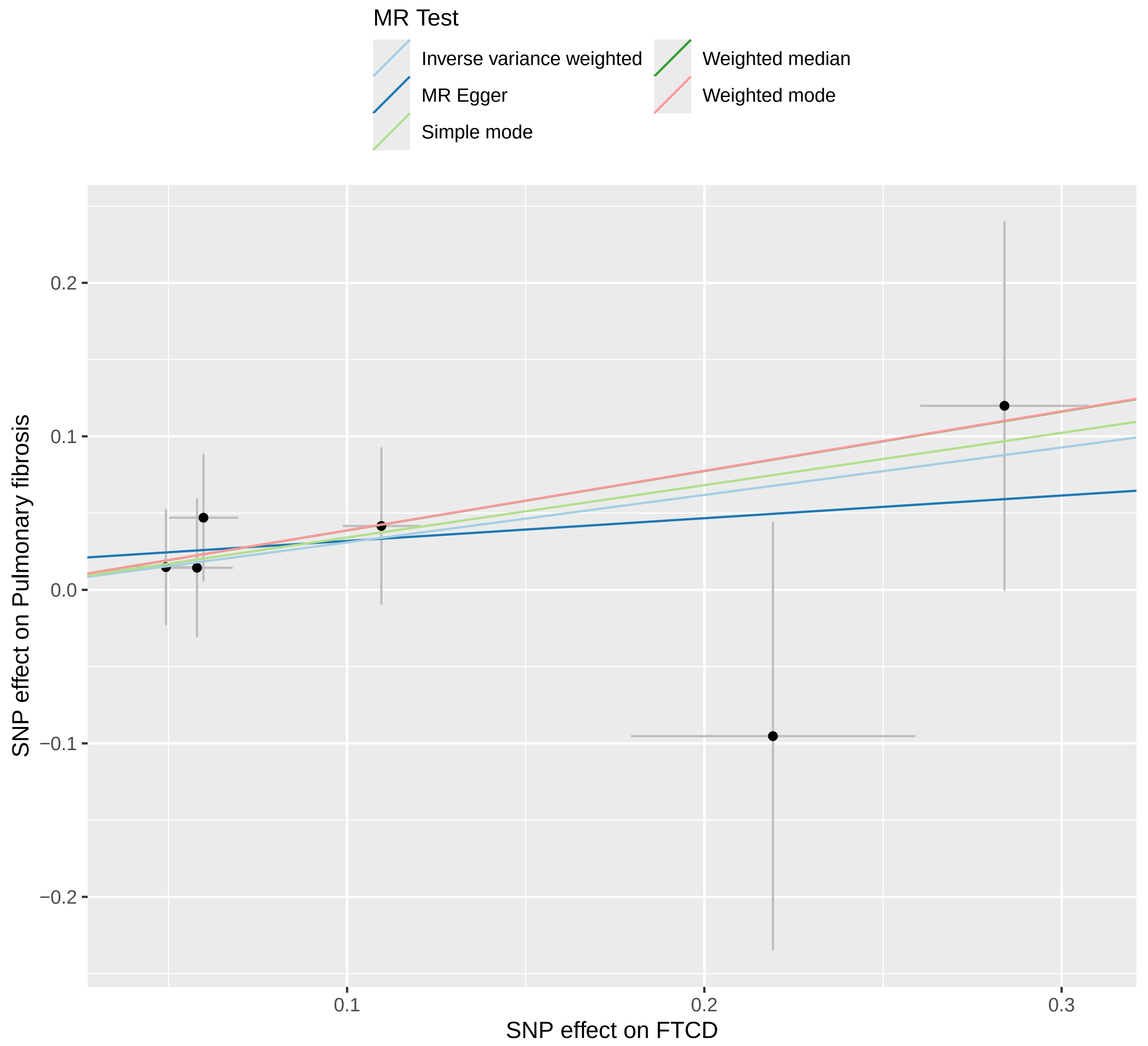

MR Test
Inverse variance weighted
MR Egger
Simple mode
Weighted median
Weighted mode
0.2
0.1
0.0
−0.1
−0.2
0.1
0.2
0.3
SNP effect on Pulmonary fibrosis
SNP effect on FTCD

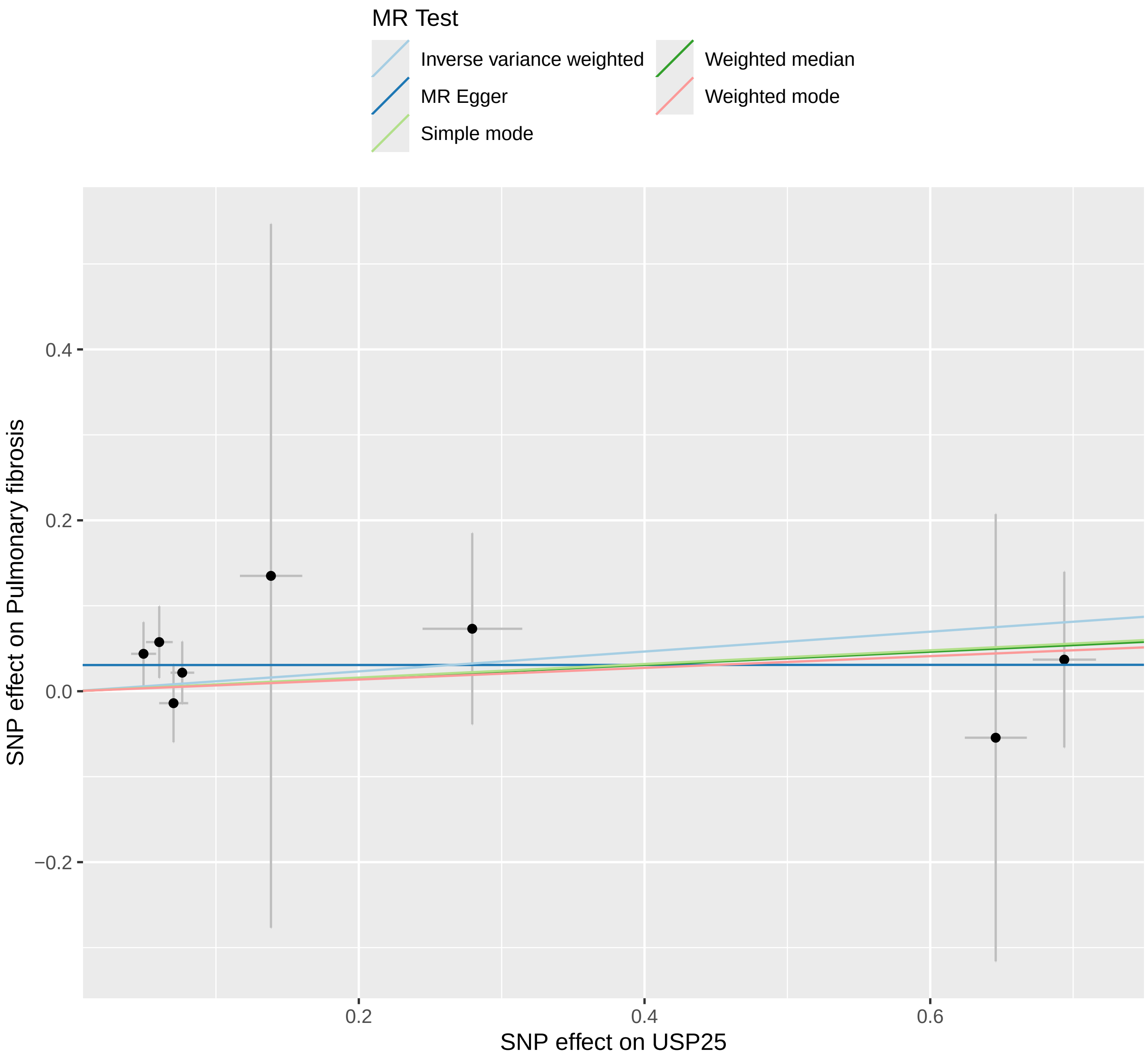
MR Test
Inverse variance weighted
MR Egger
Simple mode
Weighted median
Weighted mode
0.4
0.2
0.0
−0.2
SNP effect on Pulmonary fibrosis
0.2
0.4
0.6
SNP effect on USP25

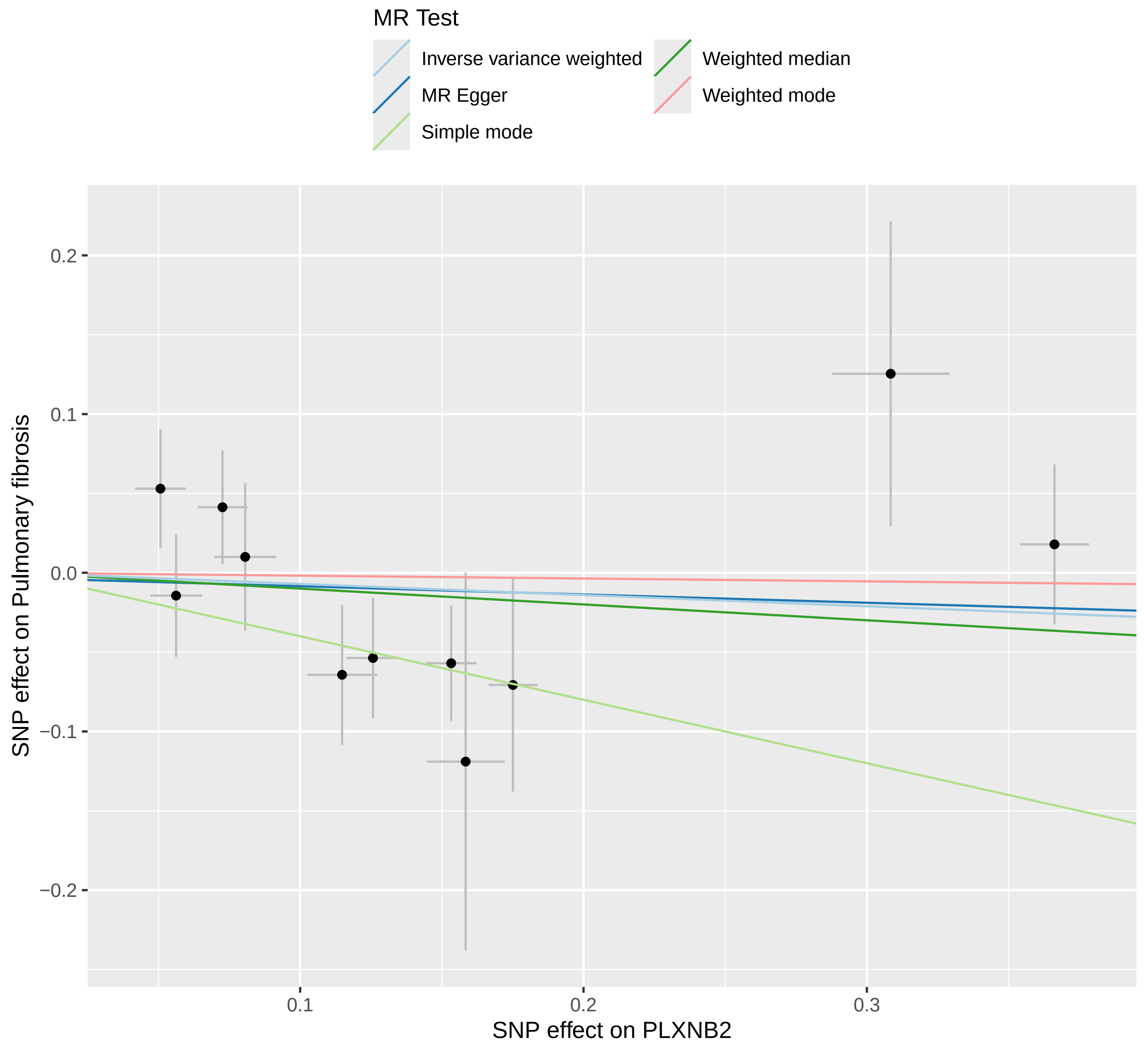
MR Test
Inverse variance weighted
MR Egger
Simple mode
Weighted median
Weighted mode
0.2
0.1
0.0
−0.1
−0.2
0.1
0.2
0.3
SNP effect on Pulmonary fibrosis
SNP effect on PLXNB2

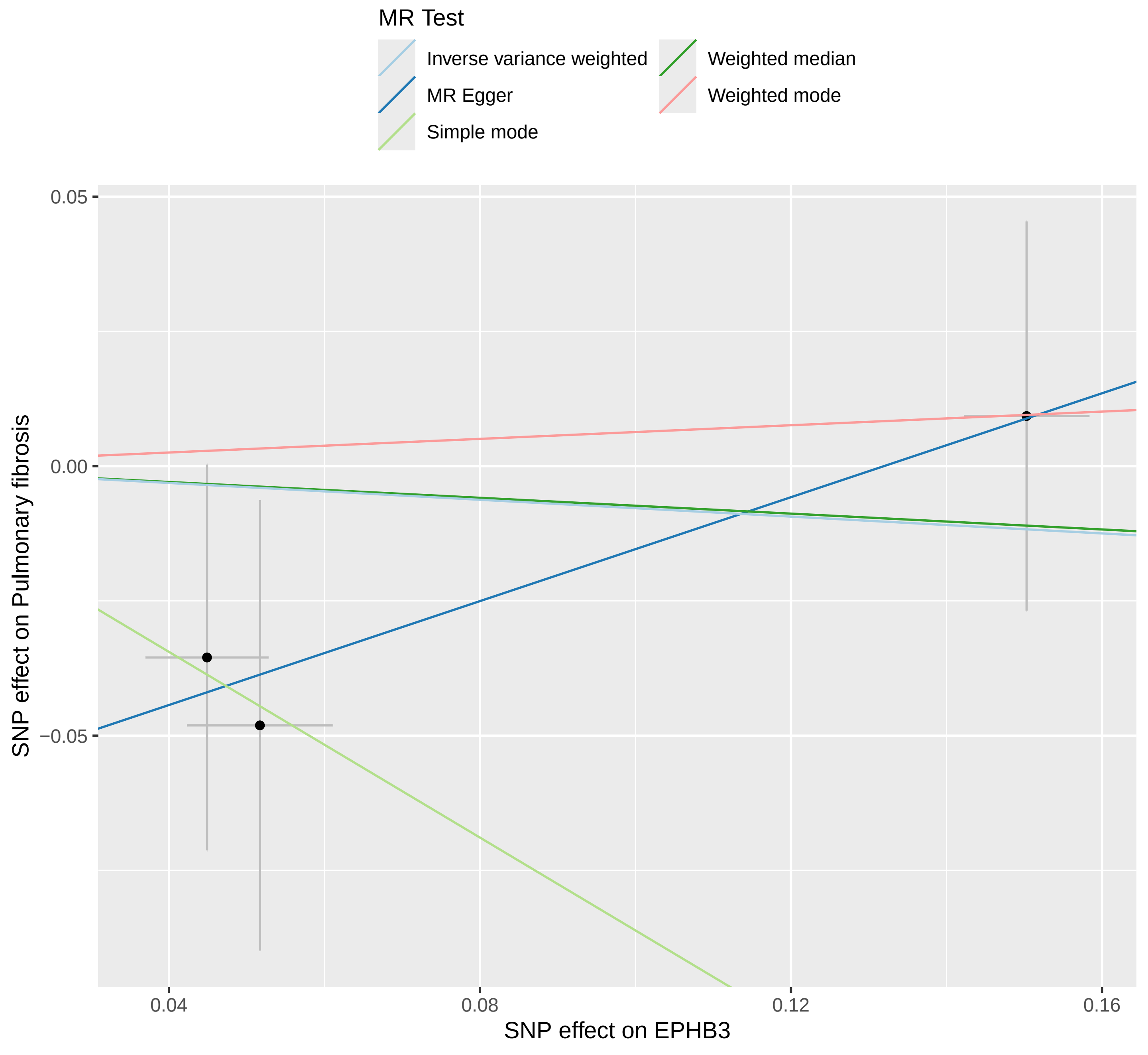

MR Test
Inverse variance weighted
MR Egger
Simple mode
Weighted median
Weighted mode
0.05
0.00
−0.05
0.04
0.08
0.12
0.16
SNP effect on EPHB3
SNP effect on Pulmonary fibrosis

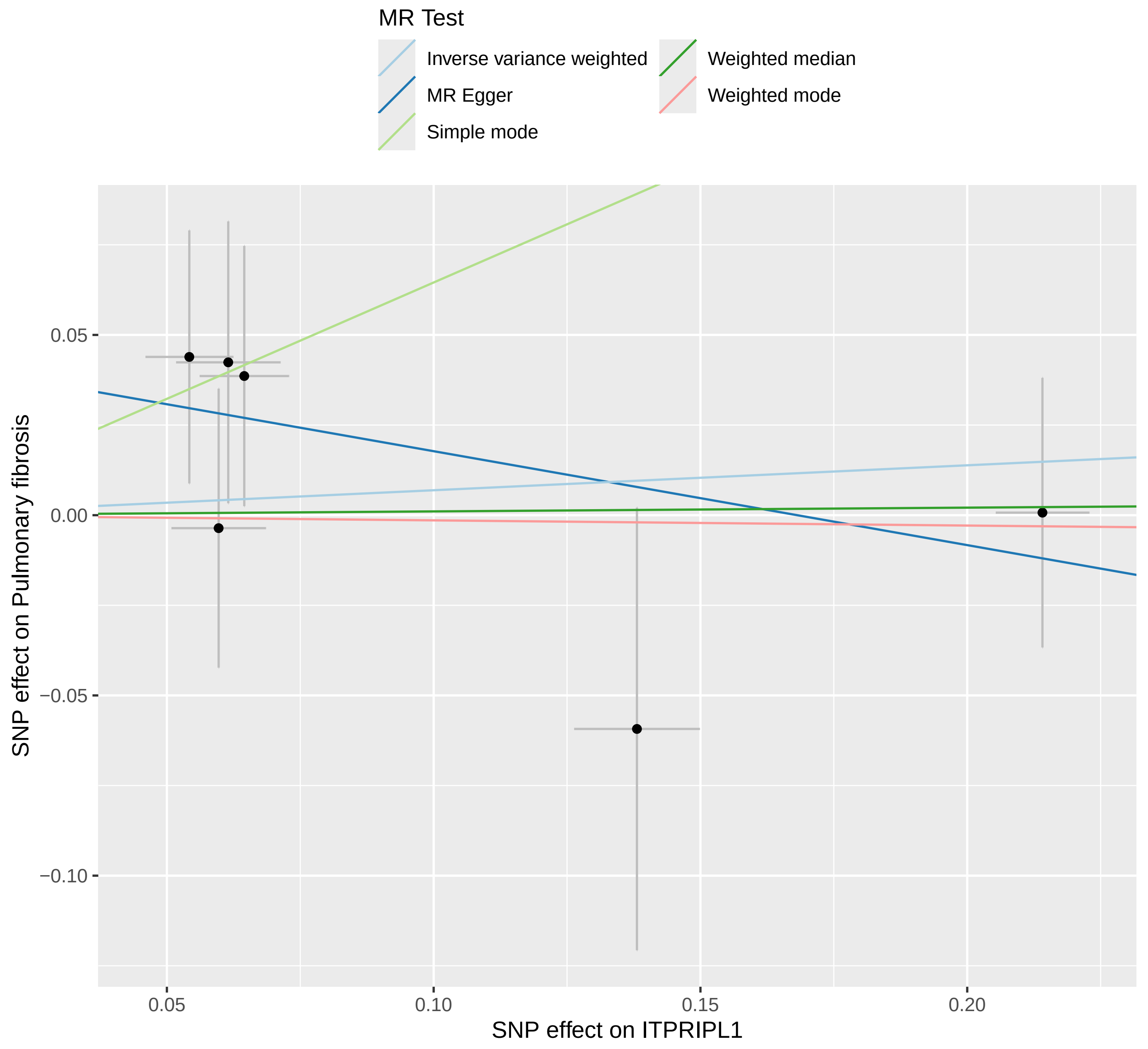
MR Test
Inverse variance weighted
MR Egger
Simple mode
Weighted median
Weighted mode
0.05
0.00
−0.05
−0.10
SNP effect on Pulmonary fibrosis
0.05
0.10
0.15
0.20
SNP effect on ITPRIPL1

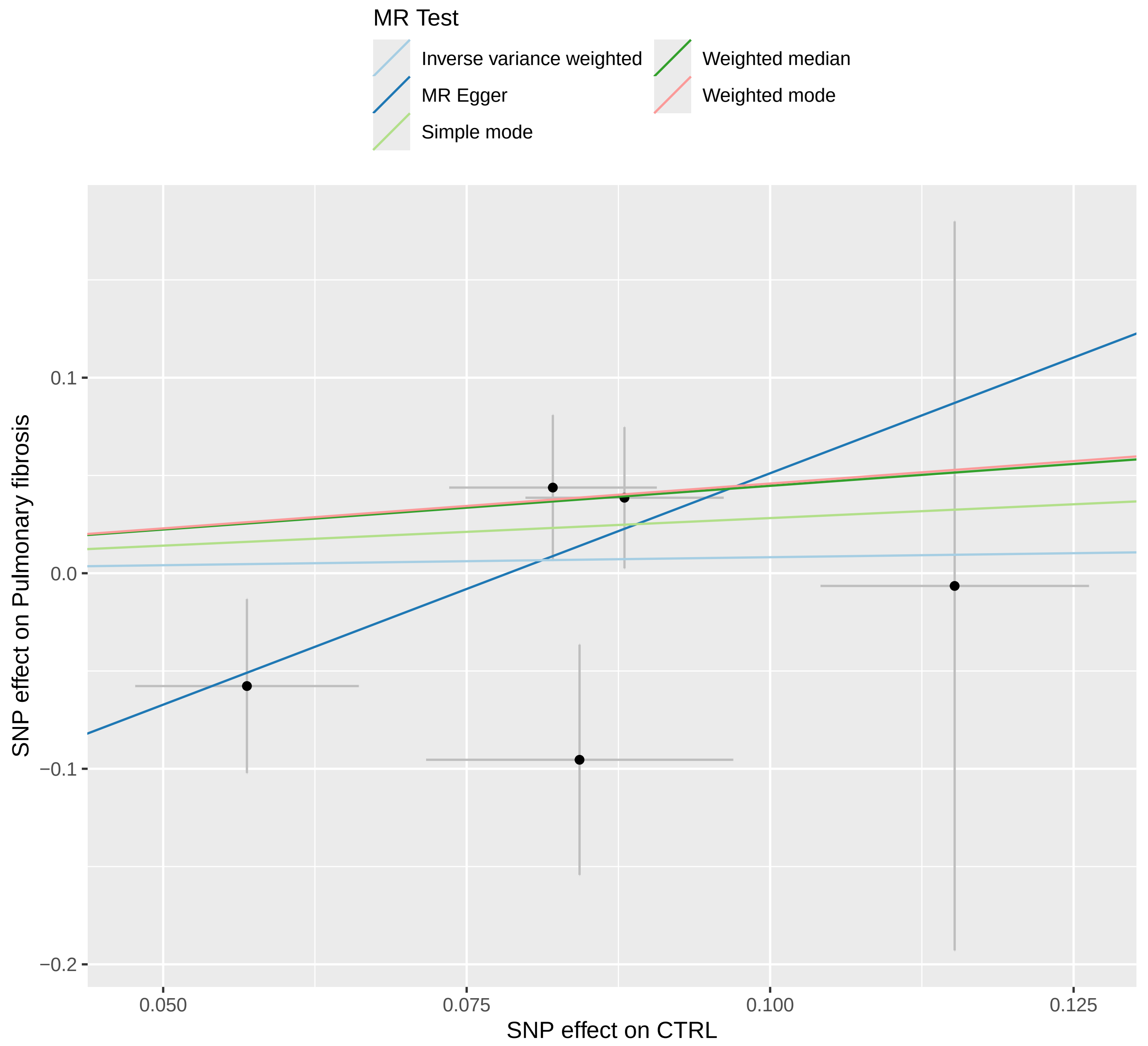
MR Test
Inverse variance weighted
MR Egger
Simple mode
Weighted median
Weighted mode
0.1
0.0
−0.1
−0.2
SNP effect on Pulmonary fibrosis
0.050
0.075
0.100
0.125
SNP effect on CTRL

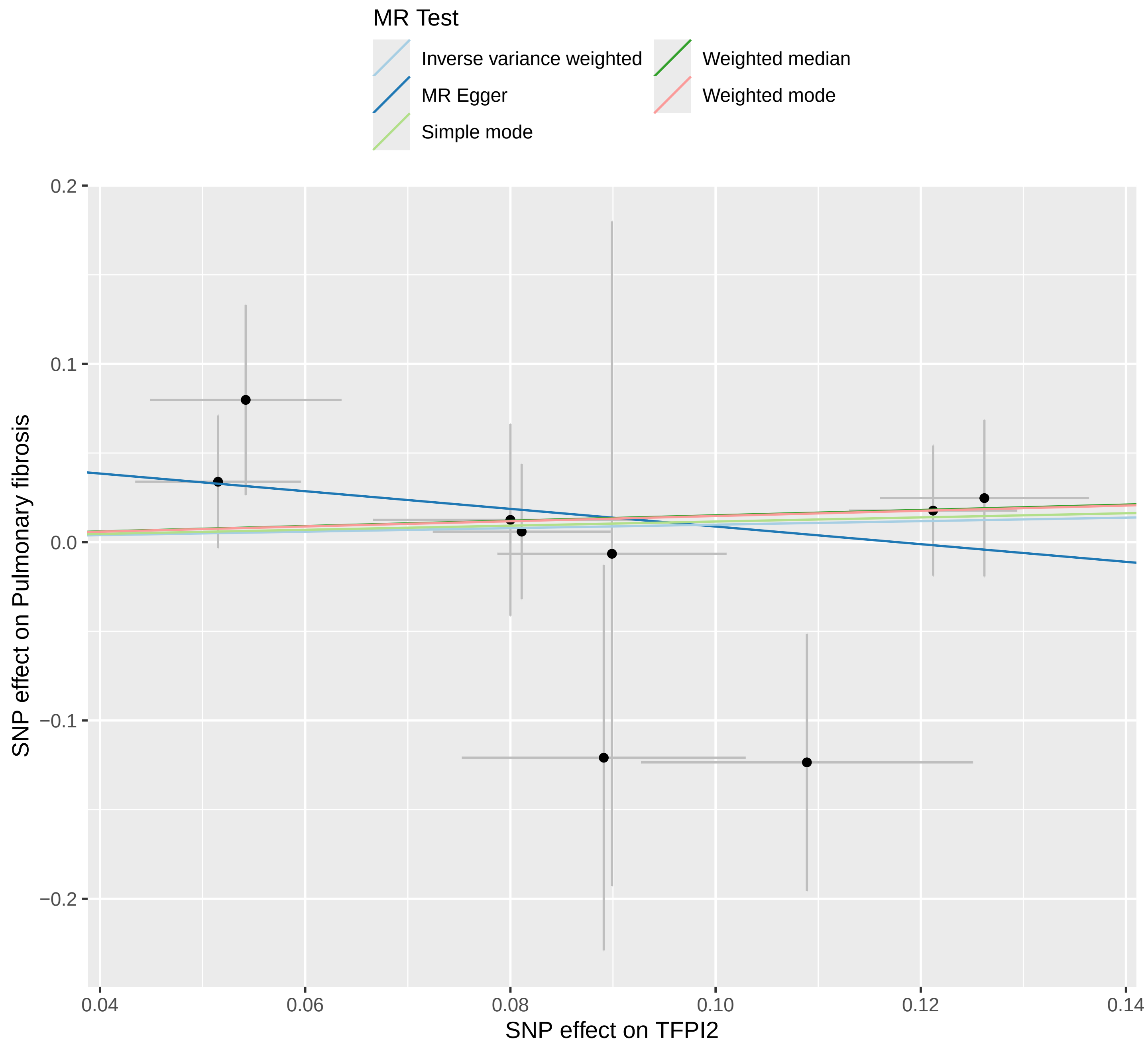

MR Test
Inverse variance weighted
MR Egger
Simple mode
Weighted median
Weighted mode
0.2
0.1
0.0
−0.1
−0.2
SNP effect on Pulmonary fibrosis
0.04
0.06
0.08
0.10
0.12
0.14
SNP effect on TFPI2

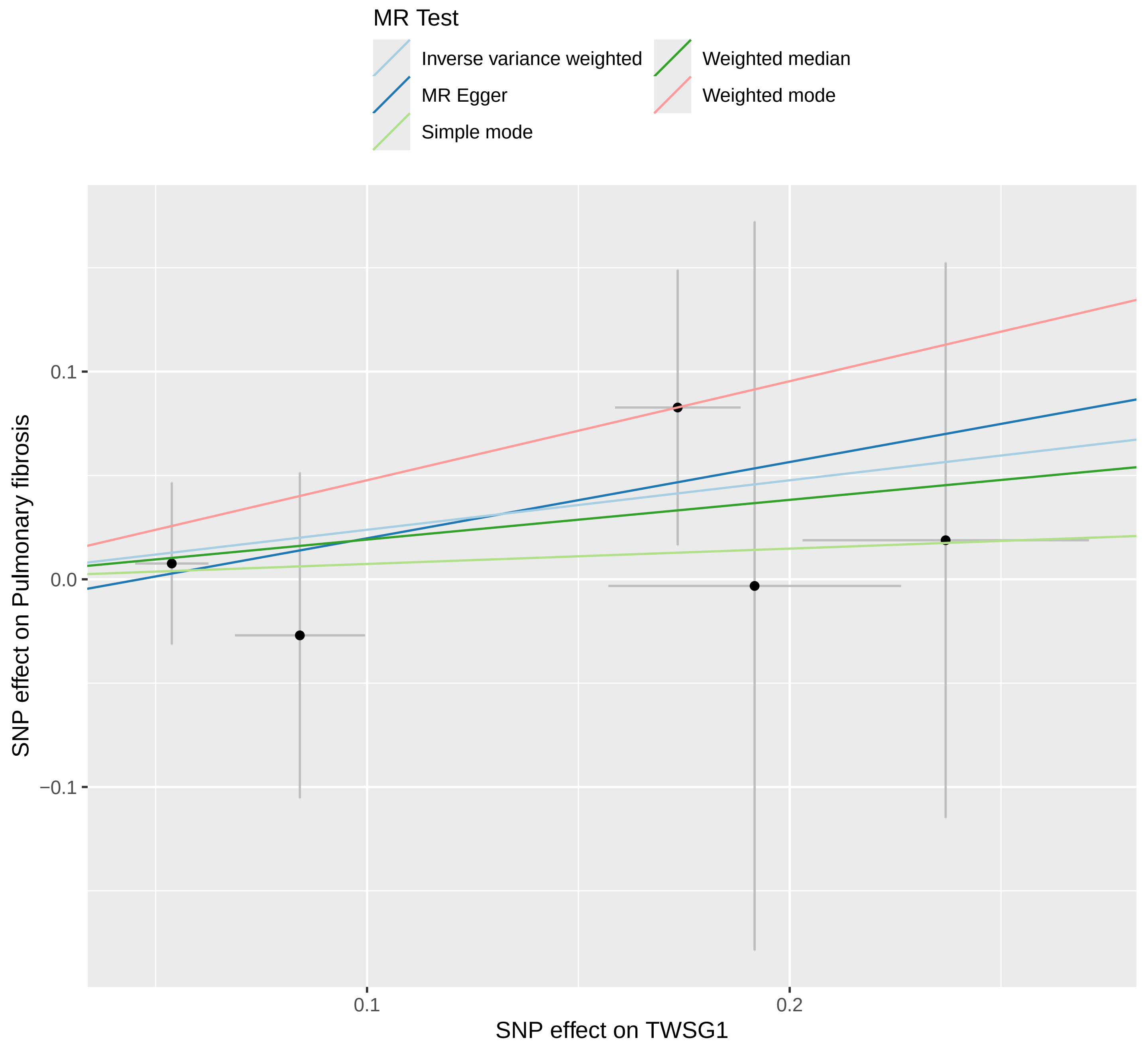
MR Test
Inverse variance weighted
MR Egger
Simple mode
Weighted median
Weighted mode
0.1
0.0
−0.1
SNP effect on Pulmonary fibrosis
0.1
0.2
SNP effect on TWSG1

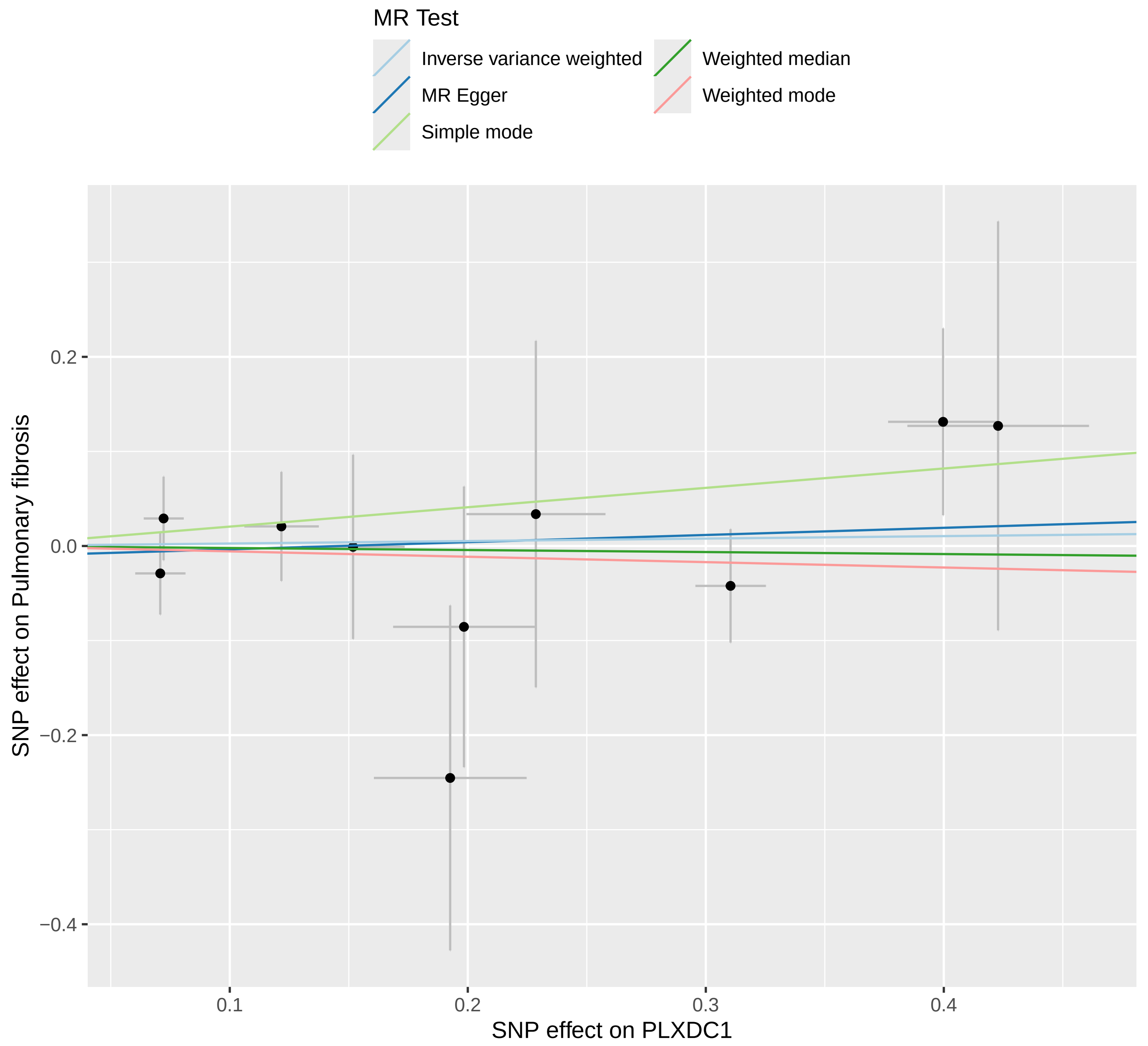

MR Test
Inverse variance weighted
MR Egger
Simple mode
Weighted median
Weighted mode
SNP effect on Pulmonary fibrosis
0.2
0.0
−0.2
−0.4
0.1
0.2
0.3
0.4
SNP effect on PLXDC1

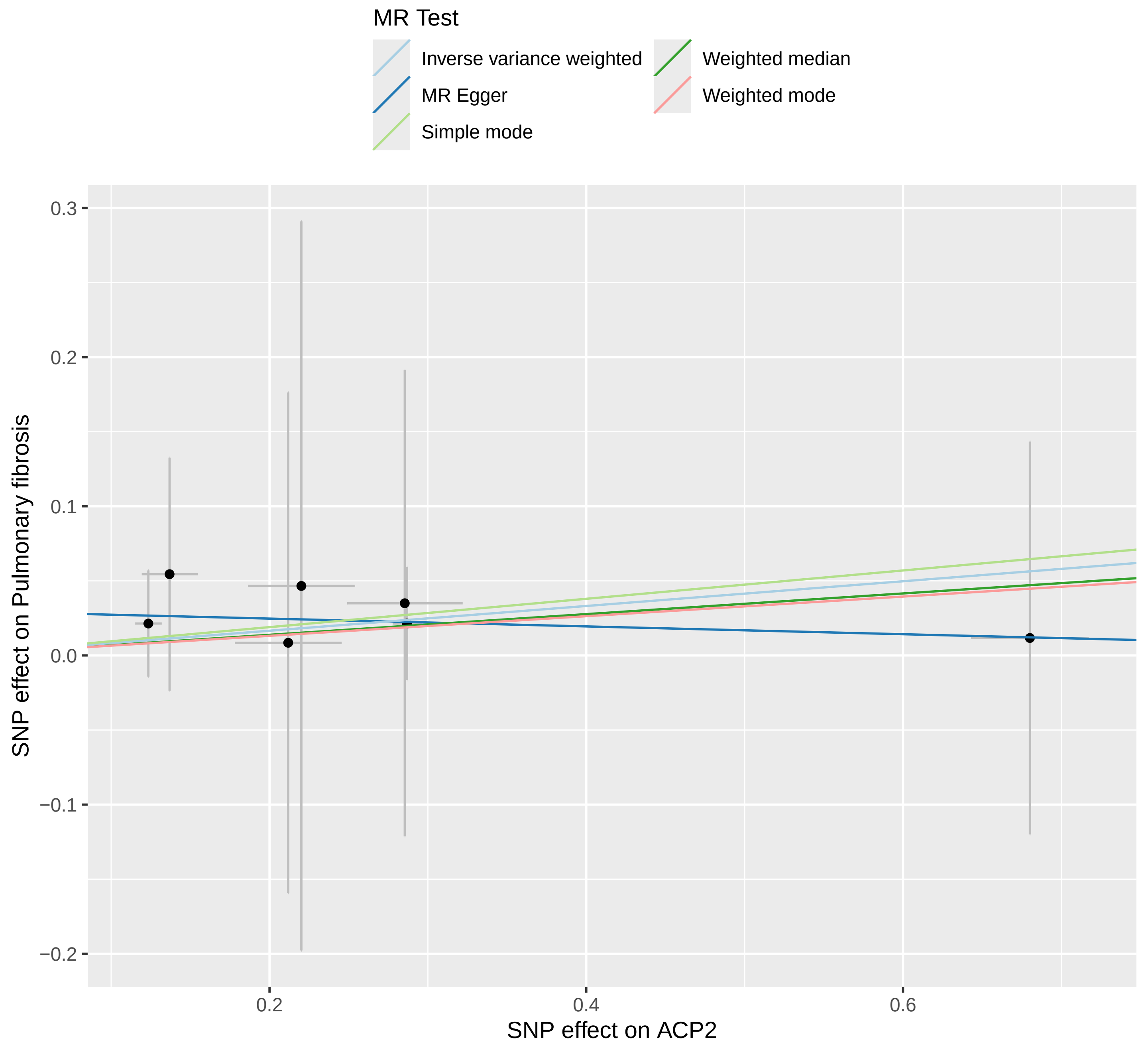

MR Test
Inverse variance weighted
MR Egger
Simple mode
Weighted median
Weighted mode
0.3
0.2
0.1
0.0
−0.1
−0.2
SNP effect on Pulmonary fibrosis
0.2
0.4
0.6
SNP effect on ACP2

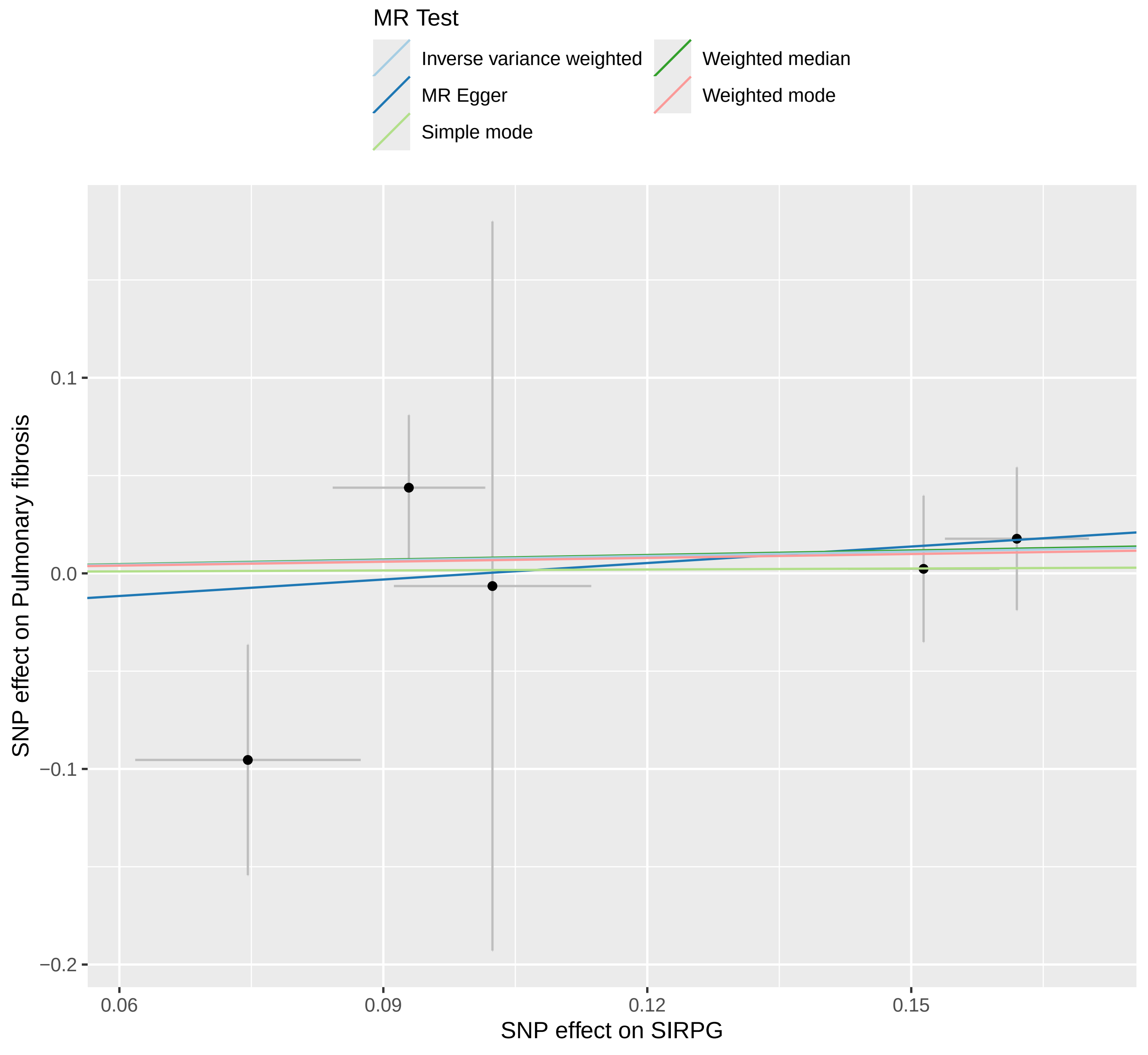
MR Test
Inverse variance weighted
MR Egger
Simple mode
Weighted median
Weighted mode
0.1
0.0
−0.1
−0.2
SNP effect on Pulmonary fibrosis
0.06
0.09
0.12
0.15
SNP effect on SIRPG

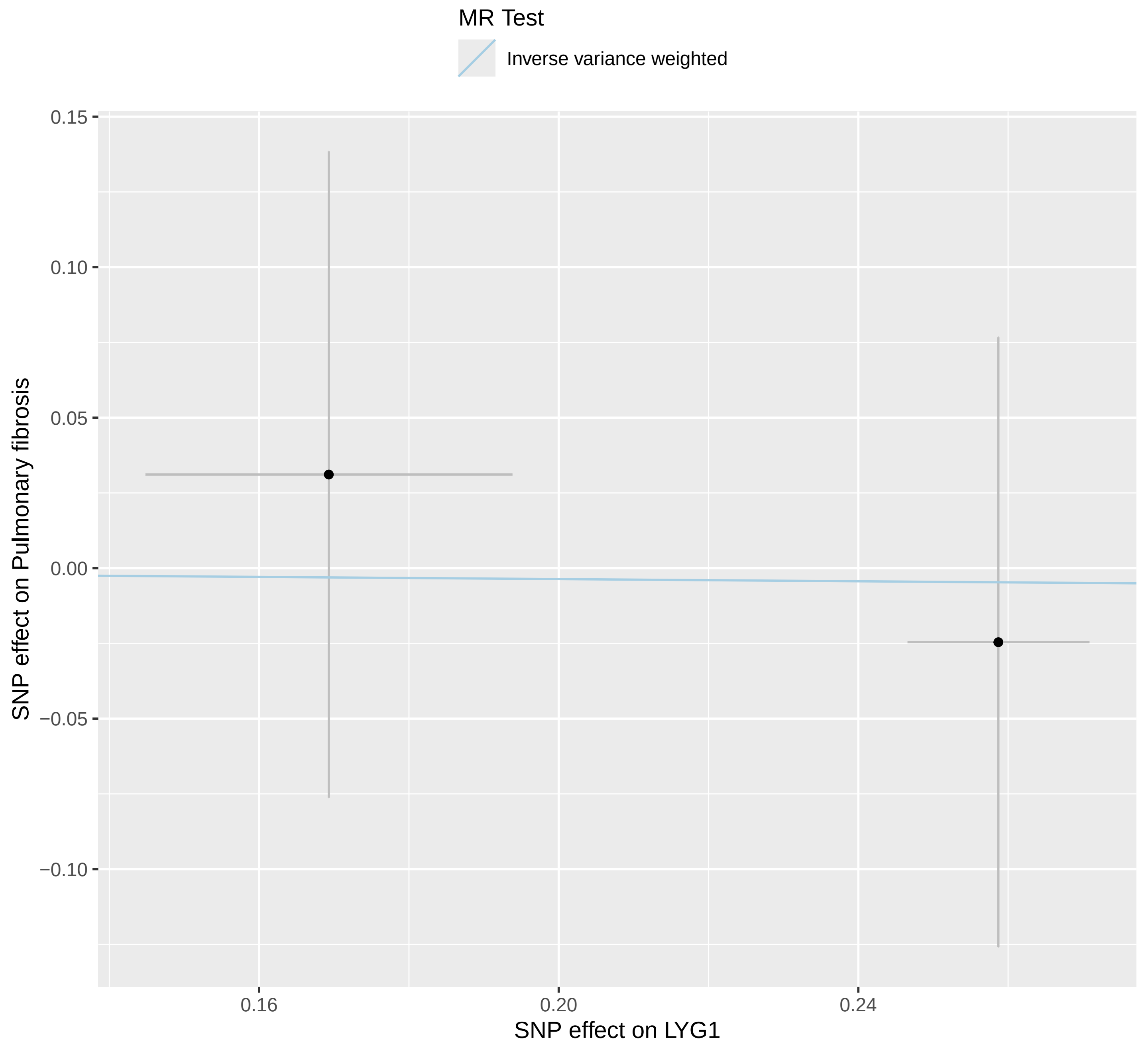
MR Test
Inverse variance weighted
0.15
0.10
0.05
0.00
−0.05
−0.10
SNP effect on Pulmonary fibrosis
0.16
0.20
0.24
SNP effect on LYG1

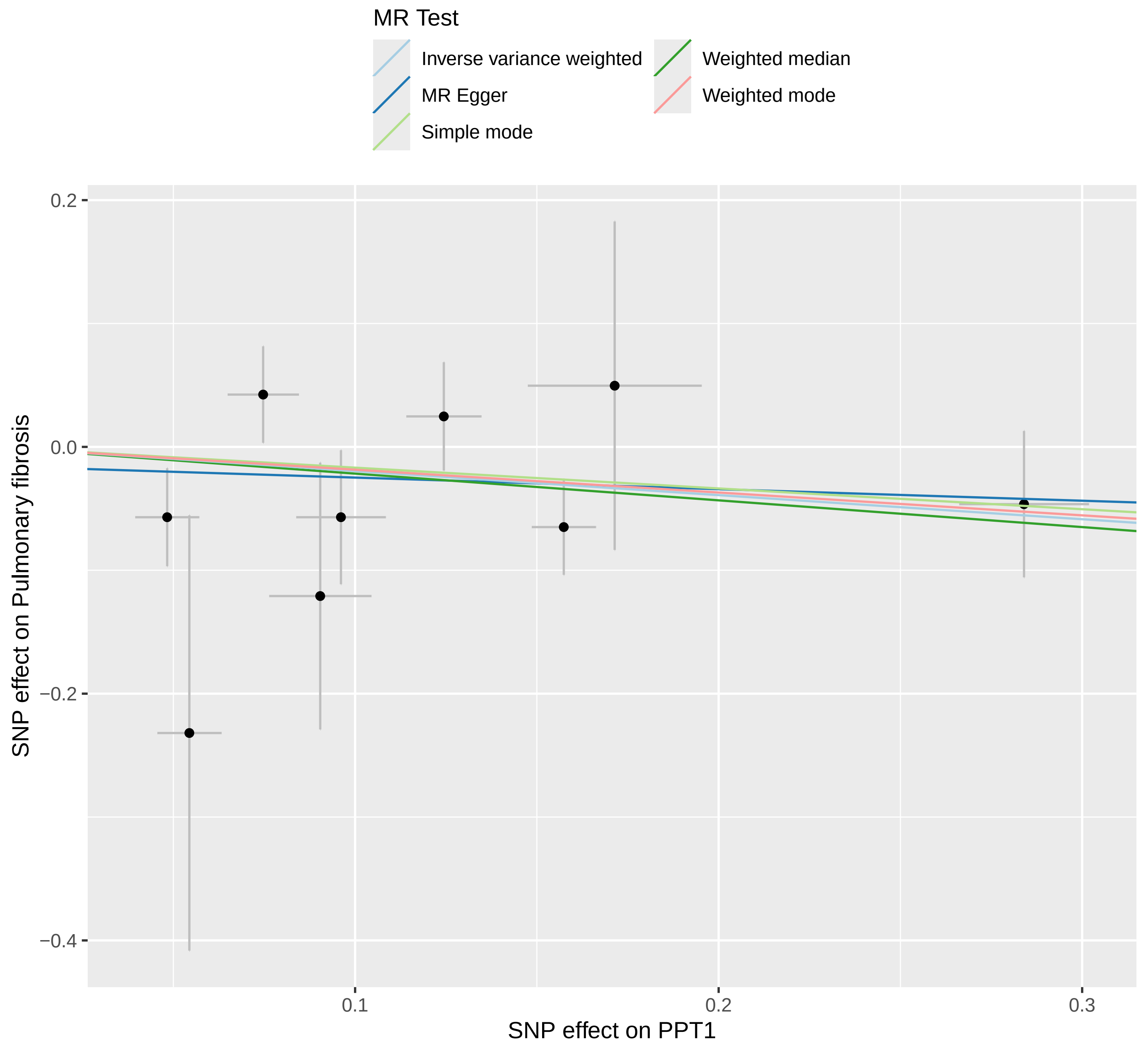

MR Test
Inverse variance weighted
MR Egger
Simple mode
Weighted median
Weighted mode
0.2
0.0
−0.2
−0.4
0.1
0.2
0.3
SNP effect on Pulmonary fibrosis
SNP effect on PPT1

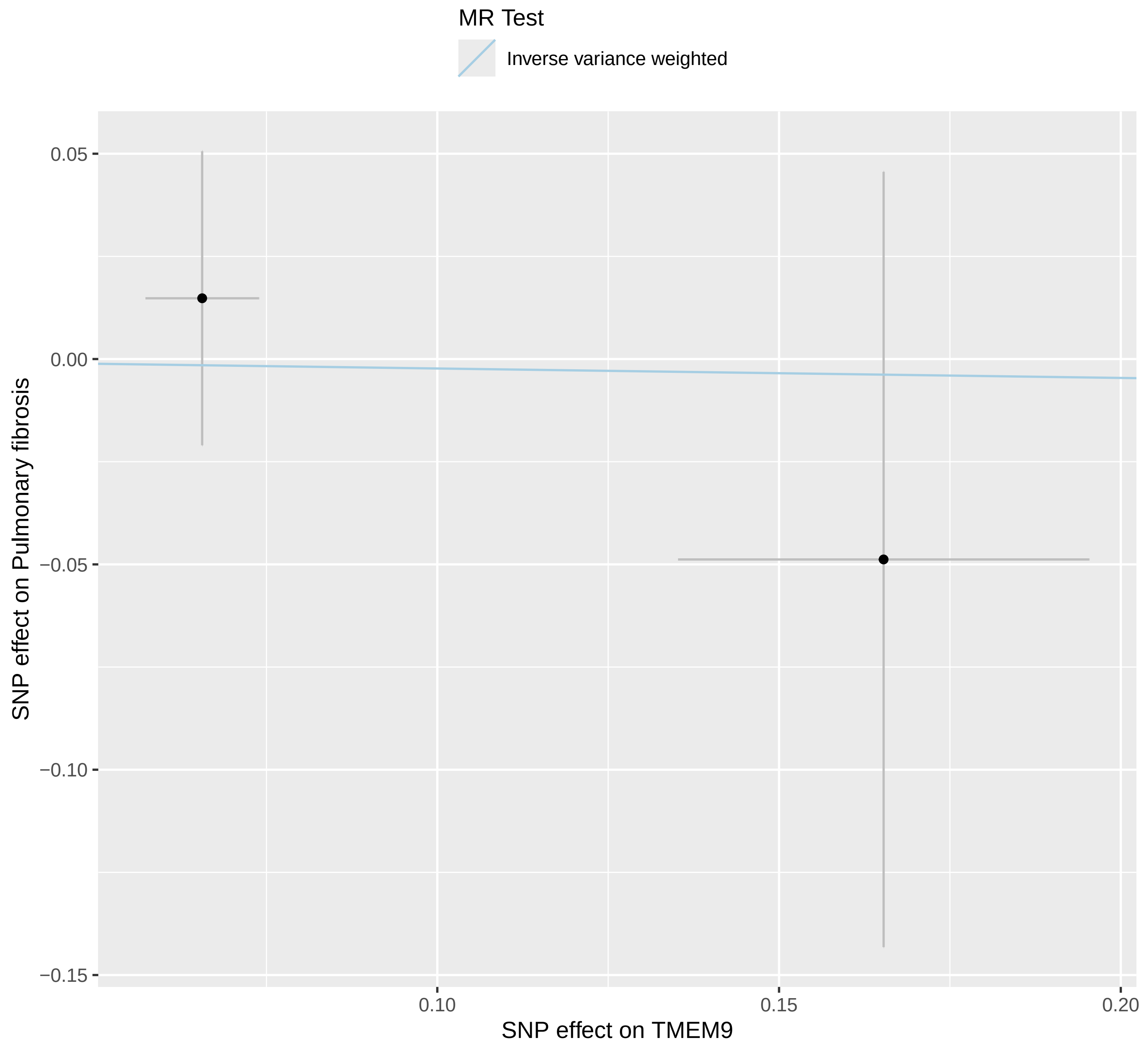
MR Test
Inverse variance weighted
0.05
0.00
−0.05
−0.10
−0.15
0.10
0.15
0.20
SNP effect on Pulmonary fibrosis
SNP effect on TMEM9

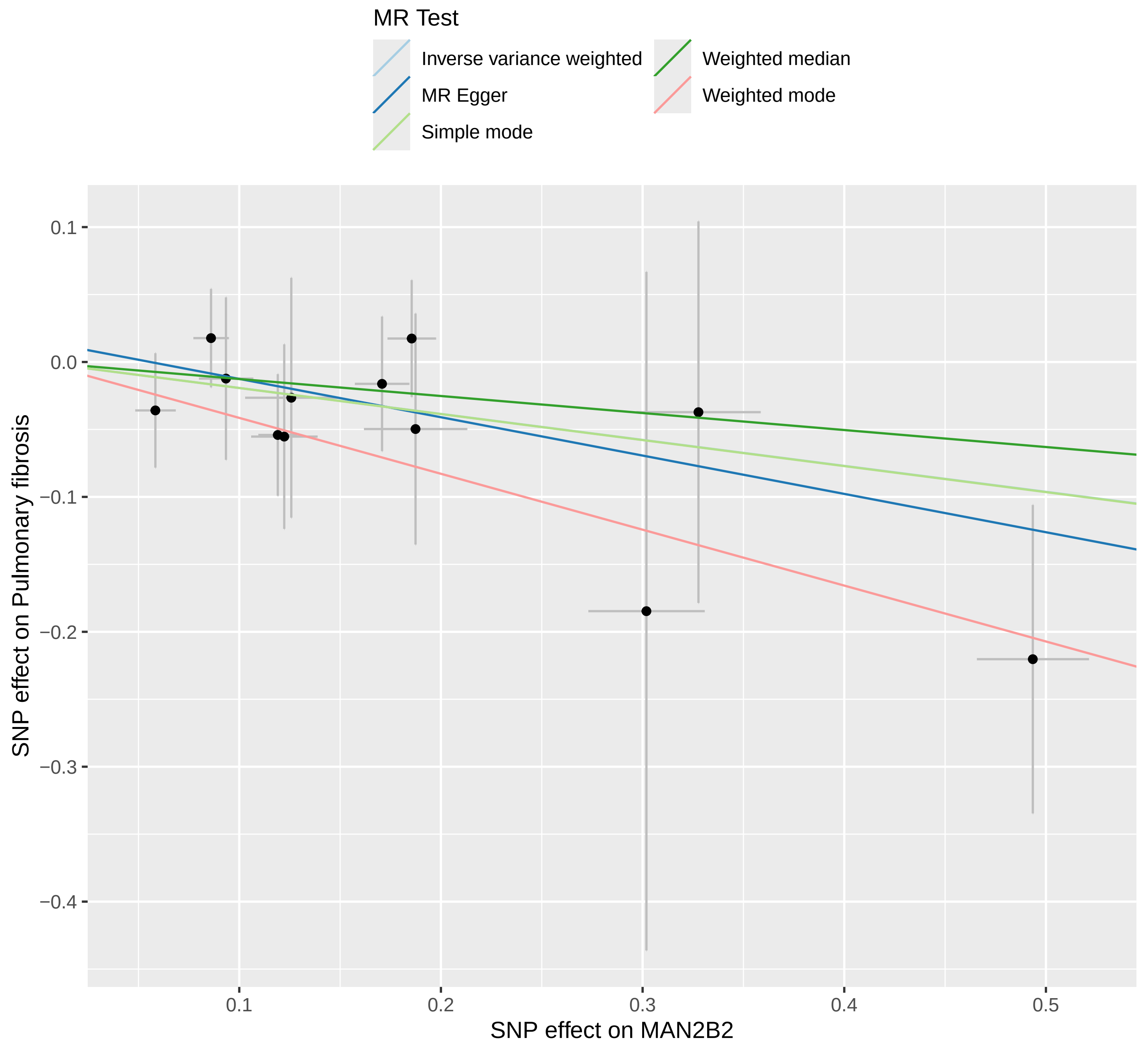
MR Test
Inverse variance weighted
MR Egger
Simple mode
Weighted median
Weighted mode
0.1
0.0
−0.1
−0.2
−0.3
−0.4
SNP effect on Pulmonary fibrosis
0.1
0.2
0.3
0.4
0.5
SNP effect on MAN2B2

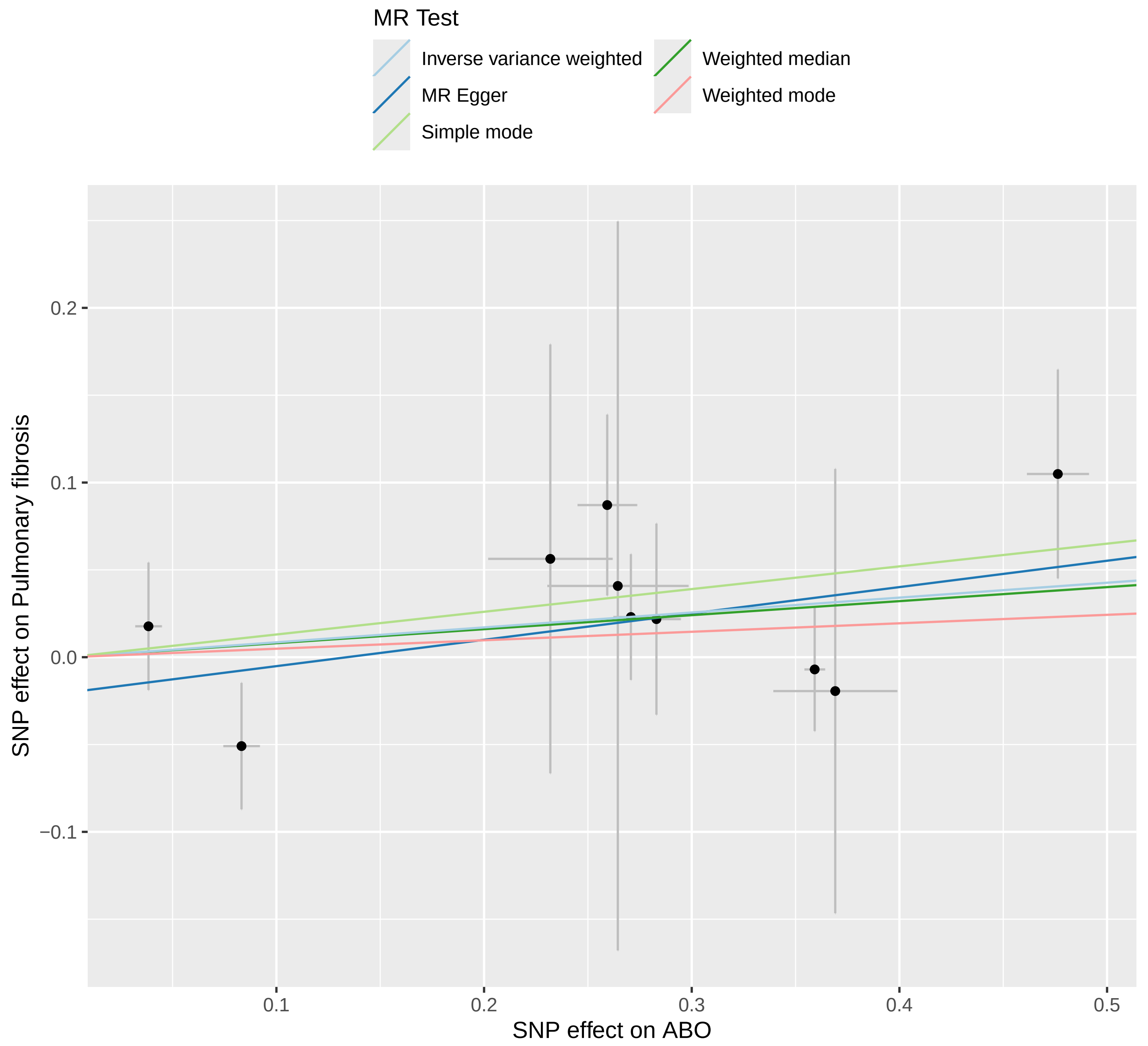
MR Test
Inverse variance weighted
MR Egger
Simple mode
Weighted median
Weighted mode
0.2
0.1
0.0
−0.1
SNP effect on Pulmonary fibrosis
0.1
0.2
0.3
0.4
0.5
SNP effect on ABO

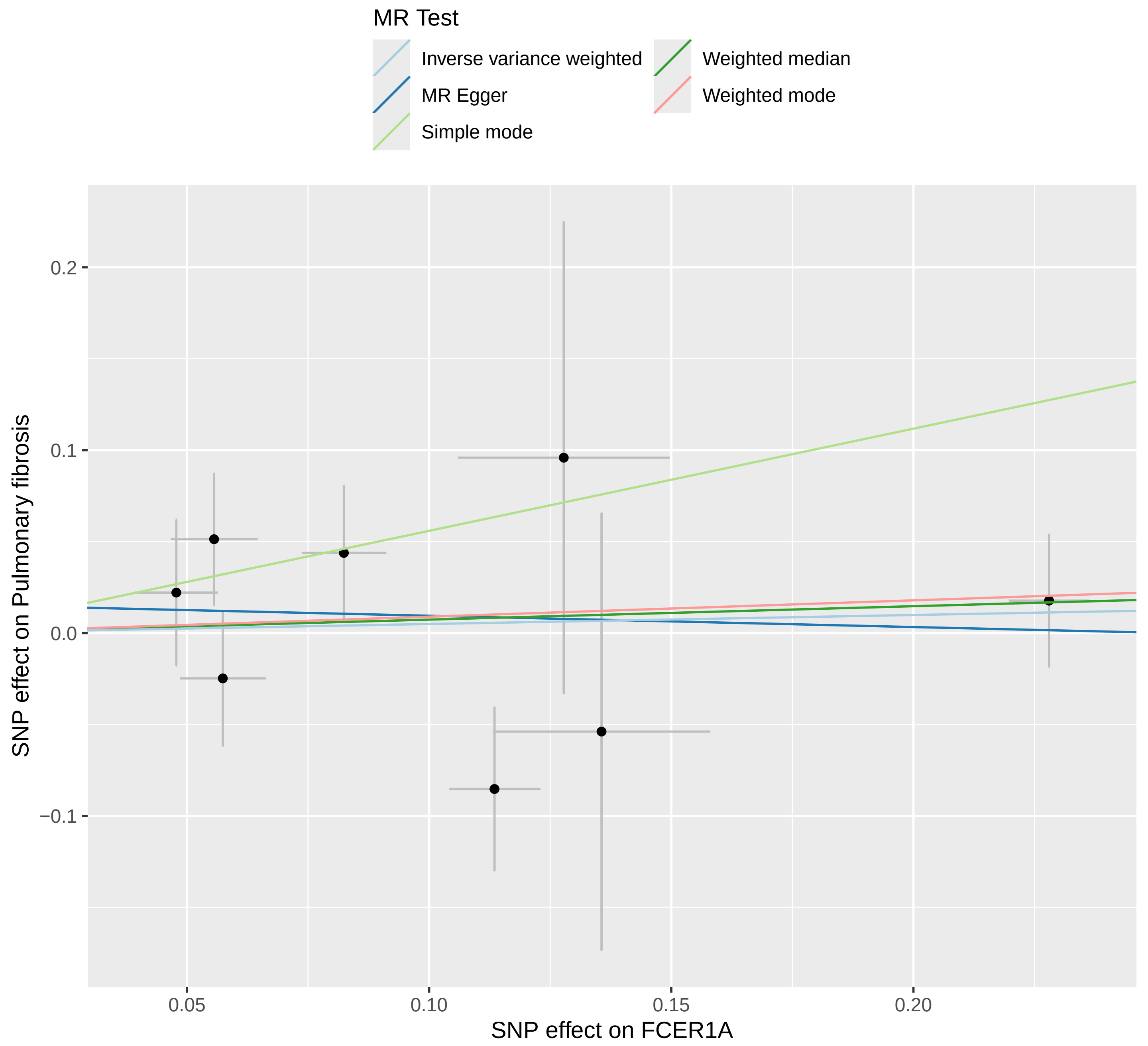

MR Test
Inverse variance weighted
MR Egger
Simple mode
Weighted median
Weighted mode
0.2
0.1
0.0
−0.1
0.05
0.10
0.15
0.20
SNP effect on FCER1A
SNP effect on Pulmonary fibrosis

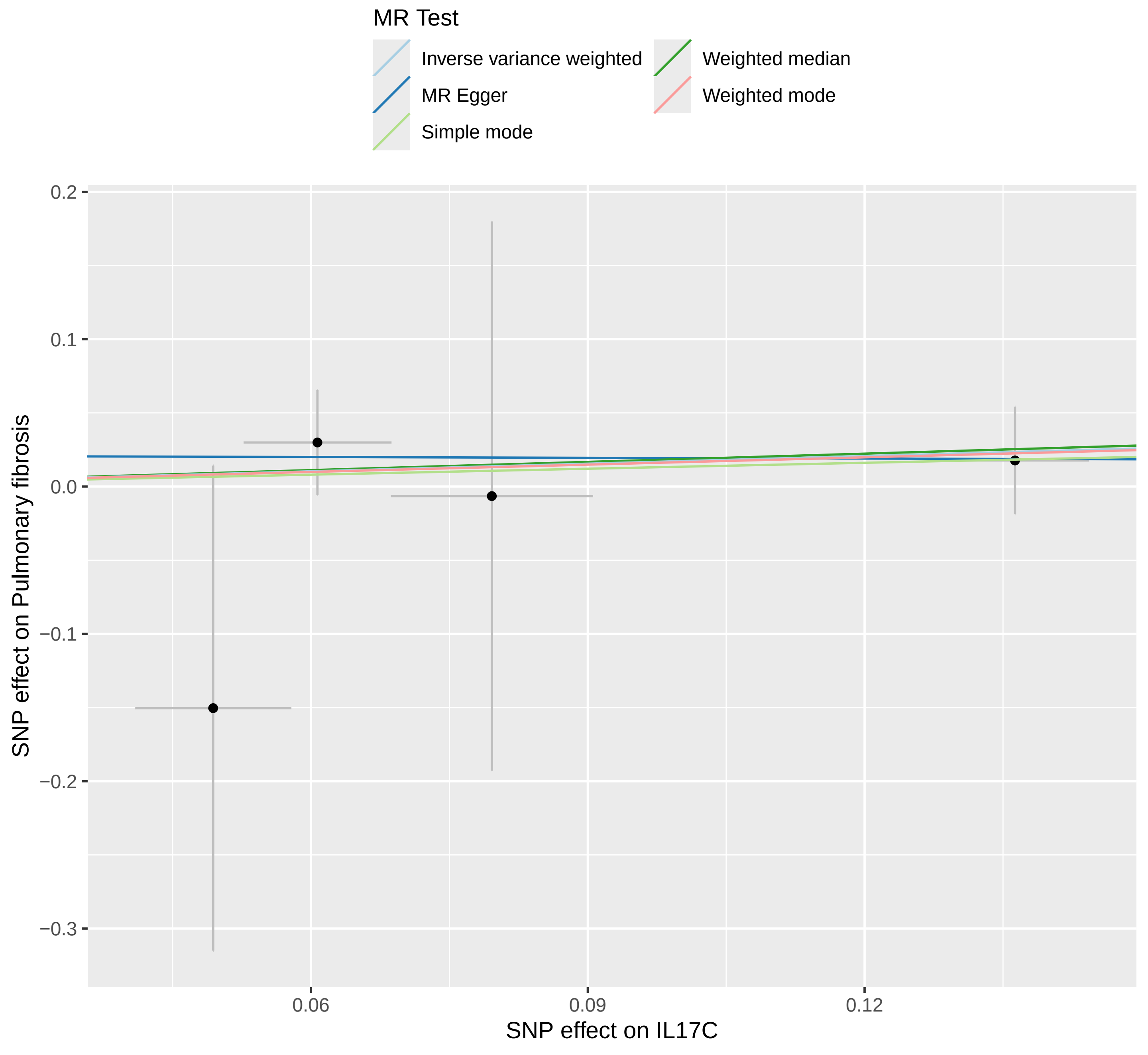
MR Test
Inverse variance weighted
MR Egger
Simple mode
Weighted median
Weighted mode
0.2
0.1
0.0
−0.1
−0.2
−0.3
SNP effect on Pulmonary fibrosis
0.06
0.09
0.12
SNP effect on IL17C

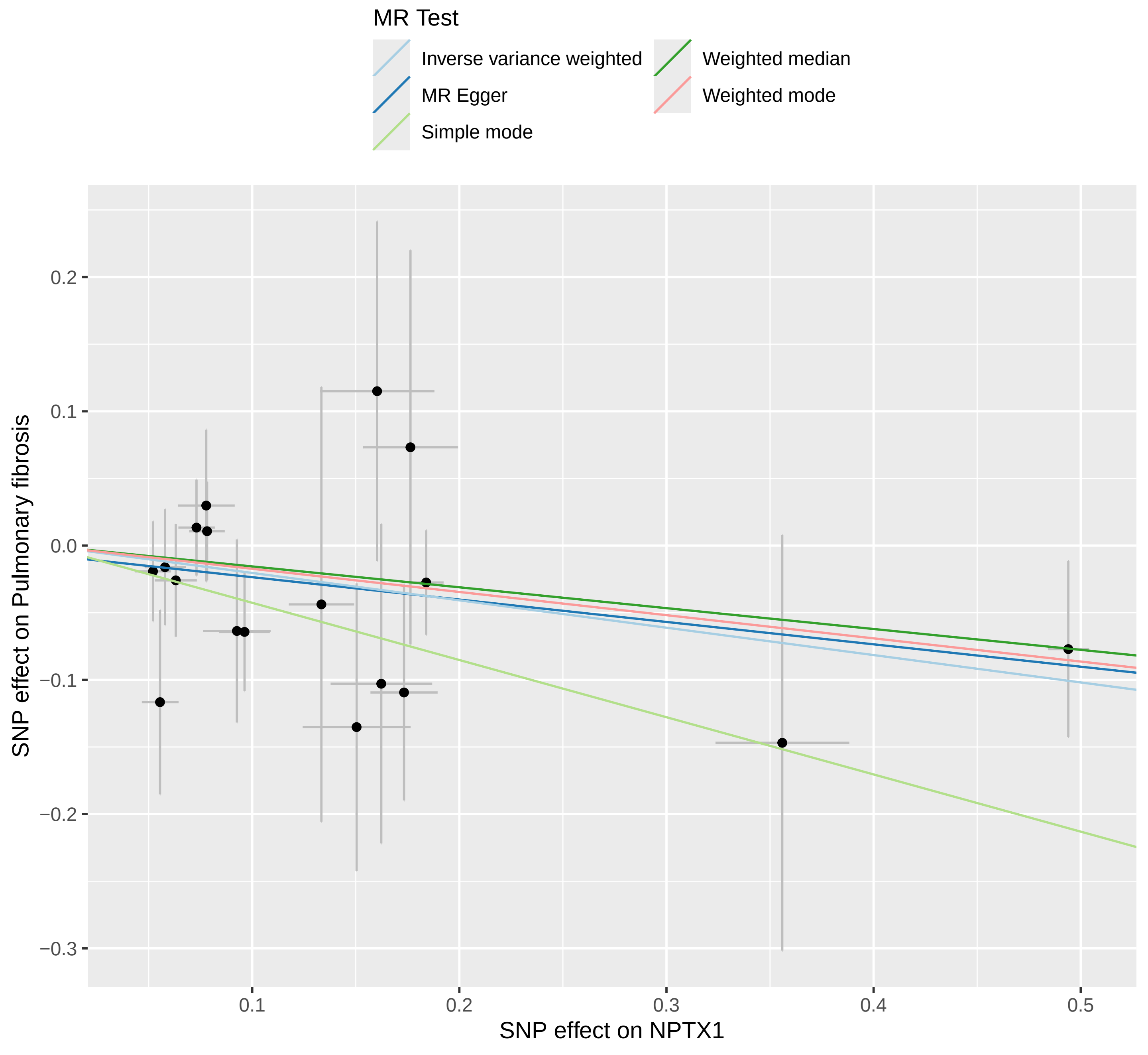
MR Test
Inverse variance weighted
MR Egger
Simple mode
Weighted median
Weighted mode
SNP effect on Pulmonary fibrosis
0.2
0.1
0.0
−0.1
−0.2
−0.3
0.1
0.2
0.3
0.4
0.5
SNP effect on NPTX1

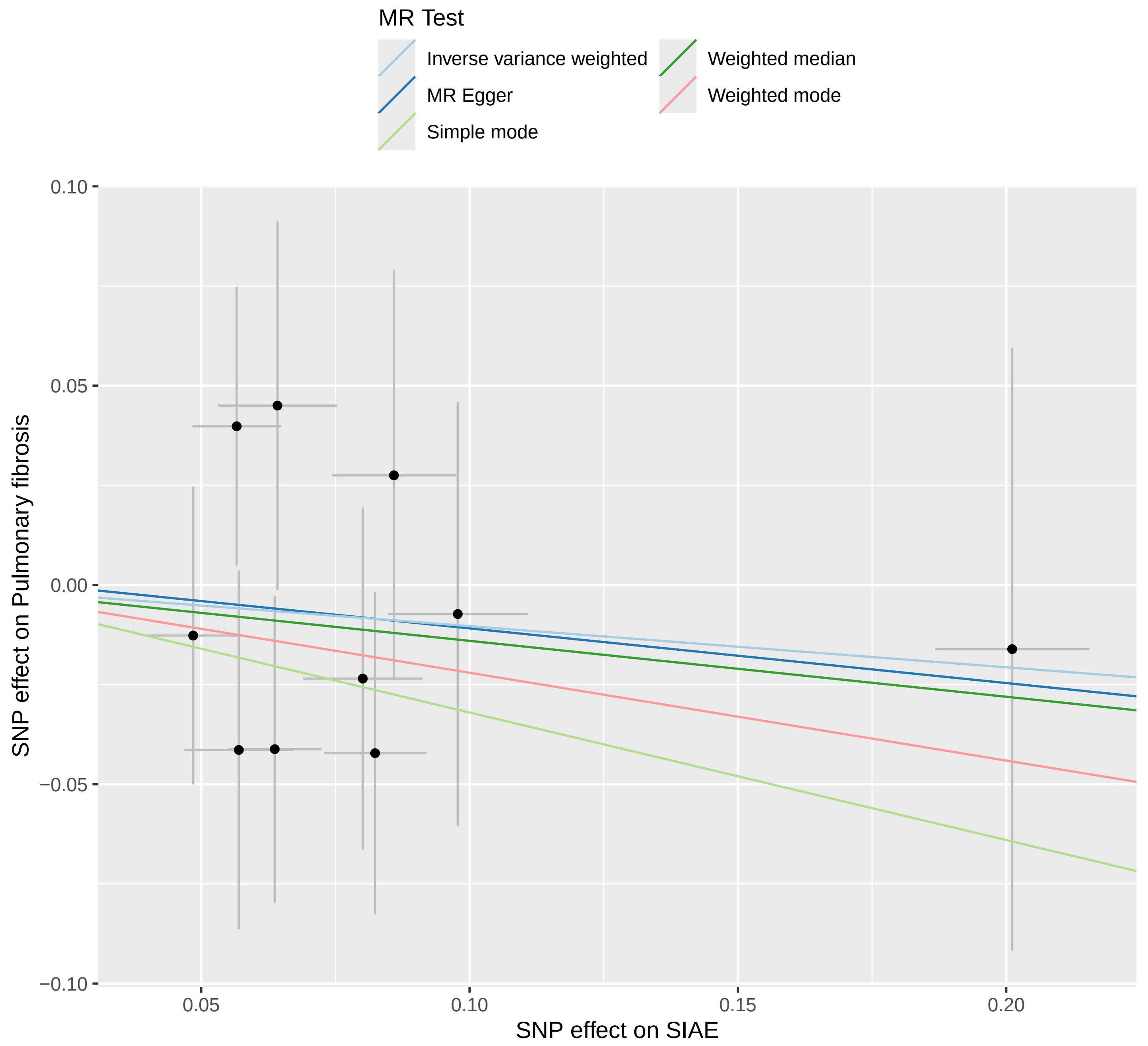

MR Test
Inverse variance weighted
MR Egger
Simple mode
Weighted median
Weighted mode
0.10
0.05
0.00
−0.05
−0.10
0.05
0.10
0.15
0.20
SNP effect on SIAE
SNP effect on Pulmonary fibrosis

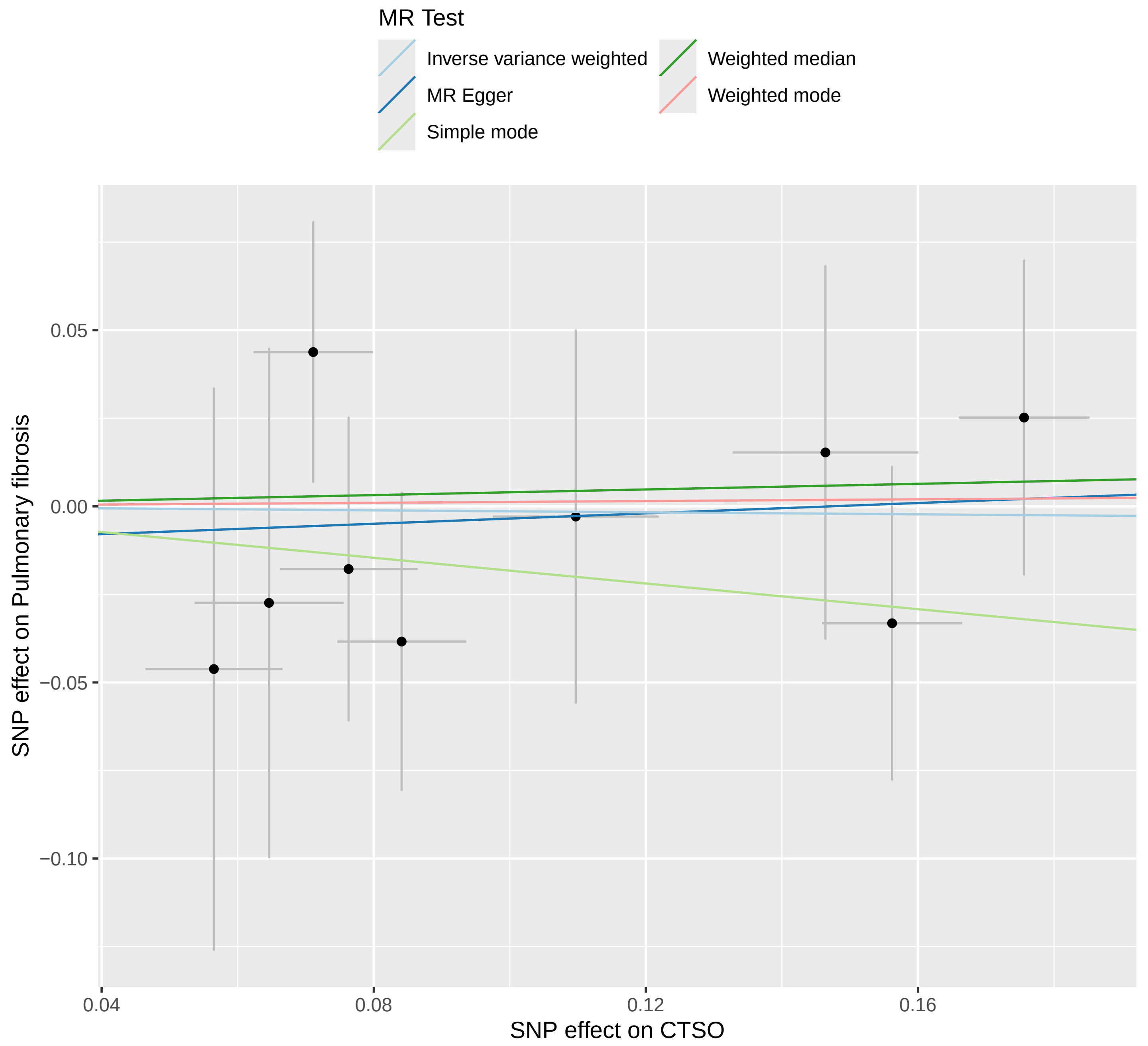

MR Test
Inverse variance weighted
MR Egger
Simple mode
Weighted median
Weighted mode
0.05
0.00
−0.05
−0.10
SNP effect on Pulmonary fibrosis
0.04
0.08
0.12
0.16
SNP effect on CTSO

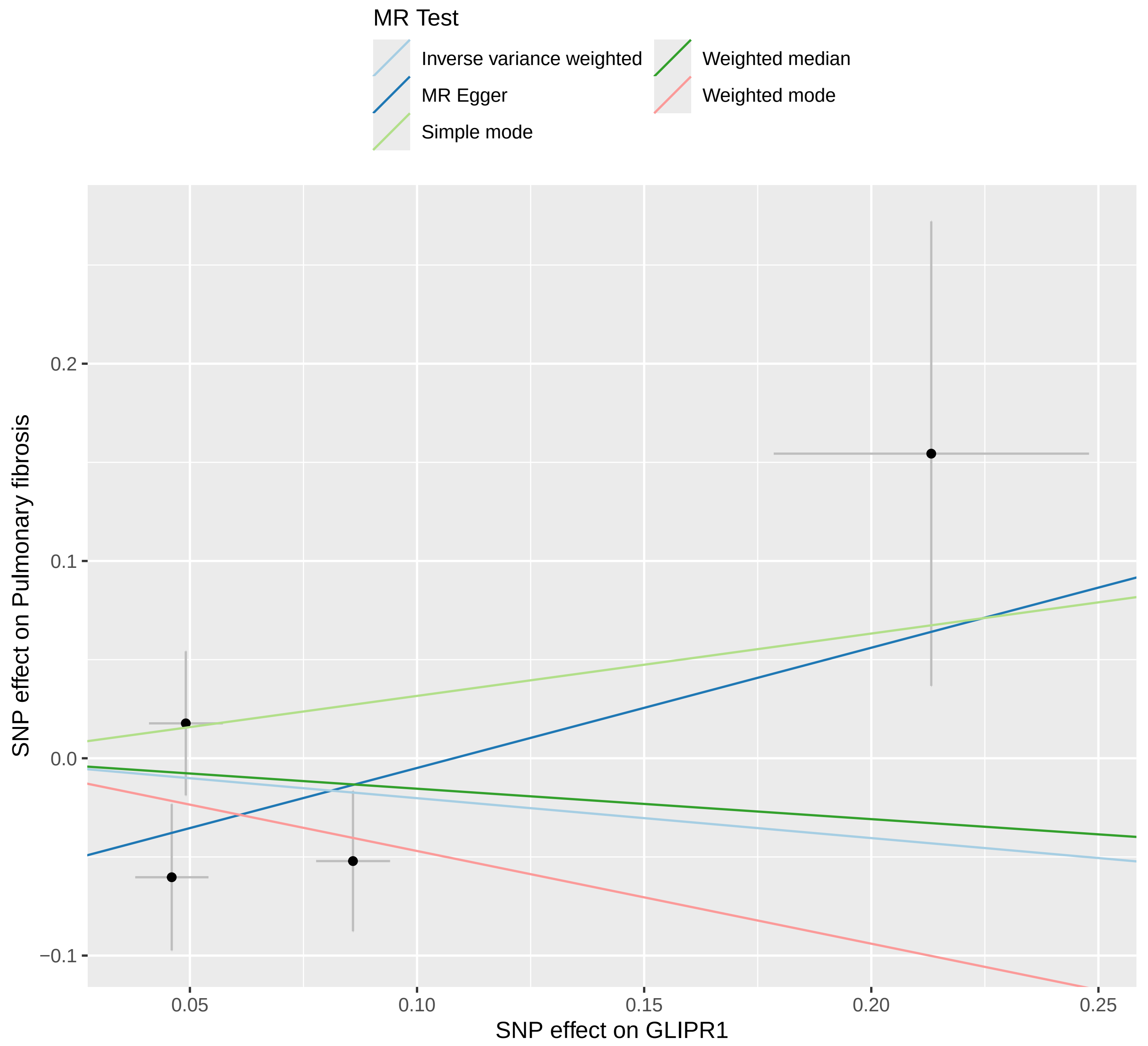
MR Test
Inverse variance weighted
MR Egger
Simple mode
Weighted median
Weighted mode
SNP effect on Pulmonary fibrosis
0.2
0.1
0.0
−0.1
0.05
0.10
0.15
0.20
0.25
SNP effect on GLIPR1

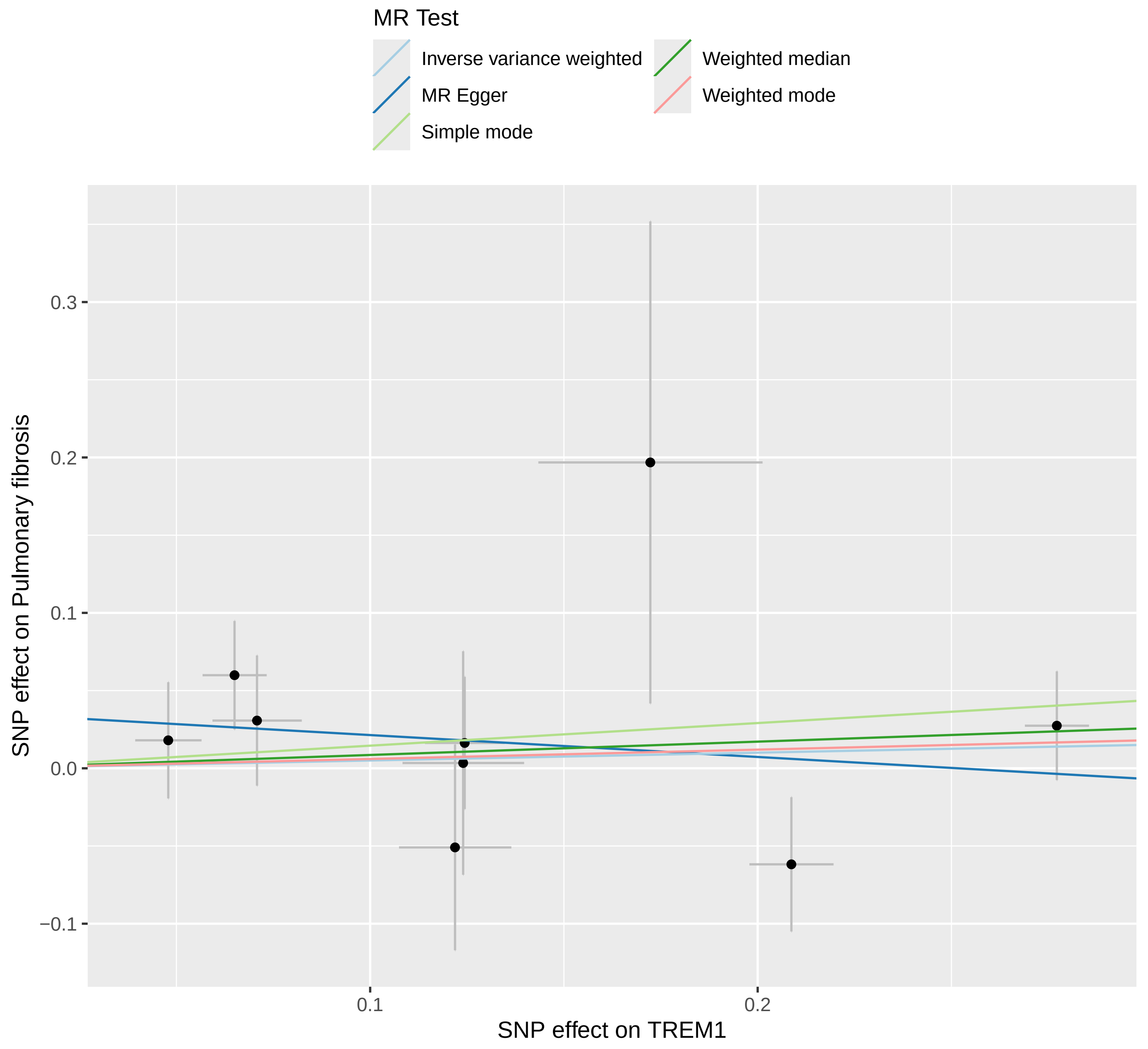

MR Test
Inverse variance weighted
MR Egger
Simple mode
Weighted median
Weighted mode
0.3
0.2
0.1
0.0
−0.1
0.1
0.2
SNP effect on Pulmonary fibrosis
SNP effect on TREM1

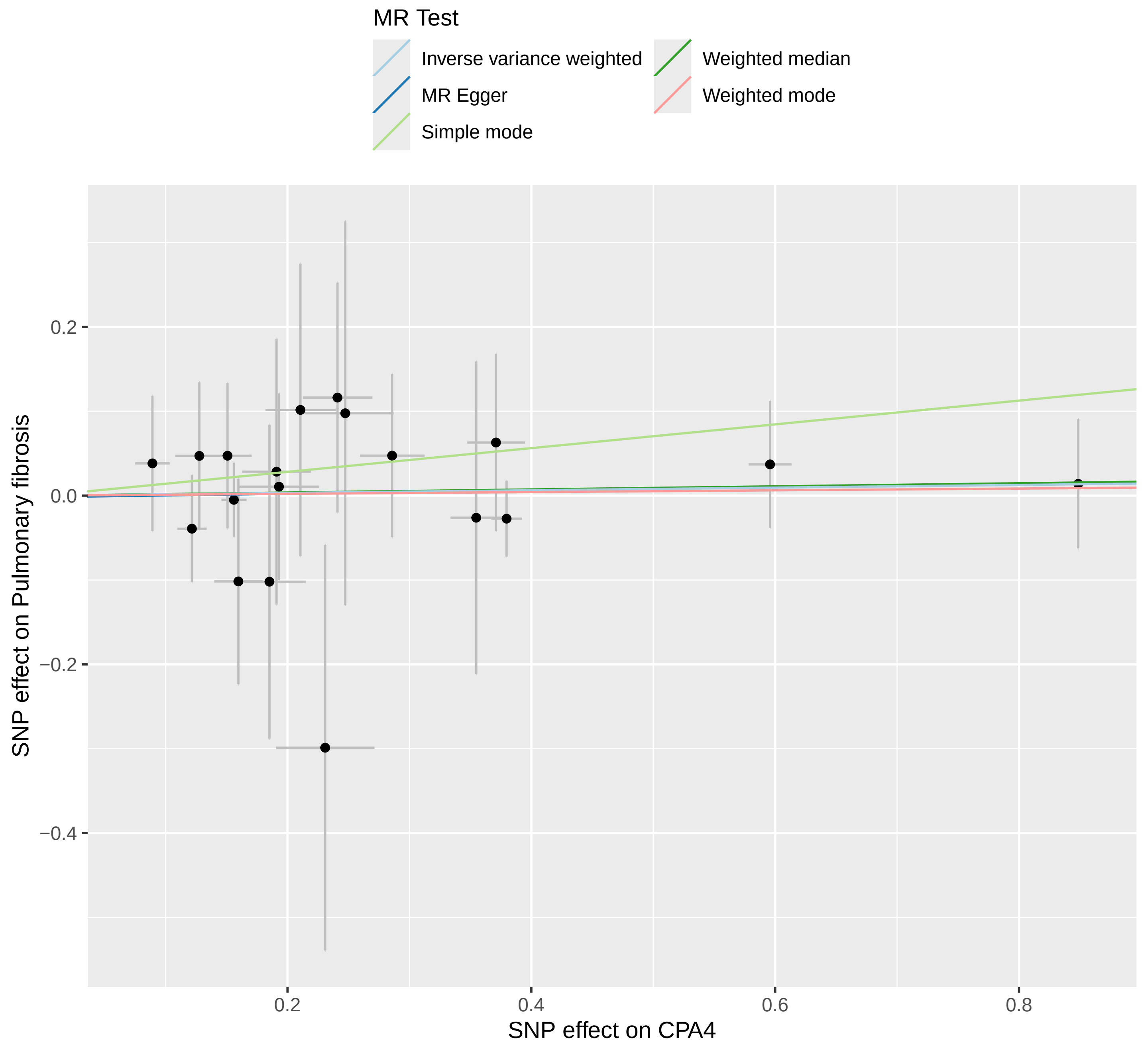

MR Test
Inverse variance weighted
MR Egger
Simple mode
Weighted median
Weighted mode
0.2
0.0
−0.2
−0.4
SNP effect on Pulmonary fibrosis
0.2
0.4
0.6
0.8
SNP effect on CPA4

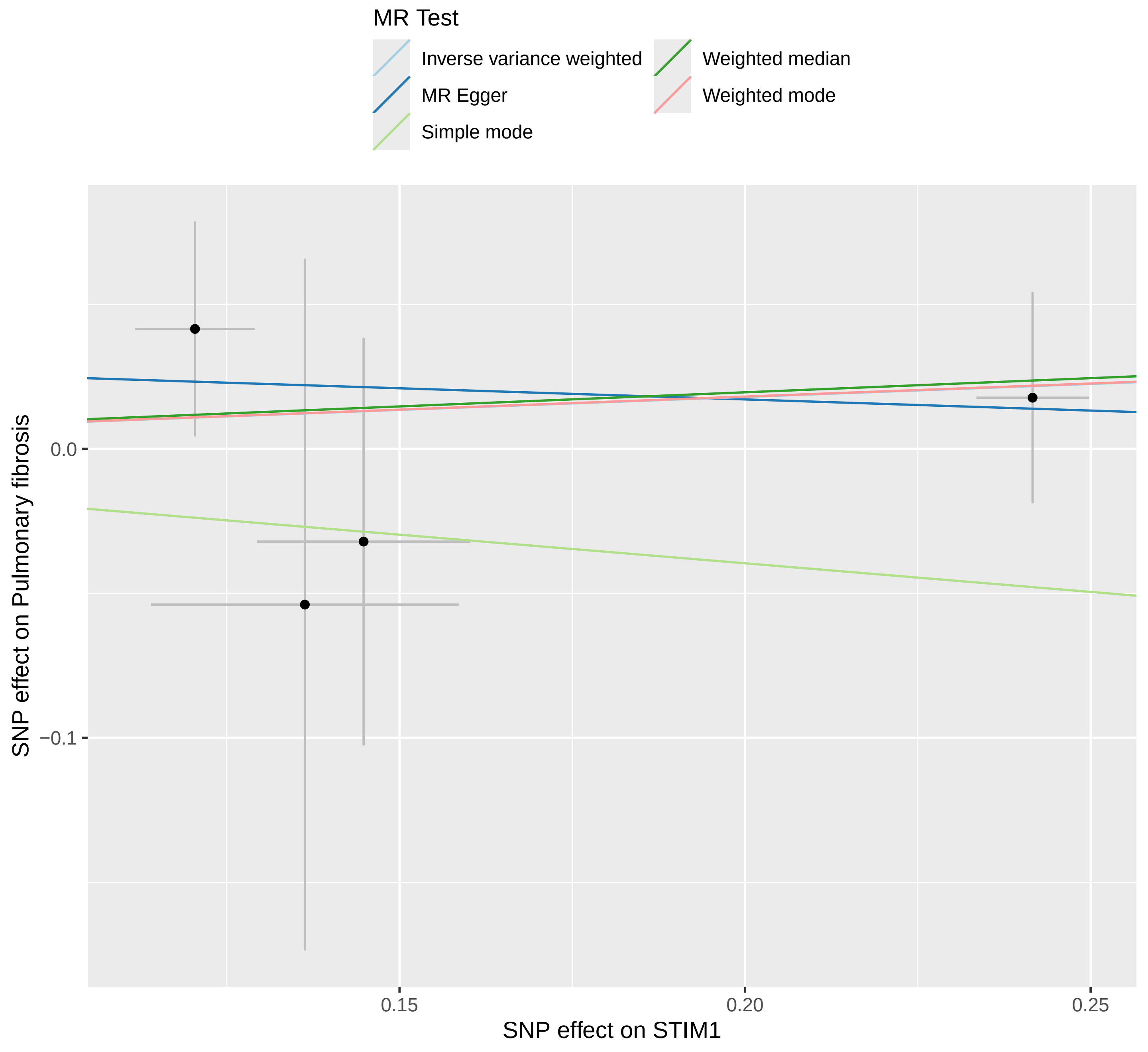
MR Test
Inverse variance weighted
MR Egger
Simple mode
Weighted median
Weighted mode
0.0
−0.1
0.15
0.20
0.25
SNP effect on STIM1
SNP effect on Pulmonary fibrosis

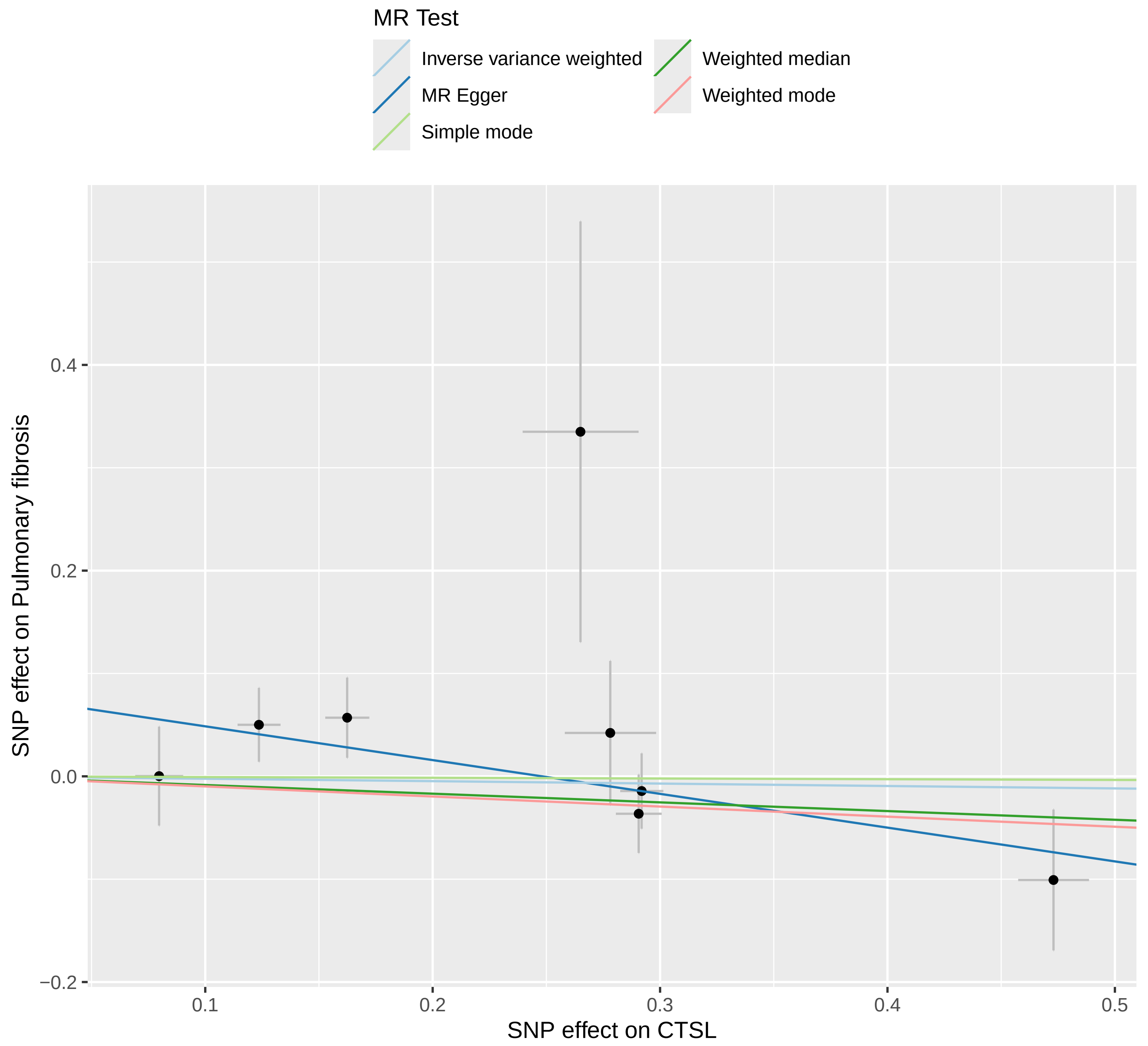

MR Test
Inverse variance weighted
MR Egger
Simple mode
Weighted median
Weighted mode
0.4
0.2
0.0
−0.2
0.1
0.2
0.3
0.4
0.5
SNP effect on Pulmonary fibrosis
SNP effect on CTSL

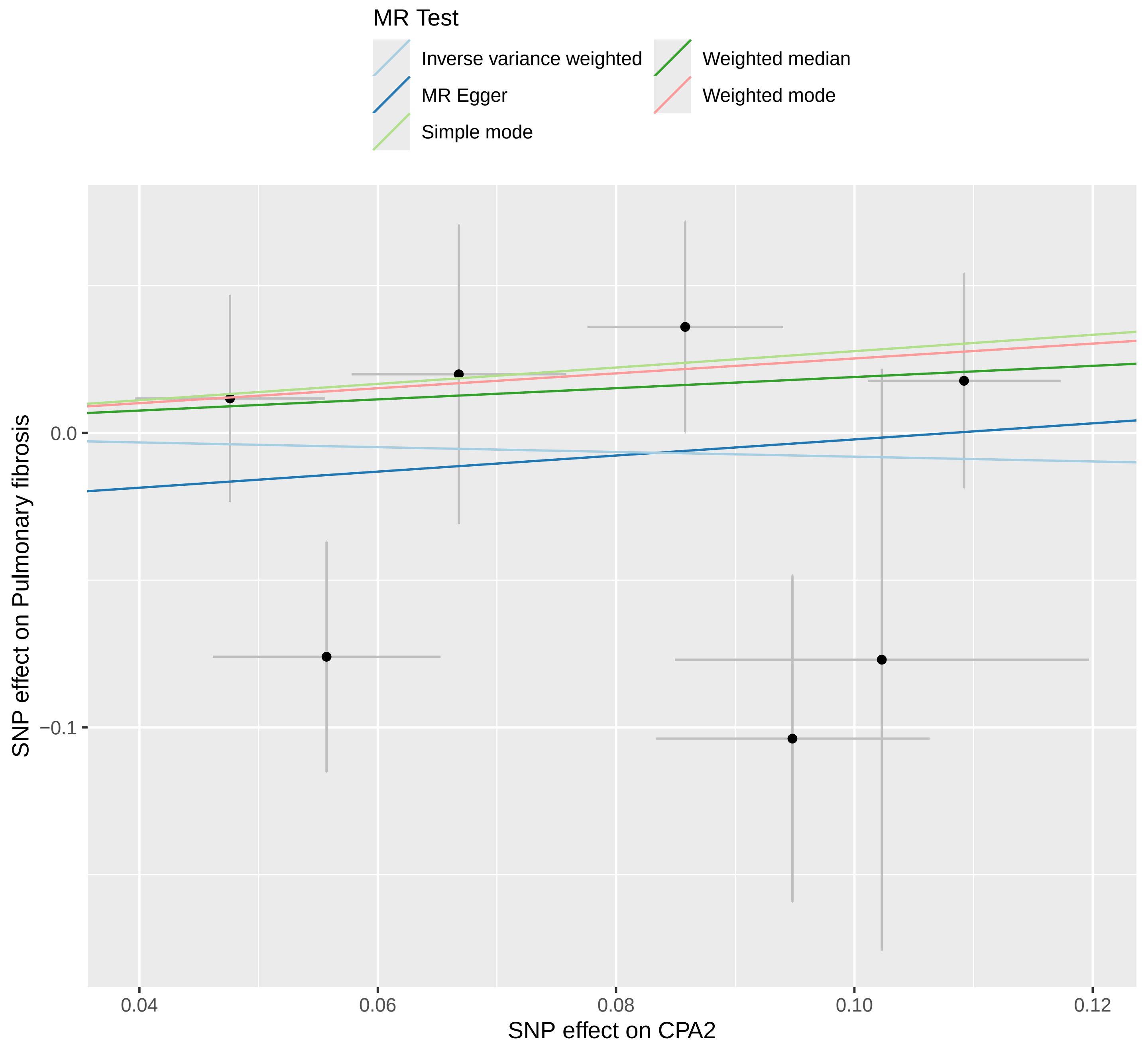
MR Test
Inverse variance weighted
MR Egger
Simple mode
Weighted median
Weighted mode
SNP effect on Pulmonary fibrosis
0.0
−0.1
0.04
0.06
0.08
0.10
0.12
SNP effect on CPA2

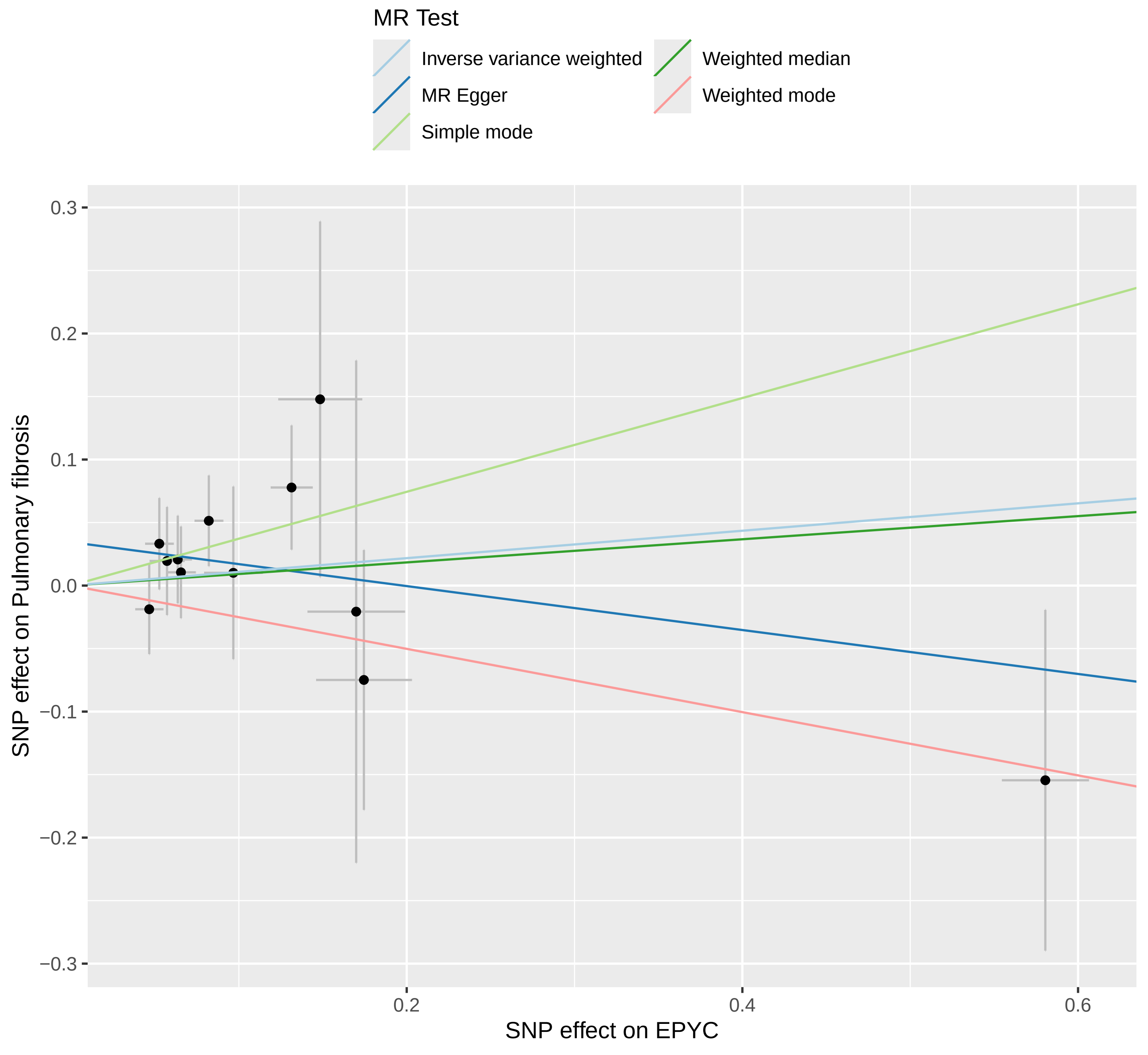
MR Test
Inverse variance weighted
MR Egger
Simple mode
Weighted median
Weighted mode
SNP effect on Pulmonary fibrosis
0.3
0.2
0.1
0.0
−0.1
−0.2
−0.3
SNP effect on EPYC
0.2
0.4
0.6

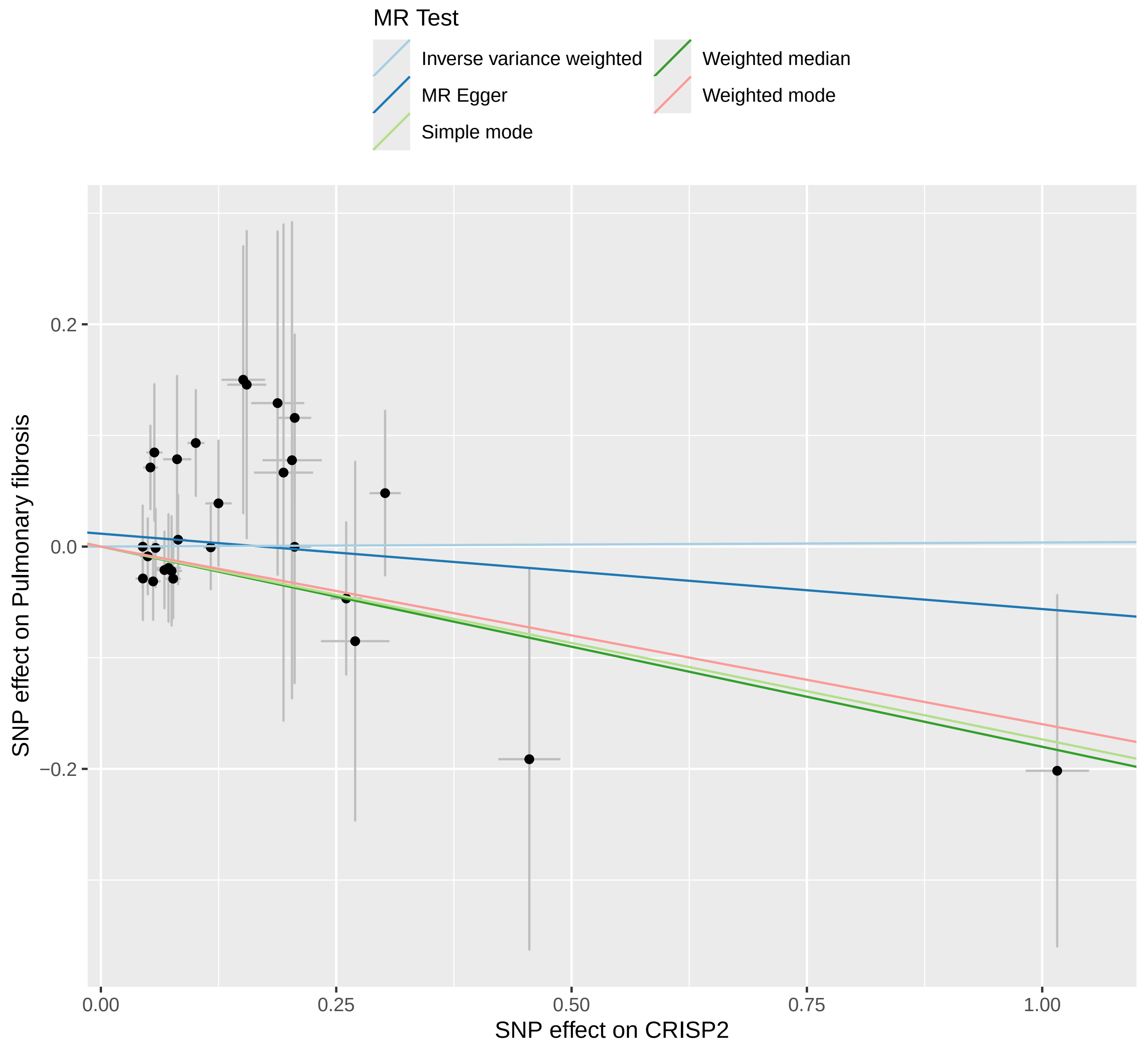

MR Test
Inverse variance weighted
MR Egger
Simple mode
Weighted median
Weighted mode
0.2
0.0
−0.2
SNP effect on Pulmonary fibrosis
0.00
0.25
0.50
0.75
1.00
SNP effect on CRISP2

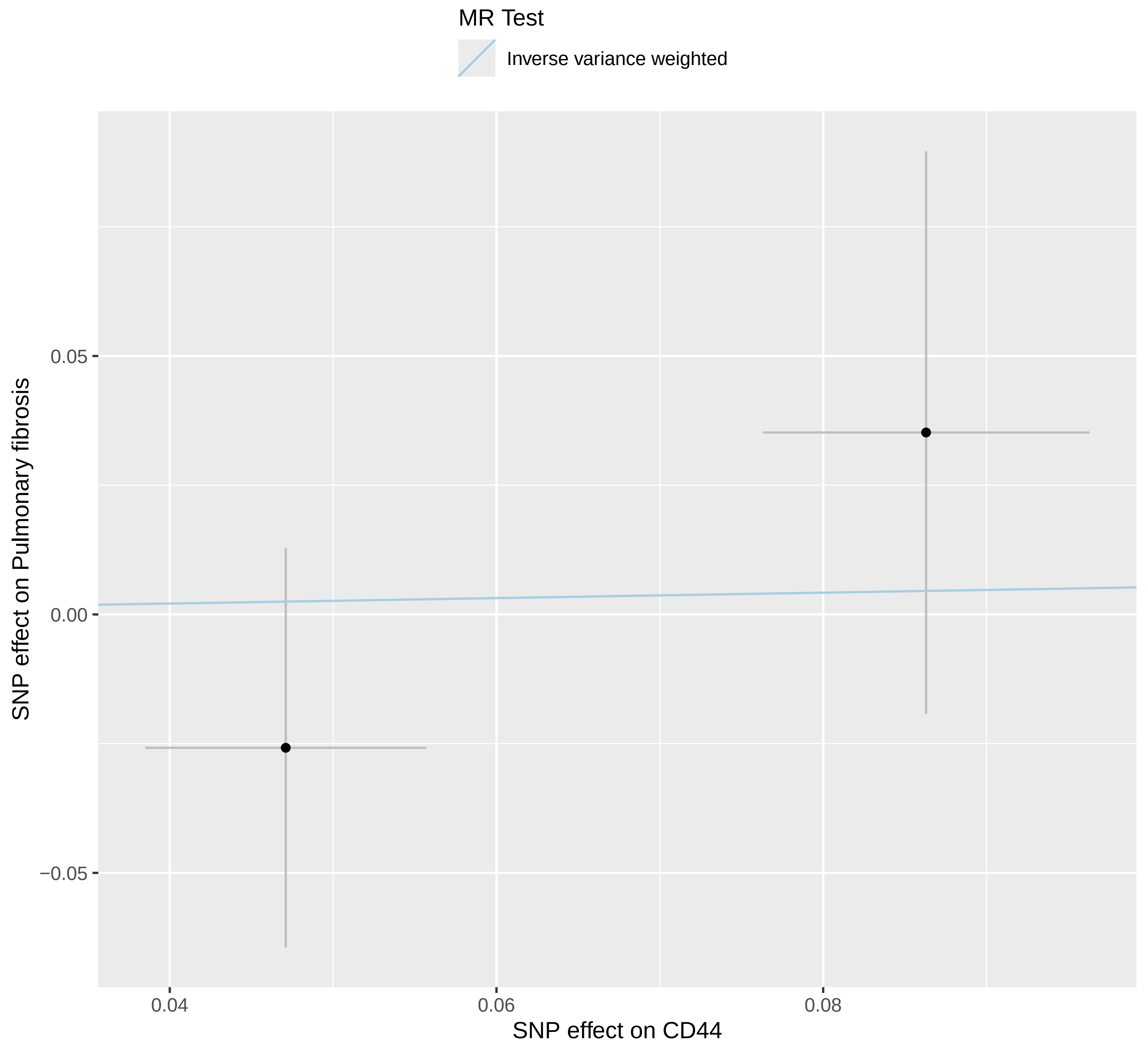
MR Test
Inverse variance weighted
SNP effect on Pulmonary fibrosis
0.05
0.00
−0.05
0.04
0.06
0.08
SNP effect on CD44

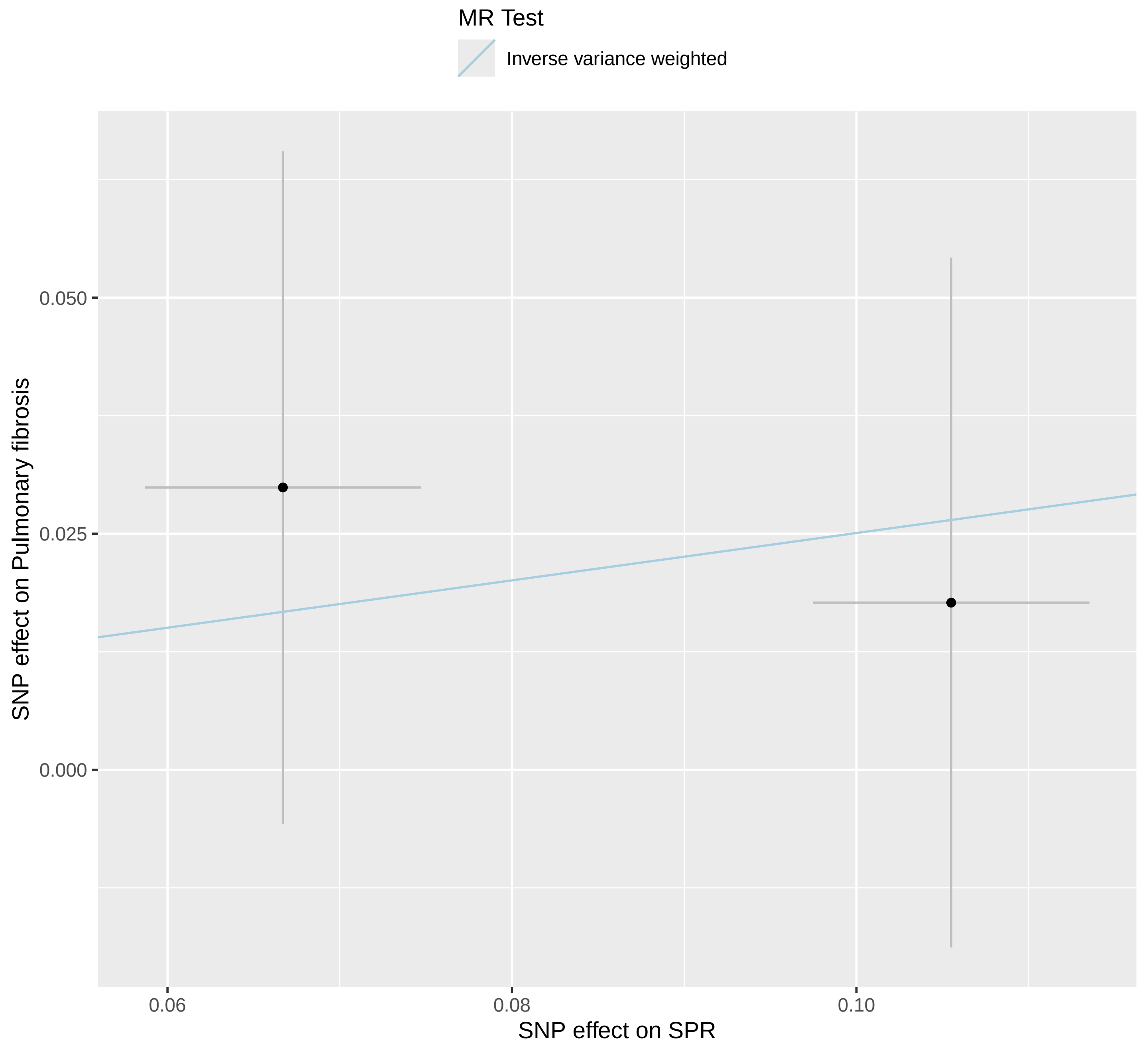

MR Test
Inverse variance weighted
0.050
0.025
0.000
0.06
0.08
0.10
SNP effect on Pulmonary fibrosis
SNP effect on SPR

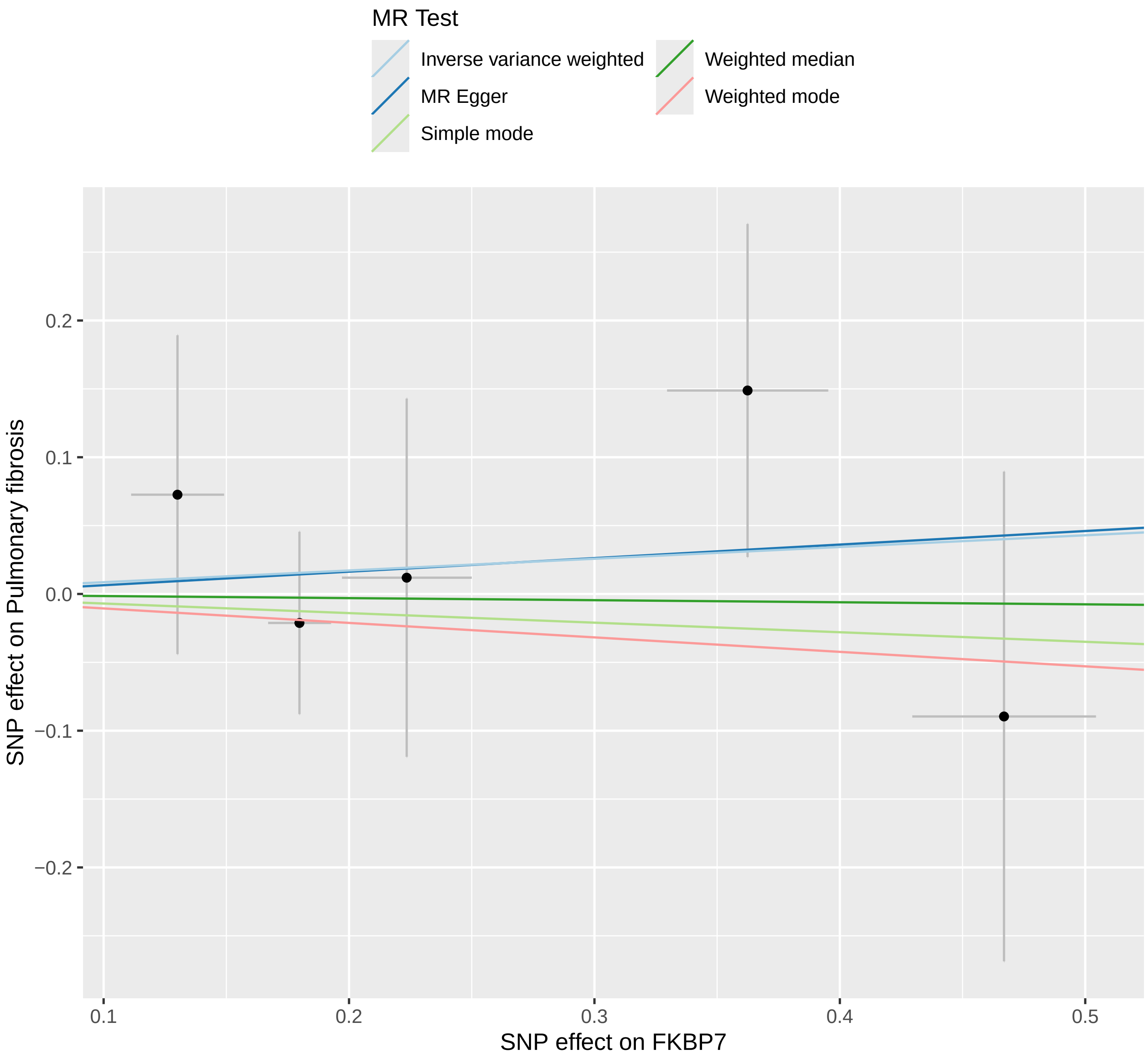
MR Test
Inverse variance weighted
MR Egger
Simple mode
Weighted median
Weighted mode
SNP effect on Pulmonary fibrosis
0.2
0.1
0.0
−0.1
−0.2
0.1
0.2
0.3
0.4
0.5
SNP effect on FKBP7

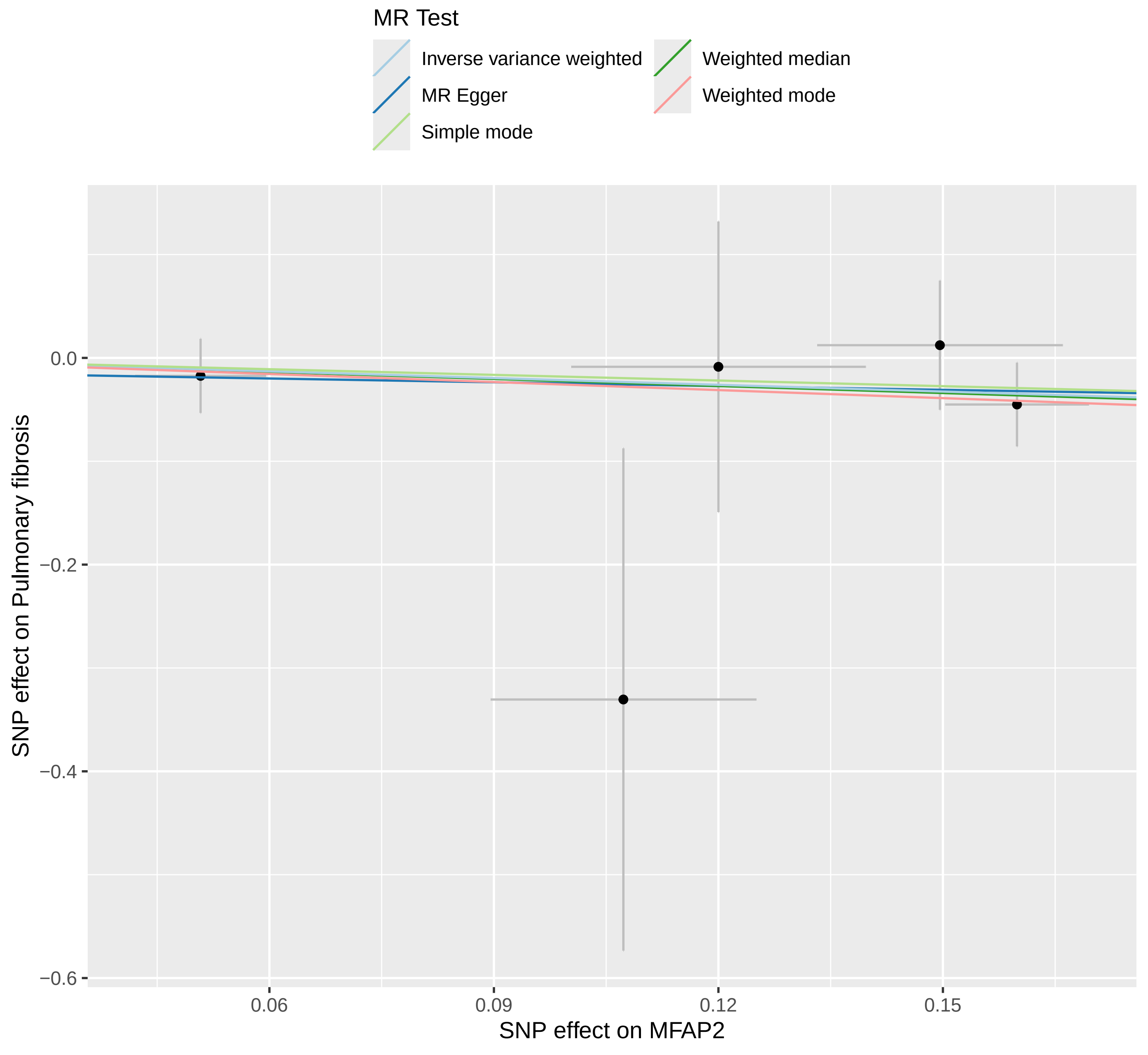

MR Test
Inverse variance weighted
MR Egger
Simple mode
Weighted median
Weighted mode
0.0
−0.2
−0.4
−0.6
0.06
0.09
0.12
0.15
SNP effect on MFAP2
SNP effect on Pulmonary fibrosis

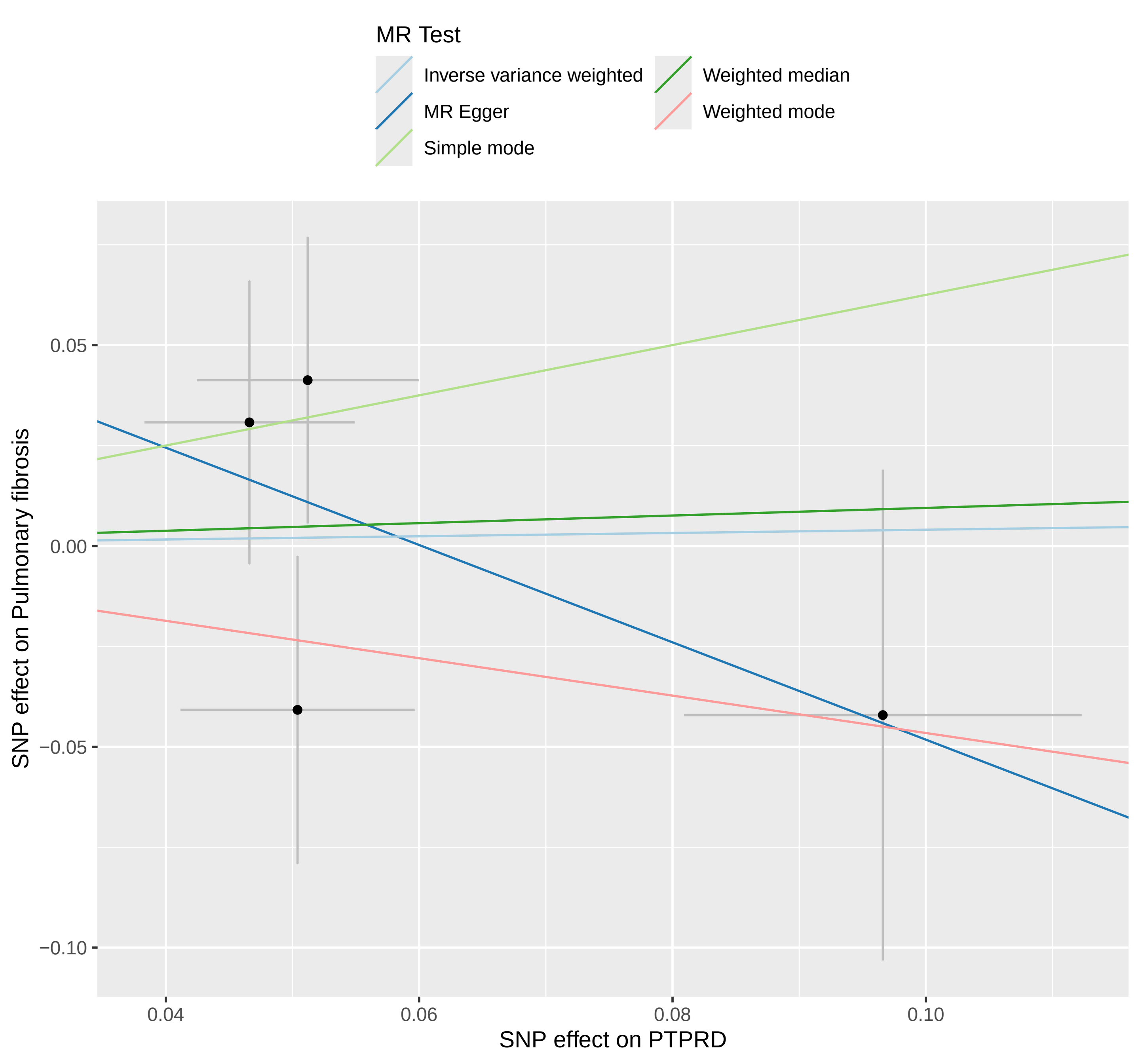
MR Test
Inverse variance weighted
MR Egger
Simple mode
Weighted median
Weighted mode
0.05
0.00
−0.05
−0.10
0.04
0.06
0.08
0.10
SNP effect on PTPRD
SNP effect on Pulmonary fibrosis

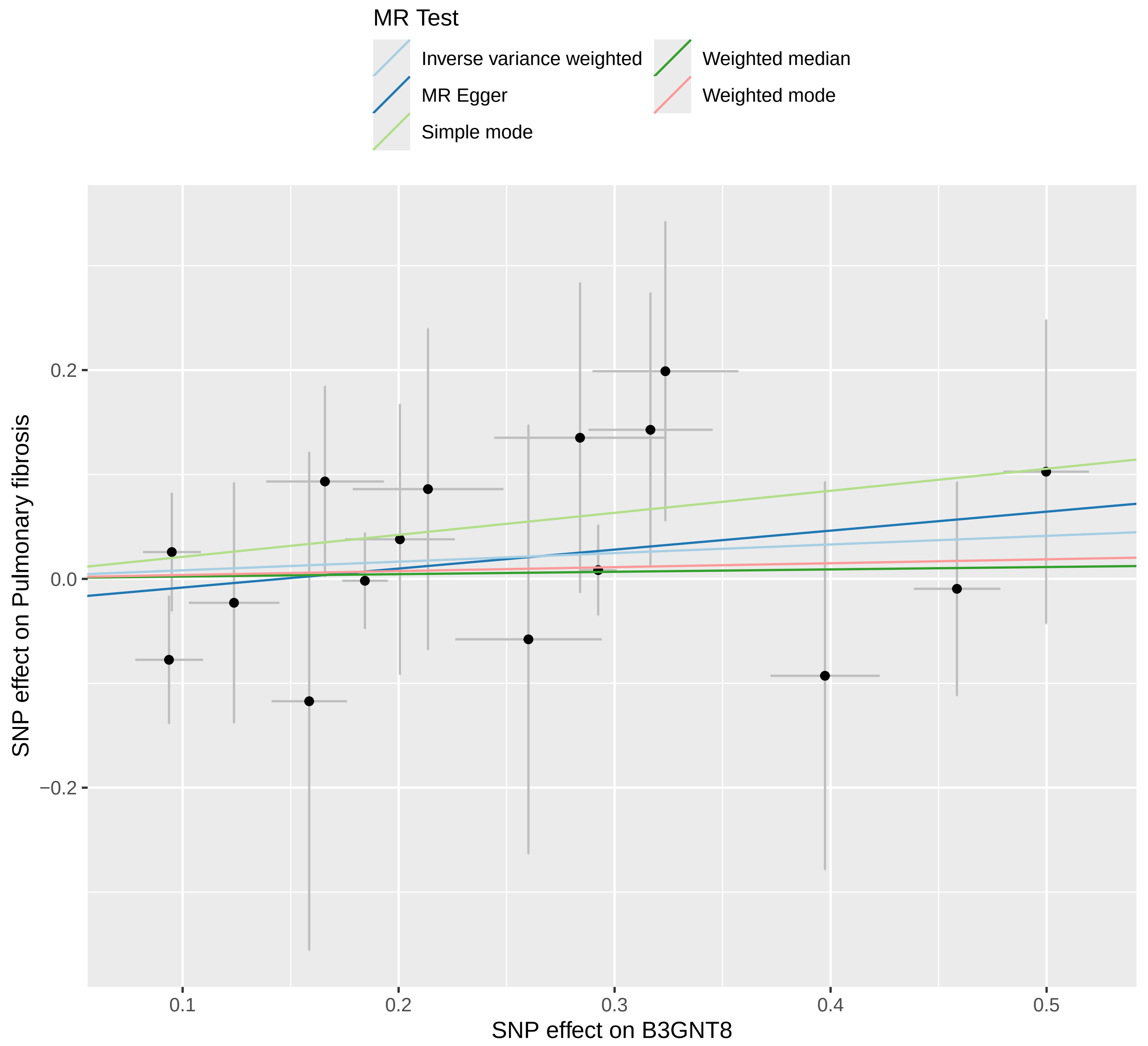

MR Test
Inverse variance weighted
MR Egger
Simple mode
Weighted median
Weighted mode
0.2
0.0
−0.2
SNP effect on Pulmonary fibrosis
0.1
0.2
0.3
0.4
0.5
SNP effect on B3GNT8

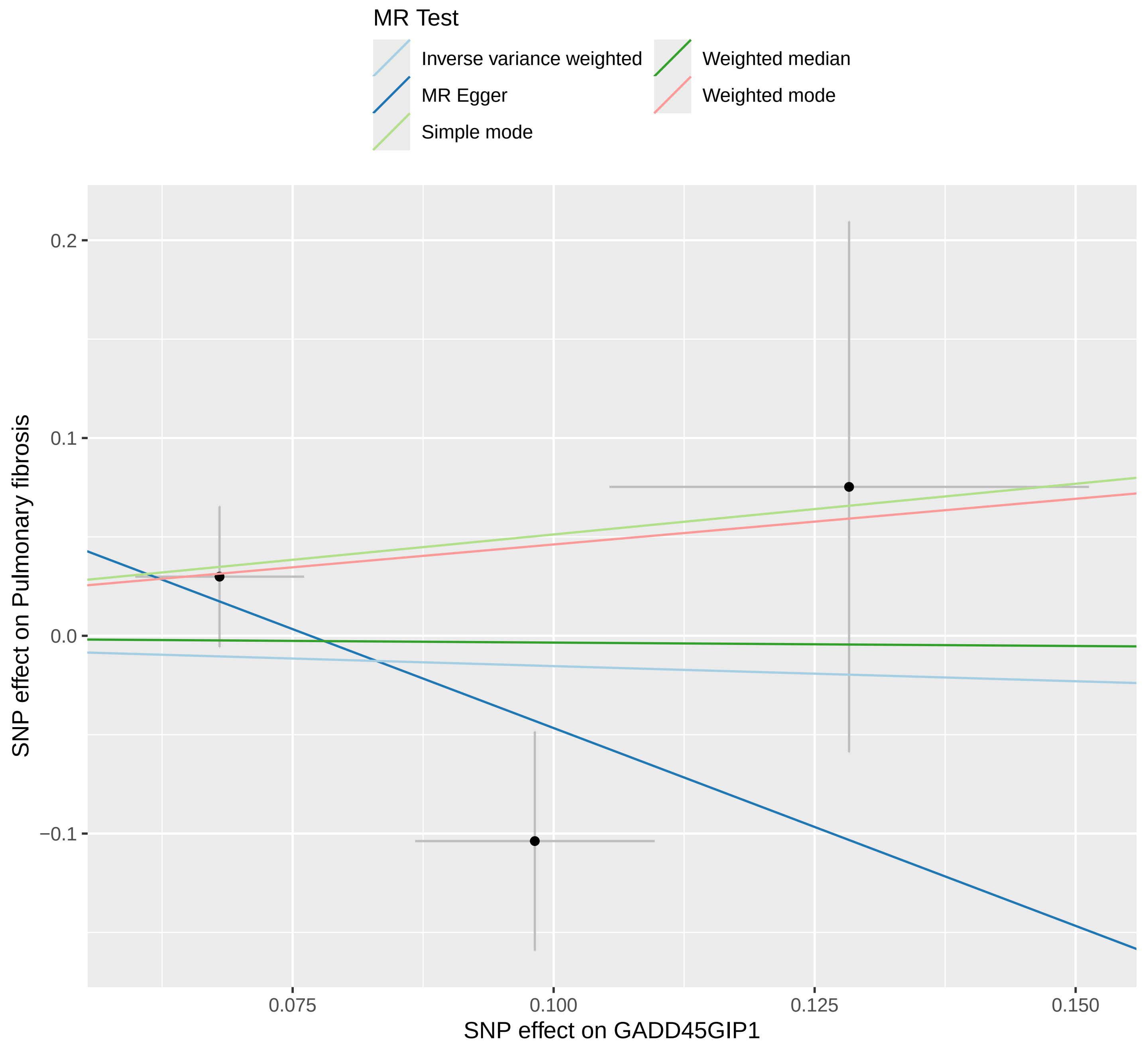

MR Test
Inverse variance weighted
MR Egger
Simple mode
Weighted median
Weighted mode
0.2
0.1
0.0
−0.1
0.075
0.100
0.125
0.150
SNP effect on Pulmonary fibrosis
SNP effect on GADD45GIP1

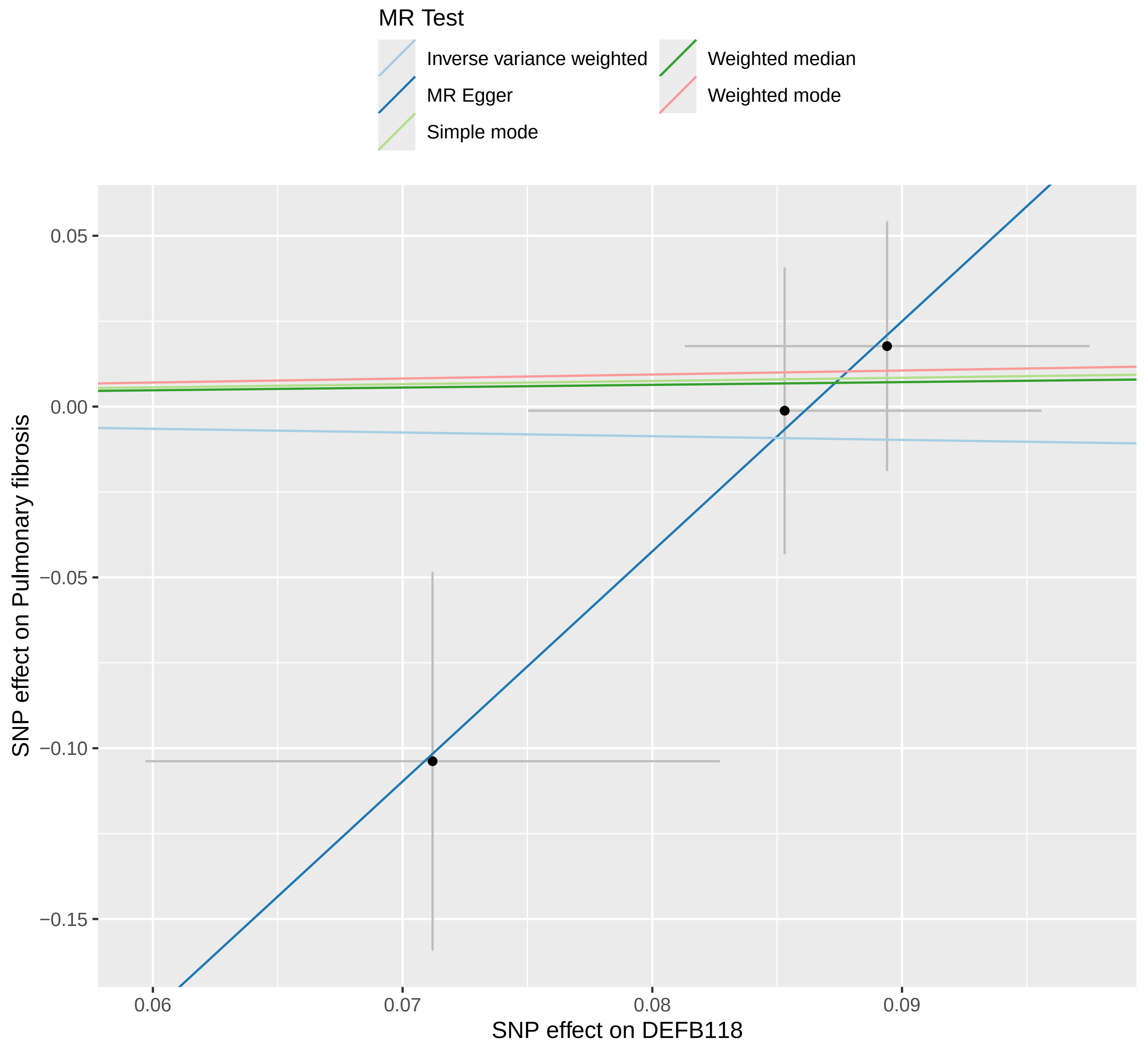

MR Test
Inverse variance weighted
MR Egger
Simple mode
Weighted median
Weighted mode
0.05
0.00
−0.05
−0.10
−0.15
SNP effect on Pulmonary fibrosis
0.06
0.07
0.08
0.09
SNP effect on DEFB118

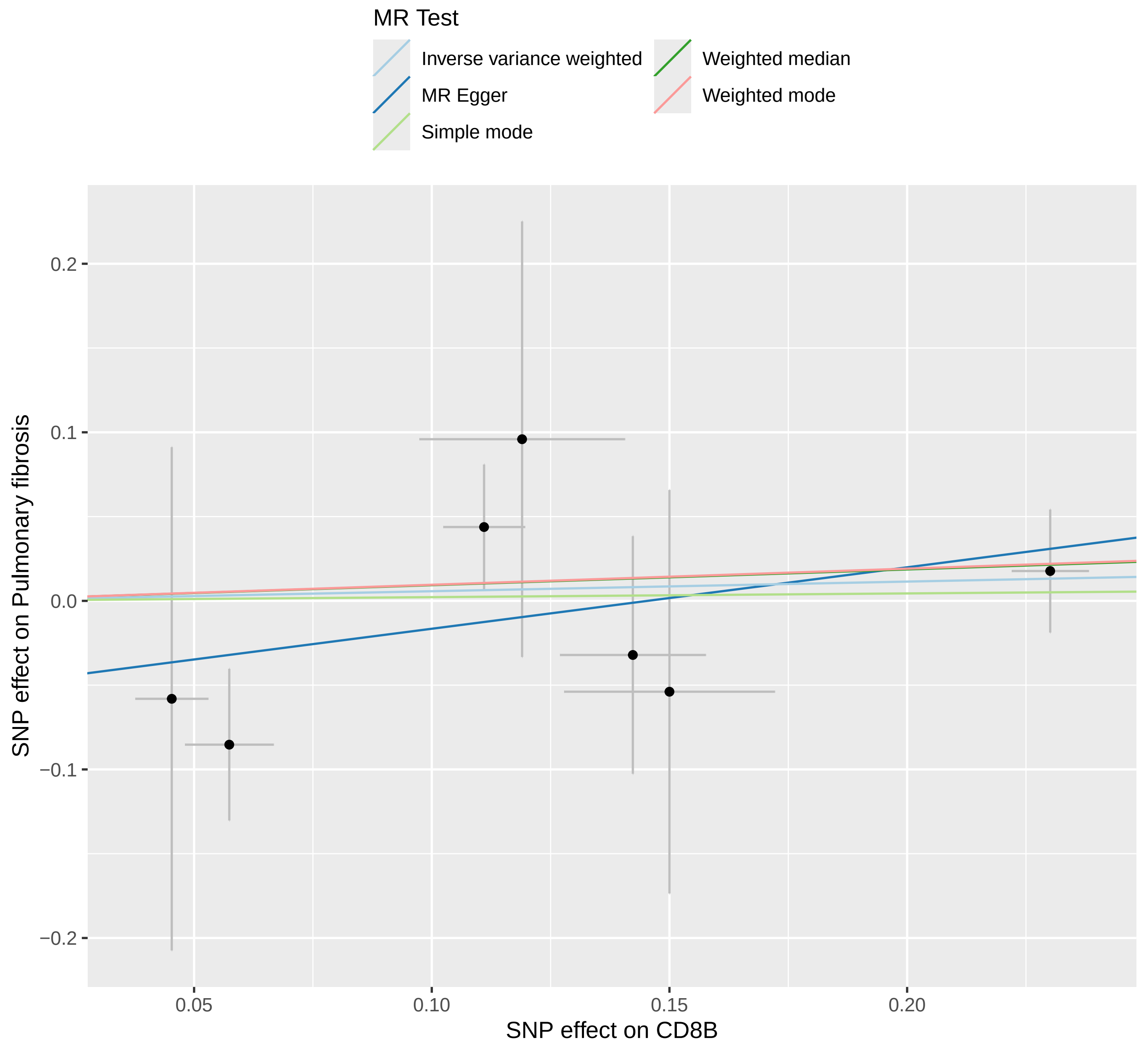

MR Test
Inverse variance weighted
MR Egger
Simple mode
Weighted median
Weighted mode
SNP effect on Pulmonary fibrosis
0.2
0.1
0.0
−0.1
−0.2
0.05
0.10
0.15
0.20
SNP effect on CD8B

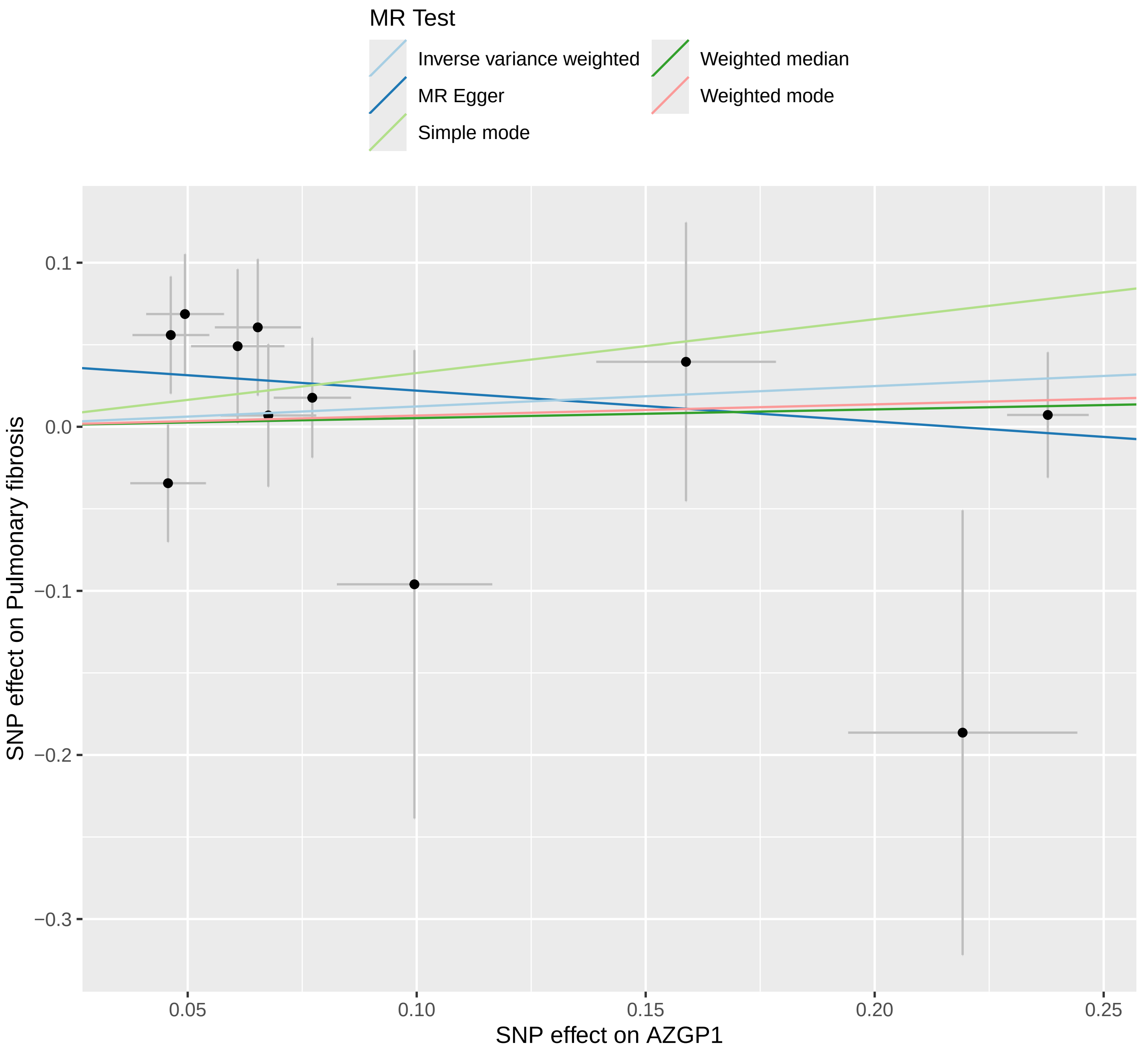

MR Test
Inverse variance weighted
MR Egger
Simple mode
Weighted median
Weighted mode
0.1
0.0
−0.1
−0.2
−0.3
SNP effect on Pulmonary fibrosis
0.05
0.10
0.15
0.20
0.25
SNP effect on AZGP1

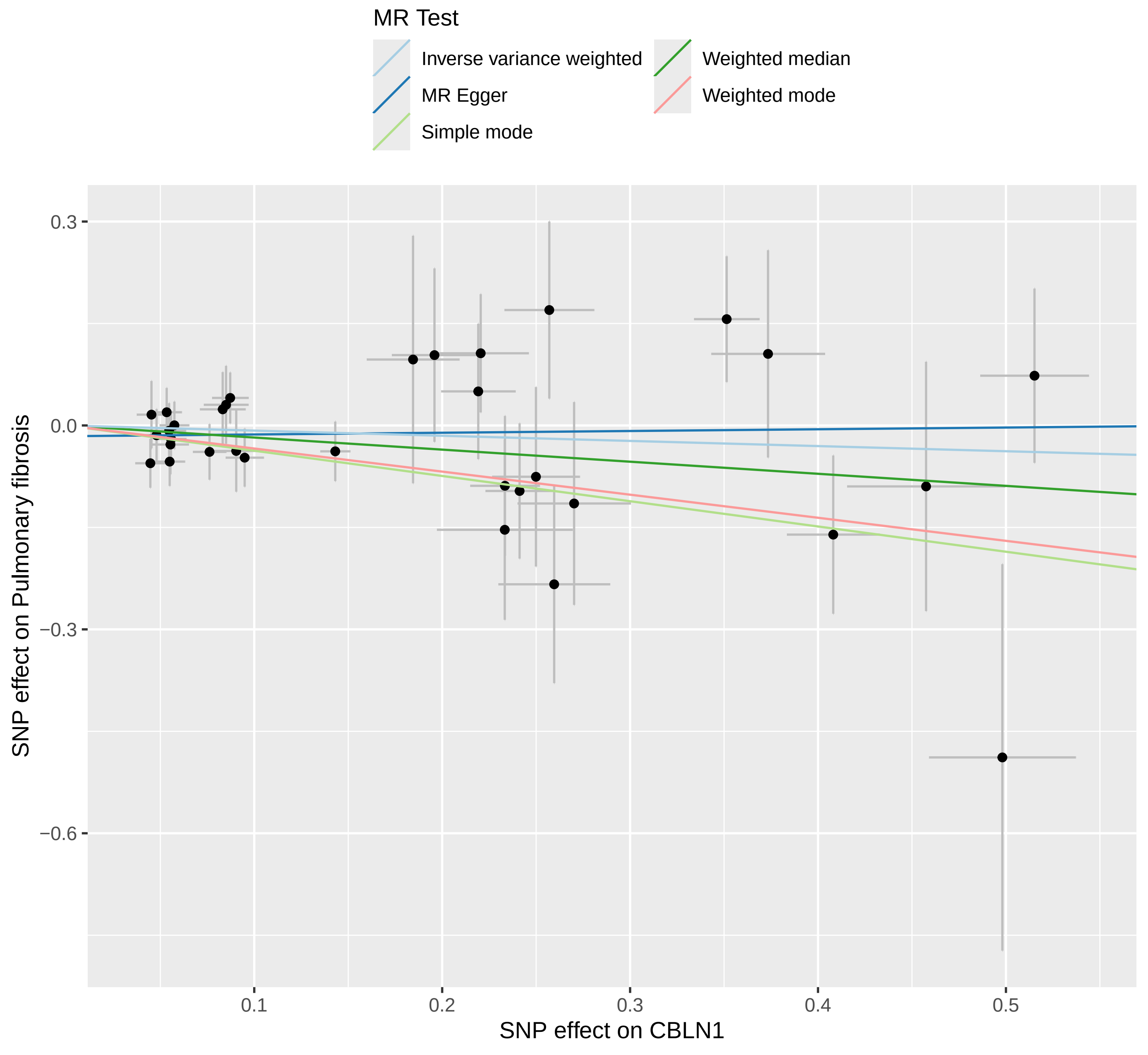
MR Test
Inverse variance weighted
MR Egger
Simple mode
Weighted median
Weighted mode
0.3
0.0
−0.3
−0.6
0.1
0.2
0.3
0.4
0.5
SNP effect on CBLN1
SNP effect on Pulmonary fibrosis

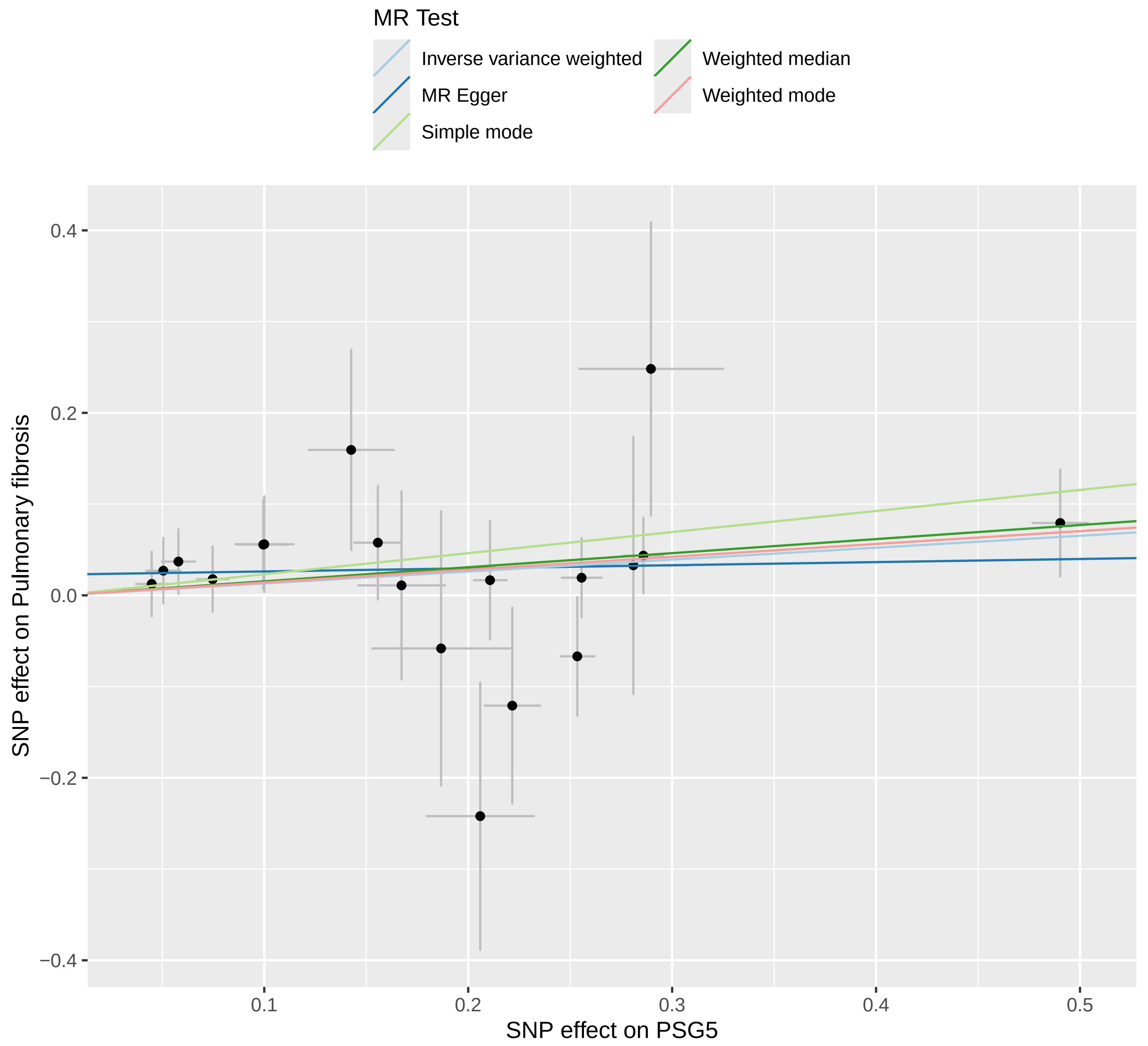
MR Test
Inverse variance weighted
MR Egger
Simple mode
Weighted median
Weighted mode
0.4
0.2
0.0
−0.2
−0.4
SNP effect on Pulmonary fibrosis
0.1
0.2
0.3
0.4
0.5
SNP effect on PSG5

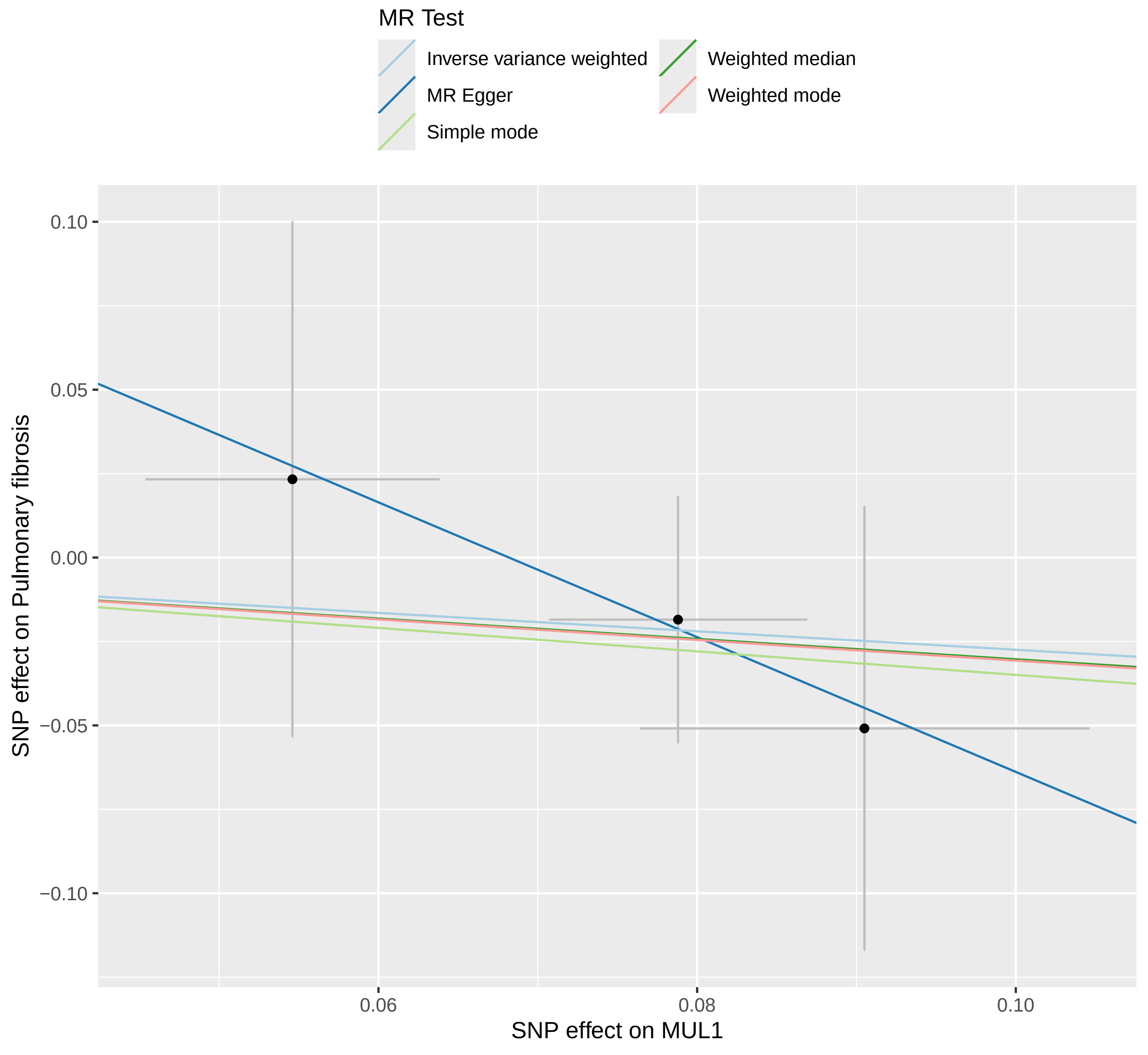
MR Test
Inverse variance weighted
MR Egger
Simple mode
Weighted median
Weighted mode
0.10
0.05
0.00
−0.05
−0.10
SNP effect on Pulmonary fibrosis
0.06
0.08
0.10
SNP effect on MUL1

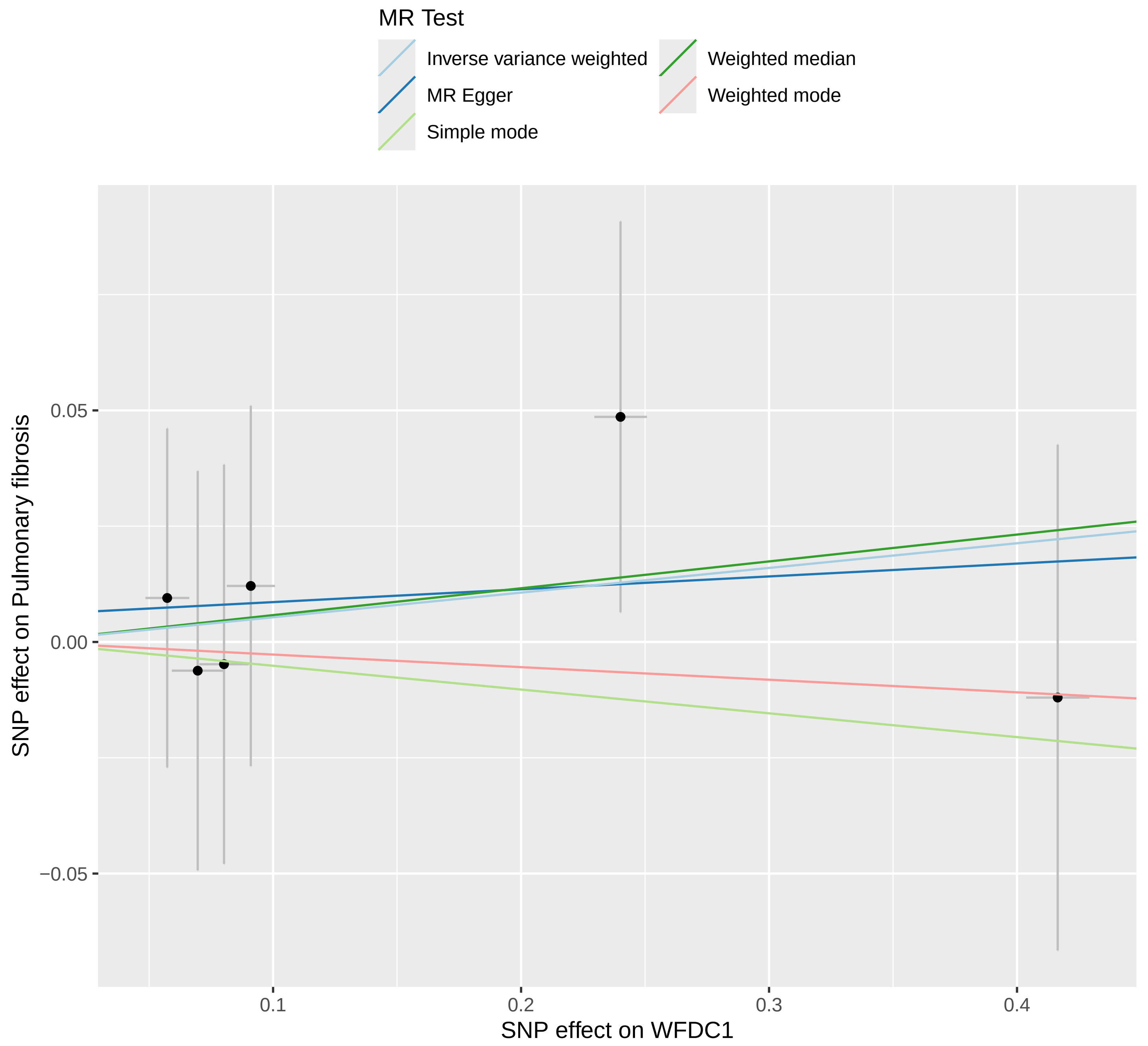

MR Test
Inverse variance weighted
MR Egger
Simple mode
Weighted median
Weighted mode
0.05
0.00
−0.05
0.1
0.2
0.3
0.4
SNP effect on Pulmonary fibrosis
SNP effect on WFDC1

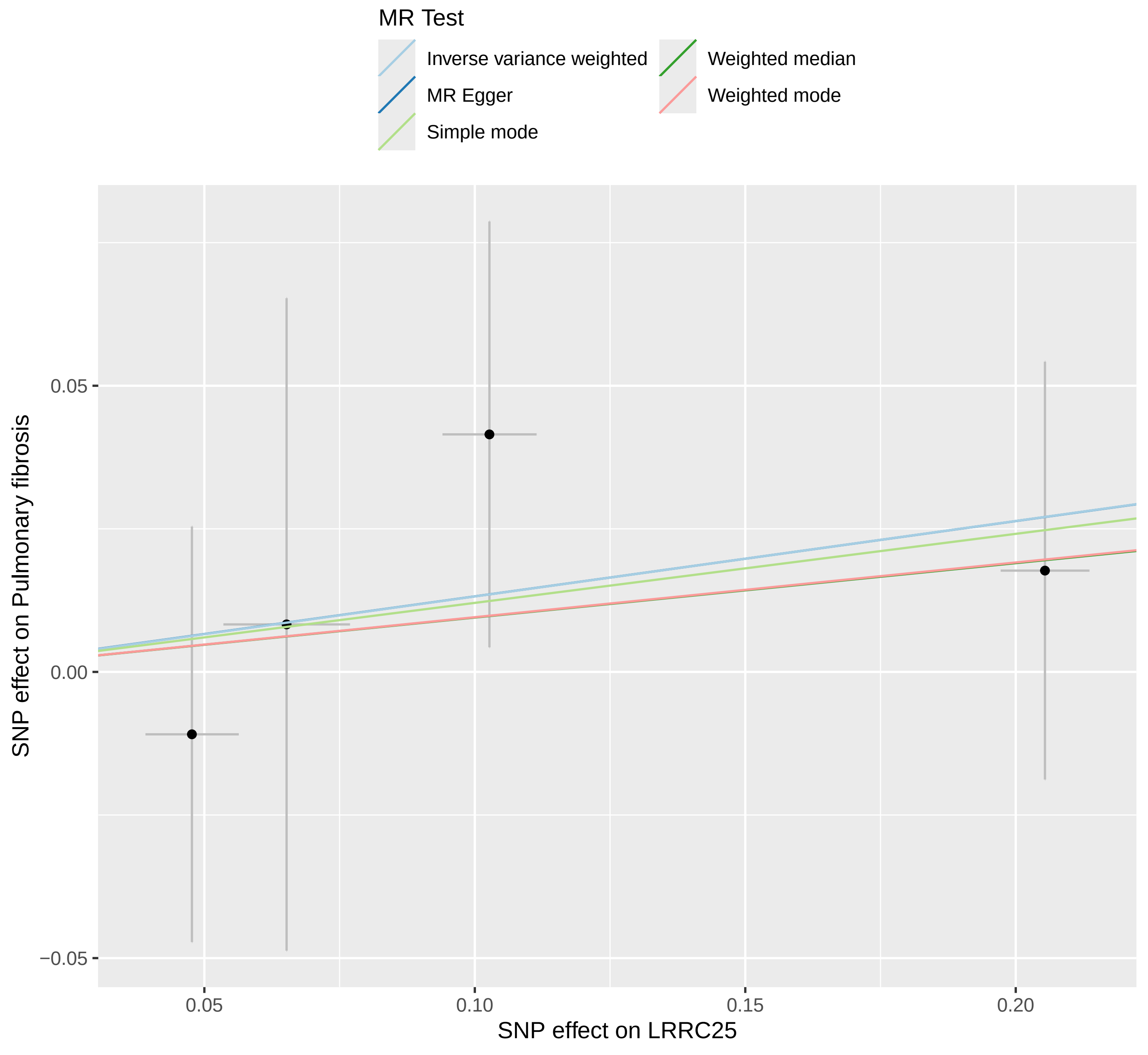
MR Test
Inverse variance weighted
MR Egger
Simple mode
Weighted median
Weighted mode
SNP effect on Pulmonary fibrosis
0.05
0.00
−0.05
0.05
0.10
0.15
0.20
SNP effect on LRRC25

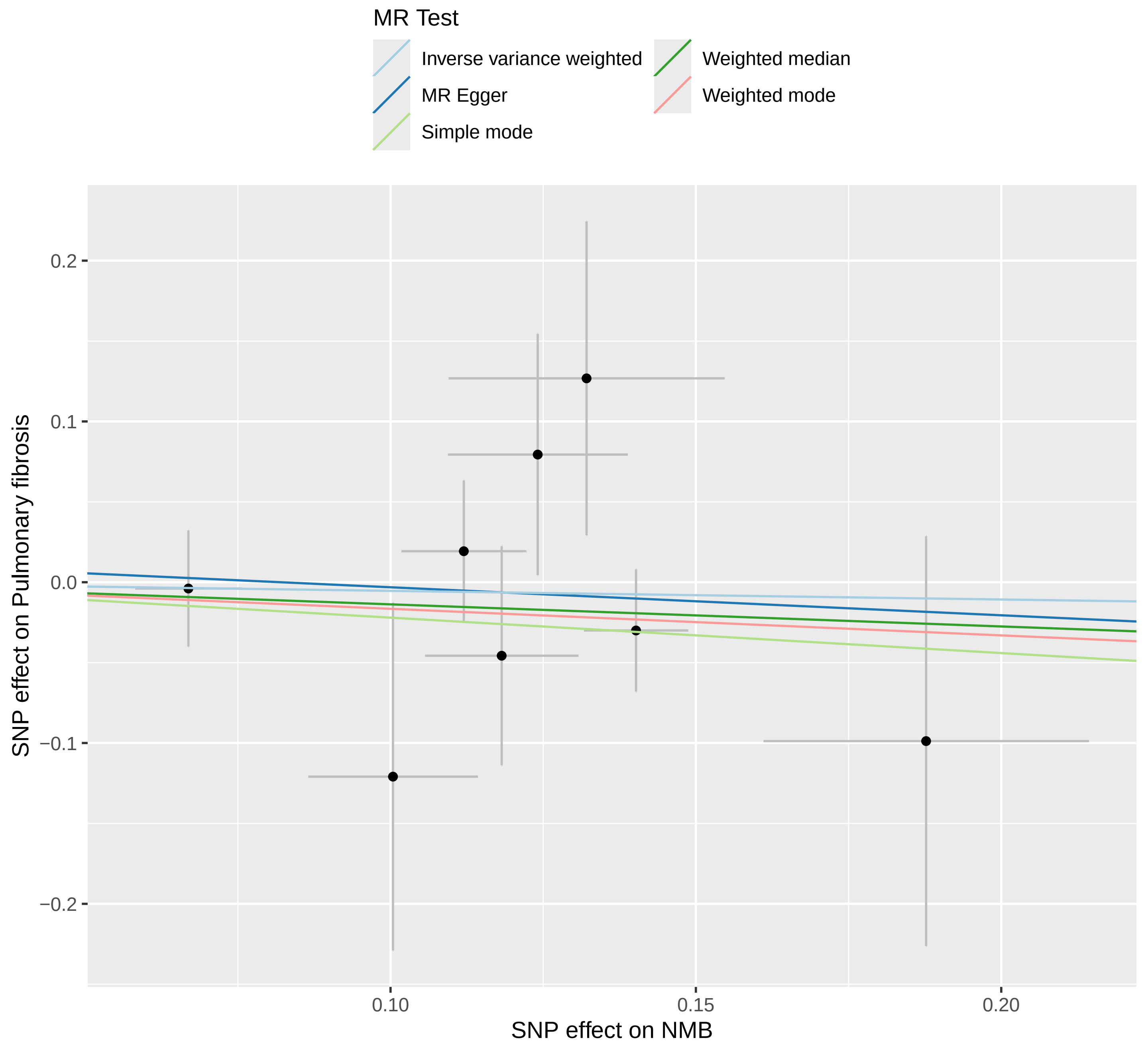
MR Test
Inverse variance weighted
MR Egger
Simple mode
Weighted median
Weighted mode
0.2
0.1
0.0
−0.1
−0.2
SNP effect on Pulmonary fibrosis
0.10
0.15
0.20
SNP effect on NMB

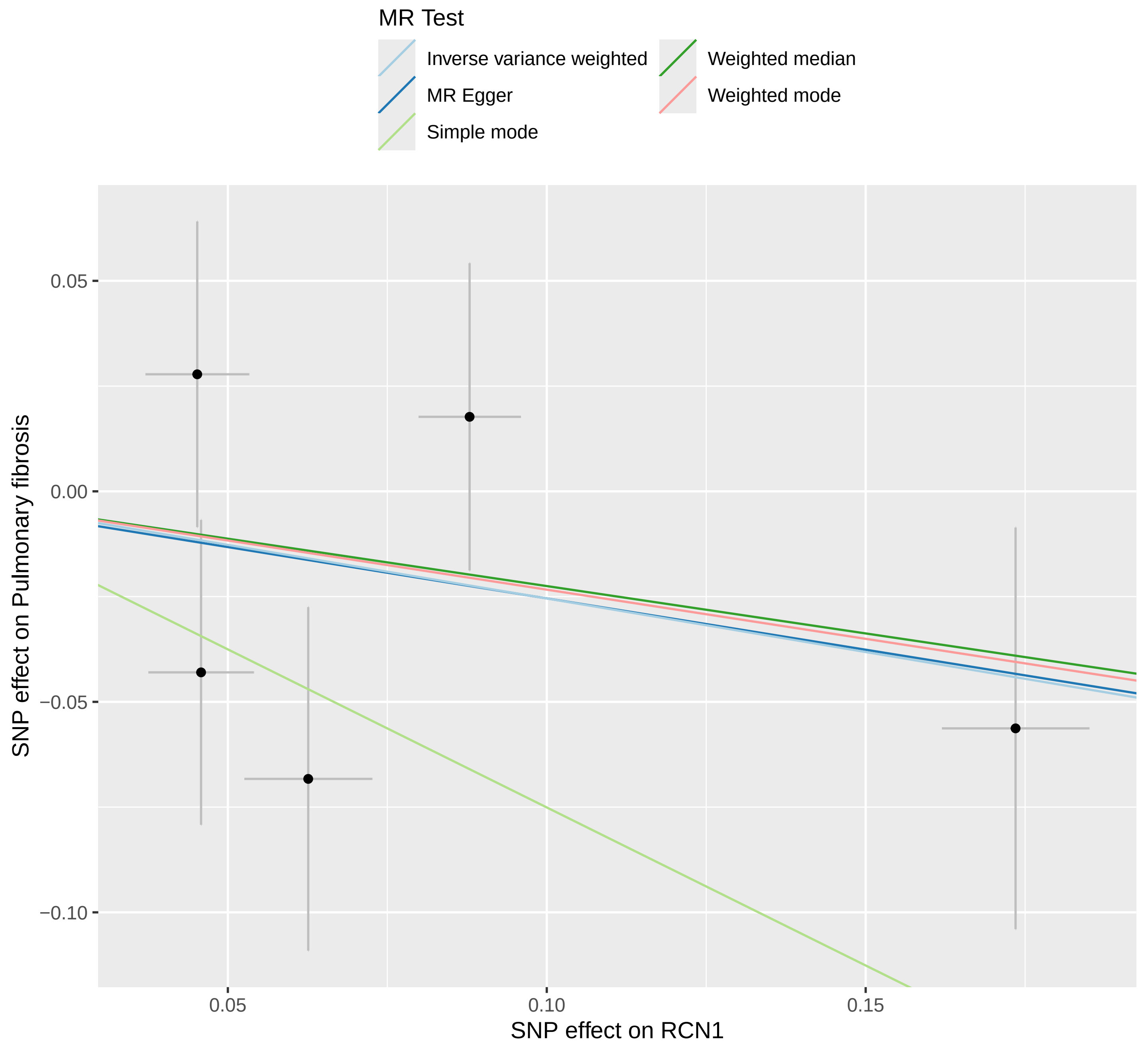
MR Test
Inverse variance weighted
MR Egger
Simple mode
Weighted median
Weighted mode
0.05
0.00
−0.05
−0.10
SNP effect on Pulmonary fibrosis
0.05
0.10
0.15
SNP effect on RCN1

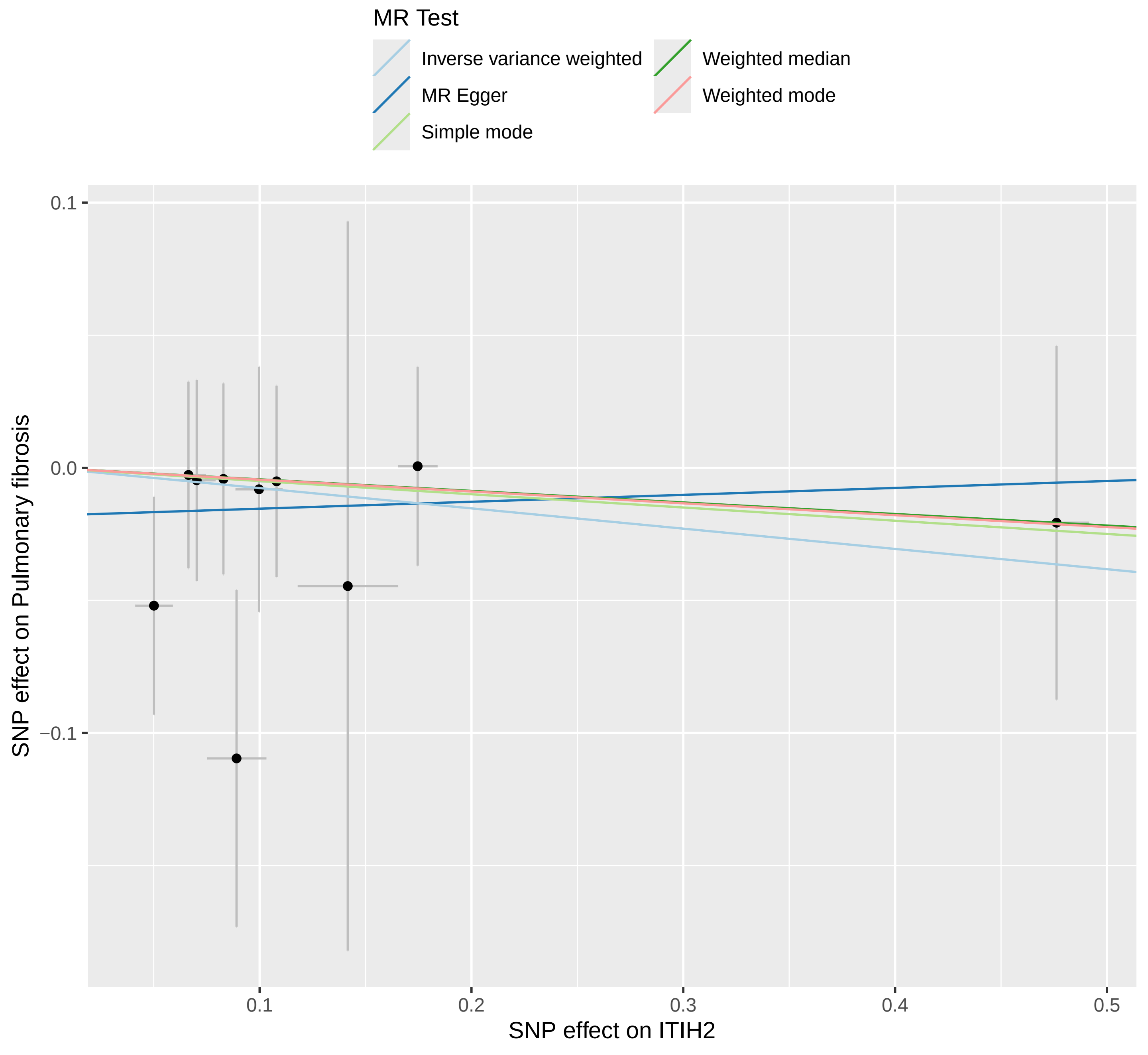
MR Test
Inverse variance weighted
MR Egger
Simple mode
Weighted median
Weighted mode
0.1
0.0
−0.1
0.1
0.2
0.3
0.4
0.5
SNP effect on Pulmonary fibrosis
SNP effect on ITIH2

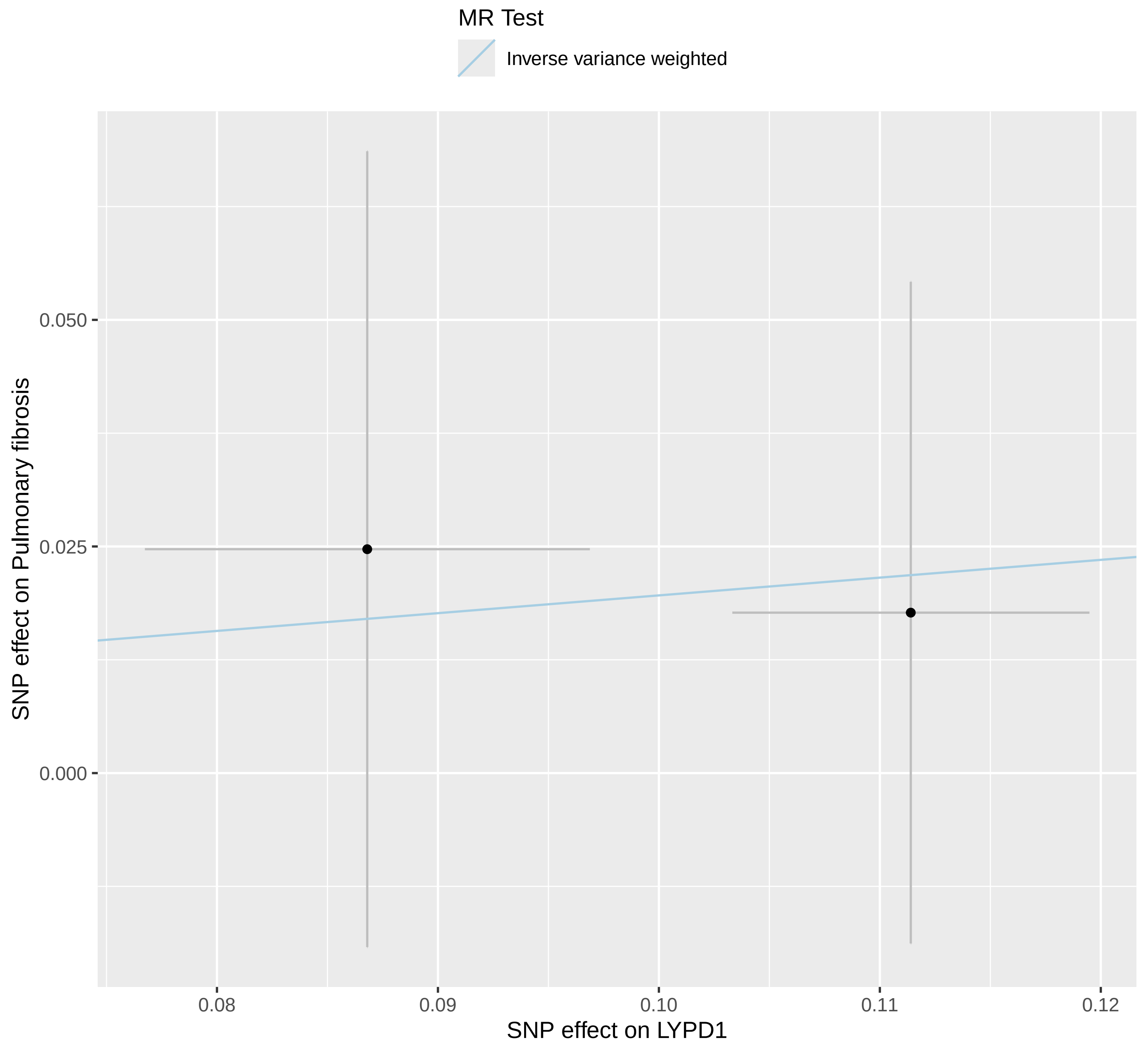

MR Test
Inverse variance weighted
0.050
0.025
0.000
0.08
0.09
0.10
0.11
0.12
SNP effect on Pulmonary fibrosis
SNP effect on LYPD1

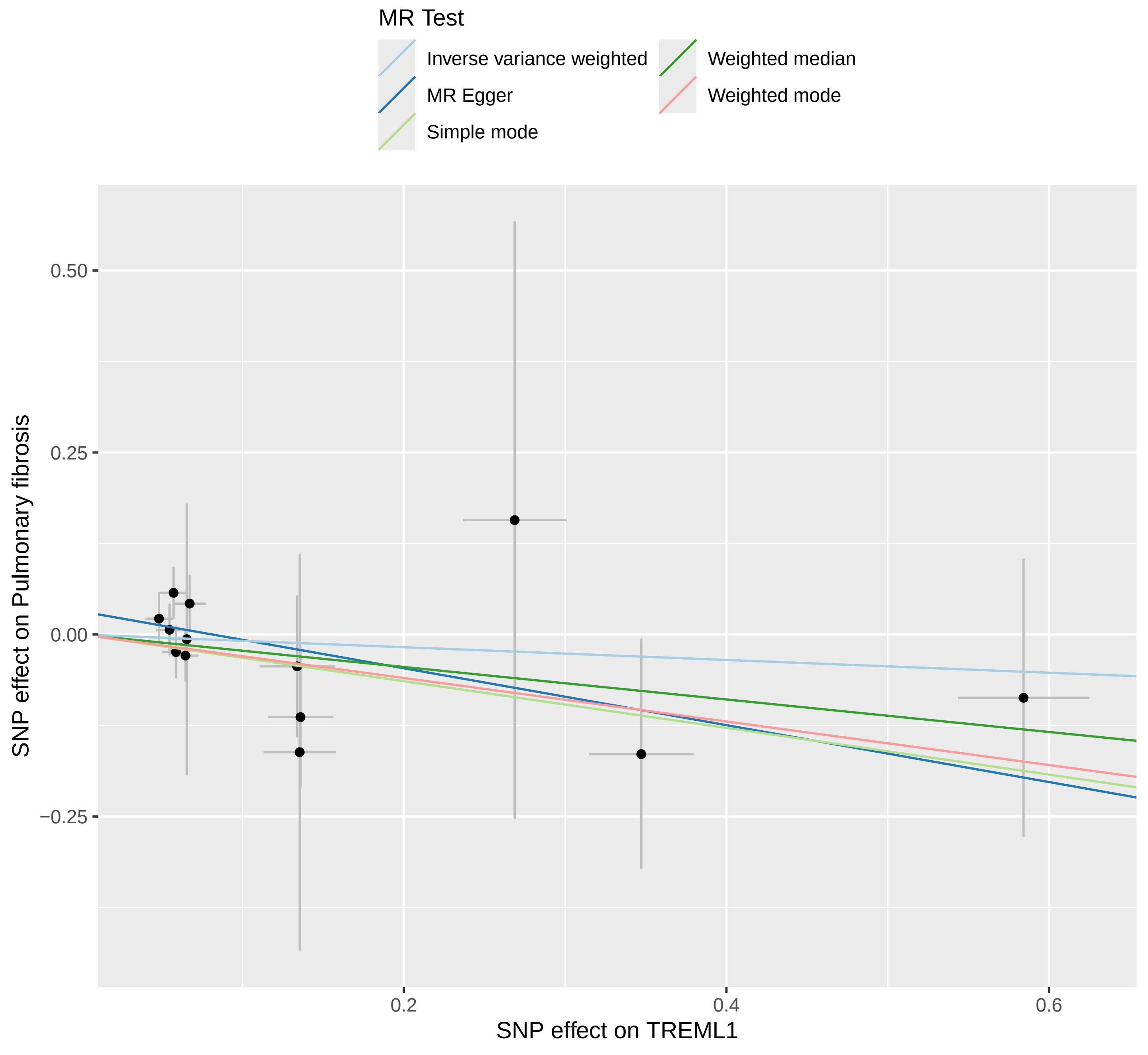
MR Test
Inverse variance weighted
MR Egger
Simple mode
Weighted median
Weighted mode
0.50
0.25
0.00
−0.25
0.2
0.4
0.6
SNP effect on Pulmonary fibrosis
SNP effect on TREML1

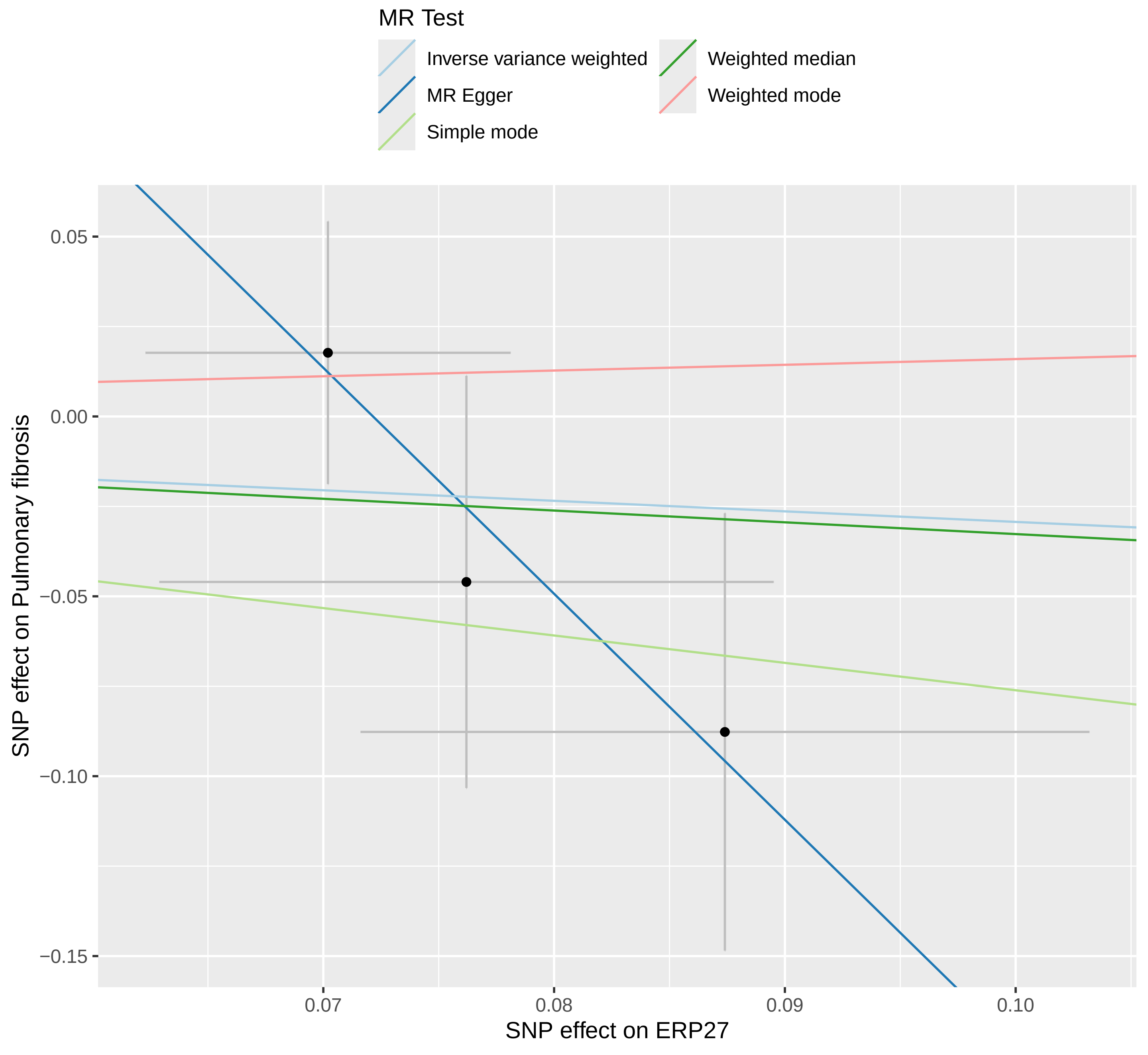

MR Test
Inverse variance weighted
MR Egger
Simple mode
Weighted median
Weighted mode
0.05
0.00
−0.05
−0.10
−0.15
0.07
0.08
0.09
0.10
SNP effect on Pulmonary fibrosis
SNP effect on ERP27

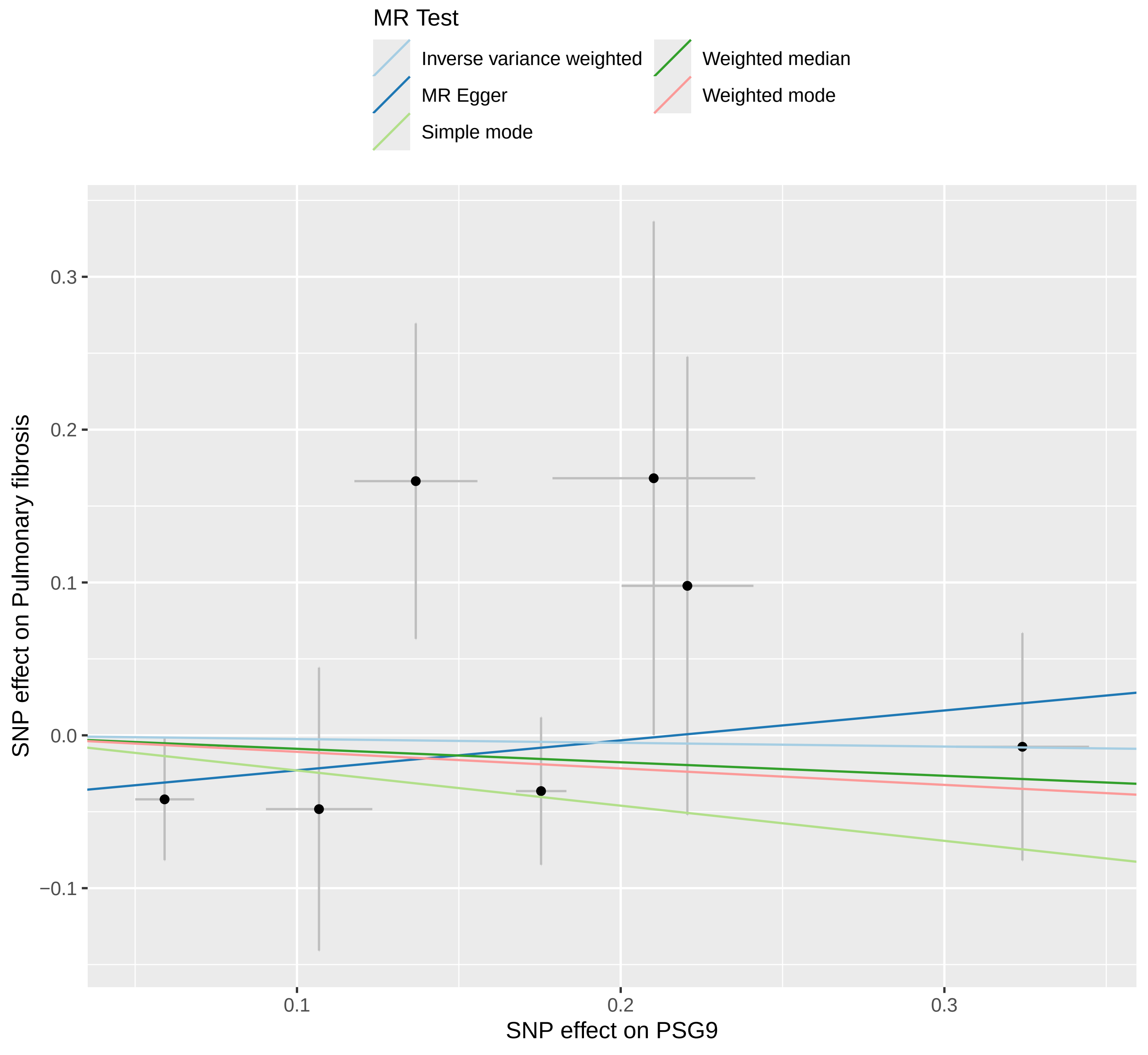
MR Test
Inverse variance weighted
MR Egger
Simple mode
Weighted median
Weighted mode
SNP effect on Pulmonary fibrosis
0.3
0.2
0.1
0.0
−0.1
0.1
0.2
0.3
SNP effect on PSG9

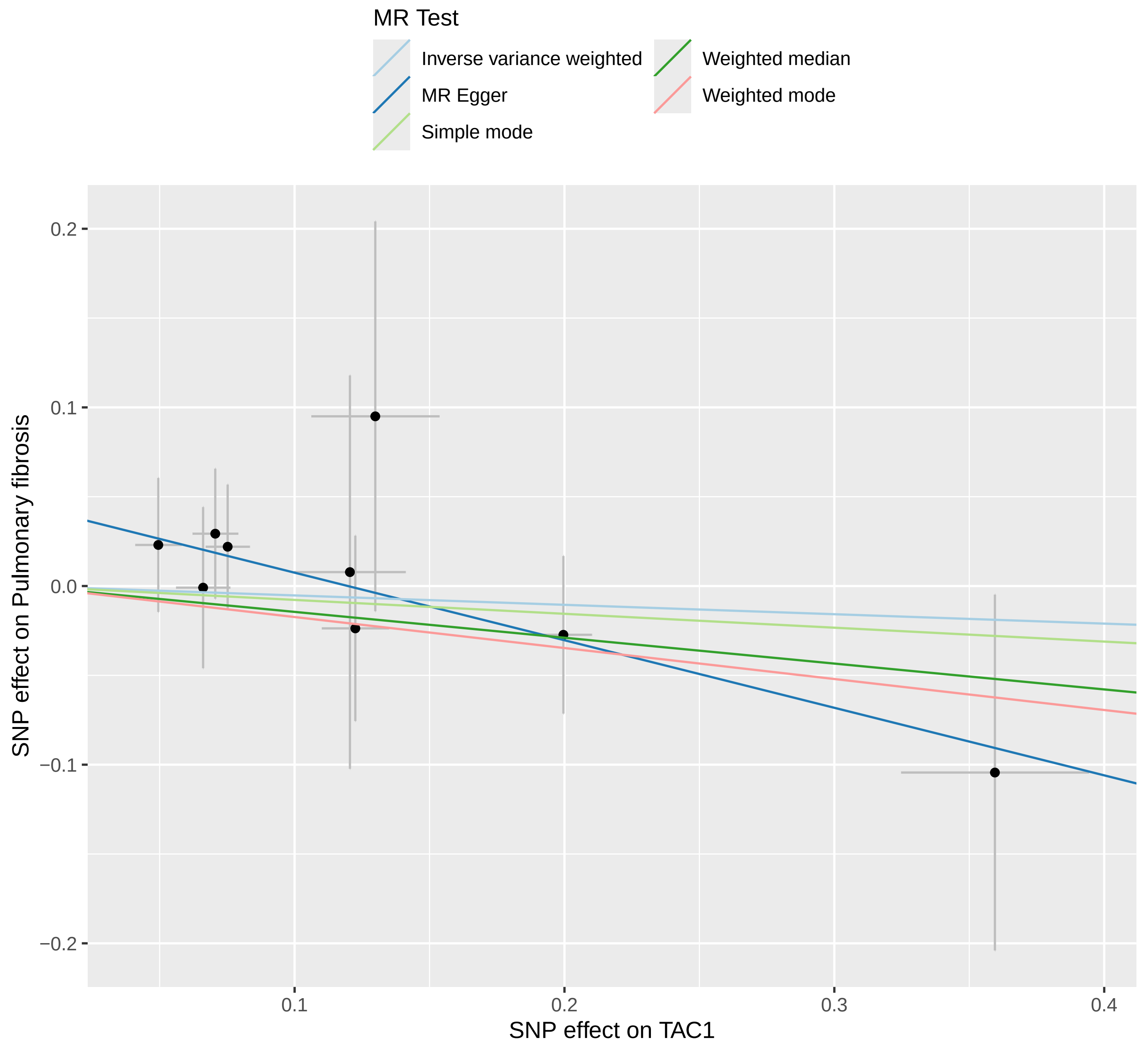
MR Test
Inverse variance weighted
MR Egger
Simple mode
Weighted median
Weighted mode
SNP effect on Pulmonary fibrosis
0.2
0.1
0.0
−0.1
−0.2
0.1
0.2
0.3
0.4
SNP effect on TAC1

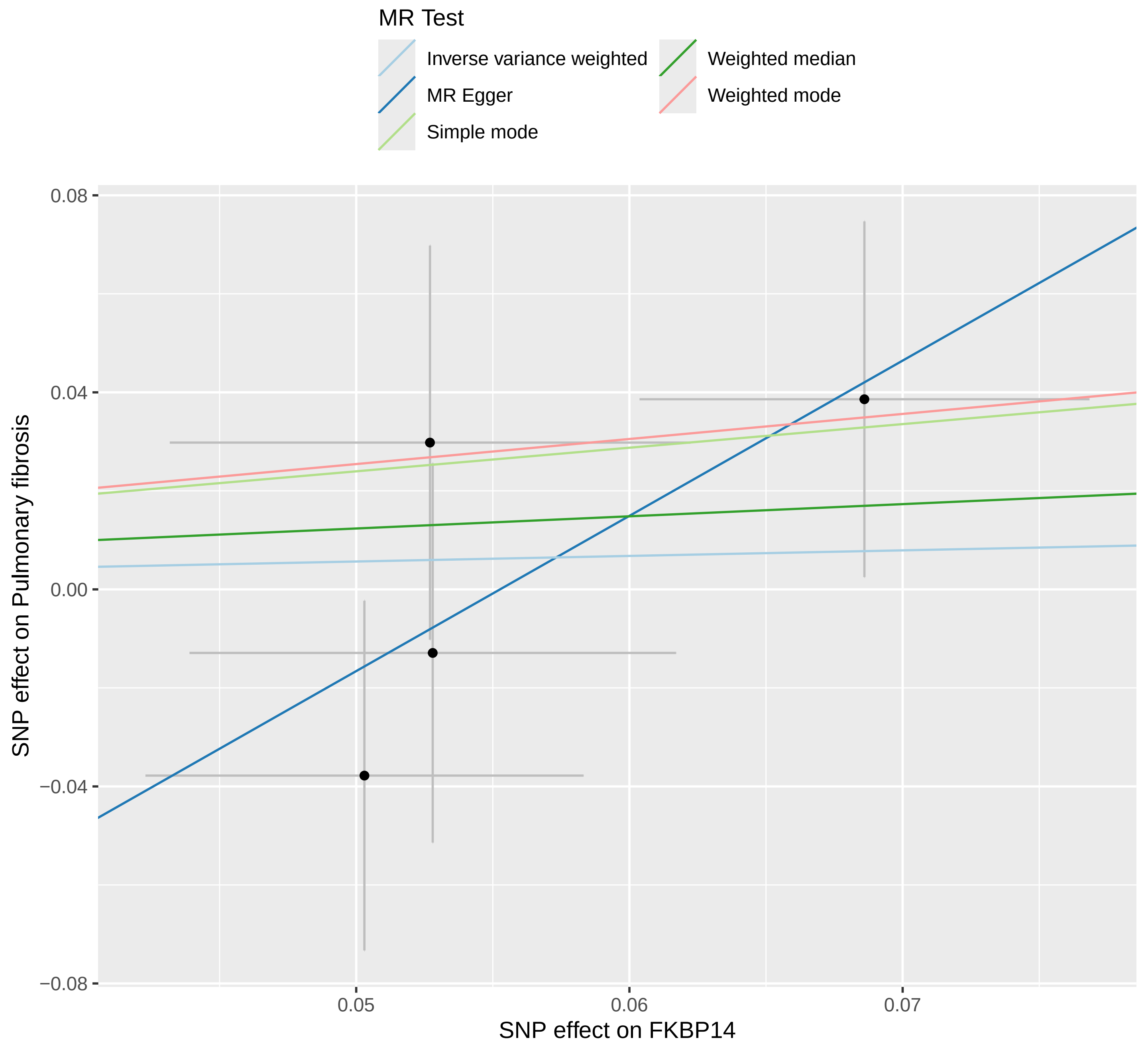
MR Test
Inverse variance weighted
MR Egger
Simple mode
Weighted median
Weighted mode
0.08
0.04
0.00
−0.04
−0.08
SNP effect on Pulmonary fibrosis
0.05
0.06
0.07
SNP effect on FKBP14

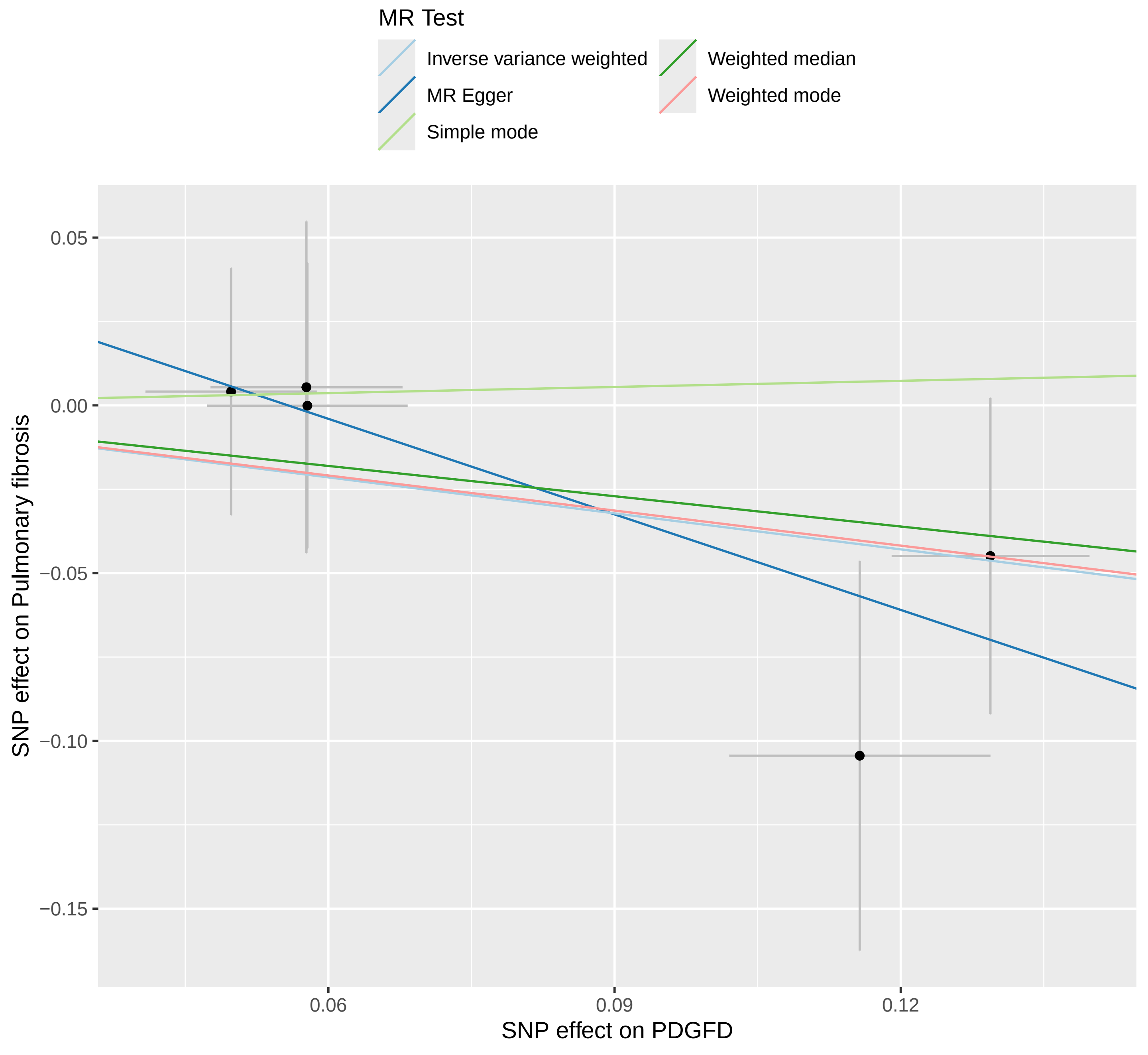
MR Test
Inverse variance weighted
MR Egger
Simple mode
Weighted median
Weighted mode
0.05
0.00
−0.05
−0.10
−0.15
SNP effect on Pulmonary fibrosis
0.06
0.09
0.12
SNP effect on PDGFD

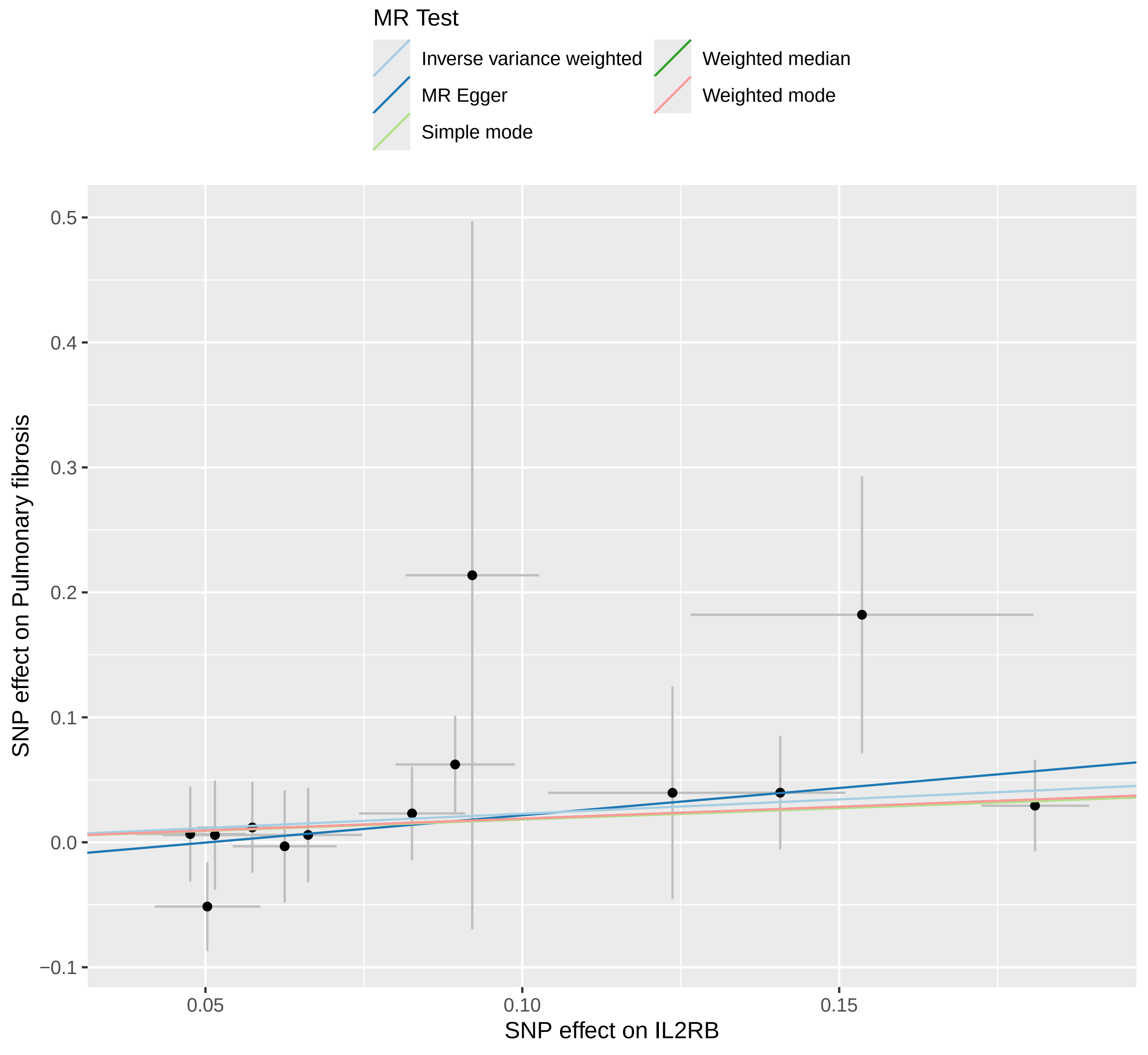

MR Test
Inverse variance weighted
MR Egger
Simple mode
Weighted median
Weighted mode
SNP effect on Pulmonary fibrosis
0.5
0.4
0.3
0.2
0.1
0.0
−0.1
0.05
0.10
0.15
SNP effect on IL2RB

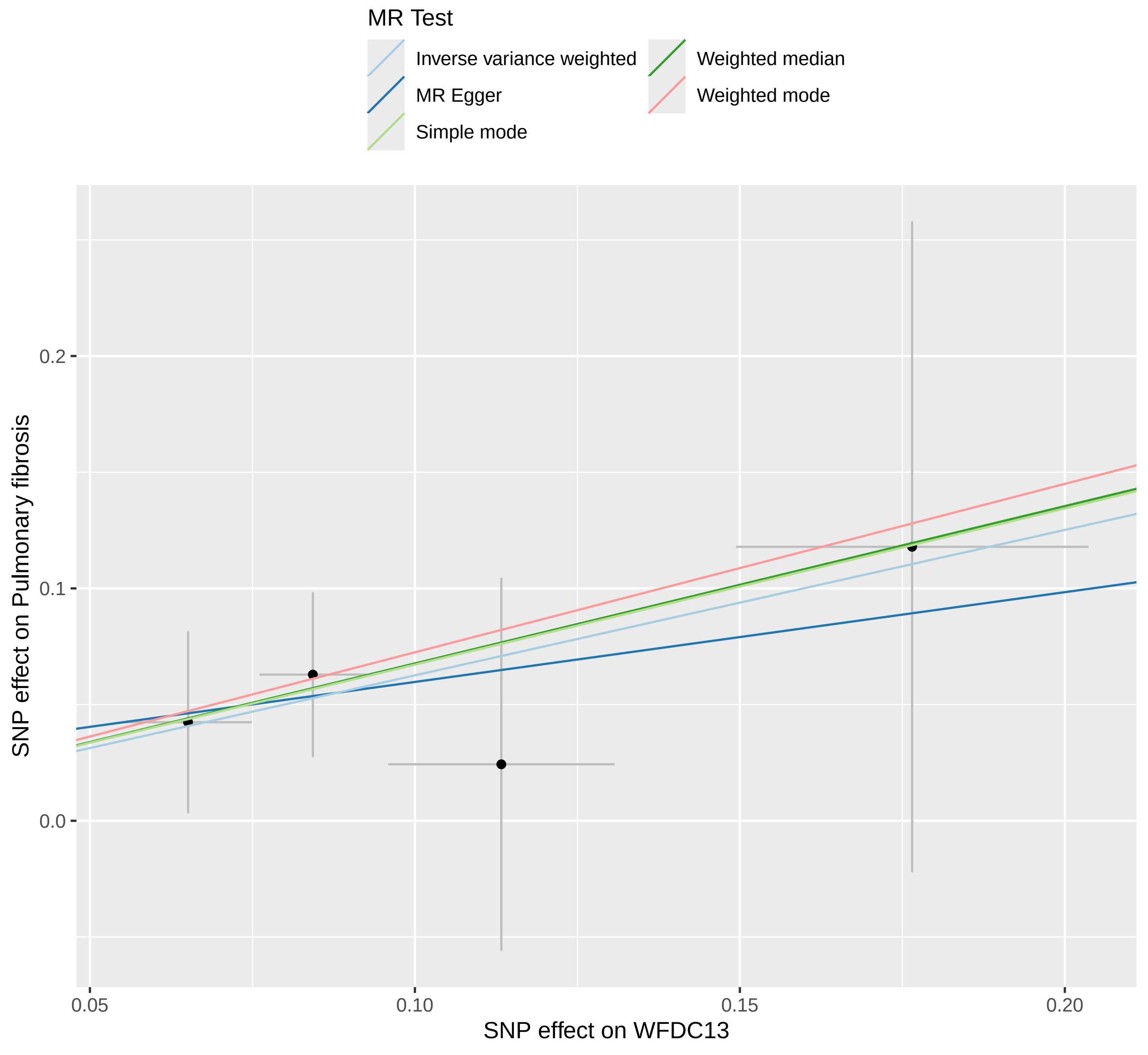
MR Test
Inverse variance weighted
MR Egger
Simple mode
Weighted median
Weighted mode
0.2
0.1
0.0
0.05
0.10
0.15
0.20
SNP effect on WFDC13
SNP effect on Pulmonary fibrosis

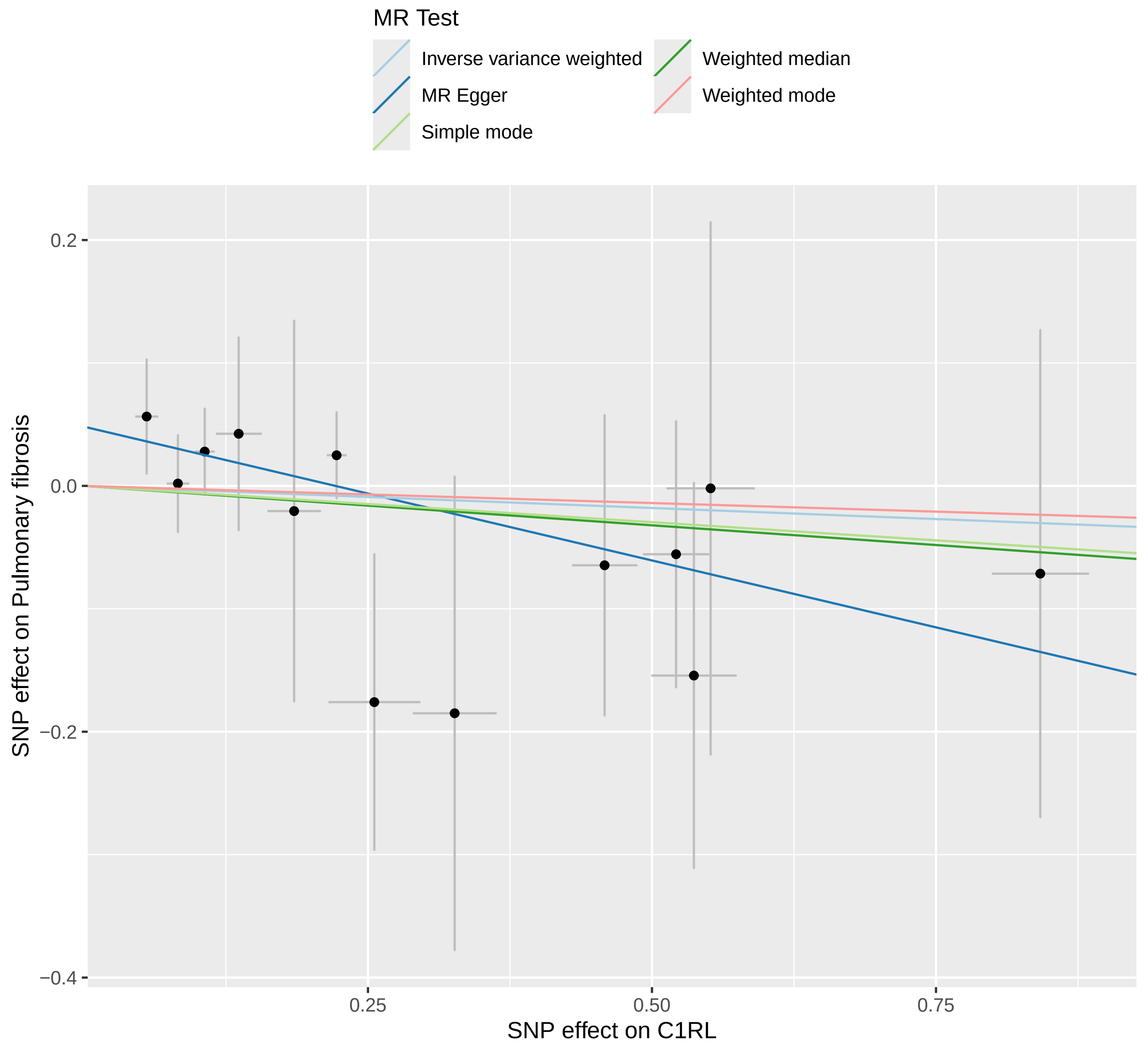
MR Test
Inverse variance weighted
MR Egger
Simple mode
Weighted median
Weighted mode
0.2
0.0
−0.2
−0.4
0.25
0.50
0.75
SNP effect on C1RL
SNP effect on Pulmonary fibrosis

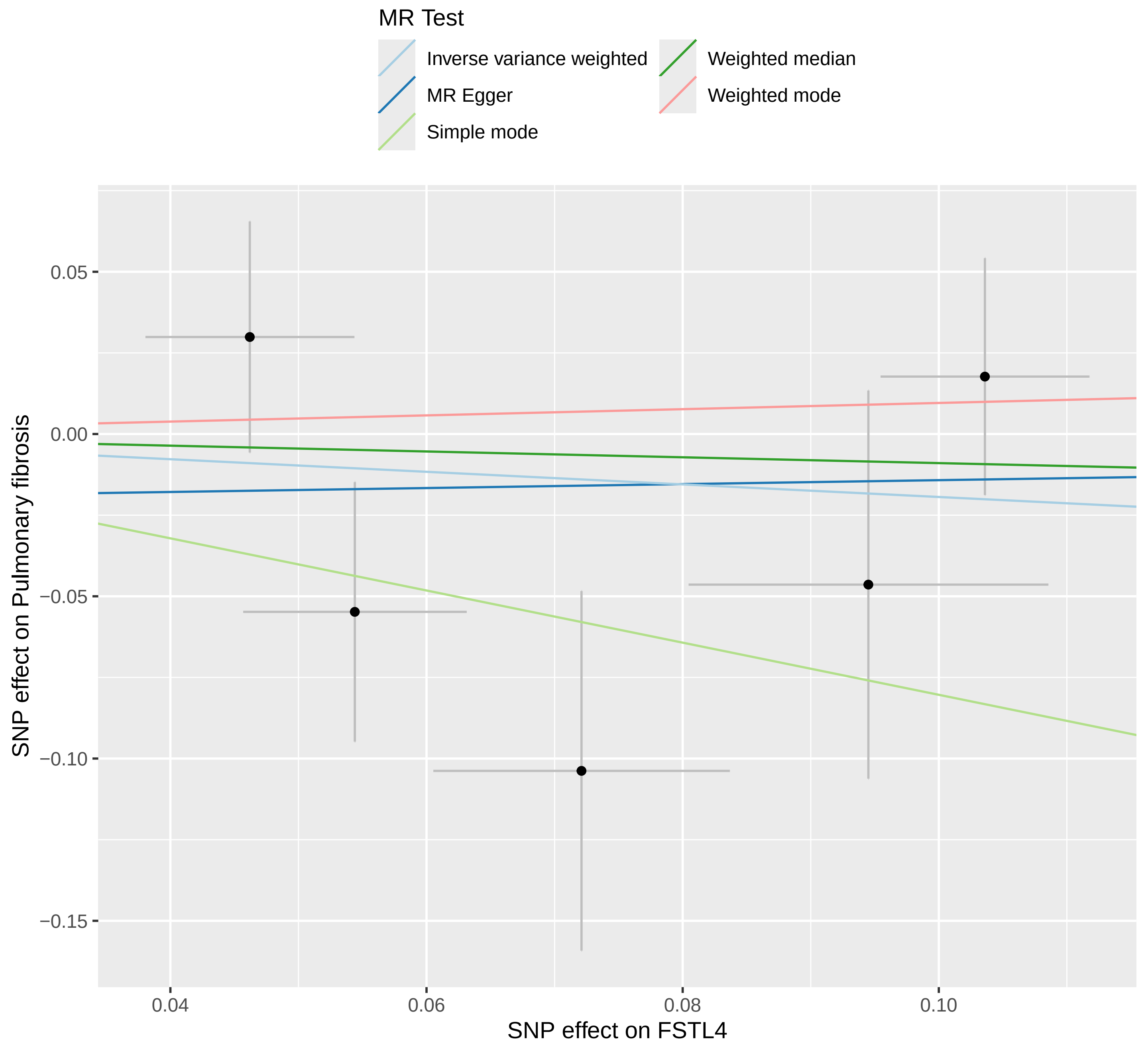
MR Test
Inverse variance weighted
MR Egger
Simple mode
Weighted median
Weighted mode
0.05
0.00
−0.05
−0.10
−0.15
0.04
0.06
0.08
0.10
SNP effect on Pulmonary fibrosis
SNP effect on FSTL4

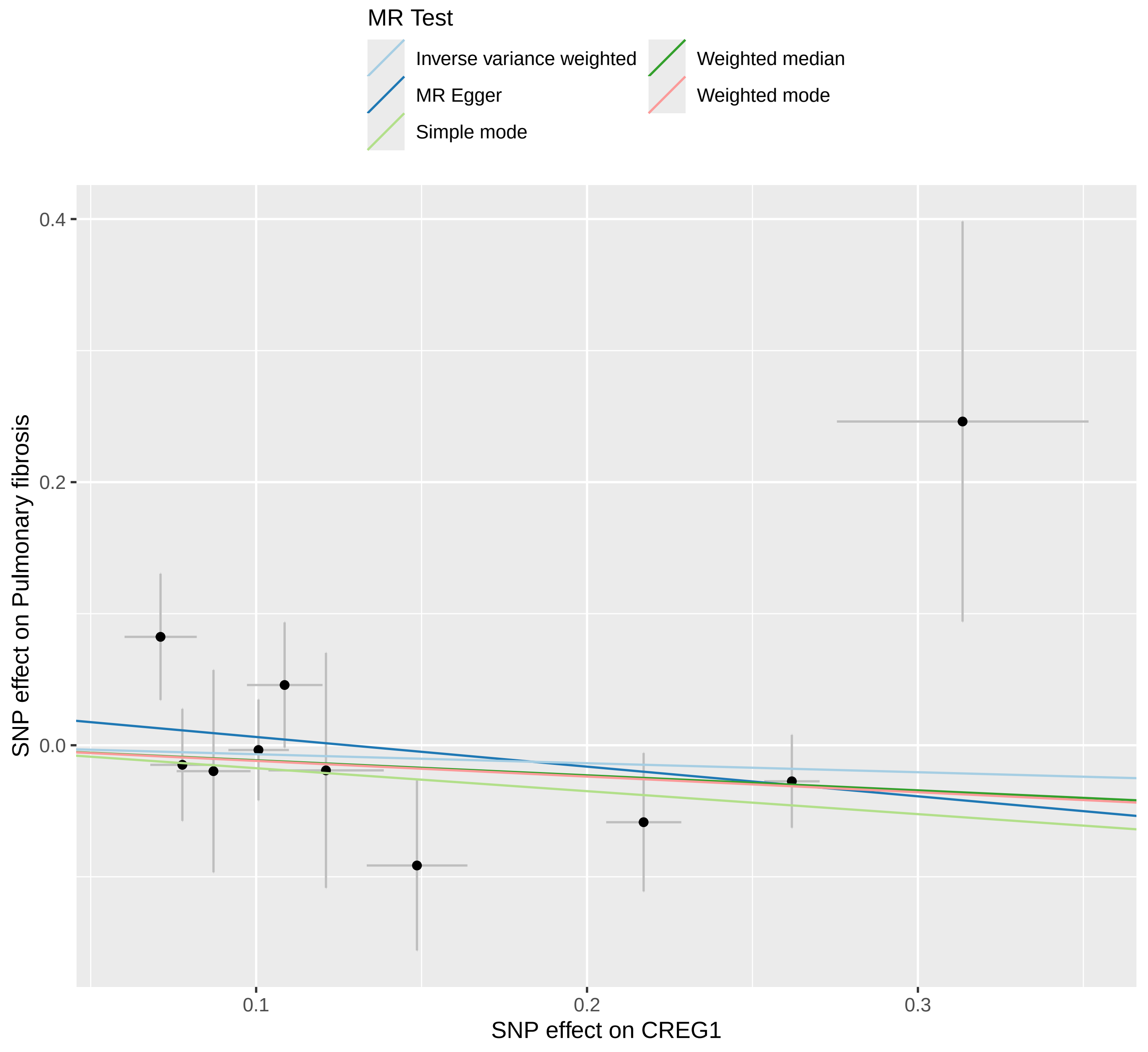

MR Test
Inverse variance weighted
MR Egger
Simple mode
Weighted median
Weighted mode
SNP effect on Pulmonary fibrosis
0.4
0.2
0.0
0.1
0.2
0.3
SNP effect on CREG1

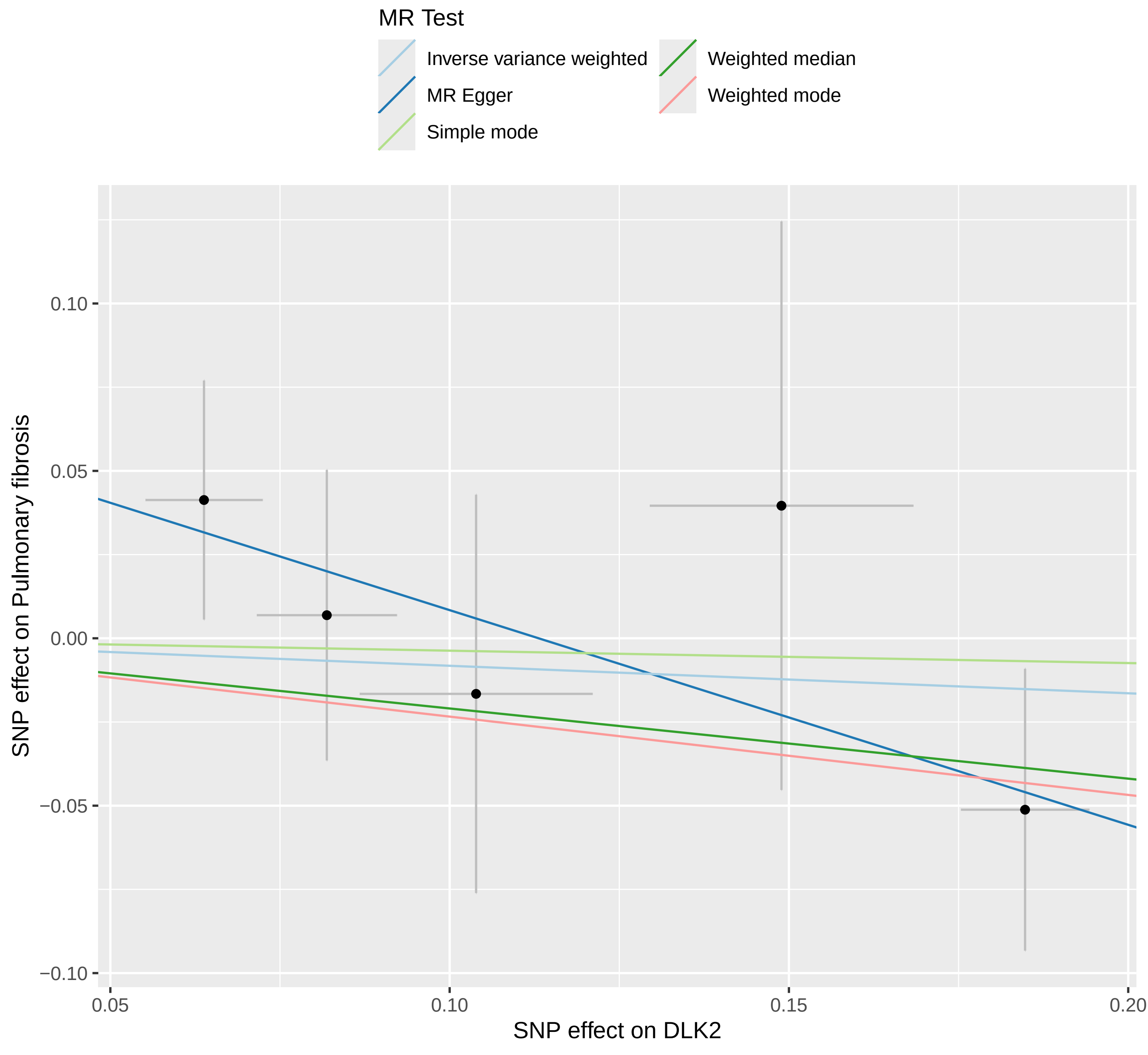

MR Test
Inverse variance weighted
MR Egger
Simple mode
Weighted median
Weighted mode
0.10
0.05
0.00
−0.05
−0.10
SNP effect on Pulmonary fibrosis
0.05
0.10
0.15
0.20
SNP effect on DLK2

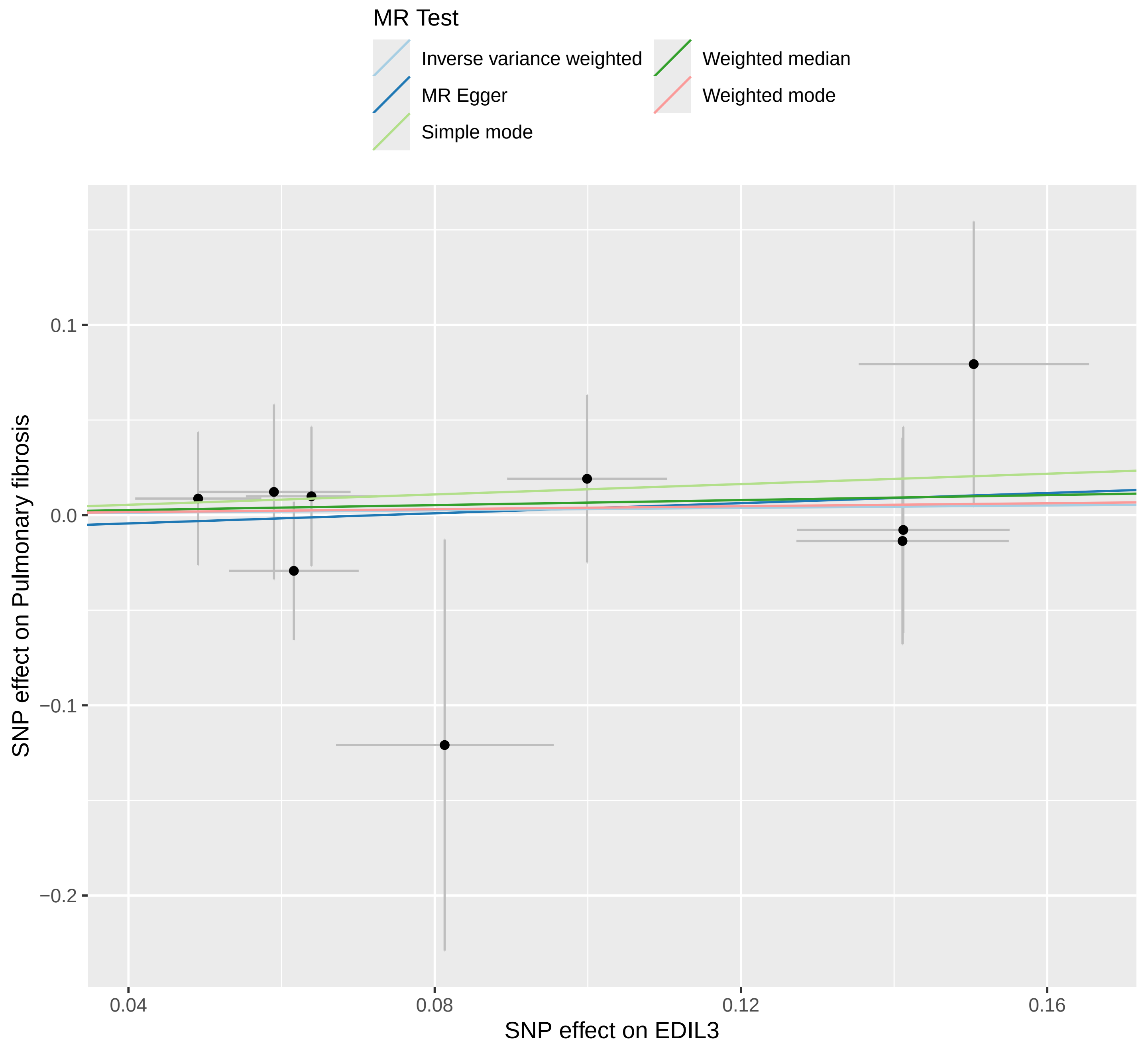

MR Test
Inverse variance weighted
MR Egger
Simple mode
Weighted median
Weighted mode
0.1
0.0
−0.1
−0.2
SNP effect on Pulmonary fibrosis
0.04
0.08
0.12
0.16
SNP effect on EDIL3

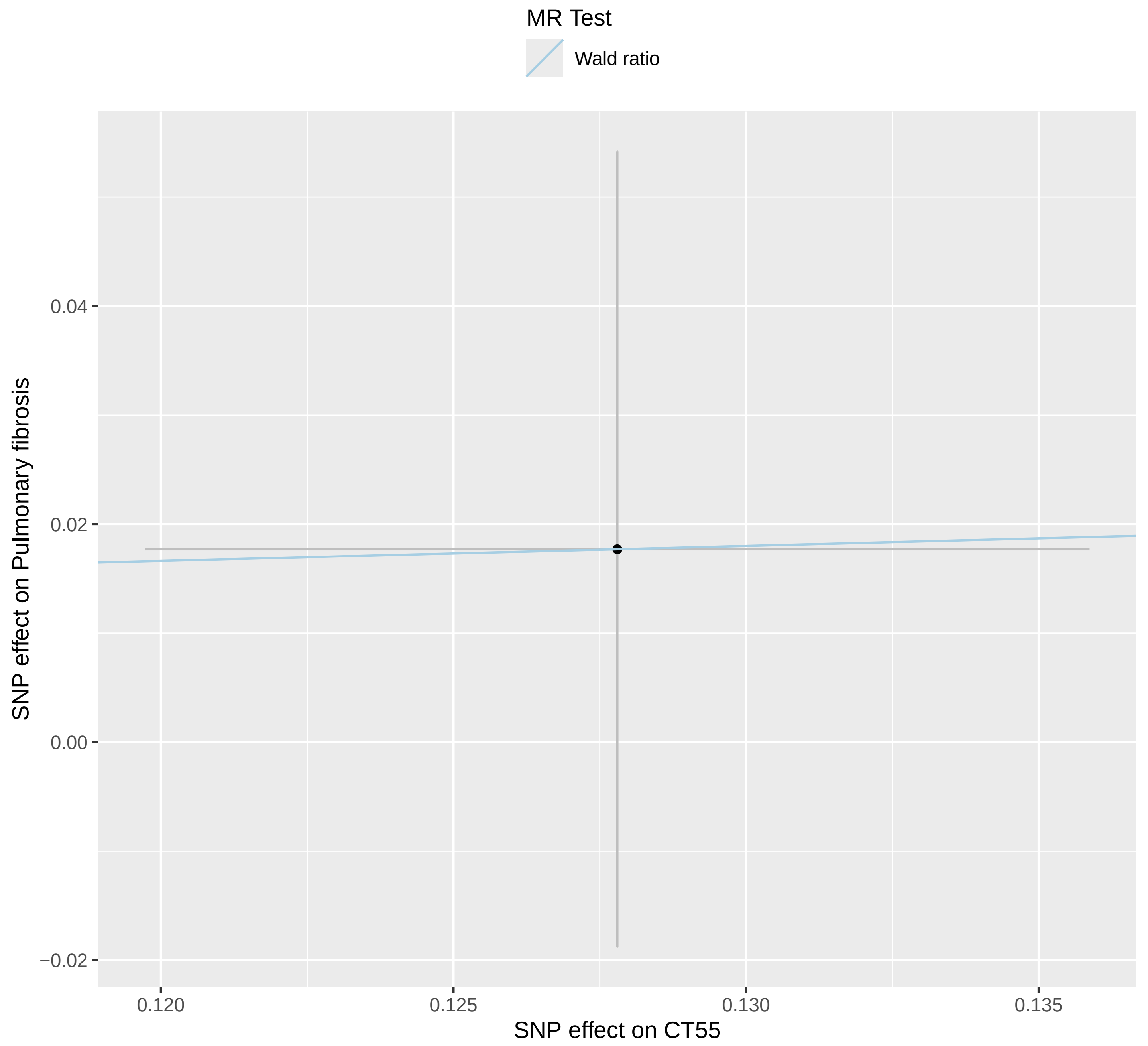
MR Test
Wald ratio
0.04
0.02
0.00
−0.02
SNP effect on Pulmonary fibrosis
0.120
0.125
0.130
0.135
SNP effect on CT55

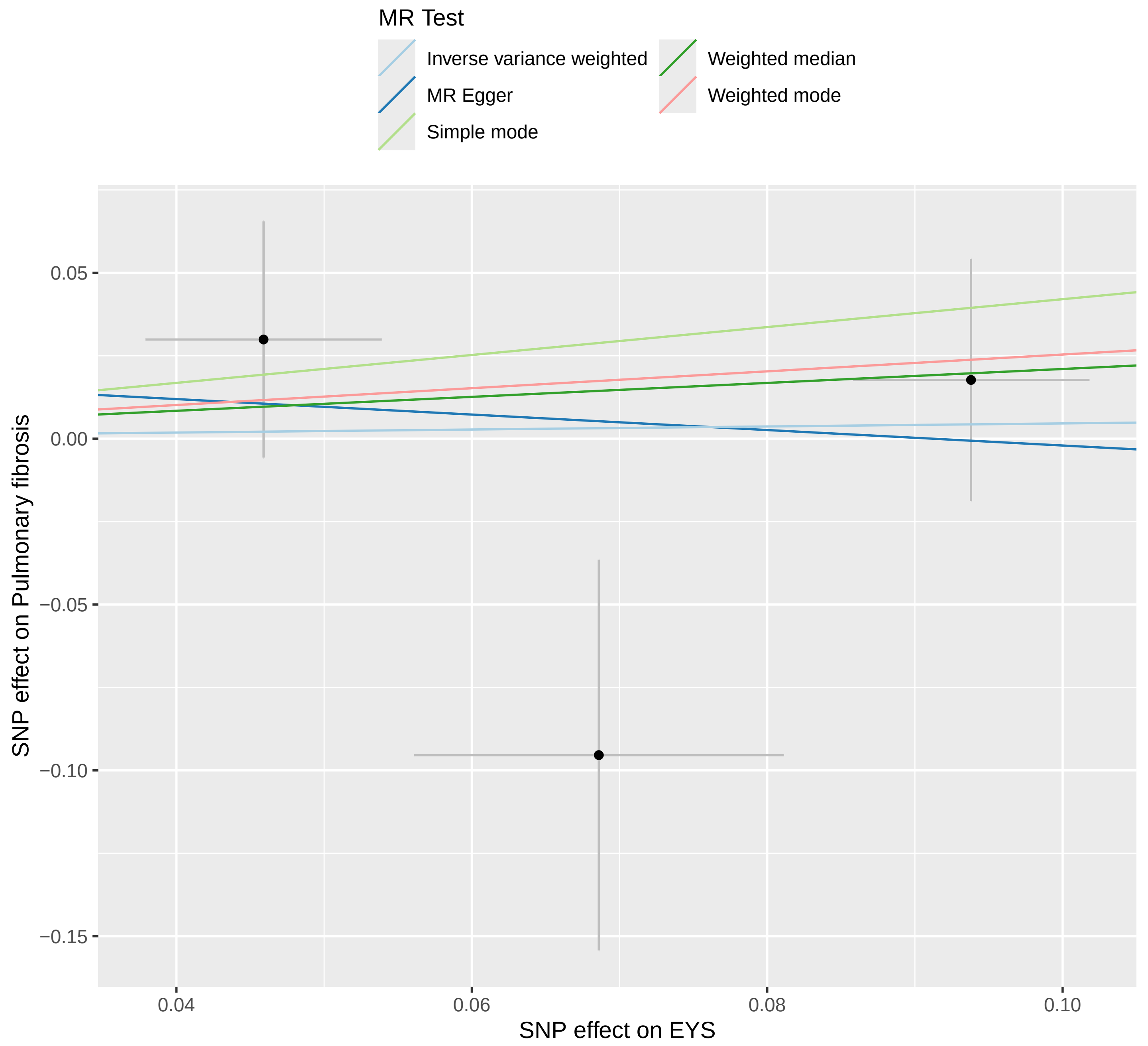

MR Test
Inverse variance weighted
MR Egger
Simple mode
Weighted median
Weighted mode
0.05
0.00
−0.05
−0.10
−0.15
SNP effect on Pulmonary fibrosis
0.04
0.06
0.08
0.10
SNP effect on EYS

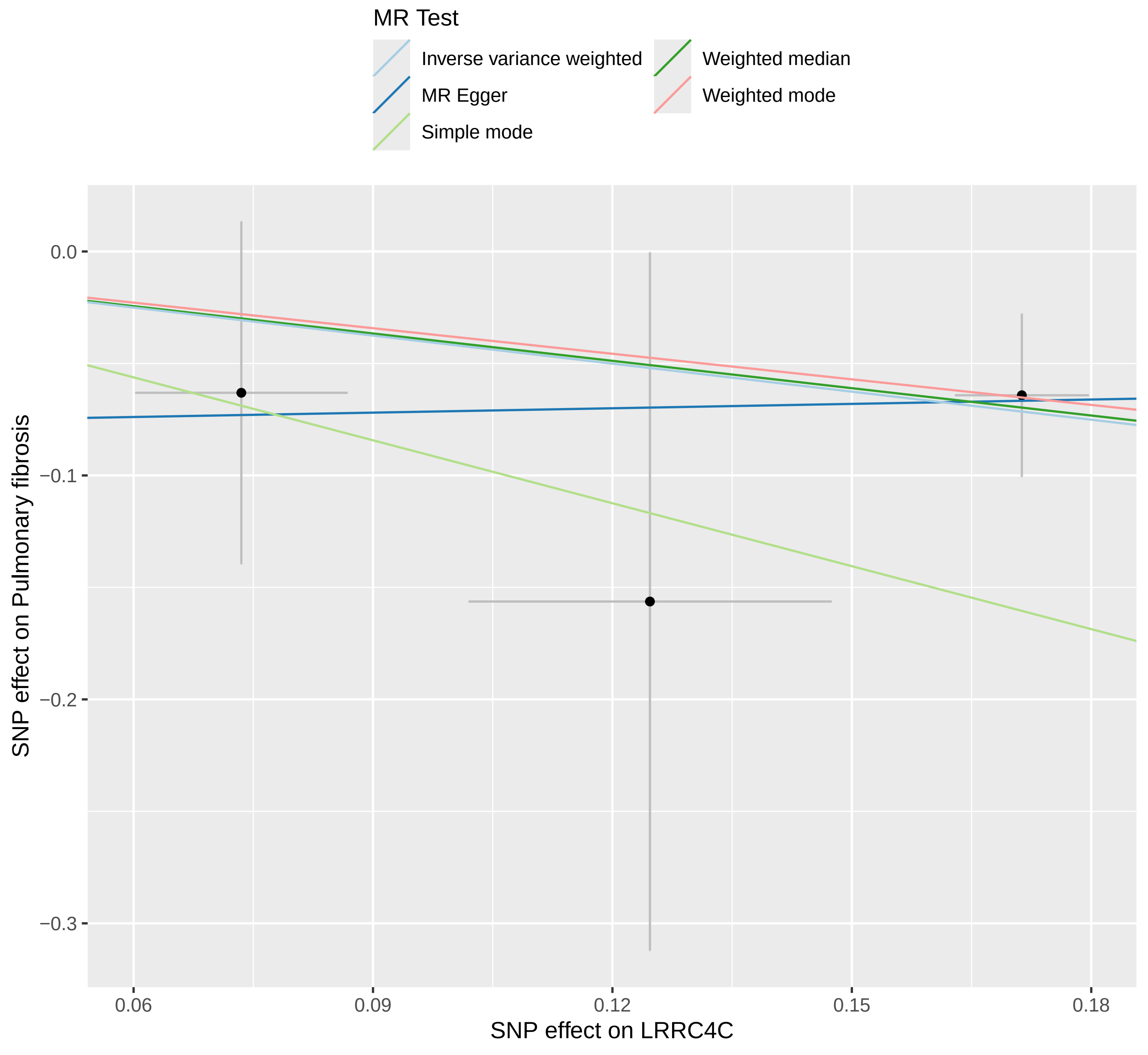
MR Test
Inverse variance weighted
MR Egger
Simple mode
Weighted median
Weighted mode
0.0
−0.1
−0.2
−0.3
0.06
0.09
0.12
0.15
0.18
SNP effect on Pulmonary fibrosis
SNP effect on LRRC4C

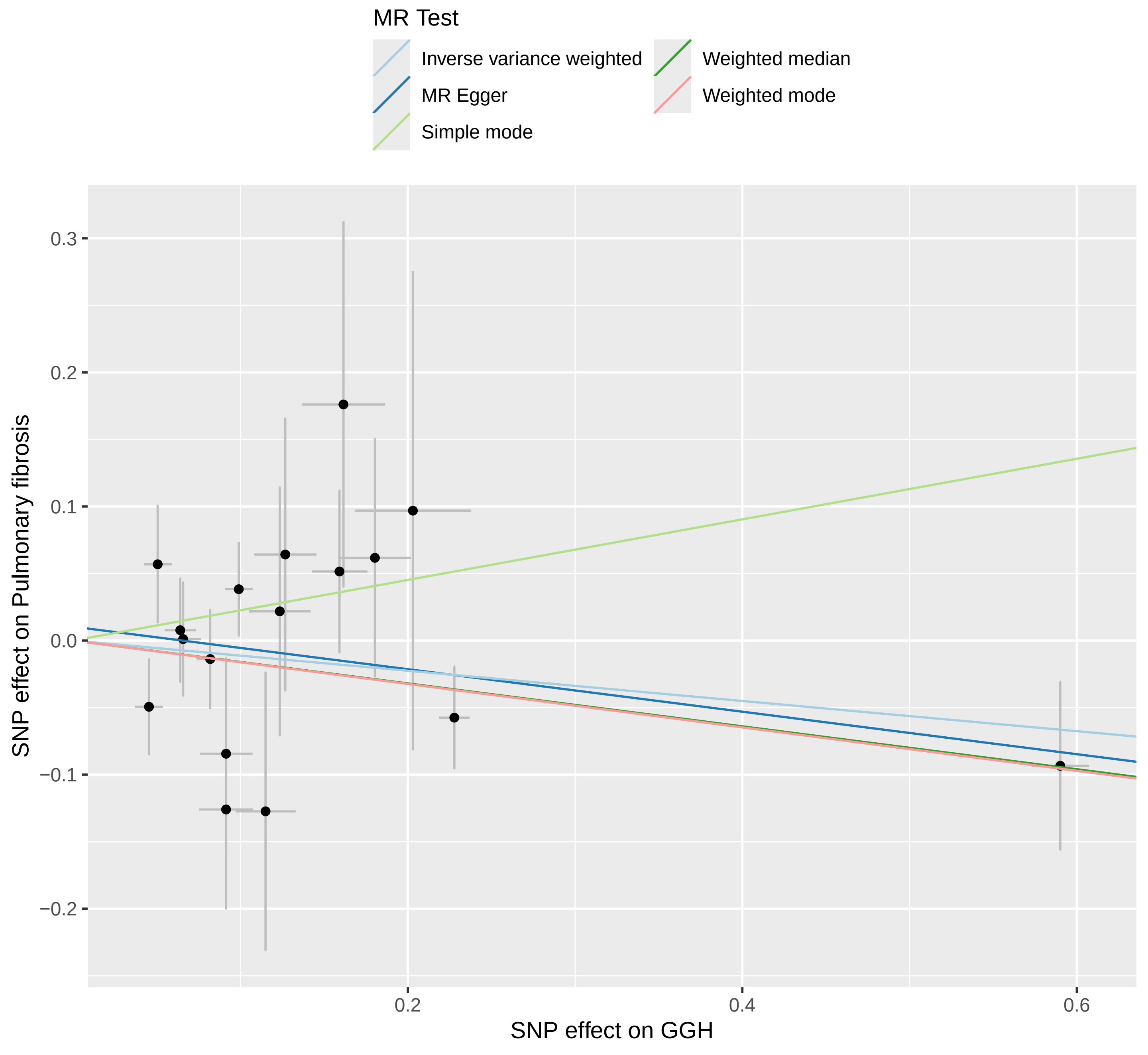
MR Test
Inverse variance weighted
MR Egger
Simple mode
Weighted median
Weighted mode
0.3
0.2
0.1
0.0
−0.1
−0.2
SNP effect on Pulmonary fibrosis
0.2
0.4
0.6
SNP effect on GGH

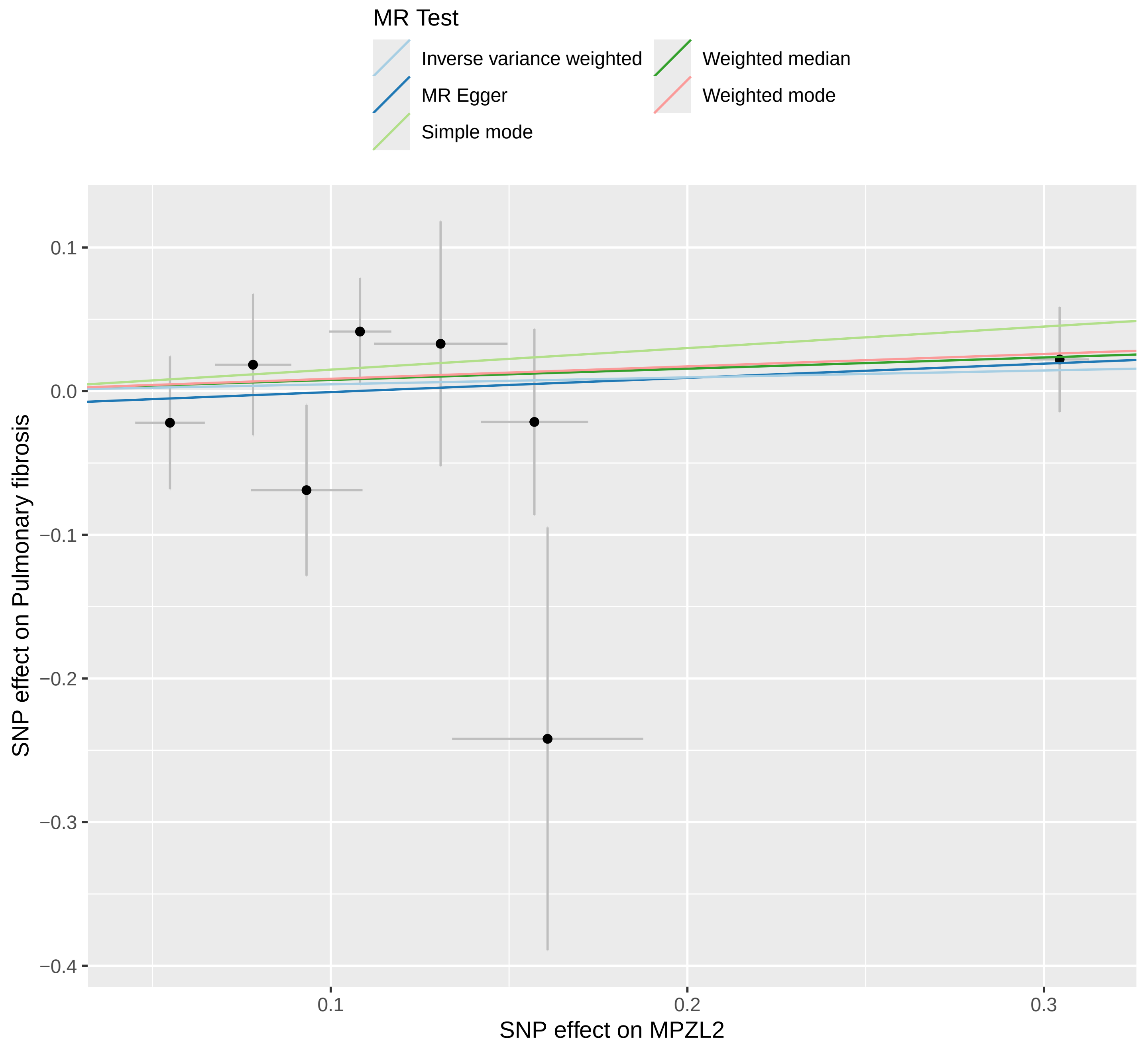

MR Test
Inverse variance weighted
MR Egger
Simple mode
Weighted median
Weighted mode
0.1
0.0
−0.1
−0.2
−0.3
−0.4
0.1
0.2
0.3
SNP effect on Pulmonary fibrosis
SNP effect on MPZL2

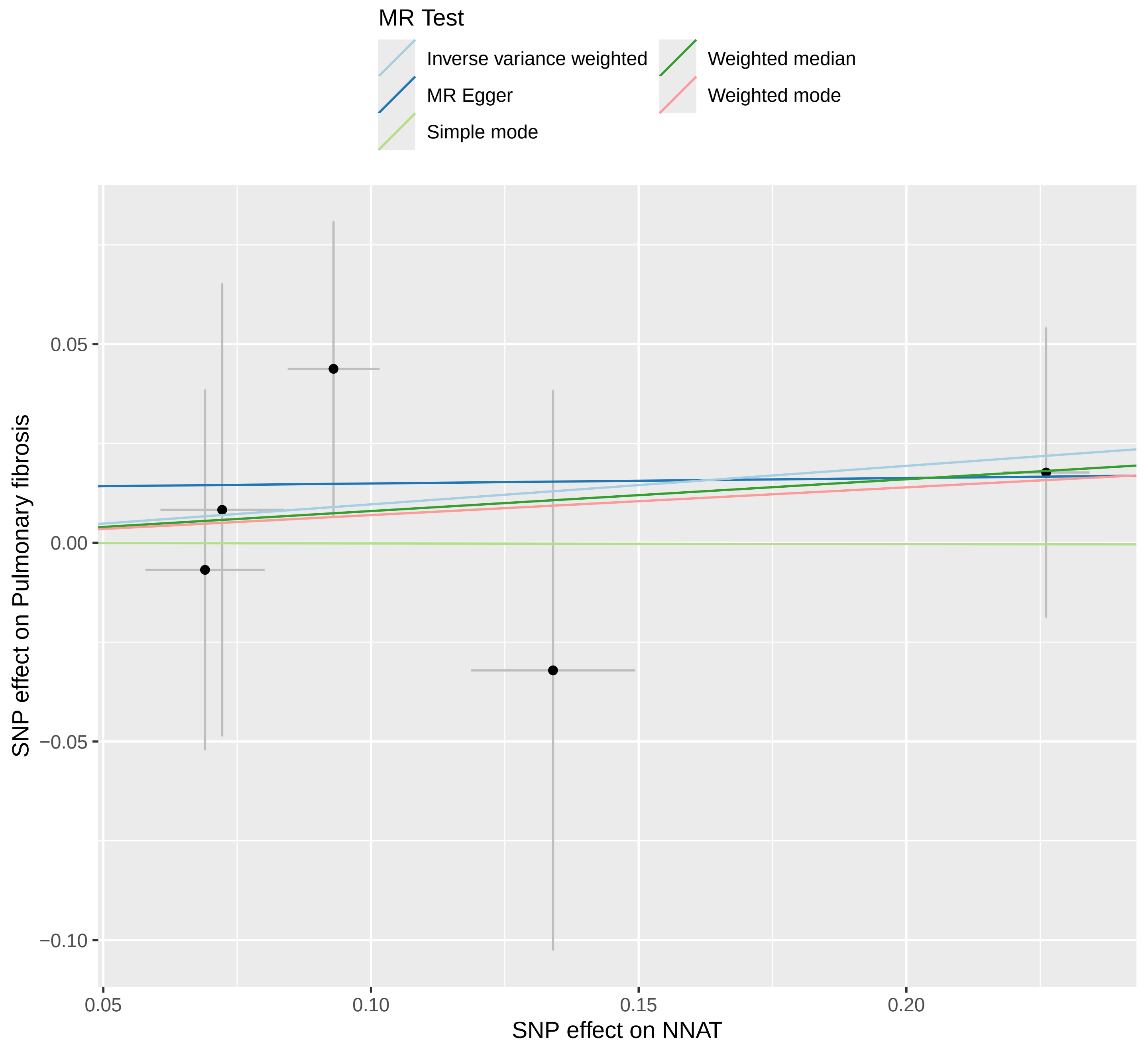

MR Test
Inverse variance weighted
MR Egger
Simple mode
Weighted median
Weighted mode
0.05
0.00
−0.05
−0.10
SNP effect on Pulmonary fibrosis
0.05
0.10
0.15
0.20
SNP effect on NNAT

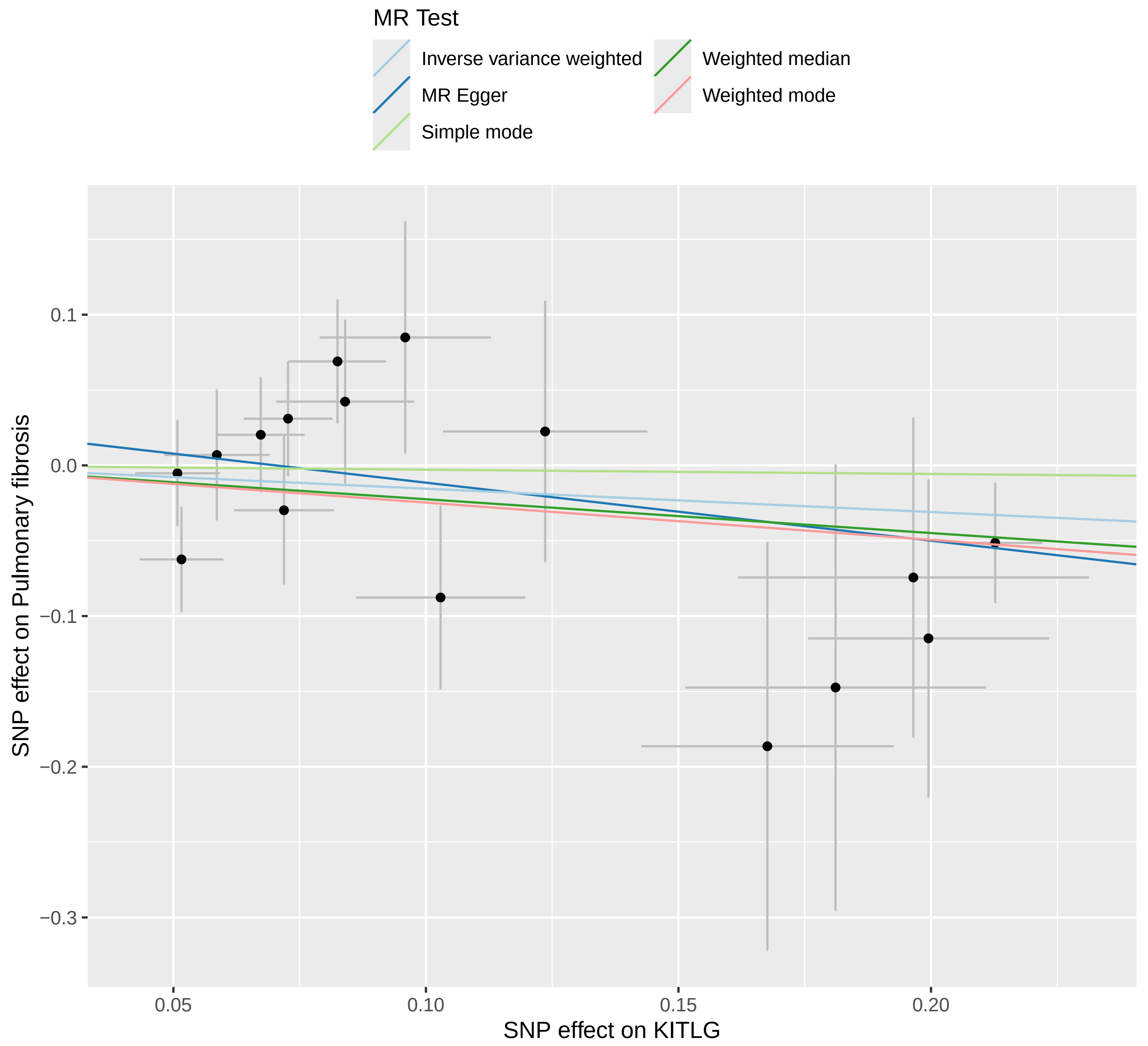

MR Test
Inverse variance weighted
MR Egger
Simple mode
Weighted median
Weighted mode
0.1
0.0
−0.1
−0.2
−0.3
SNP effect on Pulmonary fibrosis
0.05
0.10
0.15
0.20
SNP effect on KITLG

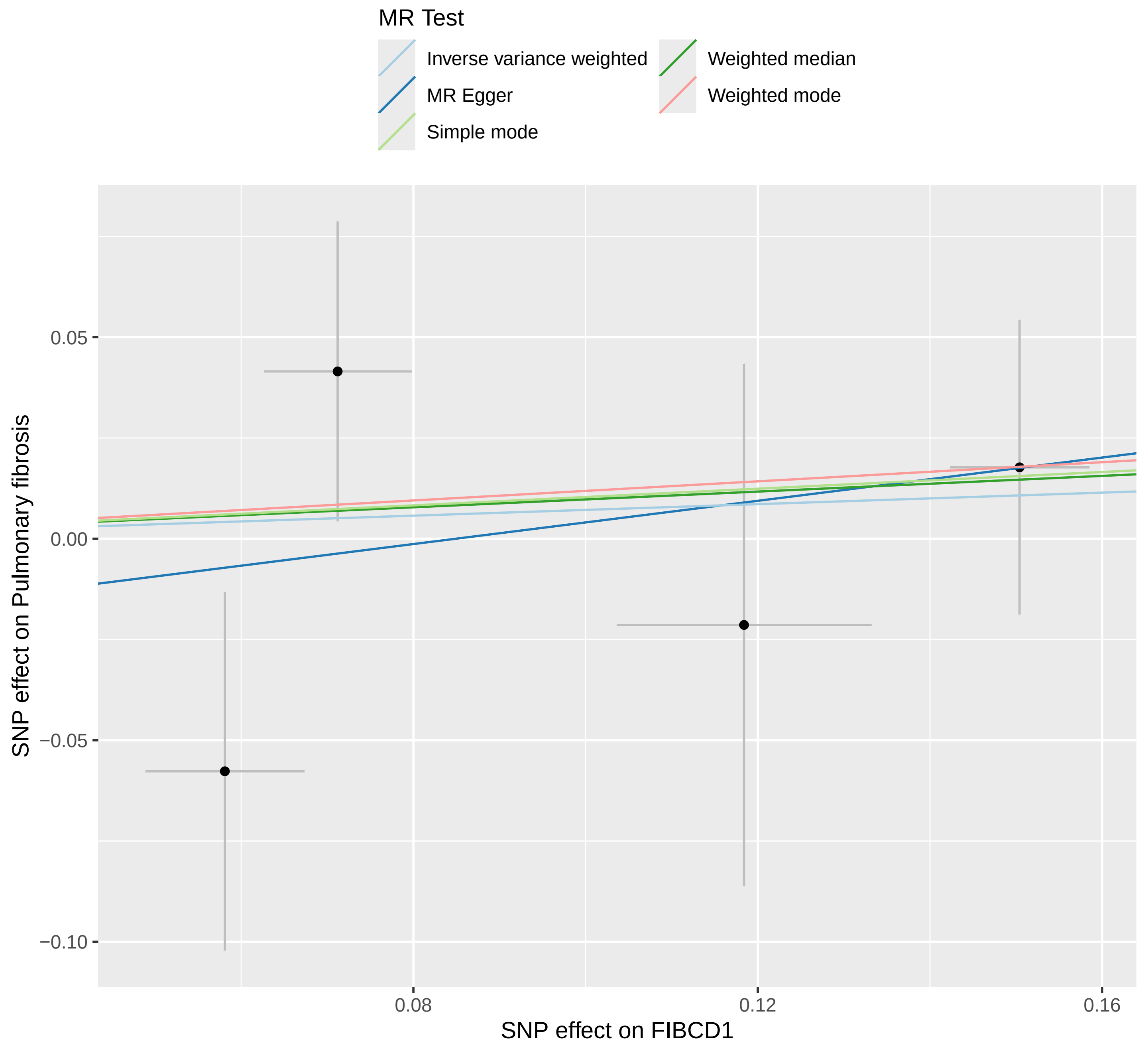

MR Test
Inverse variance weighted
MR Egger
Simple mode
Weighted median
Weighted mode
0.05
0.00
−0.05
−0.10
SNP effect on Pulmonary fibrosis
0.08
0.12
0.16
SNP effect on FIBCD1

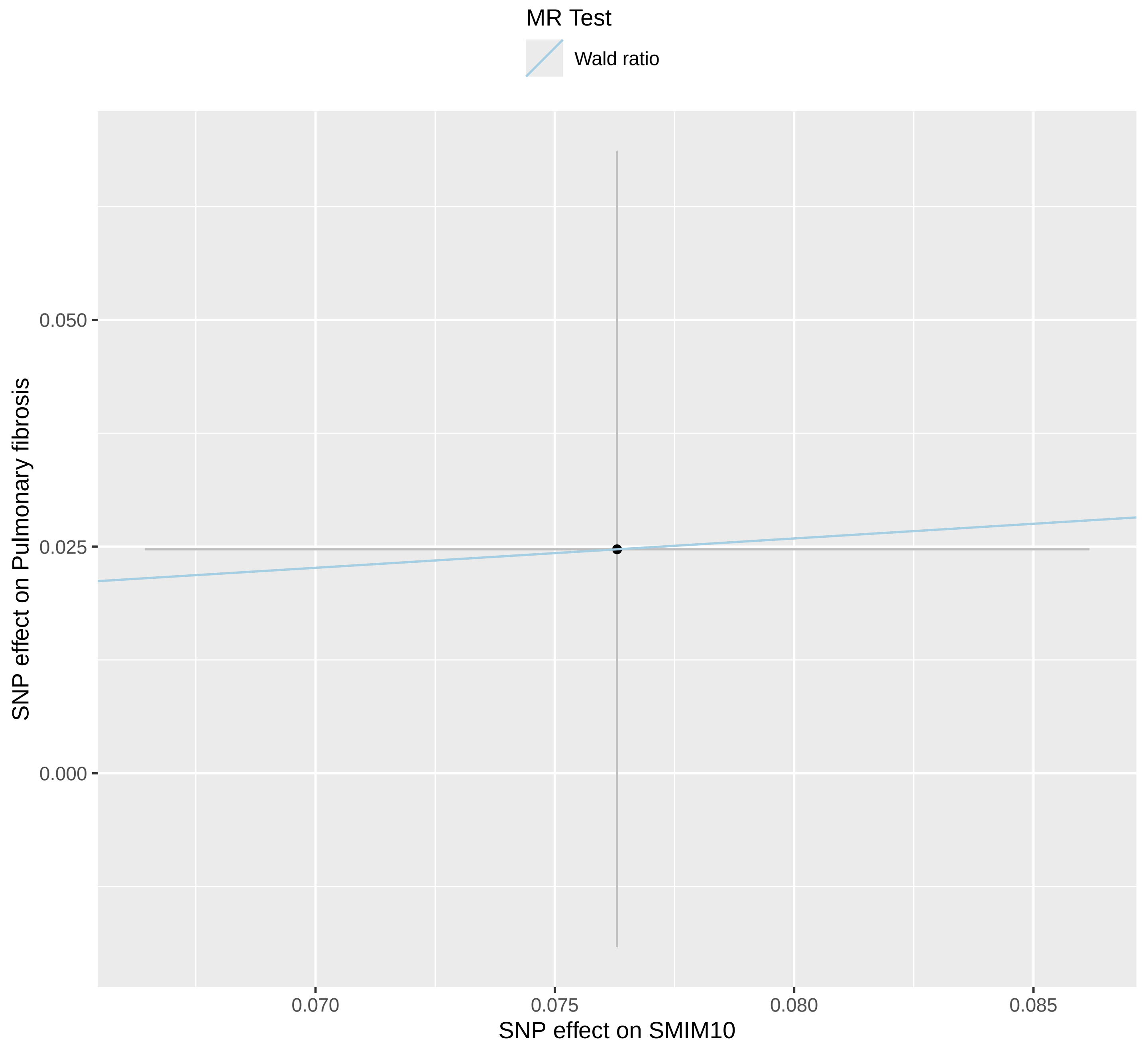

MR Test
Wald ratio
0.050
0.025
0.000
SNP effect on Pulmonary fibrosis
0.070
0.075
0.080
0.085
SNP effect on SMIM10

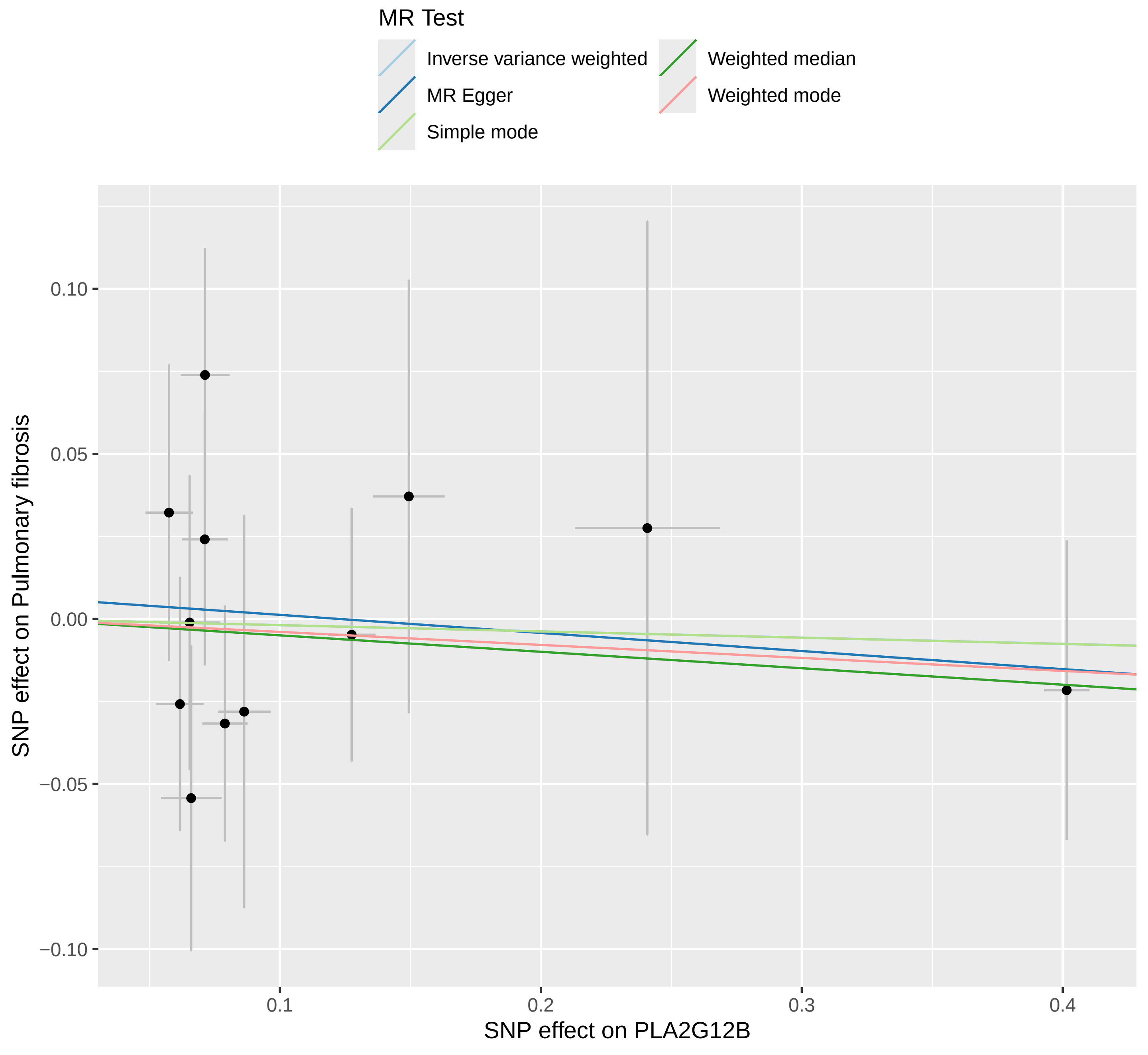
MR Test
Inverse variance weighted
MR Egger
Simple mode
Weighted median
Weighted mode
0.10
0.05
0.00
−0.05
−0.10
SNP effect on Pulmonary fibrosis
0.1
0.2
0.3
0.4
SNP effect on PLA2G12B

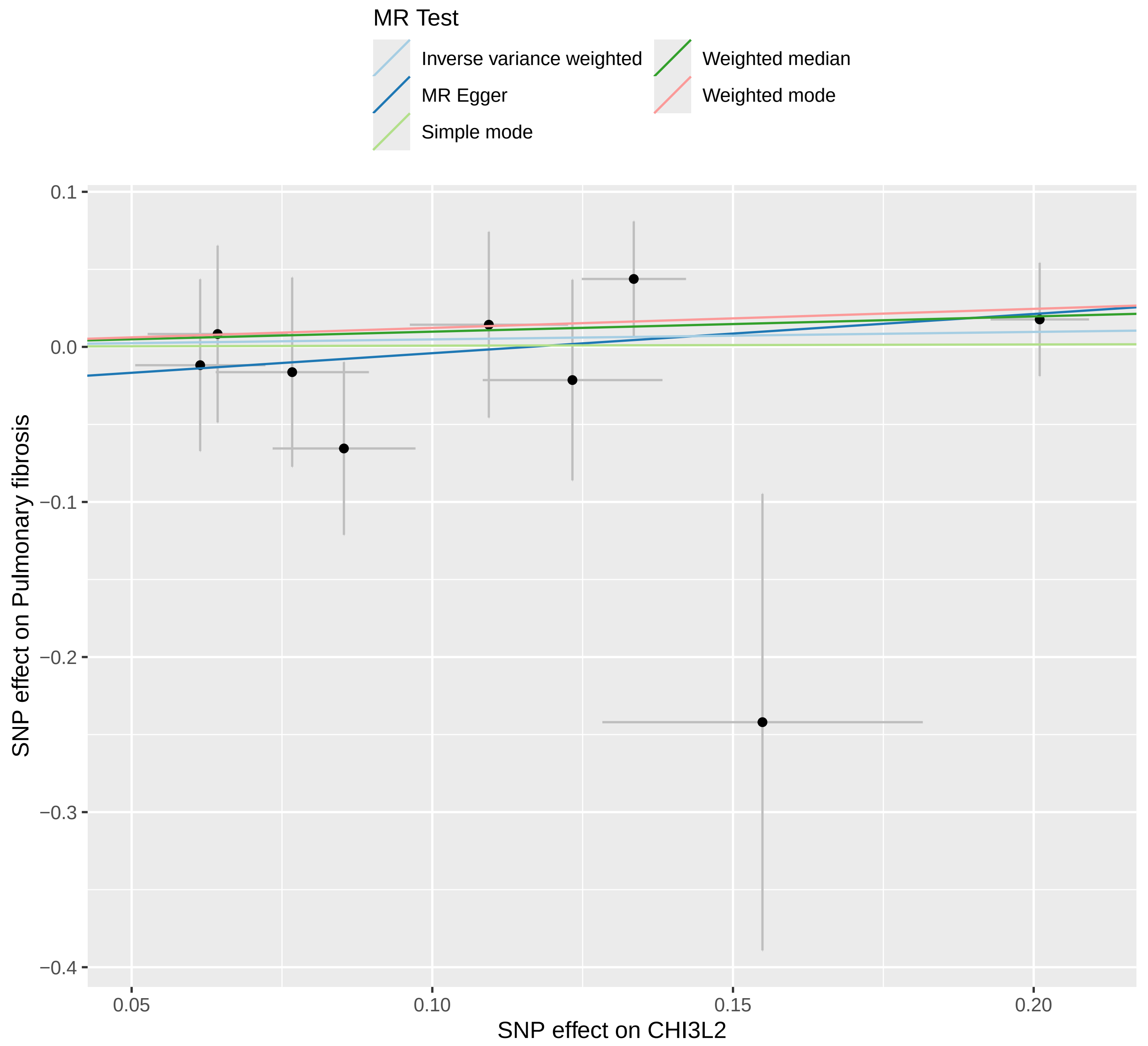
MR Test
Inverse variance weighted
MR Egger
Simple mode
Weighted median
Weighted mode
0.1
0.0
−0.1
−0.2
−0.3
−0.4
SNP effect on Pulmonary fibrosis
0.05
0.10
0.15
0.20
SNP effect on CHI3L2

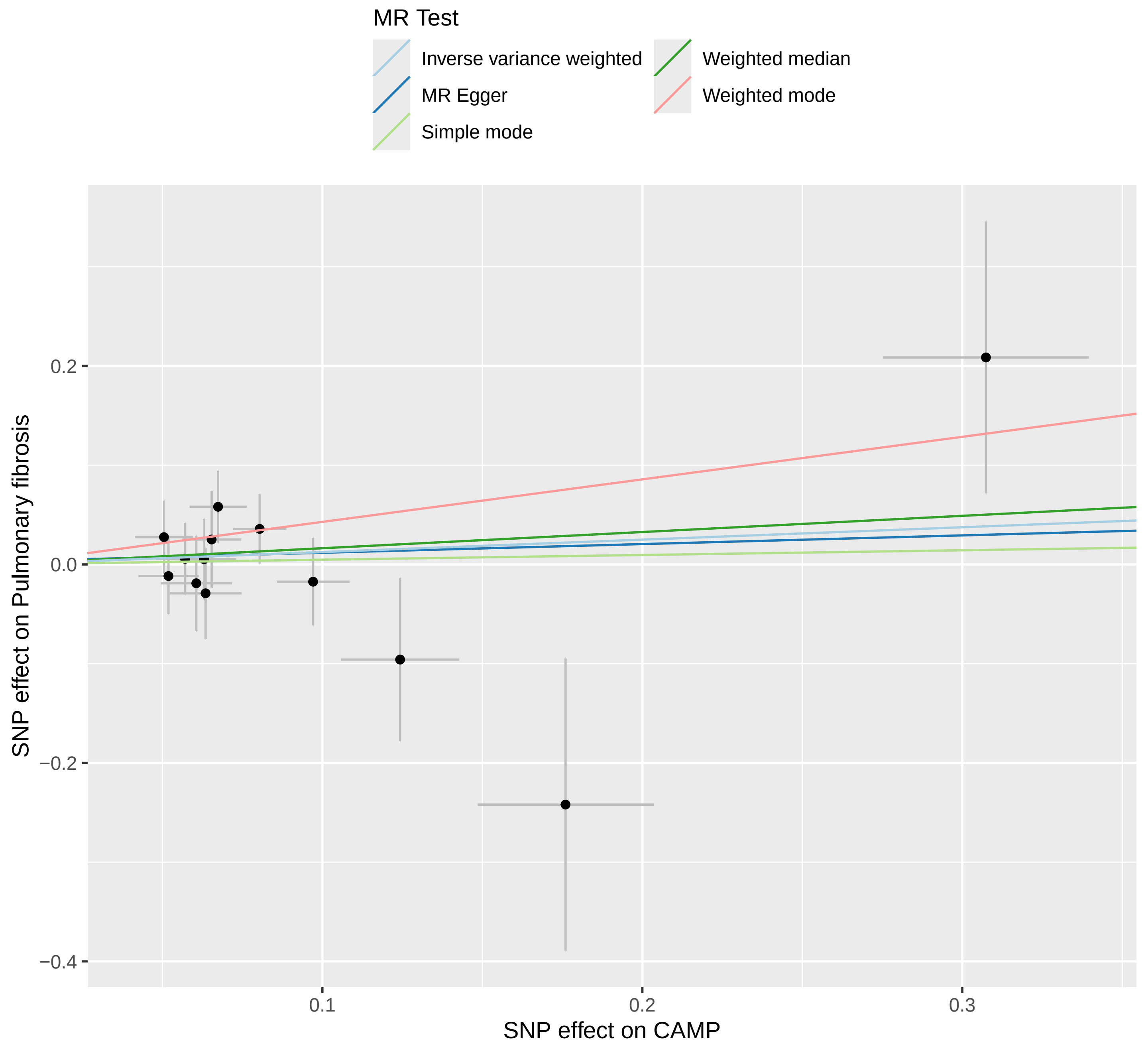

MR Test
Inverse variance weighted
MR Egger
Simple mode
Weighted median
Weighted mode
SNP effect on Pulmonary fibrosis
0.2
0.0
−0.2
−0.4
0.1
0.2
0.3
SNP effect on CAMP

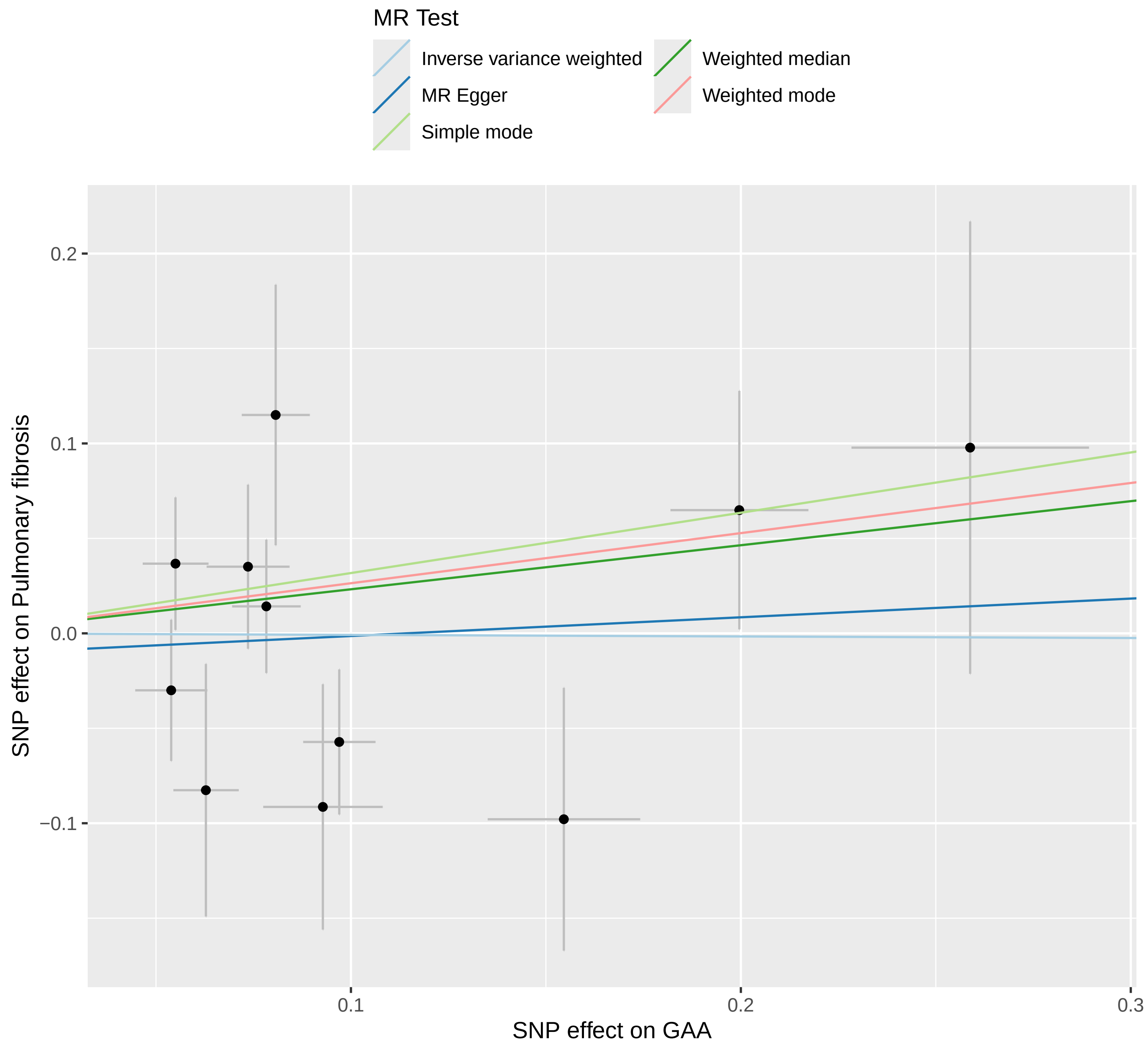
MR Test
Inverse variance weighted
MR Egger
Simple mode
Weighted median
Weighted mode
0.2
0.1
0.0
−0.1
0.1
0.2
0.3
SNP effect on Pulmonary fibrosis
SNP effect on GAA

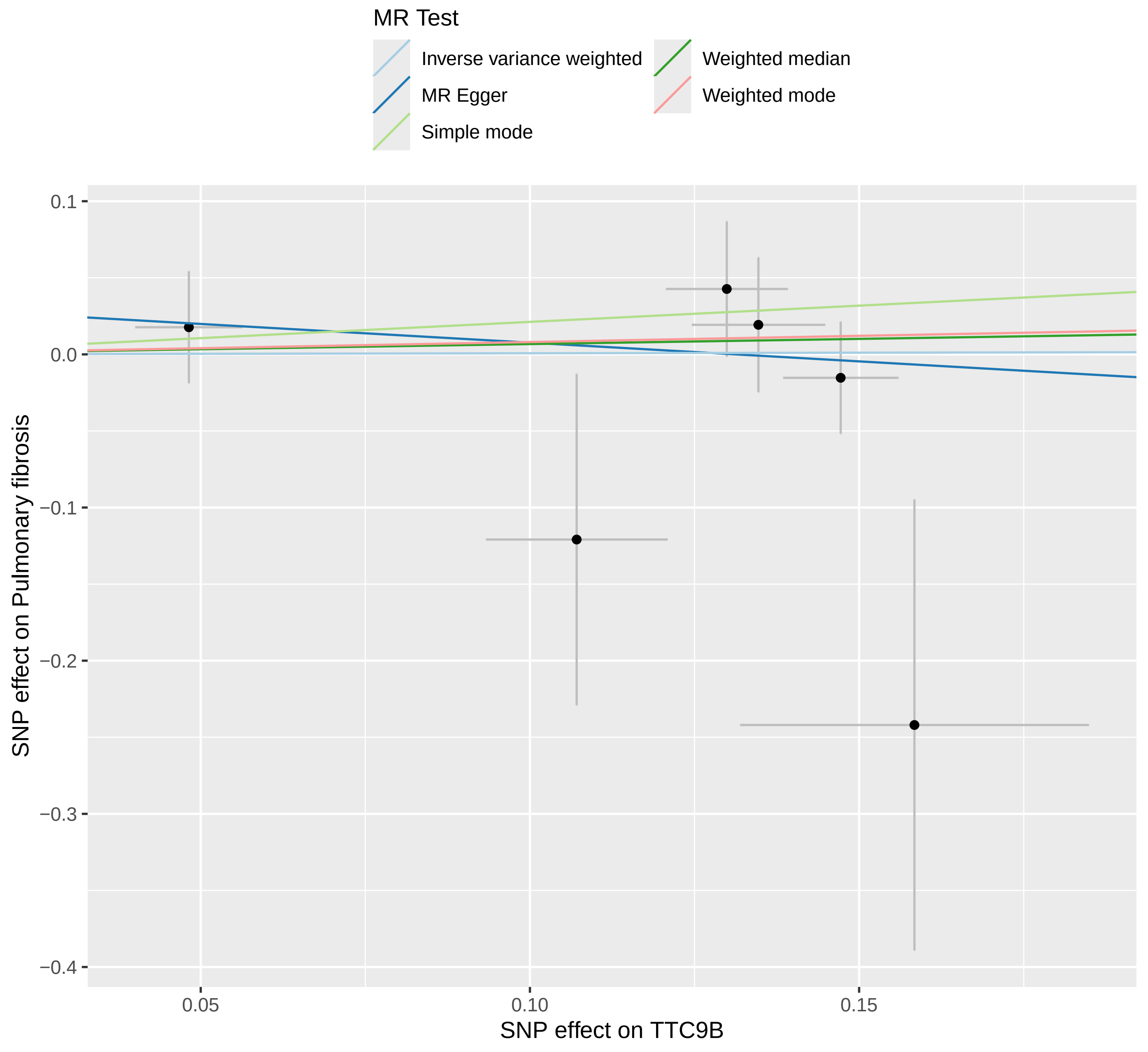

MR Test
Inverse variance weighted
MR Egger
Simple mode
Weighted median
Weighted mode
0.1
0.0
−0.1
−0.2
−0.3
−0.4
SNP effect on Pulmonary fibrosis
0.05
0.10
0.15
SNP effect on TTC9B

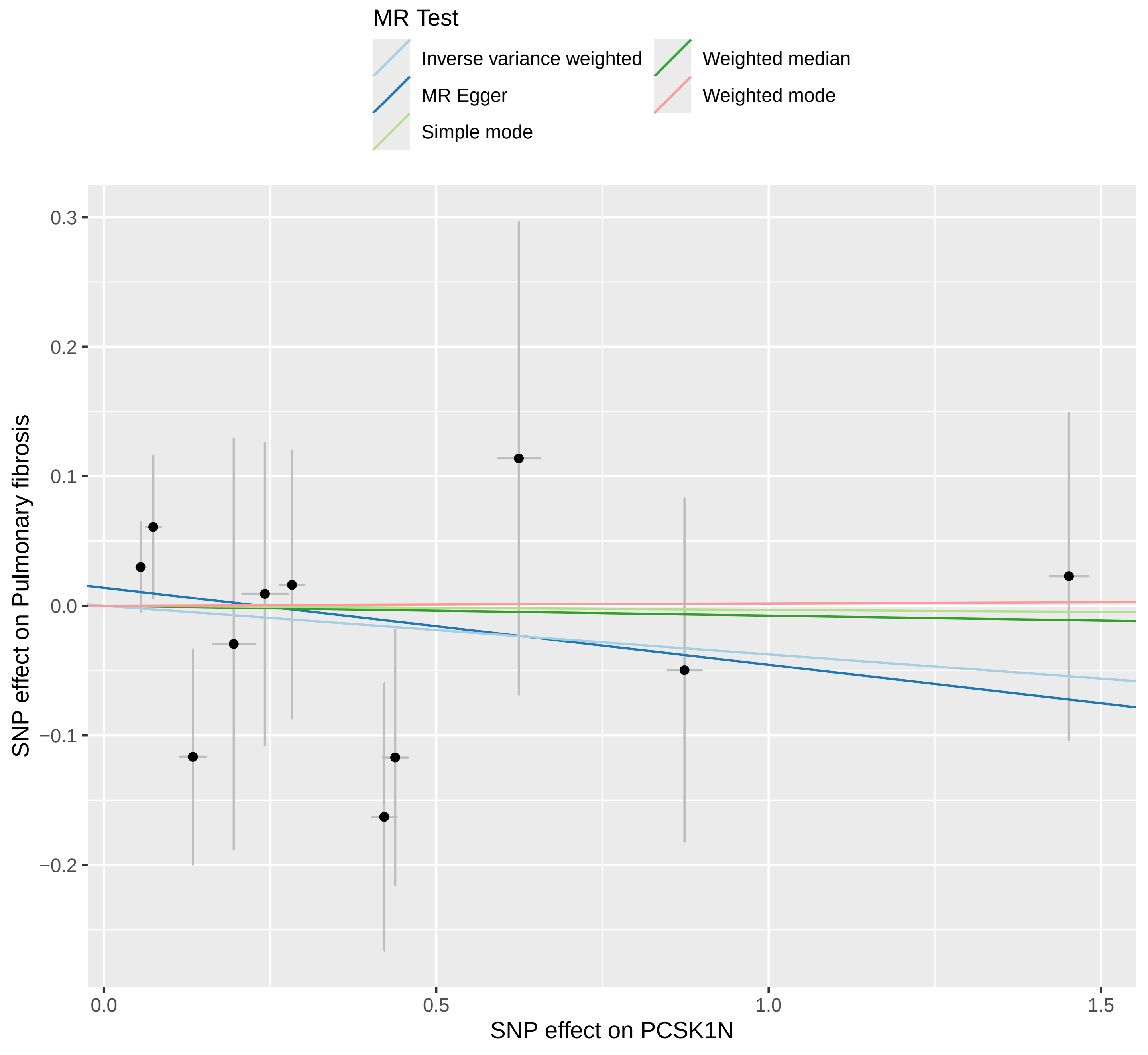
MR Test
Inverse variance weighted
MR Egger
Simple mode
Weighted median
Weighted mode
0.3
0.2
0.1
0.0
−0.1
−0.2
SNP effect on Pulmonary fibrosis
0.0
0.5
1.0
1.5
SNP effect on PCSK1N

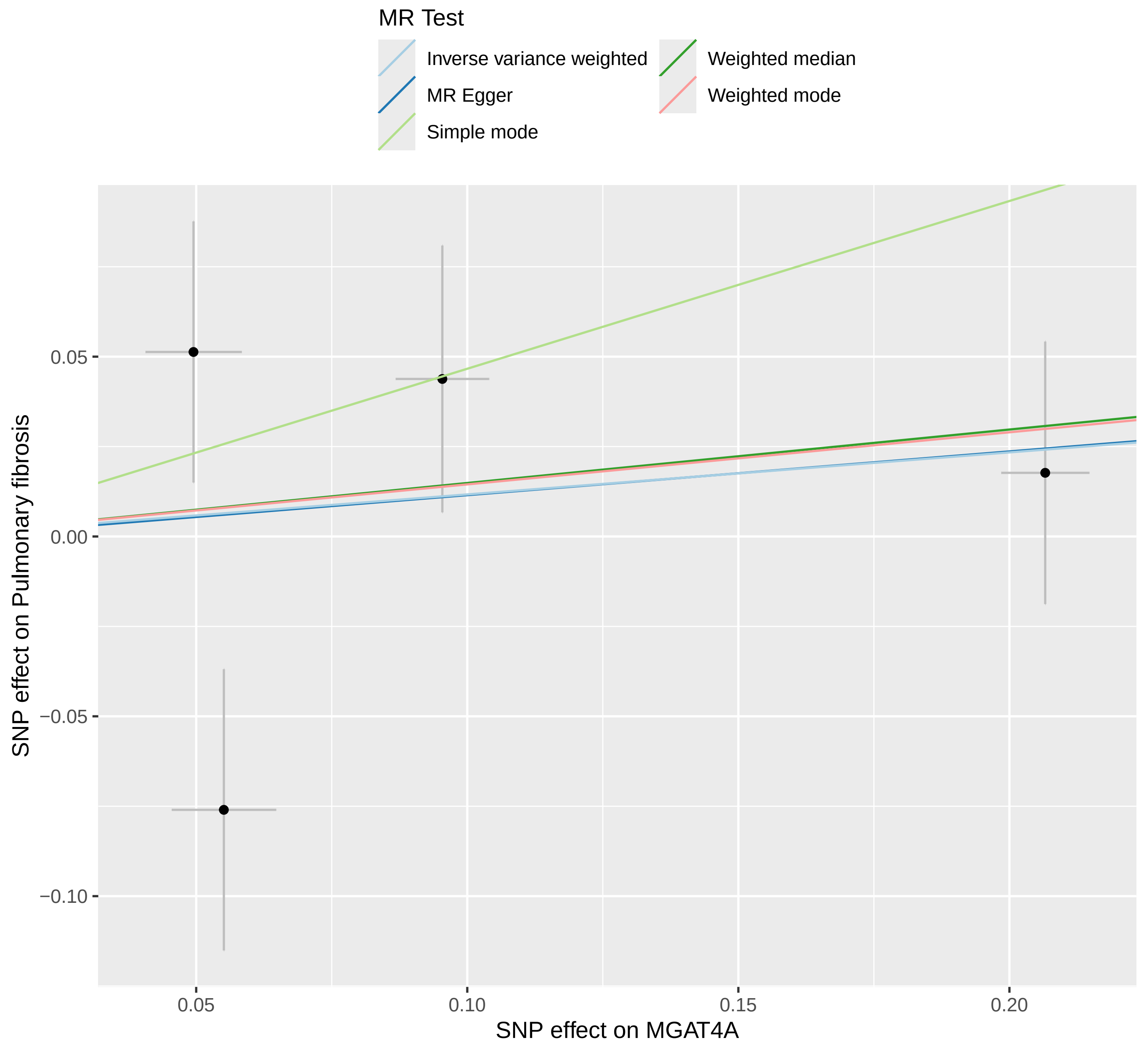

MR Test
Inverse variance weighted
MR Egger
Simple mode
Weighted median
Weighted mode
SNP effect on Pulmonary fibrosis
0.05
0.00
−0.05
−0.10
0.05
0.10
0.15
0.20
SNP effect on MGAT4A

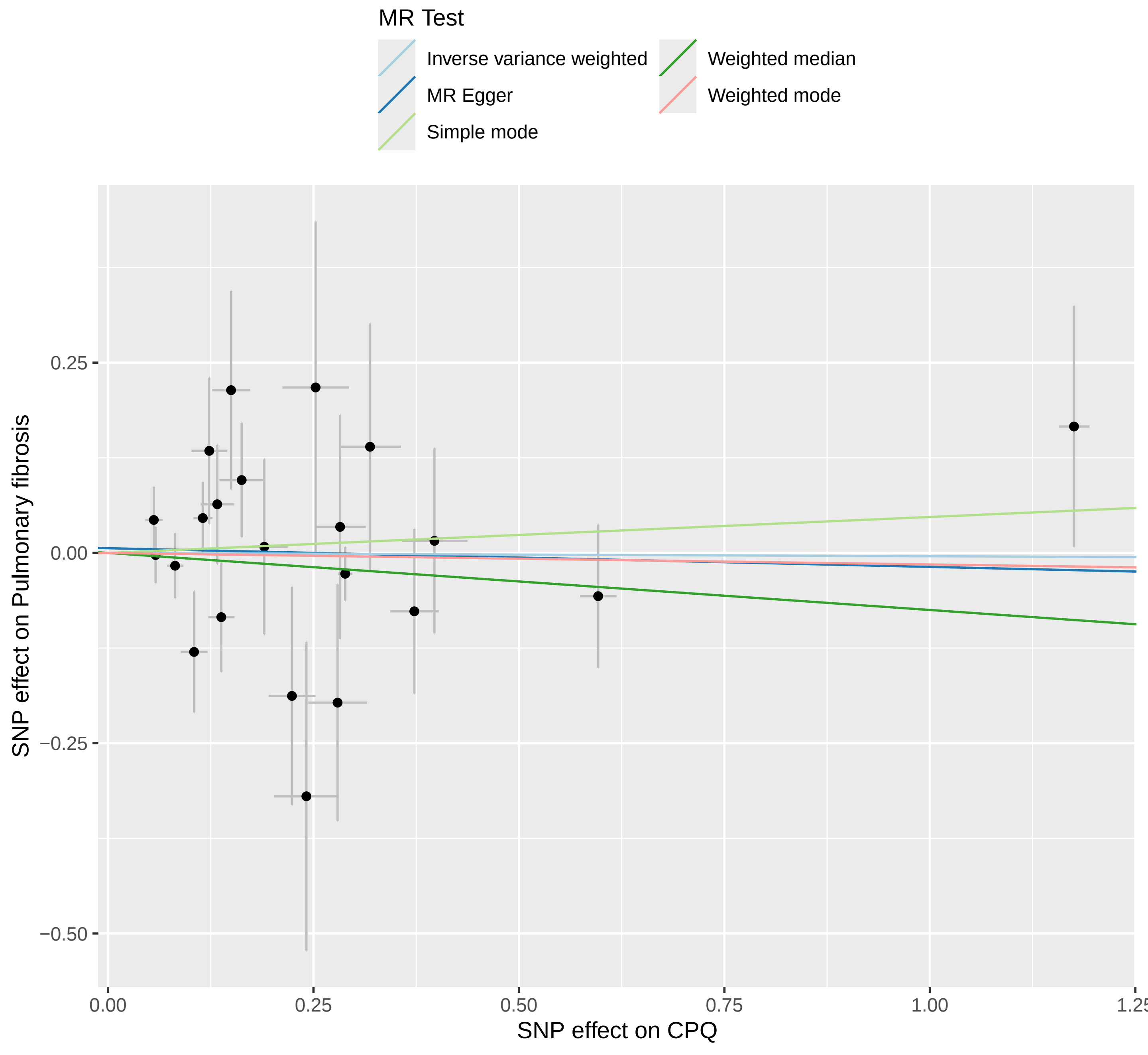
MR Test
Inverse variance weighted
MR Egger
Simple mode
Weighted median
Weighted mode
0.25
0.00
−0.25
−0.50
SNP effect on Pulmonary fibrosis
0.00
0.25
0.50
0.75
1.00
1.25
SNP effect on CPQ

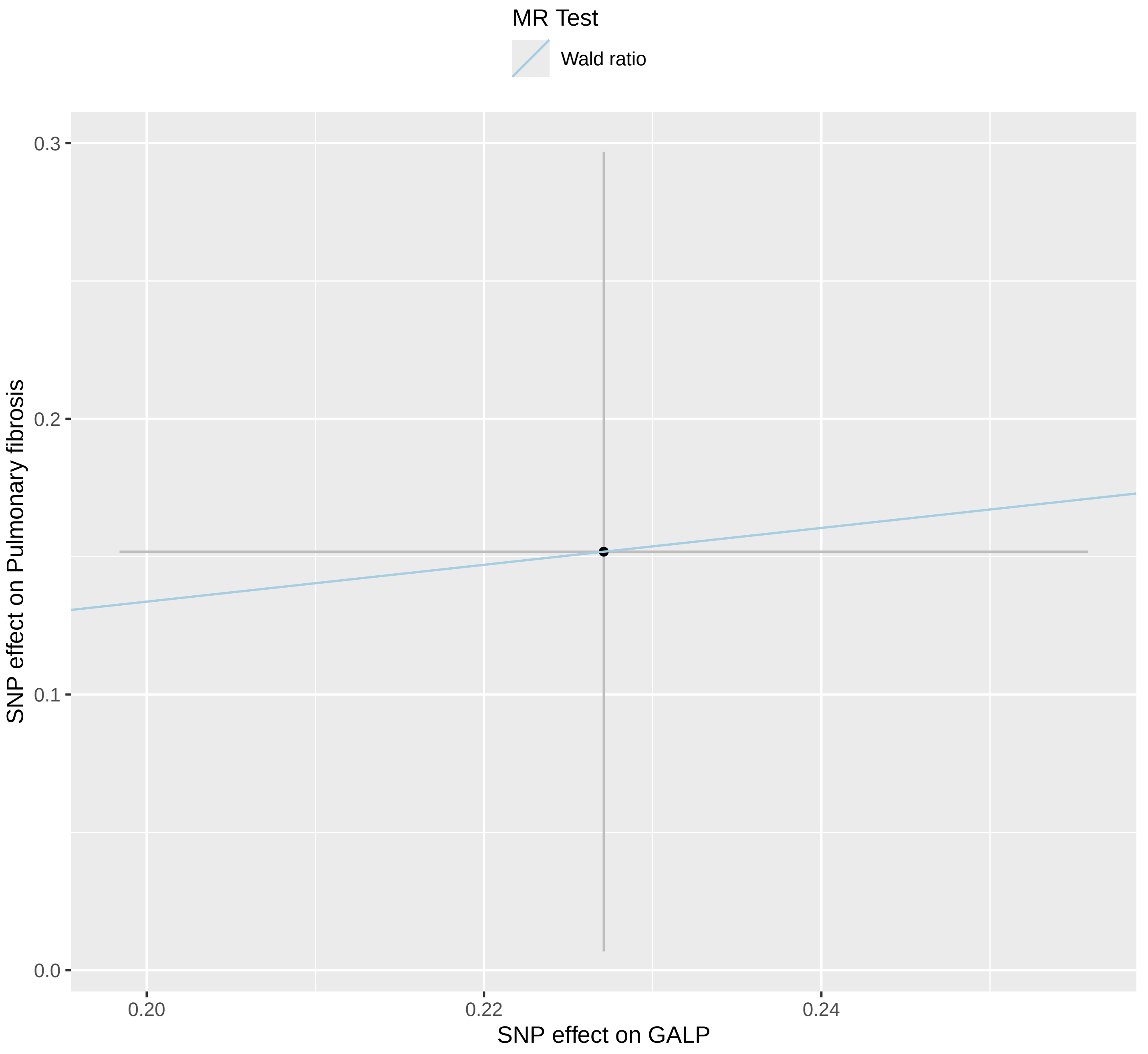
MR Test
Wald ratio
0.3
0.2
0.1
0.0
SNP effect on Pulmonary fibrosis
0.20
0.22
0.24
SNP effect on GALP

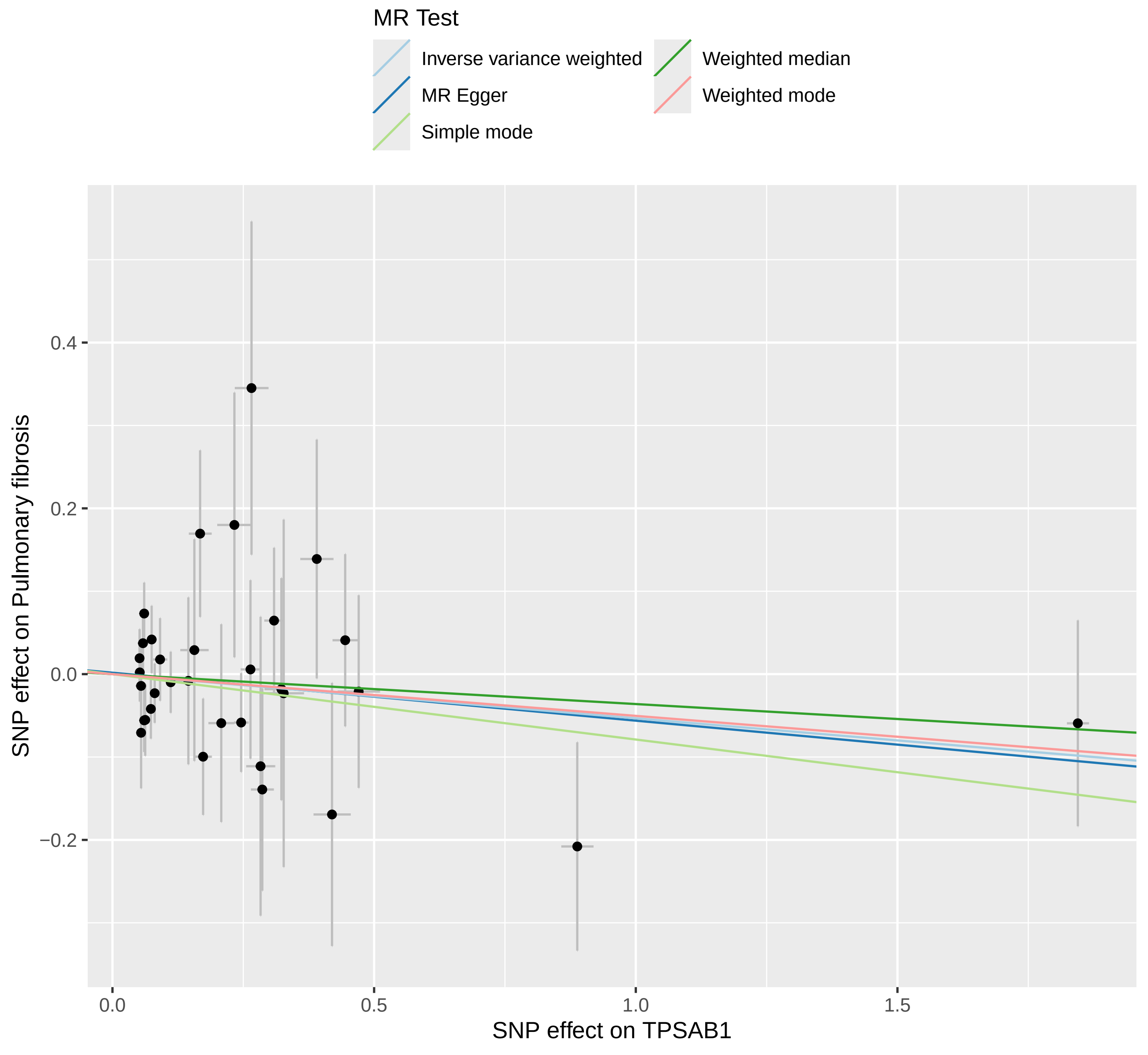

MR Test
Inverse variance weighted
MR Egger
Simple mode
Weighted median
Weighted mode
SNP effect on Pulmonary fibrosis
0.4
0.2
0.0
−0.2
0.0
0.5
1.0
1.5
SNP effect on TPSAB1

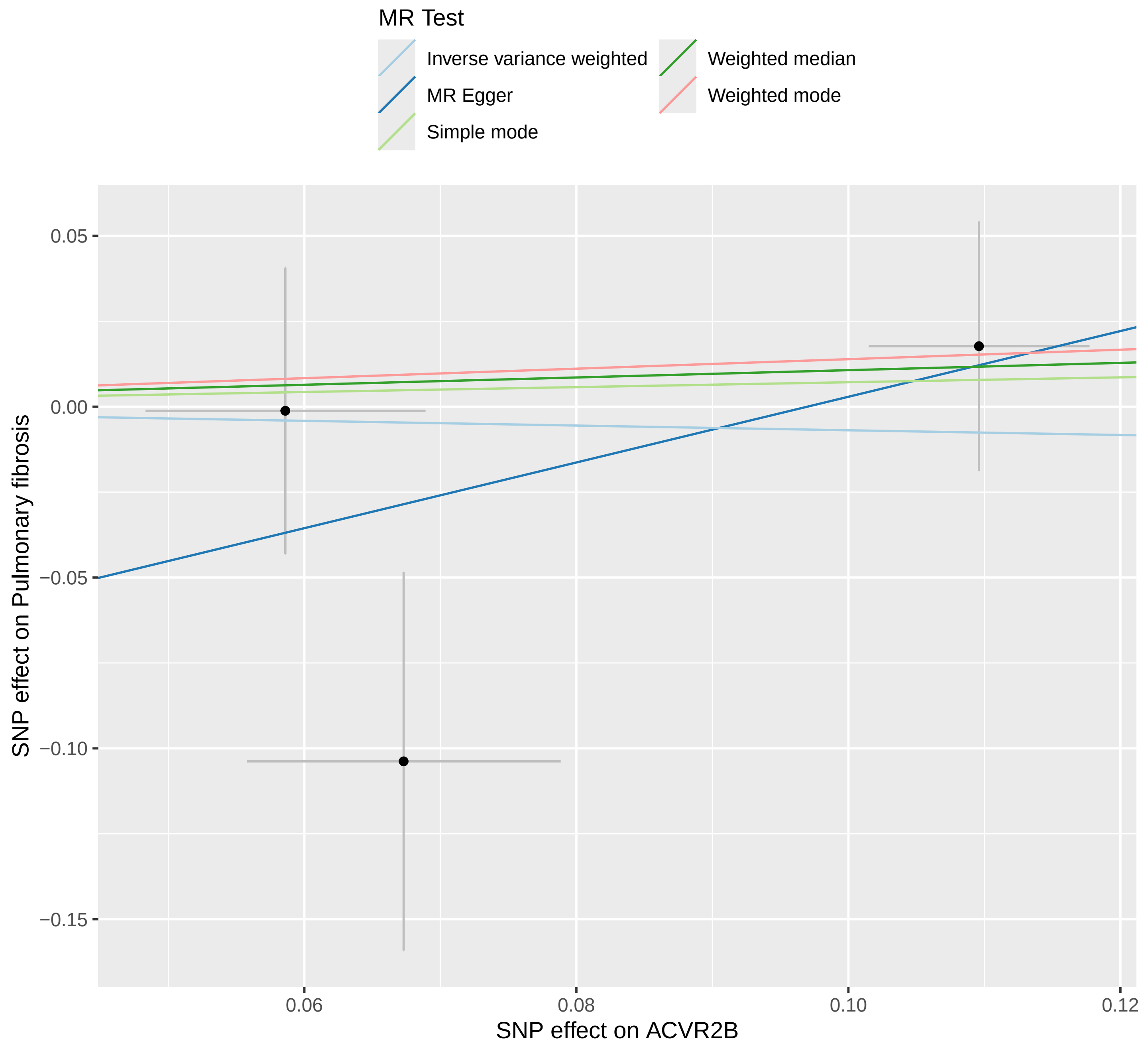
MR Test
Inverse variance weighted
MR Egger
Simple mode
Weighted median
Weighted mode
0.05
0.00
−0.05
−0.10
−0.15
SNP effect on Pulmonary fibrosis
0.06
0.08
0.10
0.12
SNP effect on ACVR2B

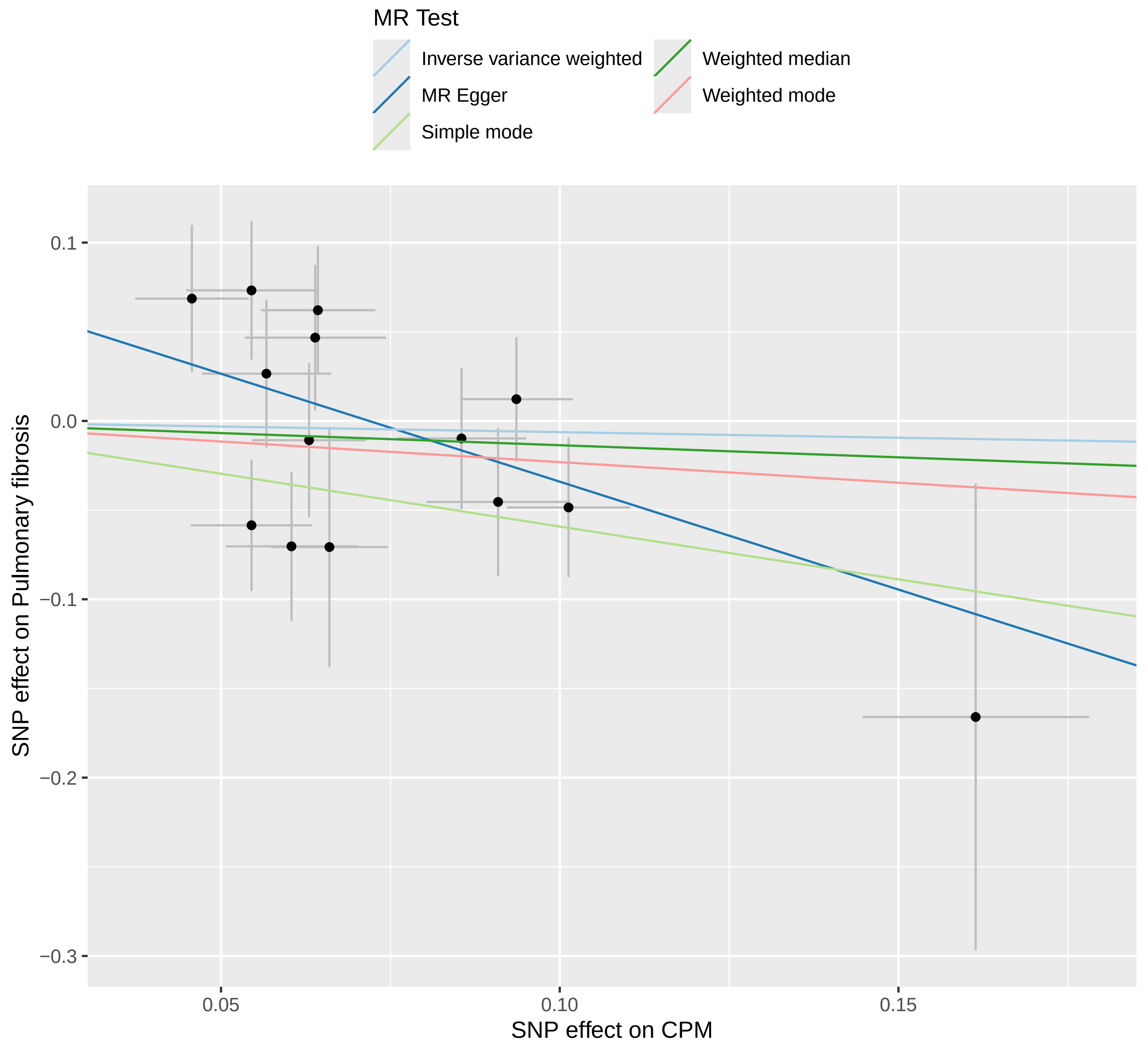

MR Test
Inverse variance weighted
MR Egger
Simple mode
Weighted median
Weighted mode
0.1
0.0
−0.1
−0.2
−0.3
SNP effect on Pulmonary fibrosis
0.05
0.10
0.15
SNP effect on CPM

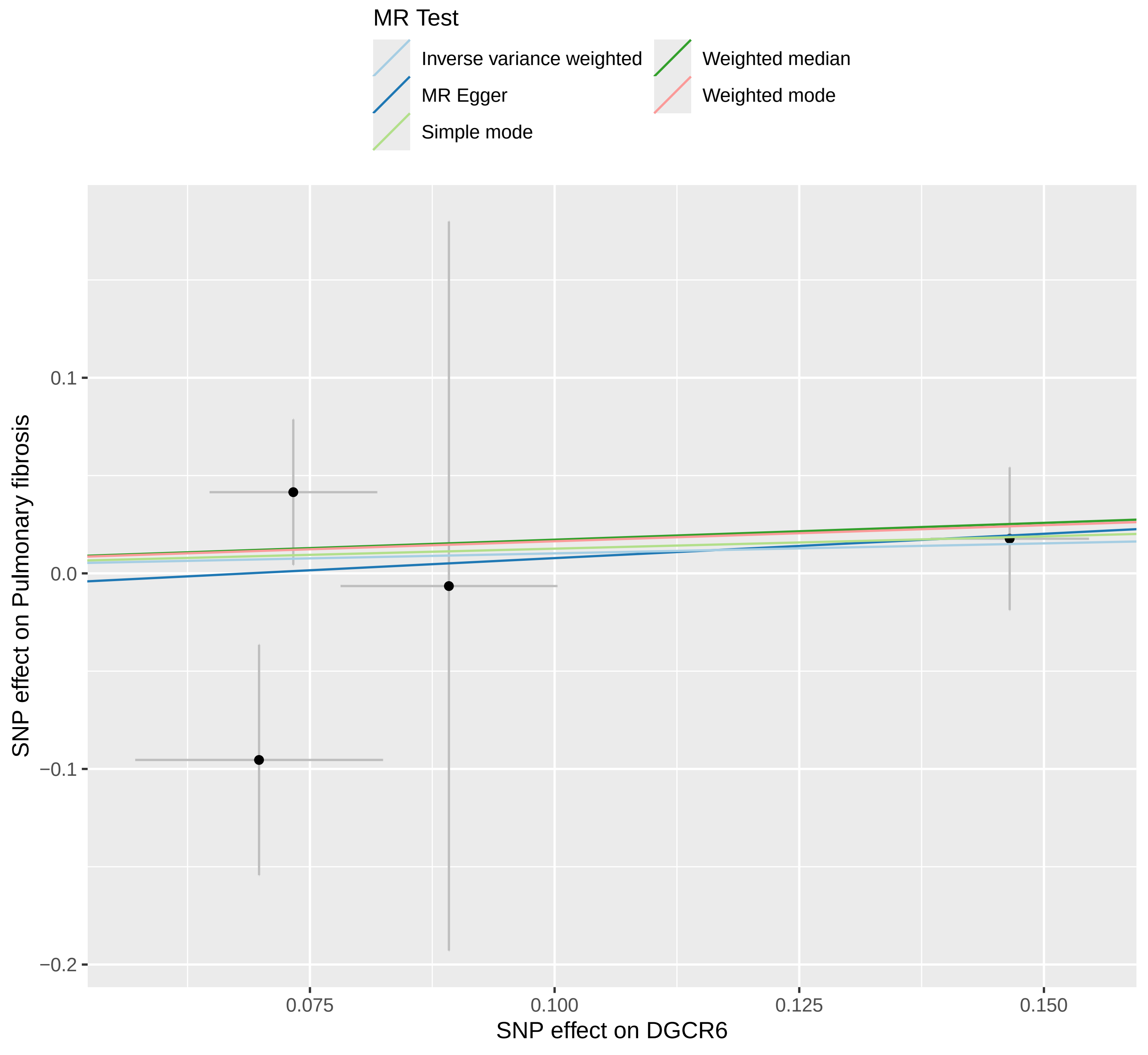
MR Test
Inverse variance weighted
MR Egger
Simple mode
Weighted median
Weighted mode
0.1
0.0
−0.1
−0.2
0.075
0.100
0.125
0.150
SNP effect on DGCR6
SNP effect on Pulmonary fibrosis

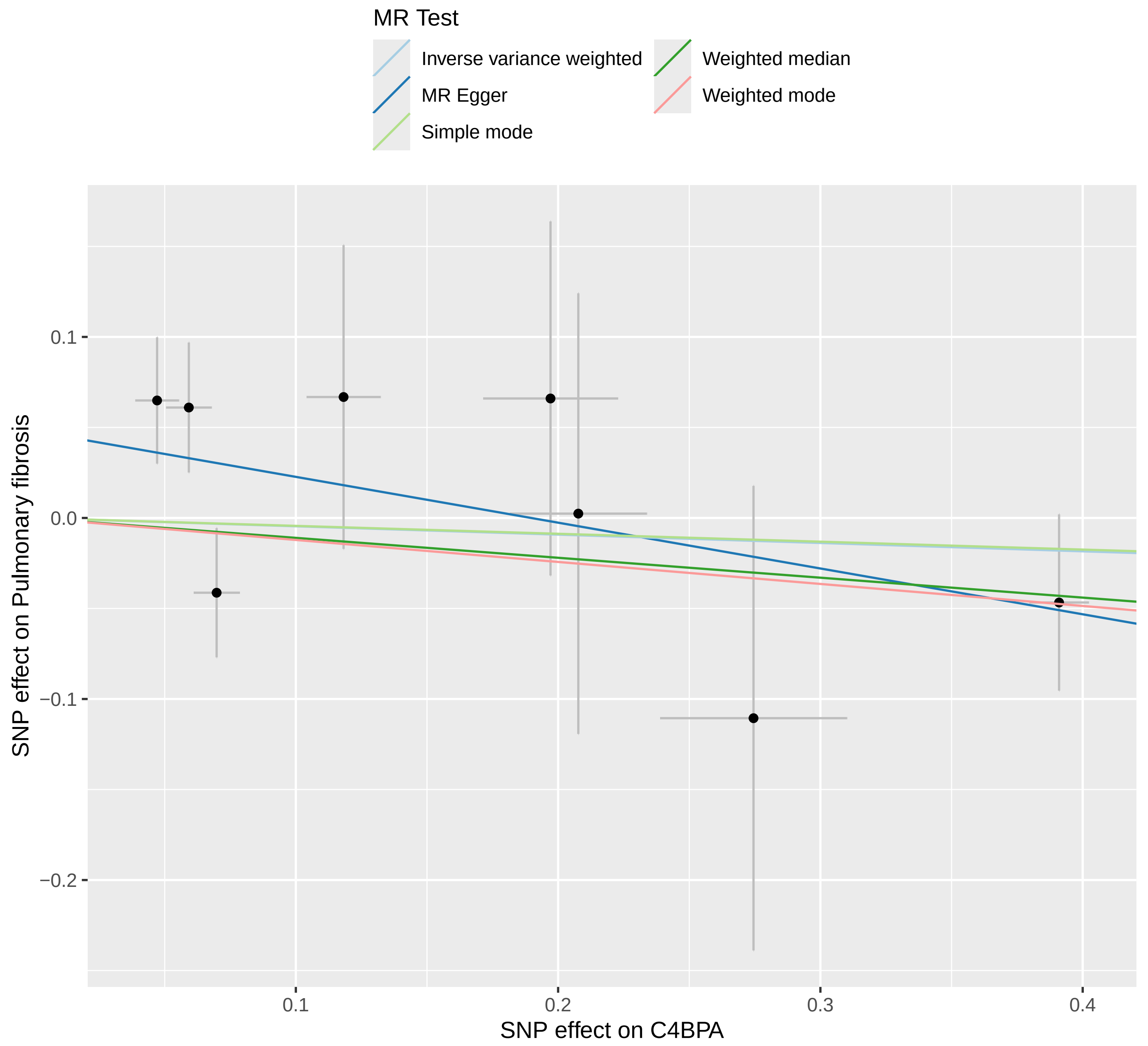
MR Test
Inverse variance weighted
MR Egger
Simple mode
Weighted median
Weighted mode
SNP effect on Pulmonary fibrosis
0.1
0.0
−0.1
−0.2
0.1
0.2
0.3
0.4
SNP effect on C4BPA

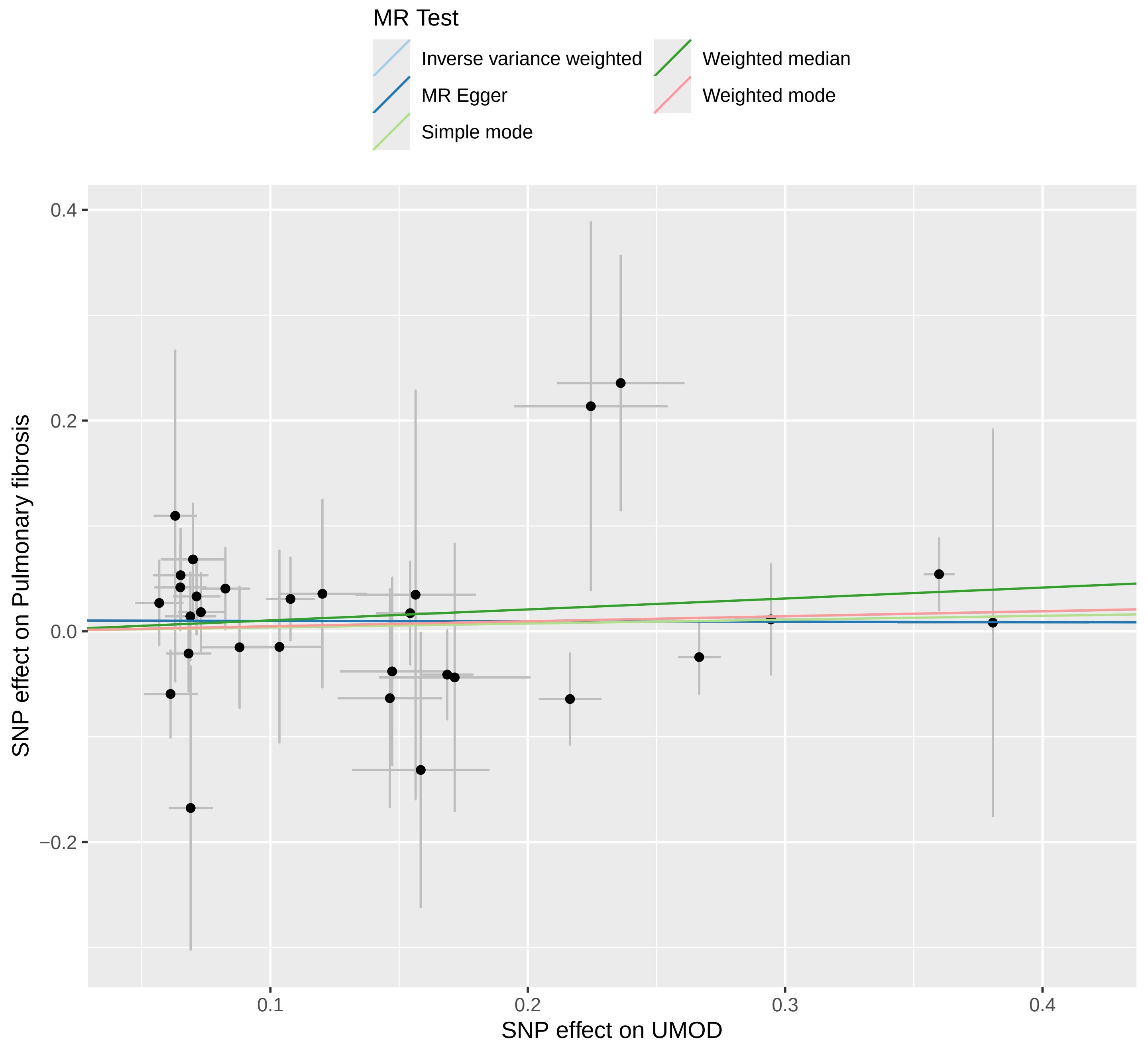

MR Test
Inverse variance weighted
MR Egger
Simple mode
Weighted median
Weighted mode
0.4
0.2
0.0
−0.2
0.1
0.2
0.3
0.4
SNP effect on Pulmonary fibrosis
SNP effect on UMOD

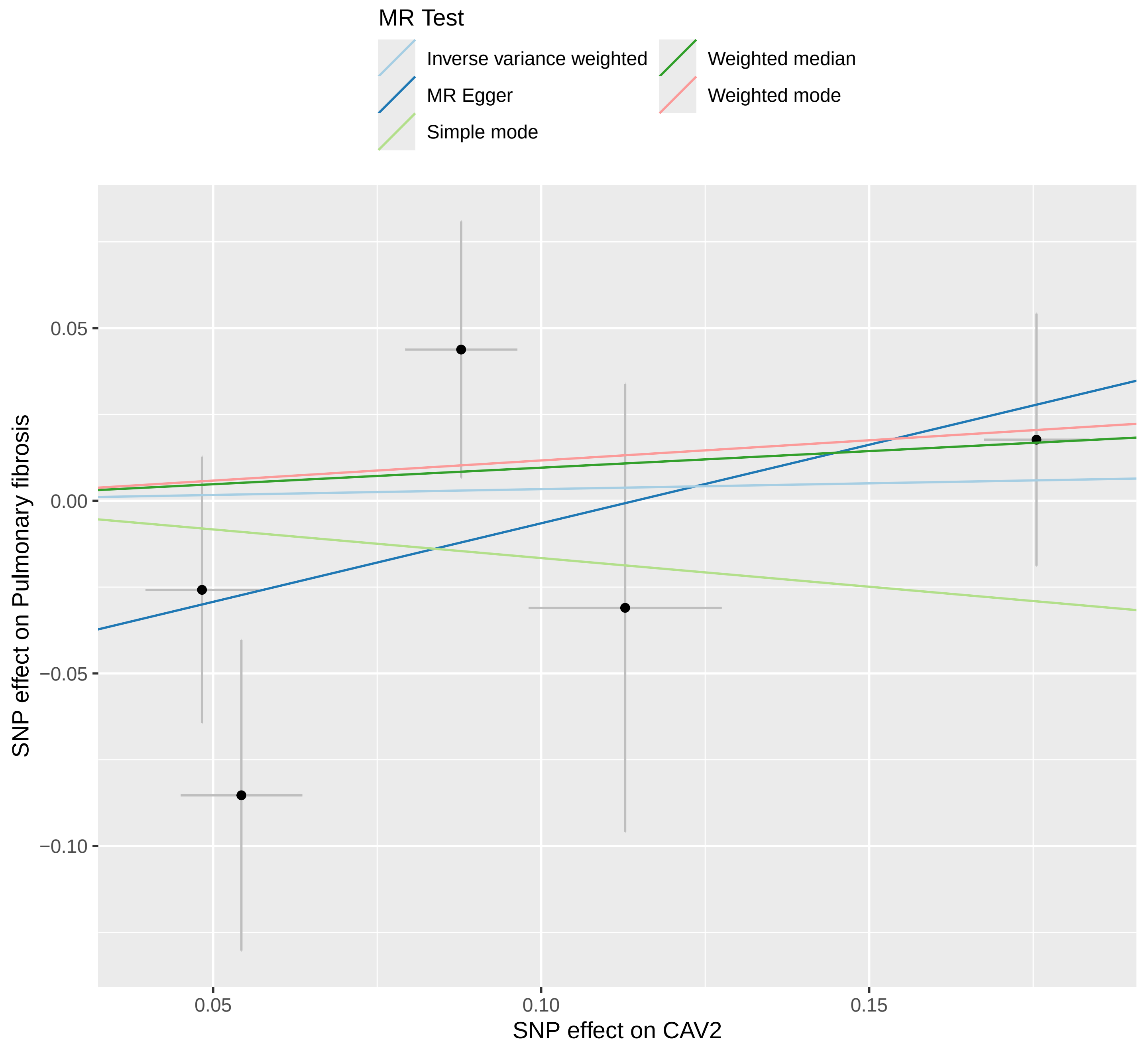
MR Test
Inverse variance weighted
MR Egger
Simple mode
Weighted median
Weighted mode
0.05
0.00
−0.05
−0.10
SNP effect on Pulmonary fibrosis
0.05
0.10
0.15
SNP effect on CAV2

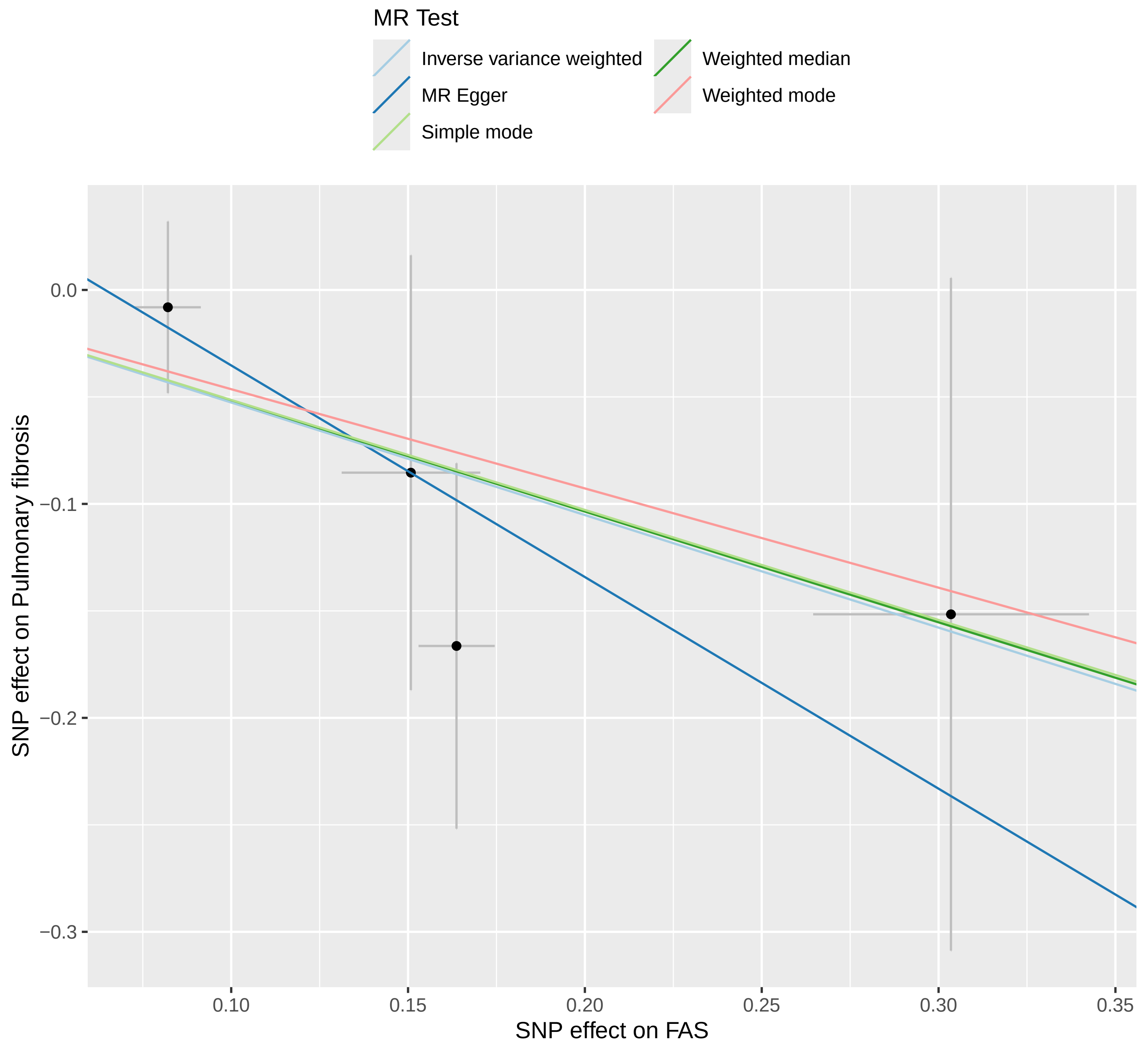
MR Test
Inverse variance weighted
MR Egger
Simple mode
Weighted median
Weighted mode
0.0
−0.1
−0.2
−0.3
SNP effect on Pulmonary fibrosis
0.10
0.15
0.20
0.25
0.30
0.35
SNP effect on FAS

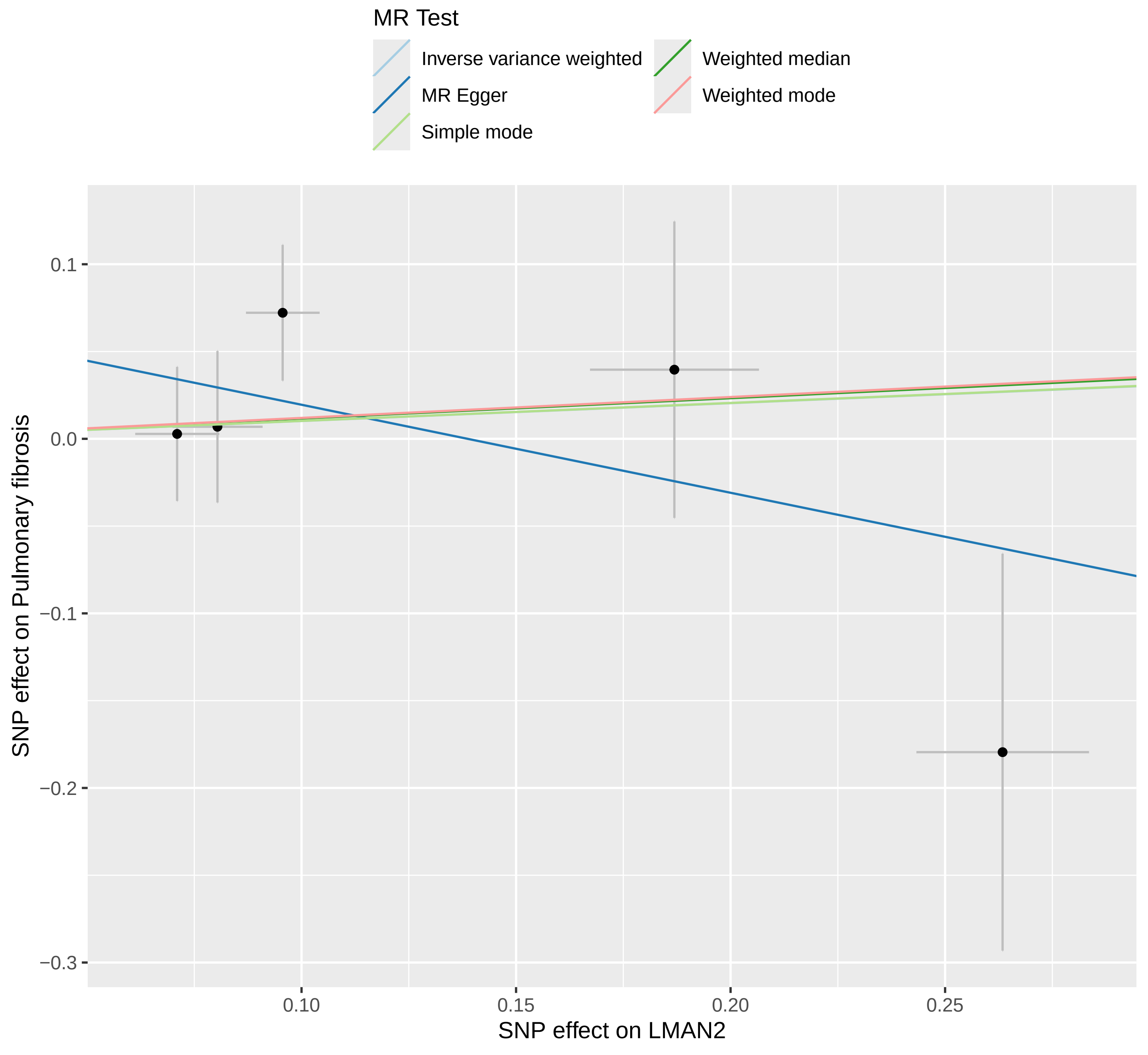
MR Test
Inverse variance weighted
MR Egger
Simple mode
Weighted median
Weighted mode
0.1
0.0
−0.1
−0.2
−0.3
SNP effect on Pulmonary fibrosis
0.10
0.15
0.20
0.25
SNP effect on LMAN2

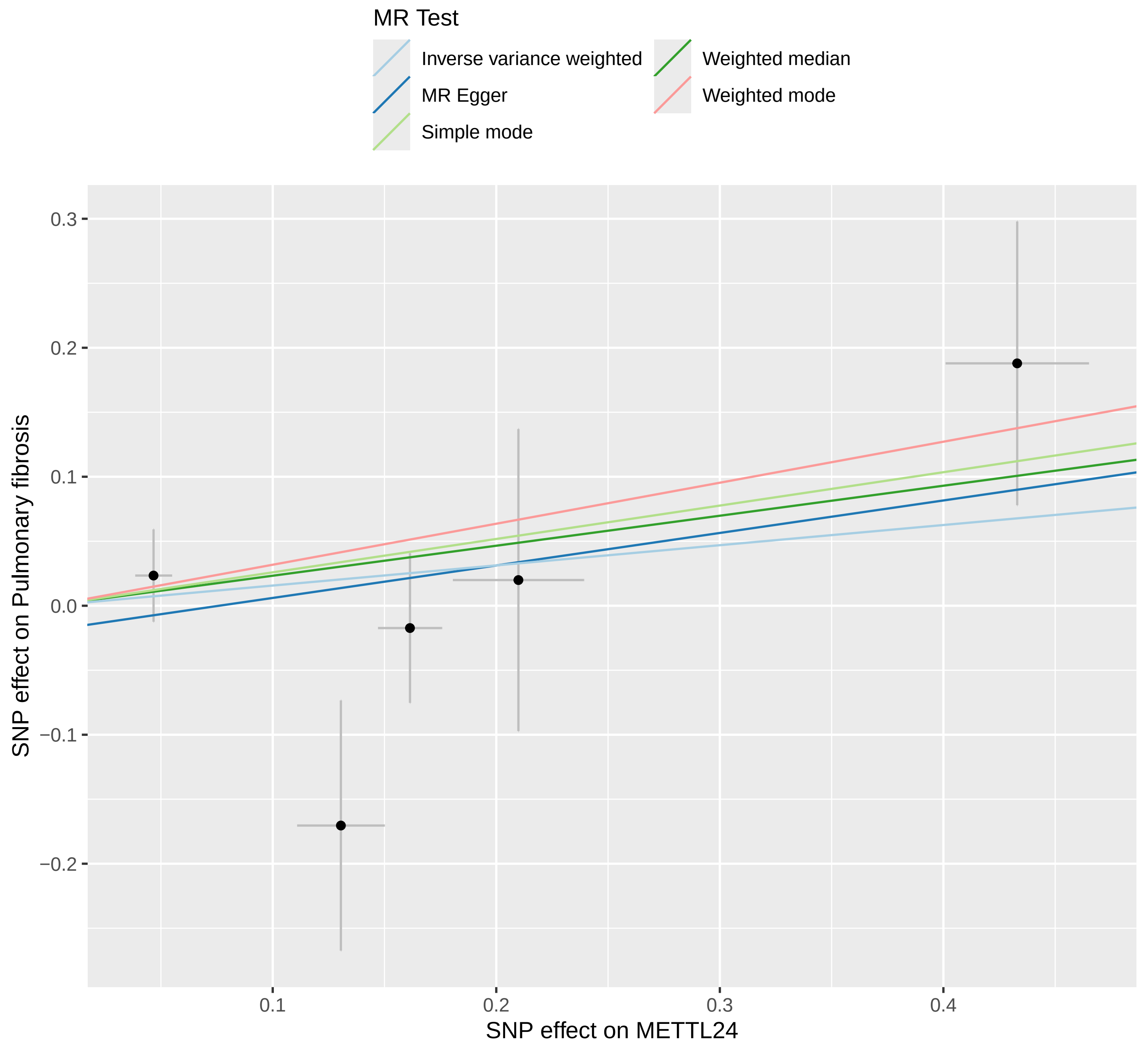
MR Test
Inverse variance weighted
MR Egger
Simple mode
Weighted median
Weighted mode
SNP effect on Pulmonary fibrosis
0.3
0.2
0.1
0.0
−0.1
−0.2
0.1
0.2
0.3
0.4
SNP effect on METTL24

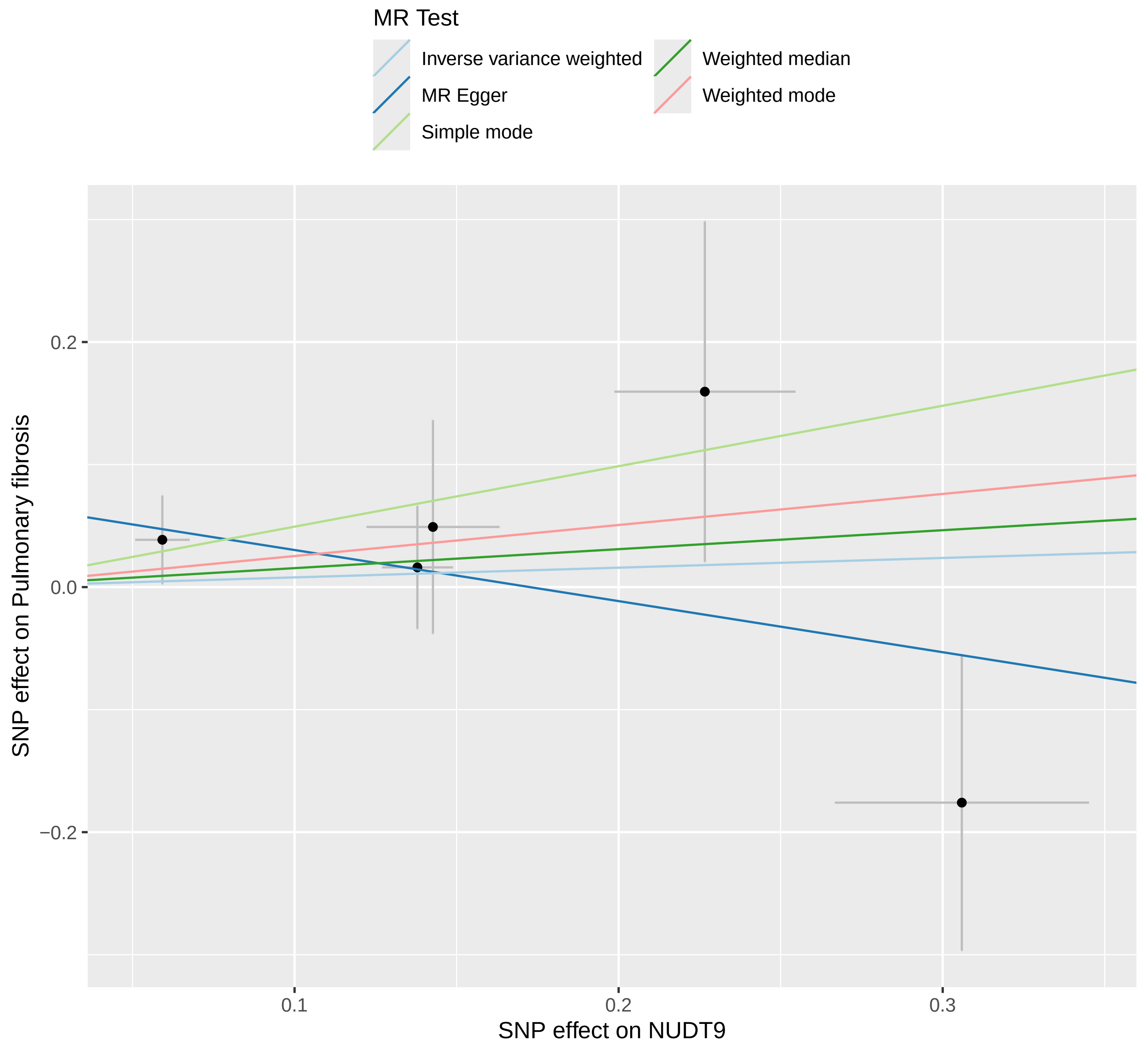

MR Test
Inverse variance weighted
MR Egger
Simple mode
Weighted median
Weighted mode
0.2
0.0
−0.2
SNP effect on Pulmonary fibrosis
0.1
0.2
0.3
SNP effect on NUDT9

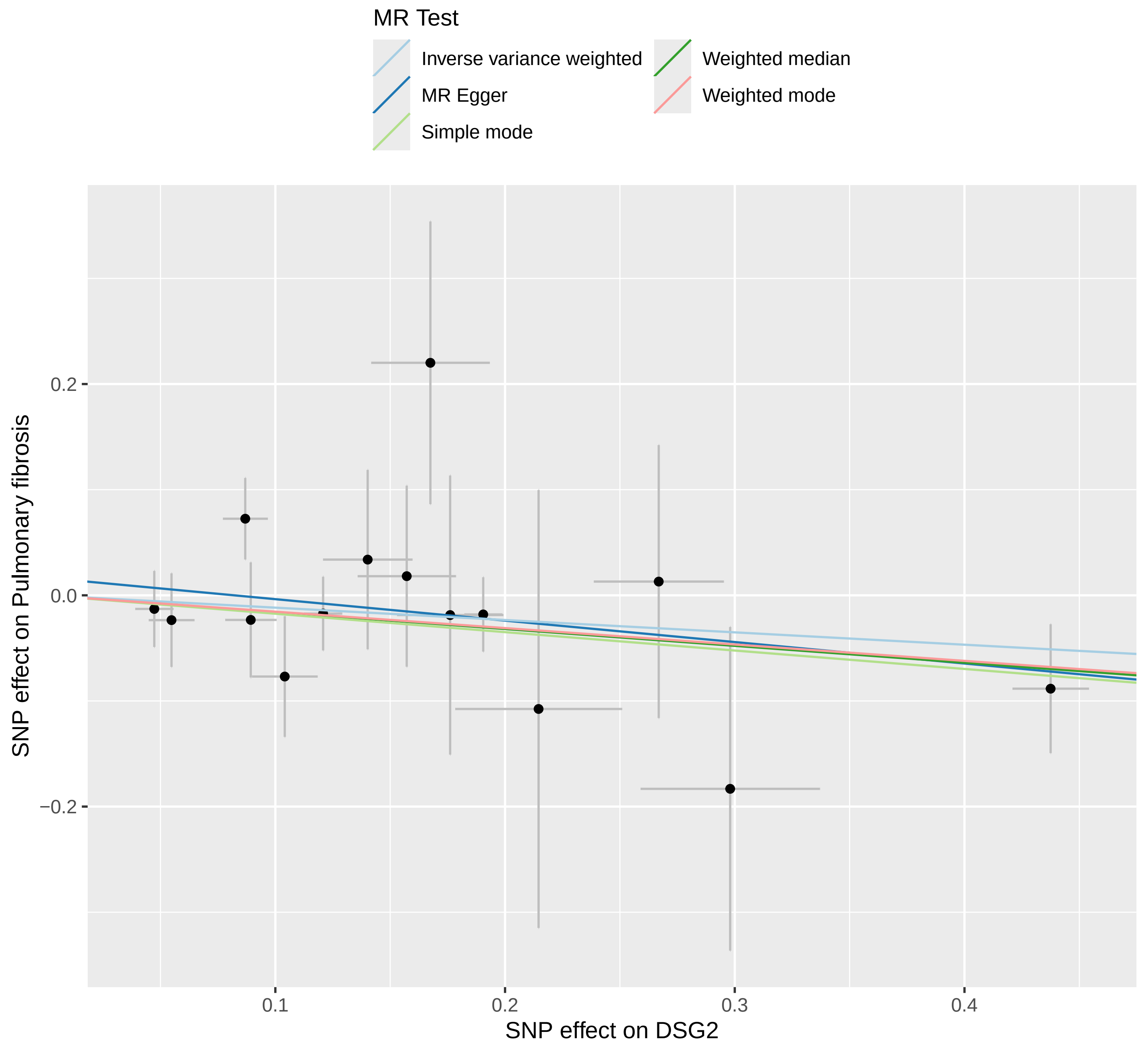

MR Test
Inverse variance weighted
MR Egger
Simple mode
Weighted median
Weighted mode
0.2
0.0
−0.2
SNP effect on Pulmonary fibrosis
0.1
0.2
0.3
0.4
SNP effect on DSG2

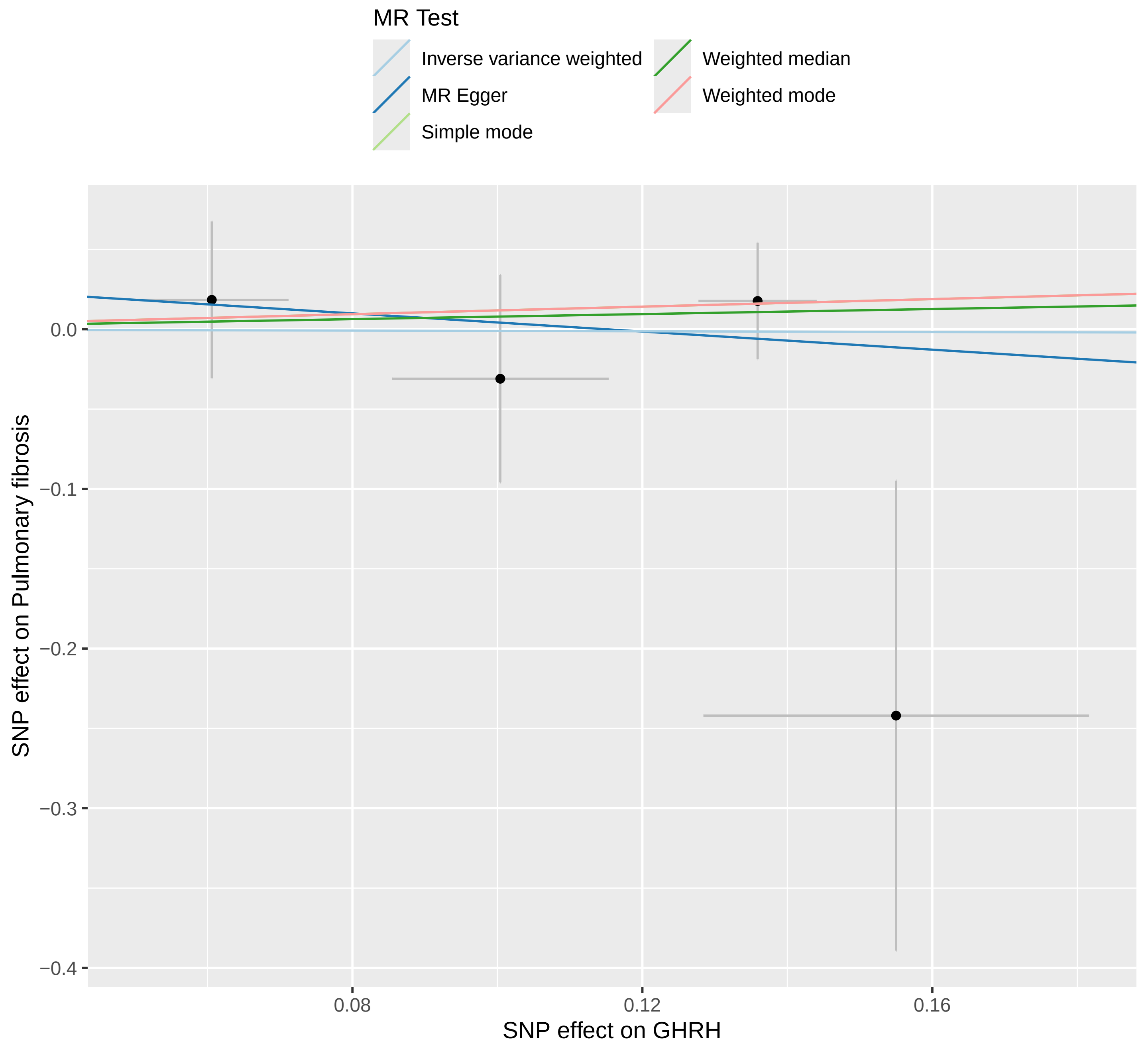

MR Test
Inverse variance weighted
MR Egger
Simple mode
Weighted median
Weighted mode
0.0
−0.1
−0.2
−0.3
−0.4
0.08
0.12
0.16
SNP effect on Pulmonary fibrosis
SNP effect on GHRH

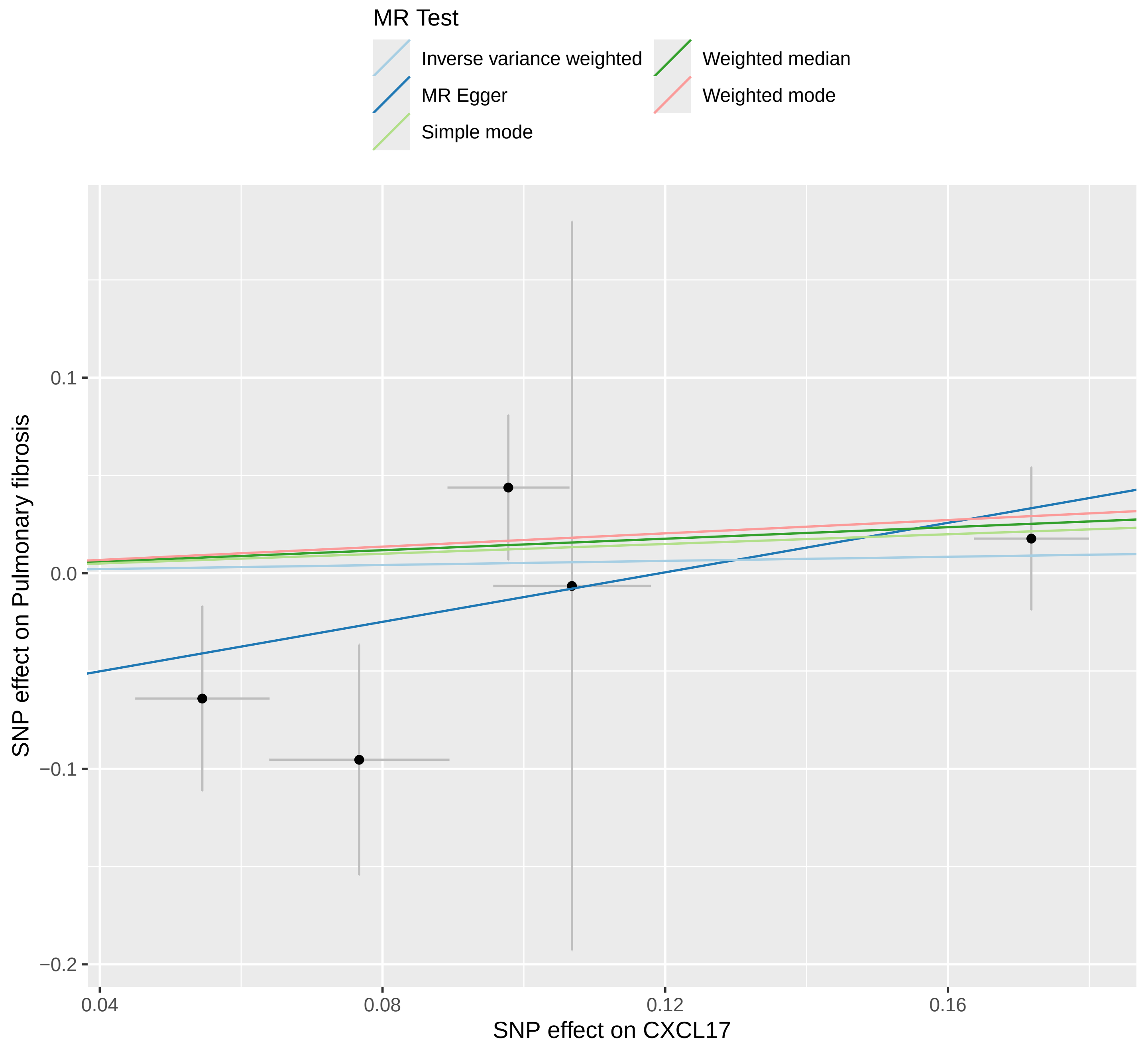

MR Test
Inverse variance weighted
MR Egger
Simple mode
Weighted median
Weighted mode
0.1
0.0
−0.1
−0.2
0.04
0.08
0.12
0.16
SNP effect on Pulmonary fibrosis
SNP effect on CXCL17

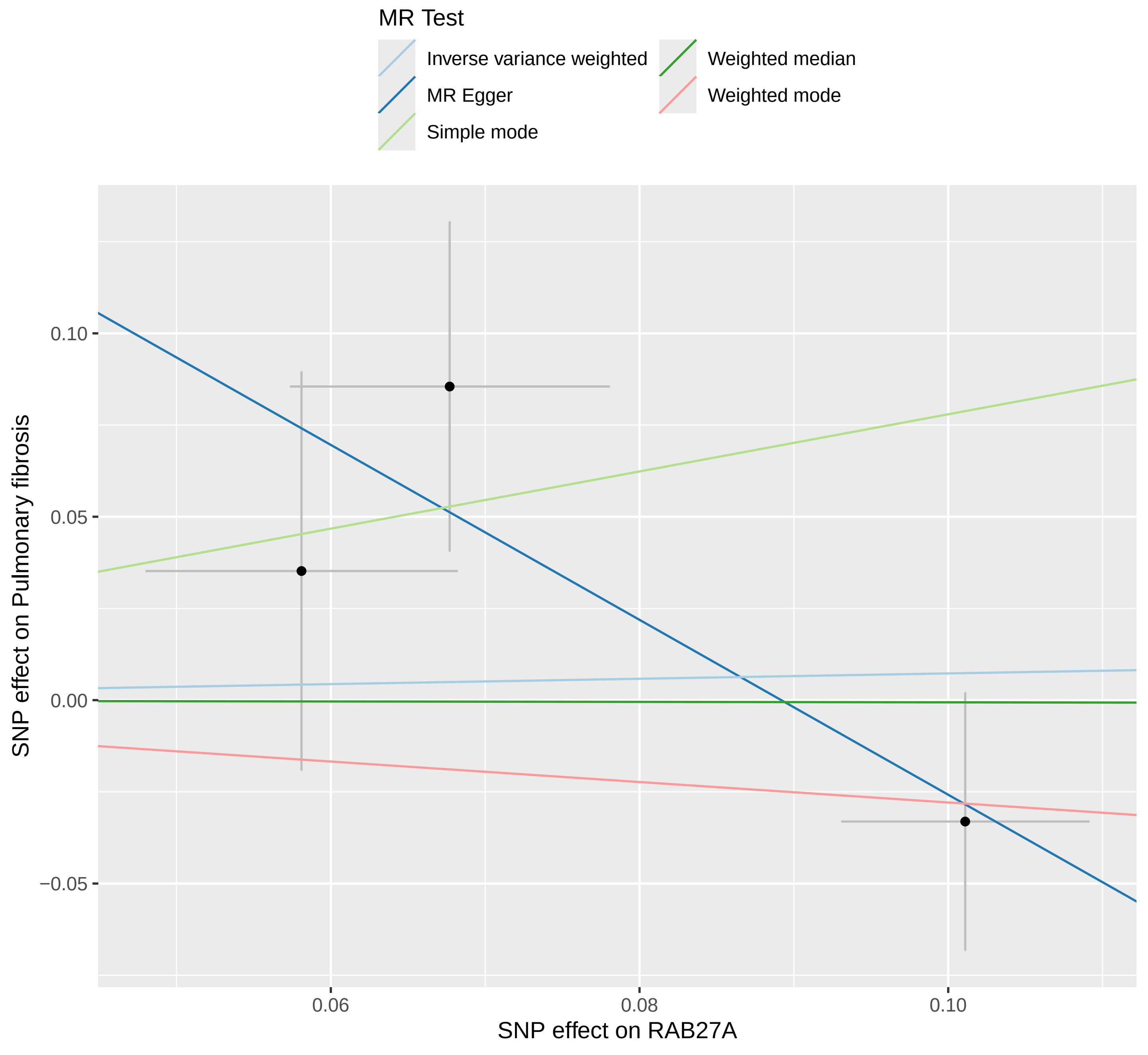

MR Test
Inverse variance weighted
MR Egger
Simple mode
Weighted median
Weighted mode
0.10
0.05
0.00
−0.05
SNP effect on Pulmonary fibrosis
0.06
0.08
0.10
SNP effect on RAB27A

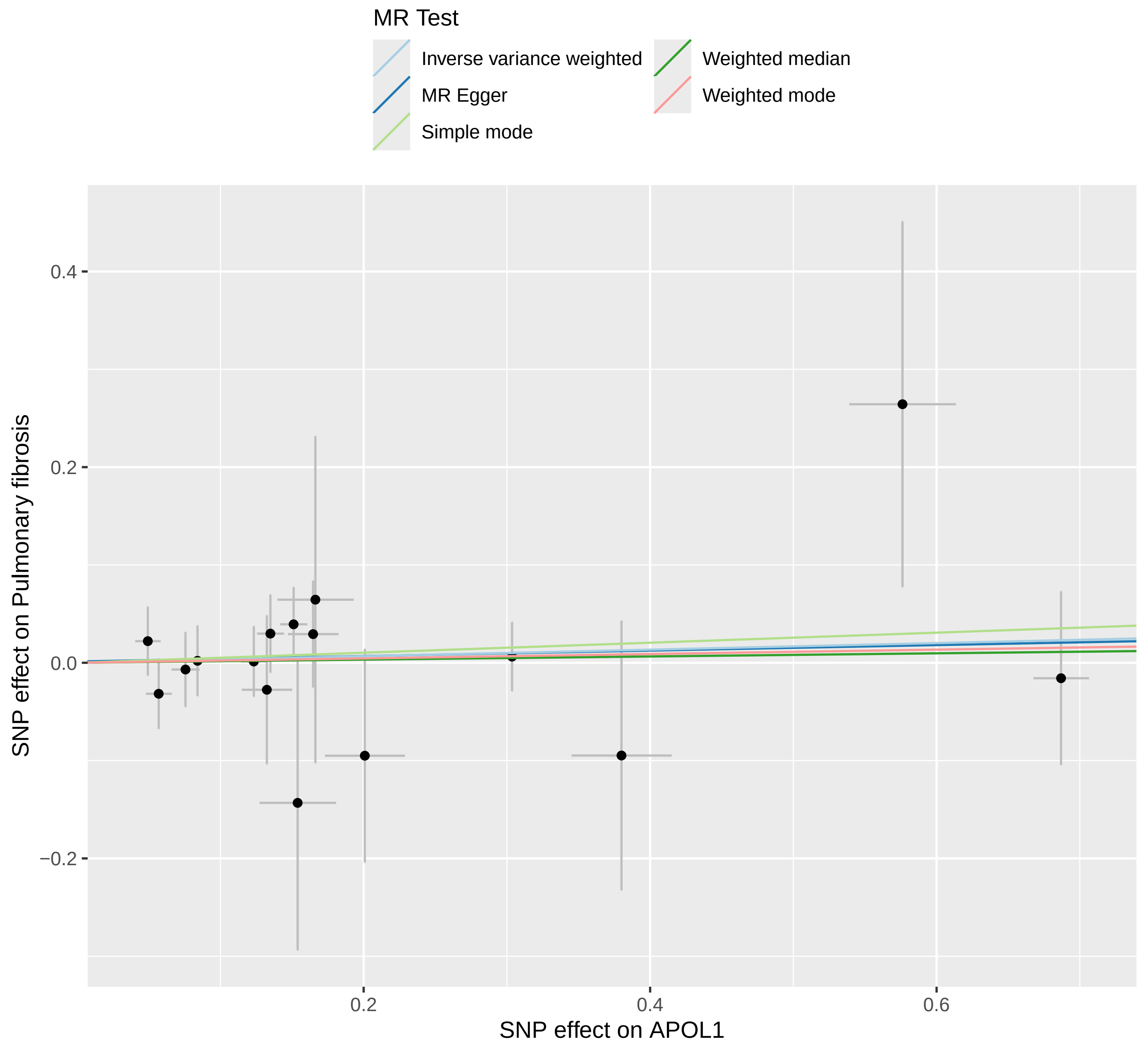

MR Test
Inverse variance weighted
MR Egger
Simple mode
Weighted median
Weighted mode
0.4
0.2
0.0
−0.2
0.2
0.4
0.6
SNP effect on APOL1
SNP effect on Pulmonary fibrosis

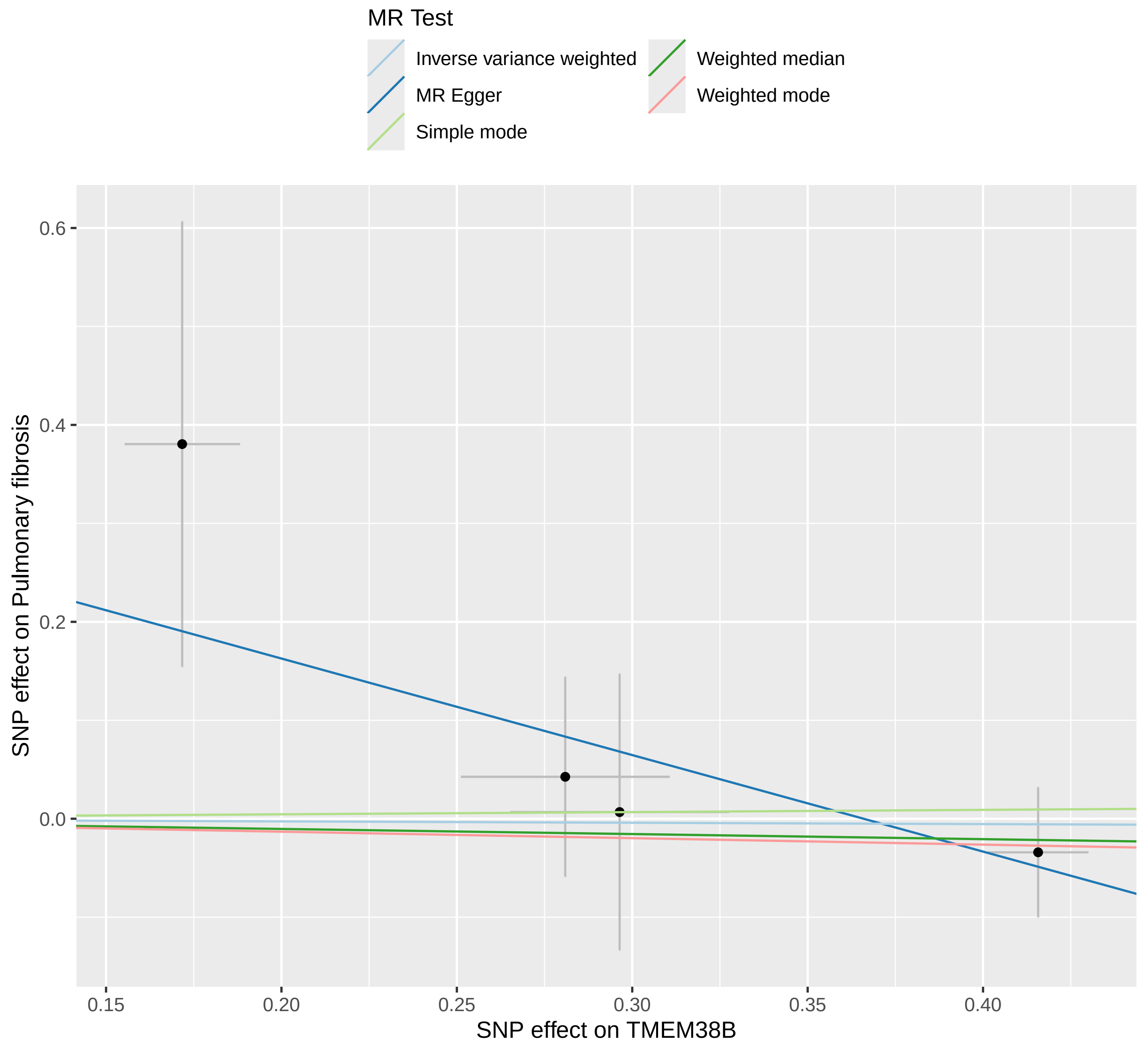

MR Test
Inverse variance weighted
MR Egger
Simple mode
Weighted median
Weighted mode
0.6
0.4
0.2
0.0
SNP effect on Pulmonary fibrosis
0.15
0.20
0.25
0.30
0.35
0.40
SNP effect on TMEM38B

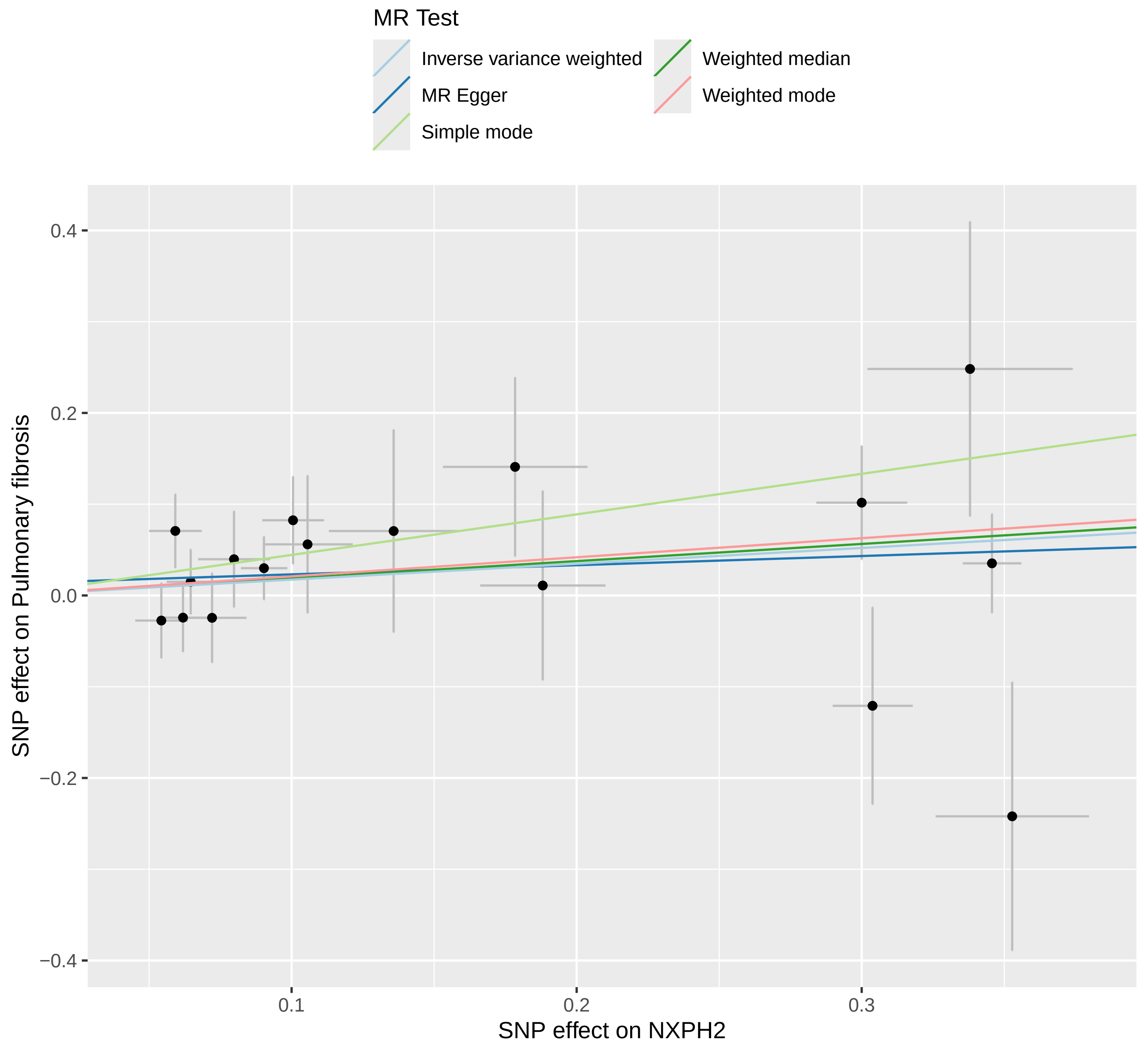

MR Test
Inverse variance weighted
MR Egger
Simple mode
Weighted median
Weighted mode
0.4
0.2
0.0
−0.2
−0.4
SNP effect on Pulmonary fibrosis
0.1
0.2
0.3
SNP effect on NXPH2

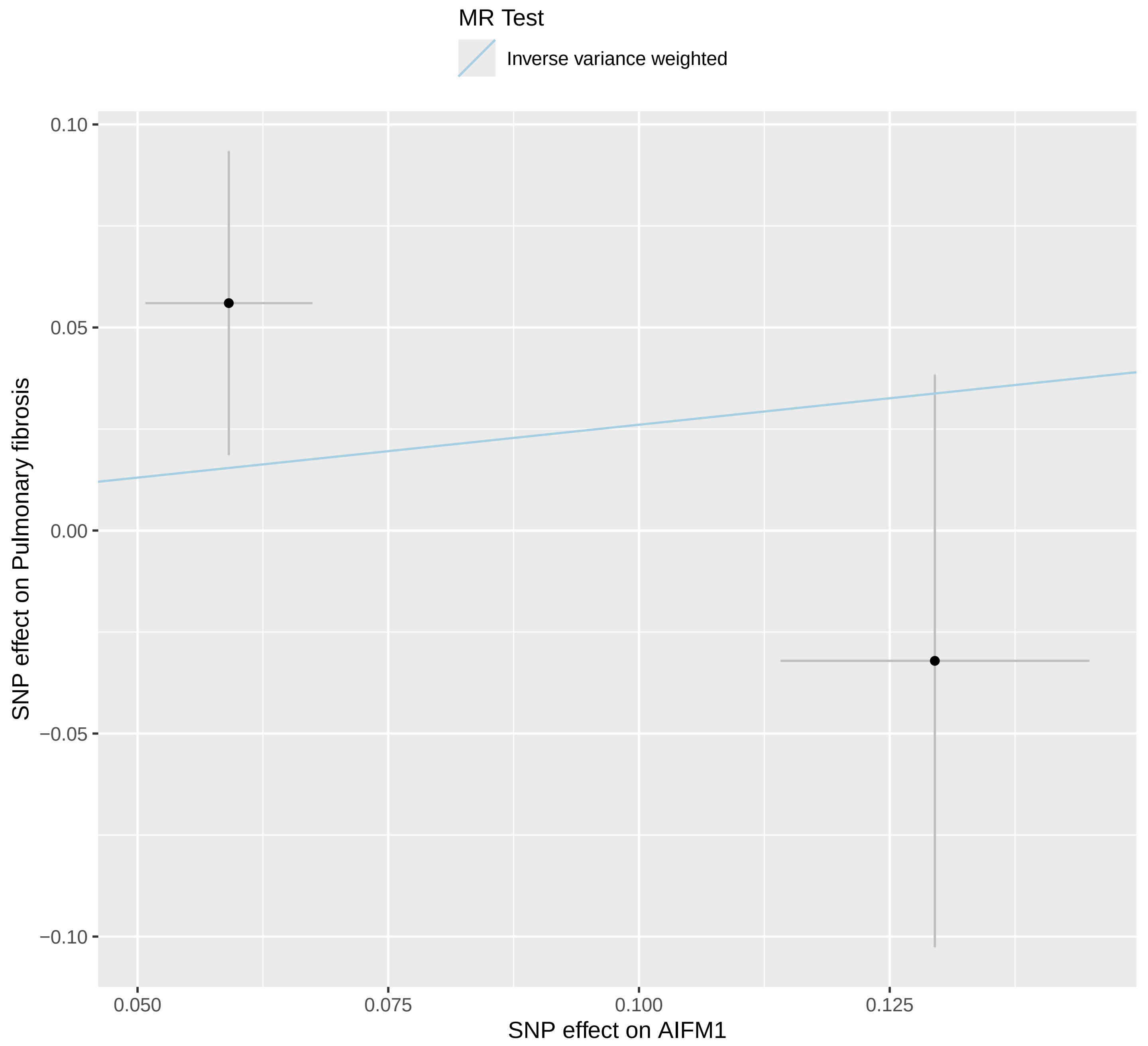
MR Test
Inverse variance weighted
0.10
0.05
0.00
−0.05
−0.10
SNP effect on Pulmonary fibrosis
0.050
0.075
0.100
0.125
SNP effect on AIFM1

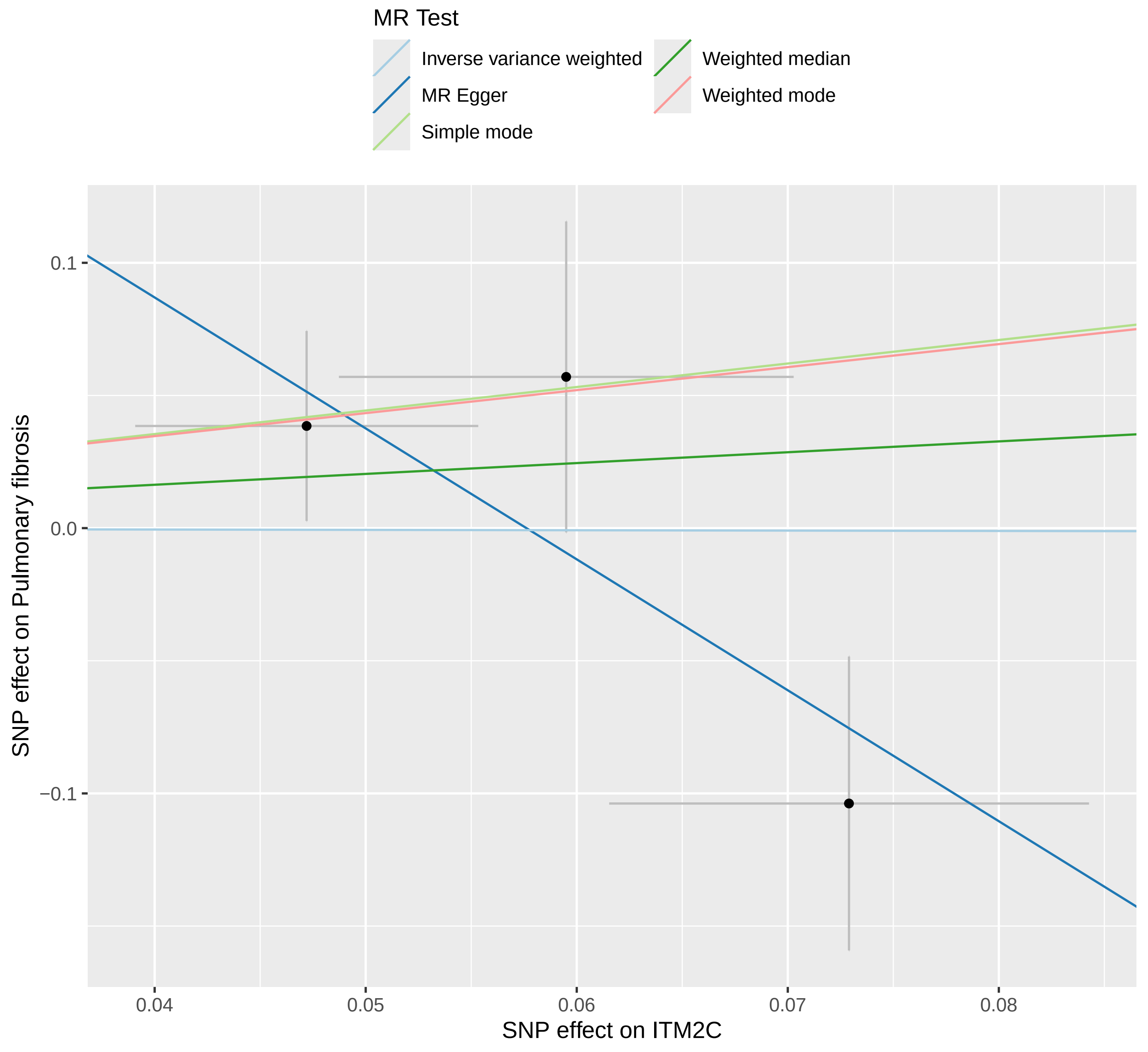
MR Test
Inverse variance weighted
MR Egger
Simple mode
Weighted median
Weighted mode
0.1
0.0
−0.1
SNP effect on Pulmonary fibrosis
0.04
0.05
0.06
0.07
0.08
SNP effect on ITM2C

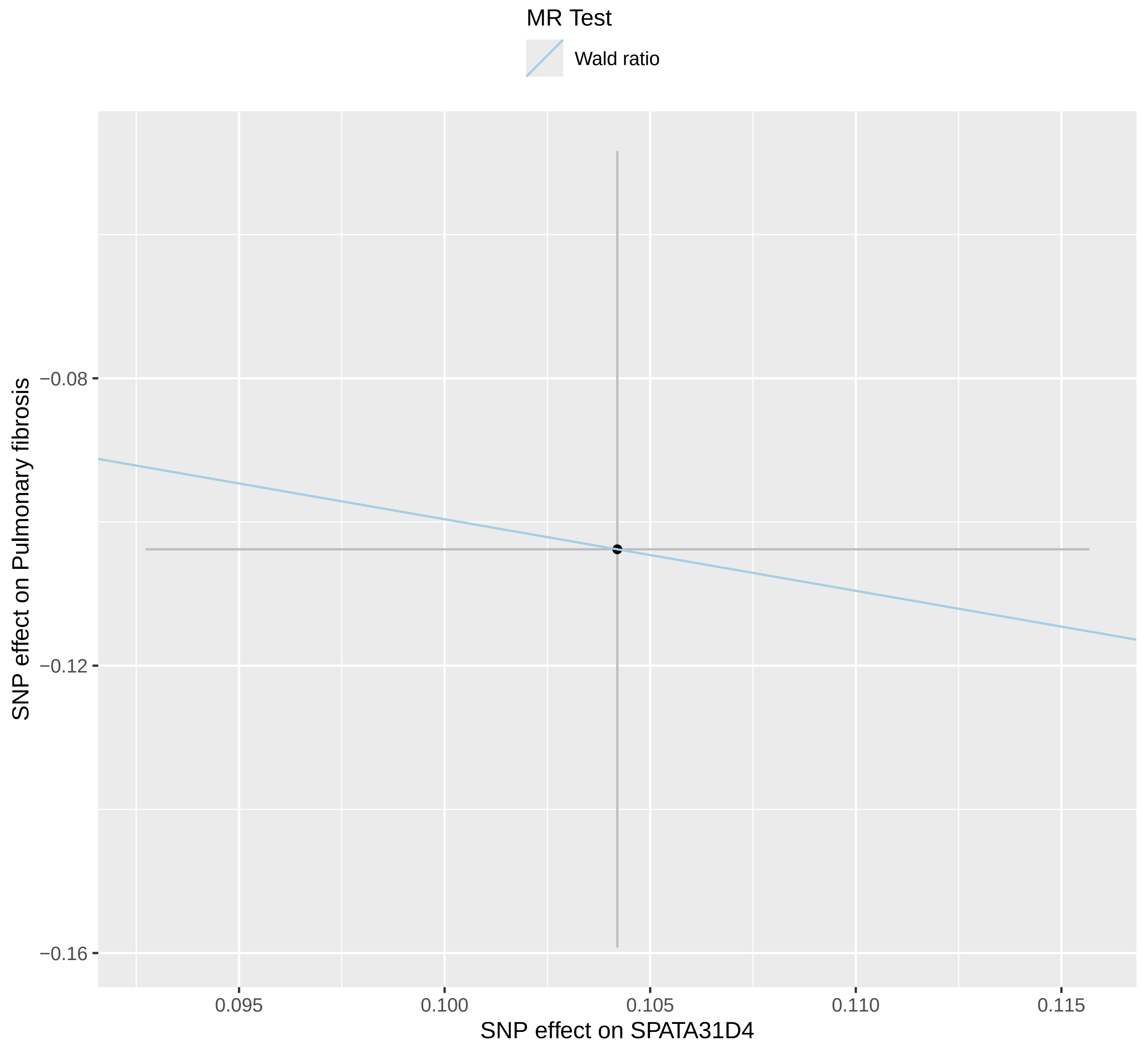

MR Test
Wald ratio
SNP effect on Pulmonary fibrosis
−0.08
−0.12
−0.16
0.095
0.100
0.105
0.110
0.115
SNP effect on SPATA31D4

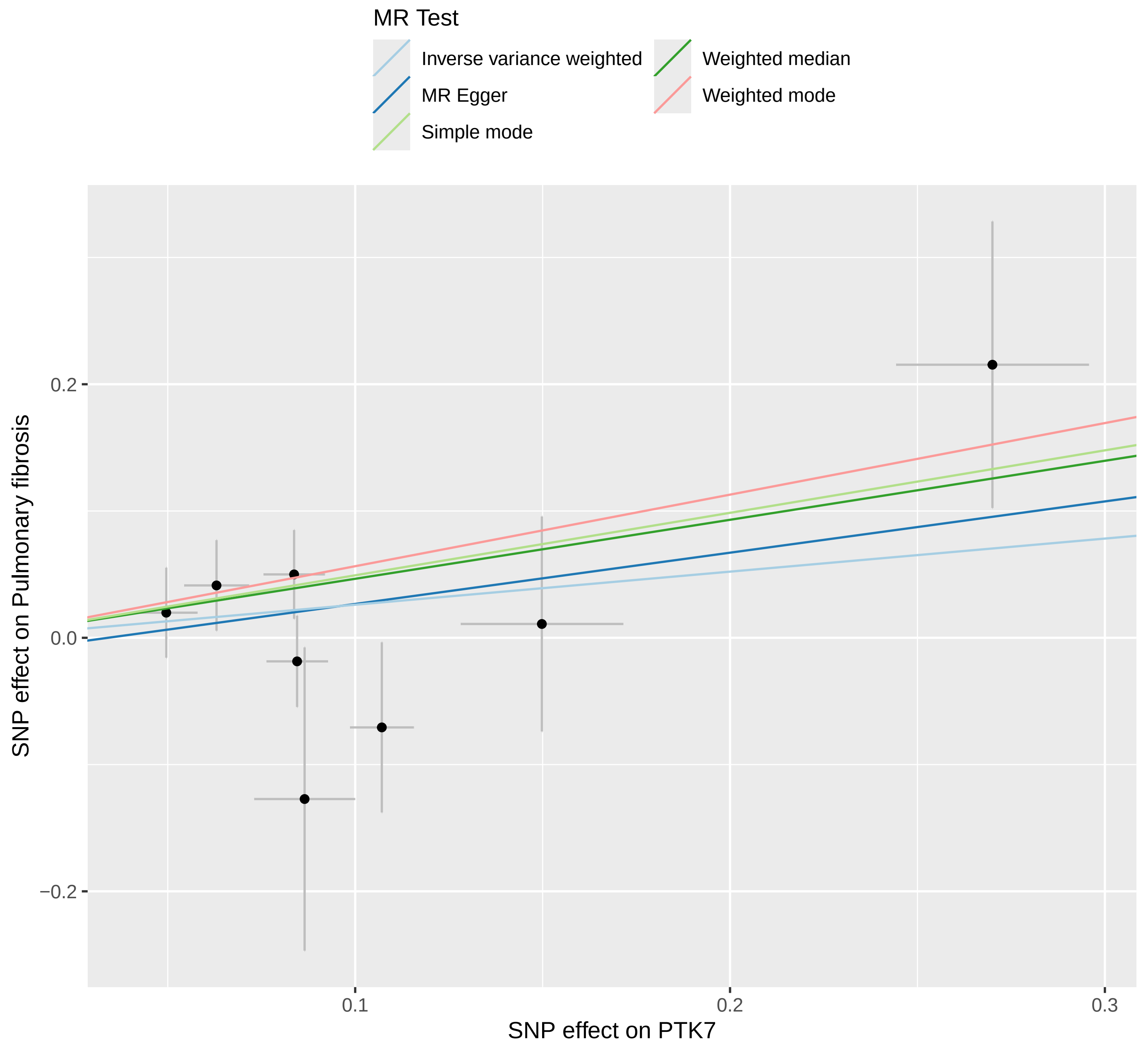
MR Test
Inverse variance weighted
MR Egger
Simple mode
Weighted median
Weighted mode
SNP effect on Pulmonary fibrosis
0.2
0.0
−0.2
0.1
0.2
0.3
SNP effect on PTK7

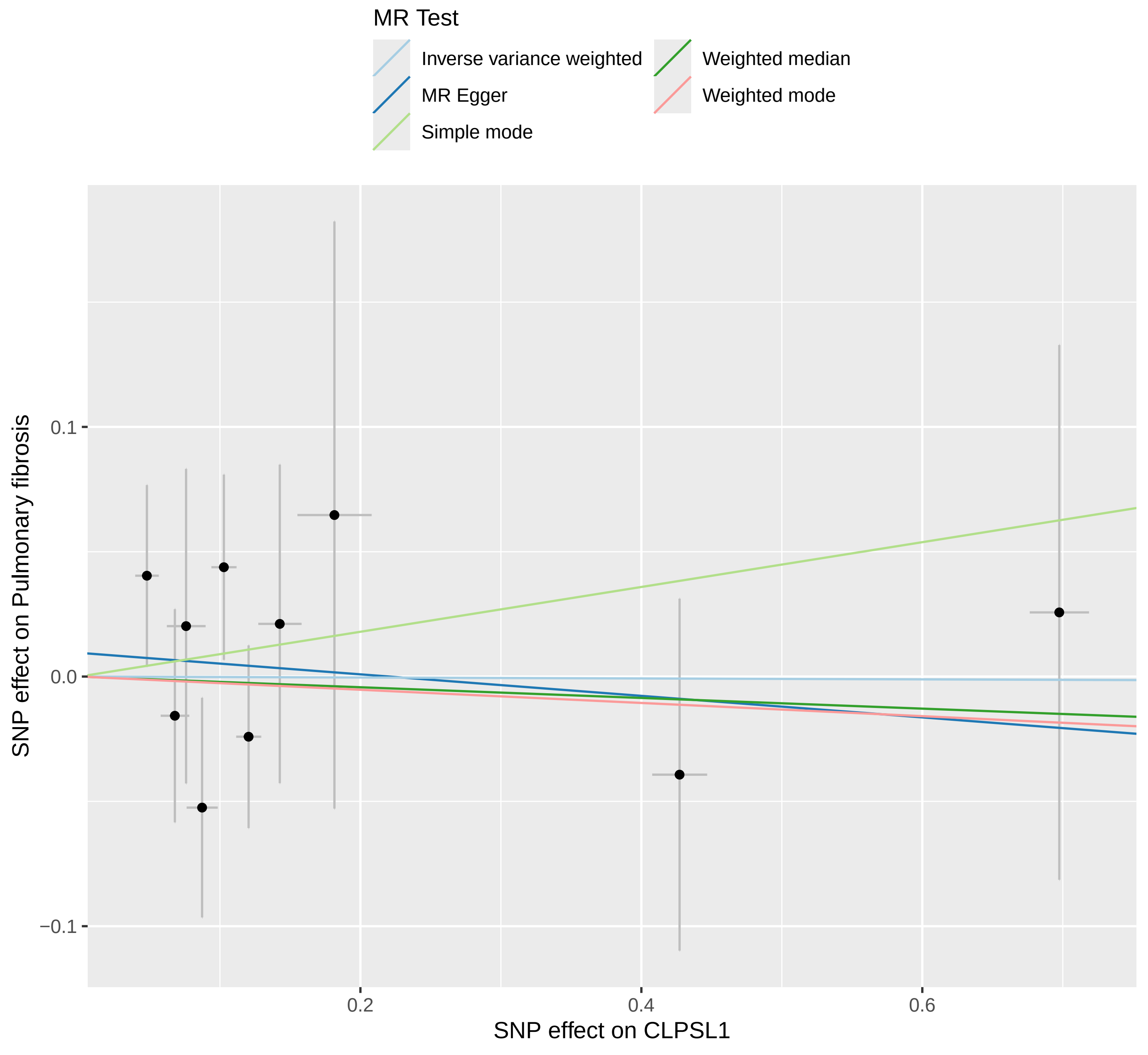

MR Test
Inverse variance weighted
MR Egger
Simple mode
Weighted median
Weighted mode
0.1
0.0
−0.1
0.2
0.4
0.6
SNP effect on Pulmonary fibrosis
SNP effect on CLPSL1

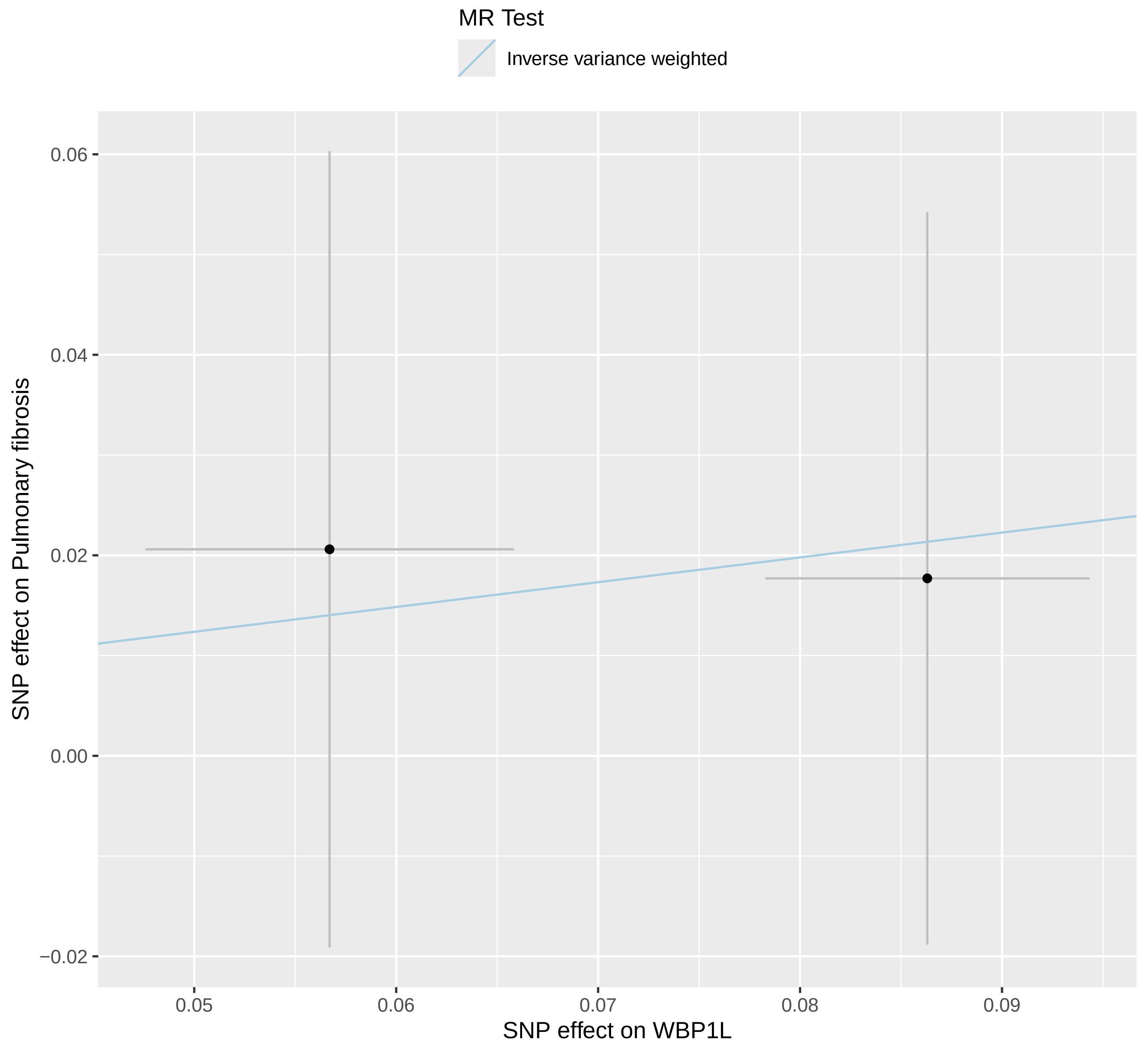

MR Test
Inverse variance weighted
0.06
0.04
0.02
0.00
−0.02
SNP effect on Pulmonary fibrosis
0.05
0.06
0.07
0.08
0.09
SNP effect on WBP1L

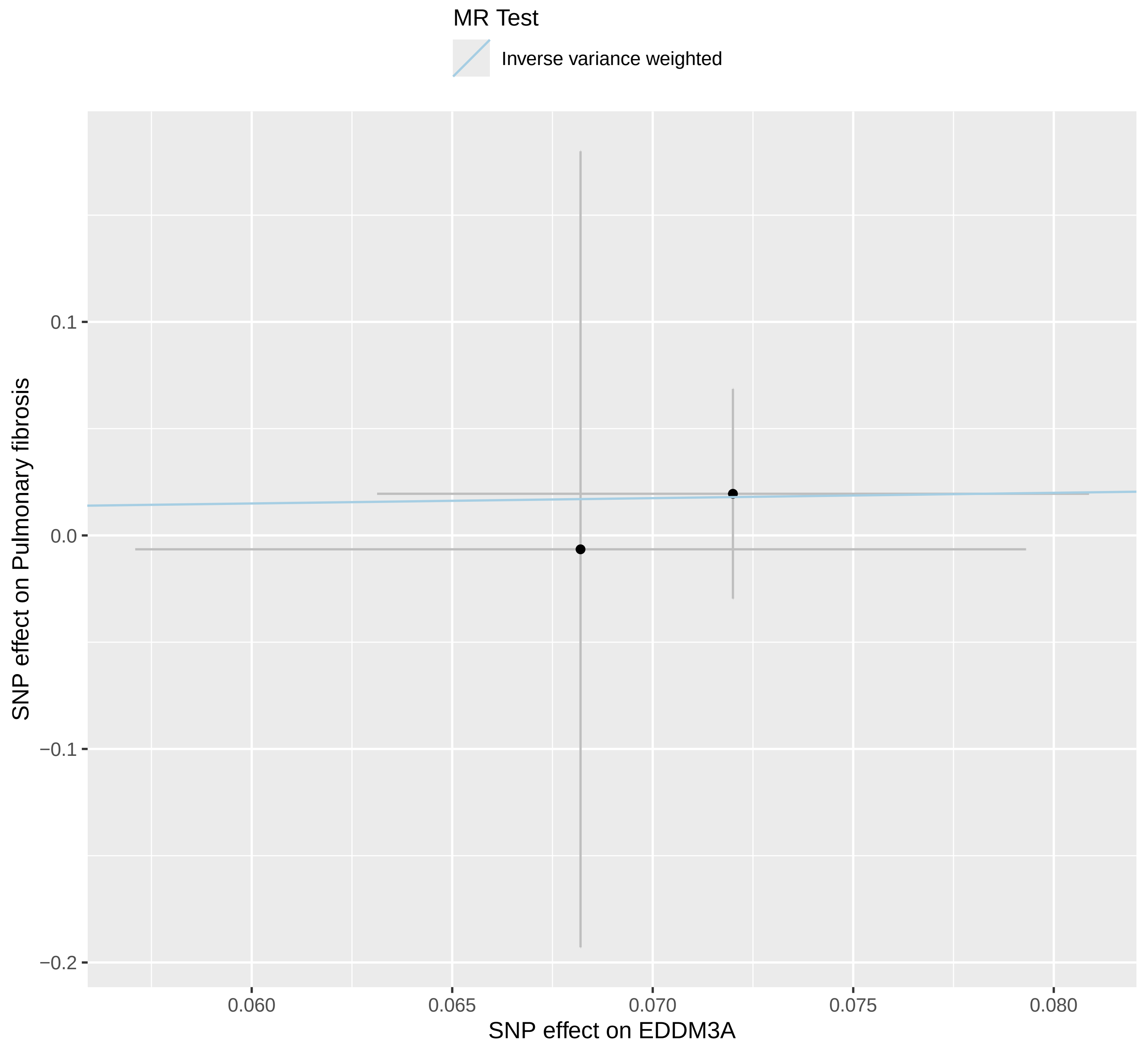
MR Test
Inverse variance weighted
0.1
0.0
−0.1
−0.2
SNP effect on Pulmonary fibrosis
0.060
0.065
0.070
0.075
0.080
SNP effect on EDDM3A

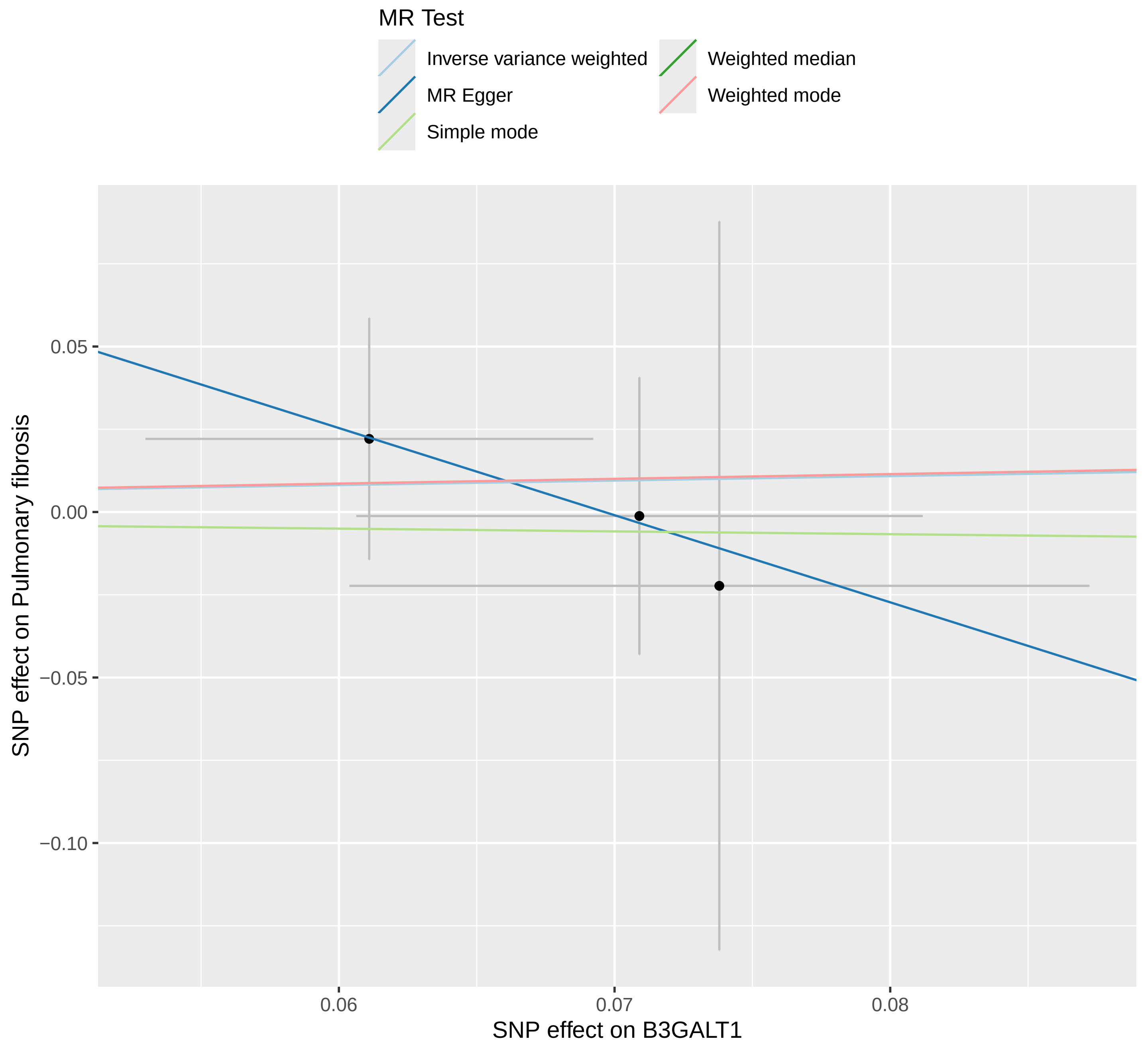

MR Test
Inverse variance weighted
MR Egger
Simple mode
Weighted median
Weighted mode
0.05
0.00
−0.05
−0.10
0.06
0.07
0.08
SNP effect on Pulmonary fibrosis
SNP effect on B3GALT1

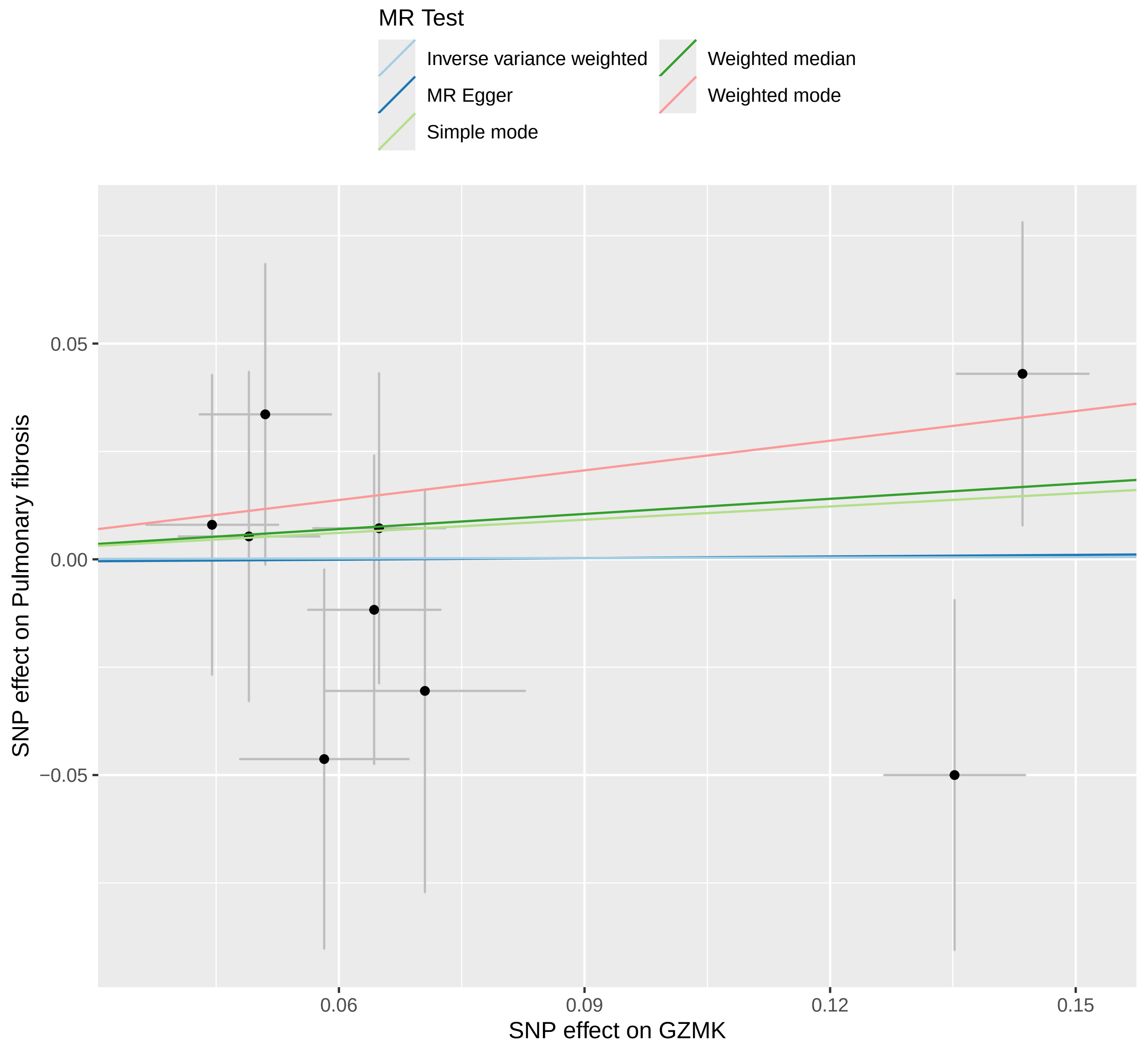
MR Test
Inverse variance weighted
MR Egger
Simple mode
Weighted median
Weighted mode
SNP effect on Pulmonary fibrosis
0.05
0.00
−0.05
0.06
0.09
0.12
0.15
SNP effect on GZMK

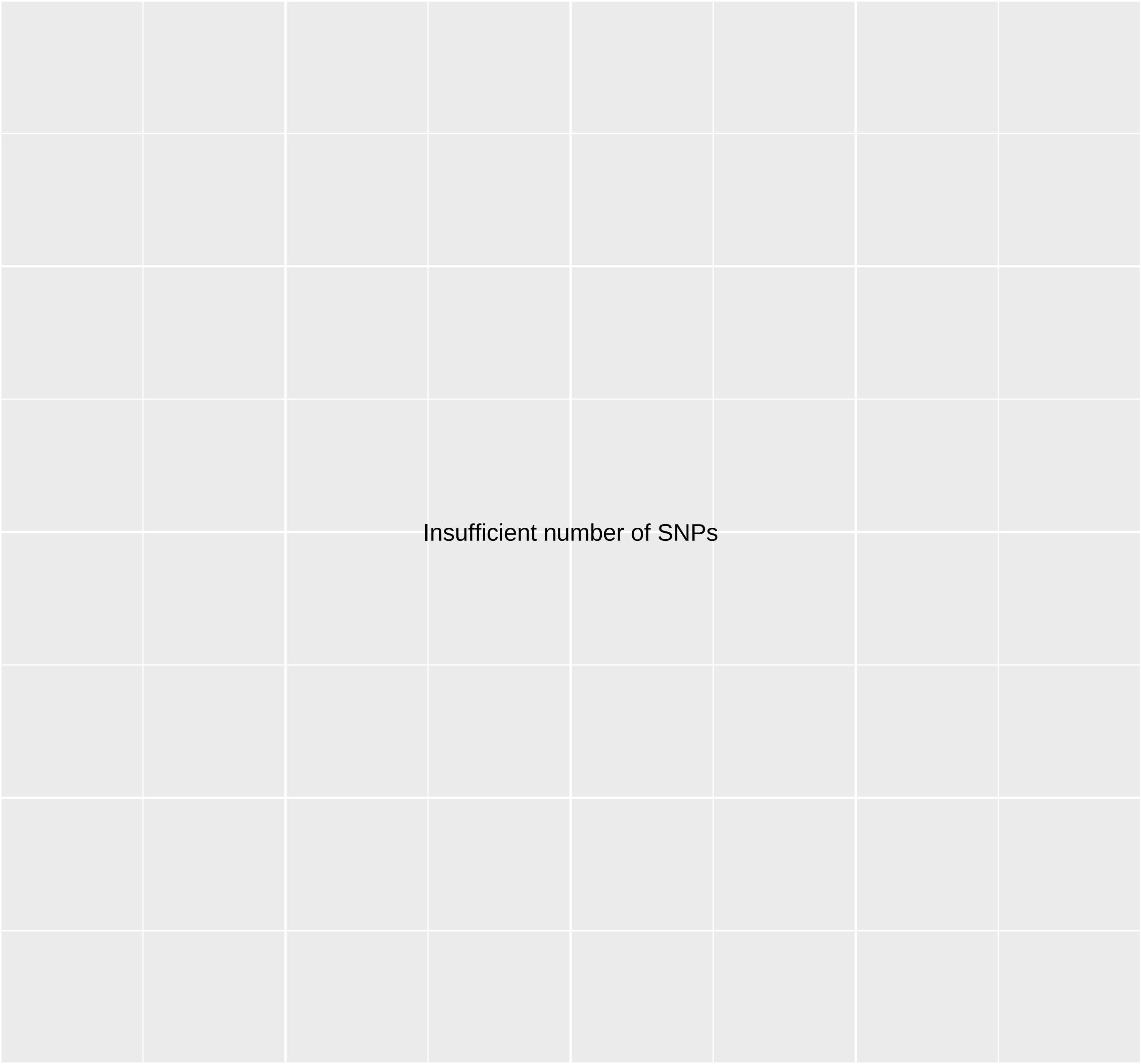
Insufficient number of SNPs

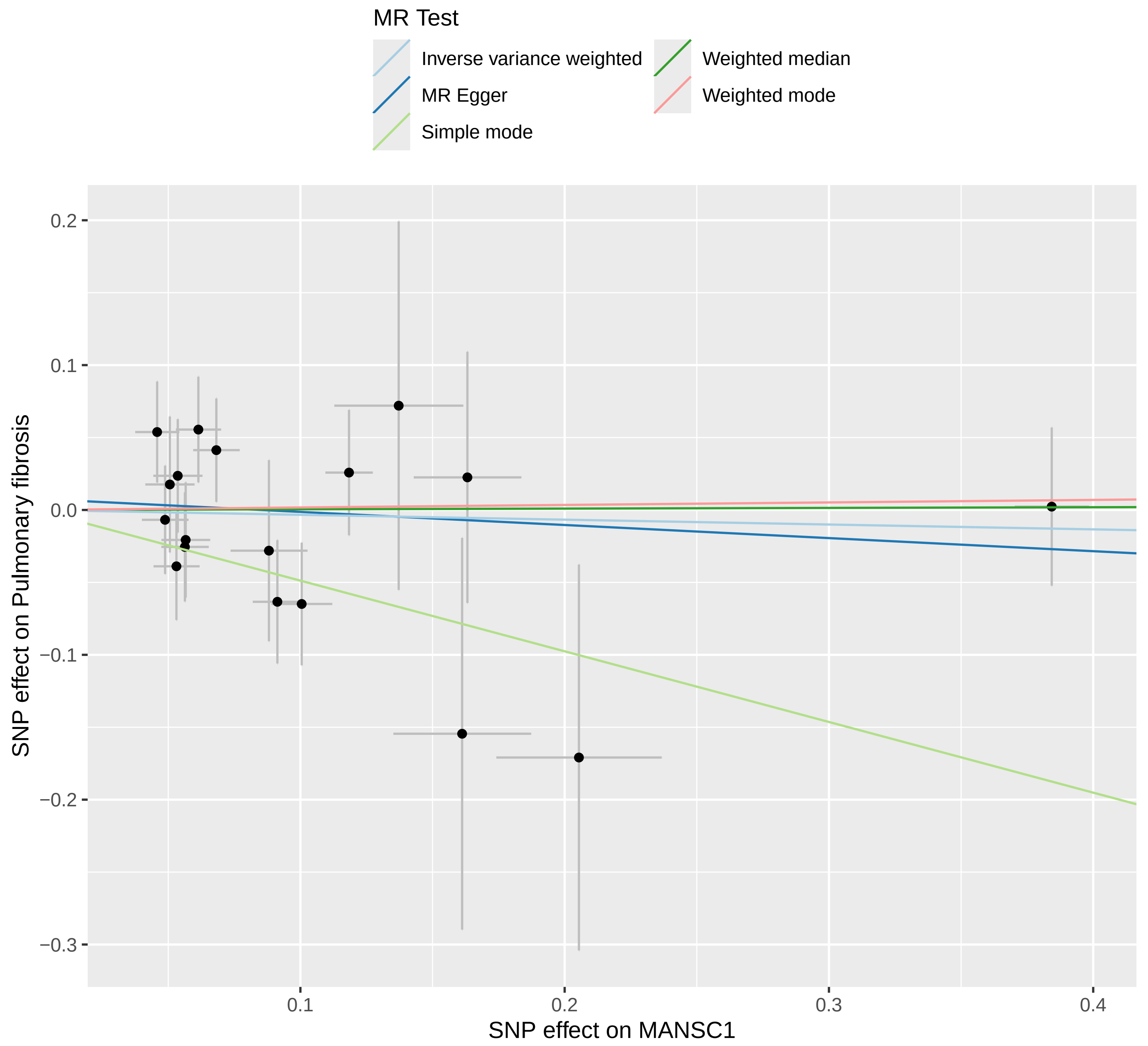

MR Test
Inverse variance weighted
Weighted median
MR Egger
Weighted mode
Simple mode
0.2
0.1
0.0
−0.1
−0.2
−0.3
SNP effect on Pulmonary fibrosis
0.1
0.2
0.3
0.4
SNP effect on MANSC1

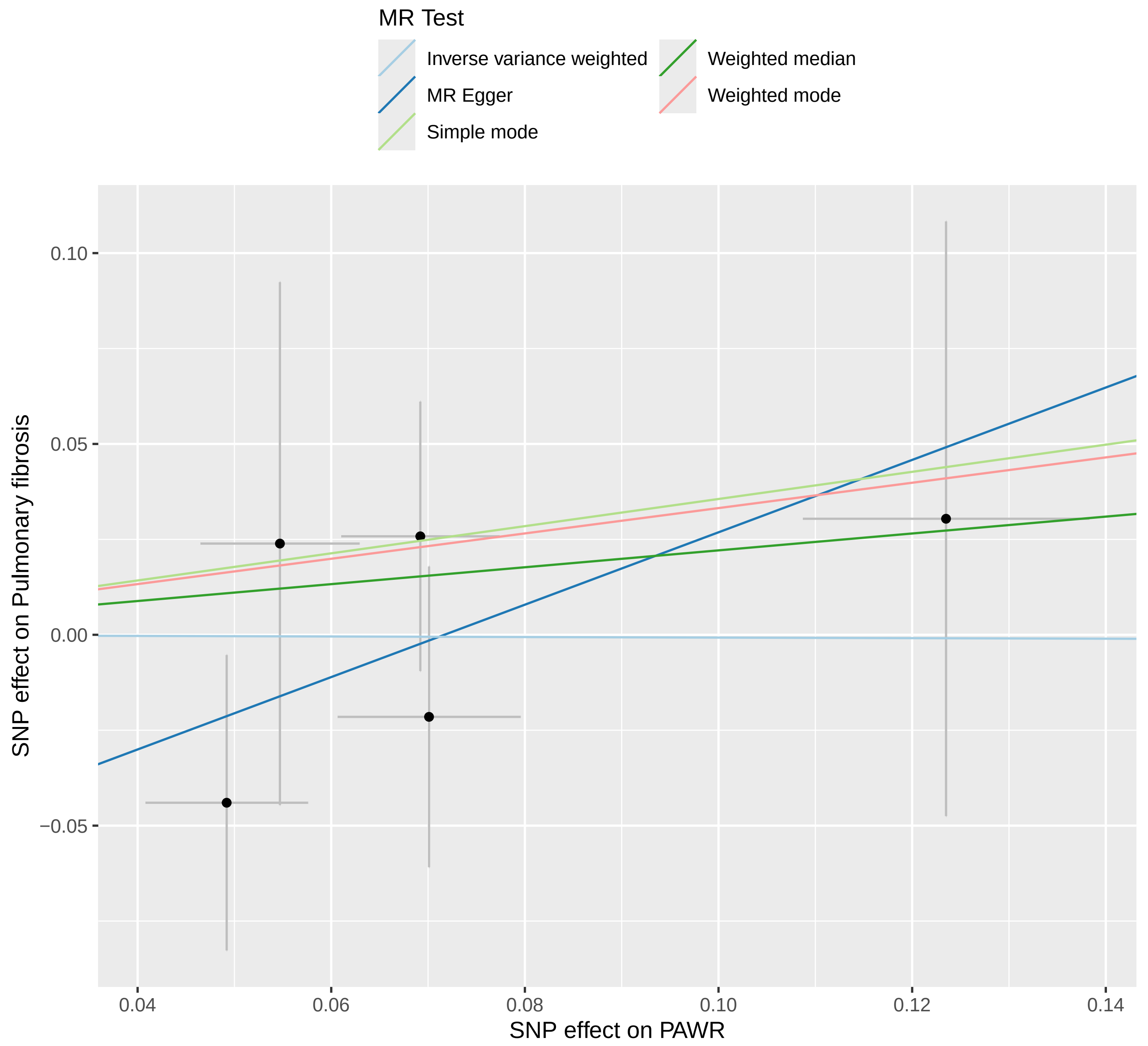
MR Test
Inverse variance weighted
MR Egger
Simple mode
Weighted median
Weighted mode
SNP effect on Pulmonary fibrosis
0.10
0.05
0.00
−0.05
0.04
0.06
0.08
0.10
0.12
0.14
SNP effect on PAWR

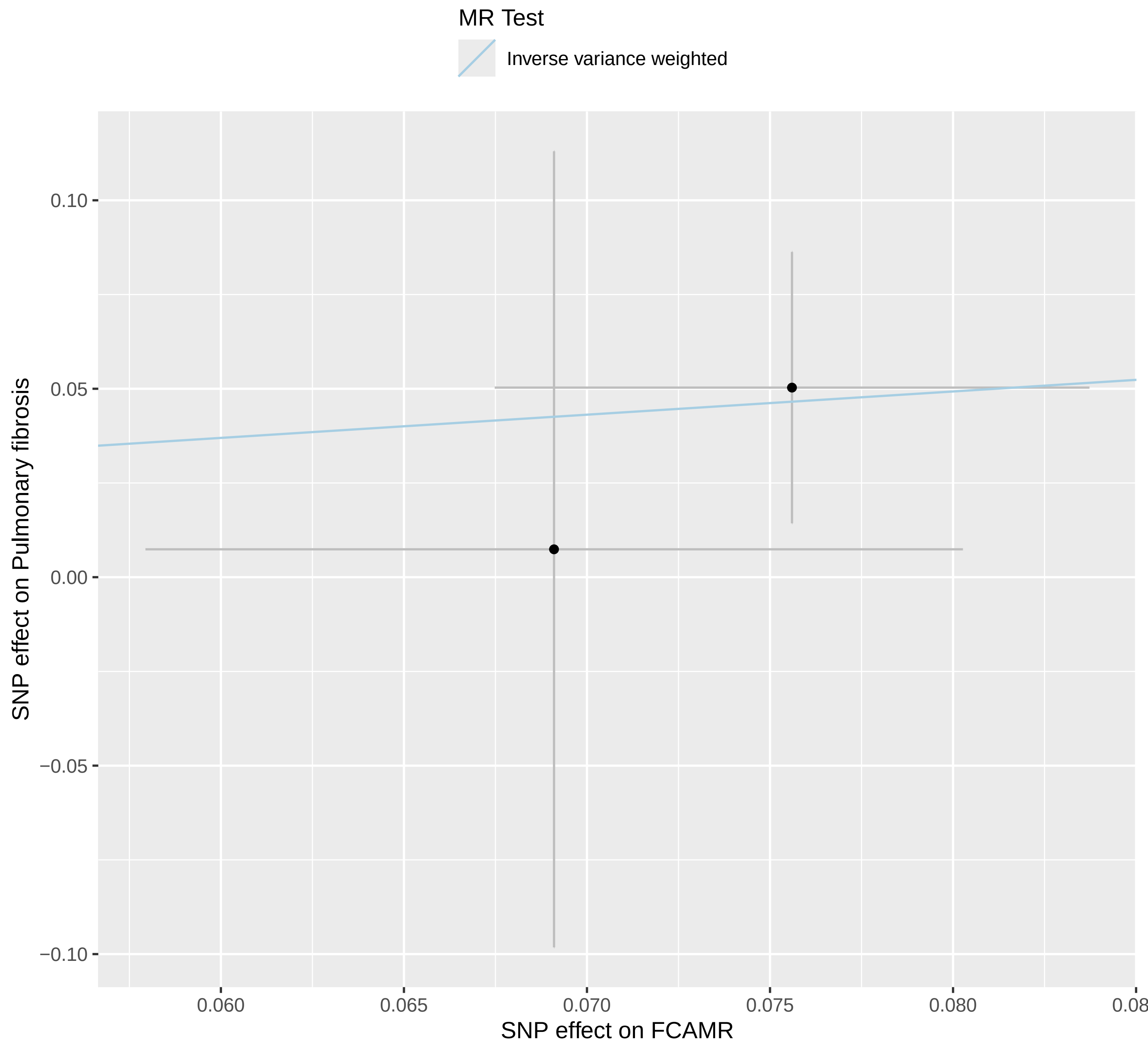

MR Test
Inverse variance weighted
0.10
0.05
0.00
−0.05
−0.10
SNP effect on Pulmonary fibrosis
0.060
0.065
0.070
0.075
0.080
SNP effect on FCAMR

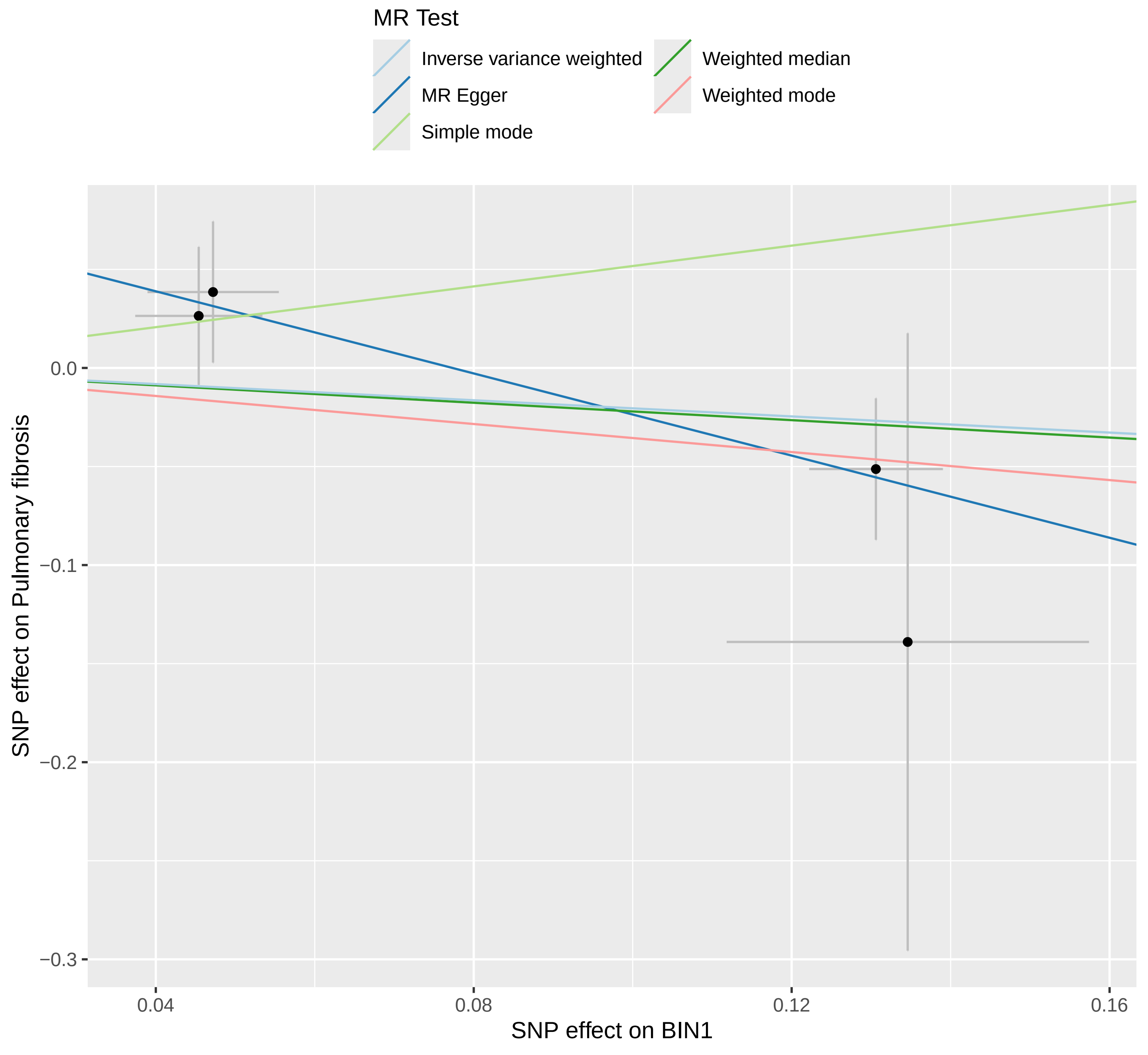

MR Test
Inverse variance weighted
MR Egger
Simple mode
Weighted median
Weighted mode
0.0
−0.1
−0.2
−0.3
SNP effect on Pulmonary fibrosis
0.04
0.08
0.12
0.16
SNP effect on BIN1

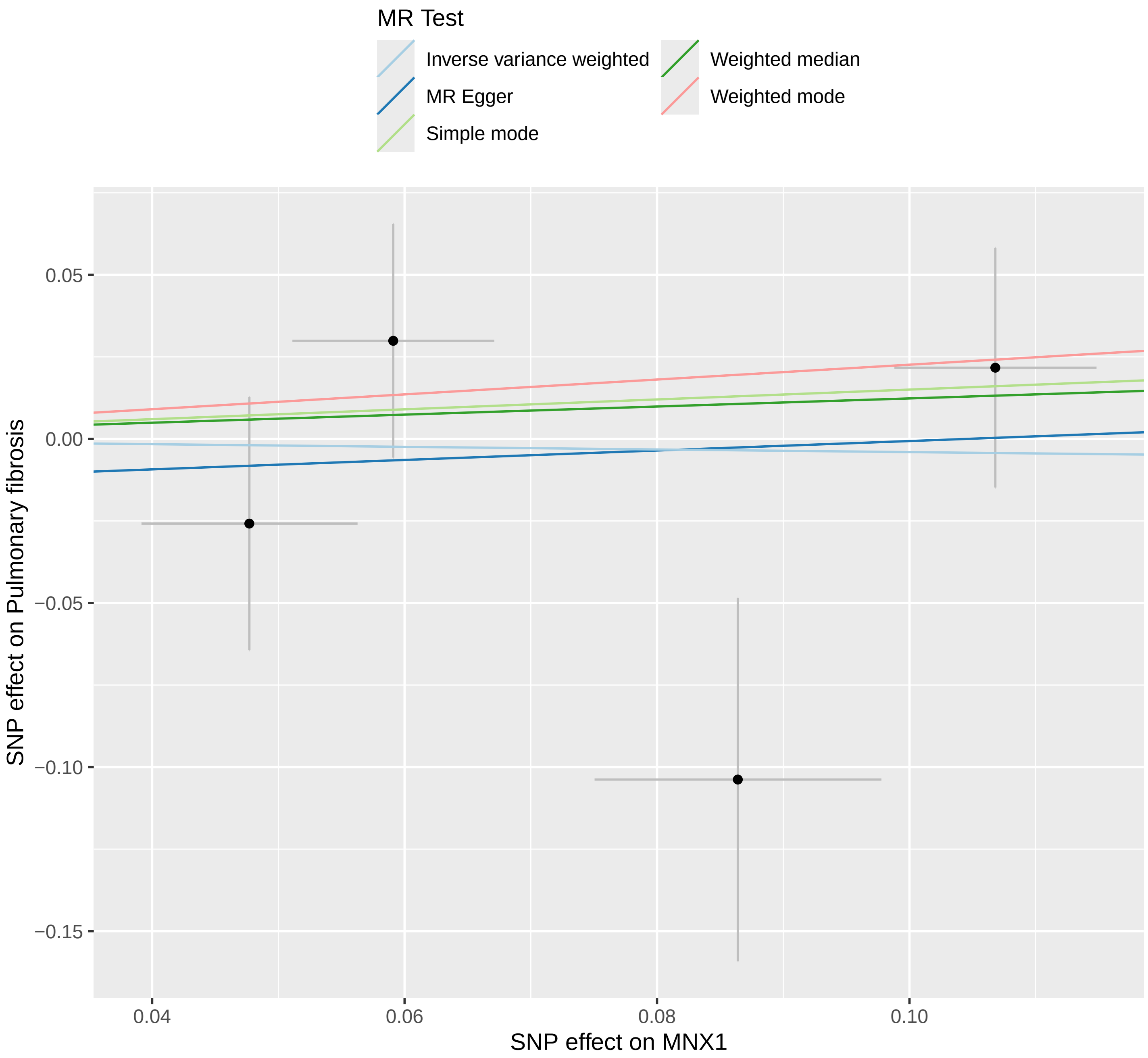

MR Test
Inverse variance weighted
MR Egger
Simple mode
Weighted median
Weighted mode
0.05
0.00
−0.05
−0.10
−0.15
SNP effect on Pulmonary fibrosis
0.04
0.06
0.08
0.10
SNP effect on MNX1

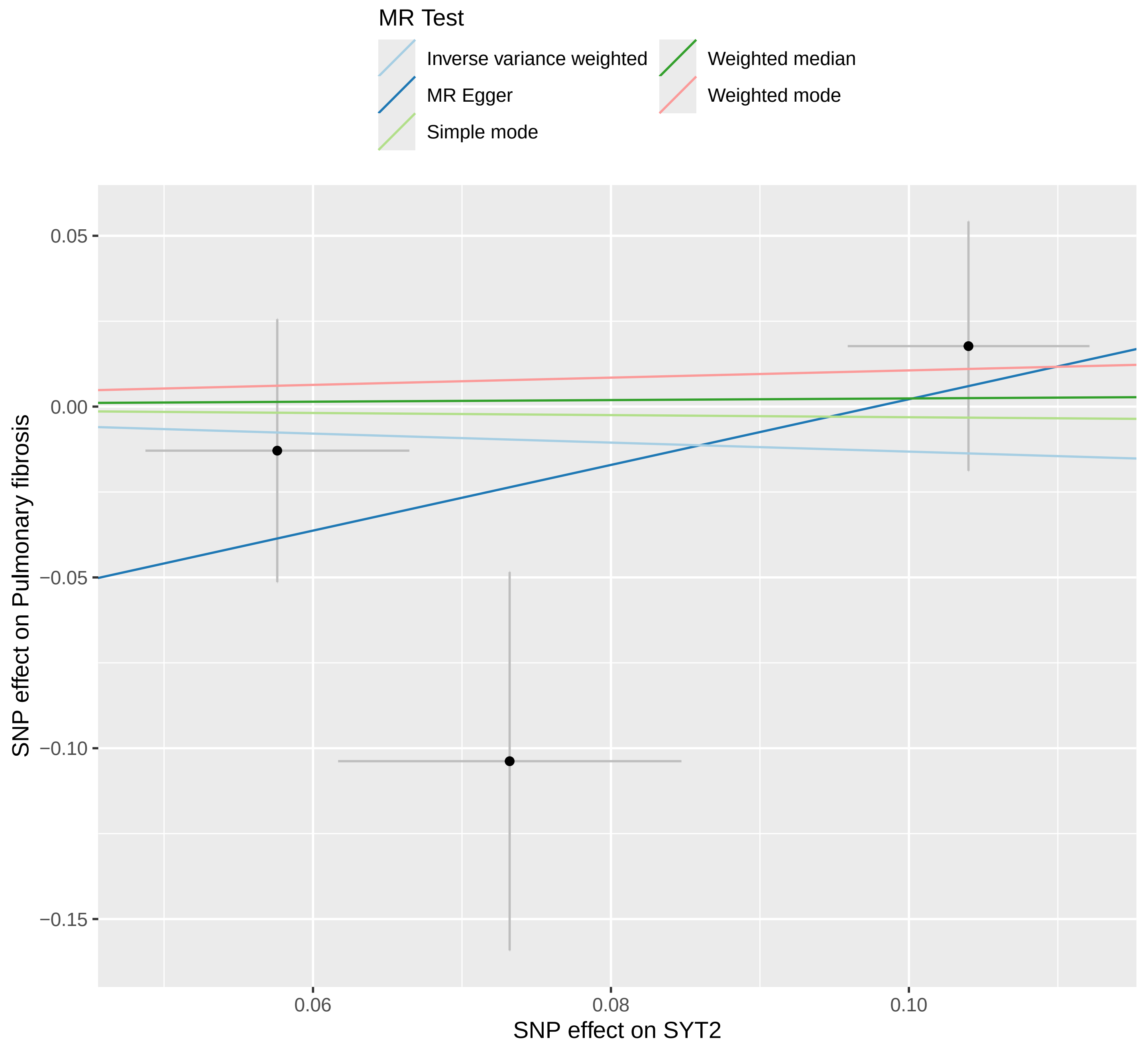
MR Test
Inverse variance weighted
MR Egger
Simple mode
Weighted median
Weighted mode
0.05
0.00
−0.05
−0.10
−0.15
SNP effect on Pulmonary fibrosis
0.06
0.08
0.10
SNP effect on SYT2

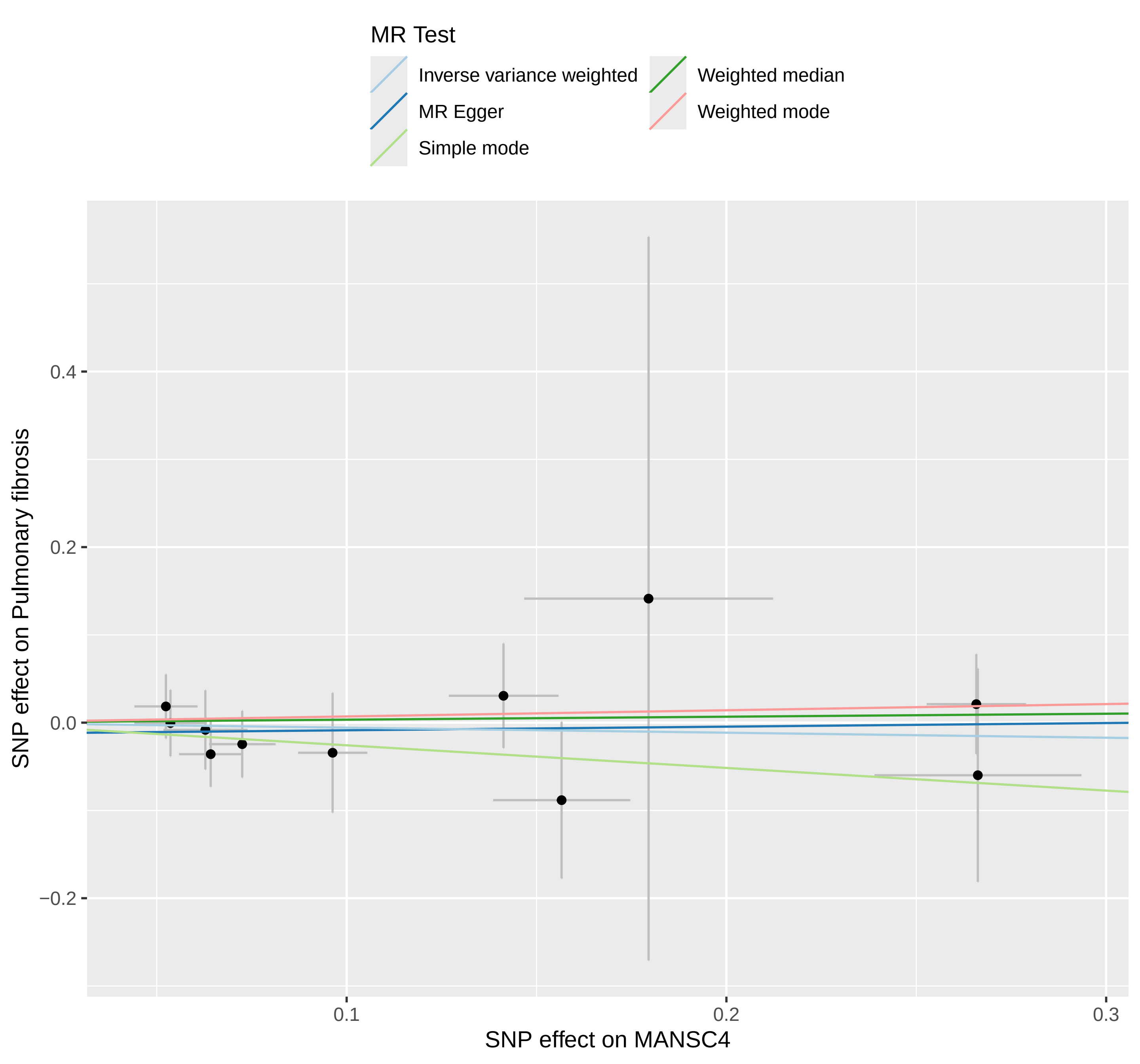

MR Test
Inverse variance weighted
MR Egger
Simple mode
Weighted median
Weighted mode
0.4
0.2
0.0
−0.2
0.1
0.2
0.3
SNP effect on Pulmonary fibrosis
SNP effect on MANSC4

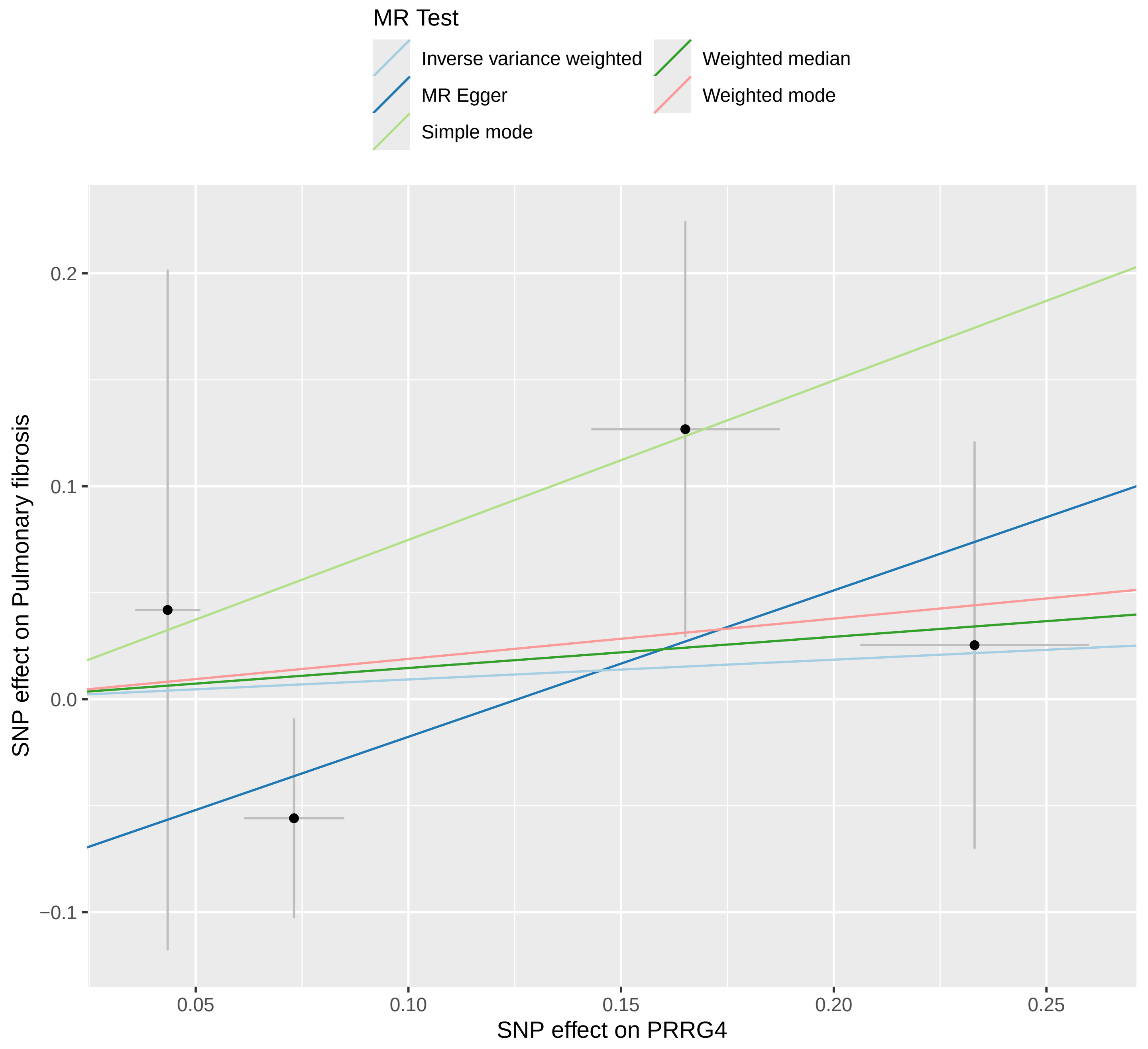
MR Test
Inverse variance weighted
MR Egger
Simple mode
Weighted median
Weighted mode
SNP effect on Pulmonary fibrosis
0.2
0.1
0.0
−0.1
0.05
0.10
0.15
0.20
0.25
SNP effect on PRRG4

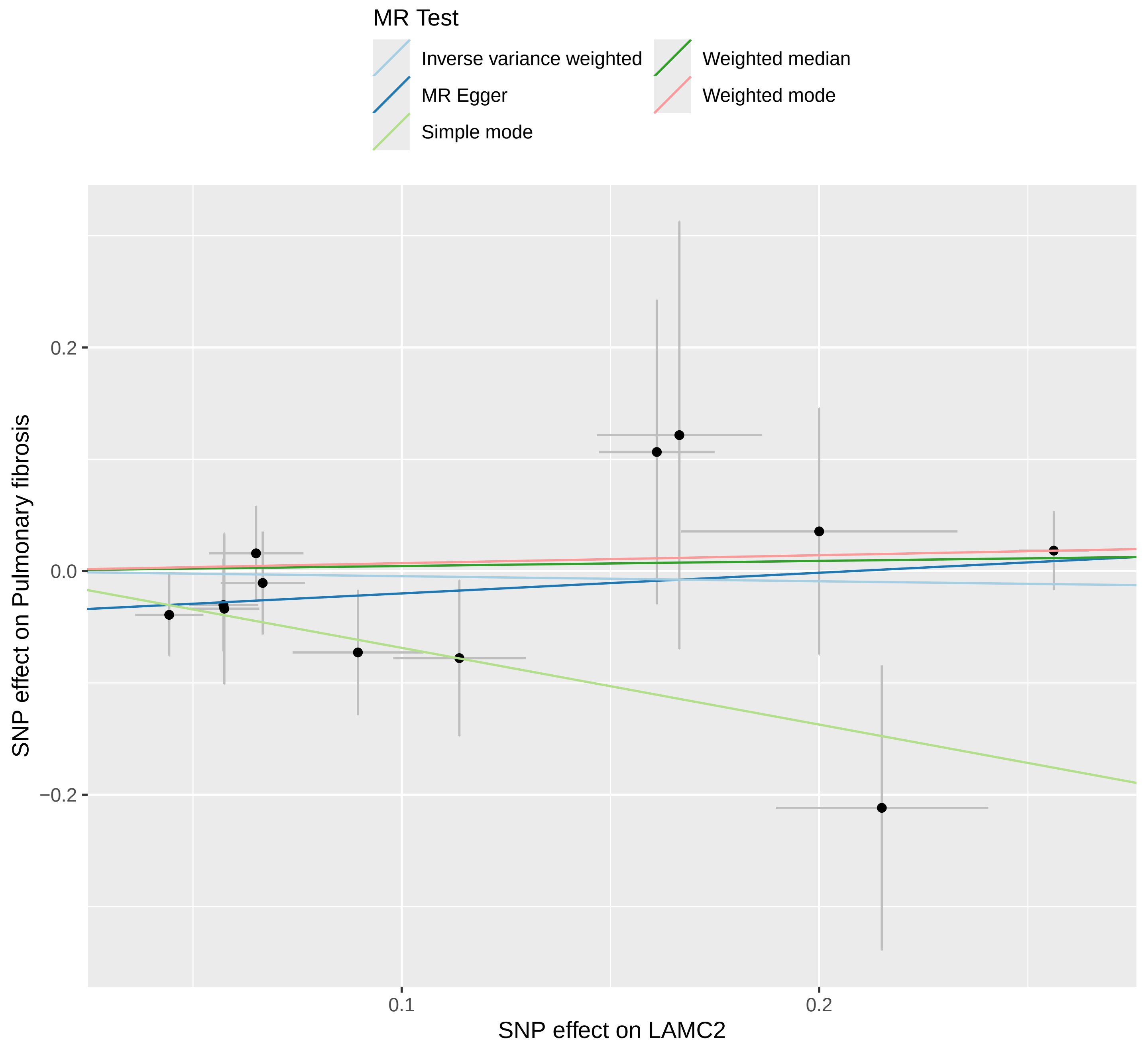

MR Test
Inverse variance weighted
MR Egger
Simple mode
Weighted median
Weighted mode
0.2
0.0
−0.2
SNP effect on Pulmonary fibrosis
0.1
0.2
SNP effect on LAMC2

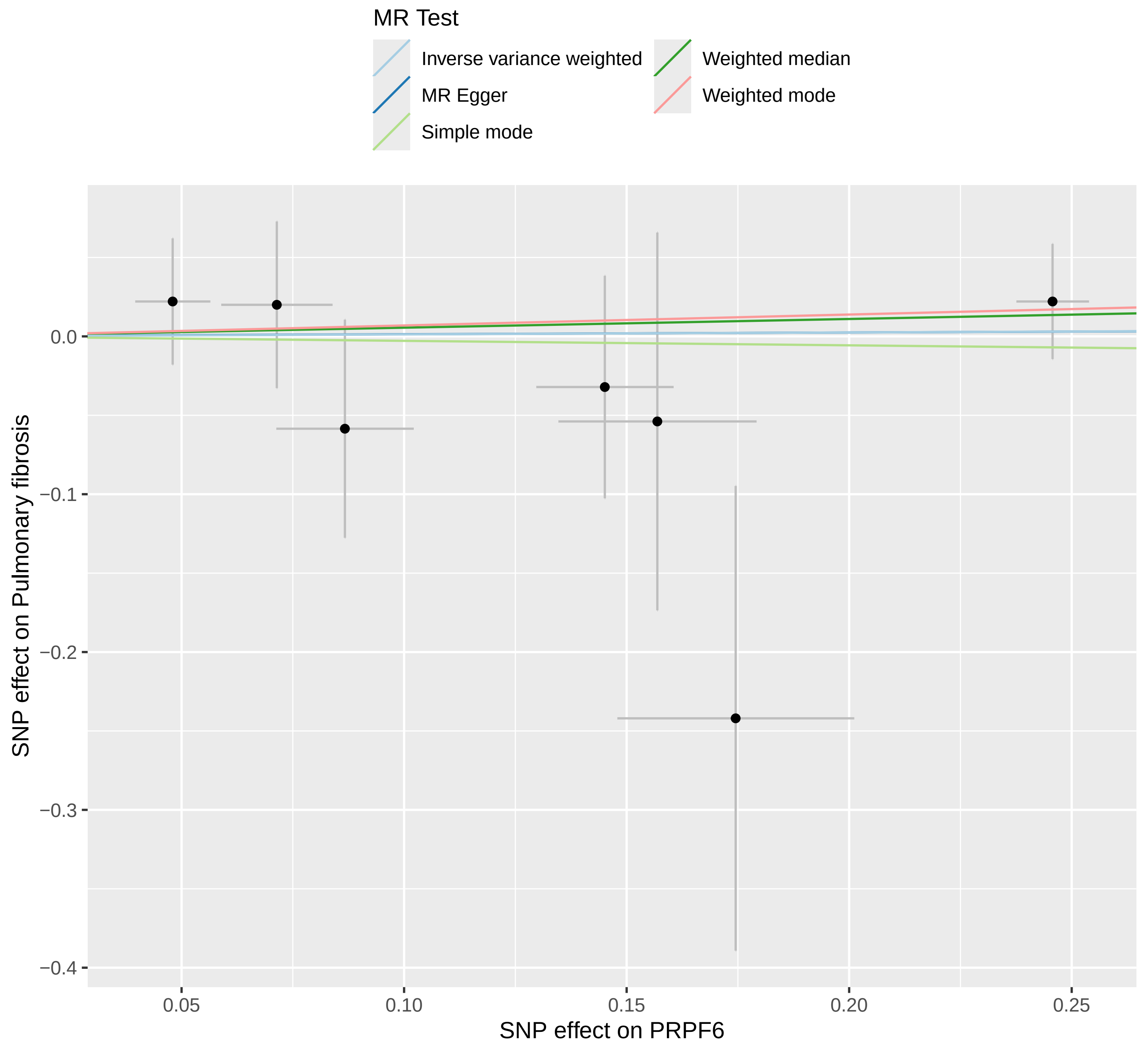
MR Test
Inverse variance weighted
MR Egger
Simple mode
Weighted median
Weighted mode
0.0
−0.1
−0.2
−0.3
−0.4
0.05
0.10
0.15
0.20
0.25
SNP effect on Pulmonary fibrosis
SNP effect on PRPF6

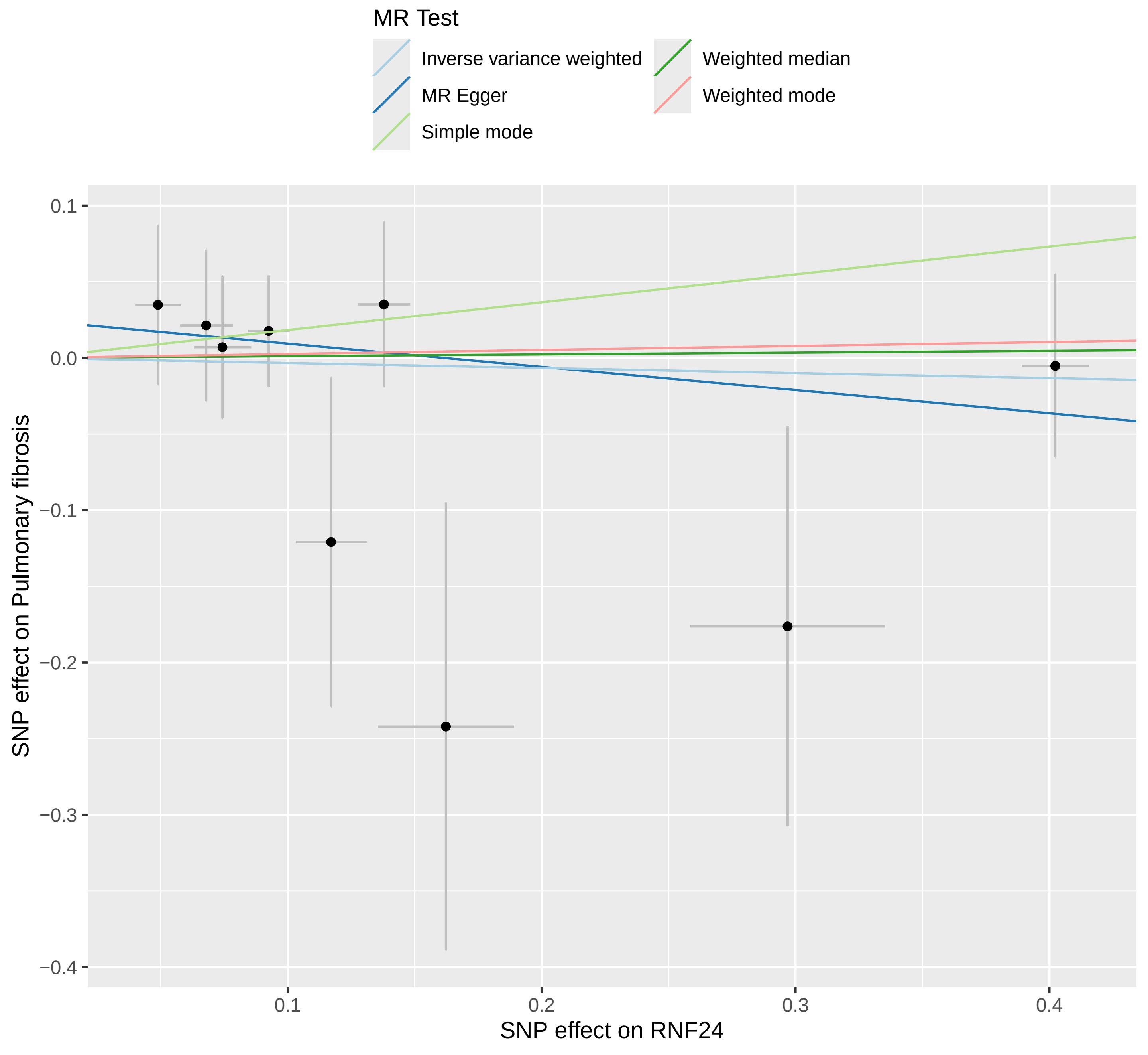
MR Test
Inverse variance weighted
MR Egger
Simple mode
Weighted median
Weighted mode
0.1
0.0
−0.1
−0.2
−0.3
−0.4
SNP effect on Pulmonary fibrosis
0.1
0.2
0.3
0.4
SNP effect on RNF24

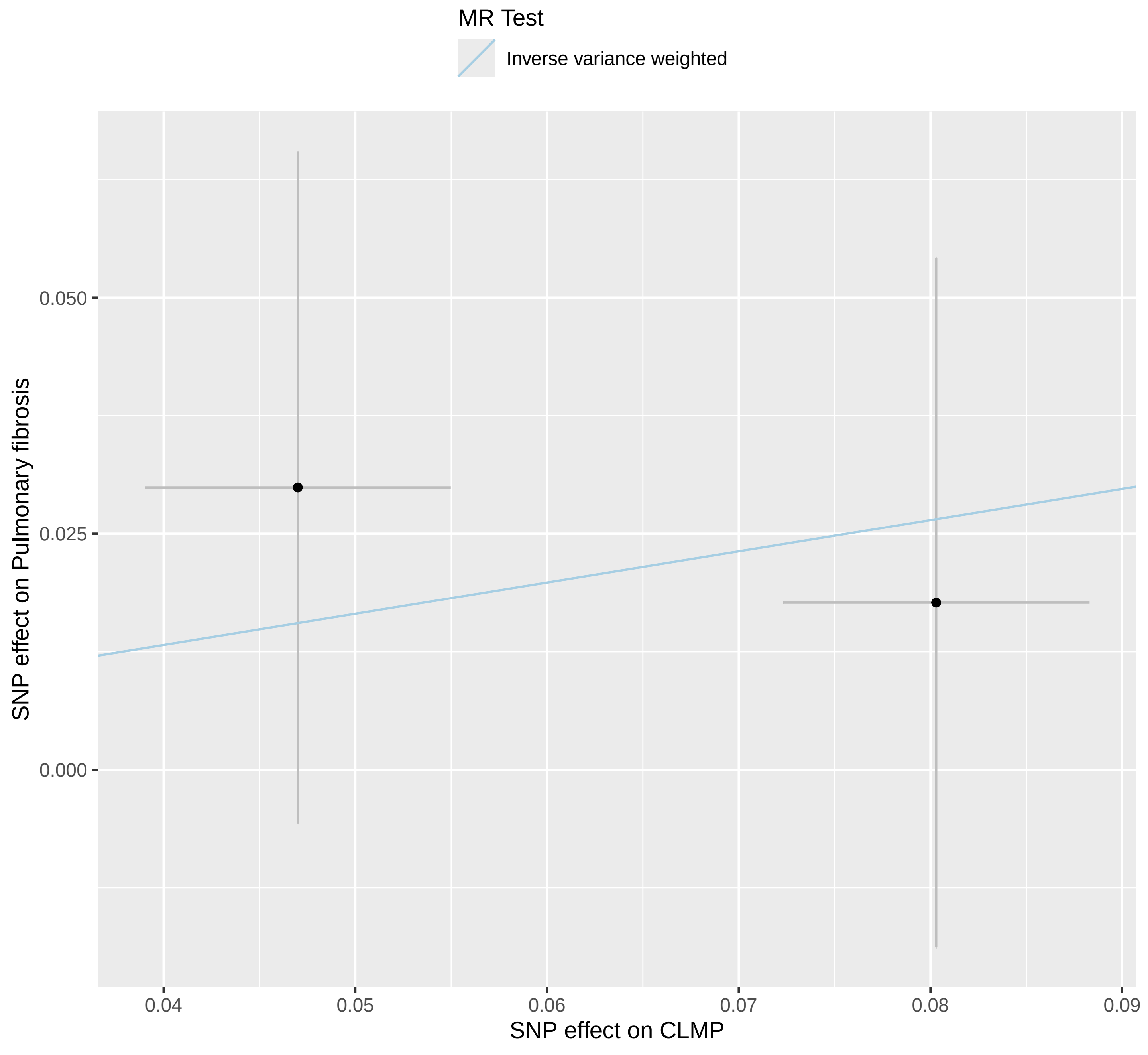
MR Test
Inverse variance weighted
0.050
0.025
0.000
0.04
0.05
0.06
0.07
0.08
0.09
SNP effect on CLMP
SNP effect on Pulmonary fibrosis

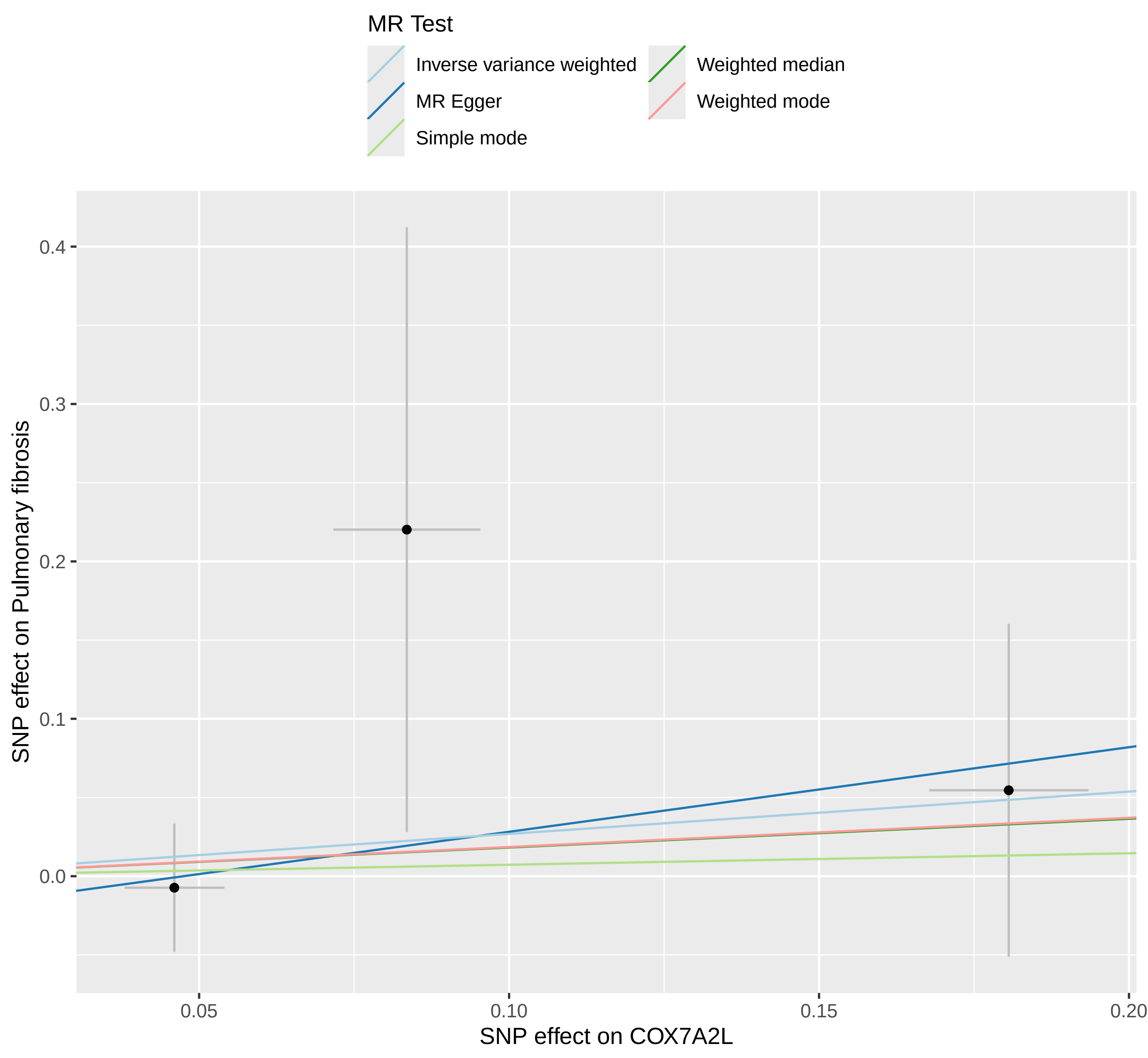
MR Test
Inverse variance weighted
MR Egger
Simple mode
Weighted median
Weighted mode
0.4
0.3
0.2
0.1
0.0
0.05
0.10
0.15
0.20
SNP effect on COX7A2L
SNP effect on Pulmonary fibrosis

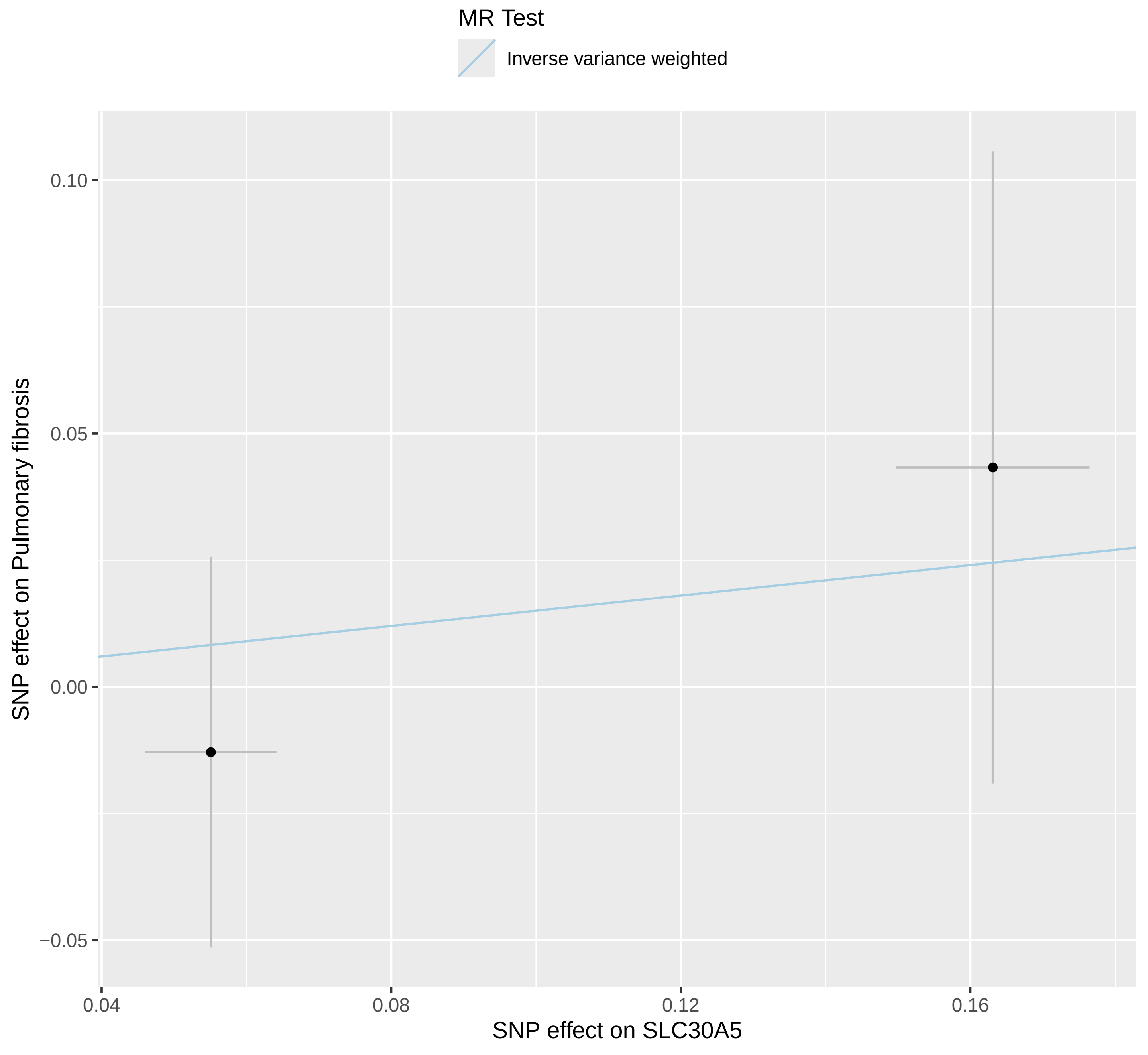

MR Test
Inverse variance weighted
SNP effect on Pulmonary fibrosis
0.10
0.05
0.00
−0.05
0.04
0.08
0.12
0.16
SNP effect on SLC30A5

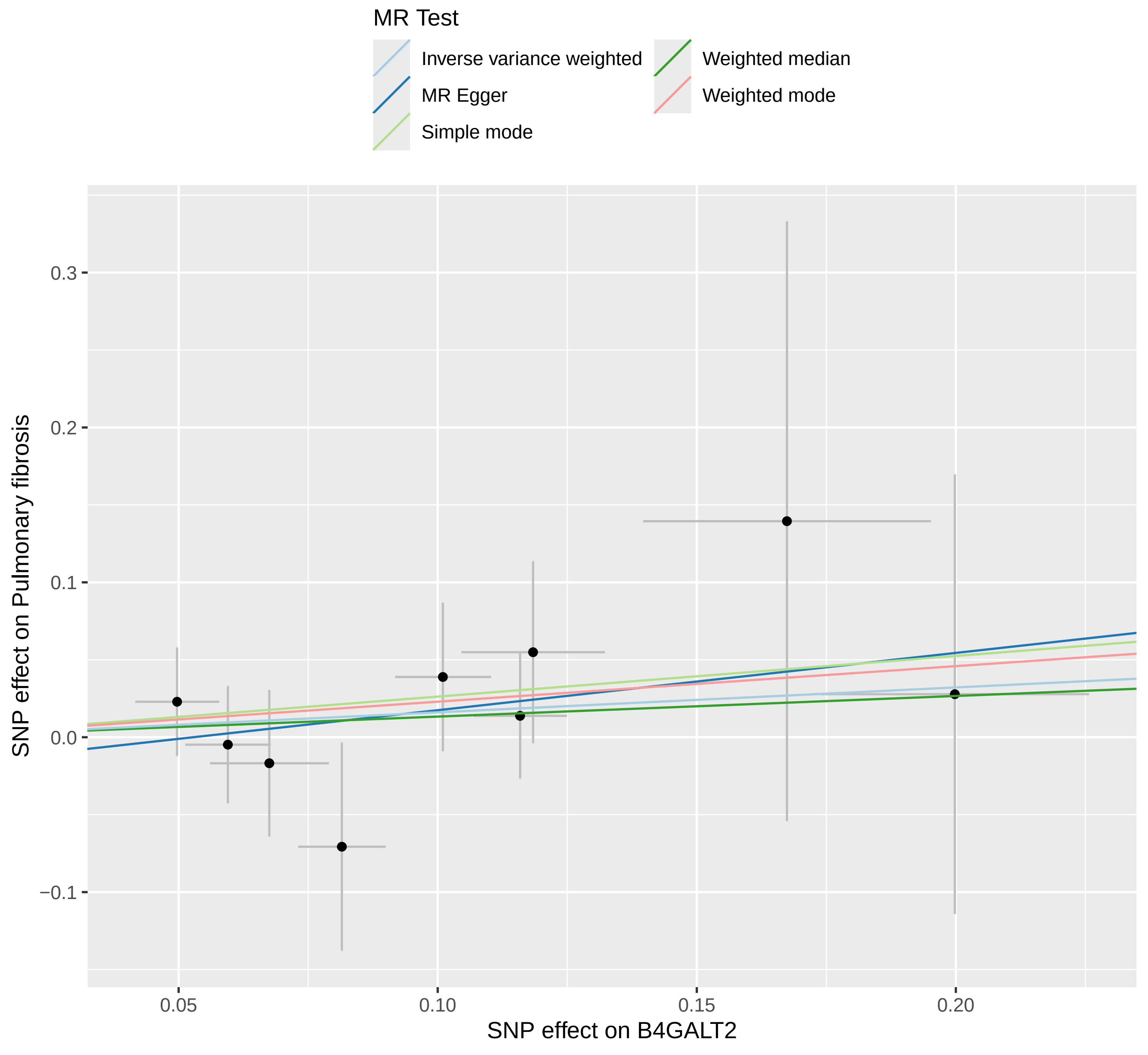

MR Test
Inverse variance weighted
MR Egger
Simple mode
Weighted median
Weighted mode
SNP effect on Pulmonary fibrosis
0.3
0.2
0.1
0.0
−0.1
0.05
0.10
0.15
0.20
SNP effect on B4GALT2

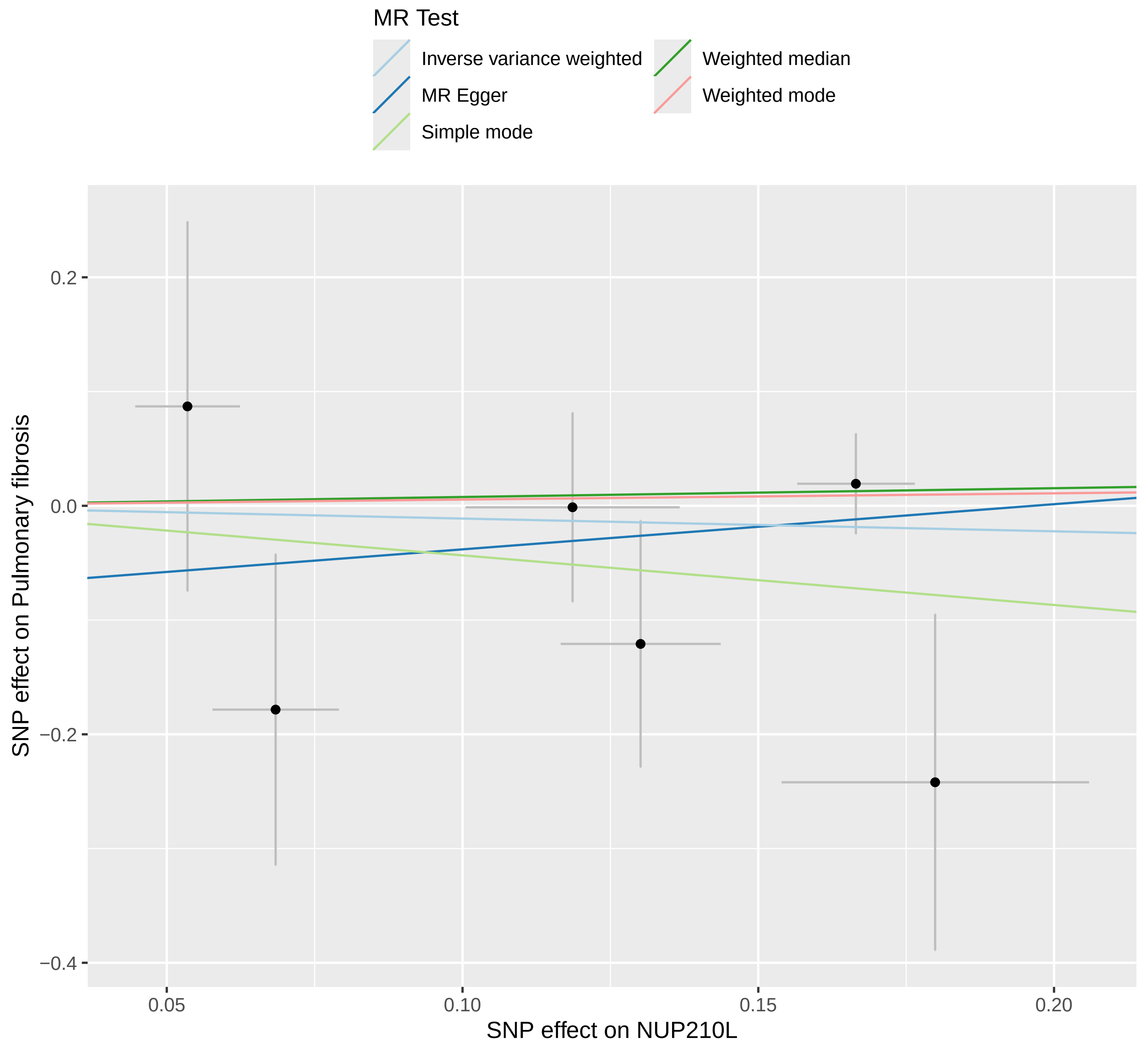
MR Test
Inverse variance weighted
MR Egger
Simple mode
Weighted median
Weighted mode
0.2
0.0
−0.2
−0.4
SNP effect on Pulmonary fibrosis
0.05
0.10
0.15
0.20
SNP effect on NUP210L

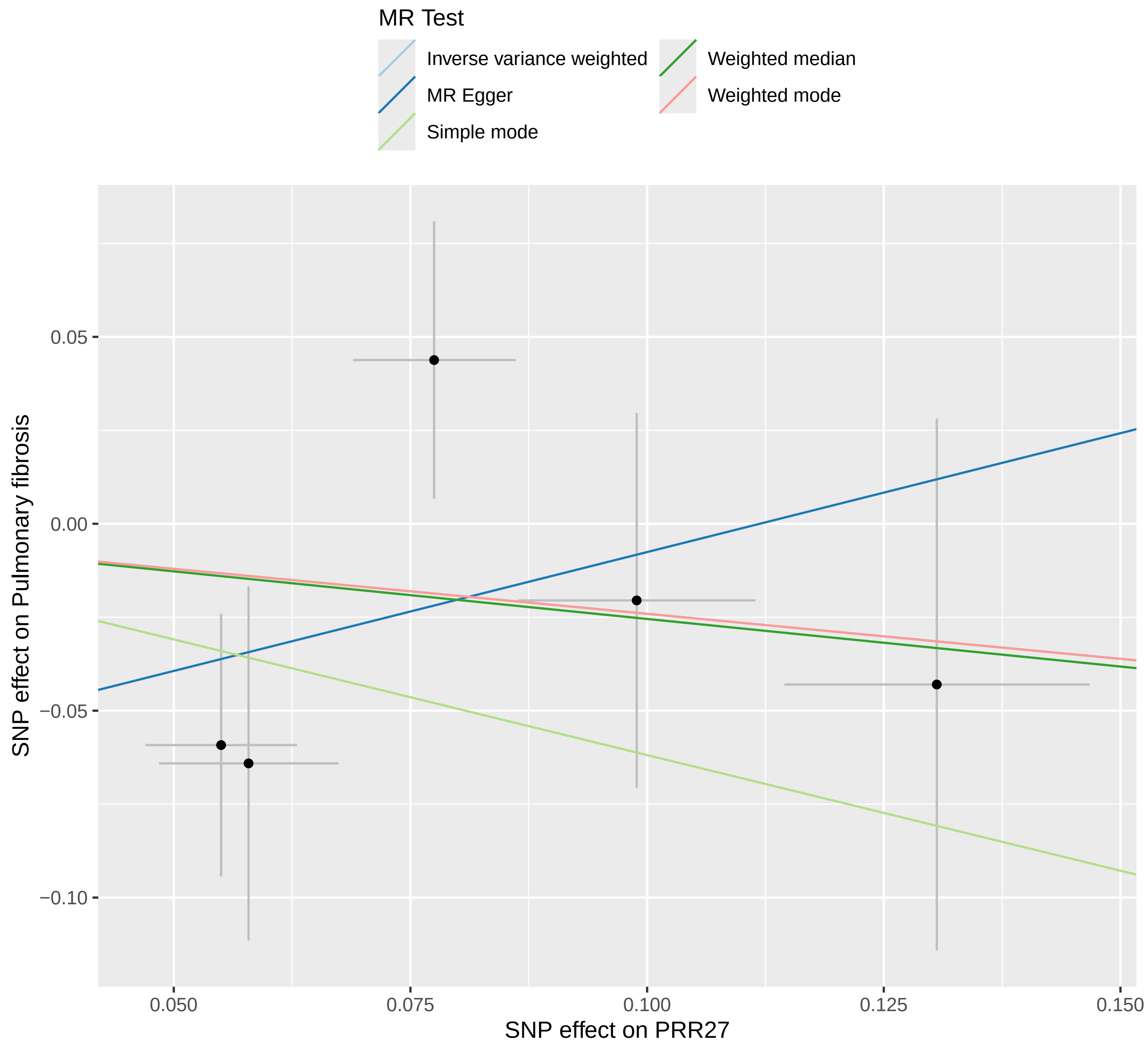

MR Test
Inverse variance weighted
MR Egger
Simple mode
Weighted median
Weighted mode
0.05
0.00
−0.05
−0.10
SNP effect on Pulmonary fibrosis
0.050
0.075
0.100
0.125
0.150
SNP effect on PRR27

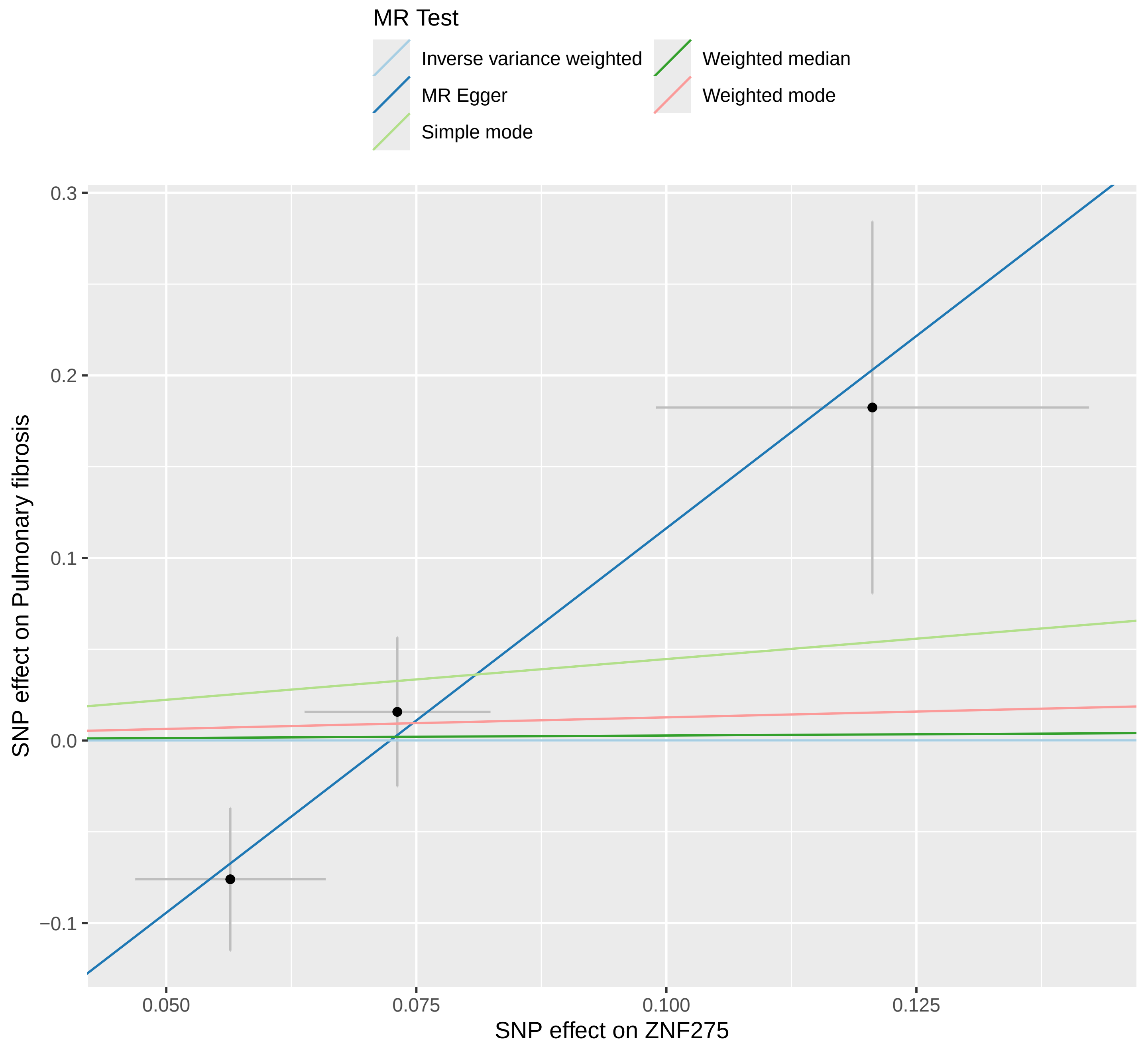

MR Test
Inverse variance weighted
MR Egger
Simple mode
Weighted median
Weighted mode
0.3
0.2
0.1
0.0
−0.1
0.050
0.075
0.100
0.125
SNP effect on ZNF275
SNP effect on Pulmonary fibrosis

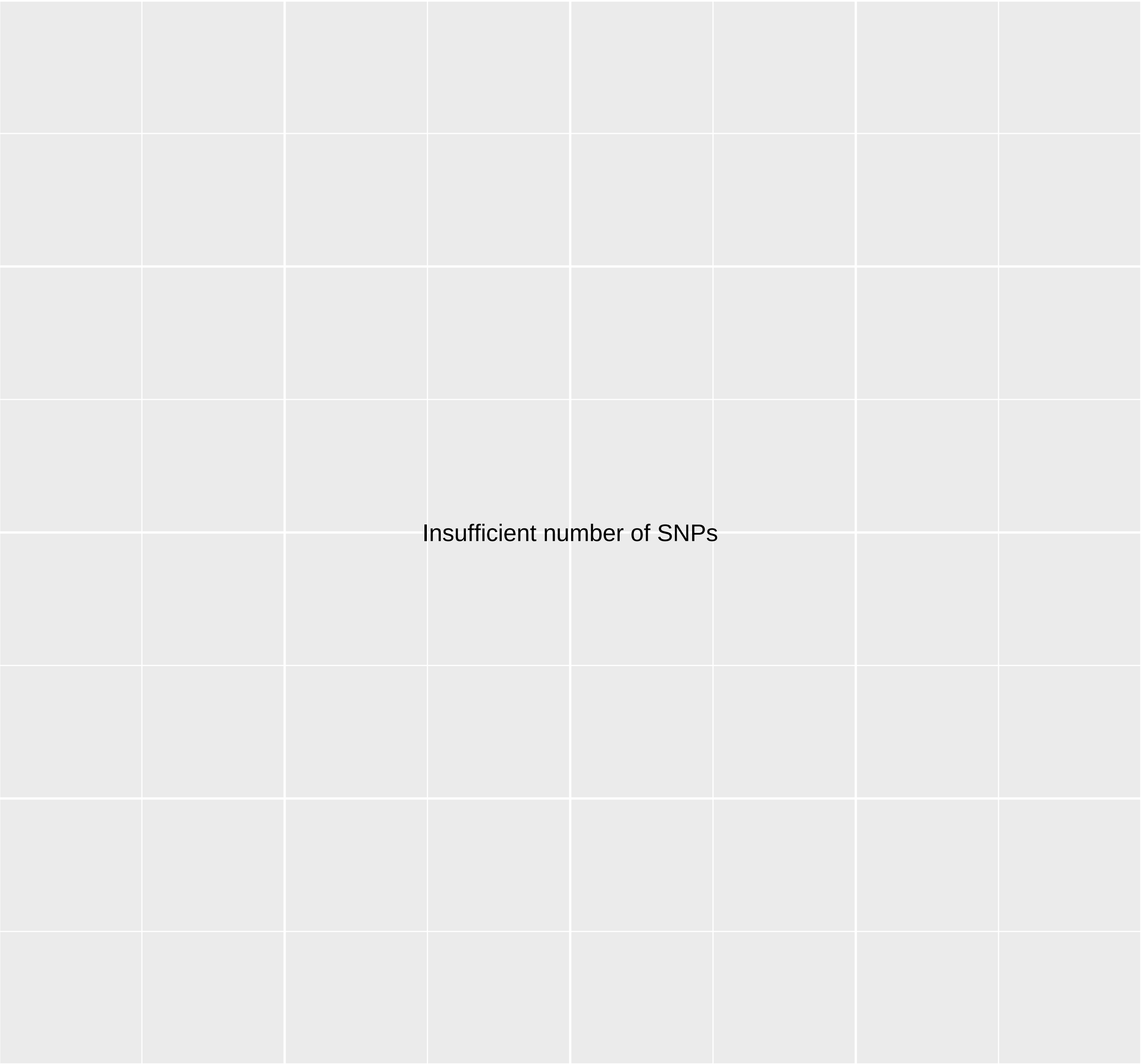
Insufficient number of SNPs

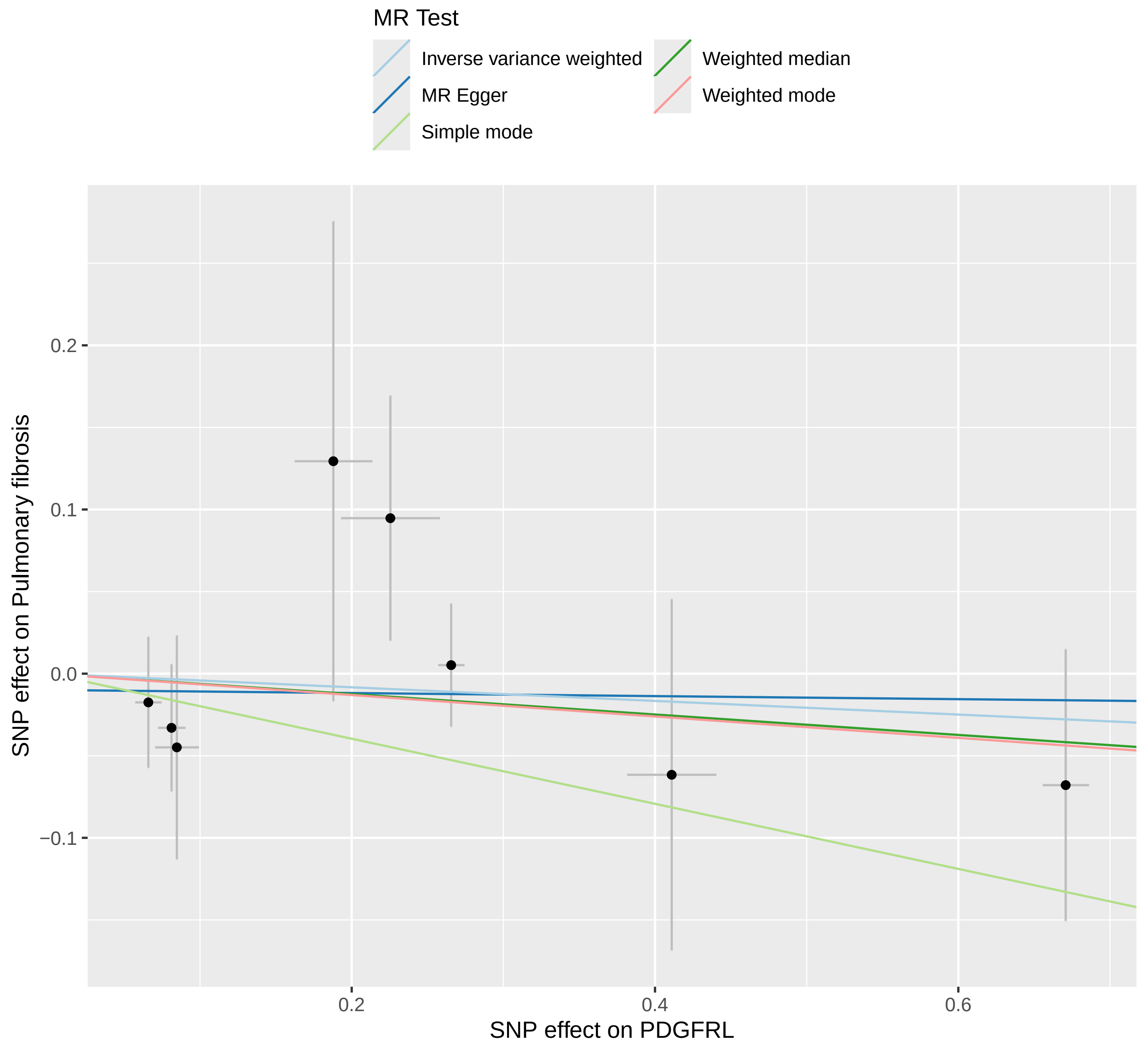
MR Test
Inverse variance weighted
MR Egger
Simple mode
Weighted median
Weighted mode
0.2
0.1
0.0
−0.1
SNP effect on Pulmonary fibrosis
0.2
0.4
0.6
SNP effect on PDGFRL

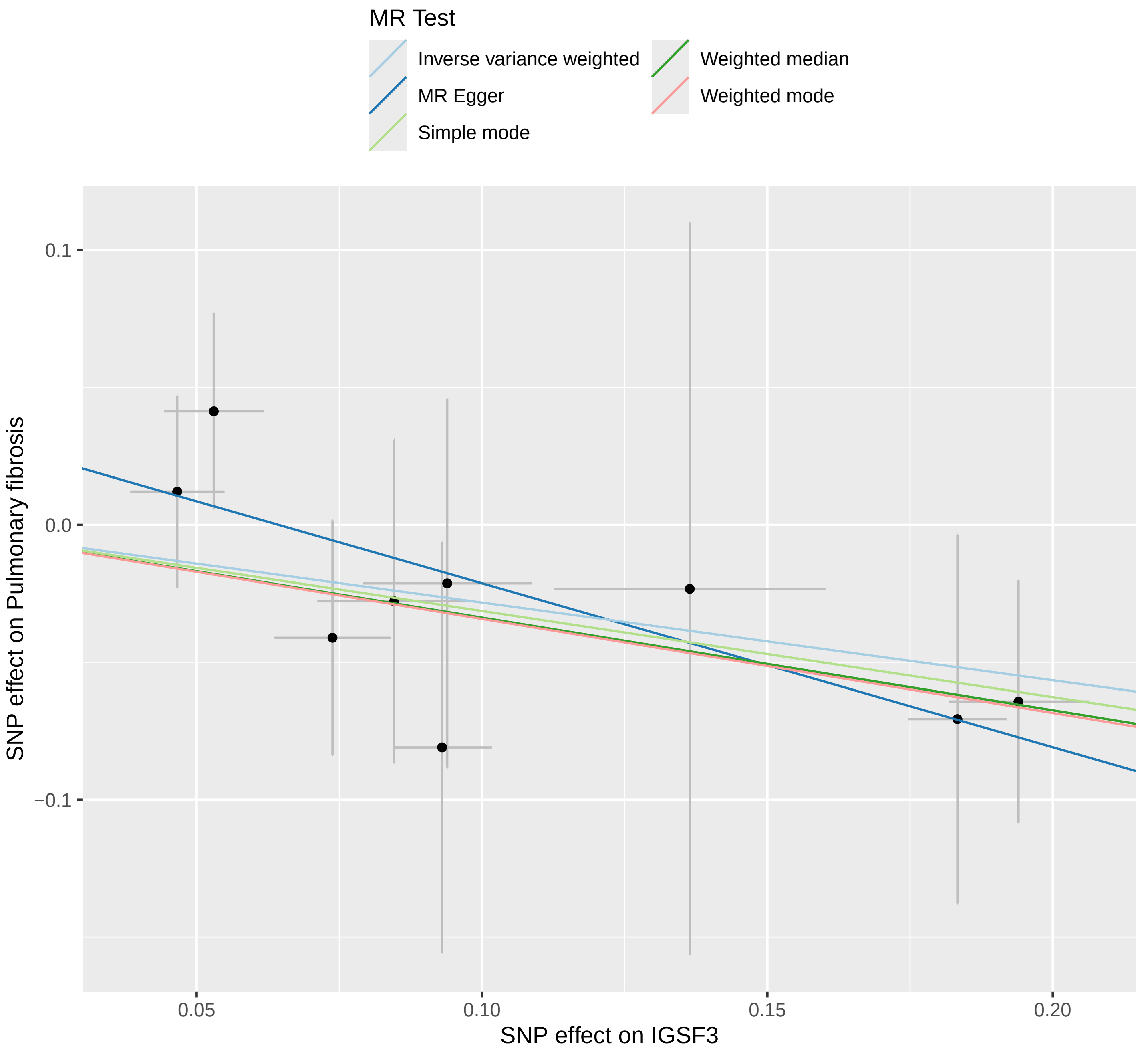
MR Test
Inverse variance weighted
MR Egger
Simple mode
Weighted median
Weighted mode
0.1
0.0
−0.1
SNP effect on Pulmonary fibrosis
0.05
0.10
0.15
0.20
SNP effect on IGSF3

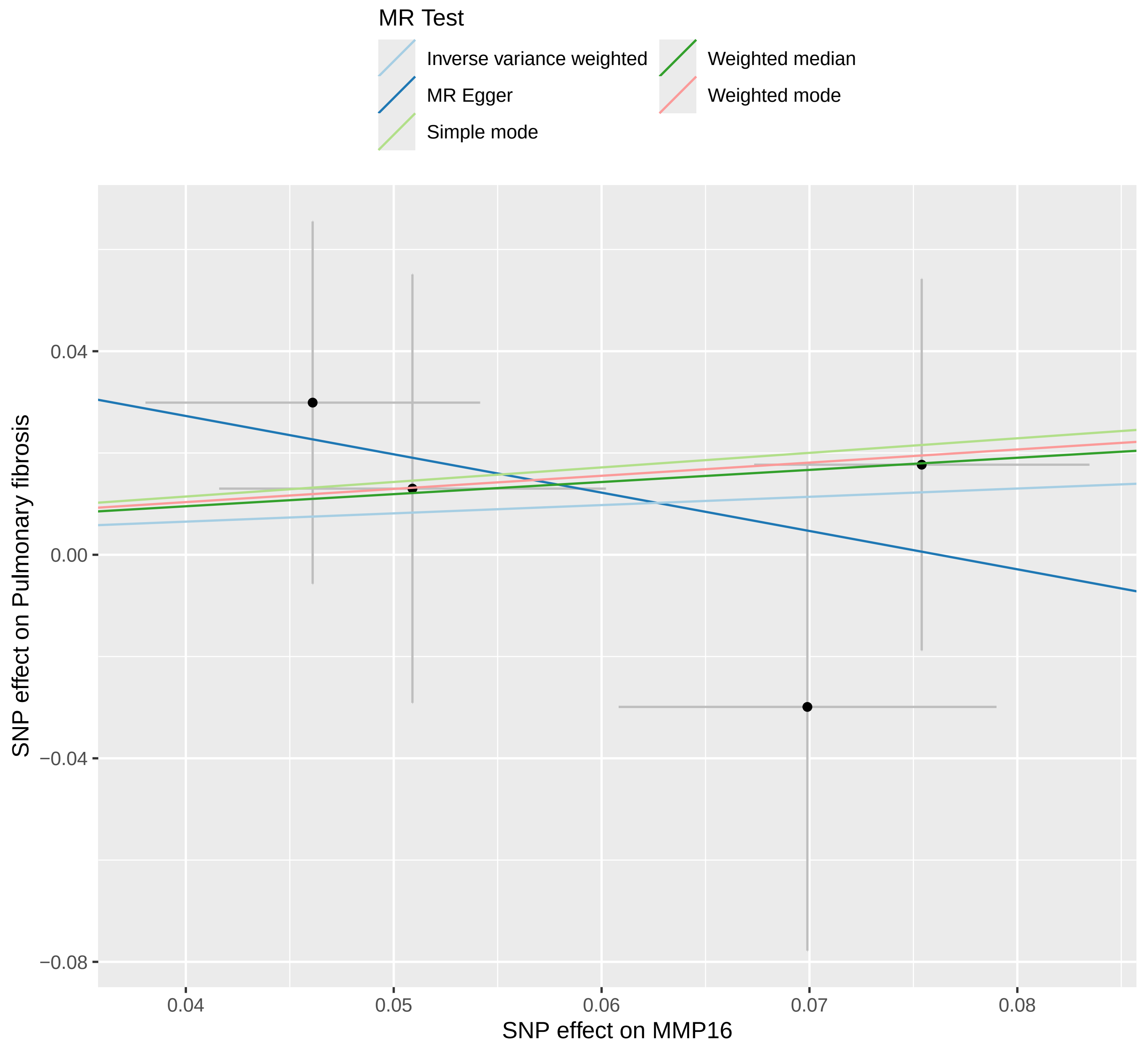
MR Test
Inverse variance weighted
MR Egger
Simple mode
Weighted median
Weighted mode
SNP effect on Pulmonary fibrosis
0.04
0.00
−0.04
−0.08
0.04
0.05
0.06
0.07
0.08
SNP effect on MMP16

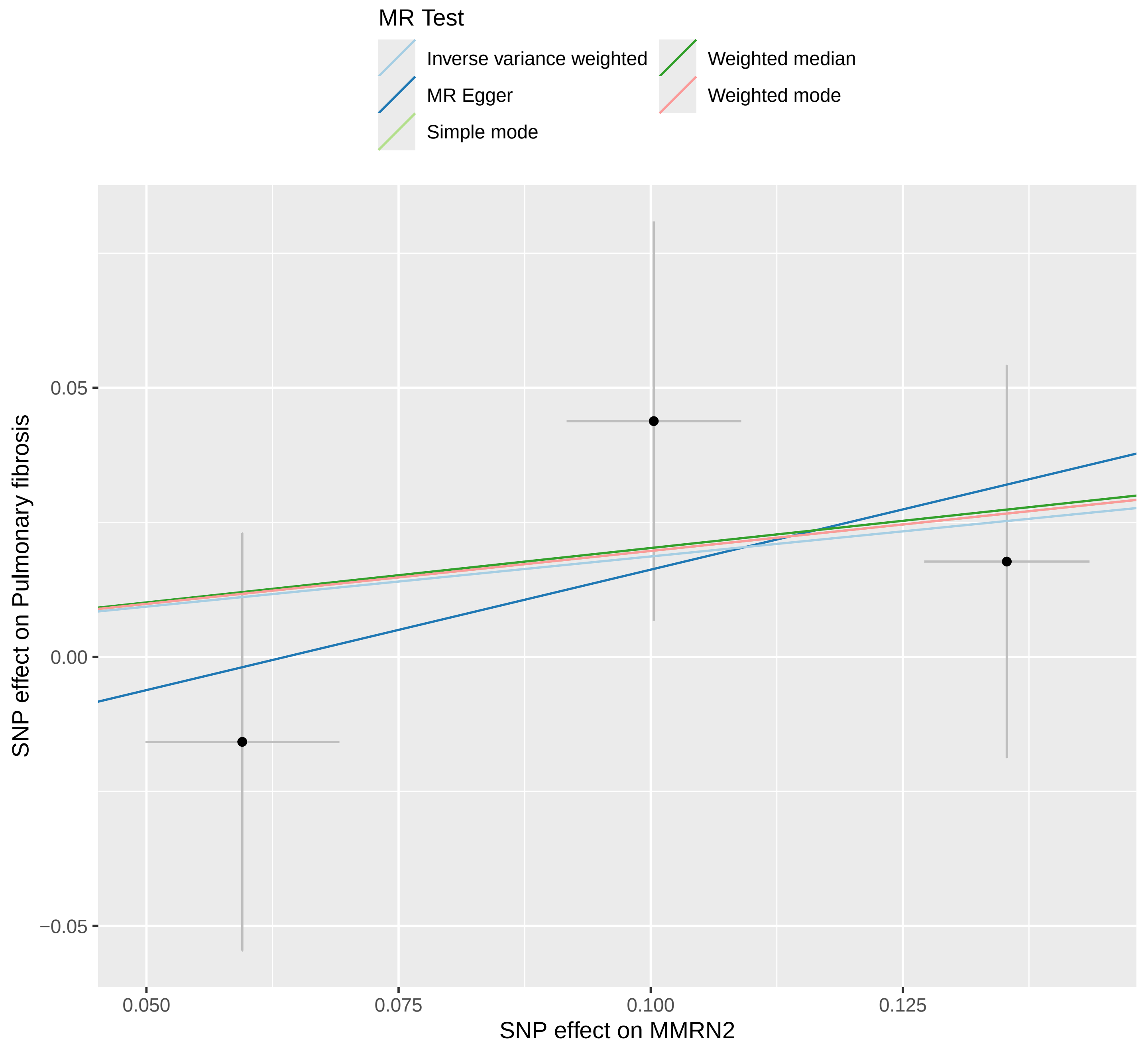
MR Test
Inverse variance weighted
MR Egger
Simple mode
Weighted median
Weighted mode
0.05
0.00
−0.05
0.050
0.075
0.100
0.125
SNP effect on MMRN2
SNP effect on Pulmonary fibrosis

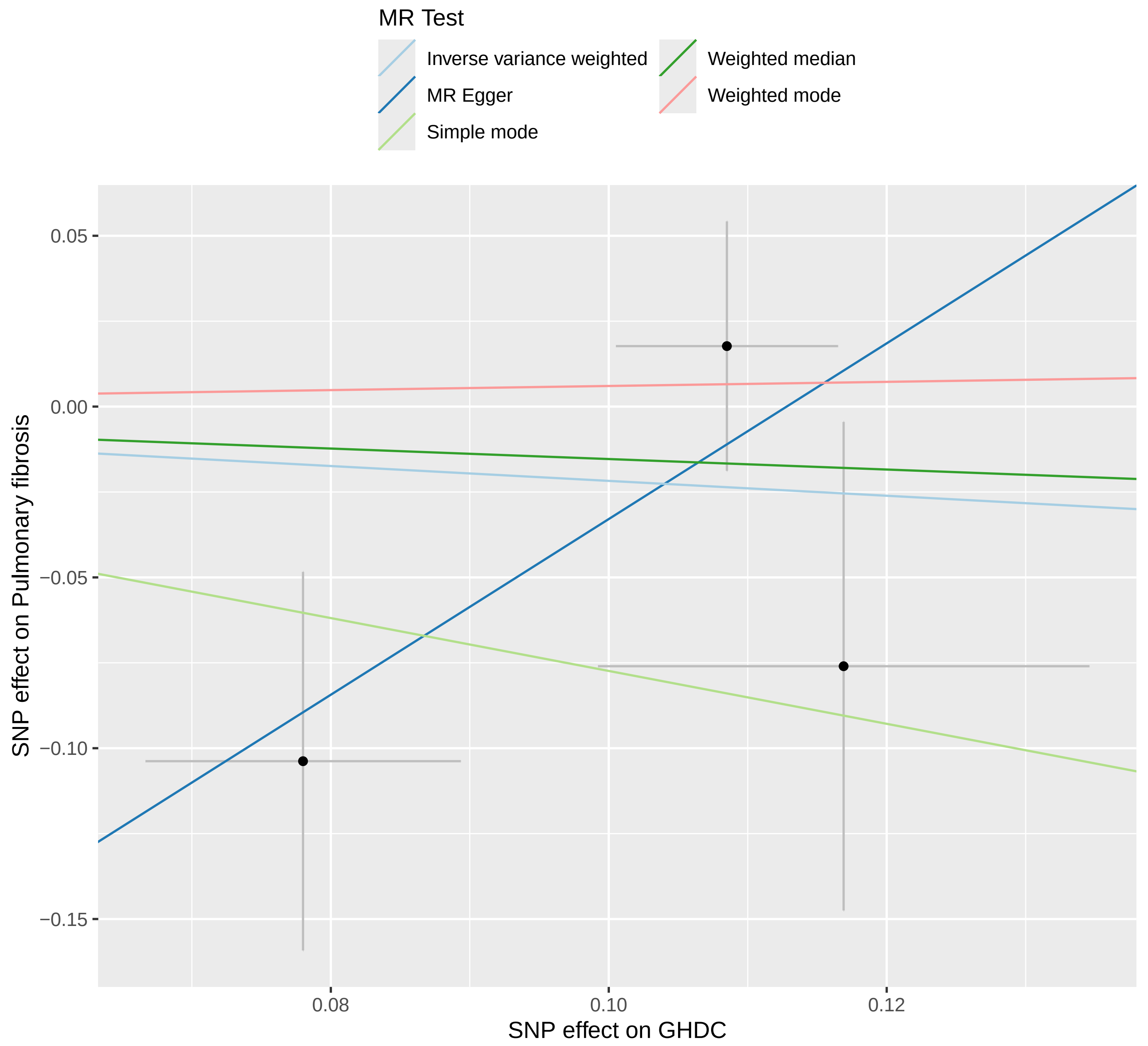

MR Test
Inverse variance weighted
MR Egger
Simple mode
Weighted median
Weighted mode
0.05
0.00
−0.05
−0.10
−0.15
SNP effect on Pulmonary fibrosis
0.08
0.10
0.12
SNP effect on GHDC

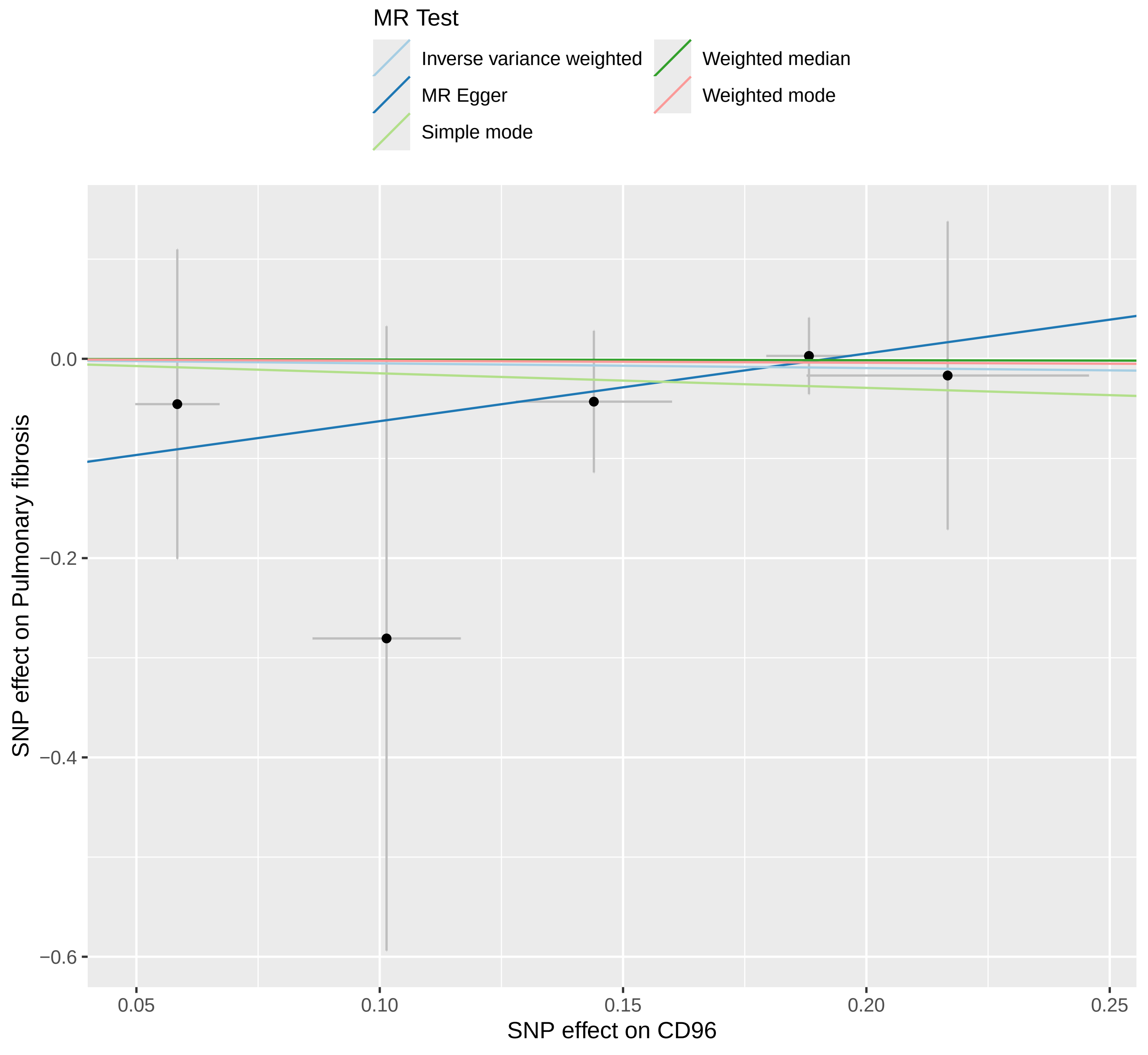

MR Test
Inverse variance weighted
MR Egger
Simple mode
Weighted median
Weighted mode
0.0
−0.2
−0.4
−0.6
SNP effect on Pulmonary fibrosis
0.05
0.10
0.15
0.20
0.25
SNP effect on CD96

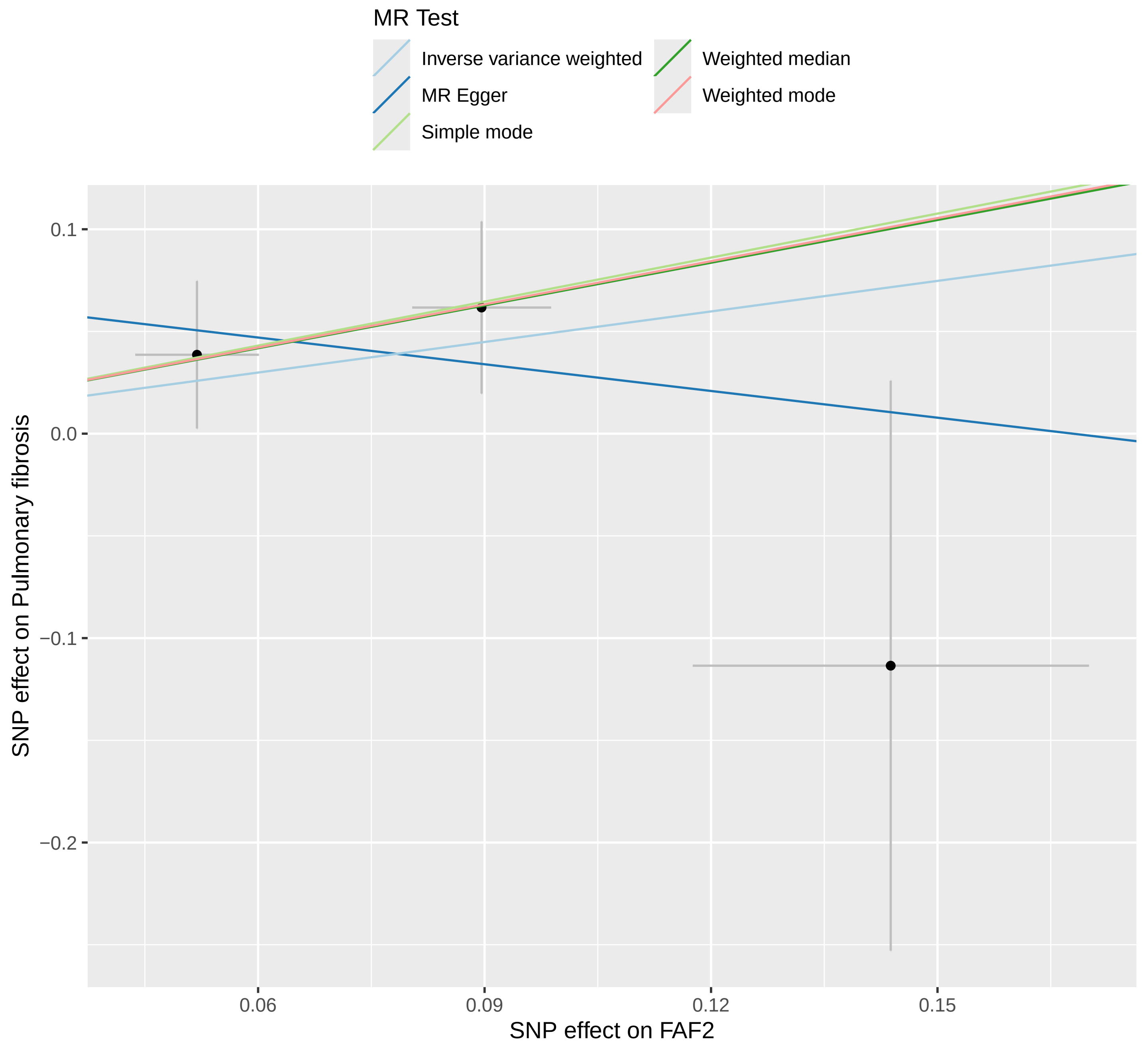
MR Test
Inverse variance weighted
MR Egger
Simple mode
Weighted median
Weighted mode
0.1
0.0
−0.1
−0.2
0.06
0.09
0.12
0.15
SNP effect on Pulmonary fibrosis
SNP effect on FAF2

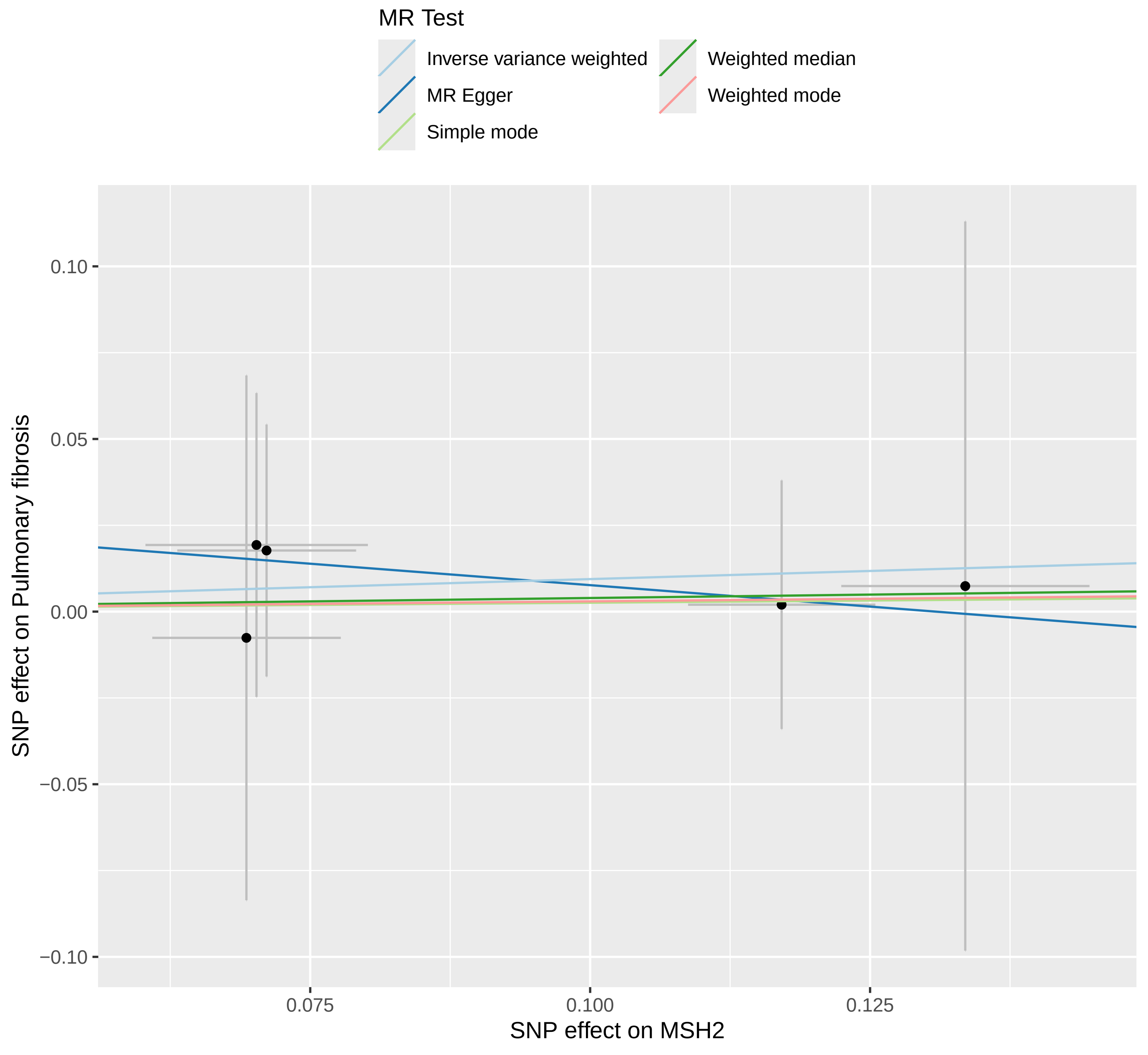

MR Test
Inverse variance weighted
MR Egger
Simple mode
Weighted median
Weighted mode
0.10
0.05
0.00
−0.05
−0.10
SNP effect on Pulmonary fibrosis
0.075
0.100
0.125
SNP effect on MSH2

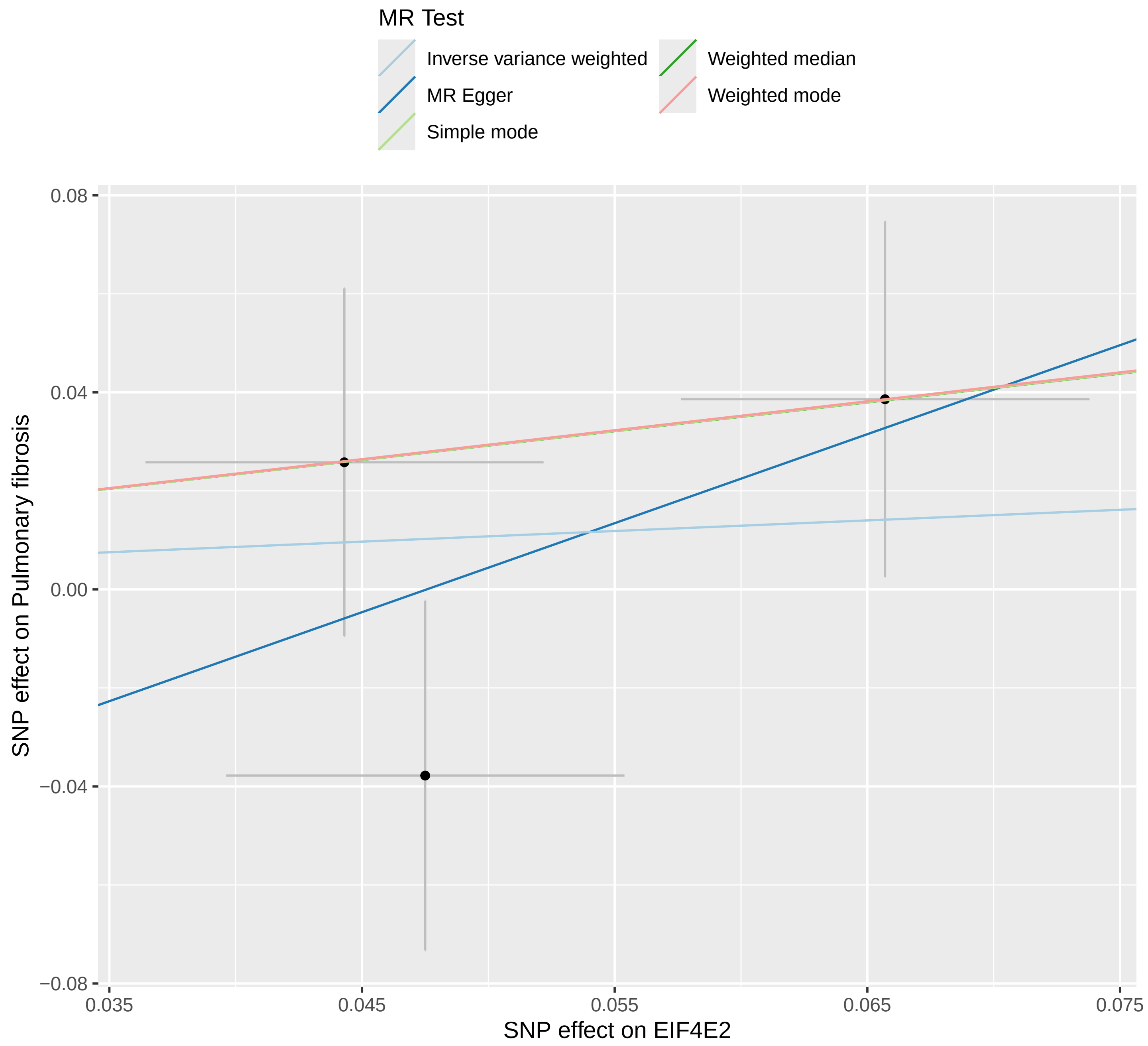
MR Test
Inverse variance weighted
MR Egger
Simple mode
Weighted median
Weighted mode
0.08
0.04
0.00
−0.04
−0.08
SNP effect on Pulmonary fibrosis
0.035
0.045
0.055
0.065
0.075
SNP effect on EIF4E2

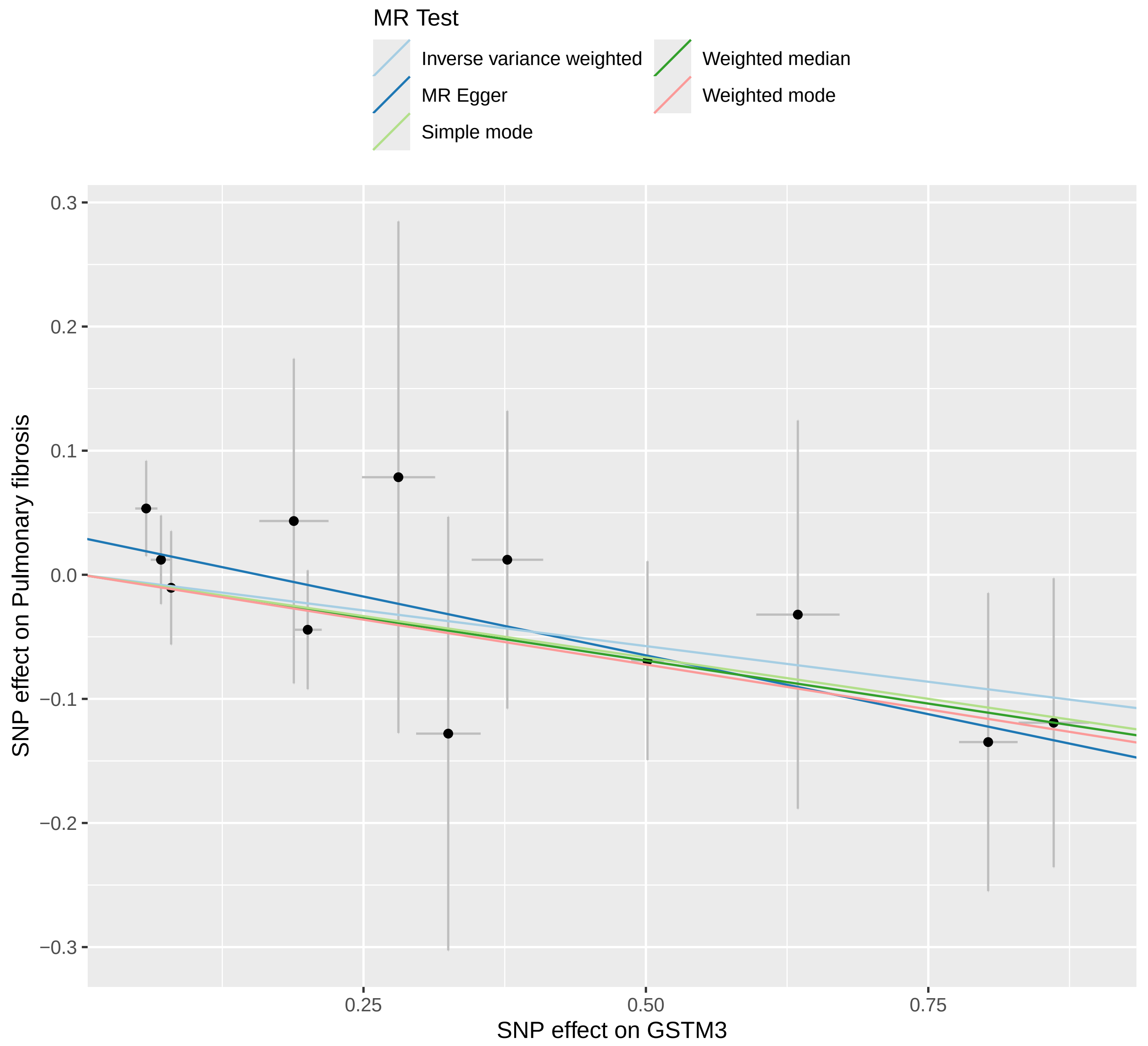
MR Test
Inverse variance weighted
MR Egger
Simple mode
Weighted median
Weighted mode
0.3
0.2
0.1
0.0
−0.1
−0.2
−0.3
0.25
0.50
0.75
SNP effect on GSTM3
SNP effect on Pulmonary fibrosis

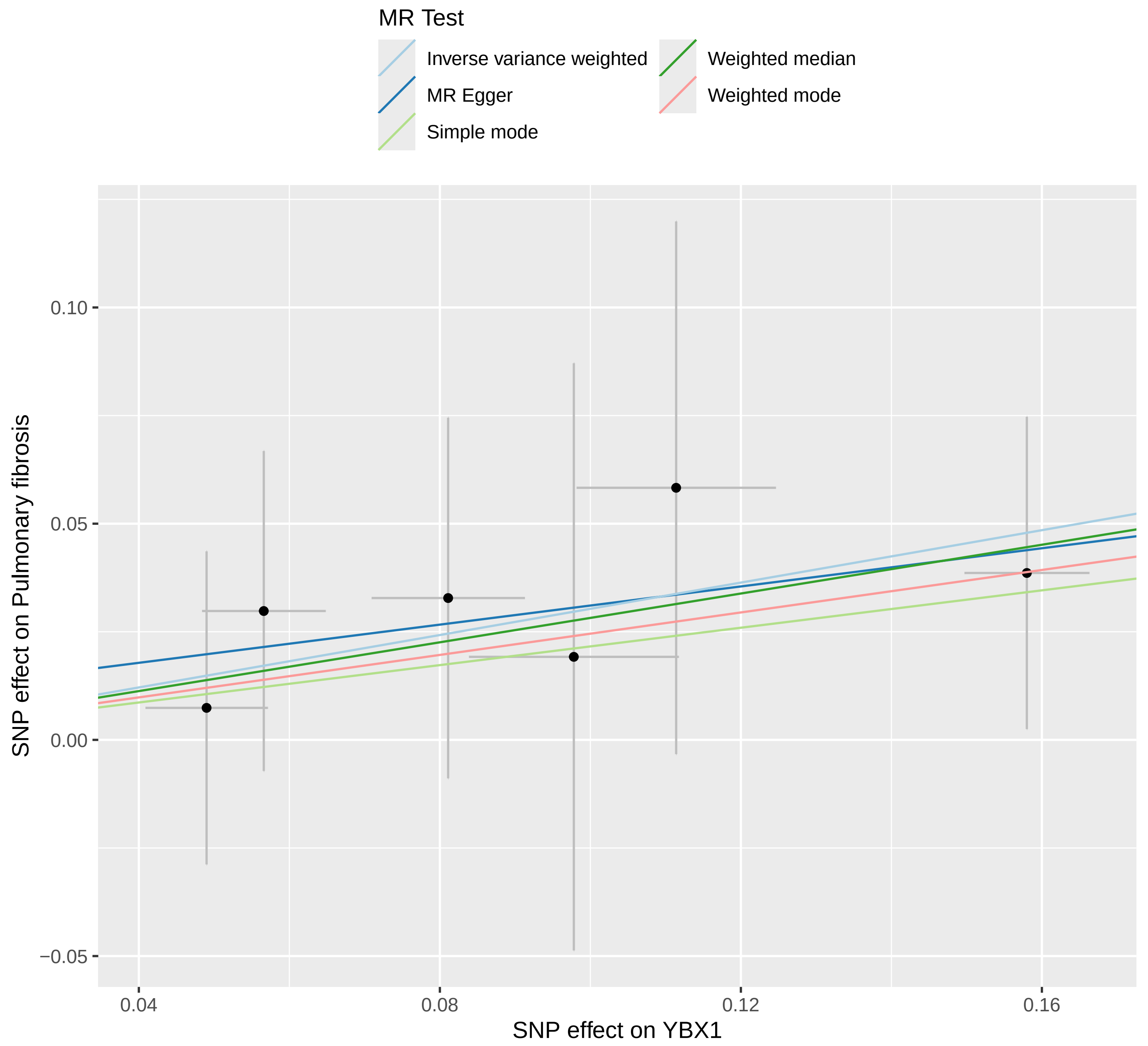

MR Test
Inverse variance weighted
MR Egger
Simple mode
Weighted median
Weighted mode
0.10
0.05
0.00
−0.05
0.04
0.08
0.12
0.16
SNP effect on Pulmonary fibrosis
SNP effect on YBX1

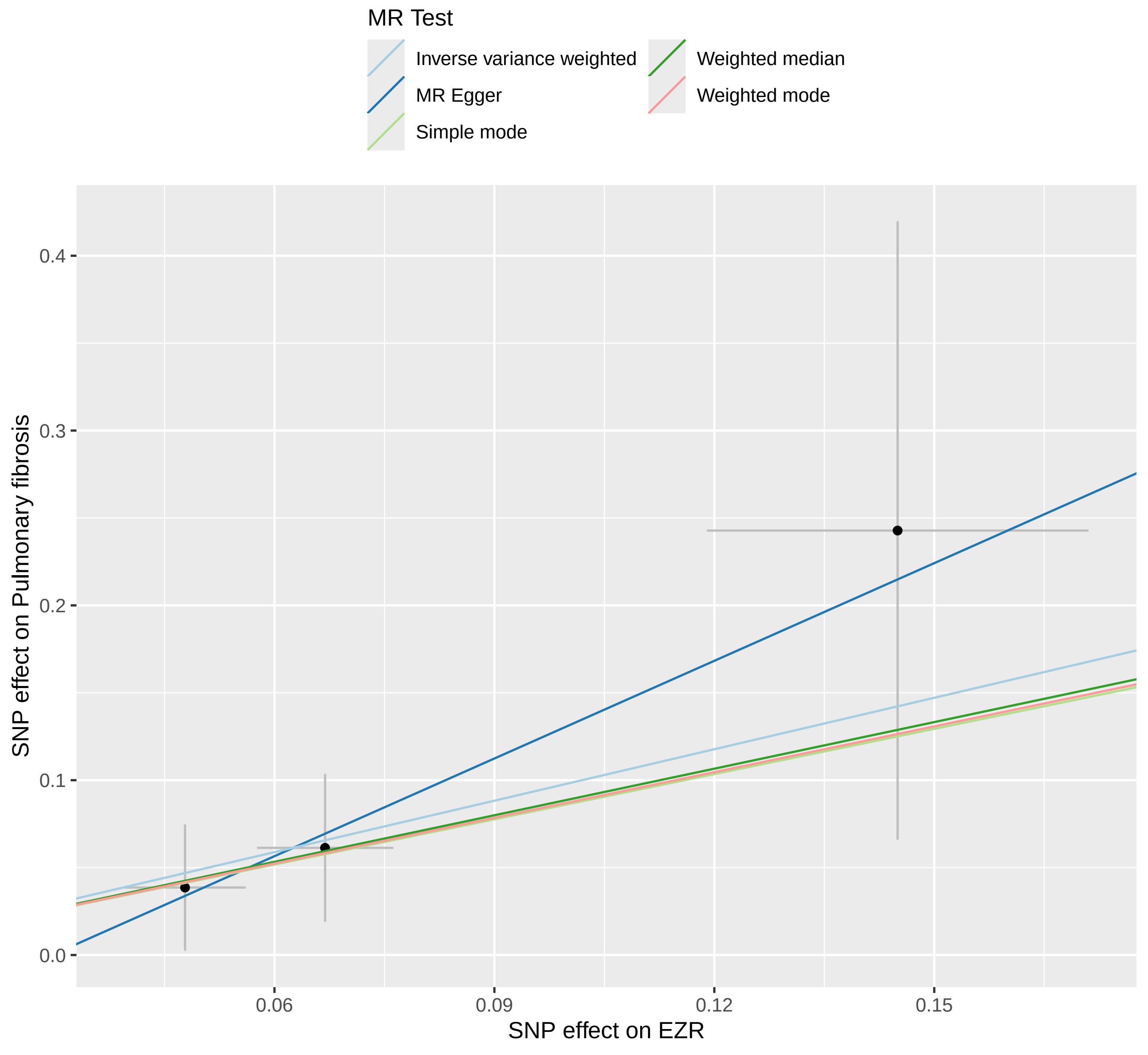

MR Test
Inverse variance weighted
MR Egger
Simple mode
Weighted median
Weighted mode
SNP effect on Pulmonary fibrosis
0.0
0.1
0.2
0.3
0.4
0.06
0.09
0.12
0.15
SNP effect on EZR

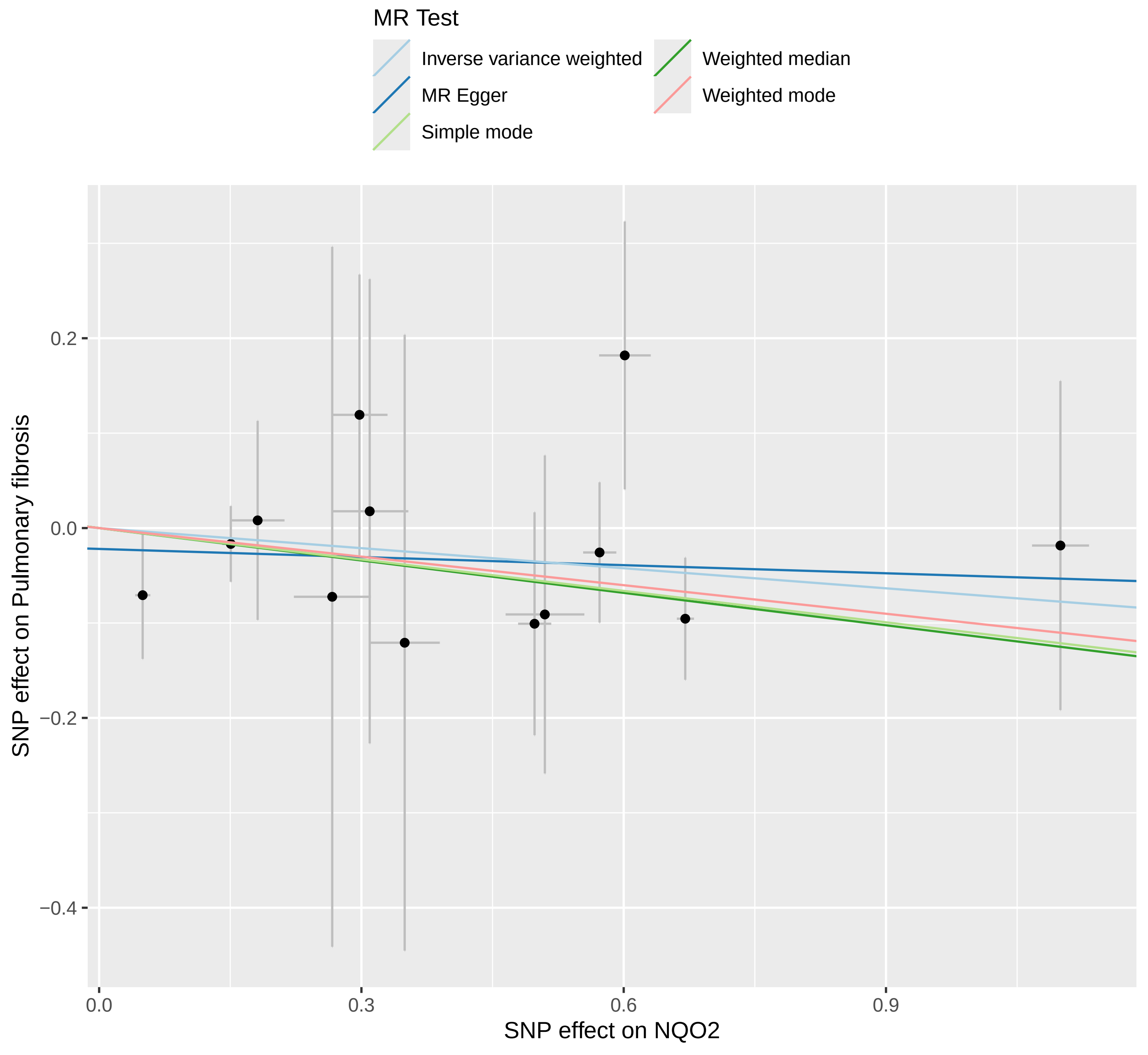
MR Test
Inverse variance weighted
MR Egger
Simple mode
Weighted median
Weighted mode
0.2
0.0
−0.2
−0.4
0.0
0.3
0.6
0.9
SNP effect on Pulmonary fibrosis
SNP effect on NQO2

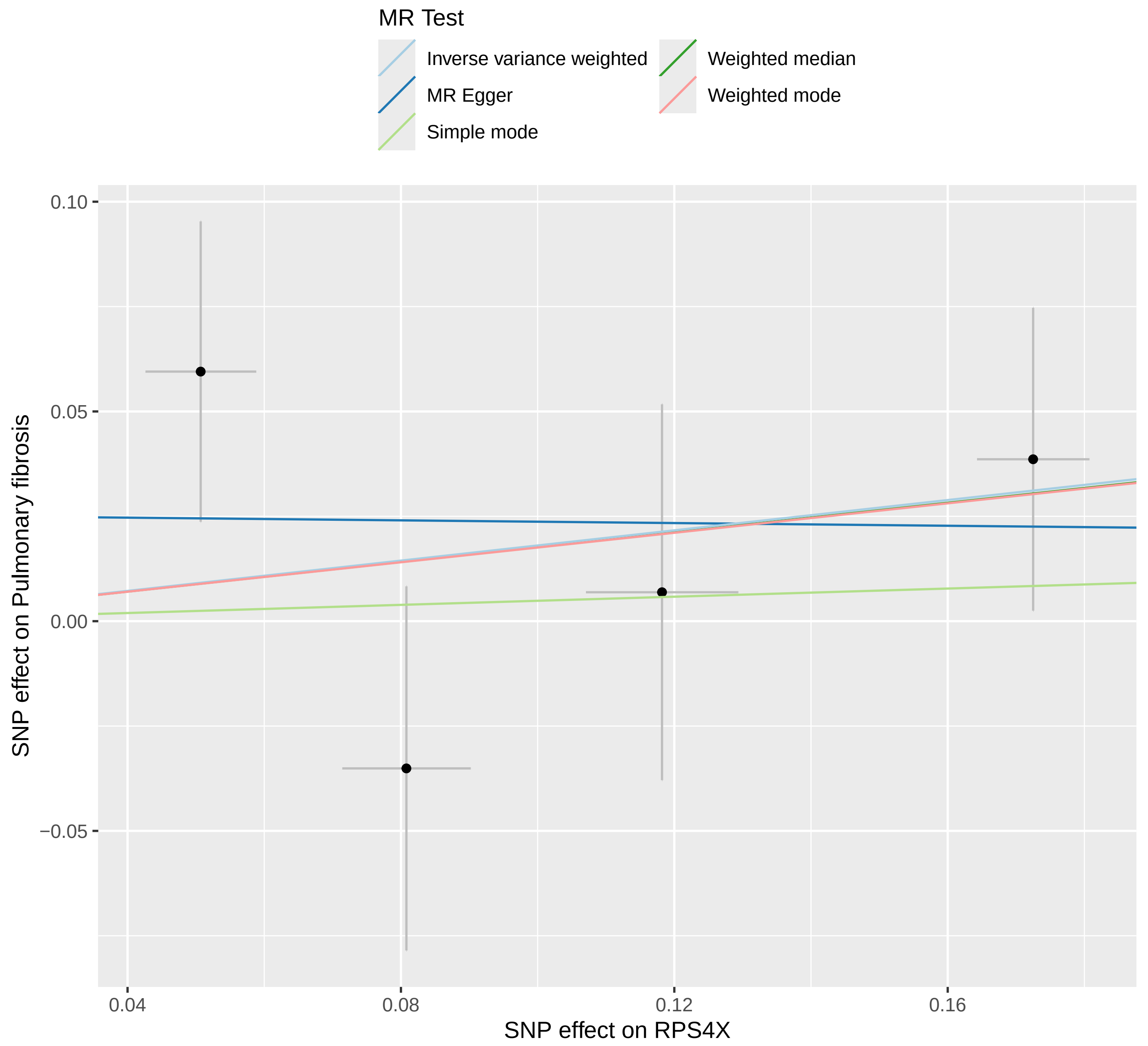
MR Test
Inverse variance weighted
MR Egger
Simple mode
Weighted median
Weighted mode
0.10
0.05
0.00
−0.05
SNP effect on Pulmonary fibrosis
0.04
0.08
0.12
0.16
SNP effect on RPS4X

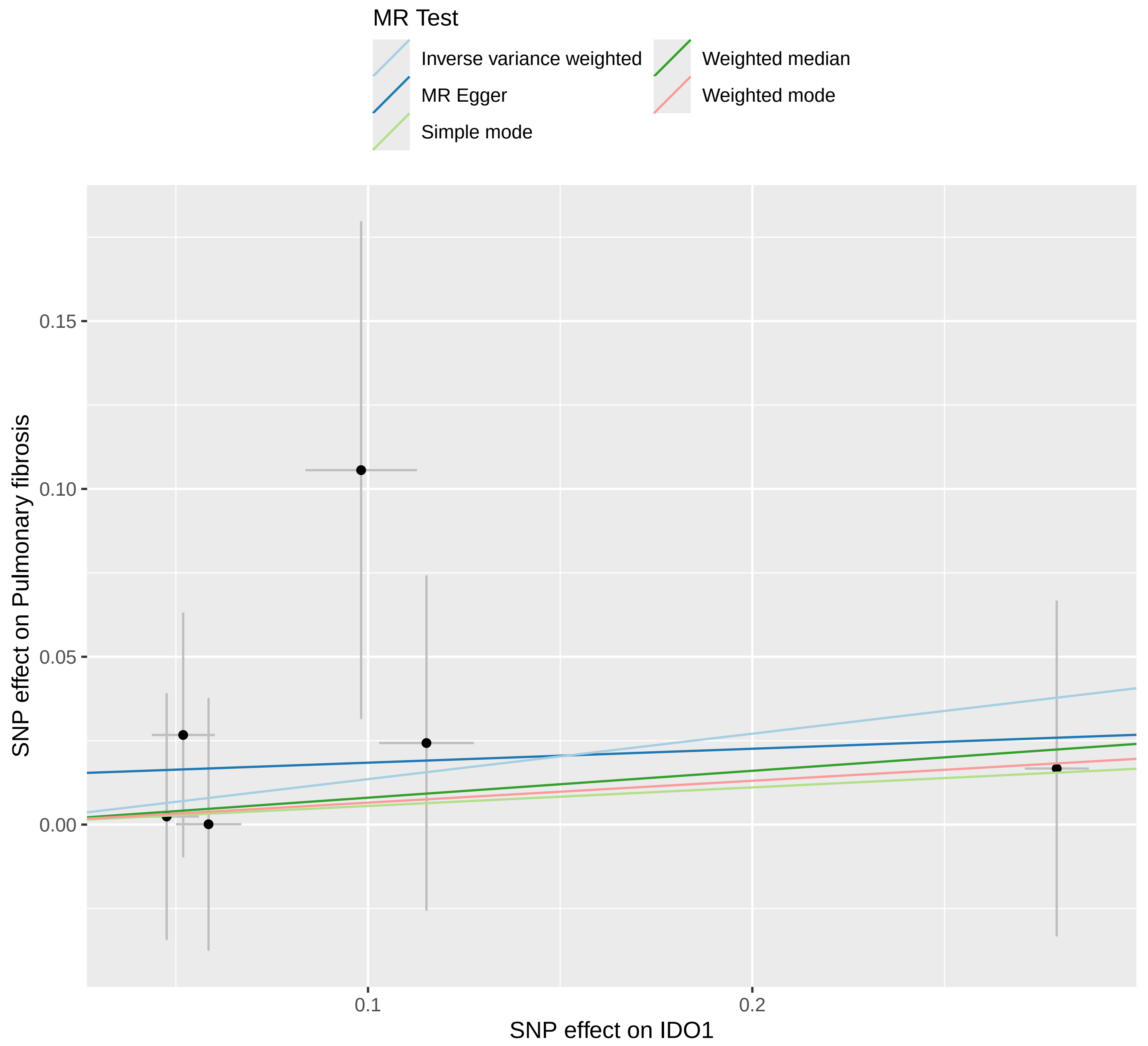
MR Test
Inverse variance weighted
MR Egger
Simple mode
Weighted median
Weighted mode
0.15
0.10
0.05
0.00
SNP effect on Pulmonary fibrosis
0.1
0.2
SNP effect on IDO1

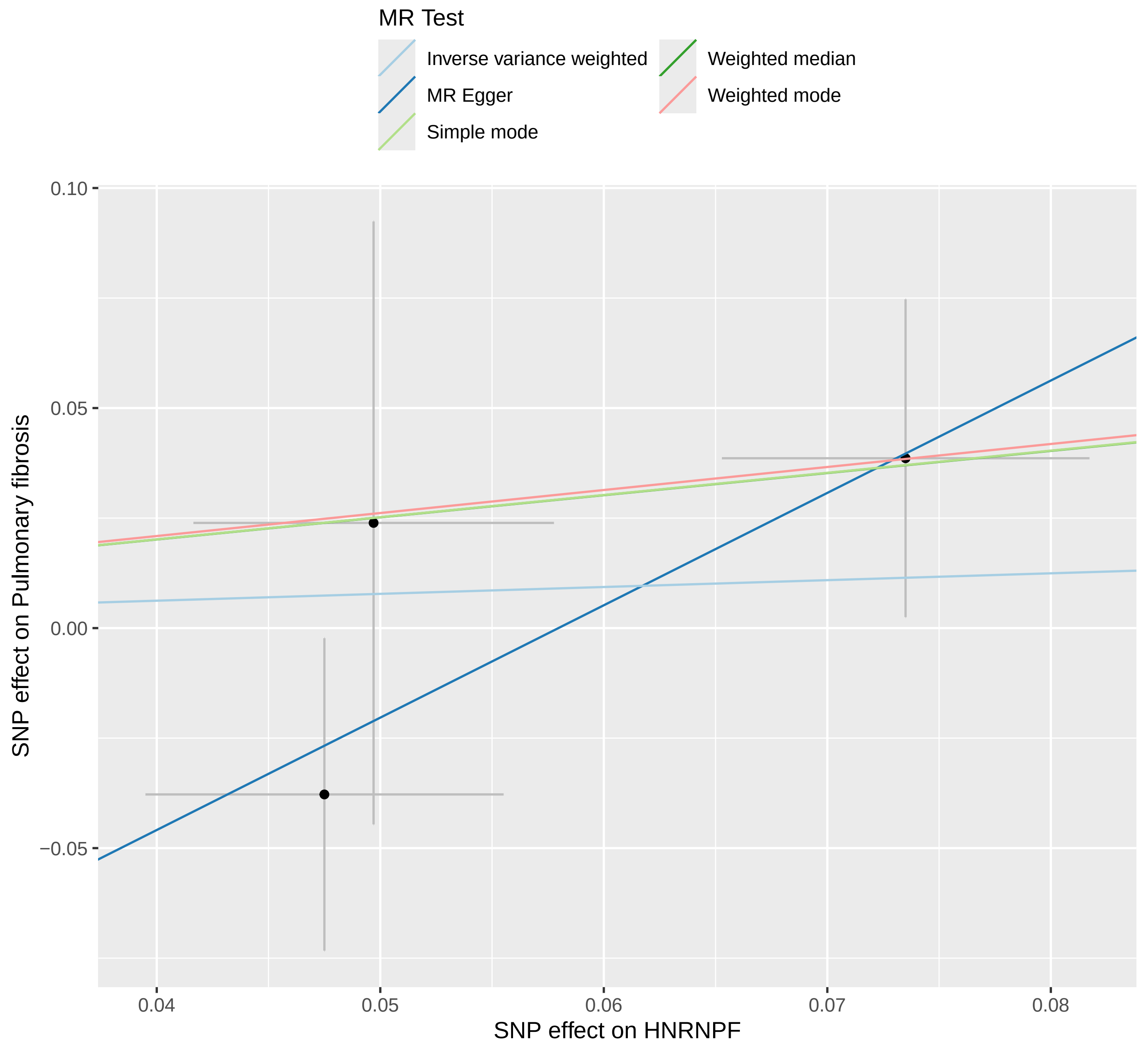

MR Test
Inverse variance weighted
MR Egger
Simple mode
Weighted median
Weighted mode
0.10
0.05
0.00
−0.05
SNP effect on Pulmonary fibrosis
0.04
0.05
0.06
0.07
0.08
SNP effect on HNRNPF

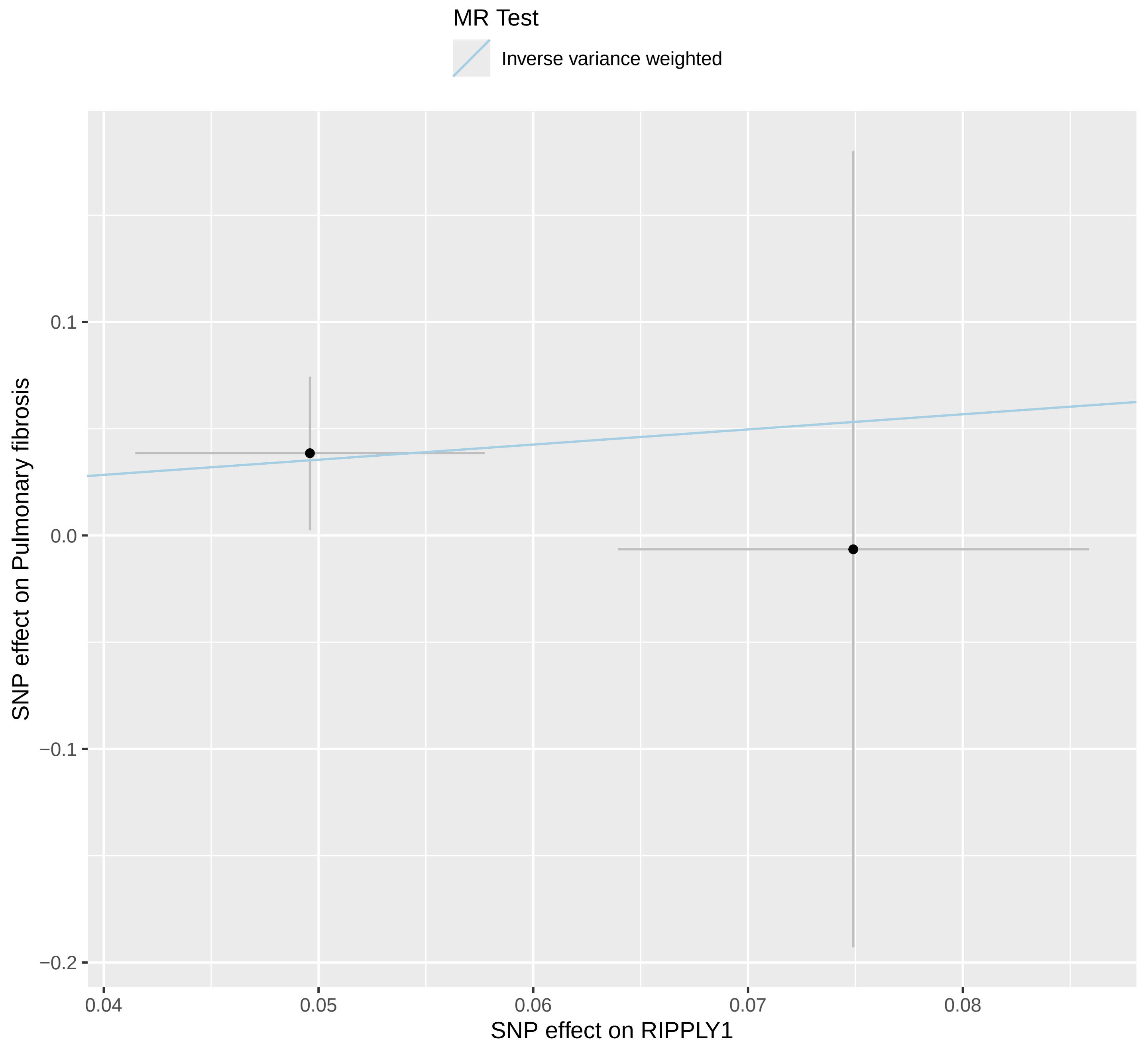
MR Test
Inverse variance weighted
SNP effect on Pulmonary fibrosis
0.1
0.0
−0.1
−0.2
0.04
0.05
0.06
0.07
0.08
SNP effect on RIPPLY1

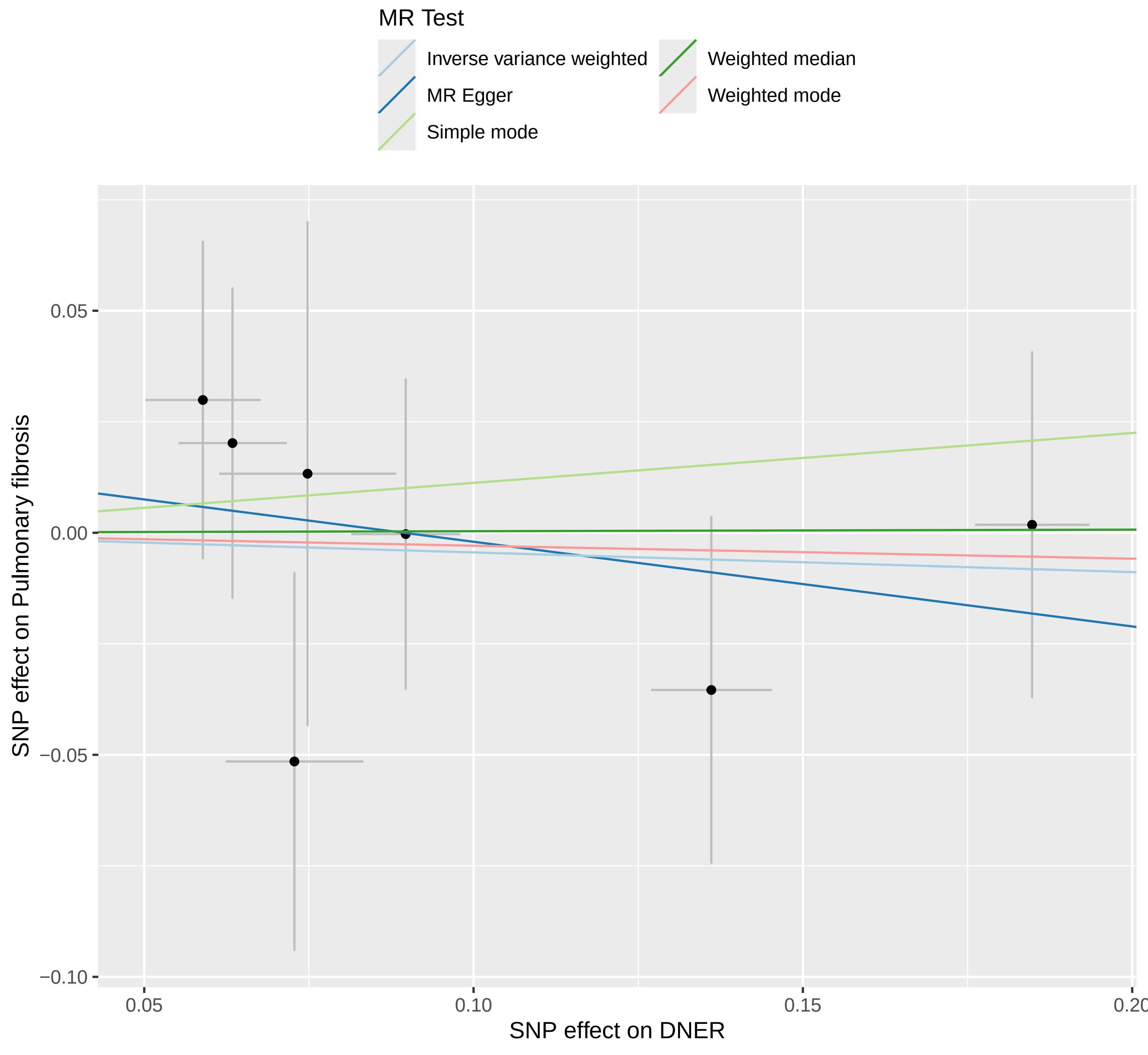

MR Test
Inverse variance weighted
MR Egger
Simple mode
Weighted median
Weighted mode
0.05
0.00
−0.05
−0.10
0.05
0.10
0.15
0.20
SNP effect on DNER
SNP effect on Pulmonary fibrosis

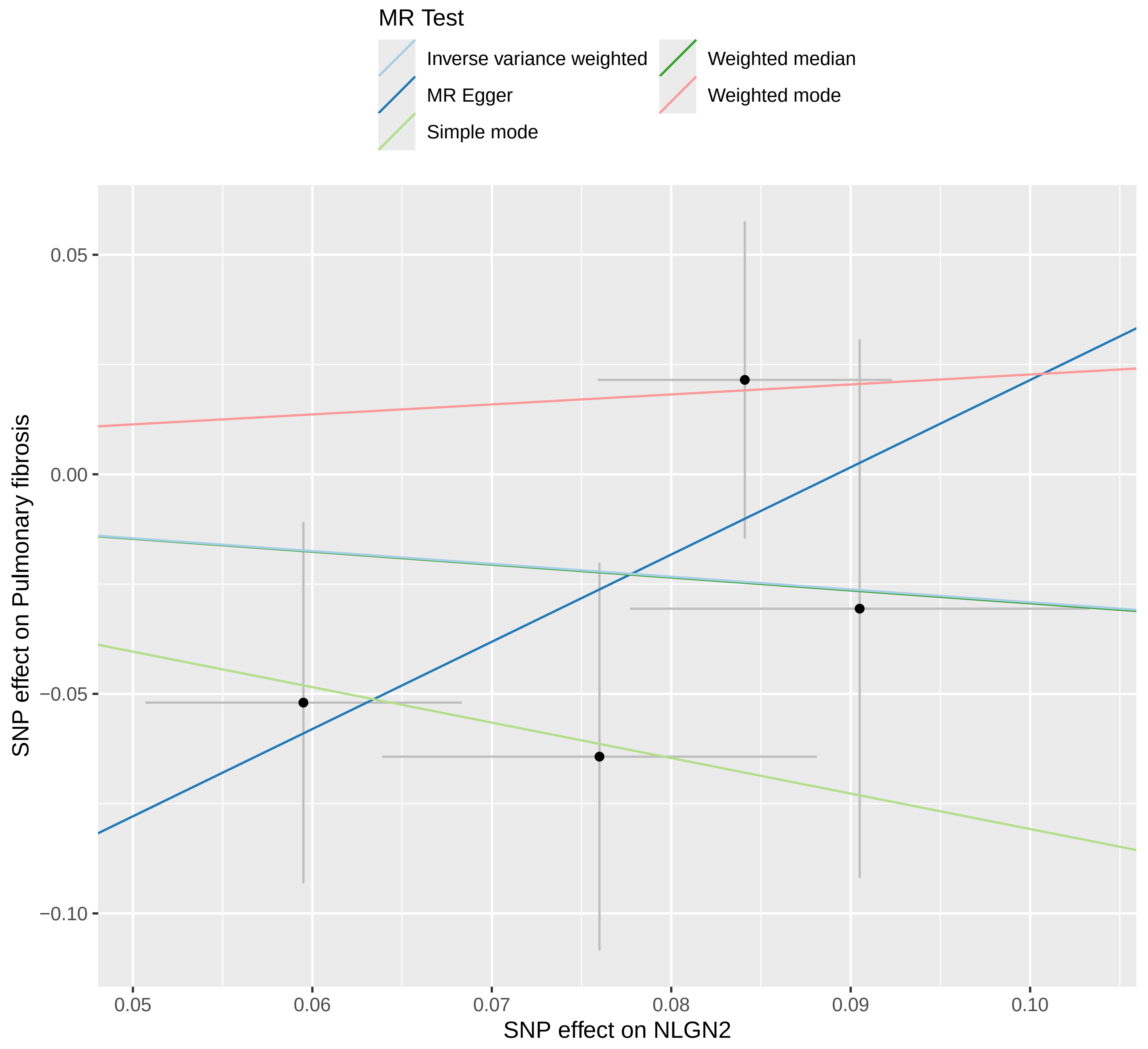
MR Test
Inverse variance weighted
MR Egger
Simple mode
Weighted median
Weighted mode
0.05
0.00
−0.05
−0.10
SNP effect on Pulmonary fibrosis
0.05
0.06
0.07
0.08
0.09
0.10
SNP effect on NLGN2

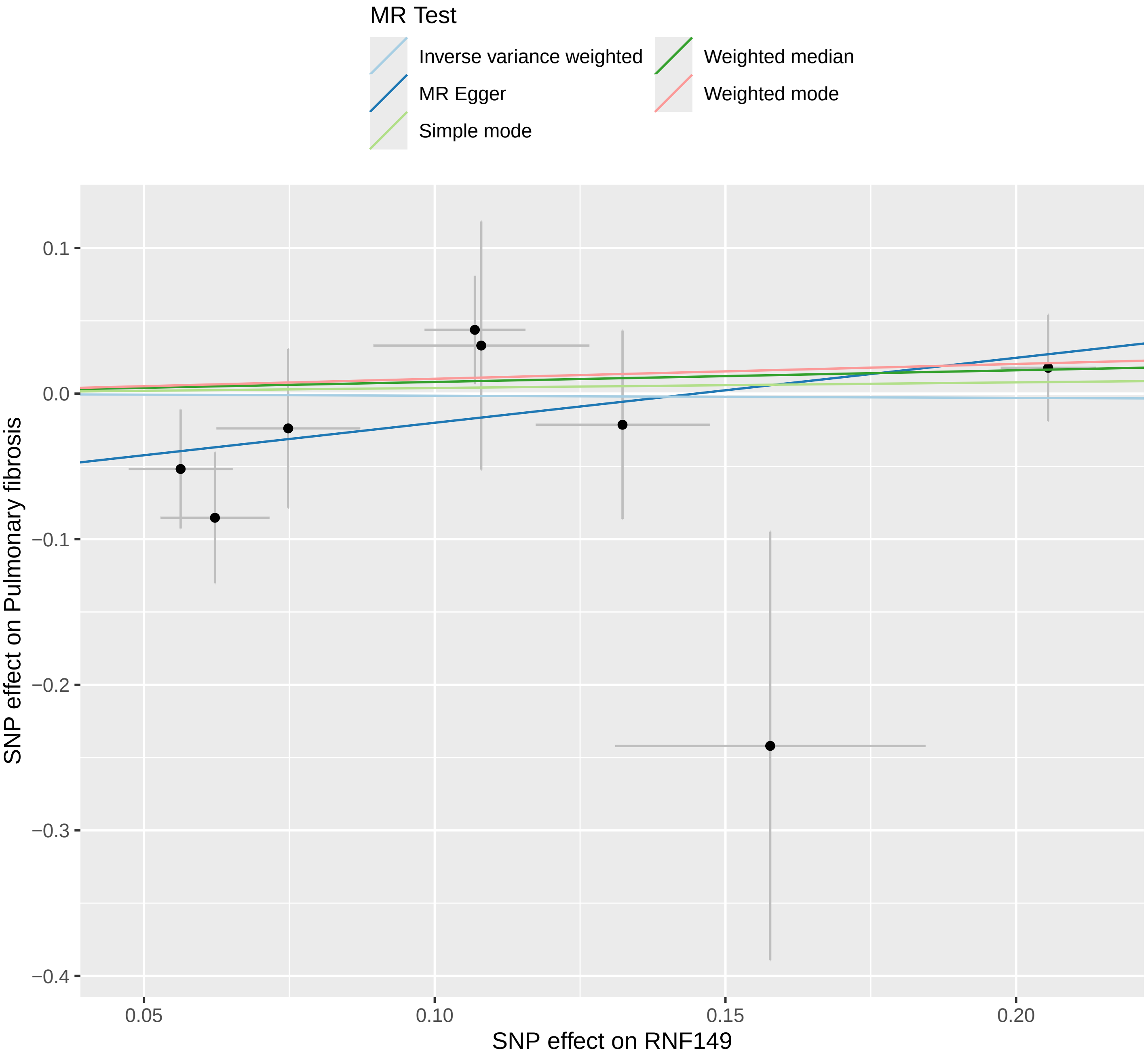
MR Test
Inverse variance weighted
MR Egger
Simple mode
Weighted median
Weighted mode
SNP effect on Pulmonary fibrosis
0.1
0.0
−0.1
−0.2
−0.3
−0.4
0.05
0.10
0.15
0.20
SNP effect on RNF149

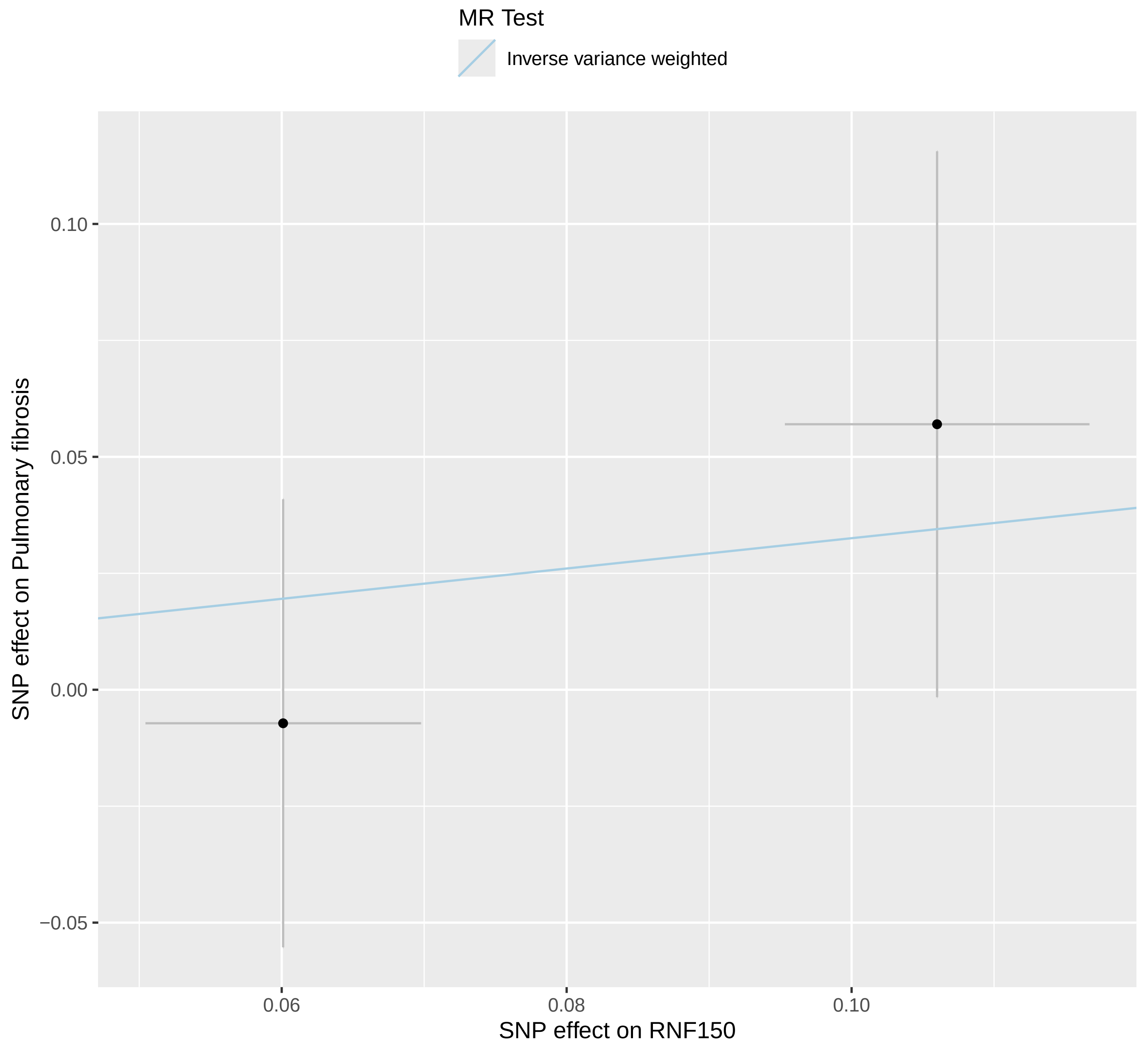
MR Test
Inverse variance weighted
0.10
0.05
0.00
−0.05
SNP effect on Pulmonary fibrosis
0.06
0.08
0.10
SNP effect on RNF150

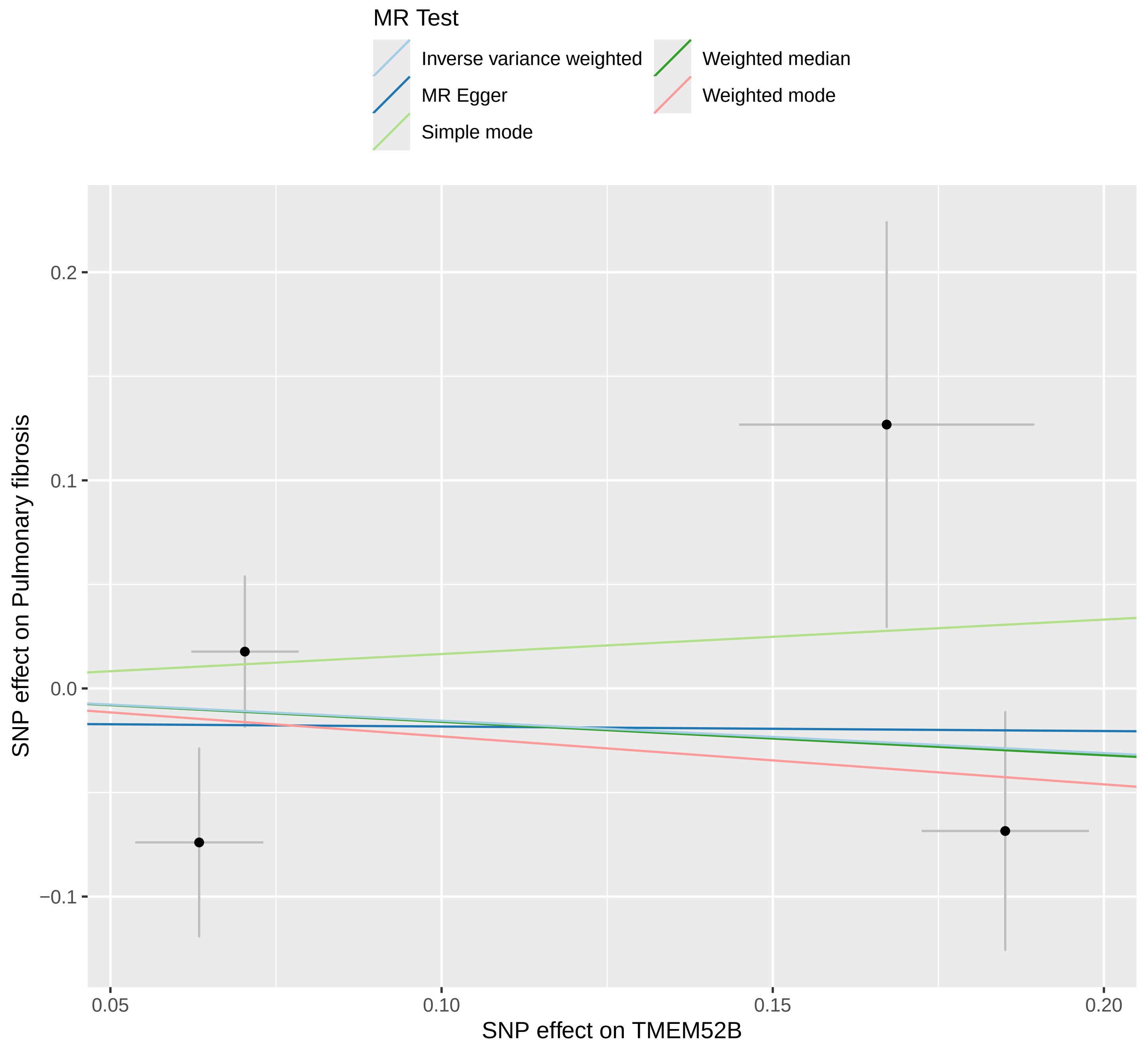

MR Test
Inverse variance weighted
MR Egger
Simple mode
Weighted median
Weighted mode
SNP effect on Pulmonary fibrosis
0.2
0.1
0.0
−0.1
0.05
0.10
0.15
0.20
SNP effect on TMEM52B

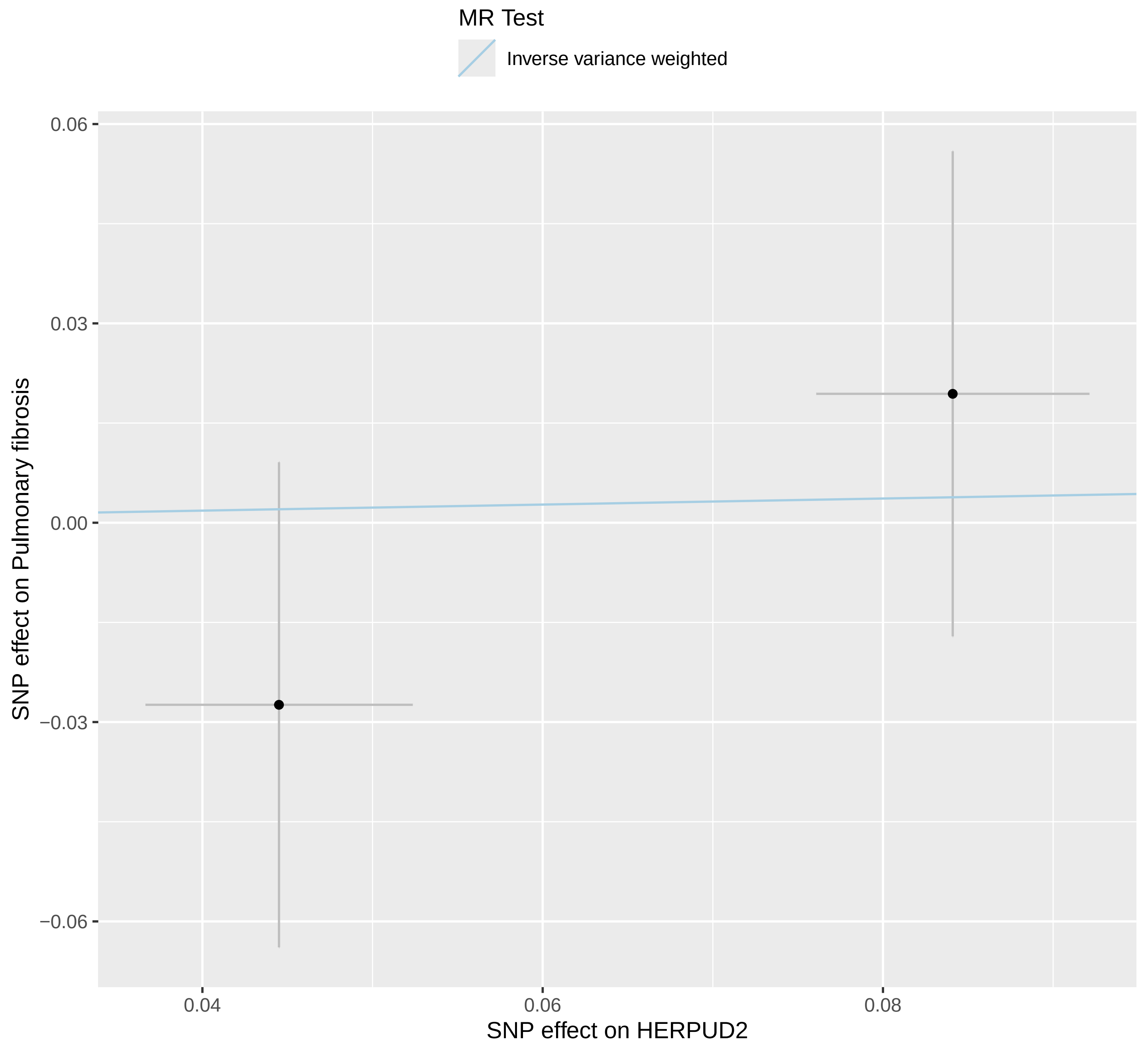

MR Test
Inverse variance weighted
0.06
0.03
0.00
−0.03
−0.06
SNP effect on Pulmonary fibrosis
0.04
0.06
0.08
SNP effect on HERPUD2

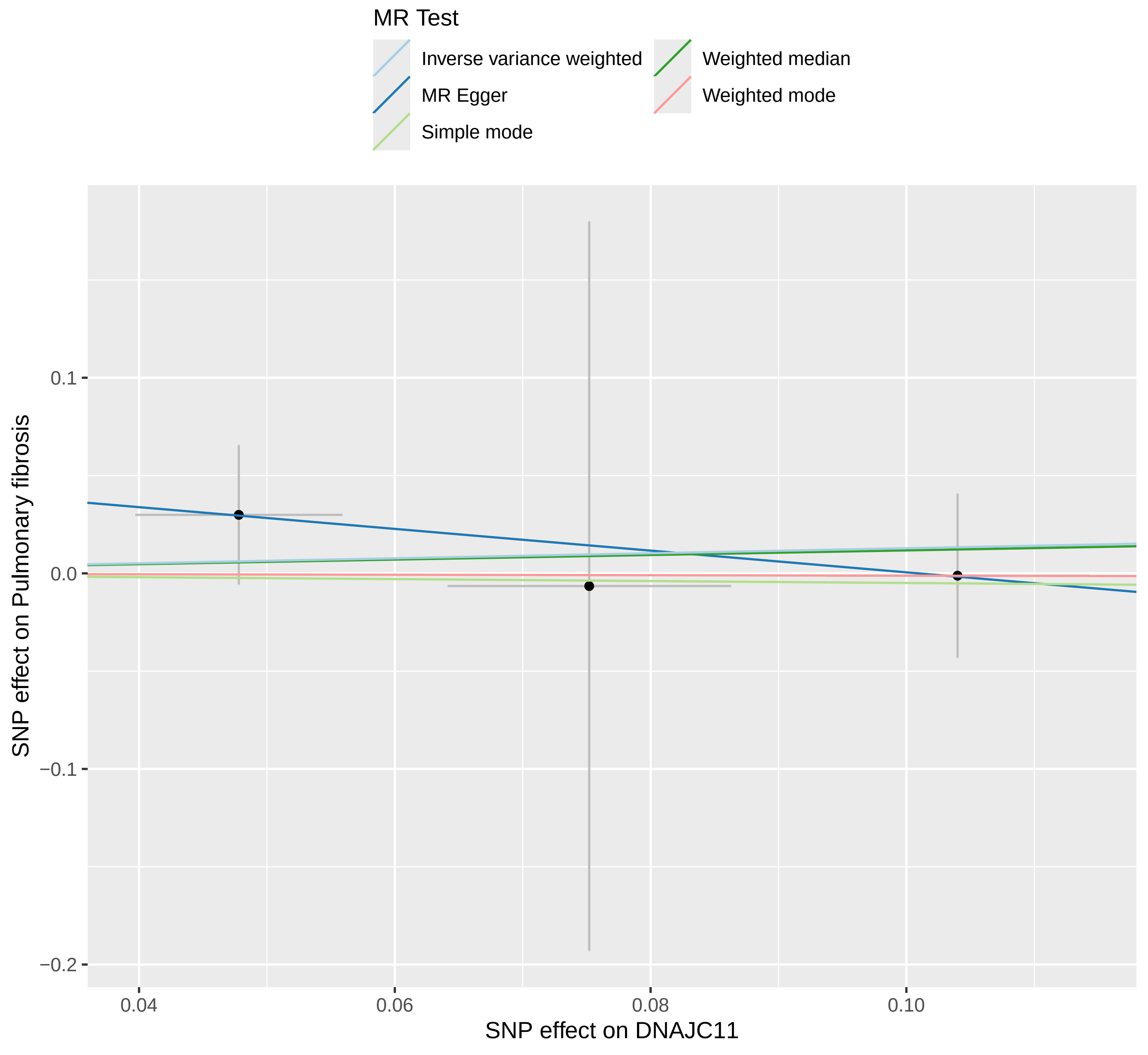

MR Test
Inverse variance weighted
MR Egger
Simple mode
Weighted median
Weighted mode
0.1
0.0
−0.1
−0.2
SNP effect on Pulmonary fibrosis
0.04
0.06
0.08
0.10
SNP effect on DNAJC11

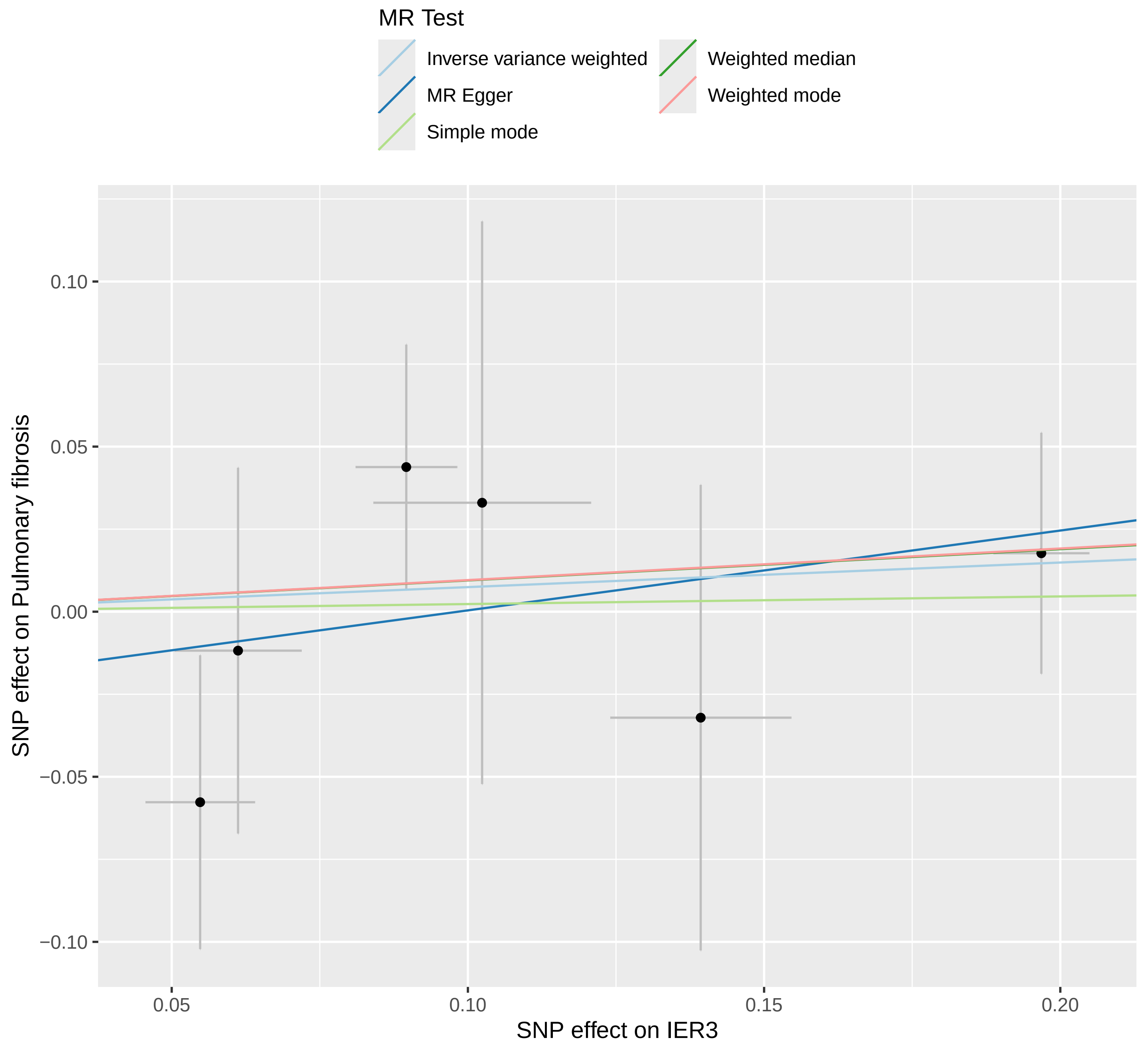
MR Test
Inverse variance weighted
MR Egger
Simple mode
Weighted median
Weighted mode
0.10
0.05
0.00
−0.05
−0.10
SNP effect on Pulmonary fibrosis
0.05
0.10
0.15
0.20
SNP effect on IER3

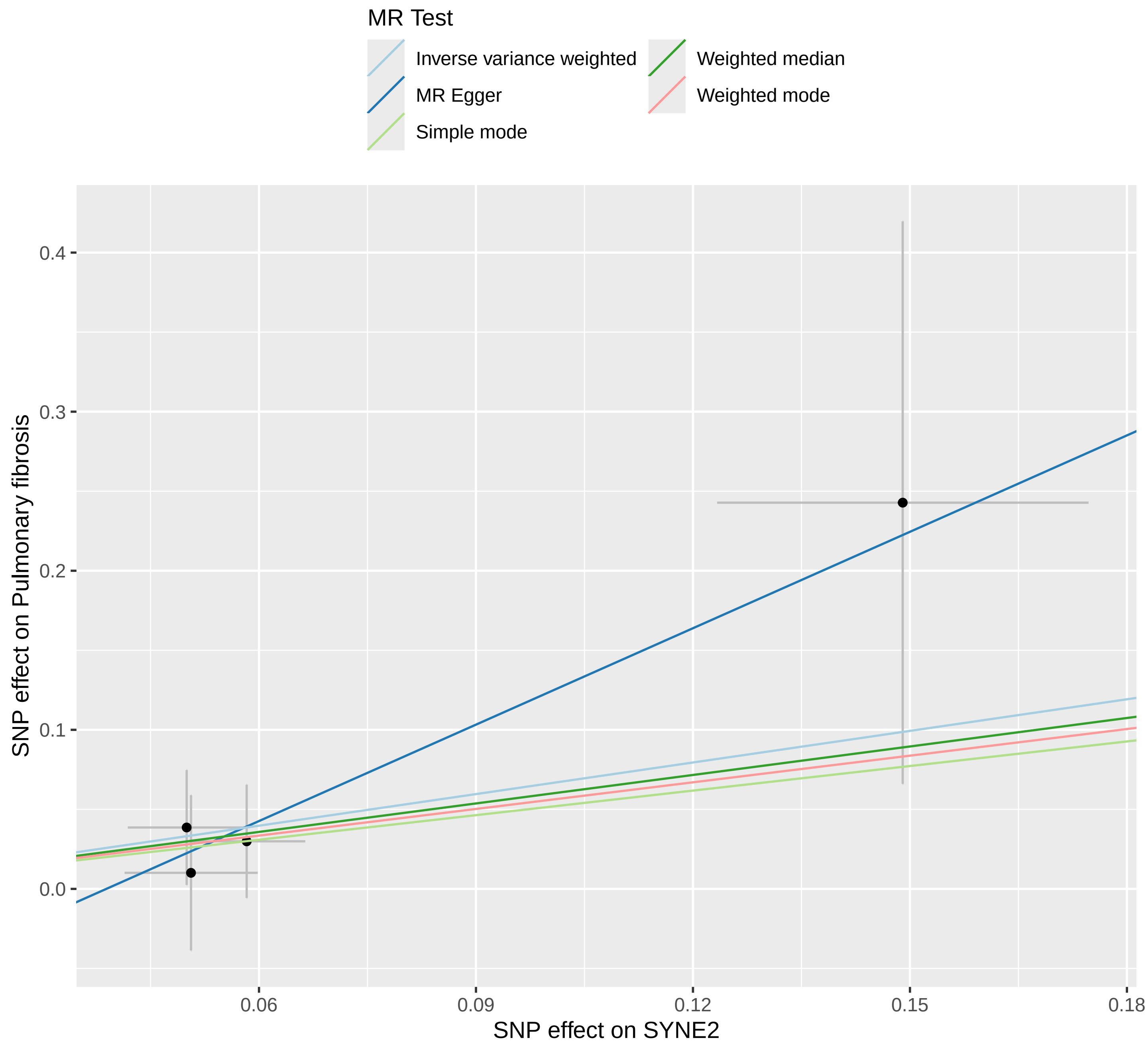
MR Test
Inverse variance weighted
MR Egger
Simple mode
Weighted median
Weighted mode
SNP effect on Pulmonary fibrosis
0.4
0.3
0.2
0.1
0.0
0.06
0.09
0.12
0.15
0.18
SNP effect on SYNE2

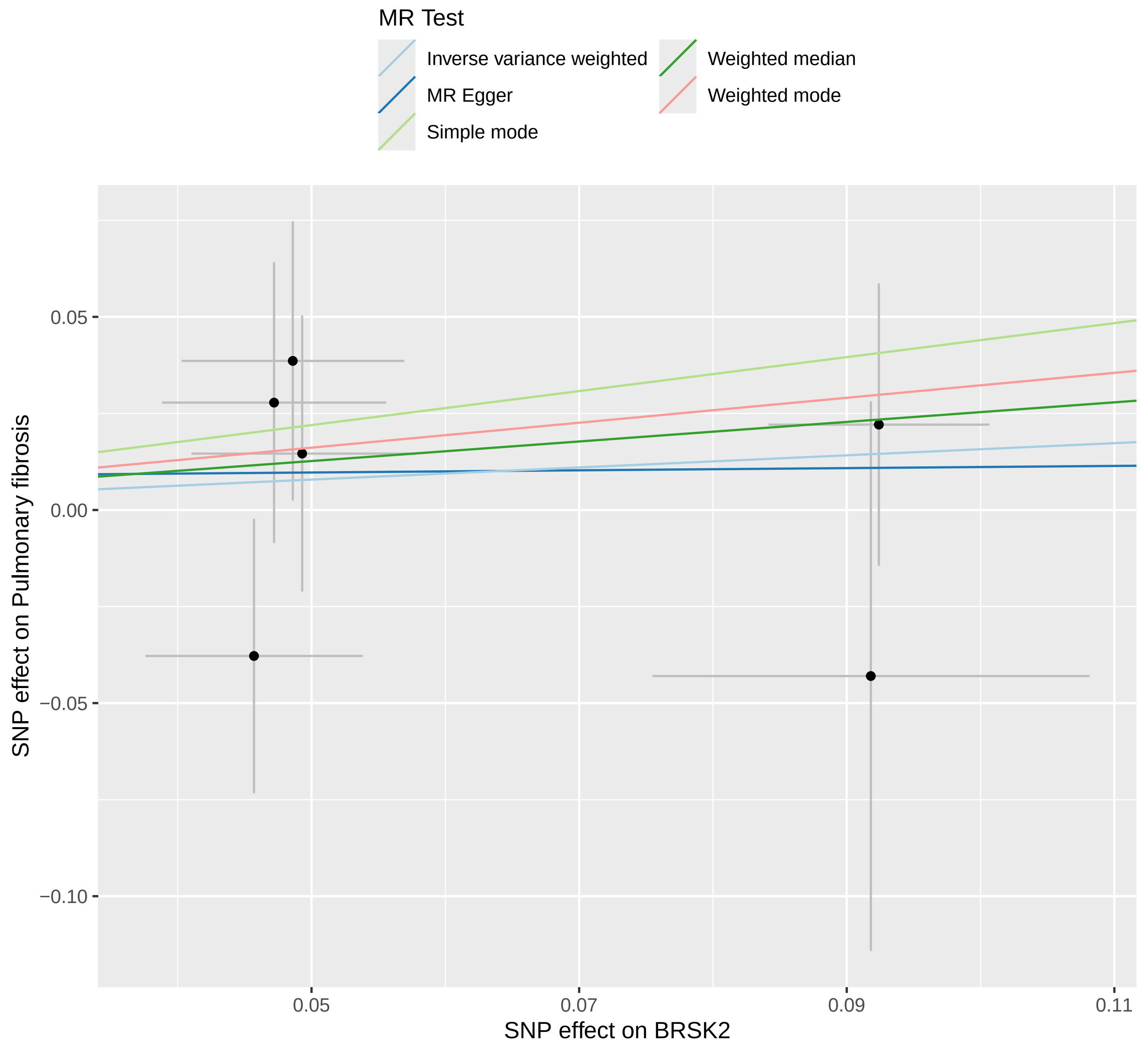
MR Test
Inverse variance weighted
MR Egger
Simple mode
Weighted median
Weighted mode
0.05
0.00
−0.05
−0.10
SNP effect on Pulmonary fibrosis
0.05
0.07
0.09
0.11
SNP effect on BRSK2

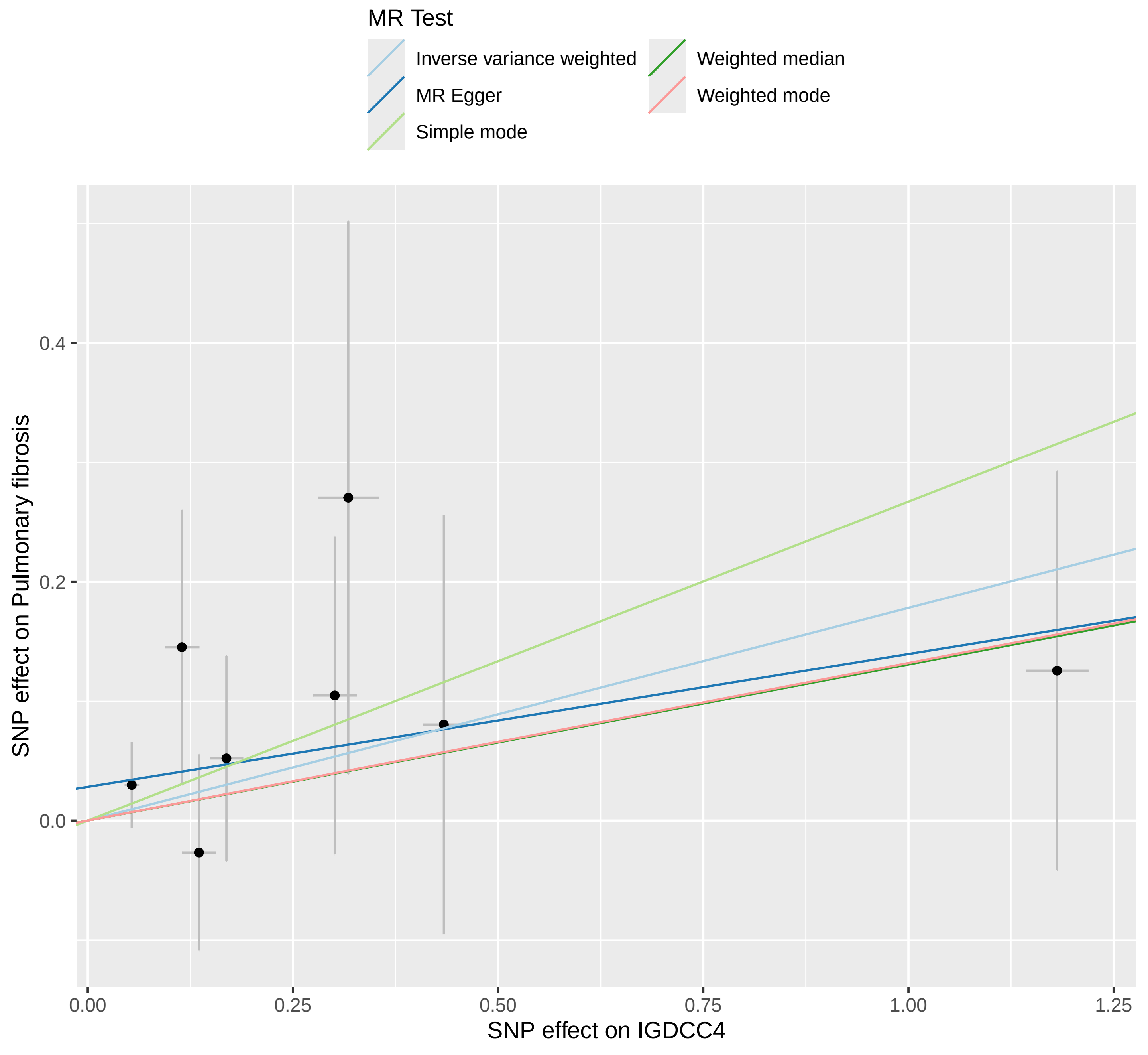
MR Test
Inverse variance weighted
MR Egger
Simple mode
Weighted median
Weighted mode
0.4
0.2
0.0
SNP effect on Pulmonary fibrosis
0.00
0.25
0.50
0.75
1.00
1.25
SNP effect on IGDCC4

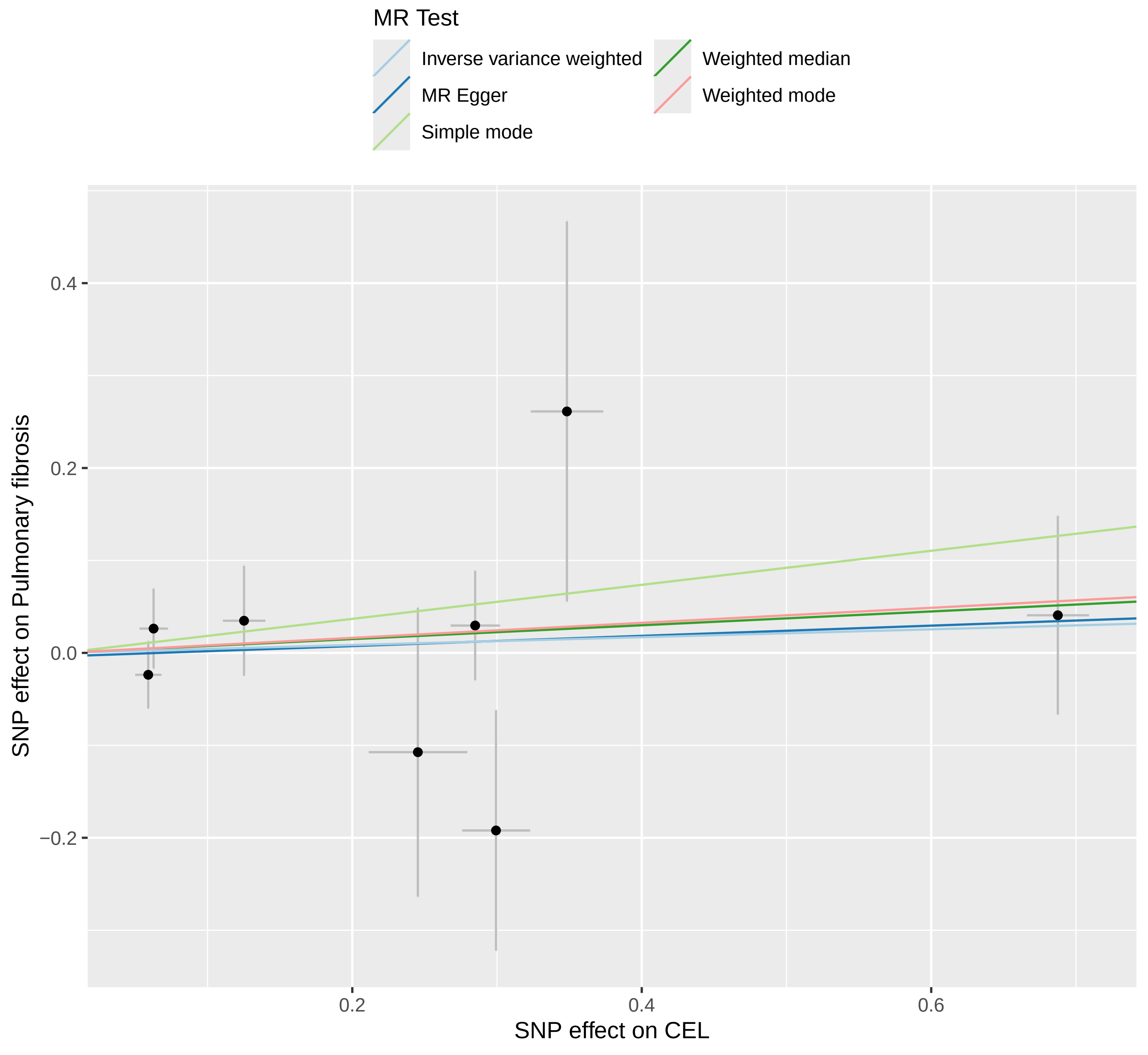
MR Test
Inverse variance weighted
MR Egger
Simple mode
Weighted median
Weighted mode
SNP effect on Pulmonary fibrosis
0.4
0.2
0.0
−0.2
0.2
0.4
0.6
SNP effect on CEL

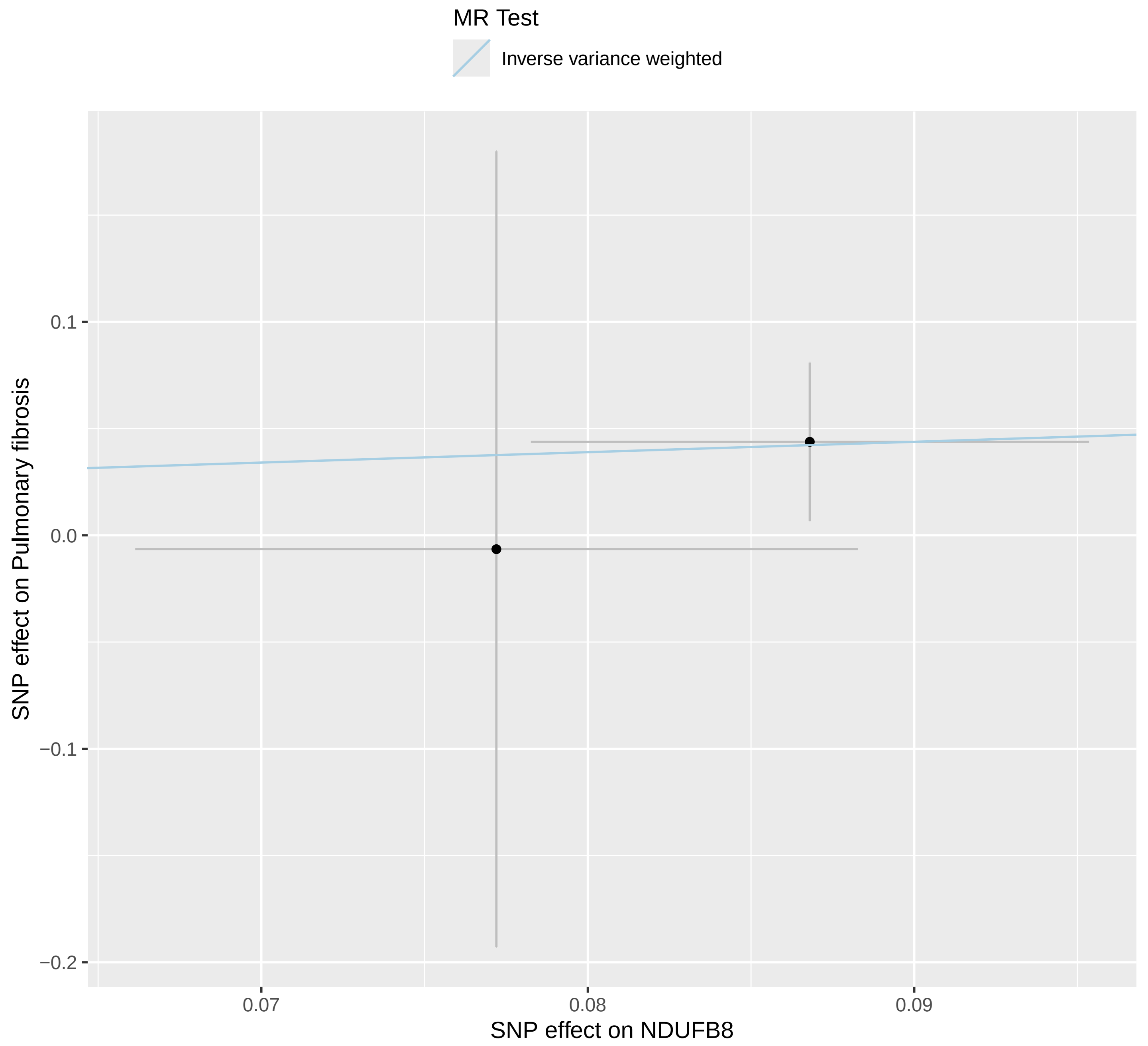

MR Test
Inverse variance weighted
0.1
0.0
−0.1
−0.2
SNP effect on Pulmonary fibrosis
0.07
0.08
0.09
SNP effect on NDUFB8

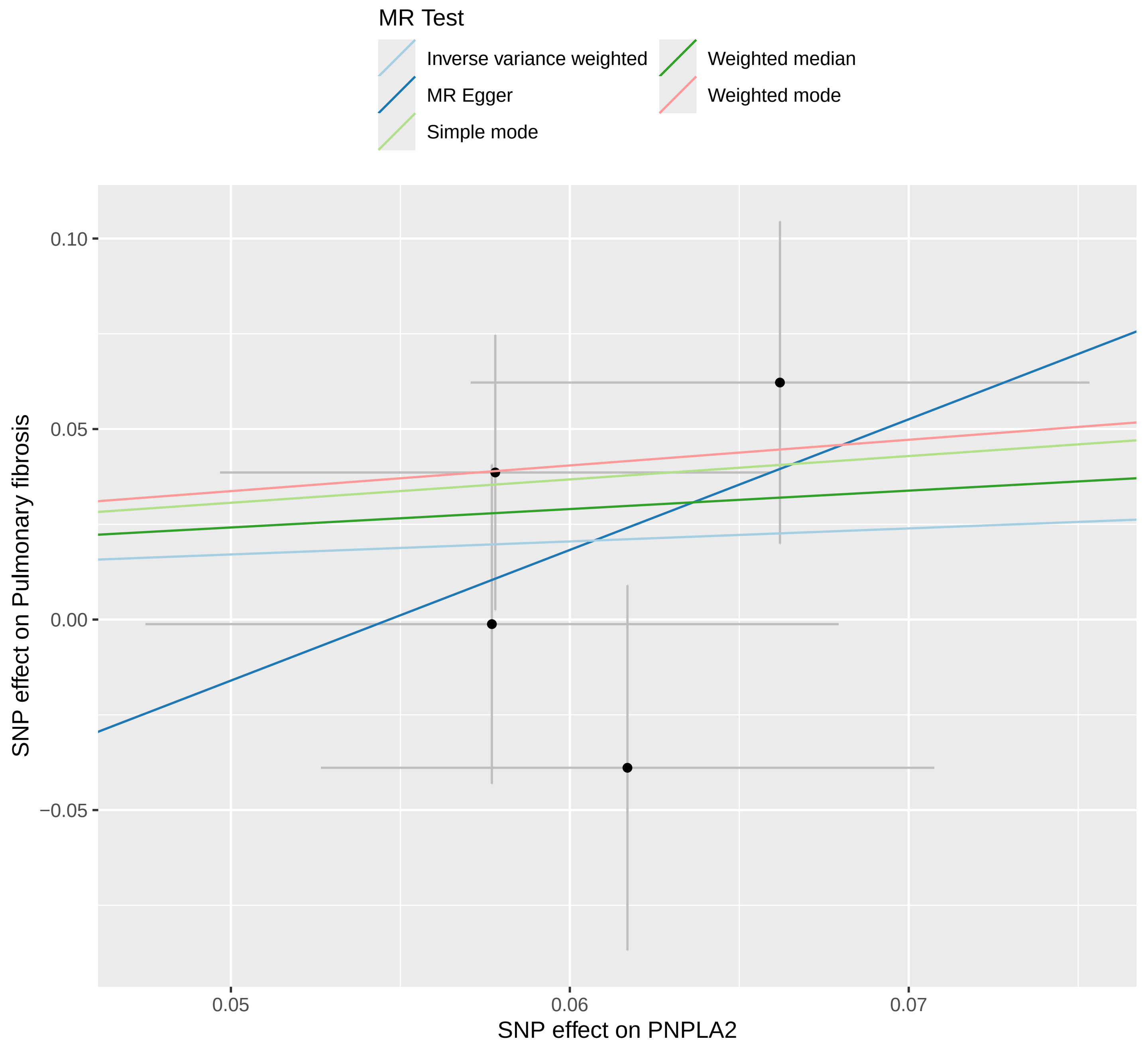
MR Test
Inverse variance weighted
MR Egger
Simple mode
Weighted median
Weighted mode
0.10
0.05
0.00
−0.05
SNP effect on Pulmonary fibrosis
0.05
0.06
0.07
SNP effect on PNPLA2

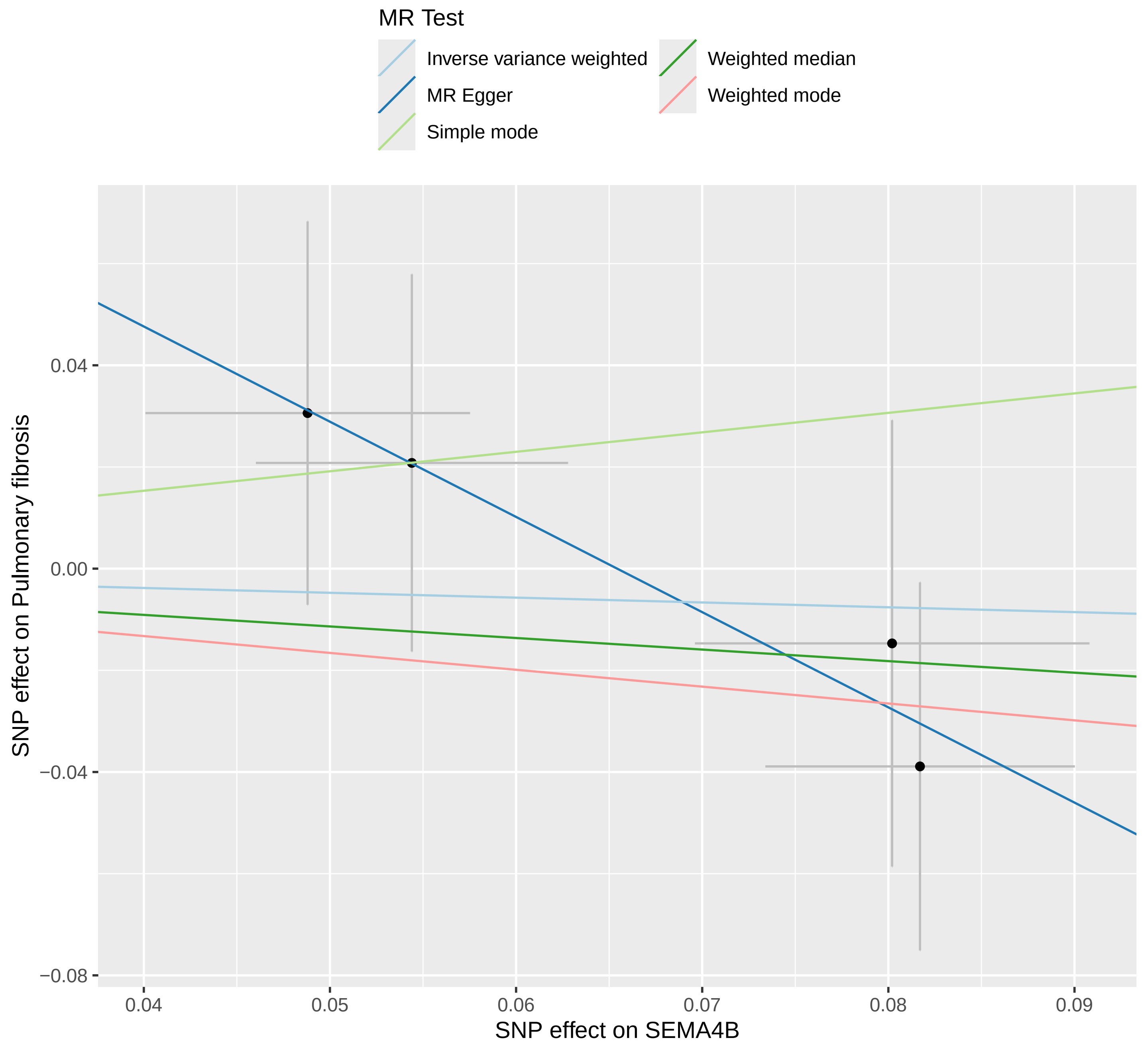
MR Test
Inverse variance weighted
MR Egger
Simple mode
Weighted median
Weighted mode
SNP effect on Pulmonary fibrosis
0.04
0.00
−0.04
−0.08
0.04
0.05
0.06
0.07
0.08
0.09
SNP effect on SEMA4B

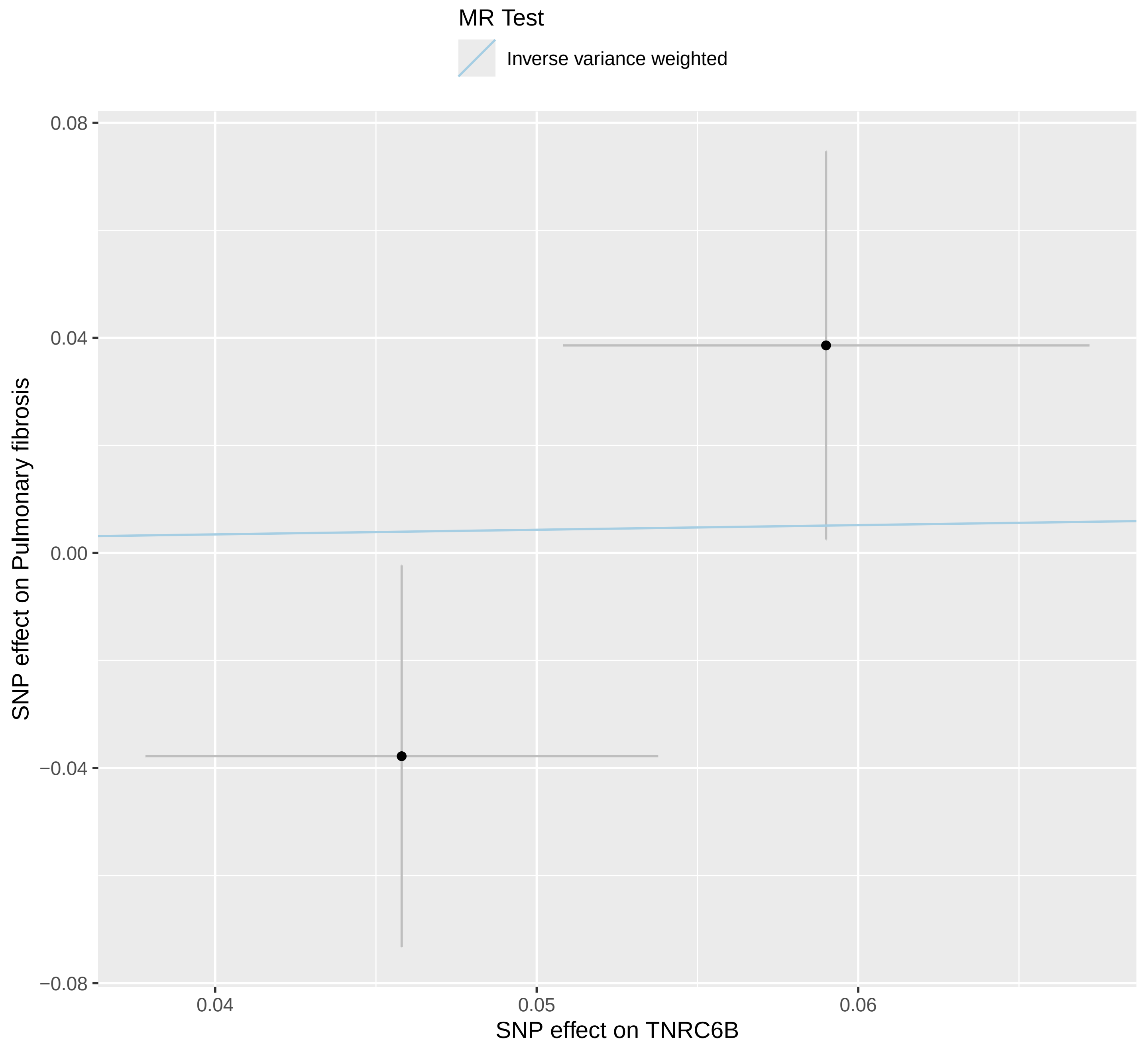

MR Test
Inverse variance weighted
0.08
0.04
0.00
−0.04
−0.08
0.04
0.05
0.06
SNP effect on Pulmonary fibrosis
SNP effect on TNRC6B

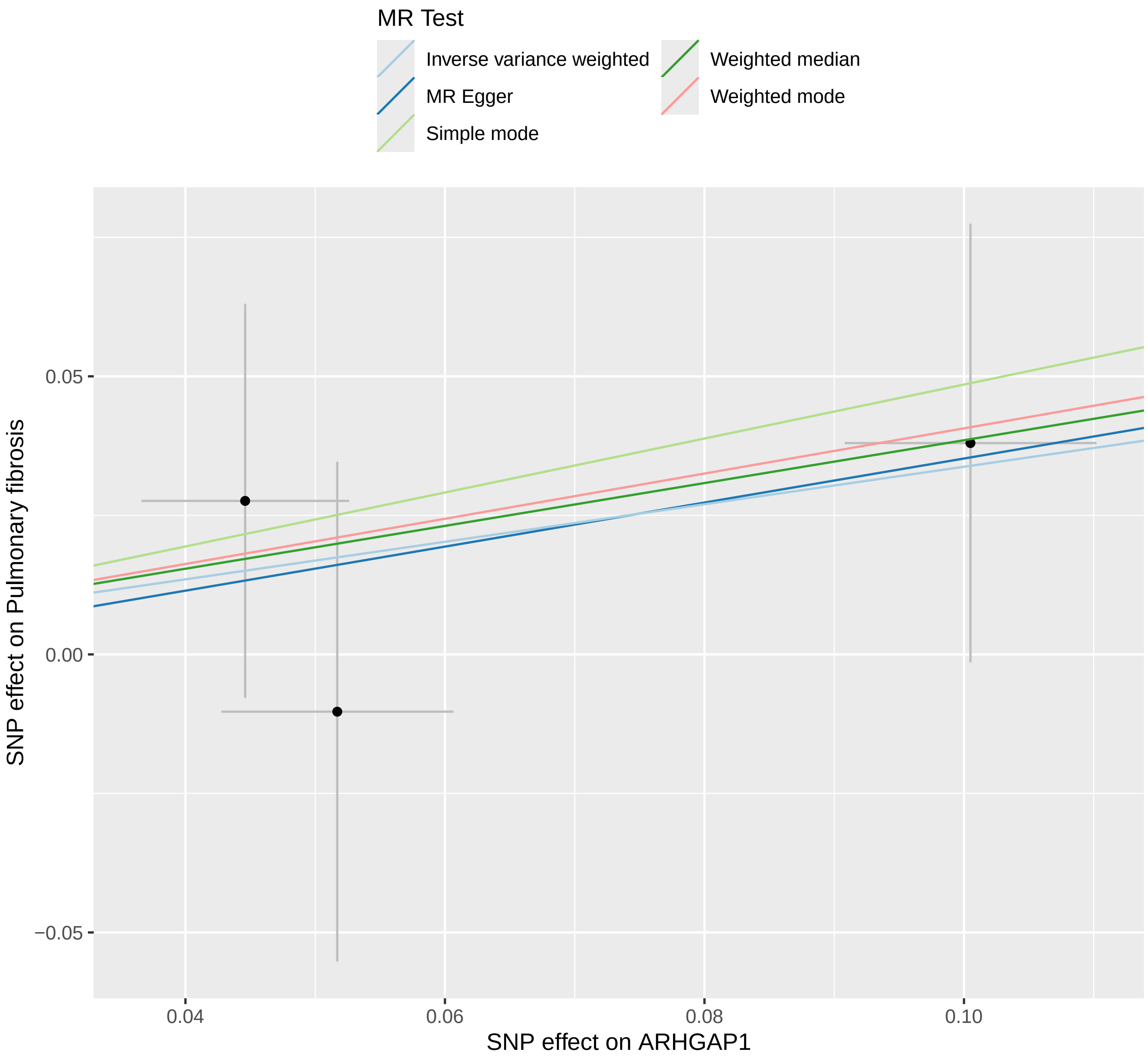
MR Test
Inverse variance weighted
MR Egger
Simple mode
Weighted median
Weighted mode
0.05
0.00
−0.05
SNP effect on Pulmonary fibrosis
0.04
0.06
0.08
0.10
SNP effect on ARHGAP1

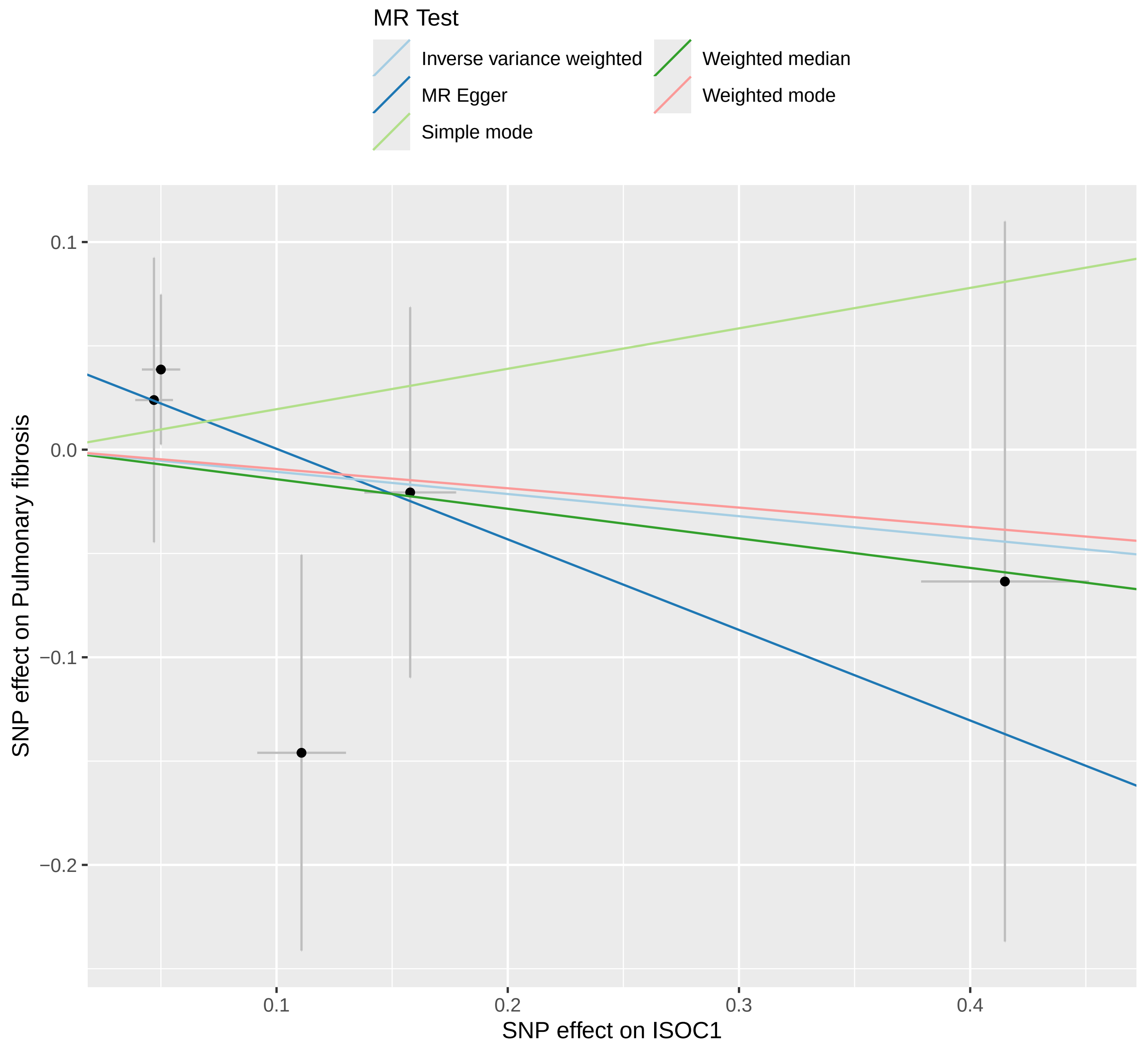

MR Test
Inverse variance weighted
MR Egger
Simple mode
Weighted median
Weighted mode
0.1
0.0
−0.1
−0.2
SNP effect on Pulmonary fibrosis
0.1
0.2
0.3
0.4
SNP effect on ISOC1

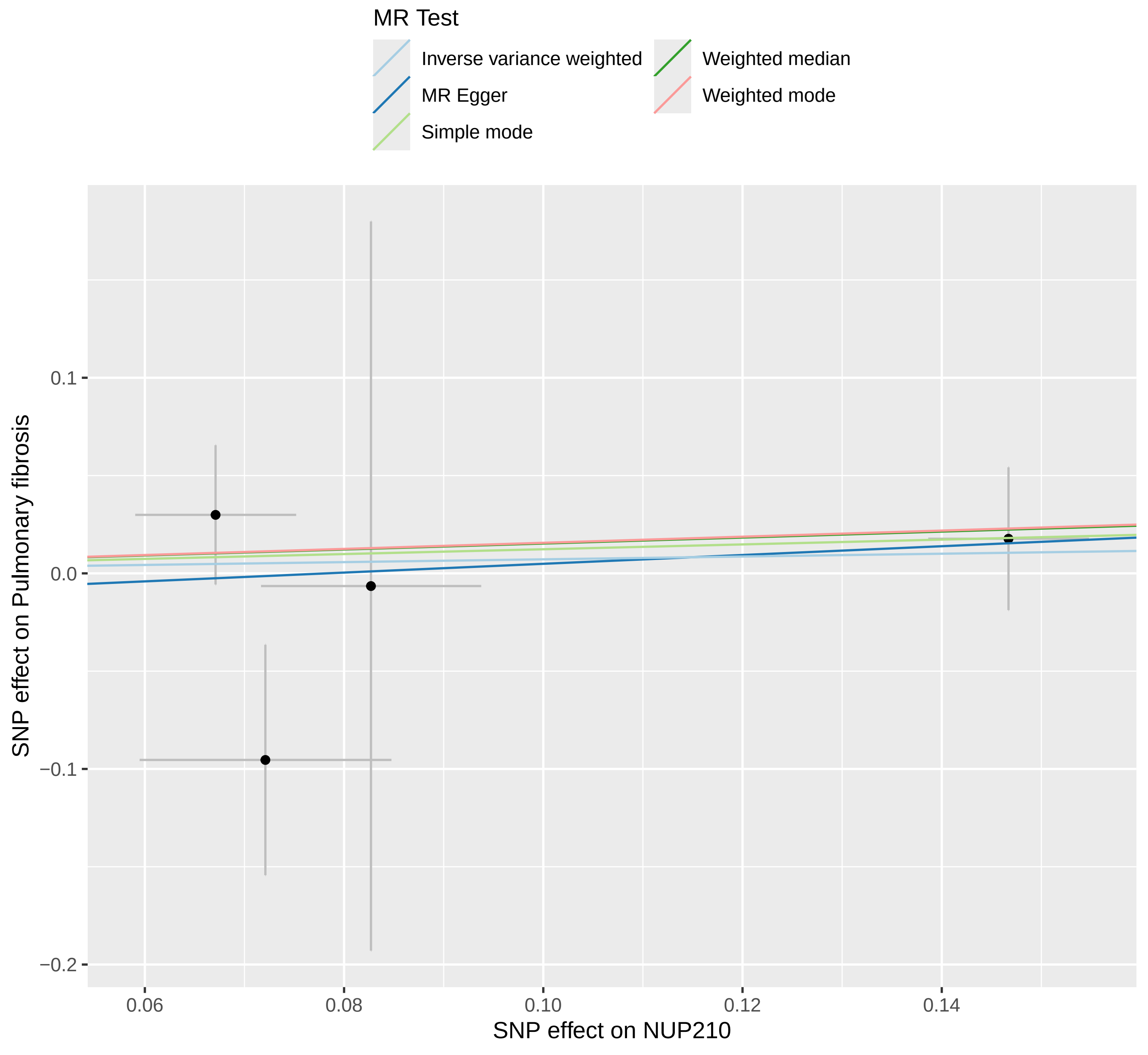
MR Test
Inverse variance weighted
MR Egger
Simple mode
Weighted median
Weighted mode
0.1
0.0
−0.1
−0.2
0.06
0.08
0.10
0.12
0.14
SNP effect on Pulmonary fibrosis
SNP effect on NUP210

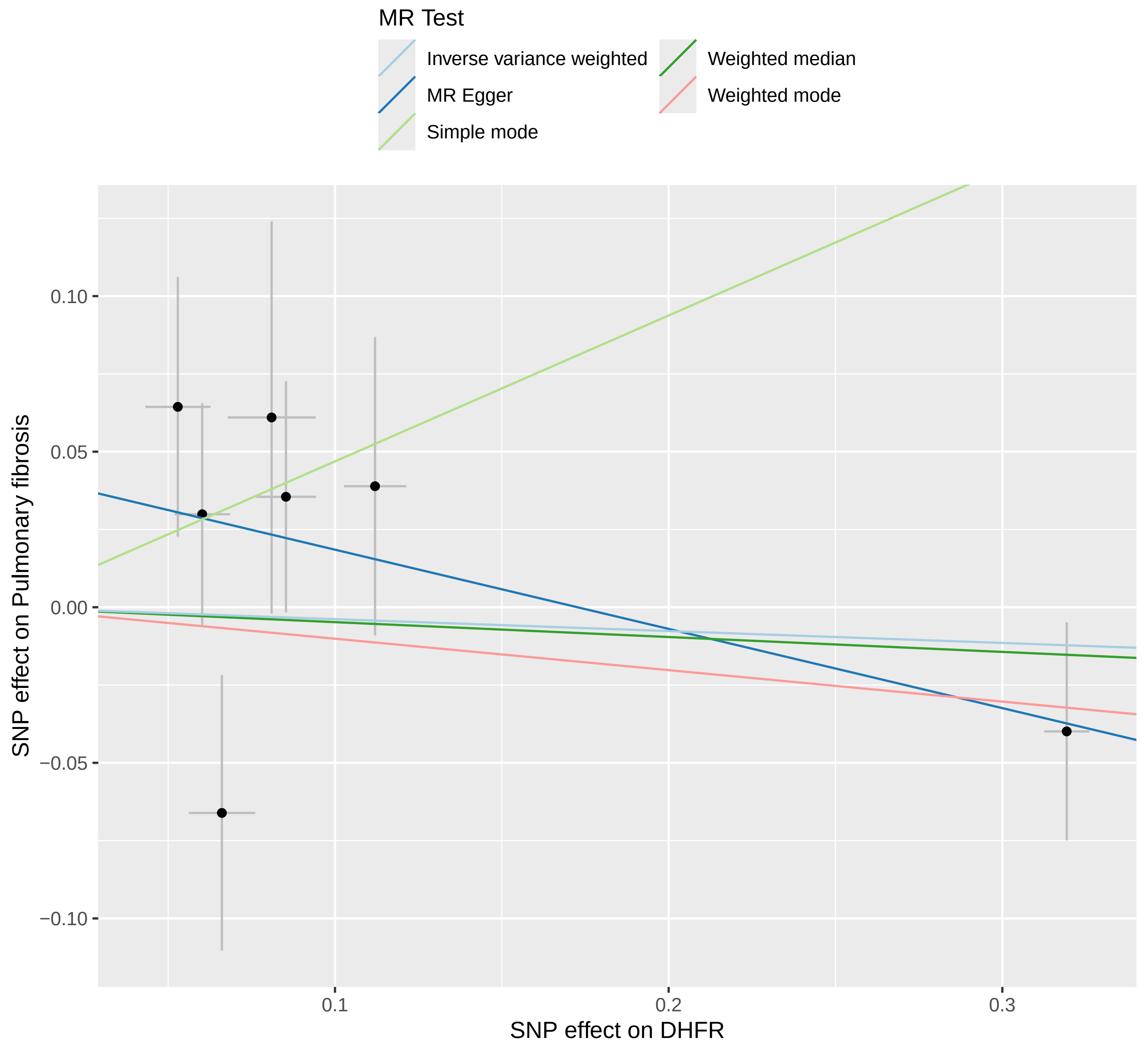

MR Test
Inverse variance weighted
MR Egger
Simple mode
Weighted median
Weighted mode
0.10
0.05
0.00
−0.05
−0.10
SNP effect on Pulmonary fibrosis
0.1
0.2
0.3
SNP effect on DHFR

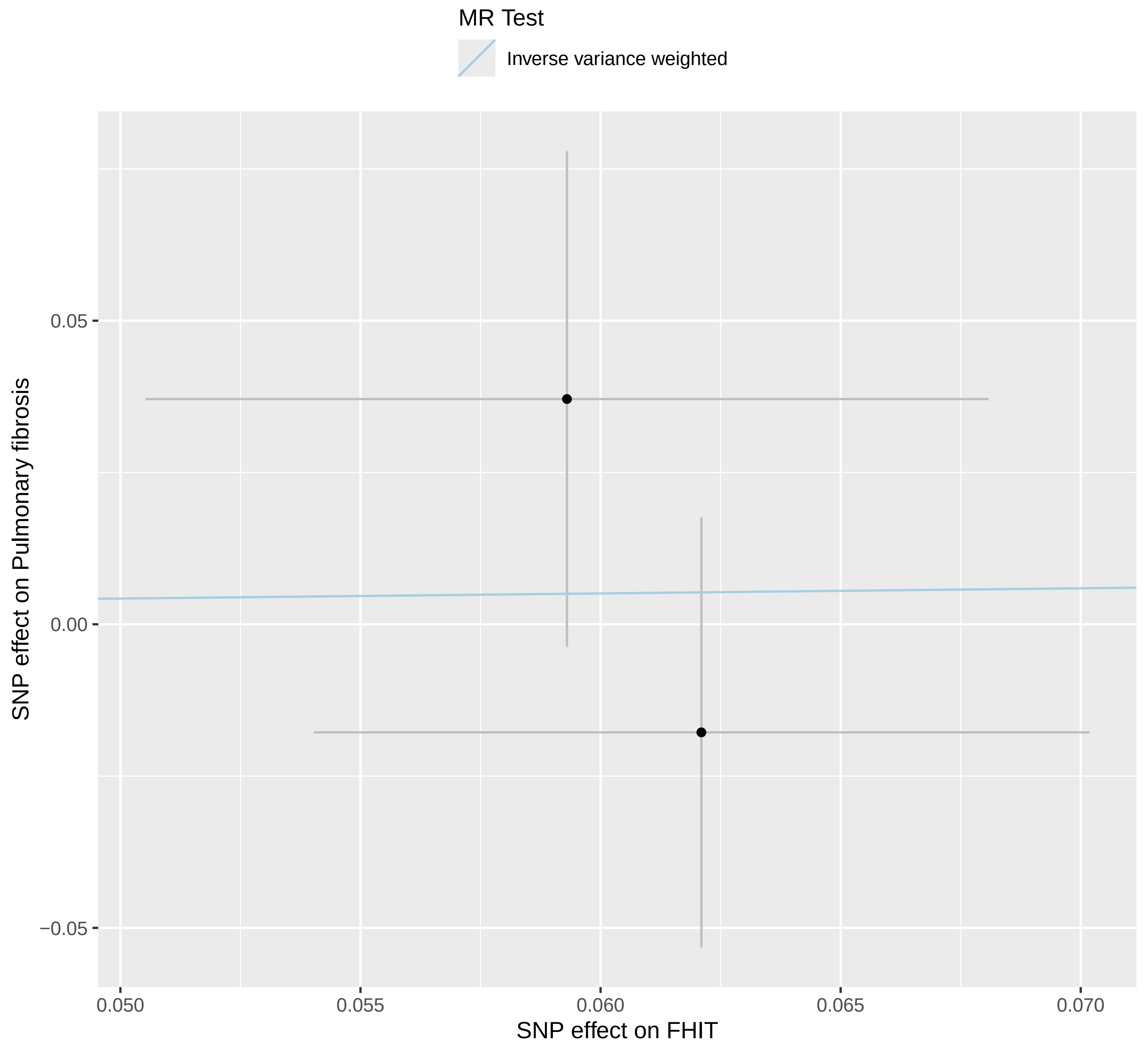
MR Test
Inverse variance weighted
0.05
0.00
−0.05
SNP effect on Pulmonary fibrosis
0.050
0.055
0.060
0.065
0.070
SNP effect on FHIT

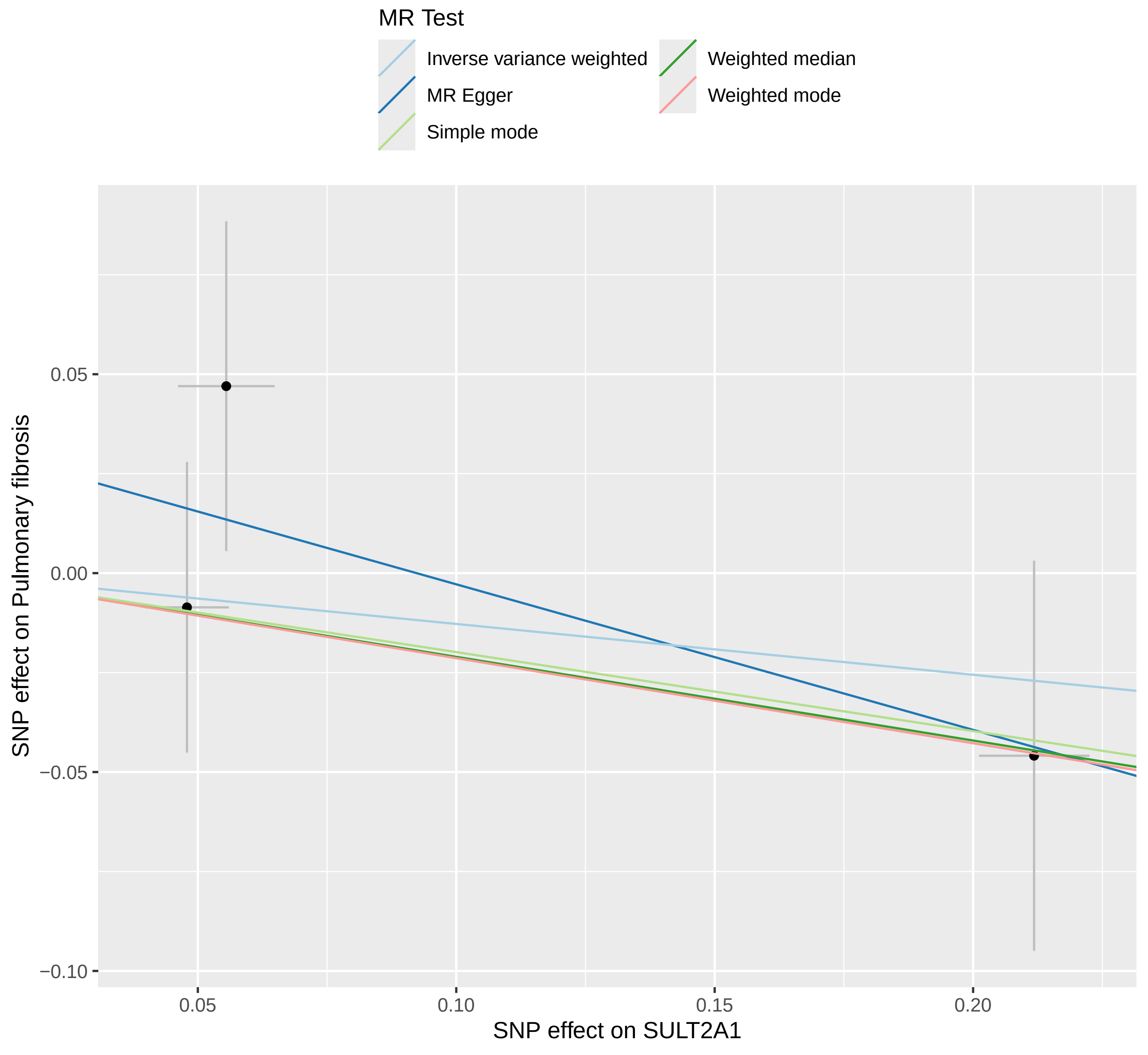

MR Test
Inverse variance weighted
MR Egger
Simple mode
Weighted median
Weighted mode
0.05
0.00
−0.05
−0.10
SNP effect on Pulmonary fibrosis
0.05
0.10
0.15
0.20
SNP effect on SULT2A1

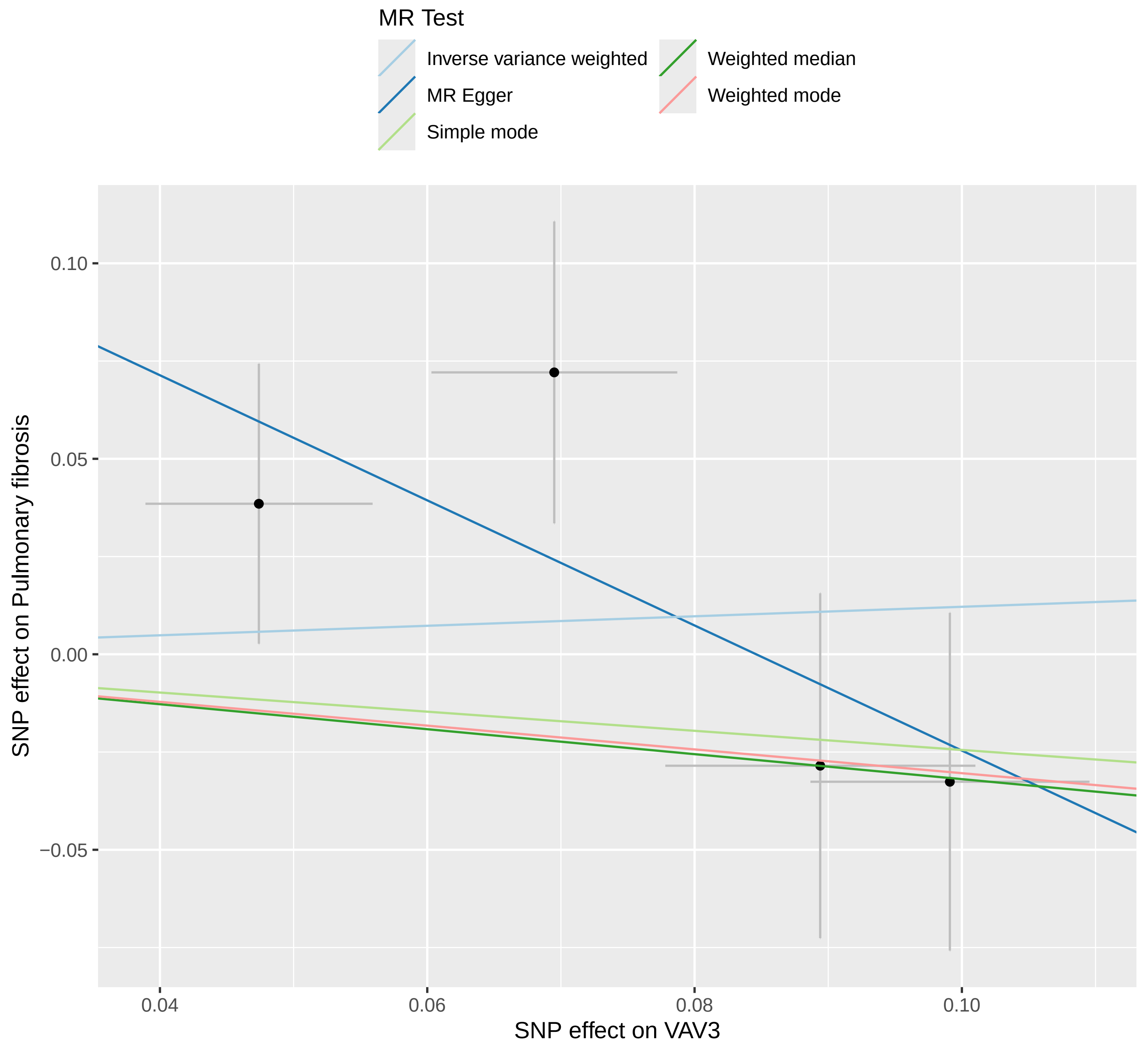
MR Test
Inverse variance weighted
MR Egger
Simple mode
Weighted median
Weighted mode
0.10
0.05
0.00
−0.05
SNP effect on Pulmonary fibrosis
0.04
0.06
0.08
0.10
SNP effect on VAV3

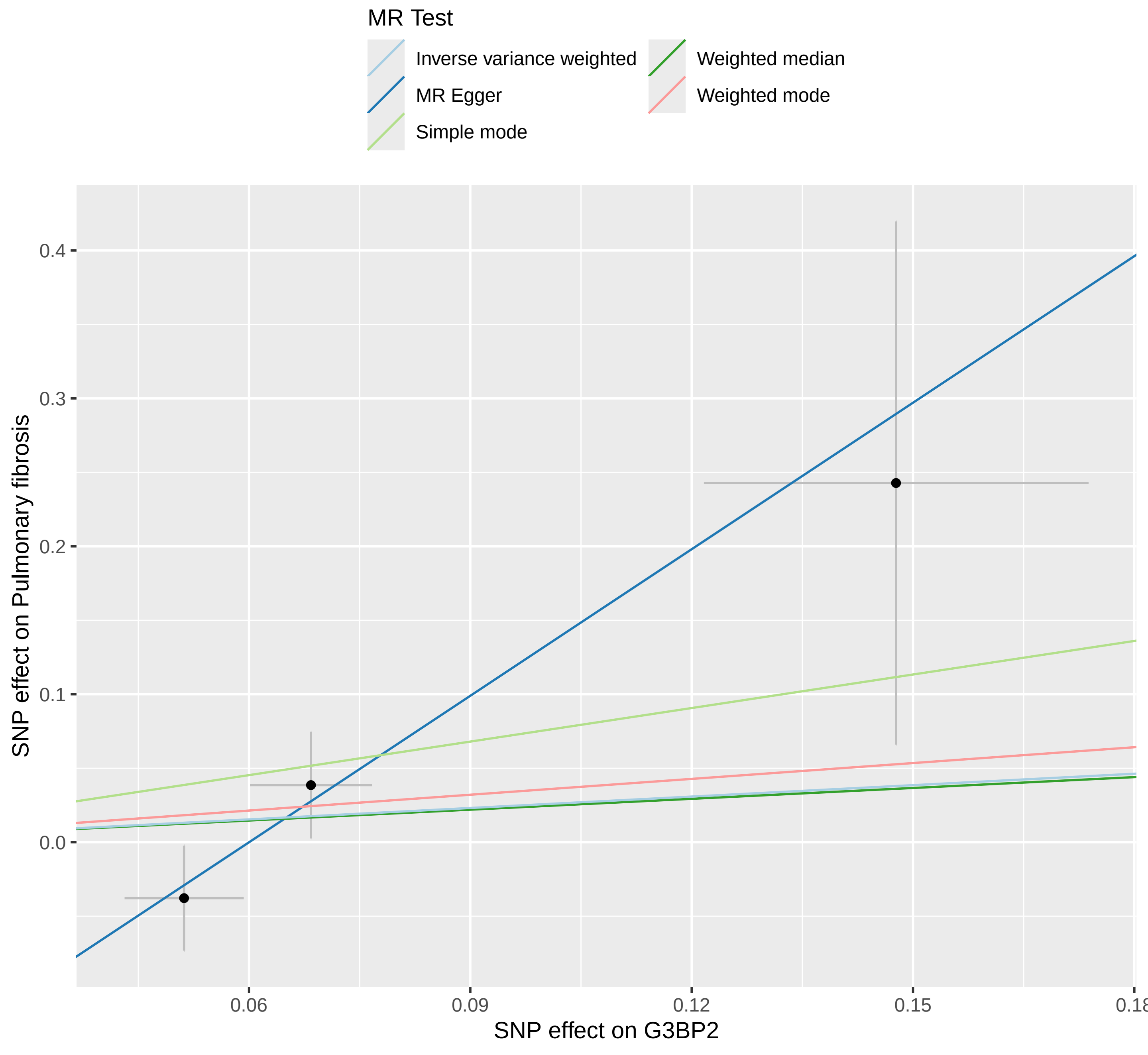
MR Test
Inverse variance weighted
MR Egger
Simple mode
Weighted median
Weighted mode
0.4
0.3
0.2
0.1
0.0
SNP effect on Pulmonary fibrosis
0.06
0.09
0.12
0.15
0.18
SNP effect on G3BP2

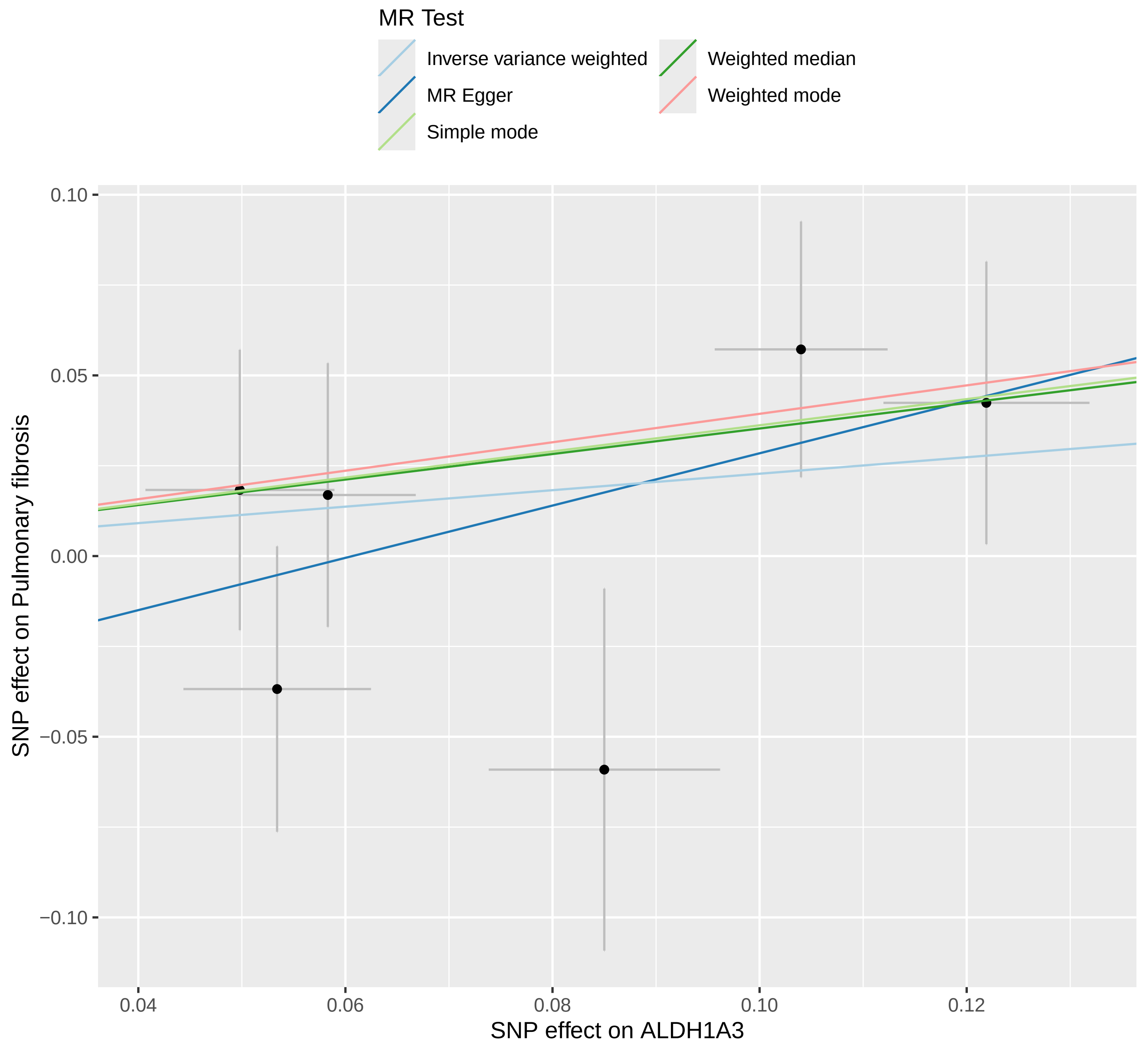
MR Test
Inverse variance weighted
MR Egger
Simple mode
Weighted median
Weighted mode
0.10
0.05
0.00
−0.05
−0.10
0.04
0.06
0.08
0.10
0.12
SNP effect on ALDH1A3
SNP effect on Pulmonary fibrosis

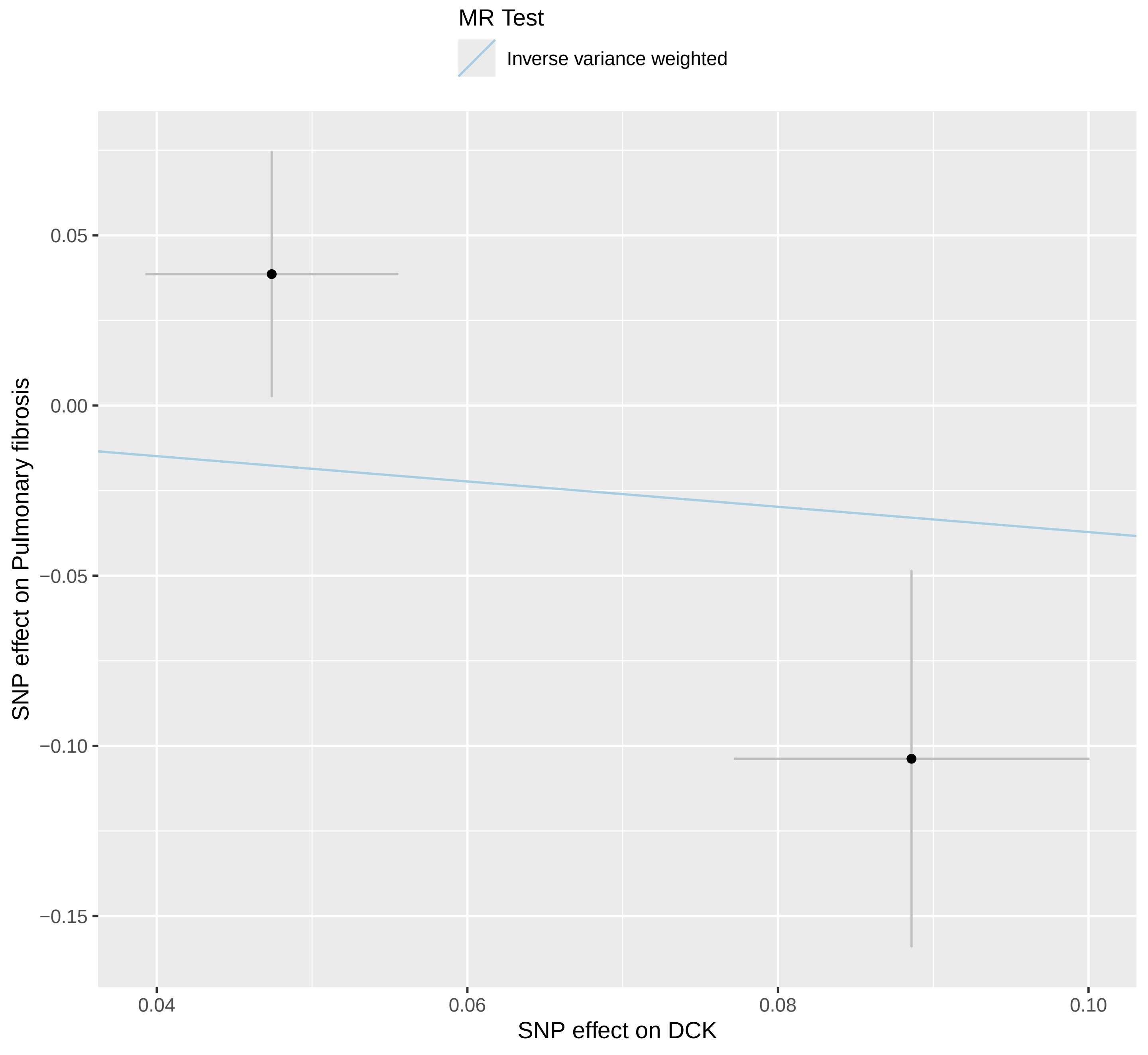
MR Test
Inverse variance weighted
0.05
0.00
−0.05
−0.10
−0.15
SNP effect on Pulmonary fibrosis
0.04
0.06
0.08
0.10
SNP effect on DCK

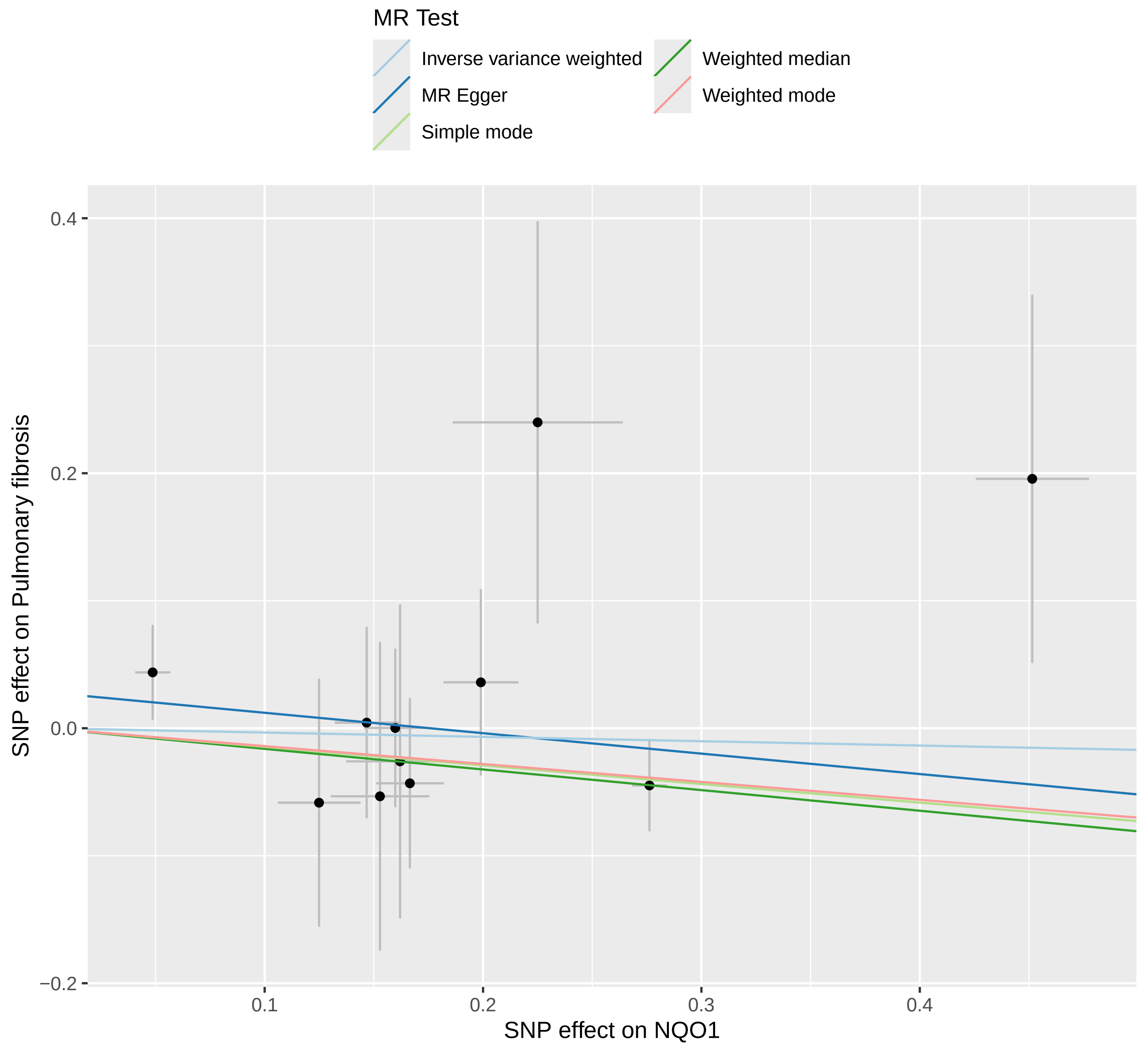

MR Test
Inverse variance weighted
MR Egger
Simple mode
Weighted median
Weighted mode
0.4
0.2
0.0
−0.2
0.1
0.2
0.3
0.4
SNP effect on NQO1
SNP effect on Pulmonary fibrosis

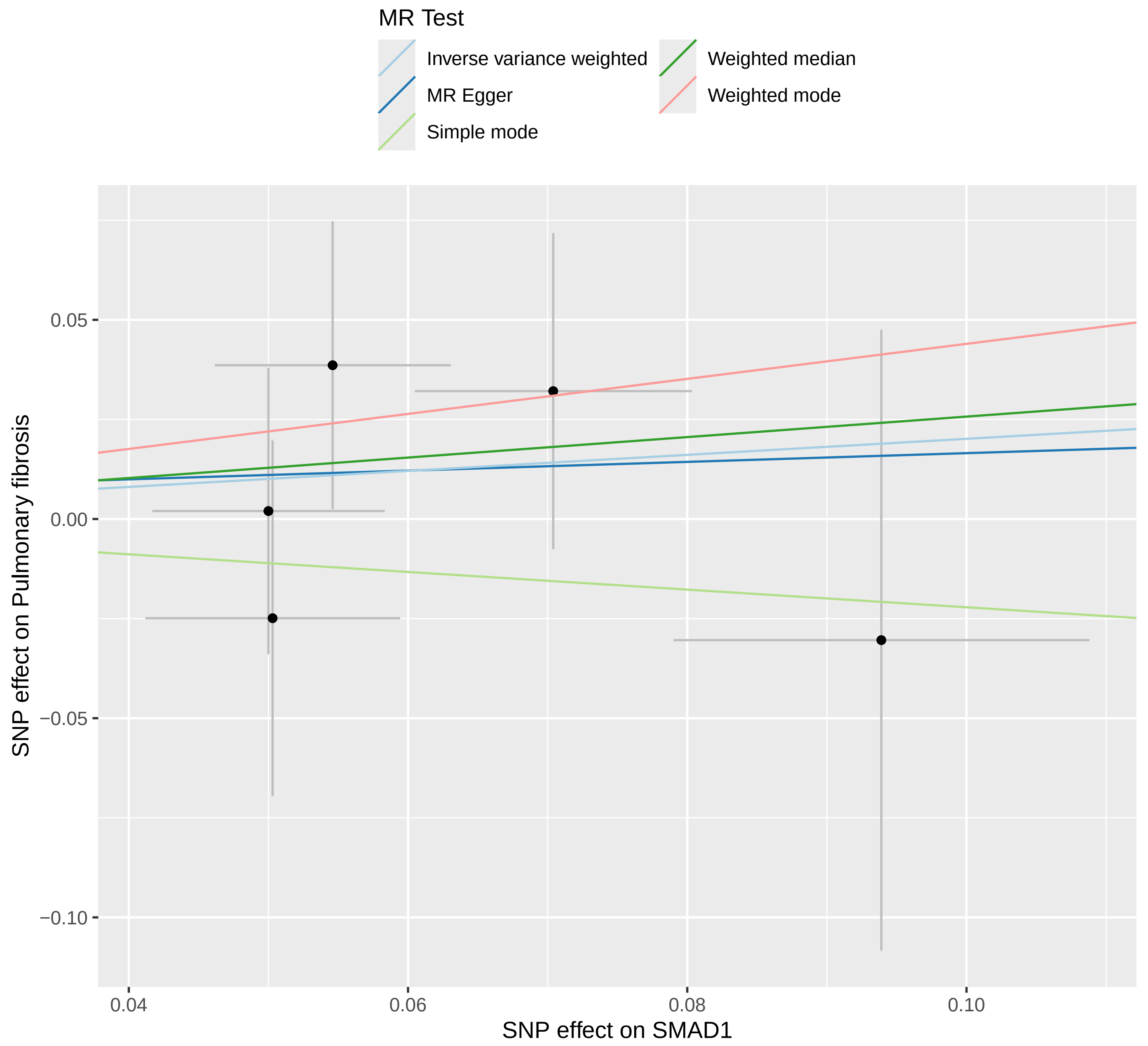

MR Test
Inverse variance weighted
MR Egger
Simple mode
Weighted median
Weighted mode
0.05
0.00
−0.05
−0.10
SNP effect on Pulmonary fibrosis
0.04
0.06
0.08
0.10
SNP effect on SMAD1

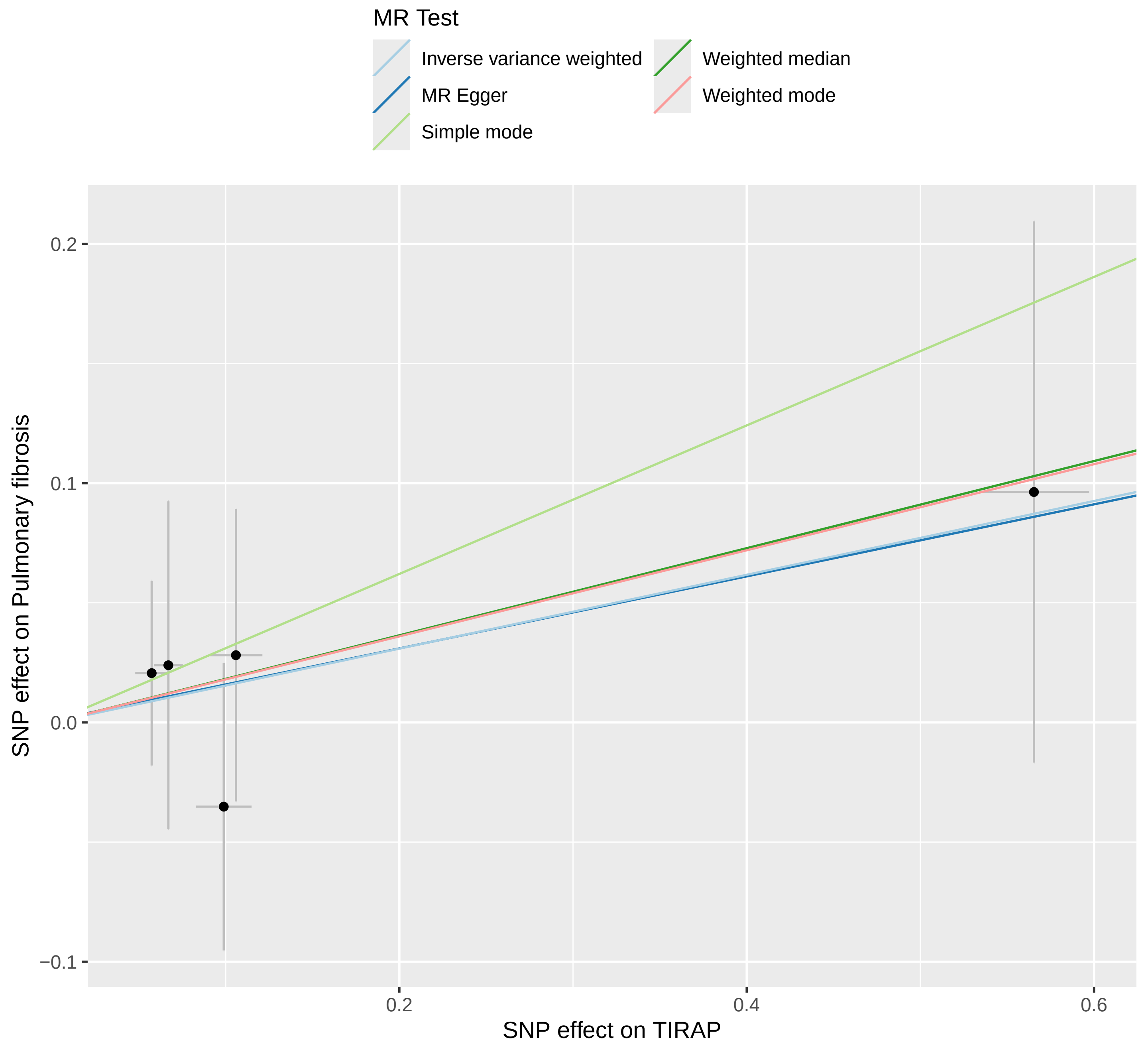
MR Test
Inverse variance weighted
MR Egger
Simple mode
Weighted median
Weighted mode
0.2
0.1
0.0
−0.1
0.2
0.4
0.6
SNP effect on Pulmonary fibrosis
SNP effect on TIRAP

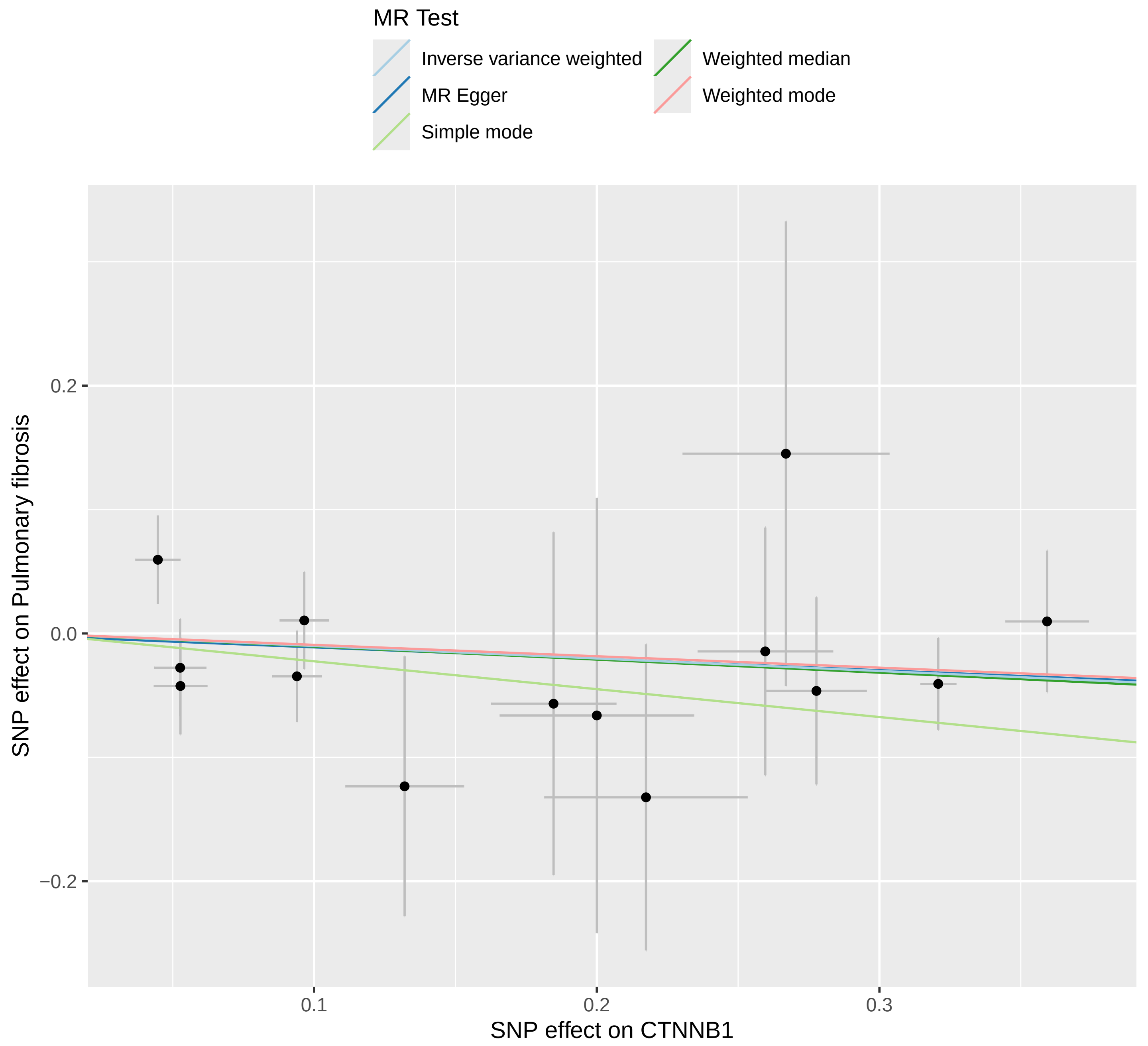
MR Test
Inverse variance weighted
MR Egger
Simple mode
Weighted median
Weighted mode
0.2
0.0
−0.2
0.1
0.2
0.3
SNP effect on Pulmonary fibrosis
SNP effect on CTNNB1

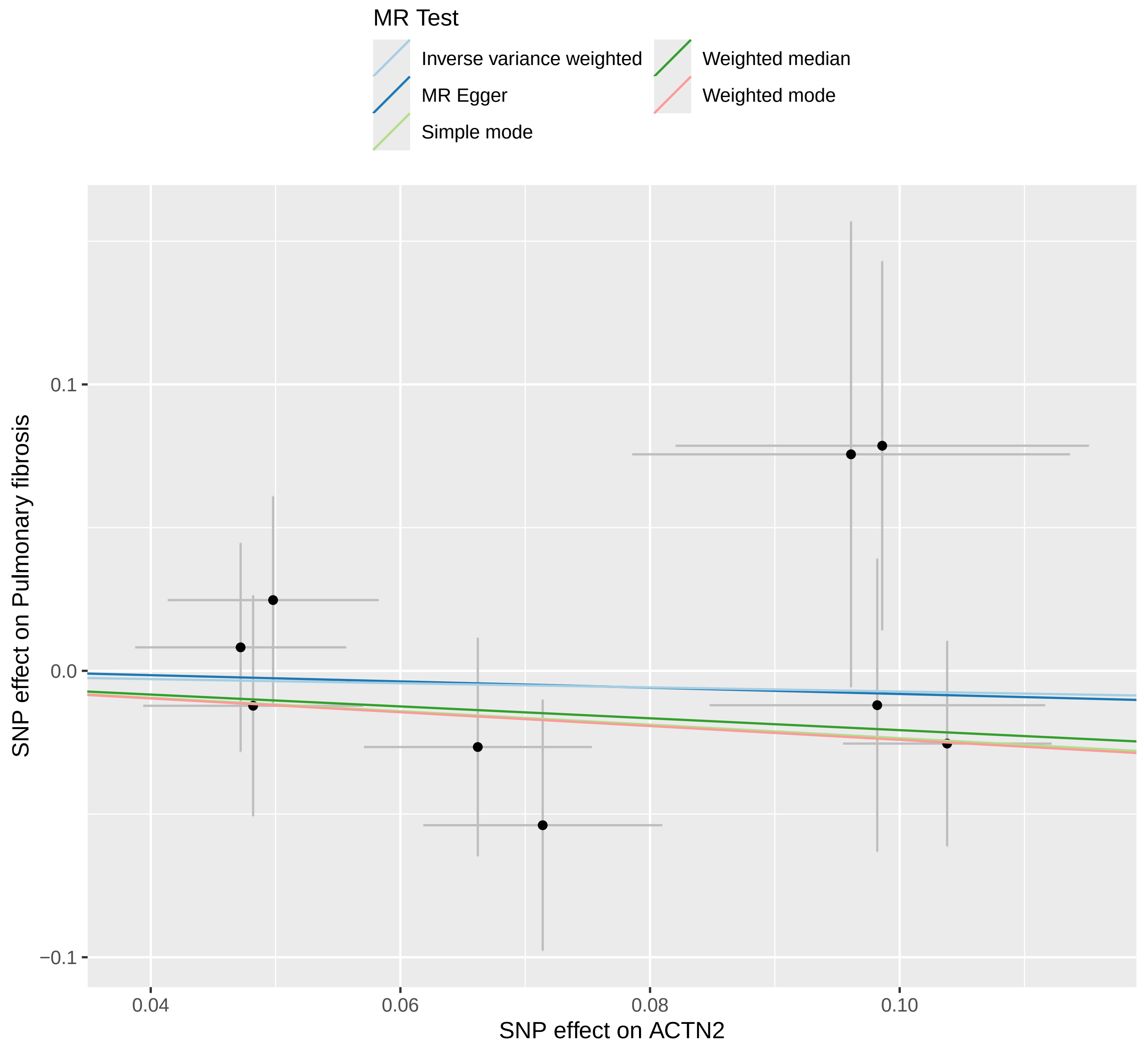

MR Test
Inverse variance weighted
MR Egger
Simple mode
Weighted median
Weighted mode
0.1
0.0
−0.1
SNP effect on Pulmonary fibrosis
0.04
0.06
0.08
0.10
SNP effect on ACTN2

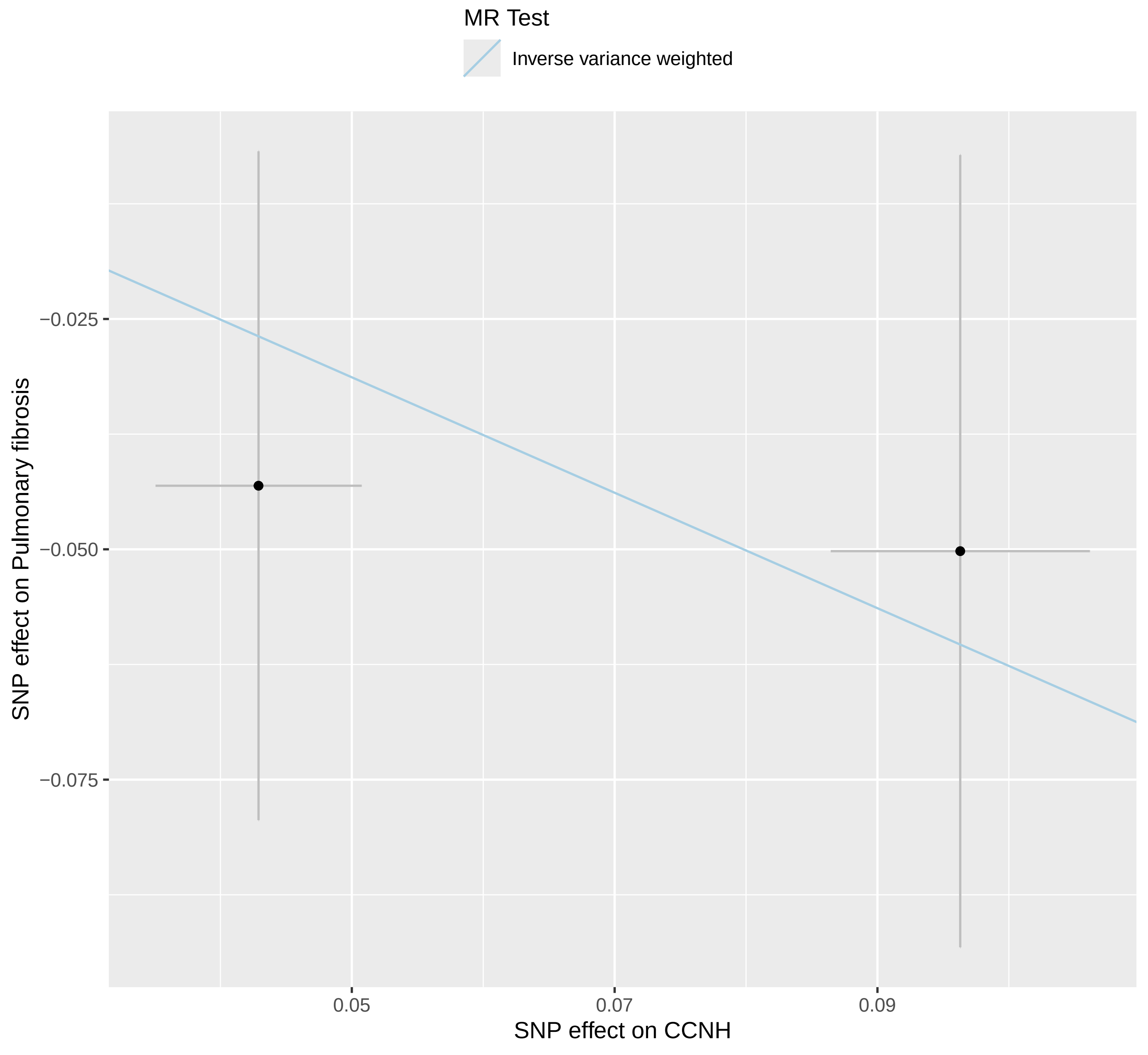

MR Test
Inverse variance weighted
−0.025
−0.050
−0.075
SNP effect on Pulmonary fibrosis
0.05
0.07
0.09
SNP effect on CCNH

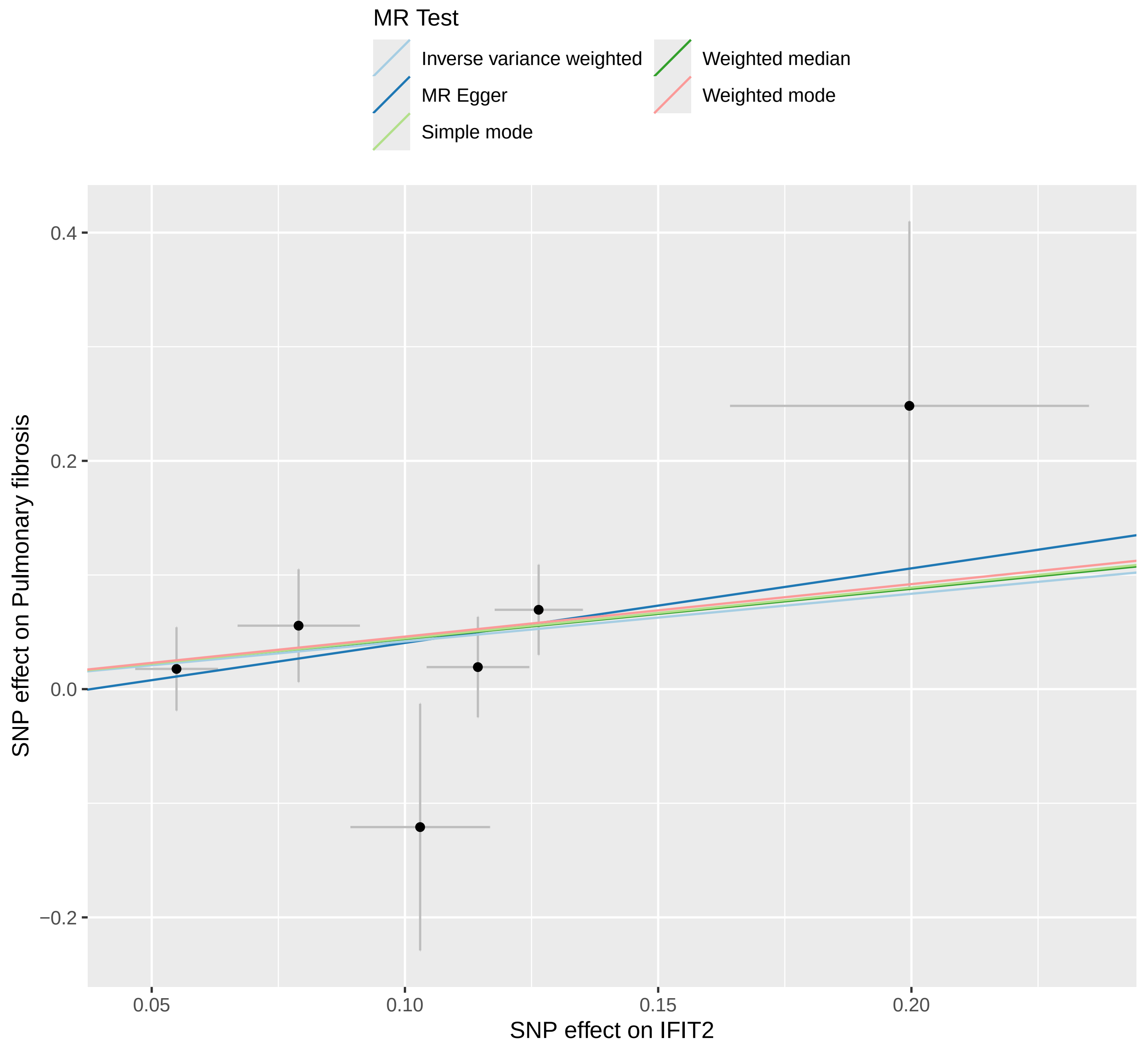
MR Test
Inverse variance weighted
MR Egger
Simple mode
Weighted median
Weighted mode
SNP effect on Pulmonary fibrosis
0.4
0.2
0.0
−0.2
0.05
0.10
0.15
0.20
SNP effect on IFIT2

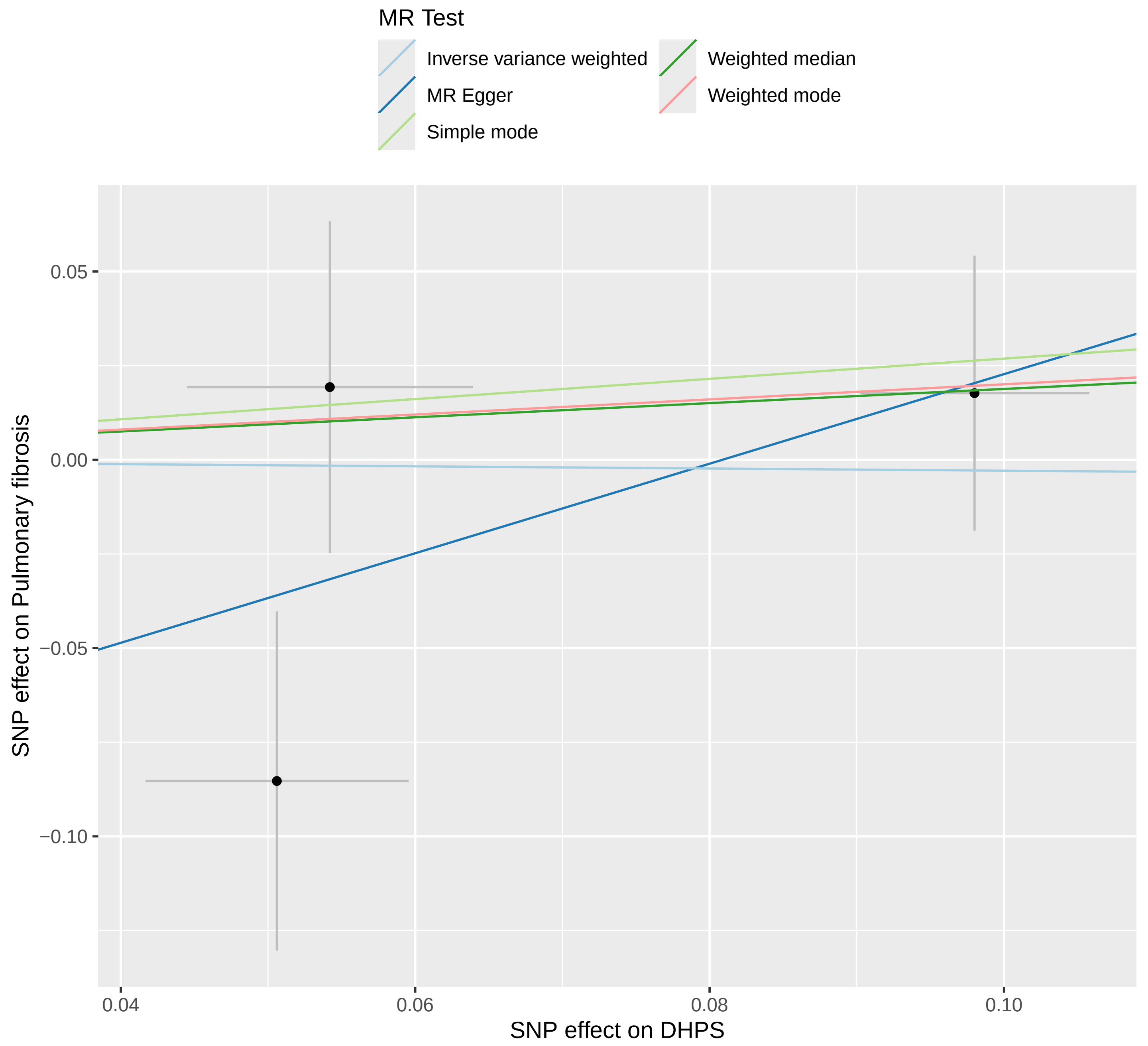

MR Test
Inverse variance weighted
MR Egger
Simple mode
Weighted median
Weighted mode
0.05
0.00
−0.05
−0.10
SNP effect on Pulmonary fibrosis
0.04
0.06
0.08
0.10
SNP effect on DHPS

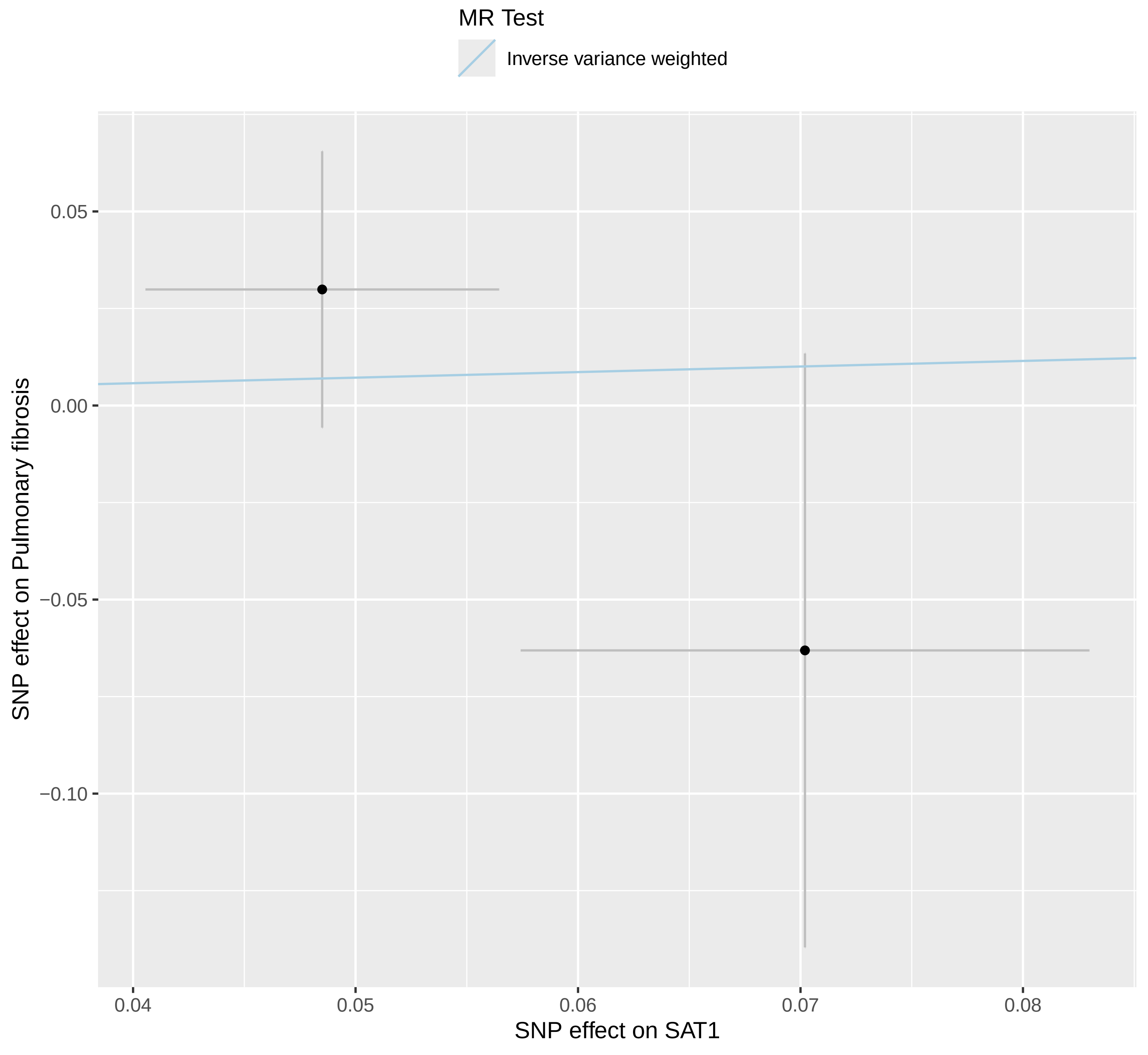
MR Test
Inverse variance weighted
0.05
0.00
−0.05
−0.10
0.04
0.05
0.06
0.07
0.08
SNP effect on SAT1
SNP effect on Pulmonary fibrosis

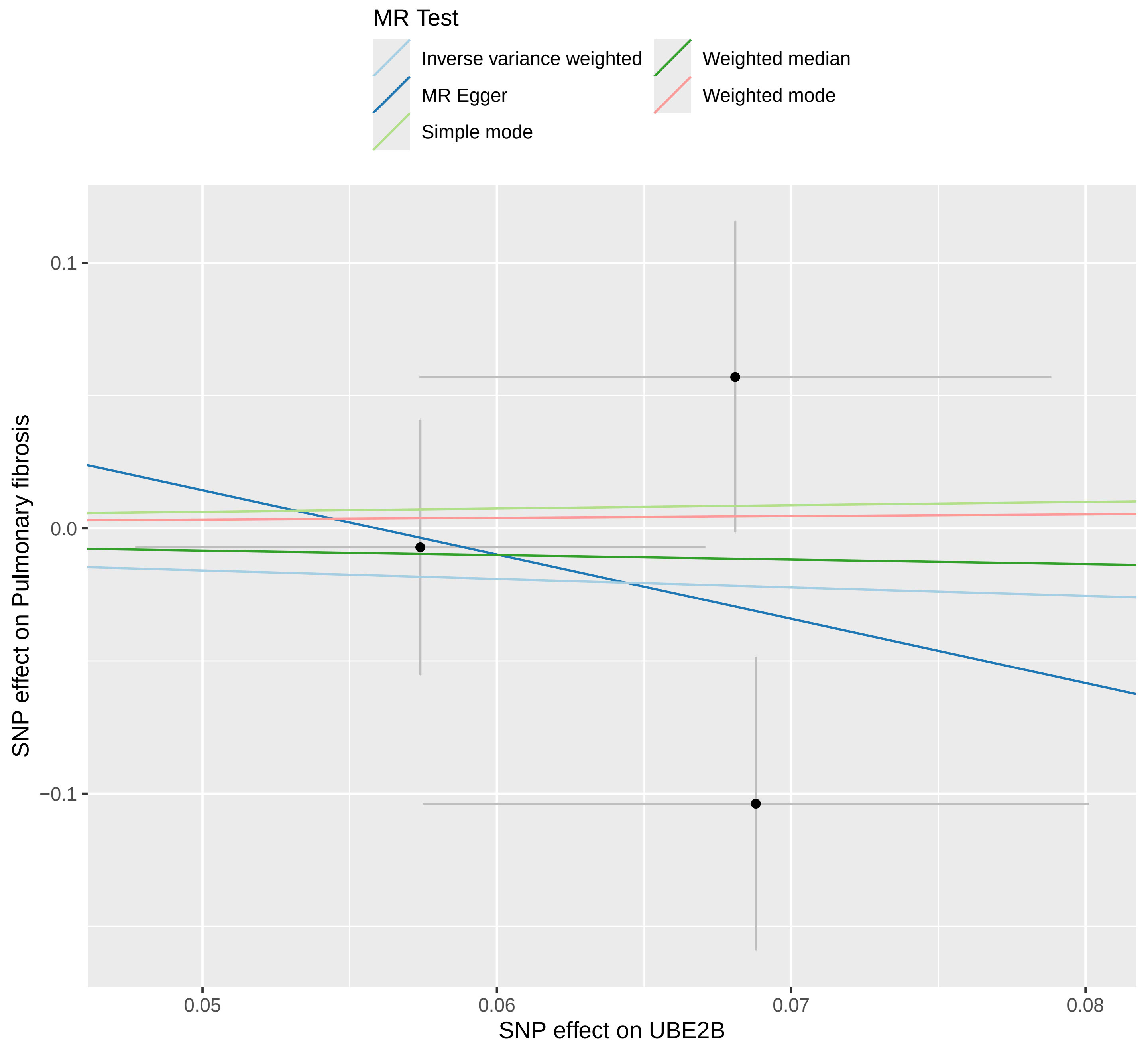

MR Test
Inverse variance weighted
MR Egger
Simple mode
Weighted median
Weighted mode
0.1
0.0
−0.1
0.05
0.06
0.07
0.08
SNP effect on Pulmonary fibrosis
SNP effect on UBE2B

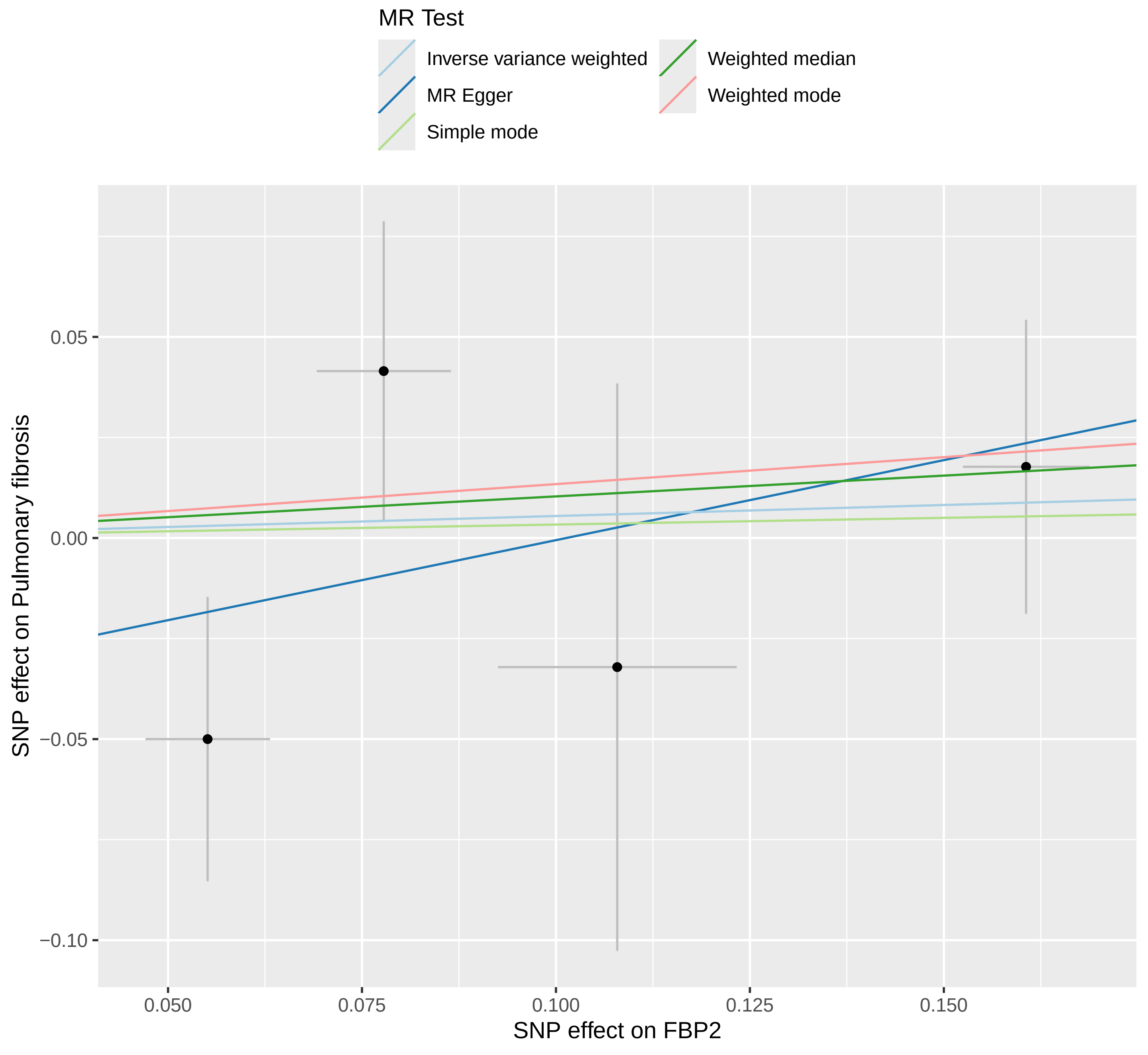

MR Test
Inverse variance weighted
MR Egger
Simple mode
Weighted median
Weighted mode
0.05
0.00
−0.05
−0.10
SNP effect on Pulmonary fibrosis
0.050
0.075
0.100
0.125
0.150
SNP effect on FBP2

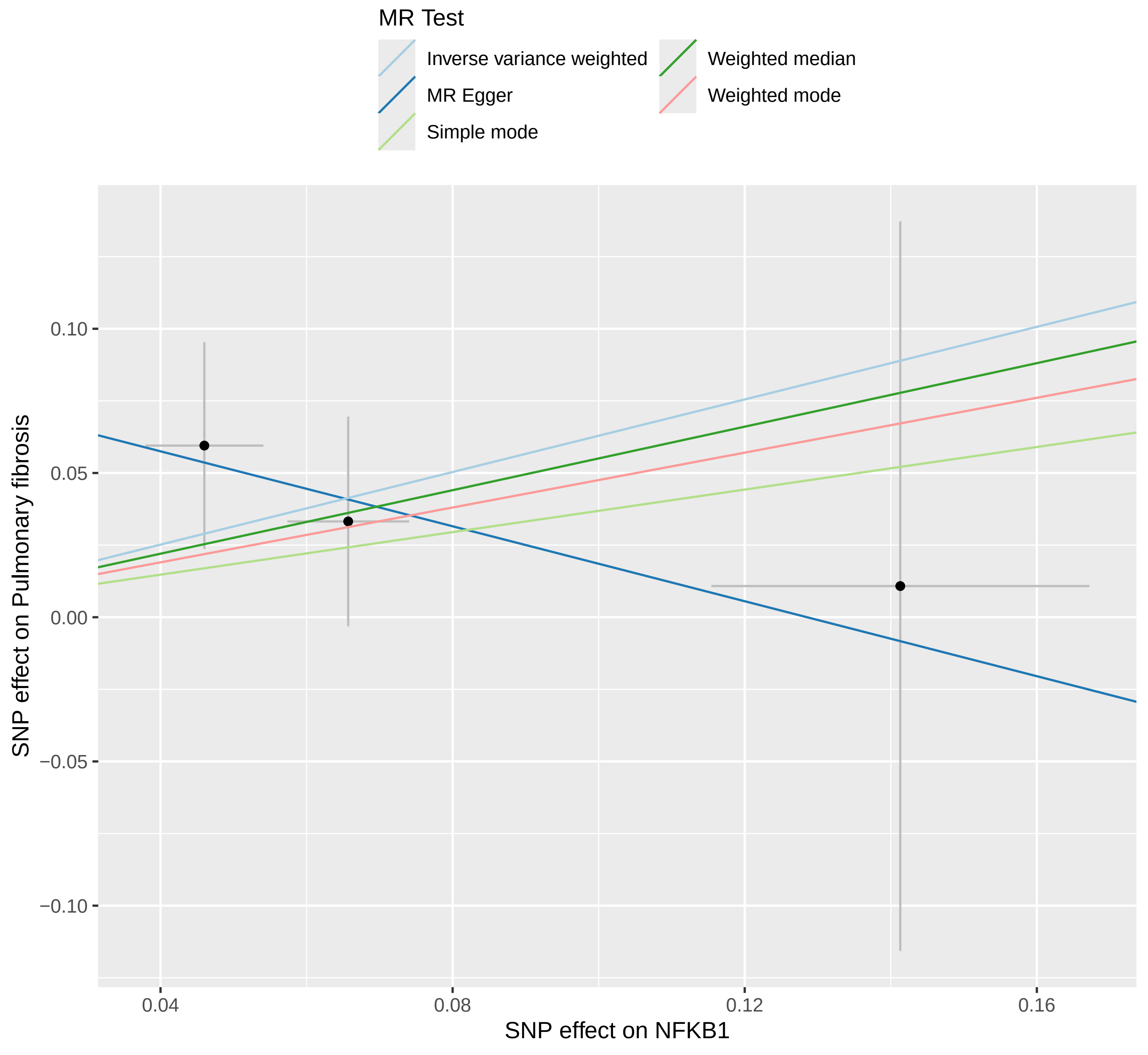
MR Test
Inverse variance weighted
MR Egger
Simple mode
Weighted median
Weighted mode
SNP effect on Pulmonary fibrosis
0.10
0.05
0.00
−0.05
−0.10
0.04
0.08
0.12
0.16
SNP effect on NFKB1

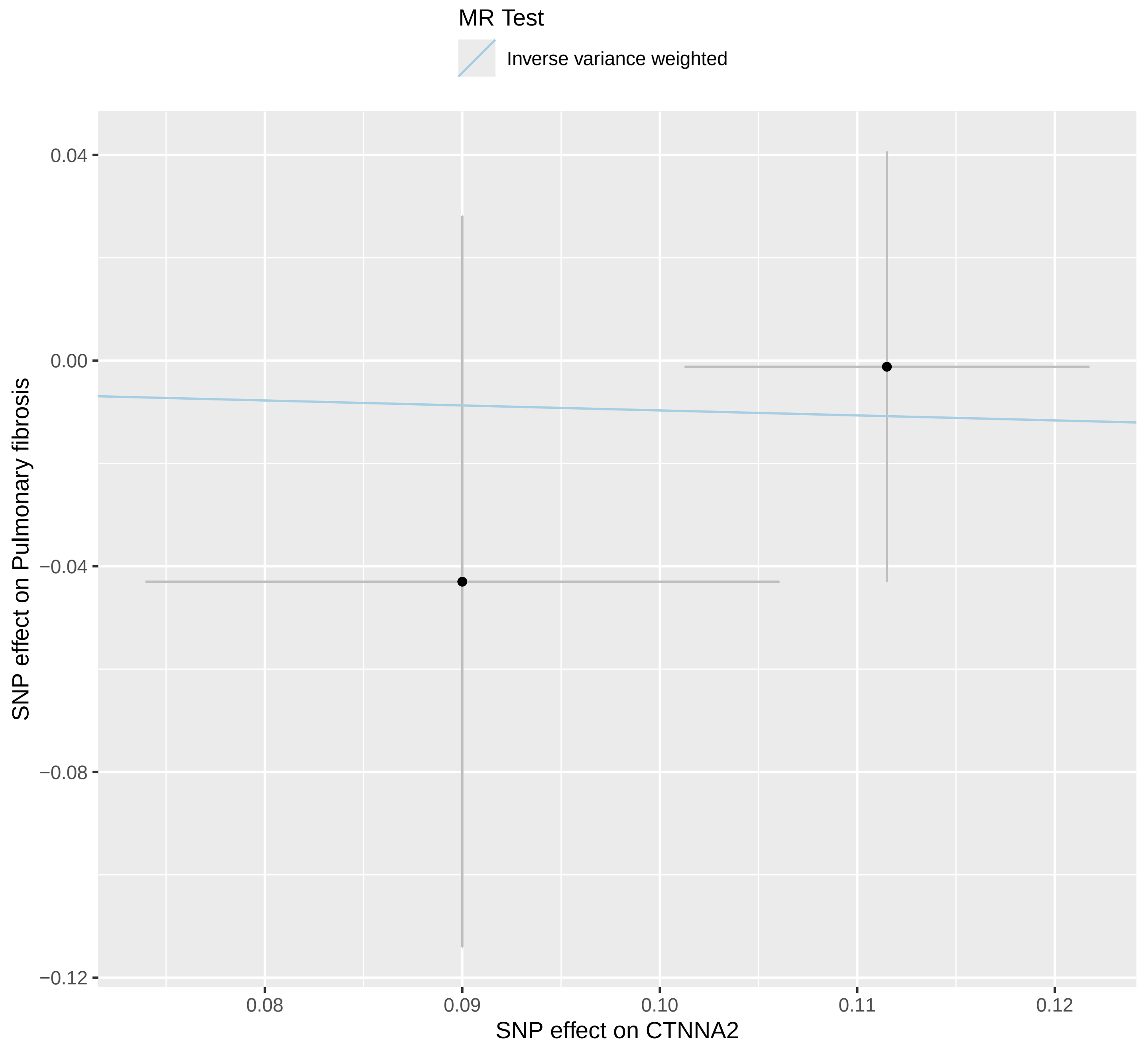

MR Test
Inverse variance weighted
0.04
0.00
−0.04
−0.08
−0.12
SNP effect on Pulmonary fibrosis
0.08
0.09
0.10
0.11
0.12
SNP effect on CTNNA2

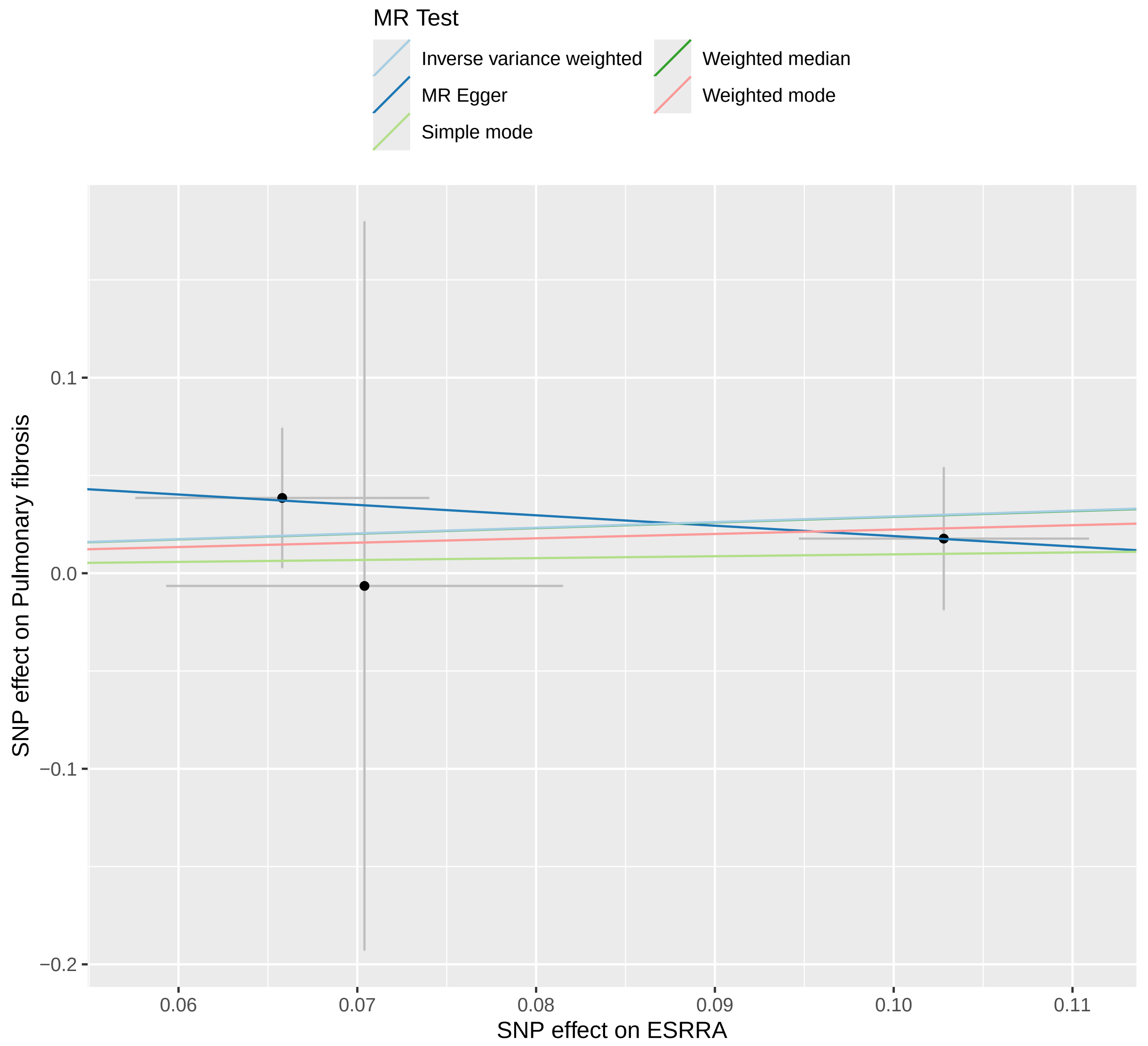
MR Test
Inverse variance weighted
MR Egger
Simple mode
Weighted median
Weighted mode
0.1
0.0
−0.1
−0.2
SNP effect on Pulmonary fibrosis
0.06
0.07
0.08
0.09
0.10
0.11
SNP effect on ESRRA

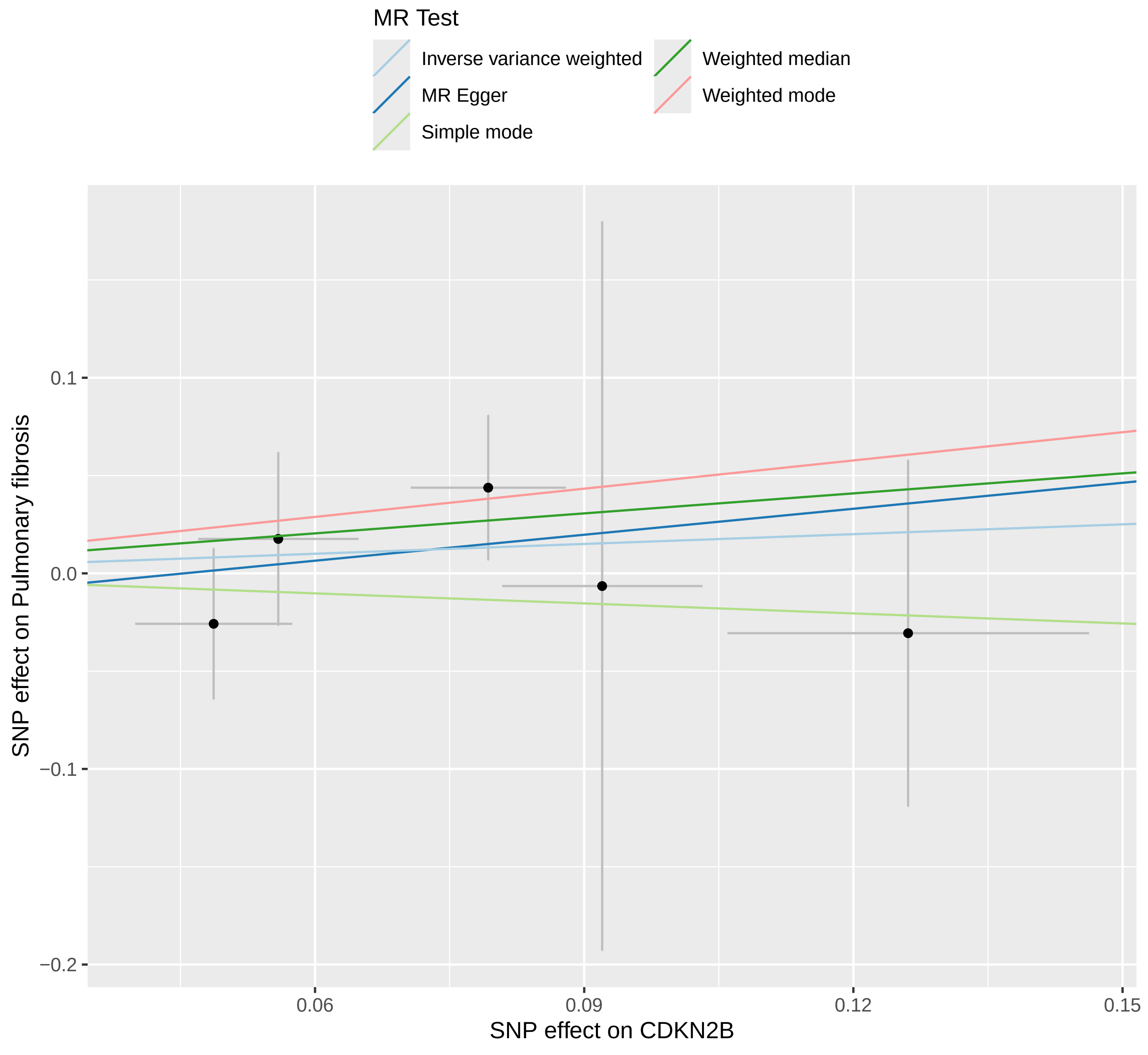

MR Test
Inverse variance weighted
MR Egger
Simple mode
Weighted median
Weighted mode
0.1
0.0
−0.1
−0.2
0.06
0.09
0.12
0.15
SNP effect on CDKN2B
SNP effect on Pulmonary fibrosis

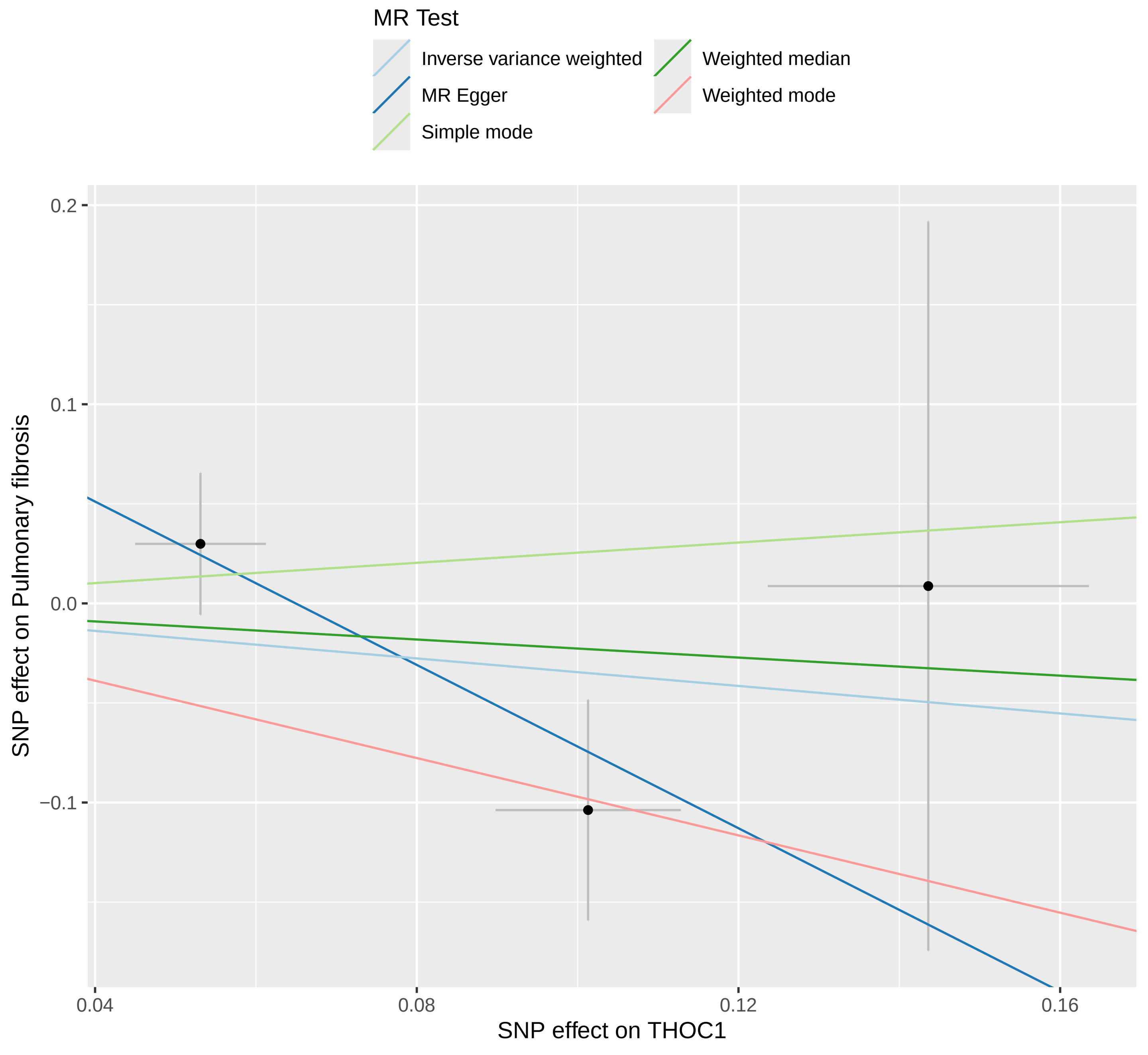

MR Test
Inverse variance weighted
MR Egger
Simple mode
Weighted median
Weighted mode
0.2
0.1
0.0
−0.1
SNP effect on Pulmonary fibrosis
0.04
0.08
0.12
0.16
SNP effect on THOC1

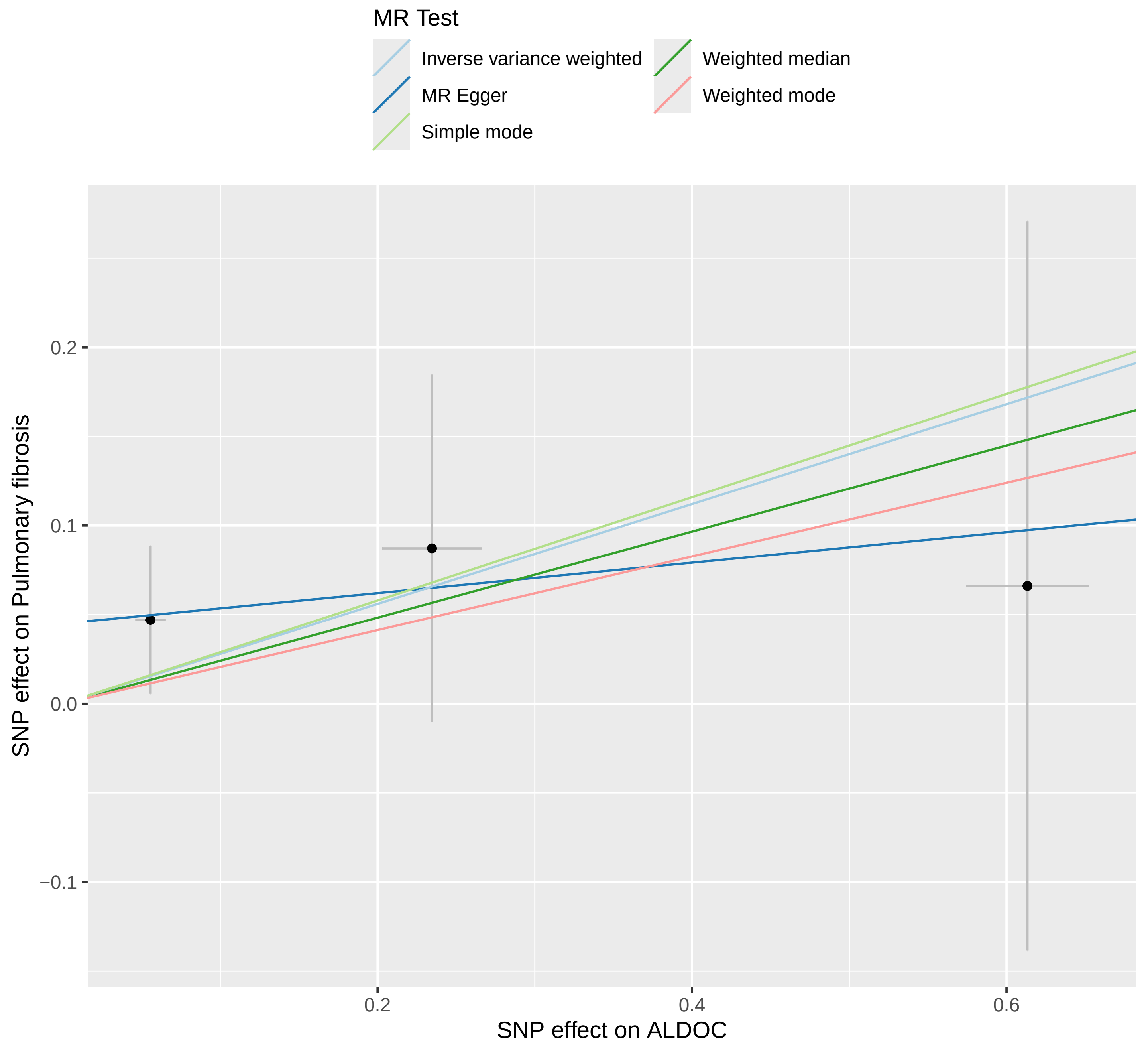

MR Test
Inverse variance weighted
MR Egger
Simple mode
Weighted median
Weighted mode
0.2
0.1
0.0
−0.1
0.2
0.4
0.6
SNP effect on Pulmonary fibrosis
SNP effect on ALDOC

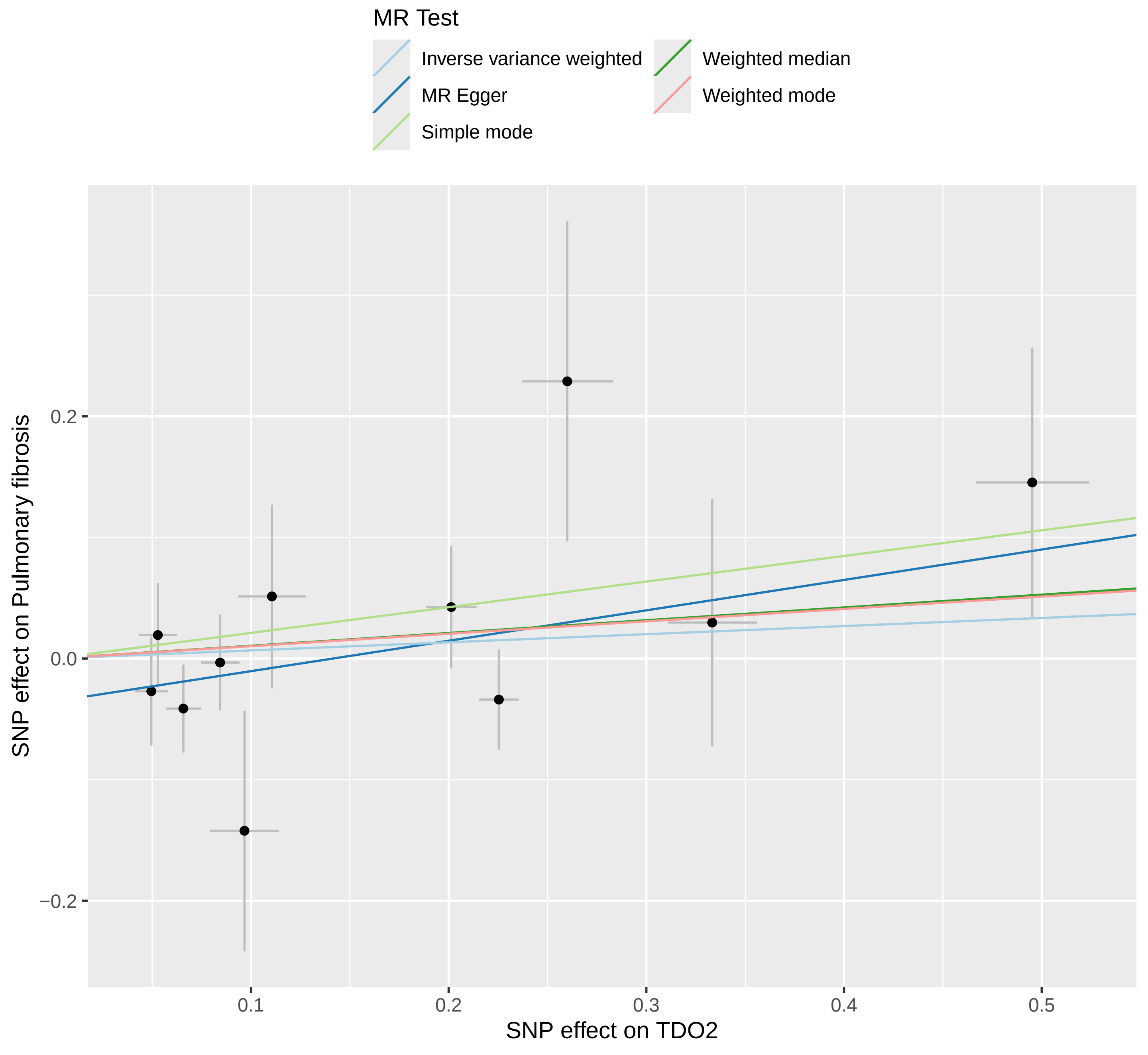

MR Test
Inverse variance weighted
MR Egger
Simple mode
Weighted median
Weighted mode
SNP effect on Pulmonary fibrosis
0.2
0.0
−0.2
0.1
0.2
0.3
0.4
0.5
SNP effect on TDO2

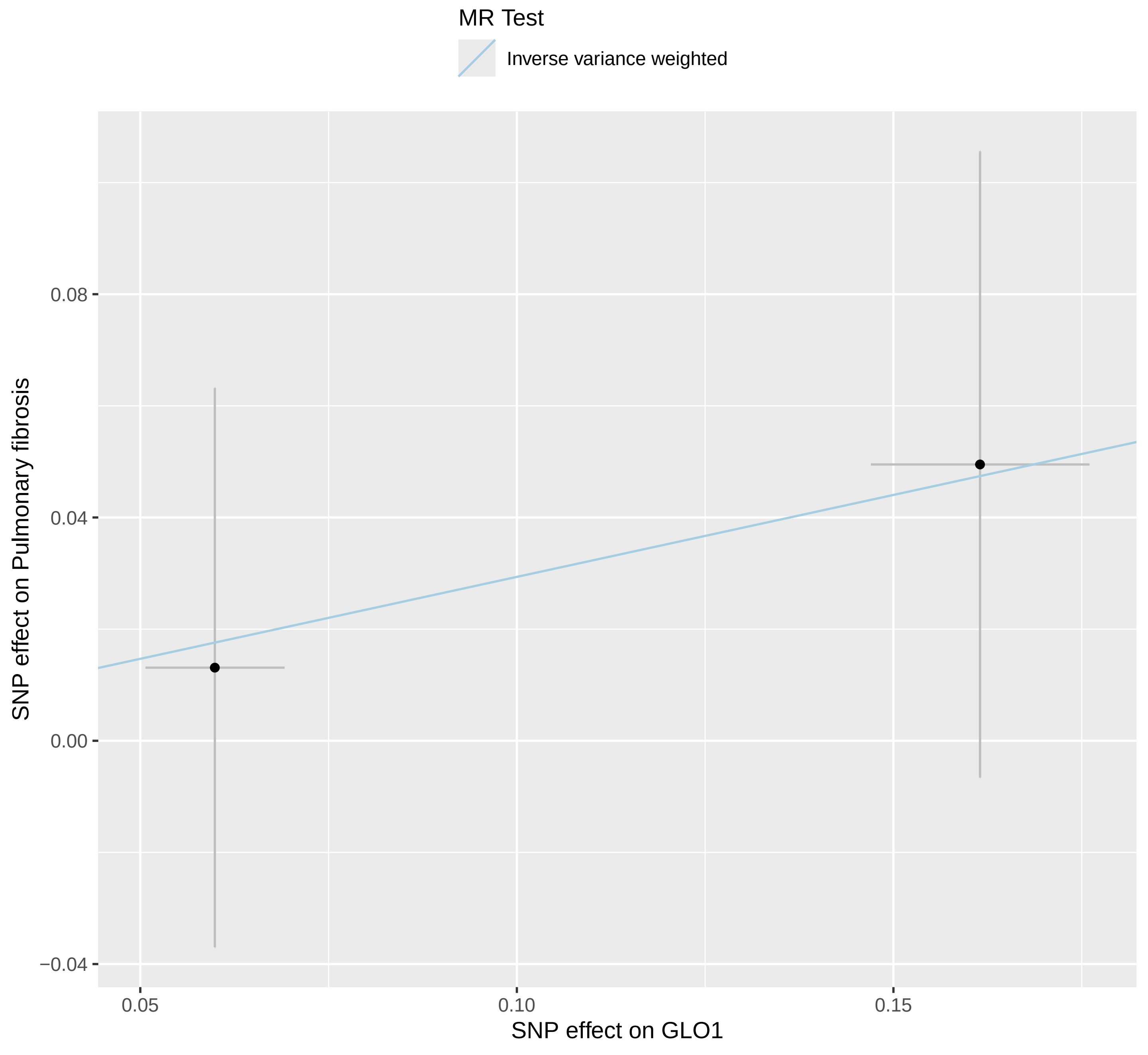

MR Test
Inverse variance weighted
0.08
0.04
0.00
−0.04
SNP effect on Pulmonary fibrosis
0.05
0.10
0.15
SNP effect on GLO1

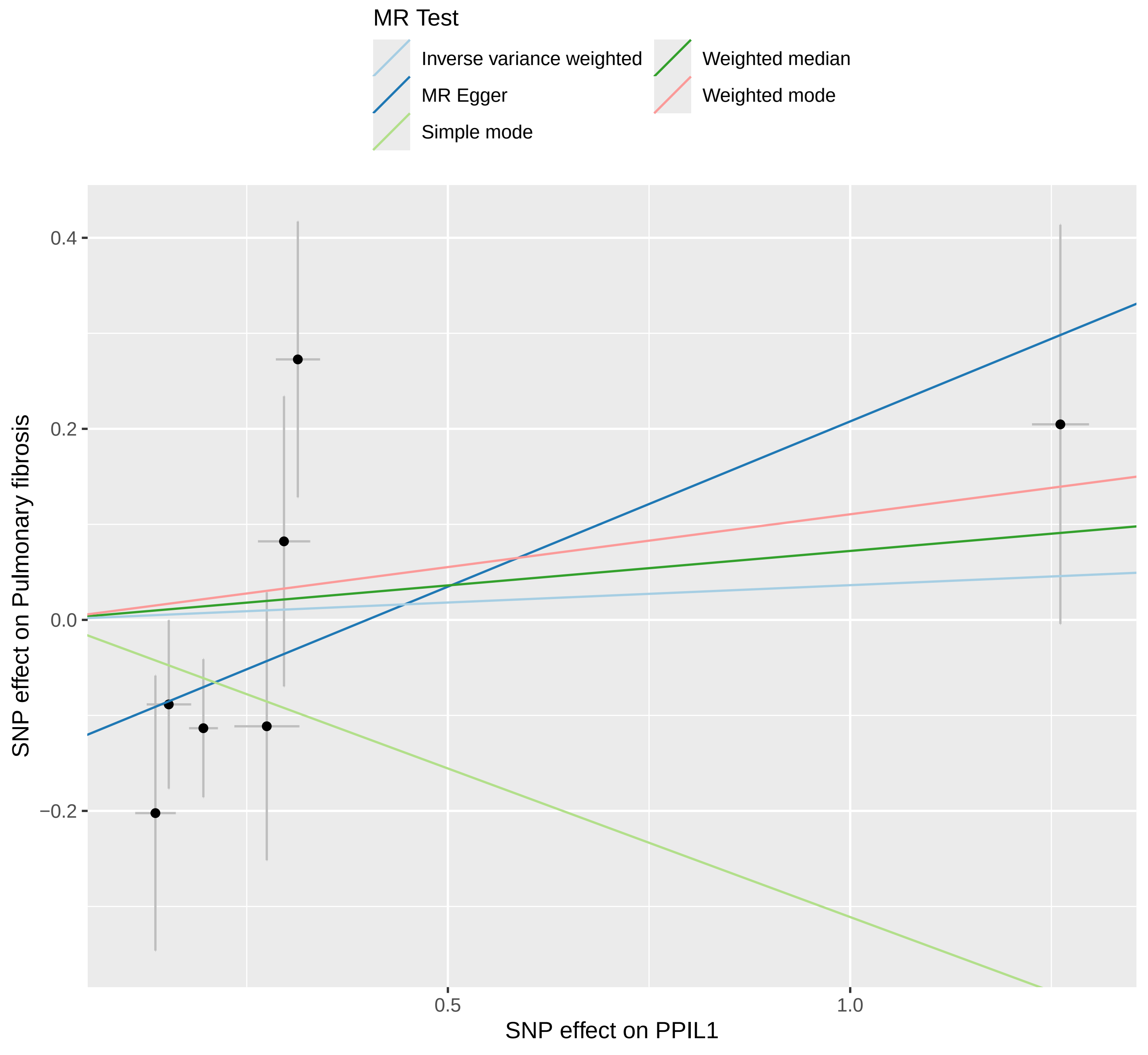
MR Test
Inverse variance weighted
MR Egger
Simple mode
Weighted median
Weighted mode
0.4
0.2
0.0
−0.2
0.5
1.0
SNP effect on Pulmonary fibrosis
SNP effect on PPIL1

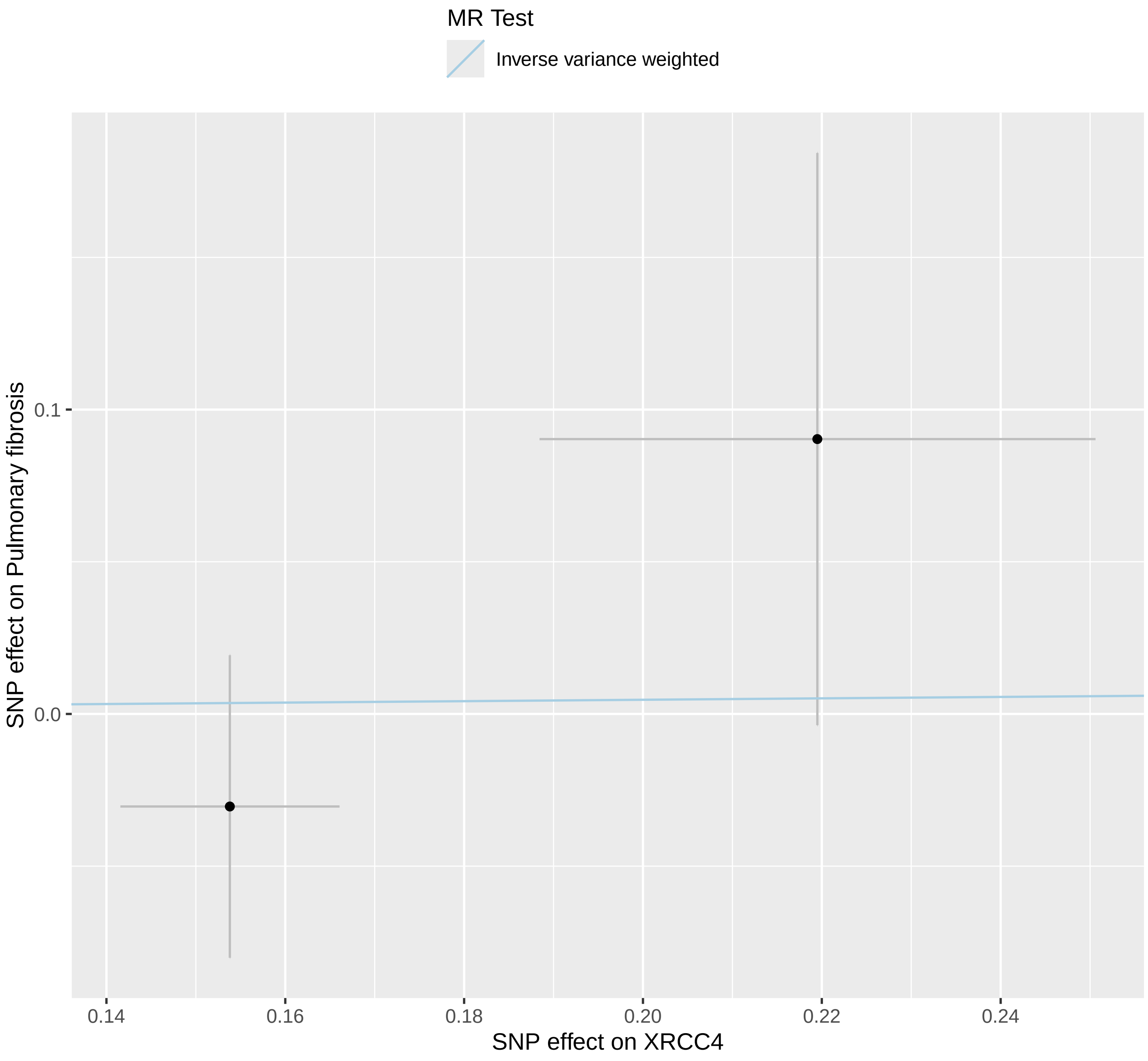
MR Test
Inverse variance weighted
SNP effect on Pulmonary fibrosis
0.1
0.0
0.14
0.16
0.18
0.20
0.22
0.24
SNP effect on XRCC4

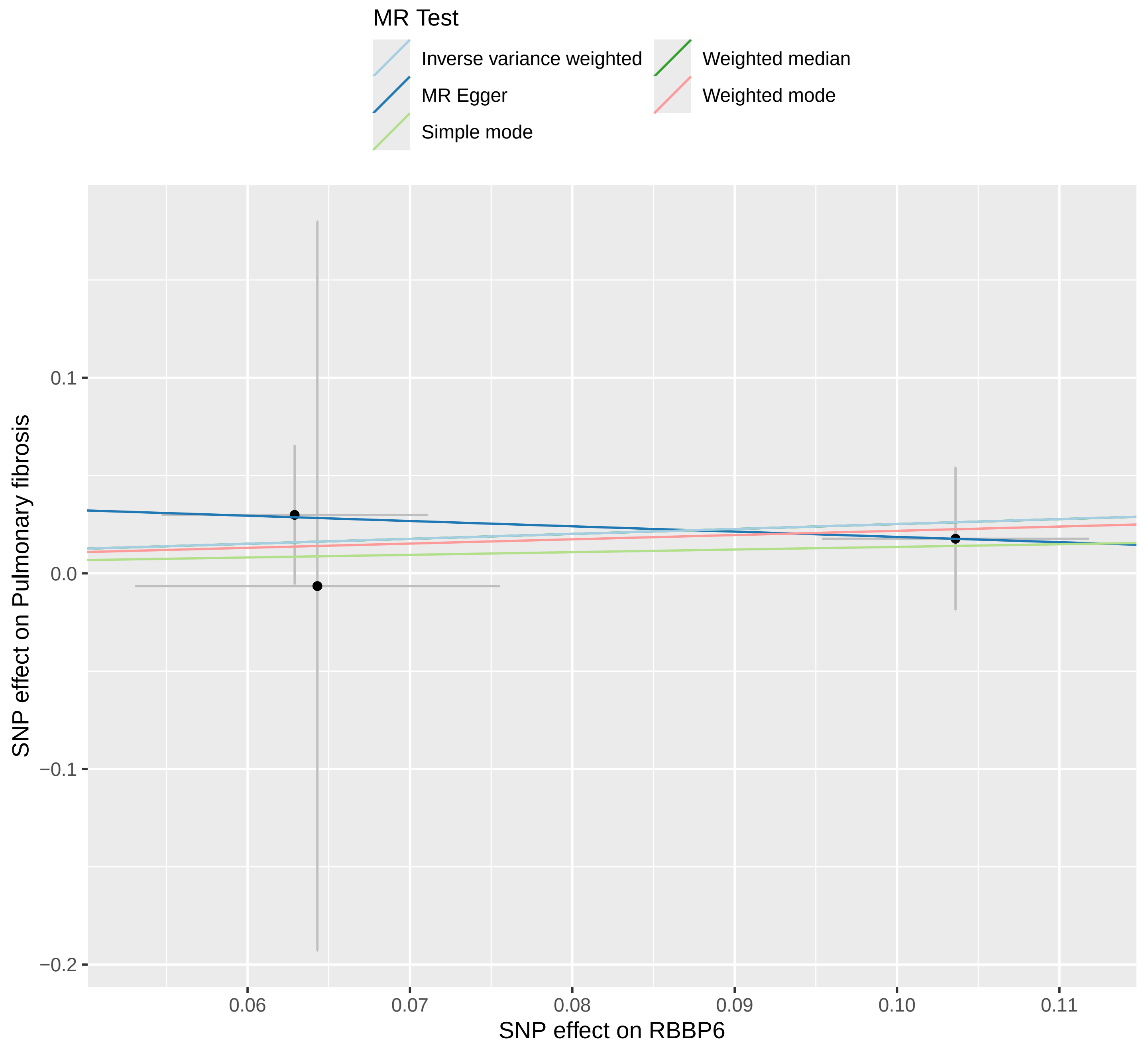

MR Test
Inverse variance weighted
MR Egger
Simple mode
Weighted median
Weighted mode
SNP effect on Pulmonary fibrosis
0.1
0.0
−0.1
−0.2
0.06
0.07
0.08
0.09
0.10
0.11
SNP effect on RBBP6

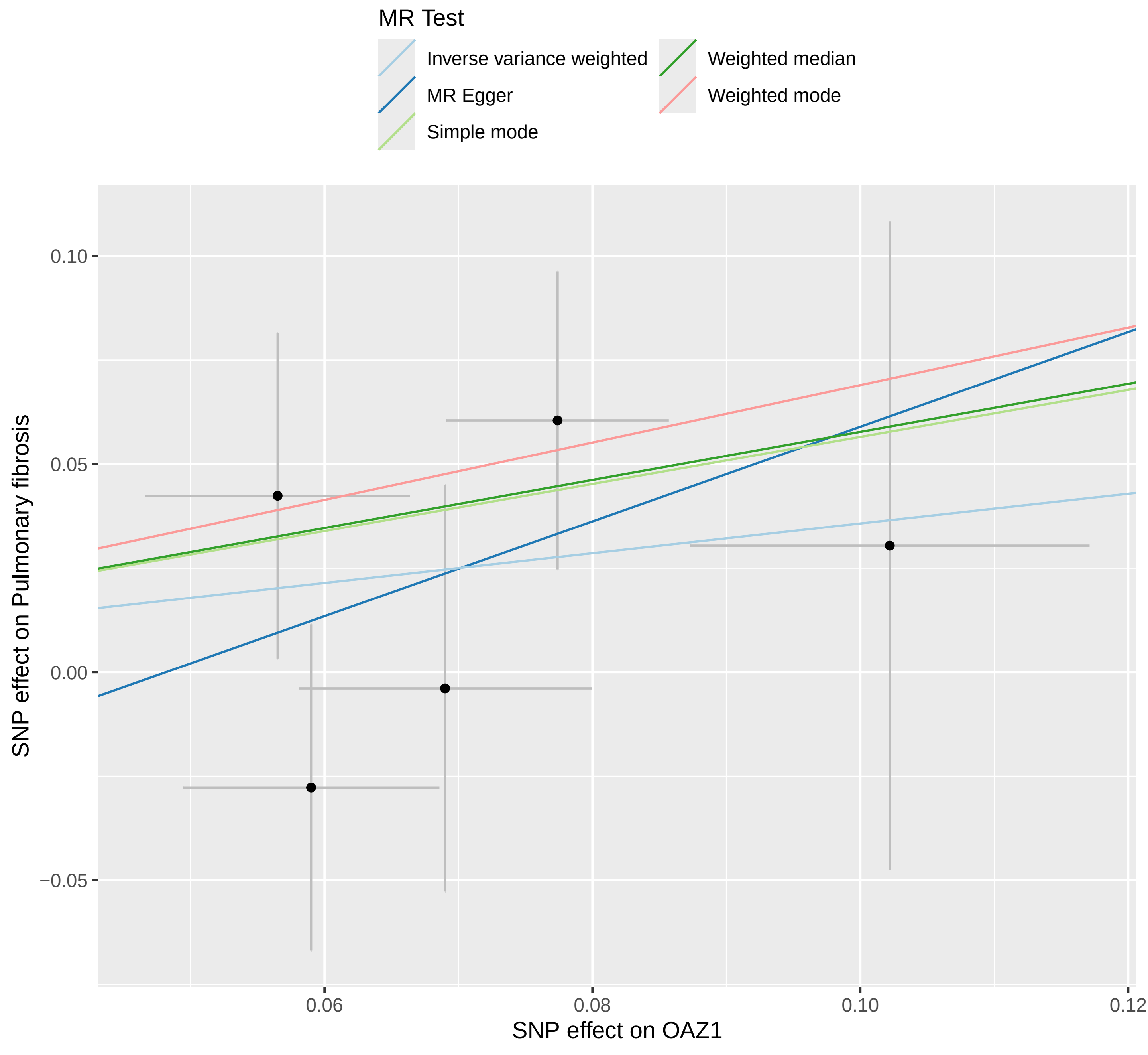

MR Test
Inverse variance weighted
MR Egger
Simple mode
Weighted median
Weighted mode
0.10
0.05
0.00
−0.05
SNP effect on Pulmonary fibrosis
0.06
0.08
0.10
0.12
SNP effect on OAZ1

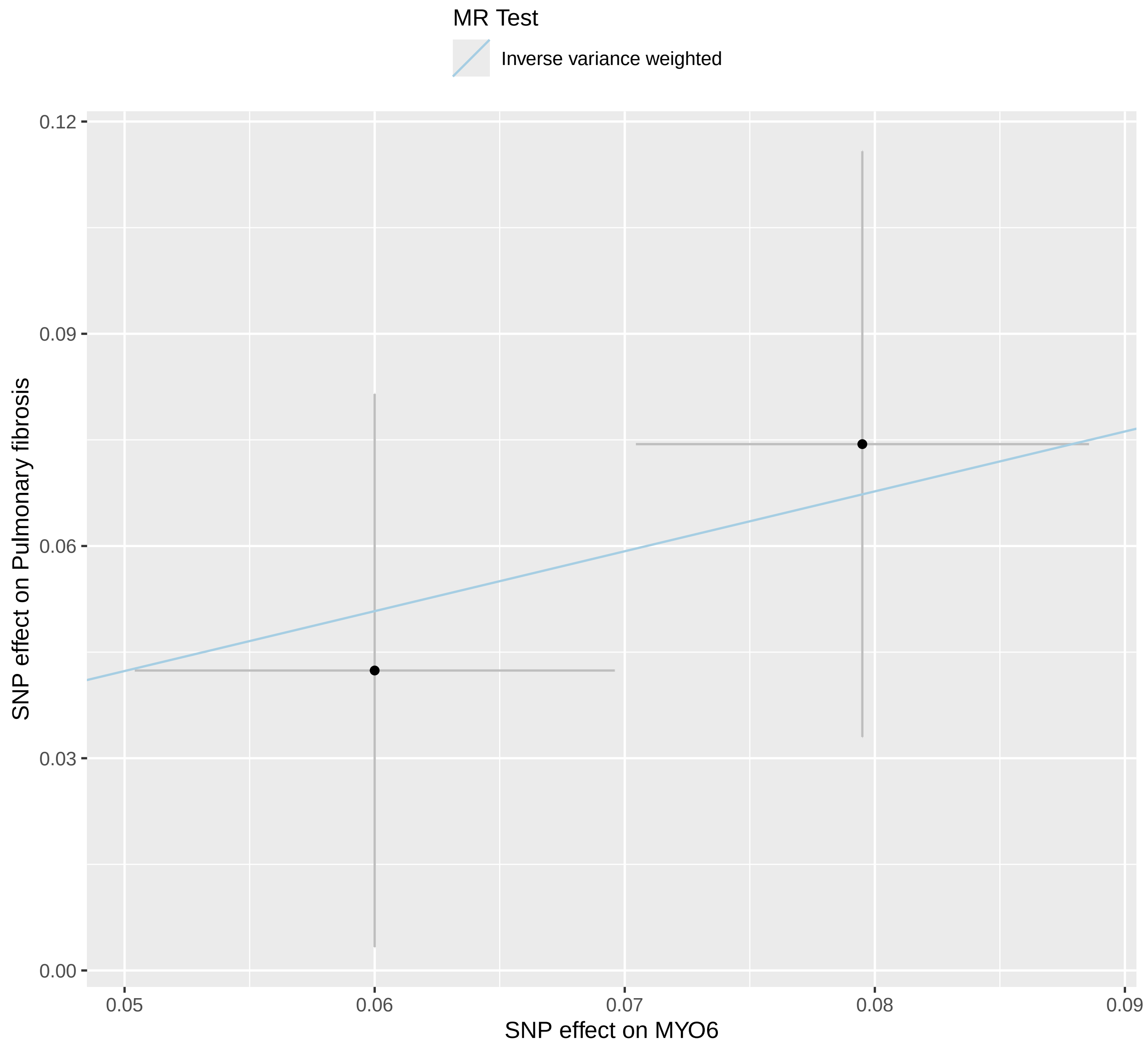
MR Test
Inverse variance weighted
0.12
0.09
0.06
0.03
0.00
0.05
0.06
0.07
0.08
0.09
SNP effect on Pulmonary fibrosis
SNP effect on MYO6

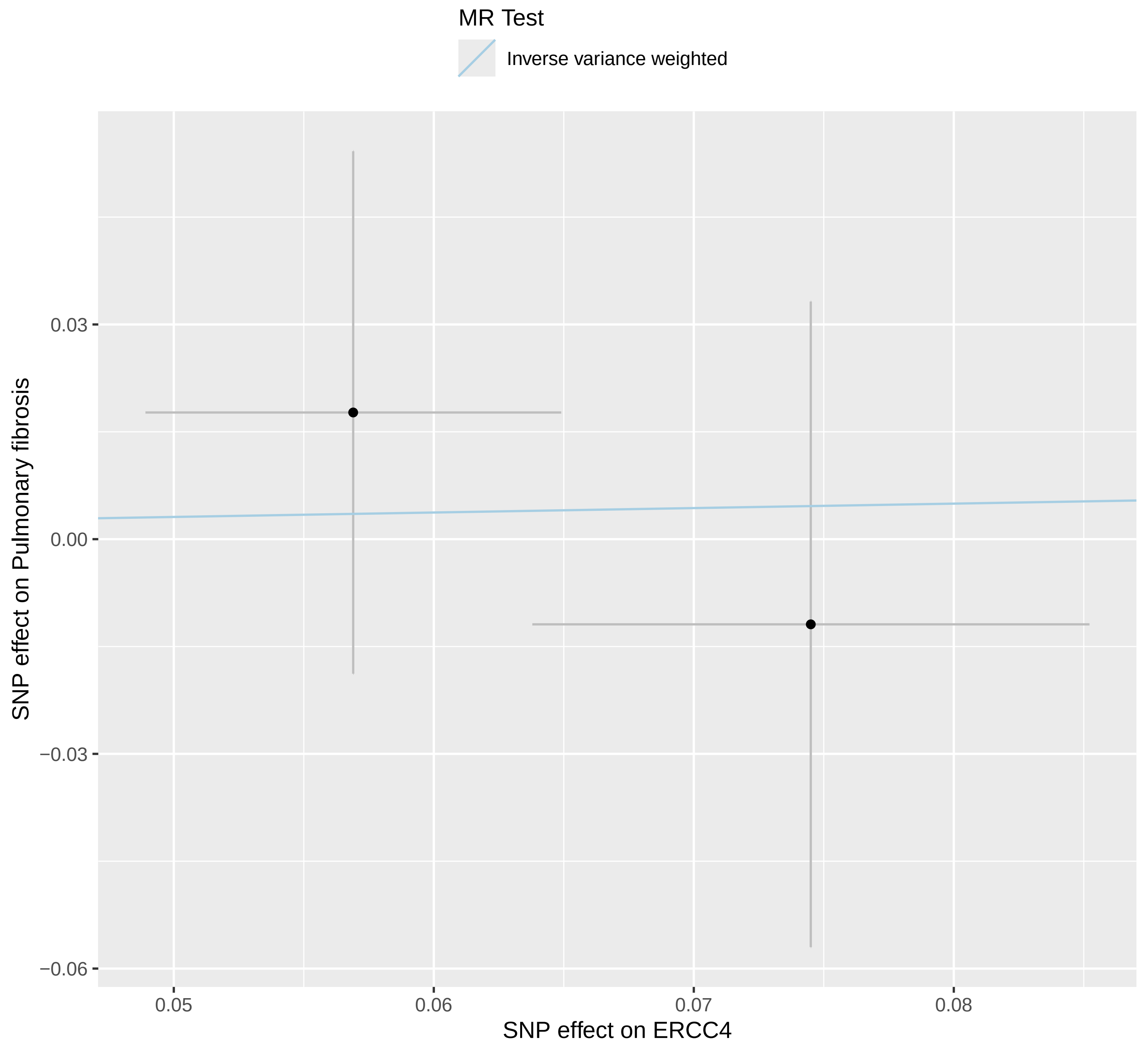

MR Test
Inverse variance weighted
SNP effect on Pulmonary fibrosis
0.03
0.00
−0.03
−0.06
0.05
0.06
0.07
0.08
SNP effect on ERCC4

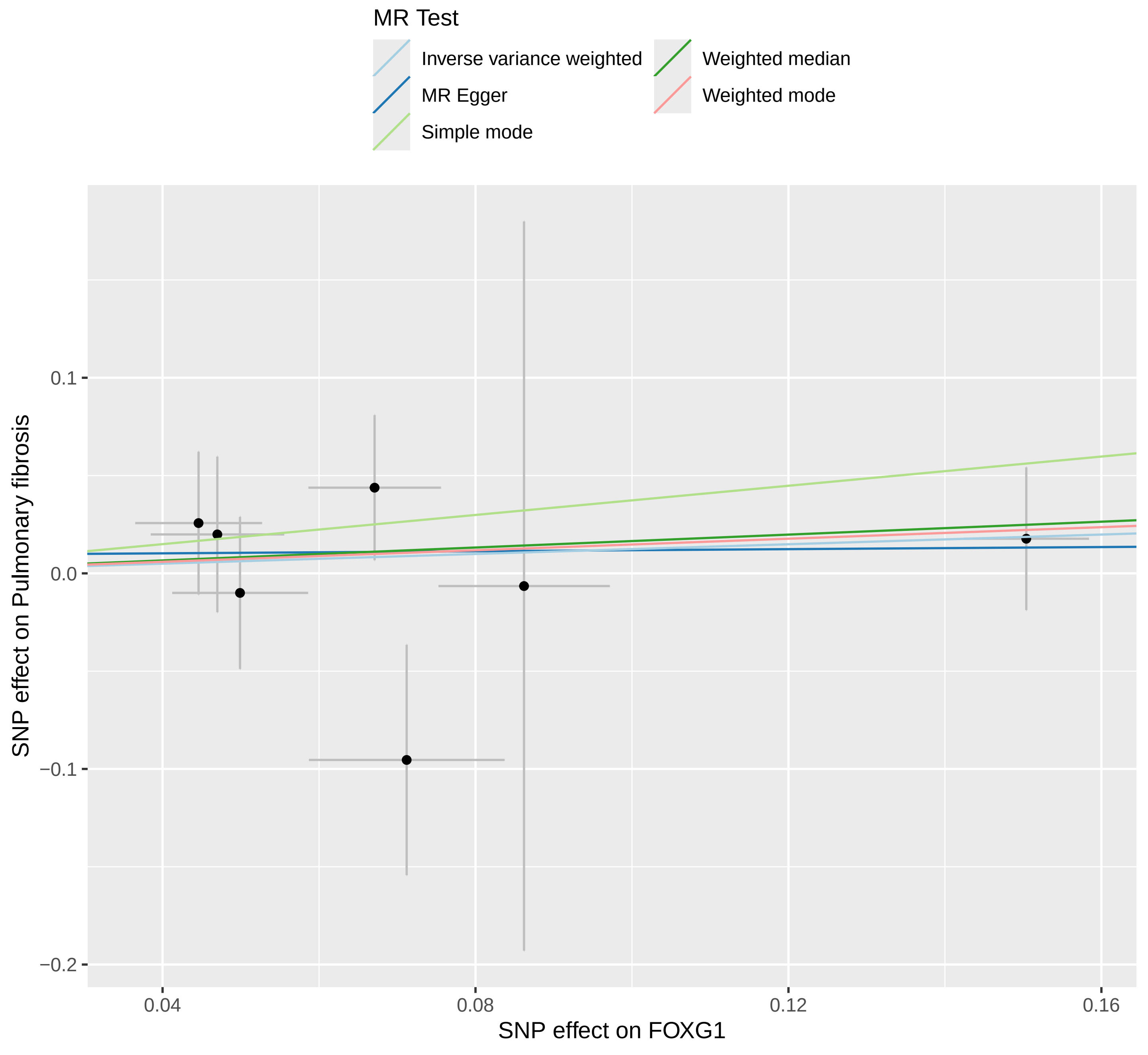

MR Test
Inverse variance weighted
MR Egger
Simple mode
Weighted median
Weighted mode
SNP effect on Pulmonary fibrosis
0.1
0.0
−0.1
−0.2
0.04
0.08
0.12
0.16
SNP effect on FOXG1

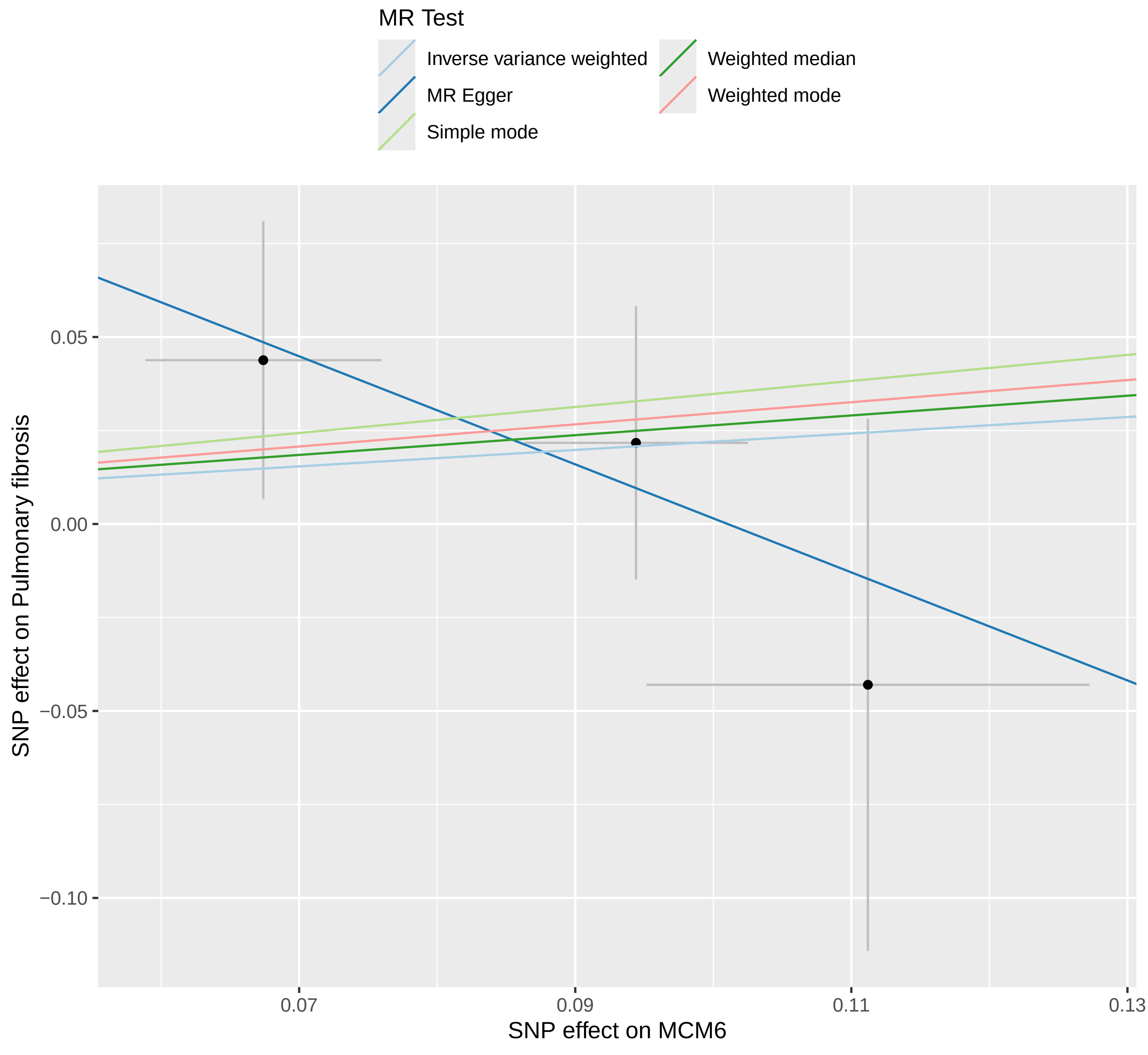
MR Test
Inverse variance weighted
MR Egger
Simple mode
Weighted median
Weighted mode
0.05
0.00
−0.05
−0.10
SNP effect on Pulmonary fibrosis
0.07
0.09
0.11
0.13
SNP effect on MCM6

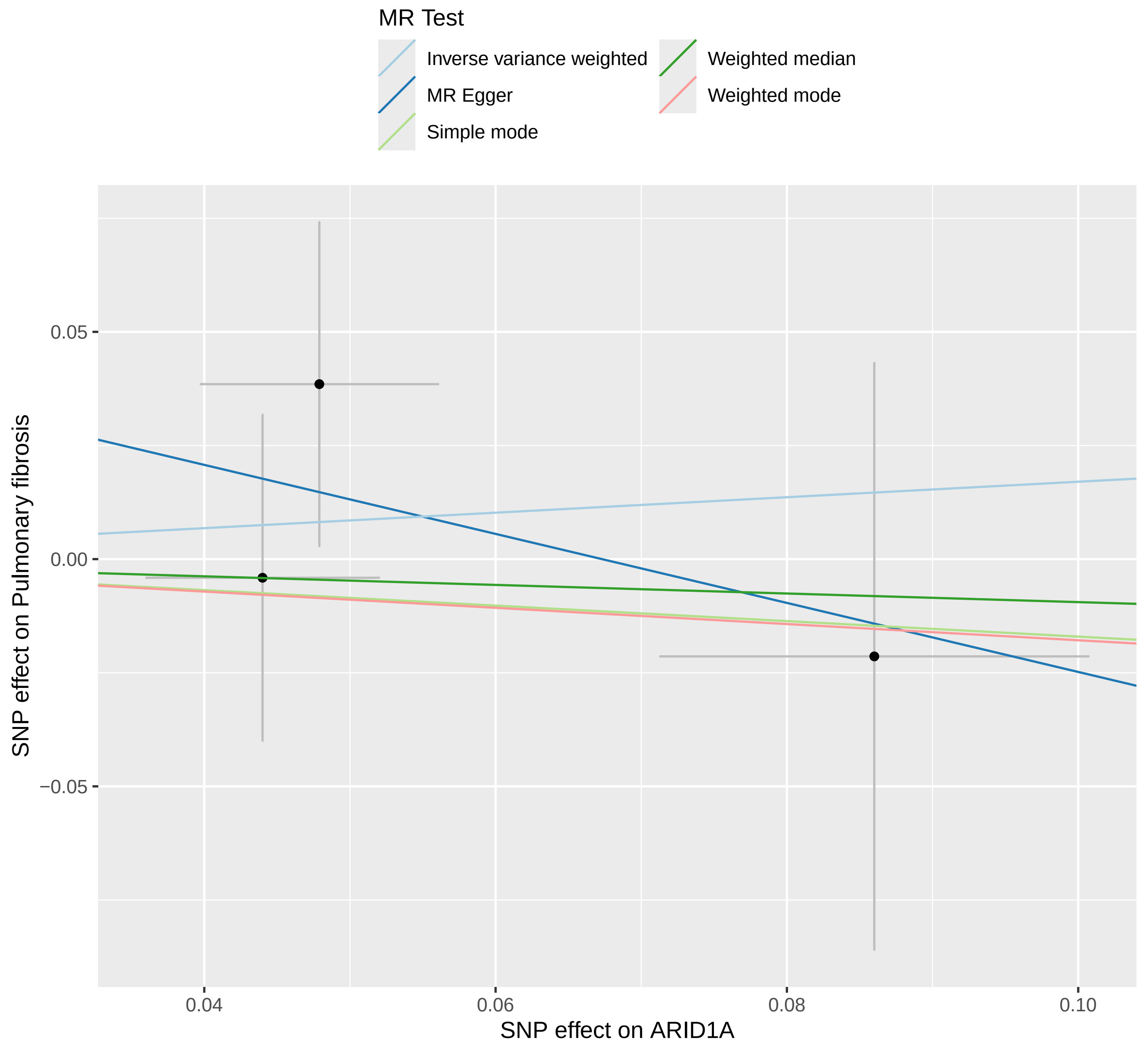

MR Test
Inverse variance weighted
MR Egger
Simple mode
Weighted median
Weighted mode
0.05
0.00
−0.05
0.04
0.06
0.08
0.10
SNP effect on Pulmonary fibrosis
SNP effect on ARID1A

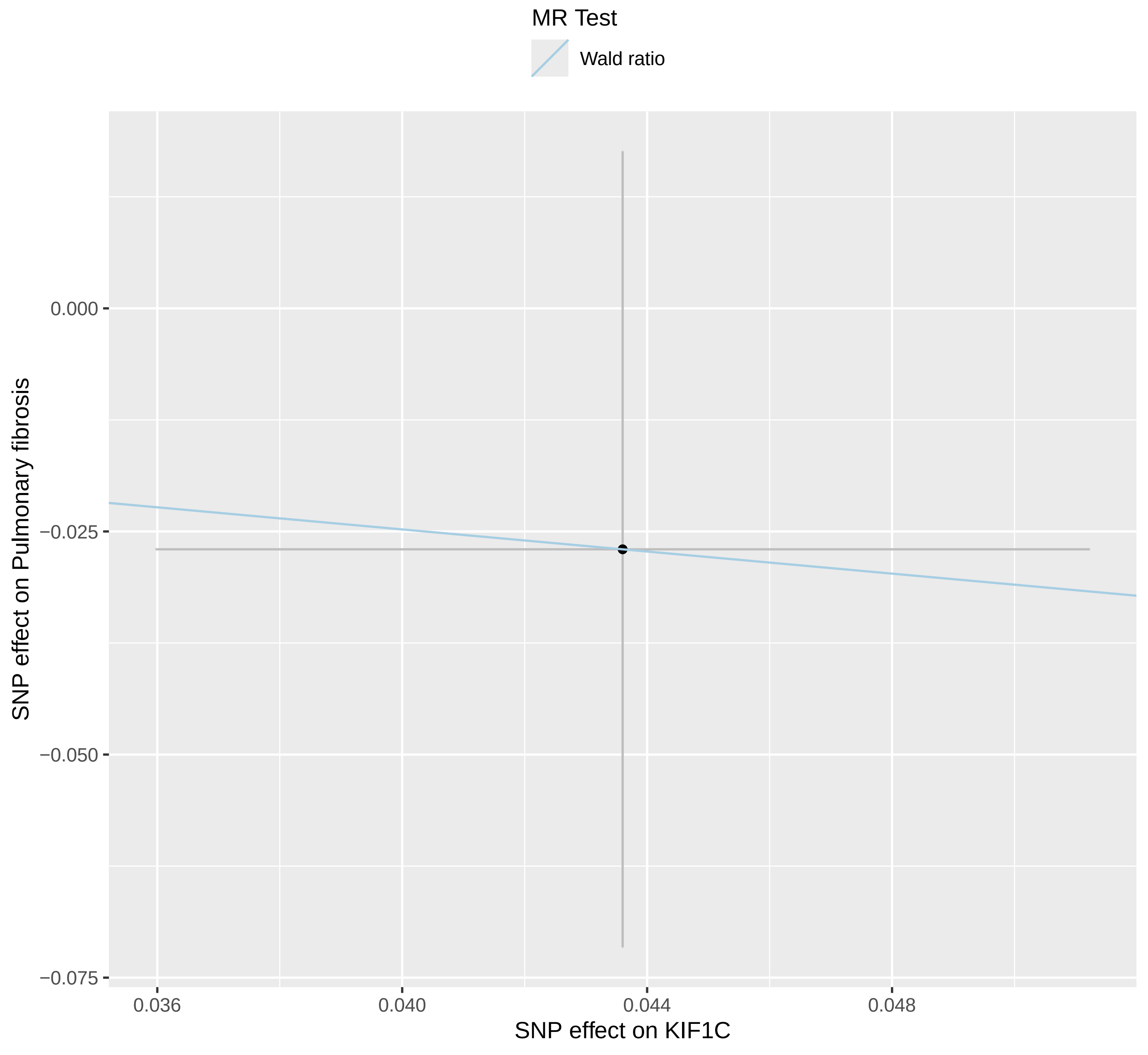
MR Test
Wald ratio
SNP effect on Pulmonary fibrosis
0.000
−0.025
−0.050
−0.075
0.036
0.040
0.044
0.048
SNP effect on KIF1C

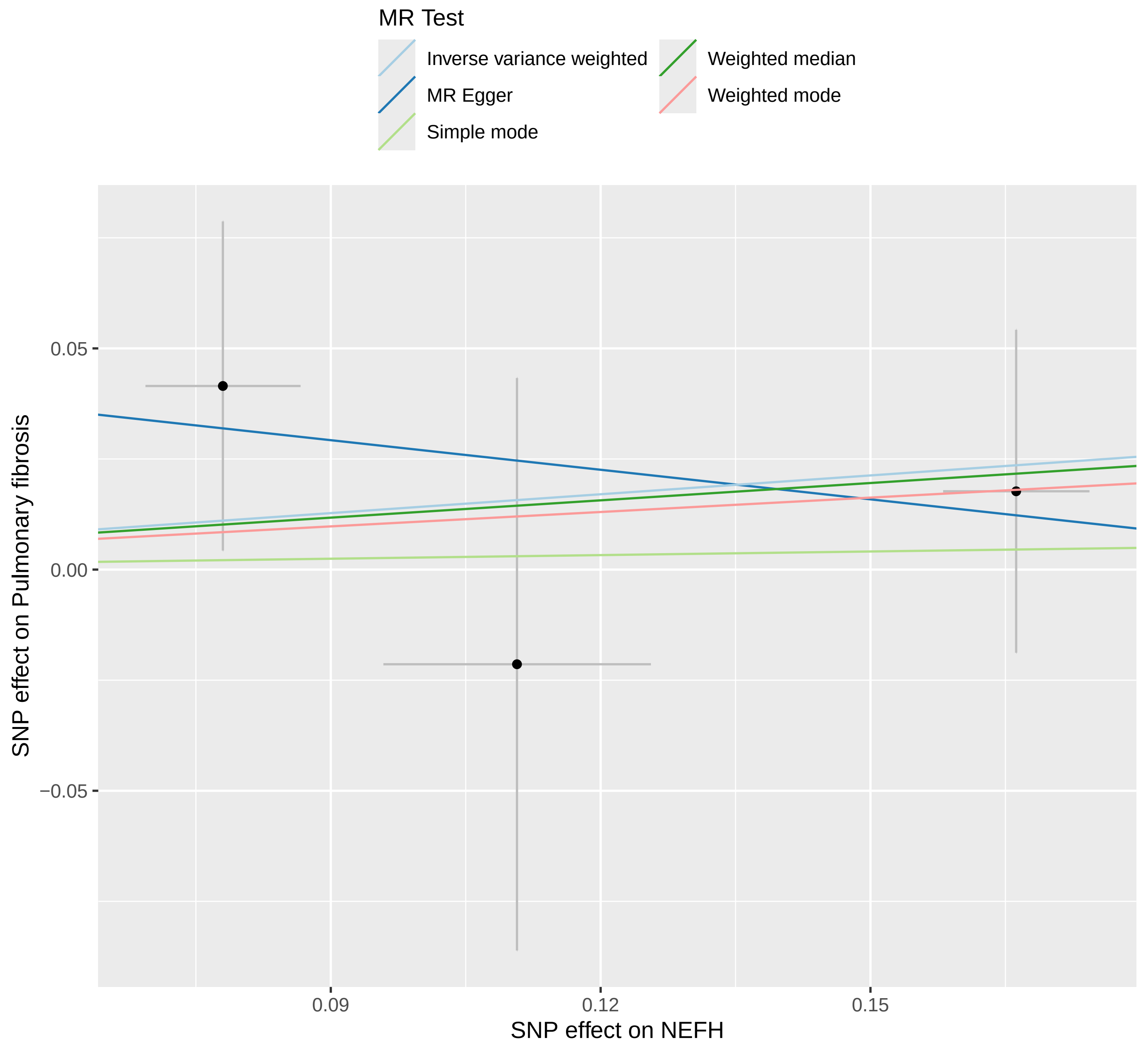
MR Test
Inverse variance weighted
MR Egger
Simple mode
Weighted median
Weighted mode
0.05
0.00
−0.05
0.09
0.12
0.15
SNP effect on NEFH
SNP effect on Pulmonary fibrosis

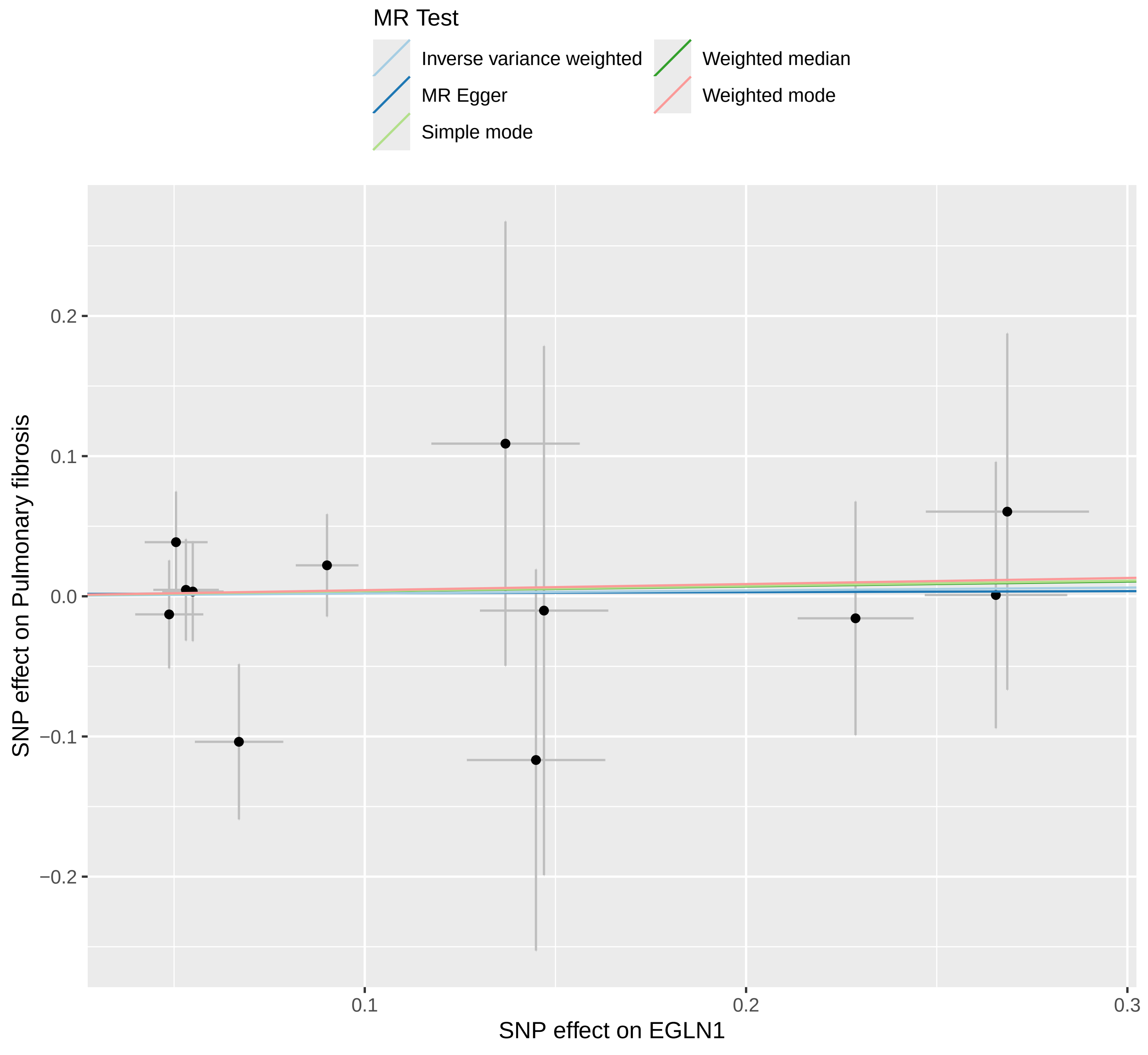
MR Test
Inverse variance weighted
MR Egger
Simple mode
Weighted median
Weighted mode
0.2
0.1
0.0
−0.1
−0.2
SNP effect on Pulmonary fibrosis
0.1
0.2
0.3
SNP effect on EGLN1

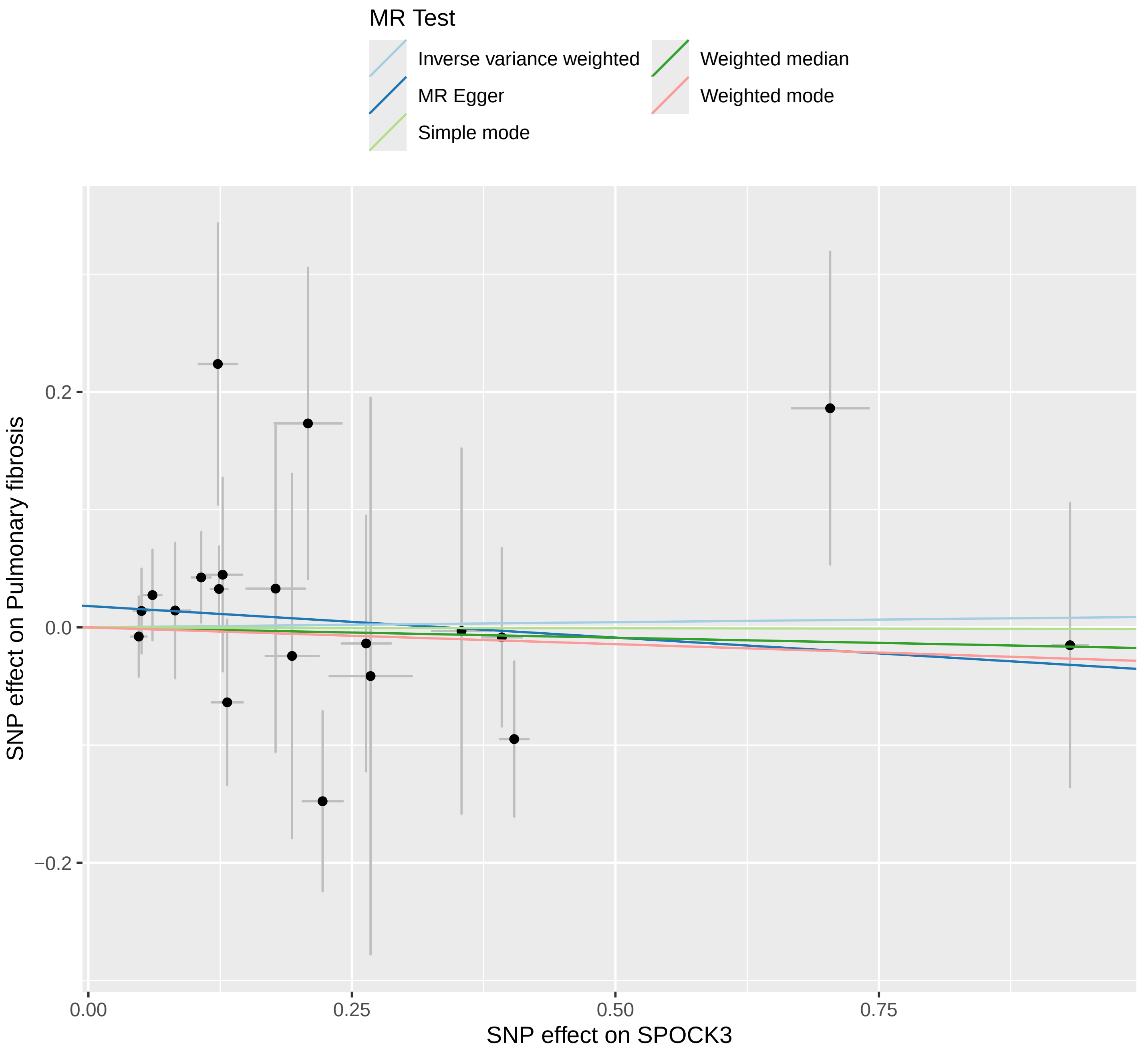
MR Test
Inverse variance weighted
MR Egger
Simple mode
Weighted median
Weighted mode
0.2
0.0
−0.2
0.00
0.25
0.50
0.75
SNP effect on Pulmonary fibrosis
SNP effect on SPOCK3

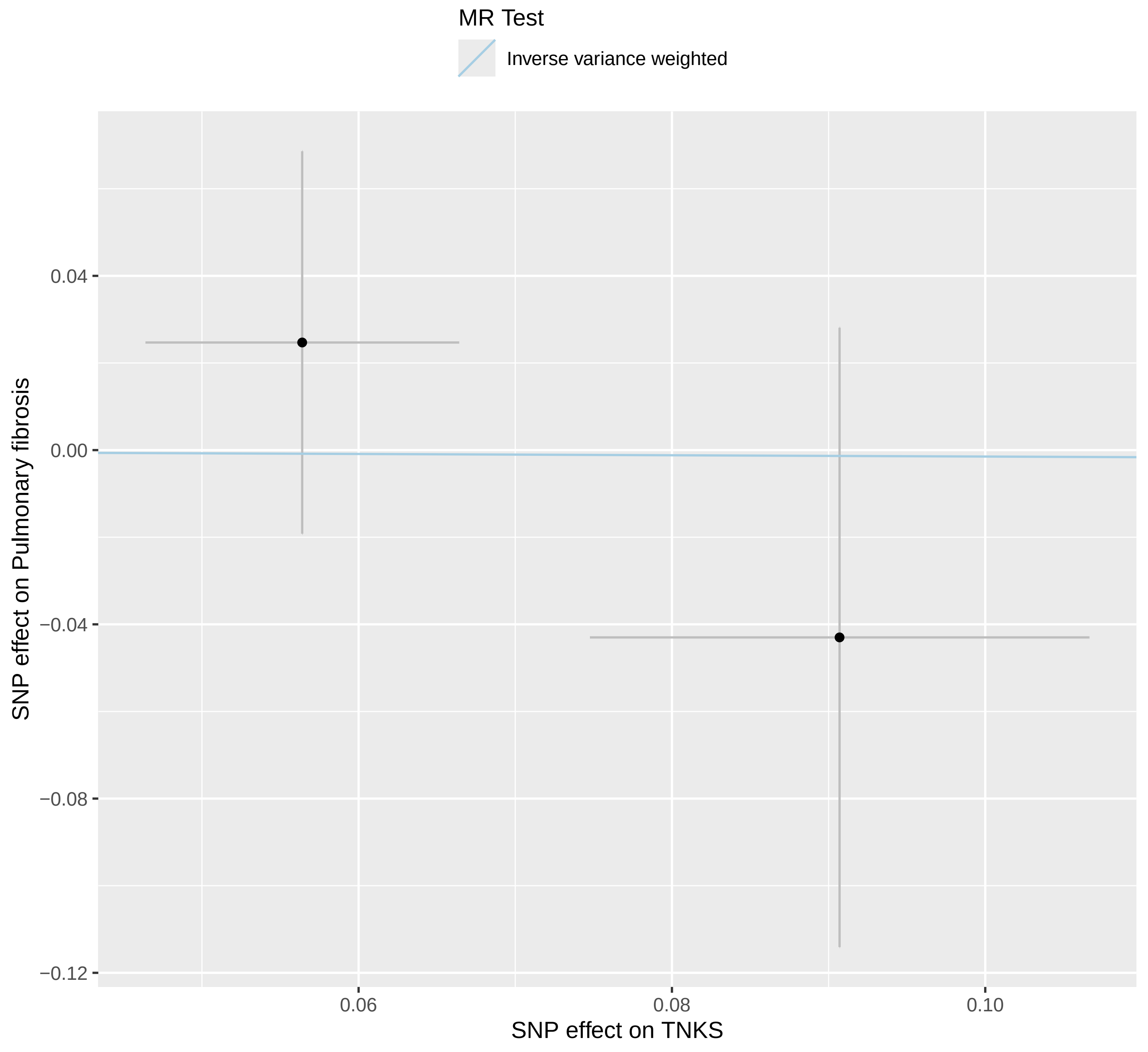

MR Test
Inverse variance weighted
SNP effect on Pulmonary fibrosis
0.04
0.00
−0.04
−0.08
−0.12
0.06
0.08
0.10
SNP effect on TNKS

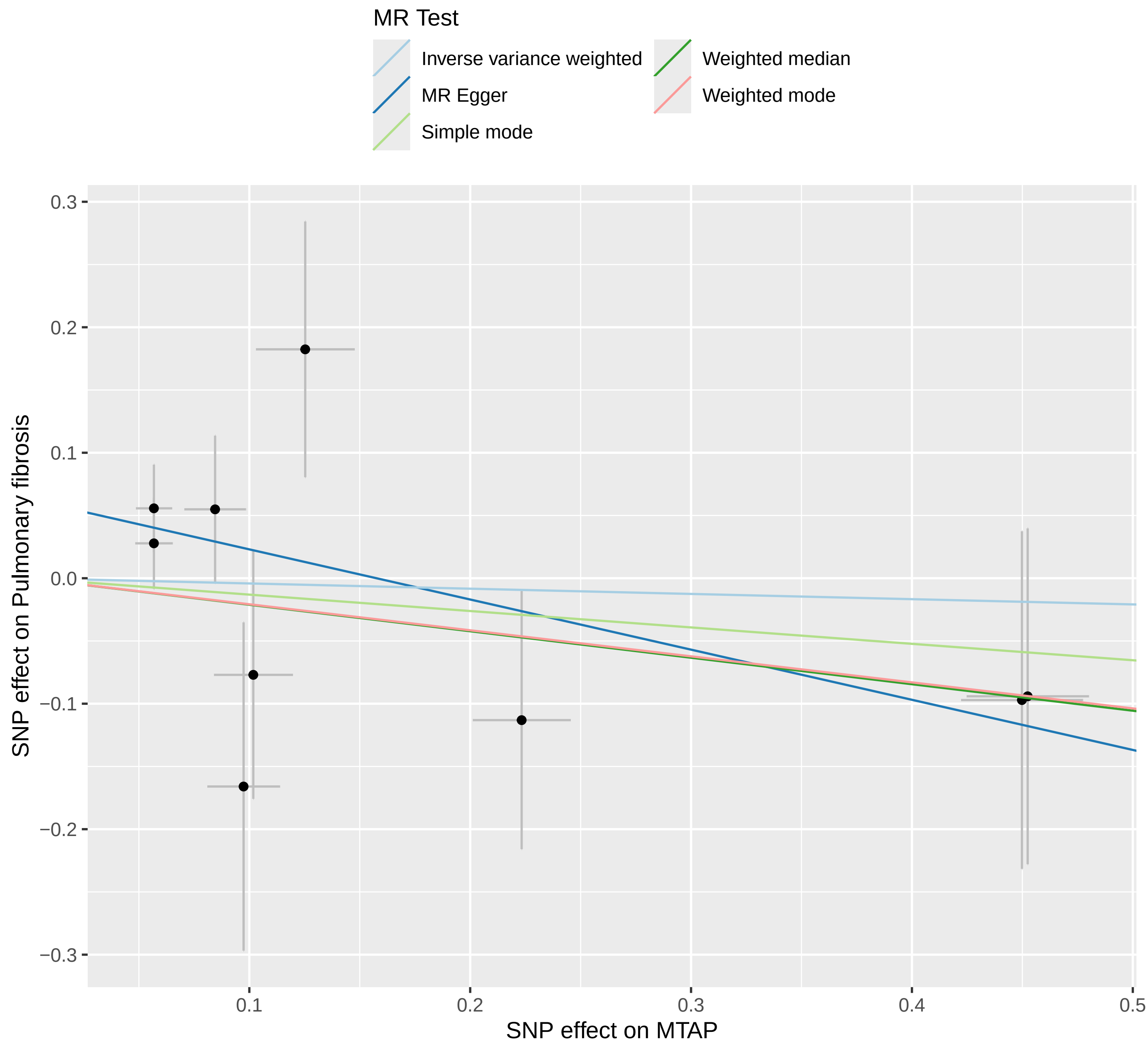
MR Test
Inverse variance weighted
MR Egger
Simple mode
Weighted median
Weighted mode
SNP effect on Pulmonary fibrosis
SNP effect on MTAP
0.3
0.2
0.1
0.0
−0.1
−0.2
−0.3
0.1
0.2
0.3
0.4
0.5

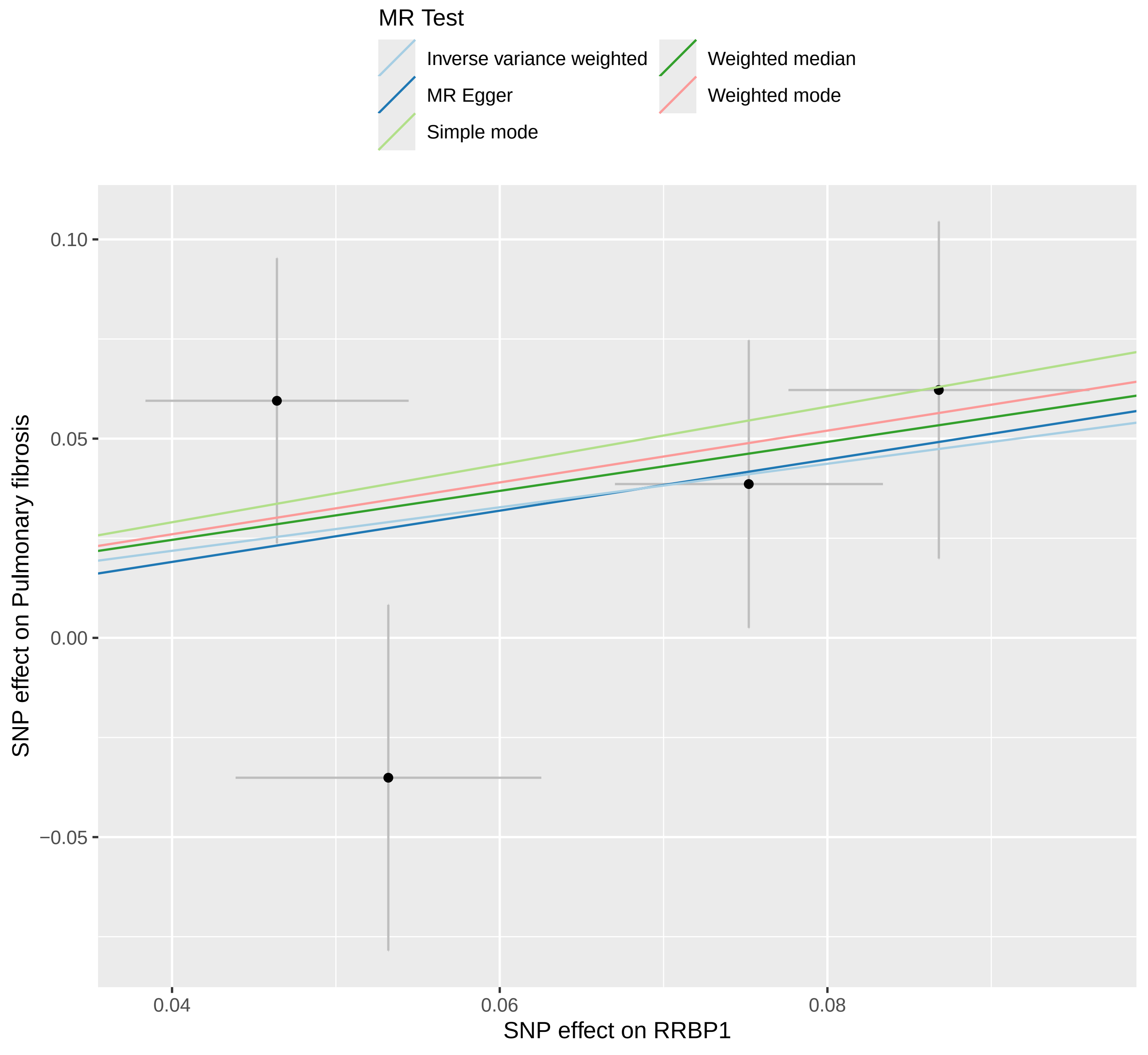

MR Test
Inverse variance weighted
MR Egger
Simple mode
Weighted median
Weighted mode
0.10
0.05
0.00
−0.05
SNP effect on Pulmonary fibrosis
0.04
0.06
0.08
SNP effect on RRBP1

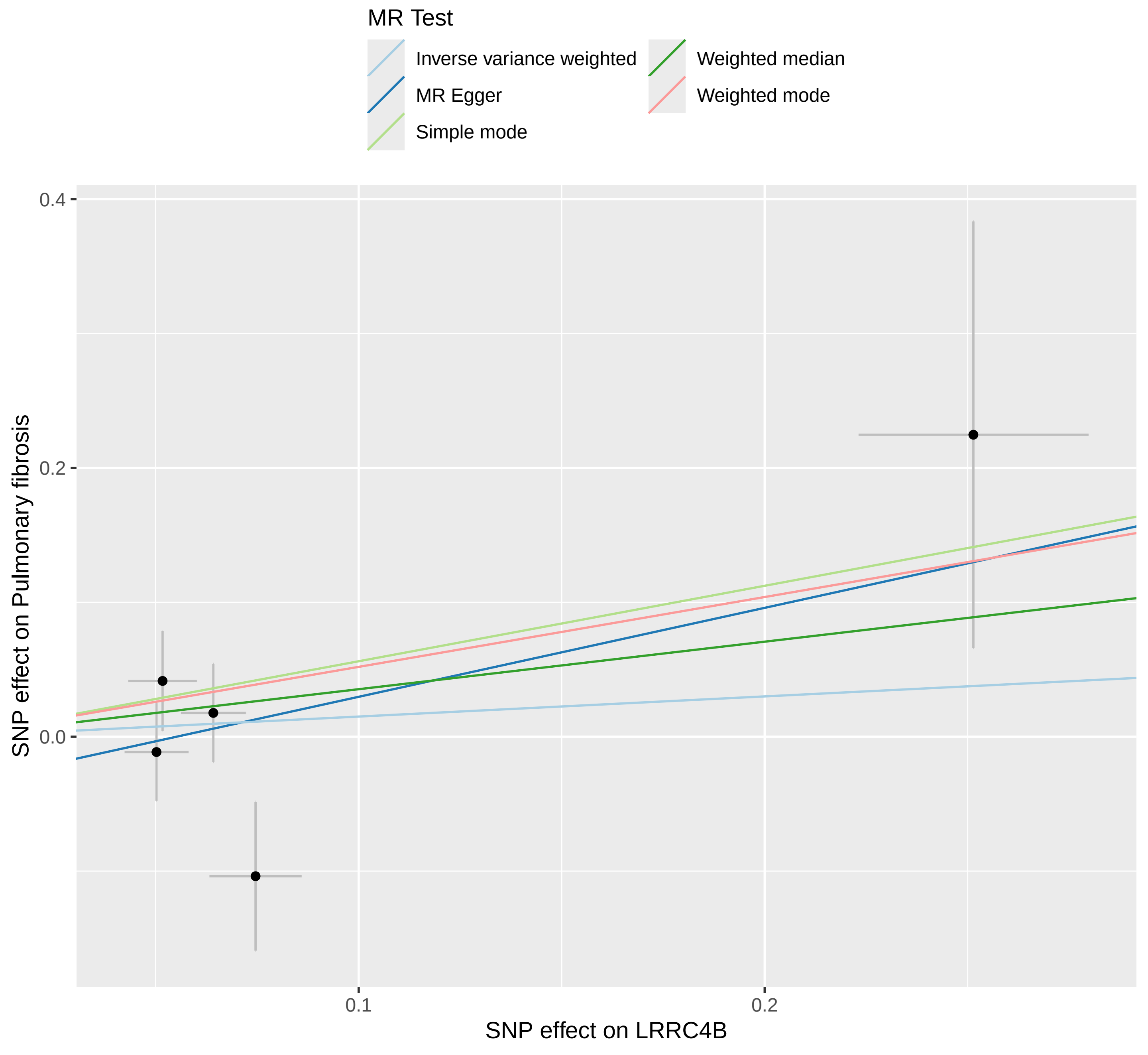
MR Test
Inverse variance weighted
MR Egger
Simple mode
Weighted median
Weighted mode
0.4
0.2
0.0
0.1
0.2
SNP effect on Pulmonary fibrosis
SNP effect on LRRC4B

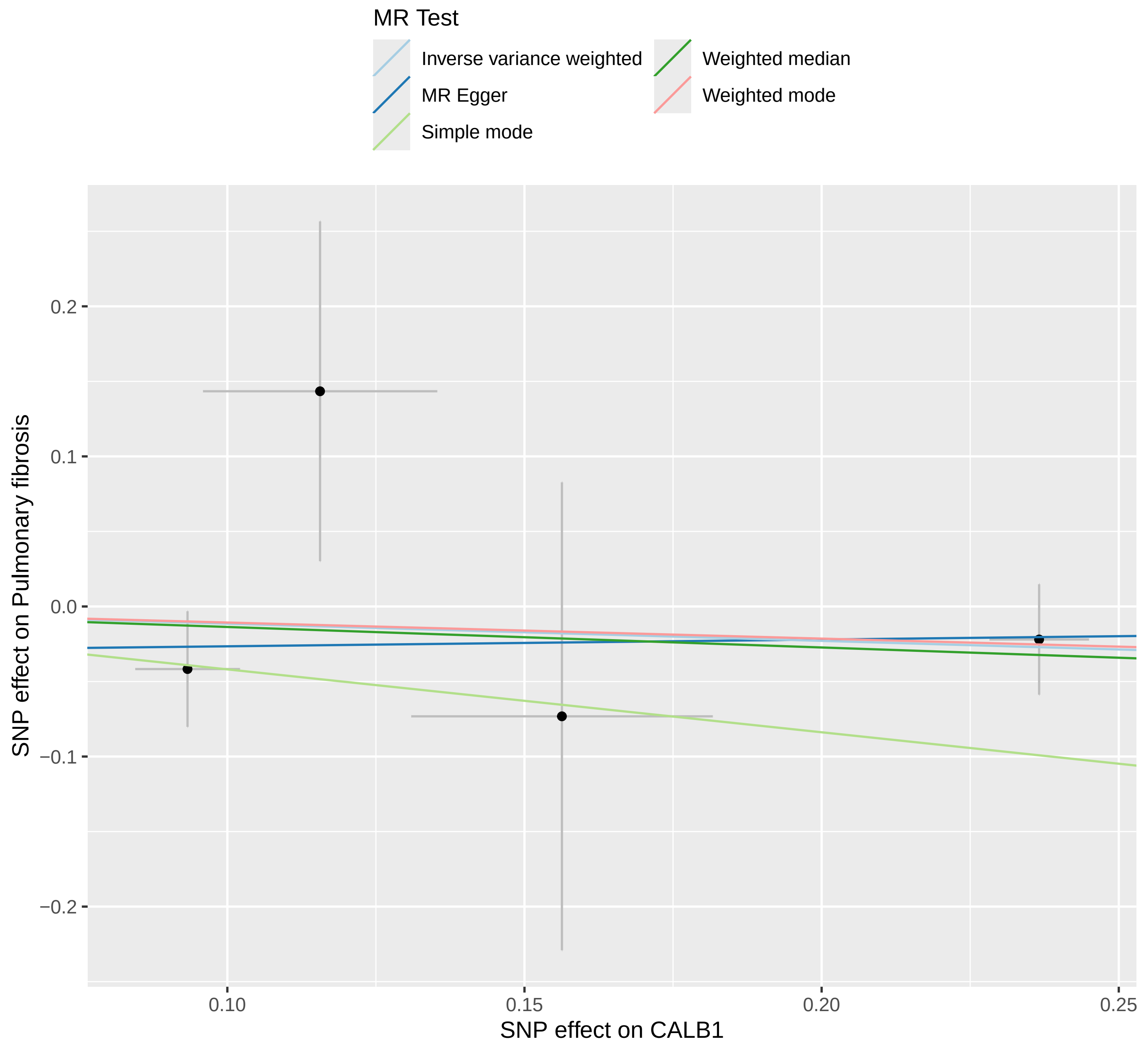

MR Test
Inverse variance weighted
MR Egger
Simple mode
Weighted median
Weighted mode
0.2
0.1
0.0
−0.1
−0.2
SNP effect on Pulmonary fibrosis
0.10
0.15
0.20
0.25
SNP effect on CALB1

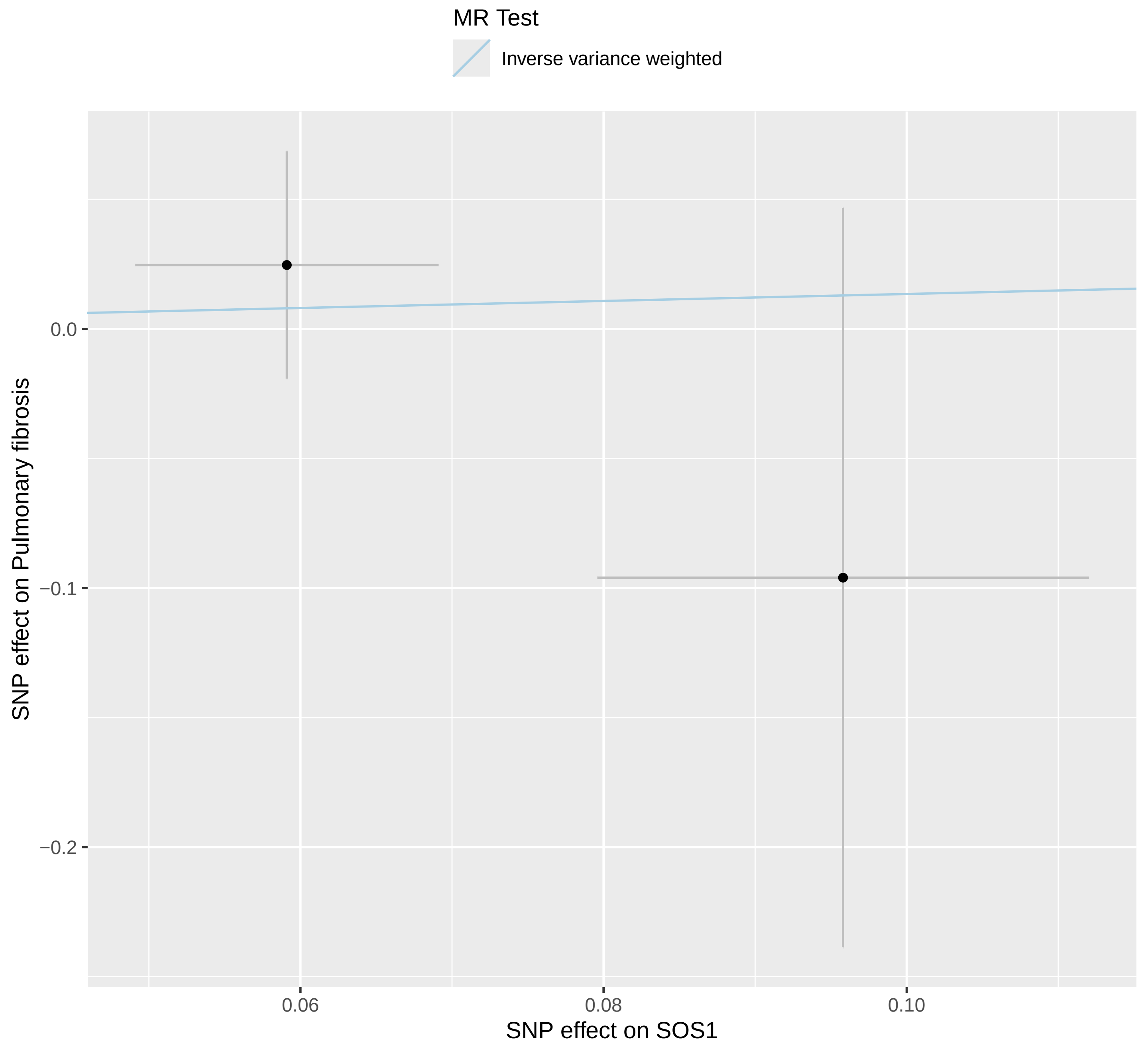

MR Test
Inverse variance weighted
0.0
−0.1
−0.2
0.06
0.08
0.10
SNP effect on SOS1
SNP effect on Pulmonary fibrosis

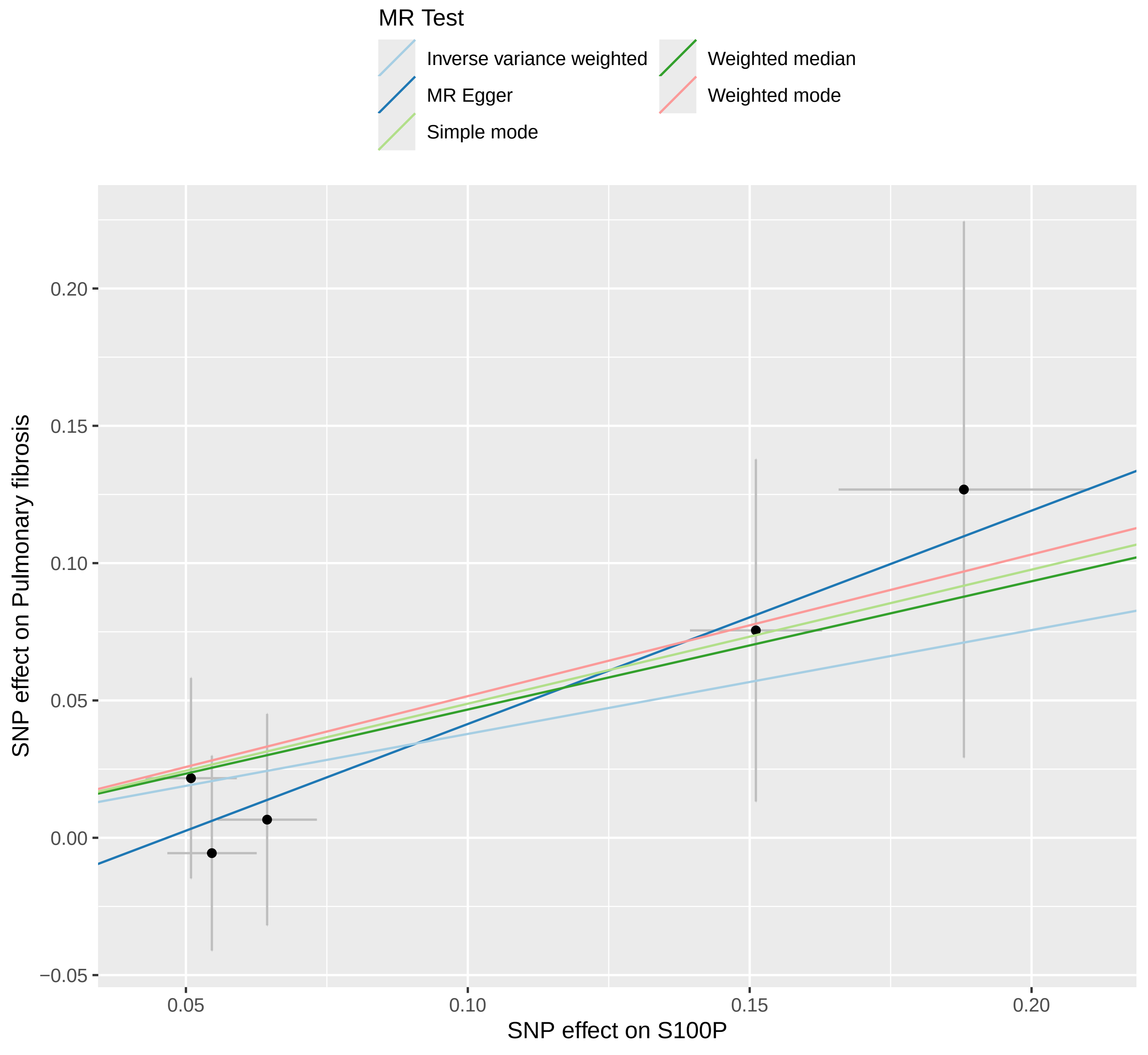

MR Test
Inverse variance weighted
MR Egger
Simple mode
Weighted median
Weighted mode
0.20
0.15
0.10
0.05
0.00
−0.05
SNP effect on Pulmonary fibrosis
0.05
0.10
0.15
0.20
SNP effect on S100P

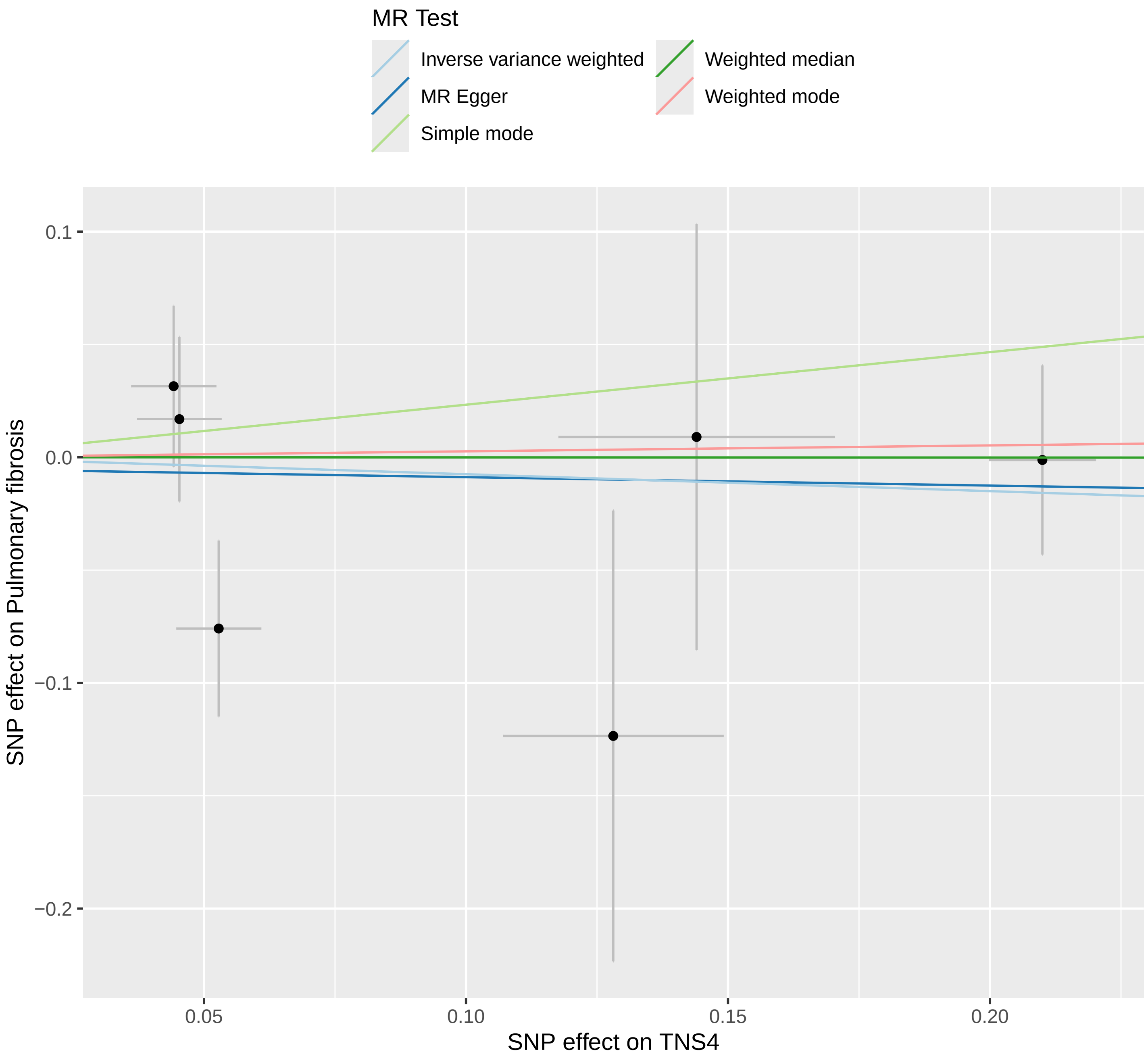
MR Test
Inverse variance weighted
MR Egger
Simple mode
Weighted median
Weighted mode
0.1
0.0
−0.1
−0.2
0.05
0.10
0.15
0.20
SNP effect on Pulmonary fibrosis
SNP effect on TNS4

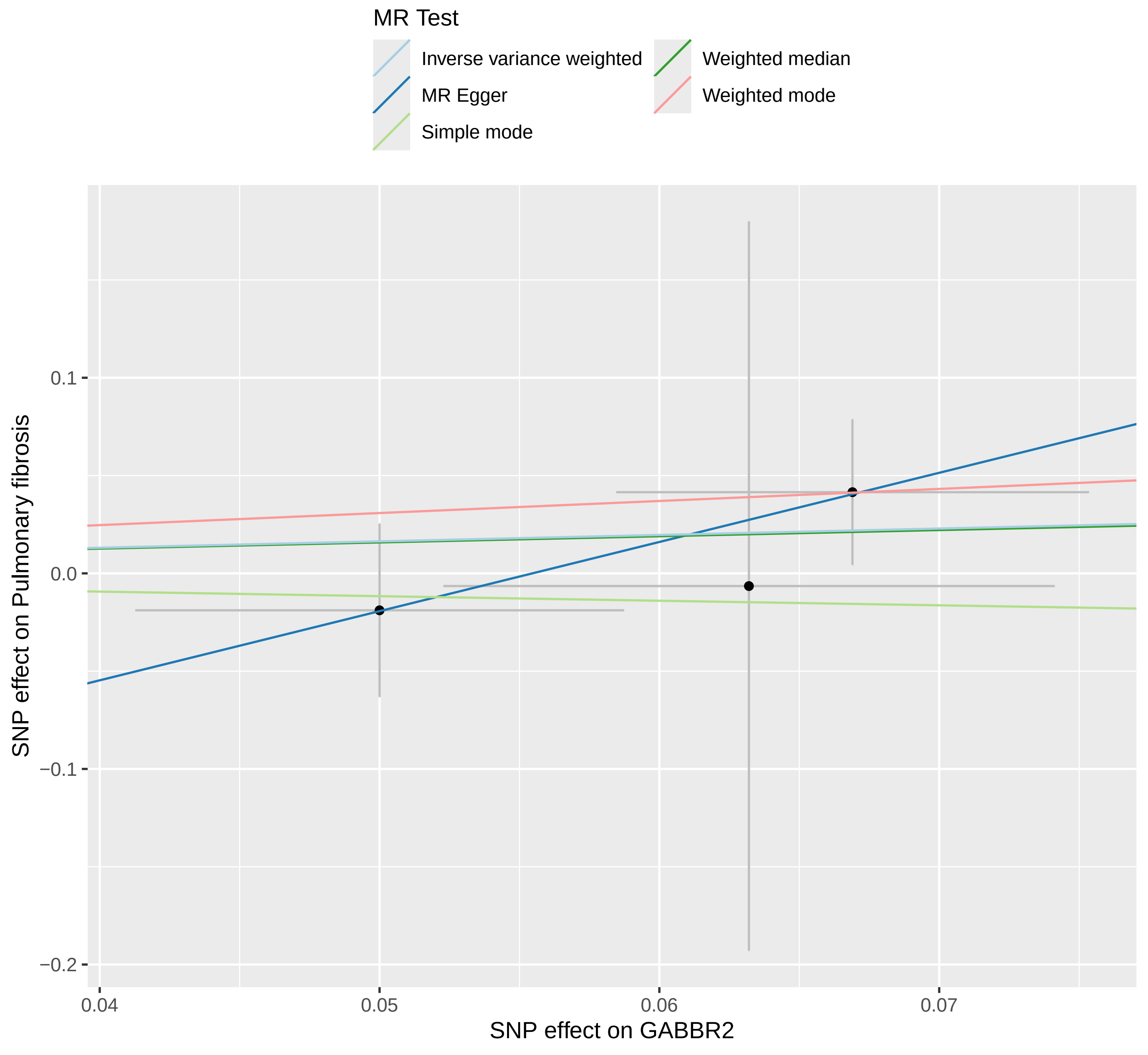

MR Test
Inverse variance weighted
MR Egger
Simple mode
Weighted median
Weighted mode
SNP effect on Pulmonary fibrosis
0.1
0.0
−0.1
−0.2
0.04
0.05
0.06
0.07
SNP effect on GABBR2

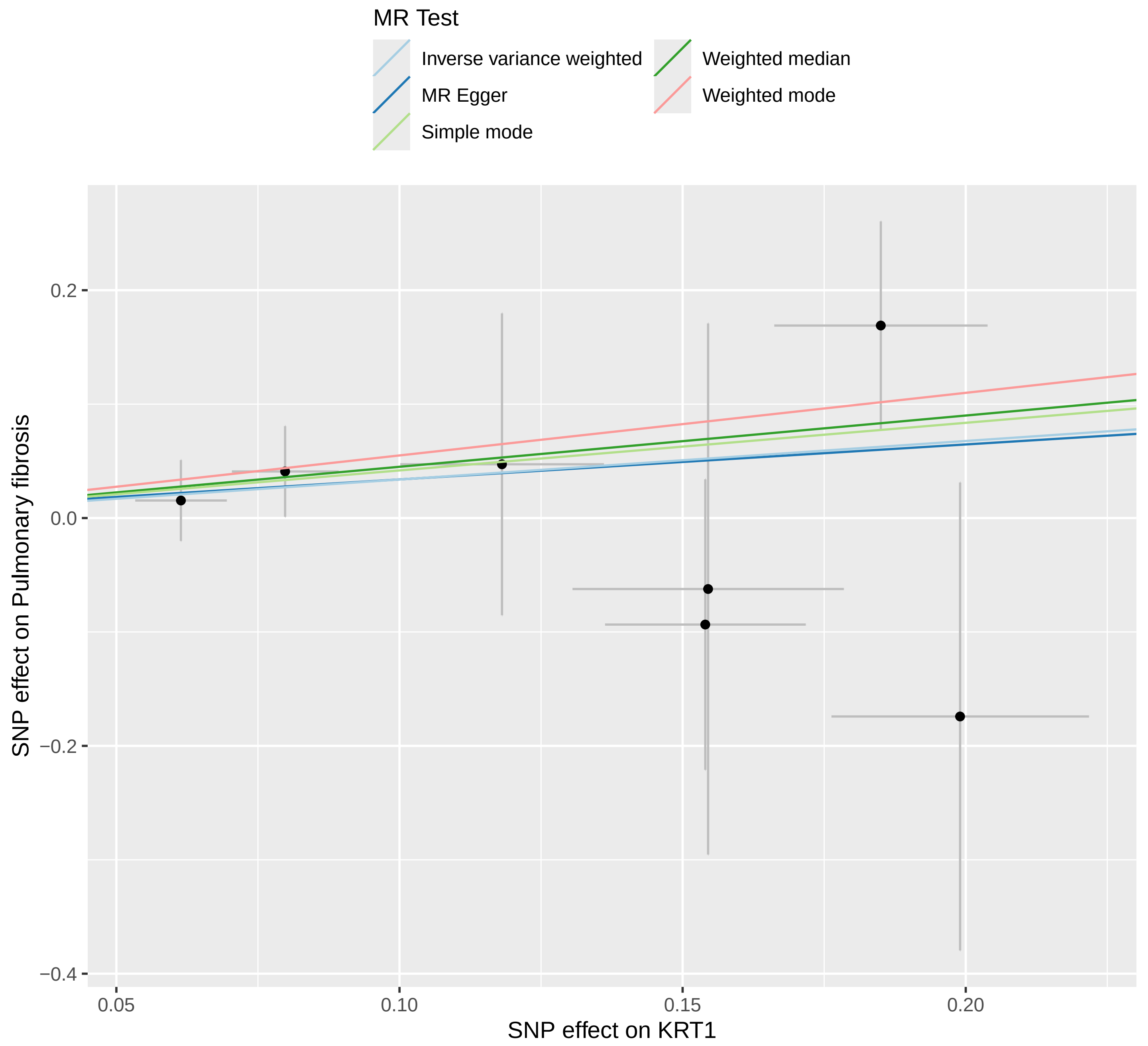

MR Test
Inverse variance weighted
MR Egger
Simple mode
Weighted median
Weighted mode
0.2
0.0
−0.2
−0.4
0.05
0.10
0.15
0.20
SNP effect on Pulmonary fibrosis
SNP effect on KRT1

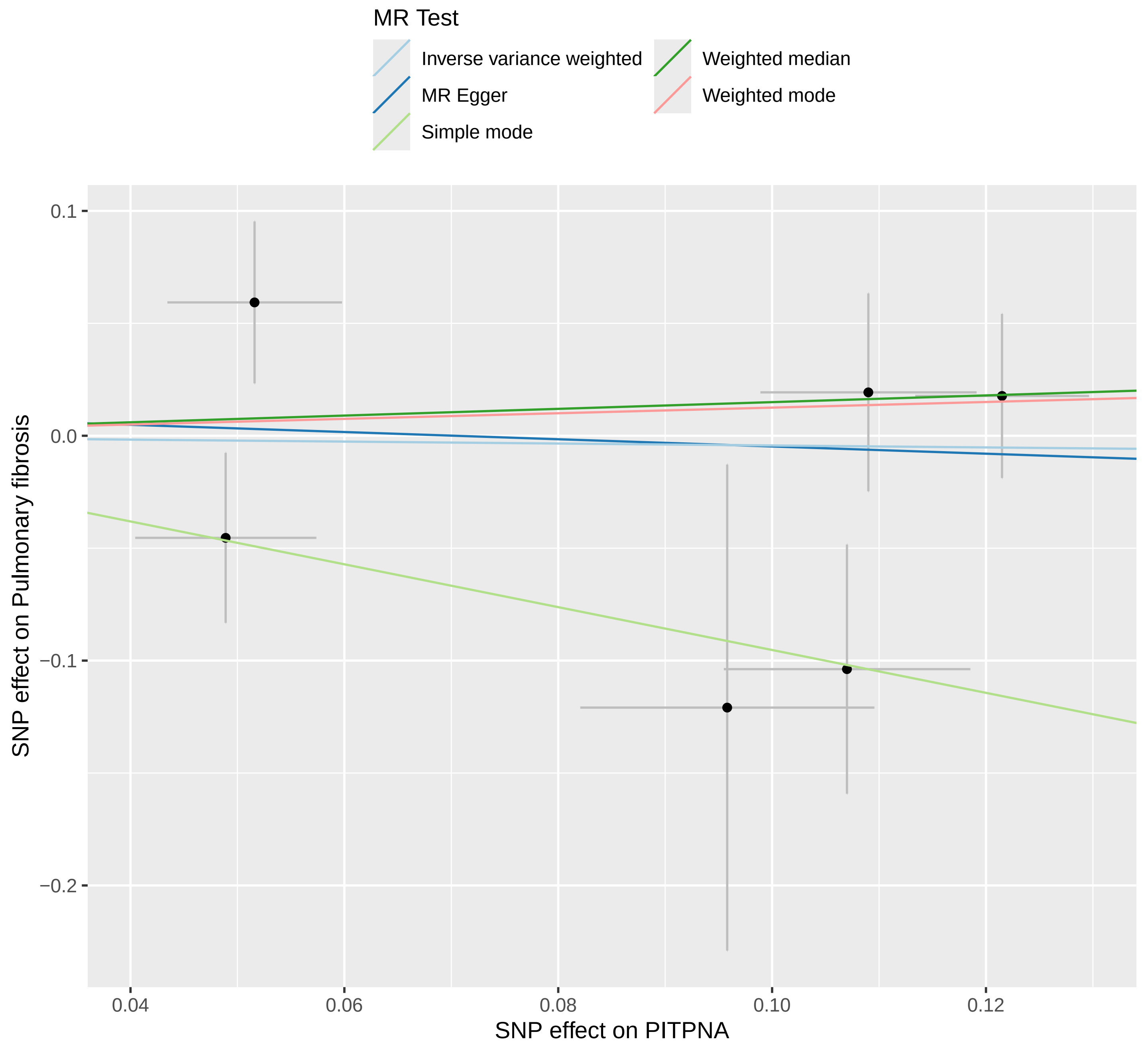

MR Test
Inverse variance weighted
Weighted median
MR Egger
Weighted mode
Simple mode
0.1
0.0
−0.1
−0.2
SNP effect on Pulmonary fibrosis
0.04
0.06
0.08
0.10
0.12
SNP effect on PITPNA

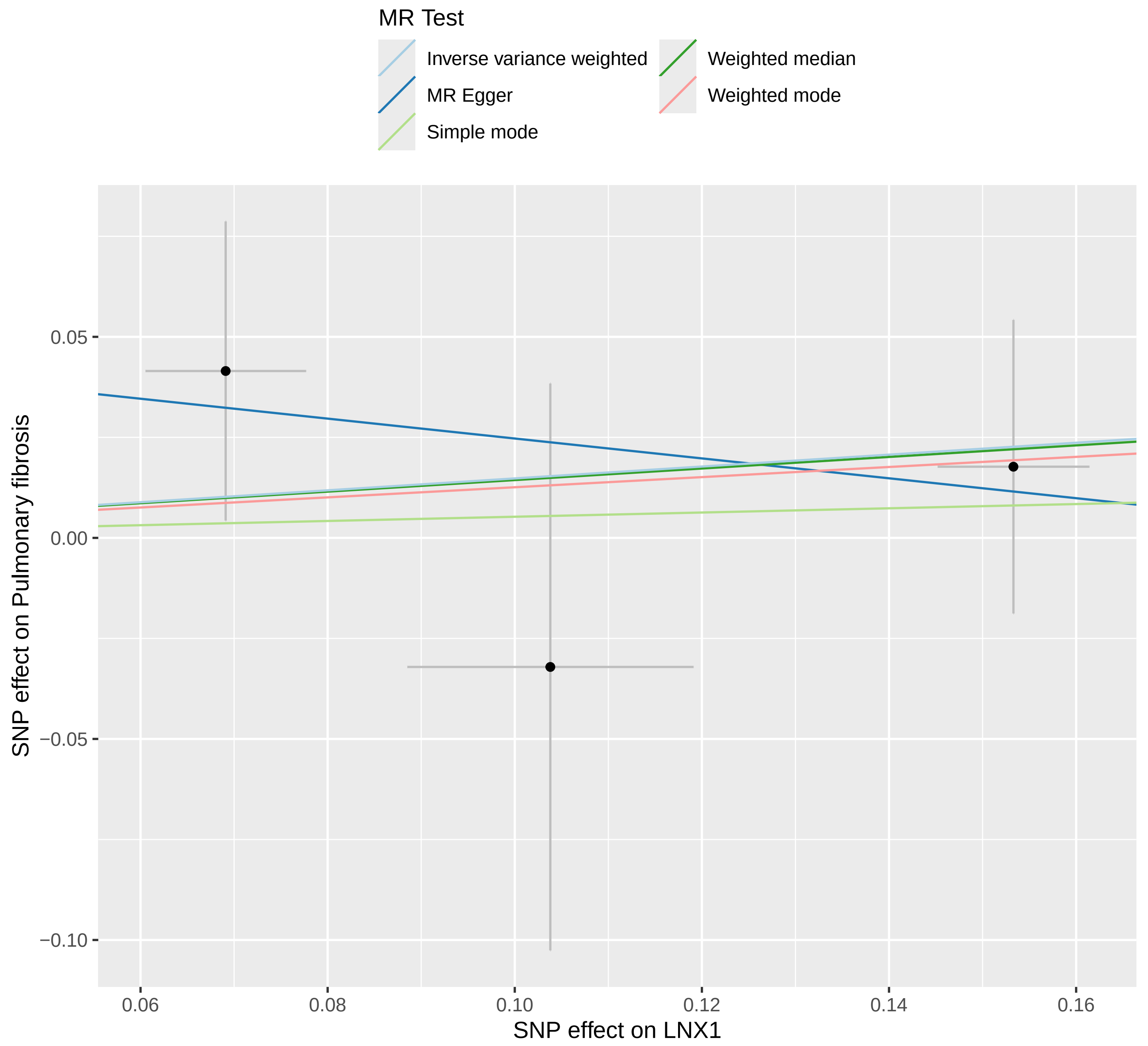
MR Test
Inverse variance weighted
MR Egger
Simple mode
Weighted median
Weighted mode
0.05
0.00
−0.05
−0.10
SNP effect on Pulmonary fibrosis
0.06
0.08
0.10
0.12
0.14
0.16
SNP effect on LNX1

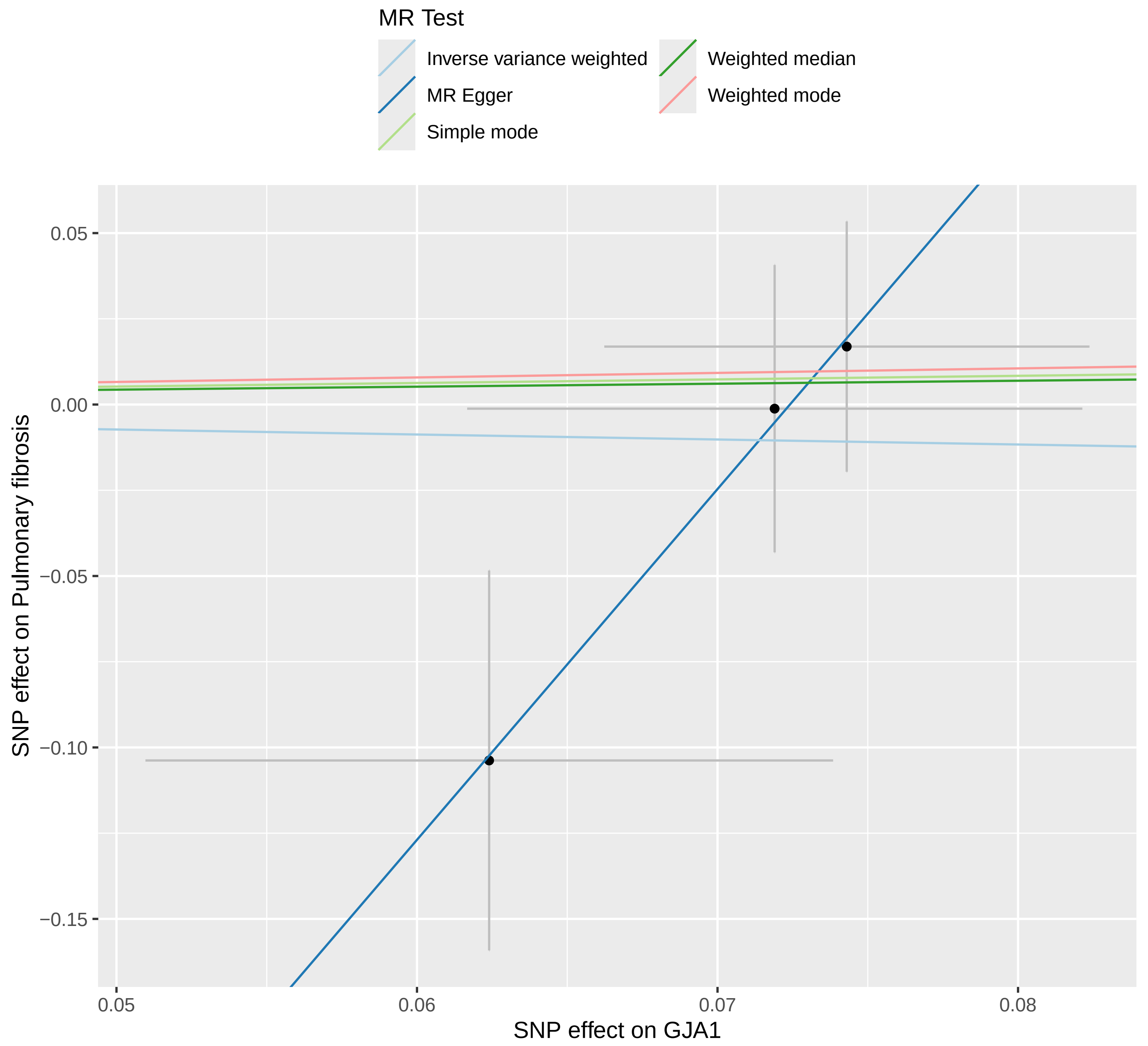

MR Test
Inverse variance weighted
MR Egger
Simple mode
Weighted median
Weighted mode
0.05
0.00
−0.05
−0.10
−0.15
SNP effect on Pulmonary fibrosis
0.05
0.06
0.07
0.08
SNP effect on GJA1

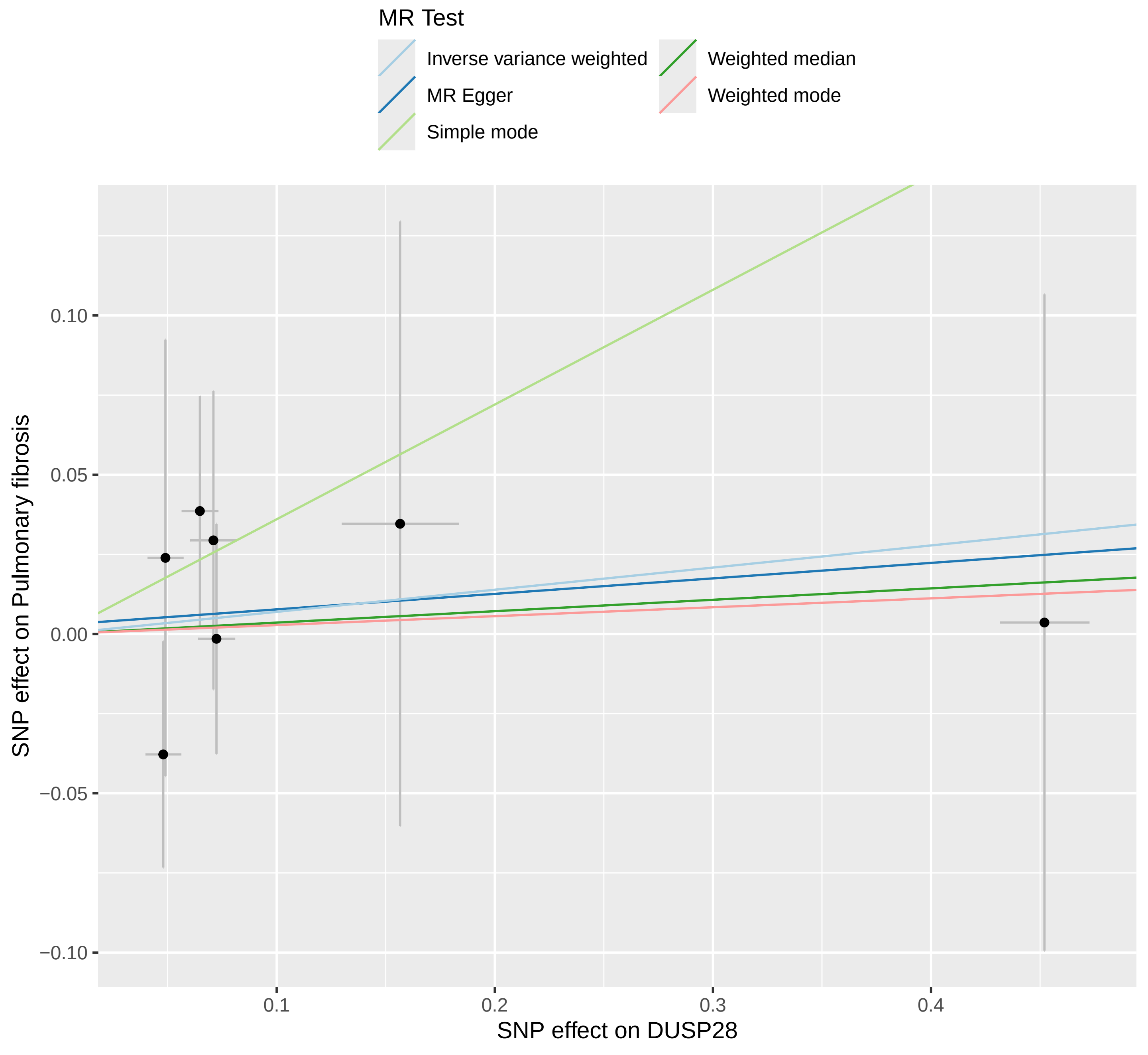
MR Test
Inverse variance weighted
MR Egger
Simple mode
Weighted median
Weighted mode
SNP effect on Pulmonary fibrosis
0.10
0.05
0.00
−0.05
−0.10
0.1
0.2
0.3
0.4
SNP effect on DUSP28

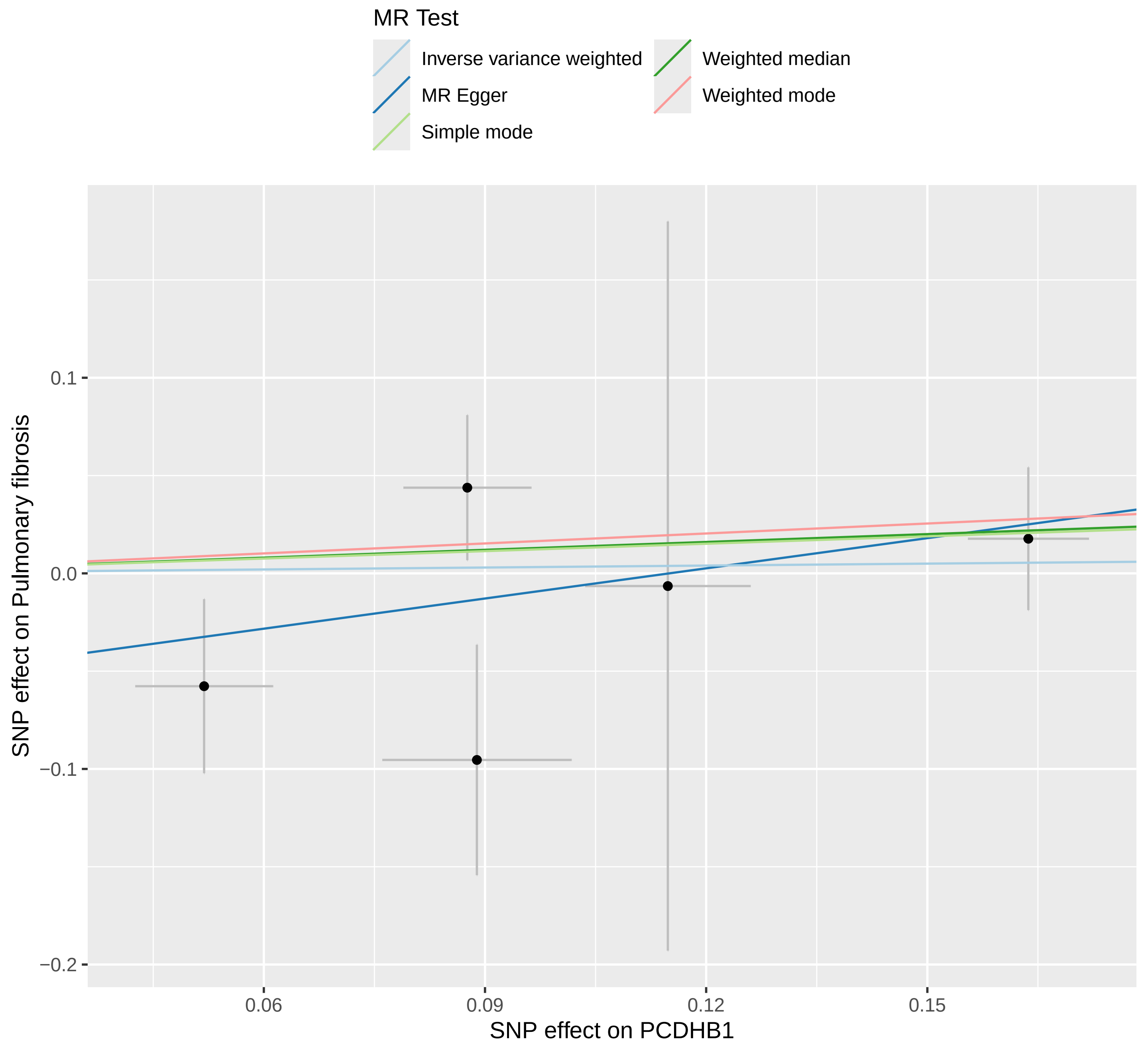

MR Test
Inverse variance weighted
MR Egger
Simple mode
Weighted median
Weighted mode
0.1
0.0
−0.1
−0.2
0.06
0.09
0.12
0.15
SNP effect on PCDHB1
SNP effect on Pulmonary fibrosis

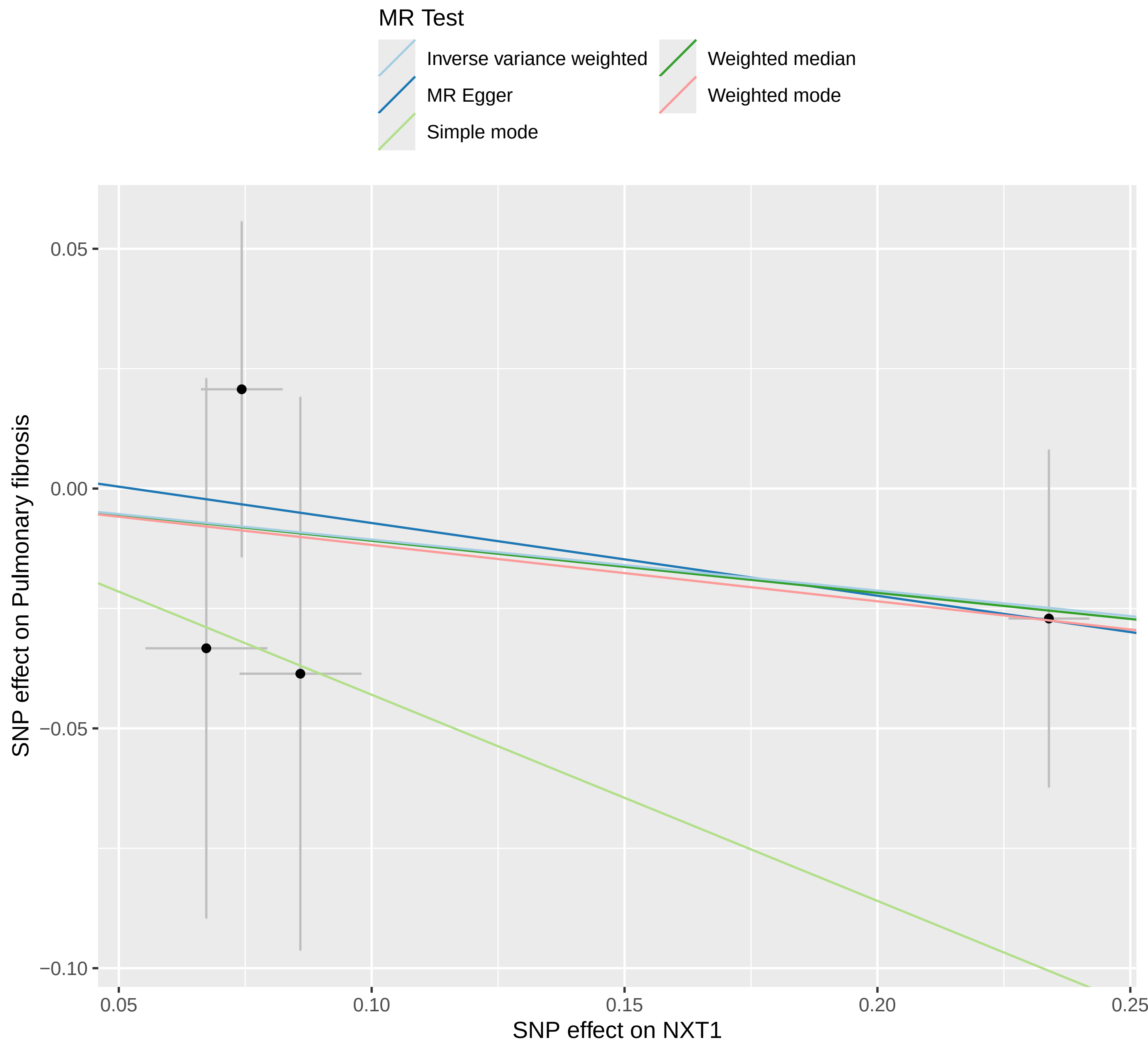

MR Test
Inverse variance weighted
MR Egger
Simple mode
Weighted median
Weighted mode
0.05
0.00
−0.05
−0.10
0.05
0.10
0.15
0.20
0.25
SNP effect on Pulmonary fibrosis
SNP effect on NXT1

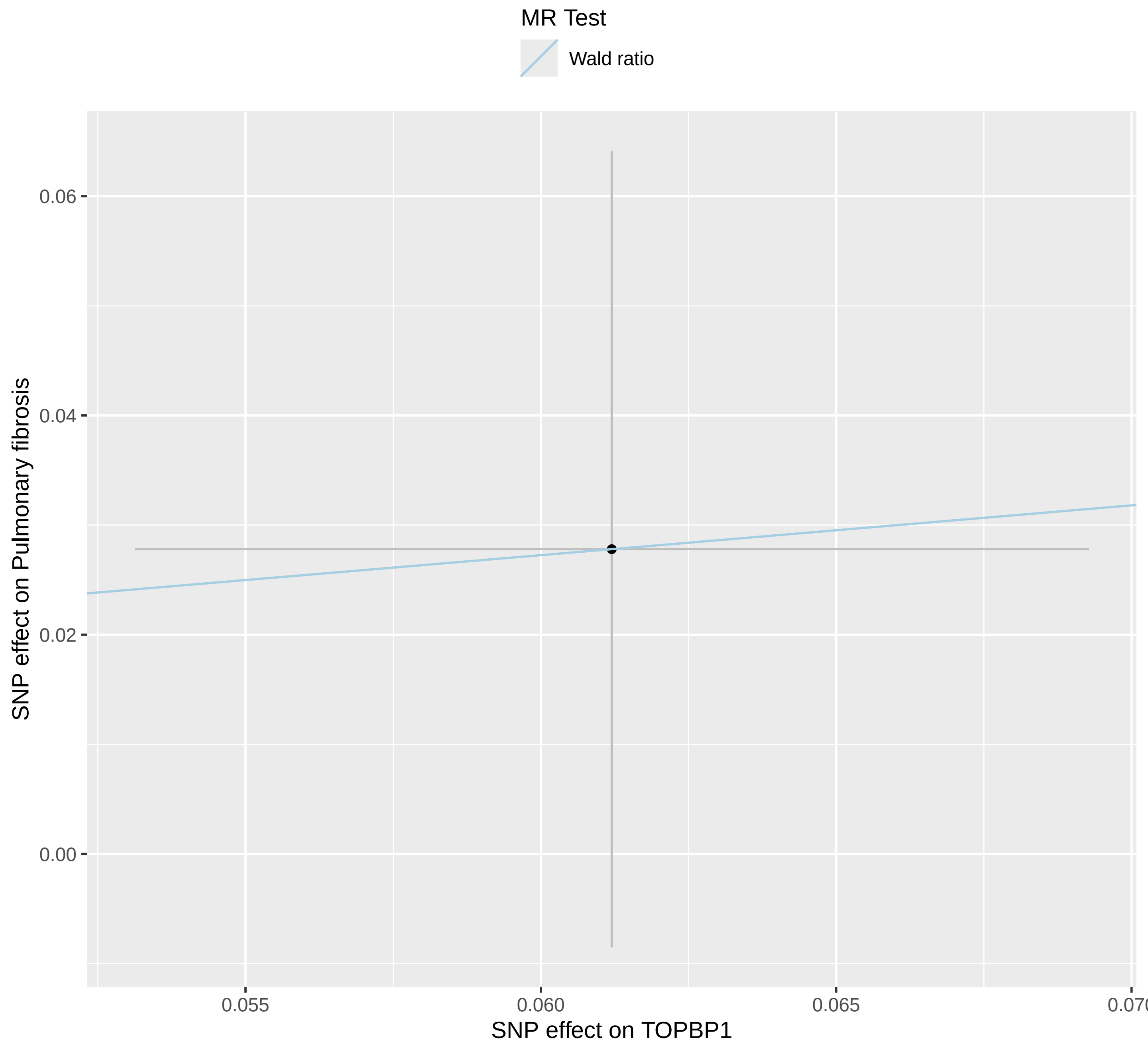
MR Test
Wald ratio
0.06
0.04
0.02
0.00
SNP effect on Pulmonary fibrosis
0.055
0.060
0.065
0.070
SNP effect on TOPBP1

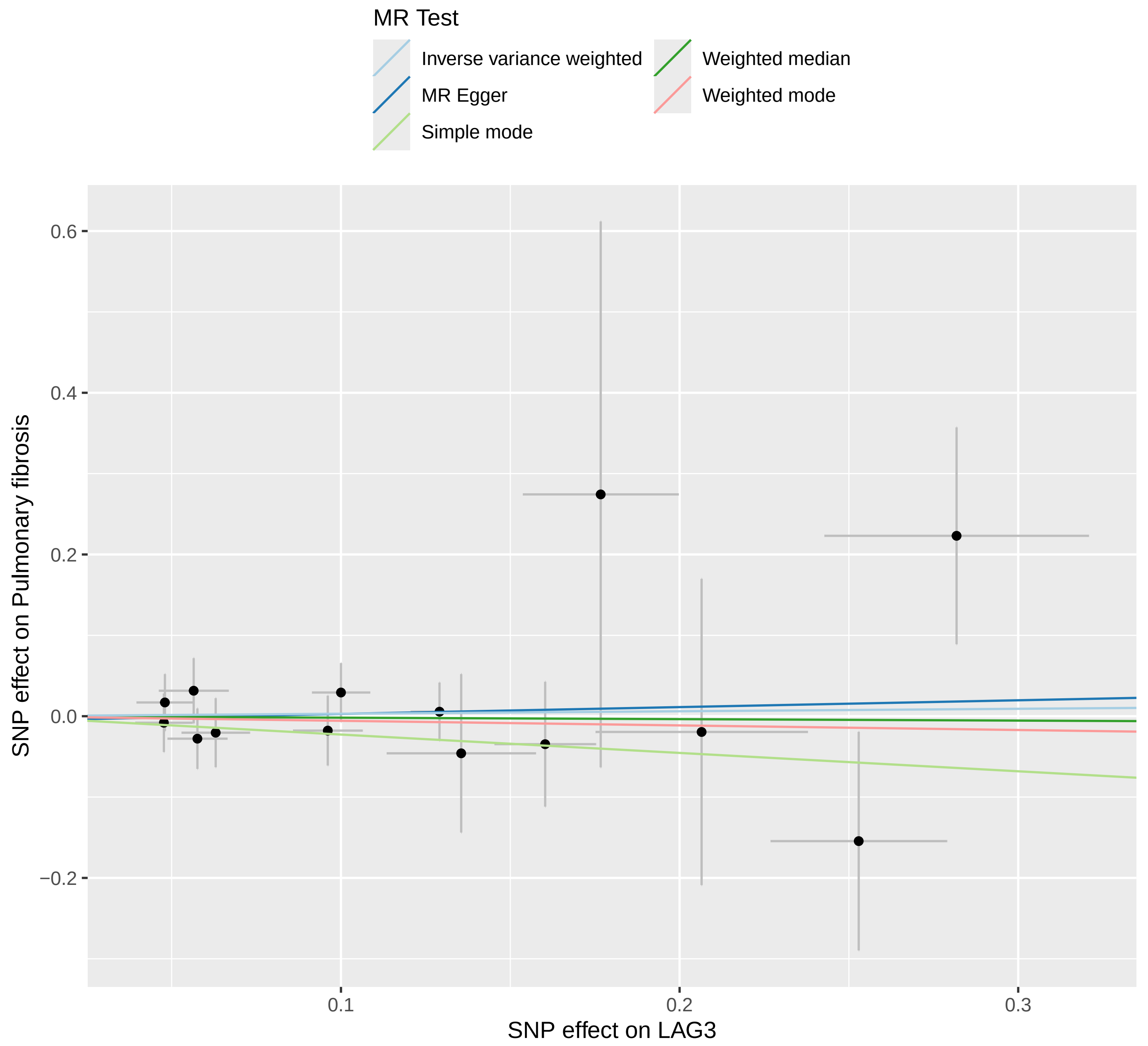
MR Test
Inverse variance weighted
MR Egger
Simple mode
Weighted median
Weighted mode
0.6
0.4
0.2
0.0
−0.2
SNP effect on Pulmonary fibrosis
0.1
0.2
0.3
SNP effect on LAG3

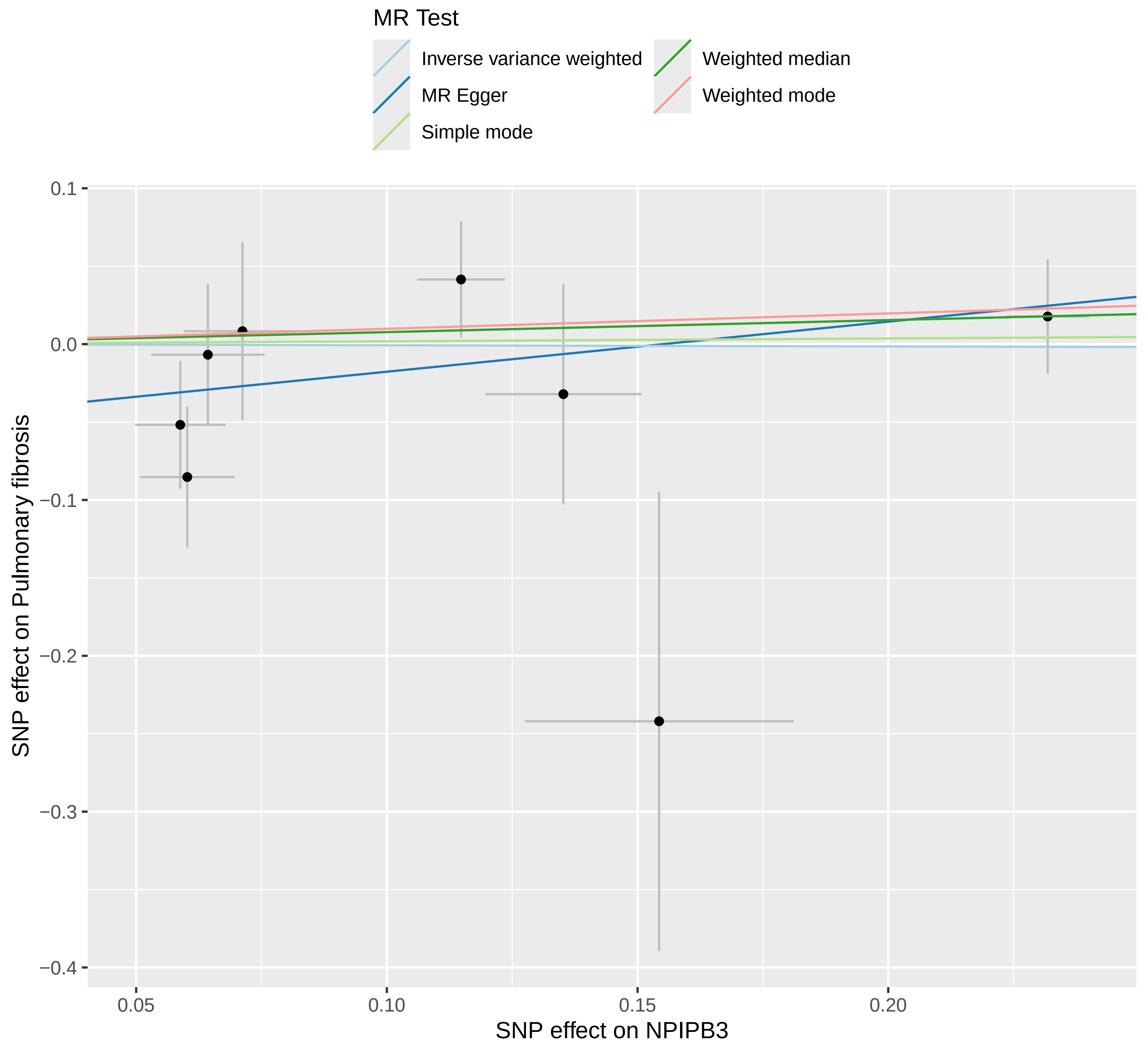
MR Test
Inverse variance weighted
MR Egger
Simple mode
Weighted median
Weighted mode
0.1
0.0
−0.1
−0.2
−0.3
−0.4
SNP effect on Pulmonary fibrosis
0.05
0.10
0.15
0.20
SNP effect on NPIPB3

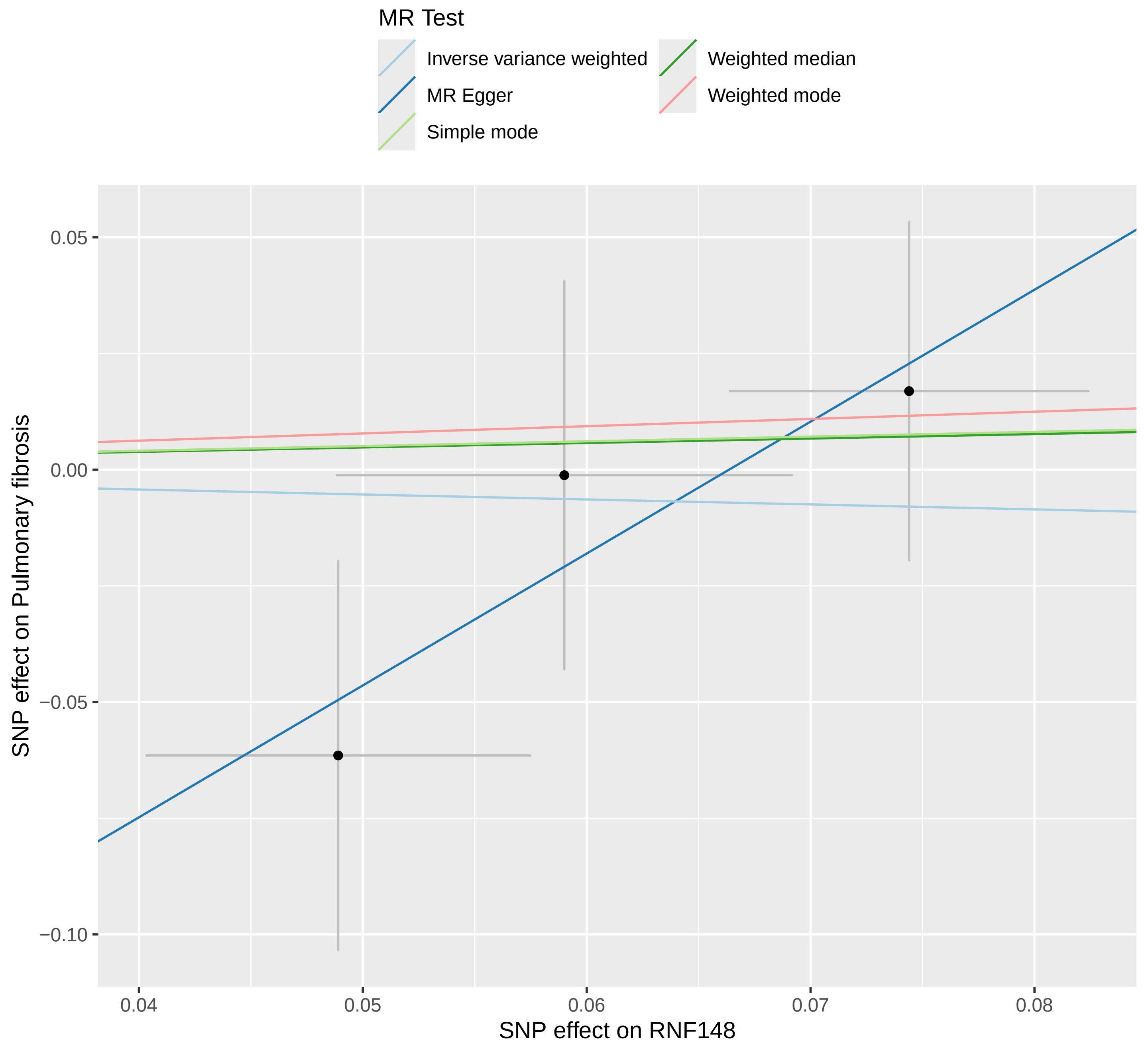

MR Test
Inverse variance weighted
MR Egger
Simple mode
Weighted median
Weighted mode
0.05
0.00
−0.05
−0.10
0.04
0.05
0.06
0.07
0.08
SNP effect on Pulmonary fibrosis
SNP effect on RNF148

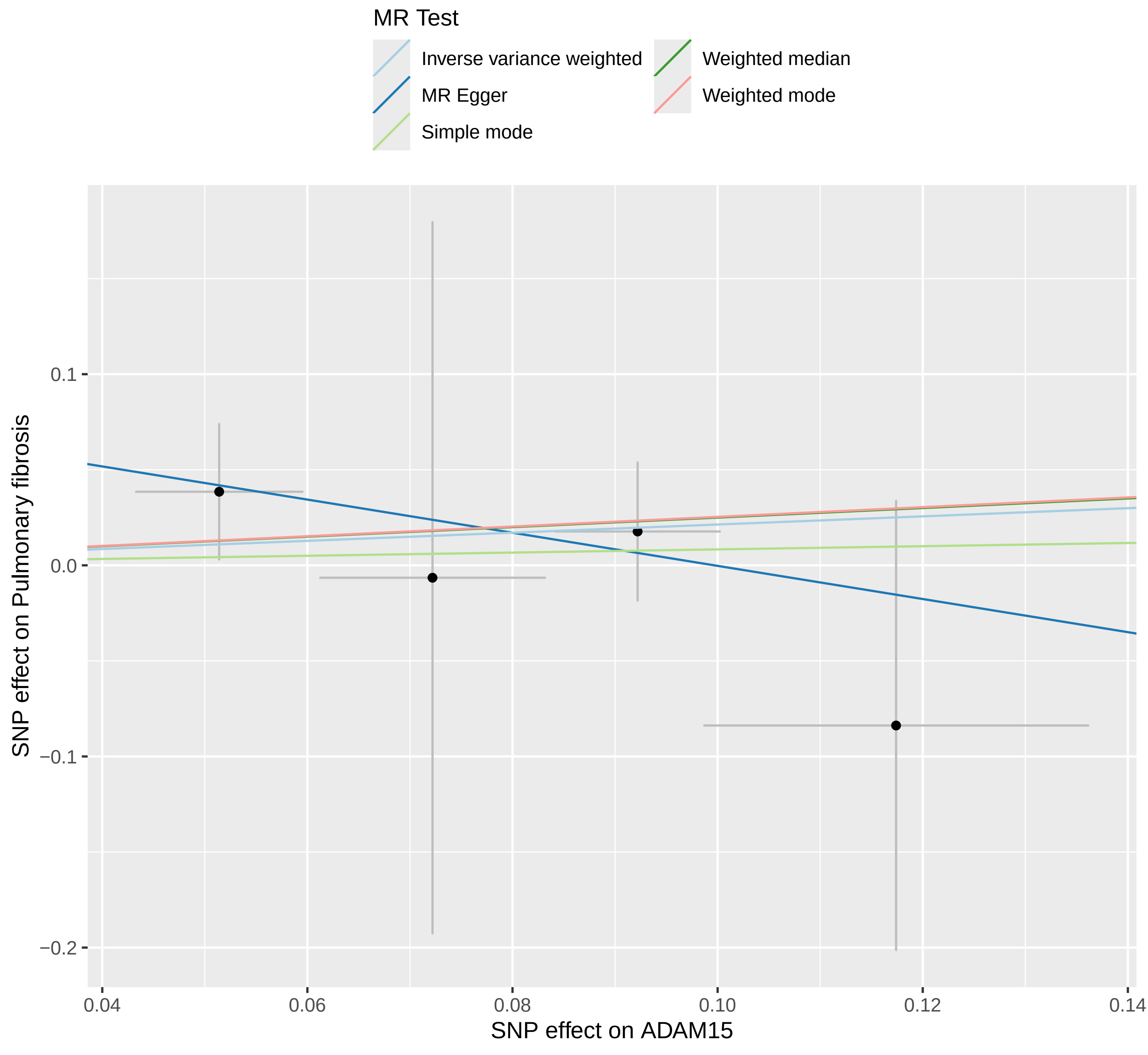

MR Test
Inverse variance weighted
MR Egger
Simple mode
Weighted median
Weighted mode
SNP effect on Pulmonary fibrosis
0.1
0.0
−0.1
−0.2
0.04
0.06
0.08
0.10
0.12
0.14
SNP effect on ADAM15

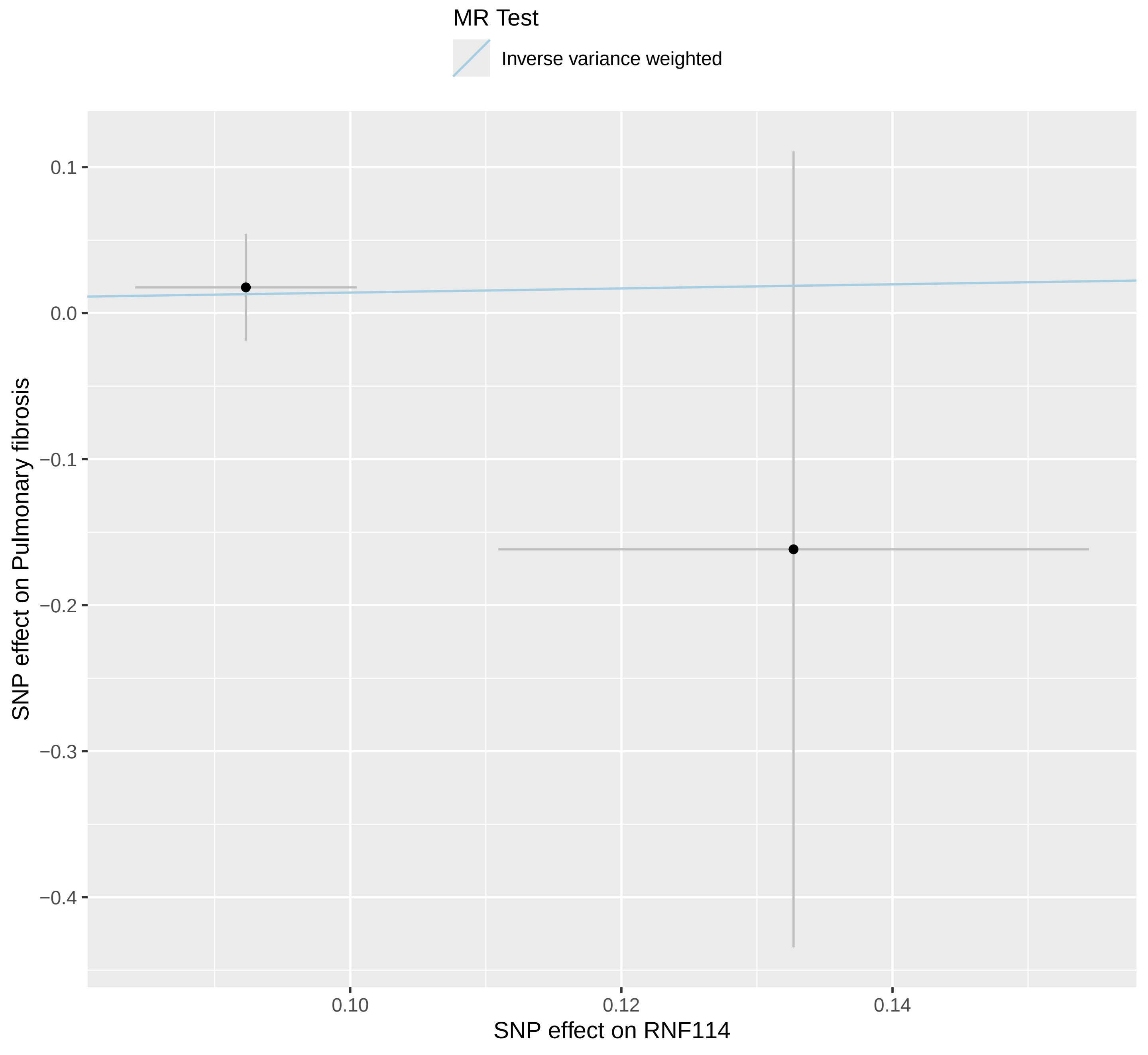
MR Test
Inverse variance weighted
0.1
0.0
−0.1
−0.2
−0.3
−0.4
0.10
0.12
0.14
SNP effect on RNF114
SNP effect on Pulmonary fibrosis

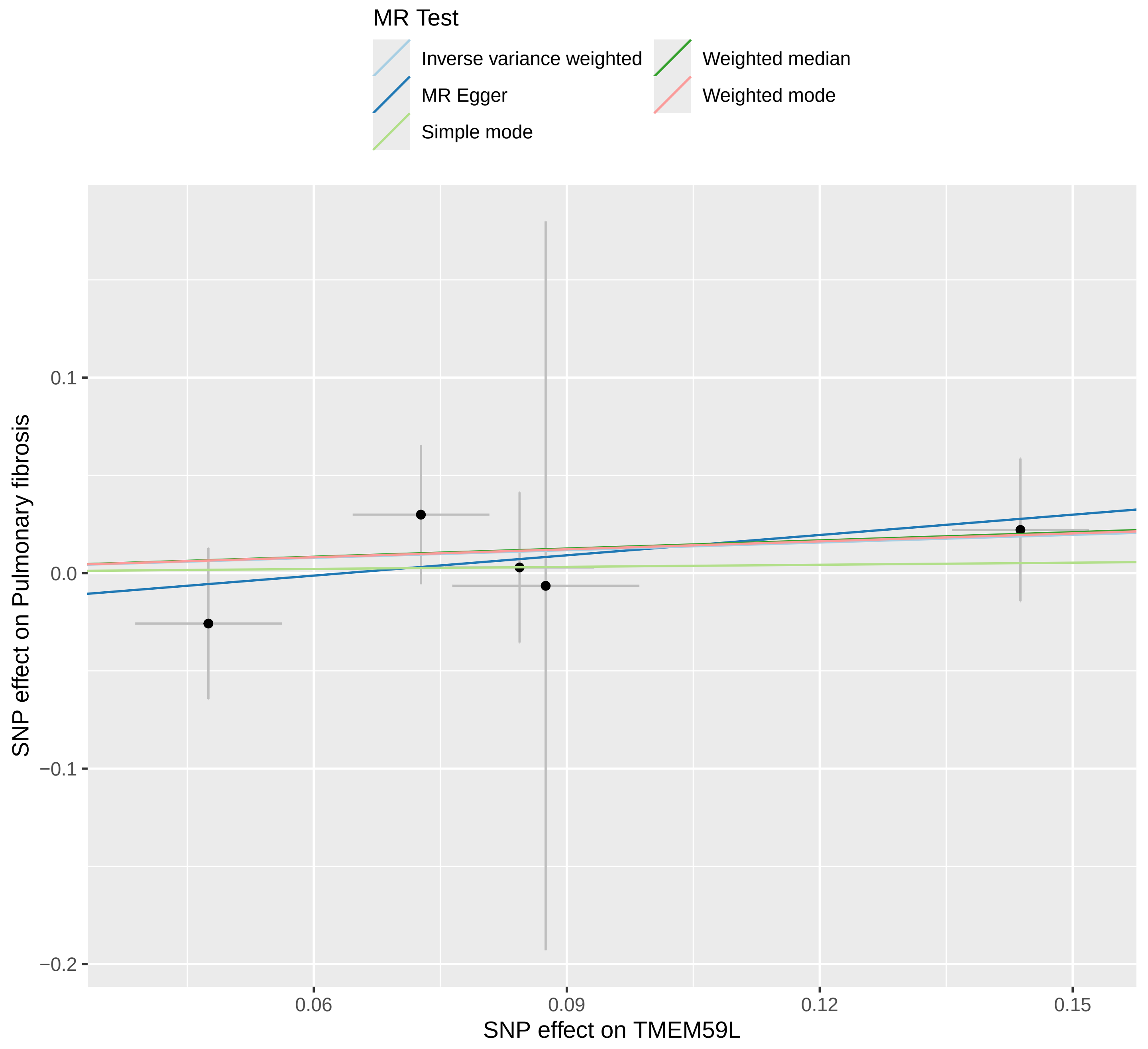
MR Test
Inverse variance weighted
MR Egger
Simple mode
Weighted median
Weighted mode
0.1
0.0
−0.1
−0.2
0.06
0.09
0.12
0.15
SNP effect on Pulmonary fibrosis
SNP effect on TMEM59L

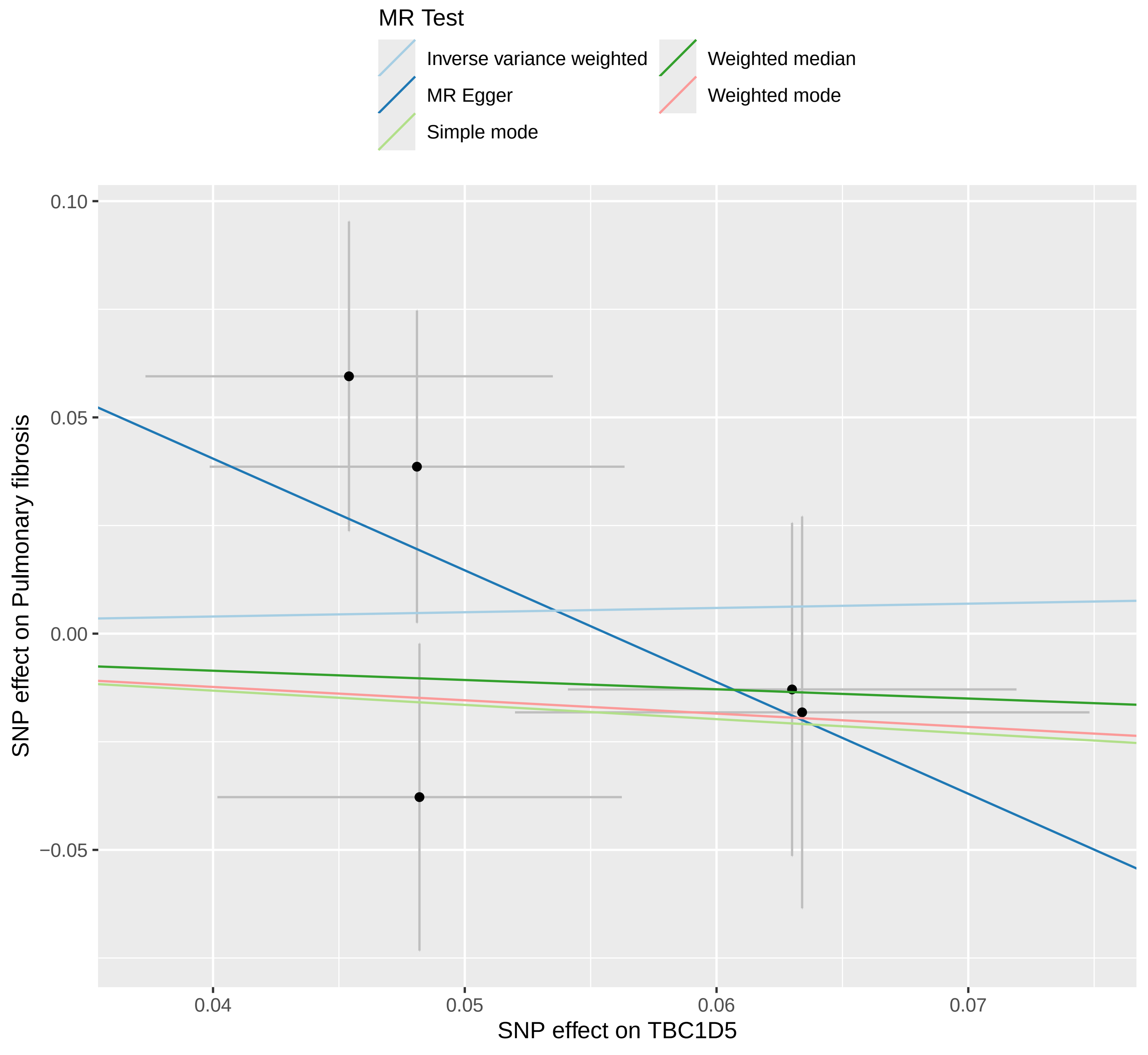

MR Test
Inverse variance weighted
MR Egger
Simple mode
Weighted median
Weighted mode
0.10
0.05
0.00
−0.05
SNP effect on Pulmonary fibrosis
0.04
0.05
0.06
0.07
SNP effect on TBC1D5

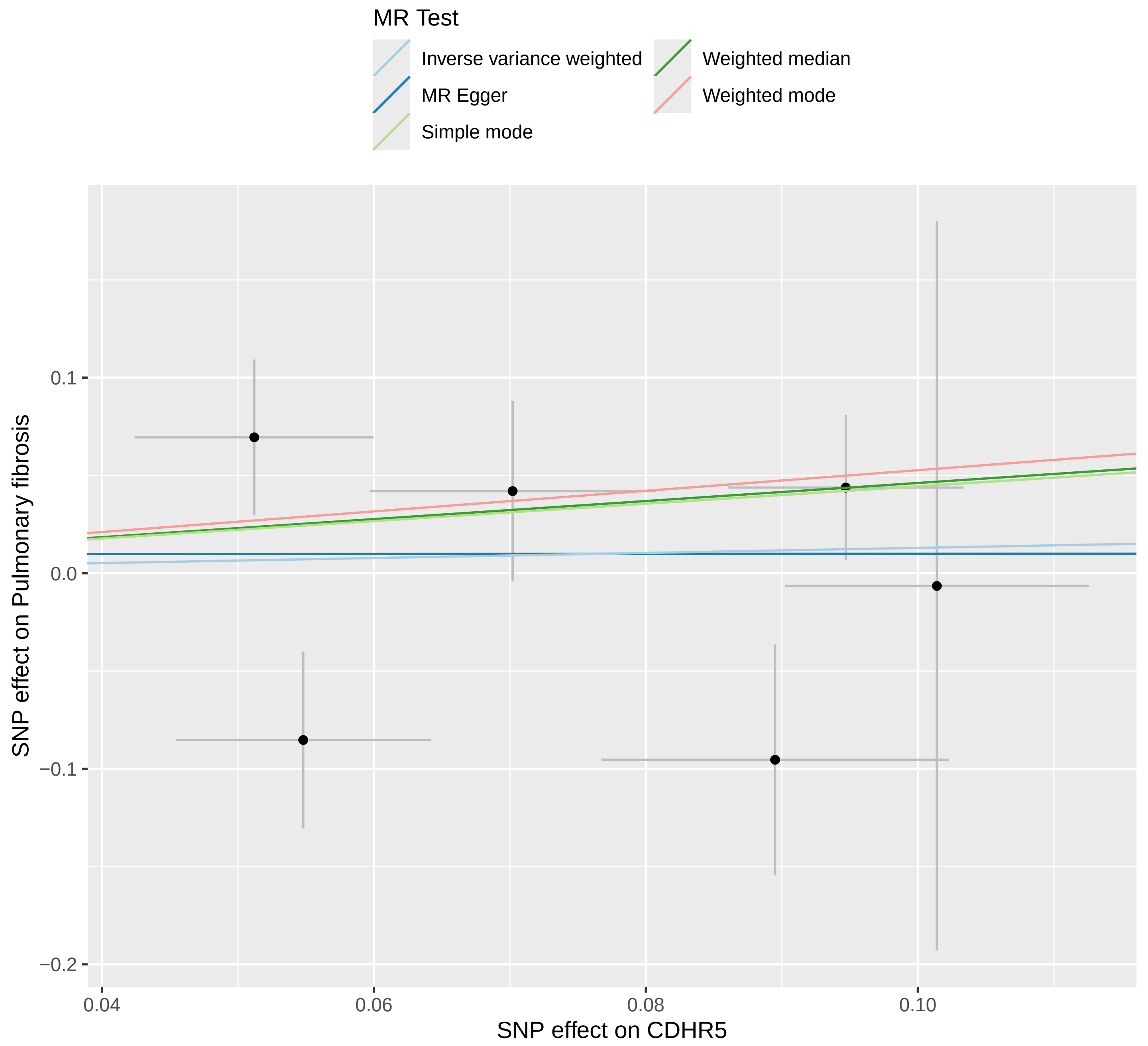
MR Test
Inverse variance weighted
MR Egger
Simple mode
Weighted median
Weighted mode
0.1
0.0
−0.1
−0.2
SNP effect on Pulmonary fibrosis
0.04
0.06
0.08
0.10
SNP effect on CDHR5

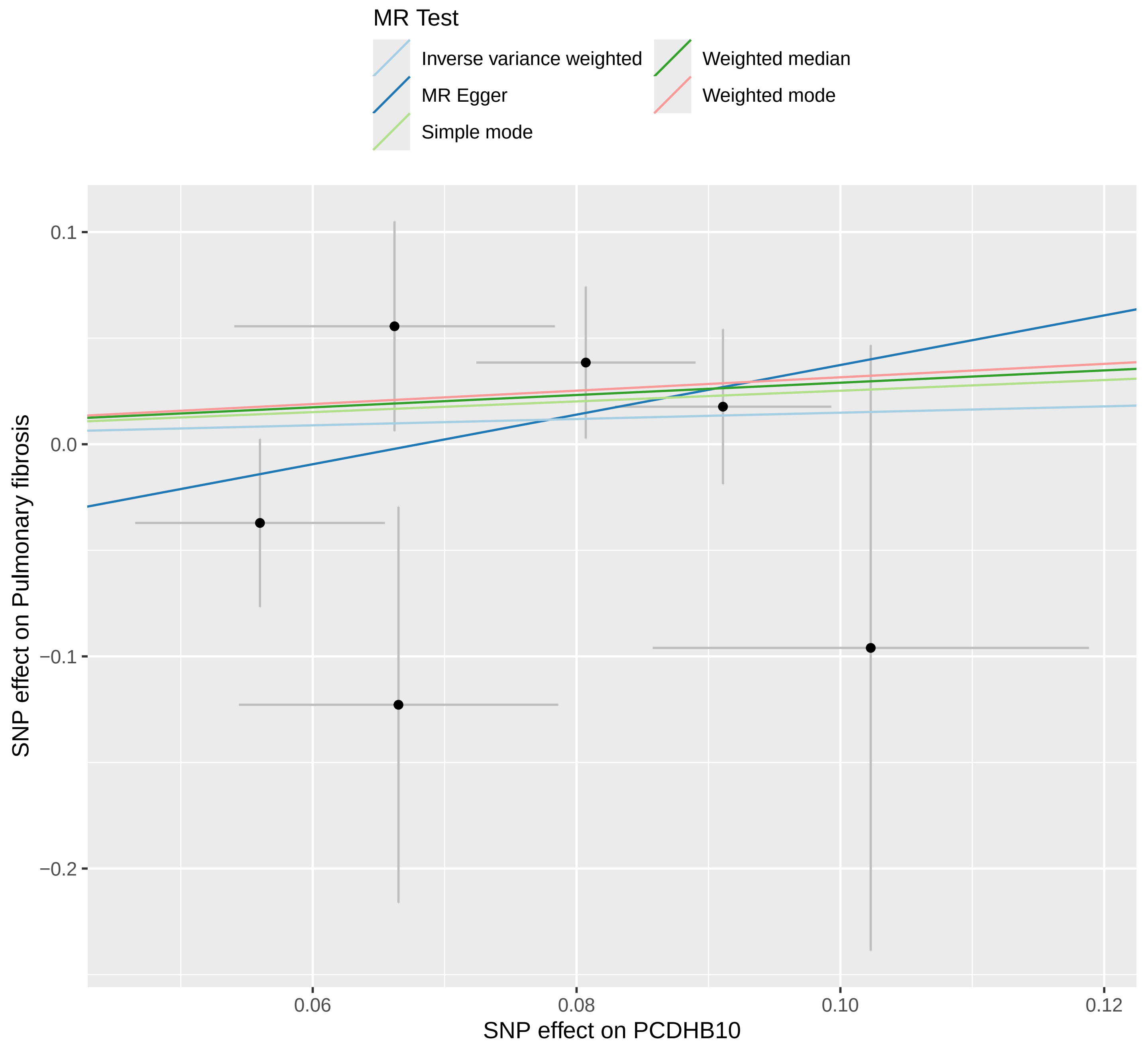

MR Test
Inverse variance weighted
MR Egger
Simple mode
Weighted median
Weighted mode
0.1
0.0
−0.1
−0.2
SNP effect on Pulmonary fibrosis
0.06
0.08
0.10
0.12
SNP effect on PCDHB10

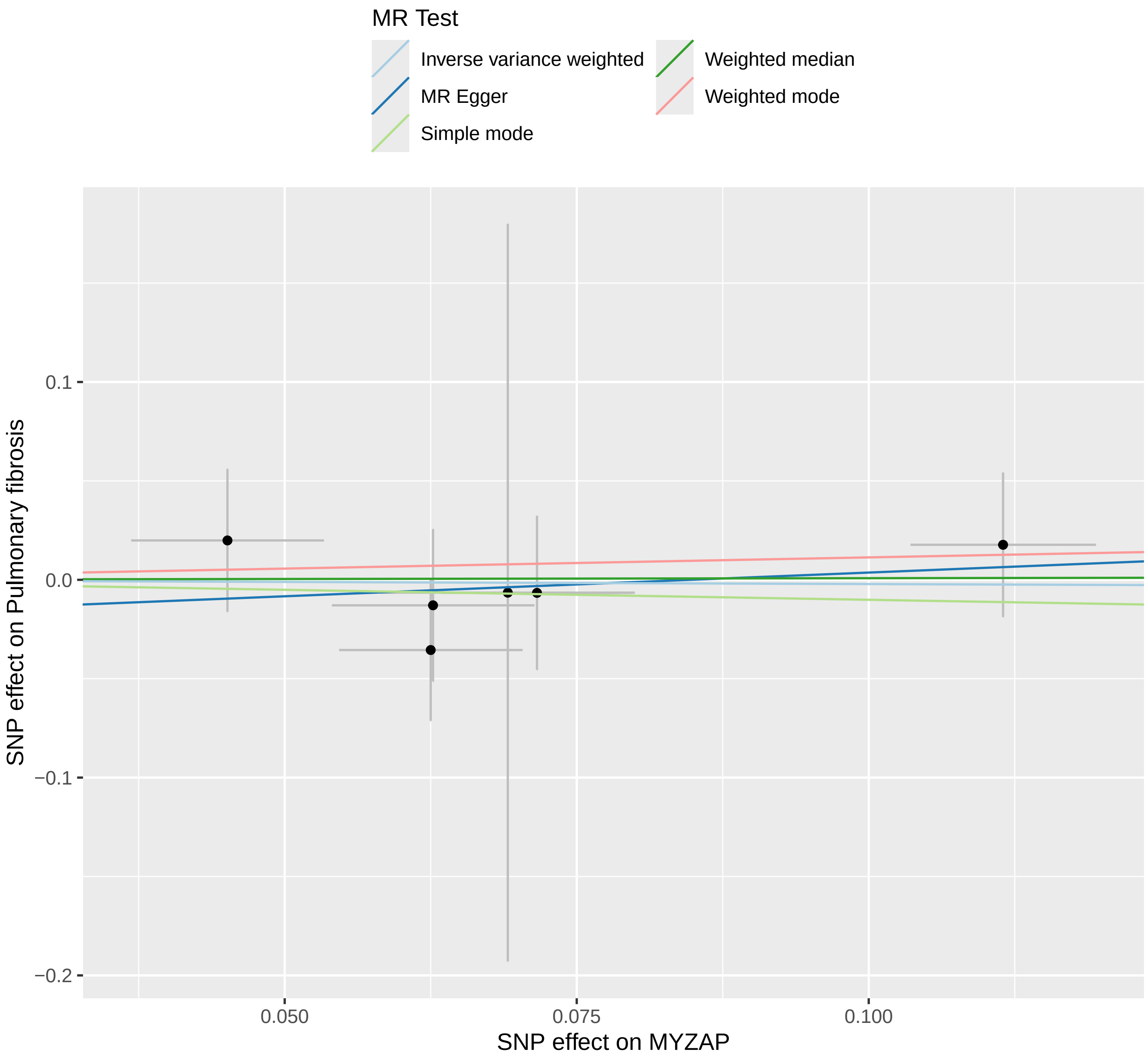

MR Test
Inverse variance weighted
MR Egger
Simple mode
Weighted median
Weighted mode
0.1
0.0
−0.1
−0.2
0.050
0.075
0.100
SNP effect on MYZAP
SNP effect on Pulmonary fibrosis

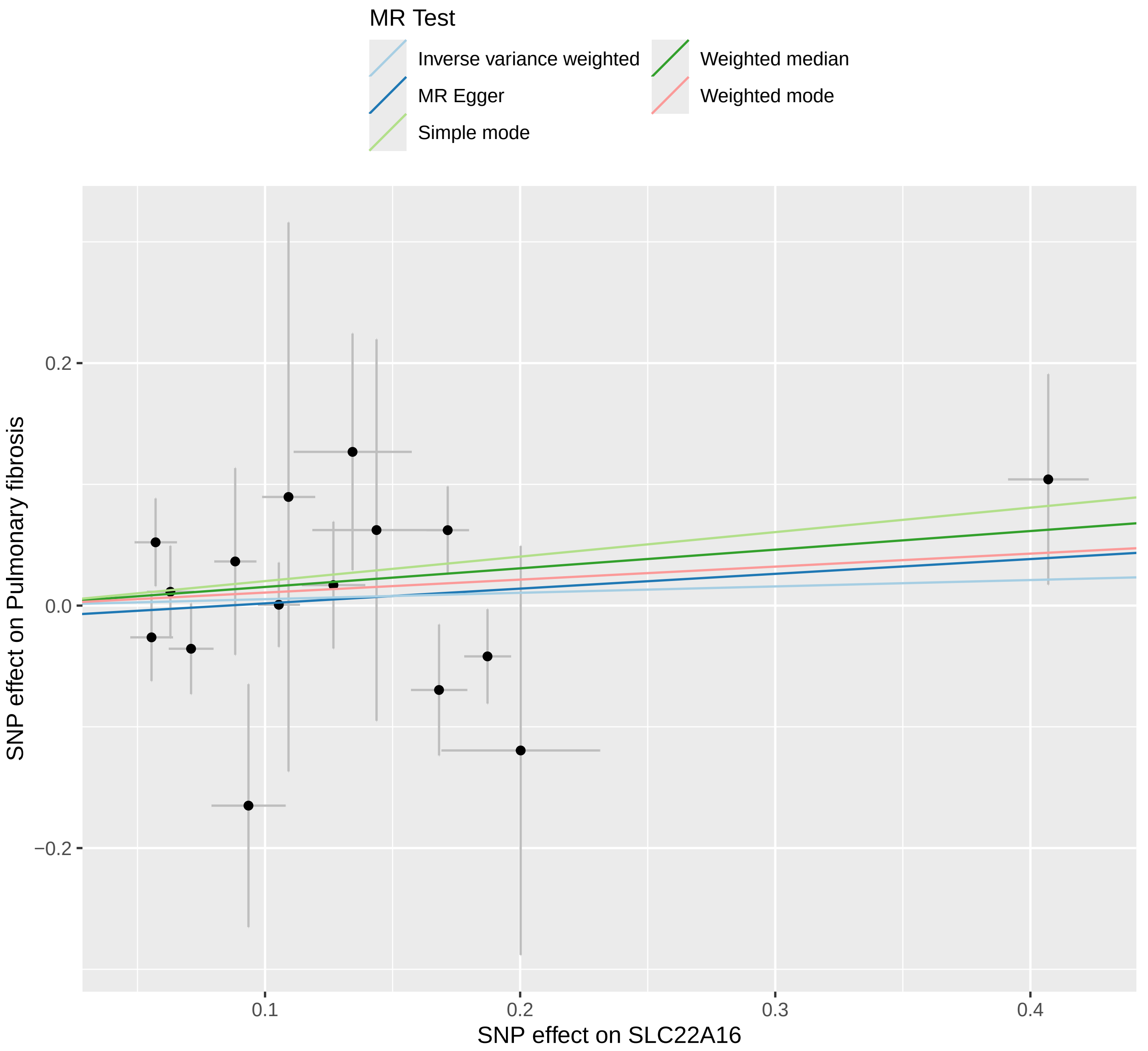
MR Test
Inverse variance weighted
MR Egger
Simple mode
Weighted median
Weighted mode
0.2
0.0
−0.2
0.1
0.2
0.3
0.4
SNP effect on SLC22A16
SNP effect on Pulmonary fibrosis

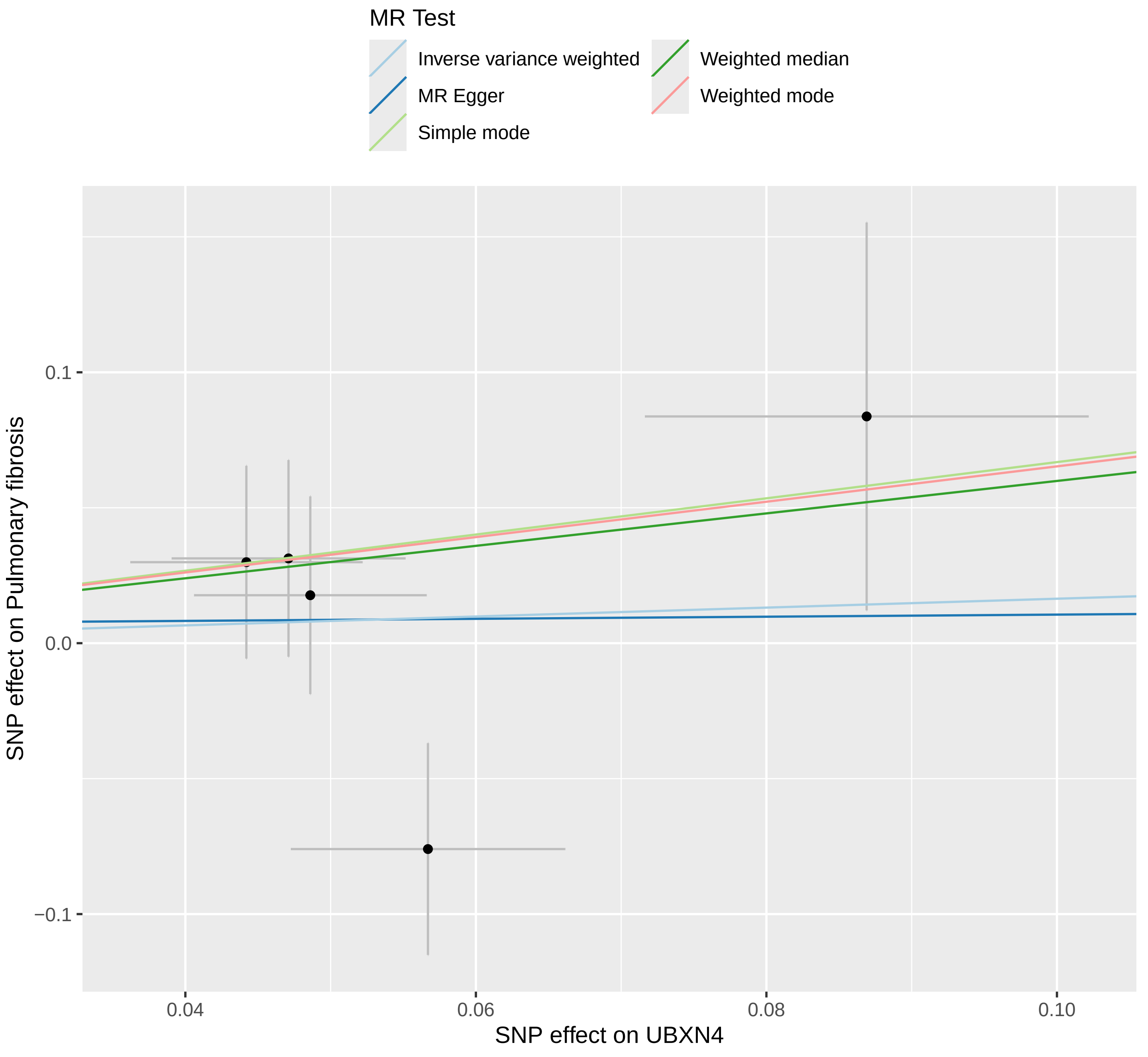

MR Test
Inverse variance weighted
MR Egger
Simple mode
Weighted median
Weighted mode
0.1
0.0
−0.1
0.04
0.06
0.08
0.10
SNP effect on UBXN4
SNP effect on Pulmonary fibrosis

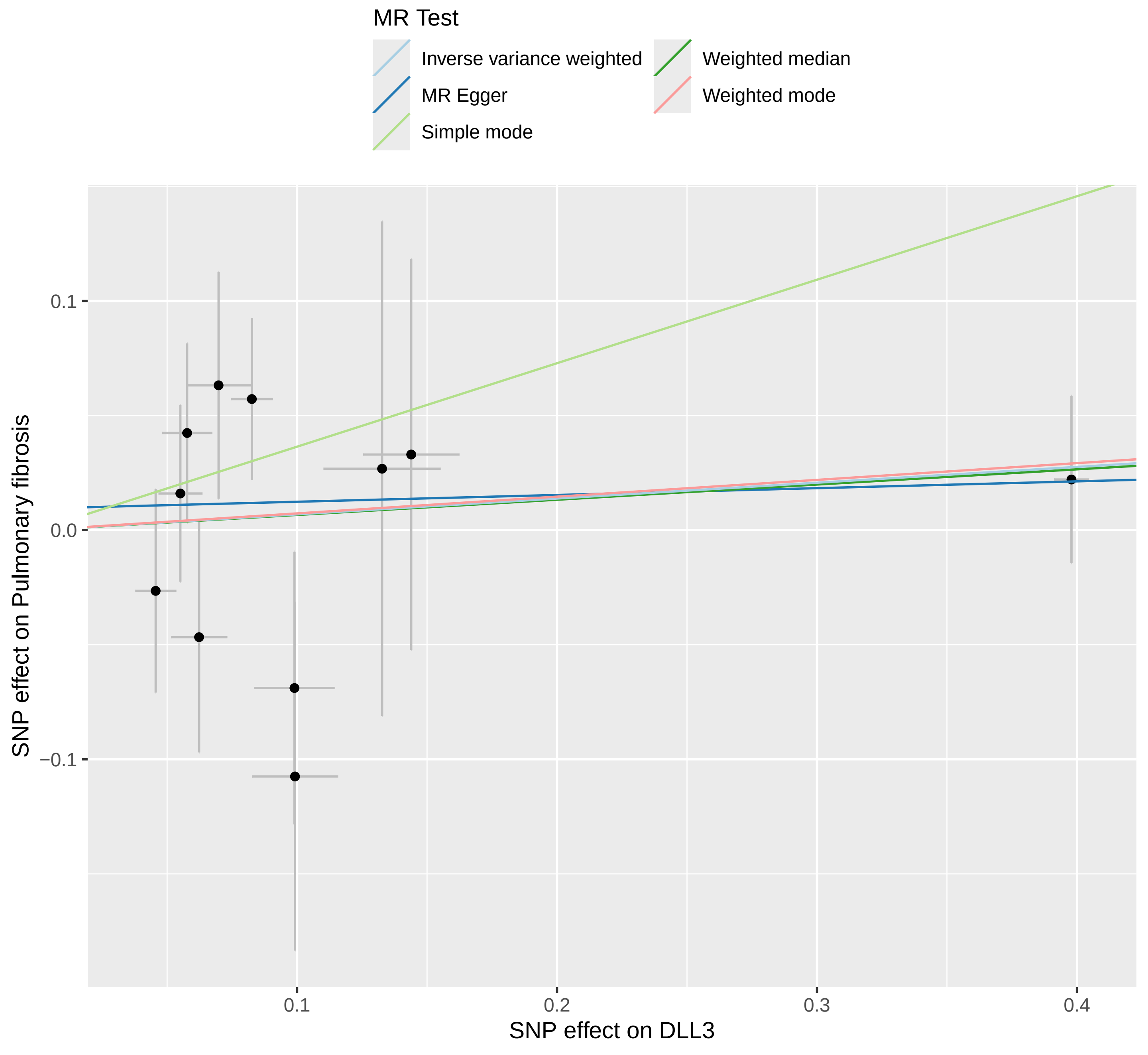

MR Test
Inverse variance weighted
MR Egger
Simple mode
Weighted median
Weighted mode
0.1
0.0
−0.1
SNP effect on Pulmonary fibrosis
0.1
0.2
0.3
0.4
SNP effect on DLL3

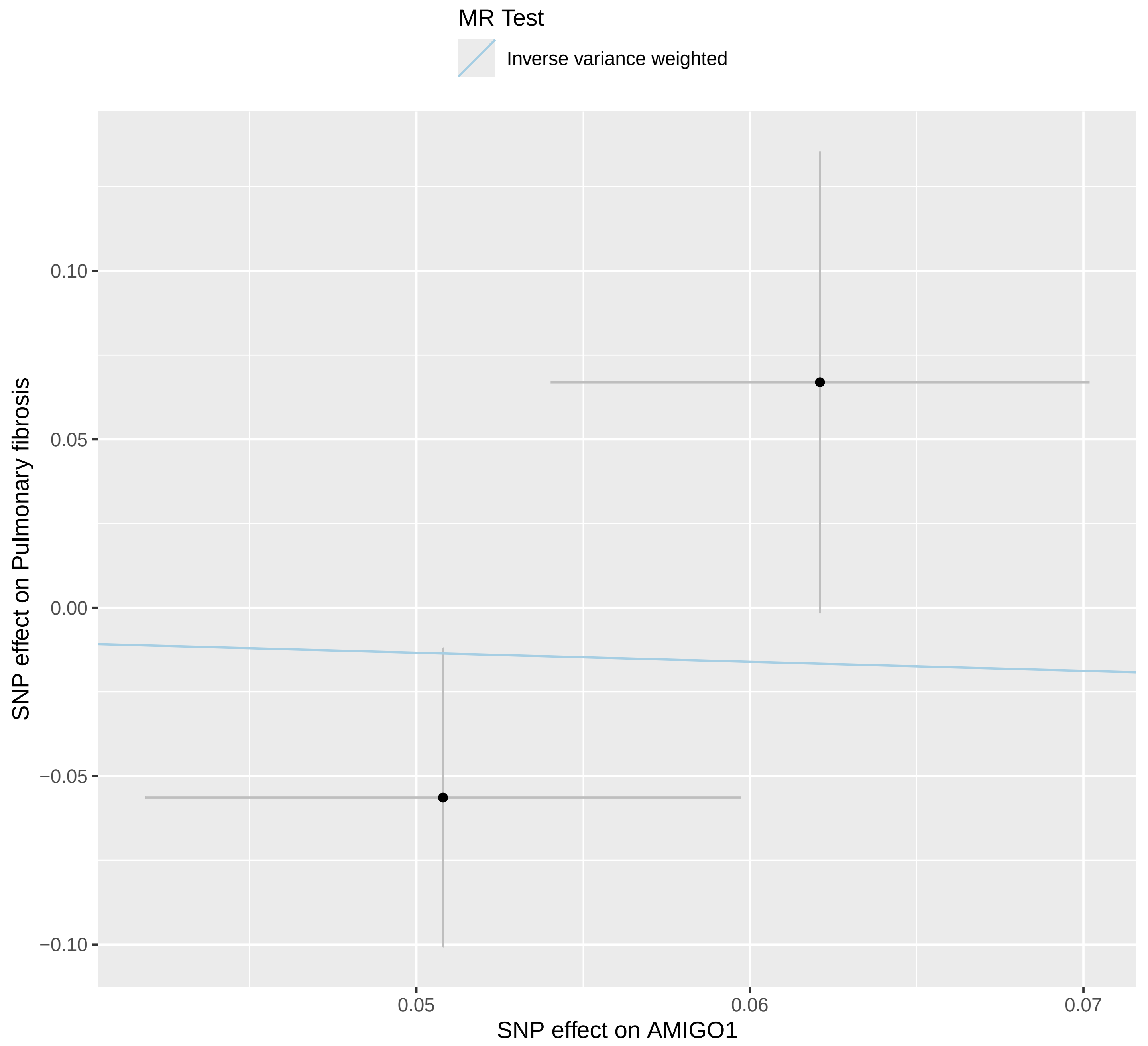
MR Test
Inverse variance weighted
SNP effect on Pulmonary fibrosis
0.10
0.05
0.00
−0.05
−0.10
0.05
0.06
0.07
SNP effect on AMIGO1

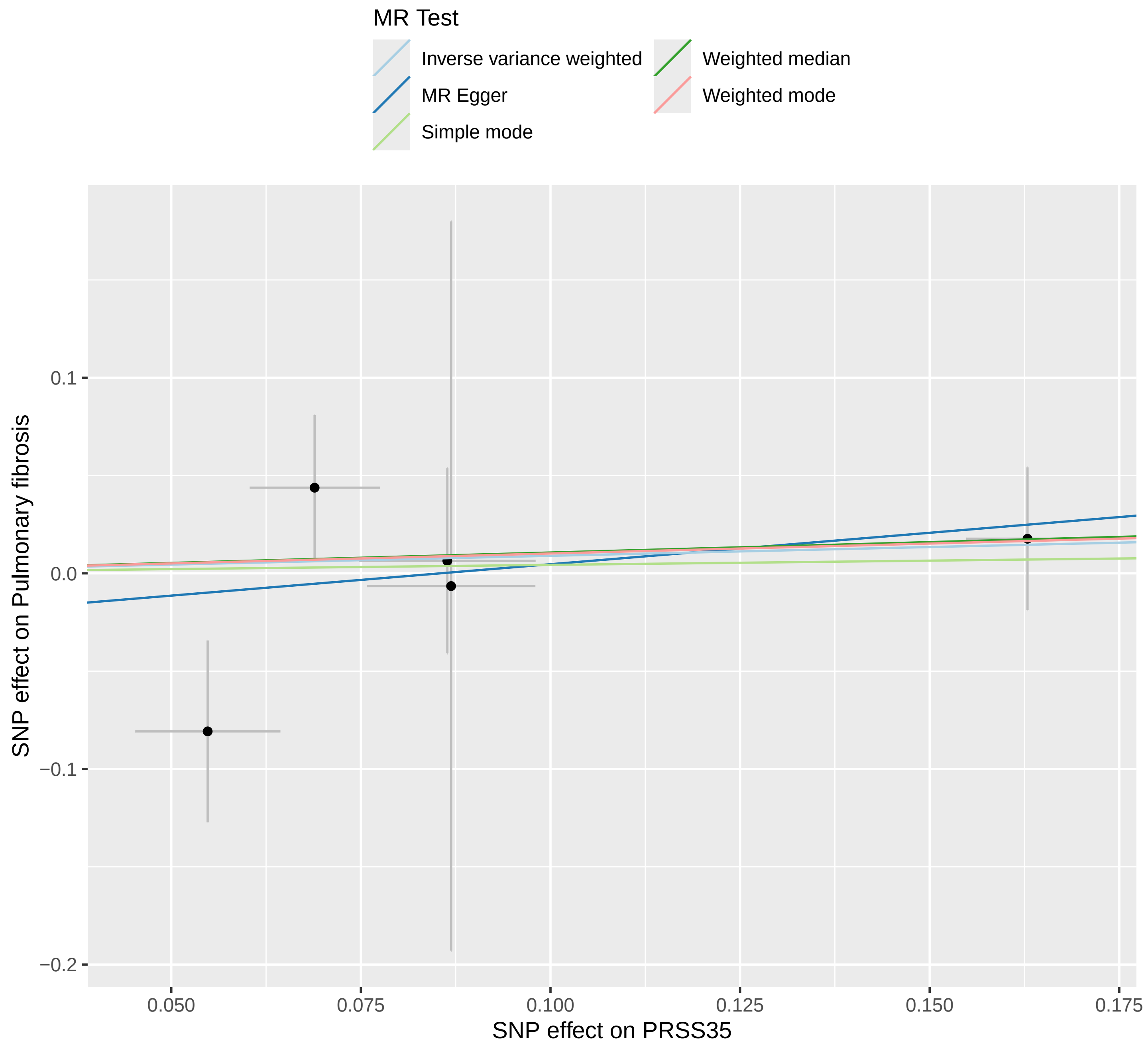
MR Test
Inverse variance weighted
MR Egger
Simple mode
Weighted median
Weighted mode
SNP effect on Pulmonary fibrosis
0.1
0.0
−0.1
−0.2
0.050
0.075
0.100
0.125
0.150
0.175
SNP effect on PRSS35

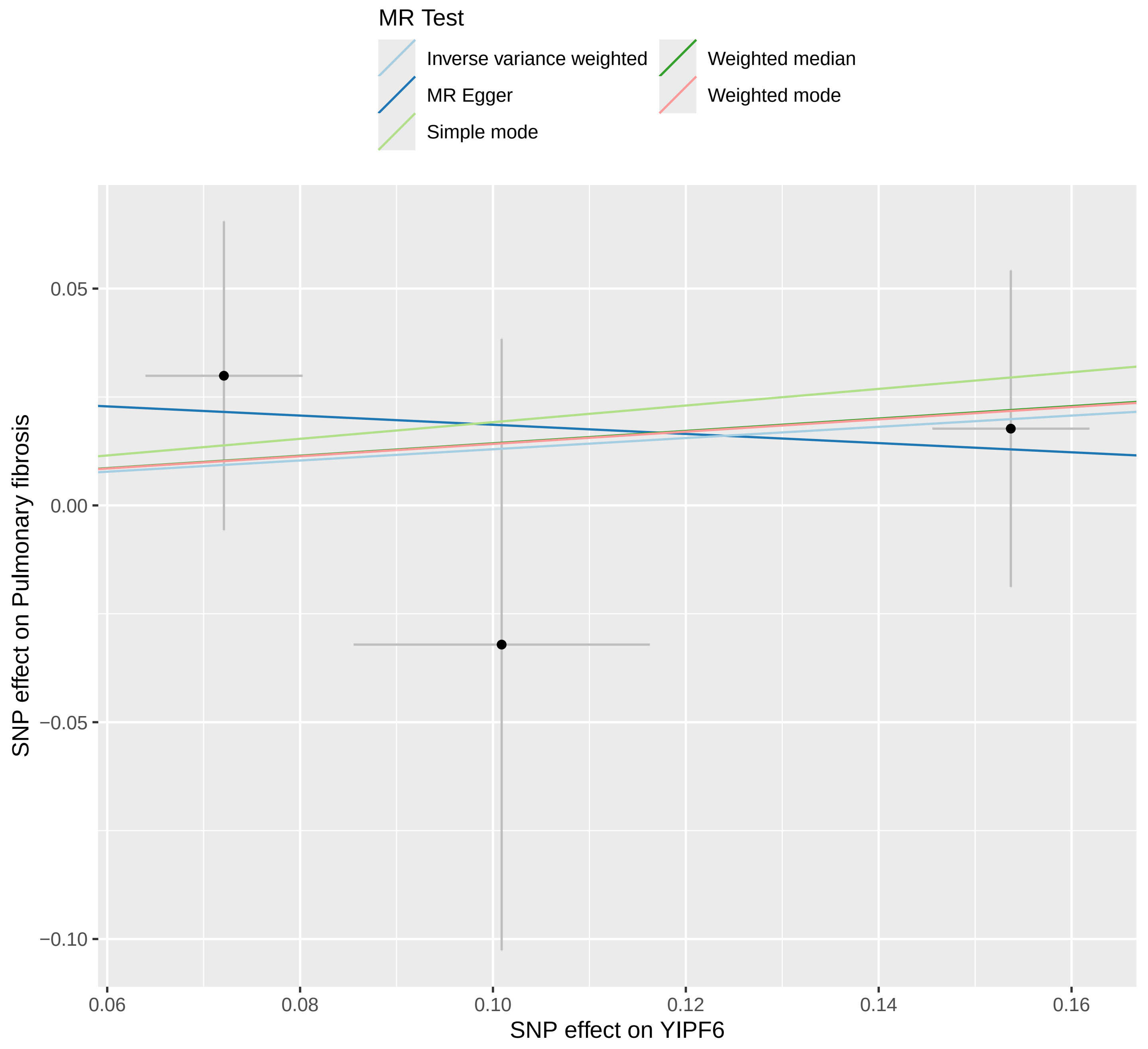
MR Test
Inverse variance weighted
MR Egger
Simple mode
Weighted median
Weighted mode
SNP effect on Pulmonary fibrosis
0.05
0.00
−0.05
−0.10
0.06
0.08
0.10
0.12
0.14
0.16
SNP effect on YIPF6

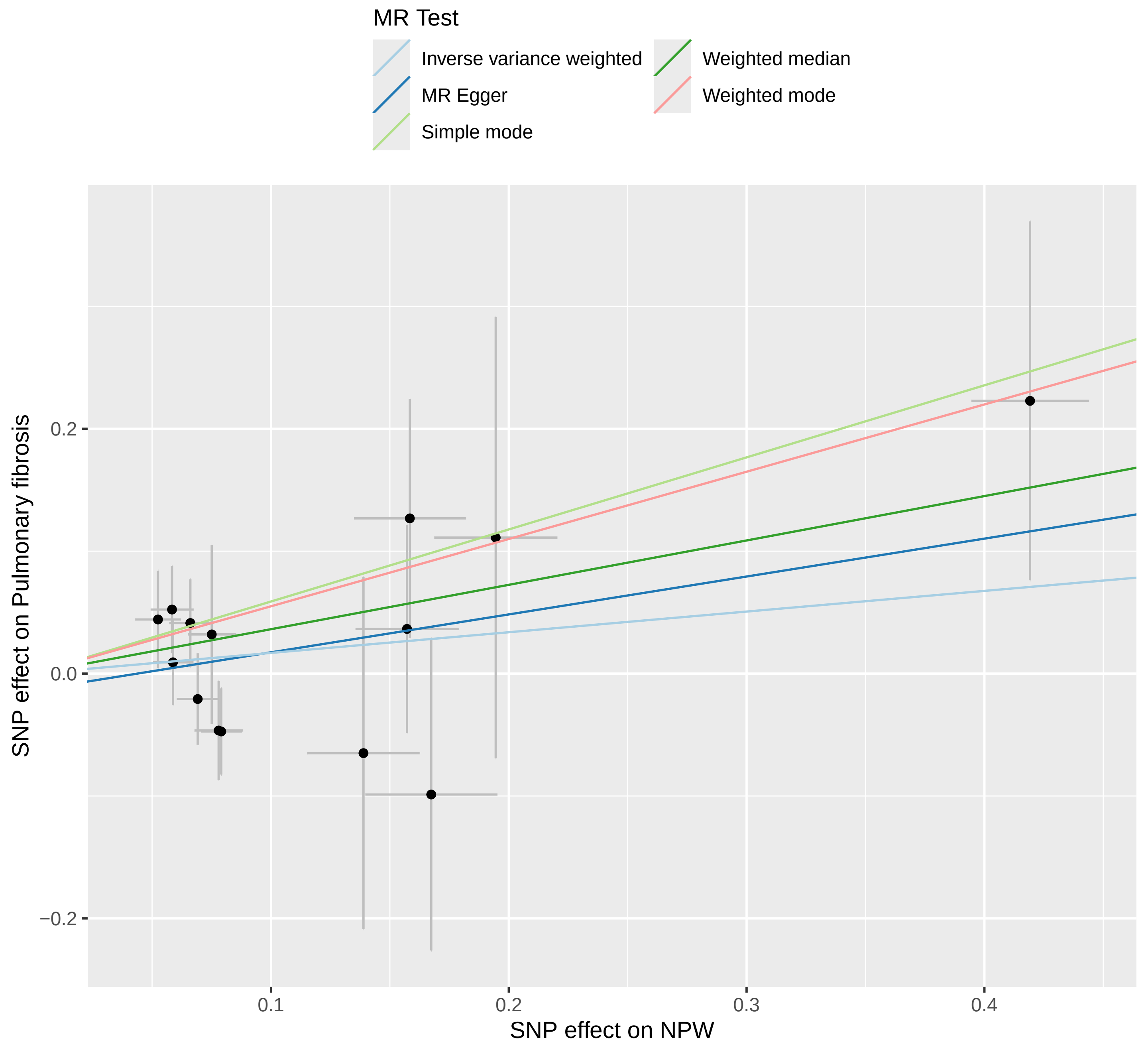

MR Test
Inverse variance weighted
MR Egger
Simple mode
Weighted median
Weighted mode
0.2
0.0
−0.2
0.1
0.2
0.3
0.4
SNP effect on Pulmonary fibrosis
SNP effect on NPW

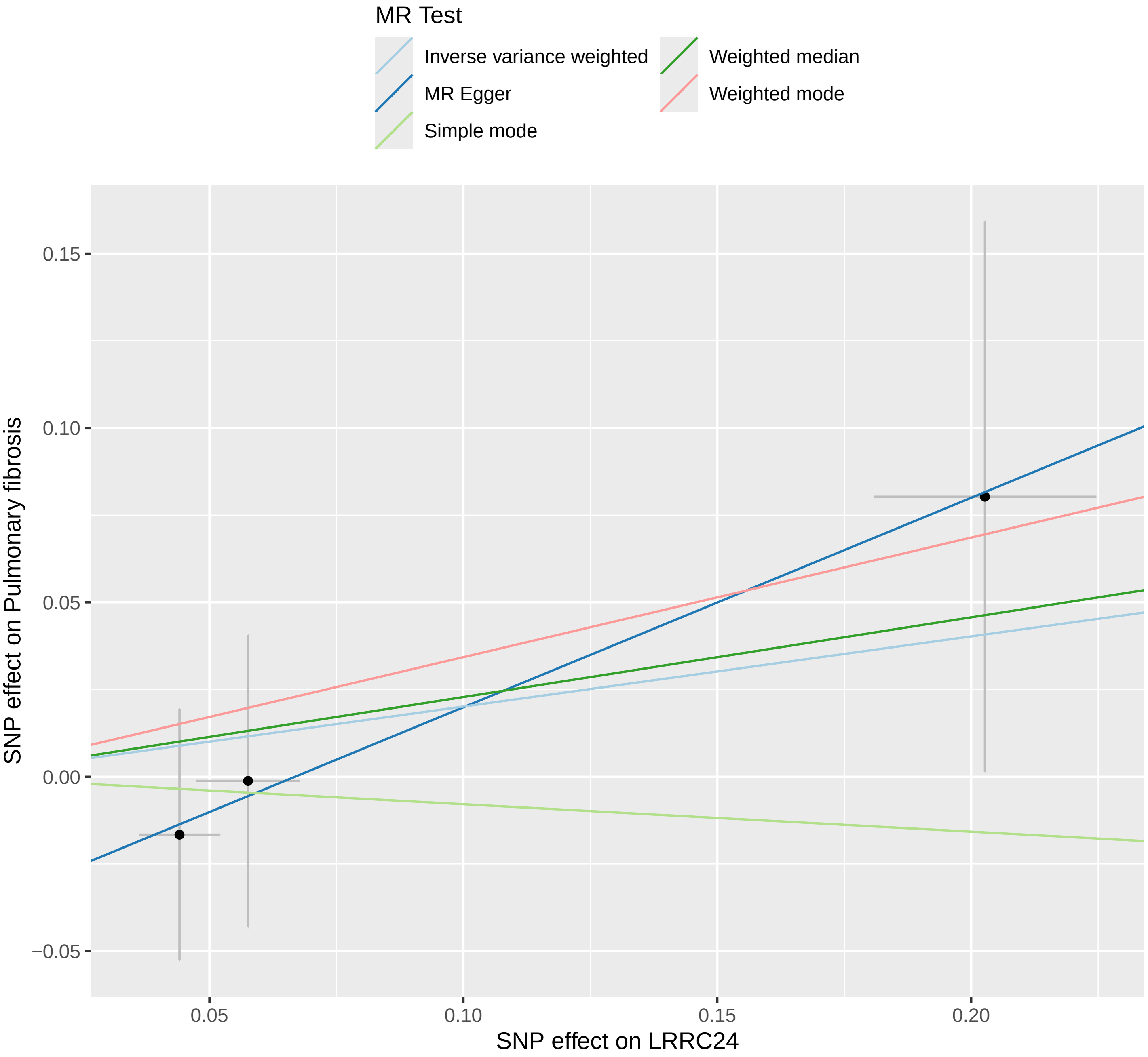
MR Test
Inverse variance weighted
MR Egger
Simple mode
Weighted median
Weighted mode
0.15
0.10
0.05
0.00
−0.05
SNP effect on Pulmonary fibrosis
0.05
0.10
0.15
0.20
SNP effect on LRRC24

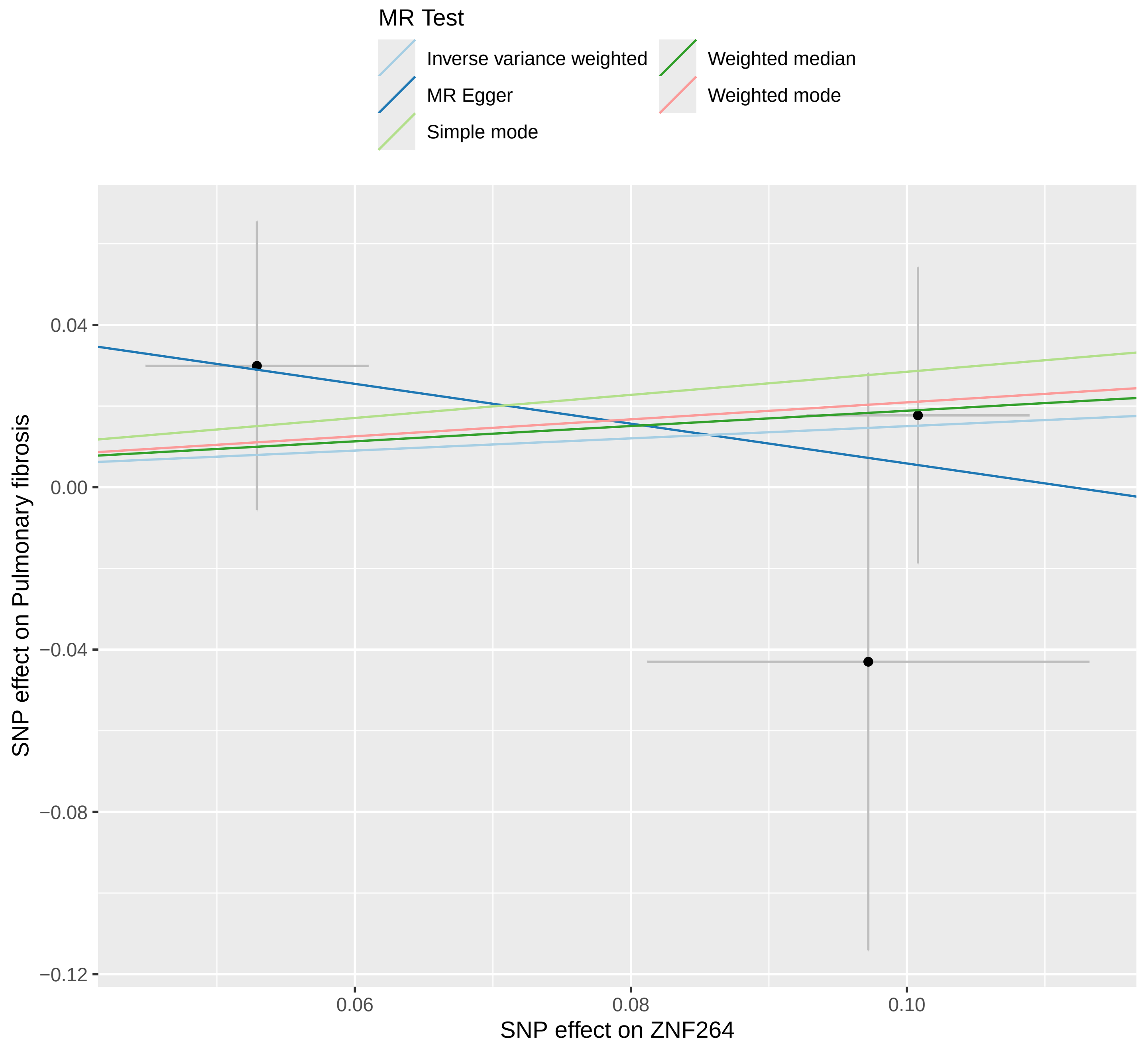

MR Test
Inverse variance weighted
MR Egger
Simple mode
Weighted median
Weighted mode
SNP effect on Pulmonary fibrosis
0.04
0.00
−0.04
−0.08
−0.12
0.06
0.08
0.10
SNP effect on ZNF264

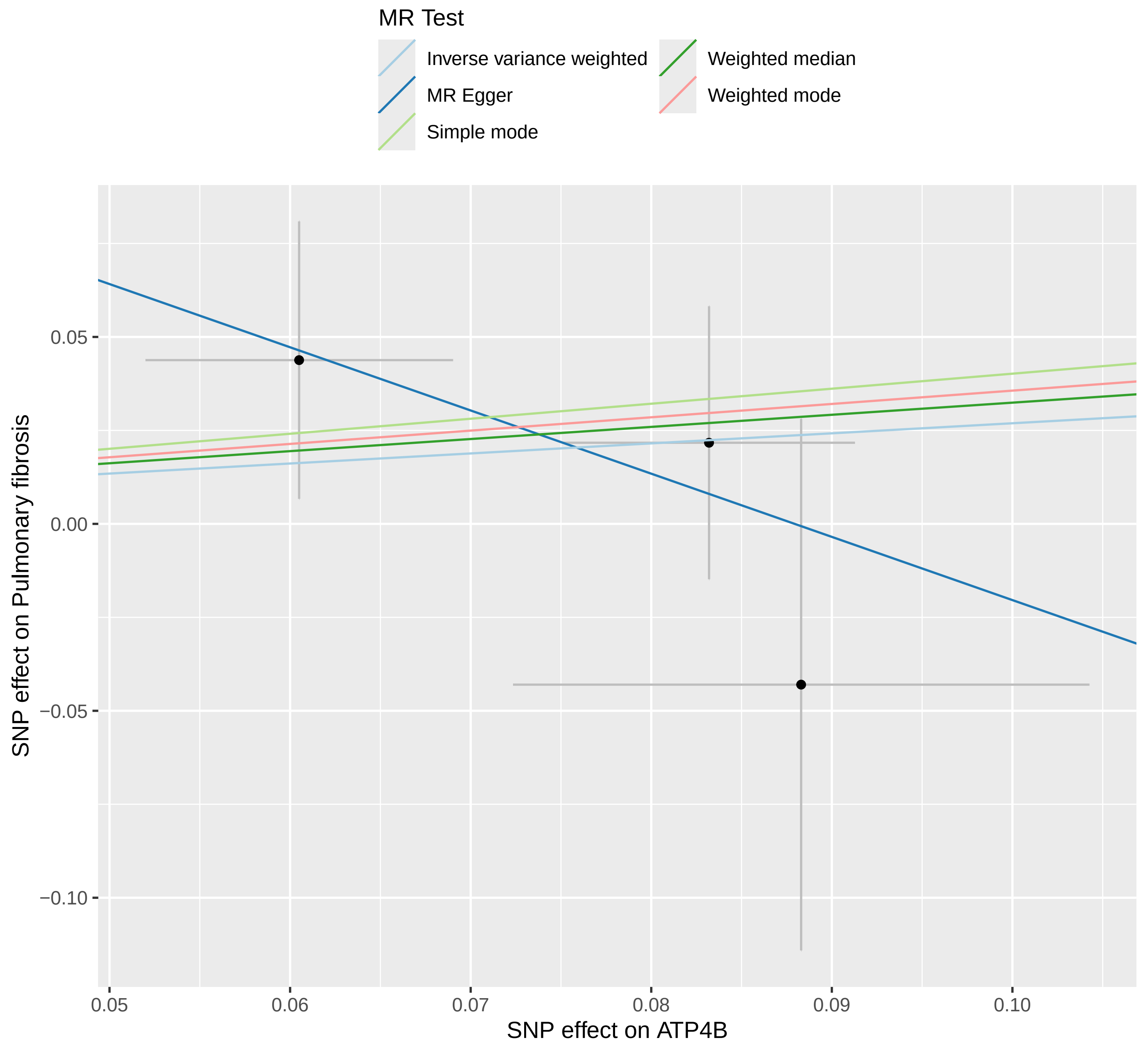

MR Test
Inverse variance weighted
MR Egger
Simple mode
Weighted median
Weighted mode
0.05
0.00
−0.05
−0.10
0.05
0.06
0.07
0.08
0.09
0.10
SNP effect on Pulmonary fibrosis
SNP effect on ATP4B

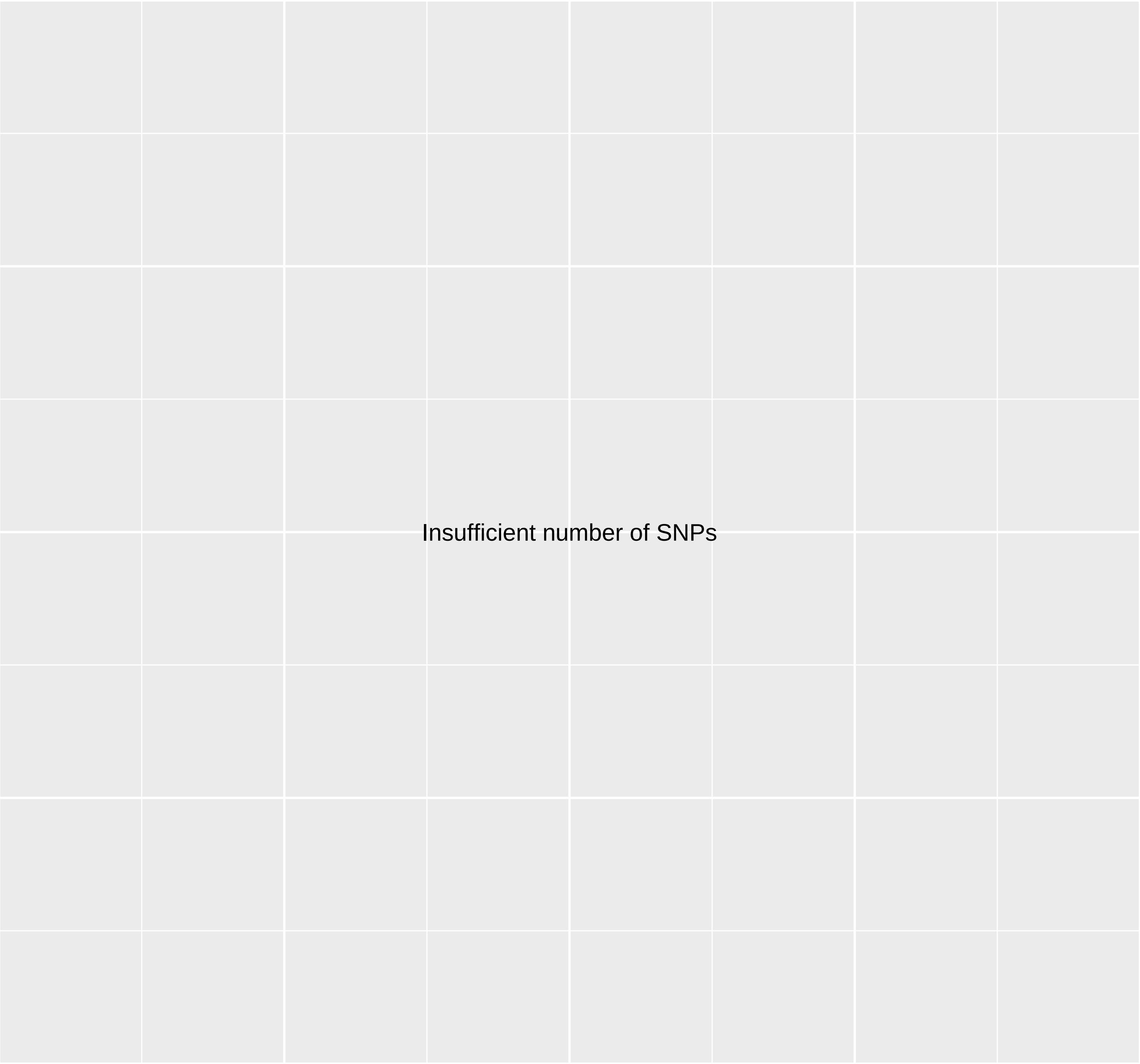
Insufficient number of SNPs
